# Supplementary material for: A genome-wide association study of mass spectrometry proteomics using a nanoparticle enrichment platform
Source: Nat Genet. 2025 Nov 27;57(12):2987–96. doi: 10.1038/s41588-025-02413-w (PMC12695657; doi:10.1038/s41588-025-02413-w)
Supplement: Supplementary file 1 — Supplementary Figs. 1–16 and Notes 1–2. [file 41588_2025_2413_MOESM1_ESM.pdf]

nature genetics

Supplementary information https://doi.org/10.1038/s41588-025-02413-w

A genome-wide association study of mass spectrometry proteomics using a nanoparticle enrichment platform

In the format provided by the authors and unedited

TABLE OF CONTENT

Supplementary Figure 1: Histogram of the highest Spearman correlation (rho) between proteins quantified in >80% of the samples in all five nanoparticle runs.

Supplementary Figure 2: Scatter plots of the first three genetic principal component (PCA) coordinates.

Supplementary Figure 3: Visualization of the Proteograph MS-proteomics data for the MST1 pQTL.

Supplementary Figure 4: Regional association plot for the GALC pQTL.

Supplementary Figure 5: Example of a pQTL with different genetic architecture between Tarkin and QMDiab.

Supplementary Figure 6: Forest plot for the meta-analysis on the peptide level.

Supplementary Figure 7: Violin plots of protein and up of to 19 peptide levels by genotype

Supplementary Figure 8: Scatterplot of the MSPA scores

Supplementary Figure 9: Scatterplot of the MSPA score against MAF

Supplementary Figure 10: Scatter plots of MSPA scores against meta-analyzed effect sizes of the peptide level associations

Supplementary Figure 11: Scatterplot of absolute protein concentrations from the Human Protein Atlas with the MSPA score

Supplementary Figure 12: Dependence of the MSPA score on the rank of the affinity pQTL.

Supplementary Figure 13: Peptide level violin plots of affinity pQTLs with power >99%, but that did not replicate, had a low MSPA scores (< 0.2), and for which PAV peptides have also been detected on the MS platform

Supplementary Figure 14: Mapping of peptides detected on nanoparticle run NP5 for ITLN1 (UniProt Q8WWA0) to the amino acid sequence of that protein.

Supplementary Figure 15: Scatterplots Tarkin versus QMDiab

Supplementary Figure 16: Regional association plots for the SPINK5 splice-QTL

Supplementary Note 1: A GWAS with protein missingness.

Supplementary Note 2: Age and sex associations were concordant between the affinity and MS based proteomics platforms.

SUPPLEMENTARY FIGURES

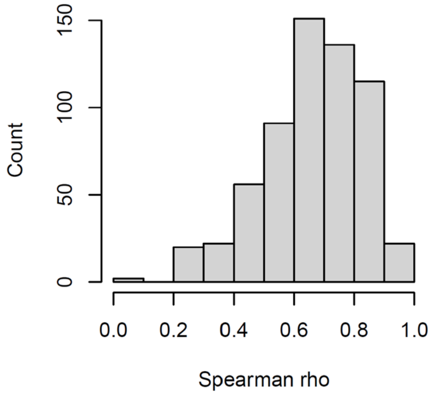

Supplementary Figure 1: Histogram of the highest Spearman correlation (rho) between proteins quantified in >80% of the samples in all five nanoparticle runs. These can be considered as quasi-technical replicates (Data in Supplementary Table 1).

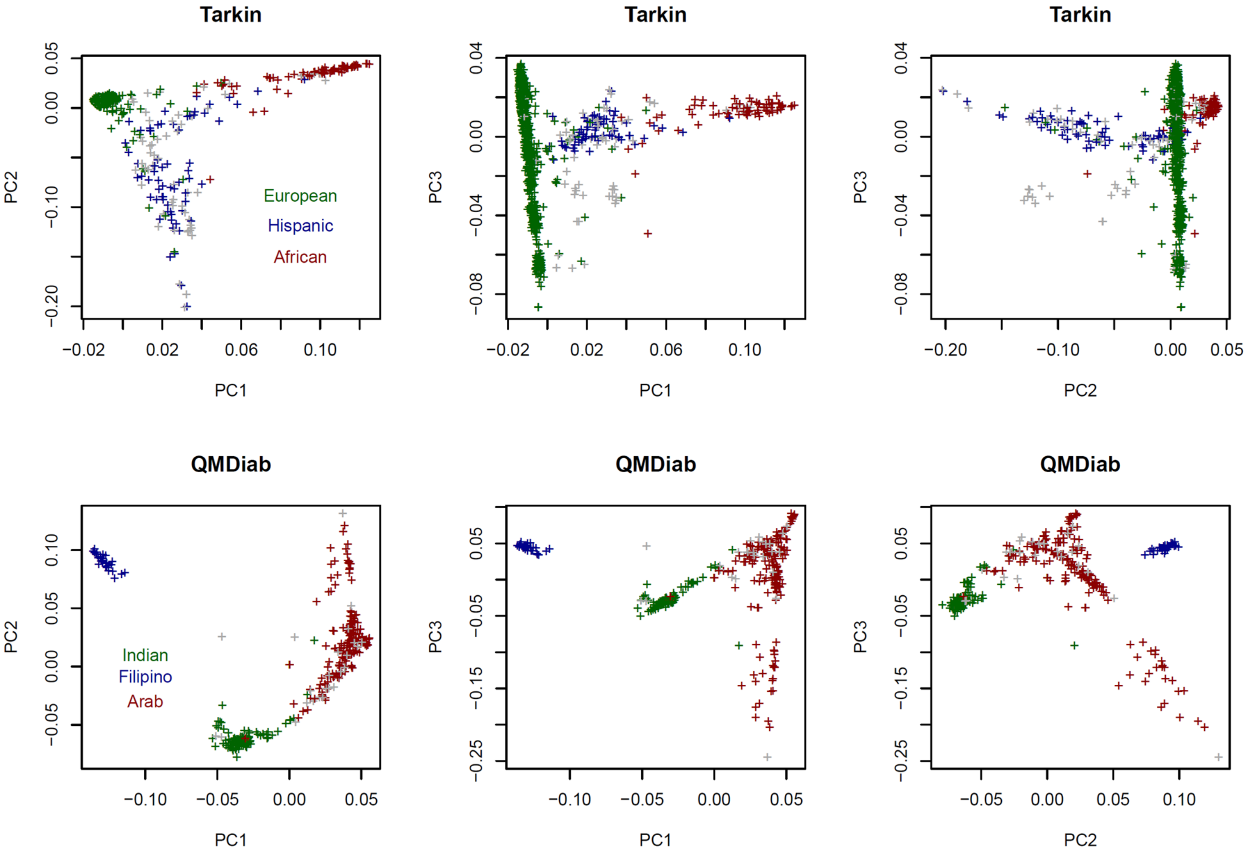

Supplementary Figure 2: Scatter plots of the first three genetic principal component (PCA) coordinates for Tarkin (top row) and QMDiab (bottom row), coloured by self-reported genetic ancestry show that genetic ancestry is well captured by these first three components. Please note that the contribution of the first ten PCA component was regressed out before the GWAS. Three proteins exhibited a residual signal due to ancestry that led to genomic inflation and were removed from the analysis.

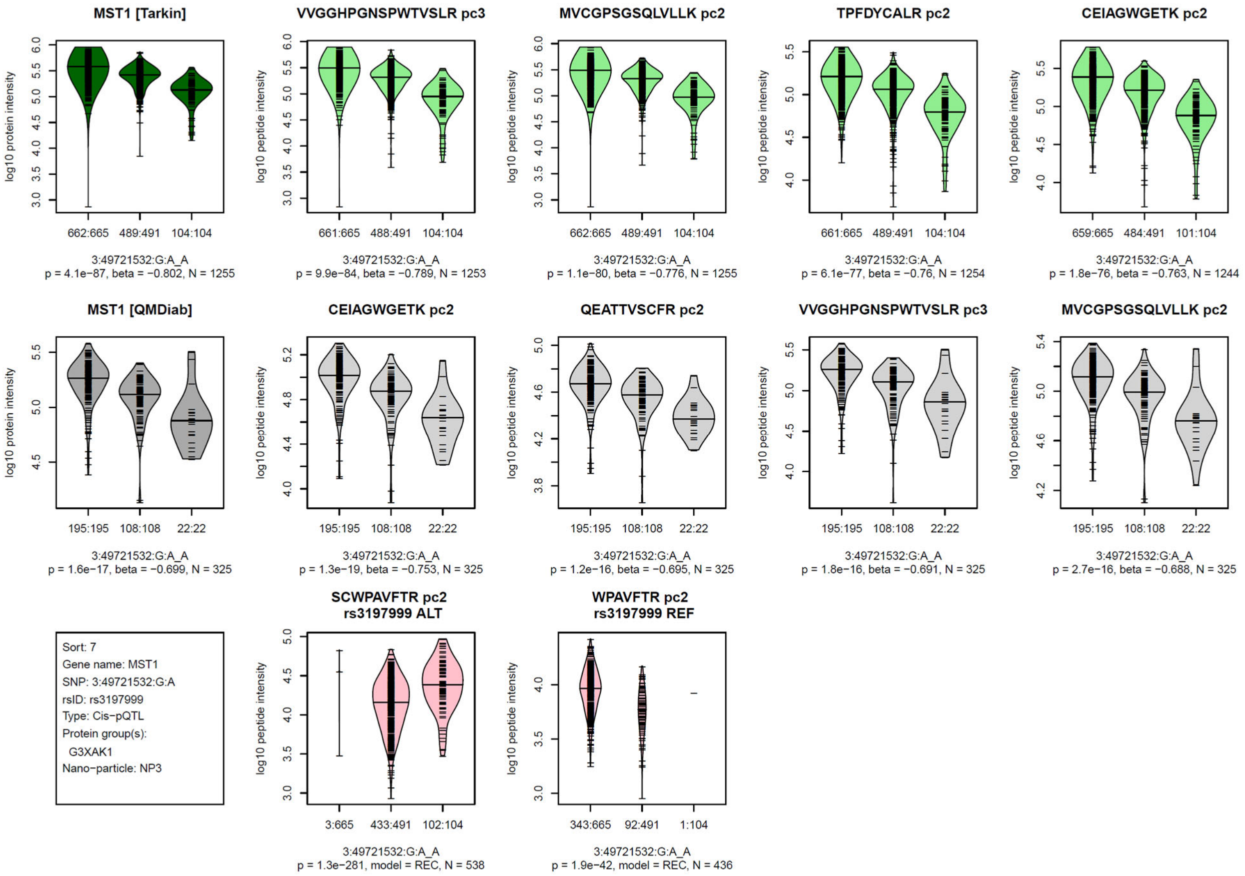

Supplementary Figure 3: Visualization of the Proteograph MS-proteomics data for the MST1 pQTL. Violin plots of log-scaled engine-normalized protein (left most column) and peptide intensities (following four columns) by genotype for the indicated protein and genetic variant. The bottom left box summarizes general information about this pQTL, with full details available in Supplementary Table 2; Top row (green): Tarkin data, using the *PAV-exclusive* library, Middle row (grey): QMDiab data, using the *PAV-exclusive* library, Bottom row (red): Tarkin data, using *PAV-inclusive* library, limited to PAV containing peptides; Titles above the peptide plots indicate the respective peptide sequences and precursor charges (pc), and additionally the PAV variant rsID and allele type (REF/ALT) for the PAV peptide plots; Summary statistics are reported below the plots and are based on linear models with residualized and inverse-normal scaled data for associations with the *PAV-exclusive* library data (top two rows) and Fisher's exact test for *PAV-inclusive* library data (bottom row); Associations with peptide data are sorted by increasing p-values from left to right and limited to a maximum of four plots; Whenever peptides were detected at multiple precursor charge values, only the strongest association was plotted; Numbers at the x-axis tick marks indicate the number of detected peptides by genotype followed by the number a samples with the corresponding genotype (e.g. 662:665); Genotypes are ordered as (1) other allele, (2) heterozygote, (3) effect allele, where the effect allele is indicated following the SNP name (chr:pos:ref:alt_eff, e.g. 3:49721532:G:A_A). Violin plots of protein and up to four peptide levels by genotype are provided as Supplementary Data 1 for 364 pQTLs identified in this study. Note that in this case the PAV rs3197999 is a C→R amino acid exchange that introduces a new trypsin cleavage site and hence leads to a shorter peptide for the alternate variant (SCWPAVFTR vs. WPAVFTR).

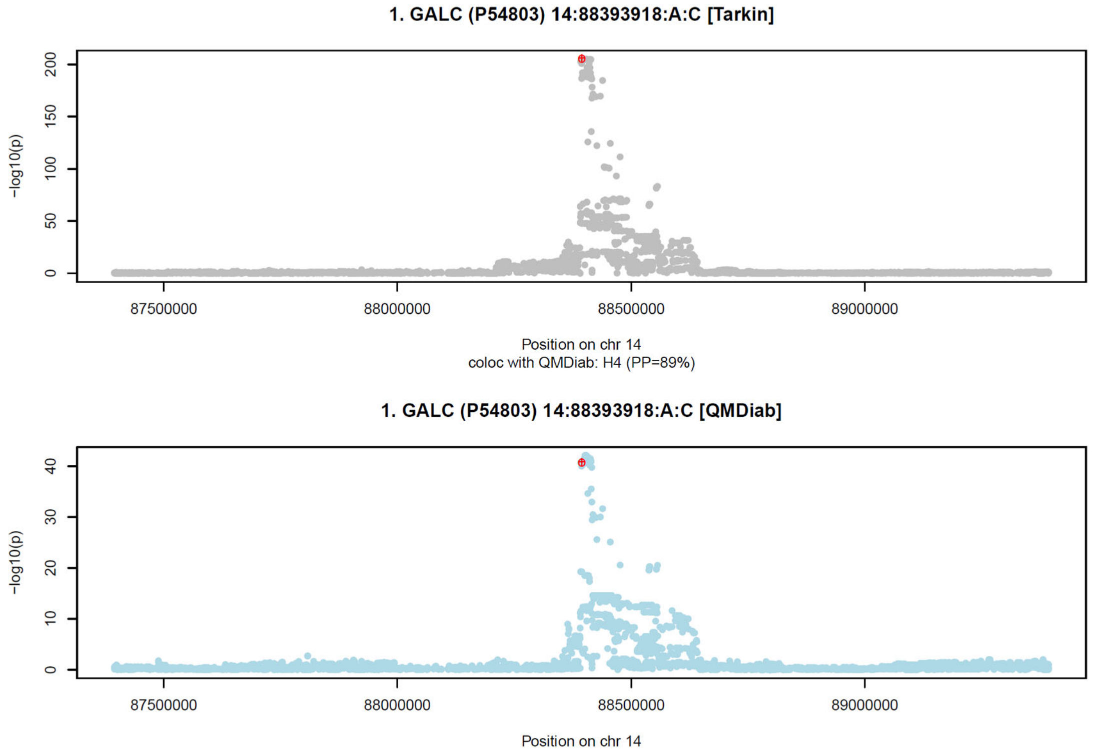

Supplementary Figure 4: Regional association plot for the GALC pQTL. Similar plots for all 364 pQTLs identified in this study are available as Supplementary Data 2. Shown here are the -log10(p-values) for the association of GALC with all variants that are available in Tarkin and QMDiab within a window size of +/-1MB around the pQTL lead variant (top row). For all pQTLs where matching SNP and protein data was available in QMDiab, a regional association plot is also provided for QMDiab (334 out of 364 pQTLs). For these cases, a coloc analysis was conducted; the corresponding supported hypothesis (H4: shared signal, H3: different signals, H2: signal in QMDiab only, H1: signal in Tarkin only, H0: no signal) and posterior probability (PP) are indicated below the upper plot; the replication SNP is indicated in red and may be different from the discovery SNP in cases where the discovery SNP was not available in QMDiab.

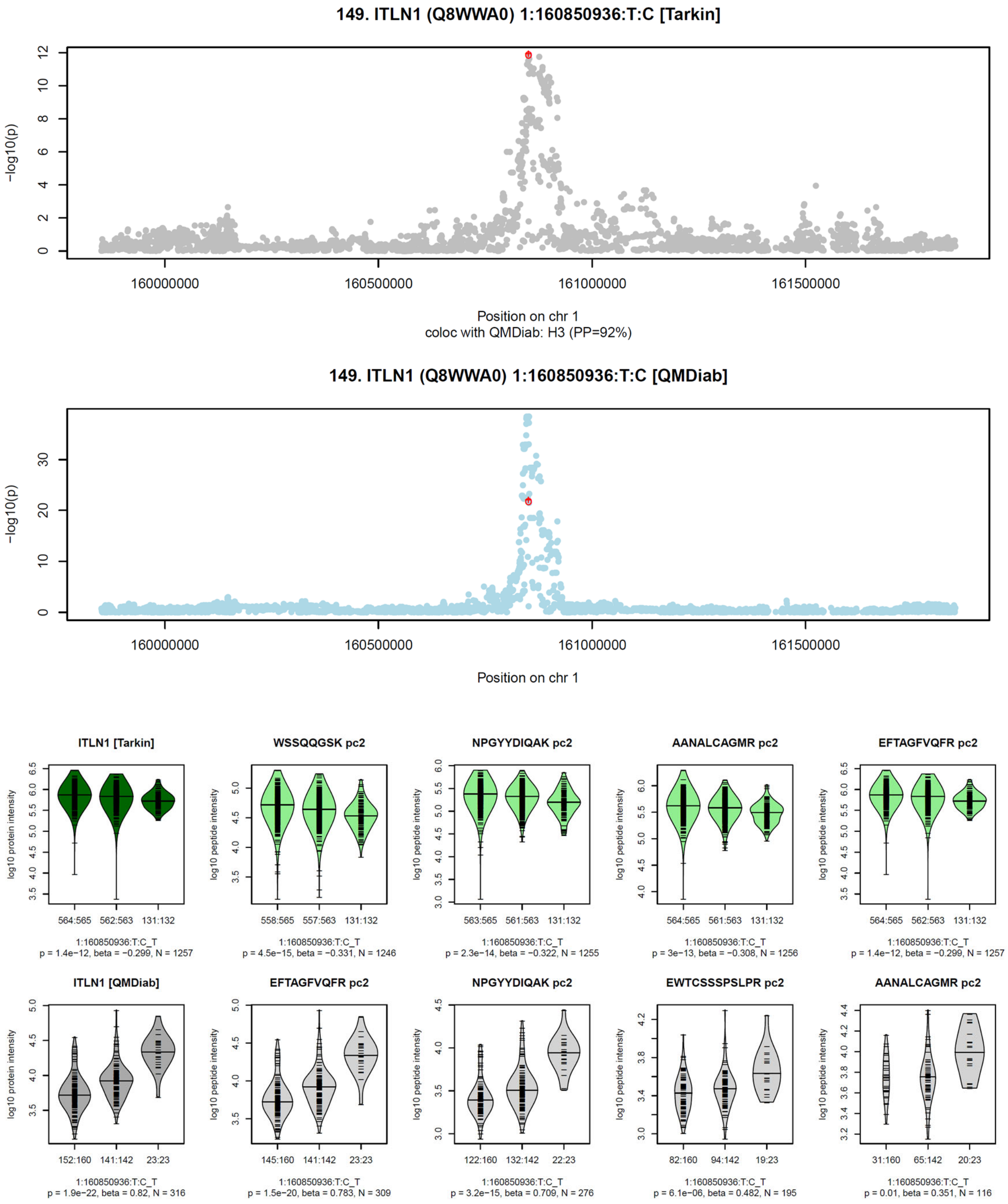

Supplementary Figure 5: Example of a pQTL with different genetic architecture between Tarkin and QMDiab. The intelectin-1 (ITLN1) pQTL is an example of a case where the genetic signal differs between the two cohorts (coloc hypothesis H3).

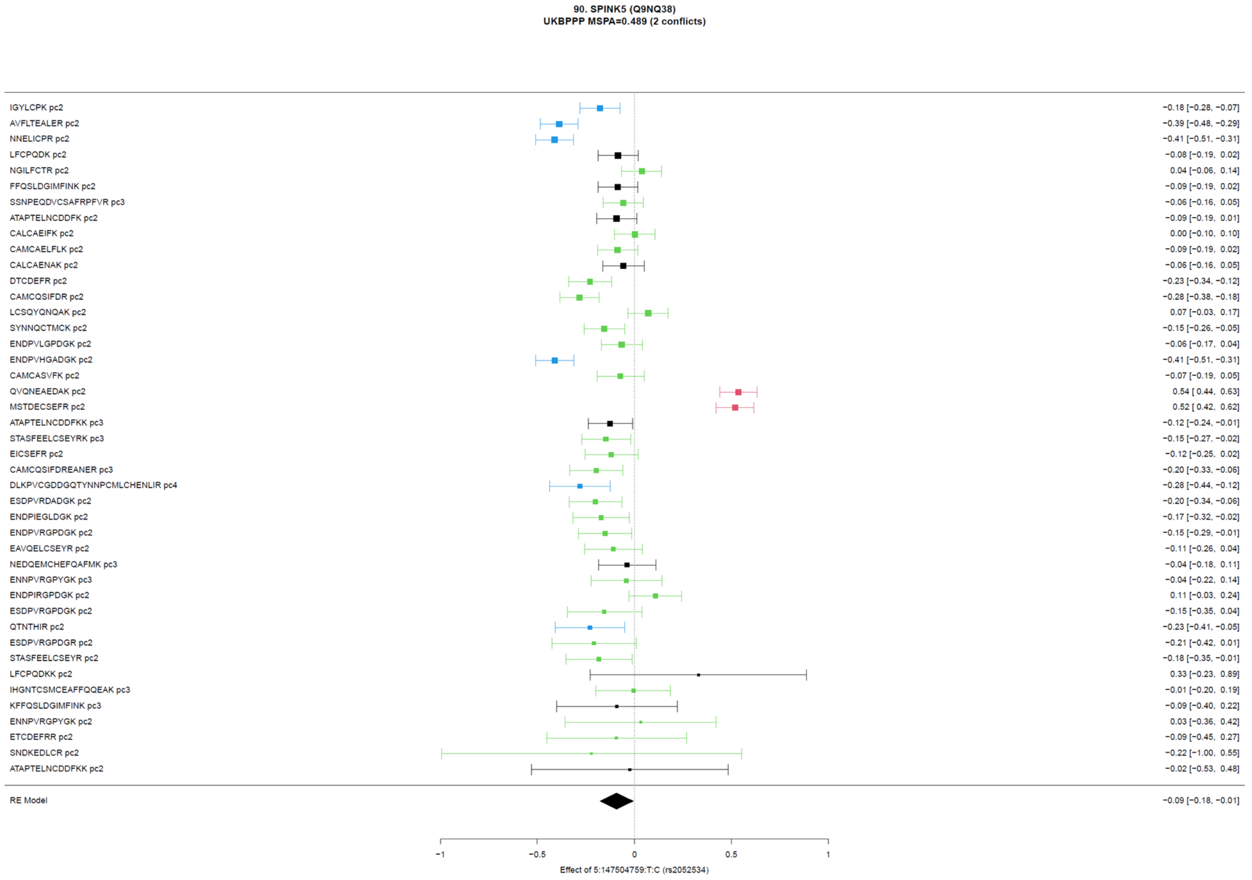

Supplementary Figure 6: Forest plot for the meta-analysis on the peptide level of the SPINK5 pQTL indicating the presence of conflicting directionality of the peptide pQTL signals. Peptides are coloured by protein group: “E5RFU9; Q9NQ38; Q9NQ38-2; Q9NQ38-3” (black), “Q9NQ38; Q9NQ38-2; Q9NQ38-3” (green), “Q9NQ38; Q9NQ38-3” (blue), “Q9NQ38-3” (red). The genetic variant is in LD with a strong GTEx splice-QTL that likely leads to a differential generation of SPINK5 isoforms (see Figure 4). The count of peptide level QTLs with conflicting directionality can be found in the supplementary tables to identify other such cases. Similar forest plots are provided for all meta-analyzed pQTLs as Supplementary Data 3-5. See also Supplementary Figure 16 for a regional association plot.

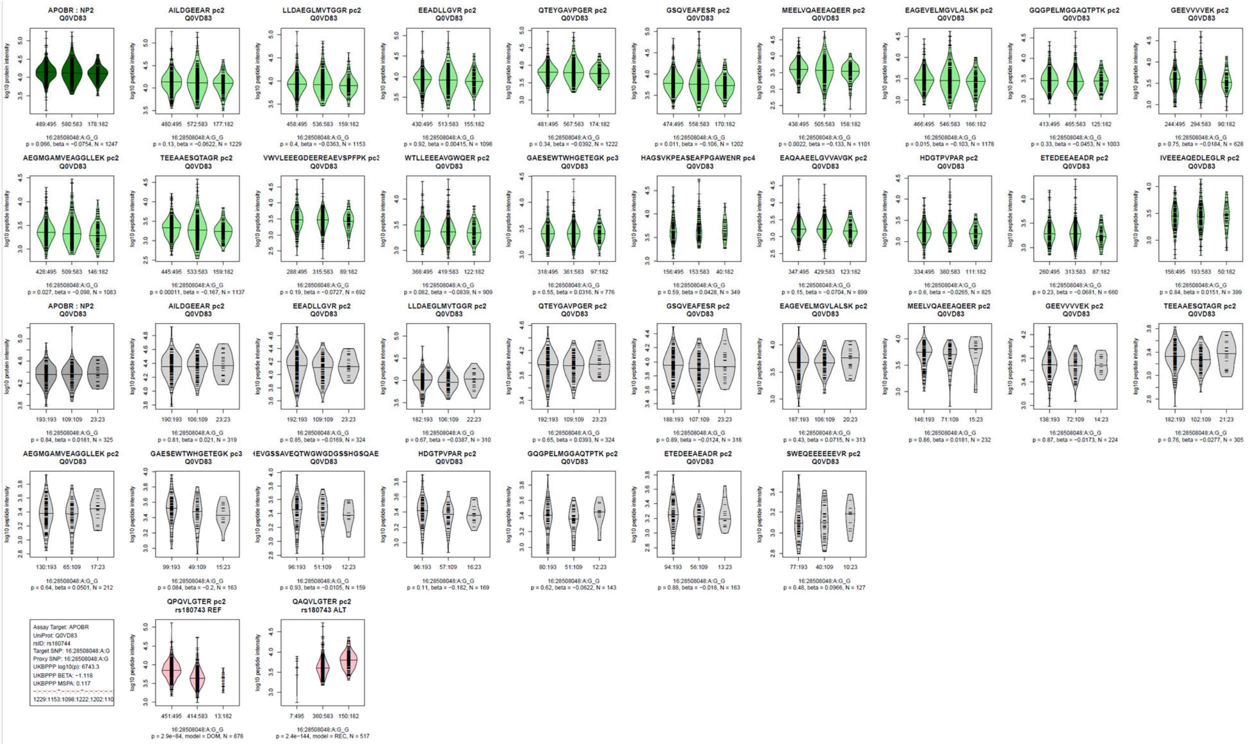

Supplementary Figure 7: Violin plots of protein and up of to 19 peptide levels by genotype are provided for 364 pQTLs identified in this study as Supplementary Data 3, for 319 cis-pQTLs identified by the deCODE SOMAscan study as Supplementary Data 4, and for 392 cis-pQTLs identified by the UKB-PPP Olink study as Supplementary Data 5 (see legend to Supplementary Figure 3 for details on the presented violin plots). The summary statistics displayed below the plots and used for the meta-analysis are provided in the respective Supplementary Tables 7-8.

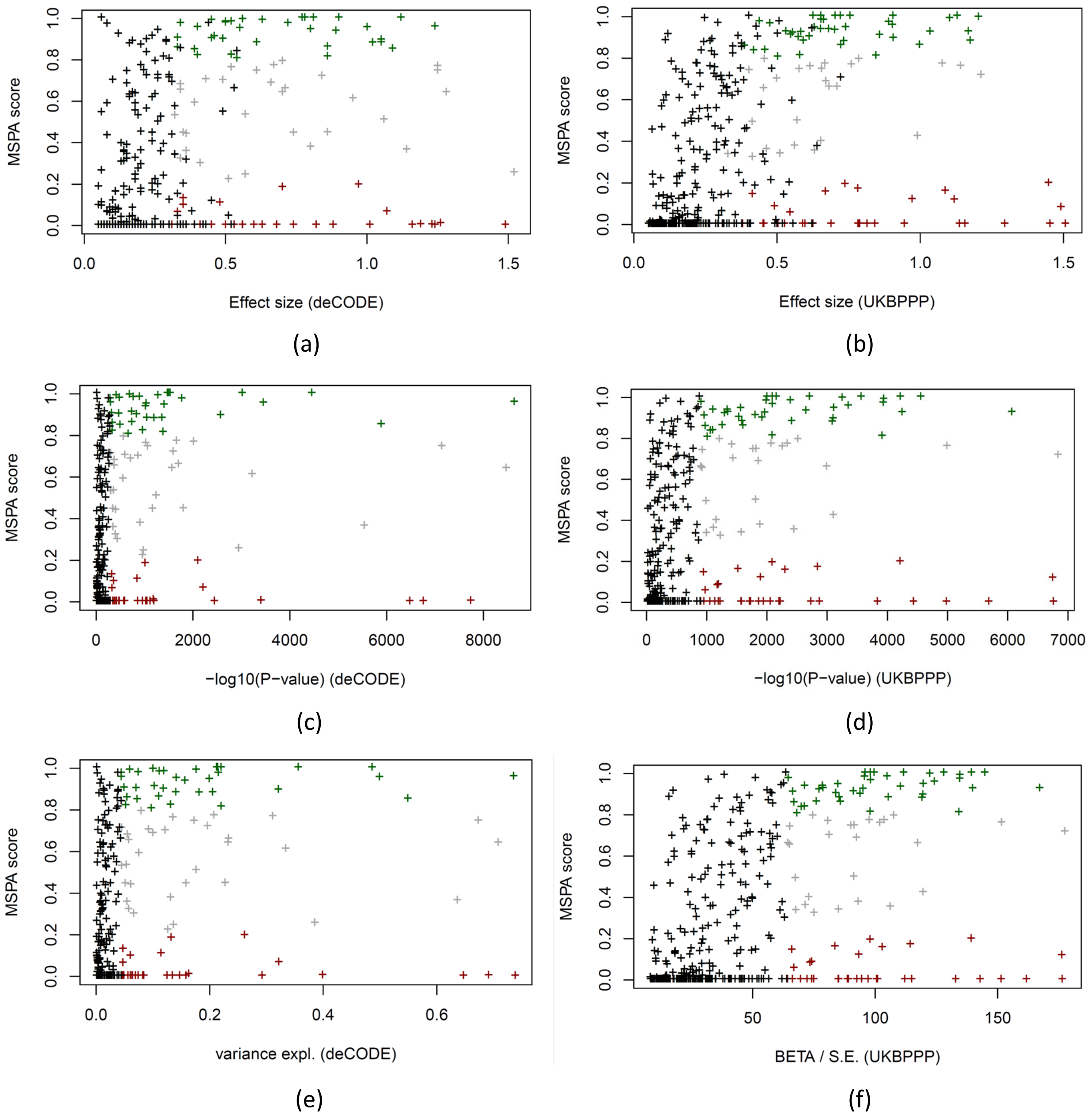

Supplementary Figure 8: Scatterplot of the MSPA scores against effect size (a,b) and -log10(p-value) (c,d) for deCODE and UKBPPP, variance explained for deCODE (e) and z-score (effect size divided by standard error) for UKBPPP (f); These plots show that these measures also discriminate to a certain extent between genuine and epitope pQTLs; The one hundred strongest pQTLs are colored in red for MSPA < 0.2, green for MSPA > 0.8, and grey otherwise, lower ranking pQTLs are in black (as in Figure 3).

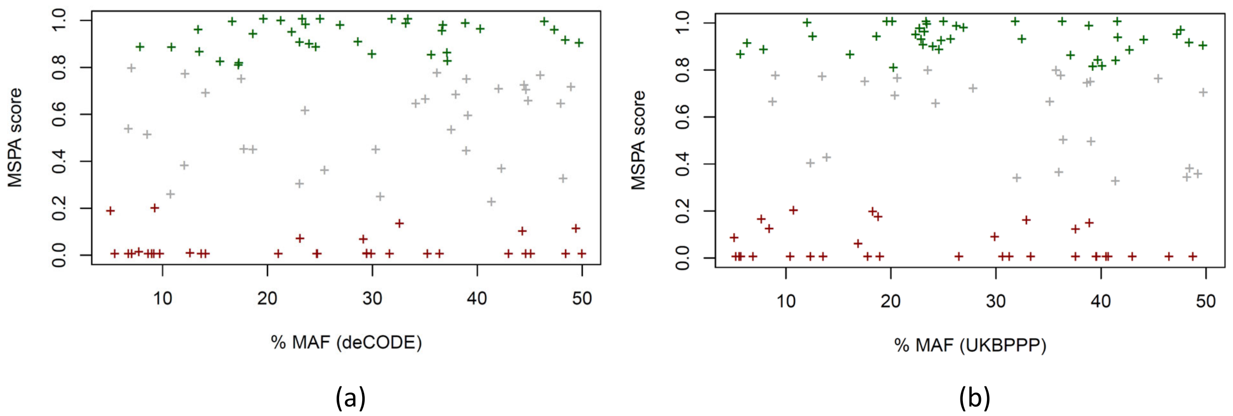

Supplementary Figure 9: Scatterplot of the MSPA score against MAF for the 100 strongest pQTLs in deCODE (a) and UKBPPP (b) indicate no dependence of the MSPA score on allele frequency. The one hundred strongest pQTLs are colored red for MSPA < 0.2, green for MSPA > 0.8, and grey otherwise.

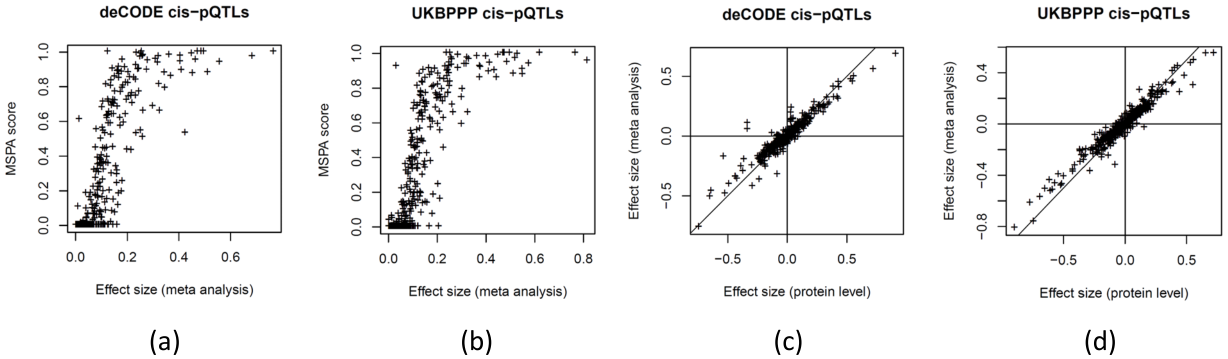

Supplementary Figure 10: Scatter plots of MSPA scores against meta-analyzed effect sizes of the peptide level associations reveal a correlation between both measures that level off at higher effect sizes (a-b); Effect sizes at the protein level and meta-analyzed effect sizes at the peptide level correlate (c-d).

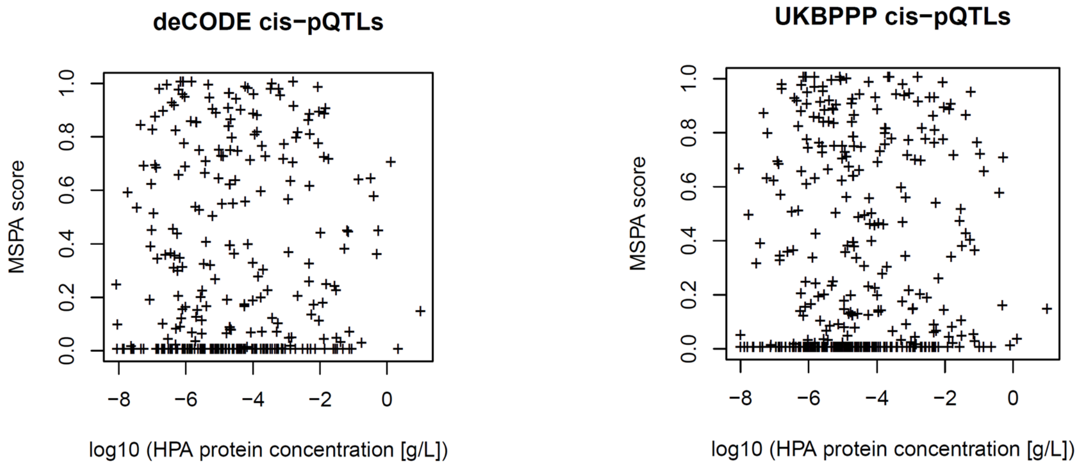

(a) (b)

Supplementary Figure 11: Scatterplot of absolute protein concentrations from the Human Protein Atlas with the MSPA score. Absolute quantification data is from The Human Protein Atlas (HPA), available for 1,629 out of the 1,980 proteins we investigate in here (https://www.proteinatlas.org/humanproteome/blood+protein). HPA provides estimated protein concentrations of proteins detected in human plasma based on immunoassays and MS-based proteomics in The Human Plasma Proteome chapter (Plot data in Supplementary Table 1). No correlation between the MSPA score of the UKBB and deCODE cis-pQTLs and the HPA protein abundance was found (Spearman p > 0.05 in both cases).

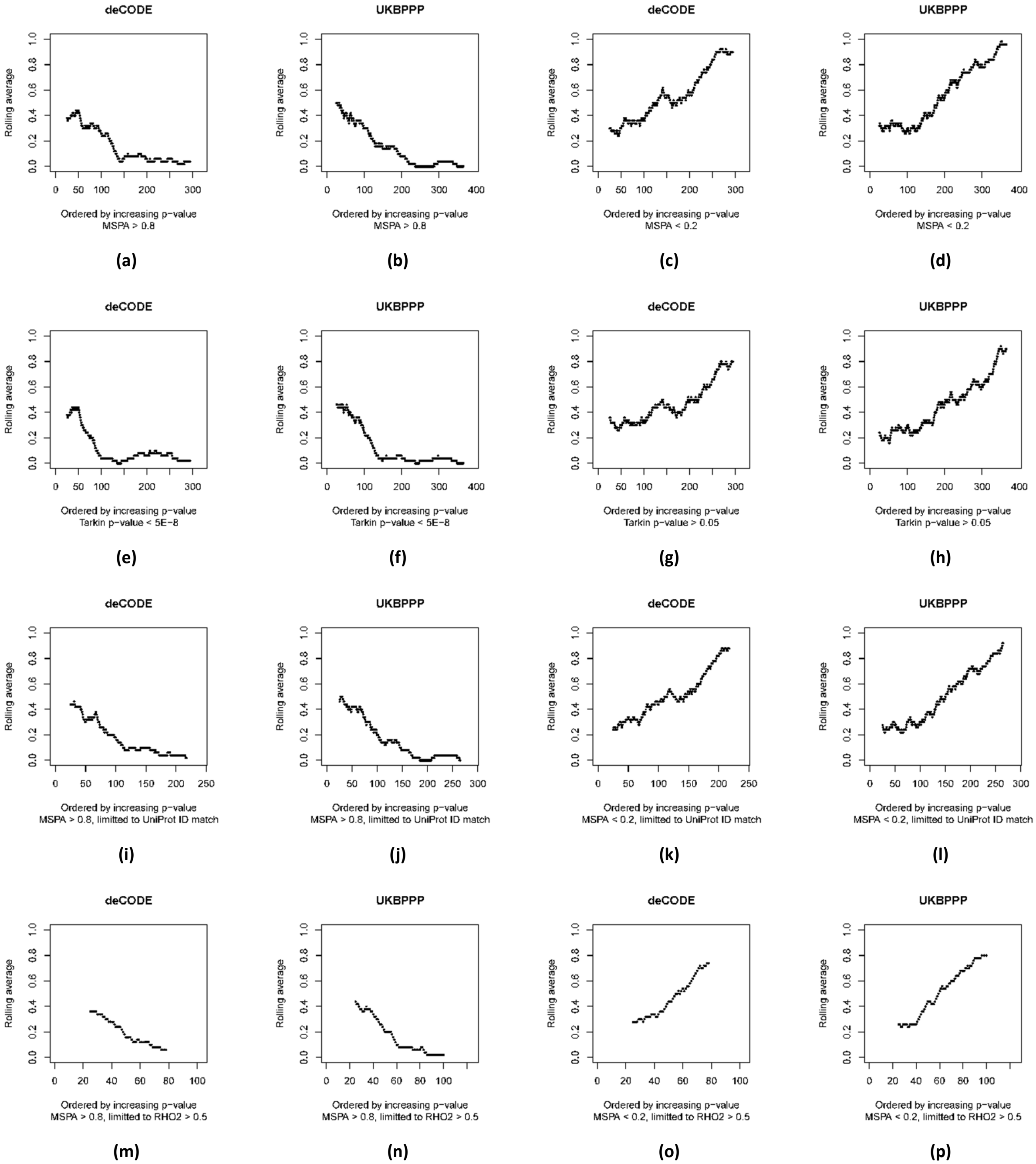

Supplementary Figure 12: Dependence of the MSPA score on the rank of the affinity pQTL. Rolling average of the fraction of pQTLs satisfying the criterion indicated below the plots, pQTLs ordered by increasing p-value, averaging window size is 50 pQTLs. Both studies display broadly similar behaviors: The fraction of pQTLs with a high MSPA (>0.8) attains ~40% and that of pQTLs with a low MSPA (<0.2) falls to

~30% for the most significant affinity pQTLs (a-d). This pattern is also observed when looking at the significance level of the pQTLs in the Tarkin study rather than the MSPA score, where power to detect genome-wide significant signals vanishes around rank 150 (e-h). These observations hold when excluding pQTLs that may potentially target different isoforms, that is, pQTLs matched on the basis of shared protein groups or on the gene name level (i-l), and also when limiting pQTLs to only proteins with high reproducibility (Spearman RHO > 0.5) between technical replicates (m-p).

Legend	Major Allele PAV	Minor Allele PAV	Non-PAV peptide QTLs
Assay Target: SERPING1<br>UniProt: P05155<br>rsID: rs11606706<br>Target SNP: 11:57616102:T:G<br>Proxy SNP: 11:57383575:G:T<br>deCODE log10(p): 1192.2<br>deCODE BETA: −0.68<br>deCODE MSPA: 0<br>-:-:-:-:-:-:-:-:-:-:-:-:-:-:-:-<br>1256:1249:1235:1253:1237:120	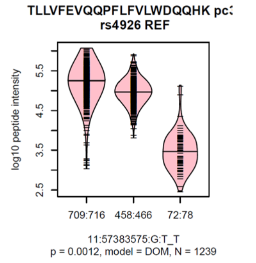	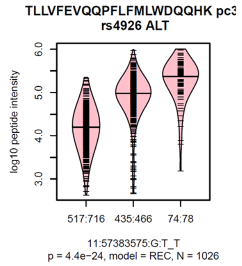	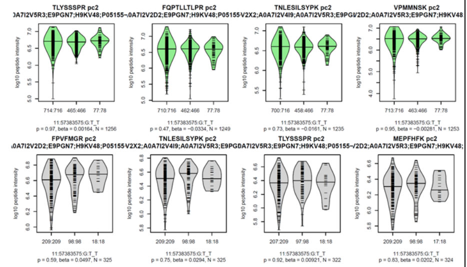
Assay Target: ADGRF5<br>UniProt: Q8IZF2<br>rsID: rs58917843<br>Target SNP: 6:46854900:G:GA<br>Proxy SNP: 6:46823695:G:A<br>deCODE log10(p): 1050.8<br>deCODE BETA: −1.49<br>deCODE MSPA: 0<br>-:-:-:-:-:-:-:NA<br>869:1055:730:331:397:247:36:2	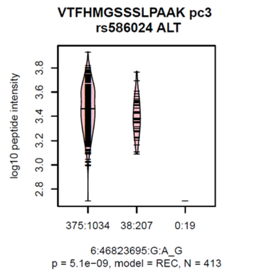		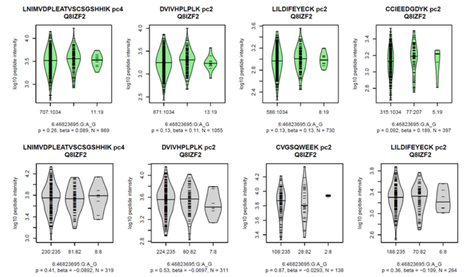
Assay Target: ITIH3<br>UniProt: Q06033<br>rsID: rs2071044<br>Target SNP: 3:52813585:T:C<br>Proxy SNP: 3:52847601:C:T<br>deCODE log10(p): 845.1<br>deCODE BETA: 0.48<br>deCODE MSPA: 0.107<br>-:-:-:-:-:-:-:-:-:-:-:-:-:-:-:-<br>1256:1225:1257:1255:1254:125		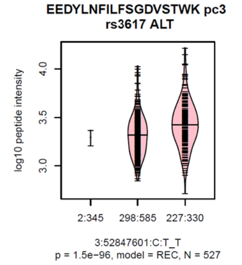	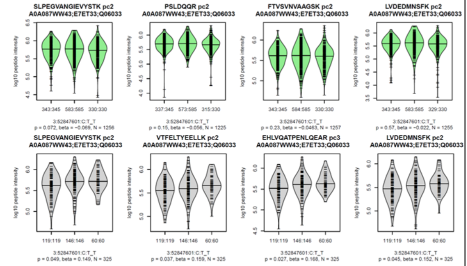
Assay Target: CPN2<br>UniProt: P22792<br>rsID: rs3732477<br>Target SNP: 3:194341790:T:C<br>Proxy SNP: 3:194062519:C:T<br>deCODE log10(p): 571.1<br>deCODE BETA: −0.5<br>deCODE MSPA: 0<br>-:-:-:-:-:-:-<br>1196:1256:1243:1254:863:728:	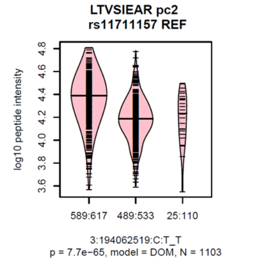	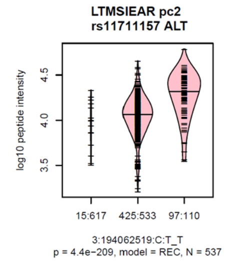	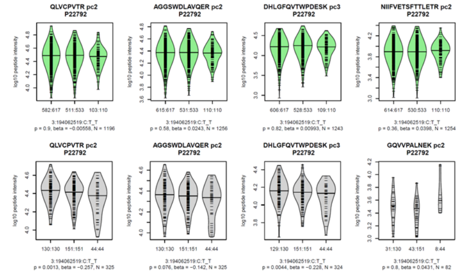
Assay Target: GDF15<br>UniProt: Q99988<br>rsID: rs1058587<br>Target SNP: 19:18388612:G:C<br>Proxy SNP: 19:18499422:C:G<br>deCODE log10(p): 476.4<br>deCODE BETA: 0.45<br>deCODE MSPA: 0<br>-:-:-:-:-:-:-:NA:-:NA:-:NA:-:-:-:NA<br>1238:124:145:162:171:87:85:15		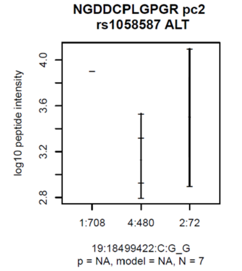	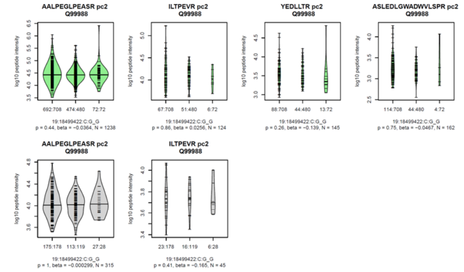
Assay Target: SERPINA1<br>UniProt: P01009<br>rsID: rs1243167<br>Target SNP: 14:94375304:A:G<br>Proxy SNP: 14:94841641:G:A<br>deCODE log10(p): 420.5<br>deCODE BETA: 0.41<br>deCODE MSPA: 0<br>-:-:-:-:-:-:-:-:-:-:-:-:-:-:-:-<br>1256:1257:1256:1256:1173:125	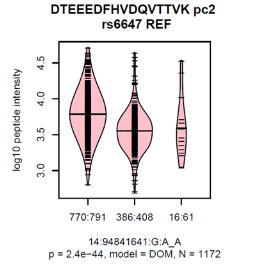	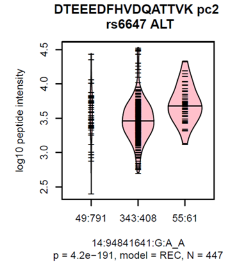	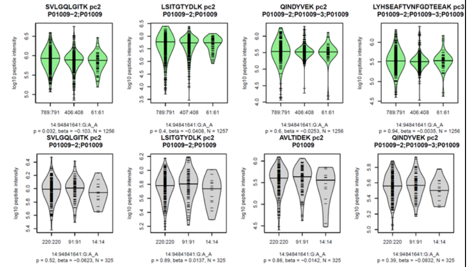

	Major Allele PAV	Minor Allele PAV	Non-PAV peptide QTLs
Assay Target: ENO3<br>UniProt: P13929<br>rsID: rs238238<br>Target SNP: 17:4953081:A:G<br>Proxy SNP: 17:4856376:A:G<br>deCODE log10(p): 325.3<br>deCODE BETA: 0.33<br>deCODE MSPA: 0.062<br>-:-:-:-:-:*:-:-:-:-:-:-:-:-:-:-:-<br>1214:1255:831:1213:1241:1223	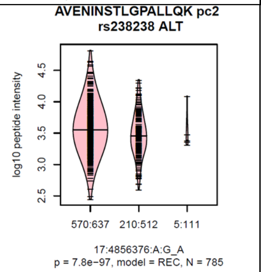	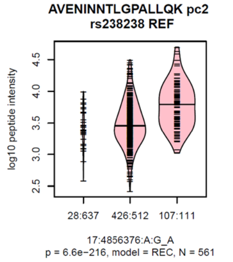	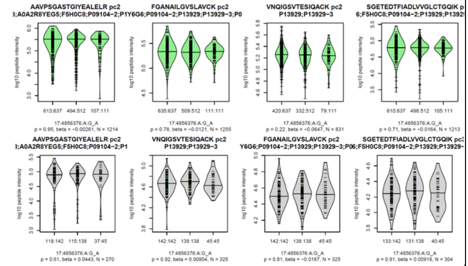
Assay Target: HDGF<br>UniProt: P51858<br>rsID: rs3806417<br>Target SNP: 1:156711623:C:A<br>Proxy SNP: 1:156711623:C:A<br>UKBPPP log10(p): 6757.8<br>UKBPPP BETA: 1.155<br>UKBPPP MSPA: 0<br>-:-:-:-:-:-:-:-:-:-:-:-:-<br>787:997:814:1132:637:893:547:	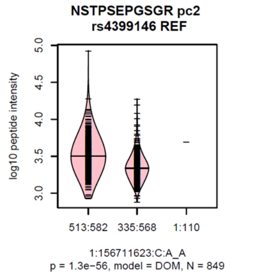	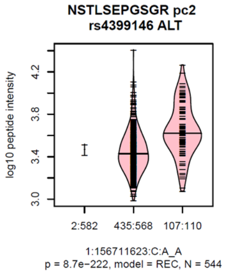	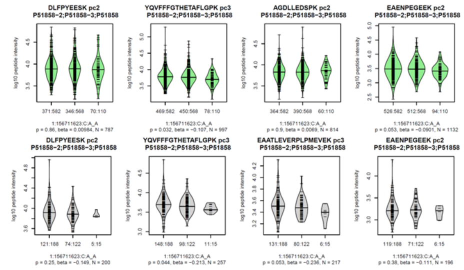
Assay Target: APOBR<br>UniProt: Q0VD83<br>rsID: rs180744<br>Target SNP: 16:28508048:A:G<br>Proxy SNP: 16:28508048:A:G<br>UKBPPP log10(p): 6743.3<br>UKBPPP BETA: −1.118<br>UKBPPP MSPA: 0.117<br>-:-:-:-:-:*:-:-:-:-:*:-:-:-:-:-:-:<br>1229:1153:1098:1222:1202:110	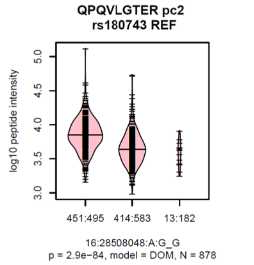	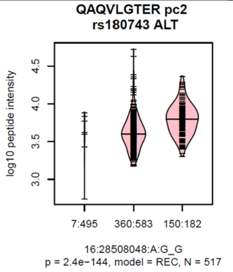	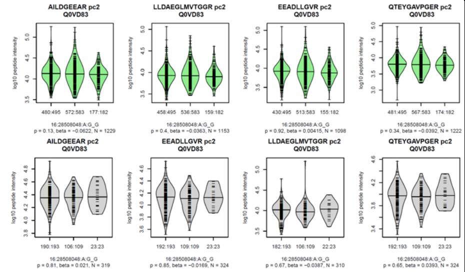
Assay Target: FCGR2A<br>UniProt: P12318<br>rsID: rs11810143<br>Target SNP: 1:161480649:A:G<br>Proxy SNP: 1:161480649:A:G<br>UKBPPP log10(p): 4980.3<br>UKBPPP BETA: −1.453<br>UKBPPP MSPA: 0<br>-:-:-:-:NA<br>842:942:592:189:12	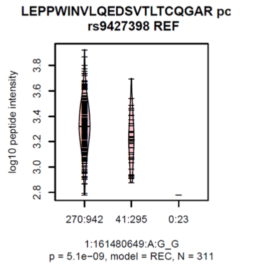		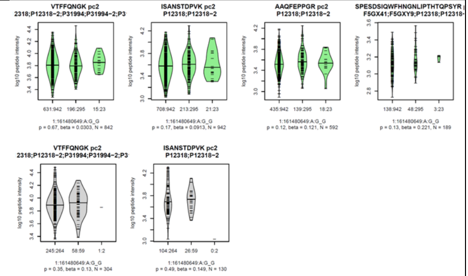
Assay Target: CHGB<br>UniProt: P05060<br>rsID: rs236153<br>Target SNP: 20:5903894:A:G<br>Proxy SNP: 20:5903894:A:G<br>UKBPPP log10(p): 2052<br>UKBPPP BETA: −0.597<br>UKBPPP MSPA: 0<br>-:-:-:-:-:-:-:-:-:-:-:-:-:-:-:-<br>1244:1255:1236:1235:1056:975	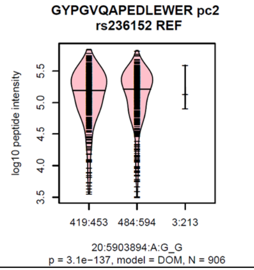		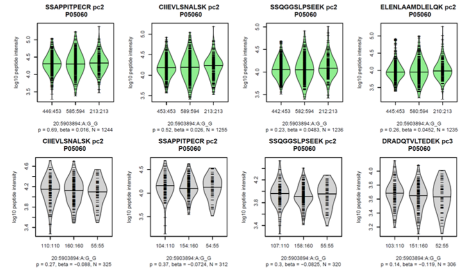
Assay Target: IGFBP3<br>UniProt: P17936<br>rsID: rs2854746<br>Target SNP: 7:45960645:G:C<br>Proxy SNP: 7:45960645:G:C<br>UKBPPP log10(p): 1220.7<br>UKBPPP BETA: 0.451<br>UKBPPP MSPA: 0<br>-:-:-:-:-:-:-:-:-:-:-:-:-:-:-:-<br>1255:1255:1249:1251:1255:125	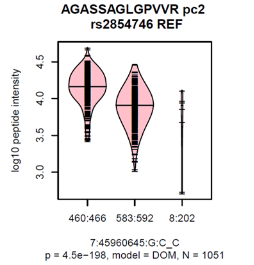	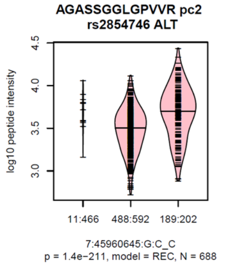	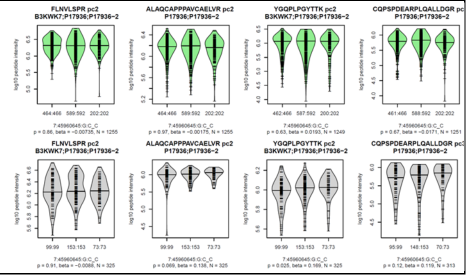

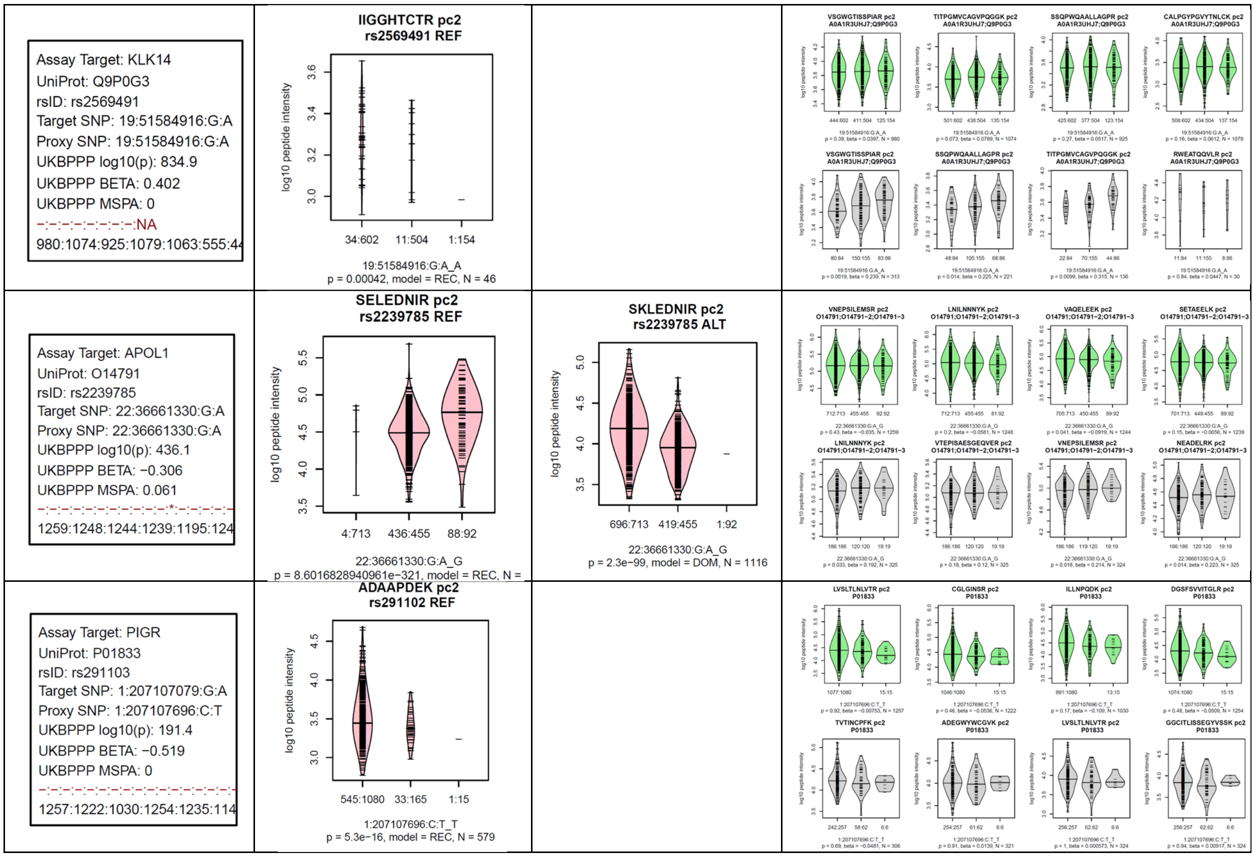

Supplementary Figure 13: Peptide level violin plots of affinity pQTLs with power >99%, but that did not replicate, had a low MSPA scores (< 0.2), and for which PAV peptides have also been detected on the MS platform (Supplementary Table 13); Violin plots are from Supplementary Data 4-5, for a legend regarding the violin plots see Supplementary Figures 3 and 7. The p-values for the PAV containing peptides are from Fisher's exact tests and represent the strongest association of the two models, REC (grouping detections for minor allele and heterozygotes) and DOM (grouping detections for major alleles and heterozygotes). These plots also demonstrate the level of information that can be drawn from MS proteomics QTLs in support or for the rejection of potential epitope pQTLs. Information about potential differential associations with different isoforms, as outlined in the SPINK5 case, can be obtained from the forest plots (provided as Supplementary Data 4-5 and summarized in Supplementary Tables 7-8). Note that the PAV containing QTLs are based on the PAV inclusive library-based analysis of the raw data (see Methods) and that peptides labeled as "ALT" would not have been detected by standard MS protocols (for details see Suhre *et al*., Nature Comm., 2024).

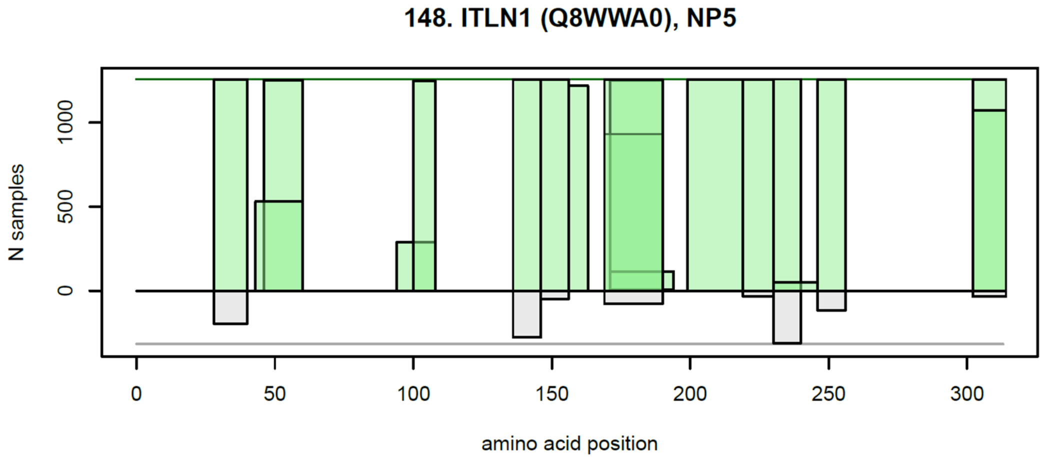

Supplementary Figure 14: Mapping of peptides detected on nanoparticle run NP5 for ITLN1 (UniProt Q8WWA0) to the amino acid sequence of that protein. The number of samples in which ITLN1 specific peptides were detected in Tarkin (green) are plotted against the amino acid position on the ITLN1 protein sequence. The number of samples in which these peptides were detected in QMDiab are plotted on the lower part of y-axis (grey). Overlapping boxes indicate peptides that were detected in multiple precursor charge states or that correspond to peptides with missed cleavages. Similar plots and the corresponding plot data (peptide level detections) are provided for all 1,980 analysed proteins as Supplementary Data 6 and Supplementary Table 14.

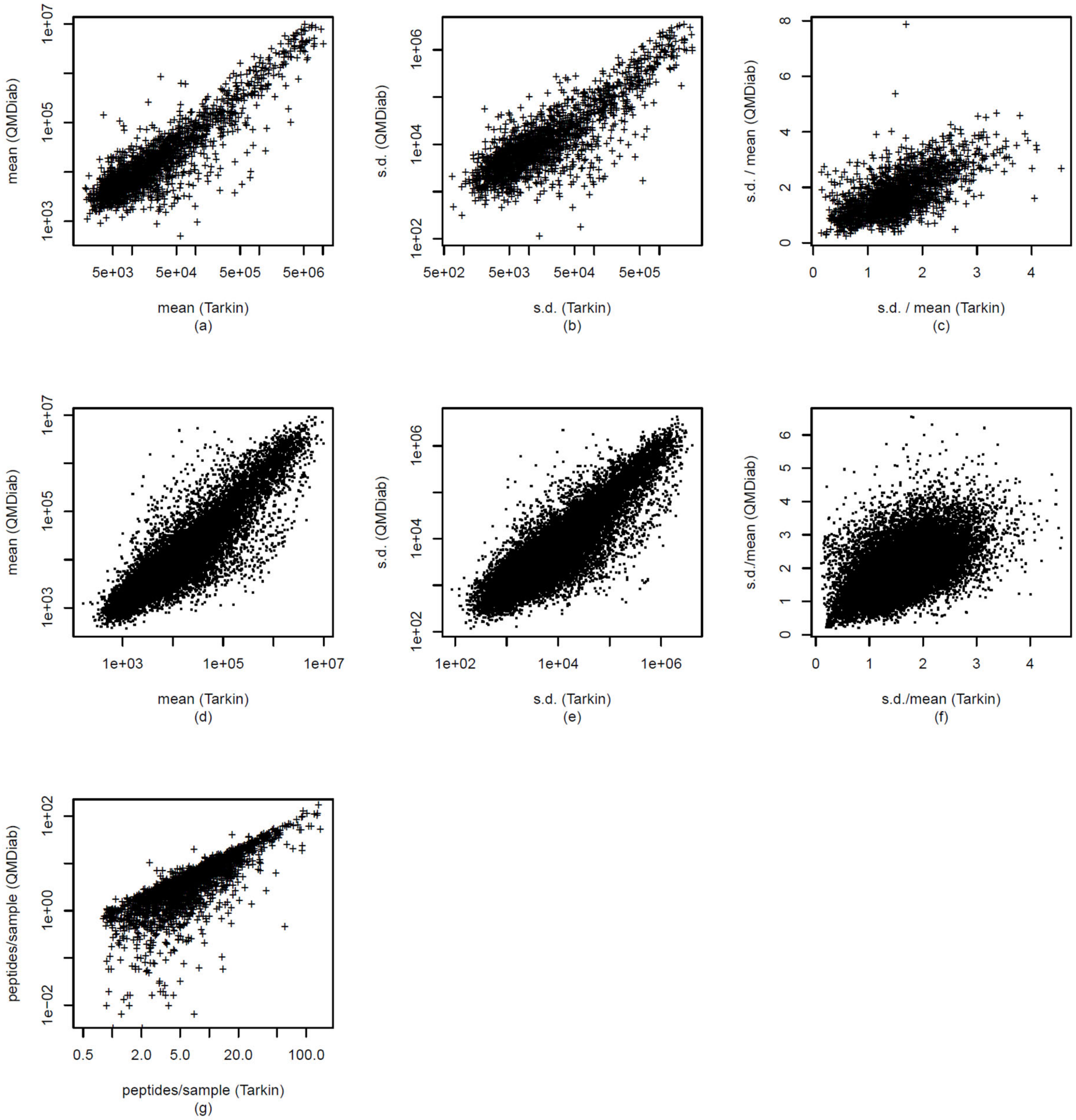

Supplementary Figure 15: Scatterplots Tarkin versus QMDiab of the intensities (mean, s.d., mean/s.d) of the 1,980 analyzed proteins (a-c) and their corresponding peptides (d-f); Scatterplot of the average number of the number of peptides detected for any given protein per sample (g).

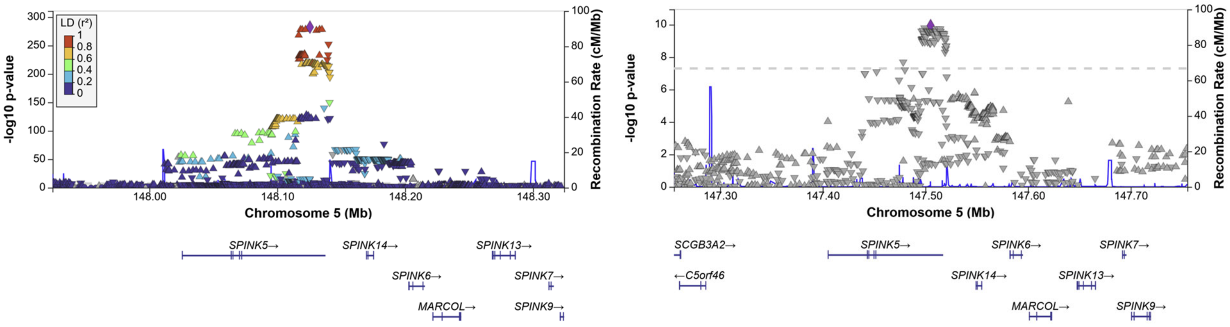

(a) (b)

Supplementary Figure 16: Regional association plots for the SPINK5 splice-QTL in Skin – Sun Exposed (Lower leg) samples from GTEx (a) and for the SPINK5 pQTL identified in Tarkin (b). Gene coordinates are with respect to build 38 for GTEx and build 37 for Tarkin.

Supplementary Note 1

A GWAS with protein missingness.

We conducted a GWAS on missingness using a Fisher exact test considering a dominant (DOM) and a recessive (REC) genetic model. A total of 28,317 proteins were detected in all nanoparticle runs. The number of proteins detected per nanoparticle run was comparable between runs (5712, 5650, 5655, 5712, 5588 for NP1 through NP5). 7,976 proteins with less than 5% detections in a nanoparticle run were excluded, leaving 20,341 protein – nanoparticle combinations for analysis. We identified 786 associations at 520 genetic loci (Supplementary Table 16). 329 of the 520 loci were led by an association with one of five proteins: MS4A14 (253 loci), HLA-C (25 loci), CD79B (20 loci), SDF2 (17 loci), and IGKV1D-13 (14). Missingness of most of these proteins were also associated with self-reported race (black vs. white), leaving 191 missingness-pQTLs that were not confounded by race. 124 of these were located in-cis, including 27 of our 177 cis-pQTLs (15.3%). For these pQTLs a potential 'epitope-like' effect can therefore not be entirely ruled out.

Supplementary Note 2

Age and sex associations were concordant between the affinity and MS based proteomics platforms.

We used the associations with sex and age to externally validate the comparability of the affinity and MS proteomics readouts to predict non-genetic outcomes. We computed the associations between all 1,980 protein readouts with age, sex, and the ten genotype principal components (Supplementary Table 3). We then asked whether the associations with age and sex were concordant between the two affinity platforms and between the affinity and the MS proteomics platforms. A total of 507 proteins were quantified on all three platforms (see Supplementary Table 4 and Venn diagrams below). The overall effect sizes were concordant for most of the proteins between all three platforms (see scatter plots below), although with some exceptions for the associations with age. Overall, 62 proteins shared significant associations with sex across all three platforms, while 110 proteins exhibited significant associations with age. Although our MS-based study was less powered compared to the two much larger affinity proteomics studies, the scatterplots of the effect sizes between the two large studies and one of the large studies and our study are largely comparable. Key signals, such as the association of sex hormone binding globulin (SHBG) with sex and leptin (LEP) with age, were significant on all platforms. There are more age-related associations with conflicting effect sizes. These may be population specific and lifestyle related effects. Noteworthy is also the higher number of positive associations with age that is present across all three platforms, which we can only speculate about.

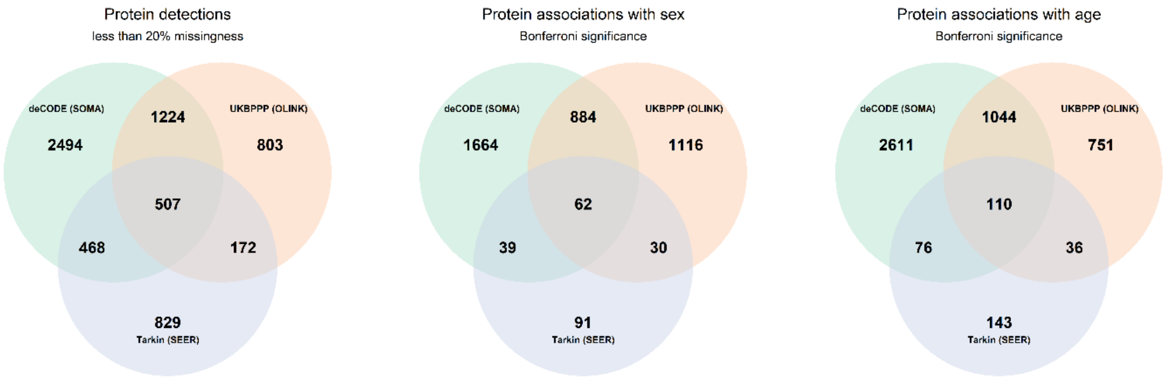

(a) (b) (c)

Venn diagrams. Proteins reported by the three studies, 1,980 proteins with <20% missingness for Tarkin, out of 5,753 proteins quantified across the discovery cohort (a); Proteins associated with sex (b) and age (c) at a significance level of 0.05 / number of proteins reported.

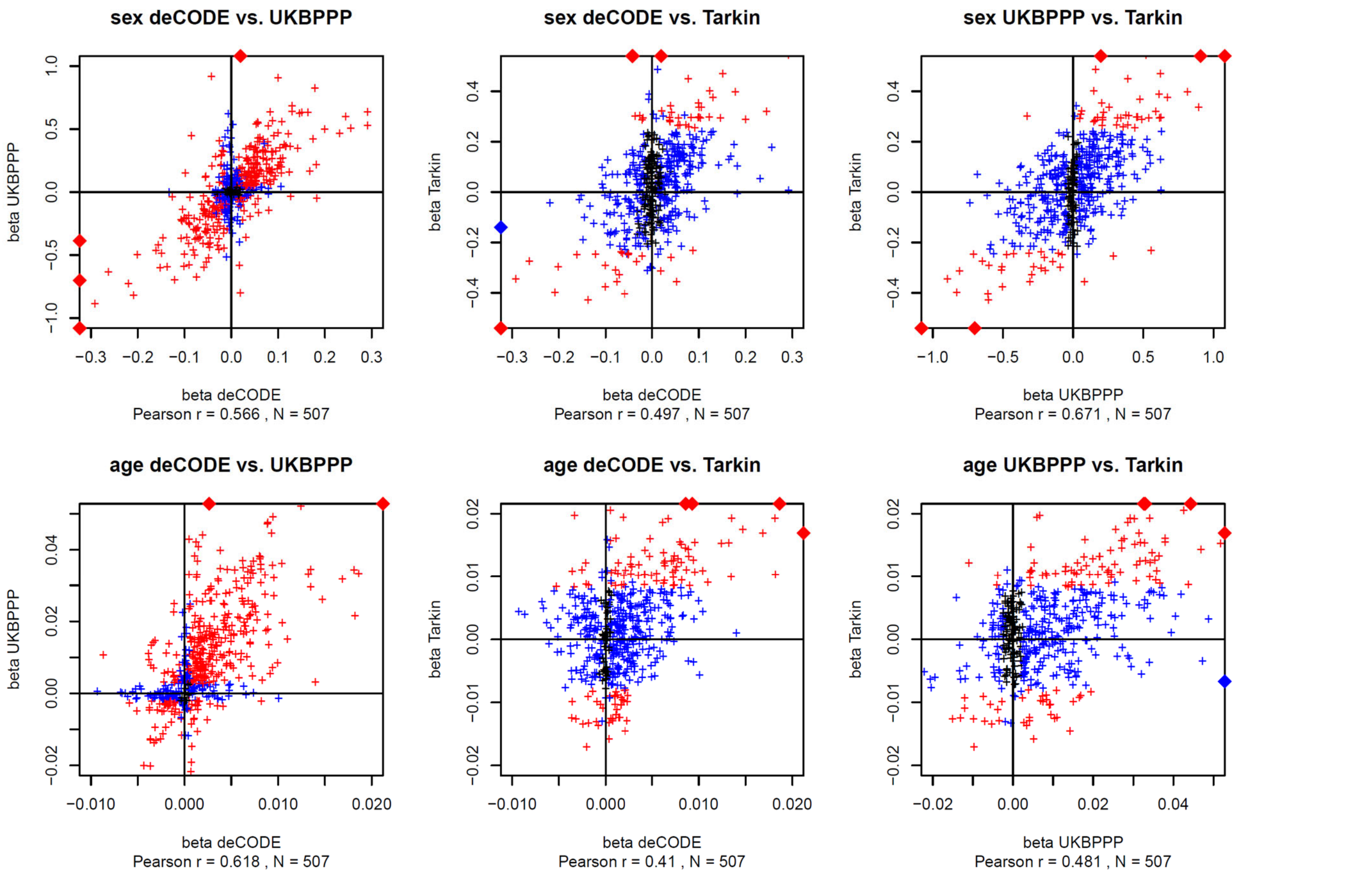

Scatterplot of the effect sizes for the protein associations with sex and age. Summary statistics for the associations with affinity proteomics were from the respective GWAS studies. Associations that reached Bonferroni significance in both respective studies are in red and in one study are in blue ($p < 0.05$ / number of reported associations). The effect sizes (beta) are reported in units of standard deviations (s.d.). Data points outside the plotting window are indicated by diamonds on the plot frames. Plot data are available in Supplementary Table 4. Scatterplots are limited to 507 unique proteins that were reported by all three studies. In cases where data for multiple affinity binders was reported, the most significant association was retained.
