# Supplementary material for: A genome-wide association study of mass spectrometry proteomics using a nanoparticle enrichment platform
Source: Nat Genet. 2025 Nov 27;57(12):2987–96. doi: 10.1038/s41588-025-02413-w (PMC12695657; doi:10.1038/s41588-025-02413-w)
Supplement: Supplementary file 2 — Reporting Summary [file 41588_2025_2413_MOESM2_ESM.pdf]

nature portfolio

Corresponding author(s): Karsten Suhre & Jessica Lasky-Su

Last updated by author(s): Sep 24, 2025

Reporting Summary

Nature Portfolio wishes to improve the reproducibility of the work that we publish. This form provides structure for consistency and transparency in reporting. For further information on Nature Portfolio policies, see our Editorial Policies and the Editorial Policy Checklist.

Statistics

For all statistical analyses, confirm that the following items are present in the figure legend, table legend, main text, or Methods section.

n/a	Confirmed	
☐	☒	The exact sample size (*n*) for each experimental group/condition, given as a discrete number and unit of measurement
☒	☐	A statement on whether measurements were taken from distinct samples or whether the same sample was measured repeatedly
☐	☒	The statistical test(s) used AND whether they are one- or two-sided<br>*Only common tests should be described solely by name; describe more complex techniques in the Methods section.*
☐	☒	A description of all covariates tested
☐	☒	A description of any assumptions or corrections, such as tests of normality and adjustment for multiple comparisons
☐	☒	A full description of the statistical parameters including central tendency (e.g. means) or other basic estimates (e.g. regression coefficient) AND variation (e.g. standard deviation) or associated estimates of uncertainty (e.g. confidence intervals)
☐	☒	For null hypothesis testing, the test statistic (e.g. *F*, *t*, *r*) with confidence intervals, effect sizes, degrees of freedom and *P* value noted<br>*Give P values as exact values whenever suitable.*
☒	☐	For Bayesian analysis, information on the choice of priors and Markov chain Monte Carlo settings
☒	☐	For hierarchical and complex designs, identification of the appropriate level for tests and full reporting of outcomes
☐	☒	Estimates of effect sizes (e.g. Cohen's *d*, Pearson's *r*), indicating how they were calculated

Our web collection on statistics for biologists contains articles on many of the points above.

Software and code

Policy information about availability of computer code

Data collection	no specific code was used for data collection
Data analysis	publicly available software was used for data analysis using simple algorithms for the computation of linear models, pre- and post-processing of data, in particular: bcftools (version 1.16), plink2 (version v2.00a5LM), DIA-NN (1.8.1), Open Targets (version 22.10), R Biostrings (version 2.60.2)

For manuscripts utilizing custom algorithms or software that are central to the research but not yet described in published literature, software must be made available to editors and reviewers. We strongly encourage code deposition in a community repository (e.g. GitHub). See the Nature Portfolio guidelines for submitting code & software for further information.

Data

Policy information about availability of data

All manuscripts must include a data availability statement. This statement should provide the following information, where applicable:

- Accession codes, unique identifiers, or web links for publicly available datasets
- A description of any restrictions on data availability
- For clinical datasets or third party data, please ensure that the statement adheres to our policy

The MS-proteomics data for the Tarkin study has been deposited with ProteomeXchange under project accession code PXD048709 and will be made public at time

of publication. The MS-proteomics data of QMDiab is already available on ProteomeXchange with identifier PXD042852. Summary statistics of the GWAS were deposited in the GWAS catalog with identifier GCP001281. Consent obtained from the study participants does not allow deposition of genetic information in public databases. Researchers affiliated with a research institution may request access to genetic data on an individual basis from the corresponding authors (Karsten Suhre, Weill Cornell Medicine - Qatar, Doha, Qatar for QMDiab and Jessica A. Lasky-Su for Tarkin). Access is subject to approval by the respective institutional research boards of Weill Cornell Medicine – Qatar and the Massachusetts General Brigham (MGB) Biobank.

Research involving human participants, their data, or biological material

Policy information about studies with human participants or human data. See also policy information about sex, gender (identity/presentation), and sexual orientation and race, ethnicity and racism.

Reporting on sex and gender	we only use the term "sex" in reference to the biological attribute
Reporting on race, ethnicity, or other socially relevant groupings	we only use the term "ethnicity" in reference to the underlying genetic diversity of the study participants
Population characteristics	no specific population characteristics are mentioned
Recruitment	participants were recruited on a random basis
Ethics oversight	The original Tarkin study was reviewed and approved by the IRCM IRB and the Mass General Brigham IRB. Participants provided written informed consent to take part in the study. The WCMQ IRB determined that use of the Tarkin data for the present project does not meet the definition of human research for this study (IRB document HRP-532). The QMDiab study was approved by the institutional research boards of Weill Cornell Medicine – Qatar under protocol #2011-0012 and of Hamad Medical Corporation under protocol #11131/11 and complies with all relevant ethical regulations. For forthgoing work with the study a non-human subject research determination was obtained. The study design and conduct complied with all relevant regulations regarding the use of human study participants and was conducted in accordance with the criteria set by the Declaration of Helsinki.

Note that full information on the approval of the study protocol must also be provided in the manuscript.

Field-specific reporting

Please select the one below that is the best fit for your research. If you are not sure, read the appropriate sections before making your selection.

☒ Life sciences ☐ Behavioural & social sciences ☐ Ecological, evolutionary & environmental sciences

For a reference copy of the document with all sections, see nature.com/documents/nr-reporting-summary-flat.pdf

Life sciences study design

All studies must disclose on these points even when the disclosure is negative.

Sample size	In GWAS, sample size determines the power to detect genetic associations. Previous GWAS with MS proteomics had significantly smaller sample size and/or smaller protein panels.
Data exclusions	Exclusion criteria were the missingness of the protein (>20%) and low minor allele frequency for the genomics data (<5%).
Replication	Full replication was provided using the QMDiab study, requiring Bonferroni significance.
Randomization	Does not apply
Blinding	Does not apply

Reporting for specific materials, systems and methods

We require information from authors about some types of materials, experimental systems and methods used in many studies. Here, indicate whether each material, system or method listed is relevant to your study. If you are not sure if a list item applies to your research, read the appropriate section before selecting a response.

Materials & experimental systems

n/a	Involved in the study
☒	☐ Antibodies
☒	☐ Eukaryotic cell lines
☒	☐ Palaeontology and archaeology
☒	☐ Animals and other organisms
☒	☐ Clinical data
☒	☐ Dual use research of concern
☒	☐ Plants

Methods

n/a	Involved in the study
☒	☐ ChIP-seq
☒	☐ Flow cytometry
☒	☐ MRI-based neuroimaging

Plants

Seed stocks	N/A
Novel plant genotypes	N/A
Authentication	N/A
