# Supplementary material for: A genome-wide association study of mass spectrometry proteomics using a nanoparticle enrichment platform
Source: Nat Genet. 2025 Nov 27;57(12):2987–96. doi: 10.1038/s41588-025-02413-w (PMC12695657; doi:10.1038/s41588-025-02413-w)
Supplement: Supplementary file 5 — Regional association plots for 364 pQTLs identified in this study. [file 41588_2025_2413_MOESM5_ESM.pdf]

1. GALC (P54803) 14:88393918:A:C [Tarkin]

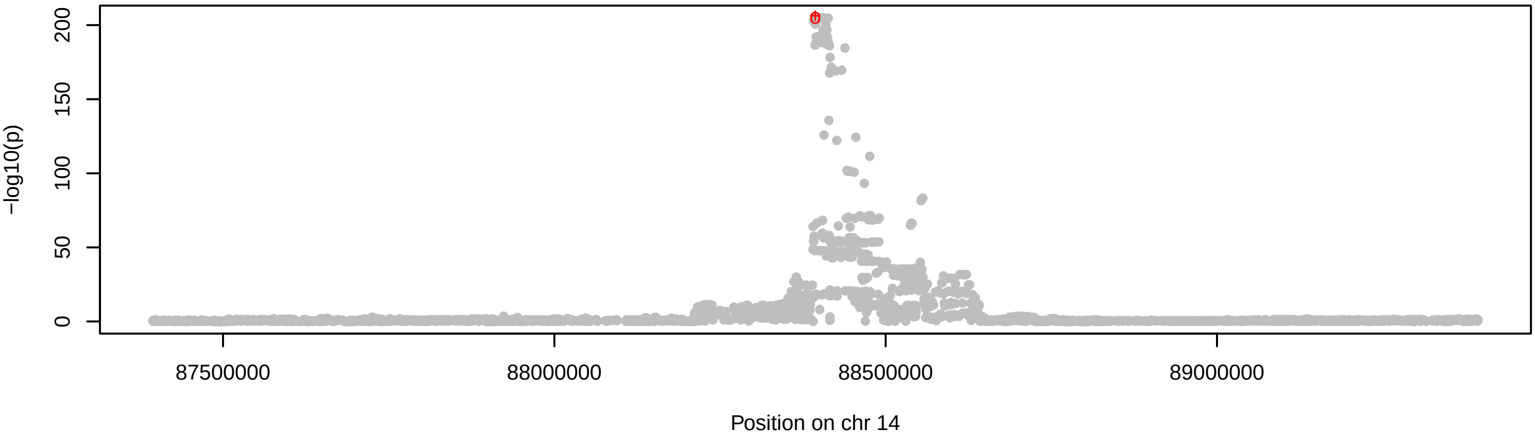

coloc with QMDiab: H4 (PP=89%)

1. GALC (P54803) 14:88393918:A:C [QMDiab]

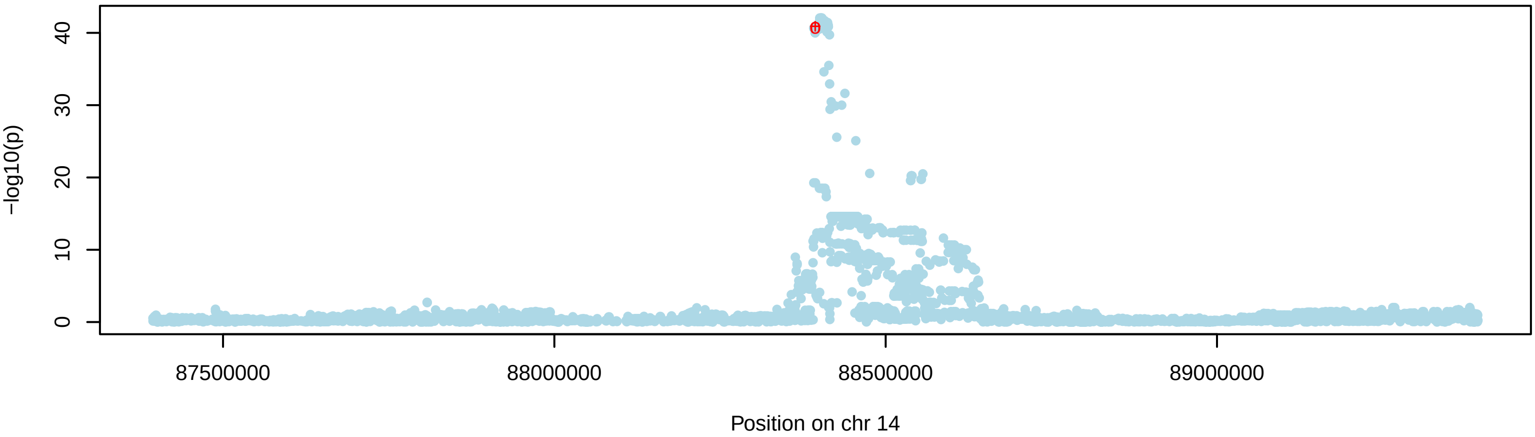

2. HEG1 (Q9ULI3) 3:124746182:C:A [Tarkin]

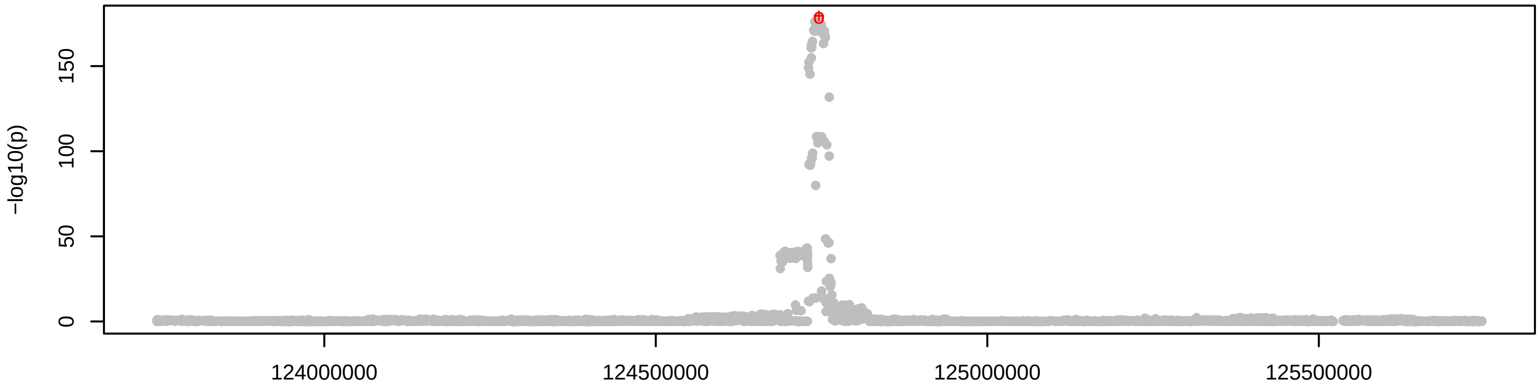

Position on chr 3
coloc with QMDiab: H4 (PP=100%)

2. HEG1 (Q9ULI3) 3:124746182:C:A [QMDiab]

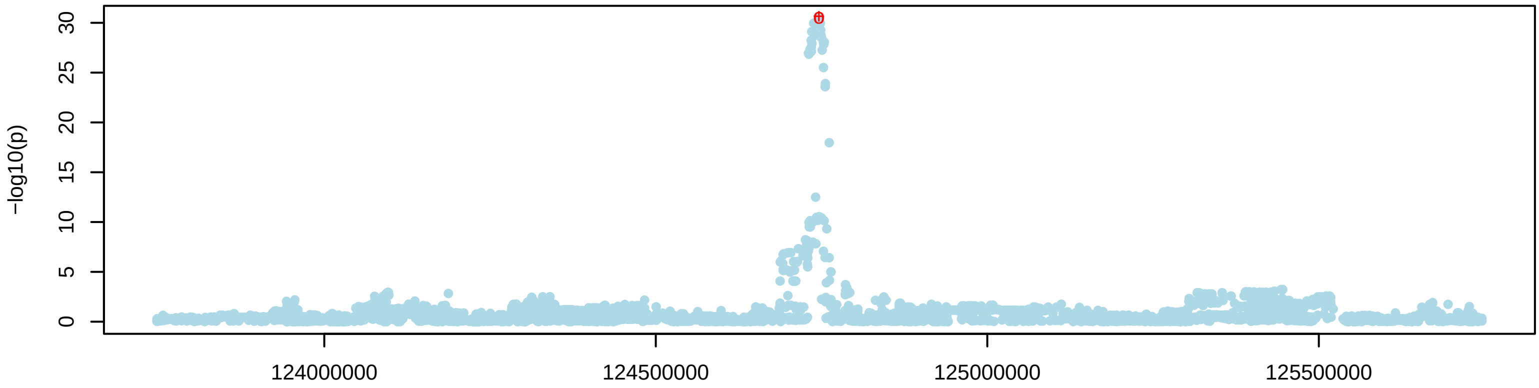

Position on chr 3

3. HSP90B1 (P14625) 12:104348430:A:G [Tarkin]

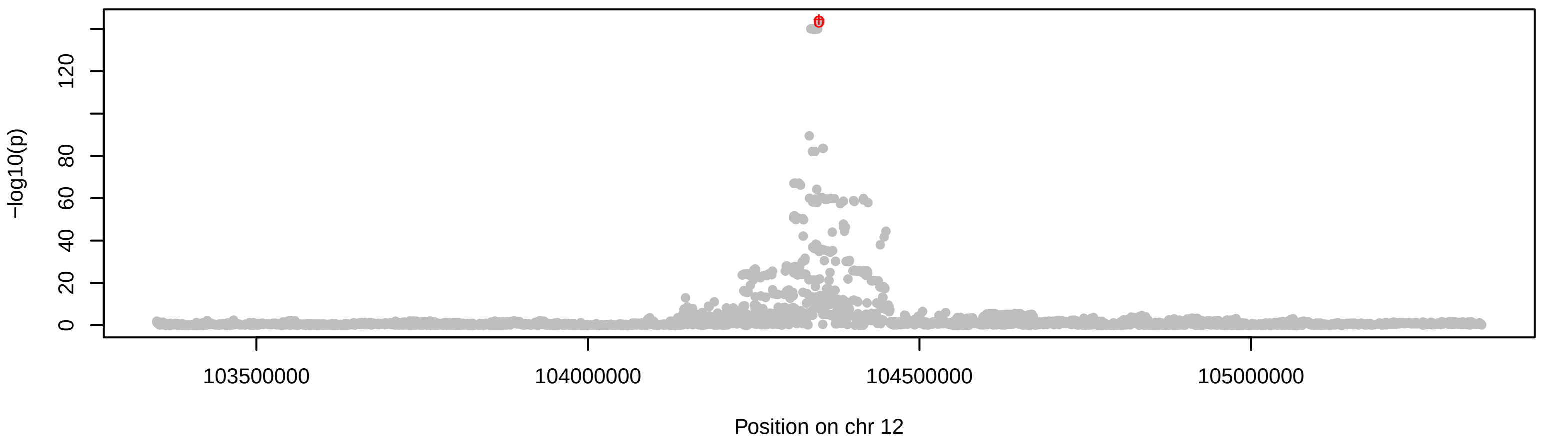

coloc with QMDiab: H4 (PP=99%)

3. HSP90B1 (P14625) 12:104348430:A:G [QMDiab]

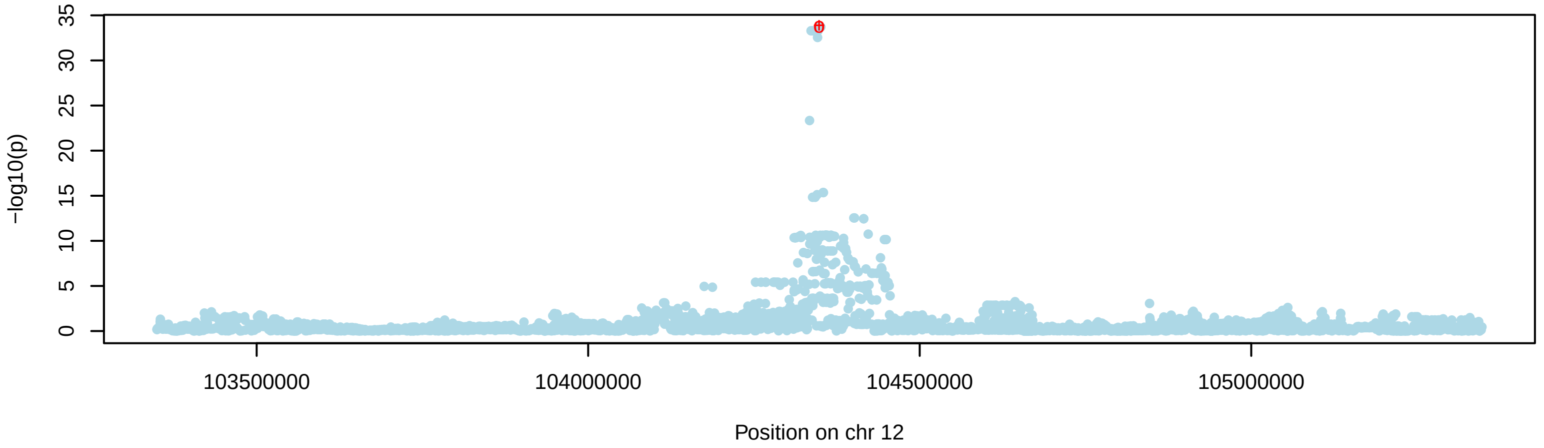

4. HP (P00738) 16:72088964:C:T [Tarkin]

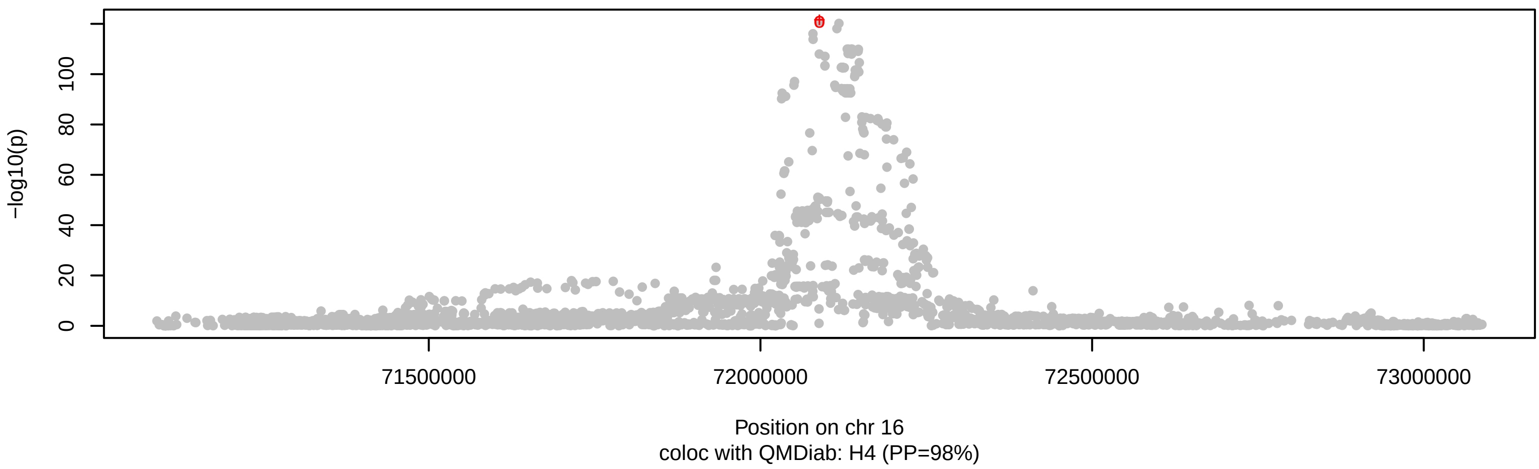

coloc with QMDiab: H4 (PP=98%)

4. HP (P00738) 16:72088964:C:T [QMDiab]

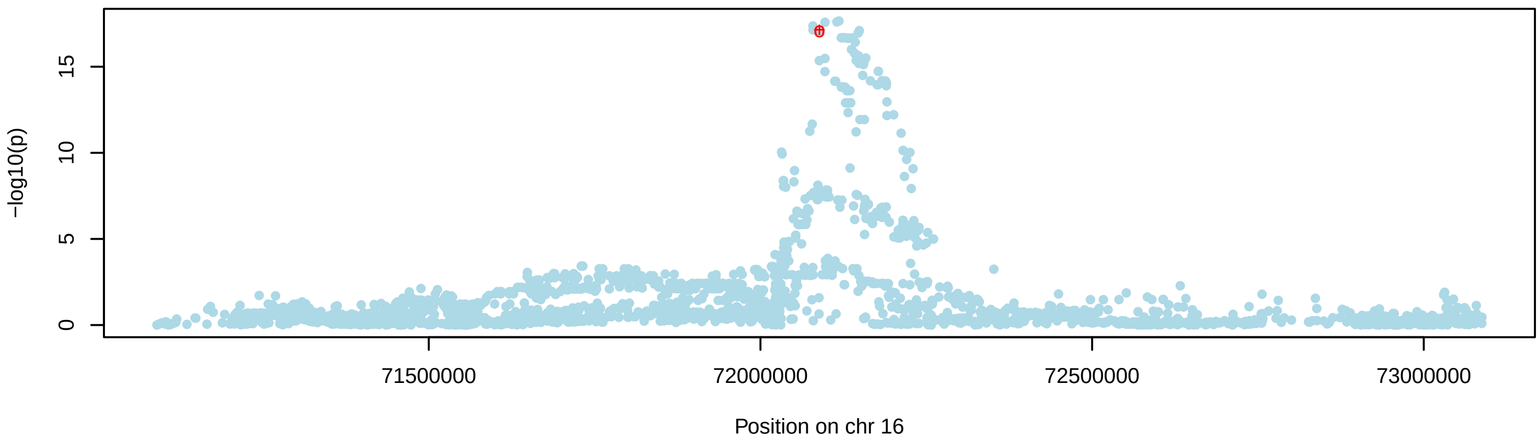

5. SFTPD (P35247) 10:81706324:A:G [Tarkin]

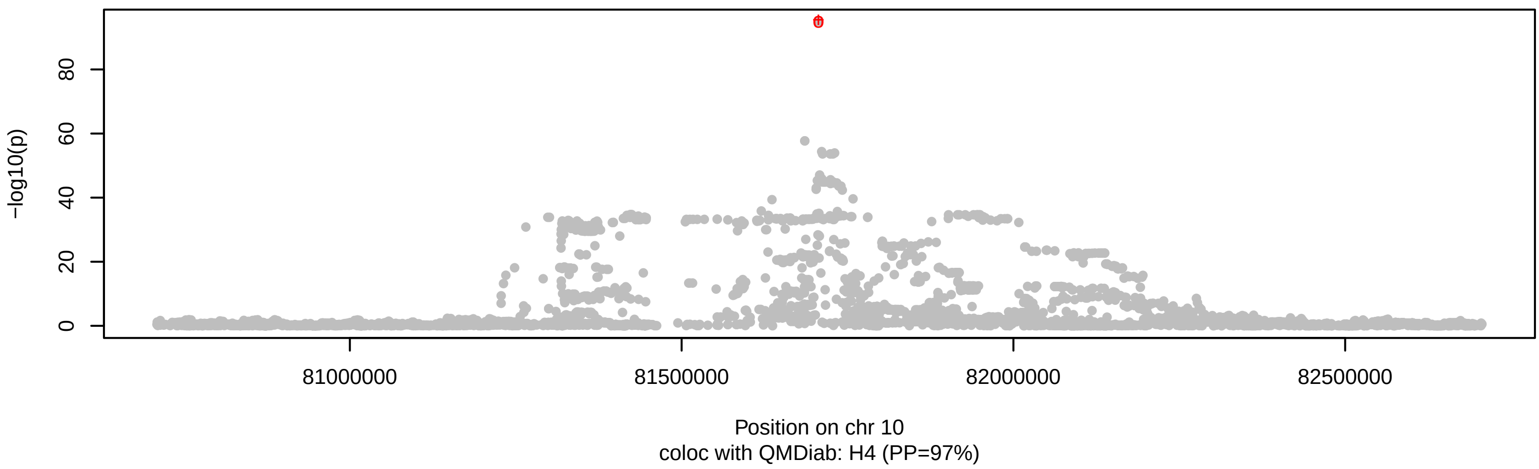

coloc with QMDiab: H4 (PP=97%)

5. SFTPD (P35247) 10:81706324:A:G [QMDiab]

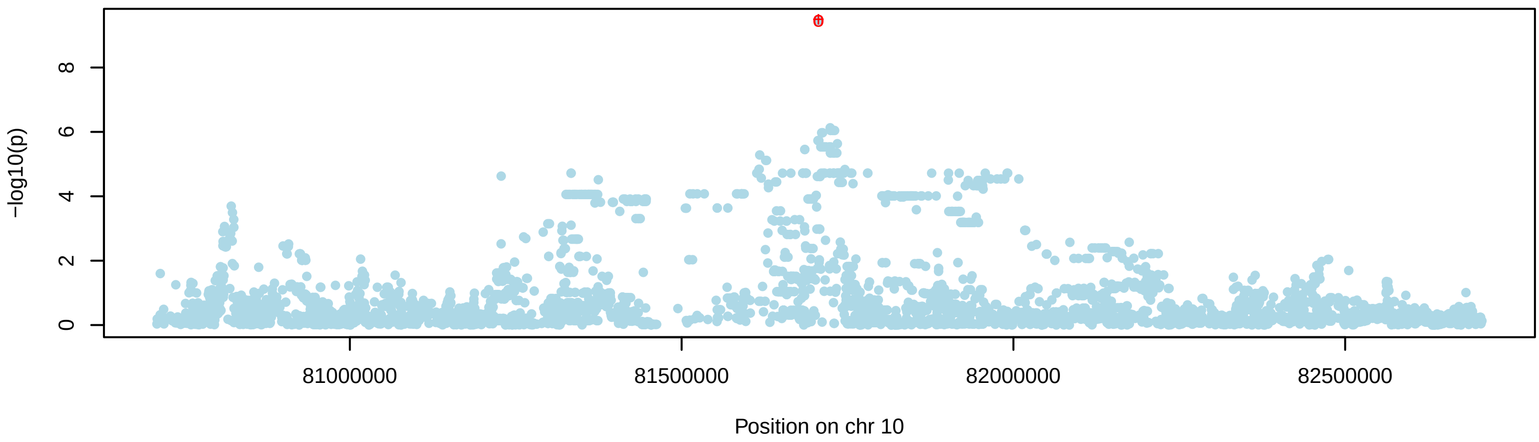

6. PON2 (A0A0J9YXF2) 7:95030632:A:C [Tarkin]

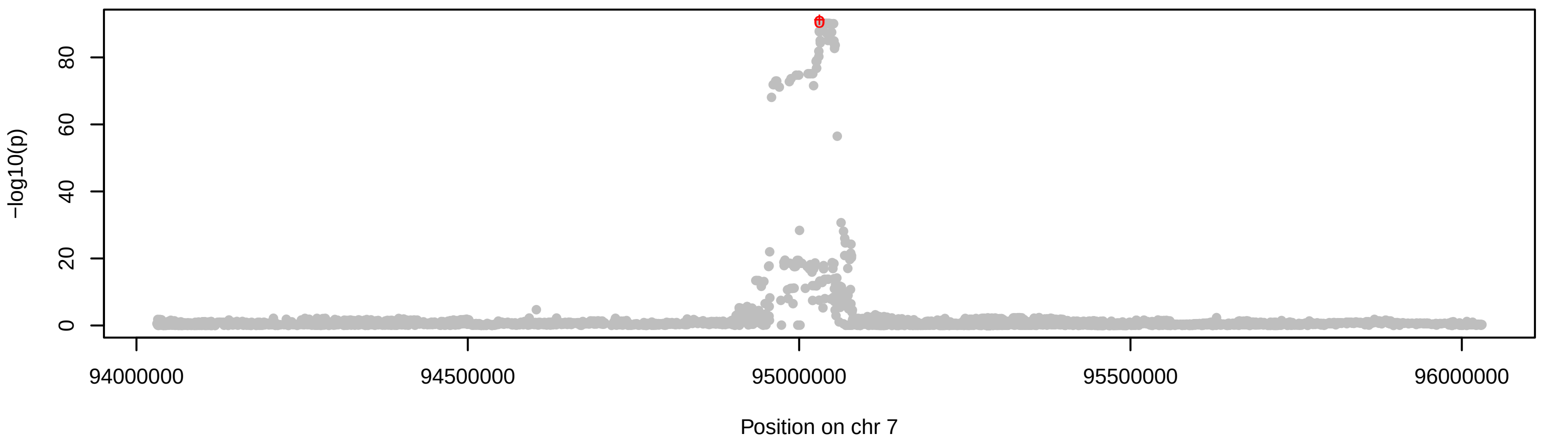

coloc with QMDiab: H4 (PP=97%)

6. PON2 (A0A0J9YXF2) 7:95030632:A:C [QMDiab]

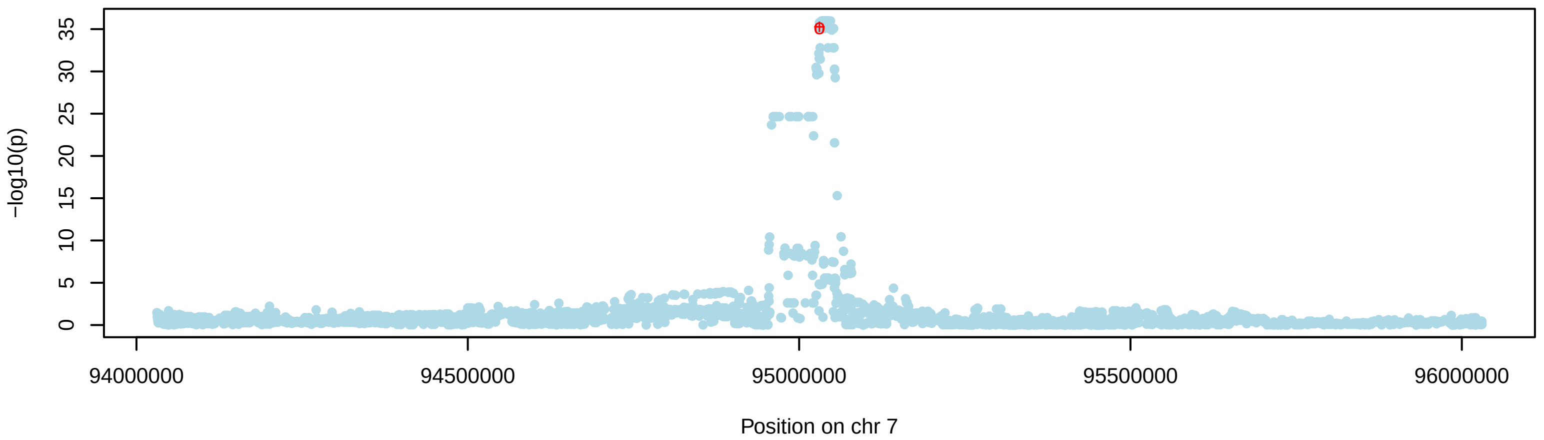

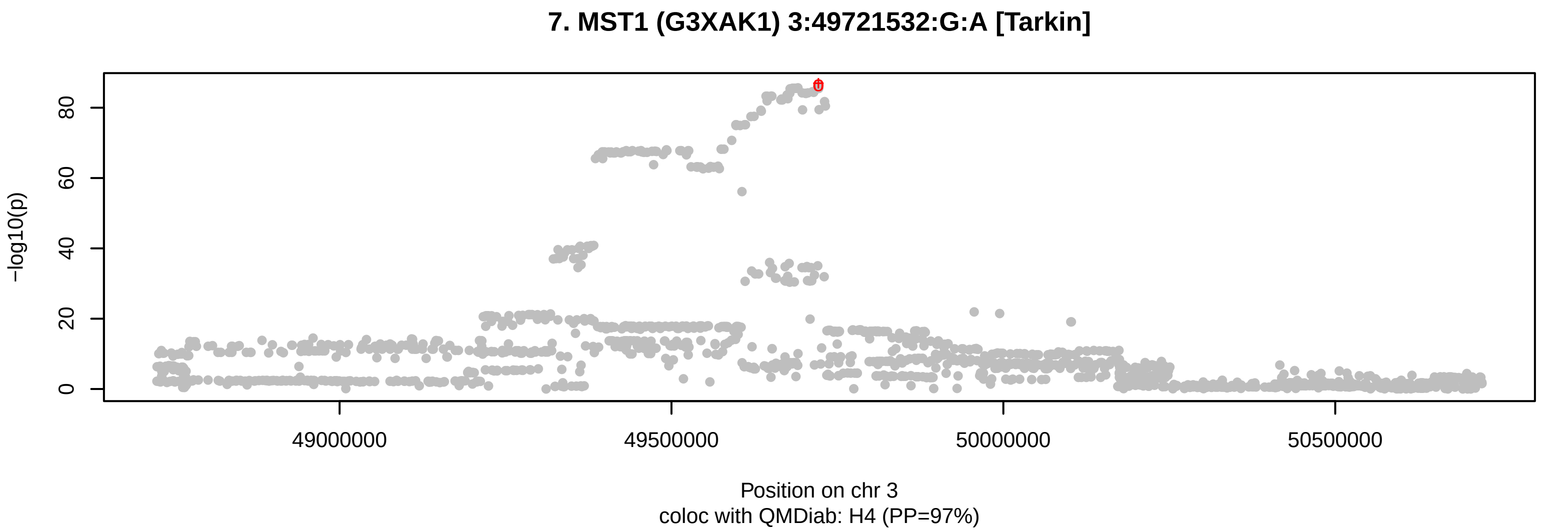

coloc with QMDiab: H4 (PP=97%)

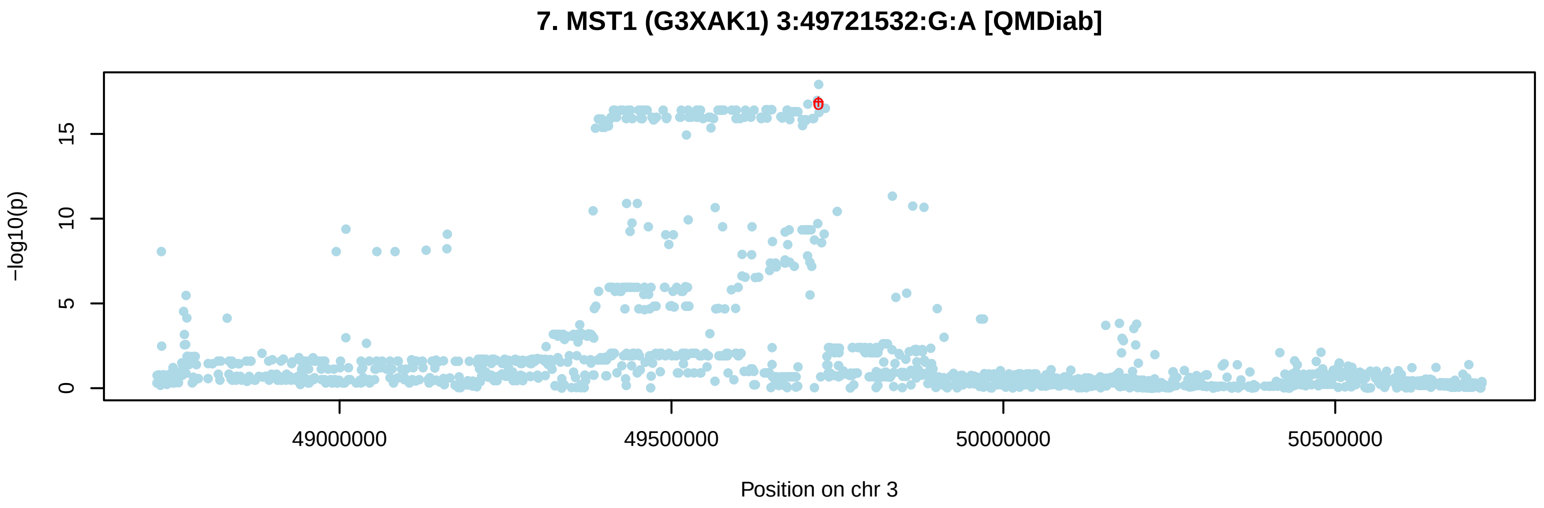

8. VMO1 (Q7Z5L0) 17:4690296:T:C [Tarkin]

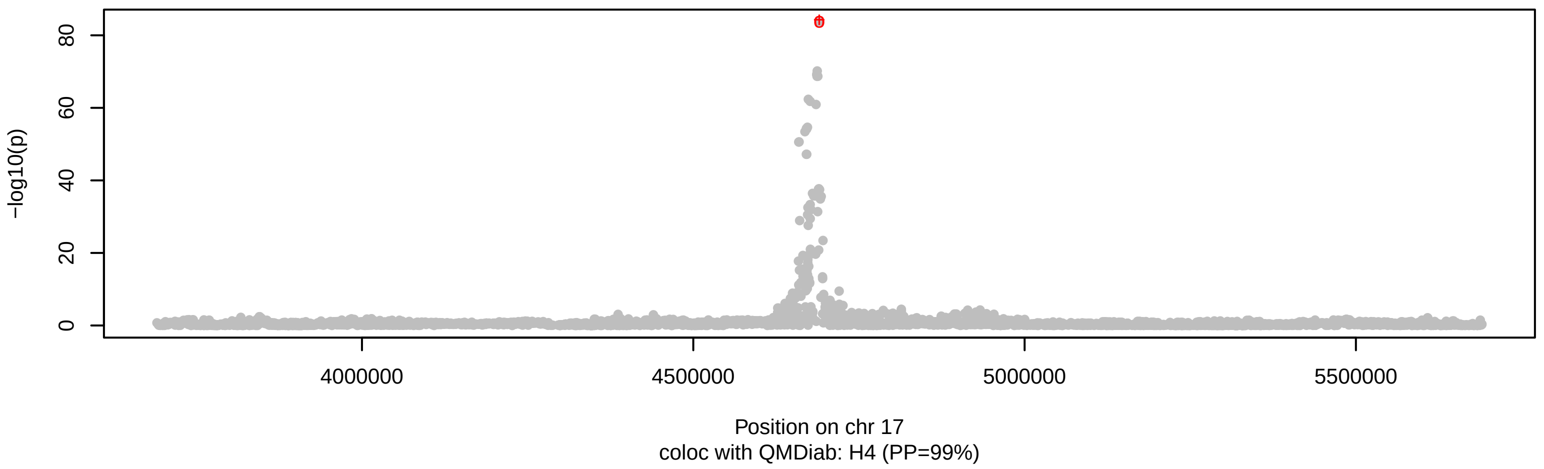

8. VMO1 (Q7Z5L0) 17:4690296:T:C [QMDiab]

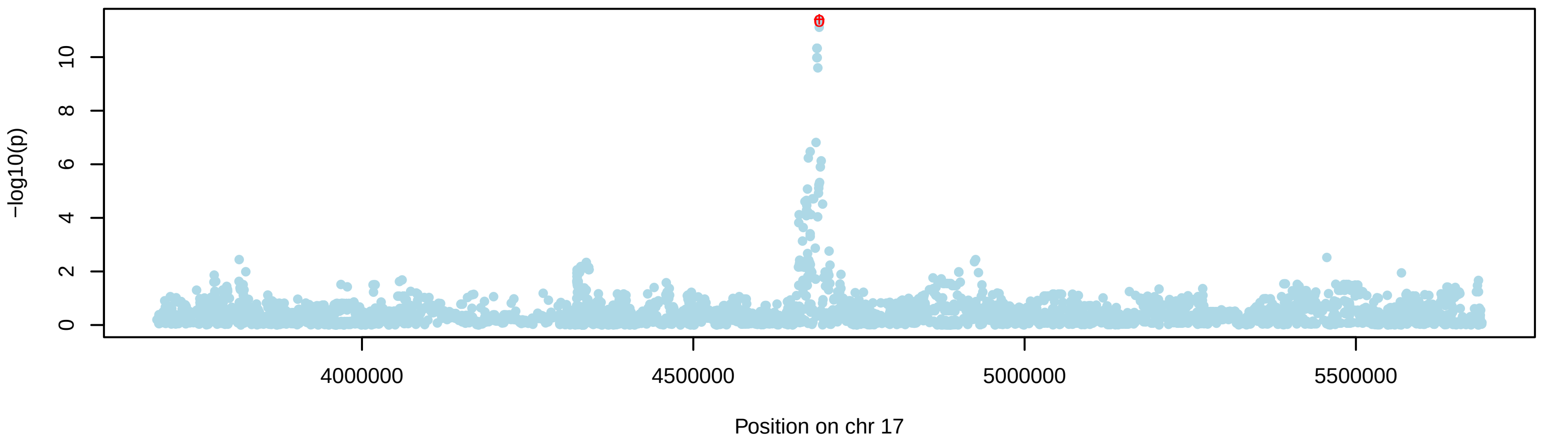

9. MBL2 (P11226) 10:54536839:T:G [Tarkin]

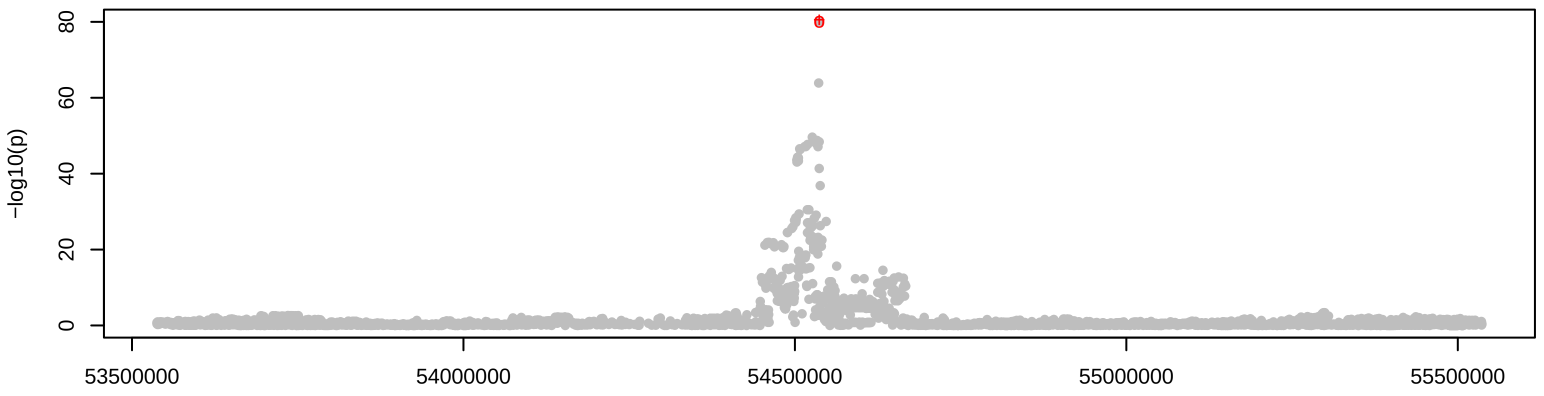

Position on chr 10
coloc with QMDiab: H4 (PP=96%)

9. MBL2 (P11226) 10:54536839:T:G [QMDiab]

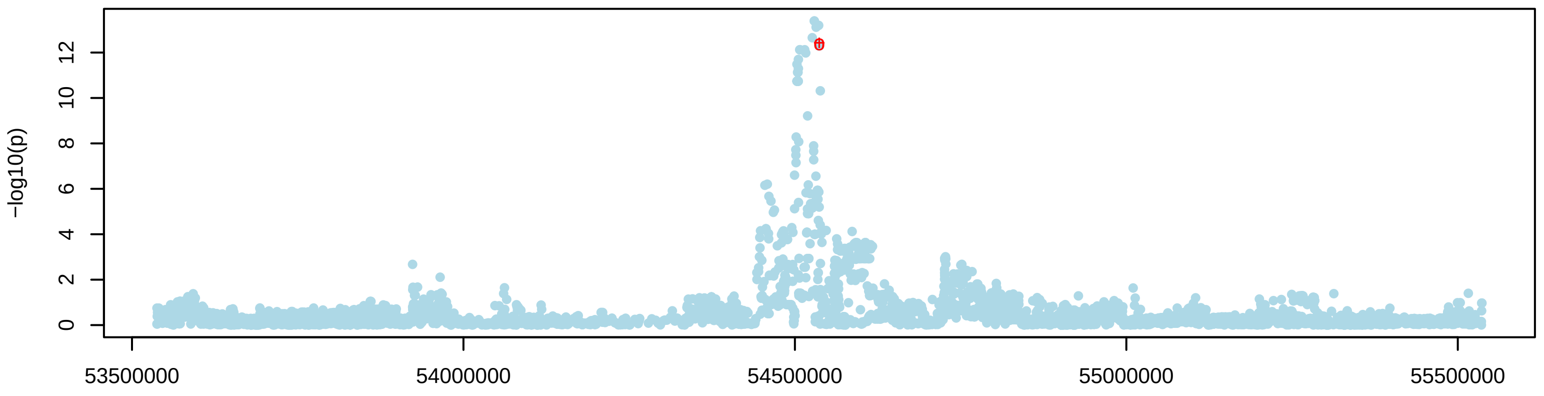

Position on chr 10

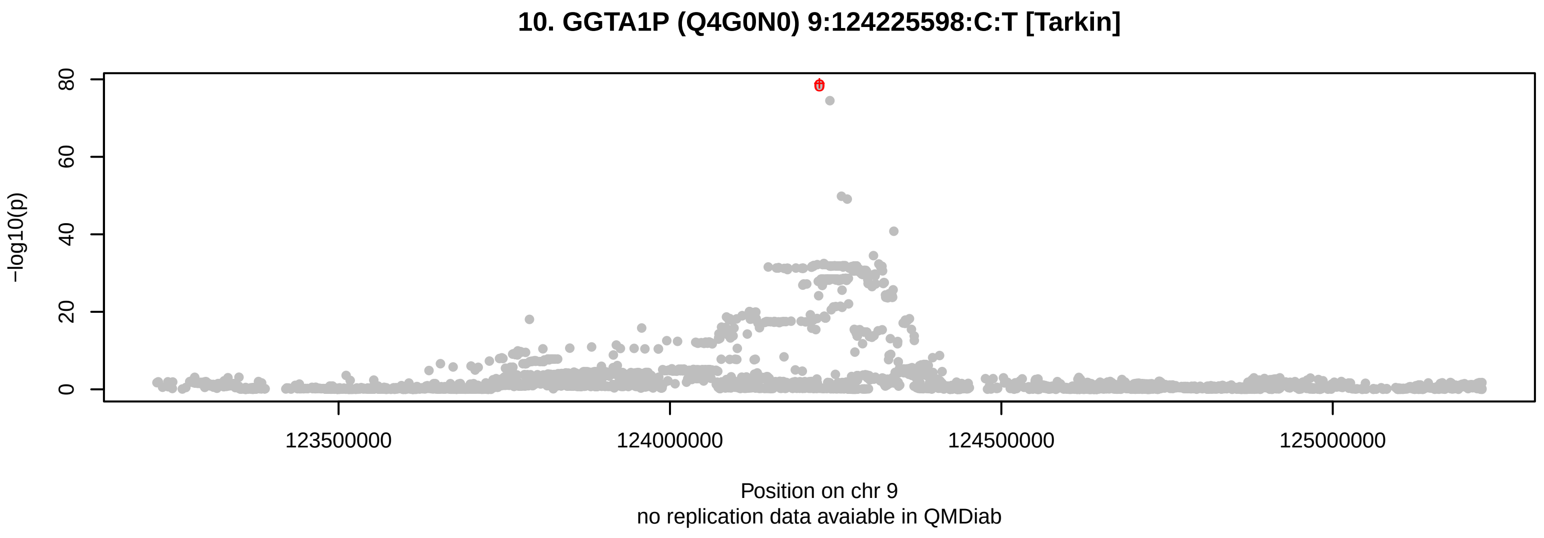
10. GGTA1P (Q4G0N0) 9:124225598:C:T [Tarkin]
−log10(p)
0
20
40
60
80
123500000
124000000
124500000
125000000
Position on chr 9
no replication data avaiable in QMDiab

11. CFHR3 (Q02985) 1:196719716:A:G [Tarkin]

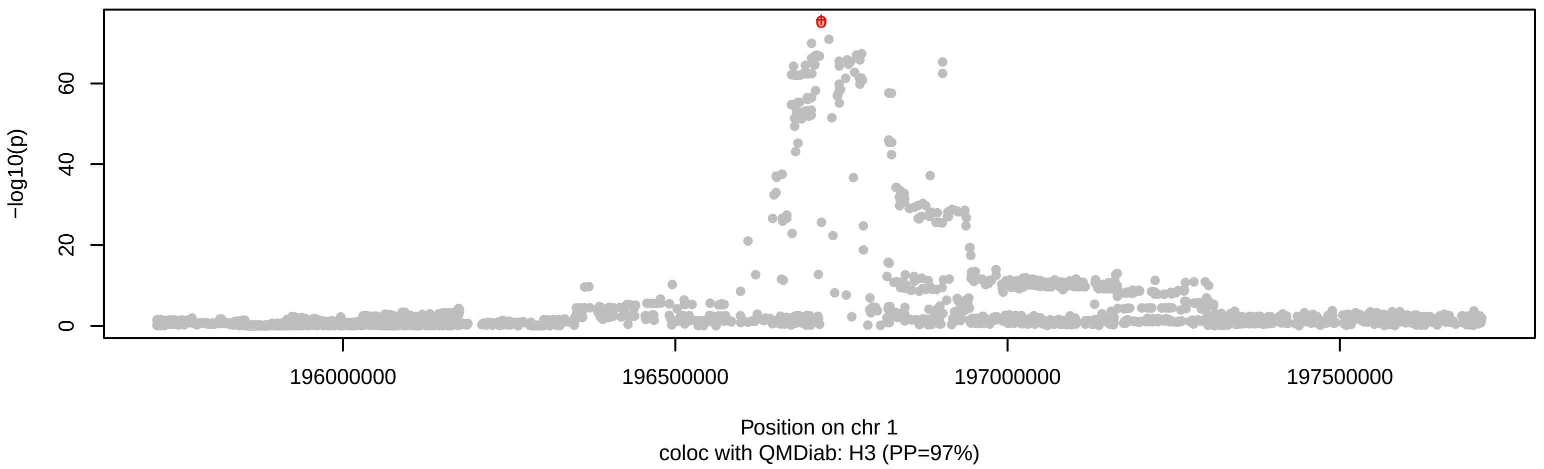

coloc with QMDiab: H3 (PP=97%)

11. CFHR3 (Q02985) 1:196719716:A:G [QMDiab]

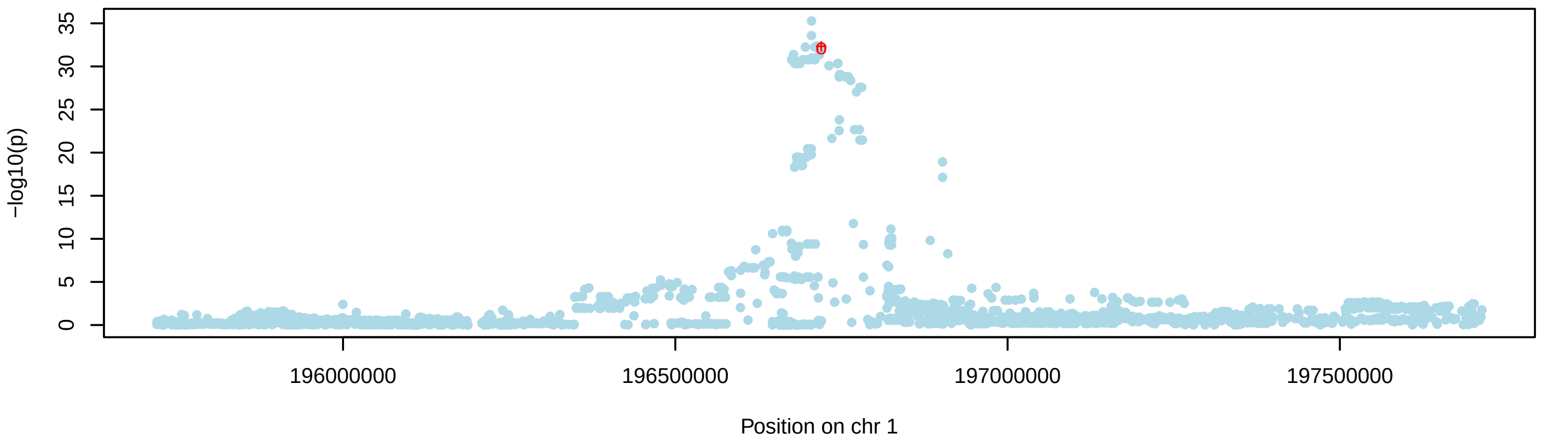

12. FN3K (Q9H479) 17:80693899:C:T [Tarkin]

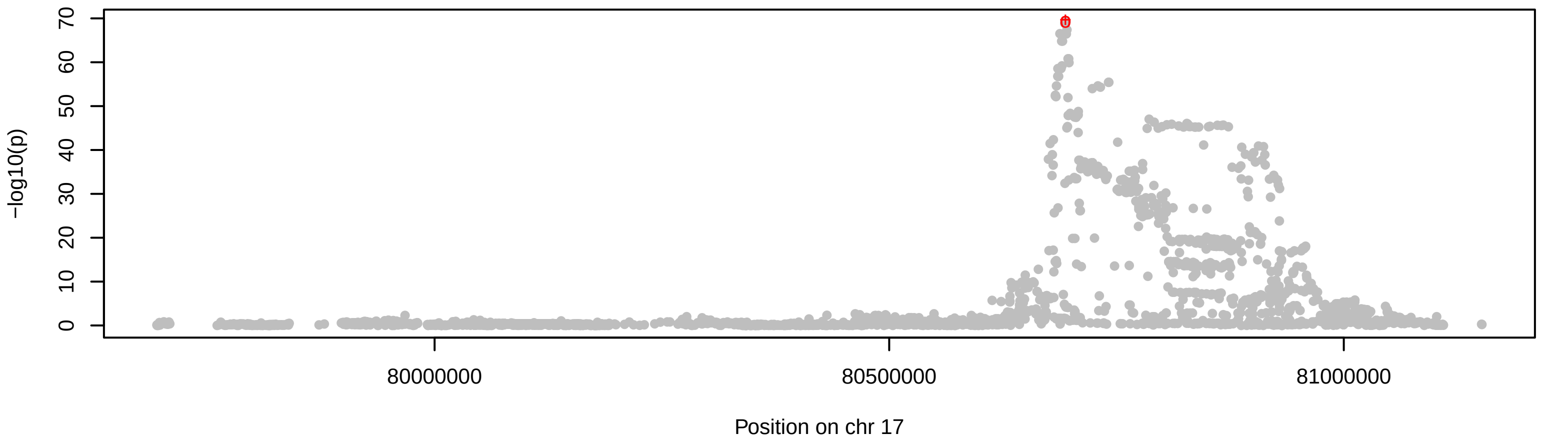

coloc with QMDiab: H4 (PP=86%)

12. FN3K (Q9H479) 17:80693899:C:T [QMDiab]

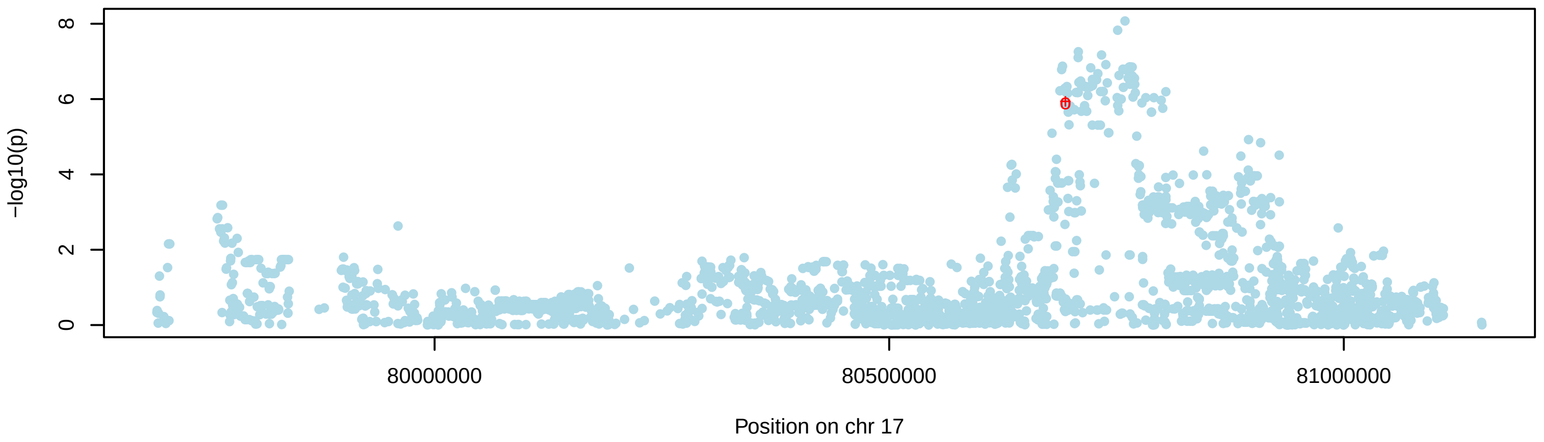

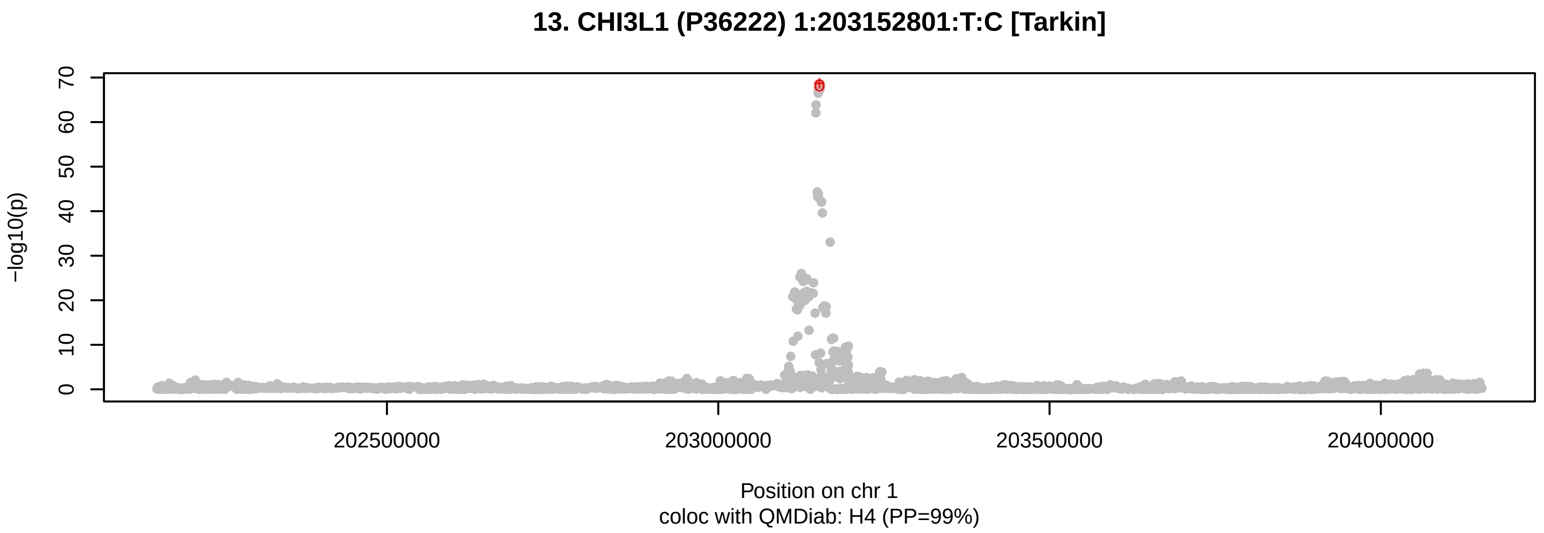
13. CHI3L1 (P36222) 1:203152801:T:C [Tarkin]
−log10(p)
0
10
20
30
40
50
60
70
202500000
203000000
203500000
204000000
Position on chr 1
coloc with QMDiab: H4 (PP=99%)

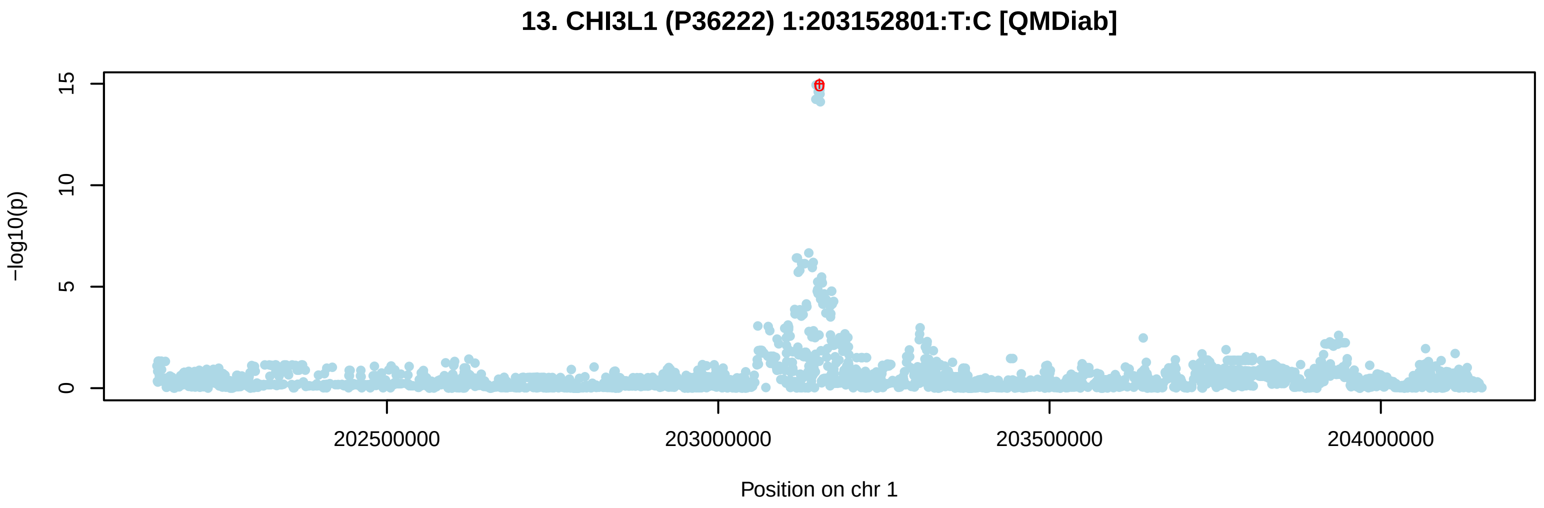
13. CHI3L1 (P36222) 1:203152801:T:C [QMDiab]
−log10(p)
0
5
10
15
202500000
203000000
203500000
204000000
Position on chr 1

14. AMBP (P02760) 3:126261345:G:A [Tarkin]

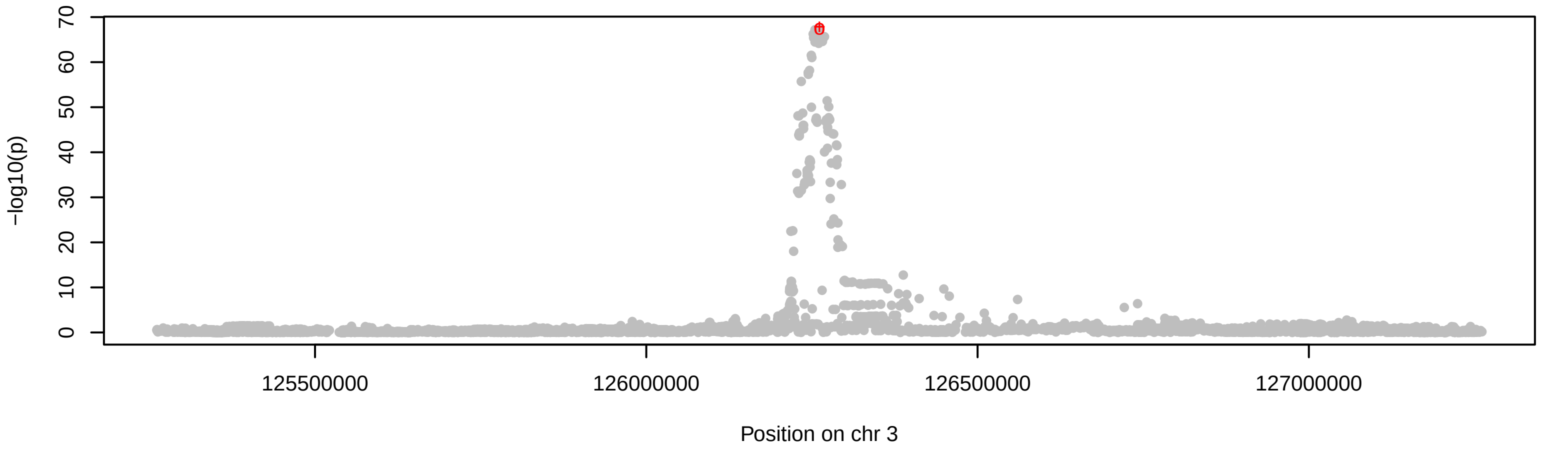

coloc with QMDiab: H4 (PP=98%)

14. AMBP (P02760) 3:126261345:G:A [QMDiab]

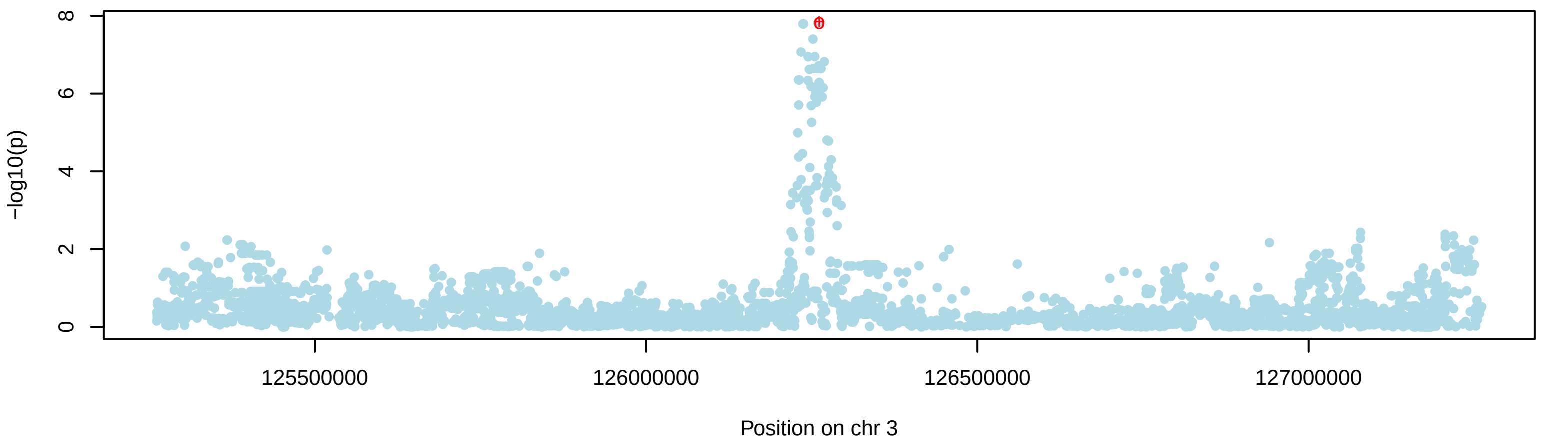

15. HLA−G (P17693;Q5RJ85) 6:29916391:A:C [Tarkin]

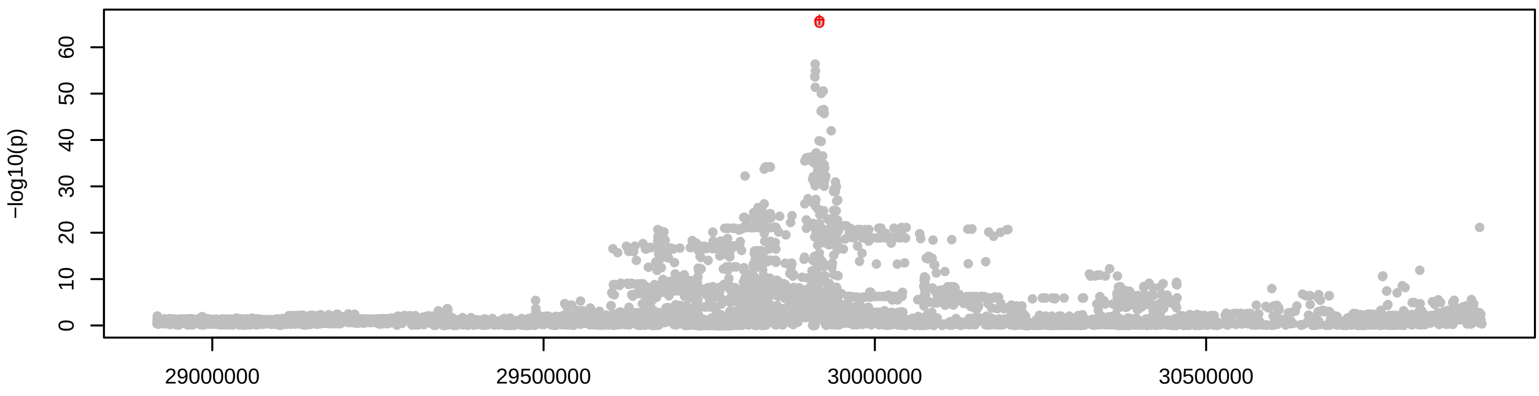

Position on chr 6
coloc with QMDiab: H4 (PP=100%)

15. HLA−G (P17693;Q5RJ85) 6:29916391:A:C [QMDiab]

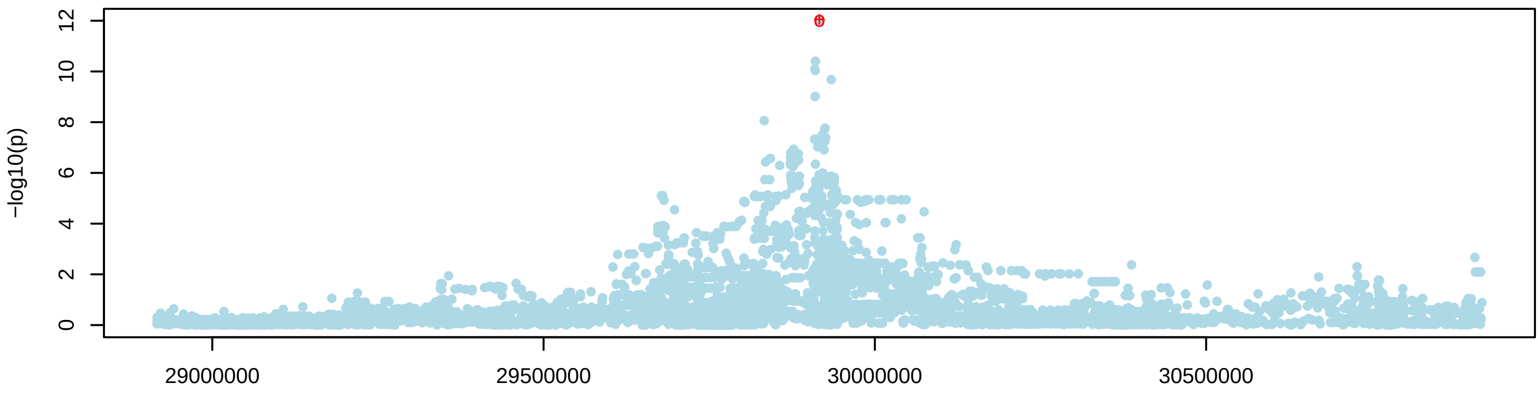

Position on chr 6

16. BRE (Q9NXR7) 1:196821380:T:G [Tarkin]

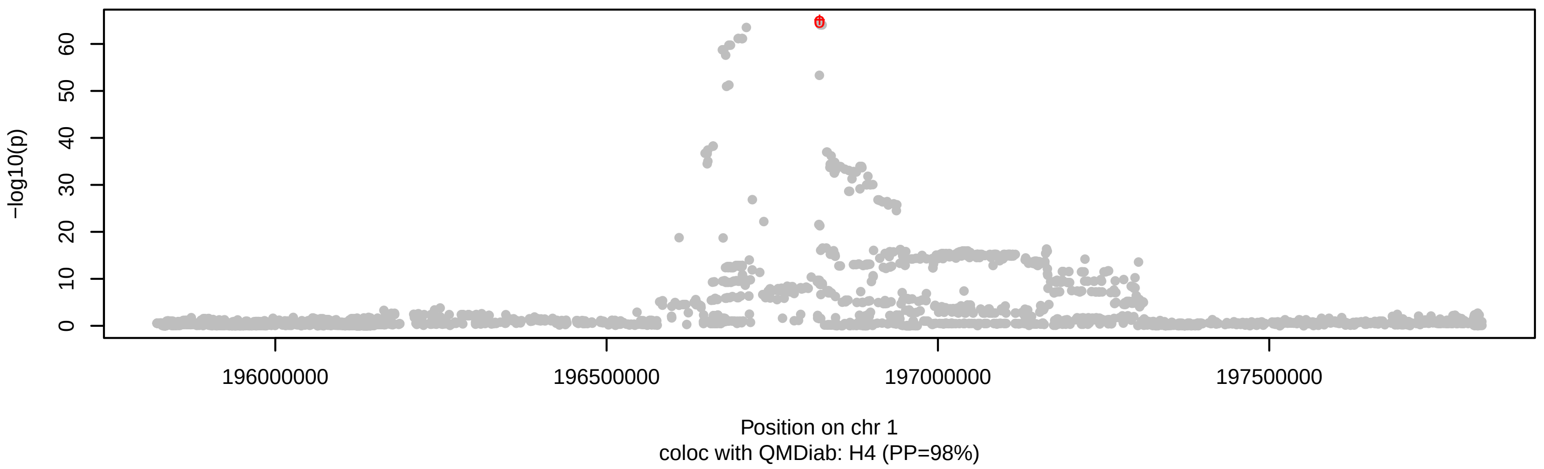

coloc with QMDiab: H4 (PP=98%)

16. BRE (Q9NXR7) 1:196821380:T:G [QMDiab]

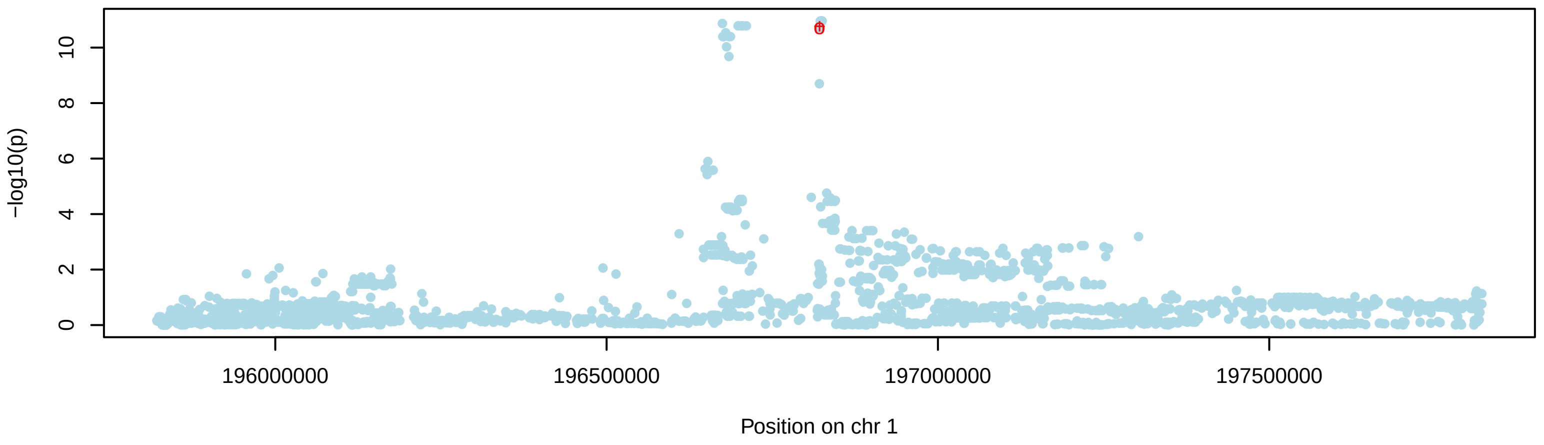

17. CFH (A0A024R962) 1:196822368:A:G [Tarkin]

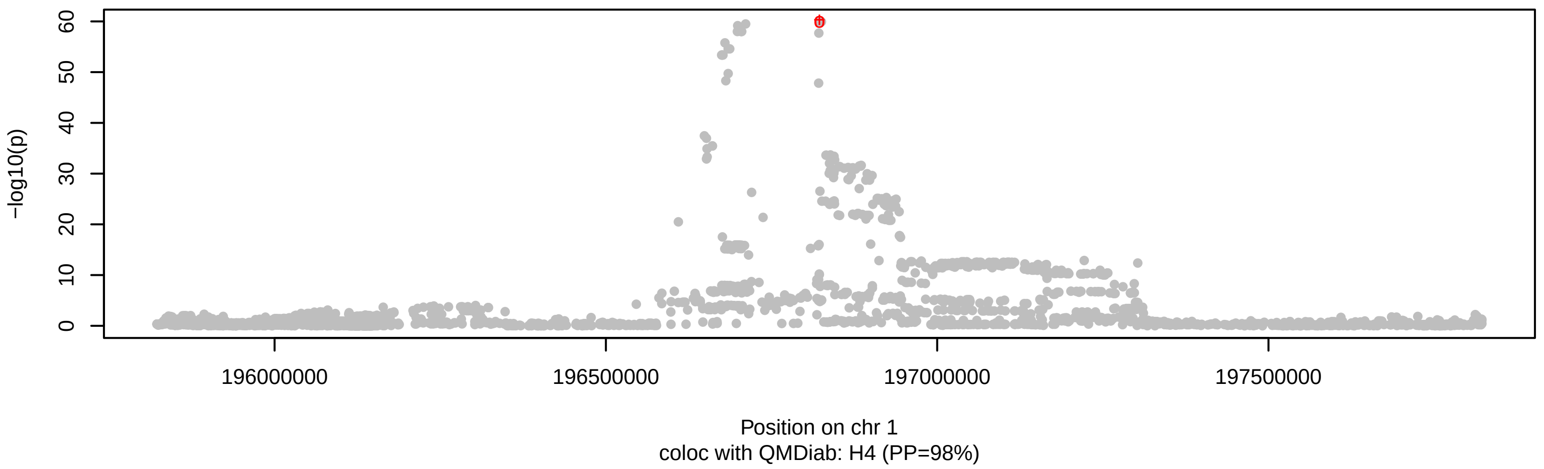

coloc with QMDiab: H4 (PP=98%)

17. CFH (A0A024R962) 1:196822368:A:G [QMDiab]

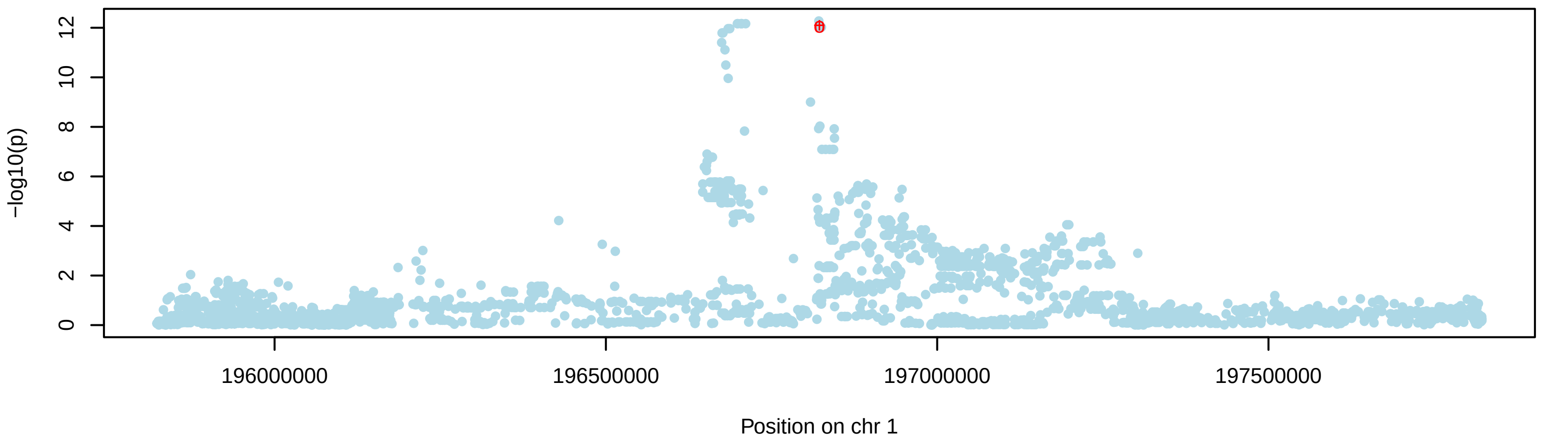

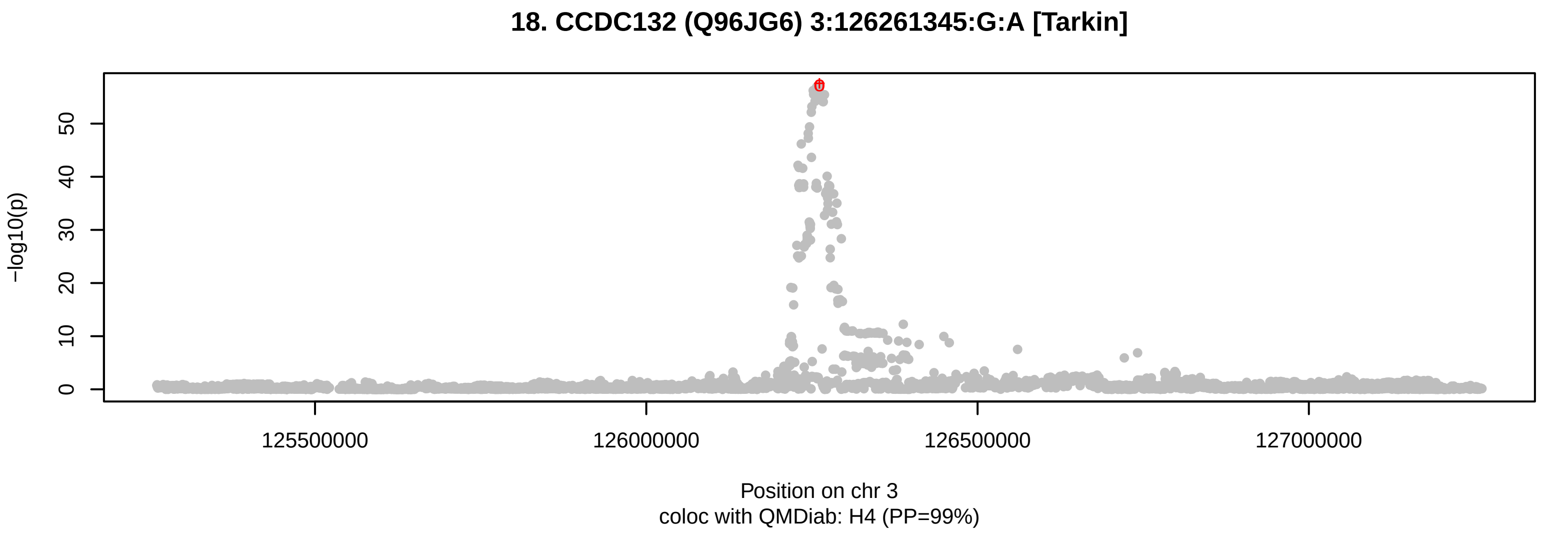
18. CCDC132 (Q96JG6) 3:126261345:G:A [Tarkin]
−log10(p)
0
10
20
30
40
50
125500000
126000000
126500000
127000000
Position on chr 3
coloc with QMDiab: H4 (PP=99%)

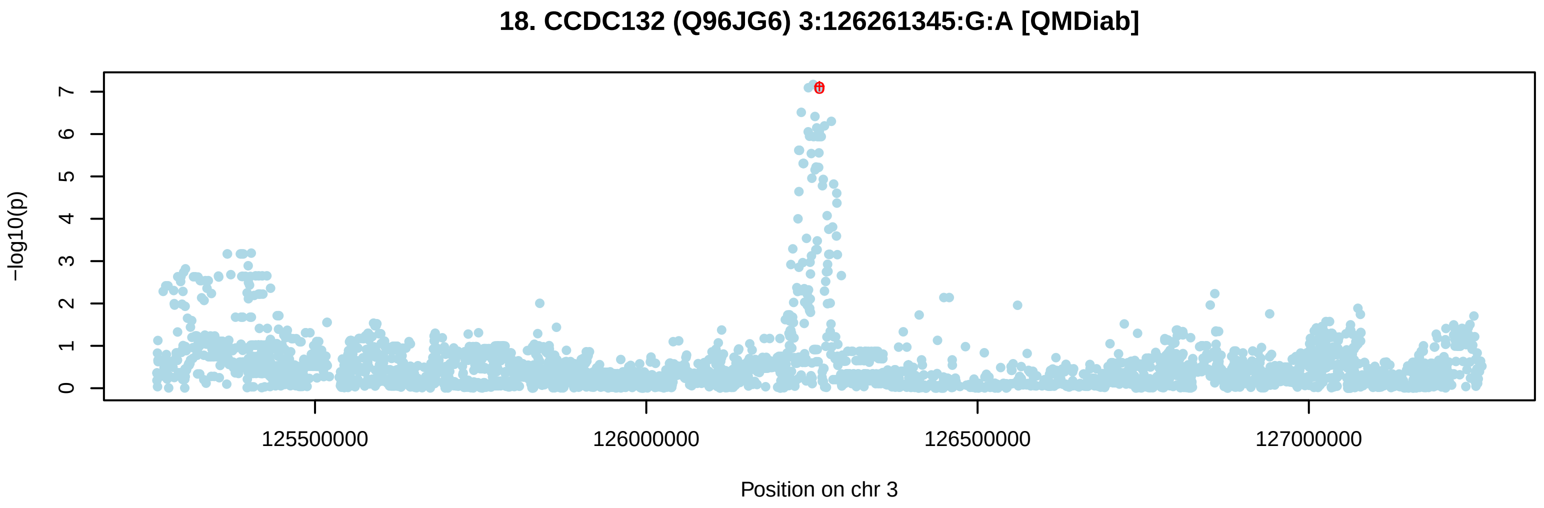
18. CCDC132 (Q96JG6) 3:126261345:G:A [QMDiab]
−log10(p)
0
1
2
3
4
5
6
7
125500000
126000000
126500000
127000000
Position on chr 3

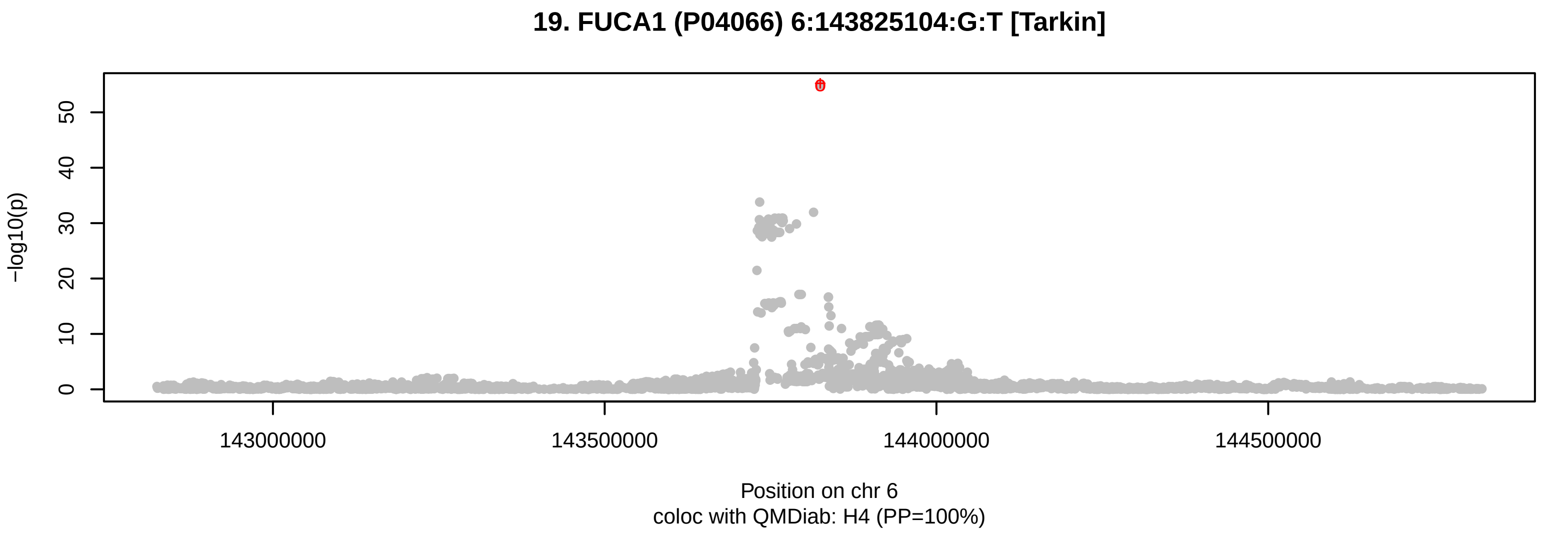
19. FUCA1 (P04066) 6:143825104:G:T [Tarkin]
−log10(p)
0
10
20
30
40
50
143000000
143500000
144000000
144500000
Position on chr 6
coloc with QMDiab: H4 (PP=100%)

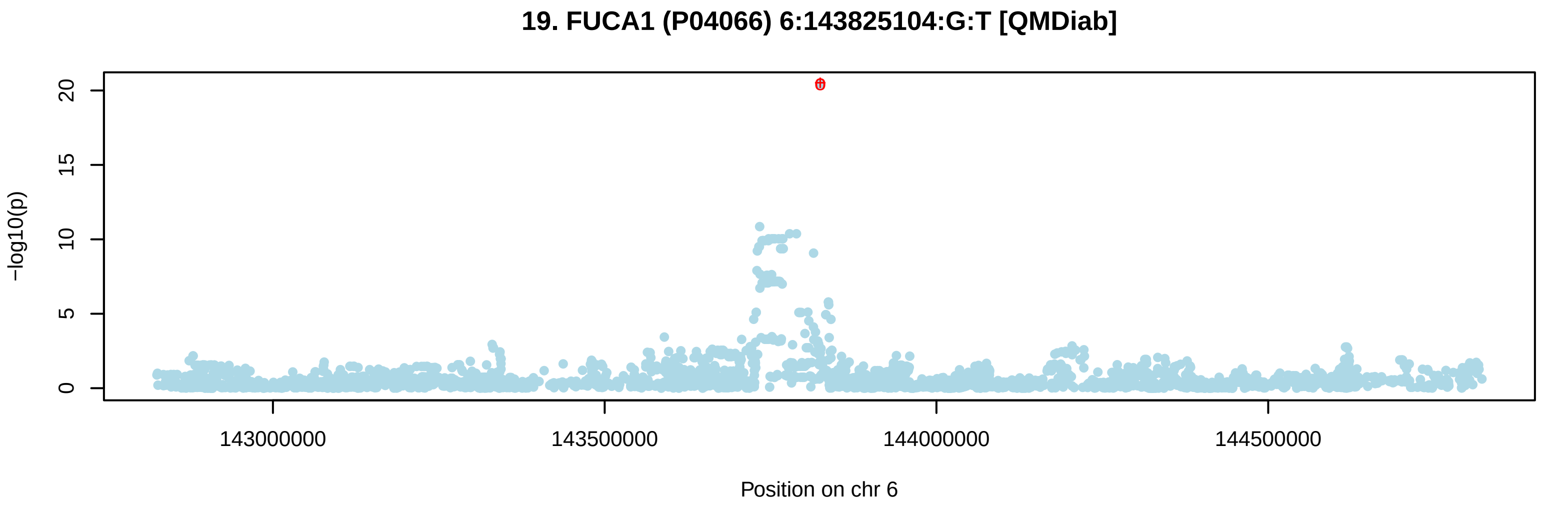
19. FUCA1 (P04066) 6:143825104:G:T [QMDiab]
−log10(p)
0
5
10
15
20
143000000
143500000
144000000
144500000
Position on chr 6

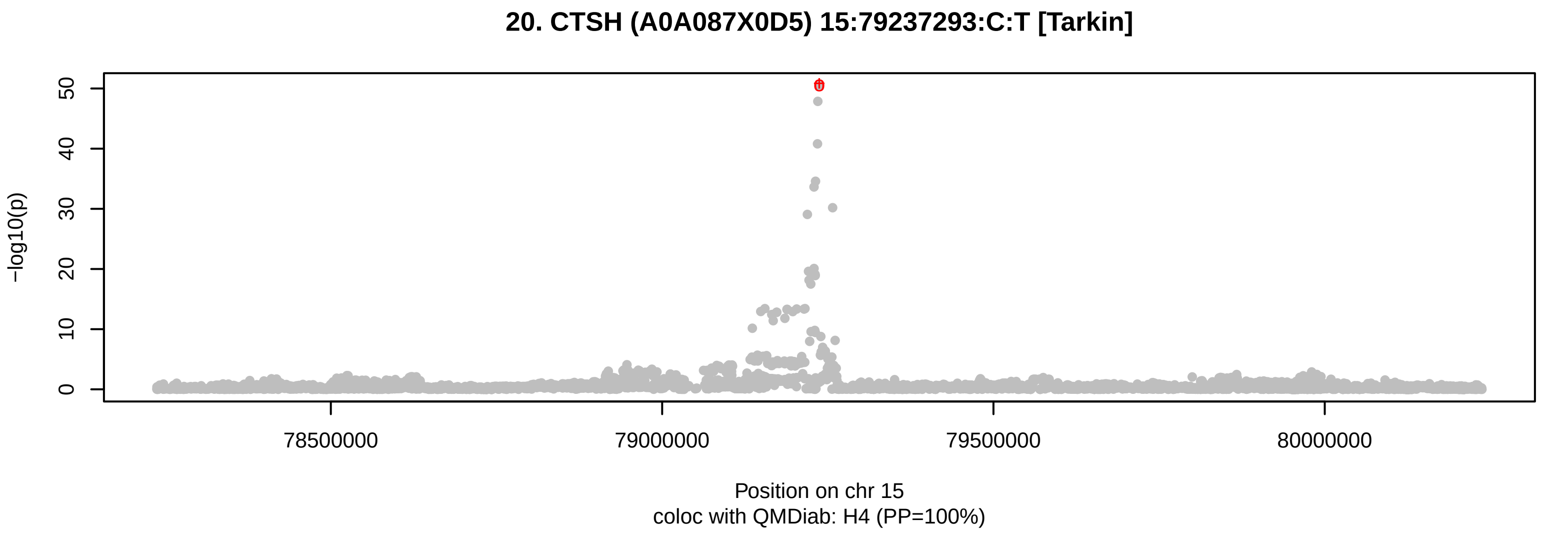
20. CTSH (A0A087X0D5) 15:79237293:C:T [Tarkin]
−log10(p)
0
10
20
30
40
50
78500000
79000000
79500000
80000000
Position on chr 15
coloc with QMDiab: H4 (PP=100%)

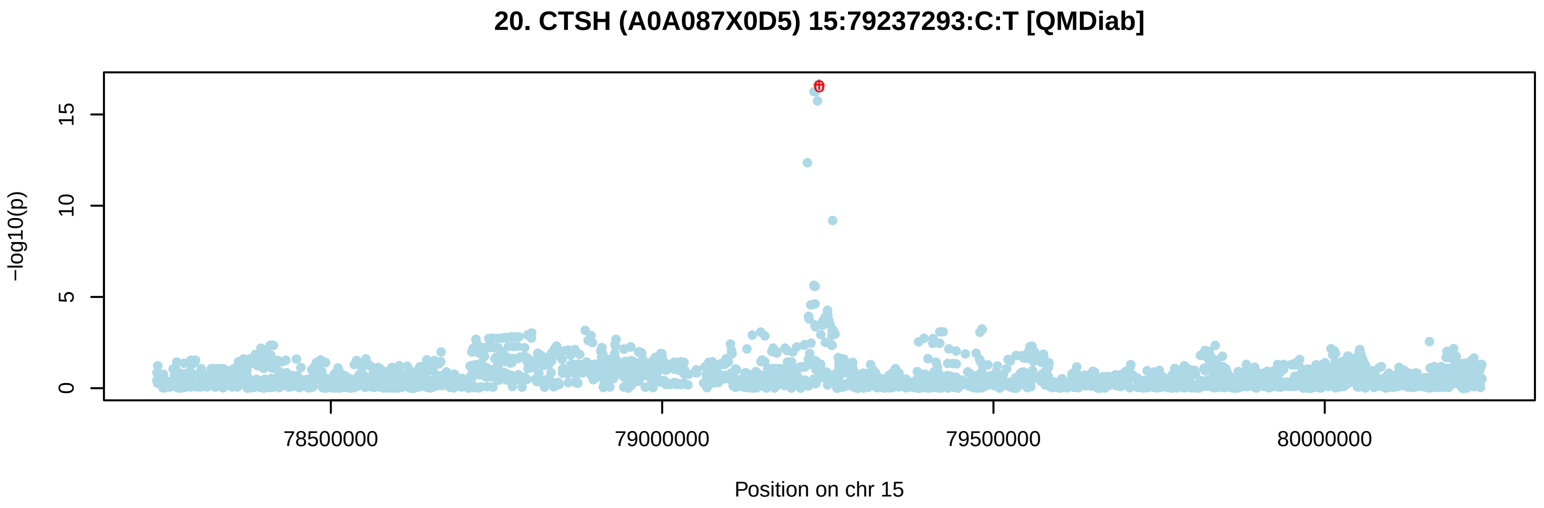
20. CTSH (A0A087X0D5) 15:79237293:C:T [QMDiab]
−log10(p)
0
5
10
15
78500000
79000000
79500000
80000000
Position on chr 15

21. HLA−G (P17693;Q5RJ85) 6:31322470:C:G [Tarkin]

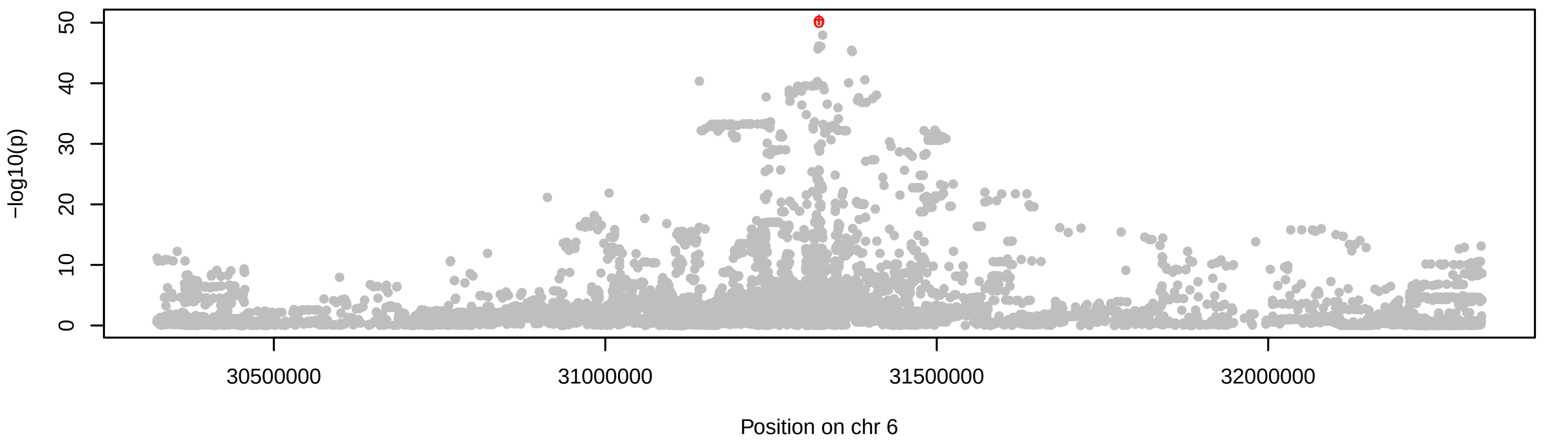

coloc with QMDiab: H3 (PP=97%)

21. HLA−G (P17693;Q5RJ85) 6:31322470:C:G [QMDiab]

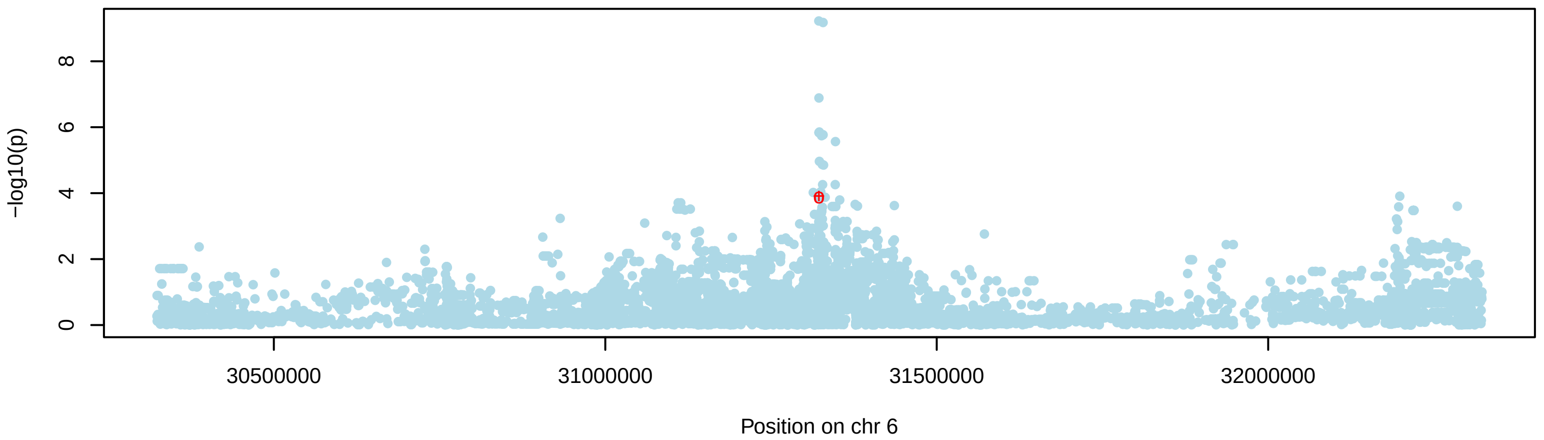

22. HLA−B (P01889) 6:31242089:A:T [Tarkin]

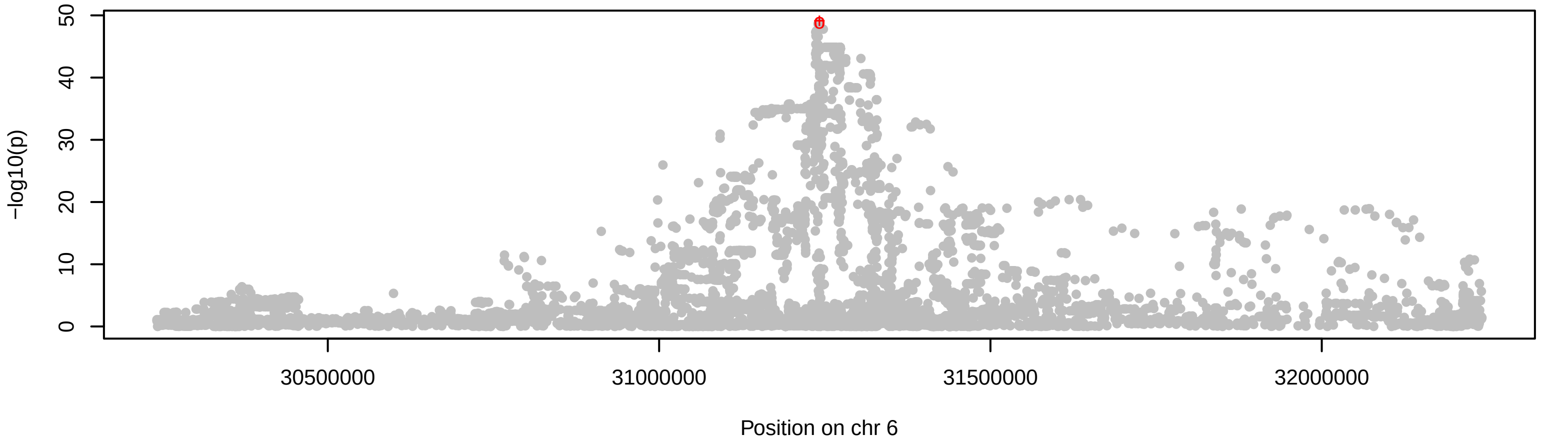

coloc with QMDiab: H3 (PP=96%)

22. HLA−B (P01889) 6:31242089:A:T [QMDiab]

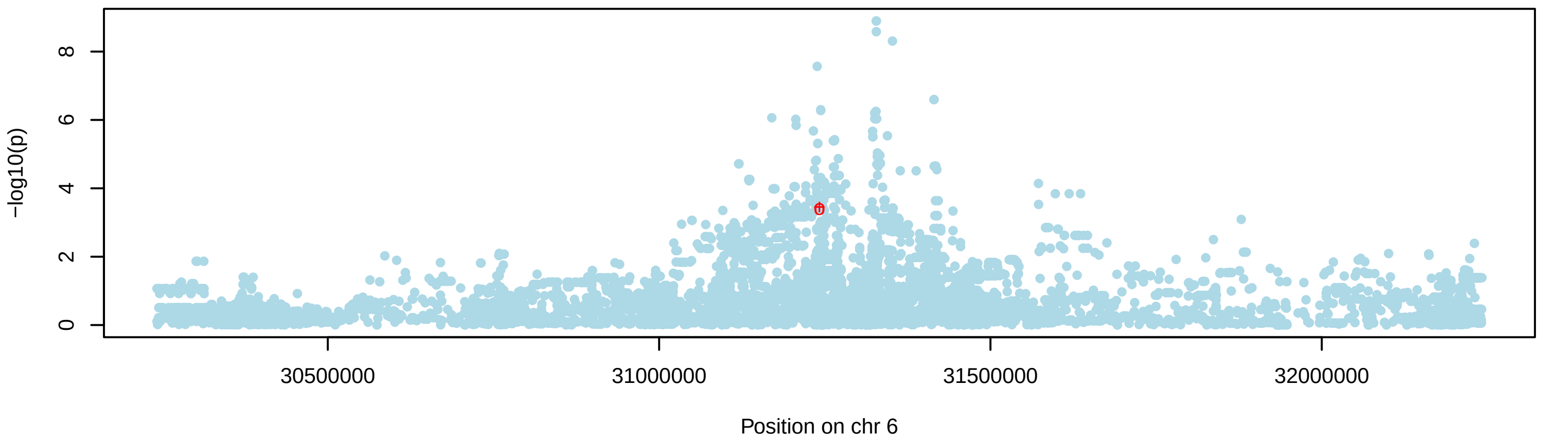

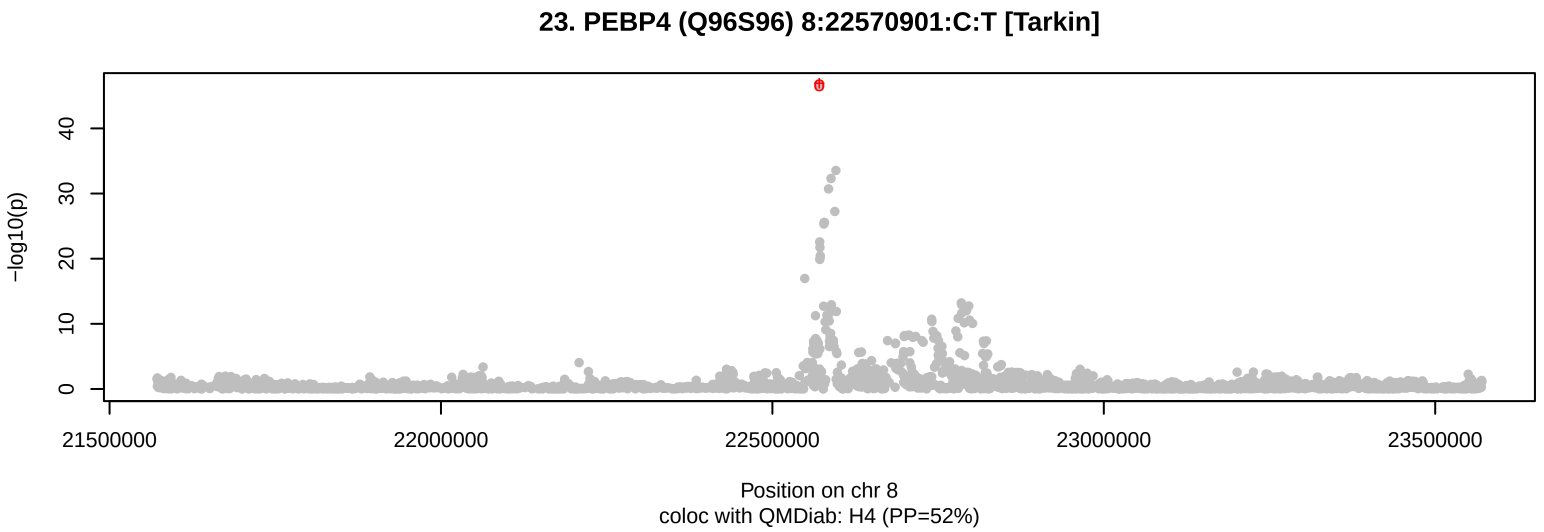

coloc with QMDiab: H4 (PP=52%)

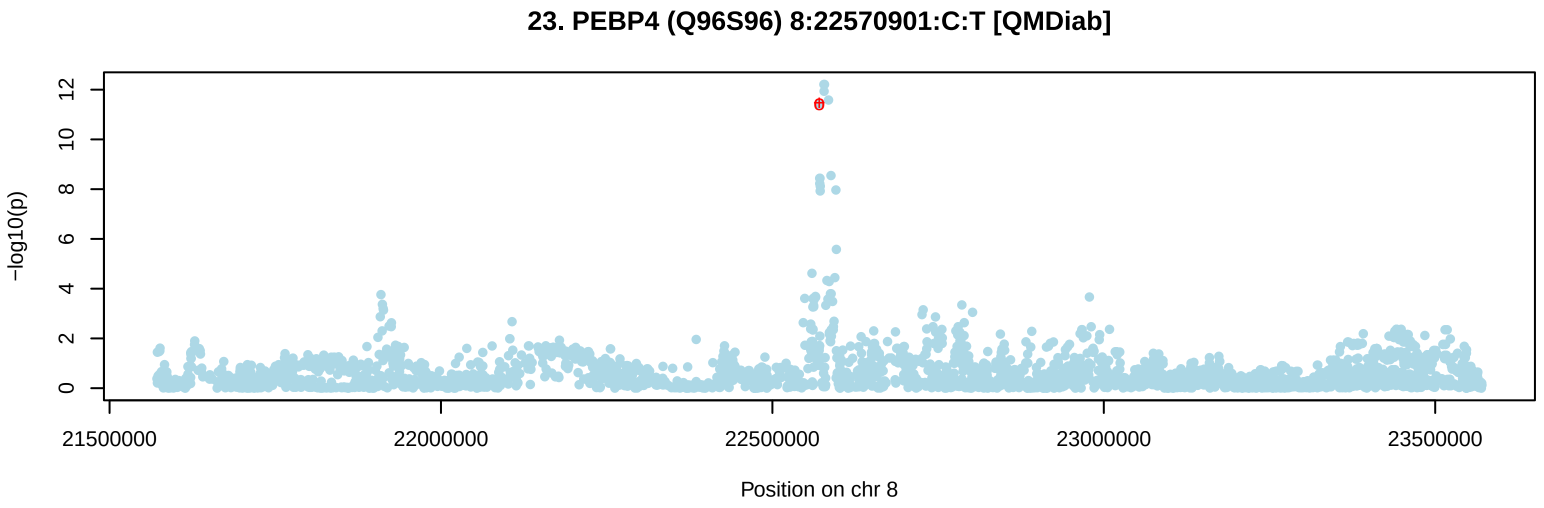

24. CCDC7 (Q96M83) 1:196822368:A:G [Tarkin]

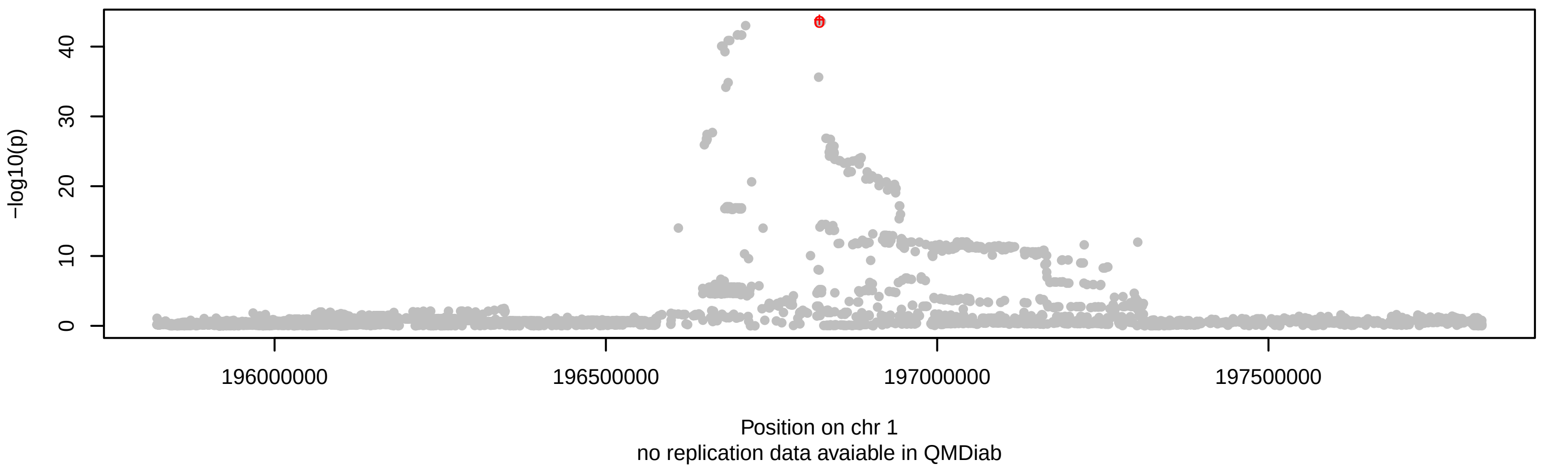

no replication data avaiable in QMDiab

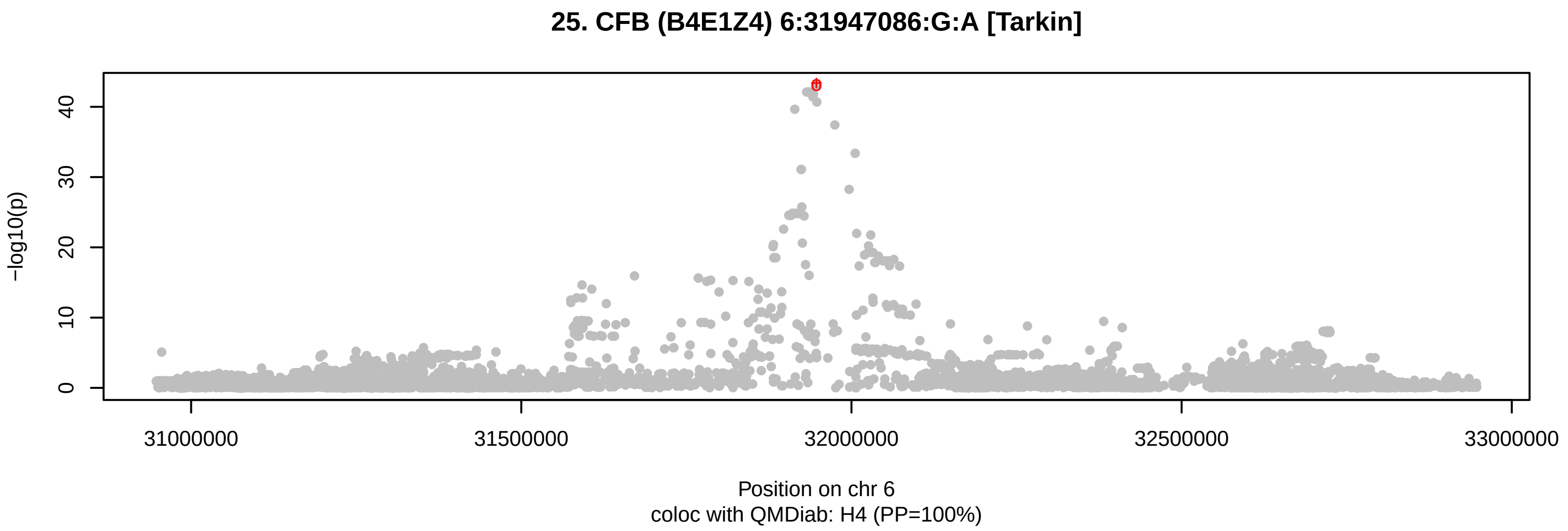
25. CFB (B4E1Z4) 6:31947086:G:A [Tarkin]
−log10(p)
0
10
20
30
40
31000000
31500000
32000000
32500000
33000000
Position on chr 6
coloc with QMDiab: H4 (PP=100%)

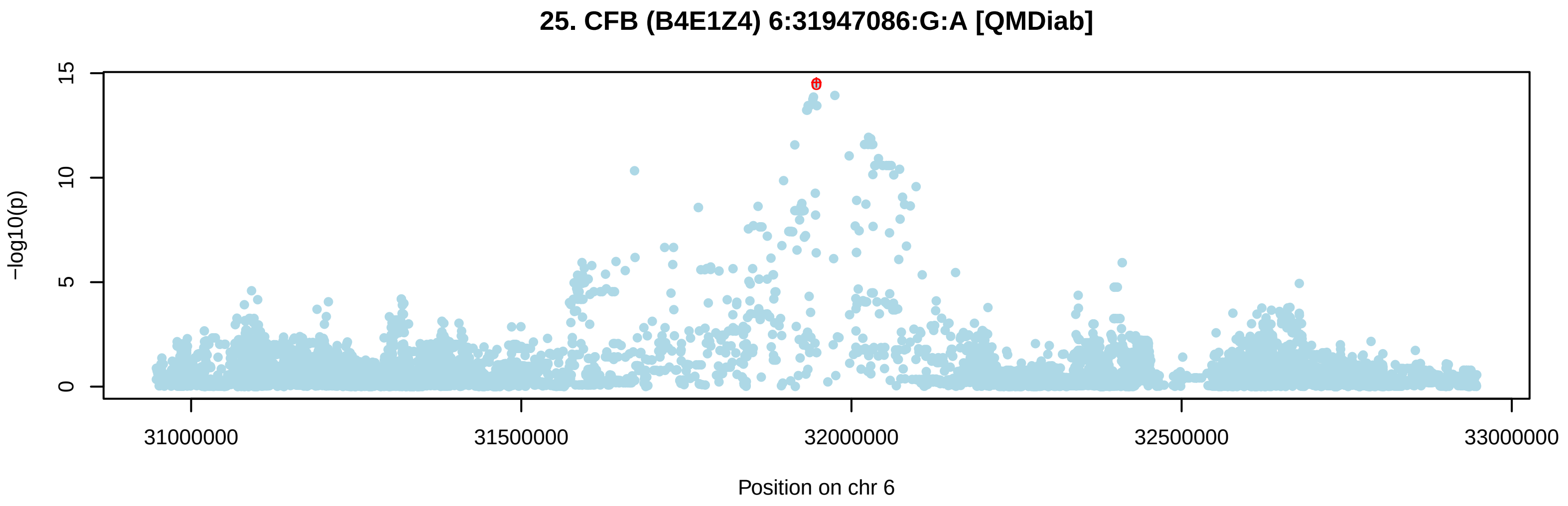
25. CFB (B4E1Z4) 6:31947086:G:A [QMDiab]
−log10(p)
0
5
10
15
31000000
31500000
32000000
32500000
33000000
Position on chr 6

26. EBI3 (Q14213) 19:4236996:G:A [Tarkin]

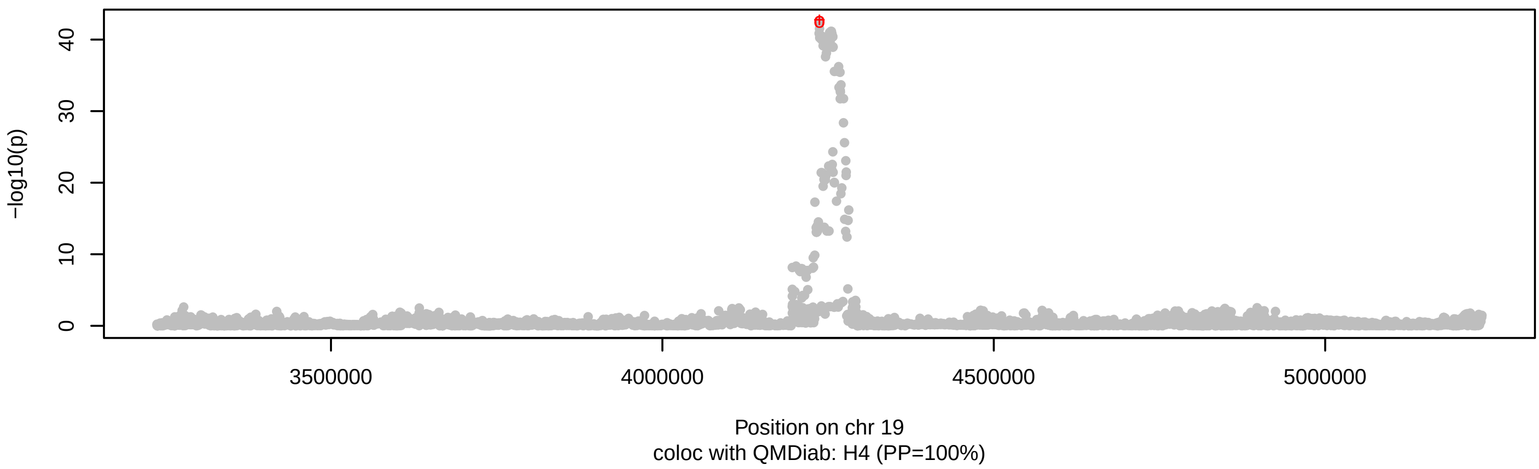

coloc with QMDiab: H4 (PP=100%)

26. EBI3 (Q14213) 19:4236996:G:A [QMDiab]

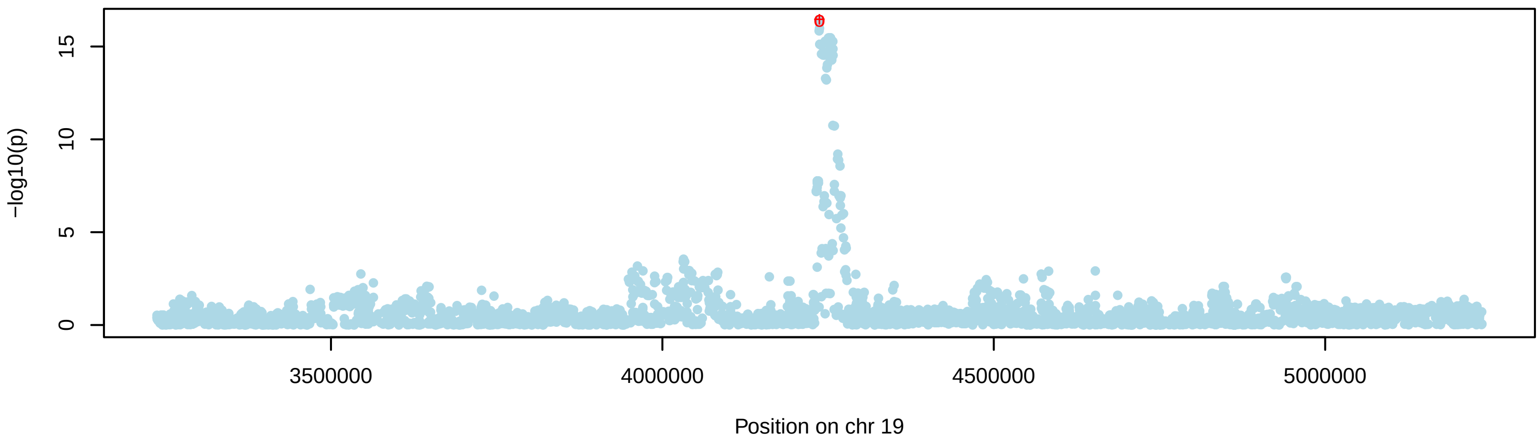

27. ERAP1 (Q9NZ08;Q9NZ08−2) 5:96147733:A:G [Tarkin]

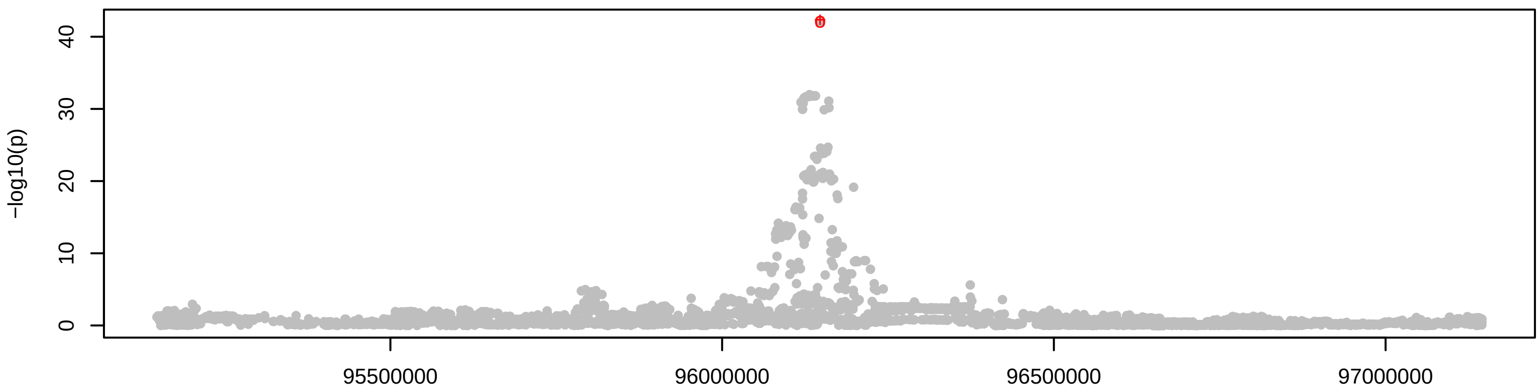

Position on chr 5
coloc with QMDiab: H1 (PP=71%)

27. ERAP1 (Q9NZ08−2) 5:96147733:A:G [QMDiab]

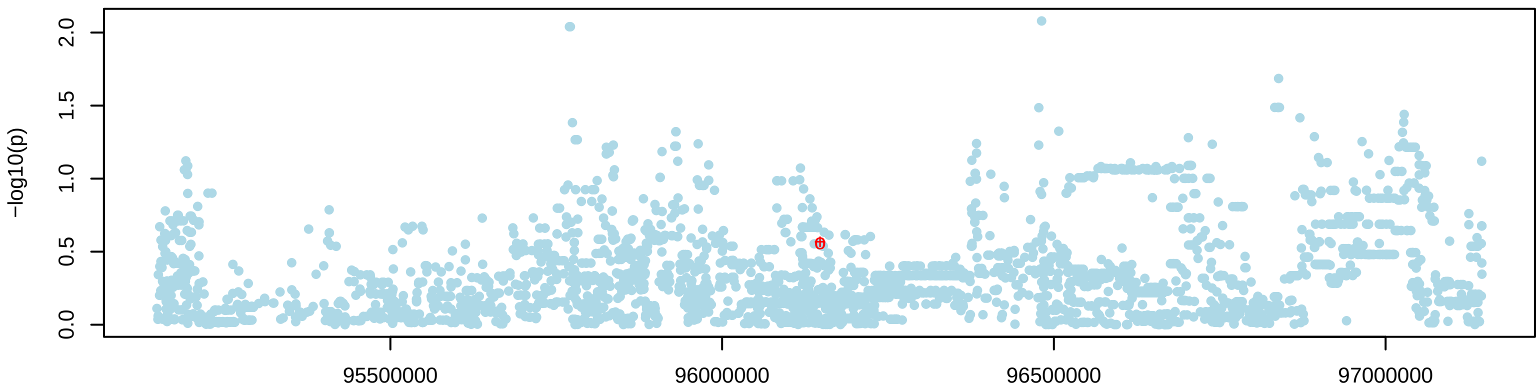

Position on chr 5

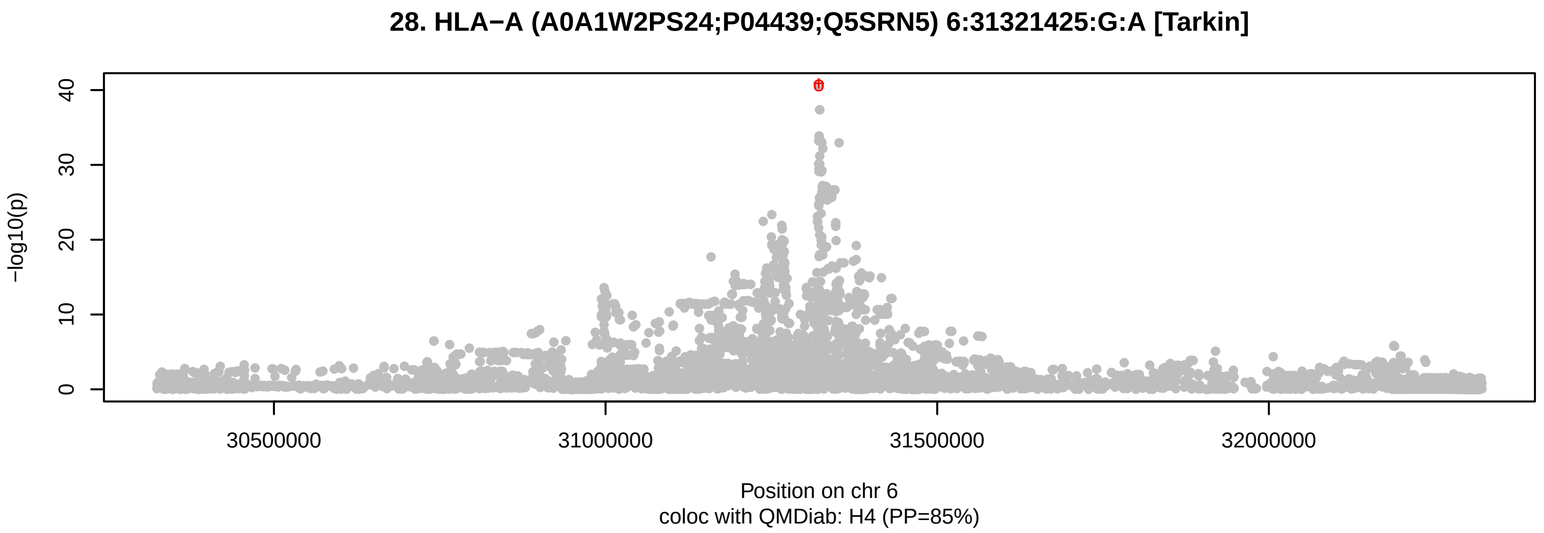
28. HLA−A (A0A1W2PS24;P04439;Q5SRN5) 6:31321425:G:A [Tarkin]
−log10(p)
0
10
20
30
40
30500000
31000000
31500000
32000000
Position on chr 6
coloc with QMDiab: H4 (PP=85%)

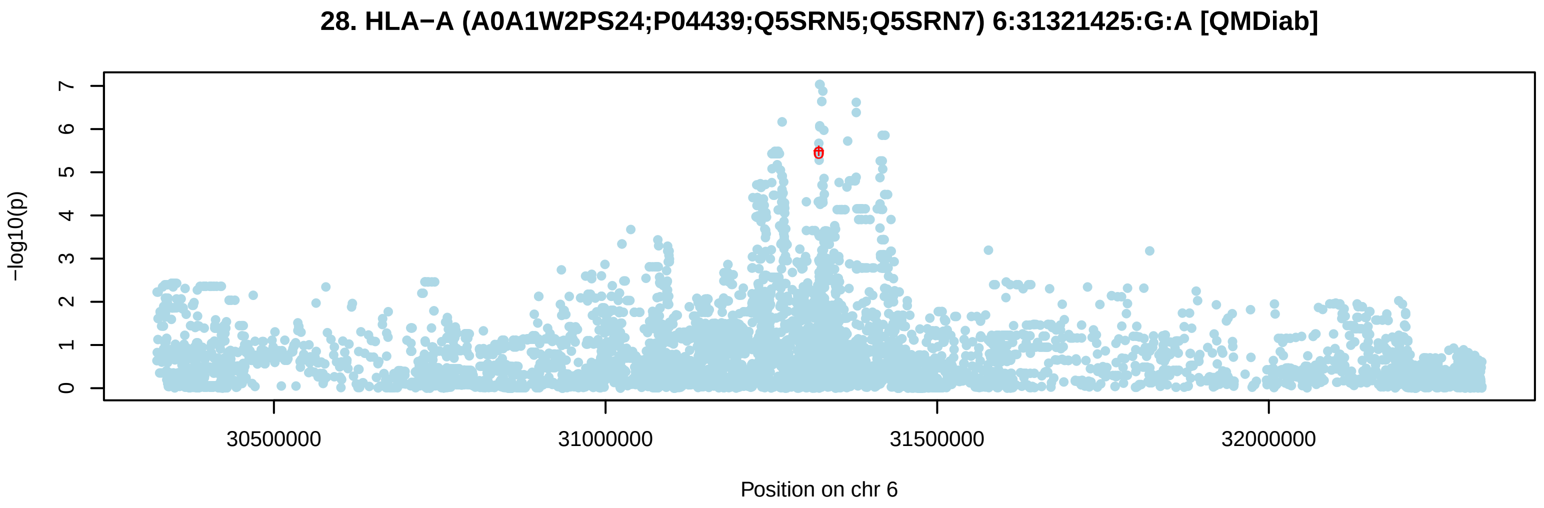
28. HLA−A (A0A1W2PS24;P04439;Q5SRN5;Q5SRN7) 6:31321425:G:A [QMDiab]
−log10(p)
0
1
2
3
4
5
6
7
30500000
31000000
31500000
32000000
Position on chr 6

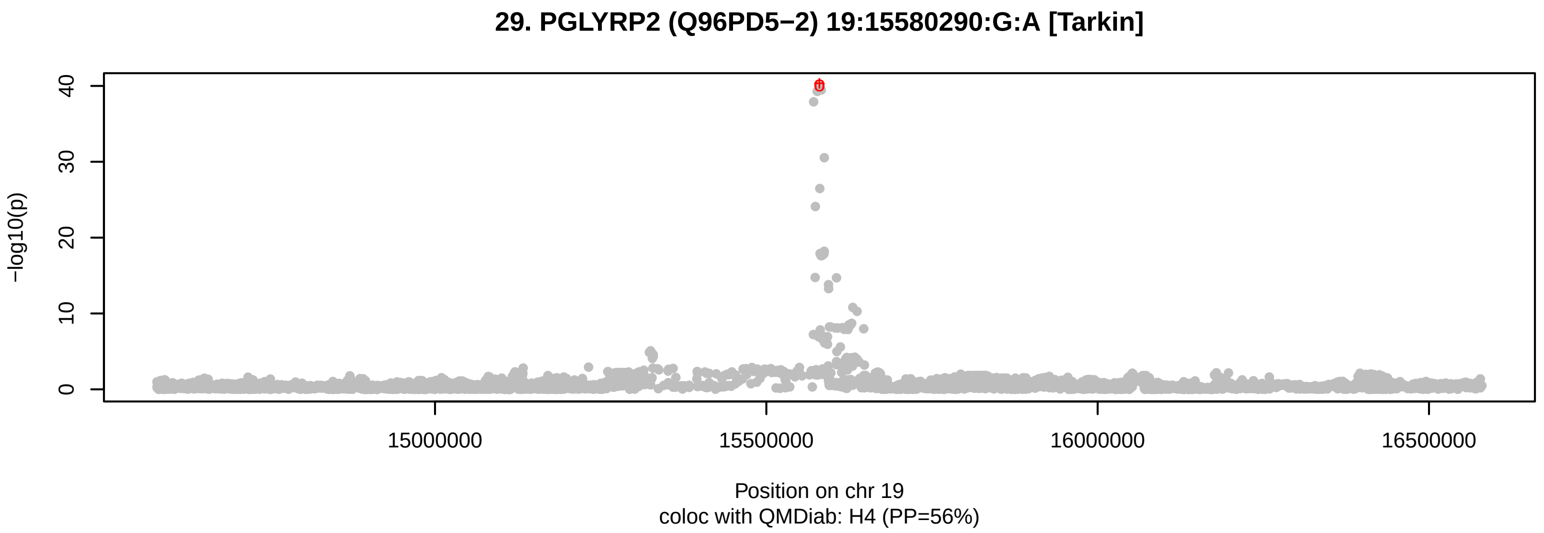

29. PGLYRP2 (Q96PD5−2) 19:15580290:G:A [Tarkin]
−log10(p)
0
10
20
30
40
15000000
15500000
16000000
16500000
Position on chr 19
coloc with QMDiab: H4 (PP=56%)

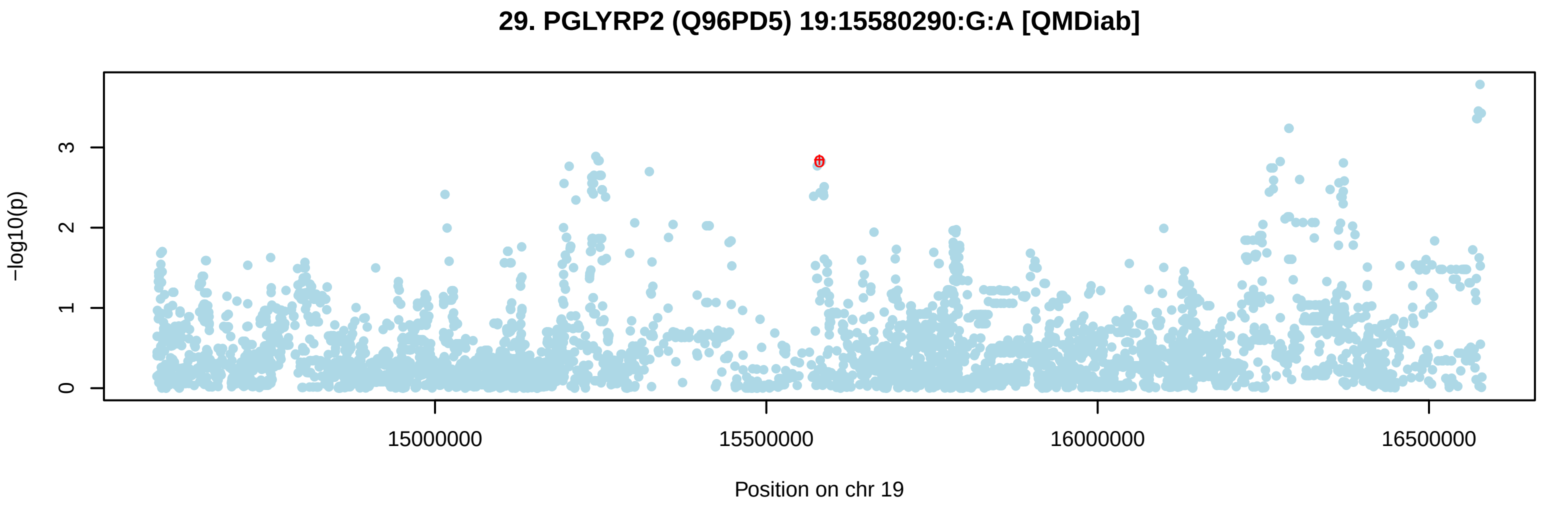

29. PGLYRP2 (Q96PD5) 19:15580290:G:A [QMDiab]
−log10(p)
0
1
2
3
15000000
15500000
16000000
16500000
Position on chr 19

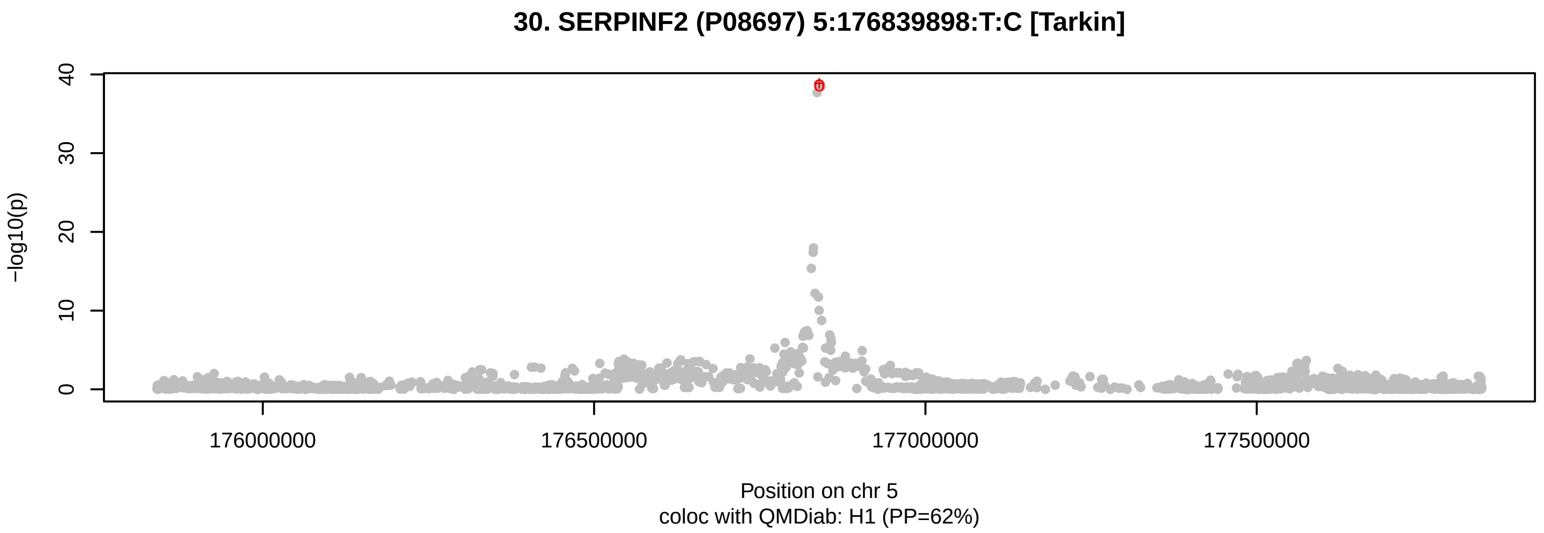
30. SERPINF2 (P08697) 5:176839898:T:C [Tarkin]
−log10(p)
0
10
20
30
40
176000000
176500000
177000000
177500000
Position on chr 5
coloc with QMDiab: H1 (PP=62%)

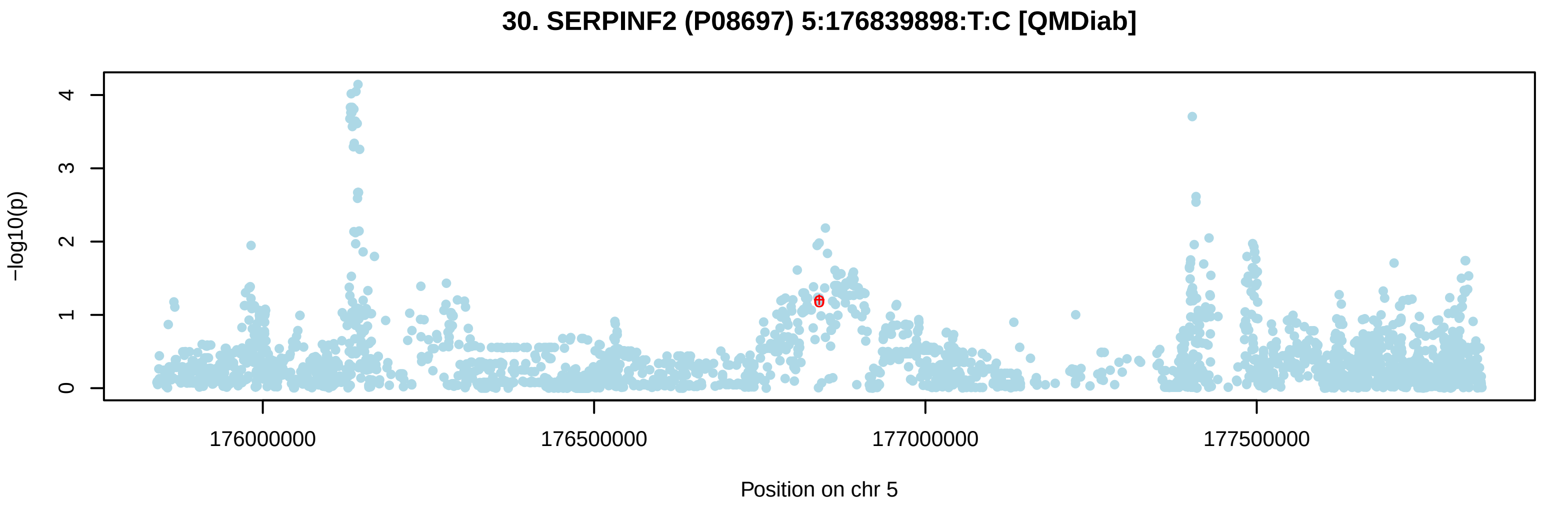
30. SERPINF2 (P08697) 5:176839898:T:C [QMDiab]
−log10(p)
0
1
2
3
4
176000000
176500000
177000000
177500000
Position on chr 5

31. IL7R (P16871) 5:35874575:C:T [Tarkin]

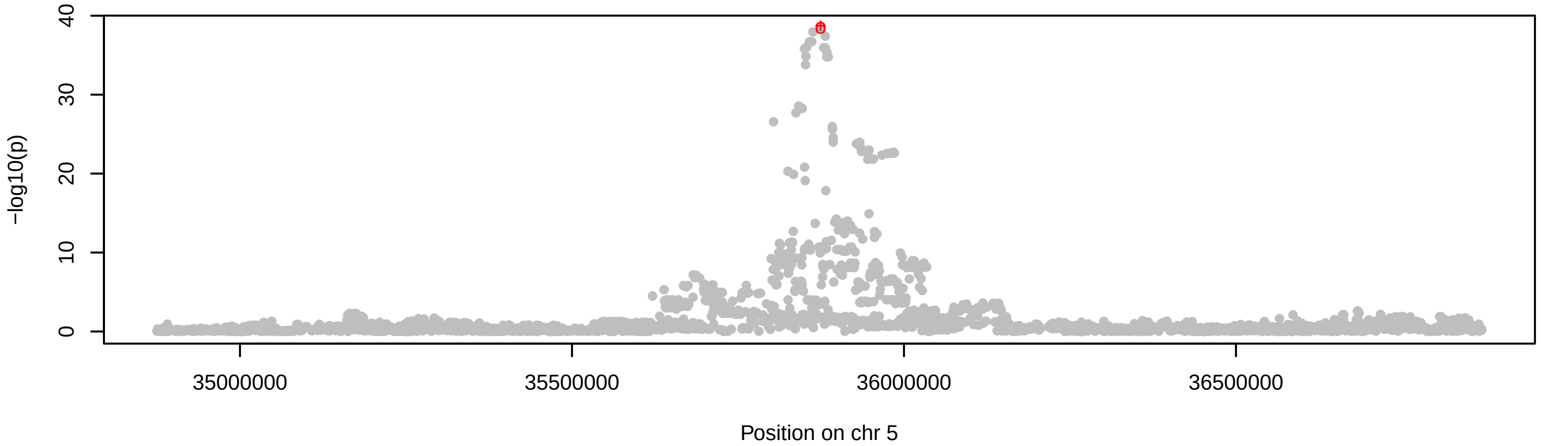

coloc with QMDiab: H4 (PP=99%)

31. IL7R (P16871−3) 5:35874575:C:T [QMDiab]

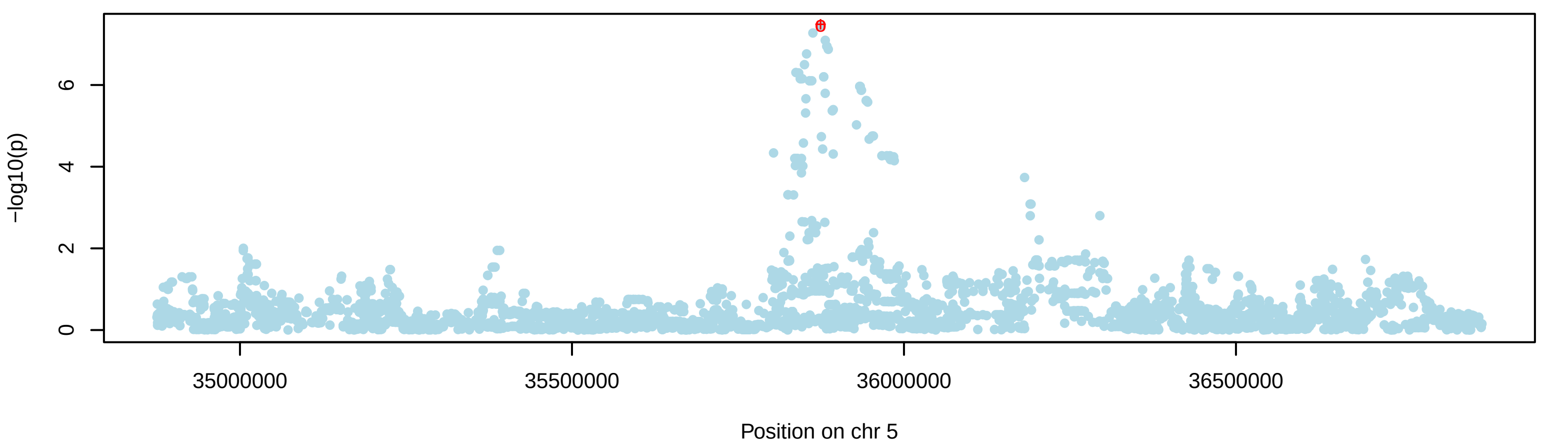

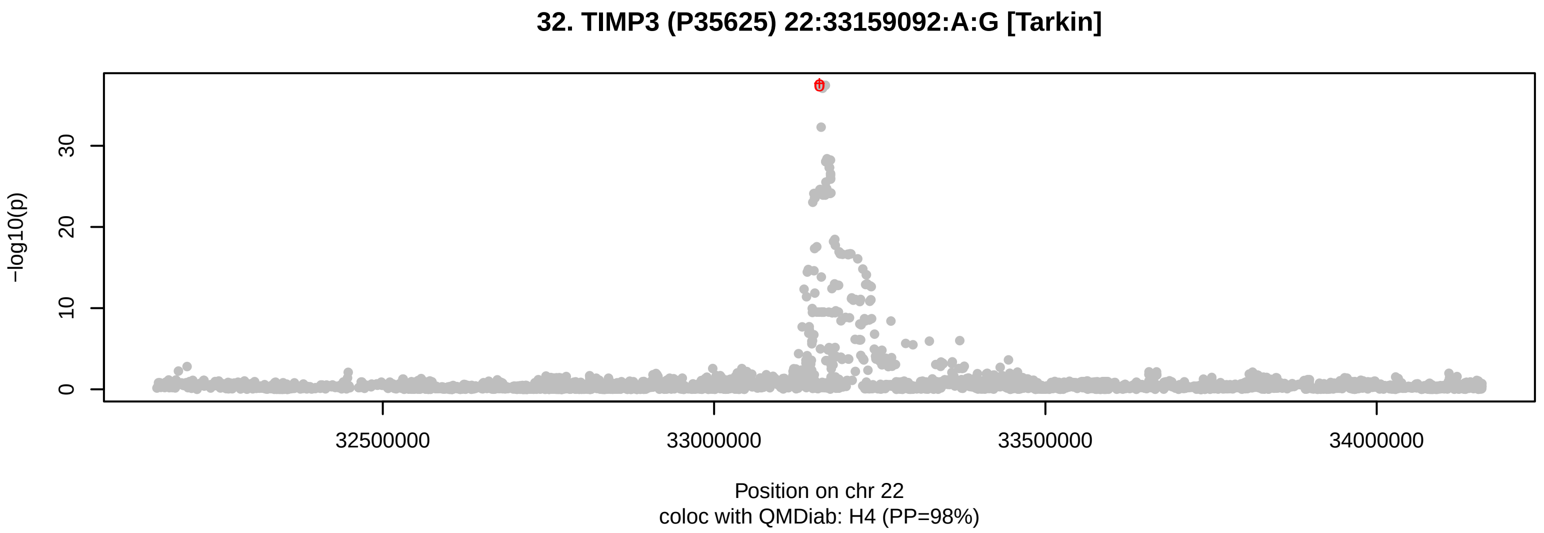
32. TIMP3 (P35625) 22:33159092:A:G [Tarkin]
−log10(p)
0
10
20
30
32500000
33000000
33500000
34000000
Position on chr 22
coloc with QMDiab: H4 (PP=98%)

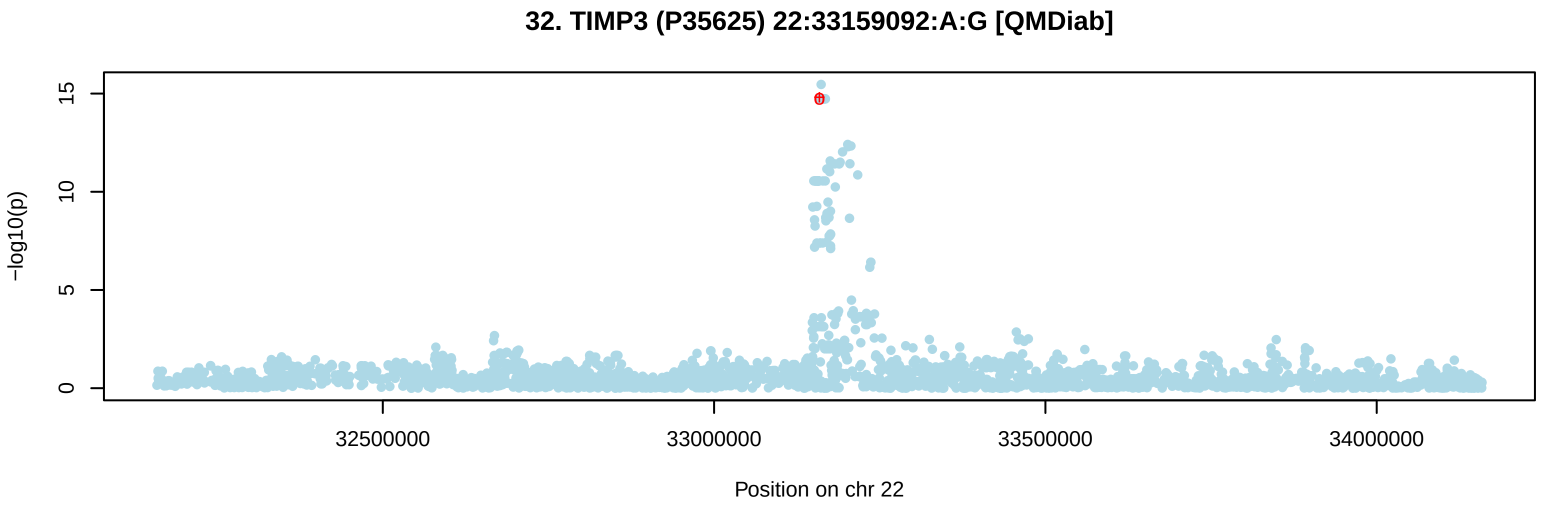
32. TIMP3 (P35625) 22:33159092:A:G [QMDiab]
−log10(p)
0
5
10
15
32500000
33000000
33500000
34000000
Position on chr 22

33. PRB1 (A0A4W8X8U3) 12:11522616:G:A [Tarkin]

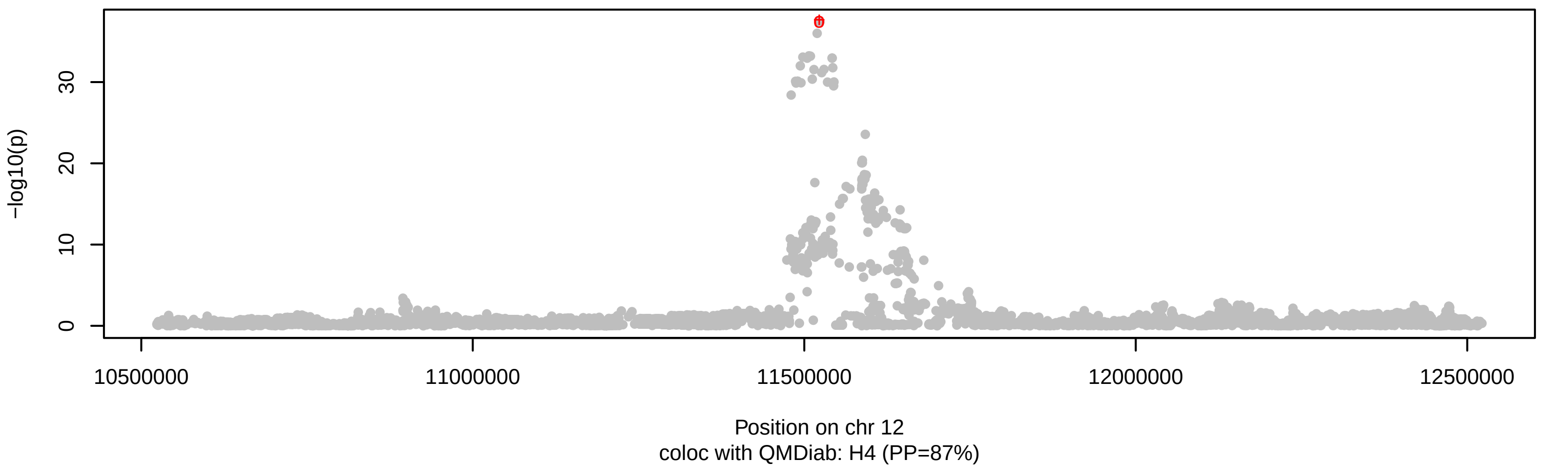

coloc with QMDiab: H4 (PP=87%)

33. PRB1 (A0A4W8X8U3;G3V1R1) 12:11522616:G:A [QMDiab]

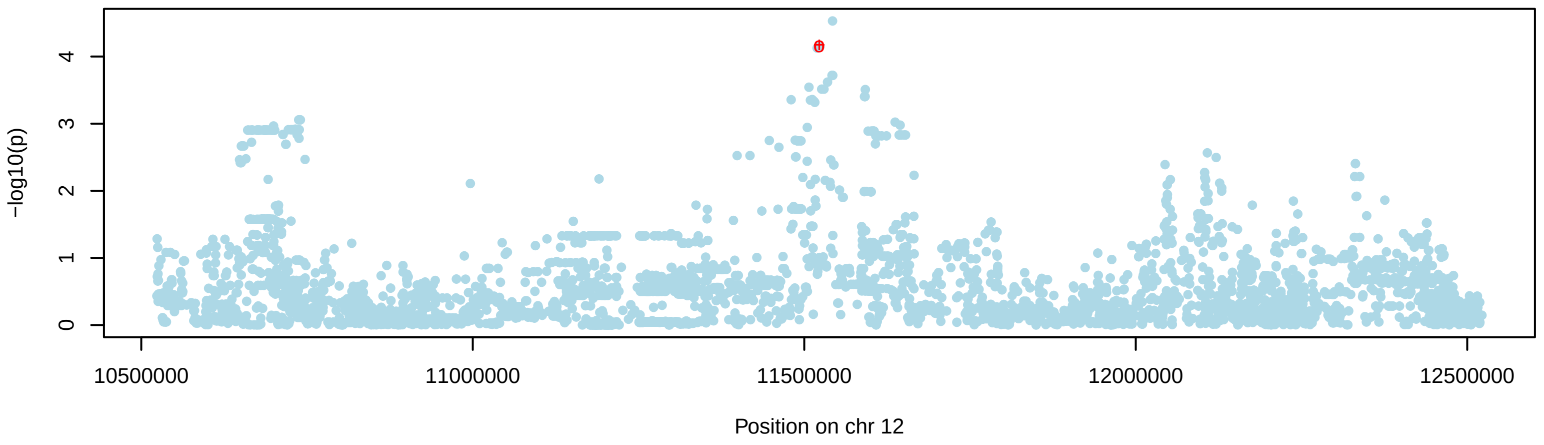

34. C2orf40 (B8ZZE5;Q9H1Z8) 2:106687456:G:C [Tarkin]

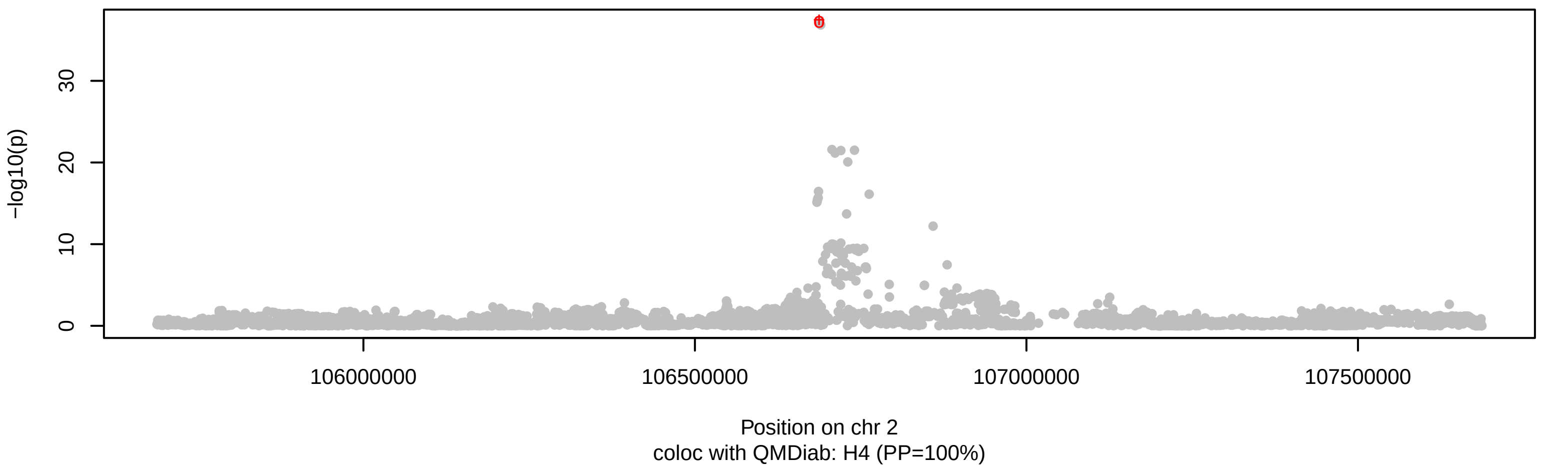

coloc with QMDiab: H4 (PP=100%)

34. C2orf40 (B8ZZE5;Q9H1Z8) 2:106687456:G:C [QMDiab]

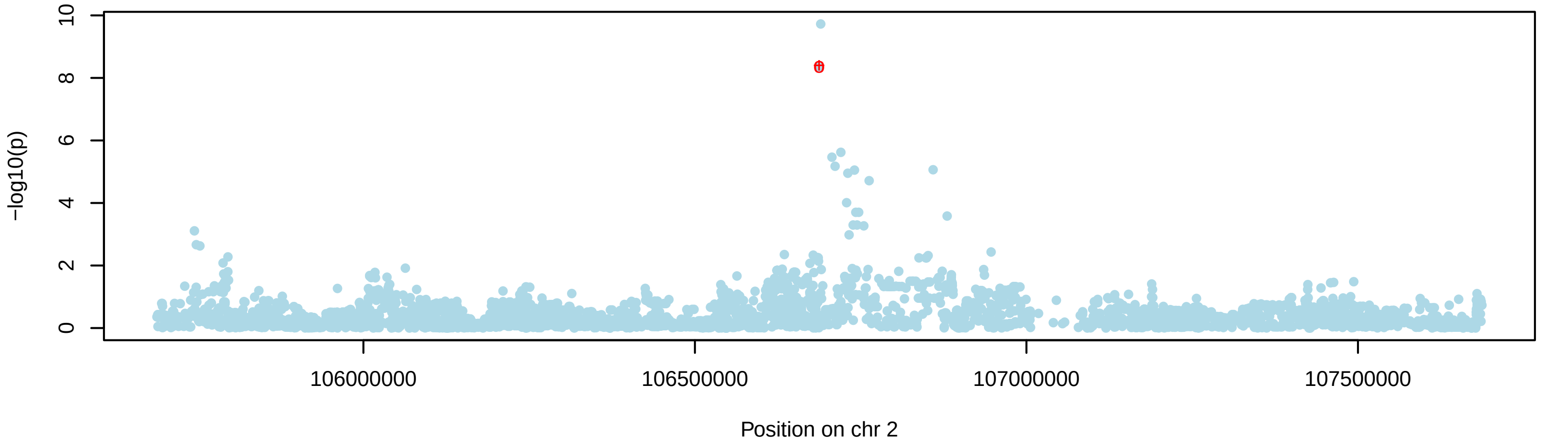

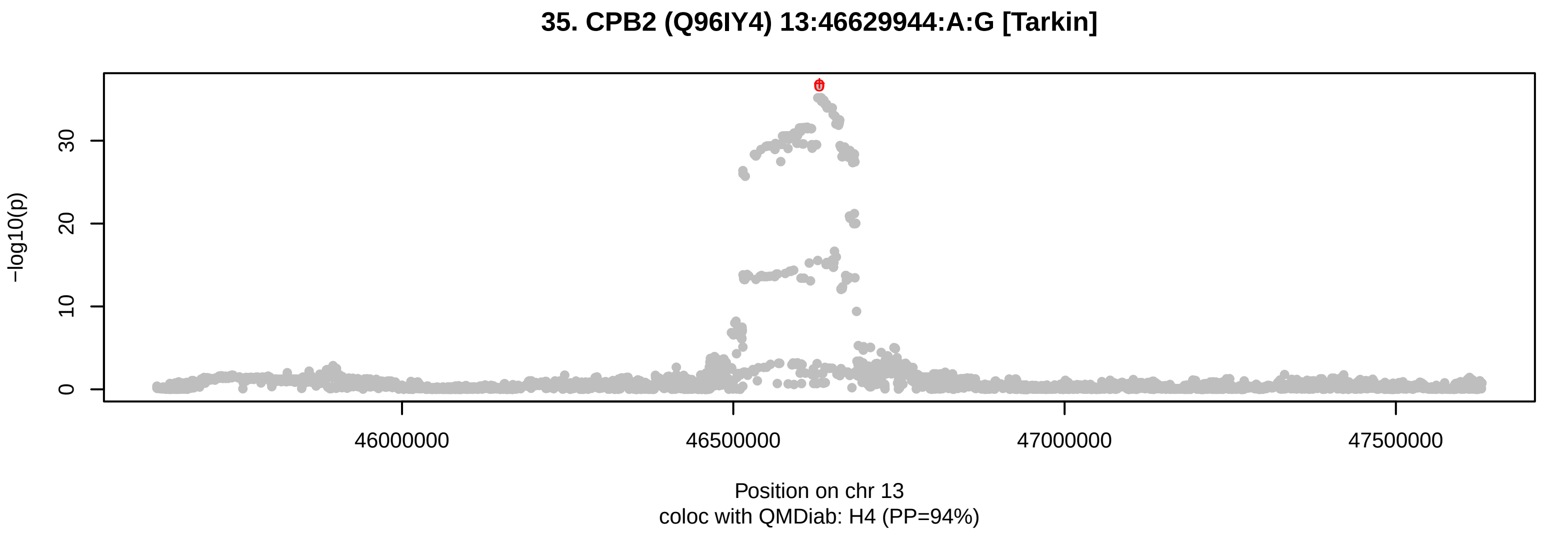
35. CPB2 (Q96IY4) 13:46629944:A:G [Tarkin]
−log10(p)
0
10
20
30
46000000
46500000
47000000
47500000
Position on chr 13
coloc with QMDiab: H4 (PP=94%)

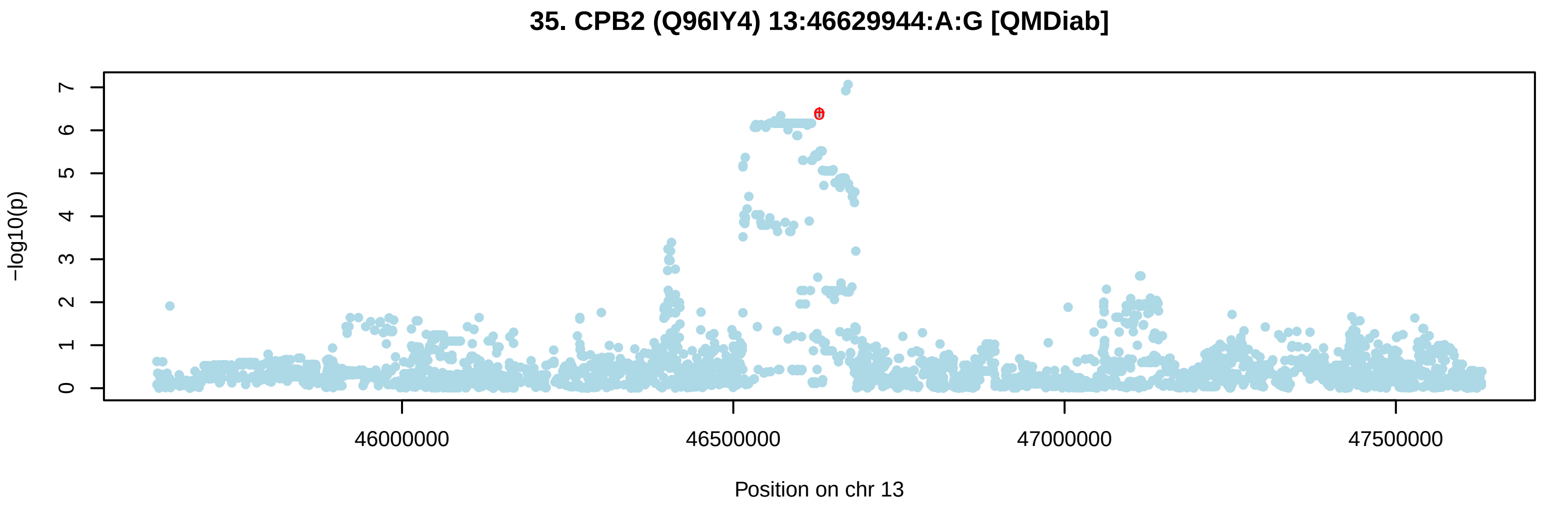
35. CPB2 (Q96IY4) 13:46629944:A:G [QMDiab]
−log10(p)
0
1
2
3
4
5
6
7
46000000
46500000
47000000
47500000
Position on chr 13

36. LEFTY1 (O75610) 1:226074563:T:G [Tarkin]

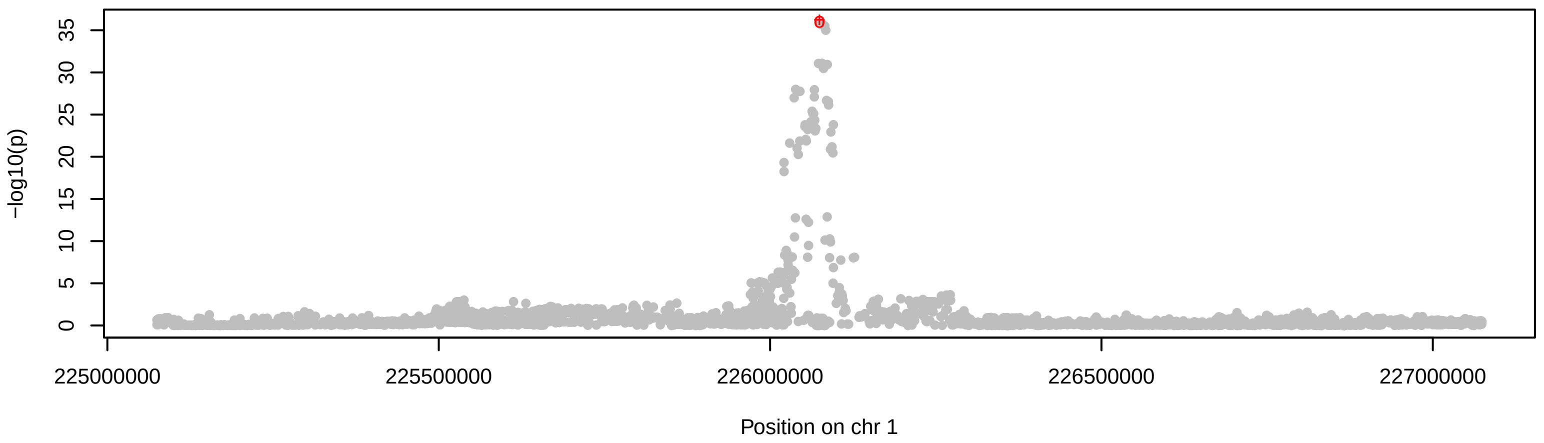

coloc with QMDiab: H4 (PP=98%)

36. LEFTY1 (O75610) 1:226074563:T:G [QMDiab]

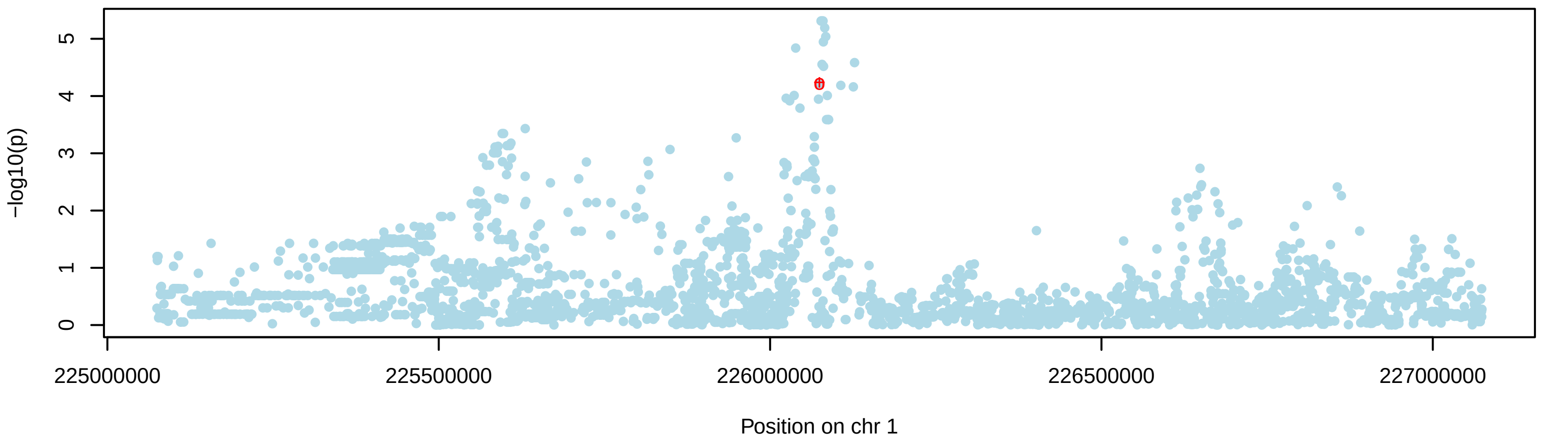

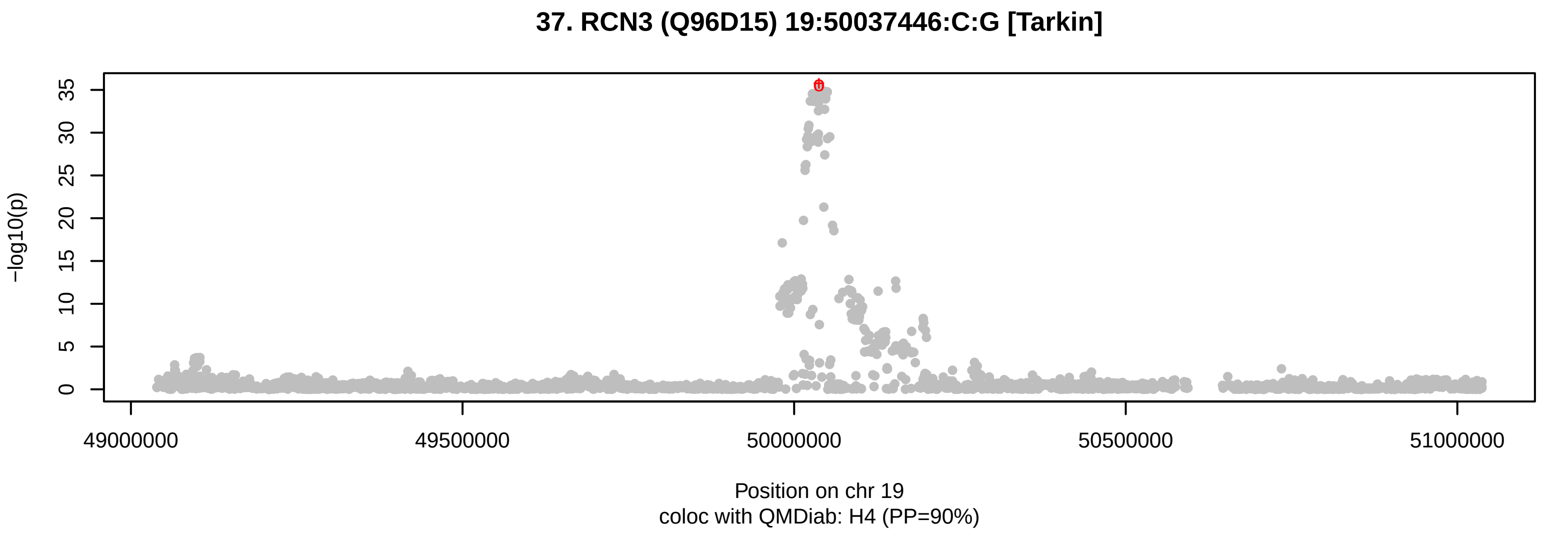
37. RCN3 (Q96D15) 19:50037446:C:G [Tarkin]
−log10(p)
0
5
10
15
20
25
30
35
49000000
49500000
50000000
50500000
51000000
Position on chr 19
coloc with QMDiab: H4 (PP=90%)

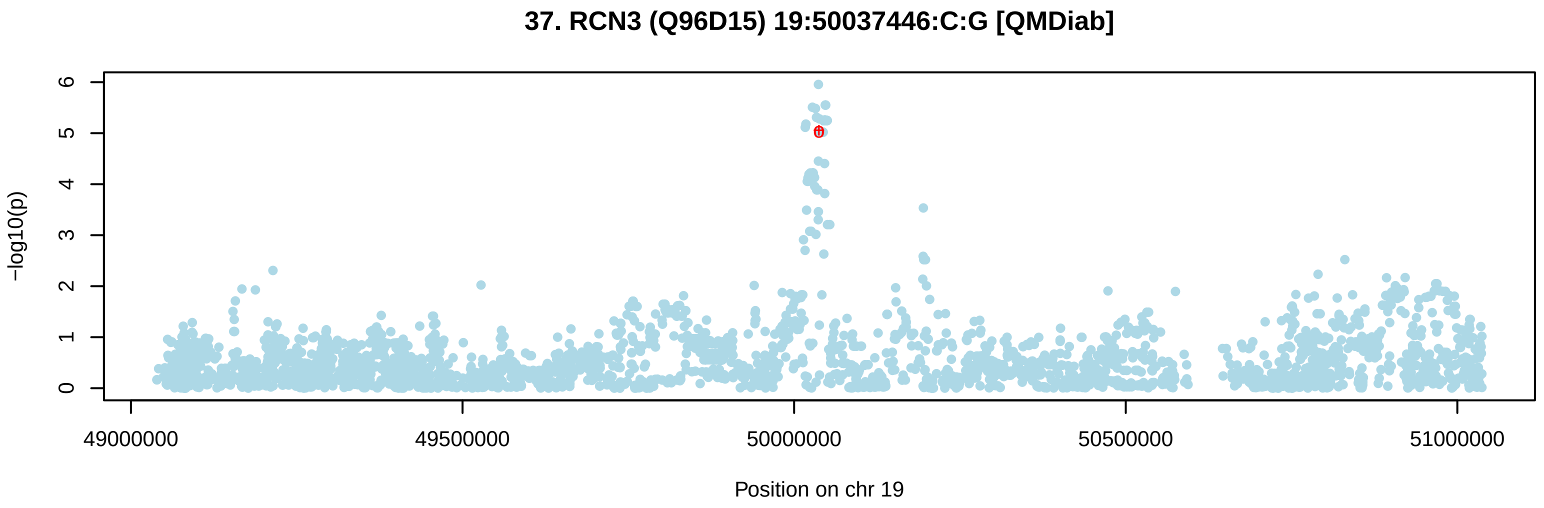
37. RCN3 (Q96D15) 19:50037446:C:G [QMDiab]
−log10(p)
0
1
2
3
4
5
6
49000000
49500000
50000000
50500000
51000000
Position on chr 19

38. C1QTNF9 (P0C862) 13:24906852:T:C [Tarkin]

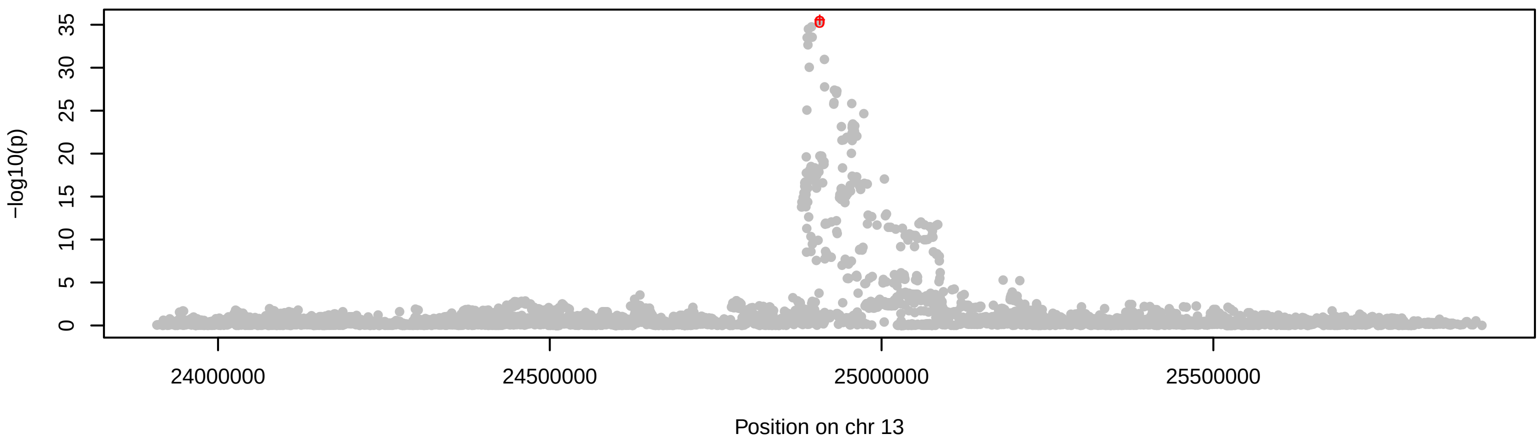

coloc with QMDiab: H1 (PP=55%)

38. C1QTNF9 (P0C862) 13:24906852:T:C [QMDiab]

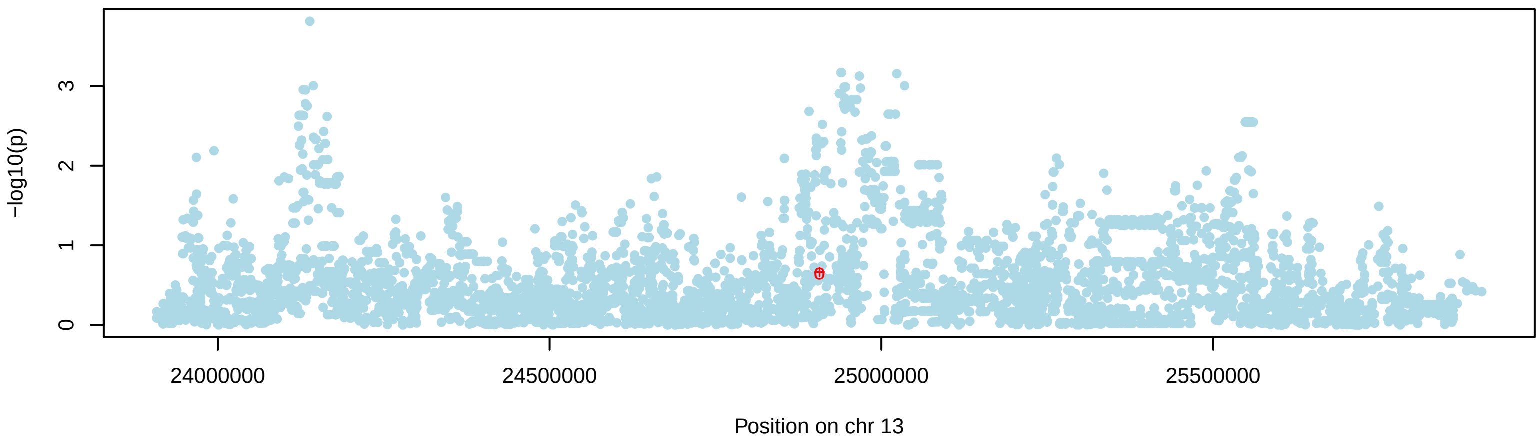

39. ITIH1 (P19827) 3:126261345:G:A [Tarkin]

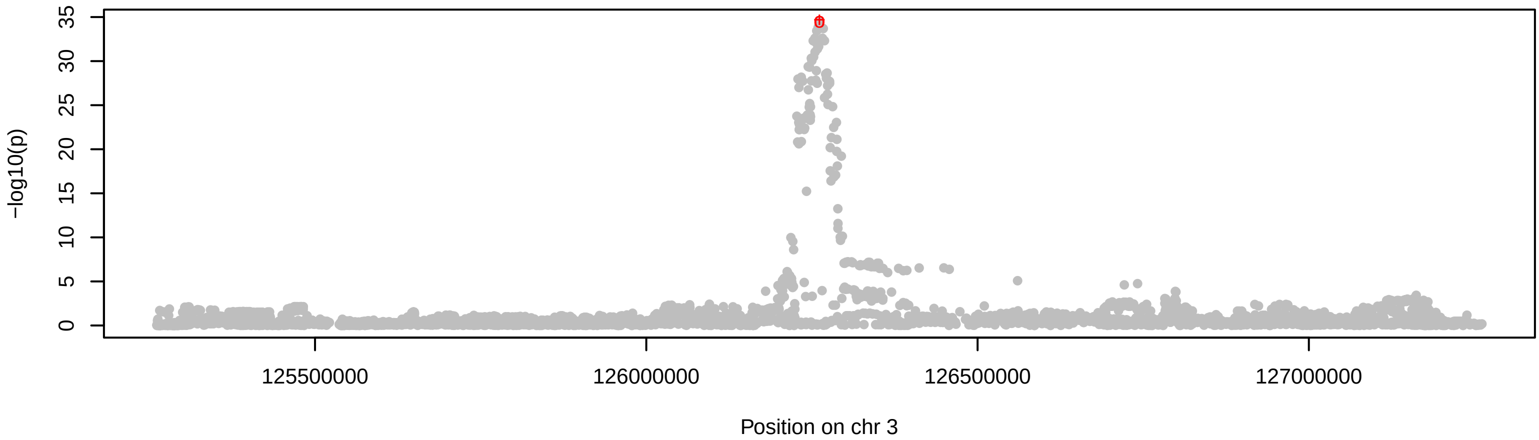

coloc with QMDiab: H4 (PP=94%)

39. ITIH1 (P19827) 3:126261345:G:A [QMDiab]

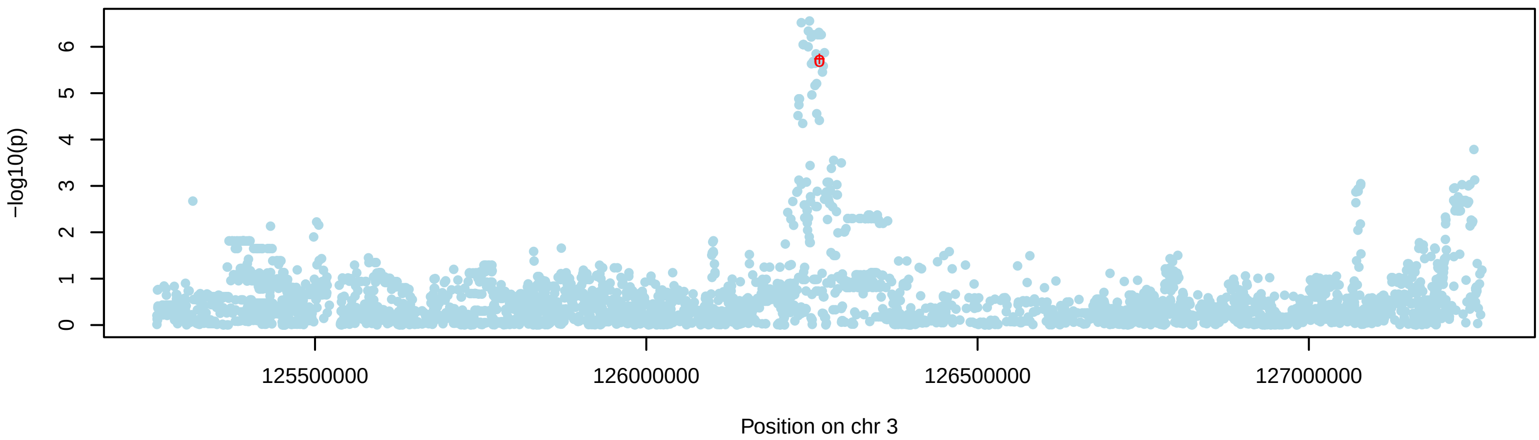

40. PGLYRP2 (Q96PD5) 19:15580290:G:A [Tarkin]

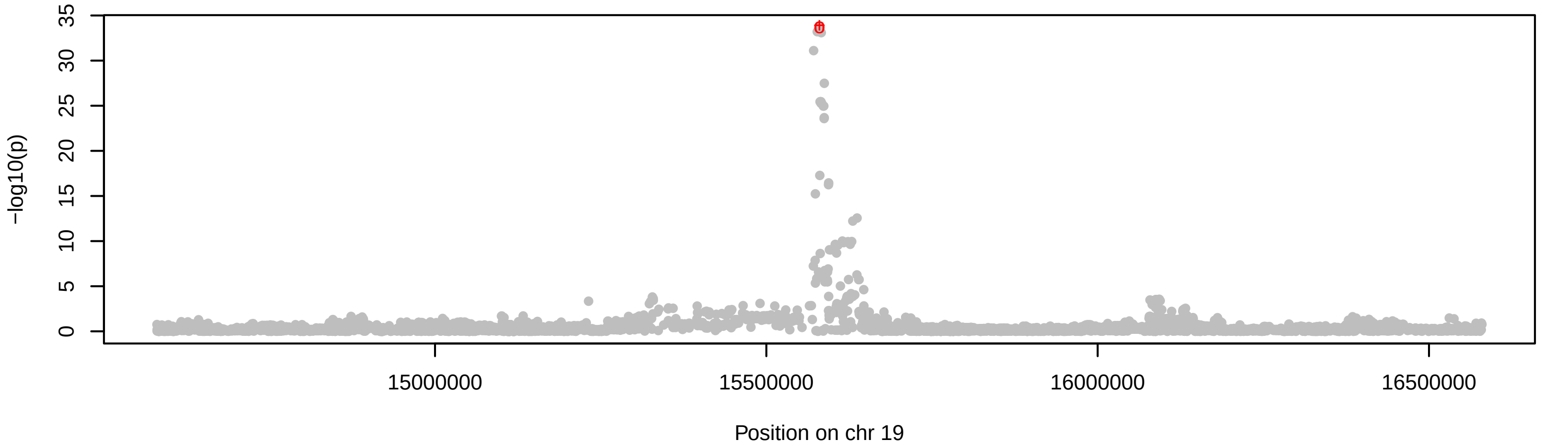

coloc with QMDiab: H4 (PP=56%)

40. PGLYRP2 (Q96PD5) 19:15580290:G:A [QMDiab]

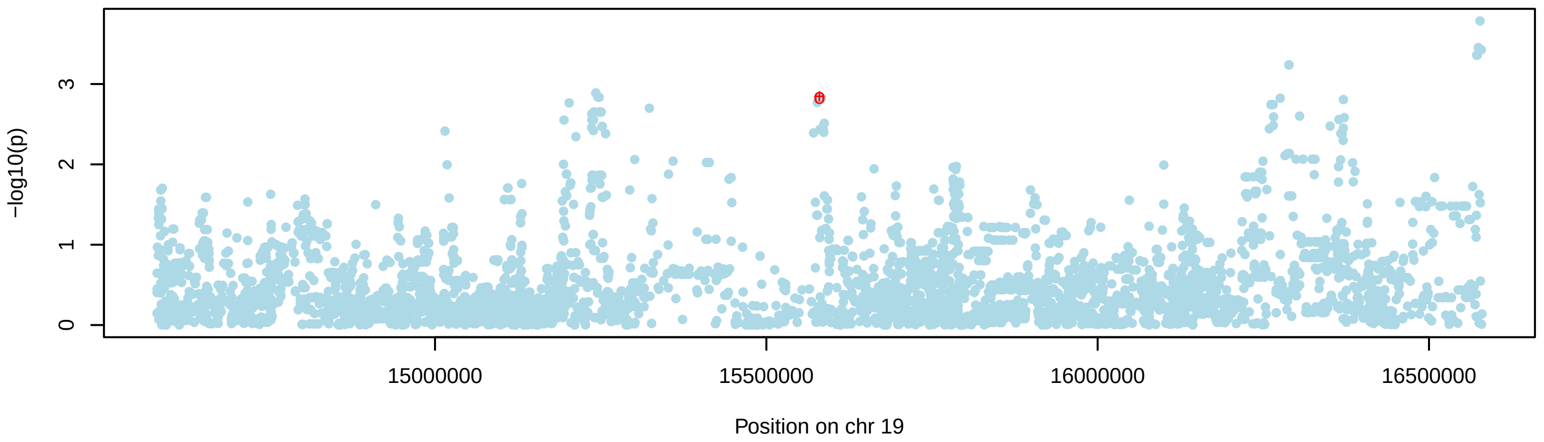

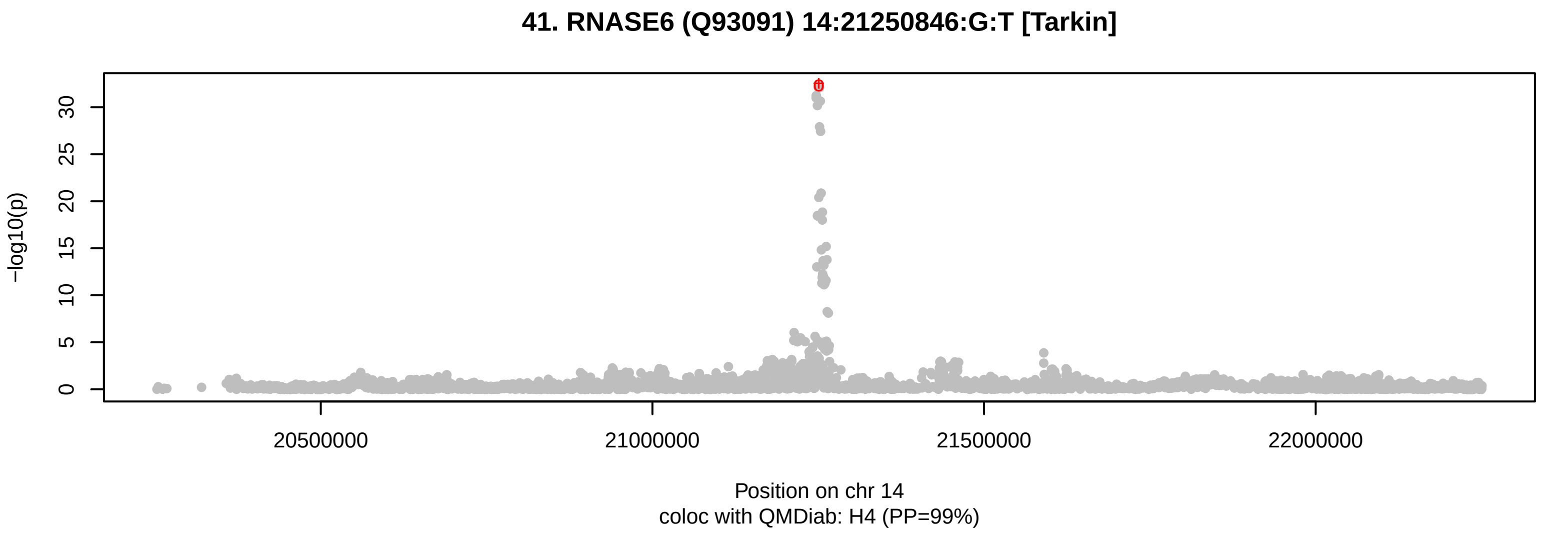
41. RNASE6 (Q93091) 14:21250846:G:T [Tarkin]
−log10(p)
0
5
10
15
20
25
30
20500000
21000000
21500000
22000000
Position on chr 14
coloc with QMDiab: H4 (PP=99%)

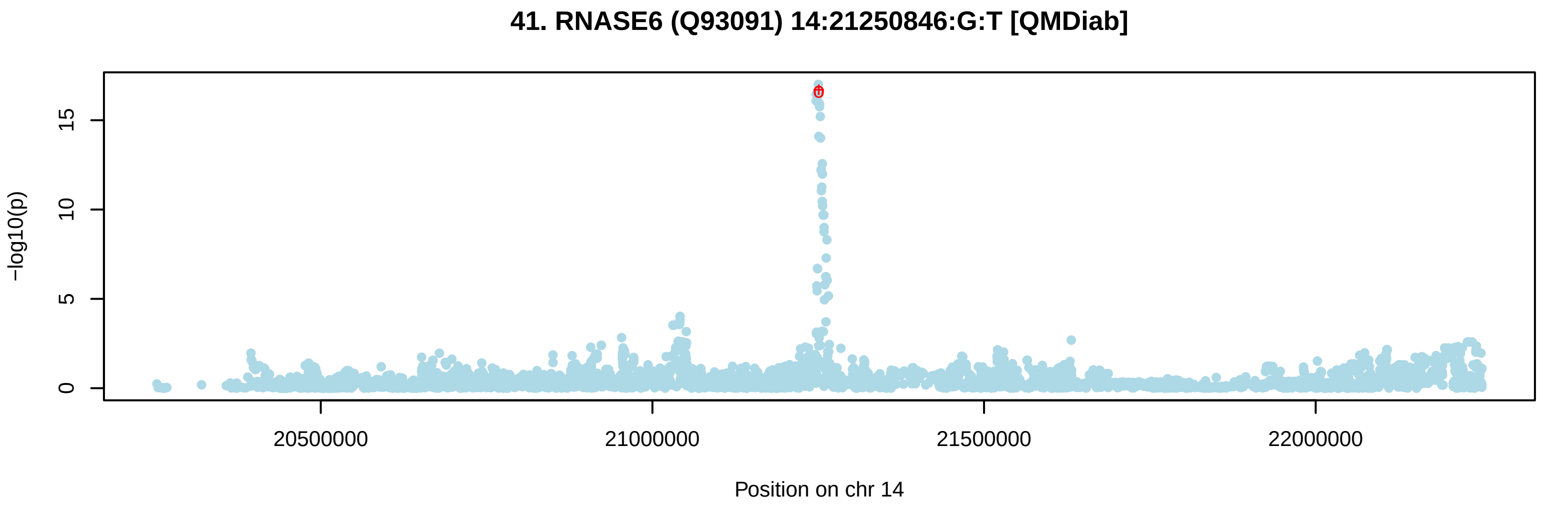
41. RNASE6 (Q93091) 14:21250846:G:T [QMDiab]
−log10(p)
0
5
10
15
20500000
21000000
21500000
22000000
Position on chr 14

42. SPON2 (Q9BUD6) 4:1165130:G:T [Tarkin]

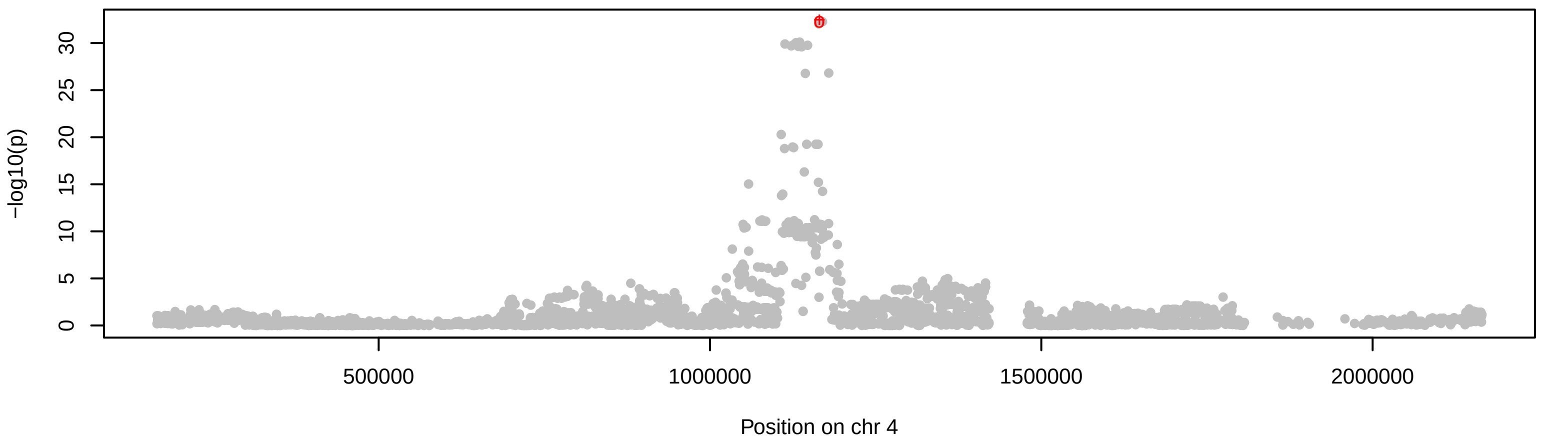

coloc with QMDiab: H4 (PP=42%)

42. SPON2 (Q9BUD6) 4:1165130:G:T [QMDiab]

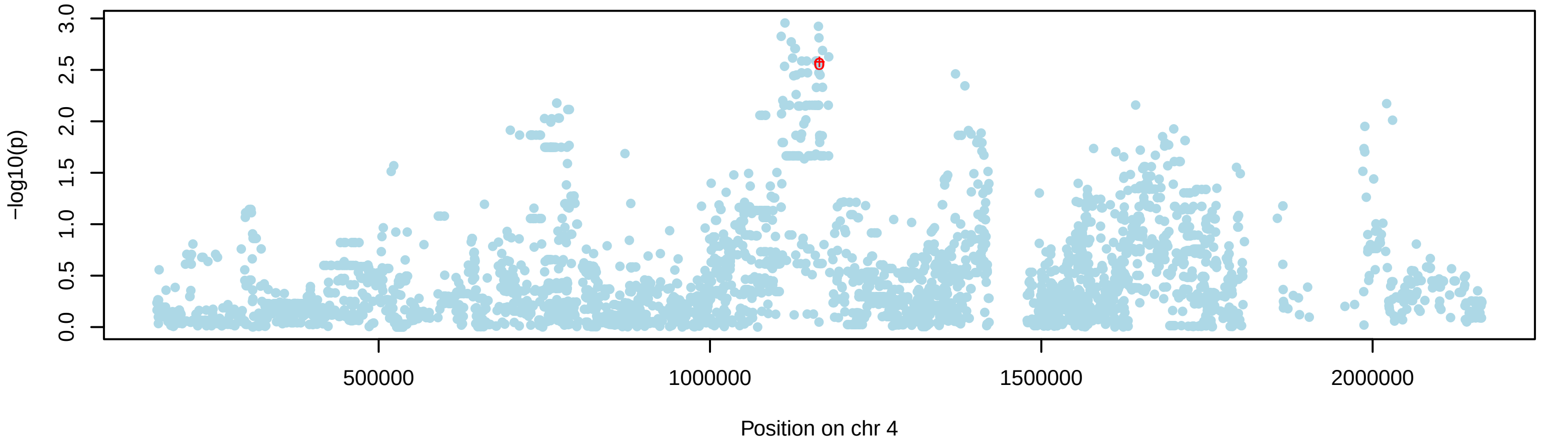

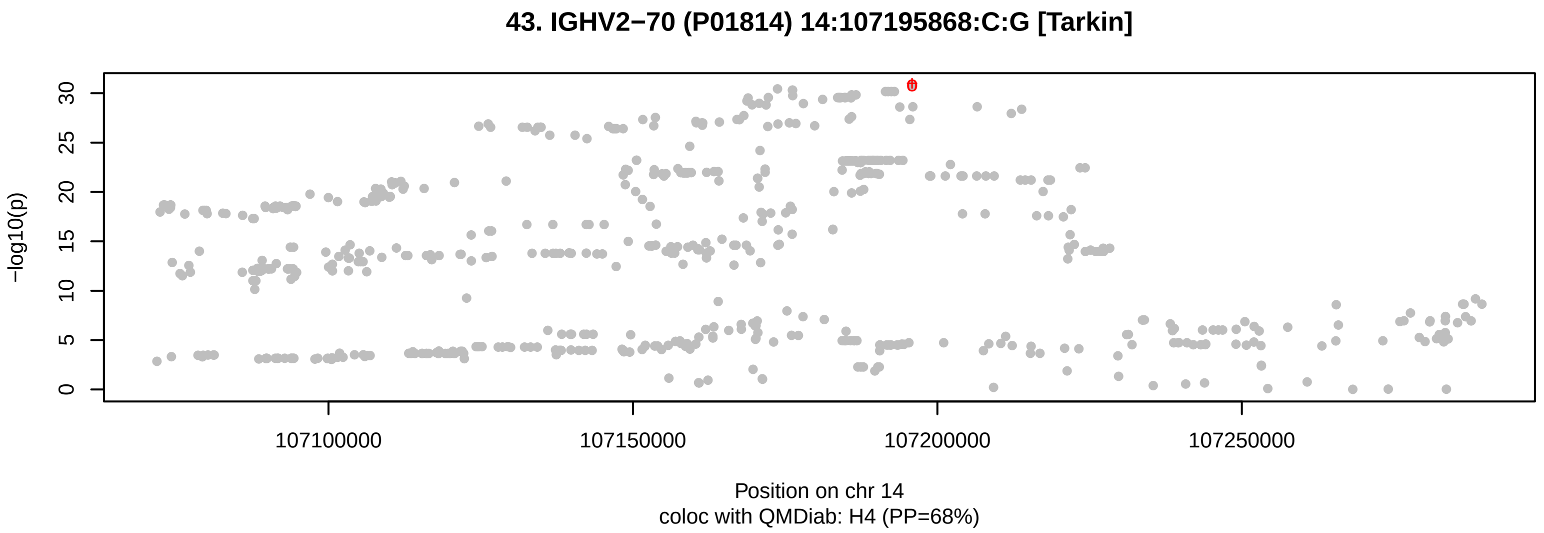
43. IGHV2−70 (P01814) 14:107195868:C:G [Tarkin]
−log10(p)
0
5
10
15
20
25
30
107100000
107150000
107200000
107250000
Position on chr 14
coloc with QMDiab: H4 (PP=68%)

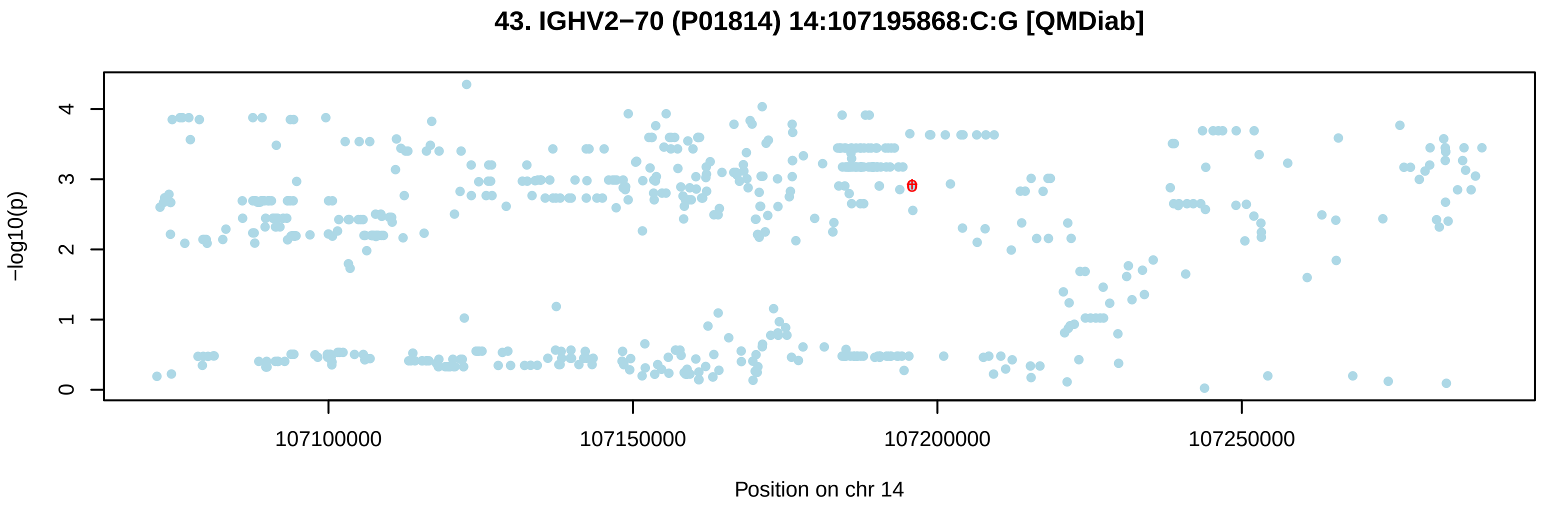
43. IGHV2−70 (P01814) 14:107195868:C:G [QMDiab]
−log10(p)
0
1
2
3
4
107100000
107150000
107200000
107250000
Position on chr 14

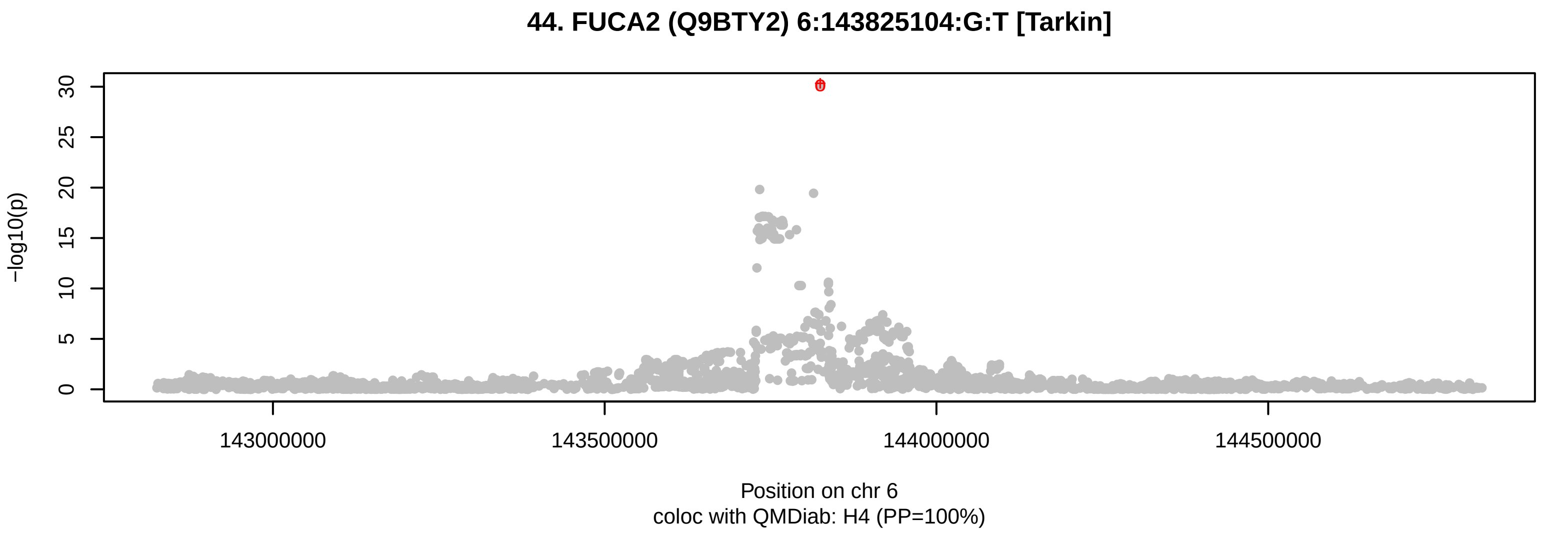
44. FUCA2 (Q9BTY2) 6:143825104:G:T [Tarkin]
−log10(p)
0
5
10
15
20
25
30
143000000
143500000
144000000
144500000
Position on chr 6
coloc with QMDiab: H4 (PP=100%)

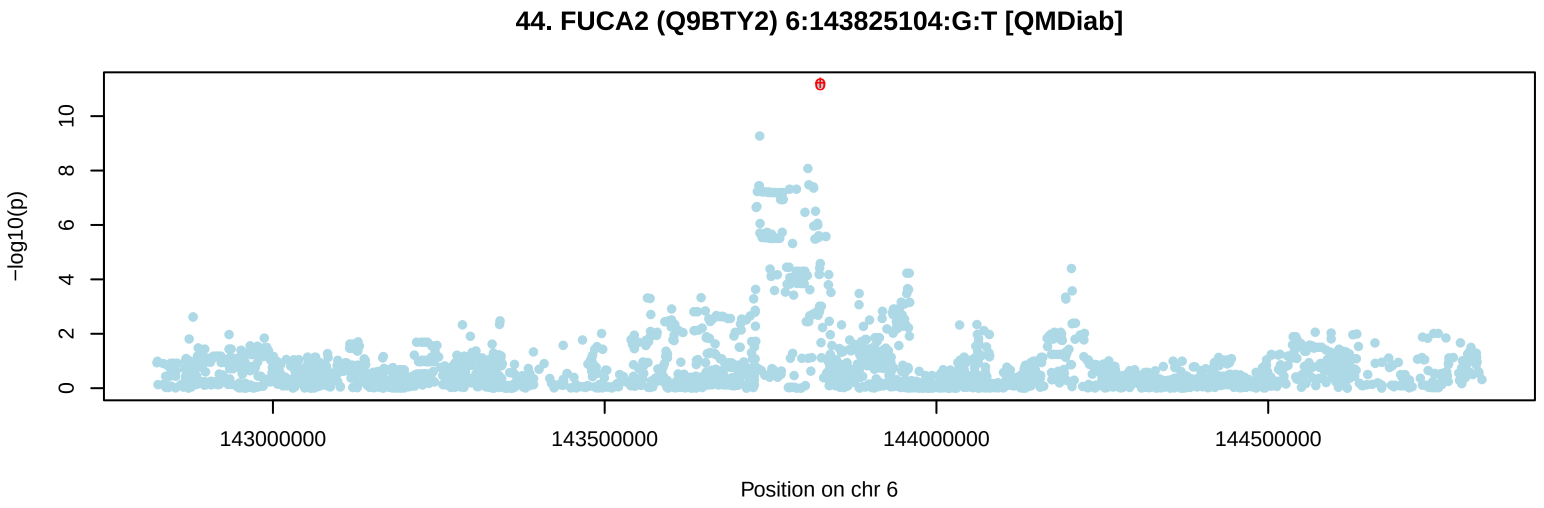
44. FUCA2 (Q9BTY2) 6:143825104:G:T [QMDiab]
−log10(p)
0
2
4
6
8
10
143000000
143500000
144000000
144500000
Position on chr 6

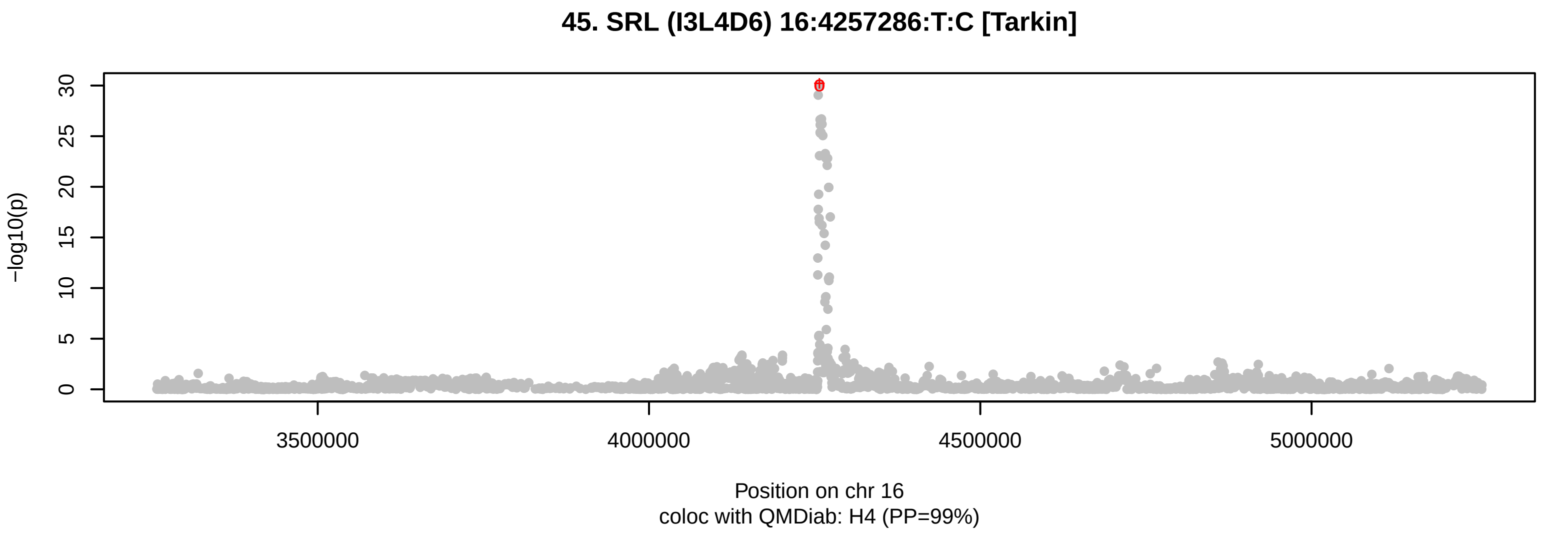

45. SRL (I3L4D6) 16:4257286:T:C [Tarkin]
−log10(p)
0
5
10
15
20
25
30
3500000
4000000
4500000
5000000
Position on chr 16
coloc with QMDiab: H4 (PP=99%)

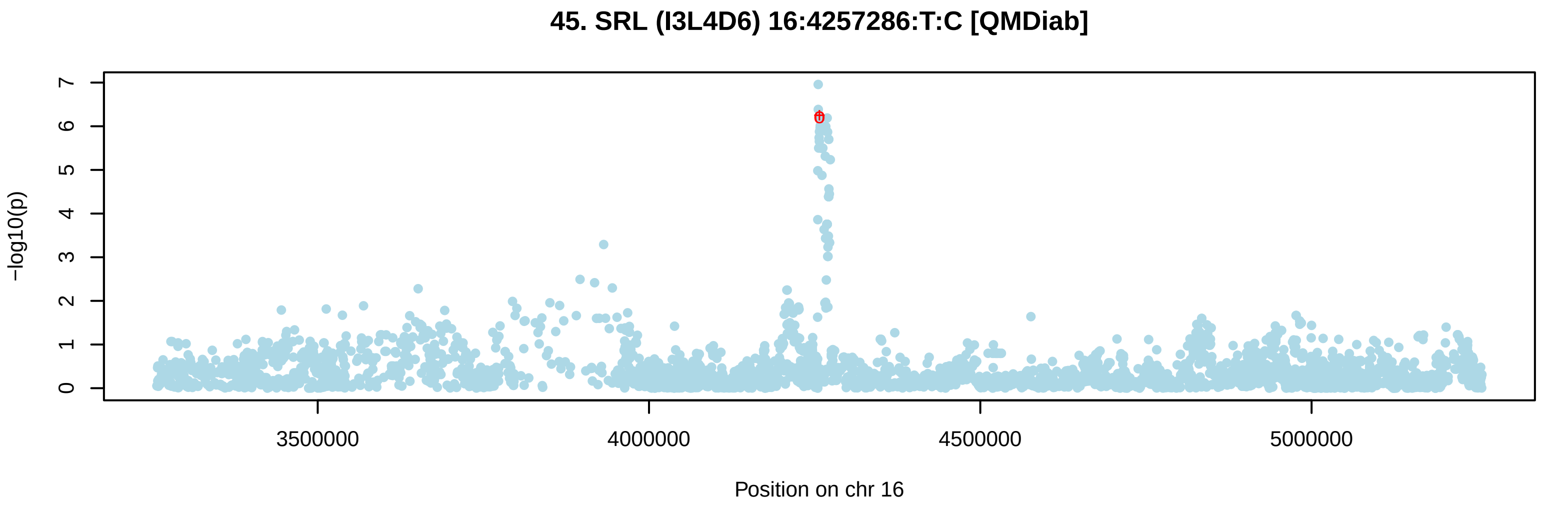

45. SRL (I3L4D6) 16:4257286:T:C [QMDiab]
−log10(p)
0
1
2
3
4
5
6
7
3500000
4000000
4500000
5000000
Position on chr 16

46. HLA−C (A2AEA2;P10321) 6:31323468:G:T [Tarkin]

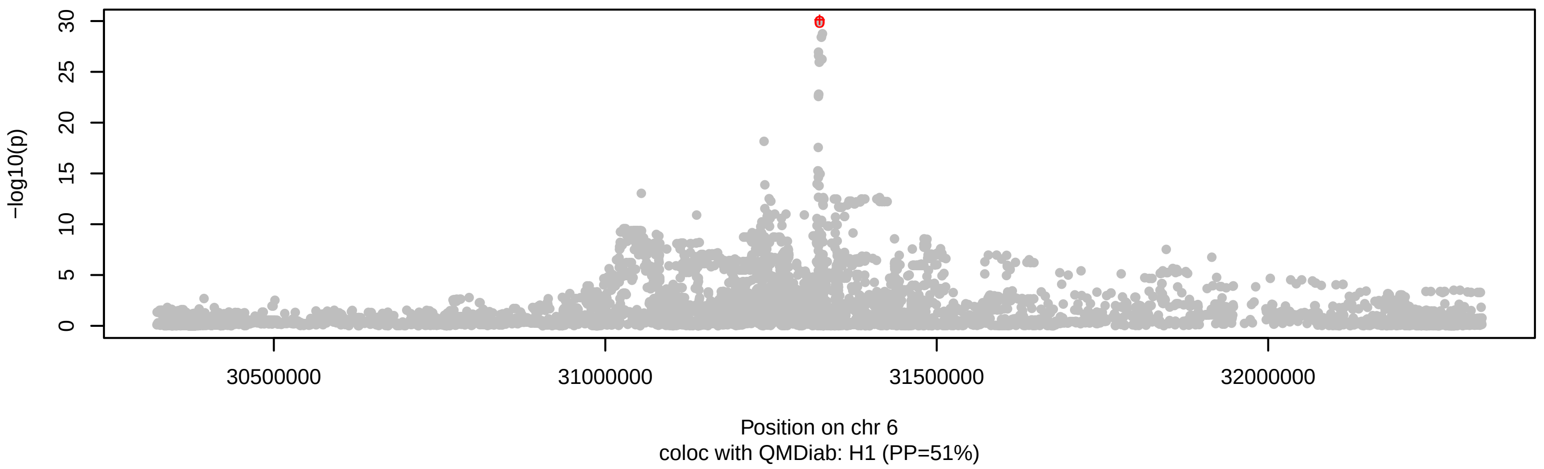

coloc with QMDiab: H1 (PP=51%)

46. HLA−C (A2AEA2;P10321) 6:31323468:G:T [QMDiab]

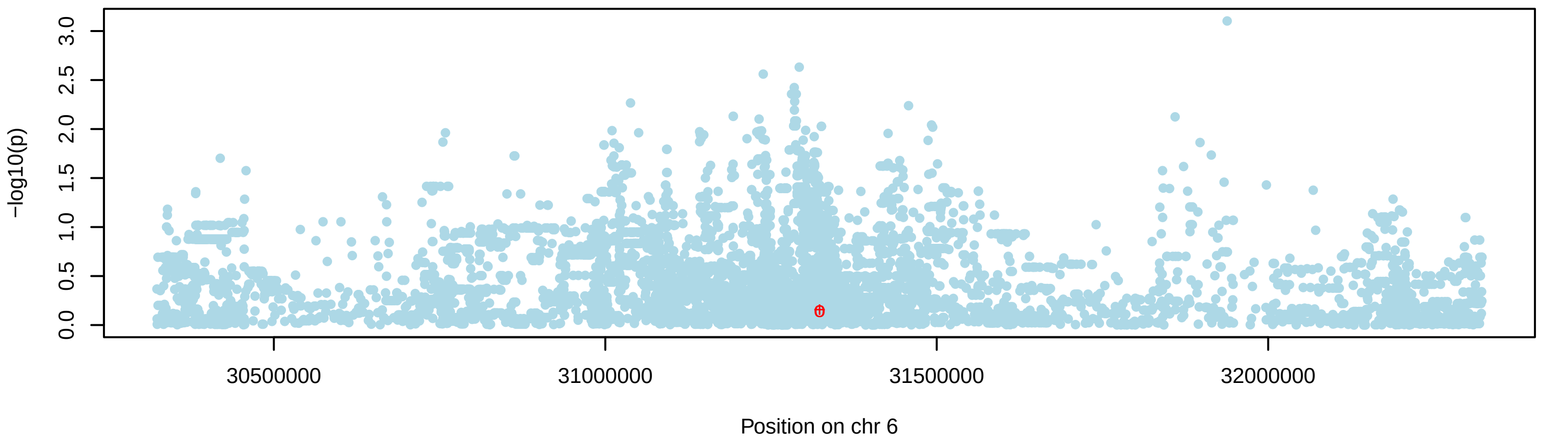

47. FGL1 (Q08830) 8:17722867:G:C [Tarkin]

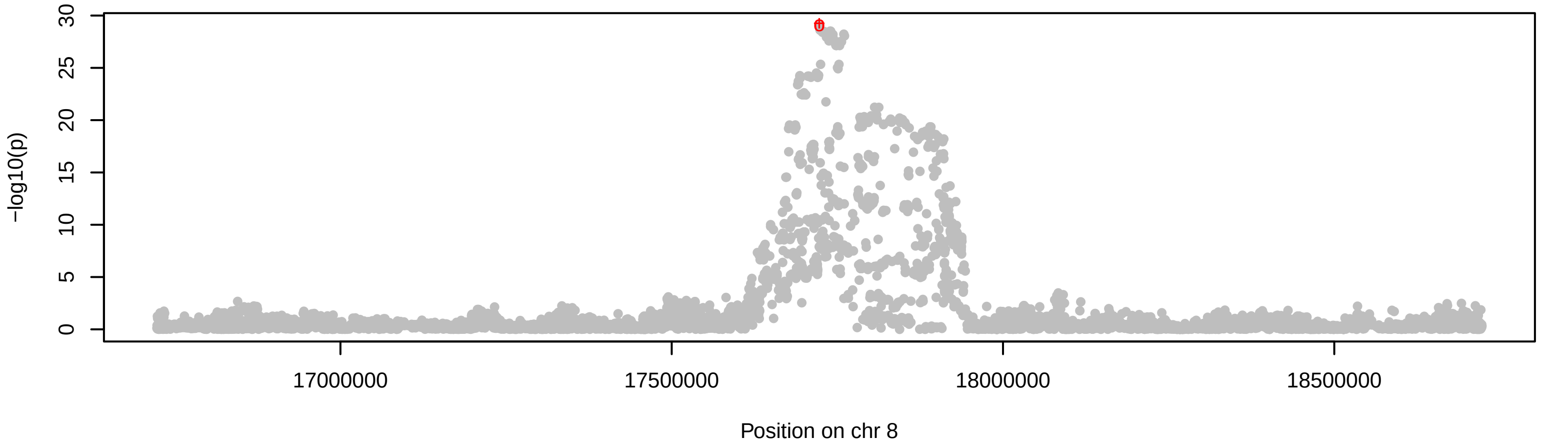

coloc with QMDiab: H4 (PP=91%)

47. FGL1 (Q08830) 8:17722867:G:C [QMDiab]

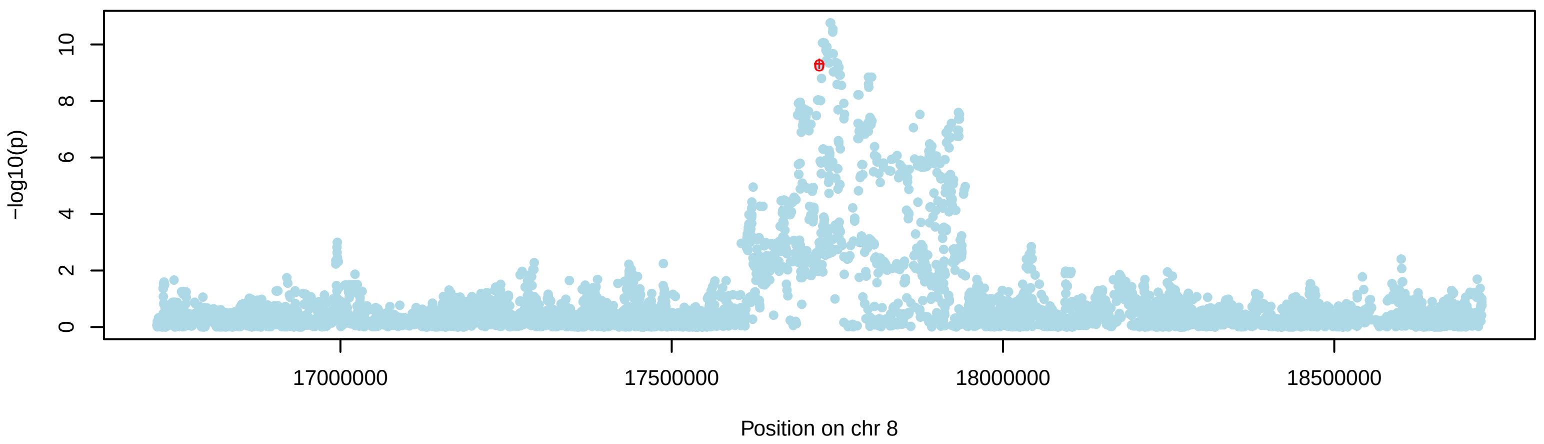

48. PLA2G2A (A0A3B3IRX2;P14555) 1:20304857:G:A [Tarkin]

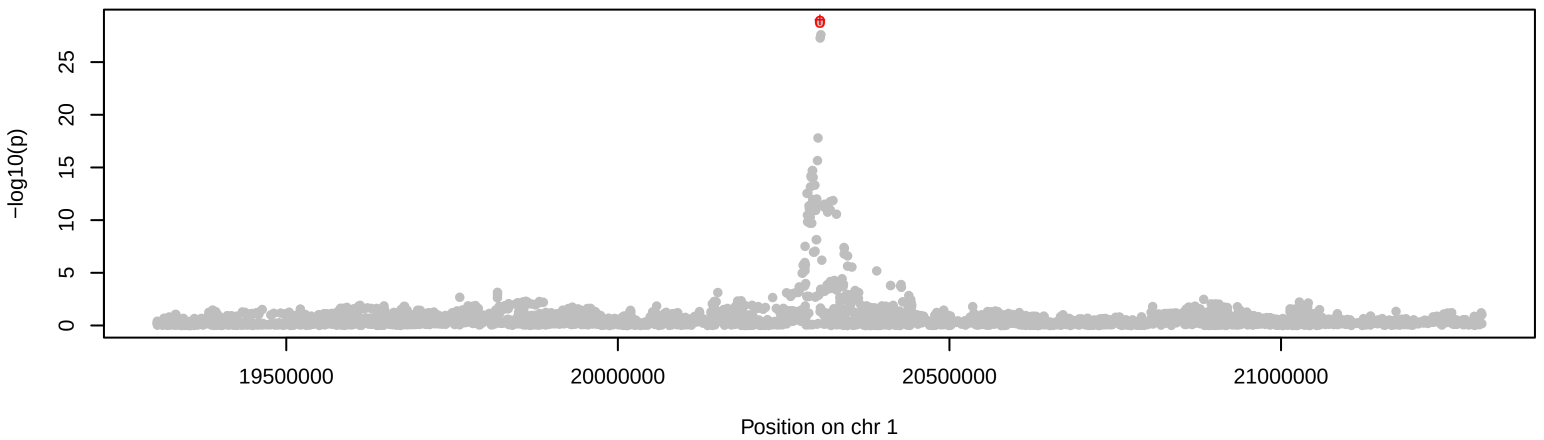

coloc with QMDiab: H4 (PP=100%)

48. PLA2G2A (A0A3B3IRX2;P14555) 1:20304857:G:A [QMDiab]

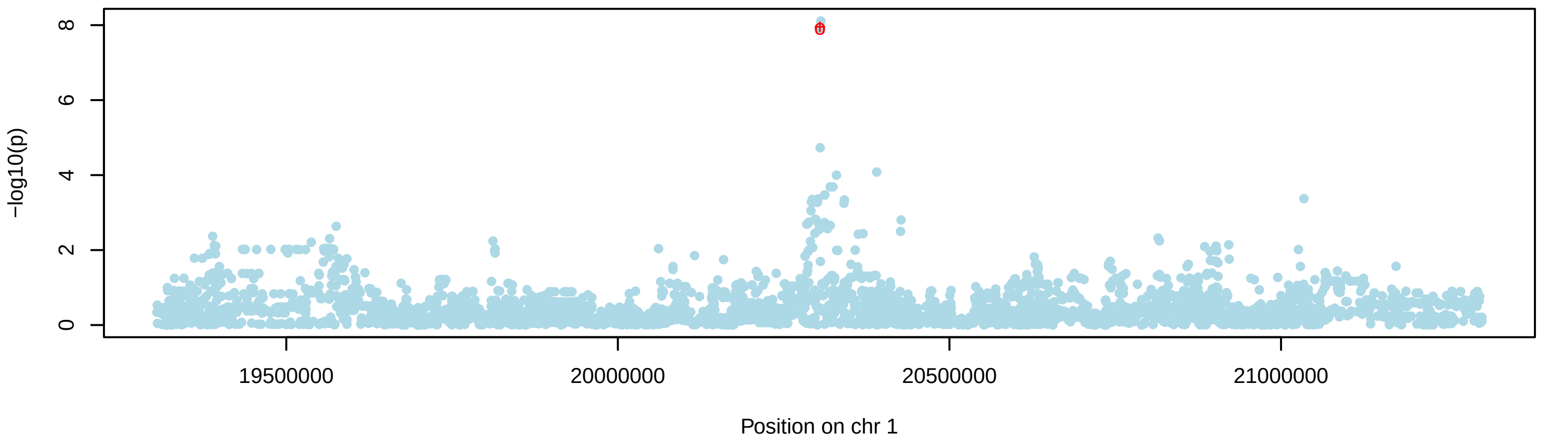

49. COL15A1 (A0A087X0K0;P39059) 9:101762528:C:T [Tarkin]

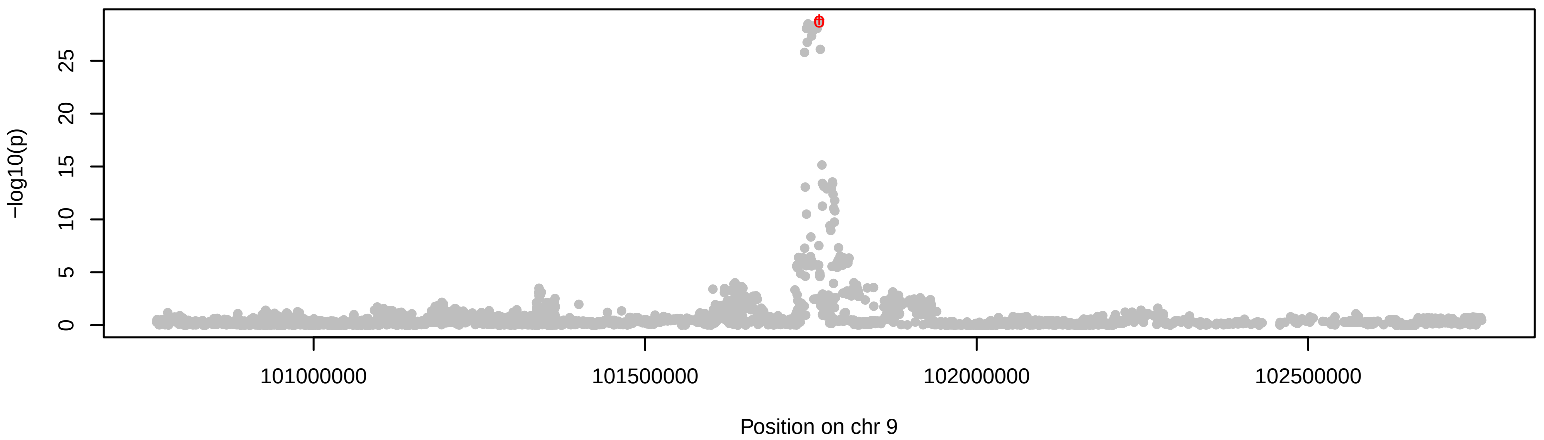

coloc with QMDiab: H4 (PP=97%)

49. COL15A1 (A0A087X0K0;P39059) 9:101762528:C:T [QMDiab]

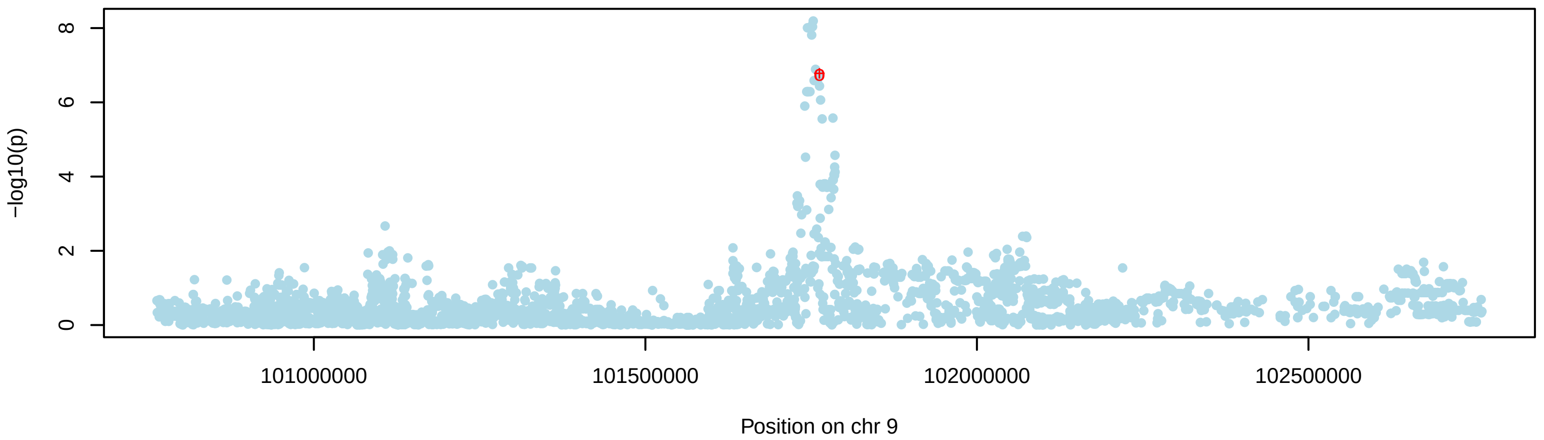

50. COLEC12 (Q5KU26) 18:334742:C:T [Tarkin]

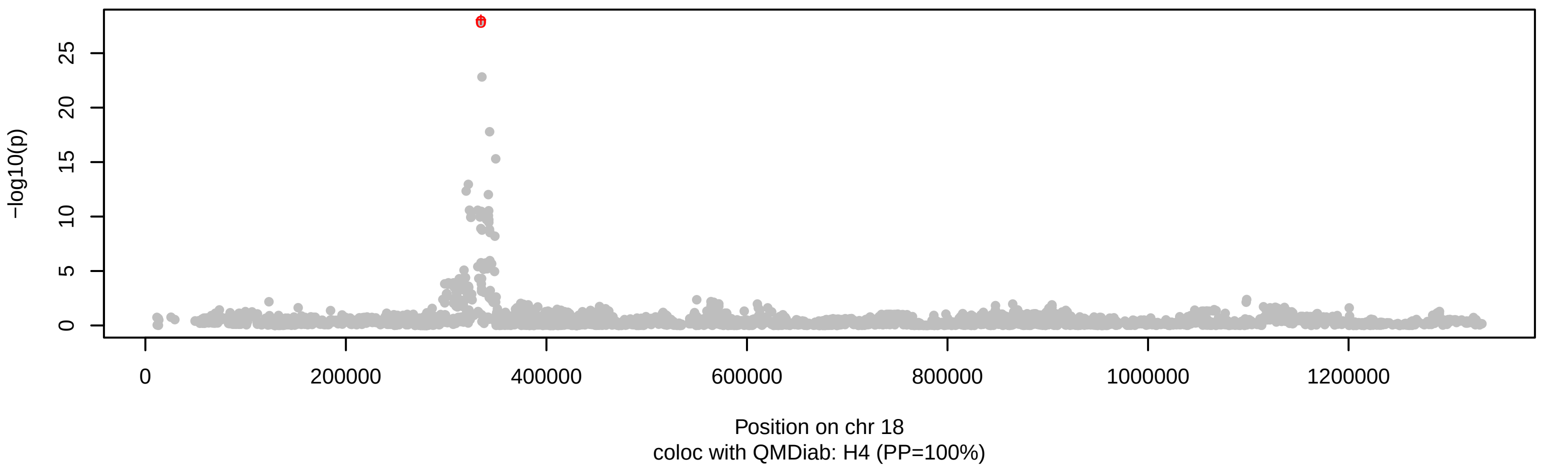

50. COLEC12 (Q5KU26) 18:334742:C:T [QMDiab]

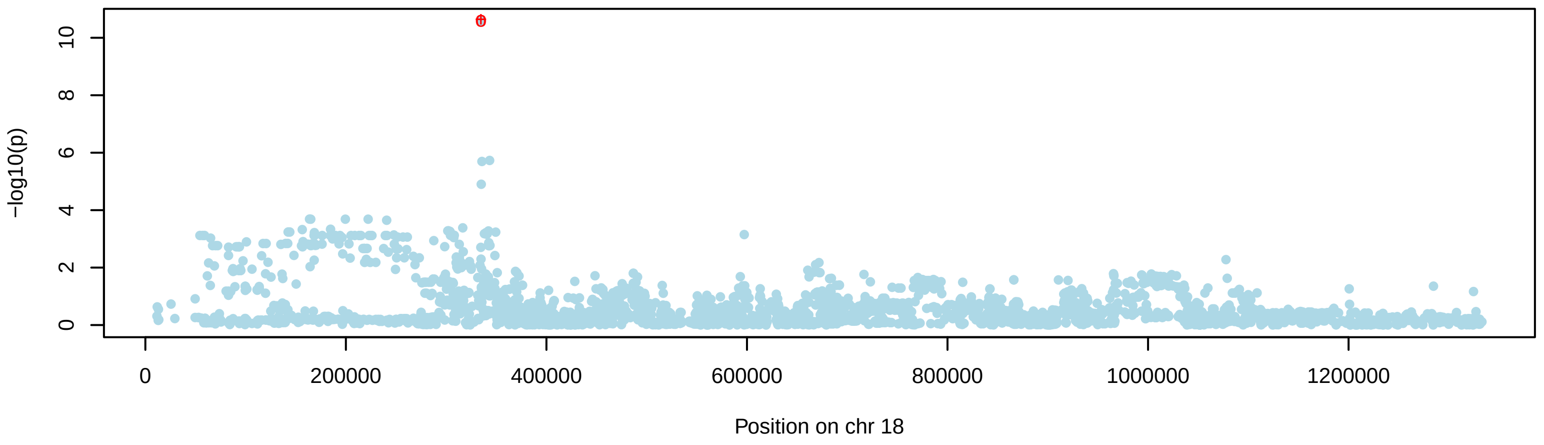

51. IGHV2−70 (A0A0C4DH43) 14:107173745:T:C [Tarkin]

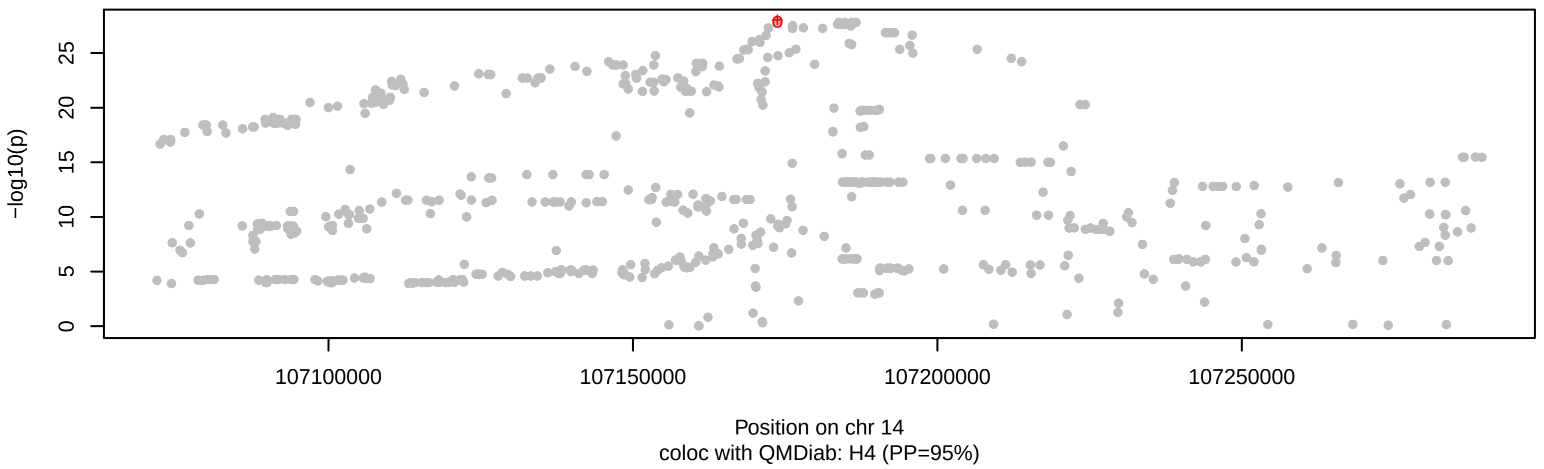

coloc with QMDiab: H4 (PP=95%)

51. IGHV2−70 (A0A0C4DH43) 14:107173745:T:C [QMDiab]

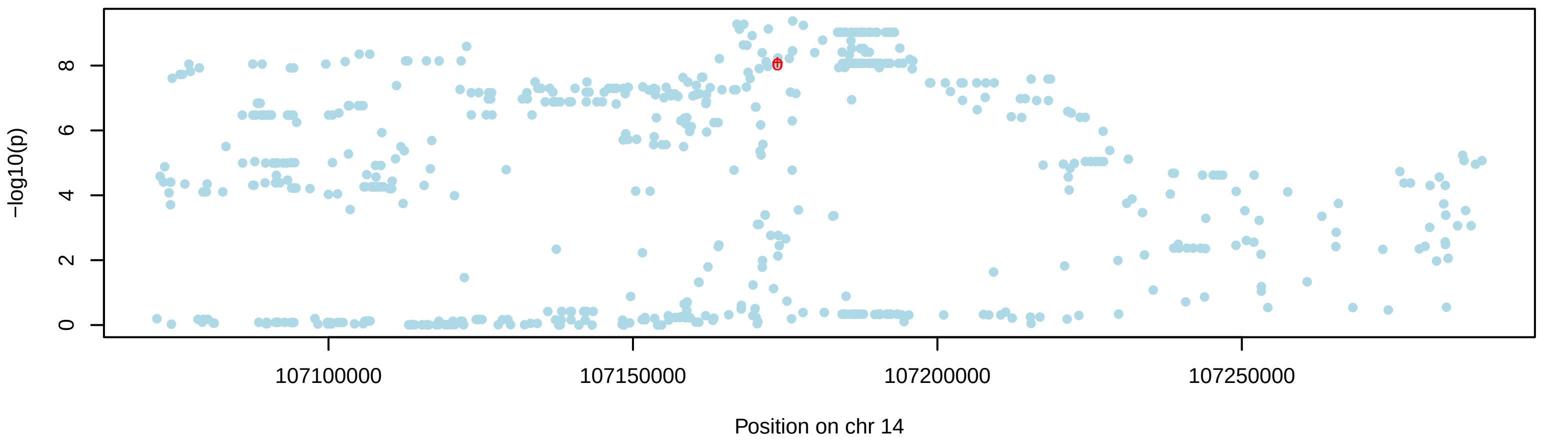

52. ITIH3 (Q06033) 3:126261202:G:A [Tarkin]

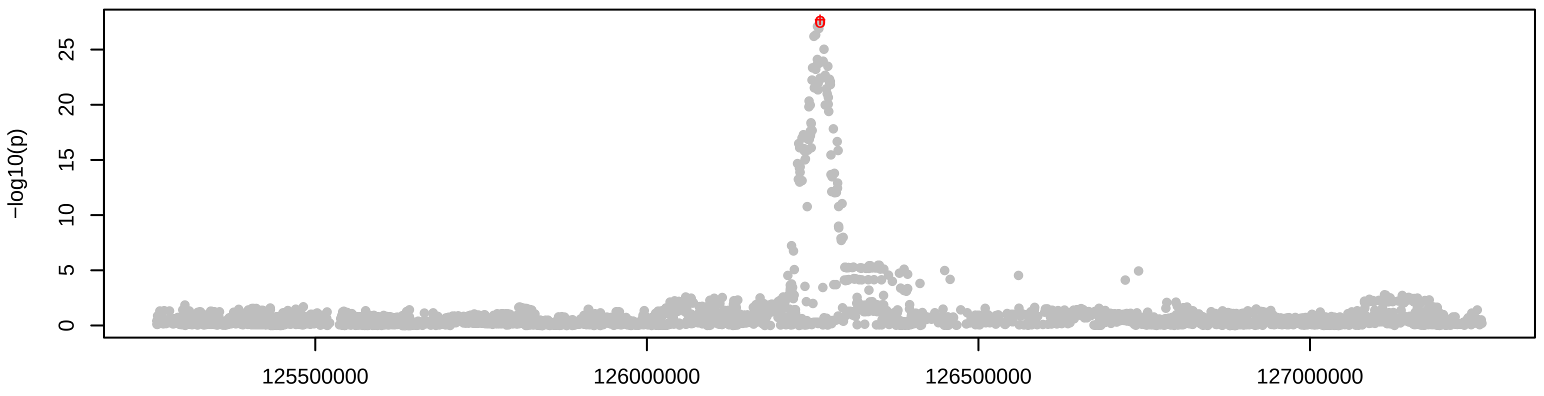

Position on chr 3
coloc with QMDiab: H4 (PP=95%)

52. ITIH3 (Q06033) 3:126261202:G:A [QMDiab]

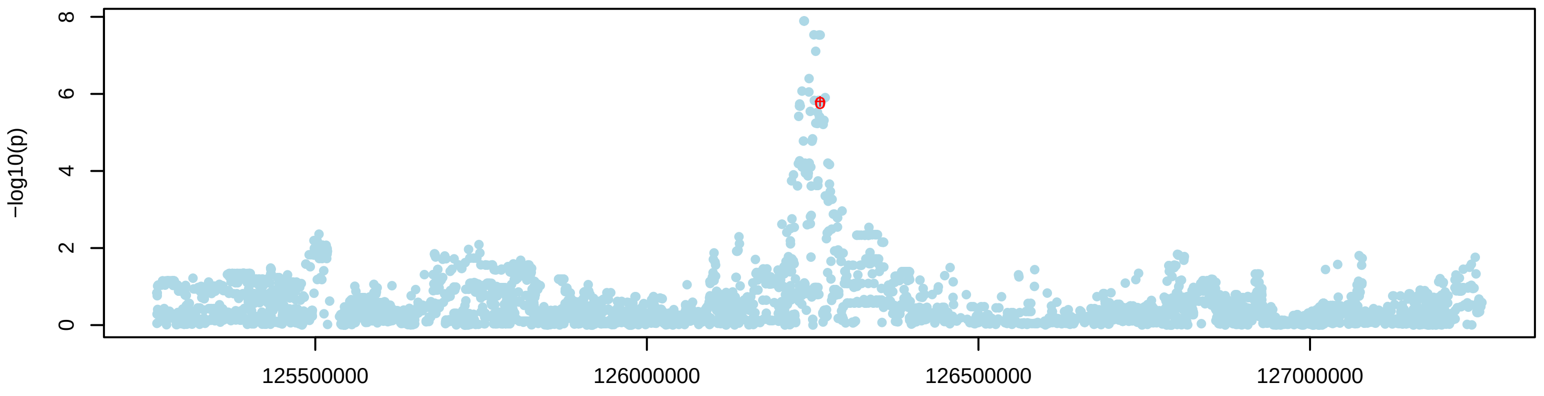

Position on chr 3

53. PDIA5 (Q14554) 3:122846881:C:T [Tarkin]

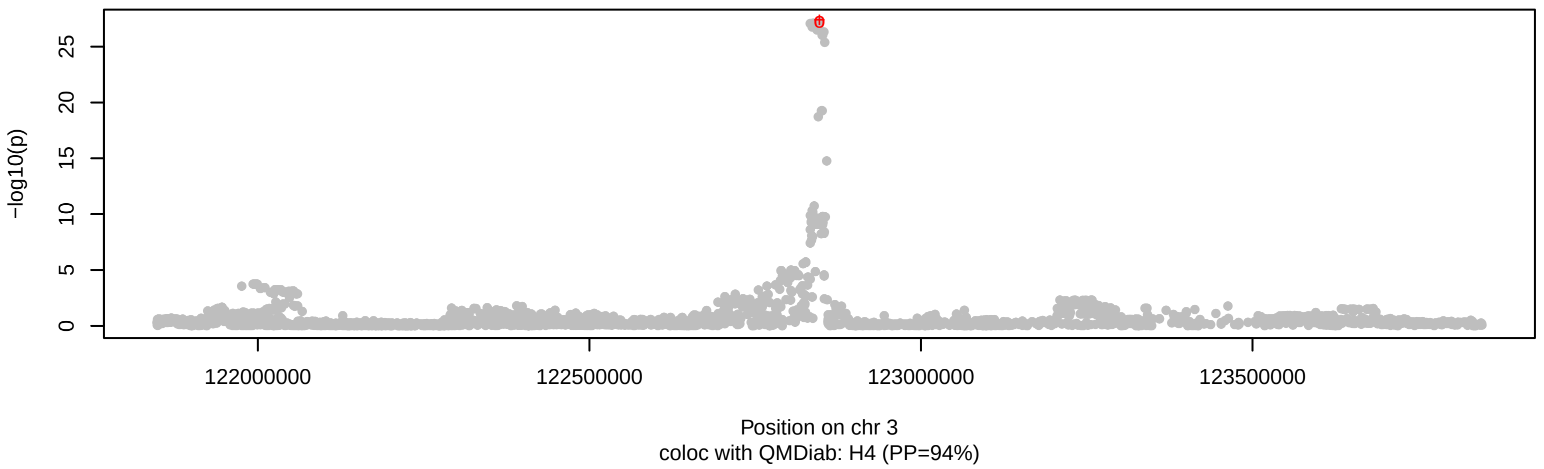

coloc with QMDiab: H4 (PP=94%)

53. PDIA5 (Q14554) 3:122846881:C:T [QMDiab]

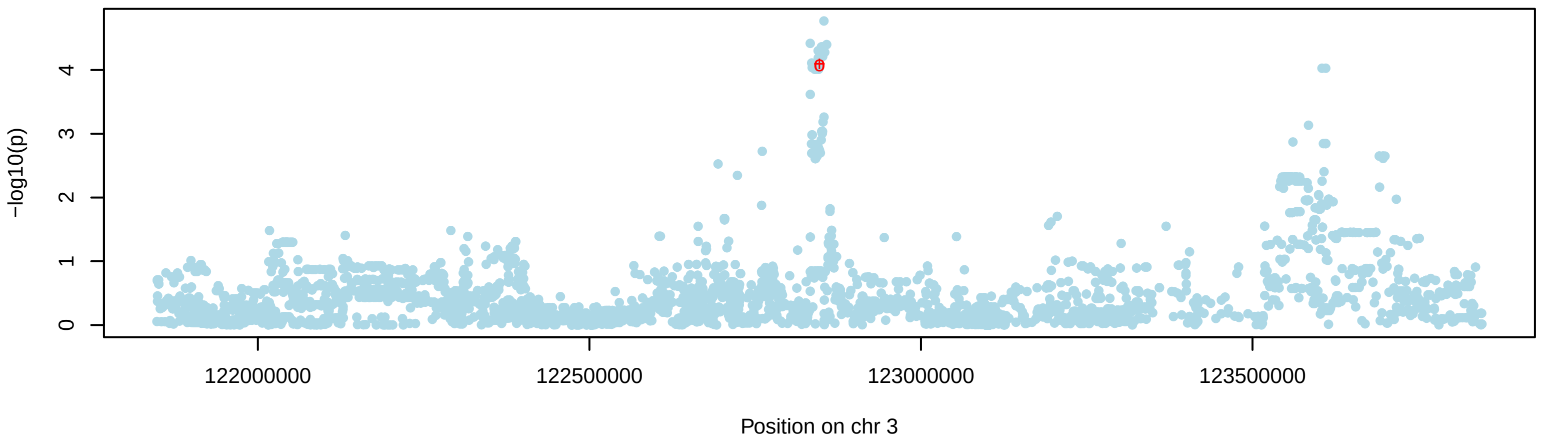

54. SERPINE2 (P07093) 2:224880498:T:C [Tarkin]

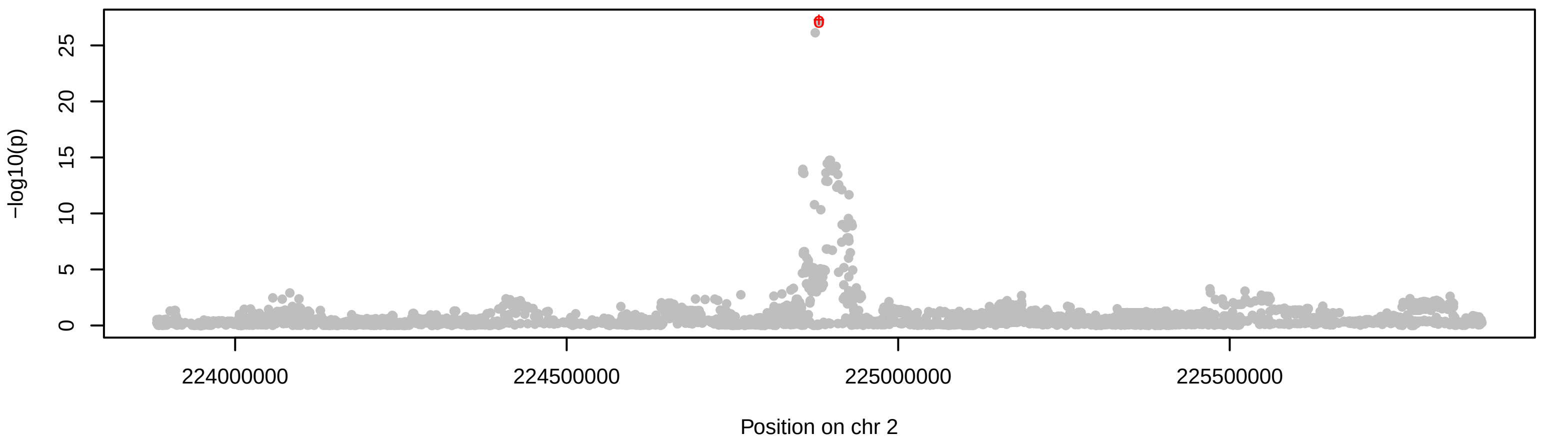

coloc with QMDiab: H1 (PP=68%)

54. SERPINE2 (P07093) 2:224880498:T:C [QMDiab]

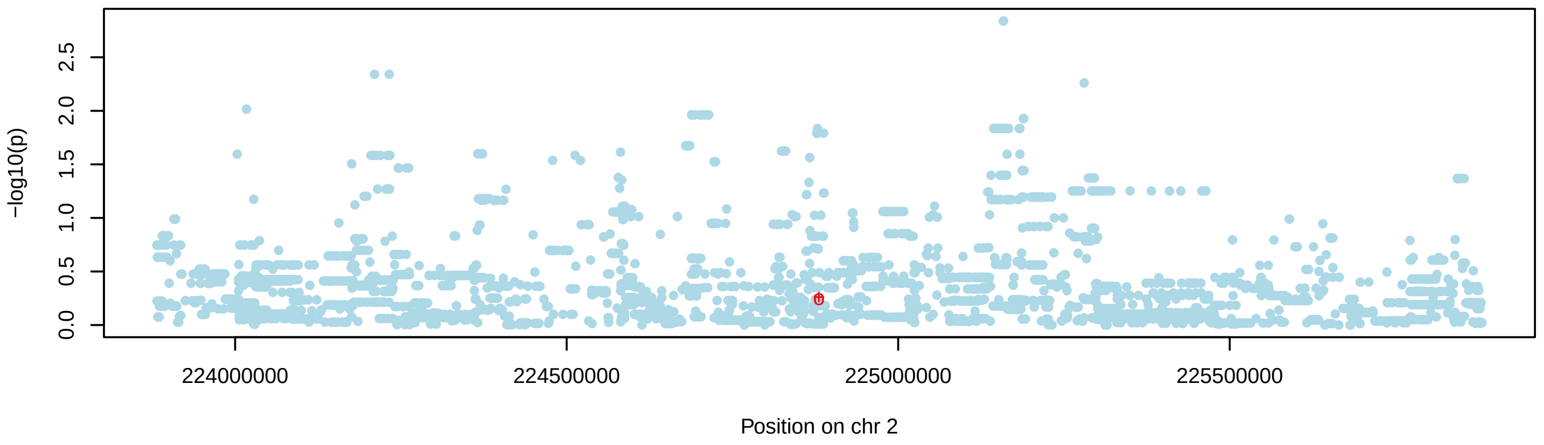

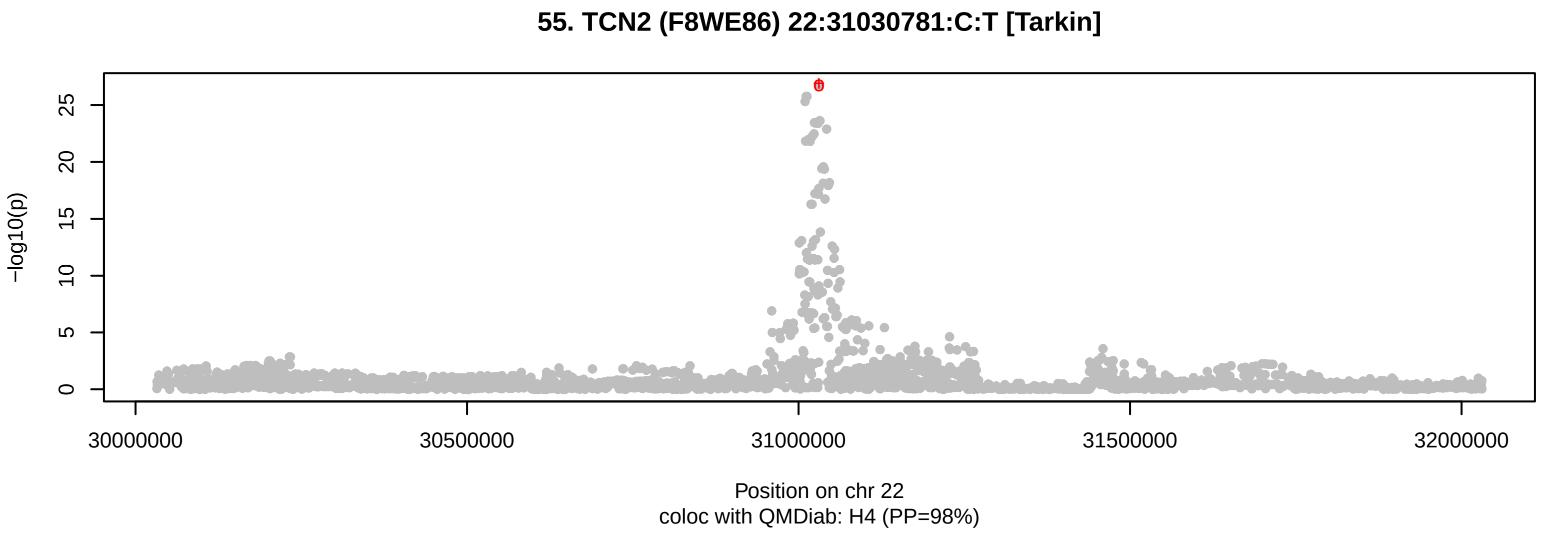
55. TCN2 (F8WE86) 22:31030781:C:T [Tarkin]
−log10(p)
0
5
10
15
20
25
30000000
30500000
31000000
31500000
32000000
Position on chr 22
coloc with QMDiab: H4 (PP=98%)

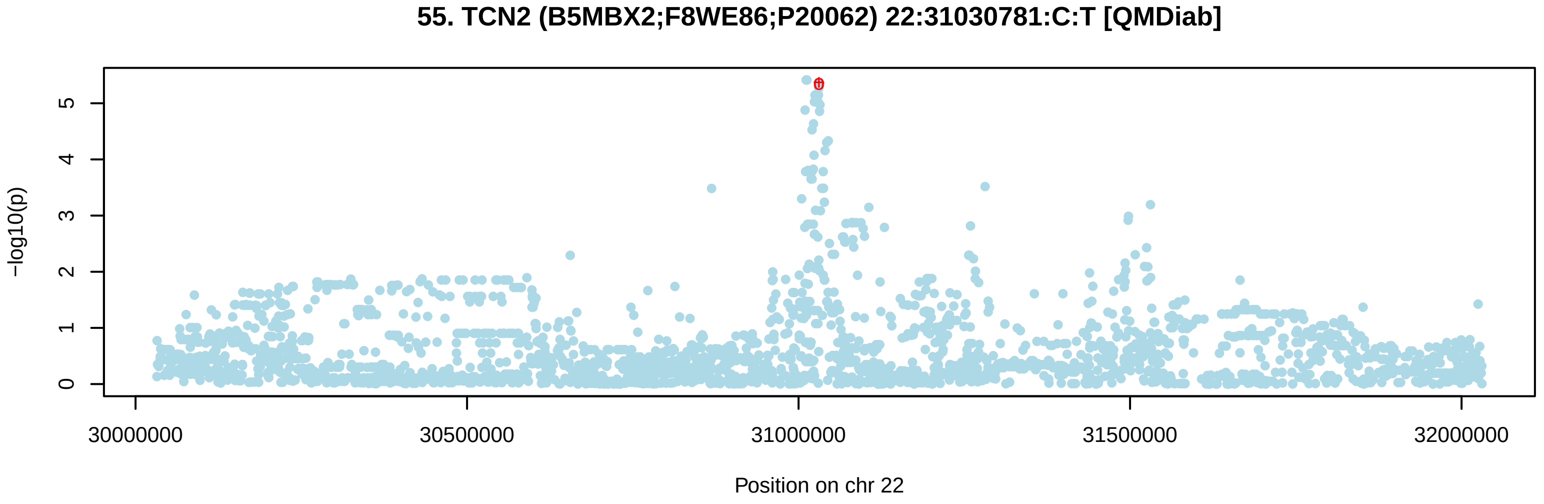
55. TCN2 (B5MBX2;F8WE86;P20062) 22:31030781:C:T [QMDiab]
−log10(p)
0
1
2
3
4
5
30000000
30500000
31000000
31500000
32000000
Position on chr 22

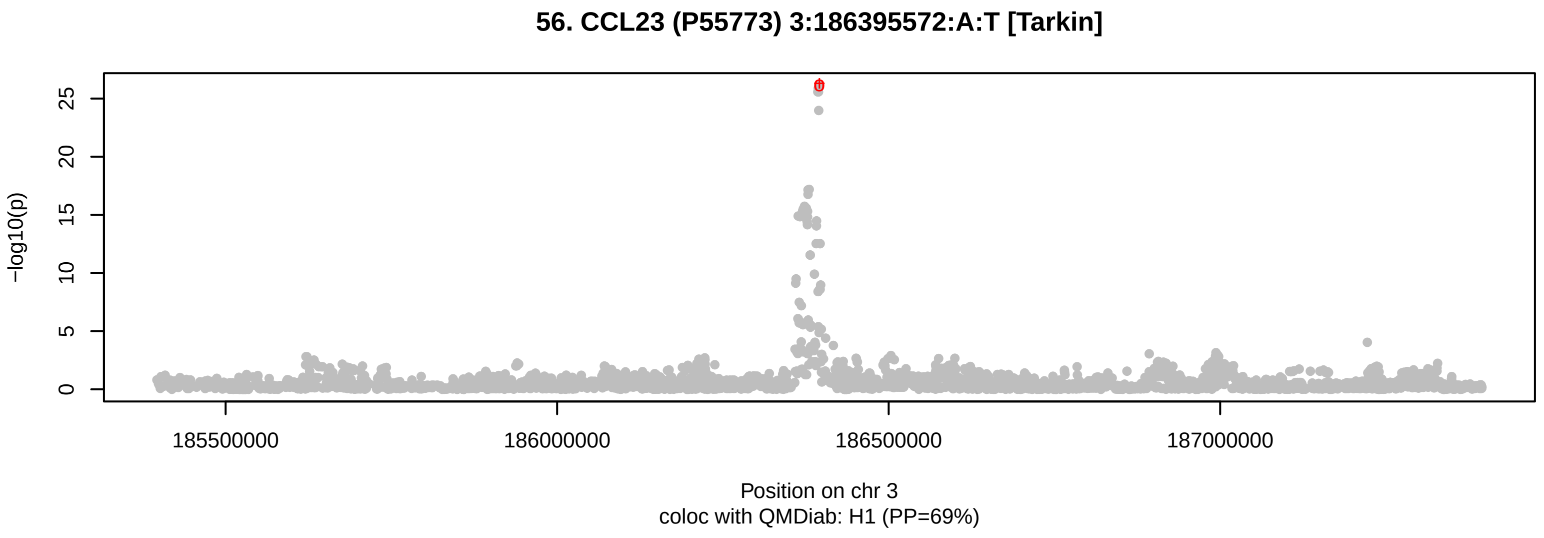
56. CCL23 (P55773) 3:186395572:A:T [Tarkin]
−log10(p)
0
5
10
15
20
25
185500000
186000000
186500000
187000000
Position on chr 3
coloc with QMDiab: H1 (PP=69%)

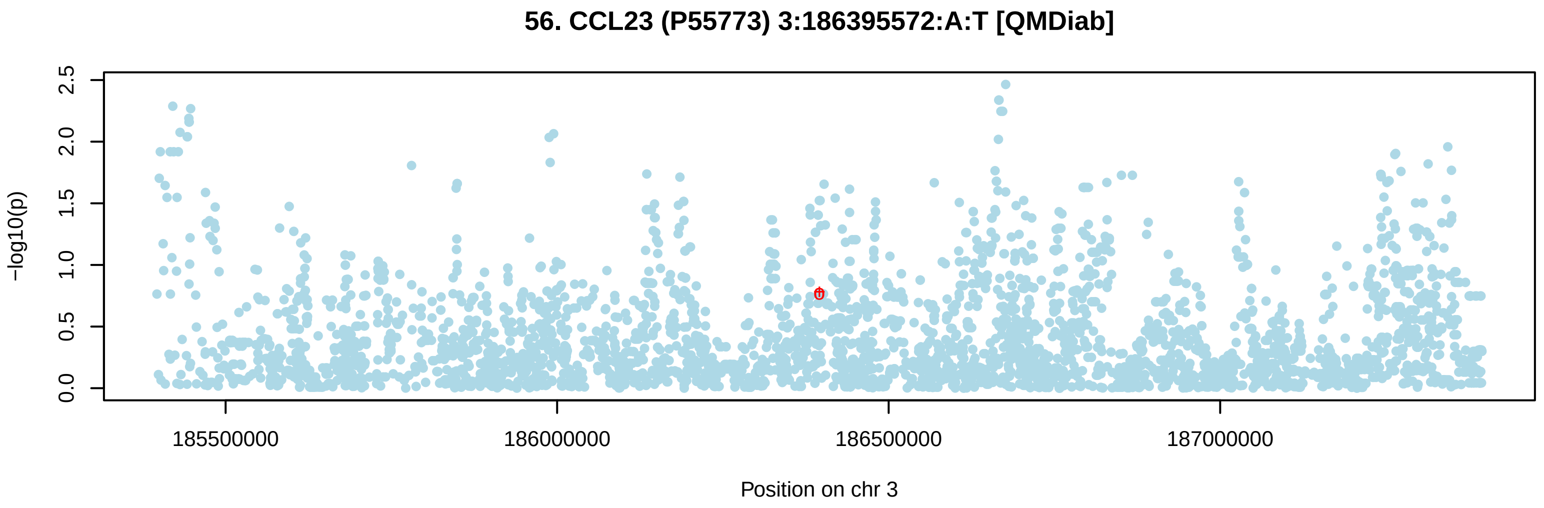
56. CCL23 (P55773) 3:186395572:A:T [QMDiab]
−log10(p)
0.0
0.5
1.0
1.5
2.0
2.5
185500000
186000000
186500000
187000000
Position on chr 3

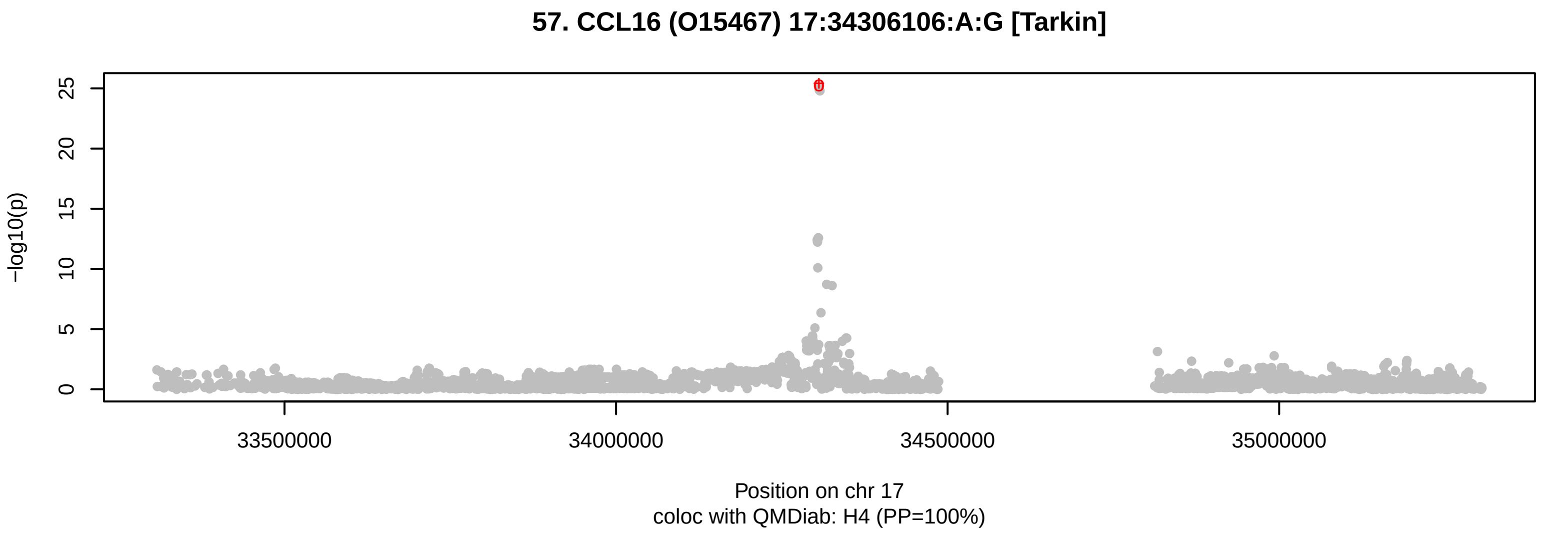
57. CCL16 (O15467) 17:34306106:A:G [Tarkin]
−log10(p)
0
5
10
15
20
25
33500000
34000000
34500000
35000000
Position on chr 17
coloc with QMDiab: H4 (PP=100%)

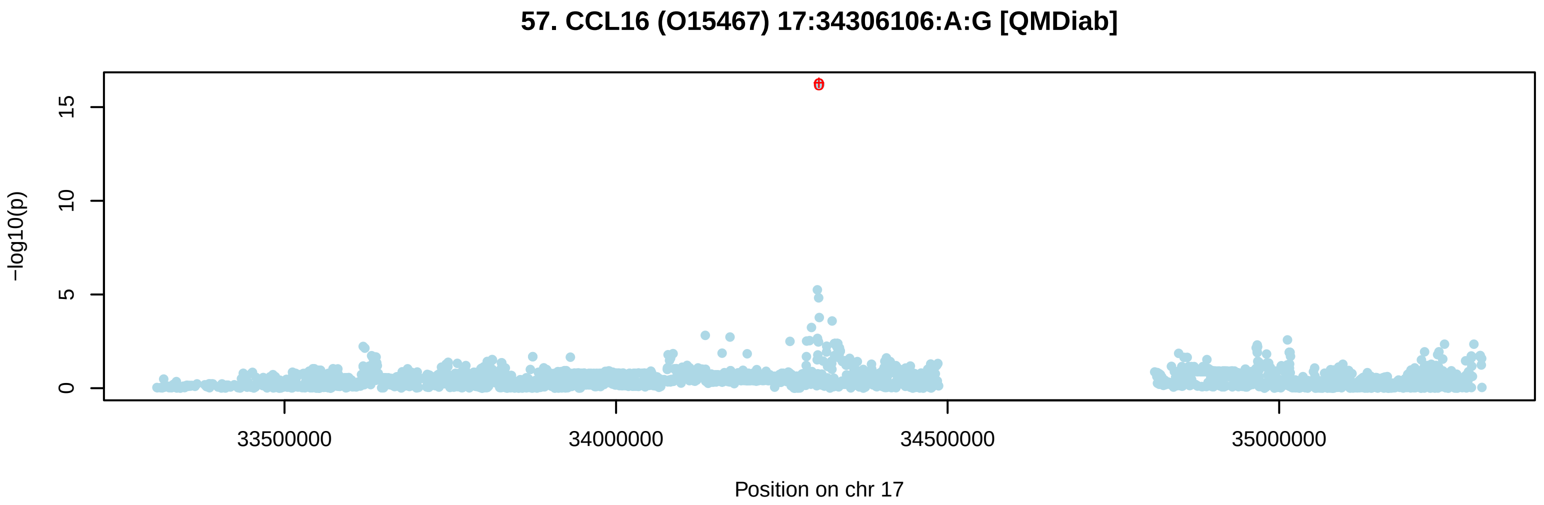
57. CCL16 (O15467) 17:34306106:A:G [QMDiab]
−log10(p)
0
5
10
15
33500000
34000000
34500000
35000000
Position on chr 17

58. SERPINA10 (G3V2W1) 13:113800622:T:C [Tarkin]

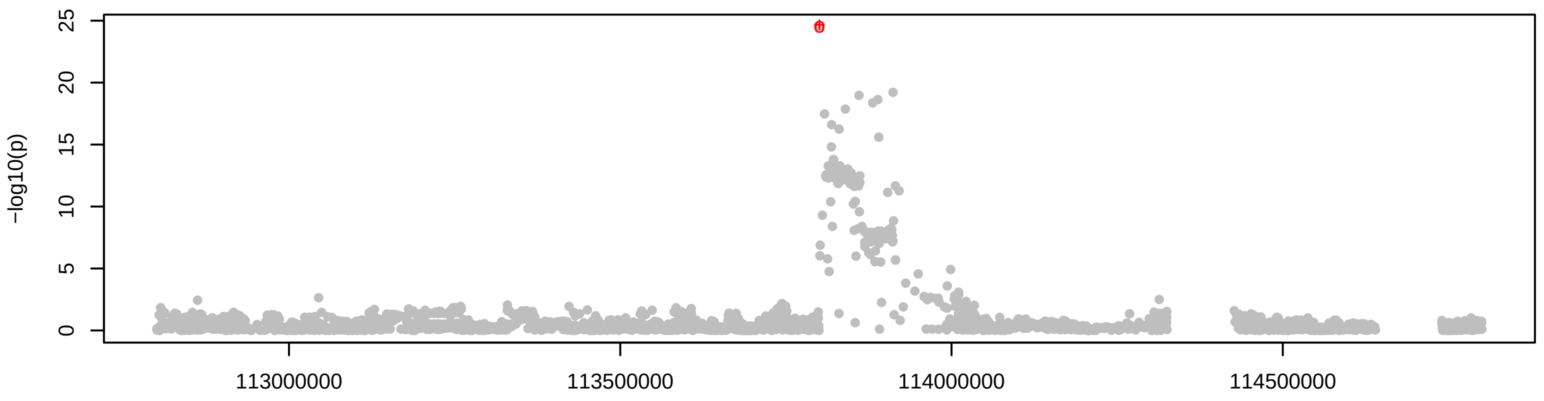

coloc with QMDiab: H4 (PP=85%)

58. SERPINA10 (G3V2W1;Q9UK55) 13:113800622:T:C [QMDiab]

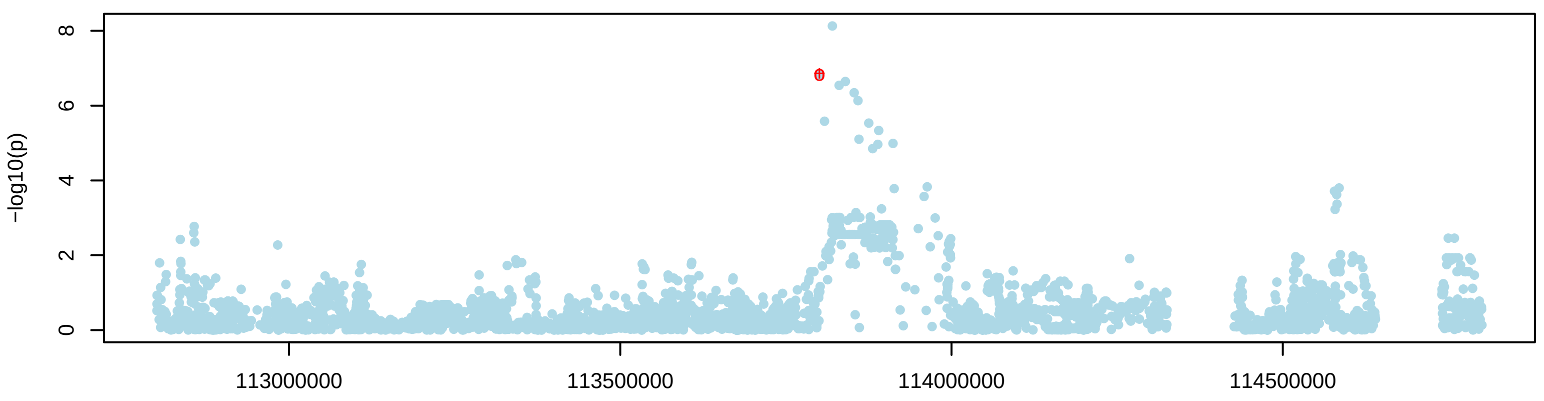

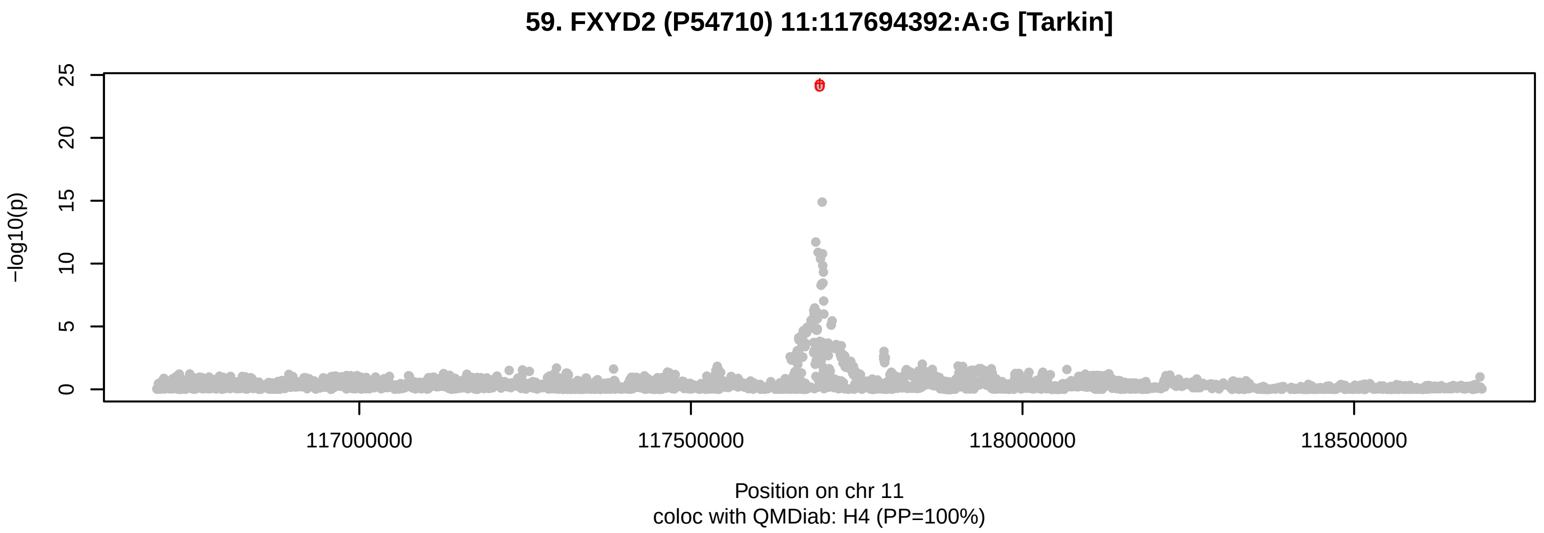
59. FXYD2 (P54710) 11:117694392:A:G [Tarkin]
−log10(p)
0
5
10
15
20
25
117000000
117500000
118000000
118500000
Position on chr 11
coloc with QMDiab: H4 (PP=100%)

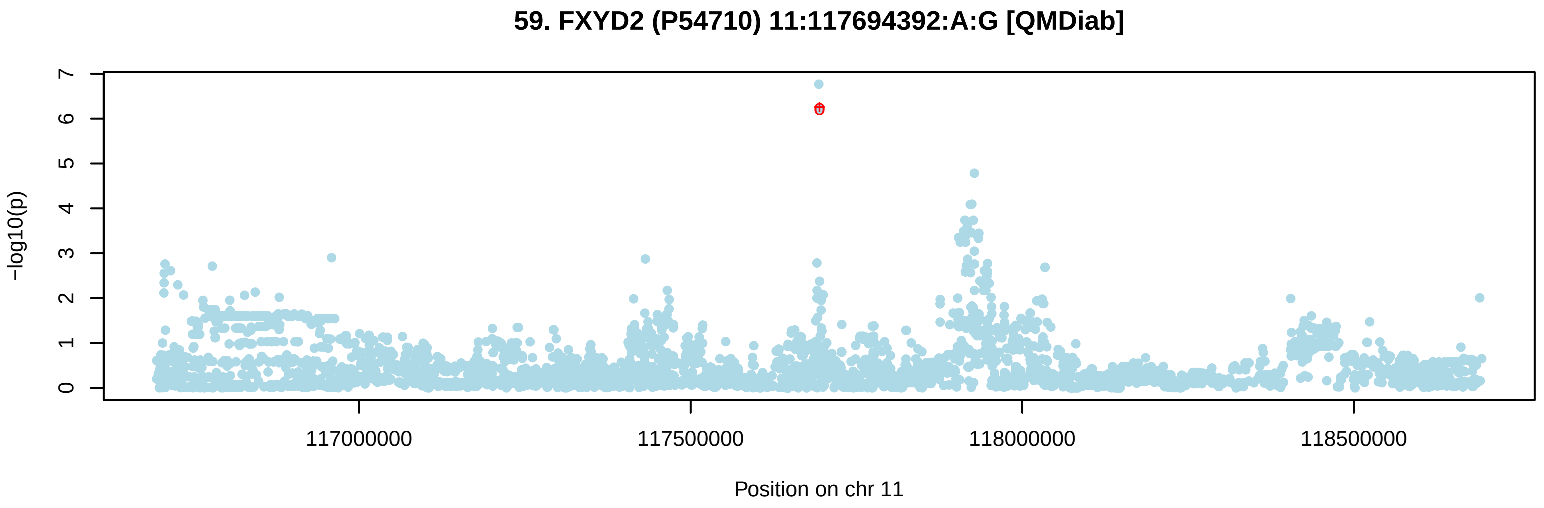
59. FXYD2 (P54710) 11:117694392:A:G [QMDiab]
−log10(p)
0
1
2
3
4
5
6
7
117000000
117500000
118000000
118500000
Position on chr 11

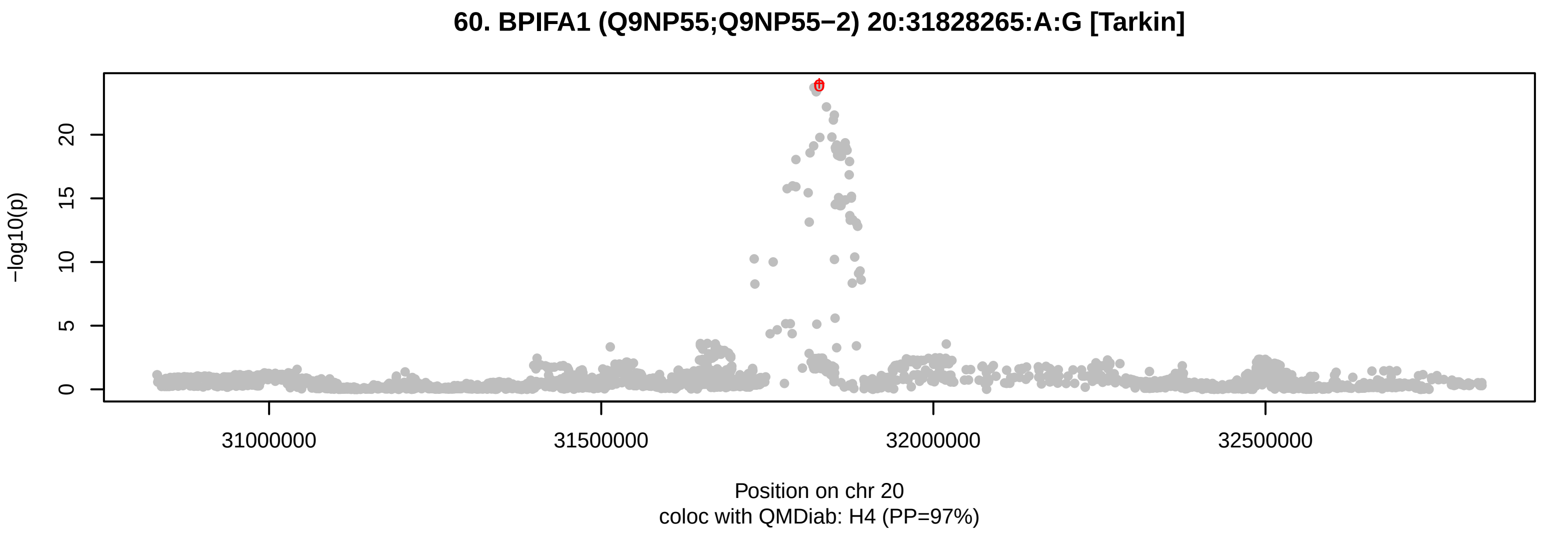
60. BPIFA1 (Q9NP55;Q9NP55−2) 20:31828265:A:G [Tarkin]
−log10(p)
0
5
10
15
20
31000000
31500000
32000000
32500000
Position on chr 20
coloc with QMDiab: H4 (PP=97%)

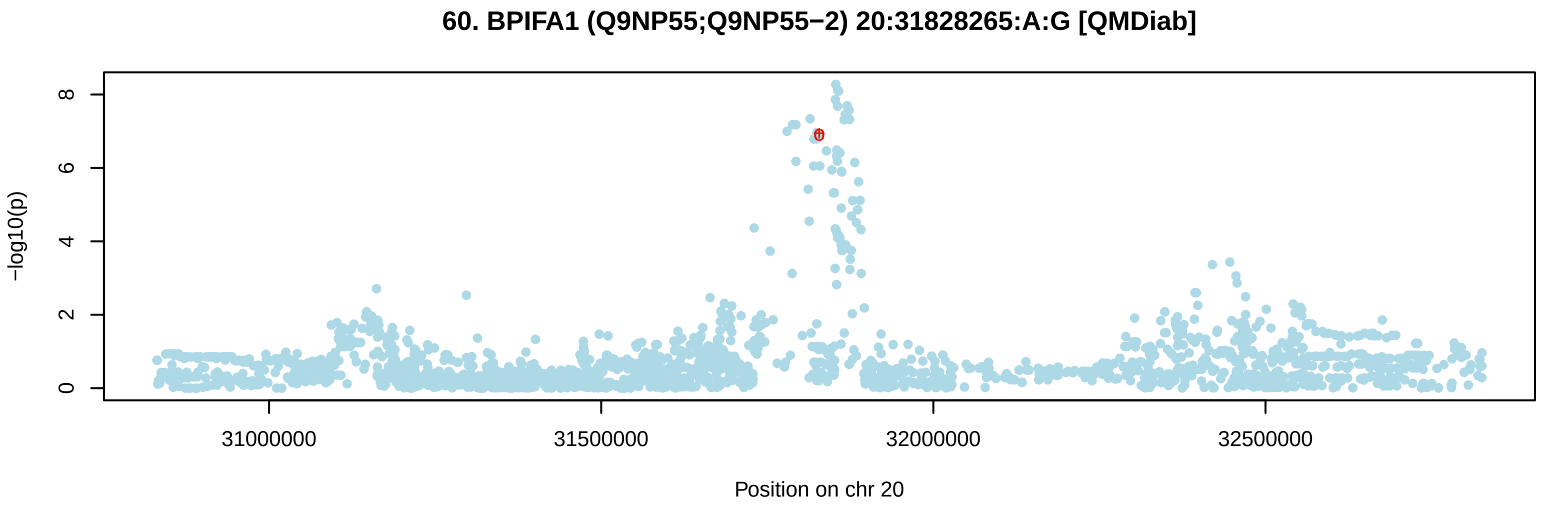
60. BPIFA1 (Q9NP55;Q9NP55−2) 20:31828265:A:G [QMDiab]
−log10(p)
0
2
4
6
8
31000000
31500000
32000000
32500000
Position on chr 20

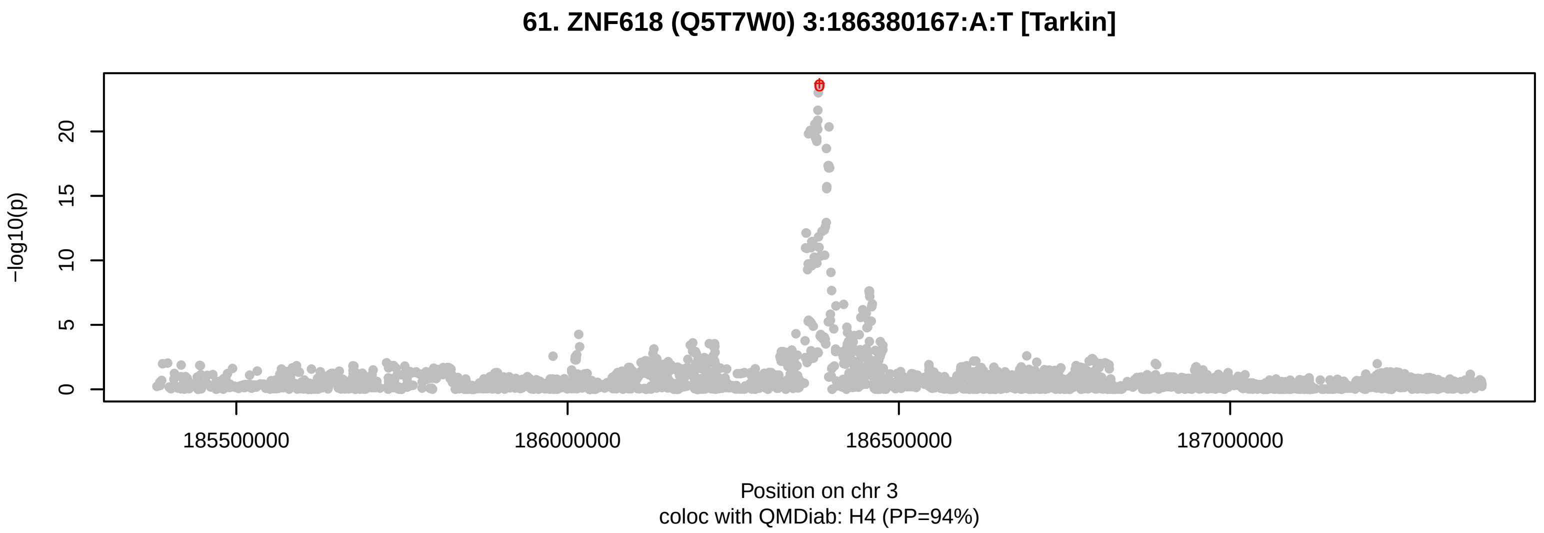
61. ZNF618 (Q5T7W0) 3:186380167:A:T [Tarkin]
−log10(p)
0
5
10
15
20
185500000
186000000
186500000
187000000
Position on chr 3
coloc with QMDiab: H4 (PP=94%)

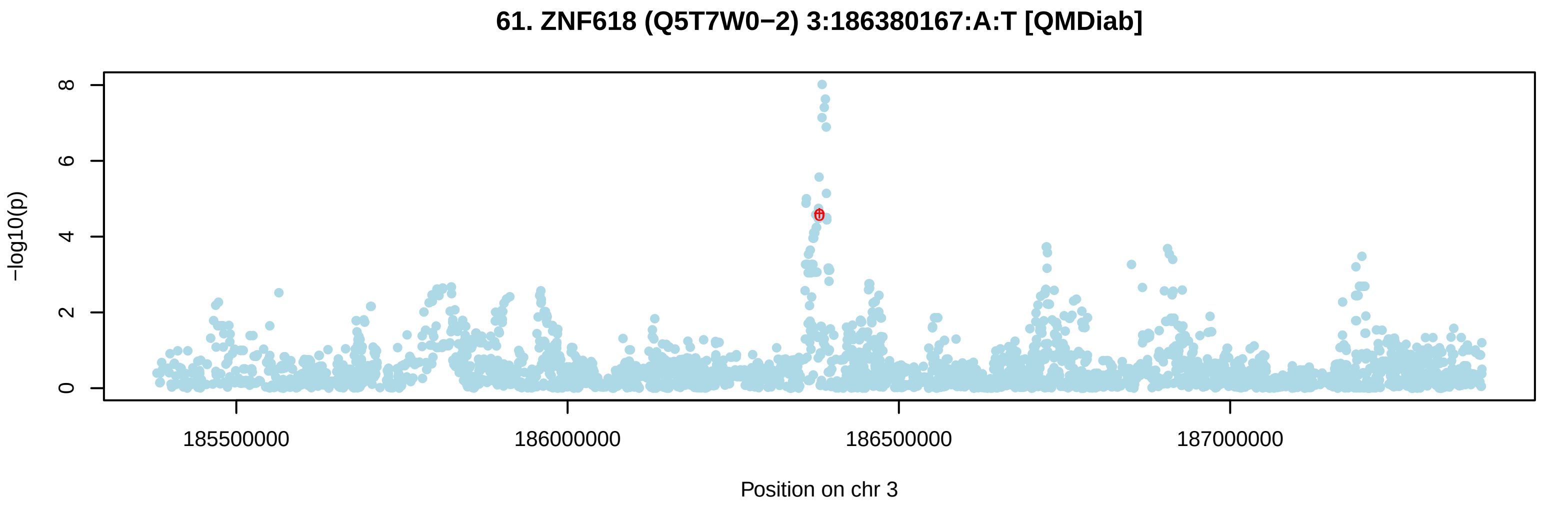
61. ZNF618 (Q5T7W0−2) 3:186380167:A:T [QMDiab]
−log10(p)
0
2
4
6
8
185500000
186000000
186500000
187000000
Position on chr 3

62. SERPINE2 (P07093−2;P07093−3) 2:224874874:G:A [Tarkin]

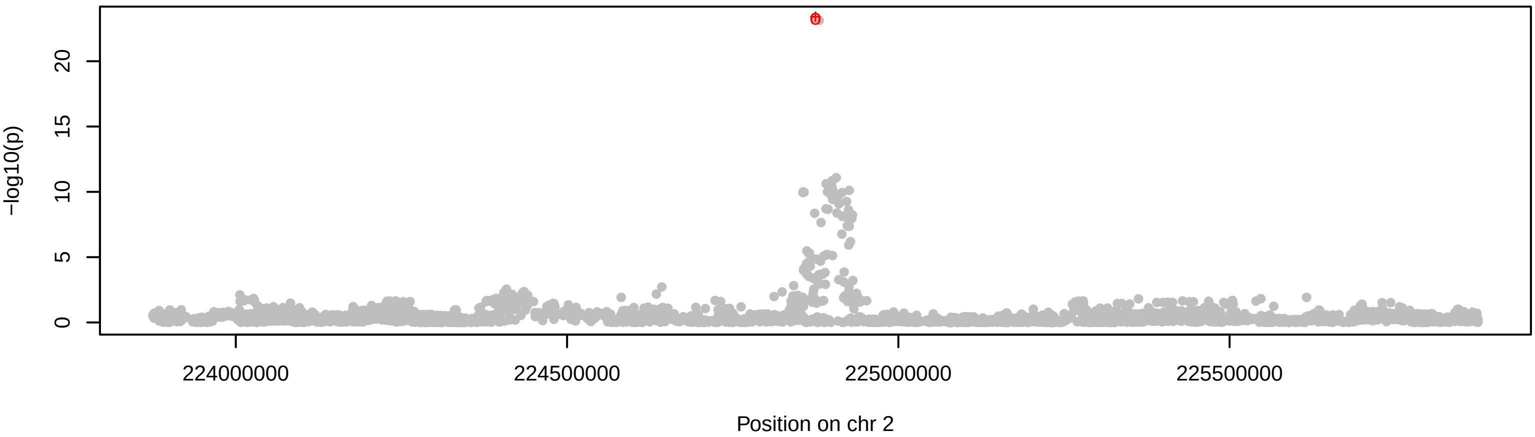

coloc with QMDiab: H4 (PP=100%)

62. SERPINE2 (P07093−2;P07093−3) 2:224874874:G:A [QMDiab]

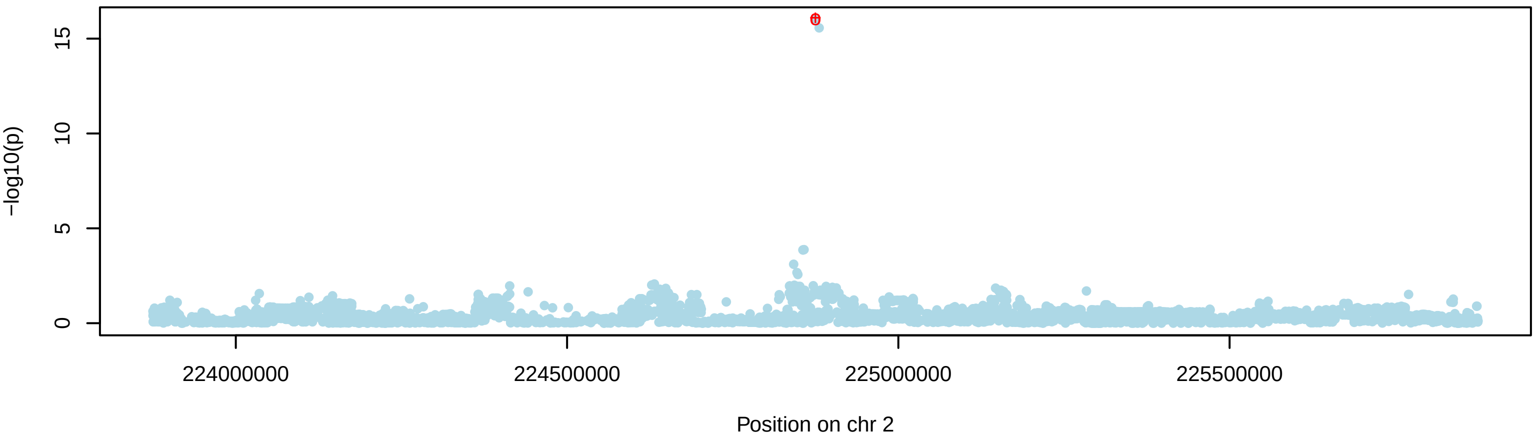

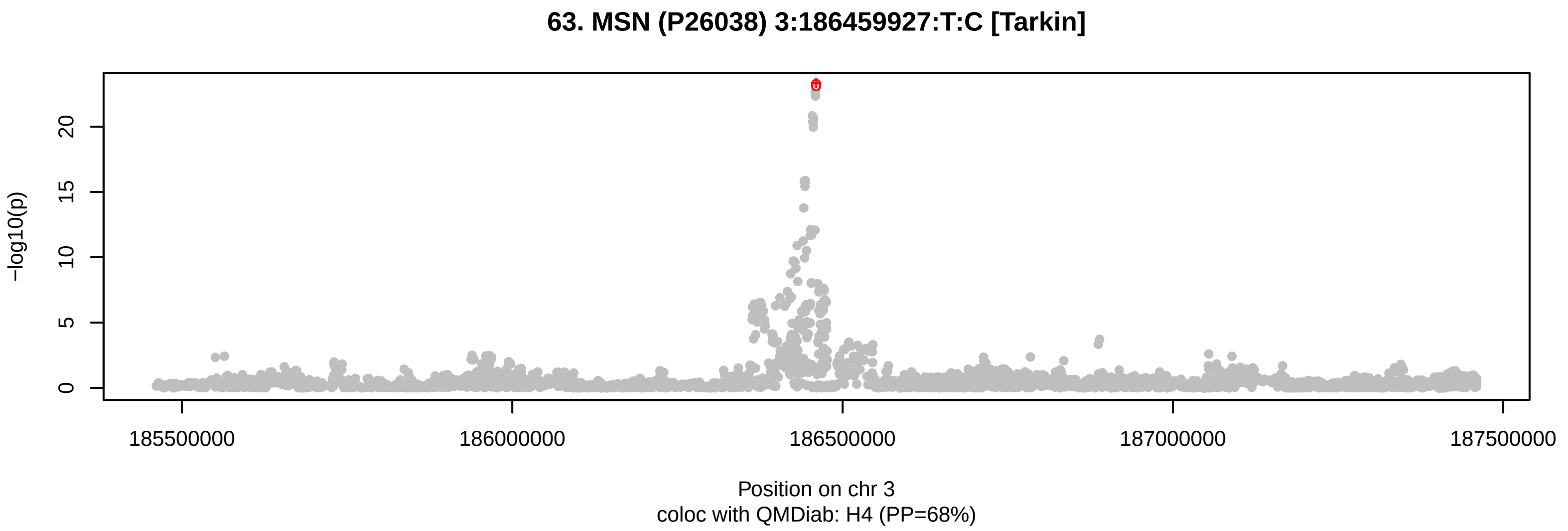

63. MSN (P26038) 3:186459927:T:C [Tarkin]
−log10(p)
0
5
10
15
20
185500000
186000000
186500000
187000000
187500000
Position on chr 3
coloc with QMDiab: H4 (PP=68%)

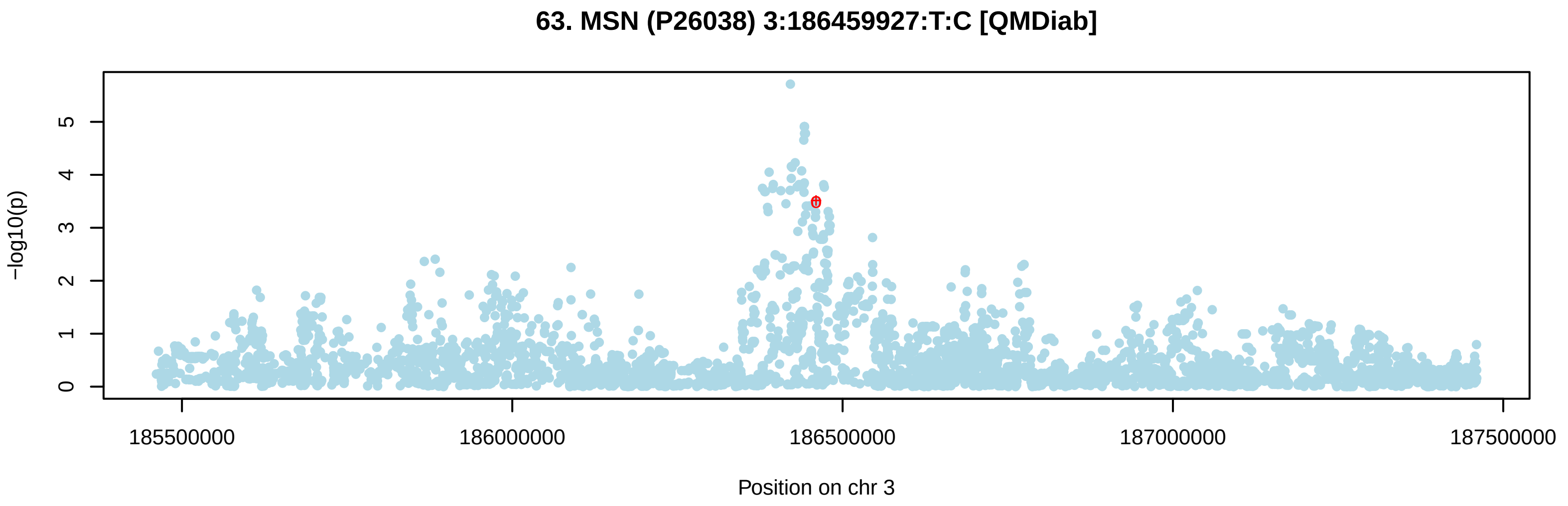

63. MSN (P26038) 3:186459927:T:C [QMDiab]
−log10(p)
0
1
2
3
4
5
185500000
186000000
186500000
187000000
187500000
Position on chr 3

64. IGLV1−51 (P01701) 22:22671670:A:G [Tarkin]

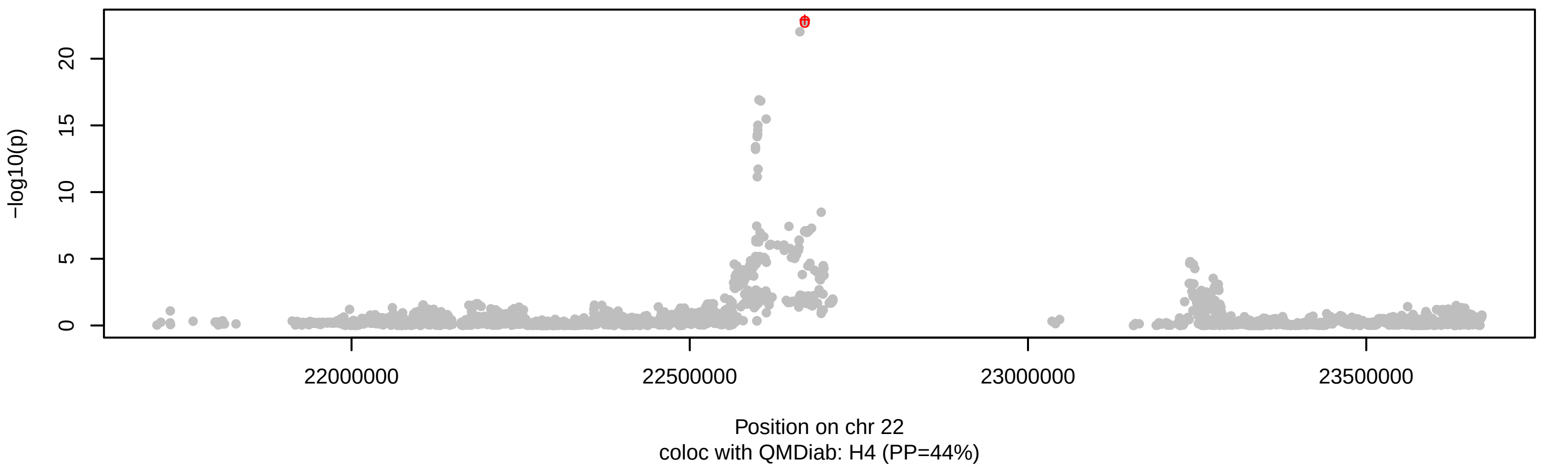

64. IGLV1−51 (P01701) 22:22671670:A:G [QMDiab]

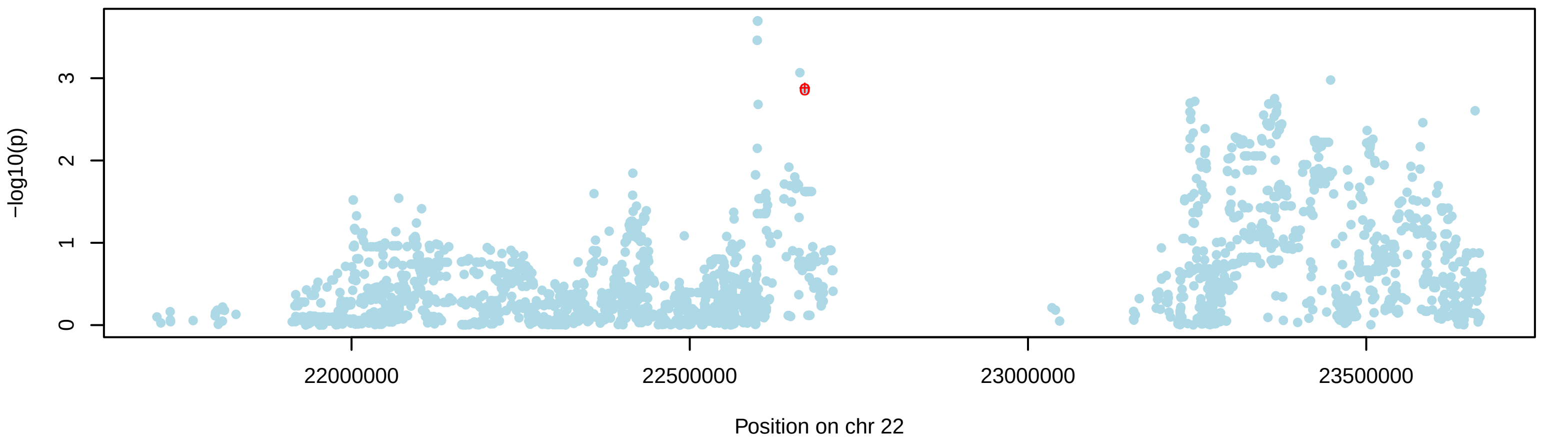

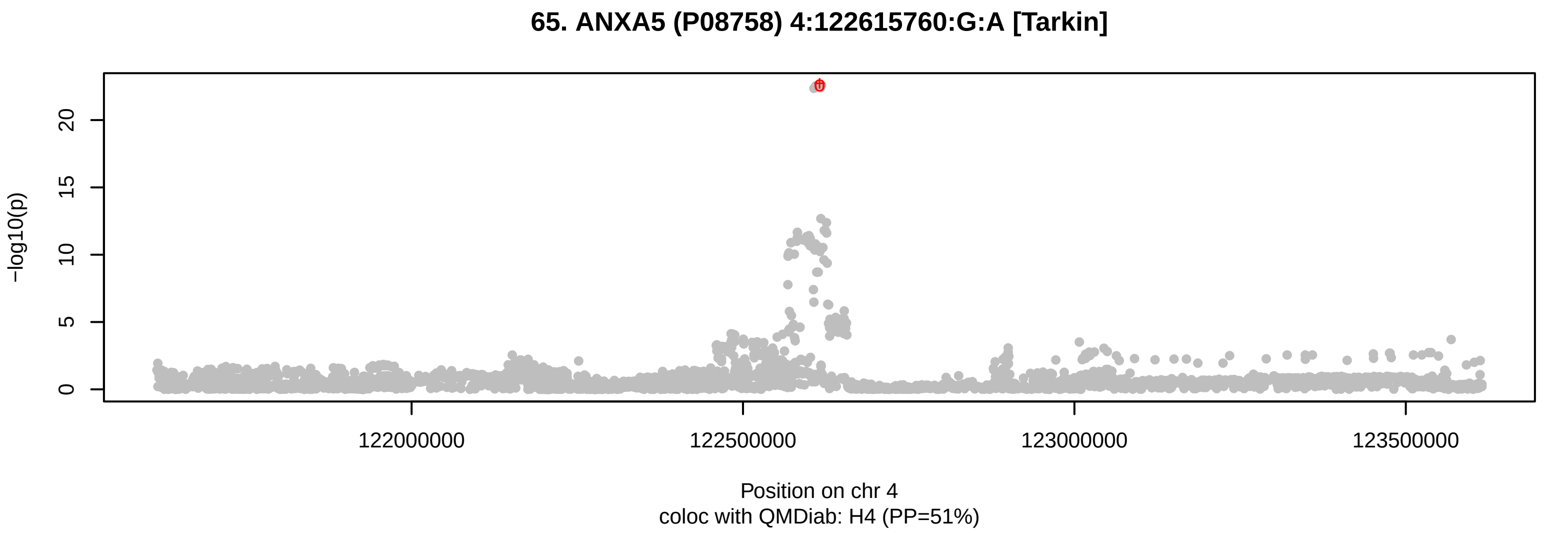
65. ANXA5 (P08758) 4:122615760:G:A [Tarkin]
−log10(p)
0
5
10
15
20
122000000
122500000
123000000
123500000
Position on chr 4
coloc with QMDiab: H4 (PP=51%)

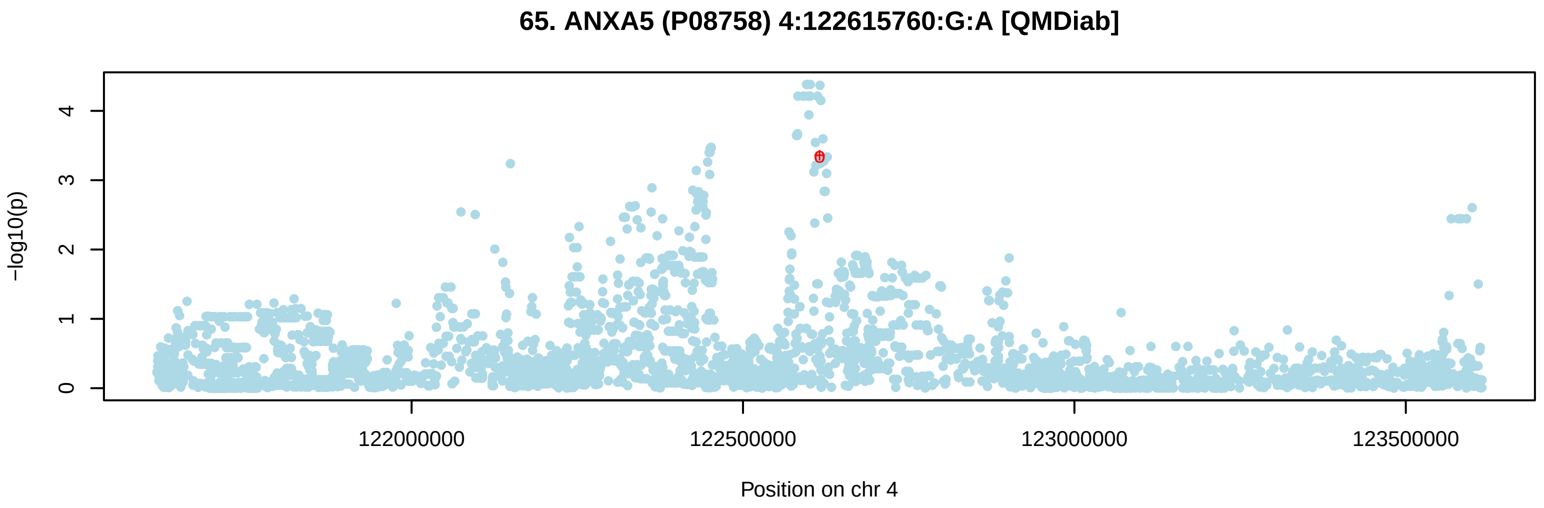
65. ANXA5 (P08758) 4:122615760:G:A [QMDiab]
−log10(p)
0
1
2
3
4
122000000
122500000
123000000
123500000
Position on chr 4

66. AKR7A2 (O43488) 1:19635011:C:T [Tarkin]

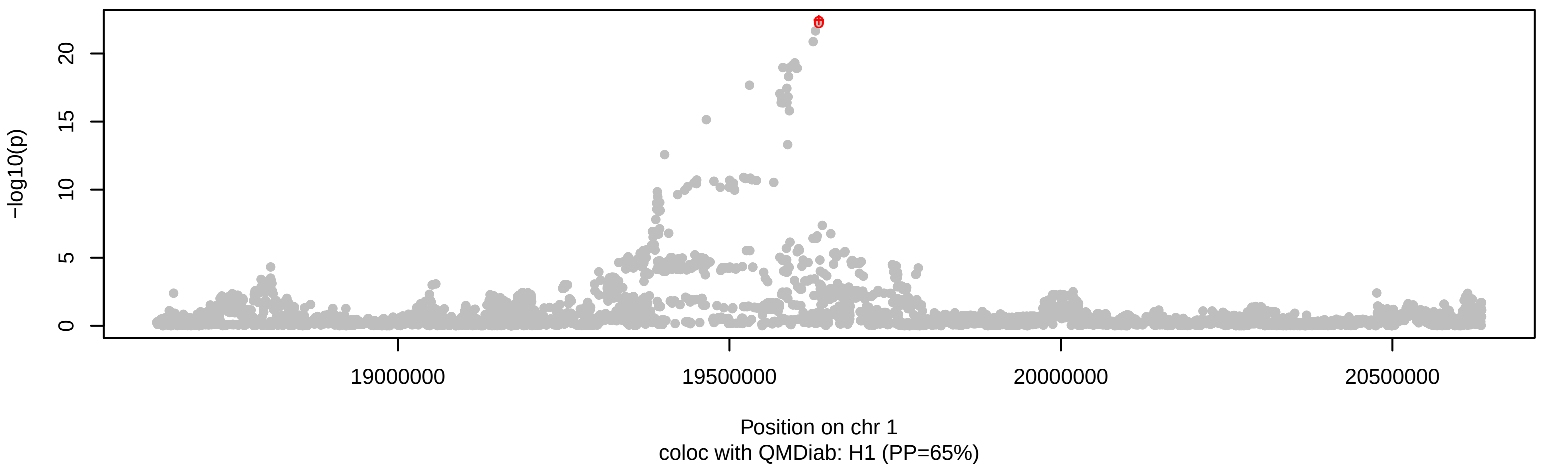

coloc with QMDiab: H1 (PP=65%)

66. AKR7A2 (O43488) 1:19635011:C:T [QMDiab]

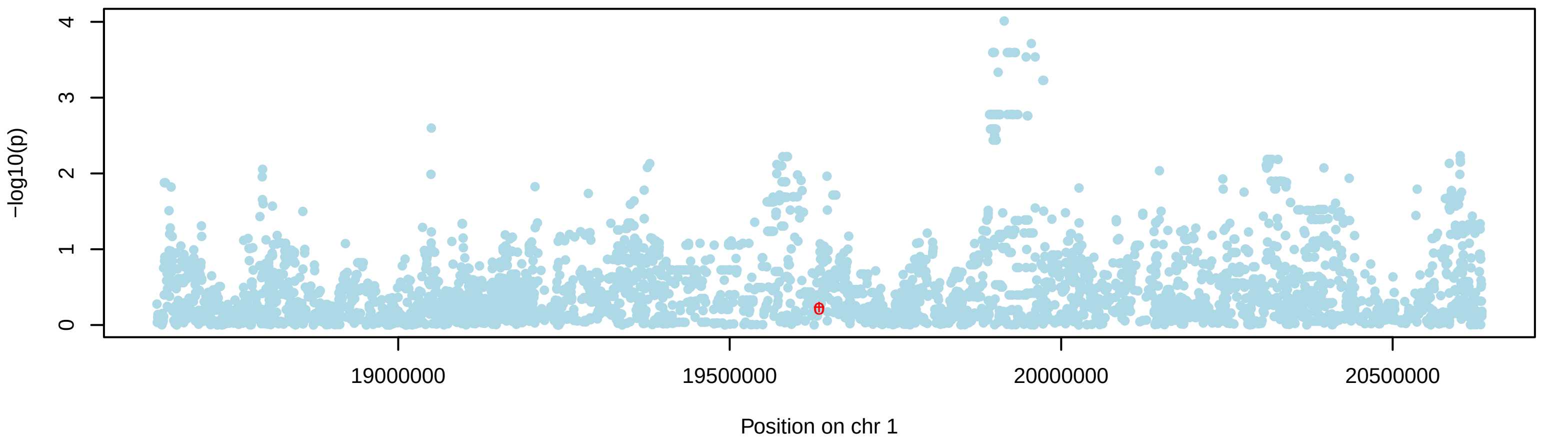

67. POSTN (Q15063−2) 1:196822368:A:G [Tarkin]

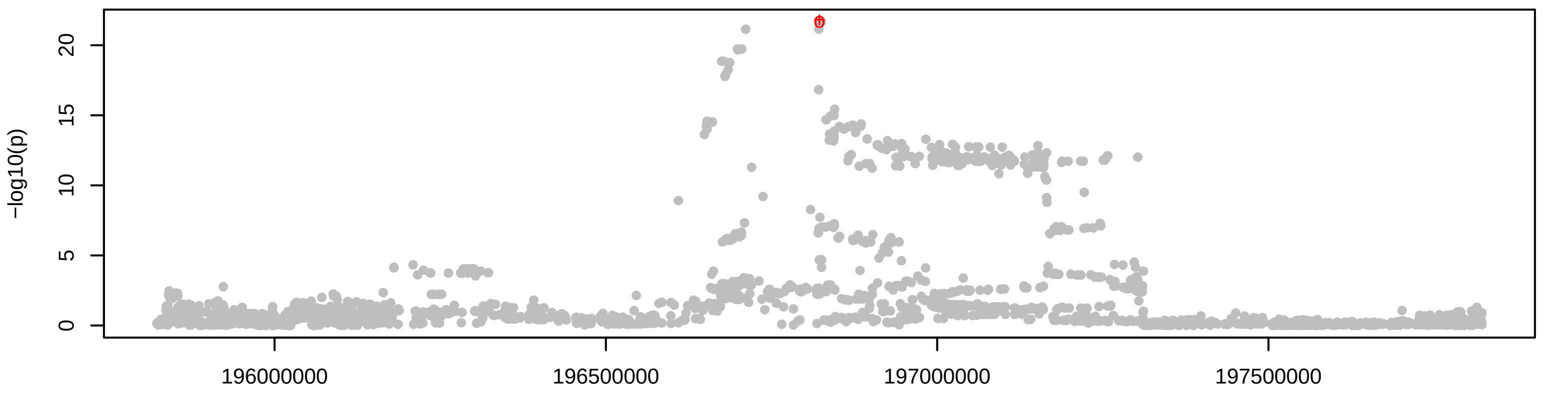

Position on chr 1
coloc with QMDiab: H1 (PP=63%)

67. POSTN (Q15063−2) 1:196822368:A:G [QMDiab]

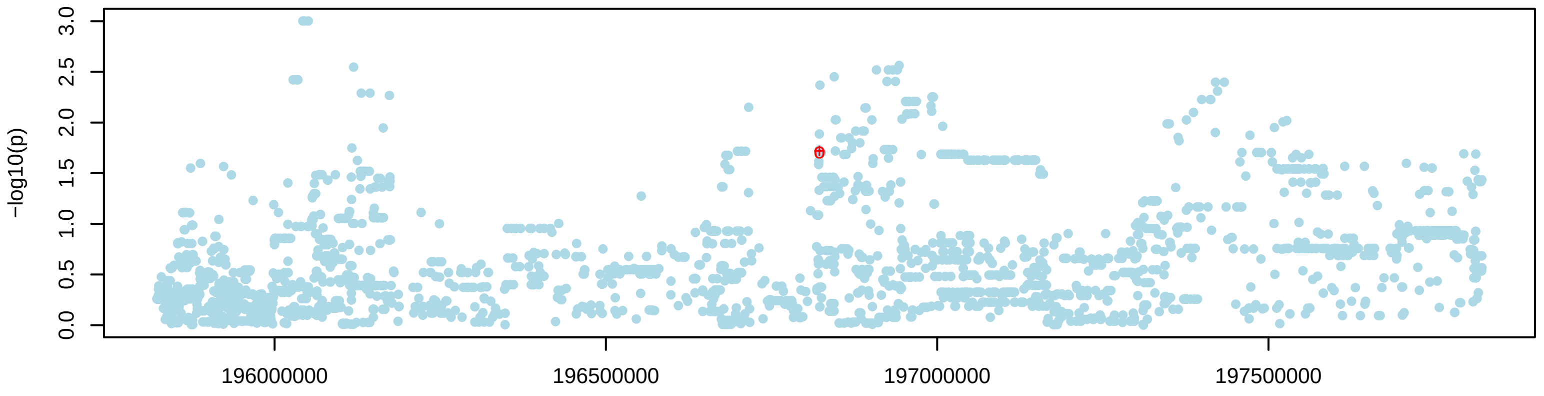

Position on chr 1

68. HSPB1 (A0A6Q8PFK8;P04792) 7:75924218:G:T [Tarkin]

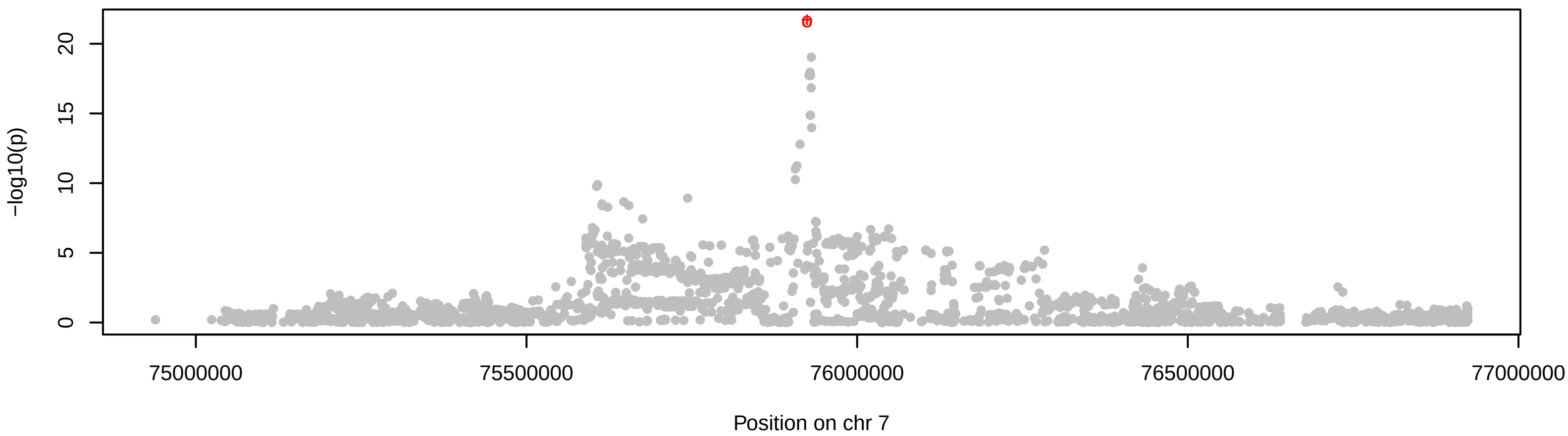

coloc with QMDiab: H4 (PP=95%)

68. HSPB1 (A0A6Q8PFK8;P04792) 7:75924218:G:T [QMDiab]

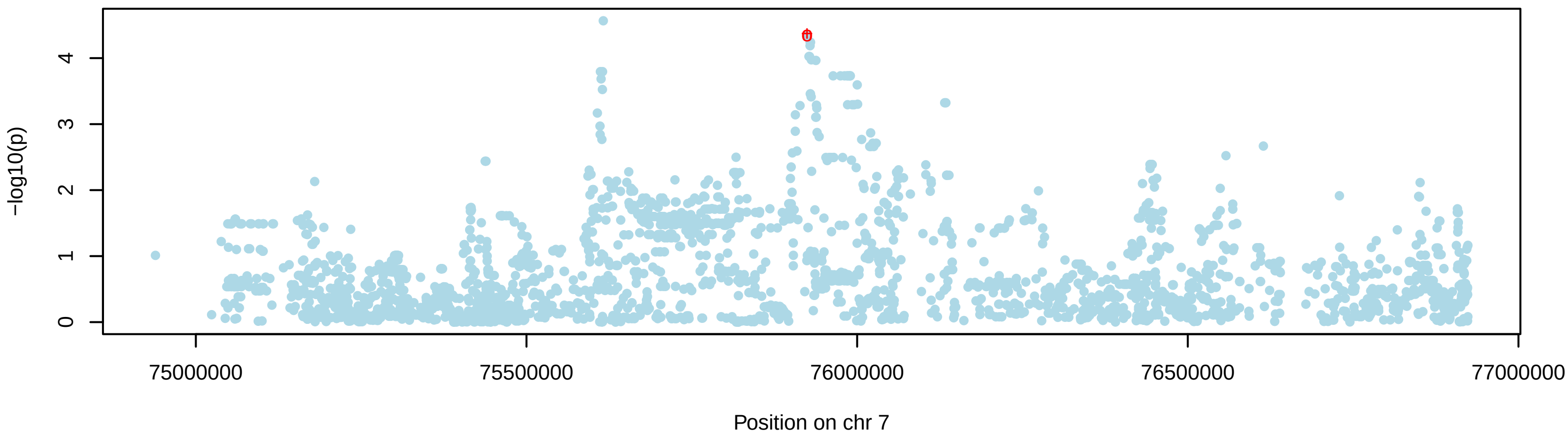

69. ITIH2 (P19823) 3:126261202:G:A [Tarkin]

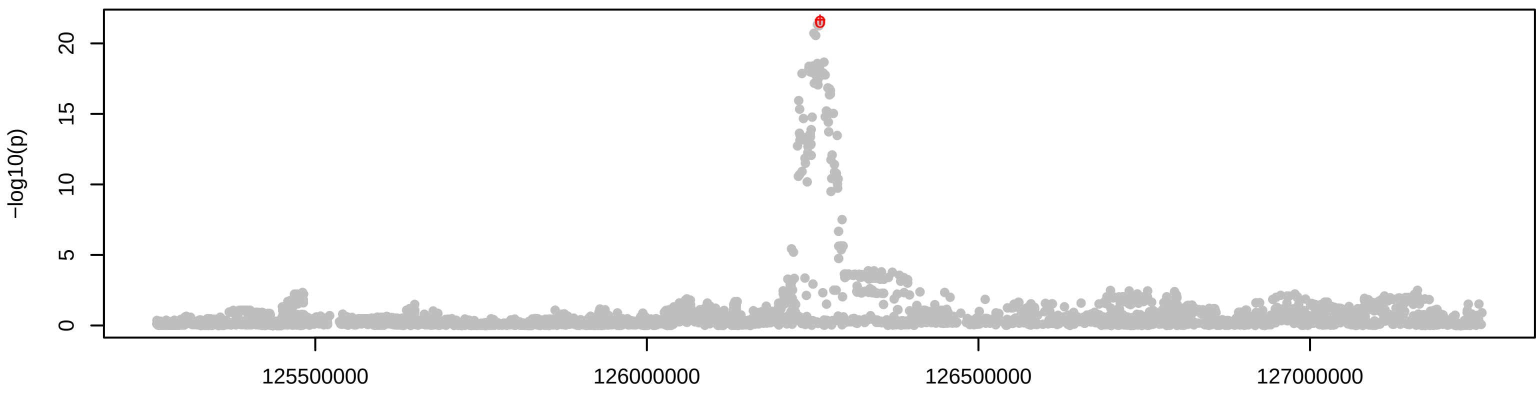

Position on chr 3
coloc with QMDiab: H4 (PP=58%)

69. ITIH2 (P19823) 3:126261202:G:A [QMDiab]

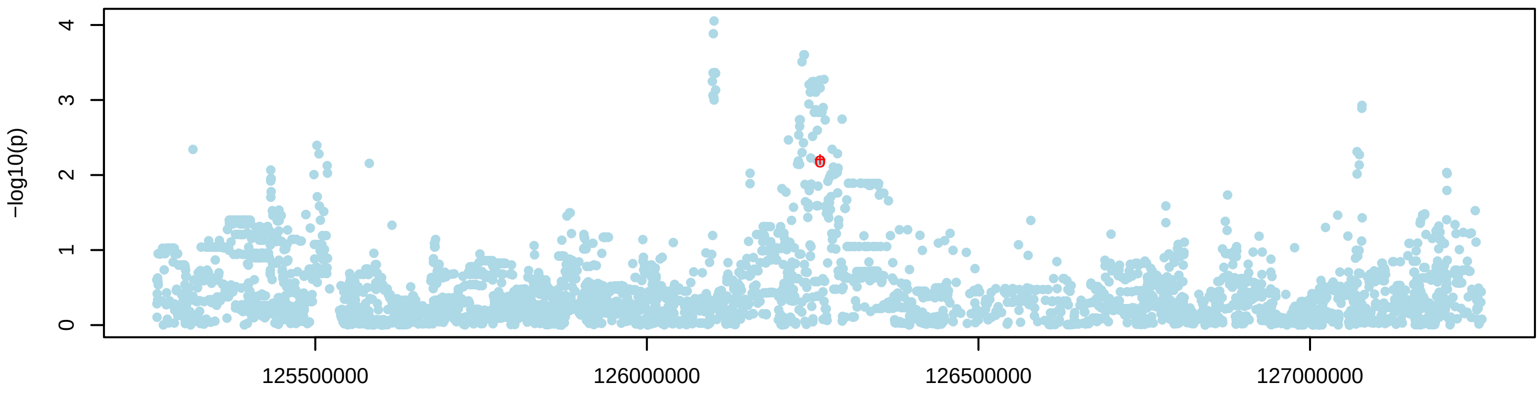

Position on chr 3

70. PROZ (P22891;P22891−2) 13:113800622:T:C [Tarkin]

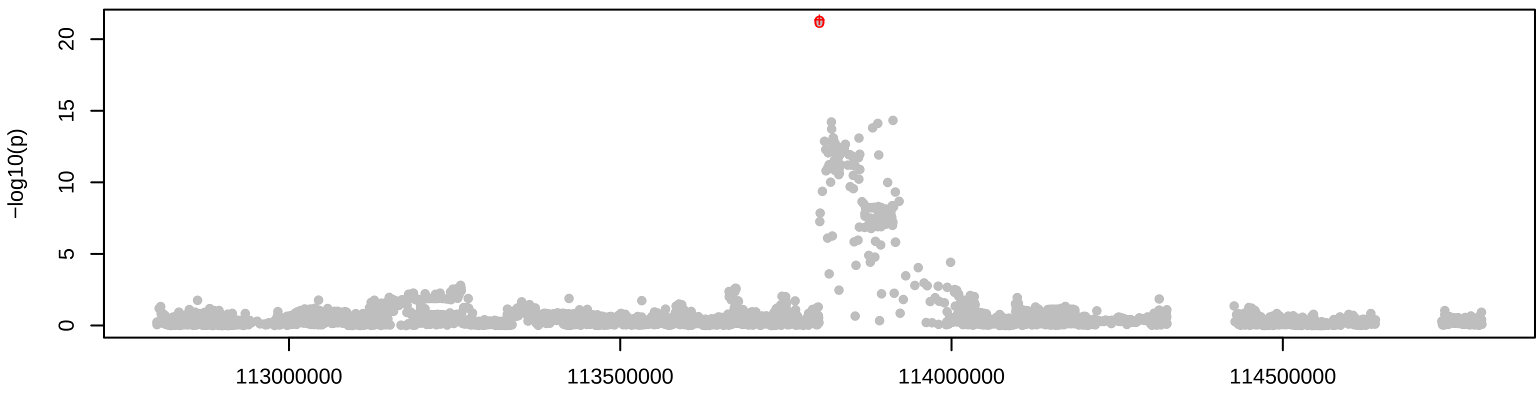

Position on chr 13
coloc with QMDiab: H3 (PP=96%)

70. PROZ (P22891;P22891−2) 13:113800622:T:C [QMDiab]

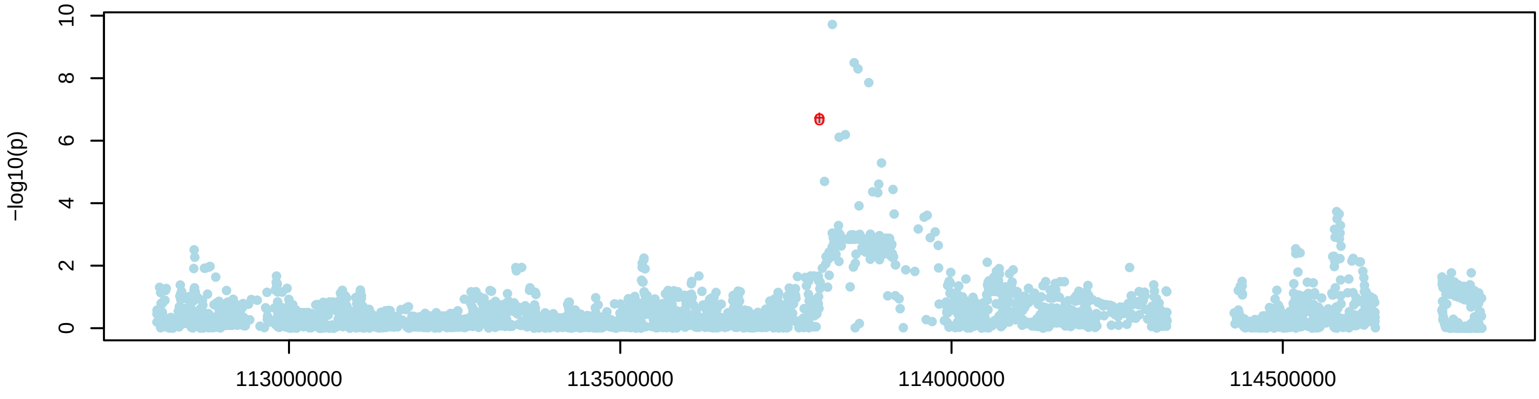

Position on chr 13

71. CSTF3 (Q12996) 20:36997655:C:T [Tarkin]

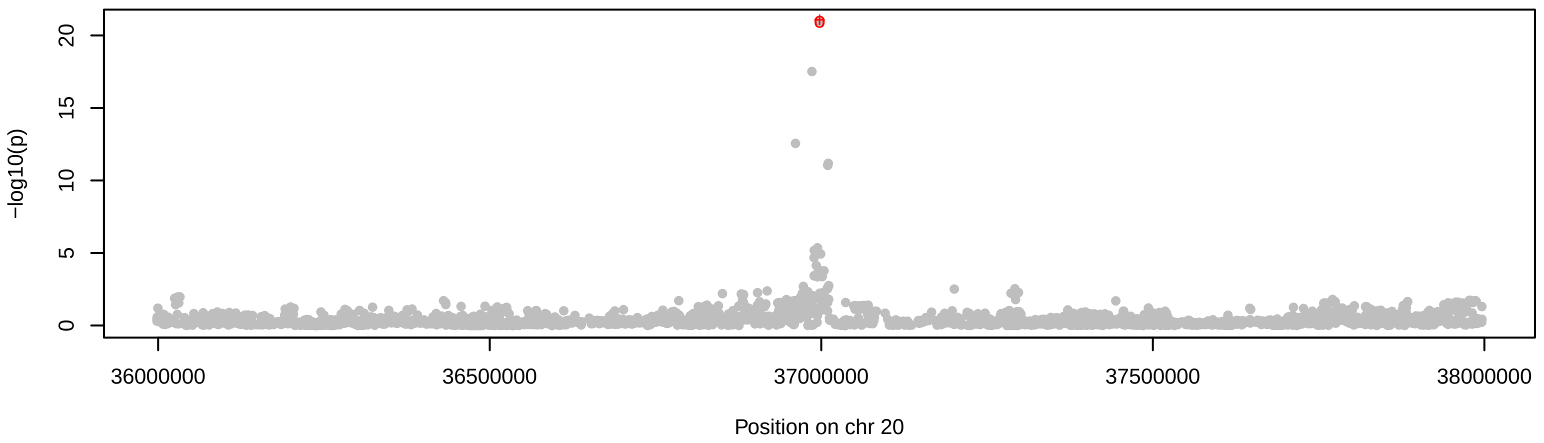

no replication data avaiable in QMDiab

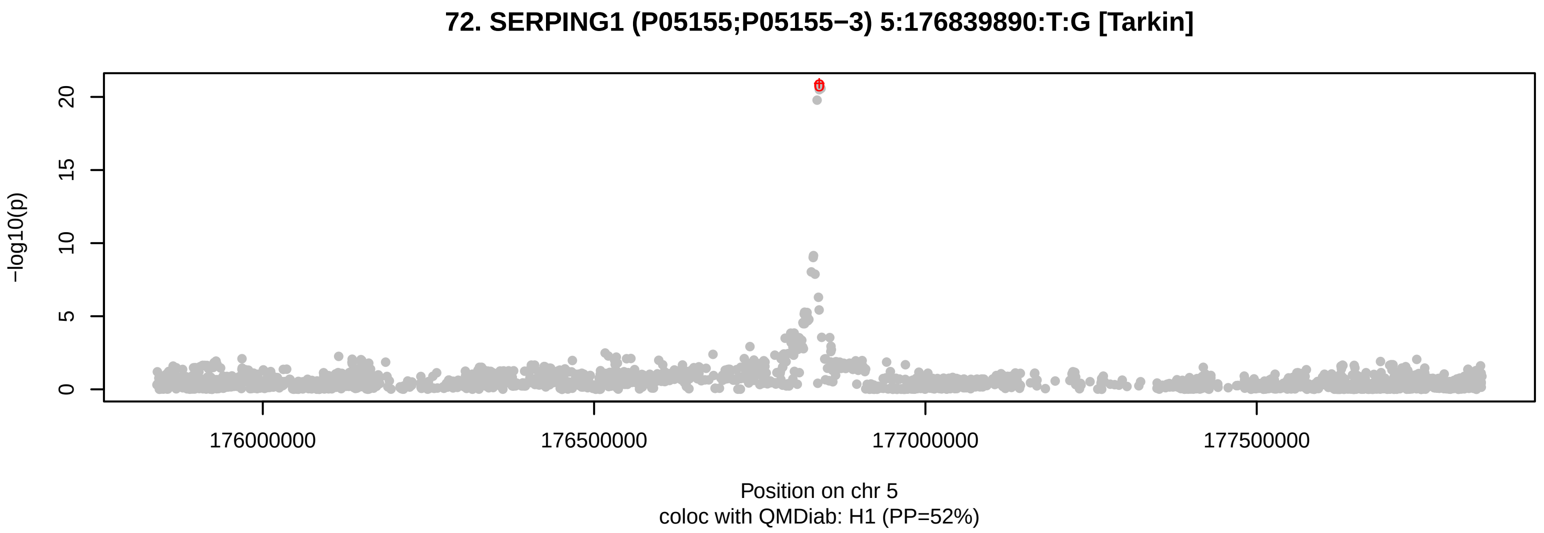
72. SERPING1 (P05155;P05155−3) 5:176839890:T:G [Tarkin]
−log10(p)
0
5
10
15
20
176000000
176500000
177000000
177500000
Position on chr 5
coloc with QMDiab: H1 (PP=52%)

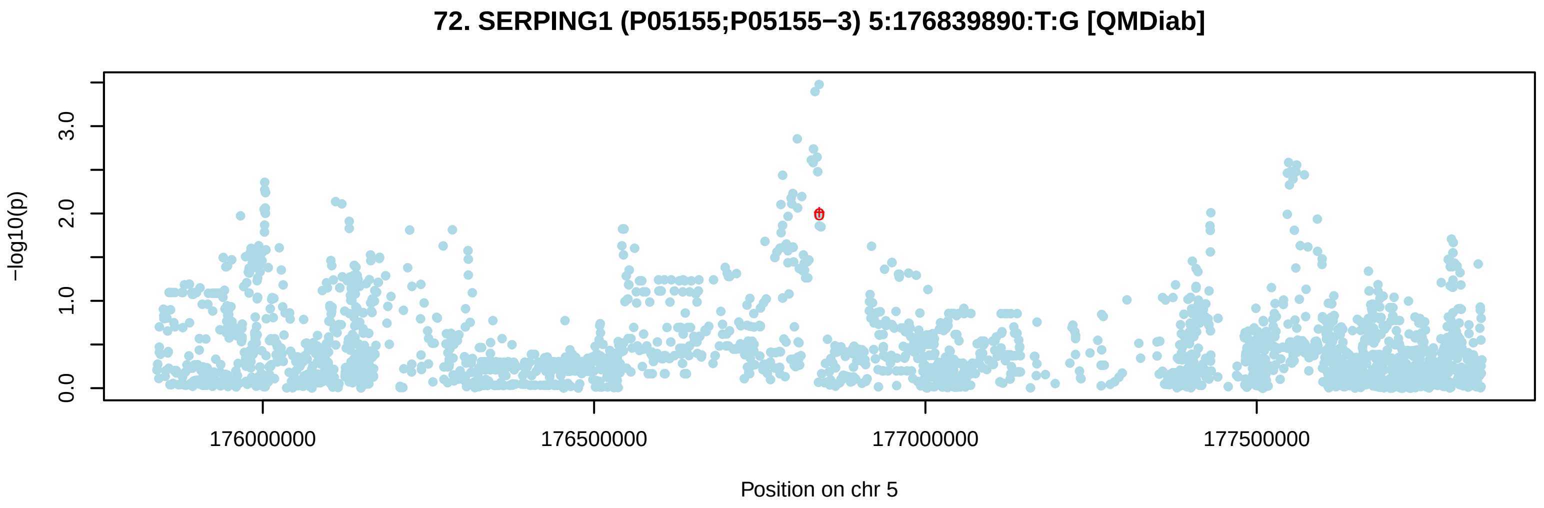
72. SERPING1 (P05155;P05155−3) 5:176839890:T:G [QMDiab]
−log10(p)
0.0
1.0
2.0
3.0
176000000
176500000
177000000
177500000
Position on chr 5

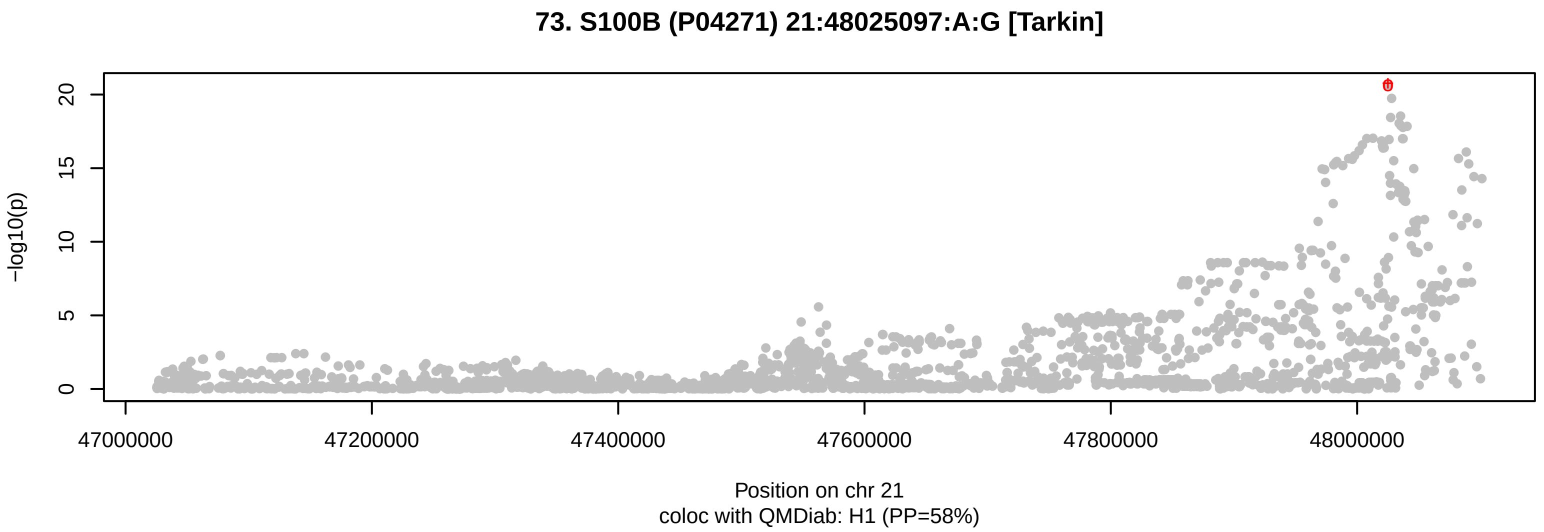

coloc with QMDiab: H1 (PP=58%)

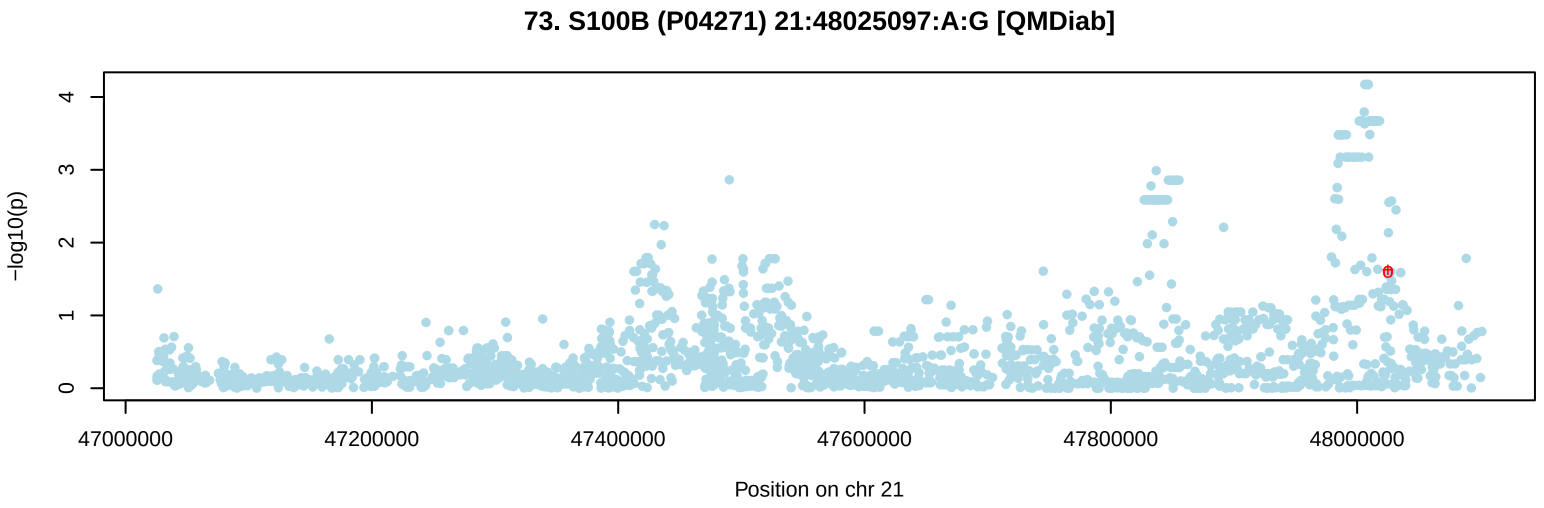

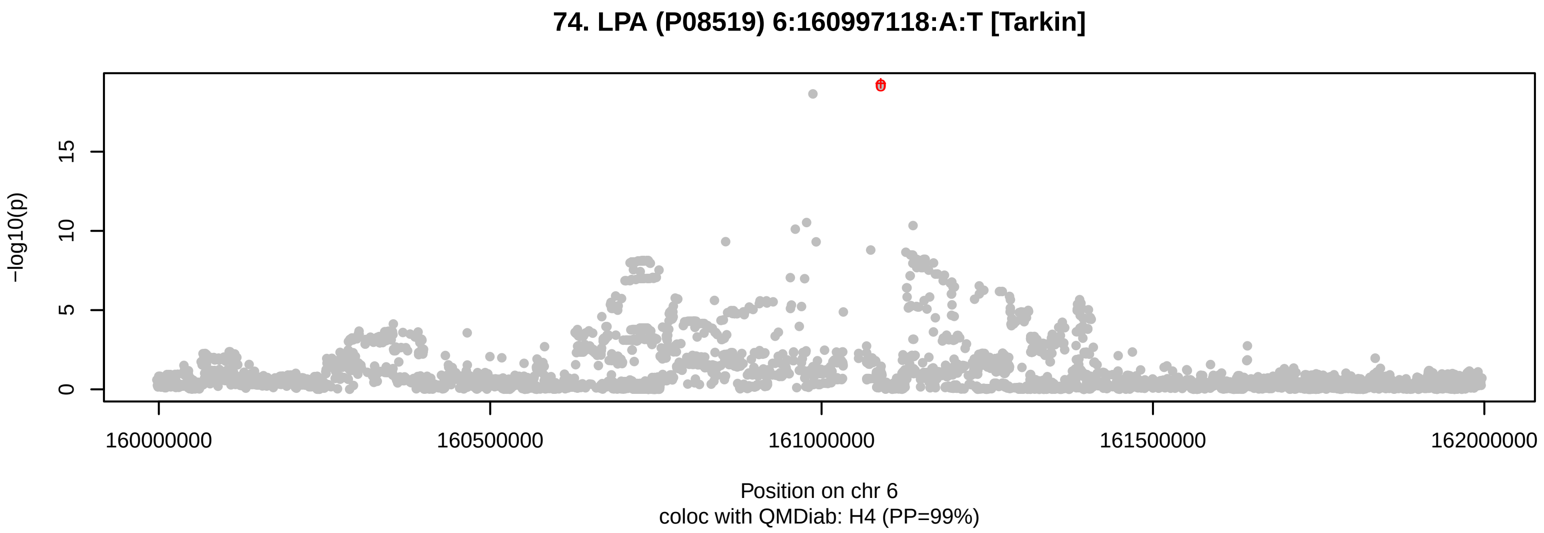
74. LPA (P08519) 6:160997118:A:T [Tarkin]
−log10(p)
0
5
10
15
160000000
160500000
161000000
161500000
162000000
Position on chr 6
coloc with QMDiab: H4 (PP=99%)

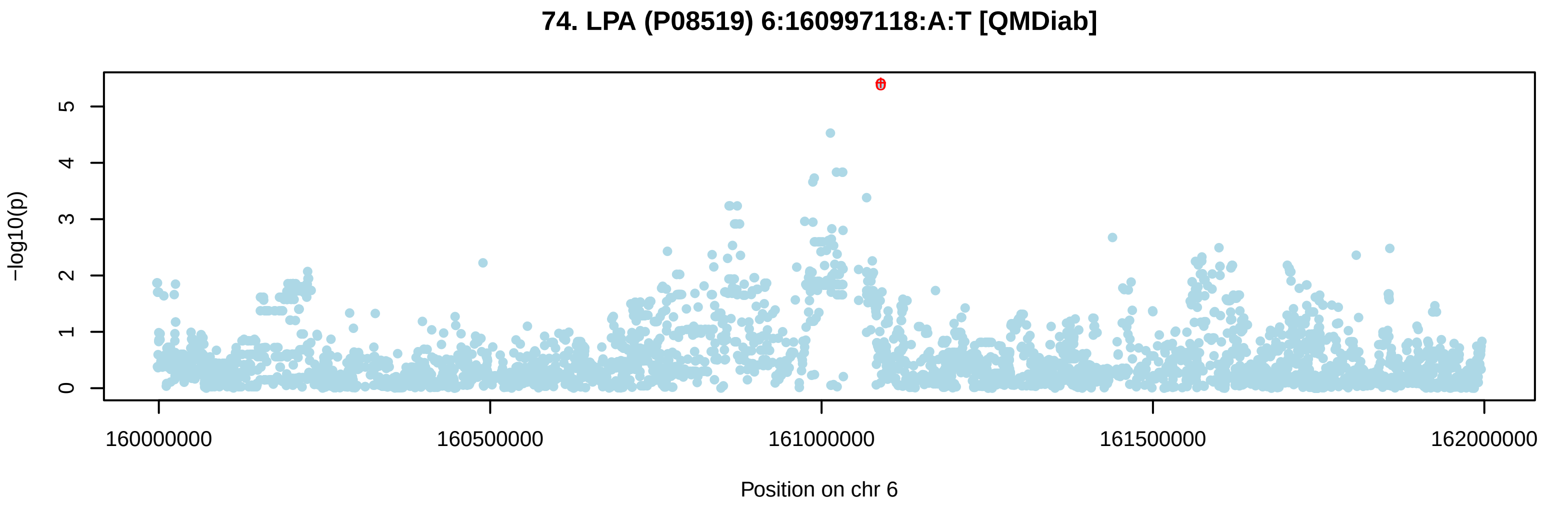
74. LPA (P08519) 6:160997118:A:T [QMDiab]
−log10(p)
0
1
2
3
4
5
160000000
160500000
161000000
161500000
162000000
Position on chr 6

75. OAF (Q86UD1) 11:120101092:A:T [Tarkin]

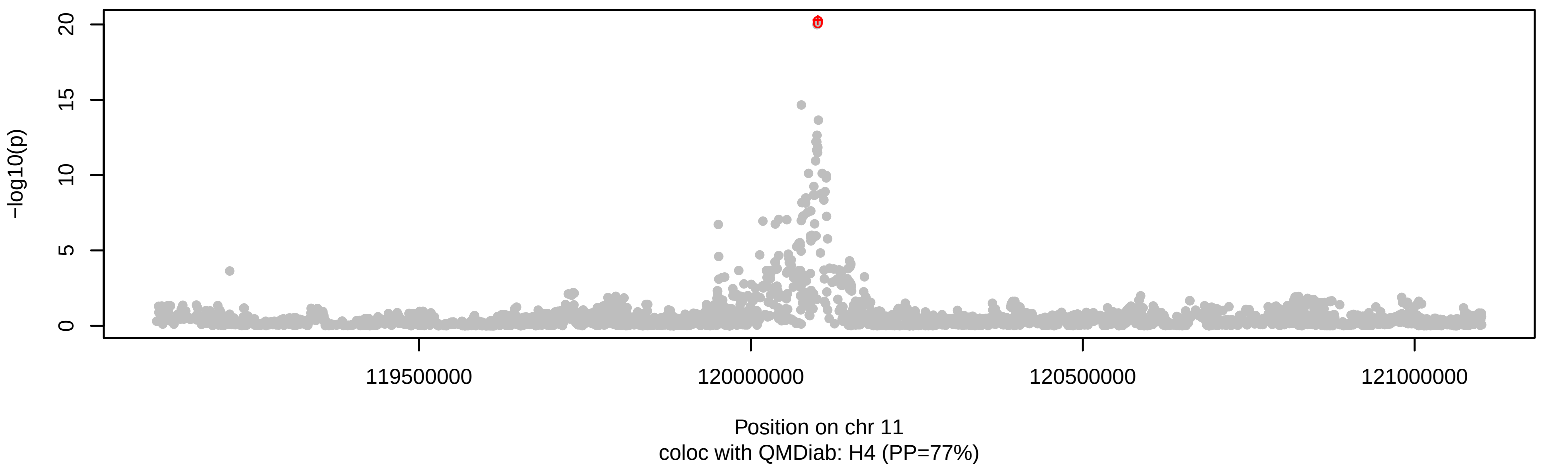

coloc with QMDiab: H4 (PP=77%)

75. OAF (Q86UD1) 11:120101092:A:T [QMDiab]

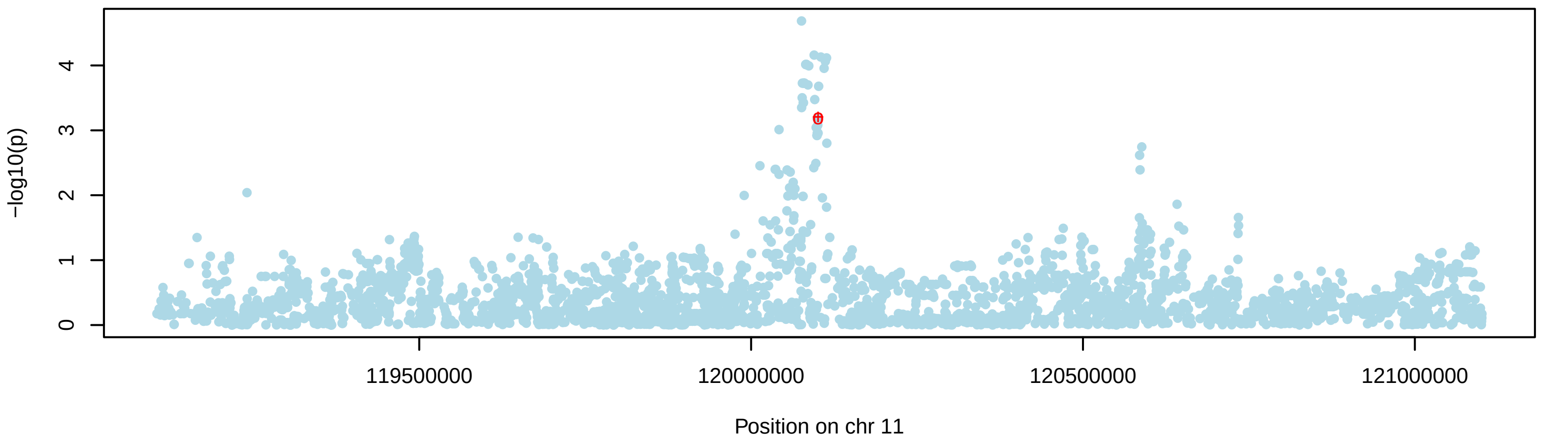

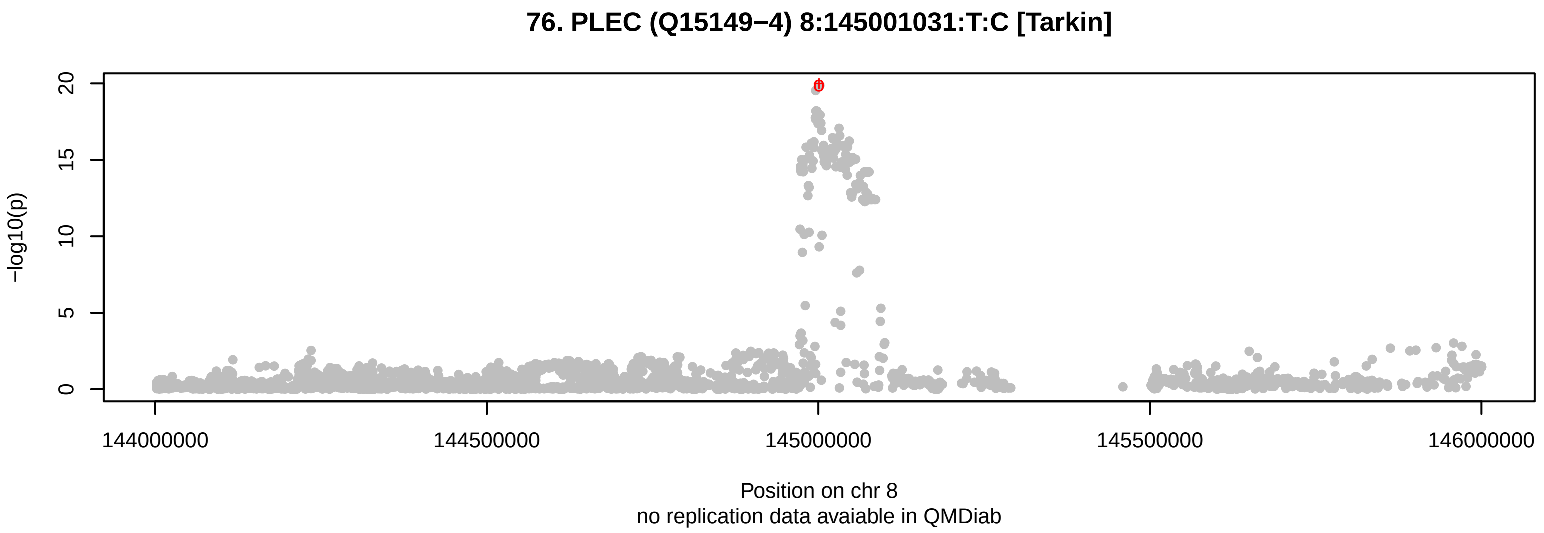

76. PLEC (Q15149−4) 8:145001031:T:C [Tarkin]
−log10(p)
0
5
10
15
20
144000000
144500000
145000000
145500000
146000000
Position on chr 8
no replication data avaiable in QMDiab

77. PON1 (P27169) 7:94953895:G:A [Tarkin]

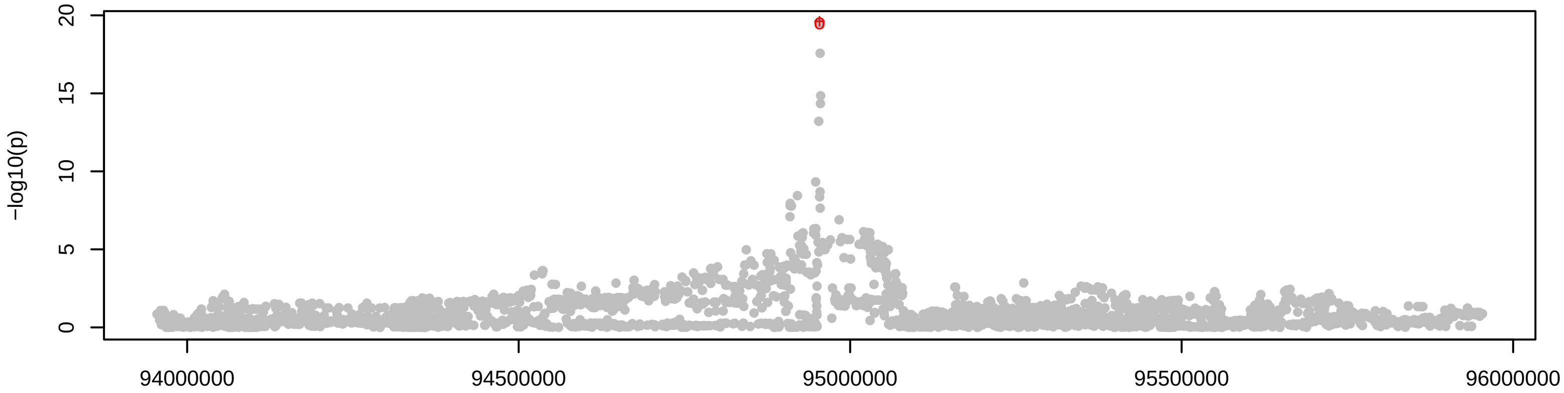

Position on chr 7
coloc with QMDiab: H3 (PP=100%)

77. PON1 (P27169) 7:94953895:G:A [QMDiab]

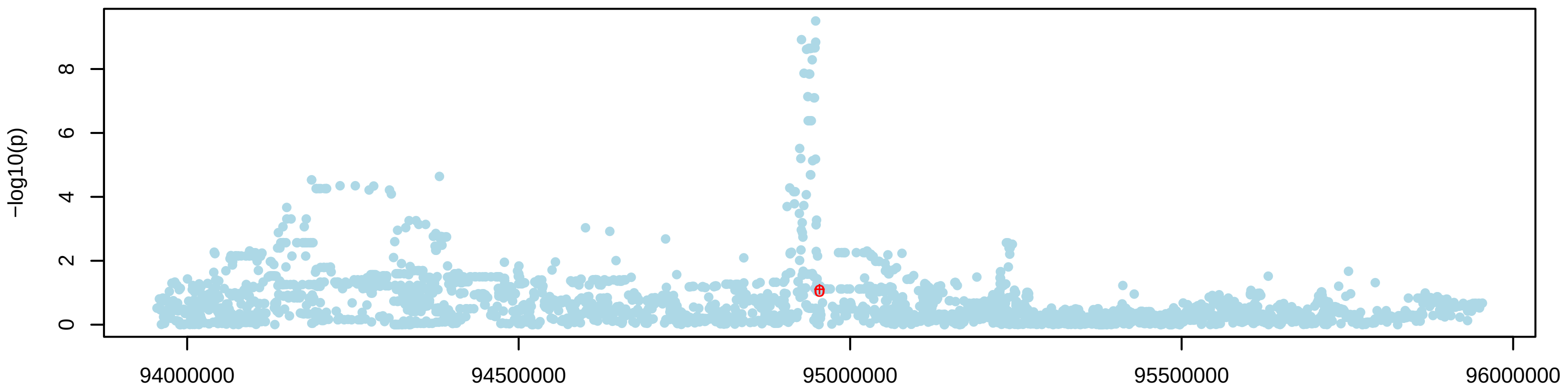

Position on chr 7

78. HEATR6 (Q6AI08) 1:196822368:A:G [Tarkin]

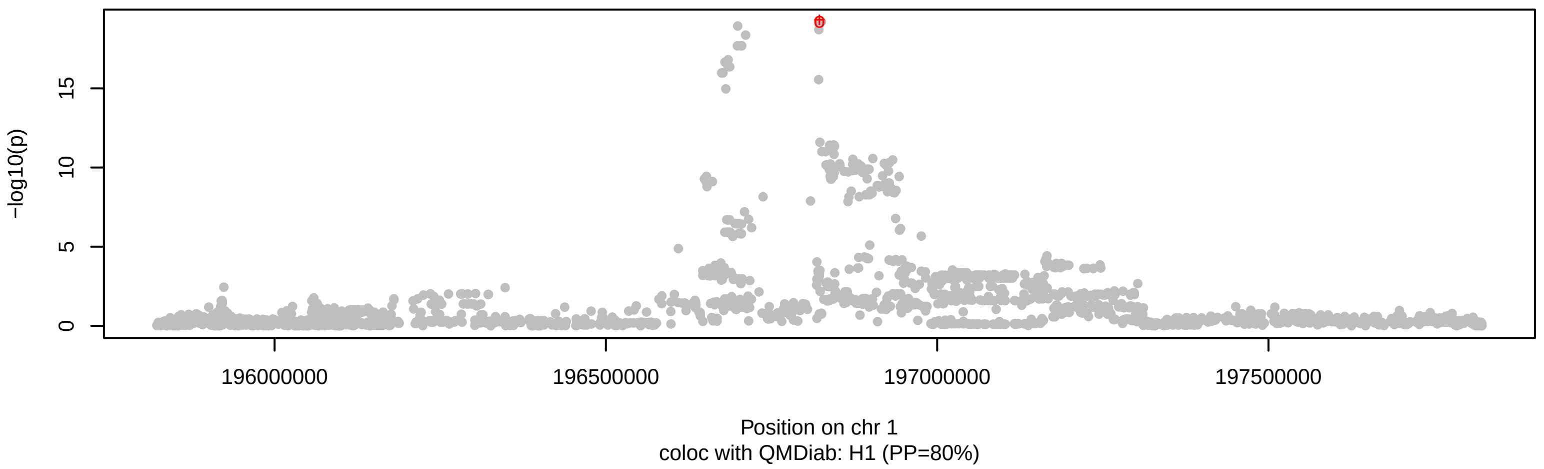

coloc with QMDiab: H1 (PP=80%)

78. HEATR6 (K7EIX2;Q6AI08) 1:196822368:A:G [QMDiab]

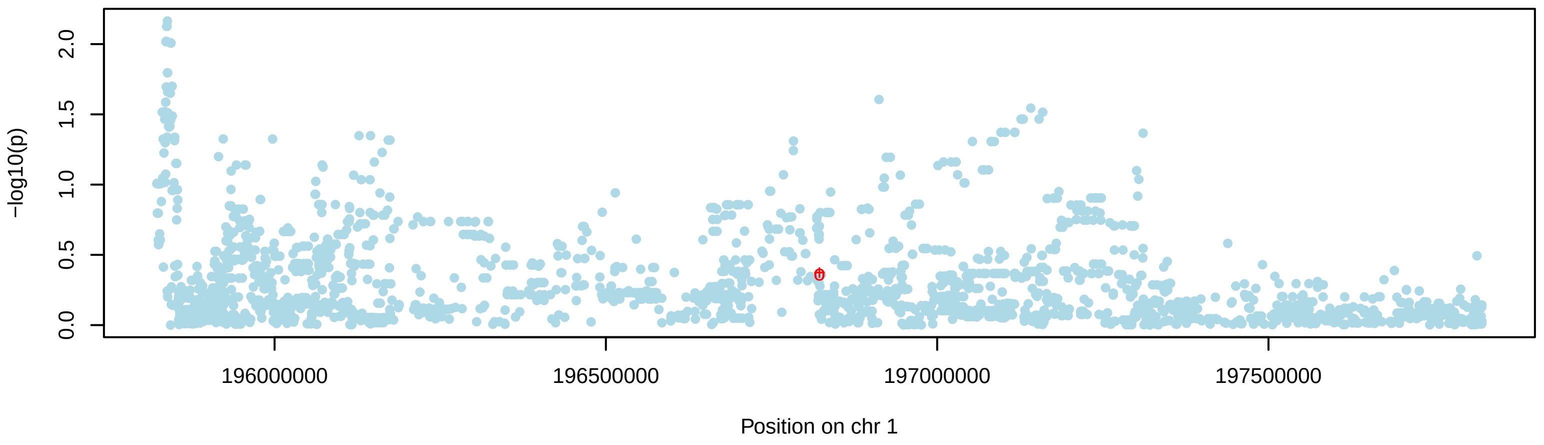

79. DYNC1H1 (Q14204) 1:248039294:G:A [Tarkin]

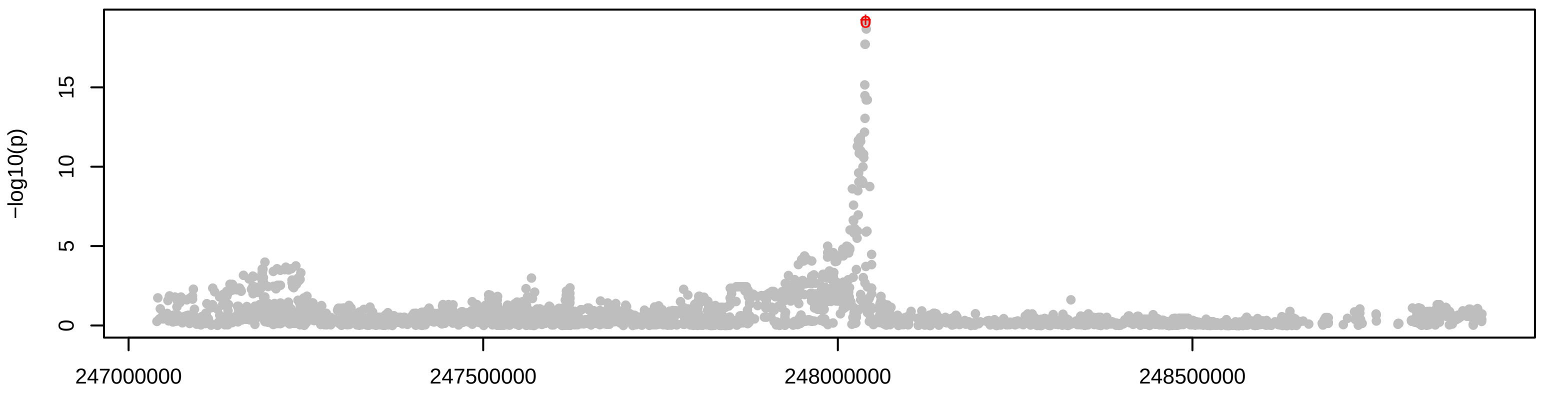

Position on chr 1
coloc with QMDiab: H4 (PP=44%)

79. DYNC1H1 (A0A2R8Y706;Q14204) 1:248039294:G:A [QMDiab]

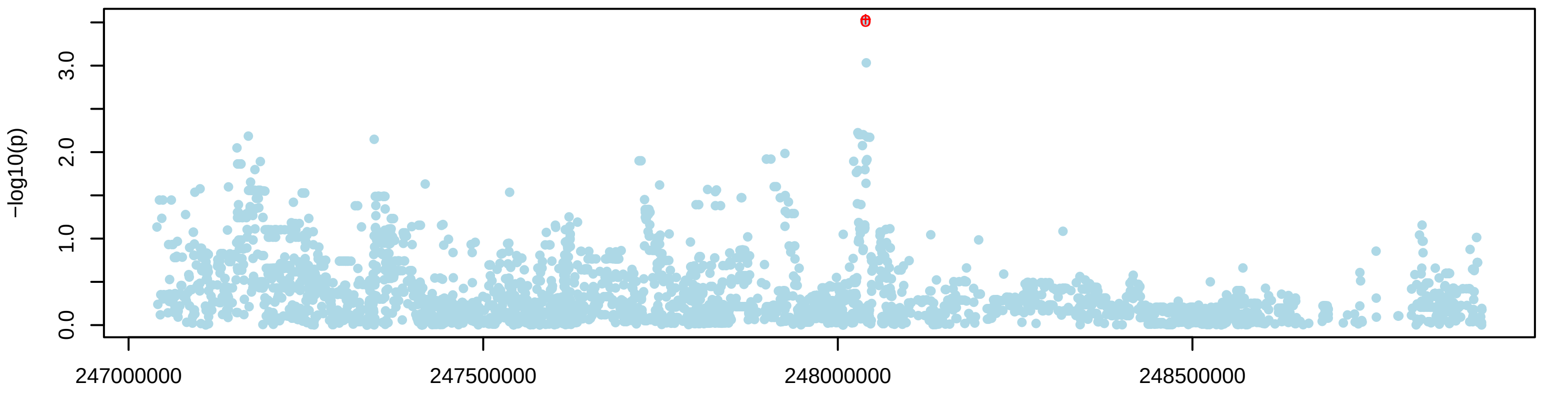

Position on chr 1

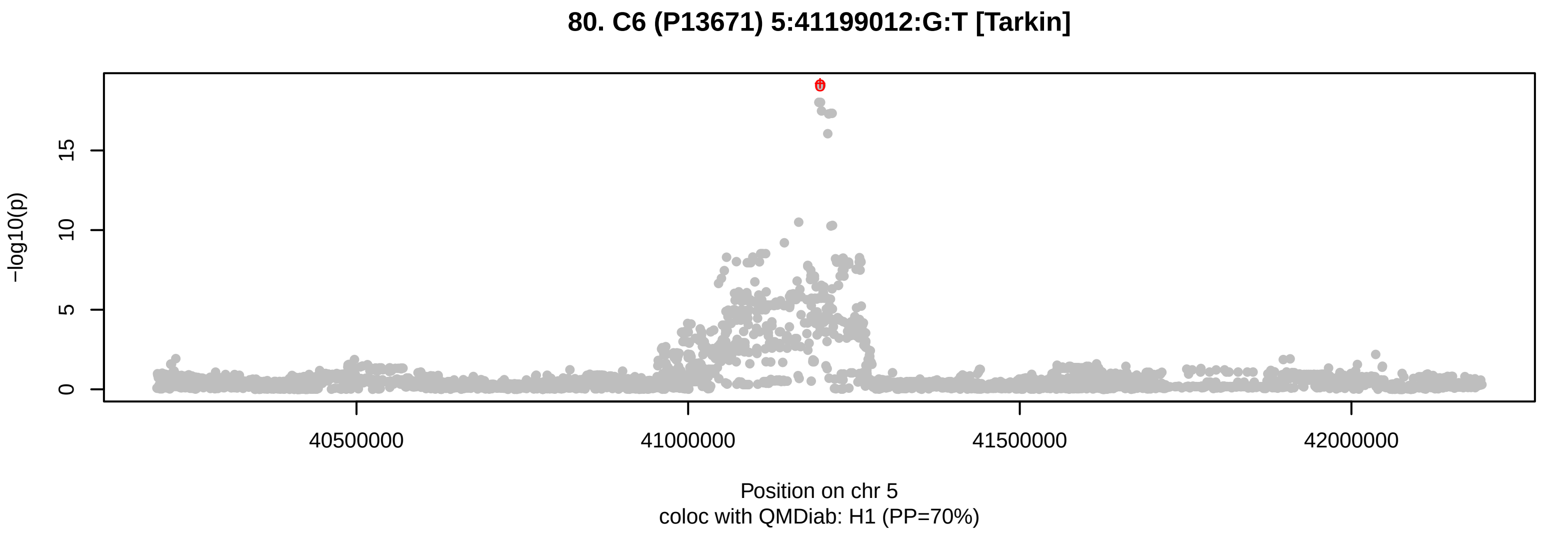
80. C6 (P13671) 5:41199012:G:T [Tarkin]
−log10(p)
0
5
10
15
40500000
41000000
41500000
42000000
Position on chr 5
coloc with QMDiab: H1 (PP=70%)

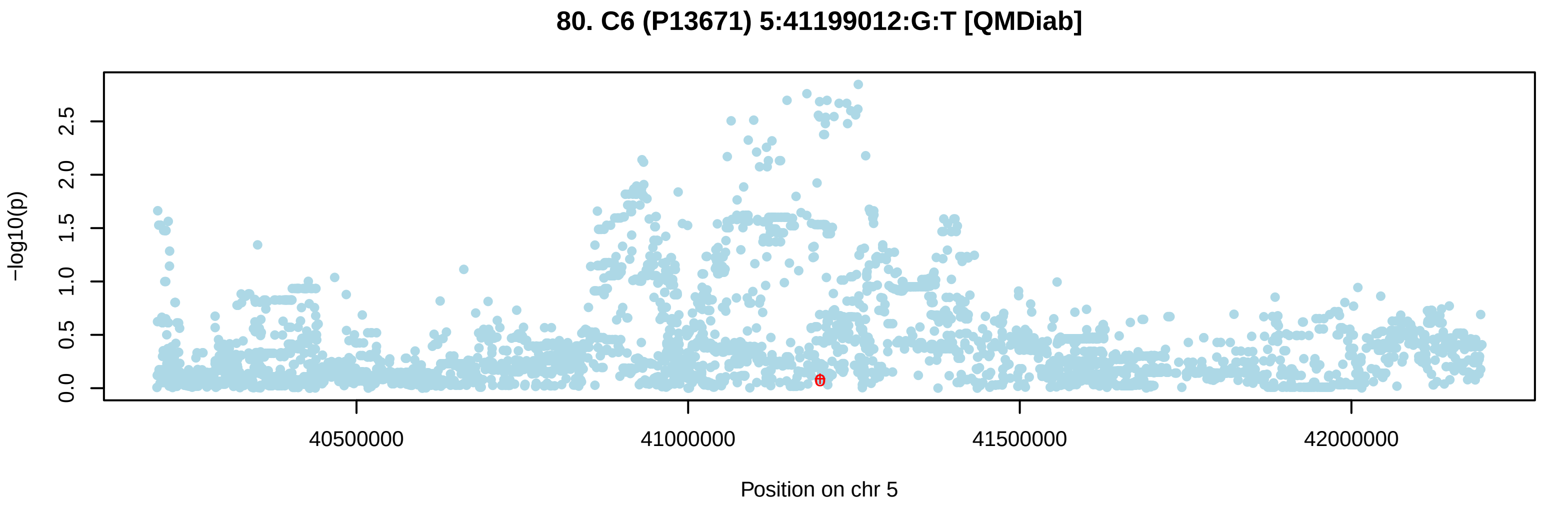
80. C6 (P13671) 5:41199012:G:T [QMDiab]
−log10(p)
0.0
0.5
1.0
1.5
2.0
2.5
40500000
41000000
41500000
42000000
Position on chr 5

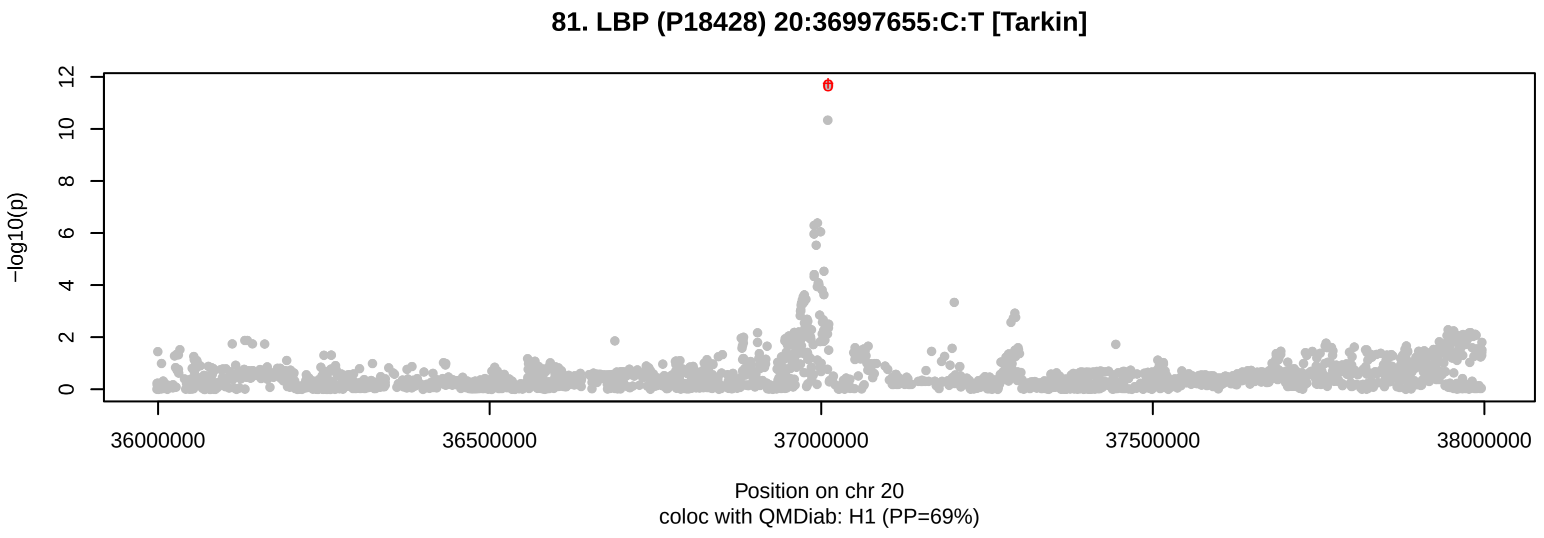
81. LBP (P18428) 20:36997655:C:T [Tarkin]
−log10(p)
0
2
4
6
8
10
12
36000000
36500000
37000000
37500000
38000000
Position on chr 20
coloc with QMDiab: H1 (PP=69%)

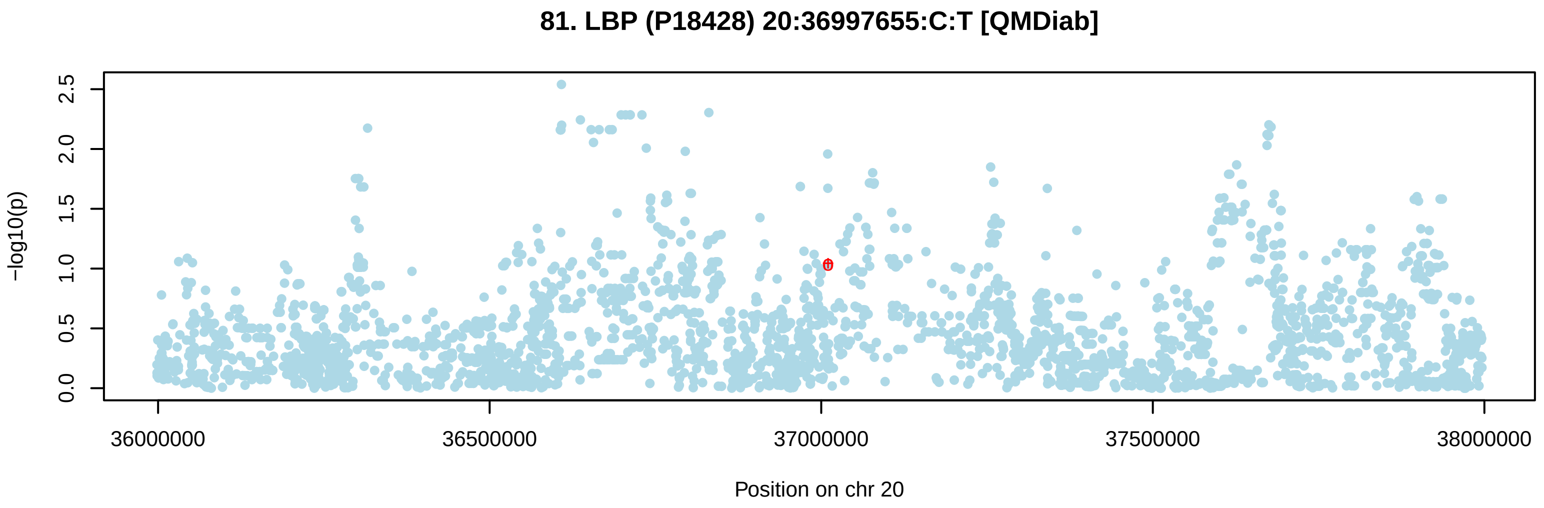
81. LBP (P18428) 20:36997655:C:T [QMDiab]
−log10(p)
0.0
0.5
1.0
1.5
2.0
2.5
36000000
36500000
37000000
37500000
38000000
Position on chr 20

82. FBXO7 (Q9Y3I1) 22:32871227:C:T [Tarkin]

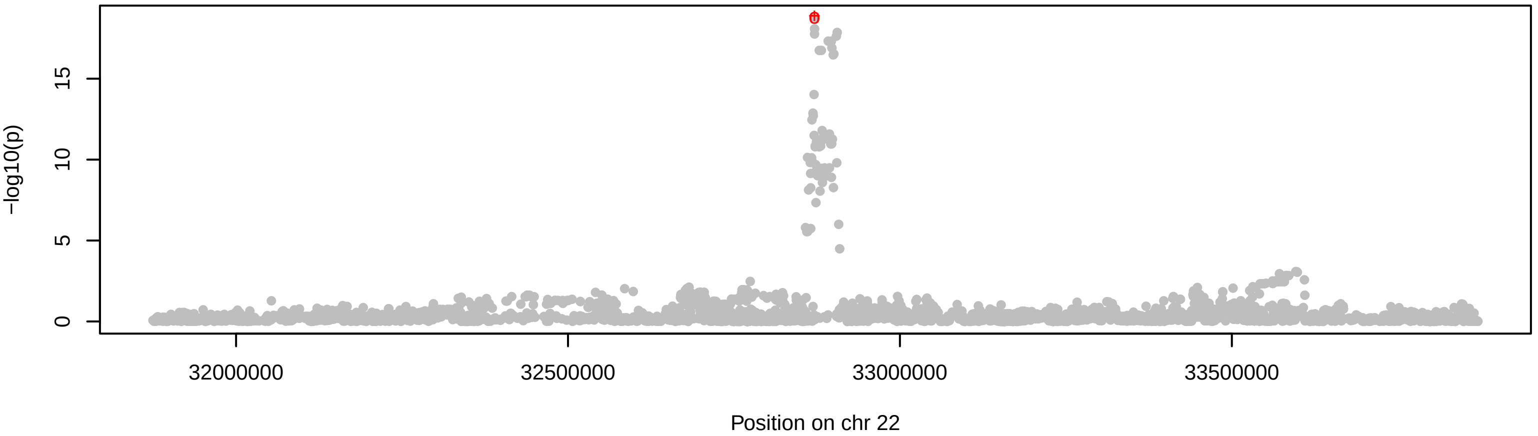

coloc with QMDiab: H1 (PP=44%)

82. FBXO7 (Q9Y3I1;Q9Y3I1−2;Q9Y3I1−3) 22:32871227:C:T [QMDiab]

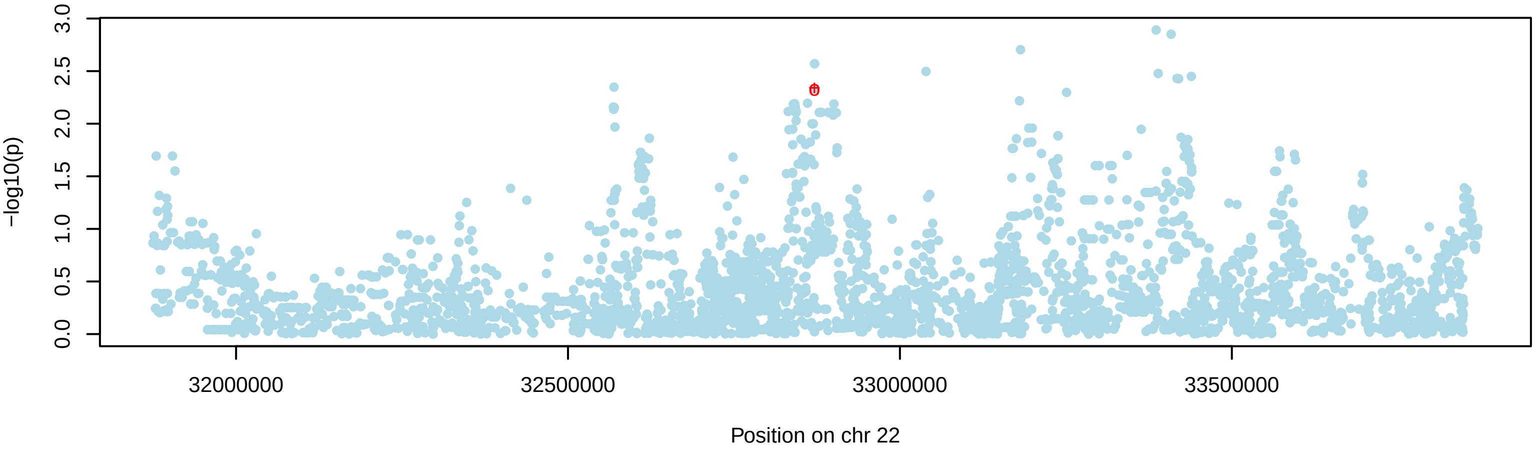

83. MASP2 (O00187−2) 1:11104845:T:C [Tarkin]

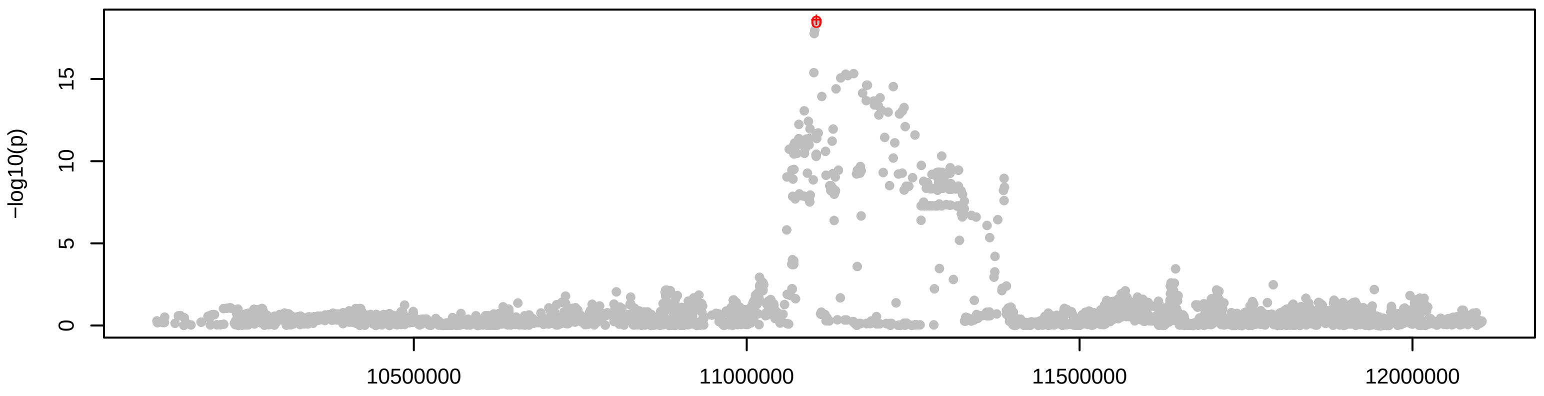

Position on chr 1
coloc with QMDiab: H1 (PP=62%)

83. MASP2 (O00187−2) 1:11104845:T:C [QMDiab]

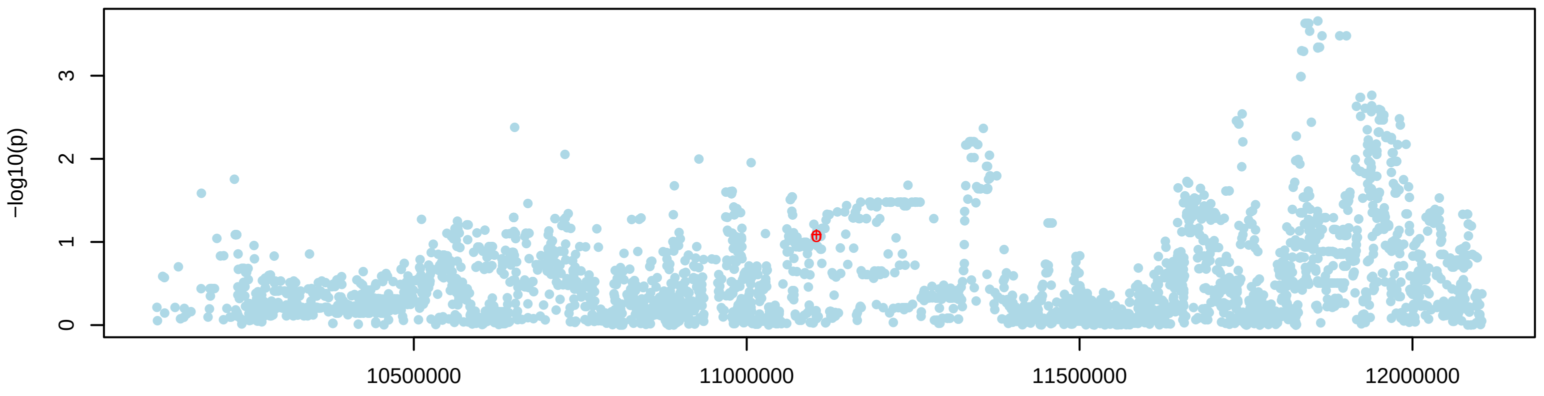

Position on chr 1

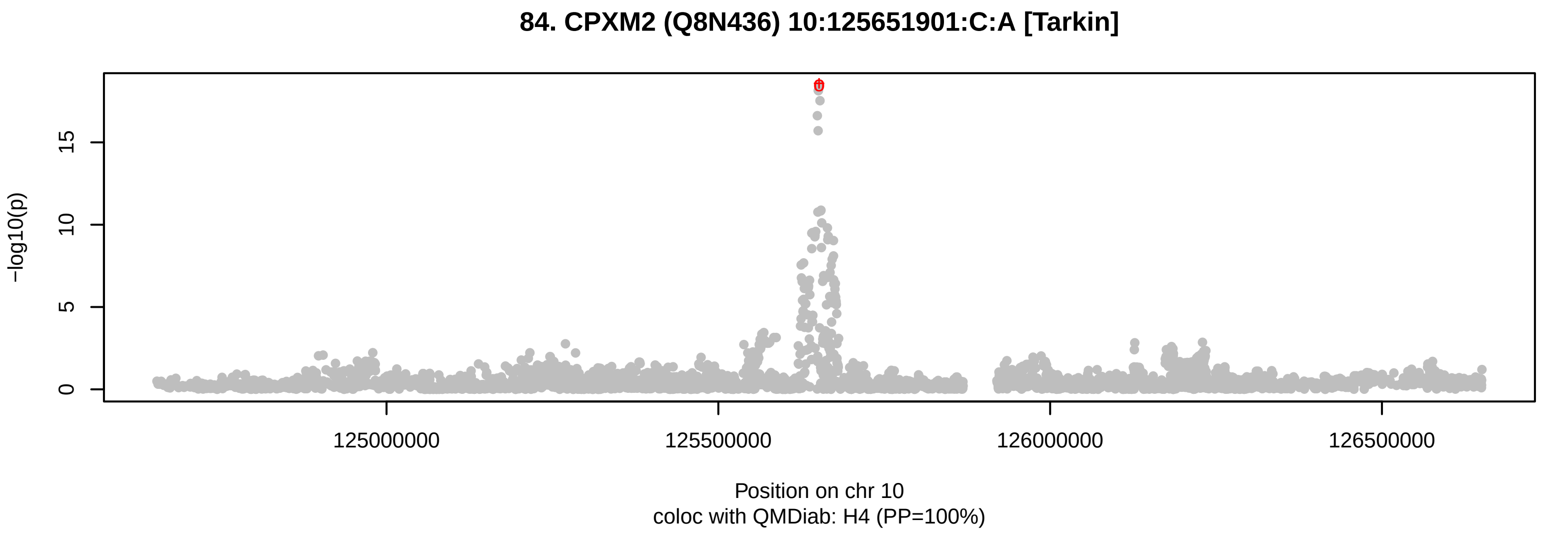
84. CPXM2 (Q8N436) 10:125651901:C:A [Tarkin]
−log10(p)
0
5
10
15
125000000
125500000
126000000
126500000
Position on chr 10
coloc with QMDiab: H4 (PP=100%)

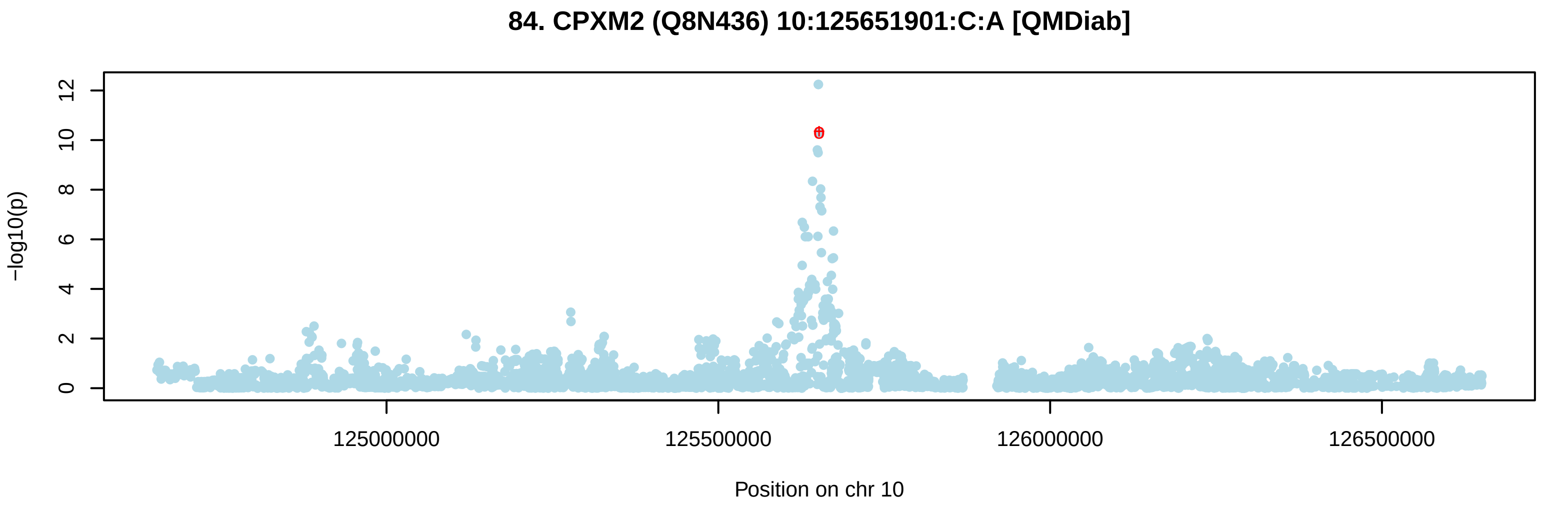
84. CPXM2 (Q8N436) 10:125651901:C:A [QMDiab]
−log10(p)
0
2
4
6
8
10
12
125000000
125500000
126000000
126500000
Position on chr 10

85. KRT13 (CONP13646−1) 13:113800622:T:C [Tarkin]

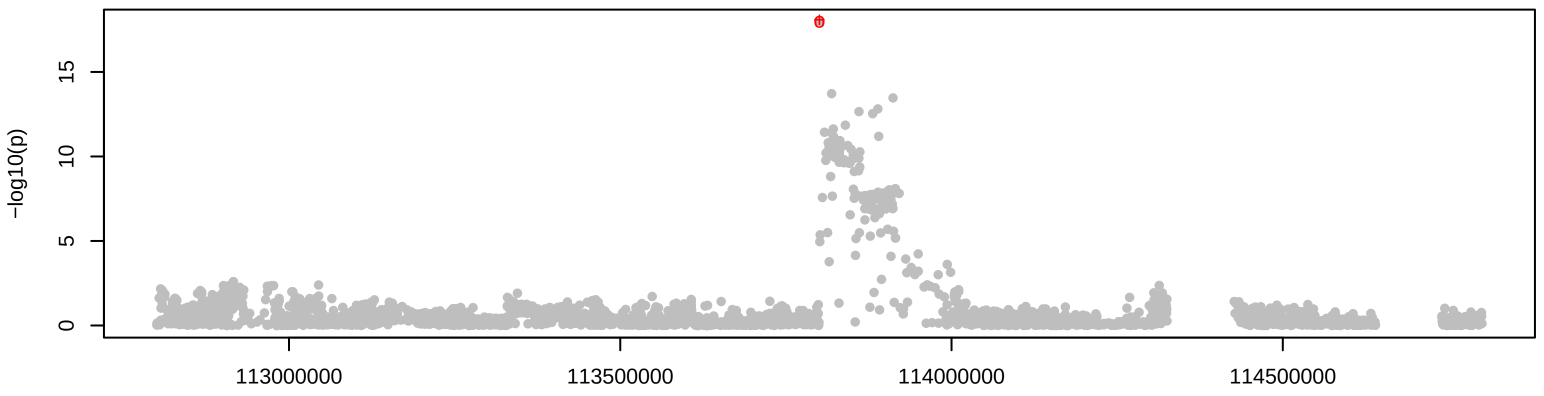

Position on chr 13
no replication data avaiable in QMDiab

86. MMP3 (P08254) 11:102687418:T:C [Tarkin]

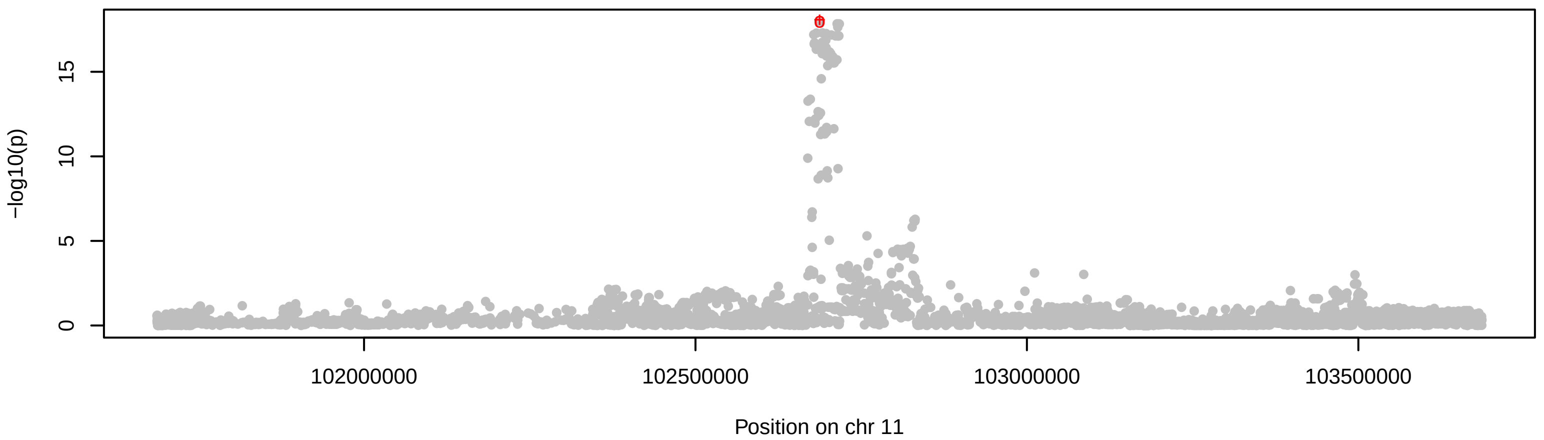

coloc with QMDiab: H1 (PP=65%)

86. MMP3 (P08254) 11:102687418:T:C [QMDiab]

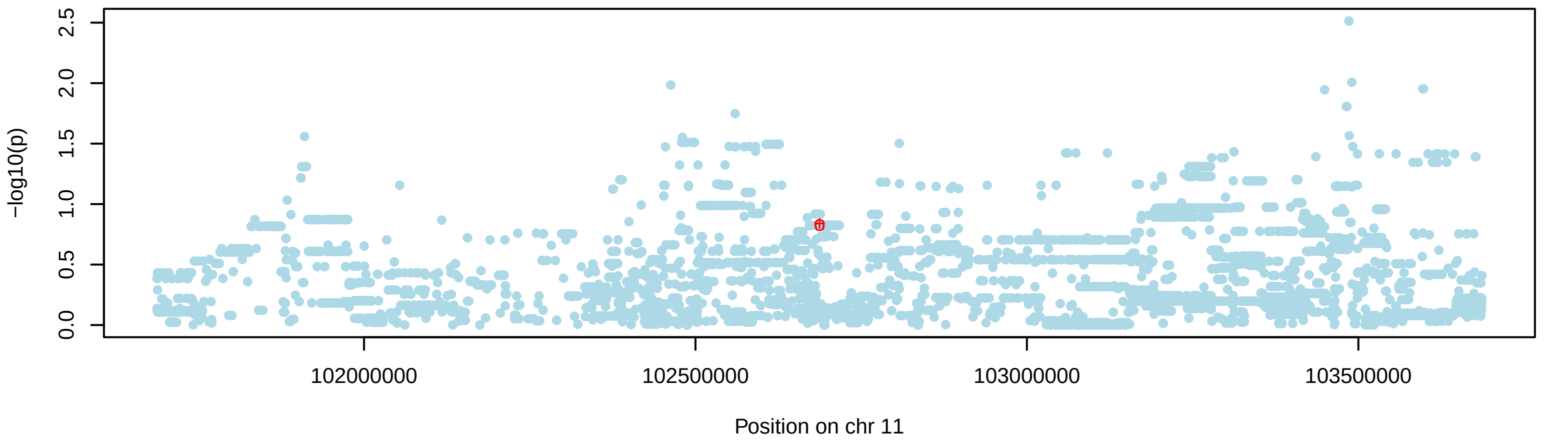

87. COLEC10 (Q9Y6Z7) 2:3640142:C:T [Tarkin]

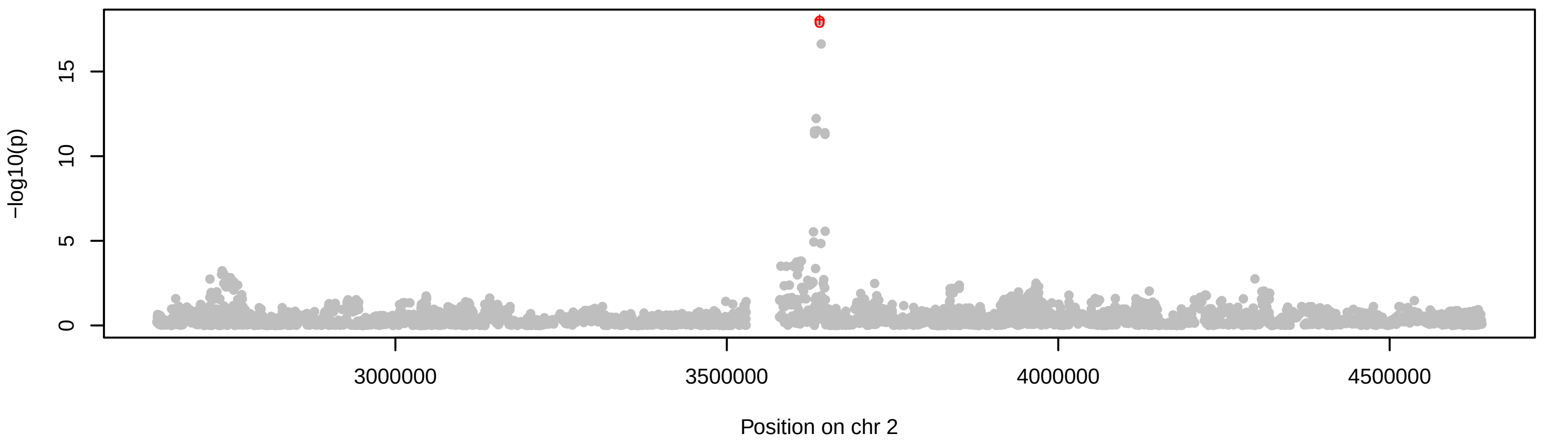

coloc with QMDiab: H4 (PP=51%)

87. COLEC10 (Q9Y6Z7) 2:3640142:C:T [QMDiab]

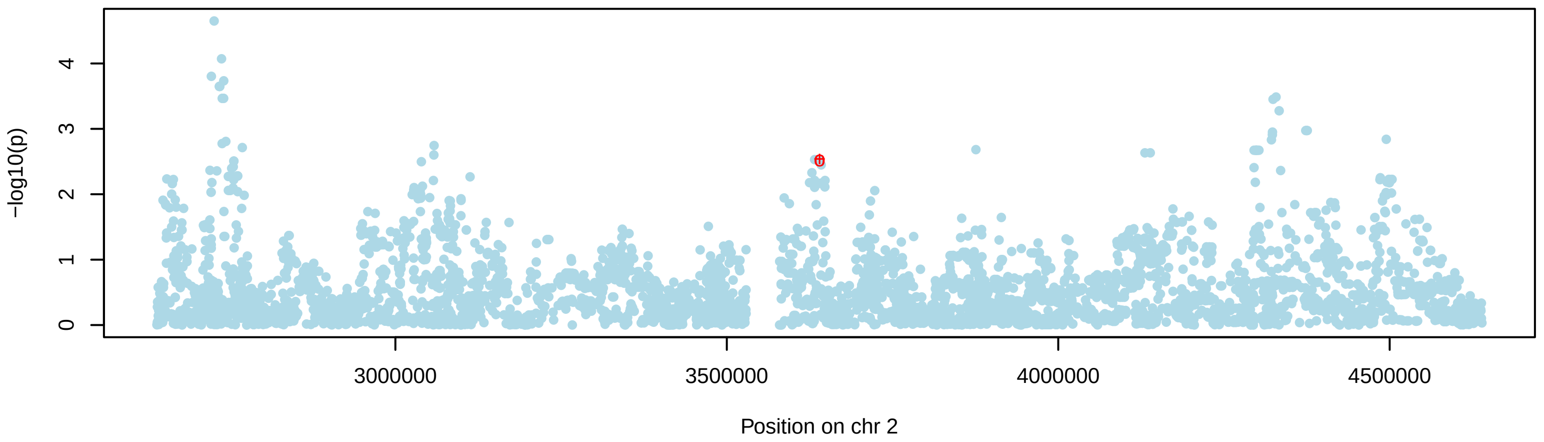

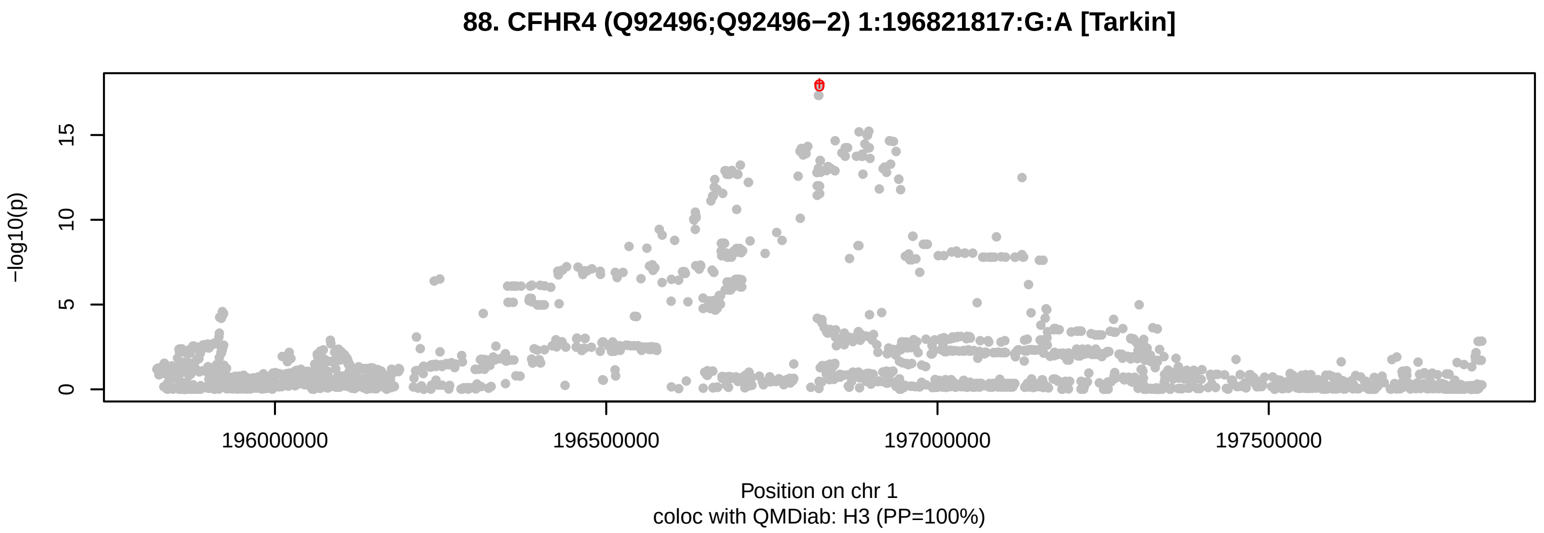
88. CFHR4 (Q92496;Q92496−2) 1:196821817:G:A [Tarkin]
−log10(p)
0
5
10
15
196000000
196500000
197000000
197500000
Position on chr 1
coloc with QMDiab: H3 (PP=100%)

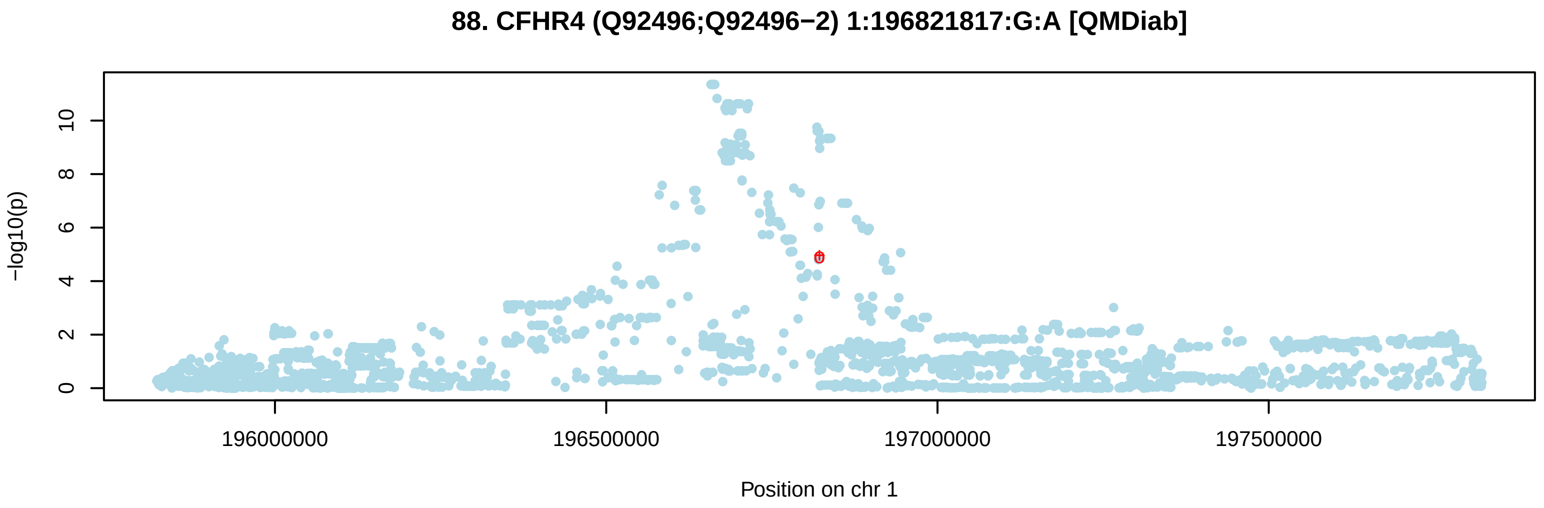
88. CFHR4 (Q92496;Q92496−2) 1:196821817:G:A [QMDiab]
−log10(p)
0
2
4
6
8
10
196000000
196500000
197000000
197500000
Position on chr 1

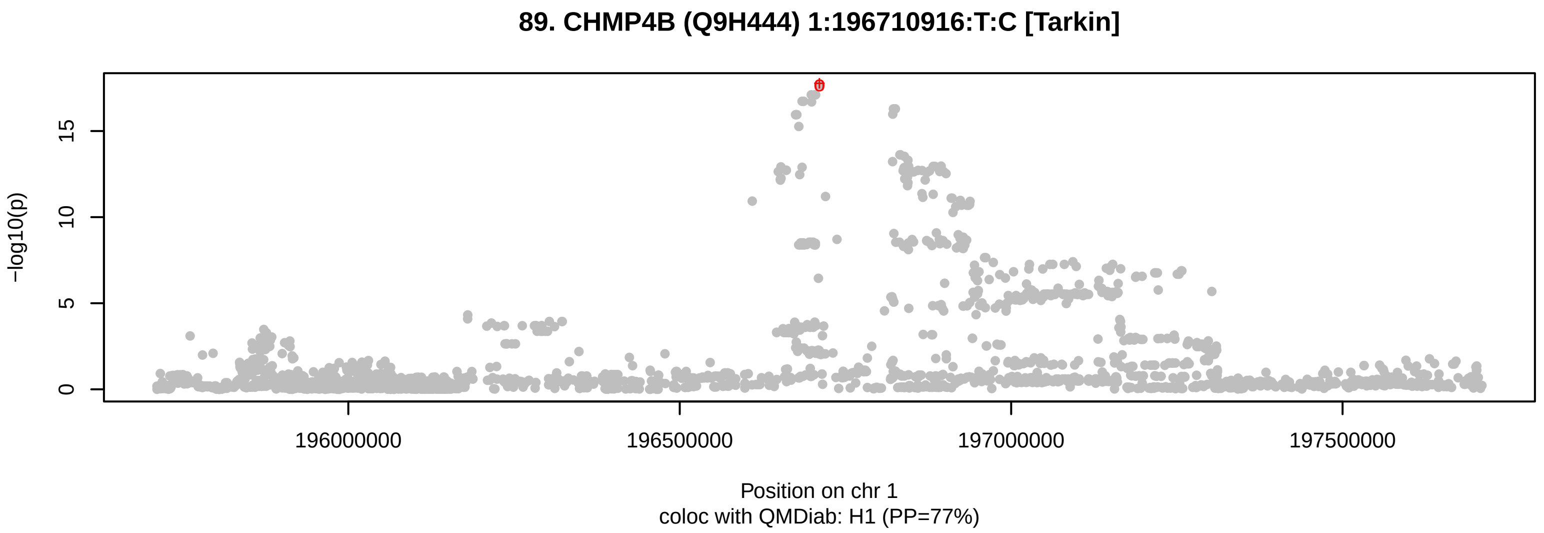
89. CHMP4B (Q9H444) 1:196710916:T:C [Tarkin]
−log10(p)
0
5
10
15
196000000
196500000
197000000
197500000
Position on chr 1
coloc with QMDiab: H1 (PP=77%)

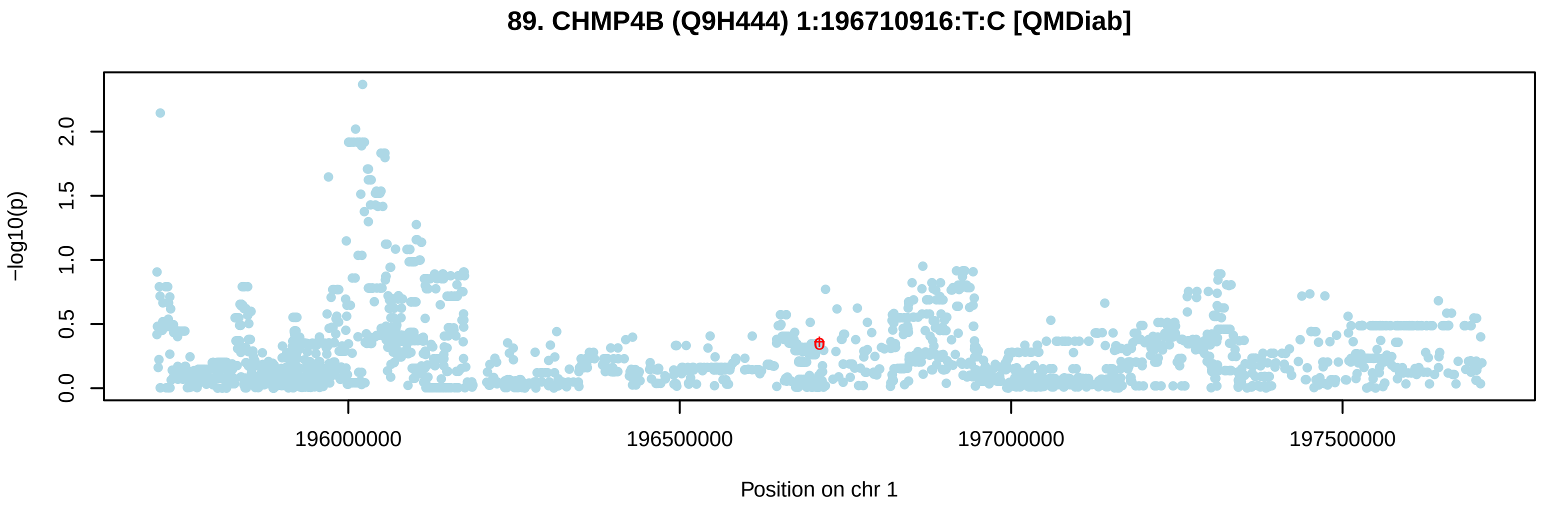
89. CHMP4B (Q9H444) 1:196710916:T:C [QMDiab]
−log10(p)
0.0
0.5
1.0
1.5
2.0
196000000
196500000
197000000
197500000
Position on chr 1

90. EMC1 (A0A8I5KW55;Q8N766;Q8N766−2) 20:36997655:C:T [Tarkin]

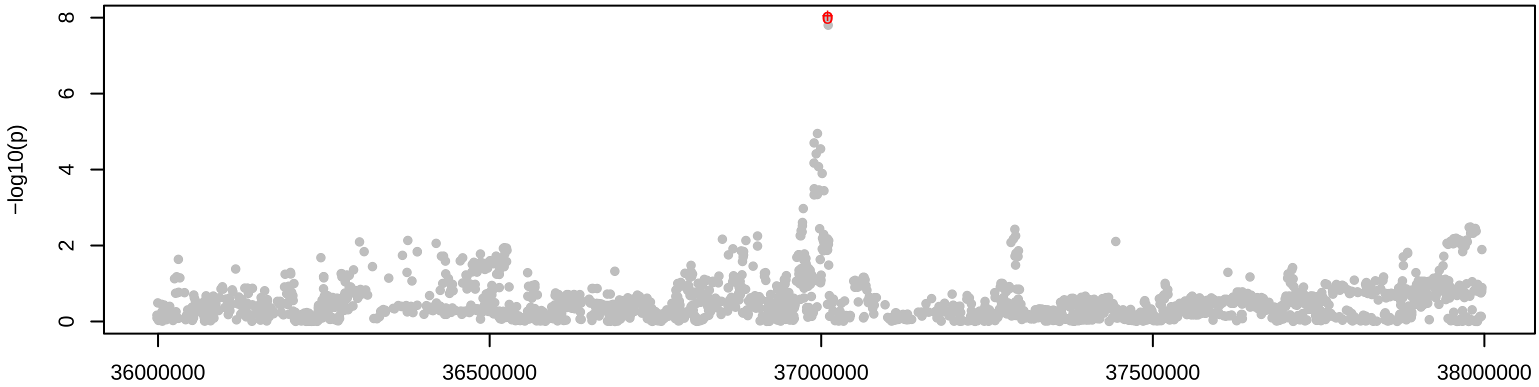

Position on chr 20
coloc with QMDiab: H1 (PP=72%)

90. EMC1 (A0A8I5KW55;H7C5A2;Q8N766;Q8N766−2) 20:36997655:C:T [QMDiab]

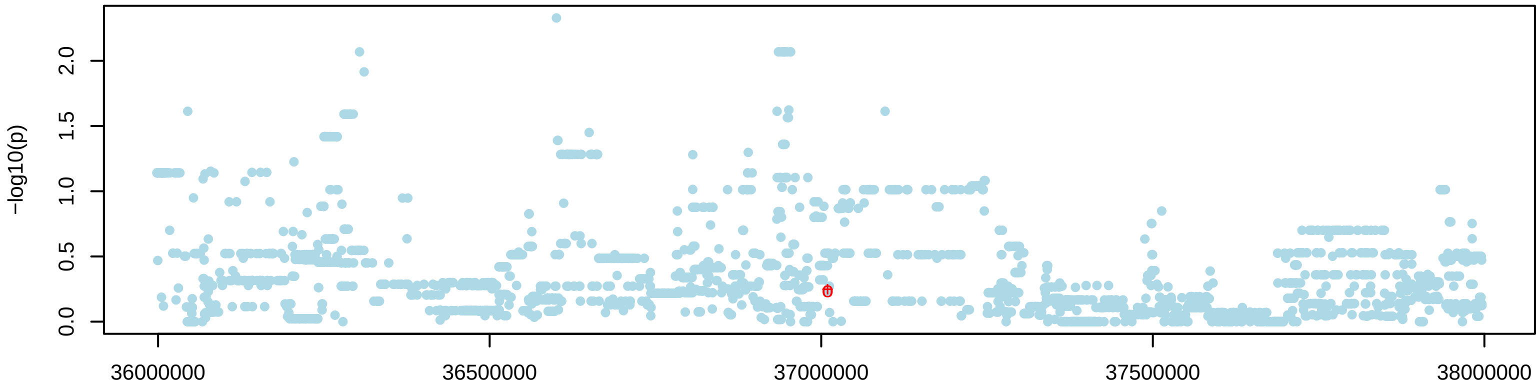

Position on chr 20

91. ADAMDEC1 (O15204) 8:24240670:T:C [Tarkin]

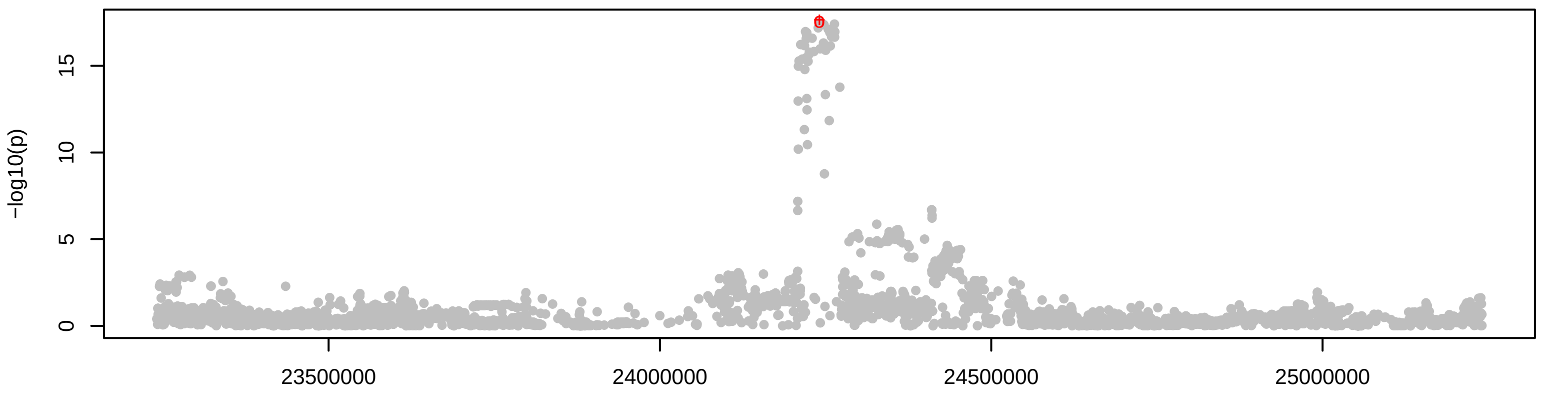

coloc with QMDiab: H1 (PP=68%)

91. ADAMDEC1 (O15204;O15204−2) 8:24240670:T:C [QMDiab]

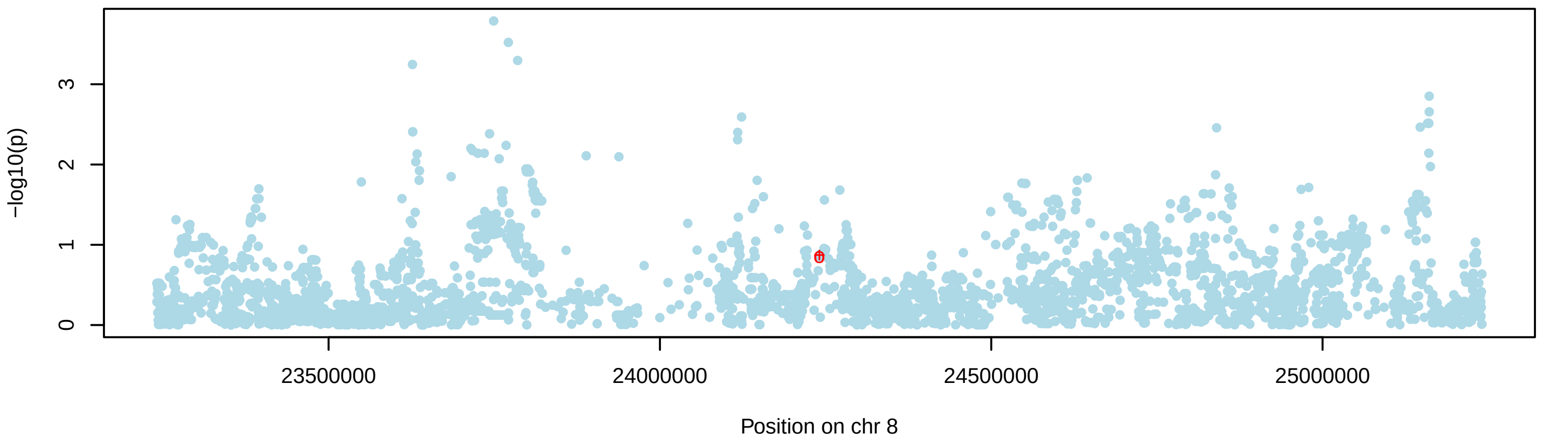

92. BPIFB1 (Q8TDL5) 20:31688868:G:C [Tarkin]

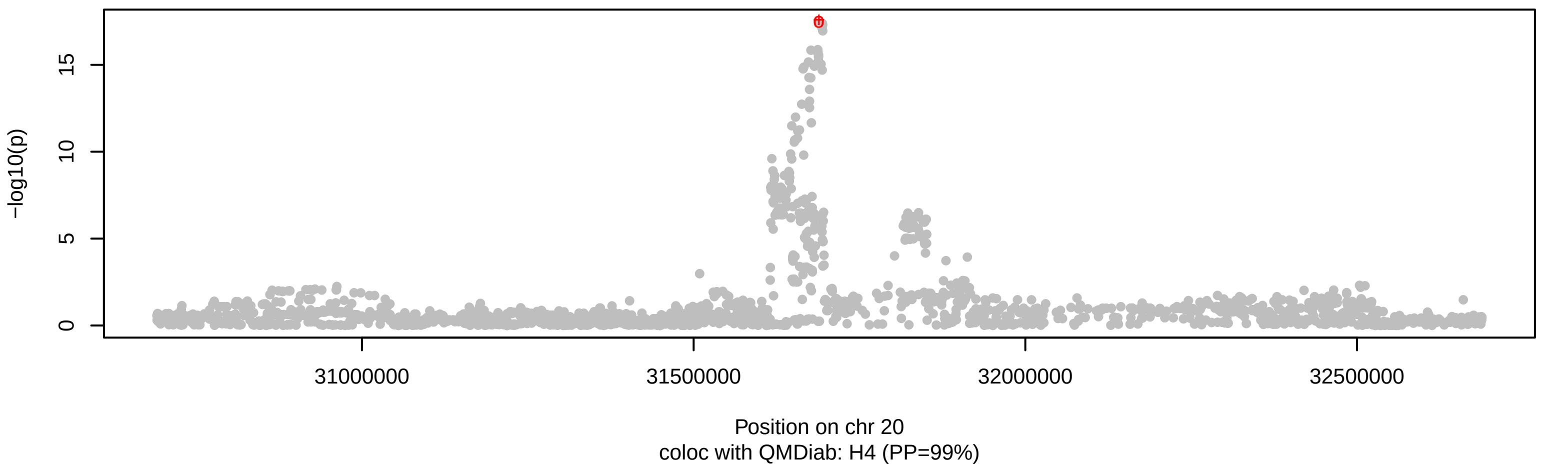

92. BPIFB1 (Q8TDL5) 20:31688868:G:C [QMDiab]

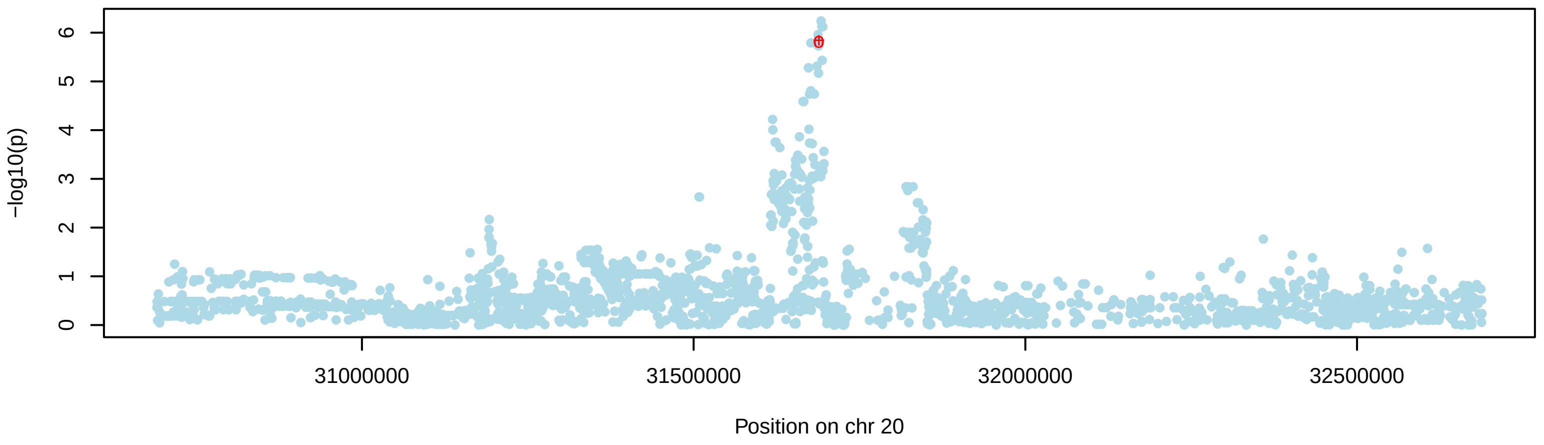

93. APMAP (Q9HDC9) 20:24949944:G:C [Tarkin]

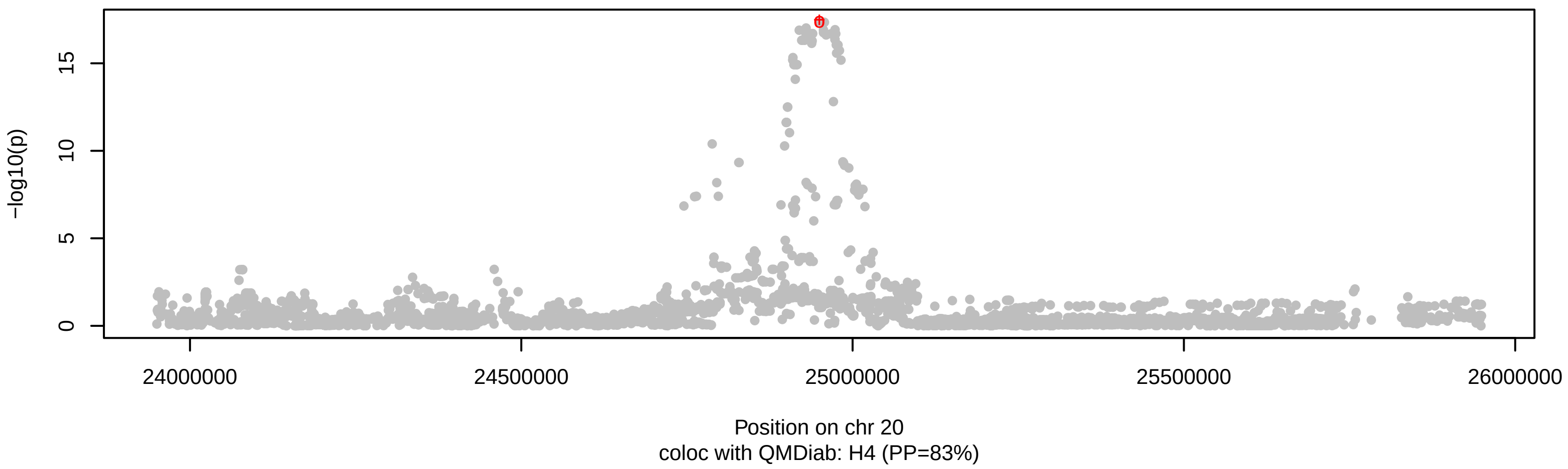

coloc with QMDiab: H4 (PP=83%)

93. APMAP (Q9HDC9) 20:24949944:G:C [QMDiab]

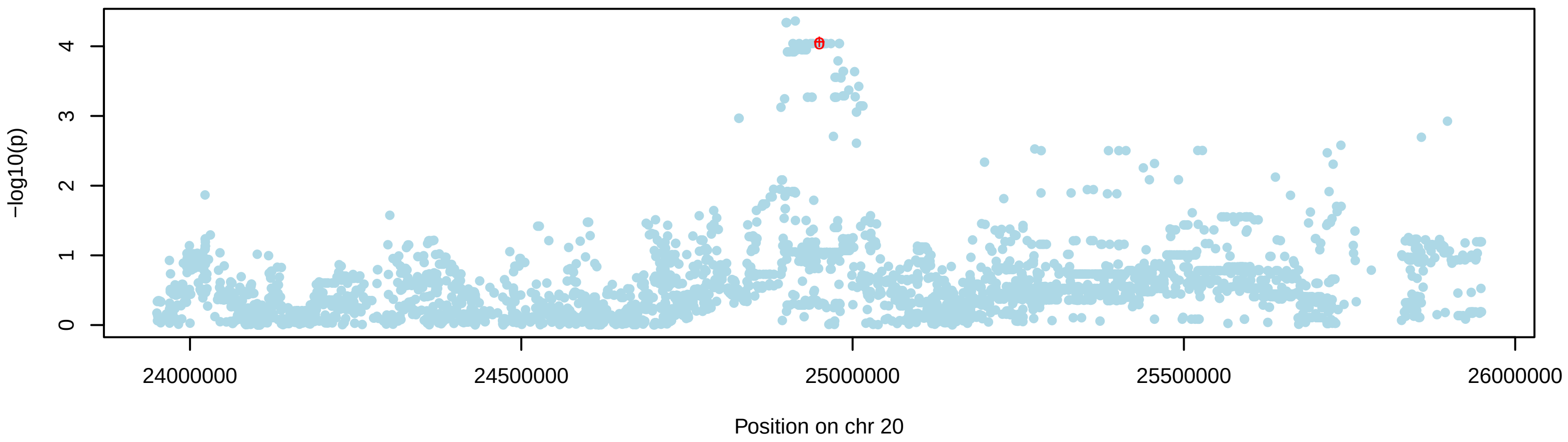

94. C19orf33 (Q9GZP8) 19:38795250:G:A [Tarkin]

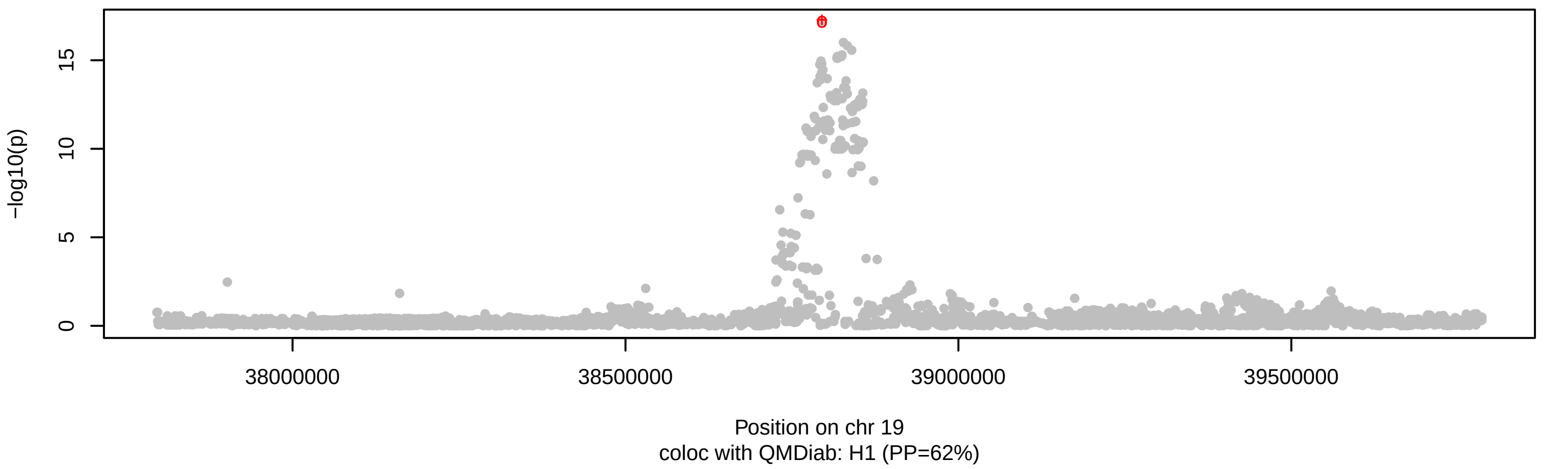

coloc with QMDiab: H1 (PP=62%)

94. C19orf33 (Q9GZP8) 19:38795250:G:A [QMDiab]

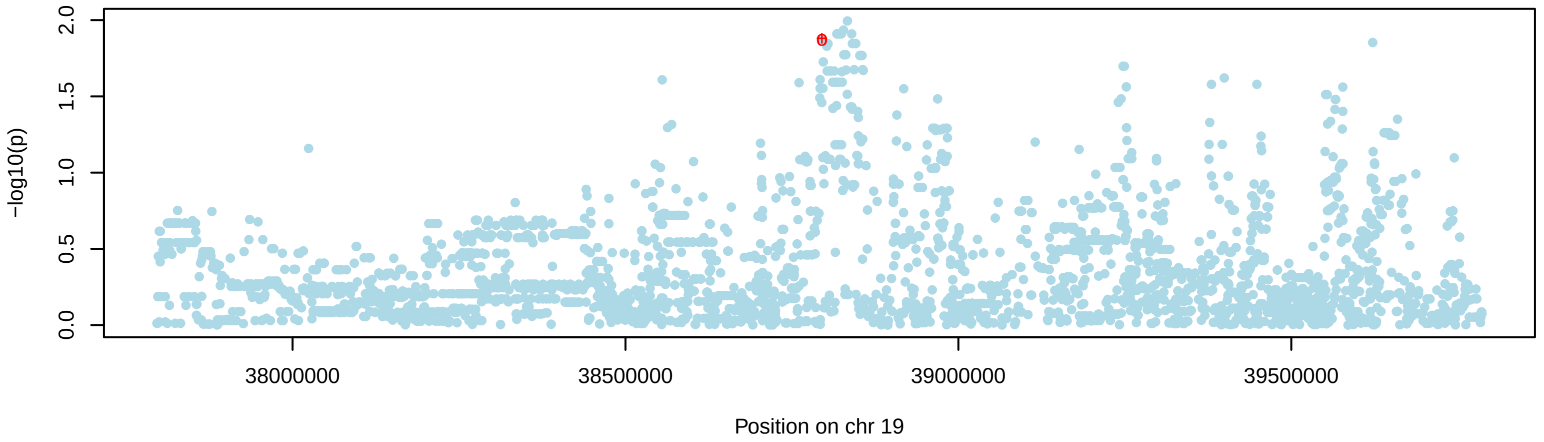

95. ITGA2 (P17301) 5:52371370:C:T [Tarkin]

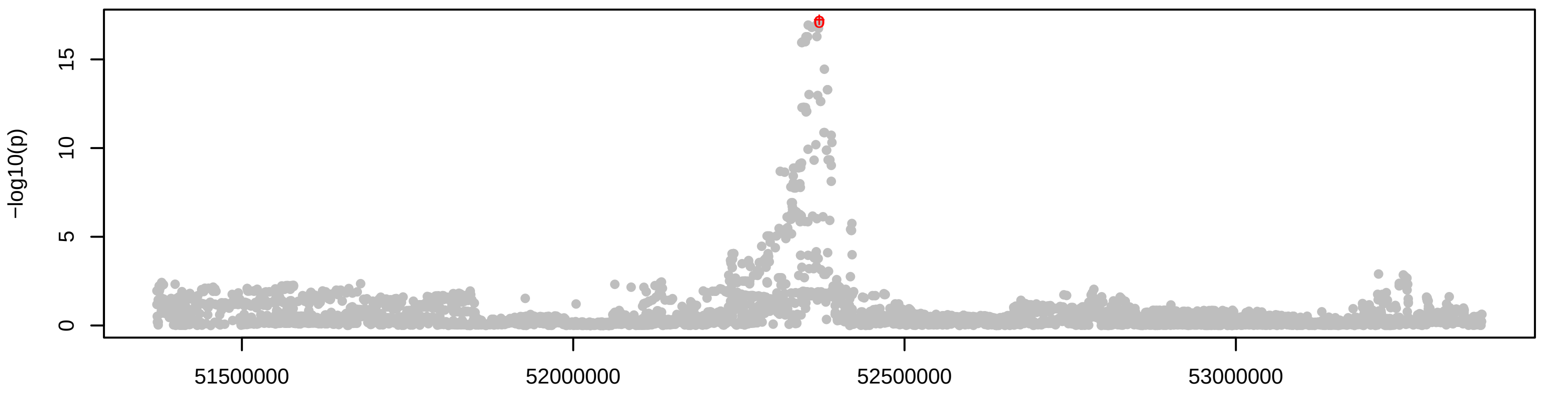

Position on chr 5
coloc with QMDiab: H1 (PP=52%)

95. ITGA2 (P17301) 5:52371370:C:T [QMDiab]

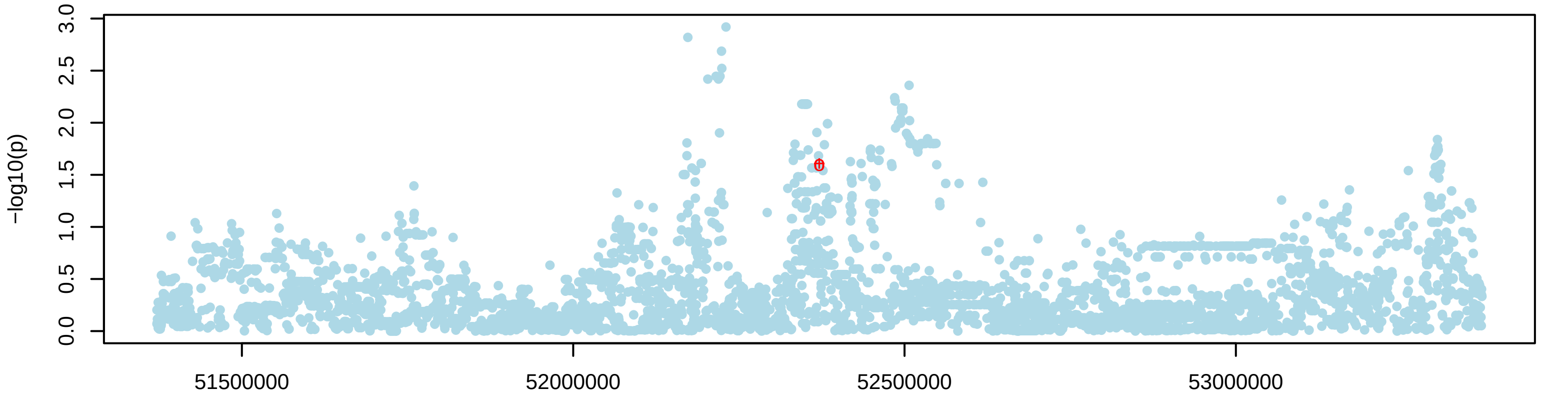

Position on chr 5

96. MAN1C1 (Q9NR34) 3:126249877:A:C [Tarkin]

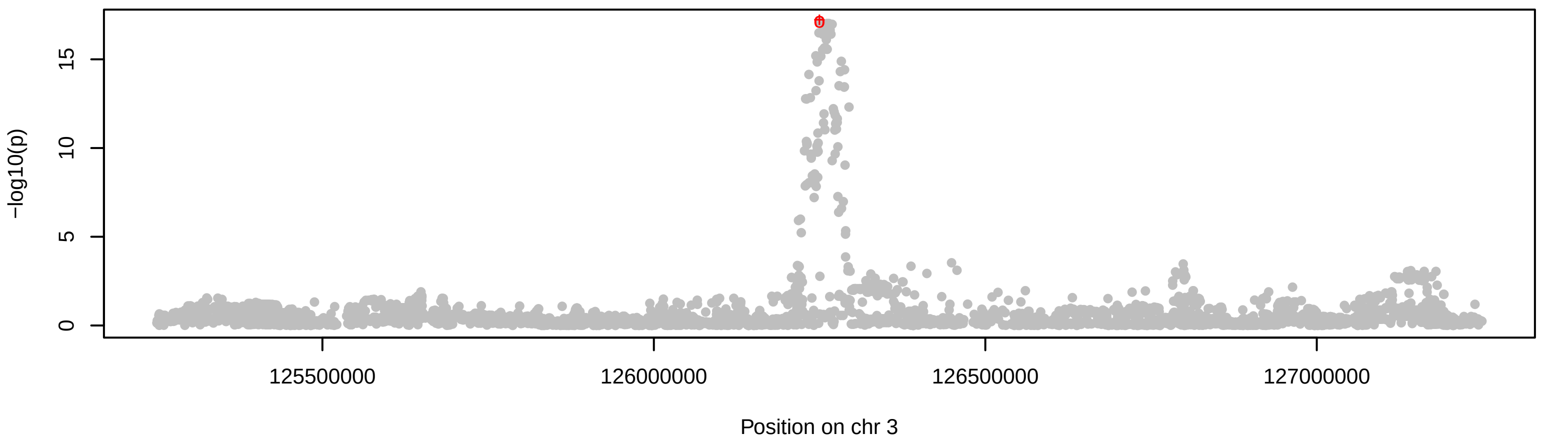

coloc with QMDiab: H4 (PP=94%)

96. MAN1C1 (A6NGN6;B1AJZ5;Q9NR34) 3:126249877:A:C [QMDiab]

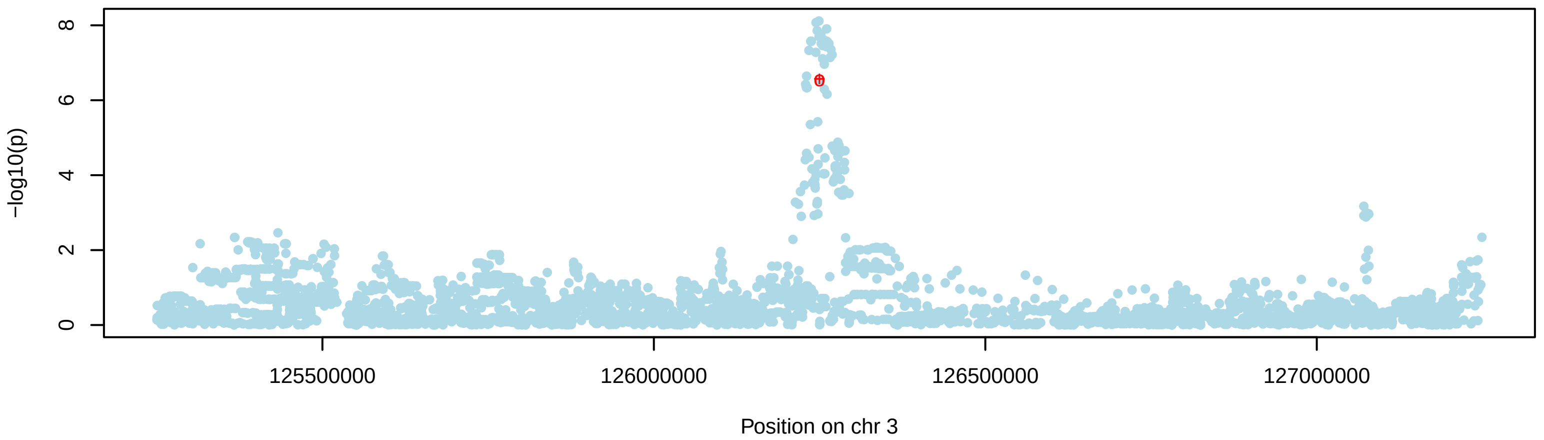

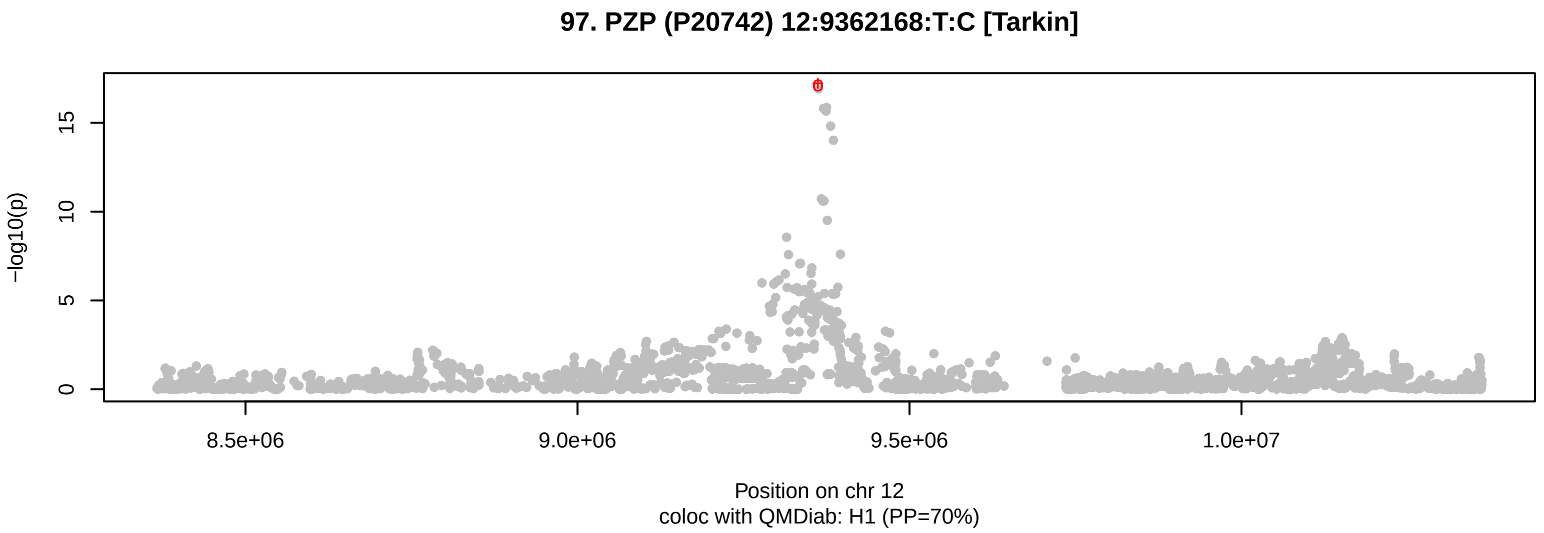
97. PZP (P20742) 12:9362168:T:C [Tarkin]
−log10(p)
0
5
10
15
8.5e+06
9.0e+06
9.5e+06
1.0e+07
Position on chr 12
coloc with QMDiab: H1 (PP=70%)

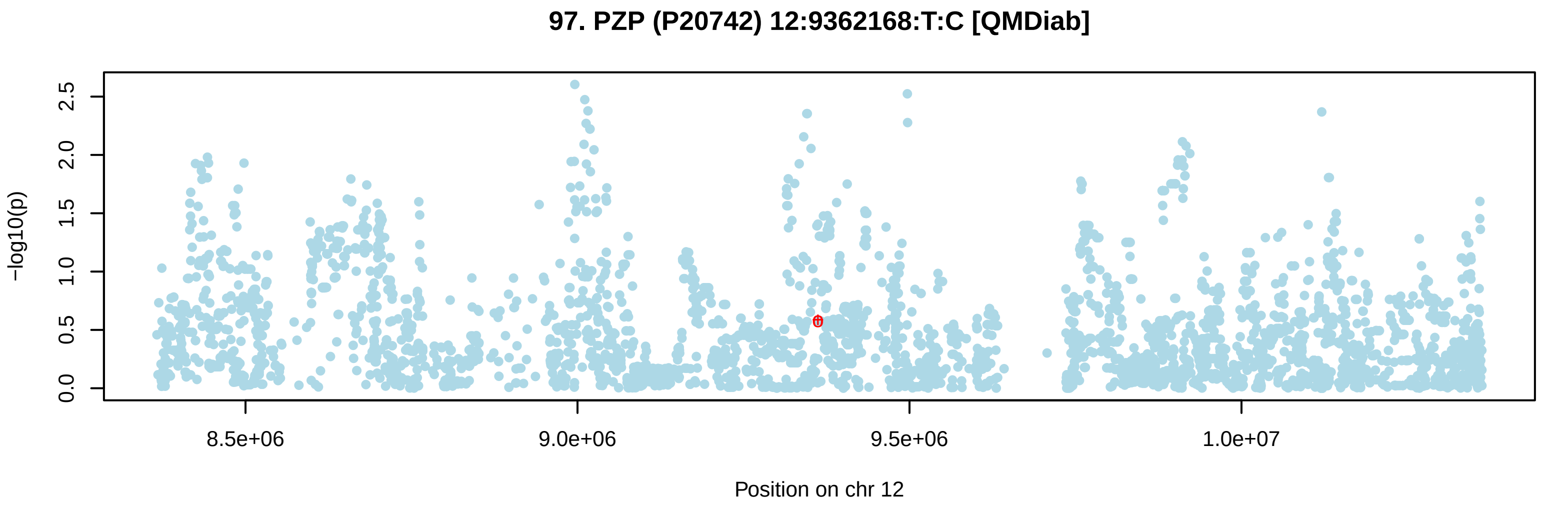
97. PZP (P20742) 12:9362168:T:C [QMDiab]
−log10(p)
0.0
0.5
1.0
1.5
2.0
2.5
8.5e+06
9.0e+06
9.5e+06
1.0e+07
Position on chr 12

98. MIA−RAB4B (W4VSR3) 19:41260831:C:T [Tarkin]

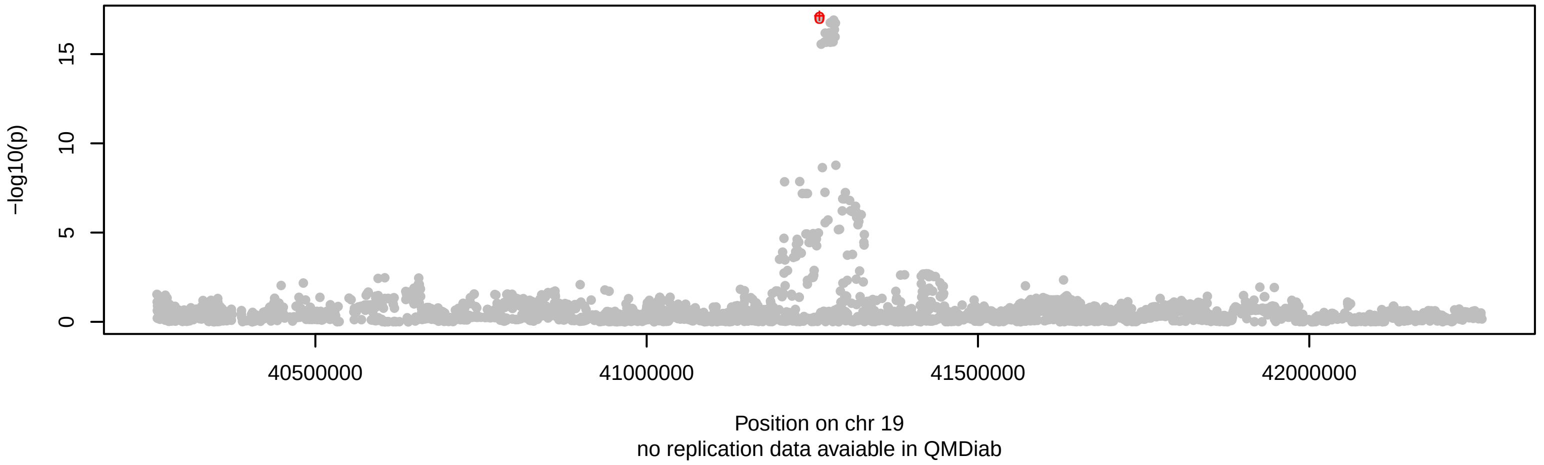

no replication data avaiable in QMDiab

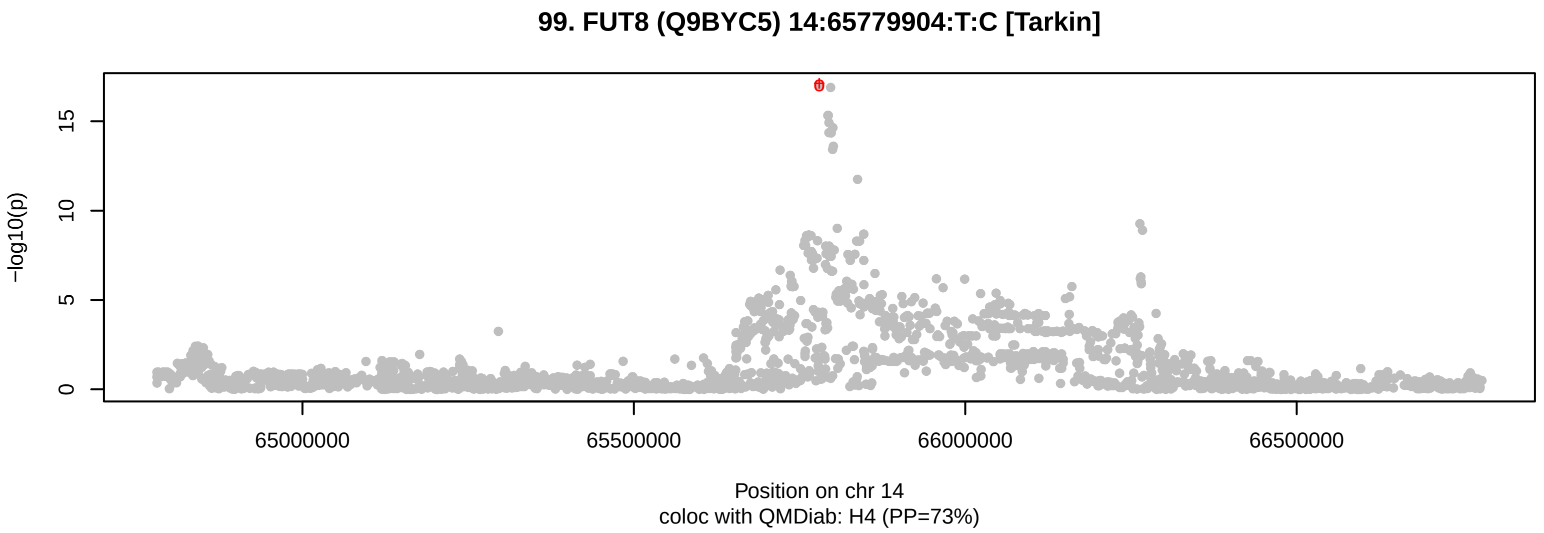
99. FUT8 (Q9BYC5) 14:65779904:T:C [Tarkin]
−log10(p)
0
5
10
15
65000000
65500000
66000000
66500000
Position on chr 14
coloc with QMDiab: H4 (PP=73%)

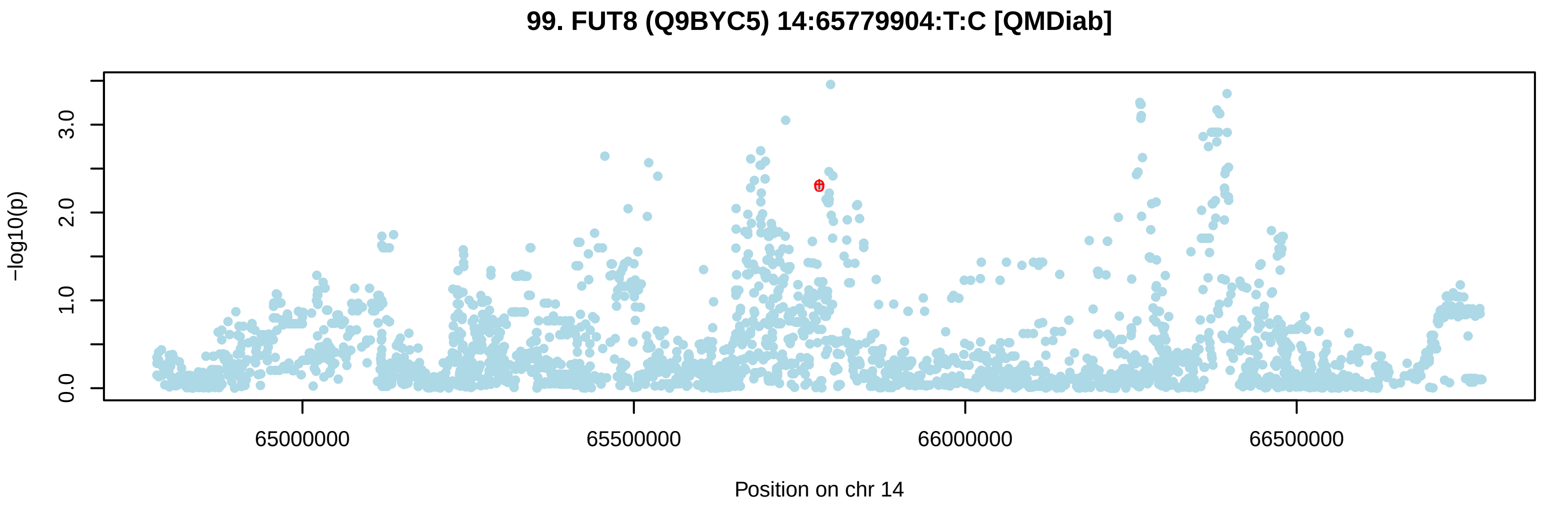
99. FUT8 (Q9BYC5) 14:65779904:T:C [QMDiab]
−log10(p)
0.0
1.0
2.0
3.0
65000000
65500000
66000000
66500000
Position on chr 14

100. FETUB (Q9UGM5) 3:186368539:C:T [Tarkin]

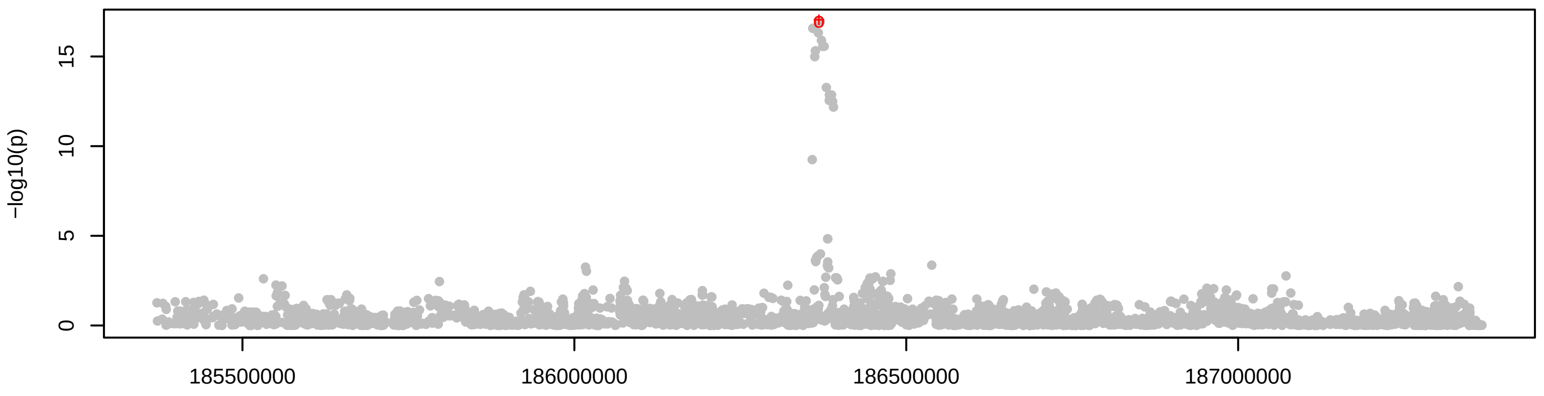

Position on chr 3
coloc with QMDiab: H1 (PP=67%)

100. FETUB (Q9UGM5) 3:186368539:C:T [QMDiab]

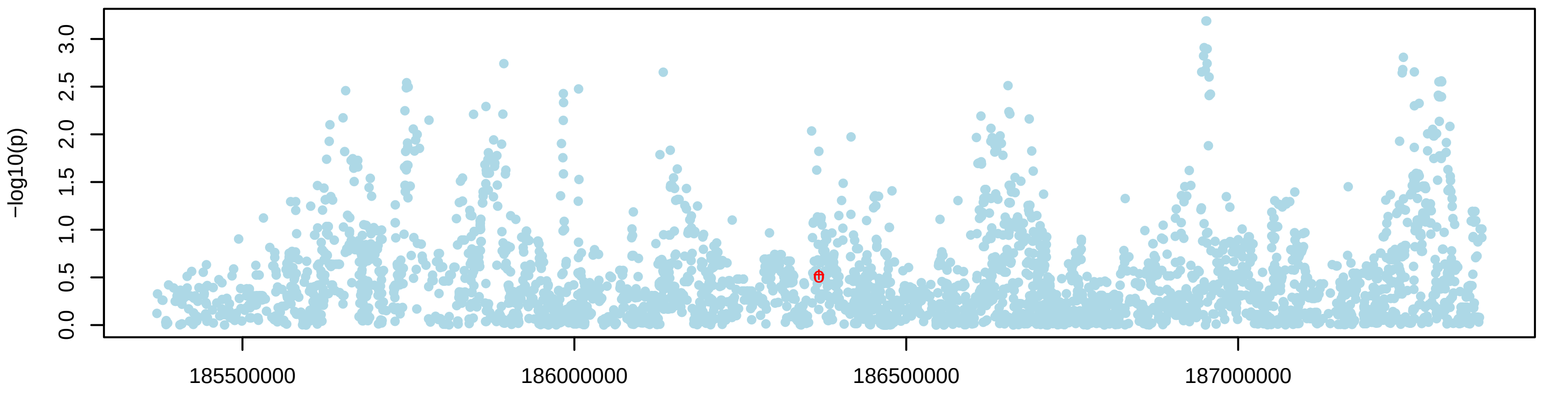

Position on chr 3

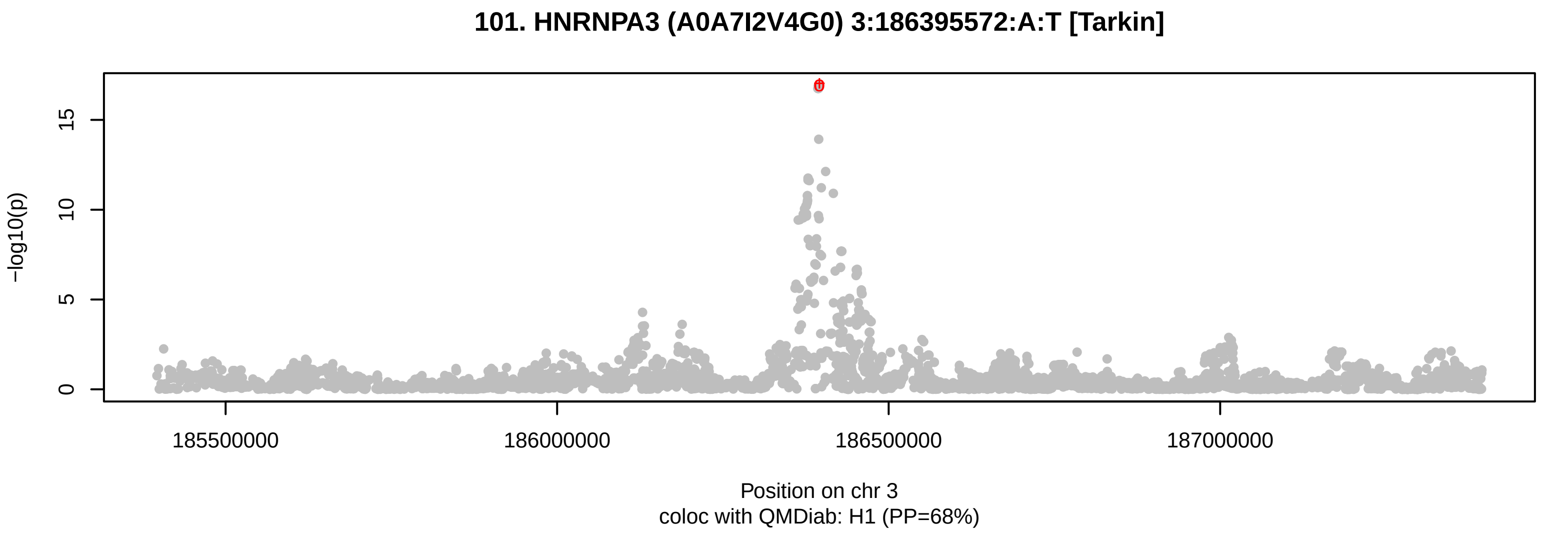
101. HNRNPA3 (A0A7I2V4G0) 3:186395572:A:T [Tarkin]
−log10(p)
0
5
10
15
185500000
186000000
186500000
187000000
Position on chr 3
coloc with QMDiab: H1 (PP=68%)

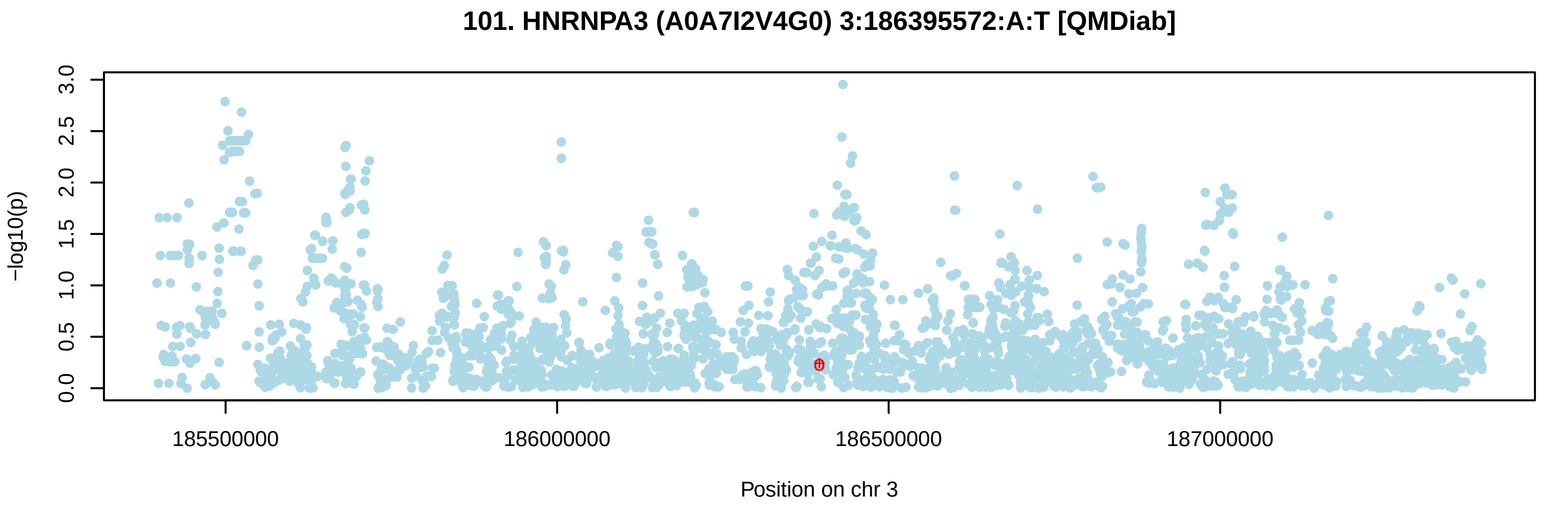
101. HNRNPA3 (A0A7I2V4G0) 3:186395572:A:T [QMDiab]
−log10(p)
0.0
0.5
1.0
1.5
2.0
2.5
3.0
185500000
186000000
186500000
187000000
Position on chr 3

102. GALNT12 (Q8IXK2) 9:101570336:A:T [Tarkin]

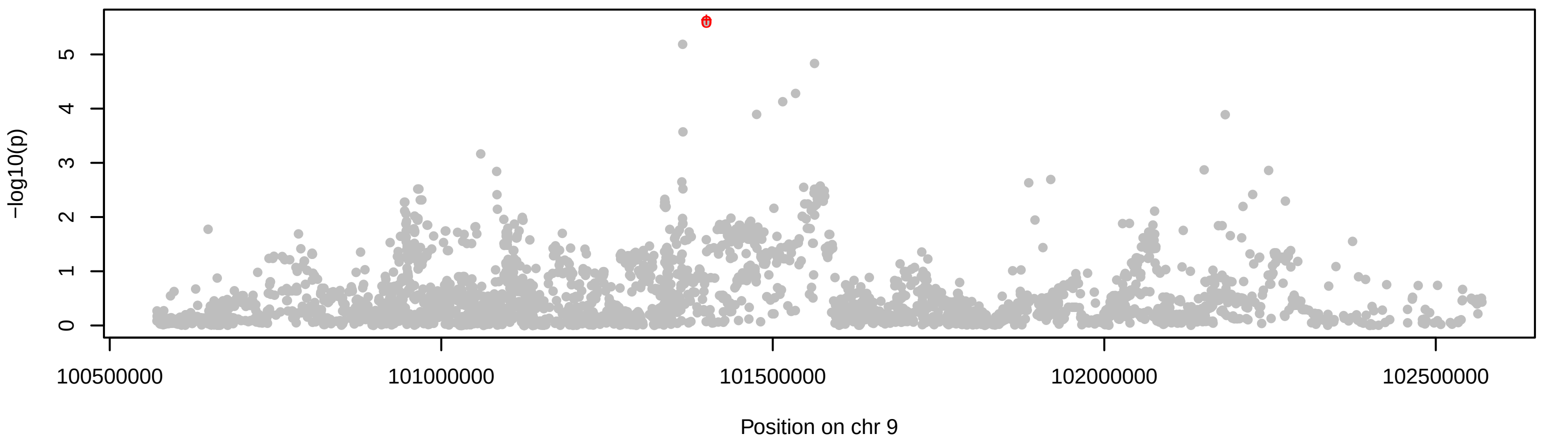

coloc with QMDiab: H1 (PP=42%)

102. GALNT12 (Q8IXK2) 9:101570336:A:T [QMDiab]

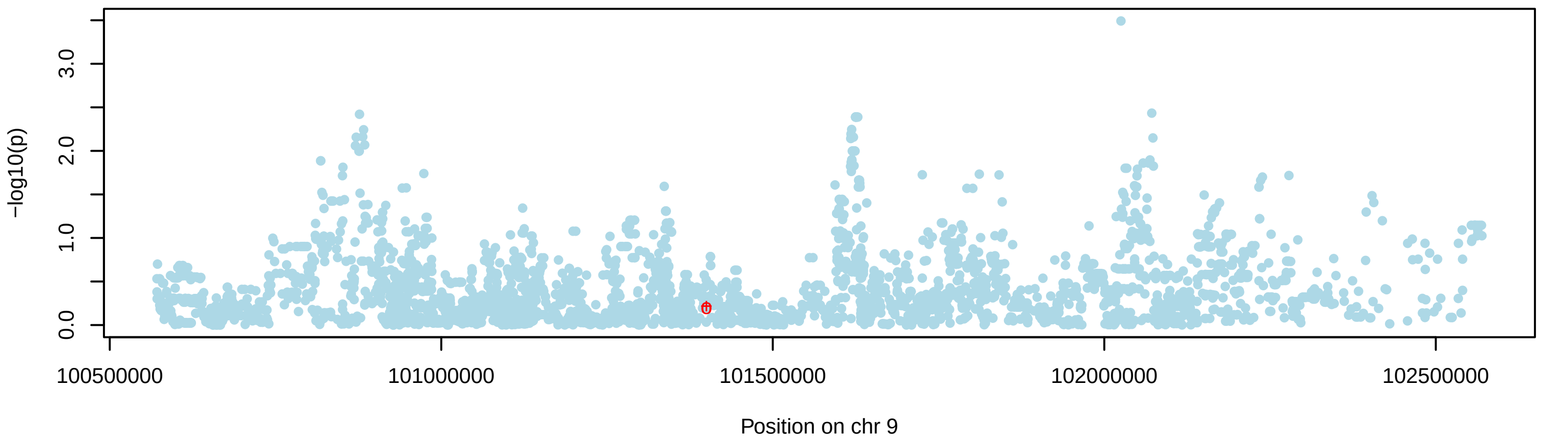

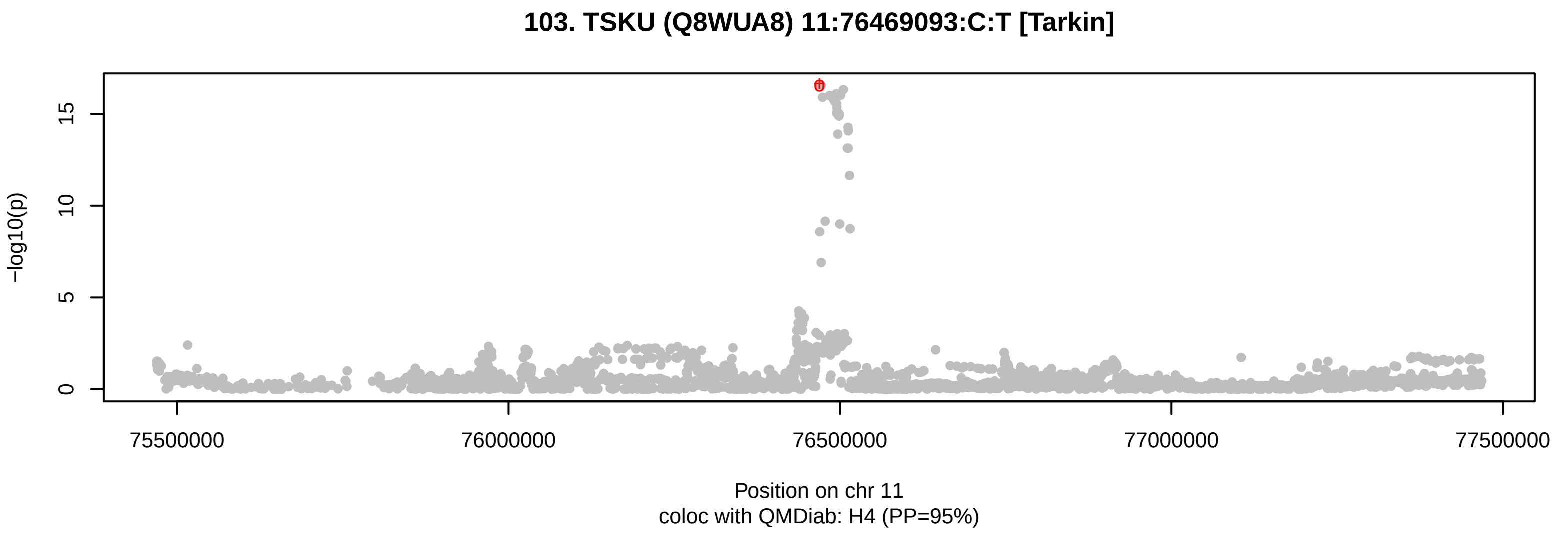
103. TSKU (Q8WUA8) 11:76469093:C:T [Tarkin]
−log10(p)
0
5
10
15
75500000
76000000
76500000
77000000
77500000
Position on chr 11
coloc with QMDiab: H4 (PP=95%)

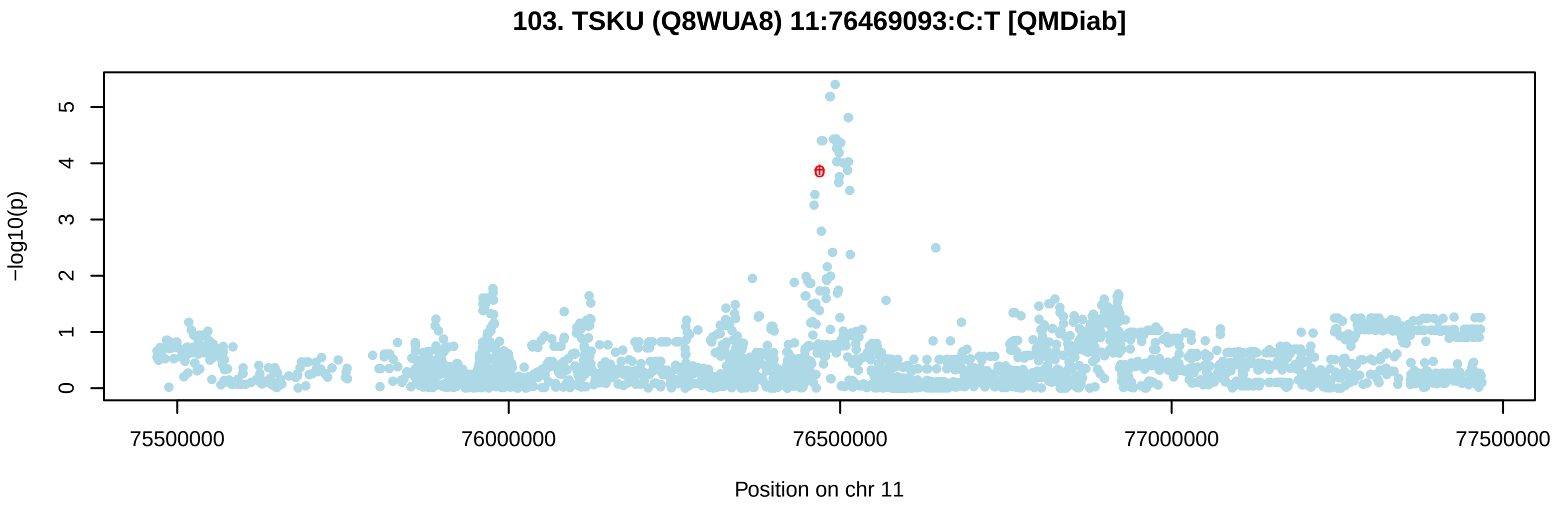
103. TSKU (Q8WUA8) 11:76469093:C:T [QMDiab]
−log10(p)
0
1
2
3
4
5
75500000
76000000
76500000
77000000
77500000
Position on chr 11

104. LTBP3 (Q9NS15) 11:65330510:C:T [Tarkin]

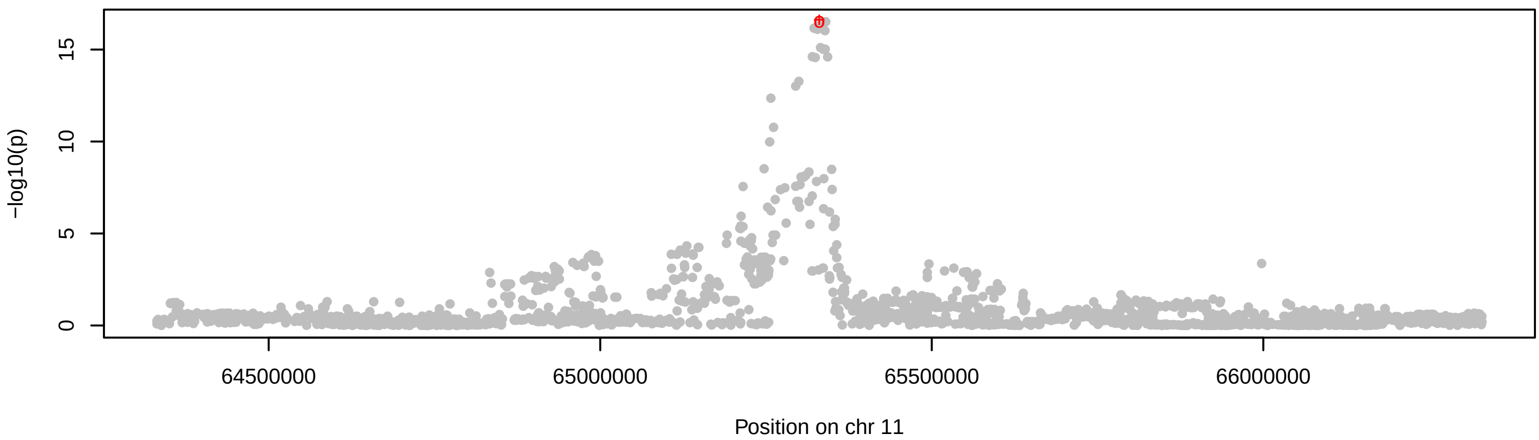

coloc with QMDiab: H4 (PP=70%)

104. LTBP3 (Q9NS15) 11:65330510:C:T [QMDiab]

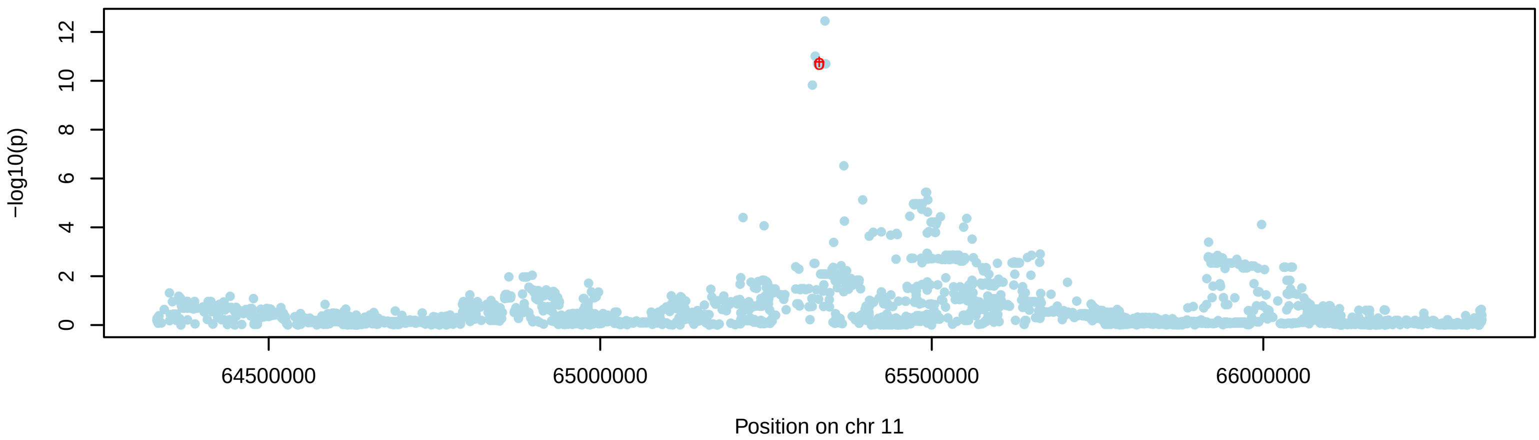

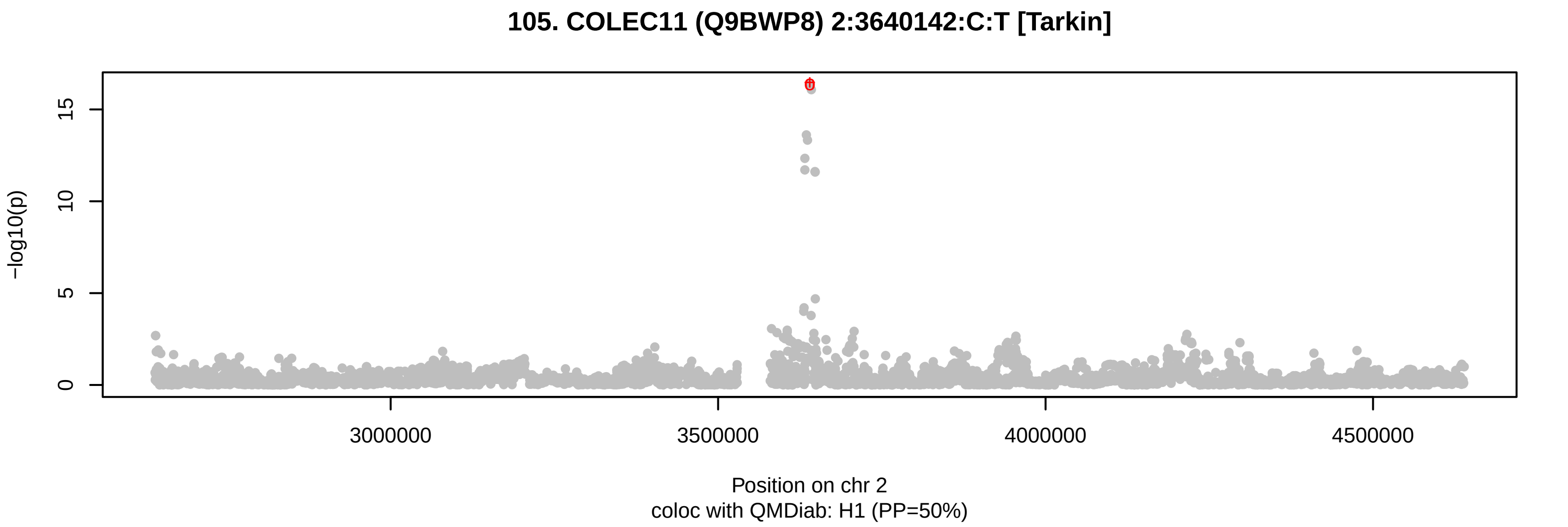

105. COLEC11 (Q9BWP8) 2:3640142:C:T [Tarkin]
−log10(p)
0
5
10
15
3000000
3500000
4000000
4500000
Position on chr 2
coloc with QMDiab: H1 (PP=50%)

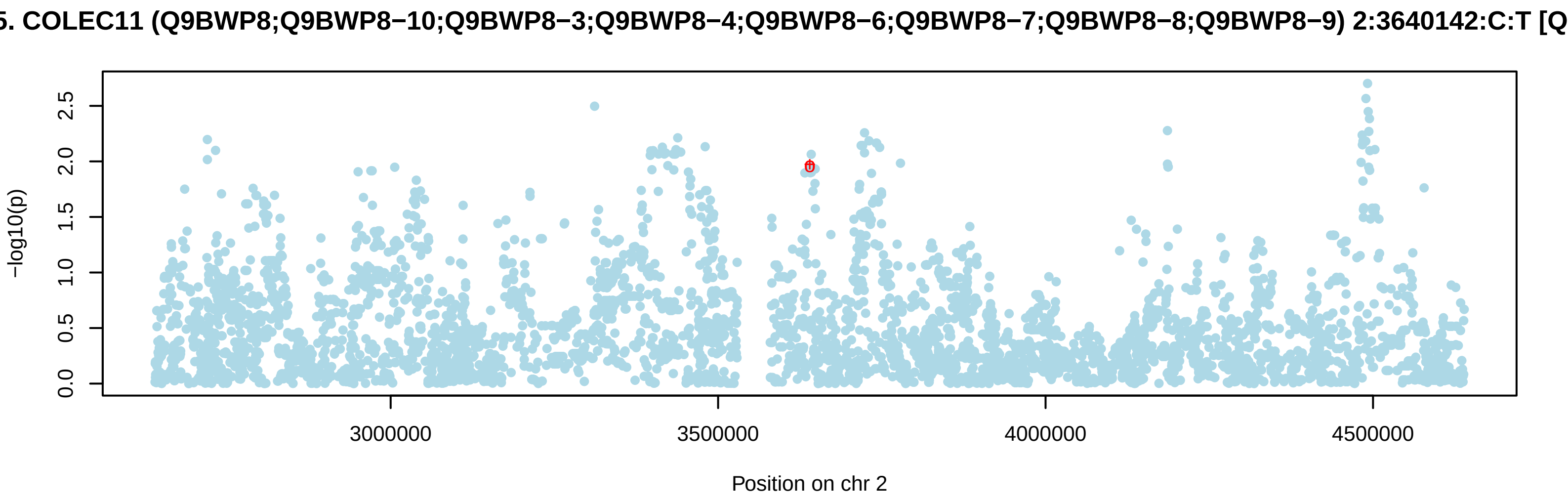

5. COLEC11 (Q9BWP8;Q9BWP8−10;Q9BWP8−3;Q9BWP8−4;Q9BWP8−6;Q9BWP8−7;Q9BWP8−8;Q9BWP8−9) 2:3640142:C:T [Q
−log10(p)
0.0
0.5
1.0
1.5
2.0
2.5
3000000
3500000
4000000
4500000
Position on chr 2

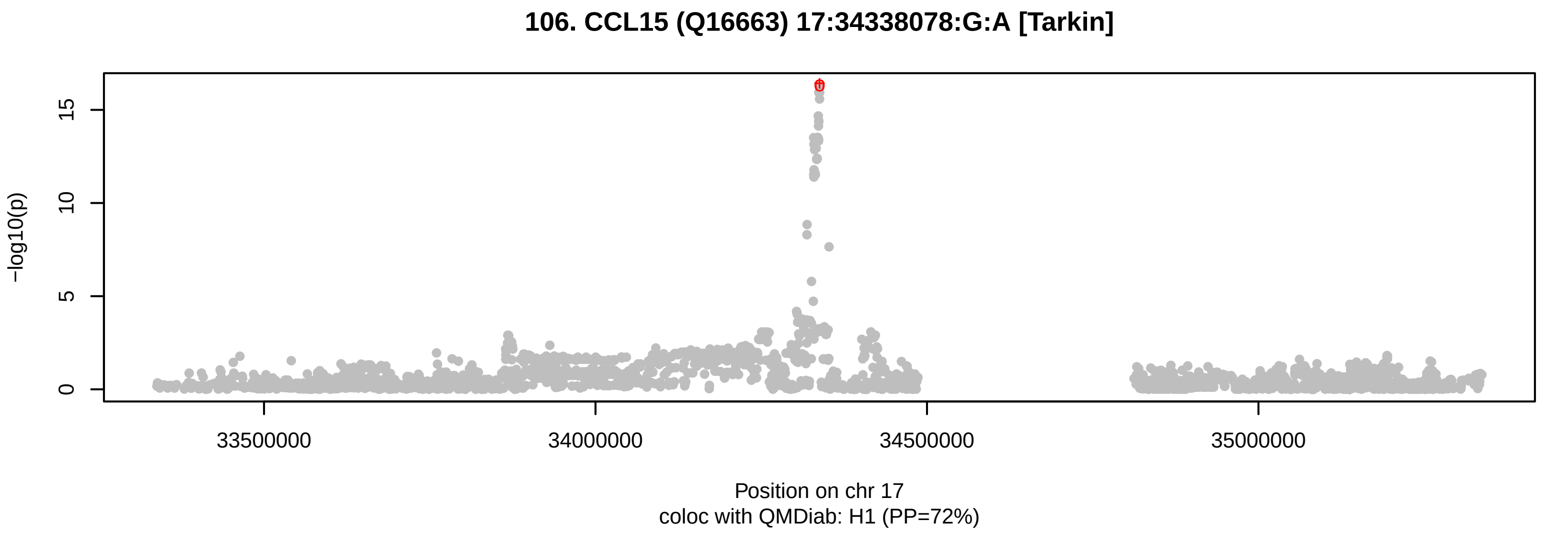

106. CCL15 (Q16663) 17:34338078:G:A [Tarkin]
−log10(p)
0
5
10
15
33500000
34000000
34500000
35000000
Position on chr 17
coloc with QMDiab: H1 (PP=72%)

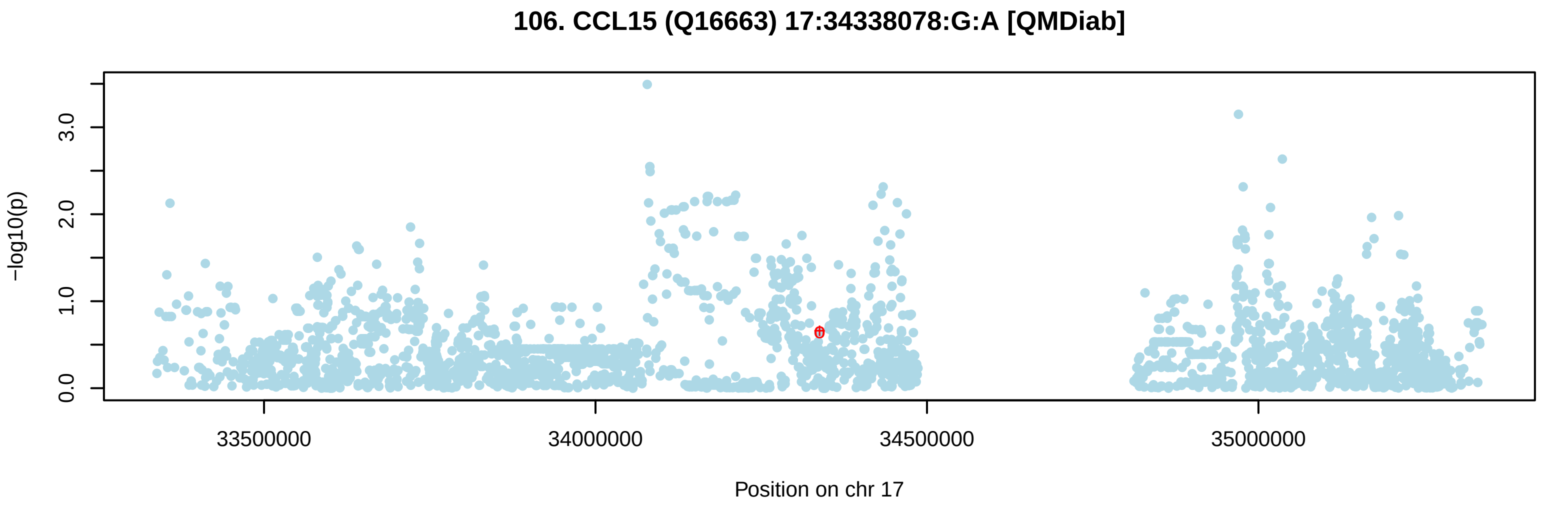

106. CCL15 (Q16663) 17:34338078:G:A [QMDiab]
−log10(p)
0.0
1.0
2.0
3.0
33500000
34000000
34500000
35000000
Position on chr 17

107. CDKL3 (E7ET86;Q8IVW4) 3:186393547:T:C [Tarkin]

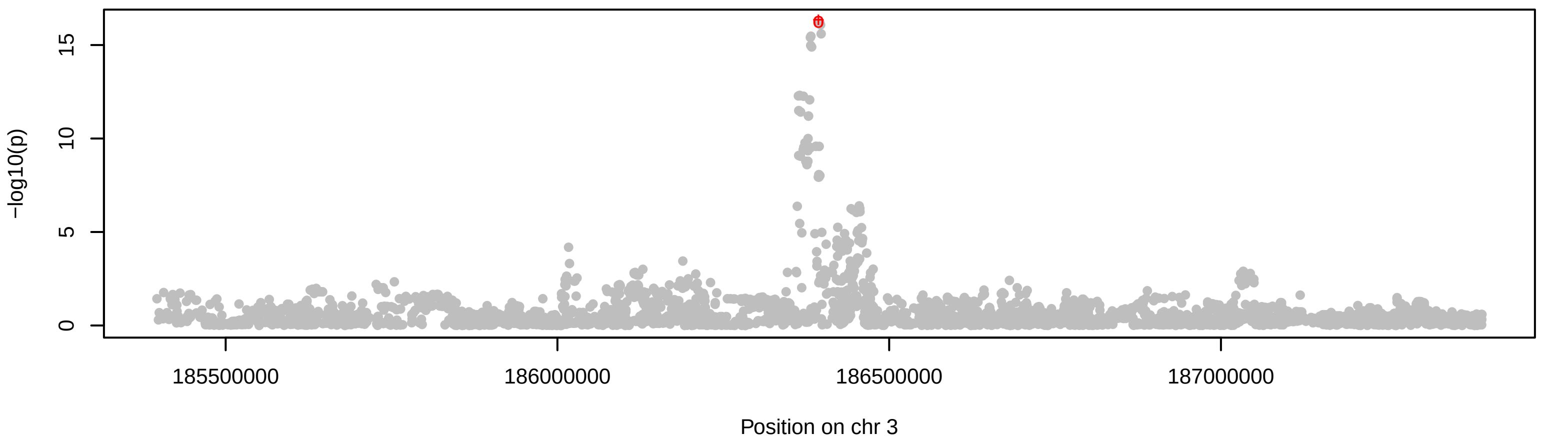

coloc with QMDiab: H1 (PP=61%)

107. CDKL3 (E7ET86;Q8IVW4) 3:186393547:T:C [QMDiab]

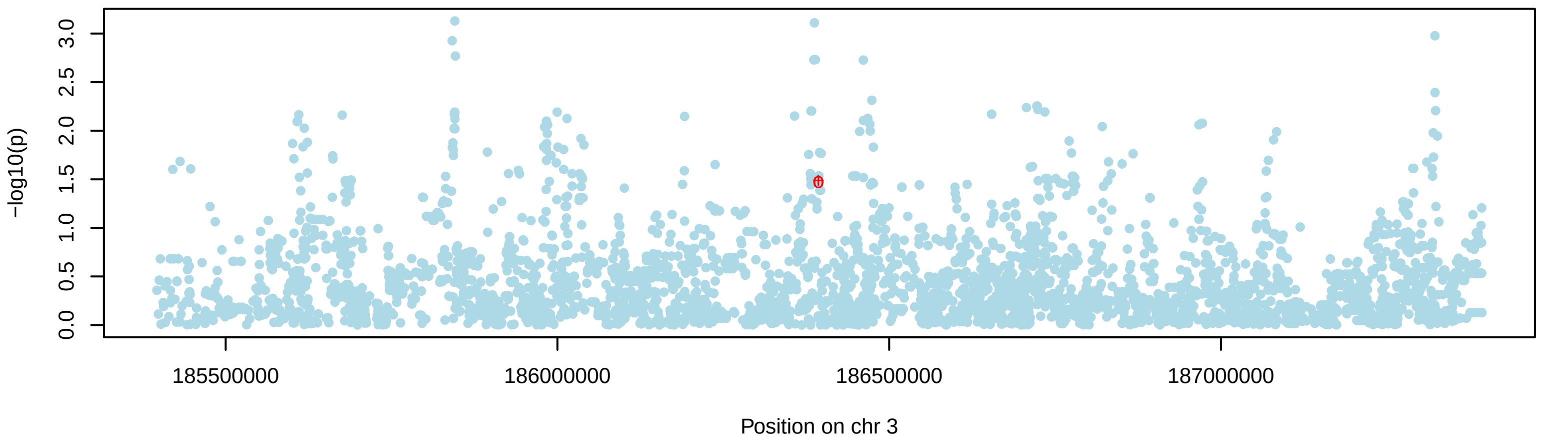

108. LGALS4 (P56470) 9:136149229:T:C [Tarkin]

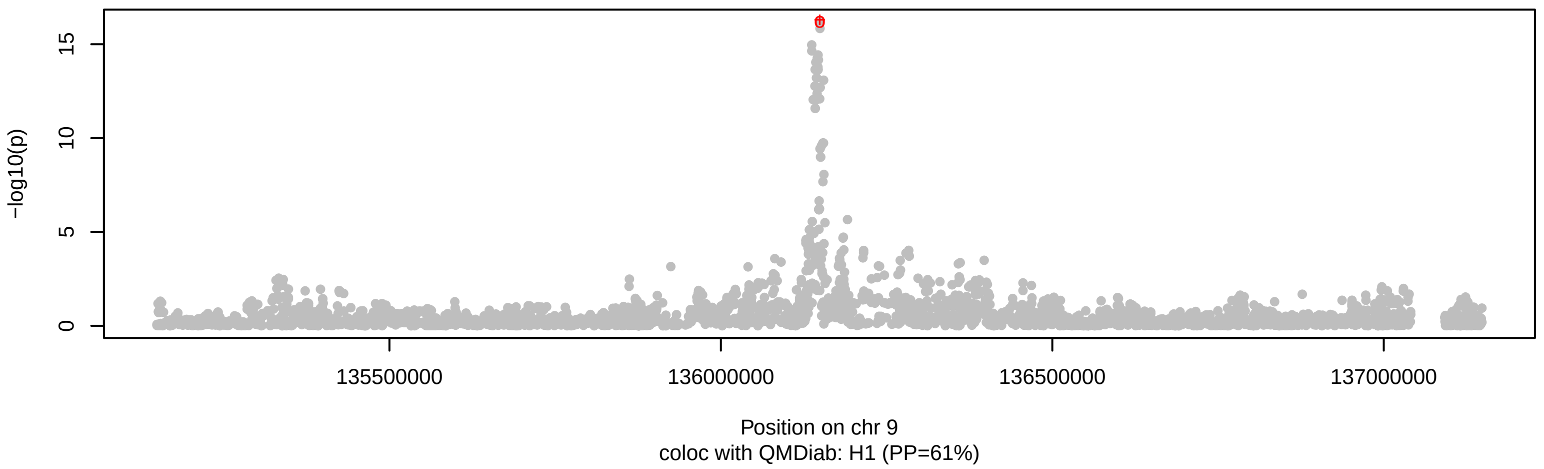

coloc with QMDiab: H1 (PP=61%)

108. LGALS4 (P56470) 9:136149229:T:C [QMDiab]

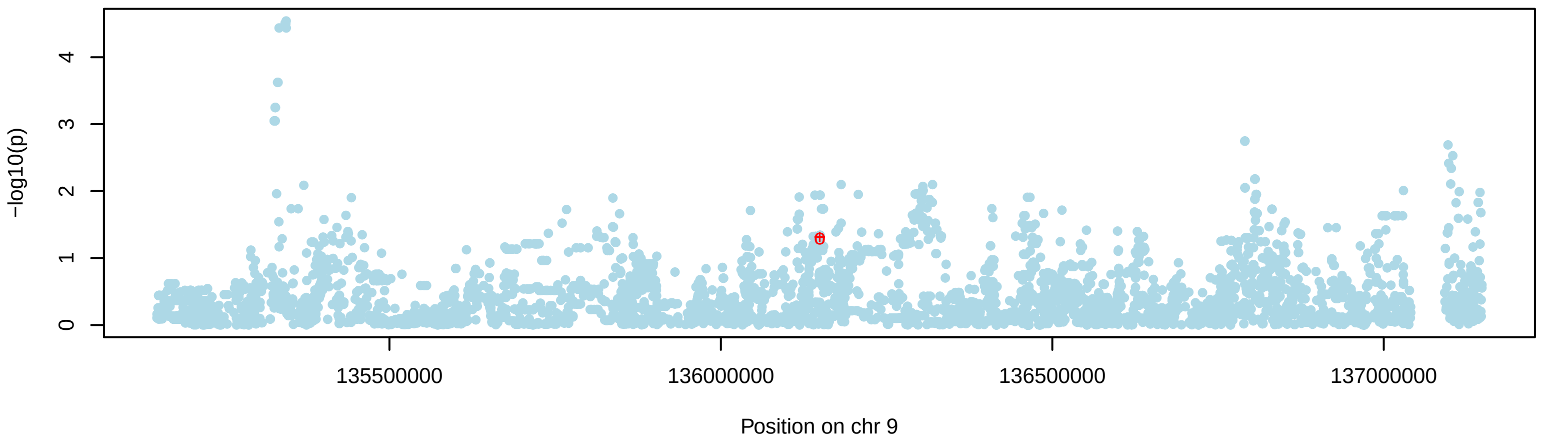

109. ICAM1 (P05362) 9:136149399:G:A [Tarkin]

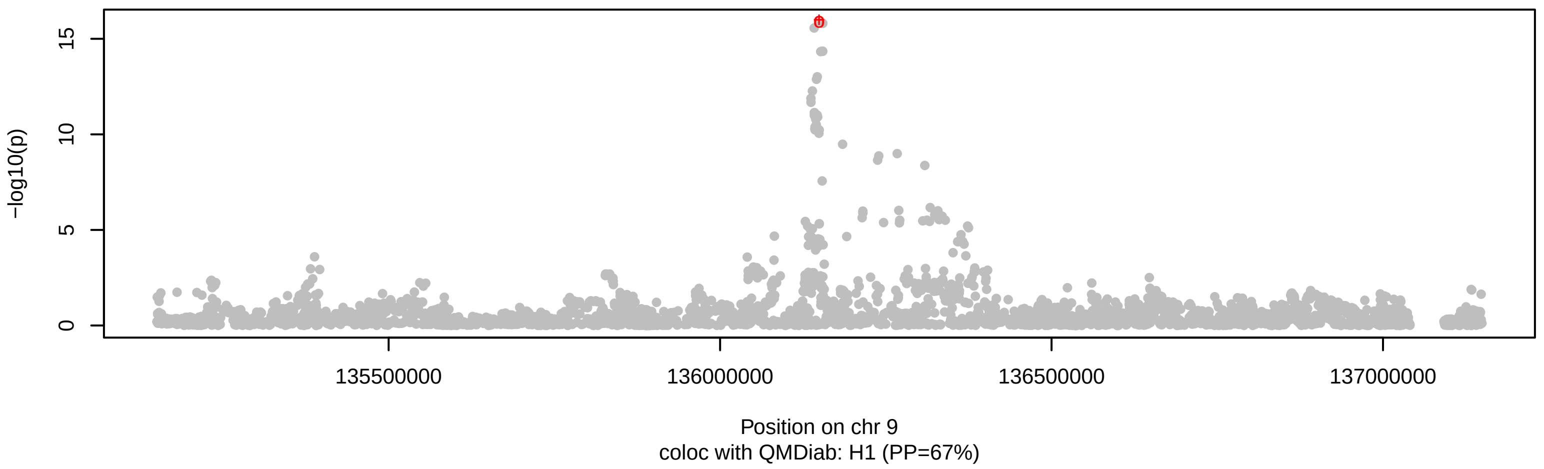

109. ICAM1 (P05362) 9:136149399:G:A [QMDiab]

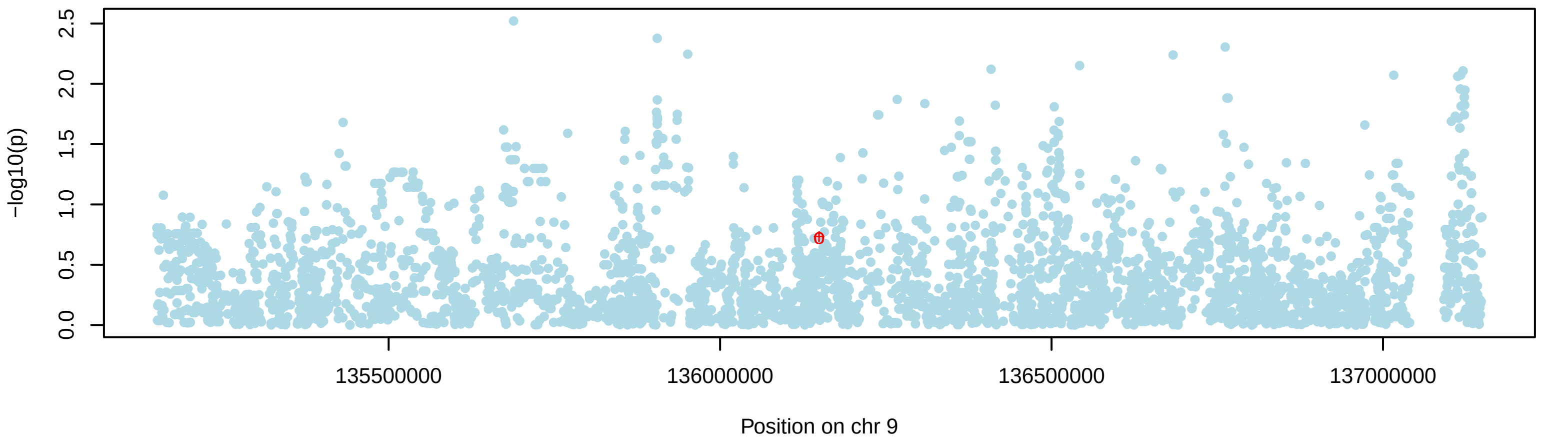

110. CDH19 (Q9H159) 18:64116504:G:C [Tarkin]

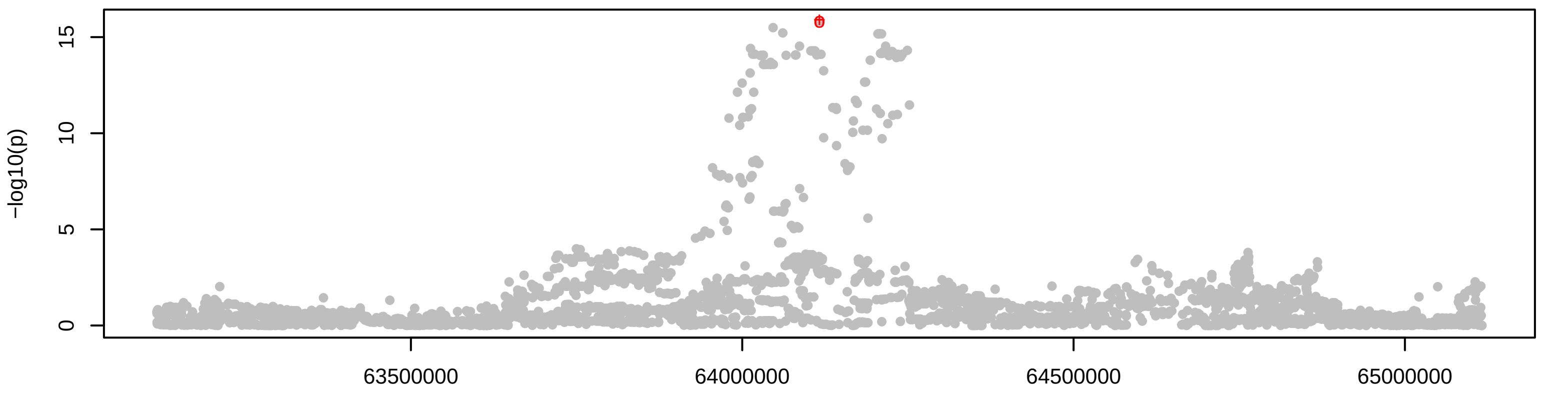

Position on chr 18
coloc with QMDiab: H4 (PP=81%)

110. CDH19 (Q9H159) 18:64116504:G:C [QMDiab]

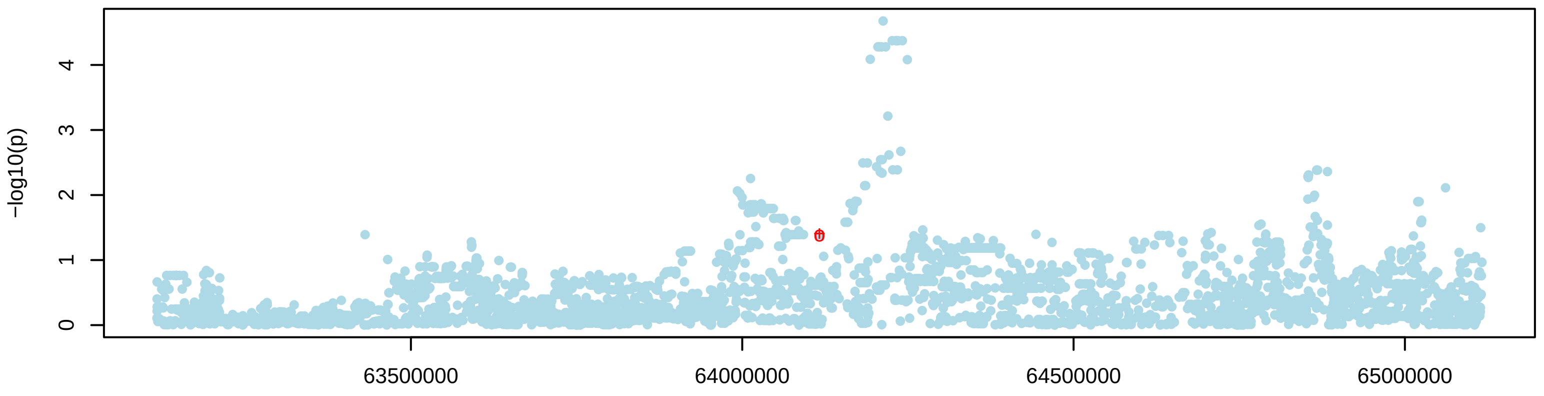

Position on chr 18

111. SAA1 (E9PQD6) 11:18278423:A:C [Tarkin]

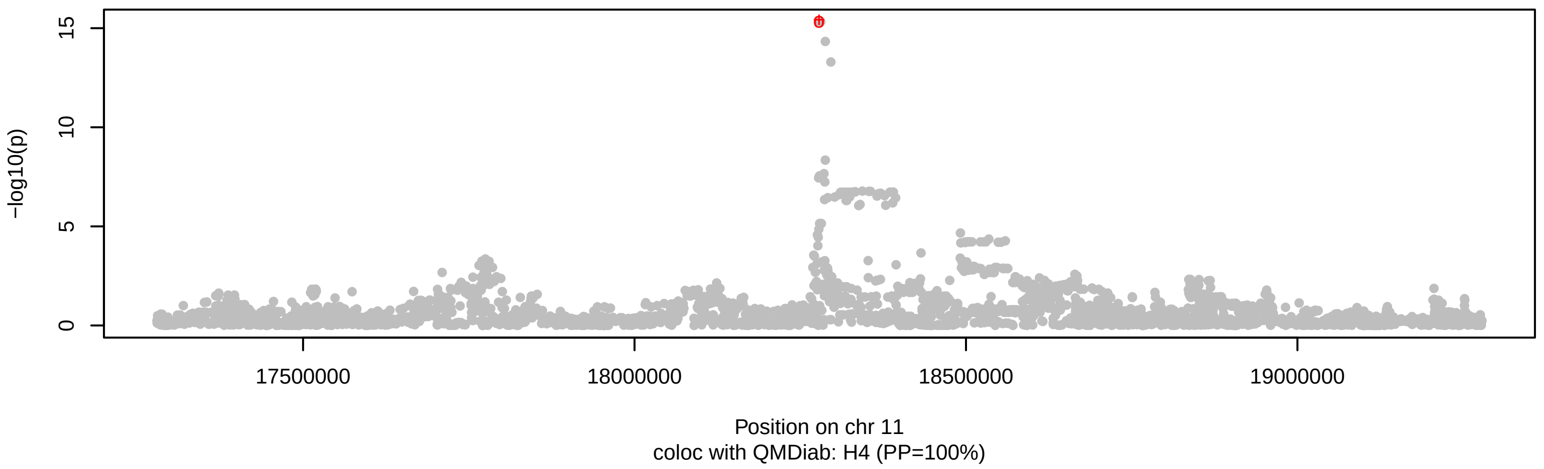

111. SAA1 (E9PQD6) 11:18278423:A:C [QMDiab]

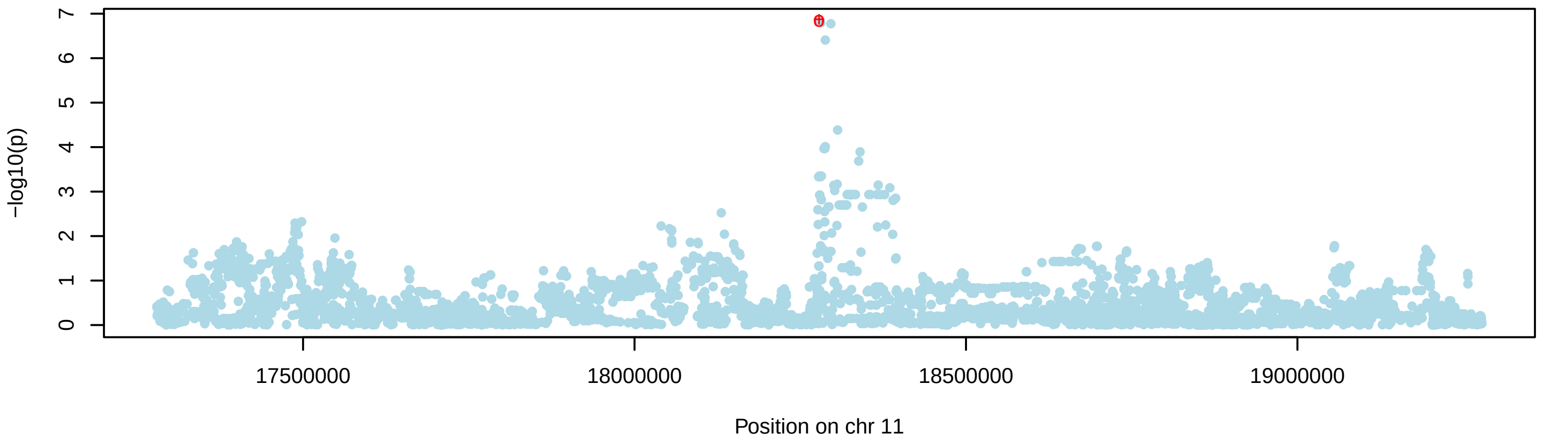

112. C1RL (Q9NZP8) 11:57381263:T:C [Tarkin]

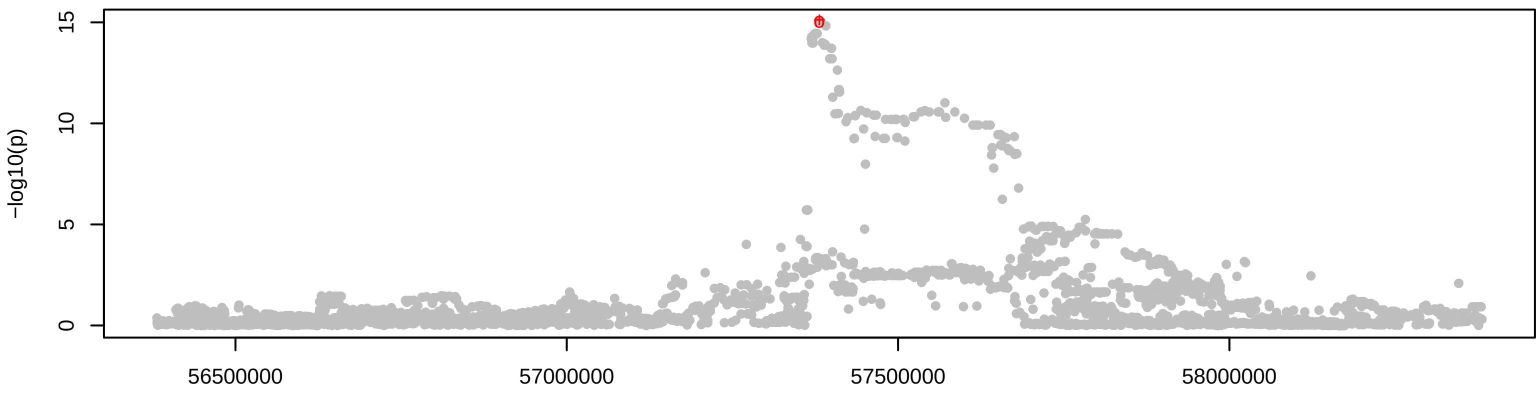

Position on chr 11
coloc with QMDiab: H1 (PP=62%)

112. C1RL (Q9NZP8) 11:57381263:T:C [QMDiab]

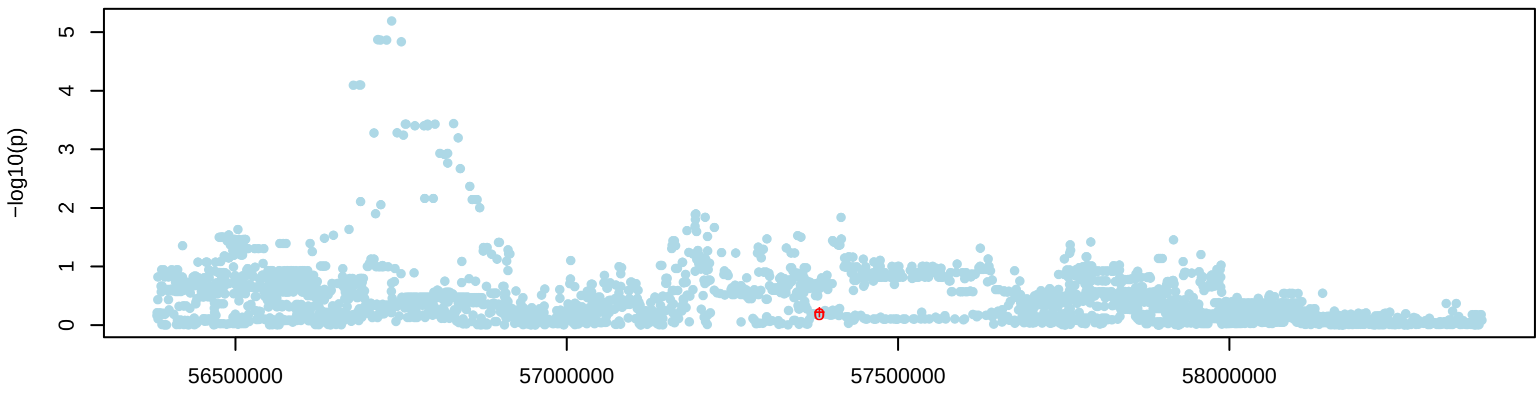

Position on chr 11

113. FAM177A1 (Q8N128;Q8N128−2) 14:35485748:G:A [Tarkin]

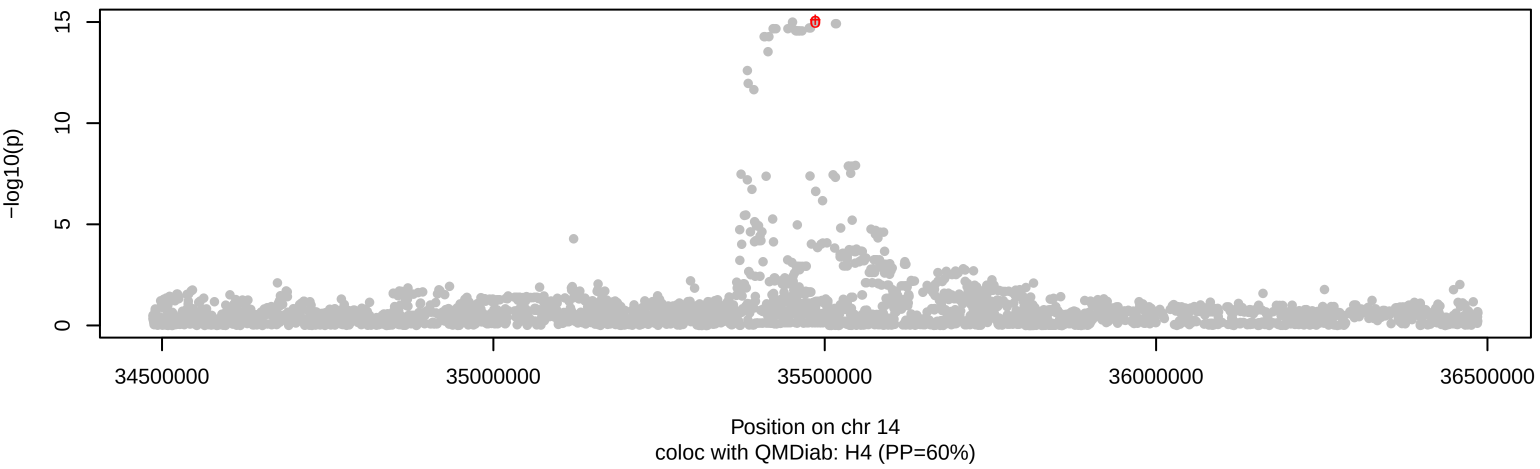

113. FAM177A1 (Q8N128;Q8N128−2) 14:35485748:G:A [QMDiab]

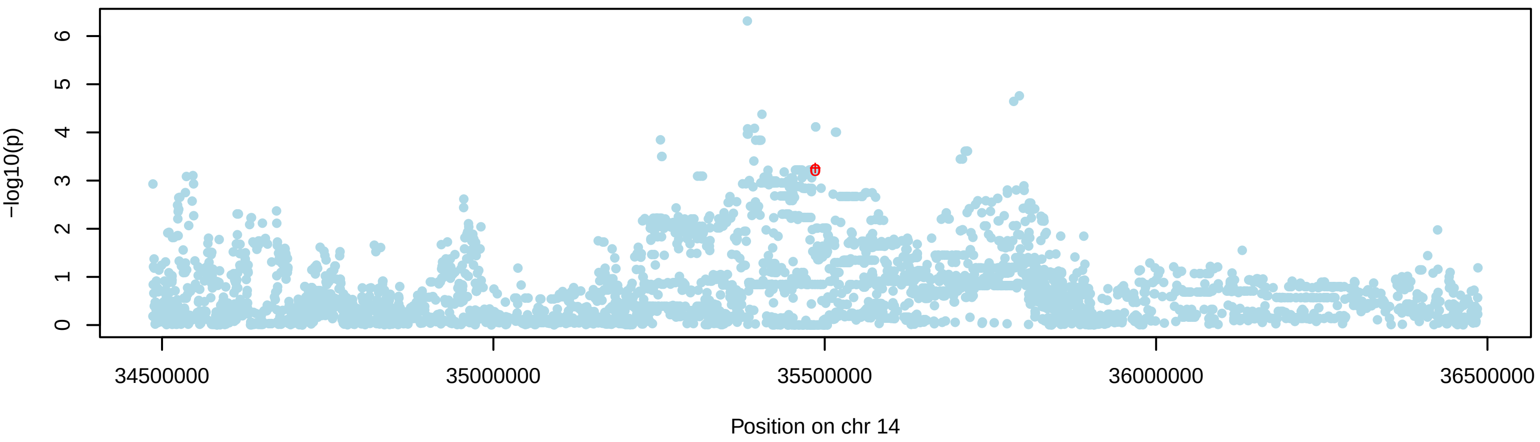

114. FCGR3A (H0Y755;P08637) 1:161588873:C:T [Tarkin]

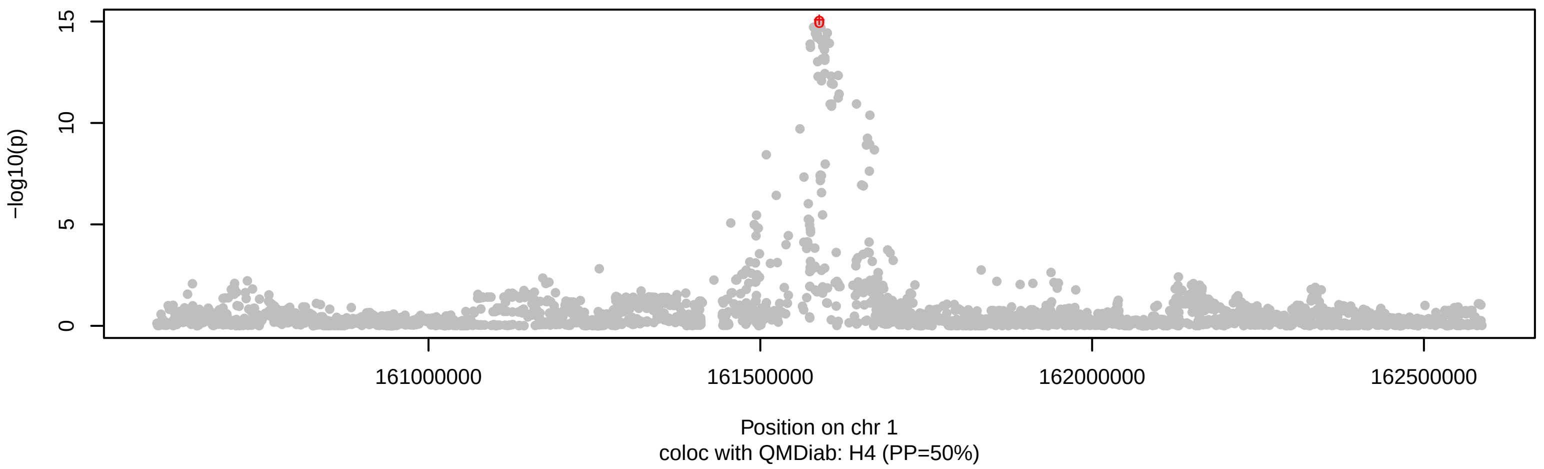

coloc with QMDiab: H4 (PP=50%)

114. FCGR3A (H0Y755;P08637) 1:161588873:C:T [QMDiab]

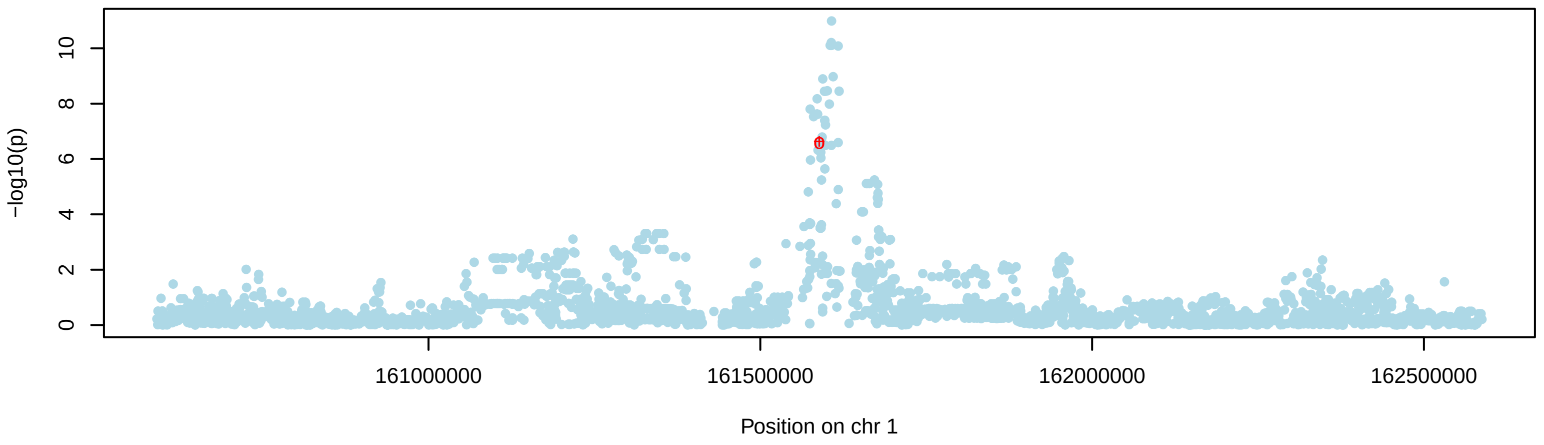

115. C3 (P01024) 1:196822368:A:G [Tarkin]

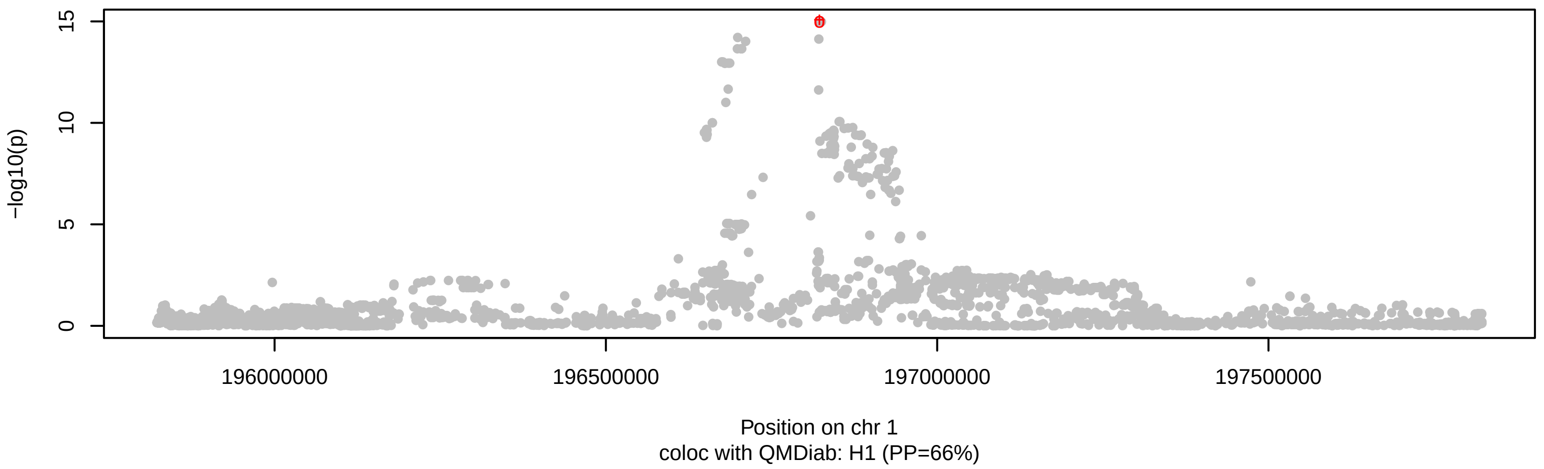

115. C3 (P01024) 1:196822368:A:G [QMDiab]

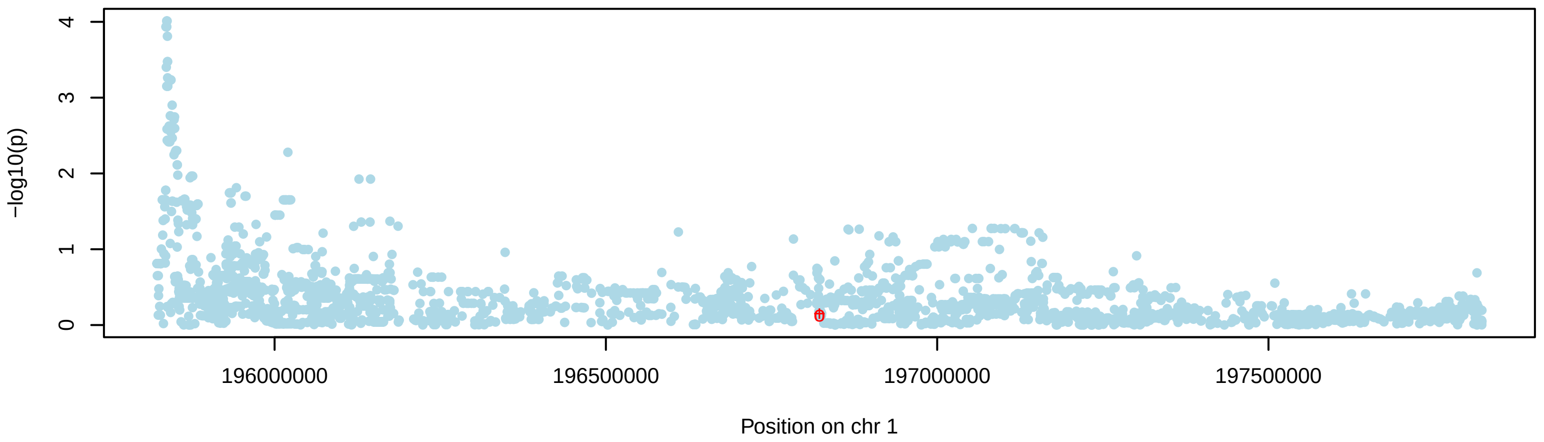

116. PLG (P00747) 3:186390627:C:T [Tarkin]

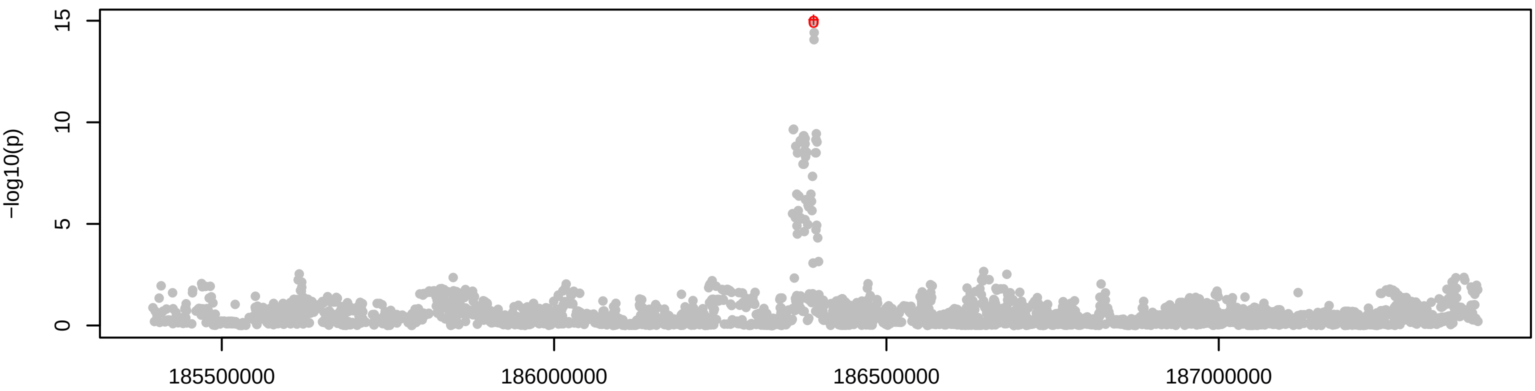

Position on chr 3
coloc with QMDiab: H1 (PP=52%)

116. PLG (P00747) 3:186390627:C:T [QMDiab]

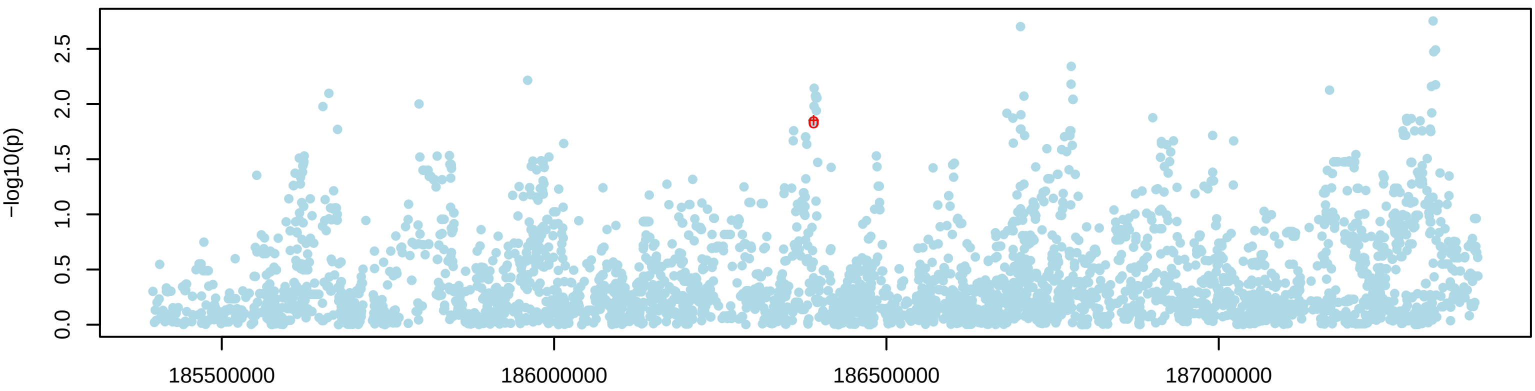

Position on chr 3

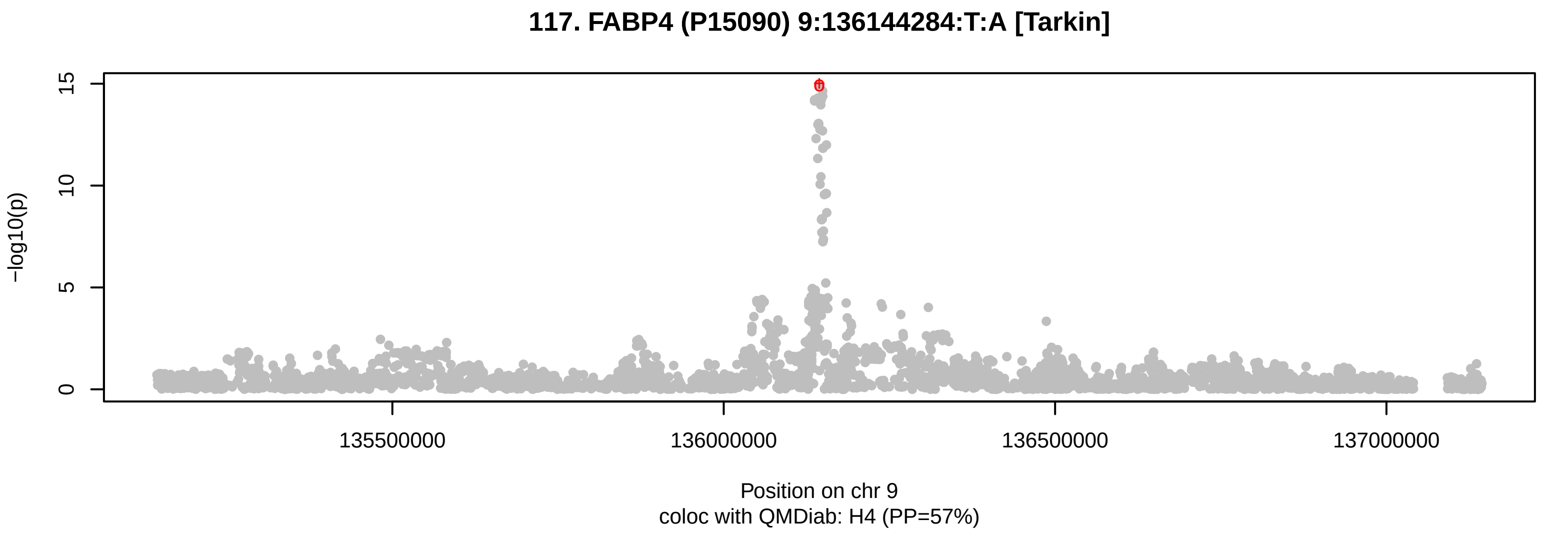
117. FABP4 (P15090) 9:136144284:T:A [Tarkin]
−log10(p)
0
5
10
15
135500000
136000000
136500000
137000000
Position on chr 9
coloc with QMDiab: H4 (PP=57%)

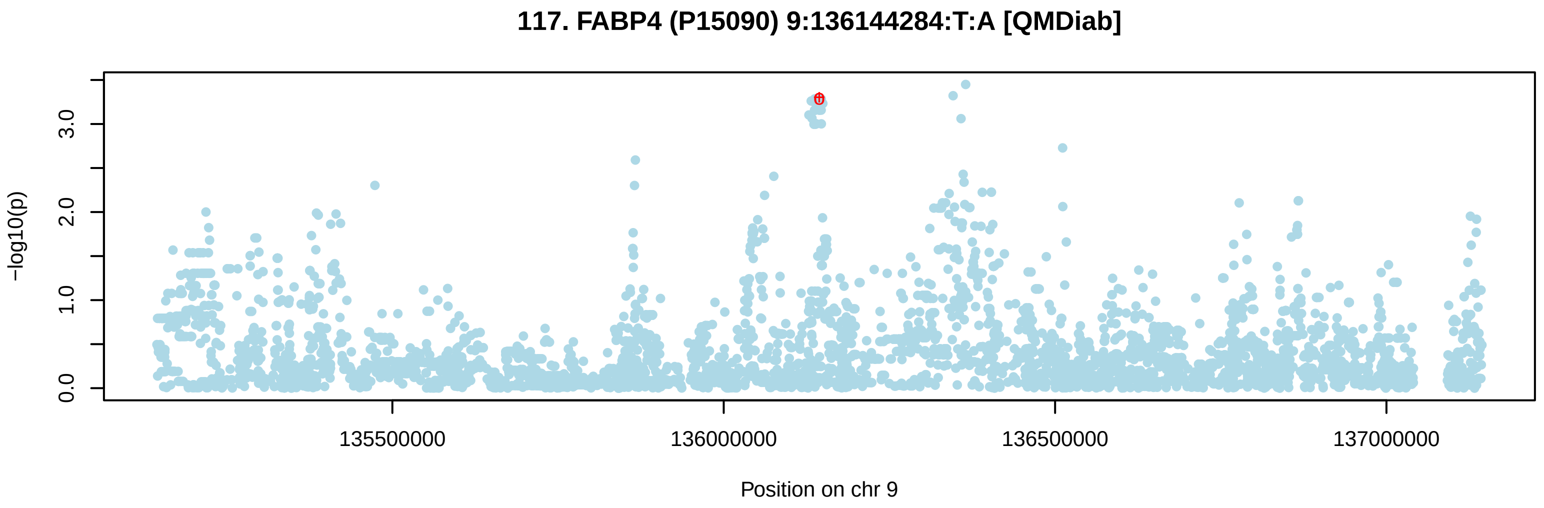
117. FABP4 (P15090) 9:136144284:T:A [QMDiab]
−log10(p)
0.0
1.0
2.0
3.0
135500000
136000000
136500000
137000000
Position on chr 9

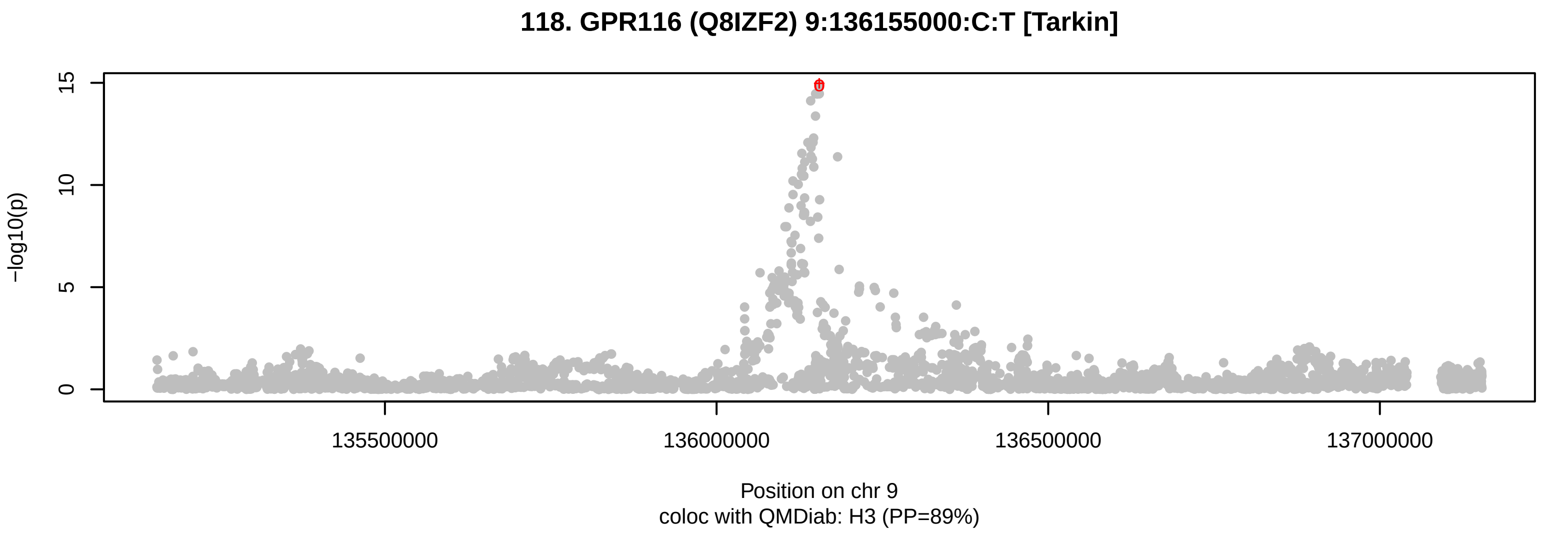
118. GPR116 (Q8IZF2) 9:136155000:C:T [Tarkin]
−log10(p)
0
5
10
15
135500000
136000000
136500000
137000000
Position on chr 9
coloc with QMDiab: H3 (PP=89%)

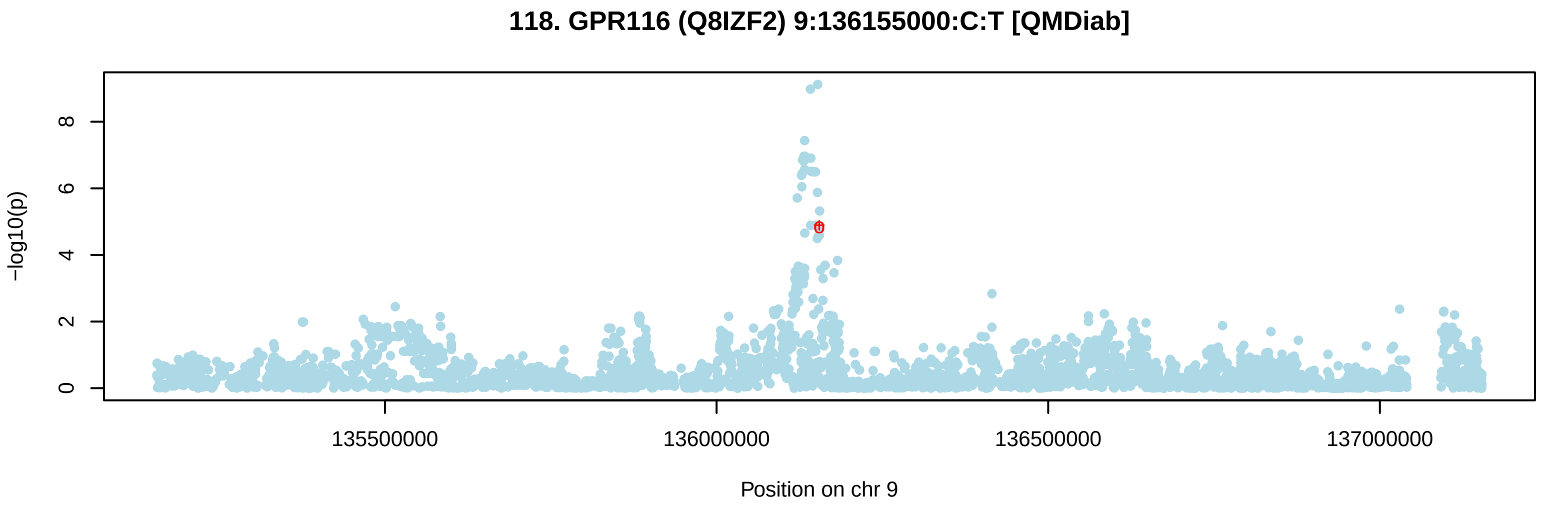
118. GPR116 (Q8IZF2) 9:136155000:C:T [QMDiab]
−log10(p)
0
2
4
6
8
135500000
136000000
136500000
137000000
Position on chr 9

119. CD34 (P28906) 9:136137065:A:G [Tarkin]

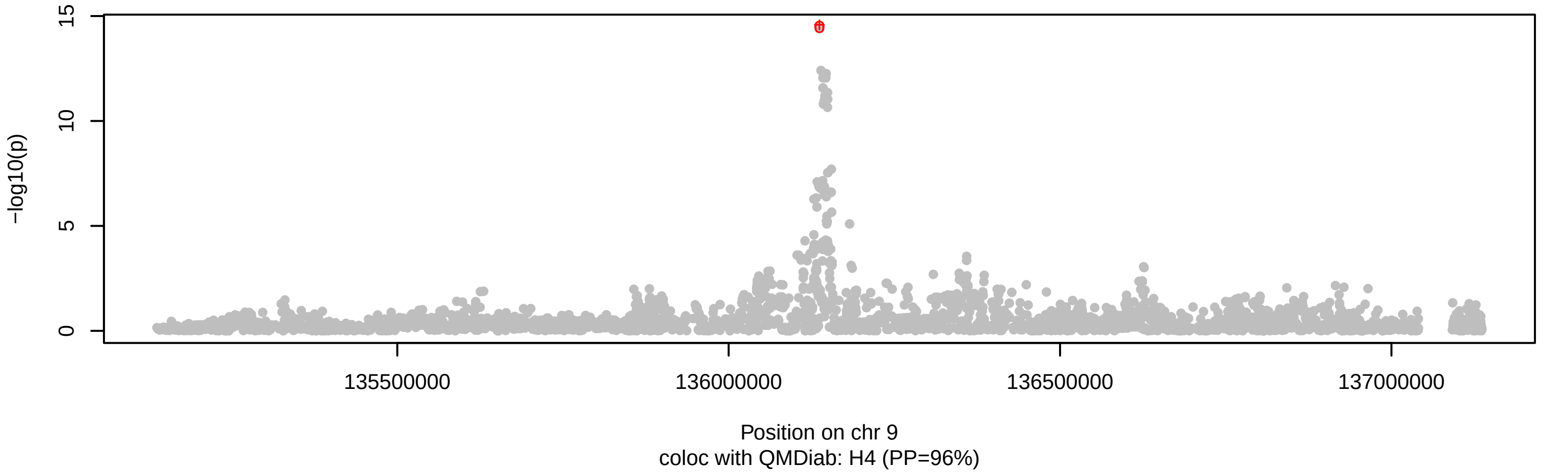

coloc with QMDiab: H4 (PP=96%)

119. CD34 (P28906) 9:136137065:A:G [QMDiab]

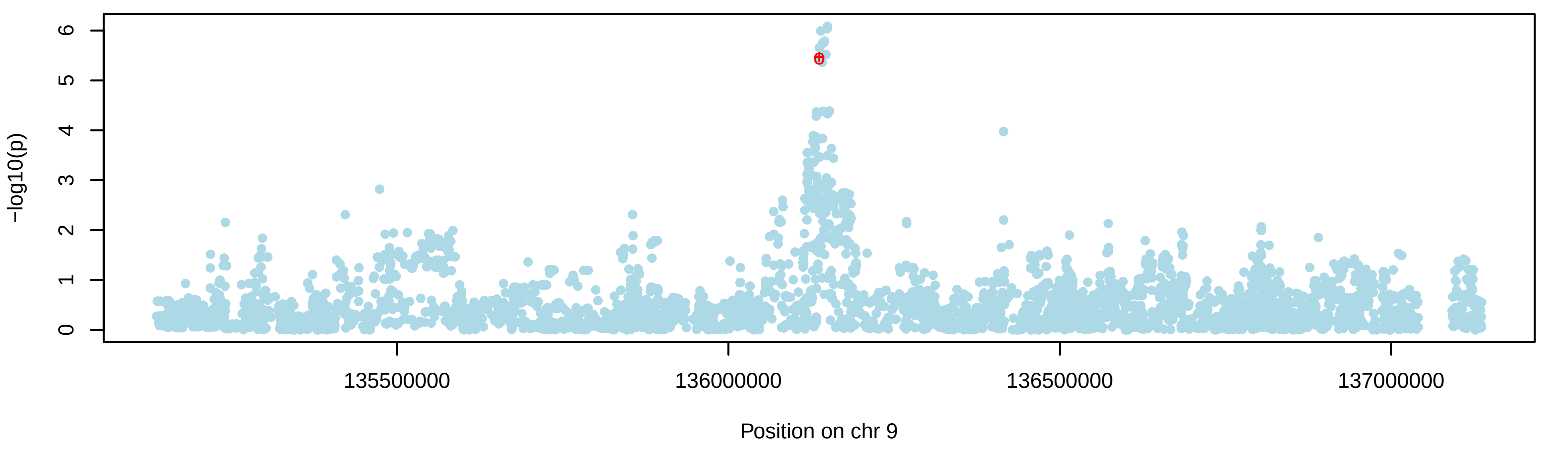

120. CTSS (P25774) 3:186454180:A:C [Tarkin]

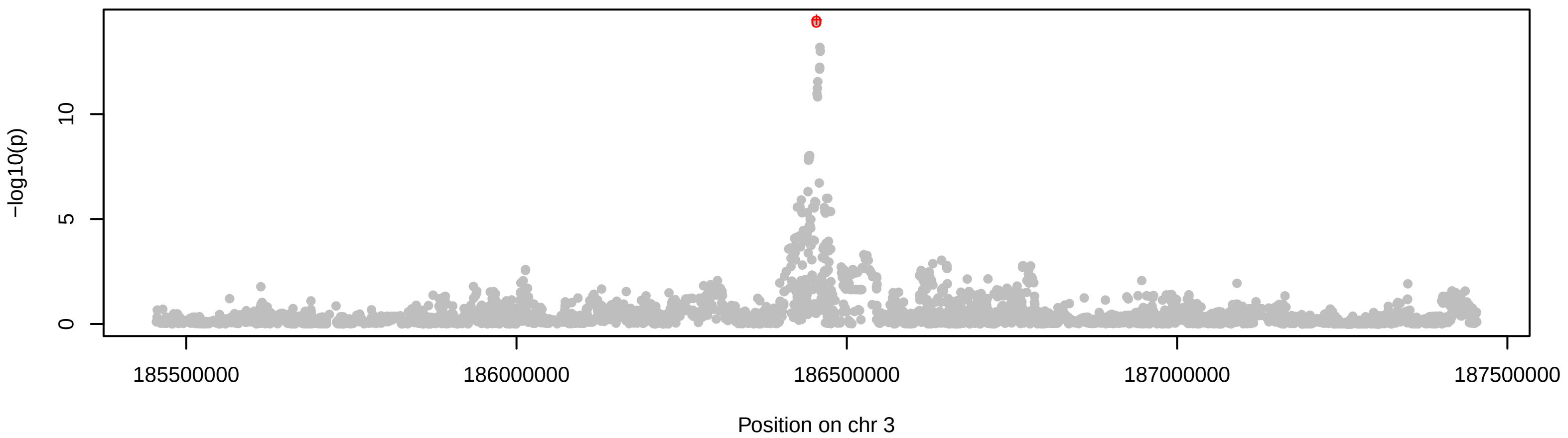

coloc with QMDiab: H4 (PP=98%)

120. CTSS (A0A7P0TAQ0;P25774) 3:186454180:A:C [QMDiab]

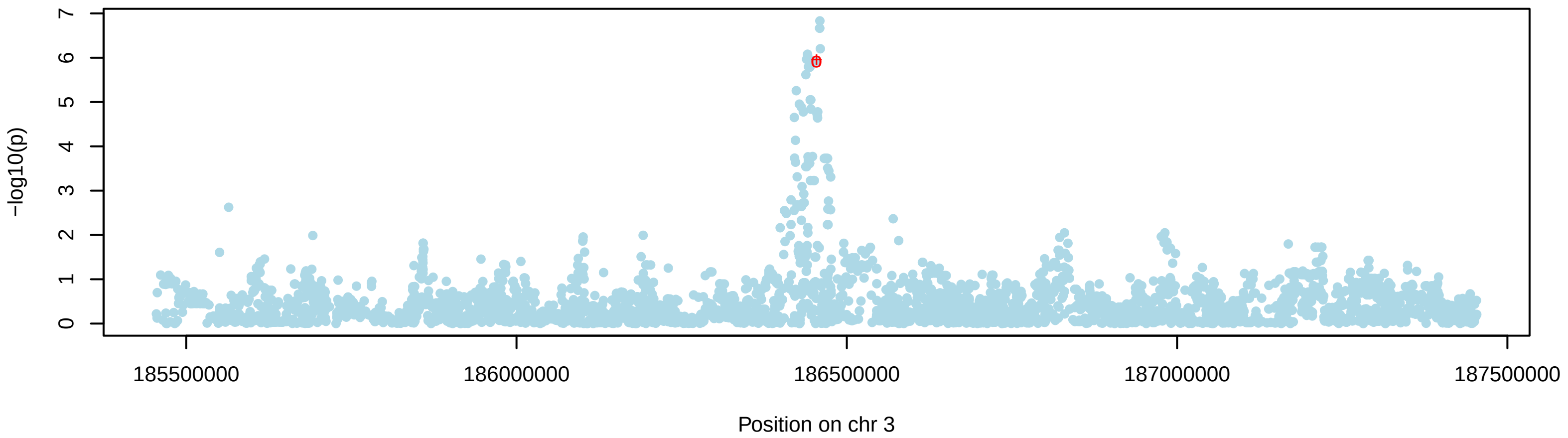

121. HSPA8 (P11142) 3:186394038:G:C [Tarkin]

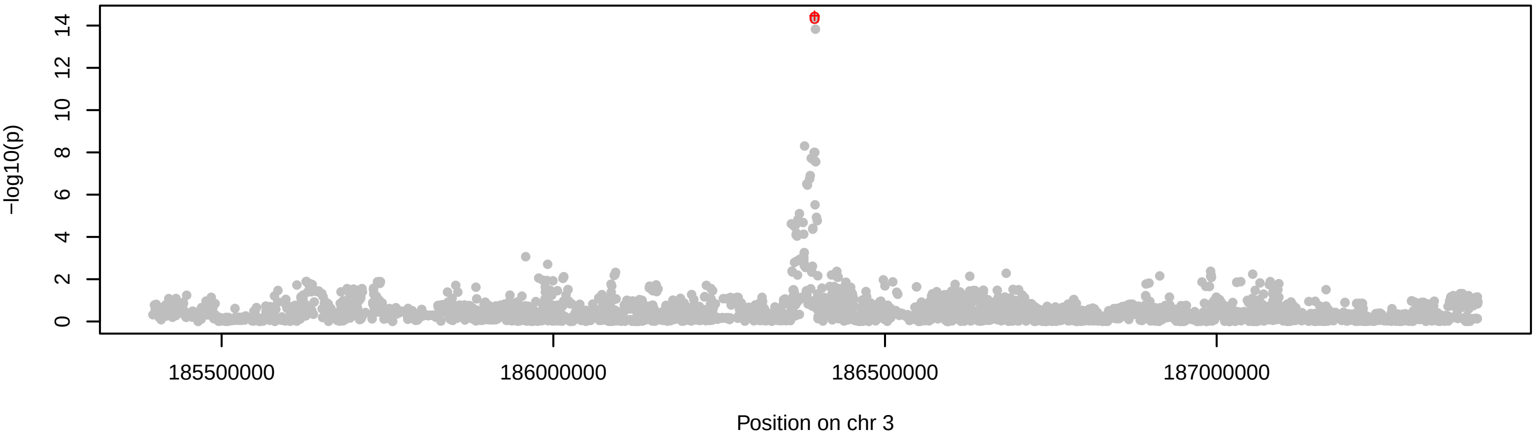

coloc with QMDiab: H3 (PP=100%)

121. HSPA8 (P11142) 3:186394038:G:C [QMDiab]

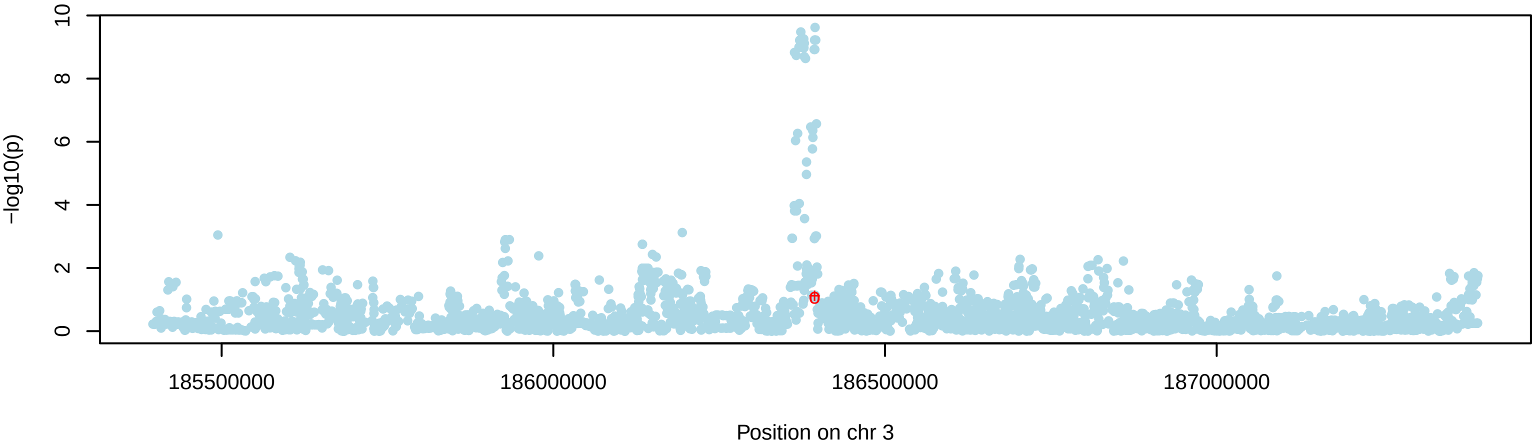

122. C8G (P07360) 9:139840471:G:T [Tarkin]

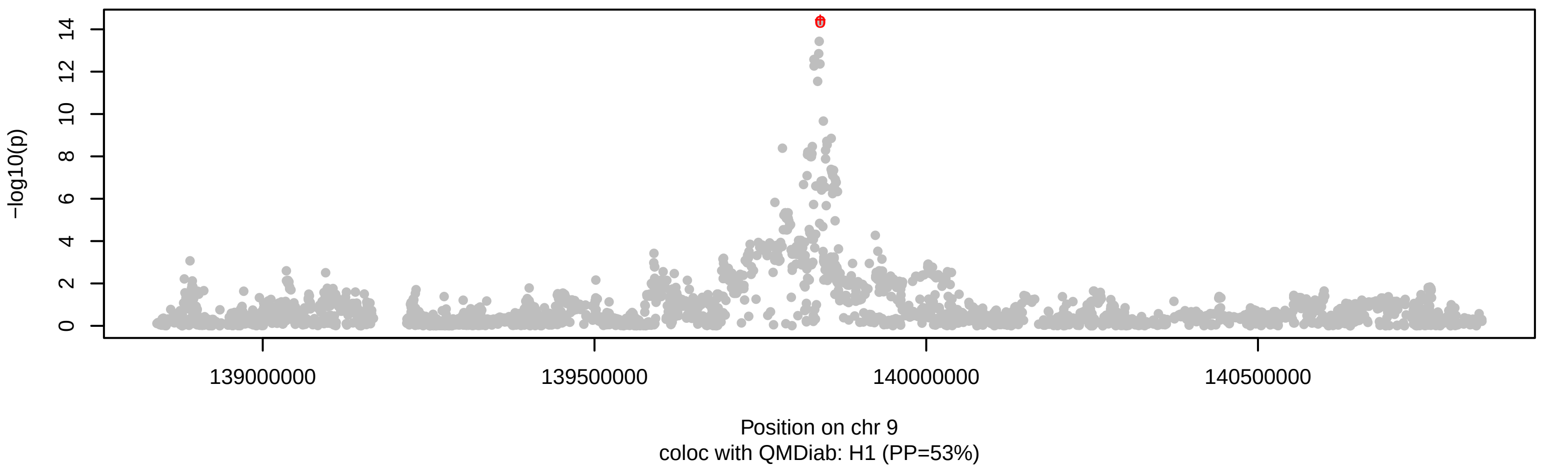

coloc with QMDiab: H1 (PP=53%)

122. C8G (P07360) 9:139840471:G:T [QMDiab]

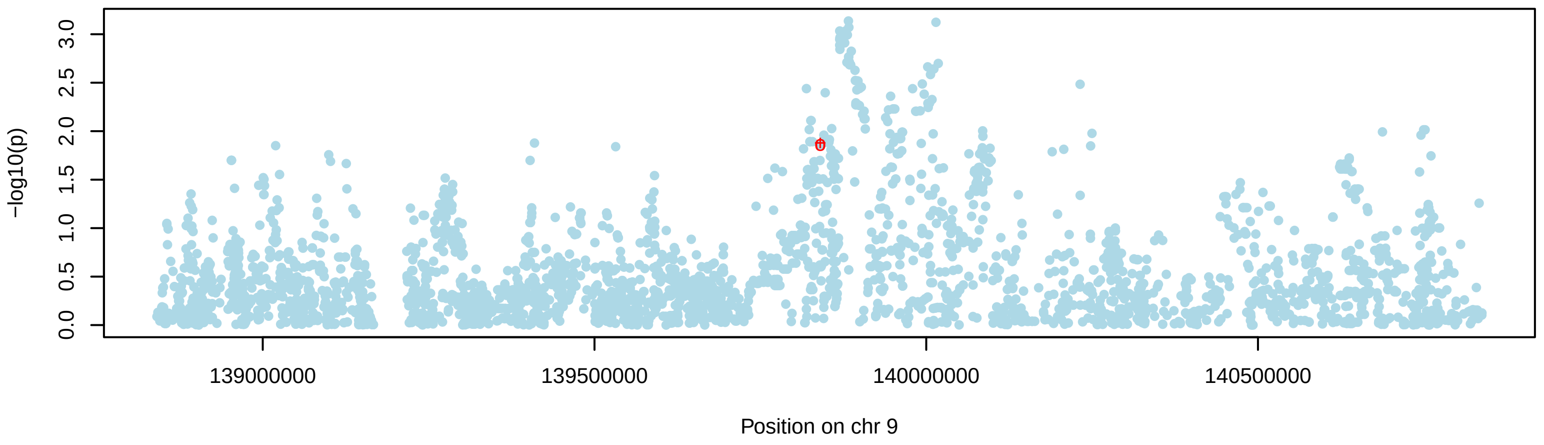

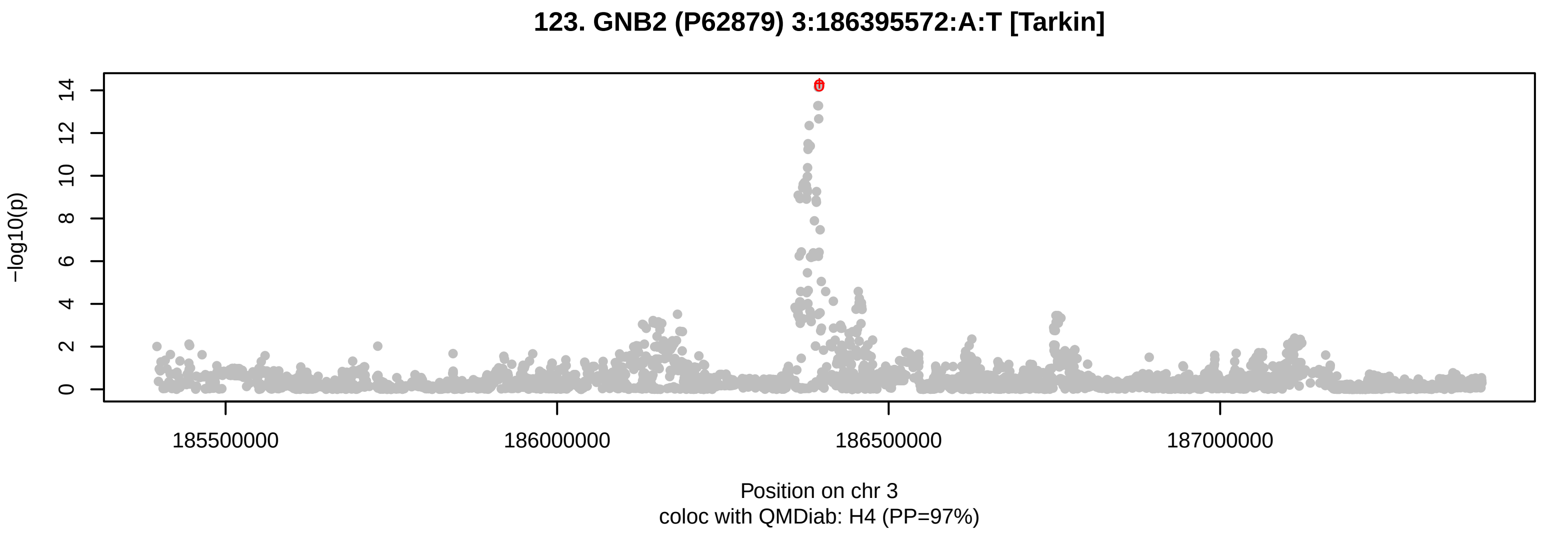
123. GNB2 (P62879) 3:186395572:A:T [Tarkin]
−log10(p)
0
2
4
6
8
10
12
14
185500000
186000000
186500000
187000000
Position on chr 3
coloc with QMDiab: H4 (PP=97%)

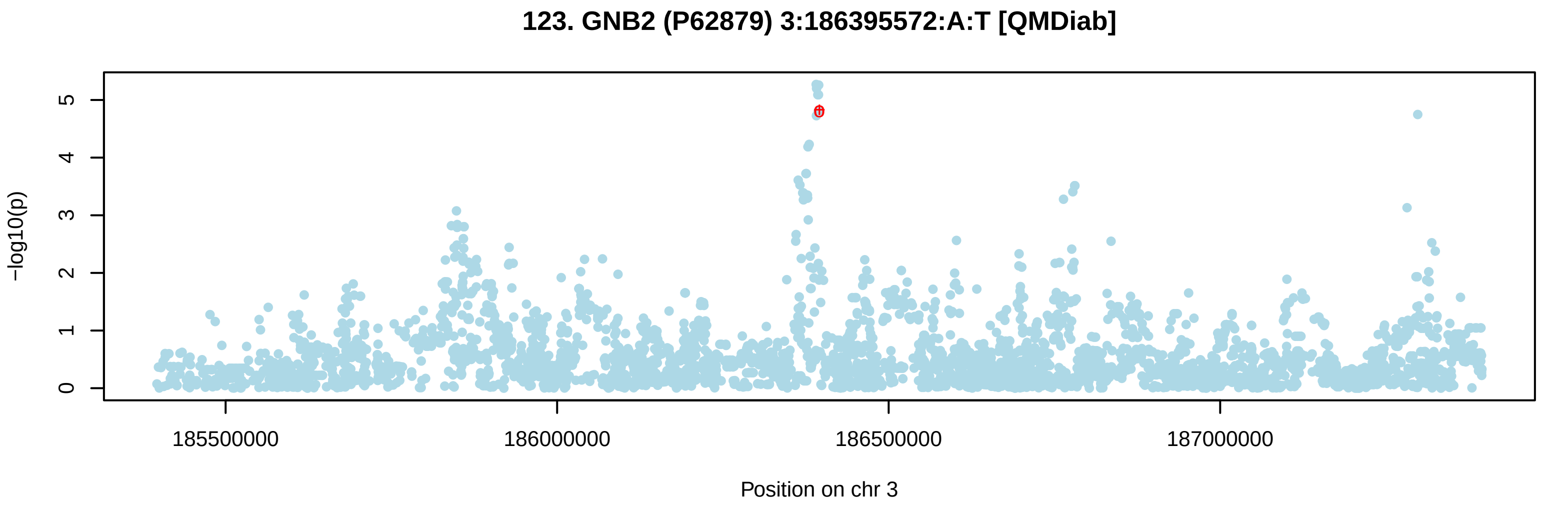
123. GNB2 (P62879) 3:186395572:A:T [QMDiab]
−log10(p)
0
1
2
3
4
5
185500000
186000000
186500000
187000000
Position on chr 3

124. DIAPH1 (O60610) 12:122216910:A:G [Tarkin]

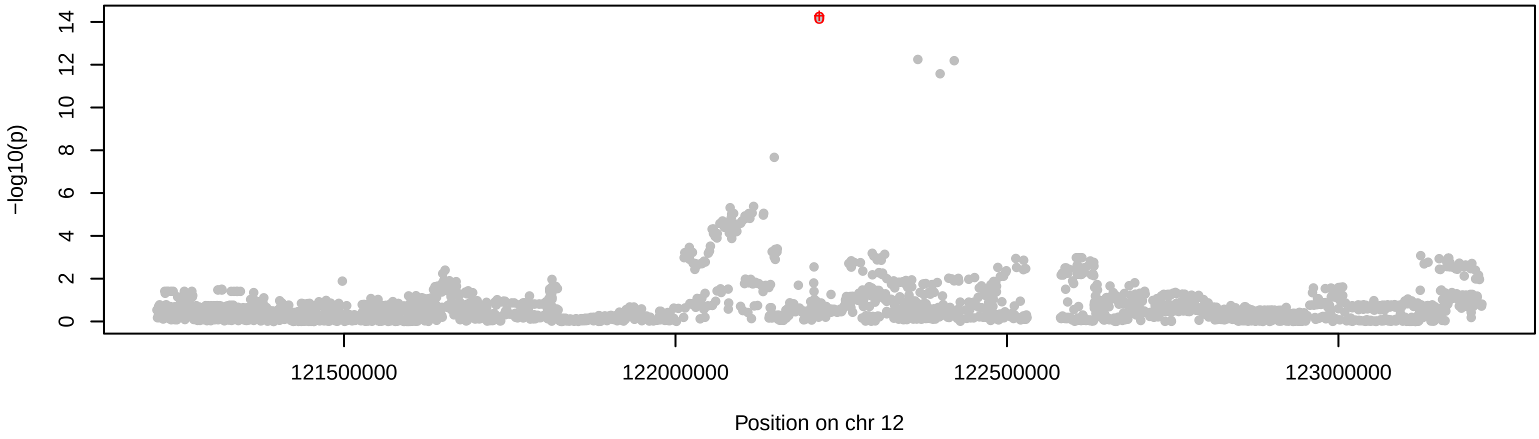

coloc with QMDiab: H1 (PP=70%)

124. DIAPH1 (O60610) 12:122216910:A:G [QMDiab]

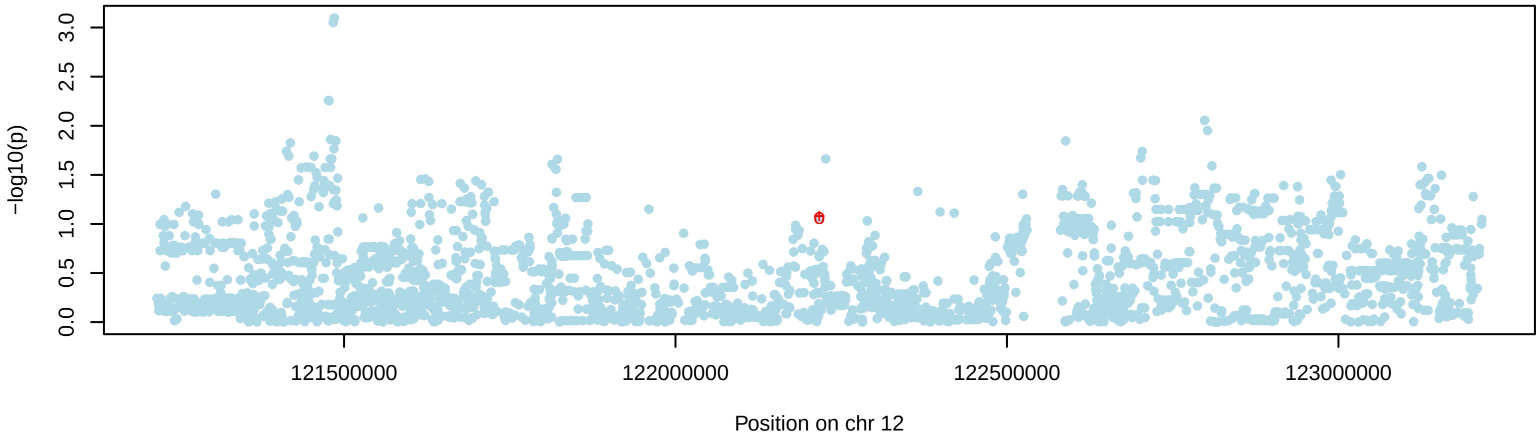

125. IGKV1−17 (P01599) 2:90104710:C:T [Tarkin]

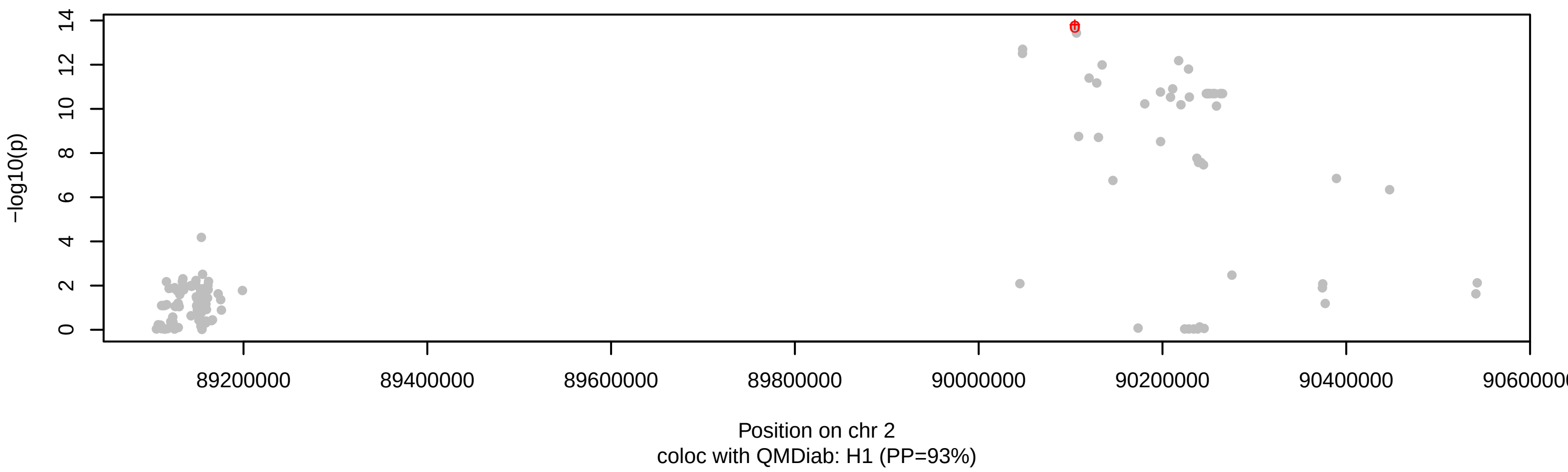

coloc with QMDiab: H1 (PP=93%)

125. IGKV1−17 (P01599) 2:90104710:C:T [QMDiab]

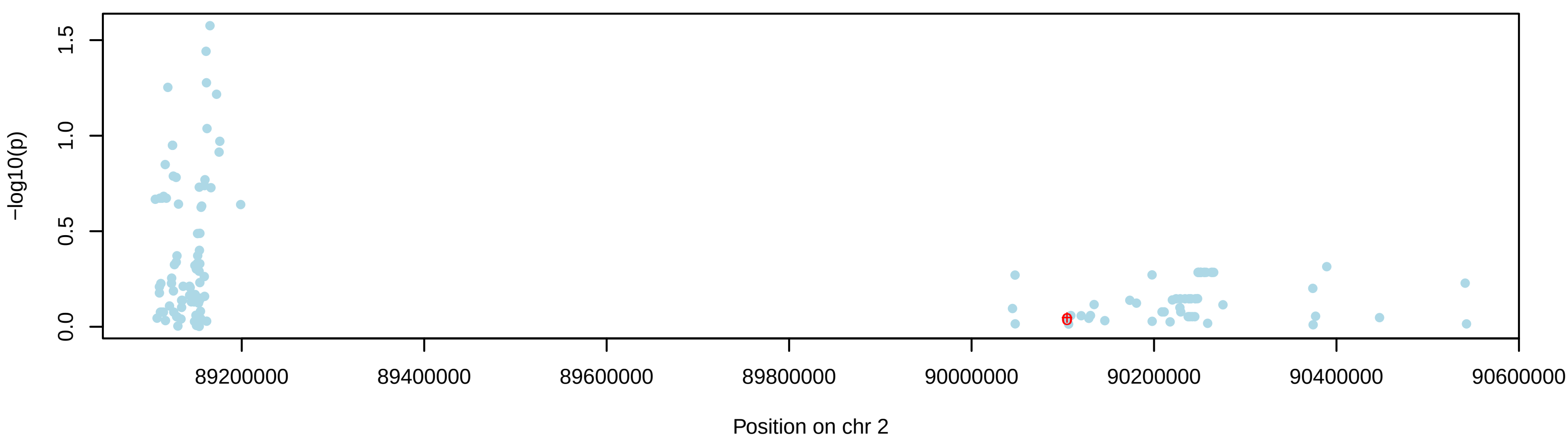

126. MAN2A2 (P49641) 15:91466158:G:A [Tarkin]

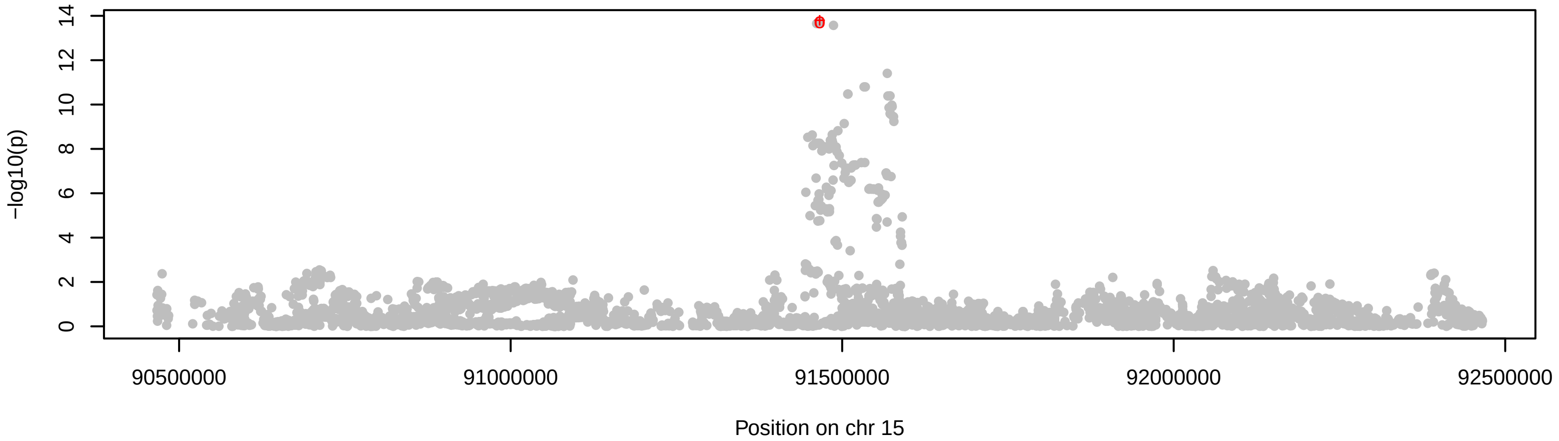

coloc with QMDiab: H1 (PP=66%)

126. MAN2A2 (P49641) 15:91466158:G:A [QMDiab]

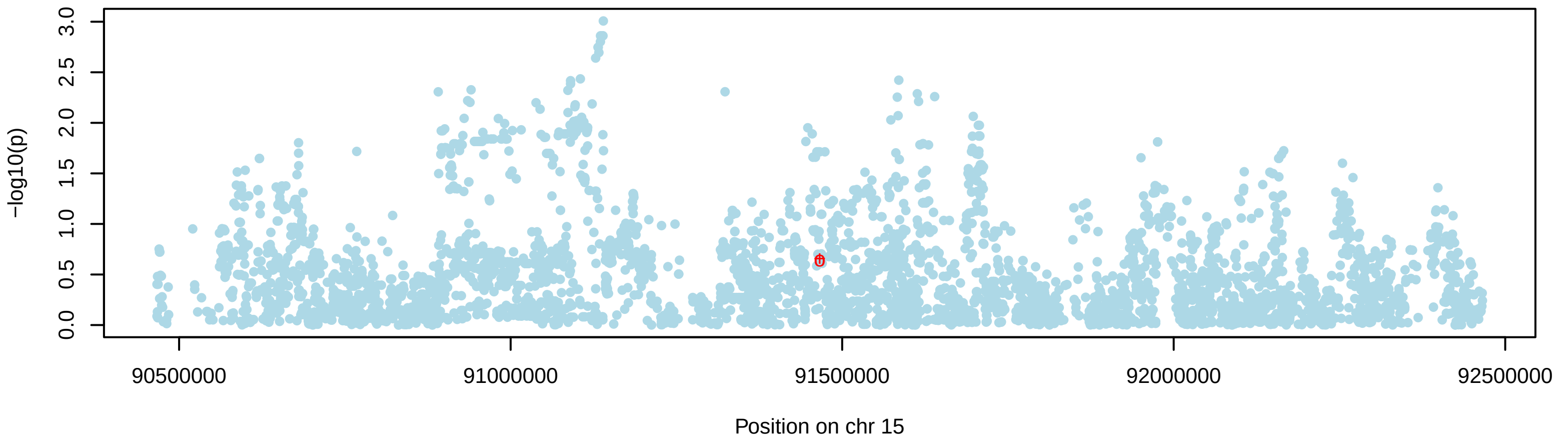

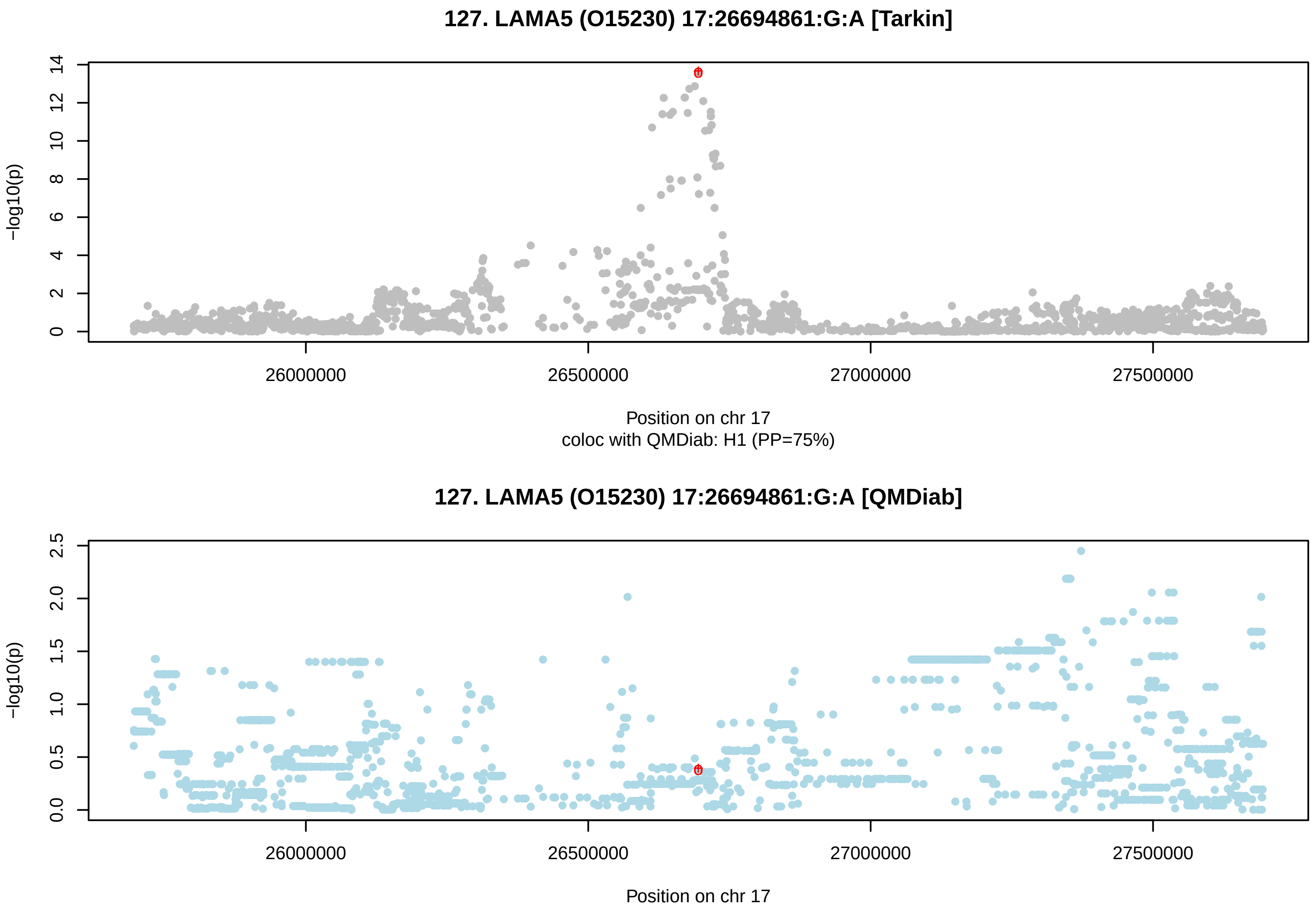
127. LAMA5 (O15230) 17:26694861:G:A [Tarkin]
−log10(p)
0
2
4
6
8
10
12
14
26000000
26500000
27000000
27500000
Position on chr 17
coloc with QMDiab: H1 (PP=75%)
127. LAMA5 (O15230) 17:26694861:G:A [QMDiab]
−log10(p)
0.0
0.5
1.0
1.5
2.0
2.5
26000000
26500000
27000000
27500000
Position on chr 17

128. SERPINA11 (Q86U17) 14:94928189:G:T [Tarkin]

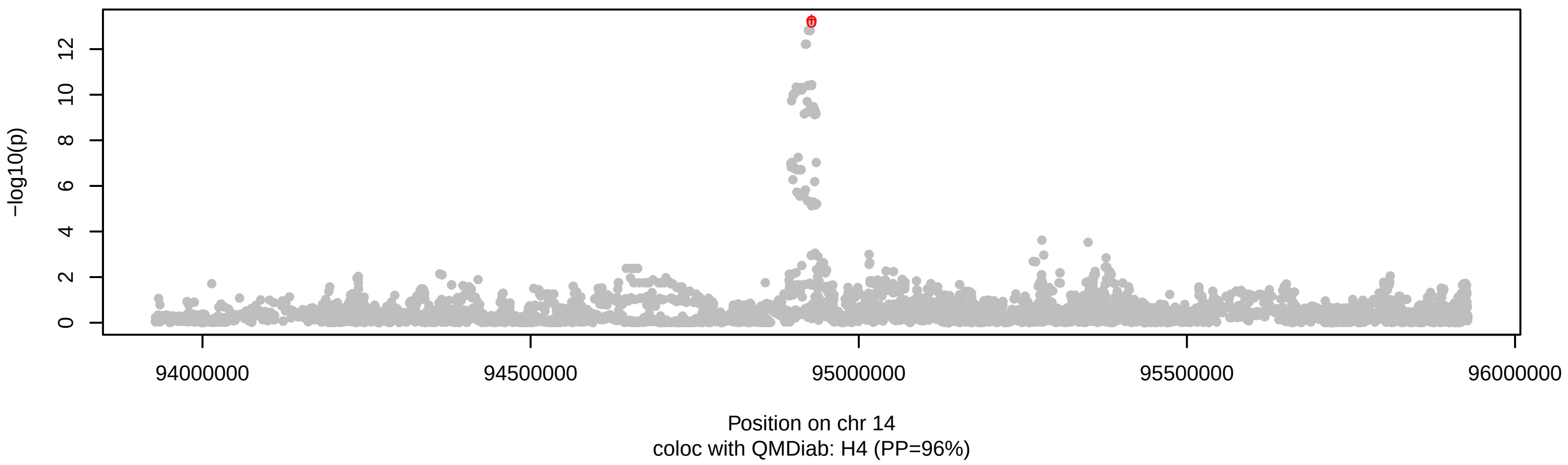

128. SERPINA11 (Q86U17) 14:94928189:G:T [QMDiab]

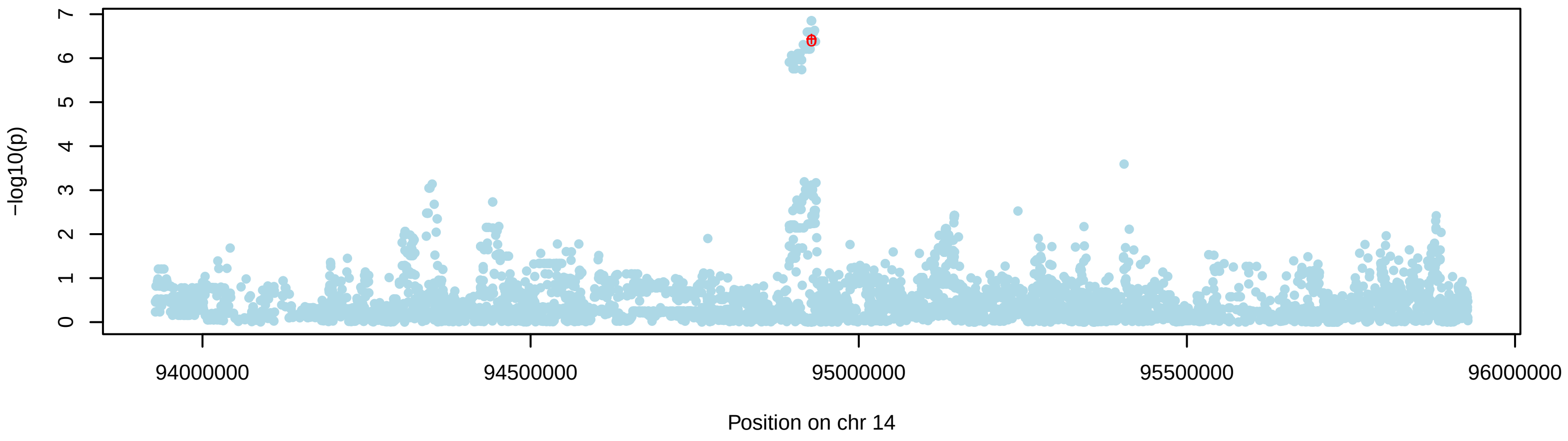

129. ORM2 (P19652) 9:117083803:C:A [Tarkin]

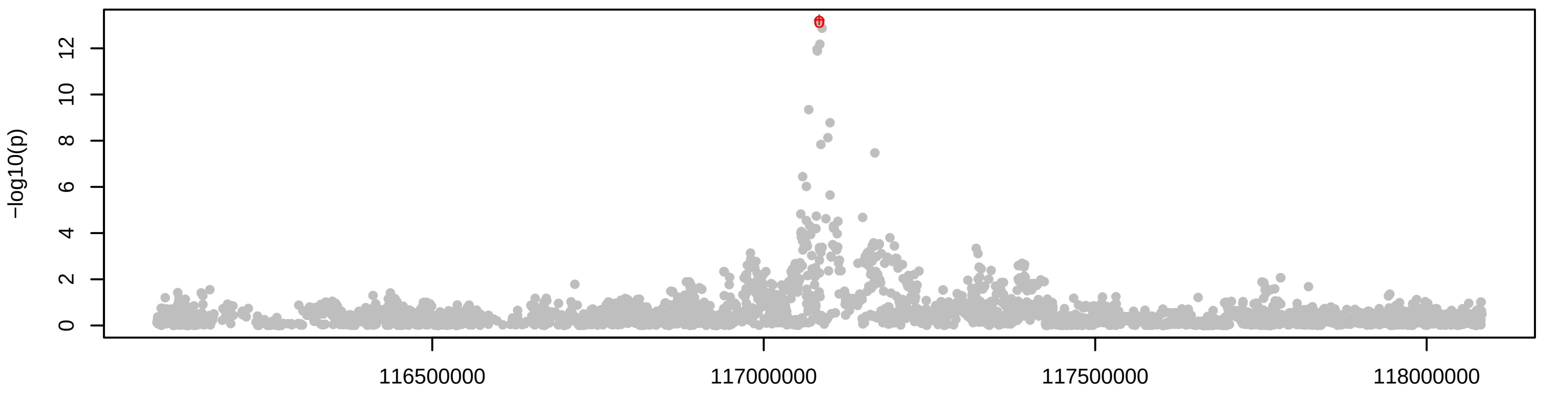

Position on chr 9
coloc with QMDiab: H1 (PP=68%)

129. ORM2 (P19652) 9:117083803:C:A [QMDiab]

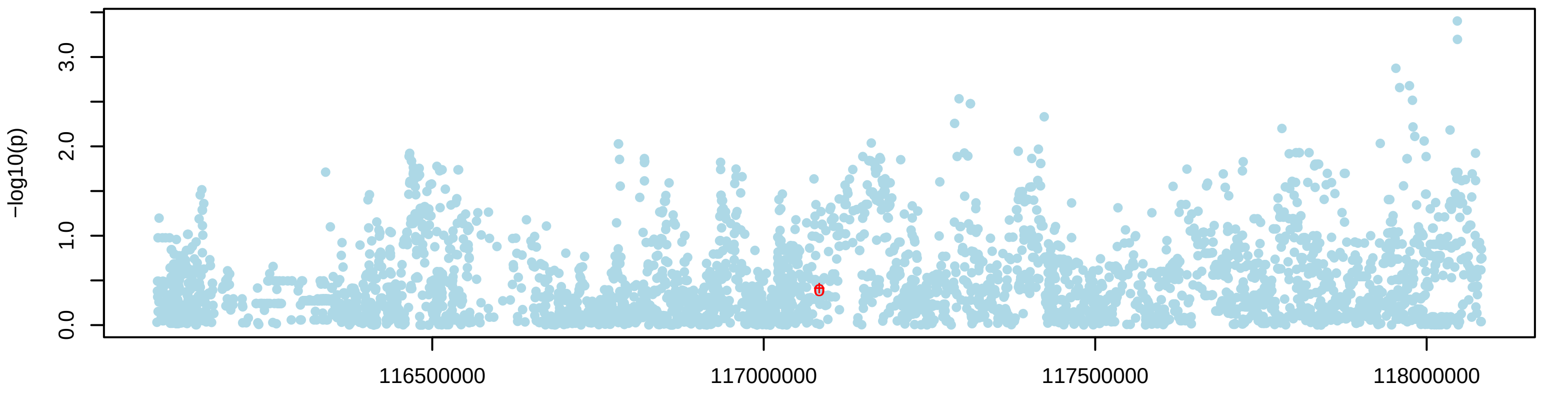

Position on chr 9

130. TNFAIP6 (P98066) 2:152142088:T:C [Tarkin]

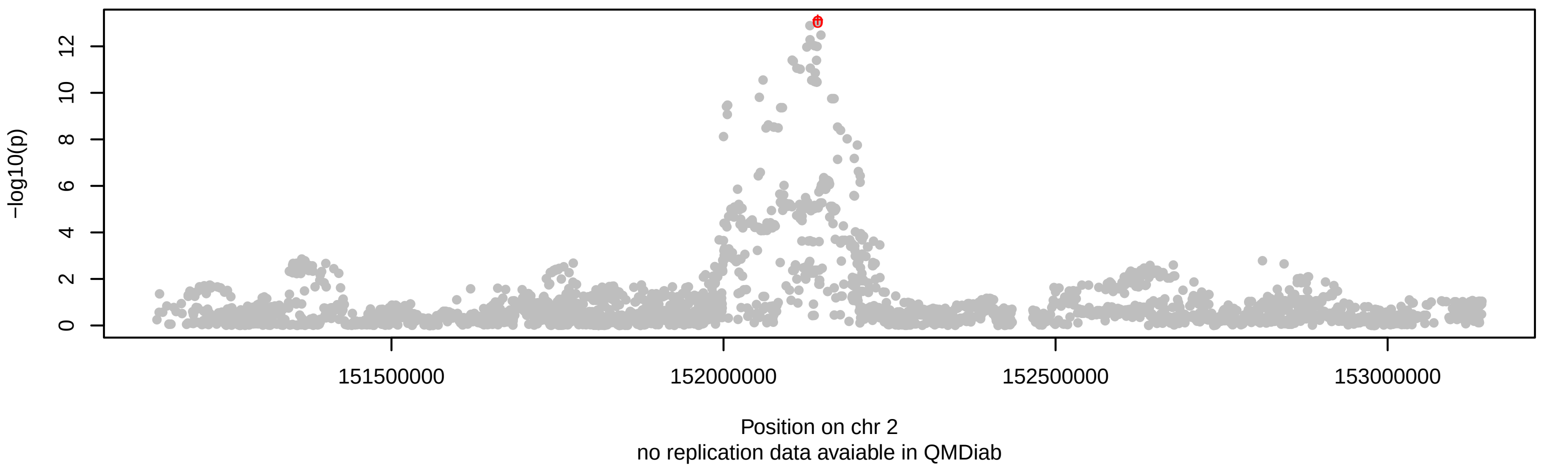

131. SSC5D (A1L4H1) 19:55999142:C:G [Tarkin]

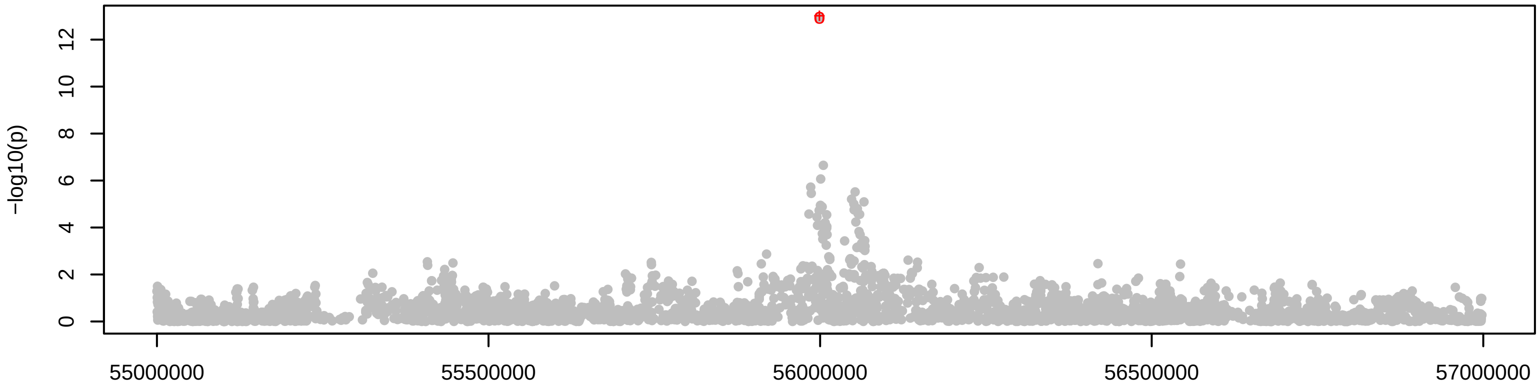

Position on chr 19
coloc with QMDiab: H1 (PP=63%)

131. SSC5D (A1L4H1) 19:55999142:C:G [QMDiab]

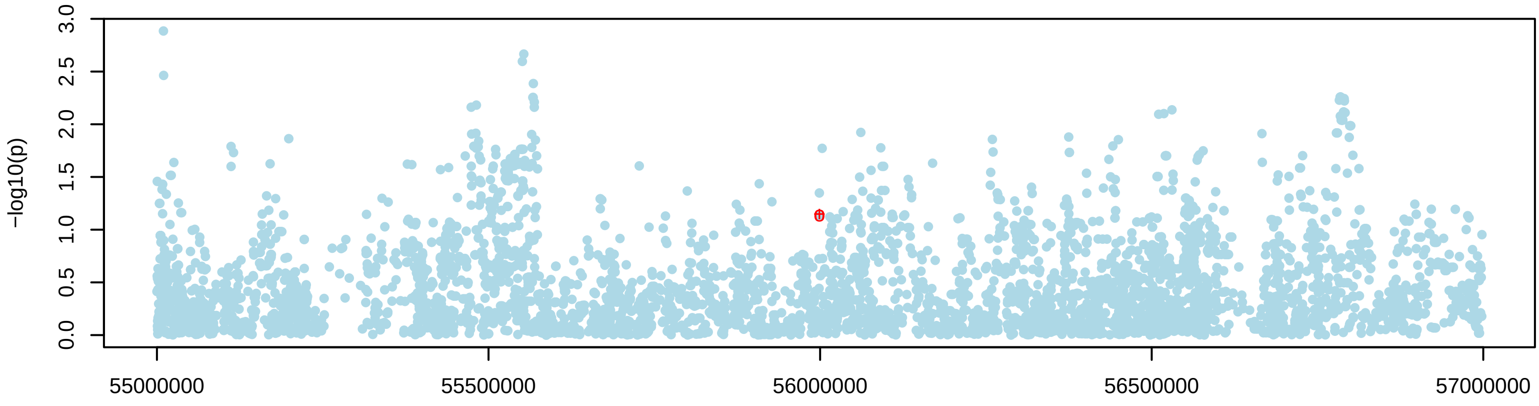

Position on chr 19

132. TPSAB1 (A0A087WUI4;A0A087X1U0;A0A140VJT7;J3QTS8;Q15661;Q15661−2) 3:186391274:G:A [Tarkin]

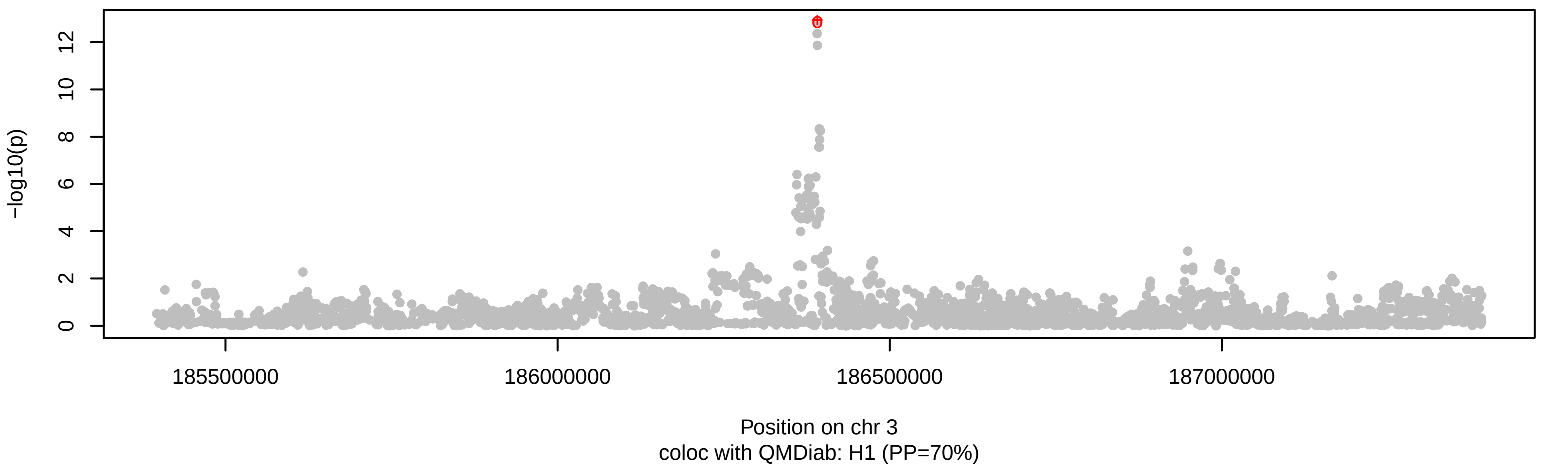

coloc with QMDiab: H1 (PP=70%)

132. TPSAB1 (A0A087WUI4;A0A087X1U0;A0A140VJT7;J3QTS8;Q15661;Q15661−2) 3:186391274:G:A [QMDiab]

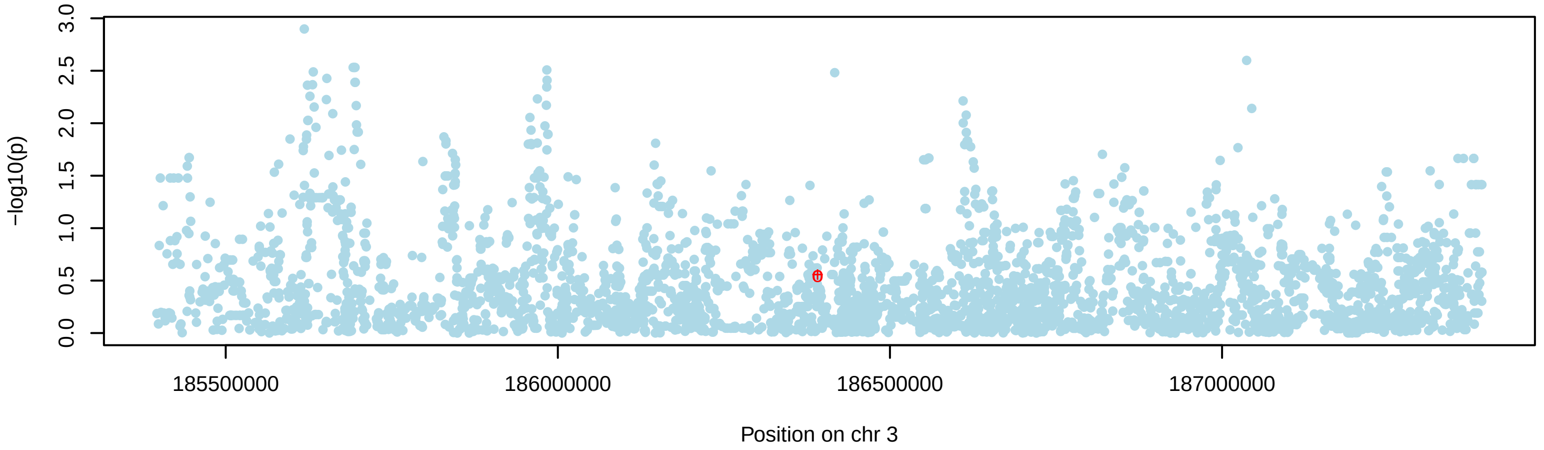

133. CCL14 (Q16627) 17:34312337:T:C [Tarkin]

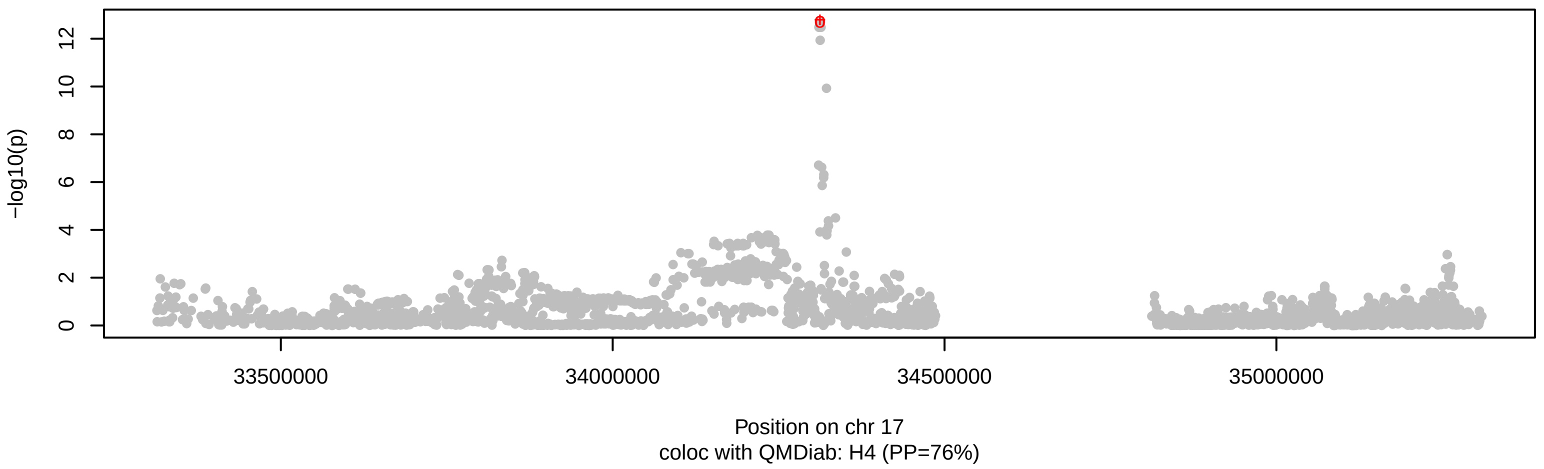

133. CCL14 (Q16627) 17:34312337:T:C [QMDiab]

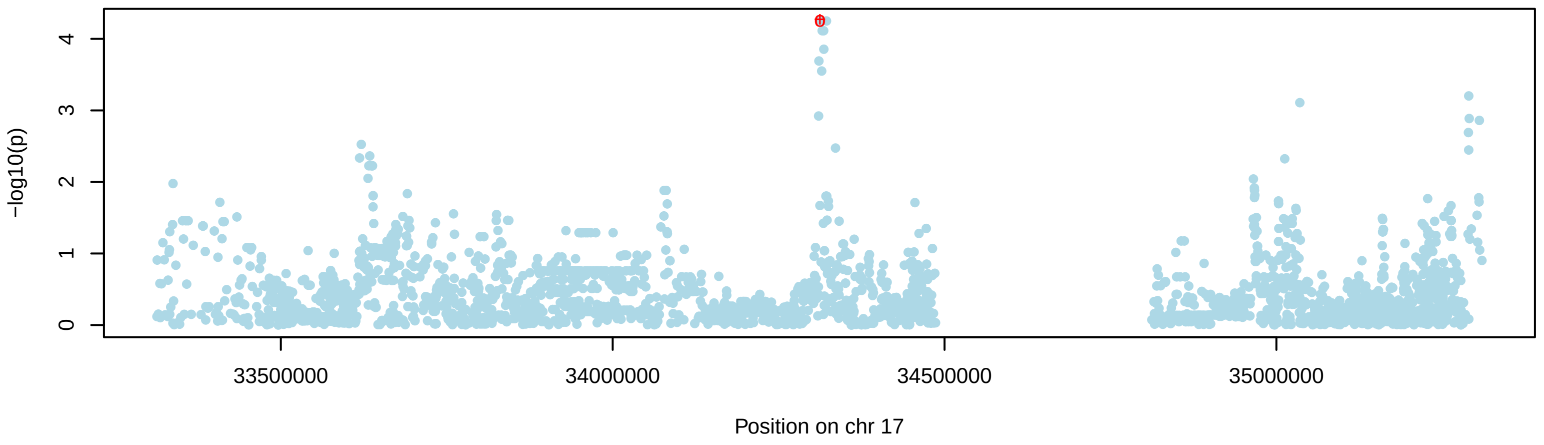

134. IL7R (P16871) 9:136153875:C:T [Tarkin]

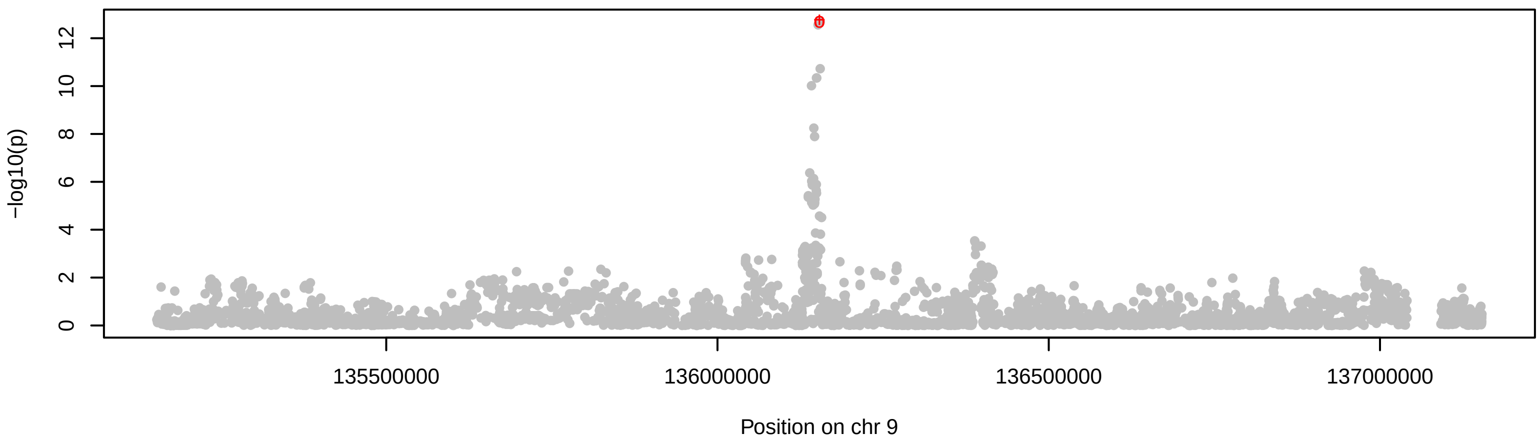

coloc with QMDiab: H1 (PP=66%)

134. IL7R (P16871−3) 9:136153875:C:T [QMDiab]

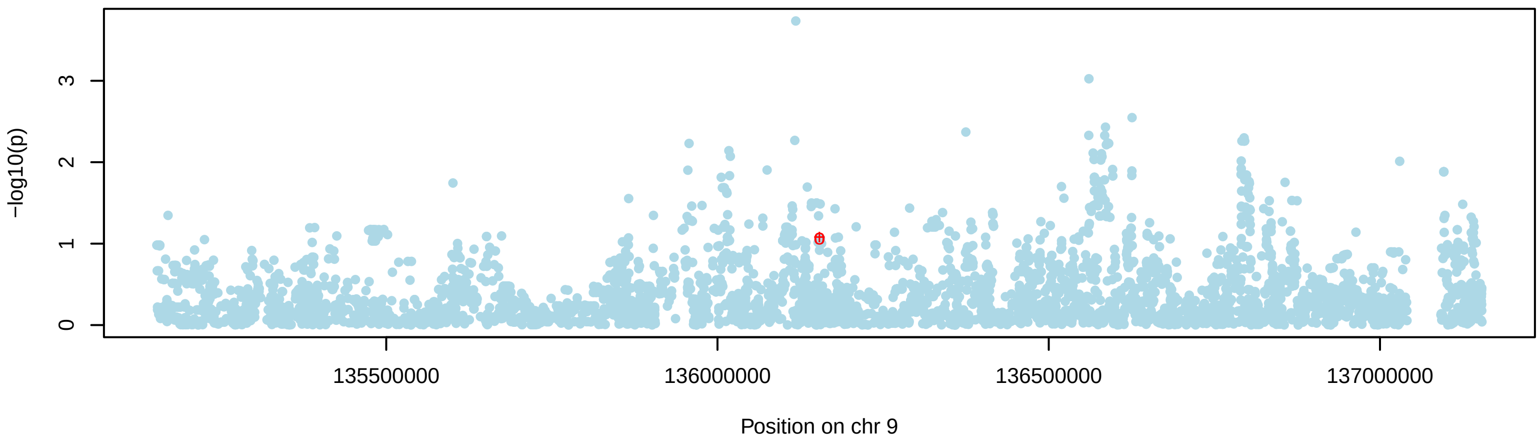

135. TAC3 (Q9UHF0−2) 12:57216590:C:T [Tarkin]

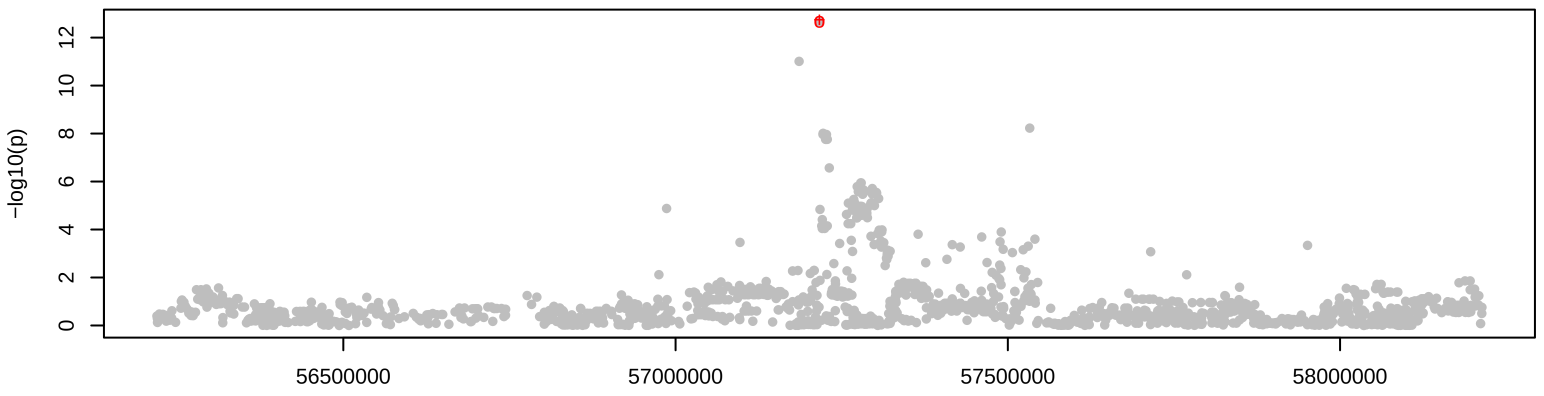

Position on chr 12
coloc with QMDiab: H1 (PP=83%)

135. TAC3 (Q9UHF0;Q9UHF0−2) 12:57216590:C:T [QMDiab]

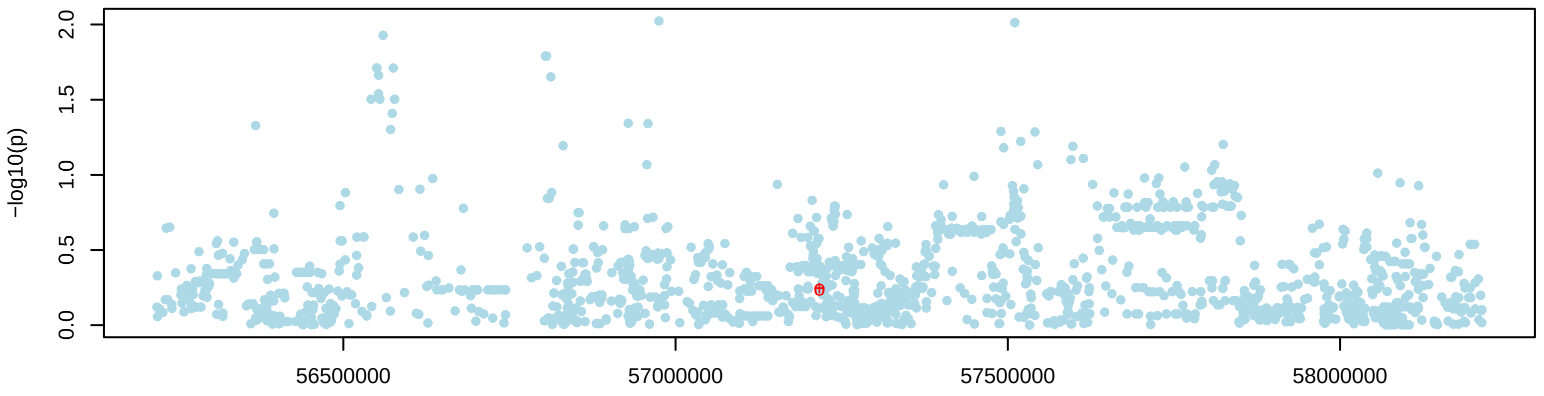

Position on chr 12

136. ECM2 (O94769) 9:95281459:A:G [Tarkin]

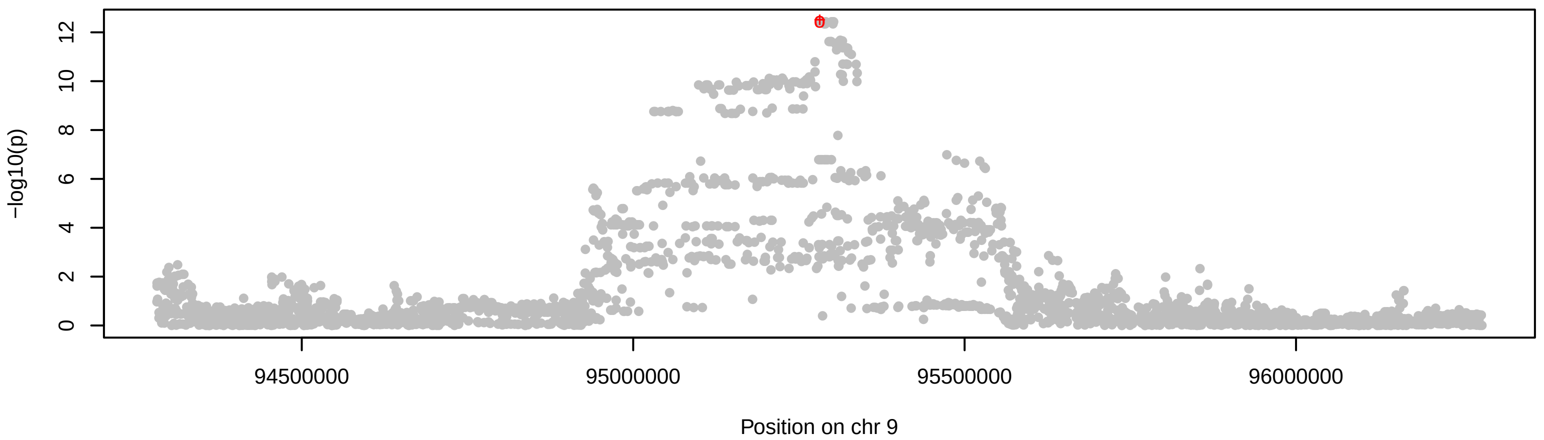

coloc with QMDiab: H4 (PP=53%)

136. ECM2 (O94769) 9:95281459:A:G [QMDiab]

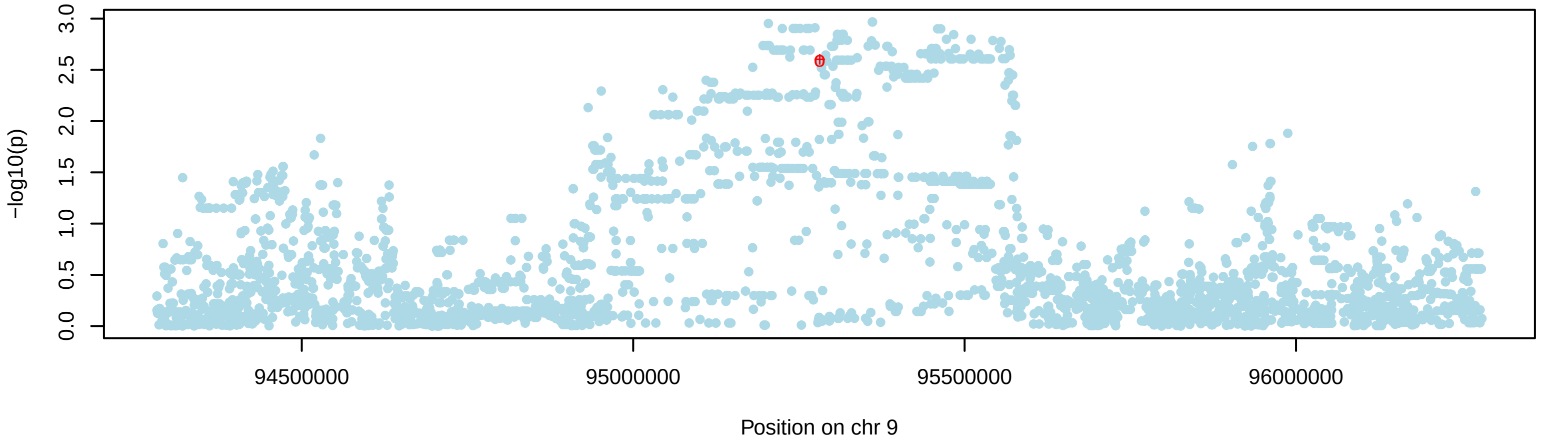

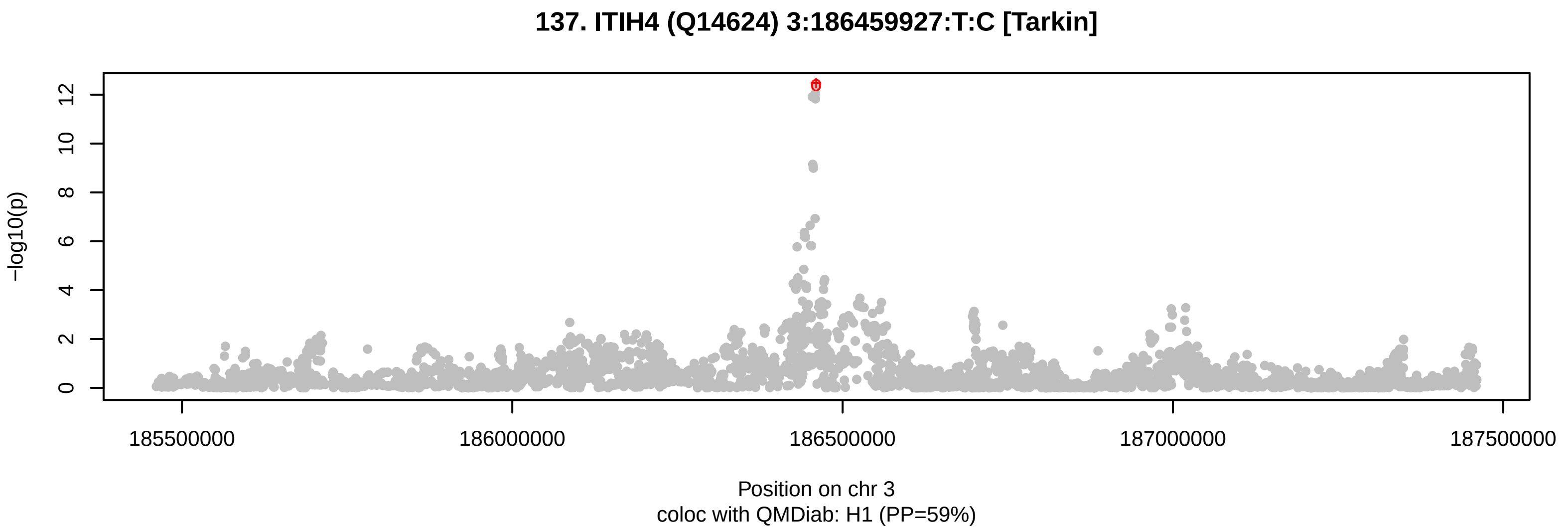
137. ITIH4 (Q14624) 3:186459927:T:C [Tarkin]
−log10(p)
0
2
4
6
8
10
12
185500000
186000000
186500000
187000000
187500000
Position on chr 3
coloc with QMDiab: H1 (PP=59%)

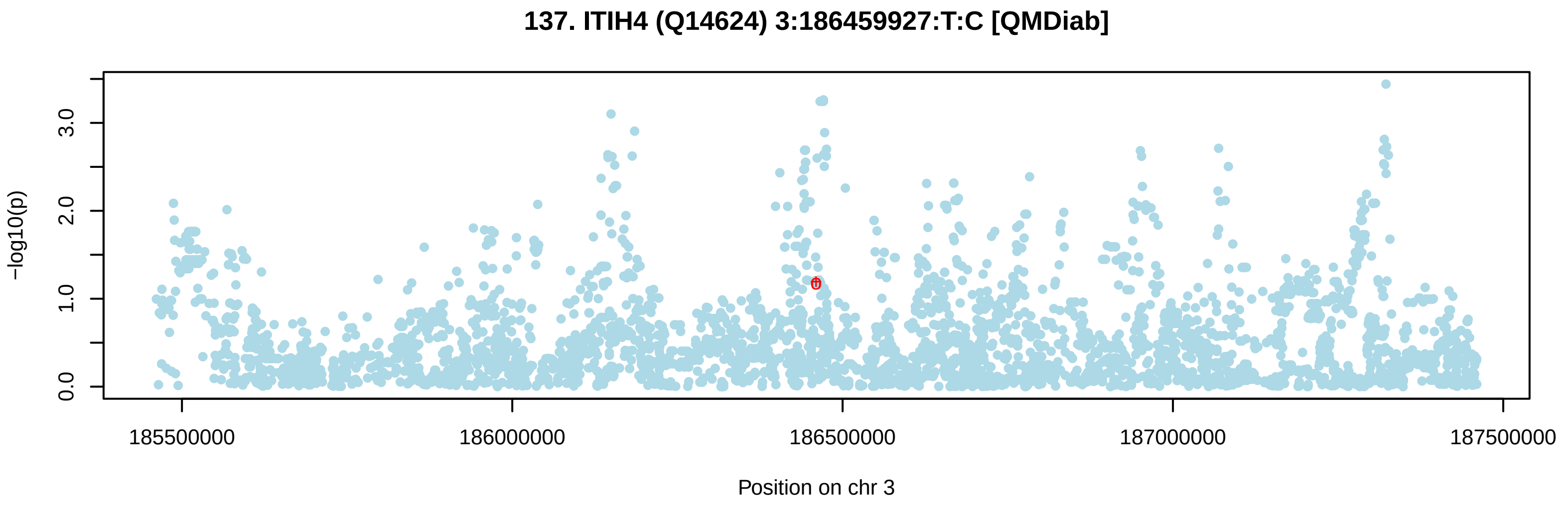
137. ITIH4 (Q14624) 3:186459927:T:C [QMDiab]
−log10(p)
0.0
1.0
2.0
3.0
185500000
186000000
186500000
187000000
187500000
Position on chr 3

138. CNDP2 (Q96KP4) 18:72176083:T:C [Tarkin]

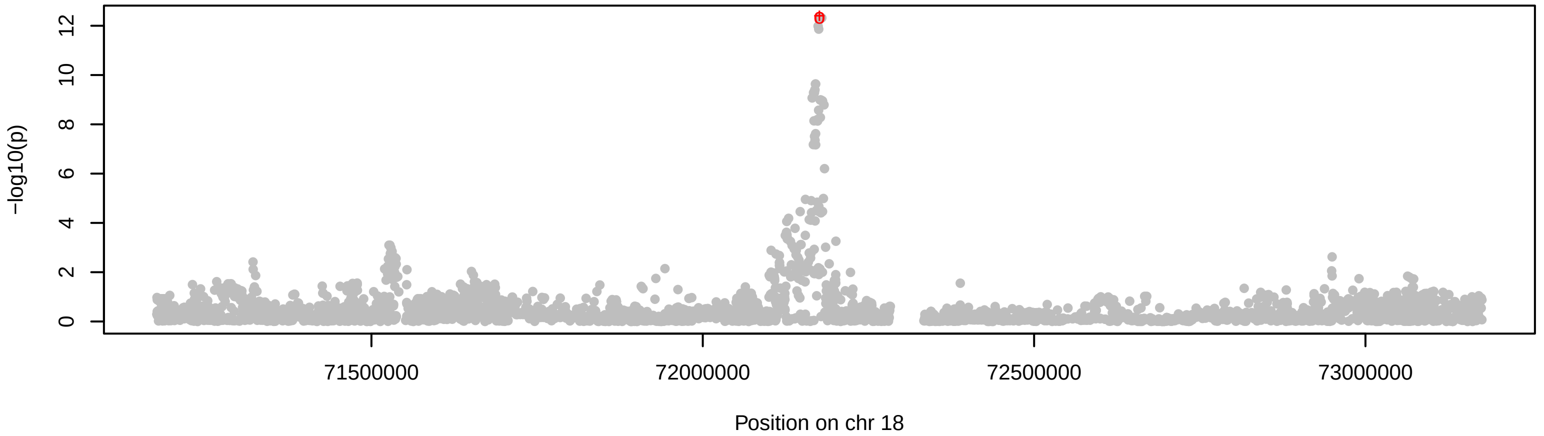

coloc with QMDiab: H1 (PP=65%)

138. CNDP2 (Q96KP4) 18:72176083:T:C [QMDiab]

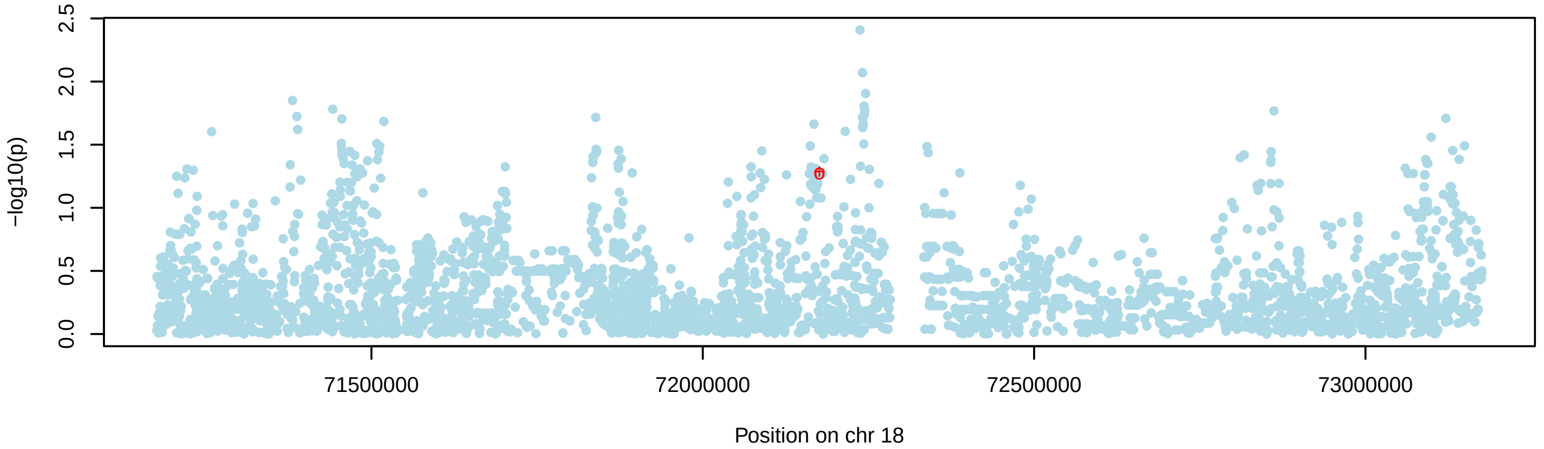

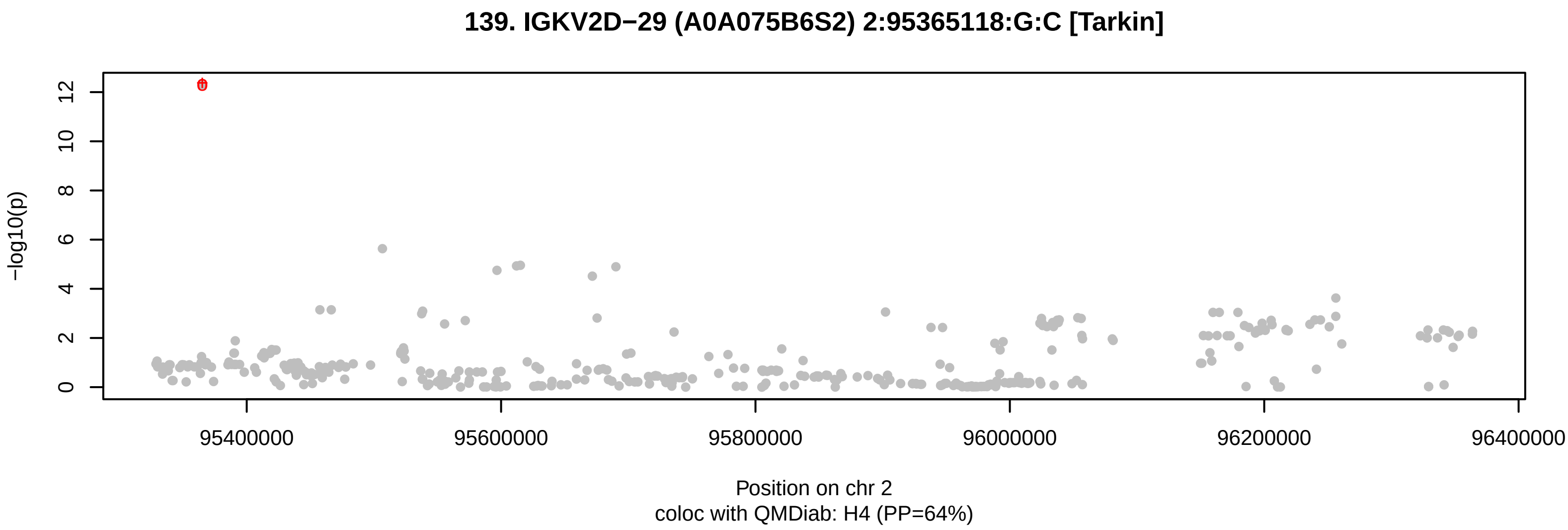
139. IGKV2D−29 (A0A075B6S2) 2:95365118:G:C [Tarkin]
−log10(p)
0
2
4
6
8
10
12
95400000
95600000
95800000
96000000
96200000
96400000
Position on chr 2
coloc with QMDiab: H4 (PP=64%)

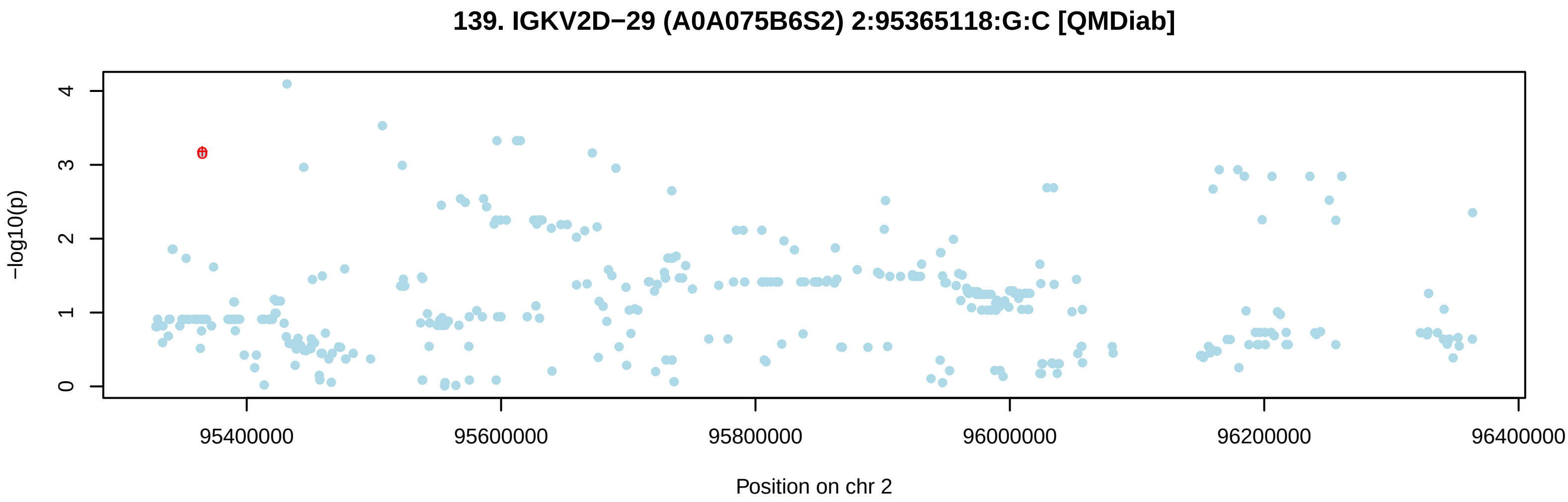
139. IGKV2D−29 (A0A075B6S2) 2:95365118:G:C [QMDiab]
−log10(p)
0
1
2
3
4
95400000
95600000
95800000
96000000
96200000
96400000
Position on chr 2

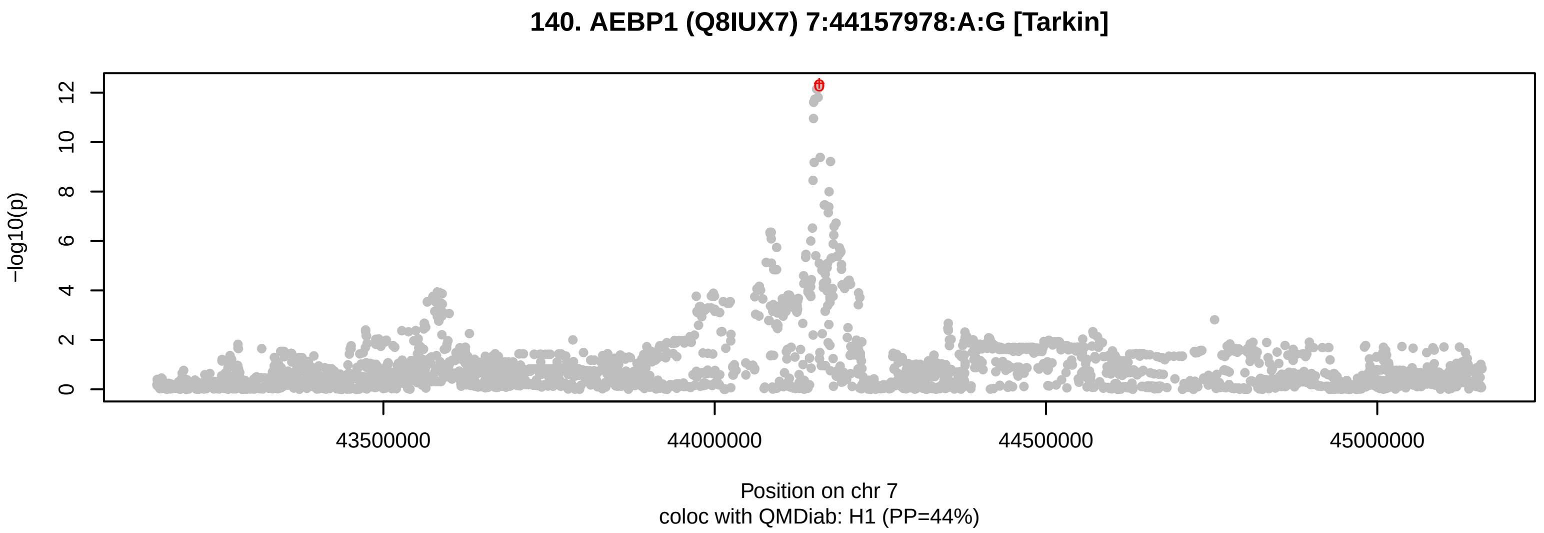

140. AEBP1 (Q8IUX7) 7:44157978:A:G [Tarkin]
−log10(p)
0
2
4
6
8
10
12
43500000
44000000
44500000
45000000
Position on chr 7
coloc with QMDiab: H1 (PP=44%)

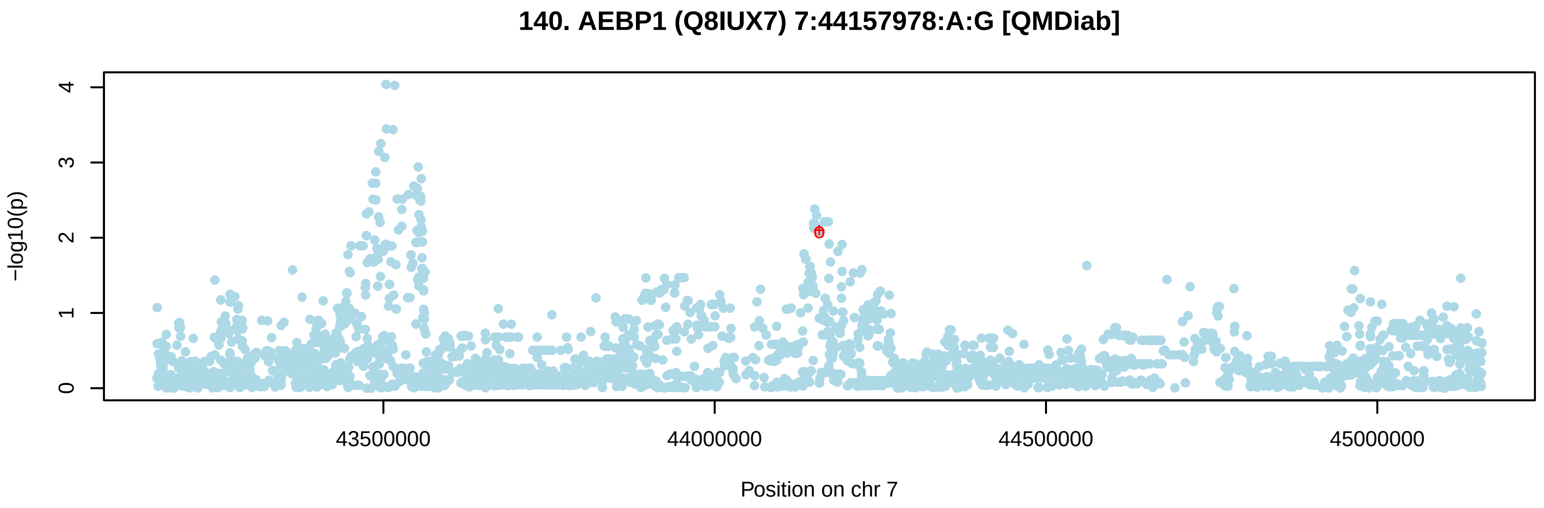

140. AEBP1 (Q8IUX7) 7:44157978:A:G [QMDiab]
−log10(p)
0
1
2
3
4
43500000
44000000
44500000
45000000
Position on chr 7

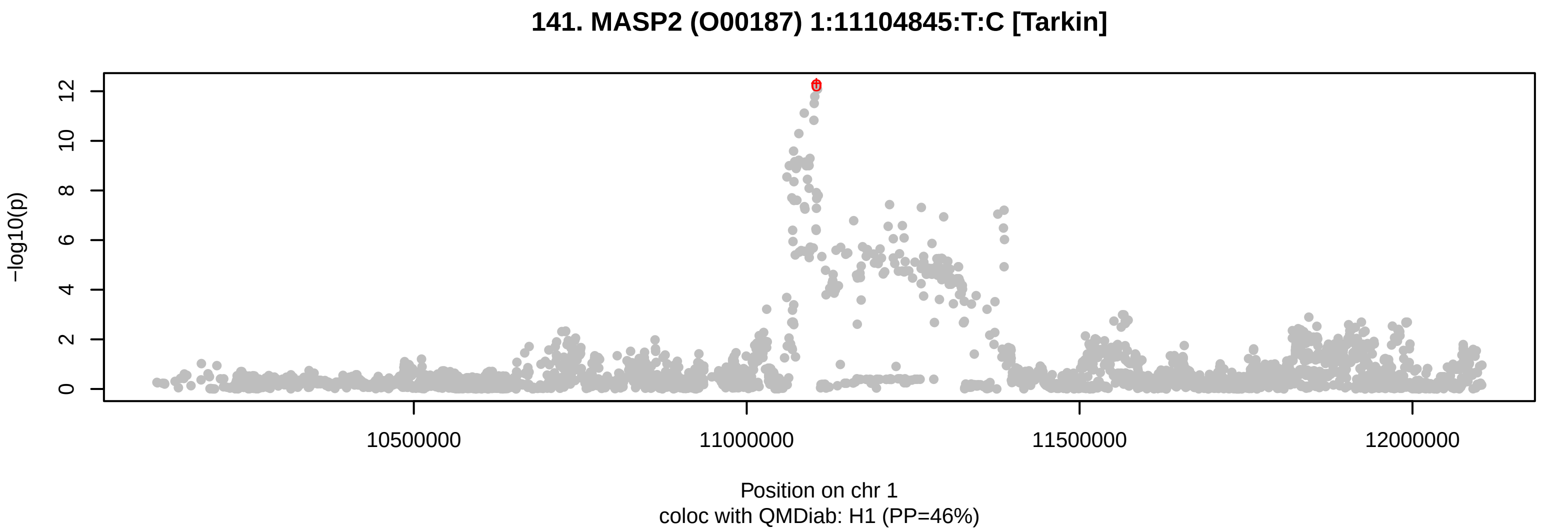

coloc with QMDiab: H1 (PP=46%)

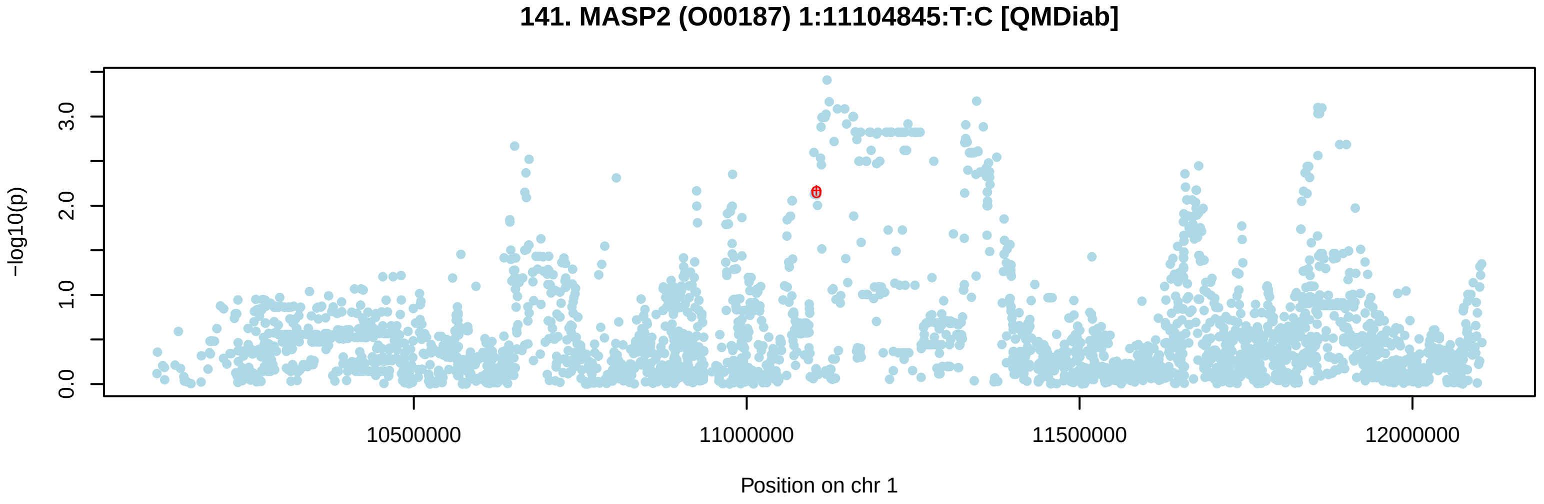

142. FCN1 (O00602) 9:137843955:T:C [Tarkin]

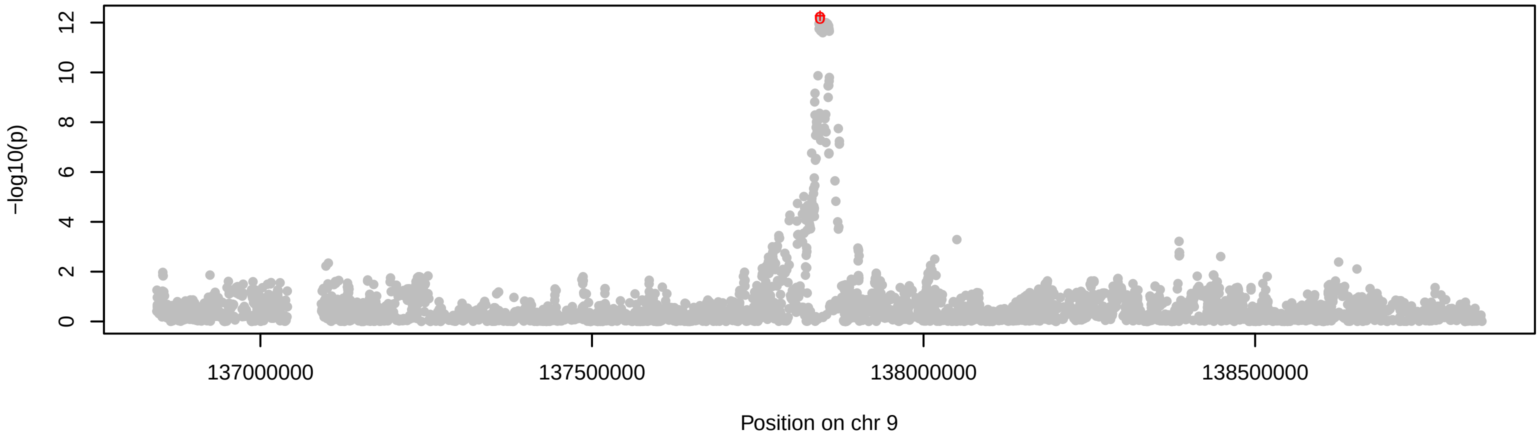

coloc with QMDiab: H1 (PP=63%)

142. FCN1 (O00602) 9:137843955:T:C [QMDiab]

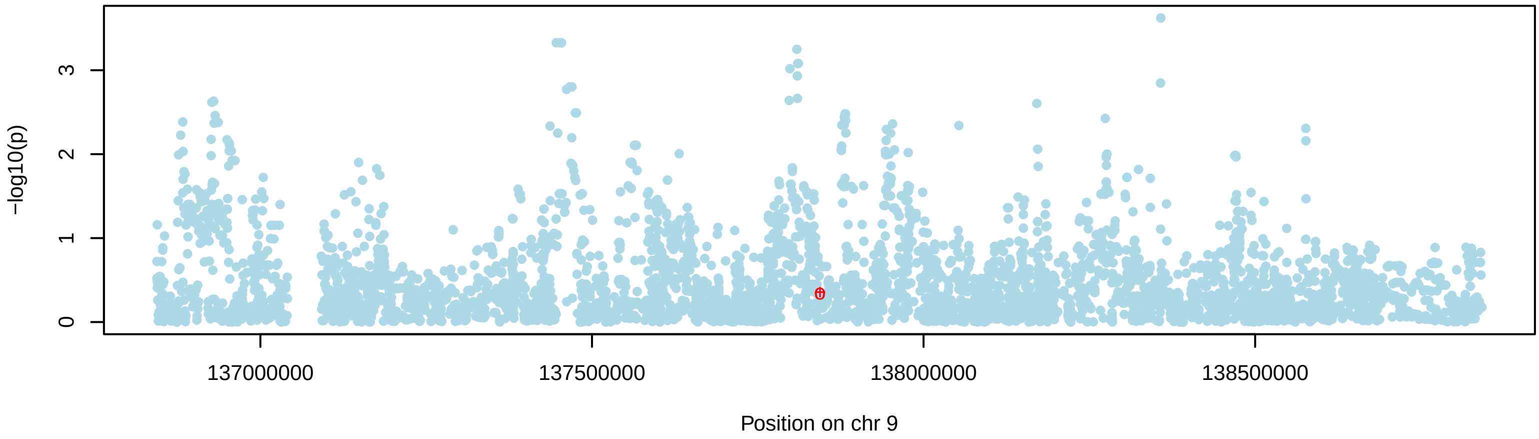

143. DEFA1 (P59665) 17:26694861:G:A [Tarkin]

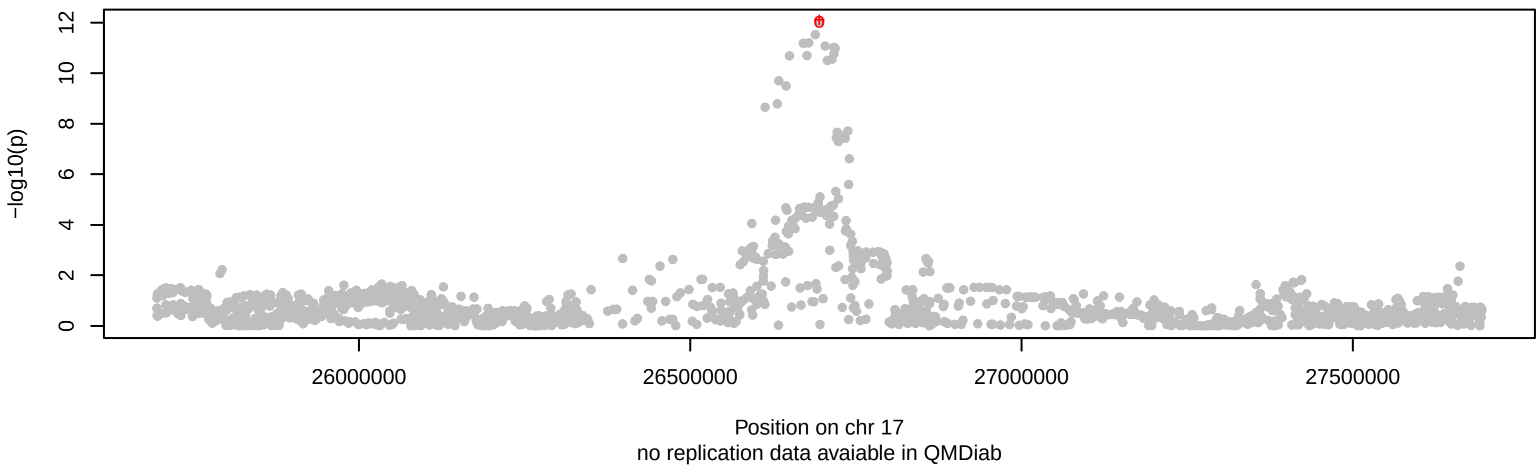

no replication data avaiable in QMDiab

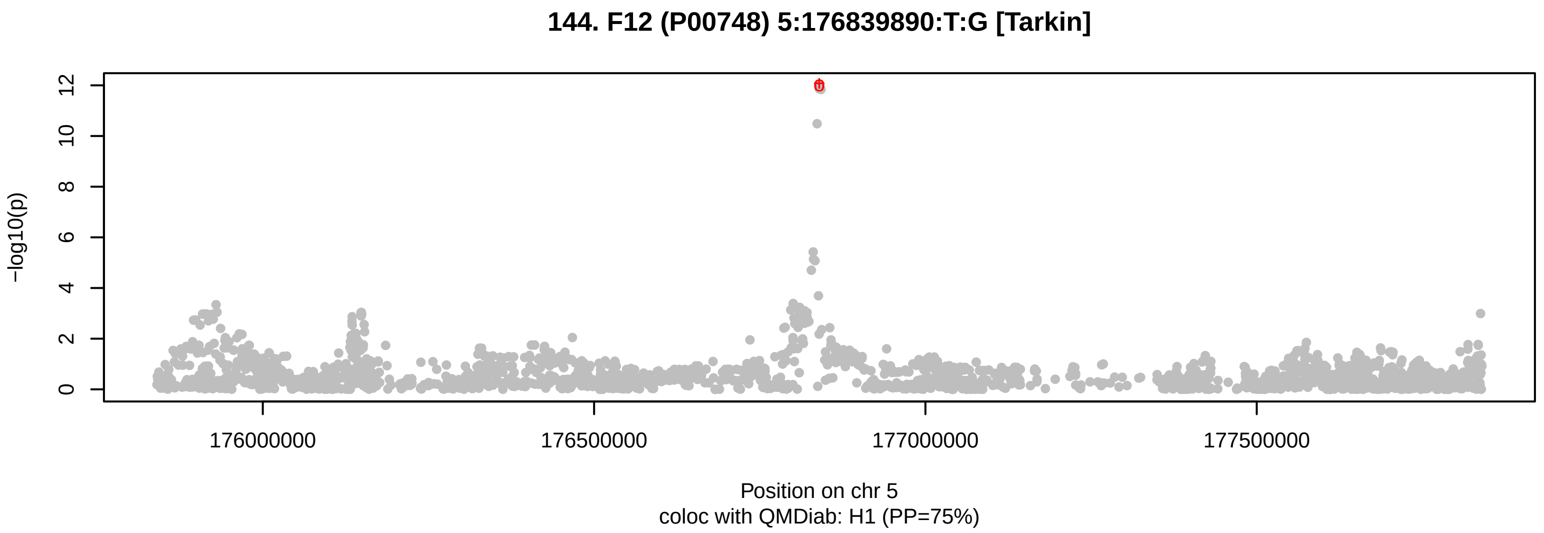

144. F12 (P00748) 5:176839890:T:G [Tarkin]
−log10(p)
0
2
4
6
8
10
12
176000000
176500000
177000000
177500000
Position on chr 5
coloc with QMDiab: H1 (PP=75%)

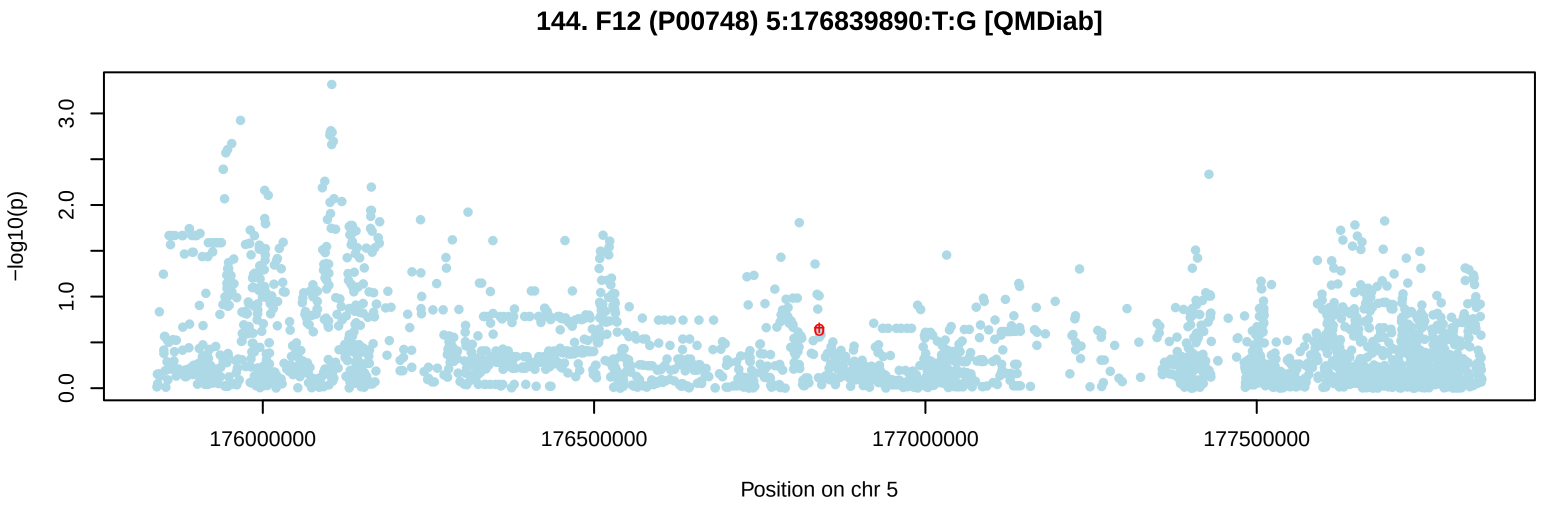

144. F12 (P00748) 5:176839890:T:G [QMDiab]
−log10(p)
0.0
1.0
2.0
3.0
176000000
176500000
177000000
177500000
Position on chr 5

145. LIPC (P11150) 15:58723426:A:G [Tarkin]

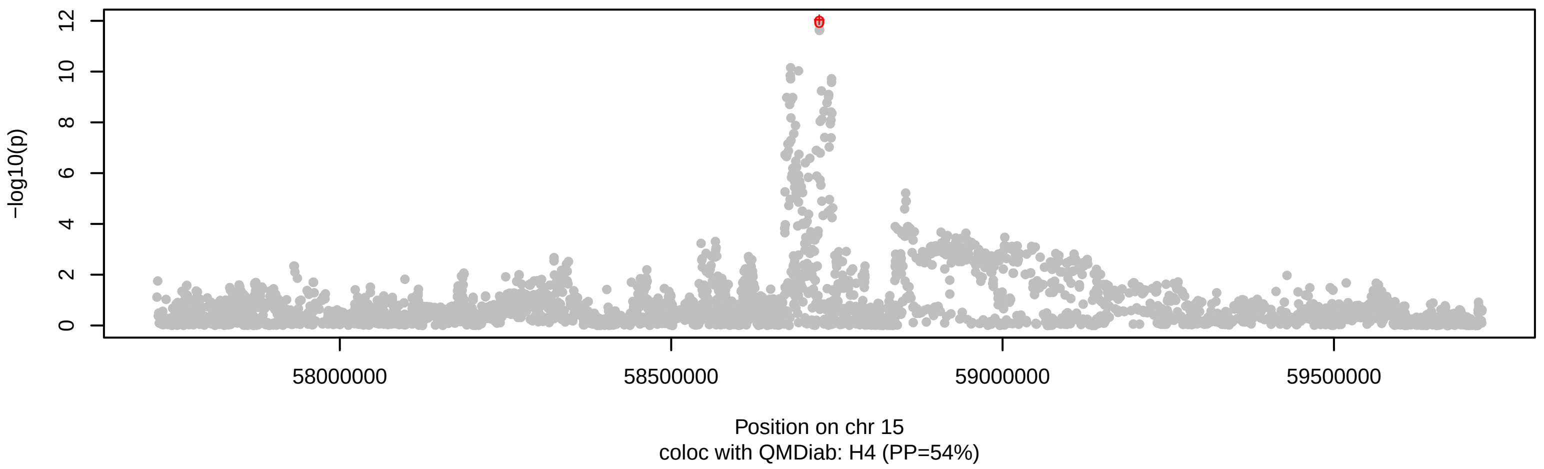

145. LIPC (P11150) 15:58723426:A:G [QMDiab]

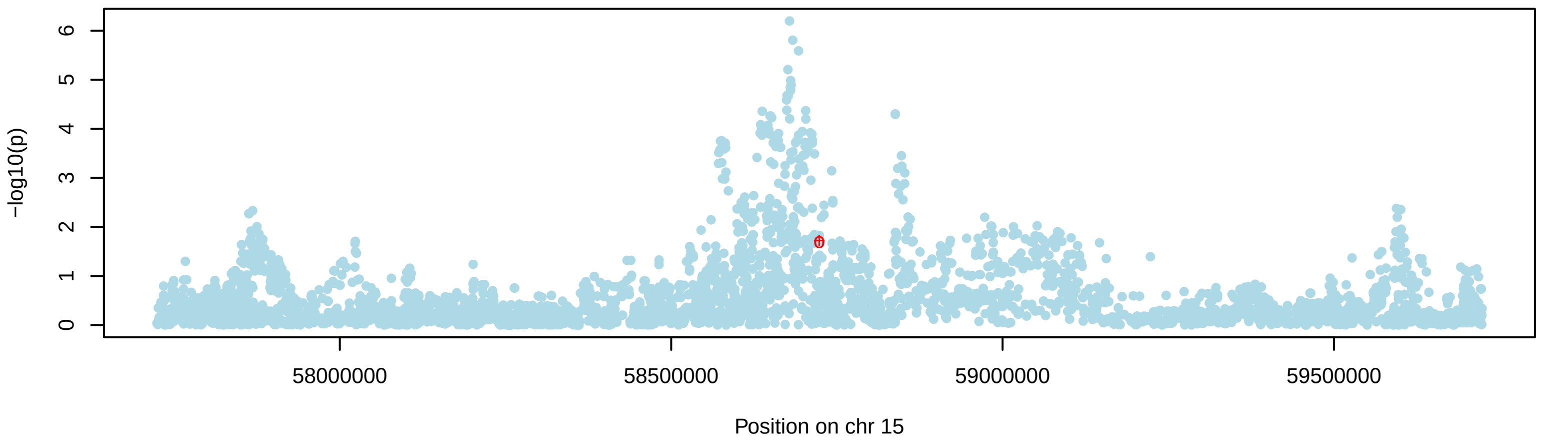

146. NUCB2 (P80303;P80303−2) 11:17285807:A:G [Tarkin]

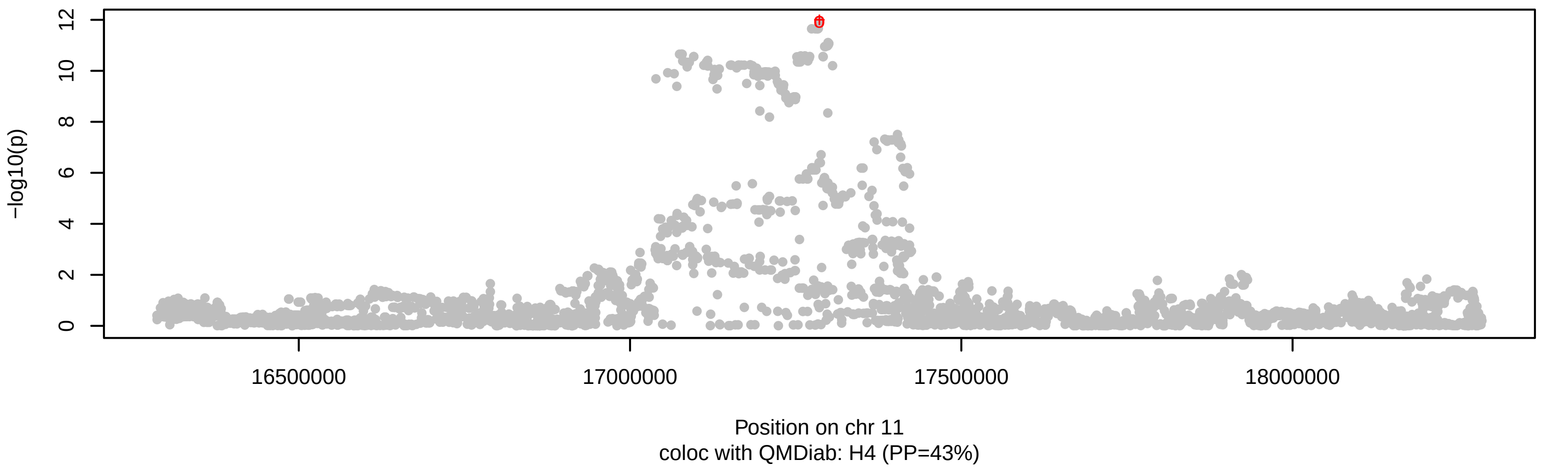

coloc with QMDiab: H4 (PP=43%)

146. NUCB2 (P80303;P80303−2) 11:17285807:A:G [QMDiab]

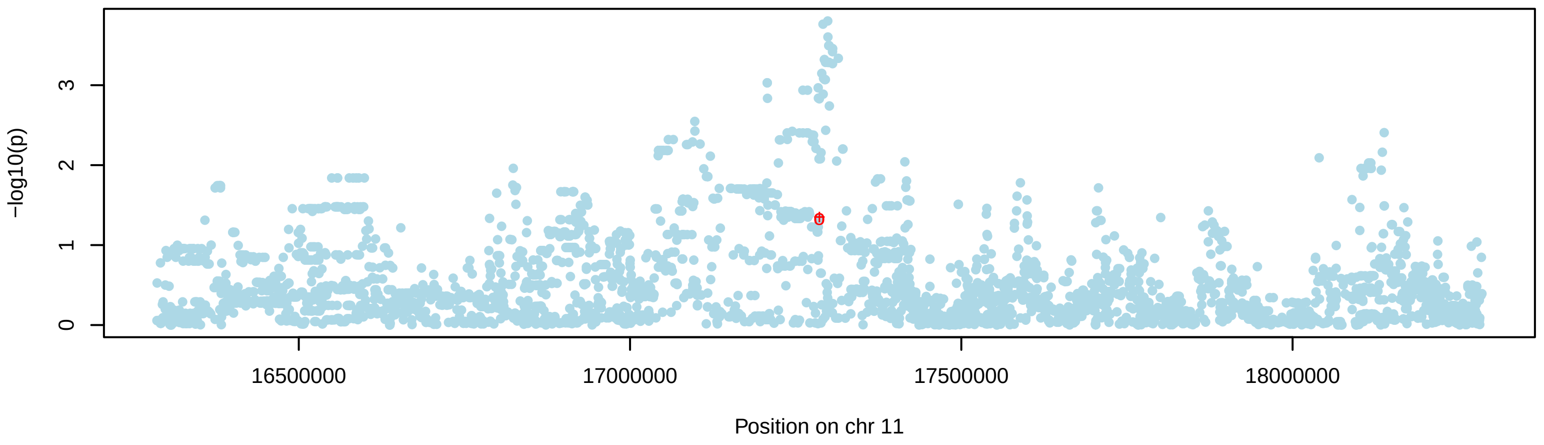

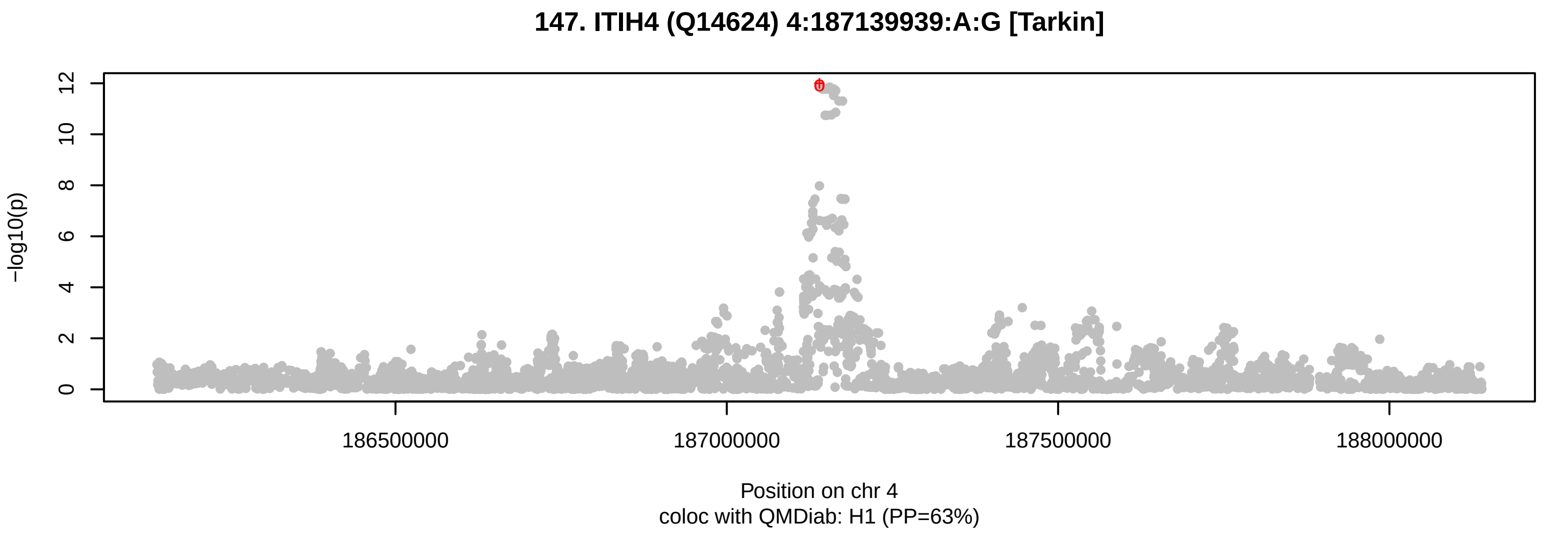
147. ITIH4 (Q14624) 4:187139939:A:G [Tarkin]
−log10(p)
0
2
4
6
8
10
12
186500000
187000000
187500000
188000000
Position on chr 4
coloc with QMDiab: H1 (PP=63%)

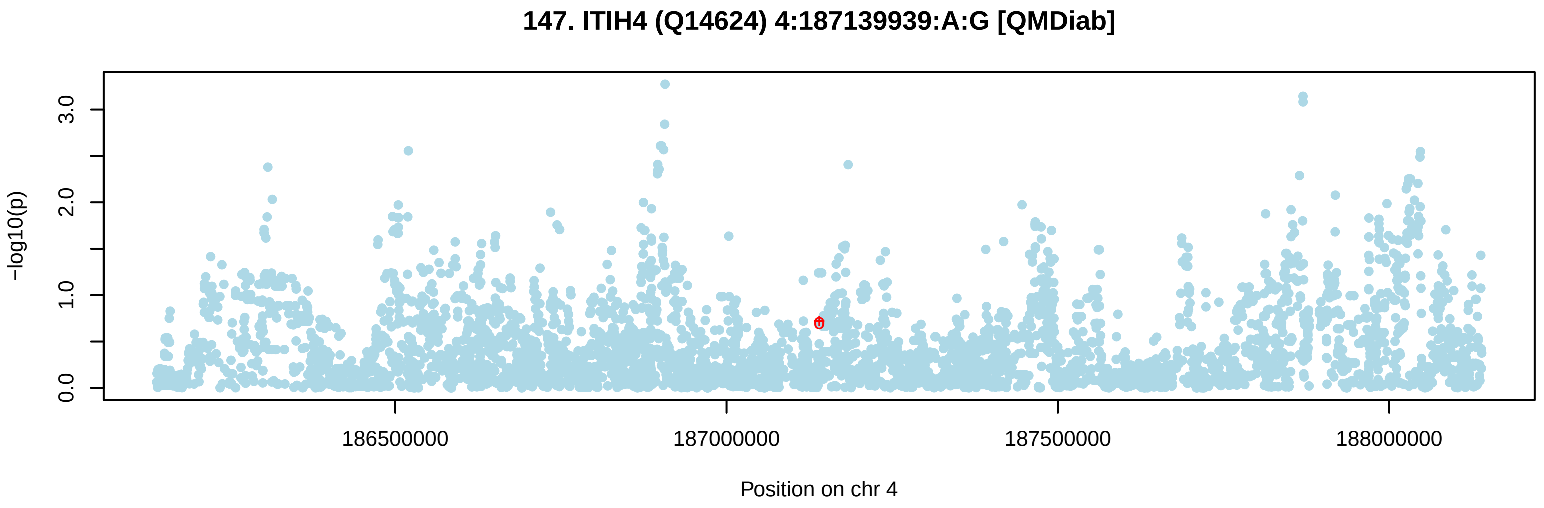
147. ITIH4 (Q14624) 4:187139939:A:G [QMDiab]
−log10(p)
0.0
1.0
2.0
3.0
186500000
187000000
187500000
188000000
Position on chr 4

148. ITIH4 (B7ZKJ8) 3:186459227:A:G [Tarkin]

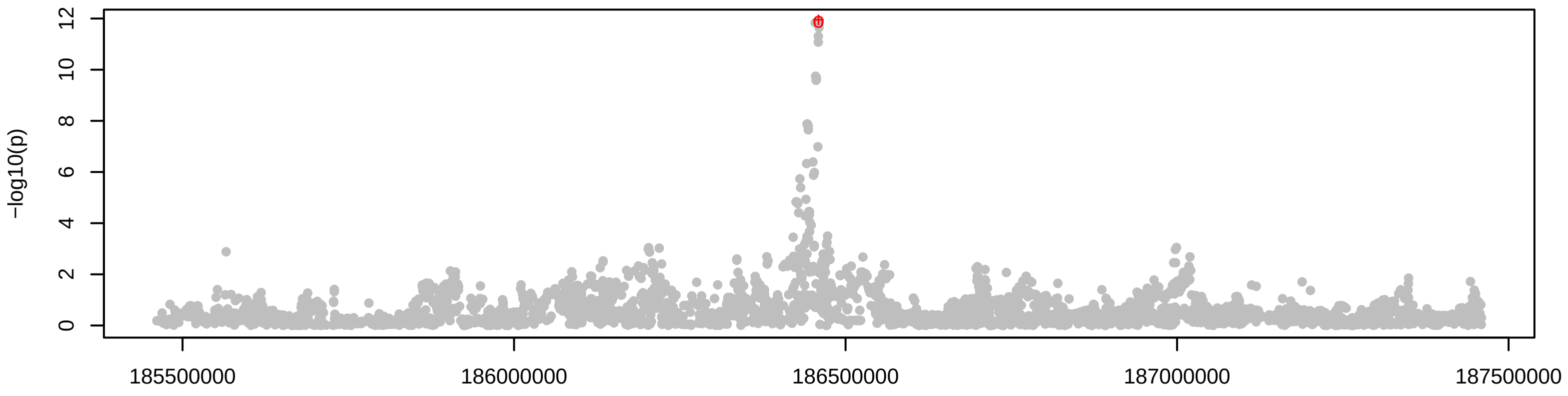

Position on chr 3
coloc with QMDiab: H4 (PP=80%)

148. ITIH4 (B7ZKJ8) 3:186459227:A:G [QMDiab]

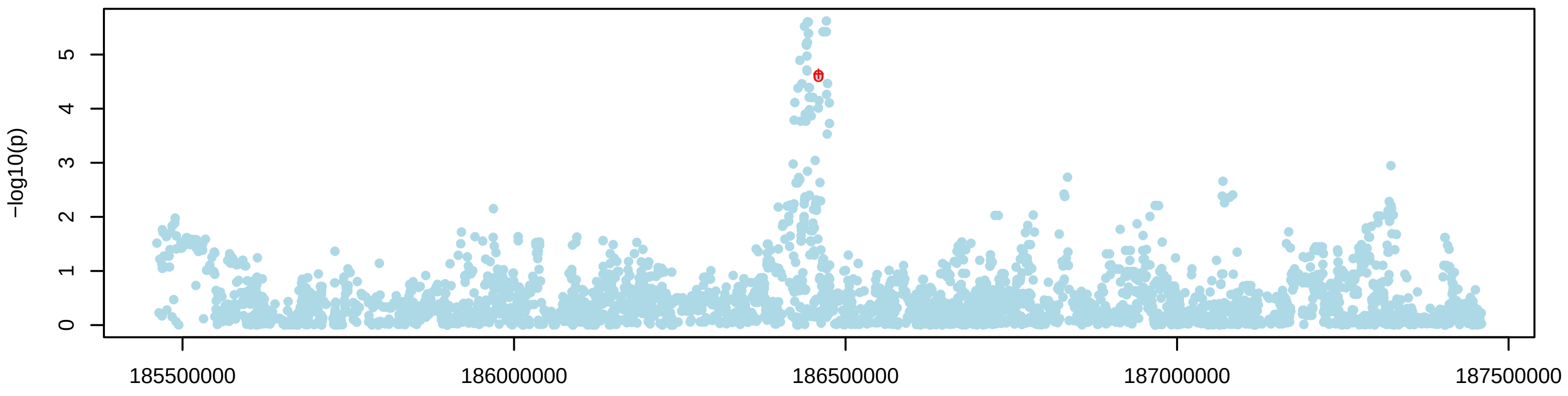

Position on chr 3

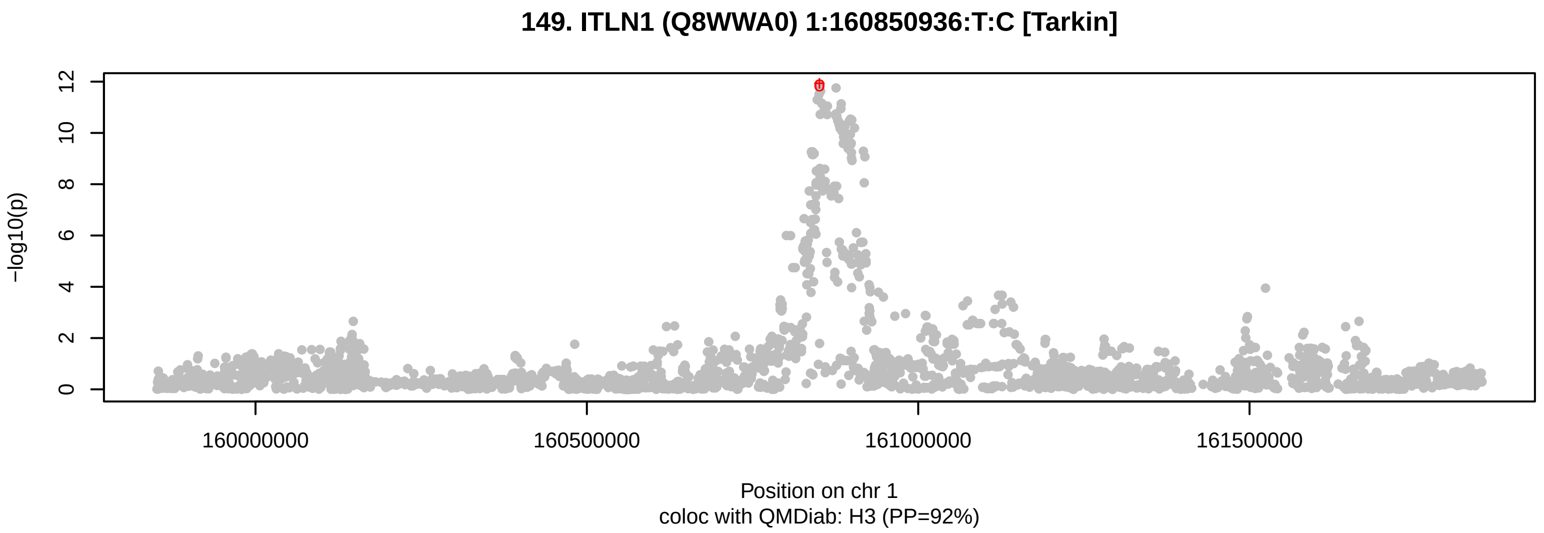

149. ITLN1 (Q8WWA0) 1:160850936:T:C [Tarkin]
−log10(p)
0
2
4
6
8
10
12
160000000
160500000
161000000
161500000
Position on chr 1
coloc with QMDiab: H3 (PP=92%)

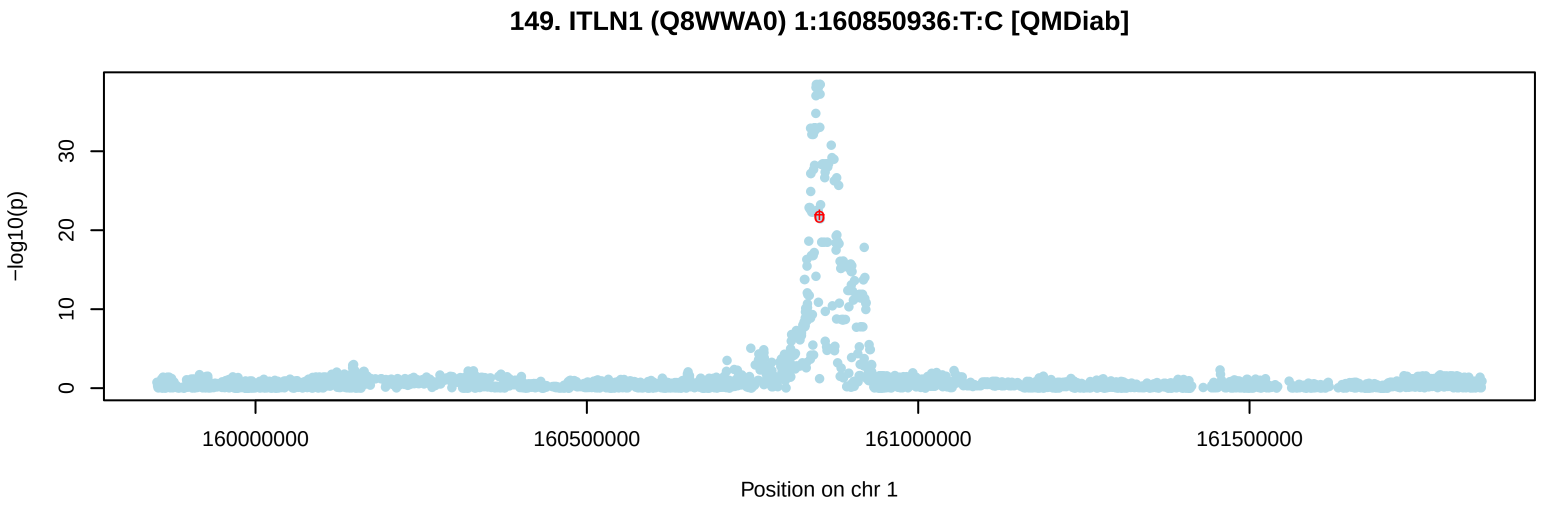

149. ITLN1 (Q8WWA0) 1:160850936:T:C [QMDiab]
−log10(p)
0
10
20
30
160000000
160500000
161000000
161500000
Position on chr 1

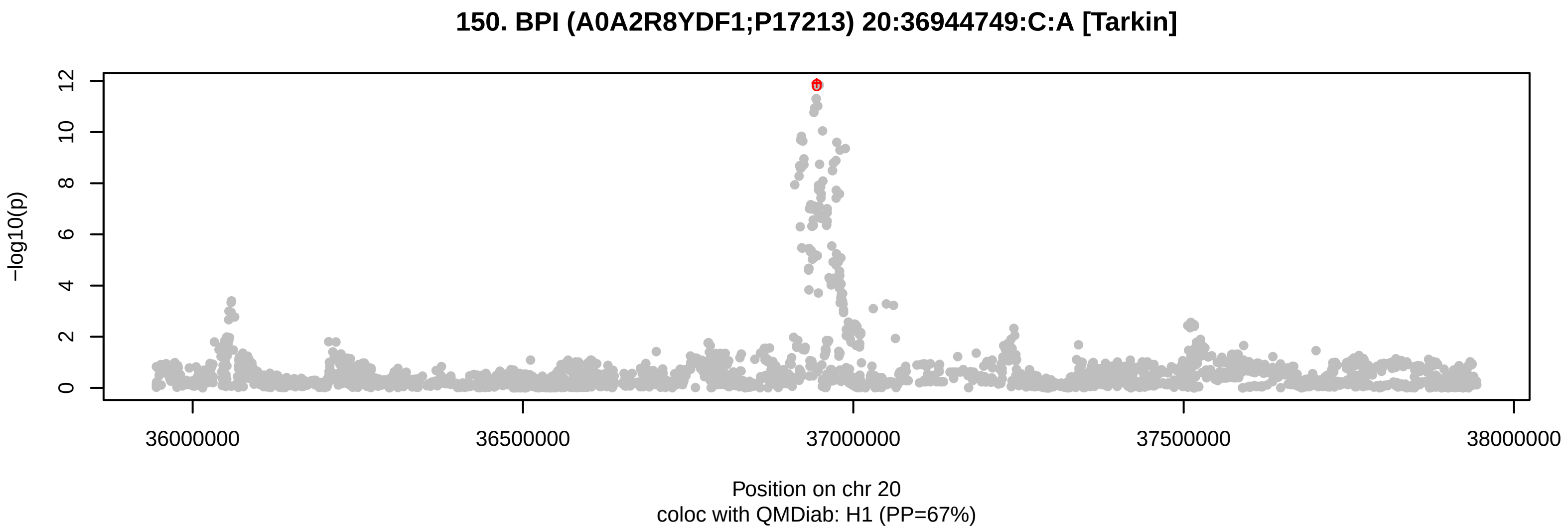
150. BPI (A0A2R8YDF1;P17213) 20:36944749:C:A [Tarkin]
−log10(p)
0
2
4
6
8
10
12
36000000
36500000
37000000
37500000
38000000
Position on chr 20
coloc with QMDiab: H1 (PP=67%)

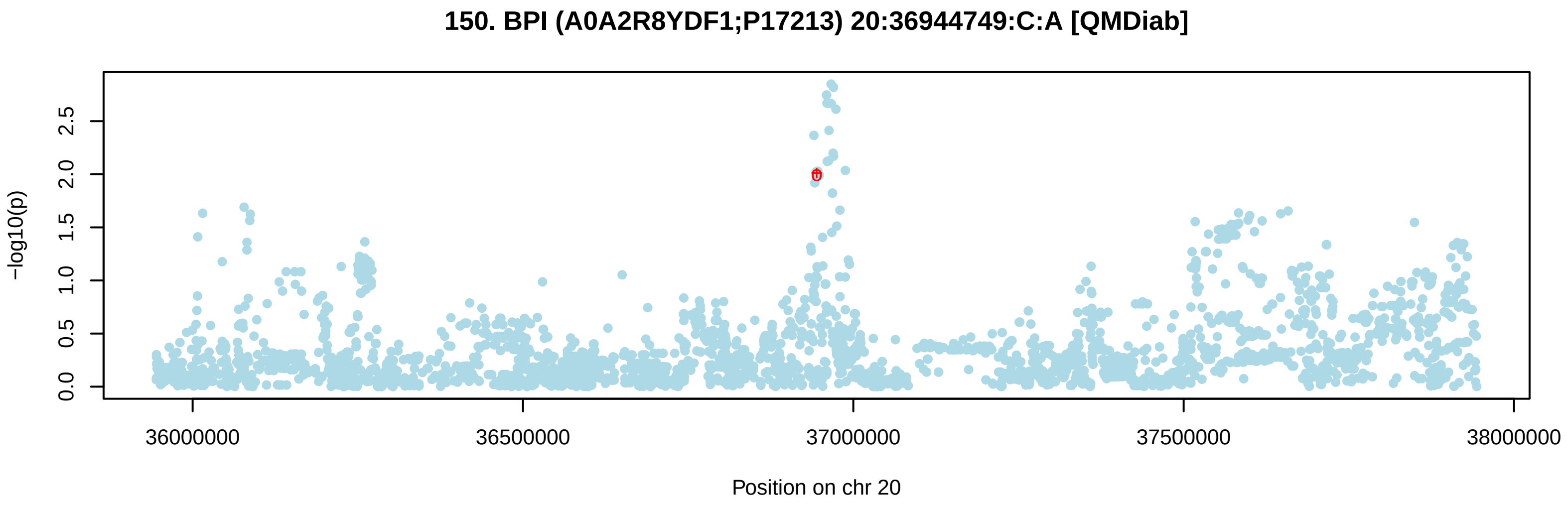
150. BPI (A0A2R8YDF1;P17213) 20:36944749:C:A [QMDiab]
−log10(p)
0.0
0.5
1.0
1.5
2.0
2.5
36000000
36500000
37000000
37500000
38000000
Position on chr 20

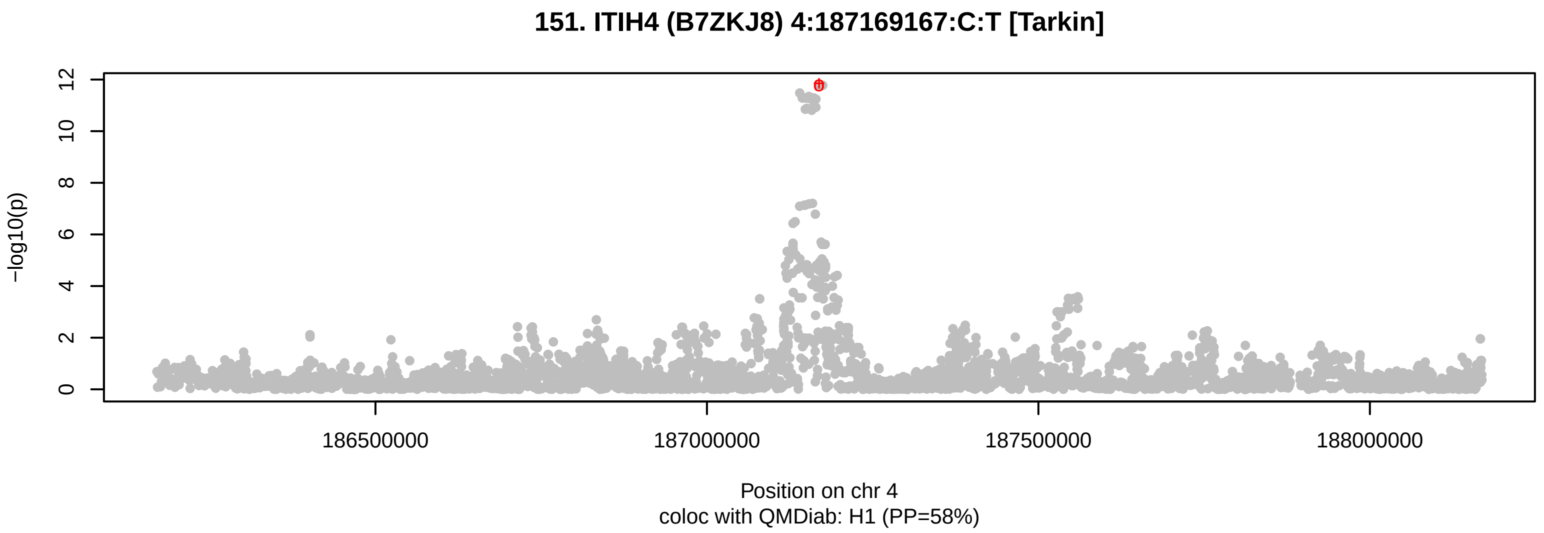
151. ITIH4 (B7ZKJ8) 4:187169167:C:T [Tarkin]
−log10(p)
0
2
4
6
8
10
12
186500000
187000000
187500000
188000000
Position on chr 4
coloc with QMDiab: H1 (PP=58%)

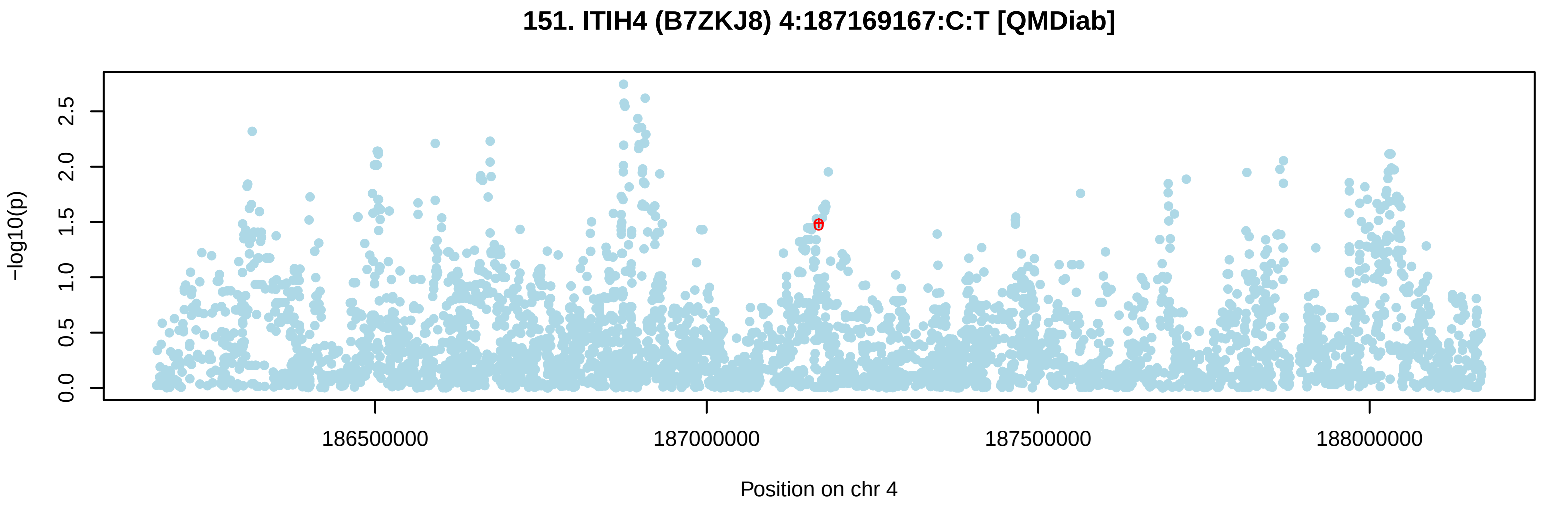
151. ITIH4 (B7ZKJ8) 4:187169167:C:T [QMDiab]
−log10(p)
0.0
0.5
1.0
1.5
2.0
2.5
186500000
187000000
187500000
188000000
Position on chr 4

152. FCN2 (Q15485) 9:137777504:G:A [Tarkin]

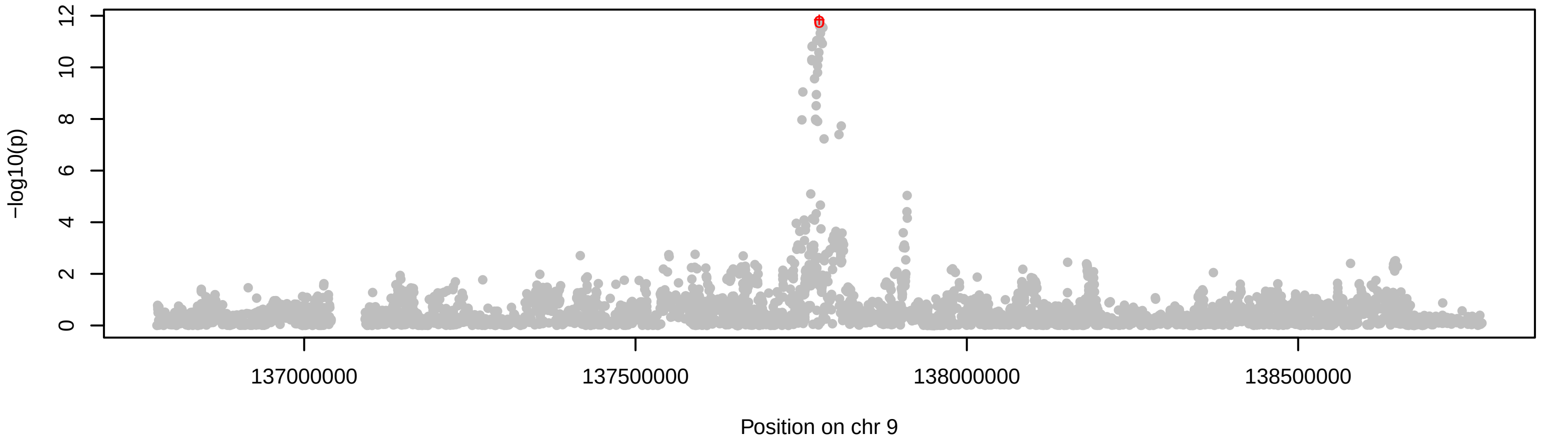

coloc with QMDiab: H4 (PP=56%)

152. FCN2 (Q15485) 9:137777504:G:A [QMDiab]

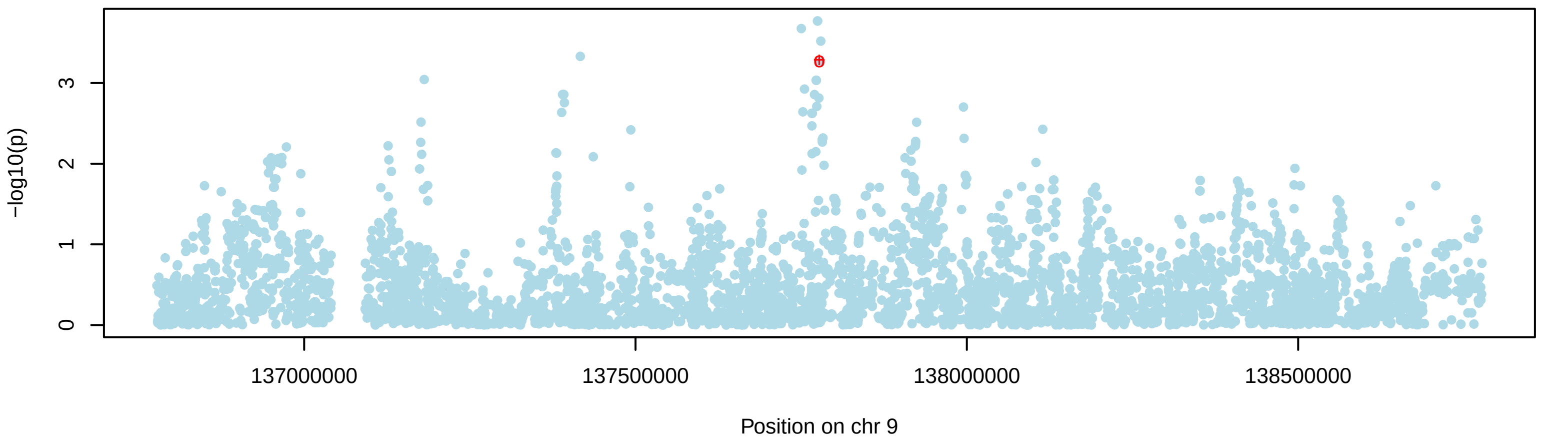

153. PDCD5 (O14737) 19:33083746:A:T [Tarkin]

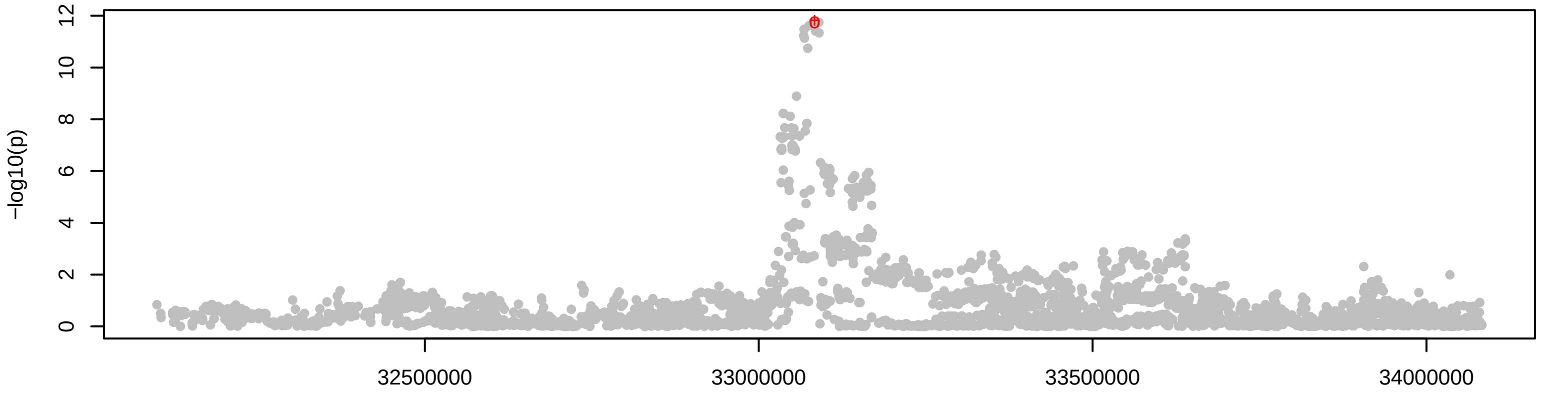

Position on chr 19
coloc with QMDiab: H1 (PP=69%)

153. PDCD5 (O14737) 19:33083746:A:T [QMDiab]

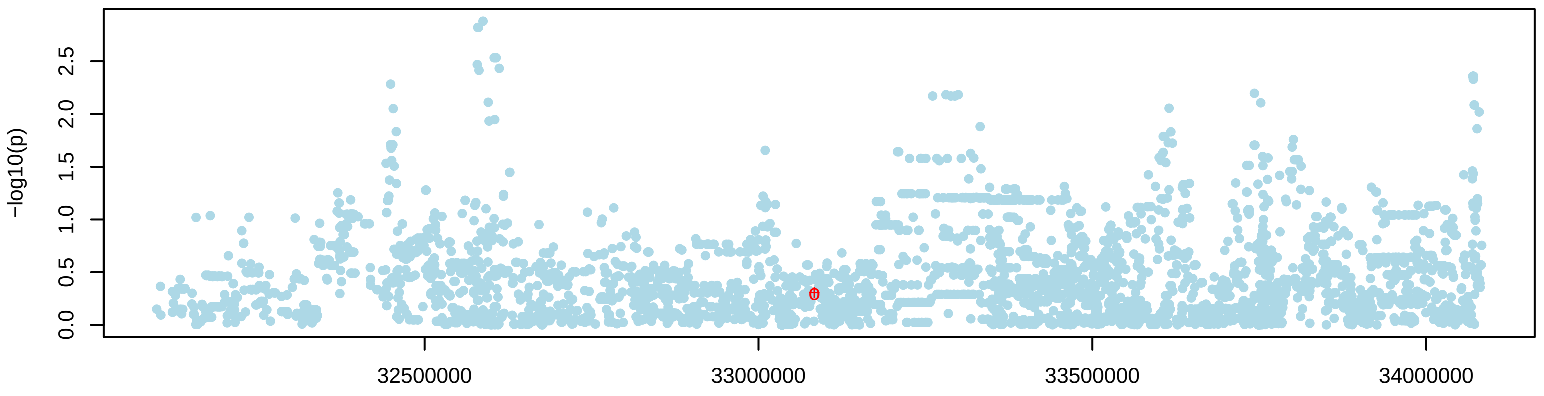

Position on chr 19

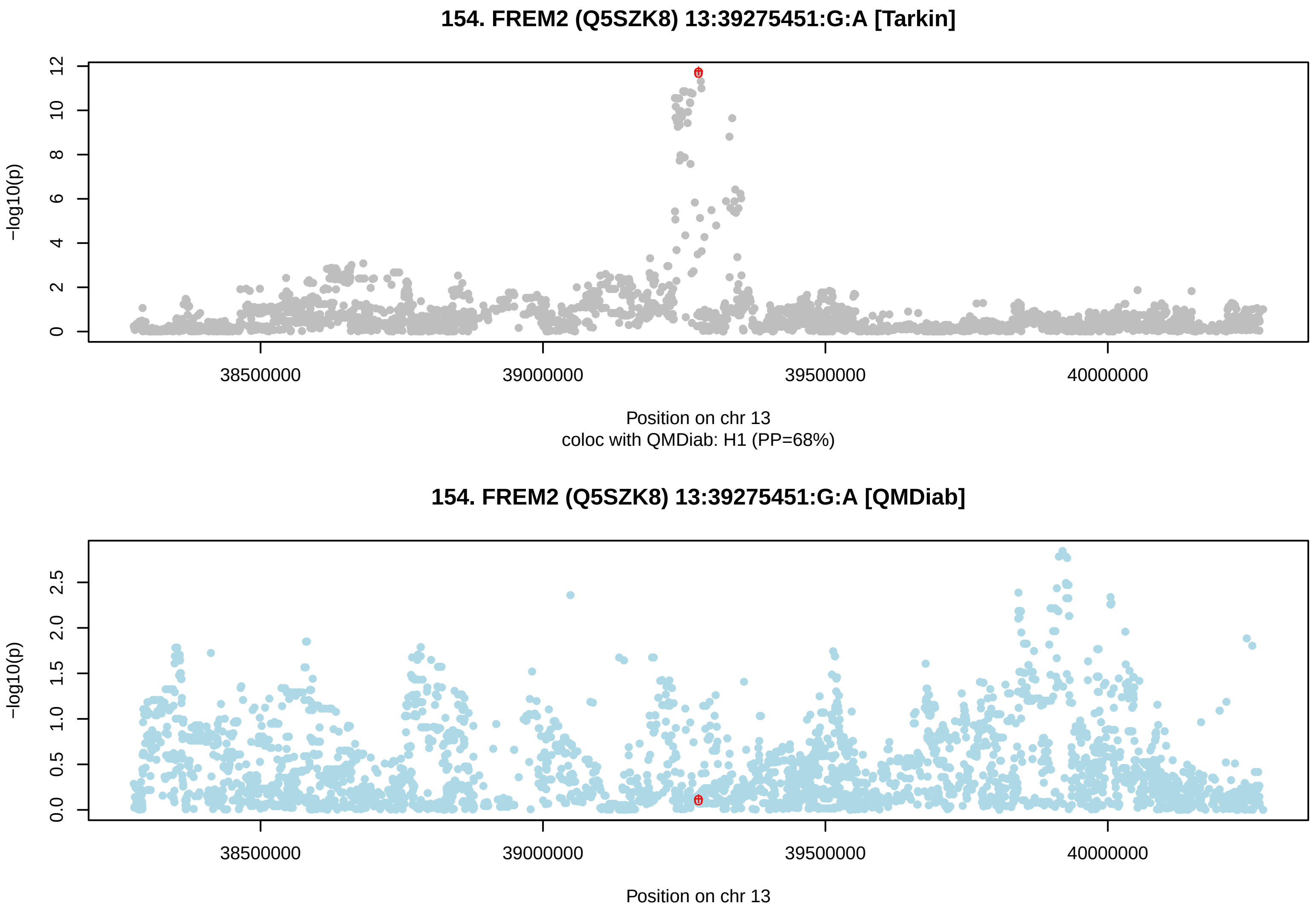
154. FREM2 (Q5SZK8) 13:39275451:G:A [Tarkin]
−log10(p)
Position on chr 13
coloc with QMDiab: H1 (PP=68%)
154. FREM2 (Q5SZK8) 13:39275451:G:A [QMDiab]
−log10(p)
Position on chr 13

155. MMRN2 (Q9H8L6) 10:88696622:C:G [Tarkin]

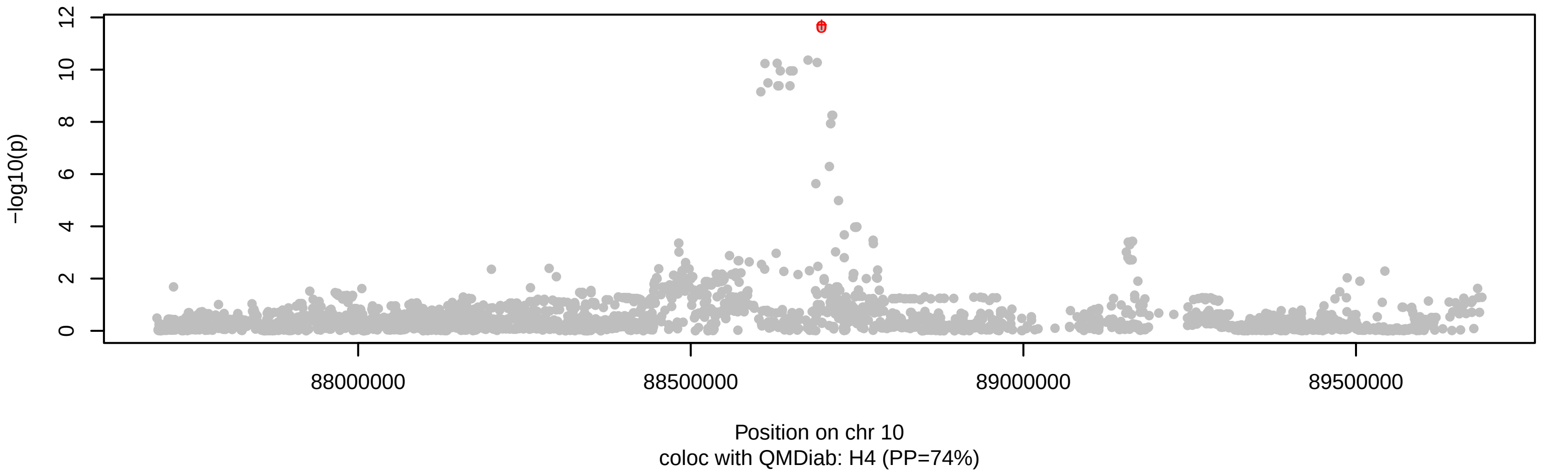

coloc with QMDiab: H4 (PP=74%)

155. MMRN2 (Q9H8L6) 10:88696622:C:G [QMDiab]

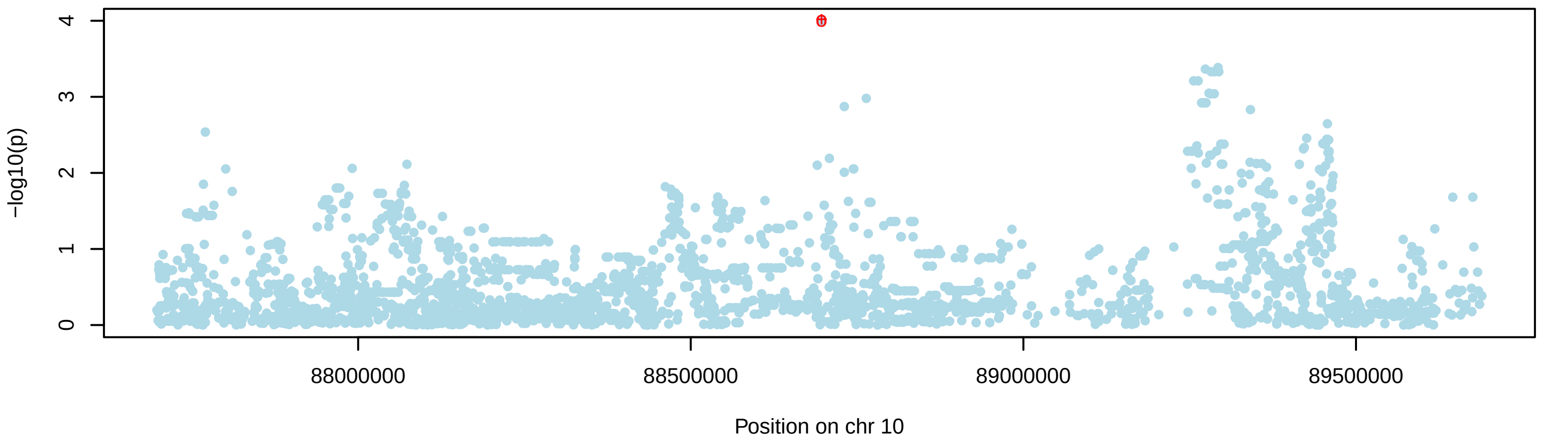

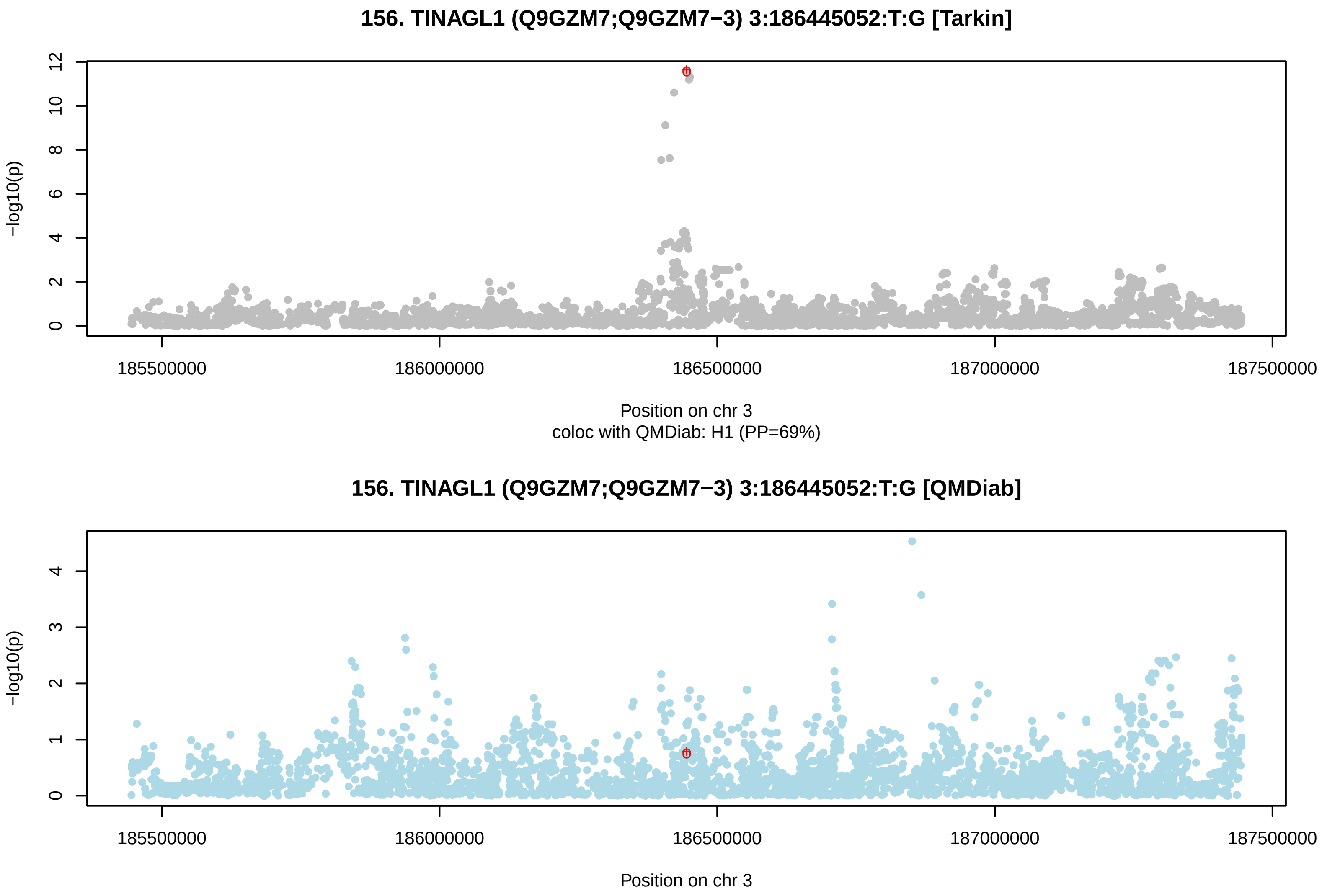
156. TINAGL1 (Q9GZM7;Q9GZM7−3) 3:186445052:T:G [Tarkin]
−log10(p)
0
2
4
6
8
10
12
185500000
186000000
186500000
187000000
187500000
Position on chr 3
coloc with QMDiab: H1 (PP=69%)
156. TINAGL1 (Q9GZM7;Q9GZM7−3) 3:186445052:T:G [QMDiab]
−log10(p)
0
1
2
3
4
185500000
186000000
186500000
187000000
187500000
Position on chr 3

157. CDH15 (P55291) 16:89199108:G:A [Tarkin]

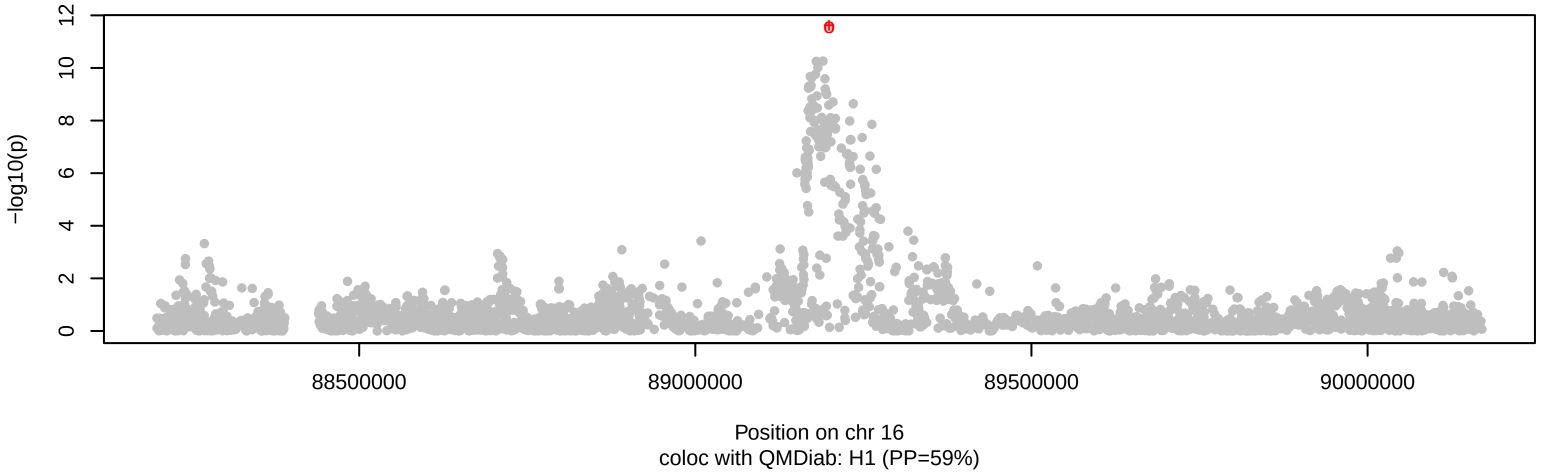

coloc with QMDiab: H1 (PP=59%)

157. CDH15 (P55291) 16:89199108:G:A [QMDiab]

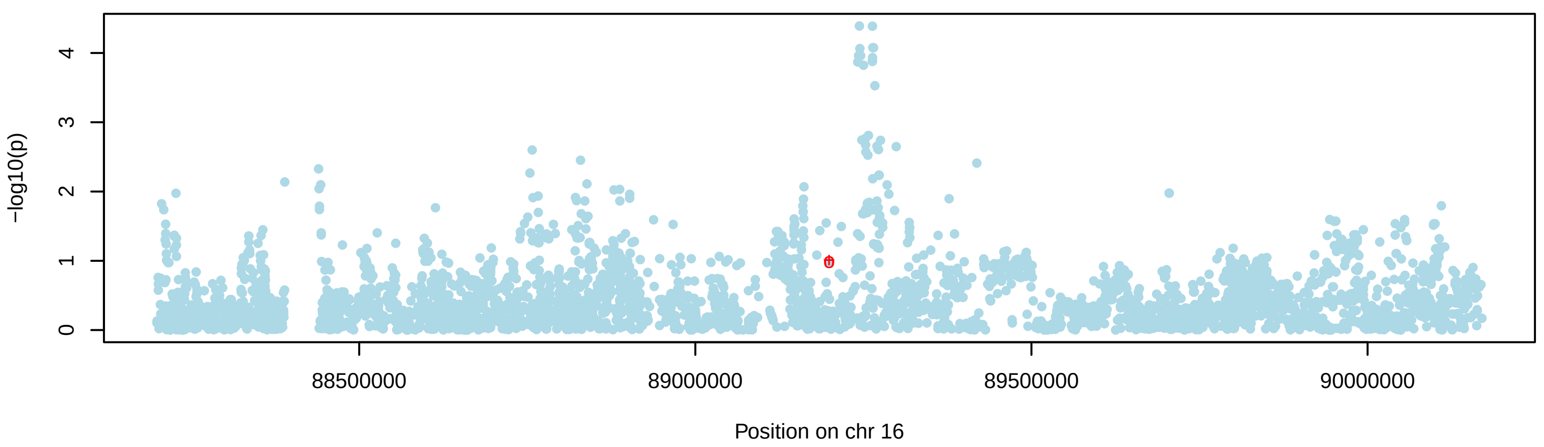

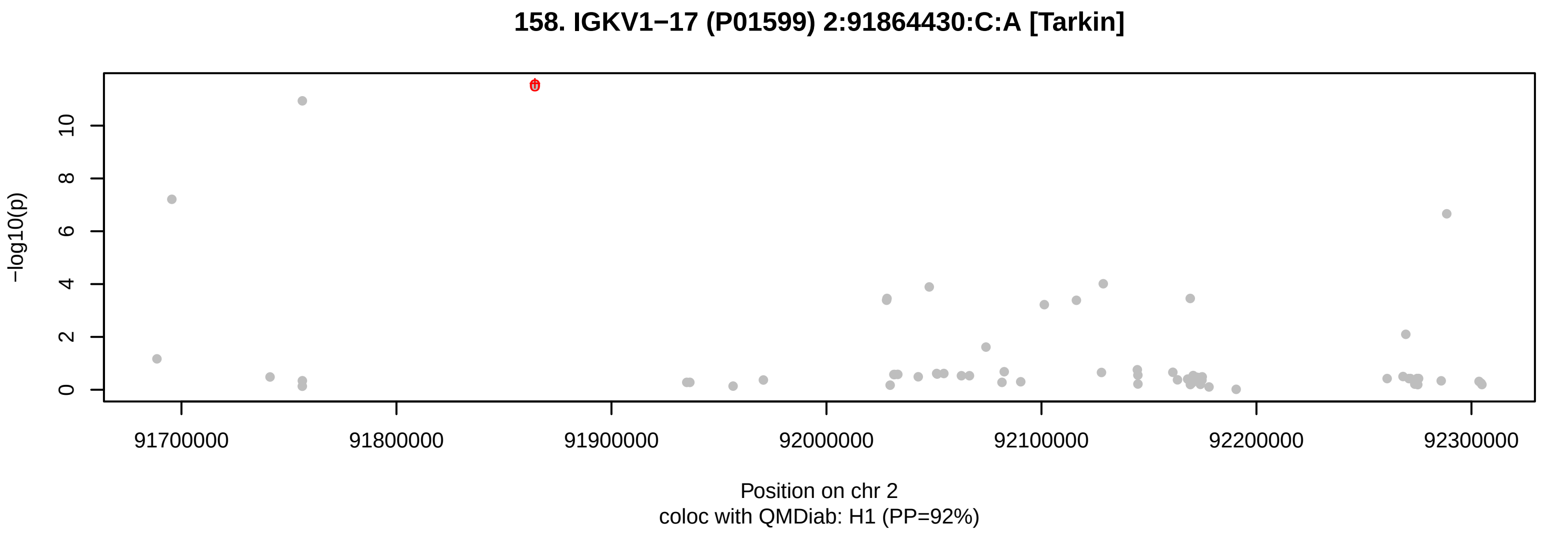
158. IGKV1−17 (P01599) 2:91864430:C:A [Tarkin]
−log10(p)
0
2
4
6
8
10
91700000
91800000
91900000
92000000
92100000
92200000
92300000
Position on chr 2
coloc with QMDiab: H1 (PP=92%)

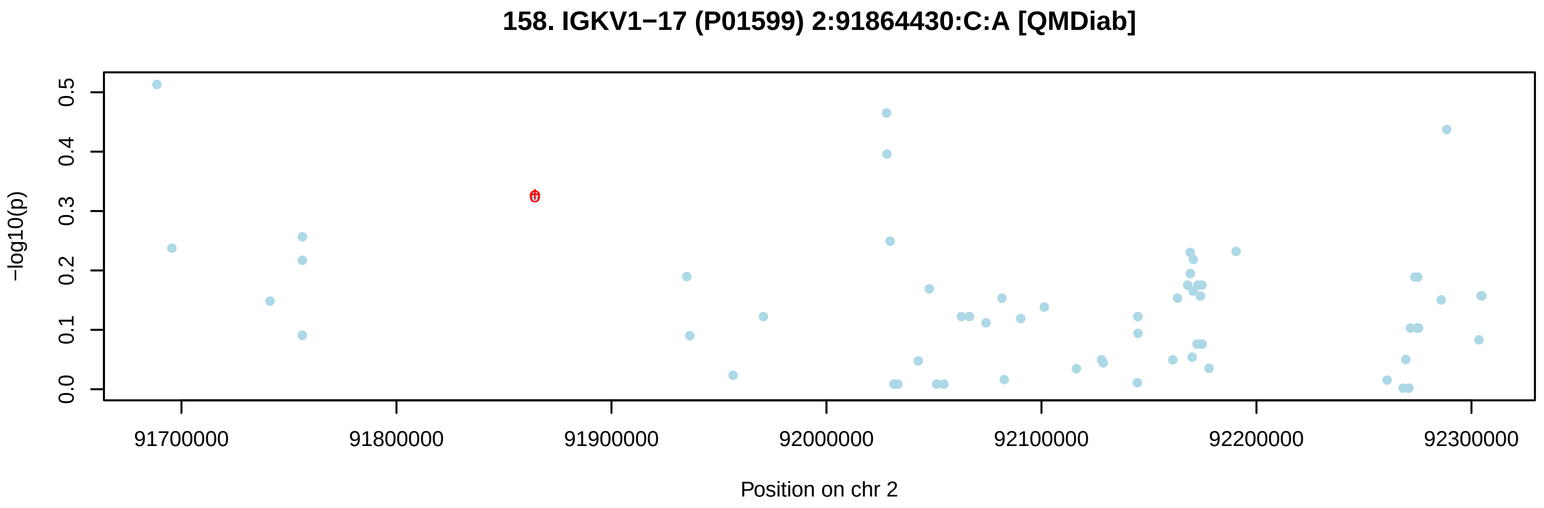
158. IGKV1−17 (P01599) 2:91864430:C:A [QMDiab]
−log10(p)
0.0
0.1
0.2
0.3
0.4
0.5
91700000
91800000
91900000
92000000
92100000
92200000
92300000
Position on chr 2

159. FCN3 (O75636) 1:27709858:G:T [Tarkin]

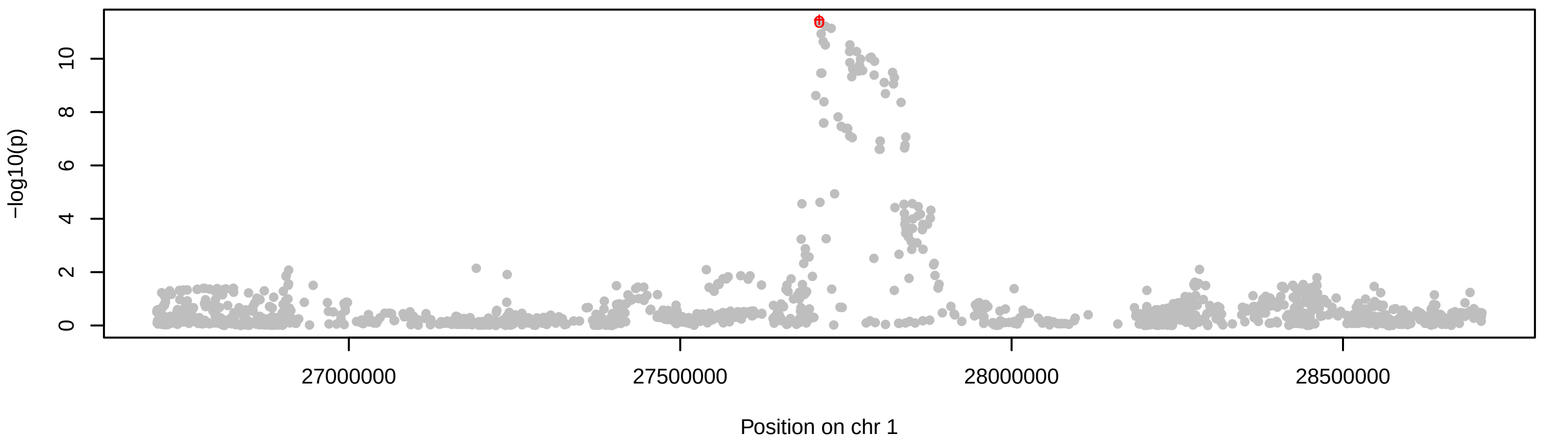

coloc with QMDiab: H1 (PP=82%)

159. FCN3 (O75636) 1:27709858:G:T [QMDiab]

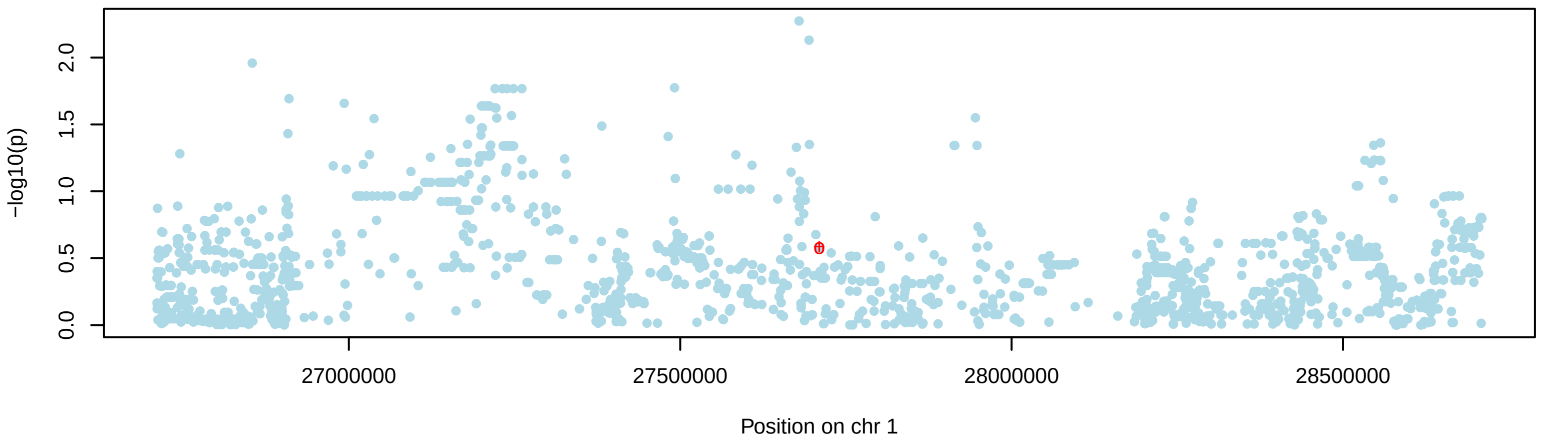

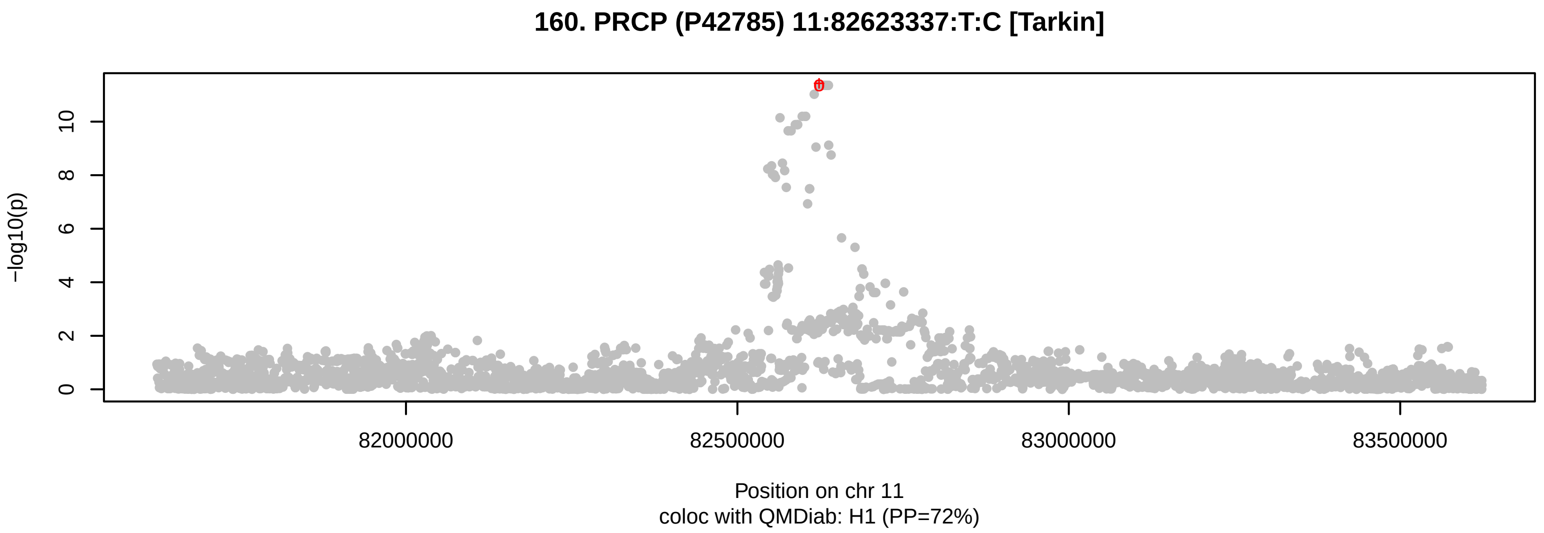
160. PRCP (P42785) 11:82623337:T:C [Tarkin]
−log10(p)
0
2
4
6
8
10
82000000
82500000
83000000
83500000
Position on chr 11
coloc with QMDiab: H1 (PP=72%)

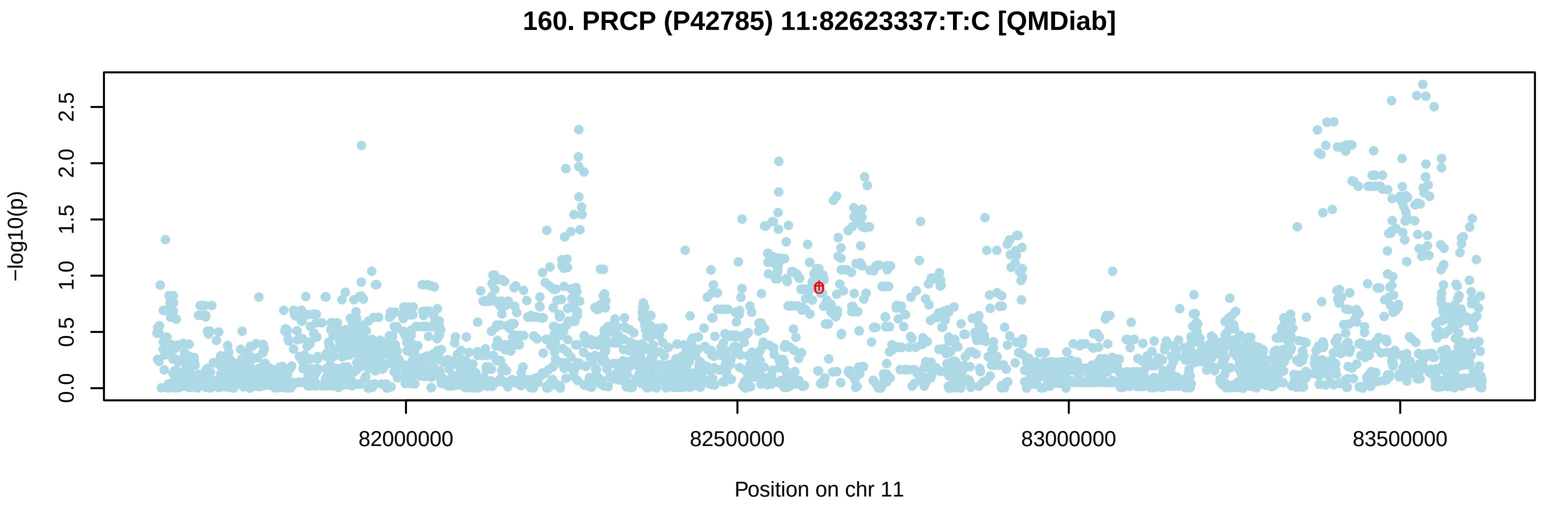
160. PRCP (P42785) 11:82623337:T:C [QMDiab]
−log10(p)
0.0
0.5
1.0
1.5
2.0
2.5
82000000
82500000
83000000
83500000
Position on chr 11

161. IGKV1−5 (P01602) 2:89481695:C:G [Tarkin]

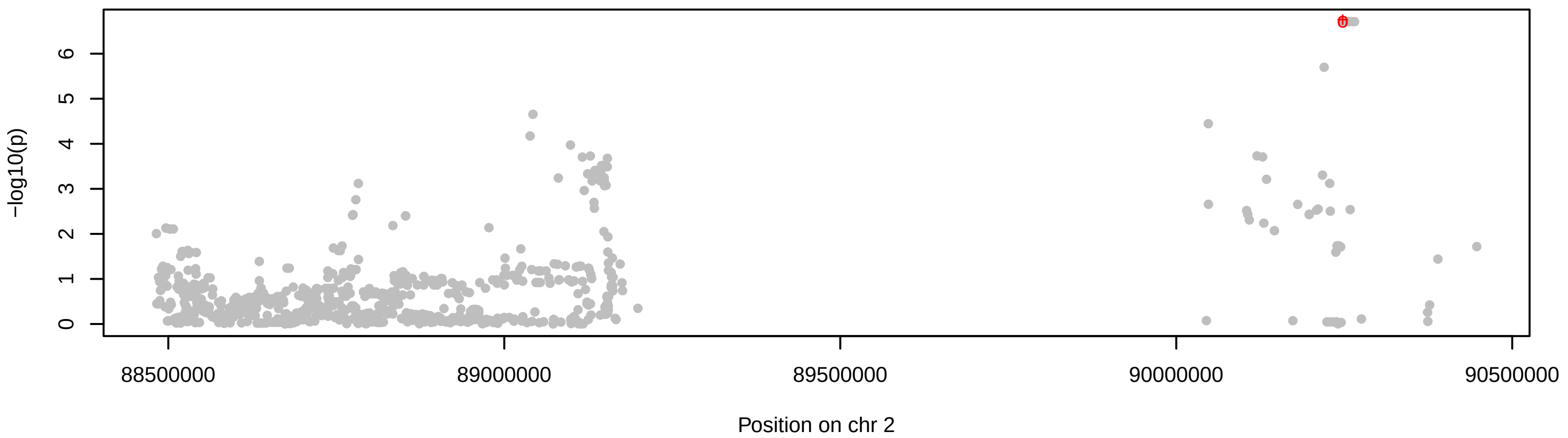

coloc with QMDiab: H4 (PP=48%)

161. IGKV1−5 (P01602) 2:89481695:C:G [QMDiab]

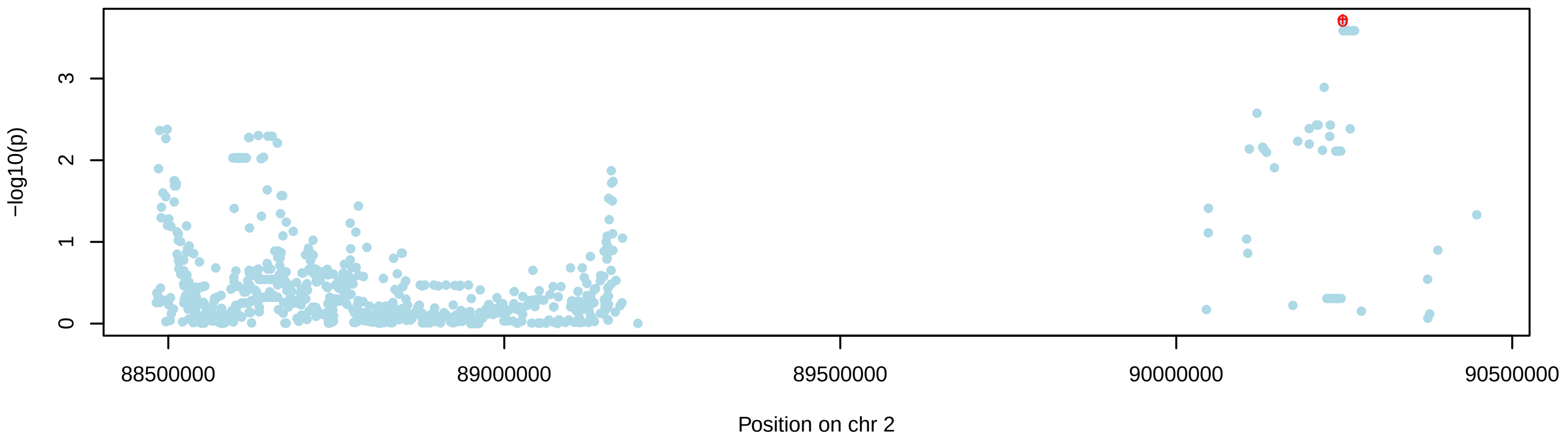

162. ANGPTL6 (Q8NI99) 2:3640142:C:T [Tarkin]

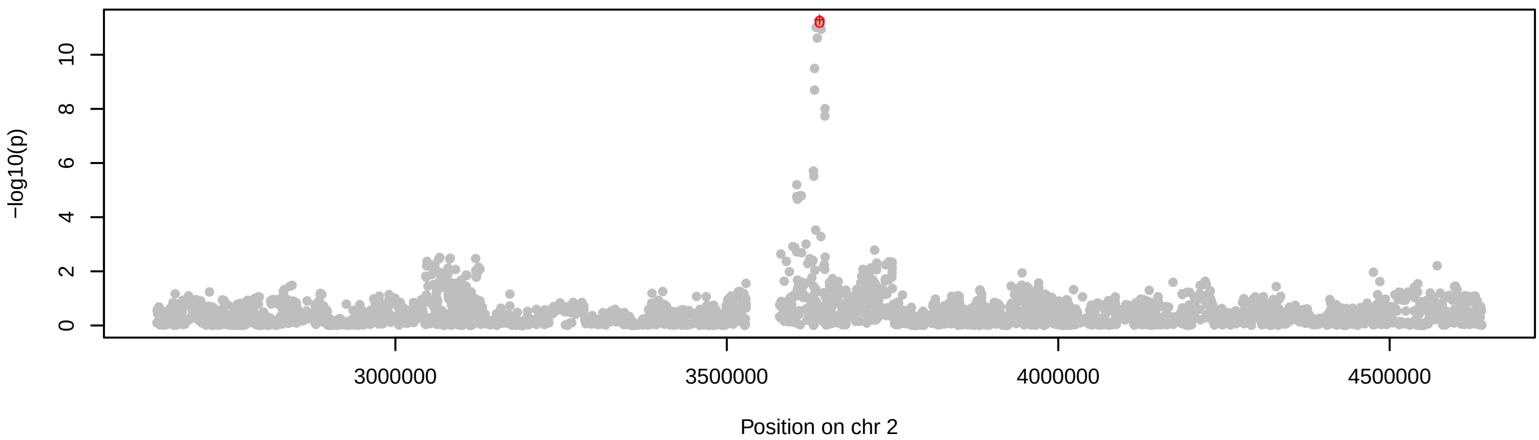

coloc with QMDiab: H4 (PP=88%)

162. ANGPTL6 (Q8NI99) 2:3640142:C:T [QMDiab]

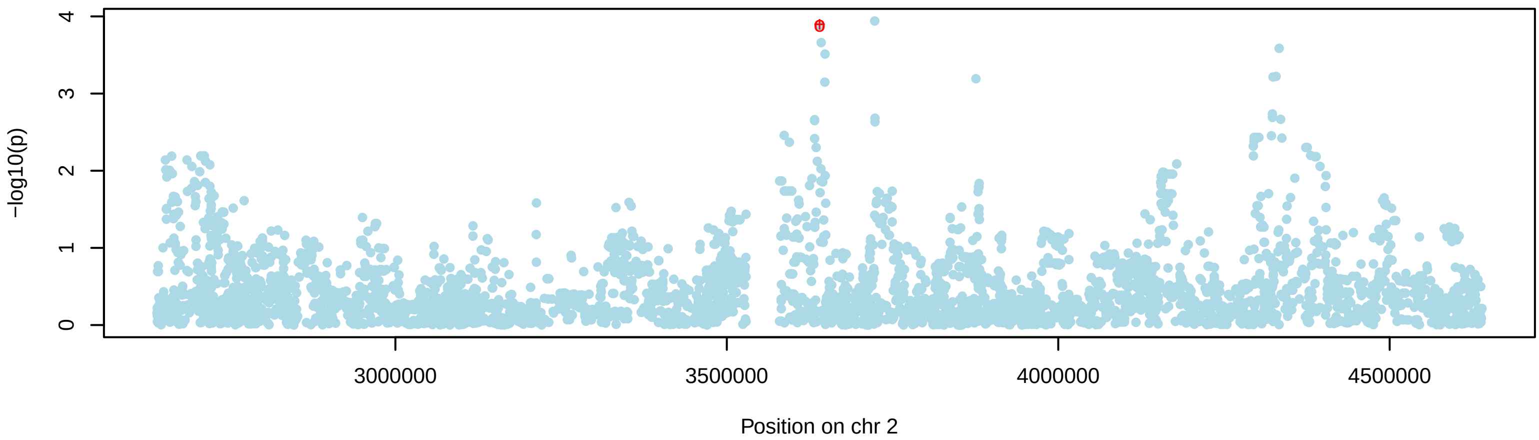

163. NID2 (Q14112) 14:52490104:C:T [Tarkin]

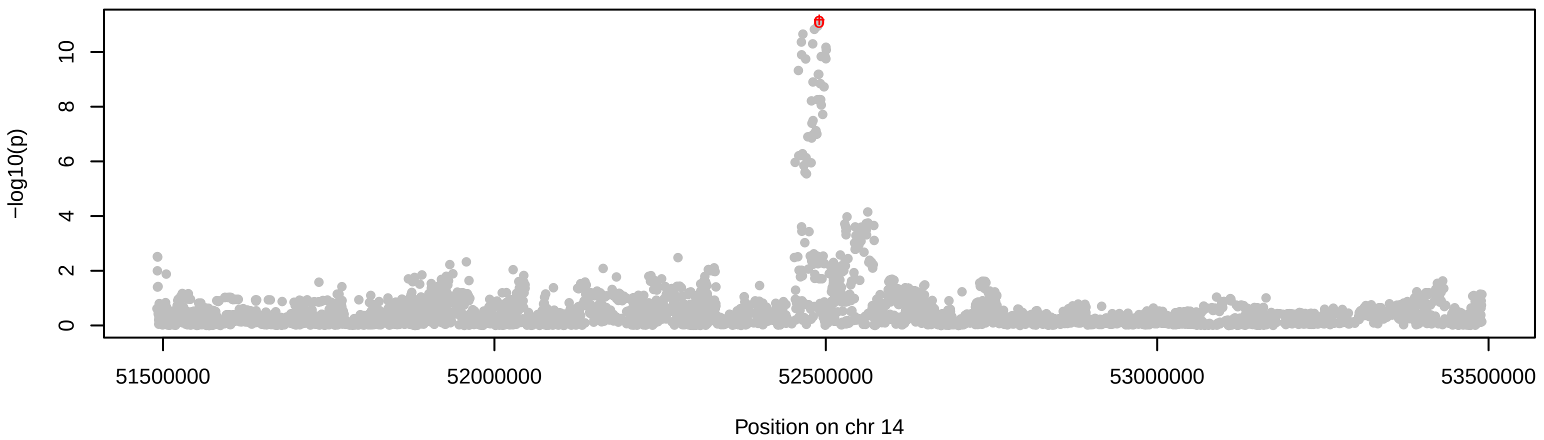

coloc with QMDiab: H4 (PP=72%)

163. NID2 (Q14112) 14:52490104:C:T [QMDiab]

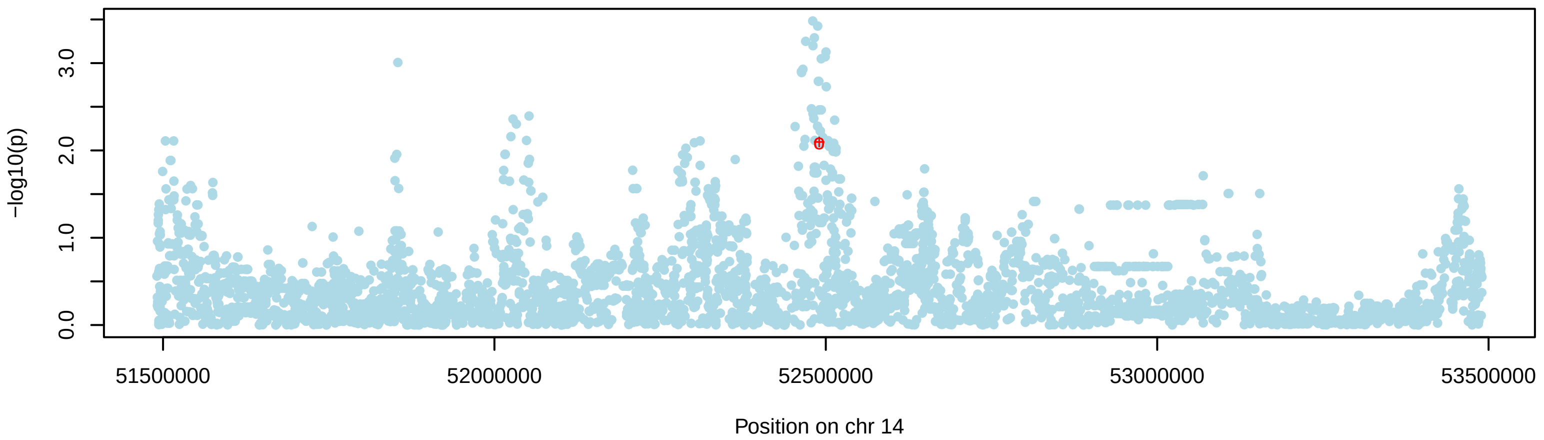

164. SRCRB4D (Q8WTU2) 7:76044757:G:T [Tarkin]

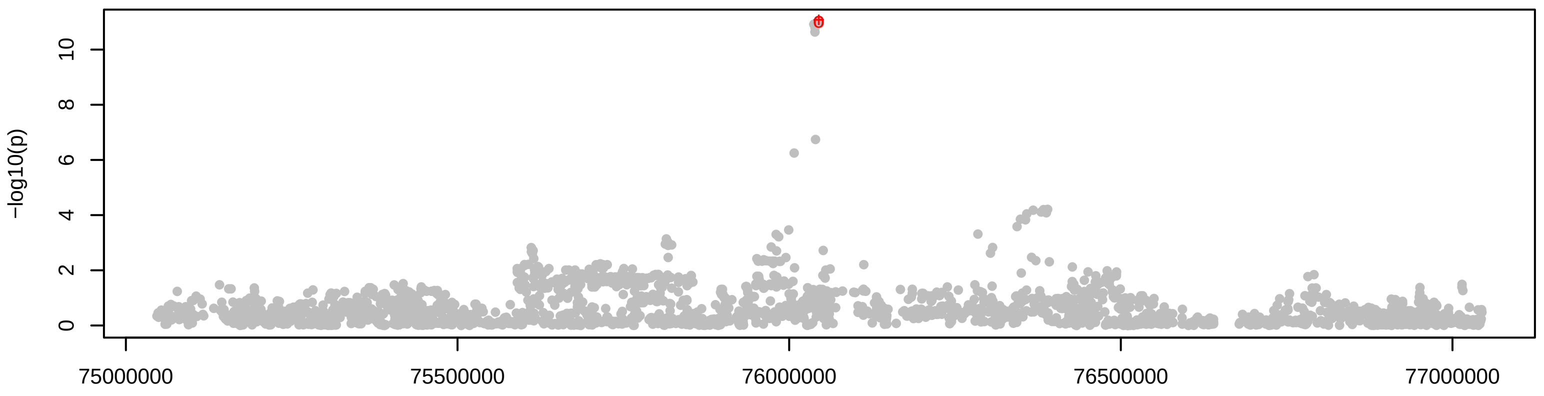

Position on chr 7
coloc with QMDiab: H4 (PP=99%)

164. SRCRB4D (Q8WTU2) 7:76044757:G:T [QMDiab]

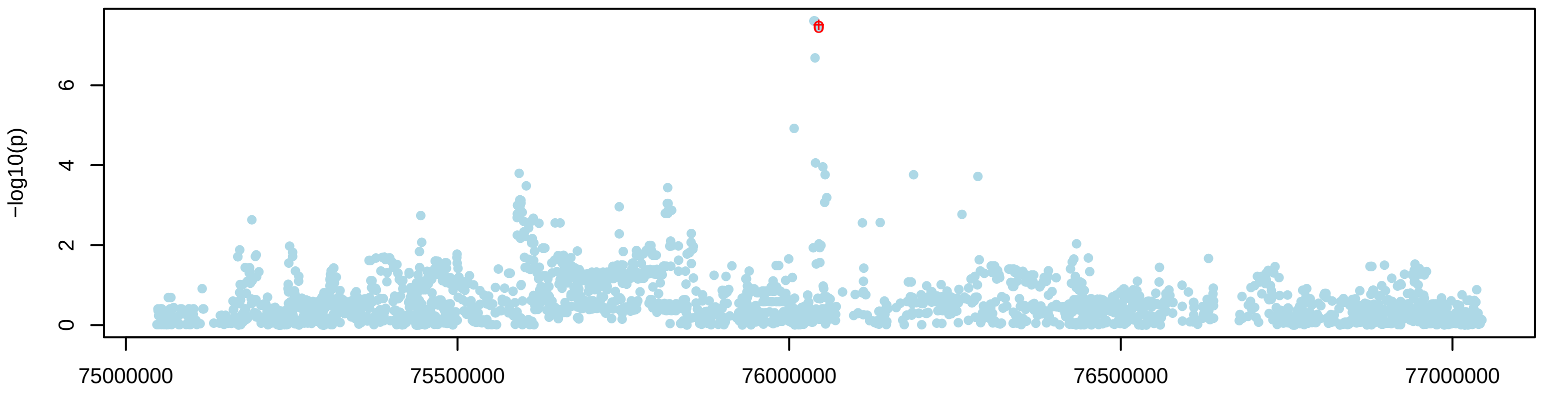

Position on chr 7

165. PADI4 (Q9UM07) 1:17650247:A:G [Tarkin]

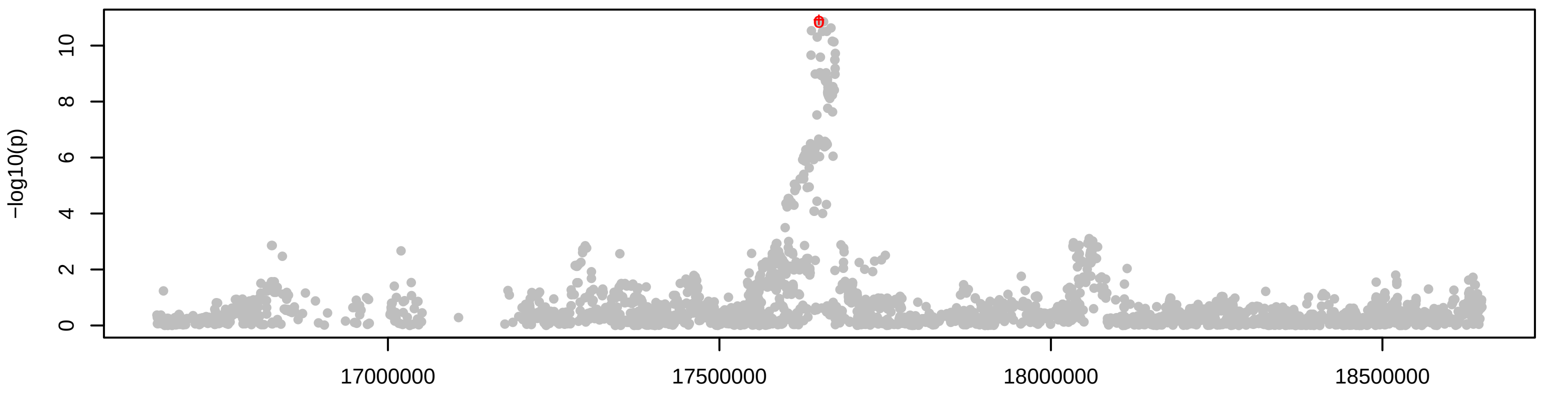

Position on chr 1
coloc with QMDiab: H1 (PP=71%)

165. PADI4 (Q9UM07) 1:17650247:A:G [QMDiab]

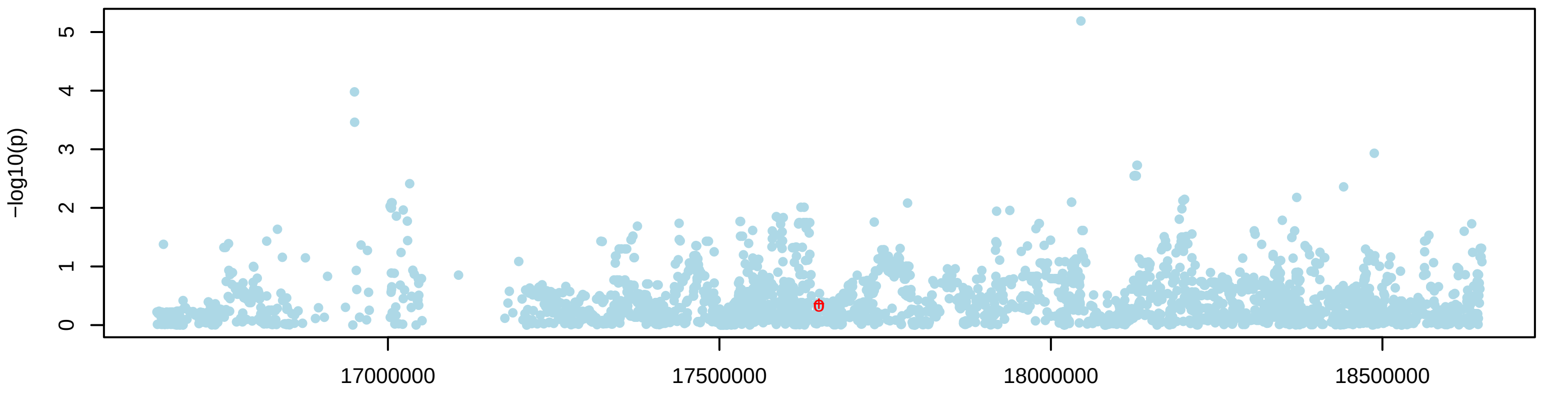

Position on chr 1

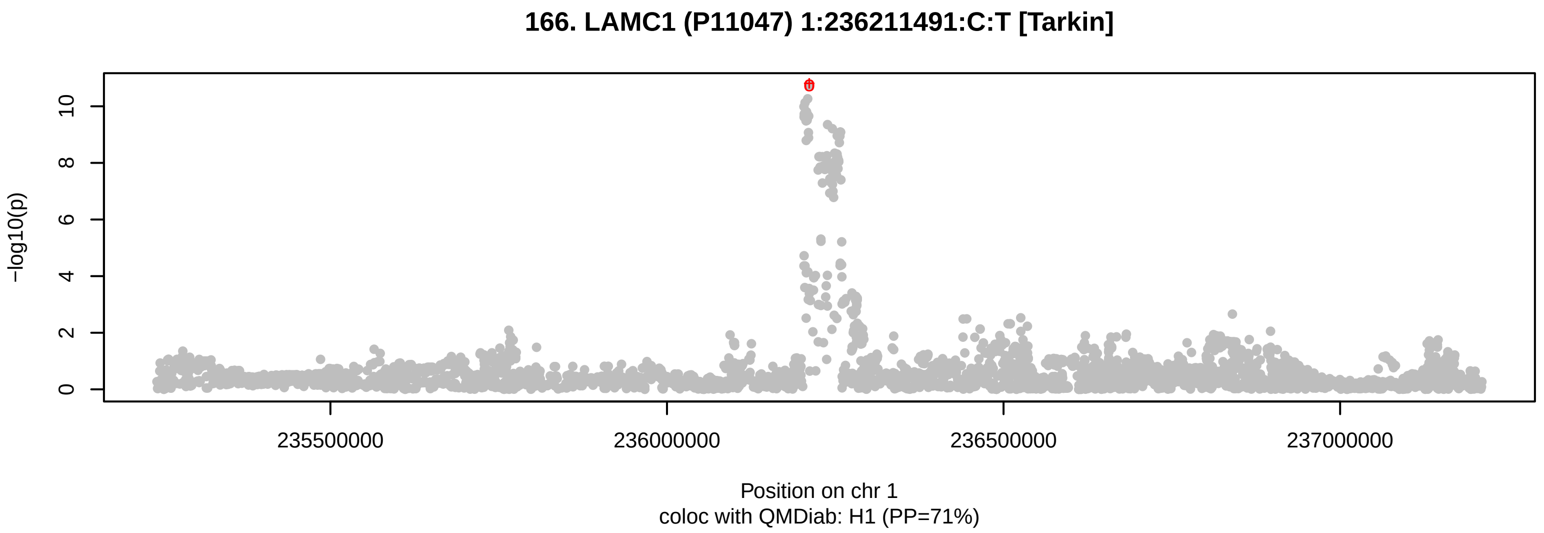
166. LAMC1 (P11047) 1:236211491:C:T [Tarkin]
−log10(p)
0
2
4
6
8
10
235500000
236000000
236500000
237000000
Position on chr 1
coloc with QMDiab: H1 (PP=71%)

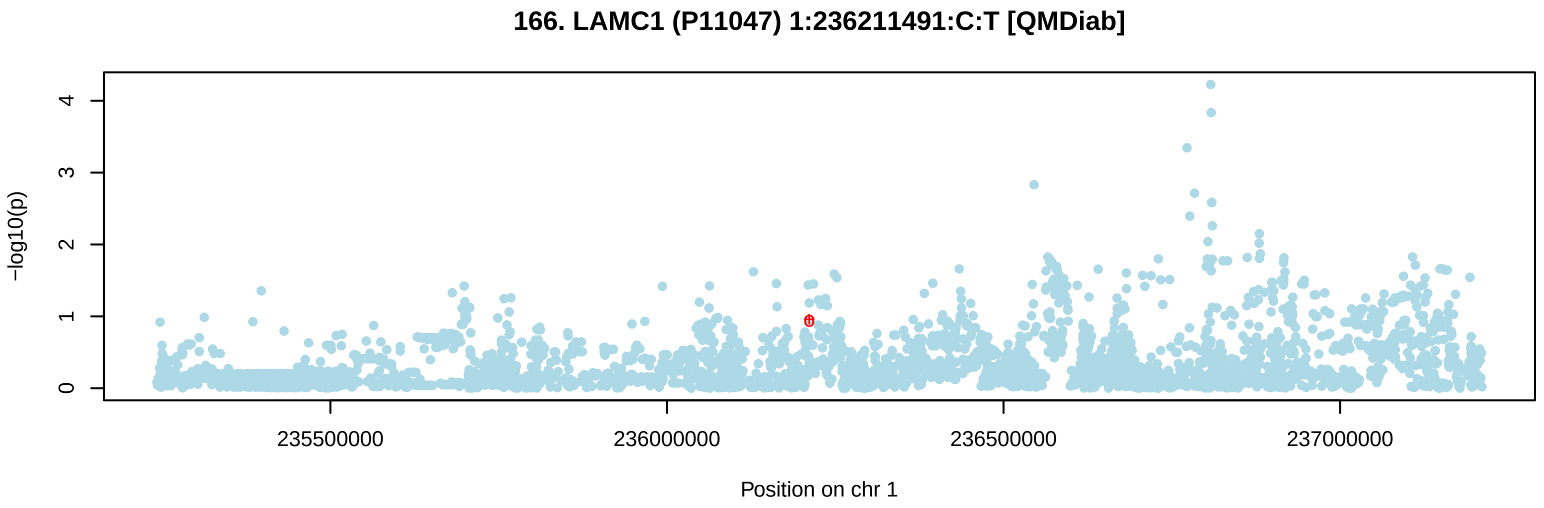
166. LAMC1 (P11047) 1:236211491:C:T [QMDiab]
−log10(p)
0
1
2
3
4
235500000
236000000
236500000
237000000
Position on chr 1

167. RNASE3 (P12724) 14:21426161:G:A [Tarkin]

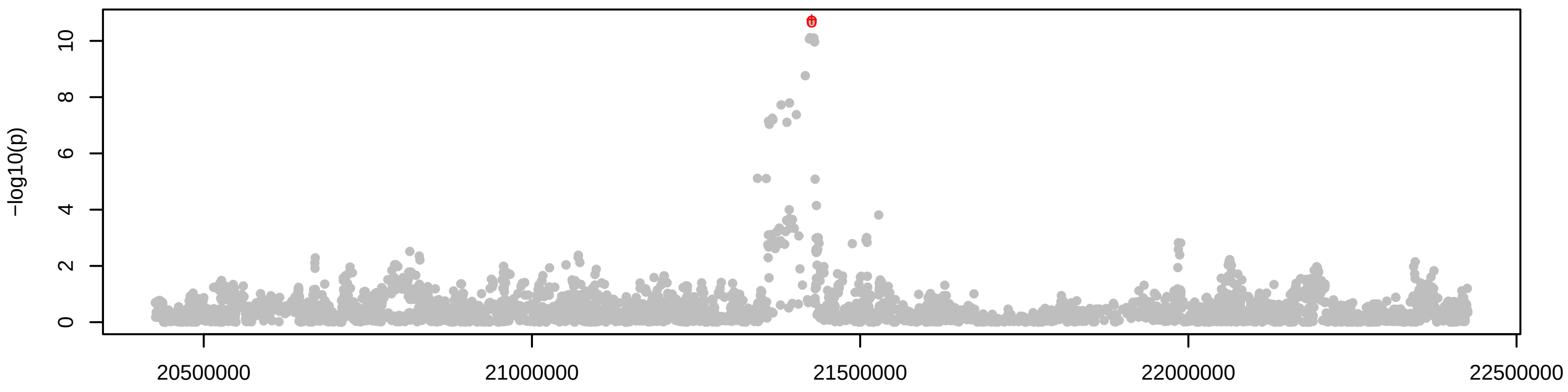

Position on chr 14
coloc with QMDiab: H4 (PP=58%)

167. RNASE3 (P12724) 14:21426161:G:A [QMDiab]

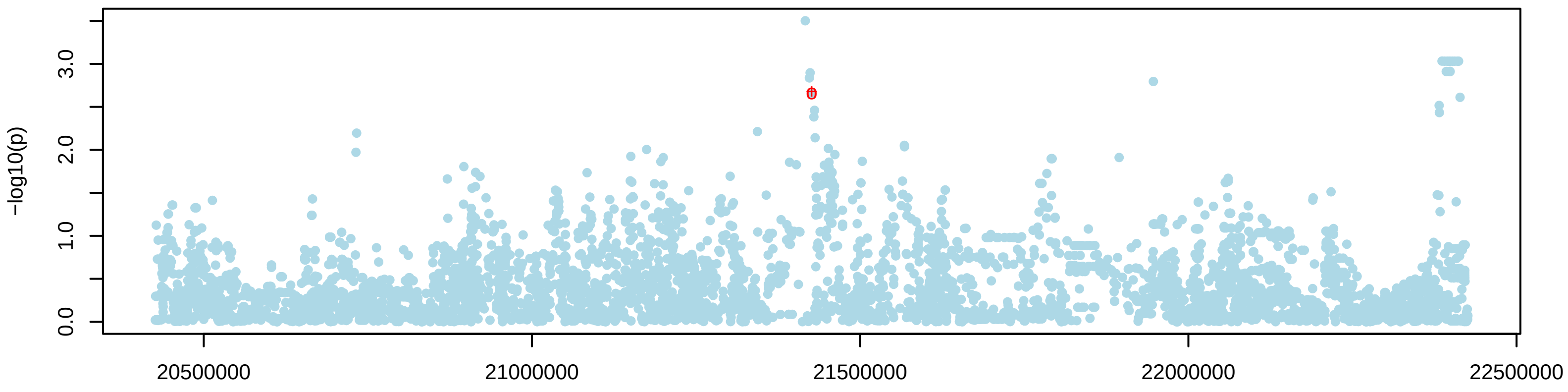

Position on chr 14

168. CCL18 (P55774) 17:34389361:G:A [Tarkin]

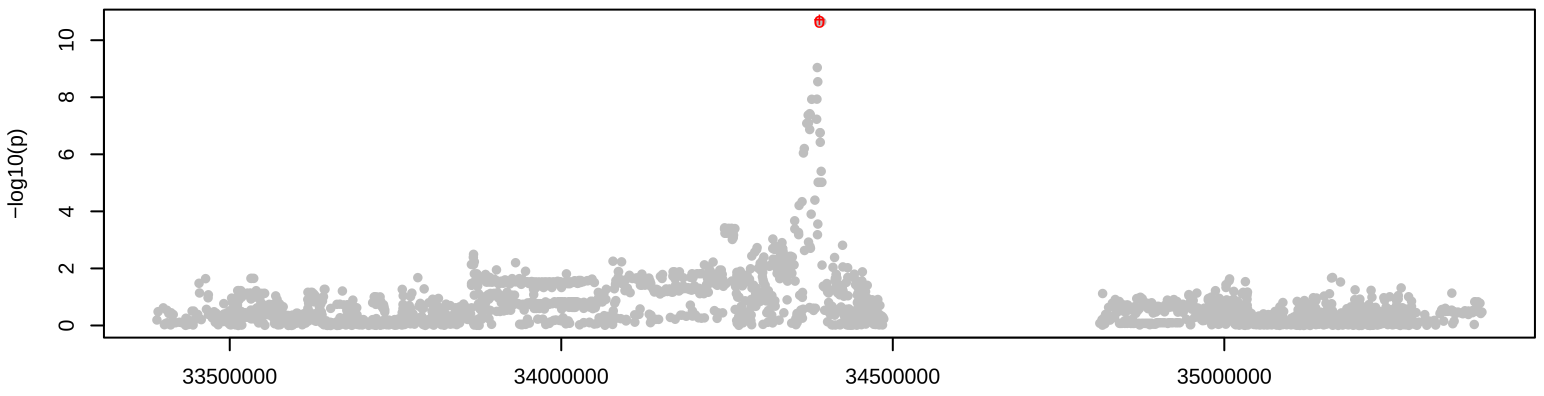

Position on chr 17
coloc with QMDiab: H4 (PP=99%)

168. CCL18 (P55774) 17:34389361:G:A [QMDiab]

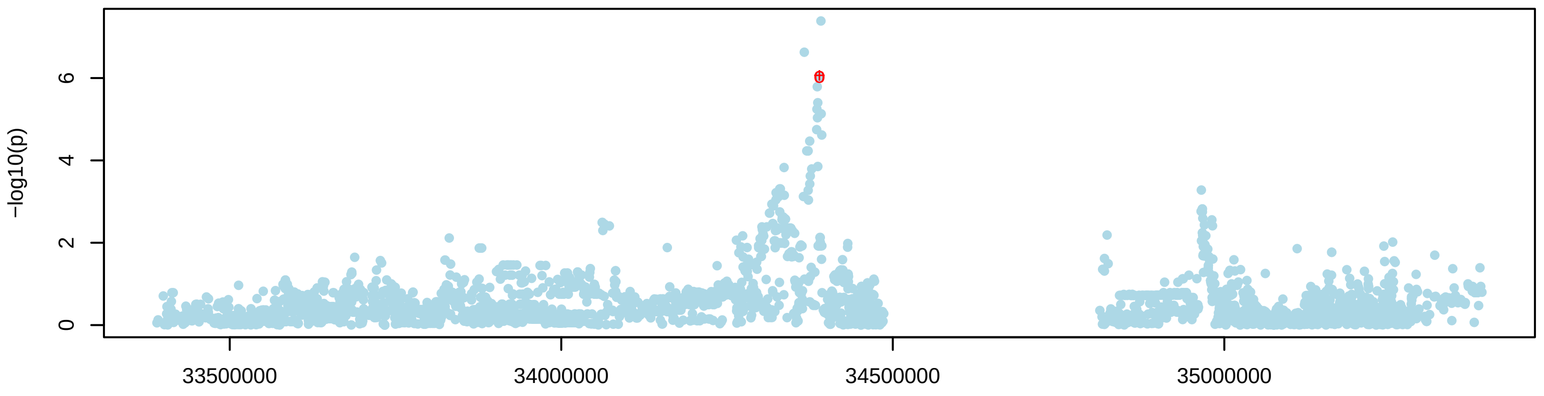

Position on chr 17

169. KNG1 (P01042−3) 3:186449122:A:G [Tarkin]

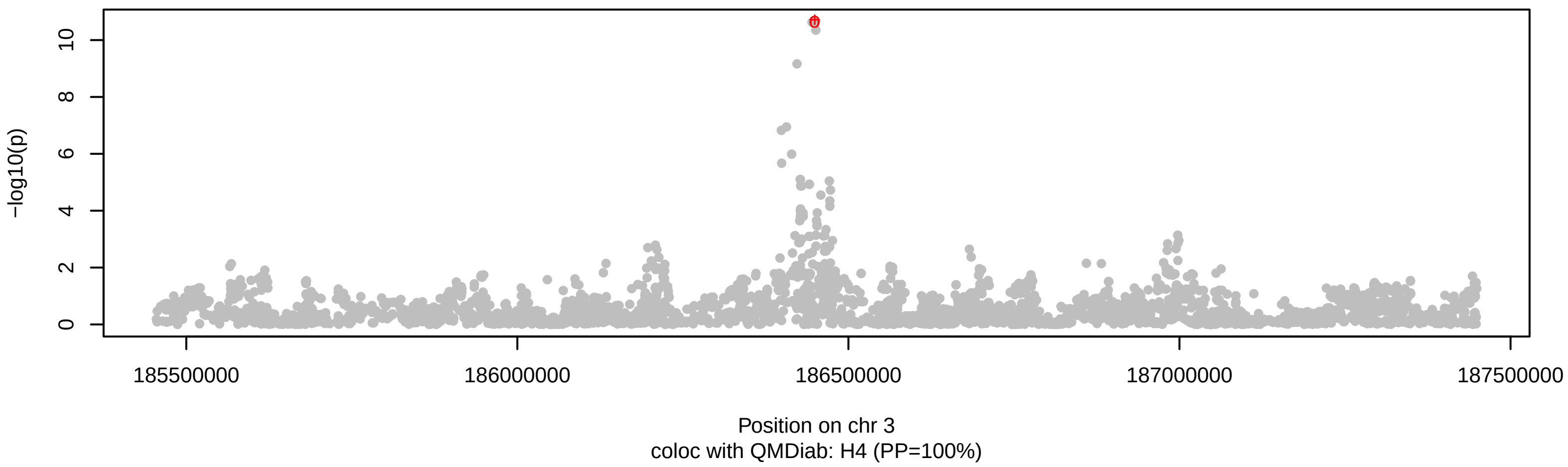

coloc with QMDiab: H4 (PP=100%)

169. KNG1 (P01042−3) 3:186449122:A:G [QMDiab]

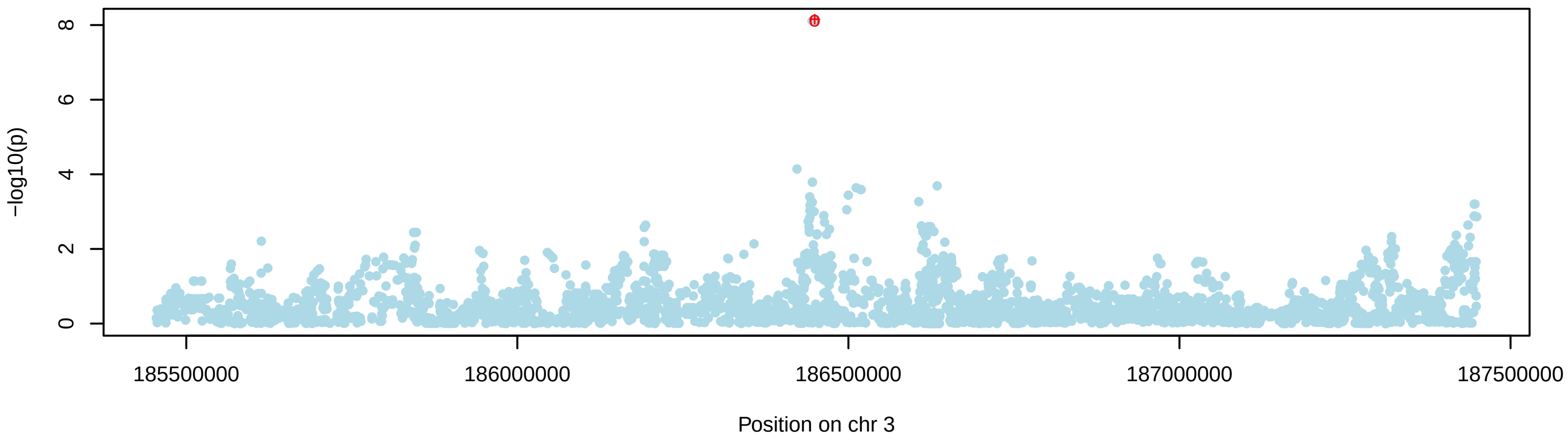

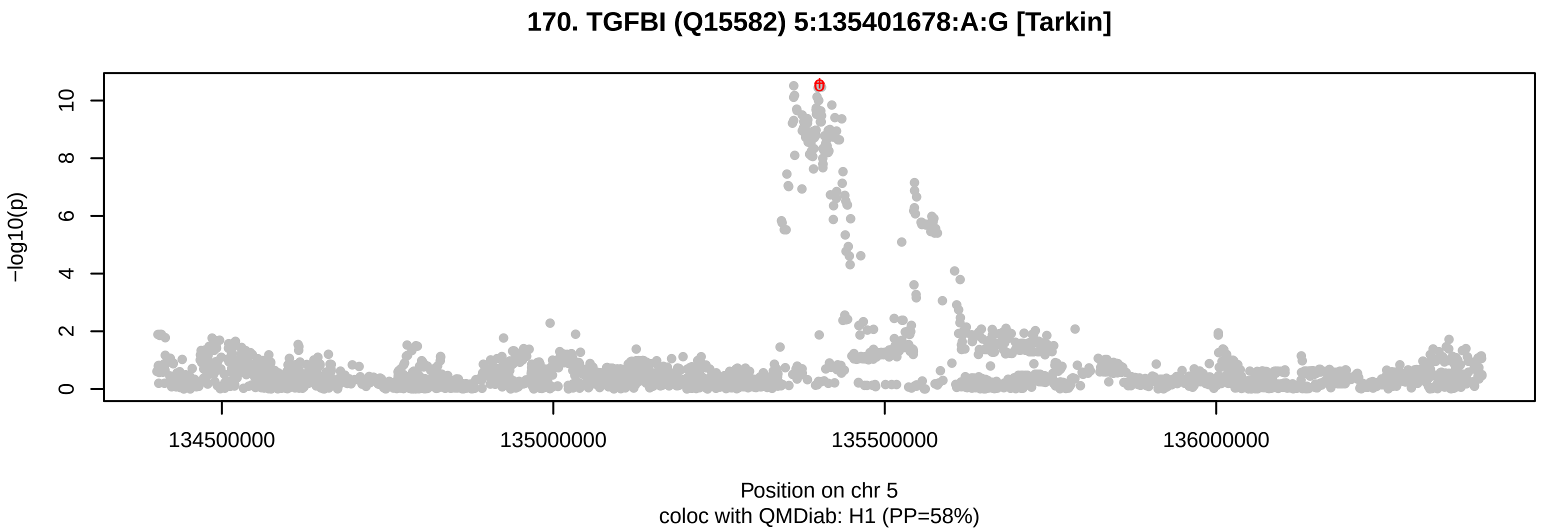

coloc with QMDiab: H1 (PP=58%)

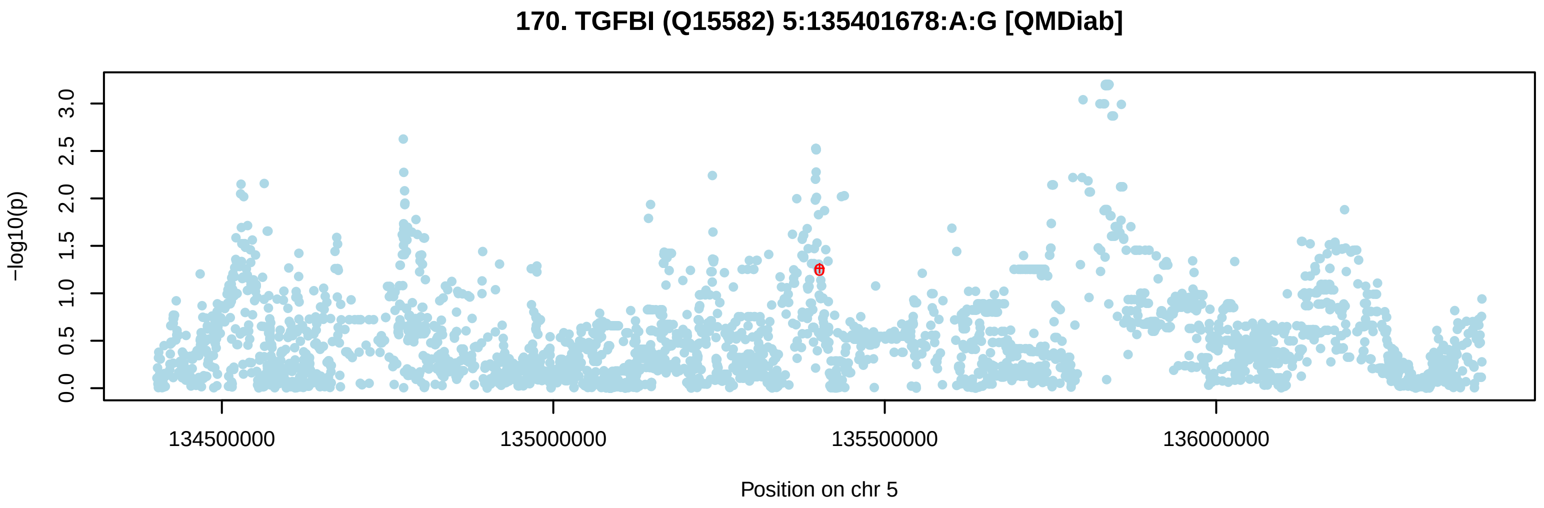

171. ANG (P03950) 14:21143334:A:G [Tarkin]

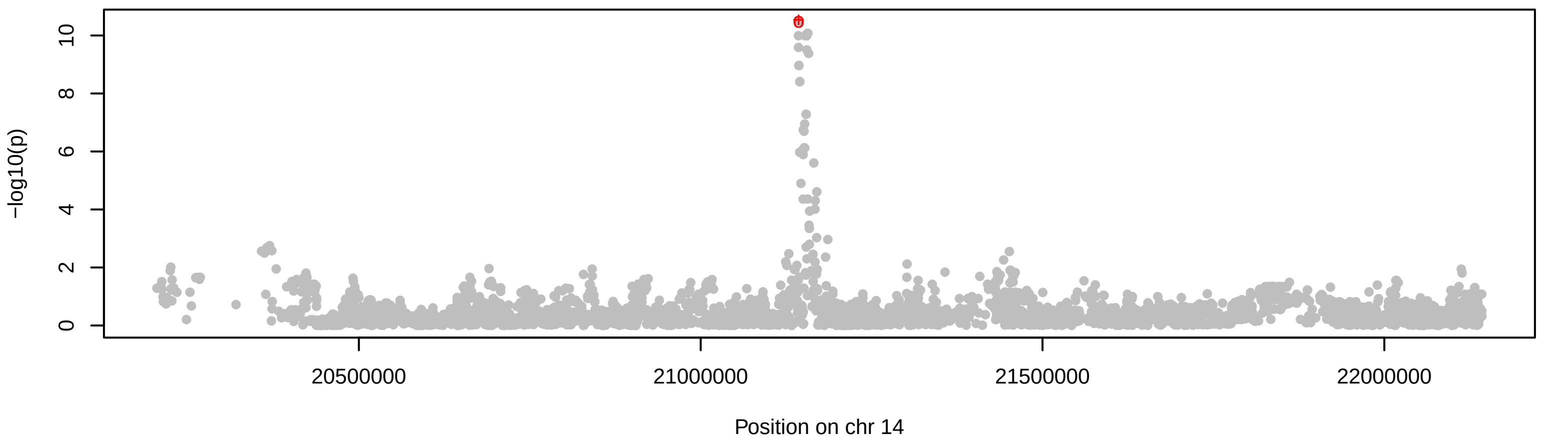

coloc with QMDiab: H4 (PP=99%)

171. ANG (P03950) 14:21143334:A:G [QMDiab]

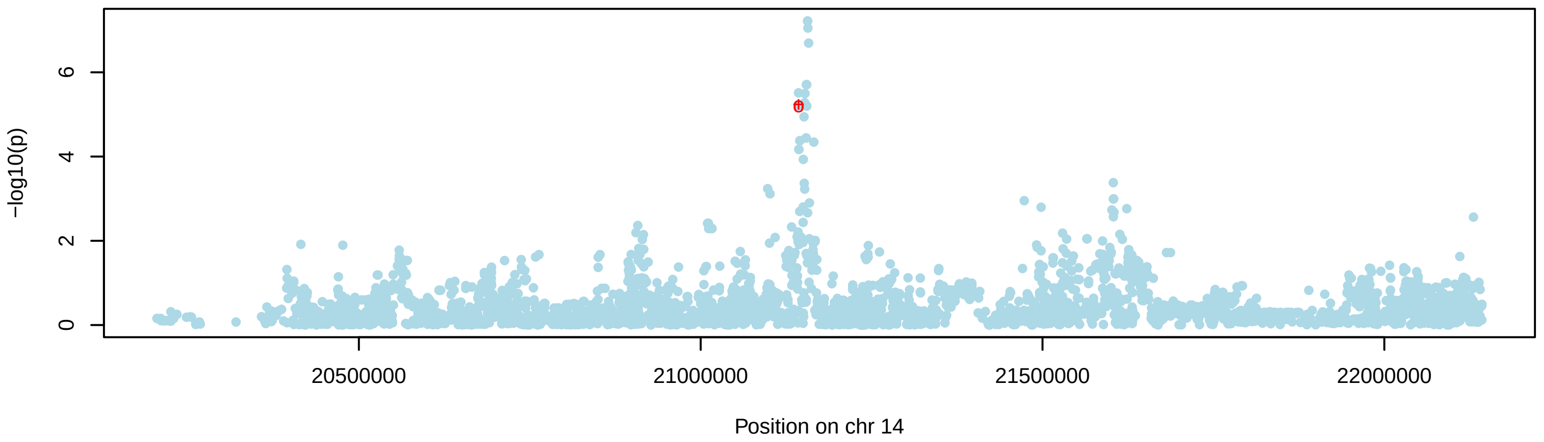

172. RHOF (Q9HBH0) 12:122216910:A:G [Tarkin]

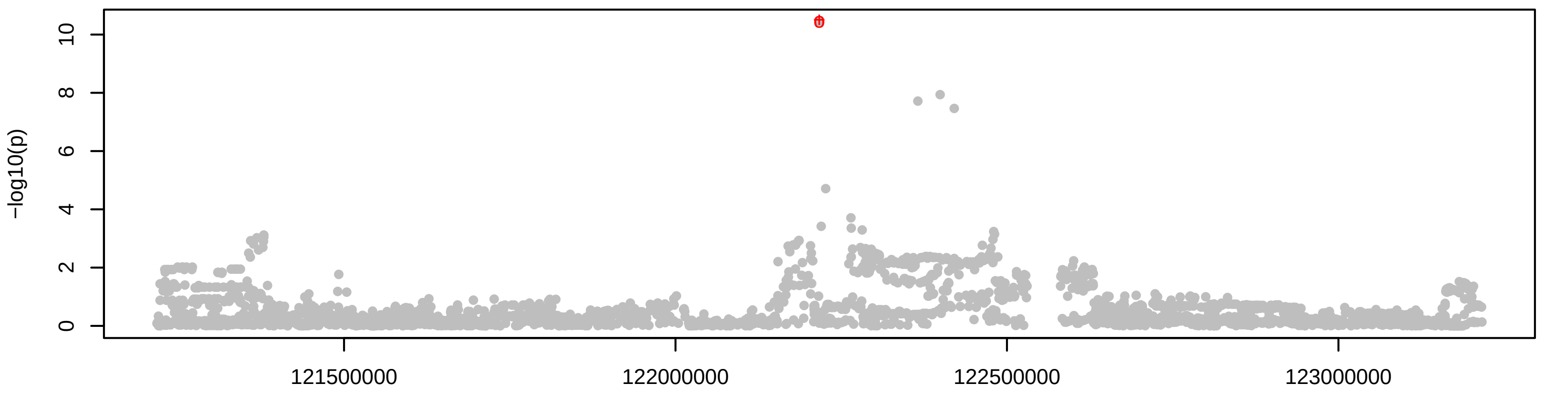

coloc with QMDiab: H1 (PP=74%)

172. RHOF (Q9HBH0) 12:122216910:A:G [QMDiab]

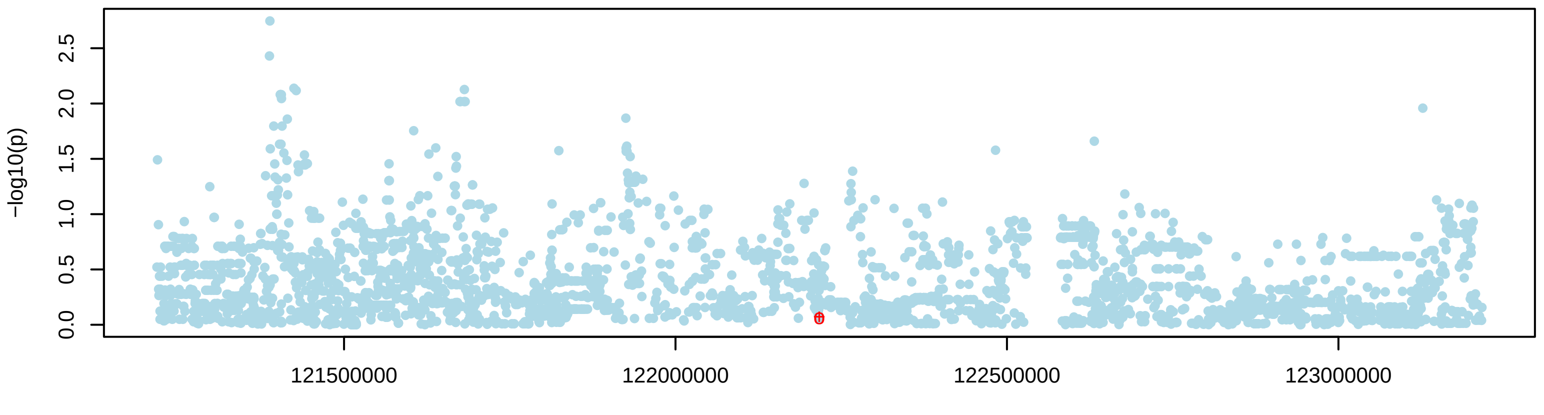

173. KLHDC1 (Q8N7A1) 20:31694060:C:T [Tarkin]

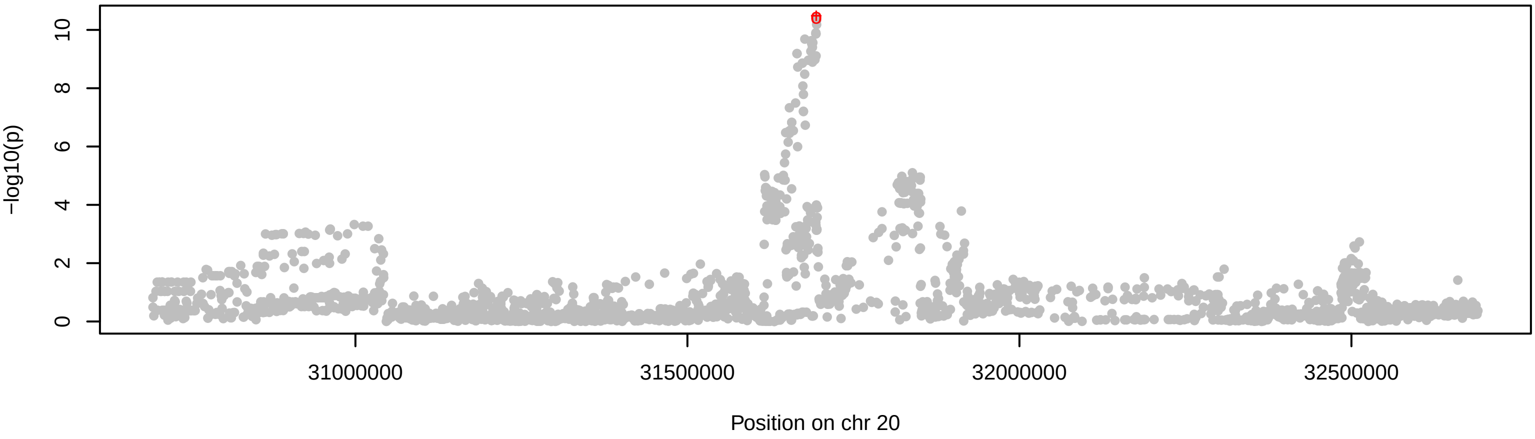

coloc with QMDiab: H4 (PP=50%)

173. KLHDC1 (Q8N7A1) 20:31694060:C:T [QMDiab]

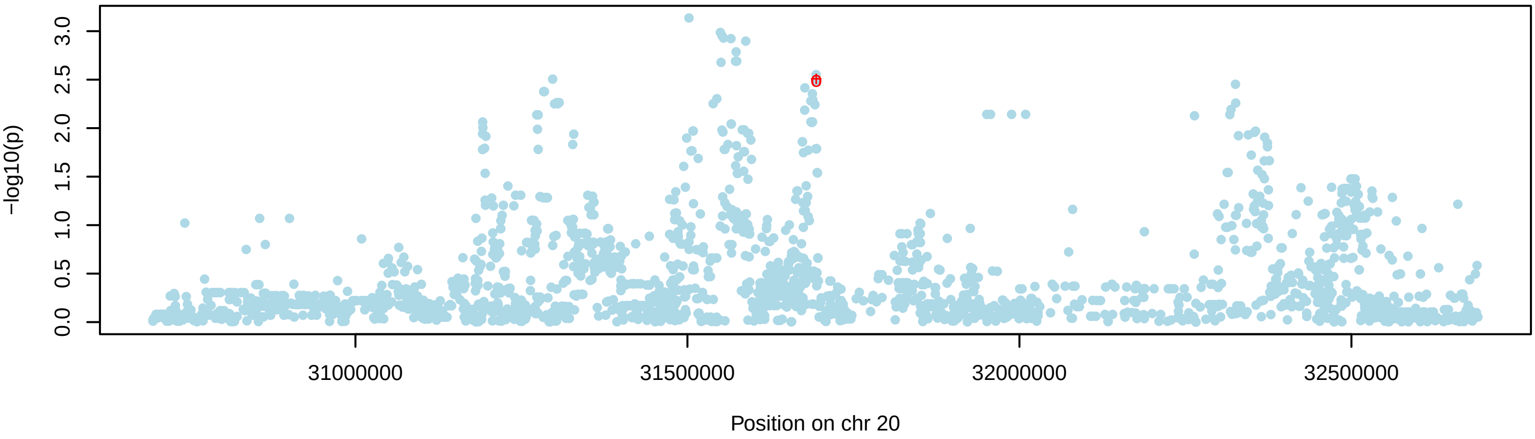

174. SPTBN1 (Q01082) 2:54834380:G:A [Tarkin]

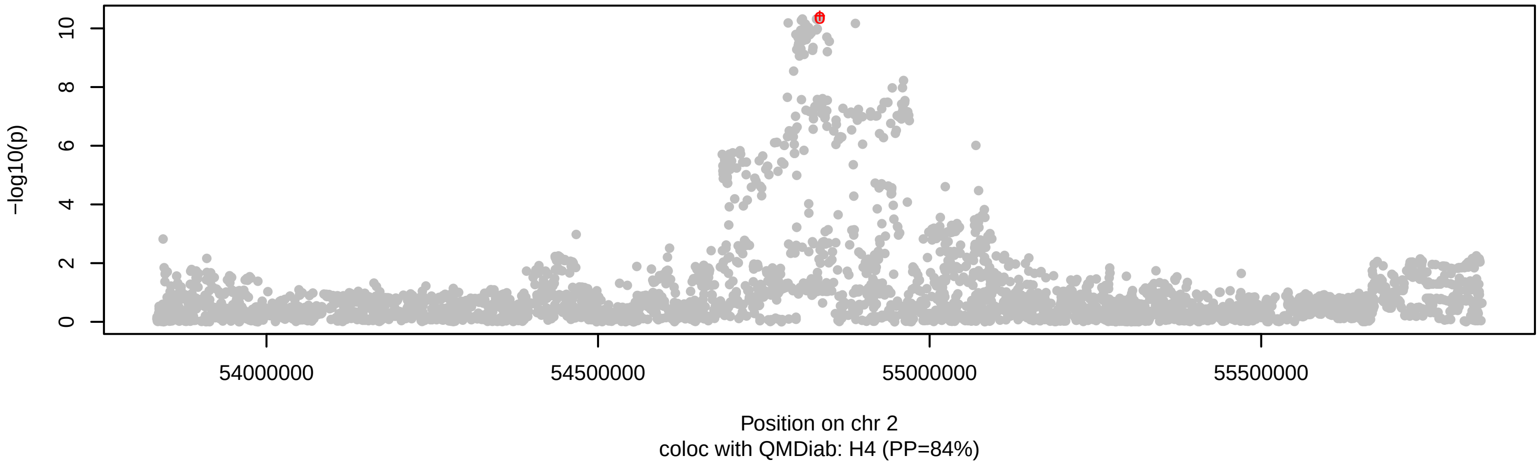

coloc with QMDiab: H4 (PP=84%)

174. SPTBN1 (Q01082) 2:54834380:G:A [QMDiab]

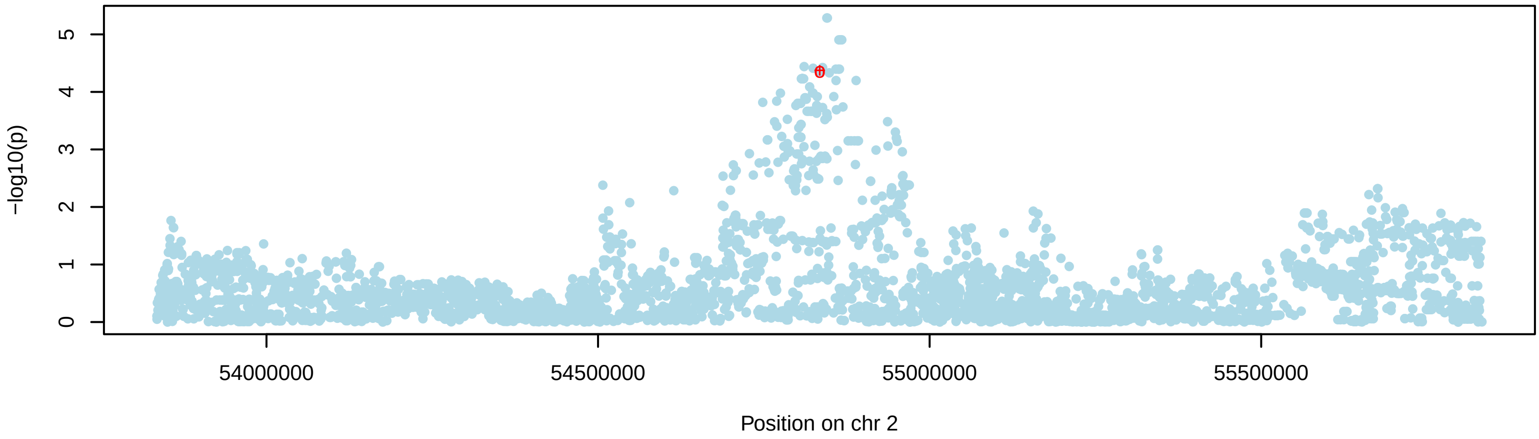

175. COL6A2 (P12110−2) 3:186393182:T:C [Tarkin]

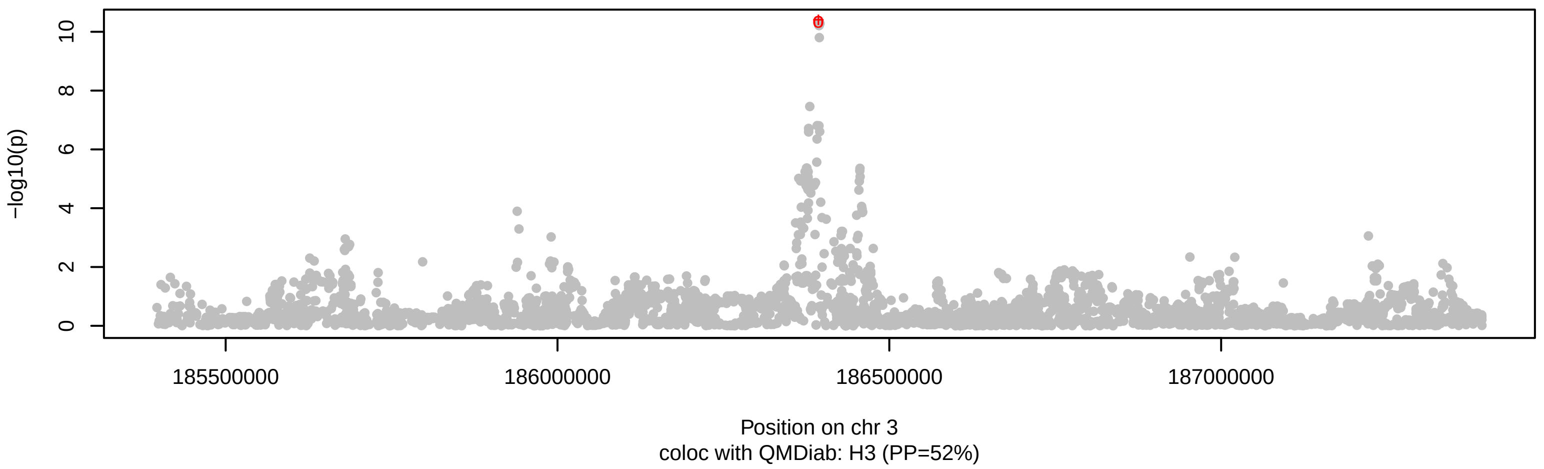

coloc with QMDiab: H3 (PP=52%)

175. COL6A2 (P12110−2) 3:186393182:T:C [QMDiab]

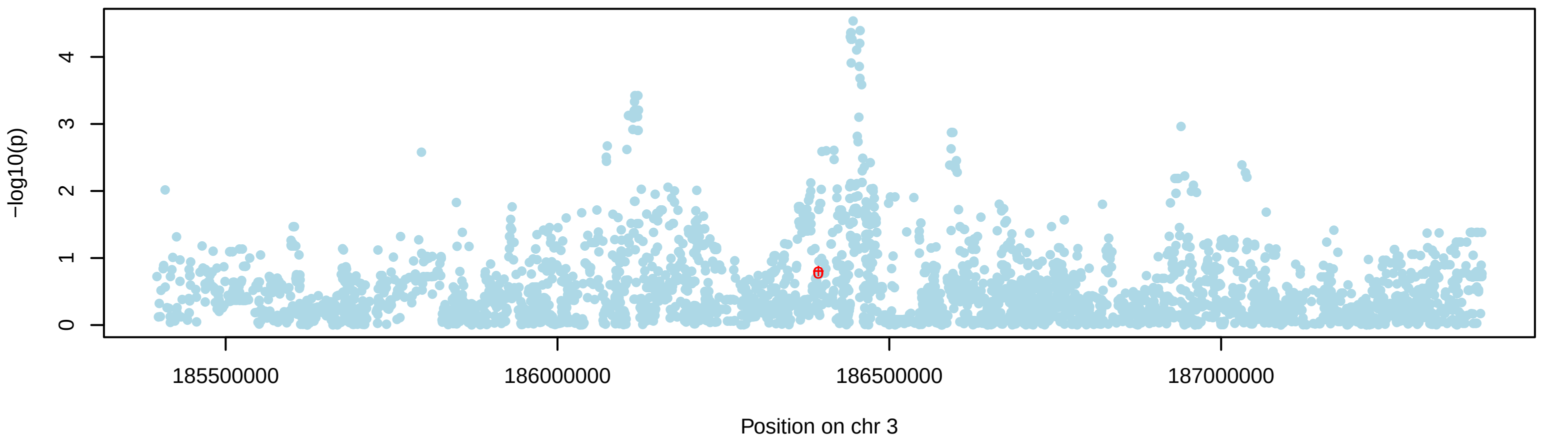

176. HHIPL1 (Q96JK4) 14:100111125:G:A [Tarkin]

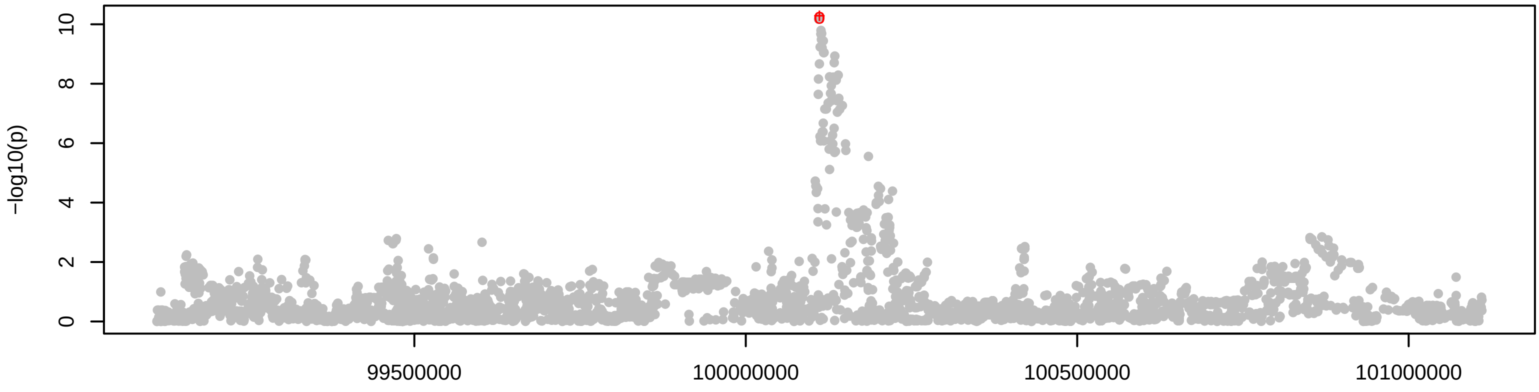

Position on chr 14
no replication data avaiable in QMDiab

177. CNRIP1 (Q96F85) 2:68579972:G:A [Tarkin]

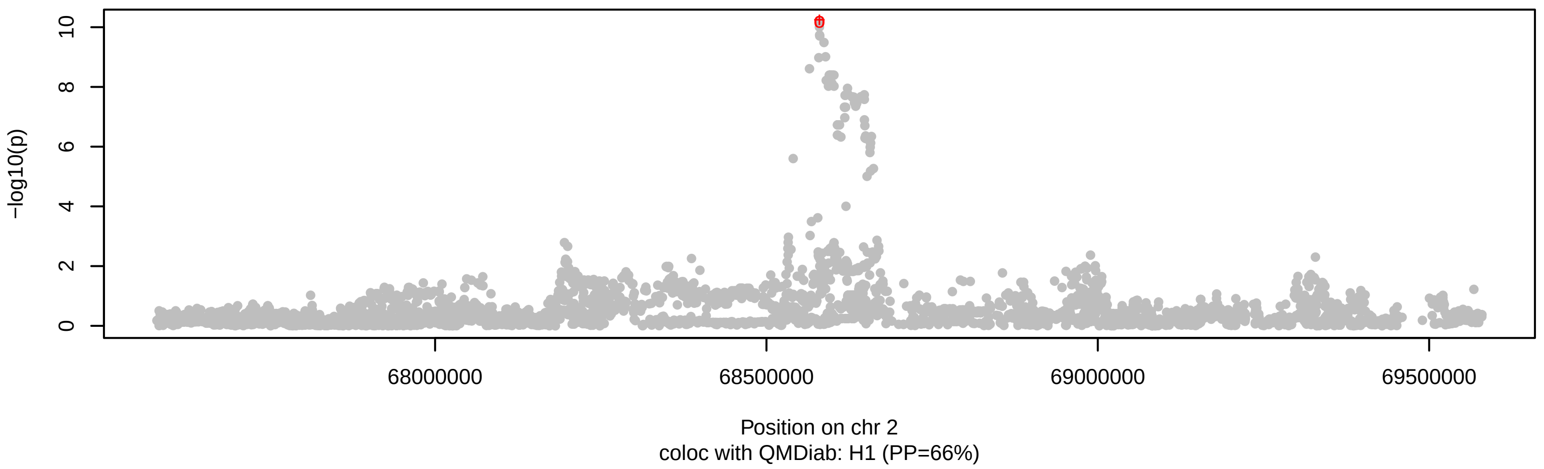

coloc with QMDiab: H1 (PP=66%)

177. CNRIP1 (Q96F85) 2:68579972:G:A [QMDiab]

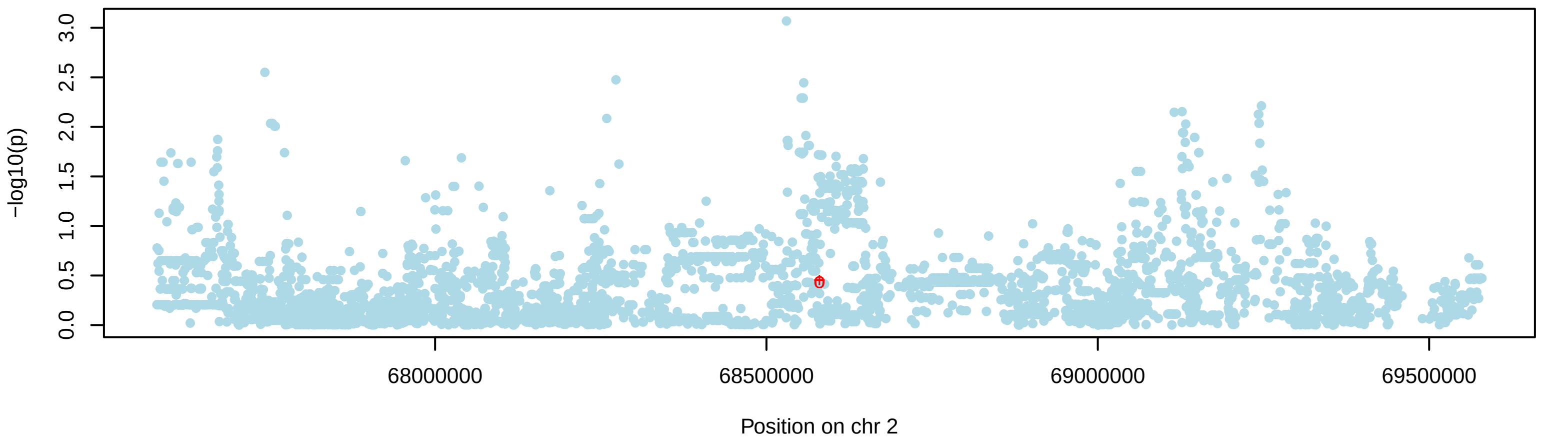

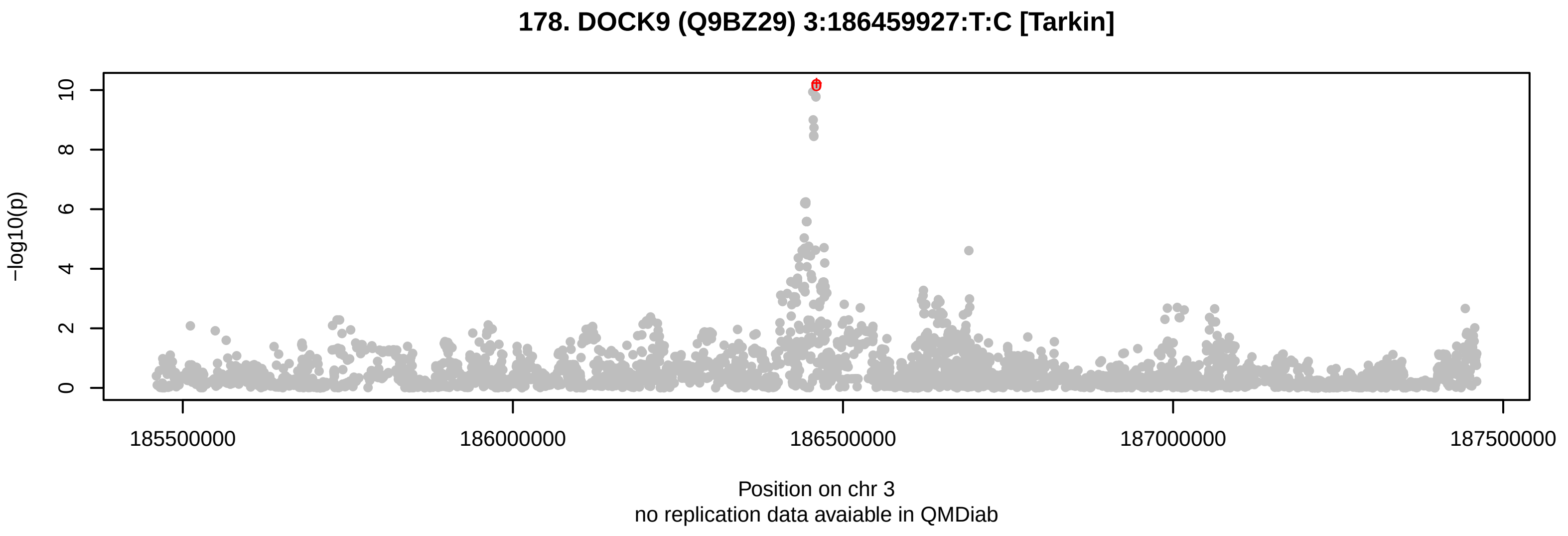
178. DOCK9 (Q9BZ29) 3:186459927:T:C [Tarkin]
−log10(p)
0
2
4
6
8
10
185500000
186000000
186500000
187000000
187500000
Position on chr 3
no replication data avaiable in QMDiab

179. CD151 (P48509) 11:837772:C:T [Tarkin]

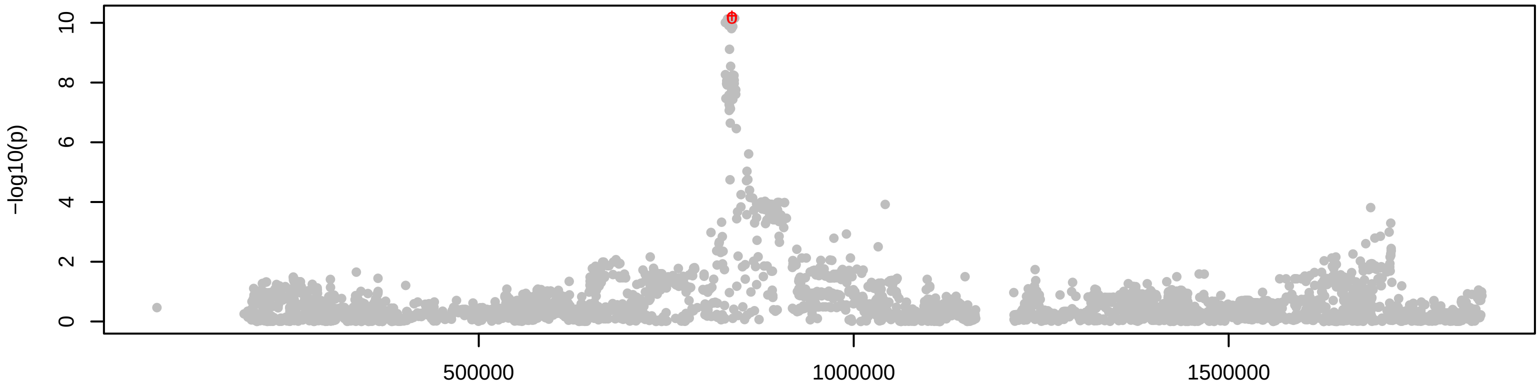

Position on chr 11
coloc with QMDiab: H1 (PP=70%)

179. CD151 (E9PMR4;P48509) 11:837772:C:T [QMDiab]

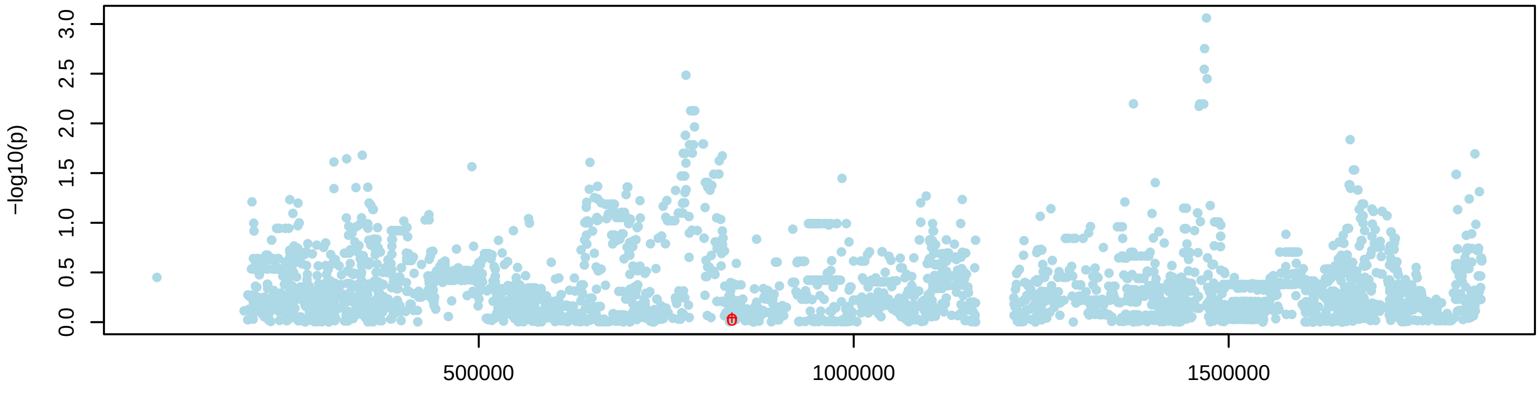

Position on chr 11

180. PON3 (Q15166) 7:94953895:G:A [Tarkin]

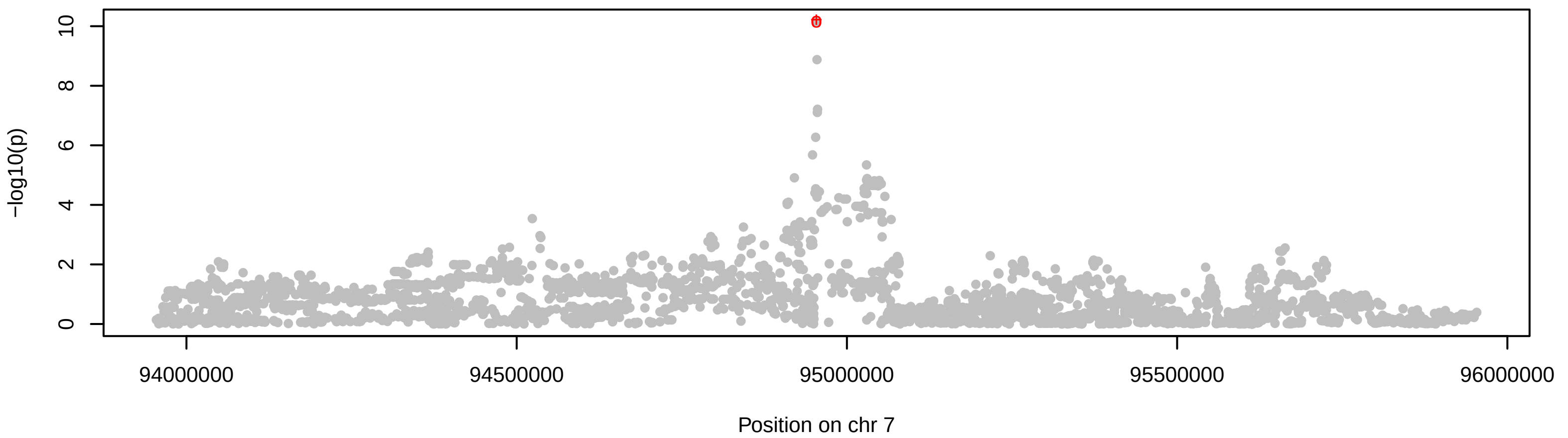

coloc with QMDiab: H3 (PP=100%)

180. PON3 (Q15166) 7:94953895:G:A [QMDiab]

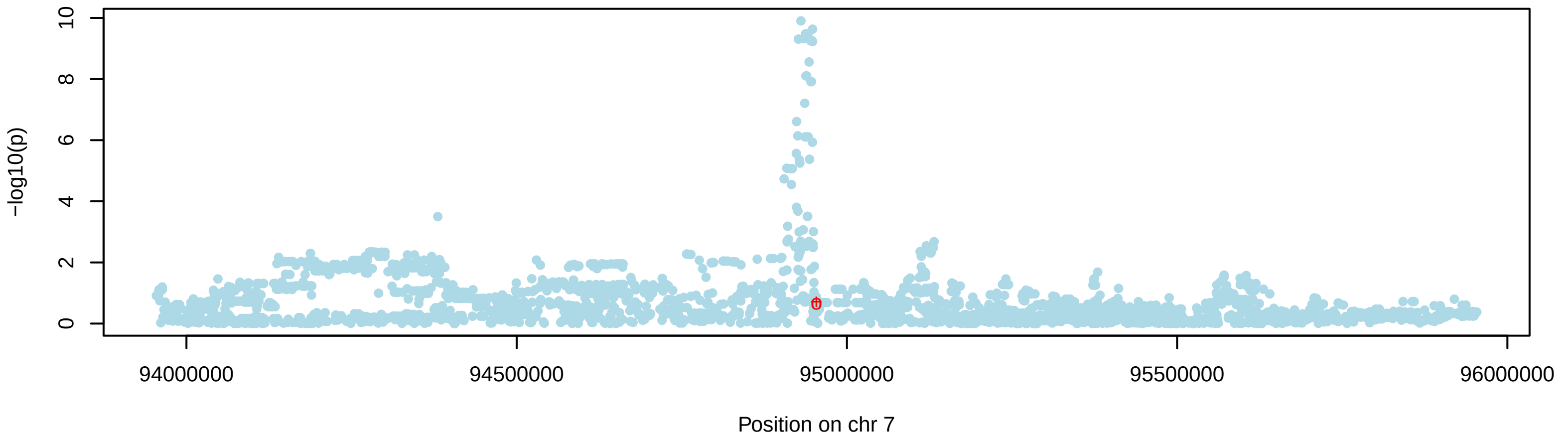

181. FABP5 (Q01469) 9:136149500:T:C [Tarkin]

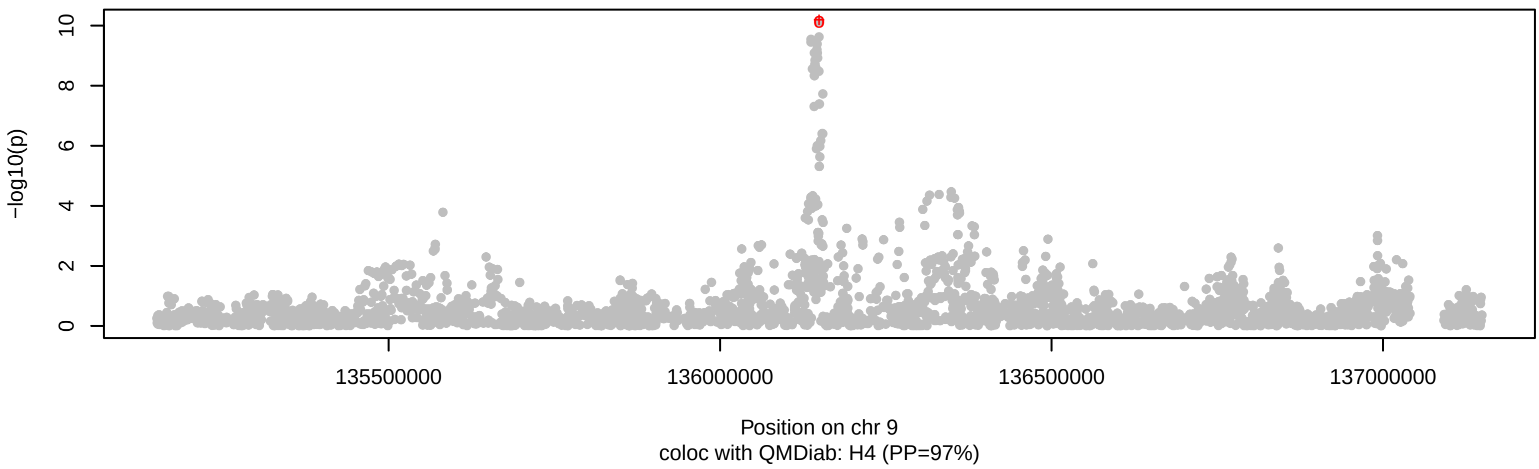

coloc with QMDiab: H4 (PP=97%)

181. FABP5 (Q01469) 9:136149500:T:C [QMDiab]

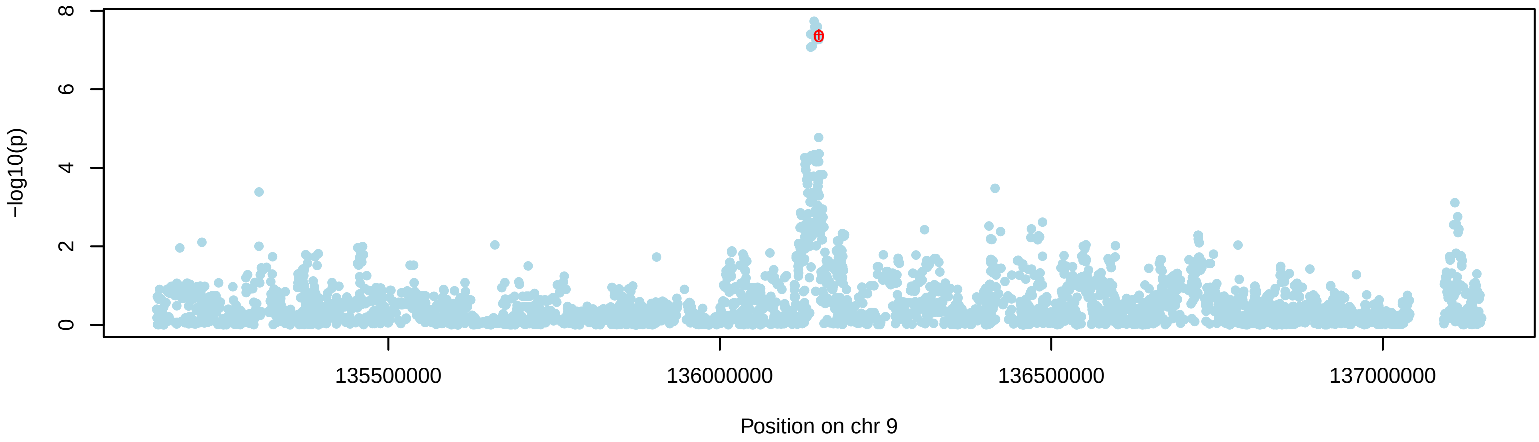

182. COCH (A0A2U3TZE7;O43405) 14:31343171:T:G [Tarkin]

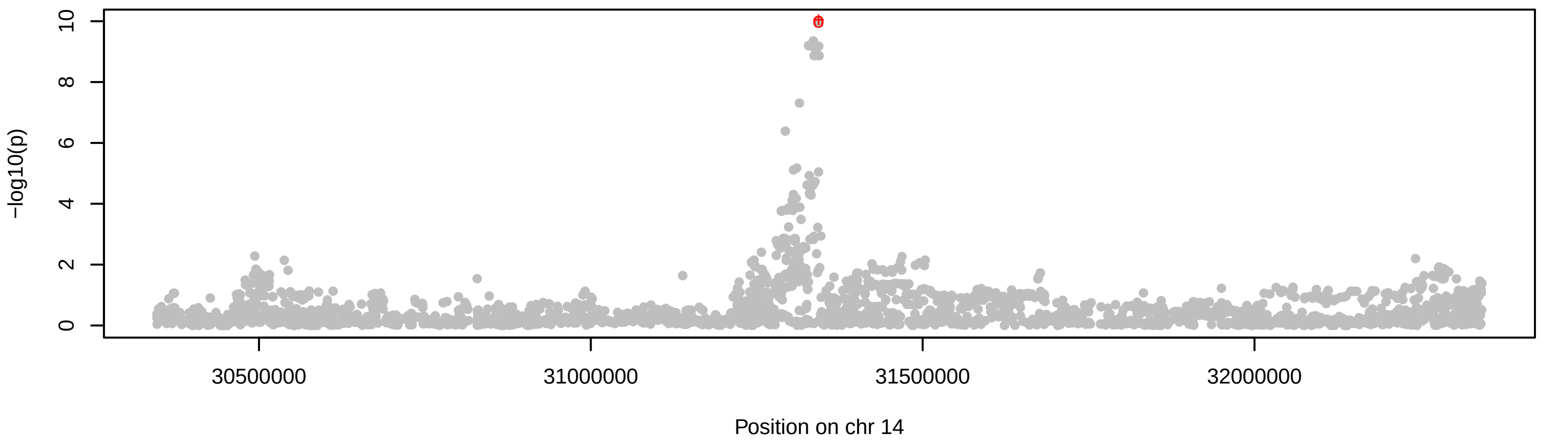

coloc with QMDiab: H1 (PP=74%)

182. COCH (A0A2U3TZE7;O43405) 14:31343171:T:G [QMDiab]

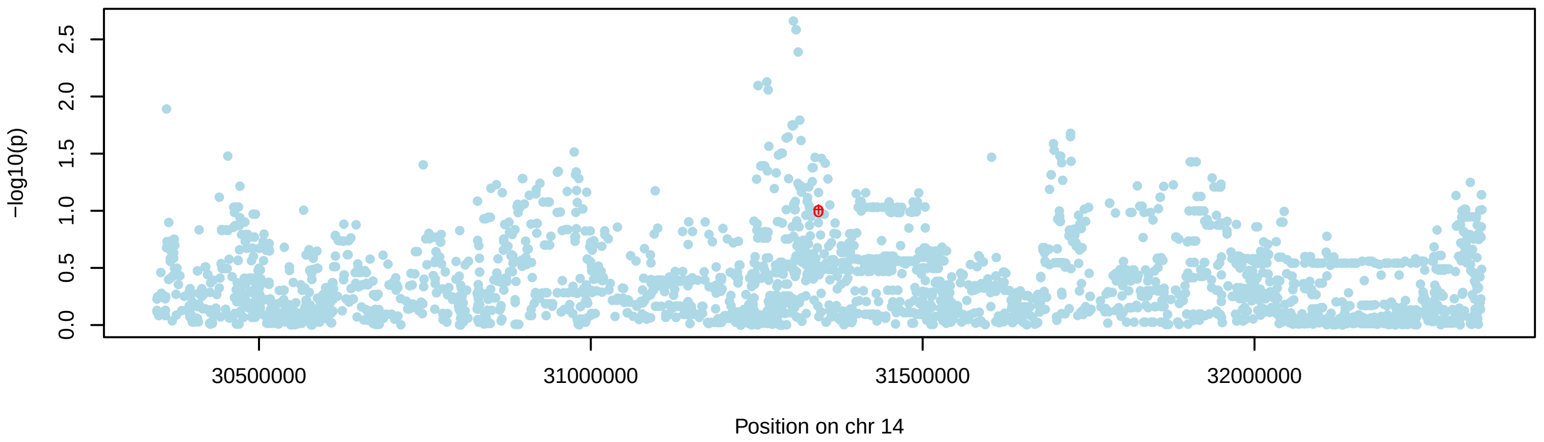

183. PAK2 (Q13177) 3:186394038:G:C [Tarkin]

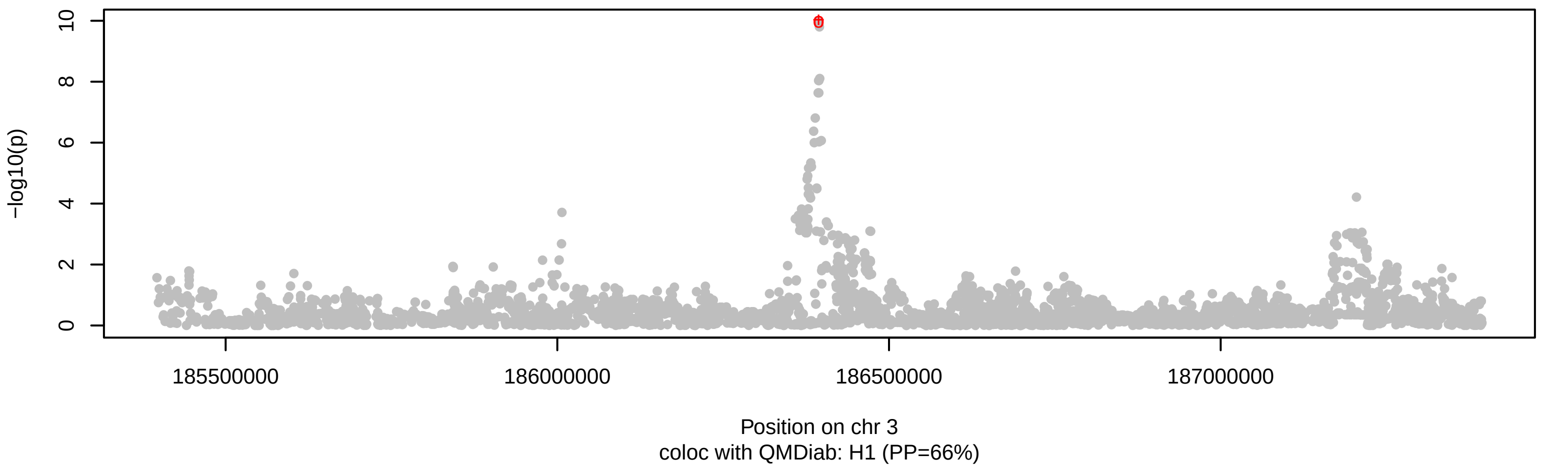

183. PAK2 (Q13177) 3:186394038:G:C [QMDiab]

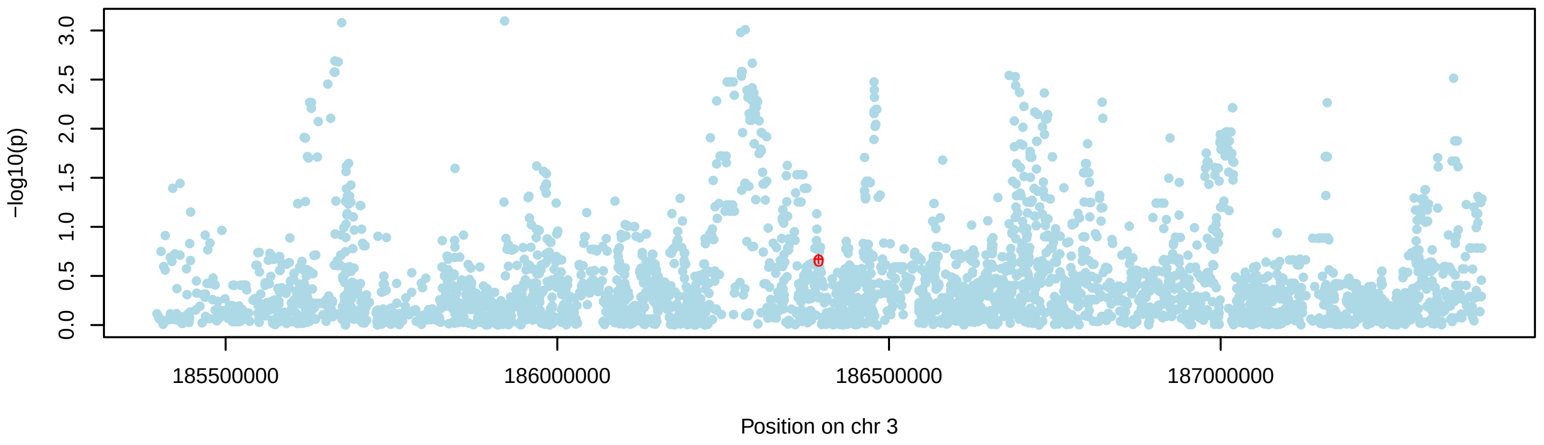

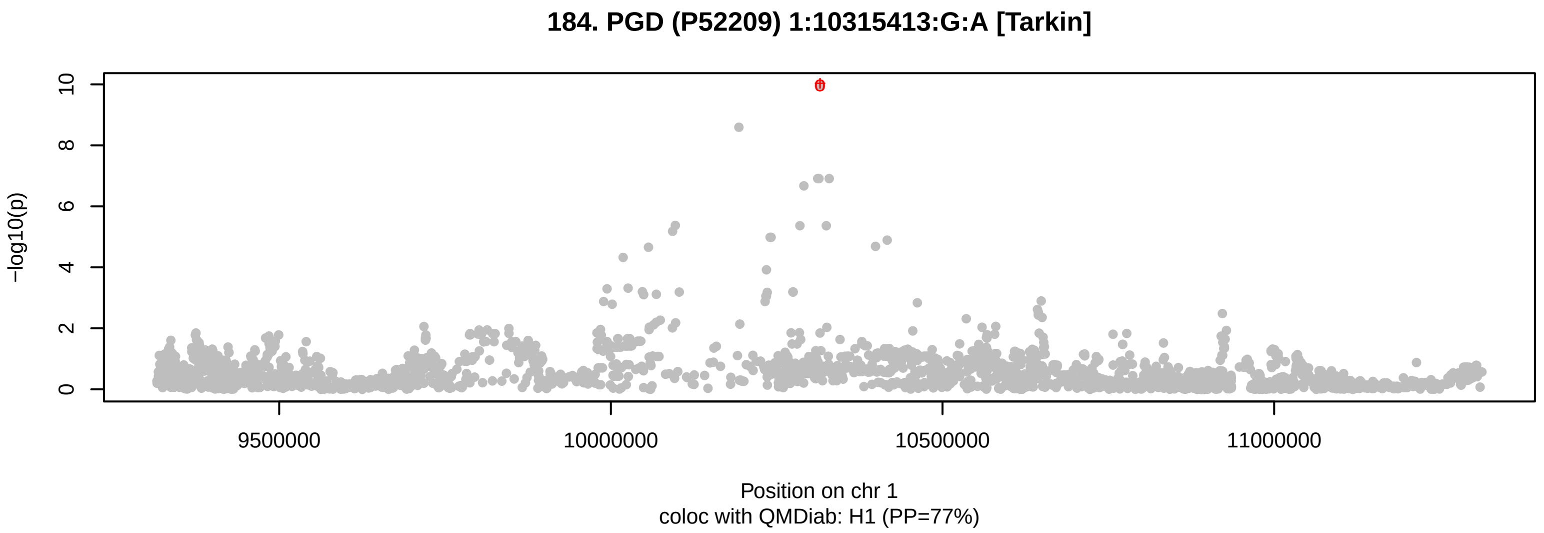

184. PGD (P52209) 1:10315413:G:A [Tarkin]
−log10(p)
0
2
4
6
8
10
9500000
10000000
10500000
11000000
Position on chr 1
coloc with QMDiab: H1 (PP=77%)

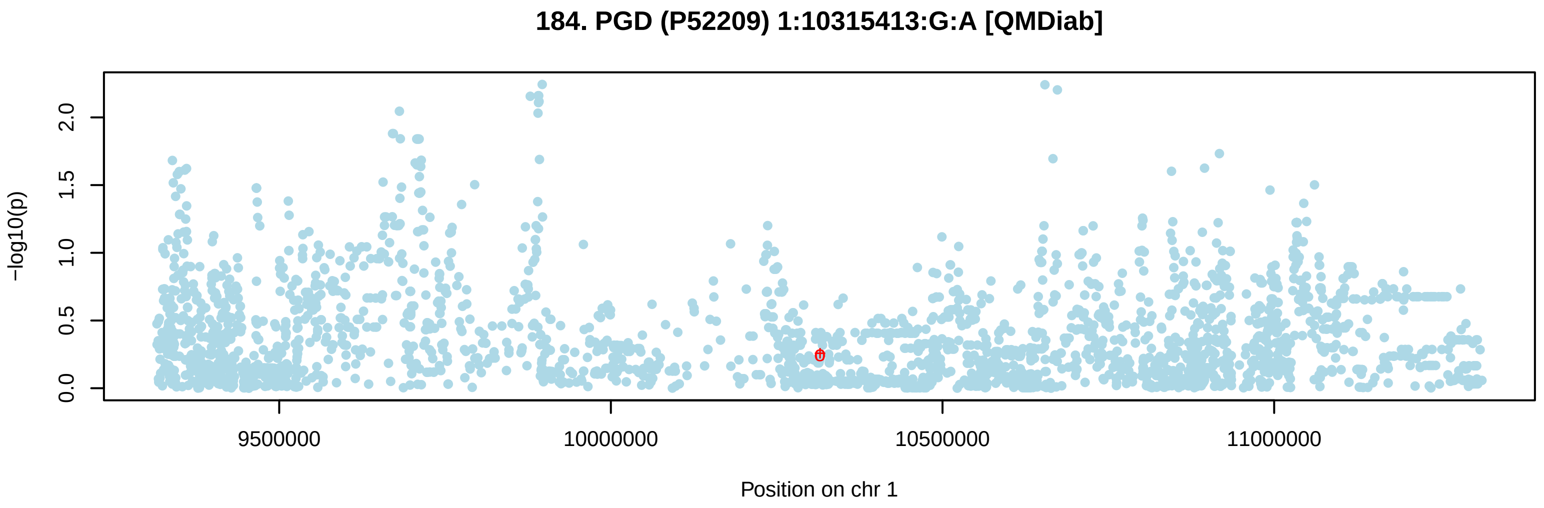

184. PGD (P52209) 1:10315413:G:A [QMDiab]
−log10(p)
0.0
0.5
1.0
1.5
2.0
9500000
10000000
10500000
11000000
Position on chr 1

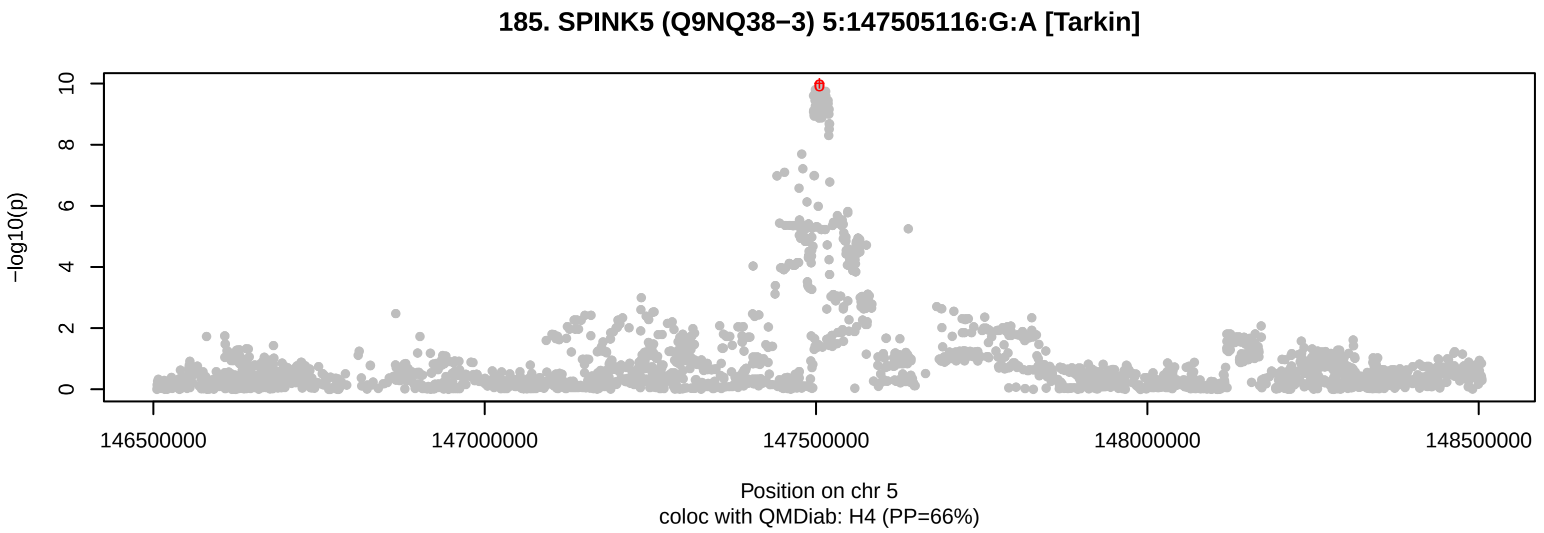
185. SPINK5 (Q9NQ38−3) 5:147505116:G:A [Tarkin]
−log10(p)
0
2
4
6
8
10
146500000
147000000
147500000
148000000
148500000
Position on chr 5
coloc with QMDiab: H4 (PP=66%)

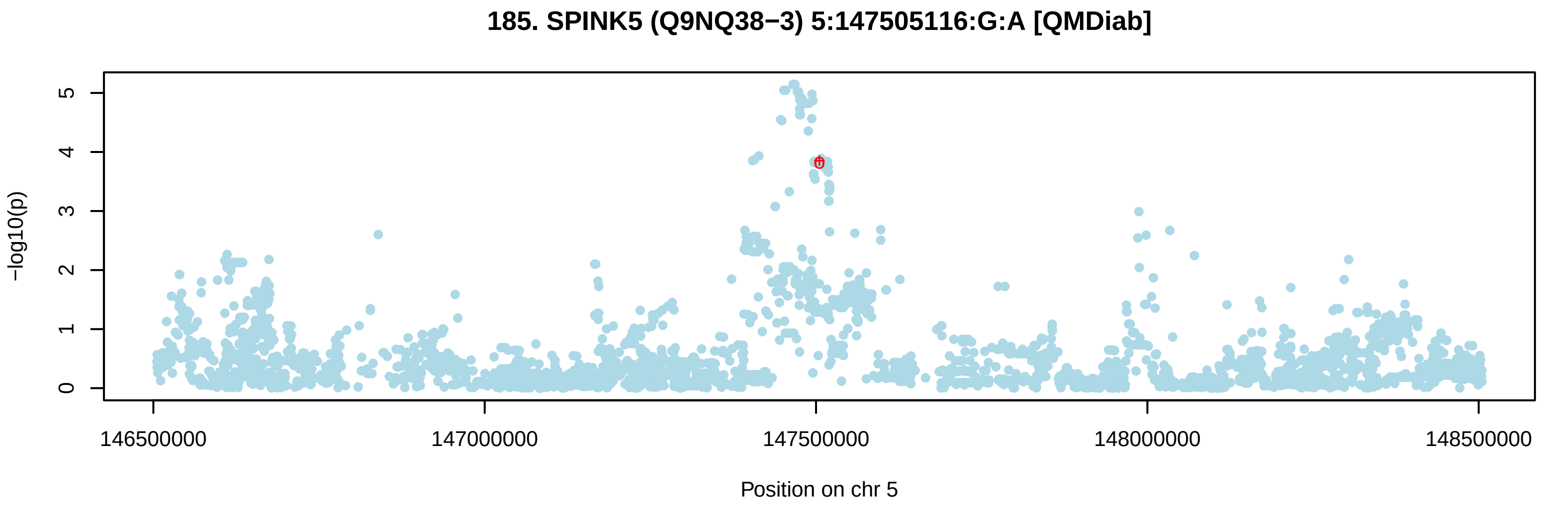
185. SPINK5 (Q9NQ38−3) 5:147505116:G:A [QMDiab]
−log10(p)
0
1
2
3
4
5
146500000
147000000
147500000
148000000
148500000
Position on chr 5

186. LINC00854 (A0A1B0GVX4) 3:186391274:G:A [Tarkin]

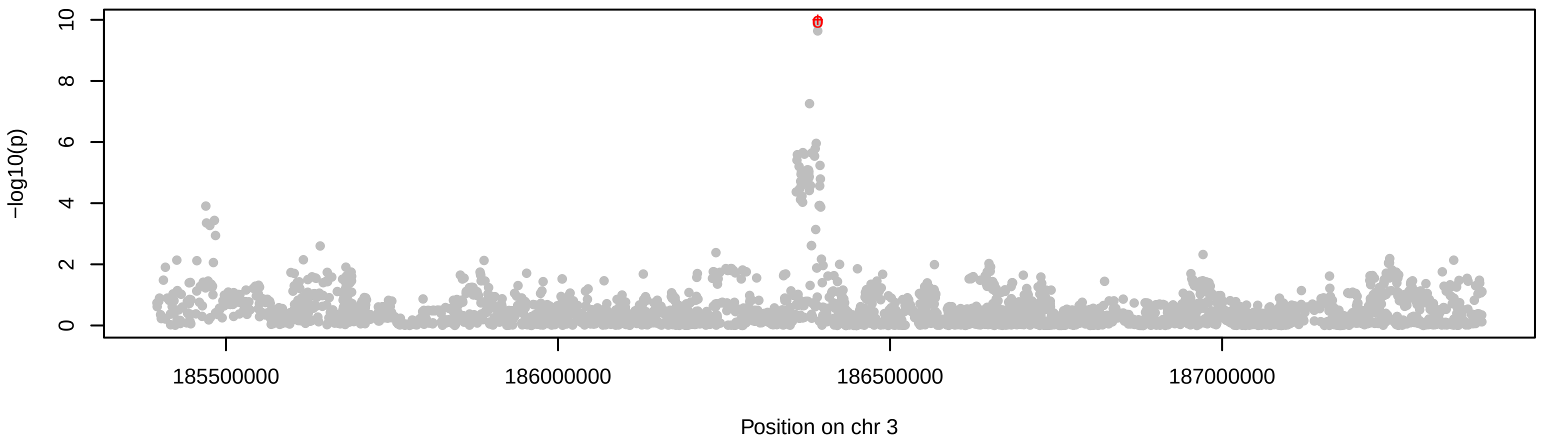

no replication data avaiable in QMDiab

187. QSOX1 (O00391) 1:180163390:A:G [Tarkin]

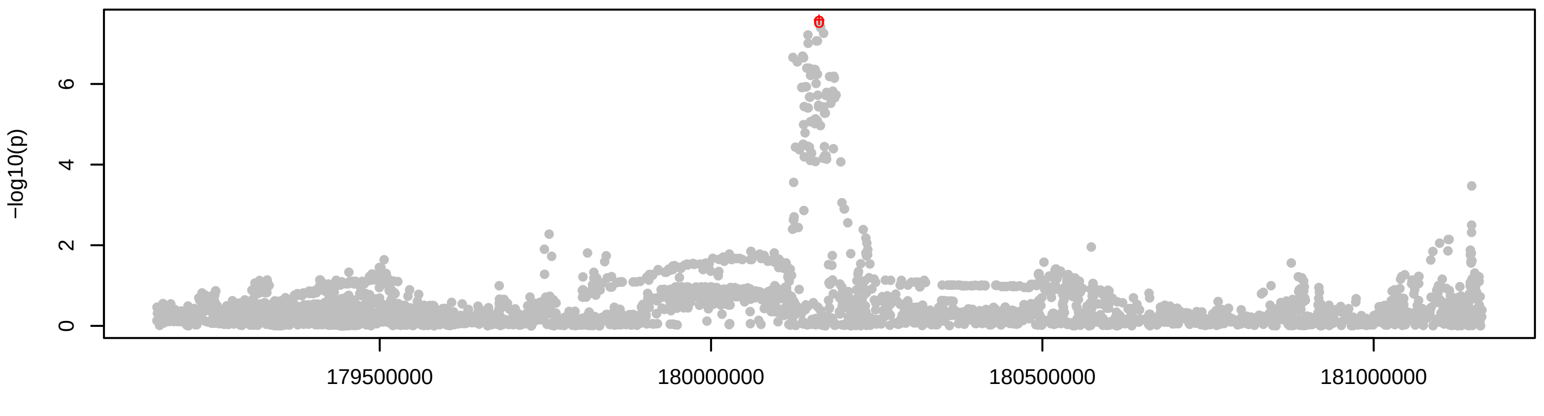

coloc with QMDiab: H1 (PP=52%)

187. QSOX1 (O00391) 1:180163390:A:G [QMDiab]

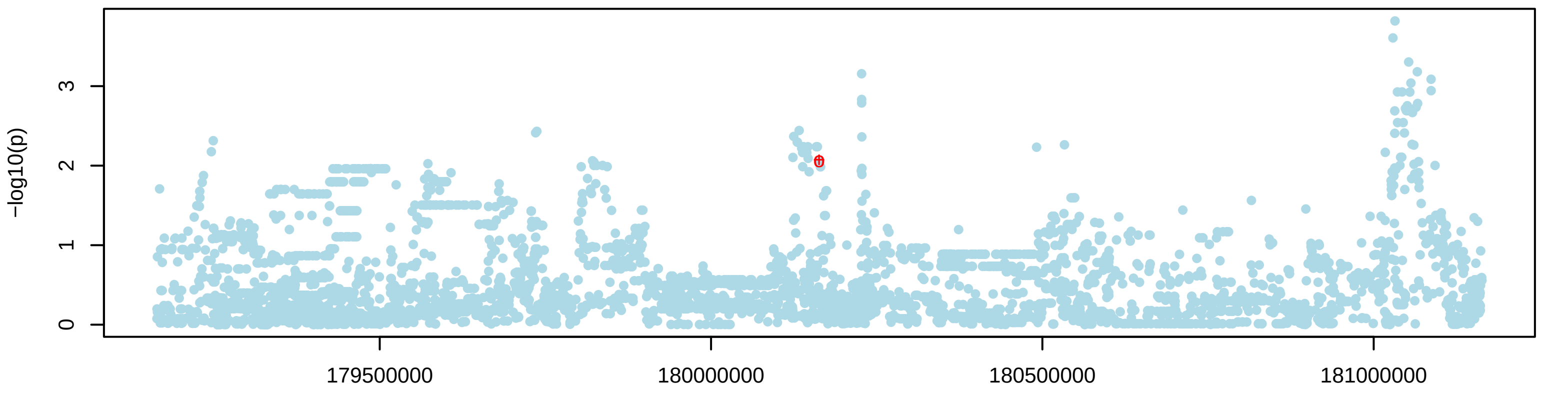

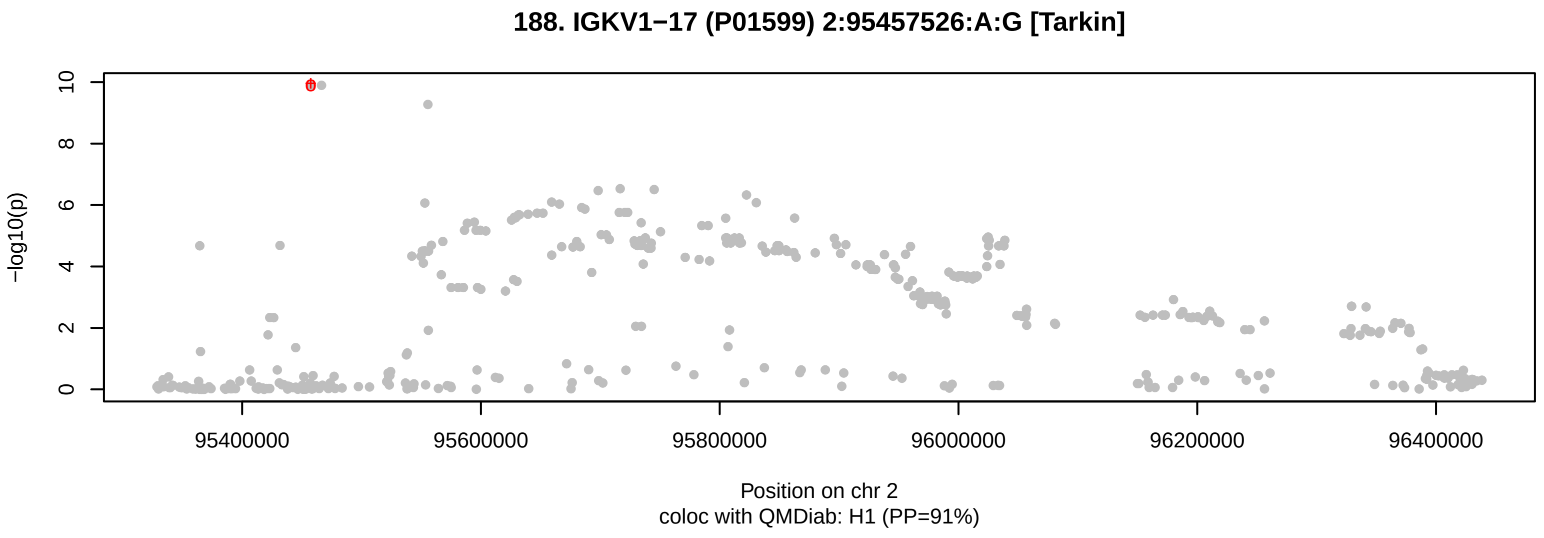
188. IGKV1−17 (P01599) 2:95457526:A:G [Tarkin]
−log10(p)
0
2
4
6
8
10
95400000
95600000
95800000
96000000
96200000
96400000
Position on chr 2
coloc with QMDiab: H1 (PP=91%)

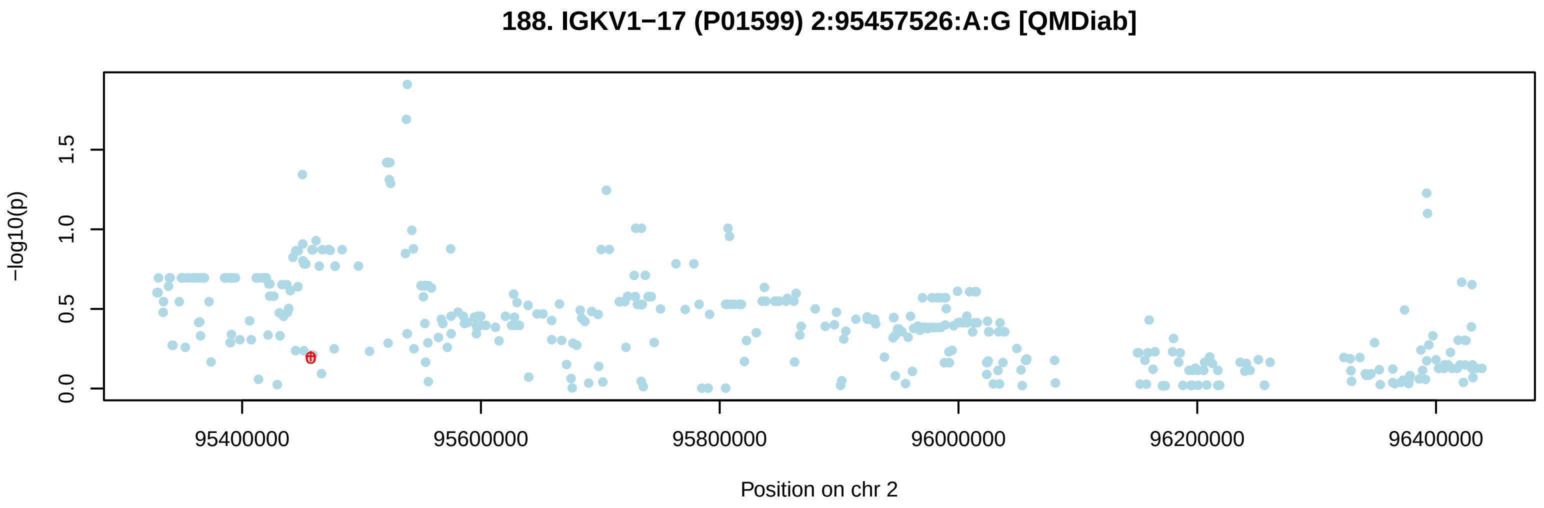
188. IGKV1−17 (P01599) 2:95457526:A:G [QMDiab]
−log10(p)
0.0
0.5
1.0
1.5
95400000
95600000
95800000
96000000
96200000
96400000
Position on chr 2

189. PGM3 (H0Y987) 6:83903626:G:C [Tarkin]

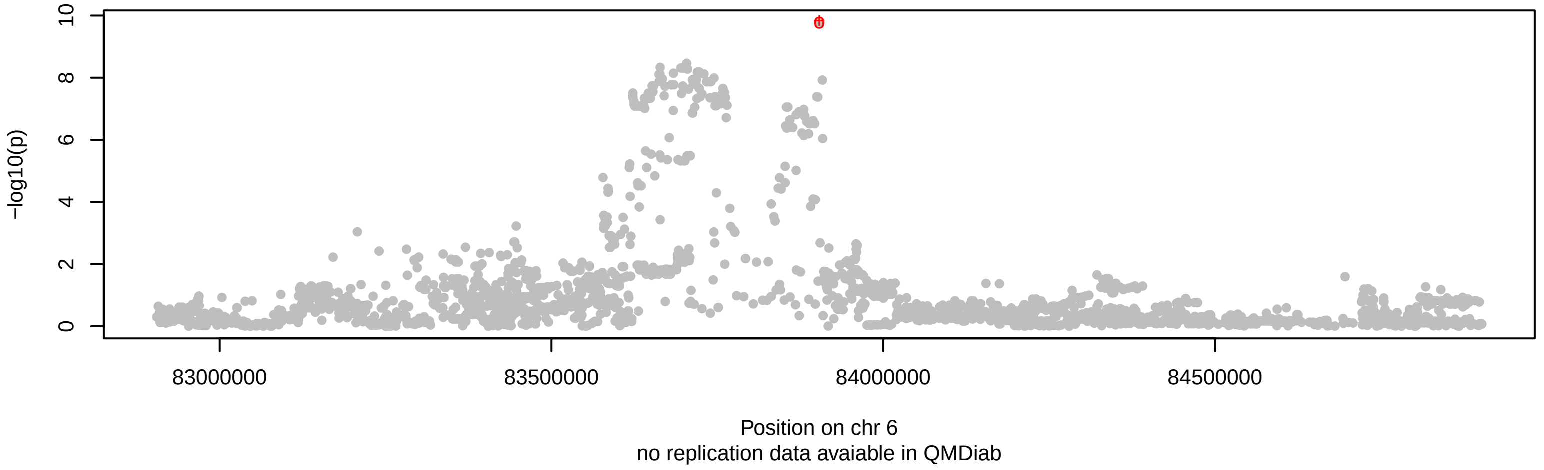

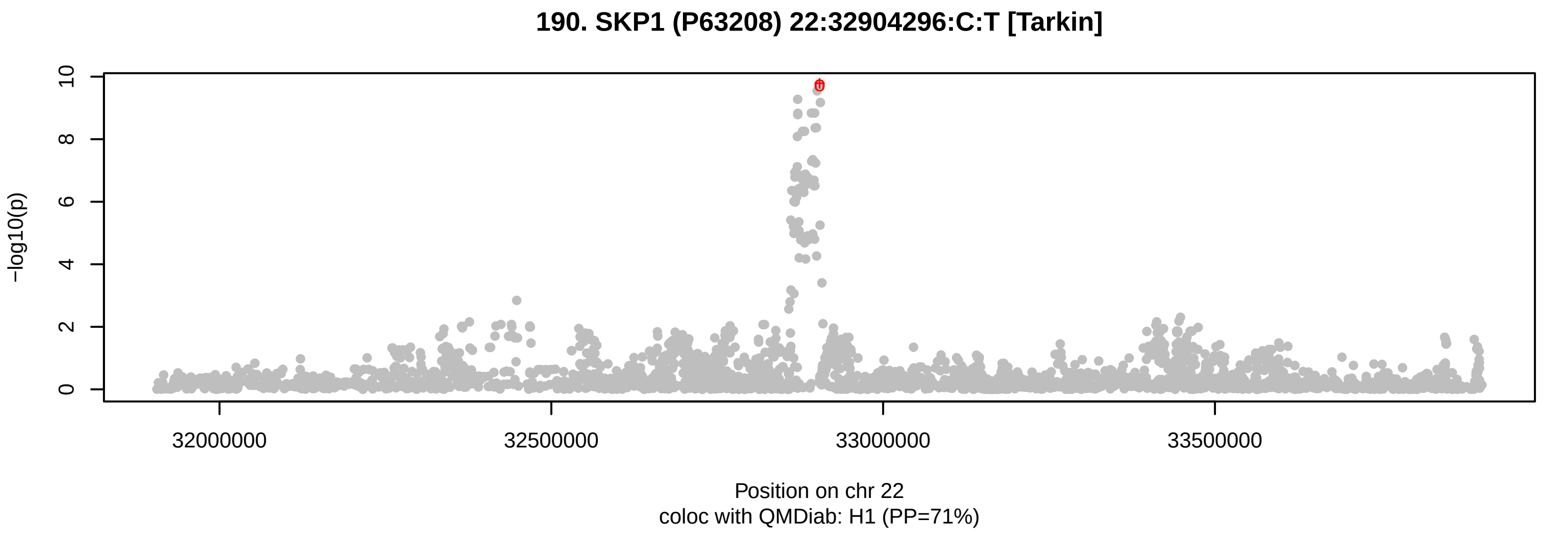
190. SKP1 (P63208) 22:32904296:C:T [Tarkin]
−log10(p)
0
2
4
6
8
10
32000000
32500000
33000000
33500000
Position on chr 22
coloc with QMDiab: H1 (PP=71%)

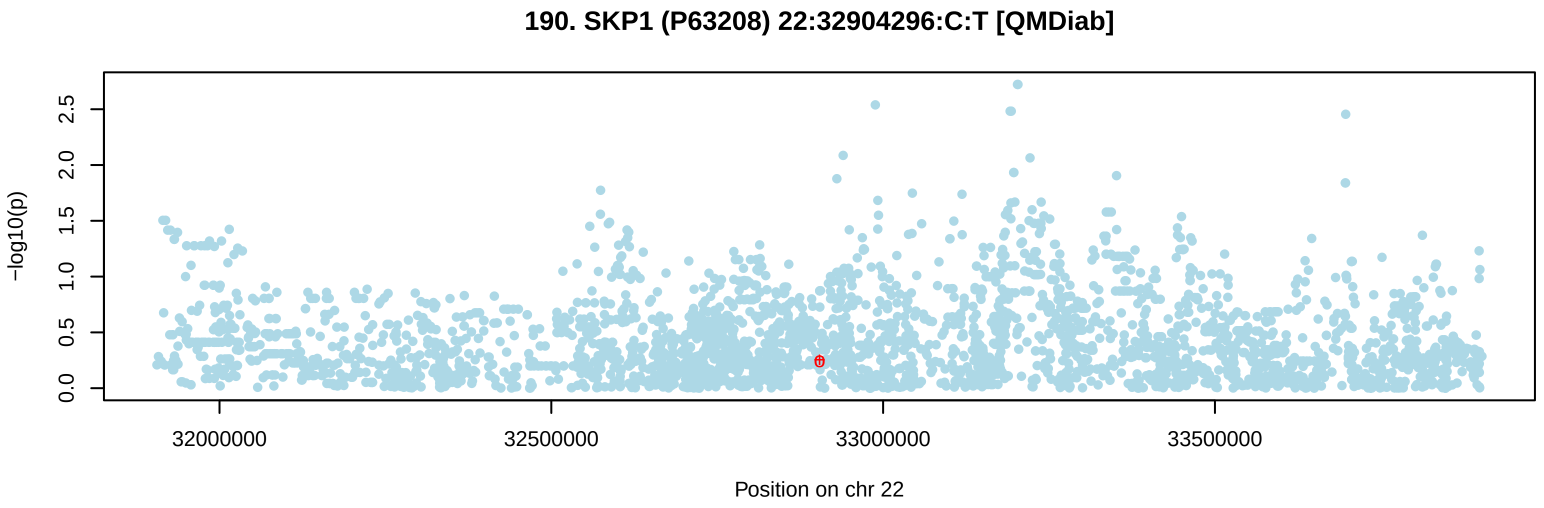
190. SKP1 (P63208) 22:32904296:C:T [QMDiab]
−log10(p)
0.0
0.5
1.0
1.5
2.0
2.5
32000000
32500000
33000000
33500000
Position on chr 22

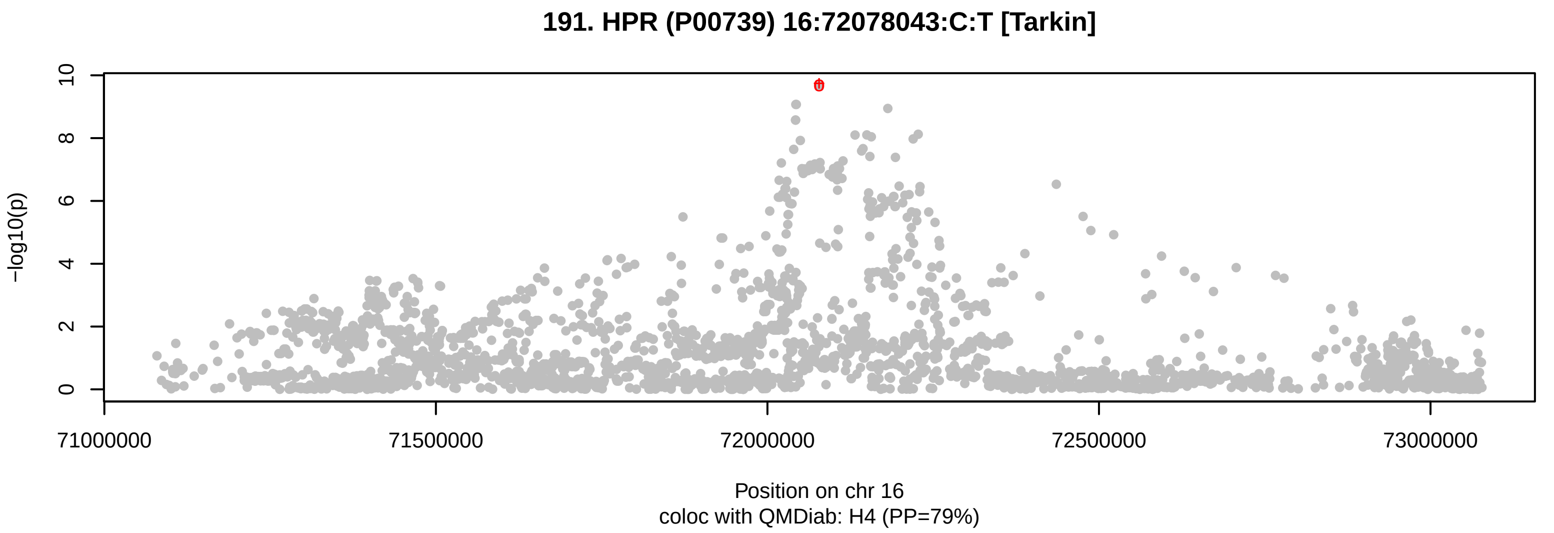

191. HPR (P00739) 16:72078043:C:T [Tarkin]
−log10(p)
0
2
4
6
8
10
71000000
71500000
72000000
72500000
73000000
Position on chr 16
coloc with QMDiab: H4 (PP=79%)

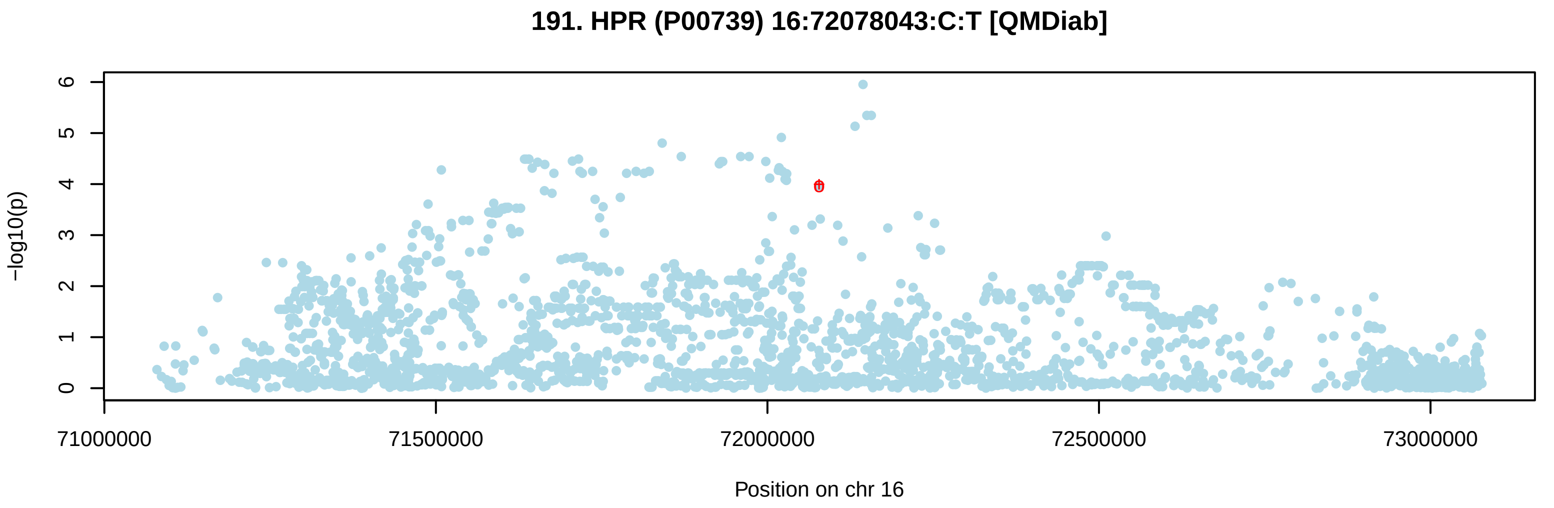

191. HPR (P00739) 16:72078043:C:T [QMDiab]
−log10(p)
0
1
2
3
4
5
6
71000000
71500000
72000000
72500000
73000000
Position on chr 16

192. IGKV1−33 (P01594) 2:89508012:G:A [Tarkin]

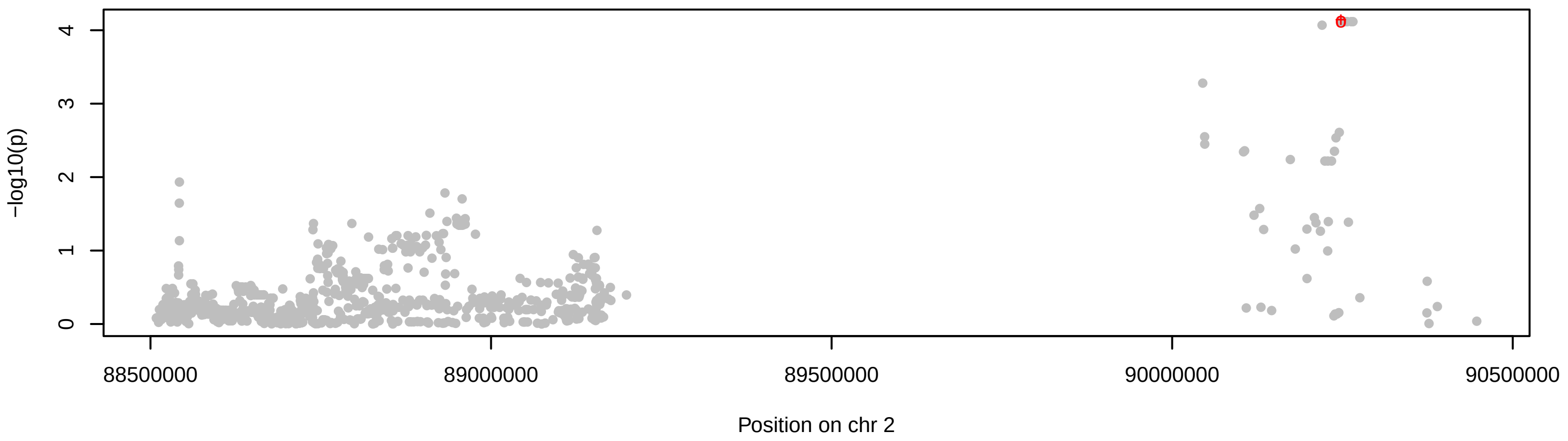

coloc with QMDiab: H0 (PP=66%)

192. IGKV1−33 (P01594) 2:89508012:G:A [QMDiab]

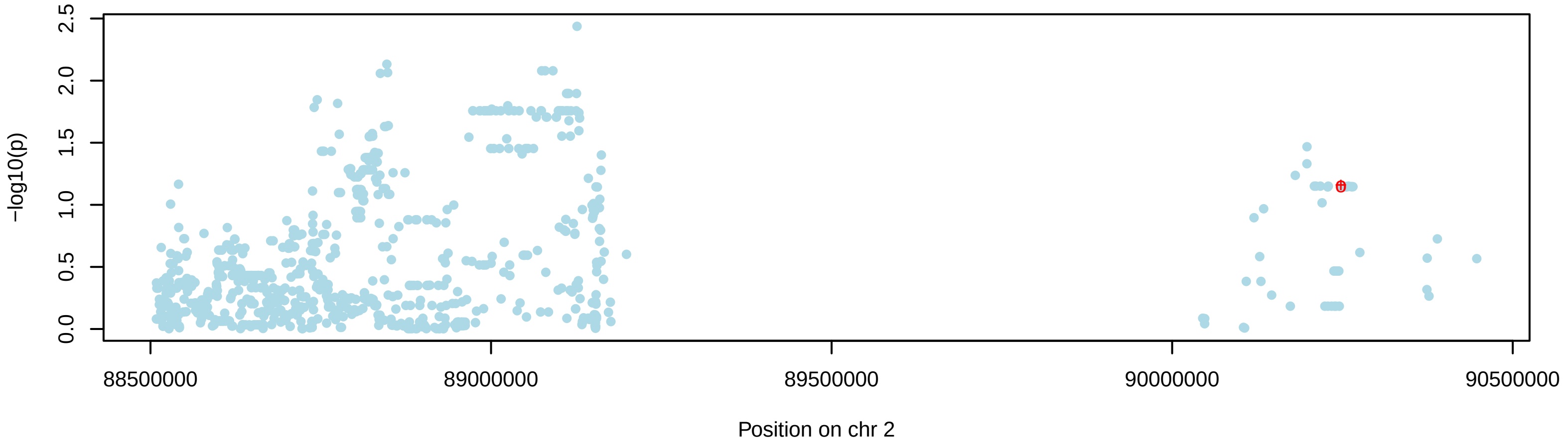

193. CTSB (E9PHZ5) 8:11702313:C:G [Tarkin]

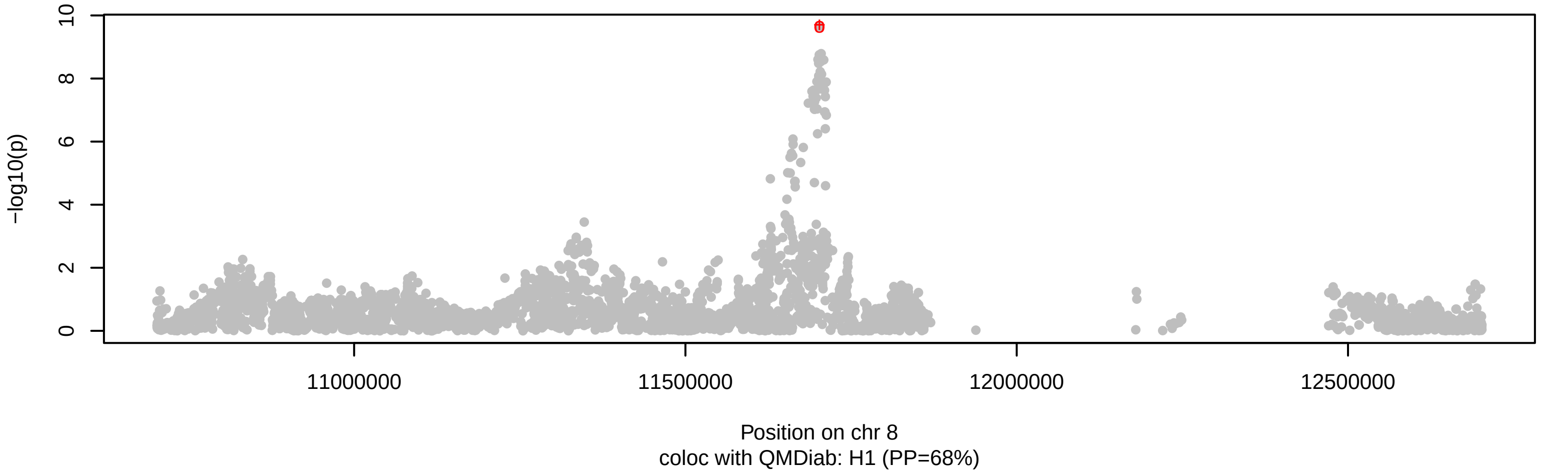

coloc with QMDiab: H1 (PP=68%)

193. CTSB (E9PHZ5;P07858) 8:11702313:C:G [QMDiab]

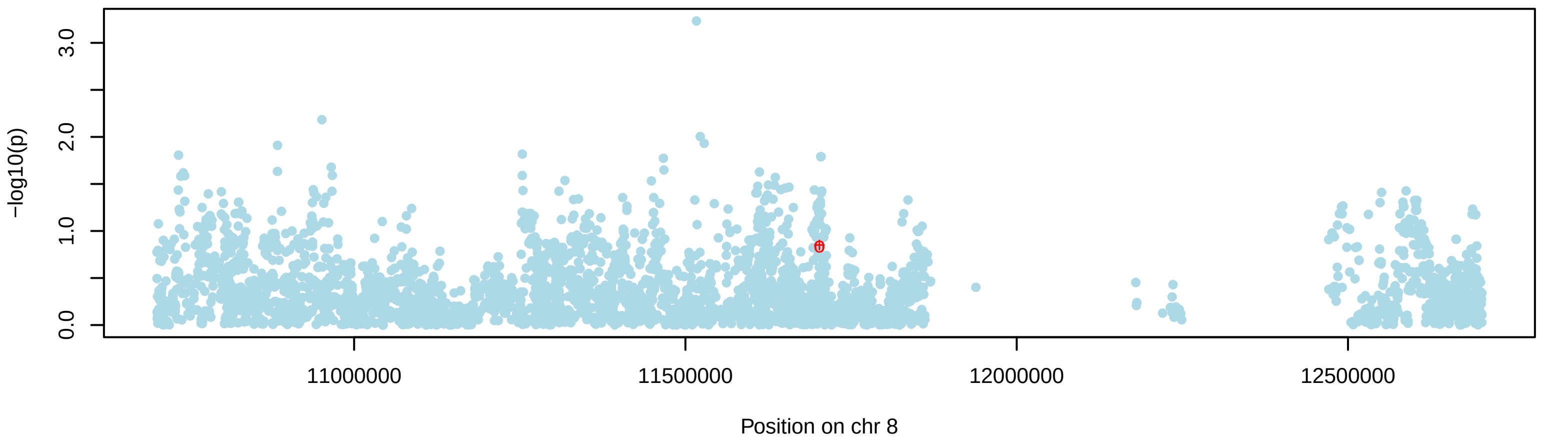

194. PPIB (A0A7P0Z497) 11:17351683:C:G [Tarkin]

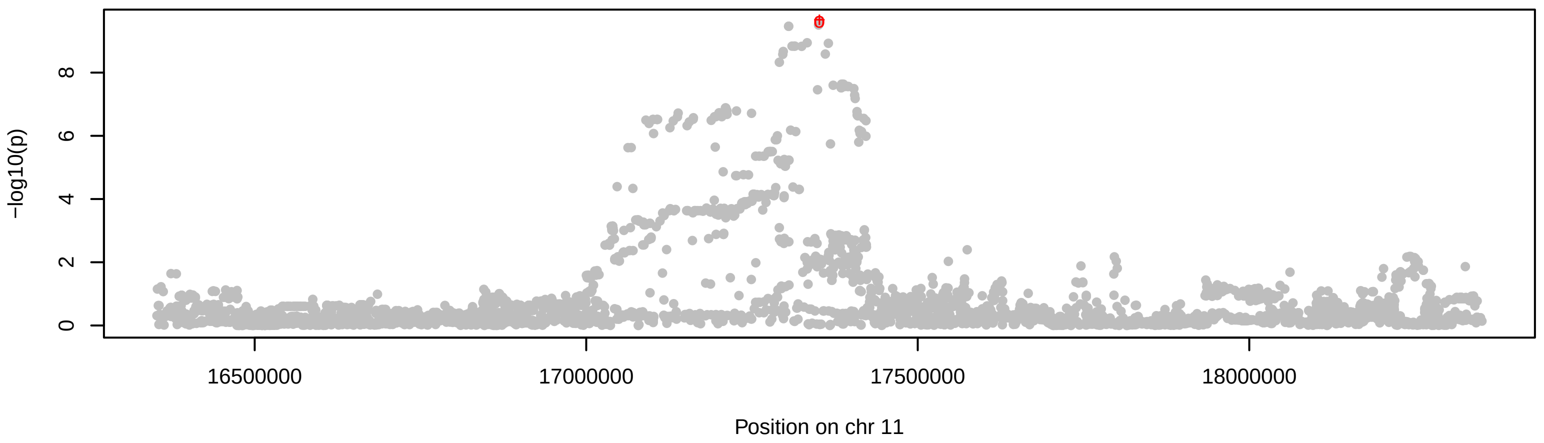

coloc with QMDiab: H3 (PP=41%)

194. PPIB (A0A7P0Z497;P23284) 11:17351683:C:G [QMDiab]

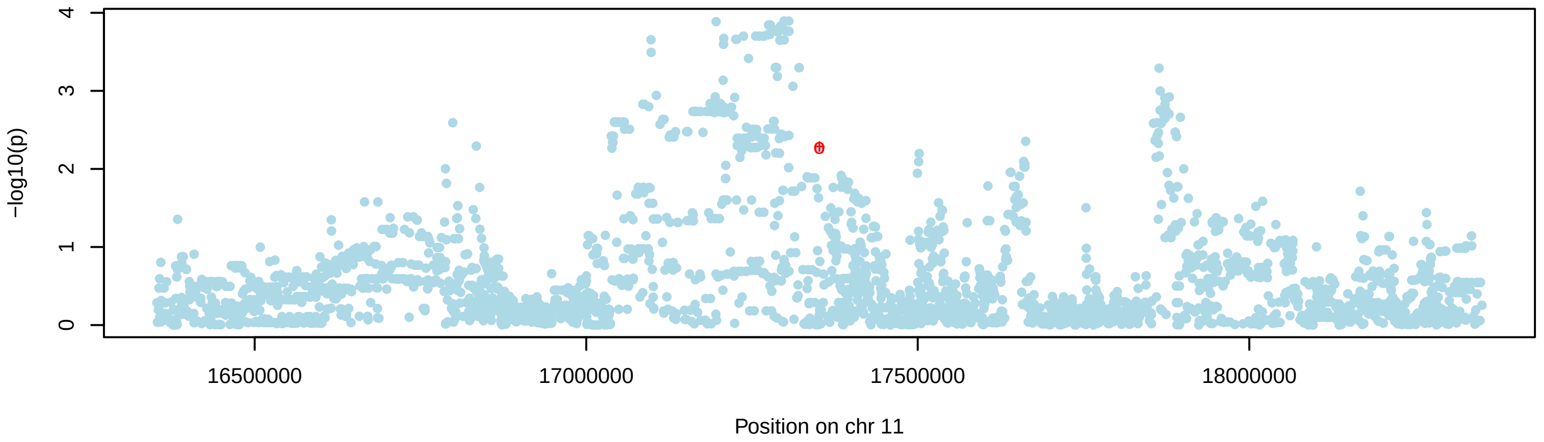

195. DNAJC10 (A0A7P0Z431;Q8IXB1) 2:183658360:A:G [Tarkin]

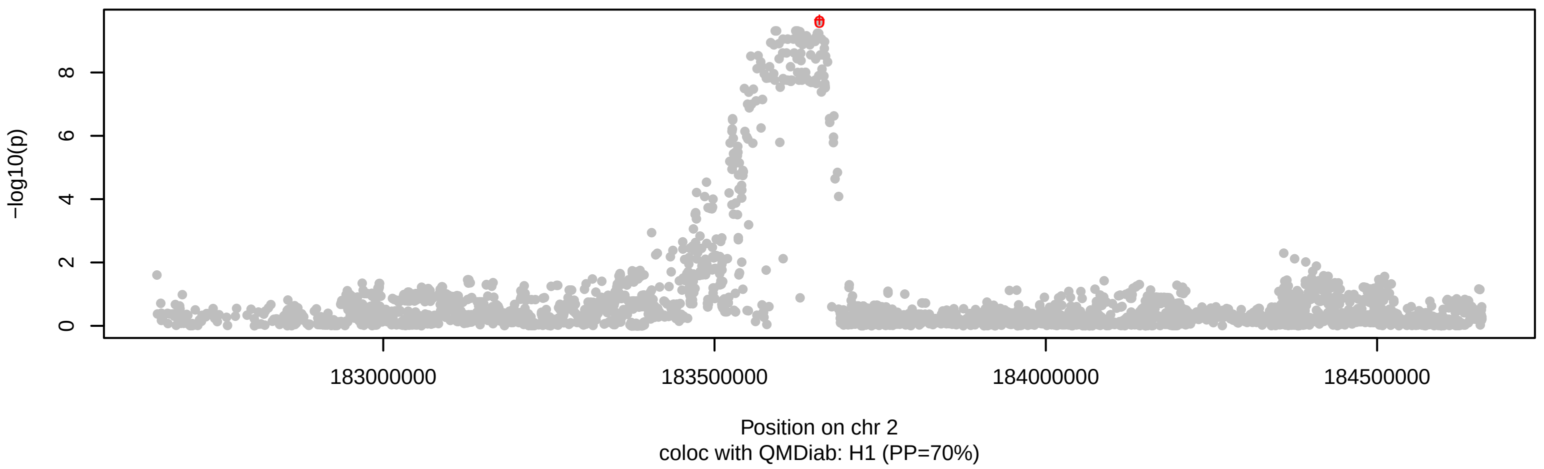

coloc with QMDiab: H1 (PP=70%)

195. DNAJC10 (A0A7P0TAQ9;A0A7P0Z431;Q8IXB1;Q8IXB1−2) 2:183658360:A:G [QMDiab]

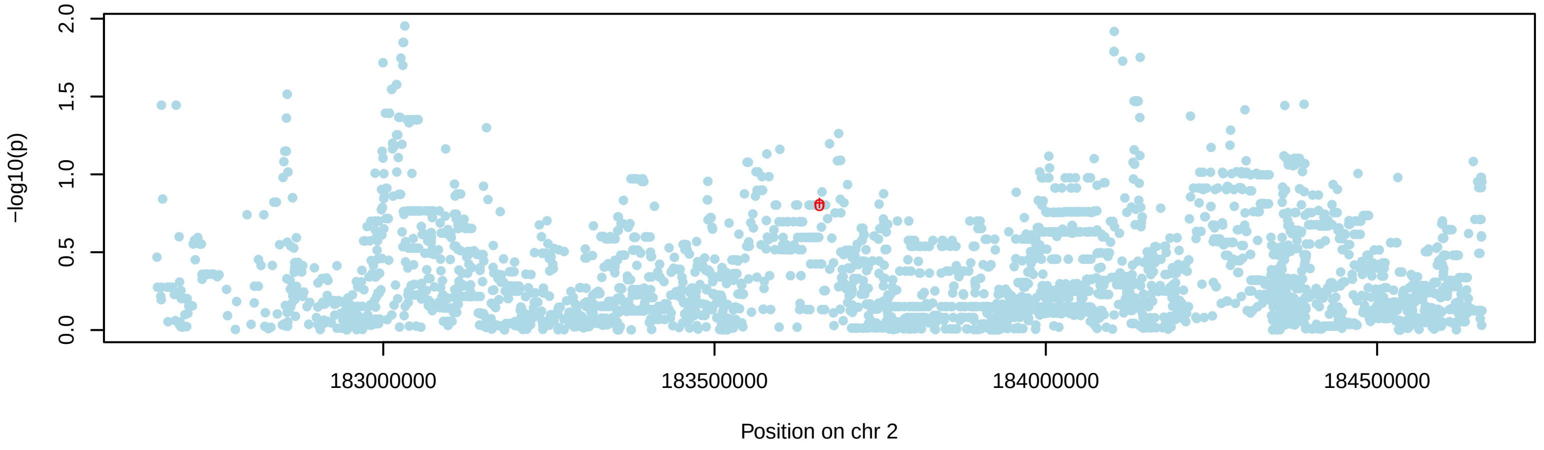

196. ST14 (Q9Y5Y6) 11:130046388:G:A [Tarkin]

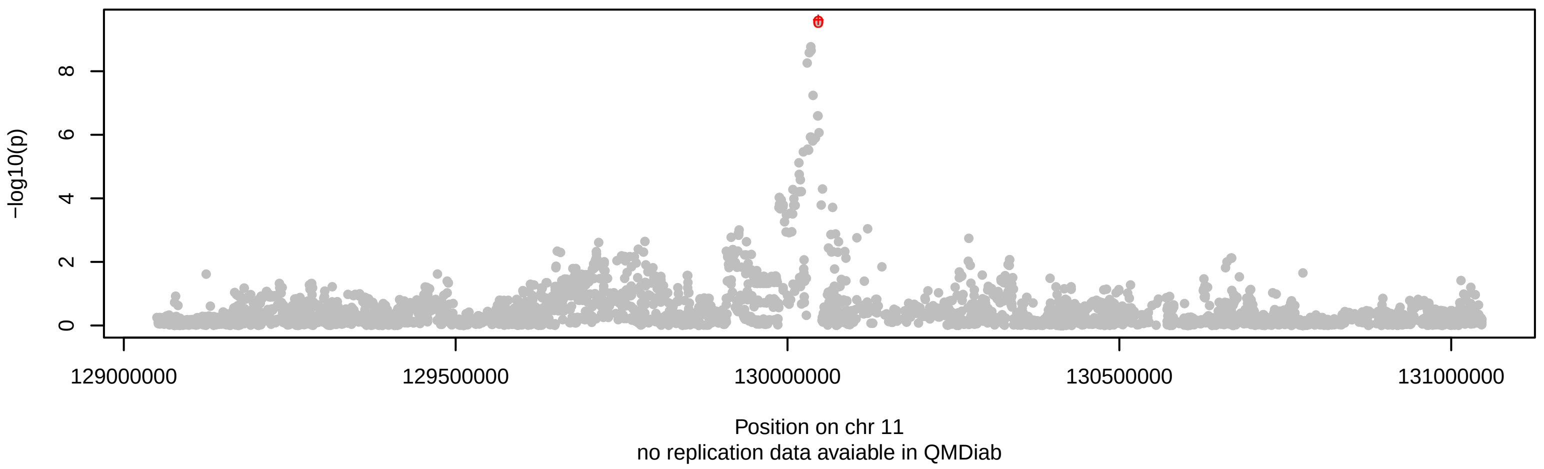

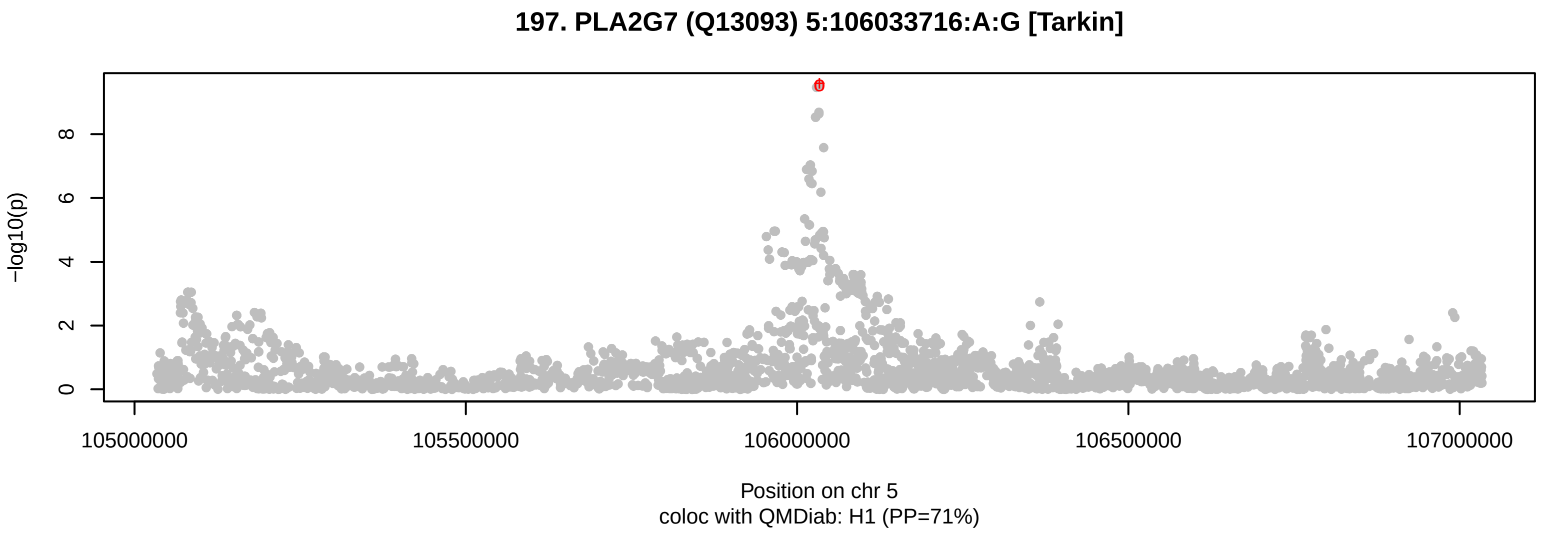
197. PLA2G7 (Q13093) 5:106033716:A:G [Tarkin]
−log10(p)
0
2
4
6
8
105000000
105500000
106000000
106500000
107000000
Position on chr 5
coloc with QMDiab: H1 (PP=71%)

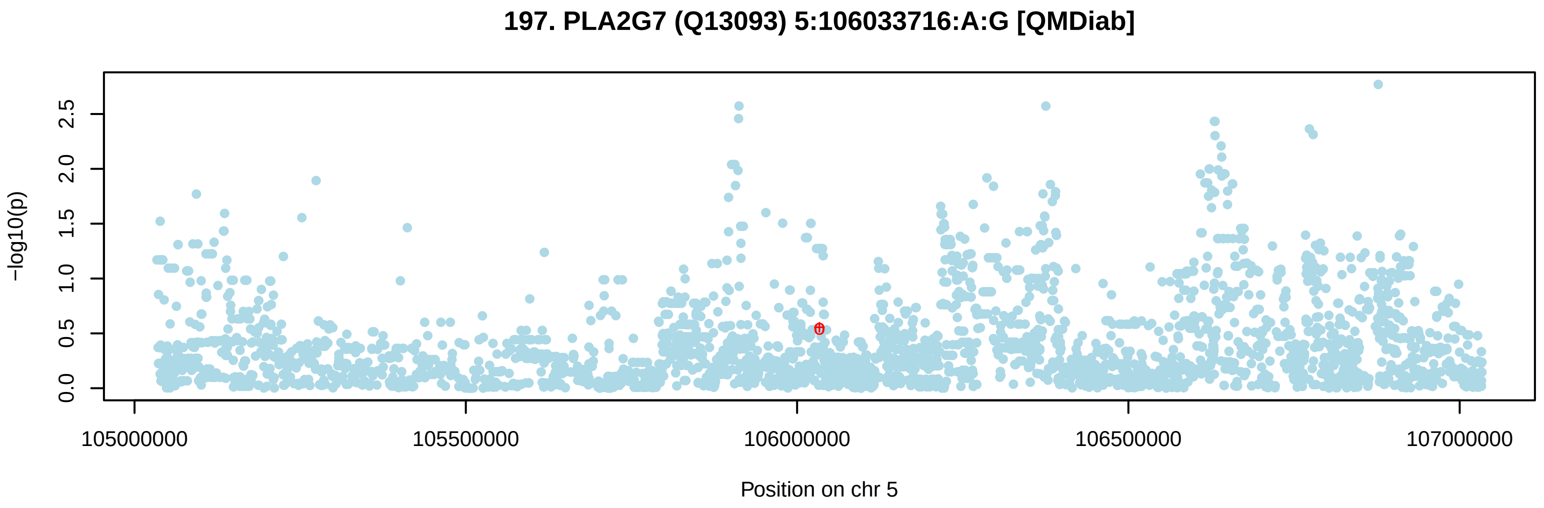
197. PLA2G7 (Q13093) 5:106033716:A:G [QMDiab]
−log10(p)
0.0
0.5
1.0
1.5
2.0
2.5
105000000
105500000
106000000
106500000
107000000
Position on chr 5

198. SERPINA4 (P29622) 14:95033595:A:T [Tarkin]

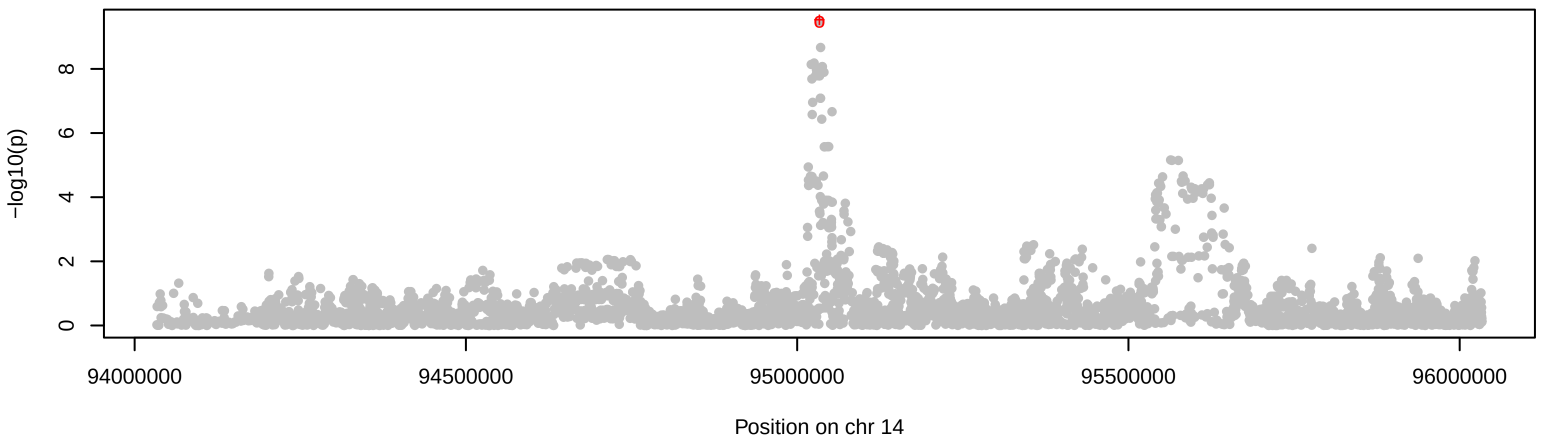

coloc with QMDiab: H4 (PP=100%)

198. SERPINA4 (P29622) 14:95033595:A:T [QMDiab]

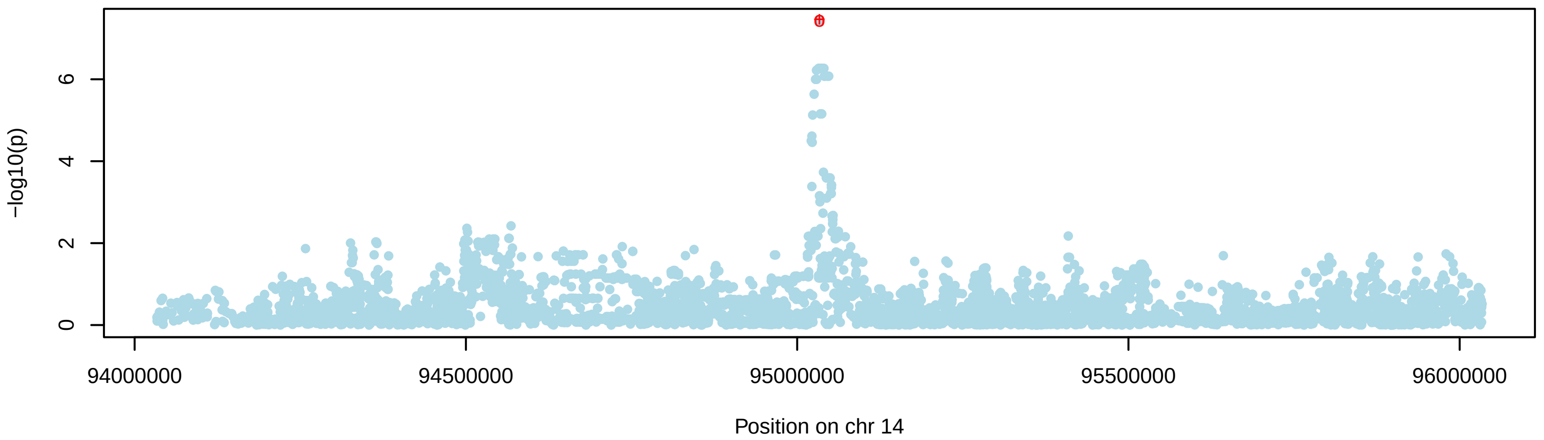

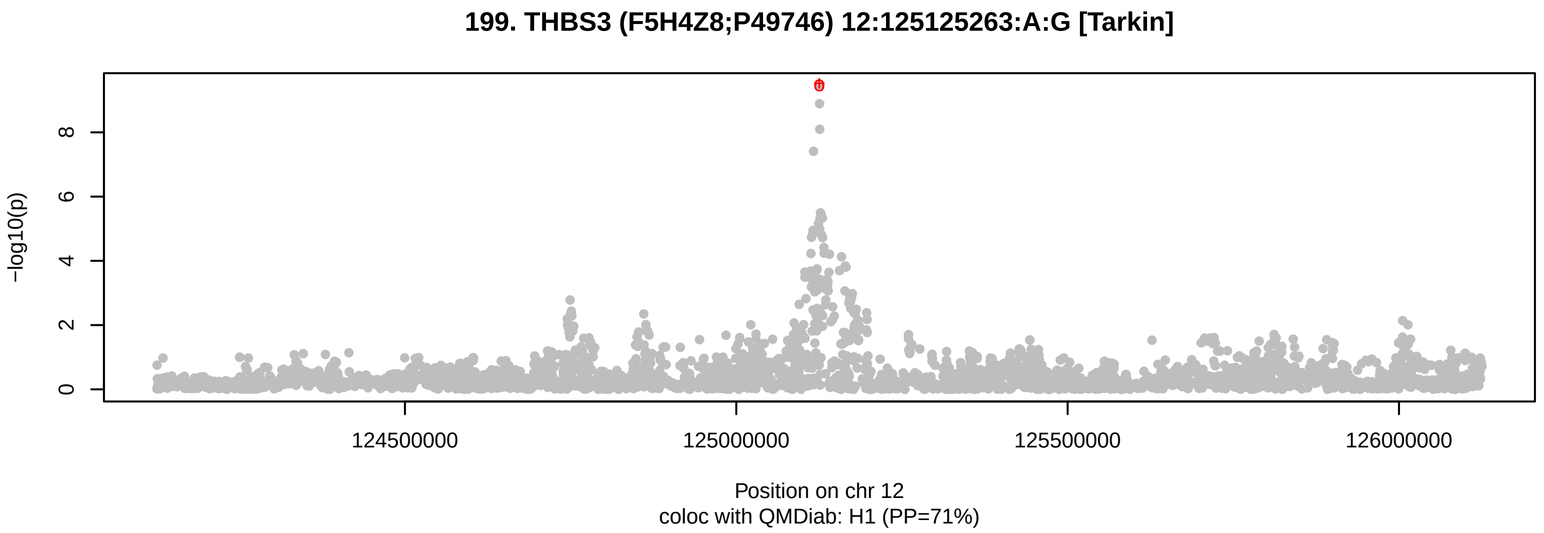
199. THBS3 (F5H4Z8;P49746) 12:125125263:A:G [Tarkin]
−log10(p)
0
2
4
6
8
124500000
125000000
125500000
126000000
Position on chr 12
coloc with QMDiab: H1 (PP=71%)

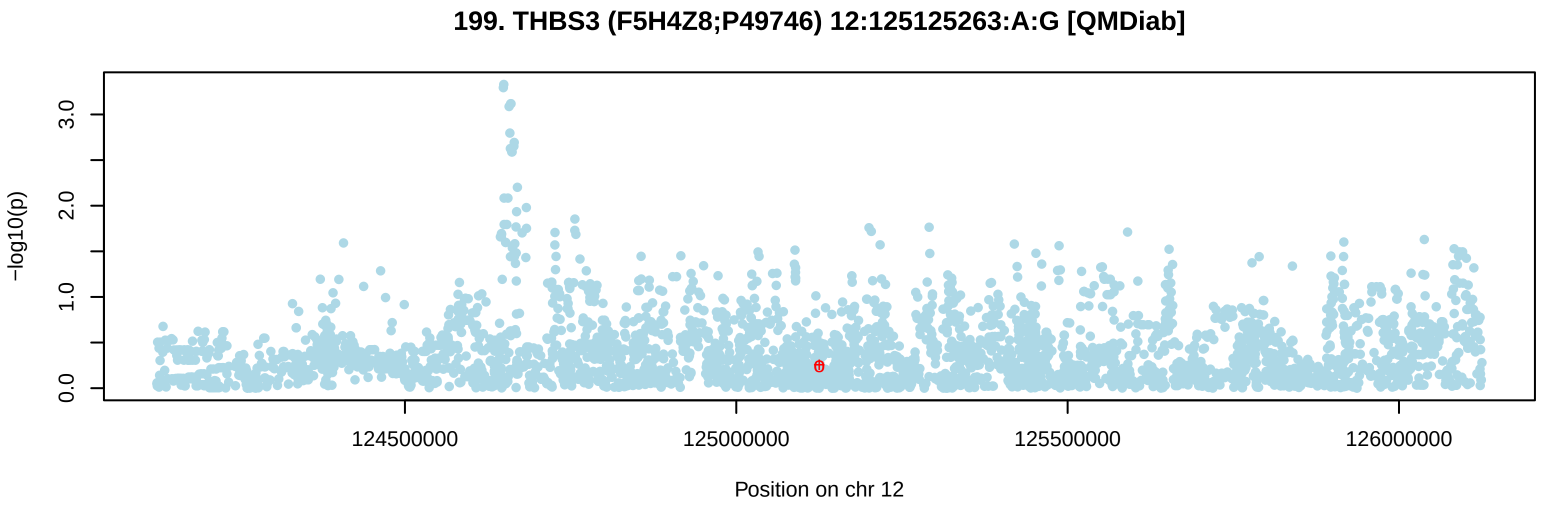
199. THBS3 (F5H4Z8;P49746) 12:125125263:A:G [QMDiab]
−log10(p)
0.0
1.0
2.0
3.0
124500000
125000000
125500000
126000000
Position on chr 12

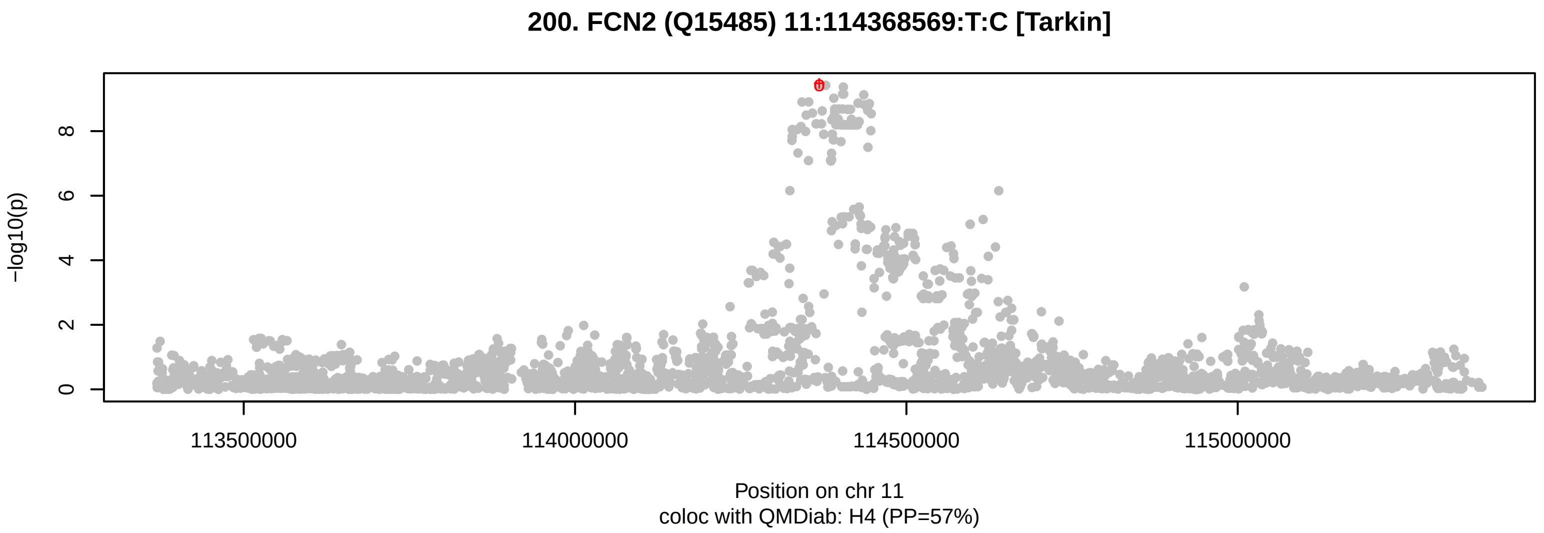
200. FCN2 (Q15485) 11:114368569:T:C [Tarkin]
−log10(p)
0
2
4
6
8
113500000
114000000
114500000
115000000
Position on chr 11
coloc with QMDiab: H4 (PP=57%)

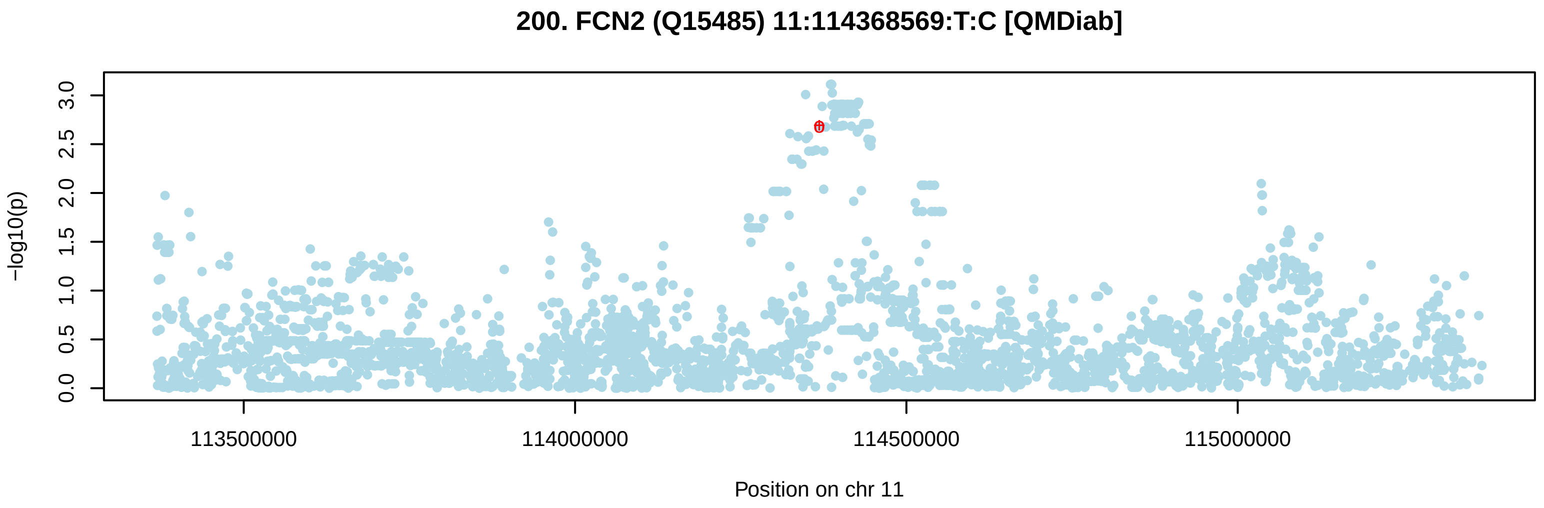
200. FCN2 (Q15485) 11:114368569:T:C [QMDiab]
−log10(p)
0.0
0.5
1.0
1.5
2.0
2.5
3.0
113500000
114000000
114500000
115000000
Position on chr 11

201. C4A (P0C0L4;P0C0L4−2) 6:31840477:C:T [Tarkin]

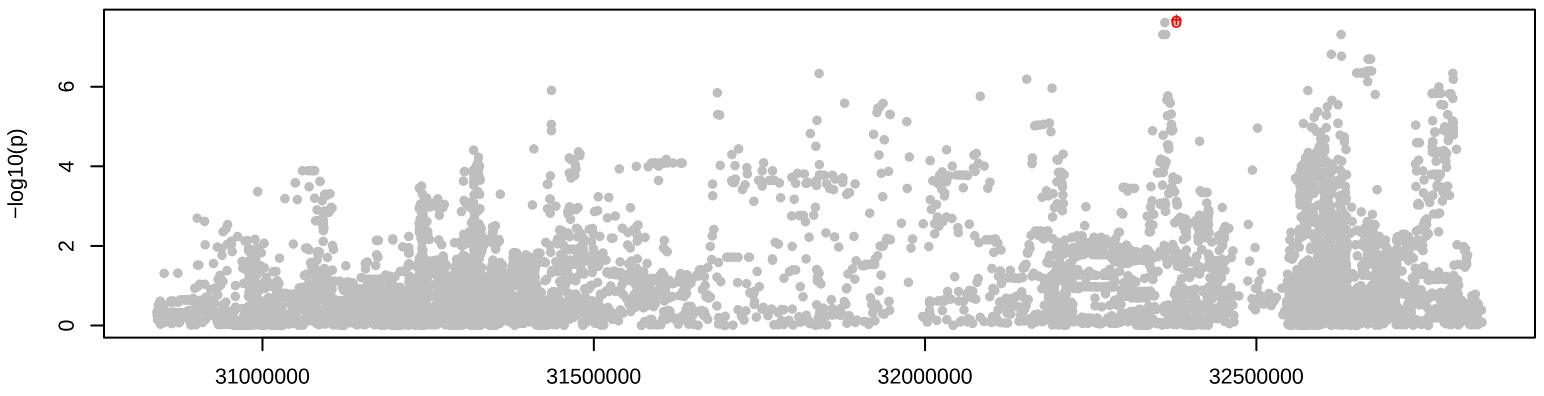

Position on chr 6
coloc with QMDiab: H3 (PP=78%)

201. C4A (P0C0L4;P0C0L4−2) 6:31840477:C:T [QMDiab]

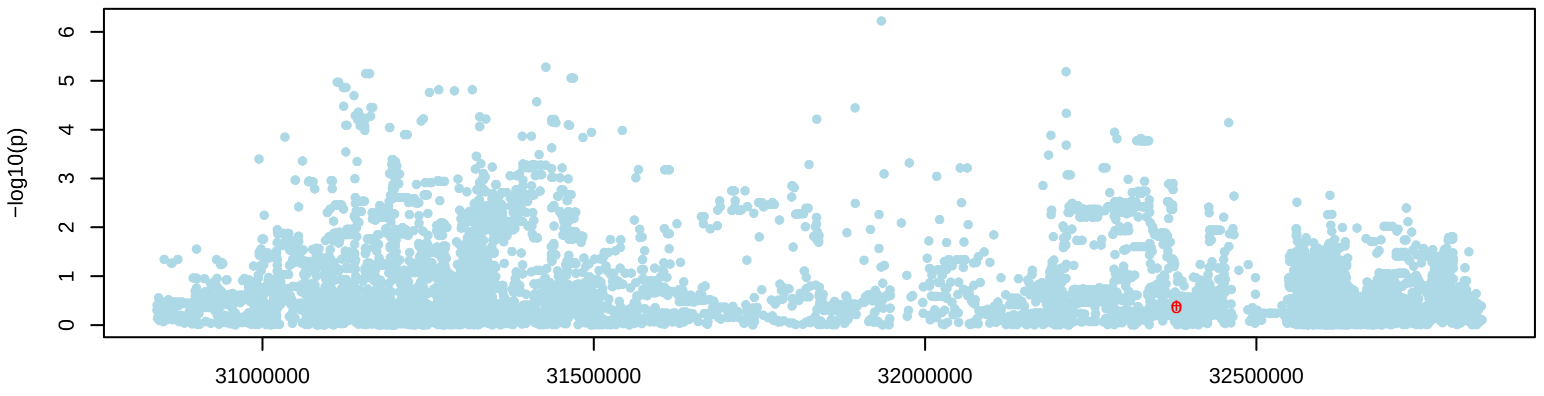

Position on chr 6

202. LAMB1 (P07942) 1:236210192:T:C [Tarkin]

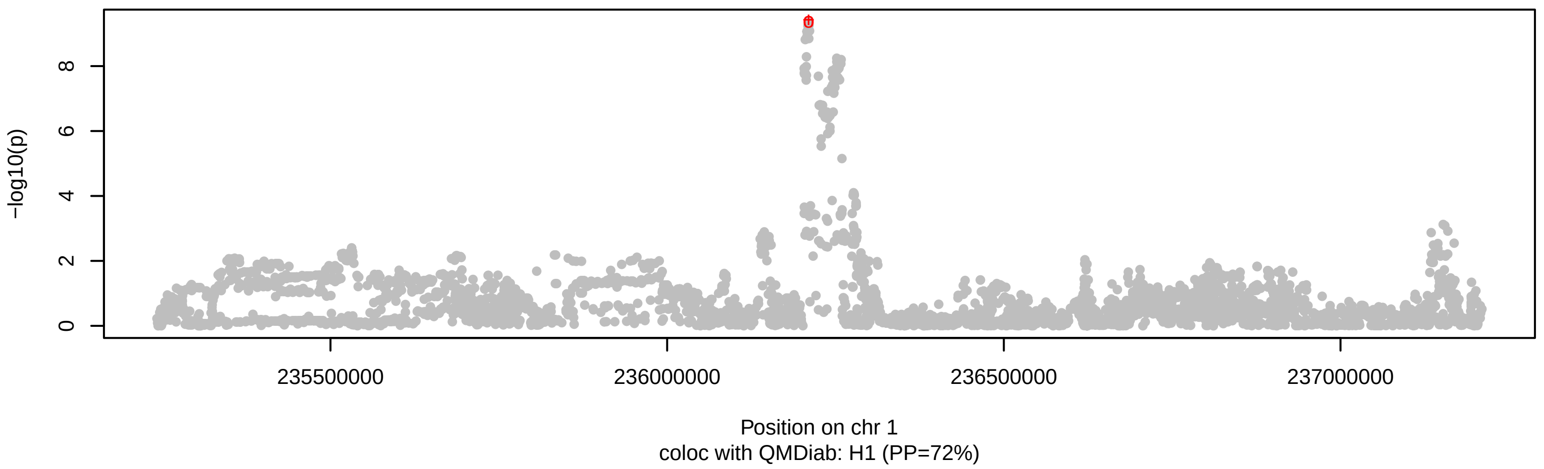

coloc with QMDiab: H1 (PP=72%)

202. LAMB1 (A0A7I2V4J9;P07942) 1:236210192:T:C [QMDiab]

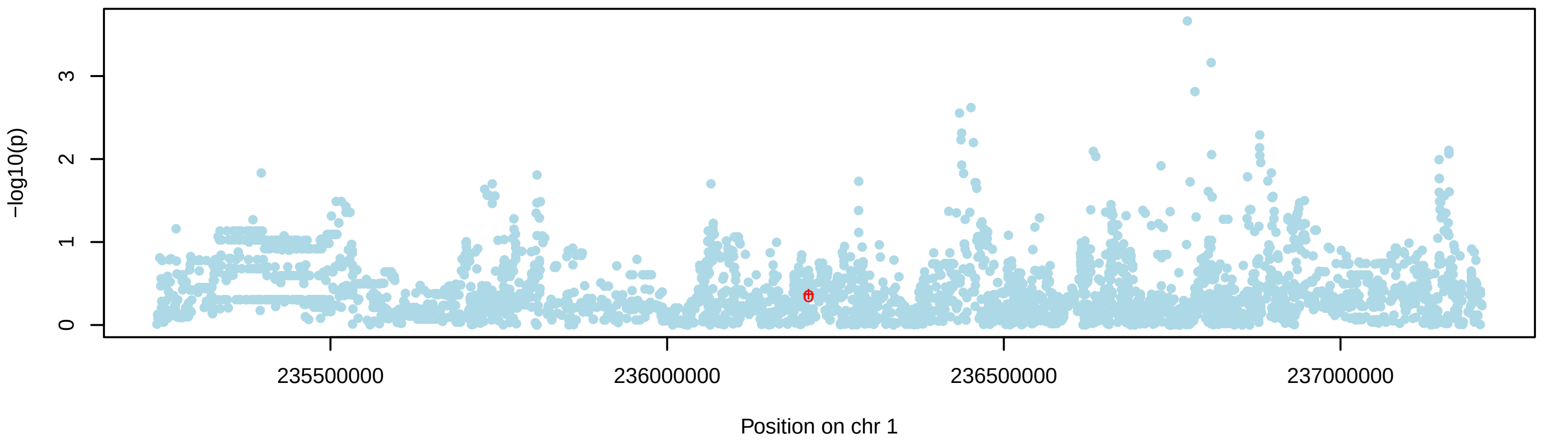

203. LTBP1 (Q14766) 1:196820924:T:G [Tarkin]

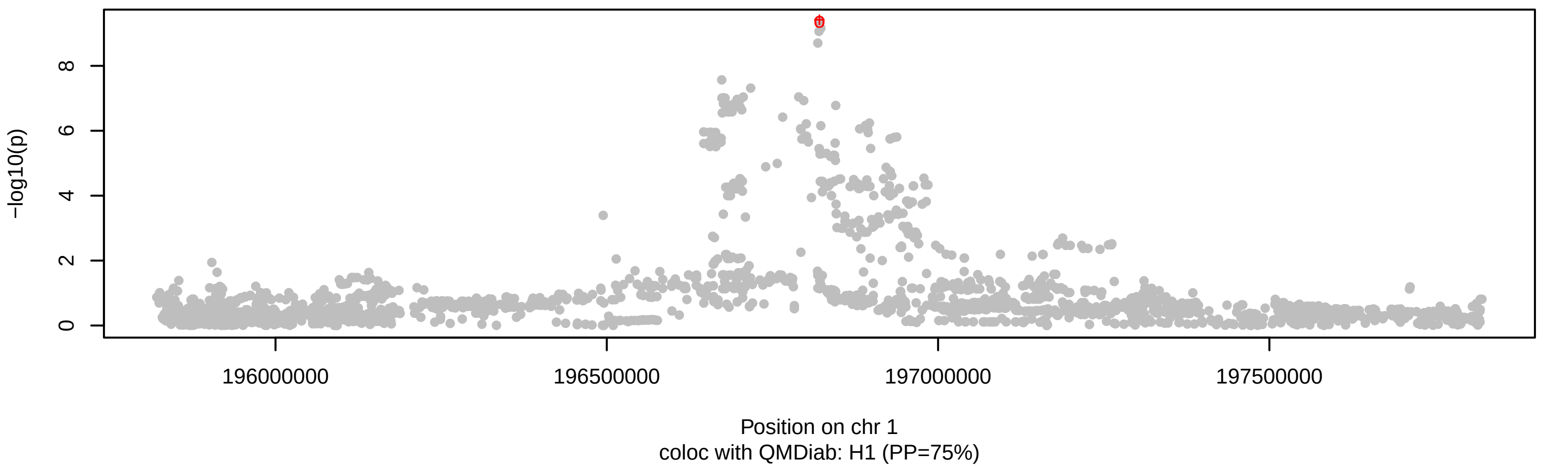

203. LTBP1 (Q14766) 1:196820924:T:G [QMDiab]

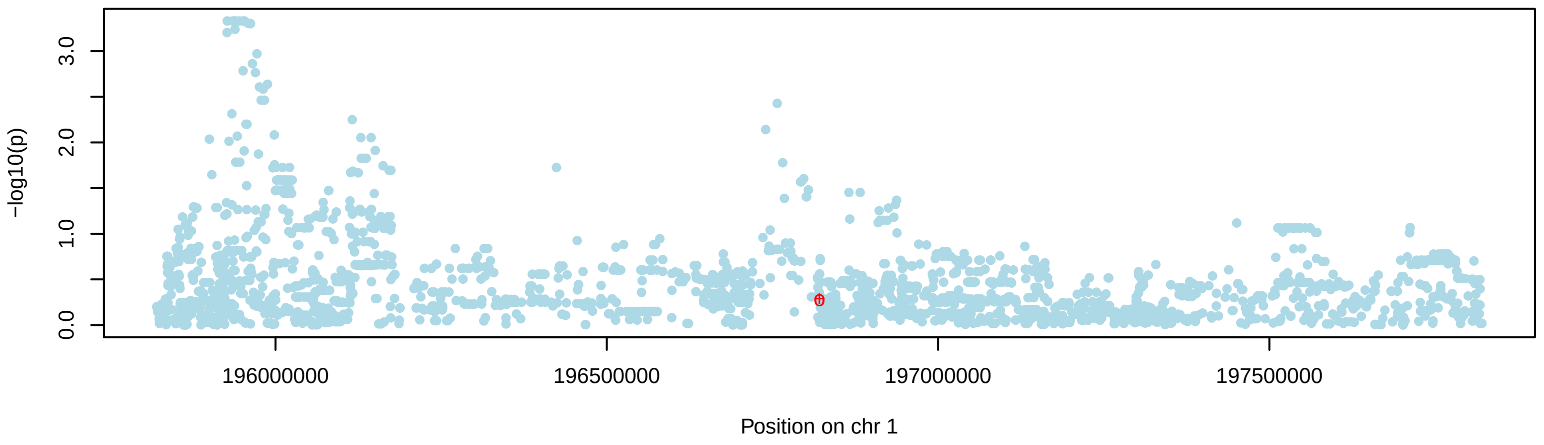

204. RAB11B (Q15907) 1:196820234:C:A [Tarkin]

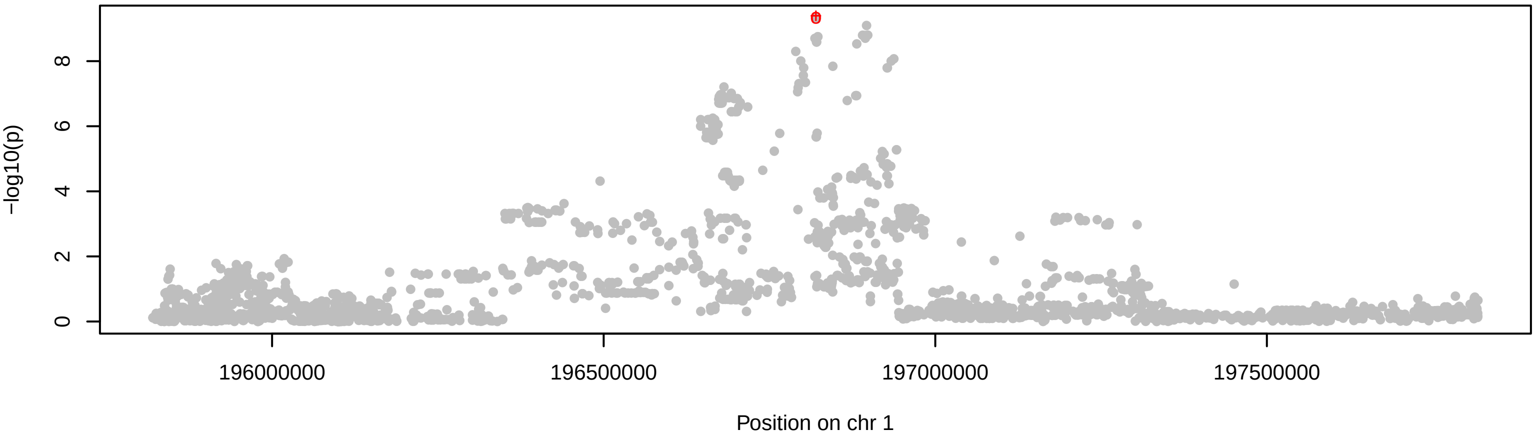

coloc with QMDiab: H1 (PP=72%)

204. RAB11B (Q15907) 1:196820234:C:A [QMDiab]

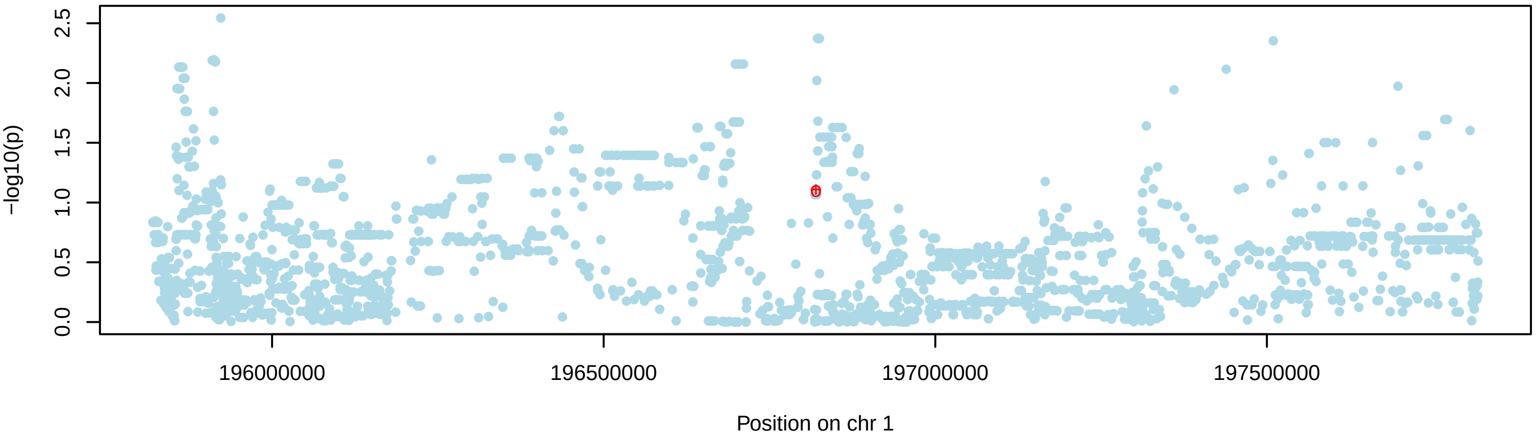

205. APOE (P02649) 19:45412079:C:T [Tarkin]

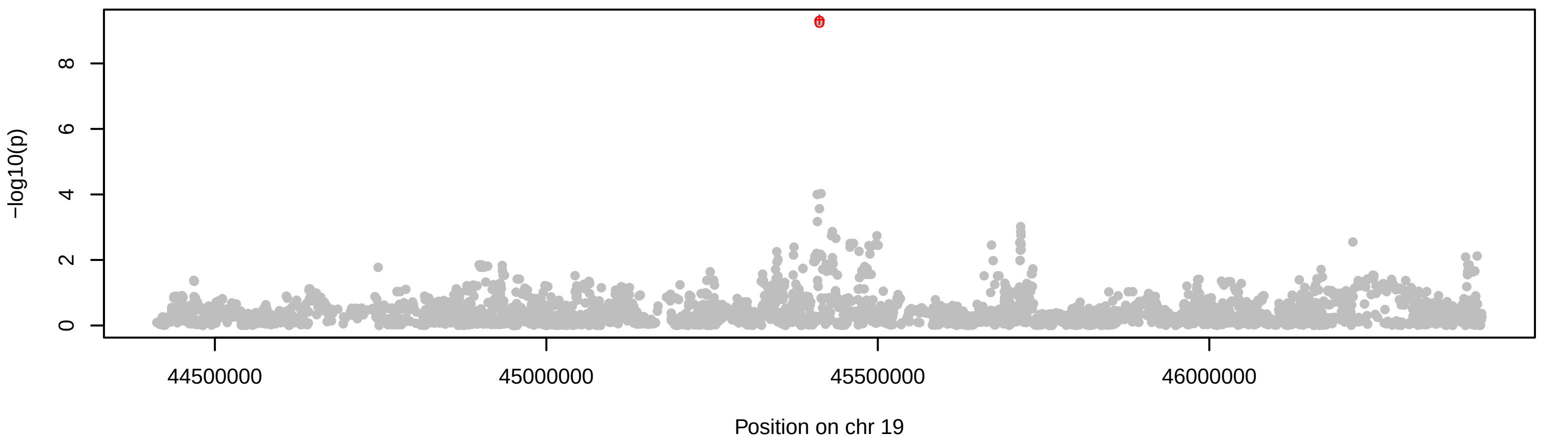

coloc with QMDiab: H1 (PP=61%)

205. APOE (P02649) 19:45412079:C:T [QMDiab]

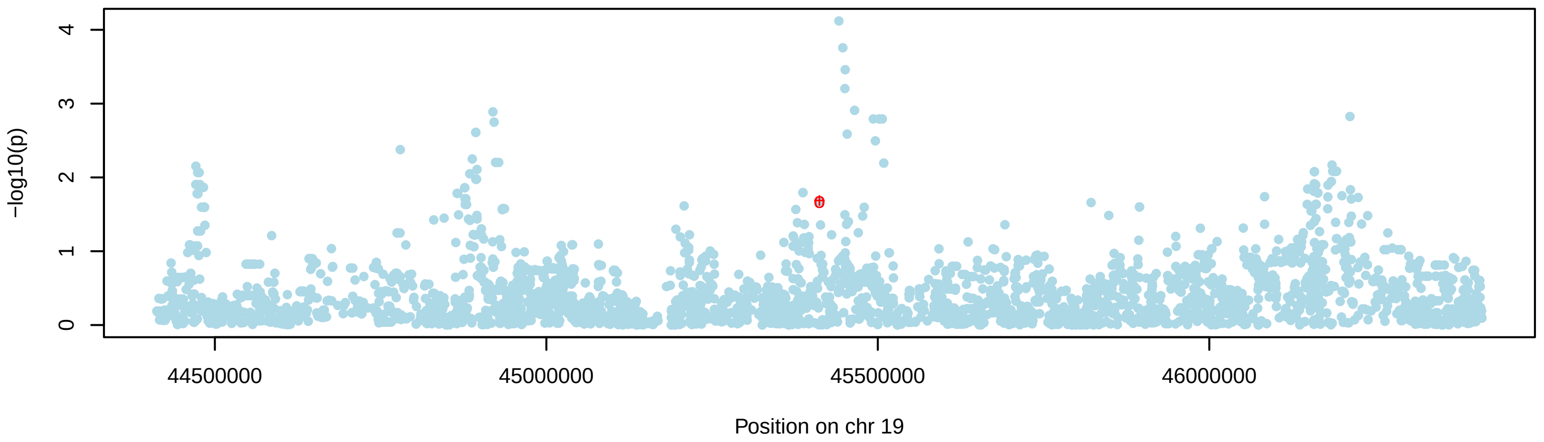

206. LYNX1 (P0DP58) 8:143861450:T:G [Tarkin]

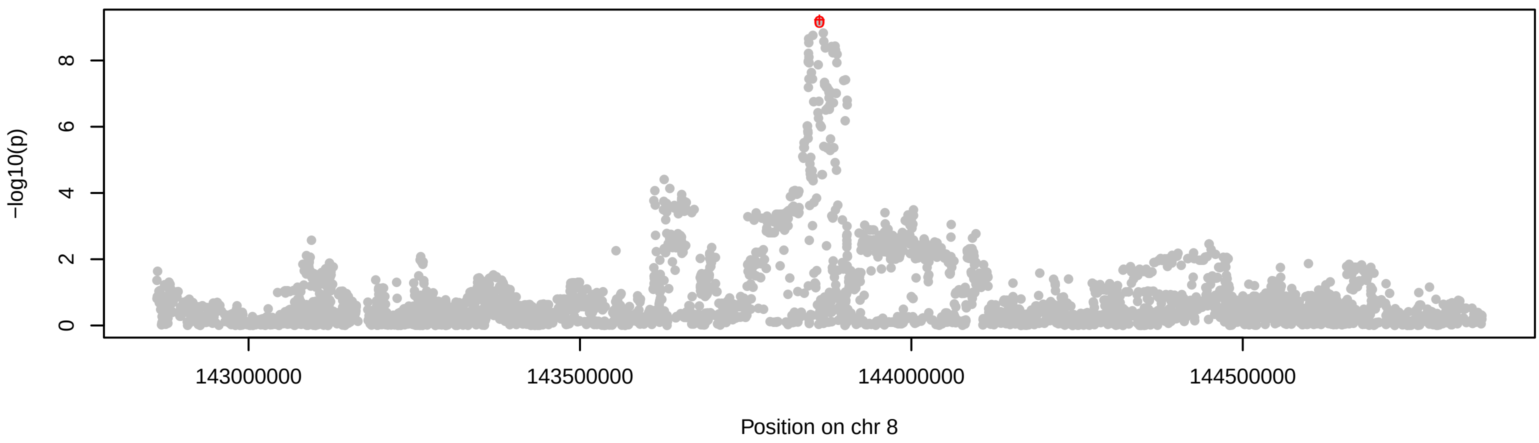

no replication data avaiable in QMDiab

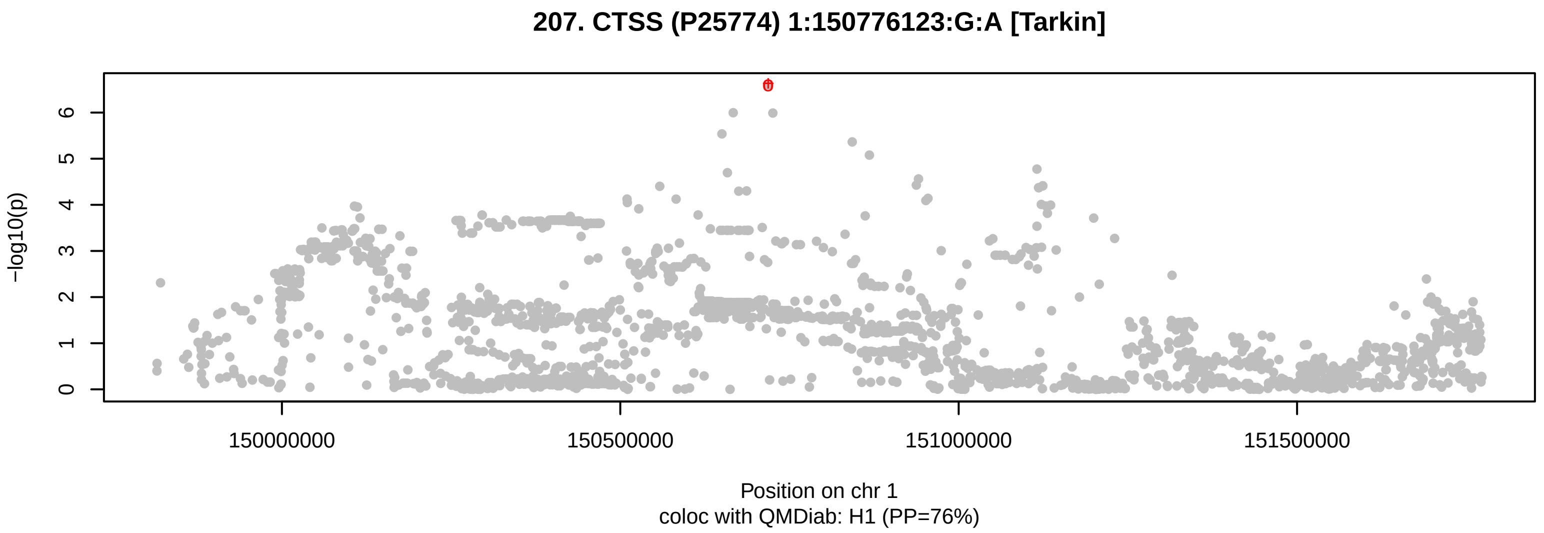
207. CTSS (P25774) 1:150776123:G:A [Tarkin]
−log10(p)
0
1
2
3
4
5
6
150000000
150500000
151000000
151500000
Position on chr 1
coloc with QMDiab: H1 (PP=76%)

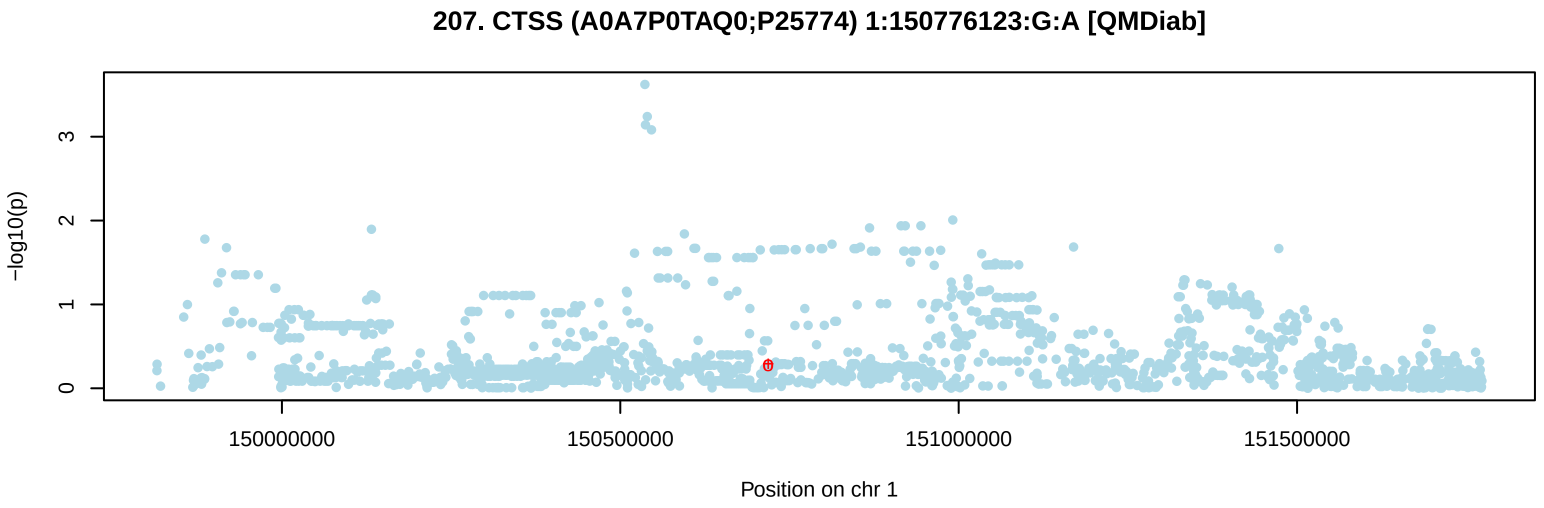
207. CTSS (A0A7P0TAQ0;P25774) 1:150776123:G:A [QMDiab]
−log10(p)
0
1
2
3
150000000
150500000
151000000
151500000
Position on chr 1

208. MAN2A1 (Q16706) 3:101473640:G:A [Tarkin]

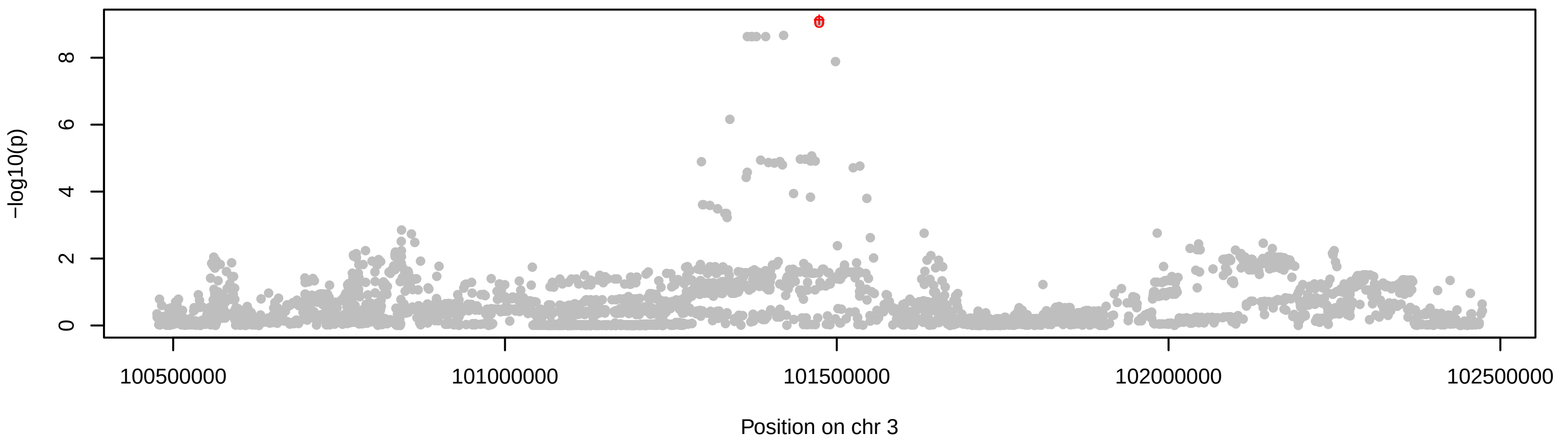

coloc with QMDiab: H1 (PP=73%)

208. MAN2A1 (Q16706) 3:101473640:G:A [QMDiab]

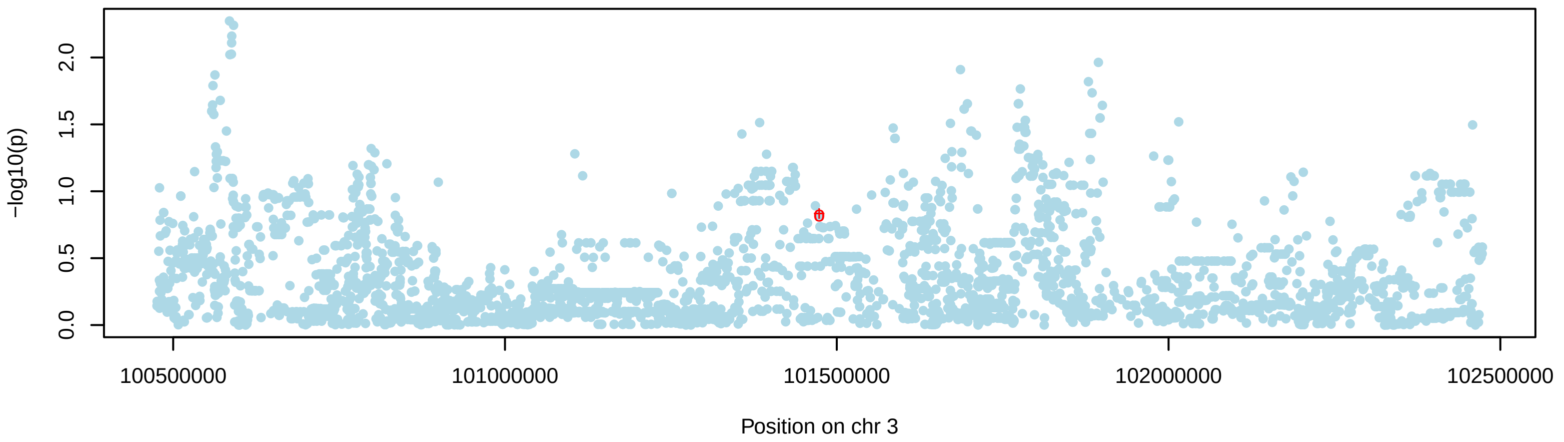

209. UBR1 (Q8IWV7) 19:45411941:T:C [Tarkin]

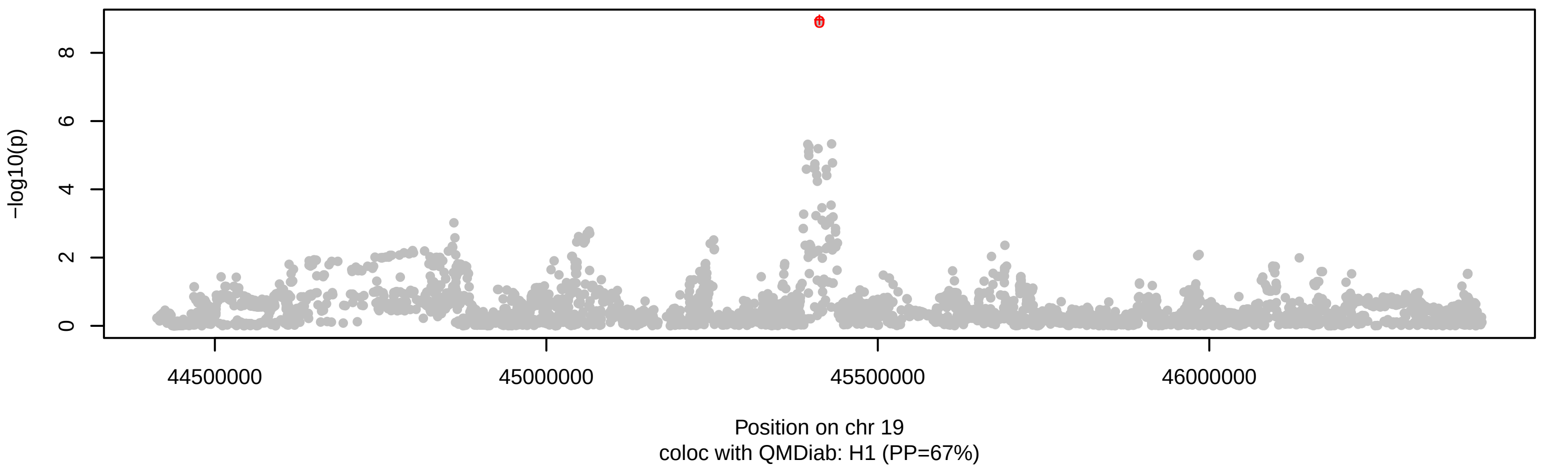

coloc with QMDiab: H1 (PP=67%)

209. UBR1 (Q8IWV7) 19:45411941:T:C [QMDiab]

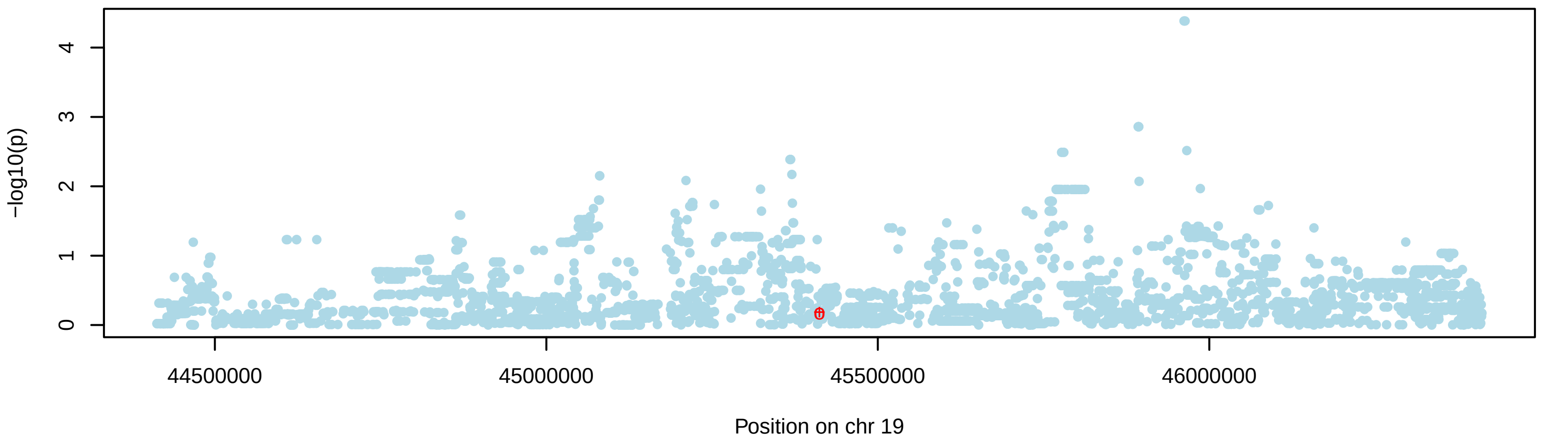

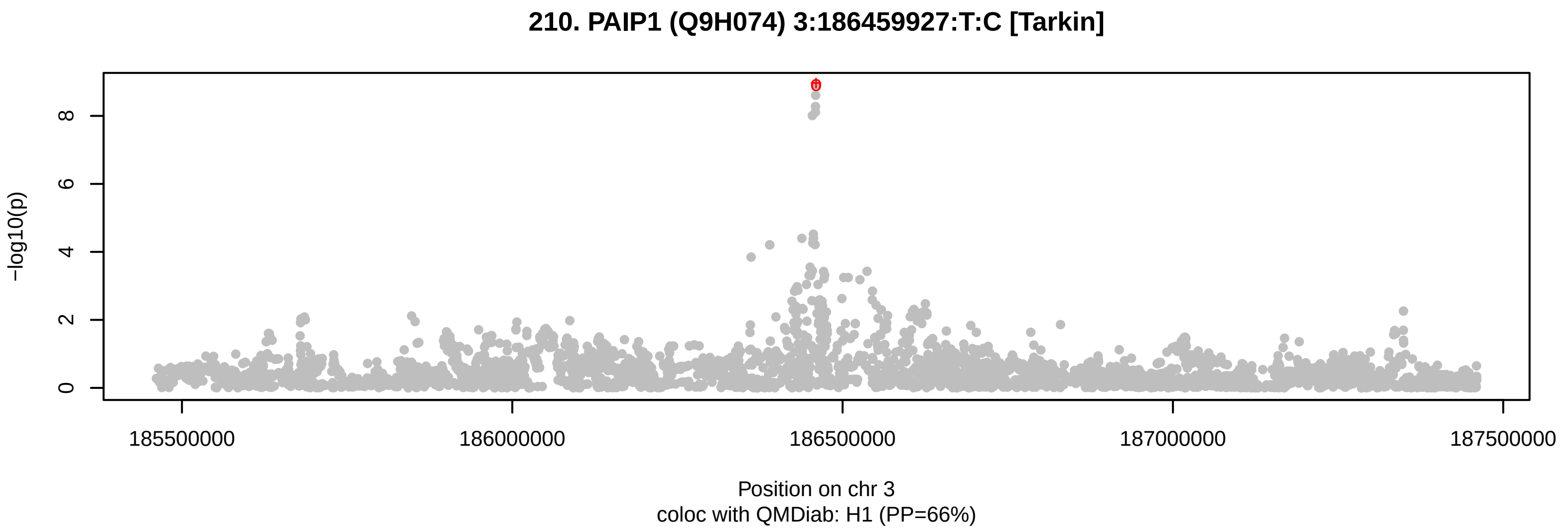

210. PAIP1 (Q9H074) 3:186459927:T:C [Tarkin]
−log10(p)
0
2
4
6
8
185500000
186000000
186500000
187000000
187500000
Position on chr 3
coloc with QMDiab: H1 (PP=66%)

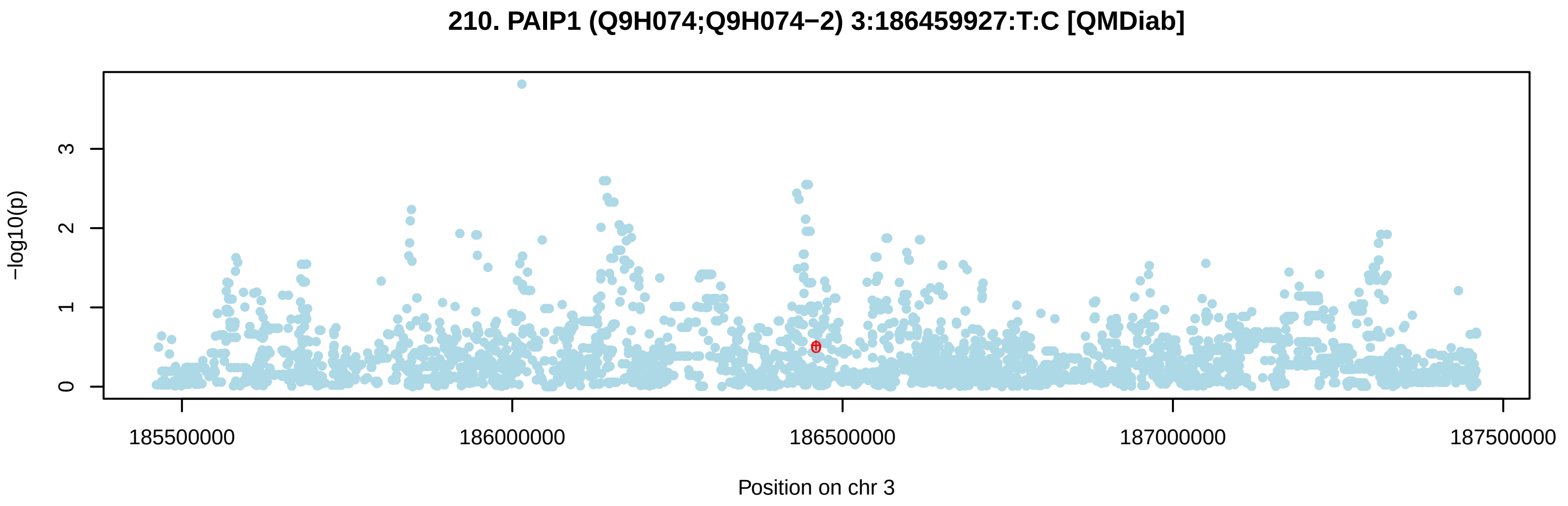

210. PAIP1 (Q9H074;Q9H074−2) 3:186459927:T:C [QMDiab]
−log10(p)
0
1
2
3
185500000
186000000
186500000
187000000
187500000
Position on chr 3

211. APOF (Q13790) 12:56726518:G:A [Tarkin]

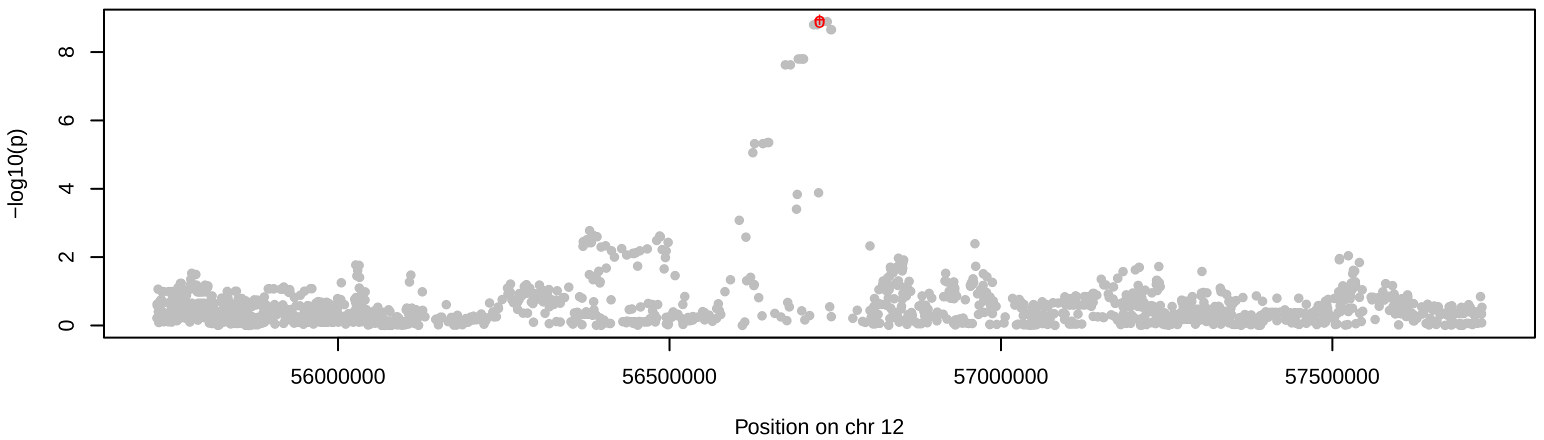

coloc with QMDiab: H1 (PP=67%)

211. APOF (Q13790) 12:56726518:G:A [QMDiab]

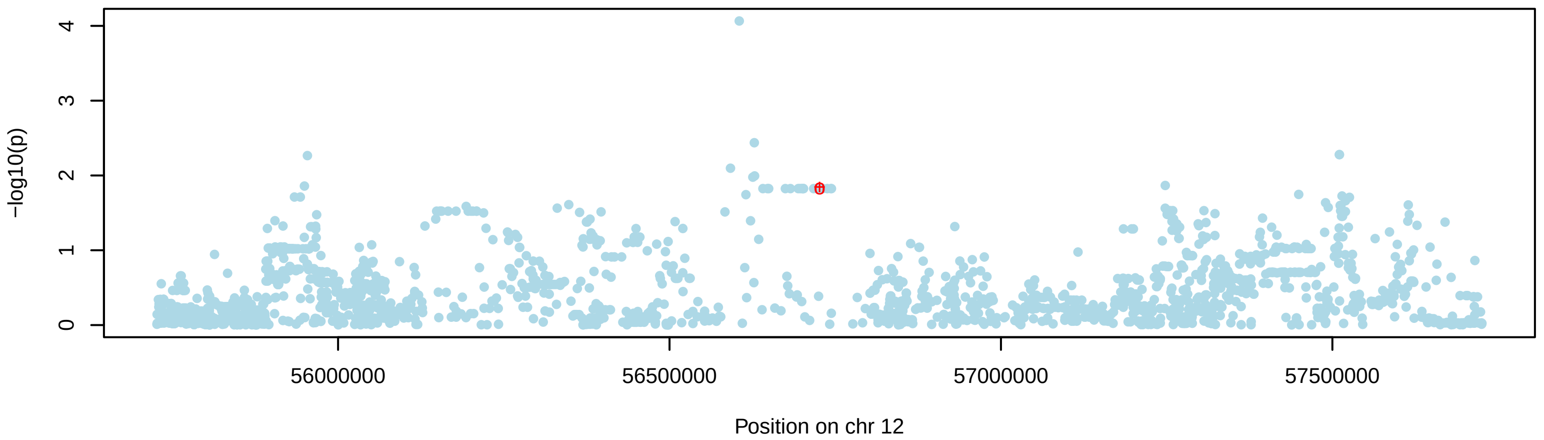

212. LECT2 (O14960) 5:135313633:T:A [Tarkin]

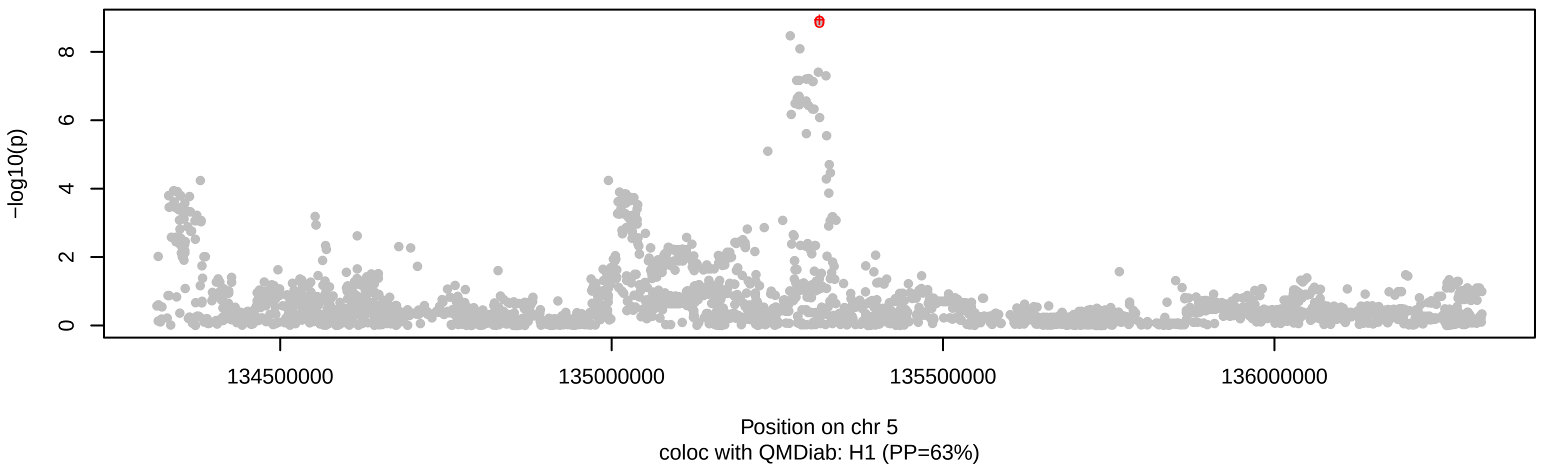

212. LECT2 (O14960) 5:135313633:T:A [QMDiab]

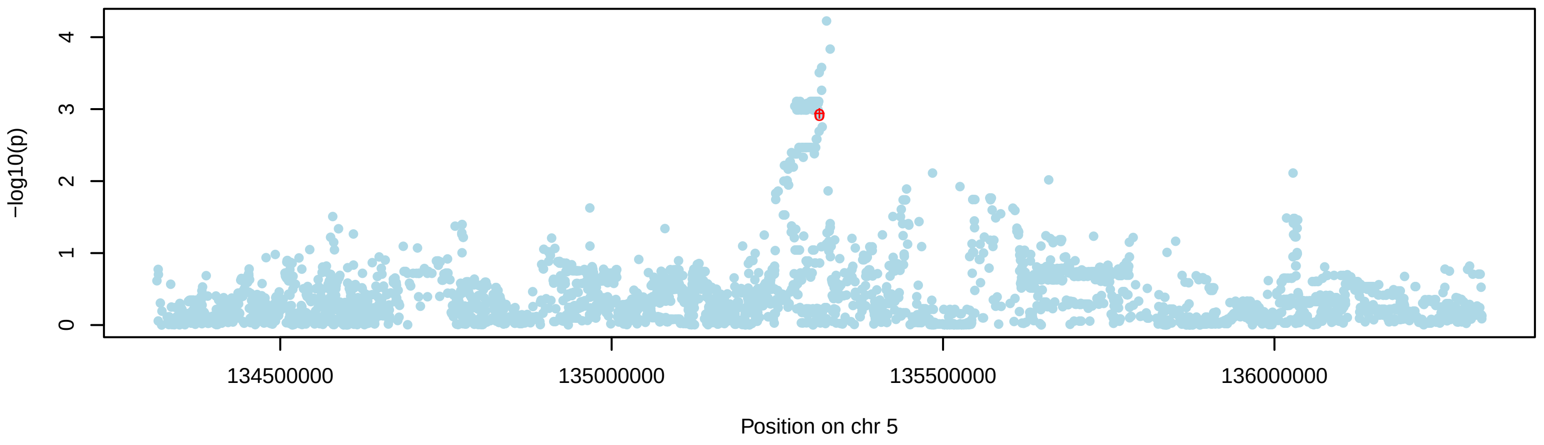

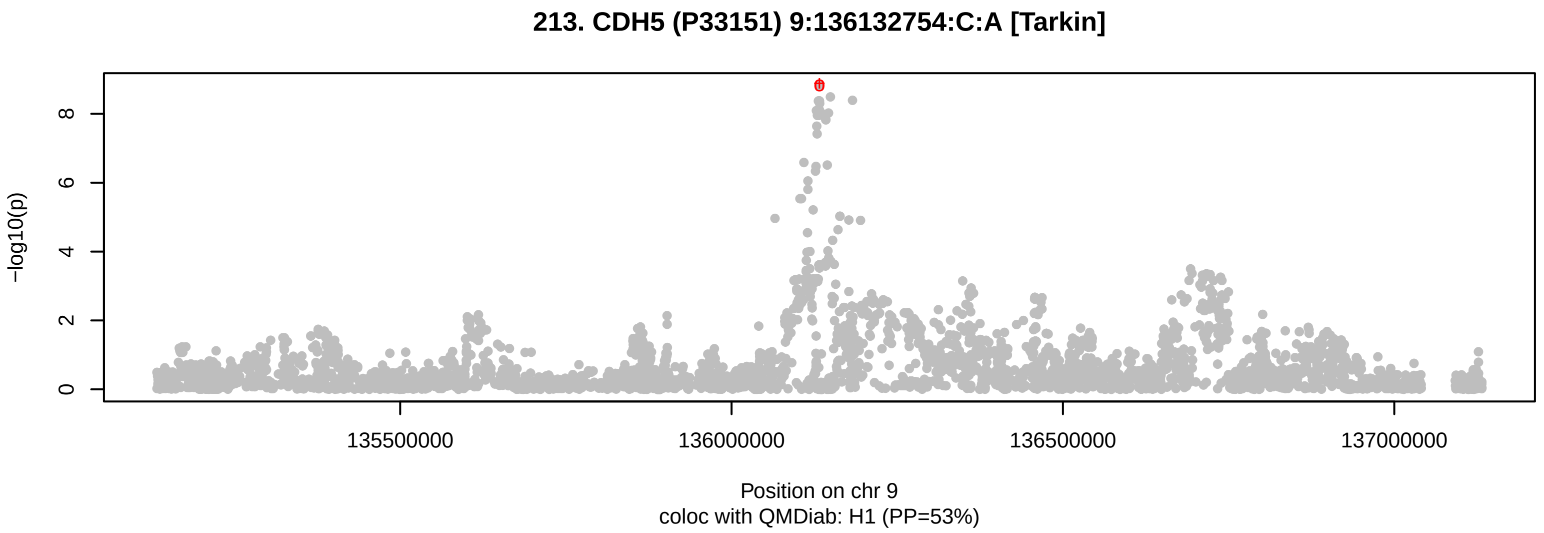

213. CDH5 (P33151) 9:136132754:C:A [Tarkin]
−log10(p)
0
2
4
6
8
135500000
136000000
136500000
137000000
Position on chr 9
coloc with QMDiab: H1 (PP=53%)

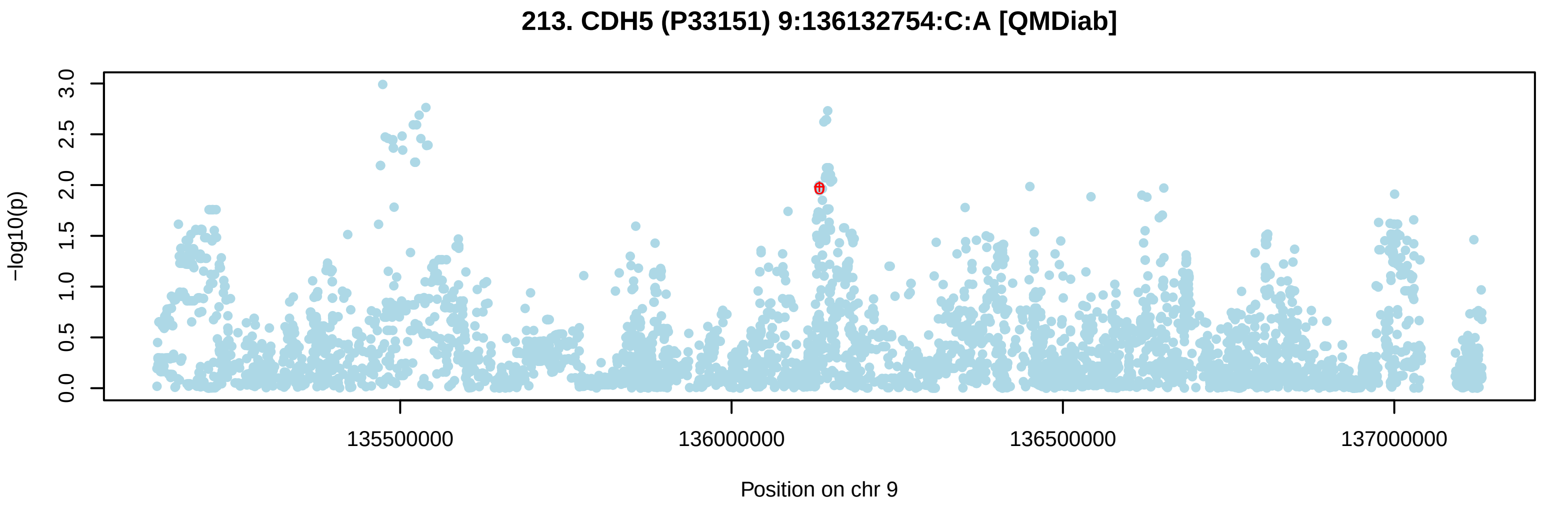

213. CDH5 (P33151) 9:136132754:C:A [QMDiab]
−log10(p)
0.0
0.5
1.0
1.5
2.0
2.5
3.0
135500000
136000000
136500000
137000000
Position on chr 9

214. CAPN1 (P07384) 3:186458910:G:A [Tarkin]

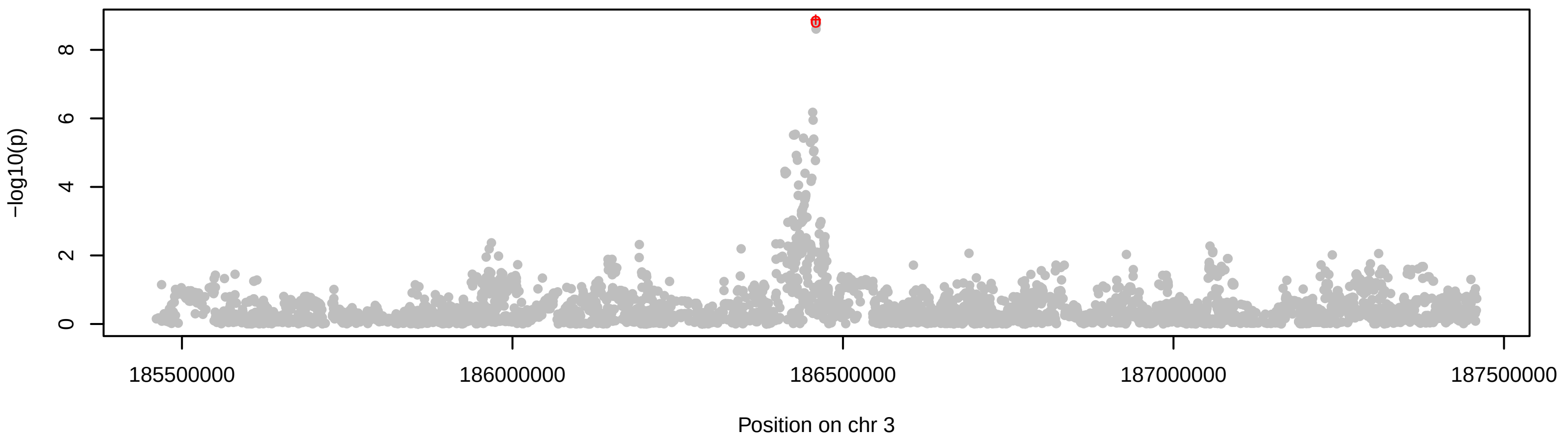

coloc with QMDiab: H1 (PP=65%)

214. CAPN1 (P07384) 3:186458910:G:A [QMDiab]

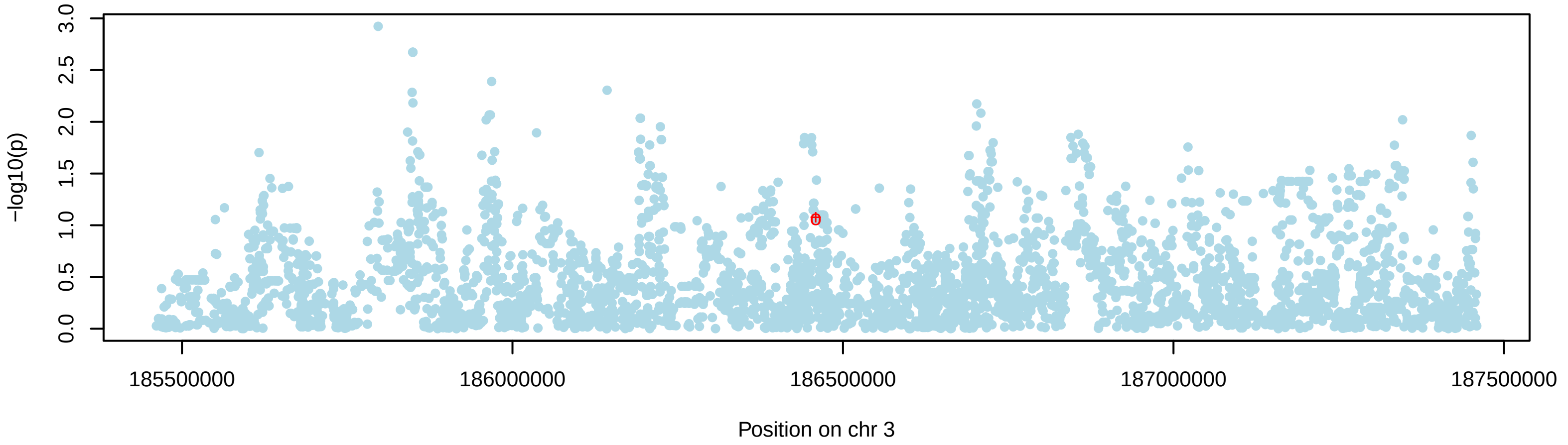

215. F8 (P00451) 9:136149098:T:A [Tarkin]

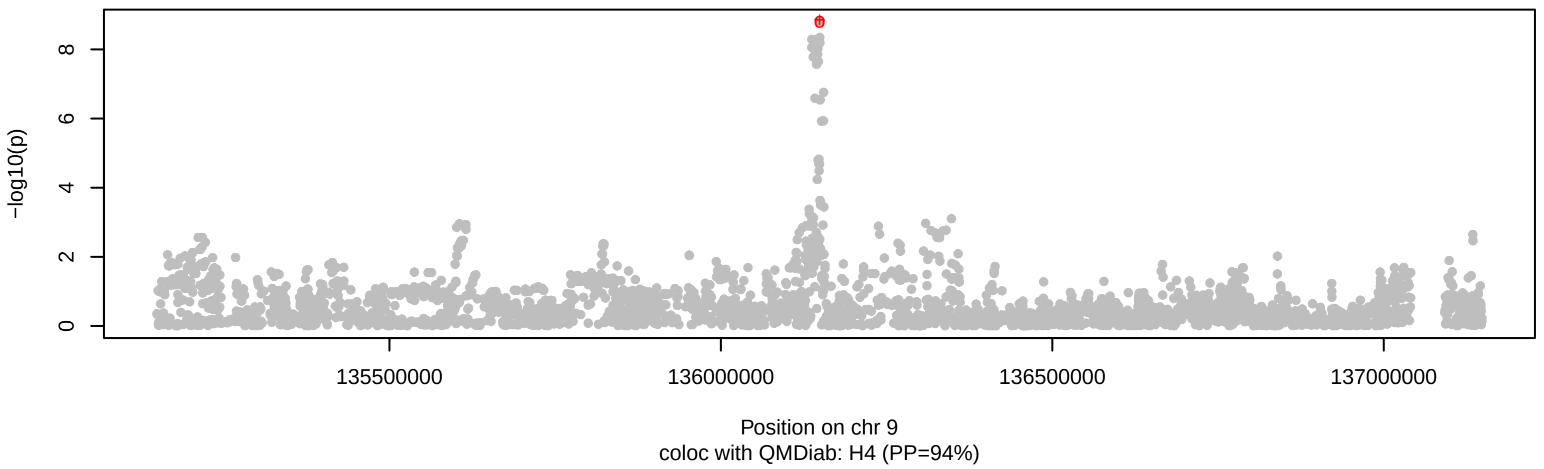

coloc with QMDiab: H4 (PP=94%)

215. F8 (P00451) 9:136149098:T:A [QMDiab]

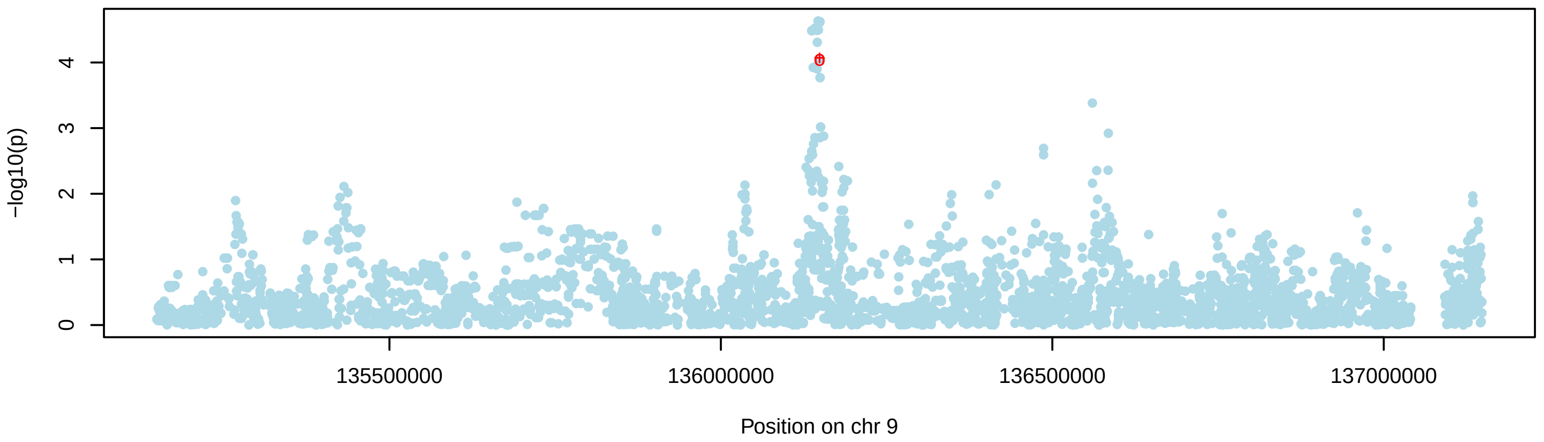

216. ALDH9A1 (P49189−3) 1:165633344:T:C [Tarkin]

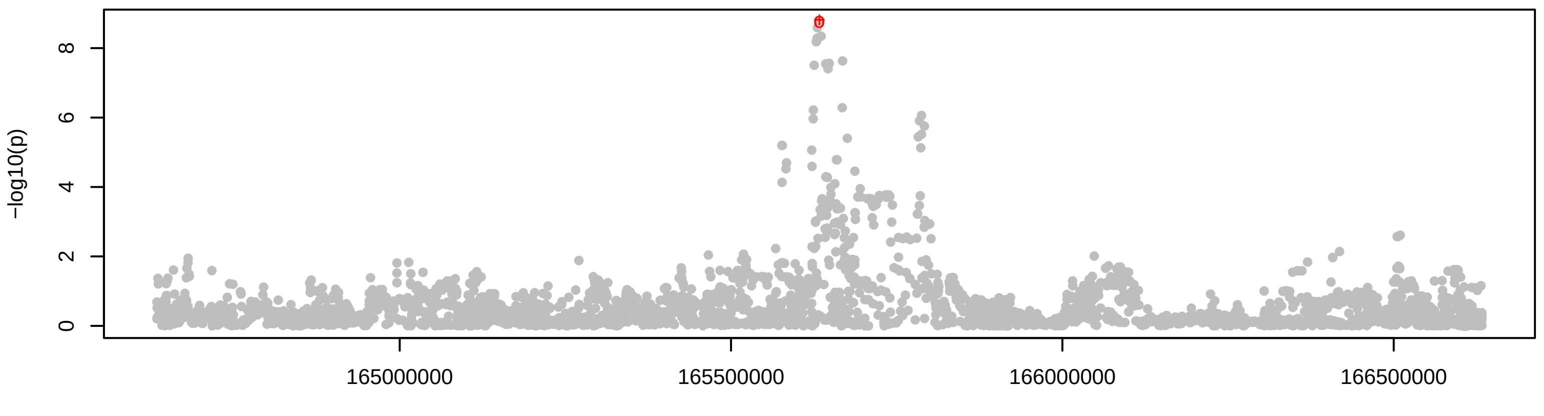

coloc with QMDiab: H1 (PP=63%)

216. ALDH9A1 (P49189−3) 1:165633344:T:C [QMDiab]

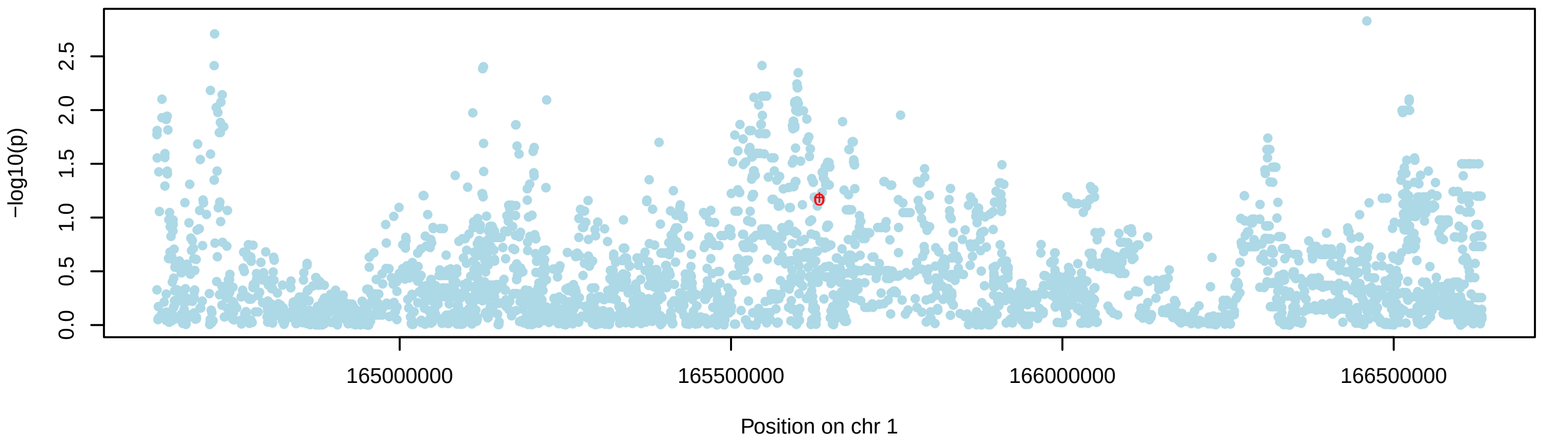

217. CTRB2 (Q6GPI1) 16:75256360:T:G [Tarkin]

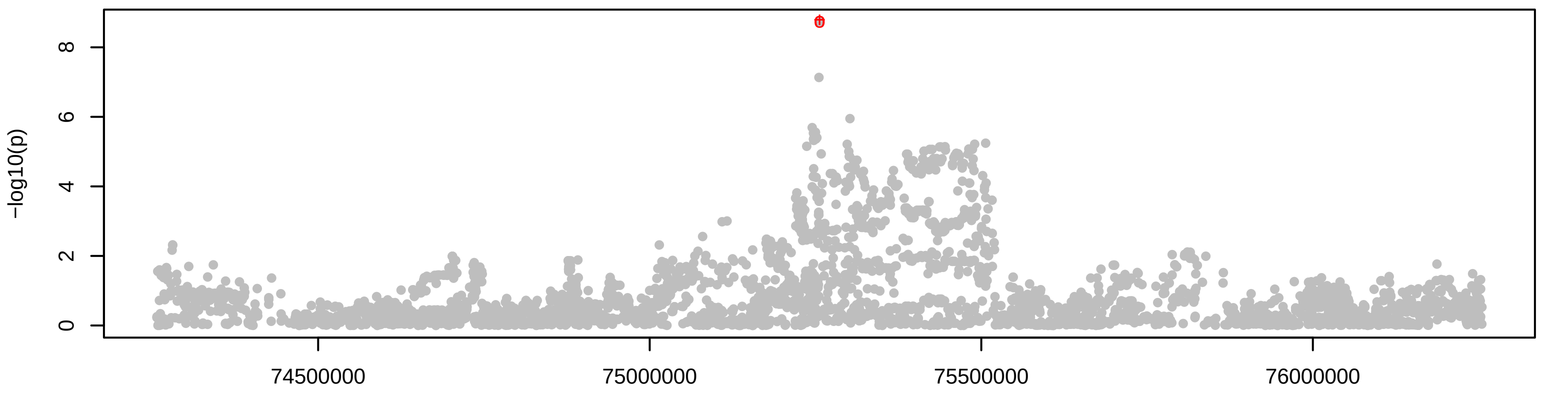

Position on chr 16
coloc with QMDiab: H1 (PP=63%)

217. CTRB2 (Q6GPI1) 16:75256360:T:G [QMDiab]

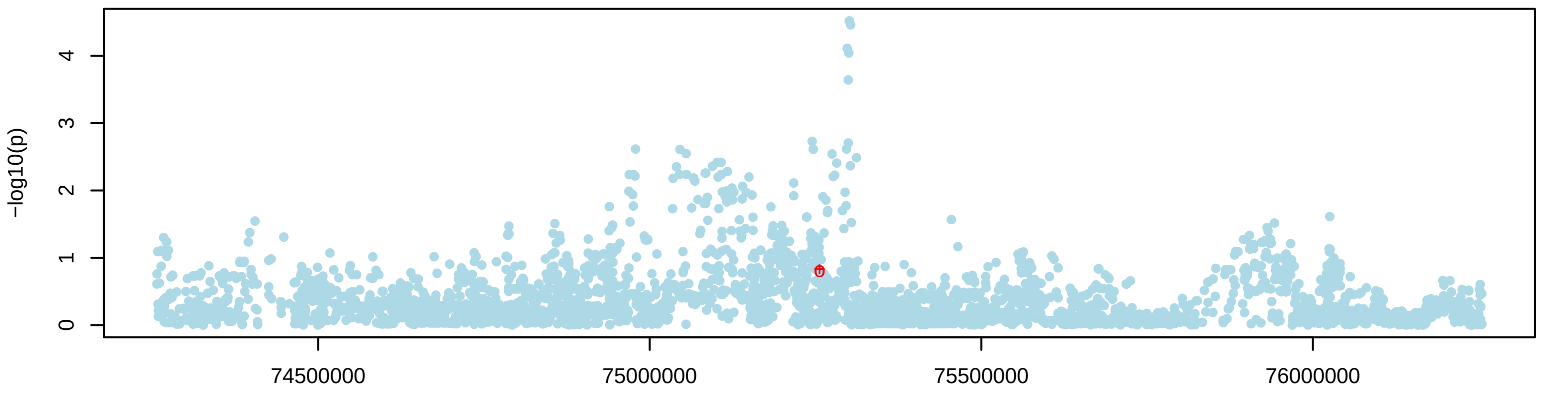

Position on chr 16

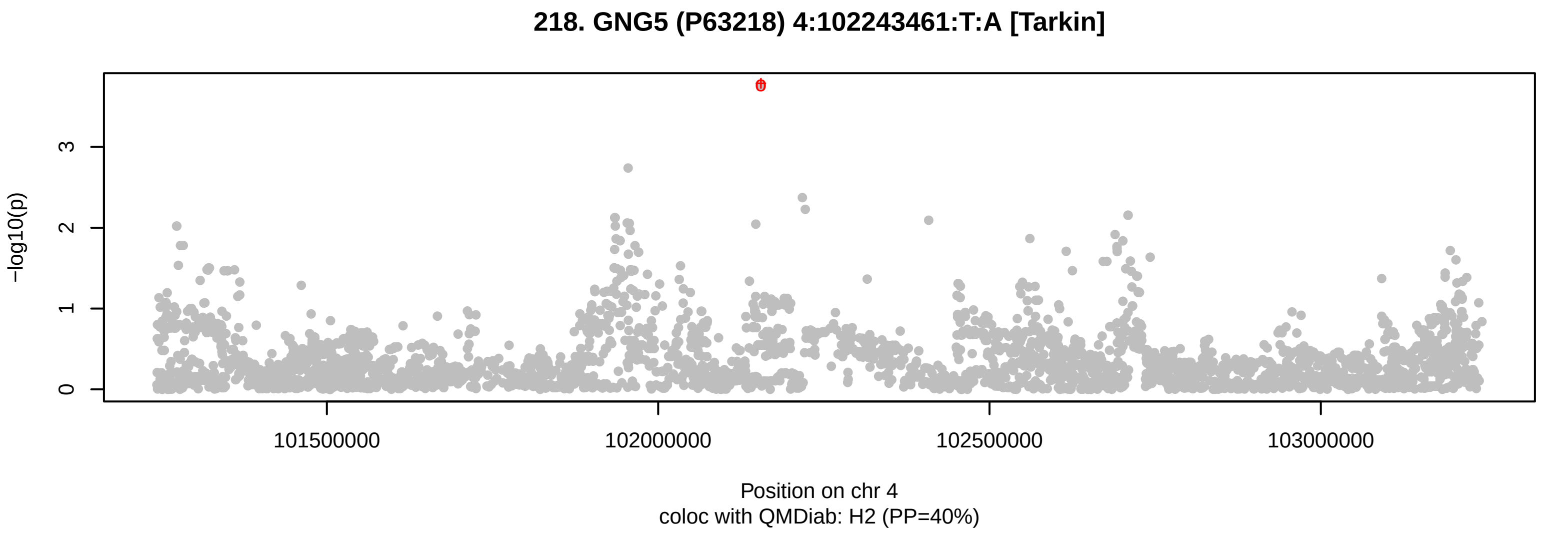

218. GNG5 (P63218) 4:102243461:T:A [Tarkin]
−log10(p)
0
1
2
3
101500000
102000000
102500000
103000000
Position on chr 4
coloc with QMDiab: H2 (PP=40%)

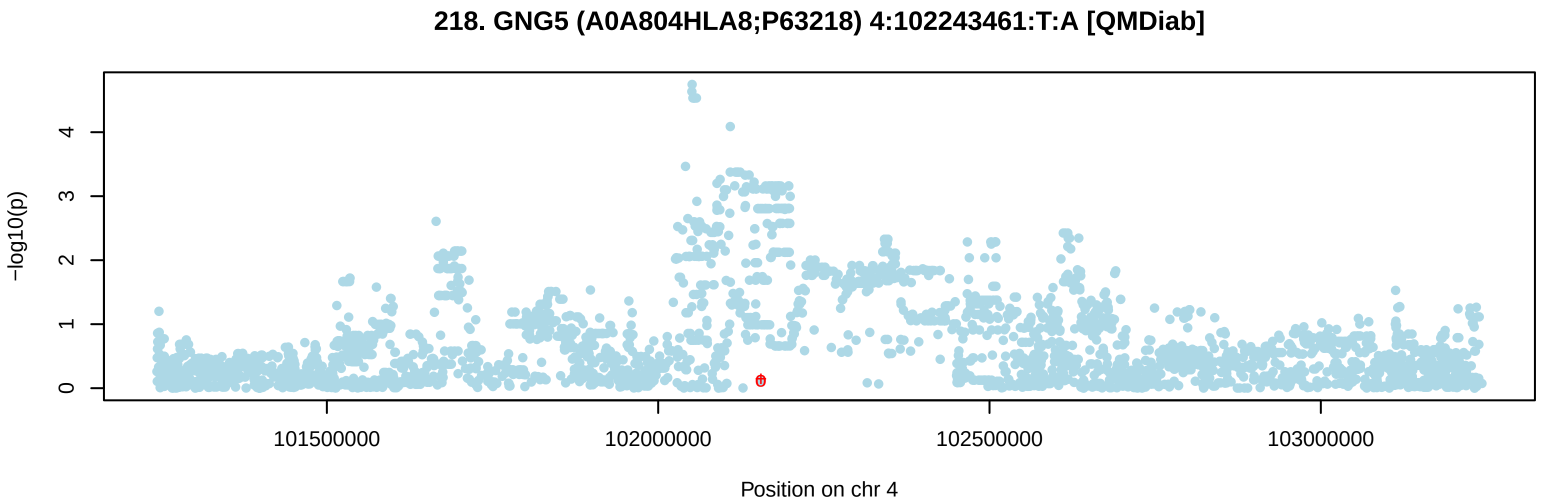

218. GNG5 (A0A804HLA8;P63218) 4:102243461:T:A [QMDiab]
−log10(p)
0
1
2
3
4
101500000
102000000
102500000
103000000
Position on chr 4

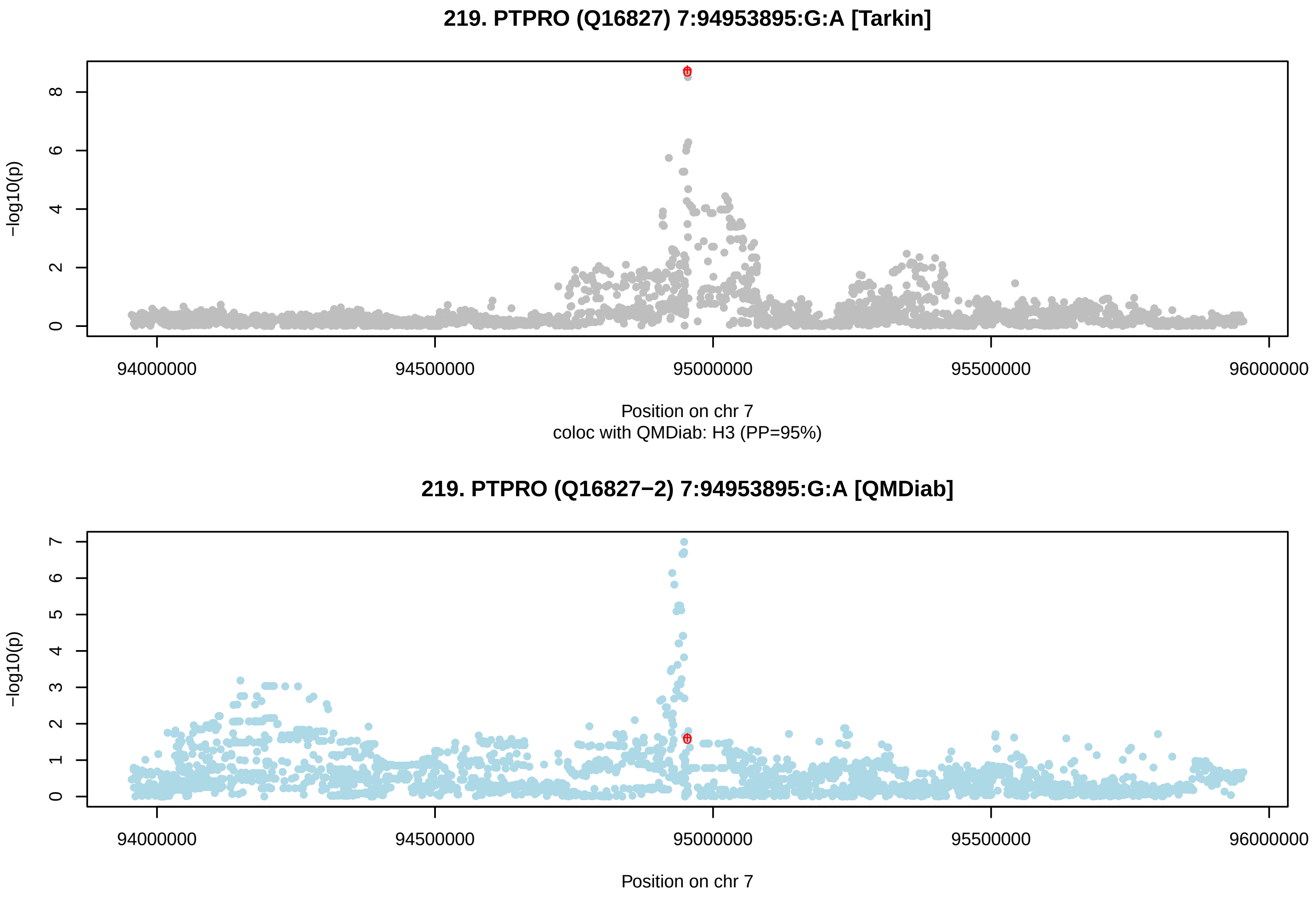
219. PTPRO (Q16827) 7:94953895:G:A [Tarkin]
−log10(p)
0
2
4
6
8
94000000
94500000
95000000
95500000
96000000
Position on chr 7
coloc with QMDiab: H3 (PP=95%)
219. PTPRO (Q16827−2) 7:94953895:G:A [QMDiab]
−log10(p)
0
1
2
3
4
5
6
7
94000000
94500000
95000000
95500000
96000000
Position on chr 7

220. CTSD (A0A1B0GVD5;A0A1B0GW44;A0A1B0GWE8;P07339) 10:13472688:G:A [Tarkin]

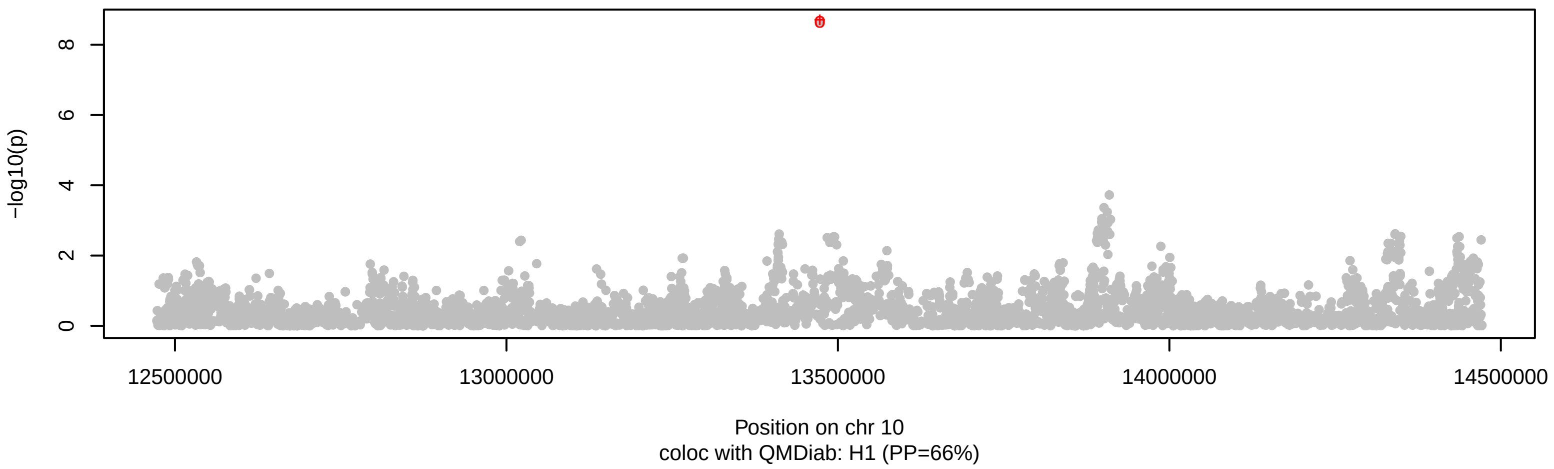

coloc with QMDiab: H1 (PP=66%)

220. CTSD (A0A1B0GVD5;A0A1B0GW44;A0A1B0GWE8;P07339) 10:13472688:G:A [QMDiab]

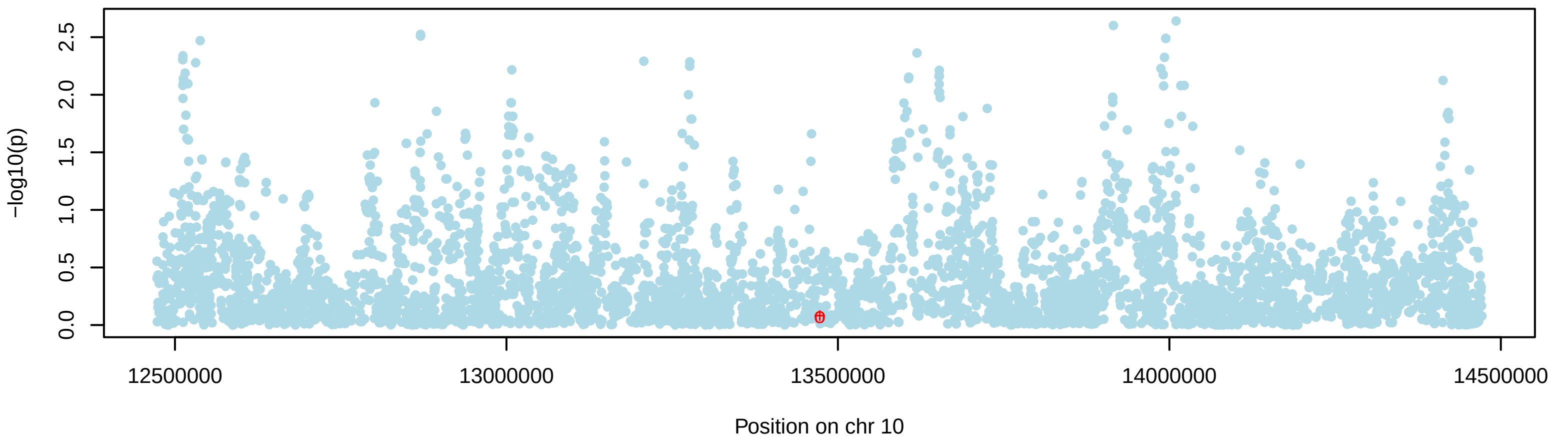

221. DKK3 (Q9UBP4) 11:11989151:C:G [Tarkin]

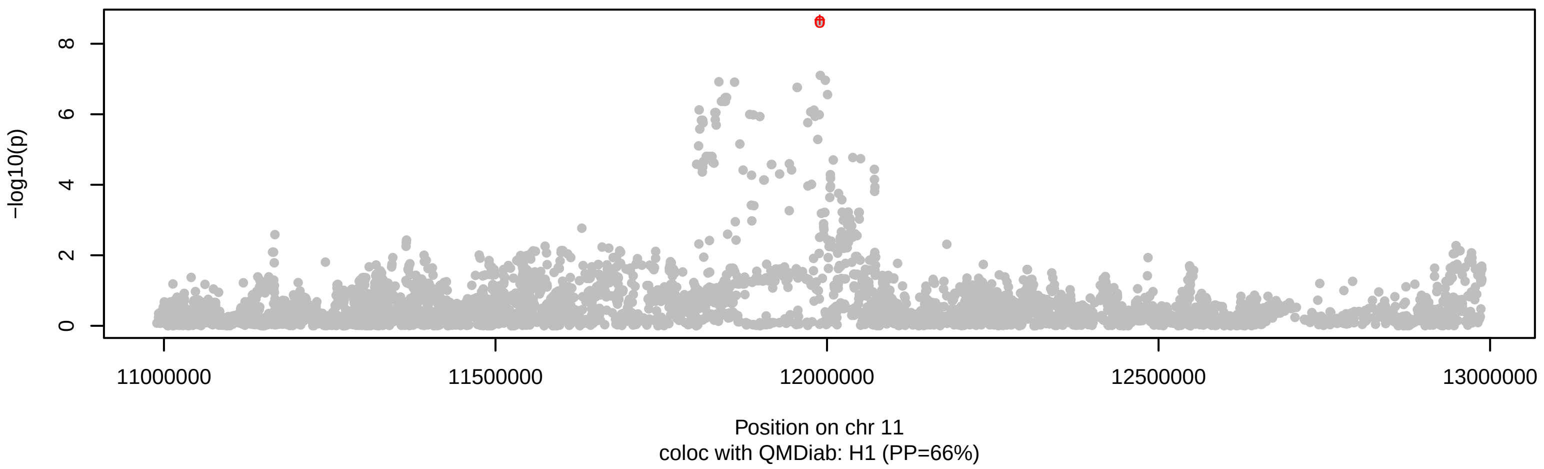

coloc with QMDiab: H1 (PP=66%)

221. DKK3 (Q9UBP4) 11:11989151:C:G [QMDiab]

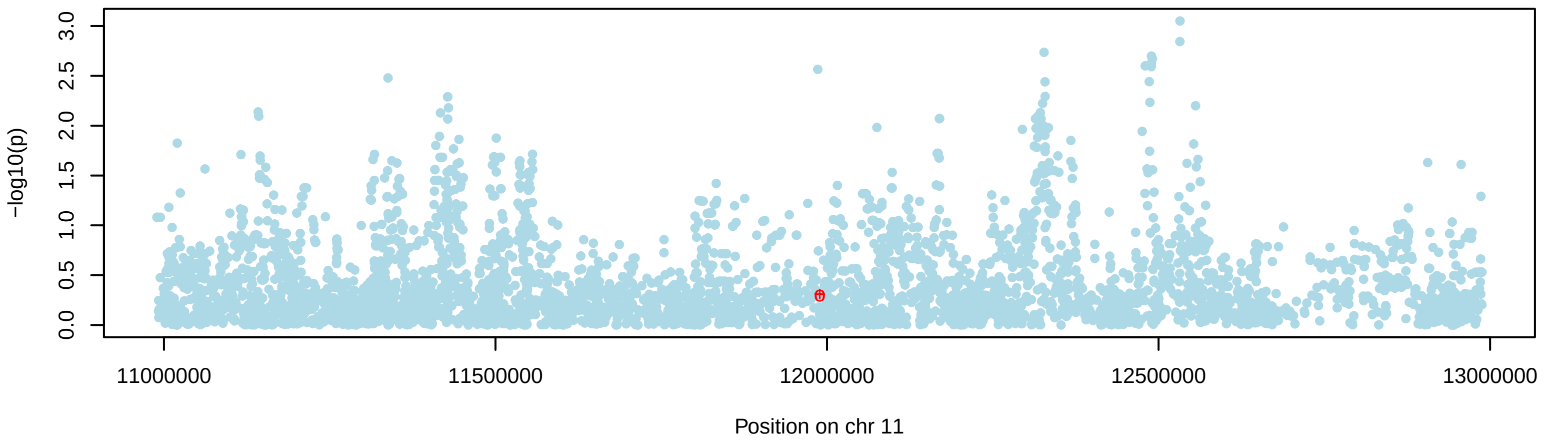

222. COL18A1 (P39060;P39060−1;P39060−2) 21:46931109:G:A [Tarkin]

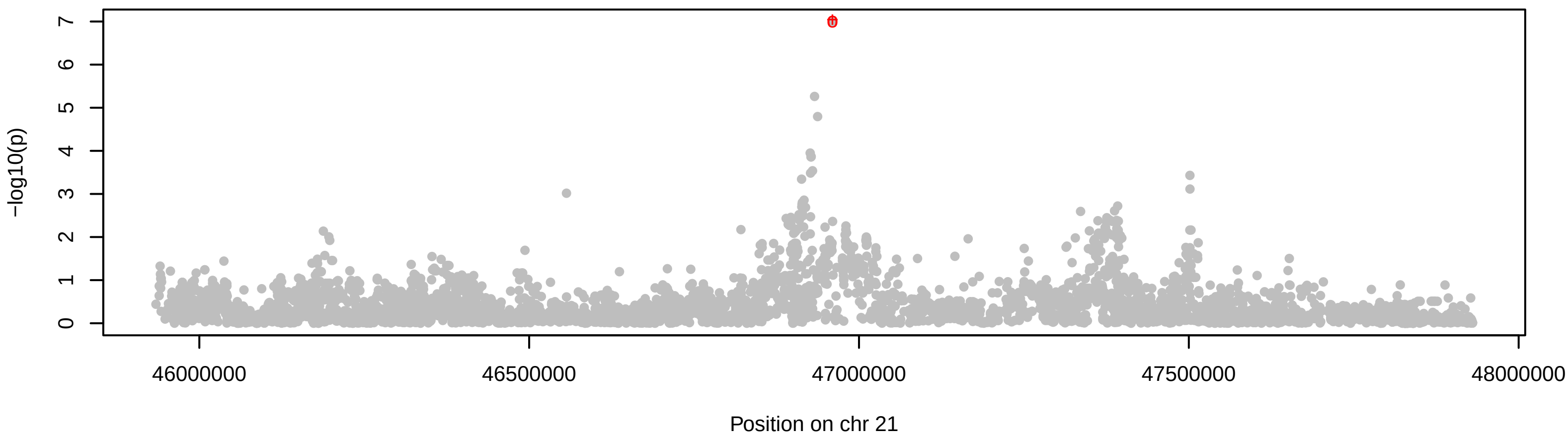

coloc with QMDiab: H4 (PP=71%)

222. COL18A1 (P39060;P39060−1;P39060−2) 21:46931109:G:A [QMDiab]

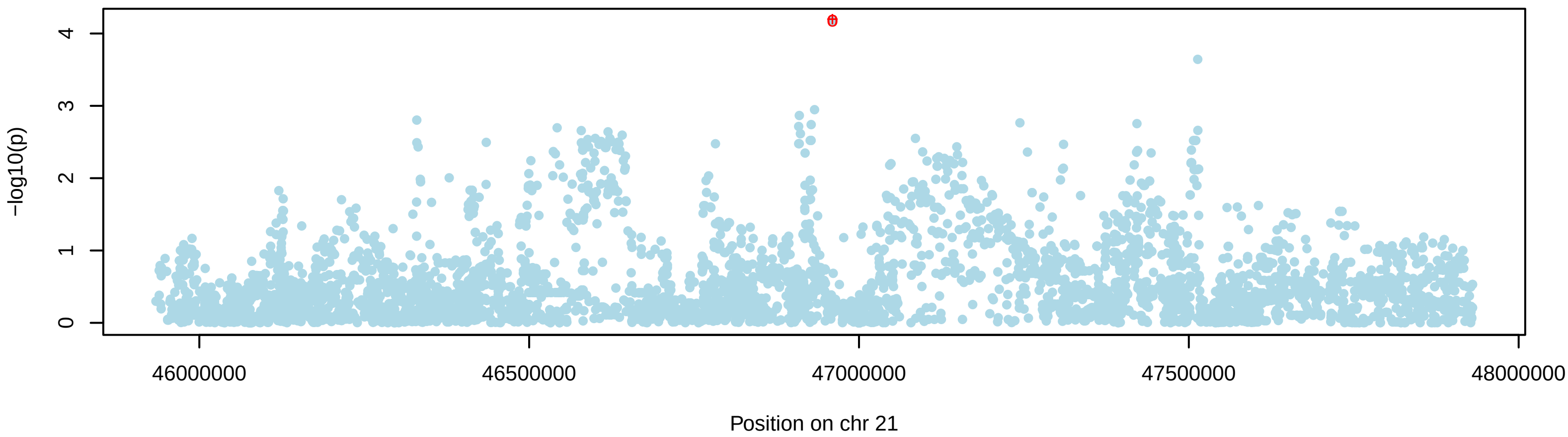

223. PAM (P19021−5) 5:102167154:C:T [Tarkin]

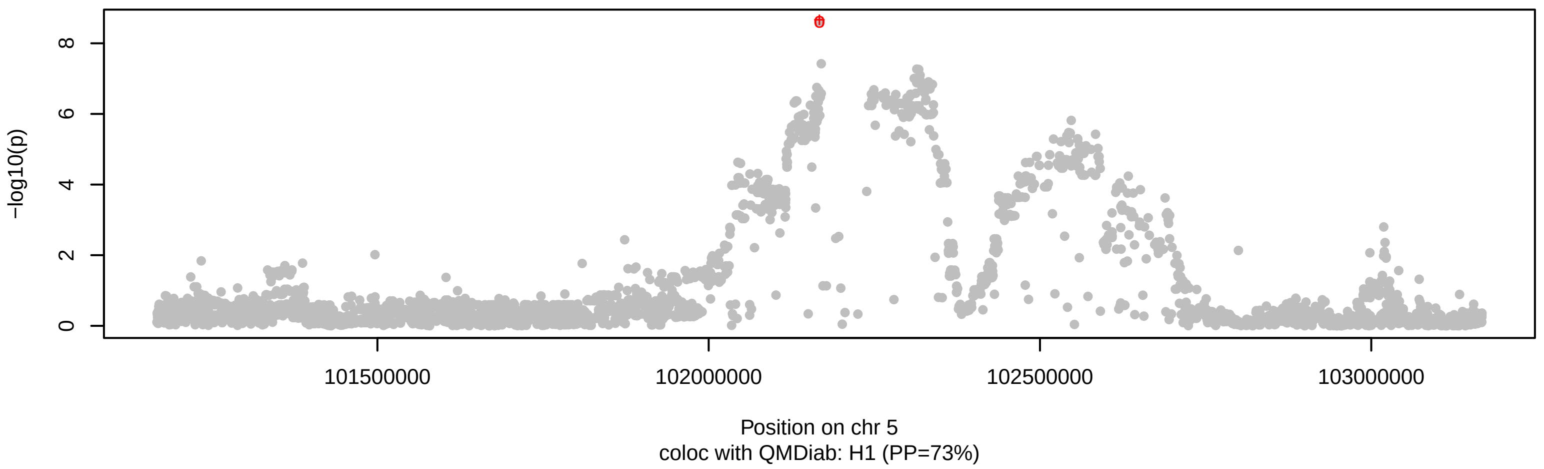

coloc with QMDiab: H1 (PP=73%)

223. PAM (A0A8C8KD64;P19021;P19021−3;P19021−4;P19021−5;P19021−6) 5:102167154:C:T [QMDiab]

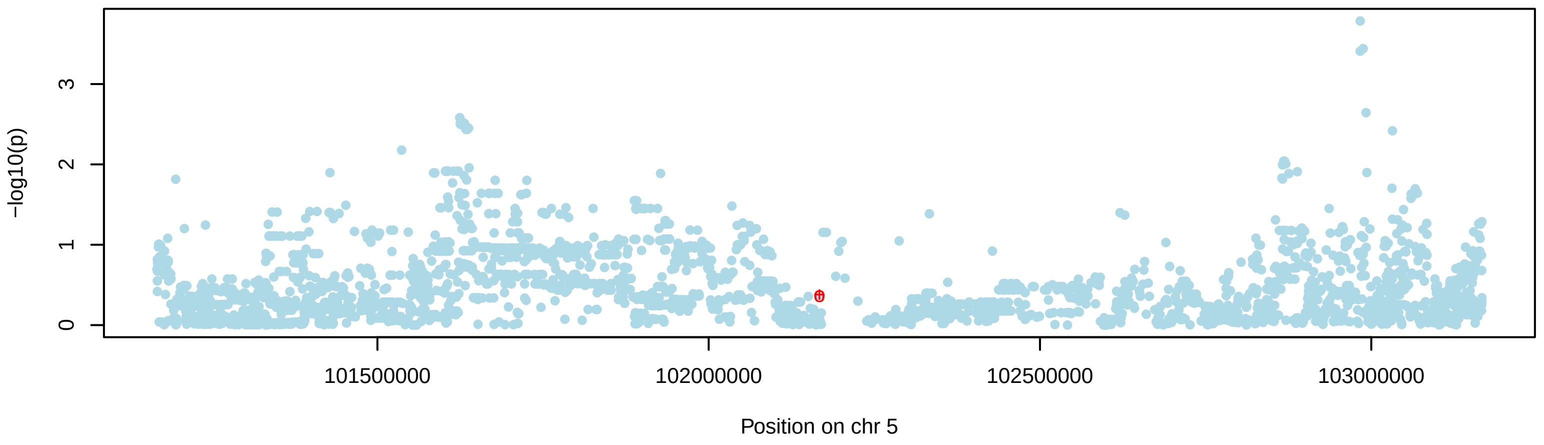

224. AZU1 (P20160) 19:829823:G:A [Tarkin]

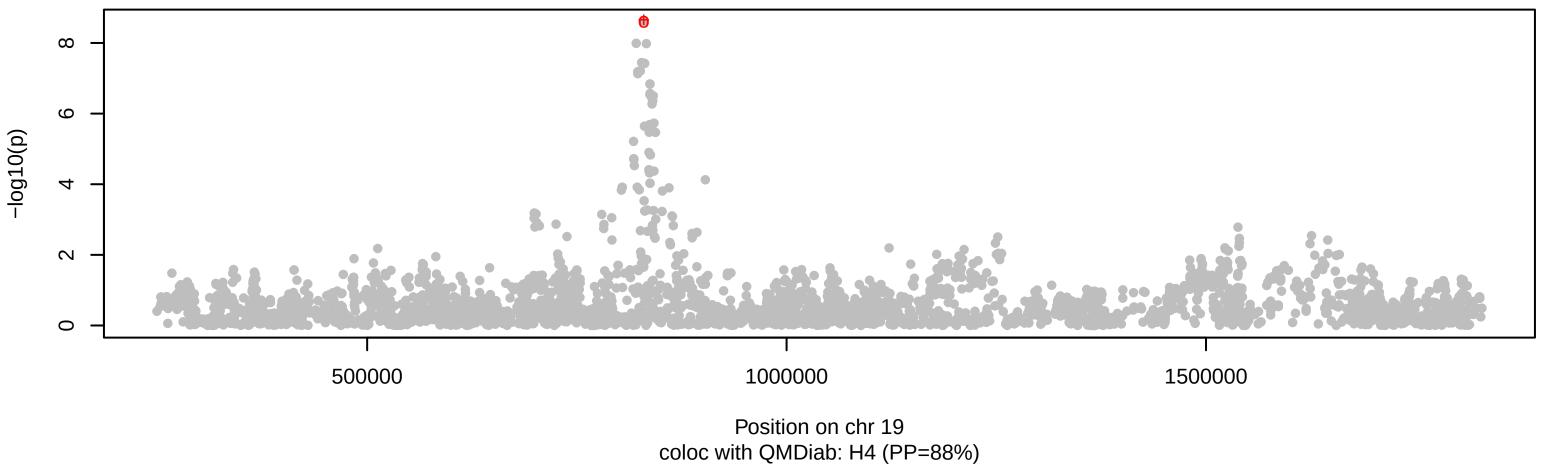

224. AZU1 (P20160) 19:829823:G:A [QMDiab]

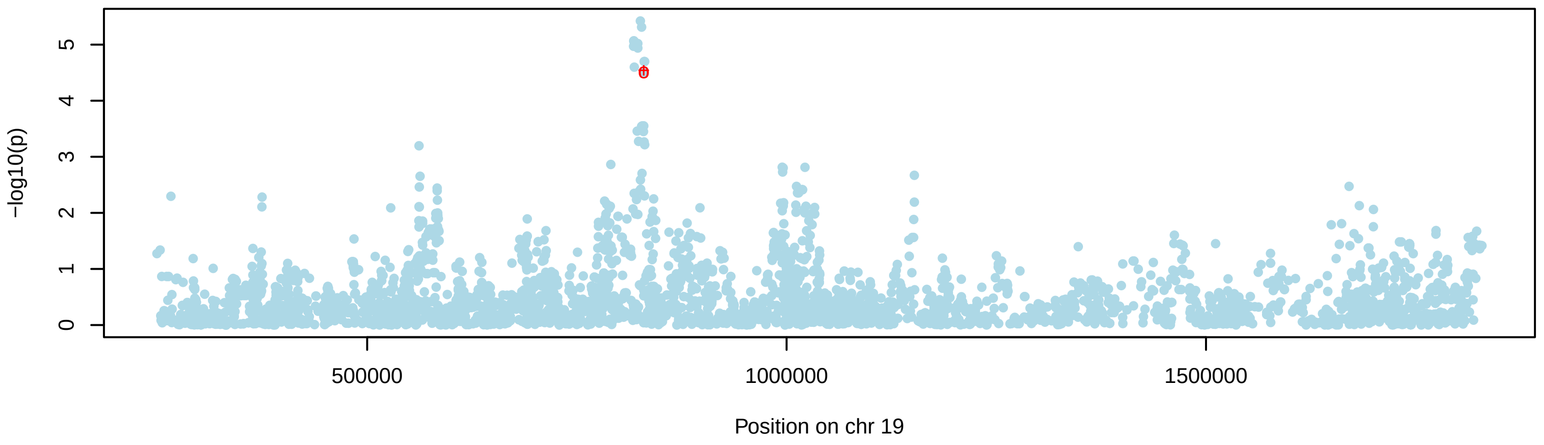

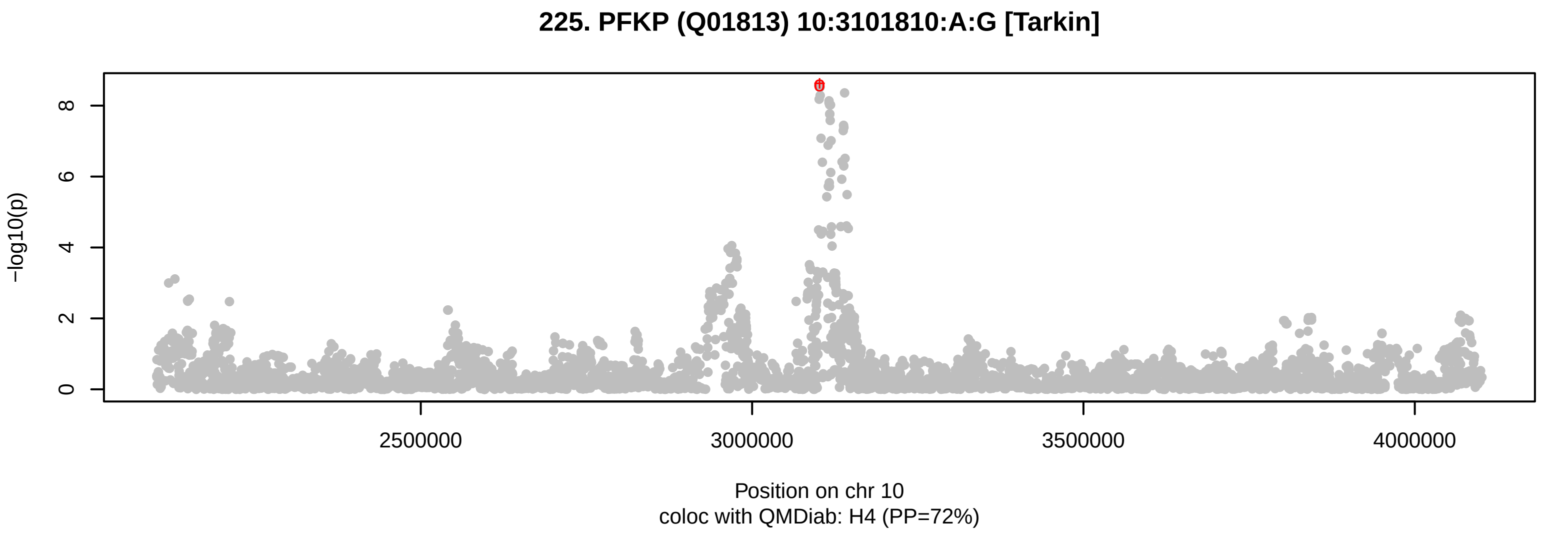

225. PFKP (Q01813) 10:3101810:A:G [Tarkin]
−log10(p)
0
2
4
6
8
2500000
3000000
3500000
4000000
Position on chr 10
coloc with QMDiab: H4 (PP=72%)

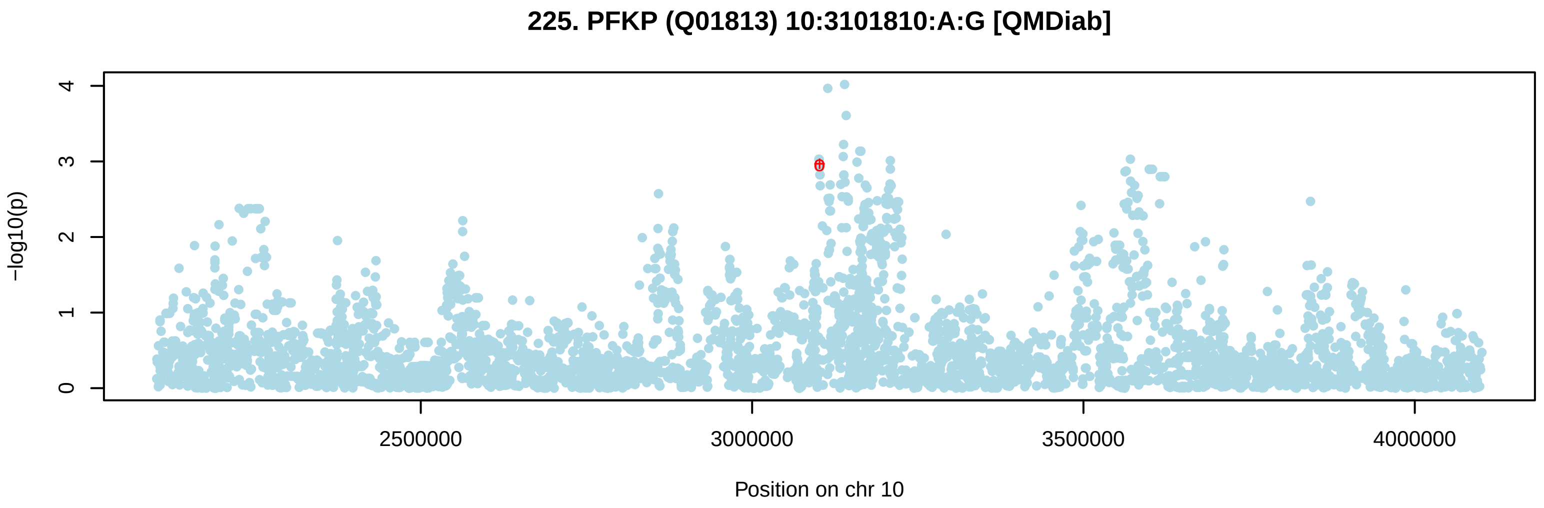

225. PFKP (Q01813) 10:3101810:A:G [QMDiab]
−log10(p)
0
1
2
3
4
2500000
3000000
3500000
4000000
Position on chr 10

226. SCP2 (P22307) 15:70138632:G:A [Tarkin]

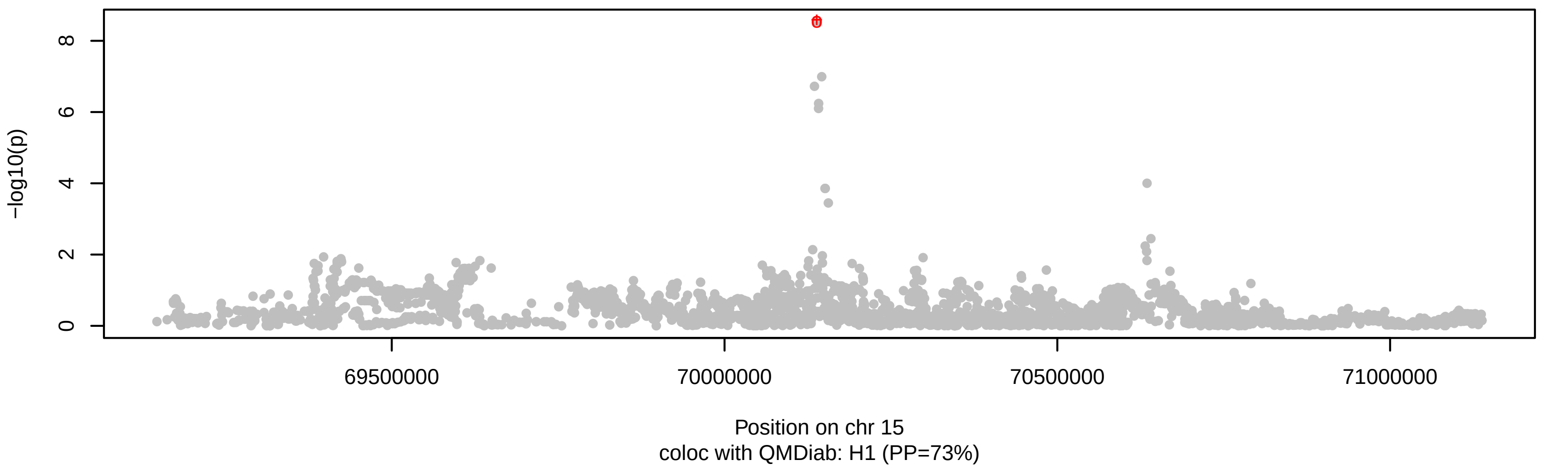

coloc with QMDiab: H1 (PP=73%)

226. SCP2 (P22307;P22307−8) 15:70138632:G:A [QMDiab]

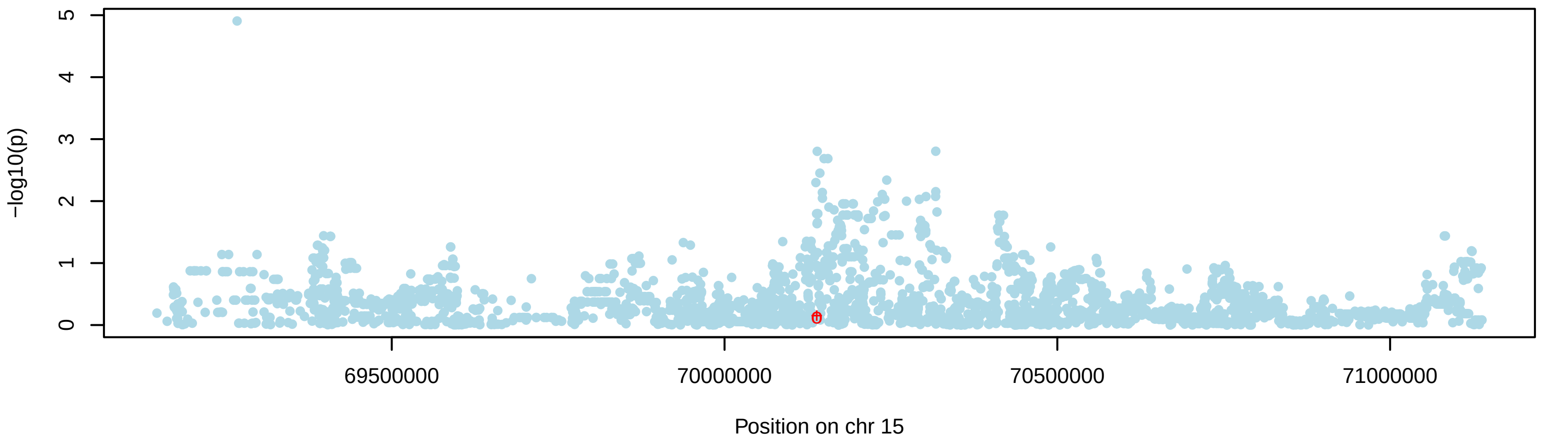

227. NCF1 (P14598) 7:75161416:G:A [Tarkin]

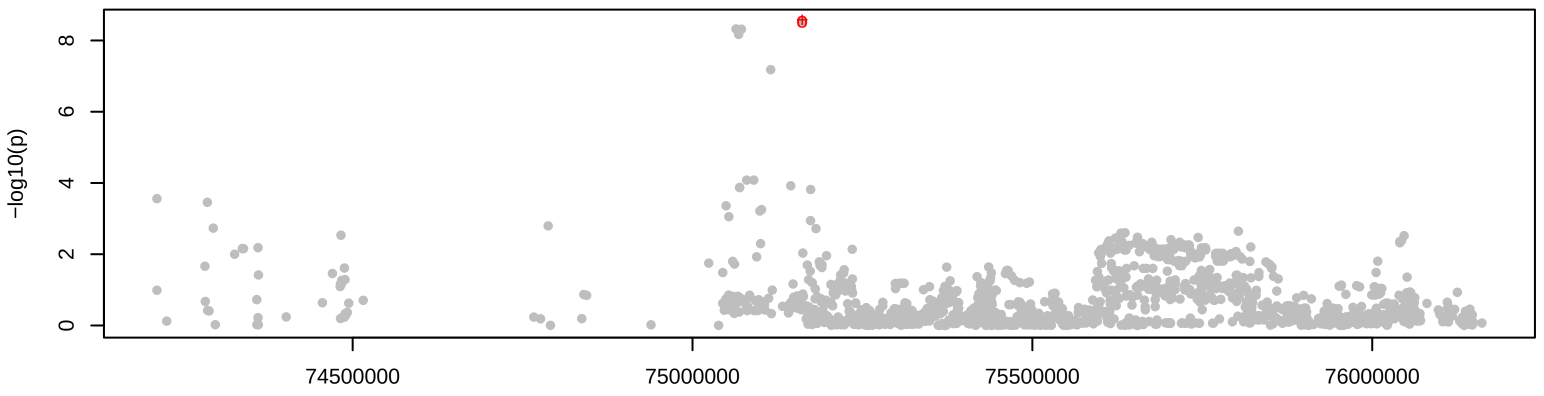

Position on chr 7
coloc with QMDiab: H1 (PP=78%)

227. NCF1 (P14598) 7:75161416:G:A [QMDiab]

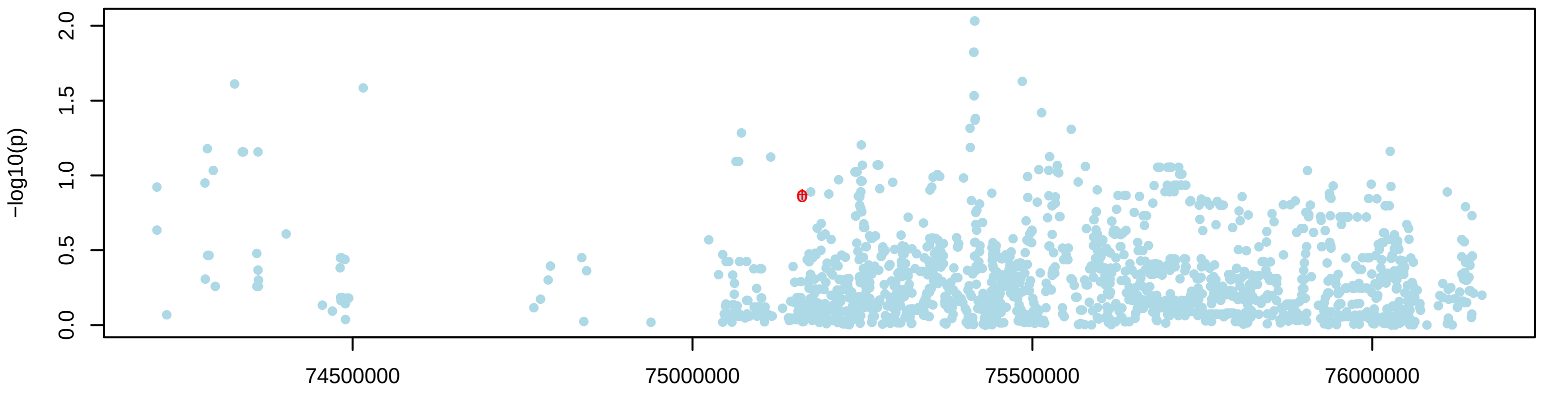

Position on chr 7

228. DPT (Q07507) 1:168697384:A:G [Tarkin]

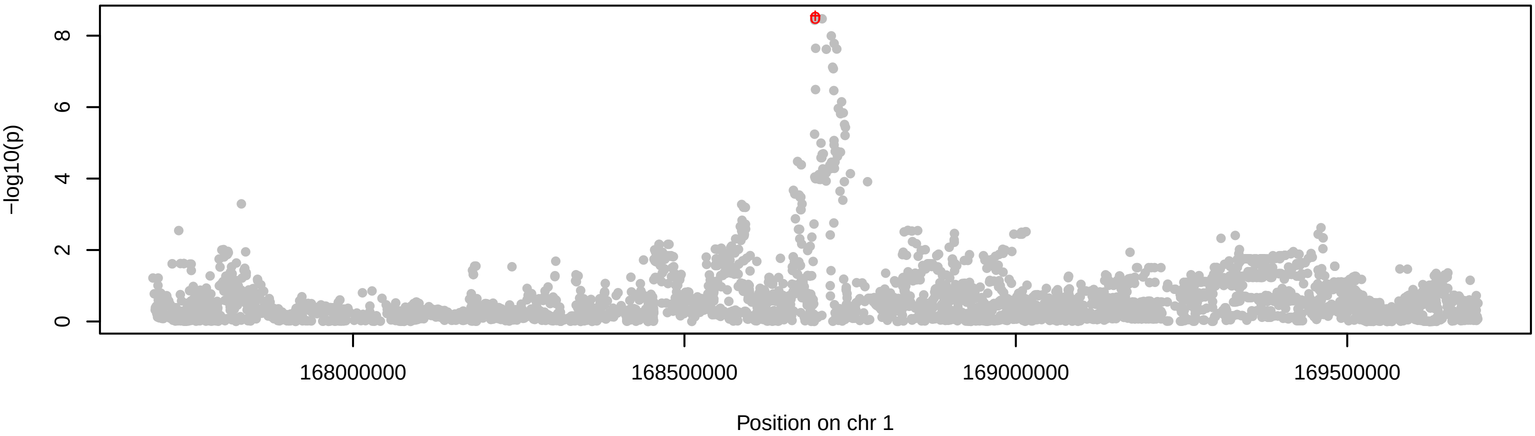

coloc with QMDiab: H1 (PP=58%)

228. DPT (Q07507) 1:168697384:A:G [QMDiab]

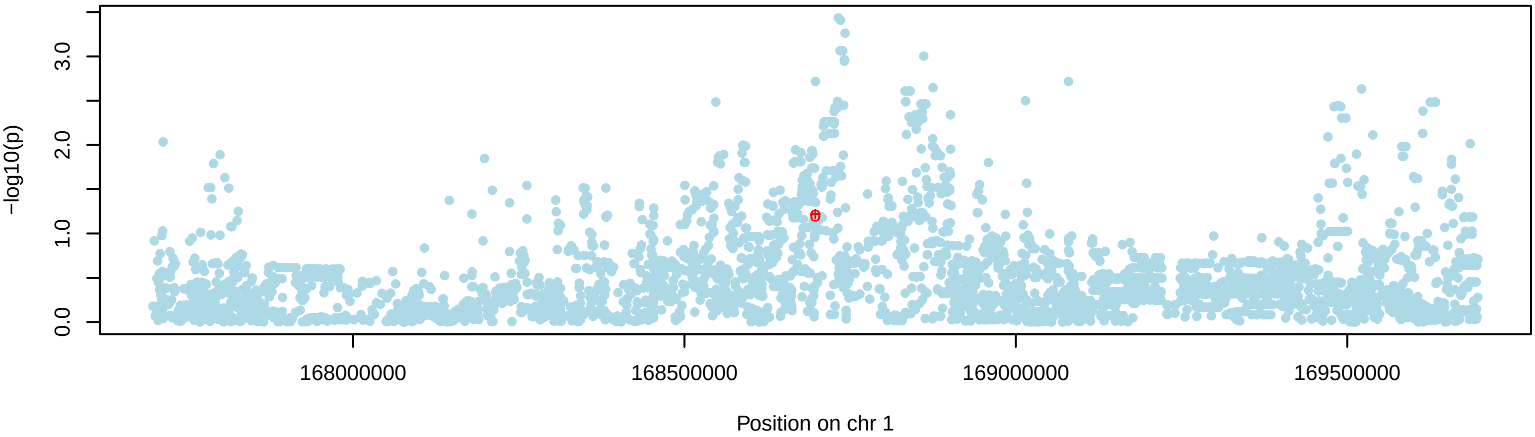

229. FCN2 (Q15485) 1:196684575:A:G [Tarkin]

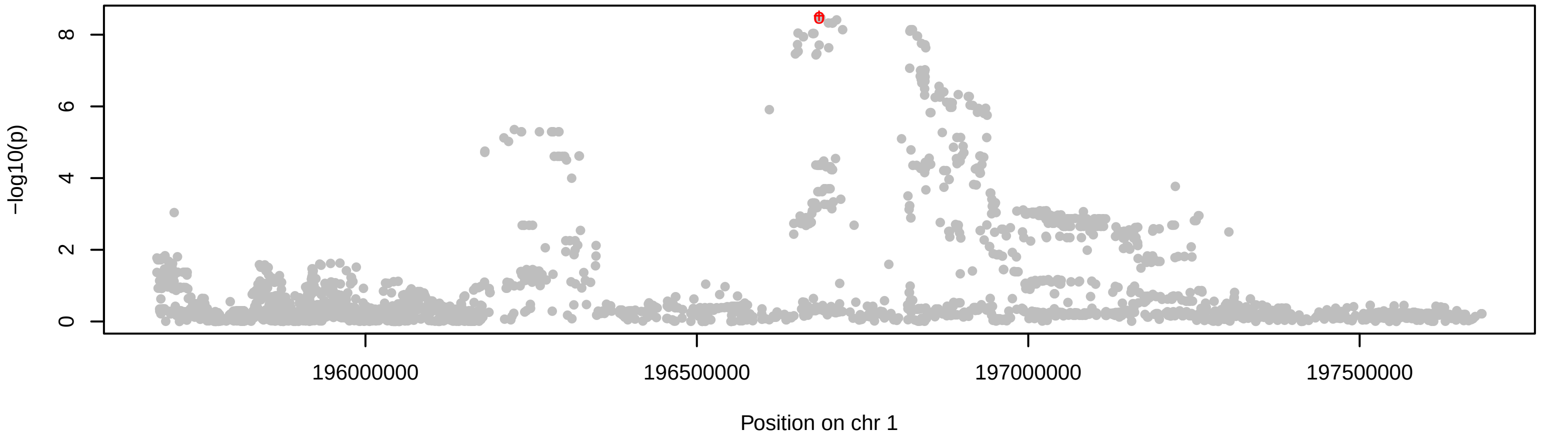

coloc with QMDiab: H4 (PP=96%)

229. FCN2 (Q15485) 1:196684575:A:G [QMDiab]

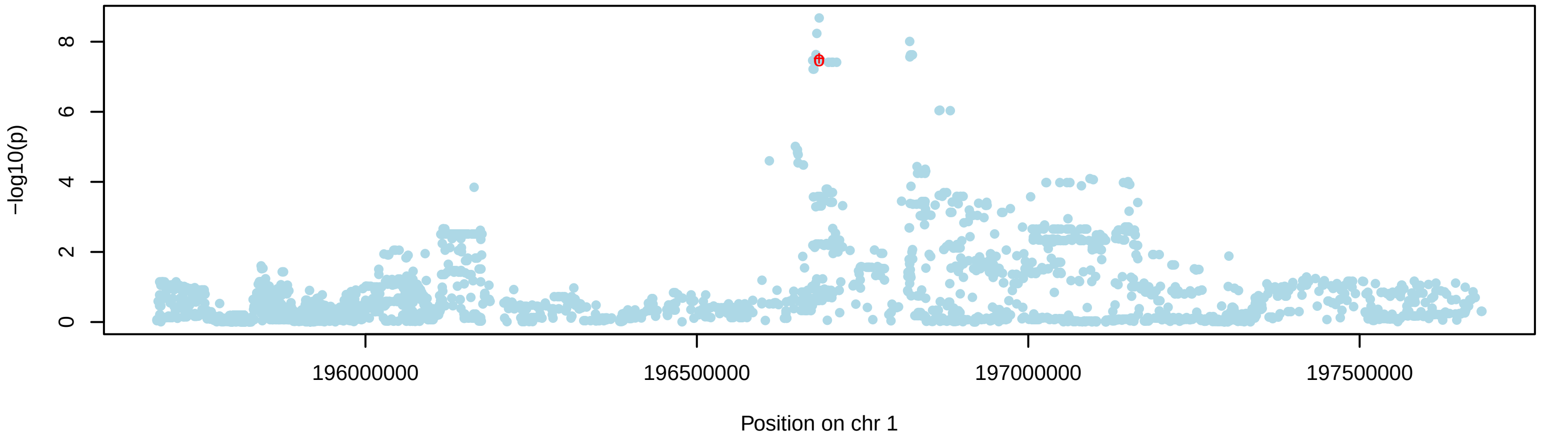

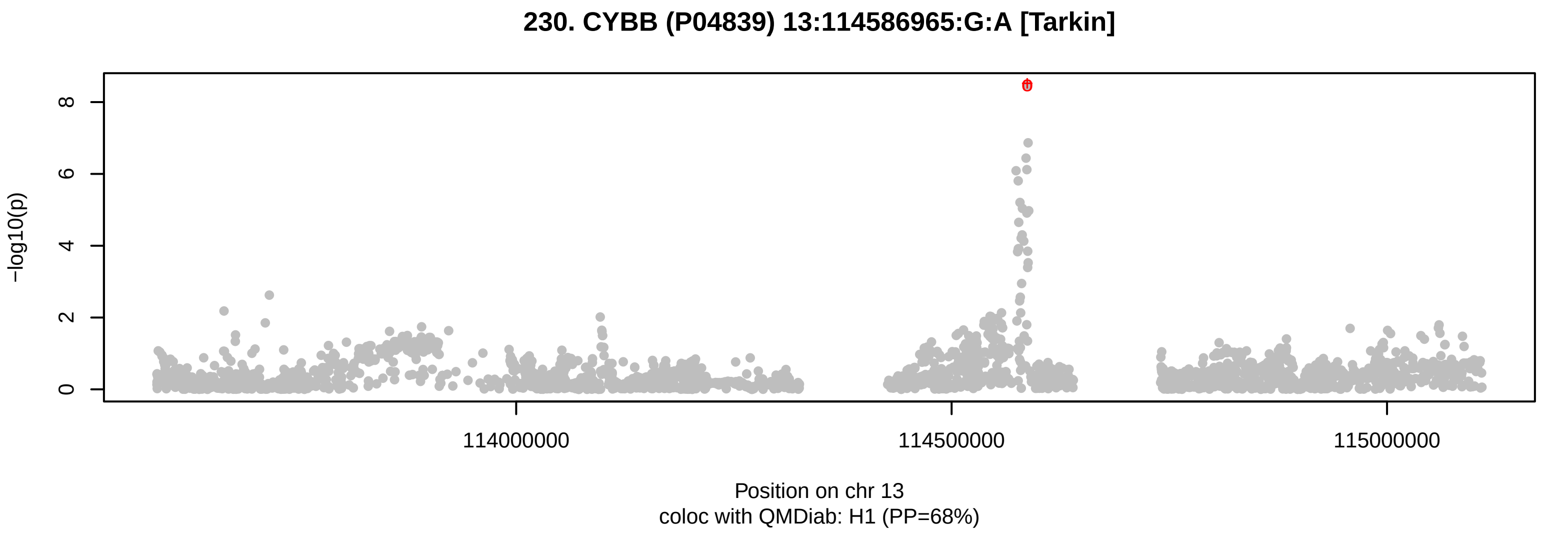
230. CYBB (P04839) 13:114586965:G:A [Tarkin]
−log10(p)
0
2
4
6
8
114000000
114500000
115000000
Position on chr 13
coloc with QMDiab: H1 (PP=68%)

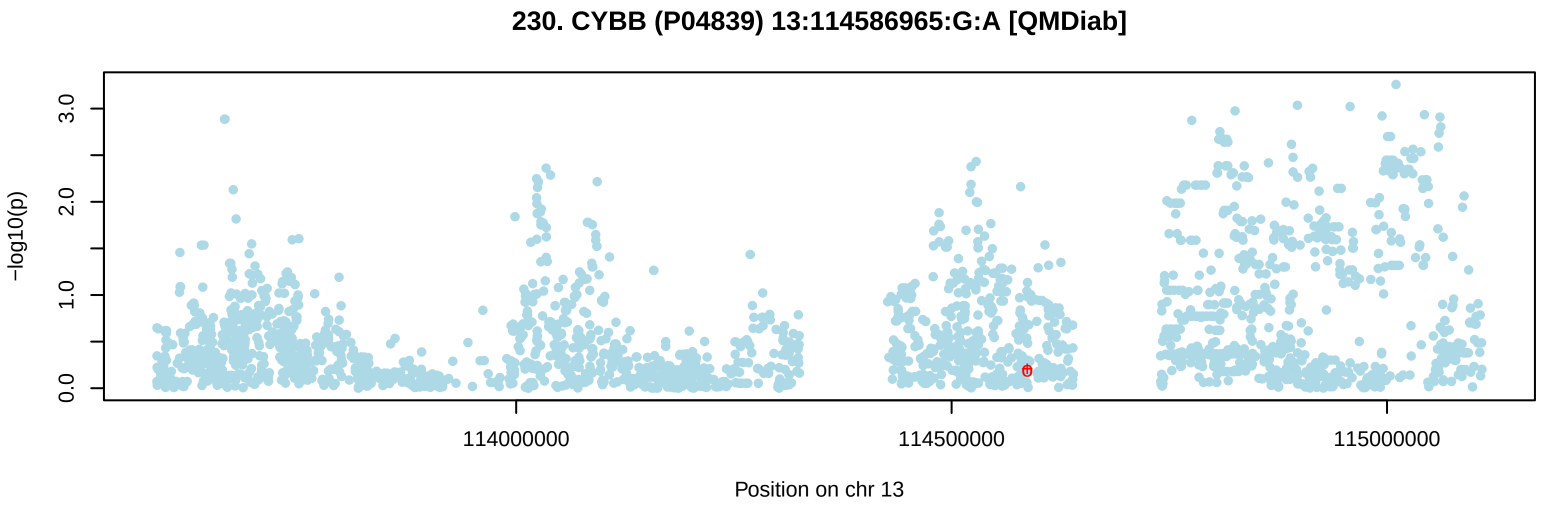
230. CYBB (P04839) 13:114586965:G:A [QMDiab]
−log10(p)
0.0
1.0
2.0
3.0
114000000
114500000
115000000
Position on chr 13

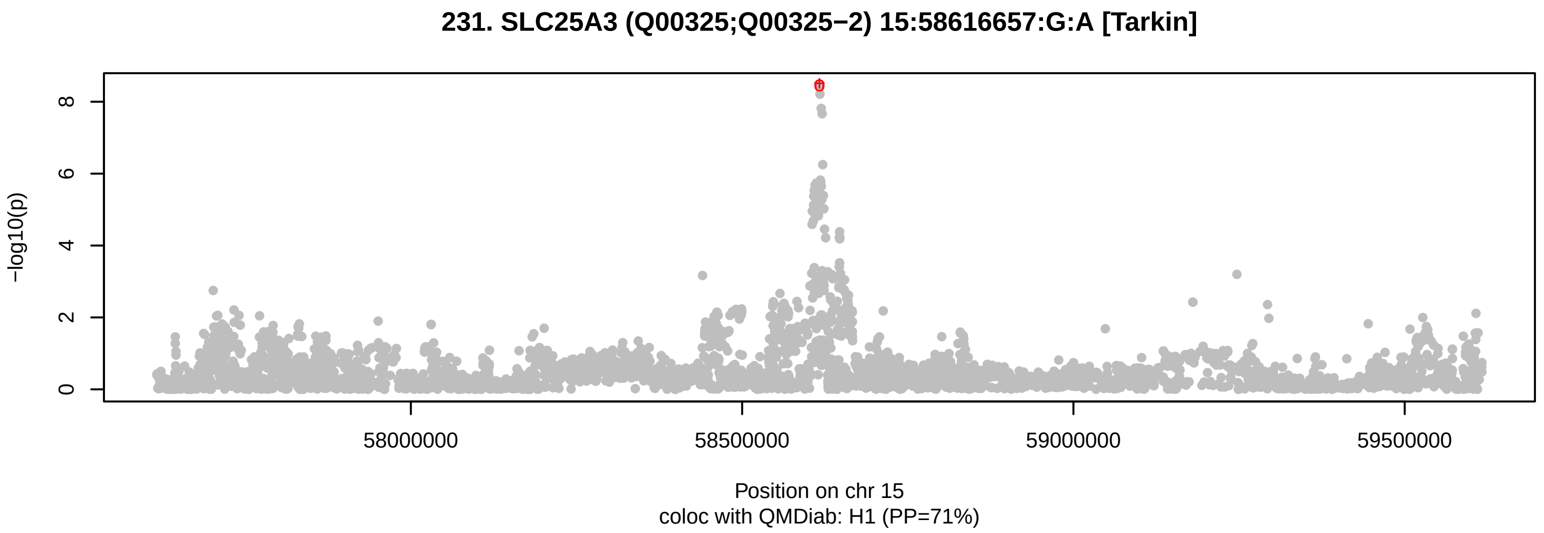

231. SLC25A3 (Q00325;Q00325−2) 15:58616657:G:A [Tarkin]
−log10(p)
0
2
4
6
8
58000000
58500000
59000000
59500000
Position on chr 15
coloc with QMDiab: H1 (PP=71%)

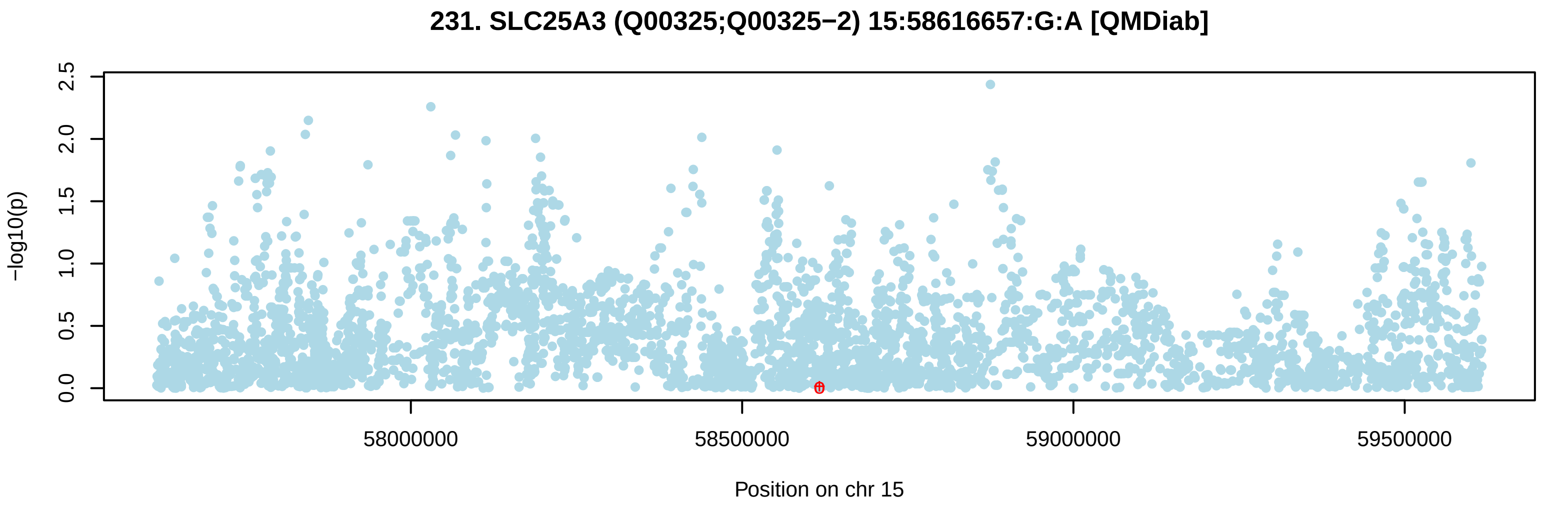

231. SLC25A3 (Q00325;Q00325−2) 15:58616657:G:A [QMDiab]
−log10(p)
0.0
0.5
1.0
1.5
2.0
2.5
58000000
58500000
59000000
59500000
Position on chr 15

232. DDX6 (P26196) 11:11391914:A:C [Tarkin]

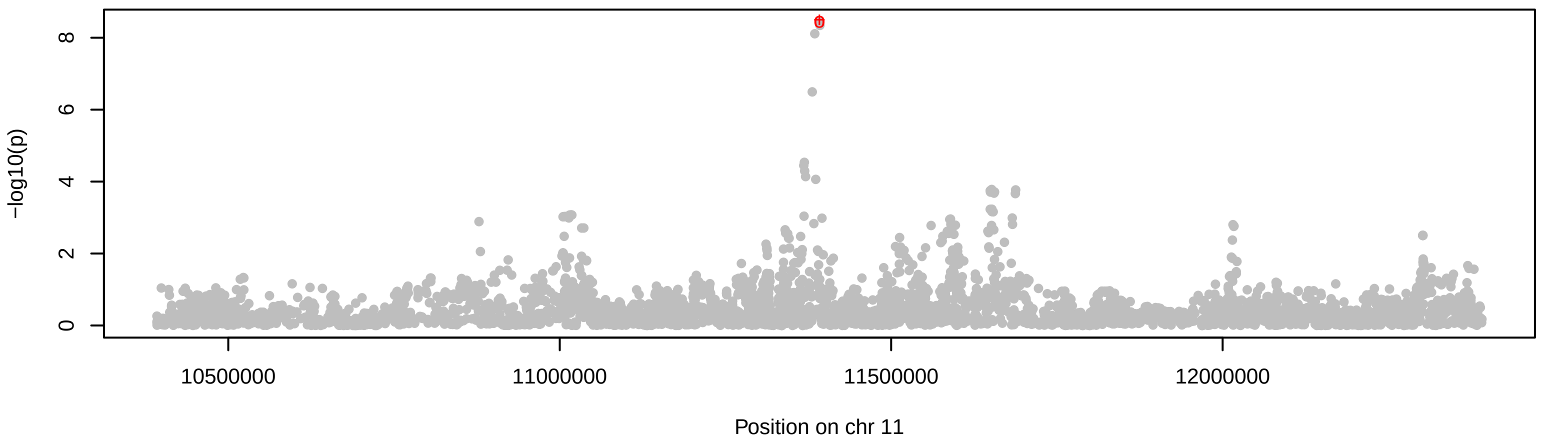

coloc with QMDiab: H1 (PP=66%)

232. DDX6 (P26196) 11:11391914:A:C [QMDiab]

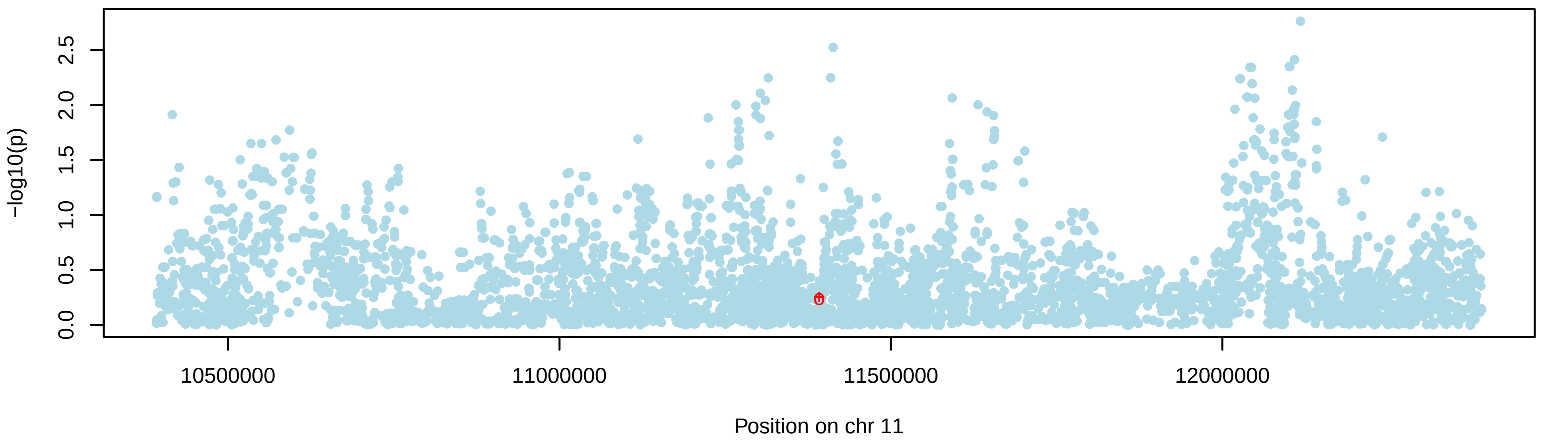

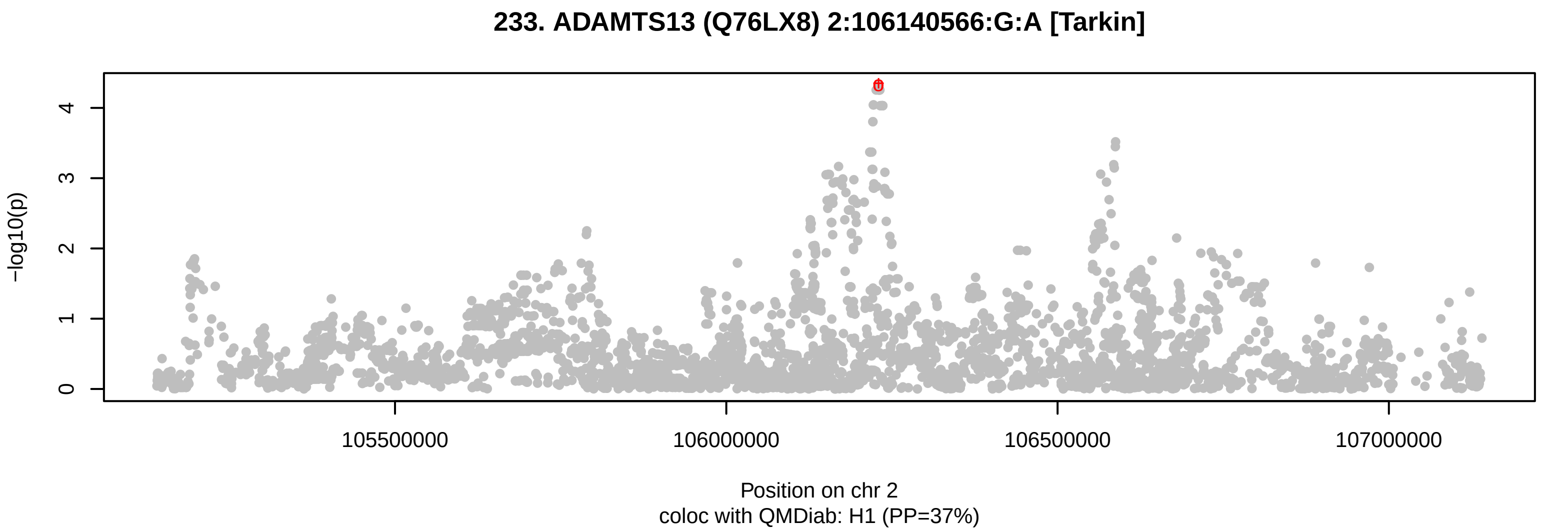

coloc with QMDiab: H1 (PP=37%)

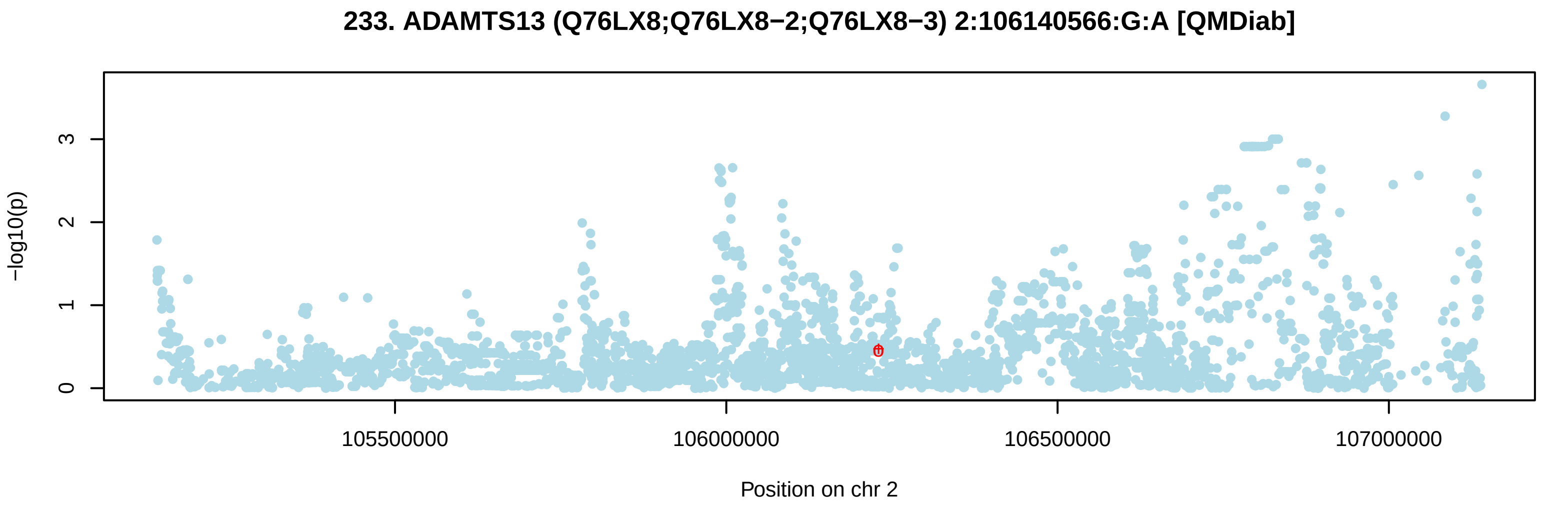

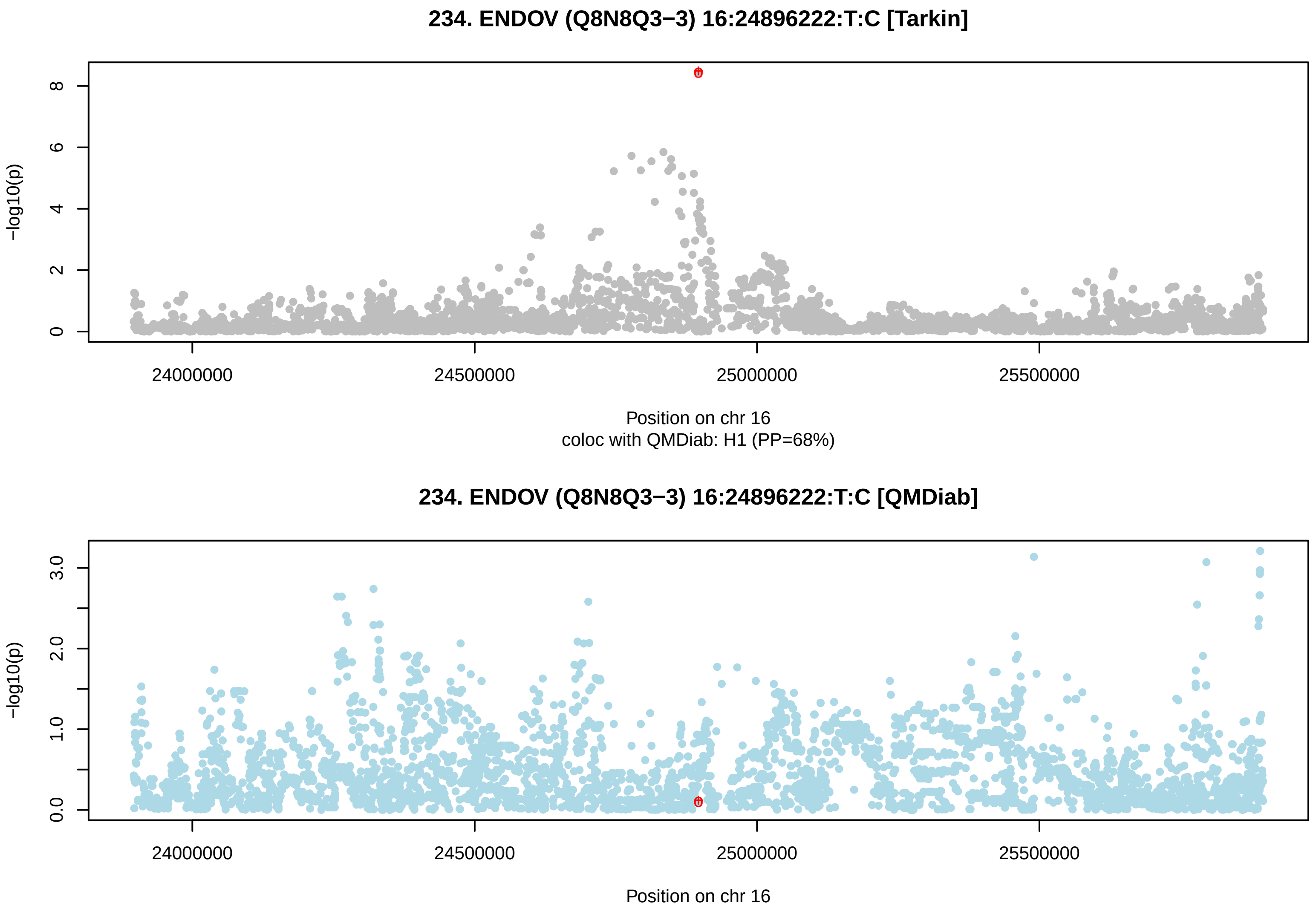
234. ENDOV (Q8N8Q3−3) 16:24896222:T:C [Tarkin]
−log10(p)
0
2
4
6
8
24000000
24500000
25000000
25500000
Position on chr 16
coloc with QMDiab: H1 (PP=68%)
234. ENDOV (Q8N8Q3−3) 16:24896222:T:C [QMDiab]
−log10(p)
0.0
1.0
2.0
3.0
24000000
24500000
25000000
25500000
Position on chr 16

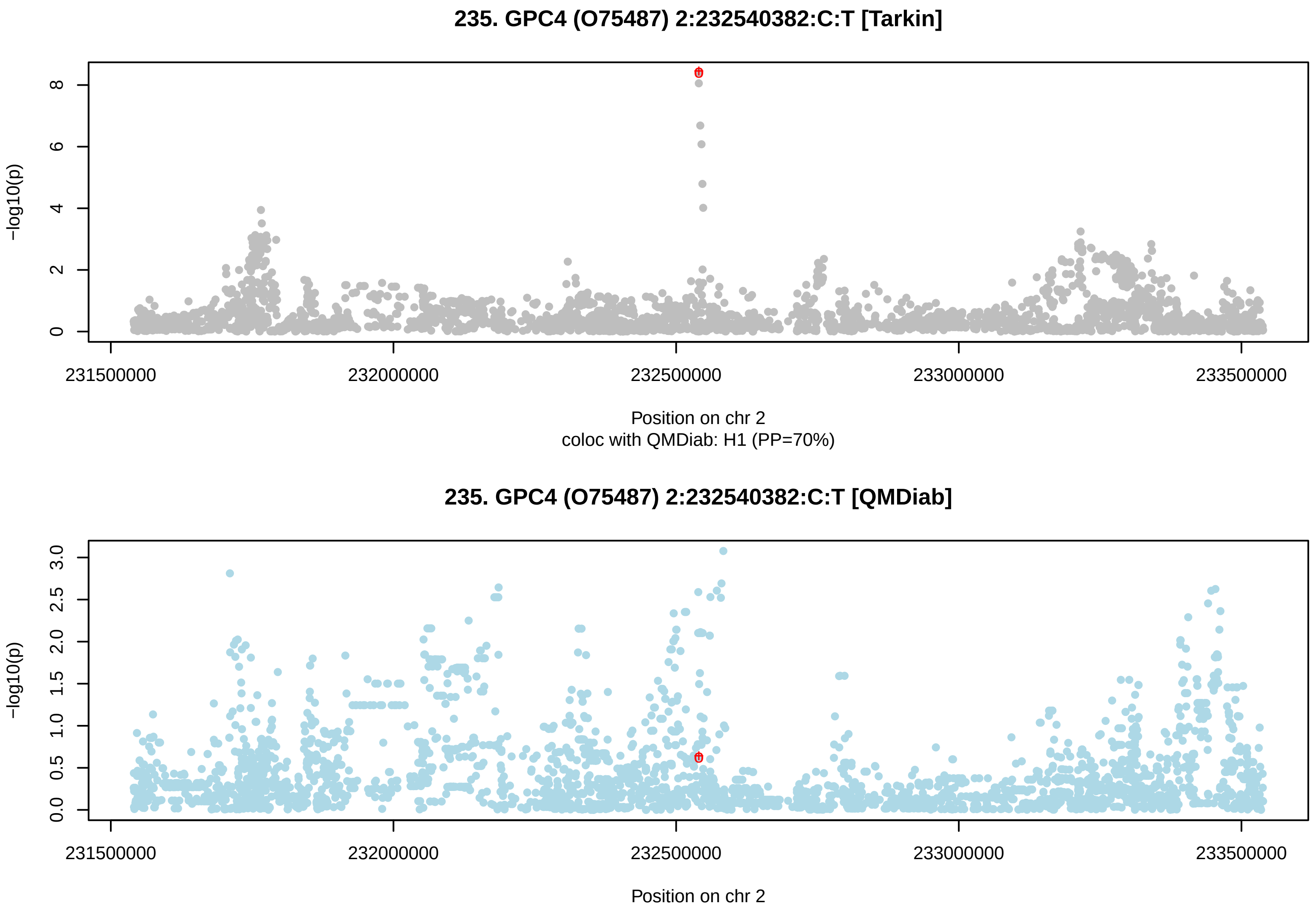
235. GPC4 (O75487) 2:232540382:C:T [Tarkin]
−log10(p)
0
2
4
6
8
231500000
232000000
232500000
233000000
233500000
Position on chr 2
coloc with QMDiab: H1 (PP=70%)
235. GPC4 (O75487) 2:232540382:C:T [QMDiab]
−log10(p)
0.0
0.5
1.0
1.5
2.0
2.5
3.0
231500000
232000000
232500000
233000000
233500000
Position on chr 2

236. HPCAL1 (P37235) 15:68223040:A:G [Tarkin]

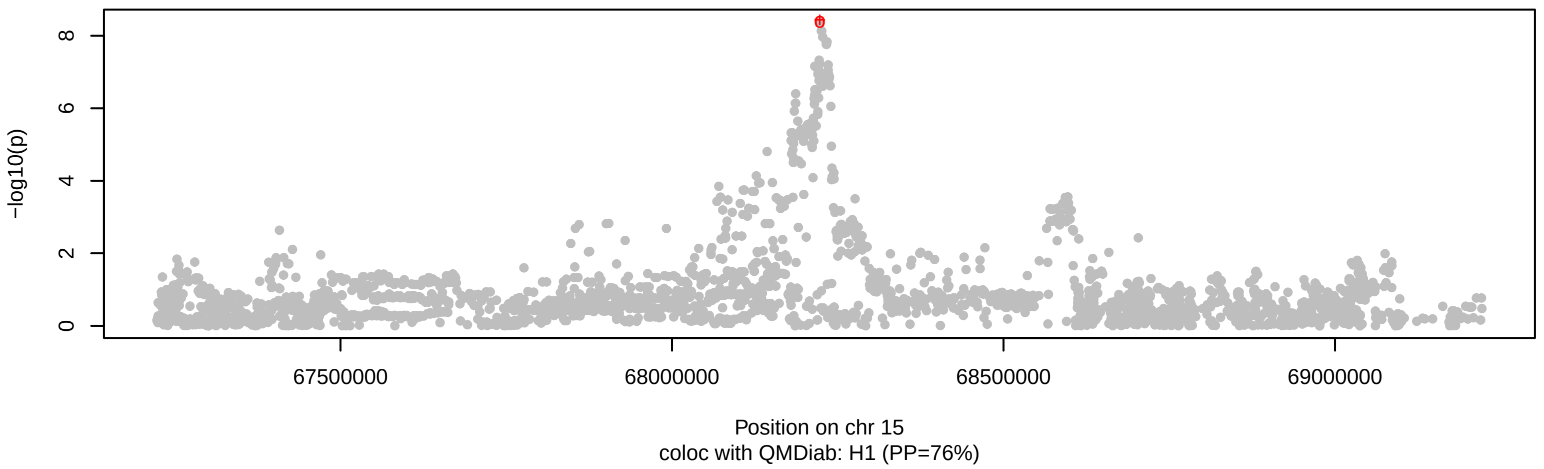

coloc with QMDiab: H1 (PP=76%)

236. HPCAL1 (P37235) 15:68223040:A:G [QMDiab]

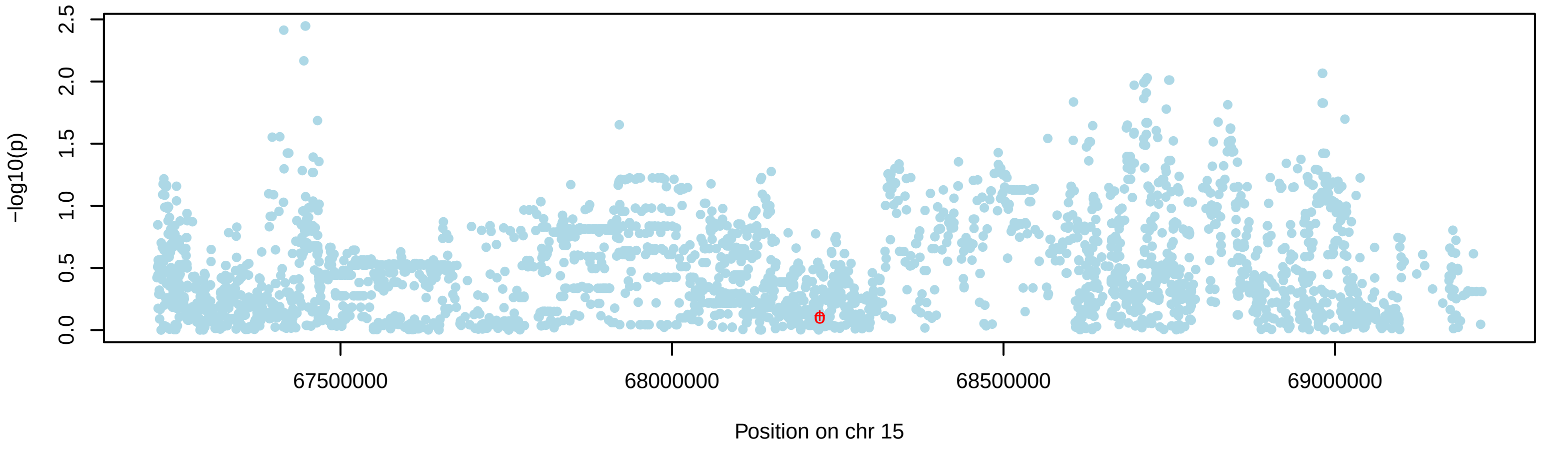

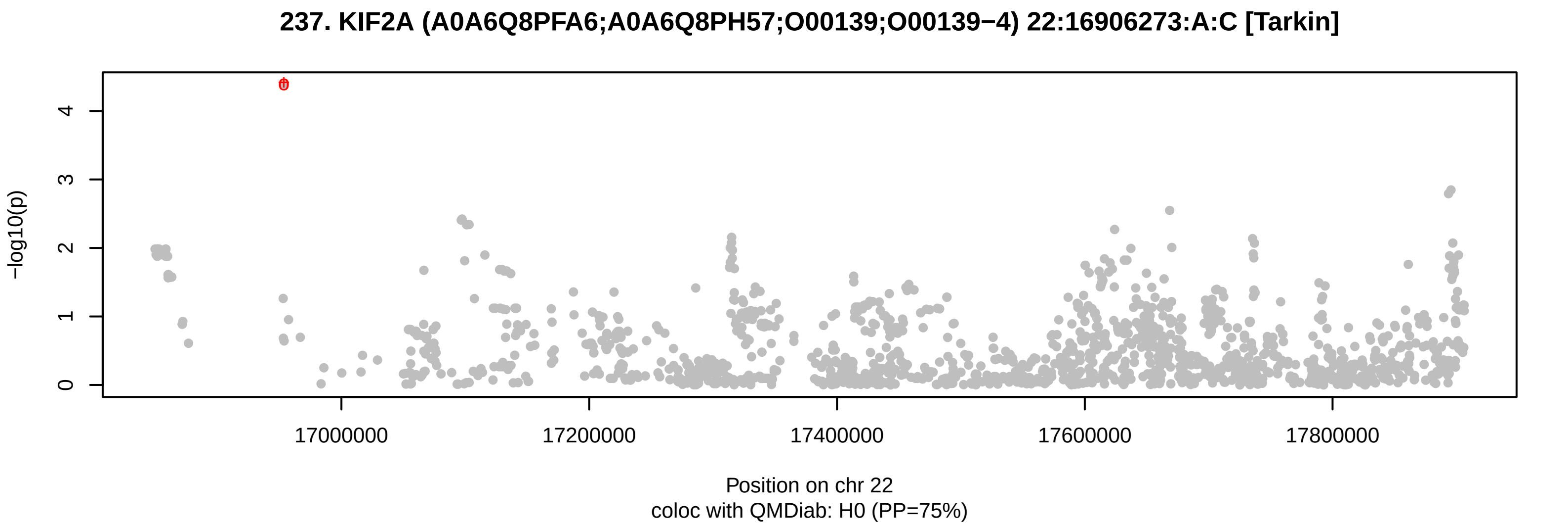
237. KIF2A (A0A6Q8PFA6;A0A6Q8PH57;O00139;O00139−4) 22:16906273:A:C [Tarkin]
−log10(p)
0
1
2
3
4
17000000
17200000
17400000
17600000
17800000
Position on chr 22
coloc with QMDiab: H0 (PP=75%)

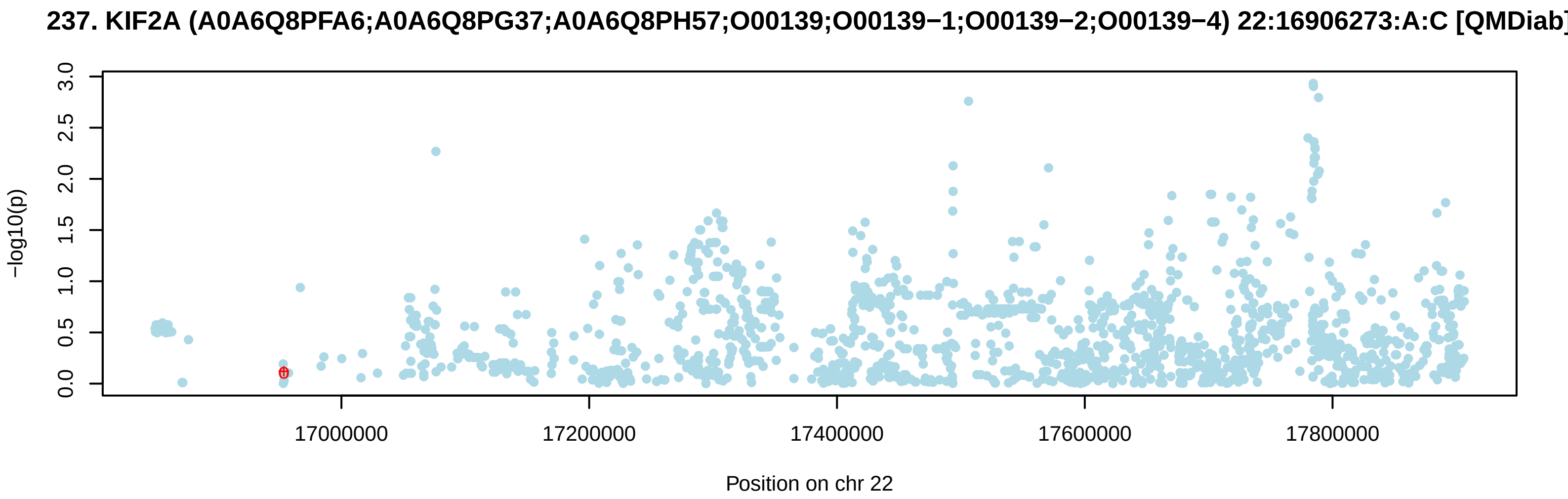
237. KIF2A (A0A6Q8PFA6;A0A6Q8PG37;A0A6Q8PH57;O00139;O00139−1;O00139−2;O00139−4) 22:16906273:A:C [QMDiab]
−log10(p)
0.0
0.5
1.0
1.5
2.0
2.5
3.0
17000000
17200000
17400000
17600000
17800000
Position on chr 22

238. FUCA1 (P04066) 4:52982614:C:T [Tarkin]

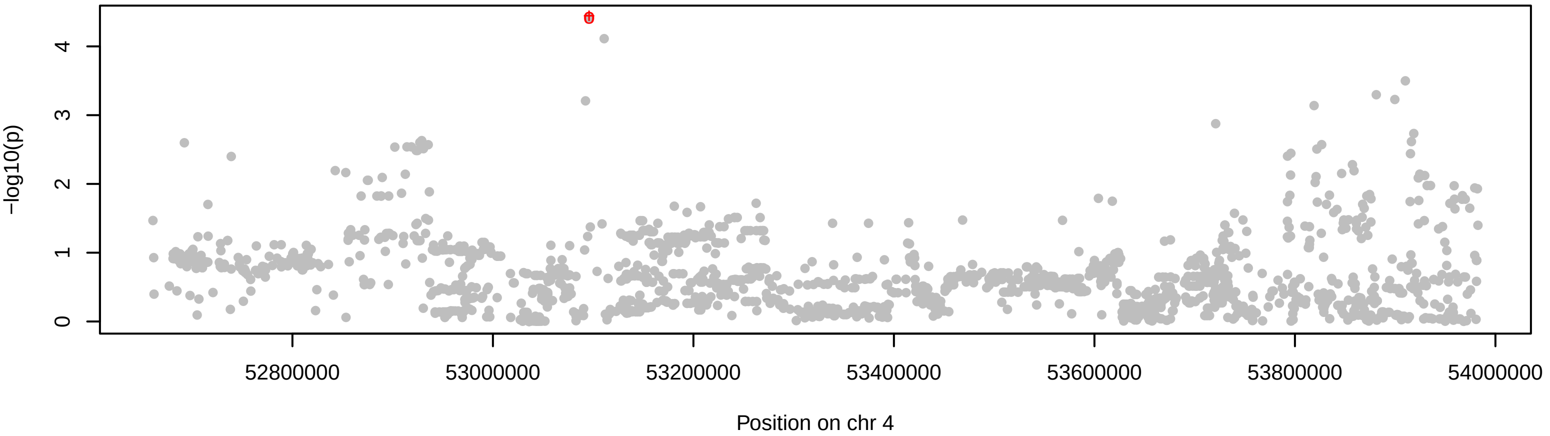

coloc with QMDiab: H0 (PP=69%)

238. FUCA1 (P04066) 4:52982614:C:T [QMDiab]

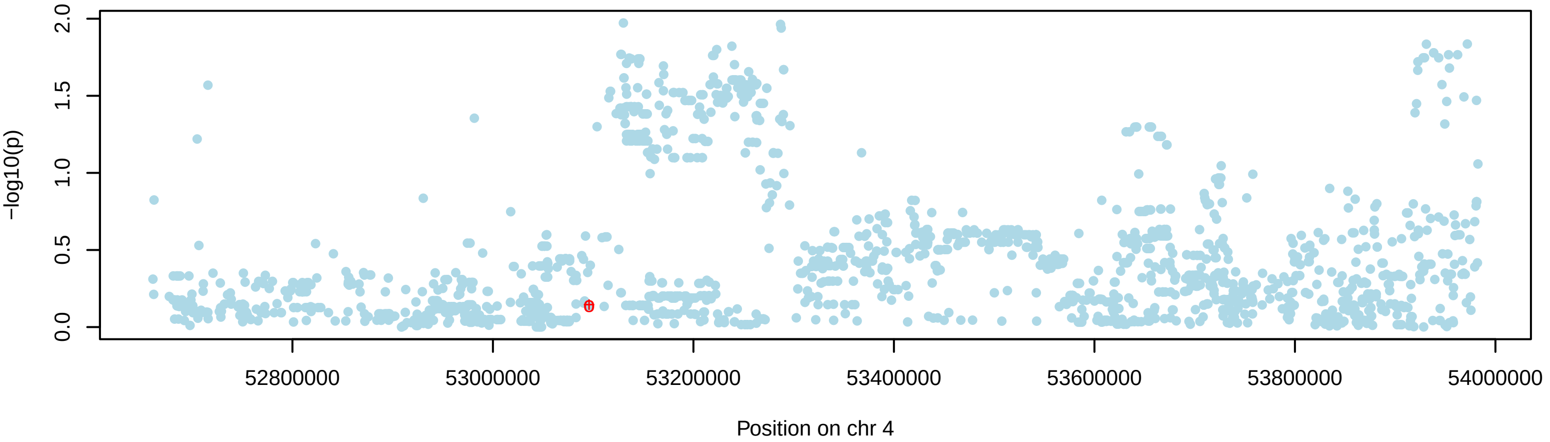

239. CDH6 (P55285) 17:26633616:C:T [Tarkin]

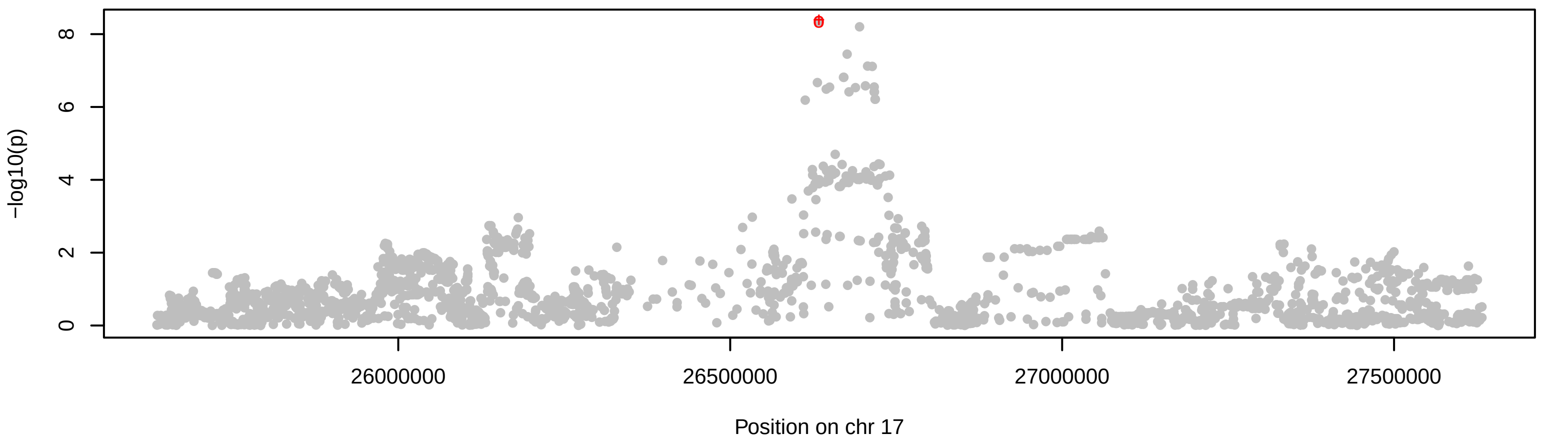

coloc with QMDiab: H1 (PP=69%)

239. CDH6 (P55285) 17:26633616:C:T [QMDiab]

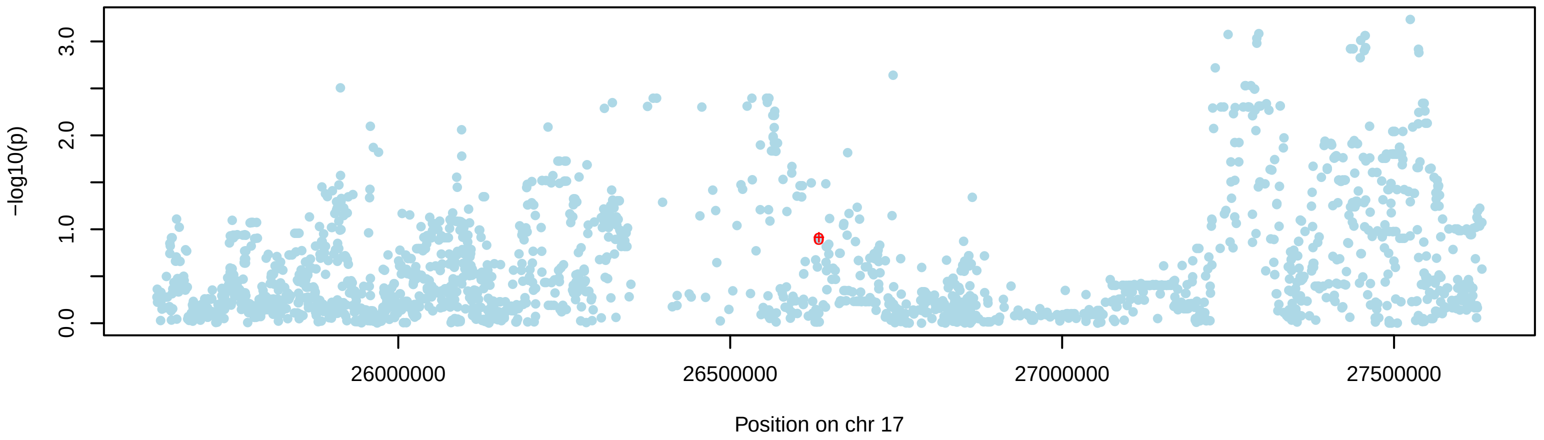

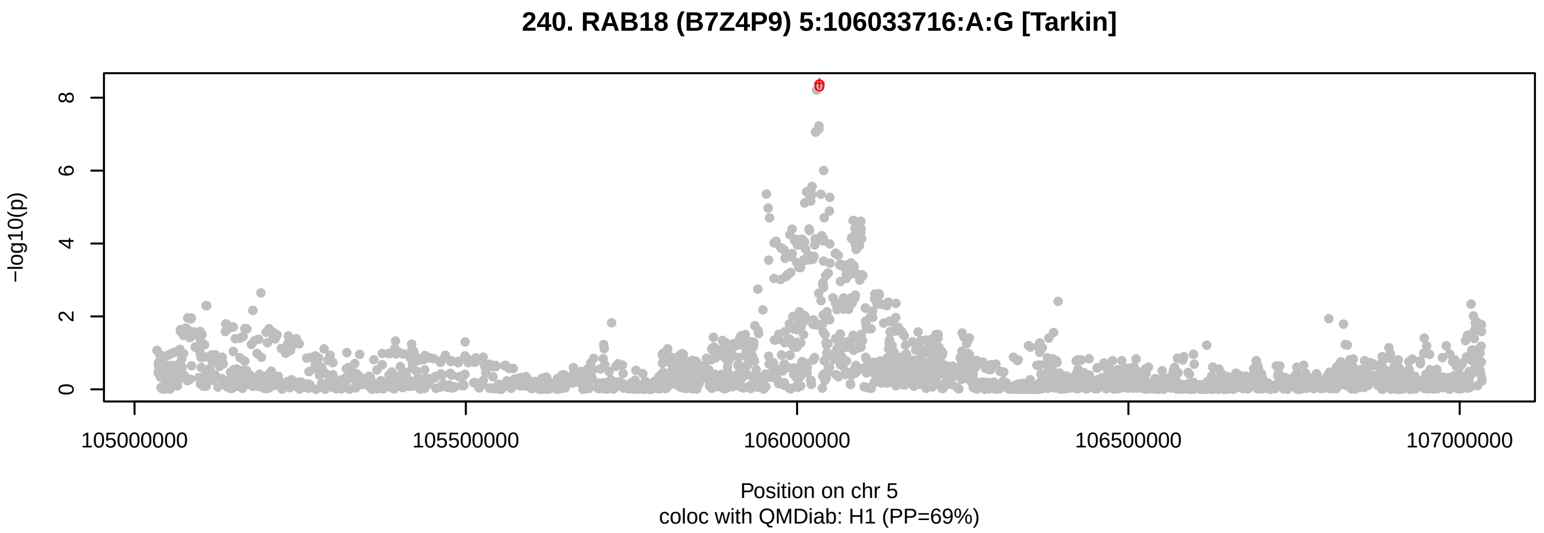
240. RAB18 (B7Z4P9) 5:106033716:A:G [Tarkin]
−log10(p)
0
2
4
6
8
105000000
105500000
106000000
106500000
107000000
Position on chr 5
coloc with QMDiab: H1 (PP=69%)

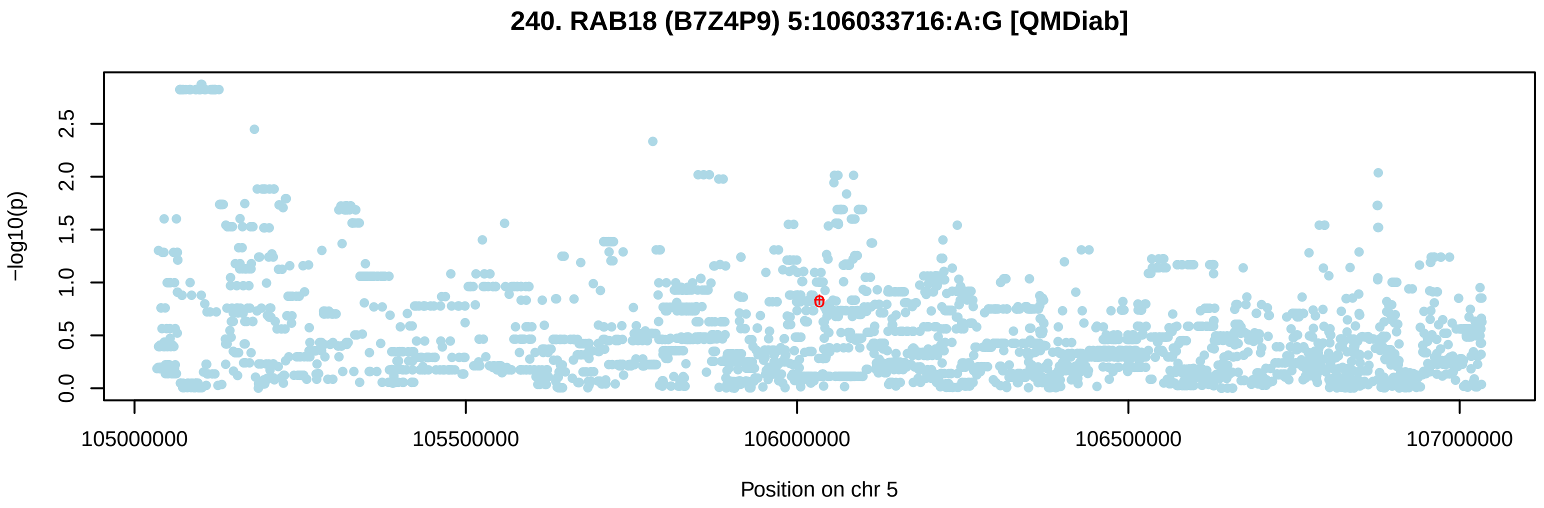
240. RAB18 (B7Z4P9) 5:106033716:A:G [QMDiab]
−log10(p)
0.0
0.5
1.0
1.5
2.0
2.5
105000000
105500000
106000000
106500000
107000000
Position on chr 5

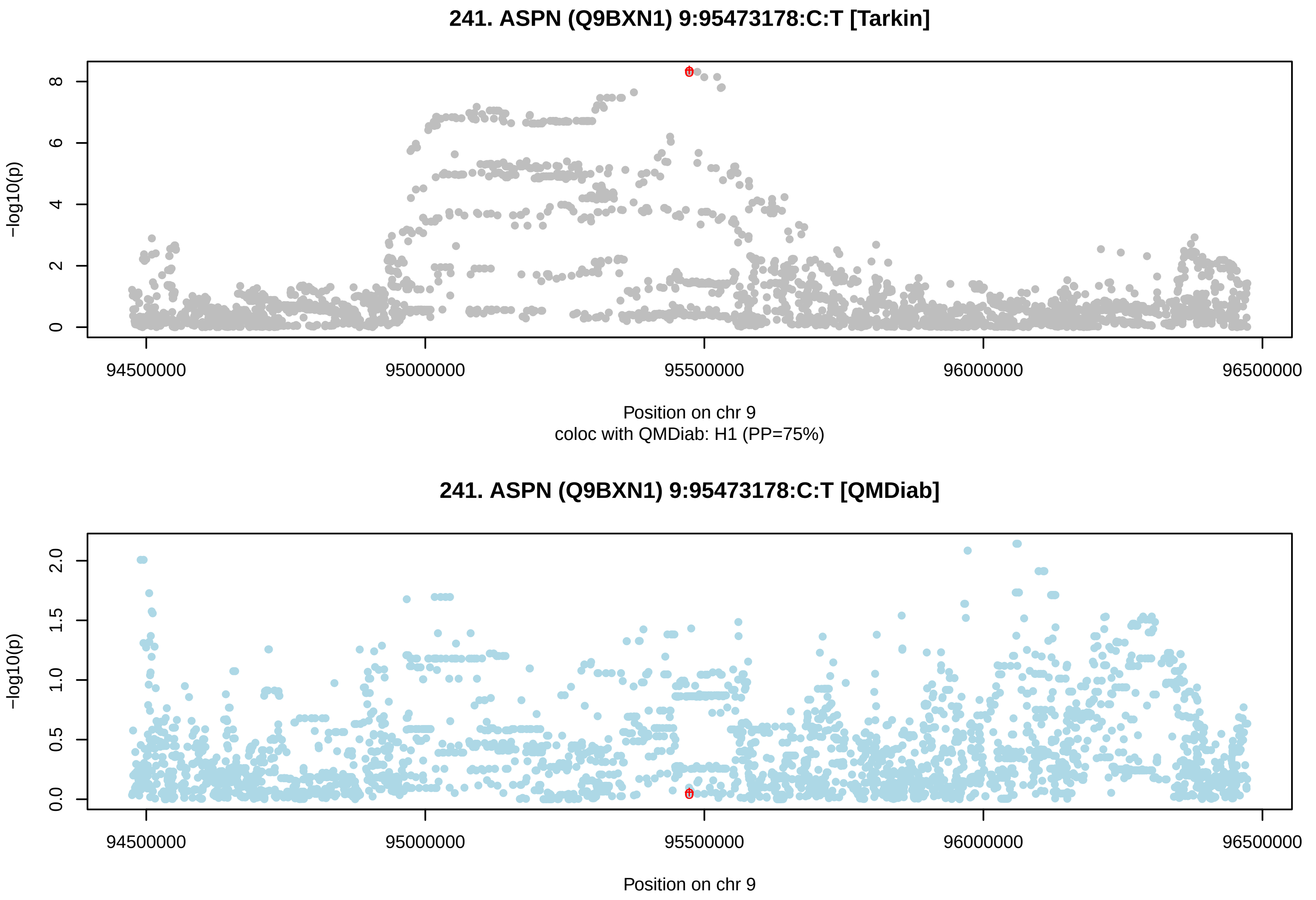
241. ASPN (Q9BXN1) 9:95473178:C:T [Tarkin]
−log10(p)
0
2
4
6
8
94500000
95000000
95500000
96000000
96500000
Position on chr 9
coloc with QMDiab: H1 (PP=75%)
241. ASPN (Q9BXN1) 9:95473178:C:T [QMDiab]
−log10(p)
0.0
0.5
1.0
1.5
2.0
94500000
95000000
95500000
96000000
96500000
Position on chr 9

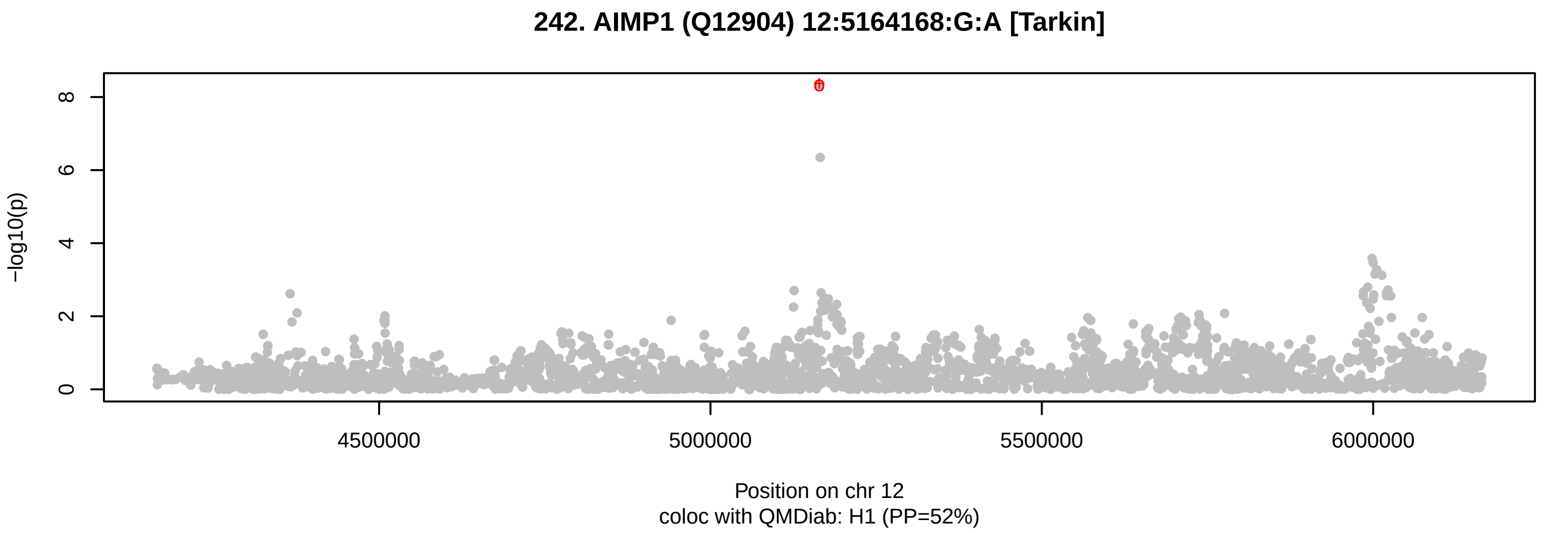
242. AIMP1 (Q12904) 12:5164168:G:A [Tarkin]
−log10(p)
0
2
4
6
8
4500000
5000000
5500000
6000000
Position on chr 12
coloc with QMDiab: H1 (PP=52%)

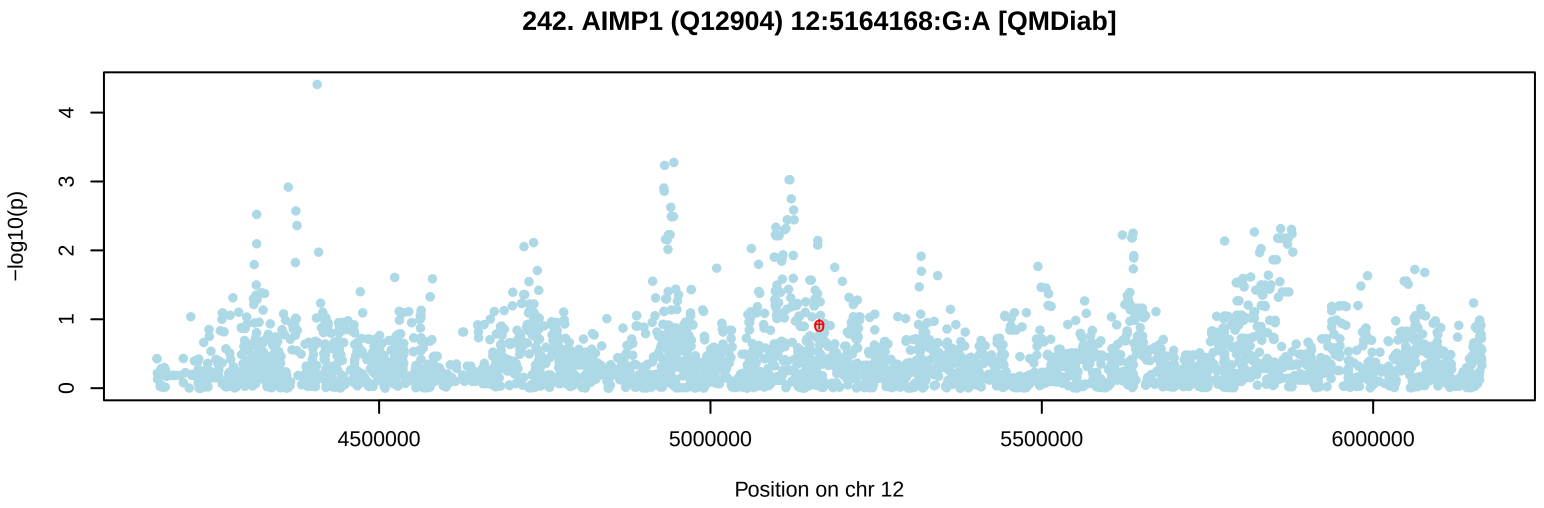
242. AIMP1 (Q12904) 12:5164168:G:A [QMDiab]
−log10(p)
0
1
2
3
4
4500000
5000000
5500000
6000000
Position on chr 12

243. BMP10 (O95393) 17:26694861:G:A [Tarkin]

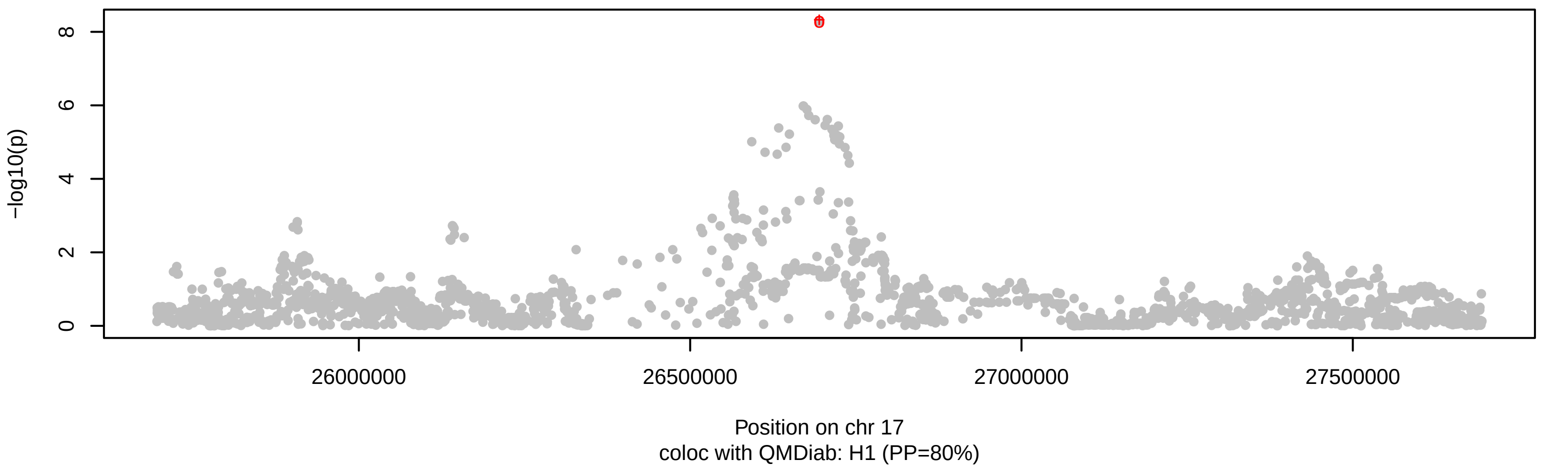

coloc with QMDiab: H1 (PP=80%)

243. BMP10 (O95393) 17:26694861:G:A [QMDiab]

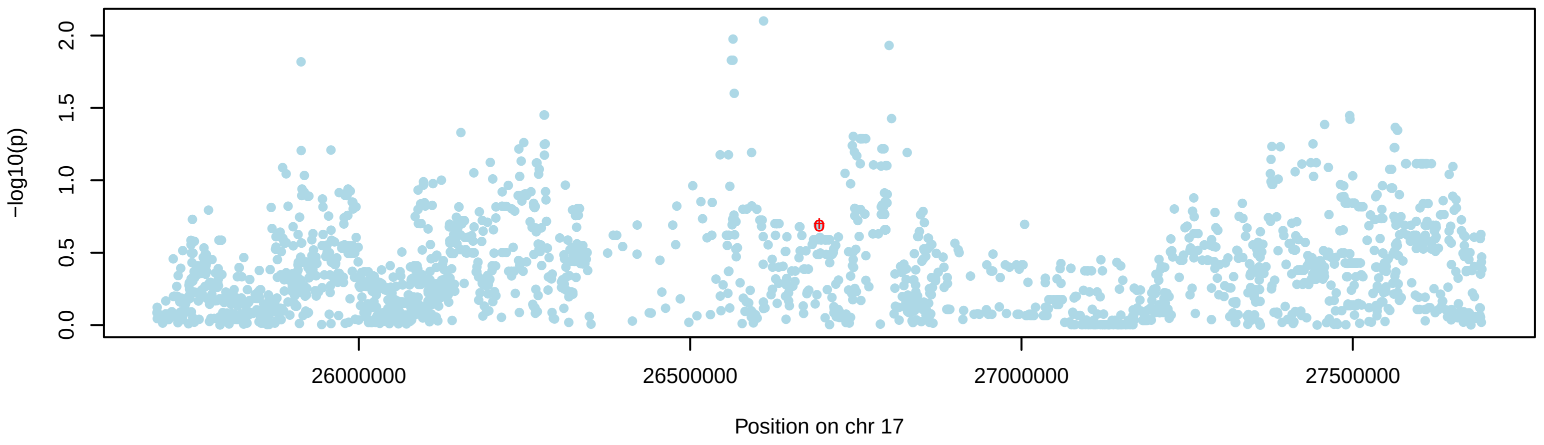

244. C19orf80 (Q6UXH0) 5:120675892:T:C [Tarkin]

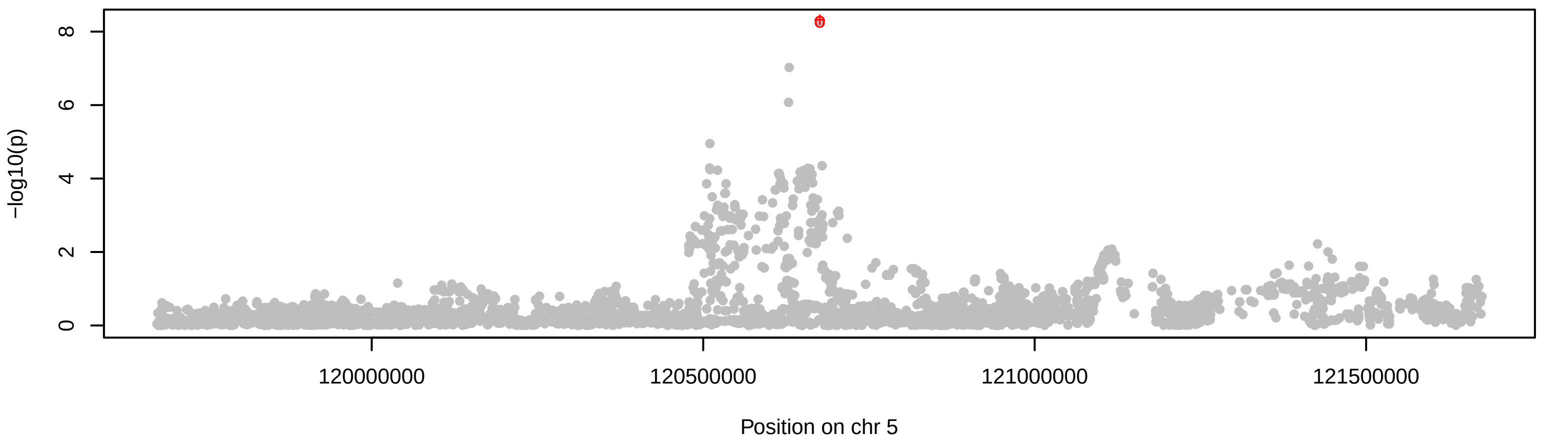

coloc with QMDiab: H1 (PP=69%)

244. C19orf80 (Q6UXH0) 5:120675892:T:C [QMDiab]

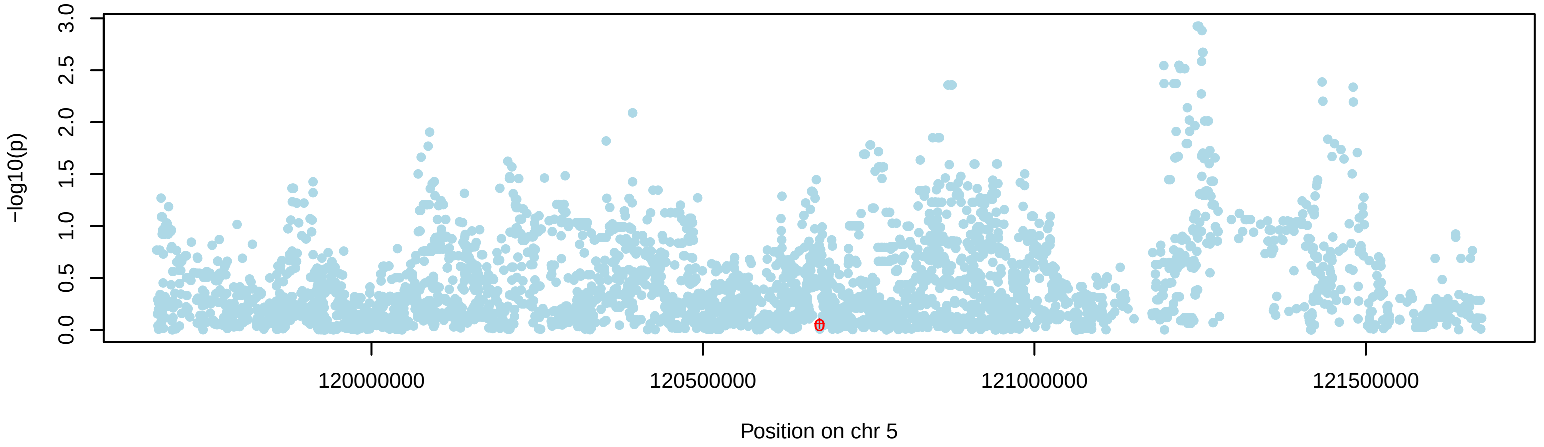

245. PSMF1 (Q5QPM7;Q92530) 20:1099294:T:C [Tarkin]

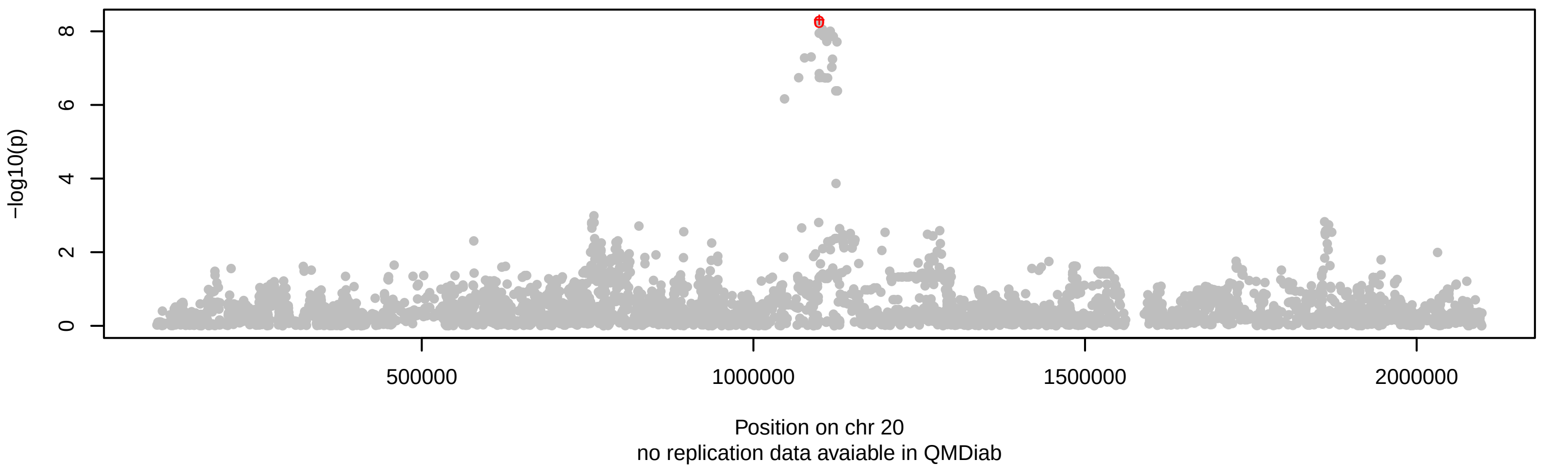

no replication data avaiable in QMDiab

246. RPS18 (P62269) 3:158595196:A:G [Tarkin]

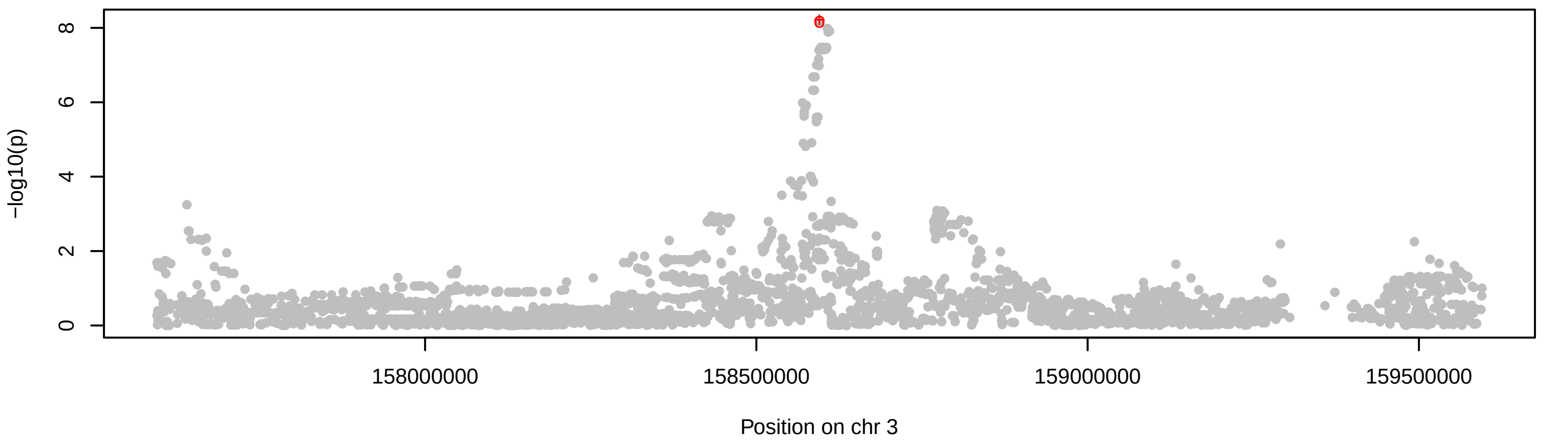

coloc with QMDiab: H1 (PP=68%)

246. RPS18 (P62269) 3:158595196:A:G [QMDiab]

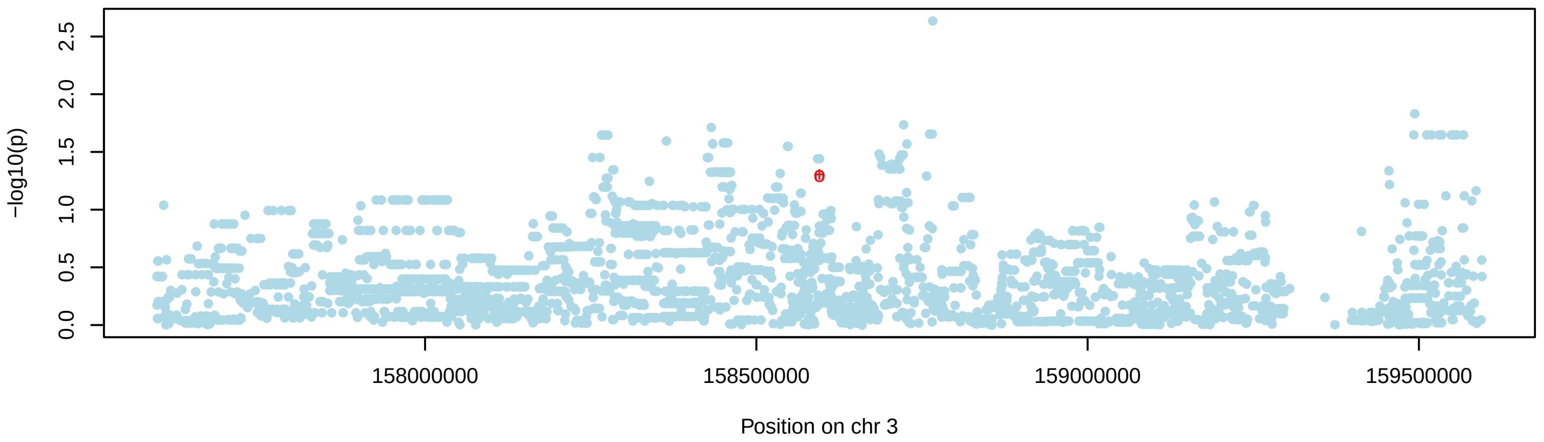

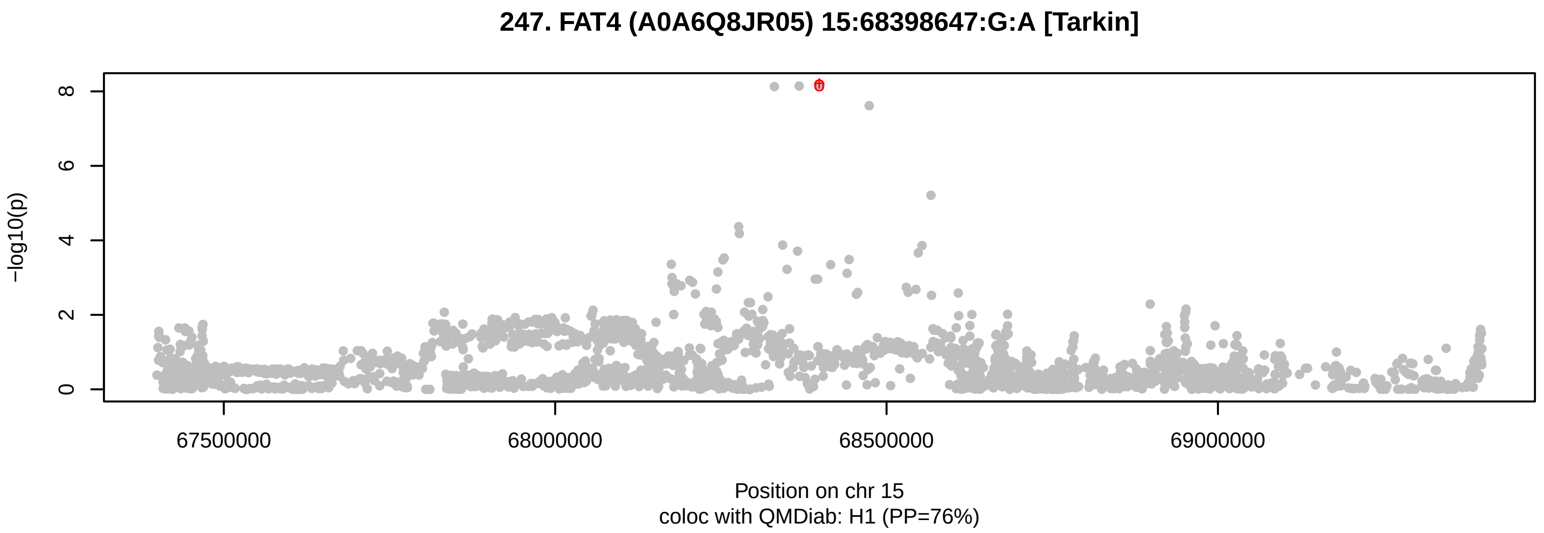
247. FAT4 (A0A6Q8JR05) 15:68398647:G:A [Tarkin]
−log10(p)
0
2
4
6
8
67500000
68000000
68500000
69000000
Position on chr 15
coloc with QMDiab: H1 (PP=76%)

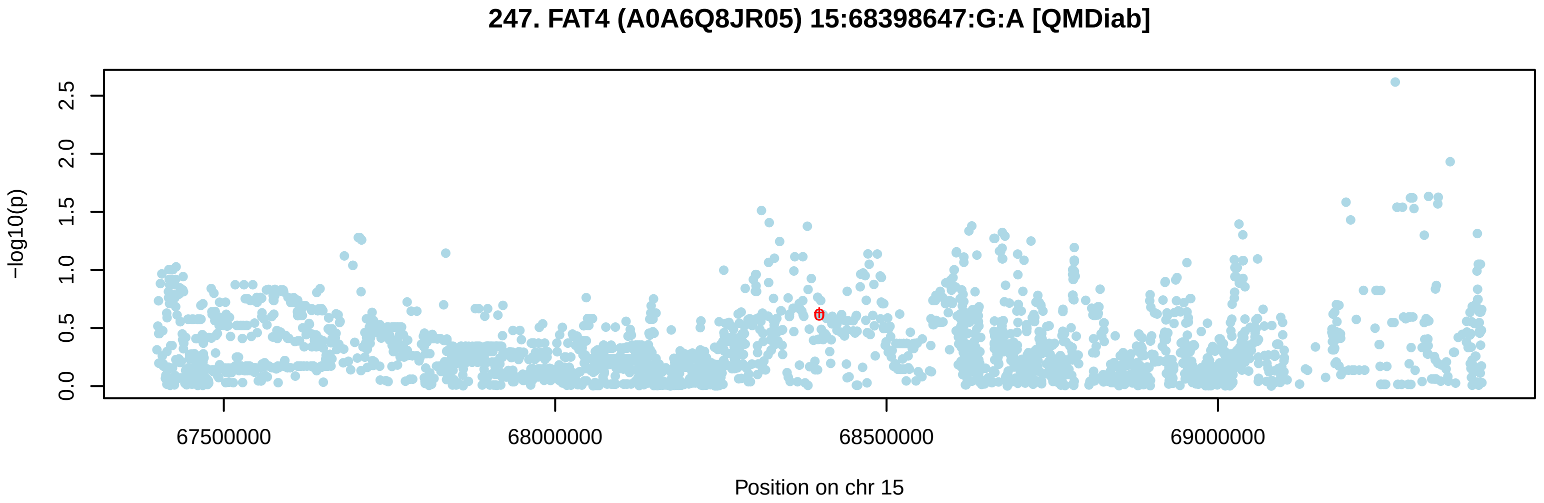
247. FAT4 (A0A6Q8JR05) 15:68398647:G:A [QMDiab]
−log10(p)
0.0
0.5
1.0
1.5
2.0
2.5
67500000
68000000
68500000
69000000
Position on chr 15

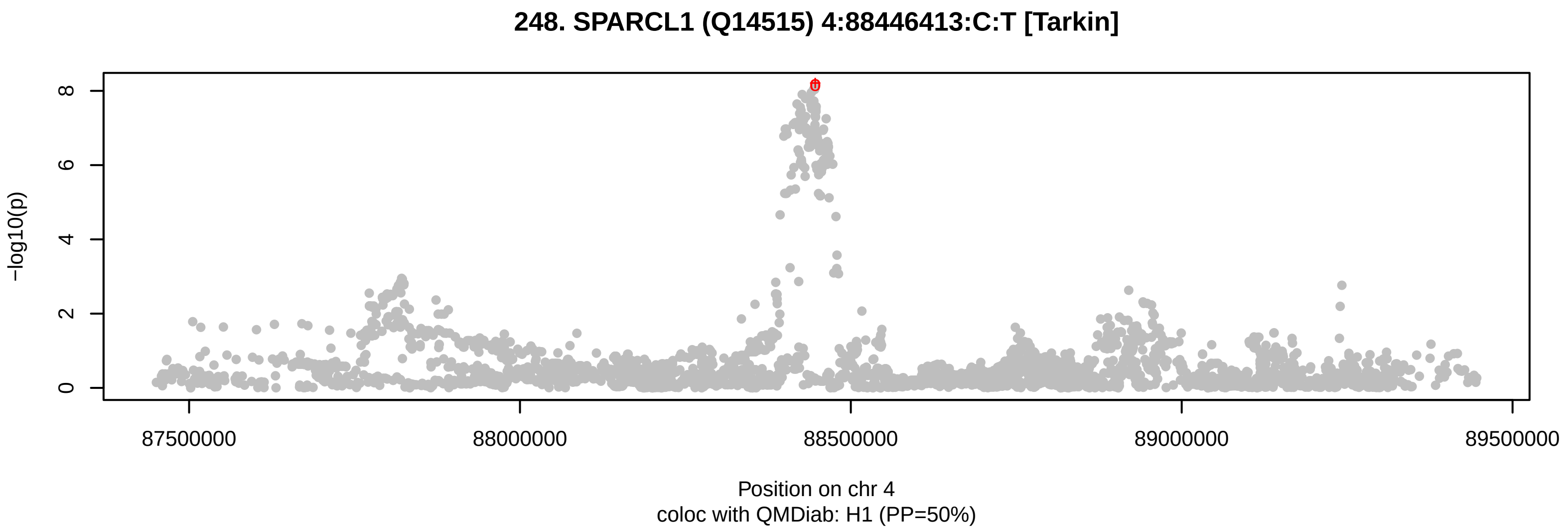
248. SPARCL1 (Q14515) 4:88446413:C:T [Tarkin]
−log10(p)
0
2
4
6
8
87500000
88000000
88500000
89000000
89500000
Position on chr 4
coloc with QMDiab: H1 (PP=50%)

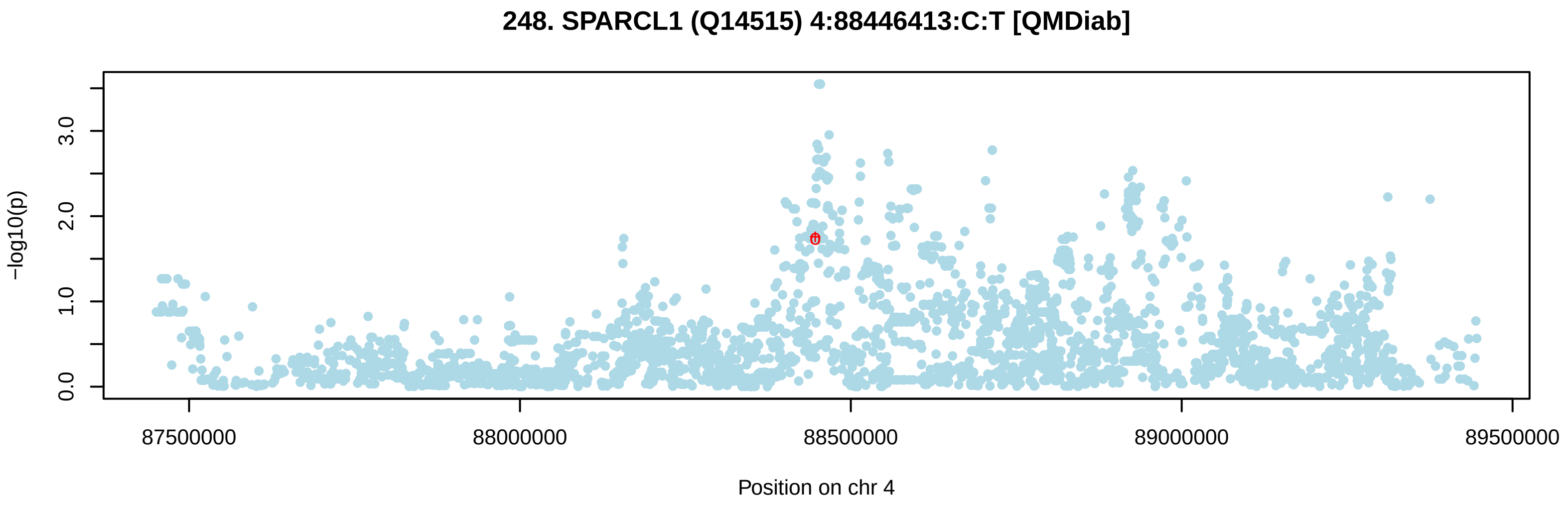
248. SPARCL1 (Q14515) 4:88446413:C:T [QMDiab]
−log10(p)
0.0
1.0
2.0
3.0
87500000
88000000
88500000
89000000
89500000
Position on chr 4

249. PGM3 (H0Y987) 20:58981121:A:G [Tarkin]

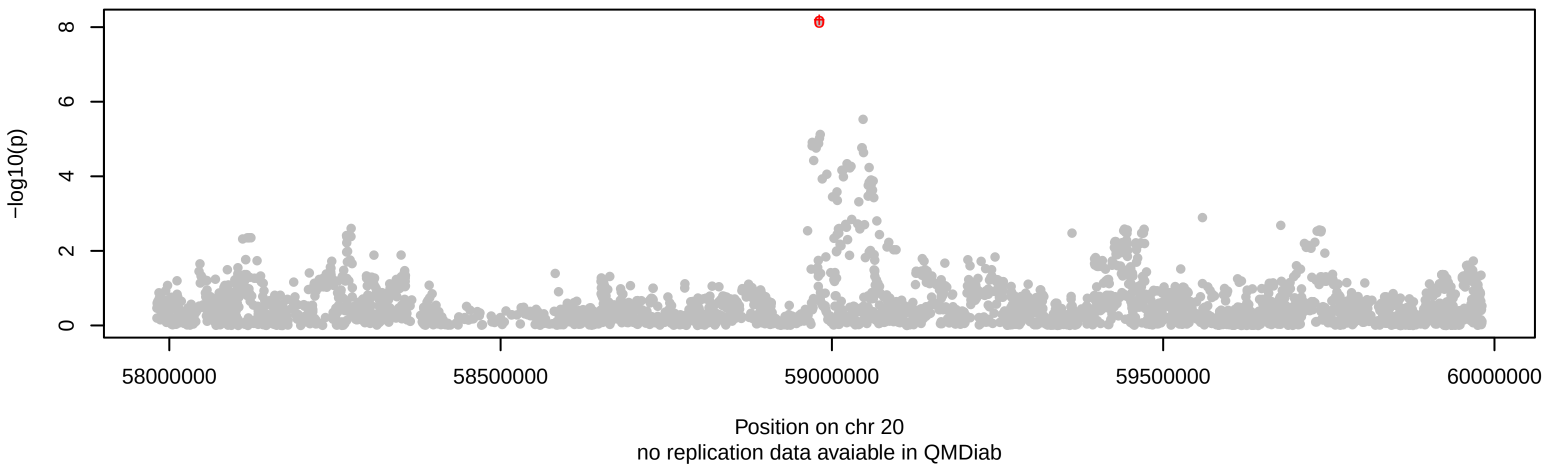

250. CALCOCO1 (Q9P1Z2;Q9P1Z2−2;Q9P1Z2−3) 9:21956078:A:G [Tarkin]

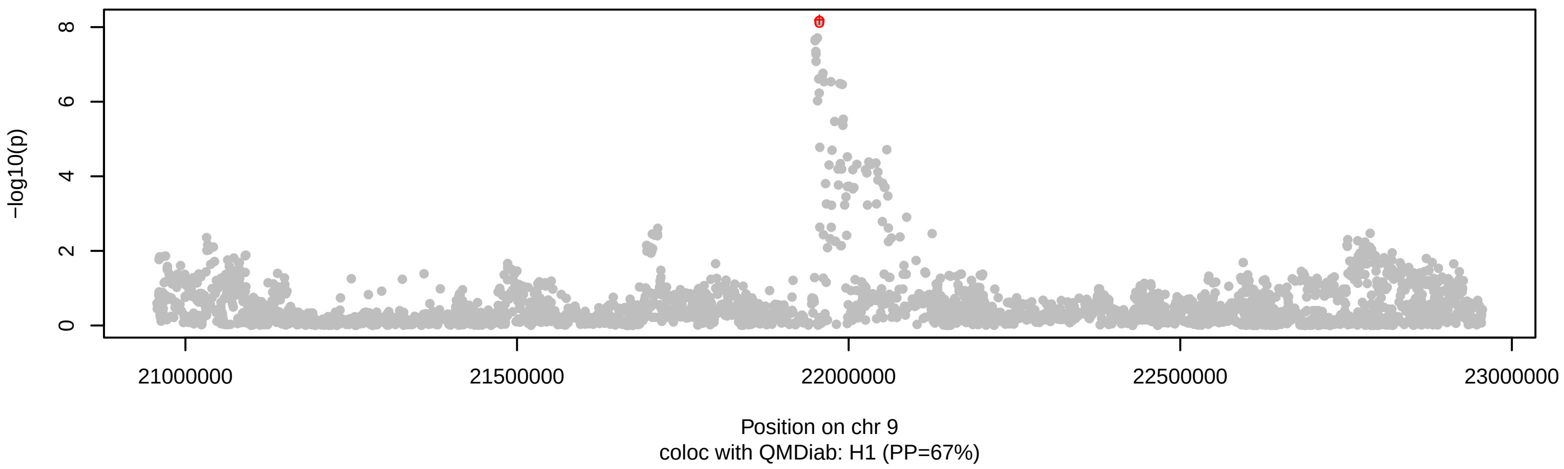

250. CALCOCO1 (Q9P1Z2;Q9P1Z2−2;Q9P1Z2−3) 9:21956078:A:G [QMDiab]

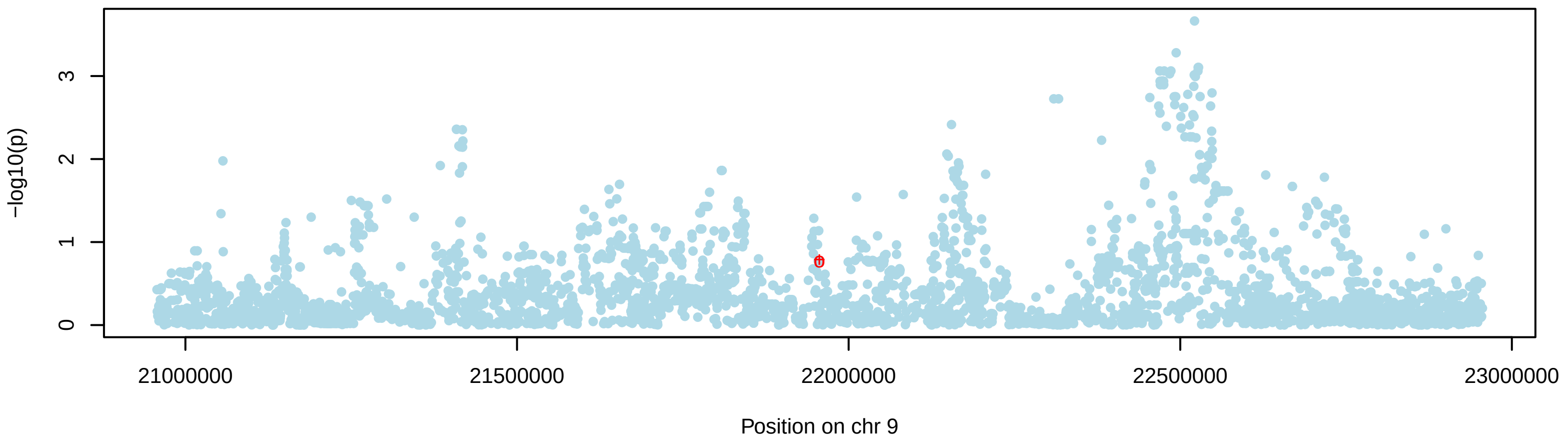

251. TTBK1 (X6R456) 17:26694861:G:A [Tarkin]

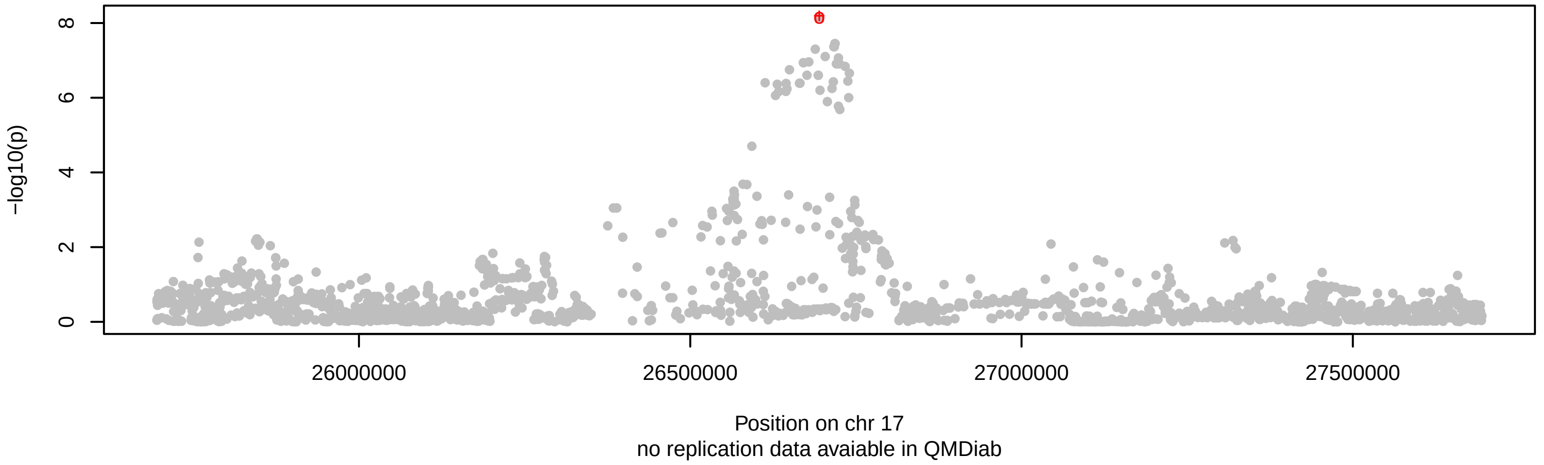

no replication data avaiable in QMDiab

252. DNAJA4 (Q8WW22−2) 6:35610681:T:C [Tarkin]

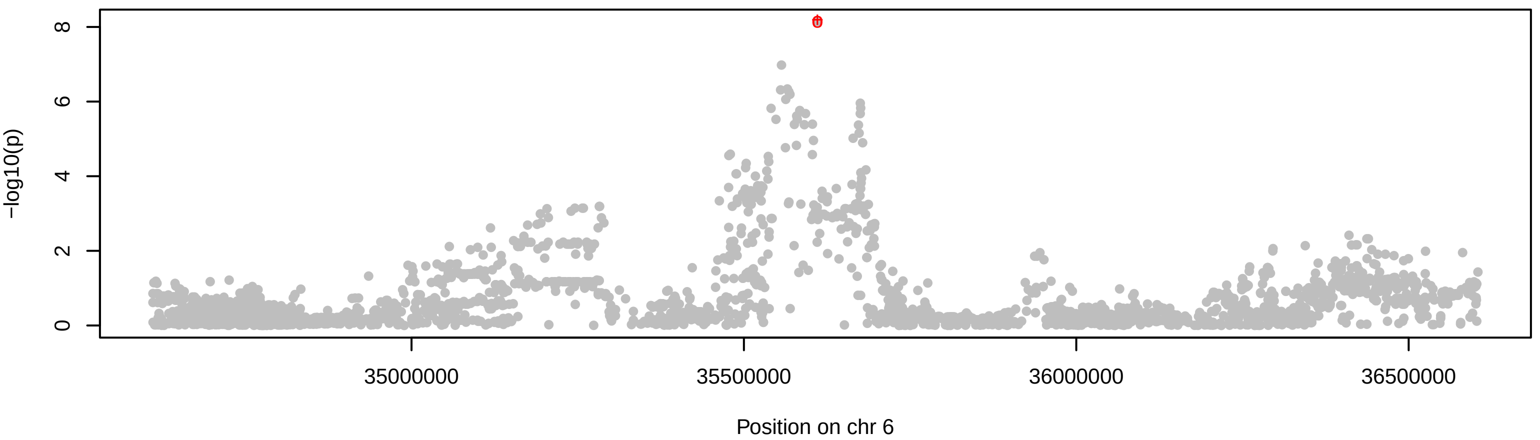

coloc with QMDiab: H1 (PP=72%)

252. DNAJA4 (Q8WW22;Q8WW22−2) 6:35610681:T:C [QMDiab]

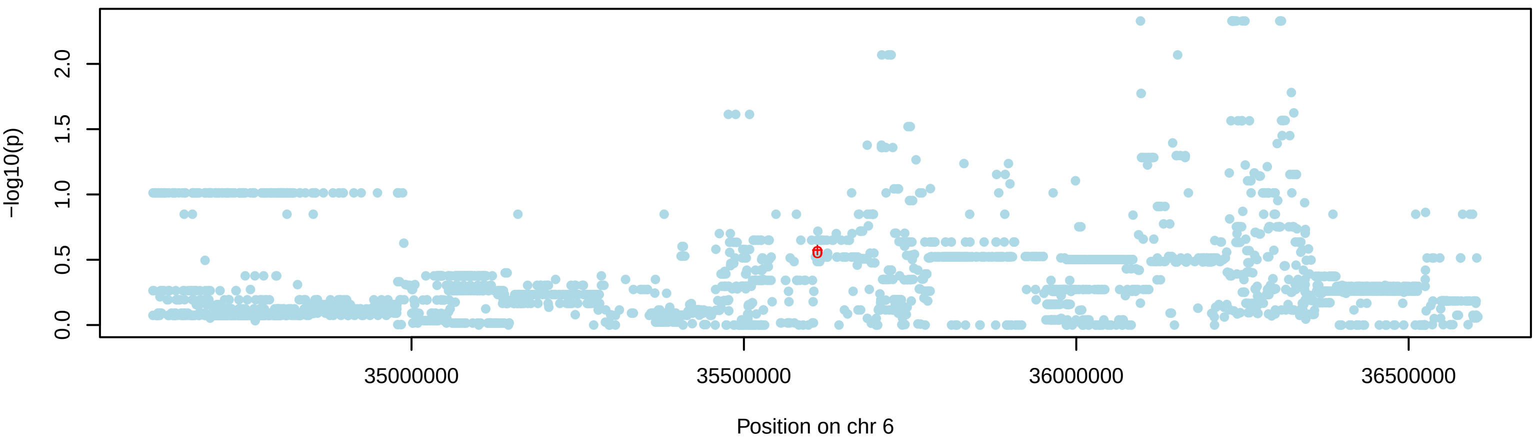

253. IGHV4−34 (P06331) 14:107117735:G:T [Tarkin]

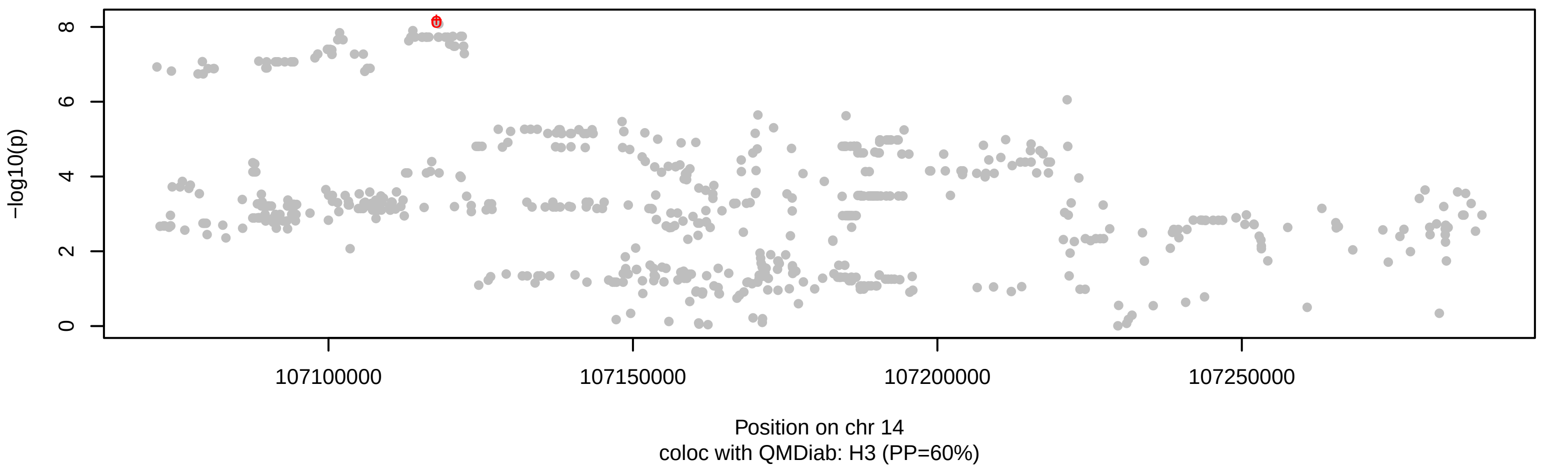

coloc with QMDiab: H3 (PP=60%)

253. IGHV4−34 (P06331) 14:107117735:G:T [QMDiab]

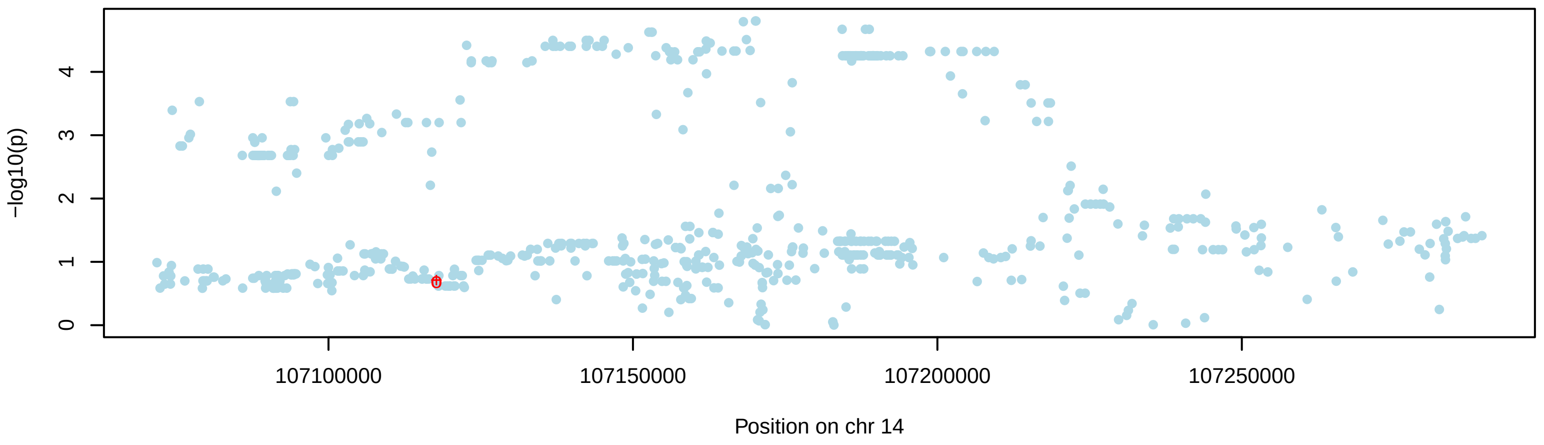

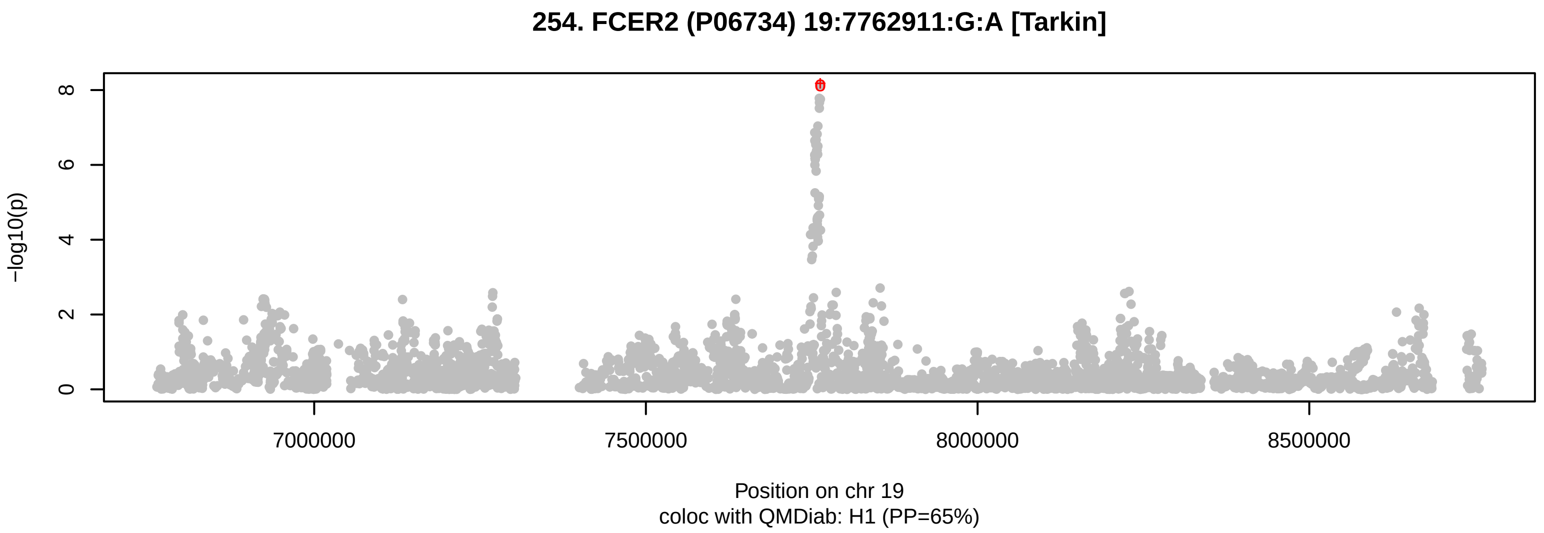

254. FCER2 (P06734) 19:7762911:G:A [Tarkin]
−log10(p)
0
2
4
6
8
7000000
7500000
8000000
8500000
Position on chr 19
coloc with QMDiab: H1 (PP=65%)

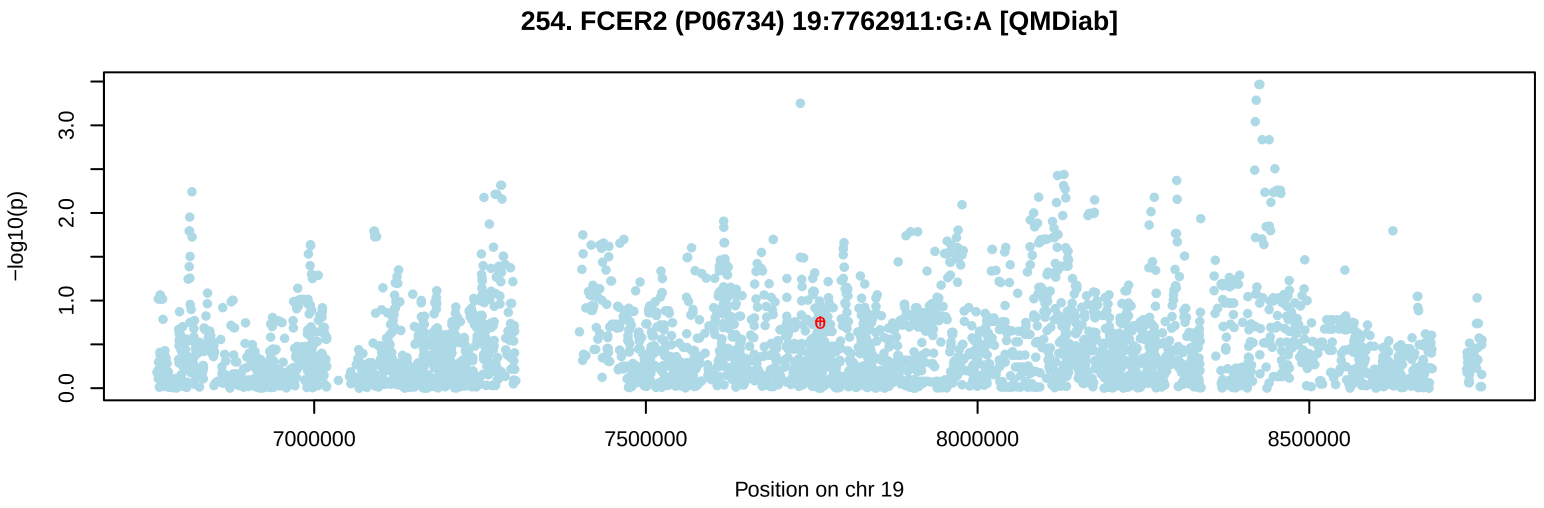

254. FCER2 (P06734) 19:7762911:G:A [QMDiab]
−log10(p)
0.0
1.0
2.0
3.0
7000000
7500000
8000000
8500000
Position on chr 19

255. STX7 (O15400;O15400−2) 6:132830944:A:G [Tarkin]

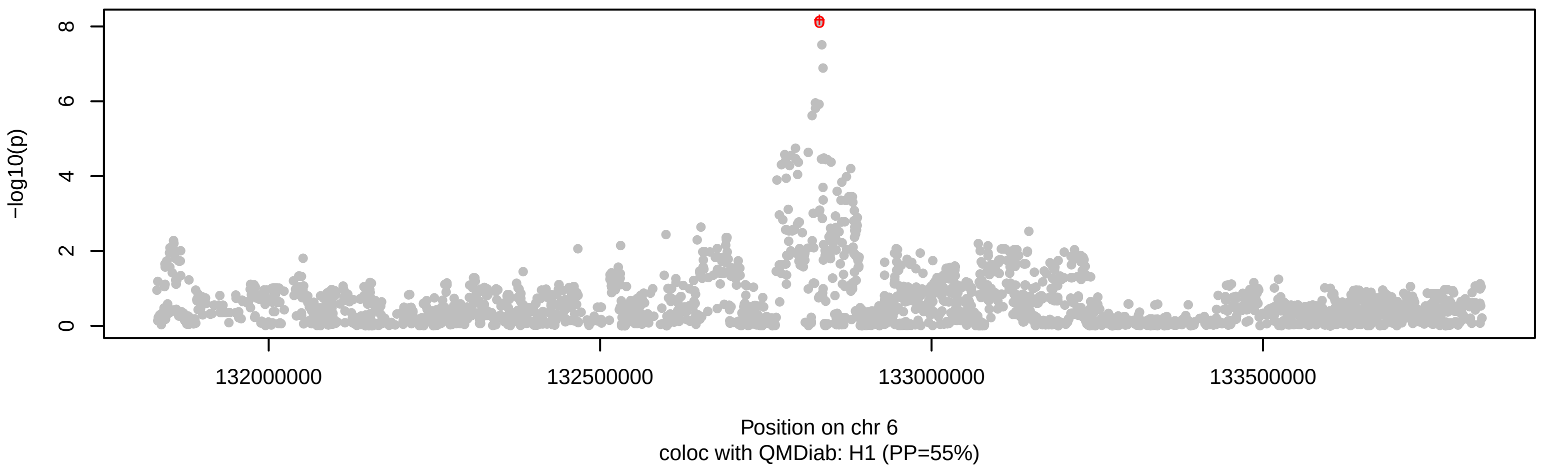

coloc with QMDiab: H1 (PP=55%)

255. STX7 (O15400;O15400−2) 6:132830944:A:G [QMDiab]

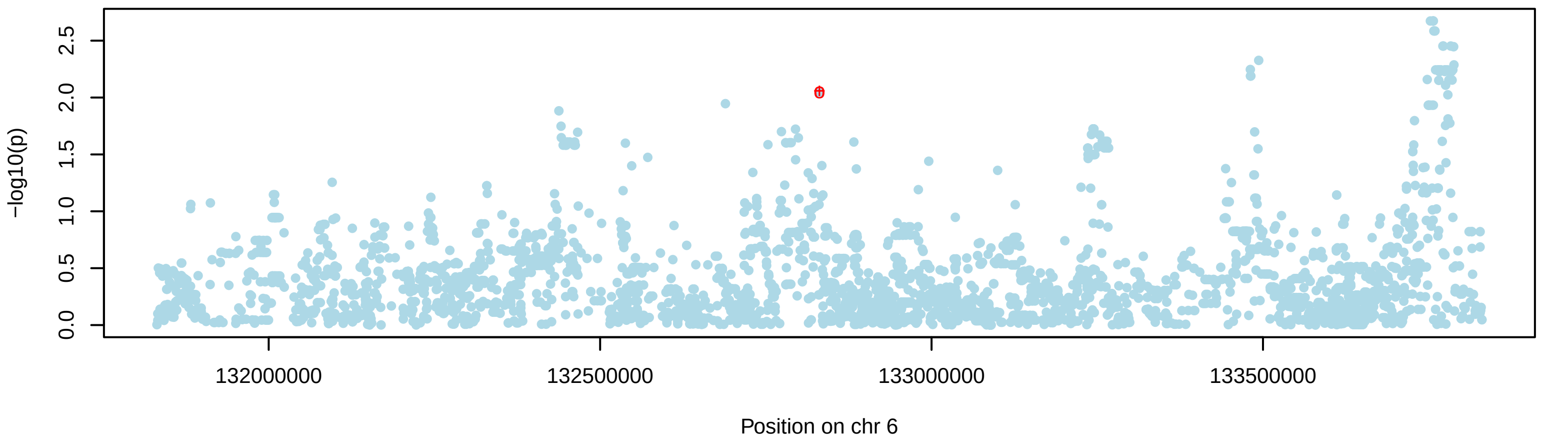

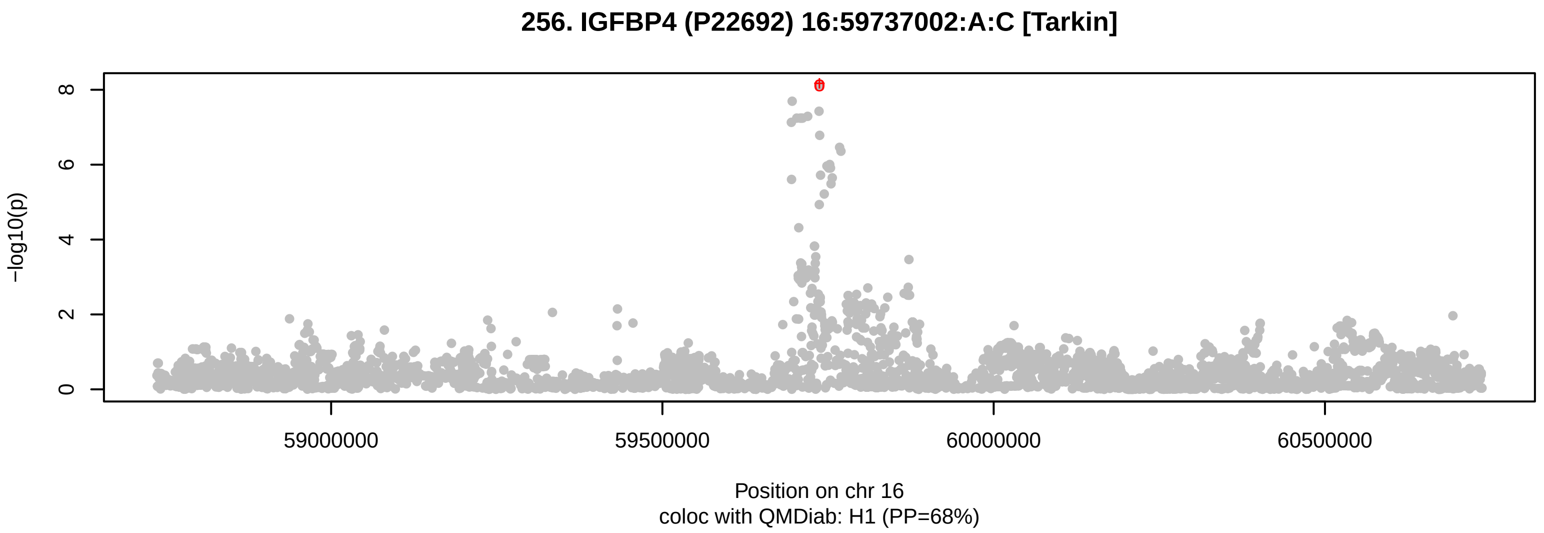
256. IGFBP4 (P22692) 16:59737002:A:C [Tarkin]
−log10(p)
0
2
4
6
8
59000000
59500000
60000000
60500000
Position on chr 16
coloc with QMDiab: H1 (PP=68%)

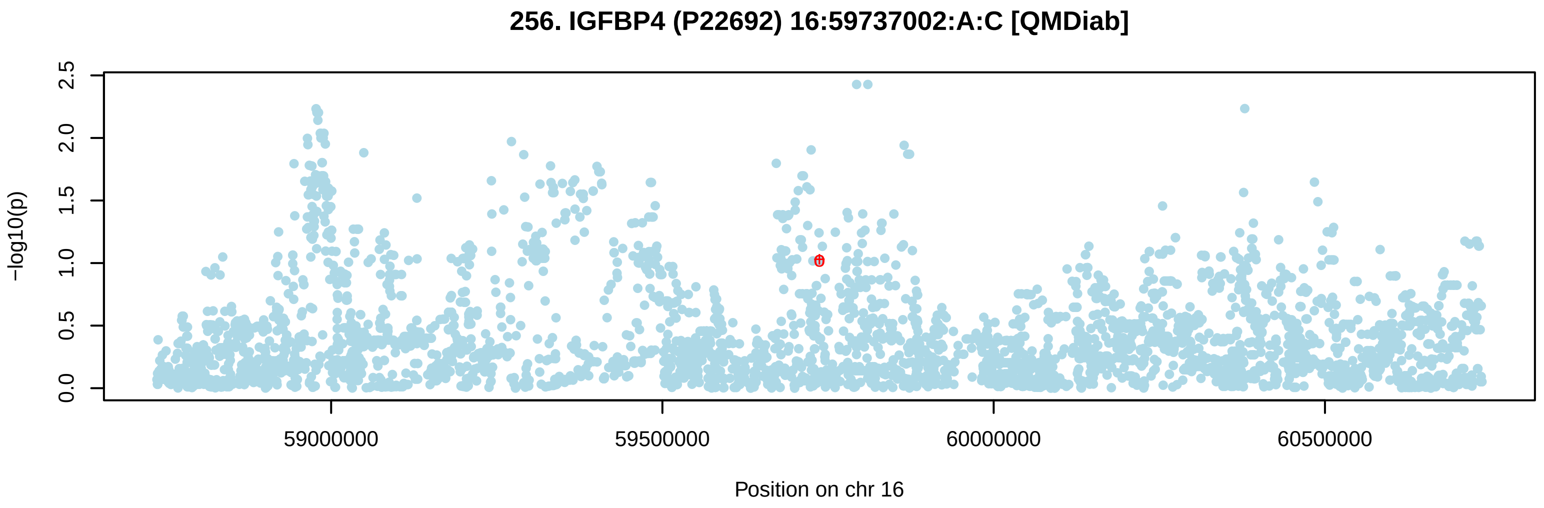
256. IGFBP4 (P22692) 16:59737002:A:C [QMDiab]
−log10(p)
0.0
0.5
1.0
1.5
2.0
2.5
59000000
59500000
60000000
60500000
Position on chr 16

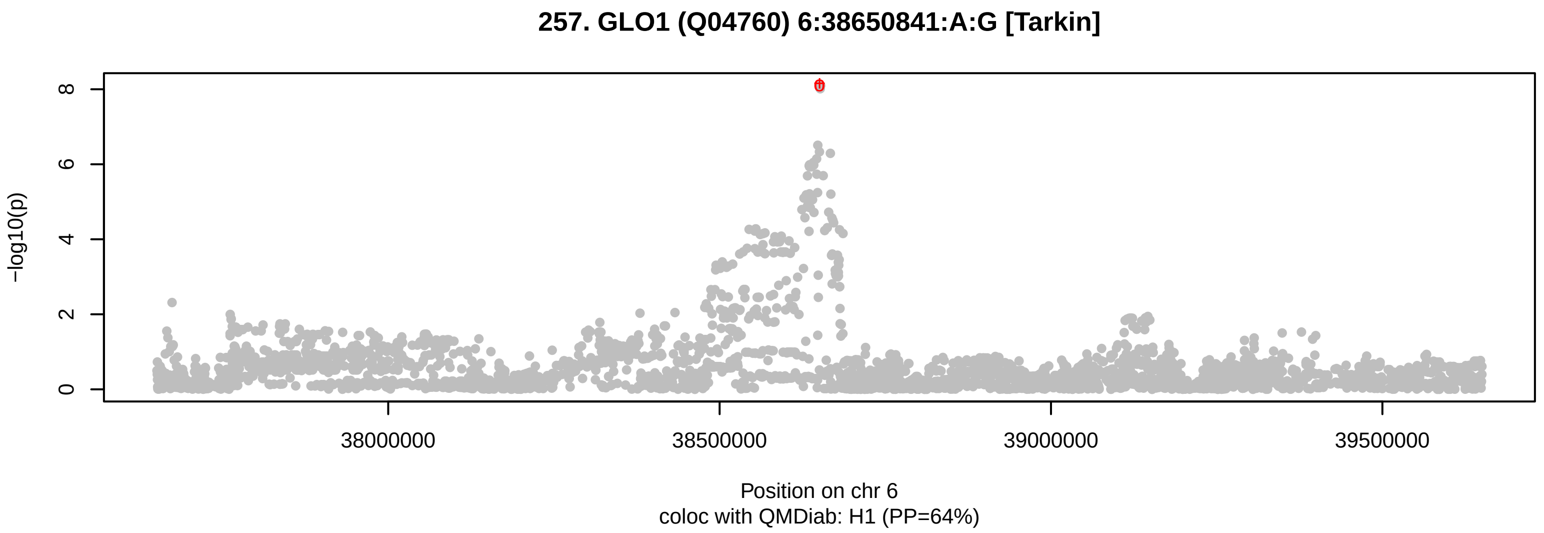
257. GLO1 (Q04760) 6:38650841:A:G [Tarkin]
−log10(p)
0
2
4
6
8
38000000
38500000
39000000
39500000
Position on chr 6
coloc with QMDiab: H1 (PP=64%)

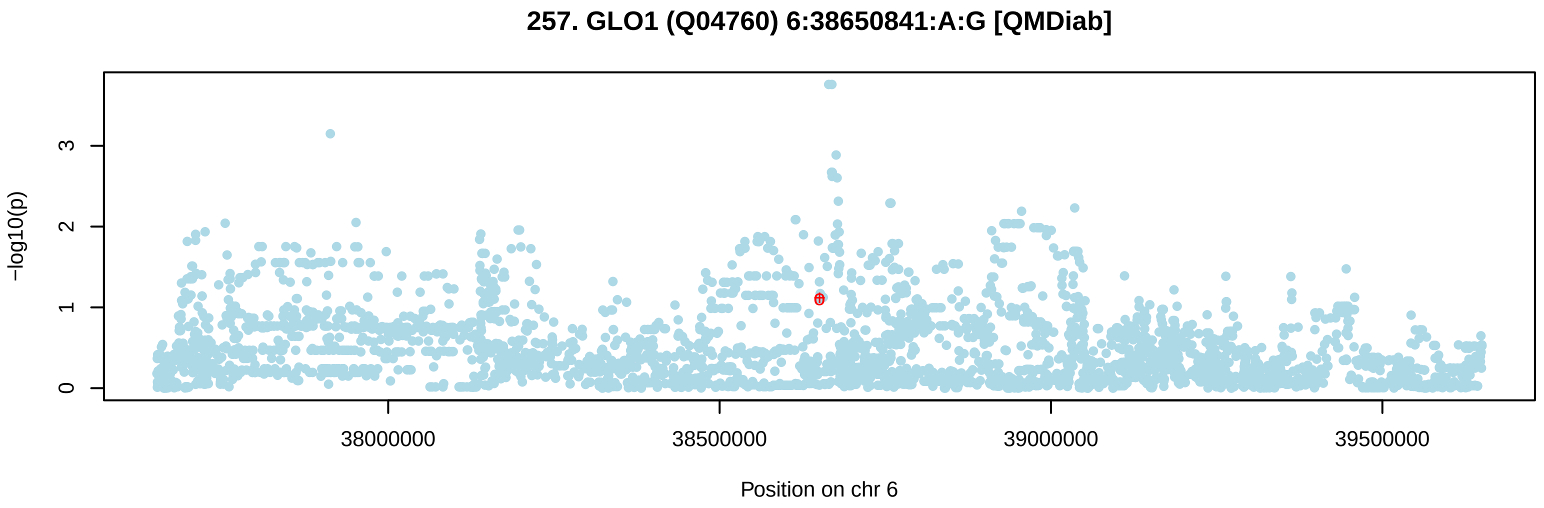
257. GLO1 (Q04760) 6:38650841:A:G [QMDiab]
−log10(p)
0
1
2
3
38000000
38500000
39000000
39500000
Position on chr 6

258. MASP1 (P48740) 17:26694861:G:A [Tarkin]

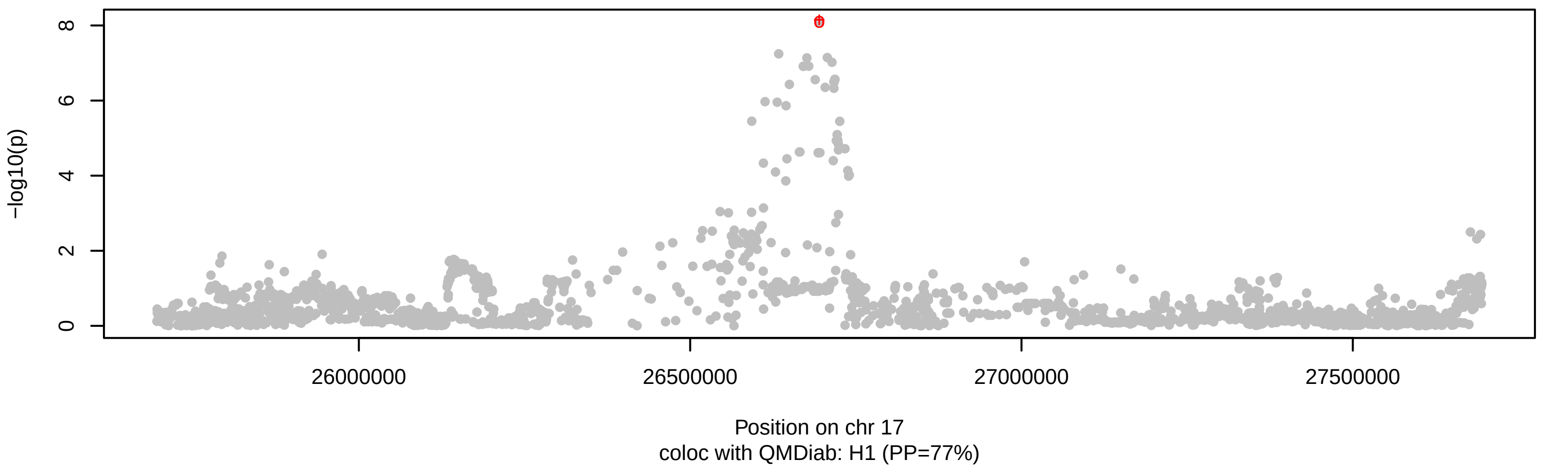

coloc with QMDiab: H1 (PP=77%)

258. MASP1 (P48740) 17:26694861:G:A [QMDiab]

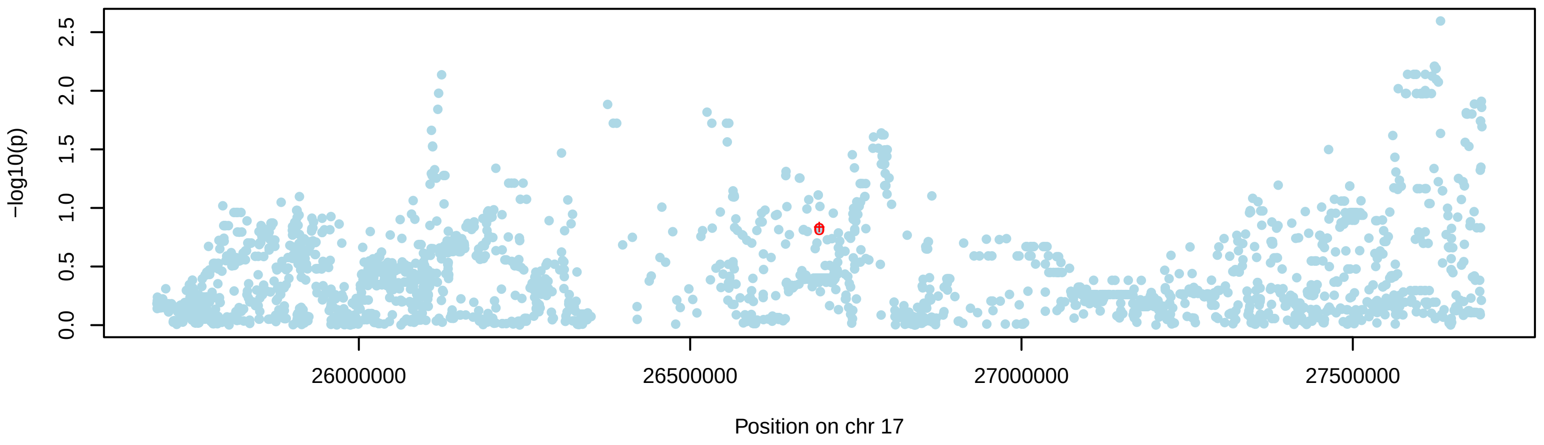

259. FUCA2 (Q9BTY2) 12:118285330:C:T [Tarkin]

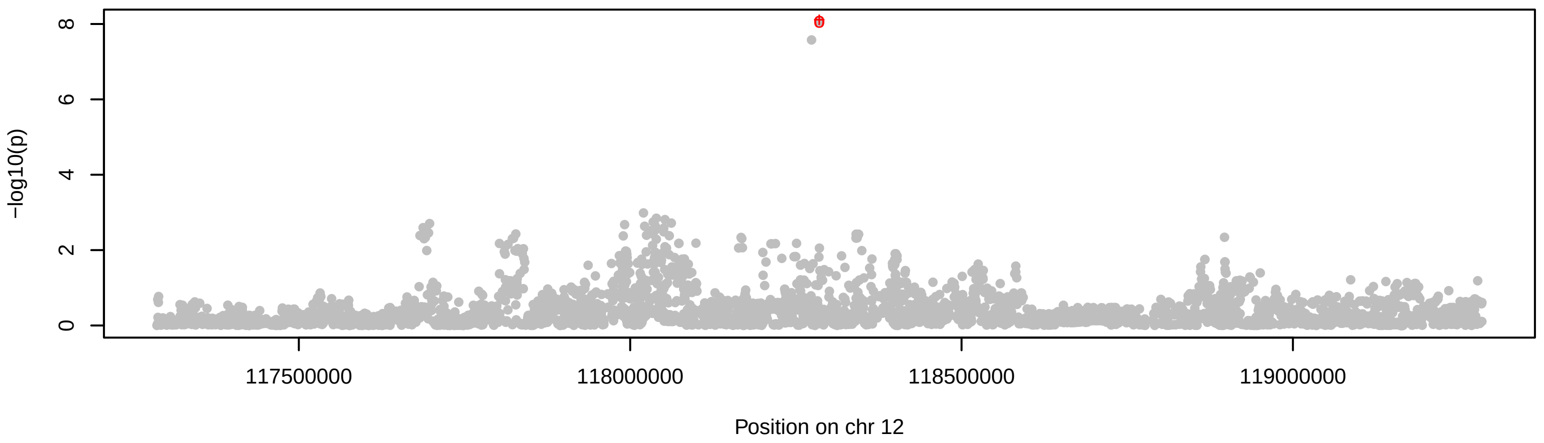

coloc with QMDiab: H1 (PP=69%)

259. FUCA2 (Q9BTY2) 12:118285330:C:T [QMDiab]

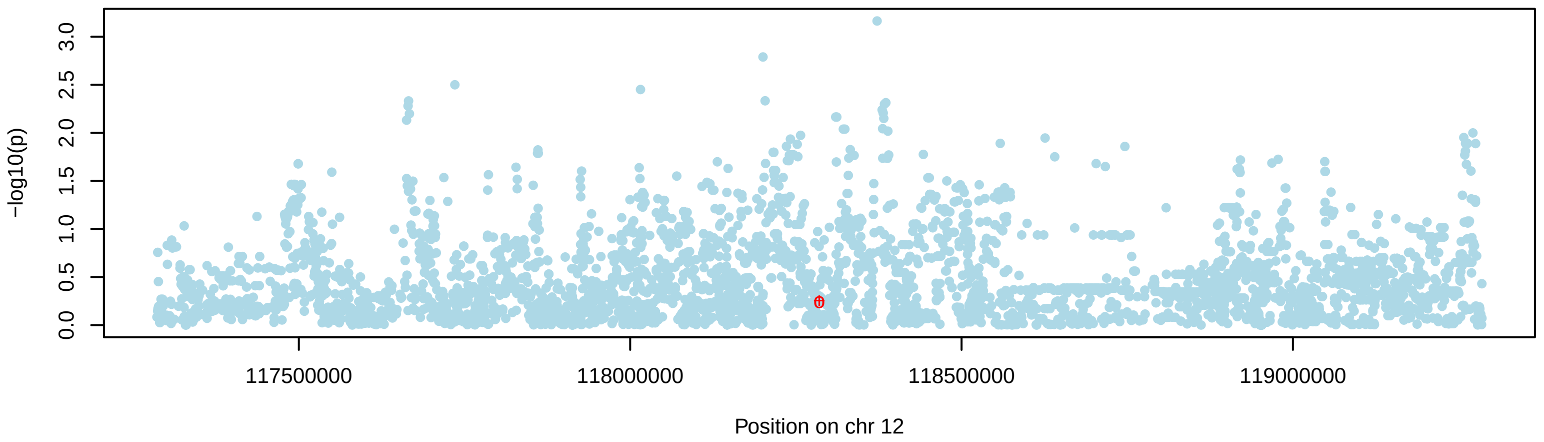

260. ETFB (P38117−2) 21:16217227:C:T [Tarkin]

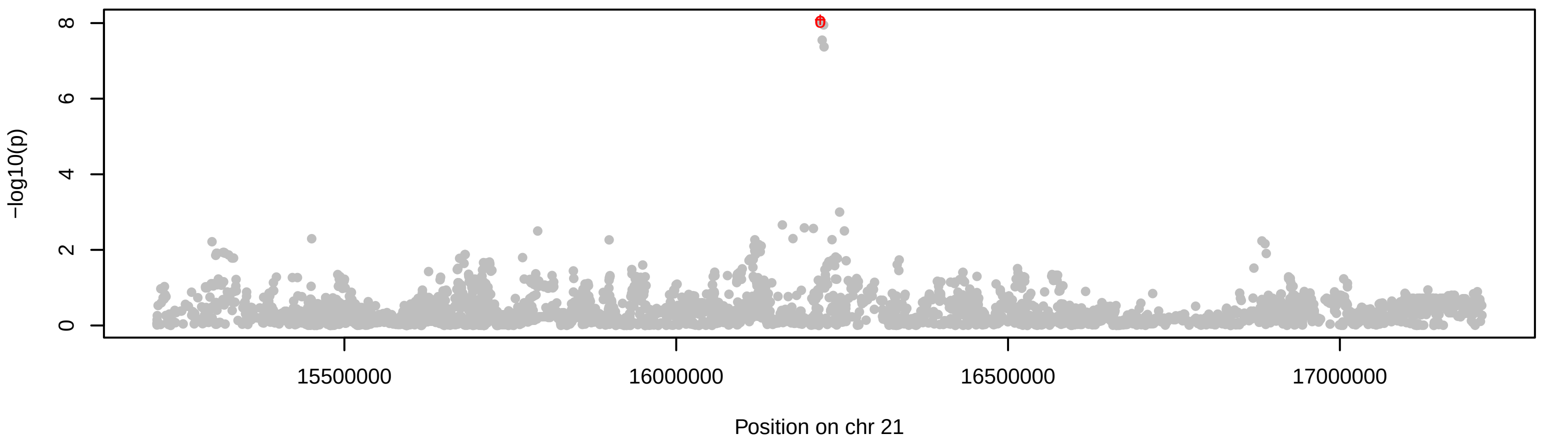

coloc with QMDiab: H1 (PP=65%)

260. ETFB (P38117;P38117−2) 21:16217227:C:T [QMDiab]

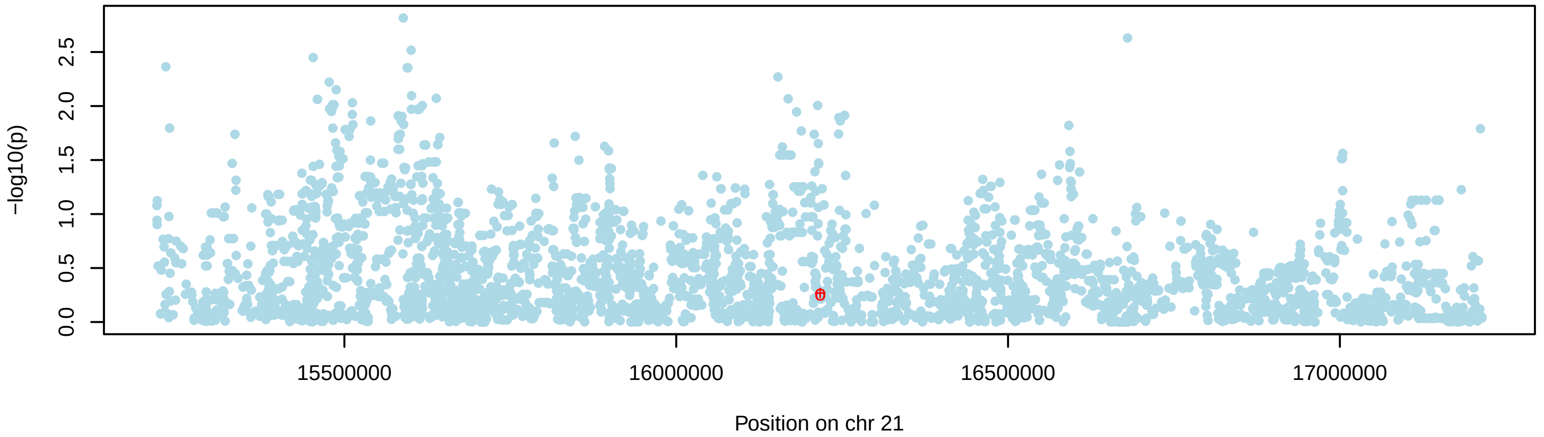

261. SVEP1 (Q4LDE5) 9:130971586:C:T [Tarkin]

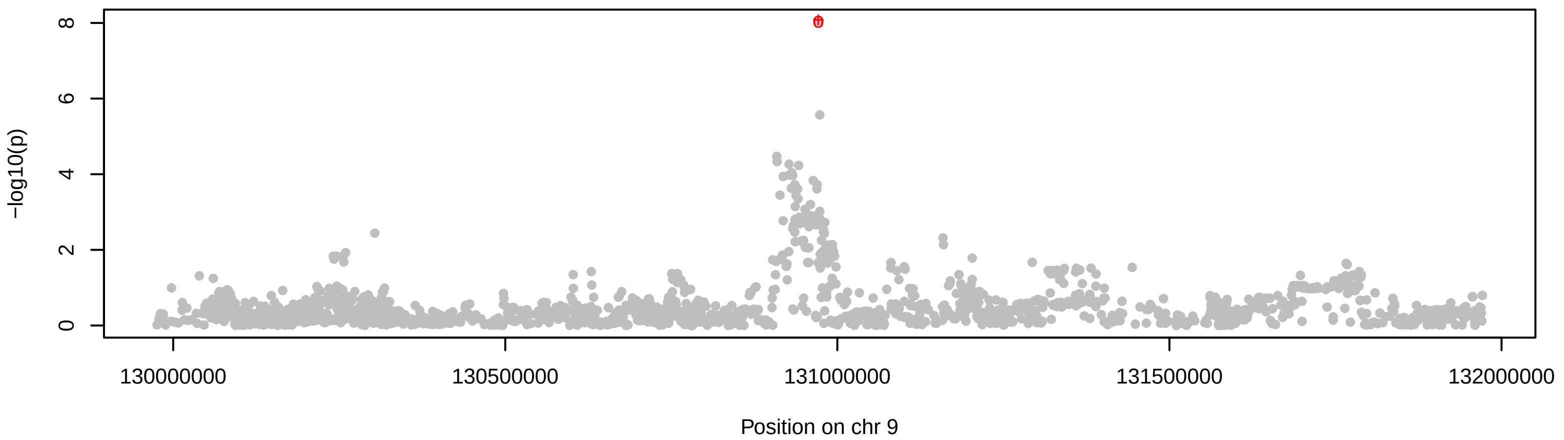

coloc with QMDiab: H1 (PP=75%)

261. SVEP1 (Q4LDE5) 9:130971586:C:T [QMDiab]

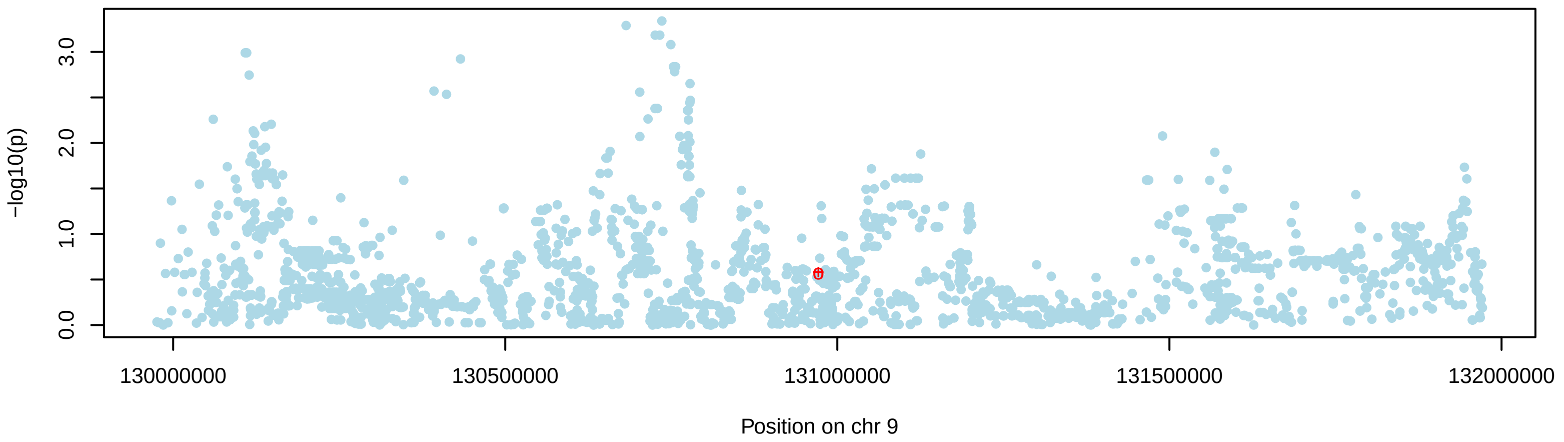

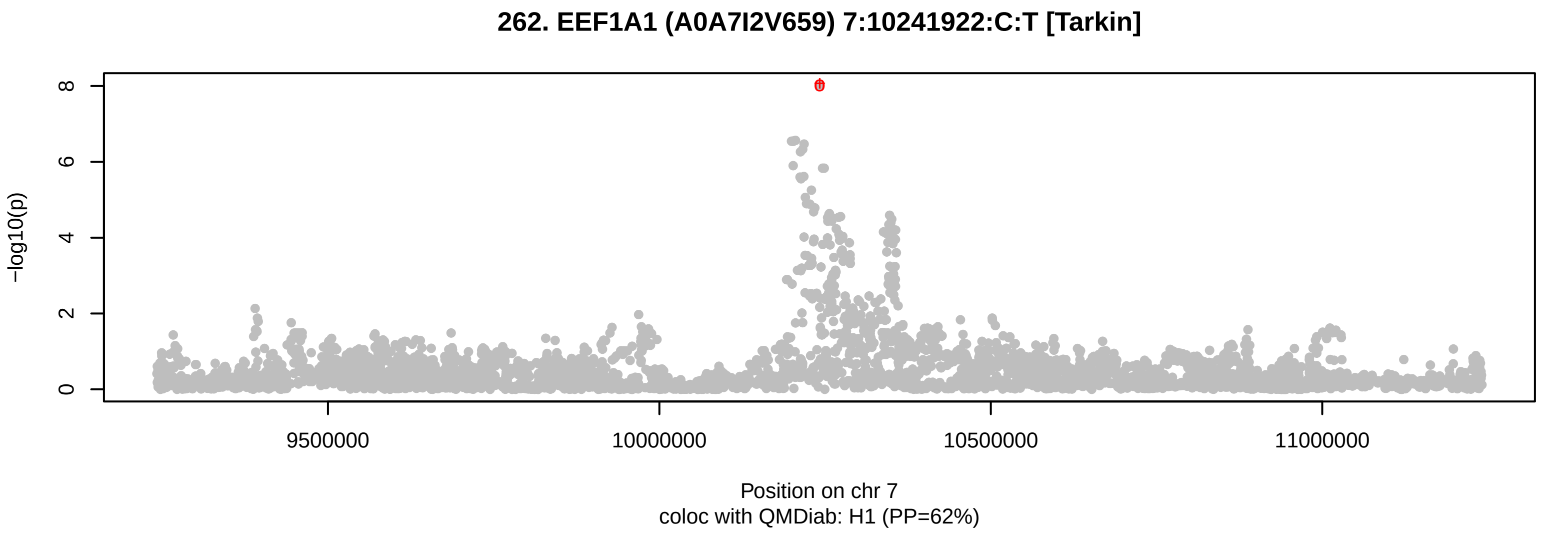

262. EEF1A1 (A0A7I2V659) 7:10241922:C:T [Tarkin]
−log10(p)
0
2
4
6
8
9500000
10000000
10500000
11000000
Position on chr 7
coloc with QMDiab: H1 (PP=62%)

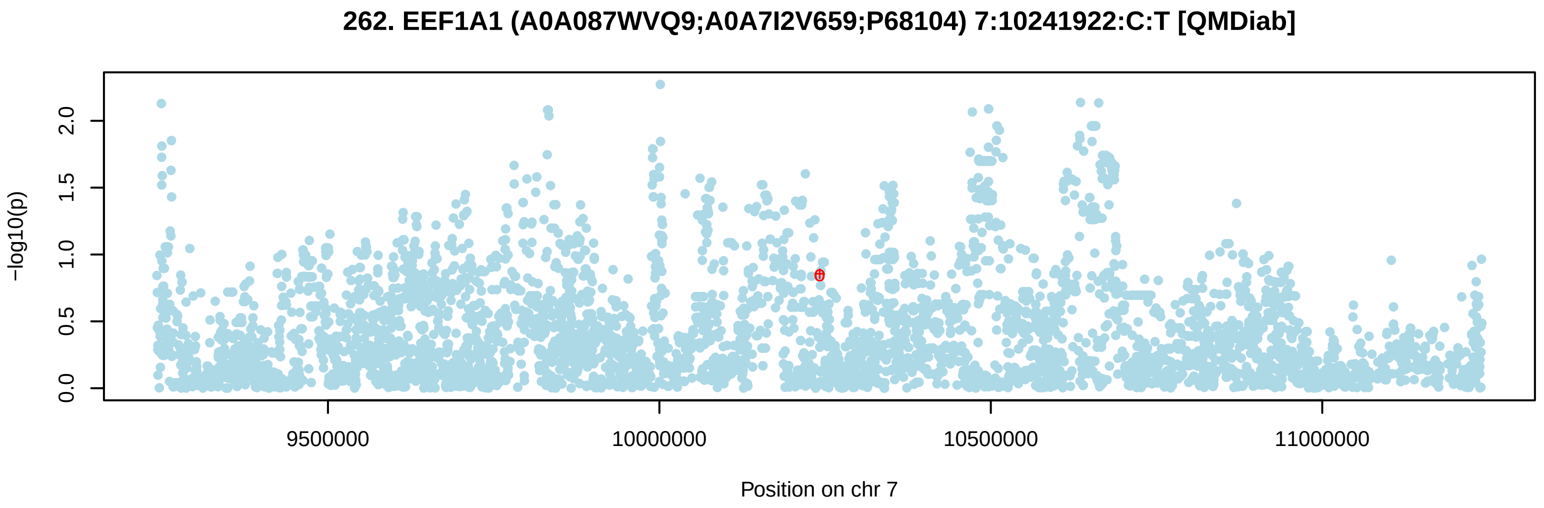

262. EEF1A1 (A0A087WVQ9;A0A7I2V659;P68104) 7:10241922:C:T [QMDiab]
−log10(p)
0.0
0.5
1.0
1.5
2.0
9500000
10000000
10500000
11000000
Position on chr 7

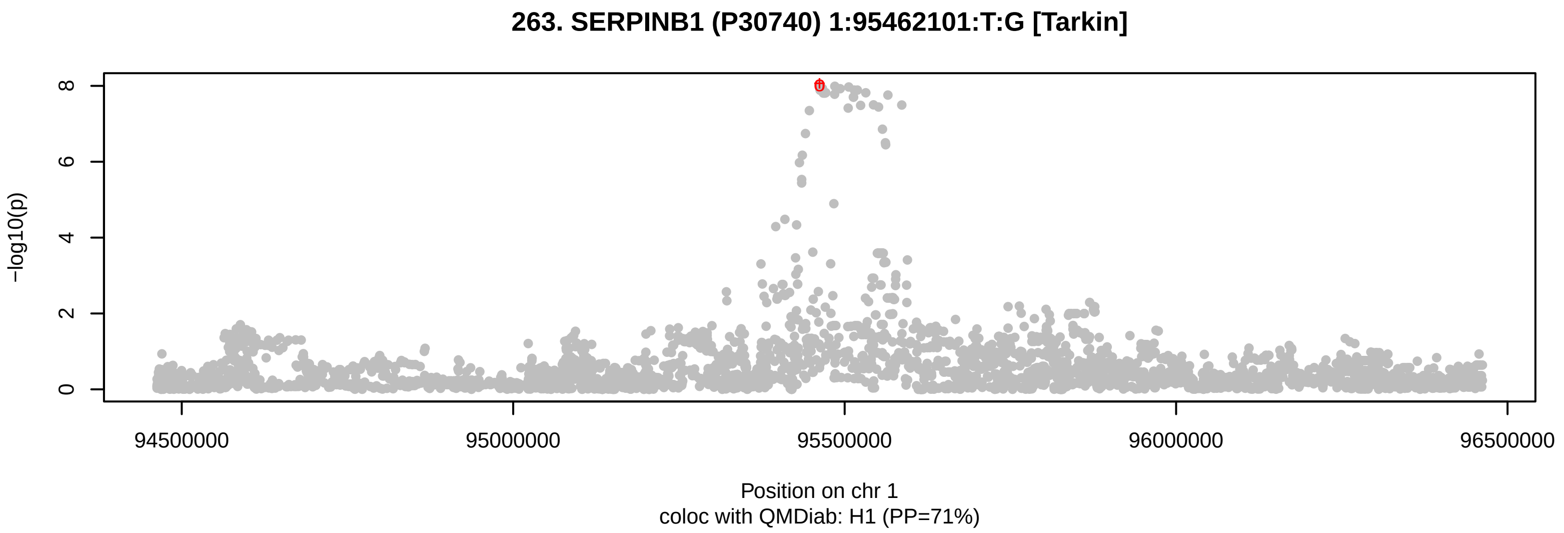
263. SERPINB1 (P30740) 1:95462101:T:G [Tarkin]
−log10(p)
0
2
4
6
8
94500000
95000000
95500000
96000000
96500000
Position on chr 1
coloc with QMDiab: H1 (PP=71%)

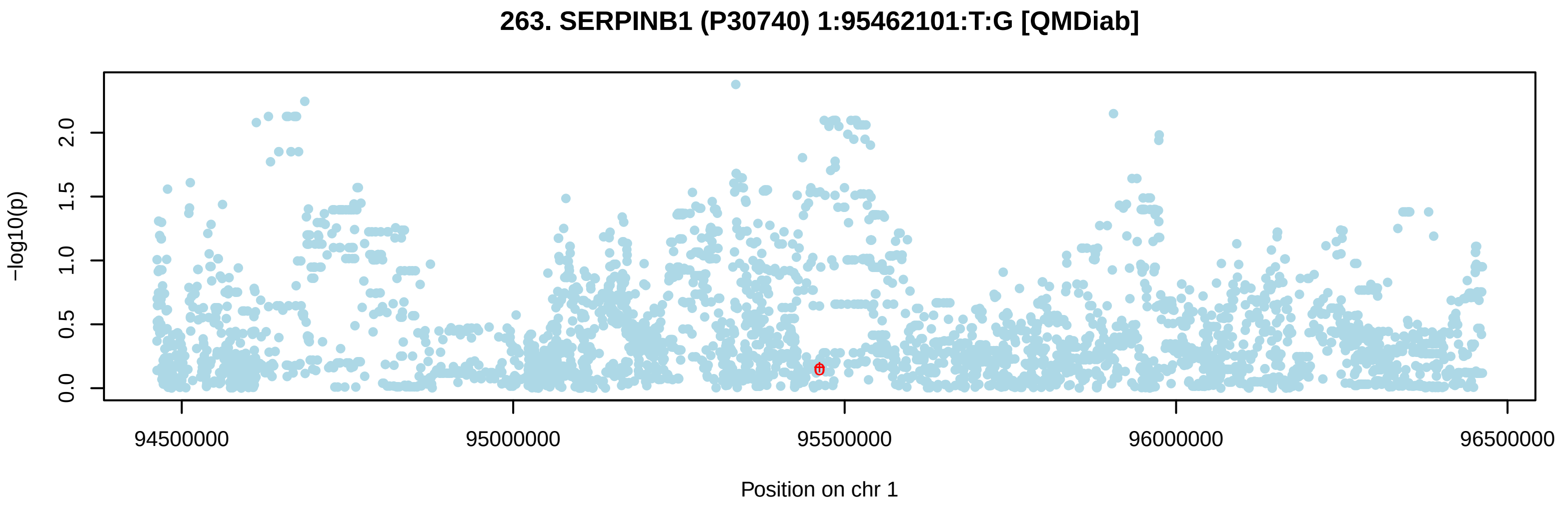
263. SERPINB1 (P30740) 1:95462101:T:G [QMDiab]
−log10(p)
0.0
0.5
1.0
1.5
2.0
94500000
95000000
95500000
96000000
96500000
Position on chr 1

264. F5 (A0A0A0MRJ7;P12259) 3:65259645:A:G [Tarkin]

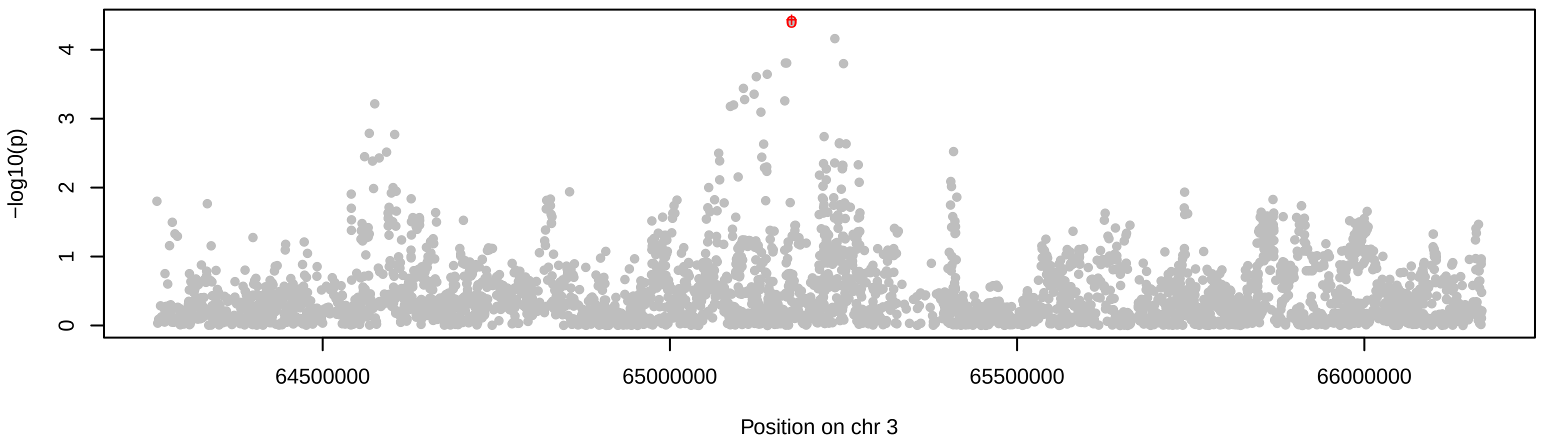

coloc with QMDiab: H0 (PP=47%)

264. F5 (A0A0A0MRJ7;P12259) 3:65259645:A:G [QMDiab]

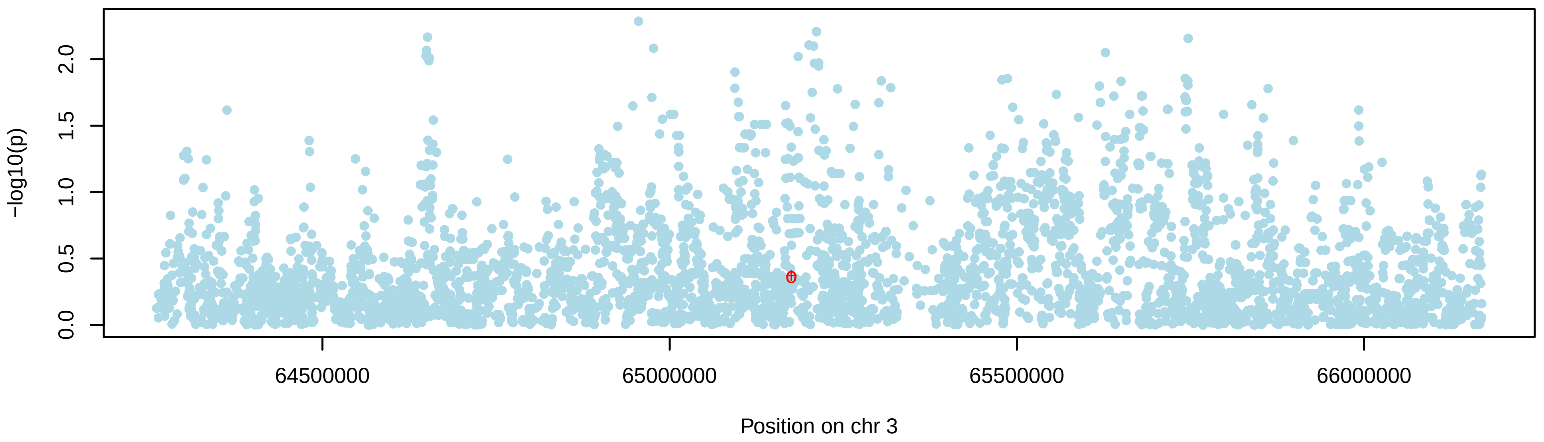

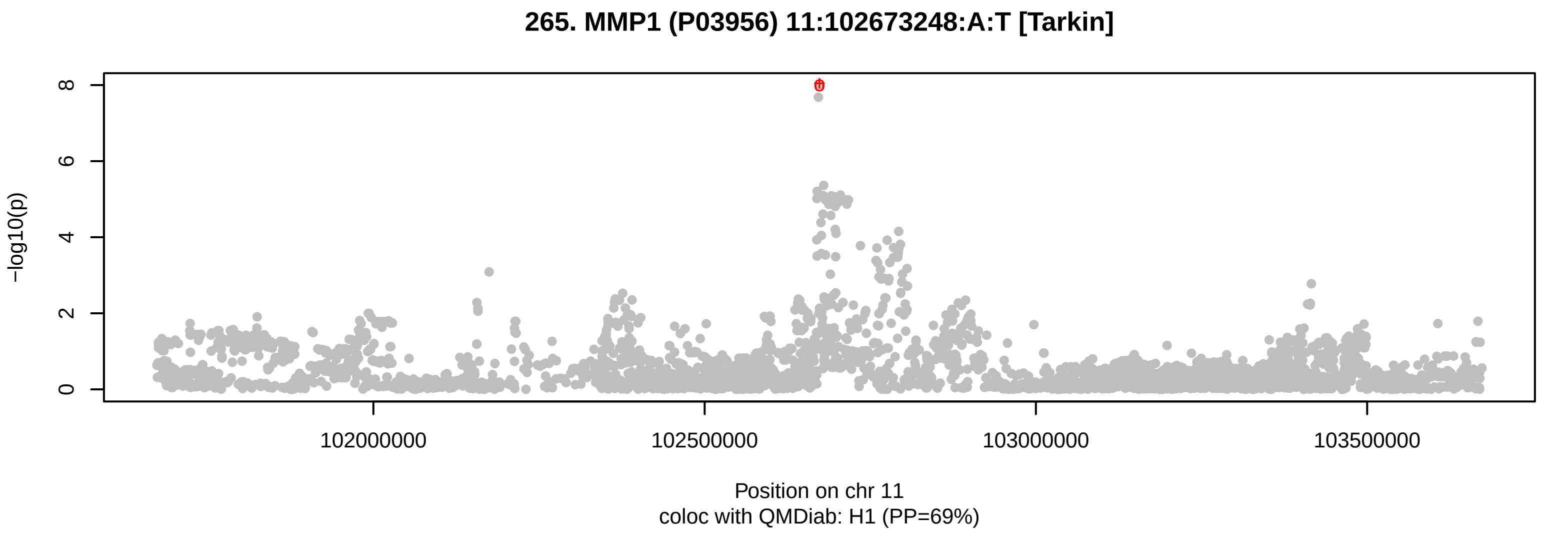
265. MMP1 (P03956) 11:102673248:A:T [Tarkin]
−log10(p)
0
2
4
6
8
102000000
102500000
103000000
103500000
Position on chr 11
coloc with QMDiab: H1 (PP=69%)

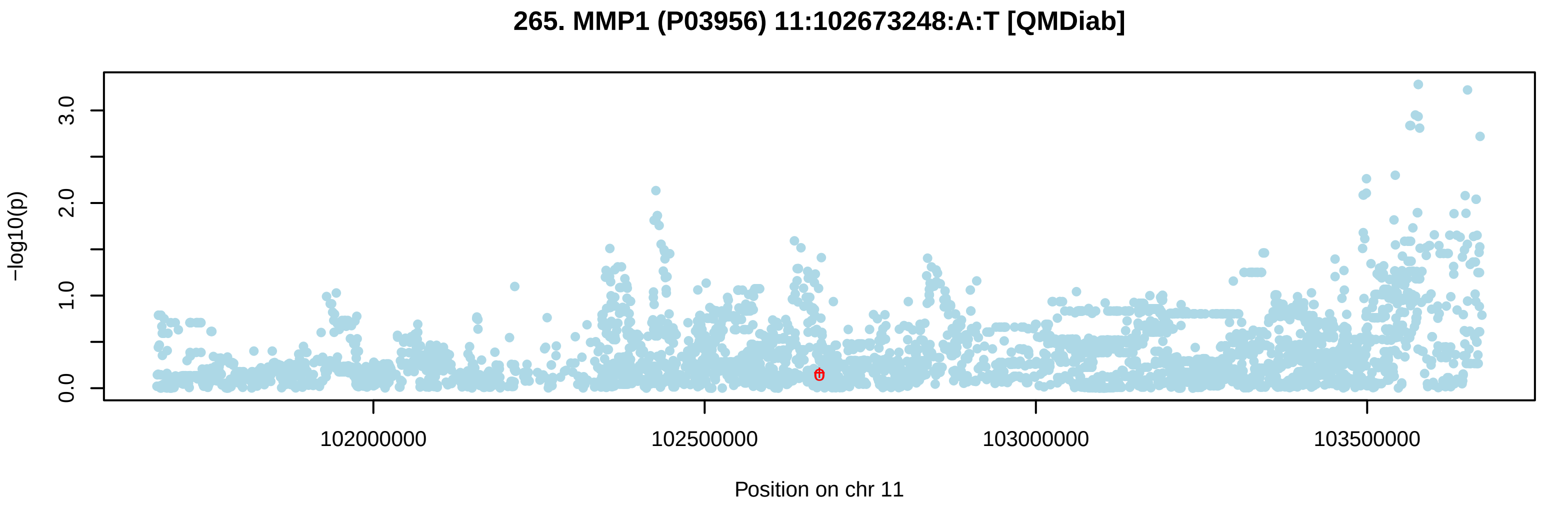
265. MMP1 (P03956) 11:102673248:A:T [QMDiab]
−log10(p)
0.0
1.0
2.0
3.0
102000000
102500000
103000000
103500000
Position on chr 11

266. PLA2G7 (Q13093) 13:24943133:C:T [Tarkin]

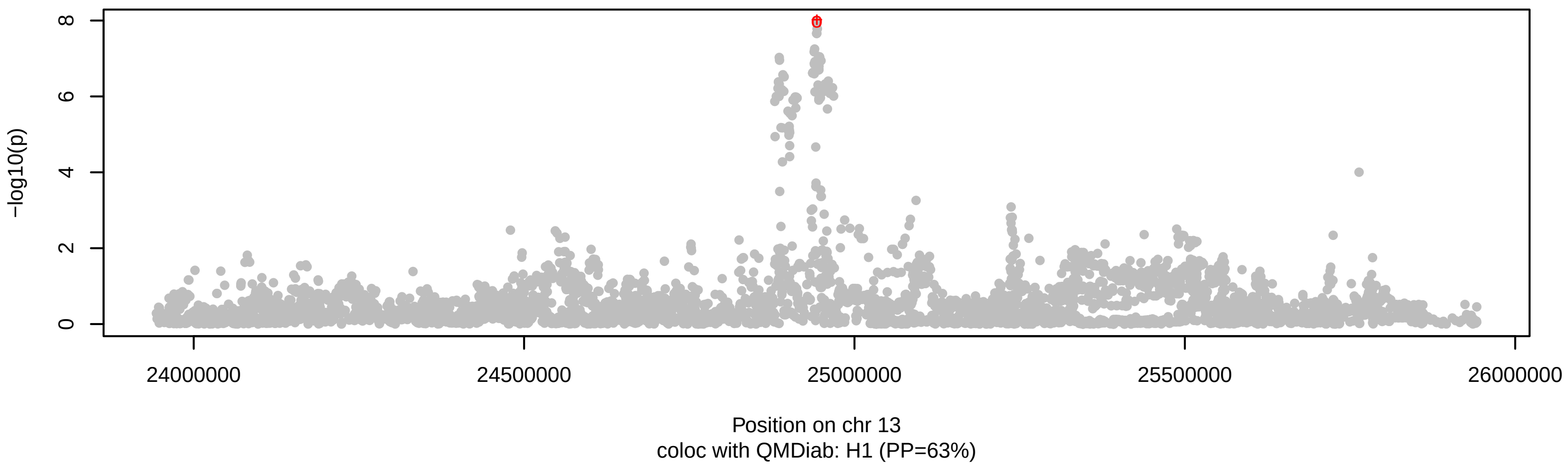

266. PLA2G7 (Q13093) 13:24943133:C:T [QMDiab]

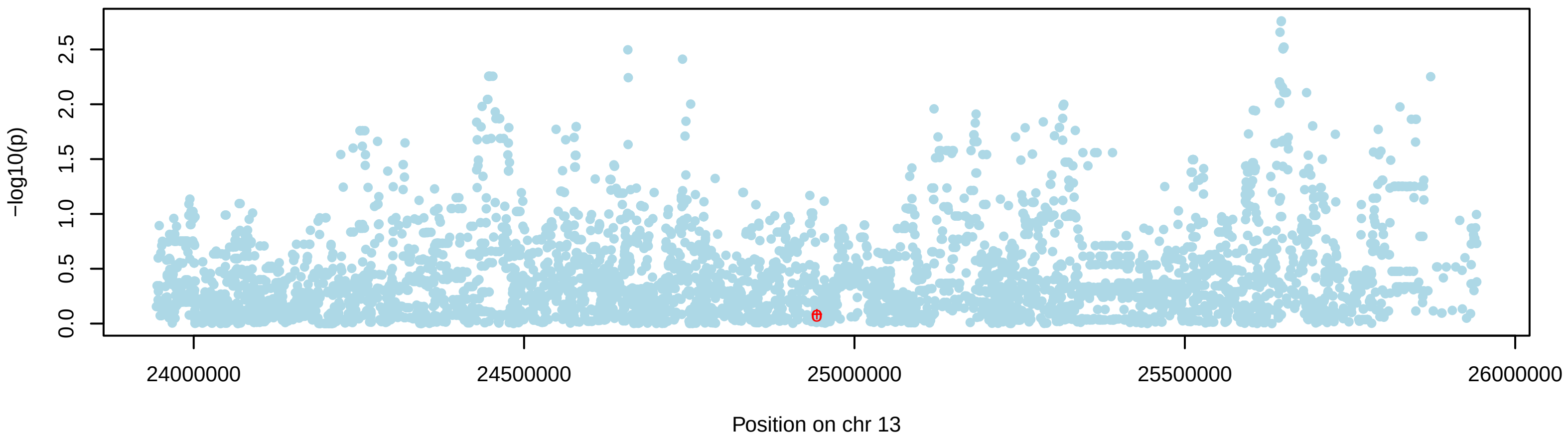

267. ERP44 (Q9BS26) 10:10862179:G:A [Tarkin]

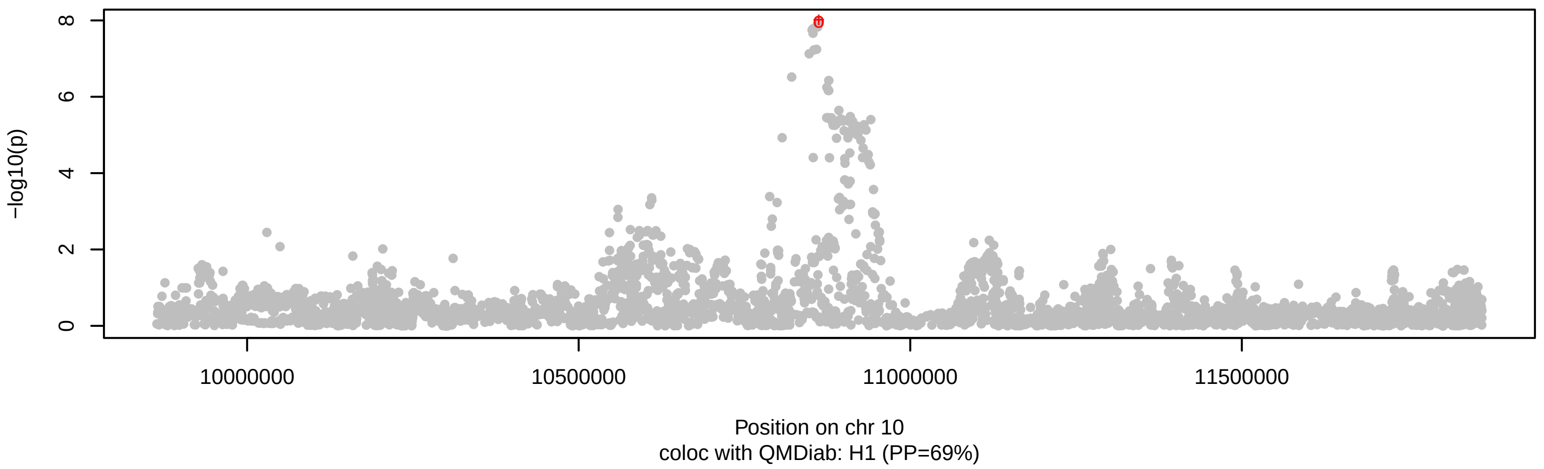

coloc with QMDiab: H1 (PP=69%)

267. ERP44 (Q9BS26) 10:10862179:G:A [QMDiab]

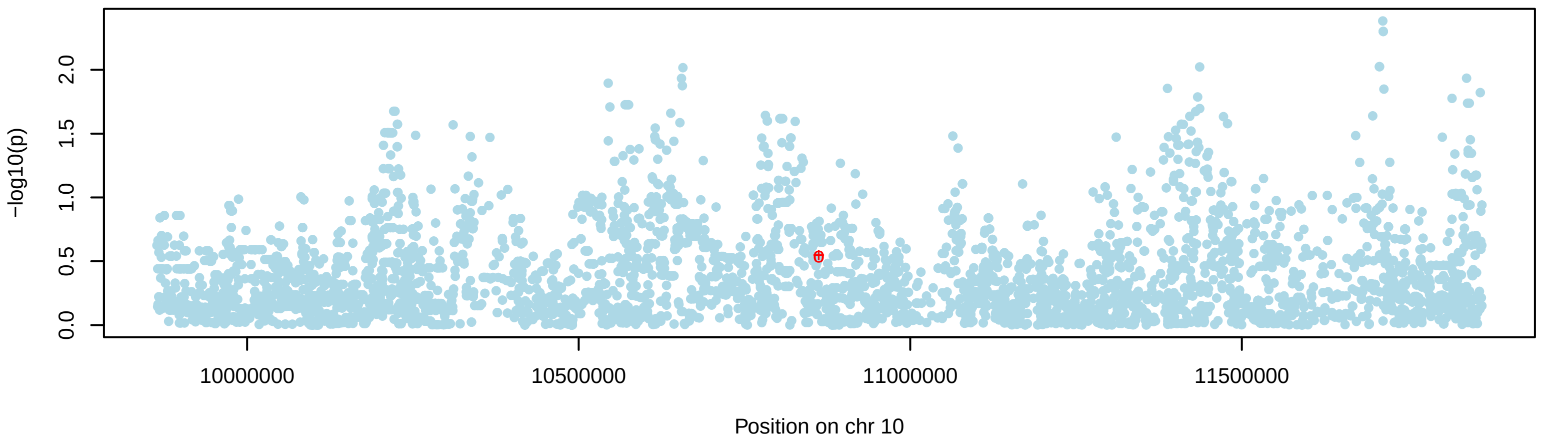

268. TFR2 (Q9UP52) 9:136155000:C:T [Tarkin]

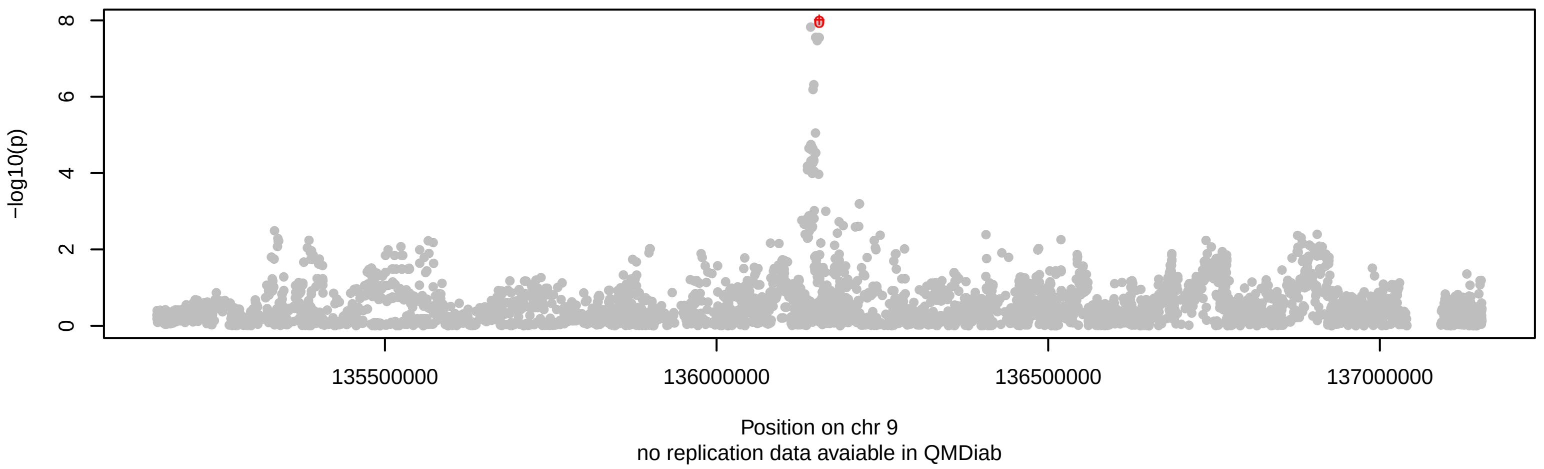

no replication data avaiable in QMDiab

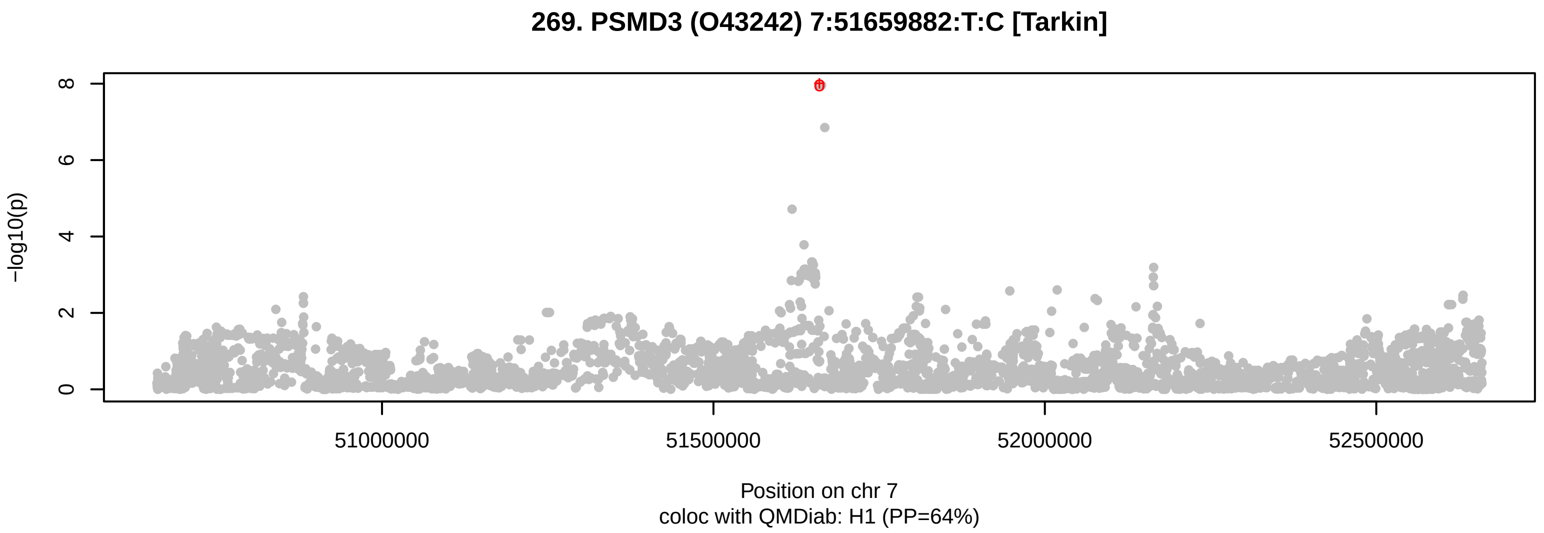
269. PSMD3 (O43242) 7:51659882:T:C [Tarkin]
−log10(p)
0
2
4
6
8
51000000
51500000
52000000
52500000
Position on chr 7
coloc with QMDiab: H1 (PP=64%)

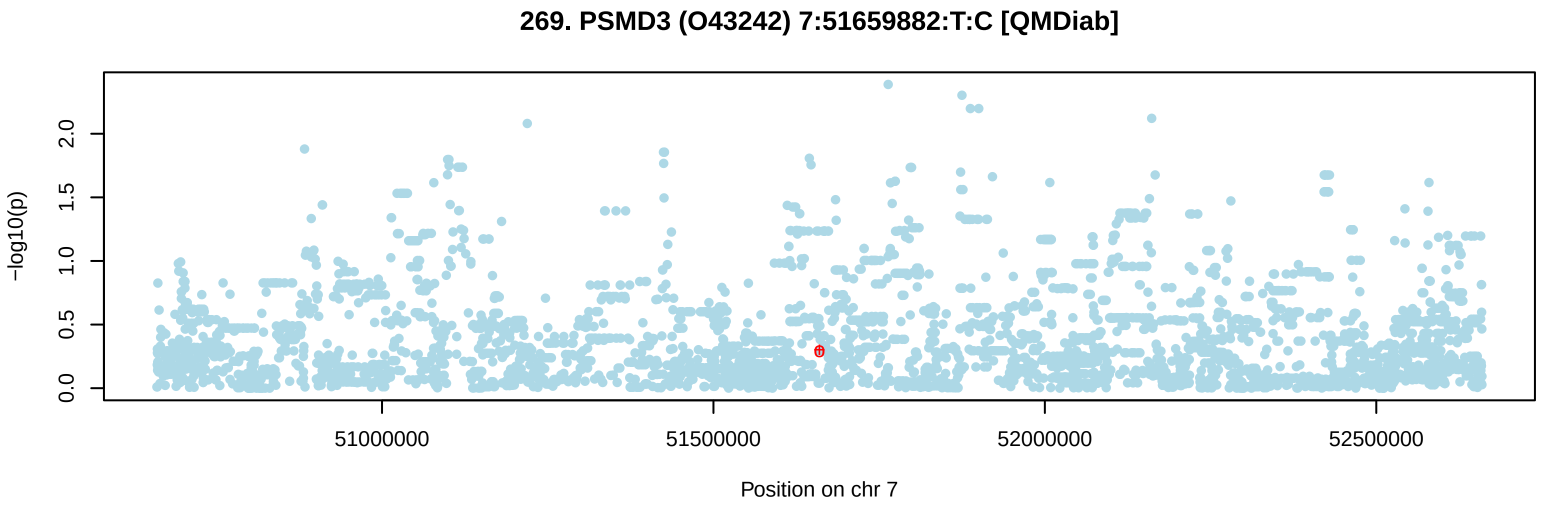
269. PSMD3 (O43242) 7:51659882:T:C [QMDiab]
−log10(p)
0.0
0.5
1.0
1.5
2.0
51000000
51500000
52000000
52500000
Position on chr 7

270. EEF2 (P13639) 4:100045141:C:T [Tarkin]

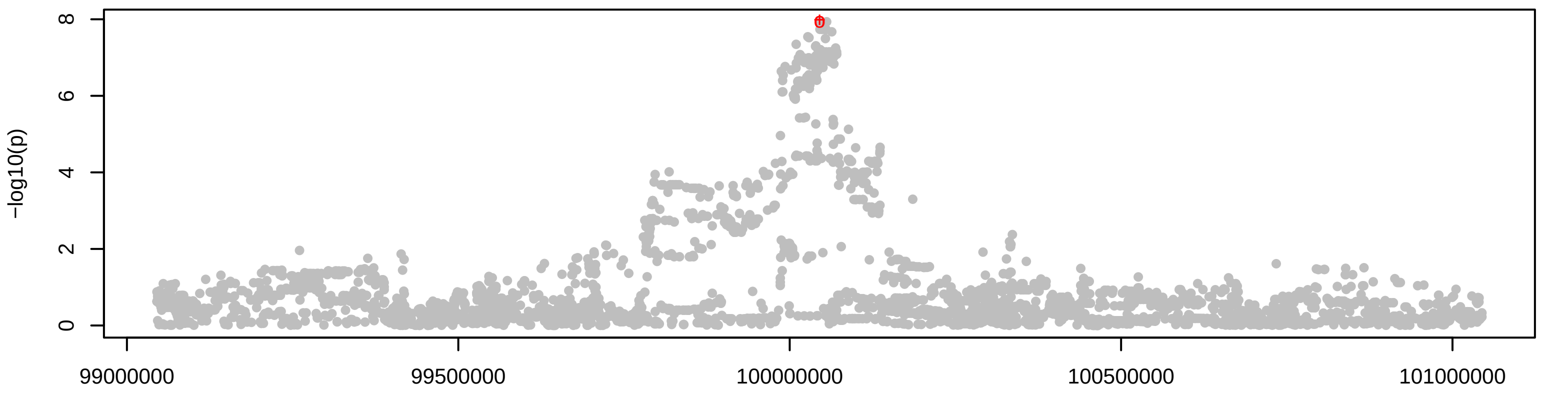

coloc with QMDiab: H1 (PP=67%)

270. EEF2 (P13639) 4:100045141:C:T [QMDiab]

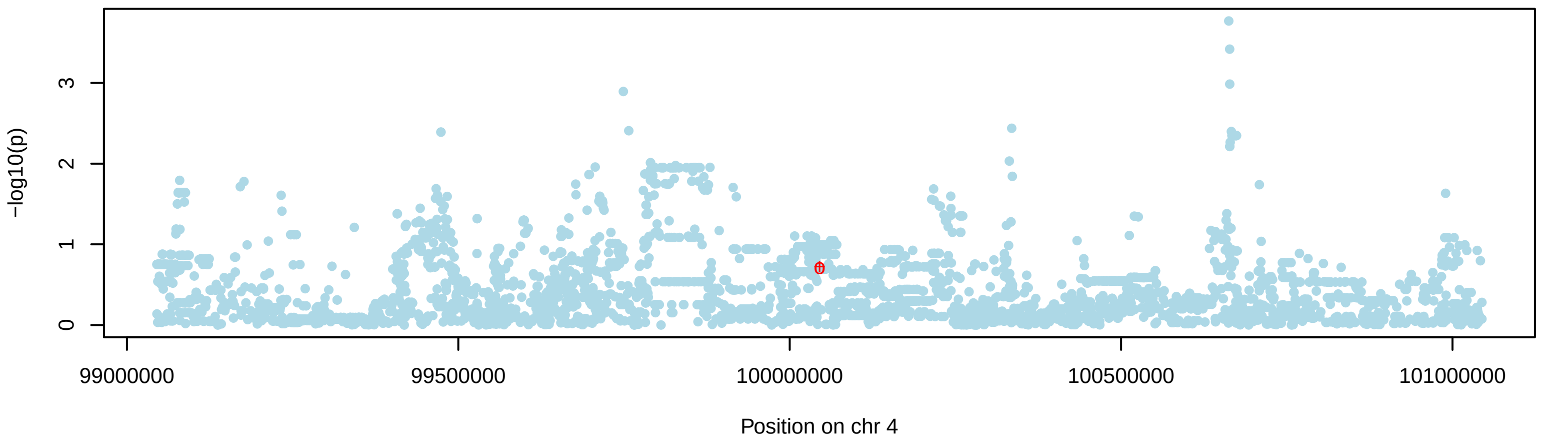

271. C1R (A0A3B3ISR2;B4DPQ0) 15:63176594:T:C [Tarkin]

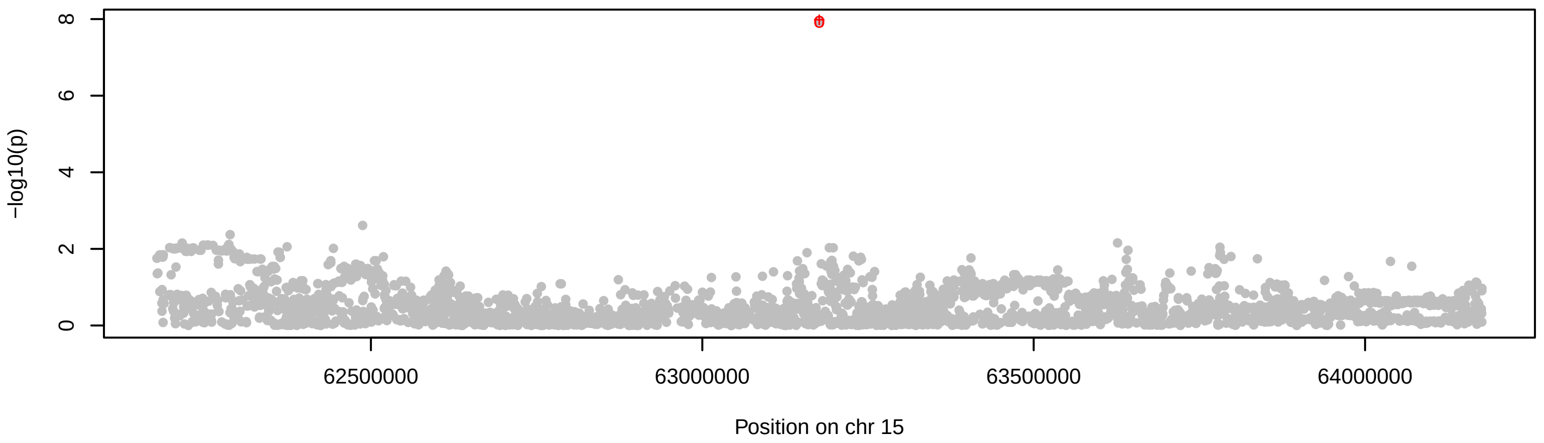

coloc with QMDiab: H1 (PP=69%)

271. C1R (A0A3B3ISR2;B4DPQ0) 15:63176594:T:C [QMDiab]

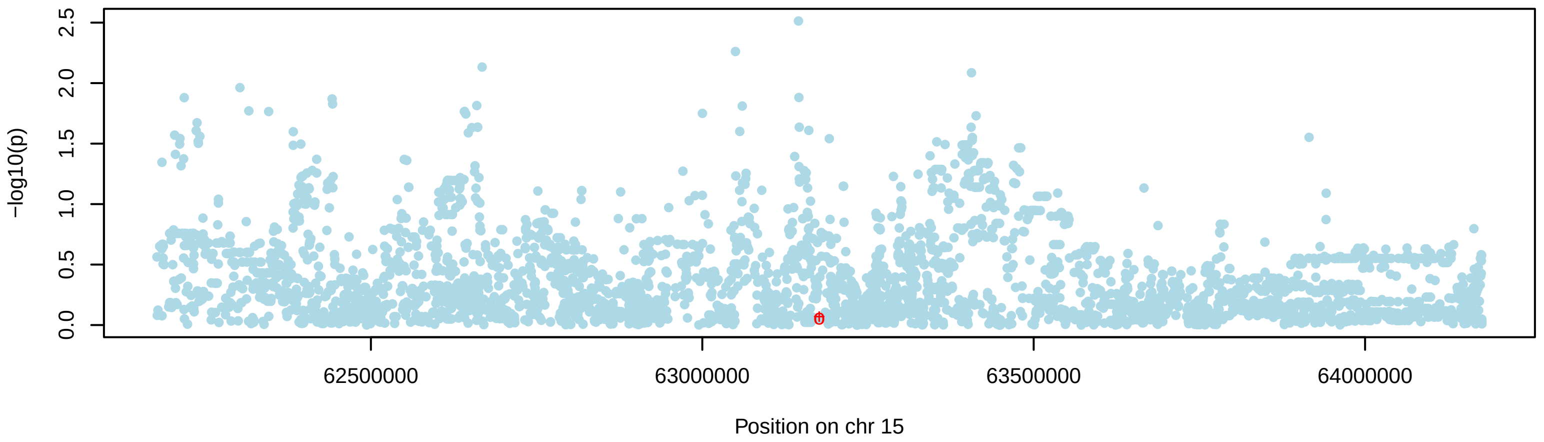

272. LCAT (P04180) 22:26520564:C:T [Tarkin]

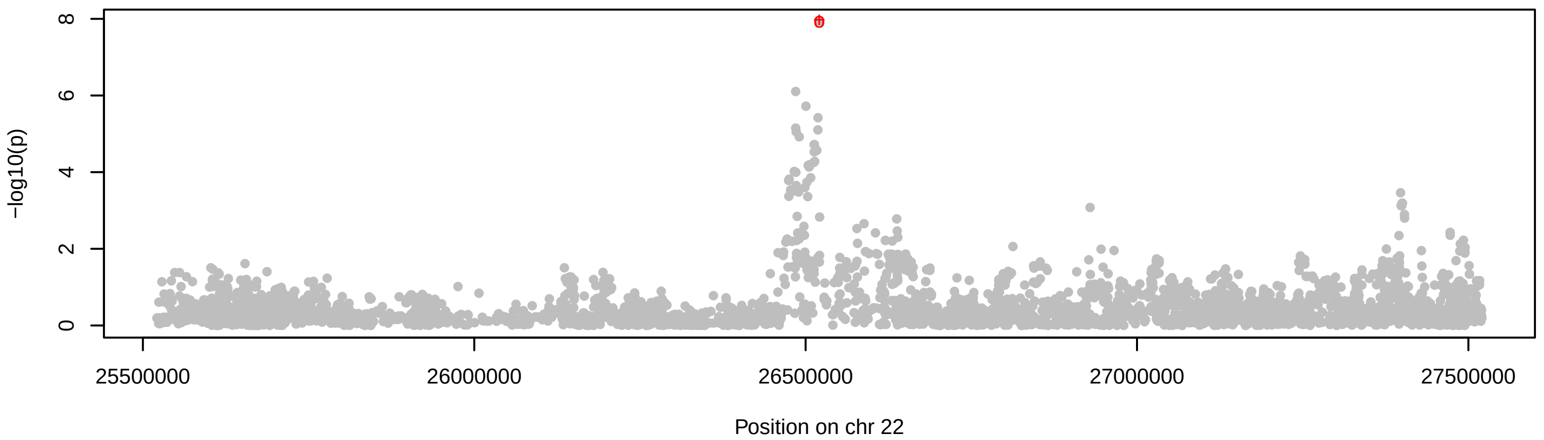

coloc with QMDiab: H1 (PP=64%)

272. LCAT (P04180) 22:26520564:C:T [QMDiab]

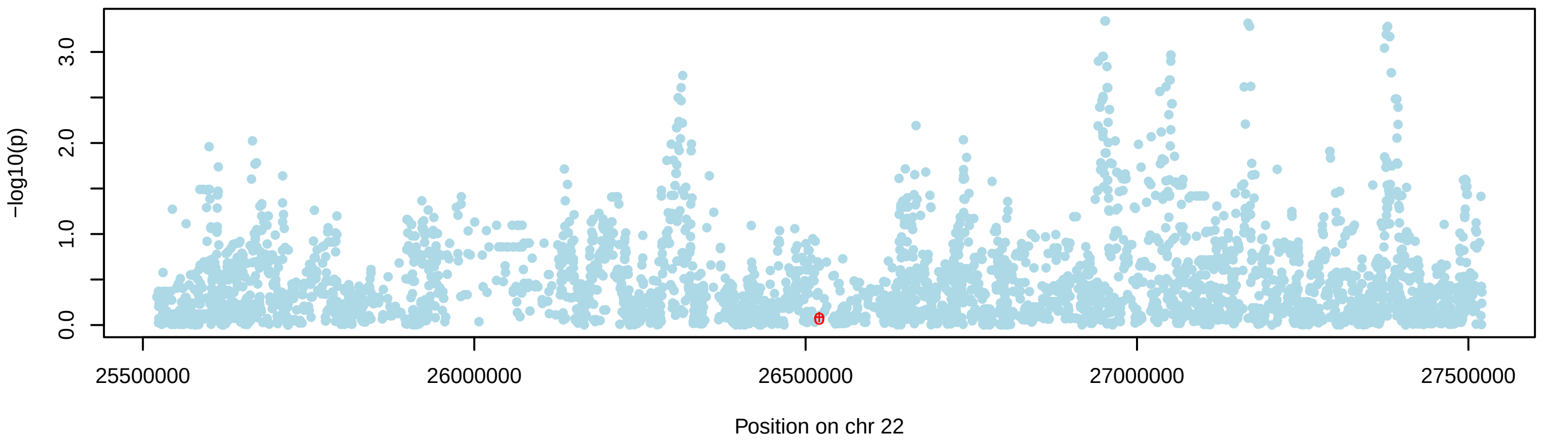

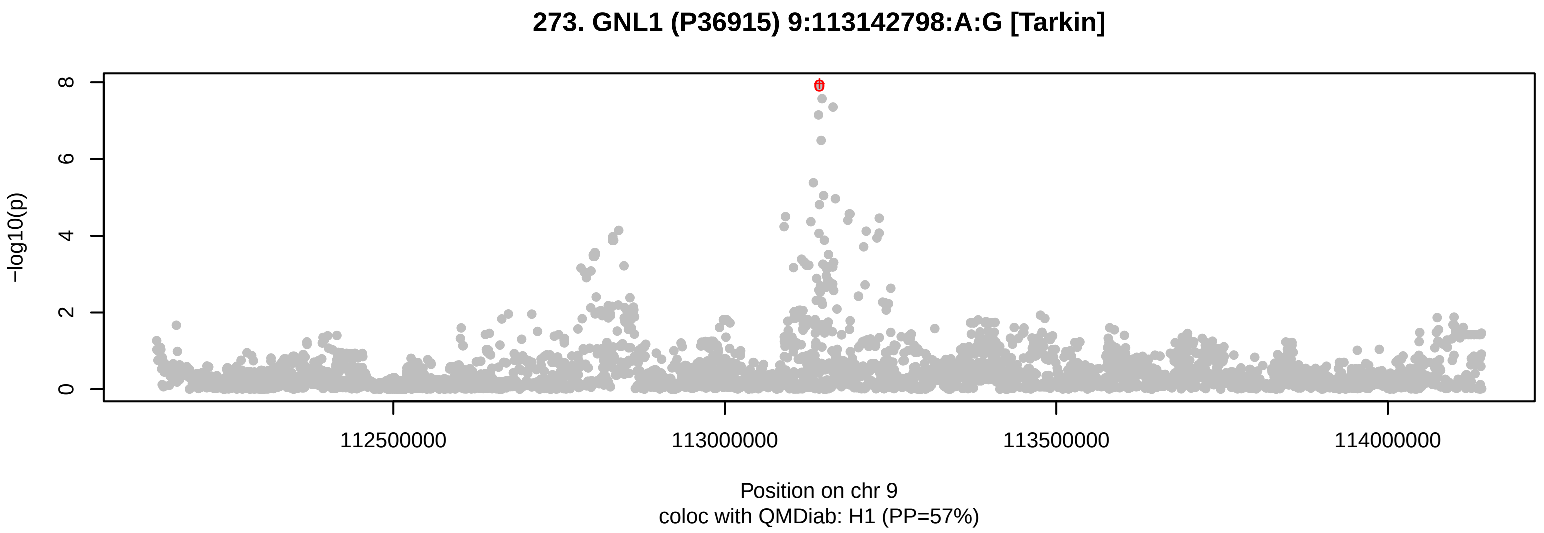
273. GNL1 (P36915) 9:113142798:A:G [Tarkin]
−log10(p)
0
2
4
6
8
112500000
113000000
113500000
114000000
Position on chr 9
coloc with QMDiab: H1 (PP=57%)

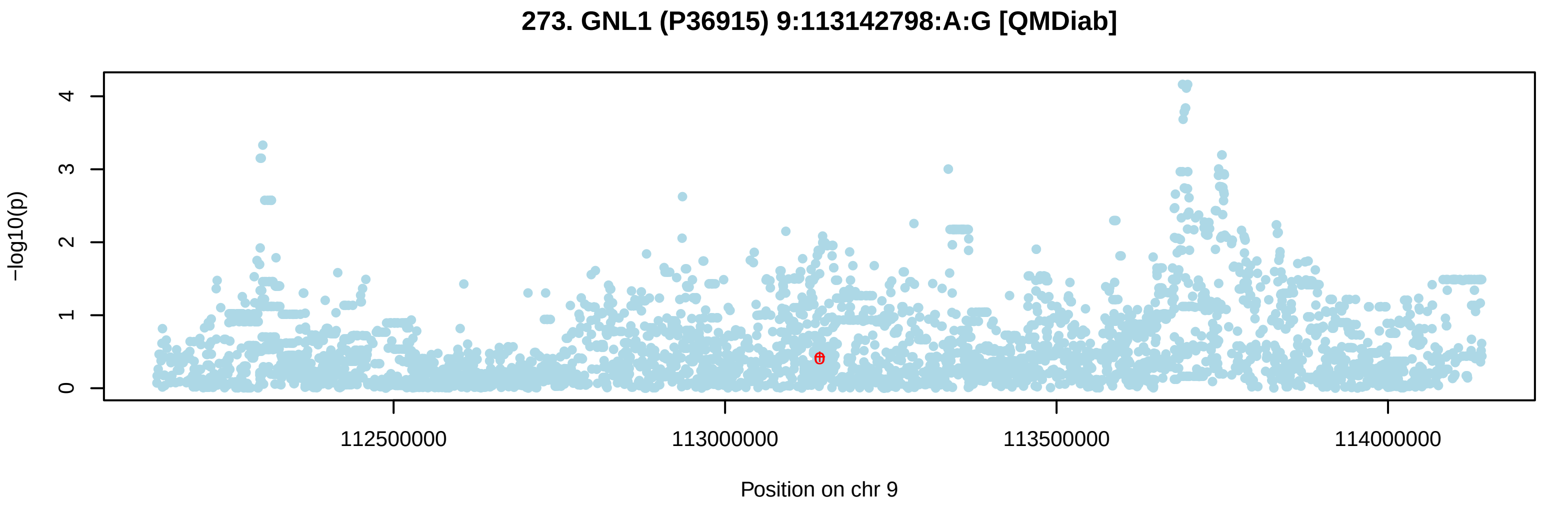
273. GNL1 (P36915) 9:113142798:A:G [QMDiab]
−log10(p)
0
1
2
3
4
112500000
113000000
113500000
114000000
Position on chr 9

274. SRGN (P10124) 19:17101403:G:A [Tarkin]

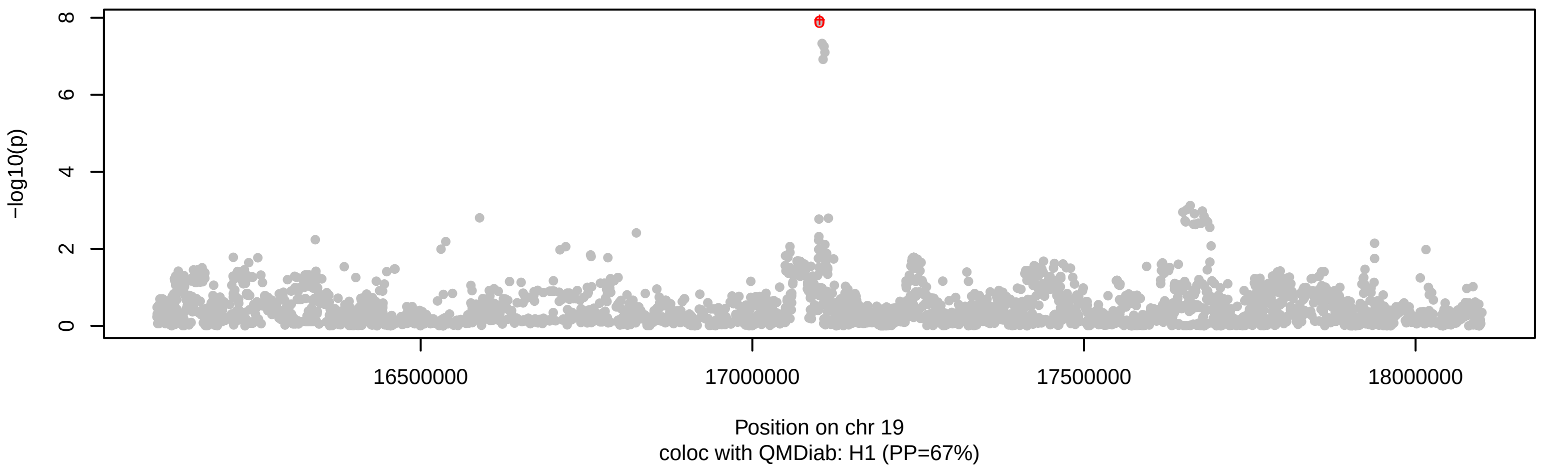

coloc with QMDiab: H1 (PP=67%)

274. SRGN (P10124) 19:17101403:G:A [QMDiab]

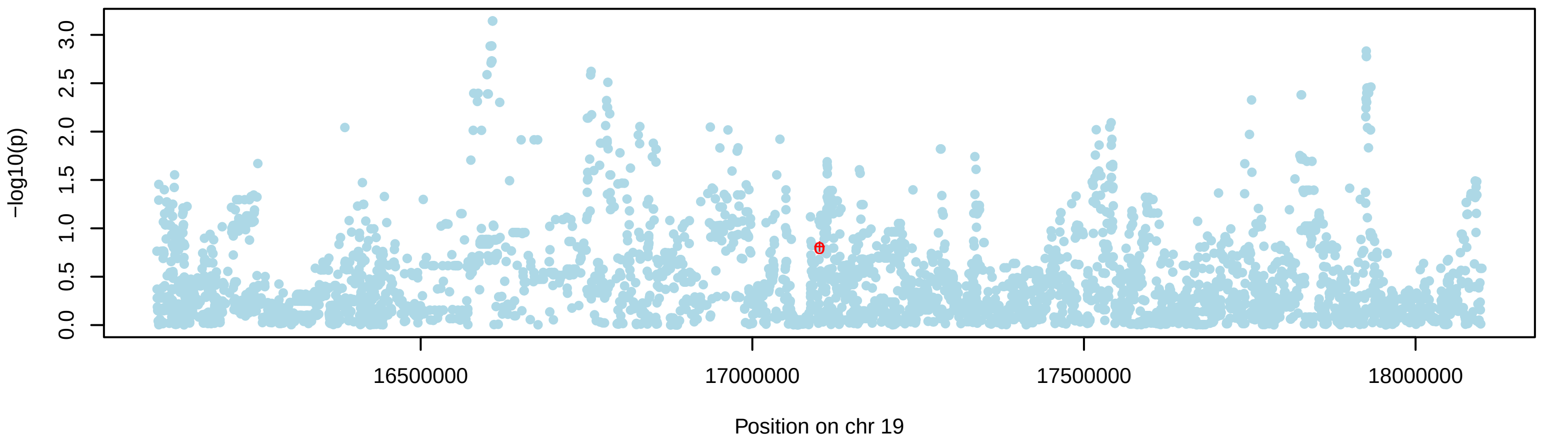

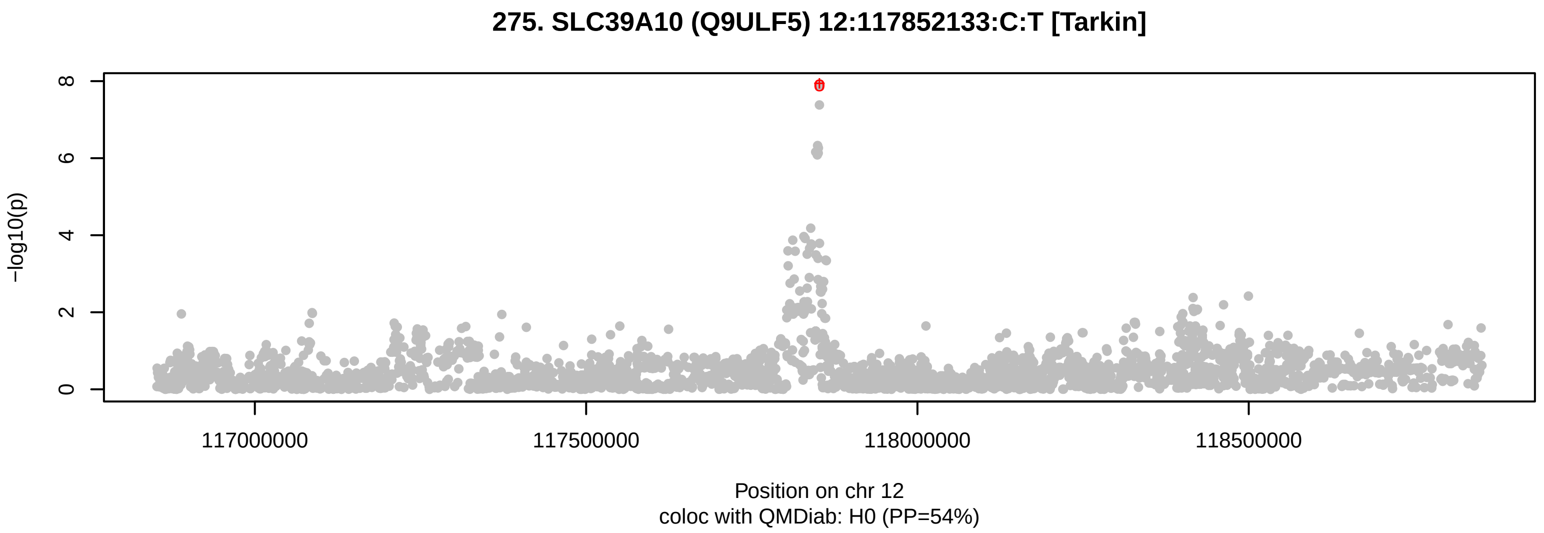
275. SLC39A10 (Q9ULF5) 12:117852133:C:T [Tarkin]
−log10(p)
0
2
4
6
8
117000000
117500000
118000000
118500000
Position on chr 12
coloc with QMDiab: H0 (PP=54%)

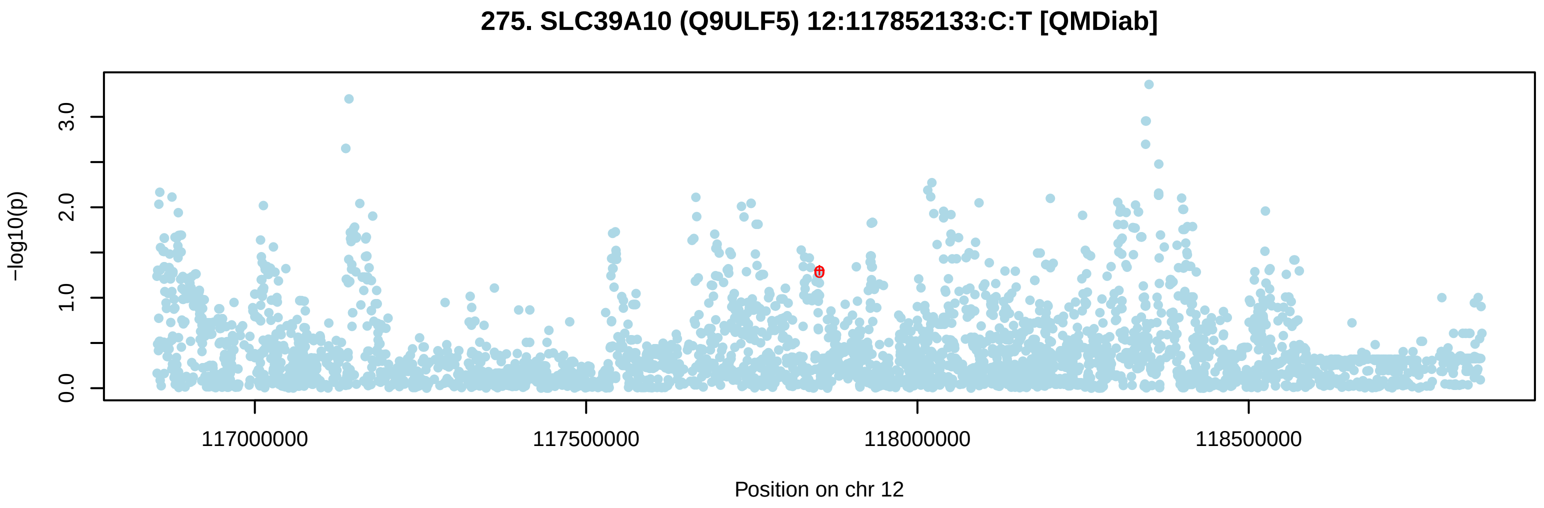
275. SLC39A10 (Q9ULF5) 12:117852133:C:T [QMDiab]
−log10(p)
0.0
1.0
2.0
3.0
117000000
117500000
118000000
118500000
Position on chr 12

276. APOM (O95445) 11:11363305:A:C [Tarkin]

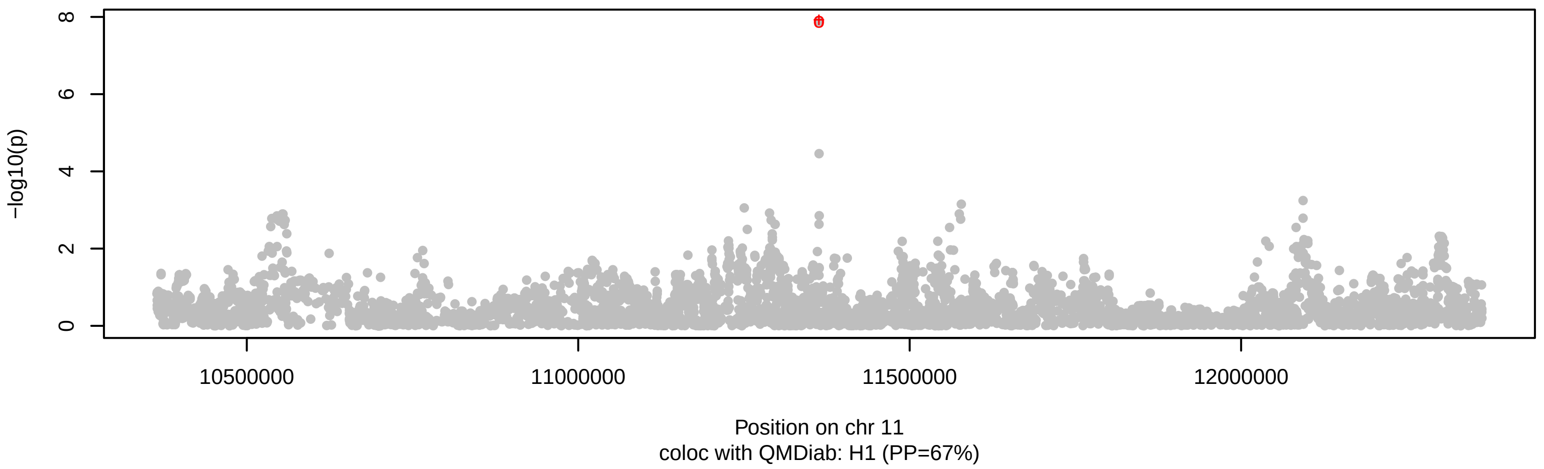

coloc with QMDiab: H1 (PP=67%)

276. APOM (O95445) 11:11363305:A:C [QMDiab]

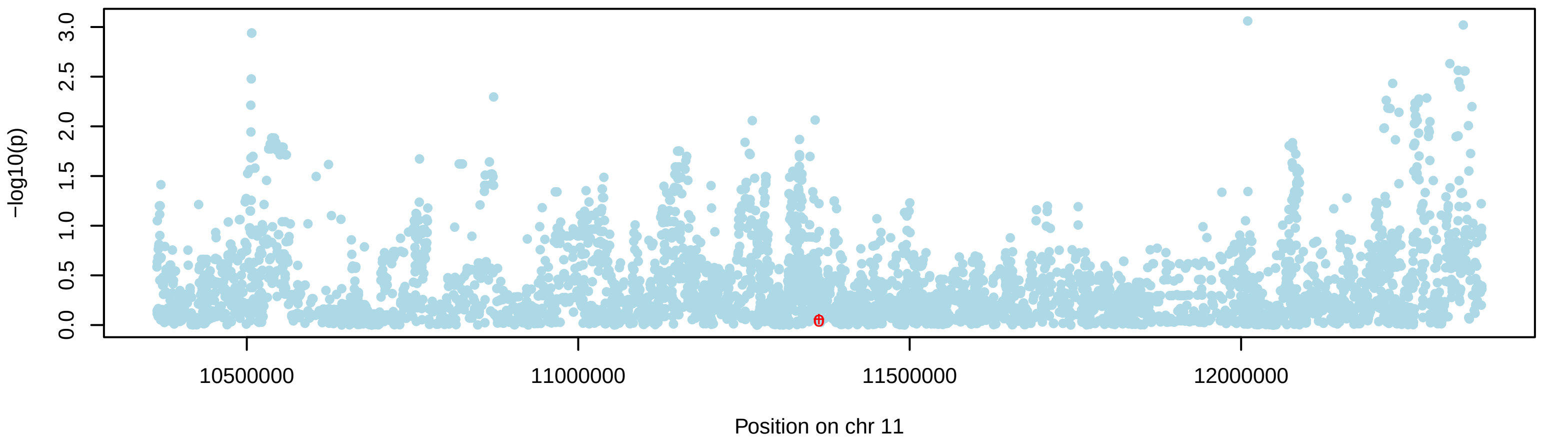

277. LAMA4 (A0A0A0MQS9;A0A0A0MTC7) 12:117062770:G:A [Tarkin]

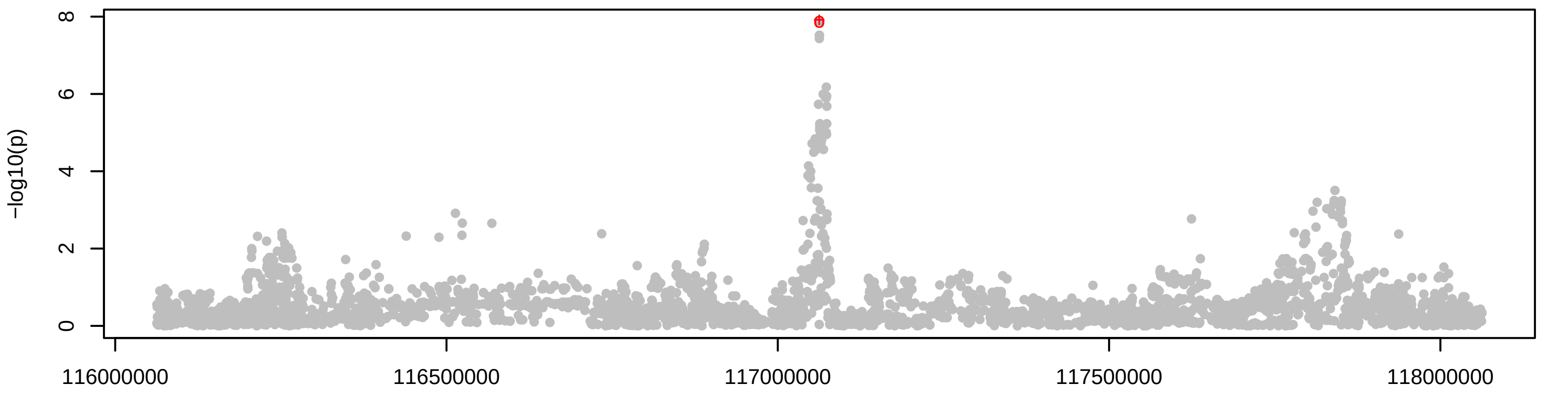

coloc with QMDiab: H1 (PP=71%)

277. LAMA4 (A0A0A0MQS9;A0A0A0MTC7) 12:117062770:G:A [QMDiab]

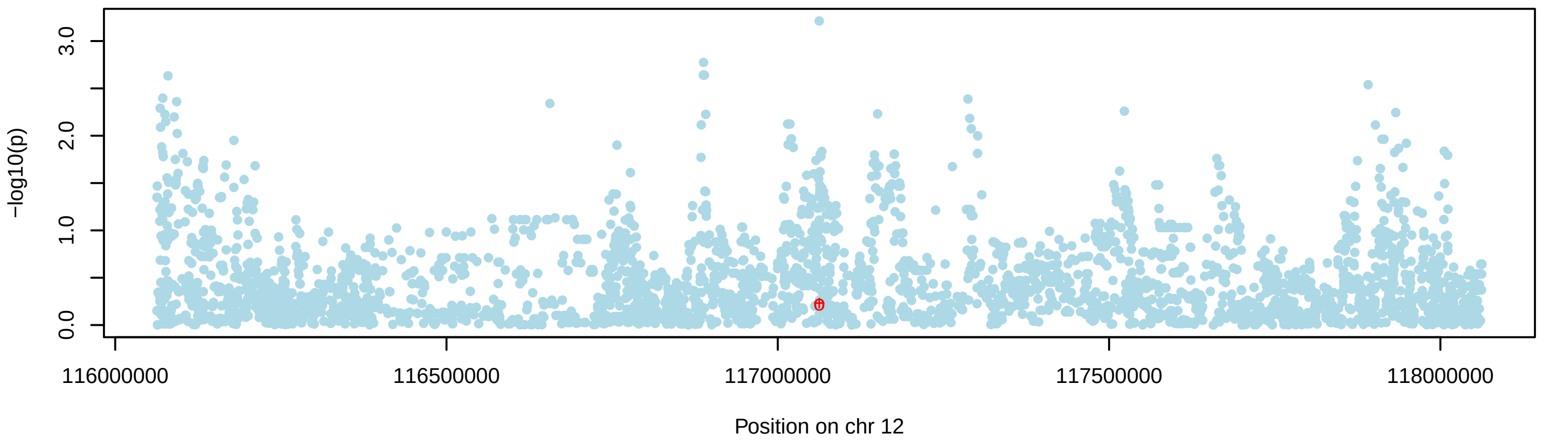

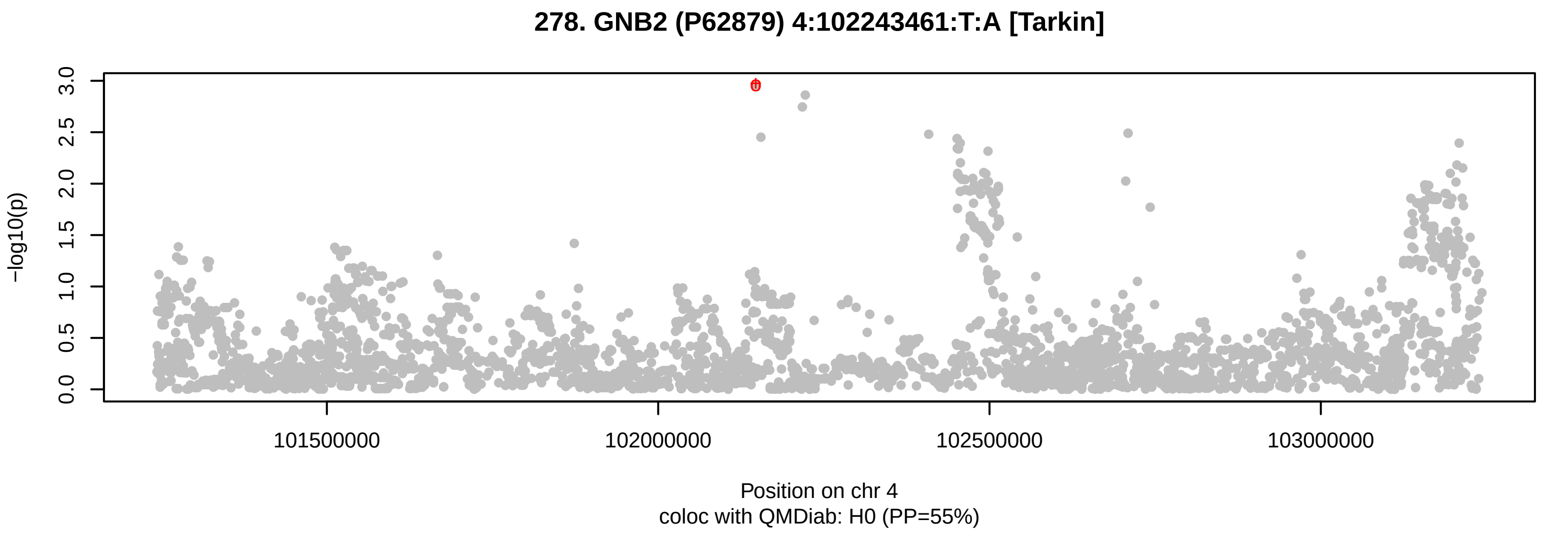

278. GNB2 (P62879) 4:102243461:T:A [Tarkin]
−log10(p)
0.0
0.5
1.0
1.5
2.0
2.5
3.0
101500000
102000000
102500000
103000000
Position on chr 4
coloc with QMDiab: H0 (PP=55%)

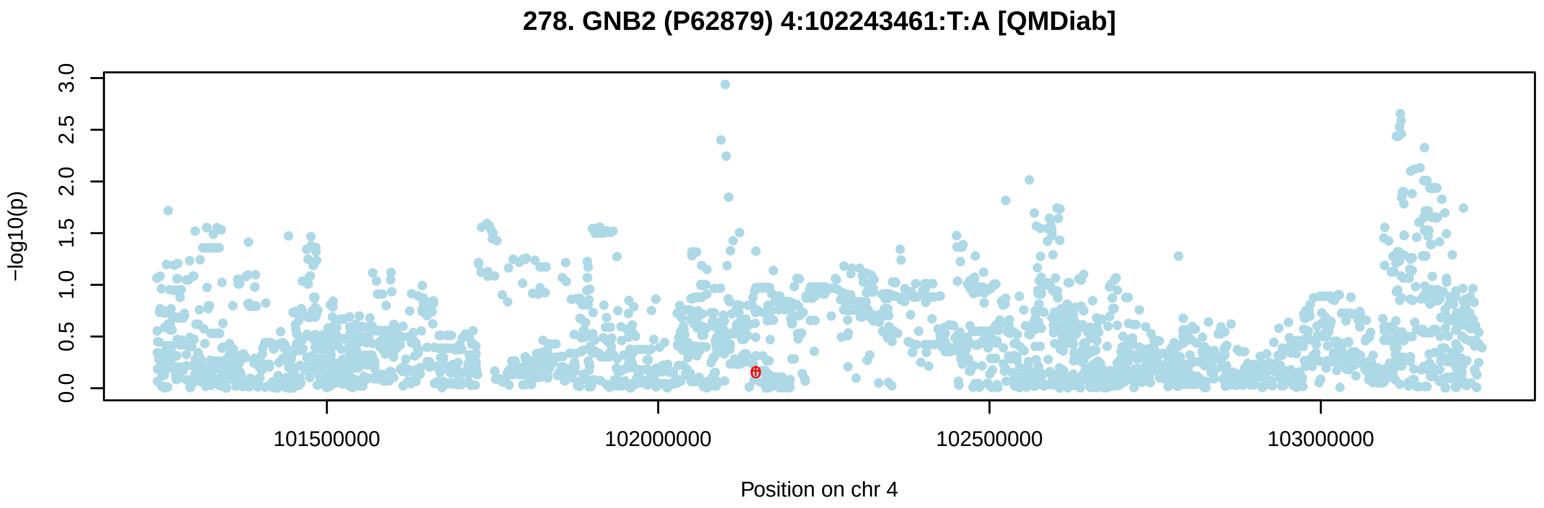

278. GNB2 (P62879) 4:102243461:T:A [QMDiab]
−log10(p)
0.0
0.5
1.0
1.5
2.0
2.5
3.0
101500000
102000000
102500000
103000000
Position on chr 4

279. RNH1 (P13489) 11:501429:G:A [Tarkin]

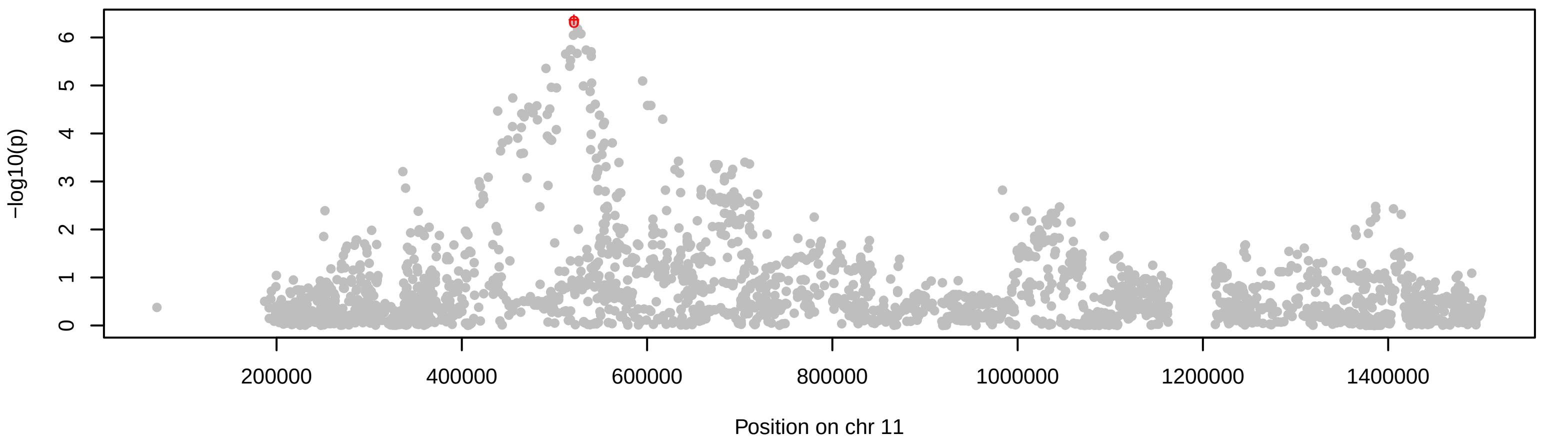

coloc with QMDiab: H1 (PP=74%)

279. RNH1 (P13489) 11:501429:G:A [QMDiab]

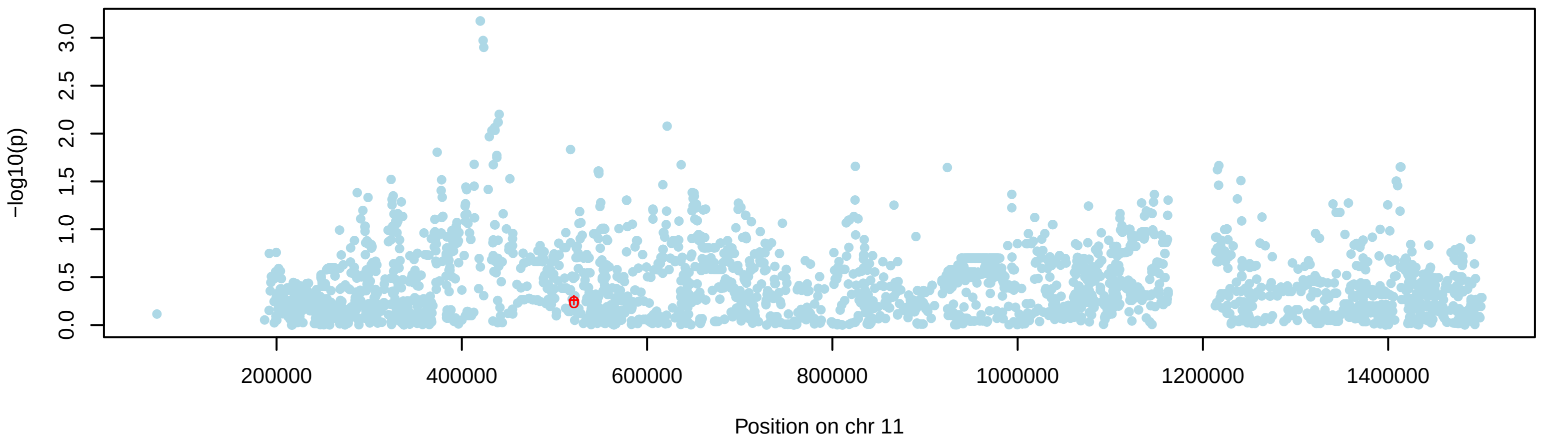

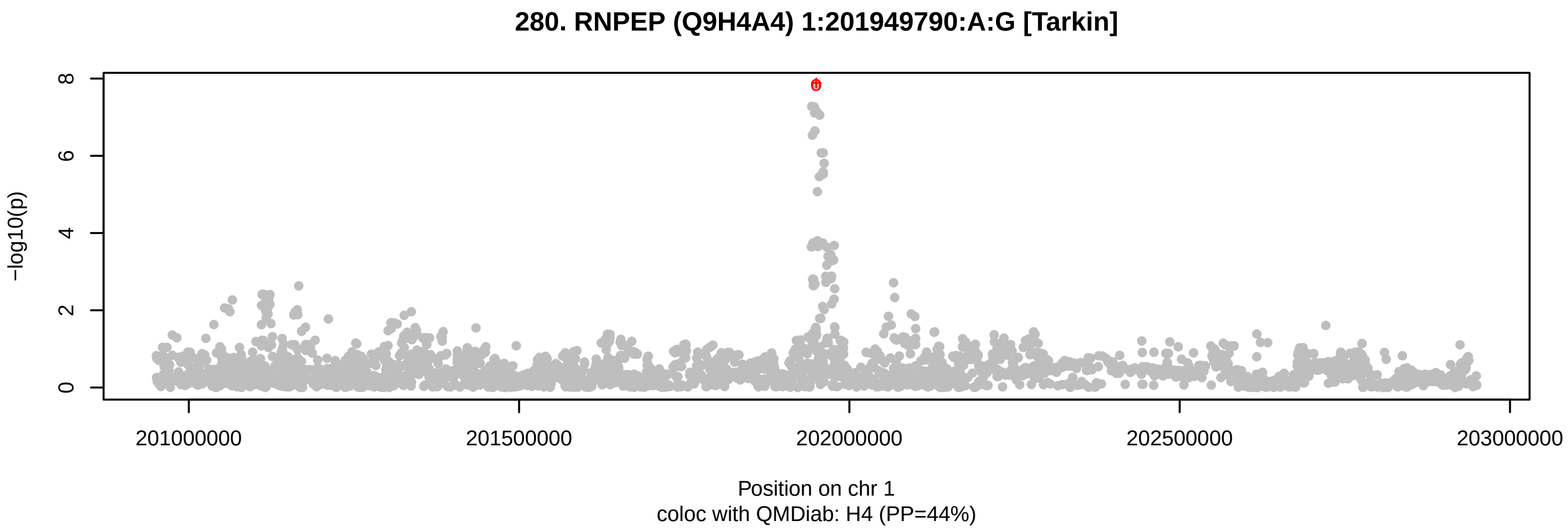

coloc with QMDiab: H4 (PP=44%)

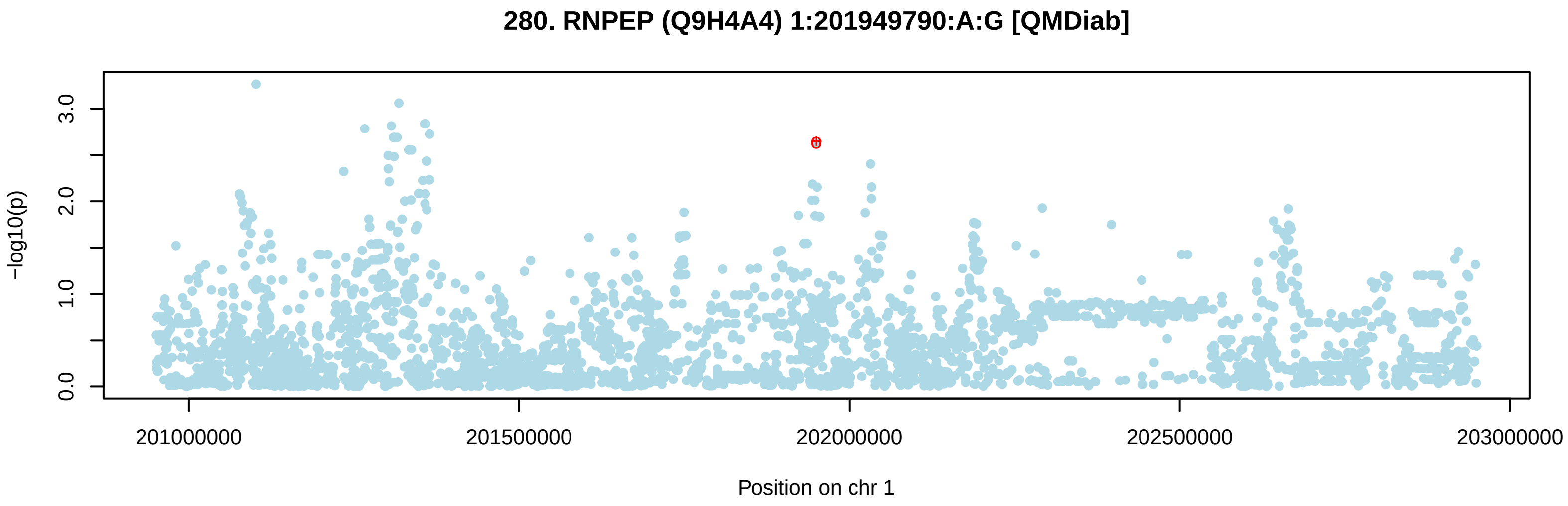

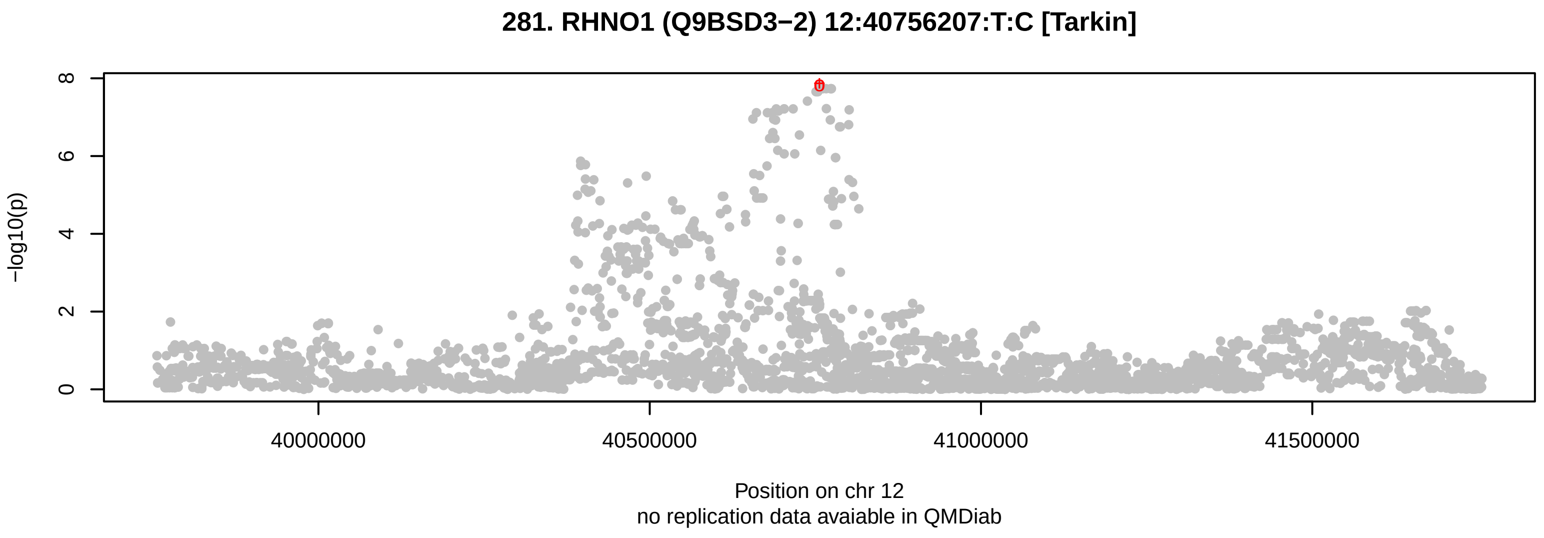
281. RHNO1 (Q9BSD3−2) 12:40756207:T:C [Tarkin]
−log10(p)
0
2
4
6
8
40000000
40500000
41000000
41500000
Position on chr 12
no replication data avaiable in QMDiab

282. MYOC (Q99972) 1:171599335:C:T [Tarkin]

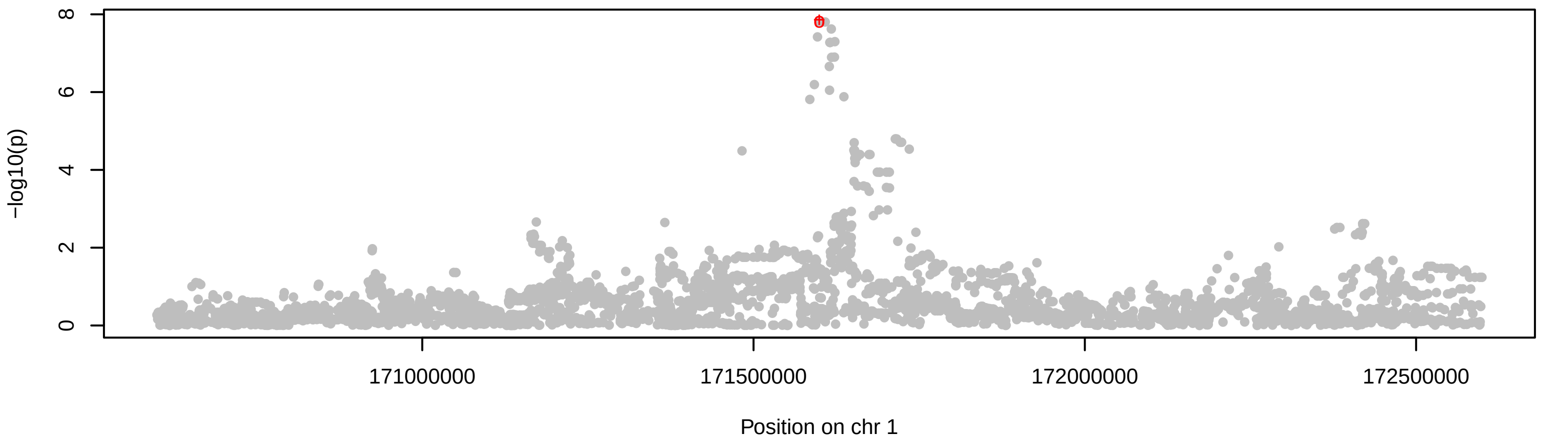

coloc with QMDiab: H4 (PP=45%)

282. MYOC (Q99972) 1:171599335:C:T [QMDiab]

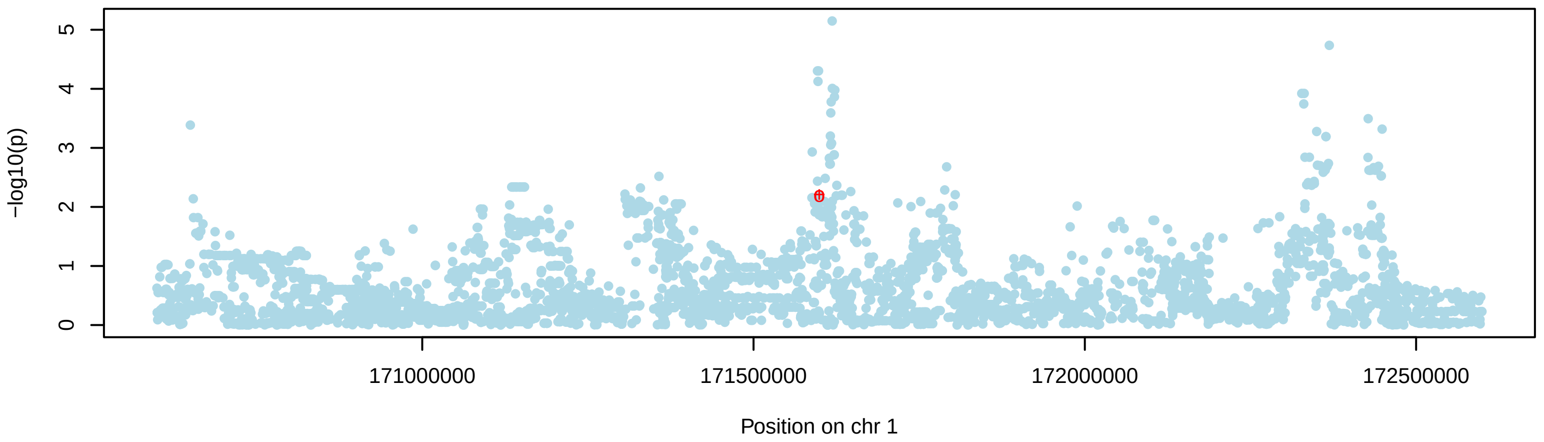

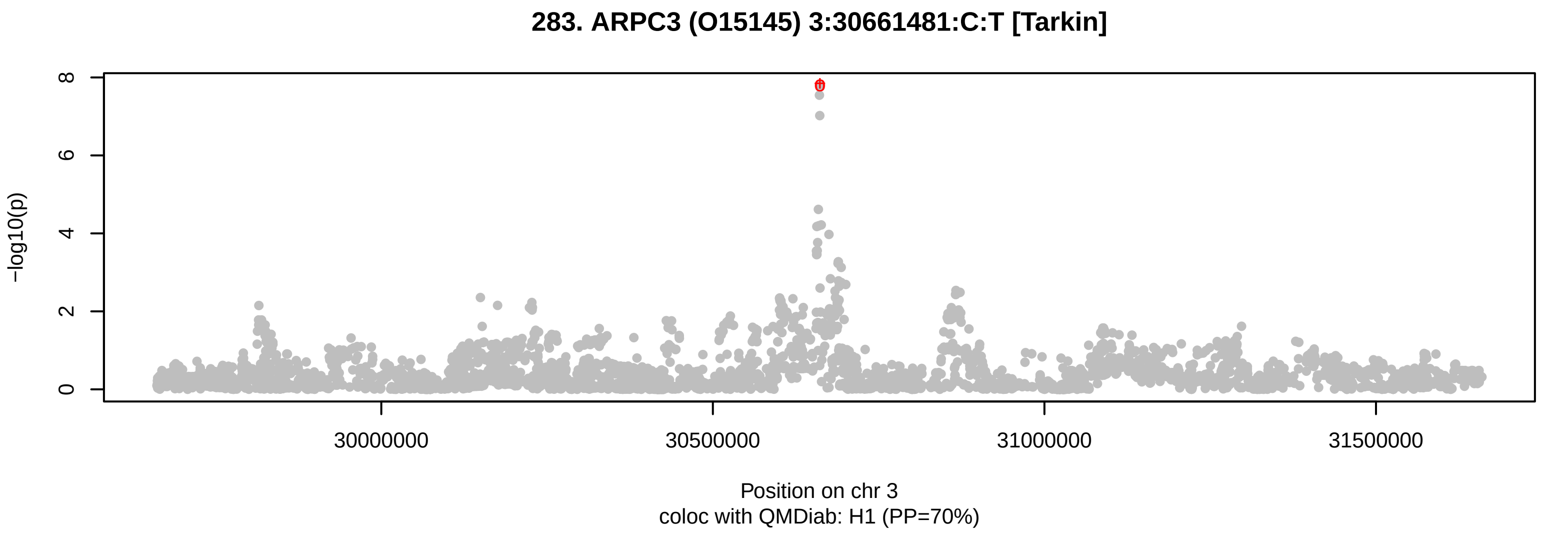
283. ARPC3 (O15145) 3:30661481:C:T [Tarkin]
−log10(p)
0
2
4
6
8
30000000
30500000
31000000
31500000
Position on chr 3
coloc with QMDiab: H1 (PP=70%)

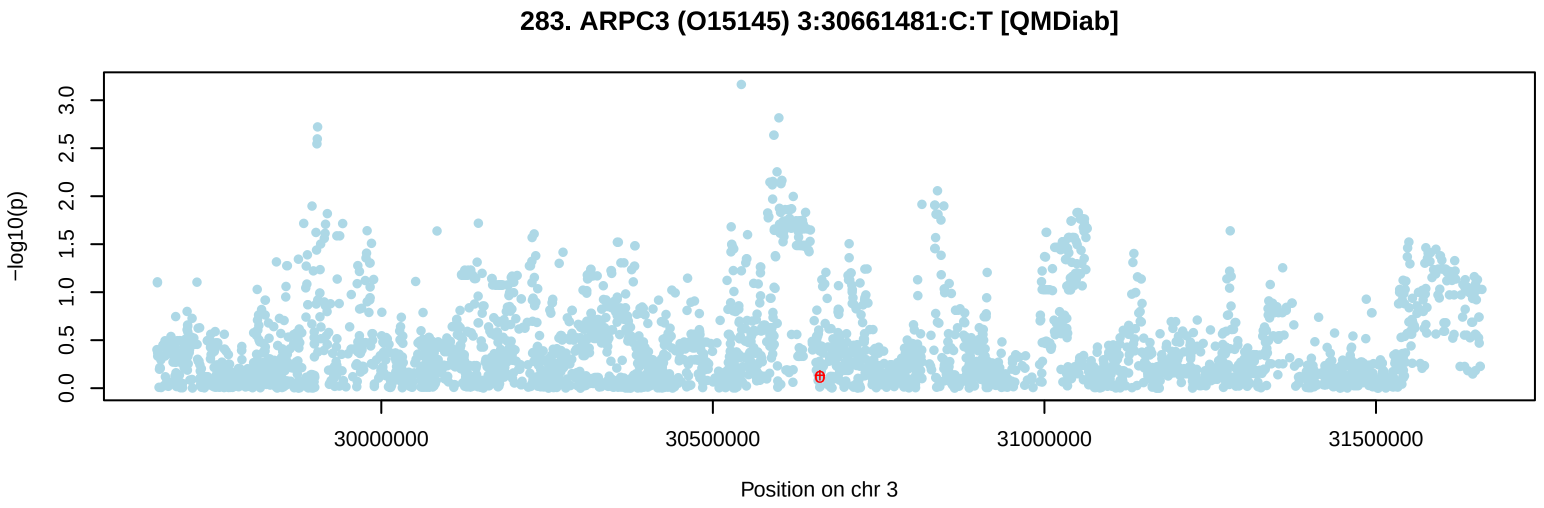
283. ARPC3 (O15145) 3:30661481:C:T [QMDiab]
−log10(p)
0.0
0.5
1.0
1.5
2.0
2.5
3.0
30000000
30500000
31000000
31500000
Position on chr 3

284. TUBB1 (Q9H4B7) 13:89081961:C:G [Tarkin]

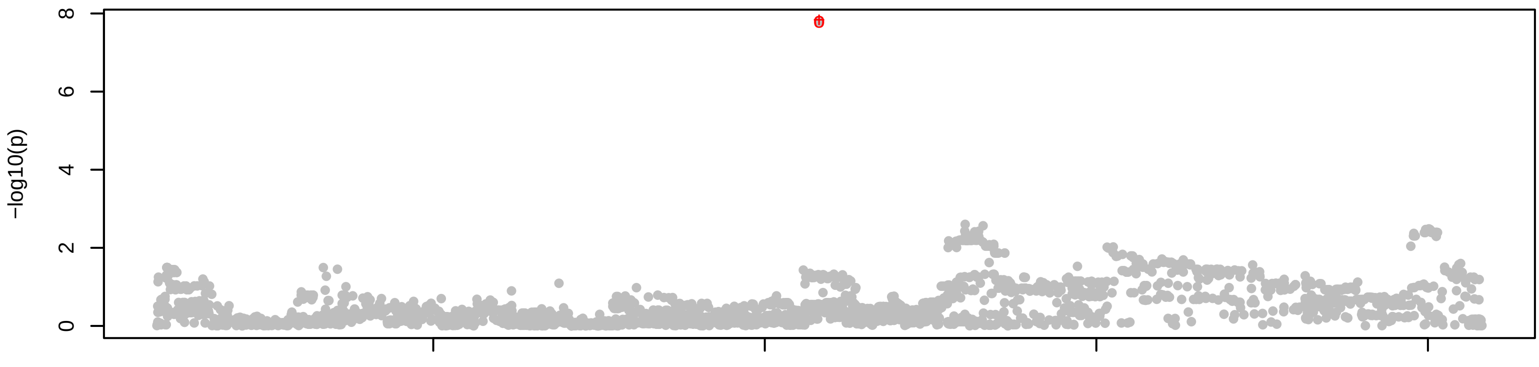

Position on chr 13
coloc with QMDiab: H1 (PP=70%)

284. TUBB1 (Q9H4B7) 13:89081961:C:G [QMDiab]

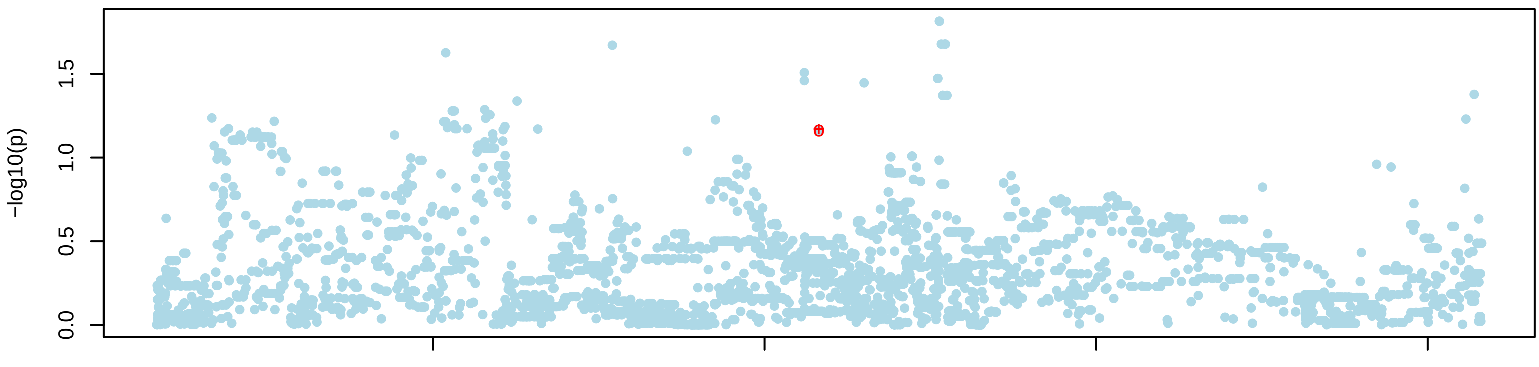

Position on chr 13

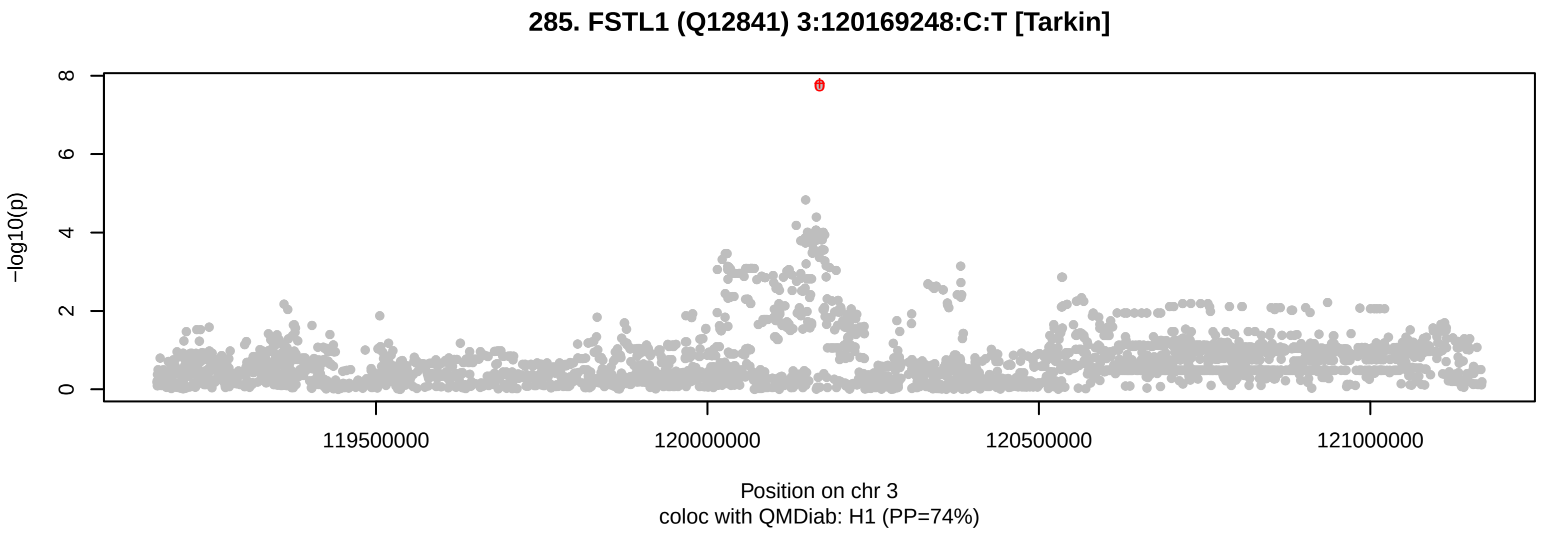

285. FSTL1 (Q12841) 3:120169248:C:T [Tarkin]
−log10(p)
0
2
4
6
8
119500000
120000000
120500000
121000000
Position on chr 3
coloc with QMDiab: H1 (PP=74%)

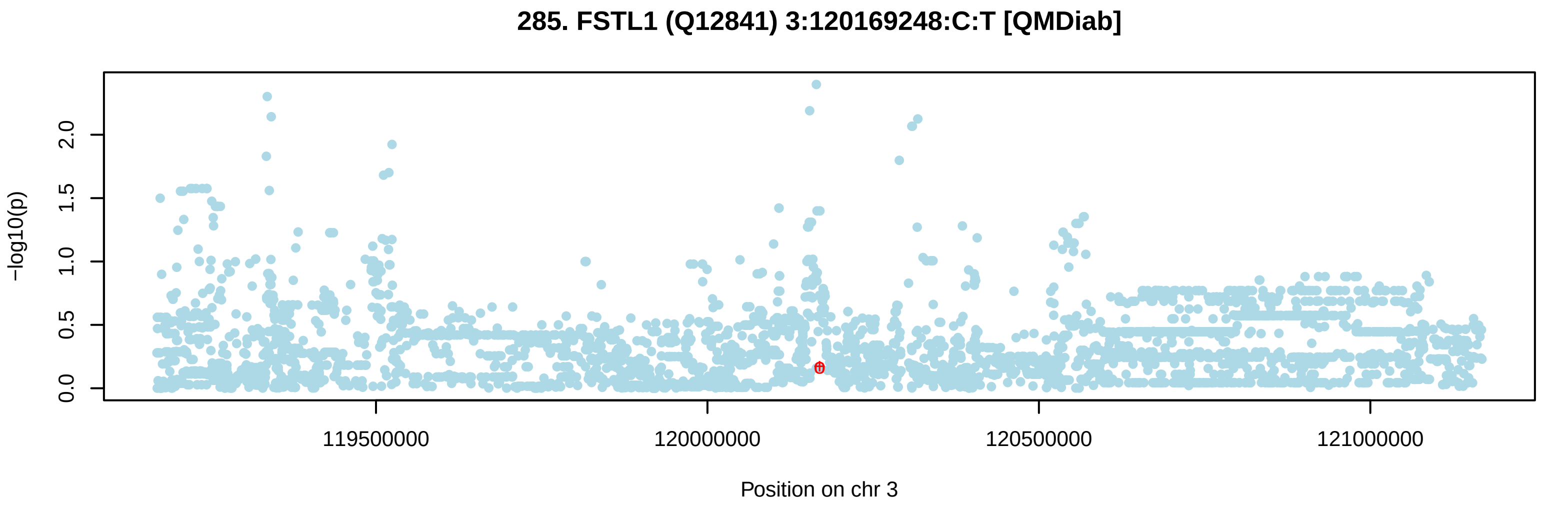

285. FSTL1 (Q12841) 3:120169248:C:T [QMDiab]
−log10(p)
0.0
0.5
1.0
1.5
2.0
119500000
120000000
120500000
121000000
Position on chr 3

286. DNAH6 (Q9C0G6) 1:196845521:T:G [Tarkin]

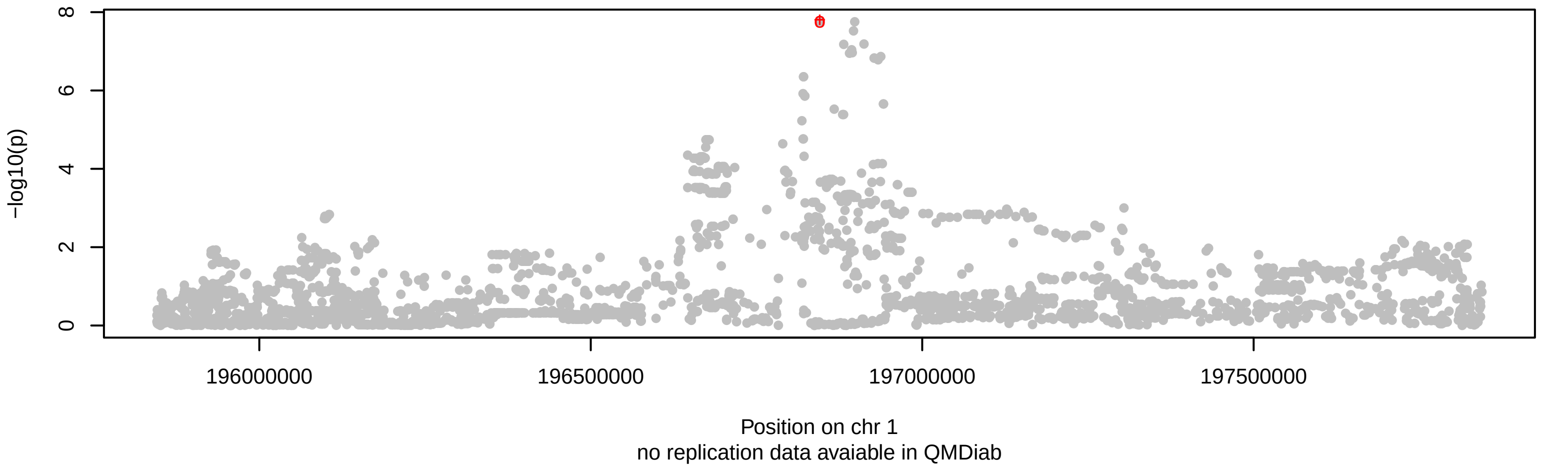

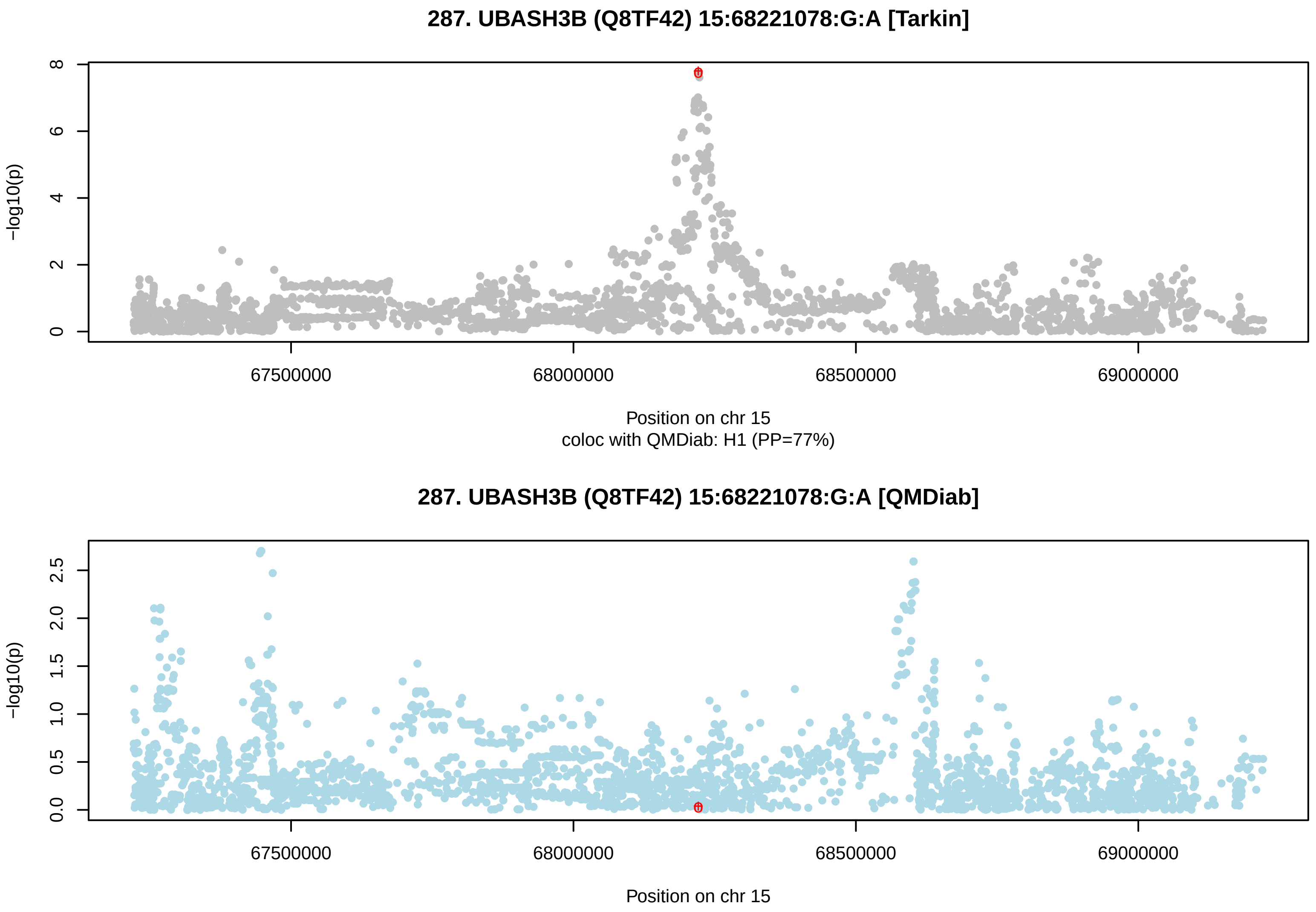
287. UBASH3B (Q8TF42) 15:68221078:G:A [Tarkin]
−log10(p)
0
2
4
6
8
67500000
68000000
68500000
69000000
Position on chr 15
coloc with QMDiab: H1 (PP=77%)
287. UBASH3B (Q8TF42) 15:68221078:G:A [QMDiab]
−log10(p)
0.0
0.5
1.0
1.5
2.0
2.5
67500000
68000000
68500000
69000000
Position on chr 15

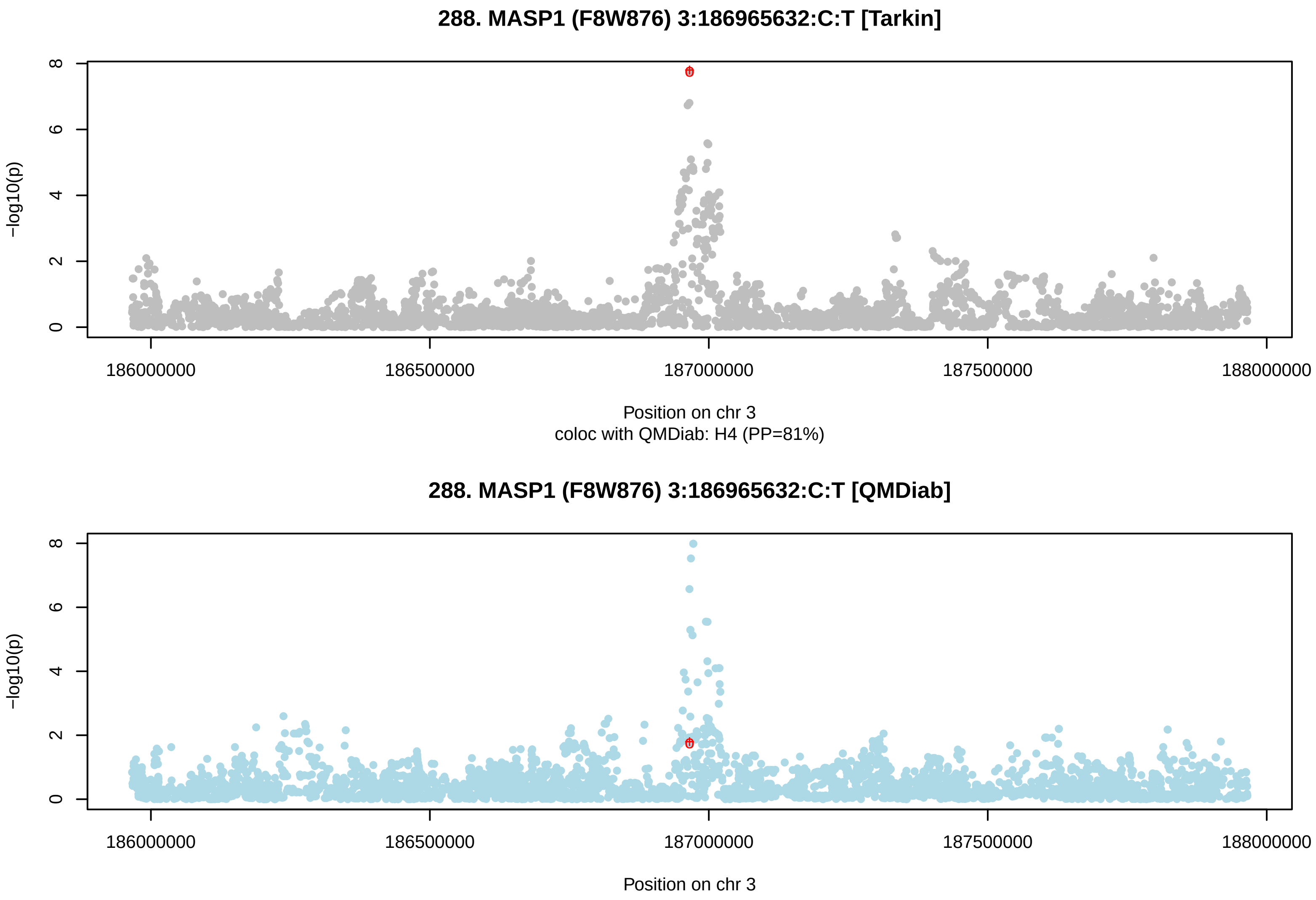
288. MASP1 (F8W876) 3:186965632:C:T [Tarkin]
−log10(p)
0
2
4
6
8
186000000
186500000
187000000
187500000
188000000
Position on chr 3
coloc with QMDiab: H4 (PP=81%)
288. MASP1 (F8W876) 3:186965632:C:T [QMDiab]
−log10(p)
0
2
4
6
8
186000000
186500000
187000000
187500000
188000000
Position on chr 3

289. POTEF (A5A3E0) 5:8473747:A:G [Tarkin]

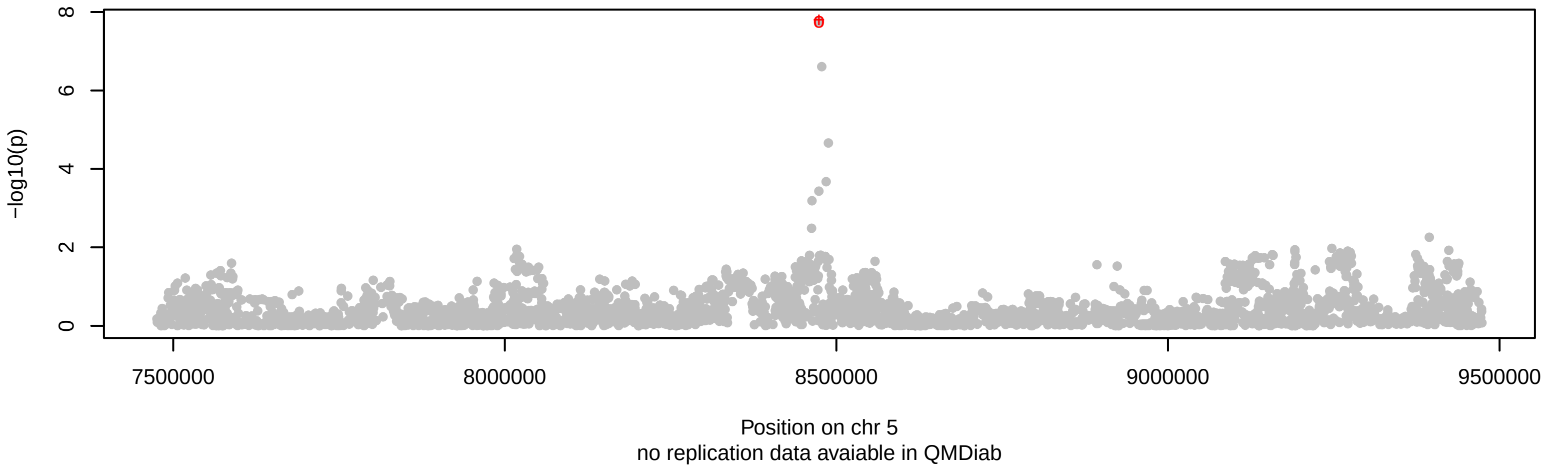

no replication data avaiable in QMDiab

290. DNAJB11 (Q9UBS4) 6:31376217:A:C [Tarkin]

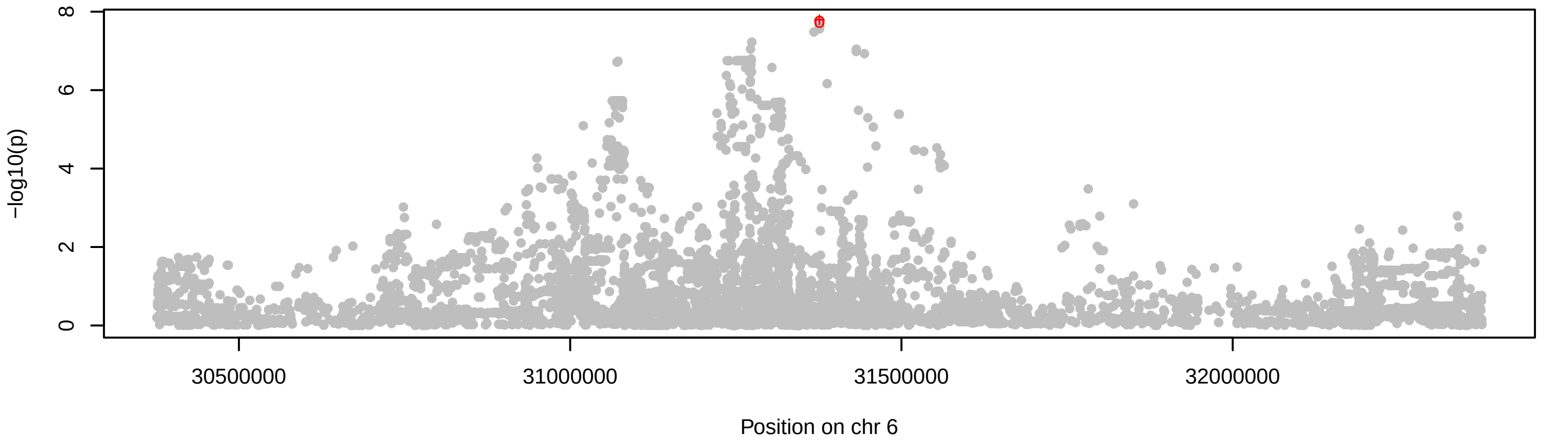

coloc with QMDiab: H1 (PP=49%)

290. DNAJB11 (Q9UBS4) 6:31376217:A:C [QMDiab]

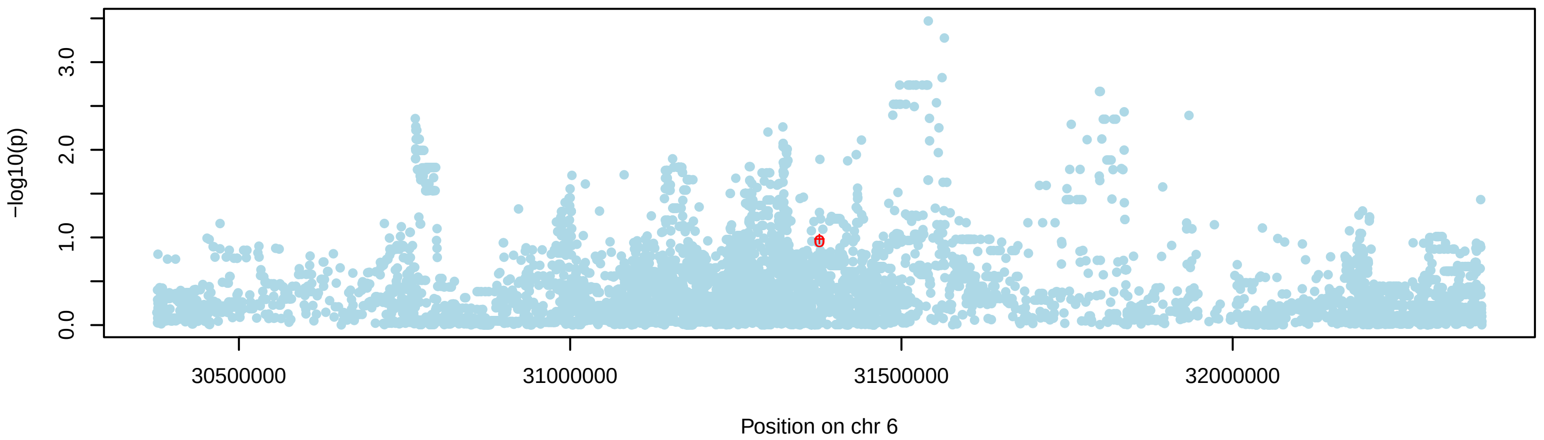

291. ATP6V1A (P38606) 7:19132687:T:C [Tarkin]

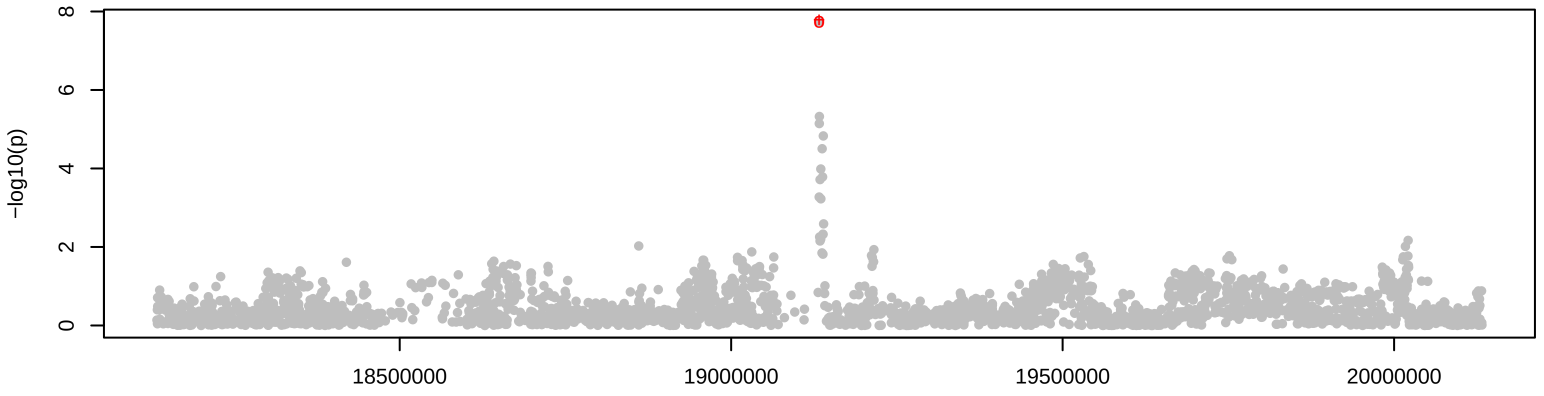

Position on chr 7
coloc with QMDiab: H1 (PP=40%)

291. ATP6V1A (P38606) 7:19132687:T:C [QMDiab]

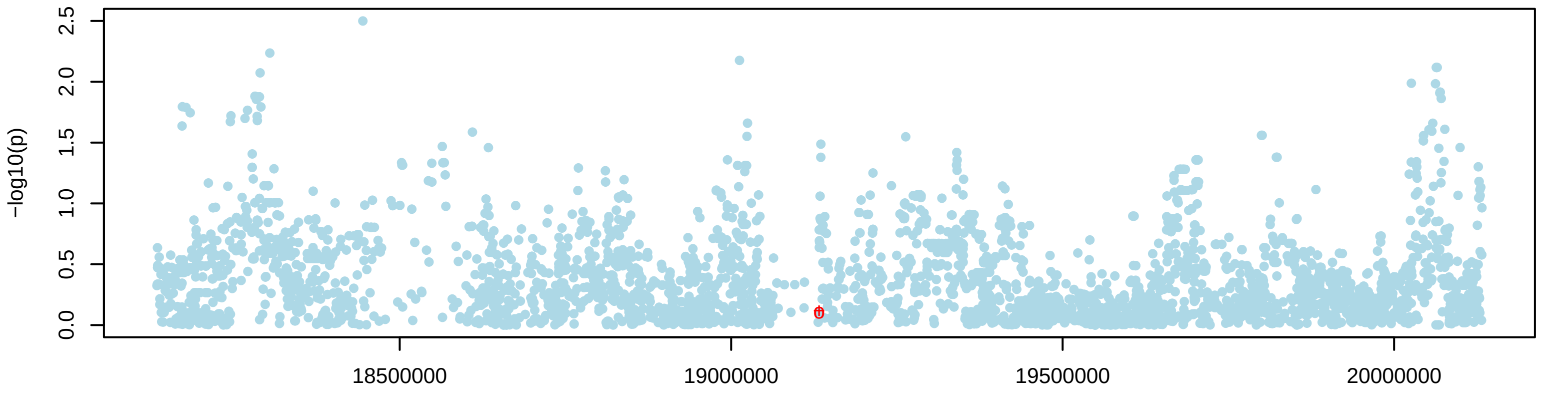

Position on chr 7

292. HNRNPF (P52597) 8:135959545:C:T [Tarkin]

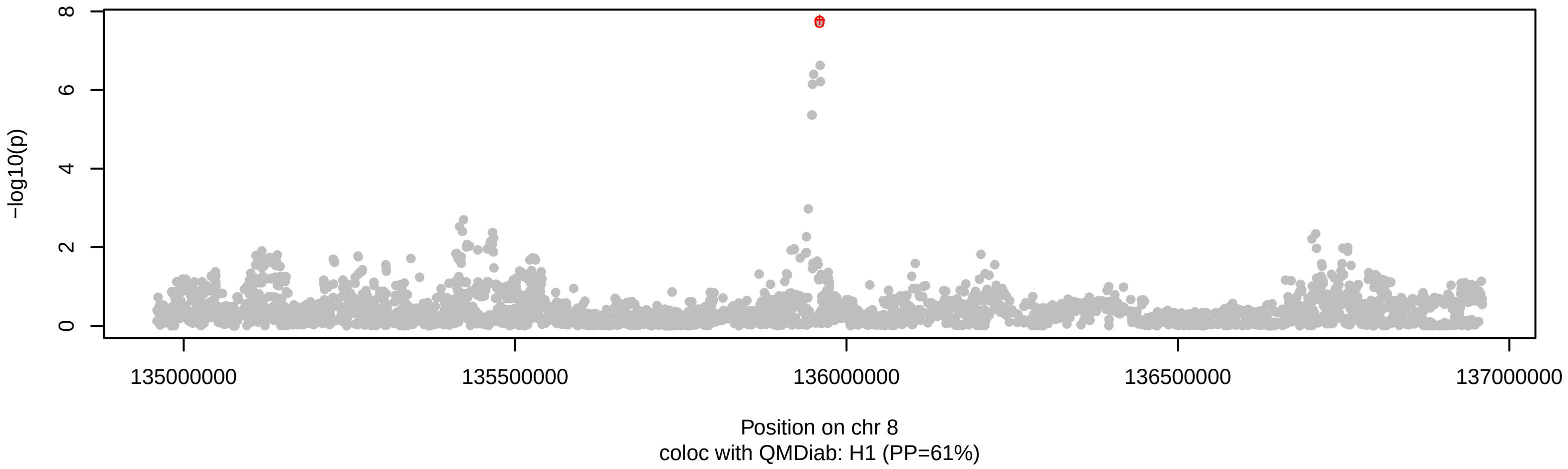

coloc with QMDiab: H1 (PP=61%)

292. HNRNPF (P52597) 8:135959545:C:T [QMDiab]

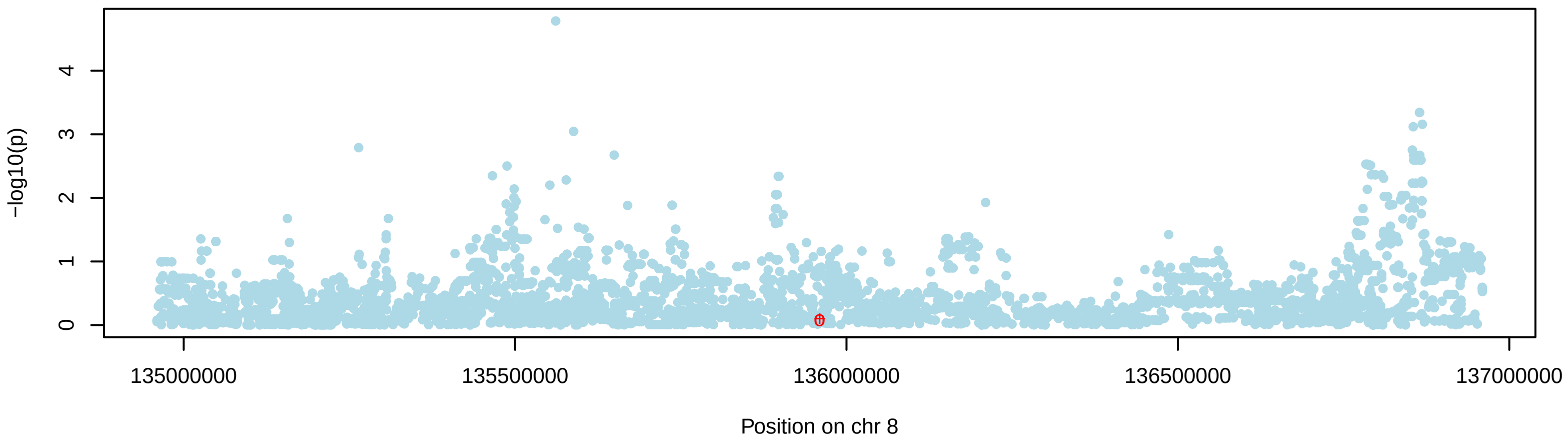

293. ACTR1A (P61163) 13:56324143:C:T [Tarkin]

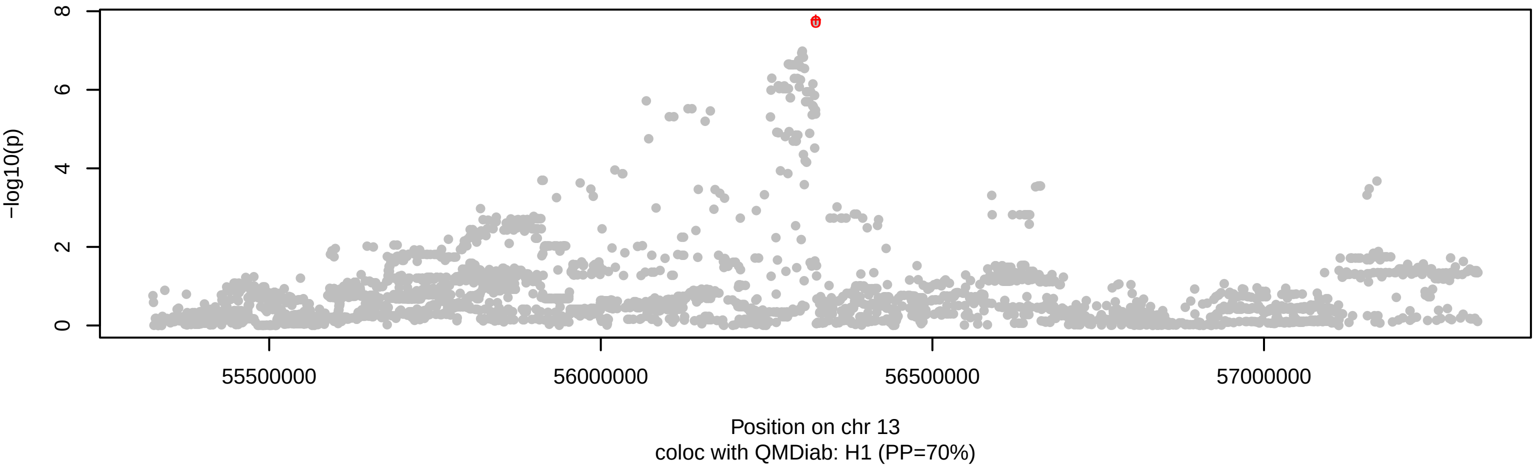

293. ACTR1A (P61163) 13:56324143:C:T [QMDiab]

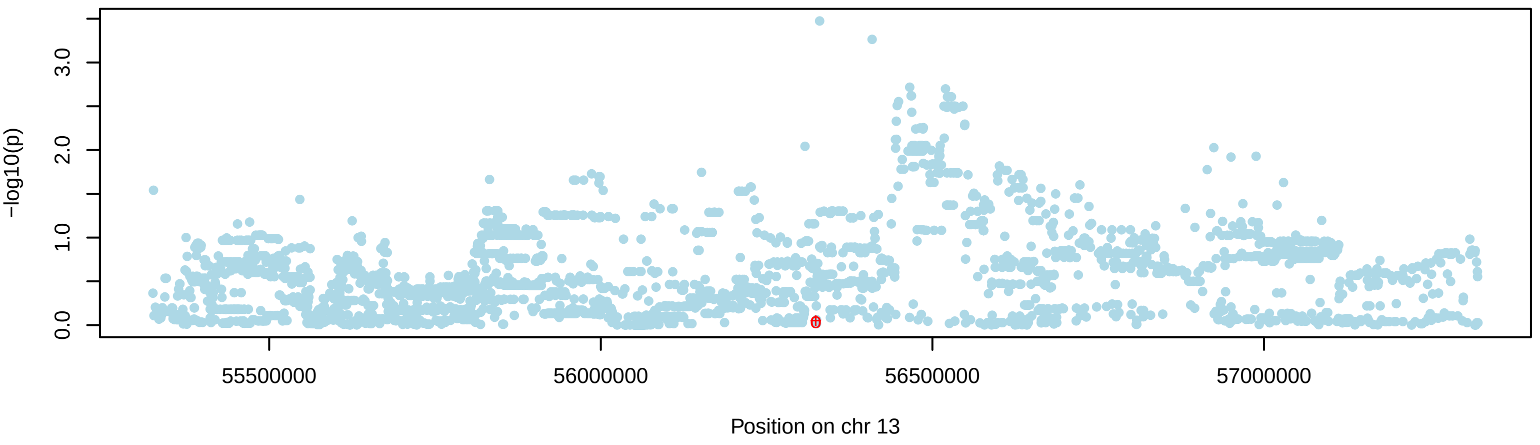

294. CHST11 (Q9NPF2;Q9NPF2−2) 10:8246475:G:A [Tarkin]

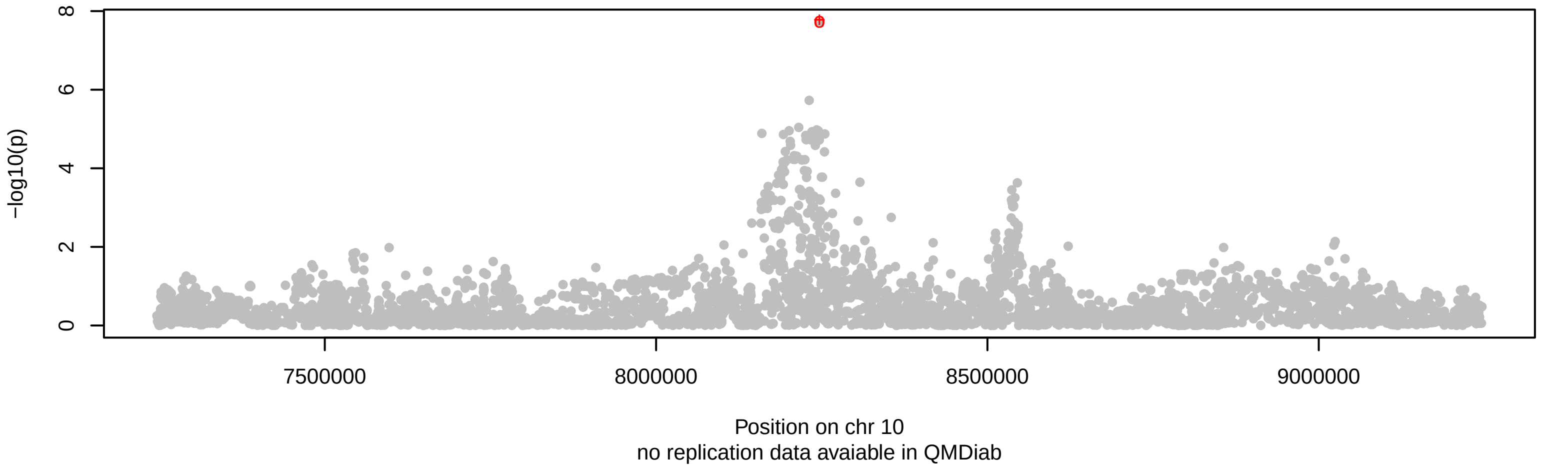

295. GNLY (B4E3H9;P22749;P22749−2) 2:85933003:C:T [Tarkin]

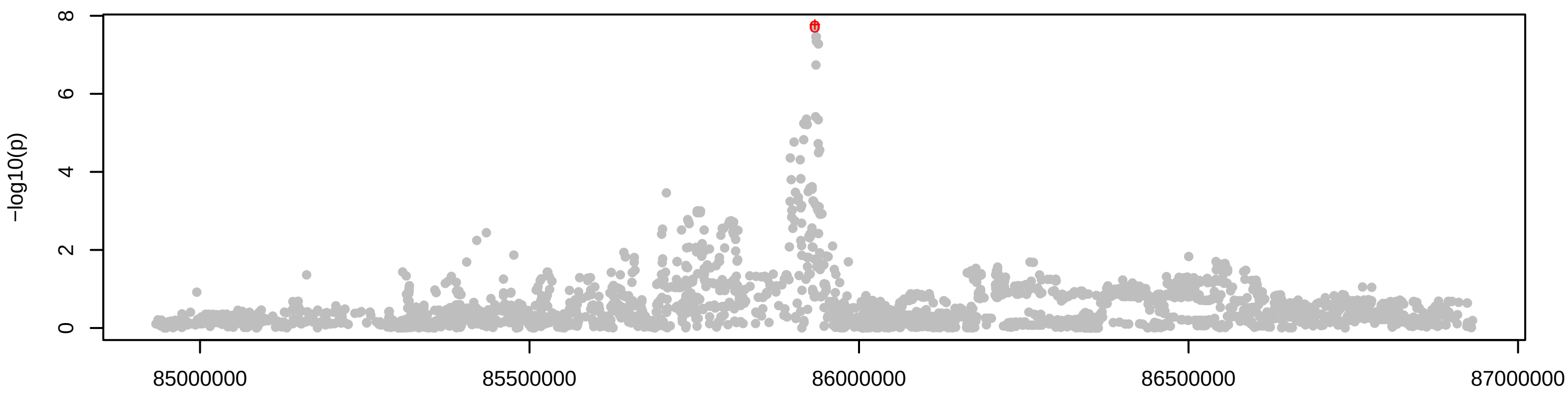

Position on chr 2
coloc with QMDiab: H1 (PP=69%)

295. GNLY (B4E3H9;P22749;P22749−2) 2:85933003:C:T [QMDiab]

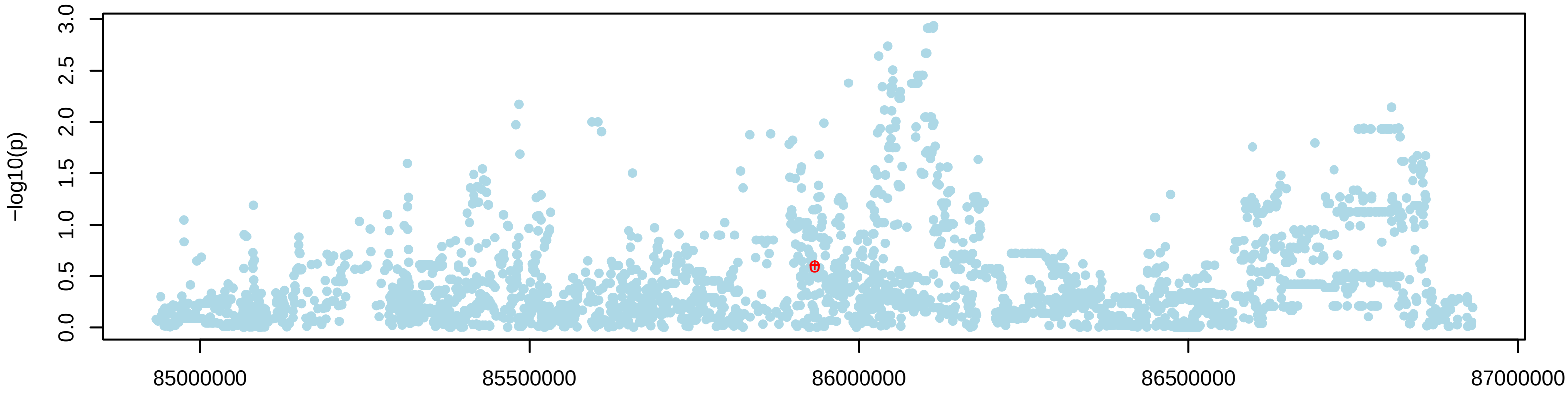

Position on chr 2

296. TARS (P26639) 7:19132687:T:C [Tarkin]

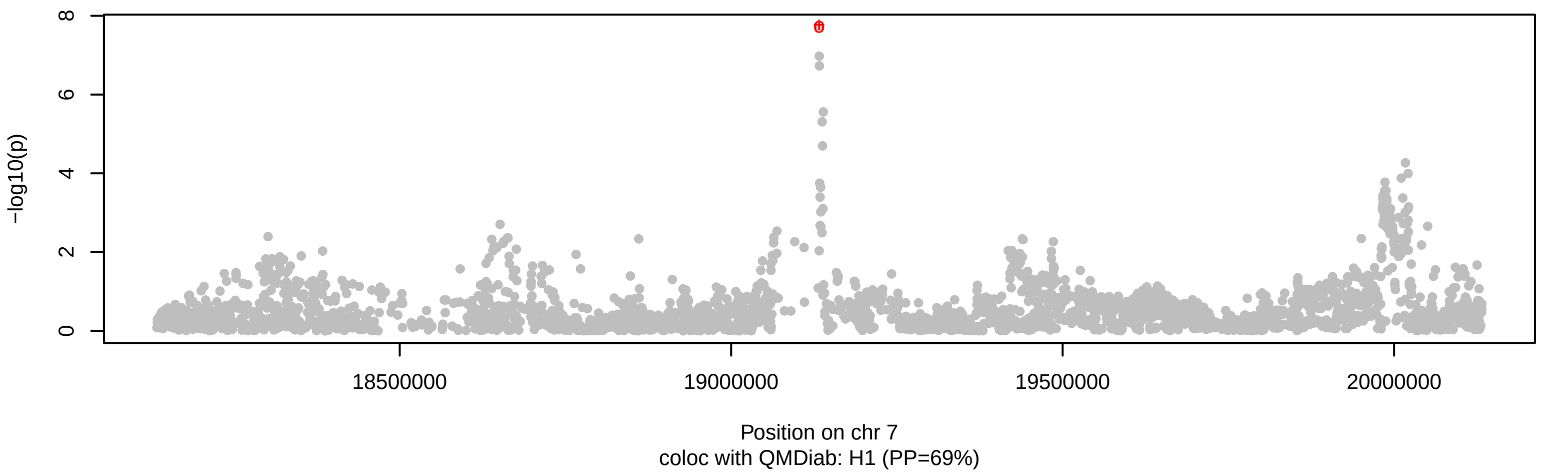

coloc with QMDiab: H1 (PP=69%)

296. TARS (P26639;P26639−2) 7:19132687:T:C [QMDiab]

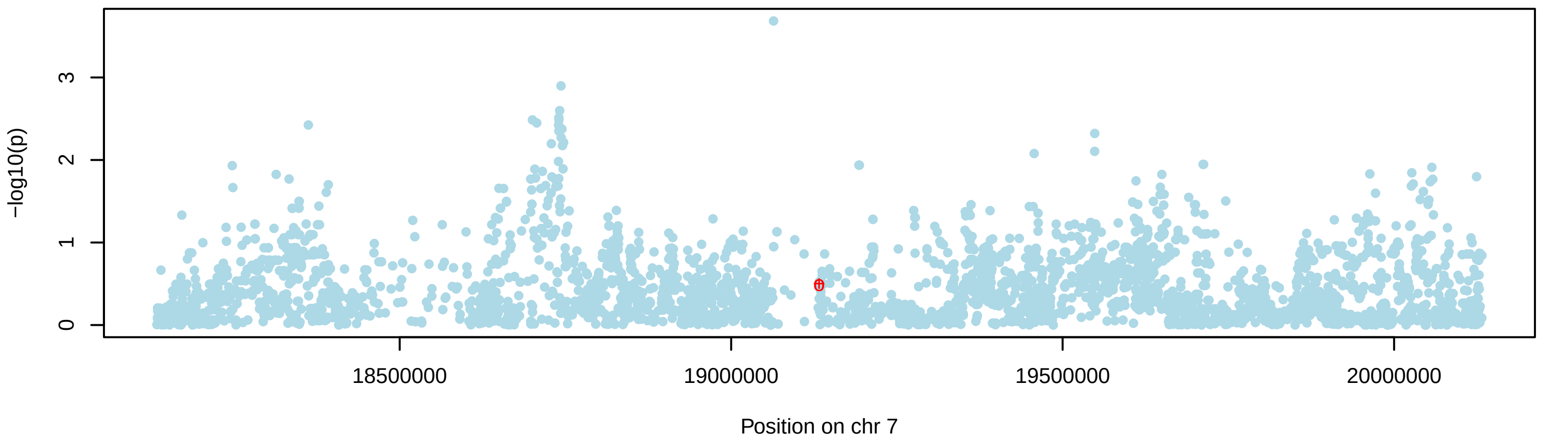

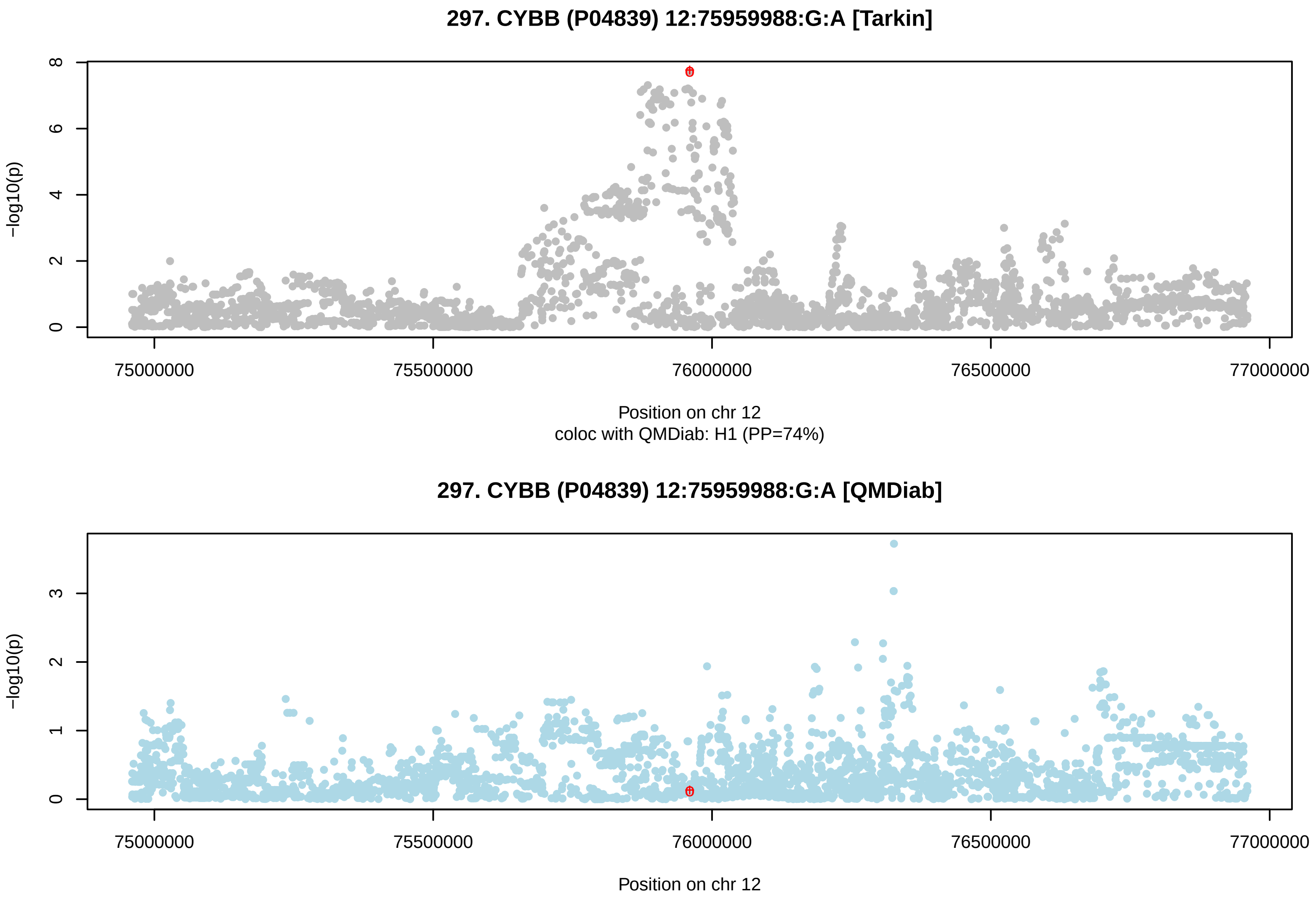
297. CYBB (P04839) 12:75959988:G:A [Tarkin]
−log10(p)
0
2
4
6
8
75000000
75500000
76000000
76500000
77000000
Position on chr 12
coloc with QMDiab: H1 (PP=74%)
297. CYBB (P04839) 12:75959988:G:A [QMDiab]
−log10(p)
0
1
2
3
75000000
75500000
76000000
76500000
77000000
Position on chr 12

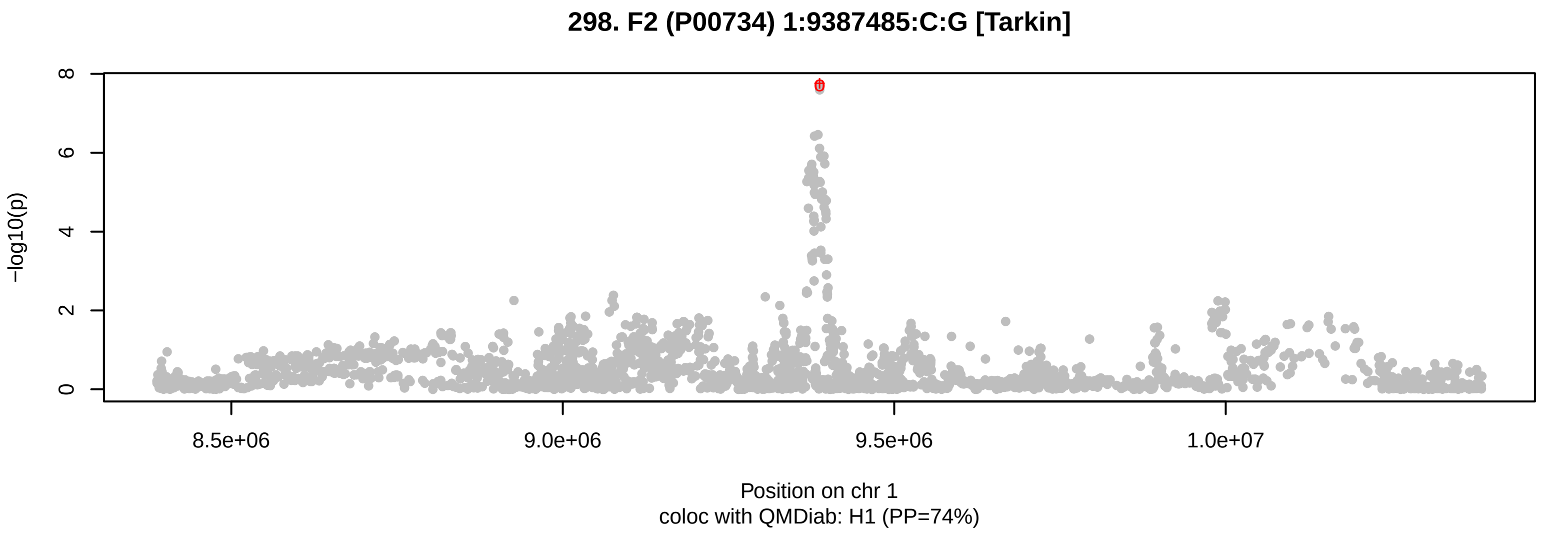
298. F2 (P00734) 1:9387485:C:G [Tarkin]
−log10(p)
0
2
4
6
8
8.5e+06
9.0e+06
9.5e+06
1.0e+07
Position on chr 1
coloc with QMDiab: H1 (PP=74%)

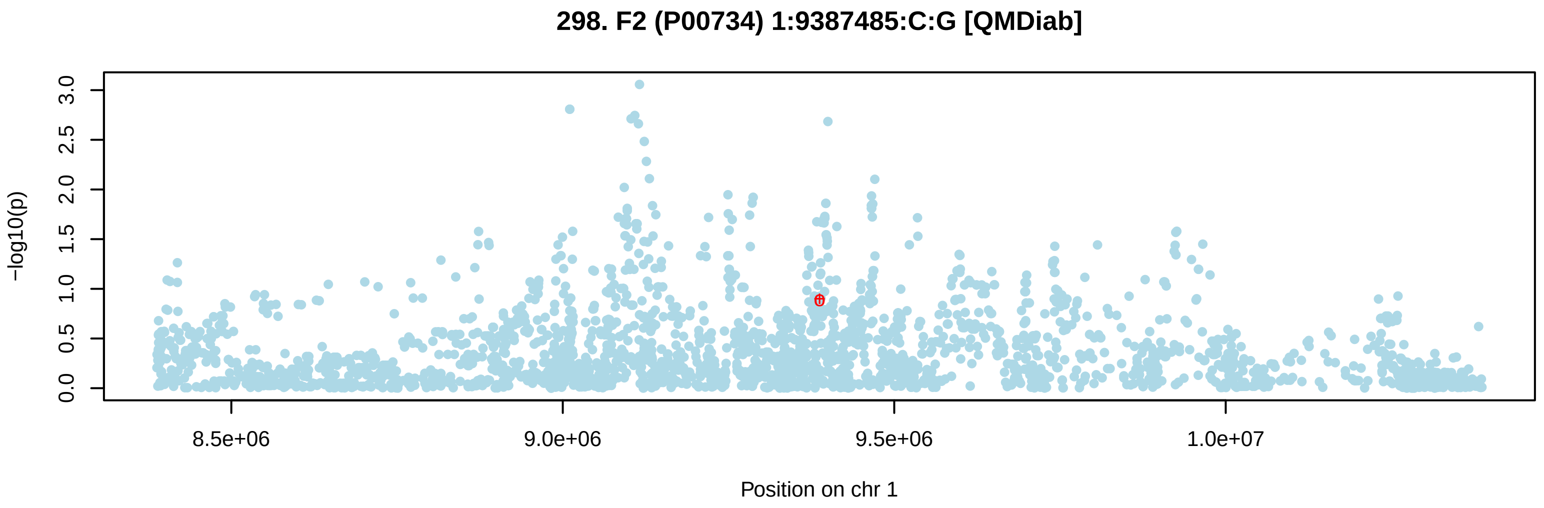
298. F2 (P00734) 1:9387485:C:G [QMDiab]
−log10(p)
0.0
0.5
1.0
1.5
2.0
2.5
3.0
8.5e+06
9.0e+06
9.5e+06
1.0e+07
Position on chr 1

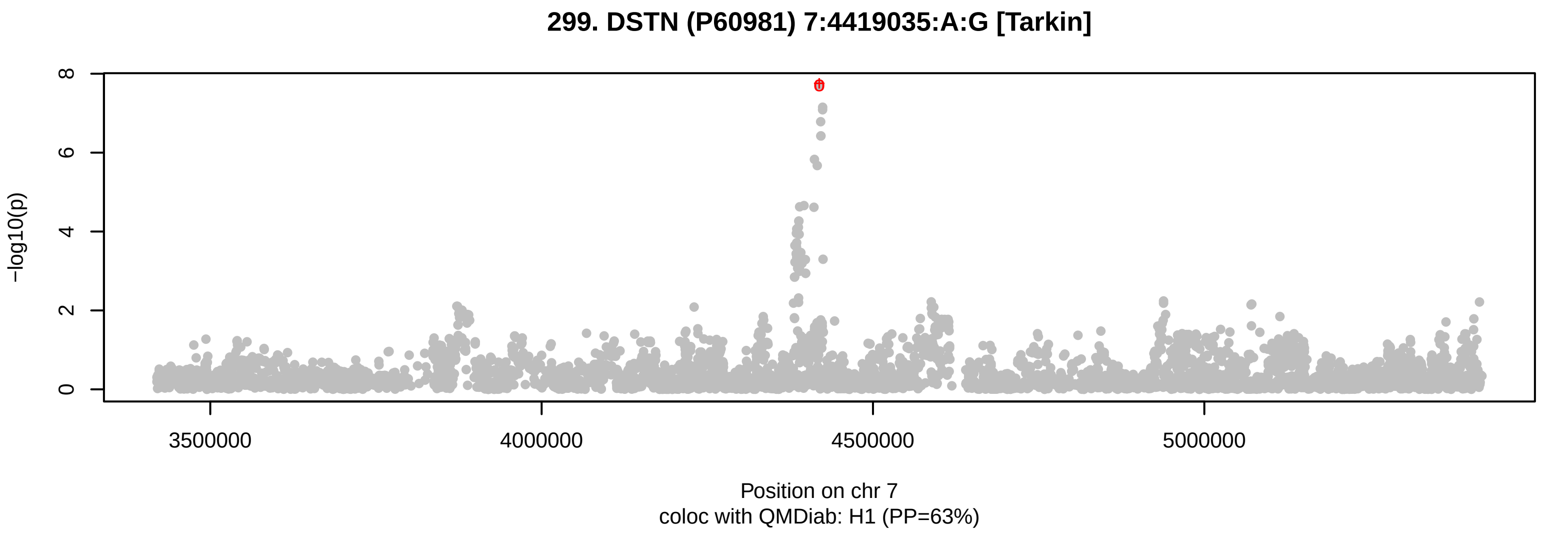
299. DSTN (P60981) 7:4419035:A:G [Tarkin]
−log10(p)
0
2
4
6
8
3500000
4000000
4500000
5000000
Position on chr 7
coloc with QMDiab: H1 (PP=63%)

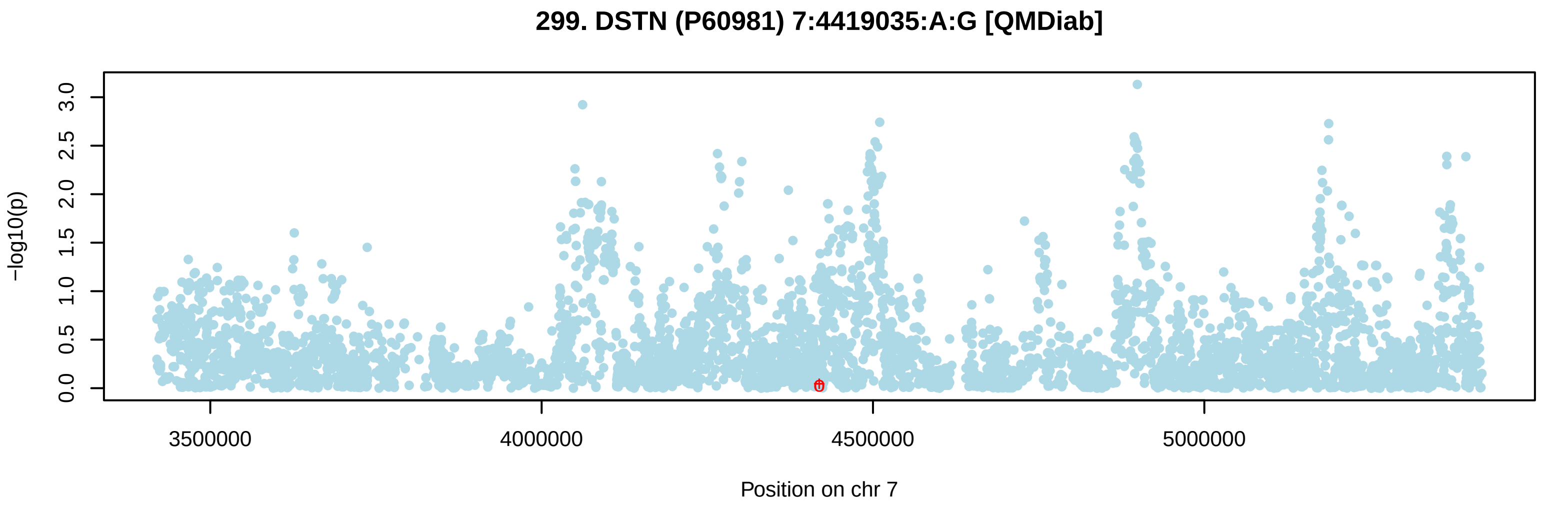
299. DSTN (P60981) 7:4419035:A:G [QMDiab]
−log10(p)
0.0
0.5
1.0
1.5
2.0
2.5
3.0
3500000
4000000
4500000
5000000
Position on chr 7

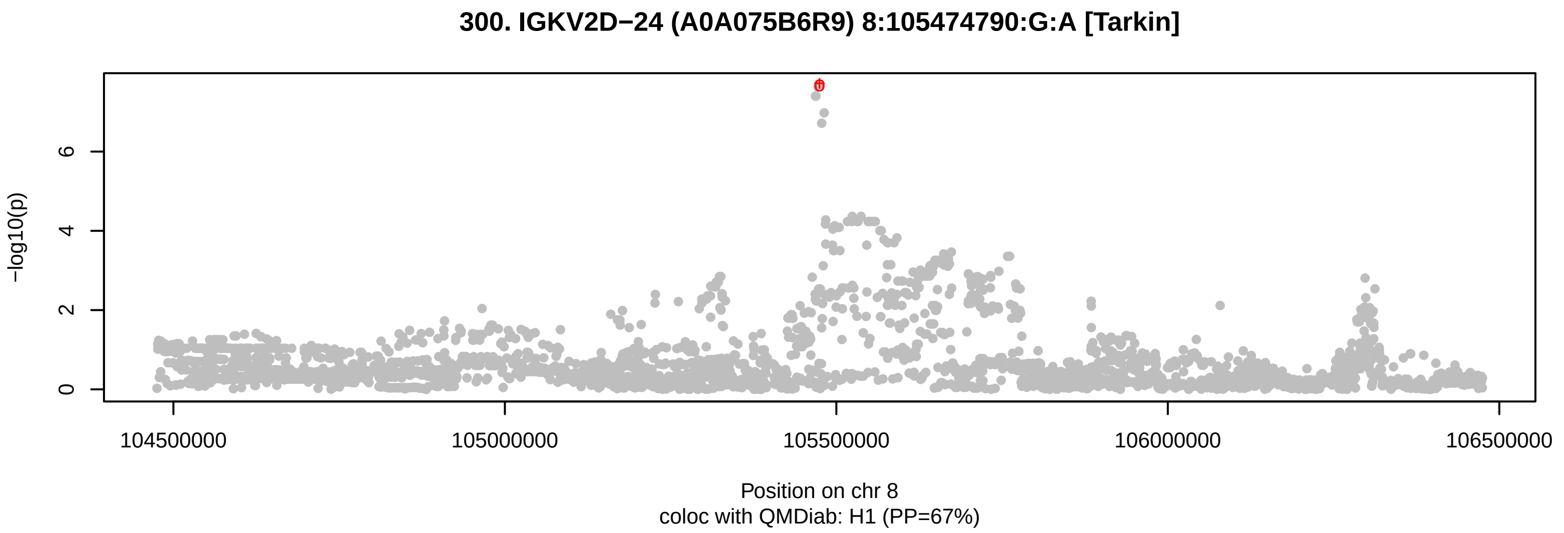
300. IGKV2D−24 (A0A075B6R9) 8:105474790:G:A [Tarkin]
−log10(p)
0
2
4
6
104500000
105000000
105500000
106000000
106500000
Position on chr 8
coloc with QMDiab: H1 (PP=67%)

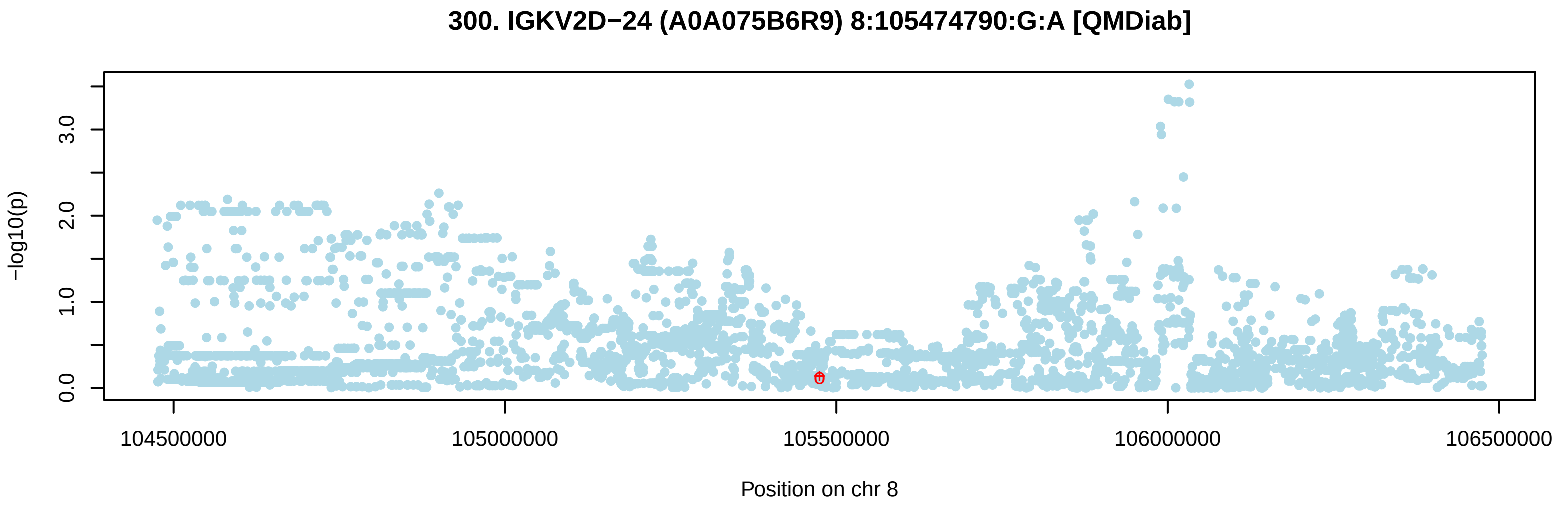
300. IGKV2D−24 (A0A075B6R9) 8:105474790:G:A [QMDiab]
−log10(p)
0.0
1.0
2.0
3.0
104500000
105000000
105500000
106000000
106500000
Position on chr 8

301. MMP9 (P14780) 11:72945341:C:T [Tarkin]

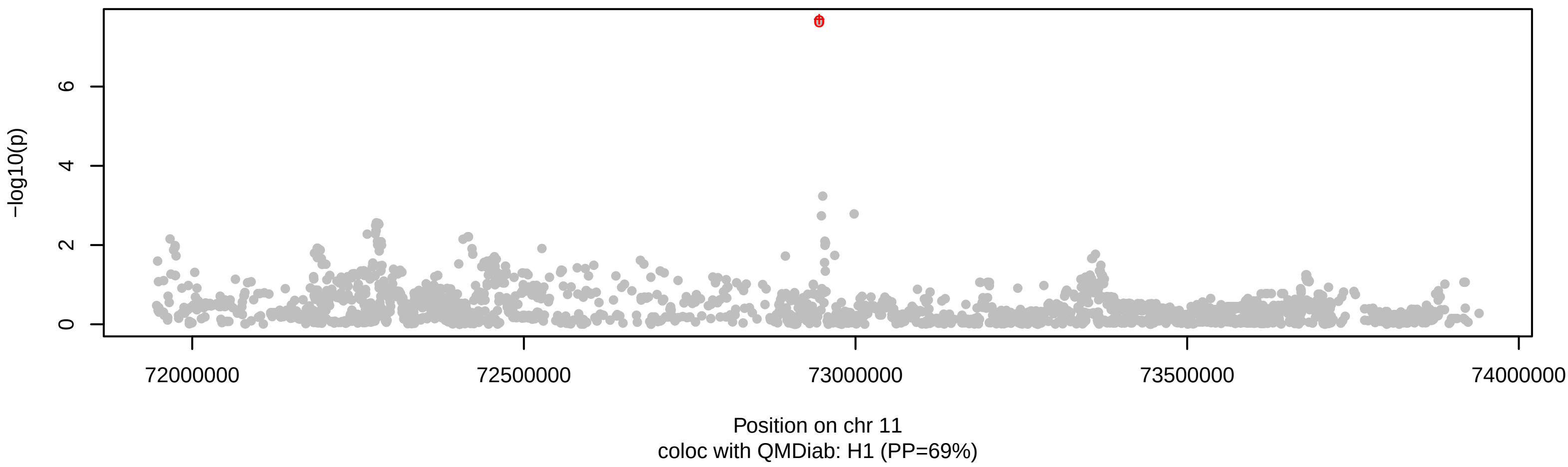

coloc with QMDiab: H1 (PP=69%)

301. MMP9 (P14780) 11:72945341:C:T [QMDiab]

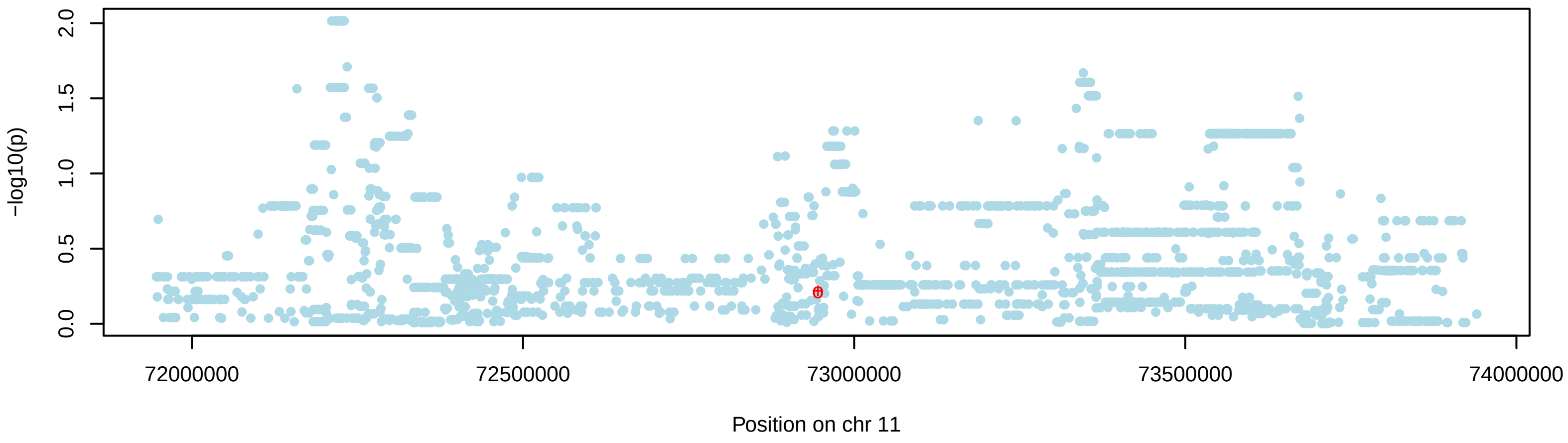

302. CPS1 (P31327−3) 8:137031710:G:T [Tarkin]

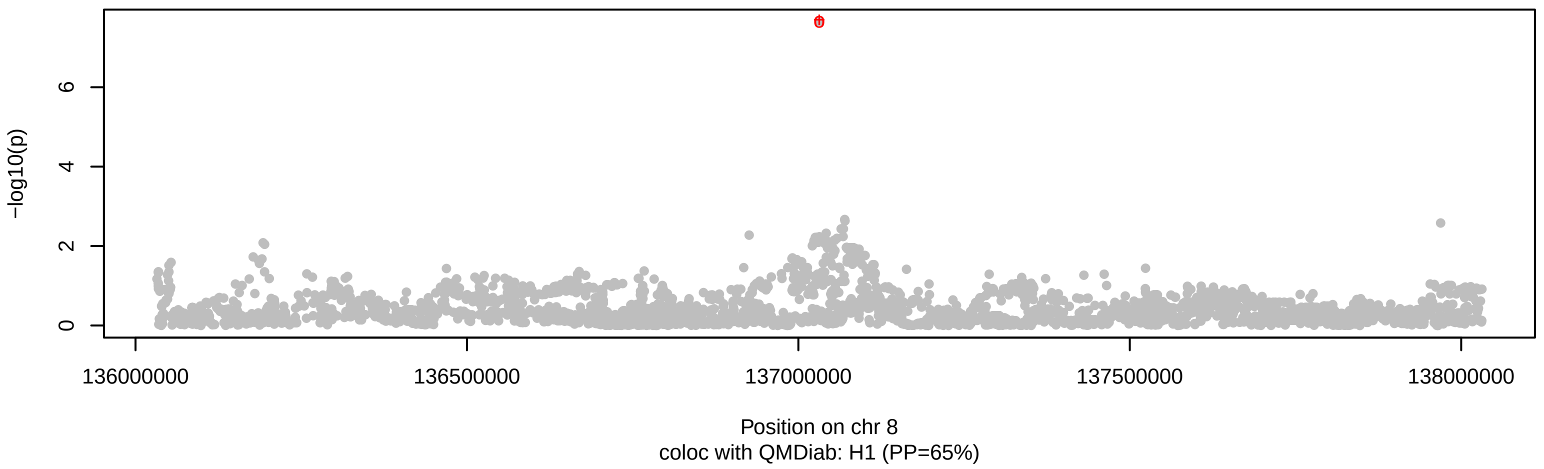

302. CPS1 (P31327;P31327−3) 8:137031710:G:T [QMDiab]

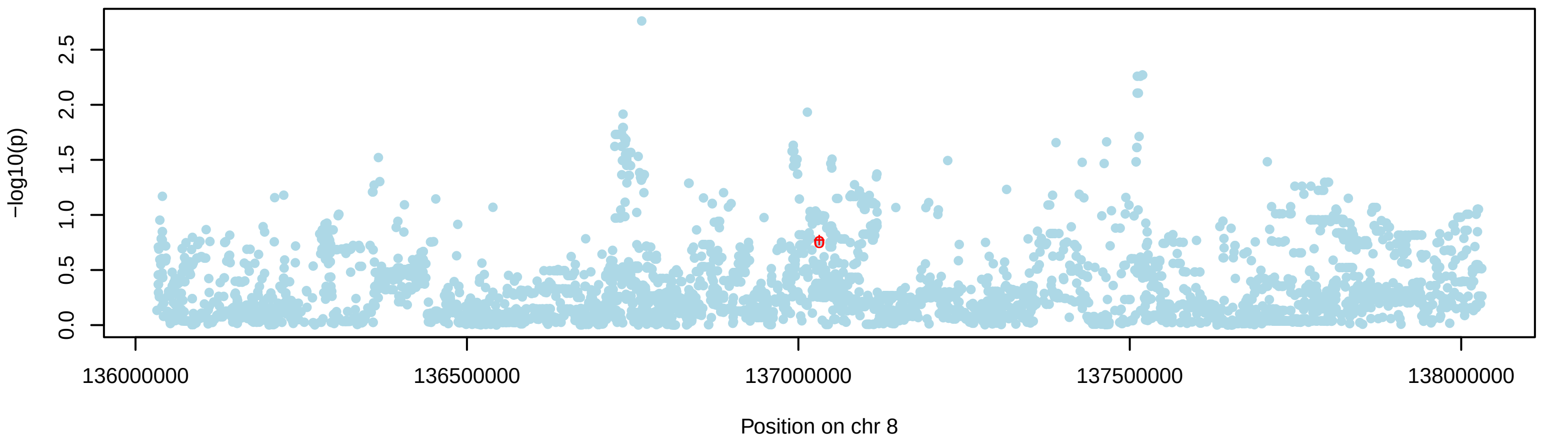

303. STAB2 (Q8WWQ8) 7:29130712:T:C [Tarkin]

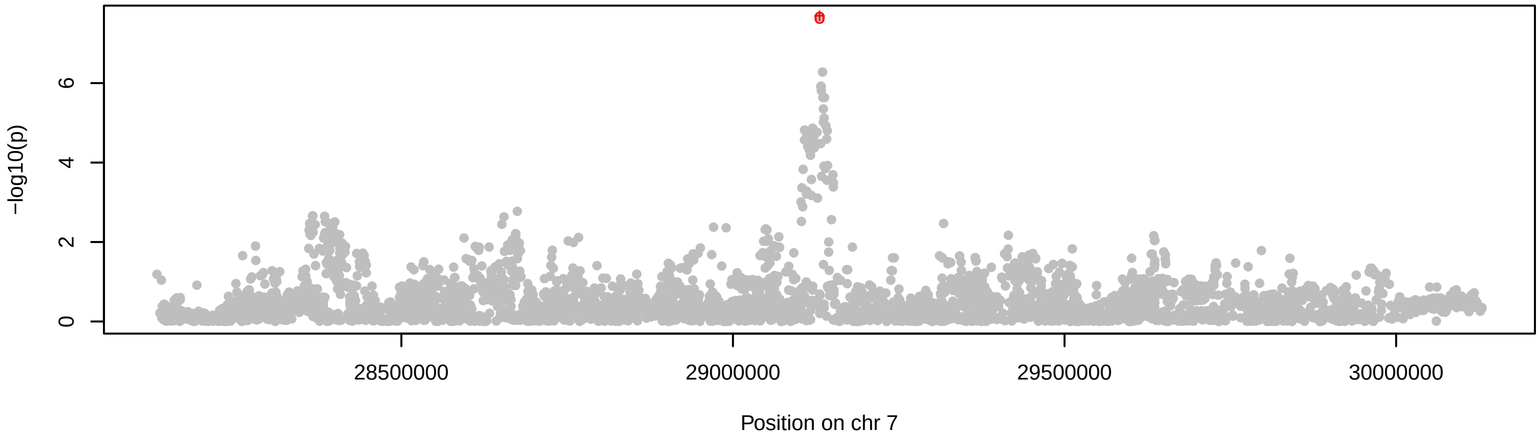

no replication data avaiable in QMDiab

304. RALA (P11233) 8:12662159:T:C [Tarkin]

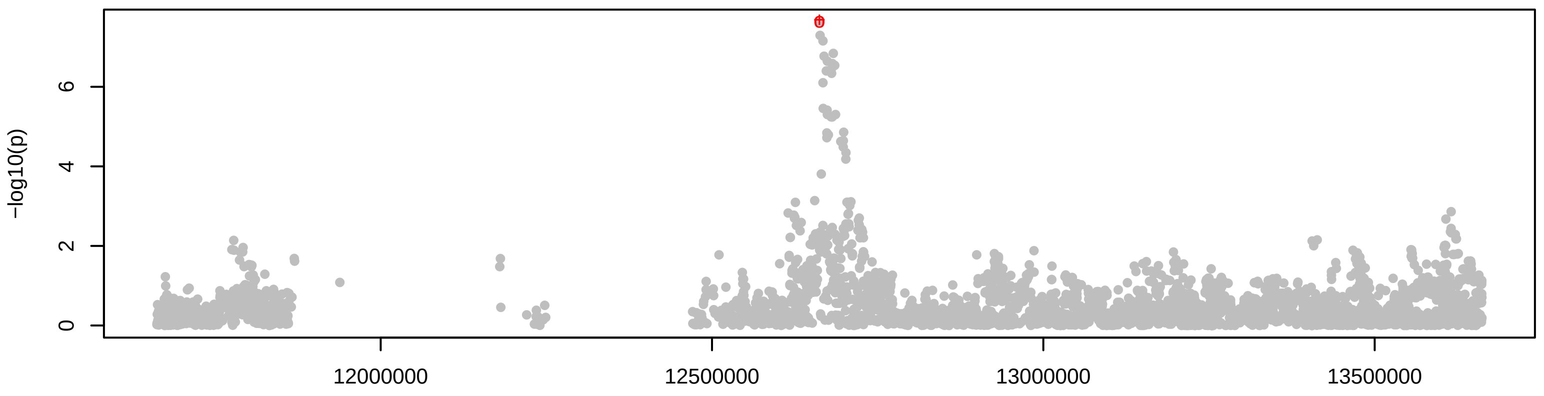

Position on chr 8
coloc with QMDiab: H1 (PP=65%)

304. RALA (P11233) 8:12662159:T:C [QMDiab]

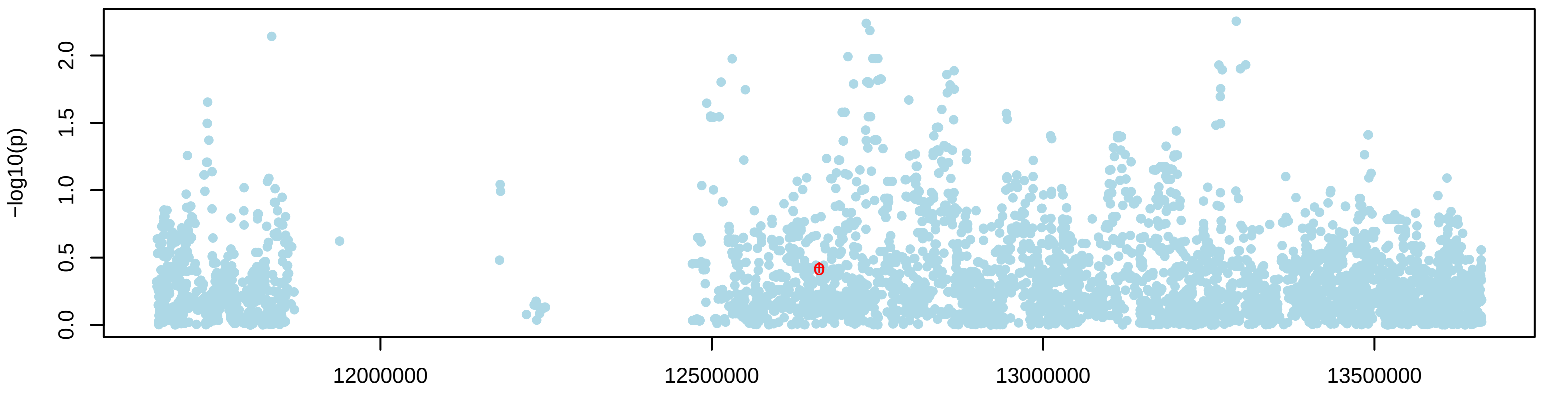

Position on chr 8

305. CDH6 (P55285) 5:30817217:C:T [Tarkin]

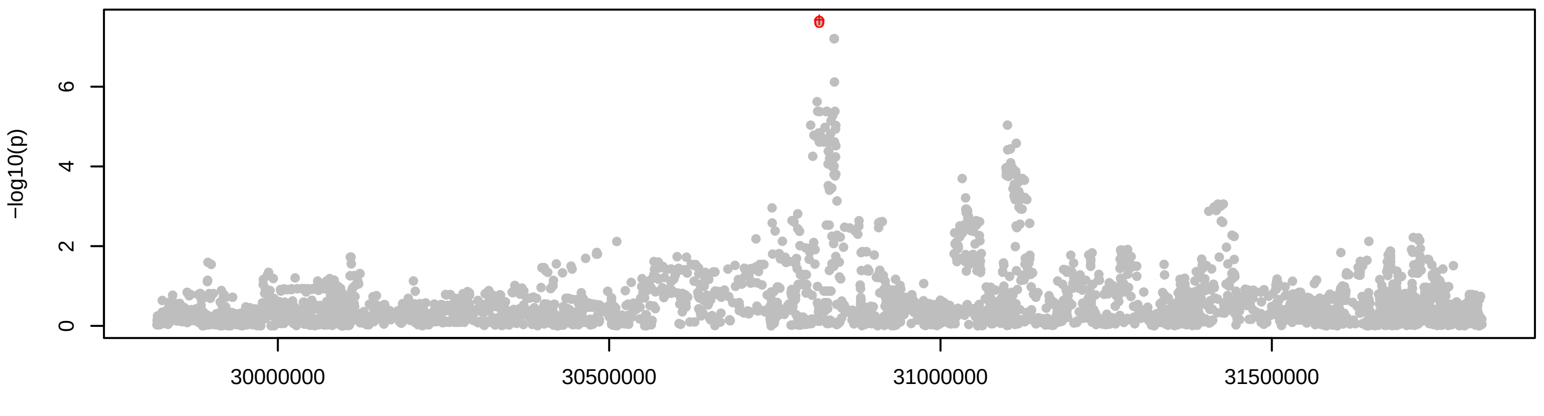

coloc with QMDiab: H1 (PP=68%)

305. CDH6 (P55285) 5:30817217:C:T [QMDiab]

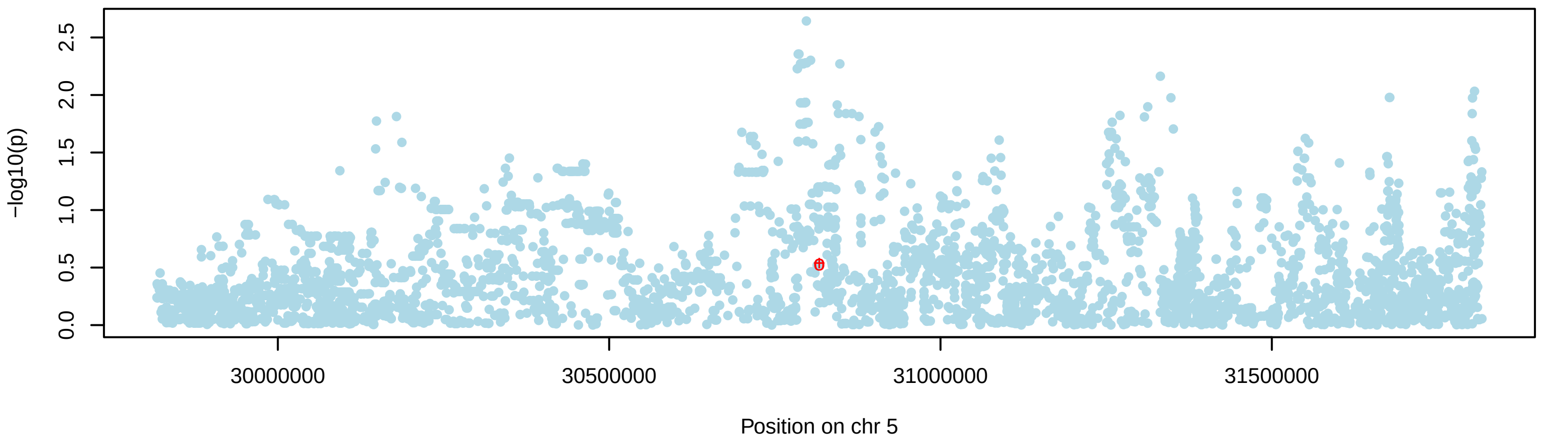

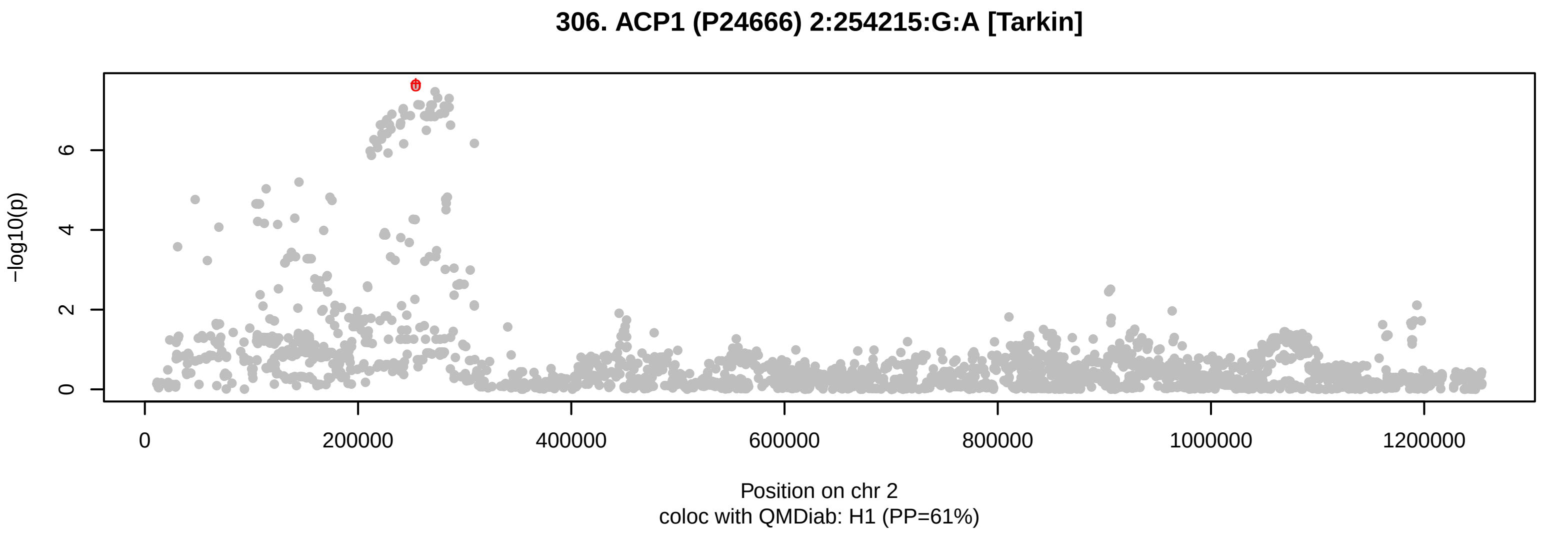
306. ACP1 (P24666) 2:254215:G:A [Tarkin]
−log10(p)
0
2
4
6
0
200000
400000
600000
800000
1000000
1200000
Position on chr 2
coloc with QMDiab: H1 (PP=61%)

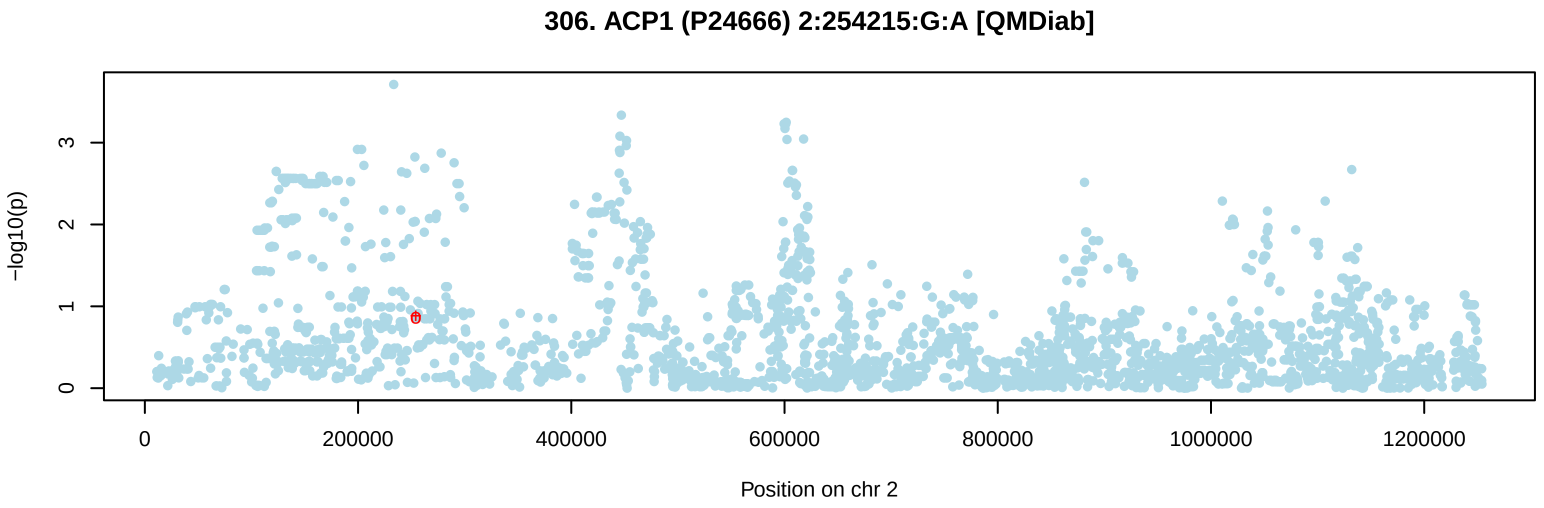
306. ACP1 (P24666) 2:254215:G:A [QMDiab]
−log10(p)
0
1
2
3
0
200000
400000
600000
800000
1000000
1200000
Position on chr 2

307. CORO1A (P31146) 18:46844240:T:C [Tarkin]

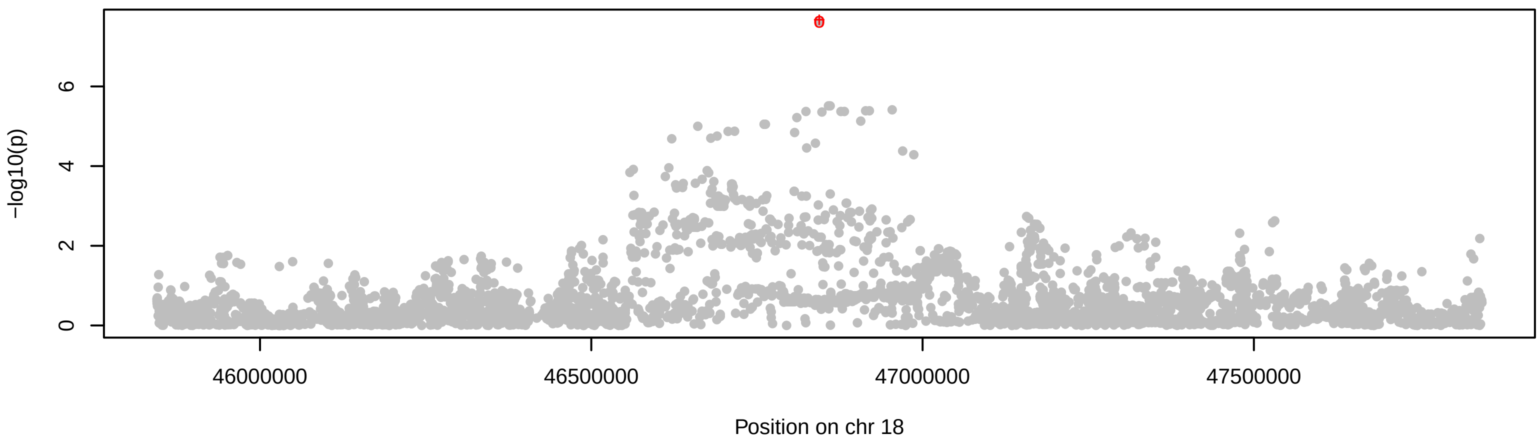

coloc with QMDiab: H1 (PP=70%)

307. CORO1A (P31146) 18:46844240:T:C [QMDiab]

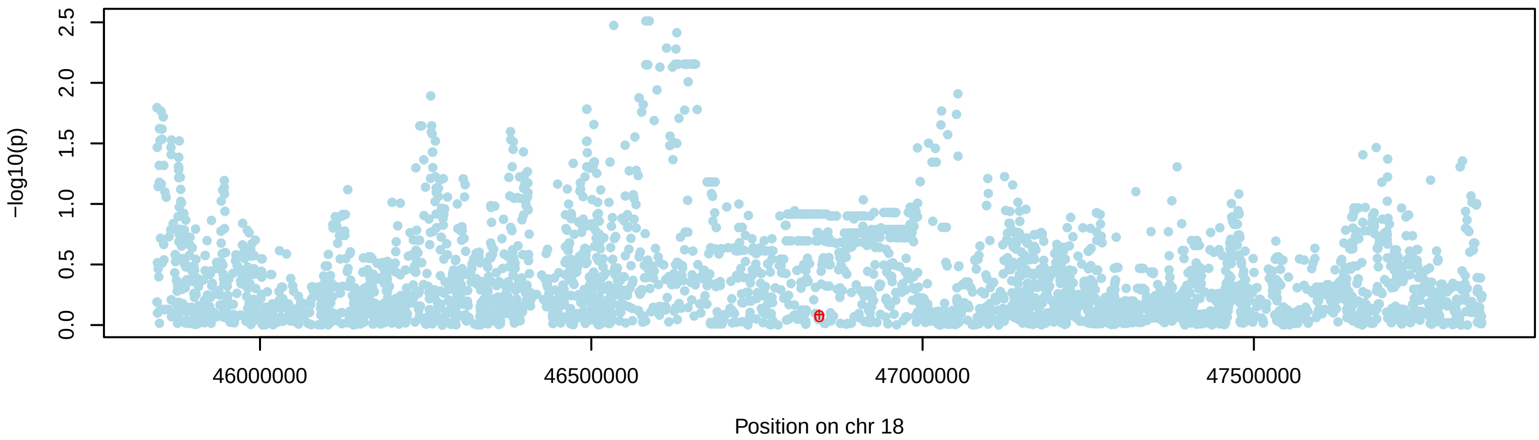

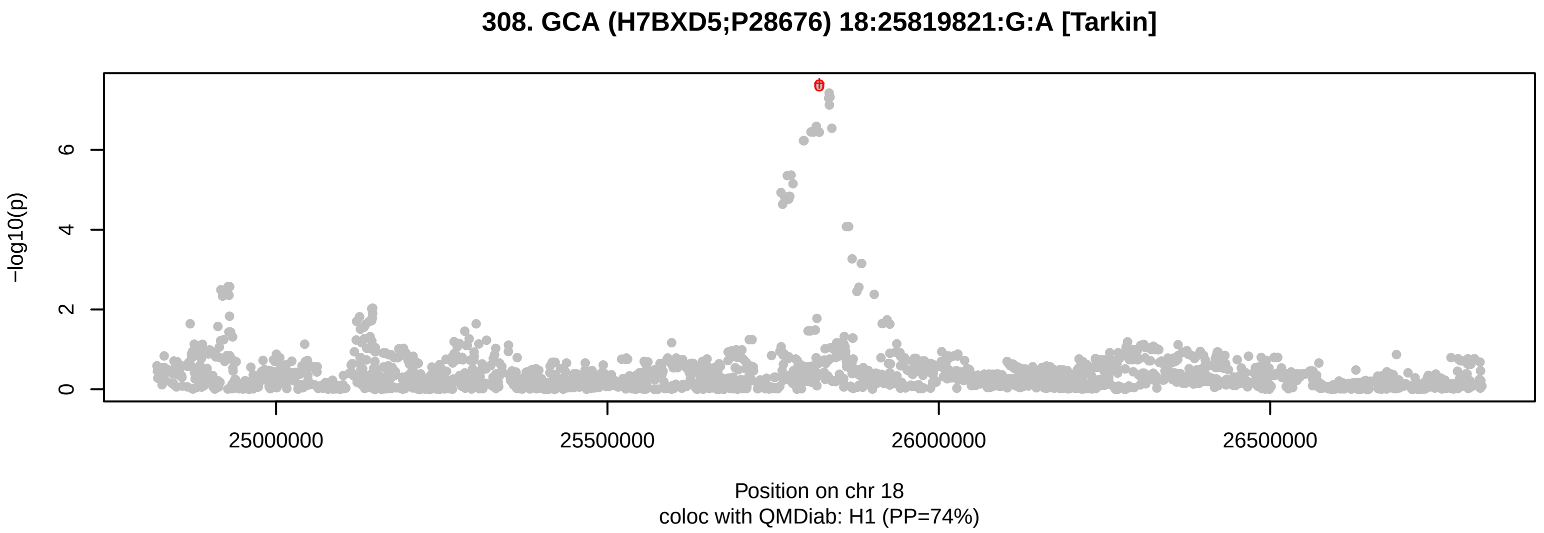
308. GCA (H7BXD5;P28676) 18:25819821:G:A [Tarkin]
−log10(p)
0
2
4
6
25000000
25500000
26000000
26500000
Position on chr 18
coloc with QMDiab: H1 (PP=74%)

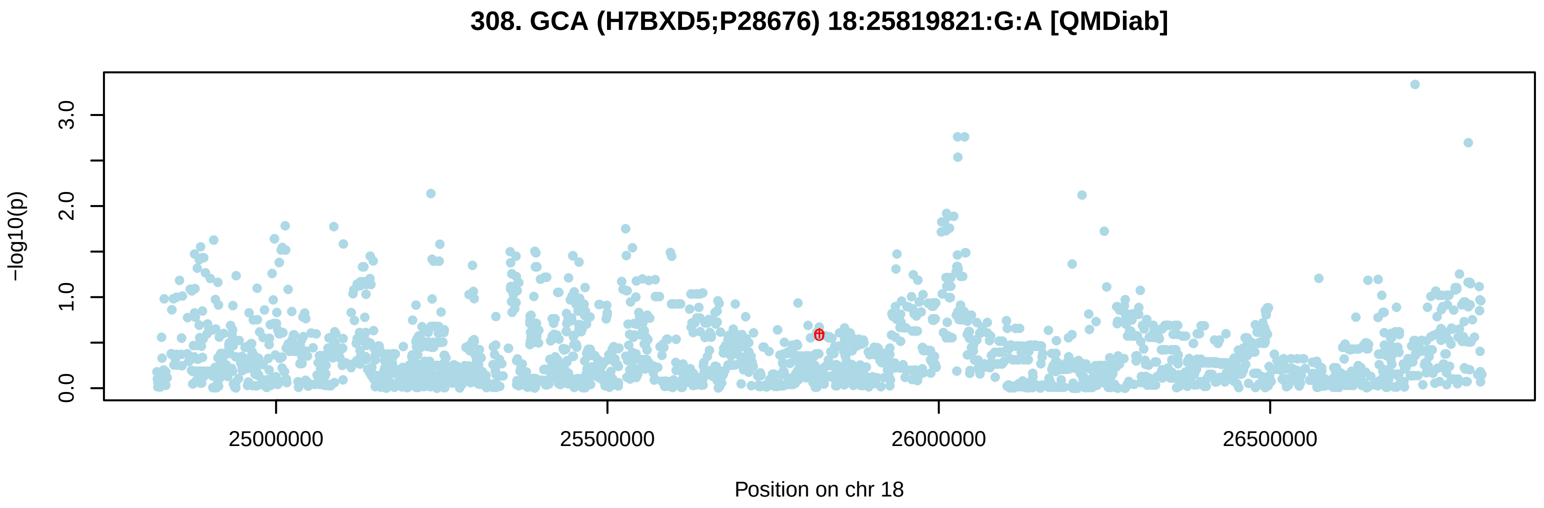
308. GCA (H7BXD5;P28676) 18:25819821:G:A [QMDiab]
−log10(p)
0.0
1.0
2.0
3.0
25000000
25500000
26000000
26500000
Position on chr 18

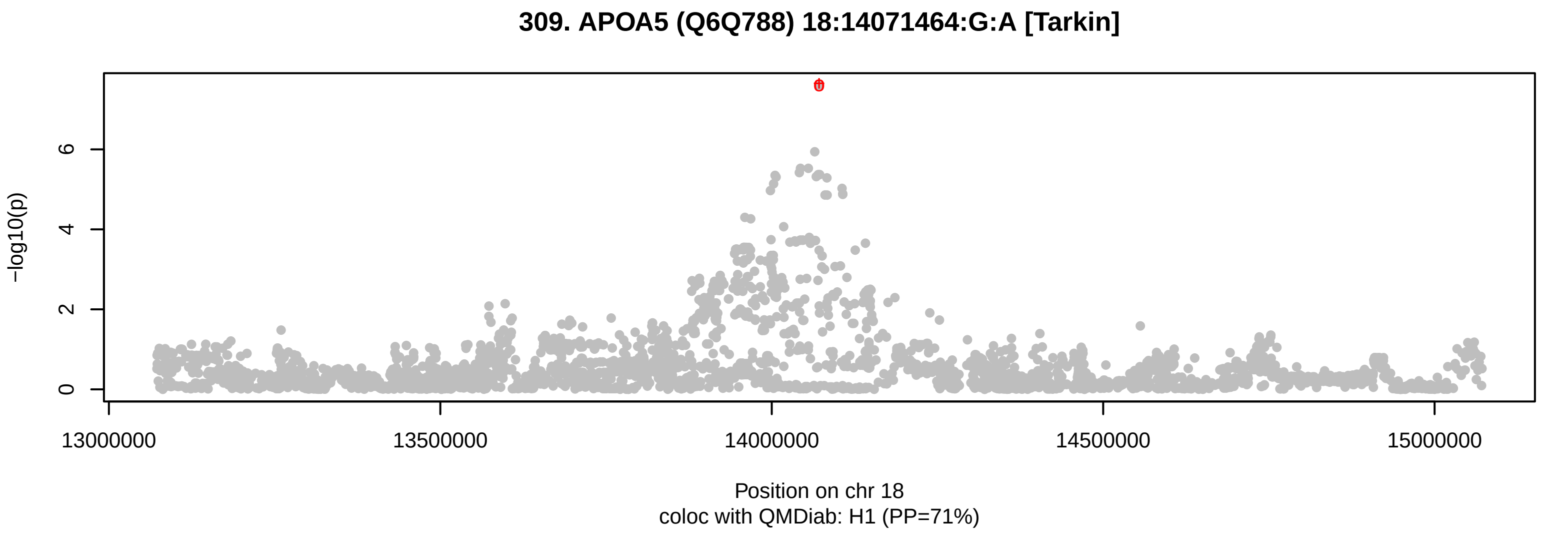
309. APOA5 (Q6Q788) 18:14071464:G:A [Tarkin]
−log10(p)
0
2
4
6
13000000
13500000
14000000
14500000
15000000
Position on chr 18
coloc with QMDiab: H1 (PP=71%)

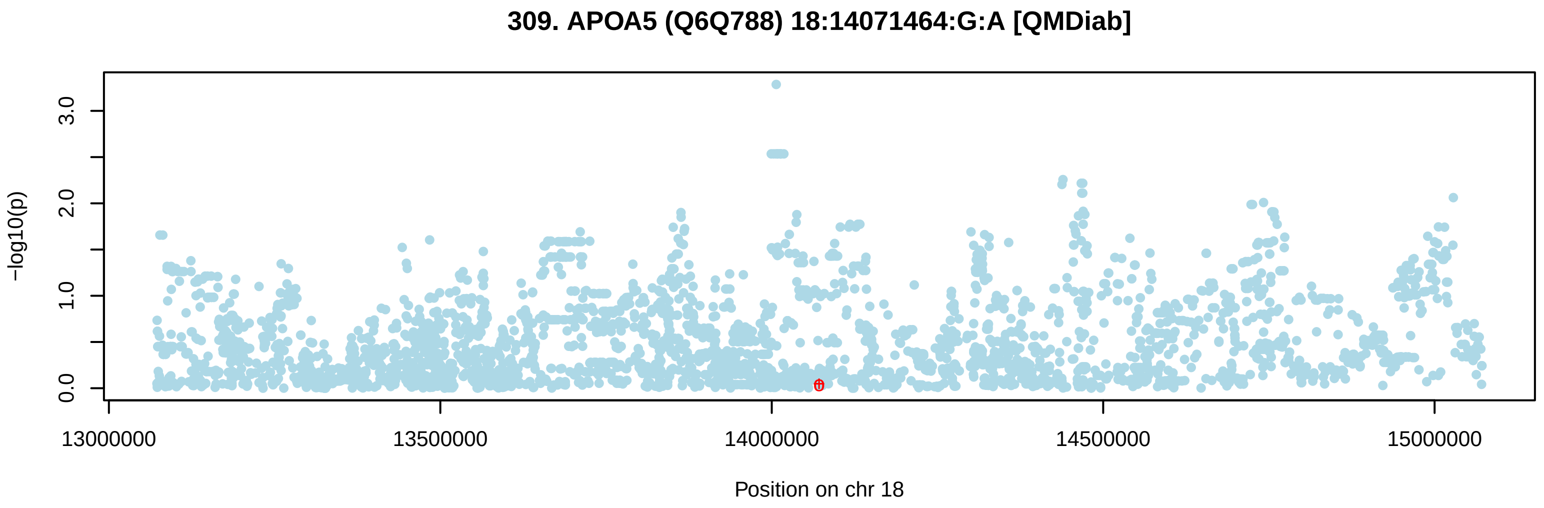
309. APOA5 (Q6Q788) 18:14071464:G:A [QMDiab]
−log10(p)
0.0
1.0
2.0
3.0
13000000
13500000
14000000
14500000
15000000
Position on chr 18

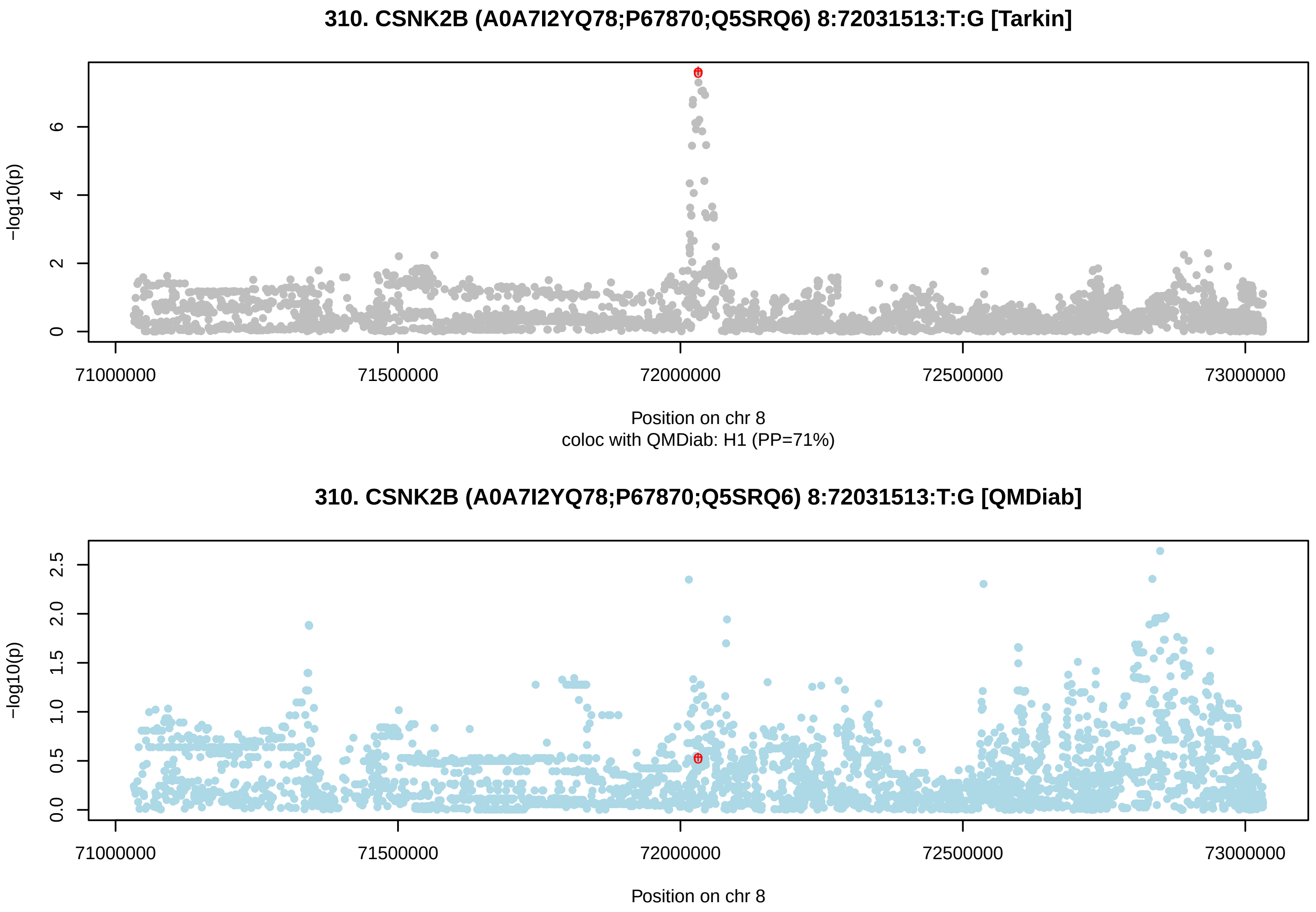
310. CSNK2B (A0A7I2YQ78;P67870;Q5SRQ6) 8:72031513:T:G [Tarkin]
−log10(p)
0
2
4
6
71000000
71500000
72000000
72500000
73000000
Position on chr 8
coloc with QMDiab: H1 (PP=71%)
310. CSNK2B (A0A7I2YQ78;P67870;Q5SRQ6) 8:72031513:T:G [QMDiab]
−log10(p)
0.0
0.5
1.0
1.5
2.0
2.5
71000000
71500000
72000000
72500000
73000000
Position on chr 8

311. C2 (P06681) 1:176816410:T:C [Tarkin]

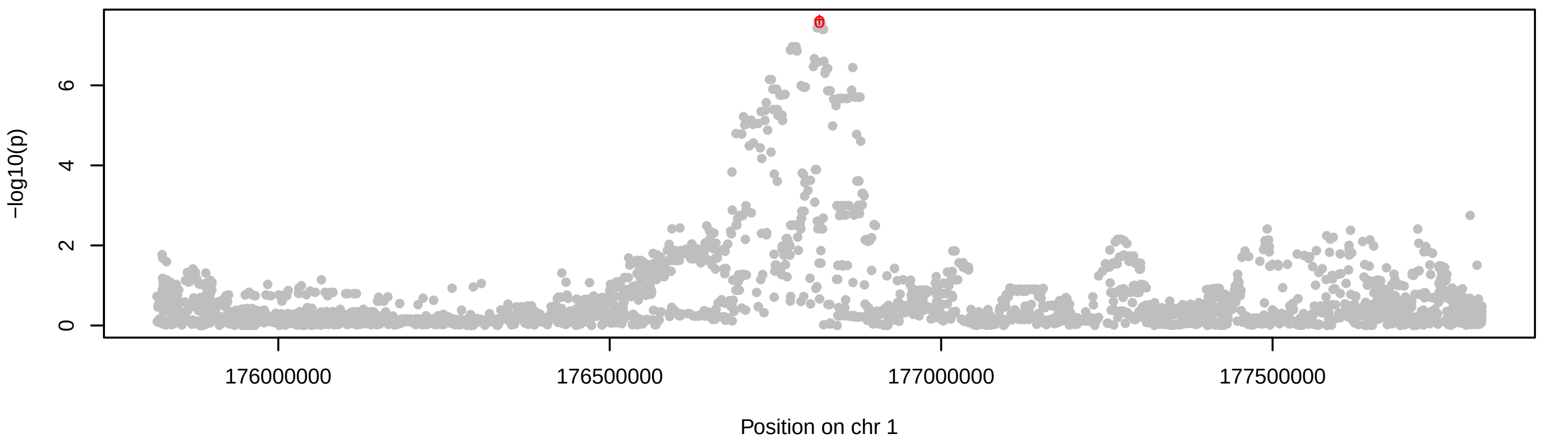

coloc with QMDiab: H1 (PP=77%)

311. C2 (P06681) 1:176816410:T:C [QMDiab]

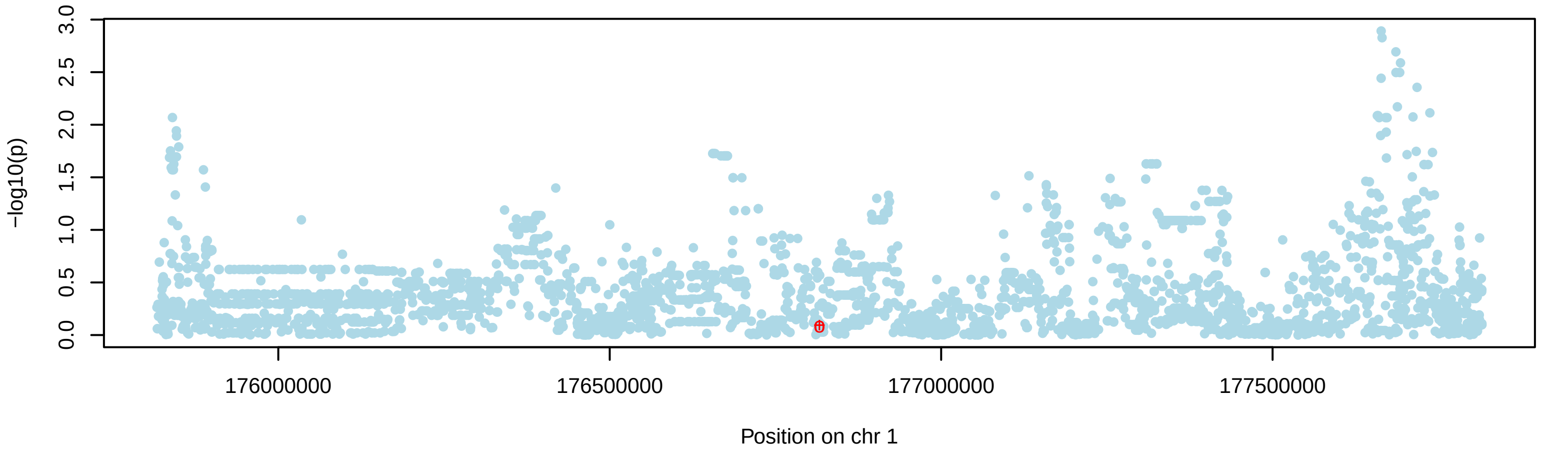

312. APOE (P02649) 6:139648918:T:C [Tarkin]

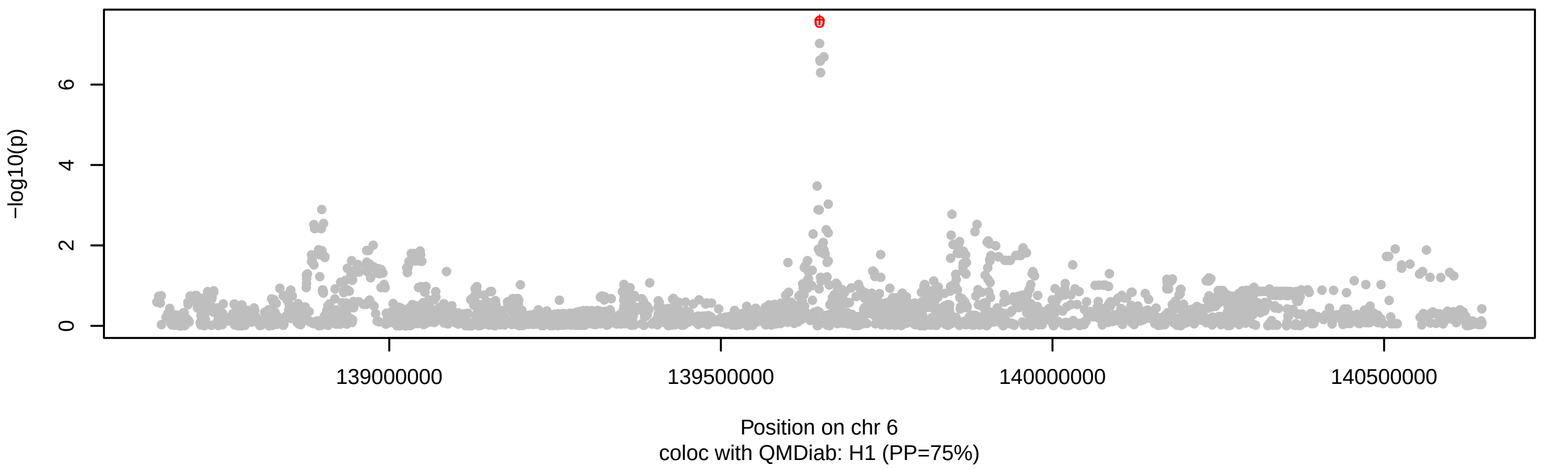

coloc with QMDiab: H1 (PP=75%)

312. APOE (P02649) 6:139648918:T:C [QMDiab]

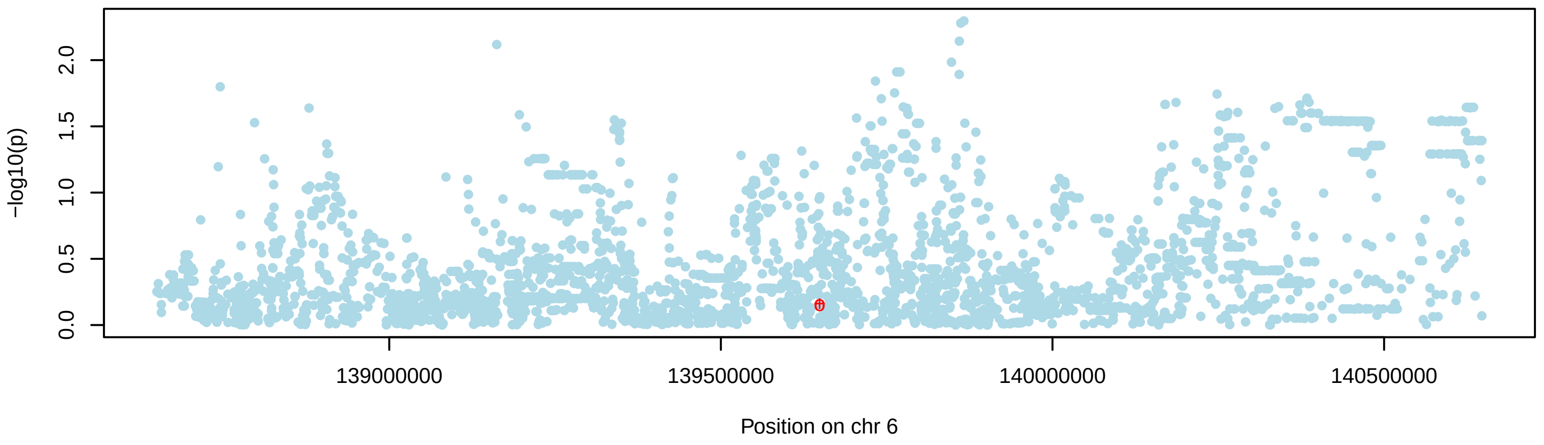

313. SGSM2 (O43147;O43147−2) 19:2250528:G:A [Tarkin]

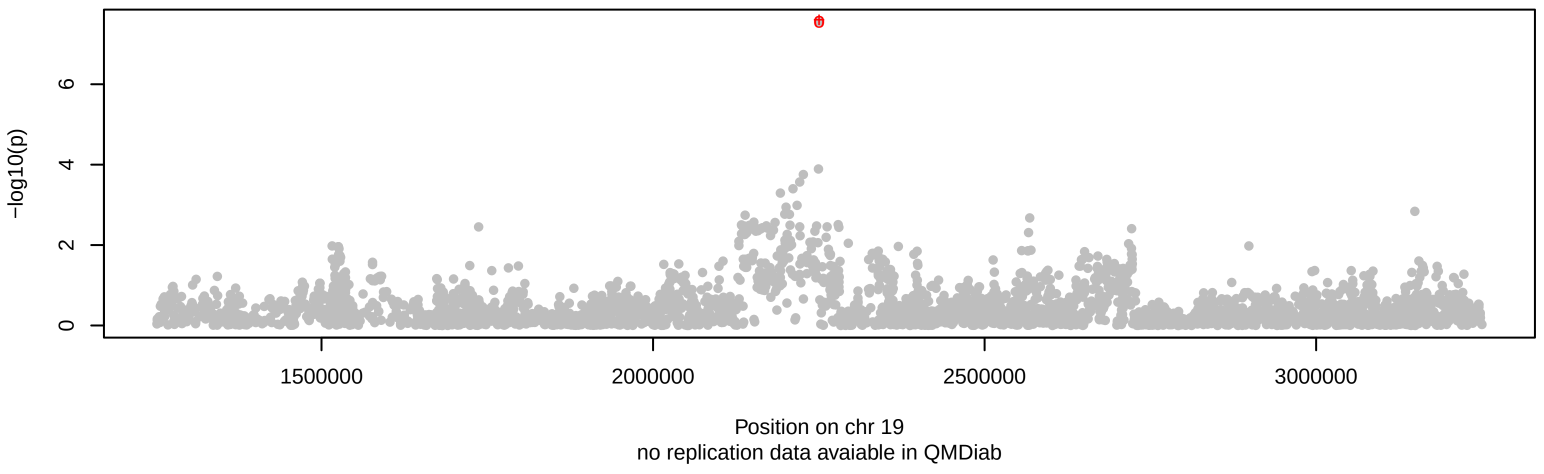

314. DNAJB11 (Q9UBS4) 14:99528084:G:A [Tarkin]

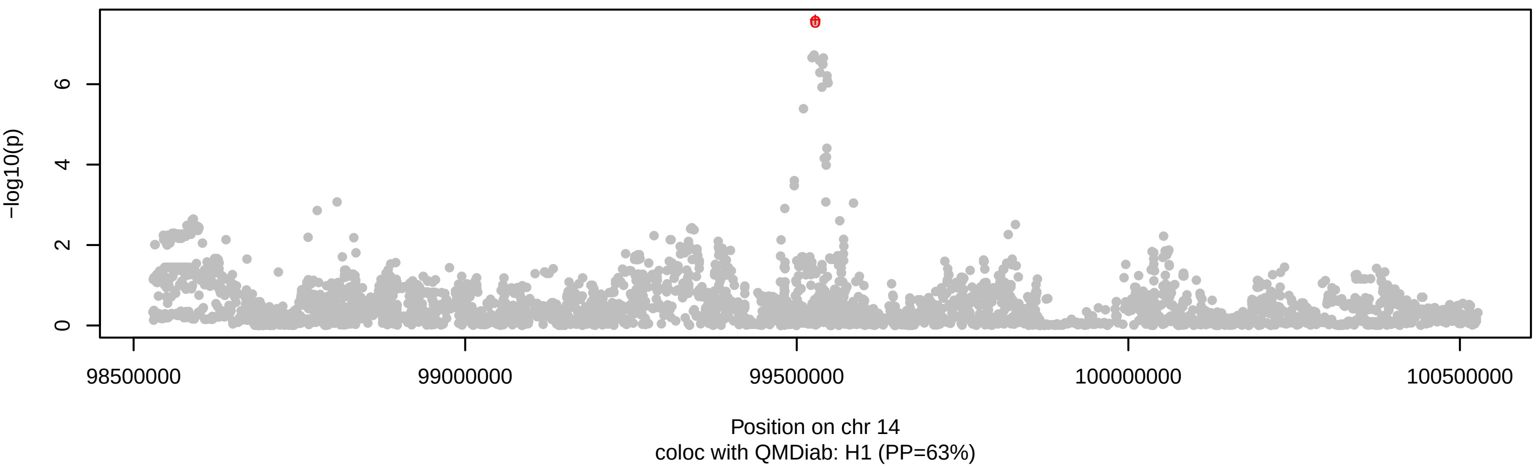

314. DNAJB11 (Q9UBS4) 14:99528084:G:A [QMDiab]

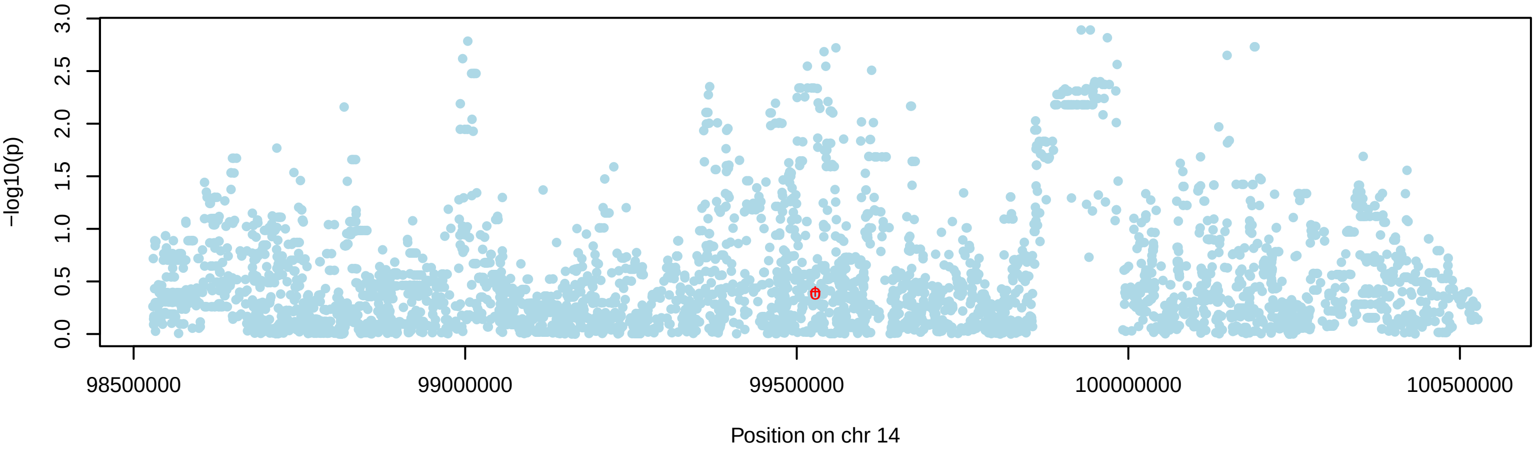

315. NID1 (P14543) 2:182343278:A:G [Tarkin]

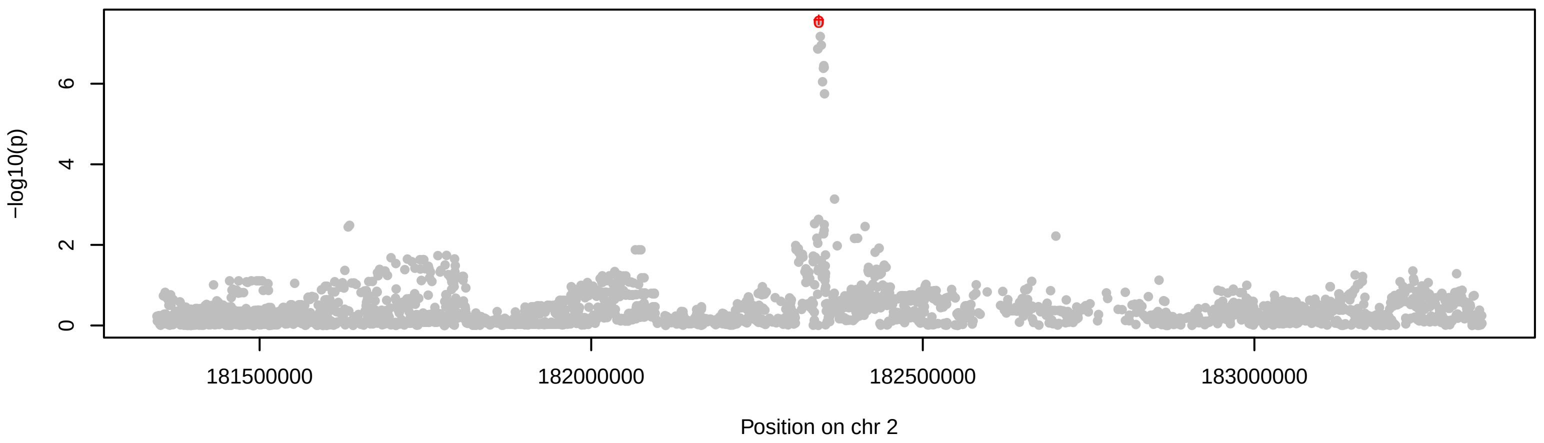

coloc with QMDiab: H1 (PP=77%)

315. NID1 (P14543) 2:182343278:A:G [QMDiab]

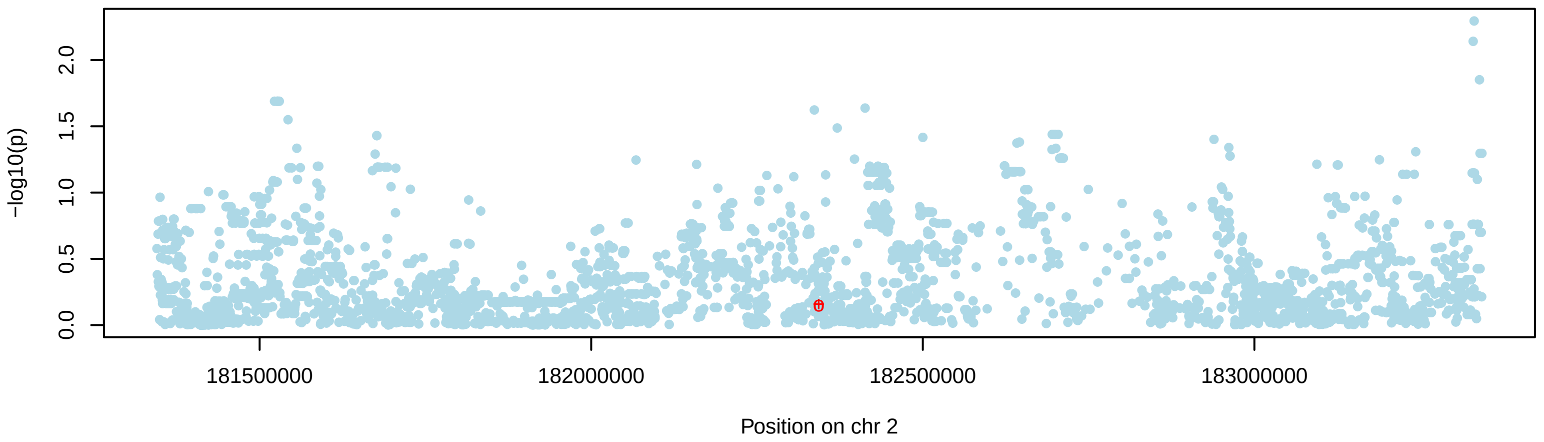

316. YWHAE (P62258) 8:69847300:A:G [Tarkin]

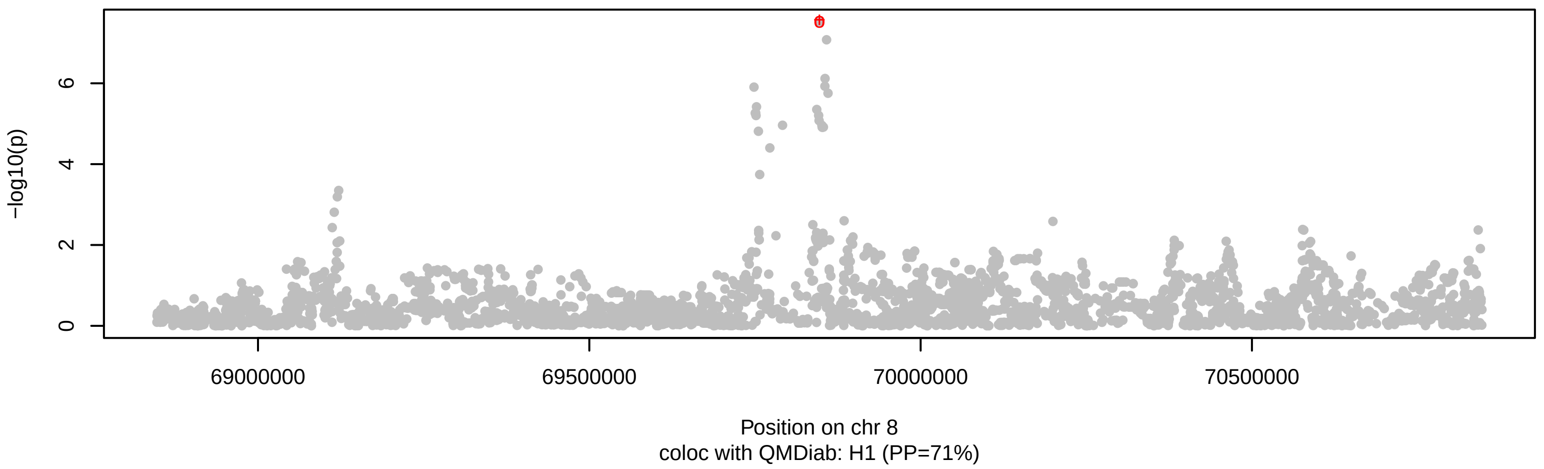

coloc with QMDiab: H1 (PP=71%)

316. YWHAE (P62258) 8:69847300:A:G [QMDiab]

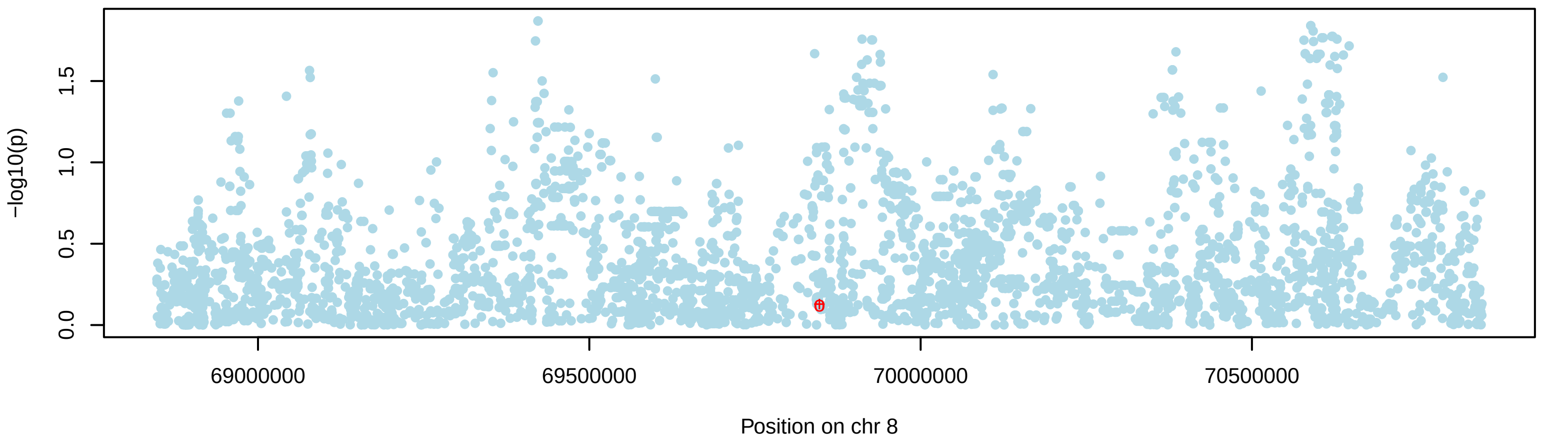

317. APOB (A8MUN2) 5:106033716:A:G [Tarkin]

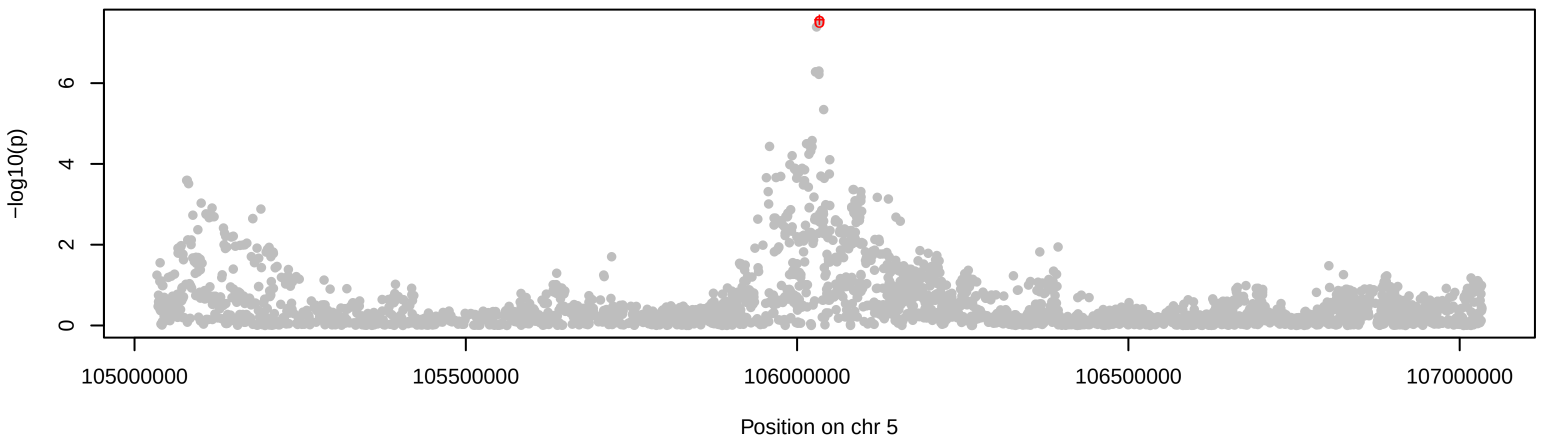

coloc with QMDiab: H1 (PP=75%)

317. APOB (A8MUN2) 5:106033716:A:G [QMDiab]

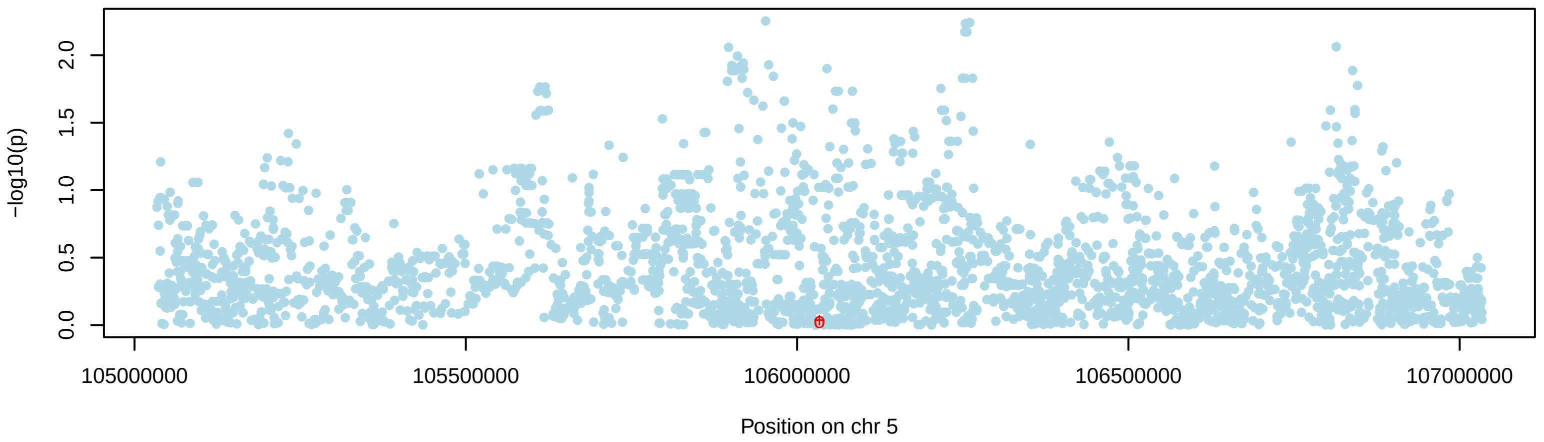

318. OLFML2A (Q68BL7) 9:127562973:G:A [Tarkin]

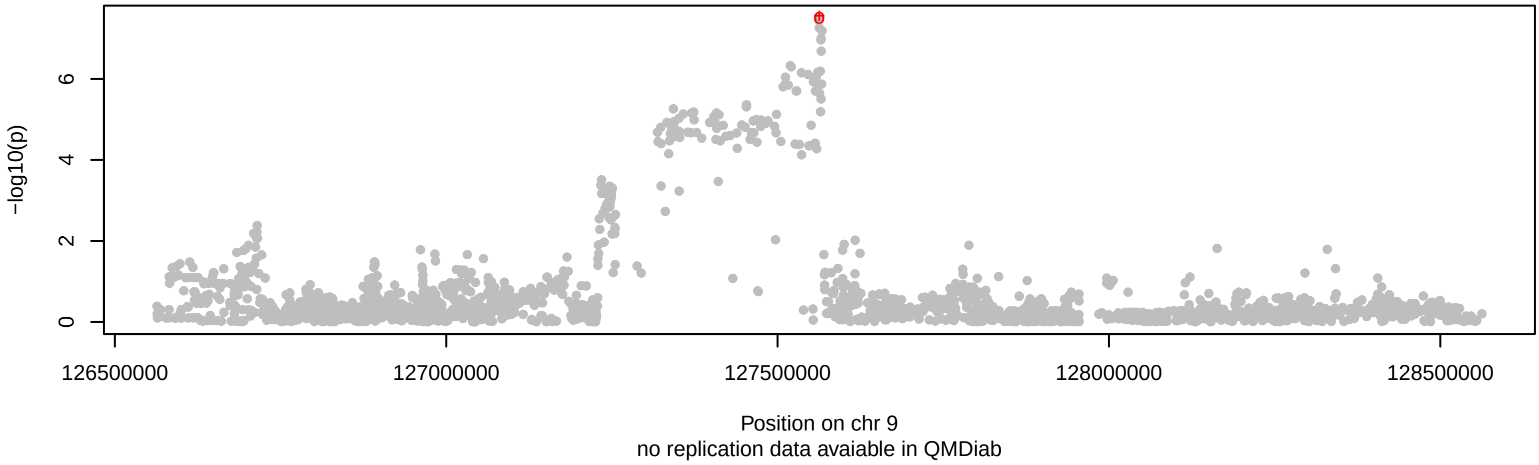

no replication data avaiable in QMDiab

319. CACYBP (Q9HB71) 3:149994553:T:C [Tarkin]

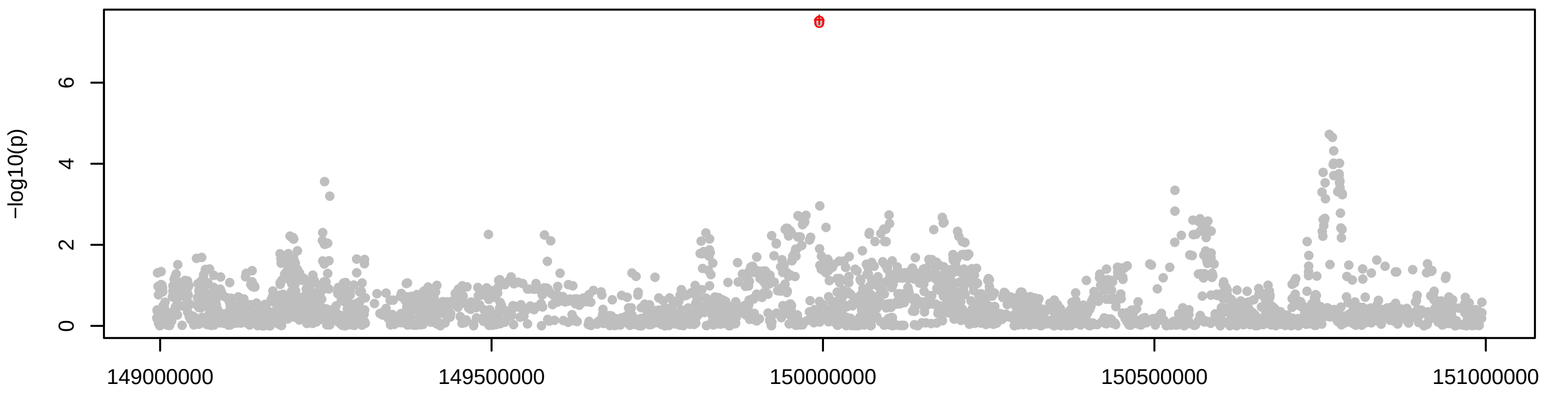

coloc with QMDiab: H1 (PP=72%)

319. CACYBP (Q9HB71) 3:149994553:T:C [QMDiab]

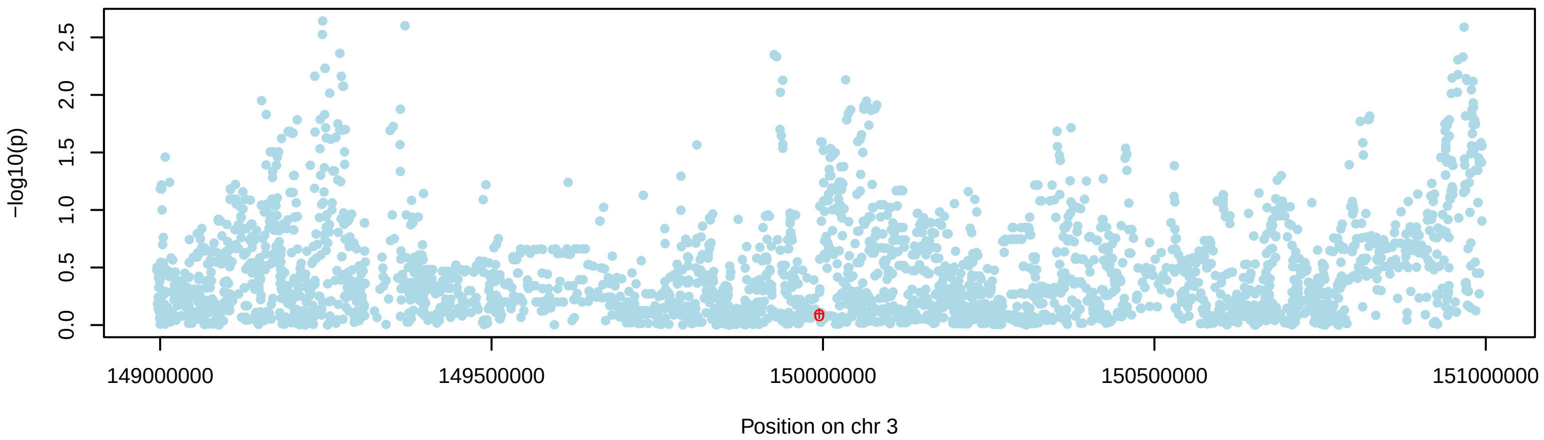

320. KPNB1 (Q14974) 17:26694861:G:A [Tarkin]

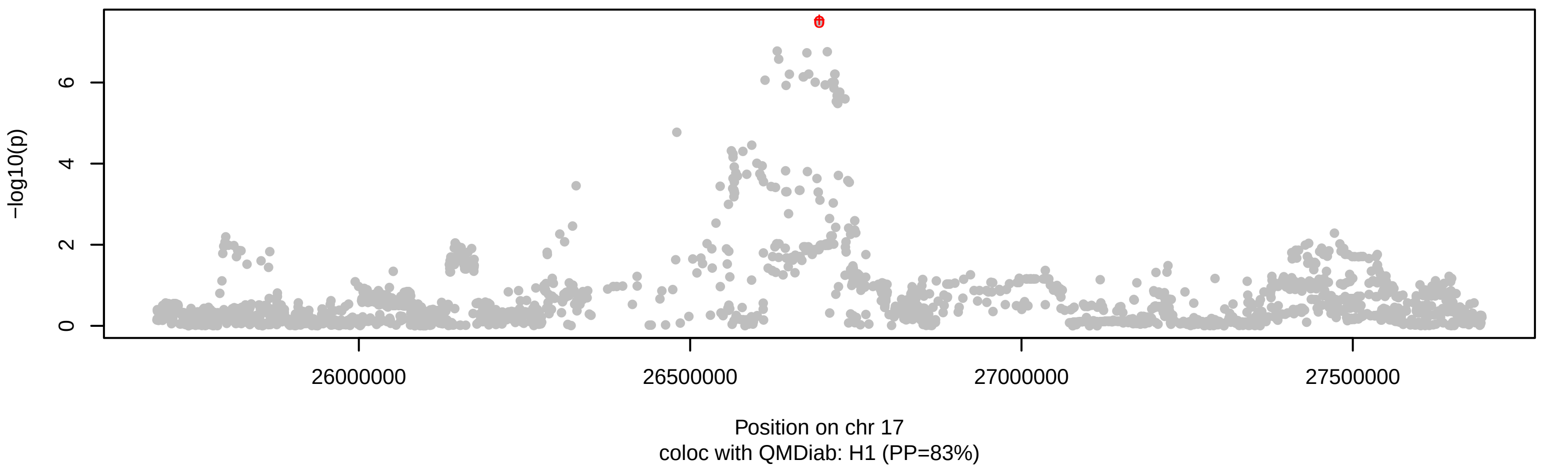

coloc with QMDiab: H1 (PP=83%)

320. KPNB1 (Q14974) 17:26694861:G:A [QMDiab]

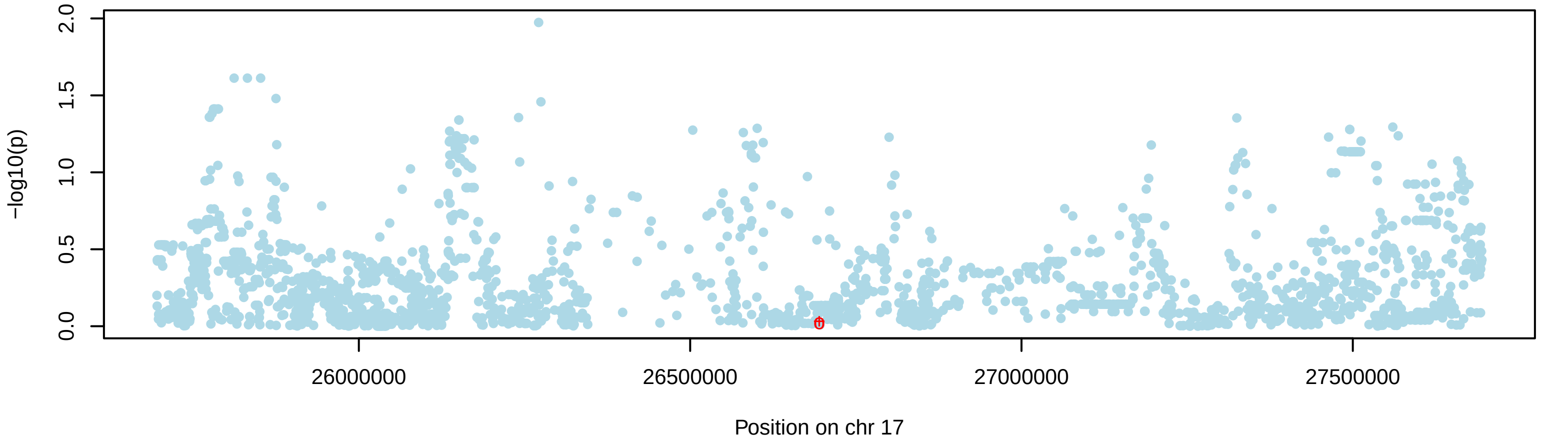

321. FABP4 (P15090) 13:51950848:C:T [Tarkin]

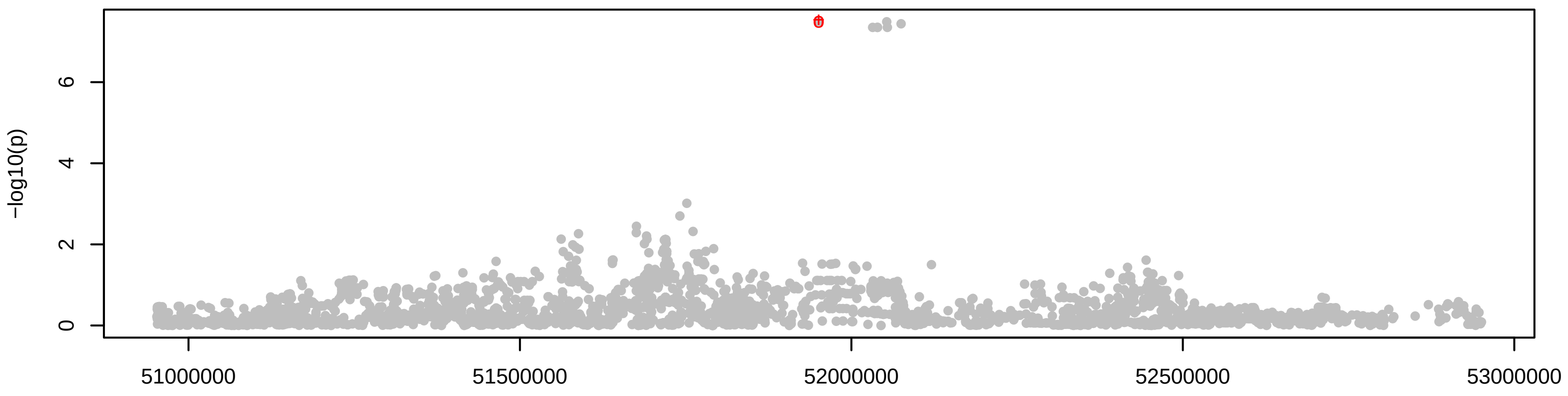

Position on chr 13
coloc with QMDiab: H1 (PP=68%)

321. FABP4 (P15090) 13:51950848:C:T [QMDiab]

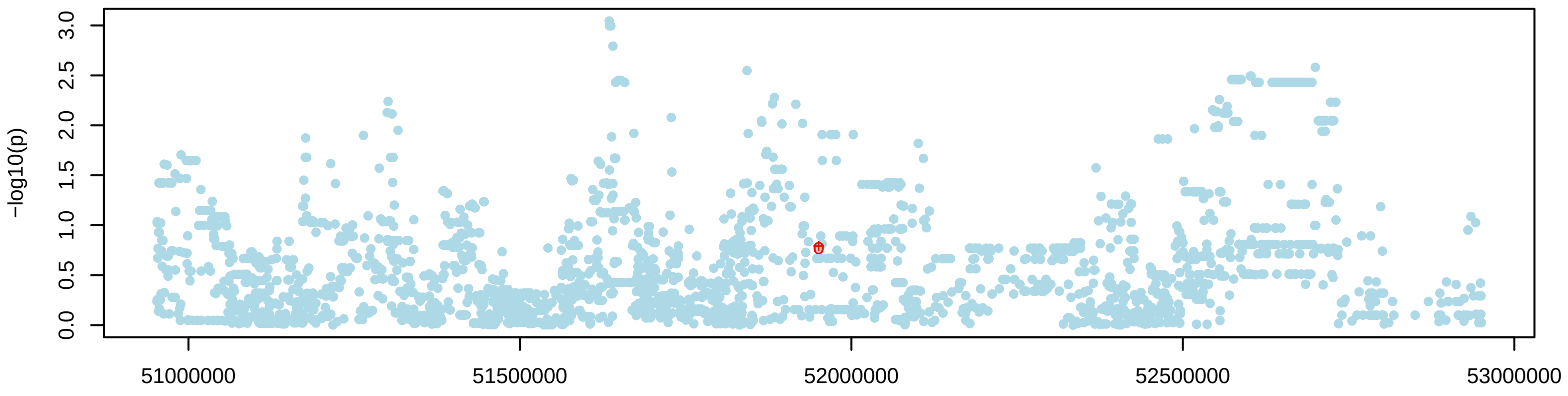

Position on chr 13

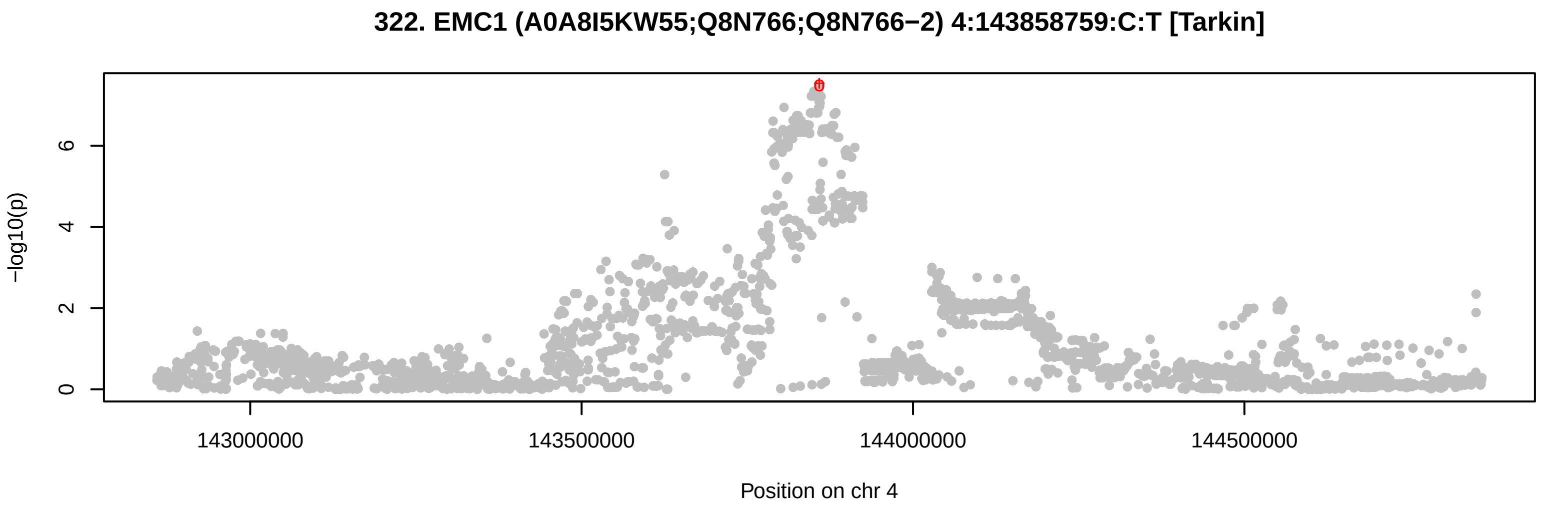

coloc with QMDiab: H1 (PP=70%)

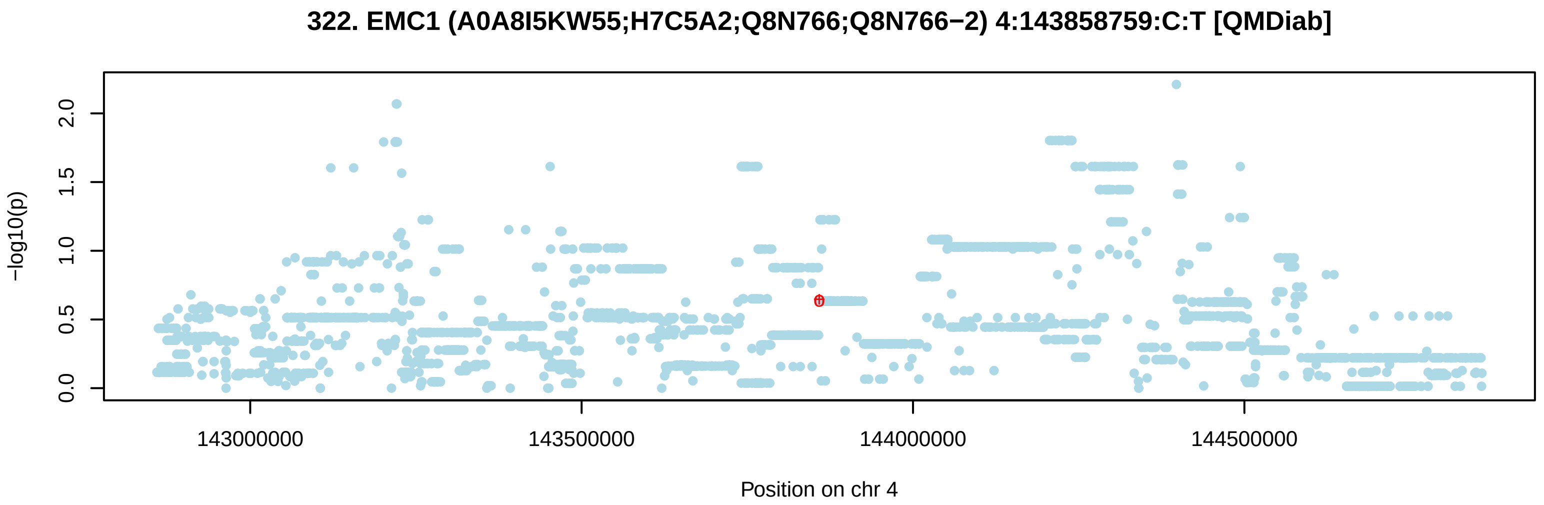

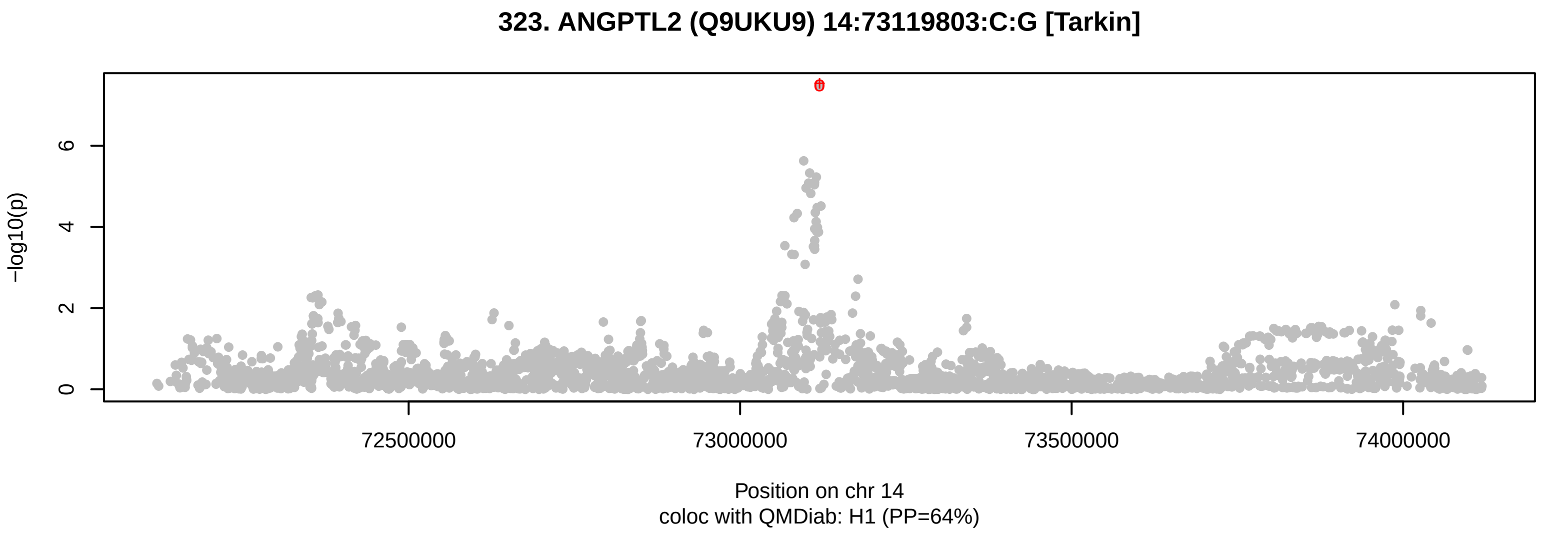
323. ANGPTL2 (Q9UKU9) 14:73119803:C:G [Tarkin]
−log10(p)
0
2
4
6
72500000
73000000
73500000
74000000
Position on chr 14
coloc with QMDiab: H1 (PP=64%)

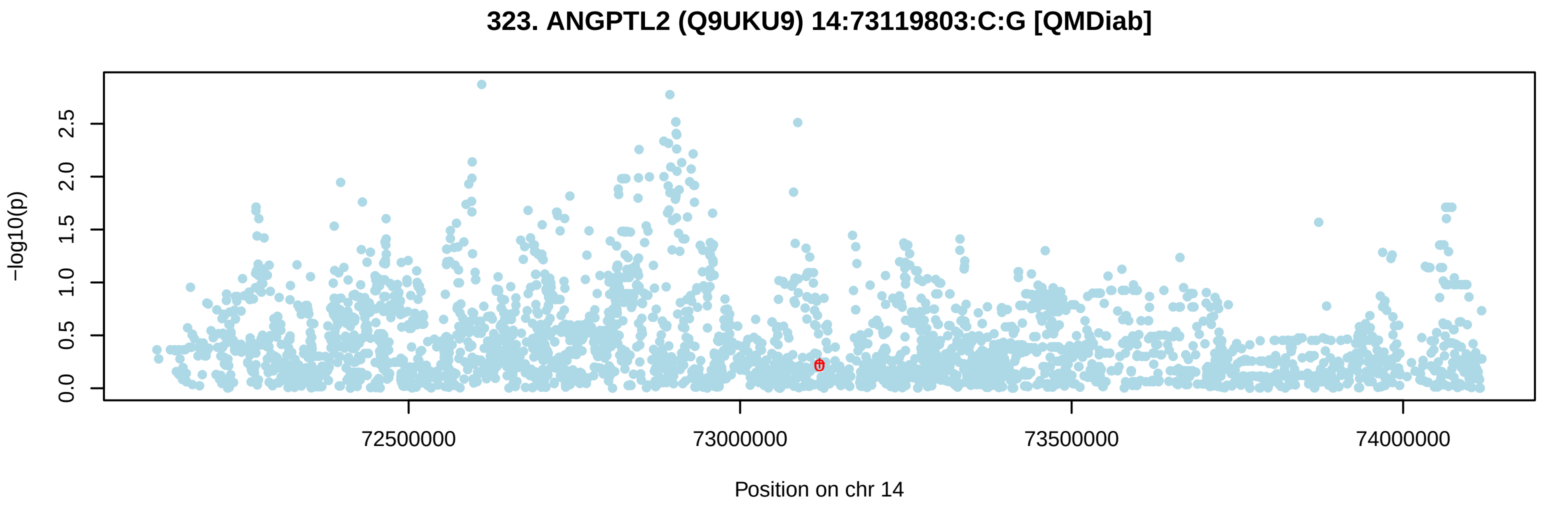
323. ANGPTL2 (Q9UKU9) 14:73119803:C:G [QMDiab]
−log10(p)
0.0
0.5
1.0
1.5
2.0
2.5
72500000
73000000
73500000
74000000
Position on chr 14

324. CHMP3 (Q9Y3E7) 19:37455948:C:G [Tarkin]

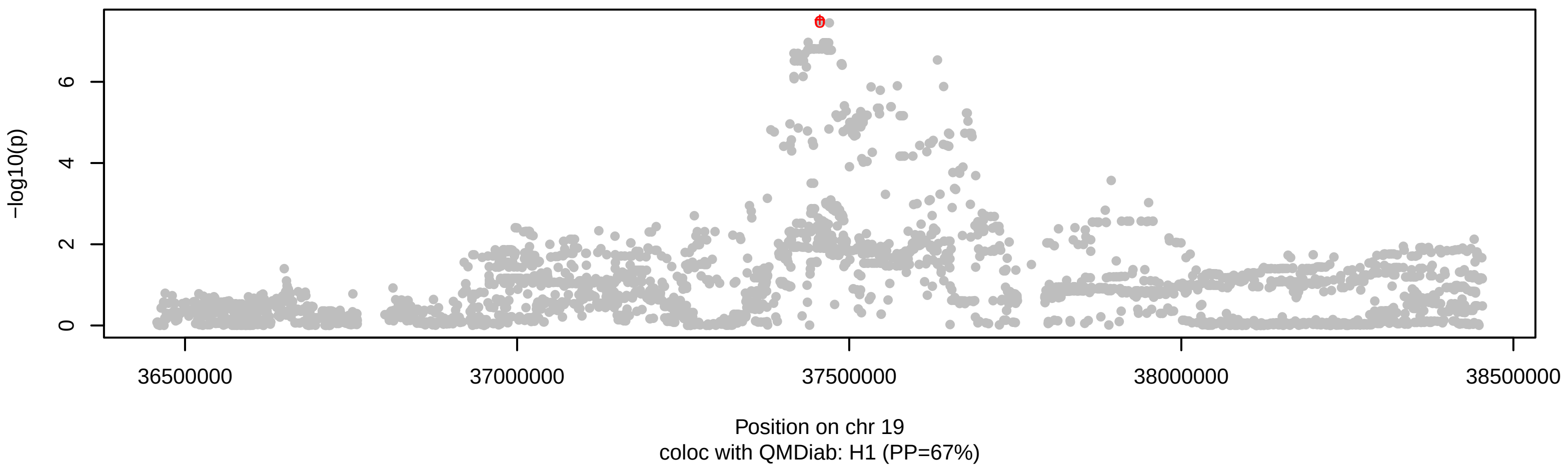

324. CHMP3 (Q9Y3E7) 19:37455948:C:G [QMDiab]

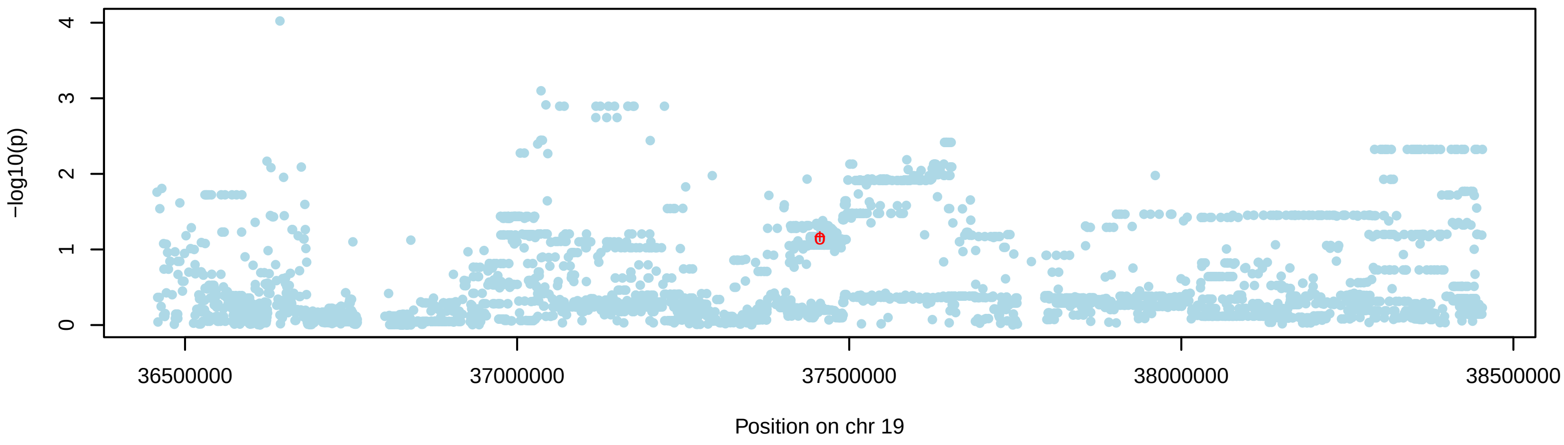

325. GBA2 (Q9HCG7) 1:9387506:G:A [Tarkin]

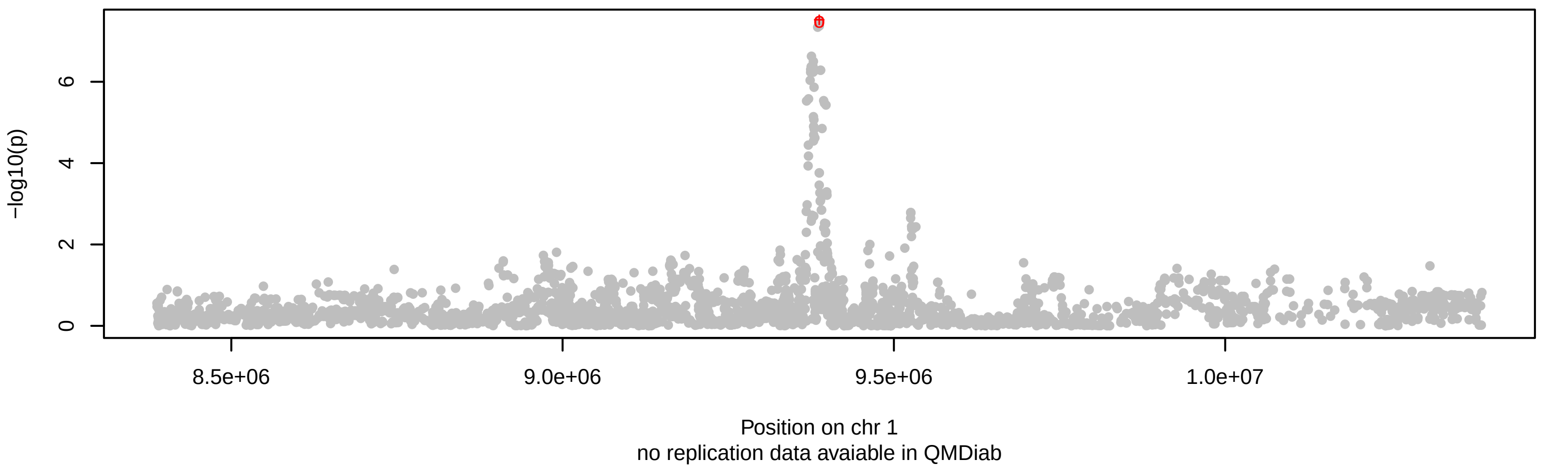

no replication data avaiable in QMDiab

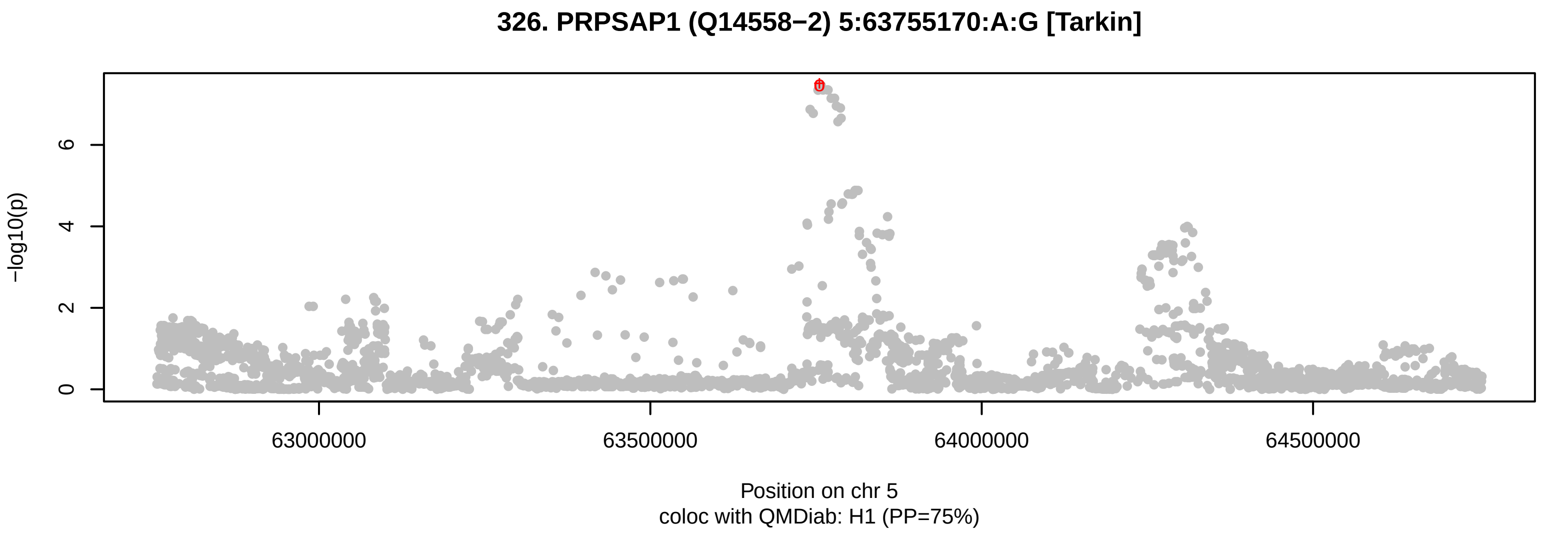
326. PRPSAP1 (Q14558−2) 5:63755170:A:G [Tarkin]
−log10(p)
0
2
4
6
63000000
63500000
64000000
64500000
Position on chr 5
coloc with QMDiab: H1 (PP=75%)

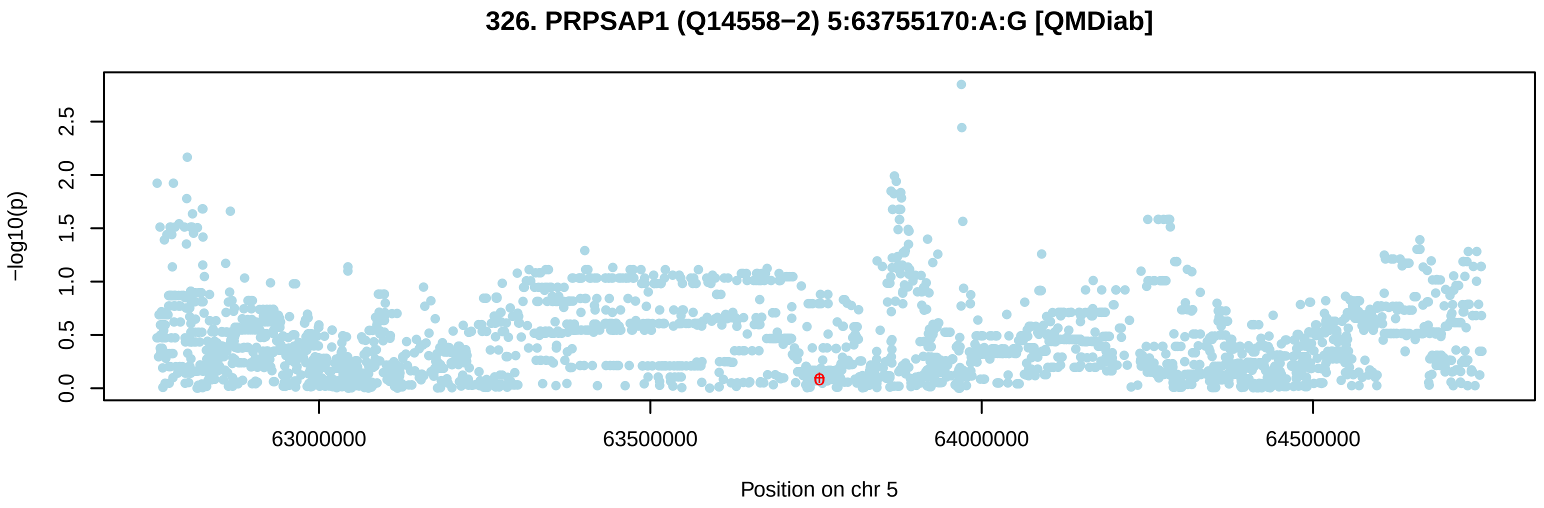
326. PRPSAP1 (Q14558−2) 5:63755170:A:G [QMDiab]
−log10(p)
0.0
0.5
1.0
1.5
2.0
2.5
63000000
63500000
64000000
64500000
Position on chr 5

327. CAPZA1 (P52907) 4:185810329:T:C [Tarkin]

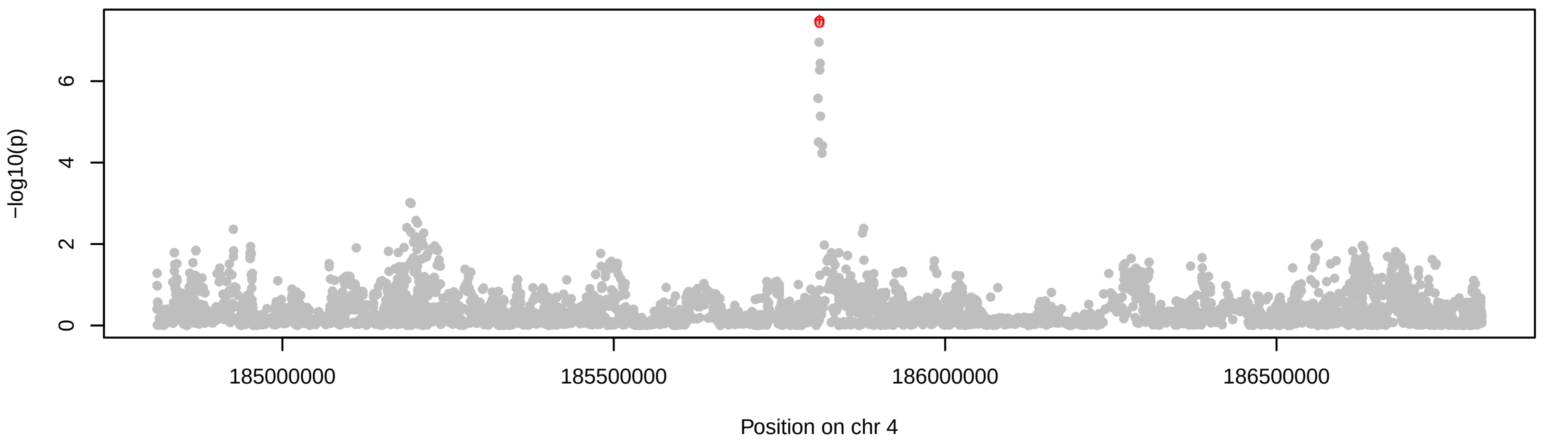

coloc with QMDiab: H1 (PP=66%)

327. CAPZA1 (P52907) 4:185810329:T:C [QMDiab]

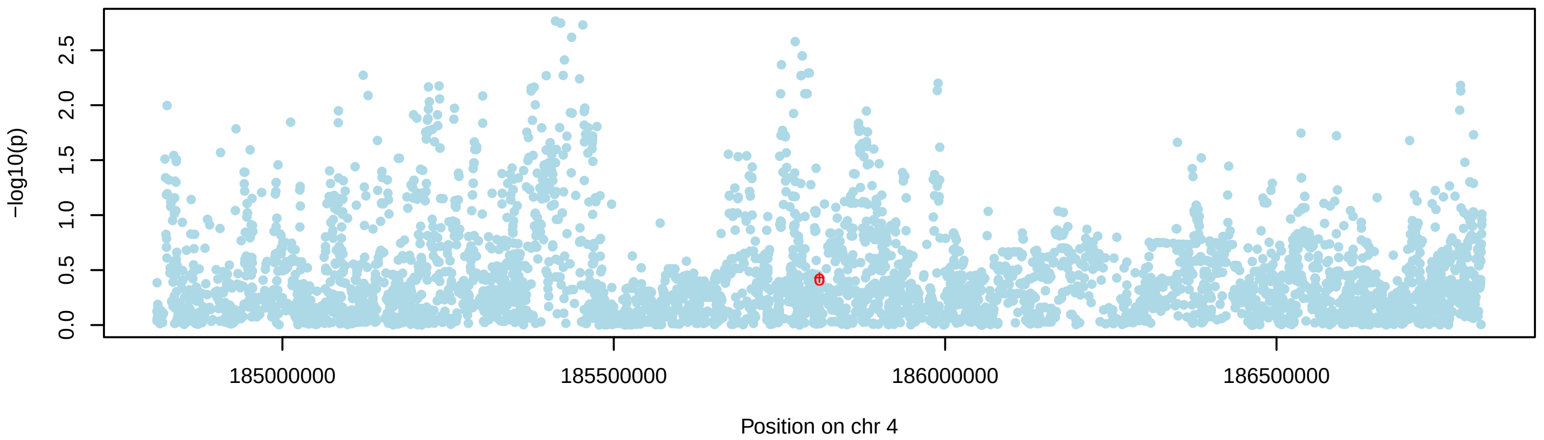

328. GPLD1 (P80108) 6:24418948:G:A [Tarkin]

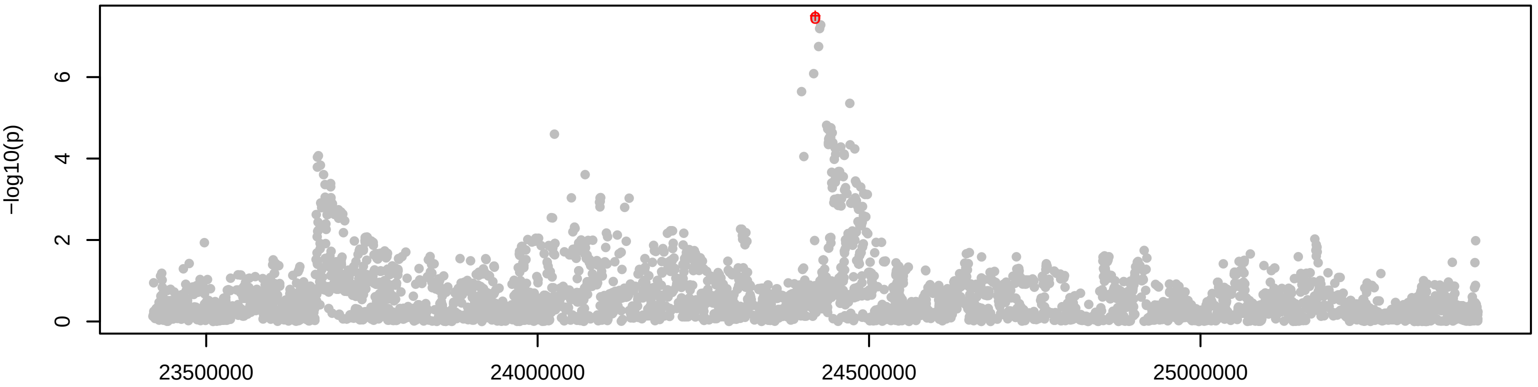

Position on chr 6
coloc with QMDiab: H1 (PP=53%)

328. GPLD1 (P80108) 6:24418948:G:A [QMDiab]

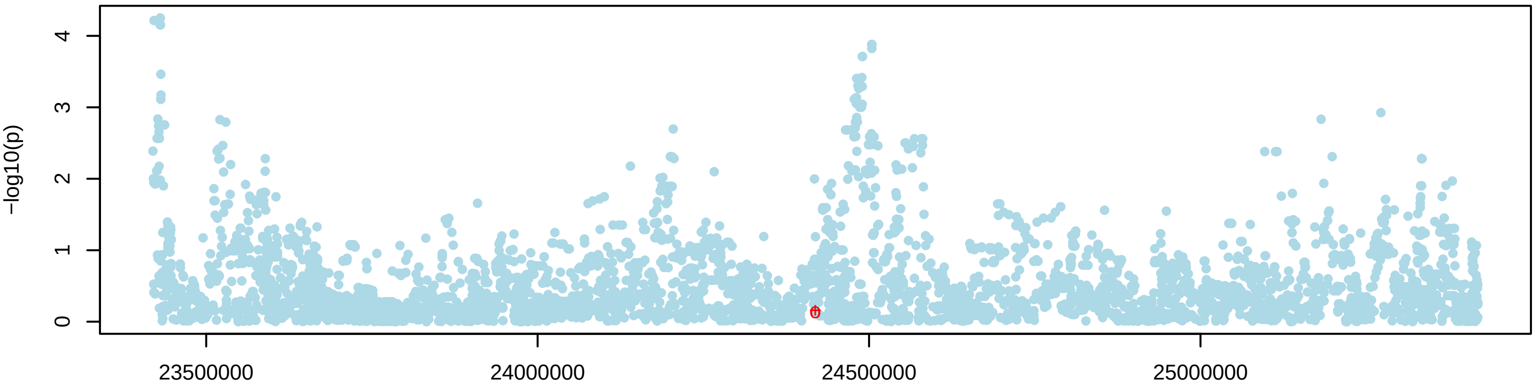

Position on chr 6

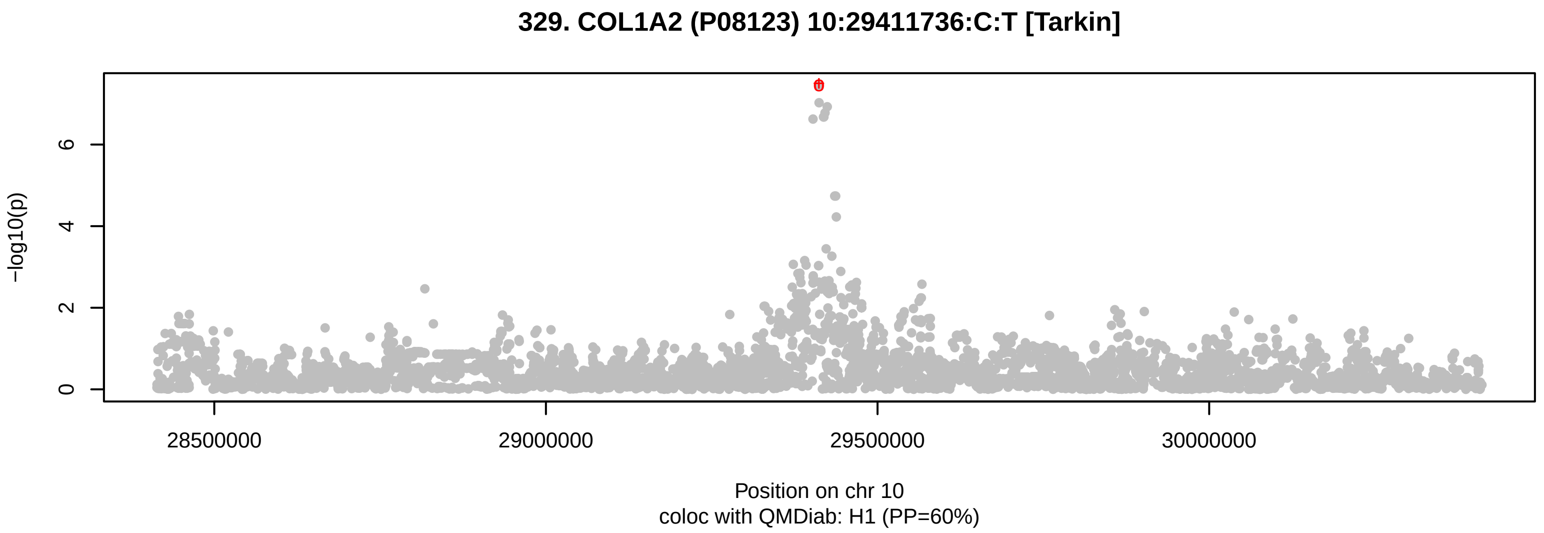
329. COL1A2 (P08123) 10:29411736:C:T [Tarkin]
−log10(p)
0
2
4
6
28500000
29000000
29500000
30000000
Position on chr 10
coloc with QMDiab: H1 (PP=60%)

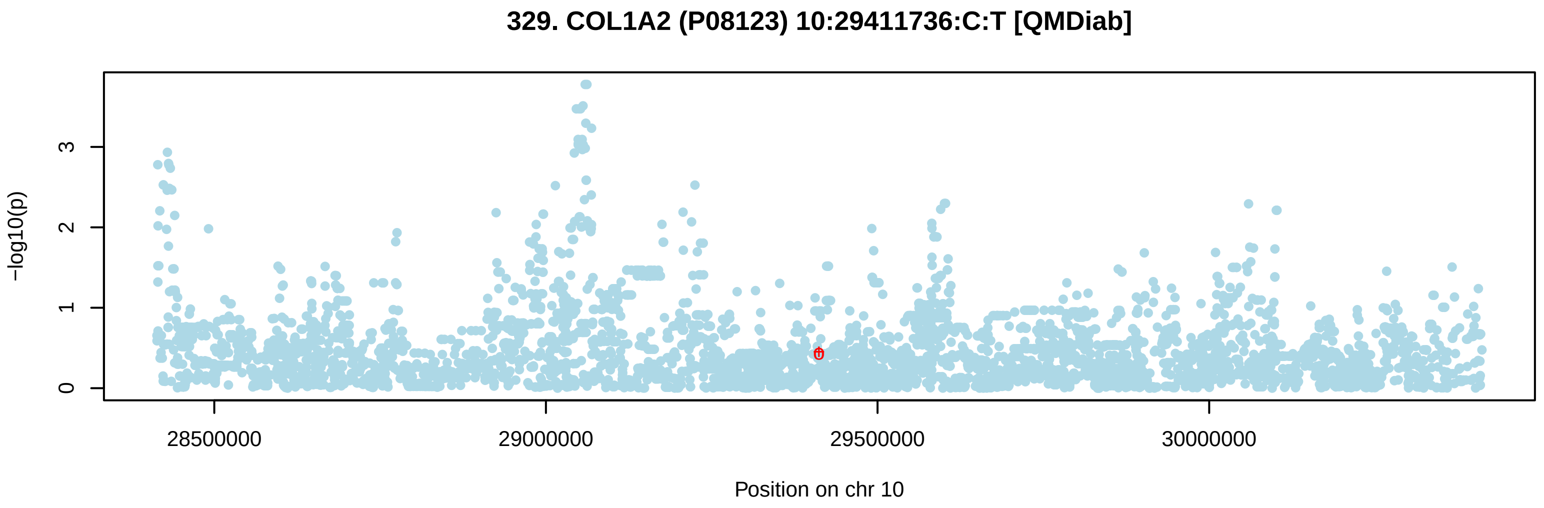
329. COL1A2 (P08123) 10:29411736:C:T [QMDiab]
−log10(p)
0
1
2
3
28500000
29000000
29500000
30000000
Position on chr 10

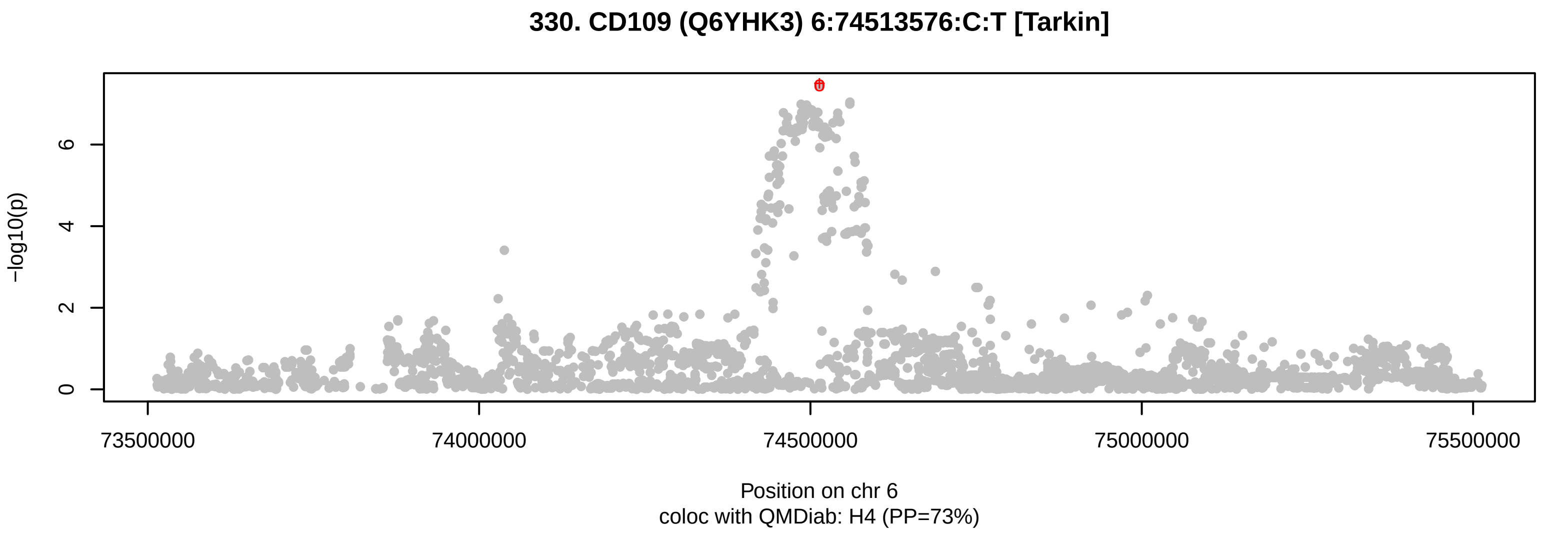
330. CD109 (Q6YHK3) 6:74513576:C:T [Tarkin]
−log10(p)
0
2
4
6
73500000
74000000
74500000
75000000
75500000
Position on chr 6
coloc with QMDiab: H4 (PP=73%)

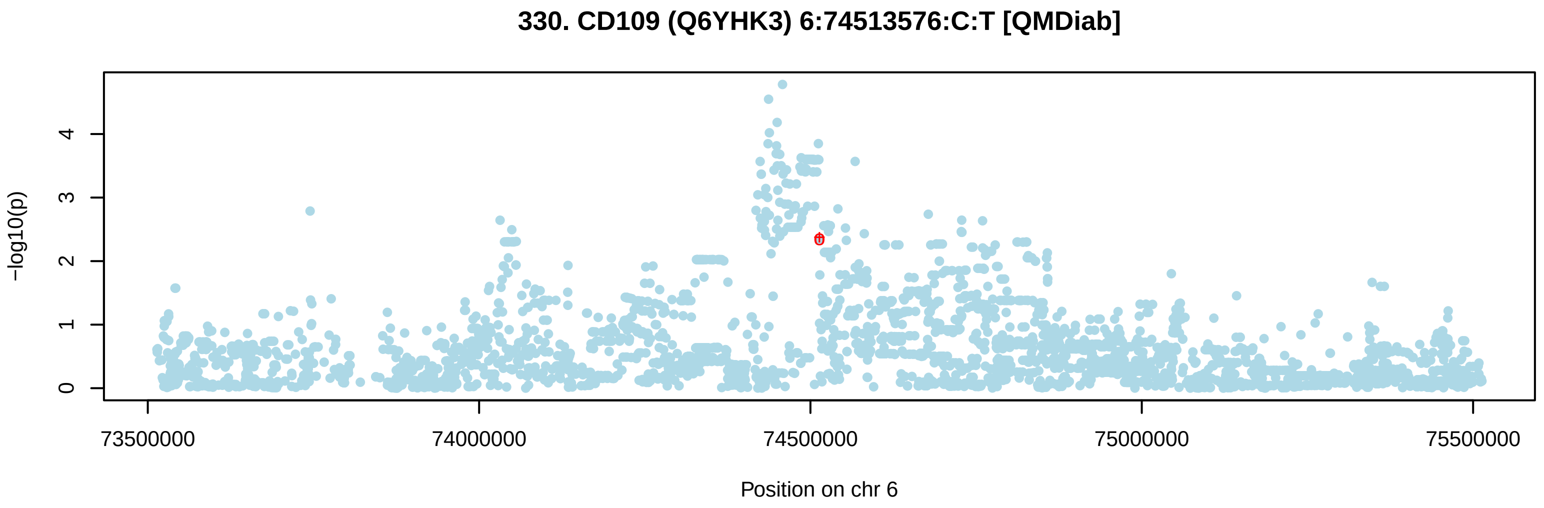
330. CD109 (Q6YHK3) 6:74513576:C:T [QMDiab]
−log10(p)
0
1
2
3
4
73500000
74000000
74500000
75000000
75500000
Position on chr 6

331. APLP1 (B7Z4G8) 4:150957395:G:C [Tarkin]

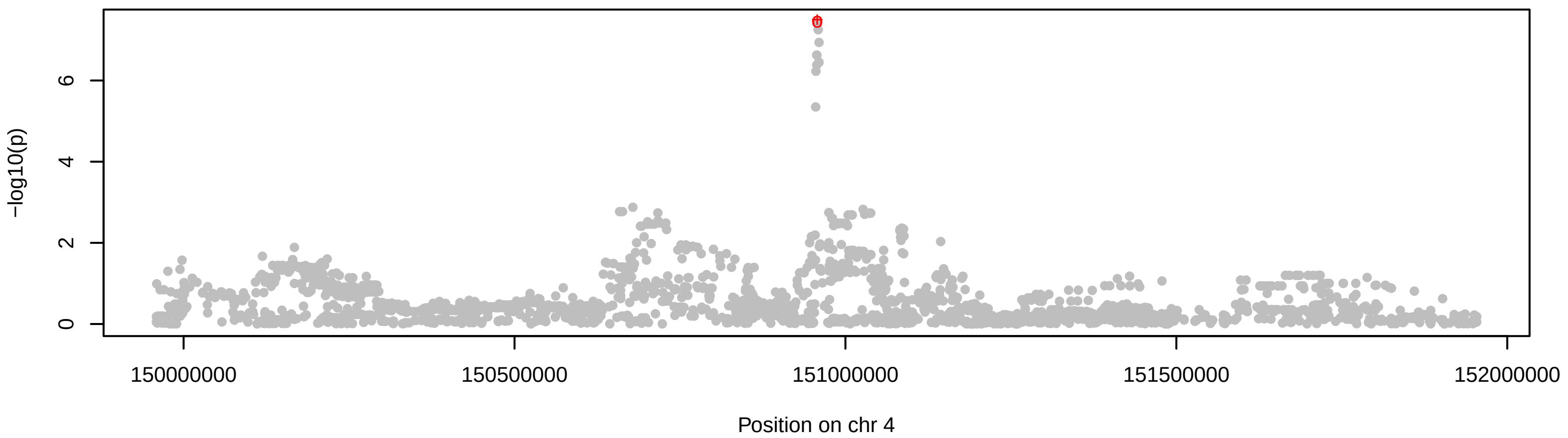

coloc with QMDiab: H1 (PP=79%)

331. APLP1 (B7Z4G8) 4:150957395:G:C [QMDiab]

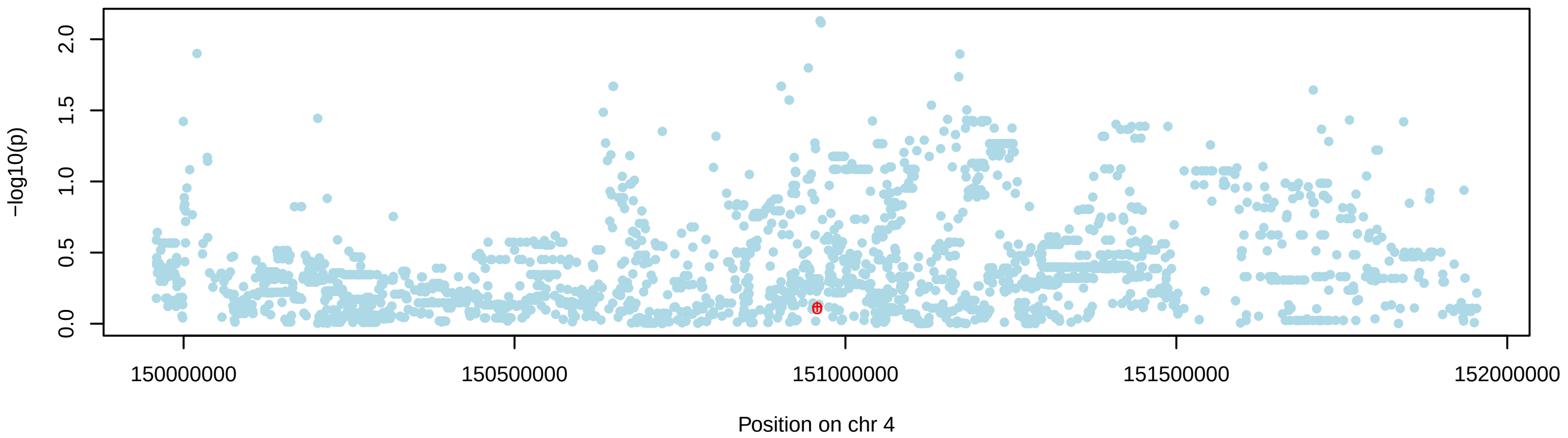

332. YKT6 (A0A7I2V4L6;O15498) 3:32882007:G:A [Tarkin]

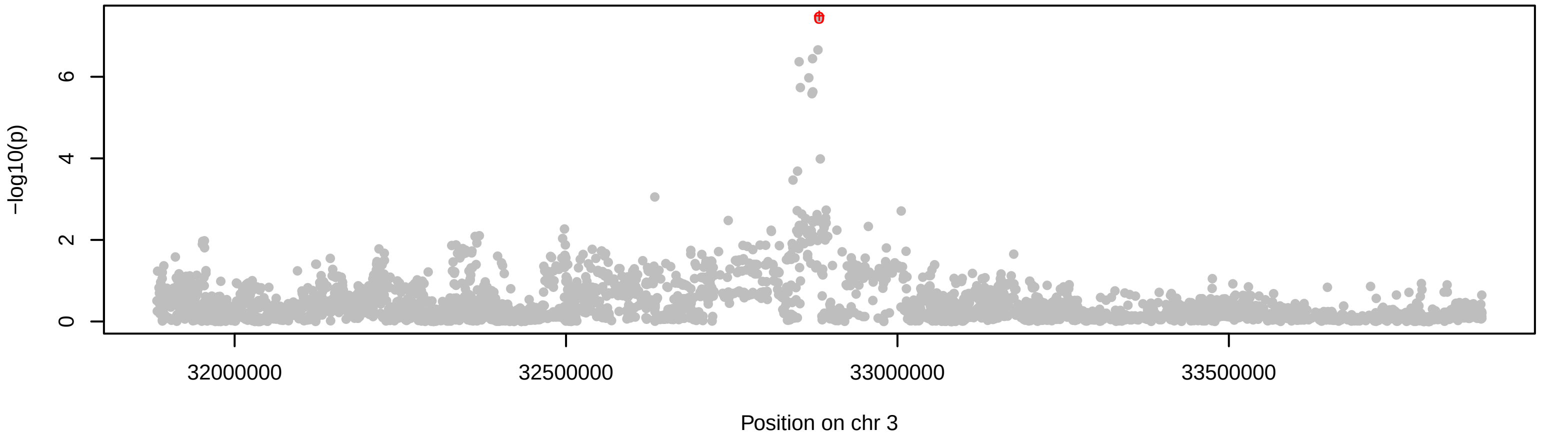

no replication data avaiable in QMDiab

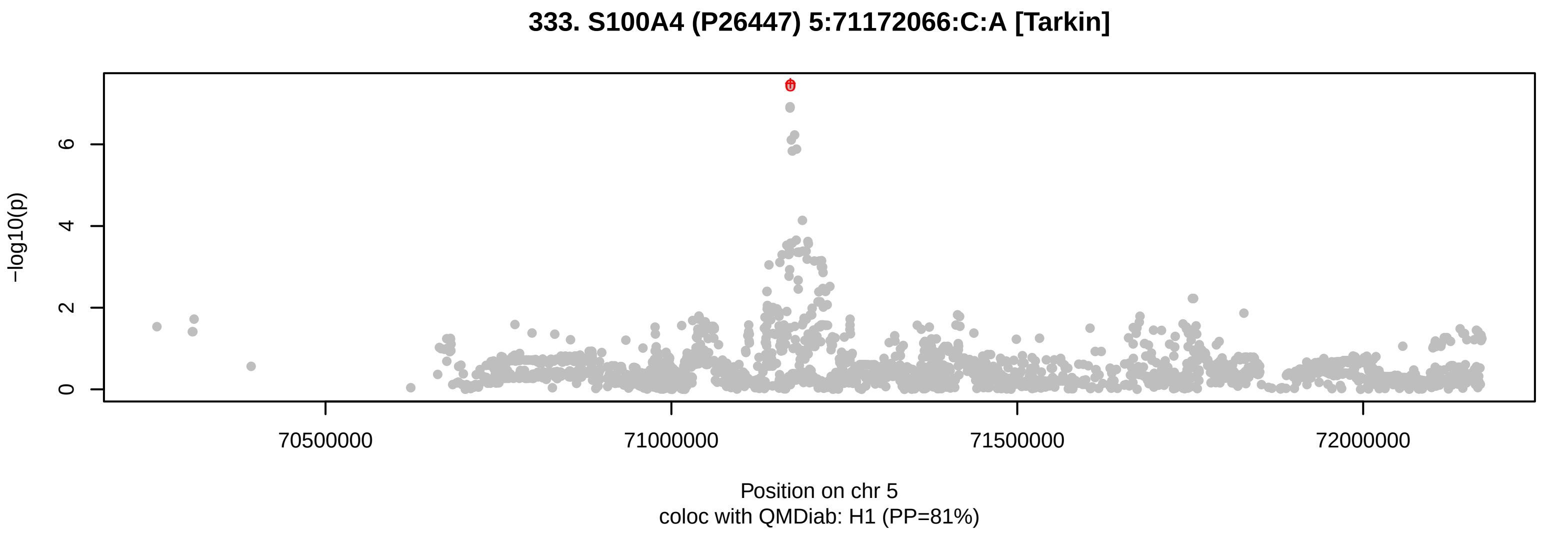

333. S100A4 (P26447) 5:71172066:C:A [Tarkin]
−log10(p)
0
2
4
6
70500000
71000000
71500000
72000000
Position on chr 5
coloc with QMDiab: H1 (PP=81%)

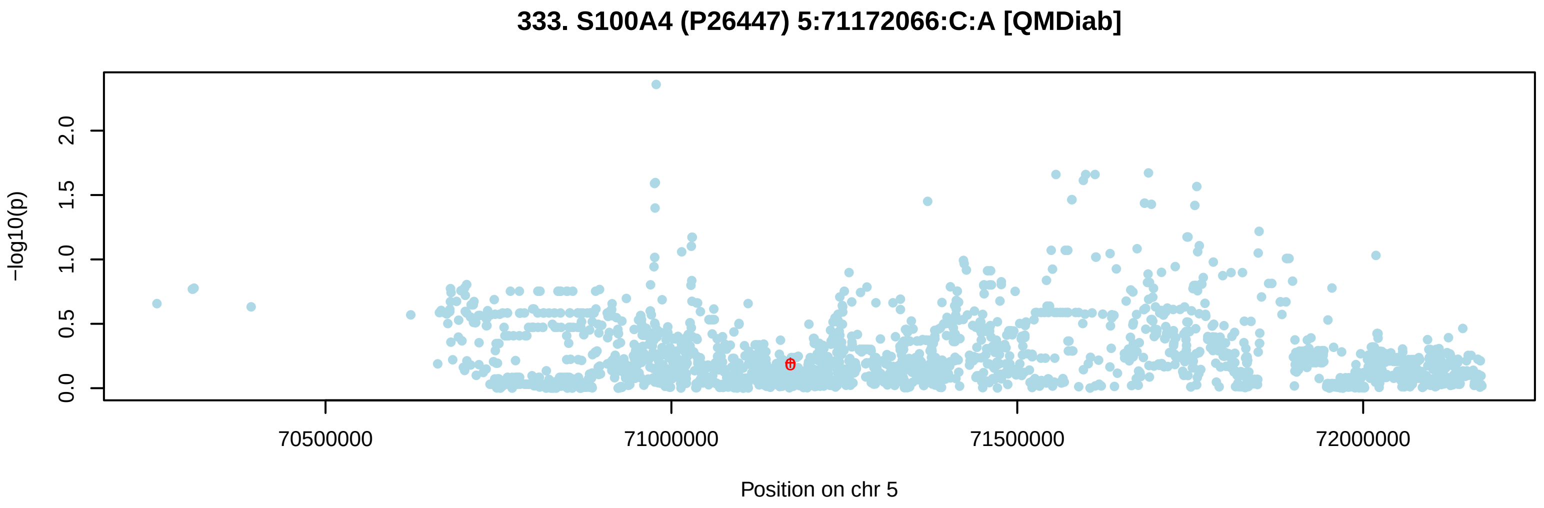

333. S100A4 (P26447) 5:71172066:C:A [QMDiab]
−log10(p)
0.0
0.5
1.0
1.5
2.0
70500000
71000000
71500000
72000000
Position on chr 5

334. MAPKAPK2 (P49137) 1:234646443:C:T [Tarkin]

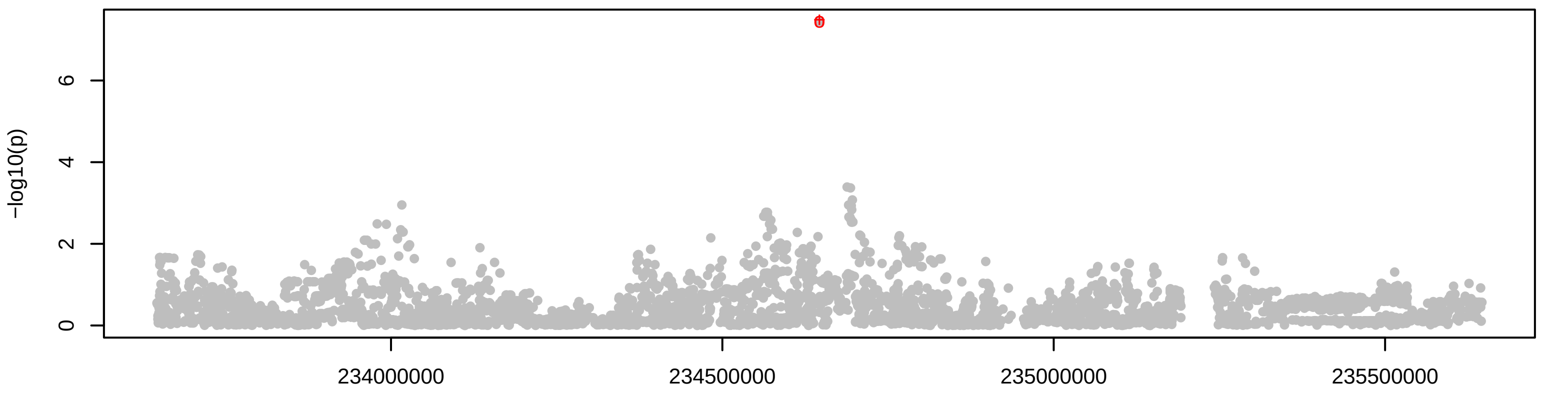

Position on chr 1
coloc with QMDiab: H0 (PP=52%)

334. MAPKAPK2 (P49137) 1:234646443:C:T [QMDiab]

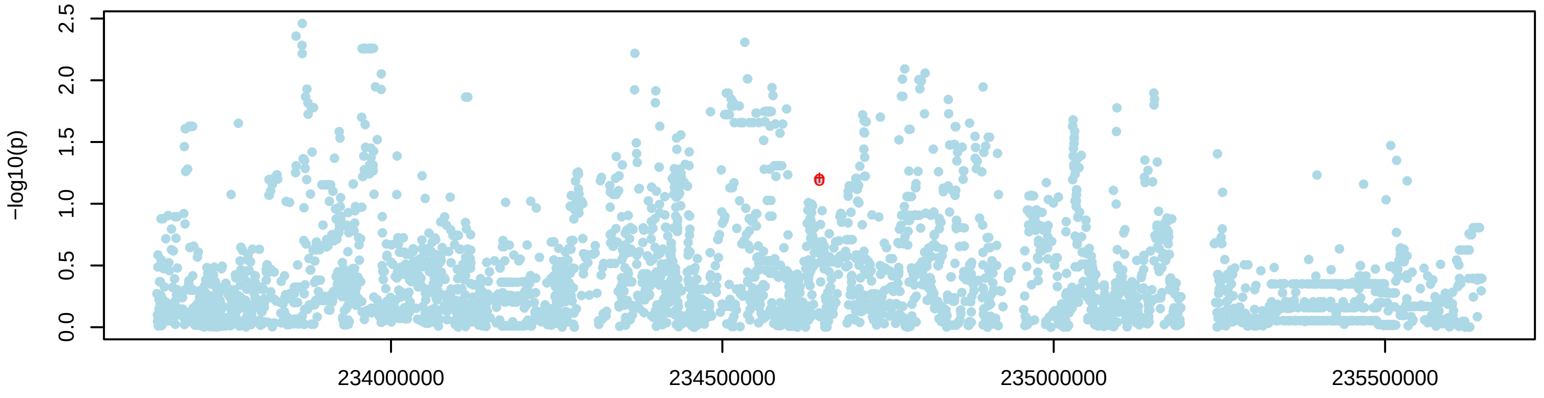

Position on chr 1

335. NACA (E9PAV3) 2:34259164:T:A [Tarkin]

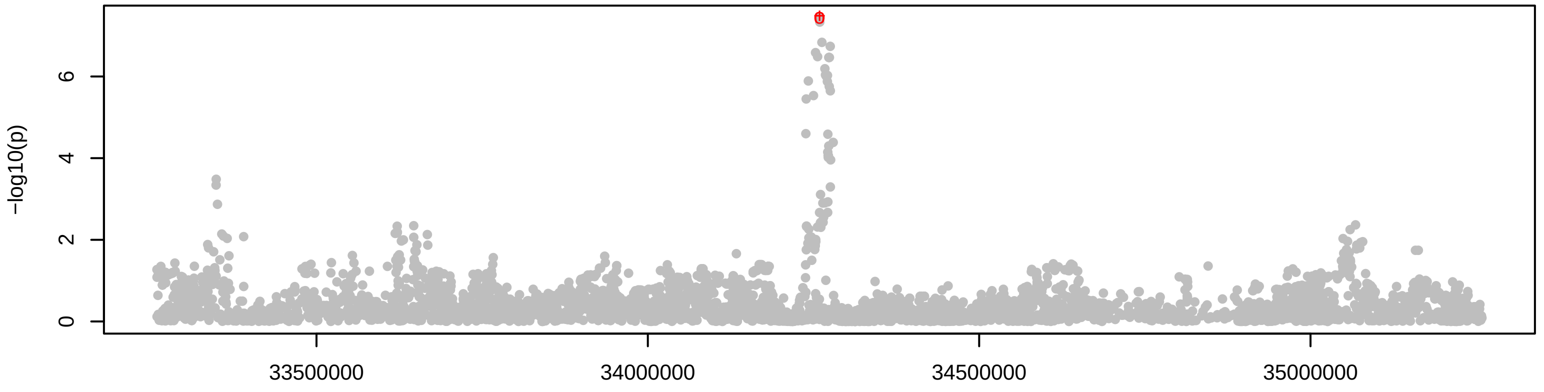

Position on chr 2
coloc with QMDiab: H1 (PP=61%)

335. NACA (E9PAV3) 2:34259164:T:A [QMDiab]

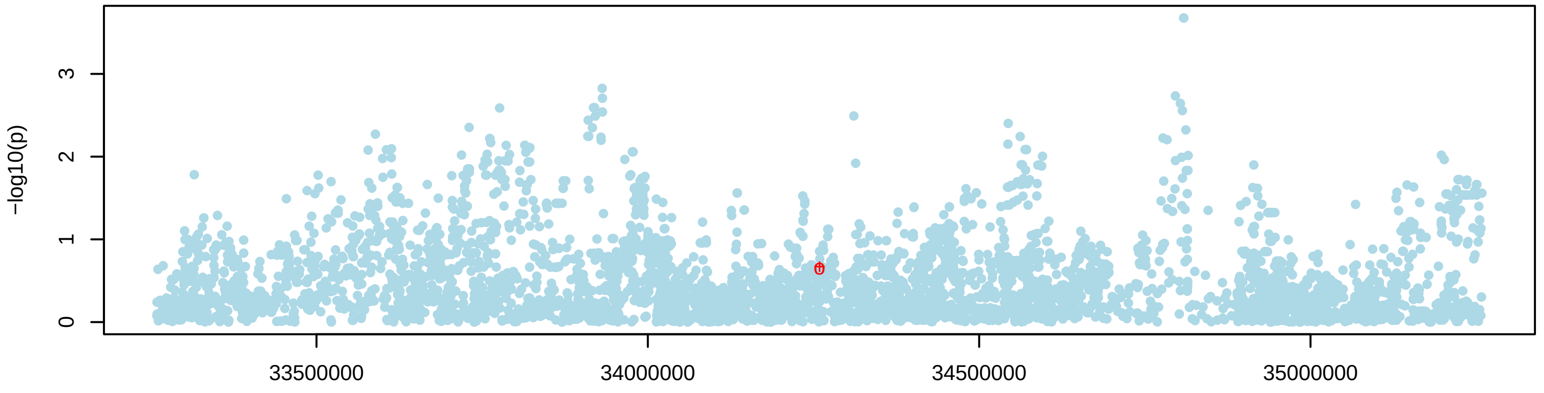

Position on chr 2

336. PLP2 (Q04941) 4:180162940:A:G [Tarkin]

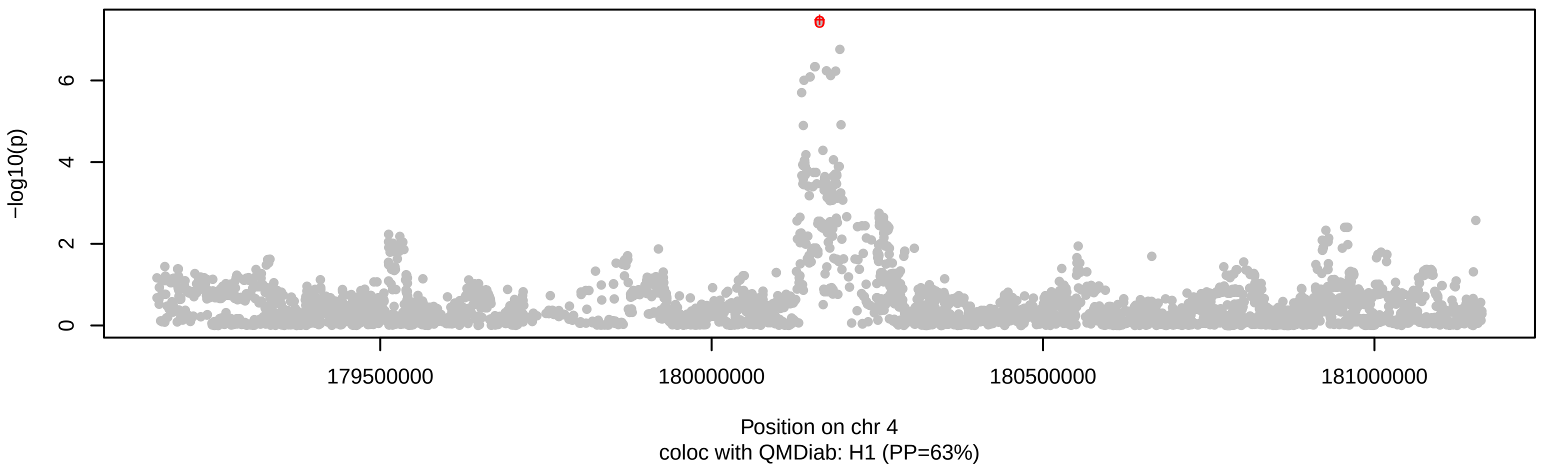

336. PLP2 (Q04941) 4:180162940:A:G [QMDiab]

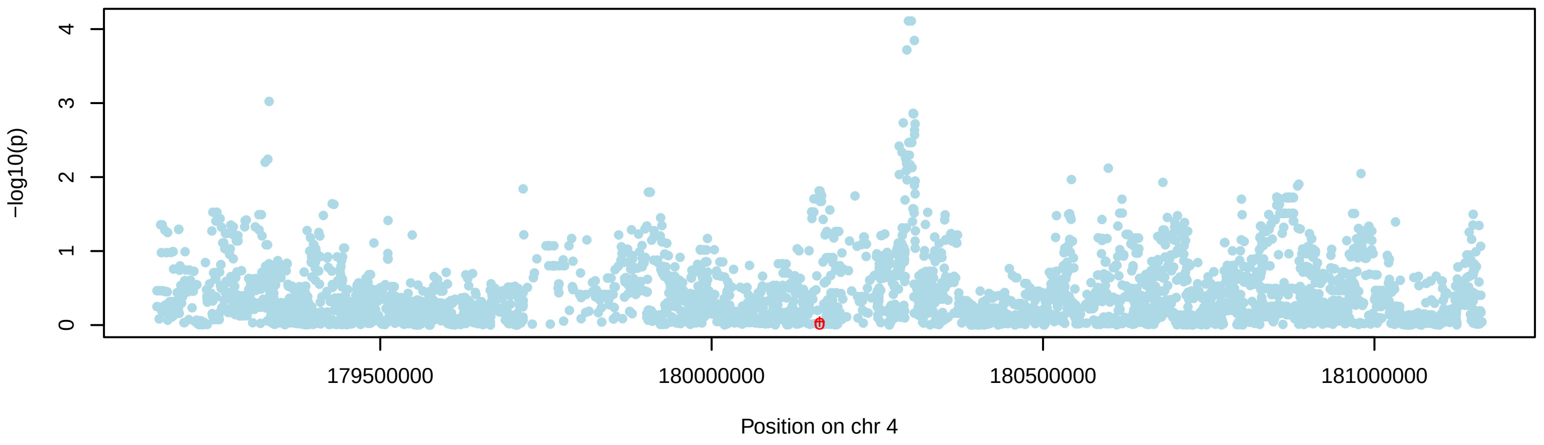

337. C8G (P07360) 14:52298040:G:A [Tarkin]

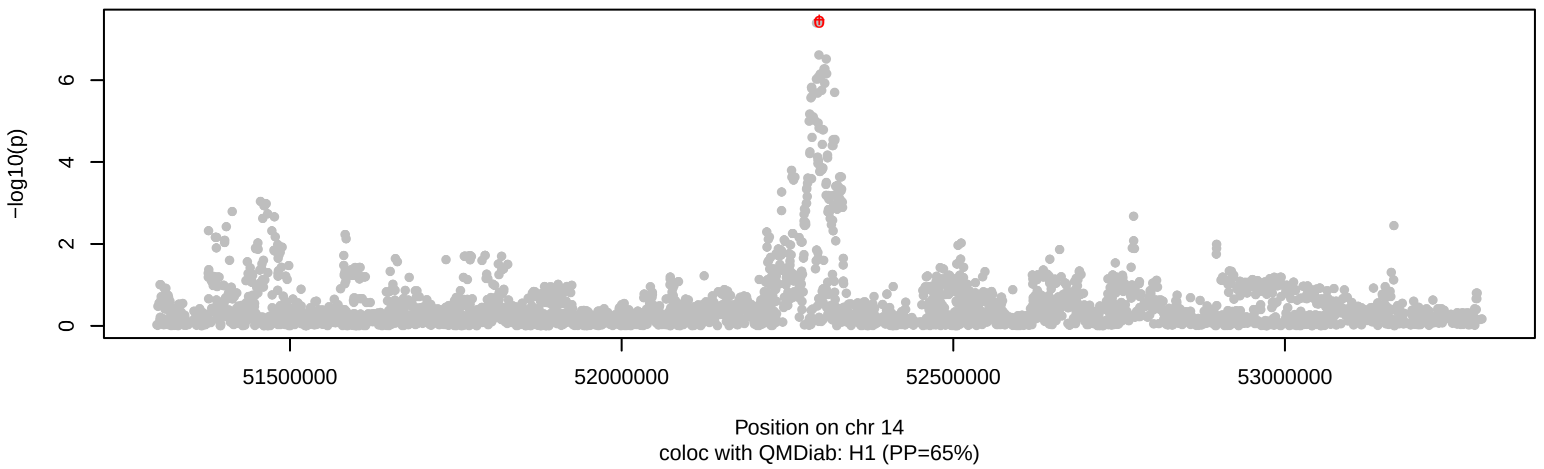

coloc with QMDiab: H1 (PP=65%)

337. C8G (P07360) 14:52298040:G:A [QMDiab]

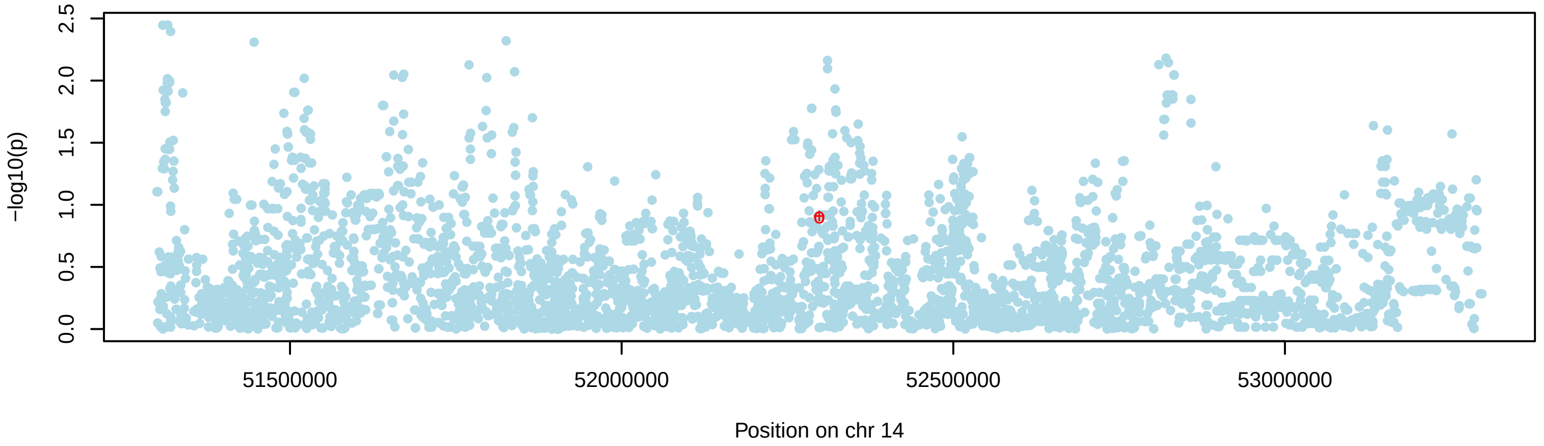

338. APOD (P05090) 3:152203443:G:A [Tarkin]

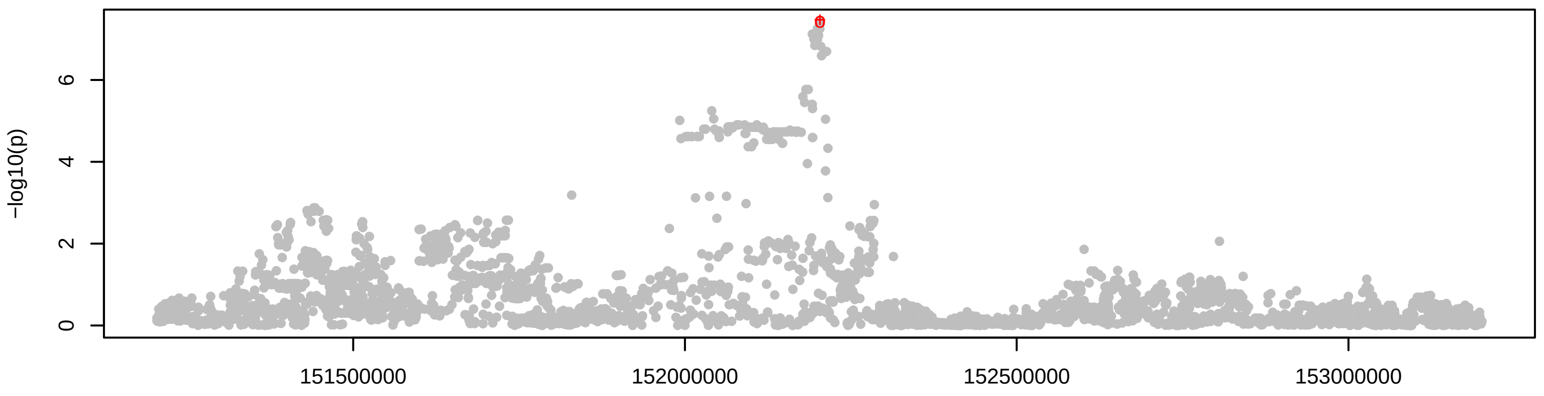

Position on chr 3
coloc with QMDiab: H1 (PP=70%)

338. APOD (P05090) 3:152203443:G:A [QMDiab]

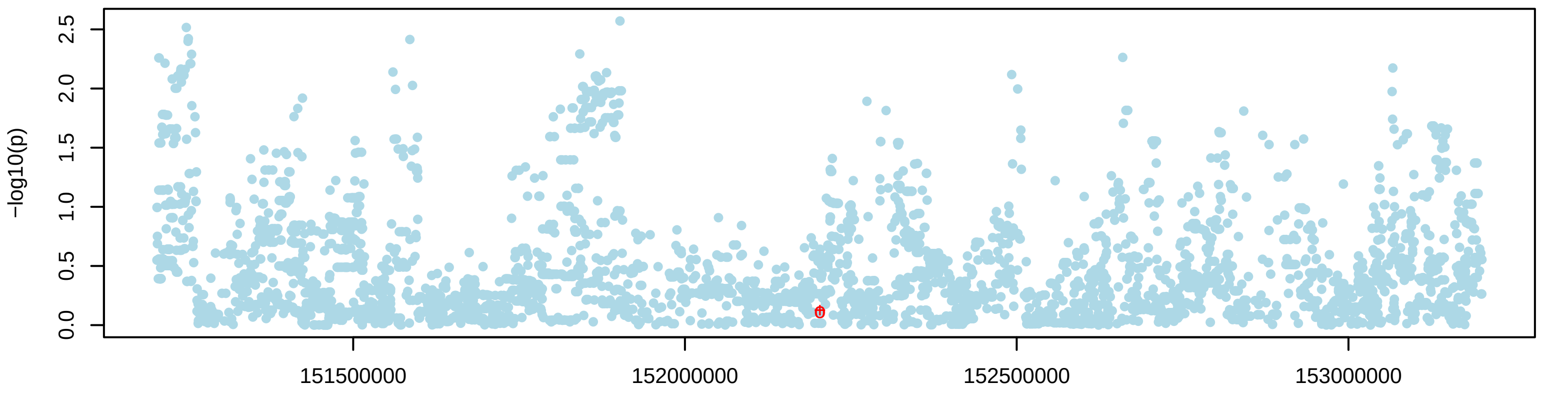

Position on chr 3

339. CCT2 (P78371) 17:76720495:G:C [Tarkin]

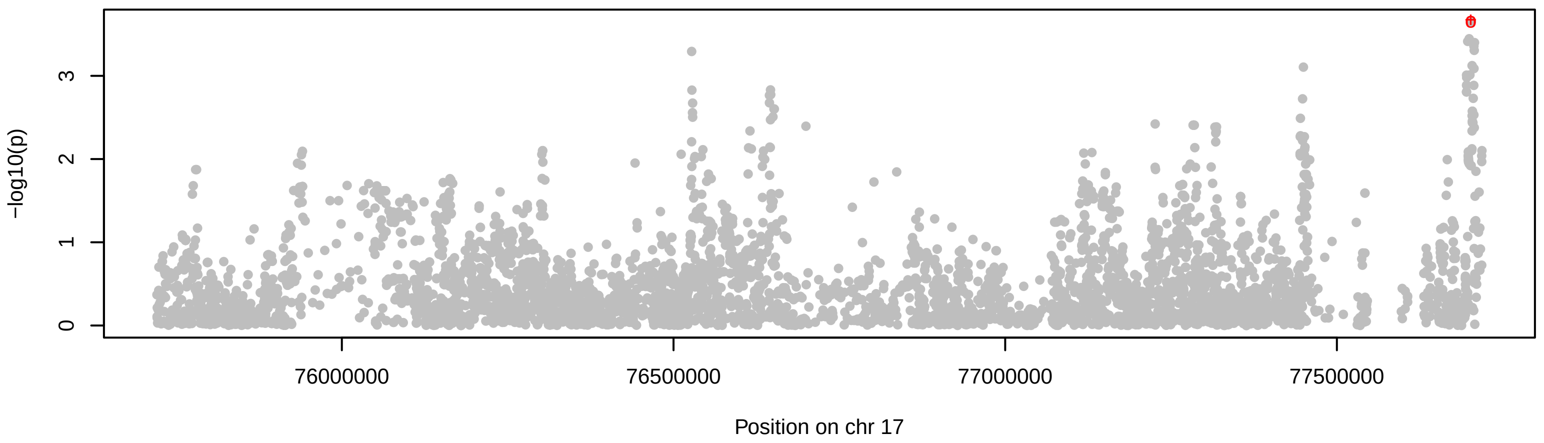

coloc with QMDiab: H0 (PP=46%)

339. CCT2 (P78371) 17:76720495:G:C [QMDiab]

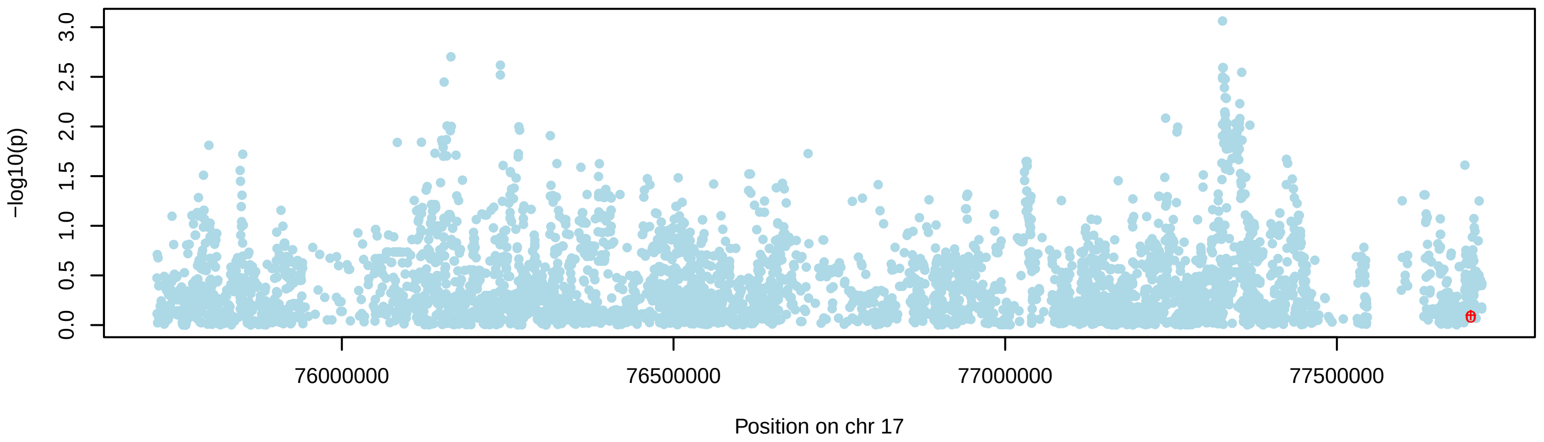

340. PPP1R14A (Q96A00) 19:38765072:C:T [Tarkin]

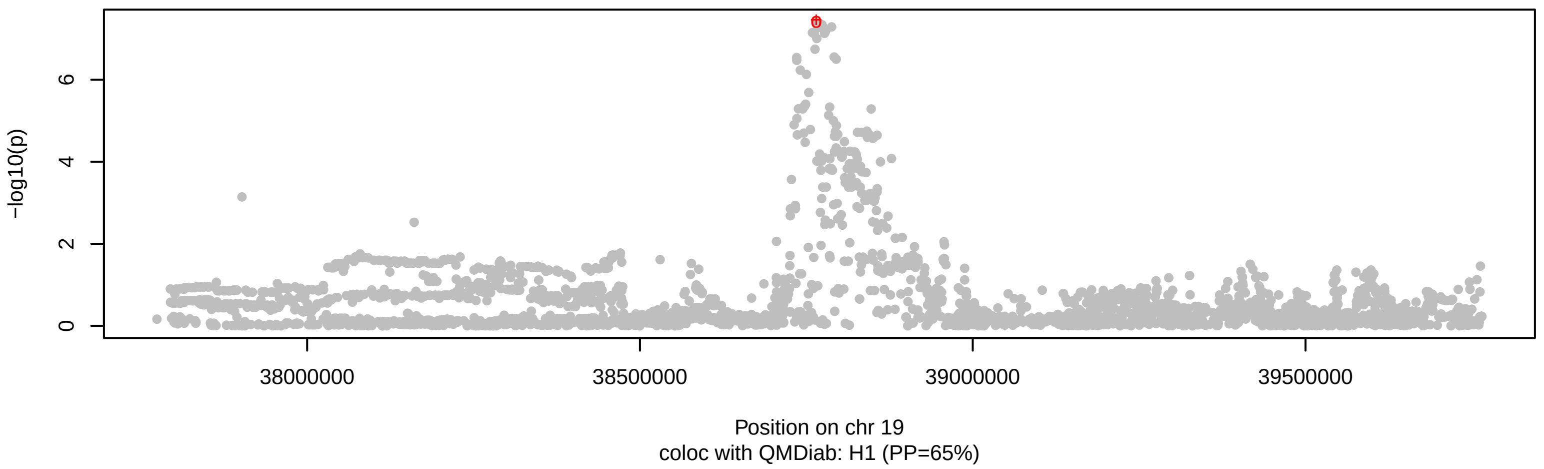

coloc with QMDiab: H1 (PP=65%)

340. PPP1R14A (Q96A00;Q96A00−2) 19:38765072:C:T [QMDiab]

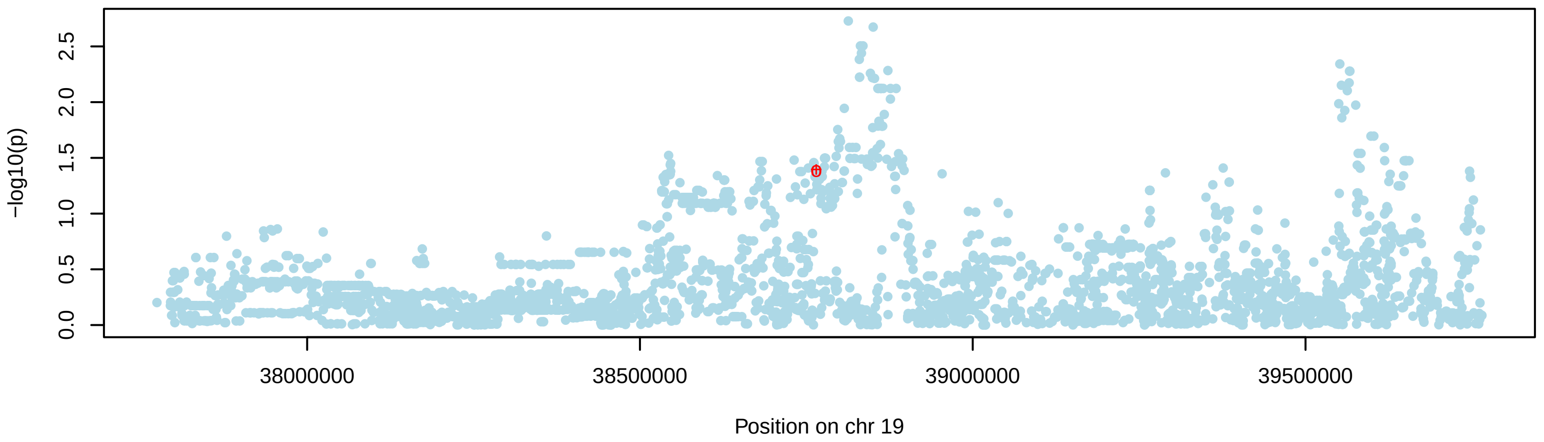

341. EIF3D (O15371) 6:126216025:G:A [Tarkin]

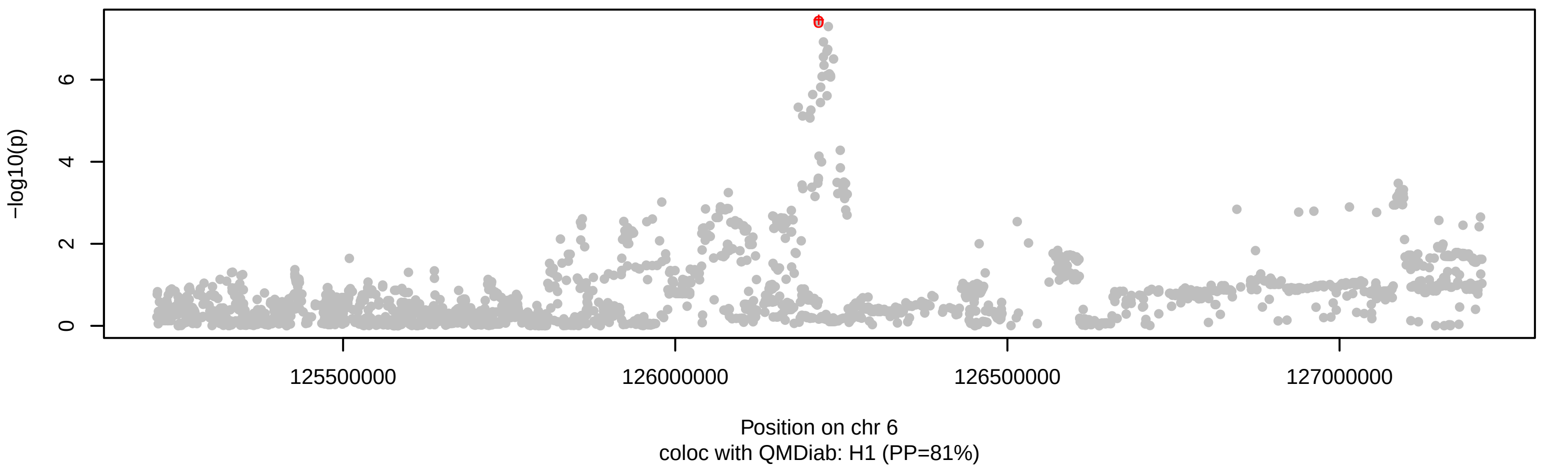

coloc with QMDiab: H1 (PP=81%)

341. EIF3D (O15371) 6:126216025:G:A [QMDiab]

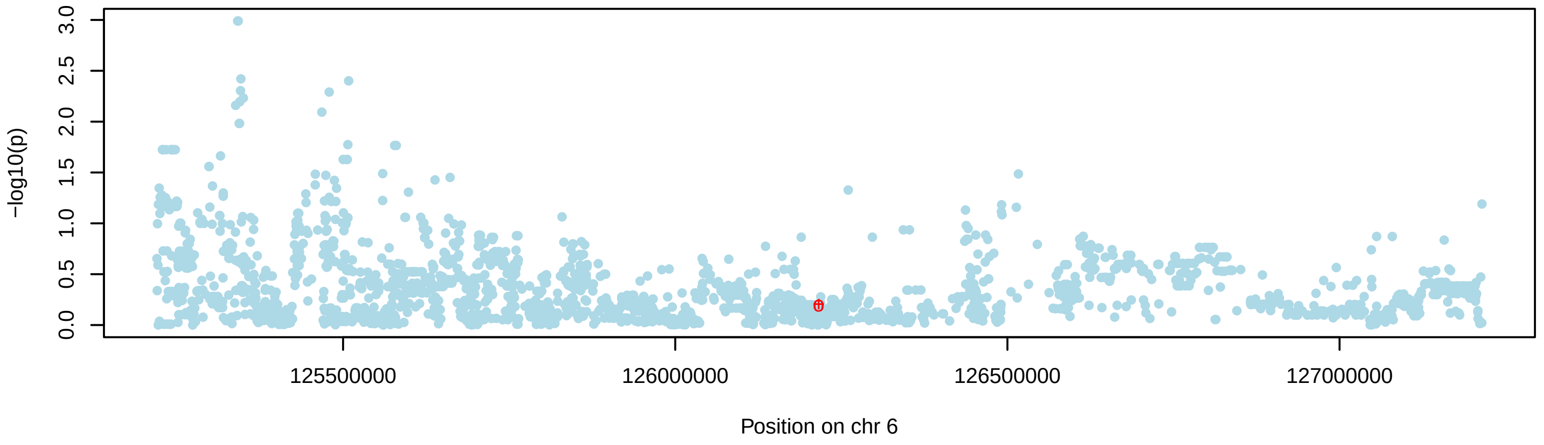

342. PCOLCE2 (Q9UKZ9) 16:24900211:G:A [Tarkin]

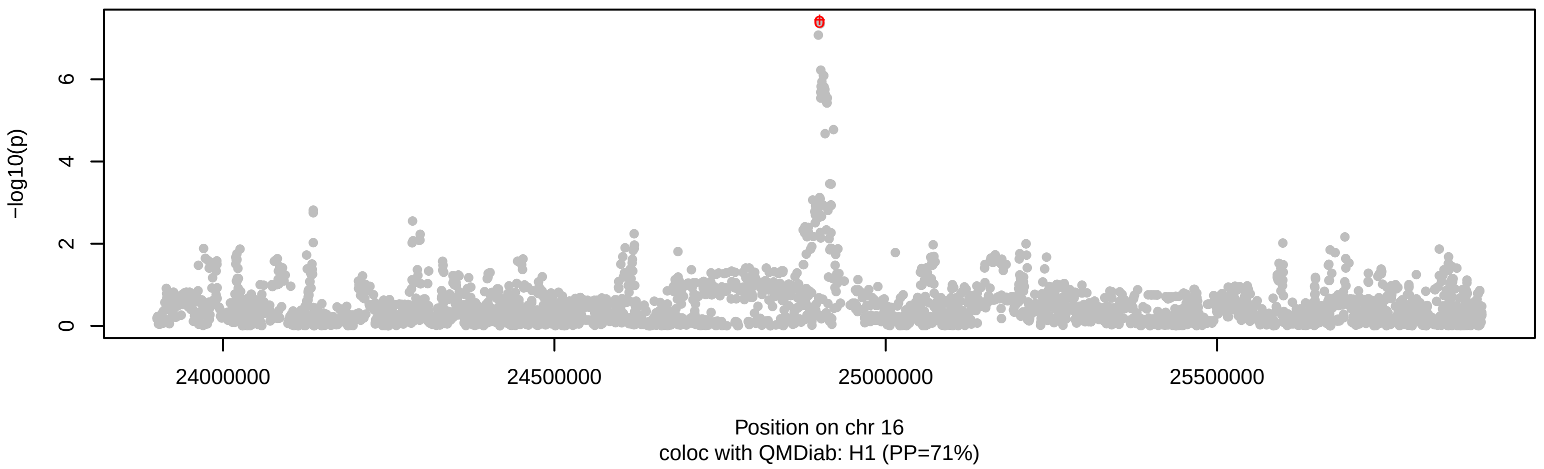

coloc with QMDiab: H1 (PP=71%)

342. PCOLCE2 (Q9UKZ9) 16:24900211:G:A [QMDiab]

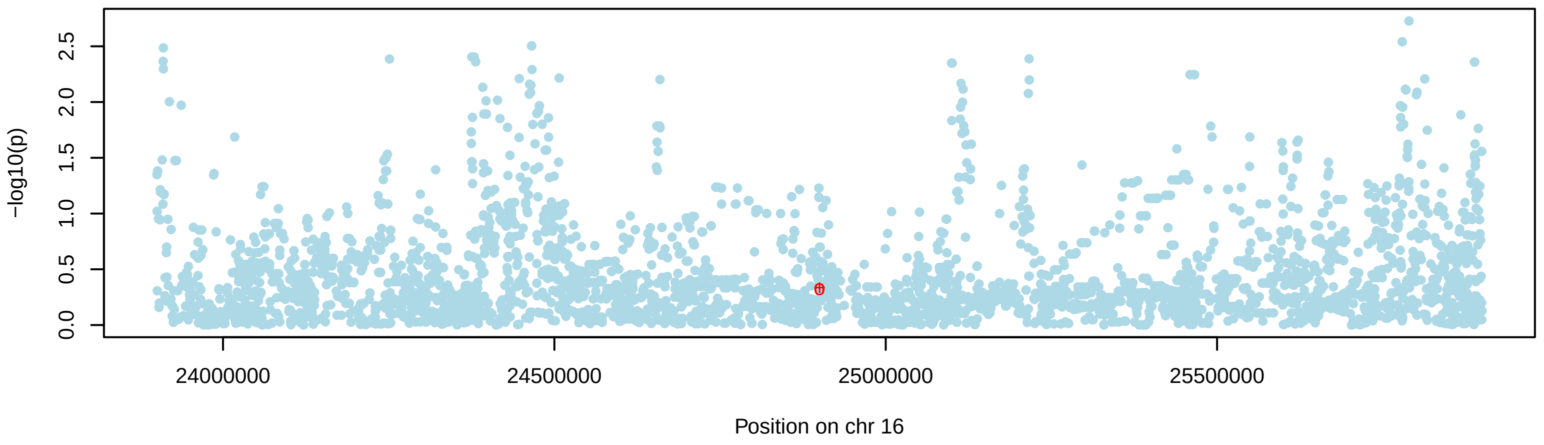

343. CALR (P27797) 21:40053079:G:C [Tarkin]

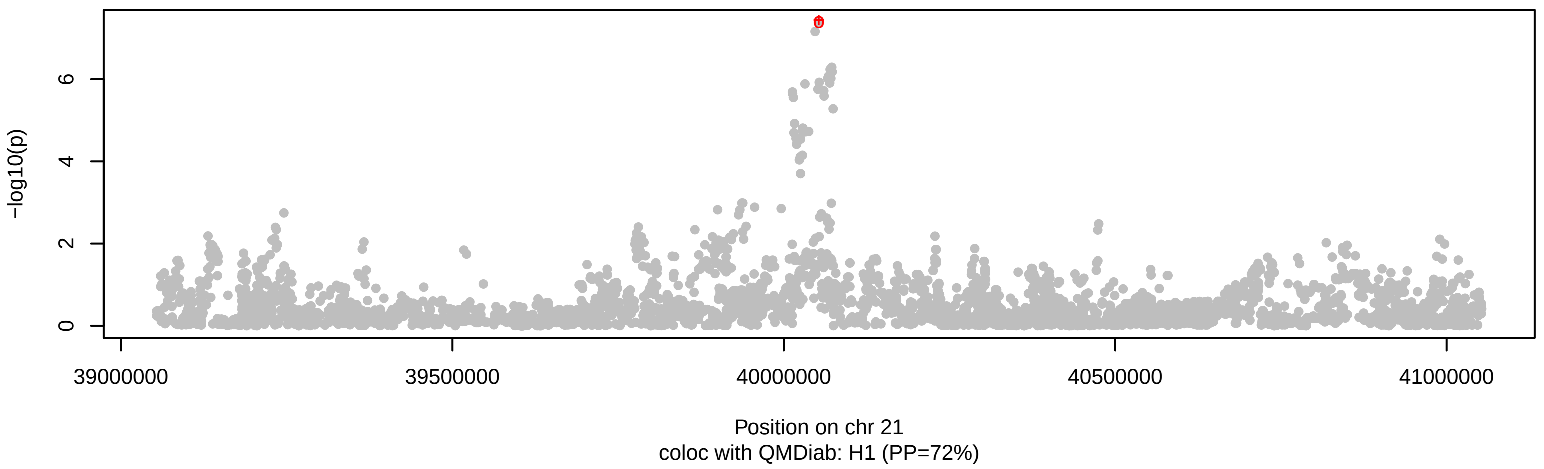

coloc with QMDiab: H1 (PP=72%)

343. CALR (P27797) 21:40053079:G:C [QMDiab]

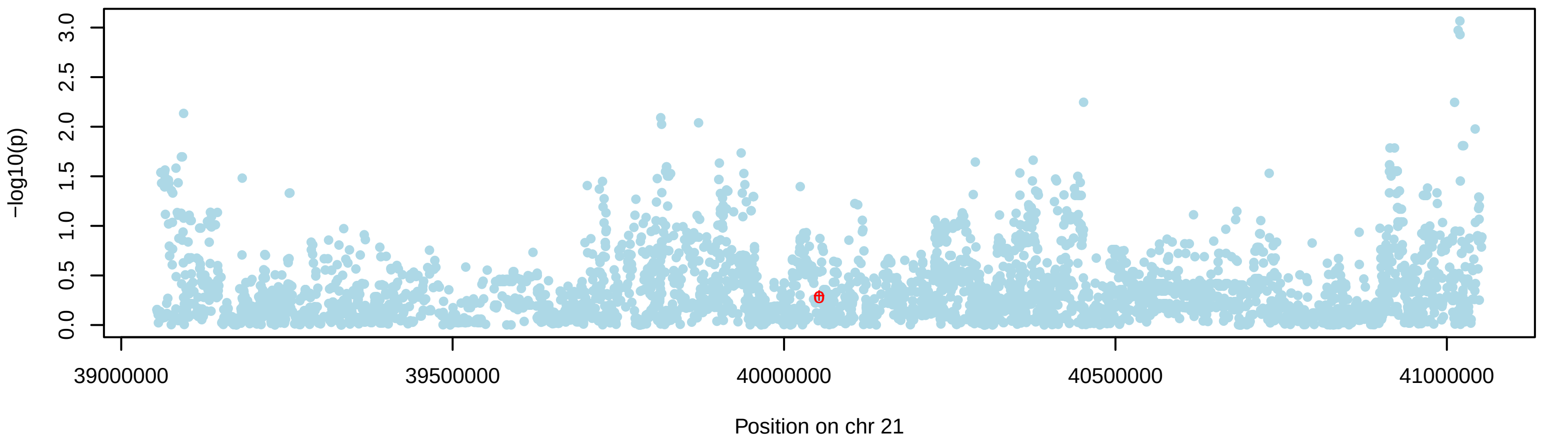

344. EPRS (P07814) 20:23503309:T:C [Tarkin]

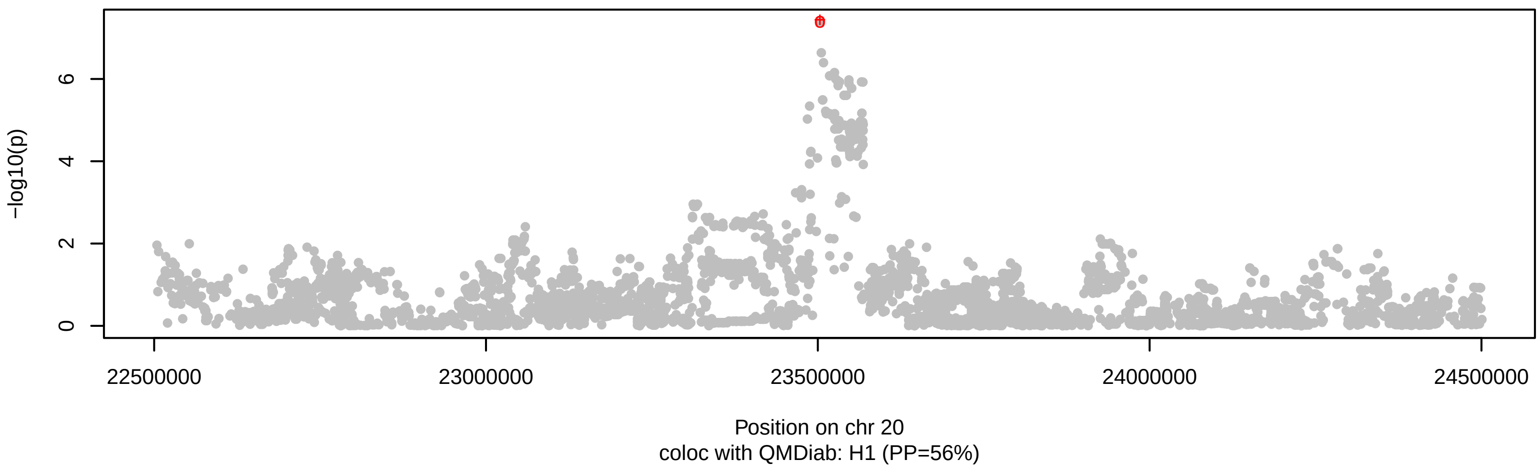

coloc with QMDiab: H1 (PP=56%)

344. EPRS (P07814) 20:23503309:T:C [QMDiab]

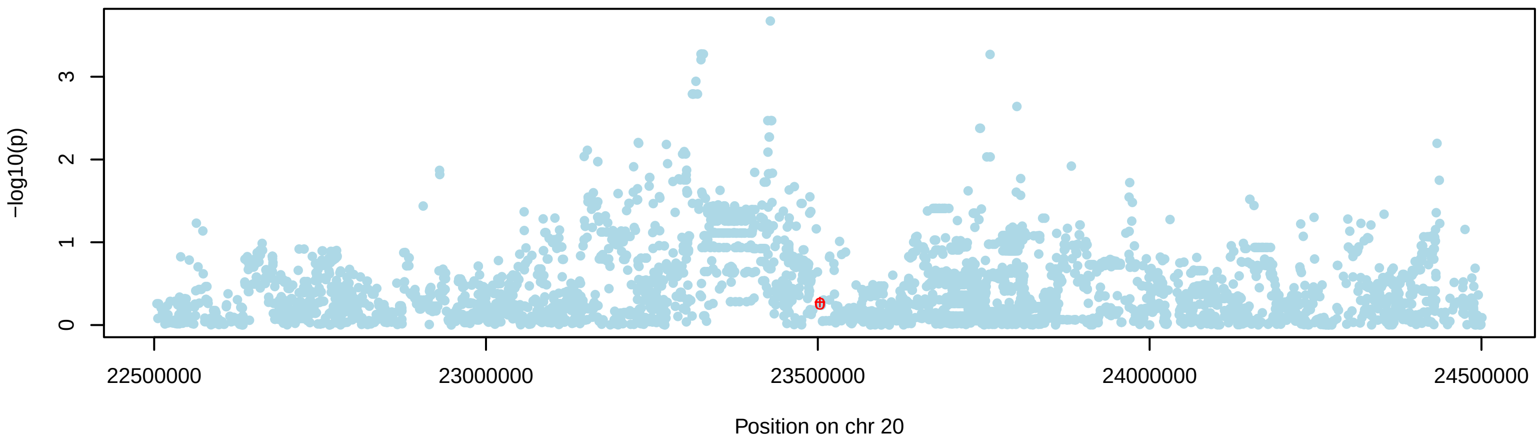

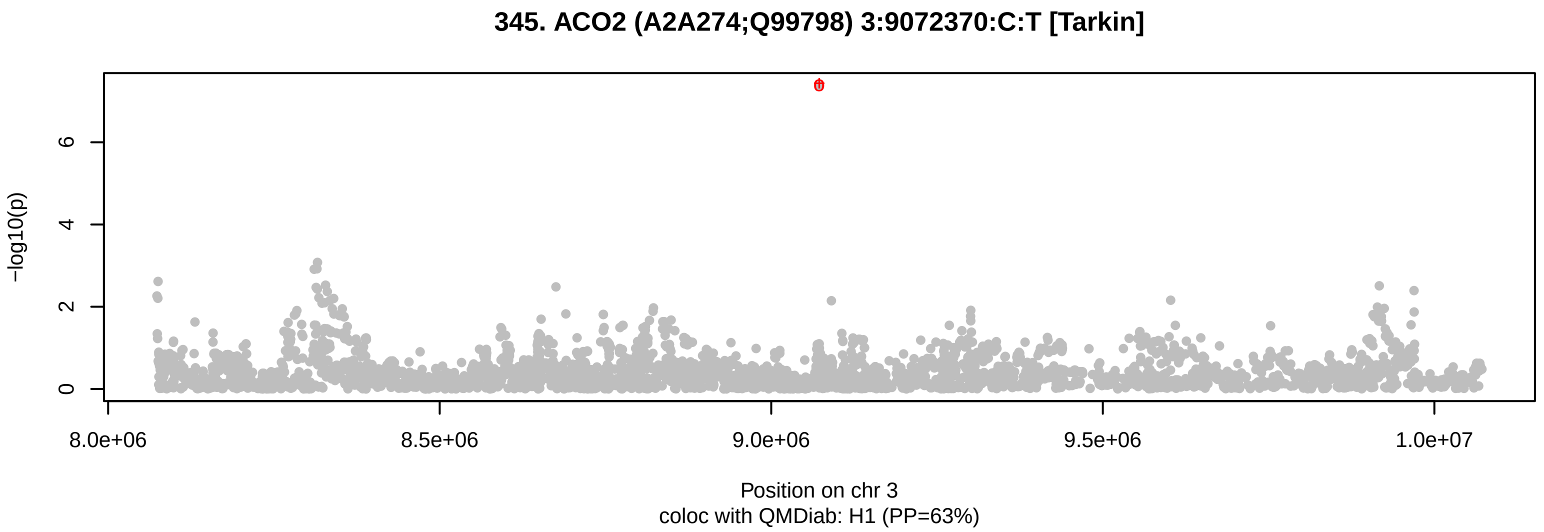

coloc with QMDiab: H1 (PP=63%)

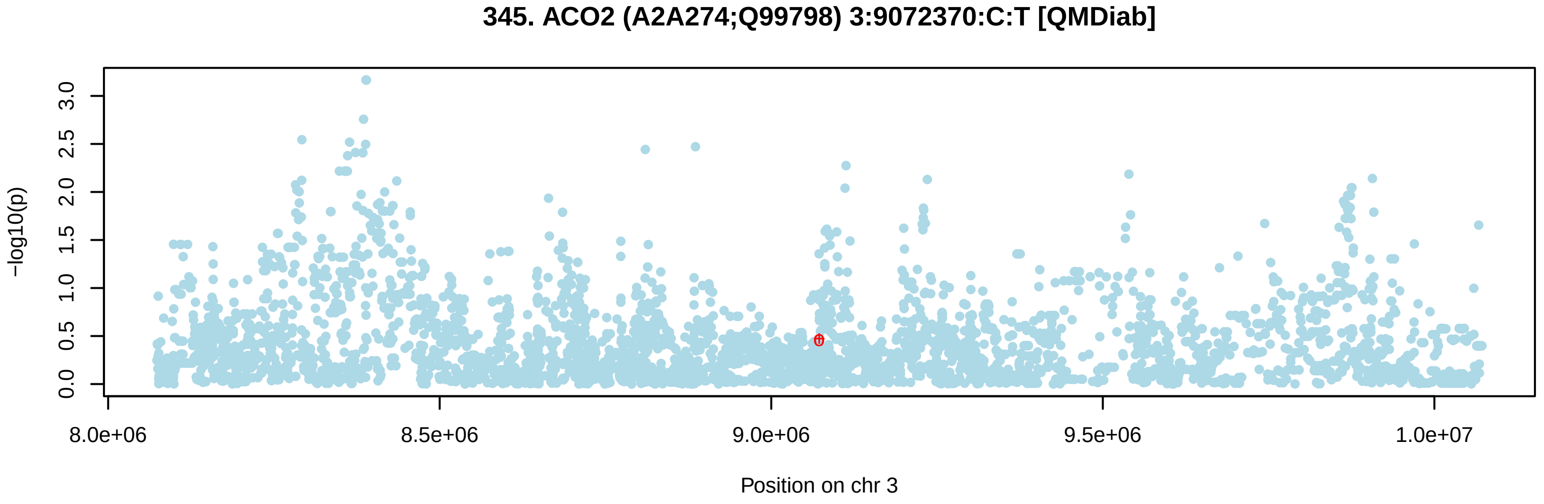

346. SLC16A3 (J3KTM6;J3QQV2;O15427) 22:44589008:T:C [Tarkin]

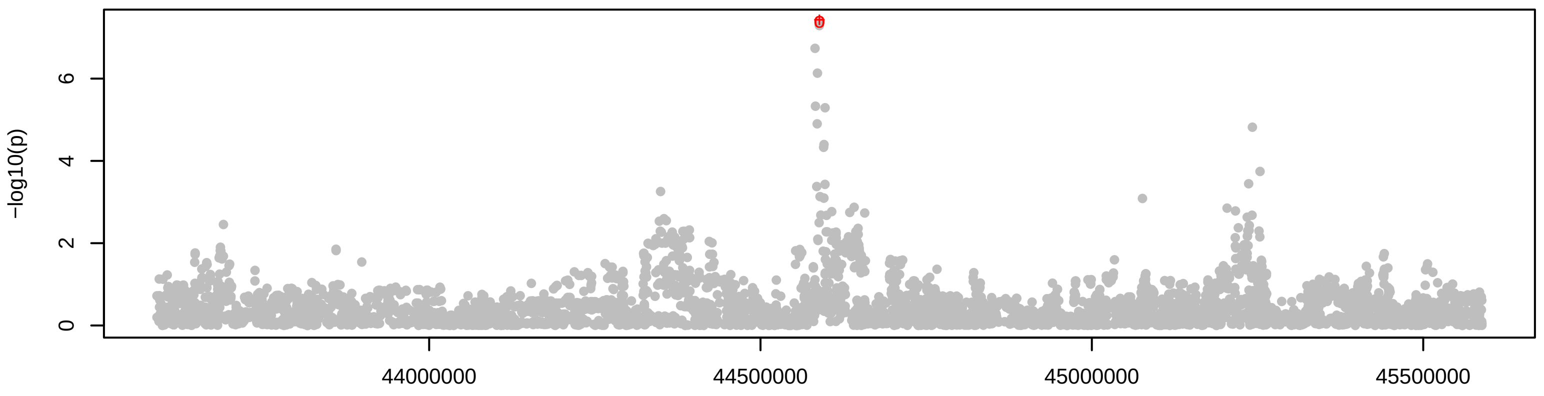

Position on chr 22
coloc with QMDiab: H1 (PP=60%)

346. SLC16A3 (J3KTM6;J3QQV2;O15427) 22:44589008:T:C [QMDiab]

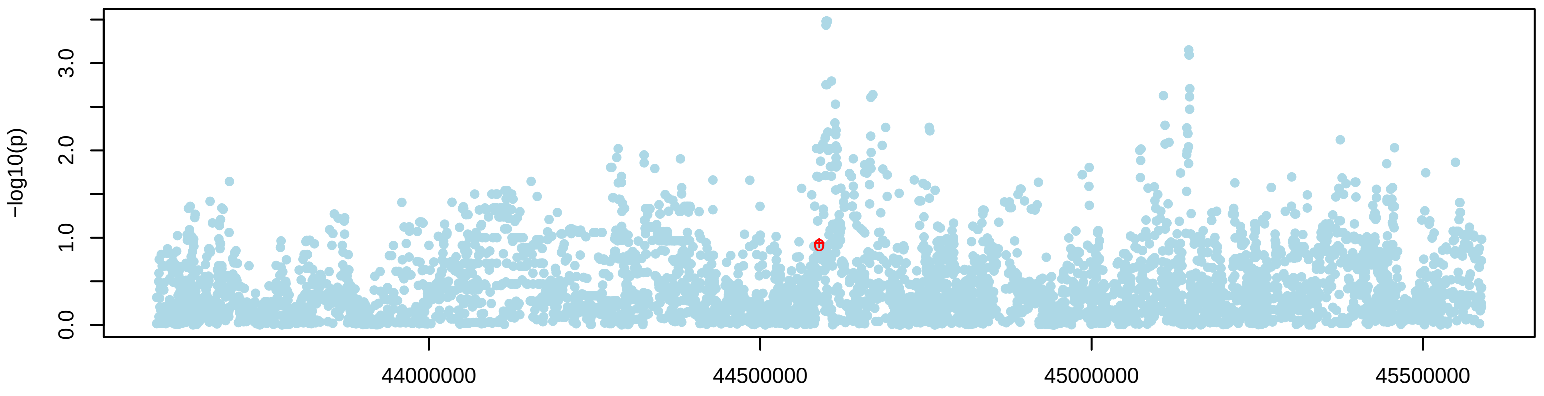

Position on chr 22

347. PDGFA (P04085) 7:47739554:G:A [Tarkin]

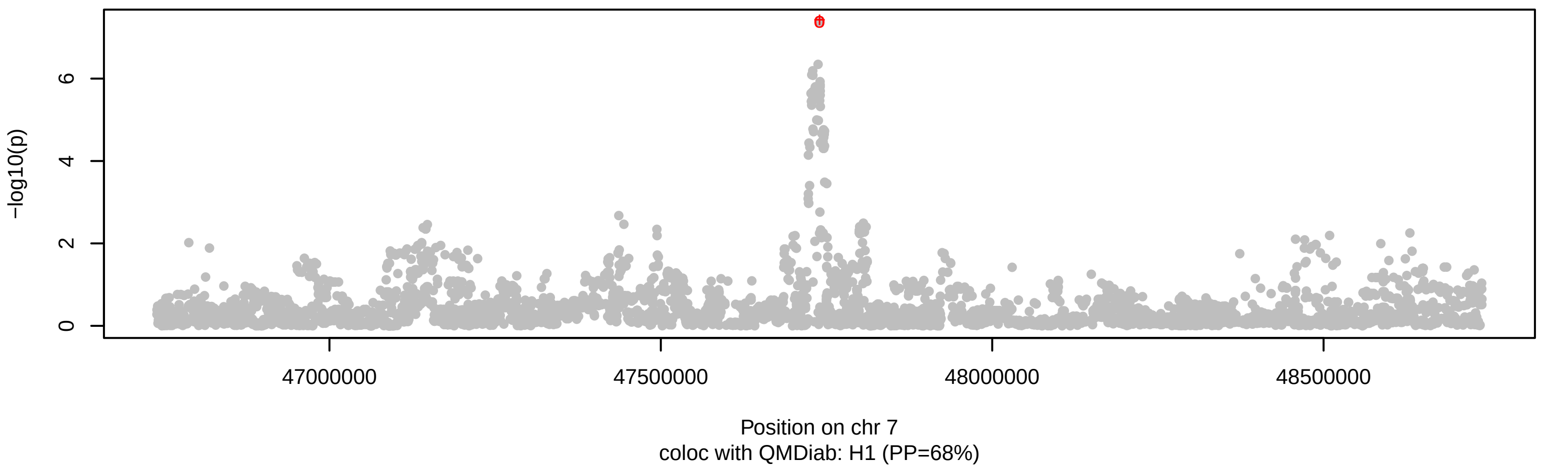

coloc with QMDiab: H1 (PP=68%)

347. PDGFA (P04085;P04085−2) 7:47739554:G:A [QMDiab]

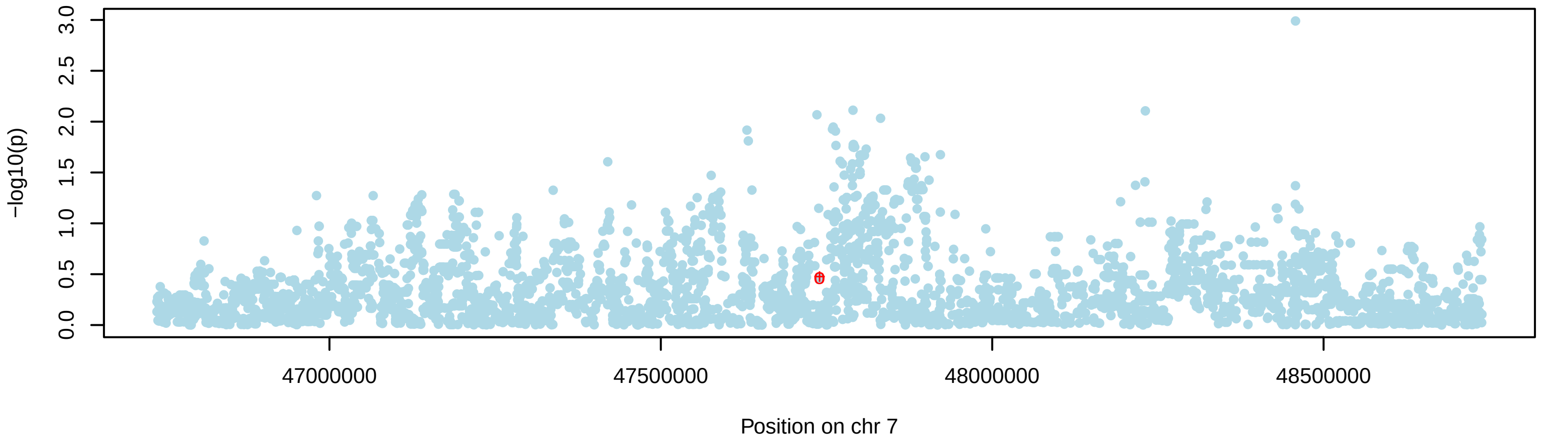

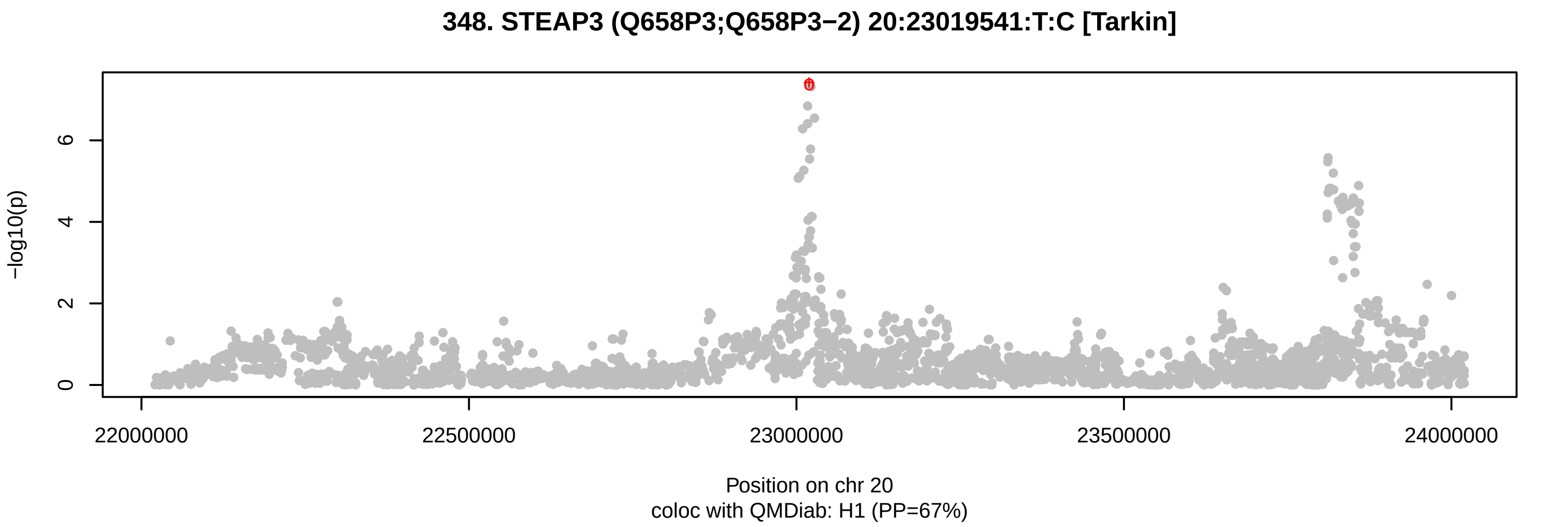
348. STEAP3 (Q658P3;Q658P3−2) 20:23019541:T:C [Tarkin]
−log10(p)
0
2
4
6
22000000
22500000
23000000
23500000
24000000
Position on chr 20
coloc with QMDiab: H1 (PP=67%)

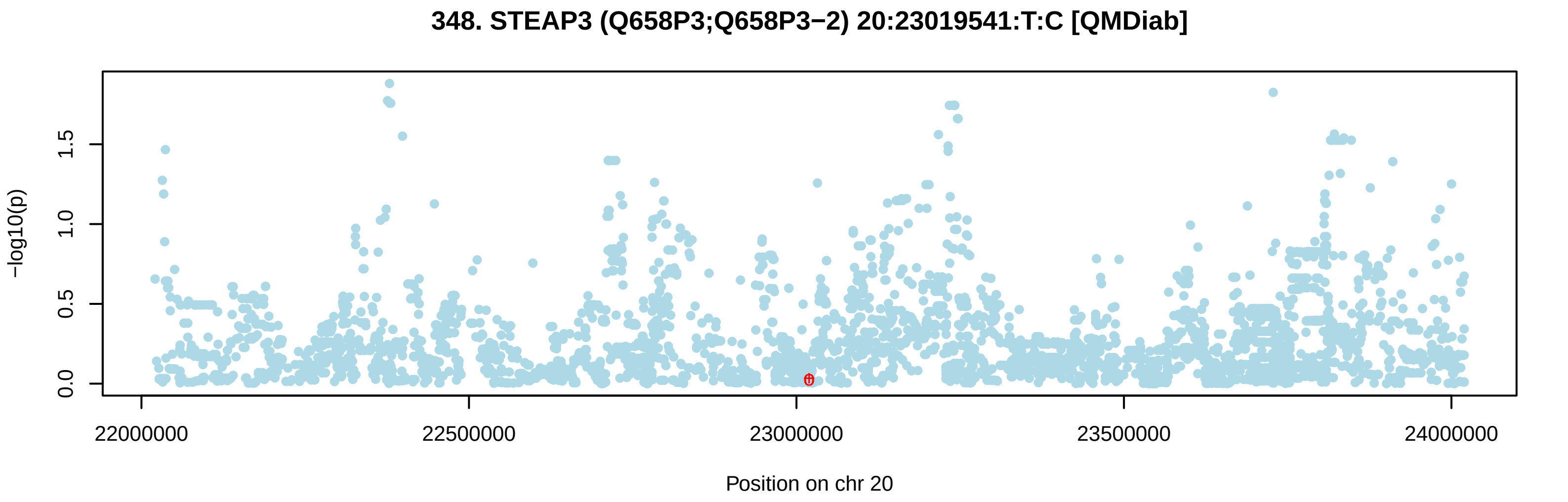
348. STEAP3 (Q658P3;Q658P3−2) 20:23019541:T:C [QMDiab]
−log10(p)
0.0
0.5
1.0
1.5
22000000
22500000
23000000
23500000
24000000
Position on chr 20

349. STK24 (B4DR80;Q9Y6E0−2) 6:169303282:T:A [Tarkin]

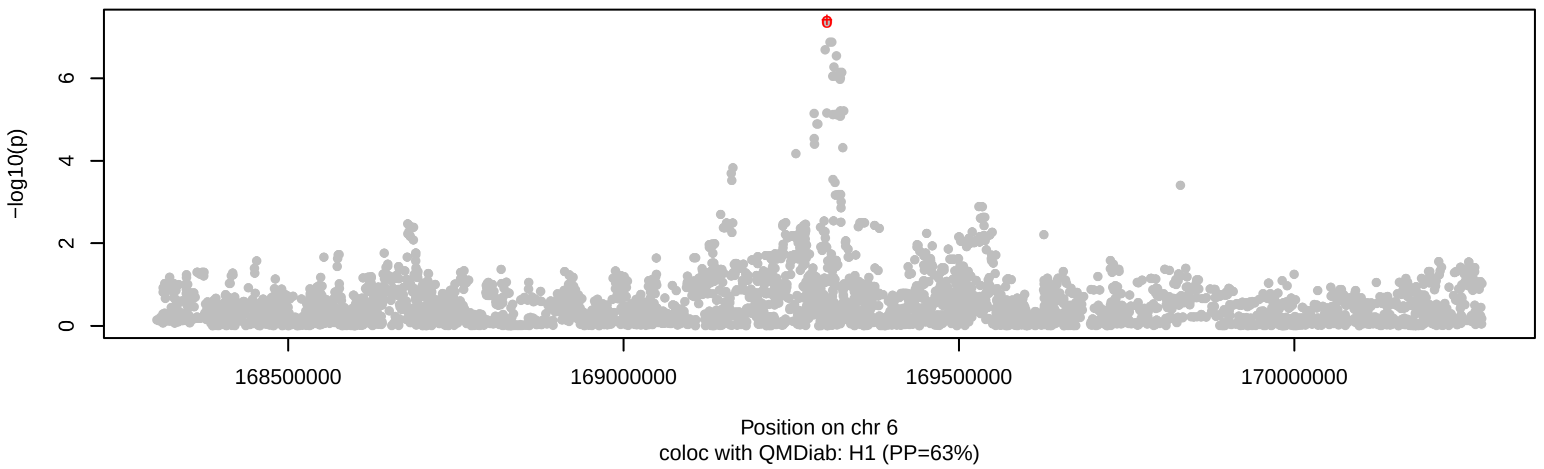

coloc with QMDiab: H1 (PP=63%)

349. STK24 (B4DR80;Q9Y6E0;Q9Y6E0−2) 6:169303282:T:A [QMDiab]

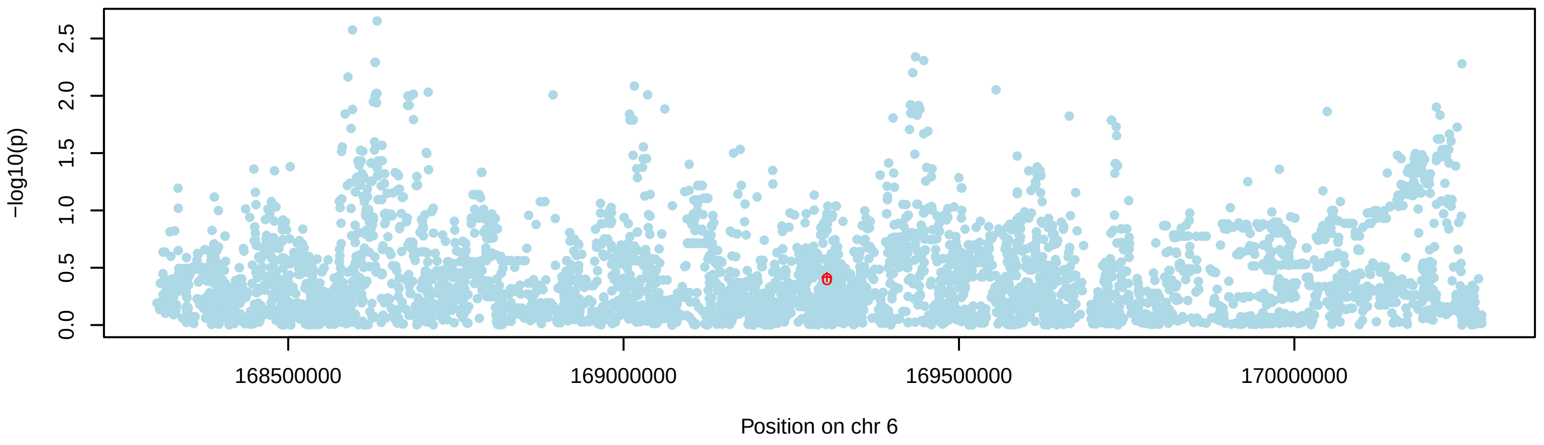

350. LCP2 (Q13094) 4:180920998:A:C [Tarkin]

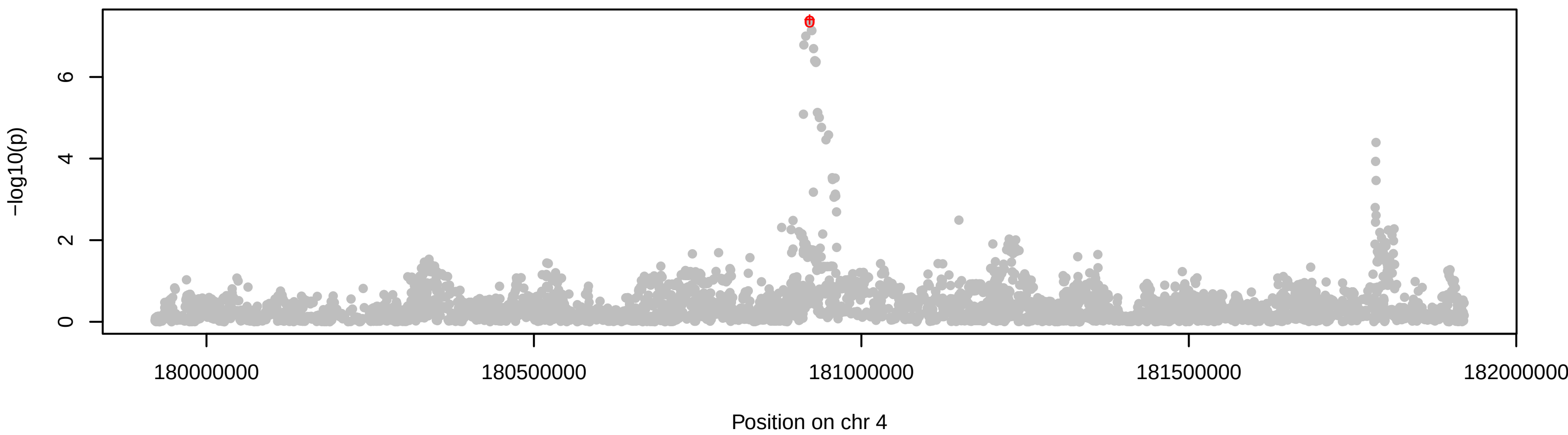

coloc with QMDiab: H1 (PP=66%)

350. LCP2 (Q13094) 4:180920998:A:C [QMDiab]

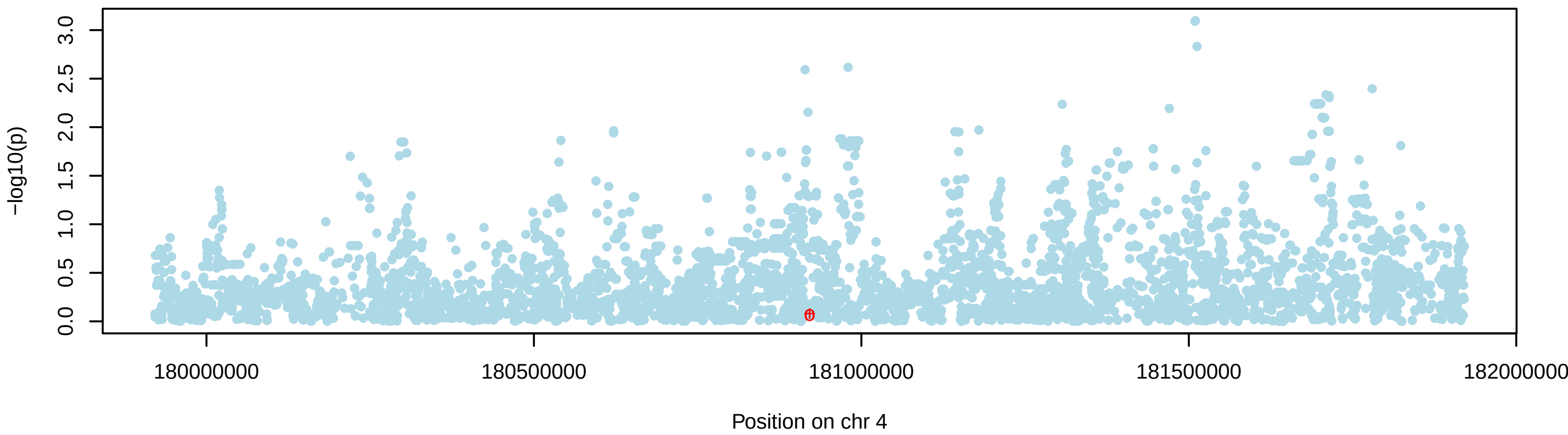

351. PLCB2 (Q00722;Q00722−2;Q00722−3) 2:79909261:G:A [Tarkin]

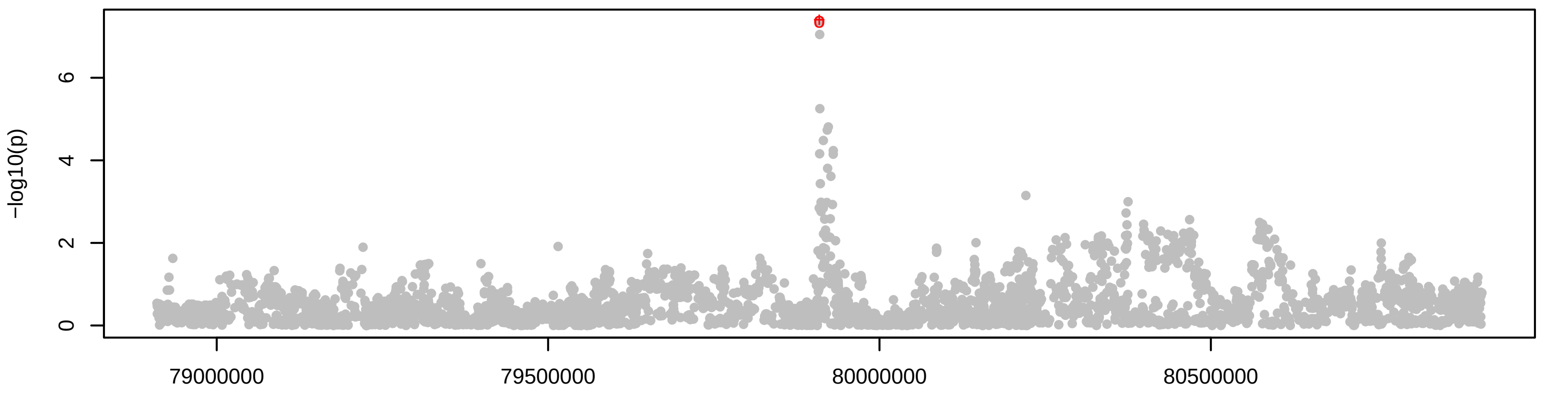

Position on chr 2
coloc with QMDiab: H1 (PP=61%)

351. PLCB2 (Q00722;Q00722−2) 2:79909261:G:A [QMDiab]

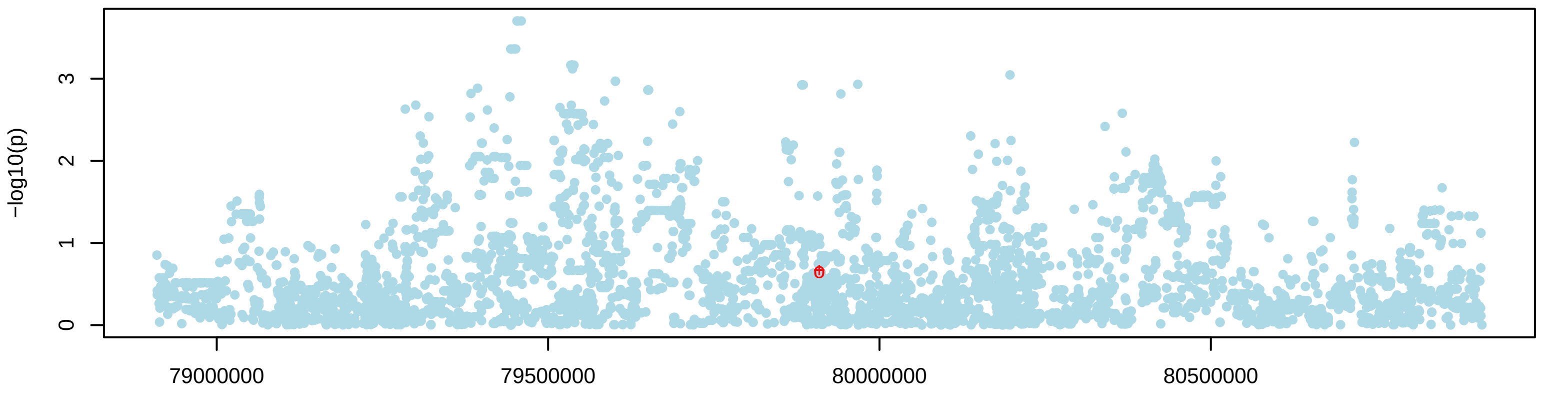

Position on chr 2

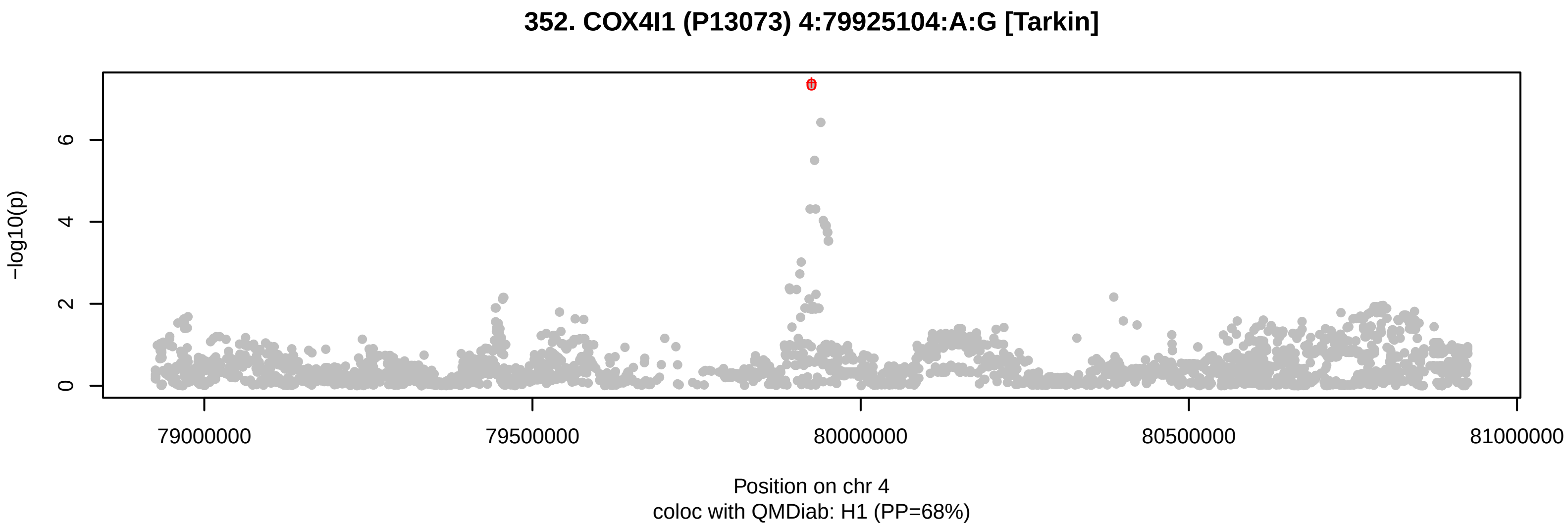
352. COX4I1 (P13073) 4:79925104:A:G [Tarkin]
−log10(p)
0
2
4
6
79000000
79500000
80000000
80500000
81000000
Position on chr 4
coloc with QMDiab: H1 (PP=68%)

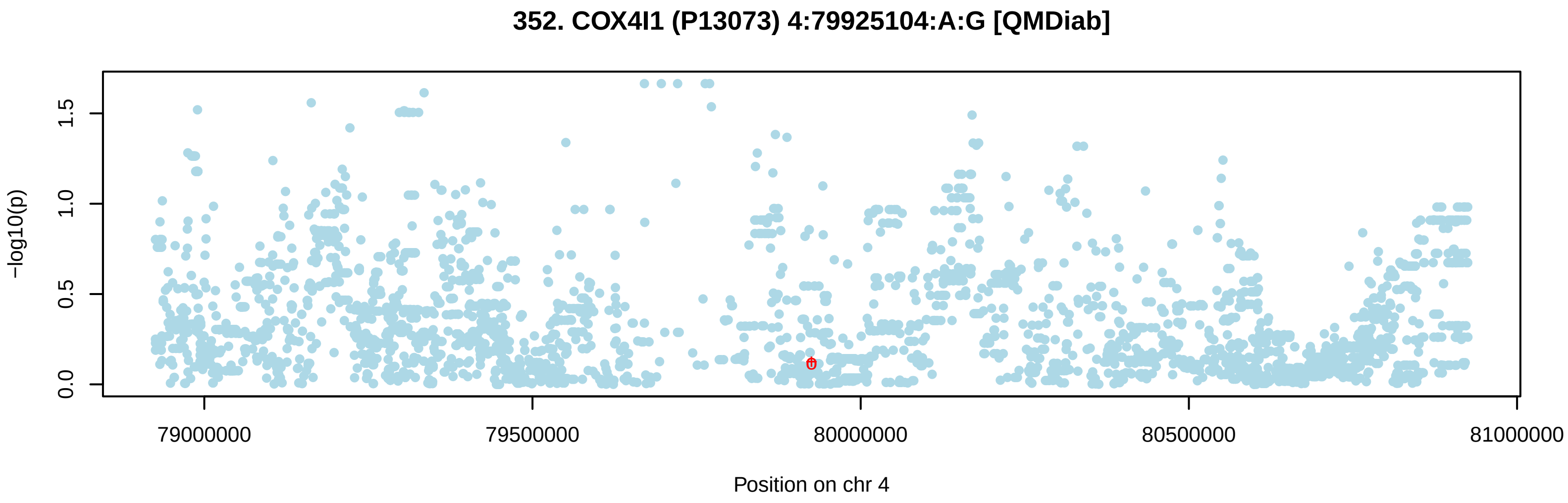
352. COX4I1 (P13073) 4:79925104:A:G [QMDiab]
−log10(p)
0.0
0.5
1.0
1.5
79000000
79500000
80000000
80500000
81000000
Position on chr 4

353. WFDC5 (Q8TCV5;Q8TCV5−2) 4:7432495:T:C [Tarkin]

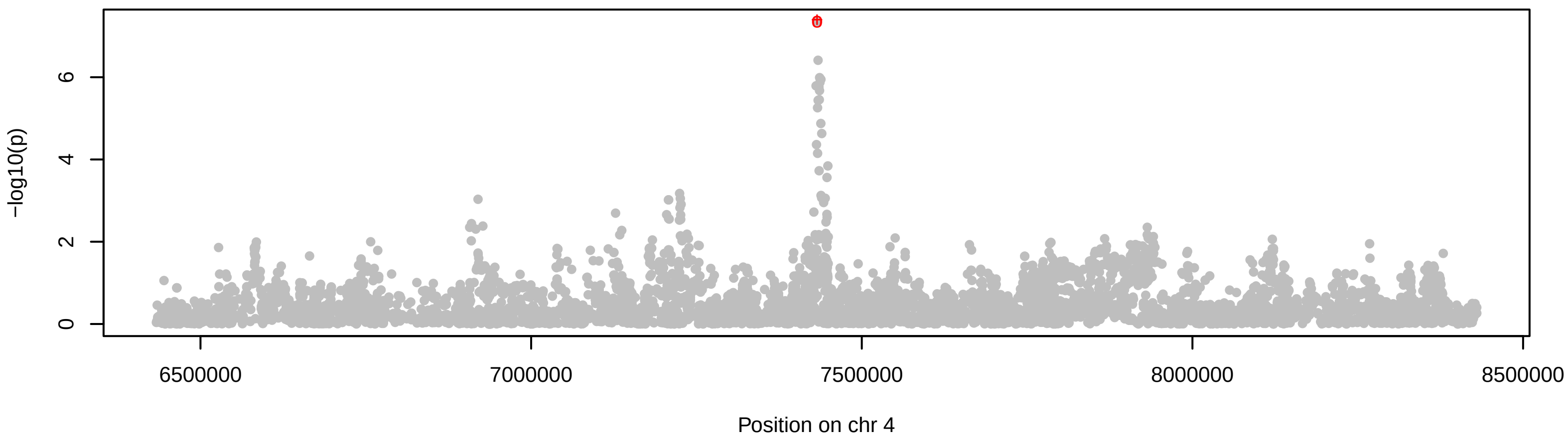

coloc with QMDiab: H1 (PP=47%)

353. WFDC5 (Q8TCV5;Q8TCV5−2) 4:7432495:T:C [QMDiab]

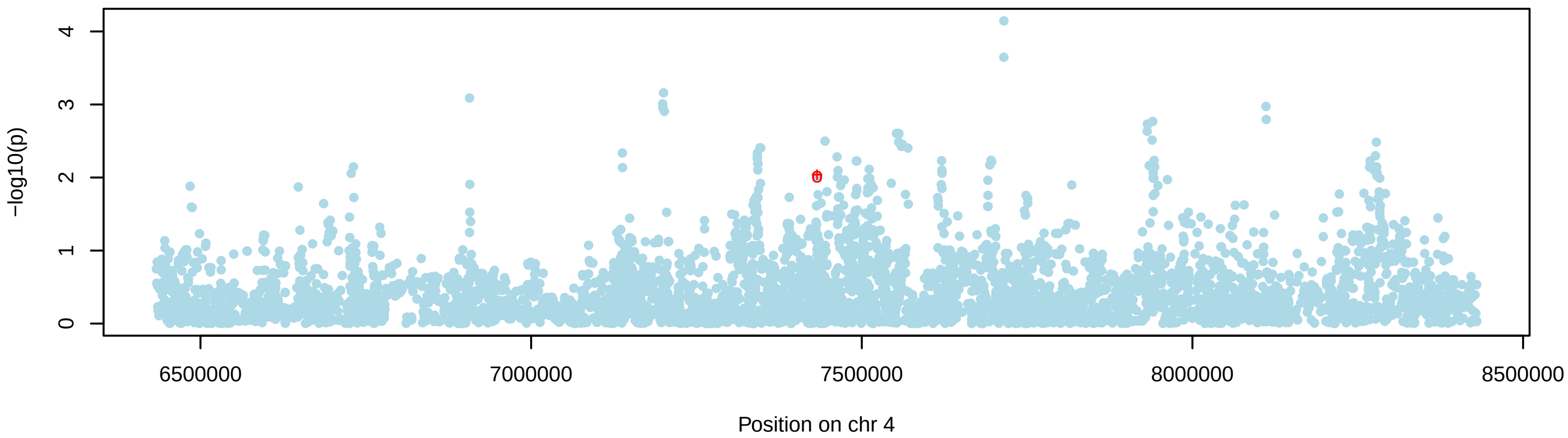

354. HNRNPAB (D6RD18) 1:151531723:G:A [Tarkin]

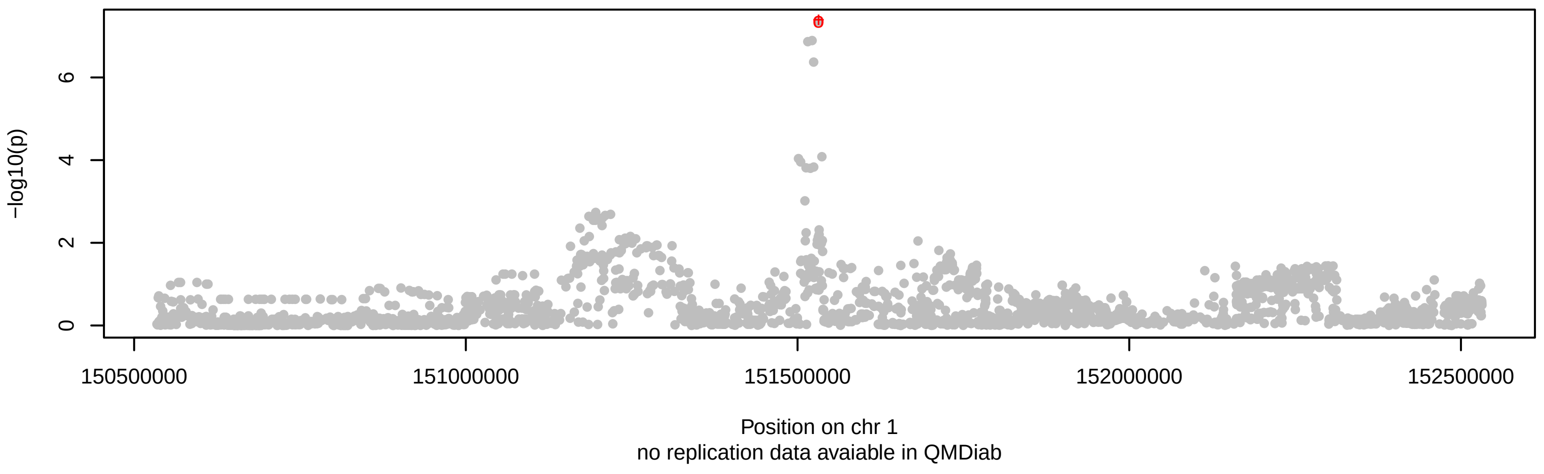

355. TST (Q16762) 19:48601350:T:C [Tarkin]

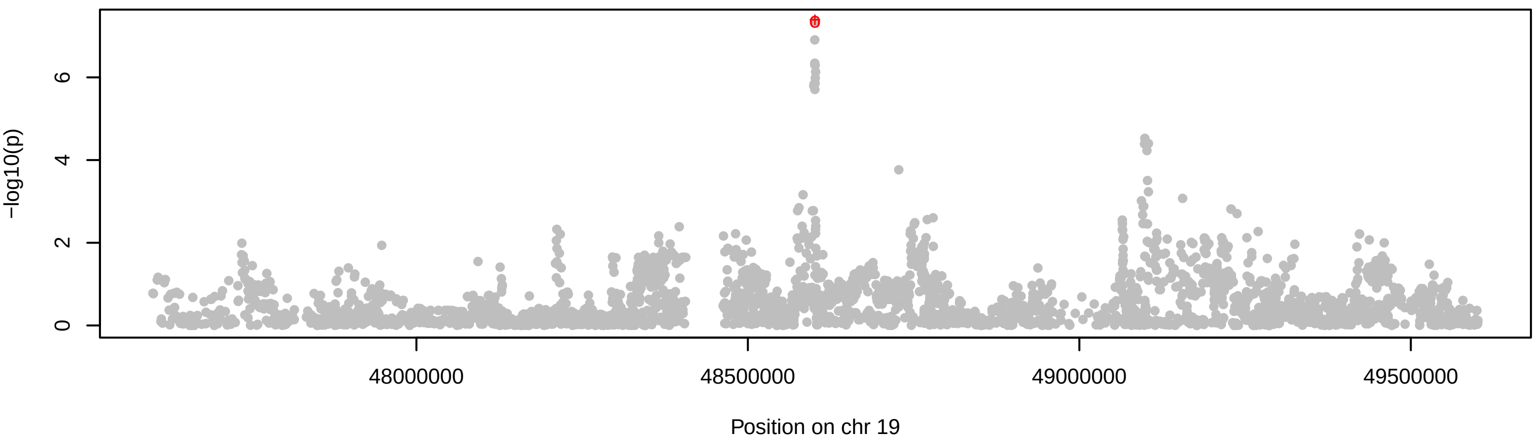

coloc with QMDiab: H1 (PP=63%)

355. TST (Q16762) 19:48601350:T:C [QMDiab]

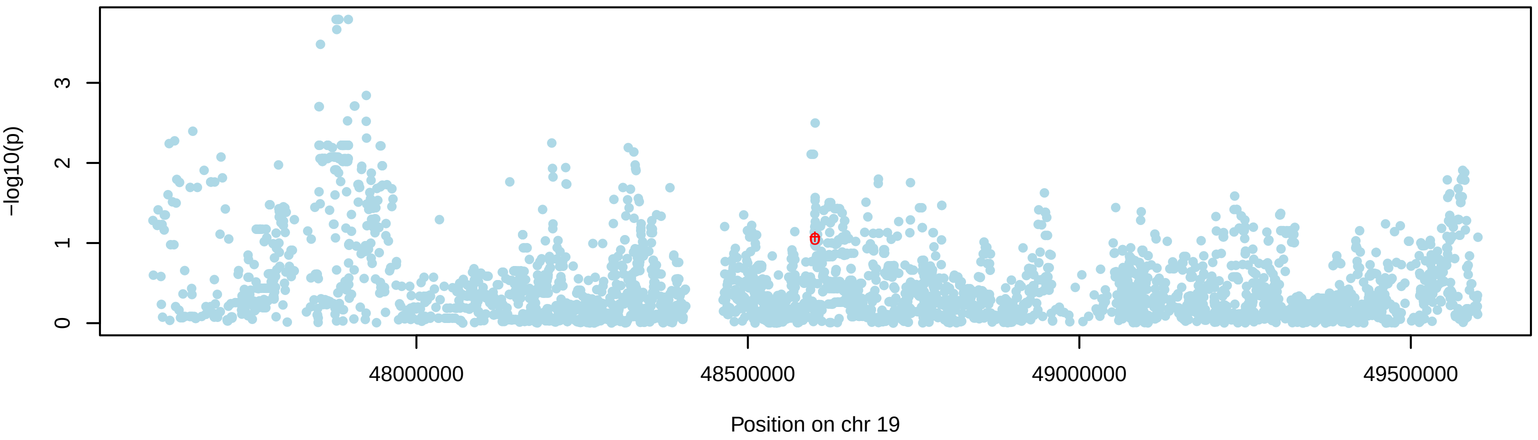

356. ESYT1 (Q9BSJ8;Q9BSJ8−2) 11:23183507:C:A [Tarkin]

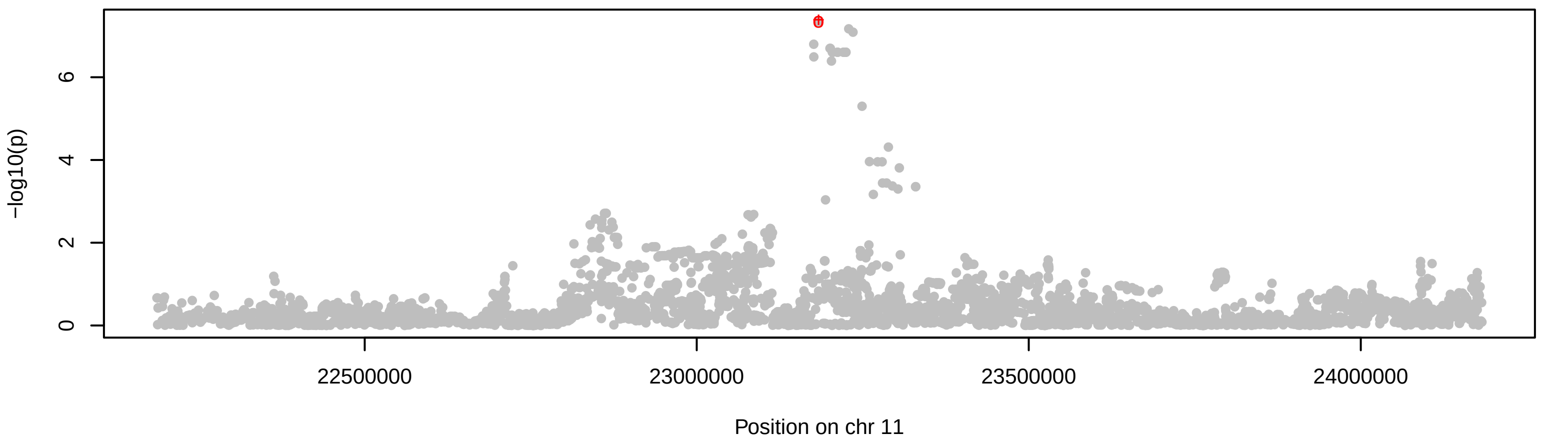

coloc with QMDiab: H1 (PP=64%)

356. ESYT1 (Q9BSJ8;Q9BSJ8−2) 11:23183507:C:A [QMDiab]

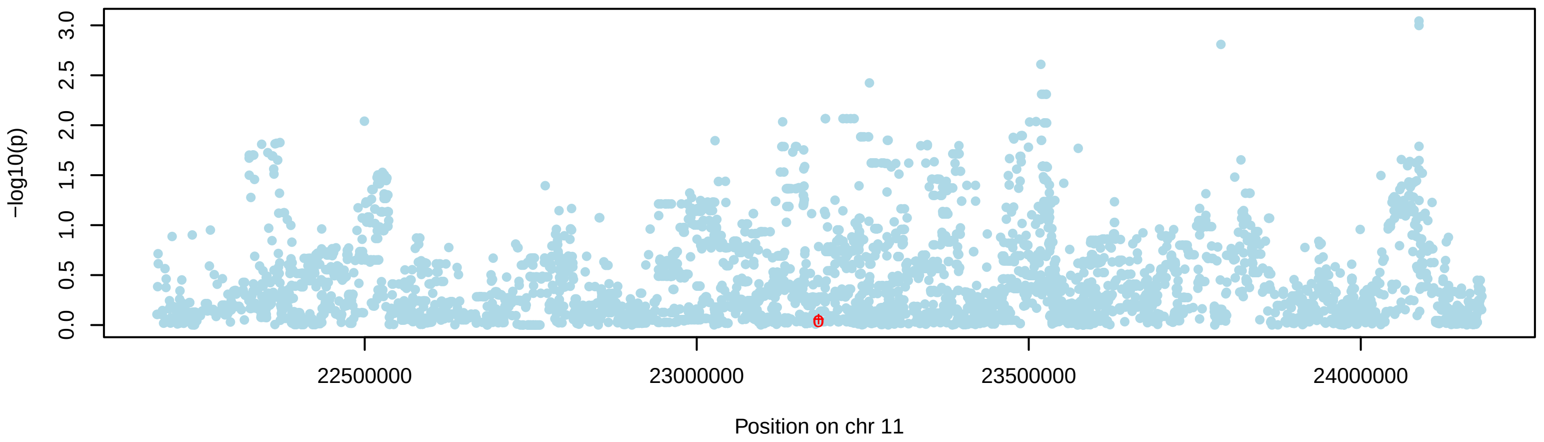

357. AP3D1 (O14617−5) 2:56061813:T:C [Tarkin]

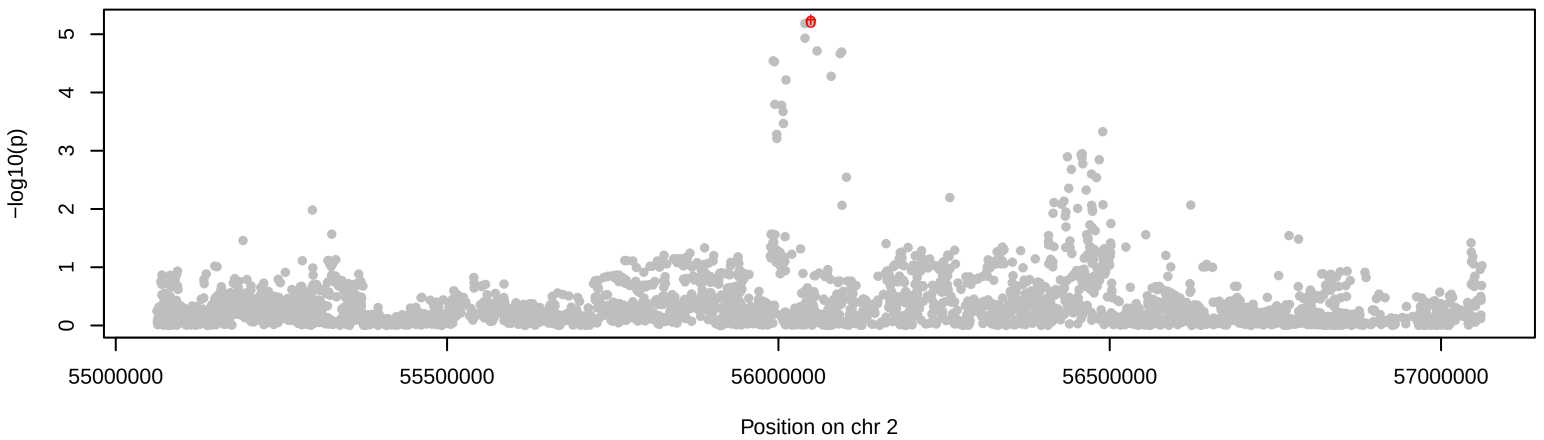

coloc with QMDiab: H1 (PP=41%)

357. AP3D1 (O14617−5) 2:56061813:T:C [QMDiab]

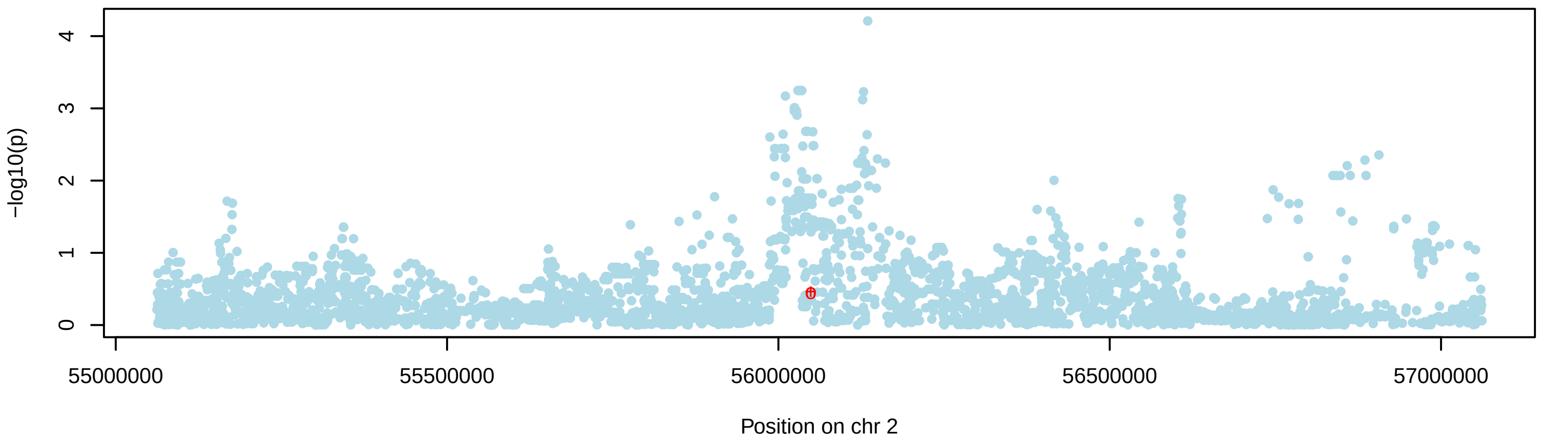

358. PROC (E7END6) 11:24439442:A:G [Tarkin]

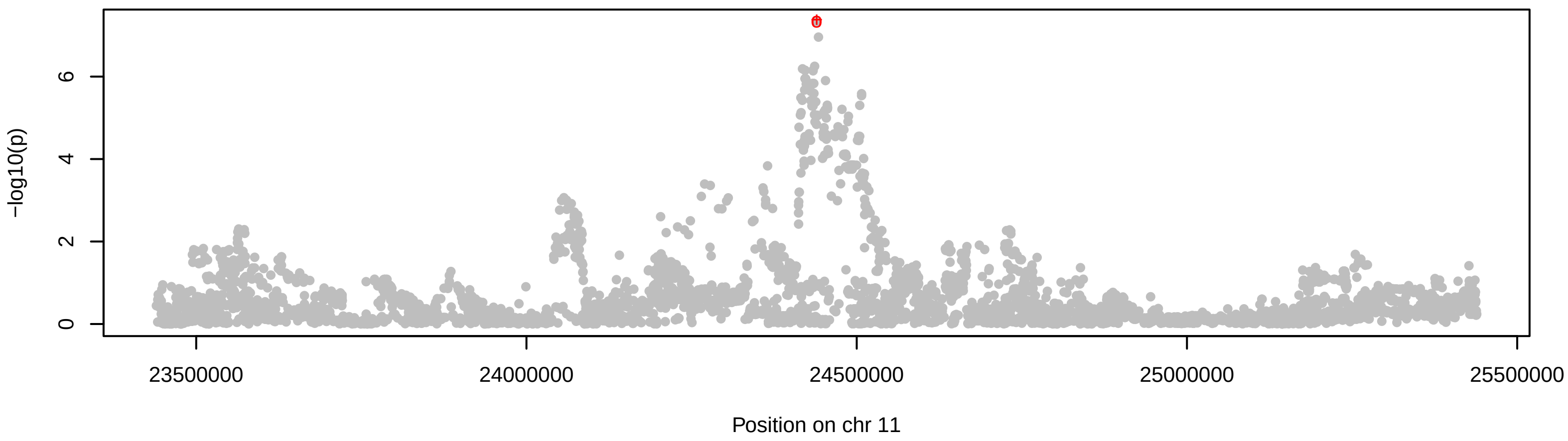

coloc with QMDiab: H1 (PP=65%)

358. PROC (E7END6) 11:24439442:A:G [QMDiab]

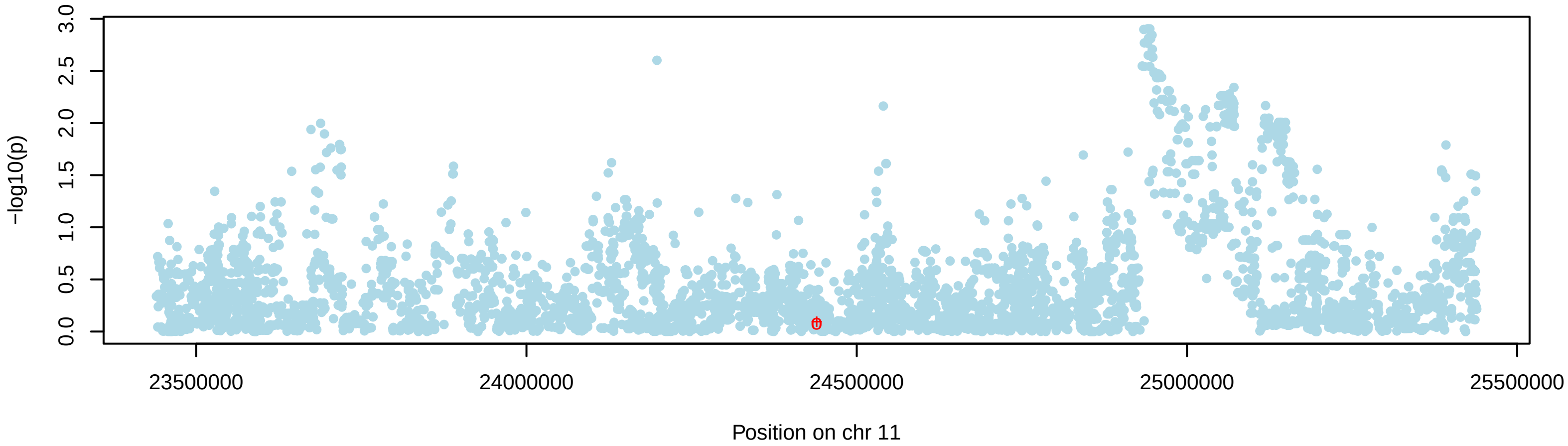

359. CTSF (A0A7I2V313) 3:155958216:T:C [Tarkin]

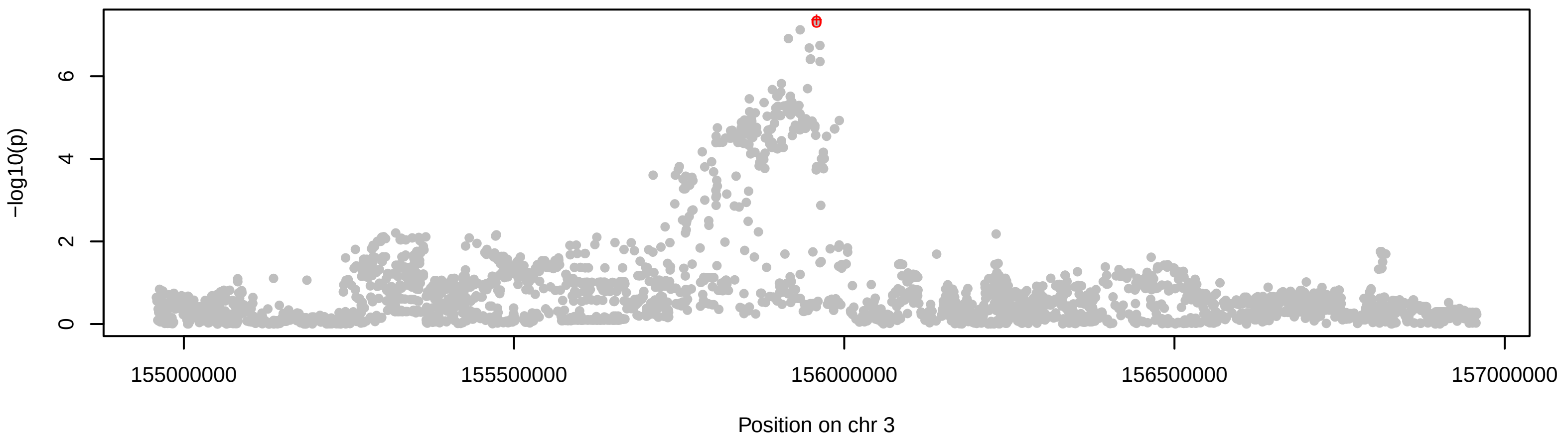

no replication data avaiable in QMDiab

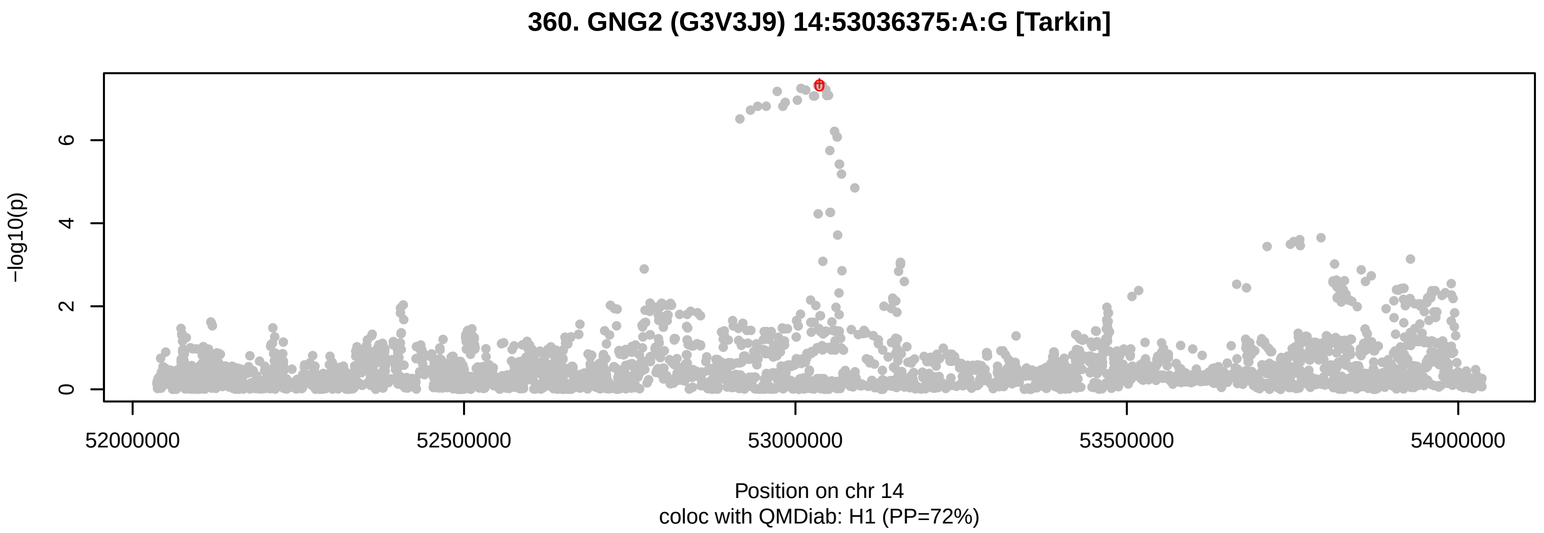
360. GNG2 (G3V3J9) 14:53036375:A:G [Tarkin]
−log10(p)
0
2
4
6
52000000
52500000
53000000
53500000
54000000
Position on chr 14
coloc with QMDiab: H1 (PP=72%)

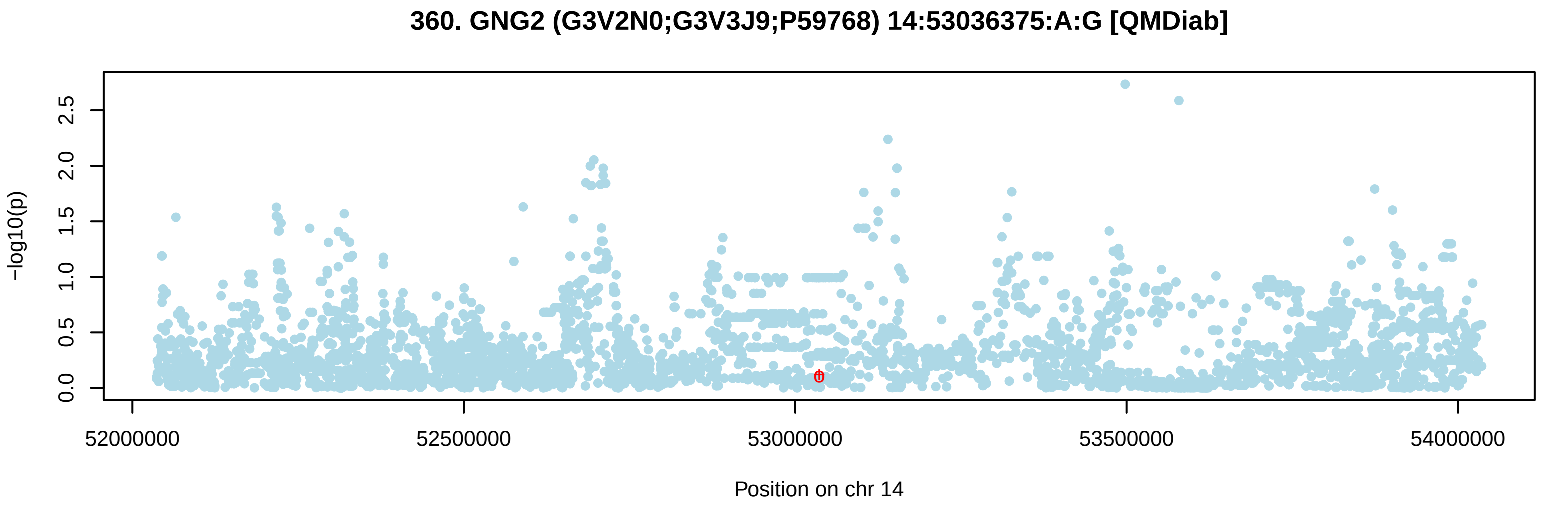
360. GNG2 (G3V2N0;G3V3J9;P59768) 14:53036375:A:G [QMDiab]
−log10(p)
0.0
0.5
1.0
1.5
2.0
2.5
52000000
52500000
53000000
53500000
54000000
Position on chr 14

361. LMNB1 (P20700) 20:60244125:A:G [Tarkin]

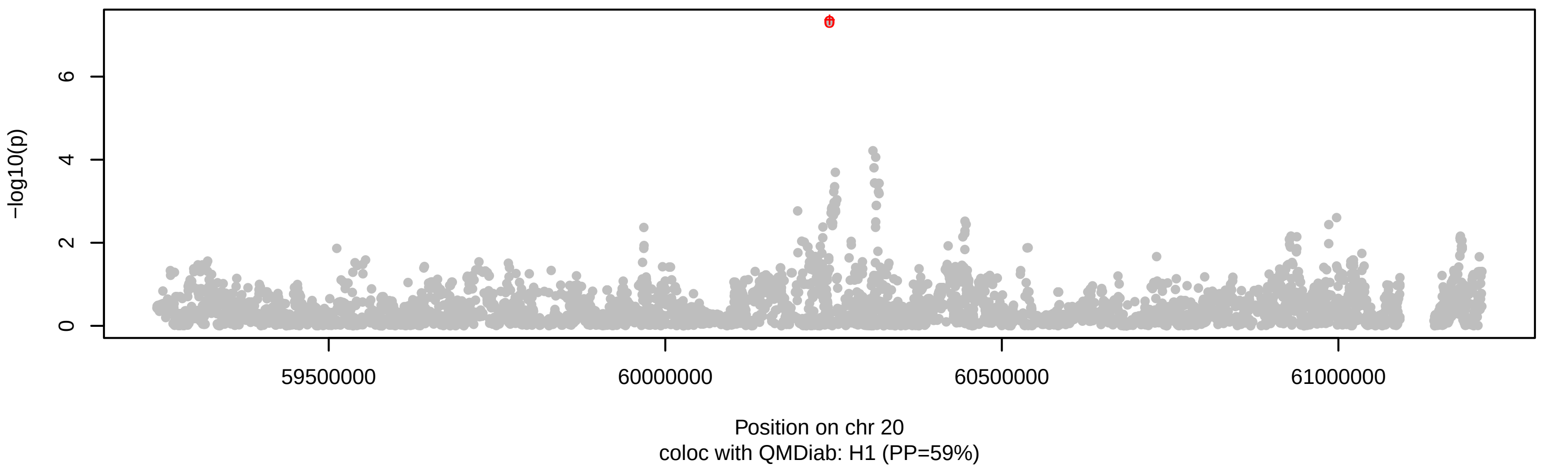

coloc with QMDiab: H1 (PP=59%)

361. LMNB1 (P20700) 20:60244125:A:G [QMDiab]

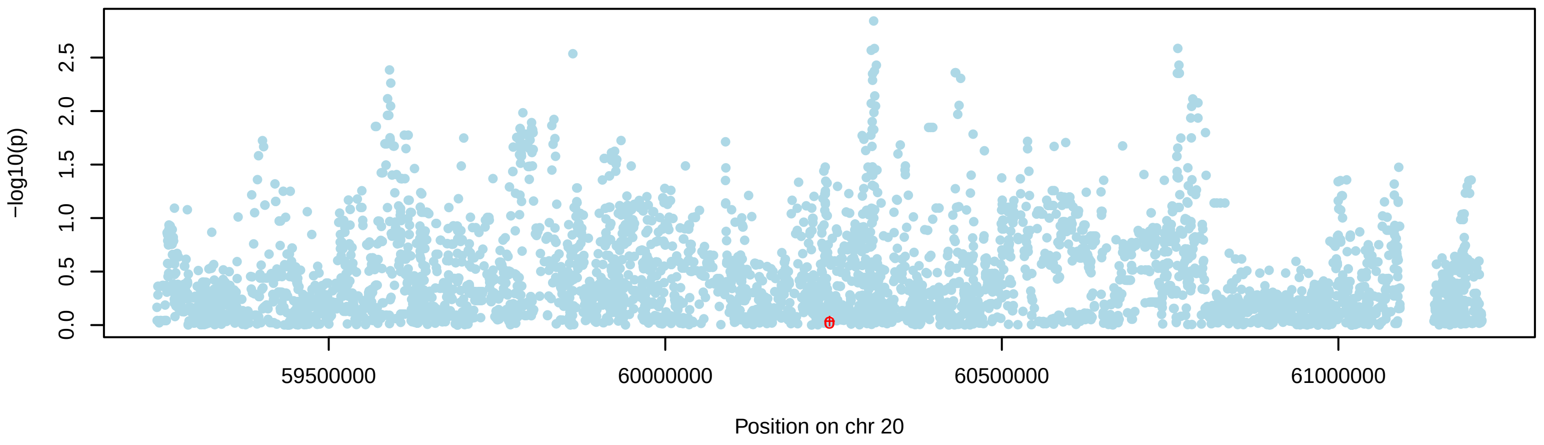

362. PLA1A (G5E9W0;Q53H76) 7:9004837:T:G [Tarkin]

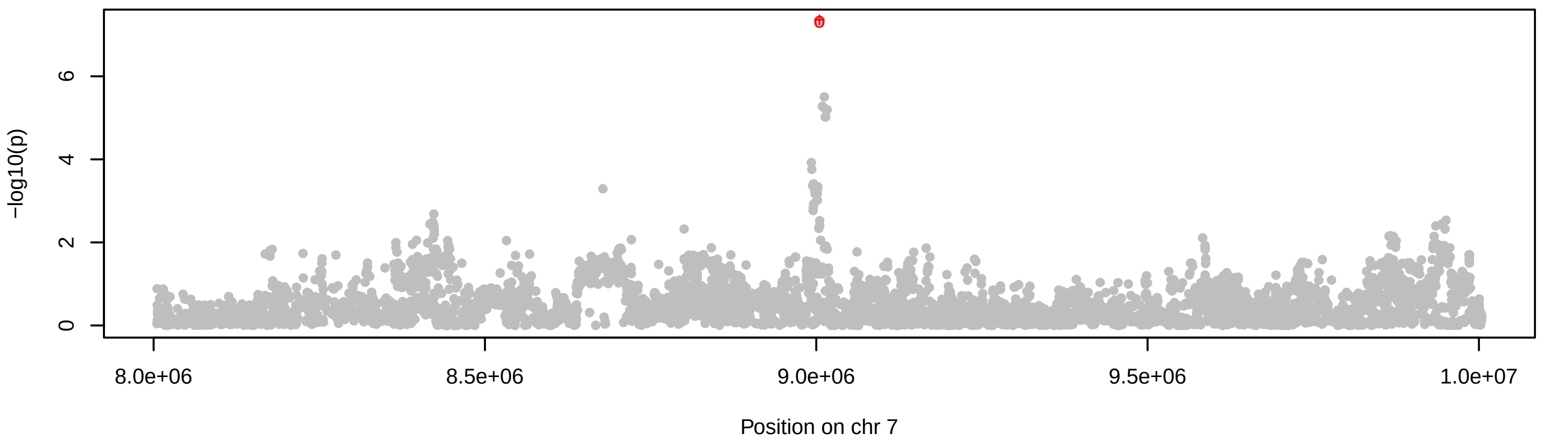

coloc with QMDiab: H1 (PP=51%)

362. PLA1A (G5E9W0;Q53H76) 7:9004837:T:G [QMDiab]

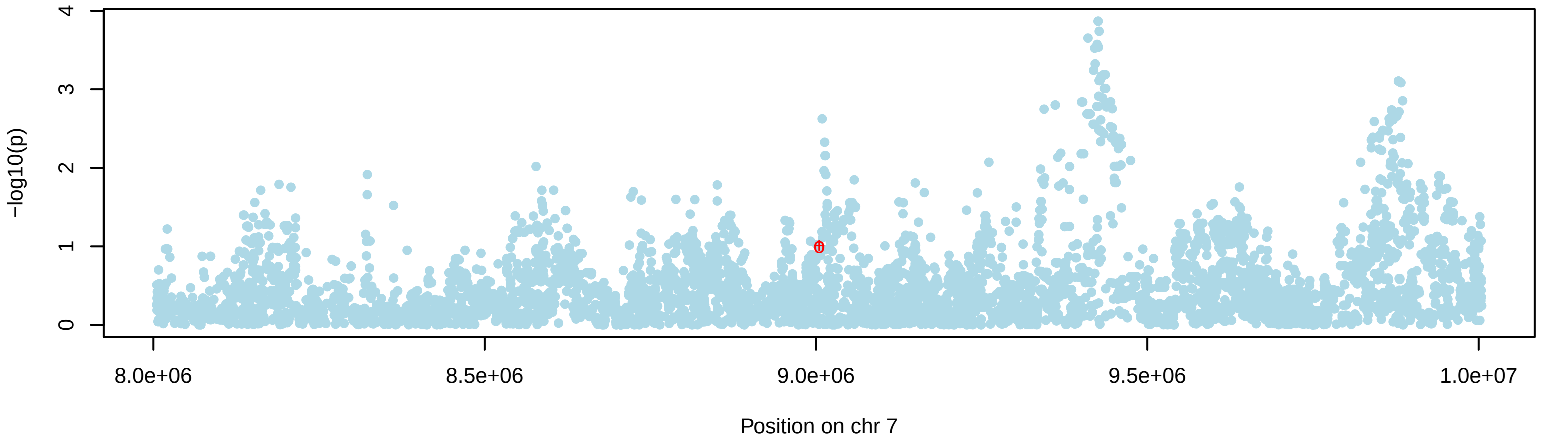

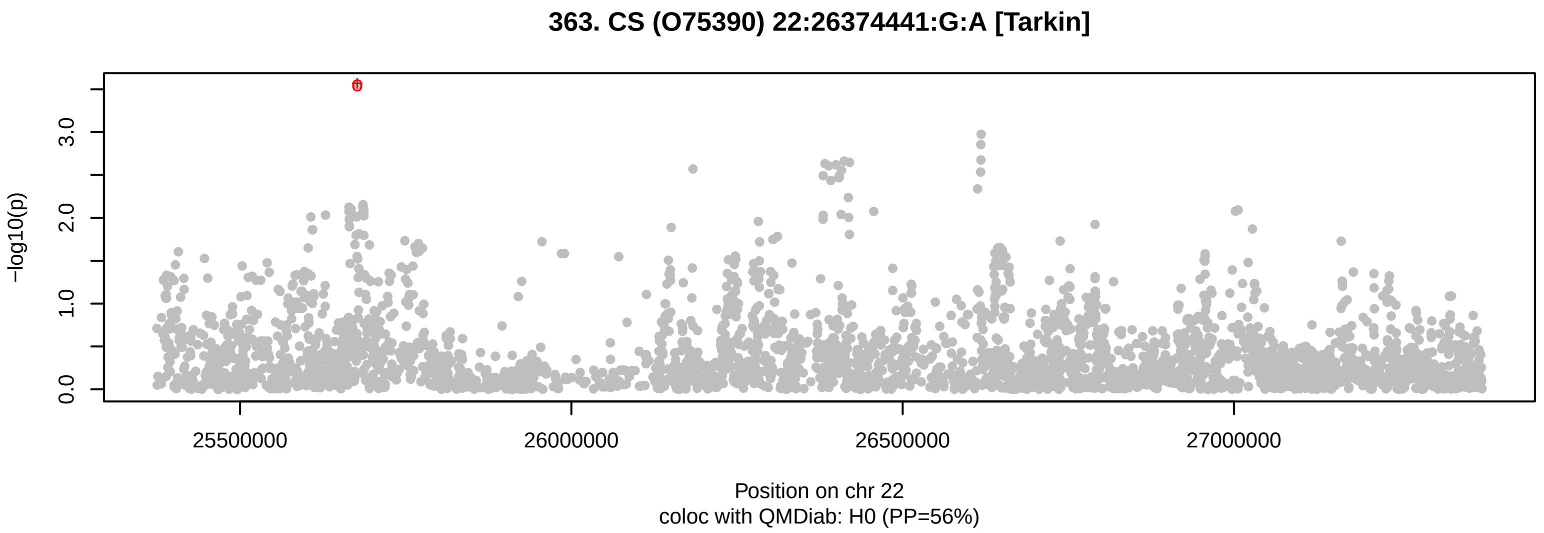

363. CS (O75390) 22:26374441:G:A [Tarkin]
−log10(p)
0.0
1.0
2.0
3.0
25500000
26000000
26500000
27000000
Position on chr 22
coloc with QMDiab: H0 (PP=56%)

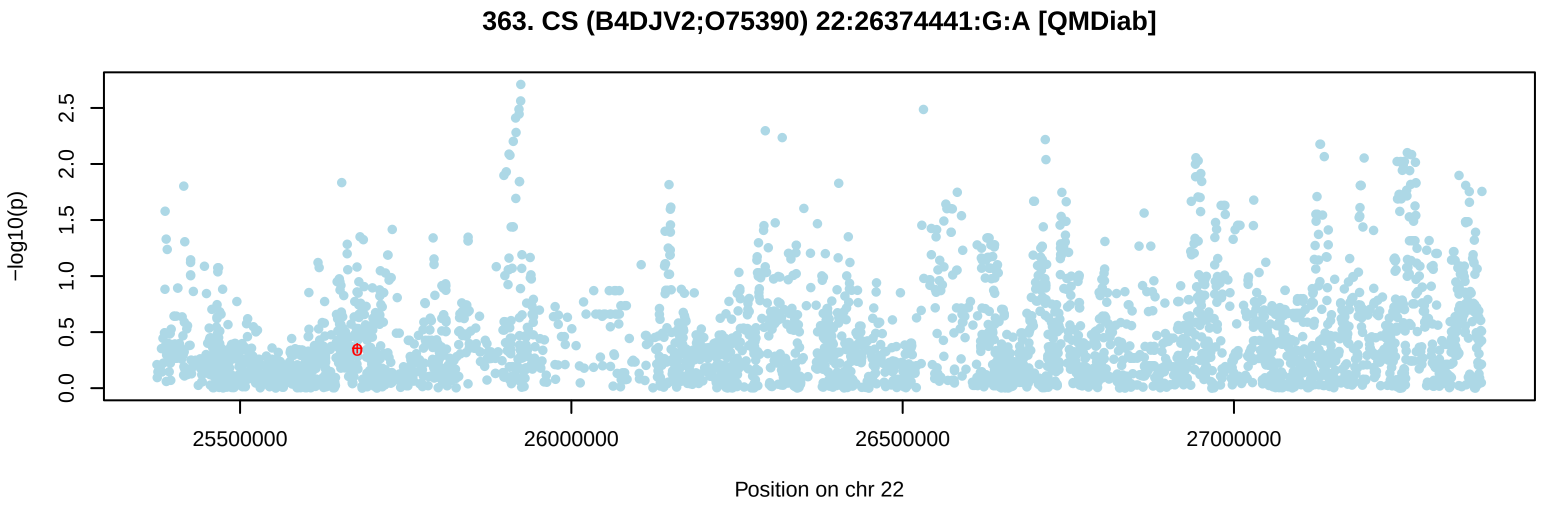

363. CS (B4DJV2;O75390) 22:26374441:G:A [QMDiab]
−log10(p)
0.0
0.5
1.0
1.5
2.0
2.5
25500000
26000000
26500000
27000000
Position on chr 22

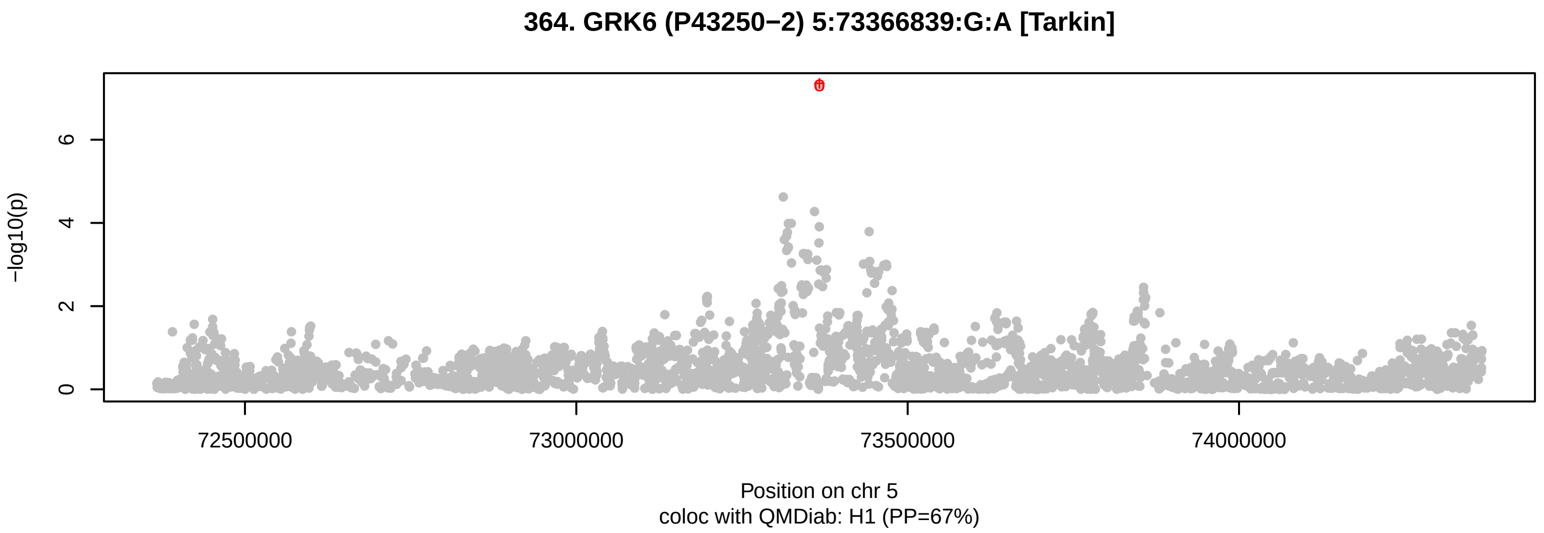
364. GRK6 (P43250−2) 5:73366839:G:A [Tarkin]
−log10(p)
0
2
4
6
72500000
73000000
73500000
74000000
Position on chr 5
coloc with QMDiab: H1 (PP=67%)

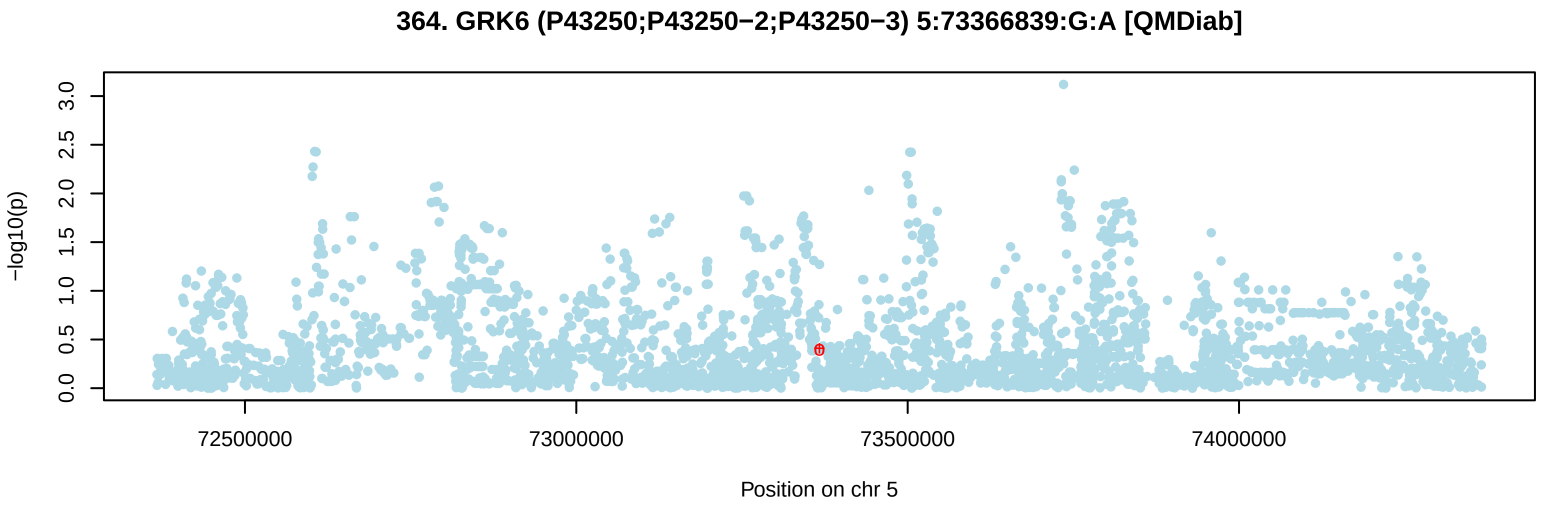
364. GRK6 (P43250;P43250−2;P43250−3) 5:73366839:G:A [QMDiab]
−log10(p)
0.0
0.5
1.0
1.5
2.0
2.5
3.0
72500000
73000000
73500000
74000000
Position on chr 5
