# Supplementary material for: A genome-wide association study of mass spectrometry proteomics using a nanoparticle enrichment platform
Source: Nat Genet. 2025 Nov 27;57(12):2987–96. doi: 10.1038/s41588-025-02413-w (PMC12695657; doi:10.1038/s41588-025-02413-w)
Supplement: Supplementary file 4 — Violin plots of protein and up to 4 peptide levels by genotype for 364 pQTLs identified in this study. [file 41588_2025_2413_MOESM4_ESM.pdf]

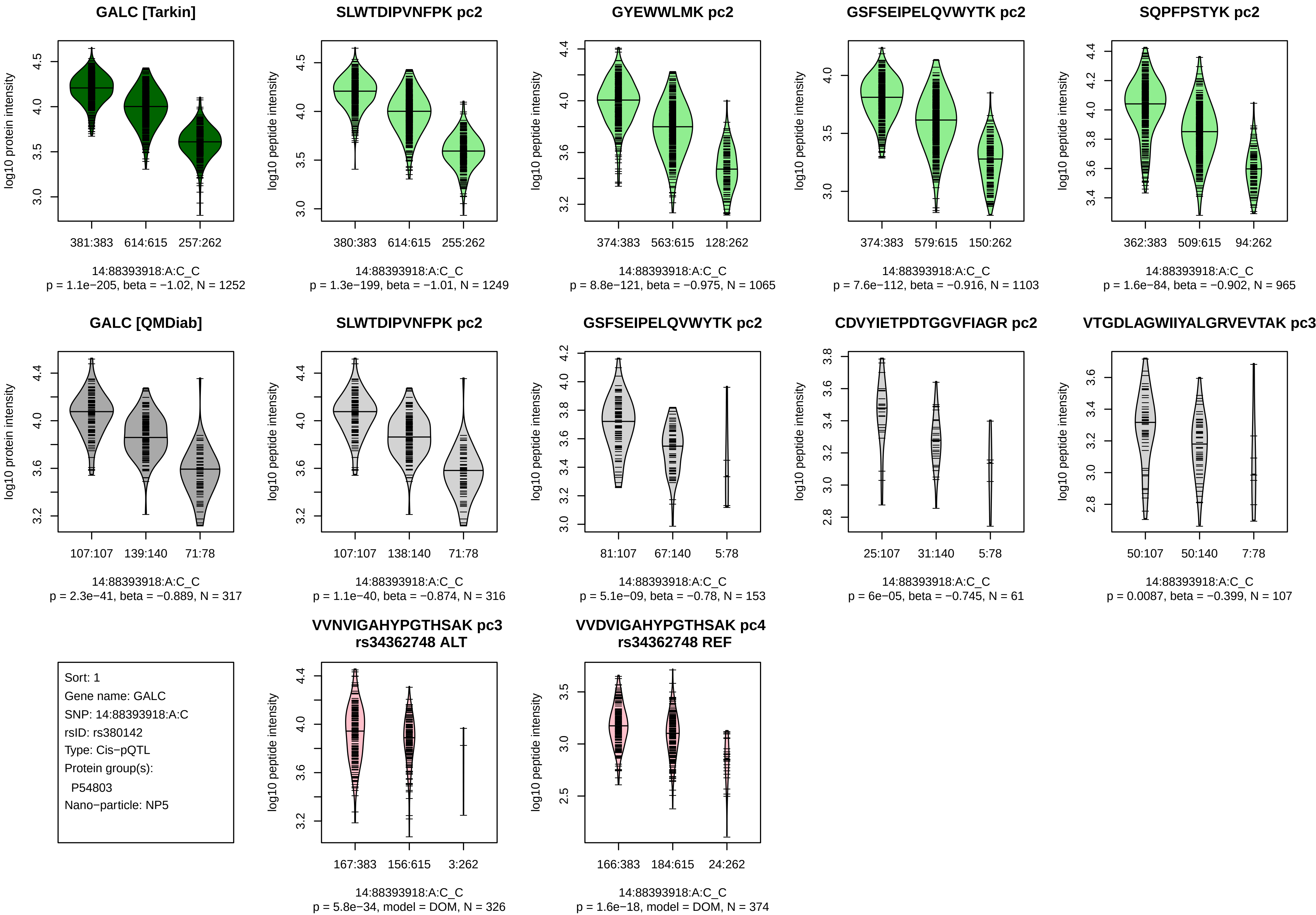
GALC [Tarkin]
381:383
614:615
257:262
14:88393918:A:C_C
p = 1.1e−205, beta = −1.02, N = 1252
SLWTDIPVNFPK pc2
380:383
614:615
255:262
14:88393918:A:C_C
p = 1.3e−199, beta = −1.01, N = 1249
GYEWWLMK pc2
374:383
563:615
128:262
14:88393918:A:C_C
p = 8.8e−121, beta = −0.975, N = 1065
GSFSEIPELQVWYTK pc2
374:383
579:615
150:262
14:88393918:A:C_C
p = 7.6e−112, beta = −0.916, N = 1103
SQPFPSTYK pc2
362:383
509:615
94:262
14:88393918:A:C_C
p = 1.6e−84, beta = −0.902, N = 965
GALC [QMDiab]
107:107
139:140
71:78
14:88393918:A:C_C
p = 2.3e−41, beta = −0.889, N = 317
SLWTDIPVNFPK pc2
107:107
138:140
71:78
14:88393918:A:C_C
p = 1.1e−40, beta = −0.874, N = 316
GSFSEIPELQVWYTK pc2
81:107
67:140
5:78
14:88393918:A:C_C
p = 5.1e−09, beta = −0.78, N = 153
CDVYIETPDTGGVFIAGR pc2
25:107
31:140
5:78
14:88393918:A:C_C
p = 6e−05, beta = −0.745, N = 61
VTGDLAGWIIYALGRVEVTAK pc3
50:107
50:140
7:78
14:88393918:A:C_C
p = 0.0087, beta = −0.399, N = 107
log10 protein intensity
log10 peptide intensity
Sort: 1
Gene name: GALC
SNP: 14:88393918:A:C
rsID: rs380142
Type: Cis−pQTL
Protein group(s):
P54803
Nano−particle: NP5
VVNVIGAHYPGTHSAK pc3
rs34362748 ALT
167:383
156:615
3:262
14:88393918:A:C_C
p = 5.8e−34, model = DOM, N = 326
VVDVIGAHYPGTHSAK pc4
rs34362748 REF
166:383
184:615
24:262
14:88393918:A:C_C
p = 1.6e−18, model = DOM, N = 374

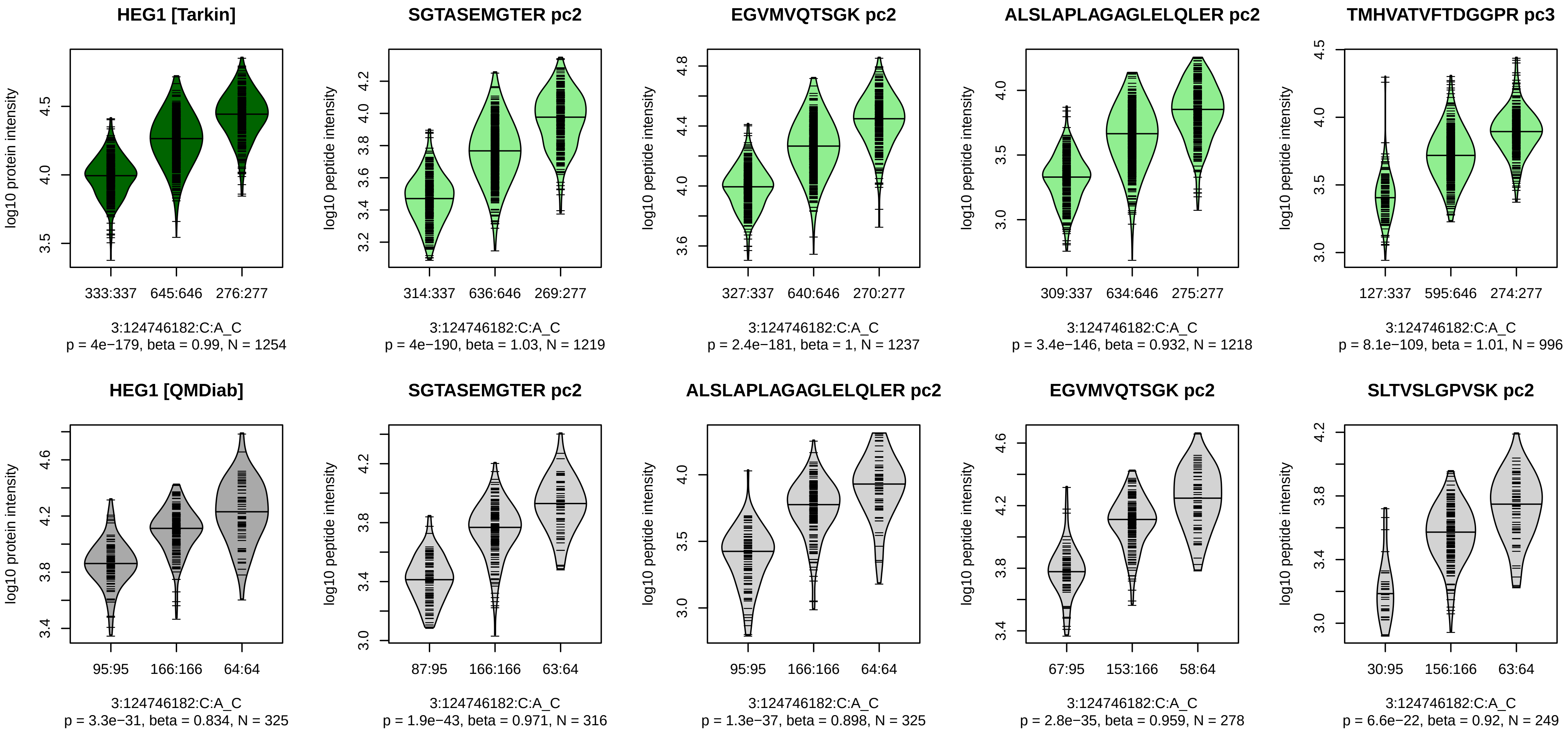

Sort: 2
Gene name: HEG1
SNP: 3:124746182:C:A
rsID: rs2333041
Type: Cis−pQTL
Protein group(s):
Q9ULI3
Nano−particle: NP2

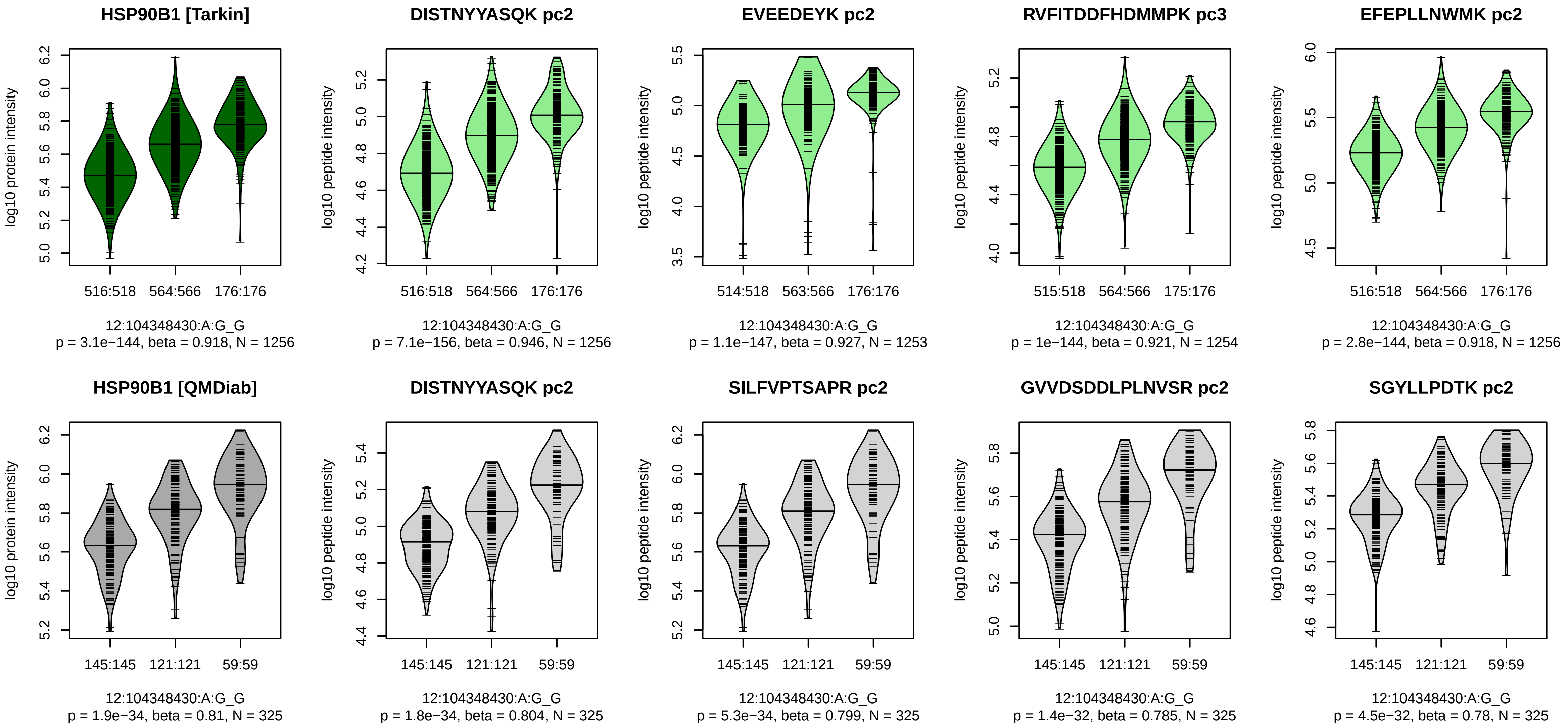

Sort: 3
Gene name: HSP90B1
SNP: 12:104348430:A:G
rsID: rs2576975
Type: Cis−pQTL
Protein group(s):
P14625
Nano−particle: NP2

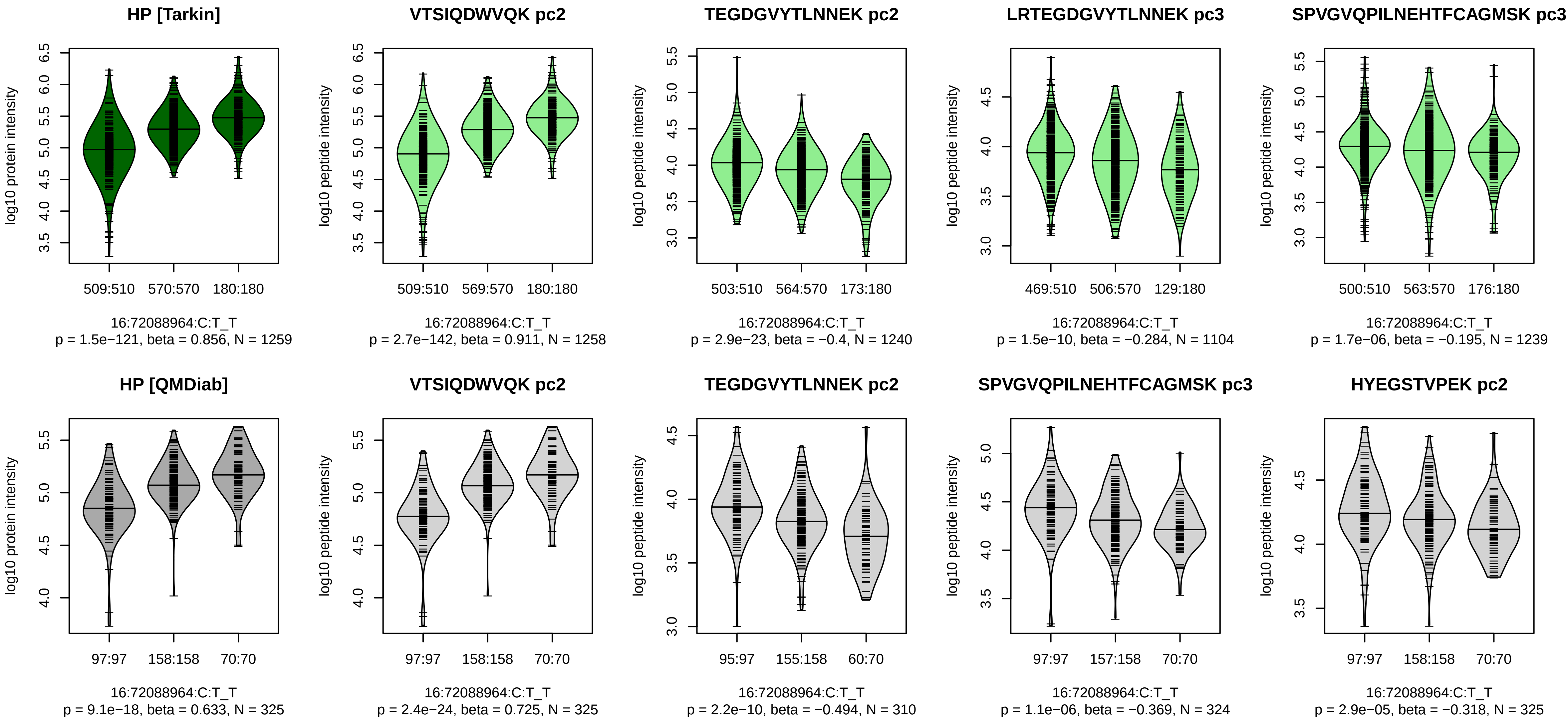

Sort: 4
Gene name: HP
SNP: 16:72088964:C:T
rsID: rs8062041
Type: Cis−pQTL
Protein group(s):
P00738
Nano−particle: NP4

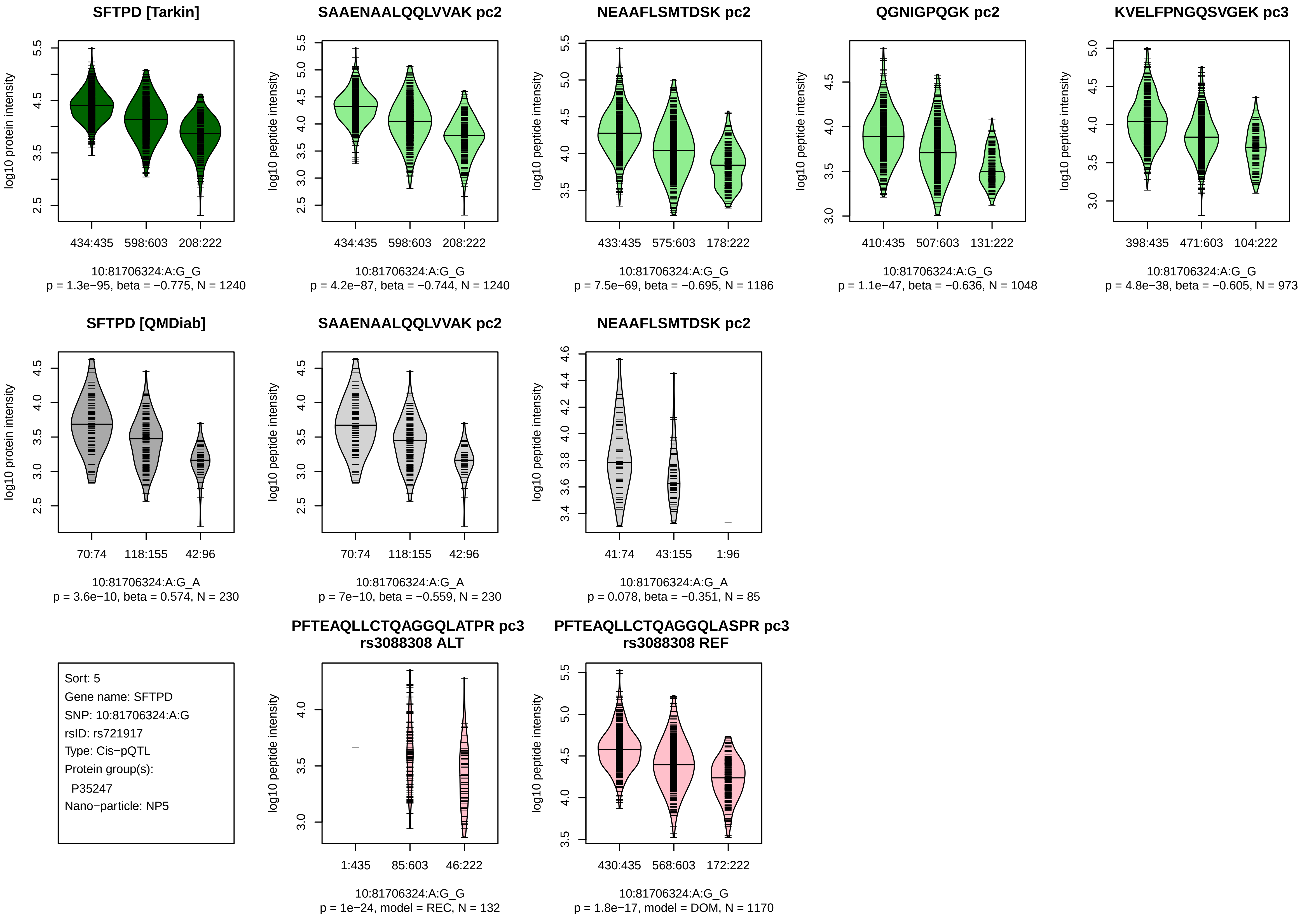
SFTPD [Tarkin]
log10 protein intensity
434:435
598:603
208:222
10:81706324:A:G_G
p = 1.3e−95, beta = −0.775, N = 1240
SAAENAALQQLVVAK pc2
log10 peptide intensity
434:435
598:603
208:222
10:81706324:A:G_G
p = 4.2e−87, beta = −0.744, N = 1240
NEAAFLSMTDSK pc2
log10 peptide intensity
433:435
575:603
178:222
10:81706324:A:G_G
p = 7.5e−69, beta = −0.695, N = 1186
QGNIGPQGK pc2
log10 peptide intensity
410:435
507:603
131:222
10:81706324:A:G_G
p = 1.1e−47, beta = −0.636, N = 1048
KVELFPNGQSVGEK pc3
log10 peptide intensity
398:435
471:603
104:222
10:81706324:A:G_G
p = 4.8e−38, beta = −0.605, N = 973
SFTPD [QMDiab]
log10 protein intensity
70:74
118:155
42:96
10:81706324:A:G_A
p = 3.6e−10, beta = 0.574, N = 230
SAAENAALQQLVVAK pc2
log10 peptide intensity
70:74
118:155
42:96
10:81706324:A:G_A
p = 7e−10, beta = −0.559, N = 230
NEAAFLSMTDSK pc2
log10 peptide intensity
41:74
43:155
1:96
10:81706324:A:G_A
p = 0.078, beta = −0.351, N = 85
Sort: 5
Gene name: SFTPD
SNP: 10:81706324:A:G
rsID: rs721917
Type: Cis−pQTL
Protein group(s):
P35247
Nano−particle: NP5
PFTEAQLLCTQAGGQLATPR pc3
rs3088308 ALT
log10 peptide intensity
1:435
85:603
46:222
10:81706324:A:G_G
p = 1e−24, model = REC, N = 132
PFTEAQLLCTQAGGQLASPR pc3
rs3088308 REF
log10 peptide intensity
430:435
568:603
172:222
10:81706324:A:G_G
p = 1.8e−17, model = DOM, N = 1170

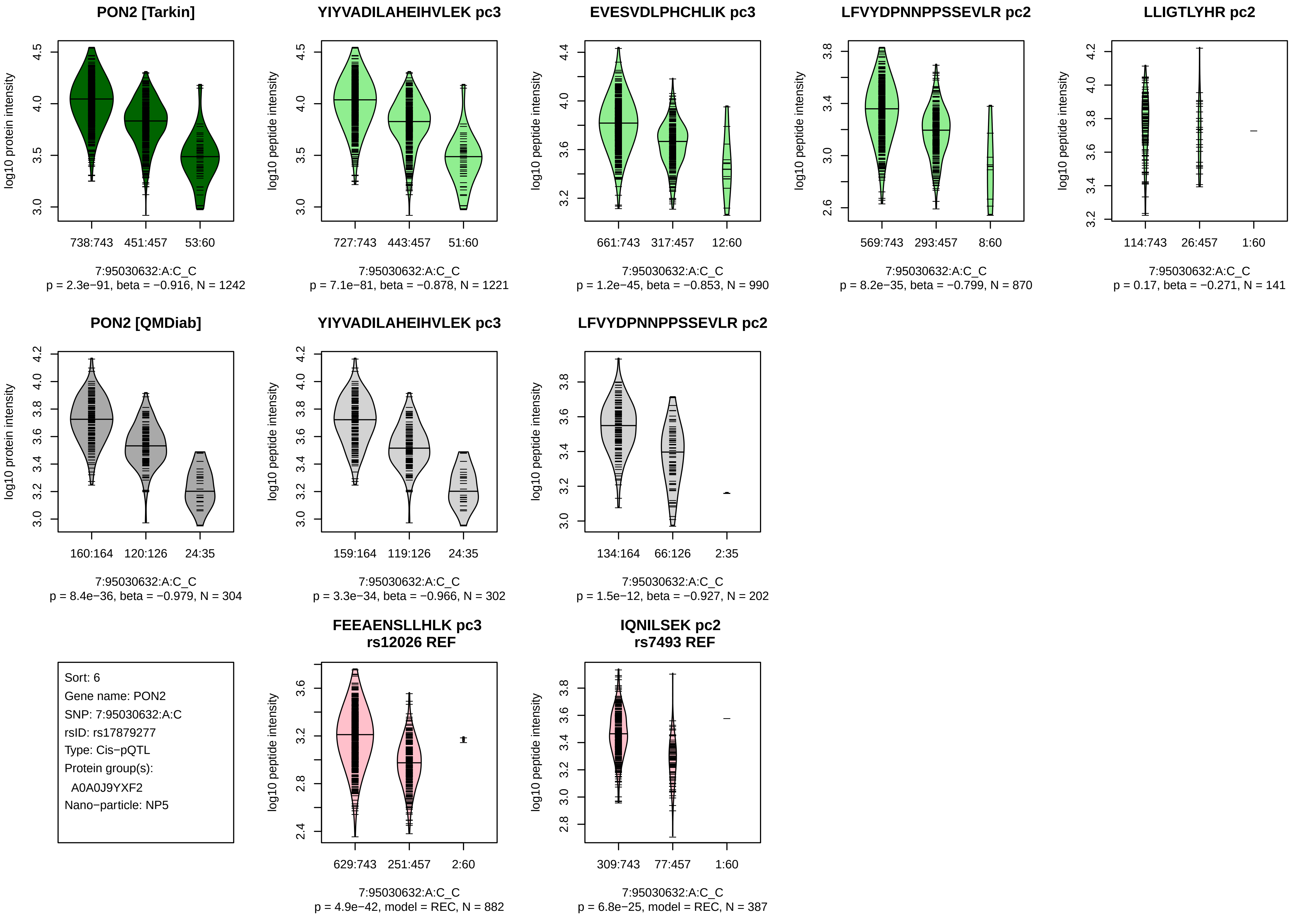
PON2 [Tarkin]
log10 protein intensity
738:743
451:457
53:60
7:95030632:A:C_C
p = 2.3e−91, beta = −0.916, N = 1242
YIYVADILAHEIHVLEK pc3
log10 peptide intensity
727:743
443:457
51:60
7:95030632:A:C_C
p = 7.1e−81, beta = −0.878, N = 1221
EVESVDLPHCHLIK pc3
log10 peptide intensity
661:743
317:457
12:60
7:95030632:A:C_C
p = 1.2e−45, beta = −0.853, N = 990
LFVYDPNNPPSSEVLR pc2
log10 peptide intensity
569:743
293:457
8:60
7:95030632:A:C_C
p = 8.2e−35, beta = −0.799, N = 870
LLIGTLYHR pc2
log10 peptide intensity
114:743
26:457
1:60
7:95030632:A:C_C
p = 0.17, beta = −0.271, N = 141
PON2 [QMDiab]
log10 protein intensity
160:164
120:126
24:35
7:95030632:A:C_C
p = 8.4e−36, beta = −0.979, N = 304
YIYVADILAHEIHVLEK pc3
log10 peptide intensity
159:164
119:126
24:35
7:95030632:A:C_C
p = 3.3e−34, beta = −0.966, N = 302
LFVYDPNNPPSSEVLR pc2
log10 peptide intensity
134:164
66:126
2:35
7:95030632:A:C_C
p = 1.5e−12, beta = −0.927, N = 202
Sort: 6
Gene name: PON2
SNP: 7:95030632:A:C
rsID: rs17879277
Type: Cis−pQTL
Protein group(s):
A0A0J9YXF2
Nano−particle: NP5
FEEAENSLLHLK pc3
rs12026 REF
log10 peptide intensity
629:743
251:457
2:60
7:95030632:A:C_C
p = 4.9e−42, model = REC, N = 882
IQNILSEK pc2
rs7493 REF
log10 peptide intensity
309:743
77:457
1:60
7:95030632:A:C_C
p = 6.8e−25, model = REC, N = 387

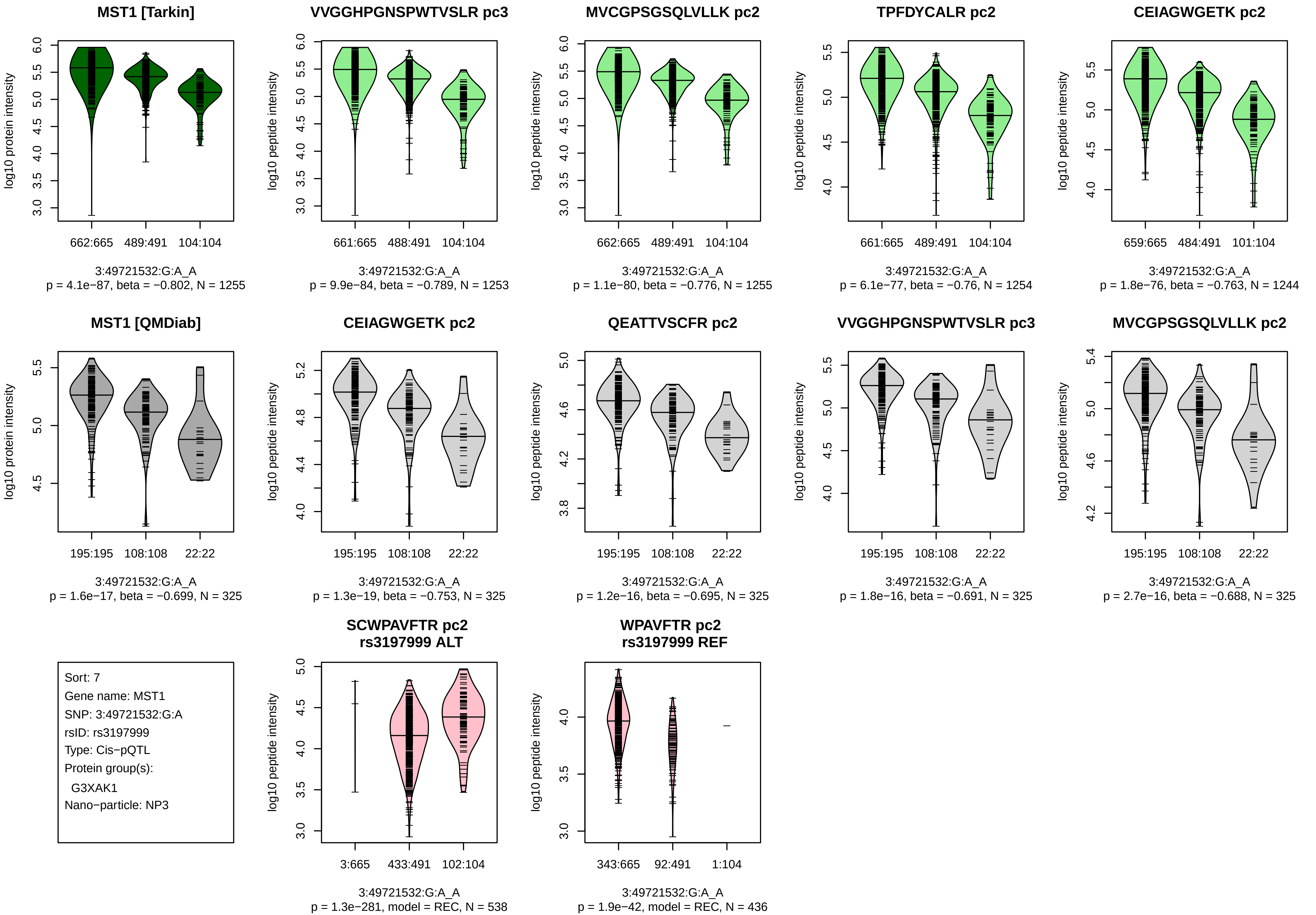
MST1 [Tarkin]
log10 protein intensity
662:665 489:491 104:104
3:49721532:G:A_A
p = 4.1e−87, beta = −0.802, N = 1255
VVGGHPGNSPWTVSLR pc3
log10 peptide intensity
661:665 488:491 104:104
3:49721532:G:A_A
p = 9.9e−84, beta = −0.789, N = 1253
MVCGPSGSQLVLLK pc2
log10 peptide intensity
662:665 489:491 104:104
3:49721532:G:A_A
p = 1.1e−80, beta = −0.776, N = 1255
TPFDYCALR pc2
log10 peptide intensity
661:665 489:491 104:104
3:49721532:G:A_A
p = 6.1e−77, beta = −0.76, N = 1254
CEIAGWGETK pc2
log10 peptide intensity
659:665 484:491 101:104
3:49721532:G:A_A
p = 1.8e−76, beta = −0.763, N = 1244
MST1 [QMDiab]
log10 protein intensity
195:195 108:108 22:22
3:49721532:G:A_A
p = 1.6e−17, beta = −0.699, N = 325
CEIAGWGETK pc2
log10 peptide intensity
195:195 108:108 22:22
3:49721532:G:A_A
p = 1.3e−19, beta = −0.753, N = 325
QEATTVSCFR pc2
log10 peptide intensity
195:195 108:108 22:22
3:49721532:G:A_A
p = 1.2e−16, beta = −0.695, N = 325
VVGGHPGNSPWTVSLR pc3
log10 peptide intensity
195:195 108:108 22:22
3:49721532:G:A_A
p = 1.8e−16, beta = −0.691, N = 325
MVCGPSGSQLVLLK pc2
log10 peptide intensity
195:195 108:108 22:22
3:49721532:G:A_A
p = 2.7e−16, beta = −0.688, N = 325
Sort: 7
Gene name: MST1
SNP: 3:49721532:G:A
rsID: rs3197999
Type: Cis−pQTL
Protein group(s):
G3XAK1
Nano−particle: NP3
SCWPAVFTR pc2
rs3197999 ALT
log10 peptide intensity
3:665 433:491 102:104
3:49721532:G:A_A
p = 1.3e−281, model = REC, N = 538
WPAVFTR pc2
rs3197999 REF
log10 peptide intensity
343:665 92:491 1:104
3:49721532:G:A_A
p = 1.9e−42, model = REC, N = 436

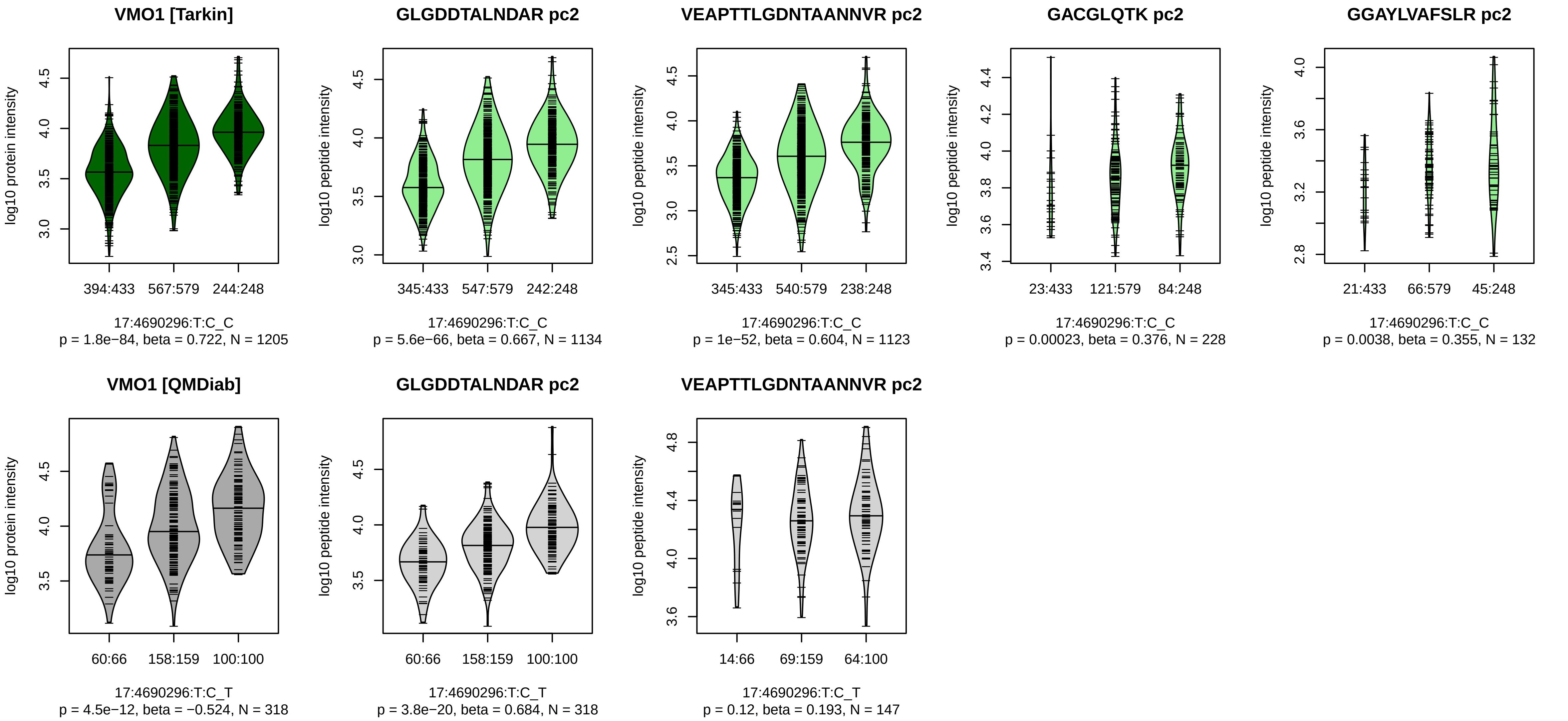

Sort: 8
Gene name: VMO1
SNP: 17:4690296:T:C
rsID: rs8068141
Type: Cis−pQTL
Protein group(s):
Q7Z5L0
Nano−particle: NP2

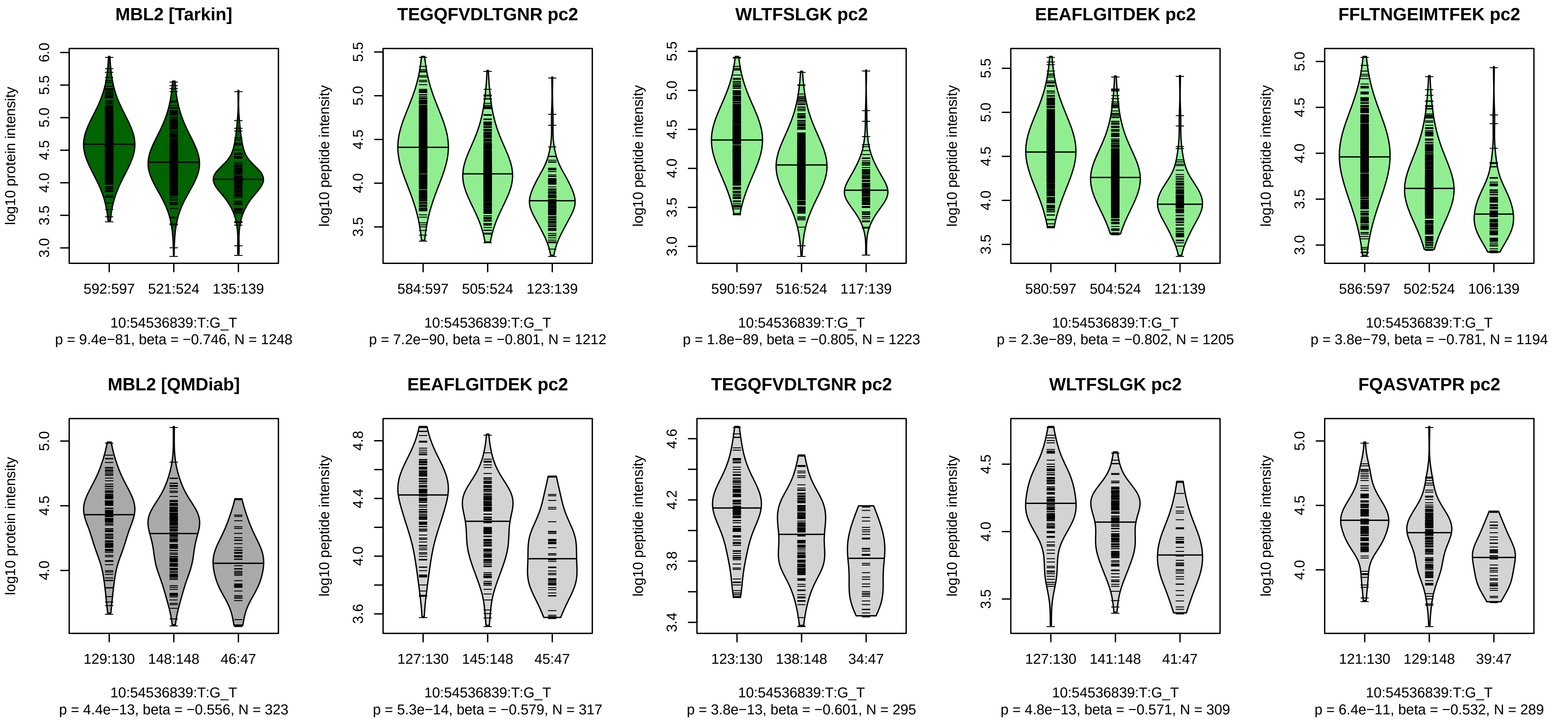

Sort: 9
Gene name: MBL2
SNP: 10:54536839:T:G
rsID: rs7899547
Type: Cis−pQTL
Protein group(s):
P11226
Nano−particle: NP2

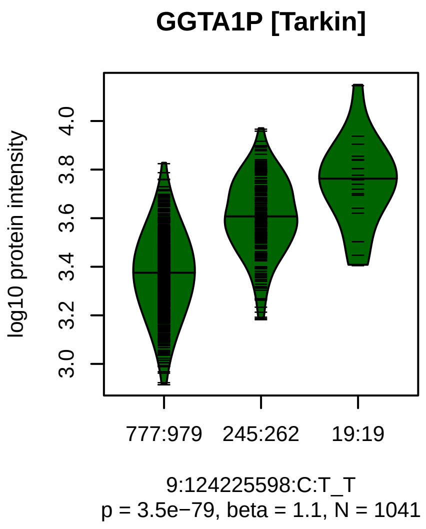

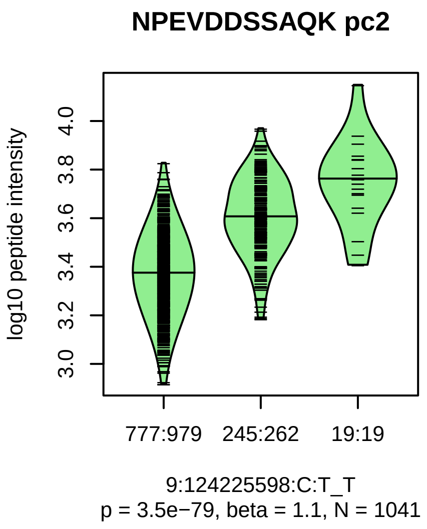

No protein data available
for replication in QMDiab

Sort: 10
Gene name: GGTA1P
SNP: 9:124225598:C:T
rsID: rs77378583
Type: Cis−pQTL
Protein group(s):
Q4G0N0
Nano−particle: NP2

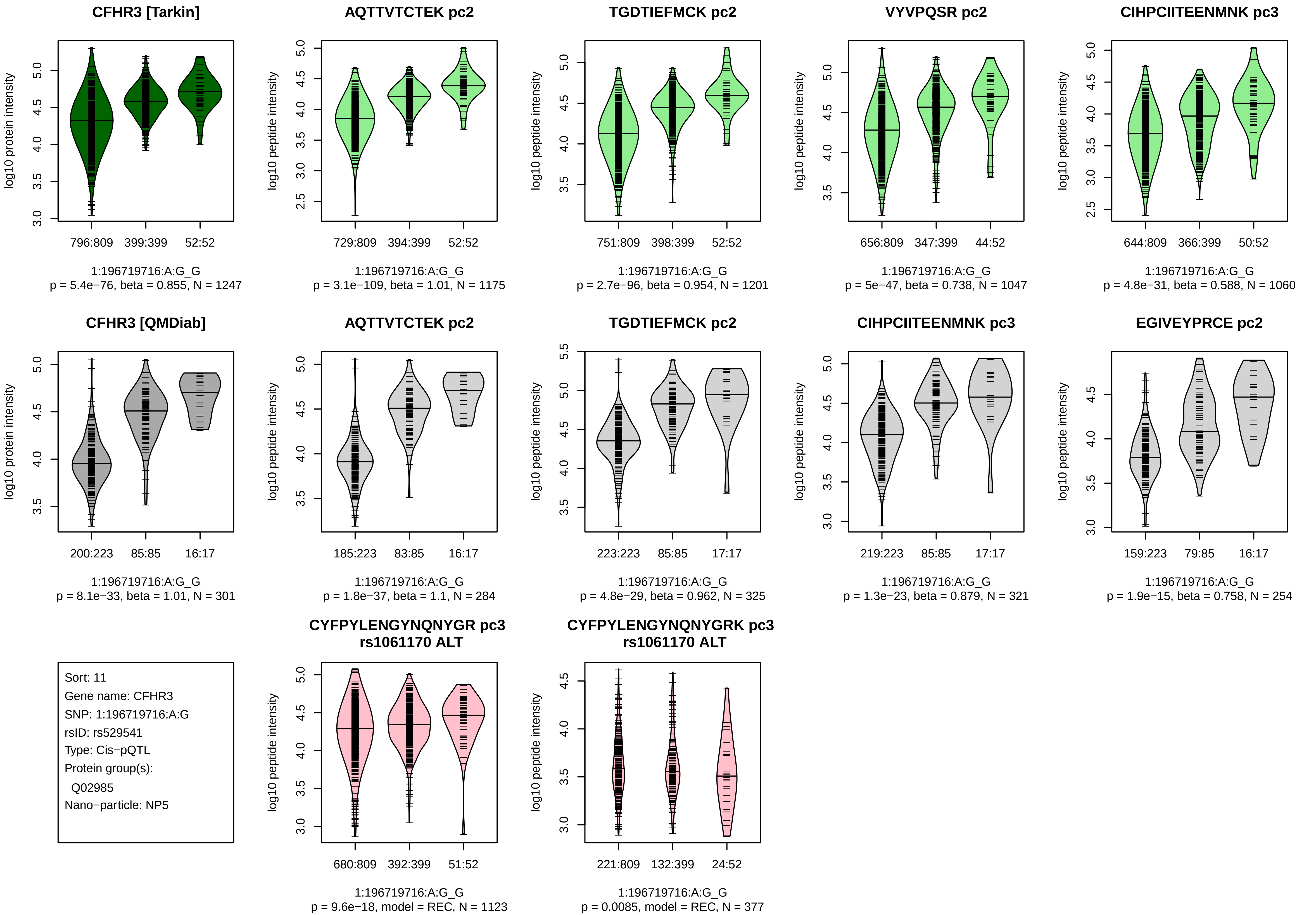
CFHR3 [Tarkin]
log10 protein intensity
796:809
399:399
52:52
1:196719716:A:G_G
p = 5.4e−76, beta = 0.855, N = 1247
AQTTVTCTEK pc2
log10 peptide intensity
729:809
394:399
52:52
1:196719716:A:G_G
p = 3.1e−109, beta = 1.01, N = 1175
TGDTIEFMCK pc2
751:809
398:399
52:52
1:196719716:A:G_G
p = 2.7e−96, beta = 0.954, N = 1201
VYVPQSR pc2
656:809
347:399
44:52
1:196719716:A:G_G
p = 5e−47, beta = 0.738, N = 1047
CIHPCIITEENMNK pc3
644:809
366:399
50:52
1:196719716:A:G_G
p = 4.8e−31, beta = 0.588, N = 1060
CFHR3 [QMDiab]
200:223
85:85
16:17
1:196719716:A:G_G
p = 8.1e−33, beta = 1.01, N = 301
AQTTVTCTEK pc2
185:223
83:85
16:17
1:196719716:A:G_G
p = 1.8e−37, beta = 1.1, N = 284
TGDTIEFMCK pc2
223:223
85:85
17:17
1:196719716:A:G_G
p = 4.8e−29, beta = 0.962, N = 325
CIHPCIITEENMNK pc3
219:223
85:85
17:17
1:196719716:A:G_G
p = 1.3e−23, beta = 0.879, N = 321
EGIVEYPRCE pc2
159:223
79:85
16:17
1:196719716:A:G_G
p = 1.9e−15, beta = 0.758, N = 254
Sort: 11
Gene name: CFHR3
SNP: 1:196719716:A:G
rsID: rs529541
Type: Cis−pQTL
Protein group(s):
Q02985
Nano−particle: NP5
CYFPYLENGYNQNYGR pc3
rs1061170 ALT
680:809
392:399
51:52
1:196719716:A:G_G
p = 9.6e−18, model = REC, N = 1123
CYFPYLENGYNQNYGRK pc3
rs1061170 ALT
221:809
132:399
24:52
1:196719716:A:G_G
p = 0.0085, model = REC, N = 377

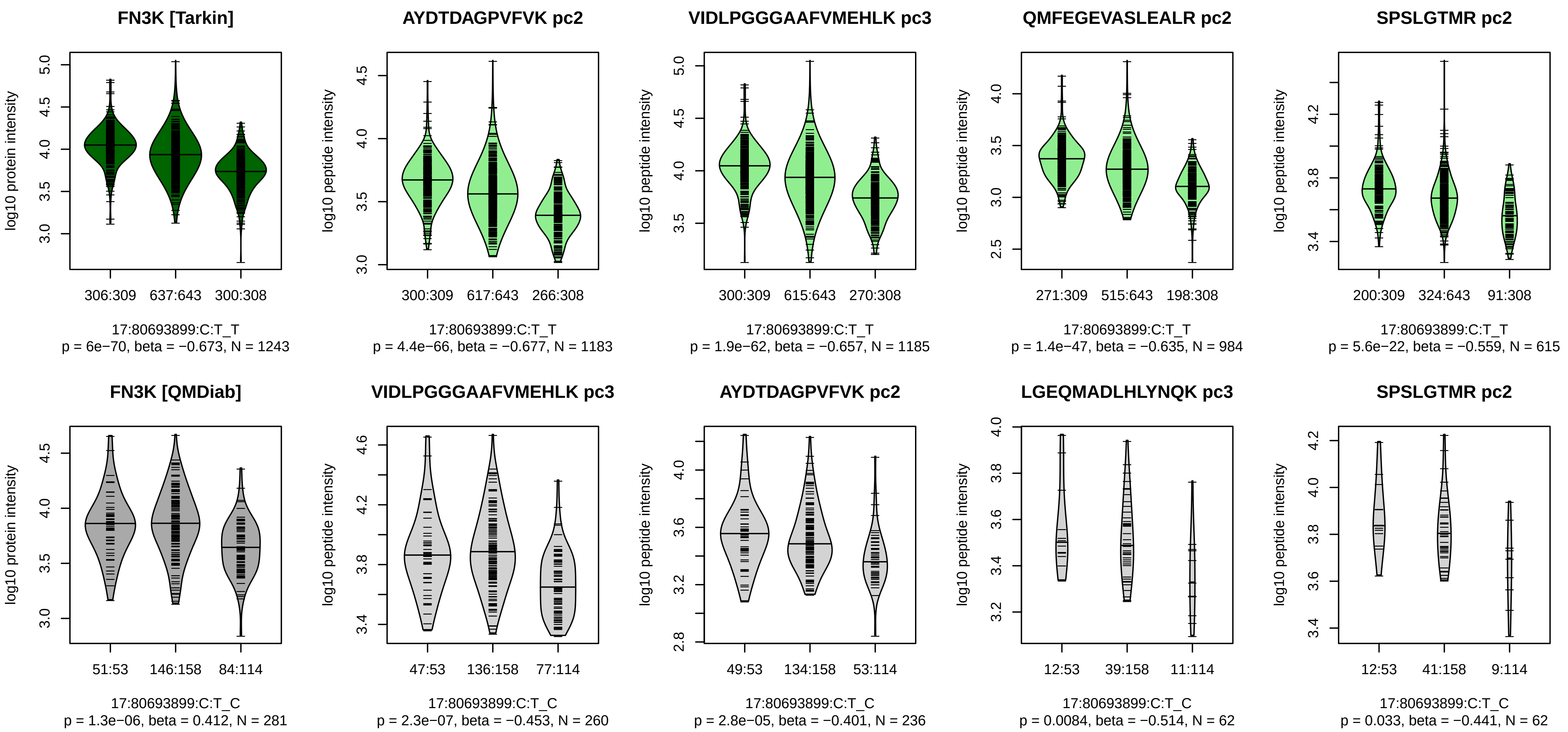

Sort: 12
Gene name: FN3K
SNP: 17:80693899:C:T
rsID: rs3848403
Type: Cis−pQTL
Protein group(s):
Q9H479
Nano−particle: NP1

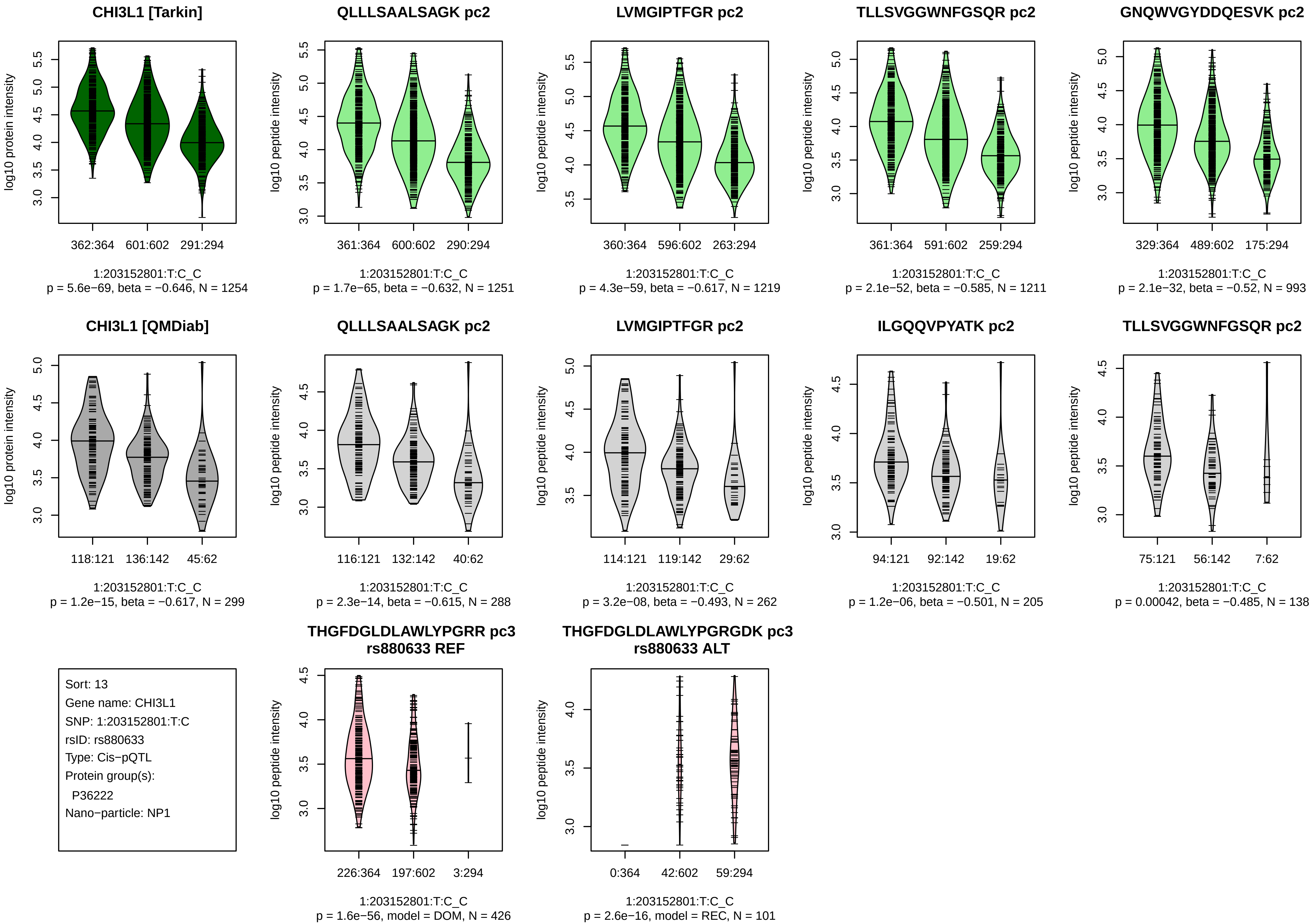
CHI3L1 [Tarkin]
log10 protein intensity
362:364 601:602 291:294
1:203152801:T:C_C
p = 5.6e−69, beta = −0.646, N = 1254
QLLLSAALSAGK pc2
log10 peptide intensity
361:364 600:602 290:294
1:203152801:T:C_C
p = 1.7e−65, beta = −0.632, N = 1251
LVMGIPTFGR pc2
log10 peptide intensity
360:364 596:602 263:294
1:203152801:T:C_C
p = 4.3e−59, beta = −0.617, N = 1219
TLLSVGGWNFGSQR pc2
log10 peptide intensity
361:364 591:602 259:294
1:203152801:T:C_C
p = 2.1e−52, beta = −0.585, N = 1211
GNQWVGYDDQESVK pc2
log10 peptide intensity
329:364 489:602 175:294
1:203152801:T:C_C
p = 2.1e−32, beta = −0.52, N = 993
CHI3L1 [QMDiab]
log10 protein intensity
118:121 136:142 45:62
1:203152801:T:C_C
p = 1.2e−15, beta = −0.617, N = 299
QLLLSAALSAGK pc2
log10 peptide intensity
116:121 132:142 40:62
1:203152801:T:C_C
p = 2.3e−14, beta = −0.615, N = 288
LVMGIPTFGR pc2
log10 peptide intensity
114:121 119:142 29:62
1:203152801:T:C_C
p = 3.2e−08, beta = −0.493, N = 262
ILGQQVPYATK pc2
log10 peptide intensity
94:121 92:142 19:62
1:203152801:T:C_C
p = 1.2e−06, beta = −0.501, N = 205
TLLSVGGWNFGSQR pc2
log10 peptide intensity
75:121 56:142 7:62
1:203152801:T:C_C
p = 0.00042, beta = −0.485, N = 138
Sort: 13
Gene name: CHI3L1
SNP: 1:203152801:T:C
rsID: rs880633
Type: Cis−pQTL
Protein group(s):
P36222
Nano−particle: NP1
THGFDGLDLAWLYPGRR pc3
rs880633 REF
log10 peptide intensity
226:364 197:602 3:294
1:203152801:T:C_C
p = 1.6e−56, model = DOM, N = 426
THGFDGLDLAWLYPGRGDK pc3
rs880633 ALT
log10 peptide intensity
0:364 42:602 59:294
1:203152801:T:C_C
p = 2.6e−16, model = REC, N = 101

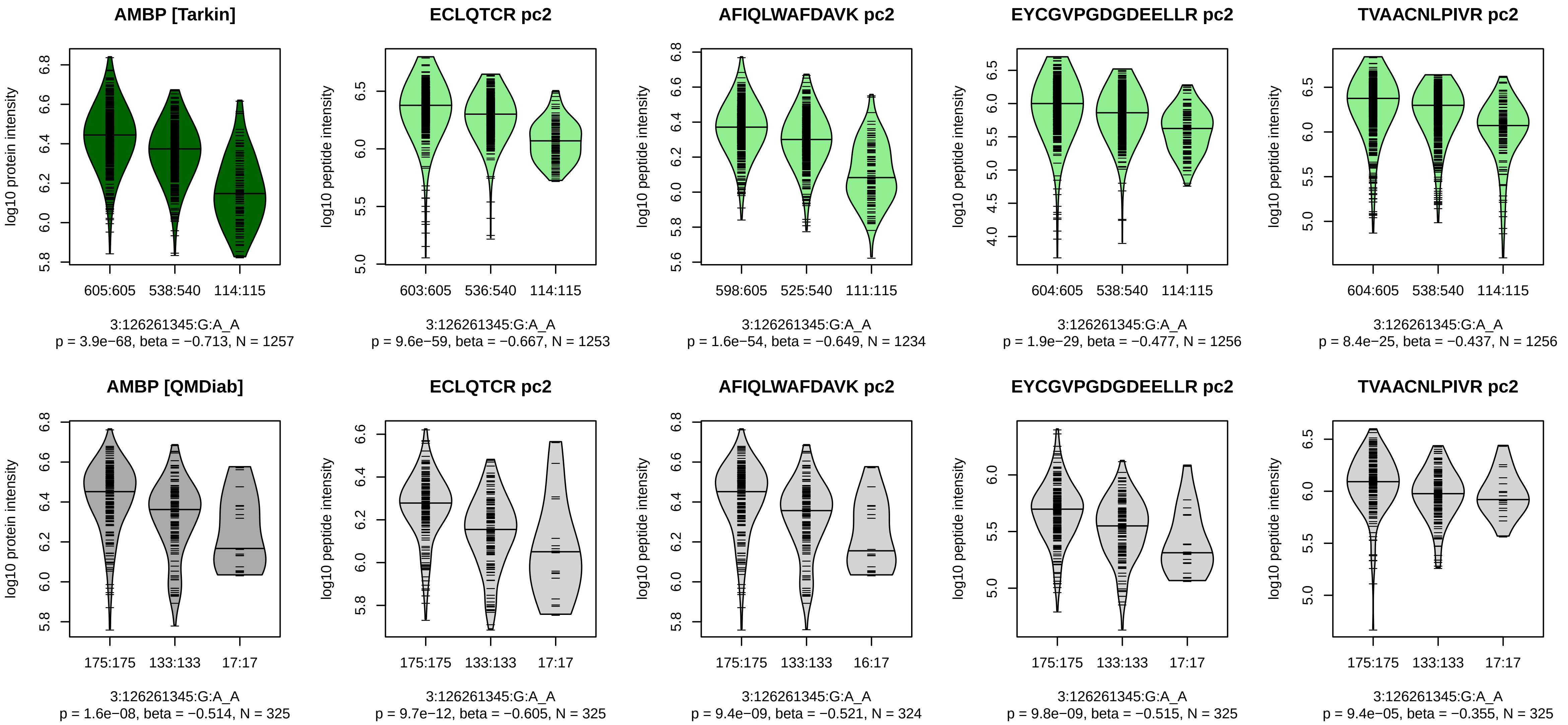

Sort: 14
Gene name: AMBP
SNP: 3:126261345:G:A
rsID: rs1056522
Type: Trans−pQTL
Protein group(s):
P02760
Nano−particle: NP2

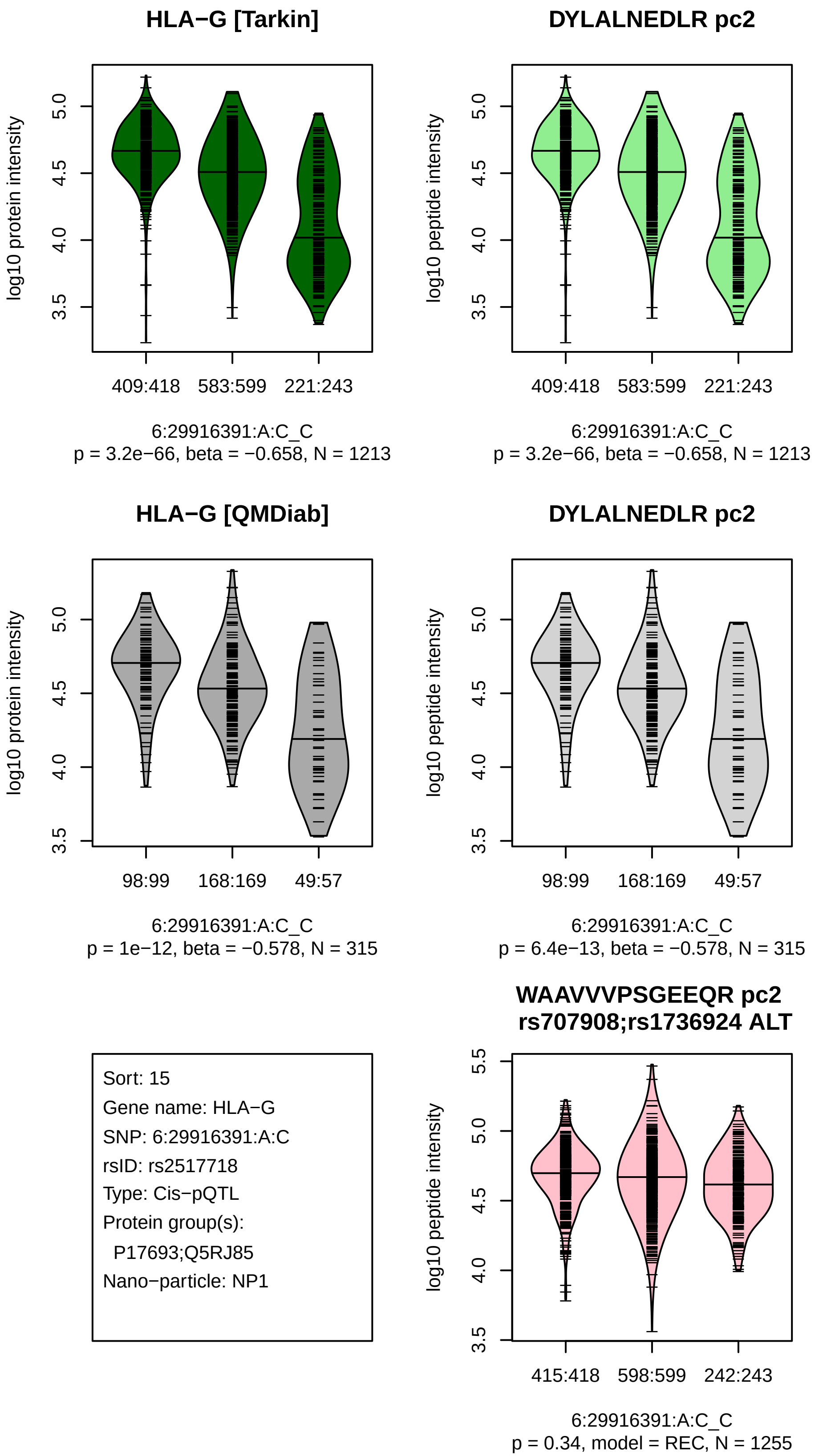
HLA−G [Tarkin]
DYLALNEDLR pc2
log10 protein intensity
log10 peptide intensity
3.5
4.0
4.5
5.0
409:418
583:599
221:243
6:29916391:A:C_C
p = 3.2e−66, beta = −0.658, N = 1213
6:29916391:A:C_C
p = 3.2e−66, beta = −0.658, N = 1213
HLA−G [QMDiab]
DYLALNEDLR pc2
98:99
168:169
49:57
6:29916391:A:C_C
p = 1e−12, beta = −0.578, N = 315
6:29916391:A:C_C
p = 6.4e−13, beta = −0.578, N = 315
Sort: 15
Gene name: HLA−G
SNP: 6:29916391:A:C
rsID: rs2517718
Type: Cis−pQTL
Protein group(s):
P17693;Q5RJ85
Nano−particle: NP1
WAAVVVPSGEEQR pc2
rs707908;rs1736924 ALT
5.5
415:418
598:599
242:243
6:29916391:A:C_C
p = 0.34, model = REC, N = 1255

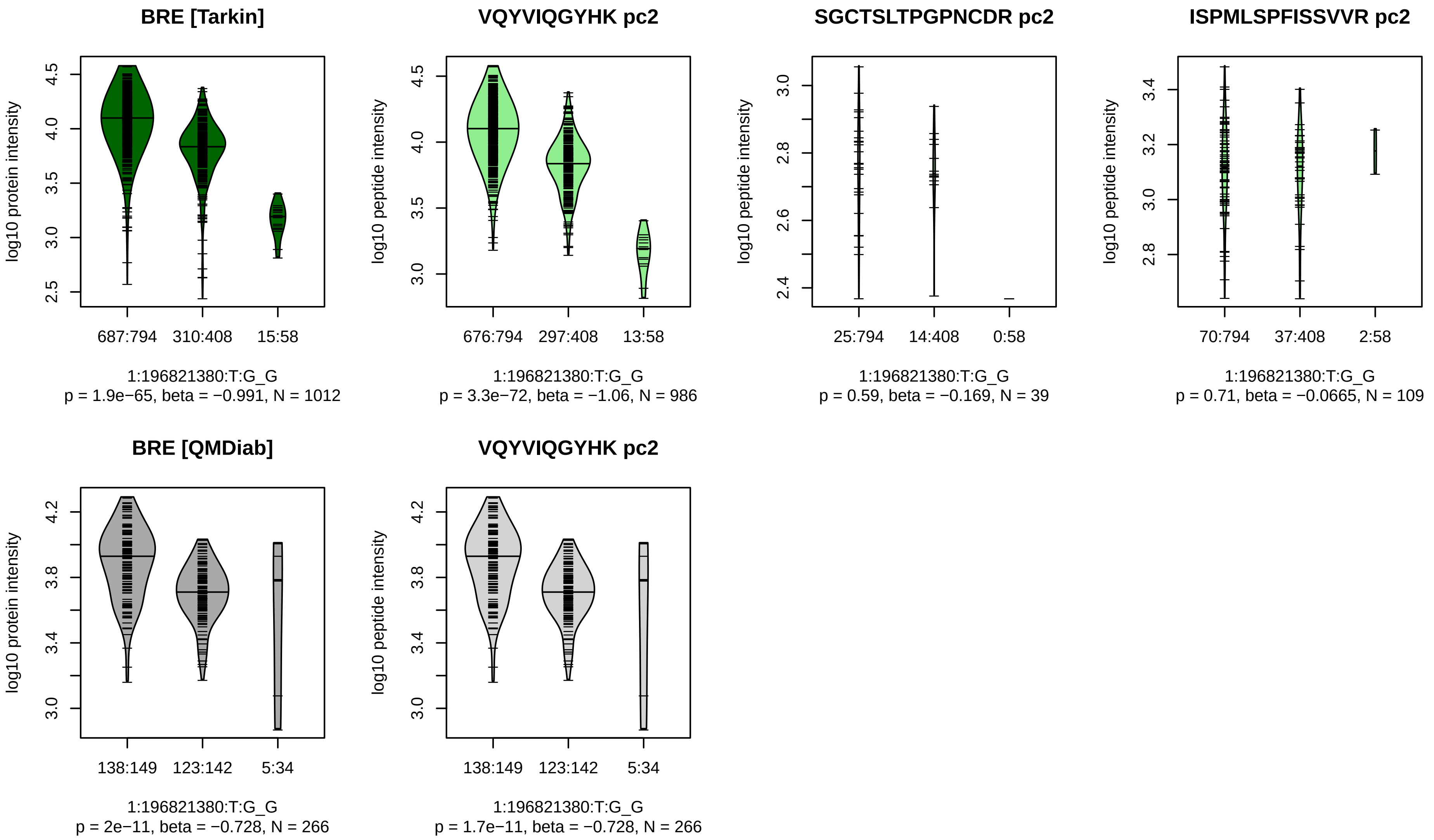

Sort: 16
Gene name: BRE
SNP: 1:196821380:T:G
rsID: rs67908756
Type: Trans−pQTL
Protein group(s):
Q9NXR7
Nano−particle: NP3

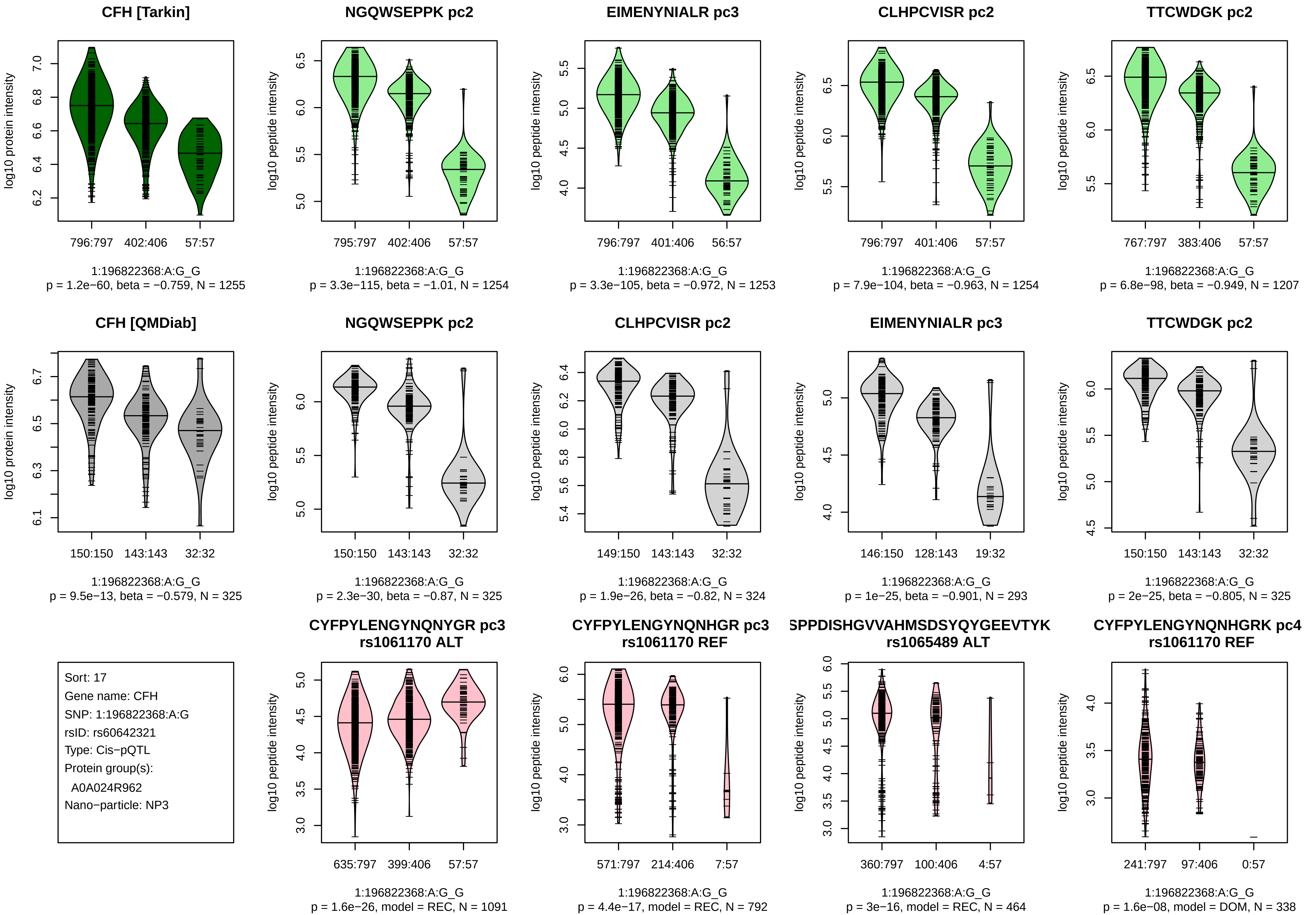

CFH [Tarkin]
log10 protein intensity
796:797 402:406 57:57
1:196822368:A:G_G
p = 1.2e−60, beta = −0.759, N = 1255
NGQWSEPPK pc2
log10 peptide intensity
795:797 402:406 57:57
1:196822368:A:G_G
p = 3.3e−115, beta = −1.01, N = 1254
EIMENYNIALR pc3
log10 peptide intensity
796:797 401:406 56:57
1:196822368:A:G_G
p = 3.3e−105, beta = −0.972, N = 1253
CLHPCVISR pc2
log10 peptide intensity
796:797 401:406 57:57
1:196822368:A:G_G
p = 7.9e−104, beta = −0.963, N = 1254
TTCWDGK pc2
log10 peptide intensity
767:797 383:406 57:57
1:196822368:A:G_G
p = 6.8e−98, beta = −0.949, N = 1207
CFH [QMDiab]
log10 protein intensity
150:150 143:143 32:32
1:196822368:A:G_G
p = 9.5e−13, beta = −0.579, N = 325
NGQWSEPPK pc2
log10 peptide intensity
150:150 143:143 32:32
1:196822368:A:G_G
p = 2.3e−30, beta = −0.87, N = 325
CLHPCVISR pc2
log10 peptide intensity
149:150 143:143 32:32
1:196822368:A:G_G
p = 1.9e−26, beta = −0.82, N = 324
EIMENYNIALR pc3
log10 peptide intensity
146:150 128:143 19:32
1:196822368:A:G_G
p = 1e−25, beta = −0.901, N = 293
TTCWDGK pc2
log10 peptide intensity
150:150 143:143 32:32
1:196822368:A:G_G
p = 2e−25, beta = −0.805, N = 325
Sort: 17
Gene name: CFH
SNP: 1:196822368:A:G
rsID: rs60642321
Type: Cis−pQTL
Protein group(s):
A0A024R962
Nano−particle: NP3
CYFPYLENGYNQNYGR pc3
rs1061170 ALT
log10 peptide intensity
635:797 399:406 57:57
1:196822368:A:G_G
p = 1.6e−26, model = REC, N = 1091
CYFPYLENGYNQNHGR pc3
rs1061170 REF
log10 peptide intensity
571:797 214:406 7:57
1:196822368:A:G_G
p = 4.4e−17, model = REC, N = 792
SPPDISHGVVAHMSDSYQYGEEVTYK
rs1065489 ALT
log10 peptide intensity
360:797 100:406 4:57
1:196822368:A:G_G
p = 3e−16, model = REC, N = 464
CYFPYLENGYNQNHGRK pc4
rs1061170 REF
log10 peptide intensity
241:797 97:406 0:57
1:196822368:A:G_G
p = 1.6e−08, model = DOM, N = 338

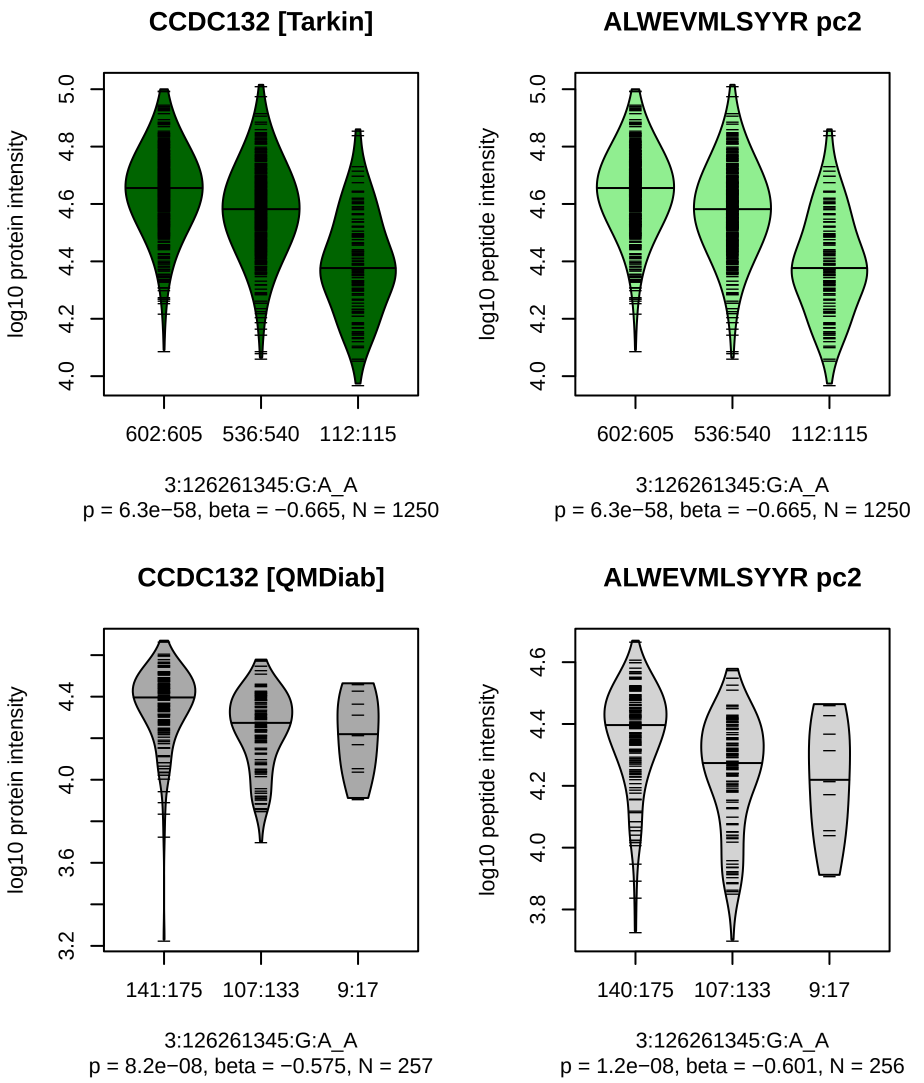

Sort: 18
Gene name: CCDC132
SNP: 3:126261345:G:A
rsID: rs1056522
Type: Trans−pQTL
Protein group(s):
Q96JG6
Nano−particle: NP2

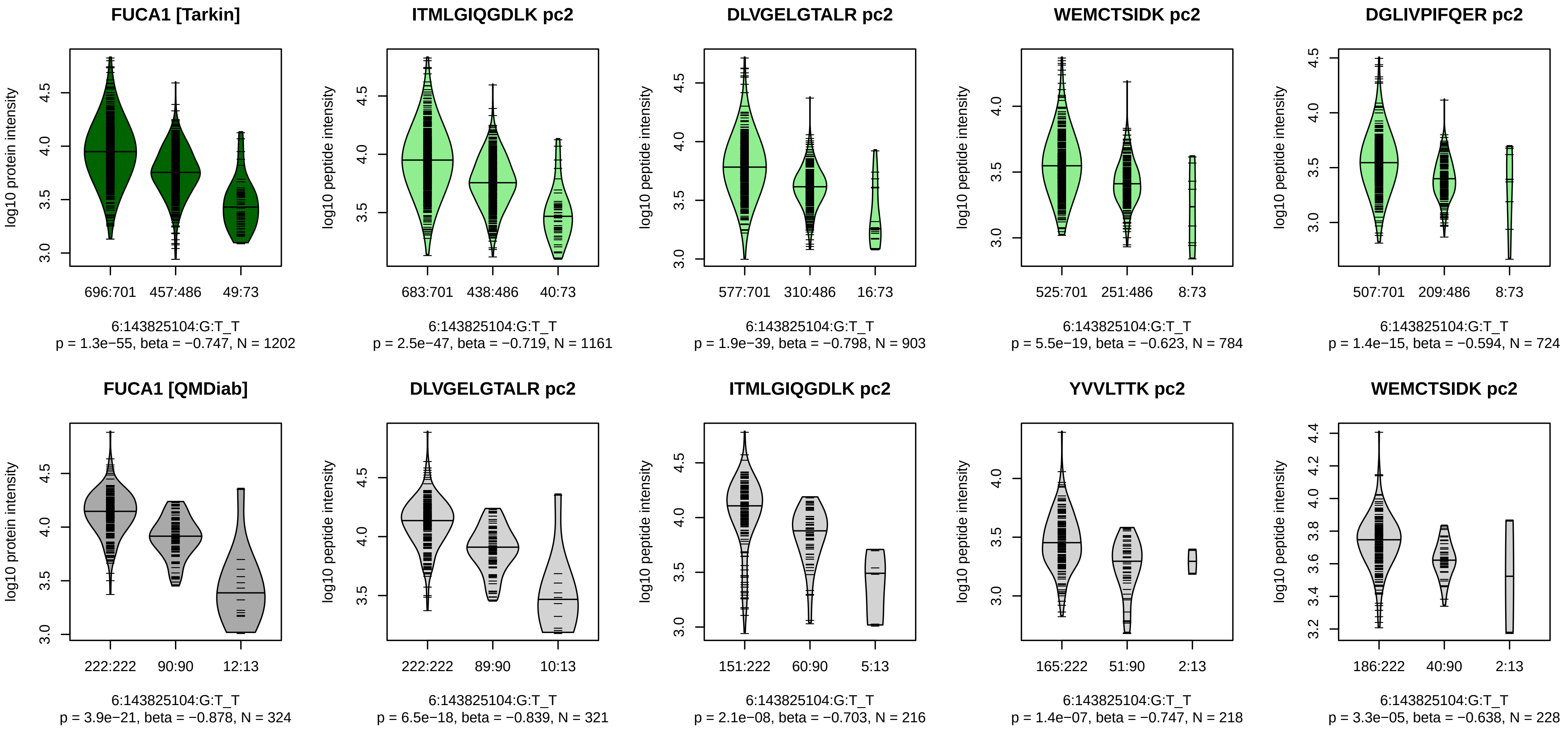

Sort: 19
Gene name: FUCA1
SNP: 6:143825104:G:T
rsID: rs11155297
Type: Trans−pQTL
Protein group(s):
P04066
Nano−particle: NP5

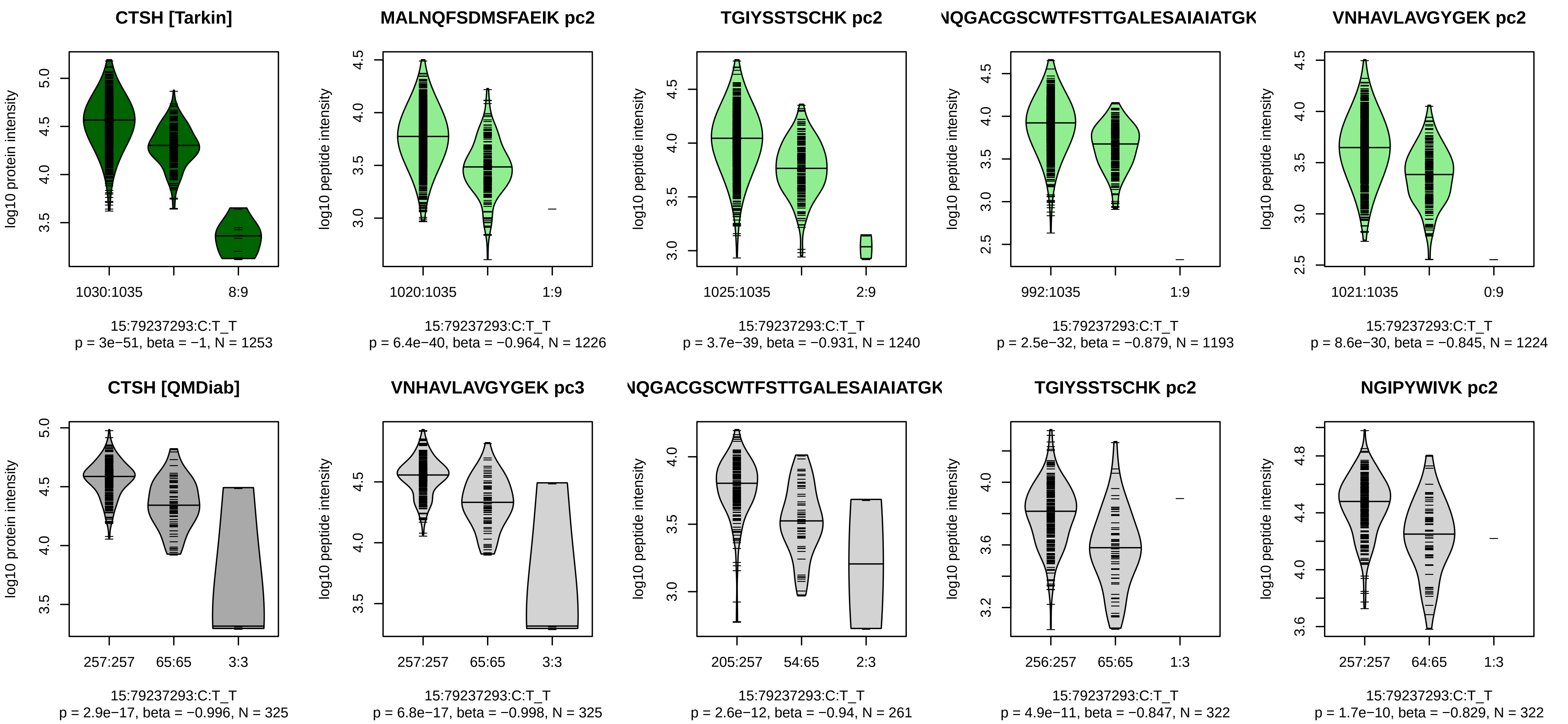

Sort: 20
Gene name: CTSH
SNP: 15:79237293:C:T
rsID: rs2289702
Type: Cis−pQTL
Protein group(s):
A0A087X0D5
Nano−particle: NP3

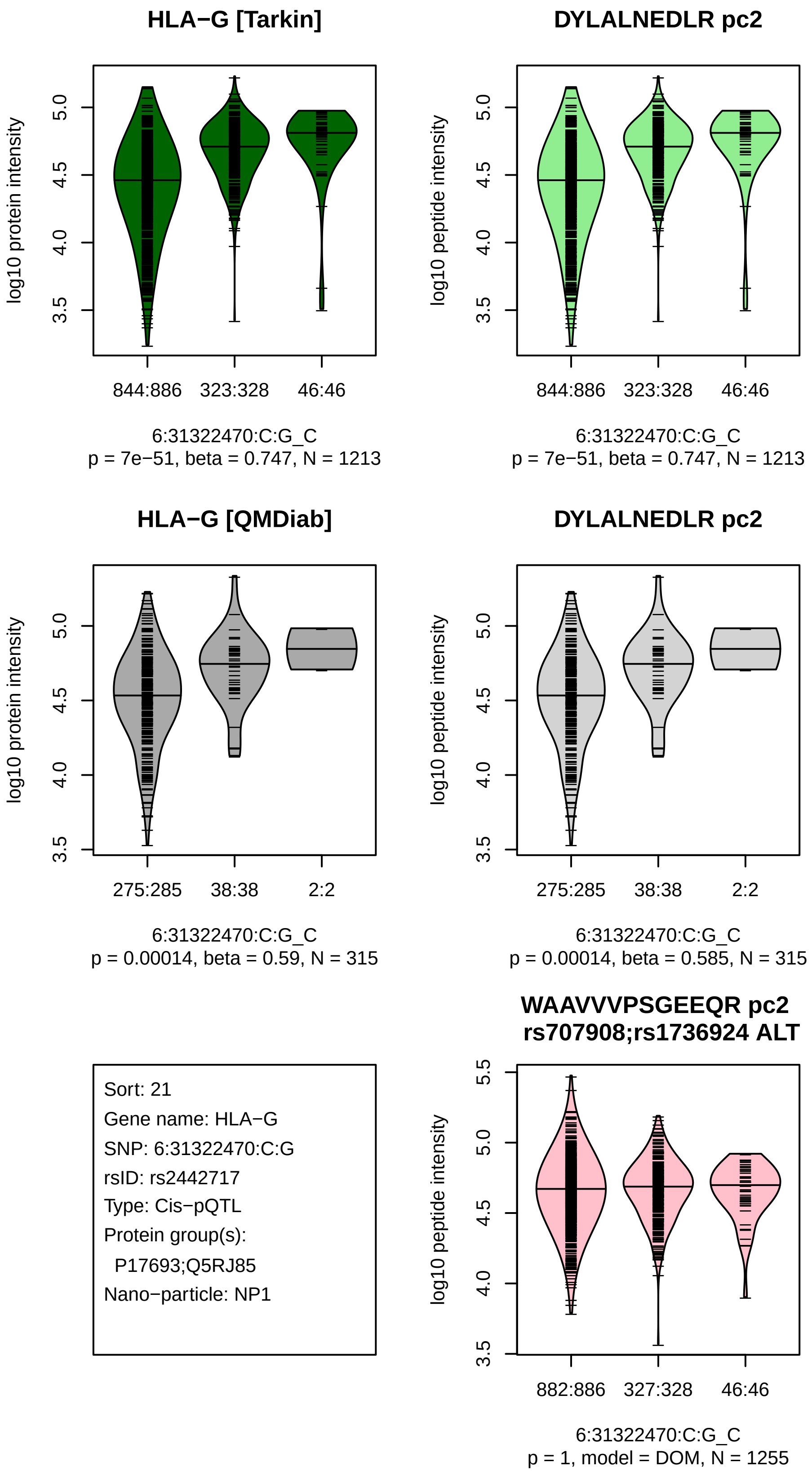
HLA−G [Tarkin]
log10 protein intensity
3.5
4.0
4.5
5.0
844:886
323:328
46:46
6:31322470:C:G_C
p = 7e−51, beta = 0.747, N = 1213
DYLALNEDLR pc2
log10 peptide intensity
3.5
4.0
4.5
5.0
844:886
323:328
46:46
6:31322470:C:G_C
p = 7e−51, beta = 0.747, N = 1213
HLA−G [QMDiab]
log10 protein intensity
3.5
4.0
4.5
5.0
275:285
38:38
2:2
6:31322470:C:G_C
p = 0.00014, beta = 0.59, N = 315
DYLALNEDLR pc2
log10 peptide intensity
3.5
4.0
4.5
5.0
275:285
38:38
2:2
6:31322470:C:G_C
p = 0.00014, beta = 0.585, N = 315
Sort: 21
Gene name: HLA−G
SNP: 6:31322470:C:G
rsID: rs2442717
Type: Cis−pQTL
Protein group(s):
P17693;Q5RJ85
Nano−particle: NP1
WAAVVVPSGEEQR pc2
rs707908;rs1736924 ALT
log10 peptide intensity
3.5
4.0
4.5
5.0
5.5
882:886
327:328
46:46
6:31322470:C:G_C
p = 1, model = DOM, N = 1255

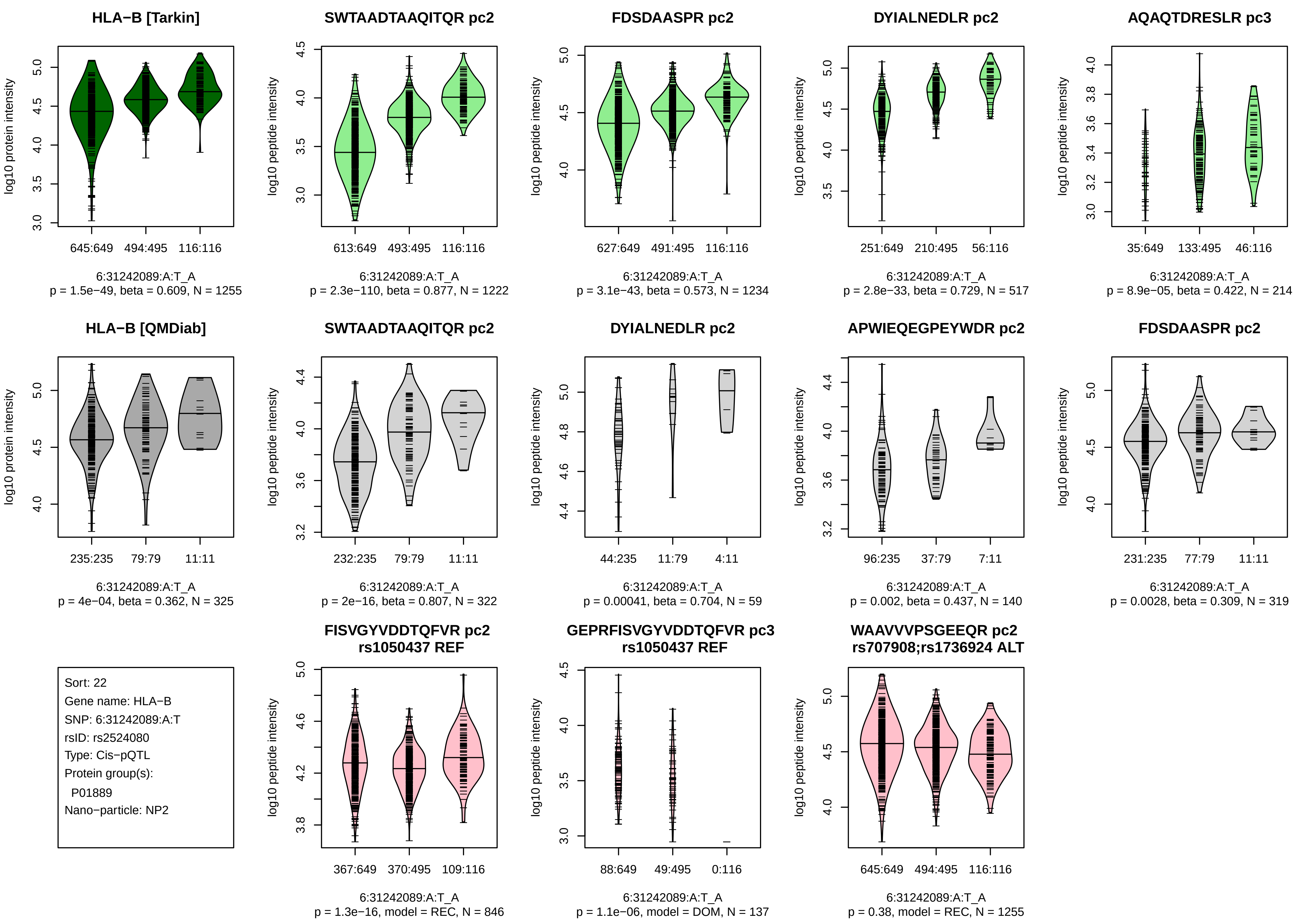
HLA−B [Tarkin]
log10 protein intensity
645:649
494:495
116:116
6:31242089:A:T_A
p = 1.5e−49, beta = 0.609, N = 1255
SWTAADTAAQITQR pc2
log10 peptide intensity
613:649
493:495
116:116
6:31242089:A:T_A
p = 2.3e−110, beta = 0.877, N = 1222
FDSDAASPR pc2
log10 peptide intensity
627:649
491:495
116:116
6:31242089:A:T_A
p = 3.1e−43, beta = 0.573, N = 1234
DYIALNEDLR pc2
log10 peptide intensity
251:649
210:495
56:116
6:31242089:A:T_A
p = 2.8e−33, beta = 0.729, N = 517
AQAQTDRESLR pc3
log10 peptide intensity
35:649
133:495
46:116
6:31242089:A:T_A
p = 8.9e−05, beta = 0.422, N = 214
HLA−B [QMDiab]
log10 protein intensity
235:235
79:79
11:11
6:31242089:A:T_A
p = 4e−04, beta = 0.362, N = 325
SWTAADTAAQITQR pc2
log10 peptide intensity
232:235
79:79
11:11
6:31242089:A:T_A
p = 2e−16, beta = 0.807, N = 322
DYIALNEDLR pc2
log10 peptide intensity
44:235
11:79
4:11
6:31242089:A:T_A
p = 0.00041, beta = 0.704, N = 59
APWIEQEGPEYWDR pc2
log10 peptide intensity
96:235
37:79
7:11
6:31242089:A:T_A
p = 0.002, beta = 0.437, N = 140
FDSDAASPR pc2
log10 peptide intensity
231:235
77:79
11:11
6:31242089:A:T_A
p = 0.0028, beta = 0.309, N = 319
Sort: 22
Gene name: HLA−B
SNP: 6:31242089:A:T
rsID: rs2524080
Type: Cis−pQTL
Protein group(s):
P01889
Nano−particle: NP2
FISVGYVDDTQFVR pc2
rs1050437 REF
log10 peptide intensity
367:649
370:495
109:116
6:31242089:A:T_A
p = 1.3e−16, model = REC, N = 846
GEPRFISVGYVDDTQFVR pc3
rs1050437 REF
log10 peptide intensity
88:649
49:495
0:116
6:31242089:A:T_A
p = 1.1e−06, model = DOM, N = 137
WAAVVVPSGEEQR pc2
rs707908;rs1736924 ALT
log10 peptide intensity
645:649
494:495
116:116
6:31242089:A:T_A
p = 0.38, model = REC, N = 1255

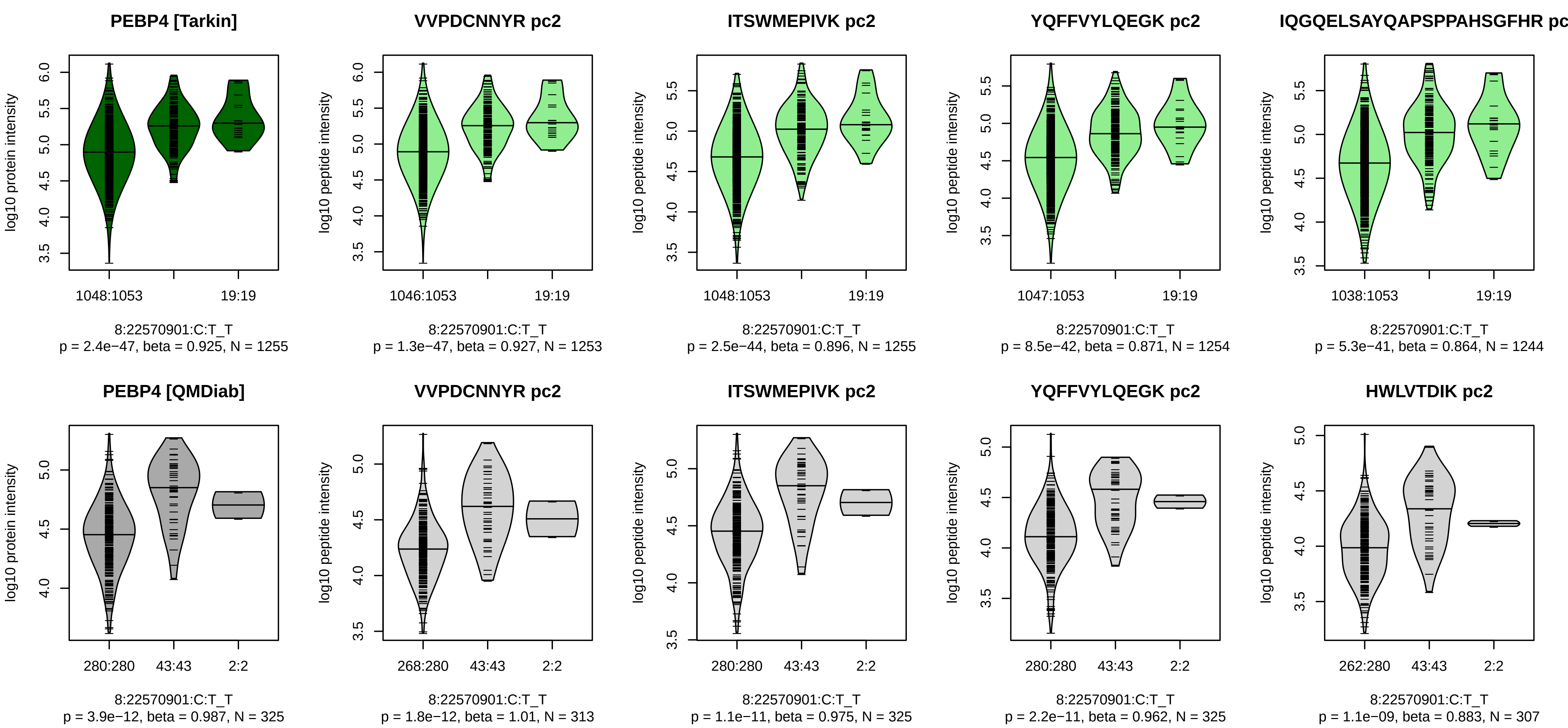

Sort: 23
Gene name: PEBP4
SNP: 8:22570901:C:T
rsID: rs3087803
Type: Cis−pQTL
Protein group(s):
Q96S96
Nano−particle: NP3

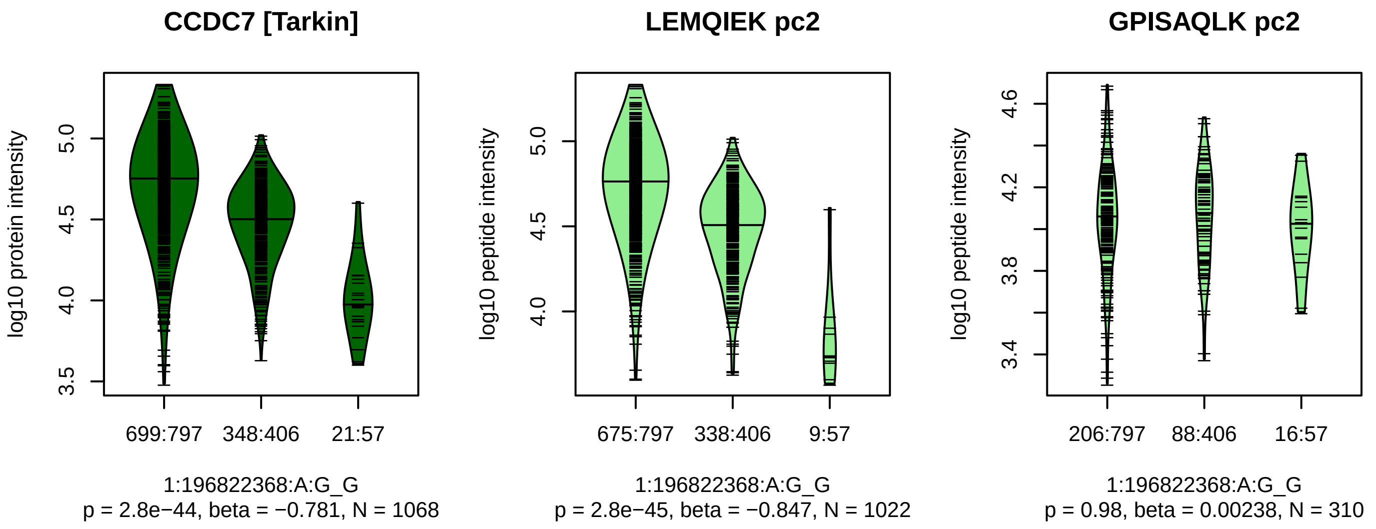

No protein data available
for replication in QMDiab

Sort: 24
Gene name: CCDC7
SNP: 1:196822368:A:G
rsID: rs60642321
Type: Trans−pQTL
Protein group(s):
Q96M83
Nano−particle: NP4

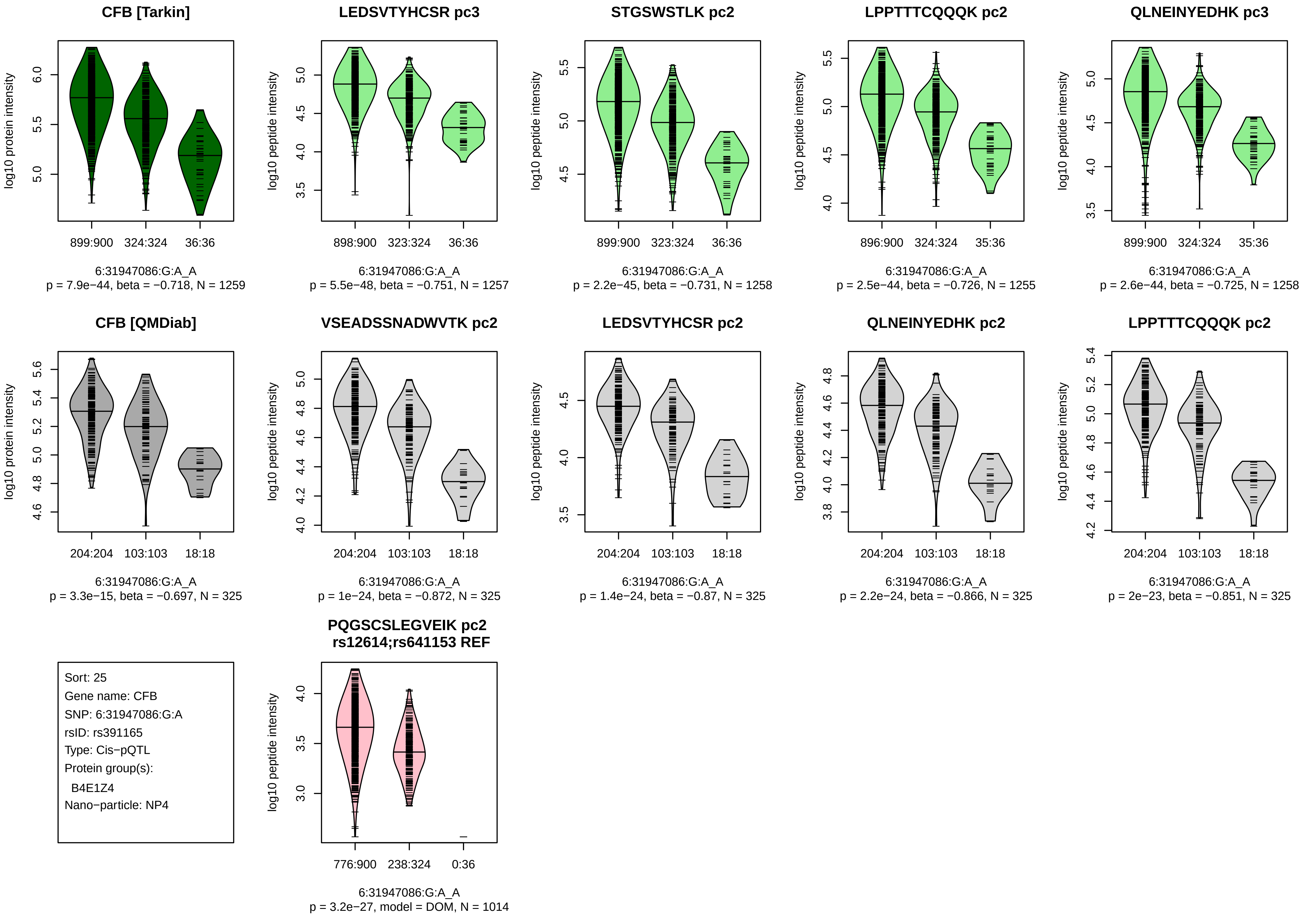
CFB [Tarkin]
log10 protein intensity
899:900 324:324 36:36
6:31947086:G:A_A
p = 7.9e−44, beta = −0.718, N = 1259
LEDSVTYHCSR pc3
log10 peptide intensity
898:900 323:324 36:36
6:31947086:G:A_A
p = 5.5e−48, beta = −0.751, N = 1257
STGSWSTLK pc2
log10 peptide intensity
899:900 323:324 36:36
6:31947086:G:A_A
p = 2.2e−45, beta = −0.731, N = 1258
LPPTTTCQQQK pc2
log10 peptide intensity
896:900 324:324 35:36
6:31947086:G:A_A
p = 2.5e−44, beta = −0.726, N = 1255
QLNEINYEDHK pc3
log10 peptide intensity
899:900 324:324 36:36
6:31947086:G:A_A
p = 2.6e−44, beta = −0.725, N = 1258
CFB [QMDiab]
log10 protein intensity
204:204 103:103 18:18
6:31947086:G:A_A
p = 3.3e−15, beta = −0.697, N = 325
VSEADSSNADWVTK pc2
log10 peptide intensity
204:204 103:103 18:18
6:31947086:G:A_A
p = 1e−24, beta = −0.872, N = 325
LEDSVTYHCSR pc2
log10 peptide intensity
204:204 103:103 18:18
6:31947086:G:A_A
p = 1.4e−24, beta = −0.87, N = 325
QLNEINYEDHK pc2
log10 peptide intensity
204:204 103:103 18:18
6:31947086:G:A_A
p = 2.2e−24, beta = −0.866, N = 325
LPPTTTCQQQK pc2
log10 peptide intensity
204:204 103:103 18:18
6:31947086:G:A_A
p = 2e−23, beta = −0.851, N = 325
Sort: 25
Gene name: CFB
SNP: 6:31947086:G:A
rsID: rs391165
Type: Cis−pQTL
Protein group(s):
B4E1Z4
Nano−particle: NP4
PQGSCSLEGVEIK pc2
rs12614;rs641153 REF
log10 peptide intensity
776:900 238:324 0:36
6:31947086:G:A_A
p = 3.2e−27, model = DOM, N = 1014

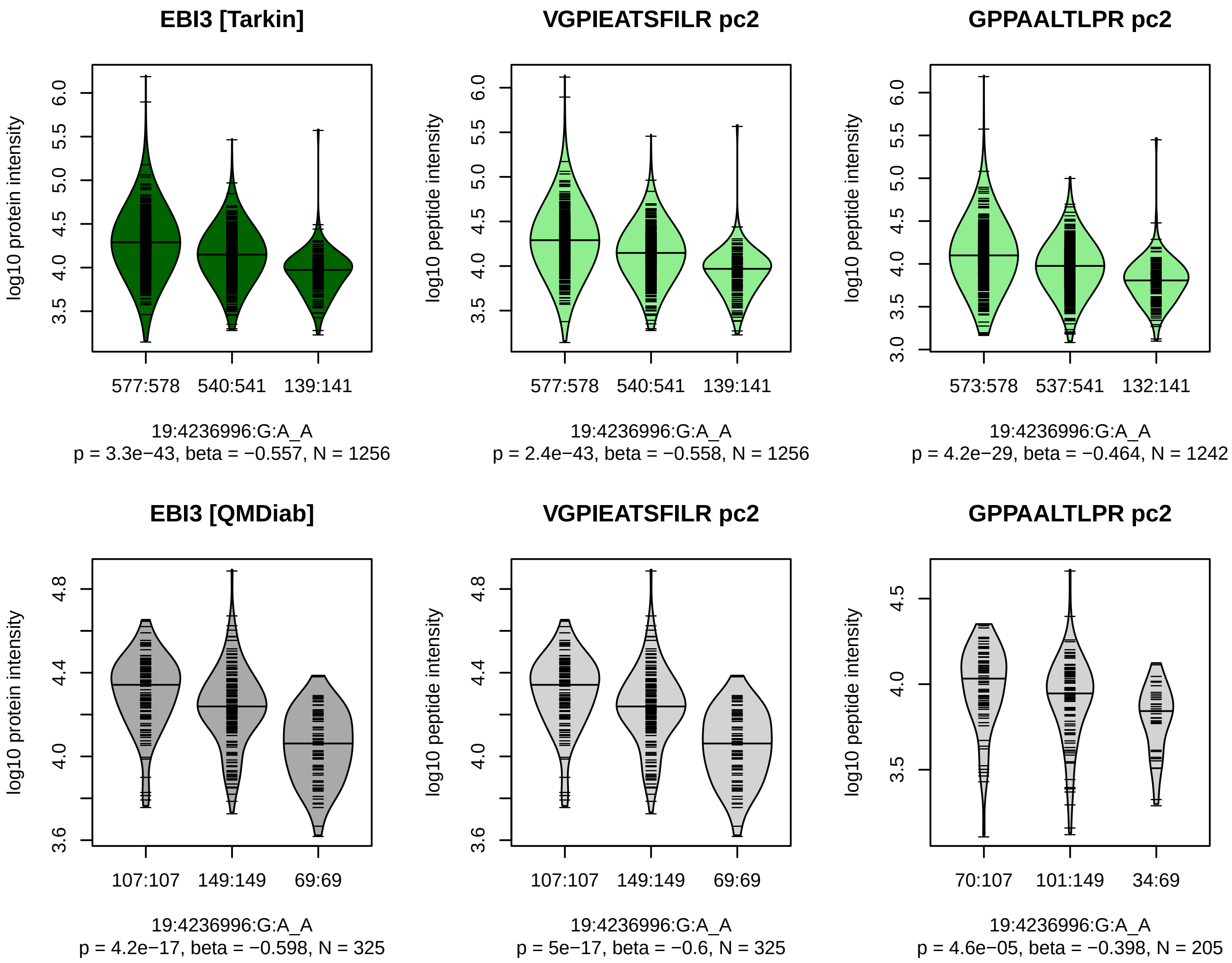

Sort: 26
Gene name: EBI3
SNP: 19:4236996:G:A
rsID: rs4740
Type: Cis−pQTL
Protein group(s):
Q14213
Nano−particle: NP4

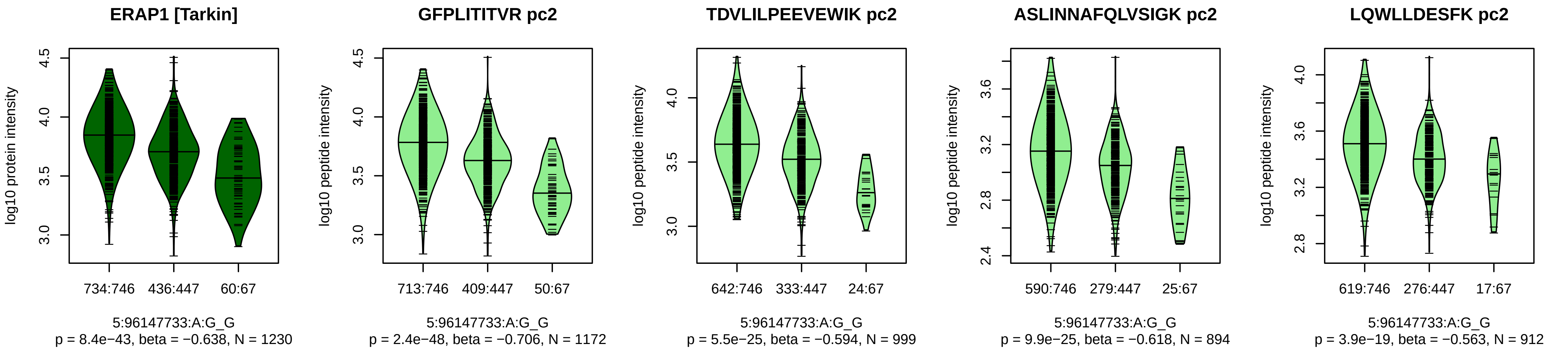

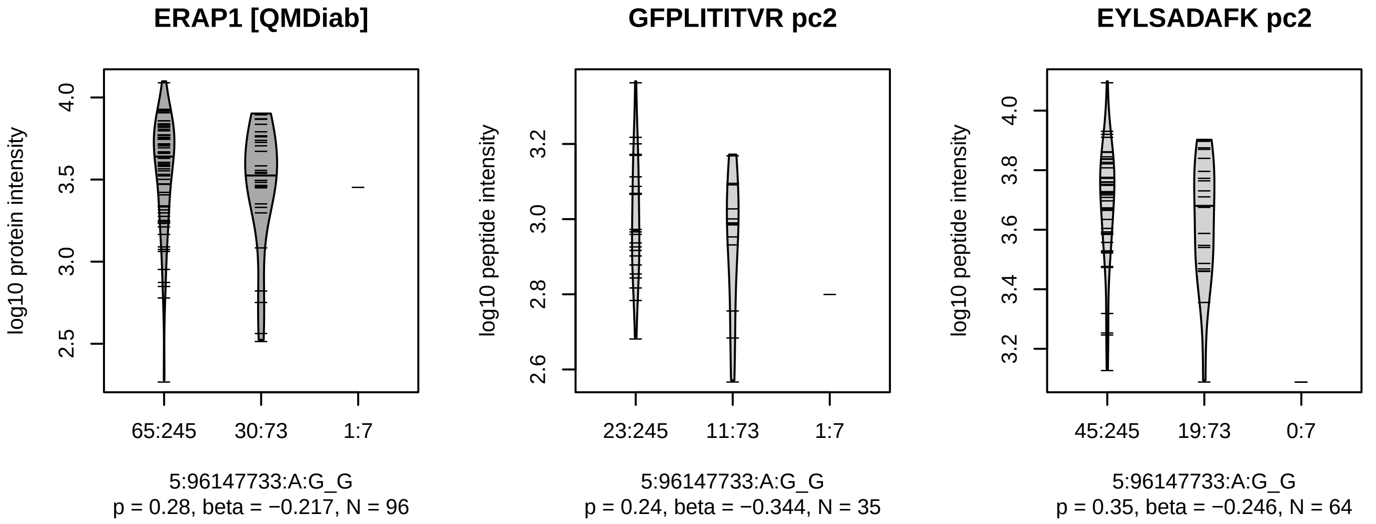

Sort: 27
Gene name: ERAP1
SNP: 5:96147733:A:G
rsID: rs10045403
Type: Cis−pQTL
Protein group(s):
T: Q9NZ08;Q9NZ08−2
Q: Q9NZ08−2
Nano−particle: NP5

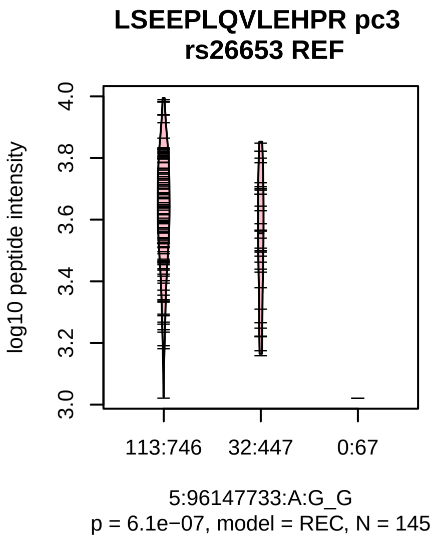

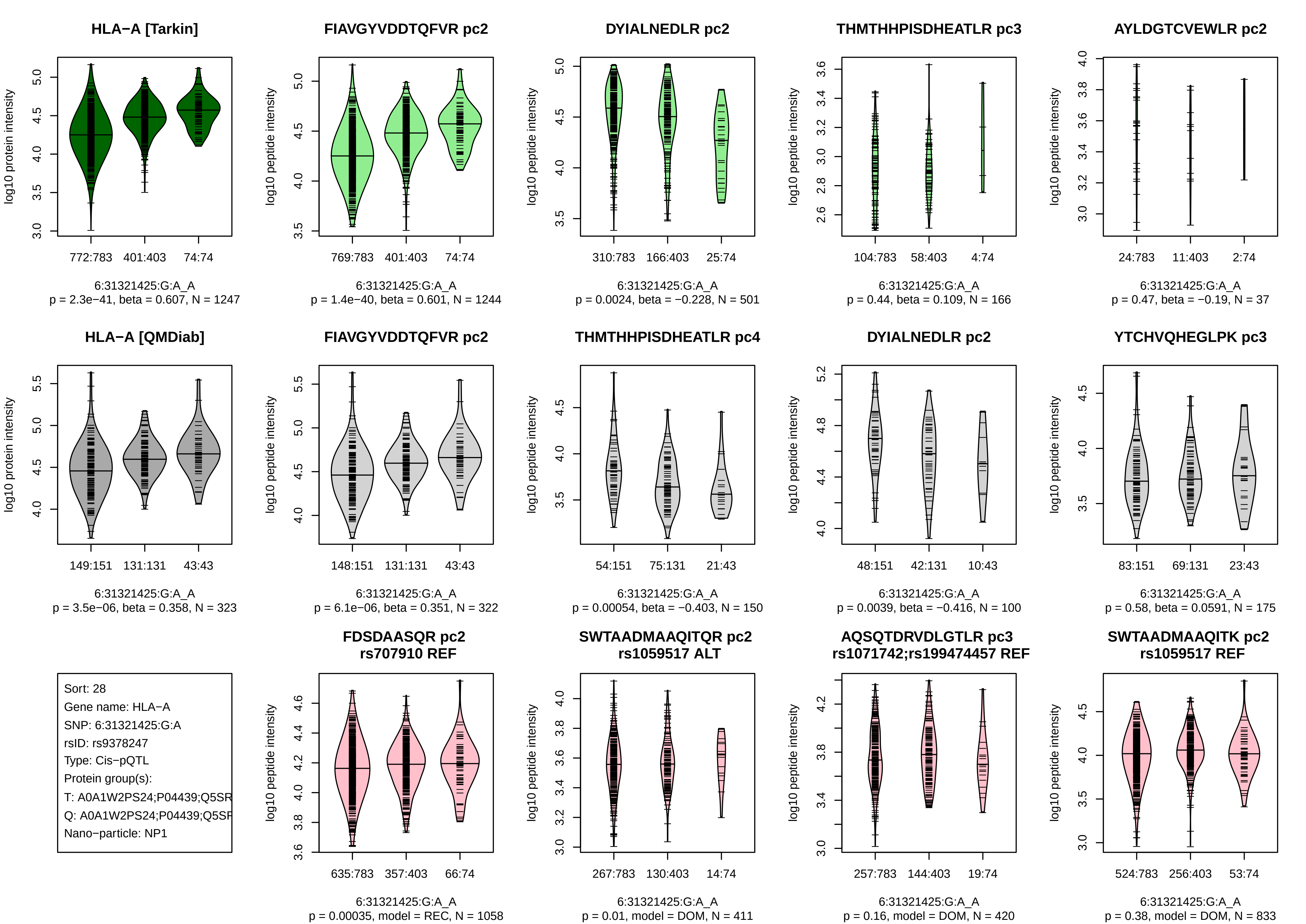

HLA−A [Tarkin]
log10 protein intensity
772:783 401:403 74:74
6:31321425:G:A_A
p = 2.3e−41, beta = 0.607, N = 1247
FIAVGYVDDTQFVR pc2
log10 peptide intensity
769:783 401:403 74:74
6:31321425:G:A_A
p = 1.4e−40, beta = 0.601, N = 1244
DYIALNEDLR pc2
log10 peptide intensity
310:783 166:403 25:74
6:31321425:G:A_A
p = 0.0024, beta = −0.228, N = 501
THMTHHPISDHEATLR pc3
log10 peptide intensity
104:783 58:403 4:74
6:31321425:G:A_A
p = 0.44, beta = 0.109, N = 166
AYLDGTCVEWLR pc2
log10 peptide intensity
24:783 11:403 2:74
6:31321425:G:A_A
p = 0.47, beta = −0.19, N = 37
HLA−A [QMDiab]
log10 protein intensity
149:151 131:131 43:43
6:31321425:G:A_A
p = 3.5e−06, beta = 0.358, N = 323
FIAVGYVDDTQFVR pc2
log10 peptide intensity
148:151 131:131 43:43
6:31321425:G:A_A
p = 6.1e−06, beta = 0.351, N = 322
THMTHHPISDHEATLR pc4
log10 peptide intensity
54:151 75:131 21:43
6:31321425:G:A_A
p = 0.00054, beta = −0.403, N = 150
DYIALNEDLR pc2
log10 peptide intensity
48:151 42:131 10:43
6:31321425:G:A_A
p = 0.0039, beta = −0.416, N = 100
YTCHVQHEGLPK pc3
log10 peptide intensity
83:151 69:131 23:43
6:31321425:G:A_A
p = 0.58, beta = 0.0591, N = 175
Sort: 28
Gene name: HLA−A
SNP: 6:31321425:G:A
rsID: rs9378247
Type: Cis−pQTL
Protein group(s):
T: A0A1W2PS24;P04439;Q5SR
Q: A0A1W2PS24;P04439;Q5SF
Nano−particle: NP1
FDSDAASQR pc2
rs707910 REF
log10 peptide intensity
635:783 357:403 66:74
6:31321425:G:A_A
p = 0.00035, model = REC, N = 1058
SWTAADMAAQITQR pc2
rs1059517 ALT
log10 peptide intensity
267:783 130:403 14:74
6:31321425:G:A_A
p = 0.01, model = DOM, N = 411
AQSQTDRVDLGTLR pc3
rs1071742;rs199474457 REF
log10 peptide intensity
257:783 144:403 19:74
6:31321425:G:A_A
p = 0.16, model = DOM, N = 420
SWTAADMAAQITK pc2
rs1059517 REF
log10 peptide intensity
524:783 256:403 53:74
6:31321425:G:A_A
p = 0.38, model = DOM, N = 833

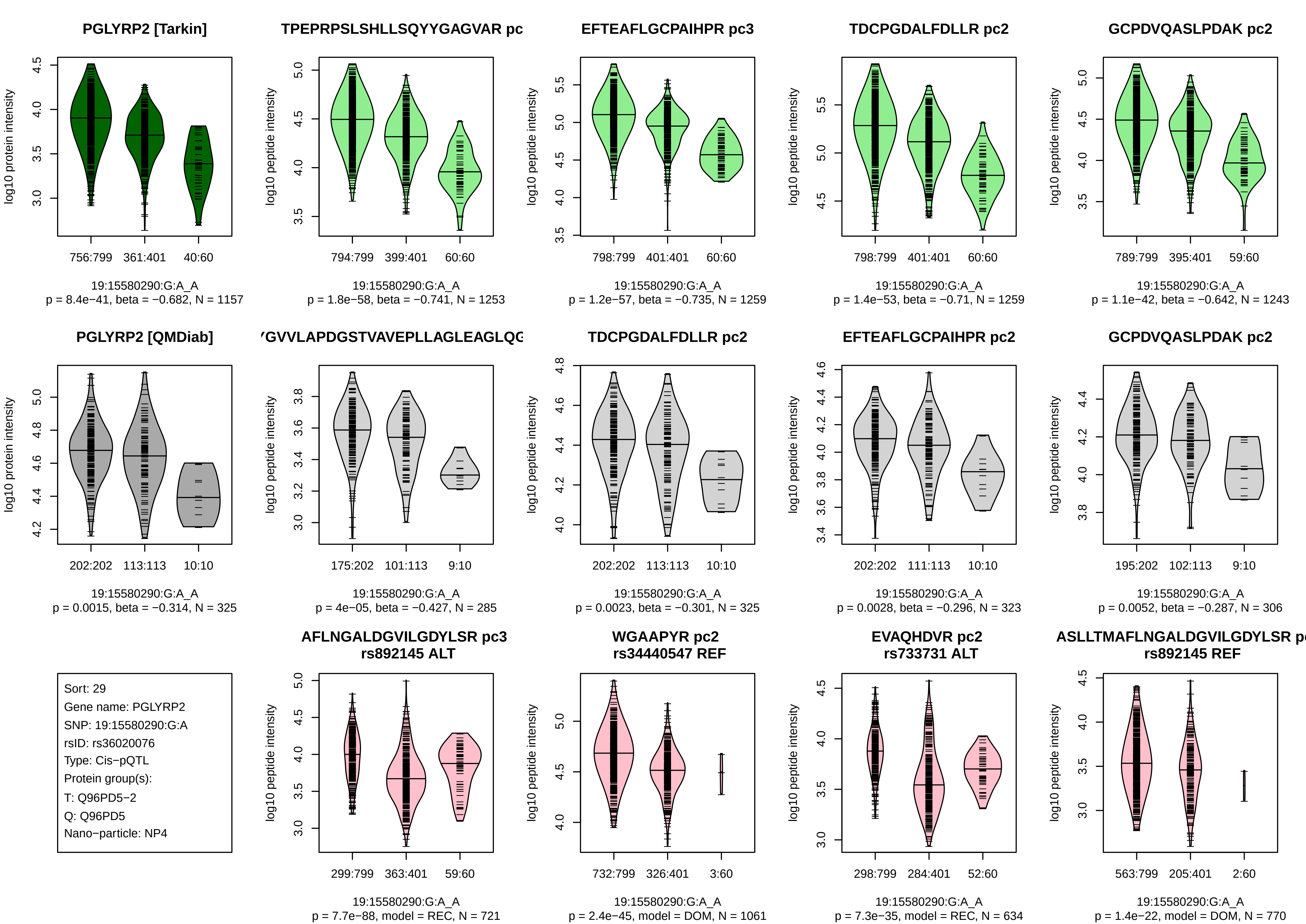

PGLYRP2 [Tarkin]
log10 protein intensity
756:799 361:401 40:60
19:15580290:G:A_A
p = 8.4e−41, beta = −0.682, N = 1157
TPEPRPSLSHLLSQYYGAGVAR pc
log10 peptide intensity
794:799 399:401 60:60
19:15580290:G:A_A
p = 1.8e−58, beta = −0.741, N = 1253
EFTEAFLGCPAIHPR pc3
log10 peptide intensity
798:799 401:401 60:60
19:15580290:G:A_A
p = 1.2e−57, beta = −0.735, N = 1259
TDCPGDALFDLLR pc2
log10 peptide intensity
798:799 401:401 60:60
19:15580290:G:A_A
p = 1.4e−53, beta = −0.71, N = 1259
GCPDVQASLPDAK pc2
log10 peptide intensity
789:799 395:401 59:60
19:15580290:G:A_A
p = 1.1e−42, beta = −0.642, N = 1243
PGLYRP2 [QMDiab]
log10 protein intensity
202:202 113:113 10:10
19:15580290:G:A_A
p = 0.0015, beta = −0.314, N = 325
'GVVLAPDGSTVAVEPLLAGLEAGLQG
log10 peptide intensity
175:202 101:113 9:10
19:15580290:G:A_A
p = 4e−05, beta = −0.427, N = 285
TDCPGDALFDLLR pc2
log10 peptide intensity
202:202 113:113 10:10
19:15580290:G:A_A
p = 0.0023, beta = −0.301, N = 325
EFTEAFLGCPAIHPR pc2
log10 peptide intensity
202:202 111:113 10:10
19:15580290:G:A_A
p = 0.0028, beta = −0.296, N = 323
GCPDVQASLPDAK pc2
log10 peptide intensity
195:202 102:113 9:10
19:15580290:G:A_A
p = 0.0052, beta = −0.287, N = 306
Sort: 29
Gene name: PGLYRP2
SNP: 19:15580290:G:A
rsID: rs36020076
Type: Cis−pQTL
Protein group(s):
T: Q96PD5−2
Q: Q96PD5
Nano−particle: NP4
AFLNGALDGVILGDYLSR pc3
rs892145 ALT
log10 peptide intensity
299:799 363:401 59:60
19:15580290:G:A_A
p = 7.7e−88, model = REC, N = 721
WGAAPYR pc2
rs34440547 REF
log10 peptide intensity
732:799 326:401 3:60
19:15580290:G:A_A
p = 2.4e−45, model = DOM, N = 1061
EVAQHDVR pc2
rs733731 ALT
log10 peptide intensity
298:799 284:401 52:60
19:15580290:G:A_A
p = 7.3e−35, model = REC, N = 634
ASLLTMAFLNGALDGVILGDYLSR pc
rs892145 REF
log10 peptide intensity
563:799 205:401 2:60
19:15580290:G:A_A
p = 1.4e−22, model = DOM, N = 770

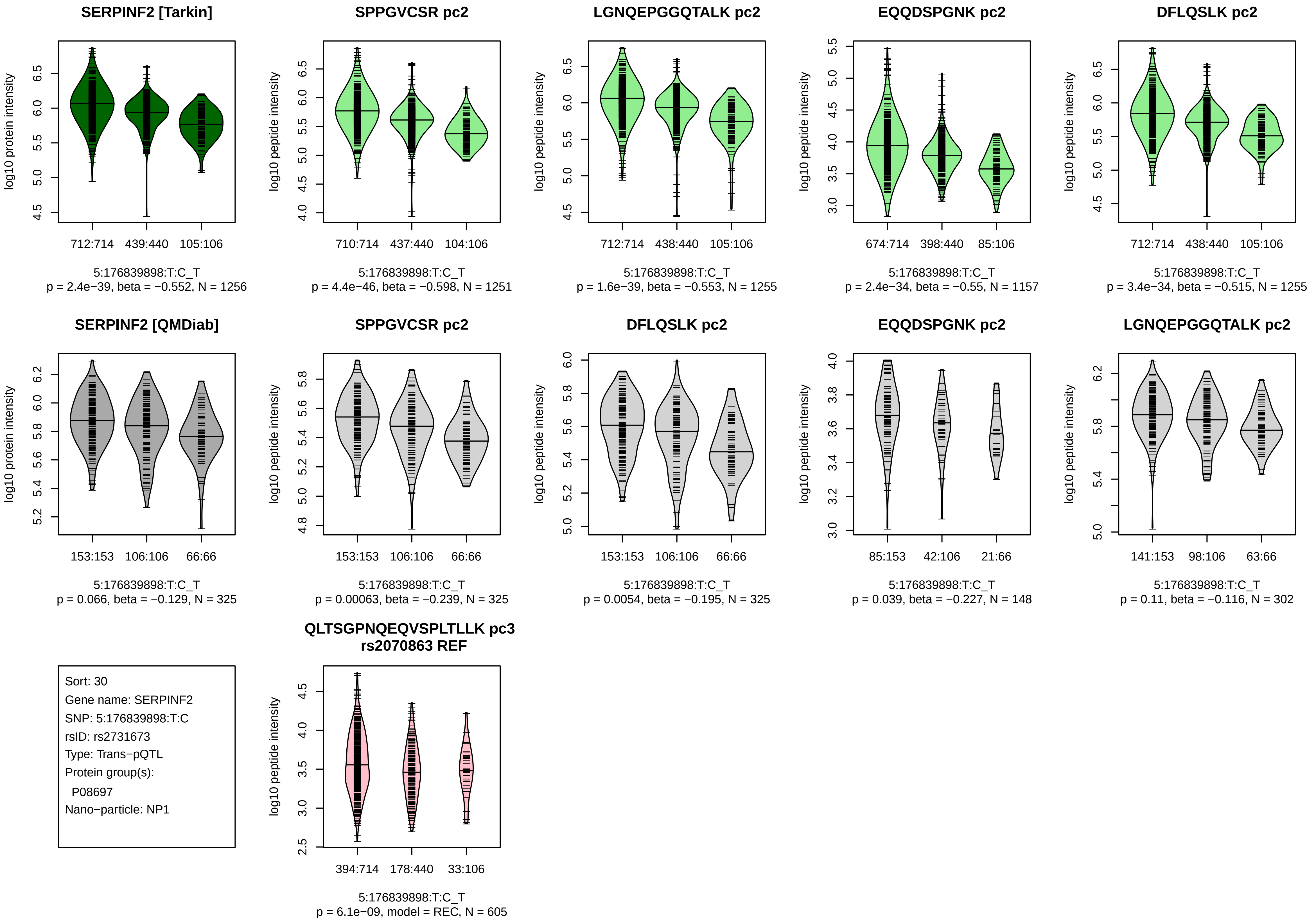
SERPINF2 [Tarkin]
log10 protein intensity
712:714
439:440
105:106
5:176839898:T:C_T
p = 2.4e−39, beta = −0.552, N = 1256
SPPGVCSR pc2
log10 peptide intensity
710:714
437:440
104:106
5:176839898:T:C_T
p = 4.4e−46, beta = −0.598, N = 1251
LGNQEPGGQTALK pc2
712:714
438:440
105:106
5:176839898:T:C_T
p = 1.6e−39, beta = −0.553, N = 1255
EQQDSPGNK pc2
674:714
398:440
85:106
5:176839898:T:C_T
p = 2.4e−34, beta = −0.55, N = 1157
DFLQSLK pc2
712:714
438:440
105:106
5:176839898:T:C_T
p = 3.4e−34, beta = −0.515, N = 1255
SERPINF2 [QMDiab]
153:153
106:106
66:66
5:176839898:T:C_T
p = 0.066, beta = −0.129, N = 325
SPPGVCSR pc2
153:153
106:106
66:66
5:176839898:T:C_T
p = 0.00063, beta = −0.239, N = 325
DFLQSLK pc2
153:153
106:106
66:66
5:176839898:T:C_T
p = 0.0054, beta = −0.195, N = 325
EQQDSPGNK pc2
85:153
42:106
21:66
5:176839898:T:C_T
p = 0.039, beta = −0.227, N = 148
LGNQEPGGQTALK pc2
141:153
98:106
63:66
5:176839898:T:C_T
p = 0.11, beta = −0.116, N = 302
Sort: 30
Gene name: SERPINF2
SNP: 5:176839898:T:C
rsID: rs2731673
Type: Trans−pQTL
Protein group(s):
P08697
Nano−particle: NP1
QLTSGPNQEQVSPLTLLK pc3
rs2070863 REF
394:714
178:440
33:106
5:176839898:T:C_T
p = 6.1e−09, model = REC, N = 605

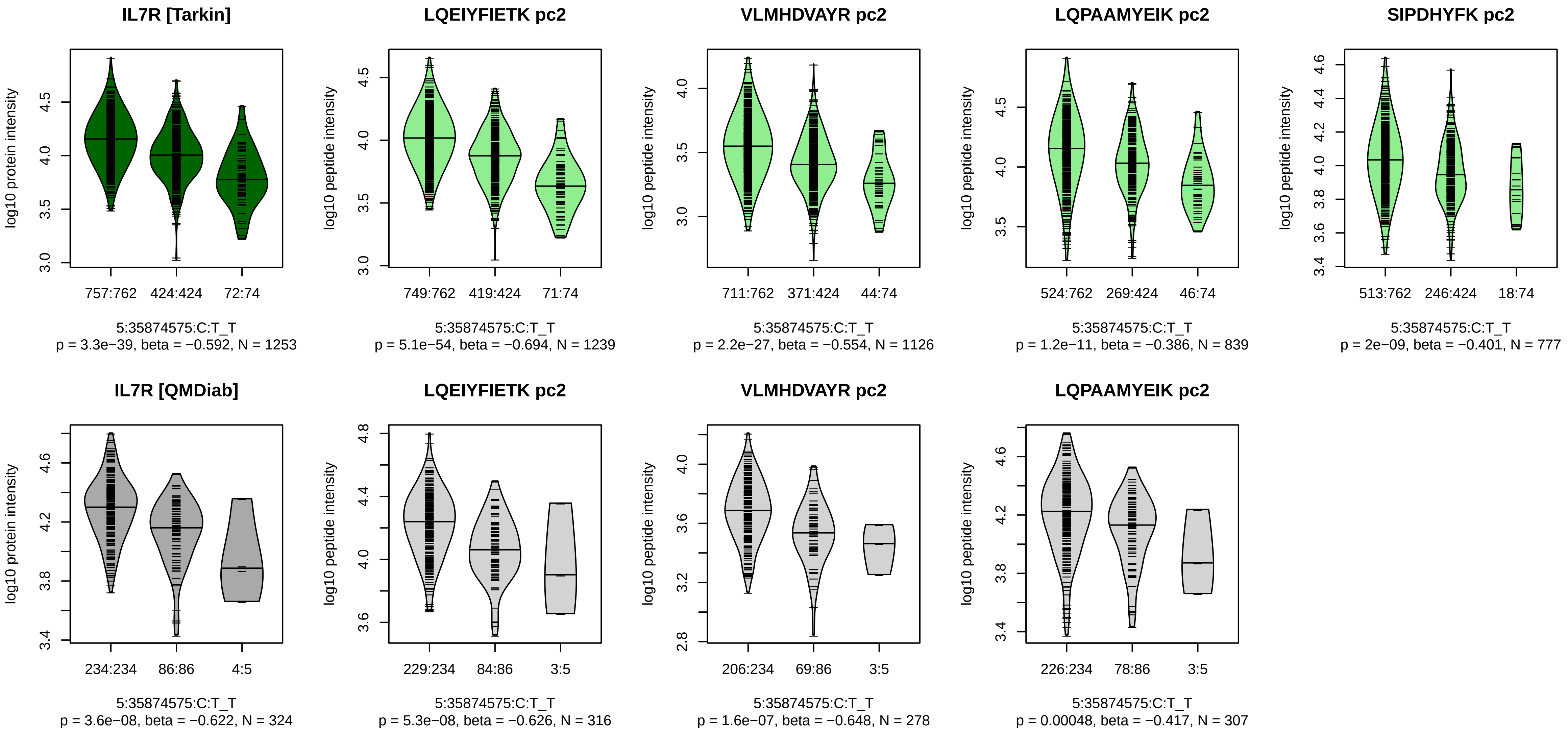

Sort: 31
Gene name: IL7R
SNP: 5:35874575:C:T
rsID: rs6897932
Type: Cis−pQTL
Protein group(s):
T: P16871
Q: P16871−3
Nano−particle: NP2

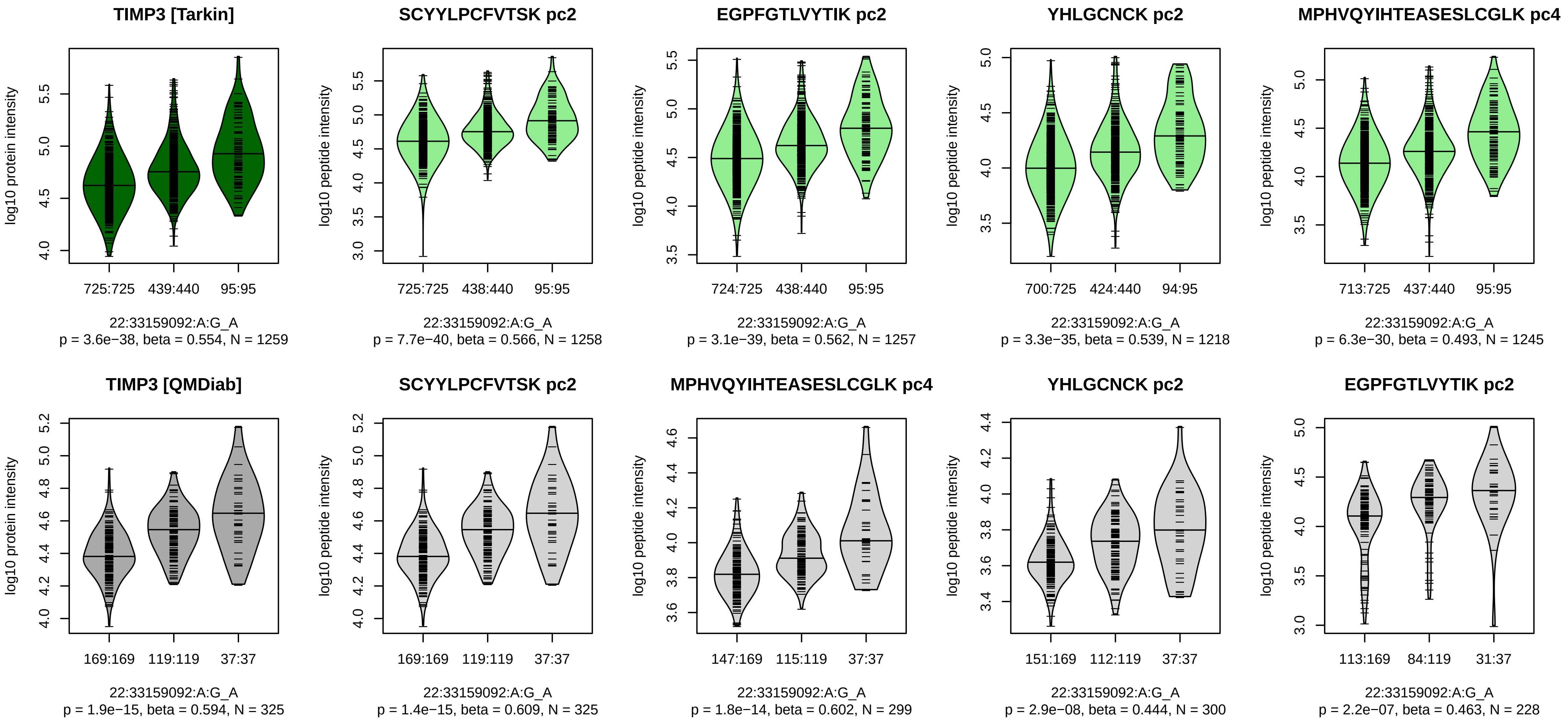

Sort: 32
Gene name: TIMP3
SNP: 22:33159092:A:G
rsID: rs4821097
Type: Cis−pQTL
Protein group(s):
P35625
Nano−particle: NP4

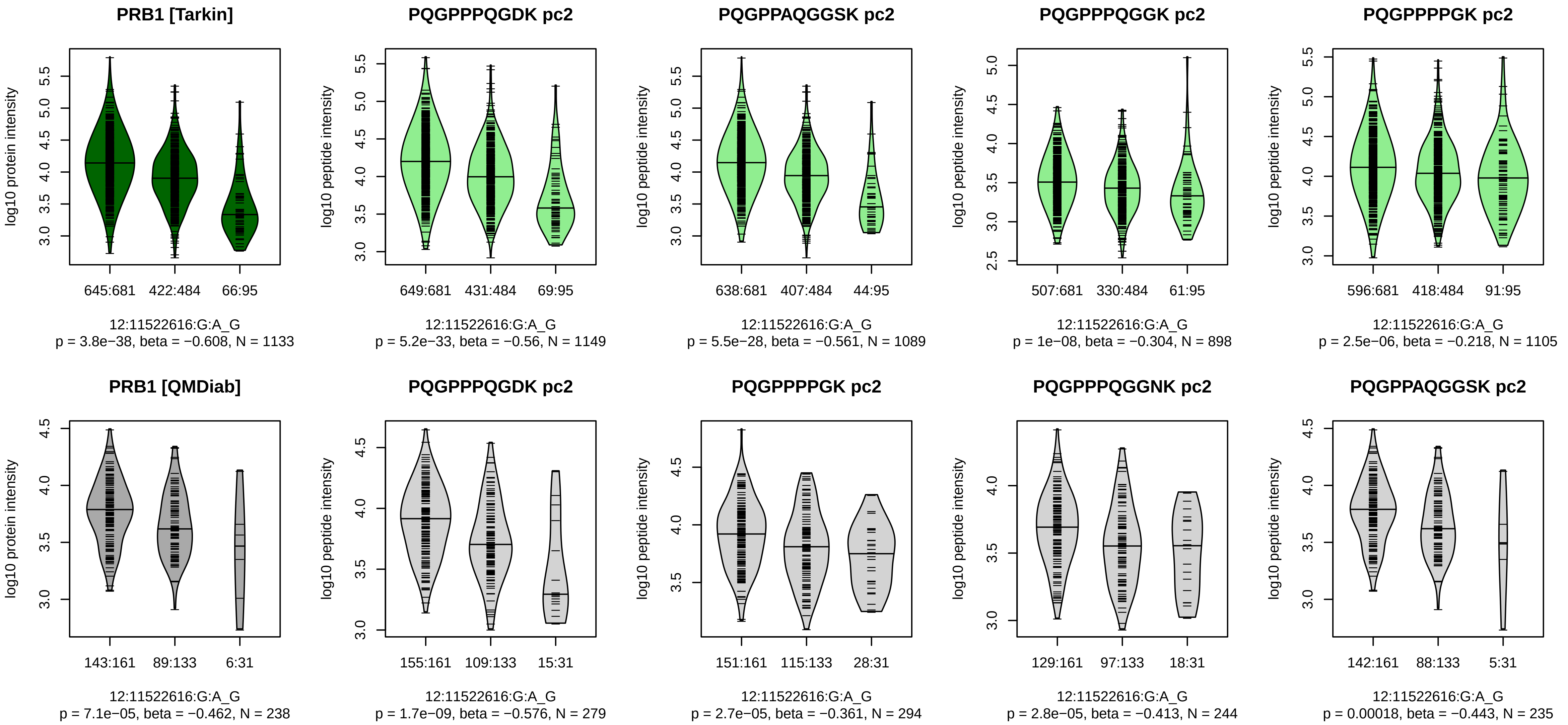

Sort: 33
Gene name: PRB1
SNP: 12:11522616:G:A
rsID: rs7966710
Type: Cis−pQTL
Protein group(s):
T: A0A4W8X8U3
Q: A0A4W8X8U3;G3V1R1
Nano−particle: NP4

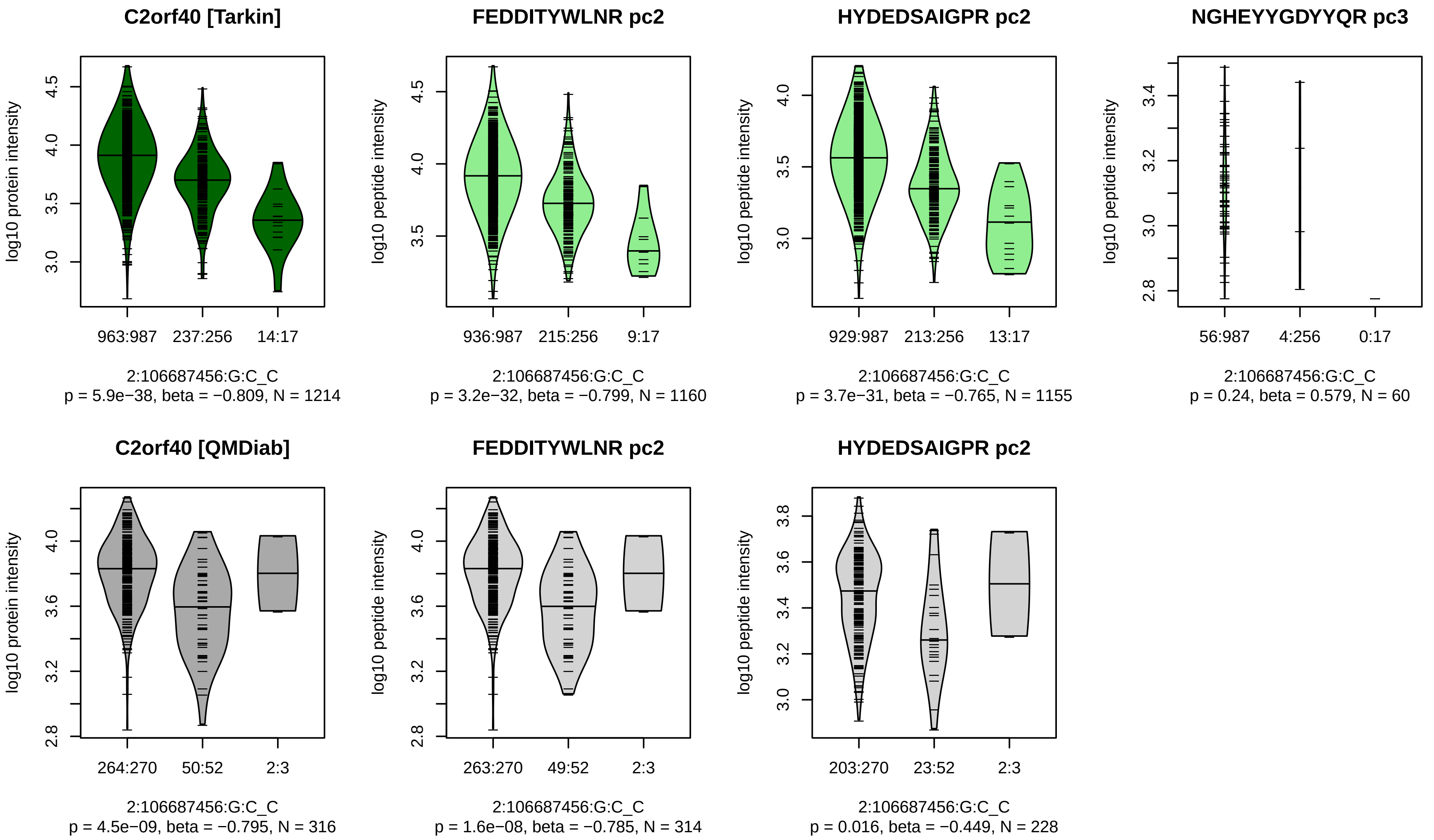

Sort: 34
Gene name: C2orf40
SNP: 2:106687456:G:C
rsID: rs13014521
Type: Cis−pQTL
Protein group(s):
B8ZZE5;Q9H1Z8
Nano−particle: NP2

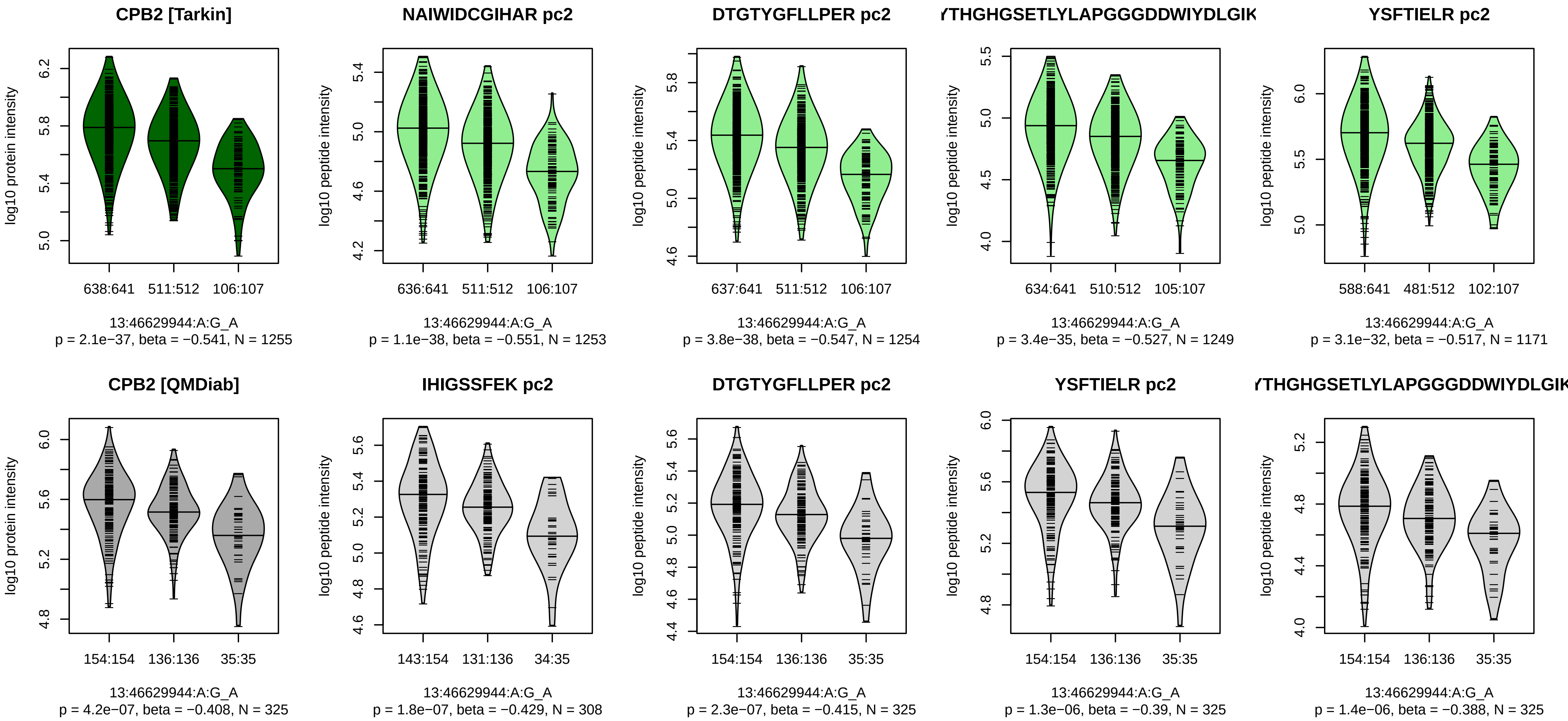

Sort: 35
Gene name: CPB2
SNP: 13:46629944:A:G
rsID: rs1926447
Type: Cis−pQTL
Protein group(s):
Q96IY4
Nano−particle: NP3

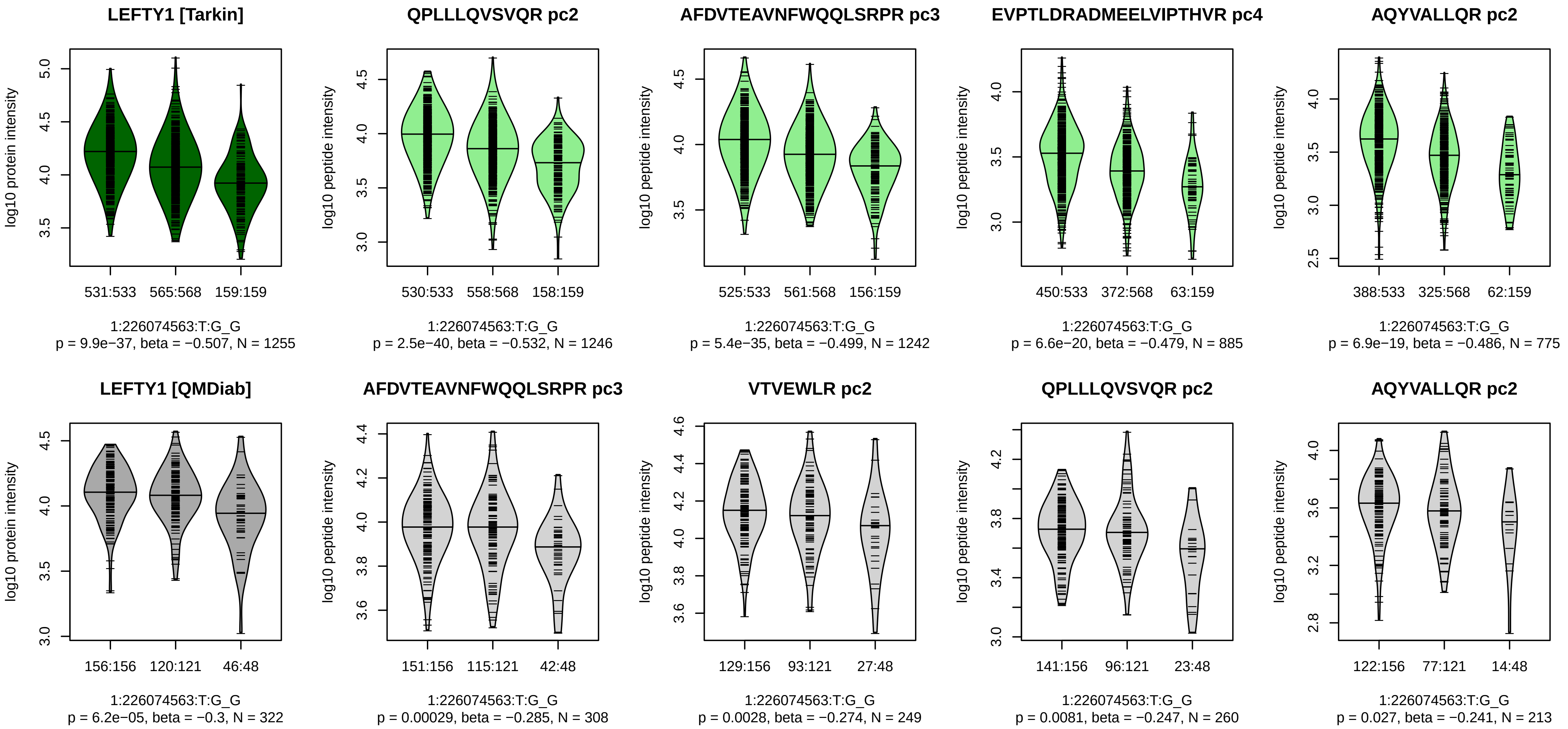

Sort: 36
Gene name: LEFTY1
SNP: 1:226074563:T:G
rsID: rs360057
Type: Cis−pQTL
Protein group(s):
O75610
Nano−particle: NP1

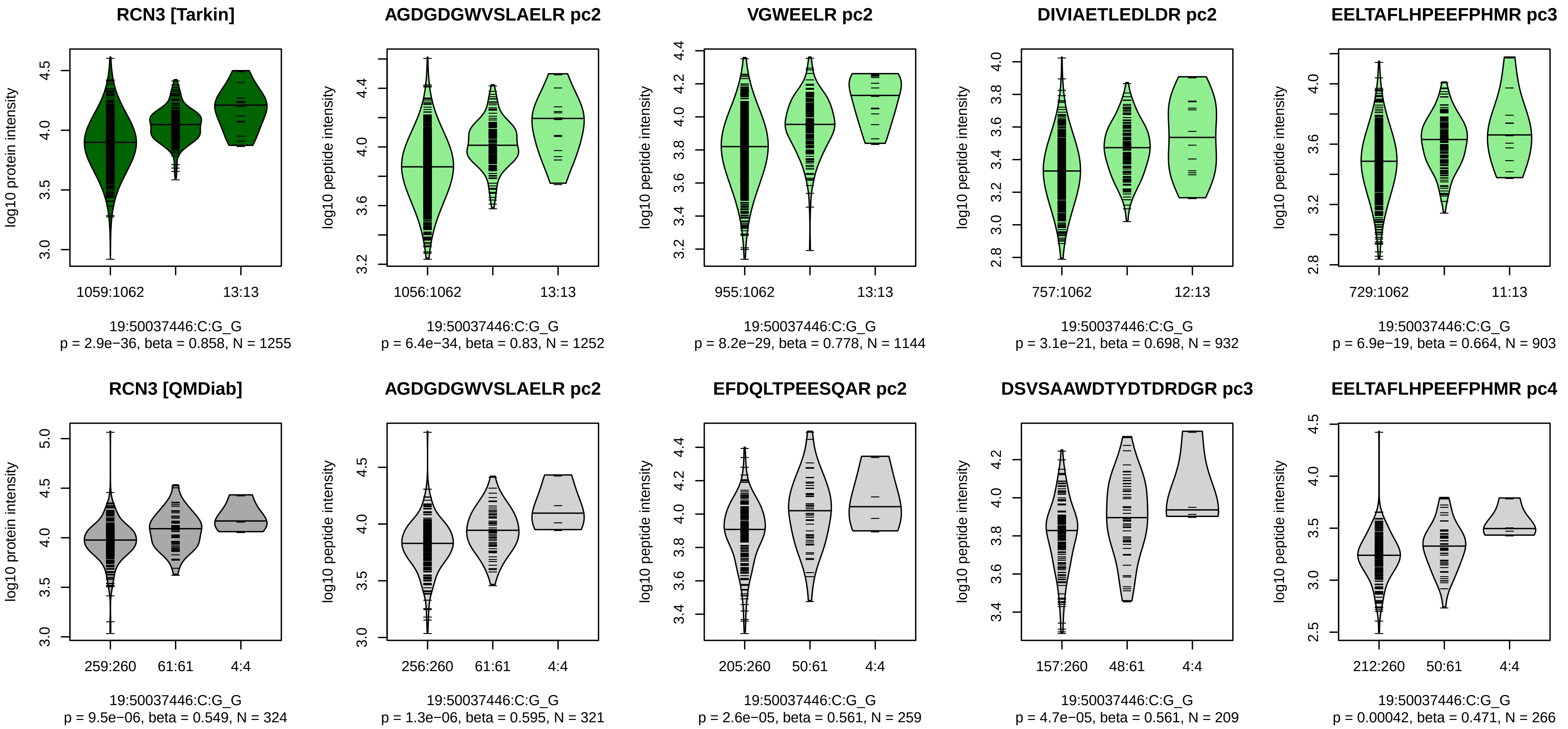

Sort: 37
Gene name: RCN3
SNP: 19:50037446:C:G
rsID: rs73582463
Type: Cis−pQTL
Protein group(s):
Q96D15
Nano−particle: NP2

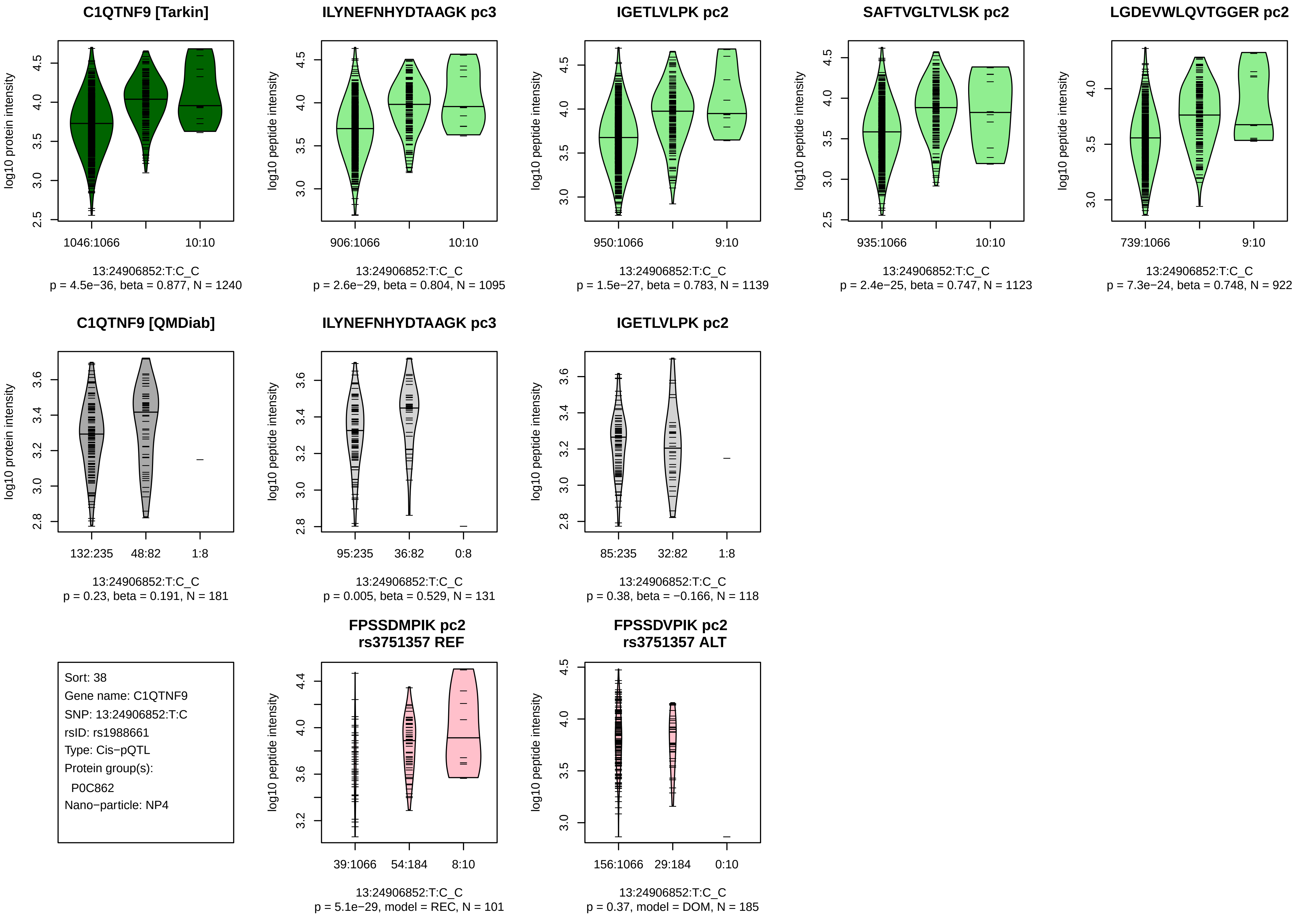
C1QTNF9 [Tarkin]
log10 protein intensity
1046:1066
10:10
13:24906852:T:C_C
p = 4.5e−36, beta = 0.877, N = 1240
ILYNEFNHYDTAAGK pc3
log10 peptide intensity
906:1066
10:10
13:24906852:T:C_C
p = 2.6e−29, beta = 0.804, N = 1095
IGETLVLPK pc2
log10 peptide intensity
950:1066
9:10
13:24906852:T:C_C
p = 1.5e−27, beta = 0.783, N = 1139
SAFTVGLTVLSK pc2
log10 peptide intensity
935:1066
10:10
13:24906852:T:C_C
p = 2.4e−25, beta = 0.747, N = 1123
LGDEVWLQVTGGER pc2
log10 peptide intensity
739:1066
9:10
13:24906852:T:C_C
p = 7.3e−24, beta = 0.748, N = 922
C1QTNF9 [QMDiab]
log10 protein intensity
132:235
48:82
1:8
13:24906852:T:C_C
p = 0.23, beta = 0.191, N = 181
ILYNEFNHYDTAAGK pc3
log10 peptide intensity
95:235
36:82
0:8
13:24906852:T:C_C
p = 0.005, beta = 0.529, N = 131
IGETLVLPK pc2
log10 peptide intensity
85:235
32:82
1:8
13:24906852:T:C_C
p = 0.38, beta = −0.166, N = 118
Sort: 38
Gene name: C1QTNF9
SNP: 13:24906852:T:C
rsID: rs1988661
Type: Cis−pQTL
Protein group(s):
P0C862
Nano−particle: NP4
FPSSDMPIK pc2
rs3751357 REF
log10 peptide intensity
39:1066
54:184
8:10
13:24906852:T:C_C
p = 5.1e−29, model = REC, N = 101
FPSSDVPIK pc2
rs3751357 ALT
log10 peptide intensity
156:1066
29:184
0:10
13:24906852:T:C_C
p = 0.37, model = DOM, N = 185

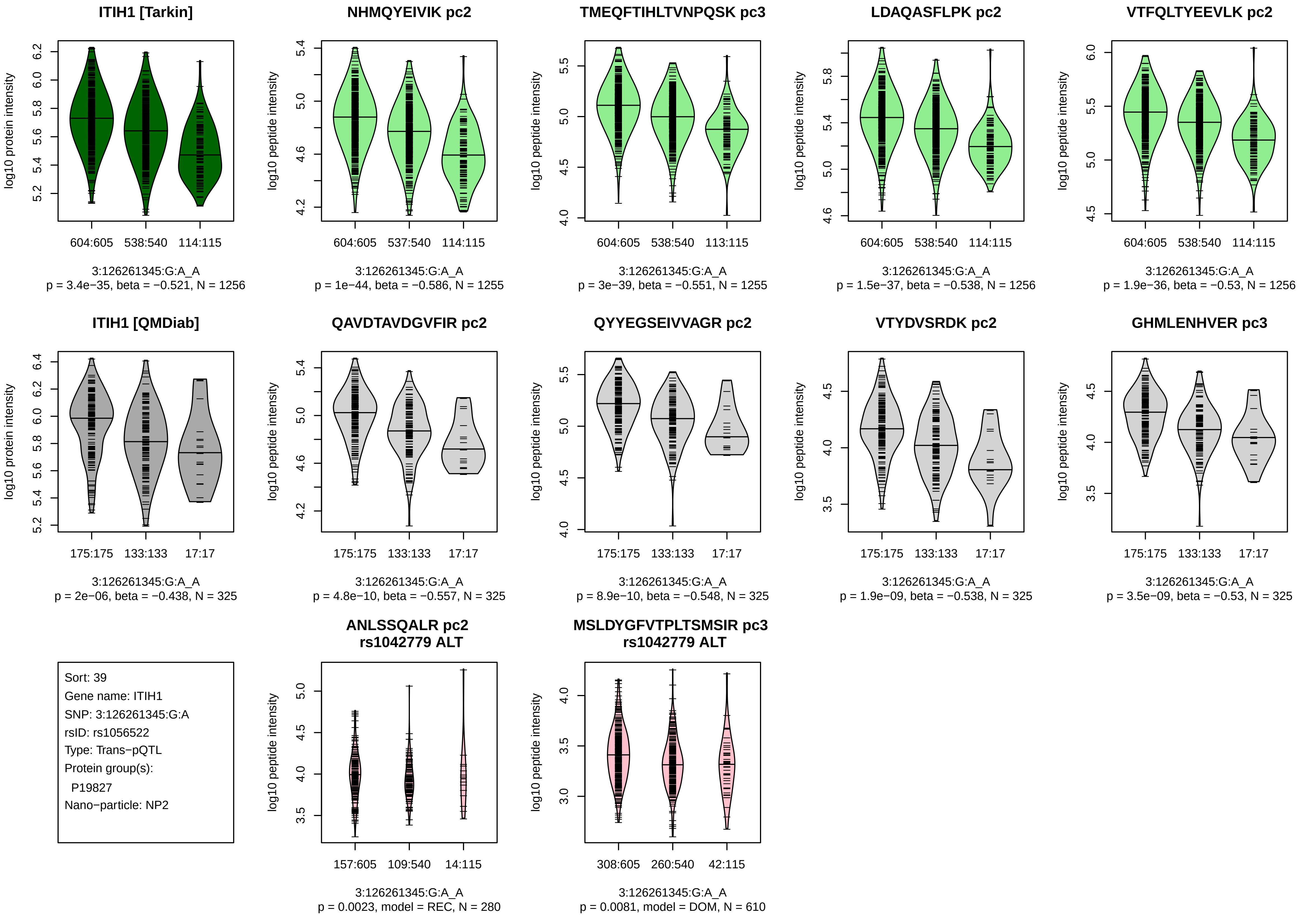
ITIH1 [Tarkin]
log10 protein intensity
604:605
538:540
114:115
3:126261345:G:A_A
p = 3.4e−35, beta = −0.521, N = 1256
NHMQYEIVIK pc2
log10 peptide intensity
604:605
537:540
114:115
3:126261345:G:A_A
p = 1e−44, beta = −0.586, N = 1255
TMEQFTIHLTVNPQSK pc3
log10 peptide intensity
604:605
538:540
113:115
3:126261345:G:A_A
p = 3e−39, beta = −0.551, N = 1255
LDAQASFLPK pc2
log10 peptide intensity
604:605
538:540
114:115
3:126261345:G:A_A
p = 1.5e−37, beta = −0.538, N = 1256
VTFQLTYEEVLK pc2
log10 peptide intensity
604:605
538:540
114:115
3:126261345:G:A_A
p = 1.9e−36, beta = −0.53, N = 1256
ITIH1 [QMDiab]
log10 protein intensity
175:175
133:133
17:17
3:126261345:G:A_A
p = 2e−06, beta = −0.438, N = 325
QAVDTAVDGVFIR pc2
log10 peptide intensity
175:175
133:133
17:17
3:126261345:G:A_A
p = 4.8e−10, beta = −0.557, N = 325
QYYEGSEIVVAGR pc2
log10 peptide intensity
175:175
133:133
17:17
3:126261345:G:A_A
p = 8.9e−10, beta = −0.548, N = 325
VTYDVSRDK pc2
log10 peptide intensity
175:175
133:133
17:17
3:126261345:G:A_A
p = 1.9e−09, beta = −0.538, N = 325
GHMLENHVER pc3
log10 peptide intensity
175:175
133:133
17:17
3:126261345:G:A_A
p = 3.5e−09, beta = −0.53, N = 325
Sort: 39
Gene name: ITIH1
SNP: 3:126261345:G:A
rsID: rs1056522
Type: Trans−pQTL
Protein group(s):
P19827
Nano−particle: NP2
ANLSSQALR pc2
rs1042779 ALT
log10 peptide intensity
157:605
109:540
14:115
3:126261345:G:A_A
p = 0.0023, model = REC, N = 280
MSLDYGFVTPLTSMSIR pc3
rs1042779 ALT
log10 peptide intensity
308:605
260:540
42:115
3:126261345:G:A_A
p = 0.0081, model = DOM, N = 610

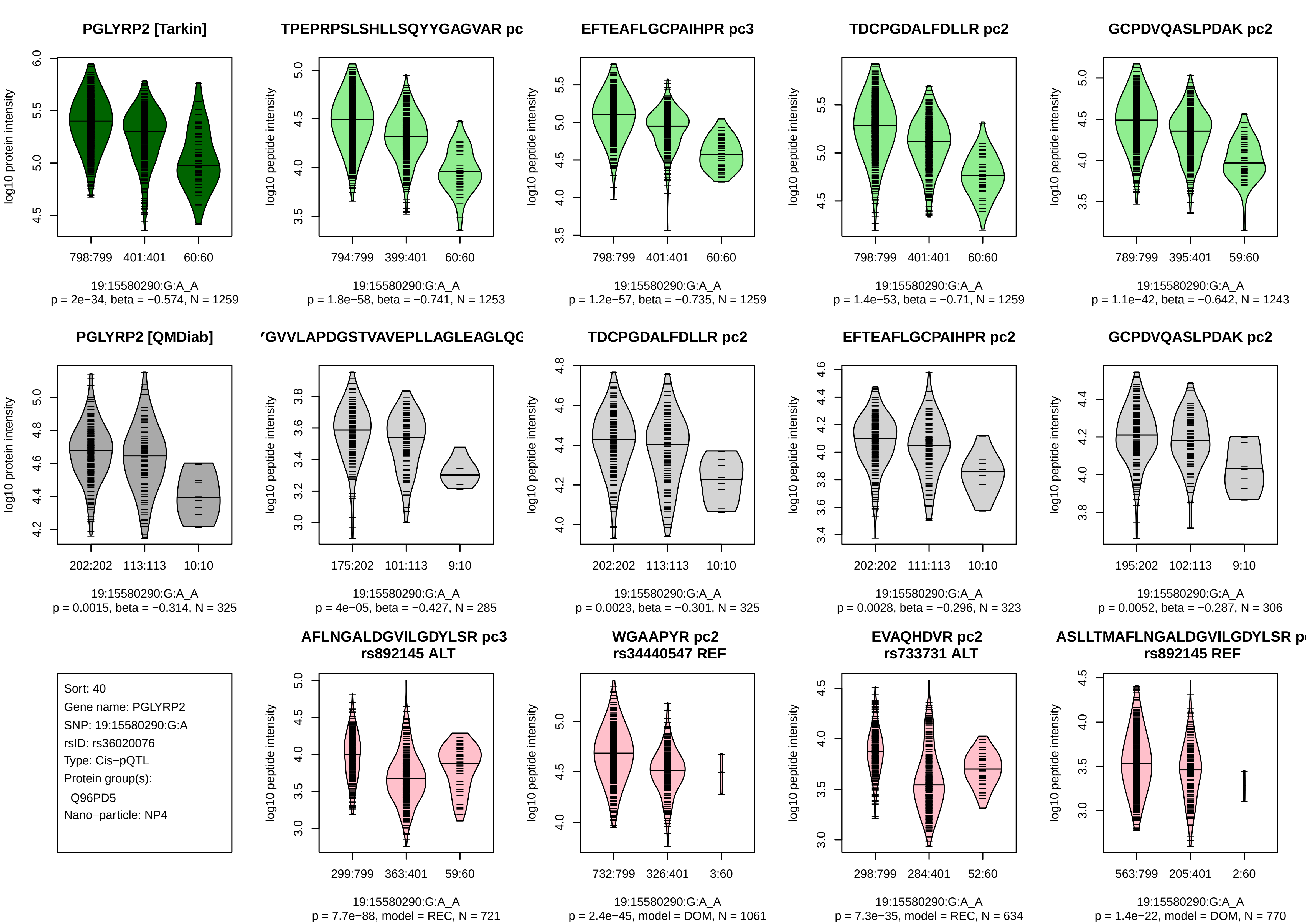
PGLYRP2 [Tarkin]
log10 protein intensity
798:799
401:401
60:60
19:15580290:G:A_A
p = 2e−34, beta = −0.574, N = 1259
TPEPRPSLSHLLSQYYGAGVAR pc
log10 peptide intensity
794:799
399:401
60:60
19:15580290:G:A_A
p = 1.8e−58, beta = −0.741, N = 1253
EFTEAFLGCPAIHPR pc3
log10 peptide intensity
798:799
401:401
60:60
19:15580290:G:A_A
p = 1.2e−57, beta = −0.735, N = 1259
TDCPGDALFDLLR pc2
log10 peptide intensity
798:799
401:401
60:60
19:15580290:G:A_A
p = 1.4e−53, beta = −0.71, N = 1259
GCPDVQASLPDAK pc2
log10 peptide intensity
789:799
395:401
59:60
19:15580290:G:A_A
p = 1.1e−42, beta = −0.642, N = 1243
PGLYRP2 [QMDiab]
log10 protein intensity
202:202
113:113
10:10
19:15580290:G:A_A
p = 0.0015, beta = −0.314, N = 325
'GVVLAPDGSTVAVEPLLAGLEAGLQG
log10 peptide intensity
175:202
101:113
9:10
19:15580290:G:A_A
p = 4e−05, beta = −0.427, N = 285
TDCPGDALFDLLR pc2
log10 peptide intensity
202:202
113:113
10:10
19:15580290:G:A_A
p = 0.0023, beta = −0.301, N = 325
EFTEAFLGCPAIHPR pc2
log10 peptide intensity
202:202
111:113
10:10
19:15580290:G:A_A
p = 0.0028, beta = −0.296, N = 323
GCPDVQASLPDAK pc2
log10 peptide intensity
195:202
102:113
9:10
19:15580290:G:A_A
p = 0.0052, beta = −0.287, N = 306
Sort: 40
Gene name: PGLYRP2
SNP: 19:15580290:G:A
rsID: rs36020076
Type: Cis−pQTL
Protein group(s):
Q96PD5
Nano−particle: NP4
AFLNGALDGVILGDYLSR pc3
rs892145 ALT
log10 peptide intensity
299:799
363:401
59:60
19:15580290:G:A_A
p = 7.7e−88, model = REC, N = 721
WGAAPYR pc2
rs34440547 REF
log10 peptide intensity
732:799
326:401
3:60
19:15580290:G:A_A
p = 2.4e−45, model = DOM, N = 1061
EVAQHDVR pc2
rs733731 ALT
log10 peptide intensity
298:799
284:401
52:60
19:15580290:G:A_A
p = 7.3e−35, model = REC, N = 634
ASLLTMAFLNGALDGVILGDYLSR pc
rs892145 REF
log10 peptide intensity
563:799
205:401
2:60
19:15580290:G:A_A
p = 1.4e−22, model = DOM, N = 770

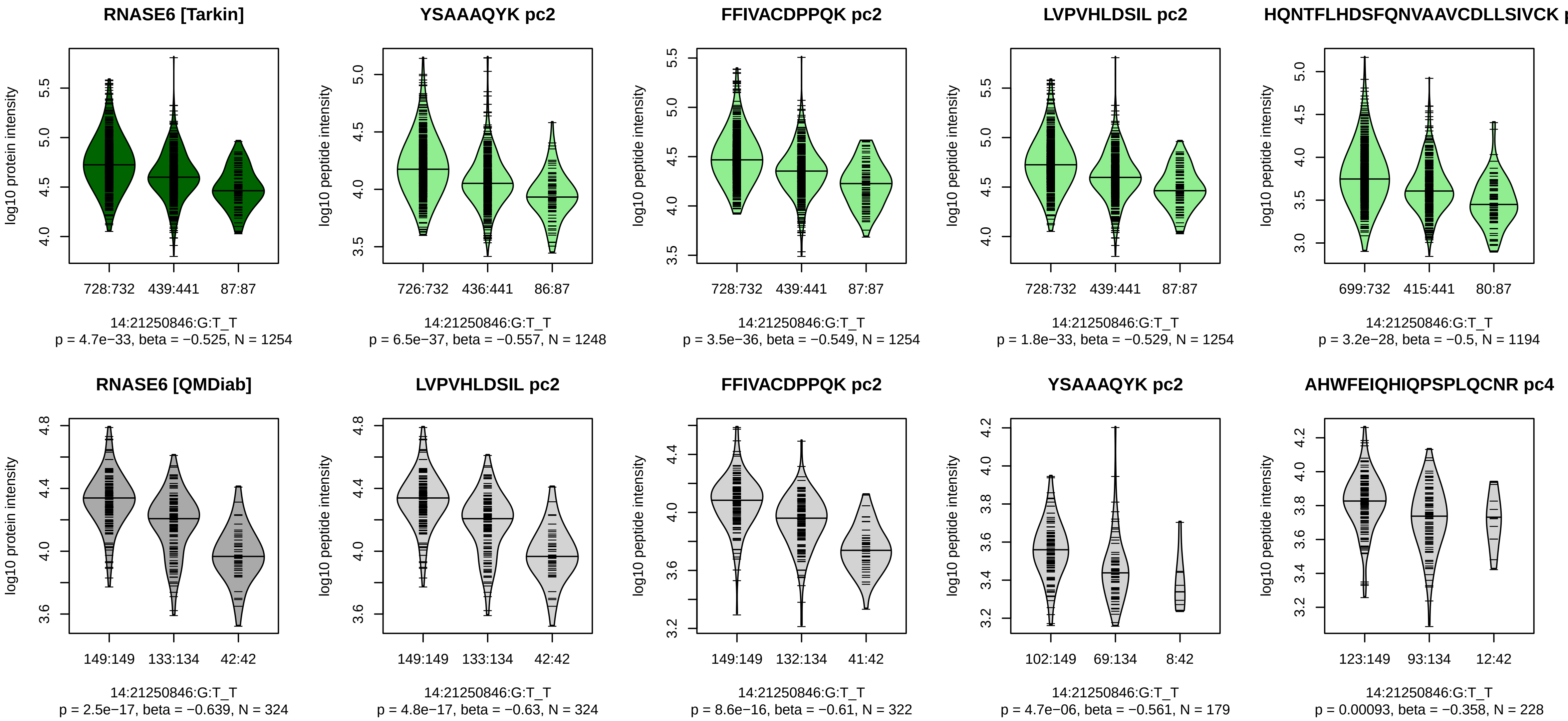

Sort: 41
Gene name: RNASE6
SNP: 14:21250846:G:T
rsID: rs11622942
Type: Cis−pQTL
Protein group(s):
Q93091
Nano−particle: NP3

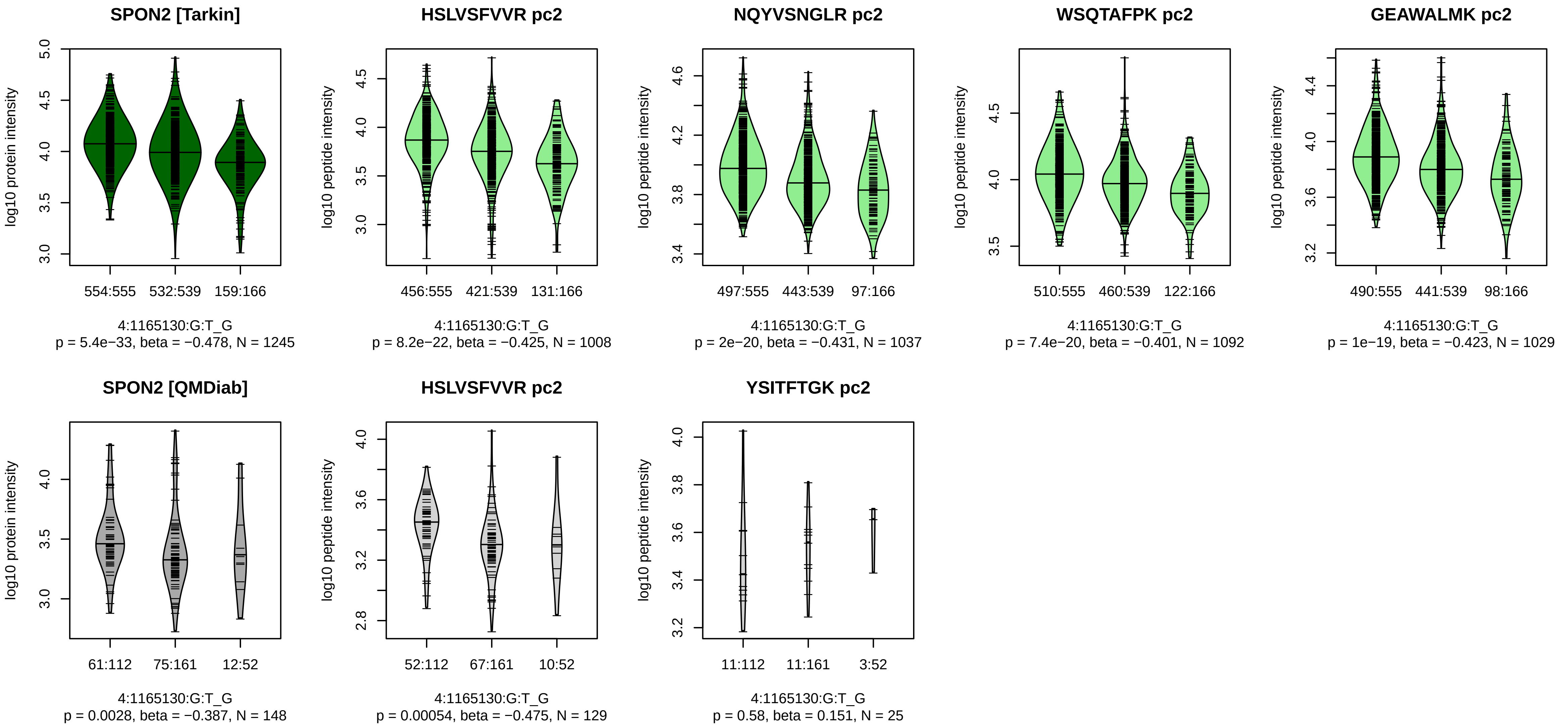

Sort: 42
Gene name: SPON2
SNP: 4:1165130:G:T
rsID: rs11247975
Type: Cis−pQTL
Protein group(s):
Q9BUD6
Nano−particle: NP5

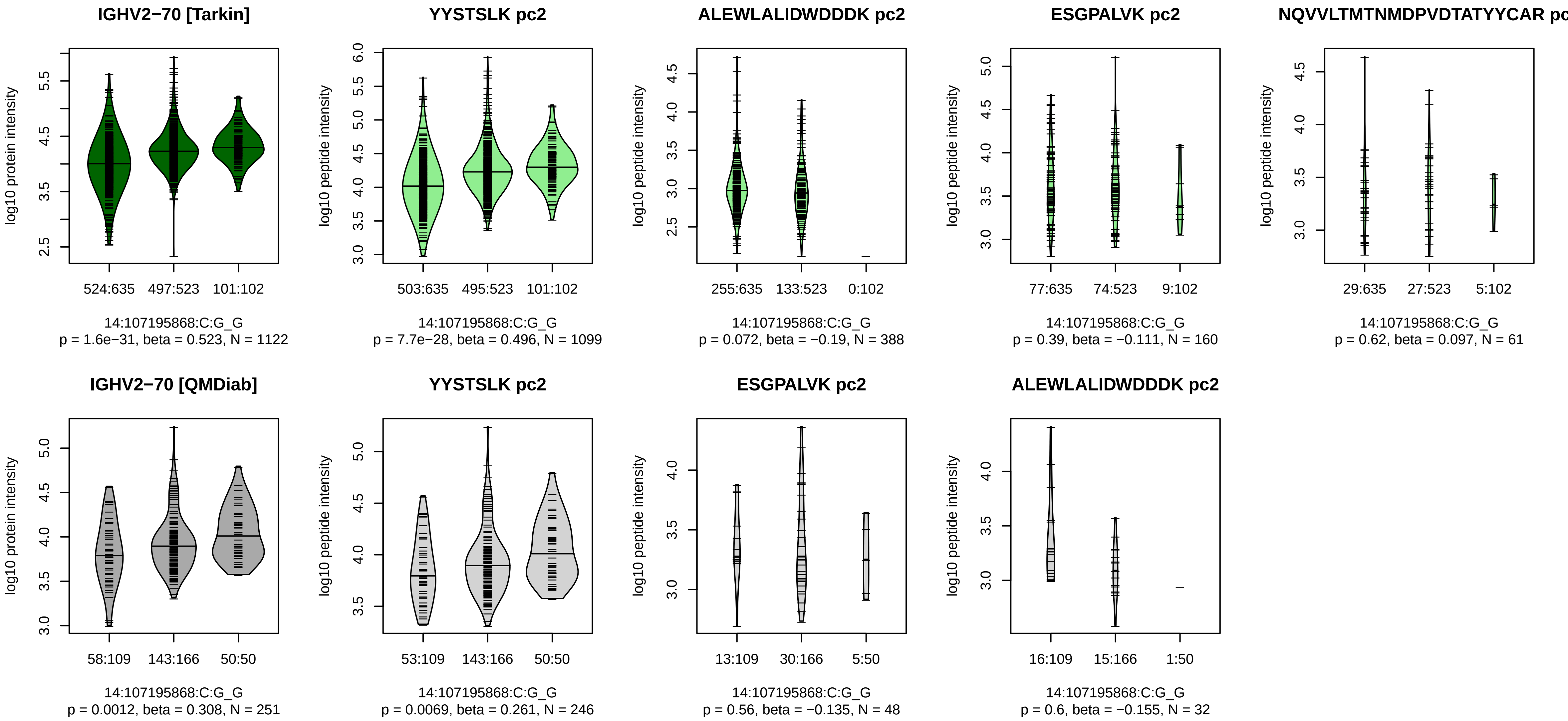

Sort: 43
Gene name: IGHV2−70
SNP: 14:107195868:C:G
rsID: rs10136560
Type: Cis−pQTL
Protein group(s):
P01814
Nano−particle: NP3

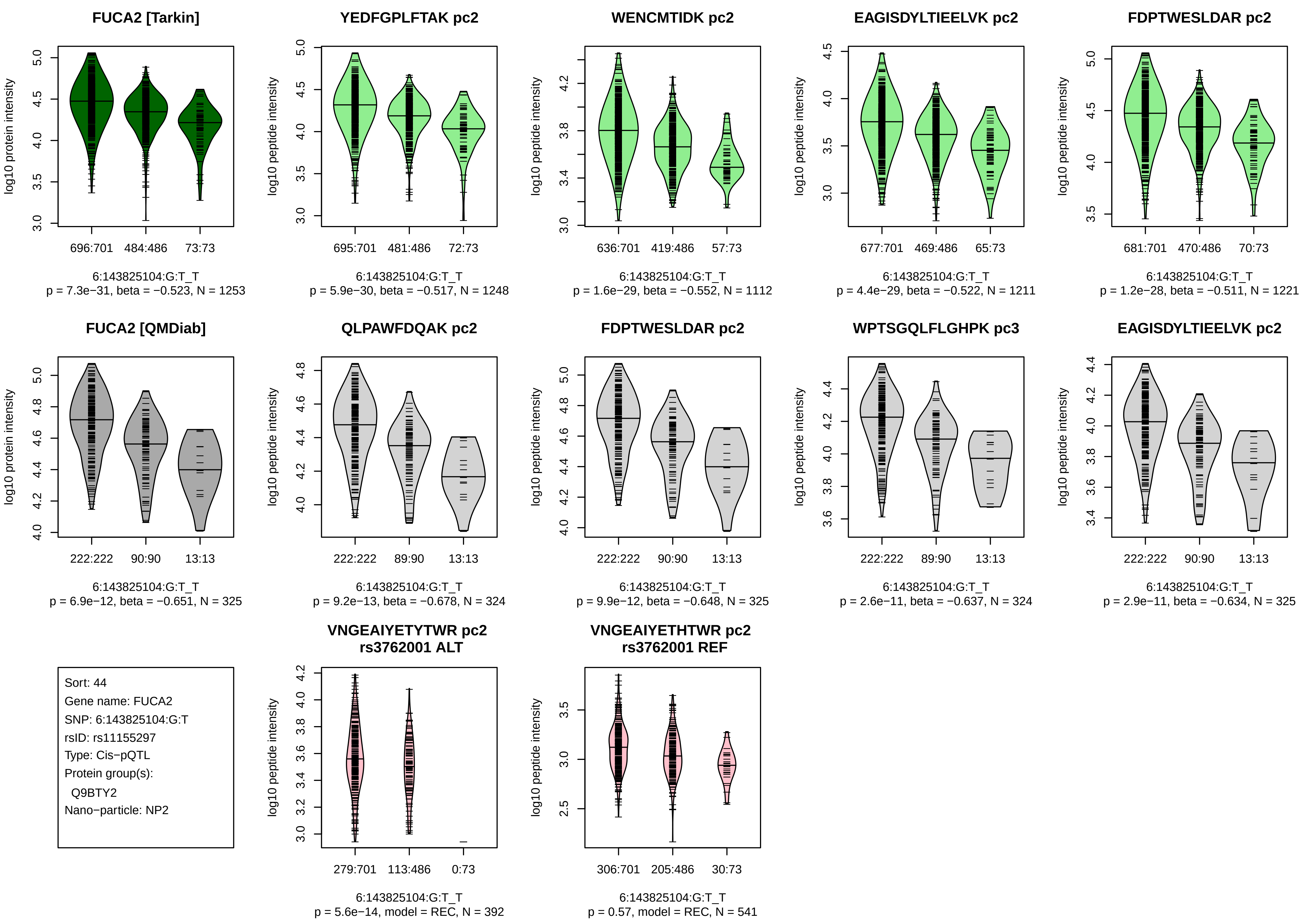
FUCA2 [Tarkin]
log10 protein intensity
696:701
484:486
73:73
6:143825104:G:T_T
p = 7.3e−31, beta = −0.523, N = 1253
YEDFGPLFTAK pc2
log10 peptide intensity
695:701
481:486
72:73
6:143825104:G:T_T
p = 5.9e−30, beta = −0.517, N = 1248
WENCMTIDK pc2
log10 peptide intensity
636:701
419:486
57:73
6:143825104:G:T_T
p = 1.6e−29, beta = −0.552, N = 1112
EAGISDYLTIEELVK pc2
log10 peptide intensity
677:701
469:486
65:73
6:143825104:G:T_T
p = 4.4e−29, beta = −0.522, N = 1211
FDPTWESLDAR pc2
log10 peptide intensity
681:701
470:486
70:73
6:143825104:G:T_T
p = 1.2e−28, beta = −0.511, N = 1221
FUCA2 [QMDiab]
log10 protein intensity
222:222
90:90
13:13
6:143825104:G:T_T
p = 6.9e−12, beta = −0.651, N = 325
QLPAWFDQAK pc2
log10 peptide intensity
222:222
89:90
13:13
6:143825104:G:T_T
p = 9.2e−13, beta = −0.678, N = 324
FDPTWESLDAR pc2
log10 peptide intensity
222:222
90:90
13:13
6:143825104:G:T_T
p = 9.9e−12, beta = −0.648, N = 325
WPTSGQLFLGHPK pc3
log10 peptide intensity
222:222
89:90
13:13
6:143825104:G:T_T
p = 2.6e−11, beta = −0.637, N = 324
EAGISDYLTIEELVK pc2
log10 peptide intensity
222:222
90:90
13:13
6:143825104:G:T_T
p = 2.9e−11, beta = −0.634, N = 325
Sort: 44
Gene name: FUCA2
SNP: 6:143825104:G:T
rsID: rs11155297
Type: Cis−pQTL
Protein group(s):
Q9BTY2
Nano−particle: NP2
VNGEAIYETYTWR pc2
rs3762001 ALT
log10 peptide intensity
279:701
113:486
0:73
6:143825104:G:T_T
p = 5.6e−14, model = REC, N = 392
VNGEAIYETHTWR pc2
rs3762001 REF
log10 peptide intensity
306:701
205:486
30:73
6:143825104:G:T_T
p = 0.57, model = REC, N = 541

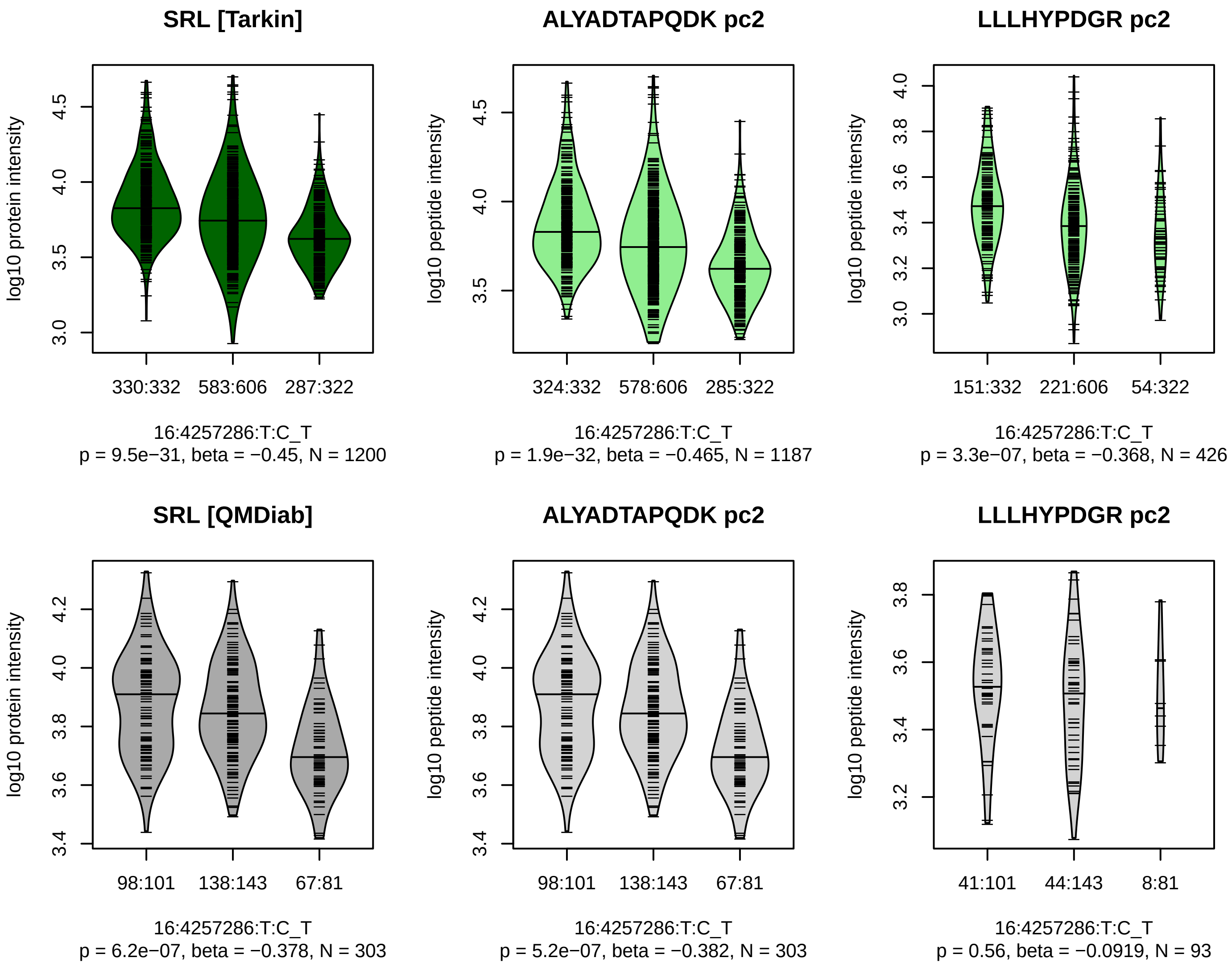

Sort: 45
Gene name: SRL
SNP: 16:4257286:T:C
rsID: rs9924822
Type: Cis−pQTL
Protein group(s):
I3L4D6
Nano−particle: NP2

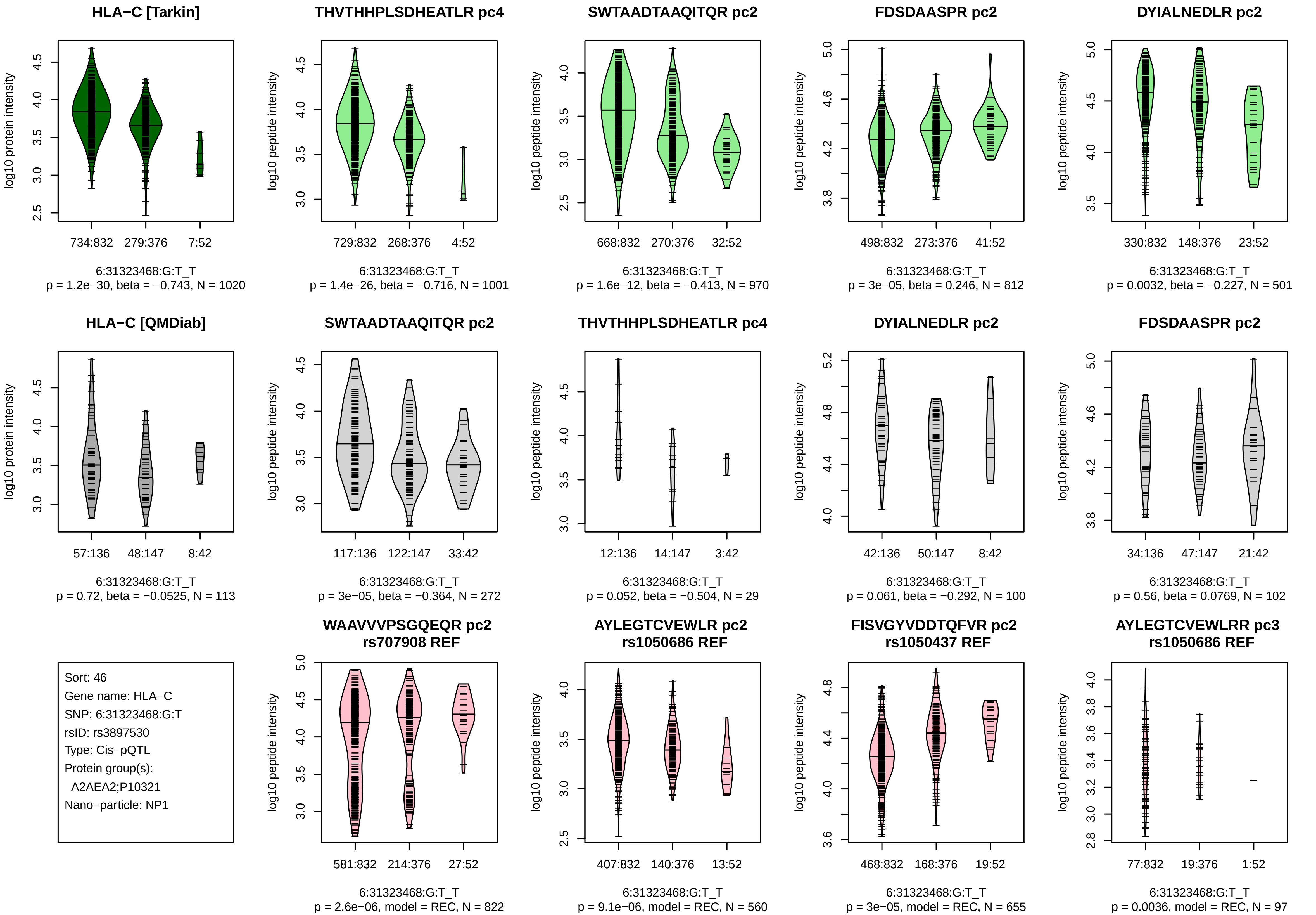
HLA−C [Tarkin]
log10 protein intensity
734:832
279:376
7:52
6:31323468:G:T_T
p = 1.2e−30, beta = −0.743, N = 1020
THVTHHPLSDHEATLR pc4
log10 peptide intensity
729:832
268:376
4:52
6:31323468:G:T_T
p = 1.4e−26, beta = −0.716, N = 1001
SWTAADTAAQITQR pc2
log10 peptide intensity
668:832
270:376
32:52
6:31323468:G:T_T
p = 1.6e−12, beta = −0.413, N = 970
FDSDAASPR pc2
log10 peptide intensity
498:832
273:376
41:52
6:31323468:G:T_T
p = 3e−05, beta = 0.246, N = 812
DYIALNEDLR pc2
log10 peptide intensity
330:832
148:376
23:52
6:31323468:G:T_T
p = 0.0032, beta = −0.227, N = 501
HLA−C [QMDiab]
log10 protein intensity
57:136
48:147
8:42
6:31323468:G:T_T
p = 0.72, beta = −0.0525, N = 113
SWTAADTAAQITQR pc2
log10 peptide intensity
117:136
122:147
33:42
6:31323468:G:T_T
p = 3e−05, beta = −0.364, N = 272
THVTHHPLSDHEATLR pc4
log10 peptide intensity
12:136
14:147
3:42
6:31323468:G:T_T
p = 0.052, beta = −0.504, N = 29
DYIALNEDLR pc2
log10 peptide intensity
42:136
50:147
8:42
6:31323468:G:T_T
p = 0.061, beta = −0.292, N = 100
FDSDAASPR pc2
log10 peptide intensity
34:136
47:147
21:42
6:31323468:G:T_T
p = 0.56, beta = 0.0769, N = 102
Sort: 46
Gene name: HLA−C
SNP: 6:31323468:G:T
rsID: rs3897530
Type: Cis−pQTL
Protein group(s):
A2AEA2;P10321
Nano−particle: NP1
WAAVVVPSGQEQR pc2
rs707908 REF
log10 peptide intensity
581:832
214:376
27:52
6:31323468:G:T_T
p = 2.6e−06, model = REC, N = 822
AYLEGTCVEWLR pc2
rs1050686 REF
log10 peptide intensity
407:832
140:376
13:52
6:31323468:G:T_T
p = 9.1e−06, model = REC, N = 560
FISVGYVDDTQFVR pc2
rs1050437 REF
log10 peptide intensity
468:832
168:376
19:52
6:31323468:G:T_T
p = 3e−05, model = REC, N = 655
AYLEGTCVEWLRR pc3
rs1050686 REF
log10 peptide intensity
77:832
19:376
1:52
6:31323468:G:T_T
p = 0.0036, model = REC, N = 97

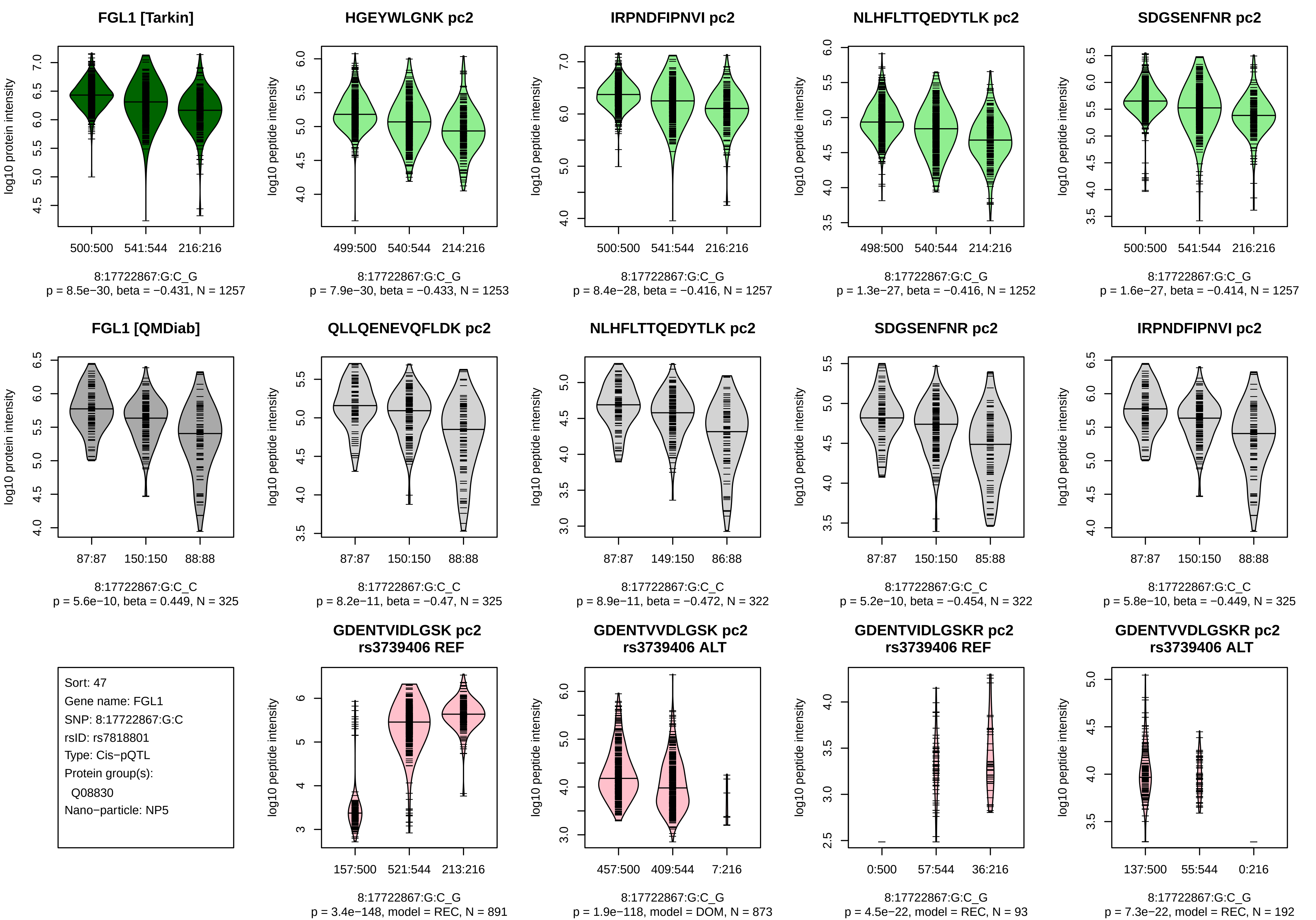
FGL1 [Tarkin]
log10 protein intensity
500:500 541:544 216:216
8:17722867:G:C_G
p = 8.5e−30, beta = −0.431, N = 1257
HGEYWLGNK pc2
log10 peptide intensity
499:500 540:544 214:216
8:17722867:G:C_G
p = 7.9e−30, beta = −0.433, N = 1253
IRPNDFIPNVI pc2
log10 peptide intensity
500:500 541:544 216:216
8:17722867:G:C_G
p = 8.4e−28, beta = −0.416, N = 1257
NLHFLTTQEDYTLK pc2
log10 peptide intensity
498:500 540:544 214:216
8:17722867:G:C_G
p = 1.3e−27, beta = −0.416, N = 1252
SDGSENFNR pc2
log10 peptide intensity
500:500 541:544 216:216
8:17722867:G:C_G
p = 1.6e−27, beta = −0.414, N = 1257
FGL1 [QMDiab]
log10 protein intensity
87:87 150:150 88:88
8:17722867:G:C_C
p = 5.6e−10, beta = 0.449, N = 325
QLLQENEVQFLDK pc2
log10 peptide intensity
87:87 150:150 88:88
8:17722867:G:C_C
p = 8.2e−11, beta = −0.47, N = 325
NLHFLTTQEDYTLK pc2
log10 peptide intensity
87:87 149:150 86:88
8:17722867:G:C_C
p = 8.9e−11, beta = −0.472, N = 322
SDGSENFNR pc2
log10 peptide intensity
87:87 150:150 85:88
8:17722867:G:C_C
p = 5.2e−10, beta = −0.454, N = 322
IRPNDFIPNVI pc2
log10 peptide intensity
87:87 150:150 88:88
8:17722867:G:C_C
p = 5.8e−10, beta = −0.449, N = 325
Sort: 47
Gene name: FGL1
SNP: 8:17722867:G:C
rsID: rs7818801
Type: Cis−pQTL
Protein group(s):
Q08830
Nano−particle: NP5
GDENTVIDLGSK pc2
rs3739406 REF
log10 peptide intensity
157:500 521:544 213:216
8:17722867:G:C_G
p = 3.4e−148, model = REC, N = 891
GDENTVVDLGSK pc2
rs3739406 ALT
log10 peptide intensity
457:500 409:544 7:216
8:17722867:G:C_G
p = 1.9e−118, model = DOM, N = 873
GDENTVIDLGSKR pc2
rs3739406 REF
log10 peptide intensity
0:500 57:544 36:216
8:17722867:G:C_G
p = 4.5e−22, model = REC, N = 93
GDENTVVDLGSKR pc2
rs3739406 ALT
log10 peptide intensity
137:500 55:544 0:216
8:17722867:G:C_G
p = 7.3e−22, model = REC, N = 192

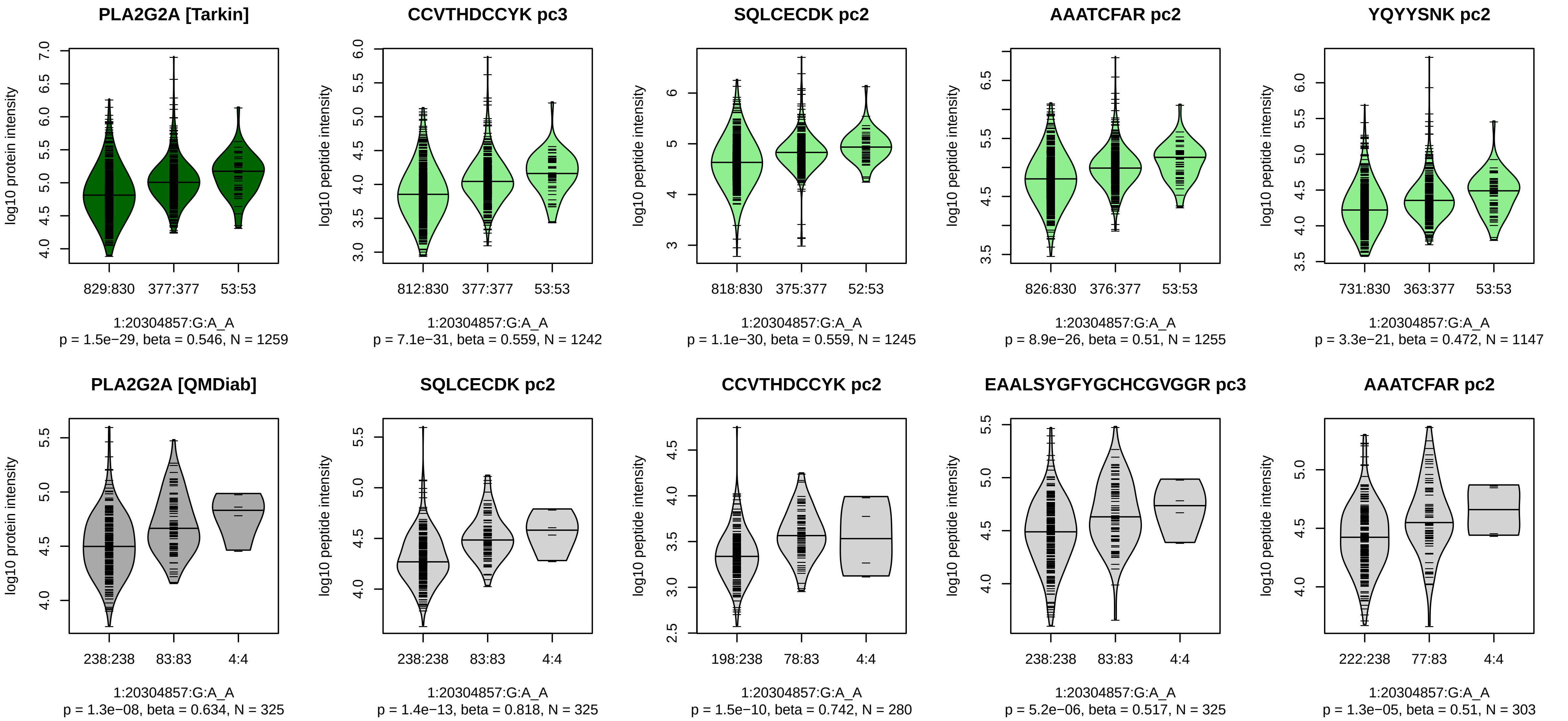

Sort: 48
Gene name: PLA2G2A
SNP: 1:20304857:G:A
rsID: rs2307246
Type: Cis−pQTL
Protein group(s):
A0A3B3IRX2;P14555
Nano−particle: NP4

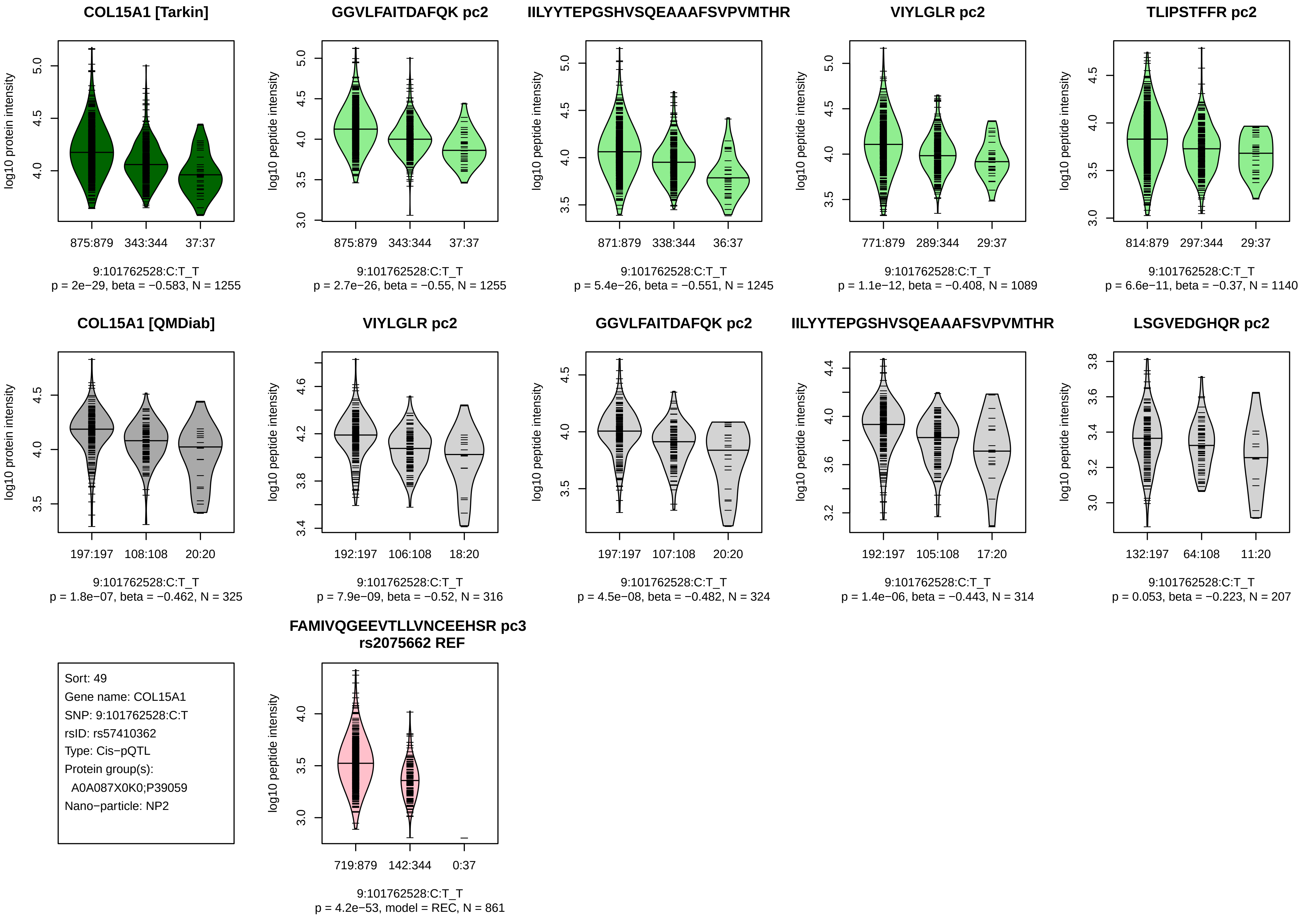
COL15A1 [Tarkin]
log10 protein intensity
875:879
343:344
37:37
9:101762528:C:T_T
p = 2e−29, beta = −0.583, N = 1255
GGVLFAITDAFQK pc2
log10 peptide intensity
875:879
343:344
37:37
9:101762528:C:T_T
p = 2.7e−26, beta = −0.55, N = 1255
IILYYTEPGSHVSQEAAAFSVPVMTHR
log10 peptide intensity
871:879
338:344
36:37
9:101762528:C:T_T
p = 5.4e−26, beta = −0.551, N = 1245
VIYLGLR pc2
log10 peptide intensity
771:879
289:344
29:37
9:101762528:C:T_T
p = 1.1e−12, beta = −0.408, N = 1089
TLIPSTFFR pc2
log10 peptide intensity
814:879
297:344
29:37
9:101762528:C:T_T
p = 6.6e−11, beta = −0.37, N = 1140
COL15A1 [QMDiab]
log10 protein intensity
197:197
108:108
20:20
9:101762528:C:T_T
p = 1.8e−07, beta = −0.462, N = 325
VIYLGLR pc2
log10 peptide intensity
192:197
106:108
18:20
9:101762528:C:T_T
p = 7.9e−09, beta = −0.52, N = 316
GGVLFAITDAFQK pc2
log10 peptide intensity
197:197
107:108
20:20
9:101762528:C:T_T
p = 4.5e−08, beta = −0.482, N = 324
IILYYTEPGSHVSQEAAAFSVPVMTHR
log10 peptide intensity
192:197
105:108
17:20
9:101762528:C:T_T
p = 1.4e−06, beta = −0.443, N = 314
LSGVEDGHQR pc2
log10 peptide intensity
132:197
64:108
11:20
9:101762528:C:T_T
p = 0.053, beta = −0.223, N = 207
Sort: 49
Gene name: COL15A1
SNP: 9:101762528:C:T
rsID: rs57410362
Type: Cis−pQTL
Protein group(s):
A0A087X0K0;P39059
Nano−particle: NP2
FAMIVQGEEVTLLVNCEEHSR pc3
rs2075662 REF
log10 peptide intensity
719:879
142:344
0:37
9:101762528:C:T_T
p = 4.2e−53, model = REC, N = 861

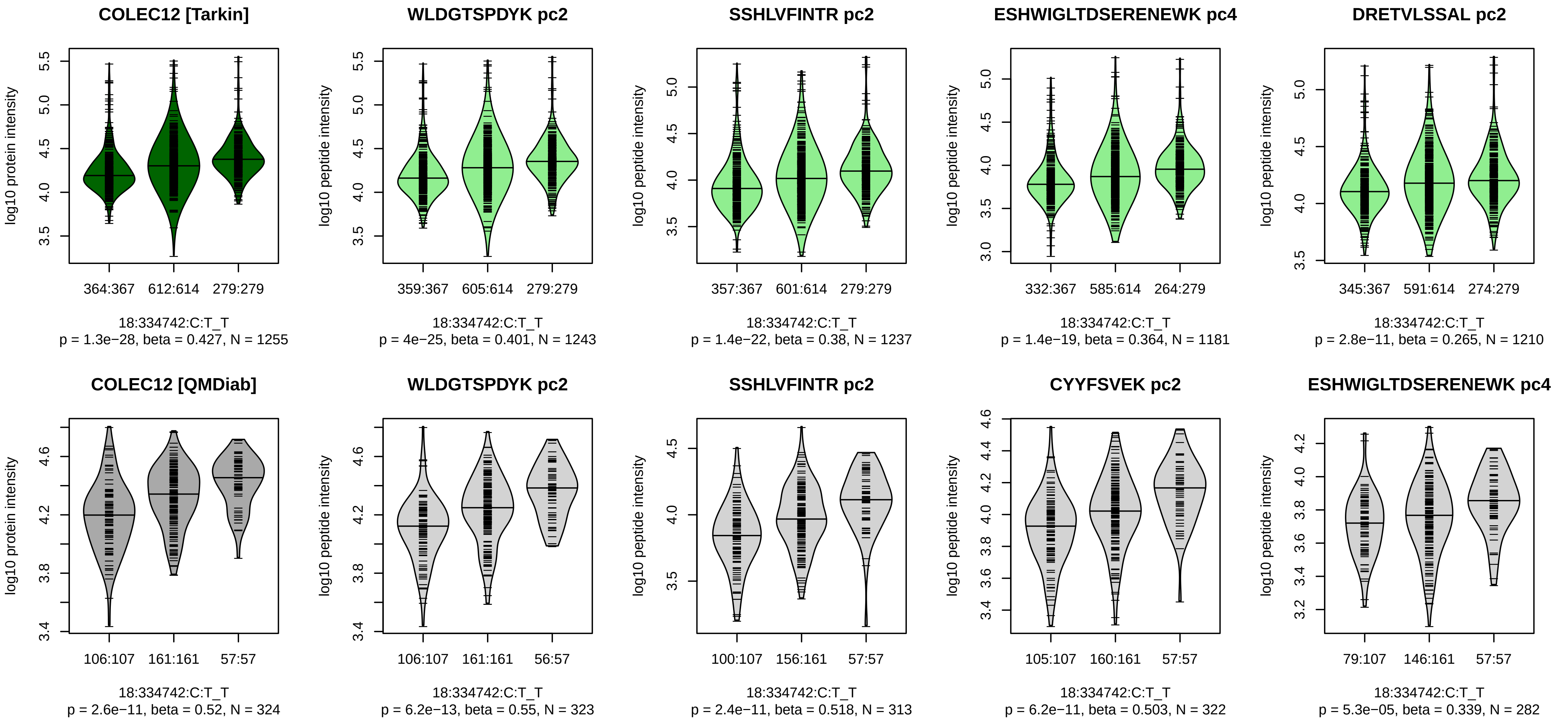

Sort: 50
Gene name: COLEC12
SNP: 18:334742:C:T
rsID: rs2305027
Type: Cis−pQTL
Protein group(s):
Q5KU26
Nano−particle: NP2

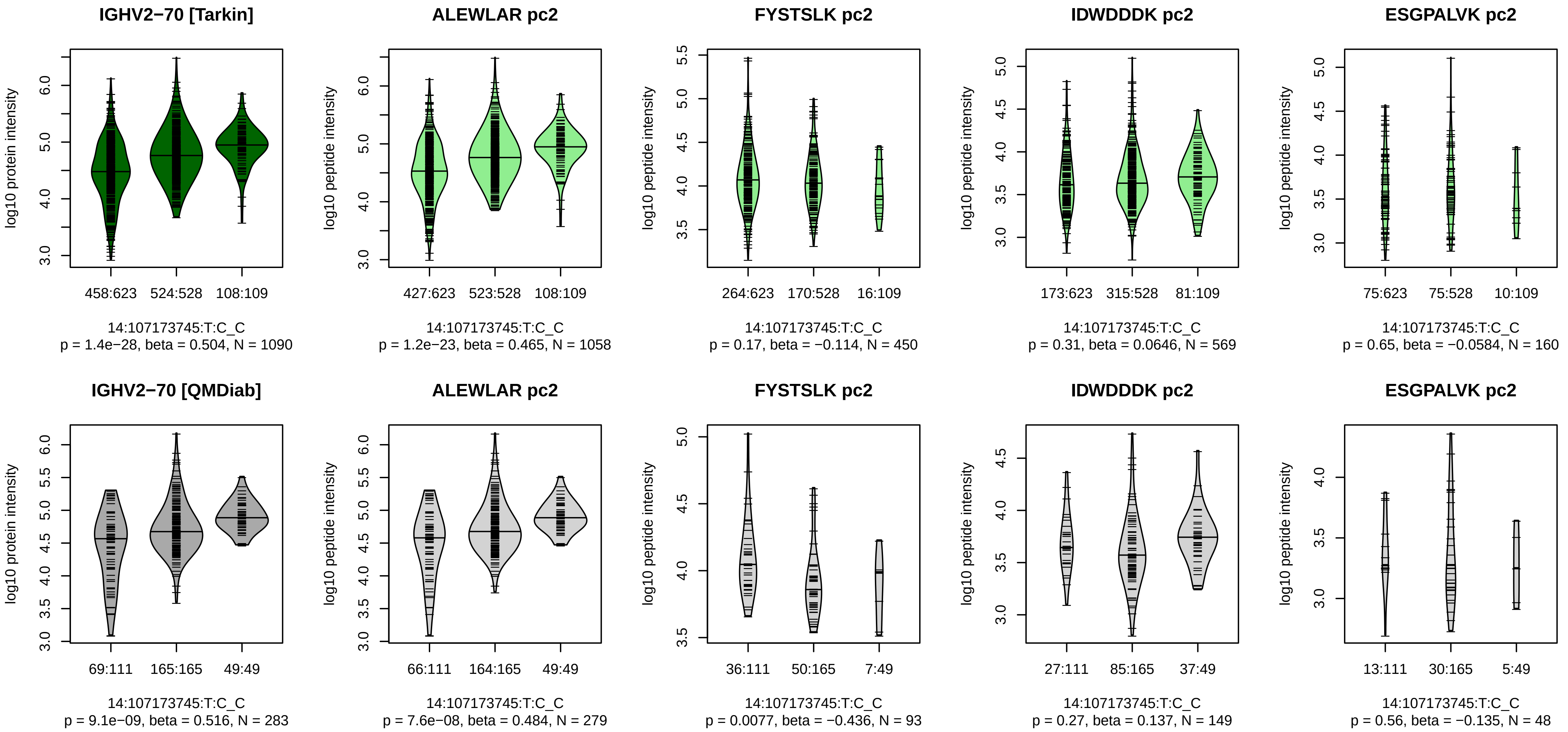

Sort: 51
Gene name: IGHV2−70
SNP: 14:107173745:T:C
rsID: rs10134517
Type: Cis−pQTL
Protein group(s):
A0A0C4DH43
Nano−particle: NP3

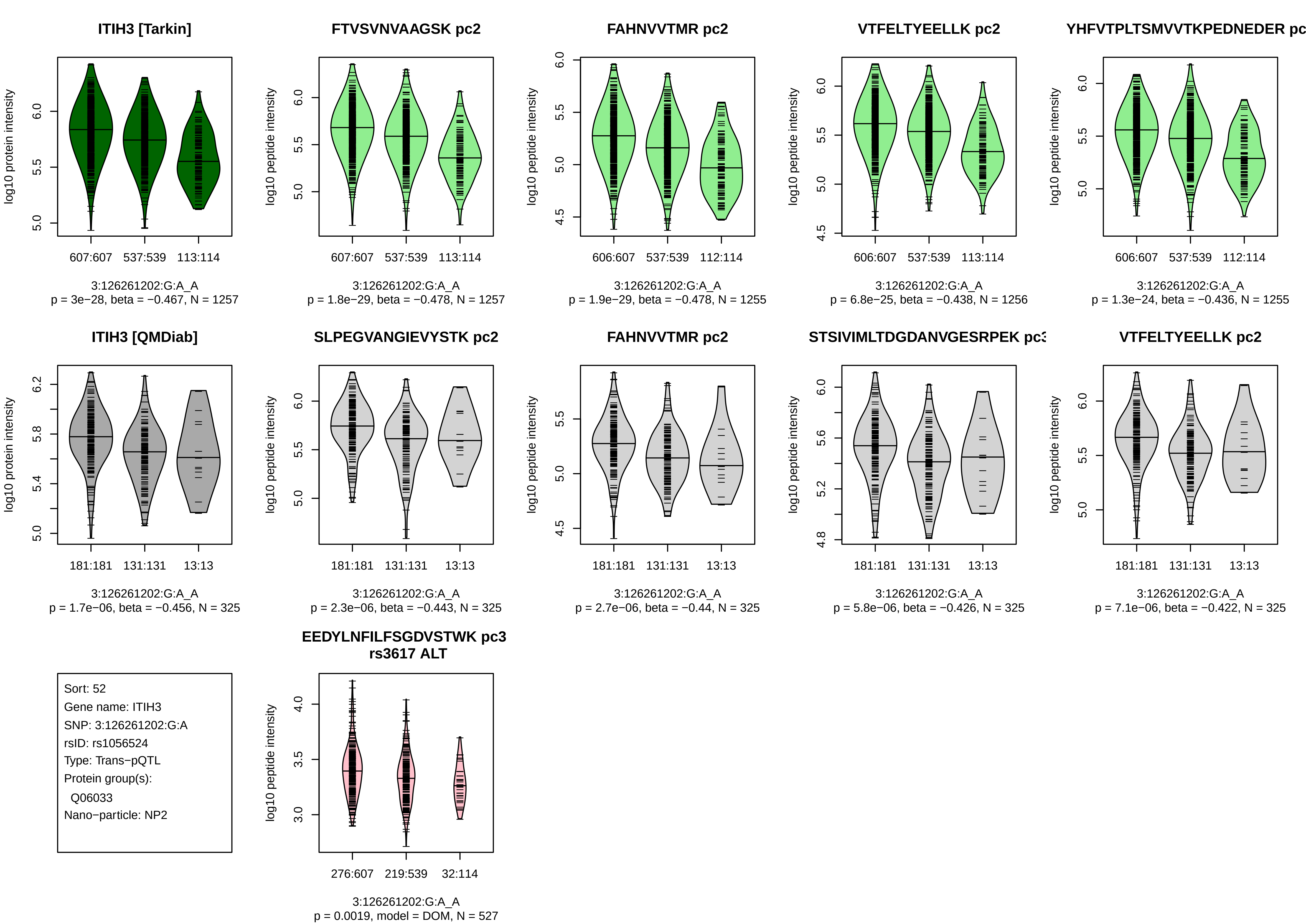
ITIH3 [Tarkin]
log10 protein intensity
607:607 537:539 113:114
3:126261202:G:A_A
p = 3e−28, beta = −0.467, N = 1257
FTVSVNVAAGSK pc2
log10 peptide intensity
607:607 537:539 113:114
3:126261202:G:A_A
p = 1.8e−29, beta = −0.478, N = 1257
FAHNVVTMR pc2
606:607 537:539 112:114
3:126261202:G:A_A
p = 1.9e−29, beta = −0.478, N = 1255
VTFELTYEELLK pc2
606:607 537:539 113:114
3:126261202:G:A_A
p = 6.8e−25, beta = −0.438, N = 1256
YHFVTPLTSMVVTKPEDNEDER pc
606:607 537:539 112:114
3:126261202:G:A_A
p = 1.3e−24, beta = −0.436, N = 1255
ITIH3 [QMDiab]
181:181 131:131 13:13
3:126261202:G:A_A
p = 1.7e−06, beta = −0.456, N = 325
SLPEGVANGIEVYSTK pc2
3:126261202:G:A_A
p = 2.3e−06, beta = −0.443, N = 325
FAHNVVTMR pc2
3:126261202:G:A_A
p = 2.7e−06, beta = −0.44, N = 325
STSIVIMLTDGDANVGESRPEK pc3
3:126261202:G:A_A
p = 5.8e−06, beta = −0.426, N = 325
VTFELTYEELLK pc2
3:126261202:G:A_A
p = 7.1e−06, beta = −0.422, N = 325
Sort: 52
Gene name: ITIH3
SNP: 3:126261202:G:A
rsID: rs1056524
Type: Trans−pQTL
Protein group(s):
Q06033
Nano−particle: NP2
EEDYLNFILFSGDVSTWK pc3
rs3617 ALT
276:607 219:539 32:114
3:126261202:G:A_A
p = 0.0019, model = DOM, N = 527

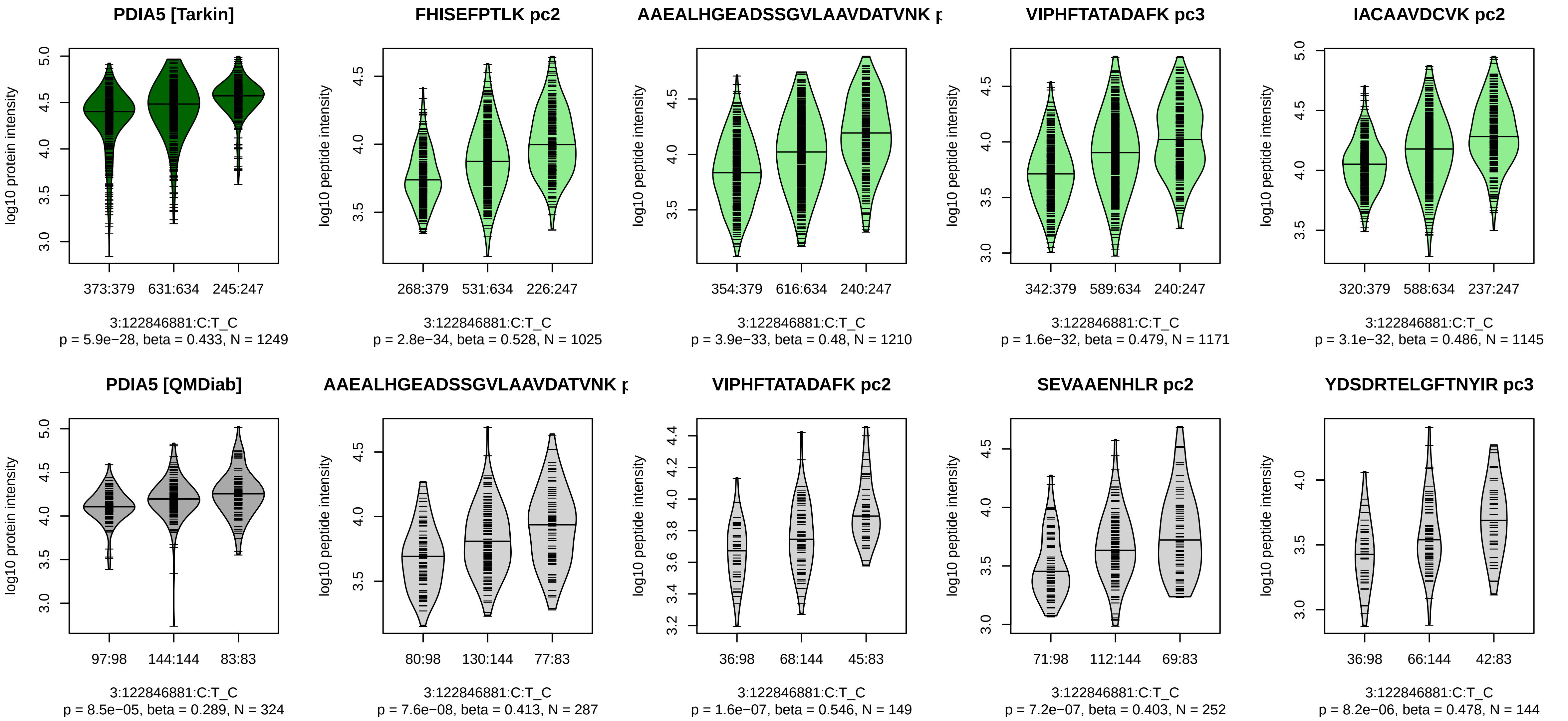

Sort: 53
Gene name: PDIA5
SNP: 3:122846881:C:T
rsID: rs9820435
Type: Cis−pQTL
Protein group(s):
Q14554
Nano−particle: NP1

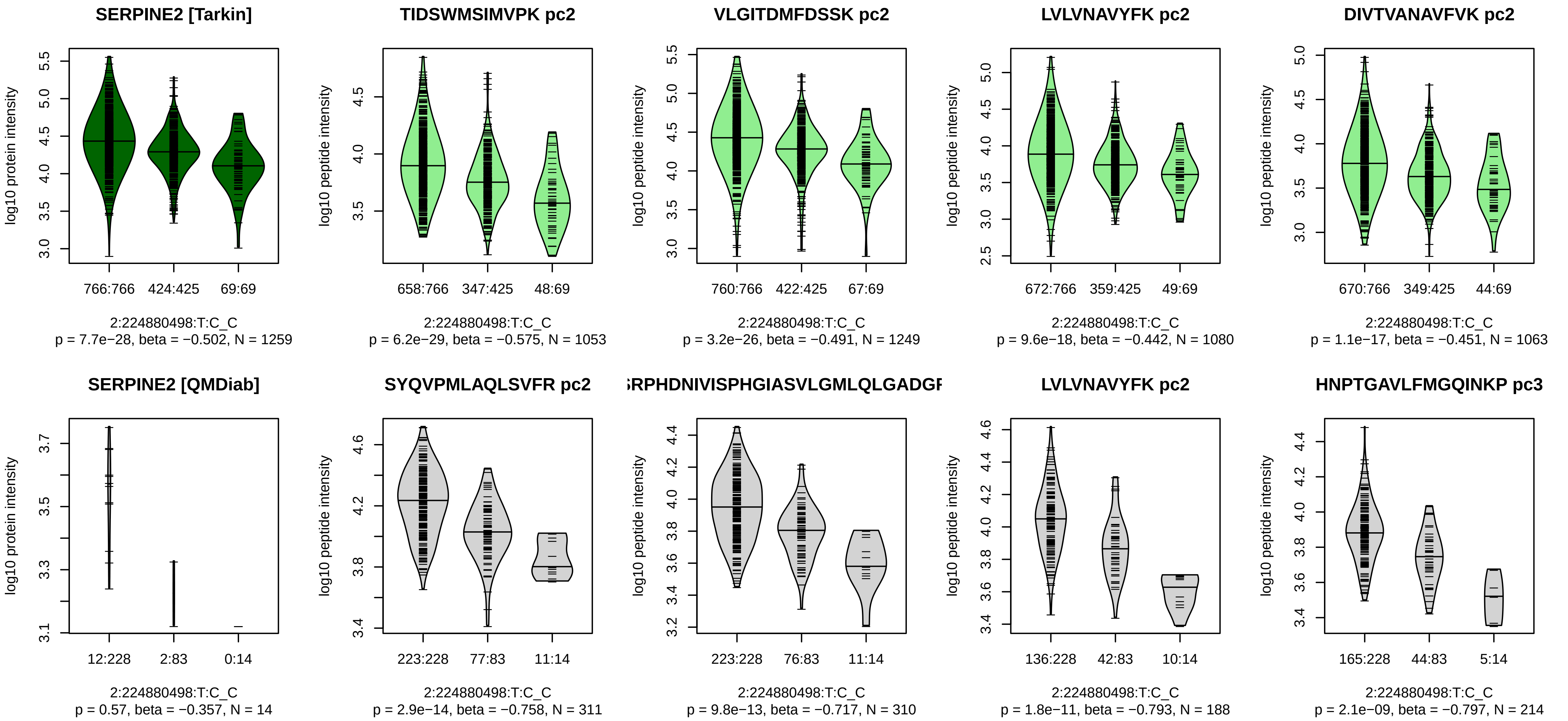

Sort: 54
Gene name: SERPINE2
SNP: 2:224880498:T:C
rsID: rs68066031
Type: Cis−pQTL
Protein group(s):
P07093
Nano−particle: NP4

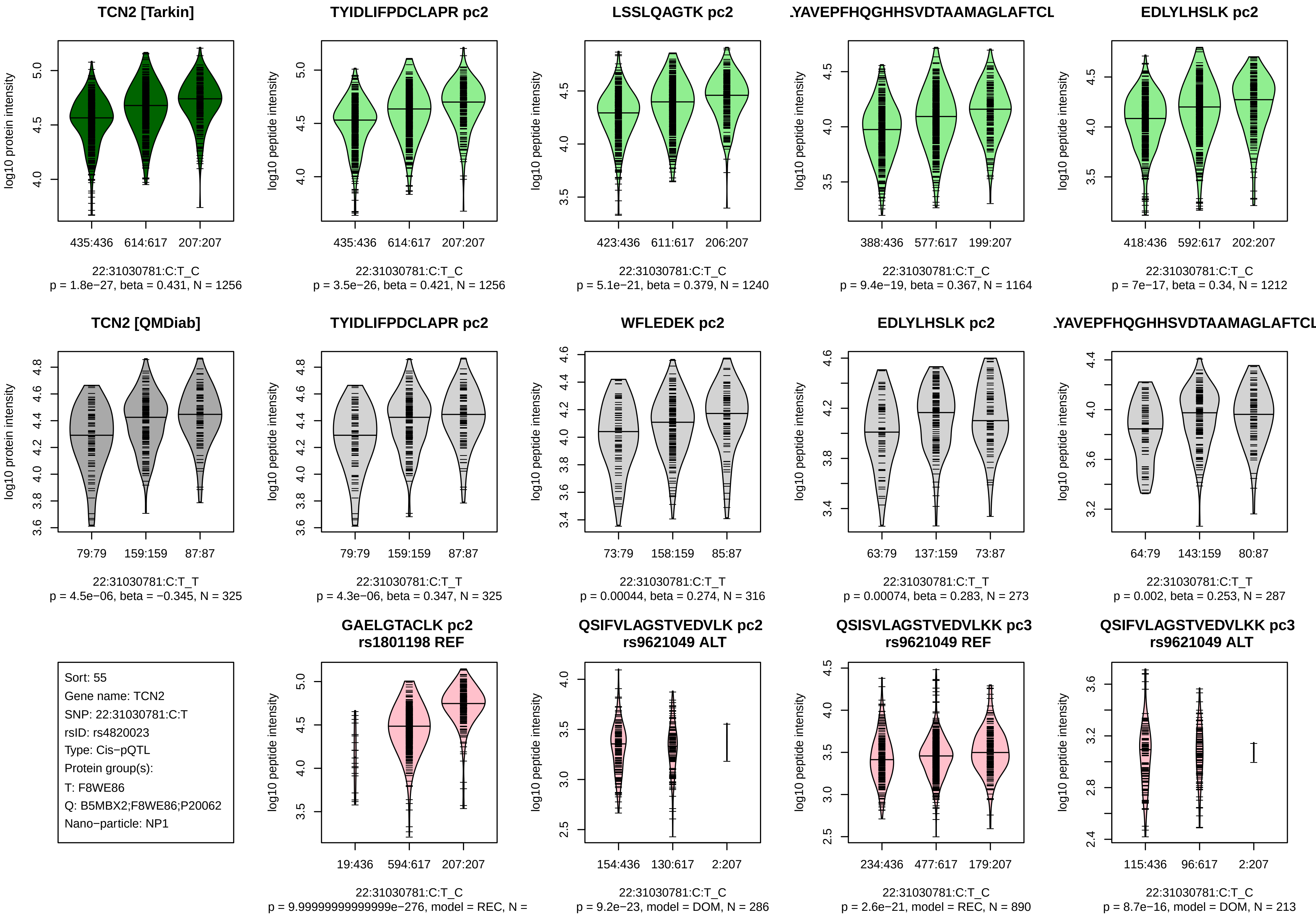
TCN2 [Tarkin]
log10 protein intensity
435:436
614:617
207:207
22:31030781:C:T_C
p = 1.8e−27, beta = 0.431, N = 1256
TYIDLIFPDCLAPR pc2
log10 peptide intensity
435:436
614:617
207:207
22:31030781:C:T_C
p = 3.5e−26, beta = 0.421, N = 1256
LSSLQAGTK pc2
log10 peptide intensity
423:436
611:617
206:207
22:31030781:C:T_C
p = 5.1e−21, beta = 0.379, N = 1240
.YAVEPFHQGHHSVDTAAMAGLAFTCL
log10 peptide intensity
388:436
577:617
199:207
22:31030781:C:T_C
p = 9.4e−19, beta = 0.367, N = 1164
EDLYLHSLK pc2
log10 peptide intensity
418:436
592:617
202:207
22:31030781:C:T_C
p = 7e−17, beta = 0.34, N = 1212
TCN2 [QMDiab]
log10 protein intensity
79:79
159:159
87:87
22:31030781:C:T_T
p = 4.5e−06, beta = −0.345, N = 325
TYIDLIFPDCLAPR pc2
log10 peptide intensity
79:79
159:159
87:87
22:31030781:C:T_T
p = 4.3e−06, beta = 0.347, N = 325
WFLEDEK pc2
log10 peptide intensity
73:79
158:159
85:87
22:31030781:C:T_T
p = 0.00044, beta = 0.274, N = 316
EDLYLHSLK pc2
log10 peptide intensity
63:79
137:159
73:87
22:31030781:C:T_T
p = 0.00074, beta = 0.283, N = 273
.YAVEPFHQGHHSVDTAAMAGLAFTCL
log10 peptide intensity
64:79
143:159
80:87
22:31030781:C:T_T
p = 0.002, beta = 0.253, N = 287
Sort: 55
Gene name: TCN2
SNP: 22:31030781:C:T
rsID: rs4820023
Type: Cis−pQTL
Protein group(s):
T: F8WE86
Q: B5MBX2;F8WE86;P20062
Nano−particle: NP1
GAELGTACLK pc2
rs1801198 REF
log10 peptide intensity
19:436
594:617
207:207
22:31030781:C:T_C
p = 9.99999999999999e−276, model = REC, N =
QSIFVLAGSTVEDVLK pc2
rs9621049 ALT
log10 peptide intensity
154:436
130:617
2:207
22:31030781:C:T_C
p = 9.2e−23, model = DOM, N = 286
QSISVLAGSTVEDVLKK pc3
rs9621049 REF
log10 peptide intensity
234:436
477:617
179:207
22:31030781:C:T_C
p = 2.6e−21, model = REC, N = 890
QSIFVLAGSTVEDVLKK pc3
rs9621049 ALT
log10 peptide intensity
115:436
96:617
2:207
22:31030781:C:T_C
p = 8.7e−16, model = DOM, N = 213

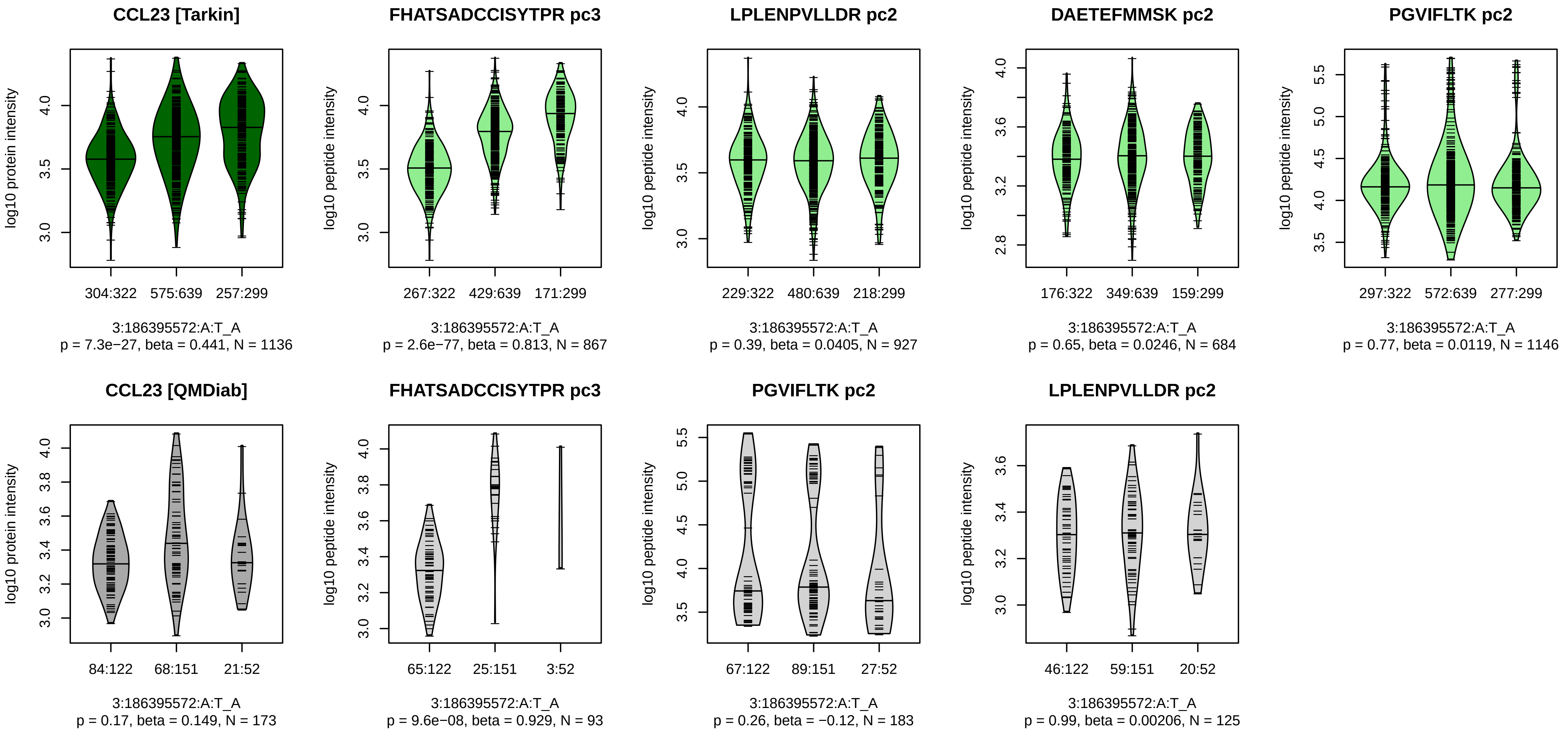

Sort: 56
Gene name: CCL23
SNP: 3:186395572:A:T
rsID: rs1042464
Type: Trans−pQTL
Protein group(s):
P55773
Nano−particle: NP3

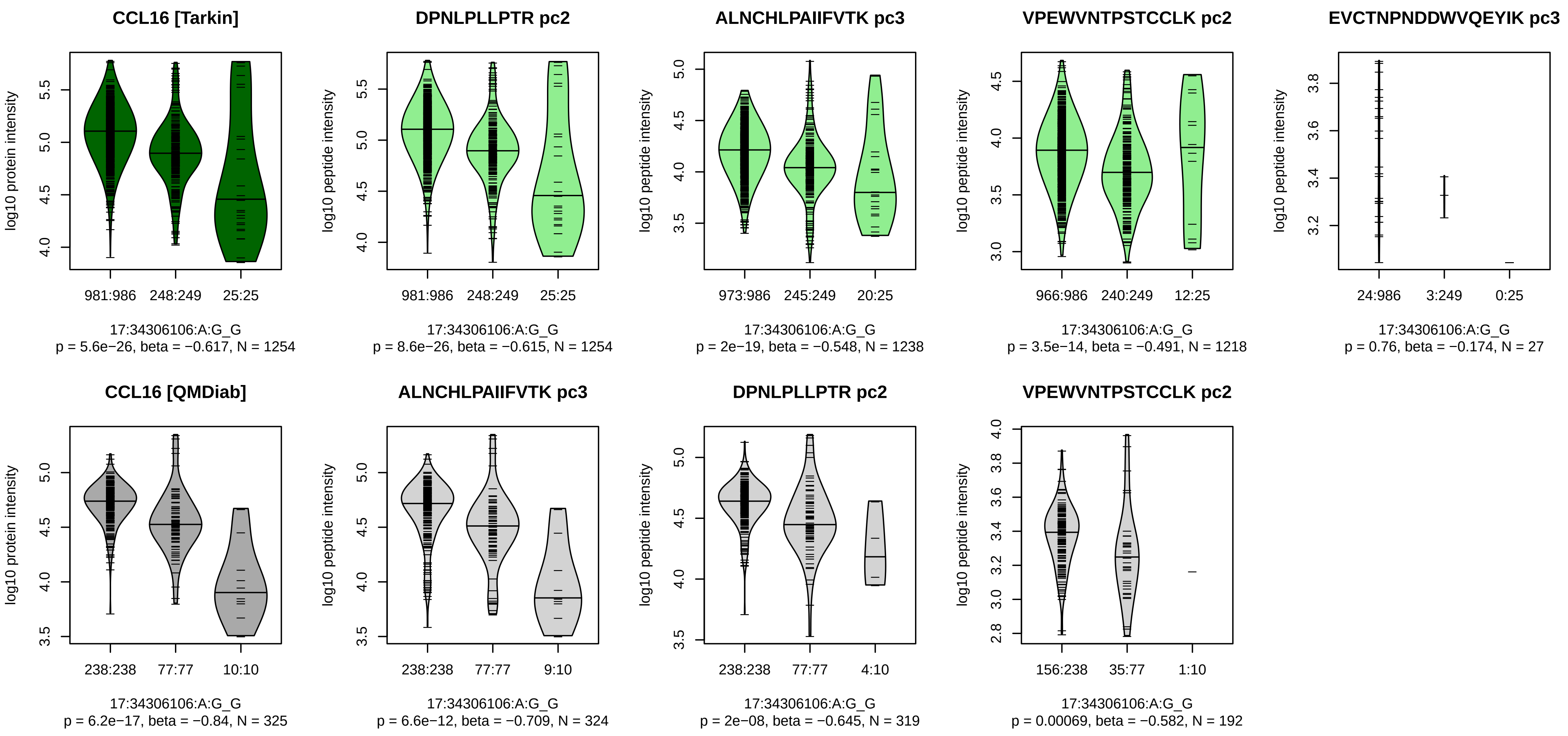

Sort: 57
Gene name: CCL16
SNP: 17:34306106:A:G
rsID: rs10445391
Type: Cis−pQTL
Protein group(s):
O15467
Nano−particle: NP3

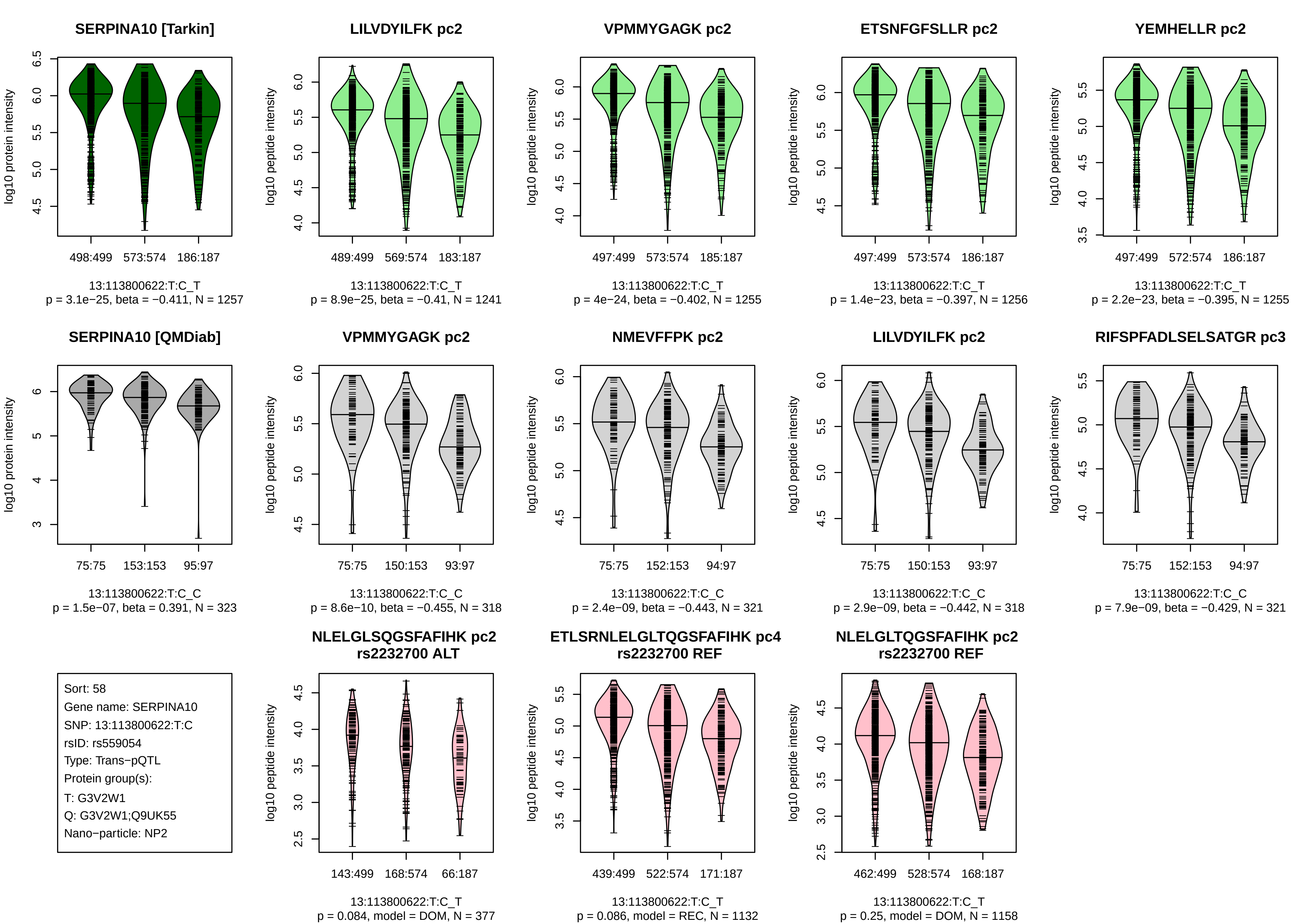
SERPINA10 [Tarkin]
log10 protein intensity
498:499 573:574 186:187
13:113800622:T:C_T
p = 3.1e−25, beta = −0.411, N = 1257
LILVDYILFK pc2
log10 peptide intensity
489:499 569:574 183:187
13:113800622:T:C_T
p = 8.9e−25, beta = −0.41, N = 1241
VPMMYGAGK pc2
log10 peptide intensity
497:499 573:574 185:187
13:113800622:T:C_T
p = 4e−24, beta = −0.402, N = 1255
ETSNFGFSLLR pc2
log10 peptide intensity
497:499 573:574 186:187
13:113800622:T:C_T
p = 1.4e−23, beta = −0.397, N = 1256
YEMHELLR pc2
log10 peptide intensity
497:499 572:574 186:187
13:113800622:T:C_T
p = 2.2e−23, beta = −0.395, N = 1255
SERPINA10 [QMDiab]
log10 protein intensity
75:75 153:153 95:97
13:113800622:T:C_C
p = 1.5e−07, beta = 0.391, N = 323
VPMMYGAGK pc2
log10 peptide intensity
75:75 150:153 93:97
13:113800622:T:C_C
p = 8.6e−10, beta = −0.455, N = 318
NMEVFFPK pc2
log10 peptide intensity
75:75 152:153 94:97
13:113800622:T:C_C
p = 2.4e−09, beta = −0.443, N = 321
LILVDYILFK pc2
log10 peptide intensity
75:75 150:153 93:97
13:113800622:T:C_C
p = 2.9e−09, beta = −0.442, N = 318
RIFSPFADLSELSATGR pc3
log10 peptide intensity
75:75 152:153 94:97
13:113800622:T:C_C
p = 7.9e−09, beta = −0.429, N = 321
Sort: 58
Gene name: SERPINA10
SNP: 13:113800622:T:C
rsID: rs559054
Type: Trans−pQTL
Protein group(s):
T: G3V2W1
Q: G3V2W1;Q9UK55
Nano−particle: NP2
NLELGLSQGSFAFIHK pc2
rs2232700 ALT
log10 peptide intensity
143:499 168:574 66:187
13:113800622:T:C_T
p = 0.084, model = DOM, N = 377
ETLSRNLELGLTQGSFAFIHK pc4
rs2232700 REF
log10 peptide intensity
439:499 522:574 171:187
13:113800622:T:C_T
p = 0.086, model = REC, N = 1132
NLELGLTQGSFAFIHK pc2
rs2232700 REF
log10 peptide intensity
462:499 528:574 168:187
13:113800622:T:C_T
p = 0.25, model = DOM, N = 1158

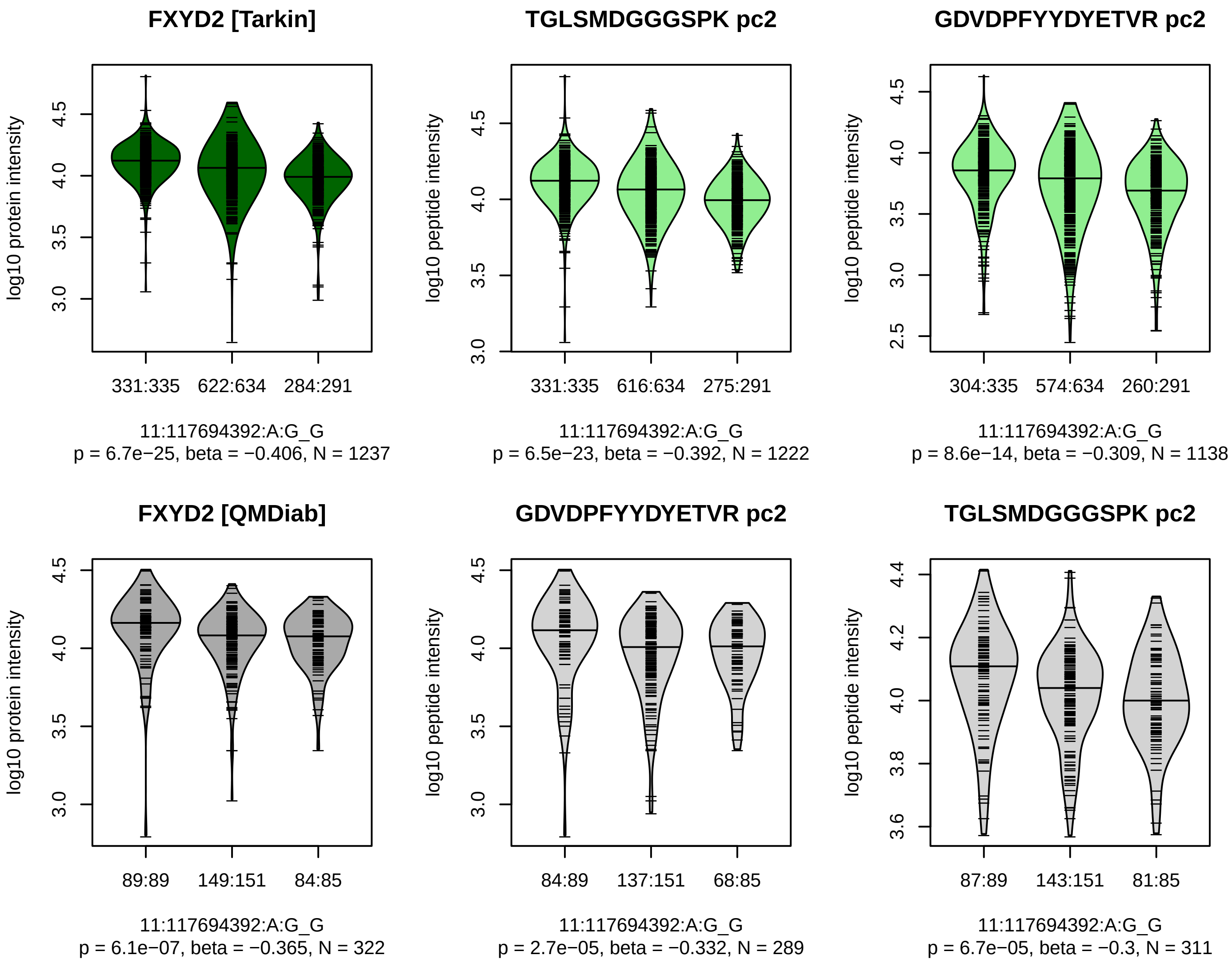

Sort: 59
Gene name: FXYD2
SNP: 11:117694392:A:G
rsID: rs4936409
Type: Cis−pQTL
Protein group(s):
P54710
Nano−particle: NP2

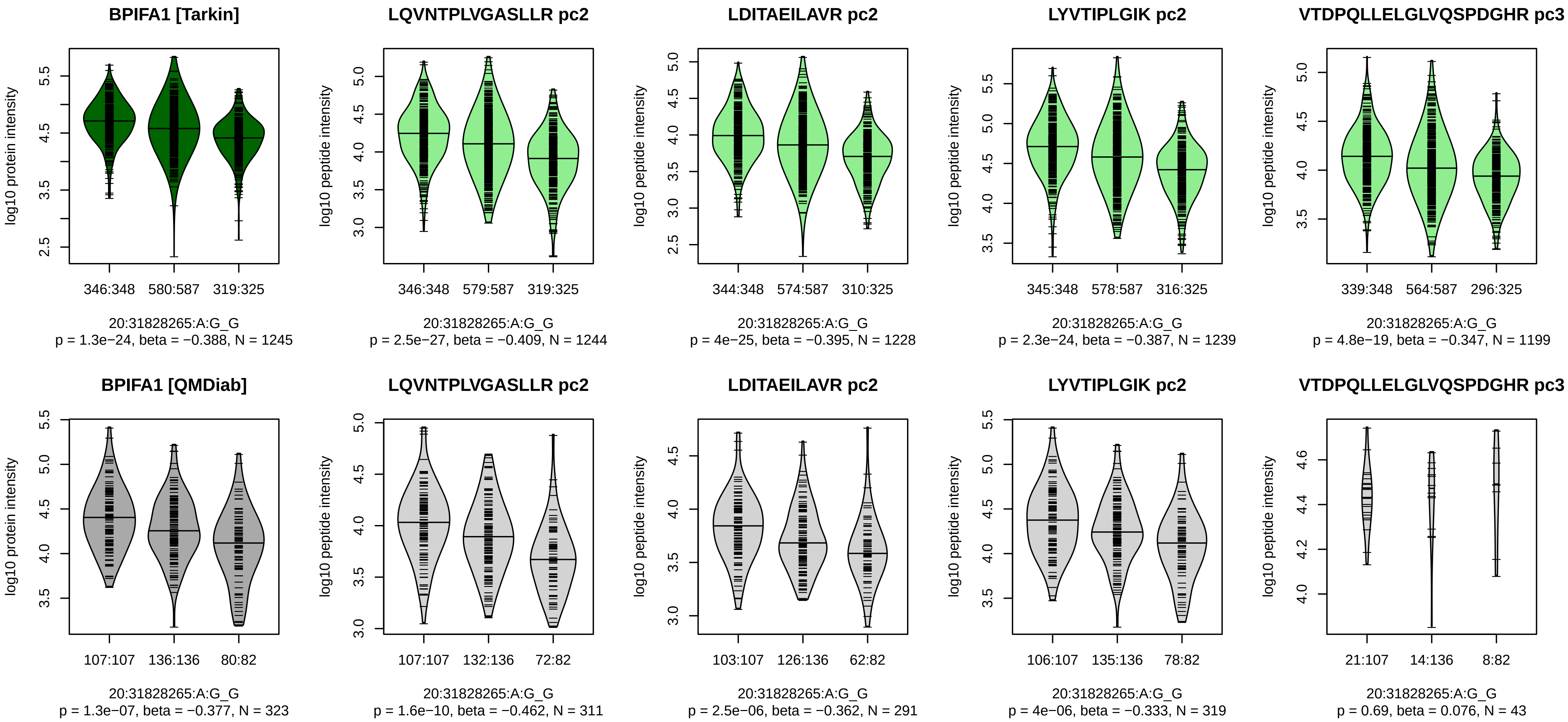

Sort: 60
Gene name: BPIFA1
SNP: 20:31828265:A:G
rsID: rs6059187
Type: Cis−pQTL
Protein group(s):
Q9NP55;Q9NP55−2
Nano−particle: NP1

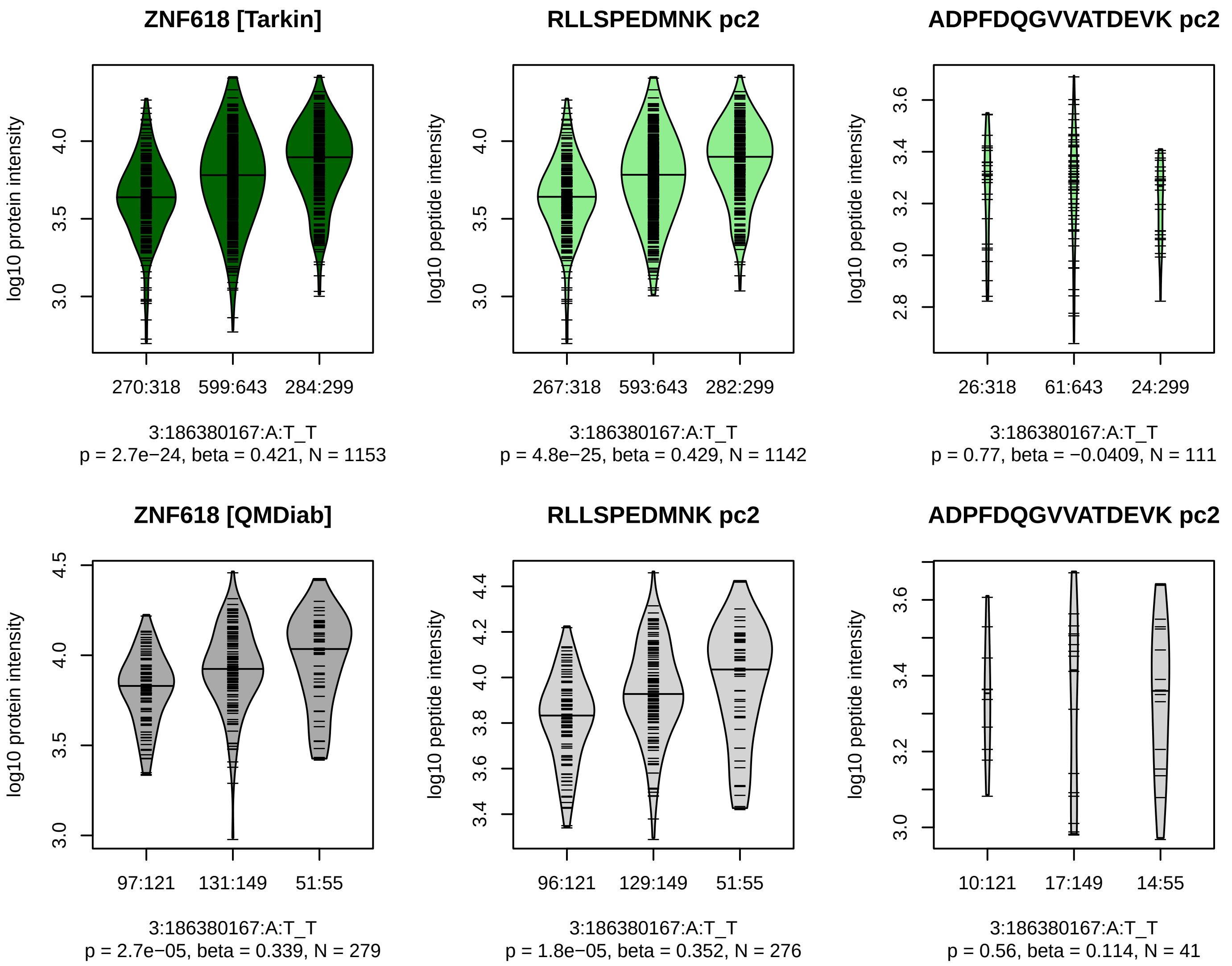

Sort: 61
Gene name: ZNF618
SNP: 3:186380167:A:T
rsID: rs9835865
Type: Trans−pQTL
Protein group(s):
T: Q5T7W0
Q: Q5T7W0−2
Nano−particle: NP1

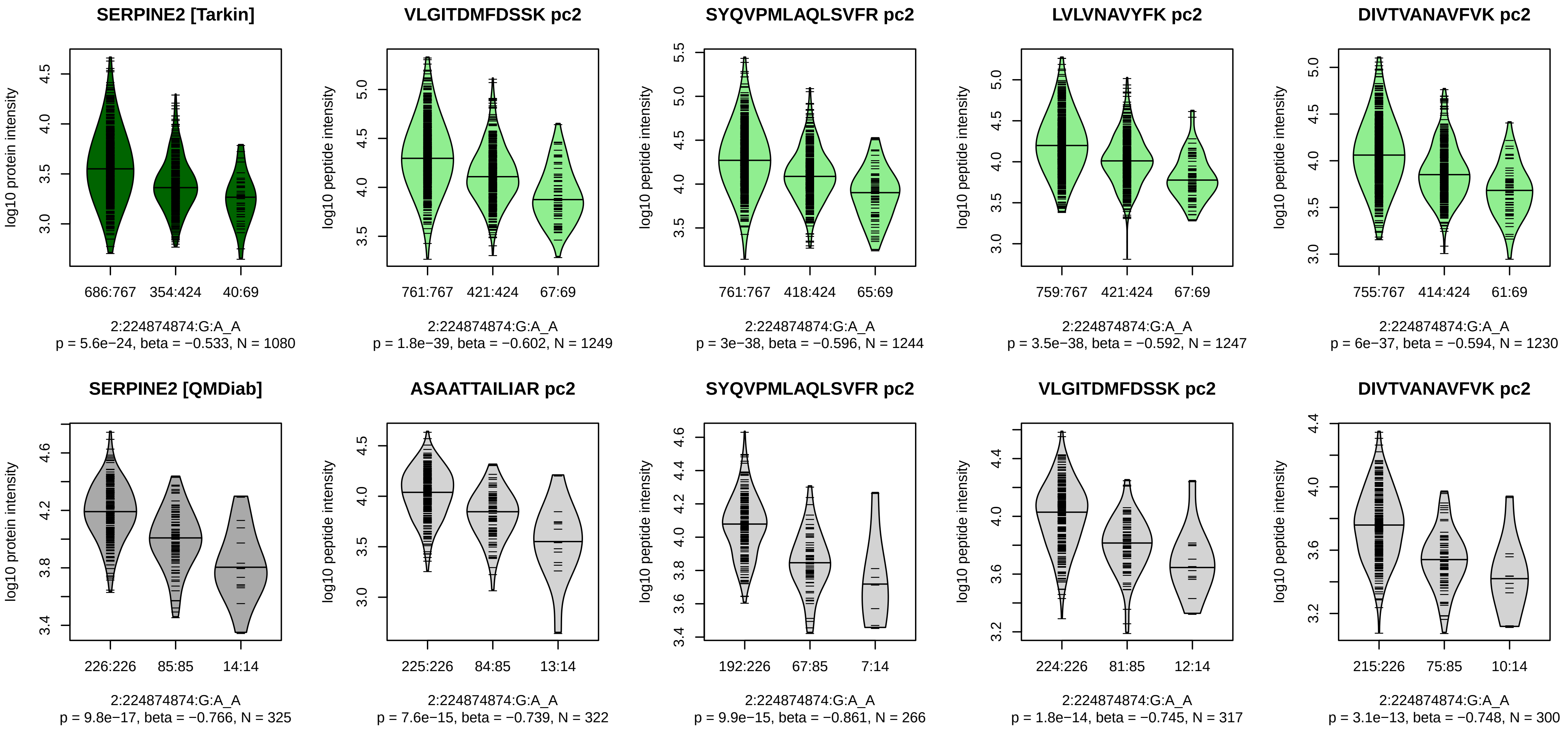

Sort: 62

Gene name: SERPINE2

SNP: 2:224874874:G:A

rsID: rs13412535

Type: Cis−pQTL

Protein group(s):

P07093−2;P07093−3

Nano−particle: NP3

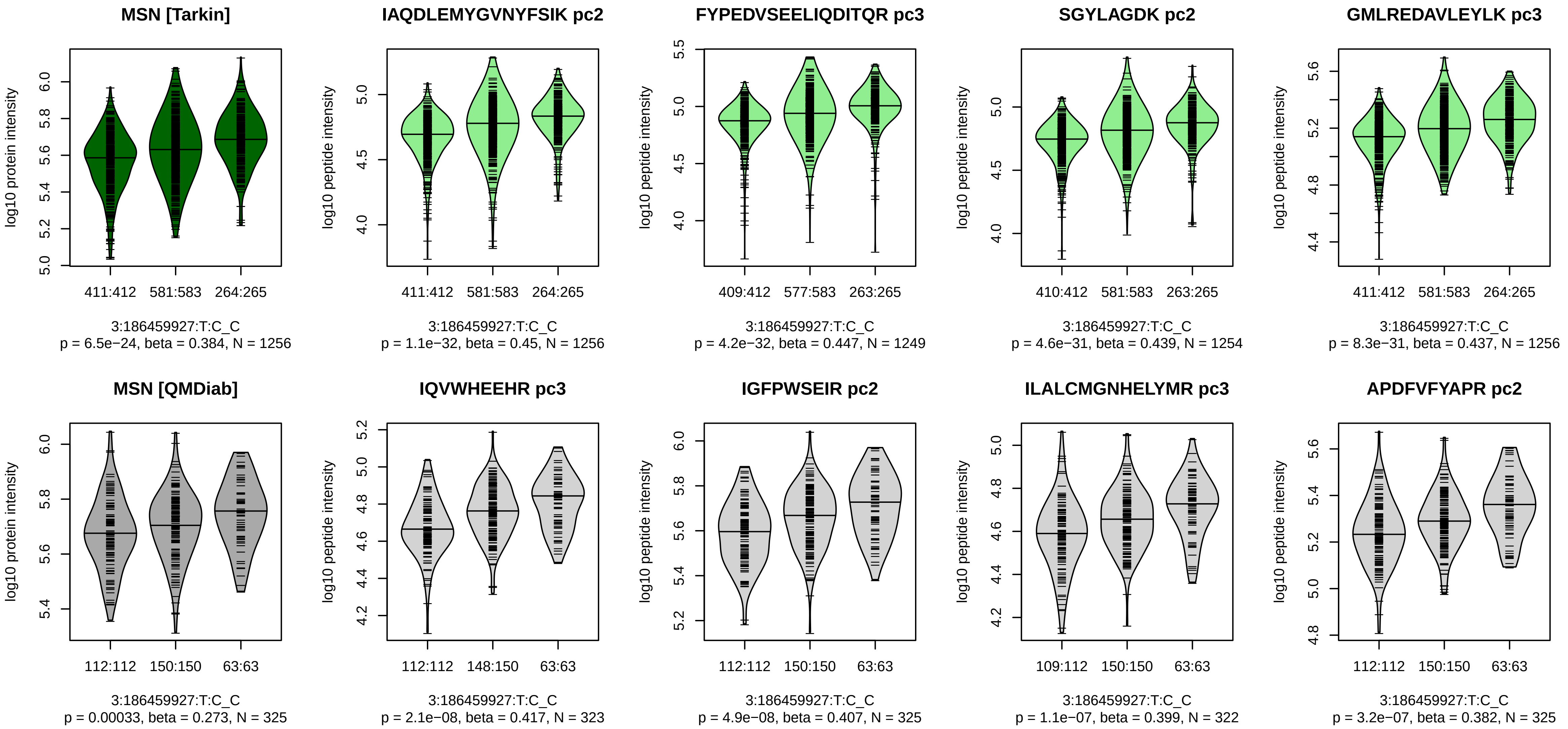

Sort: 63
Gene name: MSN
SNP: 3:186459927:T:C
rsID: rs710446
Type: Trans−pQTL
Protein group(s):
P26038
Nano−particle: NP1

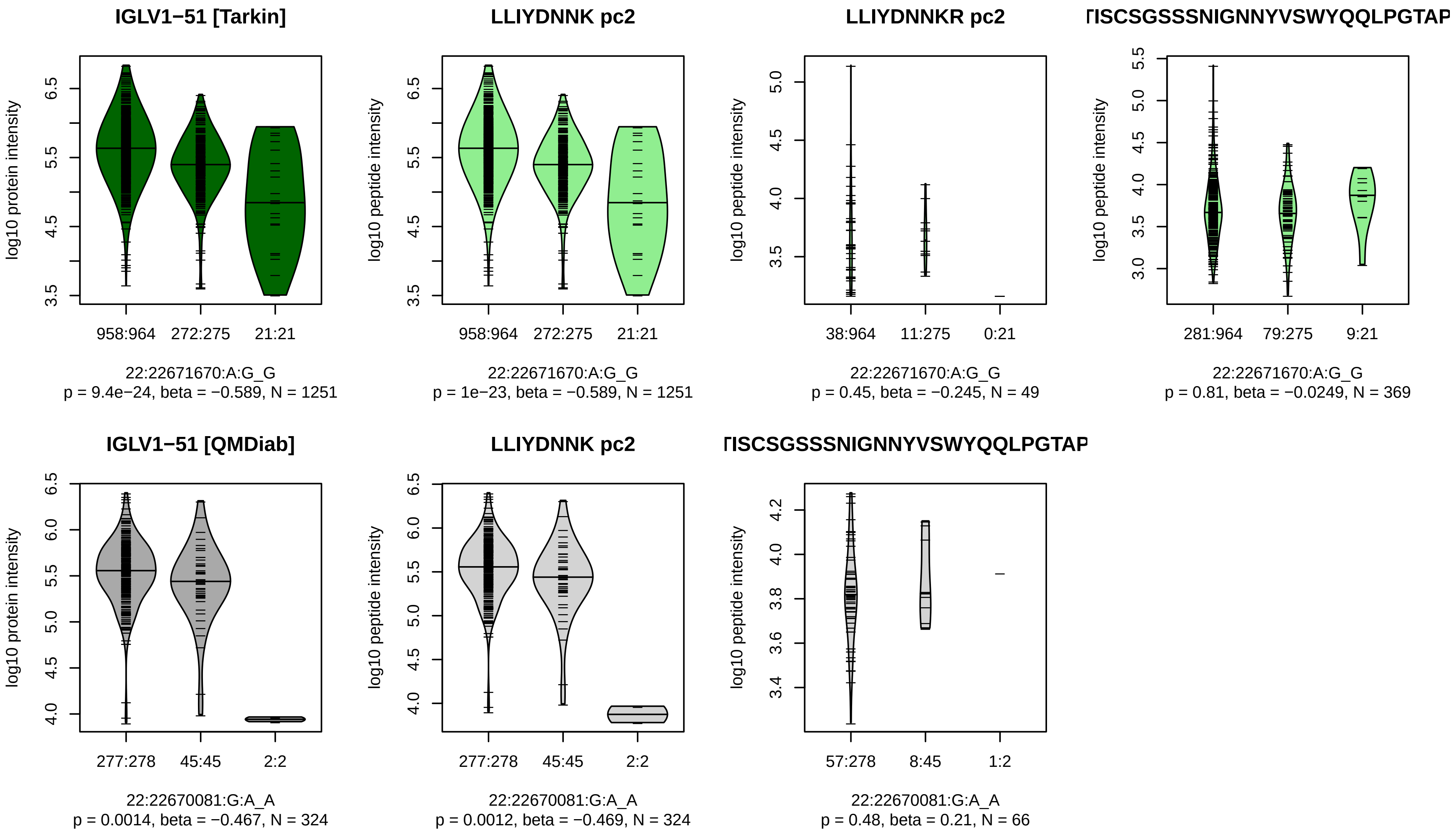

Sort: 64
Gene name: IGLV1−51
SNP: 22:22671670:A:G
rsID: rs6001756
Type: Cis−pQTL
Protein group(s):
P01701
Nano−particle: NP3

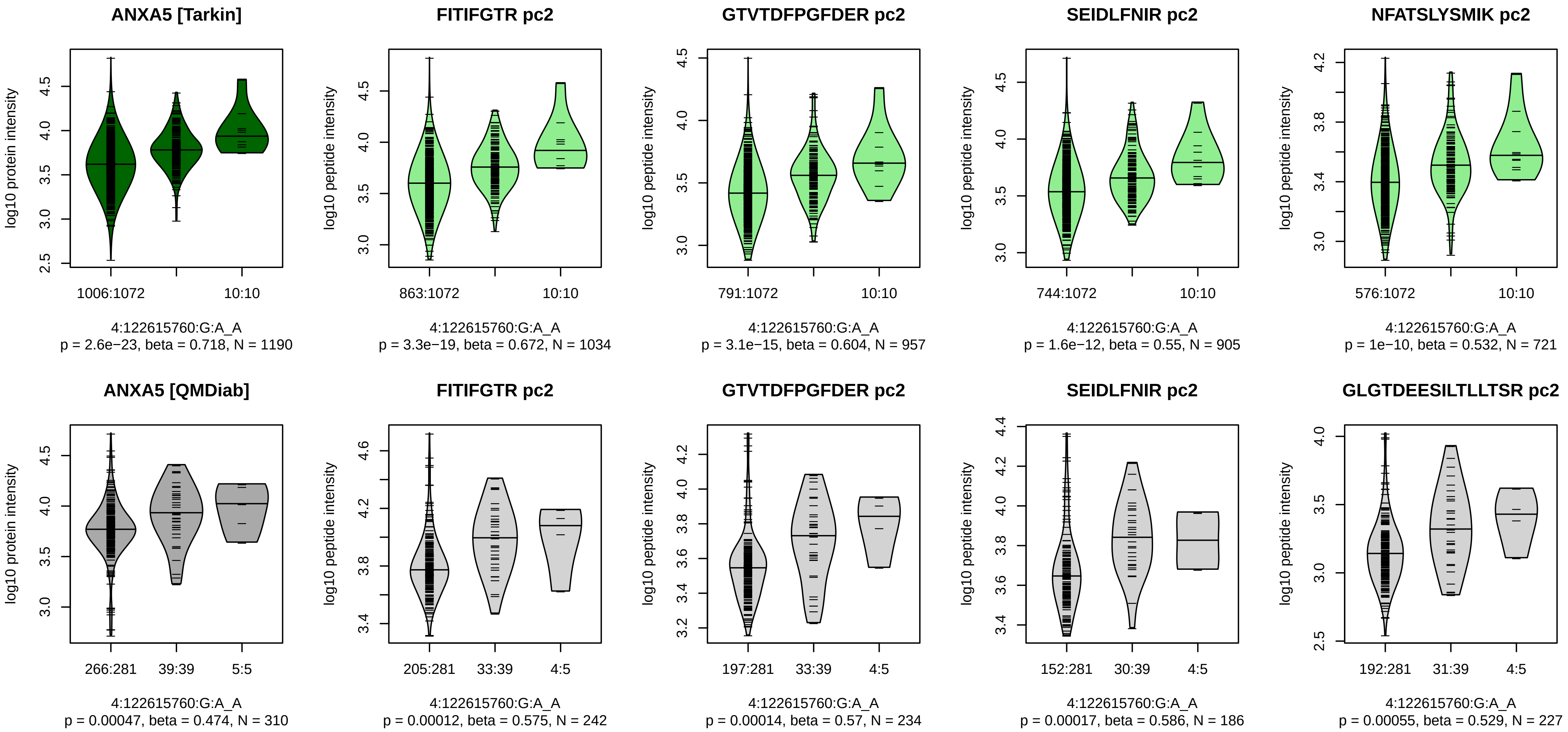

Sort: 65
Gene name: ANXA5
SNP: 4:122615760:G:A
rsID: rs9995591
Type: Cis−pQTL
Protein group(s):
P08758
Nano−particle: NP1

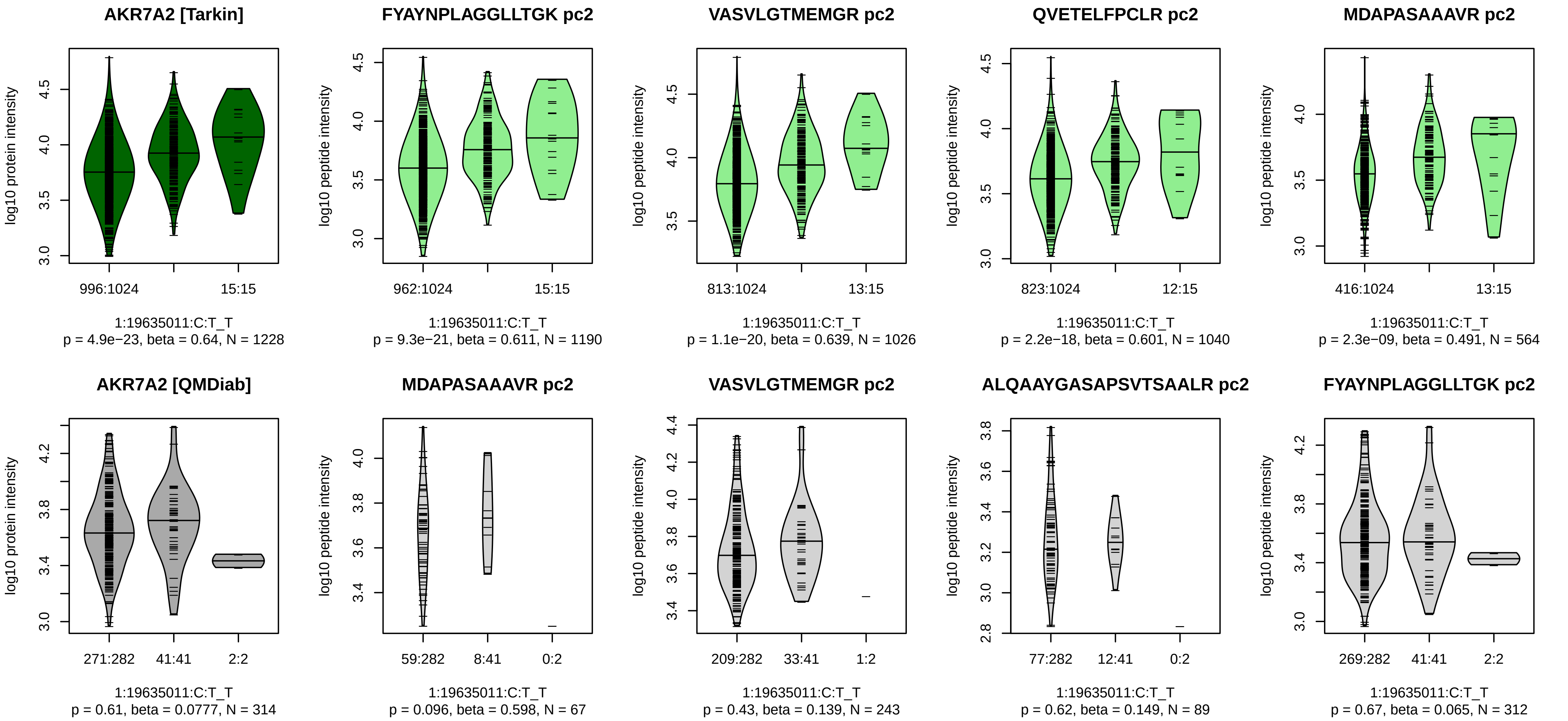

Sort: 66
Gene name: AKR7A2
SNP: 1:19635011:C:T
rsID: rs1043657
Type: Cis−pQTL
Protein group(s):
O43488
Nano−particle: NP5

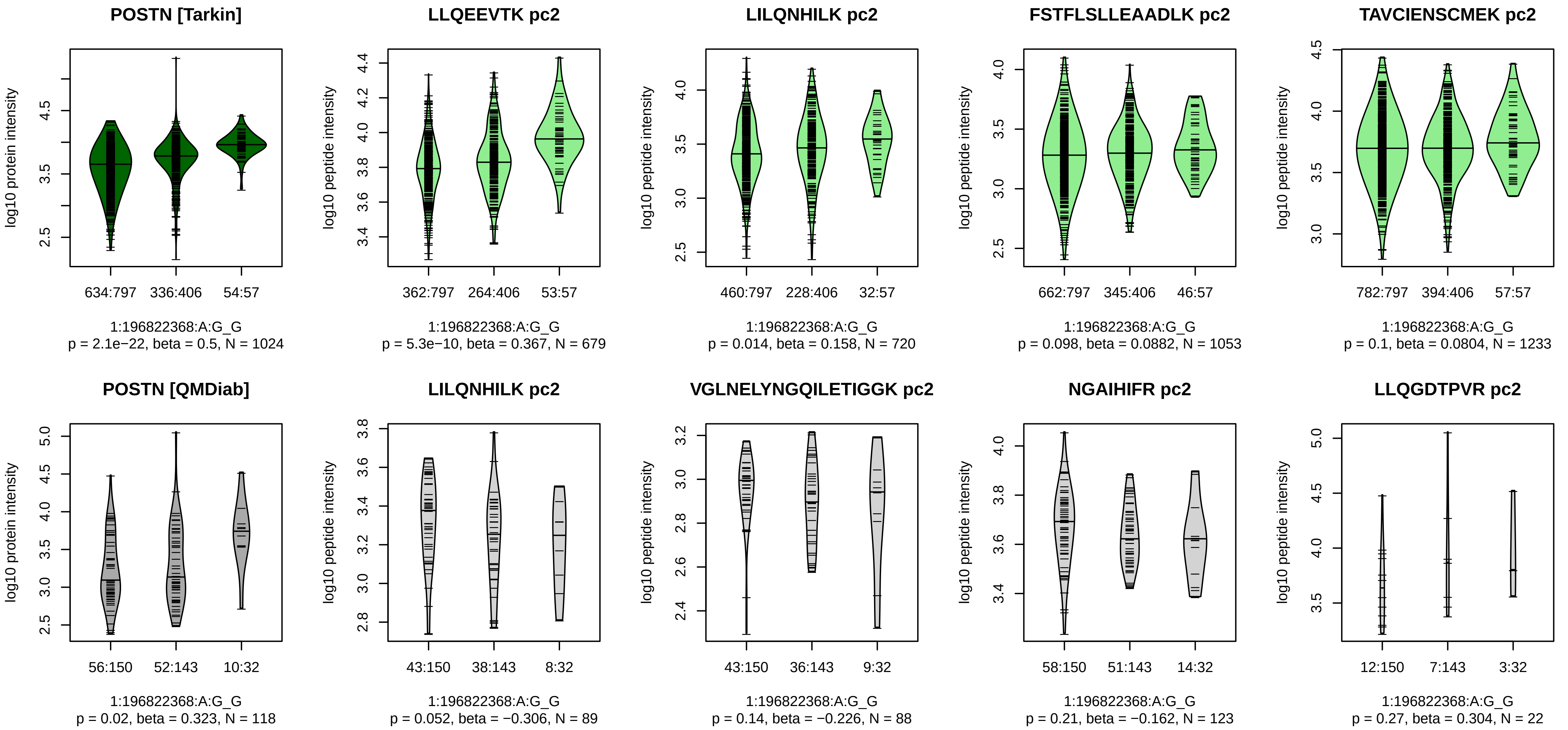

Sort: 67
Gene name: POSTN
SNP: 1:196822368:A:G
rsID: rs60642321
Type: Trans−pQTL
Protein group(s):
Q15063−2
Nano−particle: NP3

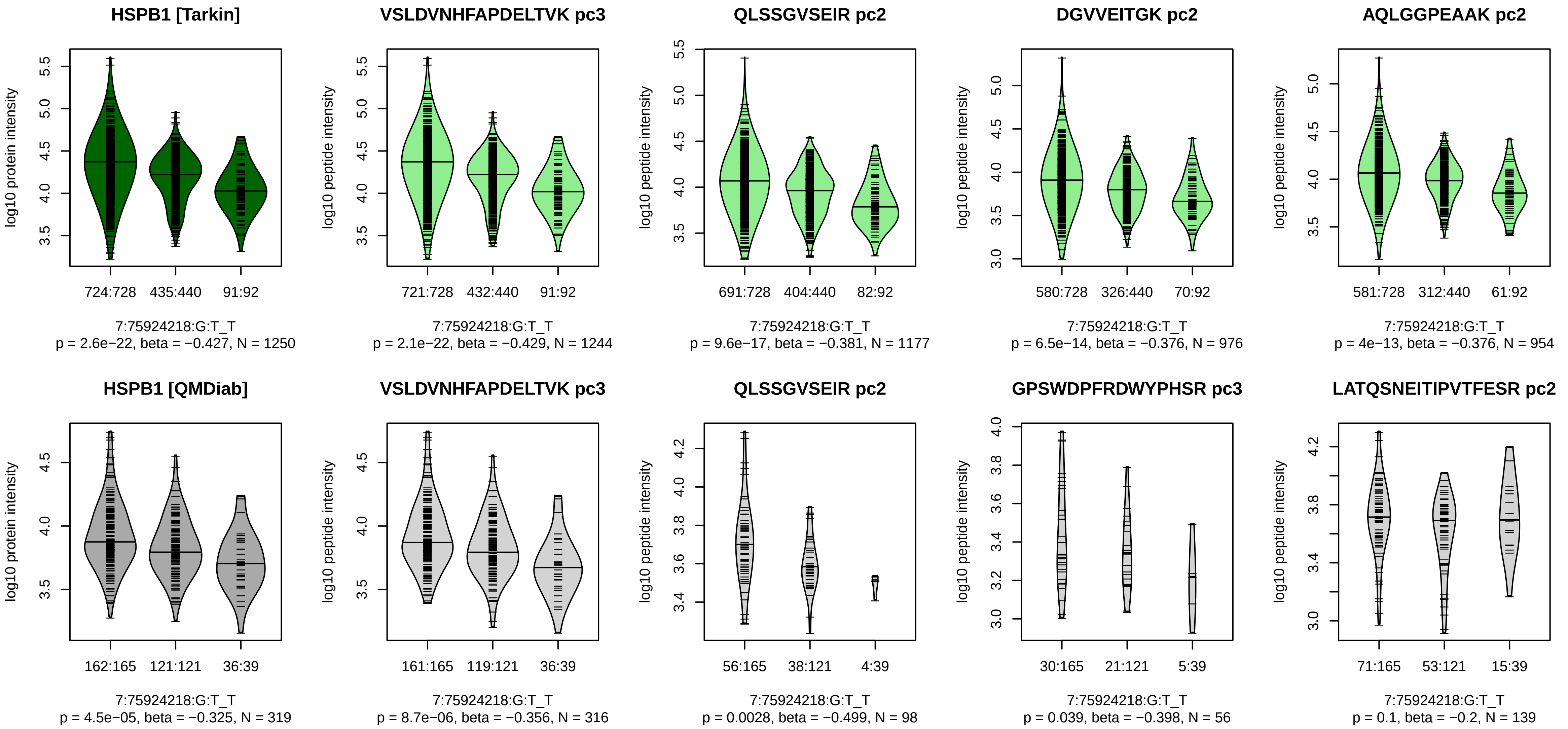

Sort: 68
Gene name: HSPB1
SNP: 7:75924218:G:T
rsID: rs2908203
Type: Cis−pQTL
Protein group(s):
A0A6Q8PFK8;P04792
Nano−particle: NP4

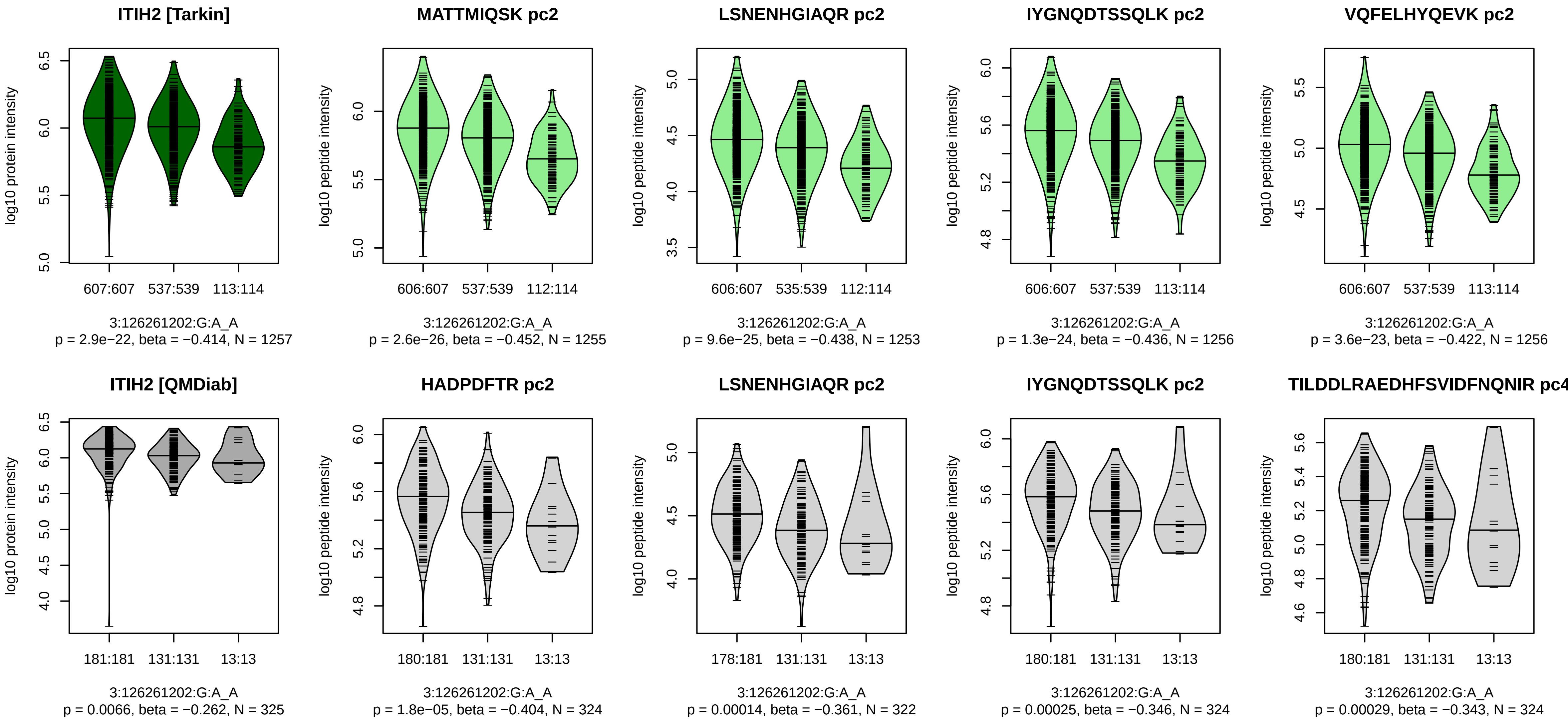

Sort: 69
Gene name: ITIH2
SNP: 3:126261202:G:A
rsID: rs1056524
Type: Trans−pQTL
Protein group(s):
P19823
Nano−particle: NP2

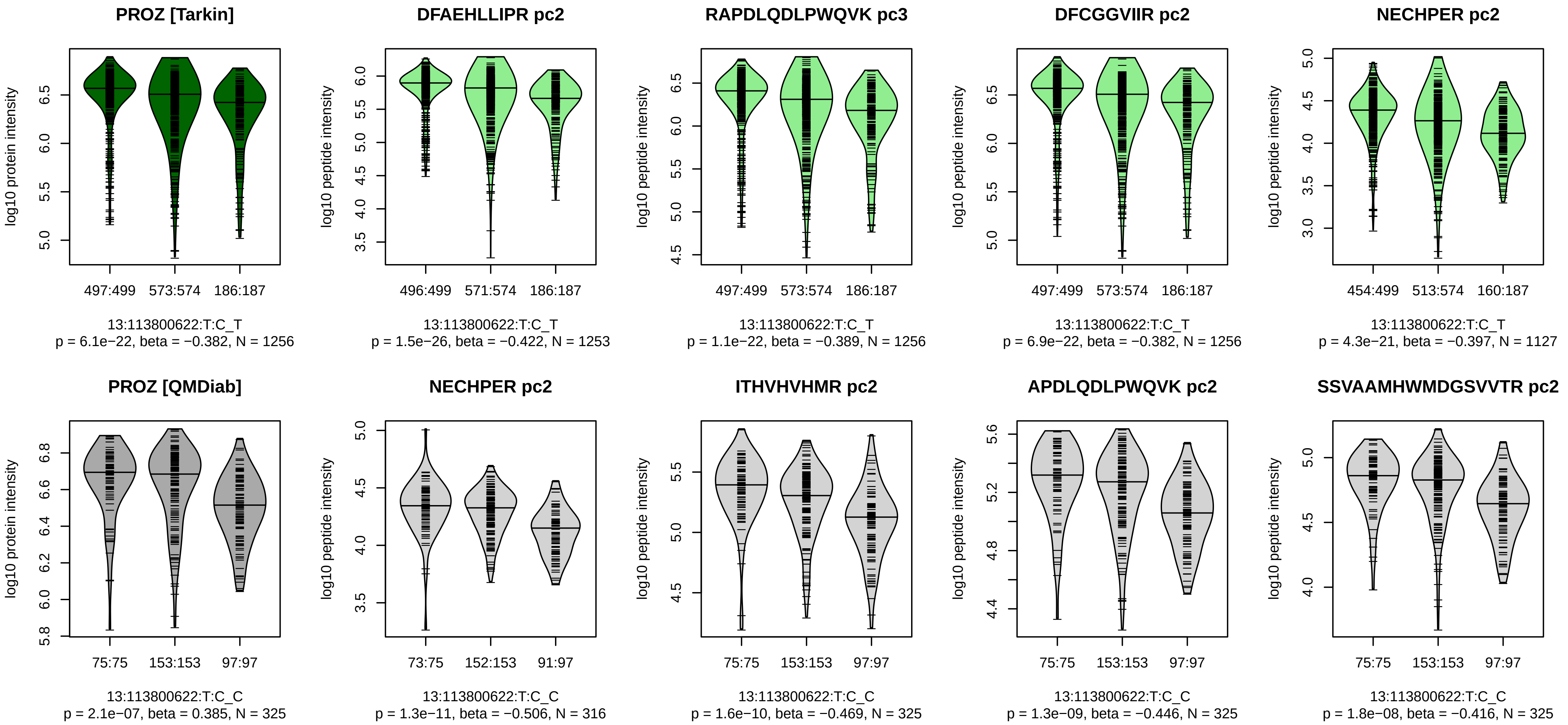

Sort: 70
Gene name: PROZ
SNP: 13:113800622:T:C
rsID: rs559054
Type: Cis−pQTL
Protein group(s):
P22891;P22891−2
Nano−particle: NP2

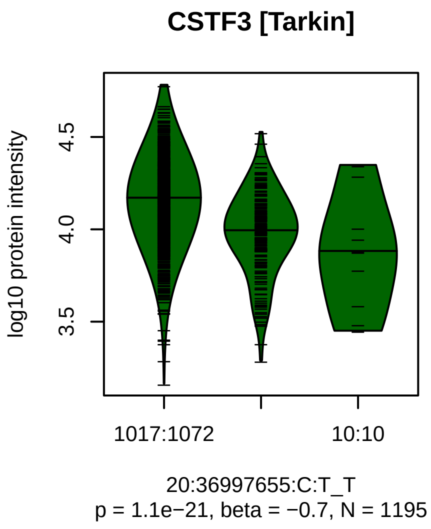

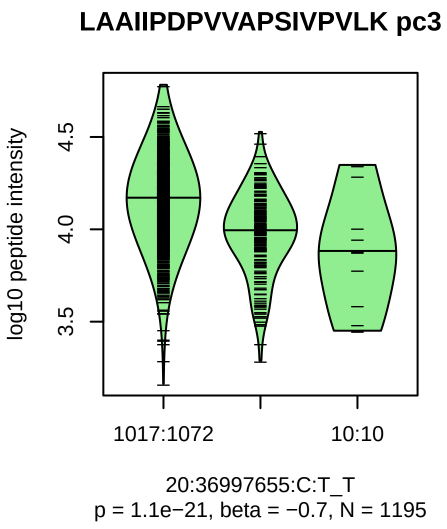

No protein data available
for replication in QMDiab

Sort: 71
Gene name: CSTF3
SNP: 20:36997655:C:T
rsID: rs2232613
Type: Trans−pQTL
Protein group(s):
Q12996
Nano−particle: NP4

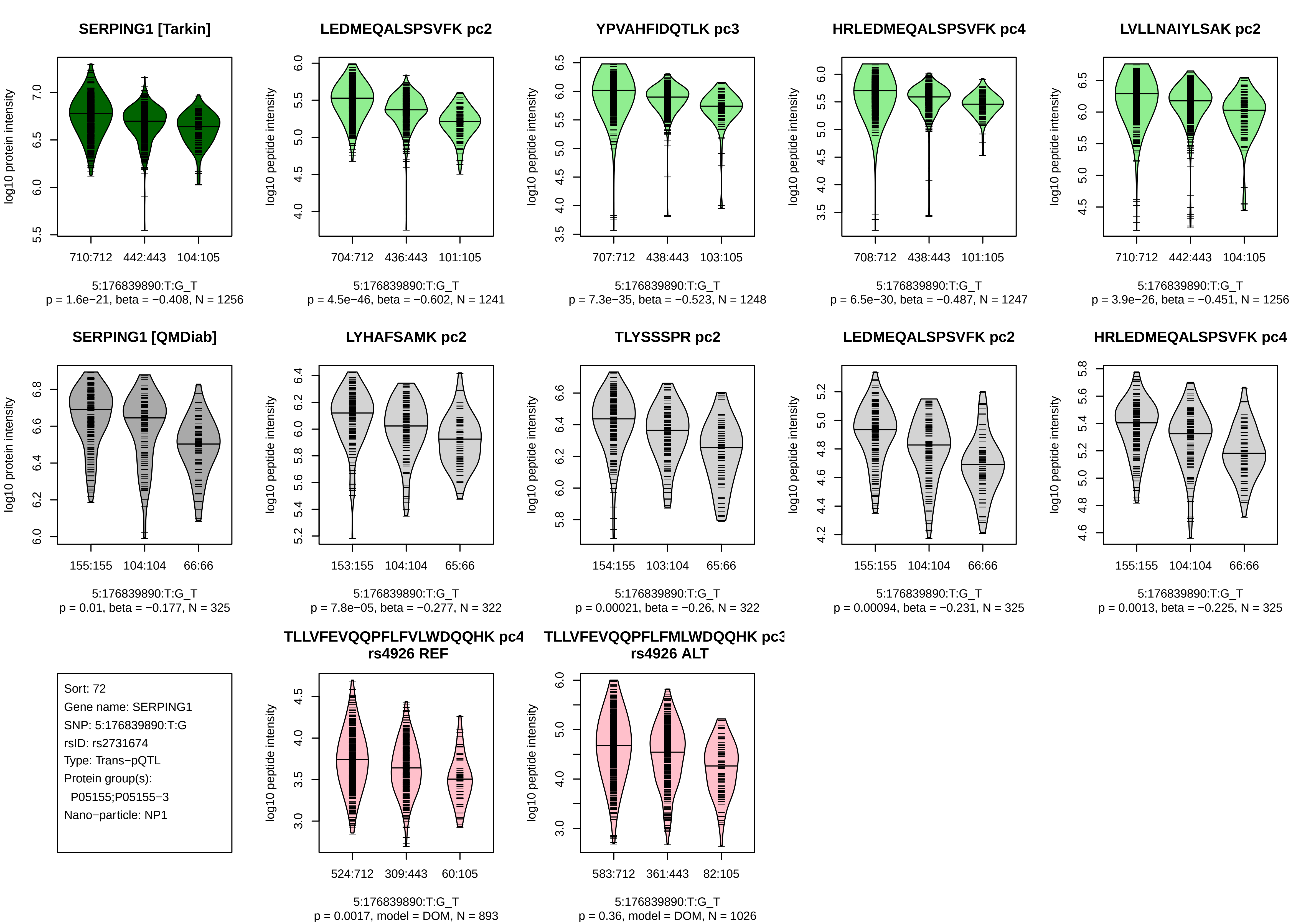
SERPING1 [Tarkin]
log10 protein intensity
710:712
442:443
104:105
5:176839890:T:G_T
p = 1.6e−21, beta = −0.408, N = 1256
LEDMEQALSPSVFK pc2
log10 peptide intensity
704:712
436:443
101:105
5:176839890:T:G_T
p = 4.5e−46, beta = −0.602, N = 1241
YPVAHFIDQTLK pc3
log10 peptide intensity
707:712
438:443
103:105
5:176839890:T:G_T
p = 7.3e−35, beta = −0.523, N = 1248
HRLEDMEQALSPSVFK pc4
log10 peptide intensity
708:712
438:443
101:105
5:176839890:T:G_T
p = 6.5e−30, beta = −0.487, N = 1247
LVLLNAIYLSAK pc2
log10 peptide intensity
710:712
442:443
104:105
5:176839890:T:G_T
p = 3.9e−26, beta = −0.451, N = 1256
SERPING1 [QMDiab]
log10 protein intensity
155:155
104:104
66:66
5:176839890:T:G_T
p = 0.01, beta = −0.177, N = 325
LYHAFSAMK pc2
log10 peptide intensity
153:155
104:104
65:66
5:176839890:T:G_T
p = 7.8e−05, beta = −0.277, N = 322
TLYSSSPR pc2
log10 peptide intensity
154:155
103:104
65:66
5:176839890:T:G_T
p = 0.00021, beta = −0.26, N = 322
LEDMEQALSPSVFK pc2
log10 peptide intensity
155:155
104:104
66:66
5:176839890:T:G_T
p = 0.00094, beta = −0.231, N = 325
HRLEDMEQALSPSVFK pc4
log10 peptide intensity
155:155
104:104
66:66
5:176839890:T:G_T
p = 0.0013, beta = −0.225, N = 325
Sort: 72
Gene name: SERPING1
SNP: 5:176839890:T:G
rsID: rs2731674
Type: Trans−pQTL
Protein group(s):
P05155;P05155−3
Nano−particle: NP1
TLLVFEVQQPFLFVLWDQQHK pc4
rs4926 REF
log10 peptide intensity
524:712
309:443
60:105
5:176839890:T:G_T
p = 0.0017, model = DOM, N = 893
TLLVFEVQQPFLFMLWDQQHK pc3
rs4926 ALT
log10 peptide intensity
583:712
361:443
82:105
5:176839890:T:G_T
p = 0.36, model = DOM, N = 1026

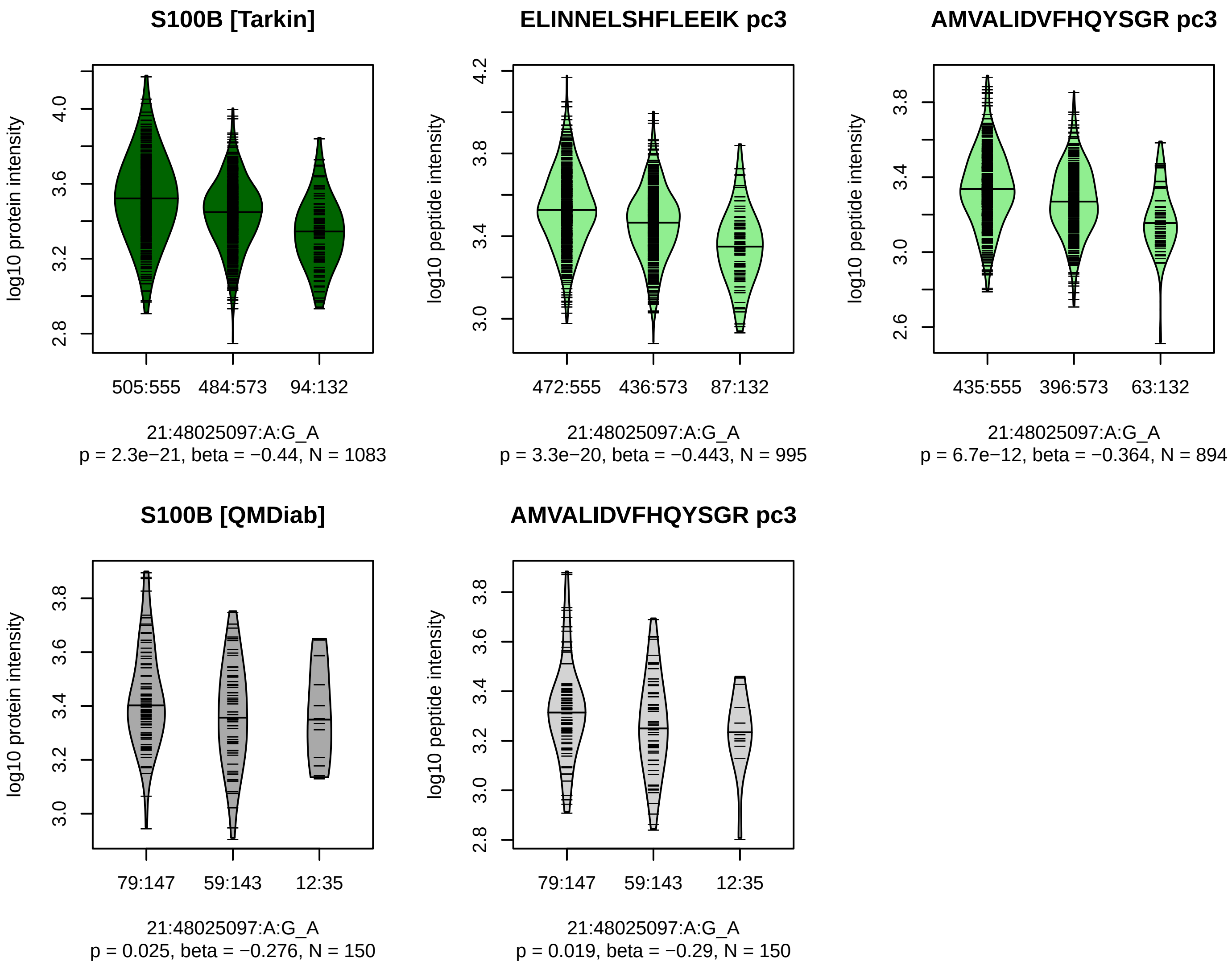

Sort: 73
Gene name: S100B
SNP: 21:48025097:A:G
rsID: rs8128872
Type: Cis−pQTL
Protein group(s):
P04271
Nano−particle: NP2

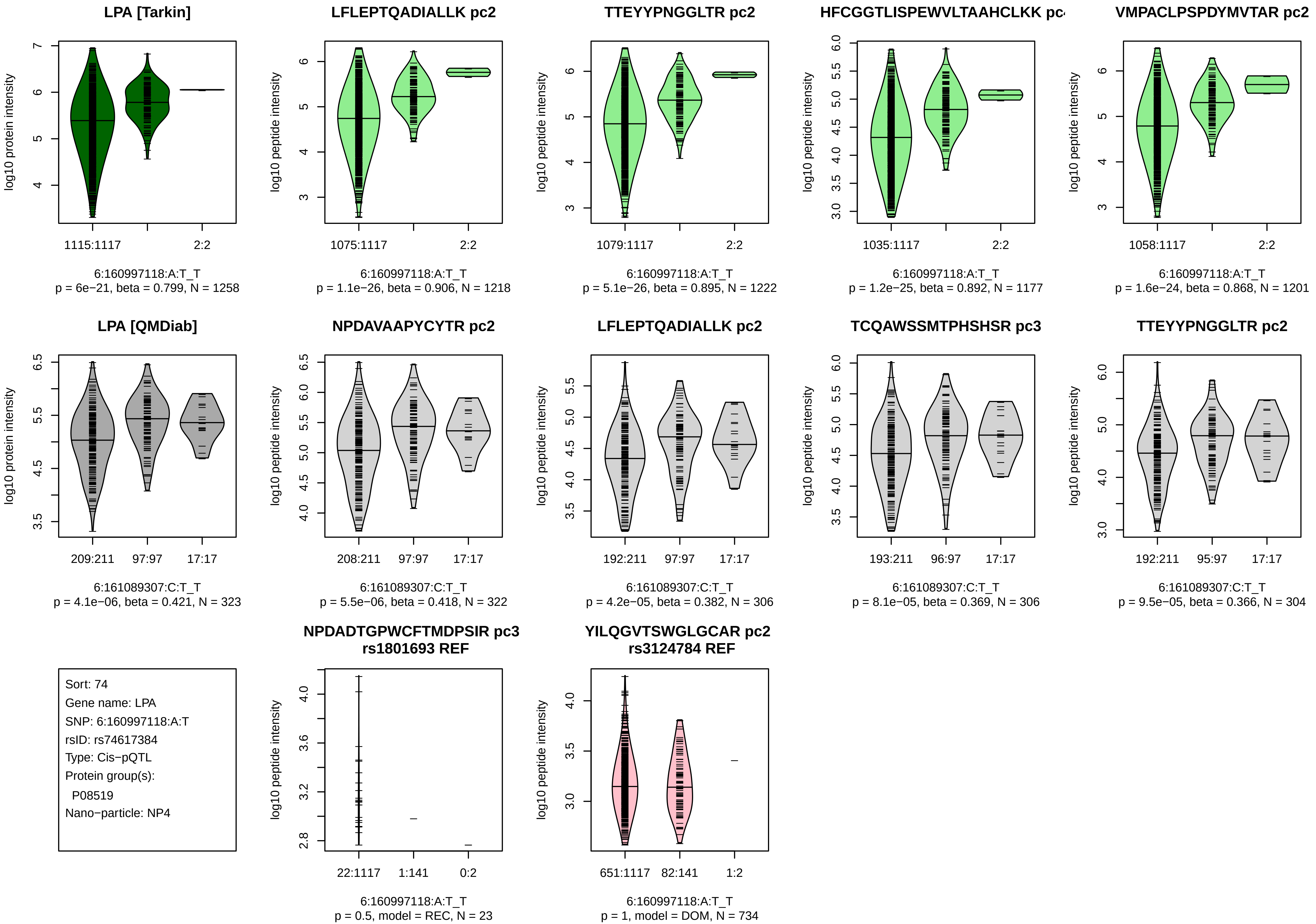
LPA [Tarkin]
6:160997118:A:T_T
p = 6e−21, beta = 0.799, N = 1258
LFLEPTQADIALLK pc2
6:160997118:A:T_T
p = 1.1e−26, beta = 0.906, N = 1218
TTEYYPNGGLTR pc2
6:160997118:A:T_T
p = 5.1e−26, beta = 0.895, N = 1222
HFCGGTLISPEWVLTAAHCLKK pc
6:160997118:A:T_T
p = 1.2e−25, beta = 0.892, N = 1177
VMPACLPSPDYMVTAR pc2
6:160997118:A:T_T
p = 1.6e−24, beta = 0.868, N = 1201
LPA [QMDiab]
6:161089307:C:T_T
p = 4.1e−06, beta = 0.421, N = 323
NPDAVAAPYCYTR pc2
6:161089307:C:T_T
p = 5.5e−06, beta = 0.418, N = 322
LFLEPTQADIALLK pc2
6:161089307:C:T_T
p = 4.2e−05, beta = 0.382, N = 306
TCQAWSSMTPHSHSR pc3
6:161089307:C:T_T
p = 8.1e−05, beta = 0.369, N = 306
TTEYYPNGGLTR pc2
6:161089307:C:T_T
p = 9.5e−05, beta = 0.366, N = 304
Sort: 74
Gene name: LPA
SNP: 6:160997118:A:T
rsID: rs74617384
Type: Cis−pQTL
Protein group(s):
P08519
Nano−particle: NP4
NPDADTGPWCFTMDPSIR pc3
rs1801693 REF
6:160997118:A:T_T
p = 0.5, model = REC, N = 23
YILQGVTSWGLGCAR pc2
rs3124784 REF
6:160997118:A:T_T
p = 1, model = DOM, N = 734

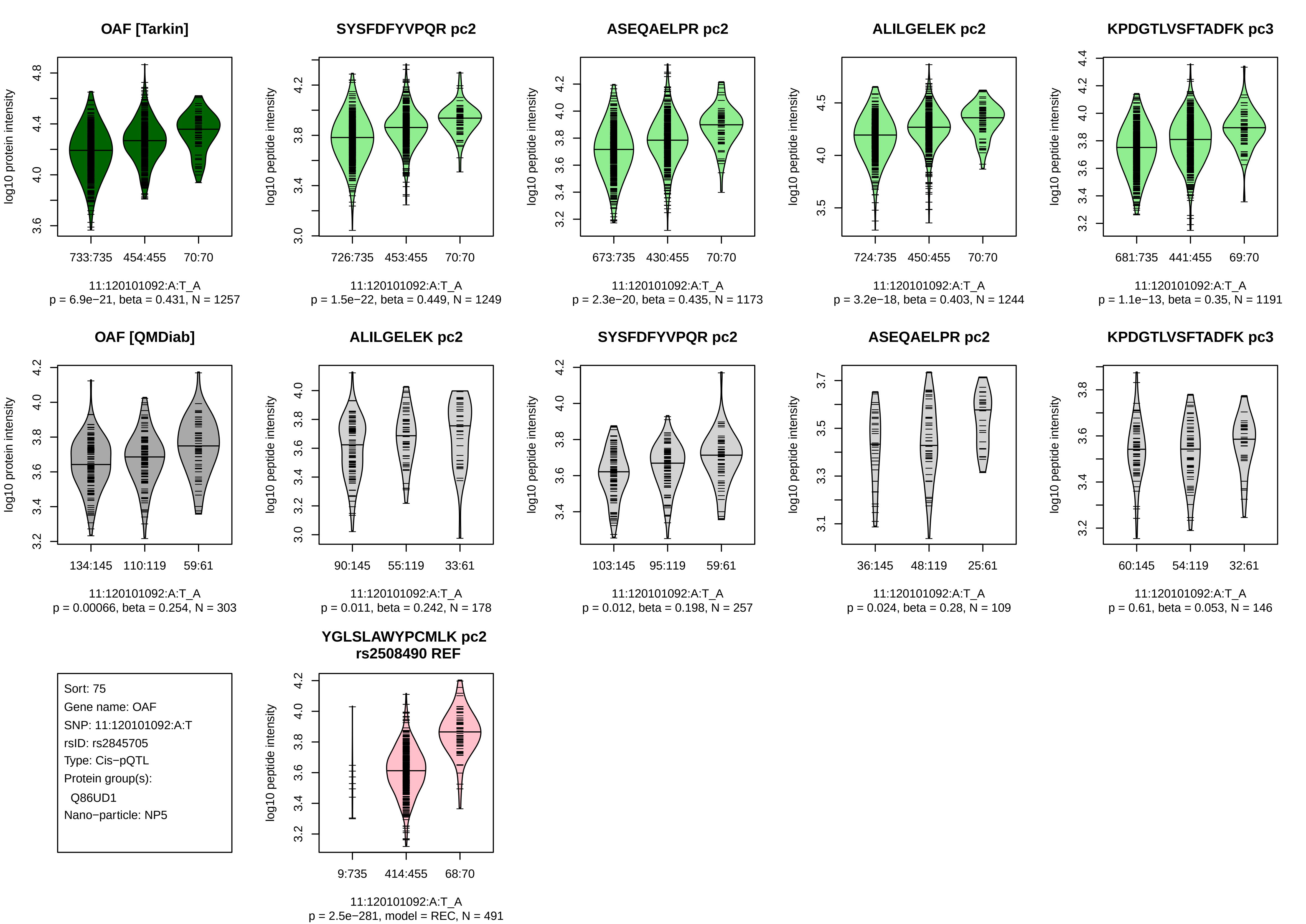
OAF [Tarkin]
log10 protein intensity
733:735
454:455
70:70
11:120101092:A:T_A
p = 6.9e−21, beta = 0.431, N = 1257
SYSFDFYVPQR pc2
log10 peptide intensity
726:735
453:455
70:70
11:120101092:A:T_A
p = 1.5e−22, beta = 0.449, N = 1249
ASEQAELPR pc2
log10 peptide intensity
673:735
430:455
70:70
11:120101092:A:T_A
p = 2.3e−20, beta = 0.435, N = 1173
ALILGELEK pc2
log10 peptide intensity
724:735
450:455
70:70
11:120101092:A:T_A
p = 3.2e−18, beta = 0.403, N = 1244
KPDGTLVSFTADFK pc3
log10 peptide intensity
681:735
441:455
69:70
11:120101092:A:T_A
p = 1.1e−13, beta = 0.35, N = 1191
OAF [QMDiab]
log10 protein intensity
134:145
110:119
59:61
11:120101092:A:T_A
p = 0.00066, beta = 0.254, N = 303
ALILGELEK pc2
log10 peptide intensity
90:145
55:119
33:61
11:120101092:A:T_A
p = 0.011, beta = 0.242, N = 178
SYSFDFYVPQR pc2
log10 peptide intensity
103:145
95:119
59:61
11:120101092:A:T_A
p = 0.012, beta = 0.198, N = 257
ASEQAELPR pc2
log10 peptide intensity
36:145
48:119
25:61
11:120101092:A:T_A
p = 0.024, beta = 0.28, N = 109
KPDGTLVSFTADFK pc3
log10 peptide intensity
60:145
54:119
32:61
11:120101092:A:T_A
p = 0.61, beta = 0.053, N = 146
Sort: 75
Gene name: OAF
SNP: 11:120101092:A:T
rsID: rs2845705
Type: Cis−pQTL
Protein group(s):
Q86UD1
Nano−particle: NP5
YGLSLAWYPCMLK pc2
rs2508490 REF
log10 peptide intensity
9:735
414:455
68:70
11:120101092:A:T_A
p = 2.5e−281, model = REC, N = 491

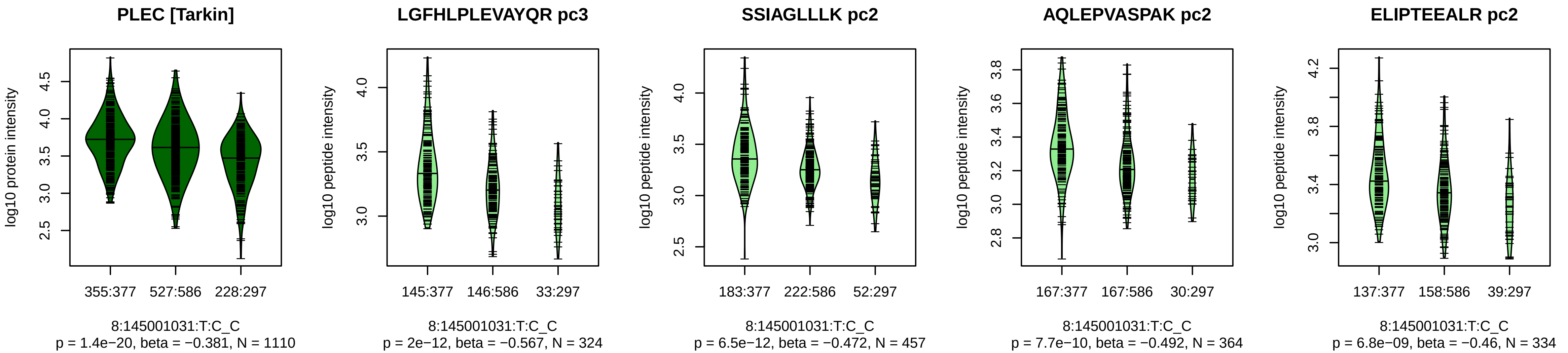

No protein data available
for replication in QMDiab

Sort: 76
Gene name: PLEC
SNP: 8:145001031:T:C
rsID: rs55895668
Type: Cis−pQTL
Protein group(s):
Q15149−4
Nano−particle: NP5

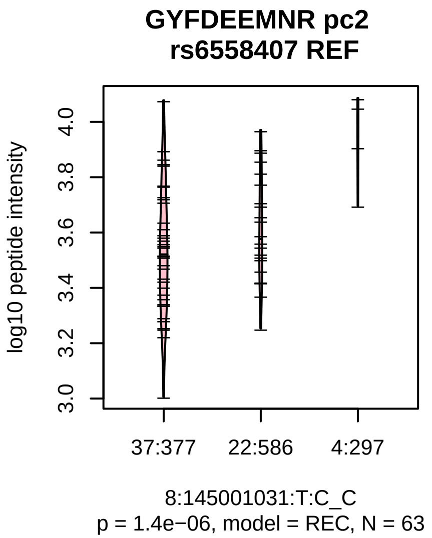

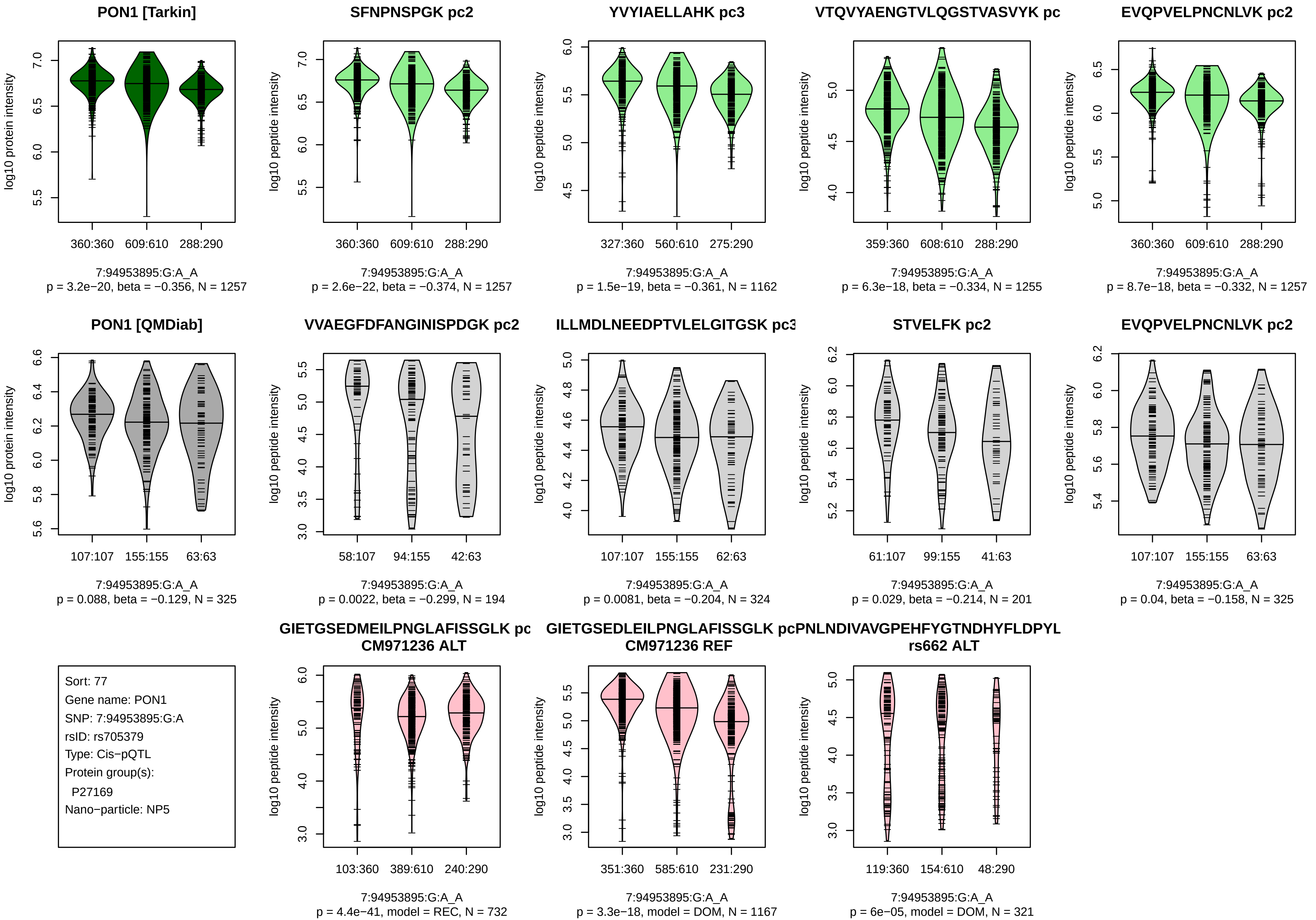
PON1 [Tarkin]
log10 protein intensity
360:360 609:610 288:290
7:94953895:G:A_A
p = 3.2e−20, beta = −0.356, N = 1257
SFNPNSPGK pc2
log10 peptide intensity
360:360 609:610 288:290
7:94953895:G:A_A
p = 2.6e−22, beta = −0.374, N = 1257
YVYIAELLAHK pc3
log10 peptide intensity
327:360 560:610 275:290
7:94953895:G:A_A
p = 1.5e−19, beta = −0.361, N = 1162
VTQVYAENGTVLQGSTVASVYK pc
log10 peptide intensity
359:360 608:610 288:290
7:94953895:G:A_A
p = 6.3e−18, beta = −0.334, N = 1255
EVQPVELPNCNLVK pc2
log10 peptide intensity
360:360 609:610 288:290
7:94953895:G:A_A
p = 8.7e−18, beta = −0.332, N = 1257
PON1 [QMDiab]
log10 protein intensity
107:107 155:155 63:63
7:94953895:G:A_A
p = 0.088, beta = −0.129, N = 325
VVAEGFDFANGINISPDGK pc2
log10 peptide intensity
58:107 94:155 42:63
7:94953895:G:A_A
p = 0.0022, beta = −0.299, N = 194
ILLMDLNEEDPTVLELGITGSK pc3
log10 peptide intensity
107:107 155:155 62:63
7:94953895:G:A_A
p = 0.0081, beta = −0.204, N = 324
STVELFK pc2
log10 peptide intensity
61:107 99:155 41:63
7:94953895:G:A_A
p = 0.029, beta = −0.214, N = 201
EVQPVELPNCNLVK pc2
log10 peptide intensity
107:107 155:155 63:63
7:94953895:G:A_A
p = 0.04, beta = −0.158, N = 325
Sort: 77
Gene name: PON1
SNP: 7:94953895:G:A
rsID: rs705379
Type: Cis−pQTL
Protein group(s):
P27169
Nano−particle: NP5
GIETGSEDMEILPNGLAFISSGLK pc
CM971236 ALT
log10 peptide intensity
103:360 389:610 240:290
7:94953895:G:A_A
p = 4.4e−41, model = REC, N = 732
GIETGSEDLEILPNGLAFISSGLK pc
CM971236 REF
log10 peptide intensity
351:360 585:610 231:290
7:94953895:G:A_A
p = 3.3e−18, model = DOM, N = 1167
PNLNDIVAVGPEHFYGTNDHYFLDPYL
rs662 ALT
log10 peptide intensity
119:360 154:610 48:290
7:94953895:G:A_A
p = 6e−05, model = DOM, N = 321

HEATR6 [Tarkin]

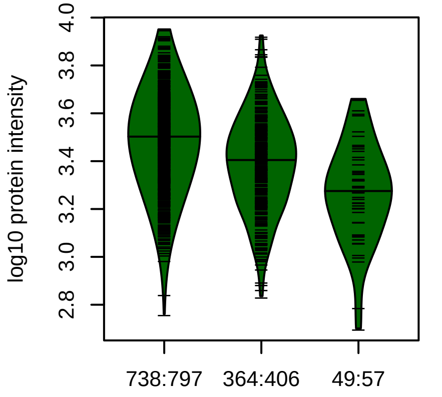

1:196822368:A:G_G
p = 6.2e−20, beta = −0.462, N = 1151

QDVIFVADAANAILMSLEDK pc3

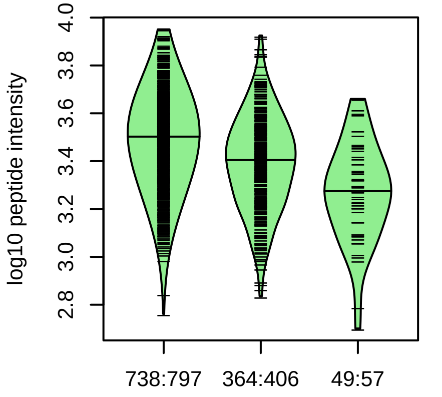

1:196822368:A:G_G
p = 6.2e−20, beta = −0.462, N = 1151

HEATR6 [QMDiab]

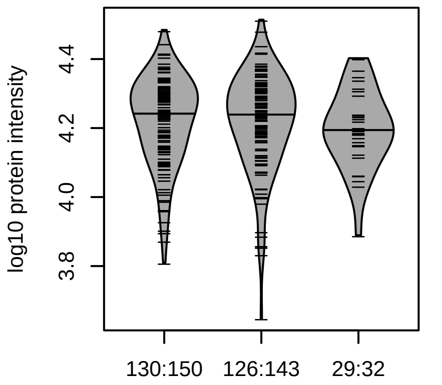

1:196822368:A:G_G
p = 0.44, beta = −0.0698, N = 285

QDVIFVADAANAILMSLEDK pc3

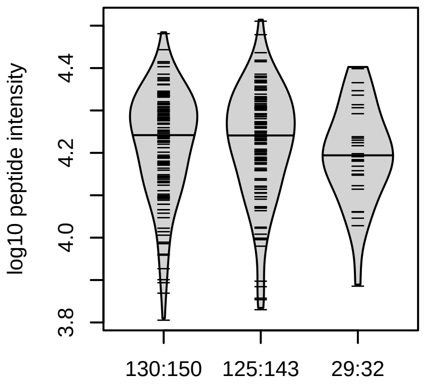

1:196822368:A:G_G
p = 0.51, beta = −0.0591, N = 284

Sort: 78
Gene name: HEATR6
SNP: 1:196822368:A:G
rsID: rs60642321
Type: Trans−pQTL
Protein group(s):
T: Q6AI08
Q: K7EIX2;Q6AI08
Nano−particle: NP4

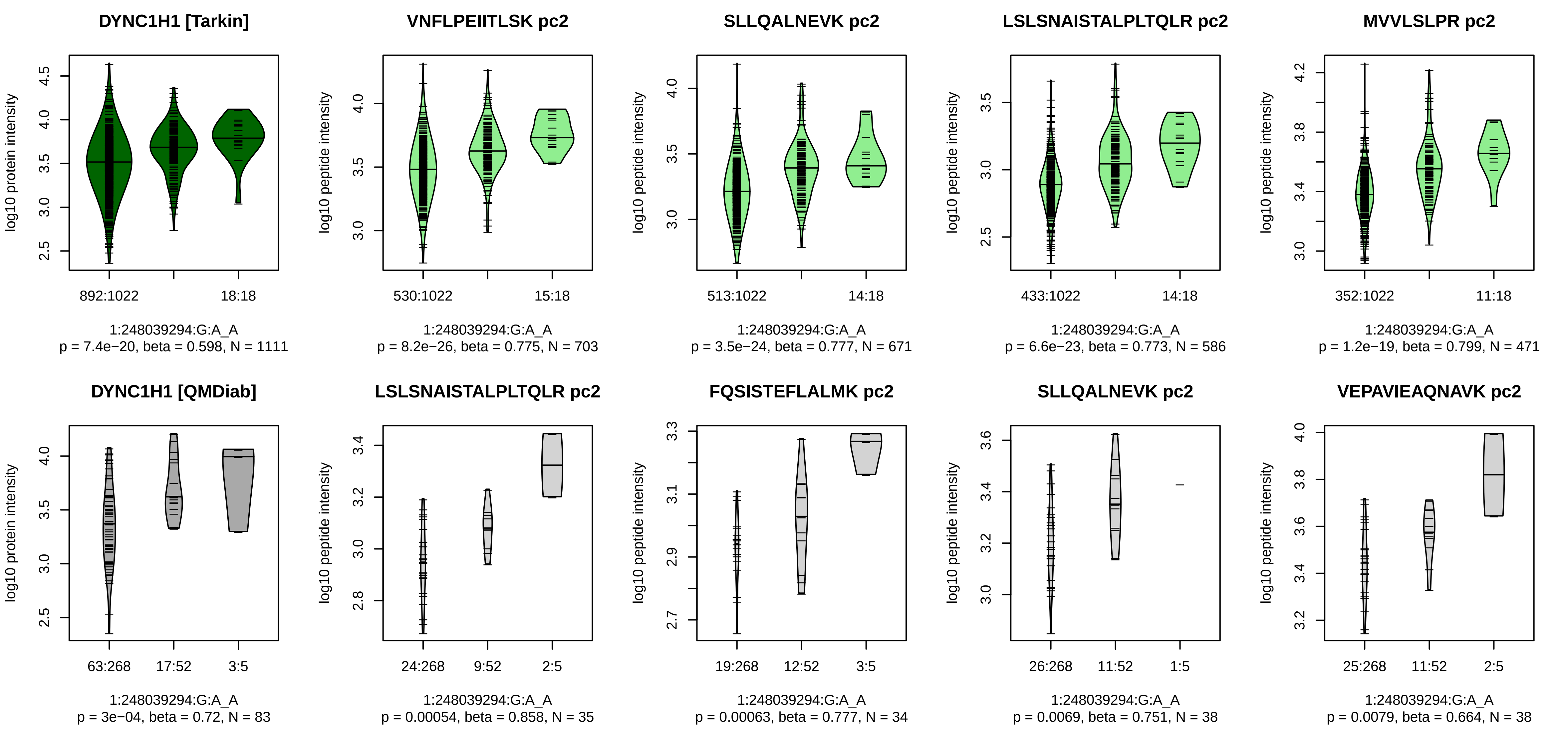

Sort: 79
Gene name: DYNC1H1
SNP: 1:248039294:G:A
rsID: rs1339847
Type: Trans−pQTL
Protein group(s):
T: Q14204
Q: A0A2R8Y706;Q14204
Nano−particle: NP4

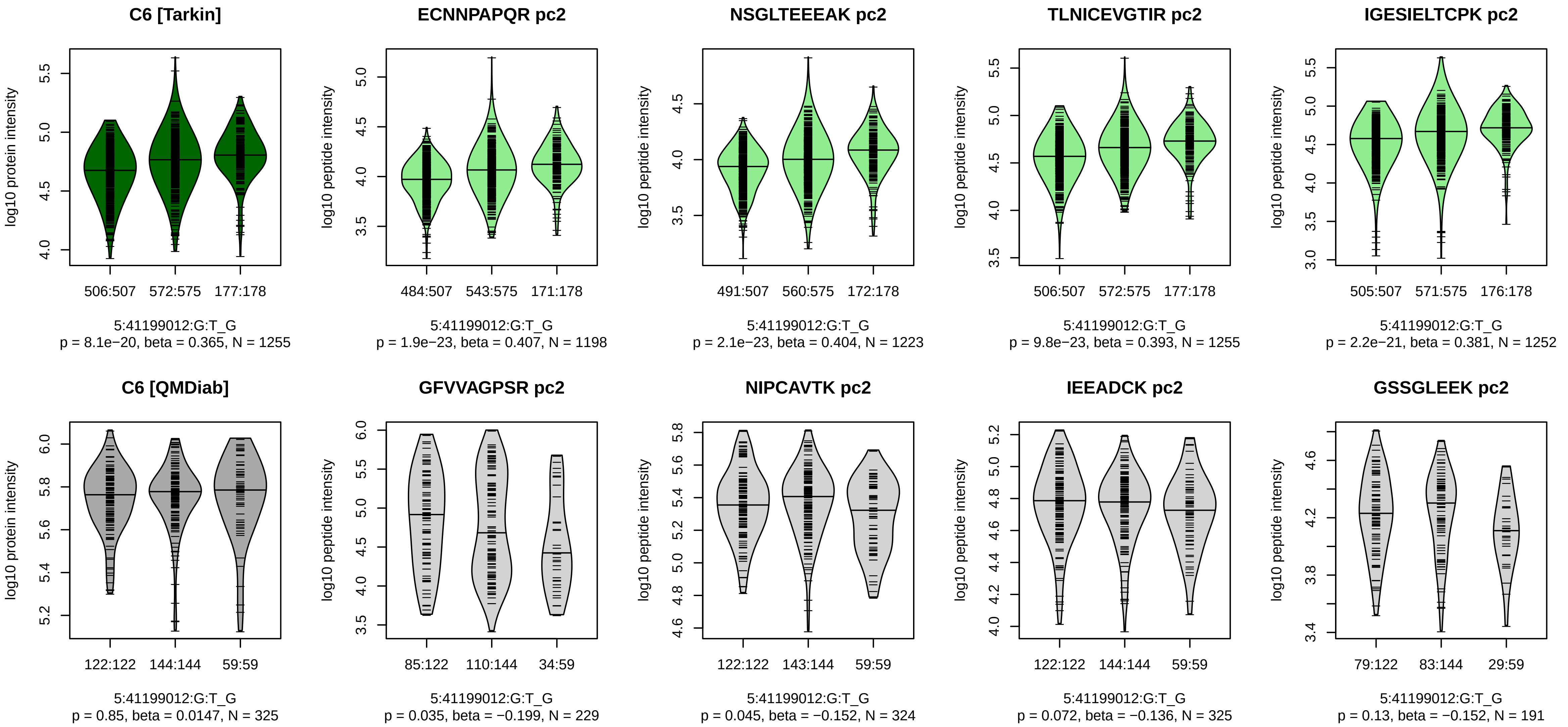

Sort: 80
Gene name: C6
SNP: 5:41199012:G:T
rsID: rs7443604
Type: Cis−pQTL
Protein group(s):
P13671
Nano−particle: NP3

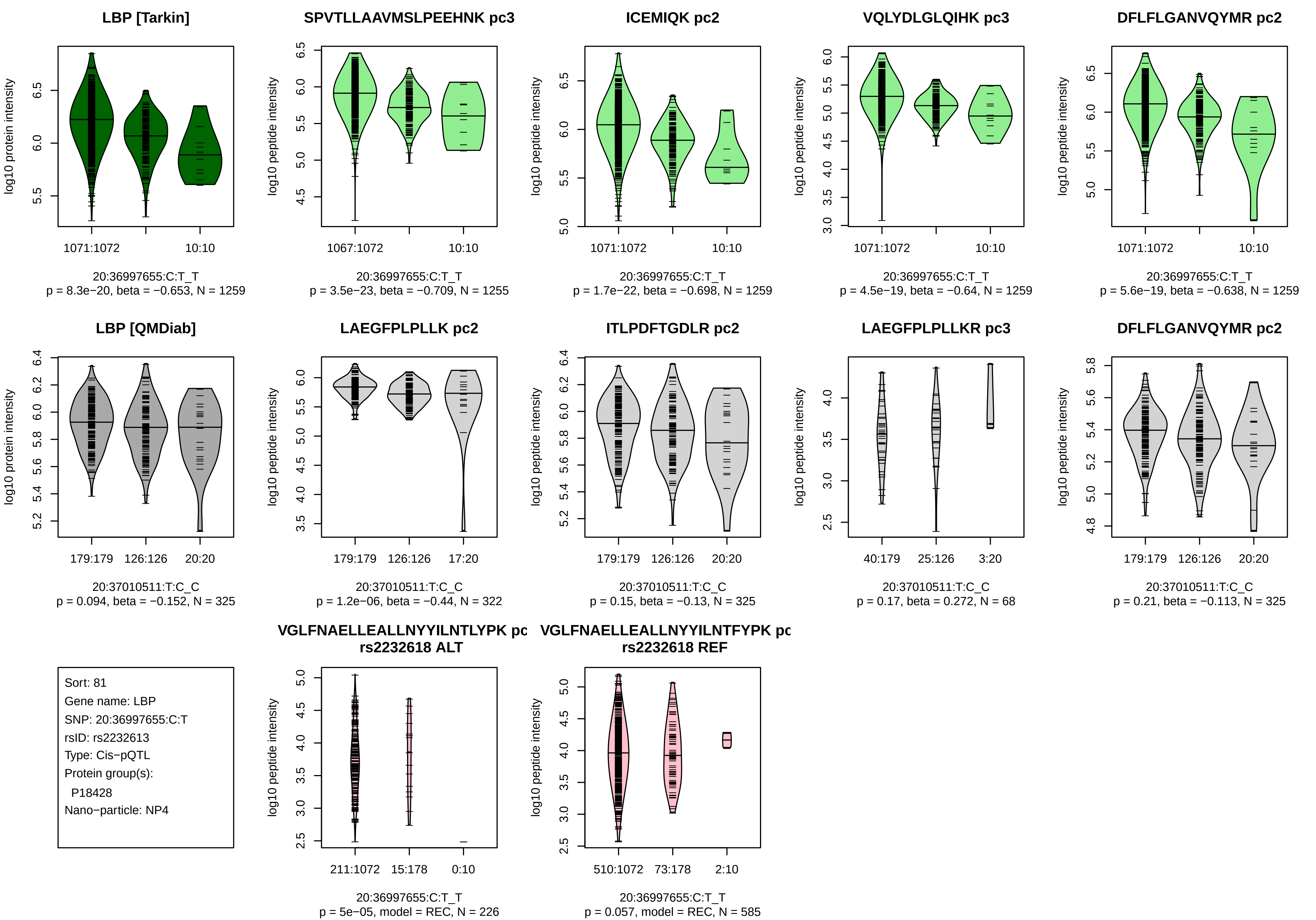

LBP [Tarkin]
log10 protein intensity
1071:1072
10:10
20:36997655:C:T_T
p = 8.3e−20, beta = −0.653, N = 1259
SPVTLLAAVMSLPEEHNK pc3
log10 peptide intensity
1067:1072
10:10
20:36997655:C:T_T
p = 3.5e−23, beta = −0.709, N = 1255
ICEMIQK pc2
log10 peptide intensity
1071:1072
10:10
20:36997655:C:T_T
p = 1.7e−22, beta = −0.698, N = 1259
VQLYDLGLQIHK pc3
log10 peptide intensity
1071:1072
10:10
20:36997655:C:T_T
p = 4.5e−19, beta = −0.64, N = 1259
DFLFLGANVQYMR pc2
log10 peptide intensity
1071:1072
10:10
20:36997655:C:T_T
p = 5.6e−19, beta = −0.638, N = 1259
LBP [QMDiab]
log10 protein intensity
179:179
126:126
20:20
20:37010511:T:C_C
p = 0.094, beta = −0.152, N = 325
LAEGFPLPLLK pc2
log10 peptide intensity
179:179
126:126
17:20
20:37010511:T:C_C
p = 1.2e−06, beta = −0.44, N = 322
ITLPDFTGDLR pc2
log10 peptide intensity
179:179
126:126
20:20
20:37010511:T:C_C
p = 0.15, beta = −0.13, N = 325
LAEGFPLPLLKR pc3
log10 peptide intensity
40:179
25:126
3:20
20:37010511:T:C_C
p = 0.17, beta = 0.272, N = 68
DFLFLGANVQYMR pc2
log10 peptide intensity
179:179
126:126
20:20
20:37010511:T:C_C
p = 0.21, beta = −0.113, N = 325
Sort: 81
Gene name: LBP
SNP: 20:36997655:C:T
rsID: rs2232613
Type: Cis−pQTL
Protein group(s):
P18428
Nano−particle: NP4
VGLFNAELLEALLNYYILNTLYPK pc
rs2232618 ALT
log10 peptide intensity
211:1072
15:178
0:10
20:36997655:C:T_T
p = 5e−05, model = REC, N = 226
VGLFNAELLEALLNYYILNTFYPK pc
rs2232618 REF
log10 peptide intensity
510:1072
73:178
2:10
20:36997655:C:T_T
p = 0.057, model = REC, N = 585

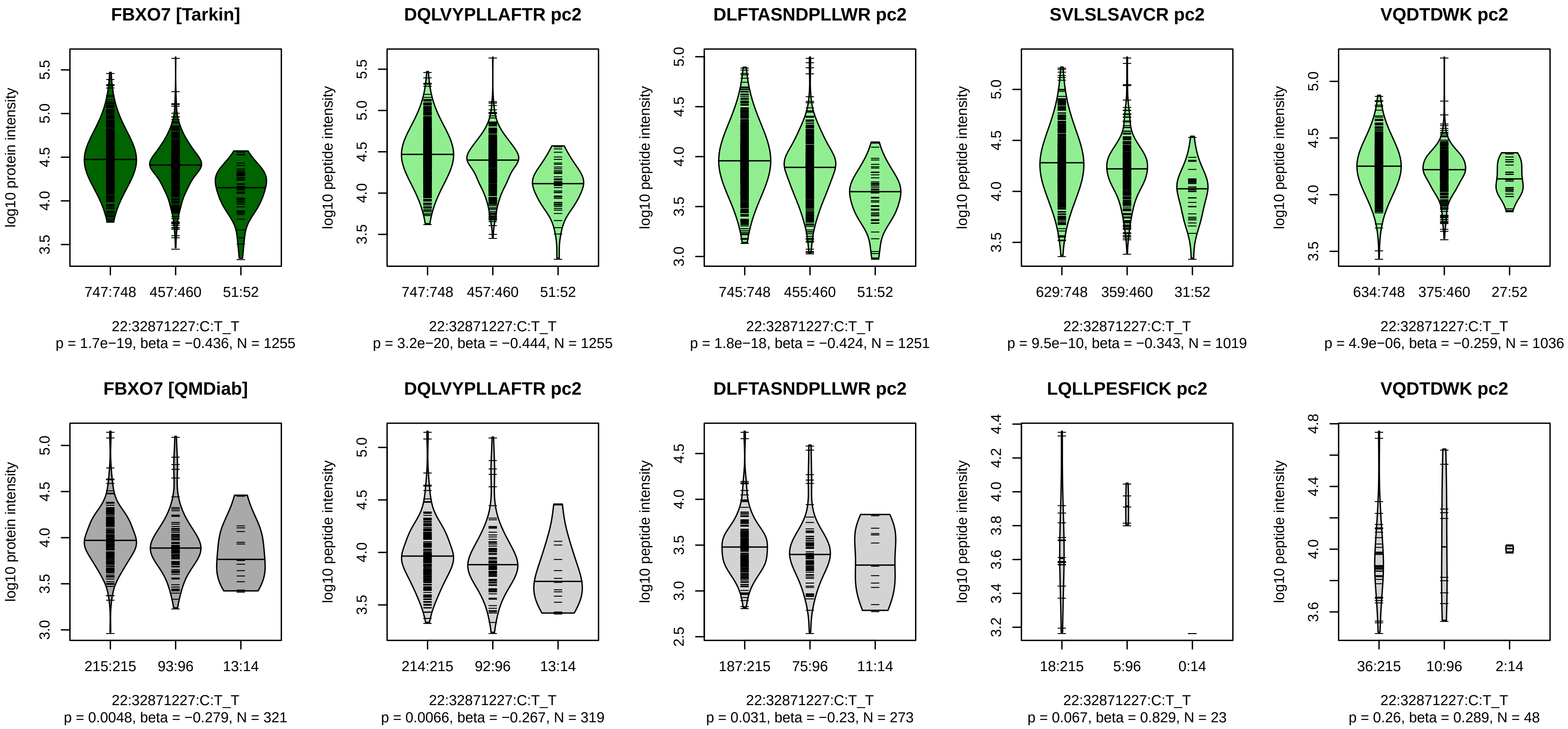

Sort: 82
Gene name: FBXO7
SNP: 22:32871227:C:T
rsID: rs8136485
Type: Cis−pQTL
Protein group(s):
T: Q9Y3I1
Q: Q9Y3I1;Q9Y3I1−2;Q9Y3I1−3
Nano−particle: NP1

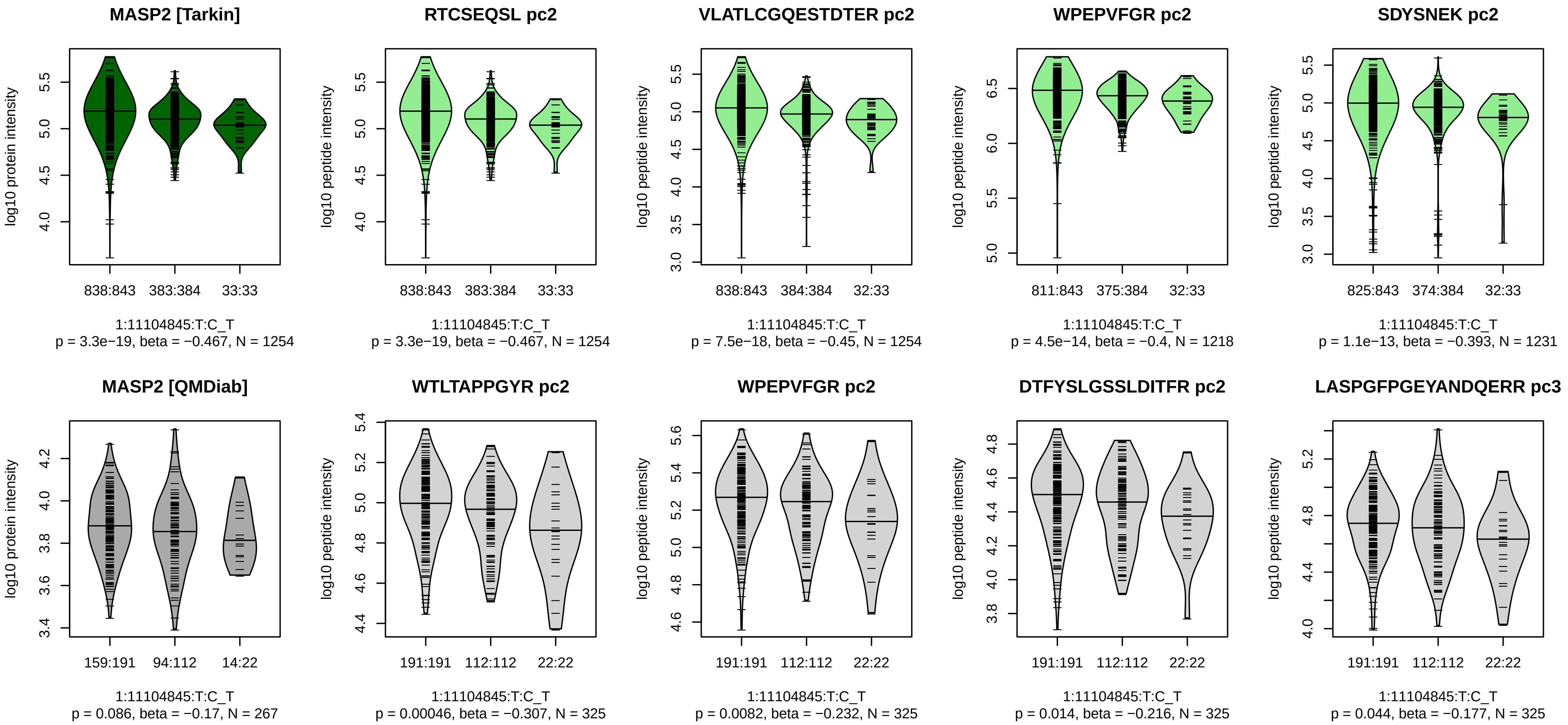

Sort: 83
Gene name: MASP2
SNP: 1:11104845:T:C
rsID: rs9430347
Type: Cis−pQTL
Protein group(s):
O00187−2
Nano−particle: NP2

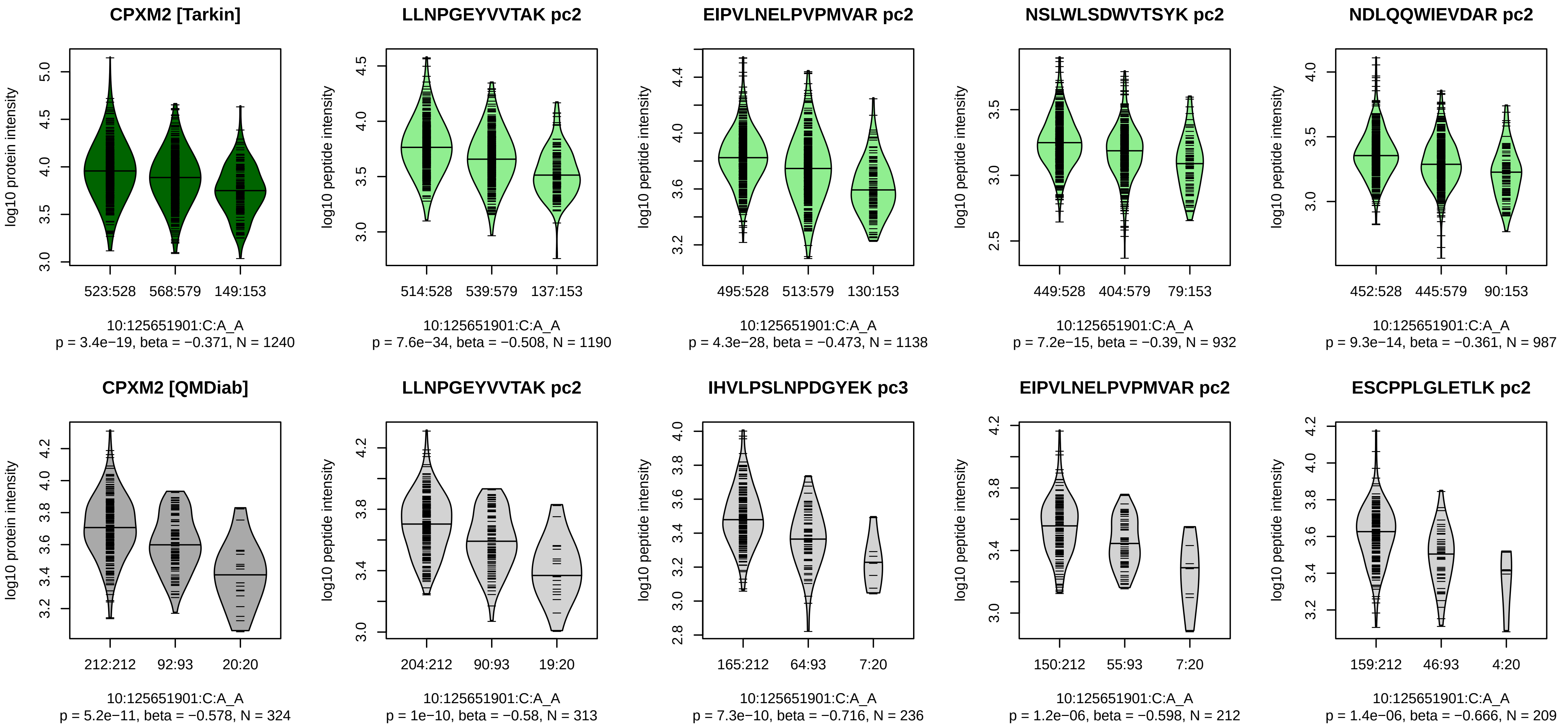

Sort: 84
Gene name: CPXM2
SNP: 10:125651901:C:A
rsID: rs3862127
Type: Cis−pQTL
Protein group(s):
Q8N436
Nano−particle: NP5

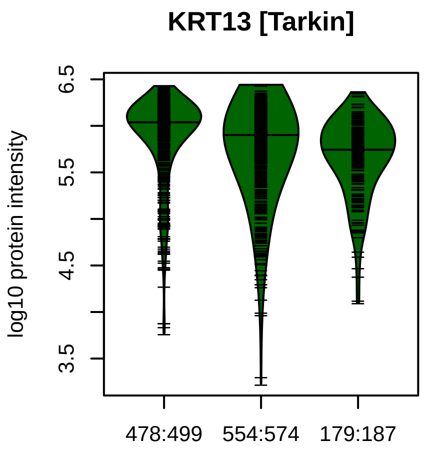

13:113800622:T:C_T
p = 1.1e−18, beta = −0.358, N = 1211

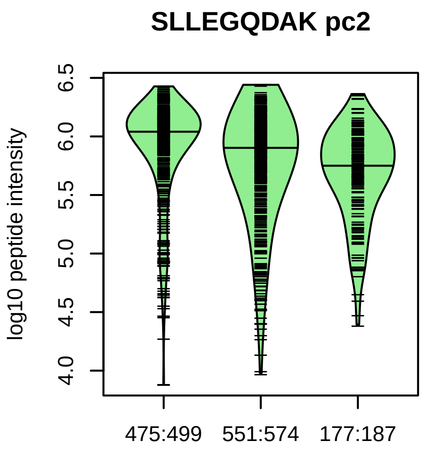

13:113800622:T:C_T
p = 2.7e−19, beta = −0.366, N = 1203

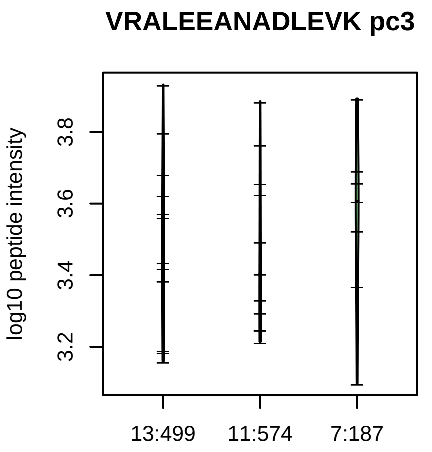

13:113800622:T:C_T
p = 0.21, beta = 0.269, N = 31

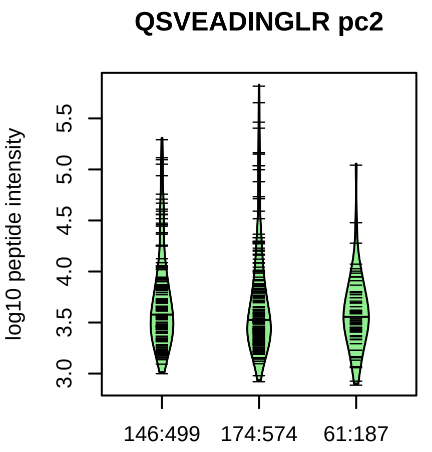

13:113800622:T:C_T
p = 0.22, beta = −0.0879, N = 381

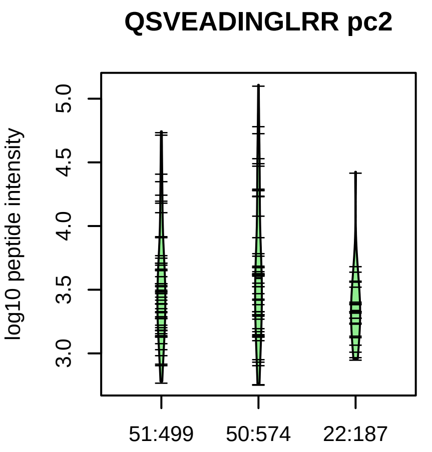

13:113800622:T:C_T
p = 0.3, beta = −0.124, N = 123

No protein data available
for replication in QMDiab

Sort: 85
Gene name: KRT13
SNP: 13:113800622:T:C
rsID: rs559054
Type: Trans−pQTL
Protein group(s):
CONP13646−1
Nano−particle: NP2

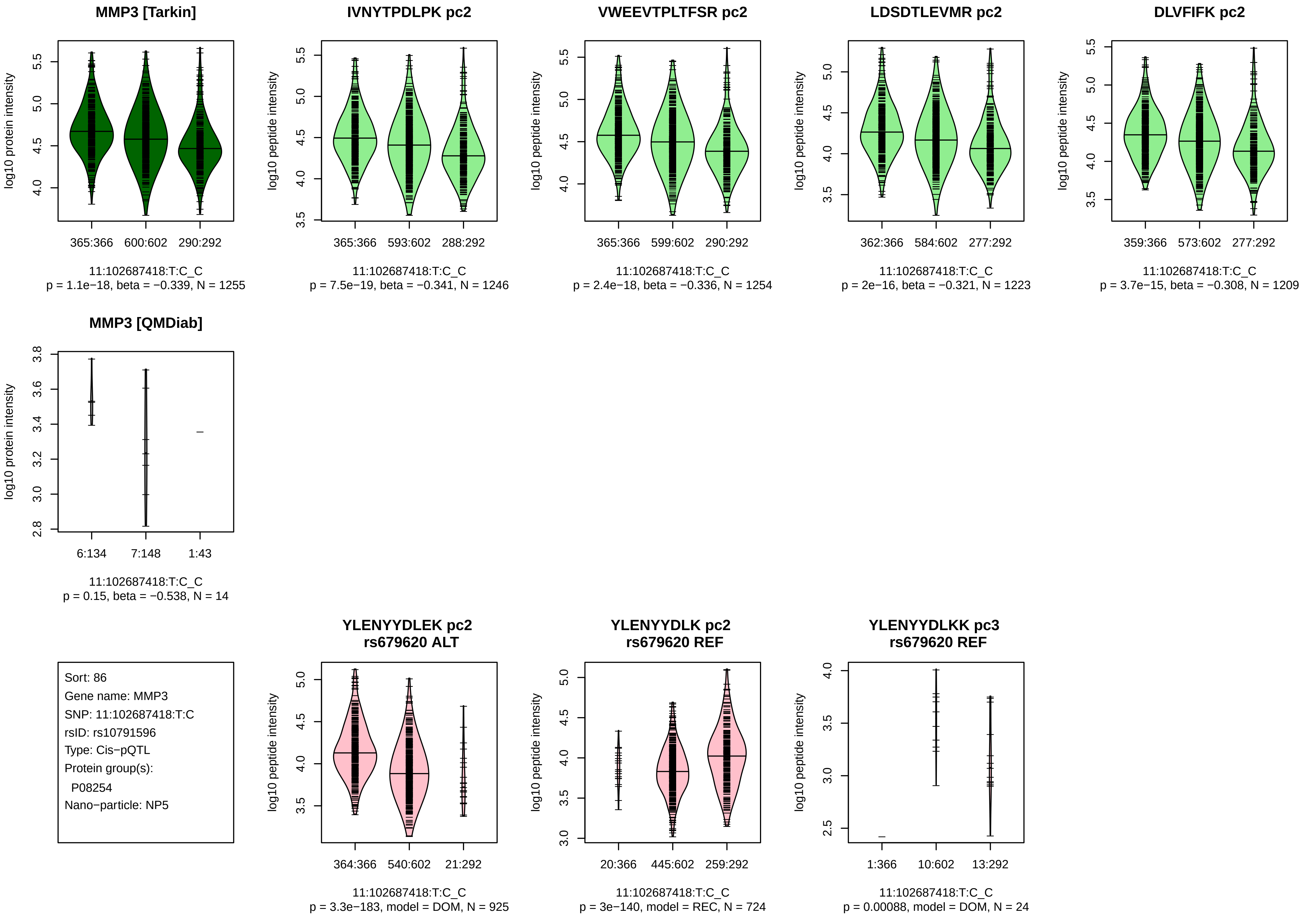
MMP3 [Tarkin]
log10 protein intensity
365:366
600:602
290:292
11:102687418:T:C_C
p = 1.1e−18, beta = −0.339, N = 1255
IVNYTPDLPK pc2
log10 peptide intensity
365:366
593:602
288:292
11:102687418:T:C_C
p = 7.5e−19, beta = −0.341, N = 1246
VWEEVTPLTFSR pc2
log10 peptide intensity
365:366
599:602
290:292
11:102687418:T:C_C
p = 2.4e−18, beta = −0.336, N = 1254
LDSDTLEVMR pc2
log10 peptide intensity
362:366
584:602
277:292
11:102687418:T:C_C
p = 2e−16, beta = −0.321, N = 1223
DLVFIFK pc2
log10 peptide intensity
359:366
573:602
277:292
11:102687418:T:C_C
p = 3.7e−15, beta = −0.308, N = 1209
MMP3 [QMDiab]
log10 protein intensity
6:134
7:148
1:43
11:102687418:T:C_C
p = 0.15, beta = −0.538, N = 14
Sort: 86
Gene name: MMP3
SNP: 11:102687418:T:C
rsID: rs10791596
Type: Cis−pQTL
Protein group(s):
P08254
Nano−particle: NP5
YLENYYDLEK pc2
rs679620 ALT
log10 peptide intensity
364:366
540:602
21:292
11:102687418:T:C_C
p = 3.3e−183, model = DOM, N = 925
YLENYYDLK pc2
rs679620 REF
log10 peptide intensity
20:366
445:602
259:292
11:102687418:T:C_C
p = 3e−140, model = REC, N = 724
YLENYYDLKK pc3
rs679620 REF
log10 peptide intensity
1:366
10:602
13:292
11:102687418:T:C_C
p = 0.00088, model = DOM, N = 24

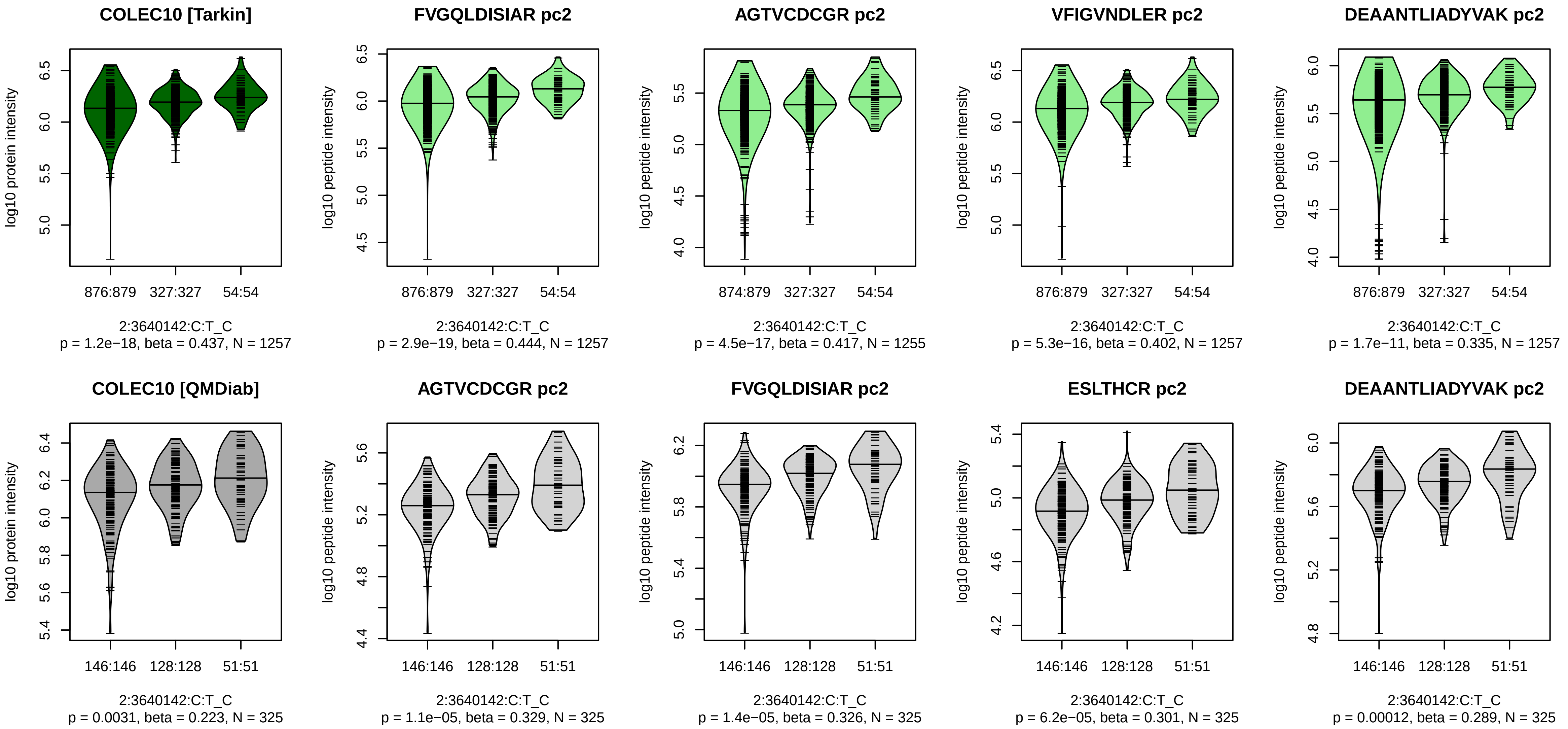

Sort: 87
Gene name: COLEC10
SNP: 2:3640142:C:T
rsID: rs6542680
Type: Trans−pQTL
Protein group(s):
Q9Y6Z7
Nano−particle: NP5

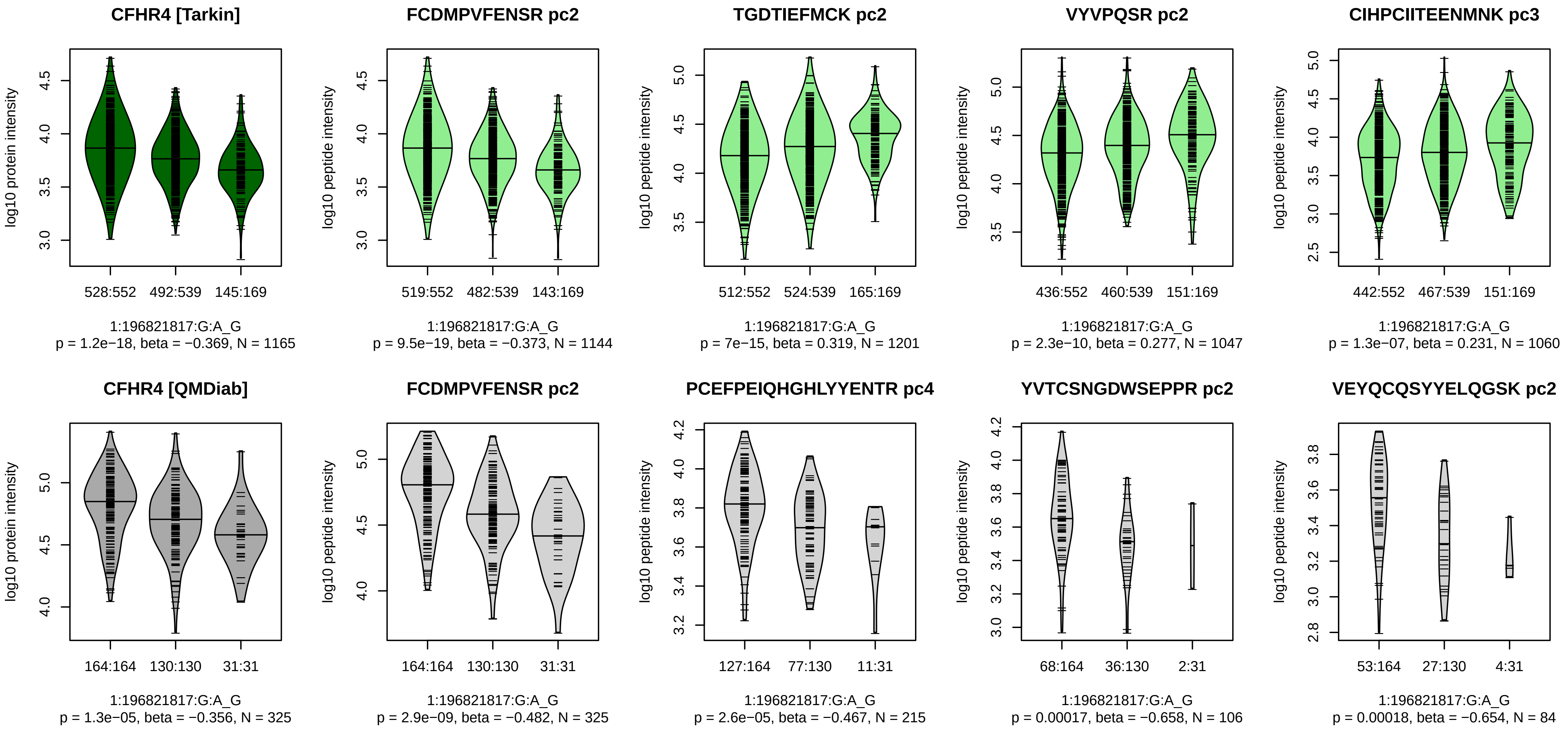

Sort: 88
Gene name: CFHR4
SNP: 1:196821817:G:A
rsID: rs12142766
Type: Cis−pQTL
Protein group(s):
Q92496;Q92496−2
Nano−particle: NP5

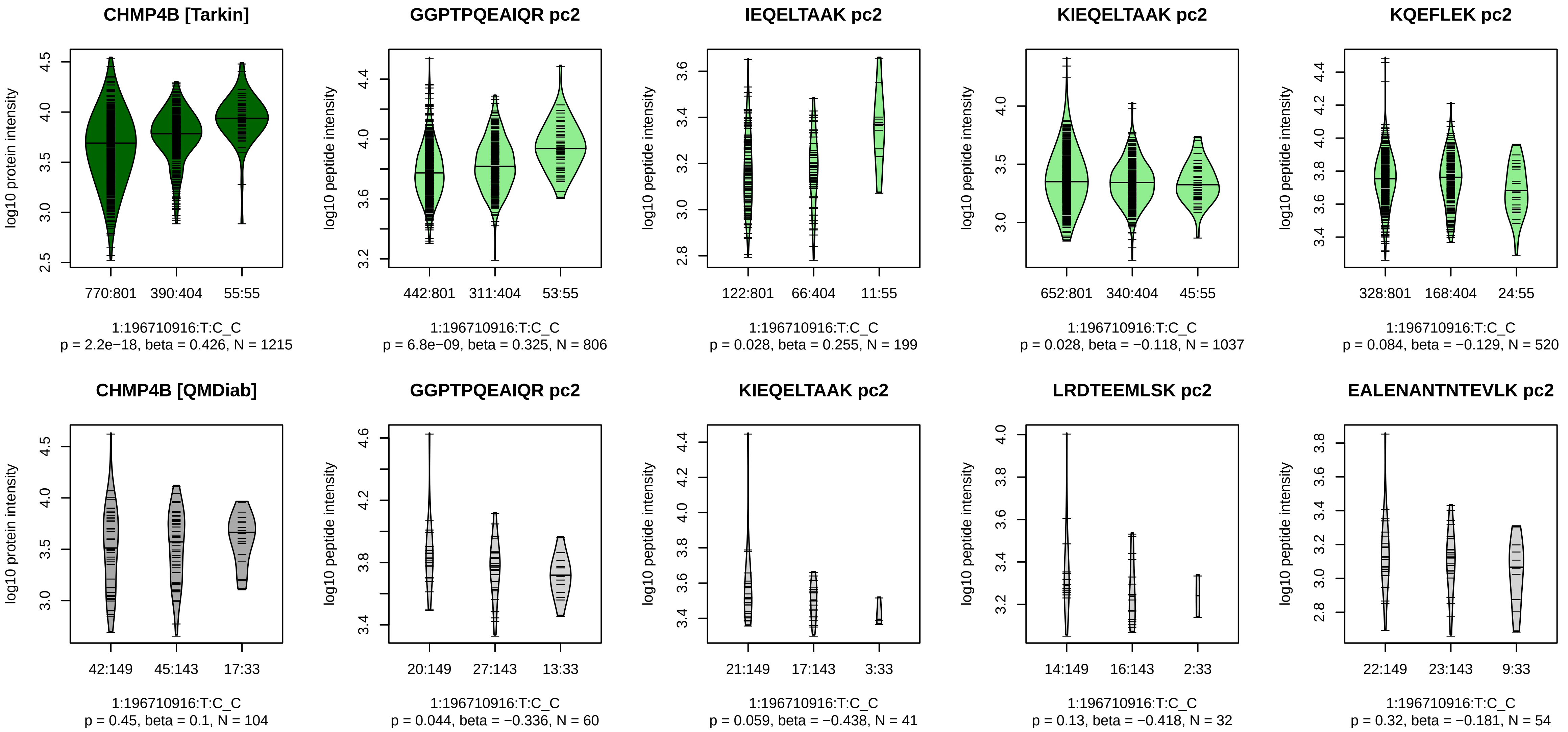

Sort: 89
Gene name: CHMP4B
SNP: 1:196710916:T:C
rsID: rs16840522
Type: Trans−pQTL
Protein group(s):
Q9H444
Nano−particle: NP4

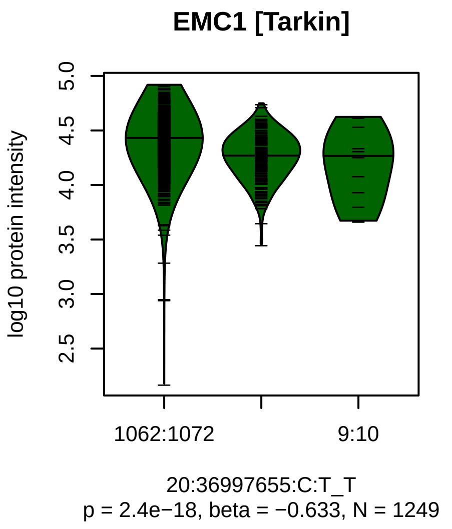

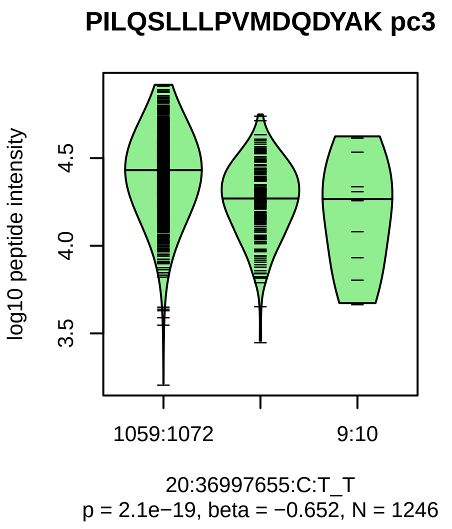

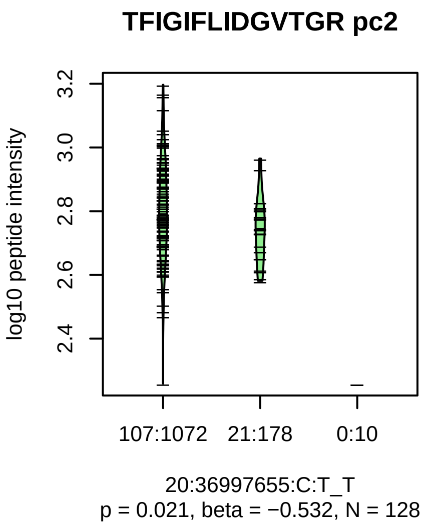

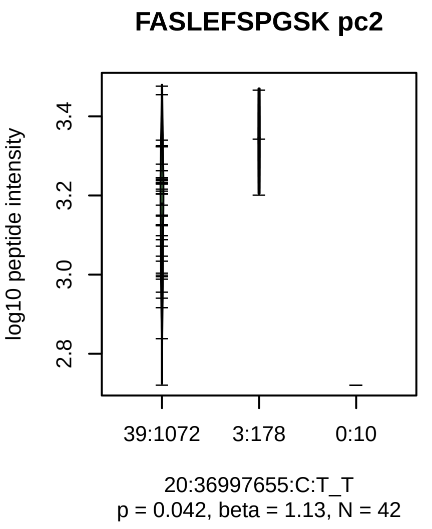

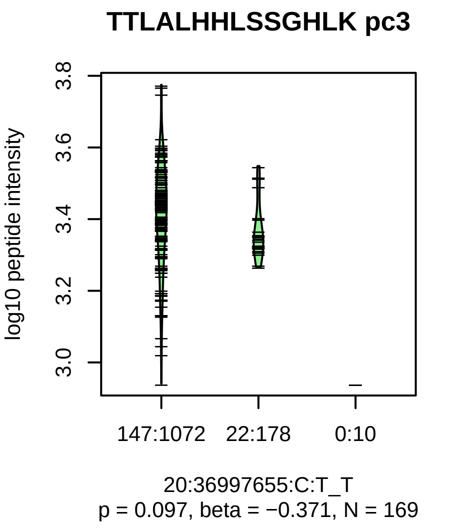

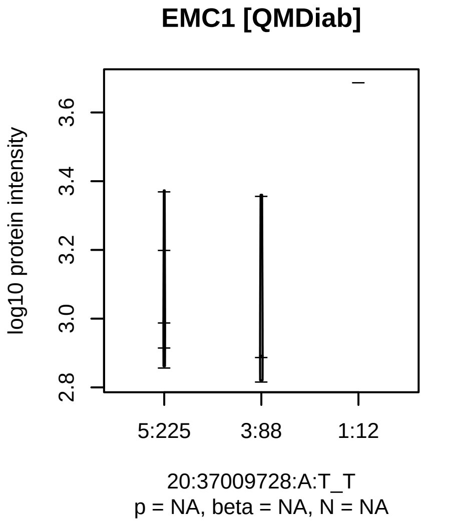

Sort: 90
Gene name: EMC1
SNP: 20:36997655:C:T
rsID: rs2232613
Type: Trans−pQTL
Protein group(s):
T: A0A8I5KW55;Q8N766;Q8N7[illegible]
Q: A0A8I5KW55;H7C5A2;Q8N7[illegible]
Nano−particle: NP4

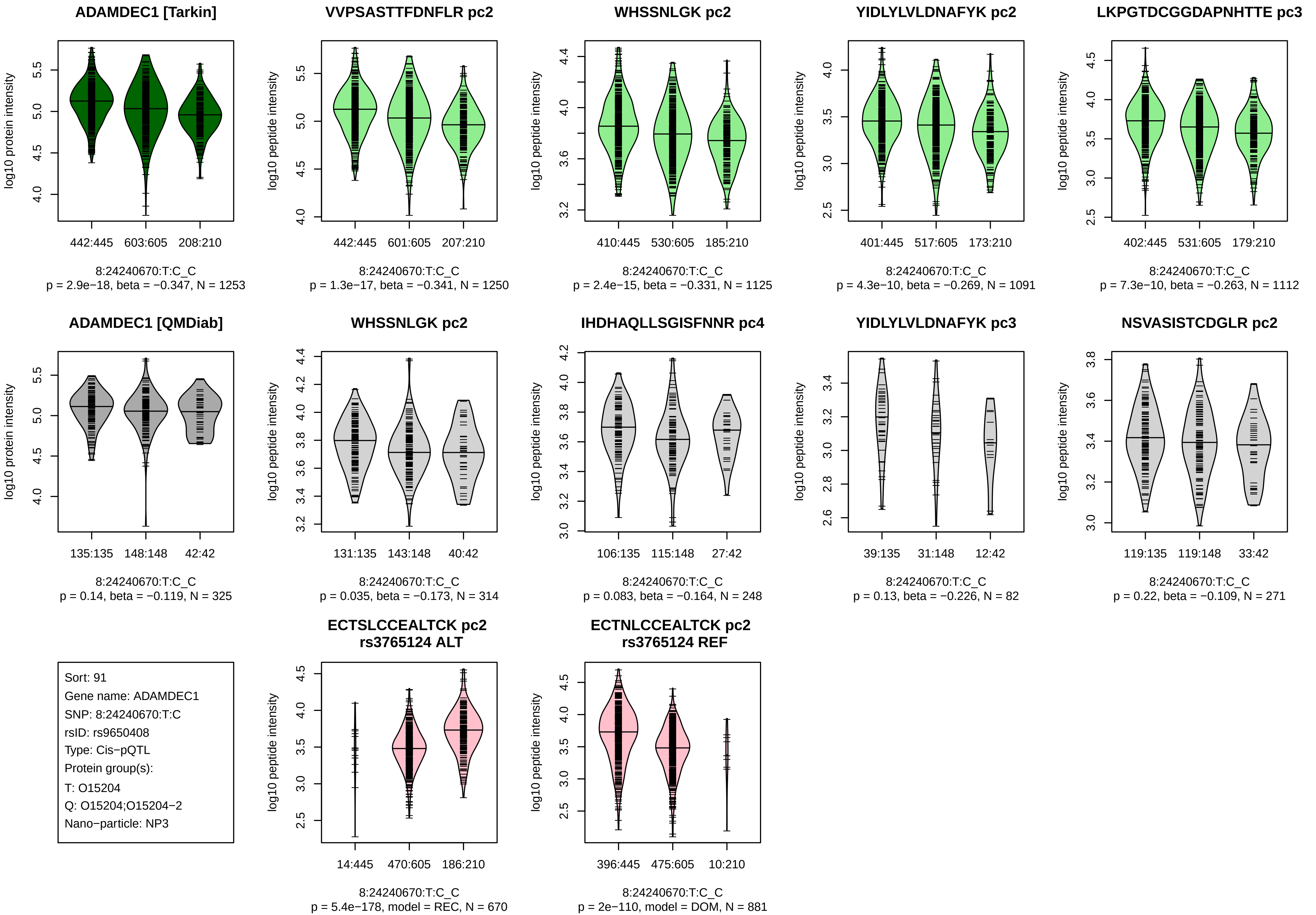

ADAMDEC1 [Tarkin]
log10 protein intensity
442:445 603:605 208:210
8:24240670:T:C_C
p = 2.9e−18, beta = −0.347, N = 1253
VVPSASTTFDNFLR pc2
log10 peptide intensity
442:445 601:605 207:210
8:24240670:T:C_C
p = 1.3e−17, beta = −0.341, N = 1250
WHSSNLGK pc2
log10 peptide intensity
410:445 530:605 185:210
8:24240670:T:C_C
p = 2.4e−15, beta = −0.331, N = 1125
YIDLYLVLDNAFYK pc2
log10 peptide intensity
401:445 517:605 173:210
8:24240670:T:C_C
p = 4.3e−10, beta = −0.269, N = 1091
LKPGTDCGGDAPNHTTE pc3
log10 peptide intensity
402:445 531:605 179:210
8:24240670:T:C_C
p = 7.3e−10, beta = −0.263, N = 1112
ADAMDEC1 [QMDiab]
log10 protein intensity
135:135 148:148 42:42
8:24240670:T:C_C
p = 0.14, beta = −0.119, N = 325
WHSSNLGK pc2
log10 peptide intensity
131:135 143:148 40:42
8:24240670:T:C_C
p = 0.035, beta = −0.173, N = 314
IHDHAQLLSGISFNNR pc4
log10 peptide intensity
106:135 115:148 27:42
8:24240670:T:C_C
p = 0.083, beta = −0.164, N = 248
YIDLYLVLDNAFYK pc3
log10 peptide intensity
39:135 31:148 12:42
8:24240670:T:C_C
p = 0.13, beta = −0.226, N = 82
NSVASISTCDGLR pc2
log10 peptide intensity
119:135 119:148 33:42
8:24240670:T:C_C
p = 0.22, beta = −0.109, N = 271
Sort: 91
Gene name: ADAMDEC1
SNP: 8:24240670:T:C
rsID: rs9650408
Type: Cis−pQTL
Protein group(s):
T: O15204
Q: O15204;O15204−2
Nano−particle: NP3
ECTSLCCEALTCK pc2
rs3765124 ALT
log10 peptide intensity
14:445 470:605 186:210
8:24240670:T:C_C
p = 5.4e−178, model = REC, N = 670
ECTNLCCEALTCK pc2
rs3765124 REF
log10 peptide intensity
396:445 475:605 10:210
8:24240670:T:C_C
p = 2e−110, model = DOM, N = 881

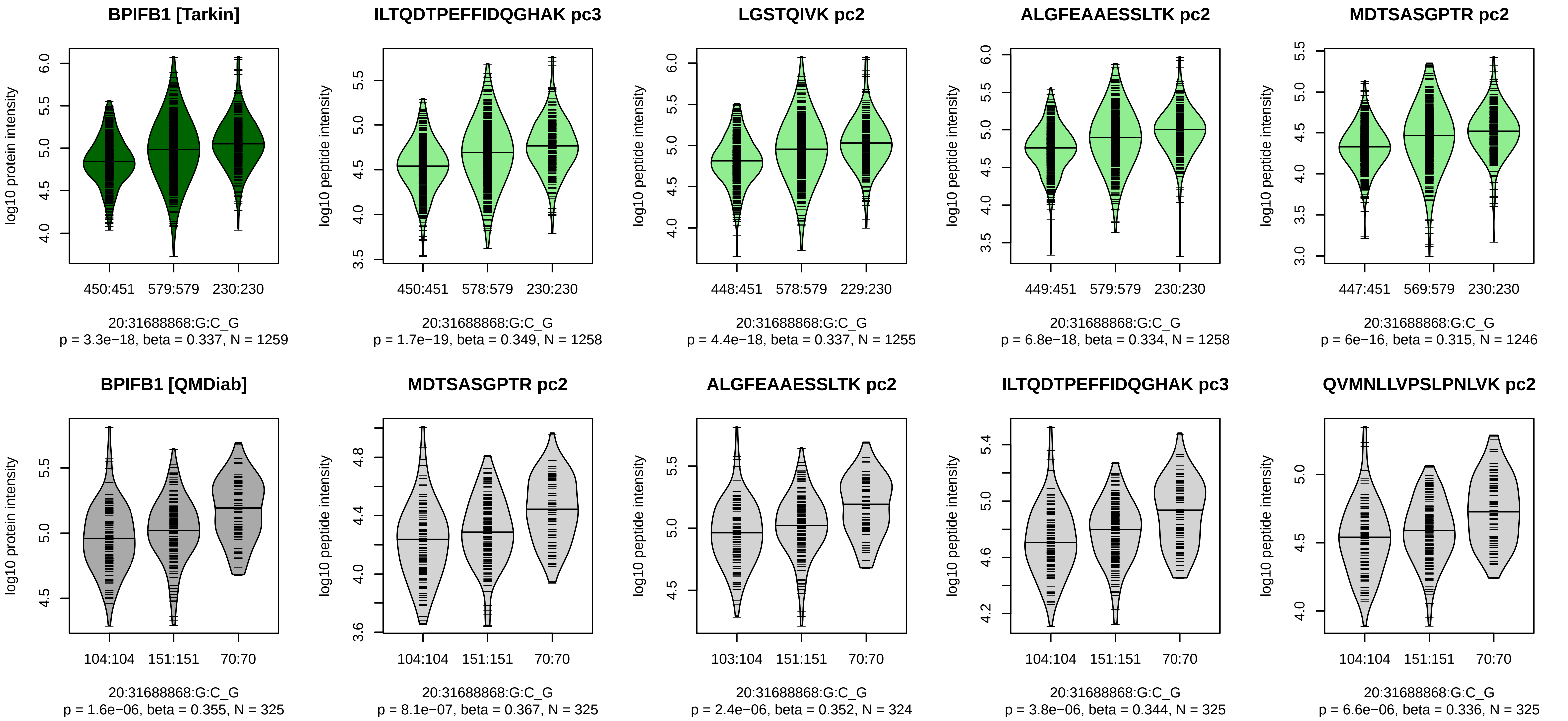

Sort: 92
Gene name: BPIFB1
SNP: 20:31688868:G:C
rsID: rs761931
Type: Cis−pQTL
Protein group(s):
Q8TDL5
Nano−particle: NP4

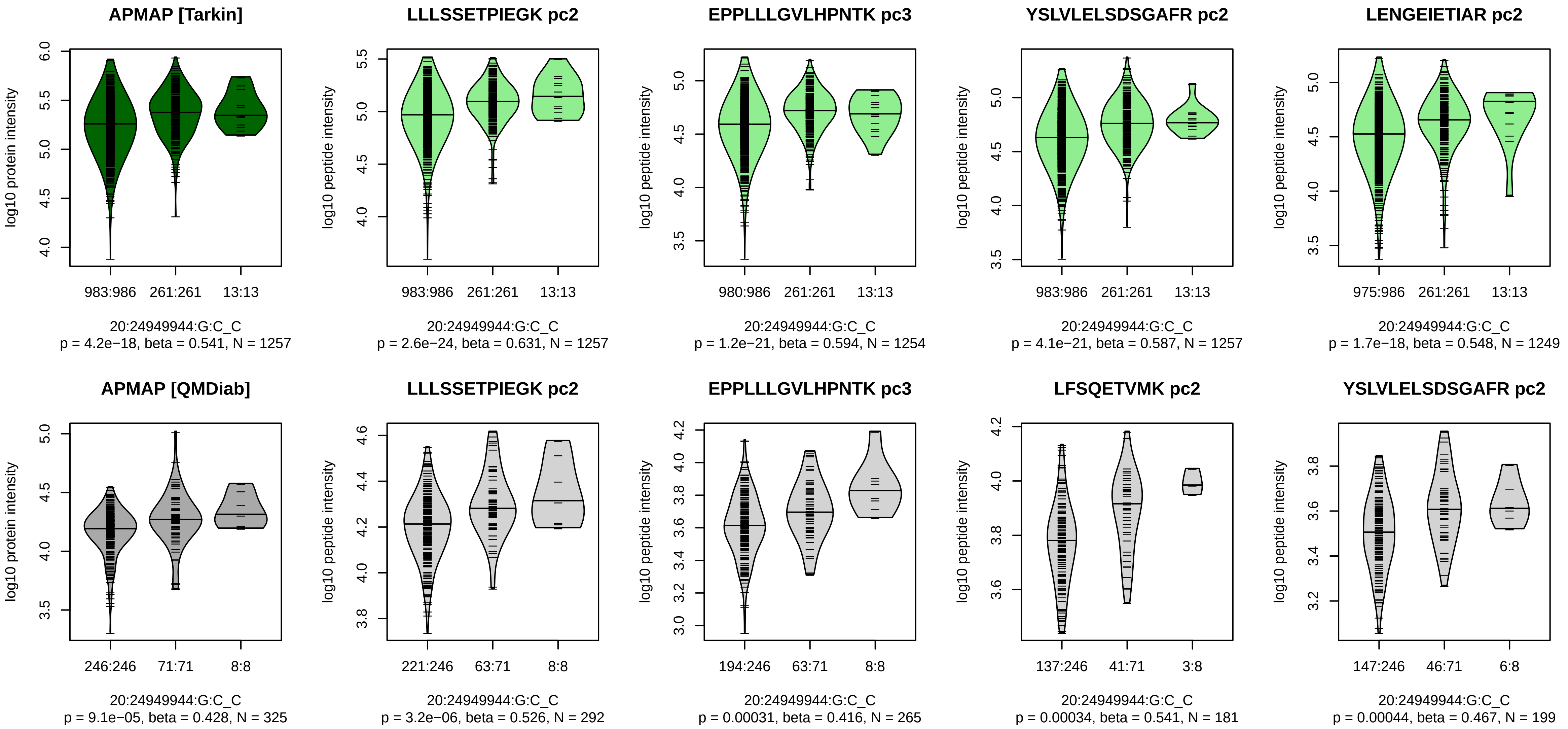

Sort: 93
Gene name: APMAP
SNP: 20:24949944:G:C
rsID: rs6138438
Type: Cis−pQTL
Protein group(s):
Q9HDC9
Nano−particle: NP5

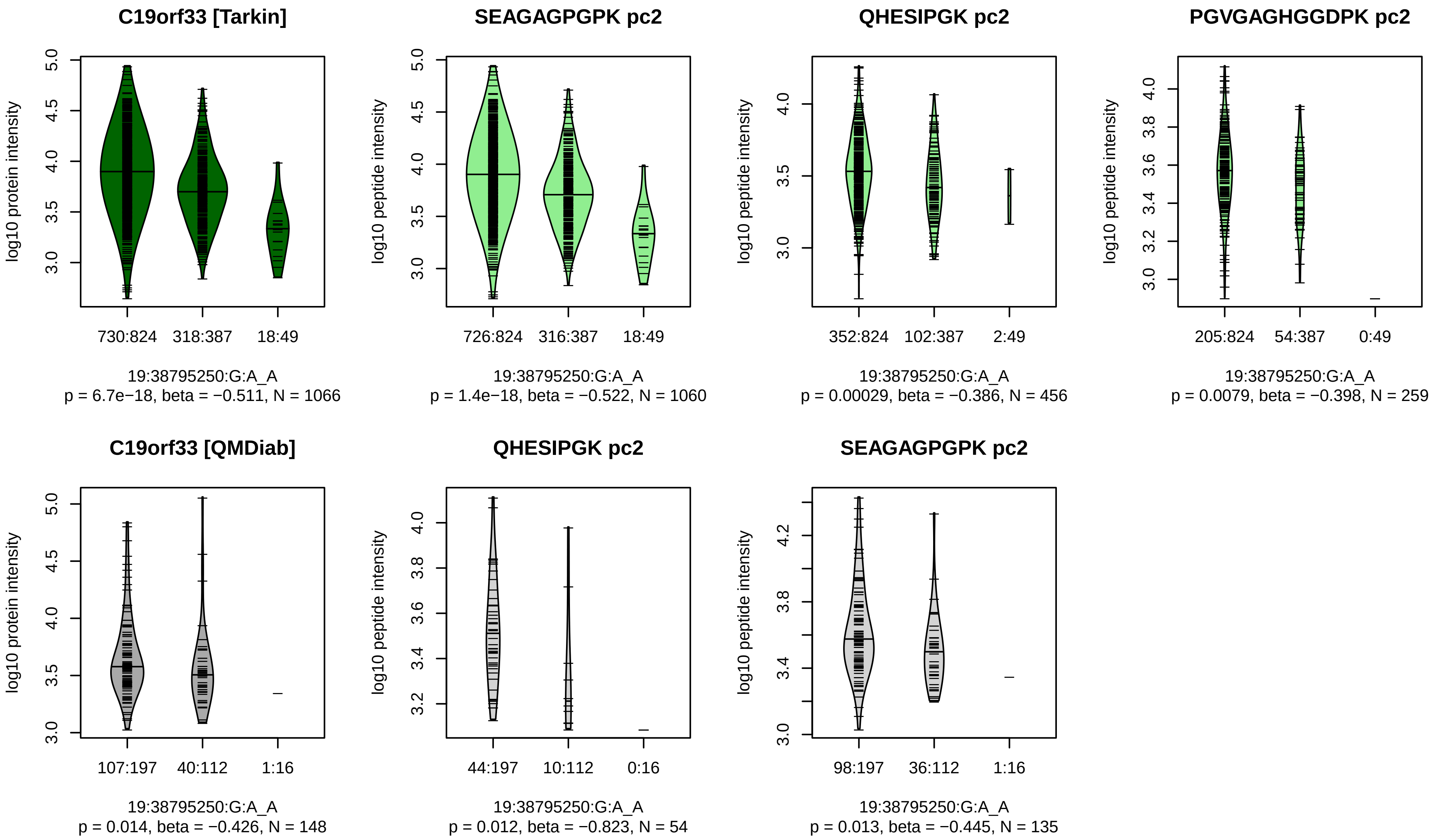

Sort: 94
Gene name: C19orf33
SNP: 19:38795250:G:A
rsID: rs45437199
Type: Cis−pQTL
Protein group(s):
Q9GZP8
Nano−particle: NP2

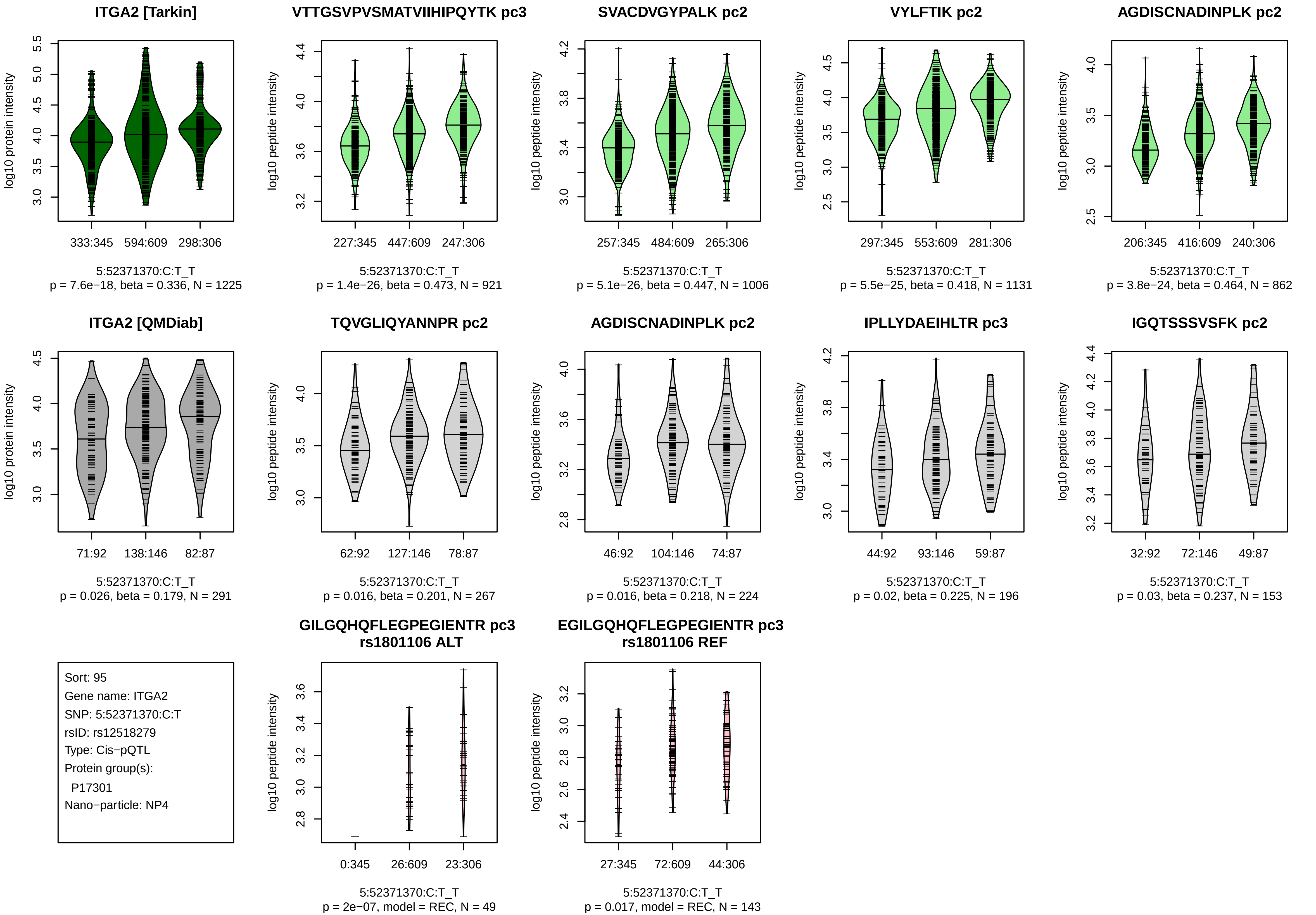
ITGA2 [Tarkin]
log10 protein intensity
333:345 594:609 298:306
5:52371370:C:T_T
p = 7.6e−18, beta = 0.336, N = 1225
VTTGSVPVSMATVIIHIPQYTK pc3
log10 peptide intensity
227:345 447:609 247:306
5:52371370:C:T_T
p = 1.4e−26, beta = 0.473, N = 921
SVACDVGYPALK pc2
log10 peptide intensity
257:345 484:609 265:306
5:52371370:C:T_T
p = 5.1e−26, beta = 0.447, N = 1006
VYLFTIK pc2
log10 peptide intensity
297:345 553:609 281:306
5:52371370:C:T_T
p = 5.5e−25, beta = 0.418, N = 1131
AGDISCNADINPLK pc2
log10 peptide intensity
206:345 416:609 240:306
5:52371370:C:T_T
p = 3.8e−24, beta = 0.464, N = 862
ITGA2 [QMDiab]
log10 protein intensity
71:92 138:146 82:87
5:52371370:C:T_T
p = 0.026, beta = 0.179, N = 291
TQVGLIQYANNPR pc2
log10 peptide intensity
62:92 127:146 78:87
5:52371370:C:T_T
p = 0.016, beta = 0.201, N = 267
AGDISCNADINPLK pc2
log10 peptide intensity
46:92 104:146 74:87
5:52371370:C:T_T
p = 0.016, beta = 0.218, N = 224
IPLLYDAEIHLTR pc3
log10 peptide intensity
44:92 93:146 59:87
5:52371370:C:T_T
p = 0.02, beta = 0.225, N = 196
IGQTSSSVSFK pc2
log10 peptide intensity
32:92 72:146 49:87
5:52371370:C:T_T
p = 0.03, beta = 0.237, N = 153
Sort: 95
Gene name: ITGA2
SNP: 5:52371370:C:T
rsID: rs12518279
Type: Cis−pQTL
Protein group(s):
P17301
Nano−particle: NP4
GILGQHQFLEGPEGIENTR pc3
rs1801106 ALT
log10 peptide intensity
0:345 26:609 23:306
5:52371370:C:T_T
p = 2e−07, model = REC, N = 49
EGILGQHQFLEGPEGIENTR pc3
rs1801106 REF
log10 peptide intensity
27:345 72:609 44:306
5:52371370:C:T_T
p = 0.017, model = REC, N = 143

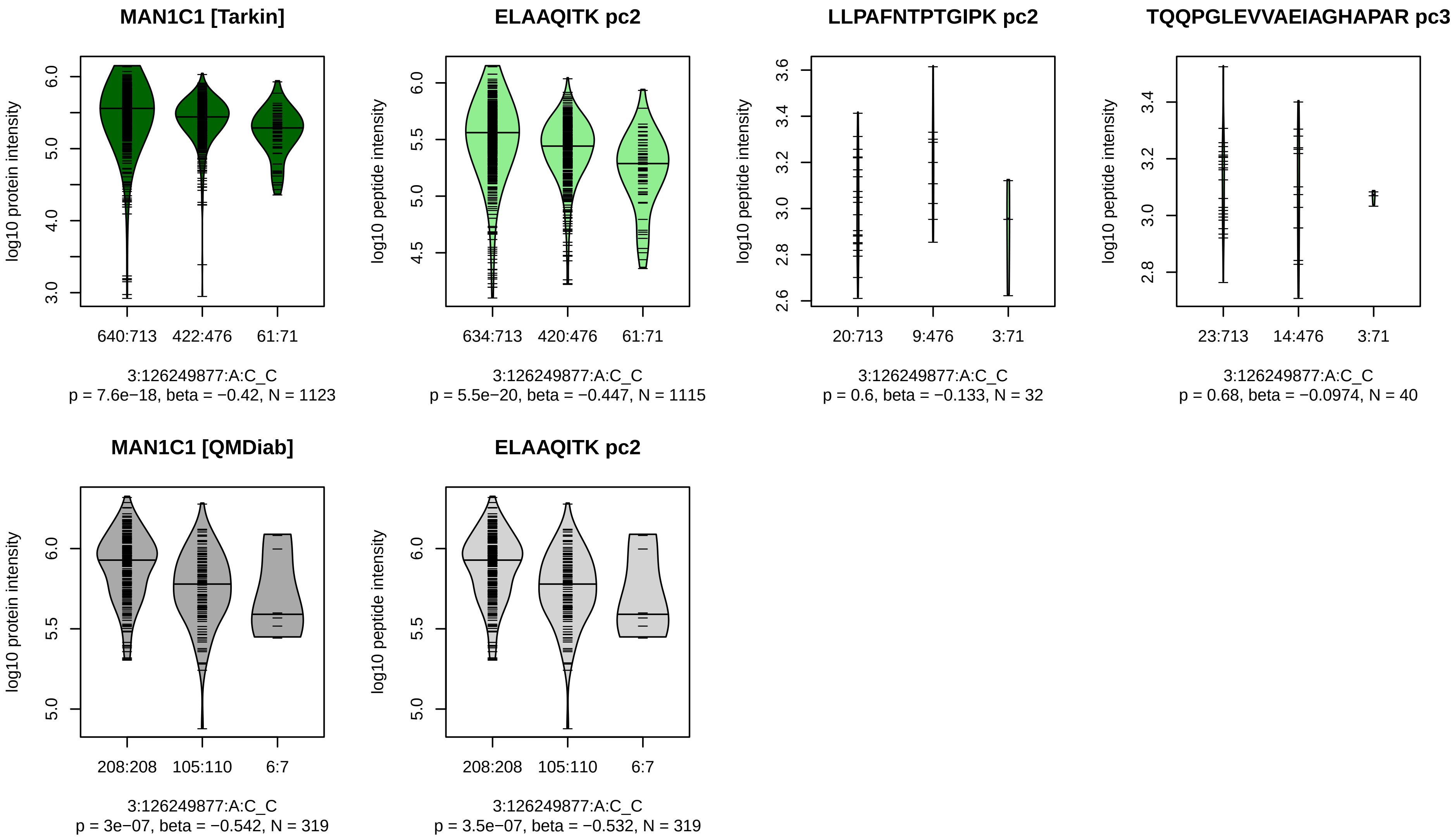

Sort: 96
Gene name: MAN1C1
SNP: 3:126249877:A:C
rsID: rs4305381
Type: Trans−pQTL
Protein group(s):
T: Q9NR34
Q: A6NGN6;B1AJZ5;Q9NR34
Nano−particle: NP2

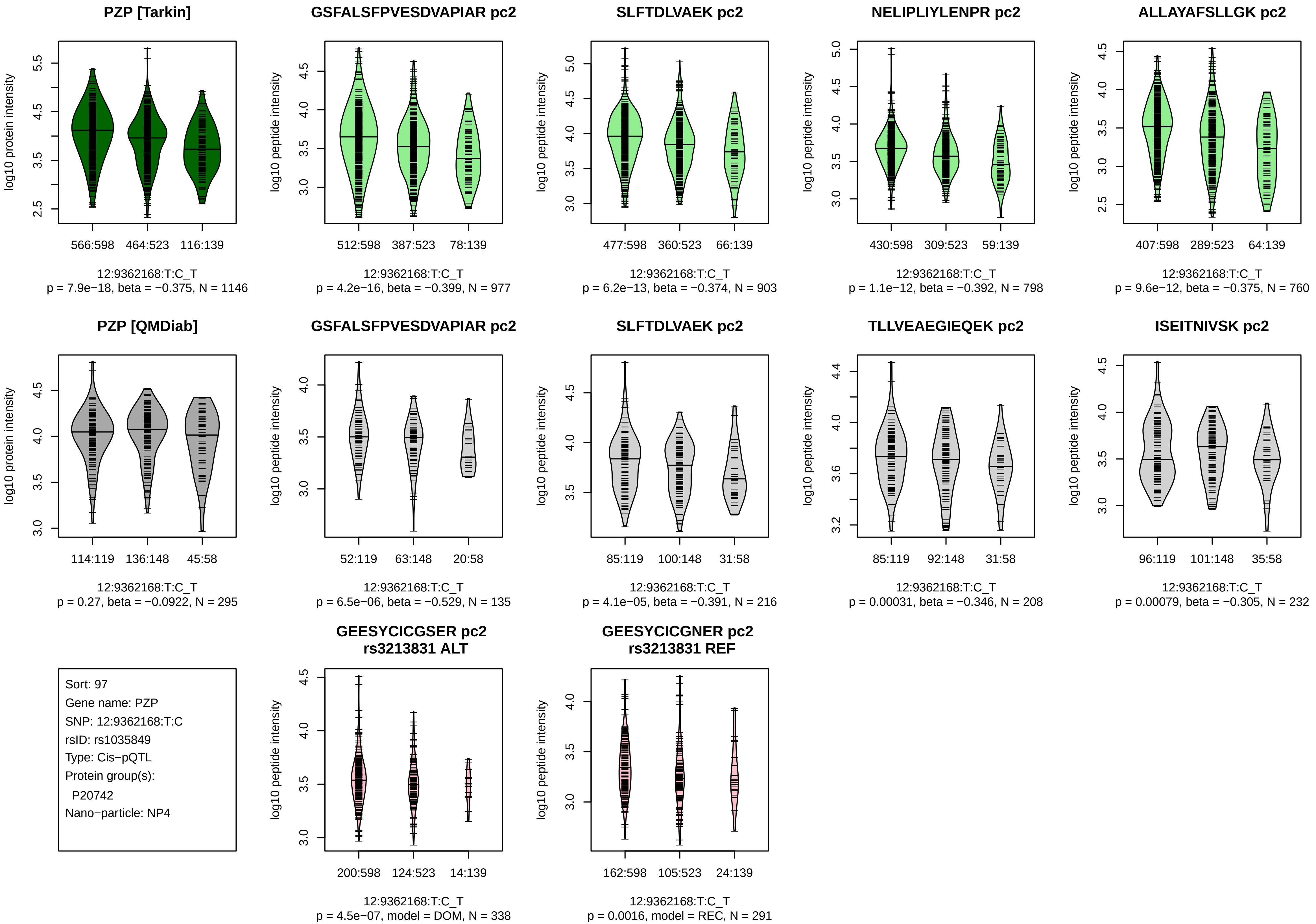
PZP [Tarkin]
log10 protein intensity
566:598
464:523
116:139
12:9362168:T:C_T
p = 7.9e−18, beta = −0.375, N = 1146
GSFALSFPVESDVAPIAR pc2
log10 peptide intensity
512:598
387:523
78:139
12:9362168:T:C_T
p = 4.2e−16, beta = −0.399, N = 977
SLFTDLVAEK pc2
log10 peptide intensity
477:598
360:523
66:139
12:9362168:T:C_T
p = 6.2e−13, beta = −0.374, N = 903
NELIPLIYLENPR pc2
log10 peptide intensity
430:598
309:523
59:139
12:9362168:T:C_T
p = 1.1e−12, beta = −0.392, N = 798
ALLAYAFSLLGK pc2
log10 peptide intensity
407:598
289:523
64:139
12:9362168:T:C_T
p = 9.6e−12, beta = −0.375, N = 760
PZP [QMDiab]
log10 protein intensity
114:119
136:148
45:58
12:9362168:T:C_T
p = 0.27, beta = −0.0922, N = 295
GSFALSFPVESDVAPIAR pc2
log10 peptide intensity
52:119
63:148
20:58
12:9362168:T:C_T
p = 6.5e−06, beta = −0.529, N = 135
SLFTDLVAEK pc2
log10 peptide intensity
85:119
100:148
31:58
12:9362168:T:C_T
p = 4.1e−05, beta = −0.391, N = 216
TLLVEAEGIEQEK pc2
log10 peptide intensity
85:119
92:148
31:58
12:9362168:T:C_T
p = 0.00031, beta = −0.346, N = 208
ISEITNIVSK pc2
log10 peptide intensity
96:119
101:148
35:58
12:9362168:T:C_T
p = 0.00079, beta = −0.305, N = 232
Sort: 97
Gene name: PZP
SNP: 12:9362168:T:C
rsID: rs1035849
Type: Cis−pQTL
Protein group(s):
P20742
Nano−particle: NP4
GEESYCICGSER pc2
rs3213831 ALT
log10 peptide intensity
200:598
124:523
14:139
12:9362168:T:C_T
p = 4.5e−07, model = DOM, N = 338
GEESYCICGNER pc2
rs3213831 REF
log10 peptide intensity
162:598
105:523
24:139
12:9362168:T:C_T
p = 0.0016, model = REC, N = 291

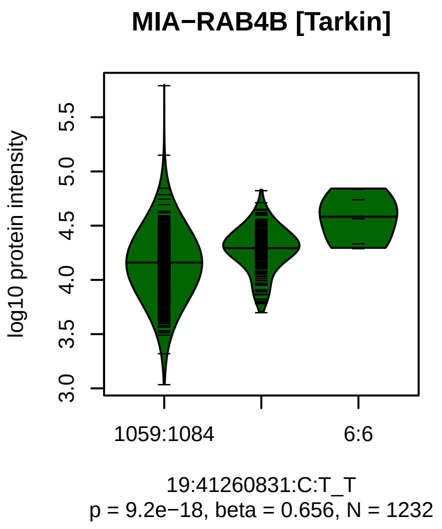

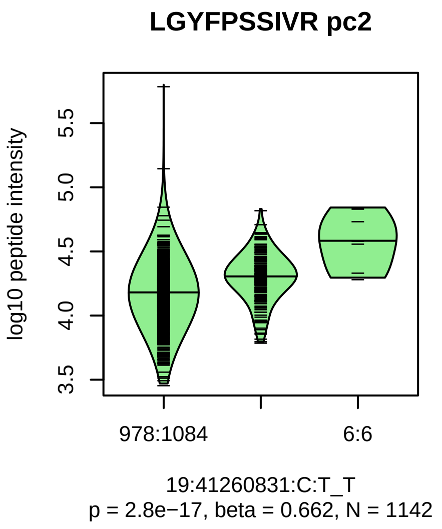

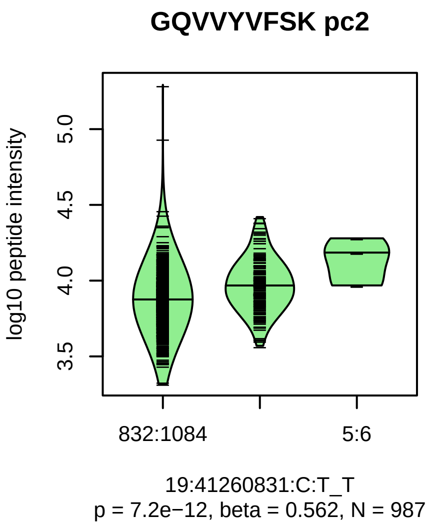

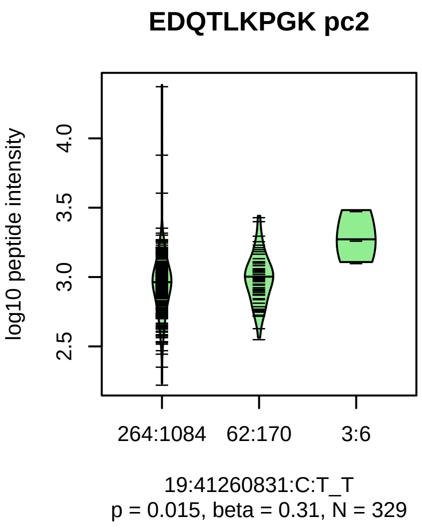

No protein data available
for replication in QMDiab

Sort: 98
Gene name: MIA−RAB4B
SNP: 19:41260831:C:T
rsID: rs2607416
Type: Cis−pQTL
Protein group(s):
W4VSR3
Nano−particle: NP3

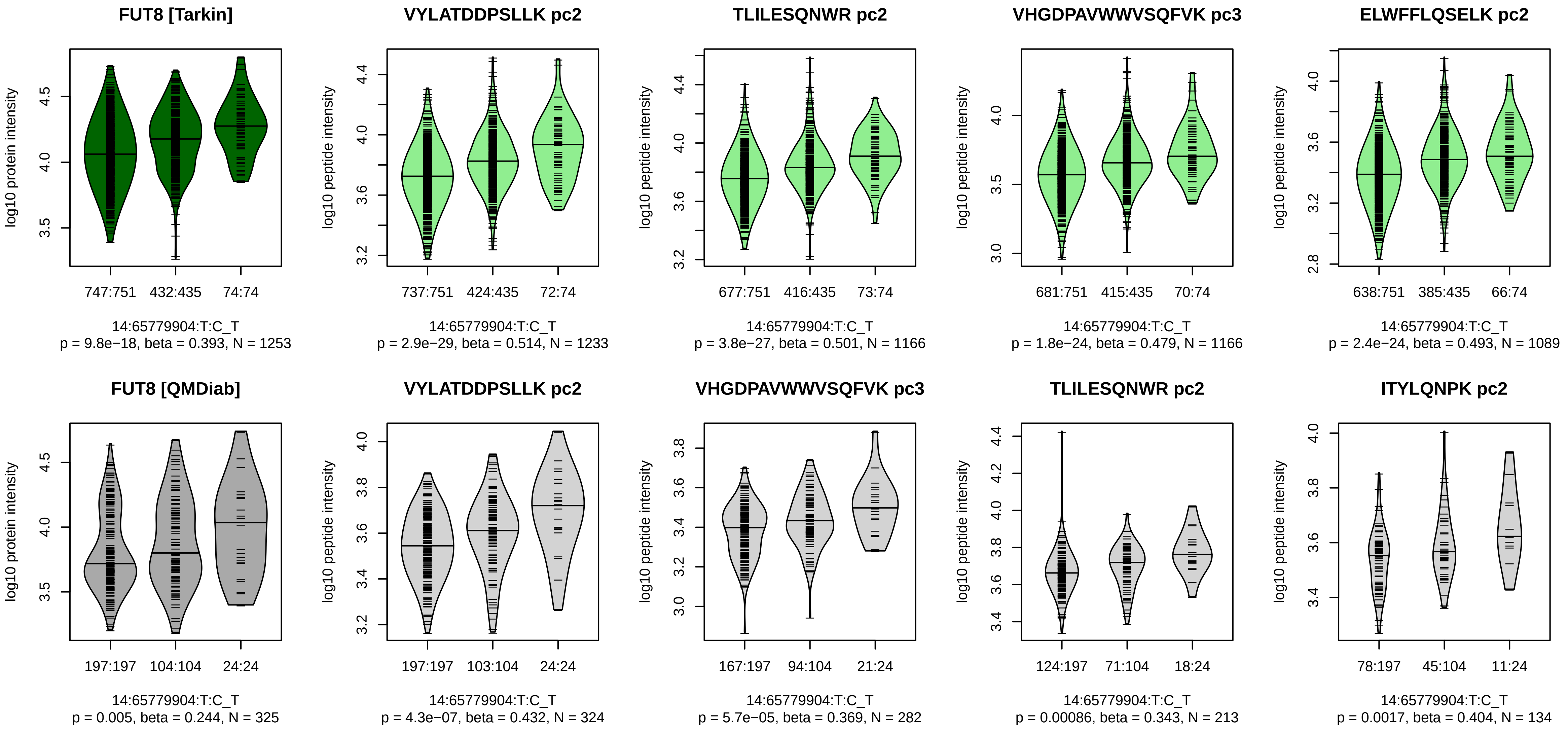

Sort: 99
Gene name: FUT8
SNP: 14:65779904:T:C
rsID: rs4899172
Type: Cis−pQTL
Protein group(s):
Q9BYC5
Nano−particle: NP3

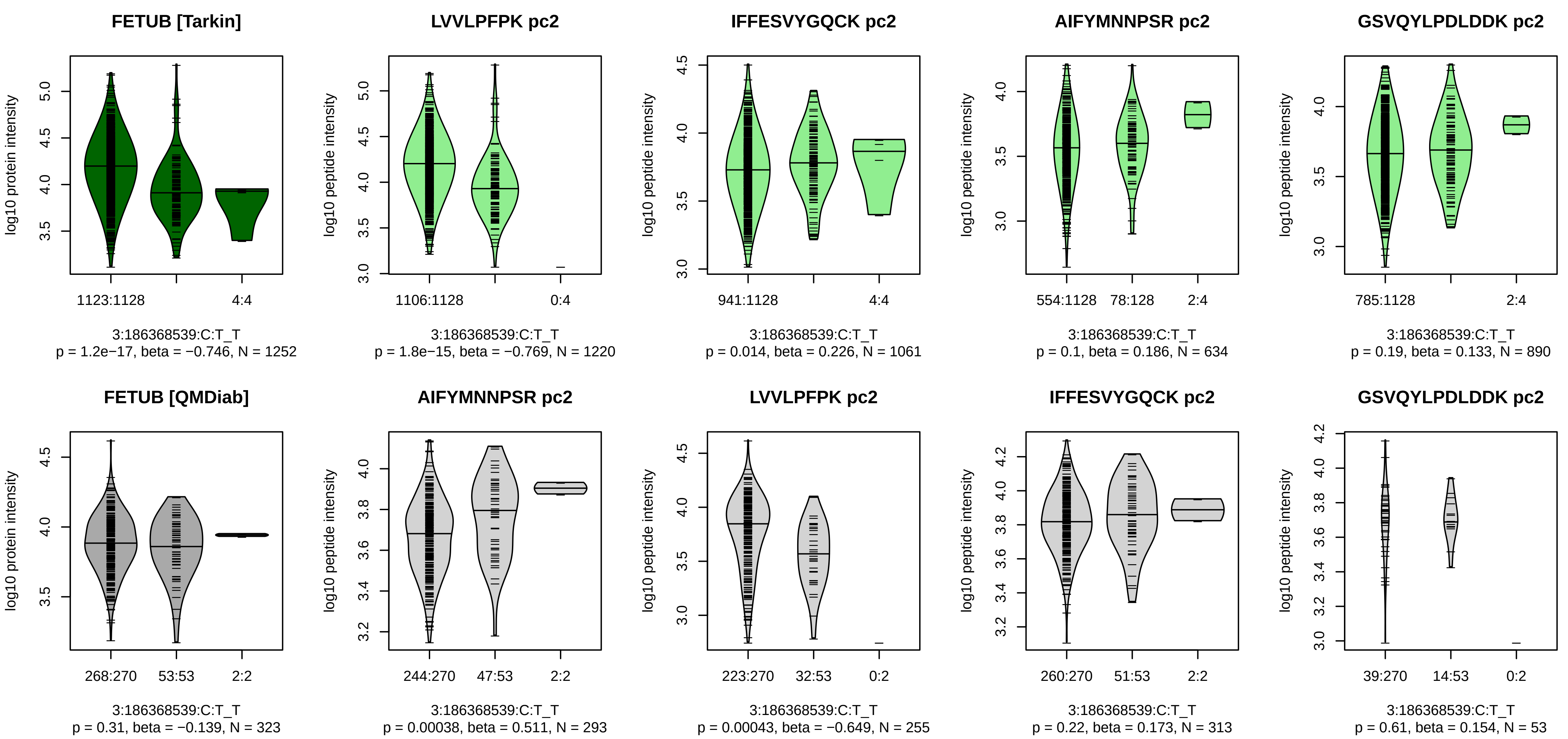

Sort: 100
Gene name: FETUB
SNP: 3:186368539:C:T
rsID: rs79014333
Type: Cis−pQTL
Protein group(s):
Q9UGM5
Nano−particle: NP4

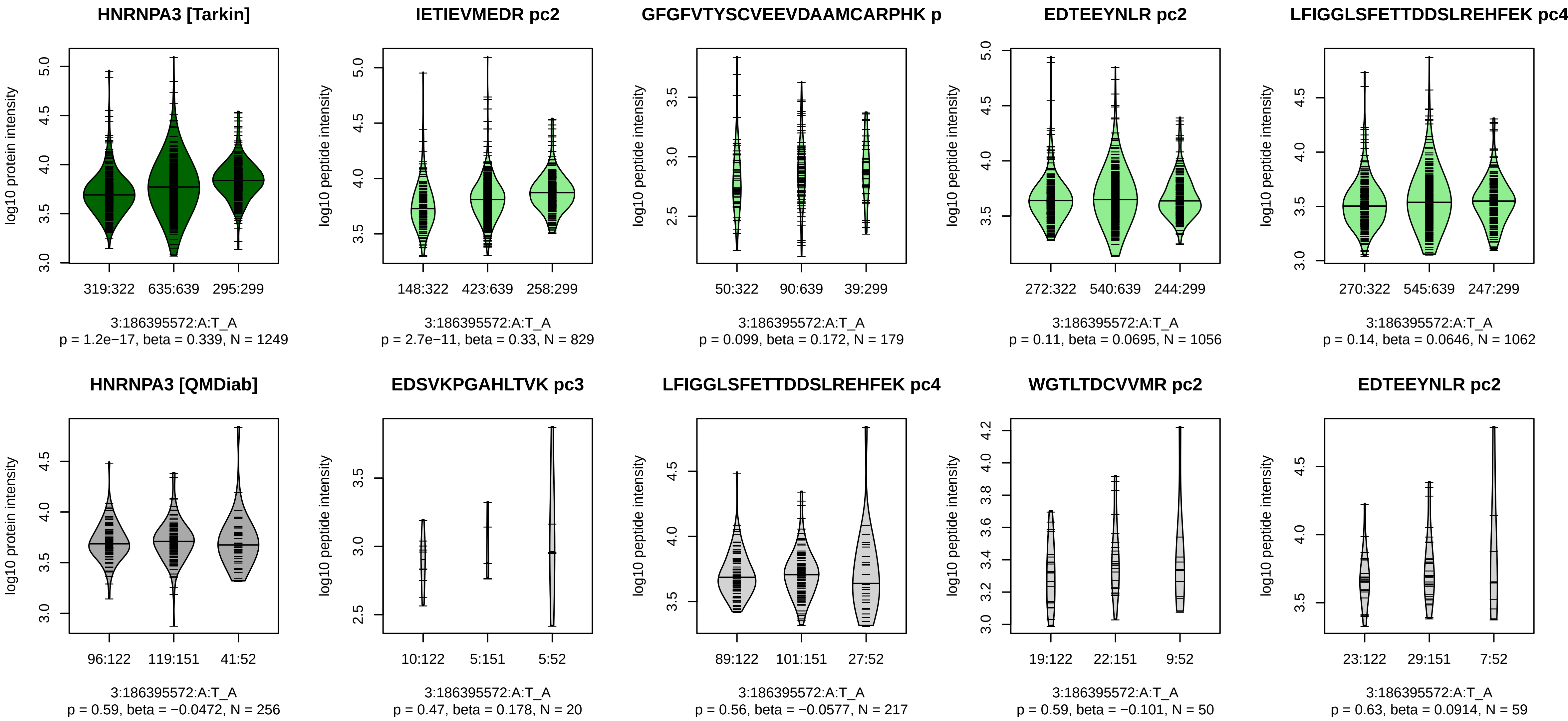

Sort: 101
Gene name: HNRNPA3
SNP: 3:186395572:A:T
rsID: rs1042464
Type: Trans−pQTL
Protein group(s):
A0A7I2V4G0
Nano−particle: NP1

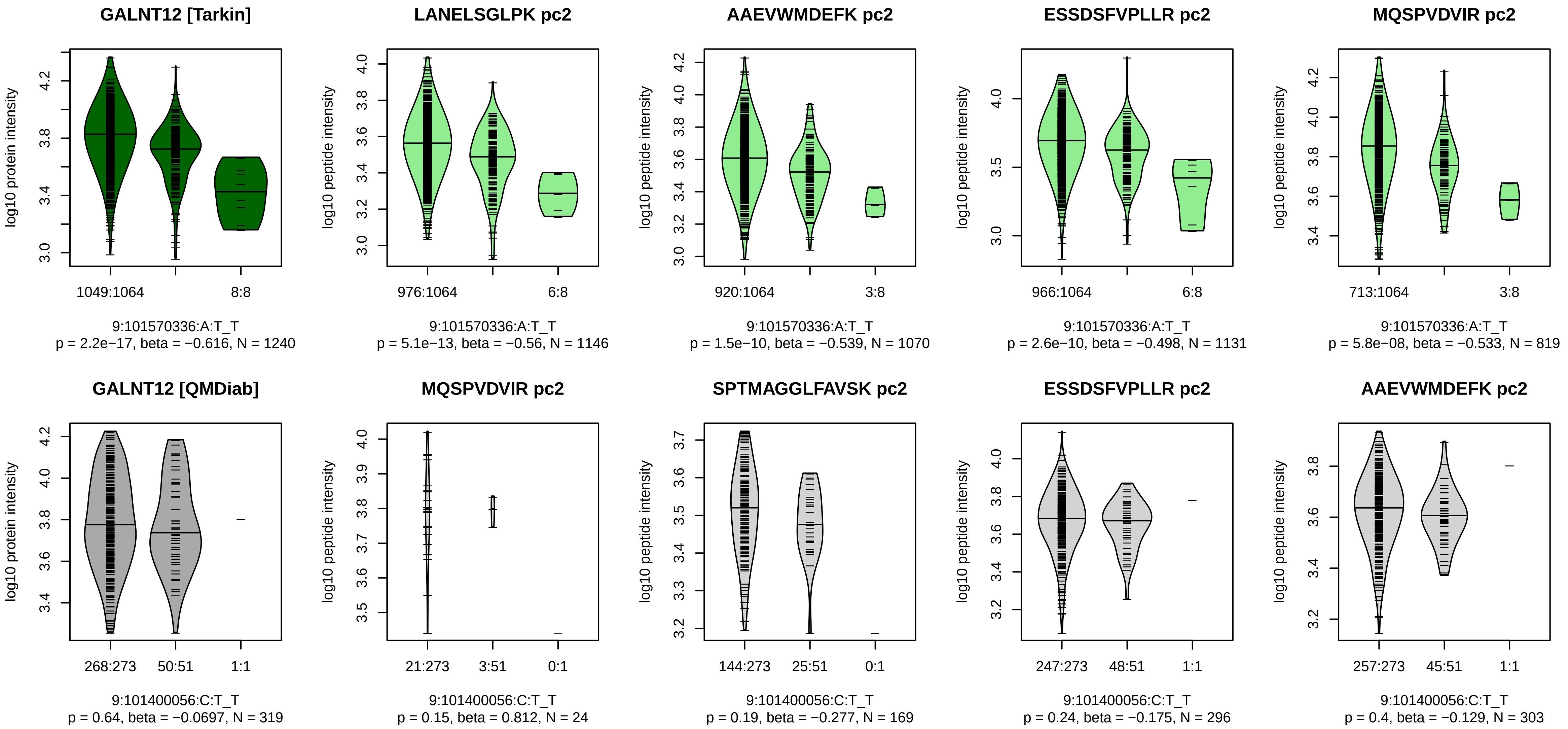

Sort: 102
Gene name: GALNT12
SNP: 9:101570336:A:T
rsID: rs1137654
Type: Cis−pQTL
Protein group(s):
Q8IXK2
Nano−particle: NP5

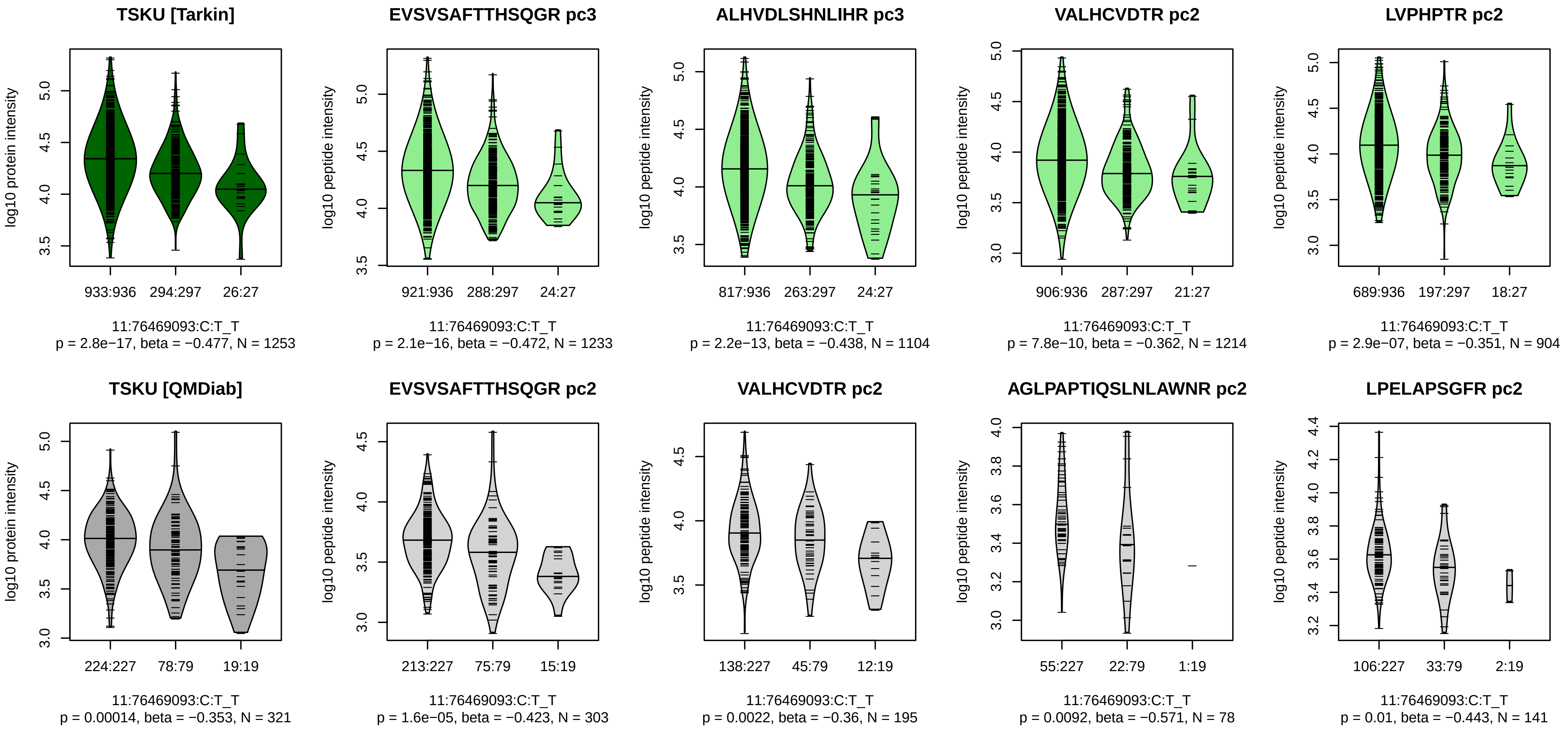

Sort: 103
Gene name: TSKU
SNP: 11:76469093:C:T
rsID: rs1149596
Type: Cis−pQTL
Protein group(s):
Q8WUA8
Nano−particle: NP5

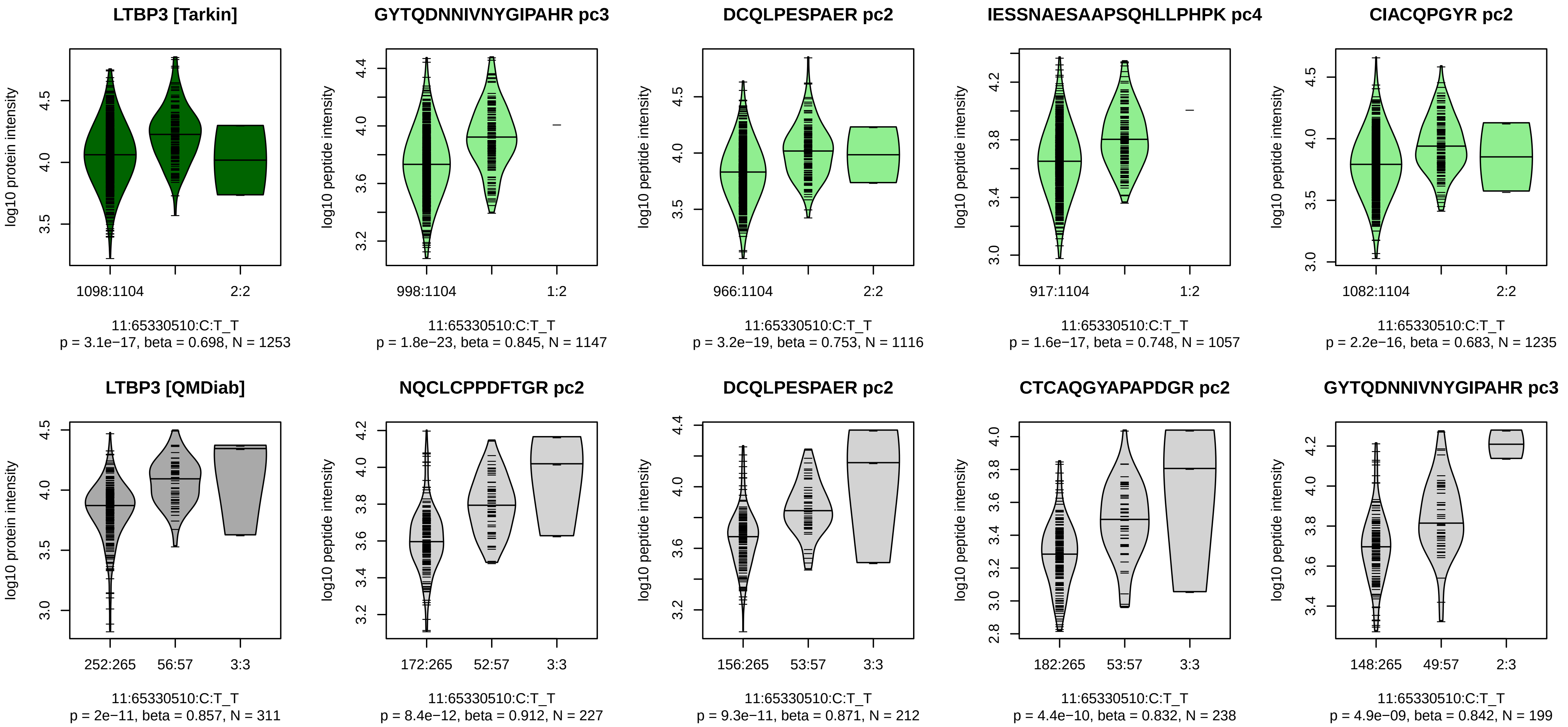

Sort: 104
Gene name: LTBP3
SNP: 11:65330510:C:T
rsID: rs11227226
Type: Cis−pQTL
Protein group(s):
Q9NS15
Nano−particle: NP5

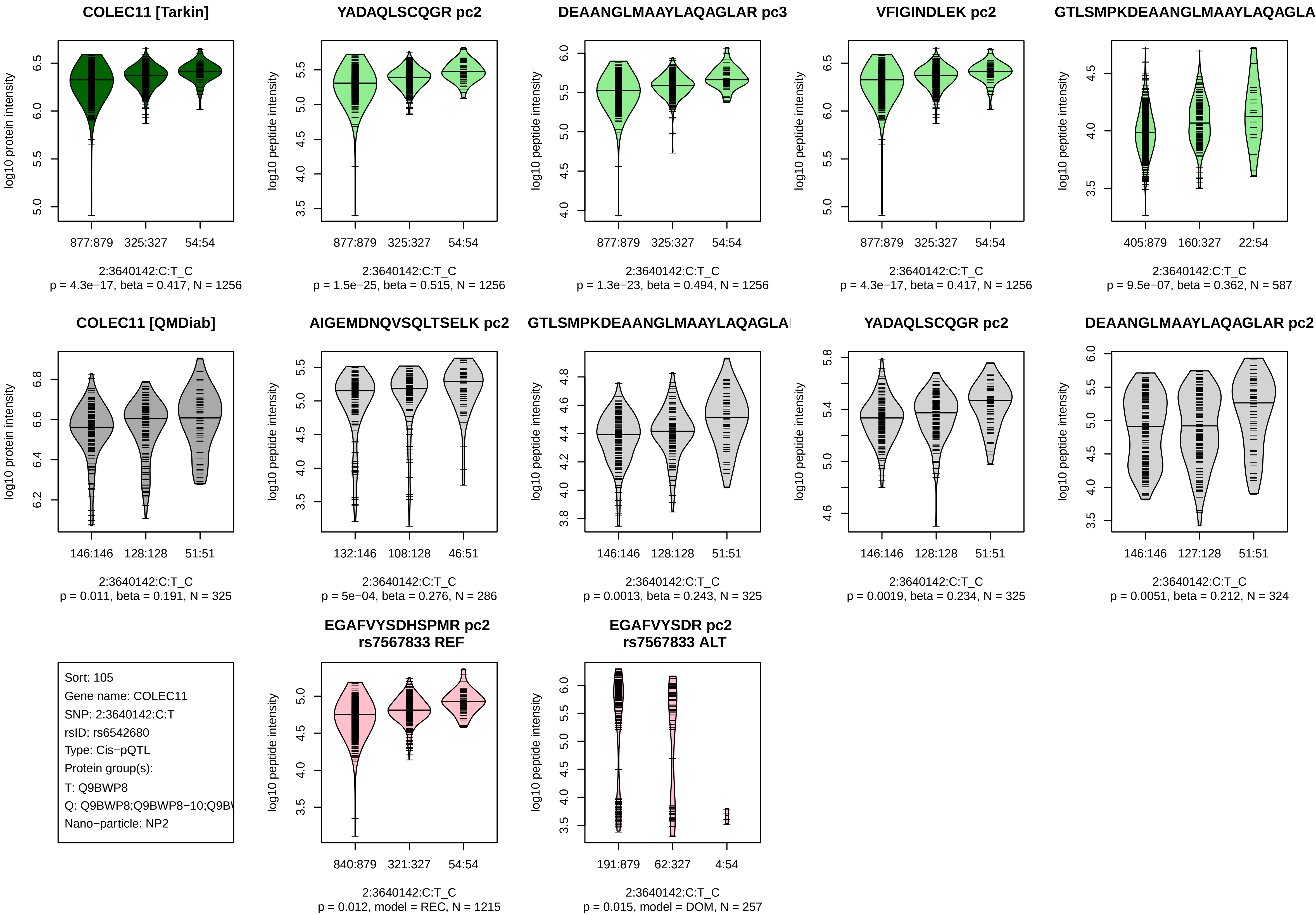
COLEC11 [Tarkin]
log10 protein intensity
877:879 325:327 54:54
2:3640142:C:T_C
p = 4.3e−17, beta = 0.417, N = 1256
YADAQLSCQGR pc2
log10 peptide intensity
877:879 325:327 54:54
2:3640142:C:T_C
p = 1.5e−25, beta = 0.515, N = 1256
DEAANGLMAAYLAQAGLAR pc3
log10 peptide intensity
877:879 325:327 54:54
2:3640142:C:T_C
p = 1.3e−23, beta = 0.494, N = 1256
VFIGINDLEK pc2
log10 peptide intensity
877:879 325:327 54:54
2:3640142:C:T_C
p = 4.3e−17, beta = 0.417, N = 1256
GTLSMPKDEAANGLMAAYLAQAGLA
log10 peptide intensity
405:879 160:327 22:54
2:3640142:C:T_C
p = 9.5e−07, beta = 0.362, N = 587
COLEC11 [QMDiab]
log10 protein intensity
146:146 128:128 51:51
2:3640142:C:T_C
p = 0.011, beta = 0.191, N = 325
AIGEMDNQVSQLTSELK pc2
log10 peptide intensity
132:146 108:128 46:51
2:3640142:C:T_C
p = 5e−04, beta = 0.276, N = 286
GTLSMPKDEAANGLMAAYLAQAGLA
log10 peptide intensity
146:146 128:128 51:51
2:3640142:C:T_C
p = 0.0013, beta = 0.243, N = 325
YADAQLSCQGR pc2
log10 peptide intensity
146:146 128:128 51:51
2:3640142:C:T_C
p = 0.0019, beta = 0.234, N = 325
DEAANGLMAAYLAQAGLAR pc2
log10 peptide intensity
146:146 127:128 51:51
2:3640142:C:T_C
p = 0.0051, beta = 0.212, N = 324
Sort: 105
Gene name: COLEC11
SNP: 2:3640142:C:T
rsID: rs6542680
Type: Cis−pQTL
Protein group(s):
T: Q9BWP8
Q: Q9BWP8;Q9BWP8−10;Q9B
Nano−particle: NP2
EGAFVYSDHSPMR pc2
rs7567833 REF
log10 peptide intensity
840:879 321:327 54:54
2:3640142:C:T_C
p = 0.012, model = REC, N = 1215
EGAFVYSDR pc2
rs7567833 ALT
log10 peptide intensity
191:879 62:327 4:54
2:3640142:C:T_C
p = 0.015, model = DOM, N = 257

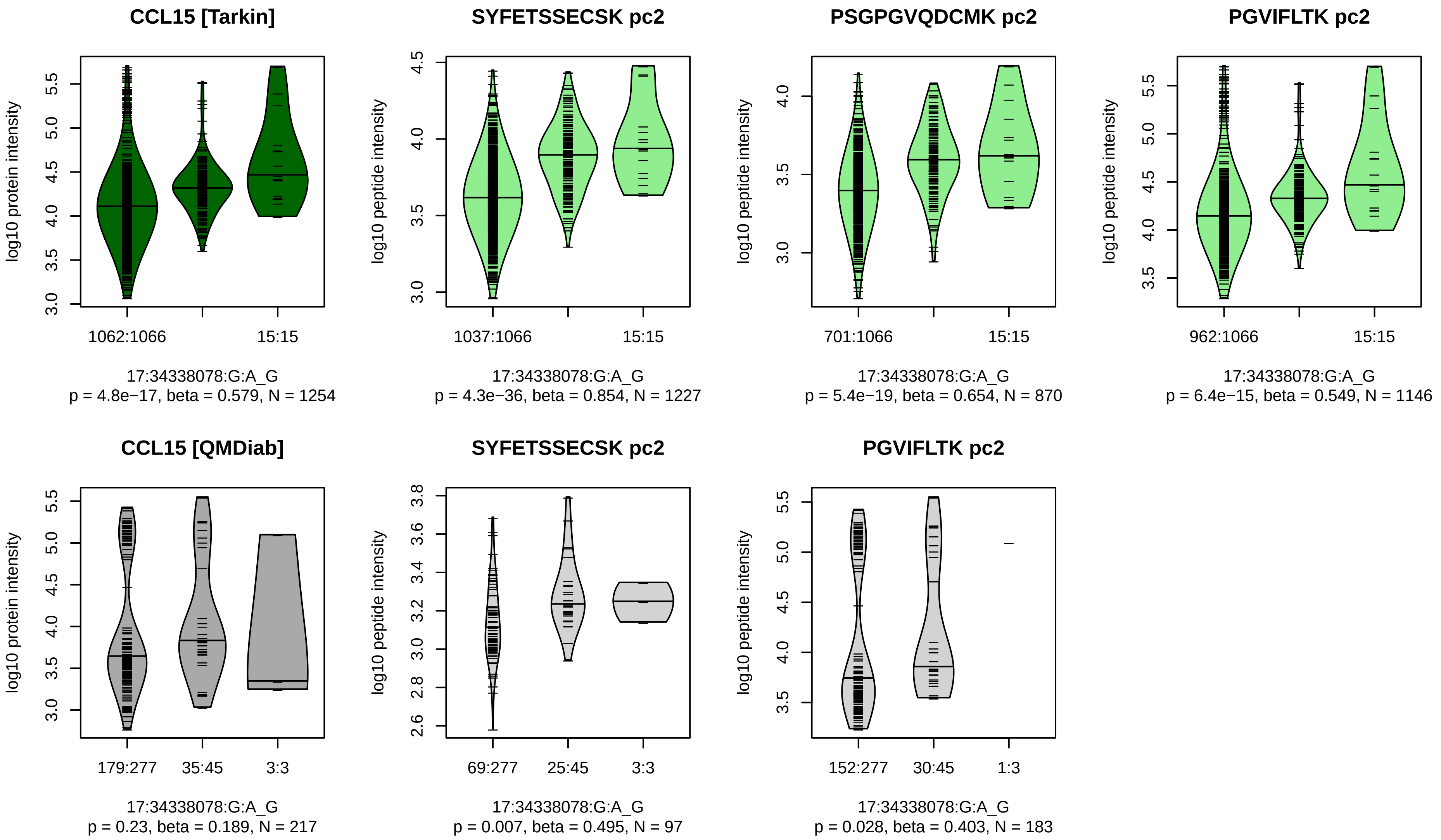

Sort: 106
Gene name: CCL15
SNP: 17:34338078:G:A
rsID: rs1719200
Type: Cis−pQTL
Protein group(s):
Q16663
Nano−particle: NP3

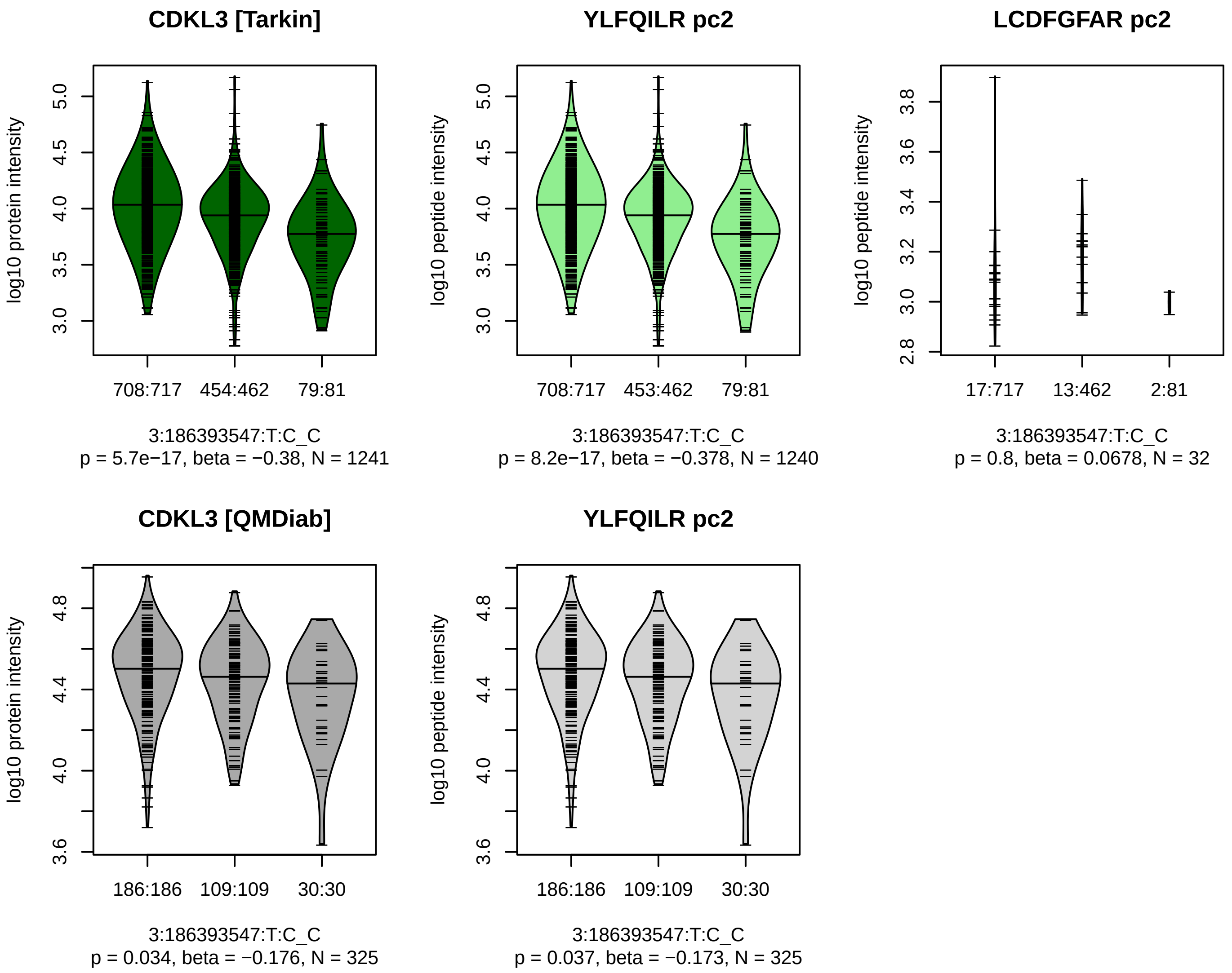

Sort: 107
Gene name: CDKL3
SNP: 3:186393547:T:C
rsID: rs7625980
Type: Trans−pQTL
Protein group(s):
E7ET86;Q8IVW4
Nano−particle: NP1

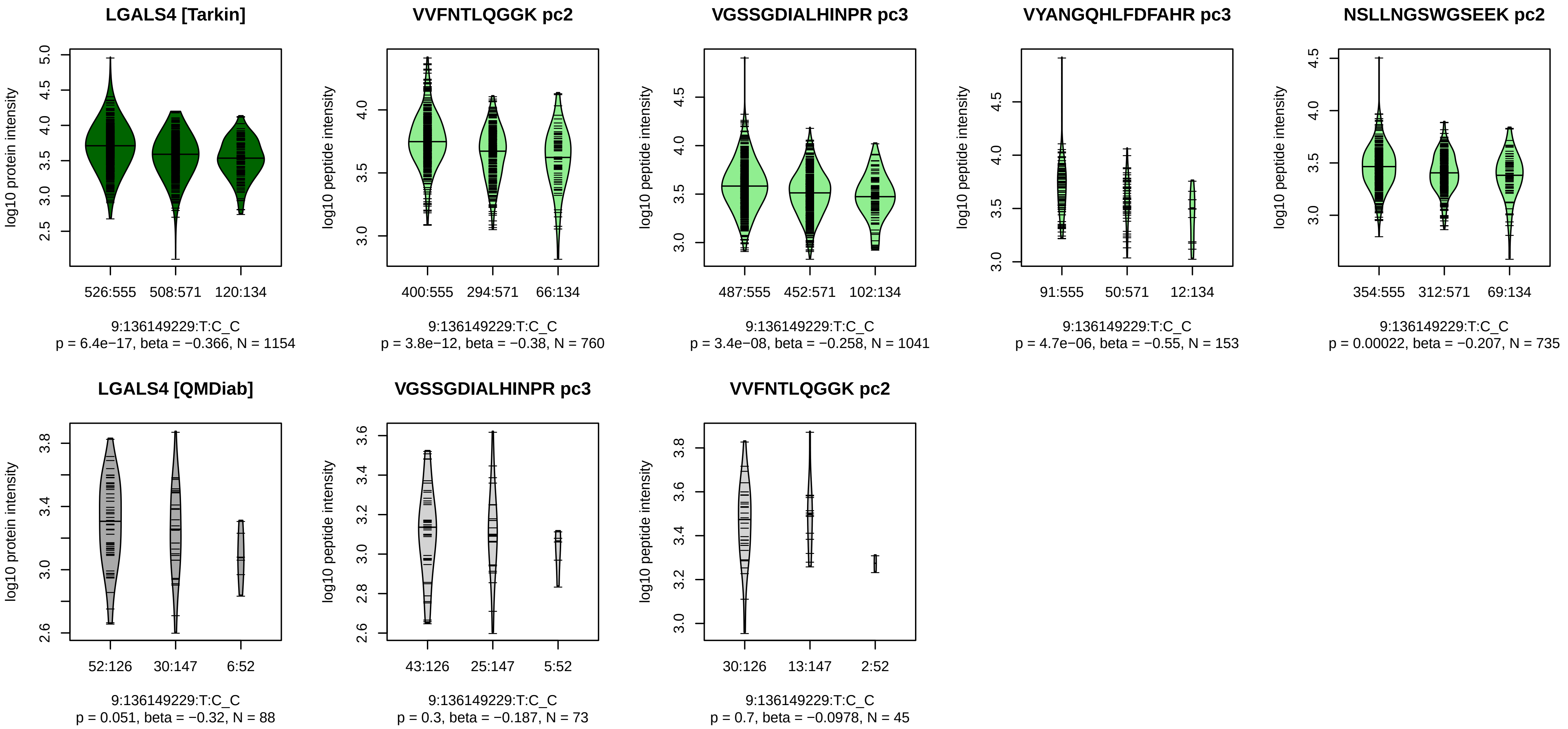

Sort: 108
Gene name: LGALS4
SNP: 9:136149229:T:C
rsID: rs505922
Type: Trans−pQTL
Protein group(s):
P56470
Nano−particle: NP3

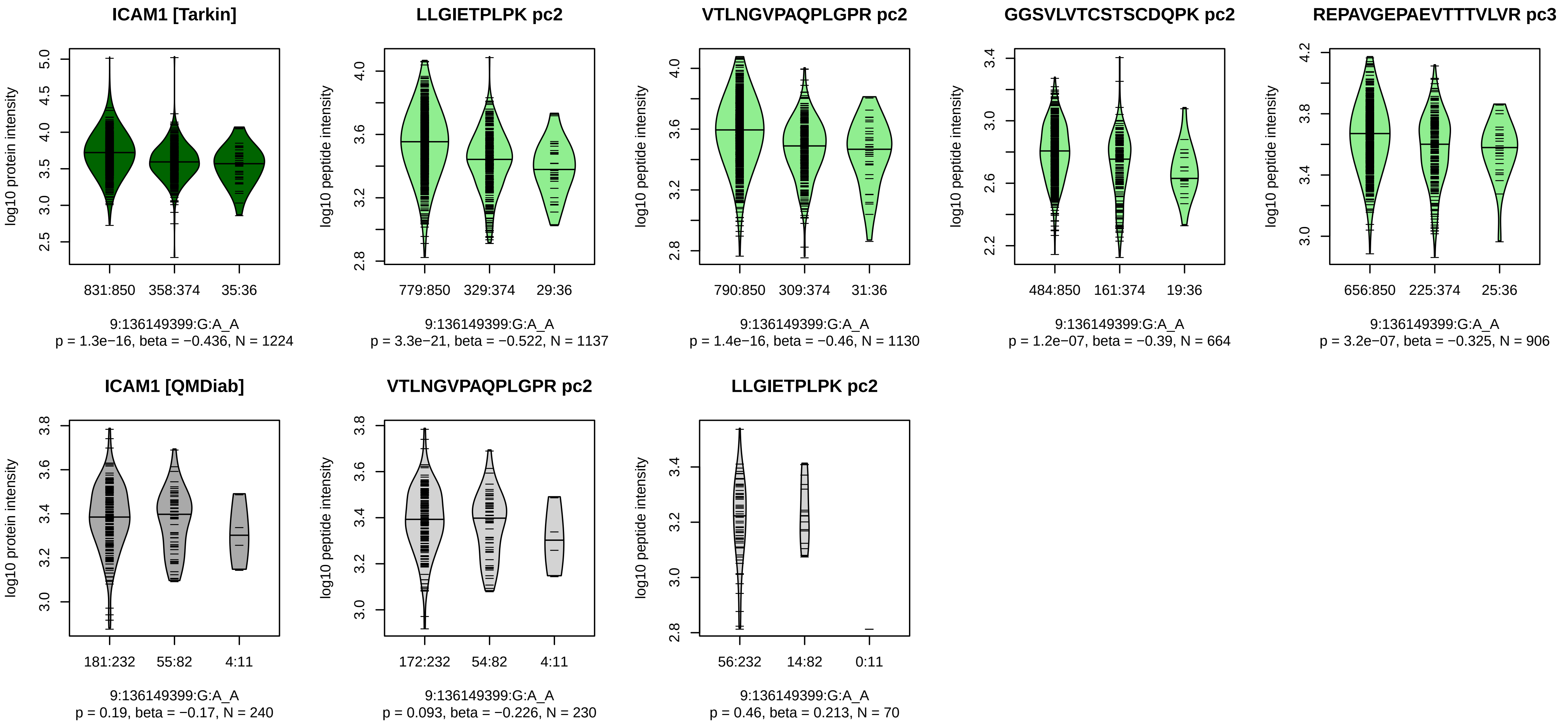

Sort: 109
Gene name: ICAM1
SNP: 9:136149399:G:A
rsID: rs507666
Type: Trans−pQTL
Protein group(s):
P05362
Nano−particle: NP4

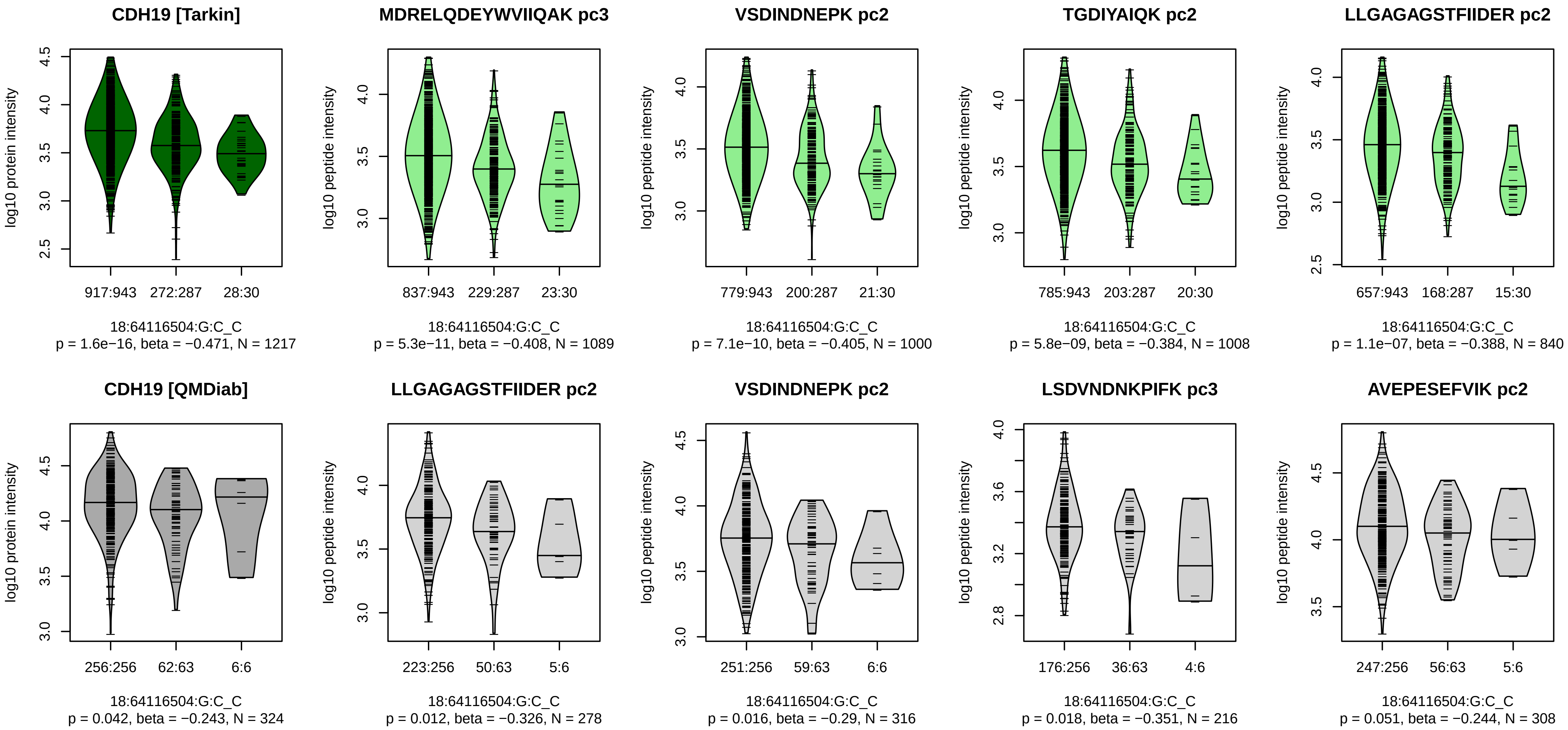

Sort: 110
Gene name: CDH19
SNP: 18:64116504:G:C
rsID: rs985088
Type: Cis−pQTL
Protein group(s):
Q9H159
Nano−particle: NP2

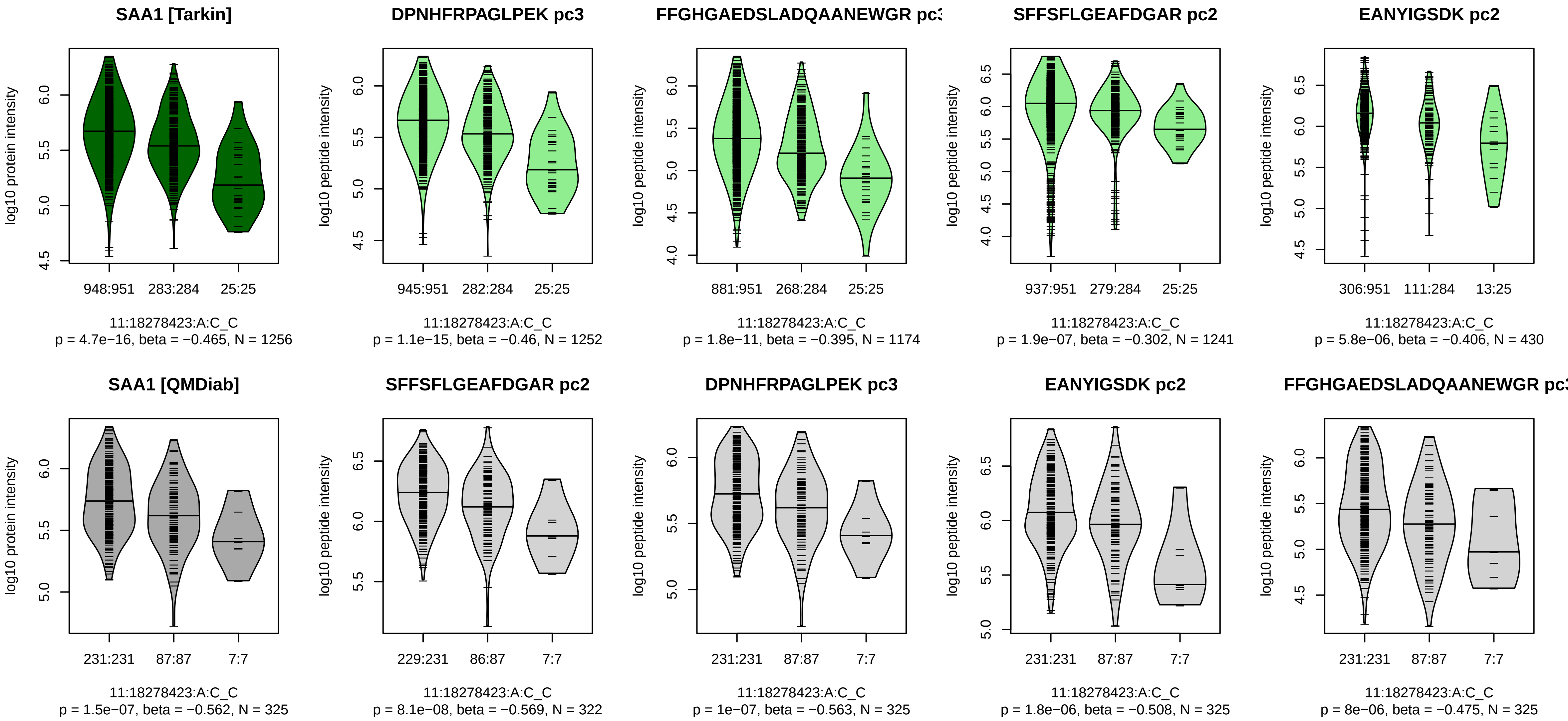

Sort: 111
Gene name: SAA1
SNP: 11:18278423:A:C
rsID: rs11024589
Type: Cis−pQTL
Protein group(s):
E9PQD6
Nano−particle: NP2

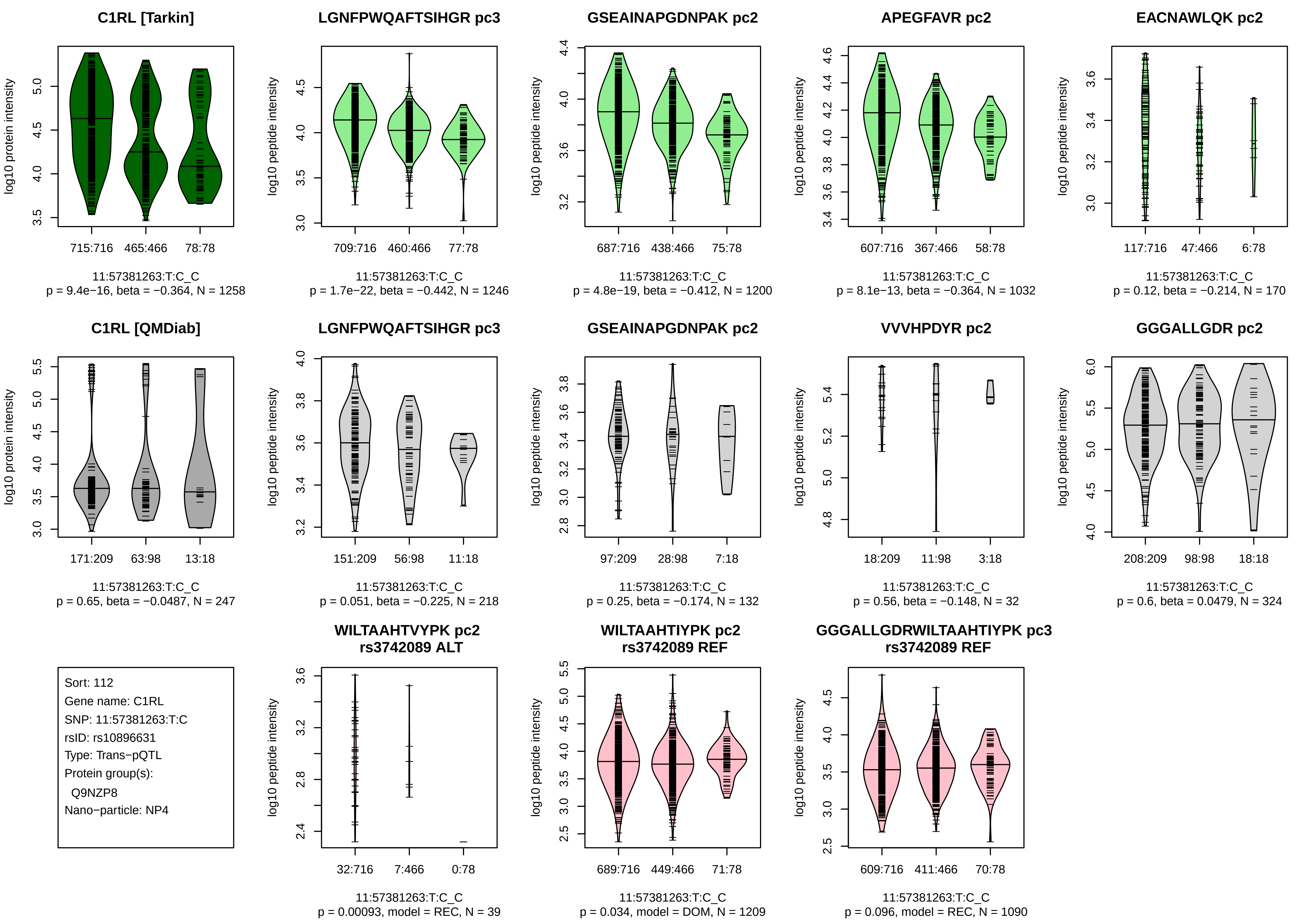
C1RL [Tarkin]
log10 protein intensity
715:716 465:466 78:78
11:57381263:T:C_C
p = 9.4e−16, beta = −0.364, N = 1258
LGNFPWQAFTSIHGR pc3
log10 peptide intensity
709:716 460:466 77:78
11:57381263:T:C_C
p = 1.7e−22, beta = −0.442, N = 1246
GSEAINAPGDNPAK pc2
log10 peptide intensity
687:716 438:466 75:78
11:57381263:T:C_C
p = 4.8e−19, beta = −0.412, N = 1200
APEGFAVR pc2
log10 peptide intensity
607:716 367:466 58:78
11:57381263:T:C_C
p = 8.1e−13, beta = −0.364, N = 1032
EACNAWLQK pc2
log10 peptide intensity
117:716 47:466 6:78
11:57381263:T:C_C
p = 0.12, beta = −0.214, N = 170
C1RL [QMDiab]
log10 protein intensity
171:209 63:98 13:18
11:57381263:T:C_C
p = 0.65, beta = −0.0487, N = 247
LGNFPWQAFTSIHGR pc3
log10 peptide intensity
151:209 56:98 11:18
11:57381263:T:C_C
p = 0.051, beta = −0.225, N = 218
GSEAINAPGDNPAK pc2
log10 peptide intensity
97:209 28:98 7:18
11:57381263:T:C_C
p = 0.25, beta = −0.174, N = 132
VVVHPDYR pc2
log10 peptide intensity
18:209 11:98 3:18
11:57381263:T:C_C
p = 0.56, beta = −0.148, N = 32
GGGALLGDR pc2
log10 peptide intensity
208:209 98:98 18:18
11:57381263:T:C_C
p = 0.6, beta = 0.0479, N = 324
Sort: 112
Gene name: C1RL
SNP: 11:57381263:T:C
rsID: rs10896631
Type: Trans−pQTL
Protein group(s):
Q9NZP8
Nano−particle: NP4
WILTAAHTVYPK pc2
rs3742089 ALT
log10 peptide intensity
32:716 7:466 0:78
11:57381263:T:C_C
p = 0.00093, model = REC, N = 39
WILTAAHTIYPK pc2
rs3742089 REF
log10 peptide intensity
689:716 449:466 71:78
11:57381263:T:C_C
p = 0.034, model = DOM, N = 1209
GGGALLGDRWILTAAHTIYPK pc3
rs3742089 REF
log10 peptide intensity
609:716 411:466 70:78
11:57381263:T:C_C
p = 0.096, model = REC, N = 1090

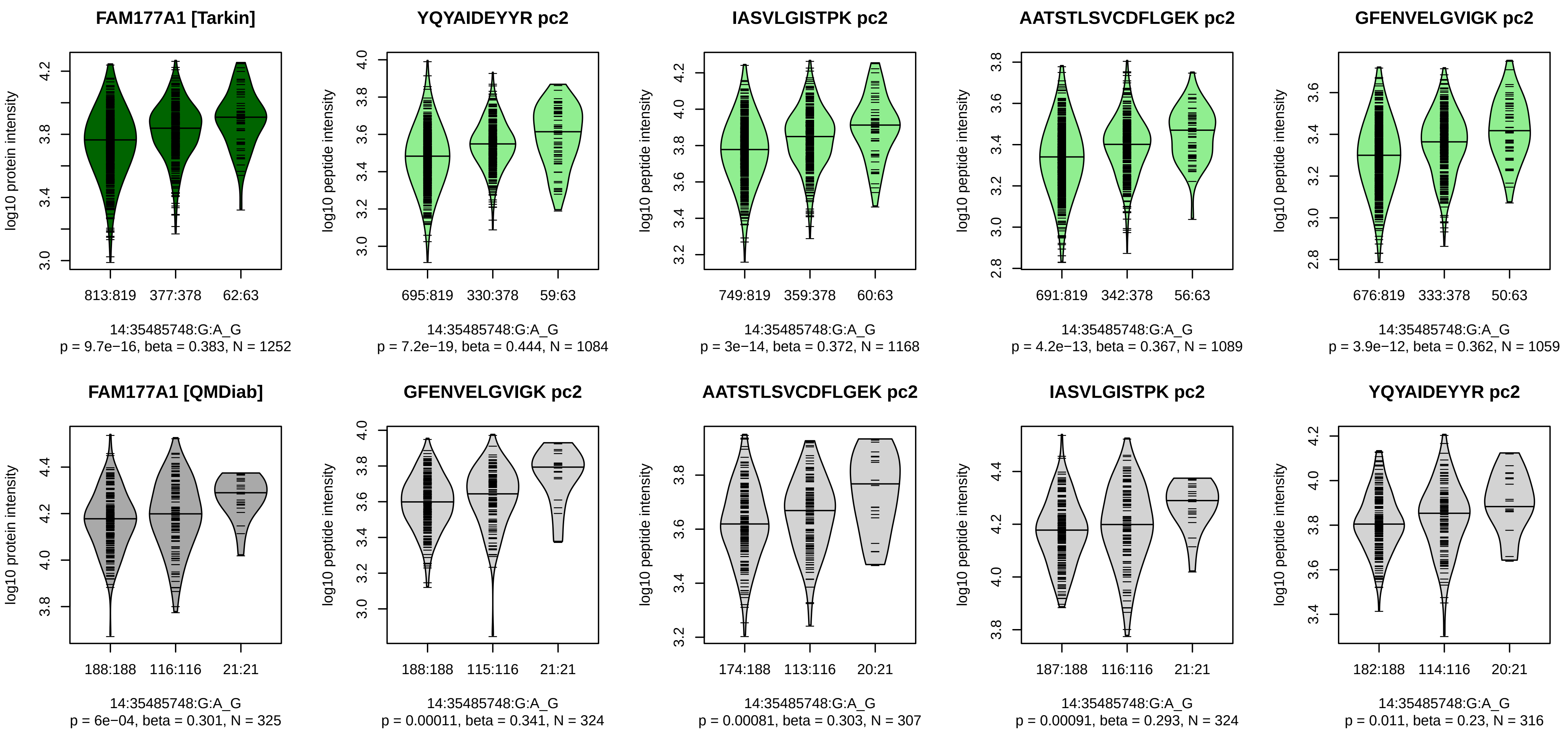

Sort: 113
Gene name: FAM177A1
SNP: 14:35485748:G:A
rsID: rs799459
Type: Cis−pQTL
Protein group(s):
Q8N128;Q8N128−2
Nano−particle: NP2

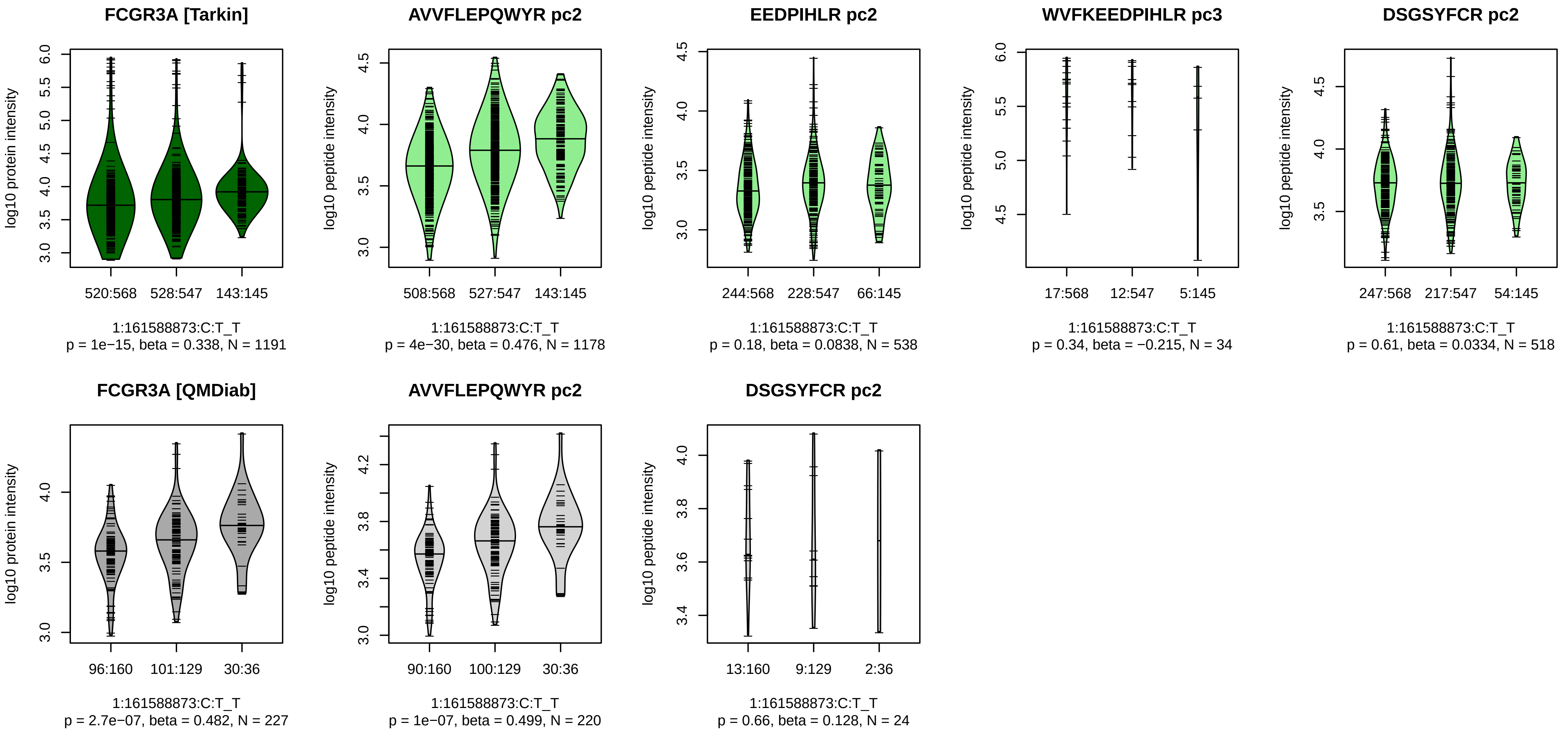

Sort: 114
Gene name: FCGR3A
SNP: 1:161588873:C:T
rsID: rs111384507
Type: Cis−pQTL
Protein group(s):
H0Y755;P08637
Nano−particle: NP1

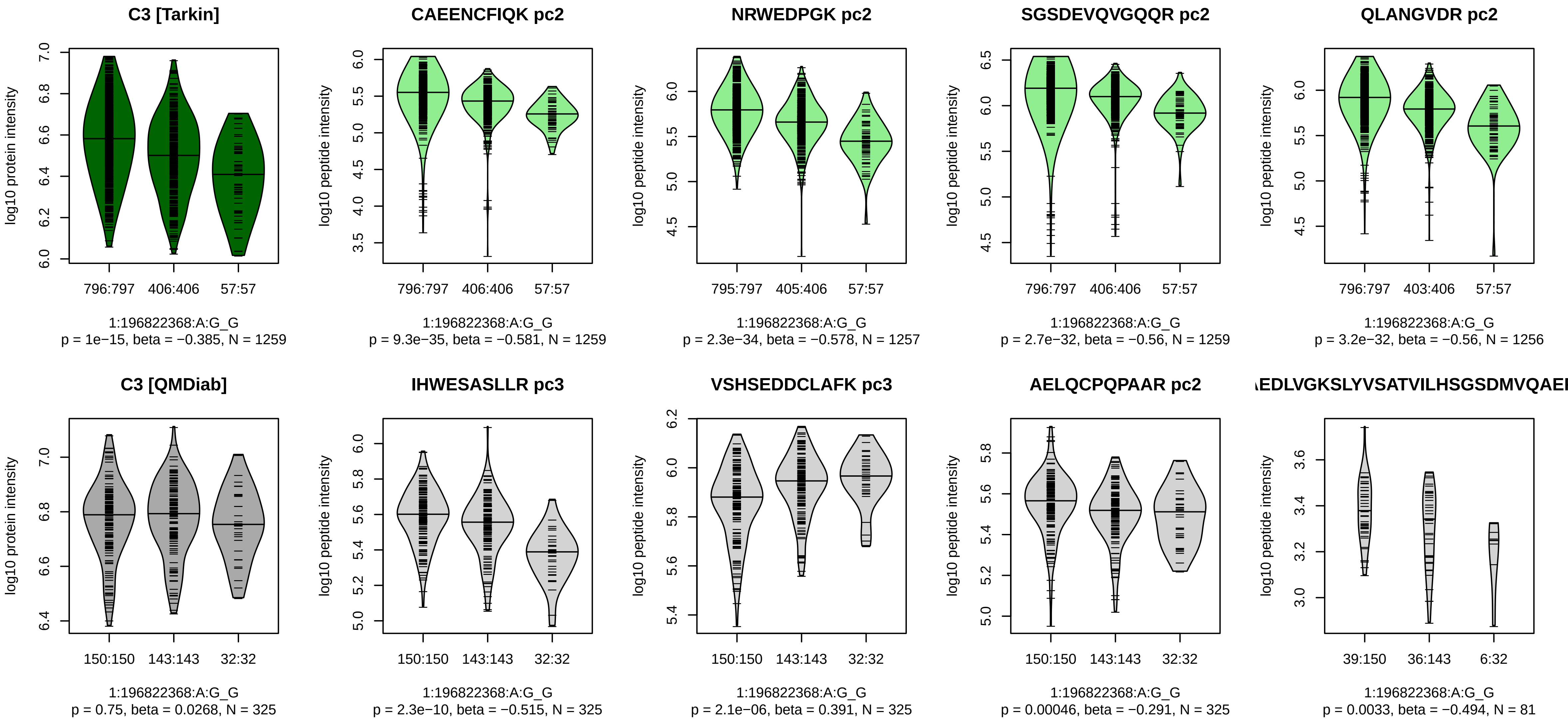

Sort: 115
Gene name: C3
SNP: 1:196822368:A:G
rsID: rs60642321
Type: Trans−pQTL
Protein group(s):
P01024
Nano−particle: NP4

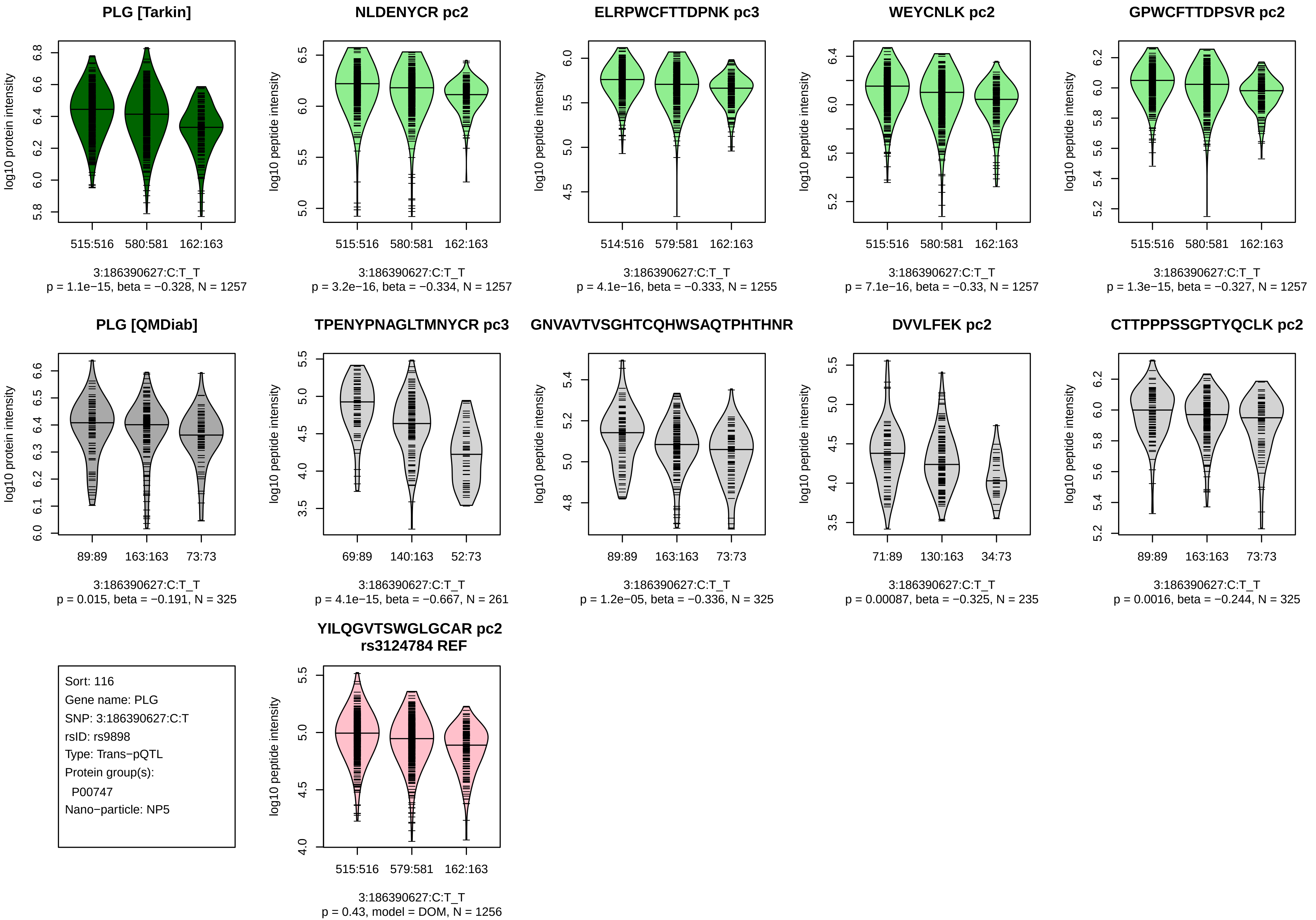
PLG [Tarkin]
log10 protein intensity
515:516
580:581
162:163
3:186390627:C:T_T
p = 1.1e−15, beta = −0.328, N = 1257
NLDENYCR pc2
log10 peptide intensity
515:516
580:581
162:163
3:186390627:C:T_T
p = 3.2e−16, beta = −0.334, N = 1257
ELRPWCFTTDPNK pc3
log10 peptide intensity
514:516
579:581
162:163
3:186390627:C:T_T
p = 4.1e−16, beta = −0.333, N = 1255
WEYCNLK pc2
log10 peptide intensity
515:516
580:581
162:163
3:186390627:C:T_T
p = 7.1e−16, beta = −0.33, N = 1257
GPWCFTTDPSVR pc2
log10 peptide intensity
515:516
580:581
162:163
3:186390627:C:T_T
p = 1.3e−15, beta = −0.327, N = 1257
PLG [QMDiab]
log10 protein intensity
89:89
163:163
73:73
3:186390627:C:T_T
p = 0.015, beta = −0.191, N = 325
TPENYPNAGLTMNYCR pc3
log10 peptide intensity
69:89
140:163
52:73
3:186390627:C:T_T
p = 4.1e−15, beta = −0.667, N = 261
GNVAVTVSGHTCQHWSAQTPHTHNR
log10 peptide intensity
89:89
163:163
73:73
3:186390627:C:T_T
p = 1.2e−05, beta = −0.336, N = 325
DVVLFEK pc2
log10 peptide intensity
71:89
130:163
34:73
3:186390627:C:T_T
p = 0.00087, beta = −0.325, N = 235
CTTPPPSSGPTYQCLK pc2
log10 peptide intensity
89:89
163:163
73:73
3:186390627:C:T_T
p = 0.0016, beta = −0.244, N = 325
Sort: 116
Gene name: PLG
SNP: 3:186390627:C:T
rsID: rs9898
Type: Trans−pQTL
Protein group(s):
P00747
Nano−particle: NP5
YILQGVTSWGLGCAR pc2
rs3124784 REF
log10 peptide intensity
515:516
579:581
162:163
3:186390627:C:T_T
p = 0.43, model = DOM, N = 1256

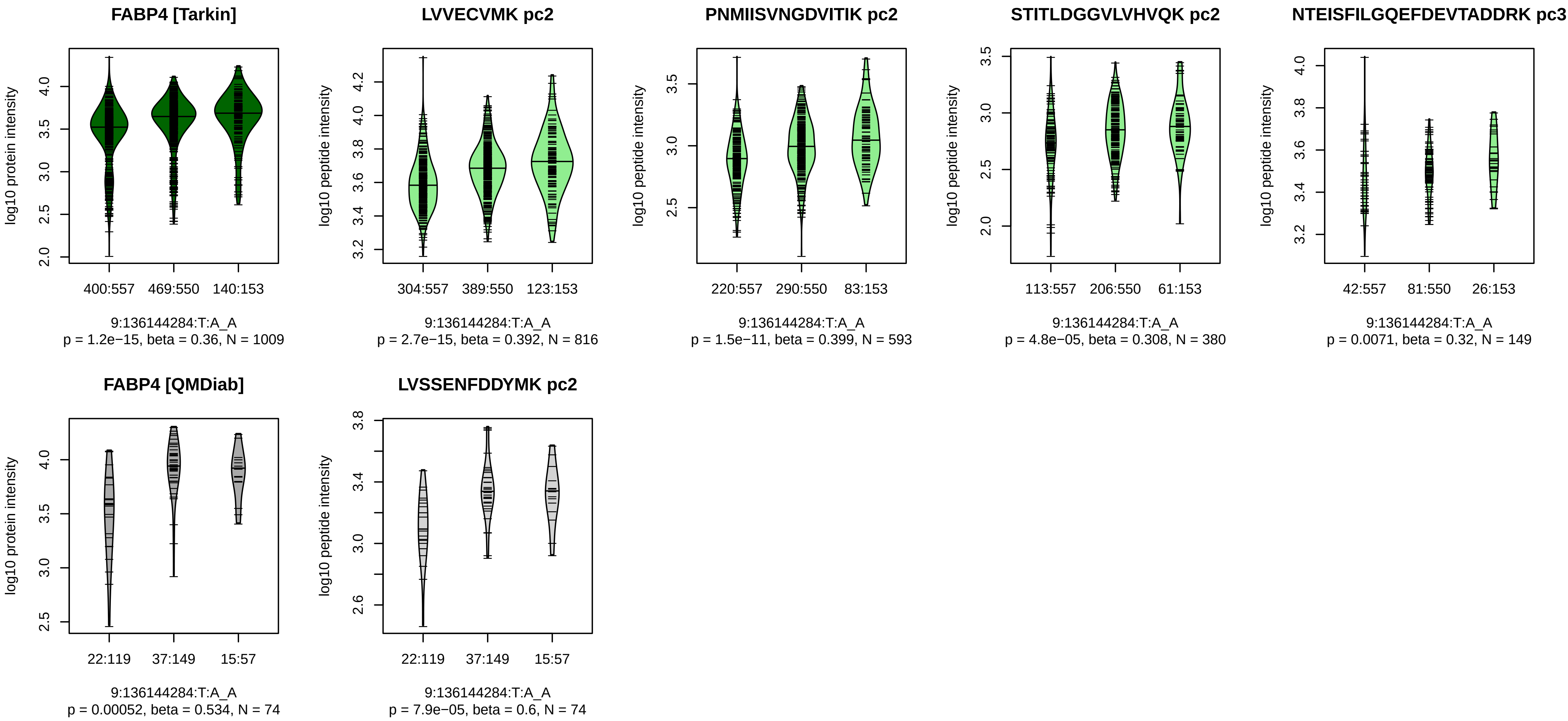

Sort: 117
Gene name: FABP4
SNP: 9:136144284:T:A
rsID: rs597988
Type: Trans−pQTL
Protein group(s):
P15090
Nano−particle: NP2

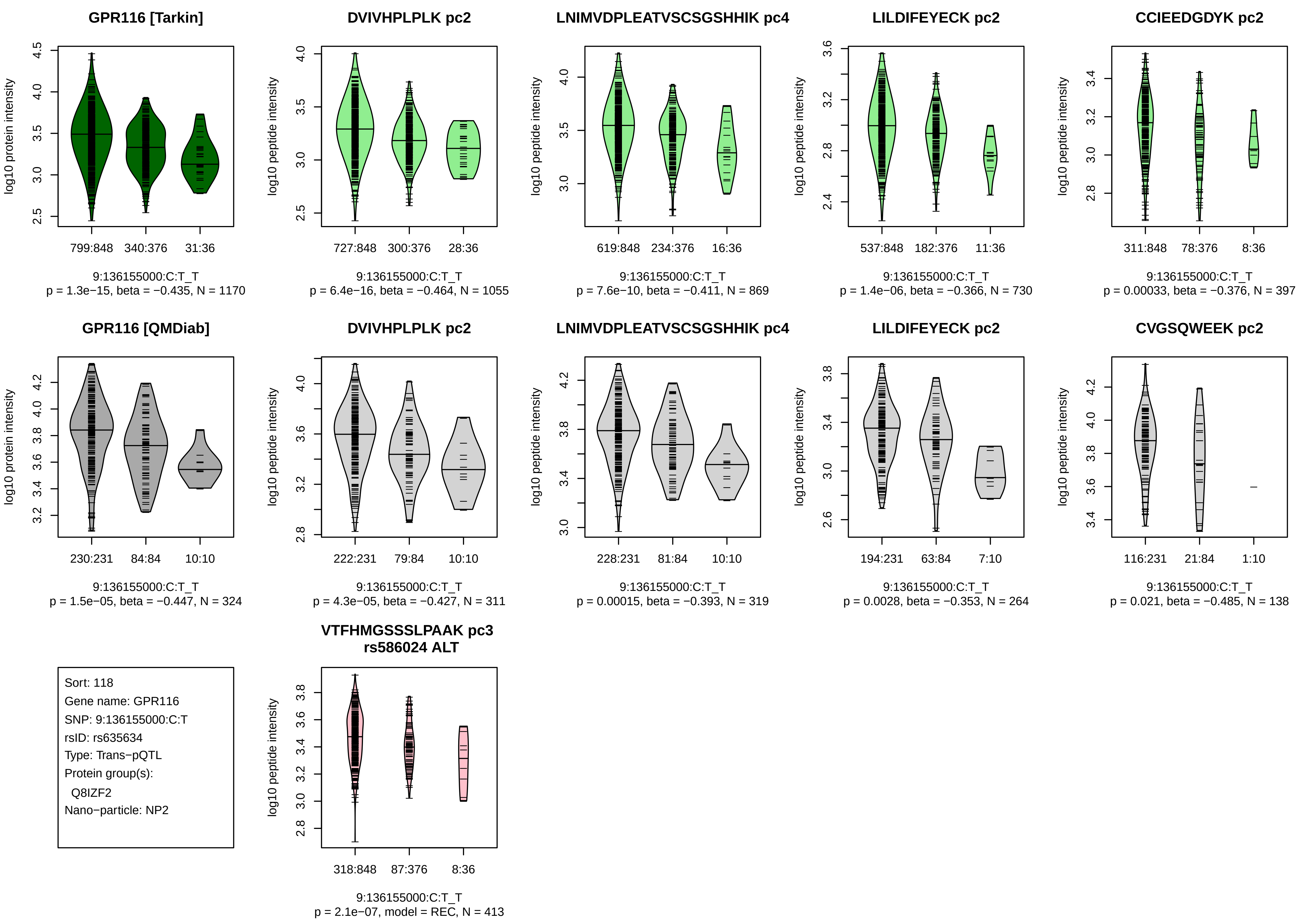
GPR116 [Tarkin]
DVIVHPLPLK pc2
LNIMVDPLEATVSCSGSHHIK pc4
LILDIFEYECK pc2
CCIEEDGDYK pc2
log10 protein intensity
log10 peptide intensity
799:848 340:376 31:36
727:848 300:376 28:36
619:848 234:376 16:36
537:848 182:376 11:36
311:848 78:376 8:36
9:136155000:C:T_T
p = 1.3e−15, beta = −0.435, N = 1170
p = 6.4e−16, beta = −0.464, N = 1055
p = 7.6e−10, beta = −0.411, N = 869
p = 1.4e−06, beta = −0.366, N = 730
p = 0.00033, beta = −0.376, N = 397
GPR116 [QMDiab]
DVIVHPLPLK pc2
LNIMVDPLEATVSCSGSHHIK pc4
LILDIFEYECK pc2
CVGSQWEEK pc2
230:231 84:84 10:10
222:231 79:84 10:10
228:231 81:84 10:10
194:231 63:84 7:10
116:231 21:84 1:10
p = 1.5e−05, beta = −0.447, N = 324
p = 4.3e−05, beta = −0.427, N = 311
p = 0.00015, beta = −0.393, N = 319
p = 0.0028, beta = −0.353, N = 264
p = 0.021, beta = −0.485, N = 138
Sort: 118
Gene name: GPR116
SNP: 9:136155000:C:T
rsID: rs635634
Type: Trans−pQTL
Protein group(s):
Q8IZF2
Nano−particle: NP2
VTFHMGSSSLPAAK pc3
rs586024 ALT
318:848 87:376 8:36
p = 2.1e−07, model = REC, N = 413

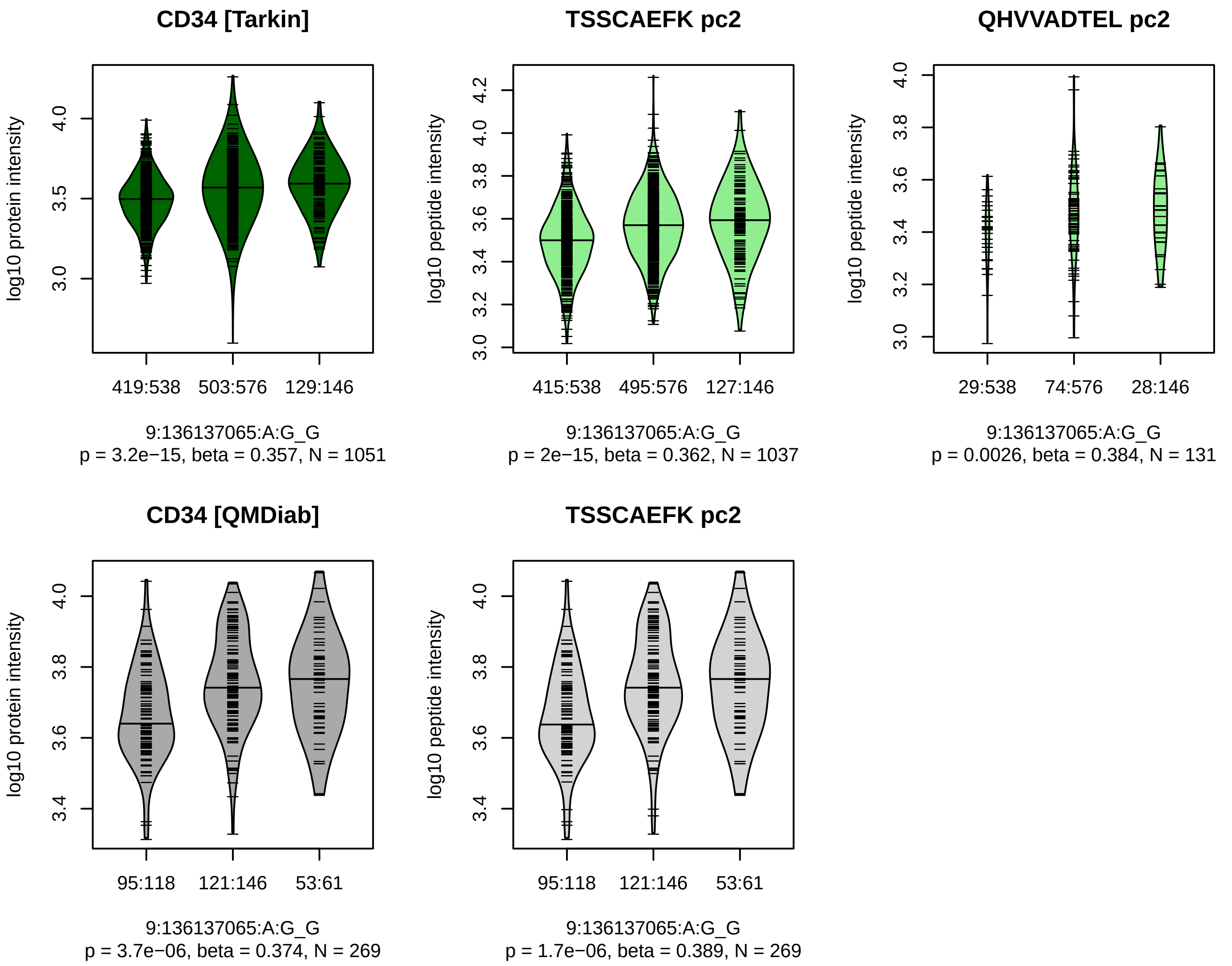

Sort: 119
Gene name: CD34
SNP: 9:136137065:A:G
rsID: rs687621
Type: Trans−pQTL
Protein group(s):
P28906
Nano−particle: NP2

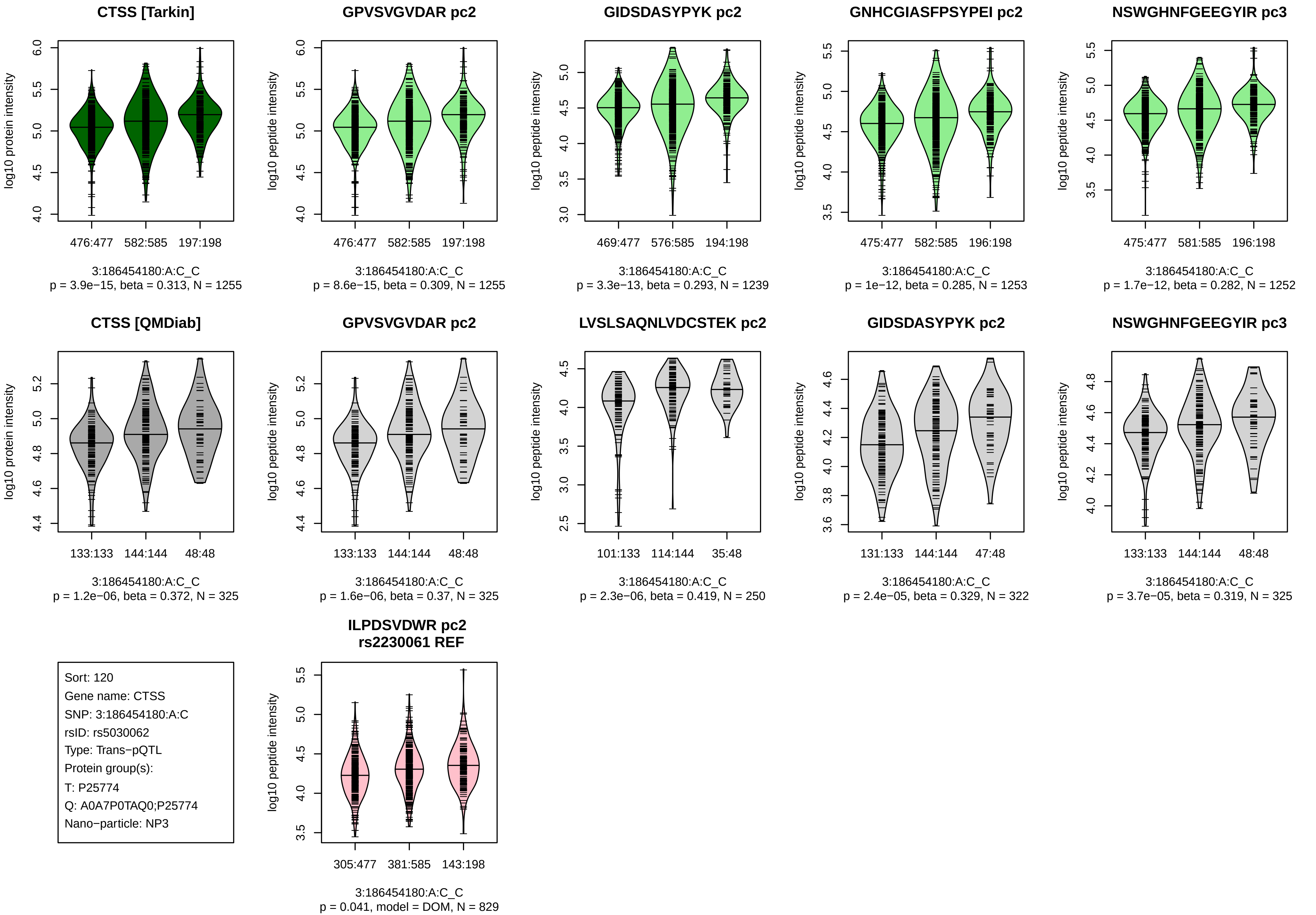

CTSS [Tarkin]
GPVSVGVDAR pc2
GIDSDASYPYK pc2
GNHCGIASFPSYPEI pc2
NSWGHNFGEEGYIR pc3
log10 protein intensity
log10 peptide intensity
476:477 582:585 197:198
469:477 576:585 194:198
475:477 582:585 196:198
475:477 581:585 196:198
3:186454180:A:C_C
p = 3.9e−15, beta = 0.313, N = 1255
p = 8.6e−15, beta = 0.309, N = 1255
p = 3.3e−13, beta = 0.293, N = 1239
p = 1e−12, beta = 0.285, N = 1253
p = 1.7e−12, beta = 0.282, N = 1252
CTSS [QMDiab]
GPVSVGVDAR pc2
LVSLSAQNLVDCSTEK pc2
GIDSDASYPYK pc2
NSWGHNFGEEGYIR pc3
133:133 144:144 48:48
101:133 114:144 35:48
131:133 144:144 47:48
p = 1.2e−06, beta = 0.372, N = 325
p = 1.6e−06, beta = 0.37, N = 325
p = 2.3e−06, beta = 0.419, N = 250
p = 2.4e−05, beta = 0.329, N = 322
p = 3.7e−05, beta = 0.319, N = 325
Sort: 120
Gene name: CTSS
SNP: 3:186454180:A:C
rsID: rs5030062
Type: Trans−pQTL
Protein group(s):
T: P25774
Q: A0A7P0TAQ0;P25774
Nano−particle: NP3
ILPDSVDWR pc2
rs2230061 REF
305:477 381:585 143:198
p = 0.041, model = DOM, N = 829

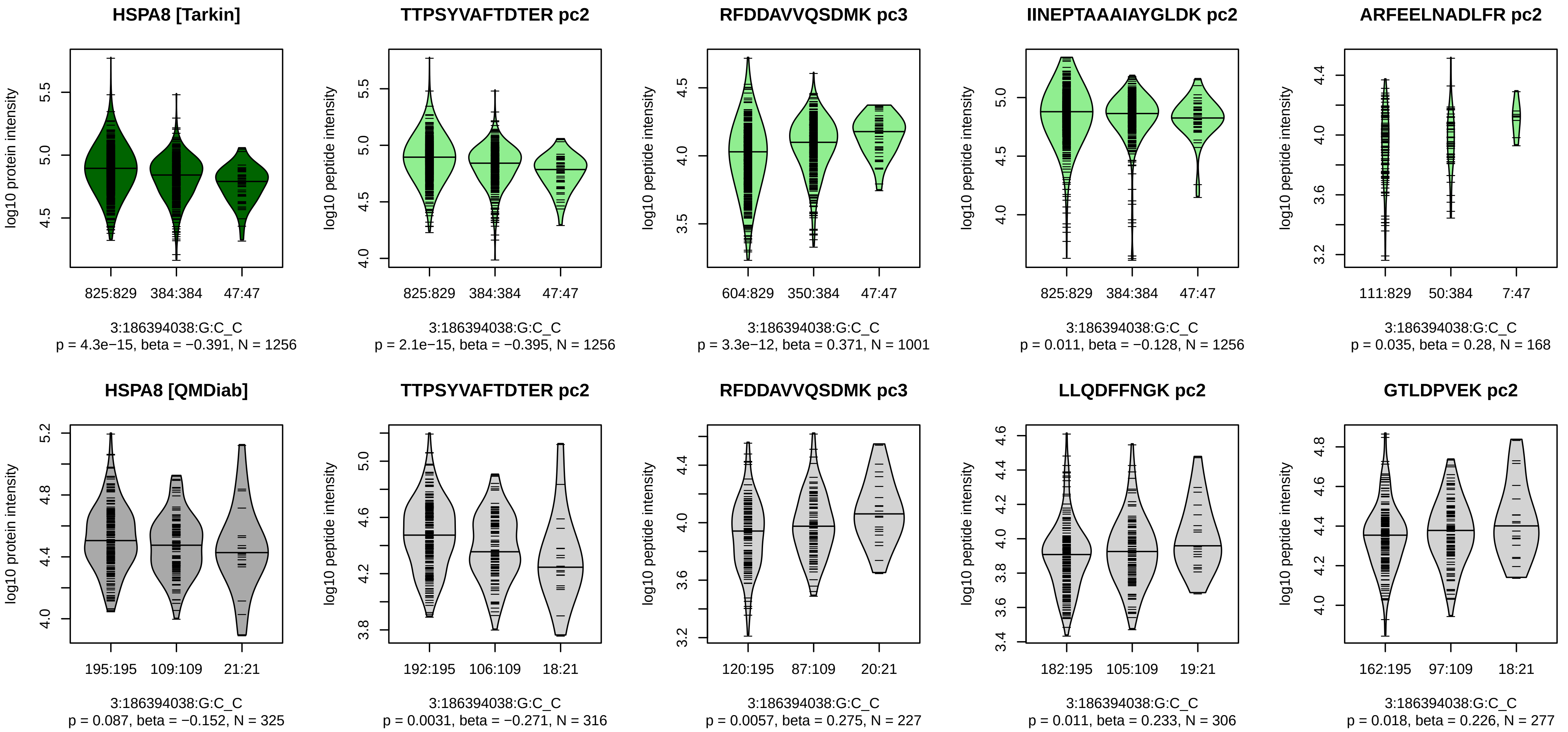

Sort: 121
Gene name: HSPA8
SNP: 3:186394038:G:C
rsID: rs16860992
Type: Trans−pQTL
Protein group(s):
P11142
Nano−particle: NP1

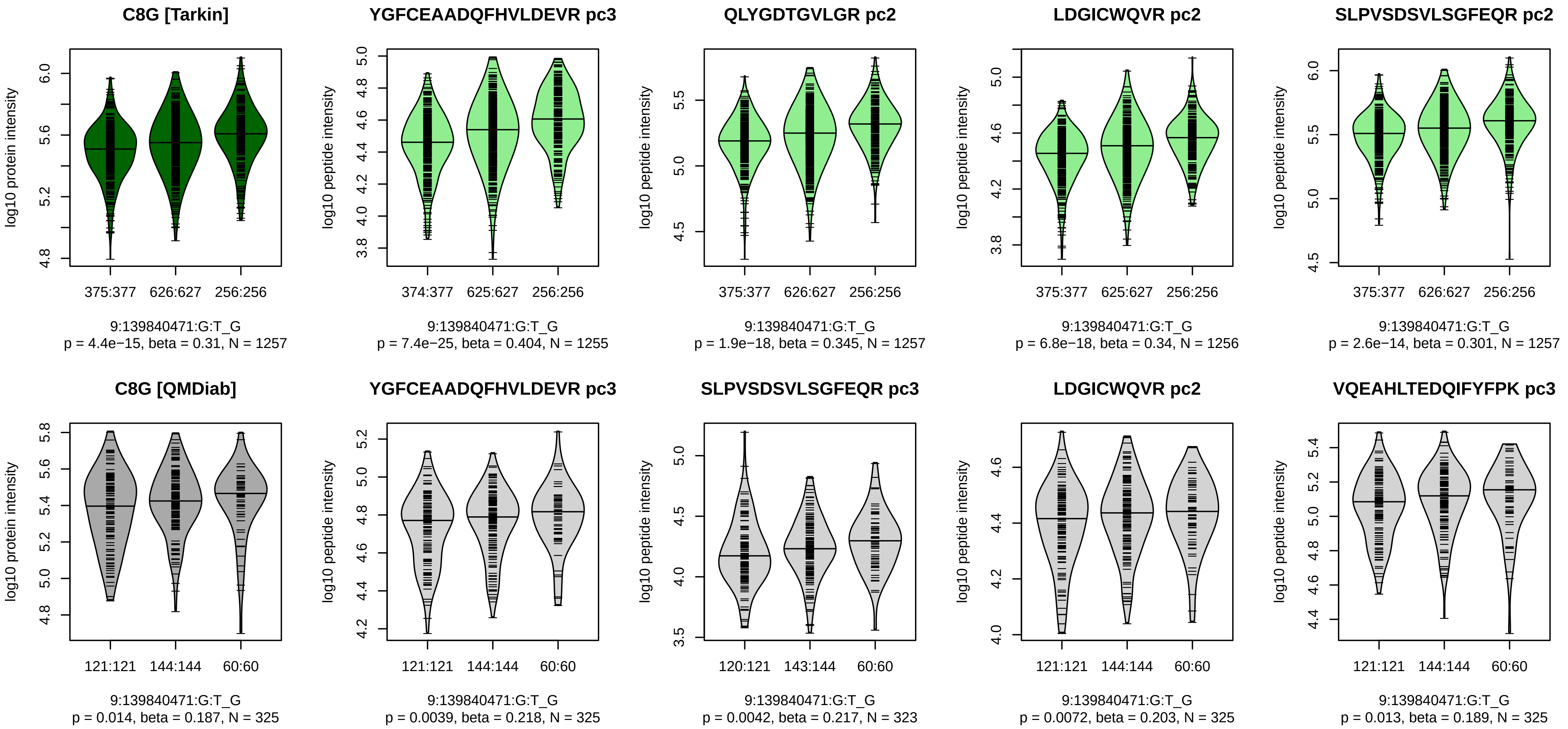

Sort: 122
Gene name: C8G
SNP: 9:139840471:G:T
rsID: rs7862602
Type: Cis−pQTL
Protein group(s):
P07360
Nano−particle: NP5

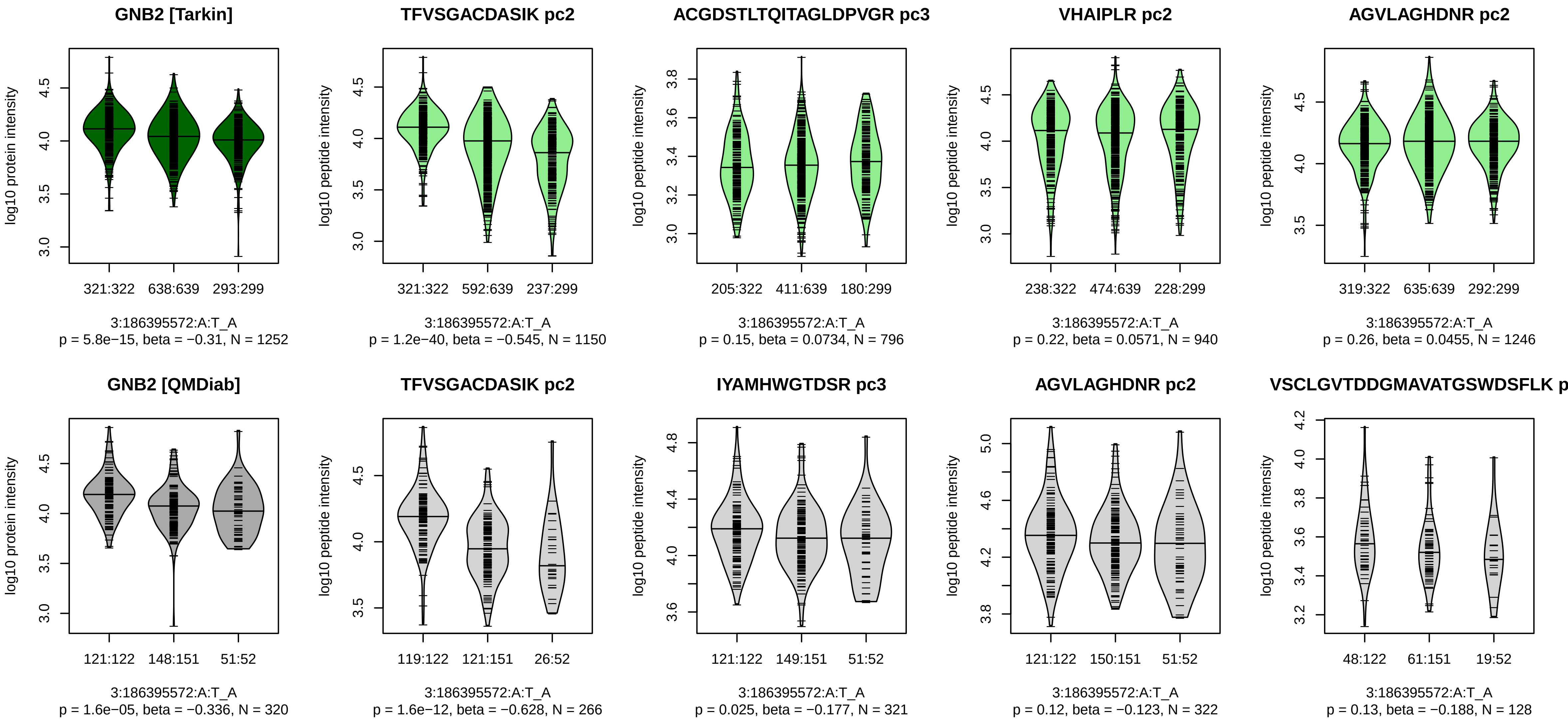

Sort: 123
Gene name: GNB2
SNP: 3:186395572:A:T
rsID: rs1042464
Type: Trans−pQTL
Protein group(s):
P62879
Nano−particle: NP1

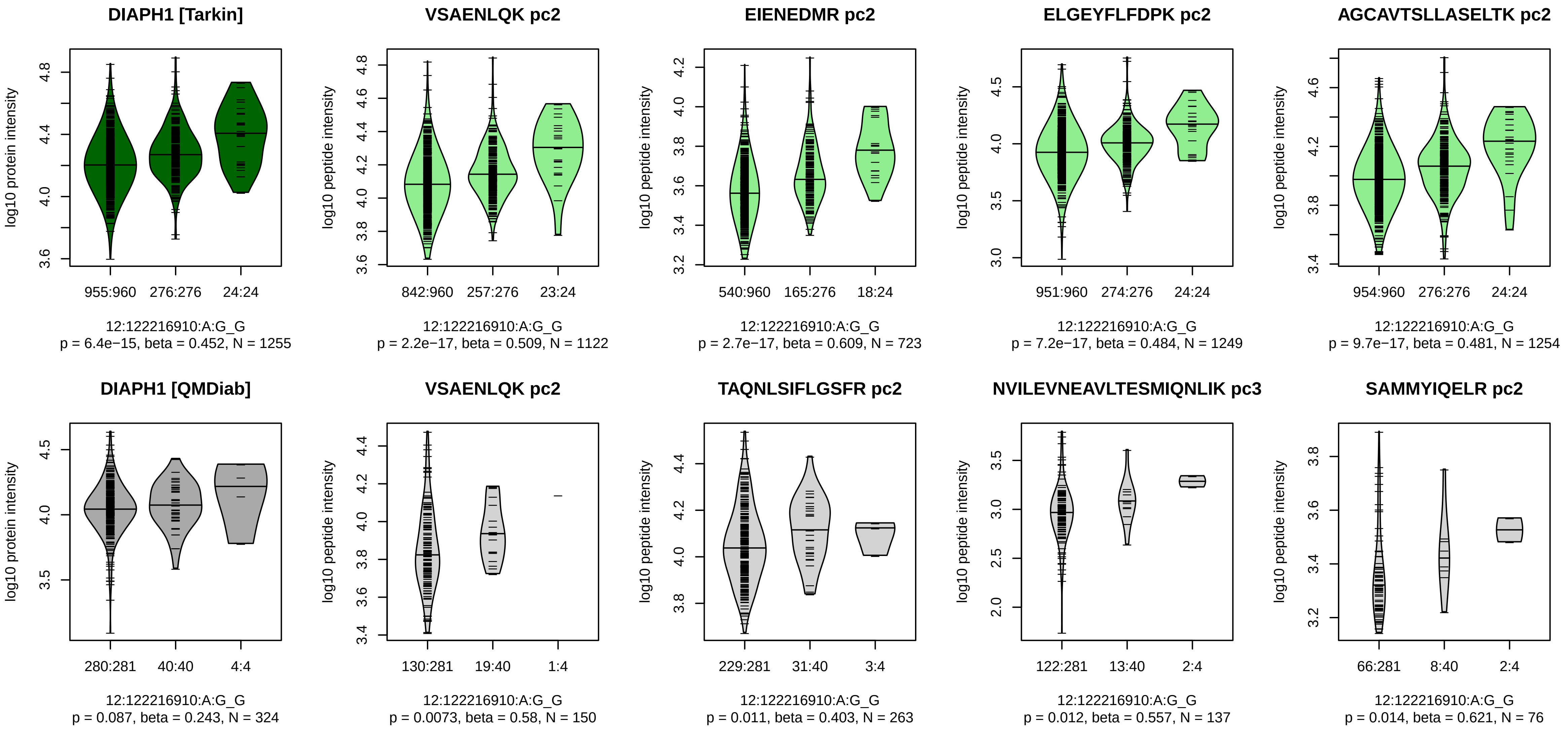

Sort: 124
Gene name: DIAPH1
SNP: 12:122216910:A:G
rsID: rs11553699
Type: Trans−pQTL
Protein group(s):
O60610
Nano−particle: NP1

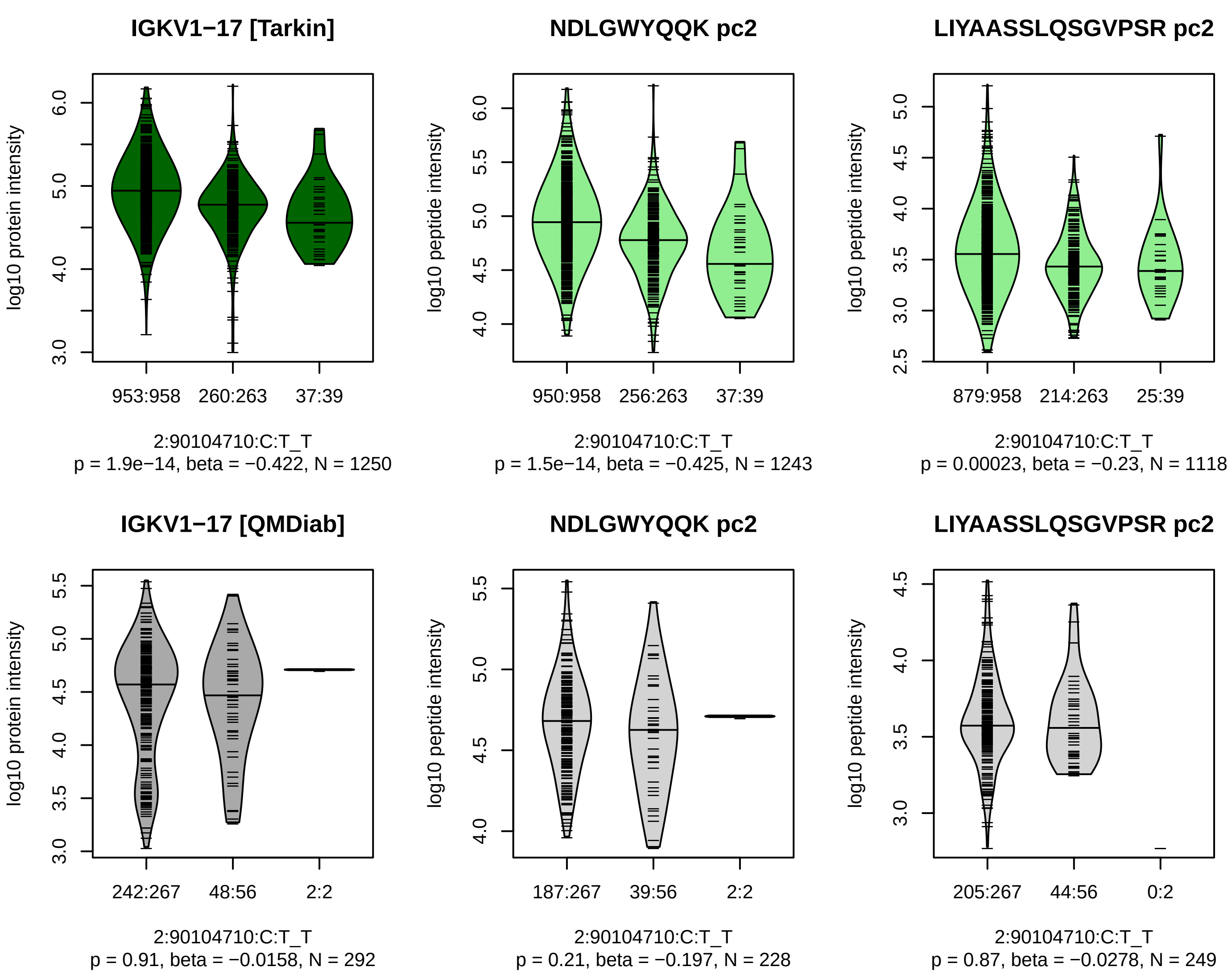

Sort: 125
Gene name: IGKV1−17
SNP: 2:90104710:C:T
rsID: rs2848139
Type: Cis−pQTL
Protein group(s):
P01599
Nano−particle: NP3

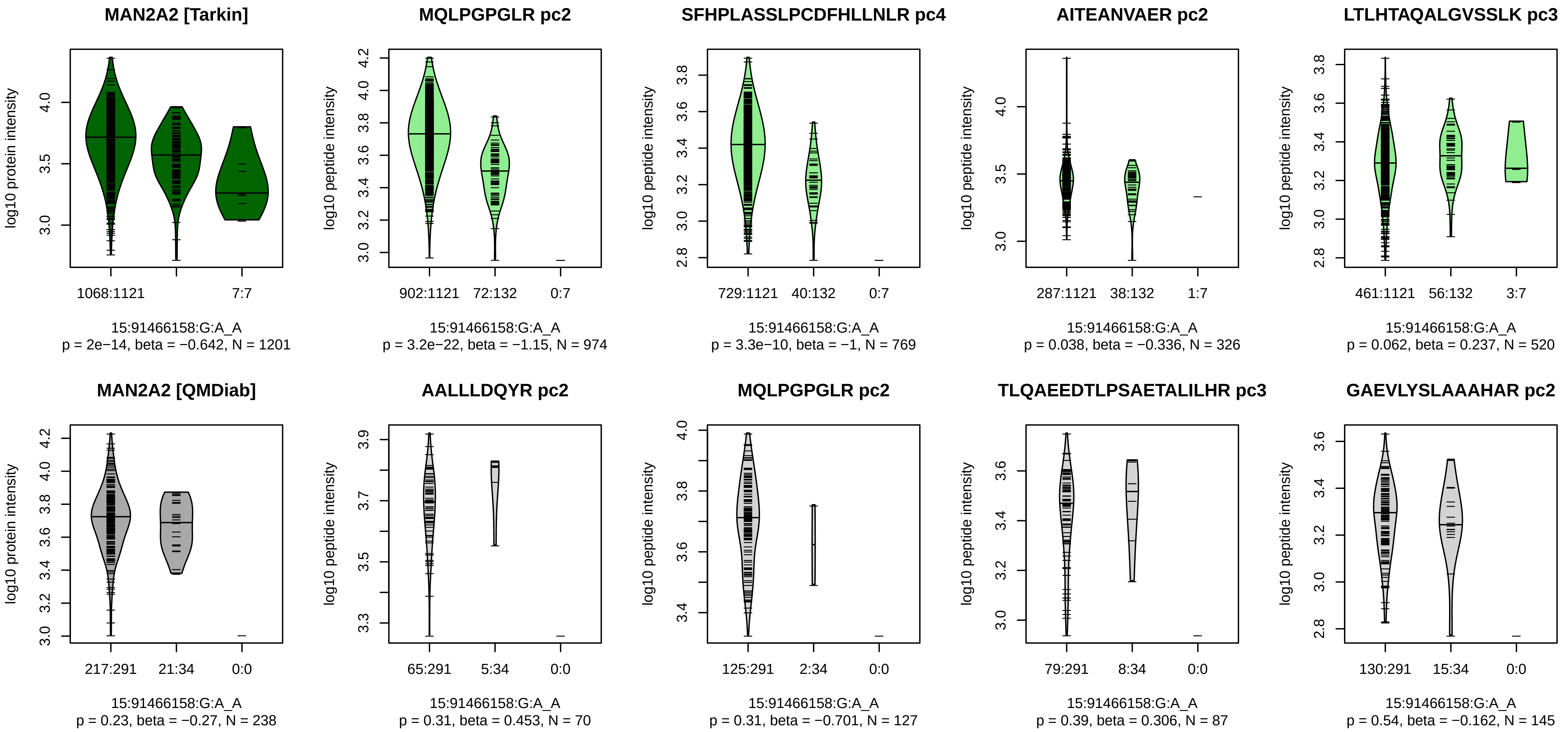

Sort: 126
Gene name: MAN2A2
SNP: 15:91466158:G:A
rsID: rs71407348
Type: Cis−pQTL
Protein group(s):
P49641
Nano−particle: NP5

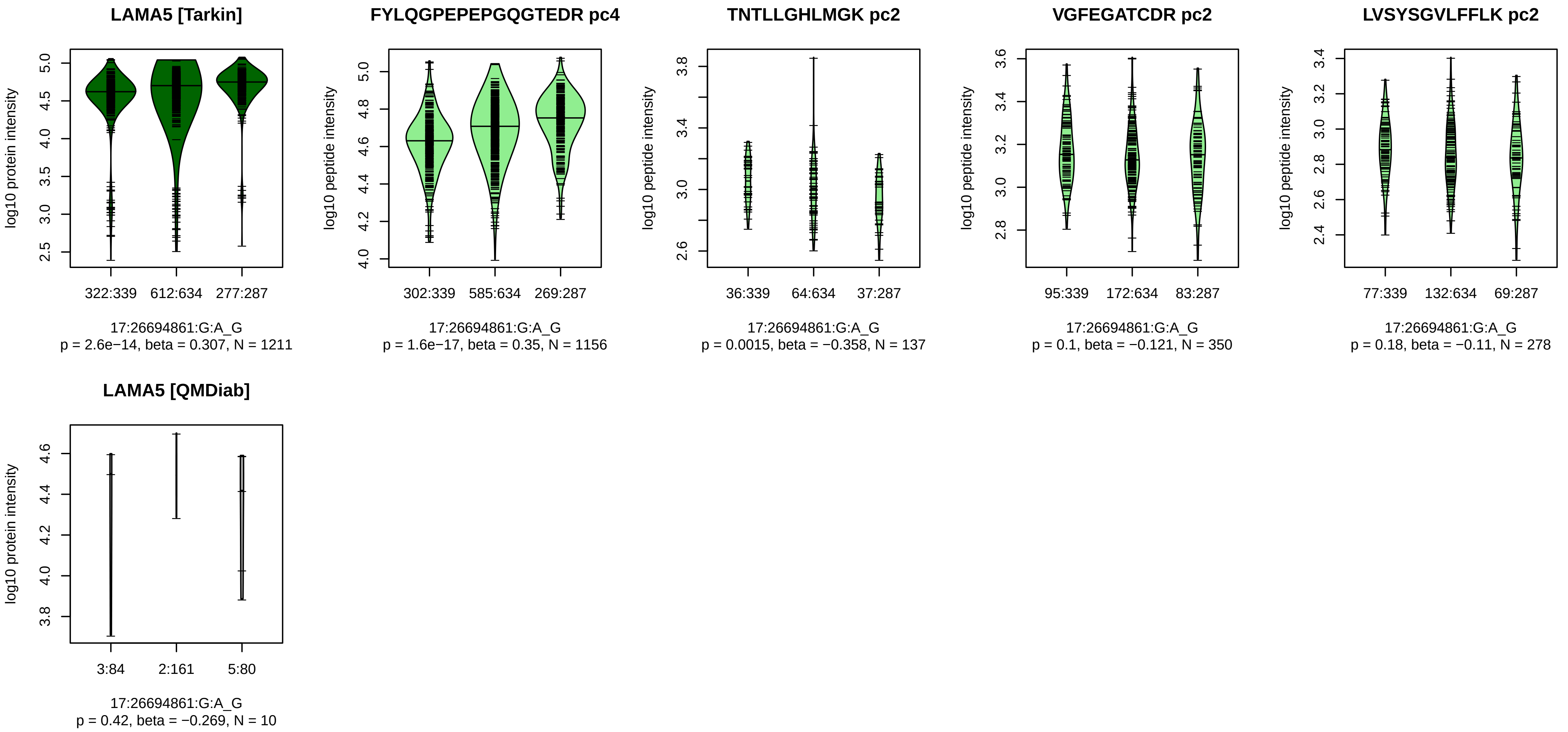

Sort: 127
Gene name: LAMA5
SNP: 17:26694861:G:A
rsID: rs704
Type: Trans−pQTL
Protein group(s):
O15230
Nano−particle: NP2

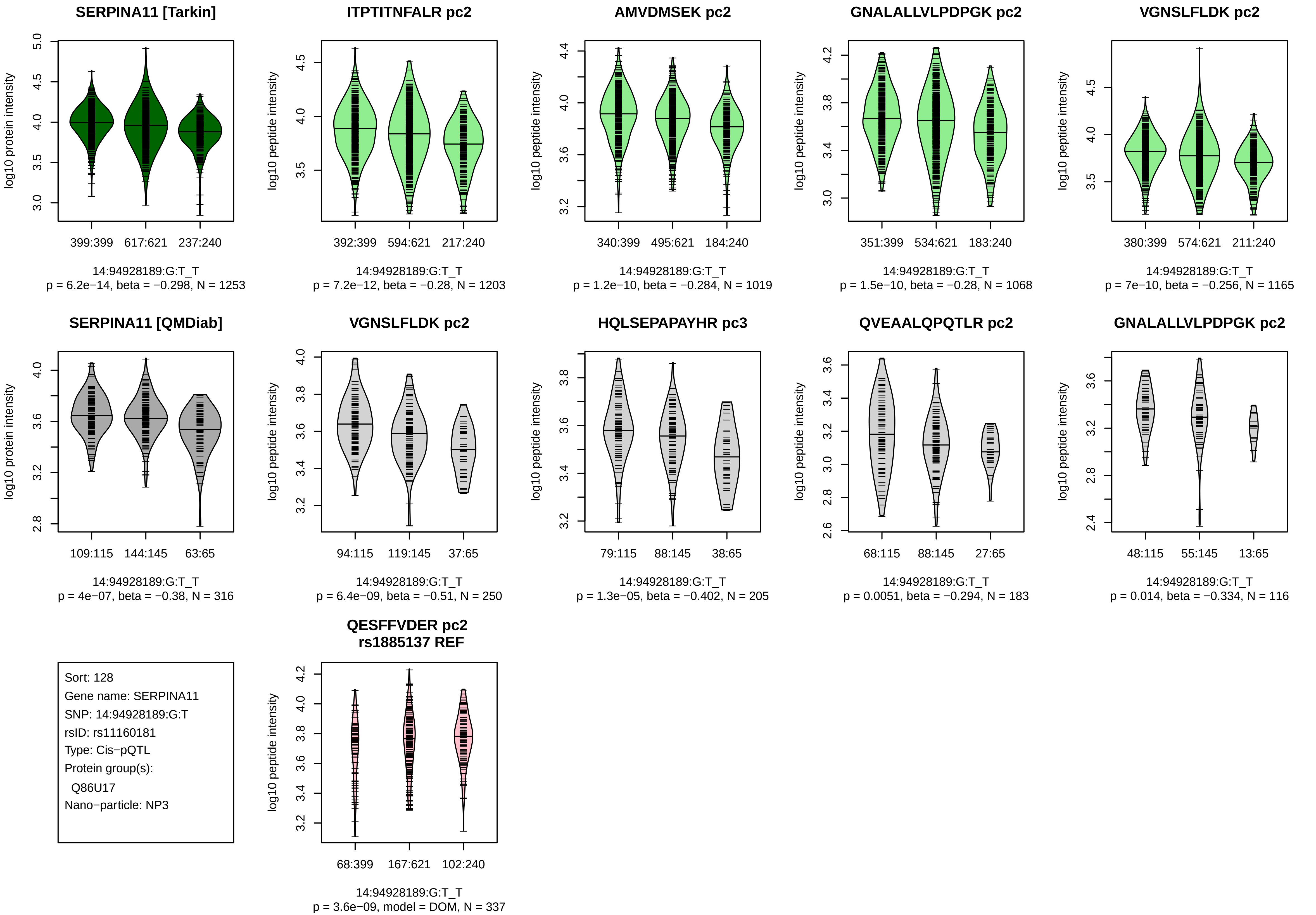
SERPINA11 [Tarkin]
log10 protein intensity
399:399 617:621 237:240
14:94928189:G:T_T
p = 6.2e−14, beta = −0.298, N = 1253
ITPTITNFALR pc2
log10 peptide intensity
392:399 594:621 217:240
14:94928189:G:T_T
p = 7.2e−12, beta = −0.28, N = 1203
AMVDMSEK pc2
log10 peptide intensity
340:399 495:621 184:240
14:94928189:G:T_T
p = 1.2e−10, beta = −0.284, N = 1019
GNALALLVLPDPGK pc2
log10 peptide intensity
351:399 534:621 183:240
14:94928189:G:T_T
p = 1.5e−10, beta = −0.28, N = 1068
VGNSLFLDK pc2
log10 peptide intensity
380:399 574:621 211:240
14:94928189:G:T_T
p = 7e−10, beta = −0.256, N = 1165
SERPINA11 [QMDiab]
log10 protein intensity
109:115 144:145 63:65
14:94928189:G:T_T
p = 4e−07, beta = −0.38, N = 316
VGNSLFLDK pc2
log10 peptide intensity
94:115 119:145 37:65
14:94928189:G:T_T
p = 6.4e−09, beta = −0.51, N = 250
HQLSEPAPAYHR pc3
log10 peptide intensity
79:115 88:145 38:65
14:94928189:G:T_T
p = 1.3e−05, beta = −0.402, N = 205
QVEAALQPQTLR pc2
log10 peptide intensity
68:115 88:145 27:65
14:94928189:G:T_T
p = 0.0051, beta = −0.294, N = 183
GNALALLVLPDPGK pc2
log10 peptide intensity
48:115 55:145 13:65
14:94928189:G:T_T
p = 0.014, beta = −0.334, N = 116
Sort: 128
Gene name: SERPINA11
SNP: 14:94928189:G:T
rsID: rs11160181
Type: Cis−pQTL
Protein group(s):
Q86U17
Nano−particle: NP3
QESFFVDER pc2
rs1885137 REF
log10 peptide intensity
68:399 167:621 102:240
14:94928189:G:T_T
p = 3.6e−09, model = DOM, N = 337

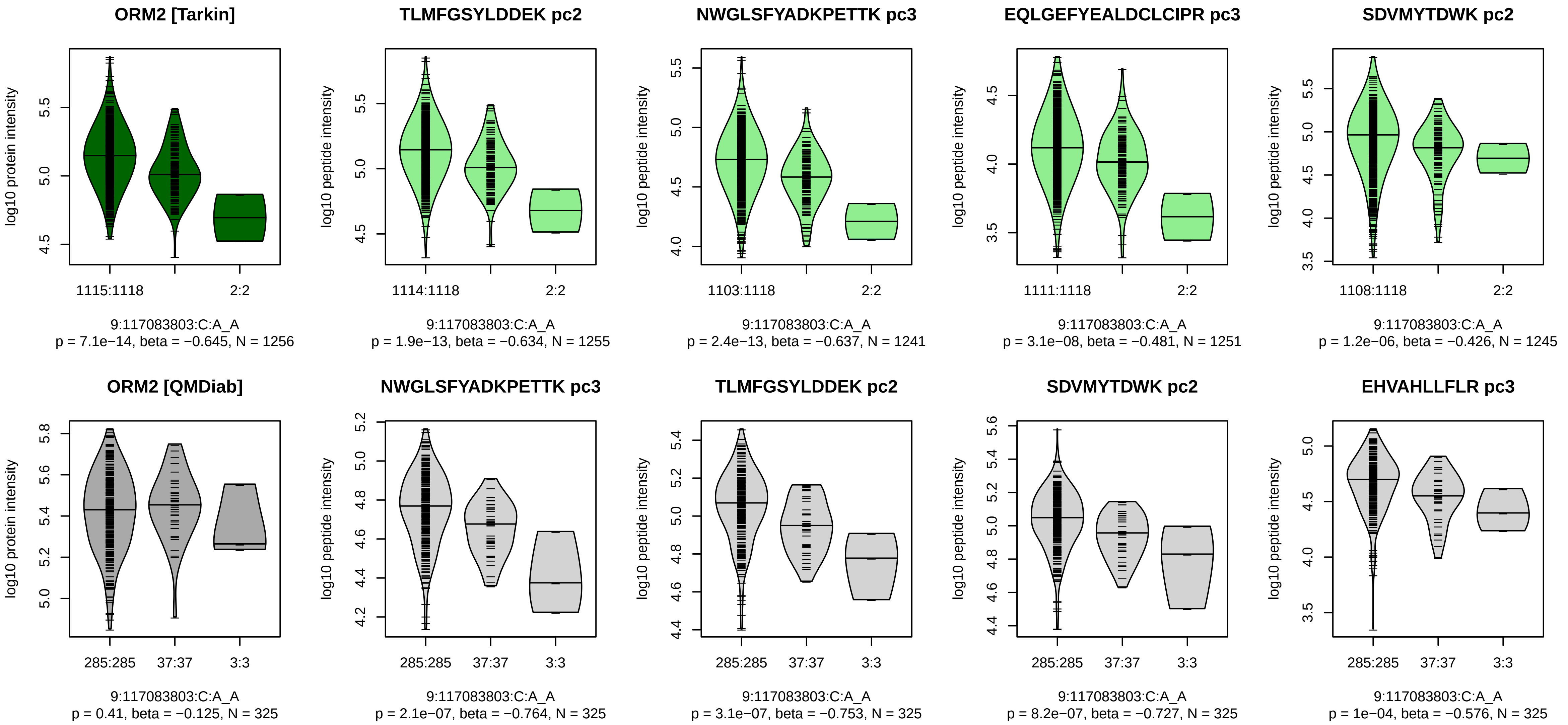

Sort: 129
Gene name: ORM2
SNP: 9:117083803:C:A
rsID: rs150611042
Type: Cis−pQTL
Protein group(s):
P19652
Nano−particle: NP2

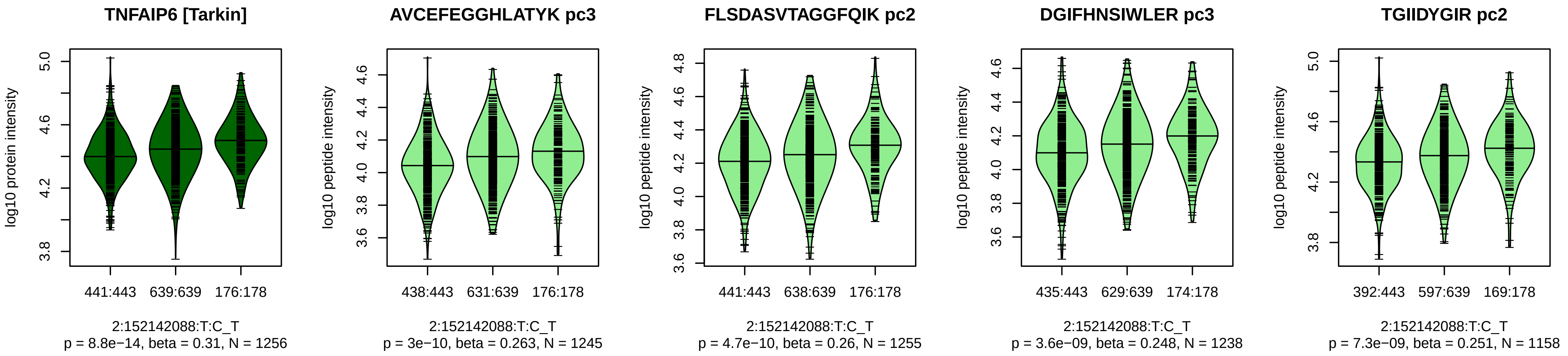

No protein data available
for replication in QMDiab

Sort: 130
Gene name: TNFAIP6
SNP: 2:152142088:T:C
rsID: rs11679466
Type: Cis−pQTL
Protein group(s):
P98066
Nano−particle: NP5

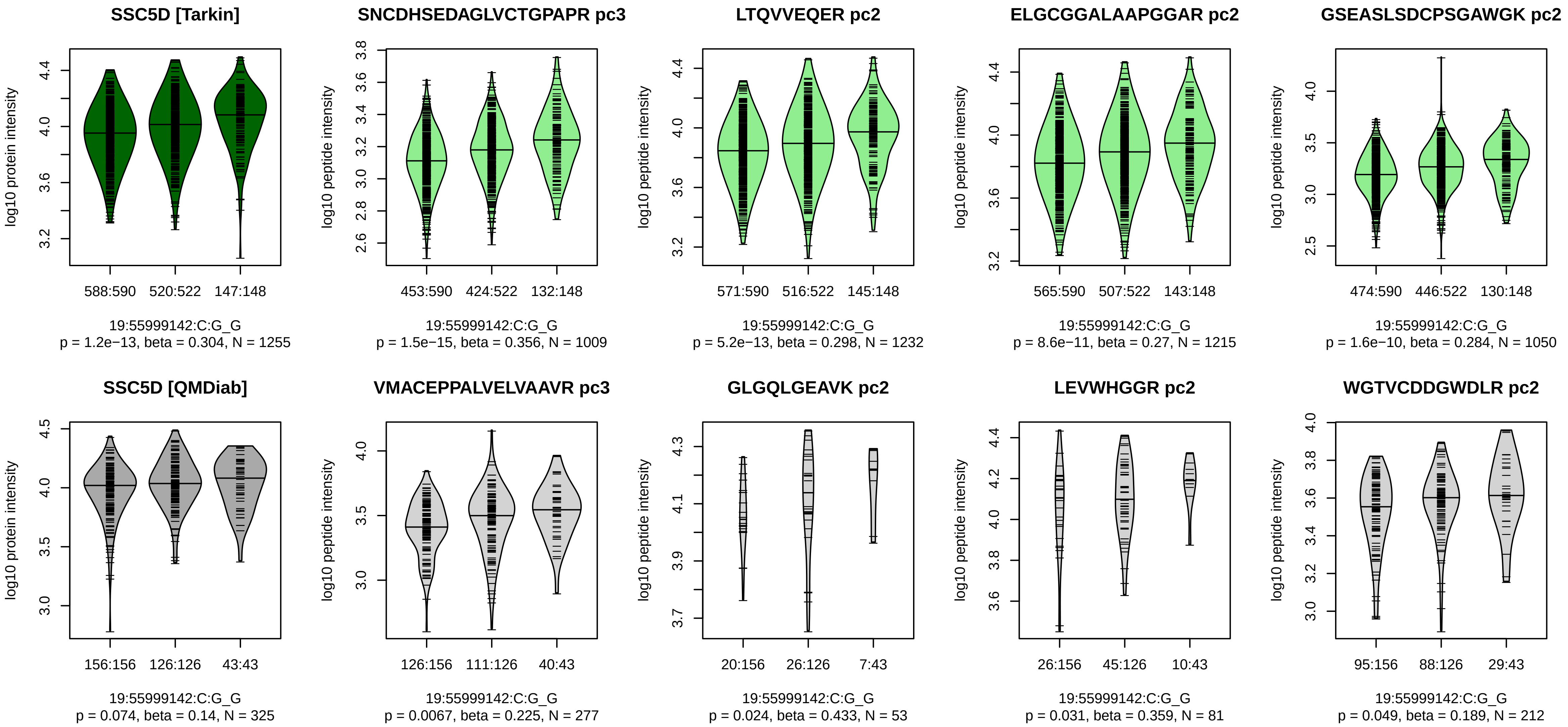

Sort: 131
Gene name: SSC5D
SNP: 19:55999142:C:G
rsID: rs8103017
Type: Cis−pQTL
Protein group(s):
A1L4H1
Nano−particle: NP2

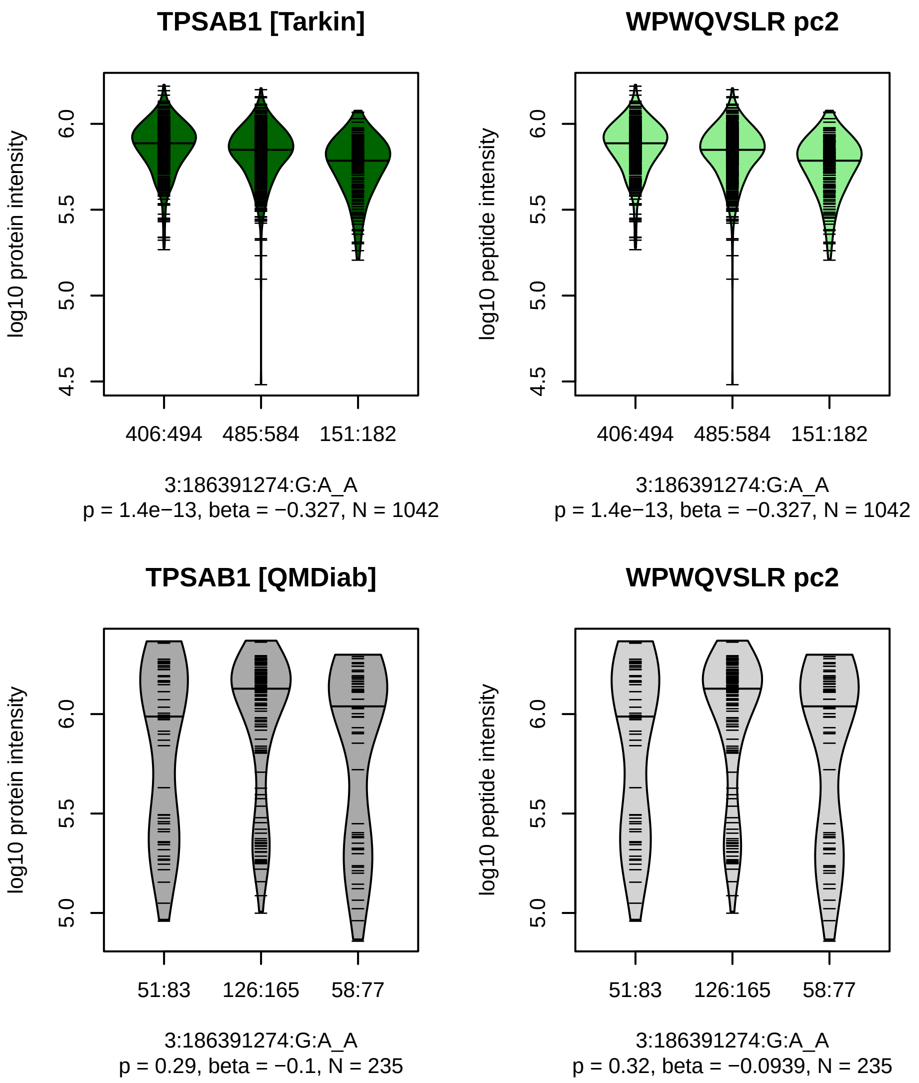

Sort: 132
Gene name: TPSAB1
SNP: 3:186391274:G:A
rsID: rs59123177
Type: Trans−pQTL
Protein group(s):
A0A087WUI4;A0A087X1U0;A0
Nano−particle: NP5

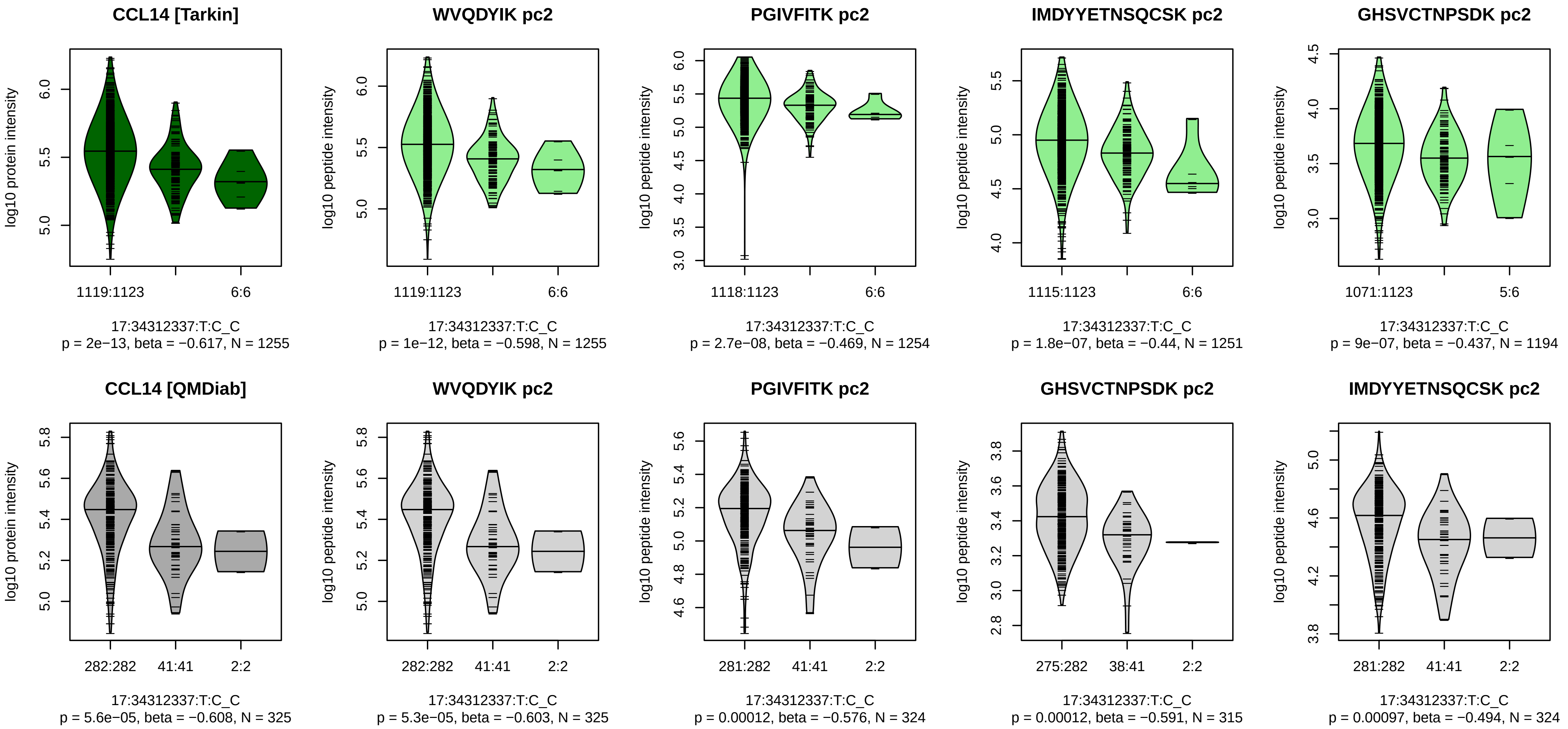

Sort: 133
Gene name: CCL14
SNP: 17:34312337:T:C
rsID: rs9903158
Type: Cis−pQTL
Protein group(s):
Q16627
Nano−particle: NP3

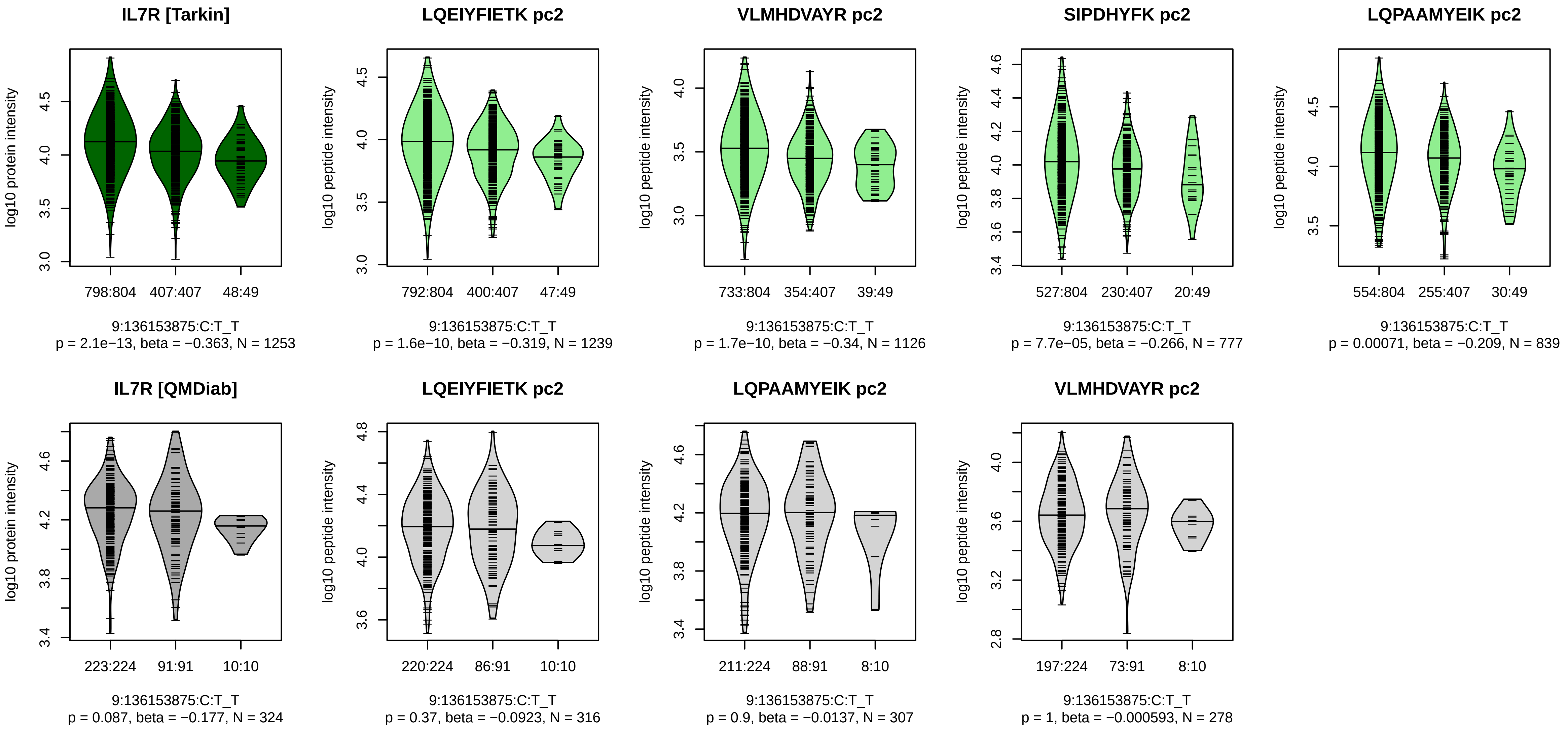

Sort: 134
Gene name: IL7R
SNP: 9:136153875:C:T
rsID: rs651007
Type: Trans−pQTL
Protein group(s):
T: P16871
Q: P16871−3
Nano−particle: NP2

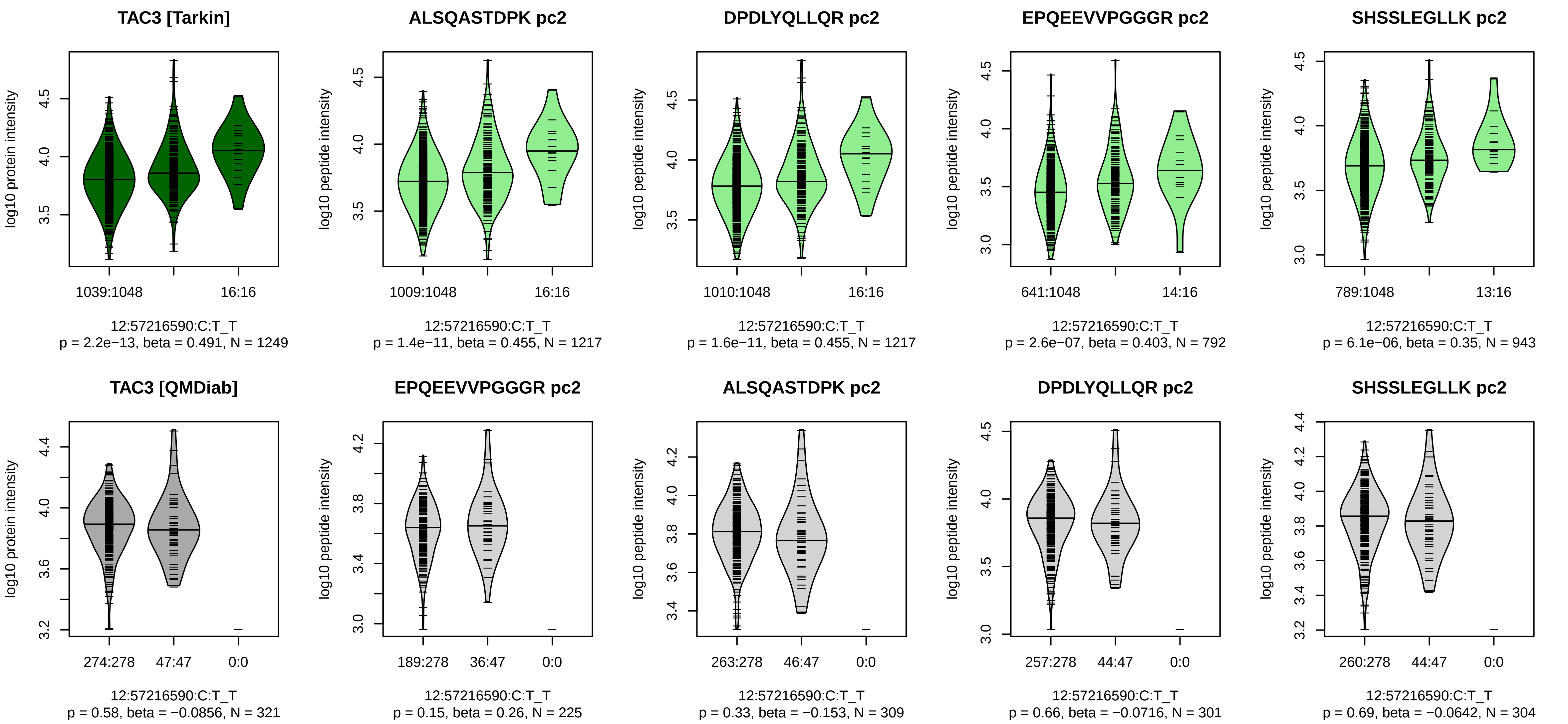

Sort: 135
Gene name: TAC3
SNP: 12:57216590:C:T
rsID: rs113940456
Type: Cis−pQTL
Protein group(s):
T: Q9UHF0−2
Q: Q9UHF0;Q9UHF0−2
Nano−particle: NP2

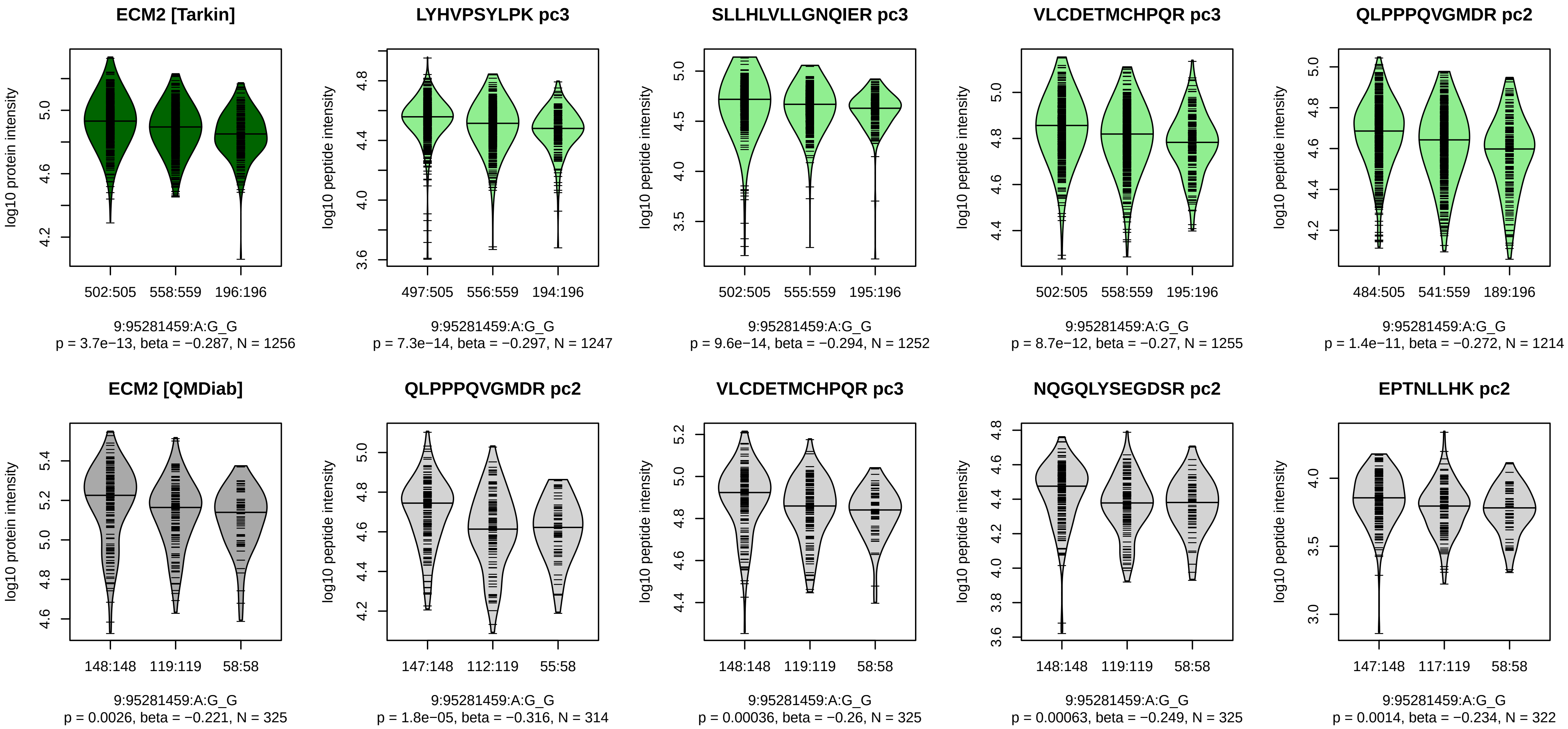

Sort: 136
Gene name: ECM2
SNP: 9:95281459:A:G
rsID: rs12338938
Type: Cis−pQTL
Protein group(s):
O94769
Nano−particle: NP2

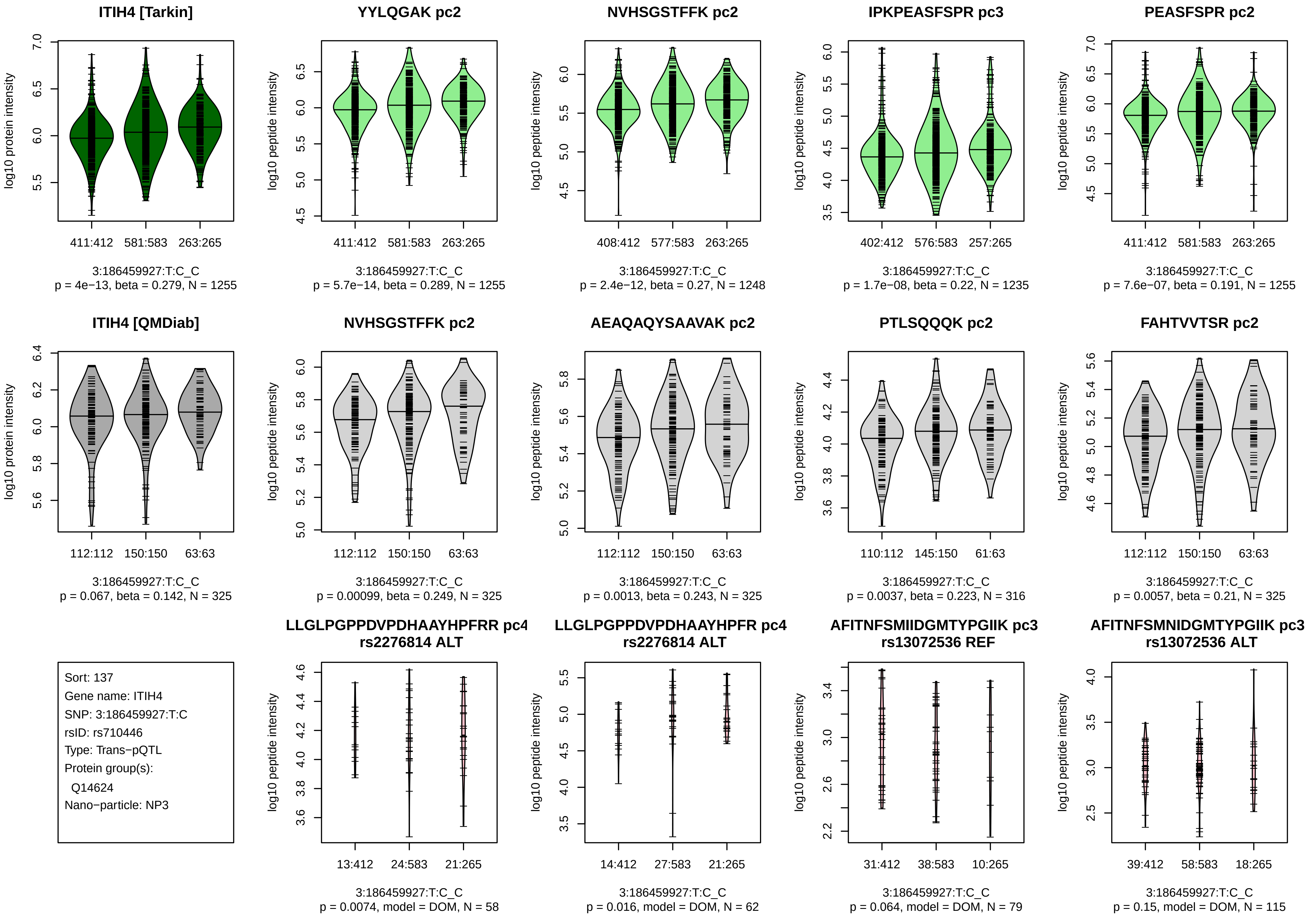
ITIH4 [Tarkin]
log10 protein intensity
411:412 581:583 263:265
3:186459927:T:C_C
p = 4e−13, beta = 0.279, N = 1255
YYLQGAK pc2
log10 peptide intensity
411:412 581:583 263:265
3:186459927:T:C_C
p = 5.7e−14, beta = 0.289, N = 1255
NVHSGSTFFK pc2
log10 peptide intensity
408:412 577:583 263:265
3:186459927:T:C_C
p = 2.4e−12, beta = 0.27, N = 1248
IPKPEASFSPR pc3
log10 peptide intensity
402:412 576:583 257:265
3:186459927:T:C_C
p = 1.7e−08, beta = 0.22, N = 1235
PEASFSPR pc2
log10 peptide intensity
411:412 581:583 263:265
3:186459927:T:C_C
p = 7.6e−07, beta = 0.191, N = 1255
ITIH4 [QMDiab]
log10 protein intensity
112:112 150:150 63:63
3:186459927:T:C_C
p = 0.067, beta = 0.142, N = 325
NVHSGSTFFK pc2
log10 peptide intensity
112:112 150:150 63:63
3:186459927:T:C_C
p = 0.00099, beta = 0.249, N = 325
AEAQAQYSAAVAK pc2
log10 peptide intensity
112:112 150:150 63:63
3:186459927:T:C_C
p = 0.0013, beta = 0.243, N = 325
PTLSQQQK pc2
log10 peptide intensity
110:112 145:150 61:63
3:186459927:T:C_C
p = 0.0037, beta = 0.223, N = 316
FAHTVVTSR pc2
log10 peptide intensity
112:112 150:150 63:63
3:186459927:T:C_C
p = 0.0057, beta = 0.21, N = 325
Sort: 137
Gene name: ITIH4
SNP: 3:186459927:T:C
rsID: rs710446
Type: Trans−pQTL
Protein group(s):
Q14624
Nano−particle: NP3
LLGLPGPPDVPDHAAYHPFRR pc4
rs2276814 ALT
log10 peptide intensity
13:412 24:583 21:265
3:186459927:T:C_C
p = 0.0074, model = DOM, N = 58
LLGLPGPPDVPDHAAYHPFR pc4
rs2276814 ALT
log10 peptide intensity
14:412 27:583 21:265
3:186459927:T:C_C
p = 0.016, model = DOM, N = 62
AFITNFSMIIDGMTYPGIIK pc3
rs13072536 REF
log10 peptide intensity
31:412 38:583 10:265
3:186459927:T:C_C
p = 0.064, model = DOM, N = 79
AFITNFSMNIDGMTYPGIIK pc3
rs13072536 ALT
log10 peptide intensity
39:412 58:583 18:265
3:186459927:T:C_C
p = 0.15, model = DOM, N = 115

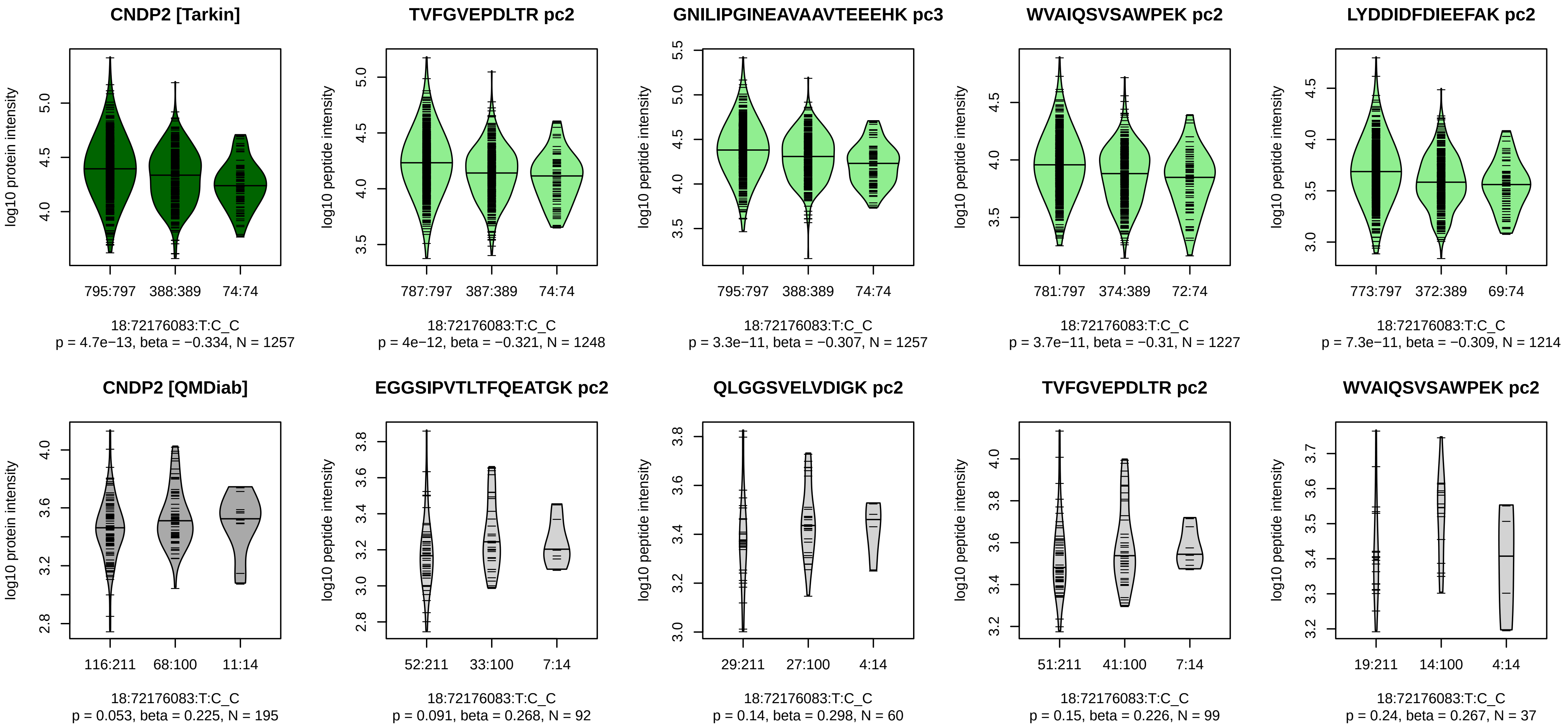

Sort: 138
Gene name: CNDP2
SNP: 18:72176083:T:C
rsID: rs2278161
Type: Cis−pQTL
Protein group(s):
Q96KP4
Nano−particle: NP5

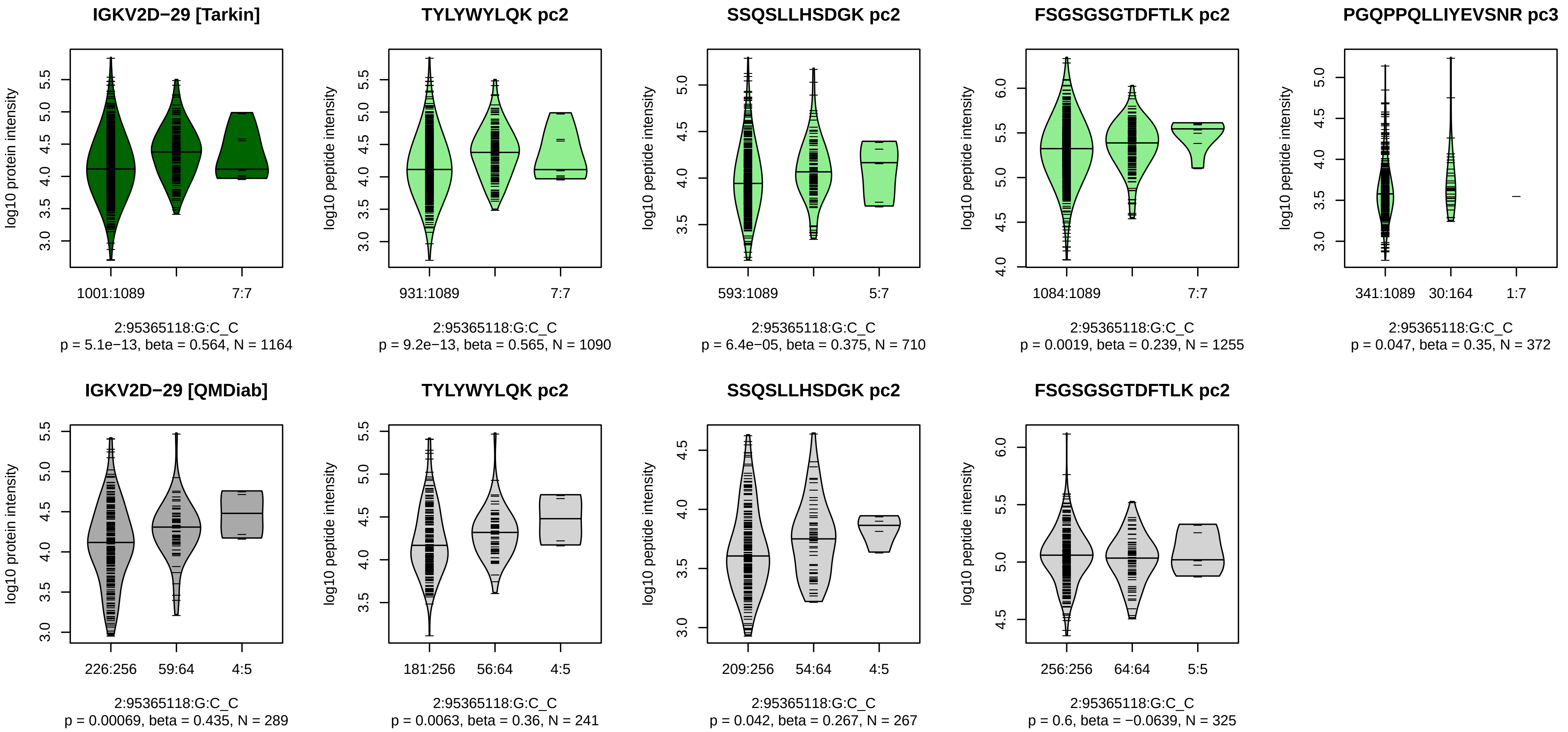

Sort: 139
Gene name: IGKV2D−29
SNP: 2:95365118:G:C
rsID: rs62148537
Type: Cis−pQTL
Protein group(s):
A0A075B6S2
Nano−particle: NP3

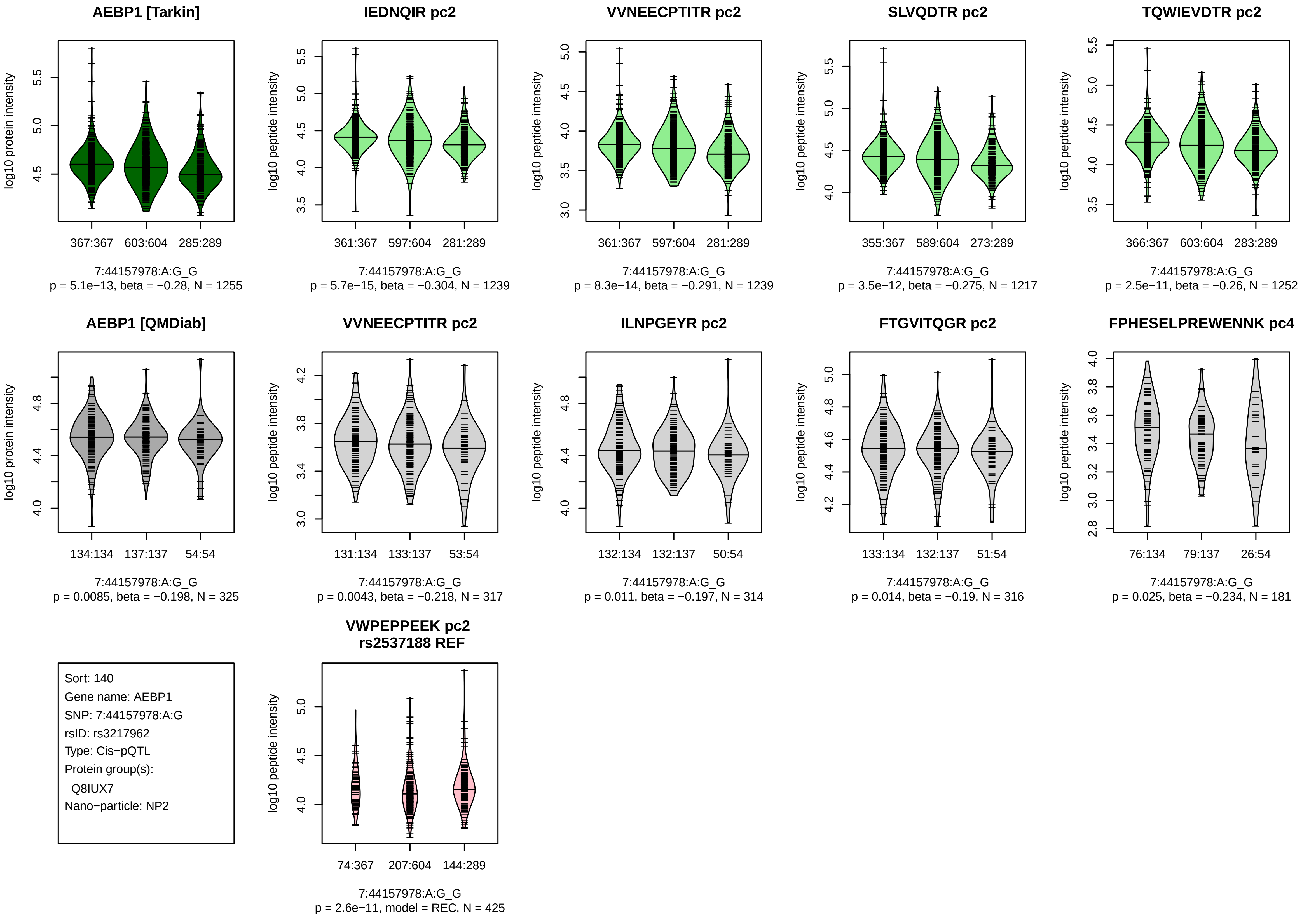

AEBP1 [Tarkin]
log10 protein intensity
367:367
603:604
285:289
7:44157978:A:G_G
p = 5.1e−13, beta = −0.28, N = 1255
IEDNQIR pc2
log10 peptide intensity
361:367
597:604
281:289
7:44157978:A:G_G
p = 5.7e−15, beta = −0.304, N = 1239
VVNEECPTITR pc2
log10 peptide intensity
361:367
597:604
281:289
7:44157978:A:G_G
p = 8.3e−14, beta = −0.291, N = 1239
SLVQDTR pc2
log10 peptide intensity
355:367
589:604
273:289
7:44157978:A:G_G
p = 3.5e−12, beta = −0.275, N = 1217
TQWIEVDTR pc2
log10 peptide intensity
366:367
603:604
283:289
7:44157978:A:G_G
p = 2.5e−11, beta = −0.26, N = 1252
AEBP1 [QMDiab]
log10 protein intensity
134:134
137:137
54:54
7:44157978:A:G_G
p = 0.0085, beta = −0.198, N = 325
VVNEECPTITR pc2
log10 peptide intensity
131:134
133:137
53:54
7:44157978:A:G_G
p = 0.0043, beta = −0.218, N = 317
ILNPGEYR pc2
log10 peptide intensity
132:134
132:137
50:54
7:44157978:A:G_G
p = 0.011, beta = −0.197, N = 314
FTGVITQGR pc2
log10 peptide intensity
133:134
132:137
51:54
7:44157978:A:G_G
p = 0.014, beta = −0.19, N = 316
FPHESELPREWENNK pc4
log10 peptide intensity
76:134
79:137
26:54
7:44157978:A:G_G
p = 0.025, beta = −0.234, N = 181
Sort: 140
Gene name: AEBP1
SNP: 7:44157978:A:G
rsID: rs3217962
Type: Cis−pQTL
Protein group(s):
Q8IUX7
Nano−particle: NP2
VWPEPPEEK pc2
rs2537188 REF
log10 peptide intensity
74:367
207:604
144:289
7:44157978:A:G_G
p = 2.6e−11, model = REC, N = 425

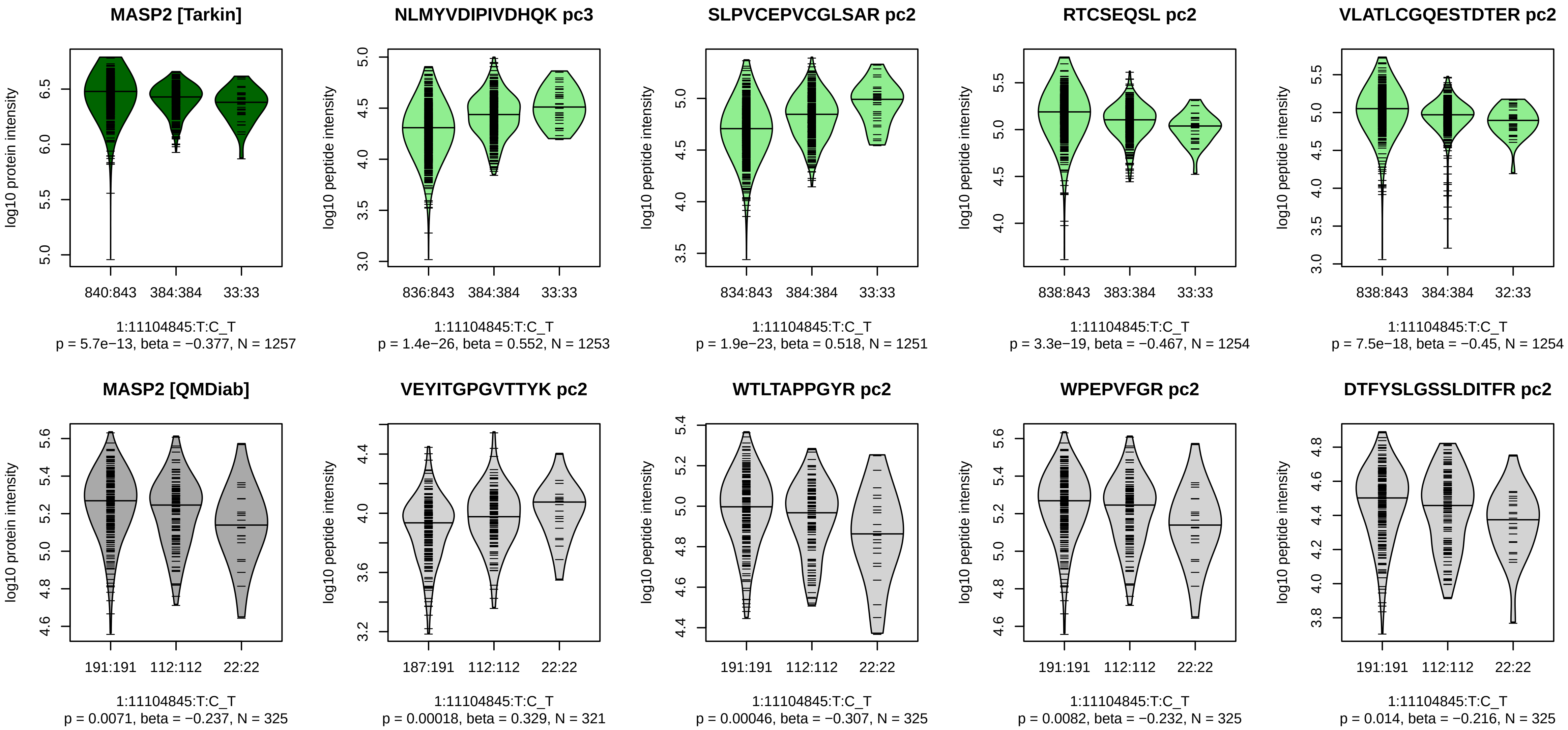

Sort: 141
Gene name: MASP2
SNP: 1:11104845:T:C
rsID: rs9430347
Type: Cis−pQTL
Protein group(s):
O00187
Nano−particle: NP2

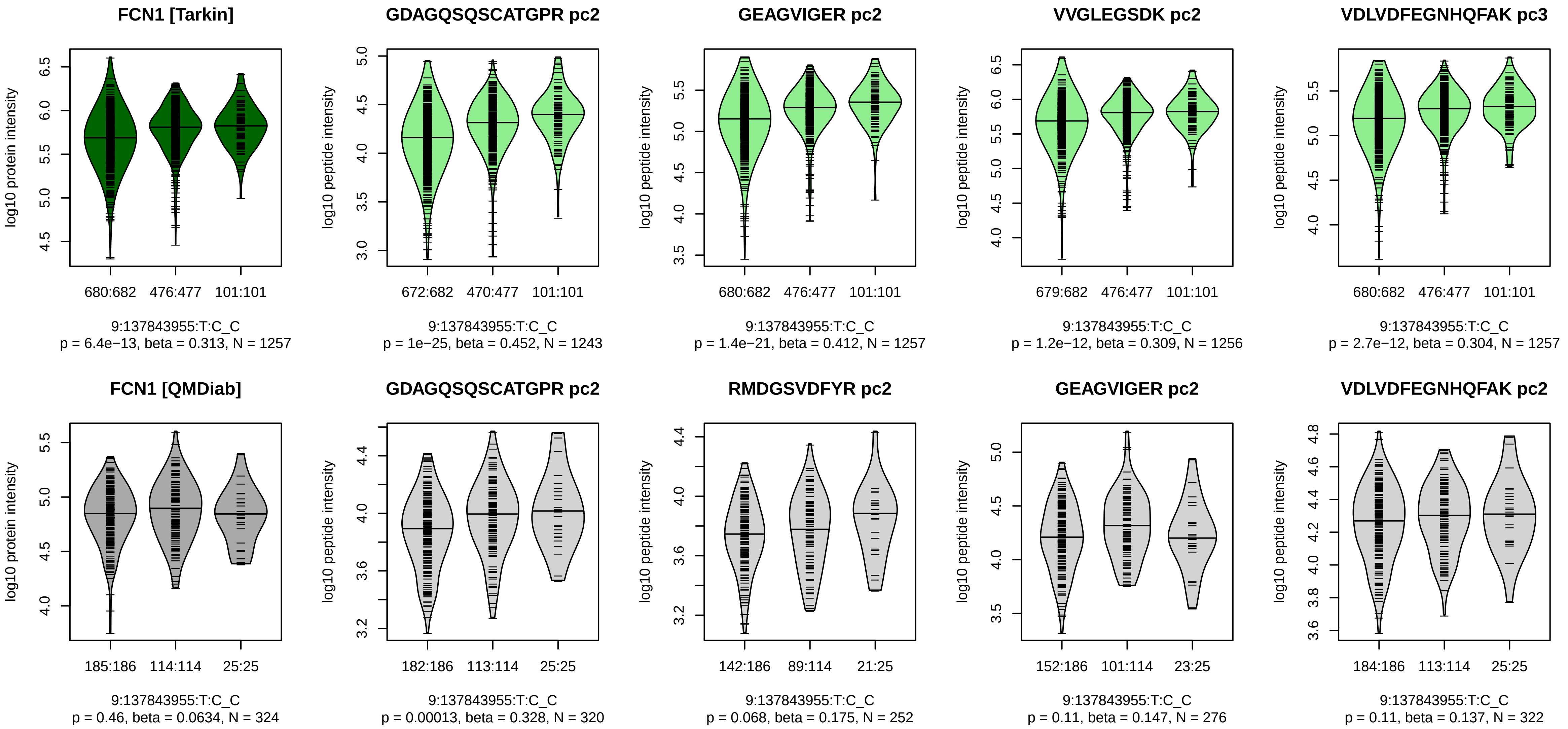

Sort: 142
Gene name: FCN1
SNP: 9:137843955:T:C
rsID: rs60008355
Type: Cis−pQTL
Protein group(s):
O00602
Nano−particle: NP5

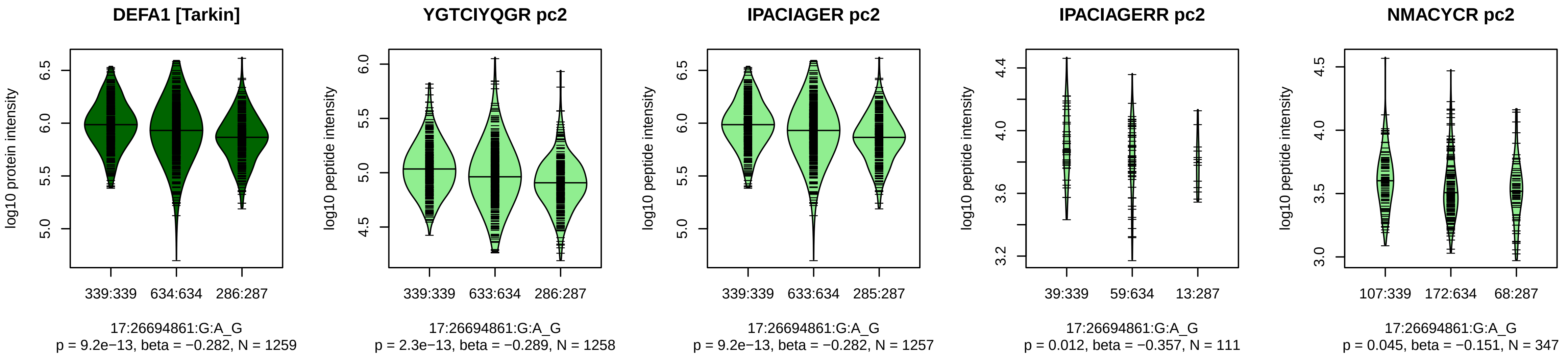

No protein data available
for replication in QMDiab

Sort: 143
Gene name: DEFA1
SNP: 17:26694861:G:A
rsID: rs704
Type: Trans−pQTL
Protein group(s):
P59665
Nano−particle: NP4

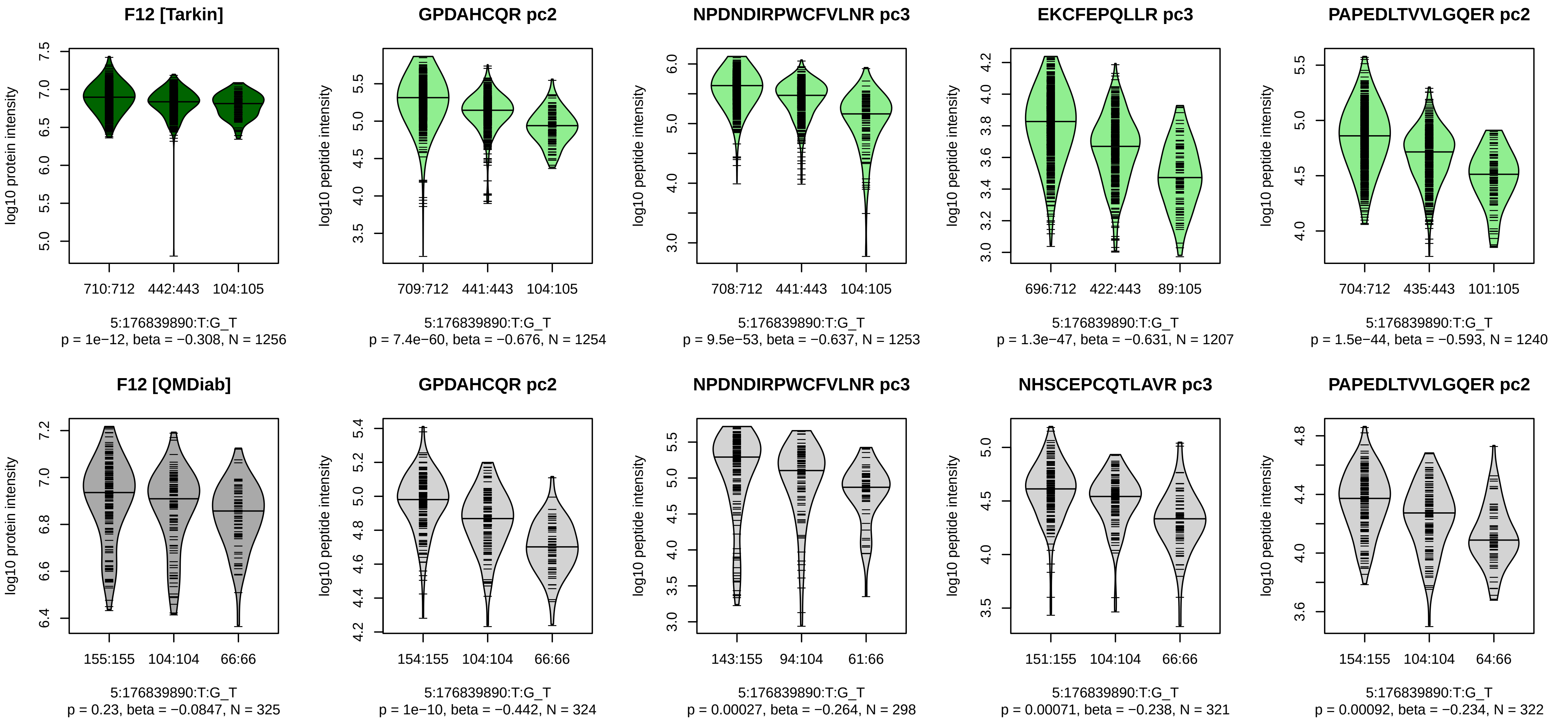

Sort: 144
Gene name: F12
SNP: 5:176839890:T:G
rsID: rs2731674
Type: Cis−pQTL
Protein group(s):
P00748
Nano−particle: NP1

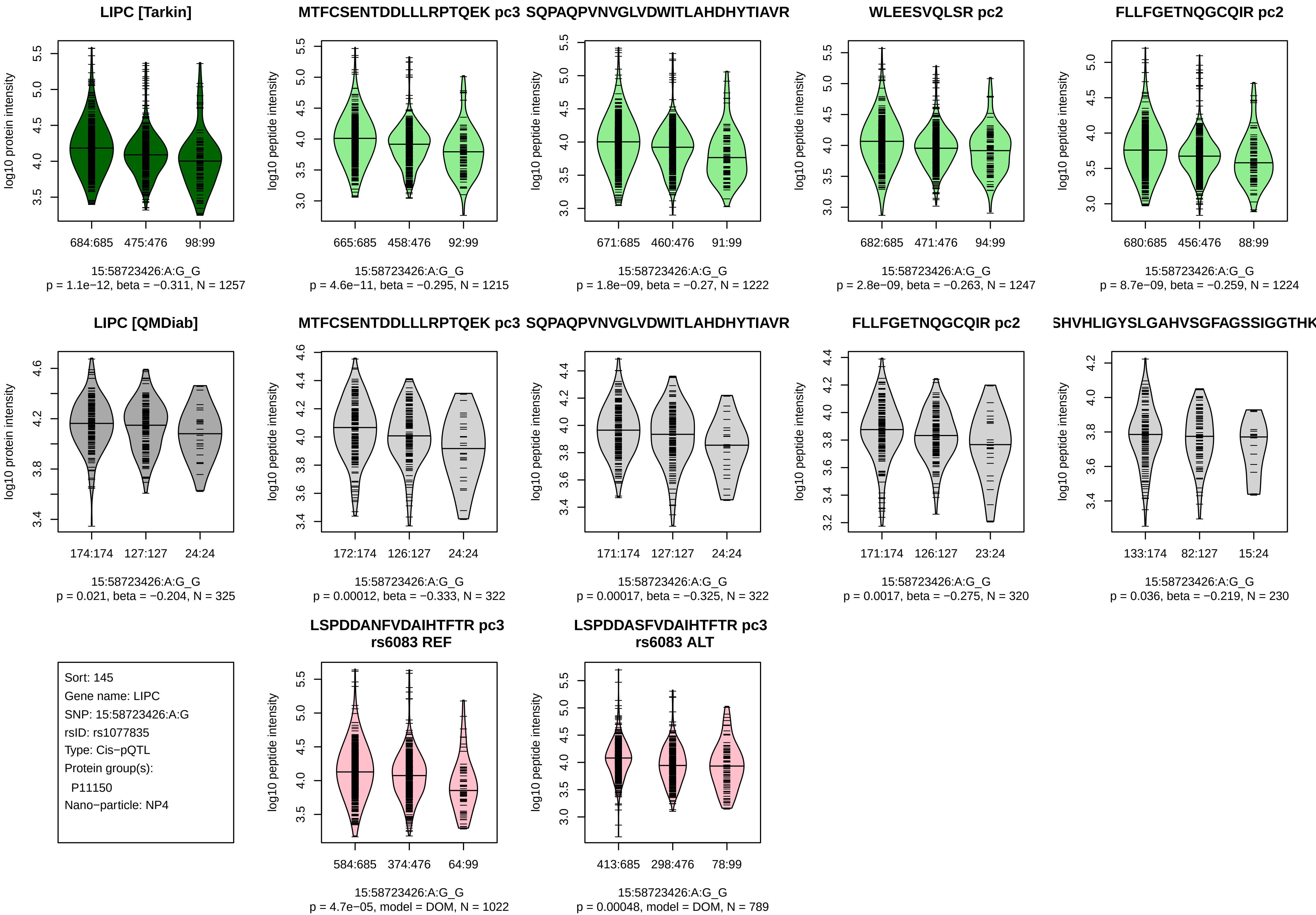
LIPC [Tarkin]
MTFCSENTDDLLLRPTQEK pc3
SQPAQPVNVGLVDWITLAHDHYTIAVR
WLEESVQLSR pc2
FLLFGETNQGCQIR pc2
log10 protein intensity
log10 peptide intensity
684:685 475:476 98:99
665:685 458:476 92:99
671:685 460:476 91:99
682:685 471:476 94:99
680:685 456:476 88:99
15:58723426:A:G_G
p = 1.1e−12, beta = −0.311, N = 1257
p = 4.6e−11, beta = −0.295, N = 1215
p = 1.8e−09, beta = −0.27, N = 1222
p = 2.8e−09, beta = −0.263, N = 1247
p = 8.7e−09, beta = −0.259, N = 1224
LIPC [QMDiab]
MTFCSENTDDLLLRPTQEK pc3
SQPAQPVNVGLVDWITLAHDHYTIAVR
FLLFGETNQGCQIR pc2
SHVHLIGYSLGAHVSGFAGSSIGGTHK
174:174 127:127 24:24
172:174 126:127 24:24
171:174 127:127 24:24
171:174 126:127 23:24
133:174 82:127 15:24
p = 0.021, beta = −0.204, N = 325
p = 0.00012, beta = −0.333, N = 322
p = 0.00017, beta = −0.325, N = 322
p = 0.0017, beta = −0.275, N = 320
p = 0.036, beta = −0.219, N = 230
Sort: 145
Gene name: LIPC
SNP: 15:58723426:A:G
rsID: rs1077835
Type: Cis−pQTL
Protein group(s):
P11150
Nano−particle: NP4
LSPDDANFVDAIHTFTR pc3
rs6083 REF
LSPDDASFVDAIHTFTR pc3
rs6083 ALT
584:685 374:476 64:99
413:685 298:476 78:99
p = 4.7e−05, model = DOM, N = 1022
p = 0.00048, model = DOM, N = 789

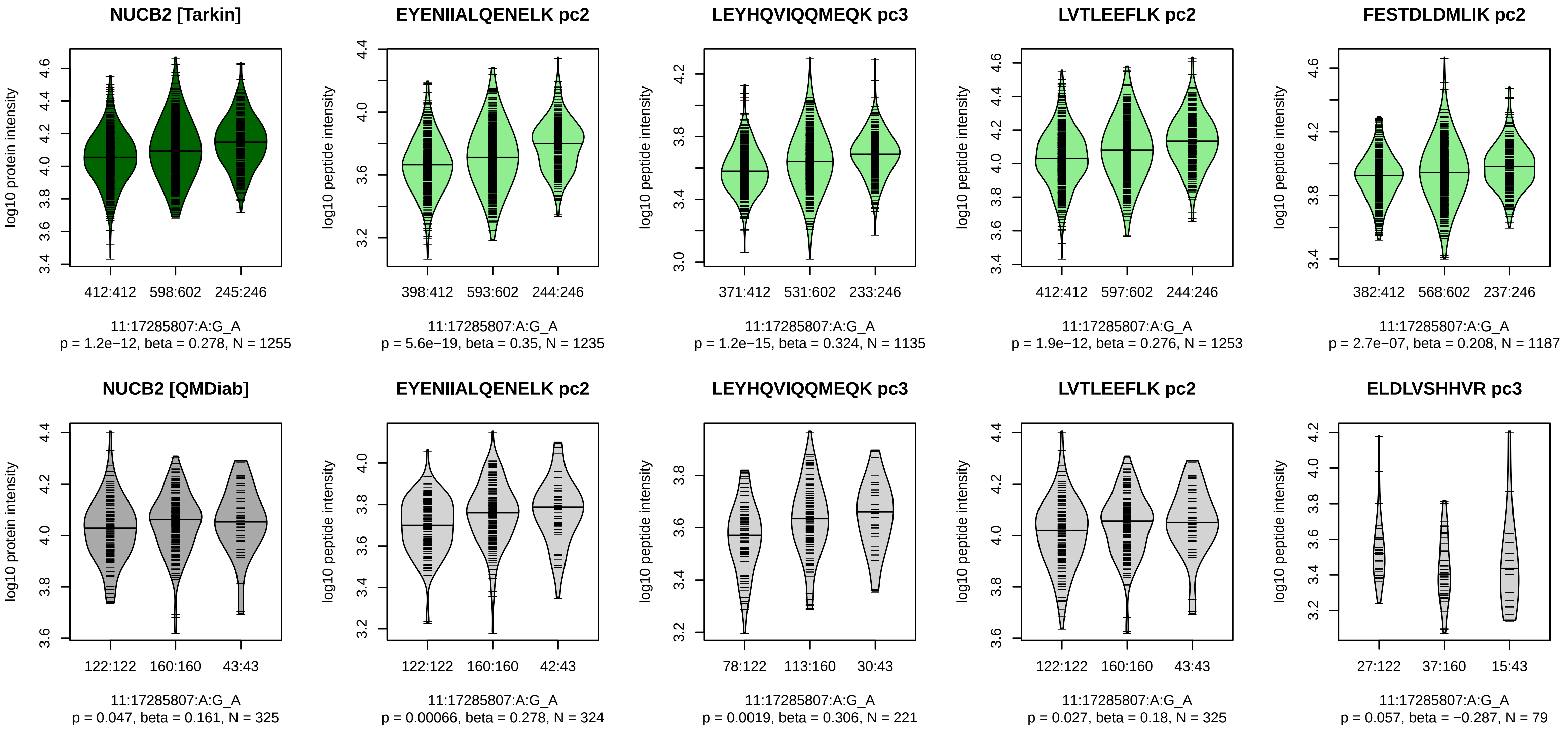

Sort: 146
Gene name: NUCB2
SNP: 11:17285807:A:G
rsID: rs7342262
Type: Cis−pQTL
Protein group(s):
P80303;P80303−2
Nano−particle: NP2

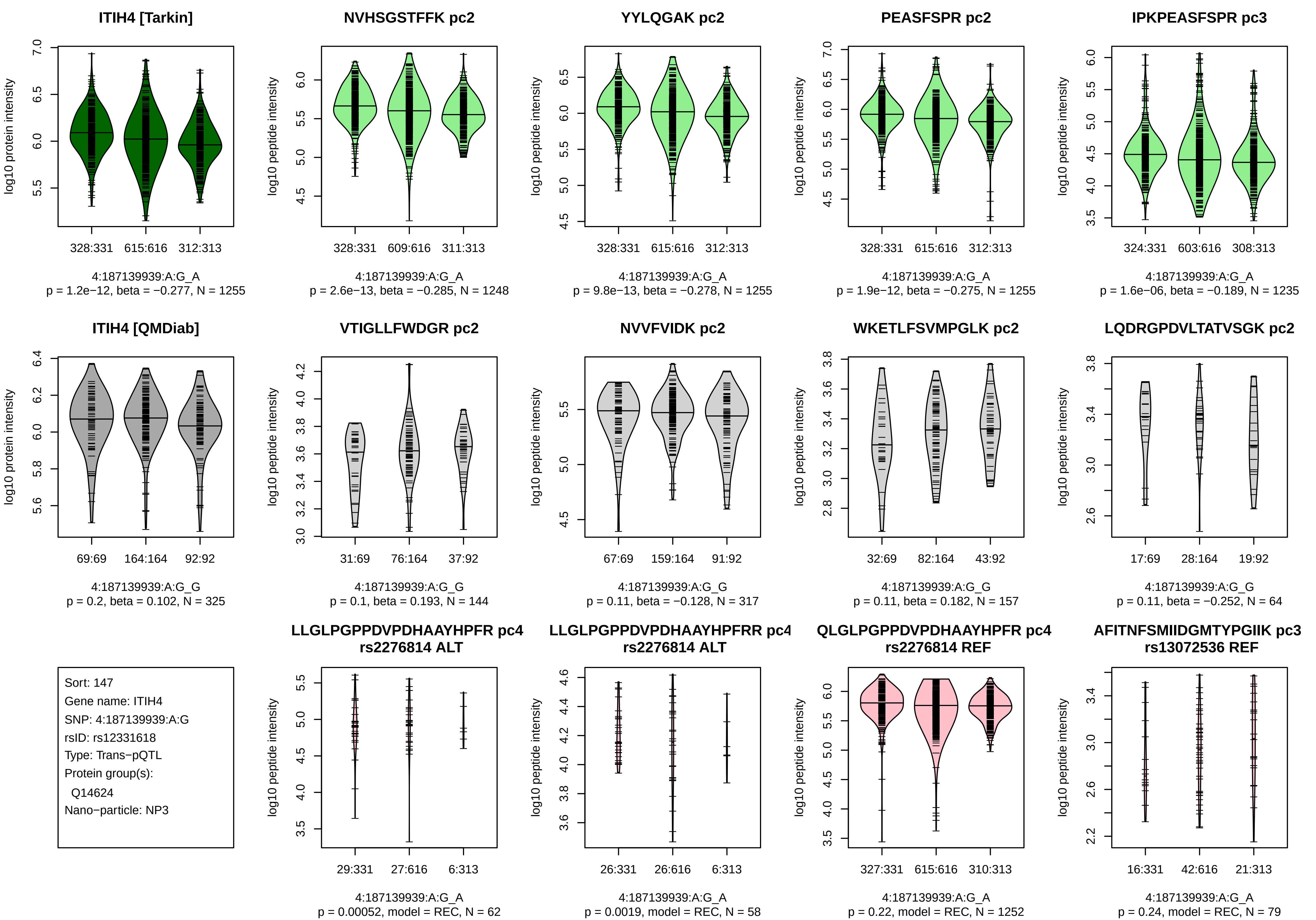
ITIH4 [Tarkin]
log10 protein intensity
328:331 615:616 312:313
4:187139939:A:G_A
p = 1.2e−12, beta = −0.277, N = 1255
NVHSGSTFFK pc2
log10 peptide intensity
328:331 609:616 311:313
4:187139939:A:G_A
p = 2.6e−13, beta = −0.285, N = 1248
YYLQGAK pc2
log10 peptide intensity
328:331 615:616 312:313
4:187139939:A:G_A
p = 9.8e−13, beta = −0.278, N = 1255
PEASFSPR pc2
log10 peptide intensity
328:331 615:616 312:313
4:187139939:A:G_A
p = 1.9e−12, beta = −0.275, N = 1255
IPKPEASFSPR pc3
log10 peptide intensity
324:331 603:616 308:313
4:187139939:A:G_A
p = 1.6e−06, beta = −0.189, N = 1235
ITIH4 [QMDiab]
log10 protein intensity
69:69 164:164 92:92
4:187139939:A:G_G
p = 0.2, beta = 0.102, N = 325
VTIGLLFWDGR pc2
log10 peptide intensity
31:69 76:164 37:92
4:187139939:A:G_G
p = 0.1, beta = 0.193, N = 144
NVVFVIDK pc2
log10 peptide intensity
67:69 159:164 91:92
4:187139939:A:G_G
p = 0.11, beta = −0.128, N = 317
WKETLFSVMPGLK pc2
log10 peptide intensity
32:69 82:164 43:92
4:187139939:A:G_G
p = 0.11, beta = 0.182, N = 157
LQDRGPDVLTATVSGK pc2
log10 peptide intensity
17:69 28:164 19:92
4:187139939:A:G_G
p = 0.11, beta = −0.252, N = 64
Sort: 147
Gene name: ITIH4
SNP: 4:187139939:A:G
rsID: rs12331618
Type: Trans−pQTL
Protein group(s):
Q14624
Nano−particle: NP3
LLGLPGPPDVPDHAAYHPFR pc4
rs2276814 ALT
log10 peptide intensity
29:331 27:616 6:313
4:187139939:A:G_A
p = 0.00052, model = REC, N = 62
LLGLPGPPDVPDHAAYHPFRR pc4
rs2276814 ALT
log10 peptide intensity
26:331 26:616 6:313
4:187139939:A:G_A
p = 0.0019, model = REC, N = 58
QLGLPGPPDVPDHAAYHPFR pc4
rs2276814 REF
log10 peptide intensity
327:331 615:616 310:313
4:187139939:A:G_A
p = 0.22, model = REC, N = 1252
AFITNFSMIIDGMTYPGIIK pc3
rs13072536 REF
log10 peptide intensity
16:331 42:616 21:313
4:187139939:A:G_A
p = 0.24, model = REC, N = 79

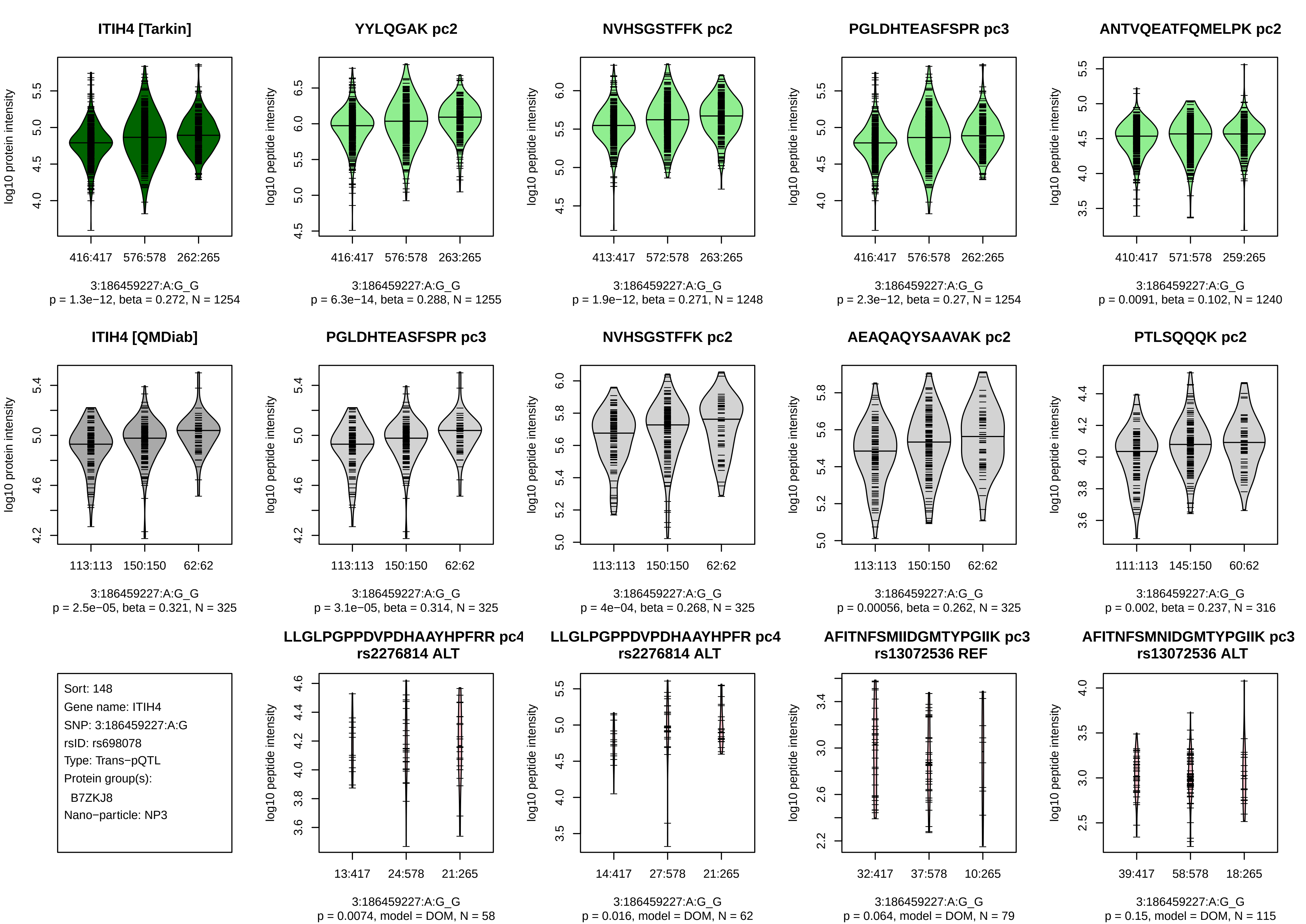
ITIH4 [Tarkin]
log10 protein intensity
416:417 576:578 262:265
3:186459227:A:G_G
p = 1.3e−12, beta = 0.272, N = 1254
YYLQGAK pc2
log10 peptide intensity
416:417 576:578 263:265
3:186459227:A:G_G
p = 6.3e−14, beta = 0.288, N = 1255
NVHSGSTFFK pc2
log10 peptide intensity
413:417 572:578 263:265
3:186459227:A:G_G
p = 1.9e−12, beta = 0.271, N = 1248
PGLDHTEASFSPR pc3
log10 peptide intensity
416:417 576:578 262:265
3:186459227:A:G_G
p = 2.3e−12, beta = 0.27, N = 1254
ANTVQEATFQMELPK pc2
log10 peptide intensity
410:417 571:578 259:265
3:186459227:A:G_G
p = 0.0091, beta = 0.102, N = 1240
ITIH4 [QMDiab]
log10 protein intensity
113:113 150:150 62:62
3:186459227:A:G_G
p = 2.5e−05, beta = 0.321, N = 325
PGLDHTEASFSPR pc3
log10 peptide intensity
113:113 150:150 62:62
3:186459227:A:G_G
p = 3.1e−05, beta = 0.314, N = 325
NVHSGSTFFK pc2
log10 peptide intensity
113:113 150:150 62:62
3:186459227:A:G_G
p = 4e−04, beta = 0.268, N = 325
AEAQAQYSAAVAK pc2
log10 peptide intensity
113:113 150:150 62:62
3:186459227:A:G_G
p = 0.00056, beta = 0.262, N = 325
PTLSQQQK pc2
log10 peptide intensity
111:113 145:150 60:62
3:186459227:A:G_G
p = 0.002, beta = 0.237, N = 316
Sort: 148
Gene name: ITIH4
SNP: 3:186459227:A:G
rsID: rs698078
Type: Trans−pQTL
Protein group(s):
B7ZKJ8
Nano−particle: NP3
LLGLPGPPDVPDHAAYHPFRR pc4
rs2276814 ALT
log10 peptide intensity
13:417 24:578 21:265
3:186459227:A:G_G
p = 0.0074, model = DOM, N = 58
LLGLPGPPDVPDHAAYHPFR pc4
rs2276814 ALT
log10 peptide intensity
14:417 27:578 21:265
3:186459227:A:G_G
p = 0.016, model = DOM, N = 62
AFITNFSMIIDGMTYPGIIK pc3
rs13072536 REF
log10 peptide intensity
32:417 37:578 10:265
3:186459227:A:G_G
p = 0.064, model = DOM, N = 79
AFITNFSMNIDGMTYPGIIK pc3
rs13072536 ALT
log10 peptide intensity
39:417 58:578 18:265
3:186459227:A:G_G
p = 0.15, model = DOM, N = 115

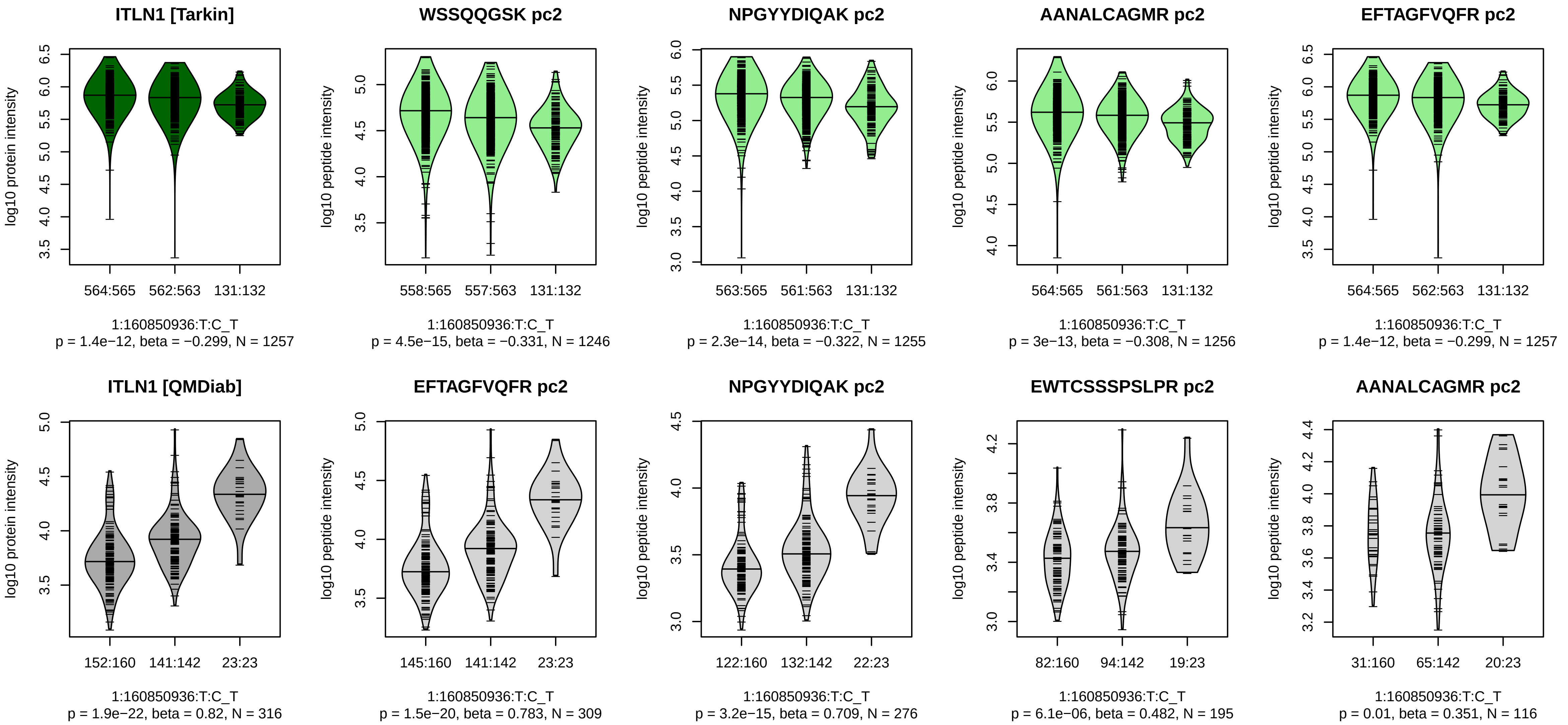

Sort: 149

Gene name: ITLN1

SNP: 1:160850936:T:C

rsID: rs2236515

Type: Cis−pQTL

Protein group(s):

Q8WWA0

Nano−particle: NP5

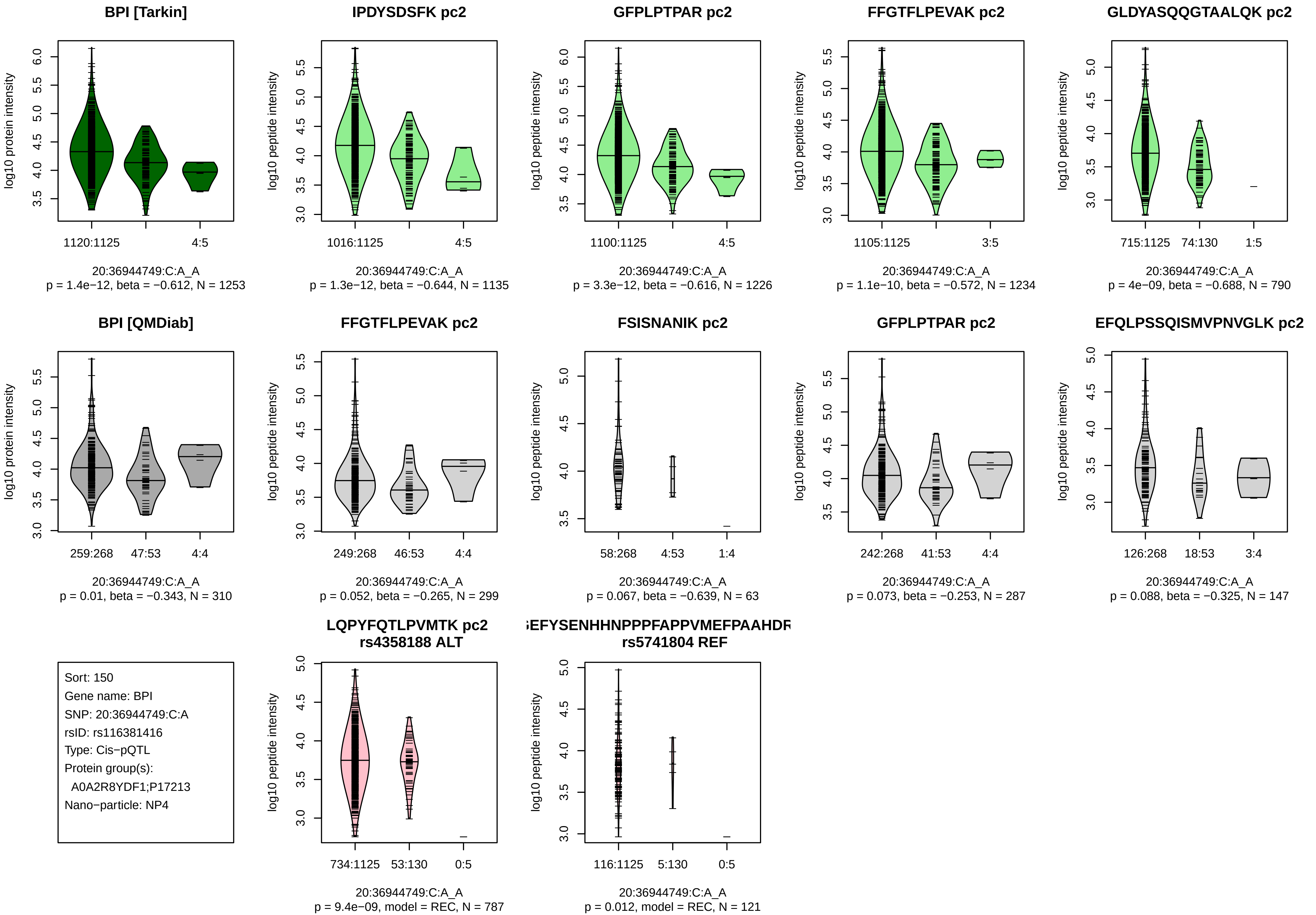
BPI [Tarkin]
log10 protein intensity
1120:1125
4:5
20:36944749:C:A_A
p = 1.4e−12, beta = −0.612, N = 1253
IPDYSDSFK pc2
log10 peptide intensity
1016:1125
4:5
20:36944749:C:A_A
p = 1.3e−12, beta = −0.644, N = 1135
GFPLPTPAR pc2
log10 peptide intensity
1100:1125
4:5
20:36944749:C:A_A
p = 3.3e−12, beta = −0.616, N = 1226
FFGTFLPEVAK pc2
log10 peptide intensity
1105:1125
3:5
20:36944749:C:A_A
p = 1.1e−10, beta = −0.572, N = 1234
GLDYASQQGTAALQK pc2
log10 peptide intensity
715:1125
74:130
1:5
20:36944749:C:A_A
p = 4e−09, beta = −0.688, N = 790
BPI [QMDiab]
log10 protein intensity
259:268
47:53
4:4
20:36944749:C:A_A
p = 0.01, beta = −0.343, N = 310
FFGTFLPEVAK pc2
log10 peptide intensity
249:268
46:53
4:4
20:36944749:C:A_A
p = 0.052, beta = −0.265, N = 299
FSISNANIK pc2
log10 peptide intensity
58:268
4:53
1:4
20:36944749:C:A_A
p = 0.067, beta = −0.639, N = 63
GFPLPTPAR pc2
log10 peptide intensity
242:268
41:53
4:4
20:36944749:C:A_A
p = 0.073, beta = −0.253, N = 287
EFQLPSSQISMVPNVGLK pc2
log10 peptide intensity
126:268
18:53
3:4
20:36944749:C:A_A
p = 0.088, beta = −0.325, N = 147
Sort: 150
Gene name: BPI
SNP: 20:36944749:C:A
rsID: rs116381416
Type: Cis−pQTL
Protein group(s):
A0A2R8YDF1;P17213
Nano−particle: NP4
LQPYFQTLPVMTK pc2
rs4358188 ALT
log10 peptide intensity
734:1125
53:130
0:5
20:36944749:C:A_A
p = 9.4e−09, model = REC, N = 787
EFYSENHHNPPPFAPPVMEFPAAHDR
rs5741804 REF
log10 peptide intensity
116:1125
5:130
0:5
20:36944749:C:A_A
p = 0.012, model = REC, N = 121

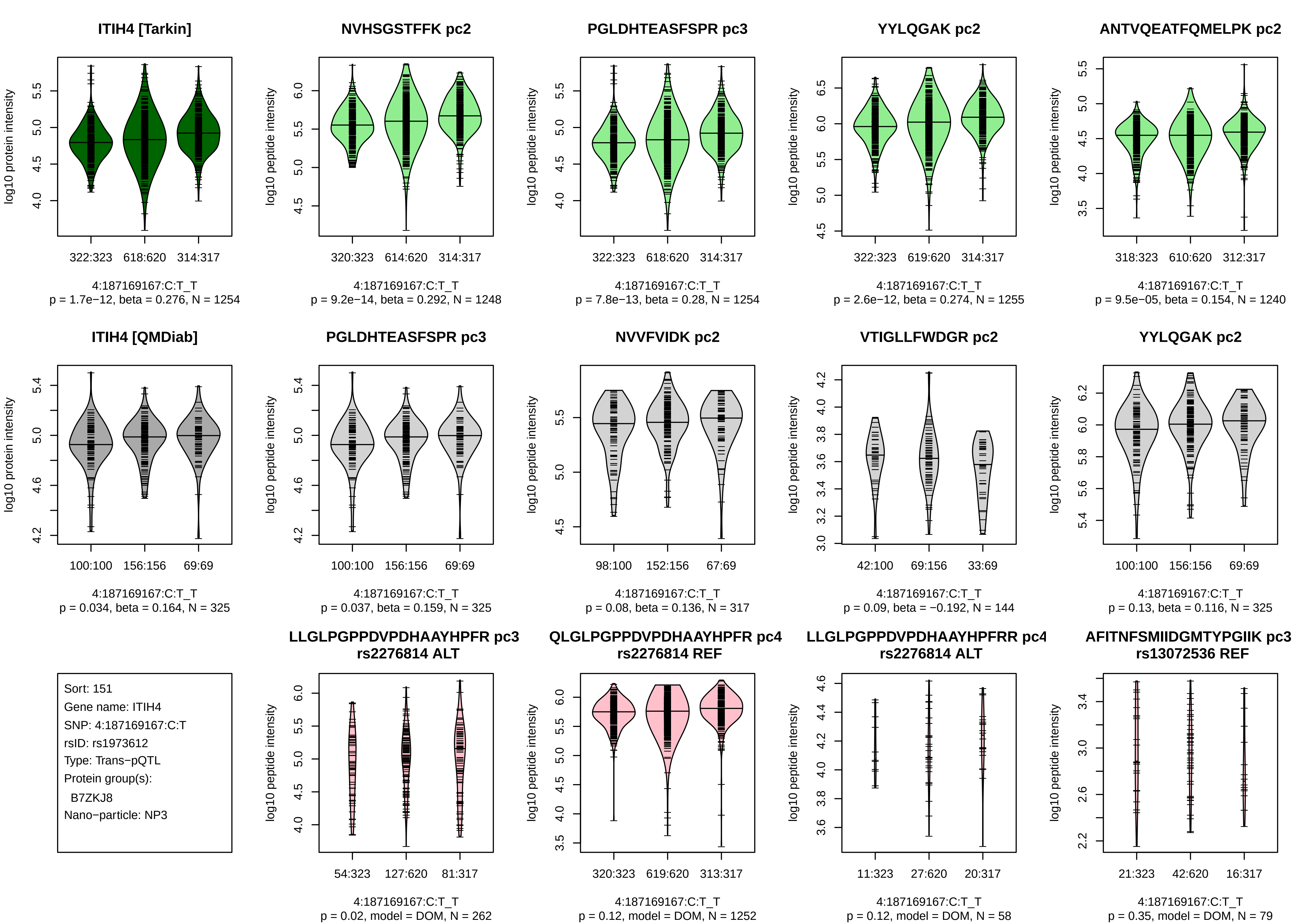
ITIH4 [Tarkin]
log10 protein intensity
322:323 618:620 314:317
4:187169167:C:T_T
p = 1.7e−12, beta = 0.276, N = 1254
NVHSGSTFFK pc2
log10 peptide intensity
320:323 614:620 314:317
4:187169167:C:T_T
p = 9.2e−14, beta = 0.292, N = 1248
PGLDHTEASFSPR pc3
log10 peptide intensity
322:323 618:620 314:317
4:187169167:C:T_T
p = 7.8e−13, beta = 0.28, N = 1254
YYLQGAK pc2
log10 peptide intensity
322:323 619:620 314:317
4:187169167:C:T_T
p = 2.6e−12, beta = 0.274, N = 1255
ANTVQEATFQMELPK pc2
log10 peptide intensity
318:323 610:620 312:317
4:187169167:C:T_T
p = 9.5e−05, beta = 0.154, N = 1240
ITIH4 [QMDiab]
log10 protein intensity
100:100 156:156 69:69
4:187169167:C:T_T
p = 0.034, beta = 0.164, N = 325
PGLDHTEASFSPR pc3
log10 peptide intensity
100:100 156:156 69:69
4:187169167:C:T_T
p = 0.037, beta = 0.159, N = 325
NVVFVIDK pc2
log10 peptide intensity
98:100 152:156 67:69
4:187169167:C:T_T
p = 0.08, beta = 0.136, N = 317
VTIGLLFWDGR pc2
log10 peptide intensity
42:100 69:156 33:69
4:187169167:C:T_T
p = 0.09, beta = −0.192, N = 144
YYLQGAK pc2
log10 peptide intensity
100:100 156:156 69:69
4:187169167:C:T_T
p = 0.13, beta = 0.116, N = 325
Sort: 151
Gene name: ITIH4
SNP: 4:187169167:C:T
rsID: rs1973612
Type: Trans−pQTL
Protein group(s):
B7ZKJ8
Nano−particle: NP3
LLGLPGPPDVPDHAAYHPFR pc3
rs2276814 ALT
log10 peptide intensity
54:323 127:620 81:317
4:187169167:C:T_T
p = 0.02, model = DOM, N = 262
QLGLPGPPDVPDHAAYHPFR pc4
rs2276814 REF
log10 peptide intensity
320:323 619:620 313:317
4:187169167:C:T_T
p = 0.12, model = DOM, N = 1252
LLGLPGPPDVPDHAAYHPFRR pc4
rs2276814 ALT
log10 peptide intensity
11:323 27:620 20:317
4:187169167:C:T_T
p = 0.12, model = DOM, N = 58
AFITNFSMIIDGMTYPGIIK pc3
rs13072536 REF
log10 peptide intensity
21:323 42:620 16:317
4:187169167:C:T_T
p = 0.35, model = DOM, N = 79

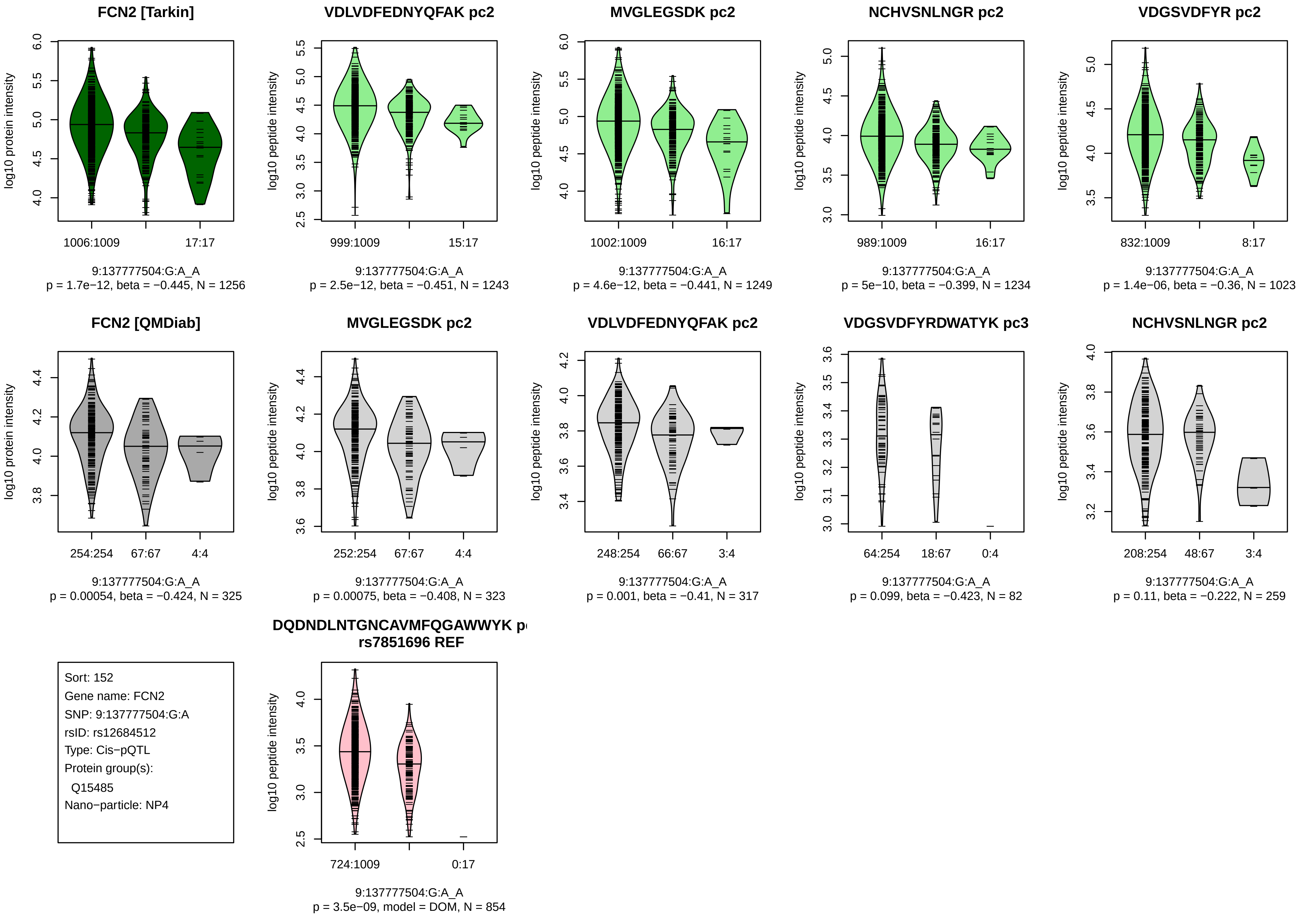
FCN2 [Tarkin]
log10 protein intensity
1006:1009
17:17
9:137777504:G:A_A
p = 1.7e−12, beta = −0.445, N = 1256
VDLVDFEDNYQFAK pc2
log10 peptide intensity
999:1009
15:17
9:137777504:G:A_A
p = 2.5e−12, beta = −0.451, N = 1243
MVGLEGSDK pc2
log10 peptide intensity
1002:1009
16:17
9:137777504:G:A_A
p = 4.6e−12, beta = −0.441, N = 1249
NCHVSNLNGR pc2
log10 peptide intensity
989:1009
16:17
9:137777504:G:A_A
p = 5e−10, beta = −0.399, N = 1234
VDGSVDFYR pc2
log10 peptide intensity
832:1009
8:17
9:137777504:G:A_A
p = 1.4e−06, beta = −0.36, N = 1023
FCN2 [QMDiab]
log10 protein intensity
254:254
67:67
4:4
9:137777504:G:A_A
p = 0.00054, beta = −0.424, N = 325
MVGLEGSDK pc2
log10 peptide intensity
252:254
67:67
4:4
9:137777504:G:A_A
p = 0.00075, beta = −0.408, N = 323
VDLVDFEDNYQFAK pc2
log10 peptide intensity
248:254
66:67
3:4
9:137777504:G:A_A
p = 0.001, beta = −0.41, N = 317
VDGSVDFYRDWATYK pc3
log10 peptide intensity
64:254
18:67
0:4
9:137777504:G:A_A
p = 0.099, beta = −0.423, N = 82
NCHVSNLNGR pc2
log10 peptide intensity
208:254
48:67
3:4
9:137777504:G:A_A
p = 0.11, beta = −0.222, N = 259
Sort: 152
Gene name: FCN2
SNP: 9:137777504:G:A
rsID: rs12684512
Type: Cis−pQTL
Protein group(s):
Q15485
Nano−particle: NP4
DQDNDLNTGNCAVMFQGAWWYK p
rs7851696 REF
log10 peptide intensity
724:1009
0:17
9:137777504:G:A_A
p = 3.5e−09, model = DOM, N = 854

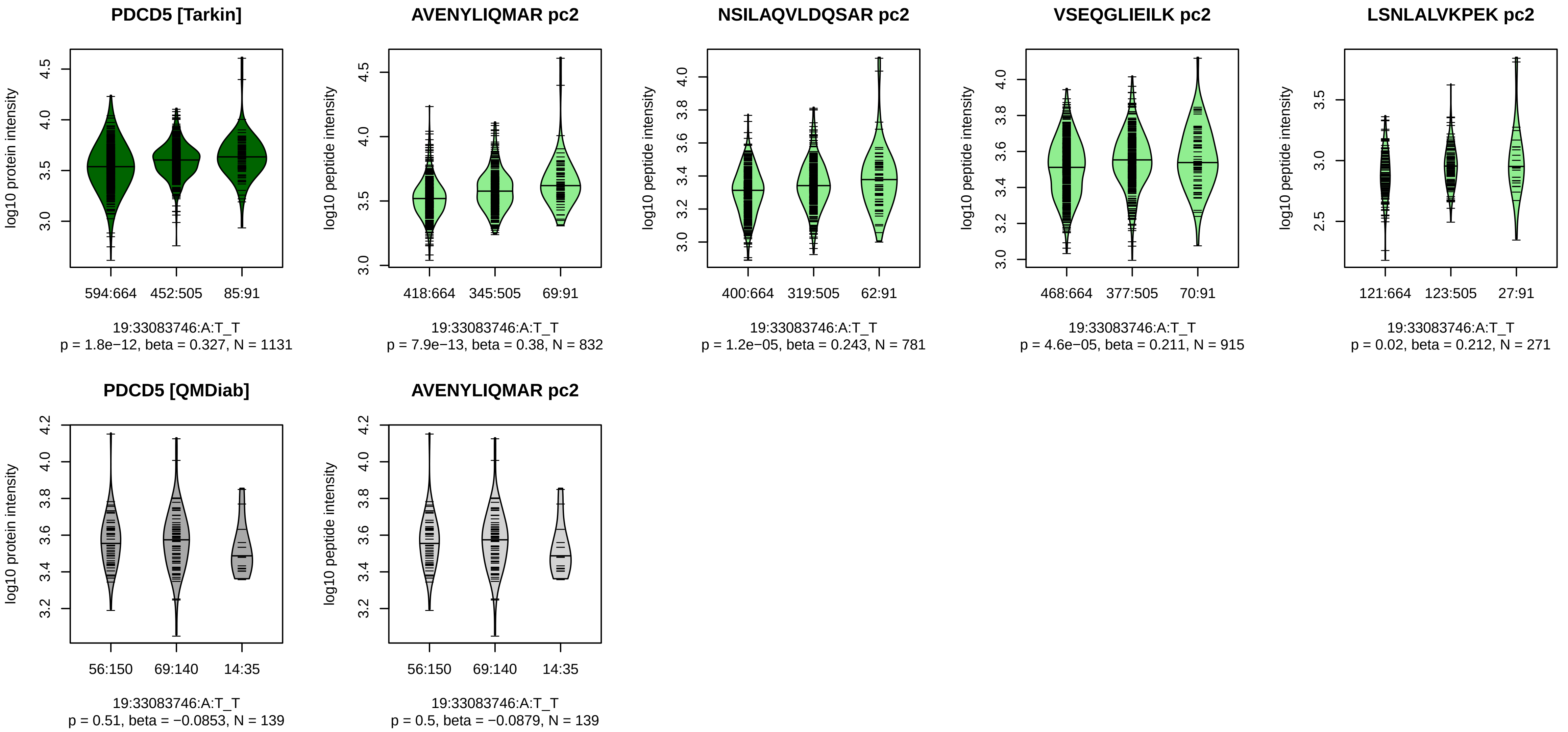

Sort: 153
Gene name: PDCD5
SNP: 19:33083746:A:T
rsID: rs11673049
Type: Cis−pQTL
Protein group(s):
O14737
Nano−particle: NP2

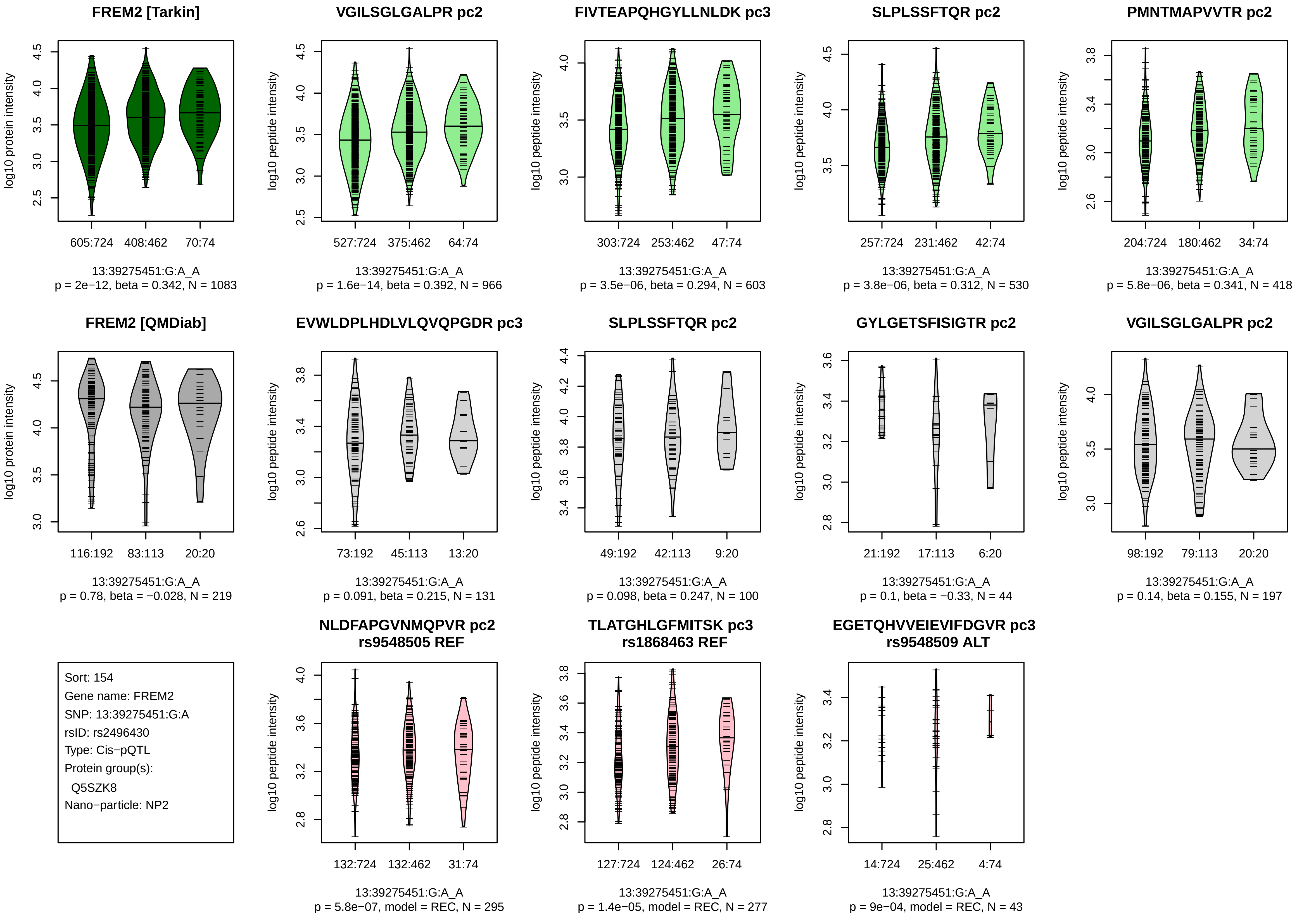
FREM2 [Tarkin]
log10 protein intensity
605:724
408:462
70:74
13:39275451:G:A_A
p = 2e−12, beta = 0.342, N = 1083
VGILSGLGALPR pc2
log10 peptide intensity
527:724
375:462
64:74
13:39275451:G:A_A
p = 1.6e−14, beta = 0.392, N = 966
FIVTEAPQHGYLLNLDK pc3
303:724
253:462
47:74
13:39275451:G:A_A
p = 3.5e−06, beta = 0.294, N = 603
SLPLSSFTQR pc2
257:724
231:462
42:74
13:39275451:G:A_A
p = 3.8e−06, beta = 0.312, N = 530
PMNTMAPVVTR pc2
204:724
180:462
34:74
13:39275451:G:A_A
p = 5.8e−06, beta = 0.341, N = 418
FREM2 [QMDiab]
116:192
83:113
20:20
13:39275451:G:A_A
p = 0.78, beta = −0.028, N = 219
EVWLDPLHDLVLQVQPGDR pc3
73:192
45:113
13:20
13:39275451:G:A_A
p = 0.091, beta = 0.215, N = 131
SLPLSSFTQR pc2
49:192
42:113
9:20
13:39275451:G:A_A
p = 0.098, beta = 0.247, N = 100
GYLGETSFISIGTR pc2
21:192
17:113
6:20
13:39275451:G:A_A
p = 0.1, beta = −0.33, N = 44
VGILSGLGALPR pc2
98:192
79:113
20:20
13:39275451:G:A_A
p = 0.14, beta = 0.155, N = 197
Sort: 154
Gene name: FREM2
SNP: 13:39275451:G:A
rsID: rs2496430
Type: Cis−pQTL
Protein group(s):
Q5SZK8
Nano−particle: NP2
NLDFAPGVNMQPVR pc2
rs9548505 REF
132:724
132:462
31:74
13:39275451:G:A_A
p = 5.8e−07, model = REC, N = 295
TLATGHLGFMITSK pc3
rs1868463 REF
127:724
124:462
26:74
13:39275451:G:A_A
p = 1.4e−05, model = REC, N = 277
EGETQHVVEIEVIFDGVR pc3
rs9548509 ALT
14:724
25:462
4:74
13:39275451:G:A_A
p = 9e−04, model = REC, N = 43

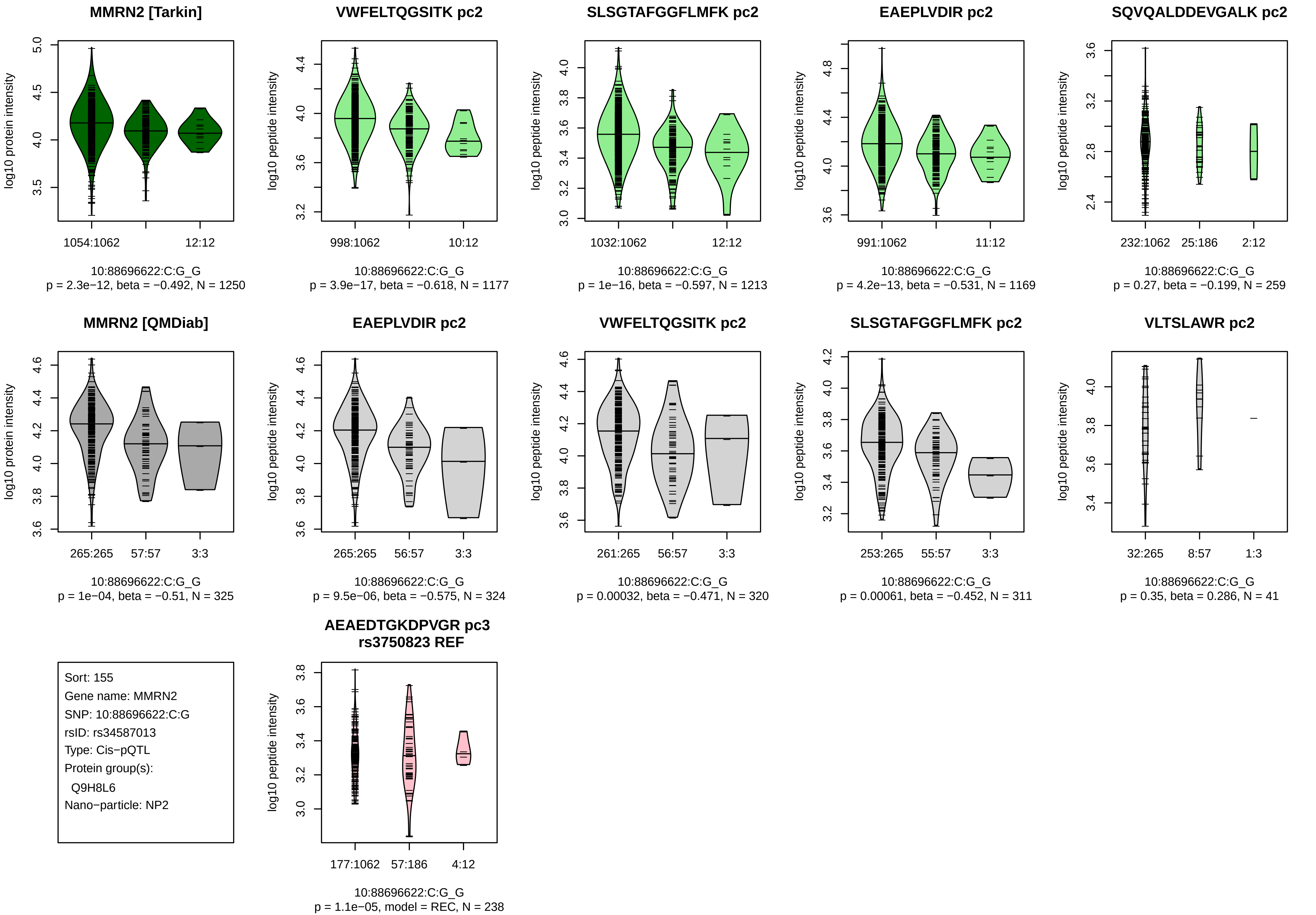
MMRN2 [Tarkin]
log10 protein intensity
1054:1062
12:12
10:88696622:C:G_G
p = 2.3e−12, beta = −0.492, N = 1250
VWFELTQGSITK pc2
log10 peptide intensity
998:1062
10:12
10:88696622:C:G_G
p = 3.9e−17, beta = −0.618, N = 1177
SLSGTAFGGFLMFK pc2
log10 peptide intensity
1032:1062
12:12
10:88696622:C:G_G
p = 1e−16, beta = −0.597, N = 1213
EAEPLVDIR pc2
log10 peptide intensity
991:1062
11:12
10:88696622:C:G_G
p = 4.2e−13, beta = −0.531, N = 1169
SQVQALDDEVGALK pc2
log10 peptide intensity
232:1062
25:186
2:12
10:88696622:C:G_G
p = 0.27, beta = −0.199, N = 259
MMRN2 [QMDiab]
log10 protein intensity
265:265
57:57
3:3
10:88696622:C:G_G
p = 1e−04, beta = −0.51, N = 325
EAEPLVDIR pc2
log10 peptide intensity
265:265
56:57
3:3
10:88696622:C:G_G
p = 9.5e−06, beta = −0.575, N = 324
VWFELTQGSITK pc2
log10 peptide intensity
261:265
56:57
3:3
10:88696622:C:G_G
p = 0.00032, beta = −0.471, N = 320
SLSGTAFGGFLMFK pc2
log10 peptide intensity
253:265
55:57
3:3
10:88696622:C:G_G
p = 0.00061, beta = −0.452, N = 311
VLTSLAWR pc2
log10 peptide intensity
32:265
8:57
1:3
10:88696622:C:G_G
p = 0.35, beta = 0.286, N = 41
Sort: 155
Gene name: MMRN2
SNP: 10:88696622:C:G
rsID: rs34587013
Type: Cis−pQTL
Protein group(s):
Q9H8L6
Nano−particle: NP2
AEAEDTGKDPVGR pc3
rs3750823 REF
log10 peptide intensity
177:1062
57:186
4:12
10:88696622:C:G_G
p = 1.1e−05, model = REC, N = 238

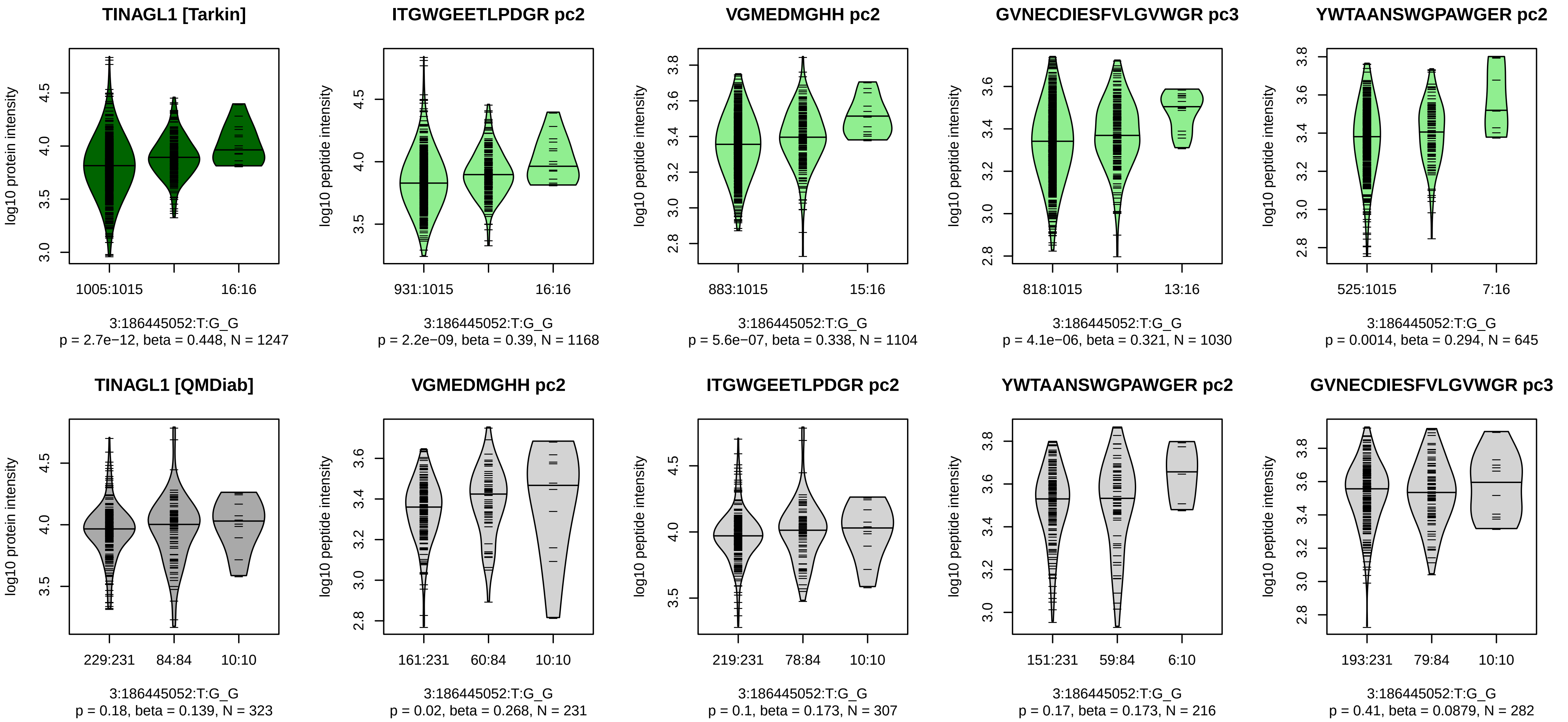

Sort: 156
Gene name: TINAGL1
SNP: 3:186445052:T:G
rsID: rs2304456
Type: Trans−pQTL
Protein group(s):
Q9GZM7;Q9GZM7−3
Nano−particle: NP2

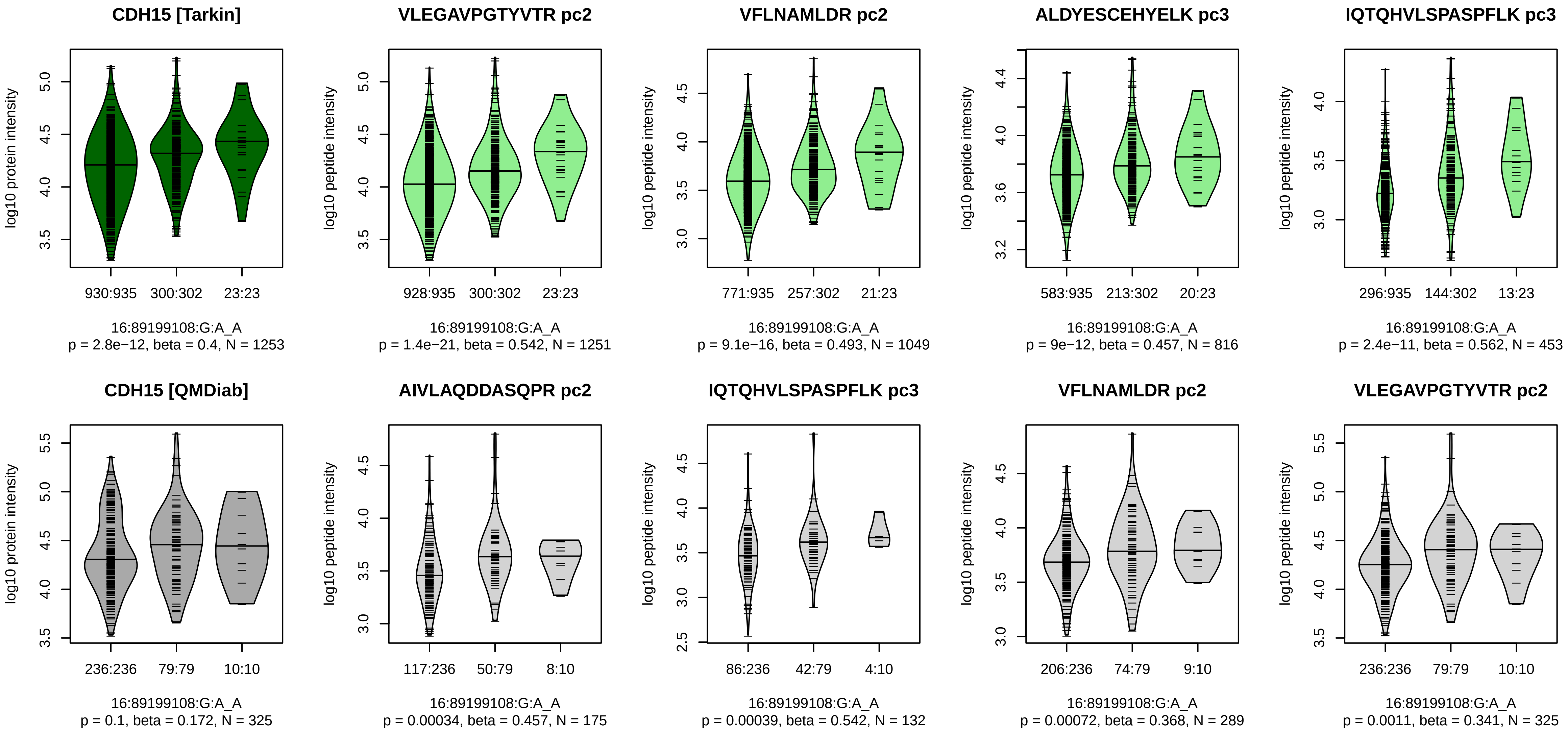

Sort: 157
Gene name: CDH15
SNP: 16:89199108:G:A
rsID: rs11646135
Type: Cis−pQTL
Protein group(s):
P55291
Nano−particle: NP2

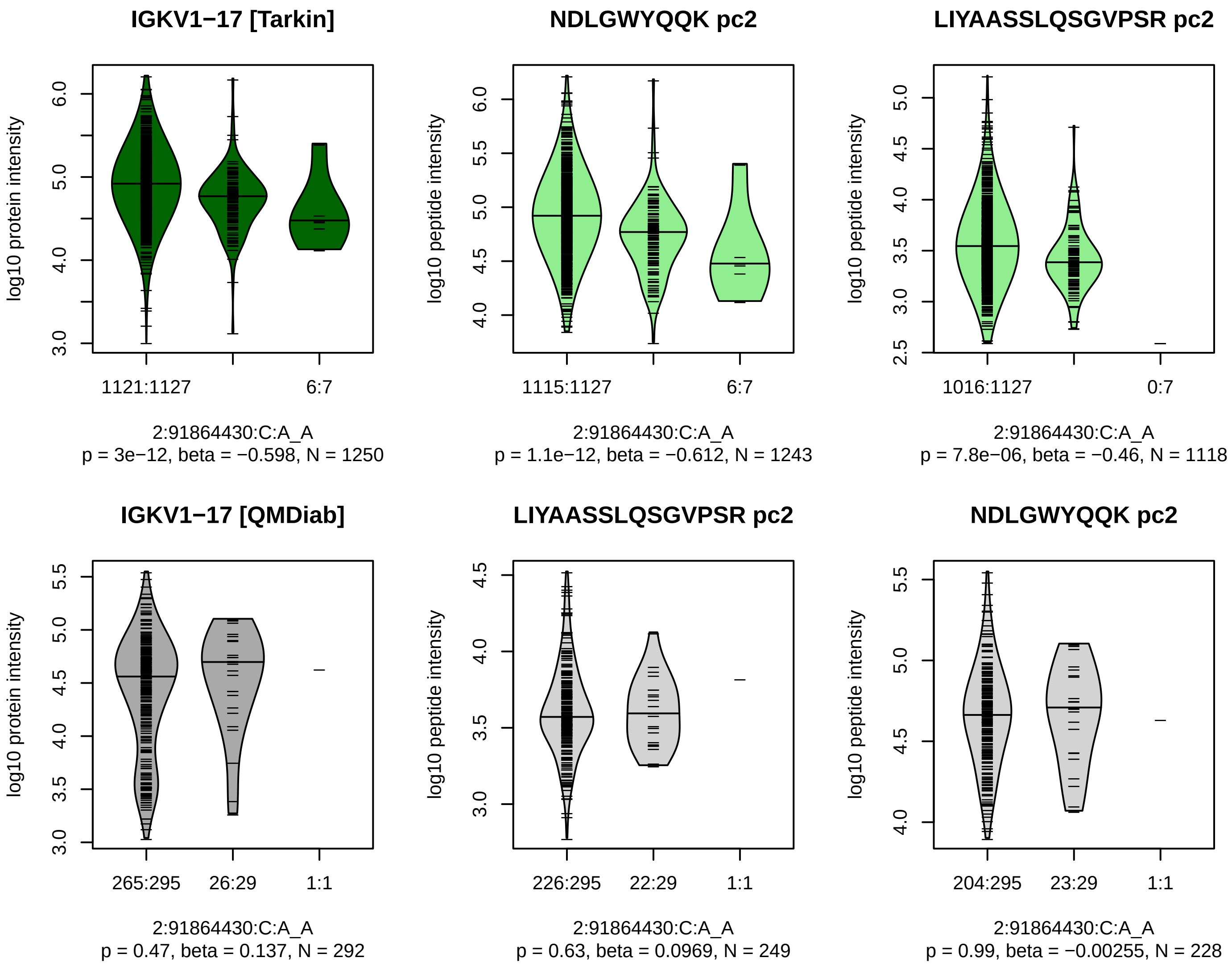

Sort: 158
Gene name: IGKV1−17
SNP: 2:91864430:C:A
rsID: rs112735595
Type: Cis−pQTL
Protein group(s):
P01599
Nano−particle: NP3

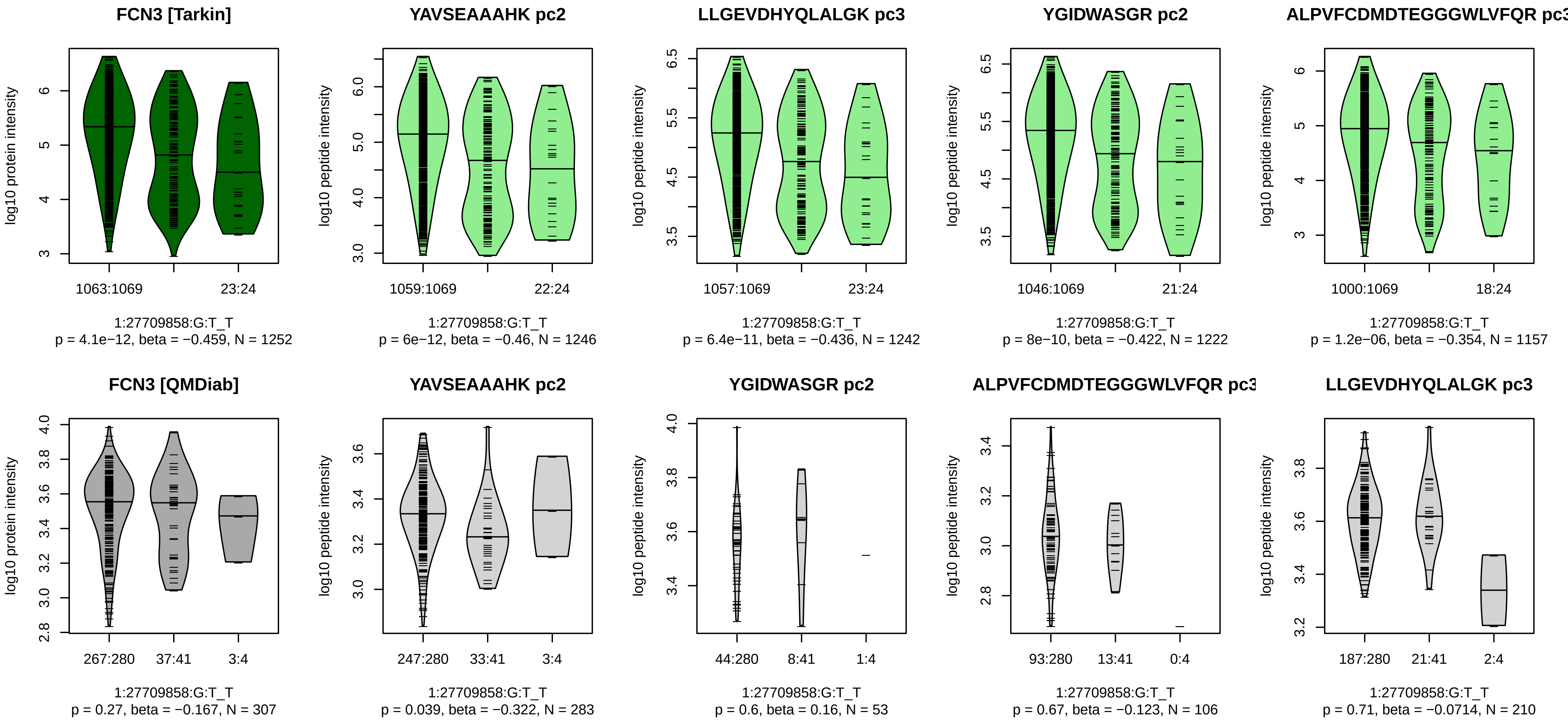

Sort: 159
Gene name: FCN3
SNP: 1:27709858:G:T
rsID: rs734725
Type: Cis−pQTL
Protein group(s):
O75636
Nano−particle: NP3

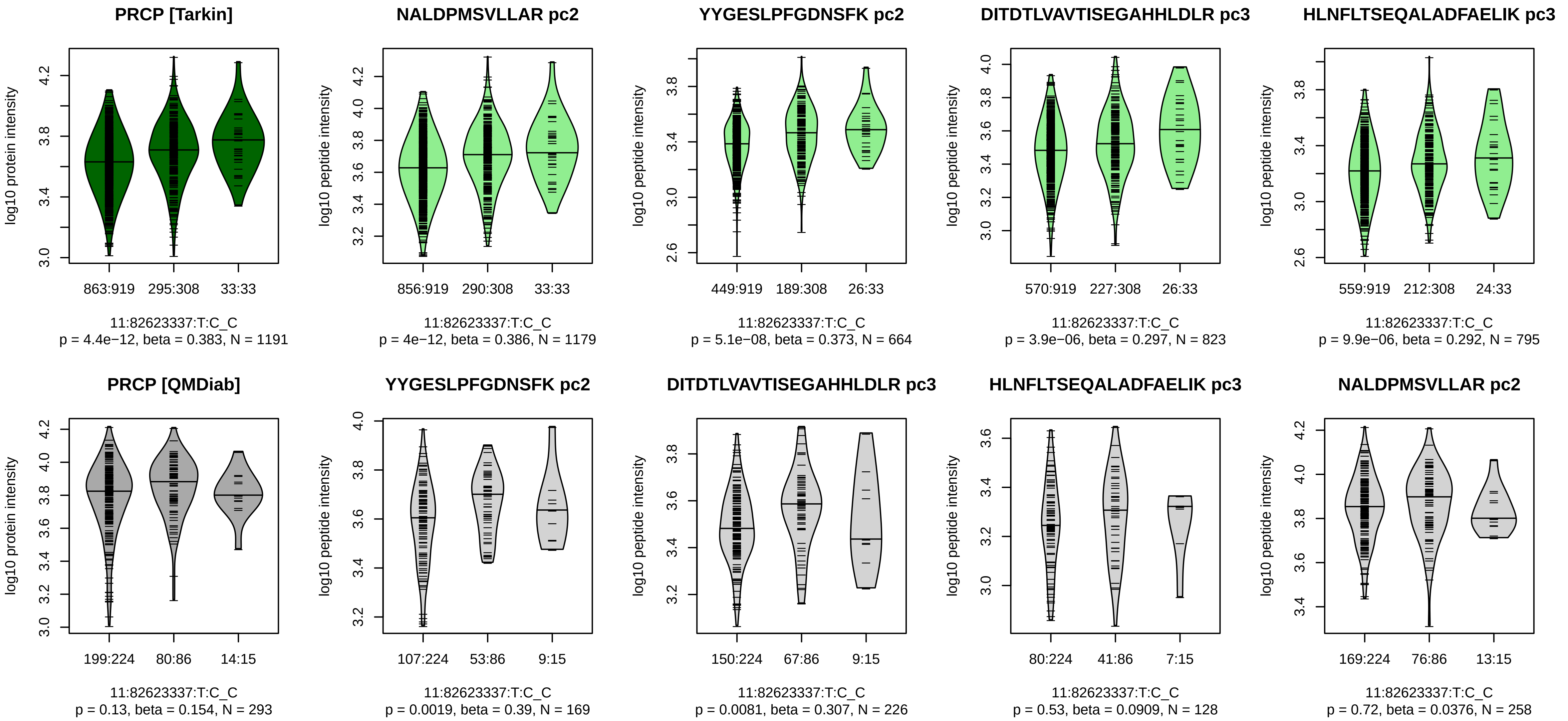

Sort: 160
Gene name: PRCP
SNP: 11:82623337:T:C
rsID: rs11233371
Type: Cis−pQTL
Protein group(s):
P42785
Nano−particle: NP5

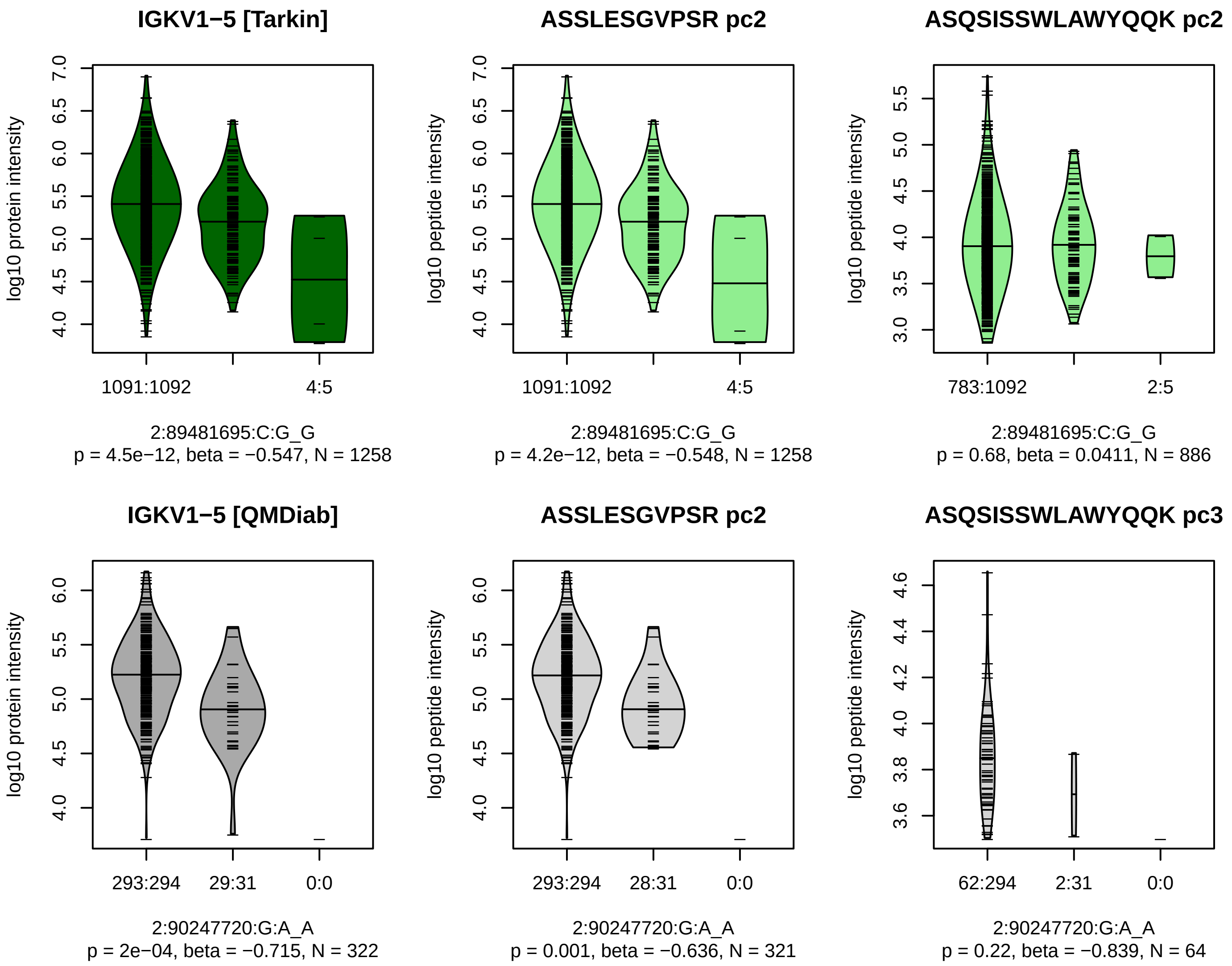

Sort: 161
Gene name: IGKV1−5
SNP: 2:89481695:C:G
rsID: rs142995009
Type: Cis−pQTL
Protein group(s):
P01602
Nano−particle: NP4

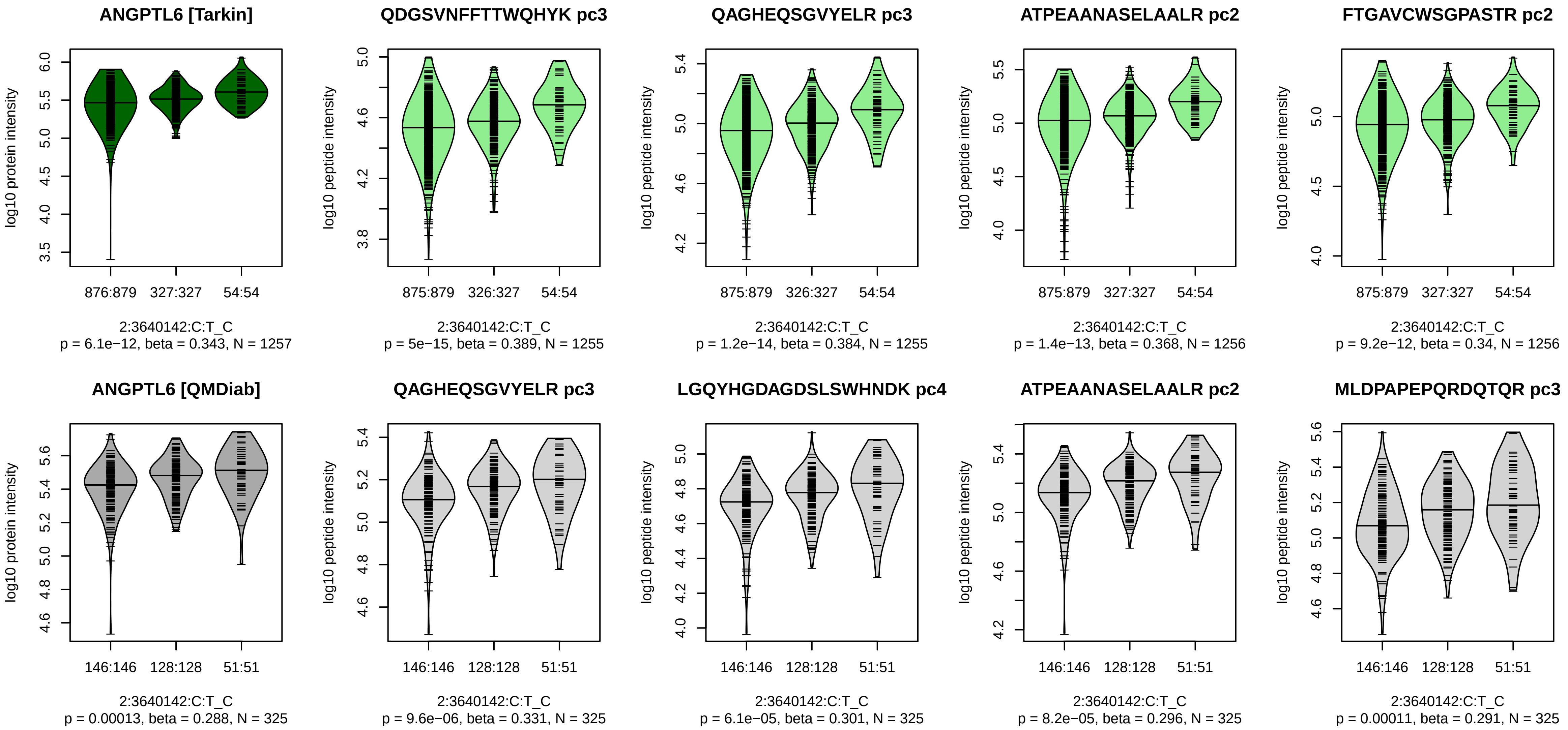

Sort: 162
Gene name: ANGPTL6
SNP: 2:3640142:C:T
rsID: rs6542680
Type: Trans−pQTL
Protein group(s):
Q8NI99
Nano−particle: NP5

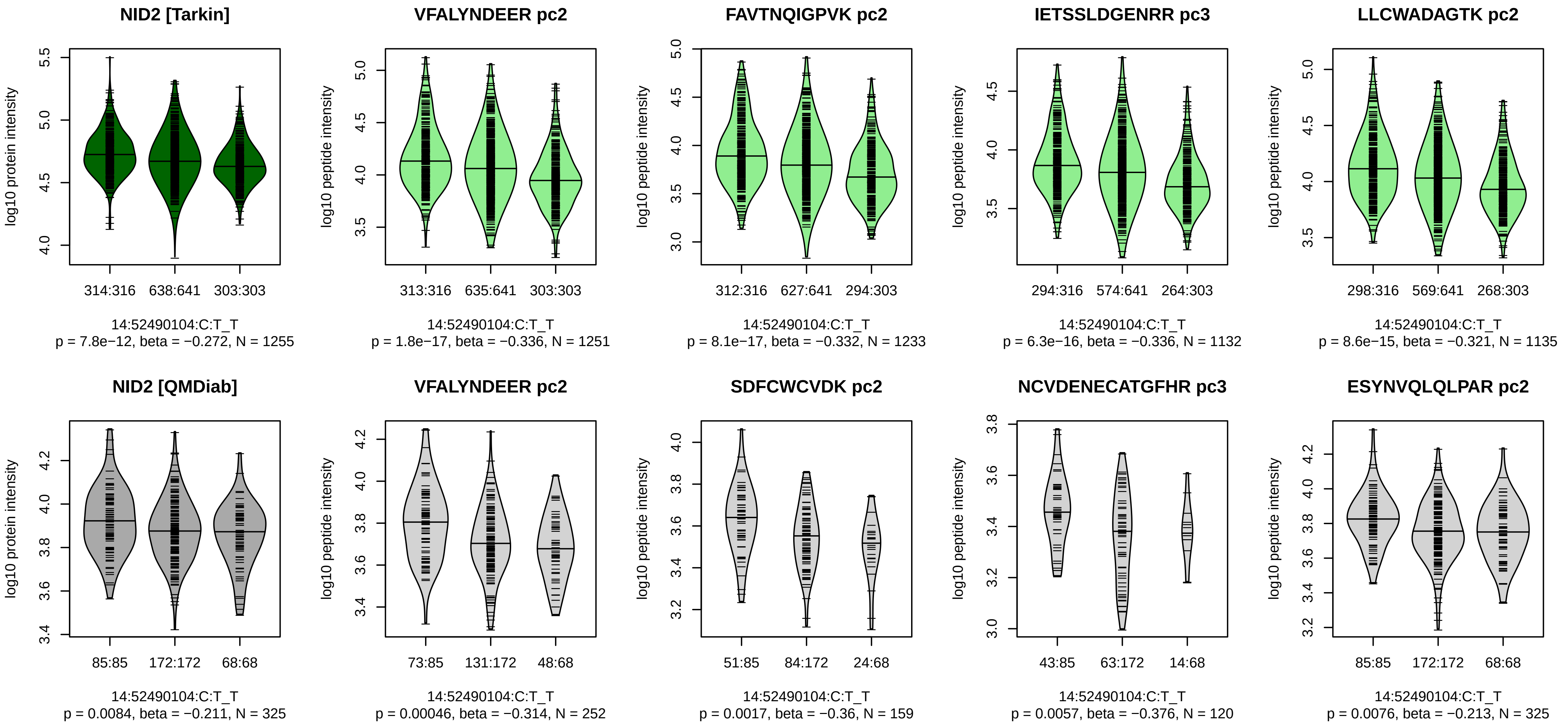

Sort: 163
Gene name: NID2
SNP: 14:52490104:C:T
rsID: rs10137847
Type: Cis−pQTL
Protein group(s):
Q14112
Nano−particle: NP2

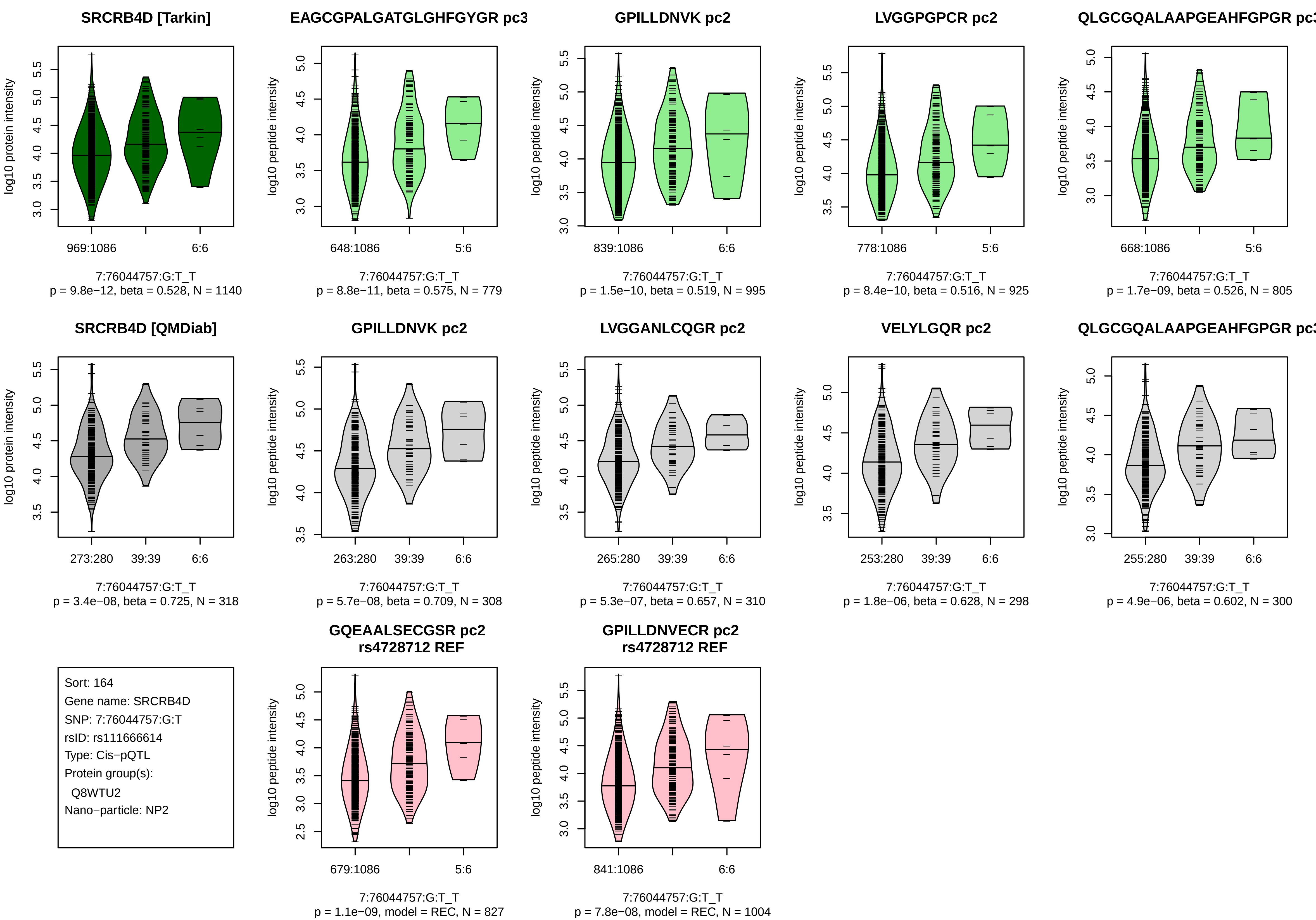
SRCRB4D [Tarkin]
log10 protein intensity
969:1086
6:6
7:76044757:G:T_T
p = 9.8e−12, beta = 0.528, N = 1140
EAGCGPALGATGLGHFGYGR pc3
log10 peptide intensity
648:1086
5:6
7:76044757:G:T_T
p = 8.8e−11, beta = 0.575, N = 779
GPILLDNVK pc2
log10 peptide intensity
839:1086
6:6
7:76044757:G:T_T
p = 1.5e−10, beta = 0.519, N = 995
LVGGPGPCR pc2
log10 peptide intensity
778:1086
5:6
7:76044757:G:T_T
p = 8.4e−10, beta = 0.516, N = 925
QLGCGQALAAPGEAHFGPGR pc3
log10 peptide intensity
668:1086
5:6
7:76044757:G:T_T
p = 1.7e−09, beta = 0.526, N = 805
SRCRB4D [QMDiab]
log10 protein intensity
273:280
39:39
6:6
7:76044757:G:T_T
p = 3.4e−08, beta = 0.725, N = 318
GPILLDNVK pc2
log10 peptide intensity
263:280
39:39
6:6
7:76044757:G:T_T
p = 5.7e−08, beta = 0.709, N = 308
LVGGANLCQGR pc2
log10 peptide intensity
265:280
39:39
6:6
7:76044757:G:T_T
p = 5.3e−07, beta = 0.657, N = 310
VELYLGQR pc2
log10 peptide intensity
253:280
39:39
6:6
7:76044757:G:T_T
p = 1.8e−06, beta = 0.628, N = 298
QLGCGQALAAPGEAHFGPGR pc3
log10 peptide intensity
255:280
39:39
6:6
7:76044757:G:T_T
p = 4.9e−06, beta = 0.602, N = 300
Sort: 164
Gene name: SRCRB4D
SNP: 7:76044757:G:T
rsID: rs111666614
Type: Cis−pQTL
Protein group(s):
Q8WTU2
Nano−particle: NP2
GQEAALSECGSR pc2
rs4728712 REF
log10 peptide intensity
679:1086
5:6
7:76044757:G:T_T
p = 1.1e−09, model = REC, N = 827
GPILLDNVECR pc2
rs4728712 REF
log10 peptide intensity
841:1086
6:6
7:76044757:G:T_T
p = 7.8e−08, model = REC, N = 1004

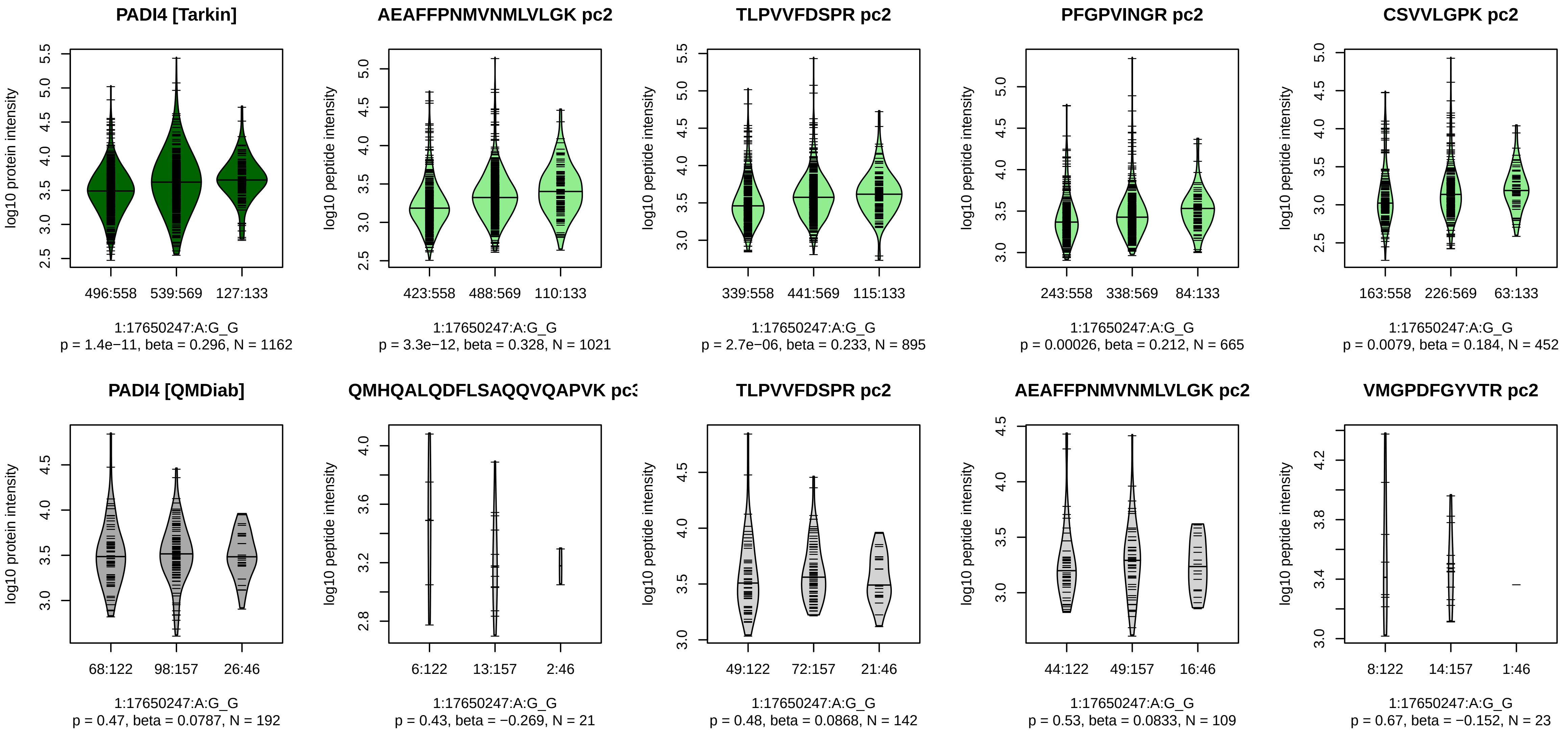

Sort: 165
Gene name: PADI4
SNP: 1:17650247:A:G
rsID: rs11203358
Type: Cis−pQTL
Protein group(s):
Q9UM07
Nano−particle: NP1

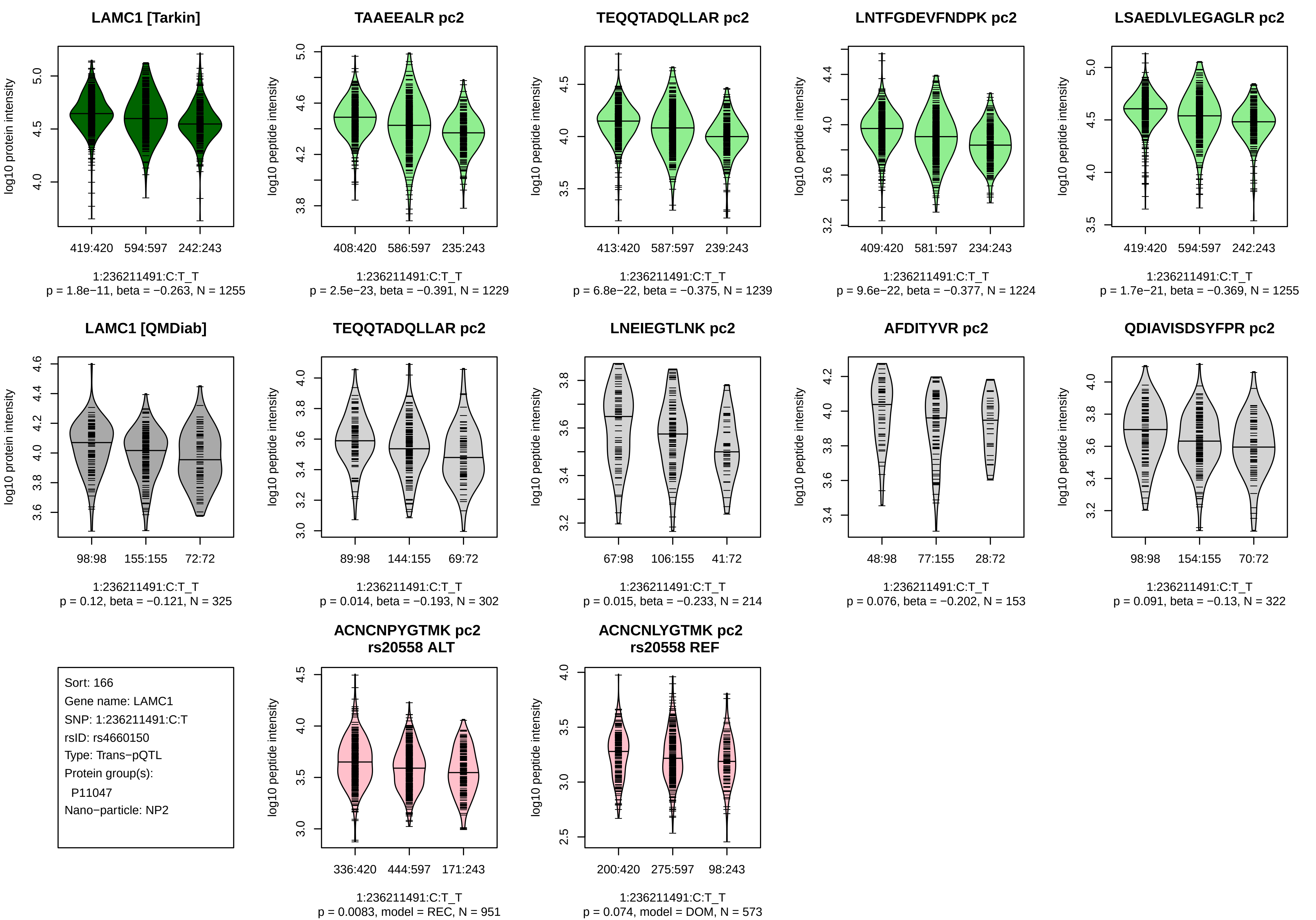
LAMC1 [Tarkin]
log10 protein intensity
419:420 594:597 242:243
1:236211491:C:T_T
p = 1.8e−11, beta = −0.263, N = 1255
TAAEEALR pc2
log10 peptide intensity
408:420 586:597 235:243
1:236211491:C:T_T
p = 2.5e−23, beta = −0.391, N = 1229
TEQQTADQLLAR pc2
log10 peptide intensity
413:420 587:597 239:243
1:236211491:C:T_T
p = 6.8e−22, beta = −0.375, N = 1239
LNTFGDEVFNDPK pc2
log10 peptide intensity
409:420 581:597 234:243
1:236211491:C:T_T
p = 9.6e−22, beta = −0.377, N = 1224
LSAEDLVLEGAGLR pc2
log10 peptide intensity
419:420 594:597 242:243
1:236211491:C:T_T
p = 1.7e−21, beta = −0.369, N = 1255
LAMC1 [QMDiab]
log10 protein intensity
98:98 155:155 72:72
1:236211491:C:T_T
p = 0.12, beta = −0.121, N = 325
TEQQTADQLLAR pc2
log10 peptide intensity
89:98 144:155 69:72
1:236211491:C:T_T
p = 0.014, beta = −0.193, N = 302
LNEIEGTLNK pc2
log10 peptide intensity
67:98 106:155 41:72
1:236211491:C:T_T
p = 0.015, beta = −0.233, N = 214
AFDITYVR pc2
log10 peptide intensity
48:98 77:155 28:72
1:236211491:C:T_T
p = 0.076, beta = −0.202, N = 153
QDIAVISDSYFPR pc2
log10 peptide intensity
98:98 154:155 70:72
1:236211491:C:T_T
p = 0.091, beta = −0.13, N = 322
Sort: 166
Gene name: LAMC1
SNP: 1:236211491:C:T
rsID: rs4660150
Type: Trans−pQTL
Protein group(s):
P11047
Nano−particle: NP2
ACNCNPYGTMK pc2
rs20558 ALT
log10 peptide intensity
336:420 444:597 171:243
1:236211491:C:T_T
p = 0.0083, model = REC, N = 951
ACNCNLYGTMK pc2
rs20558 REF
log10 peptide intensity
200:420 275:597 98:243
1:236211491:C:T_T
p = 0.074, model = DOM, N = 573

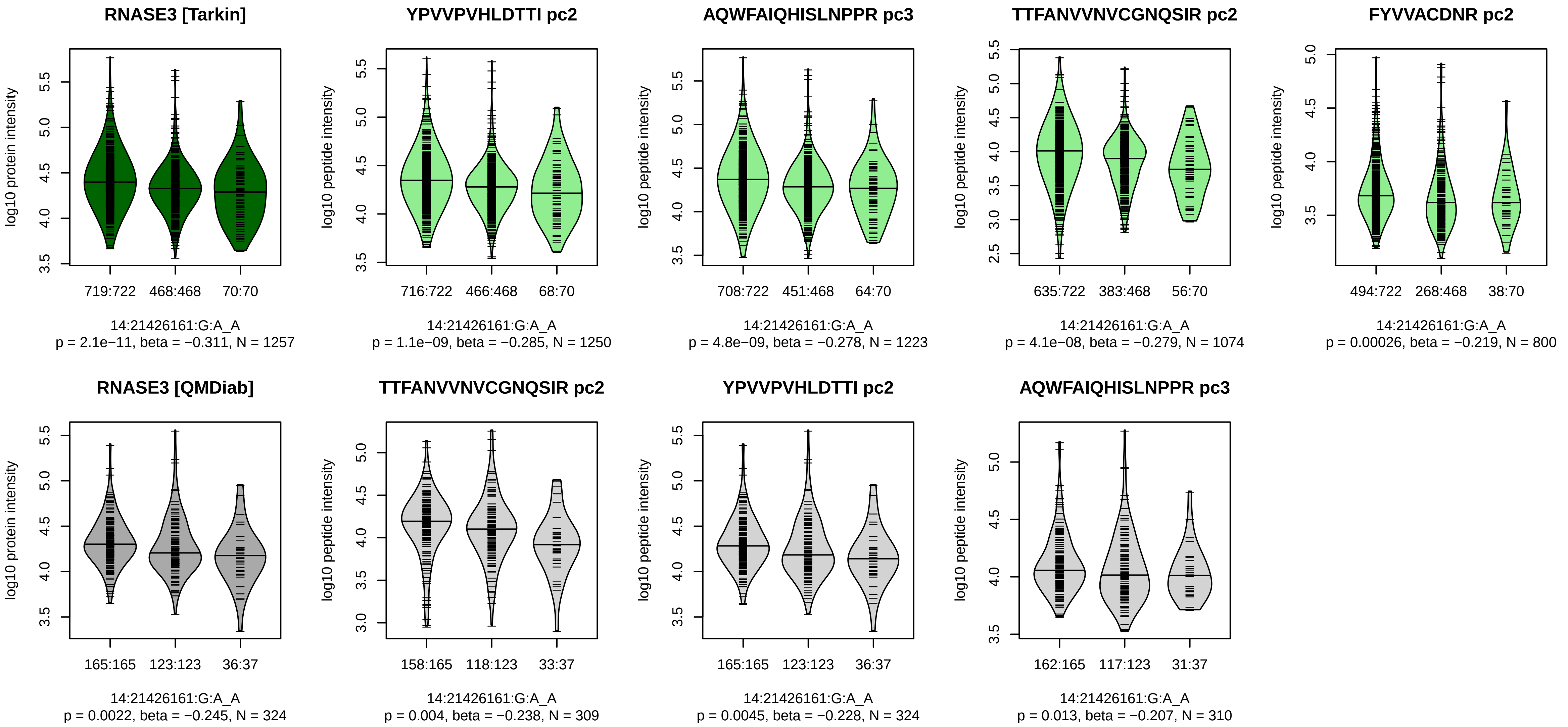

Sort: 167
Gene name: RNASE3
SNP: 14:21426161:G:A
rsID: rs2771312
Type: Cis−pQTL
Protein group(s):
P12724
Nano−particle: NP4

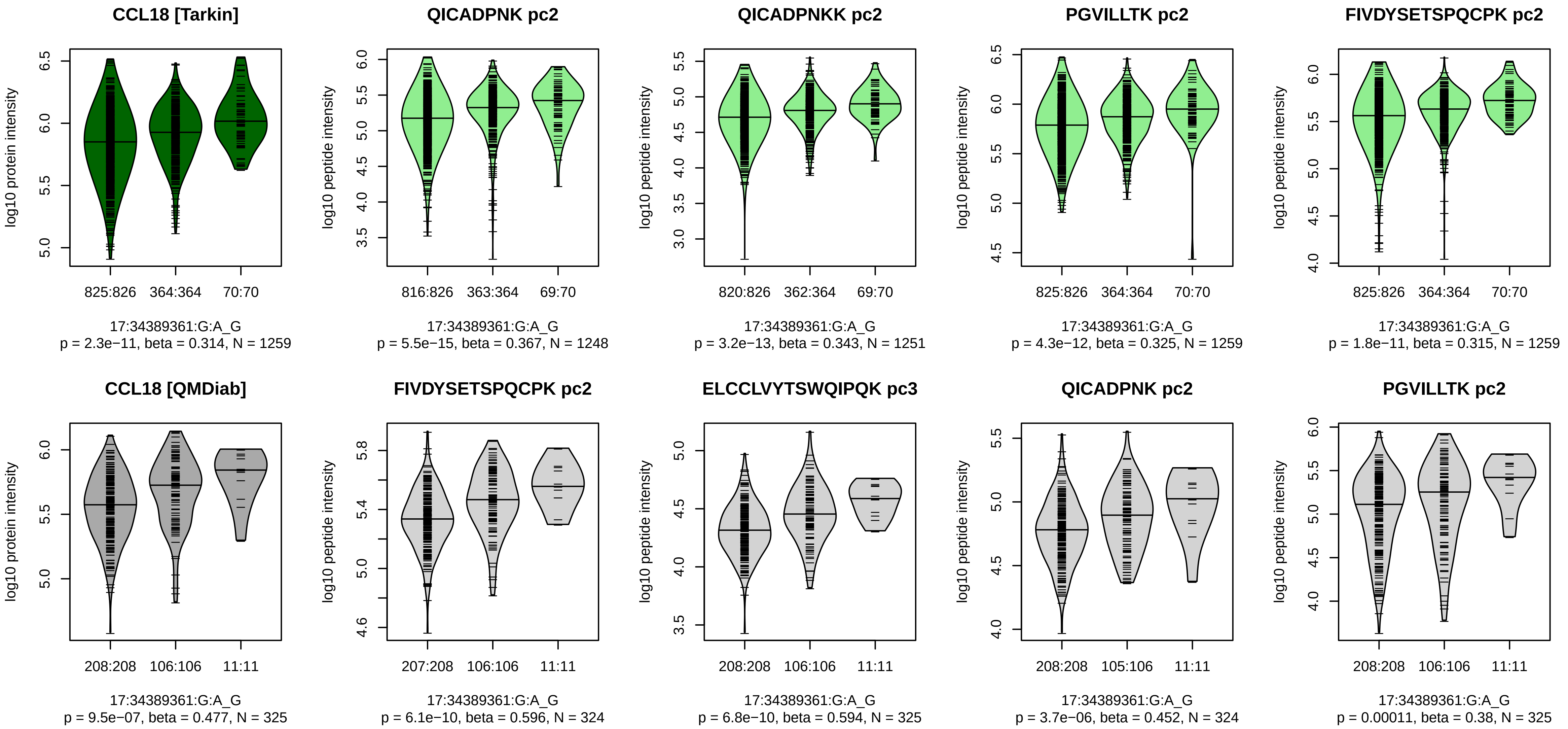

Sort: 168
Gene name: CCL18
SNP: 17:34389361:G:A
rsID: rs854469
Type: Cis−pQTL
Protein group(s):
P55774
Nano−particle: NP4

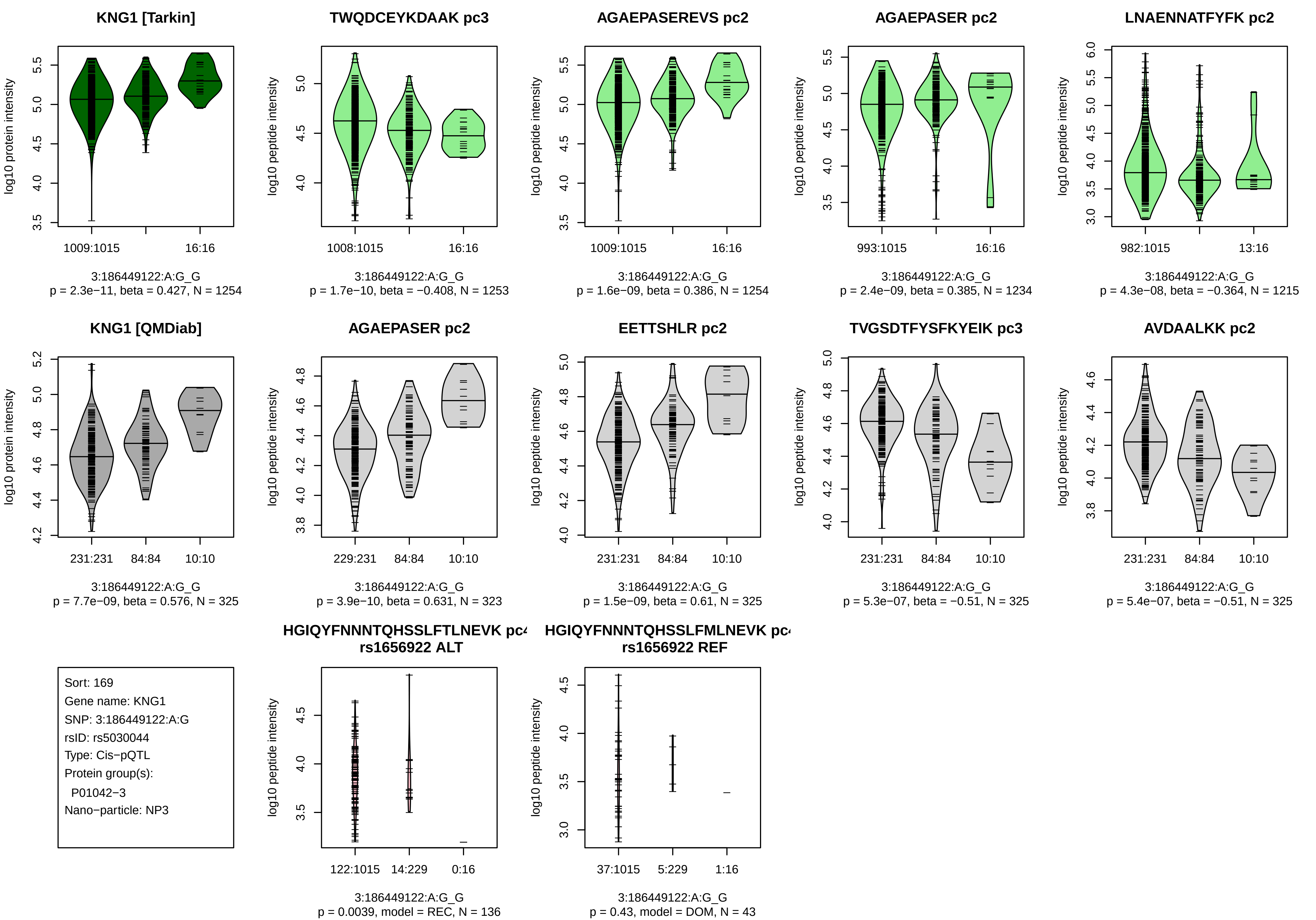
KNG1 [Tarkin]
log10 protein intensity
1009:1015
16:16
3:186449122:A:G_G
p = 2.3e−11, beta = 0.427, N = 1254
TWQDCEYKDAAK pc3
log10 peptide intensity
1008:1015
16:16
3:186449122:A:G_G
p = 1.7e−10, beta = −0.408, N = 1253
AGAEPASEREVS pc2
log10 peptide intensity
1009:1015
16:16
3:186449122:A:G_G
p = 1.6e−09, beta = 0.386, N = 1254
AGAEPASER pc2
log10 peptide intensity
993:1015
16:16
3:186449122:A:G_G
p = 2.4e−09, beta = 0.385, N = 1234
LNAENNATFYFK pc2
log10 peptide intensity
982:1015
13:16
3:186449122:A:G_G
p = 4.3e−08, beta = −0.364, N = 1215
KNG1 [QMDiab]
log10 protein intensity
231:231
84:84
10:10
3:186449122:A:G_G
p = 7.7e−09, beta = 0.576, N = 325
AGAEPASER pc2
log10 peptide intensity
229:231
84:84
10:10
3:186449122:A:G_G
p = 3.9e−10, beta = 0.631, N = 323
EETTSHLR pc2
log10 peptide intensity
231:231
84:84
10:10
3:186449122:A:G_G
p = 1.5e−09, beta = 0.61, N = 325
TVGSDTFYSFKYEIK pc3
log10 peptide intensity
231:231
84:84
10:10
3:186449122:A:G_G
p = 5.3e−07, beta = −0.51, N = 325
AVDAALKK pc2
log10 peptide intensity
231:231
84:84
10:10
3:186449122:A:G_G
p = 5.4e−07, beta = −0.51, N = 325
Sort: 169
Gene name: KNG1
SNP: 3:186449122:A:G
rsID: rs5030044
Type: Cis−pQTL
Protein group(s):
P01042-3
Nano−particle: NP3
HGIQYFNNNTQHSSLFTLNEVK pc4
rs1656922 ALT
log10 peptide intensity
122:1015
14:229
0:16
3:186449122:A:G_G
p = 0.0039, model = REC, N = 136
HGIQYFNNNTQHSSLFMLNEVK pc4
rs1656922 REF
log10 peptide intensity
37:1015
5:229
1:16
3:186449122:A:G_G
p = 0.43, model = DOM, N = 43

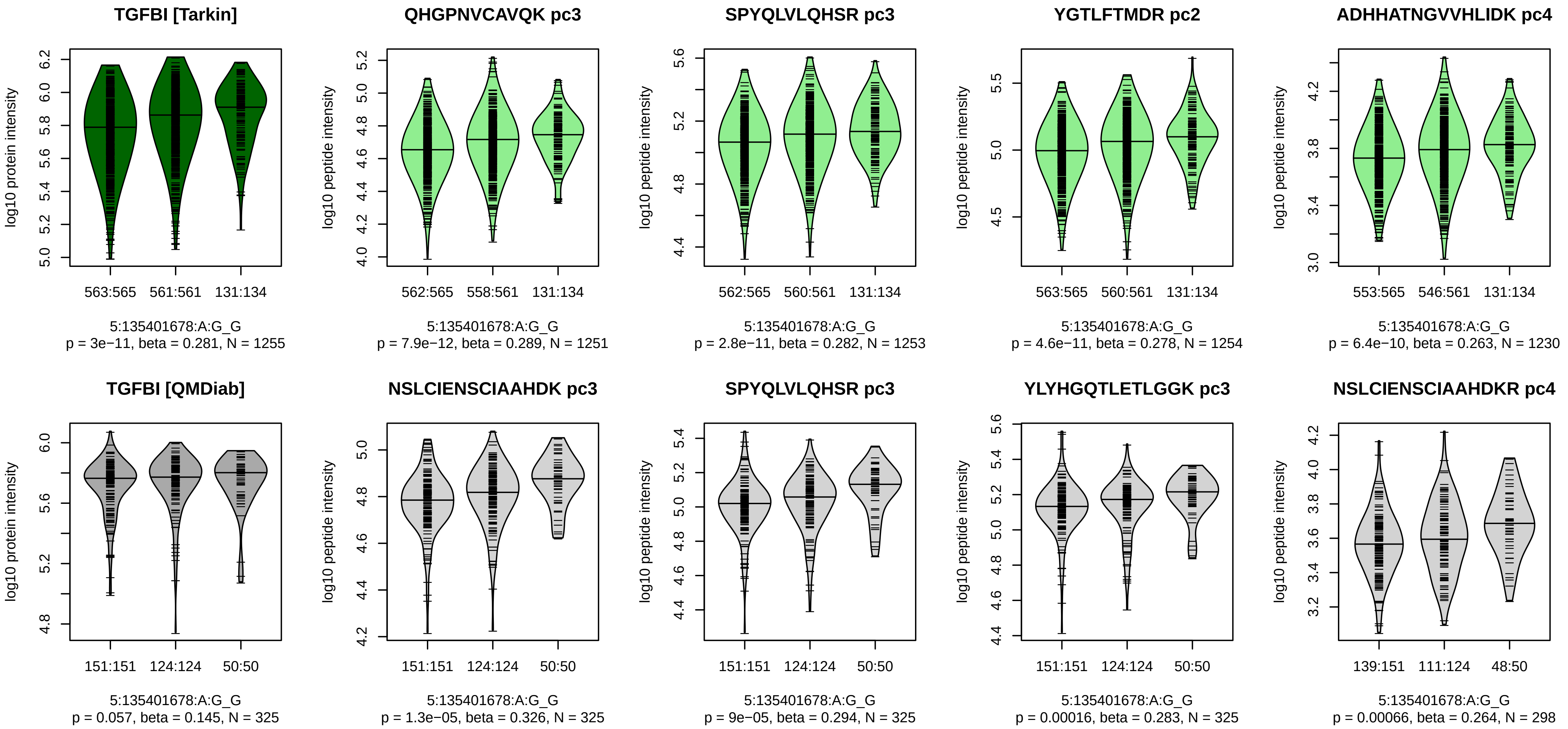

Sort: 170
Gene name: TGFBI
SNP: 5:135401678:A:G
rsID: rs17169786
Type: Cis−pQTL
Protein group(s):
Q15582
Nano−particle: NP3

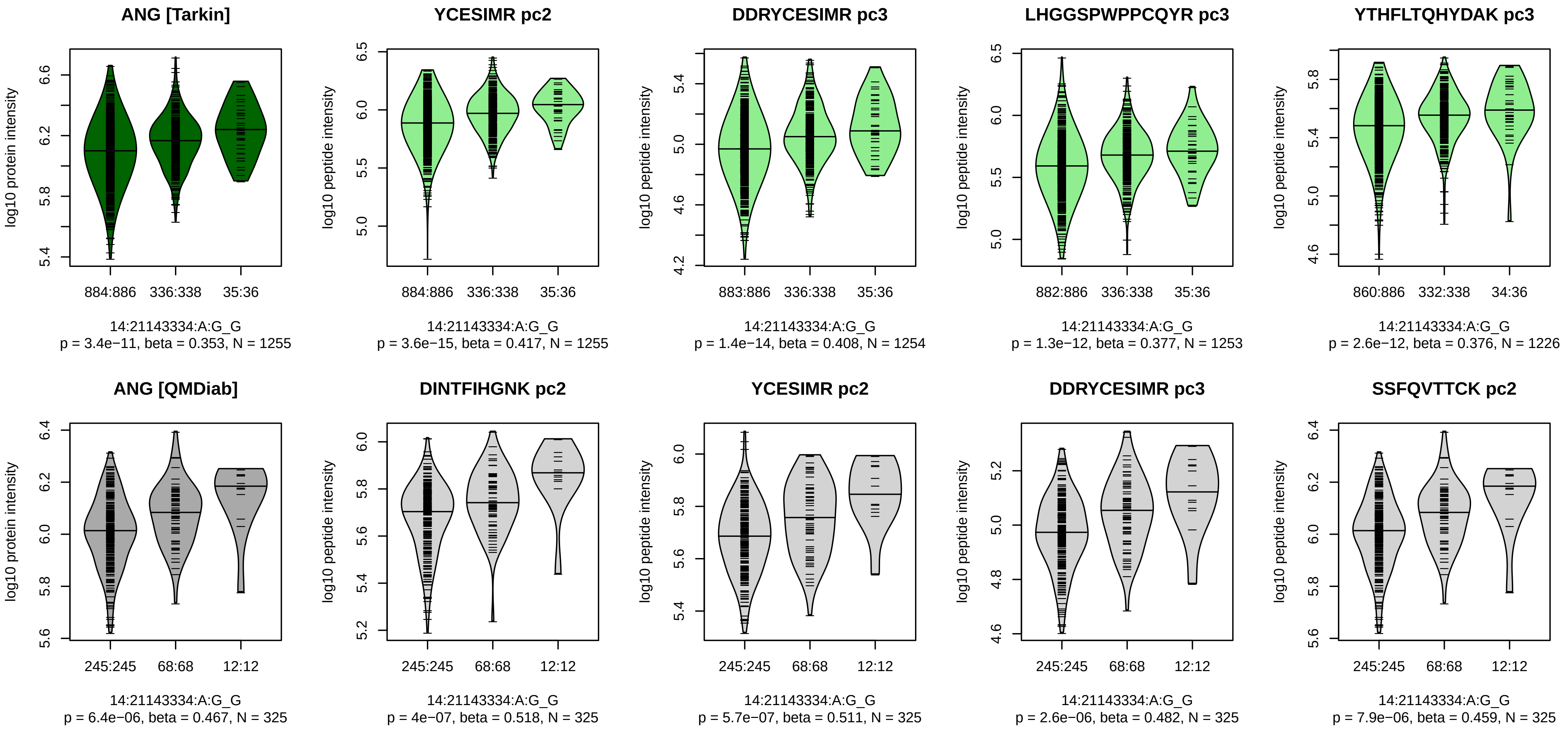

Sort: 171
Gene name: ANG
SNP: 14:21143334:A:G
rsID: rs10142601
Type: Cis−pQTL
Protein group(s):
P03950
Nano−particle: NP3

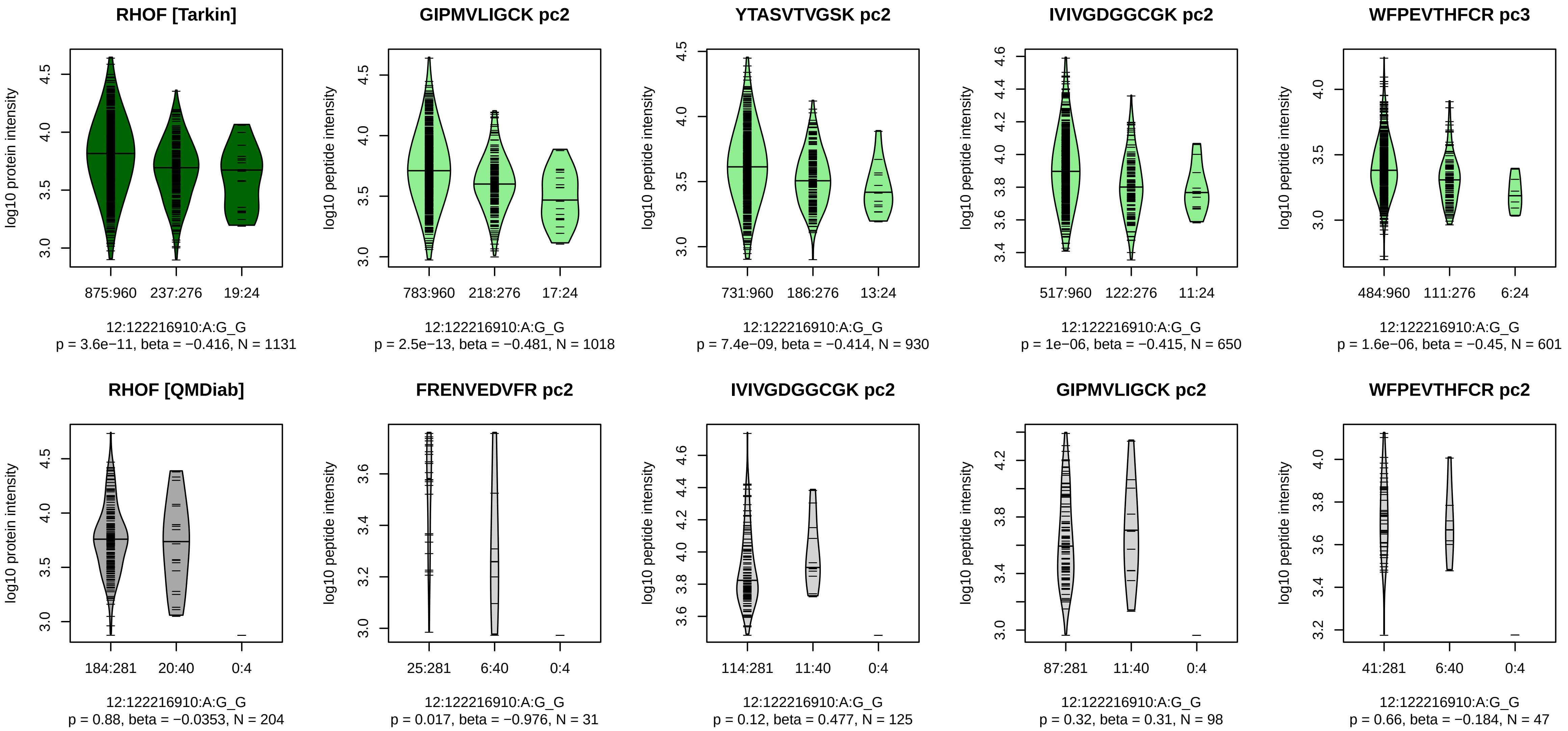

Sort: 172
Gene name: RHOF
SNP: 12:122216910:A:G
rsID: rs11553699
Type: Cis−pQTL
Protein group(s):
Q9HBH0
Nano−particle: NP4

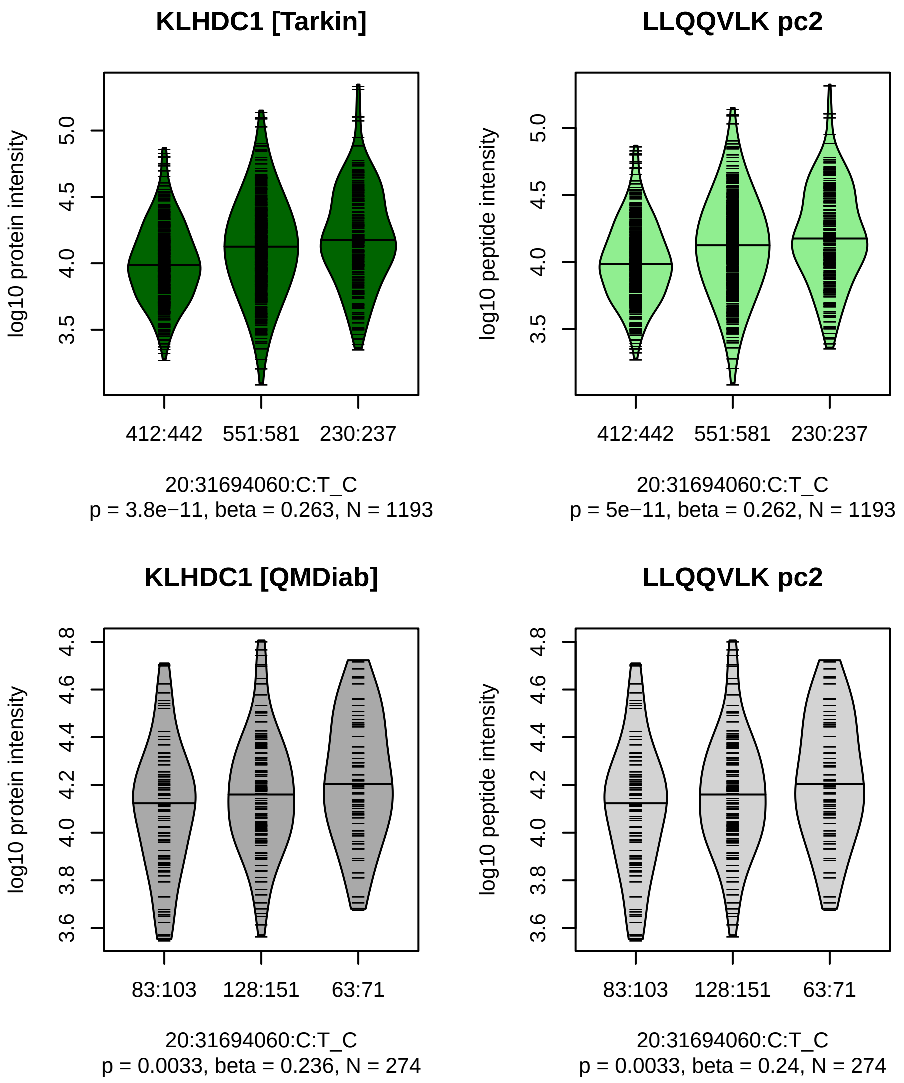

Sort: 173
Gene name: KLHDC1
SNP: 20:31694060:C:T
rsID: rs2424961
Type: Trans−pQTL
Protein group(s):
Q8N7A1
Nano−particle: NP1

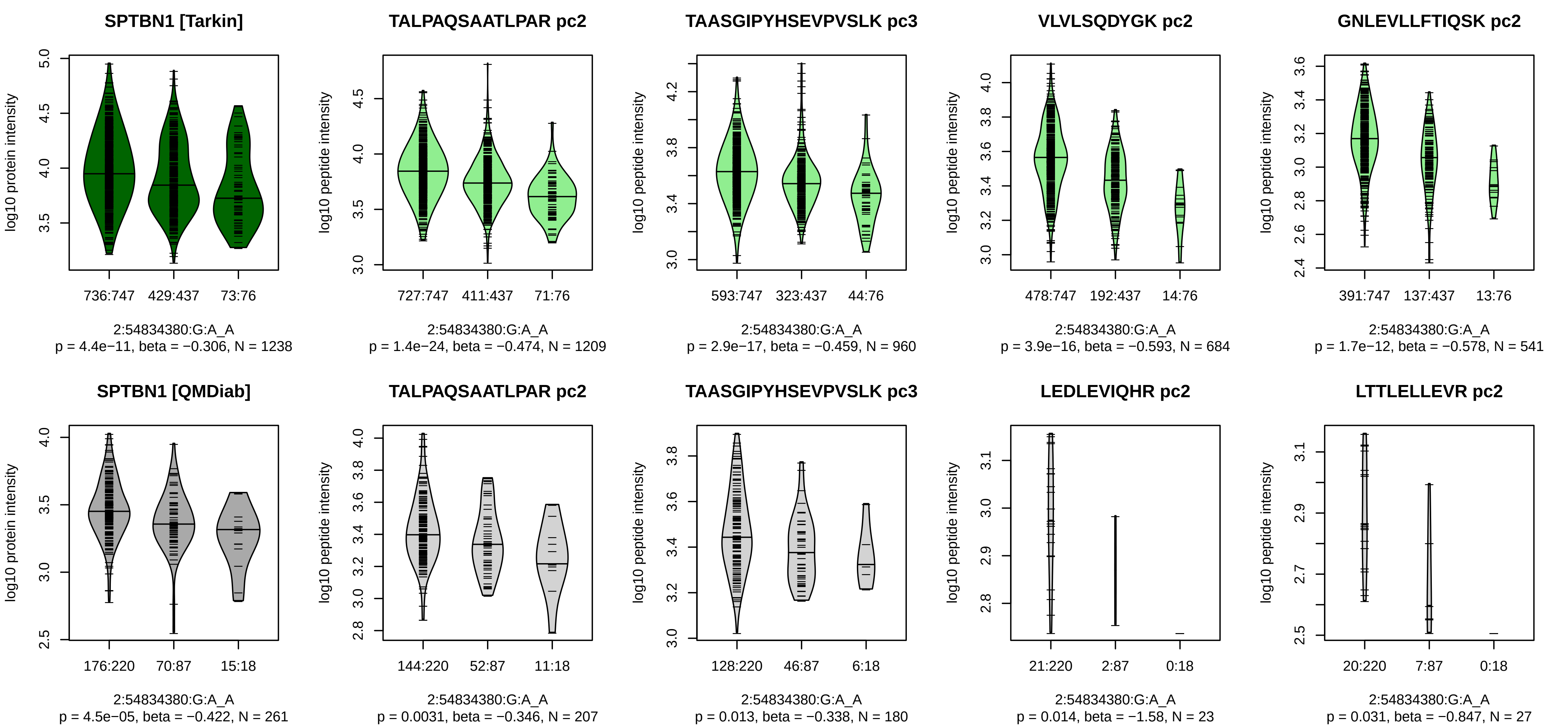

Sort: 174
Gene name: SPTBN1
SNP: 2:54834380:G:A
rsID: rs6740893
Type: Cis−pQTL
Protein group(s):
Q01082
Nano−particle: NP3

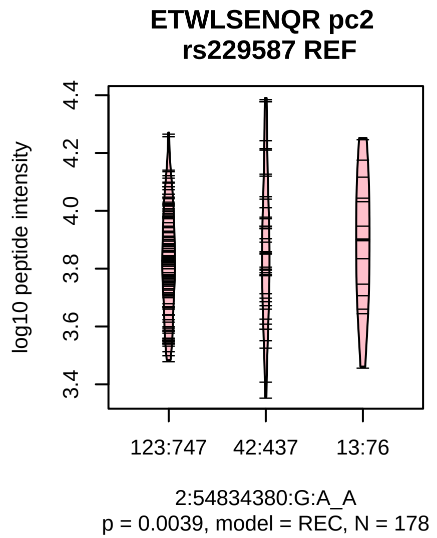

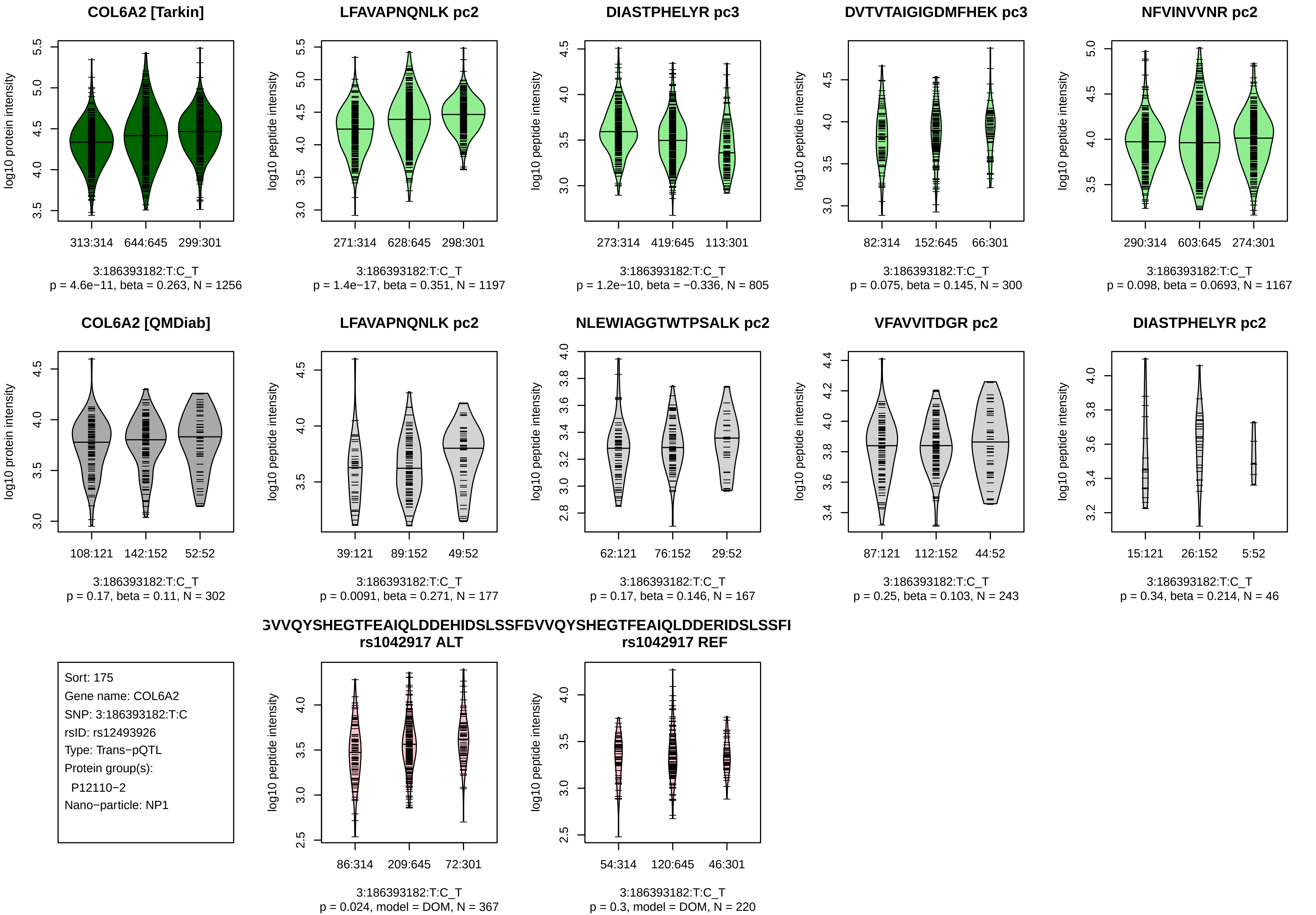

COL6A2 [Tarkin]
log10 protein intensity
313:314
644:645
299:301
3:186393182:T:C_T
p = 4.6e−11, beta = 0.263, N = 1256
LFAVAPNQNLK pc2
log10 peptide intensity
271:314
628:645
298:301
3:186393182:T:C_T
p = 1.4e−17, beta = 0.351, N = 1197
DIASTPHELYR pc3
log10 peptide intensity
273:314
419:645
113:301
3:186393182:T:C_T
p = 1.2e−10, beta = −0.336, N = 805
DVTVTAIGIGDMFHEK pc3
log10 peptide intensity
82:314
152:645
66:301
3:186393182:T:C_T
p = 0.075, beta = 0.145, N = 300
NFVINVVNR pc2
log10 peptide intensity
290:314
603:645
274:301
3:186393182:T:C_T
p = 0.098, beta = 0.0693, N = 1167
COL6A2 [QMDiab]
log10 protein intensity
108:121
142:152
52:52
3:186393182:T:C_T
p = 0.17, beta = 0.11, N = 302
LFAVAPNQNLK pc2
log10 peptide intensity
39:121
89:152
49:52
3:186393182:T:C_T
p = 0.0091, beta = 0.271, N = 177
NLEWIAGGTWTPSALK pc2
log10 peptide intensity
62:121
76:152
29:52
3:186393182:T:C_T
p = 0.17, beta = 0.146, N = 167
VFAVVITDGR pc2
log10 peptide intensity
87:121
112:152
44:52
3:186393182:T:C_T
p = 0.25, beta = 0.103, N = 243
DIASTPHELYR pc2
log10 peptide intensity
15:121
26:152
5:52
3:186393182:T:C_T
p = 0.34, beta = 0.214, N = 46
Sort: 175
Gene name: COL6A2
SNP: 3:186393182:T:C
rsID: rs12493926
Type: Trans−pQTL
Protein group(s):
P12110−2
Nano−particle: NP1
GVVQYSHEGTFEAIQLDDEHIDSLSSF
rs1042917 ALT
log10 peptide intensity
86:314
209:645
72:301
3:186393182:T:C_T
p = 0.024, model = DOM, N = 367
GVVQYSHEGTFEAIQLDDERIDSLSSFI
rs1042917 REF
log10 peptide intensity
54:314
120:645
46:301
3:186393182:T:C_T
p = 0.3, model = DOM, N = 220

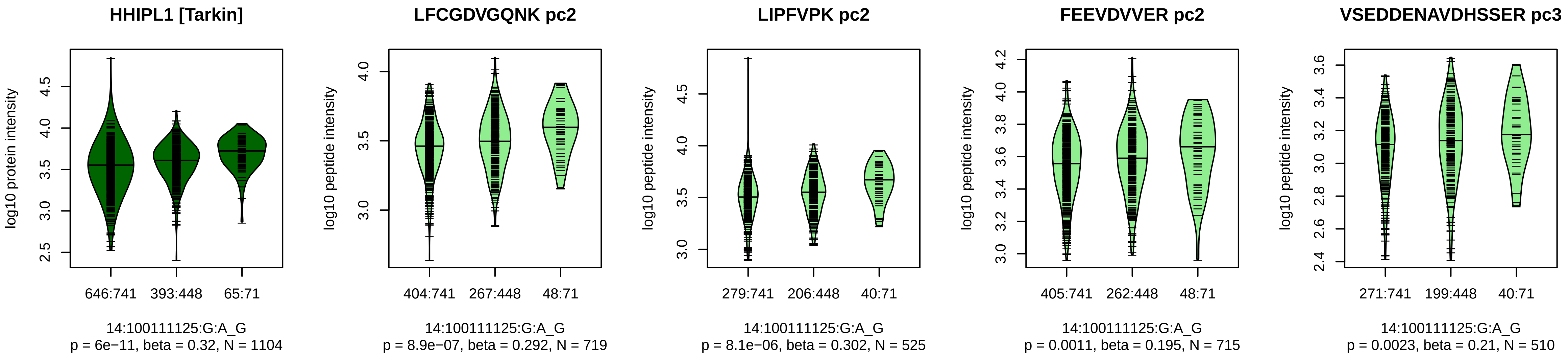

No protein data available
for replication in QMDiab

Sort: 176
Gene name: HHIPL1
SNP: 14:100111125:G:A
rsID: rs4905876
Type: Cis−pQTL
Protein group(s):
Q96JK4
Nano−particle: NP1

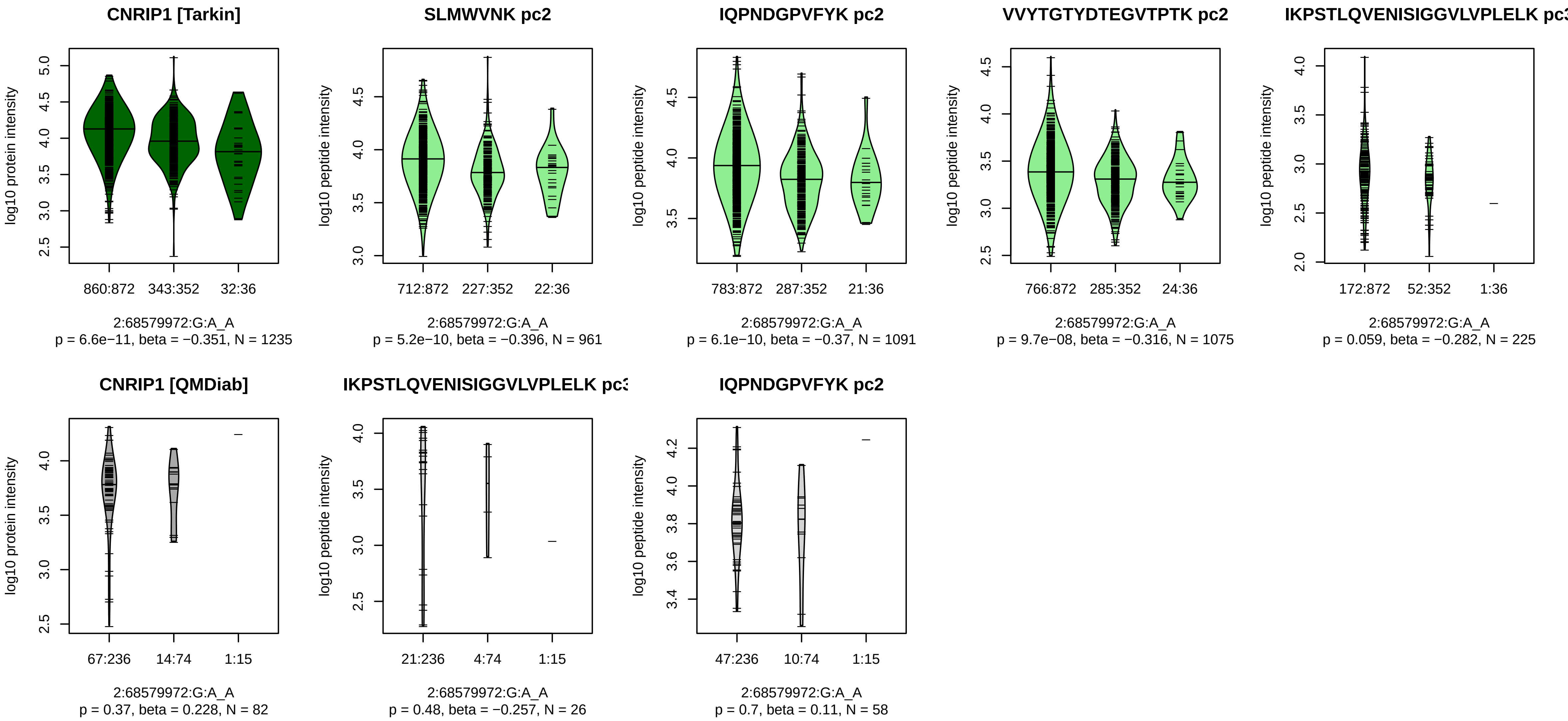

Sort: 177
Gene name: CNRIP1
SNP: 2:68579972:G:A
rsID: rs7604812
Type: Cis−pQTL
Protein group(s):
Q96F85
Nano−particle: NP3

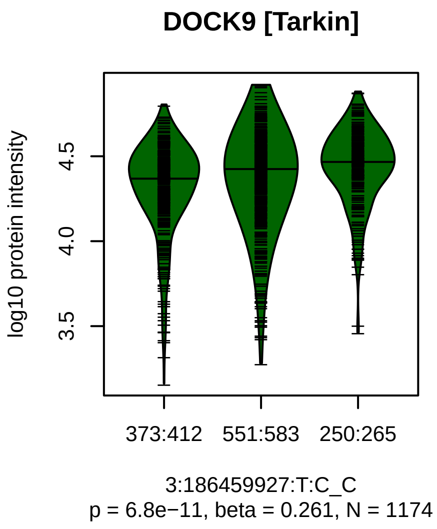

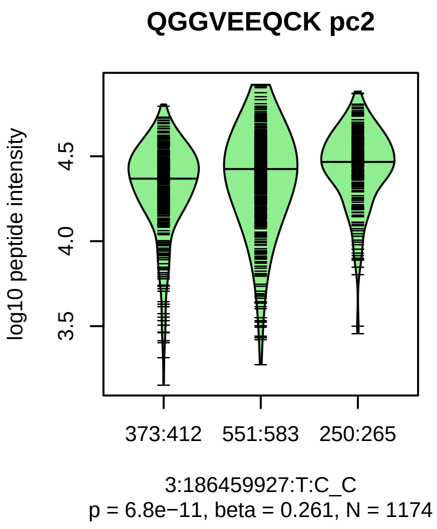

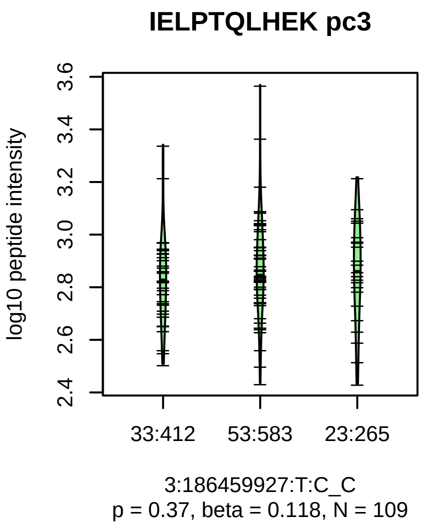

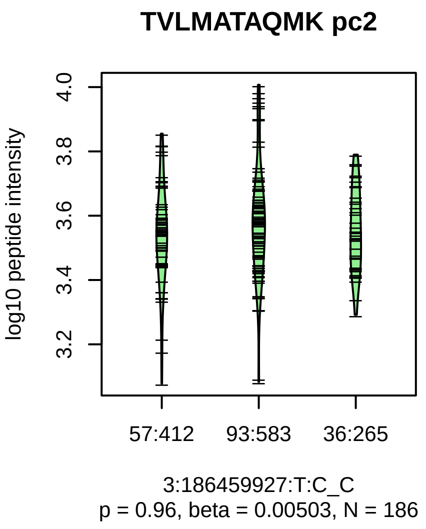

No protein data available
for replication in QMDiab

Sort: 178
Gene name: DOCK9
SNP: 3:186459927:T:C
rsID: rs710446
Type: Trans−pQTL
Protein group(s):
Q9BZ29
Nano−particle: NP3

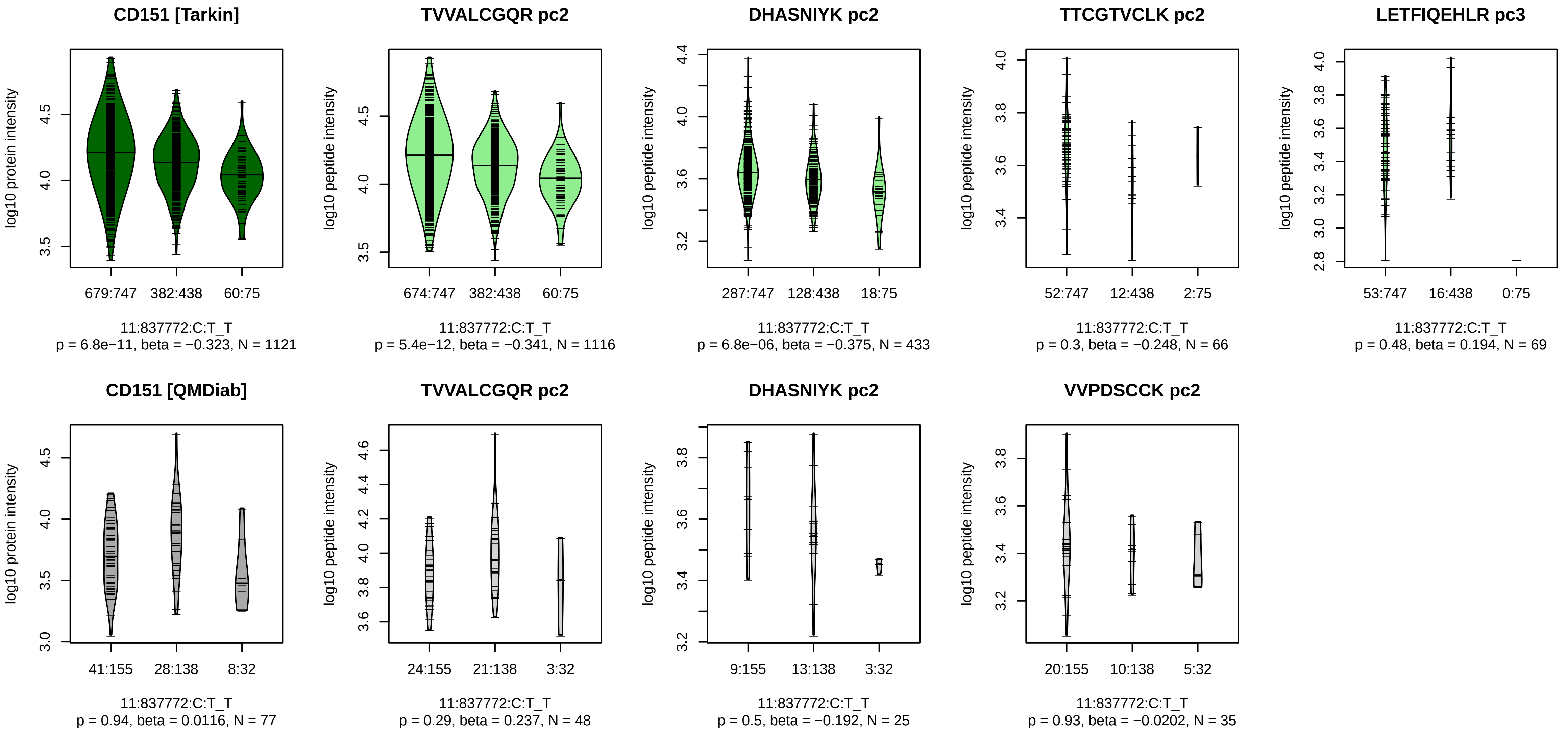

Sort: 179
Gene name: CD151
SNP: 11:837772:C:T
rsID: rs5030779
Type: Cis−pQTL
Protein group(s):
T: P48509
Q: E9PMR4;P48509
Nano−particle: NP4

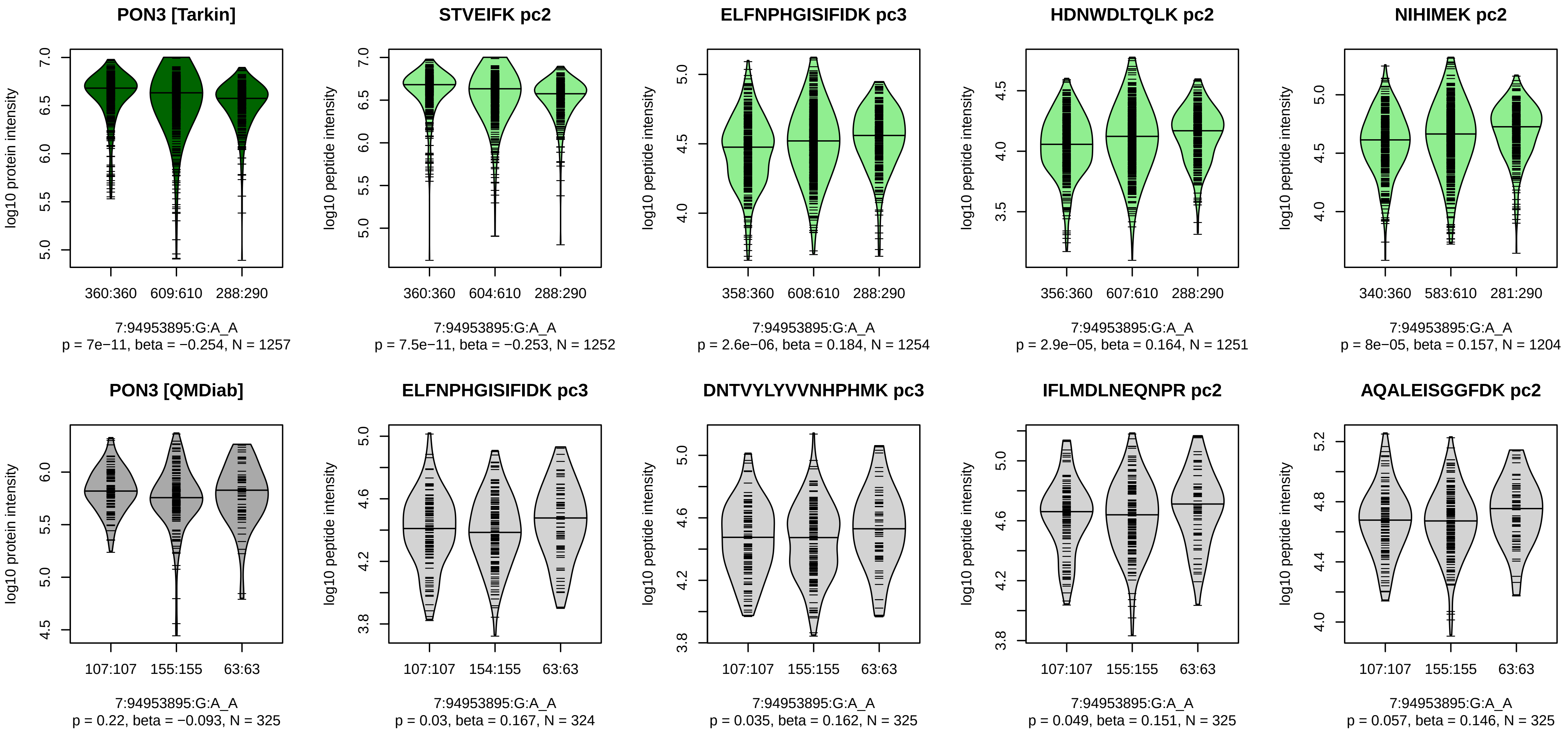

Sort: 180
Gene name: PON3
SNP: 7:94953895:G:A
rsID: rs705379
Type: Cis−pQTL
Protein group(s):
Q15166
Nano−particle: NP5

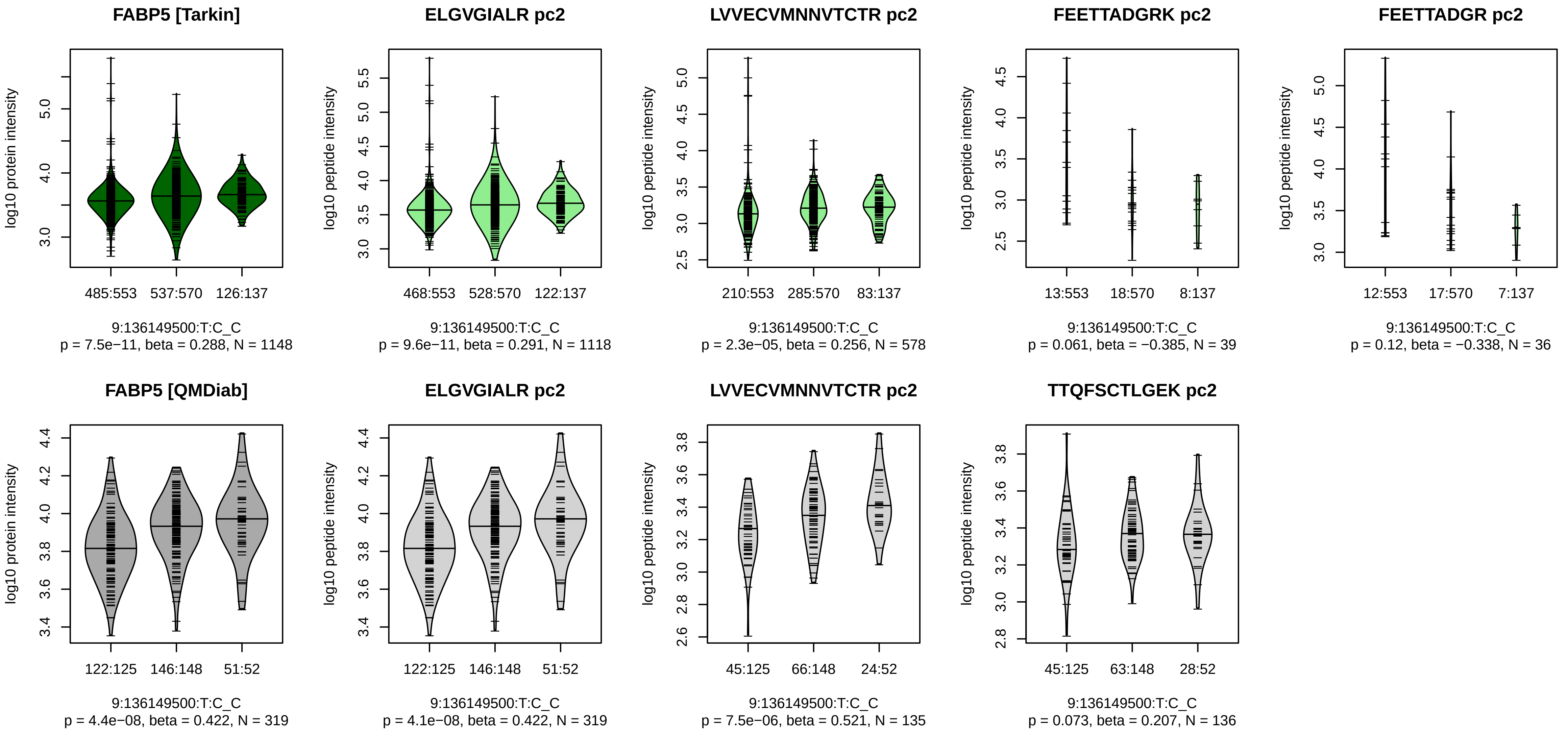

Sort: 181
Gene name: FABP5
SNP: 9:136149500:T:C
rsID: rs529565
Type: Trans−pQTL
Protein group(s):
Q01469
Nano−particle: NP2

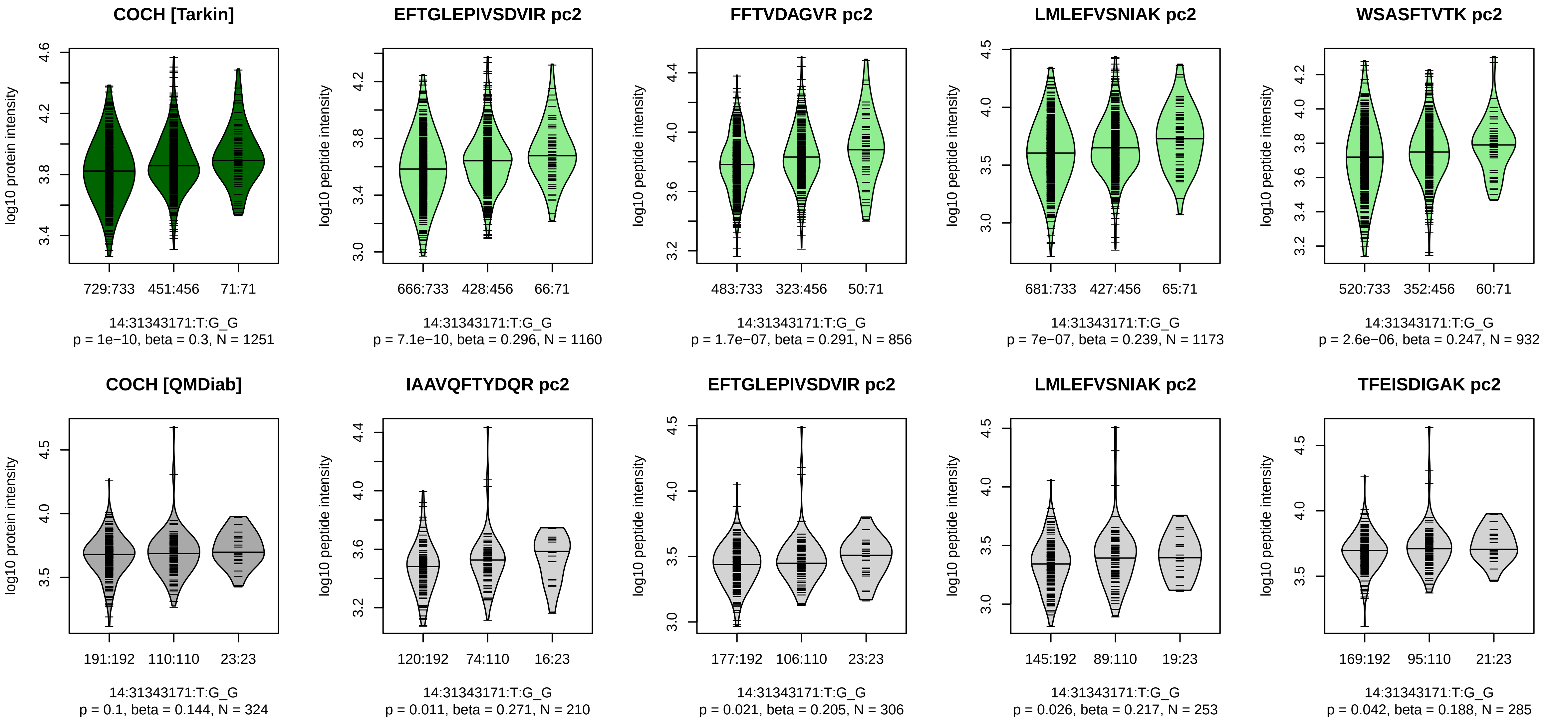

Sort: 182
Gene name: COCH
SNP: 14:31343171:T:G
rsID: rs8015095
Type: Cis−pQTL
Protein group(s):
A0A2U3TZE7;O43405
Nano−particle: NP3

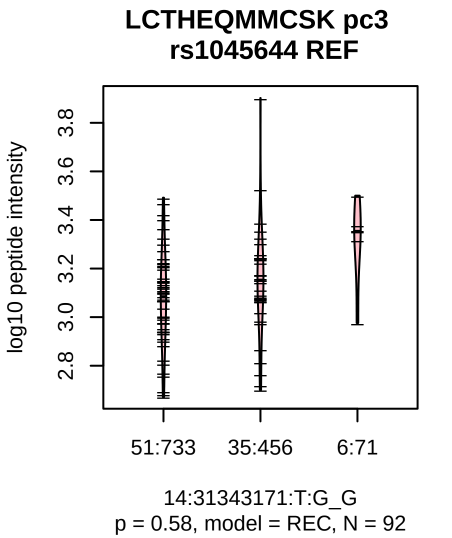

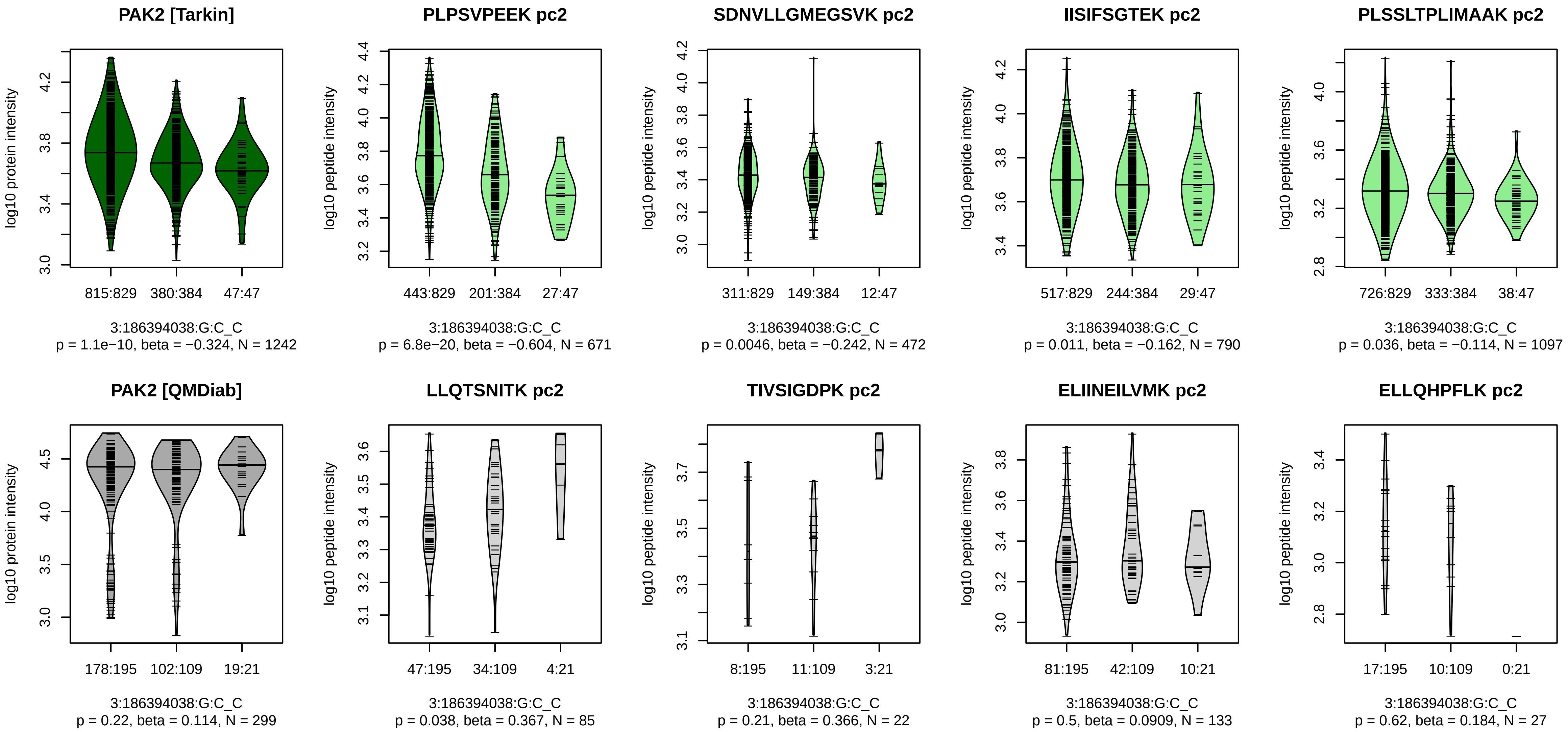

Sort: 183
Gene name: PAK2
SNP: 3:186394038:G:C
rsID: rs16860992
Type: Trans−pQTL
Protein group(s):
Q13177
Nano−particle: NP1

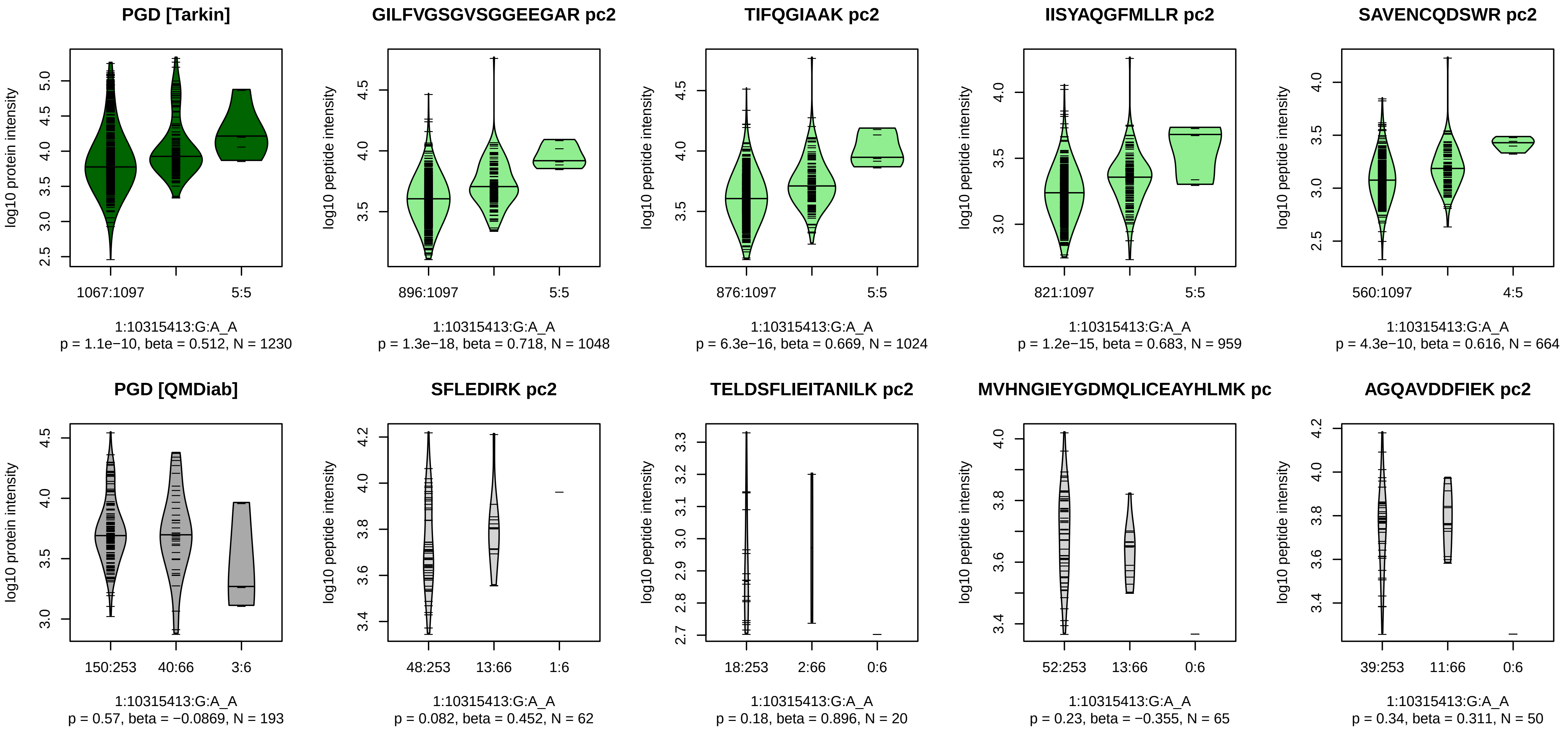

Sort: 184
Gene name: PGD
SNP: 1:10315413:G:A
rsID: rs112432872
Type: Cis−pQTL
Protein group(s):
P52209
Nano−particle: NP1

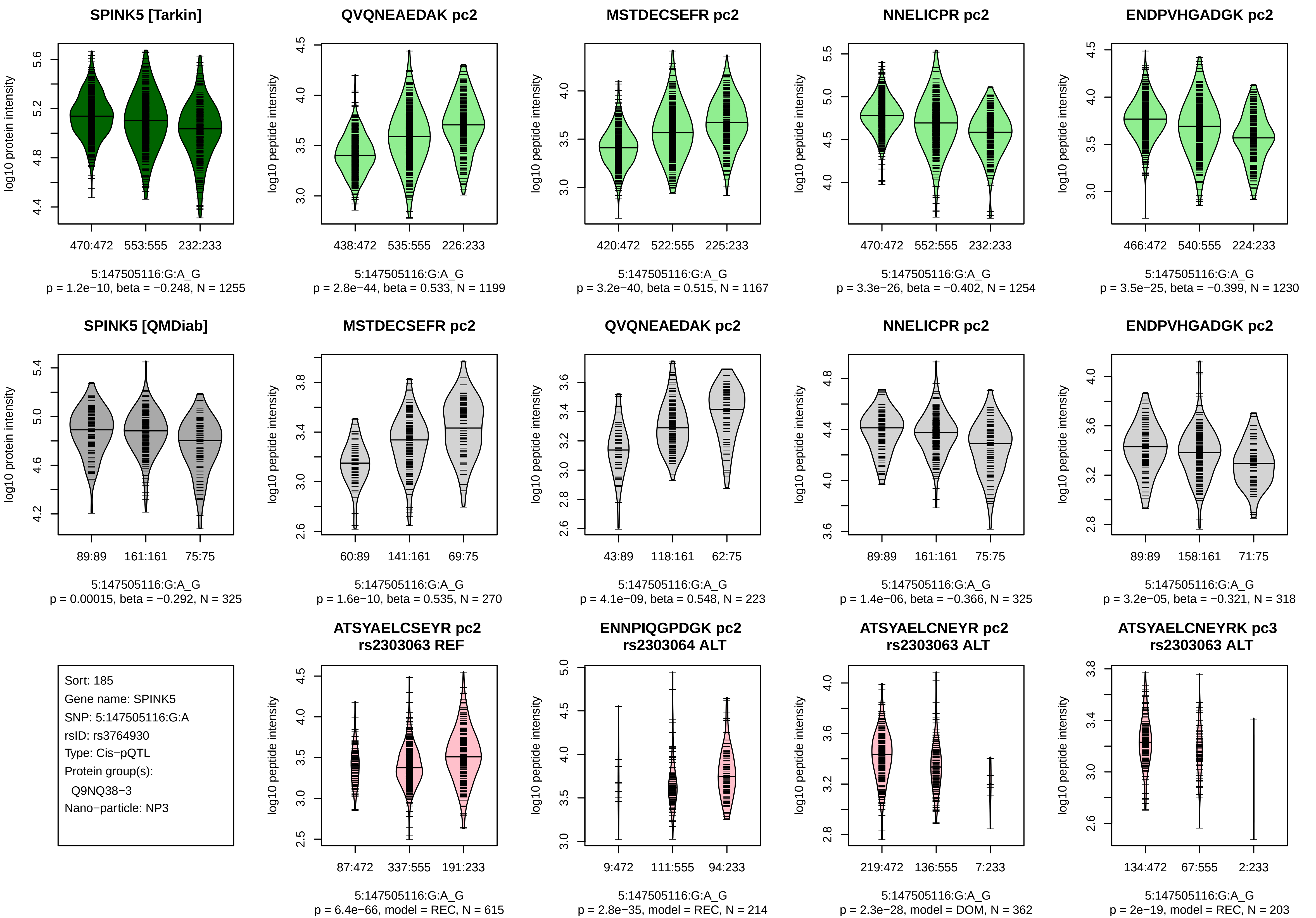
SPINK5 [Tarkin]
QVQNEAEDAK pc2
MSTDECSEFR pc2
NNELICPR pc2
ENDPVHGADGK pc2
log10 protein intensity
log10 peptide intensity
470:472 553:555 232:233
438:472 535:555 226:233
420:472 522:555 225:233
470:472 552:555 232:233
466:472 540:555 224:233
5:147505116:G:A_G
p = 1.2e−10, beta = −0.248, N = 1255
p = 2.8e−44, beta = 0.533, N = 1199
p = 3.2e−40, beta = 0.515, N = 1167
p = 3.3e−26, beta = −0.402, N = 1254
p = 3.5e−25, beta = −0.399, N = 1230
SPINK5 [QMDiab]
MSTDECSEFR pc2
QVQNEAEDAK pc2
NNELICPR pc2
ENDPVHGADGK pc2
89:89 161:161 75:75
60:89 141:161 69:75
43:89 118:161 62:75
89:89 161:161 75:75
89:89 158:161 71:75
p = 0.00015, beta = −0.292, N = 325
p = 1.6e−10, beta = 0.535, N = 270
p = 4.1e−09, beta = 0.548, N = 223
p = 1.4e−06, beta = −0.366, N = 325
p = 3.2e−05, beta = −0.321, N = 318
Sort: 185
Gene name: SPINK5
SNP: 5:147505116:G:A
rsID: rs3764930
Type: Cis−pQTL
Protein group(s):
Q9NQ38−3
Nano−particle: NP3
ATSYAELCSEYR pc2 rs2303063 REF
ENNPIQGPDGK pc2 rs2303064 ALT
ATSYAELCNEYR pc2 rs2303063 ALT
ATSYAELCNEYRK pc3 rs2303063 ALT
87:472 337:555 191:233
9:472 111:555 94:233
219:472 136:555 7:233
134:472 67:555 2:233
p = 6.4e−66, model = REC, N = 615
p = 2.8e−35, model = REC, N = 214
p = 2.3e−28, model = DOM, N = 362
p = 2e−19, model = REC, N = 203

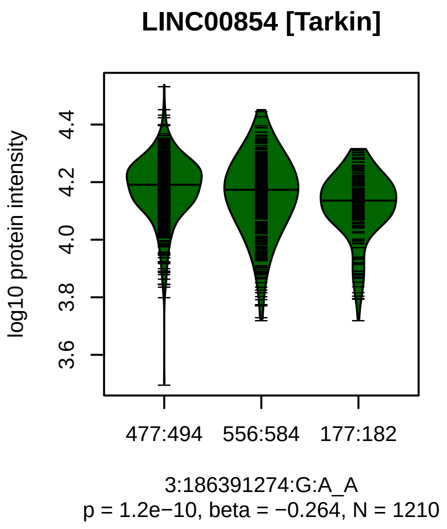

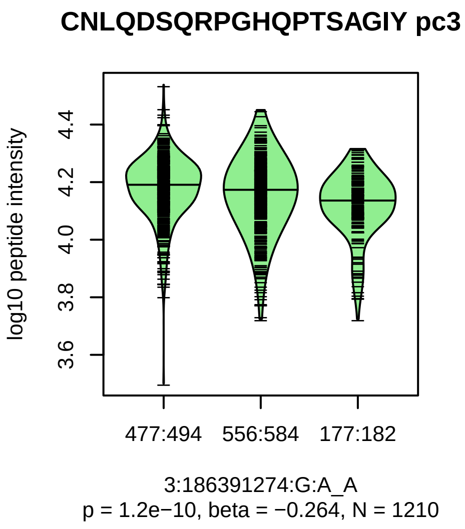

No protein data available
for replication in QMDiab

Sort: 186
Gene name: LINC00854
SNP: 3:186391274:G:A
rsID: rs59123177
Type: Trans−pQTL
Protein group(s):
A0A1B0GVX4
Nano−particle: NP5

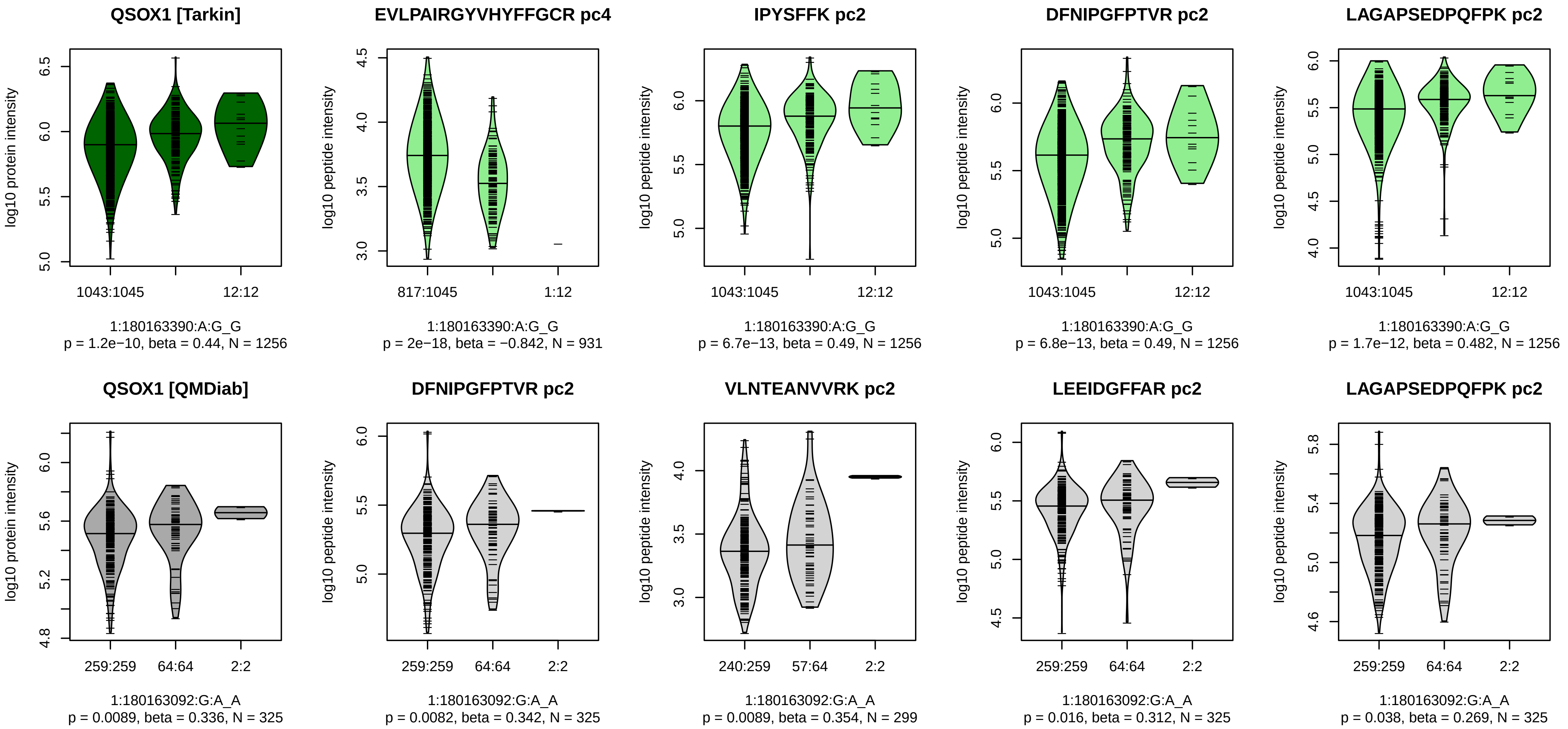

Sort: 187
Gene name: QSOX1
SNP: 1:180163390:A:G
rsID: rs12371
Type: Cis−pQTL
Protein group(s):
O00391
Nano−particle: NP1

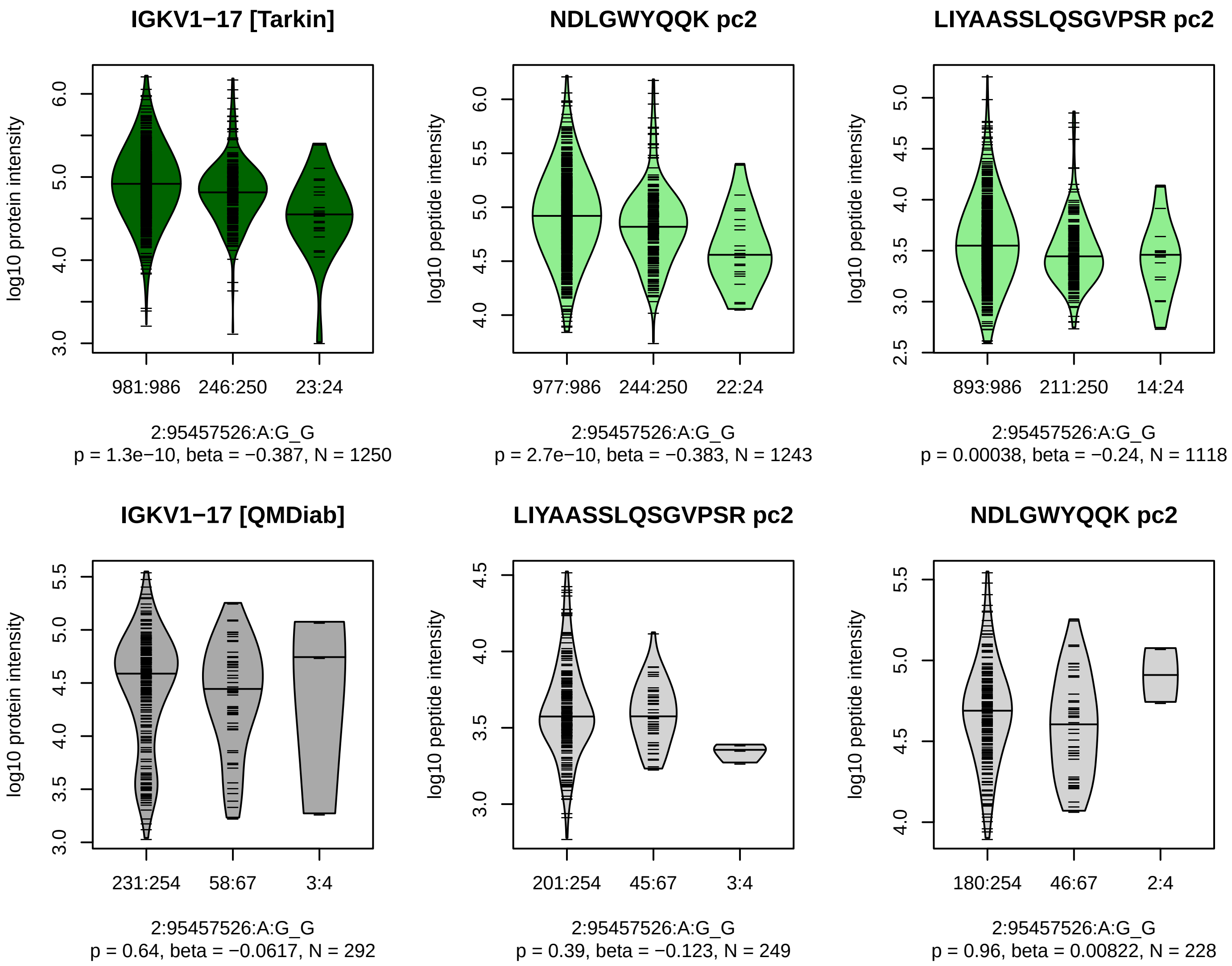

Sort: 188
Gene name: IGKV1−17
SNP: 2:95457526:A:G
rsID: rs72817642
Type: Cis−pQTL
Protein group(s):
P01599
Nano−particle: NP3

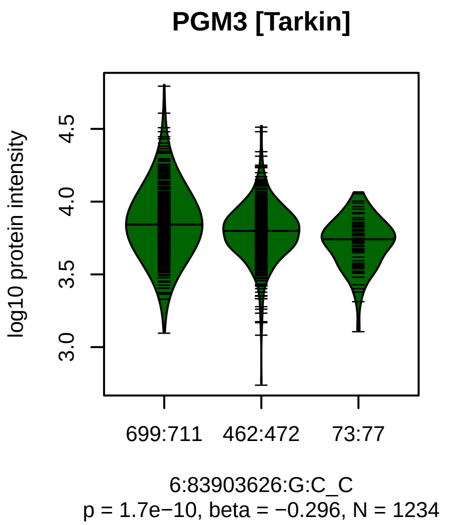

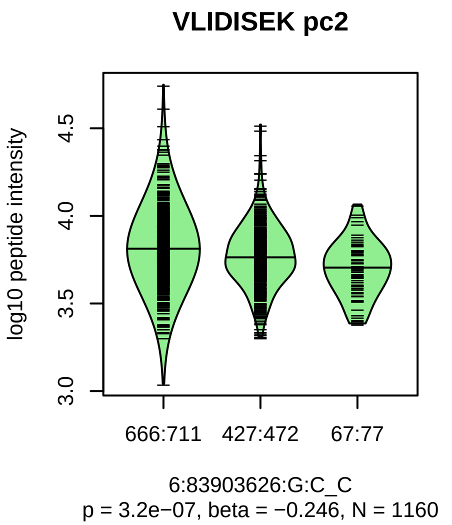

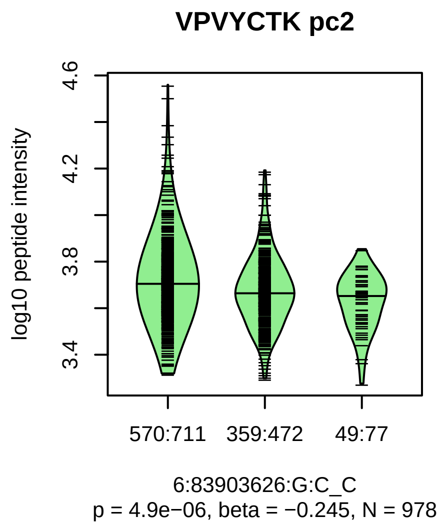

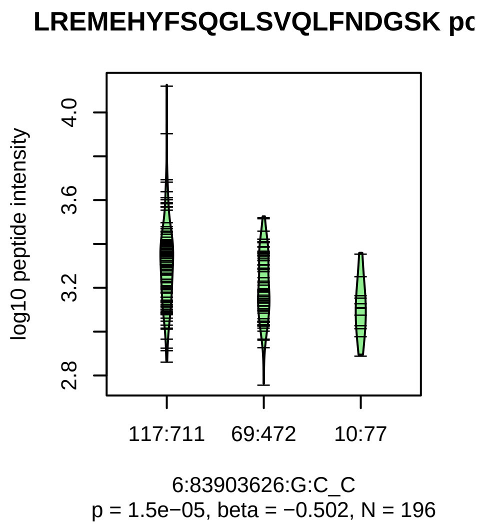

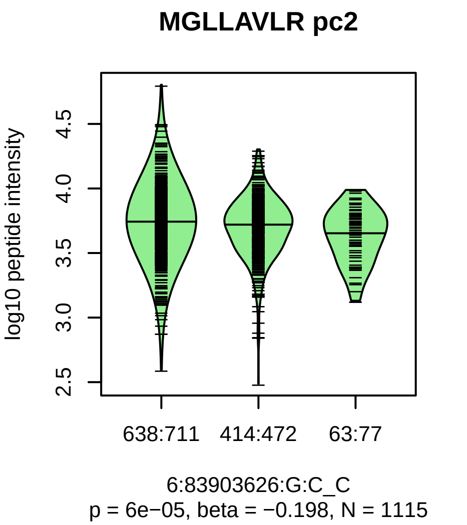

No protein data available
for replication in QMDiab

Sort: 189
Gene name: PGM3
SNP: 6:83903626:G:C
rsID: rs1180178
Type: Cis−pQTL
Protein group(s):
H0Y987
Nano−particle: NP5

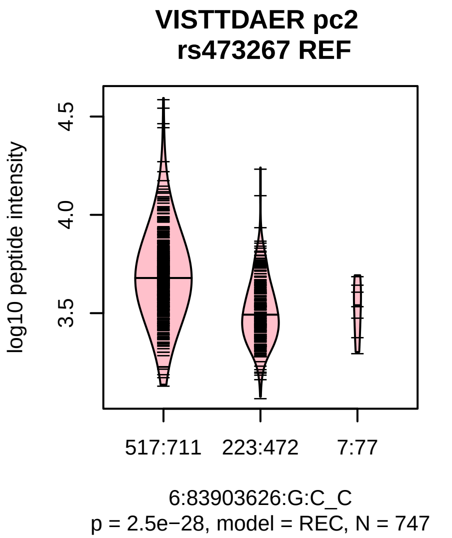

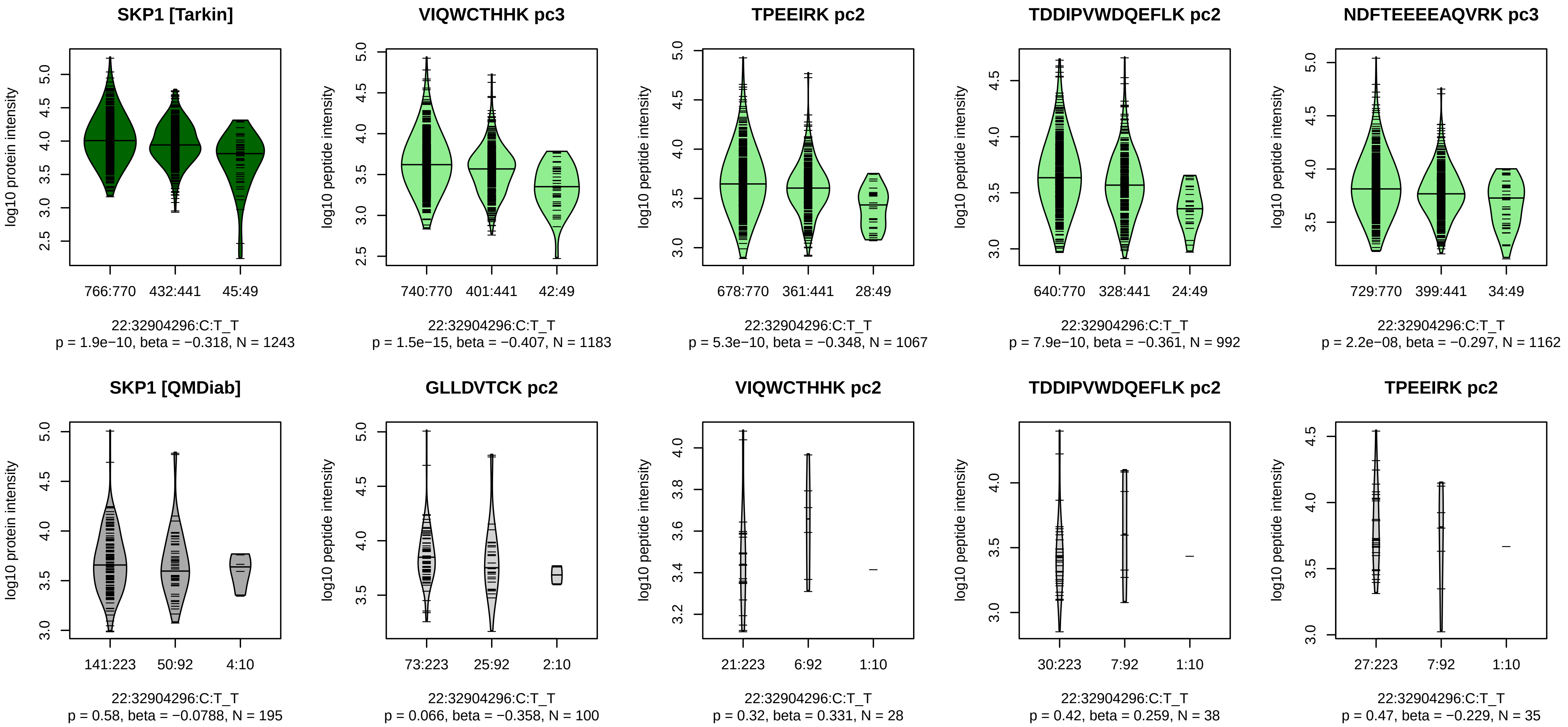

Sort: 190
Gene name: SKP1
SNP: 22:32904296:C:T
rsID: rs9609585
Type: Trans−pQTL
Protein group(s):
P63208
Nano−particle: NP1

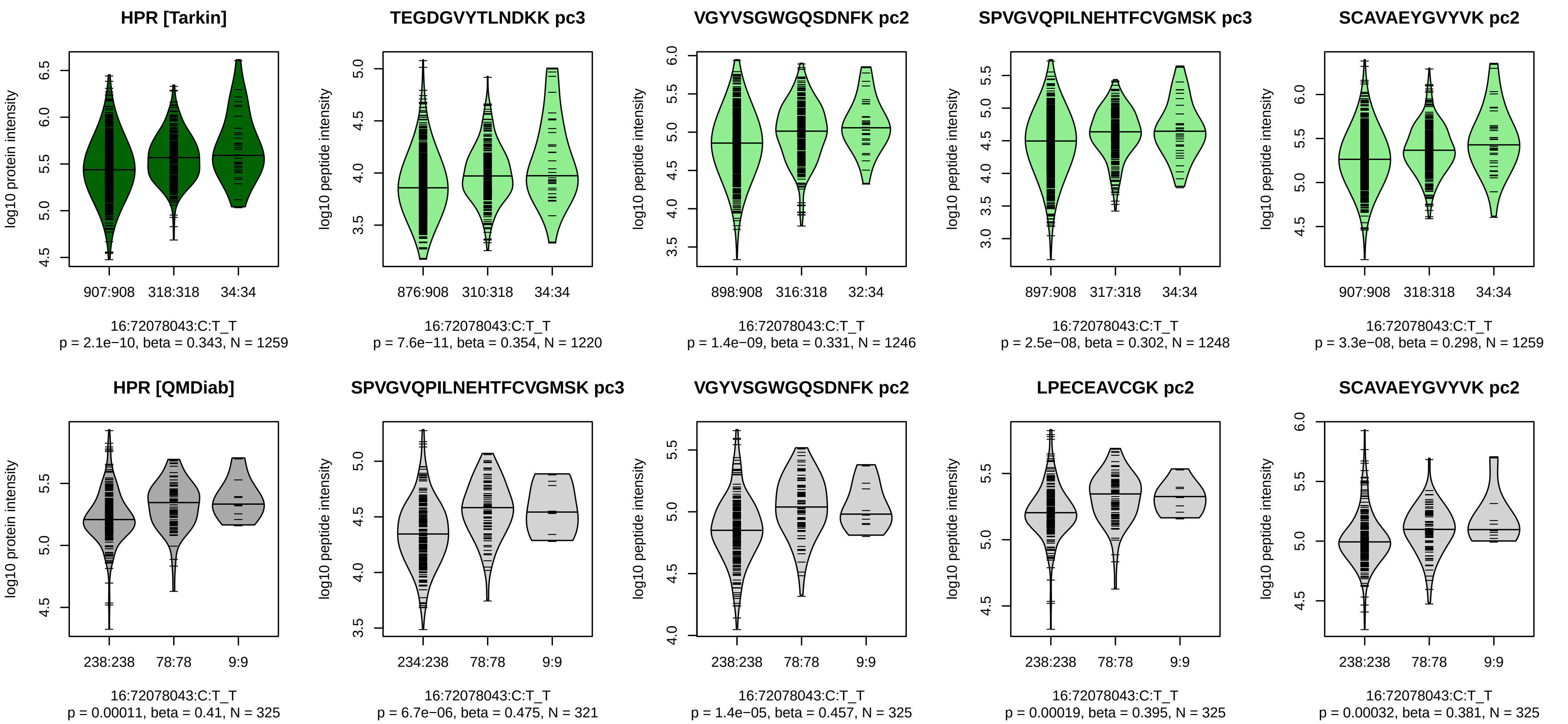

Sort: 191
Gene name: HPR
SNP: 16:72078043:C:T
rsID: rs763665
Type: Cis−pQTL
Protein group(s):
P00739
Nano−particle: NP4

IGKV1−33 [Tarkin]

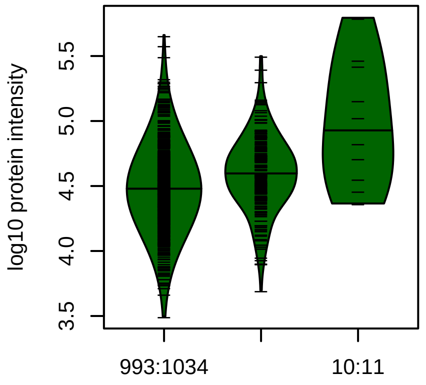

2:89508012:G:A_A
p = 2.3e−10, beta = 0.445, N = 1201

LLIYDASNLETGVPSR pc2

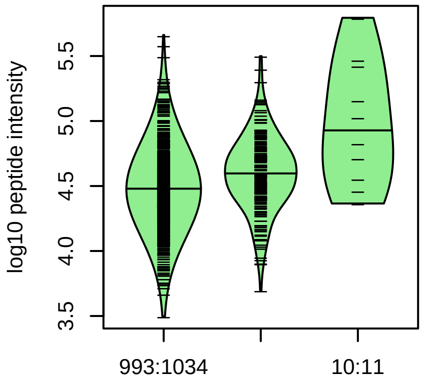

2:89508012:G:A_A
p = 2.3e−10, beta = 0.445, N = 1201

IGKV1−33 [QMDiab]

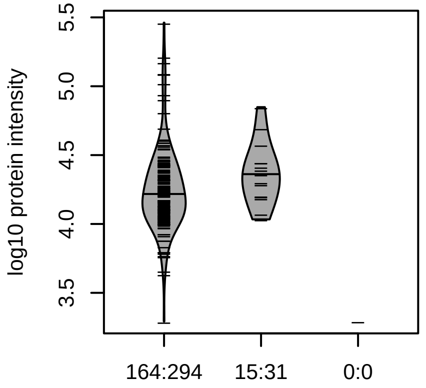

2:90247720:G:A_A
p = 0.071, beta = 0.478, N = 179

LLIYDASNLETGVPSR pc2

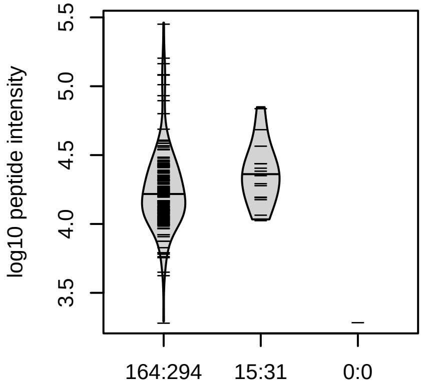

2:90247720:G:A_A
p = 0.069, beta = 0.479, N = 179

Sort: 192
Gene name: IGKV1−33
SNP: 2:89508012:G:A
rsID: rs72848969
Type: Cis−pQTL
Protein group(s):
P01594
Nano−particle: NP3

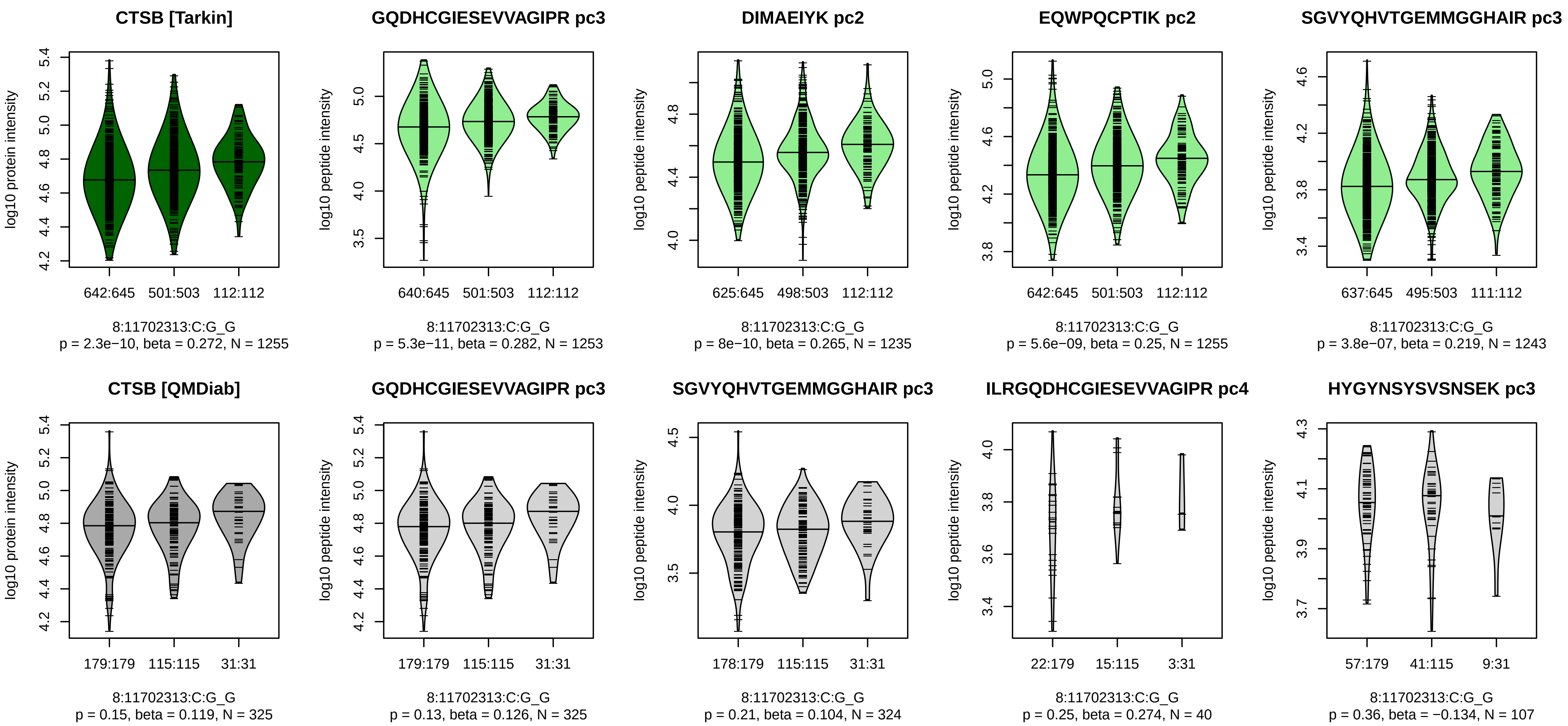

Sort: 193
Gene name: CTSB
SNP: 8:11702313:C:G
rsID: rs709822
Type: Cis−pQTL
Protein group(s):
T: E9PHZ5
Q: E9PHZ5;P07858
Nano−particle: NP2

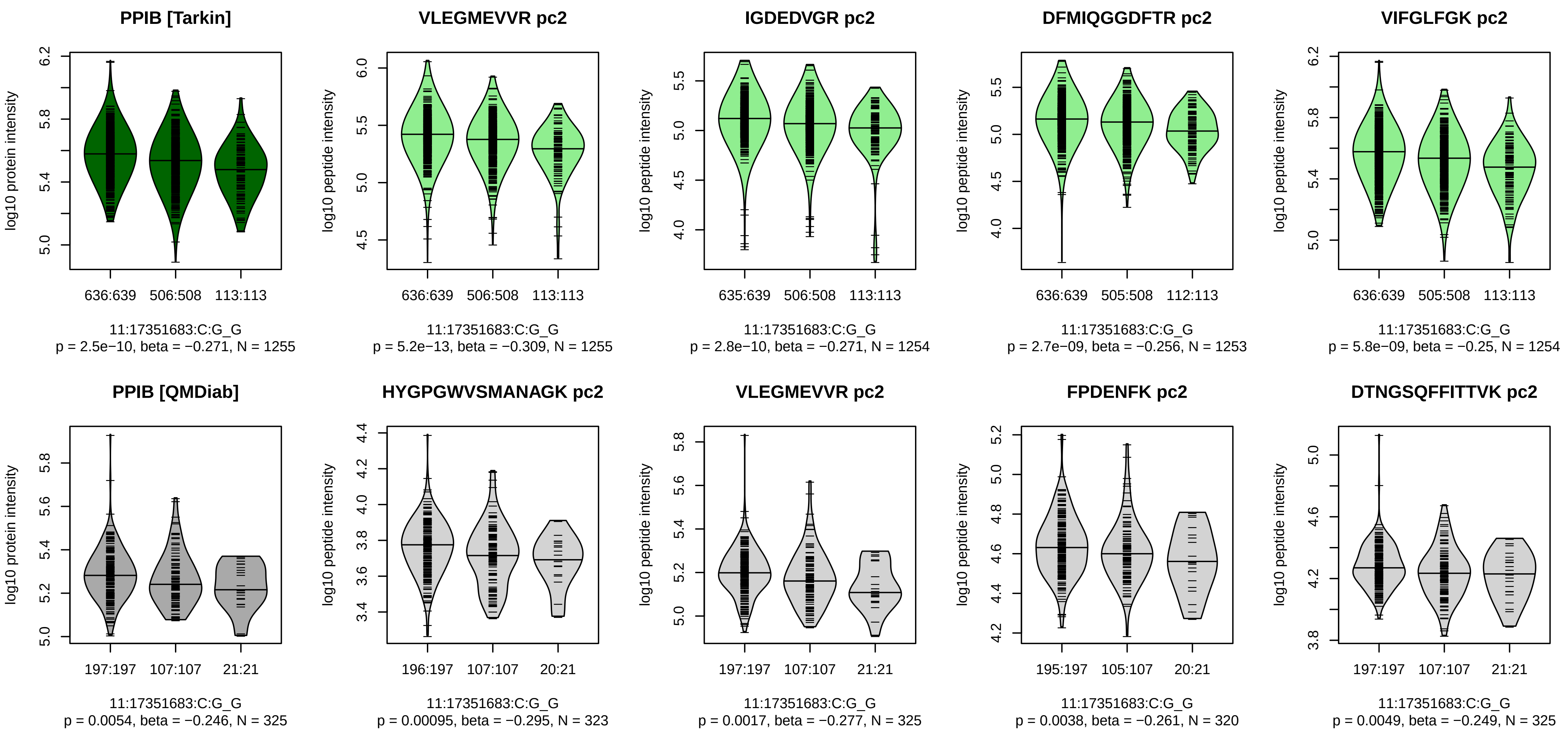

Sort: 194
Gene name: PPIB
SNP: 11:17351683:C:G
rsID: rs757081
Type: Trans−pQTL
Protein group(s):
T: A0A7P0Z497
Q: A0A7P0Z497;P23284
Nano−particle: NP3

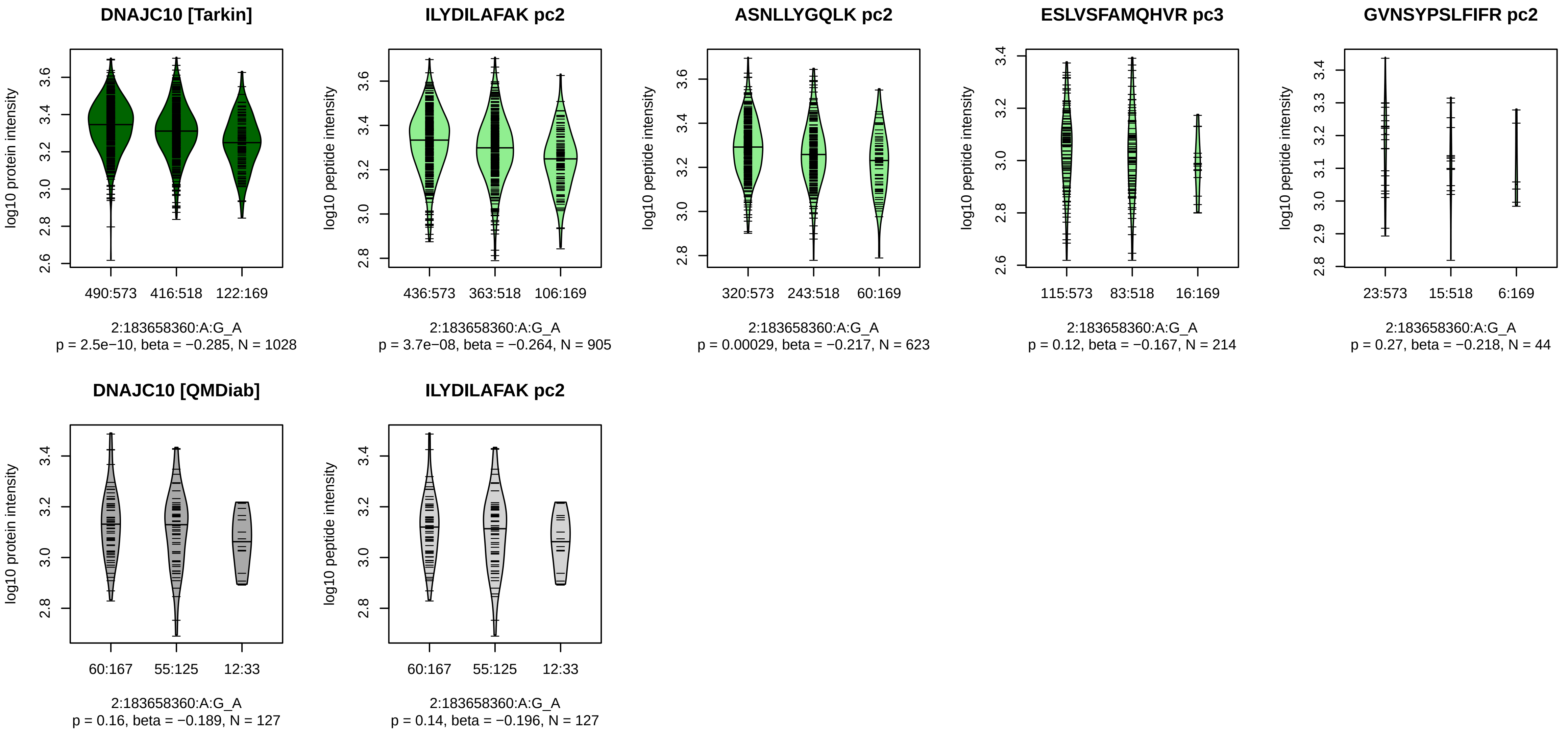

Sort: 195
Gene name: DNAJC10
SNP: 2:183658360:A:G
rsID: rs288296
Type: Cis−pQTL
Protein group(s):
T: A0A7P0Z431;Q8IXB1
Q: A0A7P0TAQ9;A0A7P0Z431;[illegible]
Nano−particle: NP3

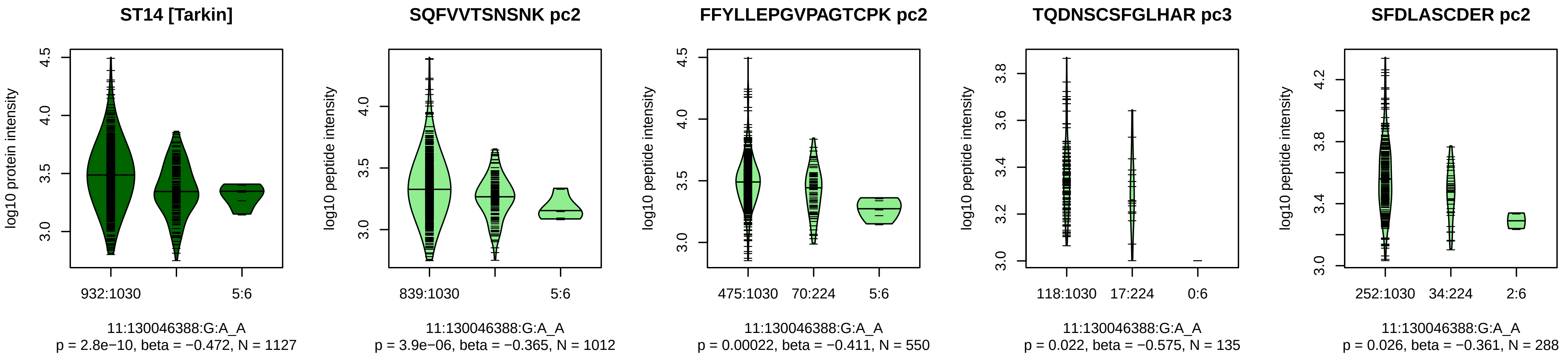

No protein data available
for replication in QMDiab

Sort: 196
Gene name: ST14
SNP: 11:130046388:G:A
rsID: rs57619715
Type: Cis−pQTL
Protein group(s):
Q9Y5Y6
Nano−particle: NP5

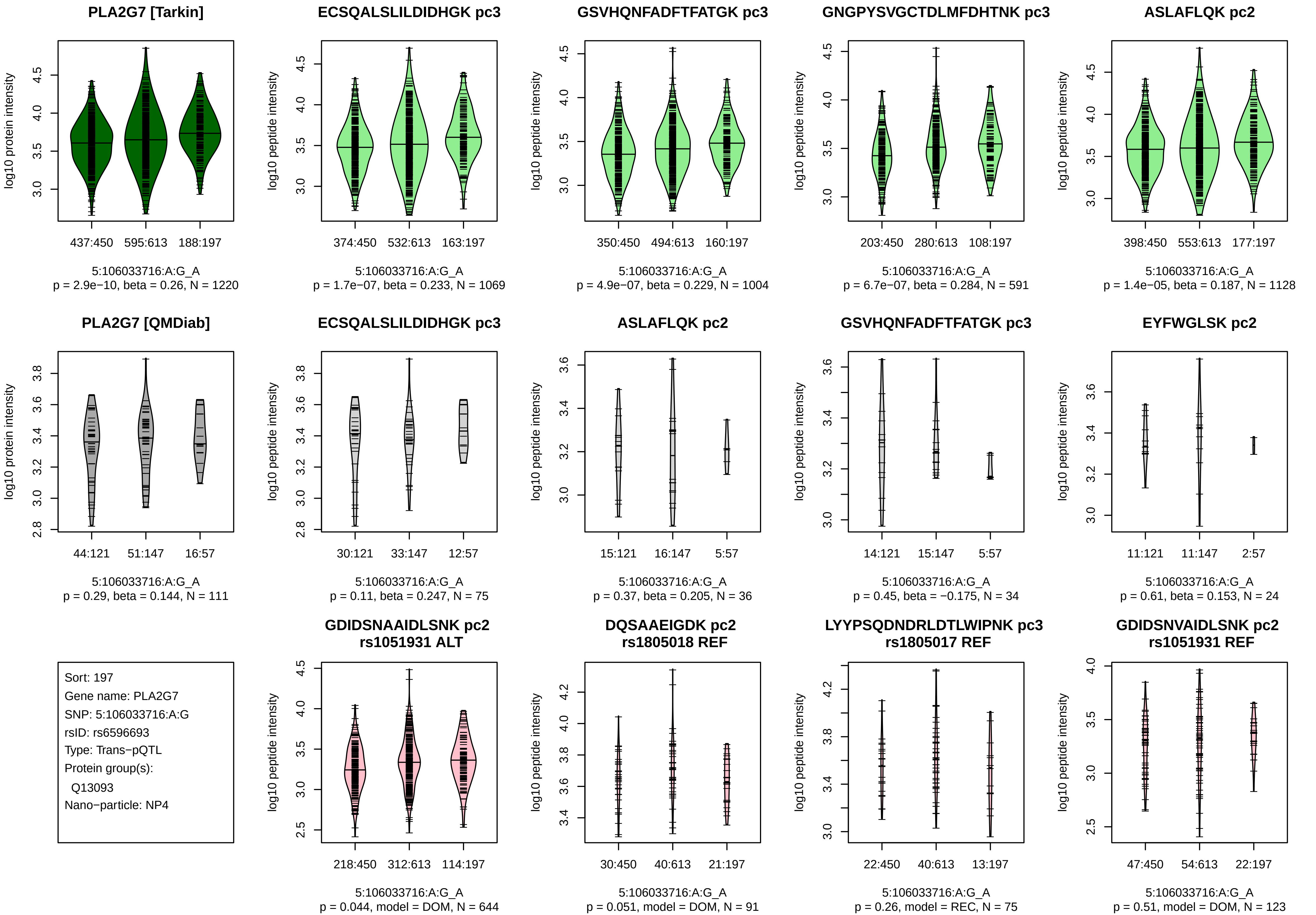
PLA2G7 [Tarkin]
log10 protein intensity
437:450
595:613
188:197
5:106033716:A:G_A
p = 2.9e−10, beta = 0.26, N = 1220
ECSQALSLILDIDHGK pc3
log10 peptide intensity
374:450
532:613
163:197
5:106033716:A:G_A
p = 1.7e−07, beta = 0.233, N = 1069
GSVHQNFADFTFATGK pc3
log10 peptide intensity
350:450
494:613
160:197
5:106033716:A:G_A
p = 4.9e−07, beta = 0.229, N = 1004
GNGPYSVGCTDLMFDHTNK pc3
log10 peptide intensity
203:450
280:613
108:197
5:106033716:A:G_A
p = 6.7e−07, beta = 0.284, N = 591
ASLAFLQK pc2
log10 peptide intensity
398:450
553:613
177:197
5:106033716:A:G_A
p = 1.4e−05, beta = 0.187, N = 1128
PLA2G7 [QMDiab]
log10 protein intensity
44:121
51:147
16:57
5:106033716:A:G_A
p = 0.29, beta = 0.144, N = 111
ECSQALSLILDIDHGK pc3
log10 peptide intensity
30:121
33:147
12:57
5:106033716:A:G_A
p = 0.11, beta = 0.247, N = 75
ASLAFLQK pc2
log10 peptide intensity
15:121
16:147
5:57
5:106033716:A:G_A
p = 0.37, beta = 0.205, N = 36
GSVHQNFADFTFATGK pc3
log10 peptide intensity
14:121
15:147
5:57
5:106033716:A:G_A
p = 0.45, beta = −0.175, N = 34
EYFWGLSK pc2
log10 peptide intensity
11:121
11:147
2:57
5:106033716:A:G_A
p = 0.61, beta = 0.153, N = 24
Sort: 197
Gene name: PLA2G7
SNP: 5:106033716:A:G
rsID: rs6596693
Type: Trans−pQTL
Protein group(s):
Q13093
Nano−particle: NP4
GDIDSNAAIDLSNK pc2
rs1051931 ALT
log10 peptide intensity
218:450
312:613
114:197
5:106033716:A:G_A
p = 0.044, model = DOM, N = 644
DQSAAEIGDK pc2
rs1805018 REF
log10 peptide intensity
30:450
40:613
21:197
5:106033716:A:G_A
p = 0.051, model = DOM, N = 91
LYYPSQDNDRLDTLWIPNK pc3
rs1805017 REF
log10 peptide intensity
22:450
40:613
13:197
5:106033716:A:G_A
p = 0.26, model = REC, N = 75
GDIDSNVAIDLSNK pc2
rs1051931 REF
log10 peptide intensity
47:450
54:613
22:197
5:106033716:A:G_A
p = 0.51, model = DOM, N = 123

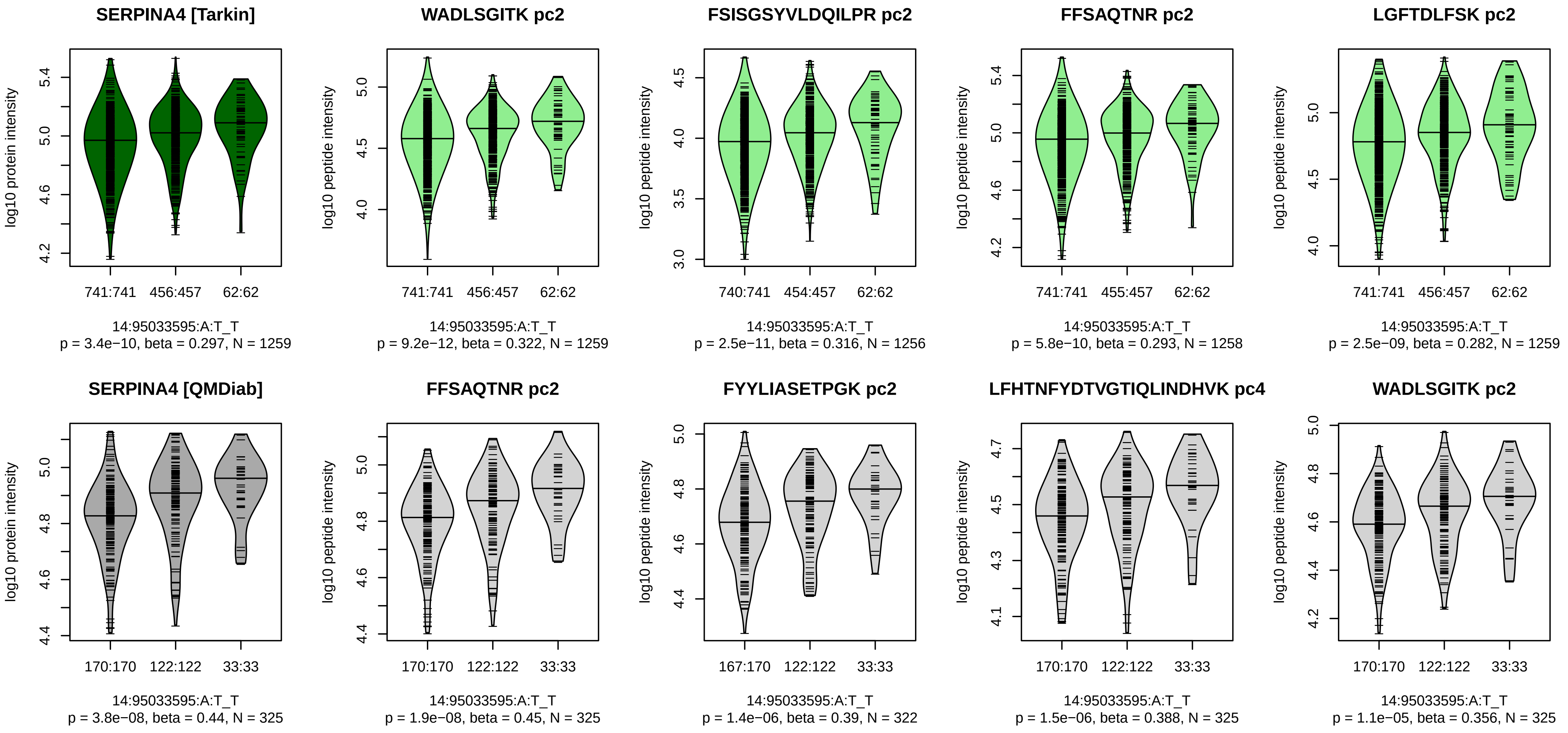

Sort: 198
Gene name: SERPINA4
SNP: 14:95033595:A:T
rsID: rs5511
Type: Cis−pQTL
Protein group(s):
P29622
Nano−particle: NP4

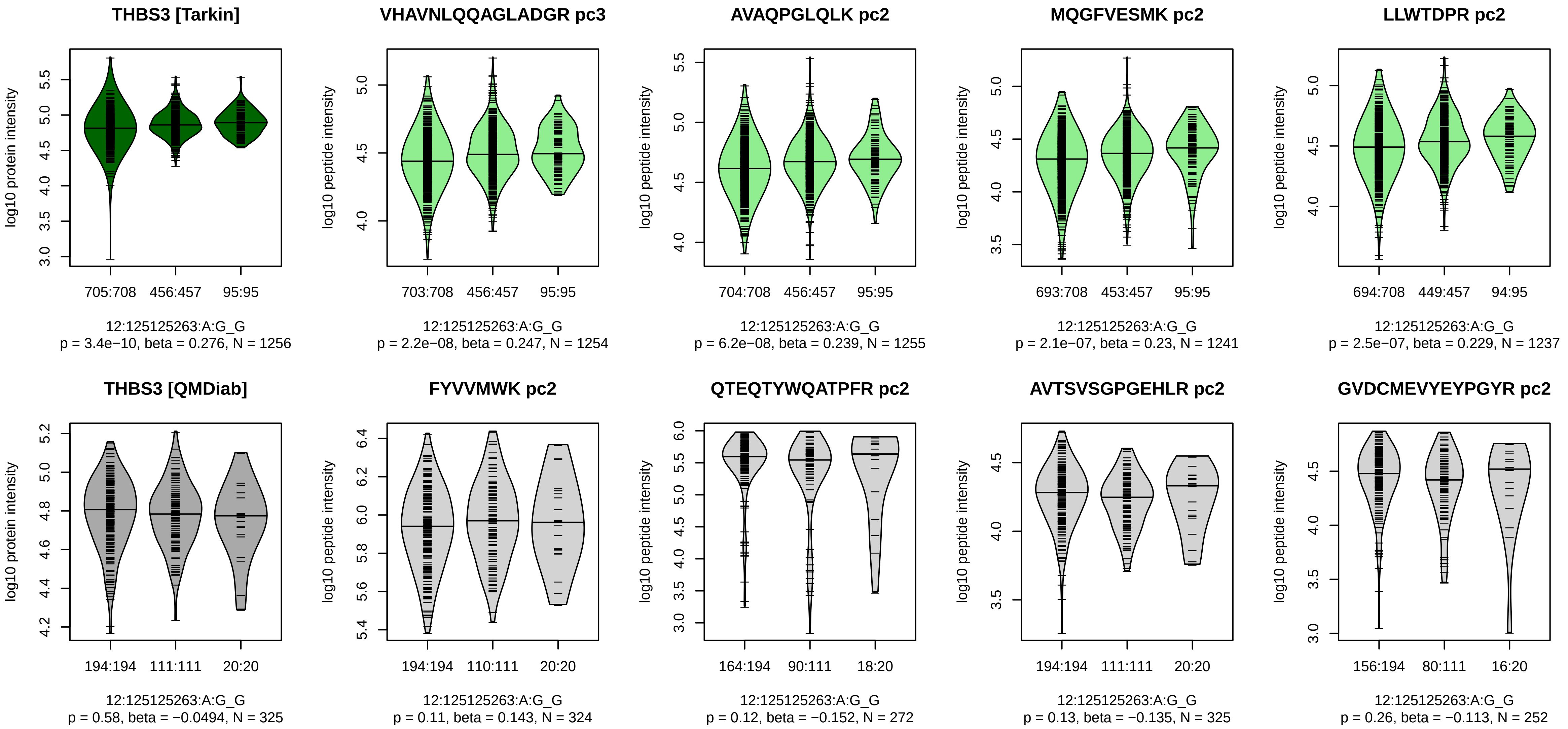

Sort: 199
Gene name: THBS3
SNP: 12:125125263:A:G
rsID: rs7952970
Type: Trans−pQTL
Protein group(s):
F5H4Z8;P49746
Nano−particle: NP2

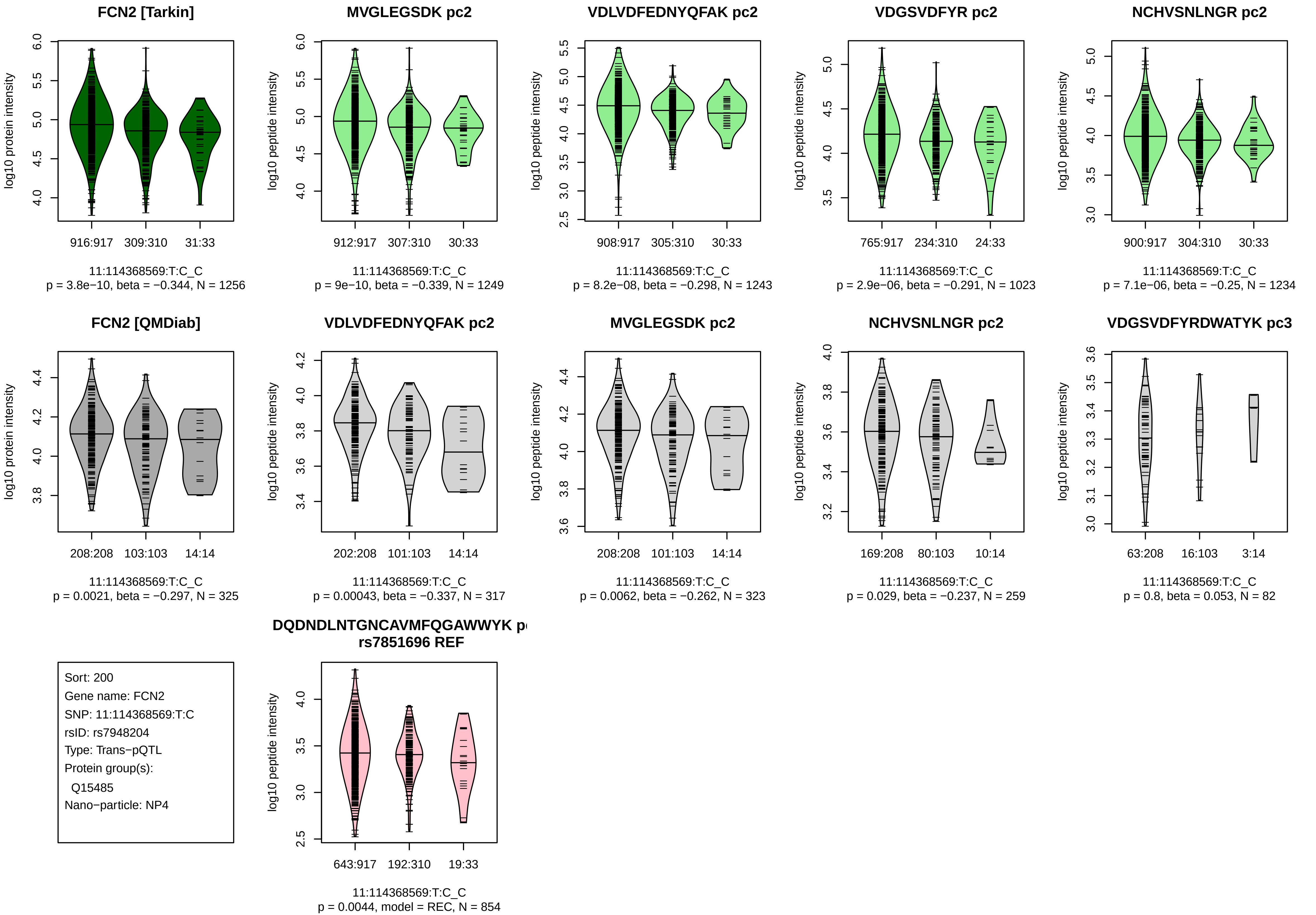
FCN2 [Tarkin]
log10 protein intensity
916:917 309:310 31:33
11:114368569:T:C_C
p = 3.8e−10, beta = −0.344, N = 1256
MVGLEGSDK pc2
log10 peptide intensity
912:917 307:310 30:33
11:114368569:T:C_C
p = 9e−10, beta = −0.339, N = 1249
VDLVDFEDNYQFAK pc2
log10 peptide intensity
908:917 305:310 30:33
11:114368569:T:C_C
p = 8.2e−08, beta = −0.298, N = 1243
VDGSVDFYR pc2
log10 peptide intensity
765:917 234:310 24:33
11:114368569:T:C_C
p = 2.9e−06, beta = −0.291, N = 1023
NCHVSNLNGR pc2
log10 peptide intensity
900:917 304:310 30:33
11:114368569:T:C_C
p = 7.1e−06, beta = −0.25, N = 1234
FCN2 [QMDiab]
log10 protein intensity
208:208 103:103 14:14
11:114368569:T:C_C
p = 0.0021, beta = −0.297, N = 325
VDLVDFEDNYQFAK pc2
log10 peptide intensity
202:208 101:103 14:14
11:114368569:T:C_C
p = 0.00043, beta = −0.337, N = 317
MVGLEGSDK pc2
log10 peptide intensity
208:208 101:103 14:14
11:114368569:T:C_C
p = 0.0062, beta = −0.262, N = 323
NCHVSNLNGR pc2
log10 peptide intensity
169:208 80:103 10:14
11:114368569:T:C_C
p = 0.029, beta = −0.237, N = 259
VDGSVDFYRDWATYK pc3
log10 peptide intensity
63:208 16:103 3:14
11:114368569:T:C_C
p = 0.8, beta = 0.053, N = 82
Sort: 200
Gene name: FCN2
SNP: 11:114368569:T:C
rsID: rs7948204
Type: Trans−pQTL
Protein group(s):
Q15485
Nano−particle: NP4
DQDNDLNTGNCAVMFQGAWWYK p
rs7851696 REF
log10 peptide intensity
643:917 192:310 19:33
11:114368569:T:C_C
p = 0.0044, model = REC, N = 854

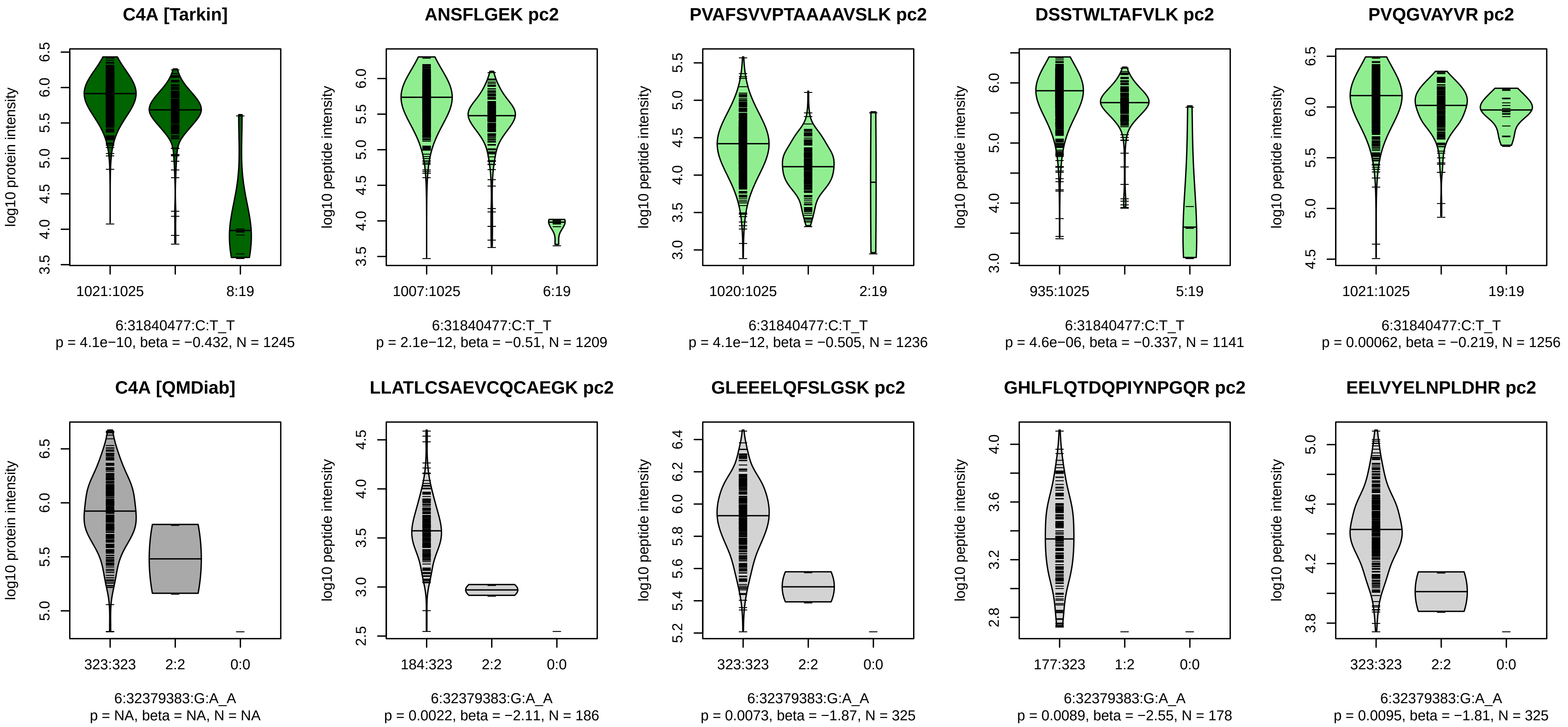

Sort: 201
Gene name: C4A
SNP: 6:31840477:C:T
rsID: rs501942
Type: Trans−pQTL
Protein group(s):
P0C0L4;P0C0L4−2
Nano−particle: NP2

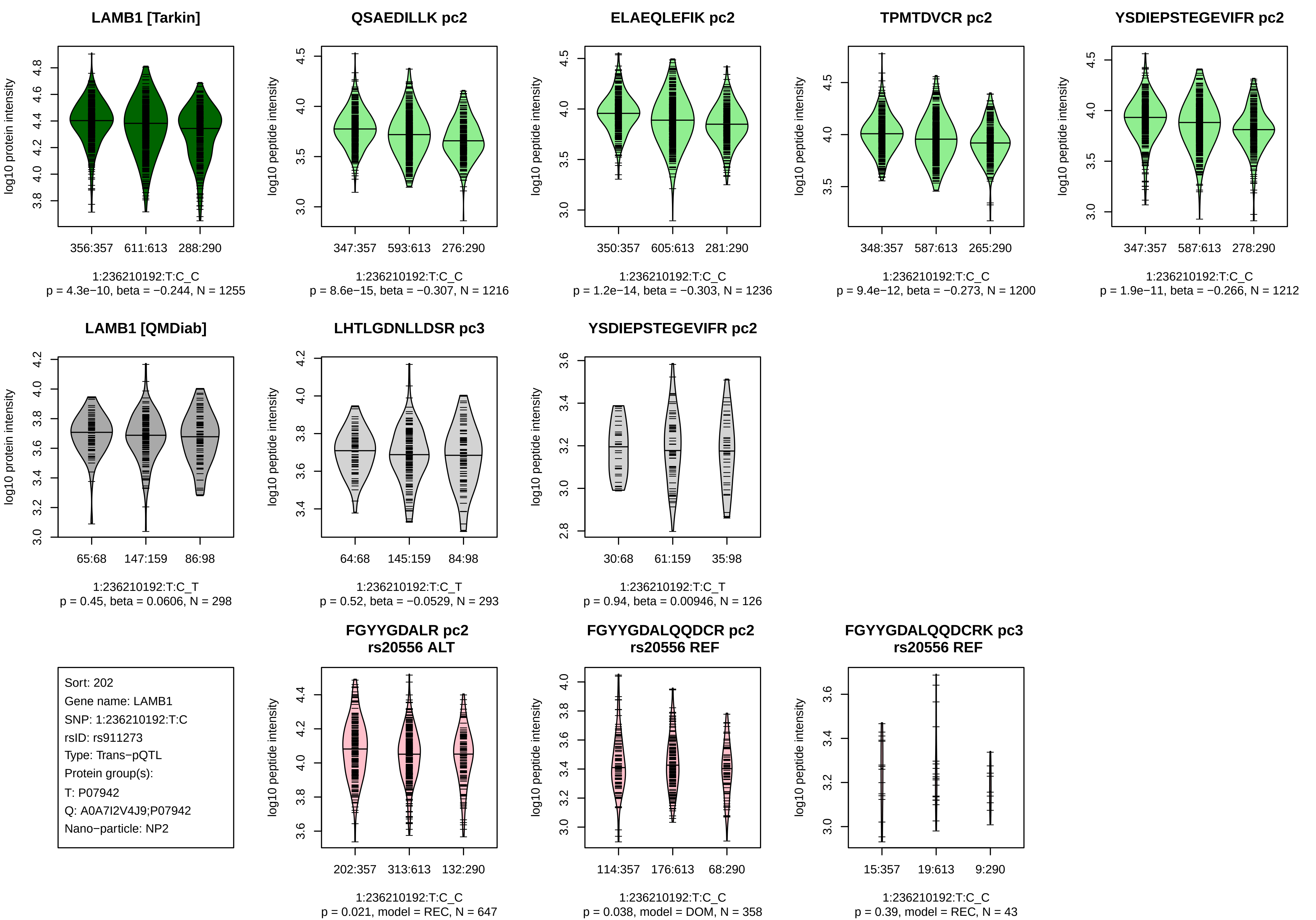

LAMB1 [Tarkin]
log10 protein intensity
356:357
611:613
288:290
1:236210192:T:C_C
p = 4.3e−10, beta = −0.244, N = 1255
QSAEDILLK pc2
log10 peptide intensity
347:357
593:613
276:290
1:236210192:T:C_C
p = 8.6e−15, beta = −0.307, N = 1216
ELAEQLEFIK pc2
log10 peptide intensity
350:357
605:613
281:290
1:236210192:T:C_C
p = 1.2e−14, beta = −0.303, N = 1236
TPMTDVCR pc2
log10 peptide intensity
348:357
587:613
265:290
1:236210192:T:C_C
p = 9.4e−12, beta = −0.273, N = 1200
YSDIEPSTEGEVIFR pc2
log10 peptide intensity
347:357
587:613
278:290
1:236210192:T:C_C
p = 1.9e−11, beta = −0.266, N = 1212
LAMB1 [QMDiab]
log10 protein intensity
65:68
147:159
86:98
1:236210192:T:C_T
p = 0.45, beta = 0.0606, N = 298
LHTLGDNLLDSR pc3
log10 peptide intensity
64:68
145:159
84:98
1:236210192:T:C_T
p = 0.52, beta = −0.0529, N = 293
YSDIEPSTEGEVIFR pc2
log10 peptide intensity
30:68
61:159
35:98
1:236210192:T:C_T
p = 0.94, beta = 0.00946, N = 126
Sort: 202
Gene name: LAMB1
SNP: 1:236210192:T:C
rsID: rs911273
Type: Trans−pQTL
Protein group(s):
T: P07942
Q: A0A7I2V4J9;P07942
Nano−particle: NP2
FGYYGDALR pc2
rs20556 ALT
log10 peptide intensity
202:357
313:613
132:290
1:236210192:T:C_C
p = 0.021, model = REC, N = 647
FGYYGDALQQDCR pc2
rs20556 REF
log10 peptide intensity
114:357
176:613
68:290
1:236210192:T:C_C
p = 0.038, model = DOM, N = 358
FGYYGDALQQDCRK pc3
rs20556 REF
log10 peptide intensity
15:357
19:613
9:290
1:236210192:T:C_C
p = 0.39, model = REC, N = 43

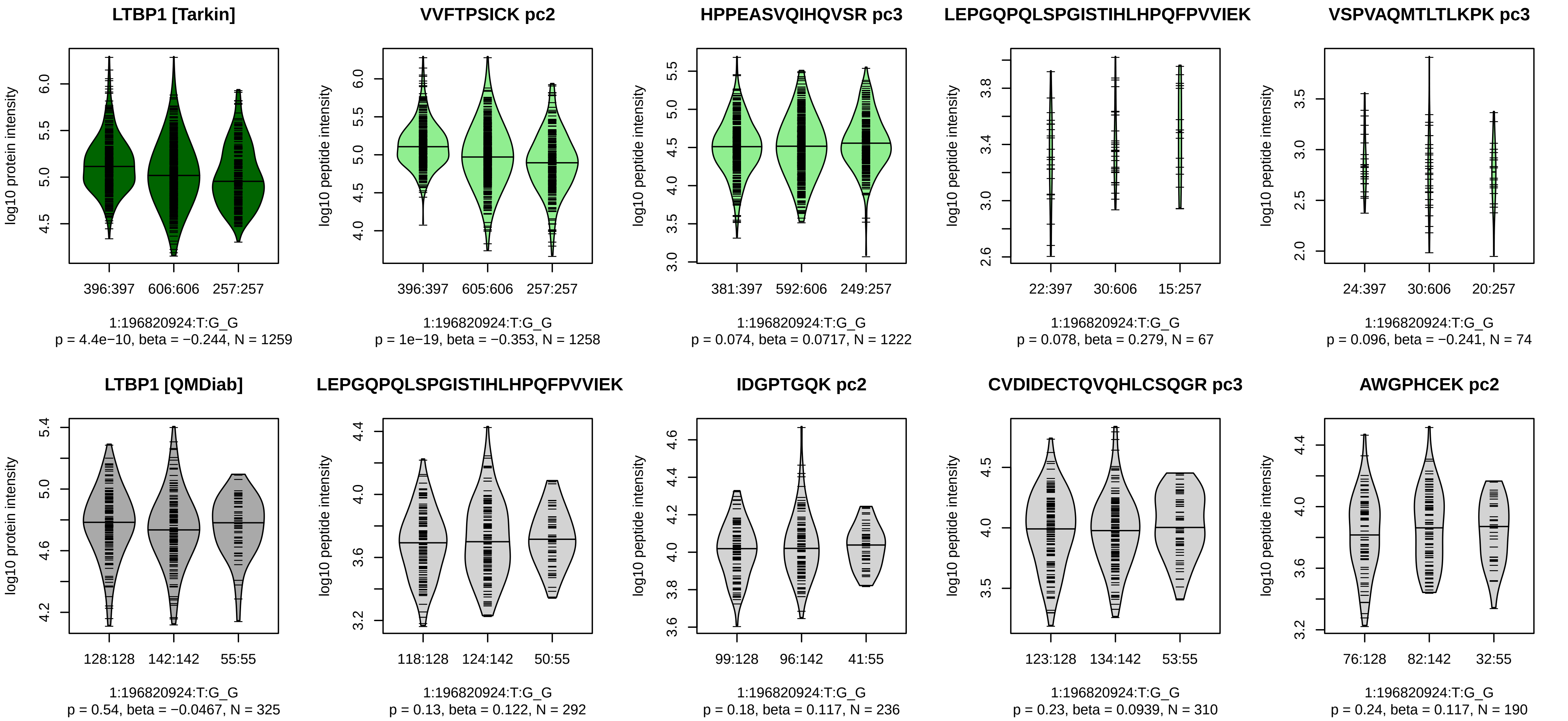

Sort: 203
Gene name: LTBP1
SNP: 1:196820924:T:G
rsID: rs10754206
Type: Trans−pQTL
Protein group(s):
Q14766
Nano−particle: NP4

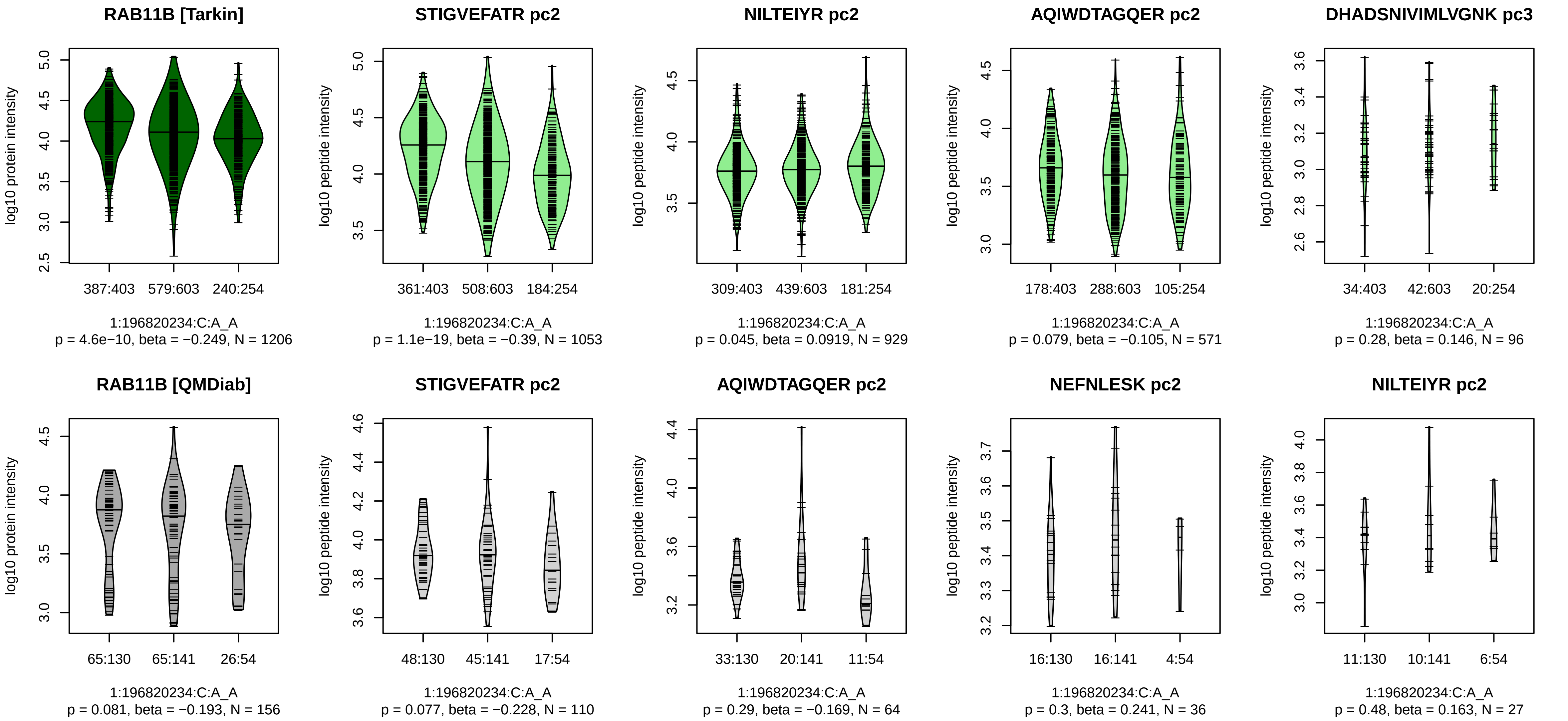

Sort: 204
Gene name: RAB11B
SNP: 1:196820234:C:A
rsID: rs6665824
Type: Trans−pQTL
Protein group(s):
Q15907
Nano−particle: NP4

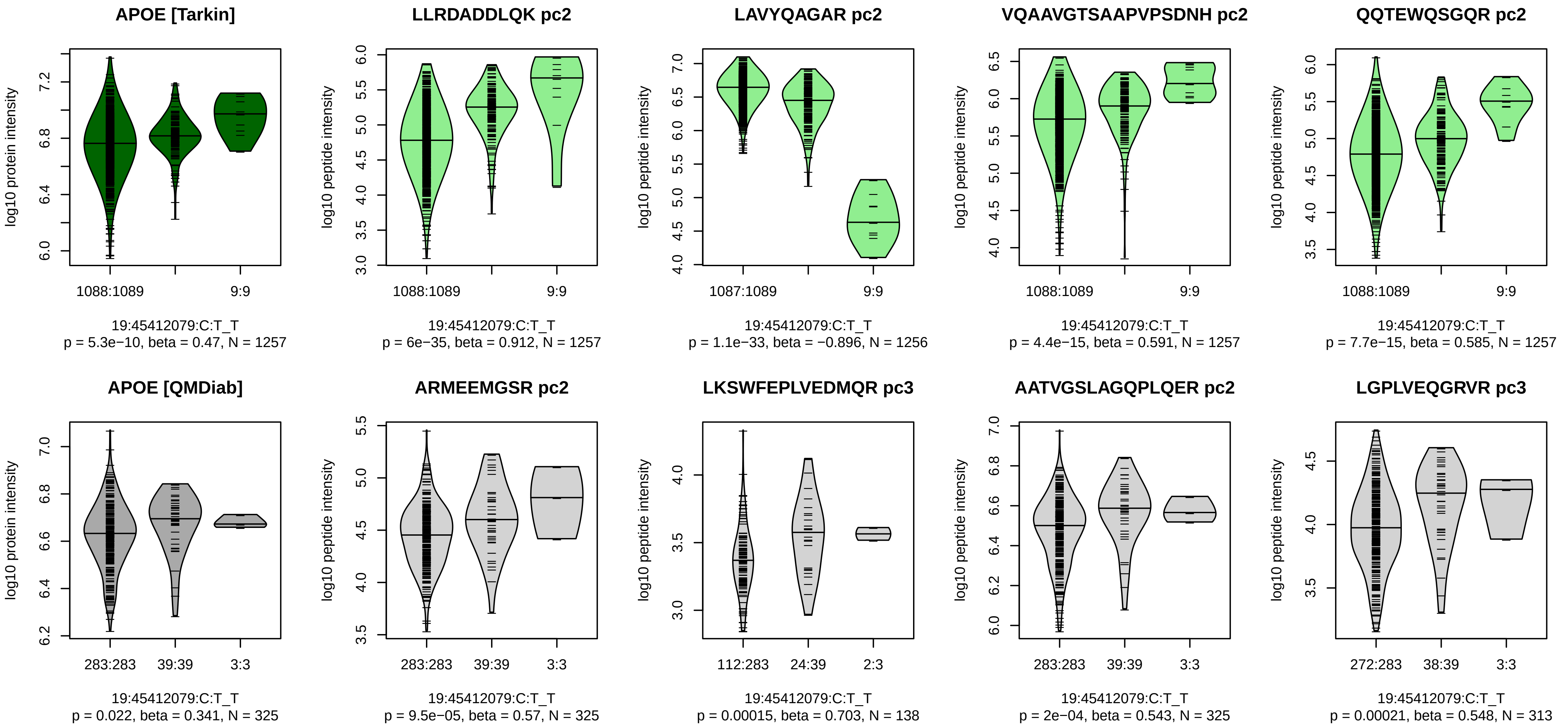

Sort: 205
Gene name: APOE
SNP: 19:45412079:C:T
rsID: rs7412
Type: Cis−pQTL
Protein group(s):
P02649
Nano−particle: NP5

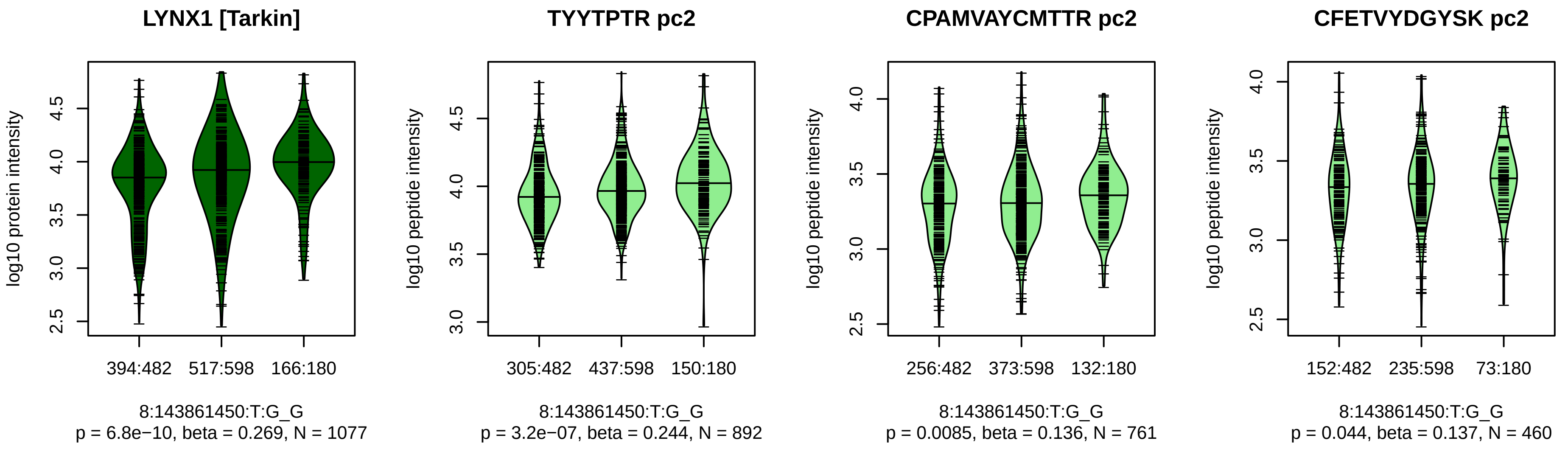

Sort: 206
Gene name: LYNX1
SNP: 8:143861450:T:G
rsID: rs55818528
Type: Cis−pQTL
Protein group(s):
T: P0DP58
Q: P0DP58−2
Nano−particle: NP3

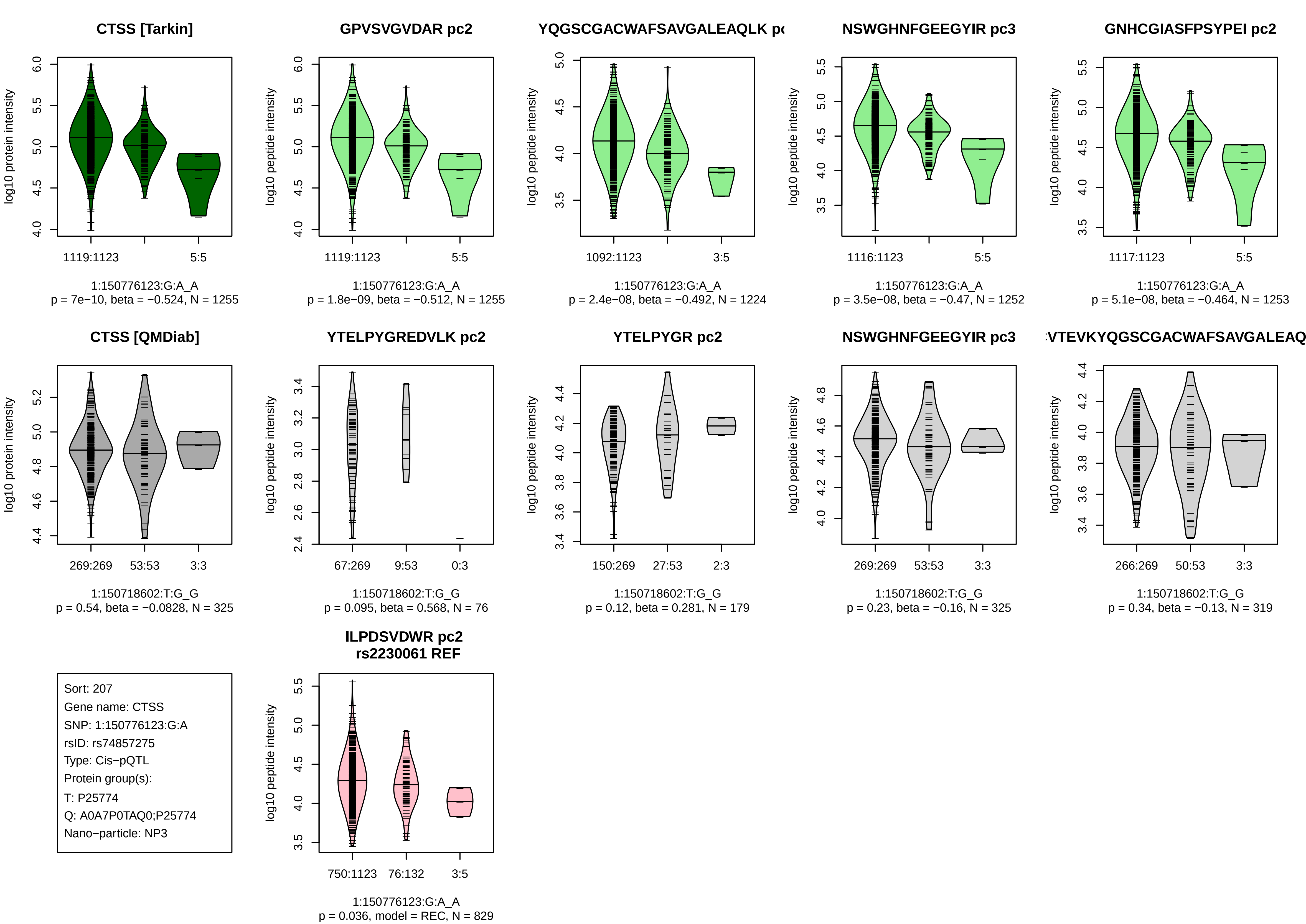
CTSS [Tarkin]
log10 protein intensity
1119:1123
5:5
1:150776123:G:A_A
p = 7e−10, beta = −0.524, N = 1255
GPVSVGVDAR pc2
log10 peptide intensity
1119:1123
5:5
1:150776123:G:A_A
p = 1.8e−09, beta = −0.512, N = 1255
YQGSCGACWAFSAVGALEAQLK pc
log10 peptide intensity
1092:1123
3:5
1:150776123:G:A_A
p = 2.4e−08, beta = −0.492, N = 1224
NSWGHNFGEEGYIR pc3
log10 peptide intensity
1116:1123
5:5
1:150776123:G:A_A
p = 3.5e−08, beta = −0.47, N = 1252
GNHCGIASFPSYPEI pc2
log10 peptide intensity
1117:1123
5:5
1:150776123:G:A_A
p = 5.1e−08, beta = −0.464, N = 1253
CTSS [QMDiab]
log10 protein intensity
269:269
53:53
3:3
1:150718602:T:G_G
p = 0.54, beta = −0.0828, N = 325
YTELPYGREDVLK pc2
log10 peptide intensity
67:269
9:53
0:3
1:150718602:T:G_G
p = 0.095, beta = 0.568, N = 76
YTELPYGR pc2
log10 peptide intensity
150:269
27:53
2:3
1:150718602:T:G_G
p = 0.12, beta = 0.281, N = 179
NSWGHNFGEEGYIR pc3
log10 peptide intensity
269:269
53:53
3:3
1:150718602:T:G_G
p = 0.23, beta = −0.16, N = 325
VTEVKYQGSCGACWAFSAVGALEAQ
log10 peptide intensity
266:269
50:53
3:3
1:150718602:T:G_G
p = 0.34, beta = −0.13, N = 319
Sort: 207
Gene name: CTSS
SNP: 1:150776123:G:A
rsID: rs74857275
Type: Cis−pQTL
Protein group(s):
T: P25774
Q: A0A7P0TAQ0;P25774
Nano−particle: NP3
ILPDSVDWR pc2
rs2230061 REF
log10 peptide intensity
750:1123
76:132
3:5
1:150776123:G:A_A
p = 0.036, model = REC, N = 829

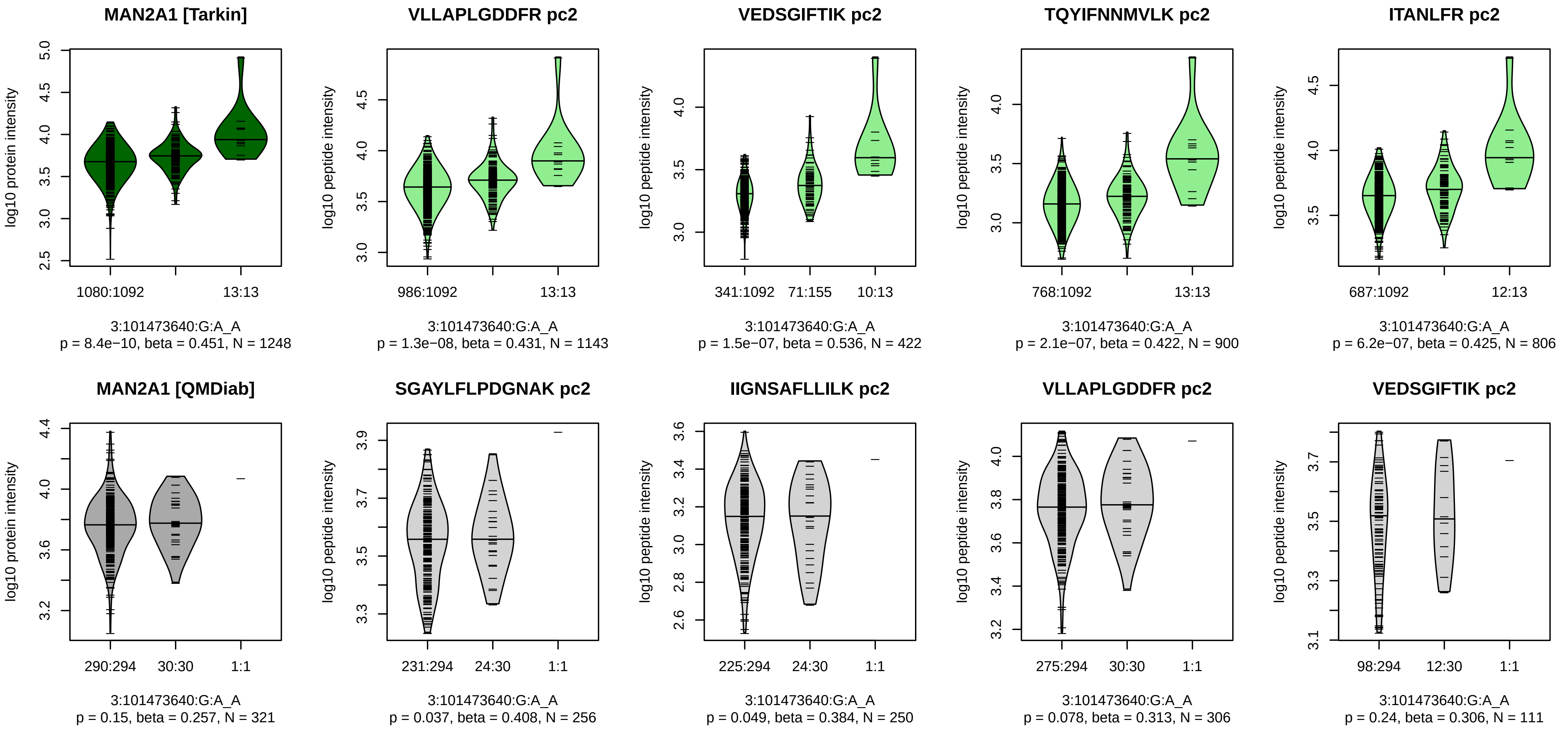

Sort: 208
Gene name: MAN2A1
SNP: 3:101473640:G:A
rsID: rs73143813
Type: Trans−pQTL
Protein group(s):
Q16706
Nano−particle: NP2

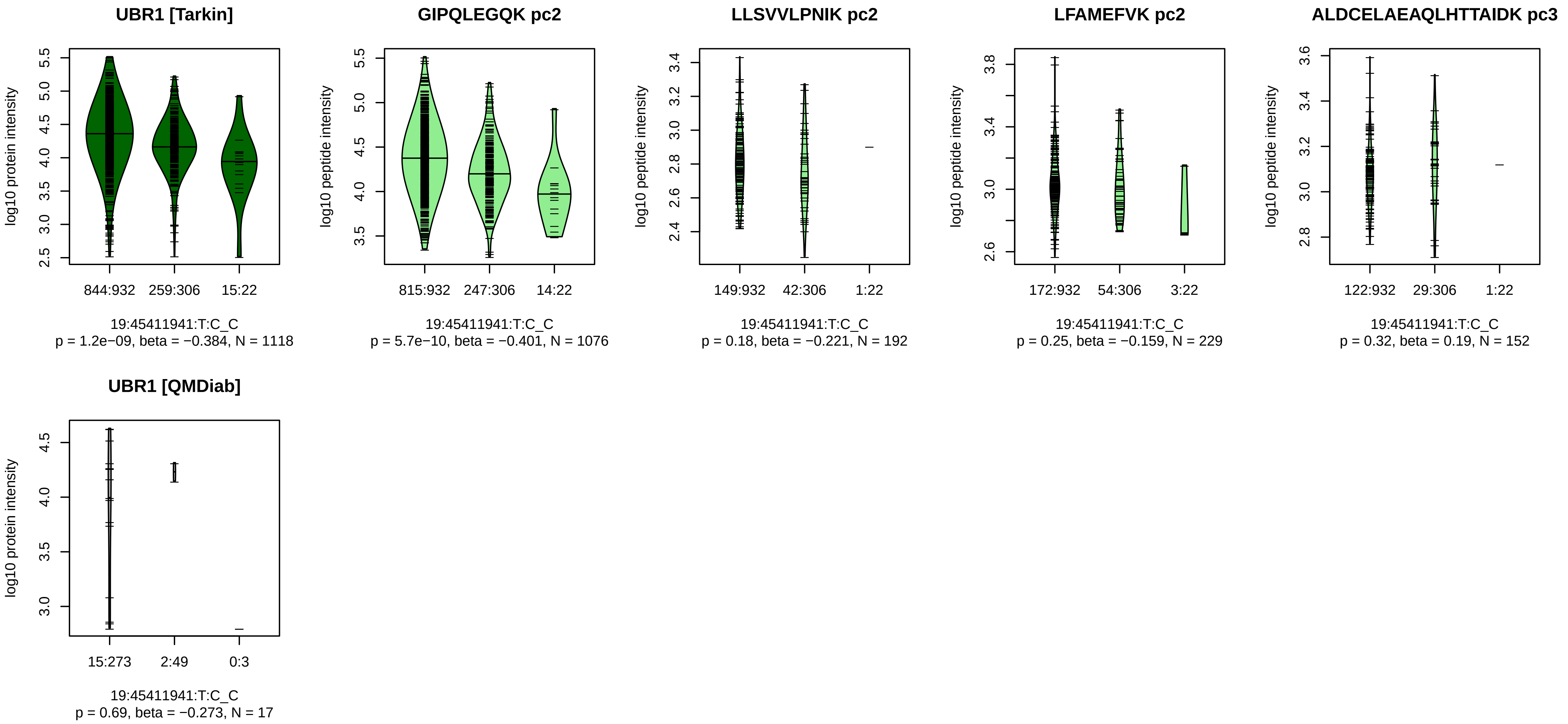

Sort: 209
Gene name: UBR1
SNP: 19:45411941:T:C
rsID: rs429358
Type: Trans−pQTL
Protein group(s):
Q8IWV7
Nano−particle: NP4

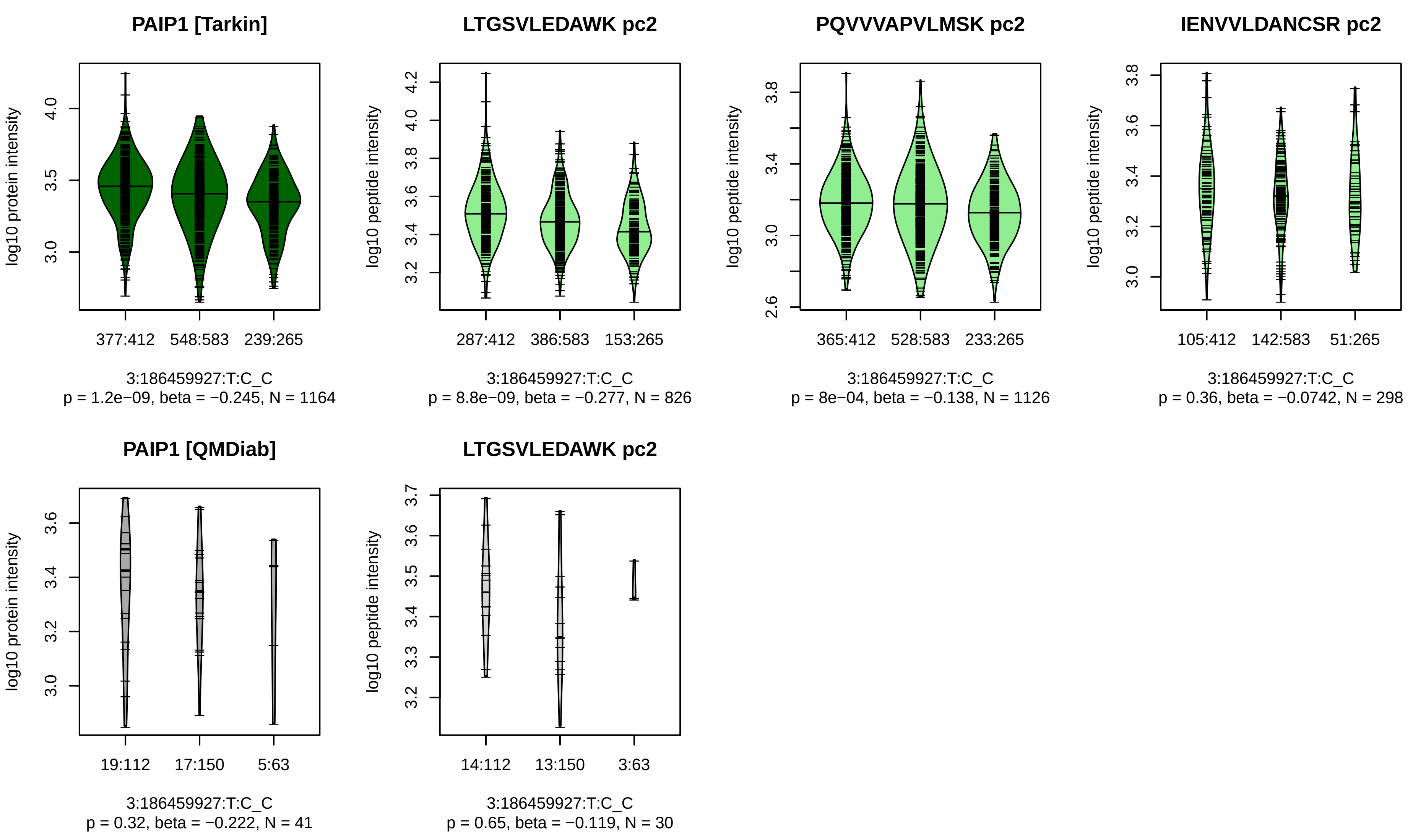

Sort: 210
Gene name: PAIP1
SNP: 3:186459927:T:C
rsID: rs710446
Type: Trans−pQTL
Protein group(s):
T: Q9H074
Q: Q9H074;Q9H074−2
Nano−particle: NP1

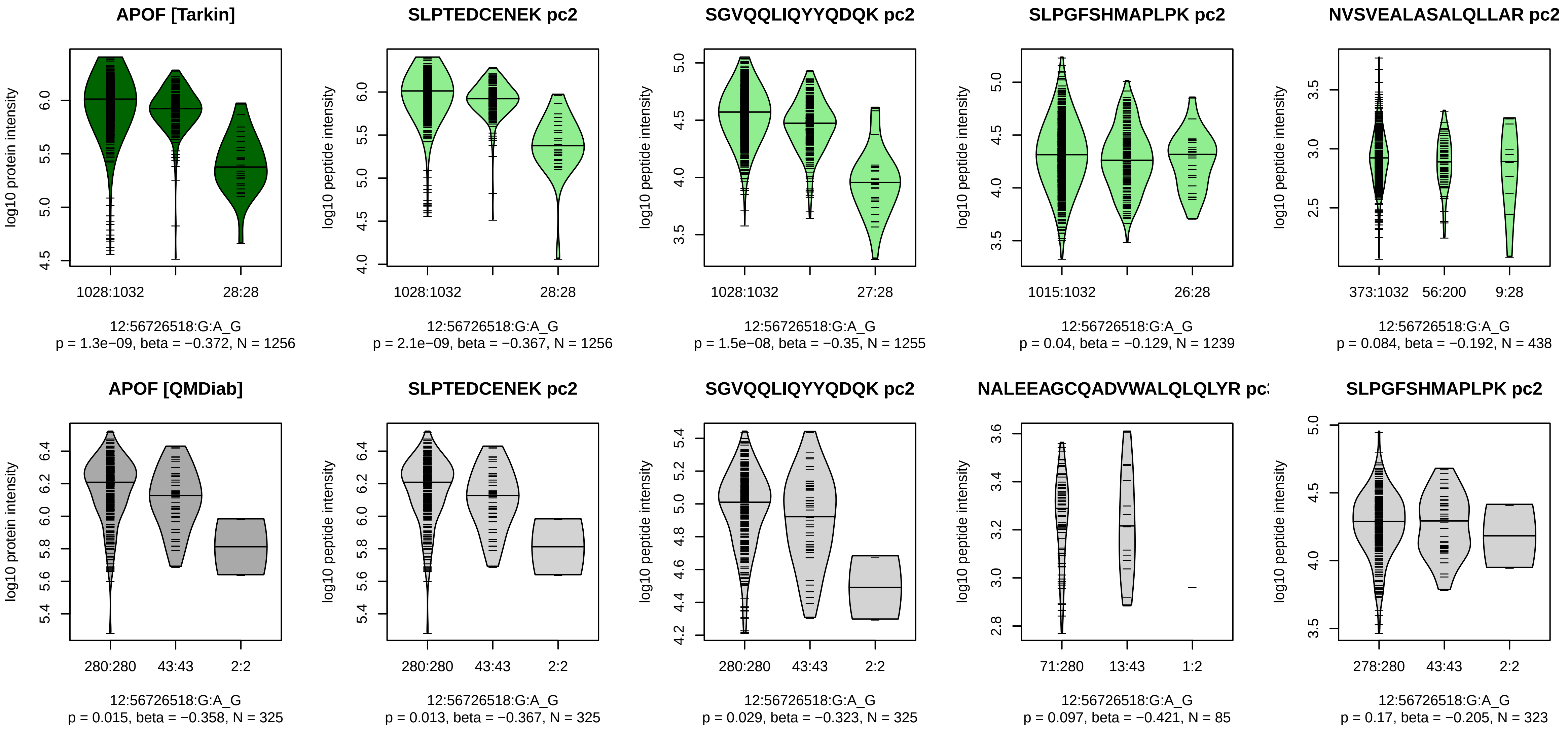

Sort: 211
Gene name: APOF
SNP: 12:56726518:G:A
rsID: rs2643626
Type: Cis−pQTL
Protein group(s):
Q13790
Nano−particle: NP2

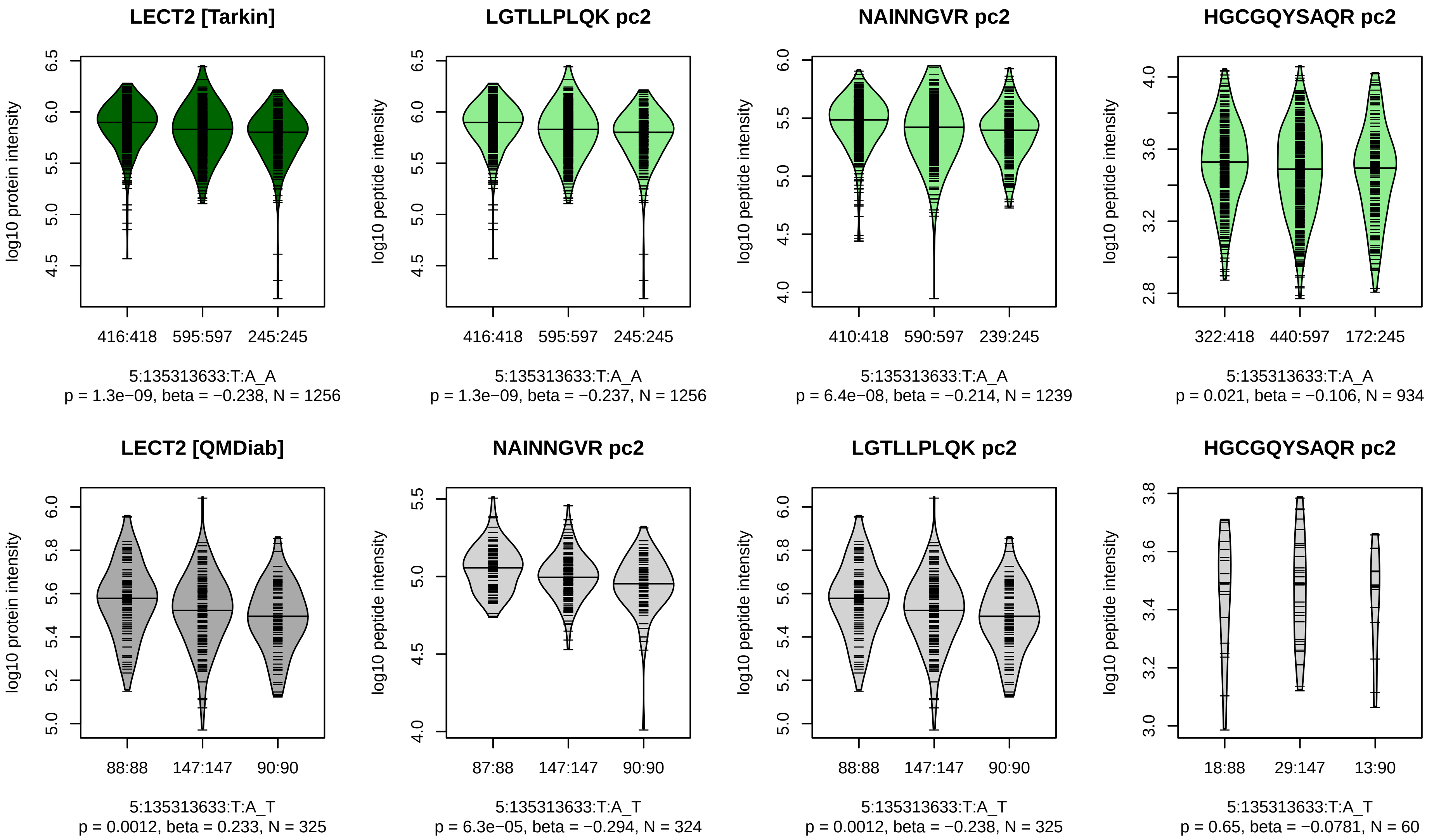

Sort: 212
Gene name: LECT2
SNP: 5:135313633:T:A
rsID: rs2428665
Type: Cis−pQTL
Protein group(s):
O14960
Nano−particle: NP1

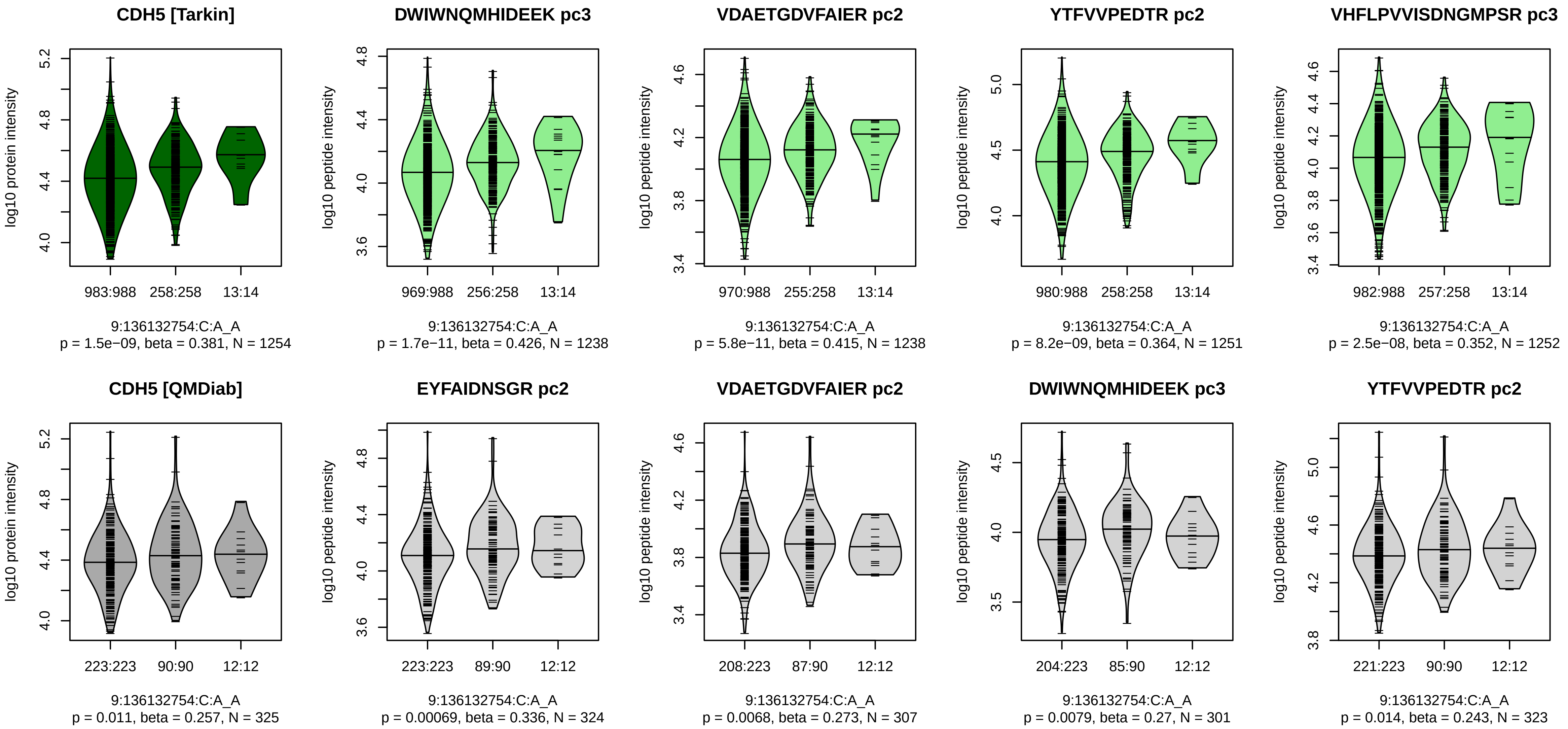

Sort: 213
Gene name: CDH5
SNP: 9:136132754:C:A
rsID: rs8176722
Type: Trans−pQTL
Protein group(s):
P33151
Nano−particle: NP2

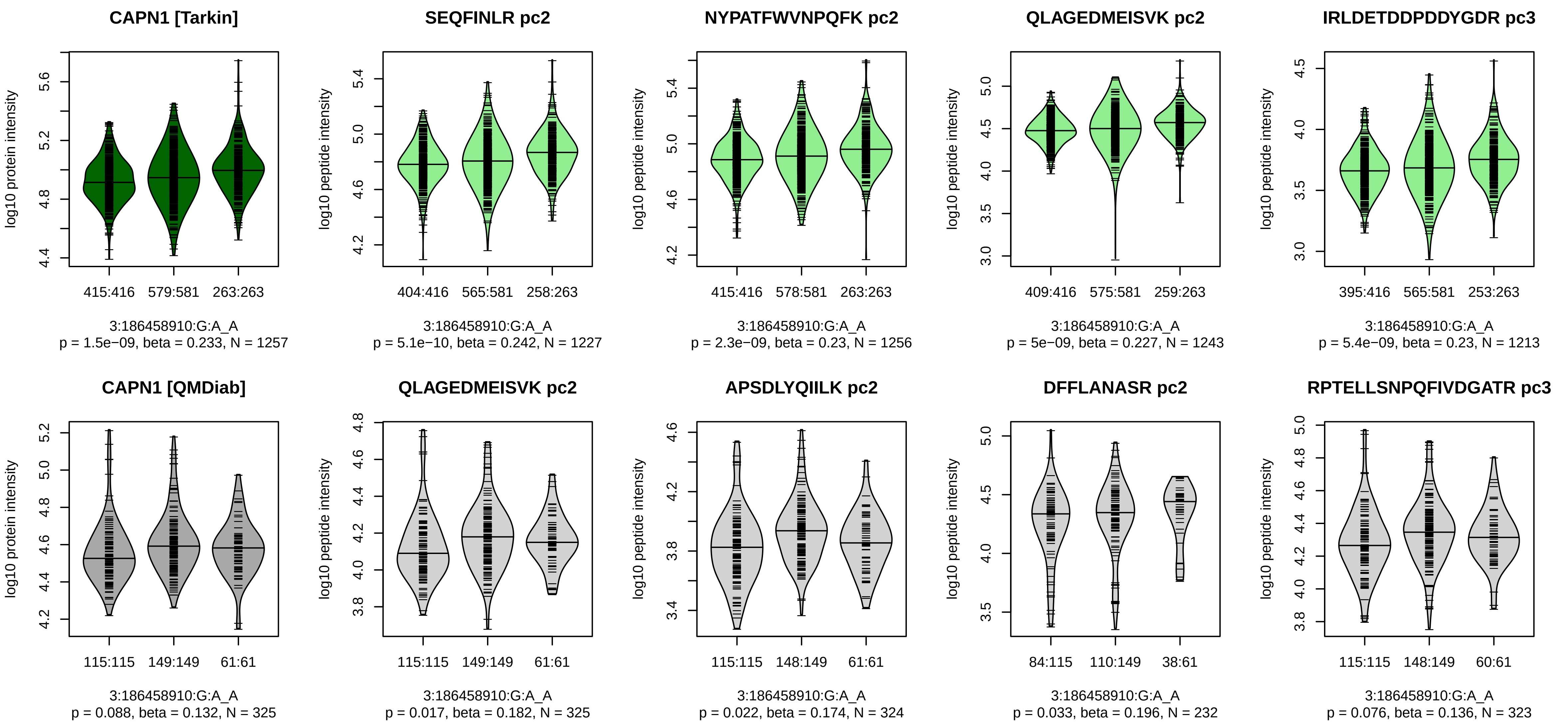

Sort: 214
Gene name: CAPN1
SNP: 3:186458910:G:A
rsID: rs5030081
Type: Trans−pQTL
Protein group(s):
P07384
Nano−particle: NP5

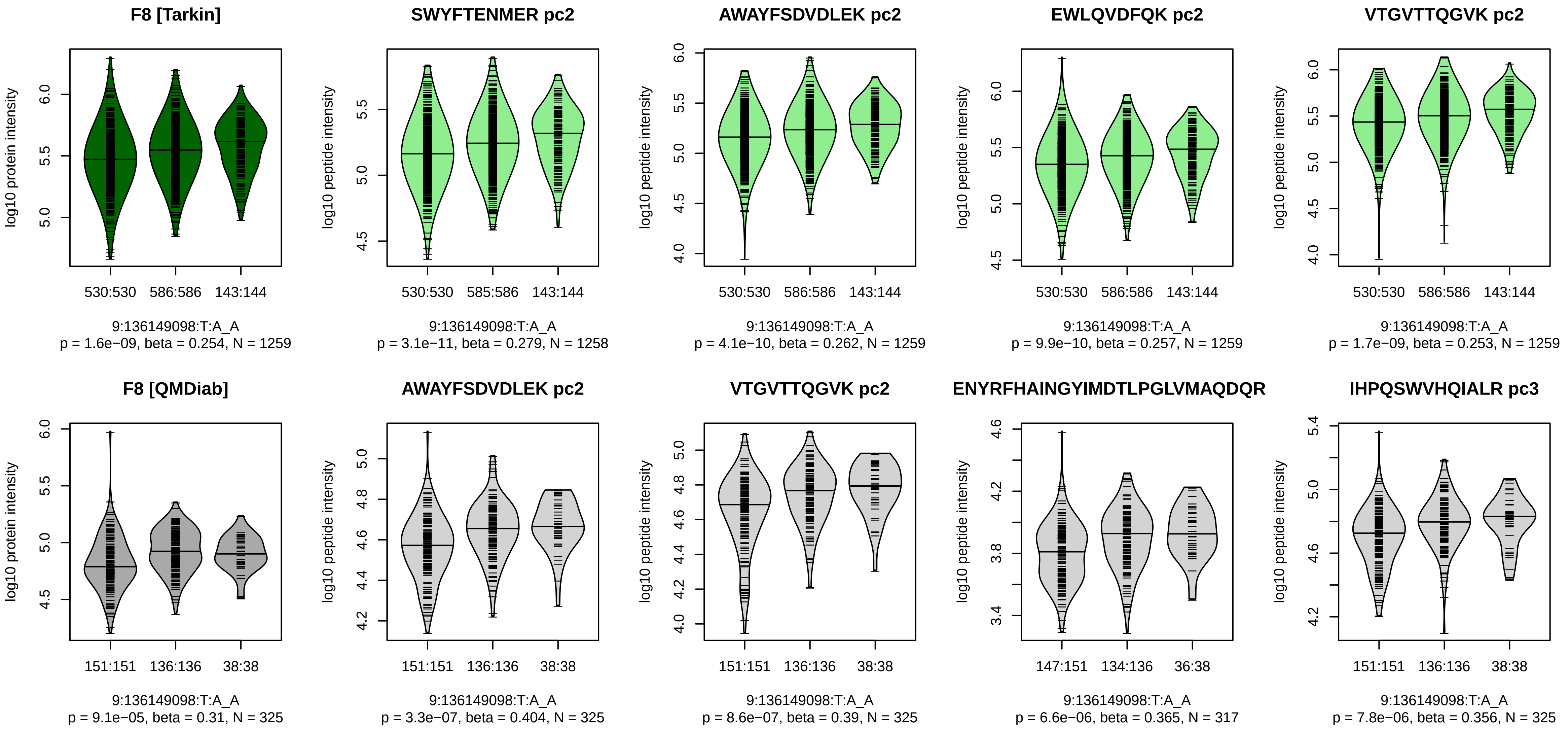

Sort: 215
Gene name: F8
SNP: 9:136149098:T:A
rsID: rs8176645
Type: Trans−pQTL
Protein group(s):
P00451
Nano−particle: NP4

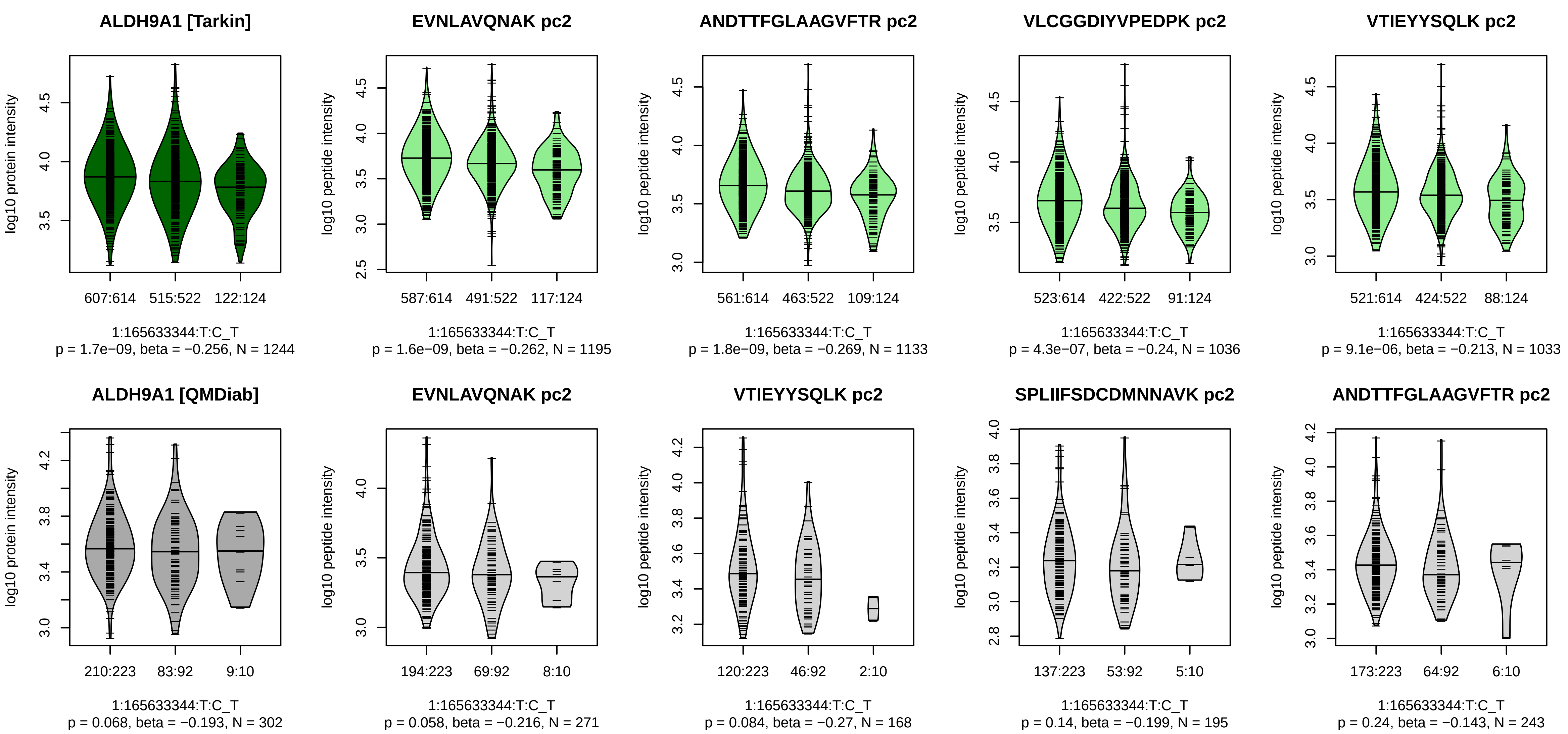

Sort: 216
Gene name: ALDH9A1
SNP: 1:165633344:T:C
rsID: rs7518869
Type: Cis−pQTL
Protein group(s):
P49189−3
Nano−particle: NP5

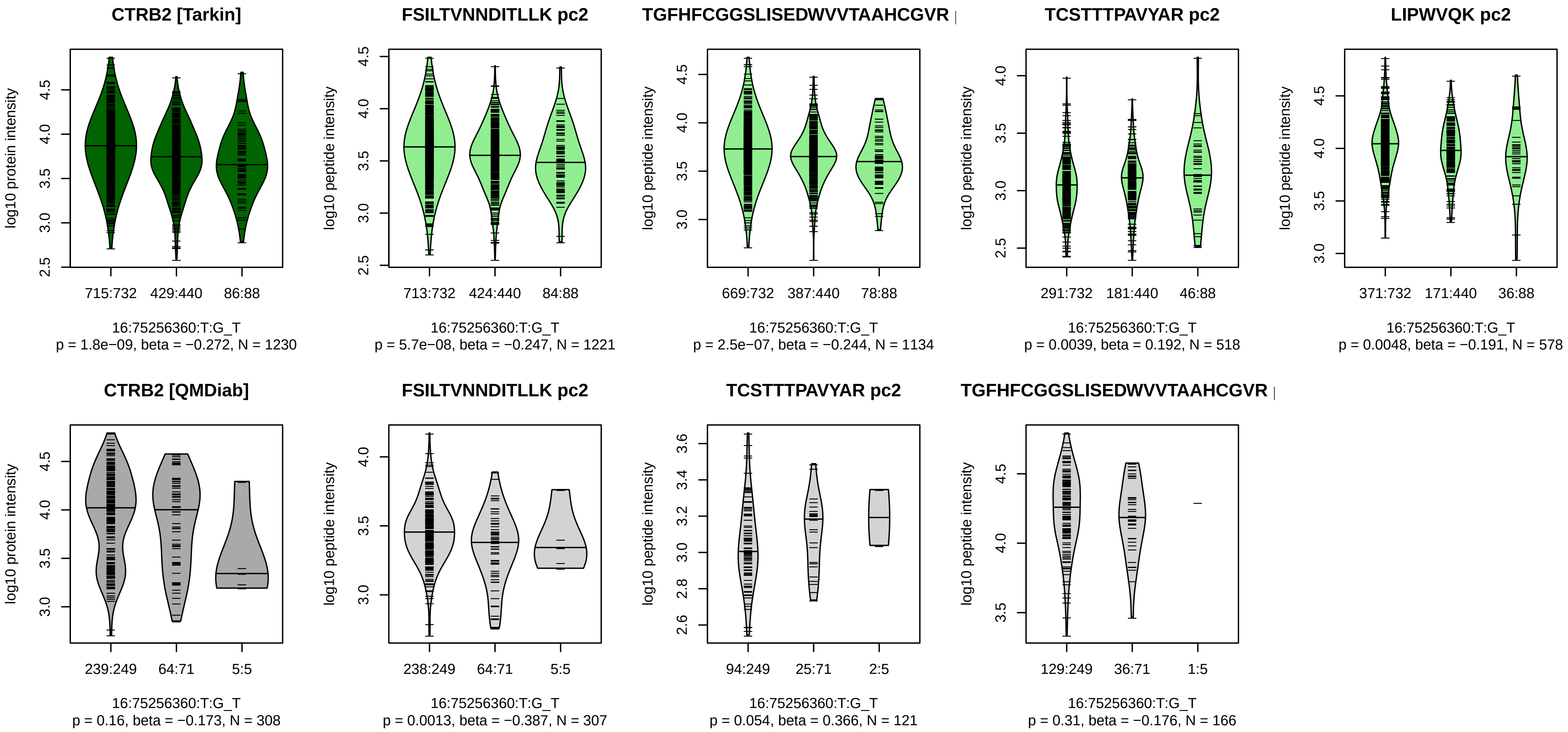

Sort: 217
Gene name: CTRB2
SNP: 16:75256360:T:G
rsID: rs8045196
Type: Cis−pQTL
Protein group(s):
Q6GPI1
Nano−particle: NP3

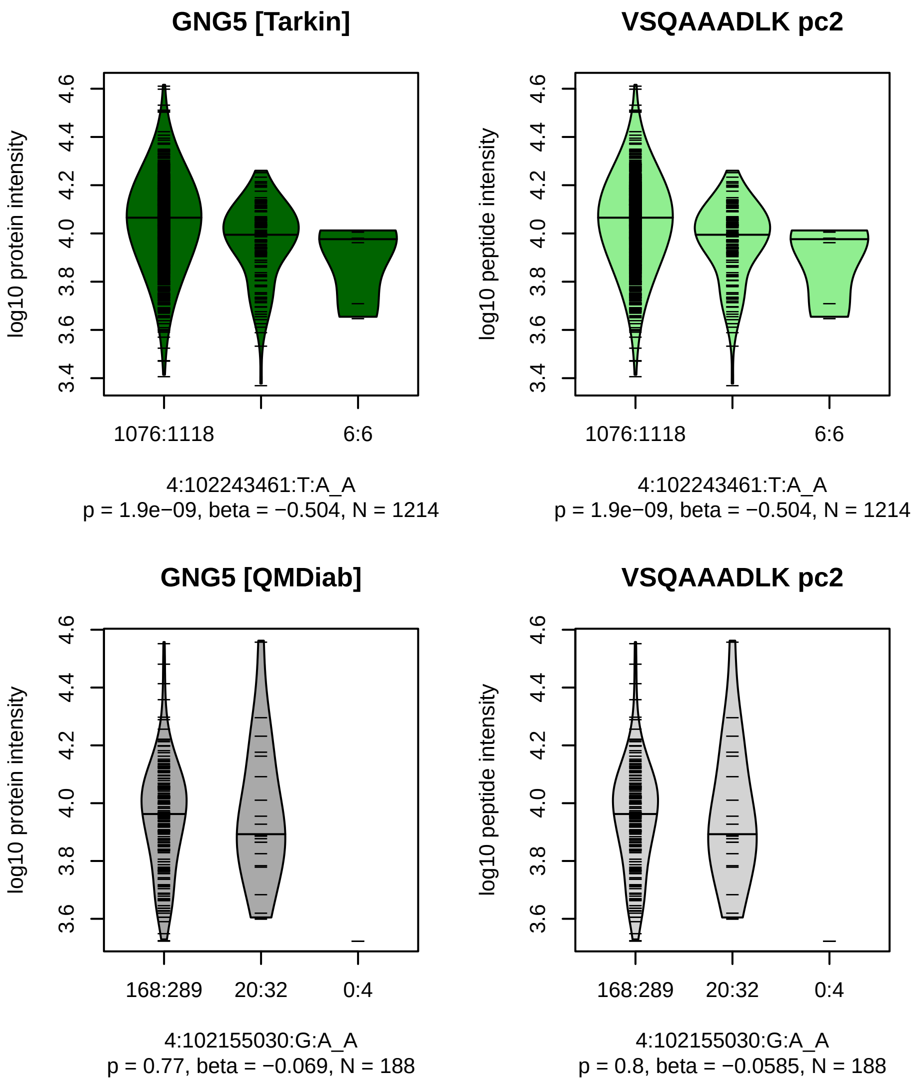

Sort: 218
Gene name: GNG5
SNP: 4:102243461:T:A
rsID: rs76455904
Type: Trans−pQTL
Protein group(s):
T: P63218
Q: A0A804HLA8;P63218
Nano−particle: NP1

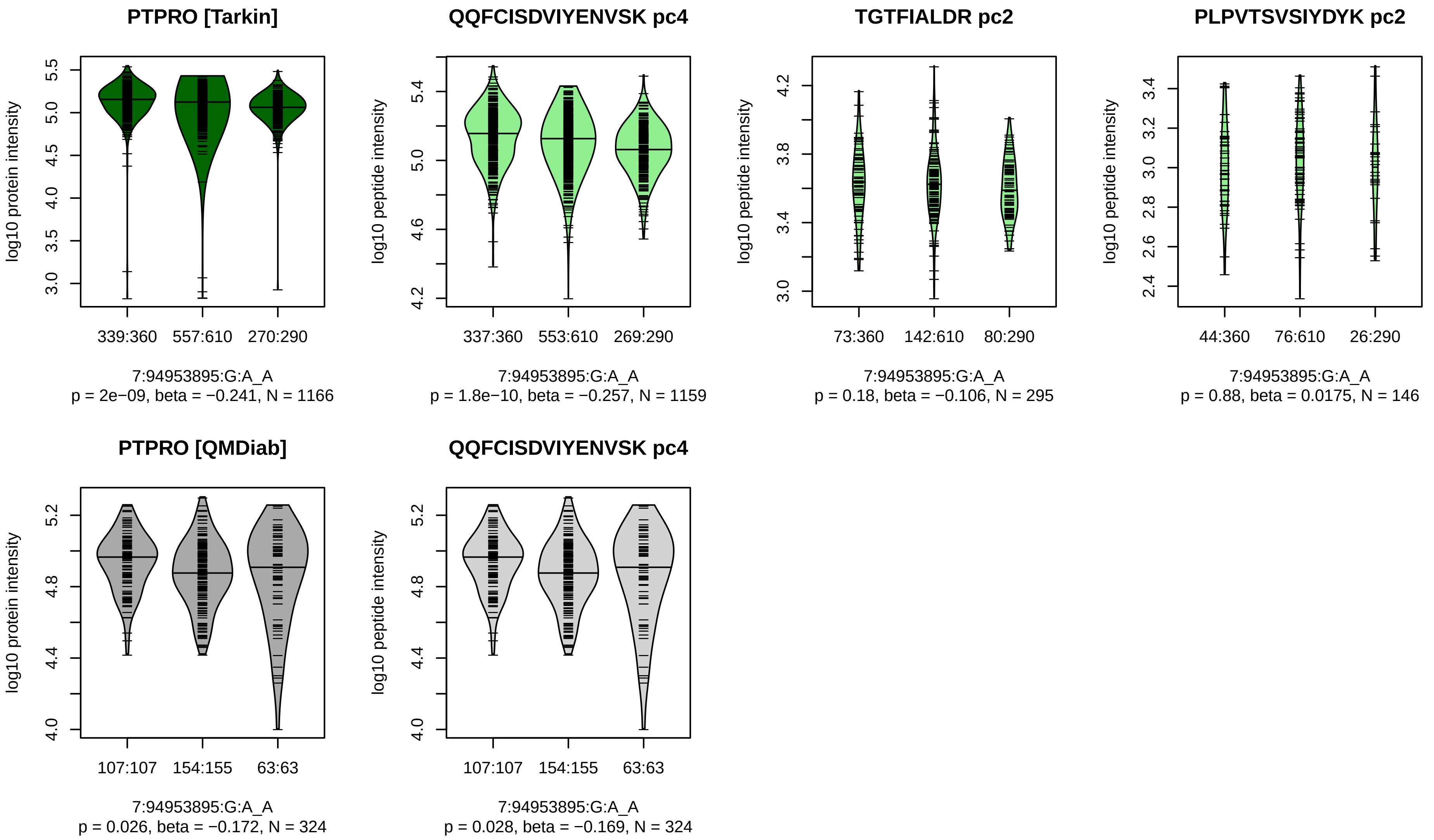

Sort: 219
Gene name: PTPRO
SNP: 7:94953895:G:A
rsID: rs705379
Type: Trans−pQTL
Protein group(s):
T: Q16827
Q: Q16827−2
Nano−particle: NP2

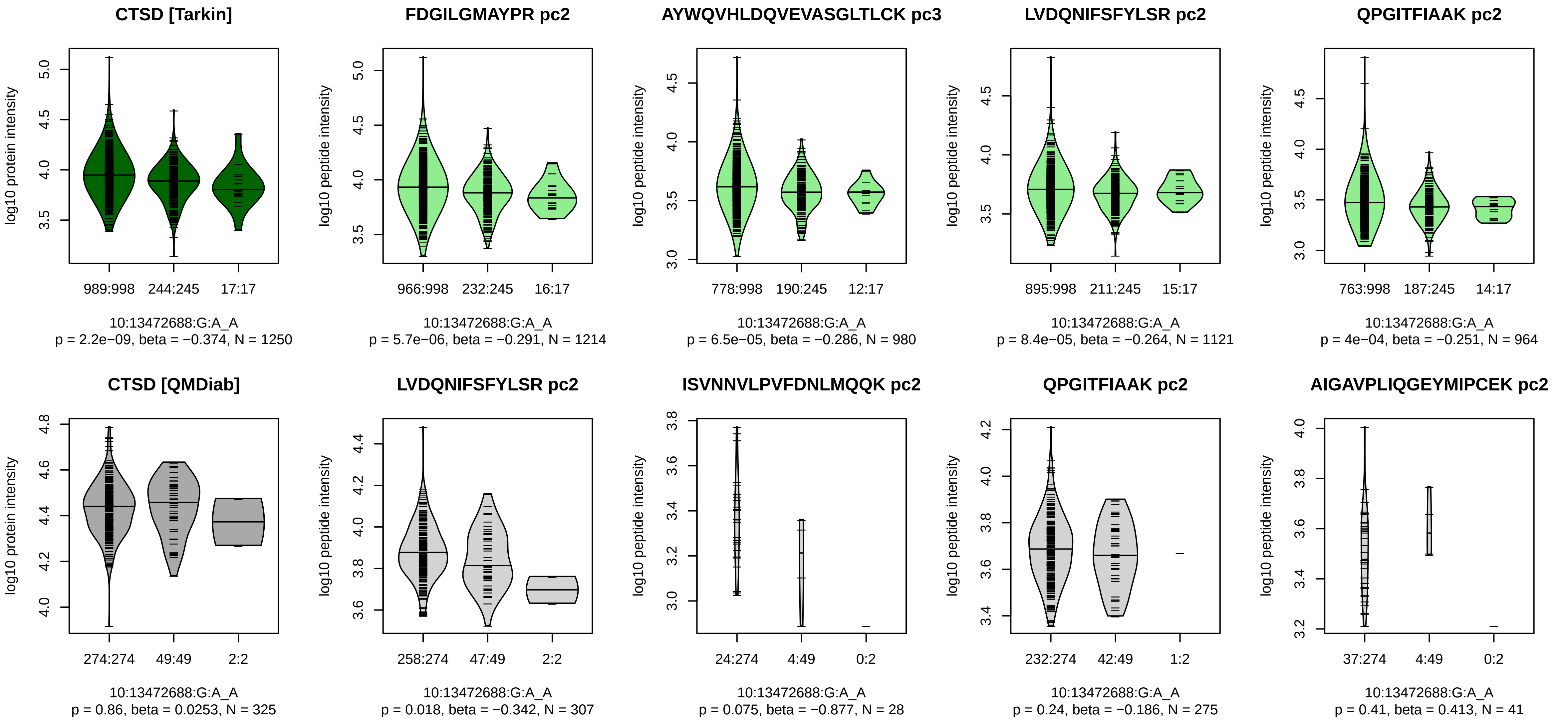

Sort: 220
Gene name: CTSD
SNP: 10:13472688:G:A
rsID: rs72783385
Type: Trans−pQTL
Protein group(s):
A0A1B0GVD5;A0A1B0GW44;/
Nano−particle: NP2

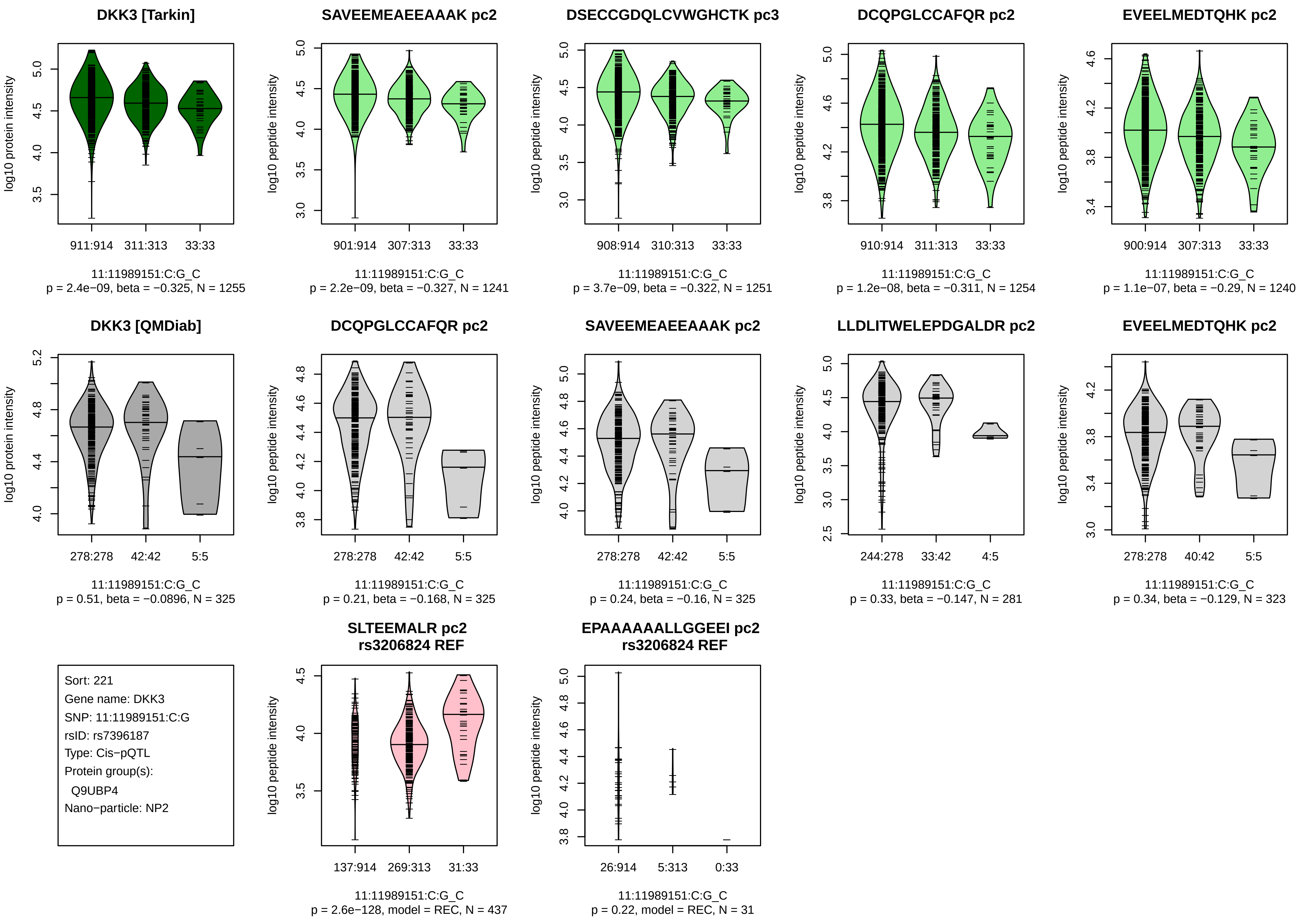
DKK3 [Tarkin]
log10 protein intensity
911:914 311:313 33:33
11:11989151:C:G_C
p = 2.4e−09, beta = −0.325, N = 1255
SAVEEMEAEEAAAK pc2
log10 peptide intensity
901:914 307:313 33:33
11:11989151:C:G_C
p = 2.2e−09, beta = −0.327, N = 1241
DSECCGDQLCVWGHCTK pc3
log10 peptide intensity
908:914 310:313 33:33
11:11989151:C:G_C
p = 3.7e−09, beta = −0.322, N = 1251
DCQPGLCCAFQR pc2
log10 peptide intensity
910:914 311:313 33:33
11:11989151:C:G_C
p = 1.2e−08, beta = −0.311, N = 1254
EVEELMEDTQHK pc2
log10 peptide intensity
900:914 307:313 33:33
11:11989151:C:G_C
p = 1.1e−07, beta = −0.29, N = 1240
DKK3 [QMDiab]
log10 protein intensity
278:278 42:42 5:5
11:11989151:C:G_C
p = 0.51, beta = −0.0896, N = 325
DCQPGLCCAFQR pc2
log10 peptide intensity
278:278 42:42 5:5
11:11989151:C:G_C
p = 0.21, beta = −0.168, N = 325
SAVEEMEAEEAAAK pc2
log10 peptide intensity
278:278 42:42 5:5
11:11989151:C:G_C
p = 0.24, beta = −0.16, N = 325
LLDLITWELEPDGALDR pc2
log10 peptide intensity
244:278 33:42 4:5
11:11989151:C:G_C
p = 0.33, beta = −0.147, N = 281
EVEELMEDTQHK pc2
log10 peptide intensity
278:278 40:42 5:5
11:11989151:C:G_C
p = 0.34, beta = −0.129, N = 323
Sort: 221
Gene name: DKK3
SNP: 11:11989151:C:G
rsID: rs7396187
Type: Cis−pQTL
Protein group(s):
Q9UBP4
Nano−particle: NP2
SLTEEMALR pc2
rs3206824 REF
log10 peptide intensity
137:914 269:313 31:33
11:11989151:C:G_C
p = 2.6e−128, model = REC, N = 437
EPAAAAAALLGGEEI pc2
rs3206824 REF
log10 peptide intensity
26:914 5:313 0:33
11:11989151:C:G_C
p = 0.22, model = REC, N = 31

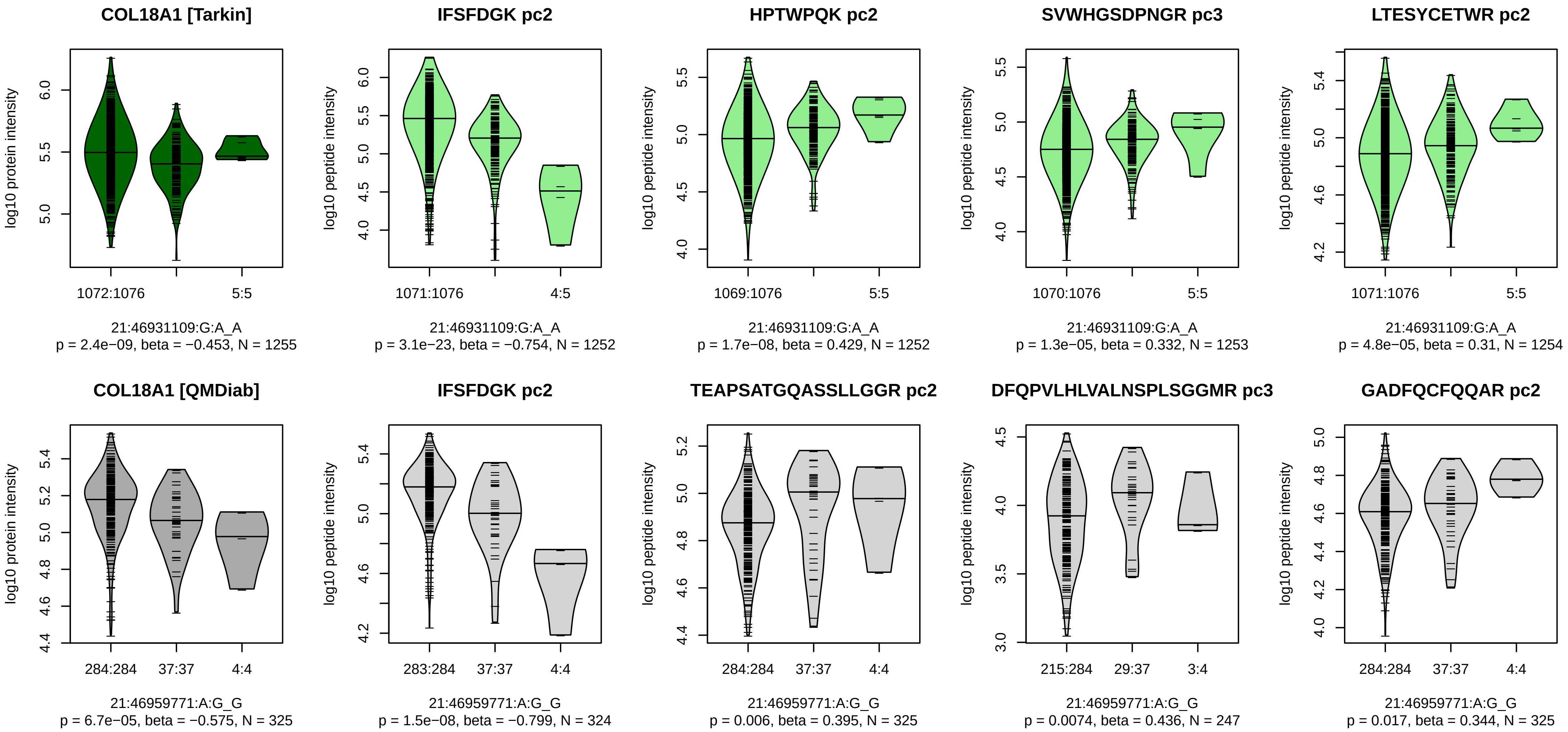

Sort: 222
Gene name: COL18A1
SNP: 21:46931109:G:A
rsID: rs12483377
Type: Cis−pQTL
Protein group(s):
P39060;P39060−1;P39060−2
Nano−particle: NP3

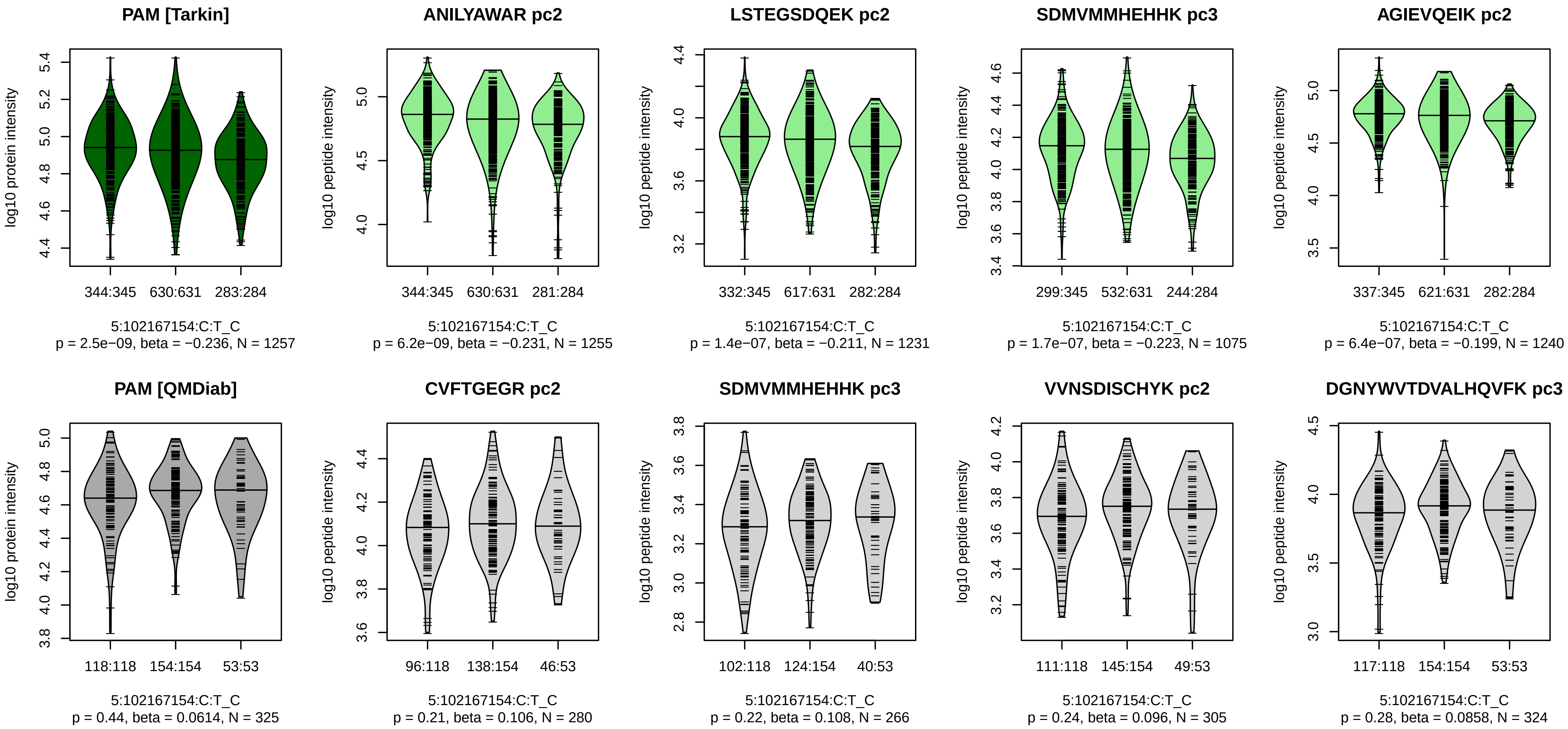

Sort: 223
Gene name: PAM
SNP: 5:102167154:C:T
rsID: rs467693
Type: Cis−pQTL
Protein group(s):
T: P19021−5
Q: A0A8C8KD64;P19021;P1902
Nano−particle: NP5

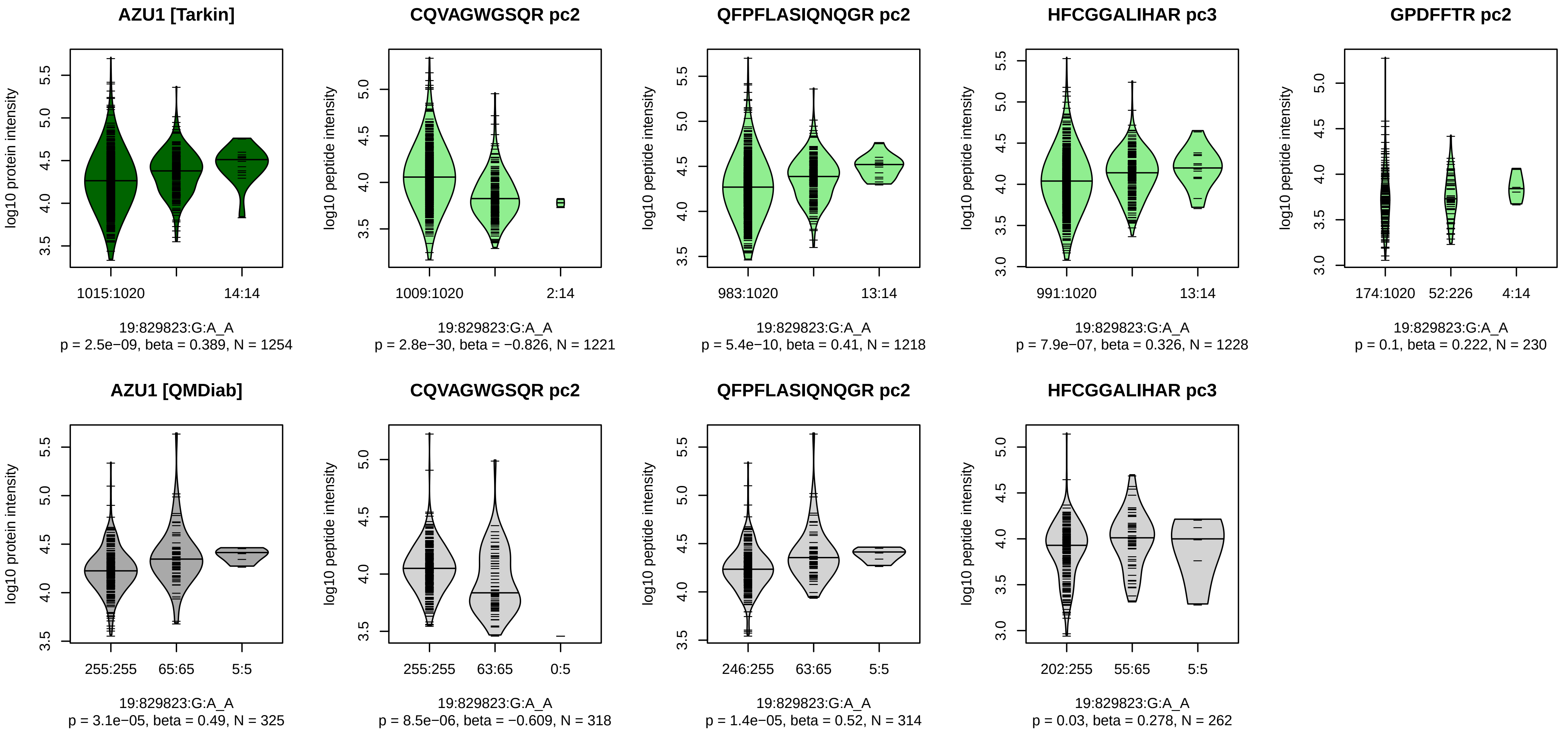

Sort: 224
Gene name: AZU1
SNP: 19:829823:G:A
rsID: rs112967183
Type: Cis−pQTL
Protein group(s):
P20160
Nano−particle: NP4

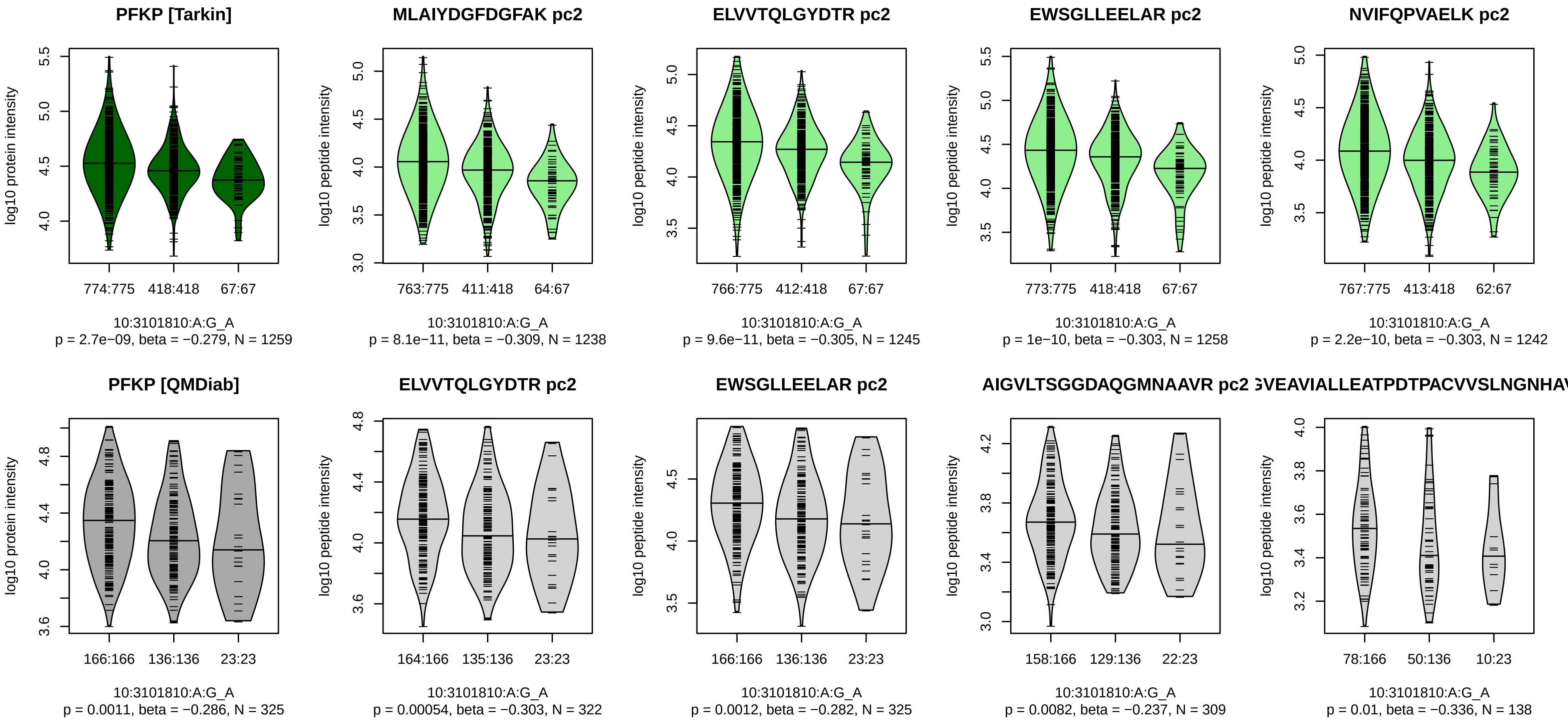

Sort: 225
Gene name: PFKP
SNP: 10:3101810:A:G
rsID: rs7920986
Type: Cis−pQTL
Protein group(s):
Q01813
Nano−particle: NP4

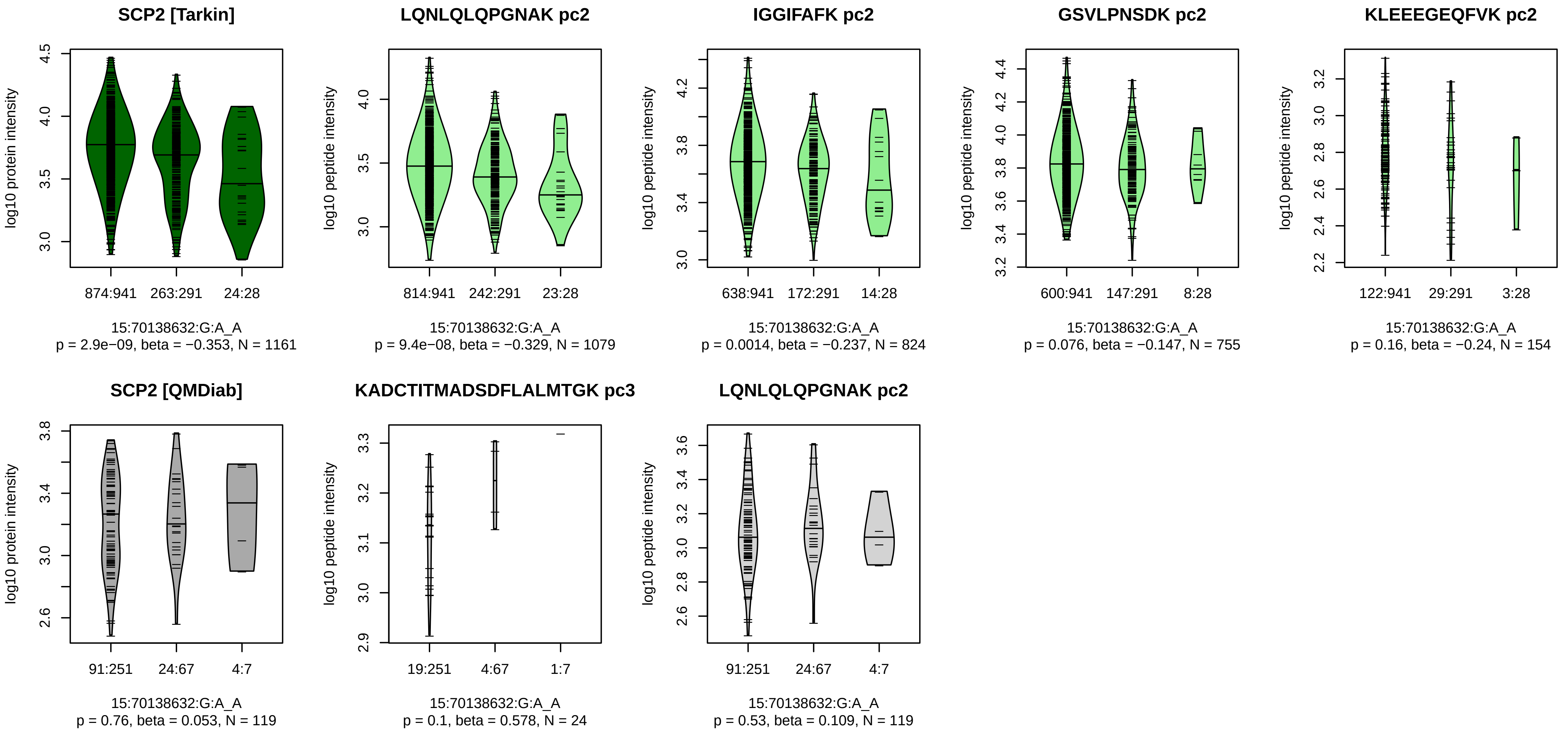

Sort: 226
Gene name: SCP2
SNP: 15:70138632:G:A
rsID: rs16953824
Type: Trans−pQTL
Protein group(s):
T: P22307
Q: P22307;P22307−8
Nano−particle: NP3

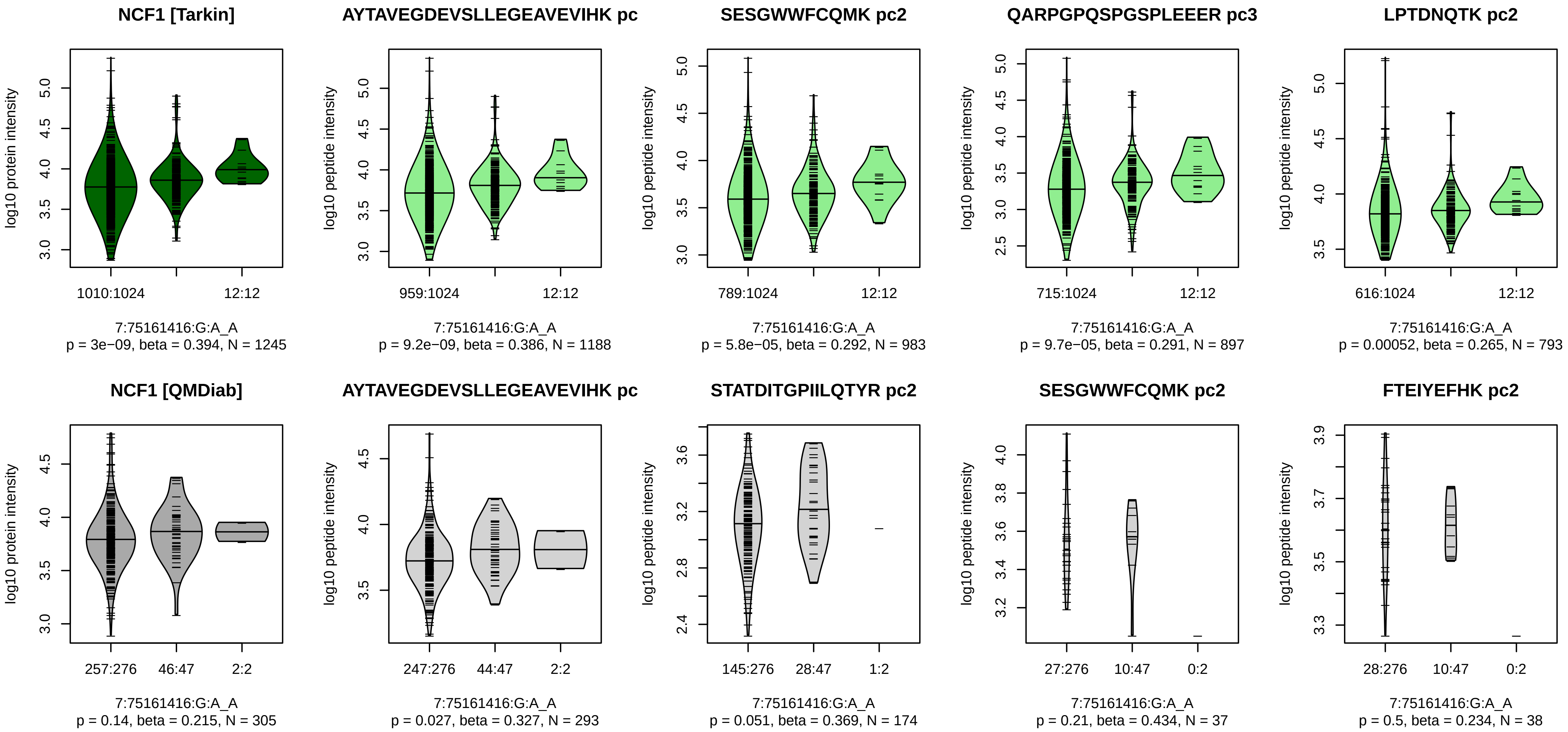

Sort: 227
Gene name: NCF1
SNP: 7:75161416:G:A
rsID: rs112661041
Type: Cis−pQTL
Protein group(s):
P14598
Nano−particle: NP1

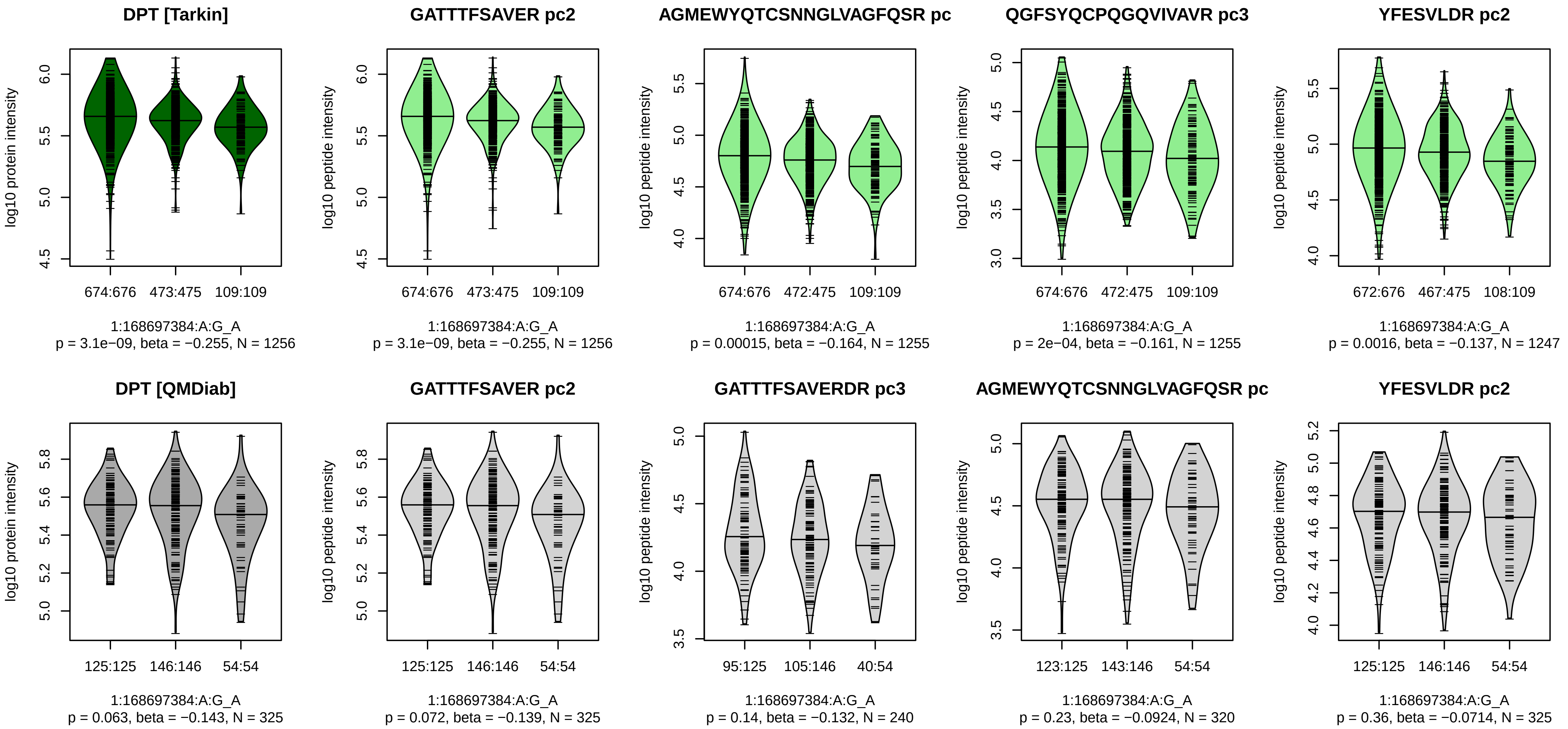

Sort: 228
Gene name: DPT
SNP: 1:168697384:A:G
rsID: rs485187
Type: Cis−pQTL
Protein group(s):
Q07507
Nano−particle: NP2

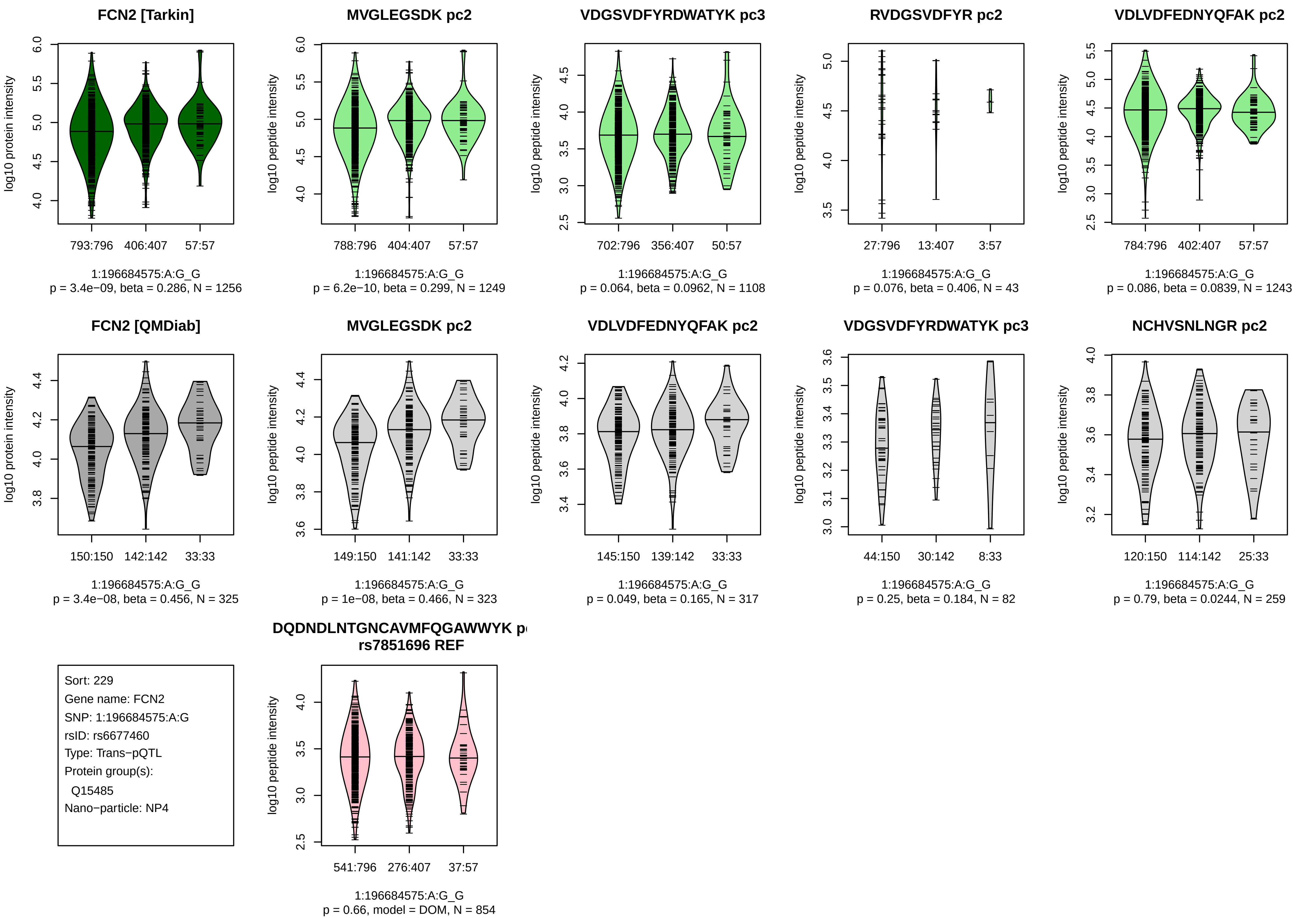
FCN2 [Tarkin]
log10 protein intensity
793:796 406:407 57:57
1:196684575:A:G_G
p = 3.4e−09, beta = 0.286, N = 1256
MVGLEGSDK pc2
log10 peptide intensity
788:796 404:407 57:57
1:196684575:A:G_G
p = 6.2e−10, beta = 0.299, N = 1249
VDGSVDFYRDWATYK pc3
log10 peptide intensity
702:796 356:407 50:57
1:196684575:A:G_G
p = 0.064, beta = 0.0962, N = 1108
RVDGSVDFYR pc2
log10 peptide intensity
27:796 13:407 3:57
1:196684575:A:G_G
p = 0.076, beta = 0.406, N = 43
VDLVDFEDNYQFAK pc2
log10 peptide intensity
784:796 402:407 57:57
1:196684575:A:G_G
p = 0.086, beta = 0.0839, N = 1243
FCN2 [QMDiab]
log10 protein intensity
150:150 142:142 33:33
1:196684575:A:G_G
p = 3.4e−08, beta = 0.456, N = 325
MVGLEGSDK pc2
log10 peptide intensity
149:150 141:142 33:33
1:196684575:A:G_G
p = 1e−08, beta = 0.466, N = 323
VDLVDFEDNYQFAK pc2
log10 peptide intensity
145:150 139:142 33:33
1:196684575:A:G_G
p = 0.049, beta = 0.165, N = 317
VDGSVDFYRDWATYK pc3
log10 peptide intensity
44:150 30:142 8:33
1:196684575:A:G_G
p = 0.25, beta = 0.184, N = 82
NCHVSNLNGR pc2
log10 peptide intensity
120:150 114:142 25:33
1:196684575:A:G_G
p = 0.79, beta = 0.0244, N = 259
Sort: 229
Gene name: FCN2
SNP: 1:196684575:A:G
rsID: rs6677460
Type: Trans−pQTL
Protein group(s):
Q15485
Nano−particle: NP4
DQDNDLNTGNCAVMFQGAWWYK p
rs7851696 REF
log10 peptide intensity
541:796 276:407 37:57
1:196684575:A:G_G
p = 0.66, model = DOM, N = 854

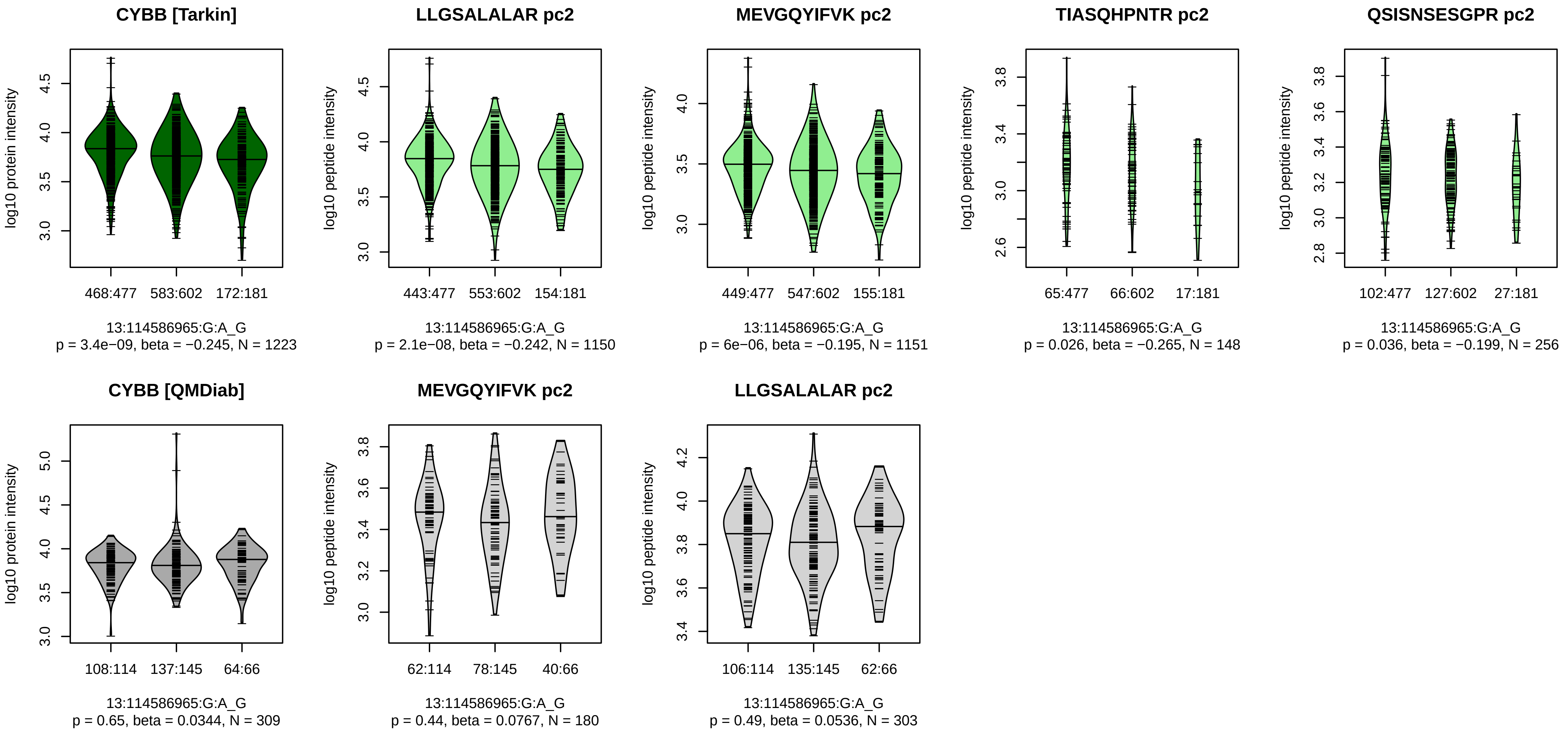

Sort: 230
Gene name: CYBB
SNP: 13:114586965:G:A
rsID: rs7996972
Type: Trans−pQTL
Protein group(s):
P04839
Nano−particle: NP1

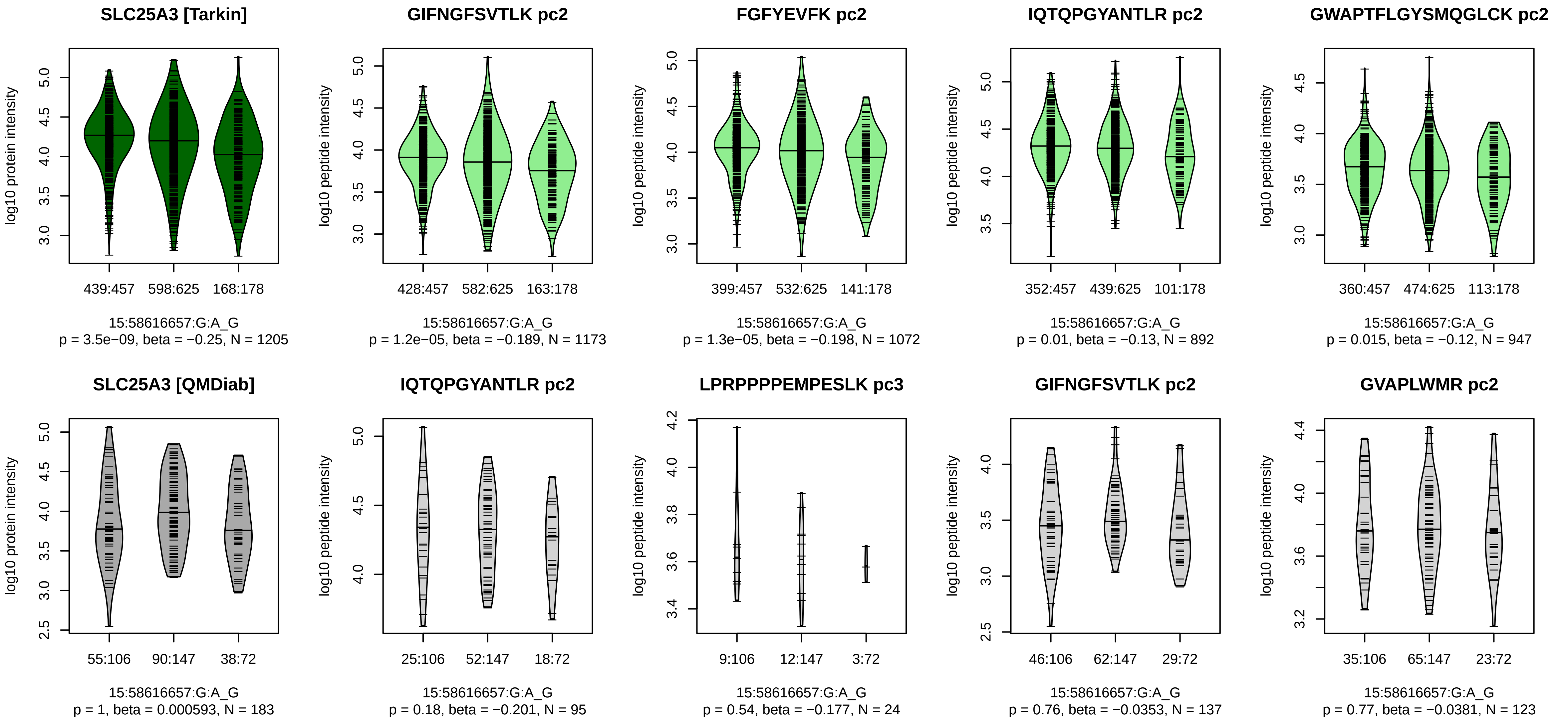

Sort: 231
Gene name: SLC25A3
SNP: 15:58616657:G:A
rsID: rs261280
Type: Trans−pQTL
Protein group(s):
Q00325;Q00325−2
Nano−particle: NP4

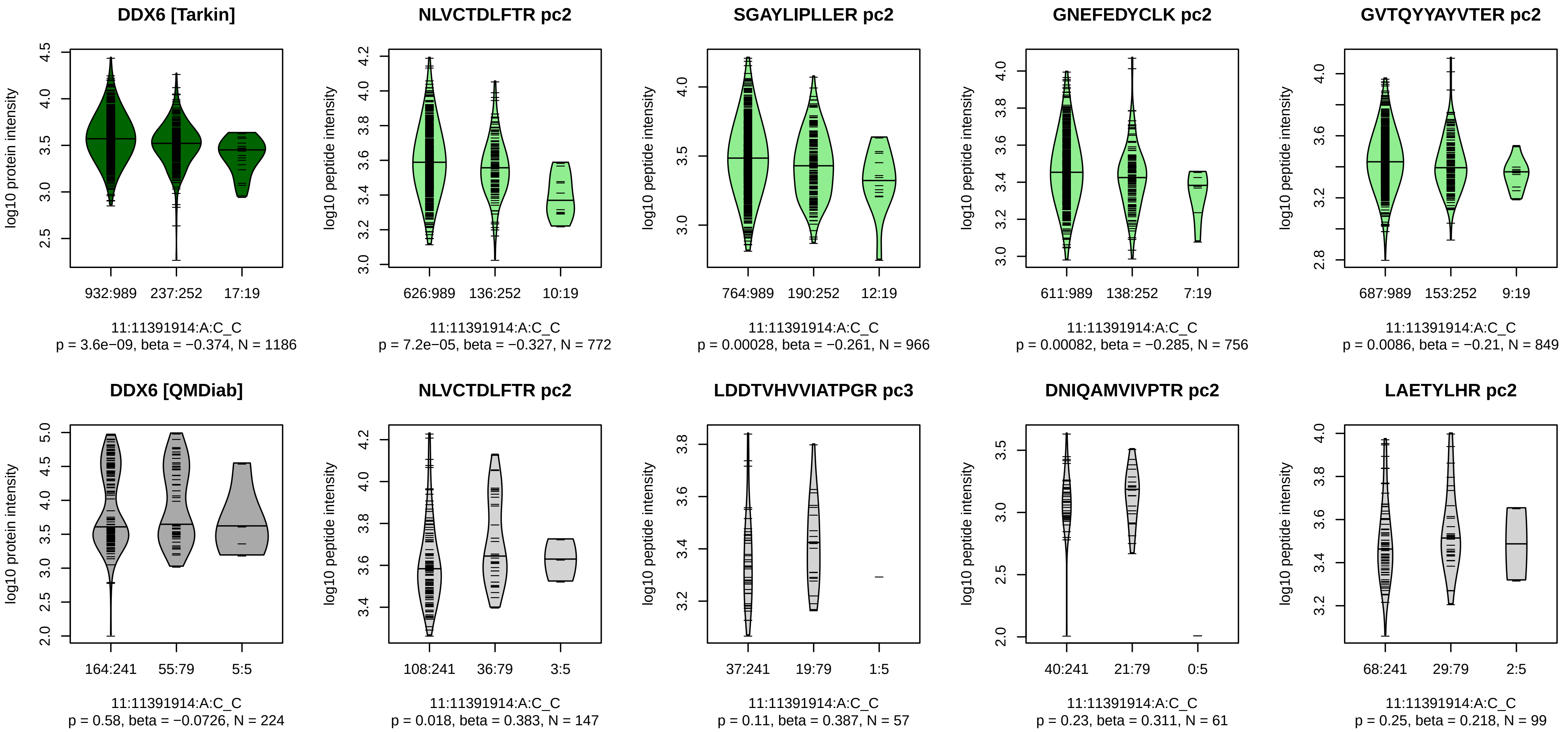

Sort: 232
Gene name: DDX6
SNP: 11:11391914:A:C
rsID: rs78516575
Type: Trans−pQTL
Protein group(s):
P26196
Nano−particle: NP1

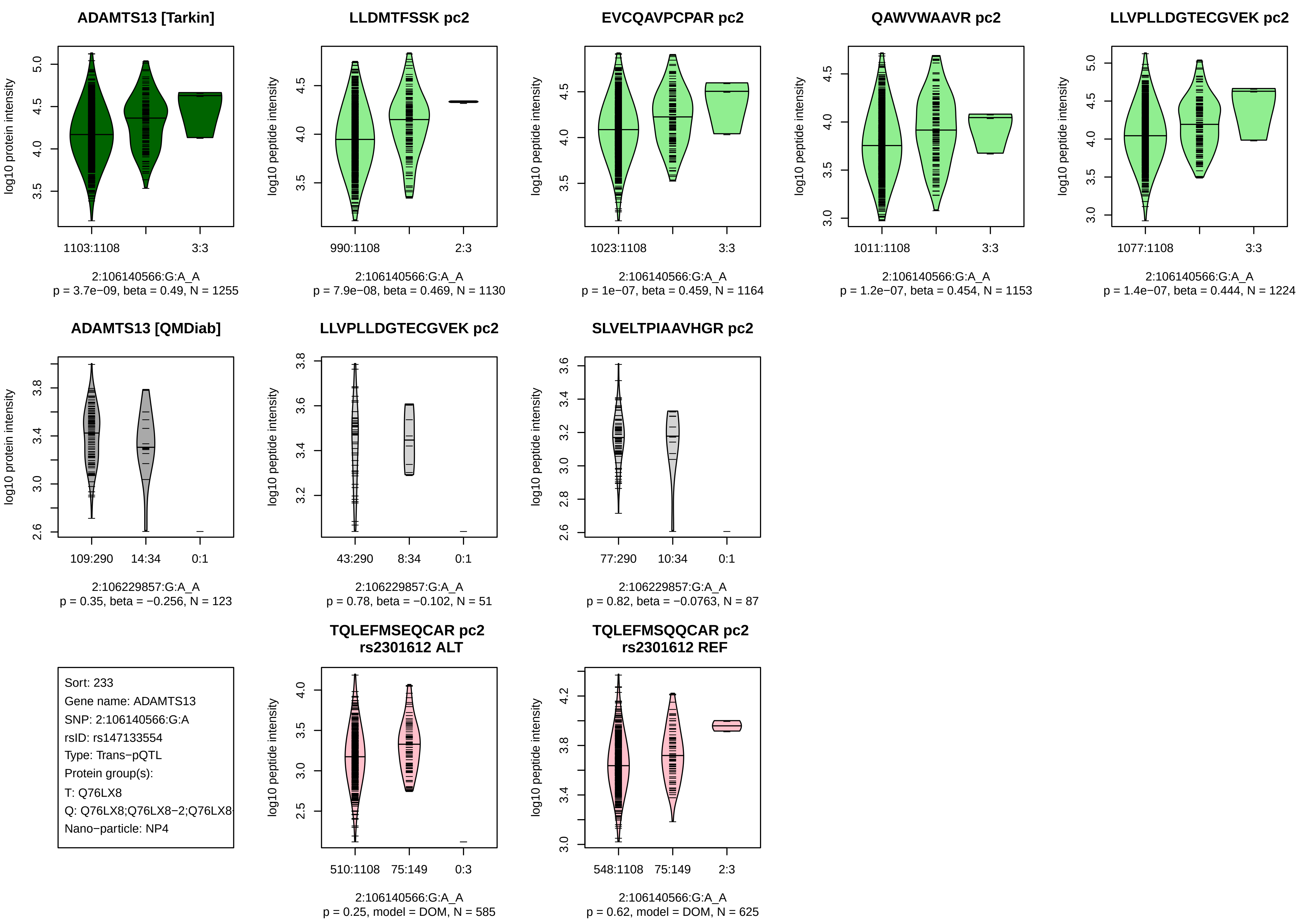
ADAMTS13 [Tarkin]
log10 protein intensity
1103:1108
3:3
2:106140566:G:A_A
p = 3.7e−09, beta = 0.49, N = 1255
LLDMTFSSK pc2
log10 peptide intensity
990:1108
2:3
2:106140566:G:A_A
p = 7.9e−08, beta = 0.469, N = 1130
EVCQAVPCPAR pc2
log10 peptide intensity
1023:1108
3:3
2:106140566:G:A_A
p = 1e−07, beta = 0.459, N = 1164
QAWVWAAVR pc2
log10 peptide intensity
1011:1108
3:3
2:106140566:G:A_A
p = 1.2e−07, beta = 0.454, N = 1153
LLVPLLDGTECGVEK pc2
log10 peptide intensity
1077:1108
3:3
2:106140566:G:A_A
p = 1.4e−07, beta = 0.444, N = 1224
ADAMTS13 [QMDiab]
log10 protein intensity
109:290
14:34
0:1
2:106229857:G:A_A
p = 0.35, beta = −0.256, N = 123
LLVPLLDGTECGVEK pc2
log10 peptide intensity
43:290
8:34
0:1
2:106229857:G:A_A
p = 0.78, beta = −0.102, N = 51
SLVELTPIAAVHGR pc2
log10 peptide intensity
77:290
10:34
0:1
2:106229857:G:A_A
p = 0.82, beta = −0.0763, N = 87
Sort: 233
Gene name: ADAMTS13
SNP: 2:106140566:G:A
rsID: rs147133554
Type: Trans−pQTL
Protein group(s):
T: Q76LX8
Q: Q76LX8;Q76LX8−2;Q76LX8
Nano−particle: NP4
TQLEFMSEQCAR pc2
rs2301612 ALT
log10 peptide intensity
510:1108
75:149
0:3
2:106140566:G:A_A
p = 0.25, model = DOM, N = 585
TQLEFMSQQCAR pc2
rs2301612 REF
log10 peptide intensity
548:1108
75:149
2:3
2:106140566:G:A_A
p = 0.62, model = DOM, N = 625

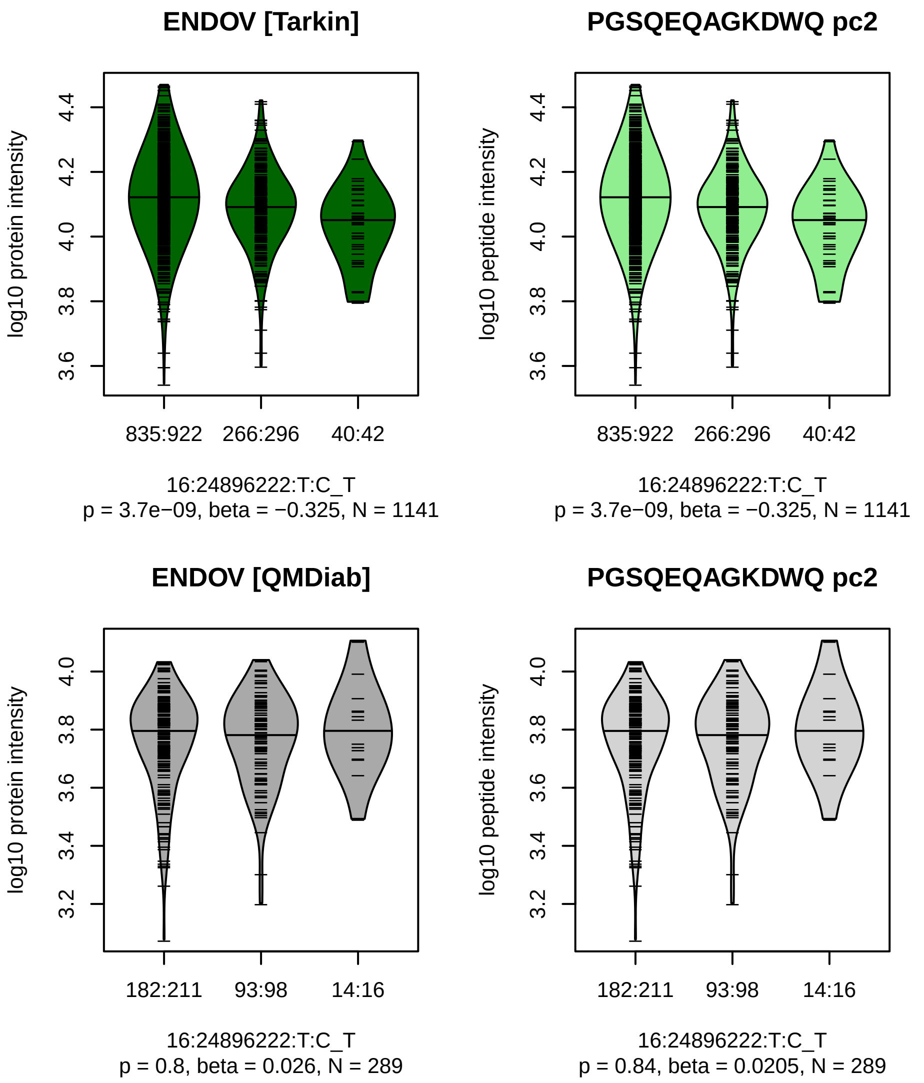

Sort: 234
Gene name: ENDOV
SNP: 16:24896222:T:C
rsID: rs8058743
Type: Trans−pQTL
Protein group(s):
Q8N8Q3−3
Nano−particle: NP2

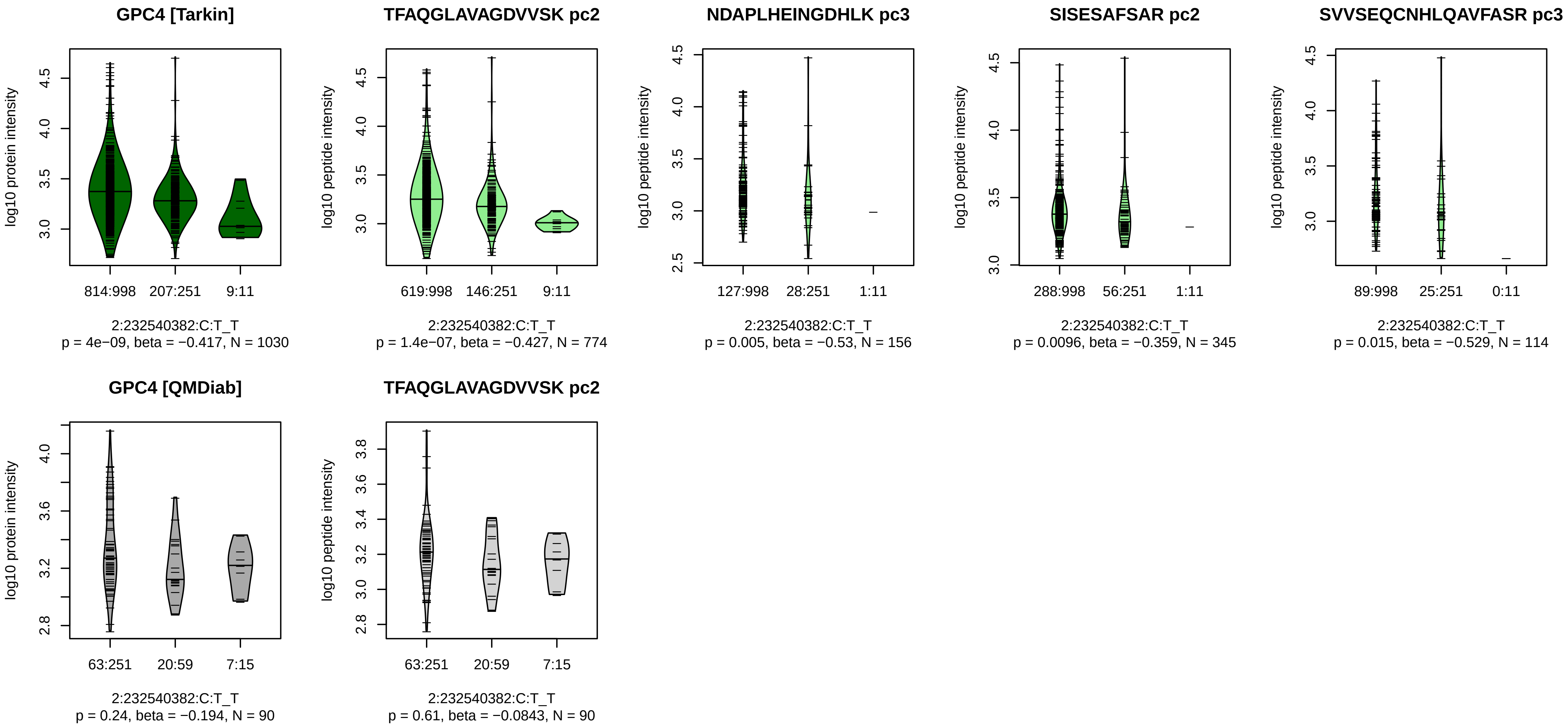

Sort: 235
Gene name: GPC4
SNP: 2:232540382:C:T
rsID: rs11677759
Type: Trans−pQTL
Protein group(s):
O75487
Nano−particle: NP2

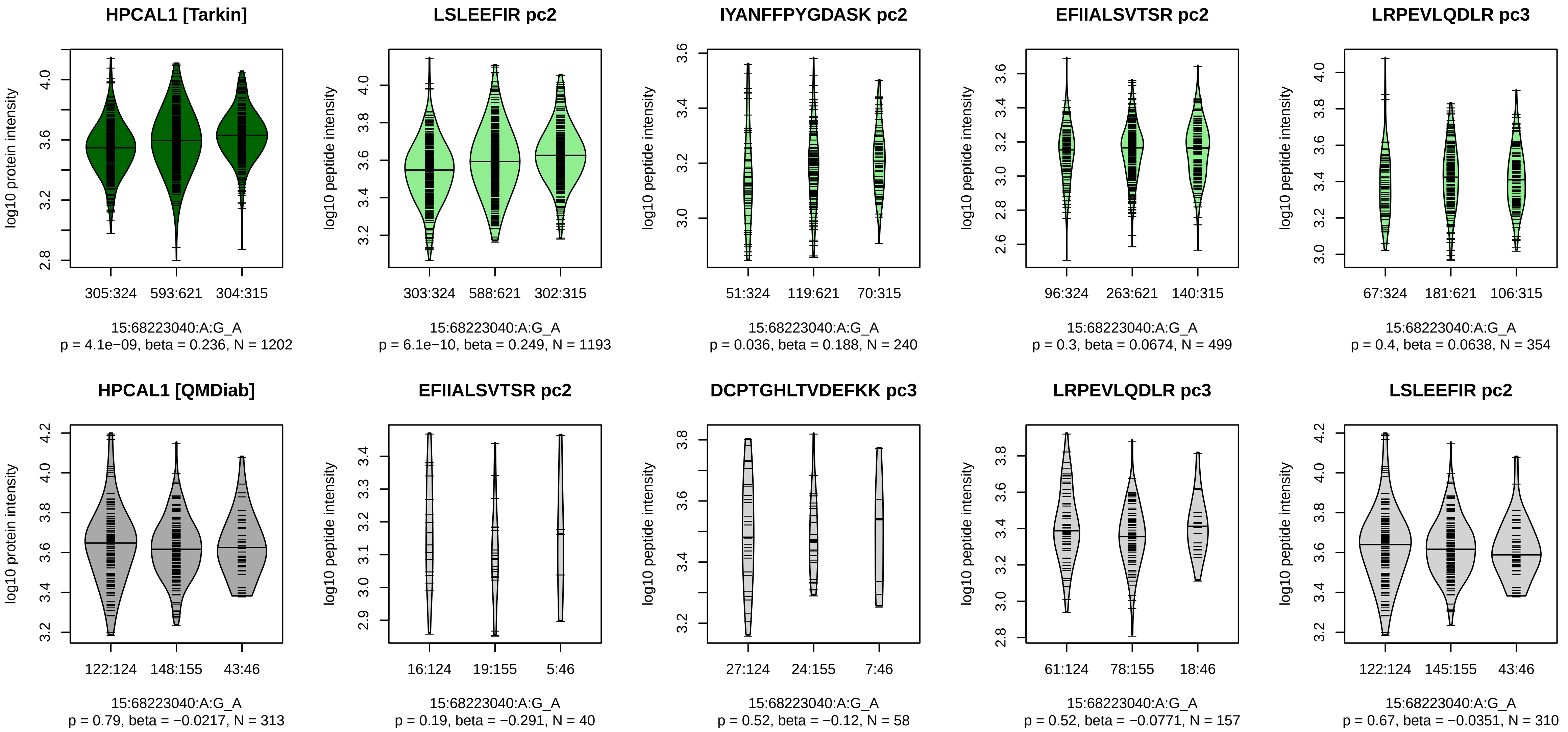

Sort: 236
Gene name: HPCAL1
SNP: 15:68223040:A:G
rsID: rs2448134
Type: Trans−pQTL
Protein group(s):
P37235
Nano−particle: NP2

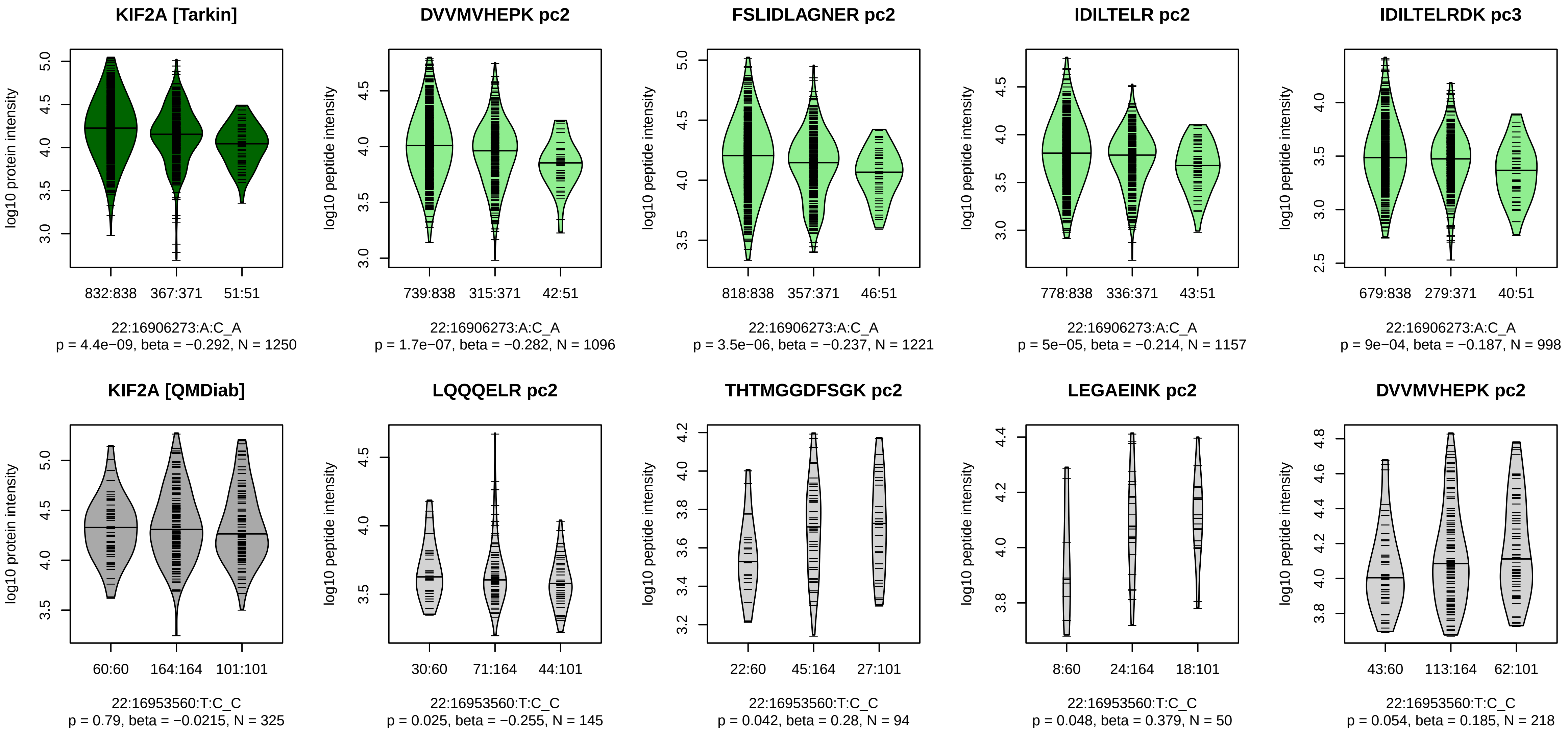

Sort: 237
Gene name: KIF2A
SNP: 22:16906273:A:C
rsID: rs3876018
Type: Trans−pQTL
Protein group(s):
T: A0A6Q8PFA6;A0A6Q8PH57;
Q: A0A6Q8PFA6;A0A6Q8PG37
Nano−particle: NP4

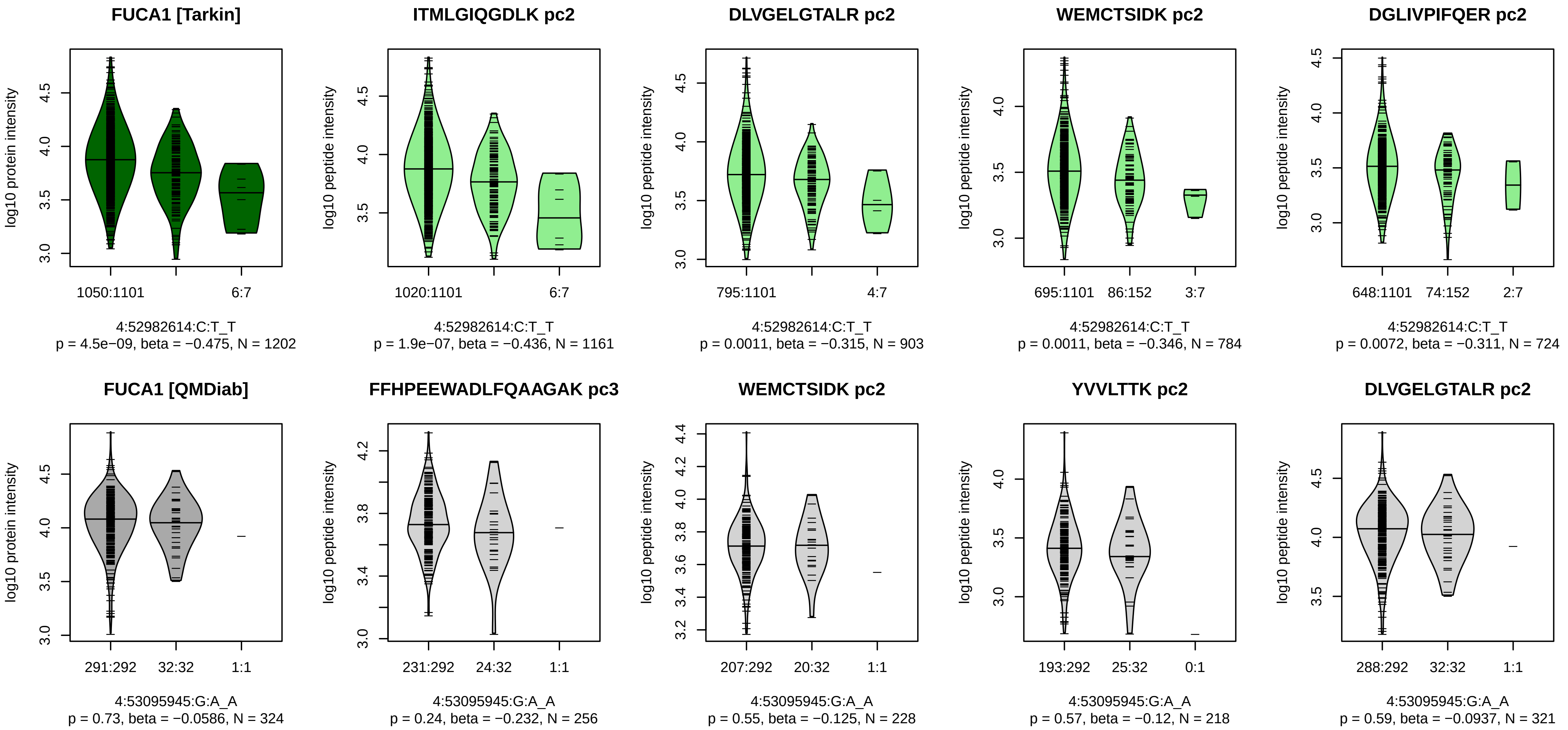

Sort: 238
Gene name: FUCA1
SNP: 4:52982614:C:T
rsID: rs75125328
Type: Trans−pQTL
Protein group(s):
P04066
Nano−particle: NP5

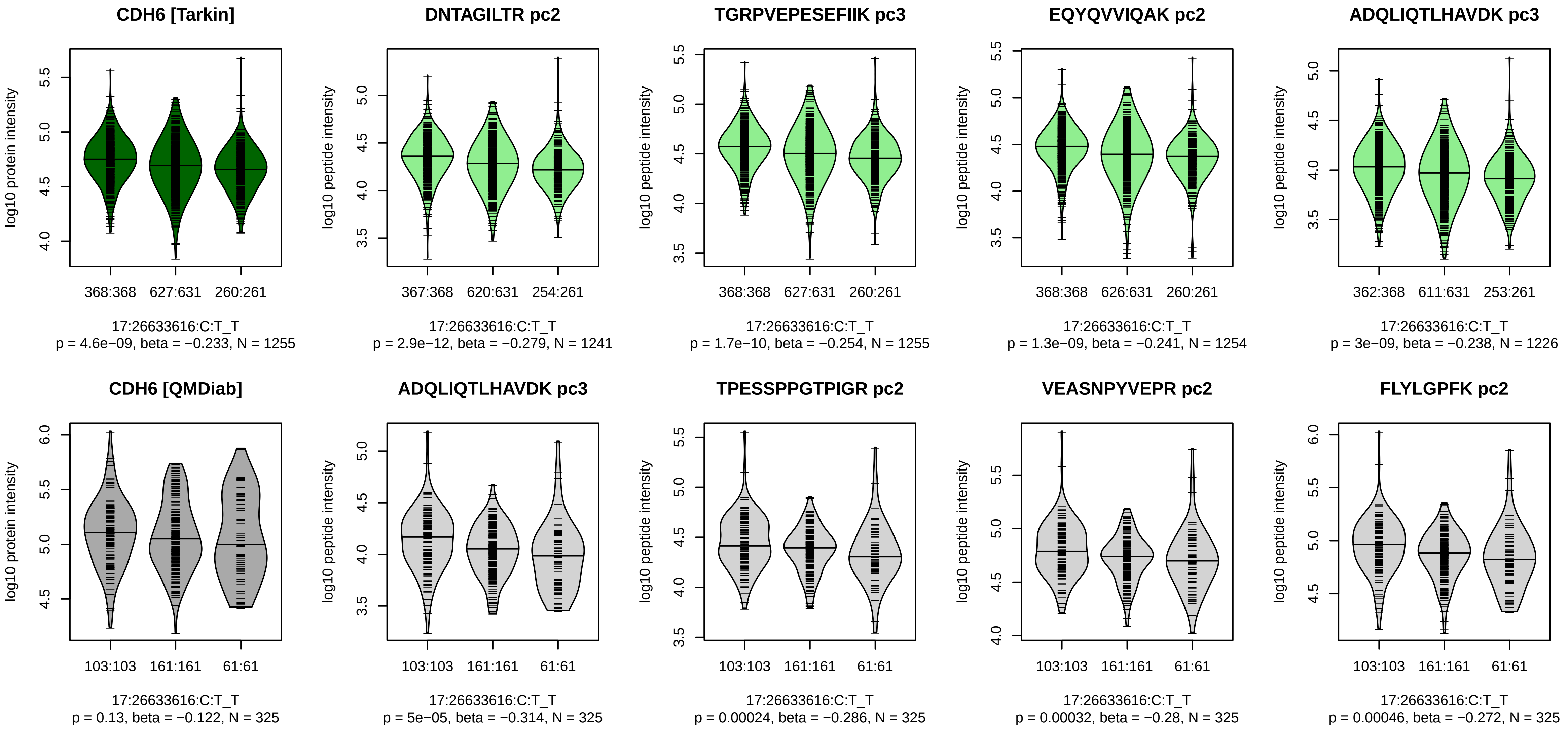

Sort: 239
Gene name: CDH6
SNP: 17:26633616:C:T
rsID: rs241777
Type: Trans−pQTL
Protein group(s):
P55285
Nano−particle: NP2

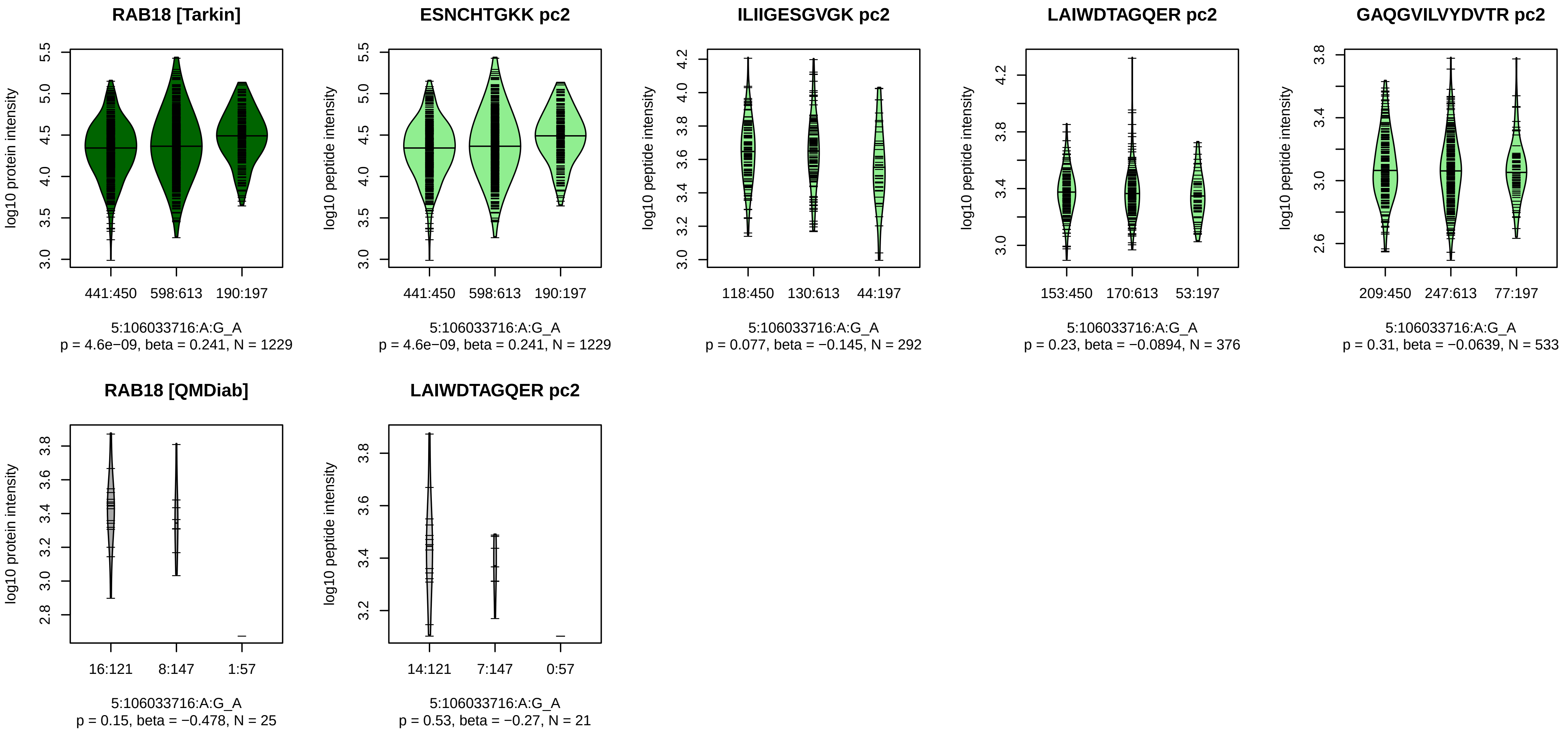

Sort: 240
Gene name: RAB18
SNP: 5:106033716:A:G
rsID: rs6596693
Type: Trans−pQTL
Protein group(s):
B7Z4P9
Nano−particle: NP4

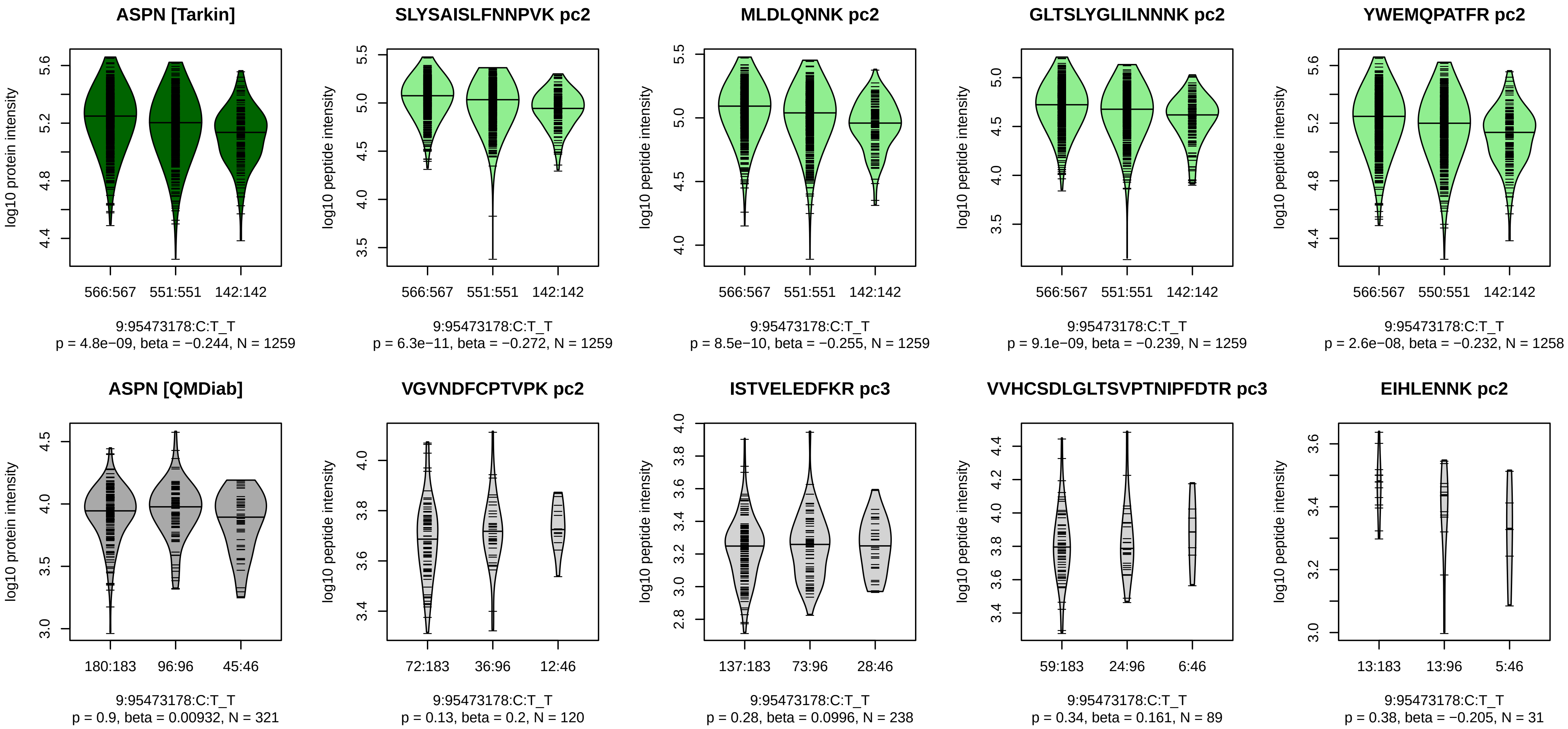

Sort: 241
Gene name: ASPN
SNP: 9:95473178:C:T
rsID: rs13286566
Type: Cis−pQTL
Protein group(s):
Q9BXN1
Nano−particle: NP4

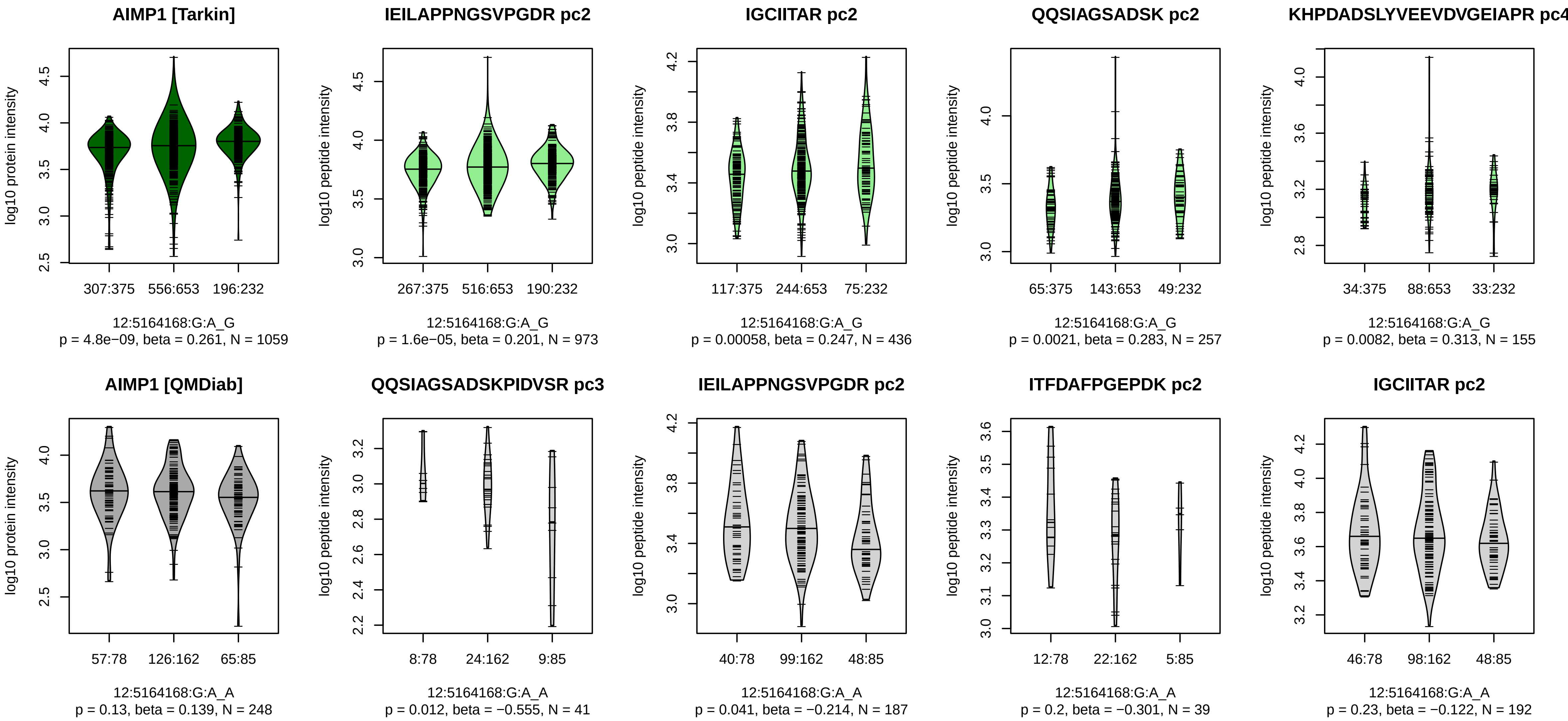

Sort: 242
Gene name: AIMP1
SNP: 12:5164168:G:A
rsID: rs10774298
Type: Trans−pQTL
Protein group(s):
Q12904
Nano−particle: NP3

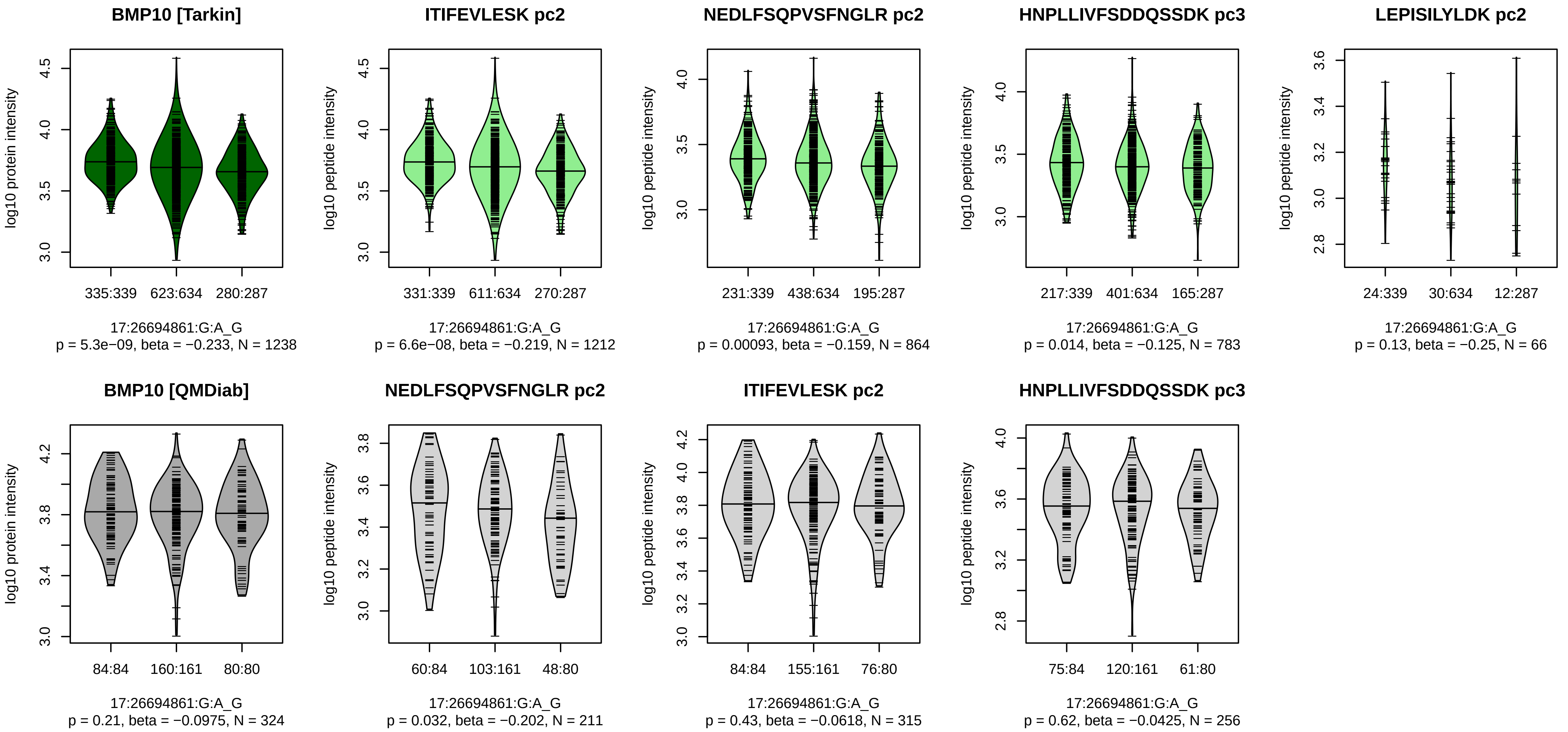

Sort: 243
Gene name: BMP10
SNP: 17:26694861:G:A
rsID: rs704
Type: Trans−pQTL
Protein group(s):
O95393
Nano−particle: NP2

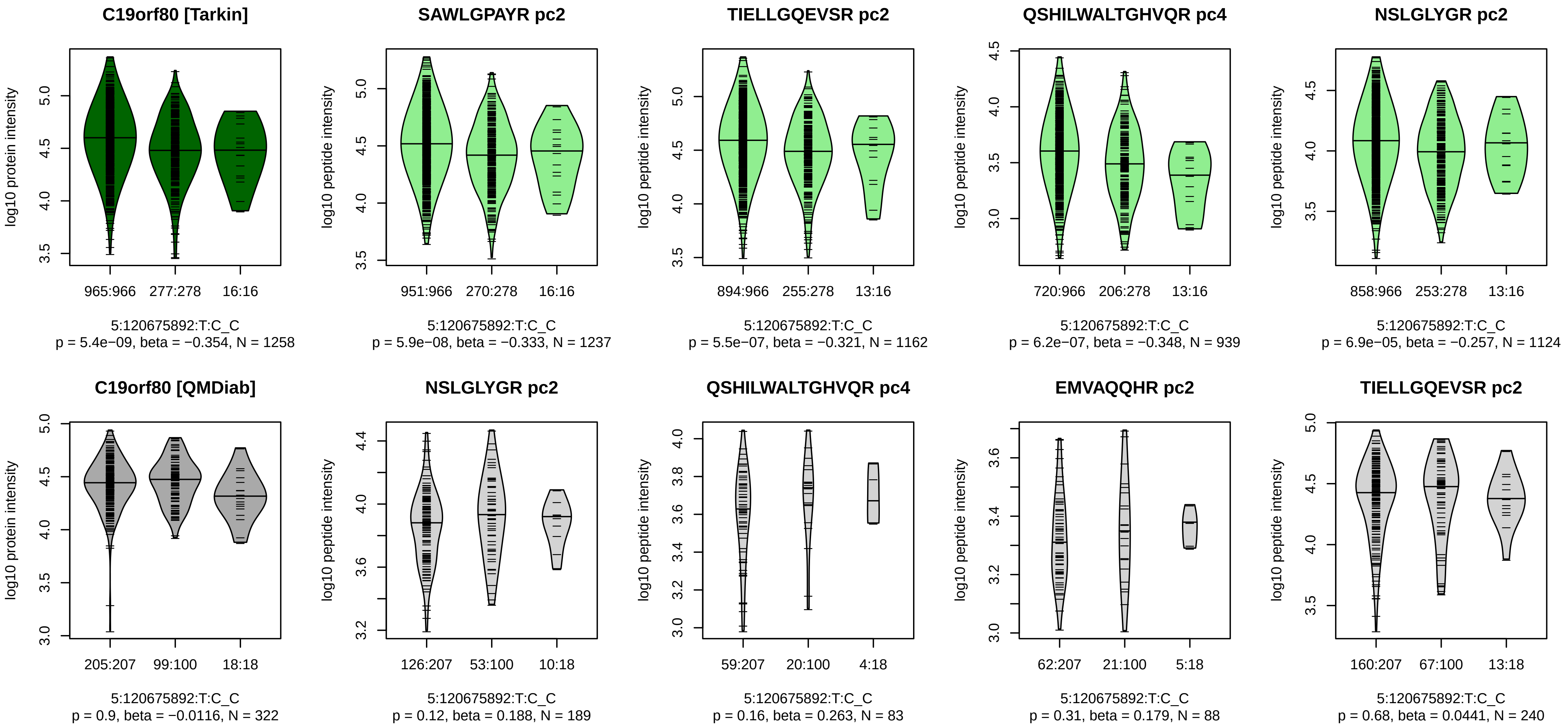

Sort: 244
Gene name: C19orf80
SNP: 5:120675892:T:C
rsID: rs67029685
Type: Trans−pQTL
Protein group(s):
Q6UXH0
Nano−particle: NP4

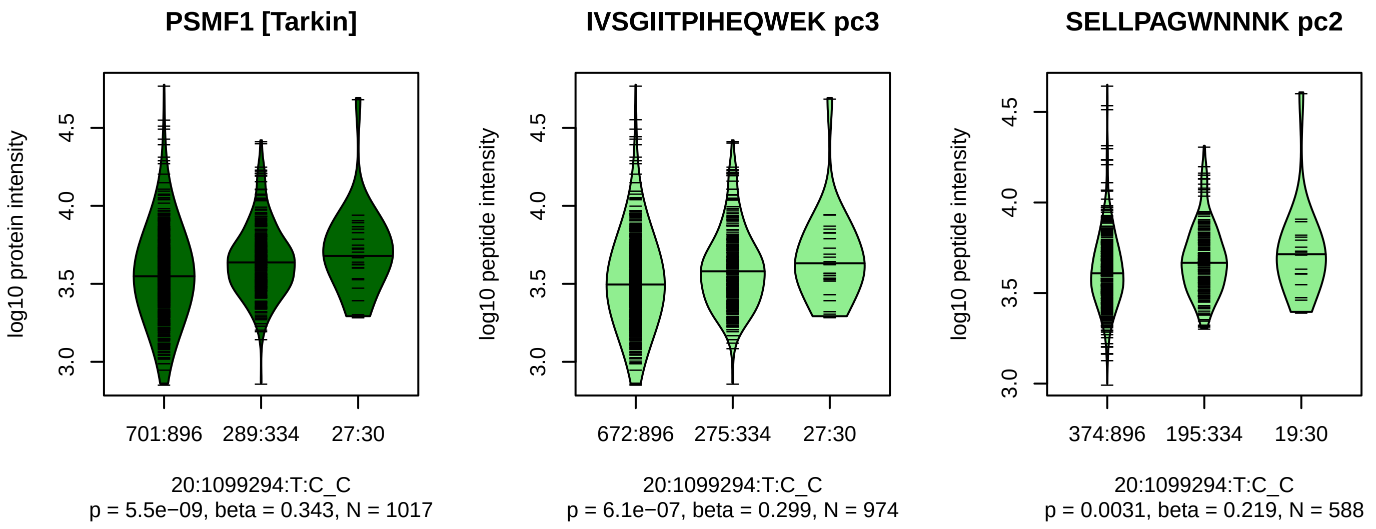

No protein data available
for replication in QMDiab

Sort: 245
Gene name: PSMF1
SNP: 20:1099294:T:C
rsID: rs2072963
Type: Cis−pQTL
Protein group(s):
Q5QPM7;Q92530
Nano−particle: NP1

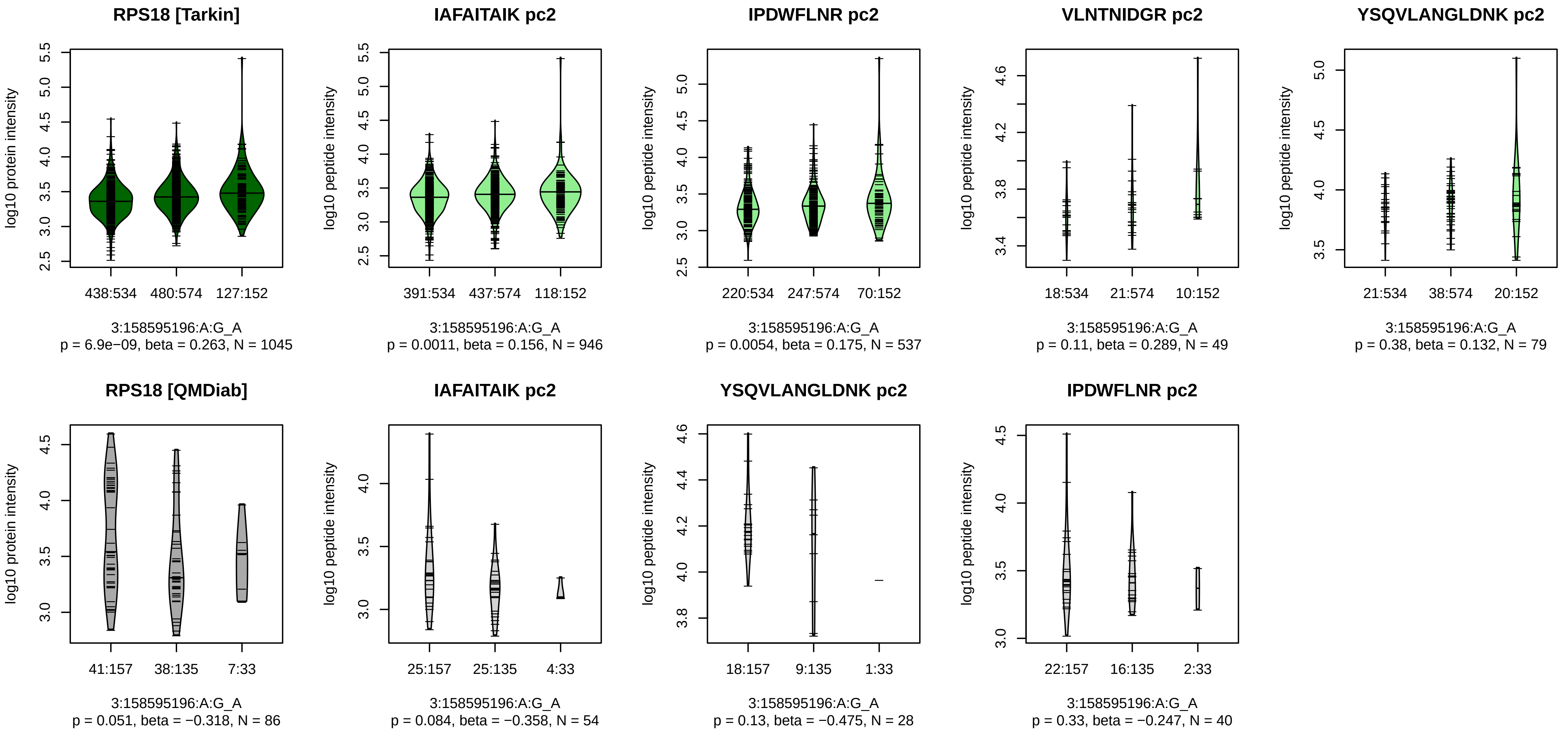

Sort: 246
Gene name: RPS18
SNP: 3:158595196:A:G
rsID: rs10804785
Type: Trans−pQTL
Protein group(s):
P62269
Nano−particle: NP4

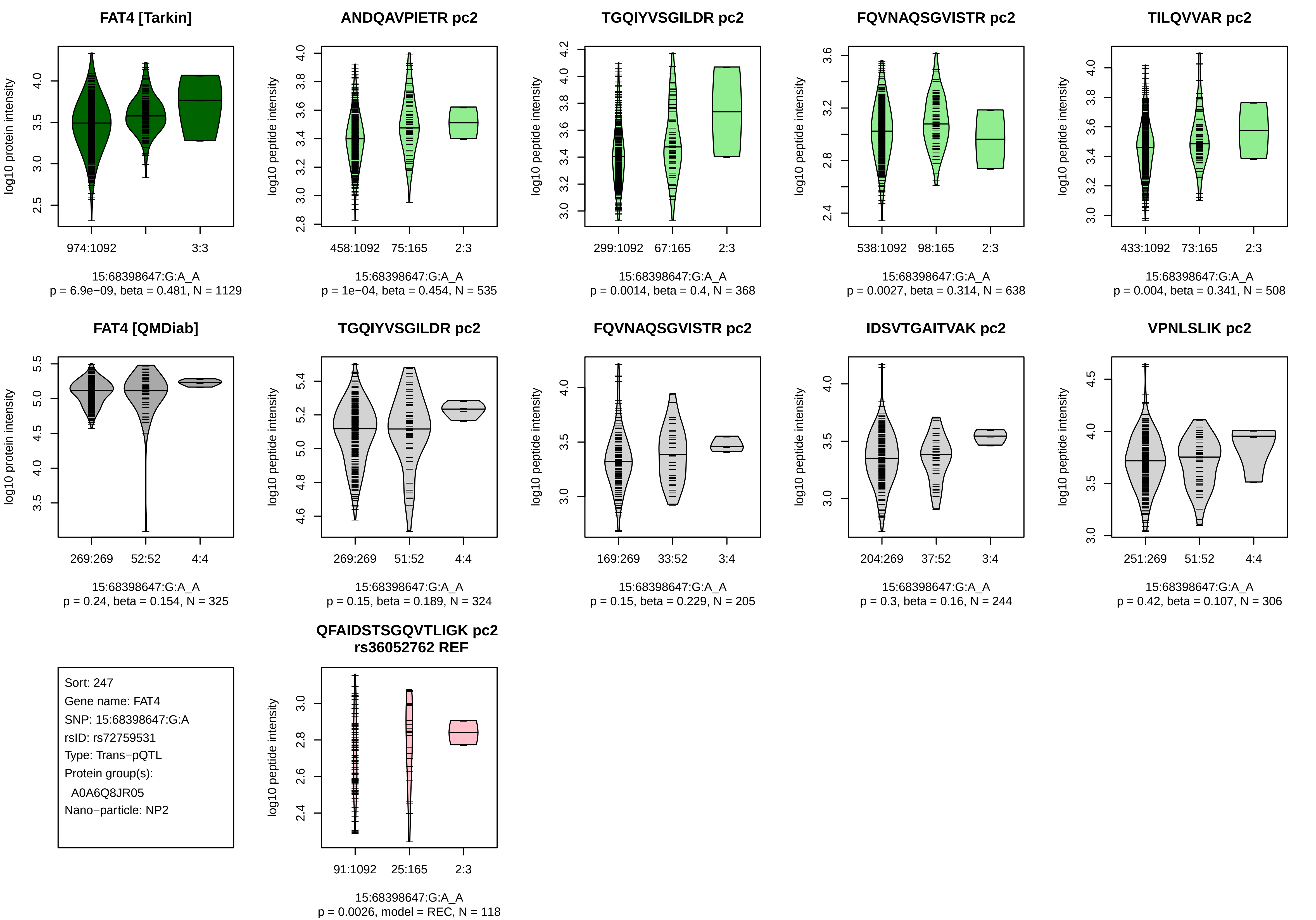
FAT4 [Tarkin]
log10 protein intensity
974:1092
3:3
15:68398647:G:A_A
p = 6.9e−09, beta = 0.481, N = 1129
ANDQAVPIETR pc2
log10 peptide intensity
458:1092 75:165 2:3
15:68398647:G:A_A
p = 1e−04, beta = 0.454, N = 535
TGQIYVSGILDR pc2
299:1092 67:165 2:3
15:68398647:G:A_A
p = 0.0014, beta = 0.4, N = 368
FQVNAQSGVISTR pc2
538:1092 98:165 2:3
15:68398647:G:A_A
p = 0.0027, beta = 0.314, N = 638
TILQVVAR pc2
433:1092 73:165 2:3
15:68398647:G:A_A
p = 0.004, beta = 0.341, N = 508
FAT4 [QMDiab]
269:269 52:52 4:4
15:68398647:G:A_A
p = 0.24, beta = 0.154, N = 325
TGQIYVSGILDR pc2
269:269 51:52 4:4
15:68398647:G:A_A
p = 0.15, beta = 0.189, N = 324
FQVNAQSGVISTR pc2
169:269 33:52 3:4
15:68398647:G:A_A
p = 0.15, beta = 0.229, N = 205
IDSVTGAITVAK pc2
204:269 37:52 3:4
15:68398647:G:A_A
p = 0.3, beta = 0.16, N = 244
VPNLSLIK pc2
251:269 51:52 4:4
15:68398647:G:A_A
p = 0.42, beta = 0.107, N = 306
Sort: 247
Gene name: FAT4
SNP: 15:68398647:G:A
rsID: rs72759531
Type: Trans−pQTL
Protein group(s):
A0A6Q8JR05
Nano−particle: NP2
QFAIDSTSGQVTLIGK pc2
rs36052762 REF
91:1092 25:165 2:3
15:68398647:G:A_A
p = 0.0026, model = REC, N = 118

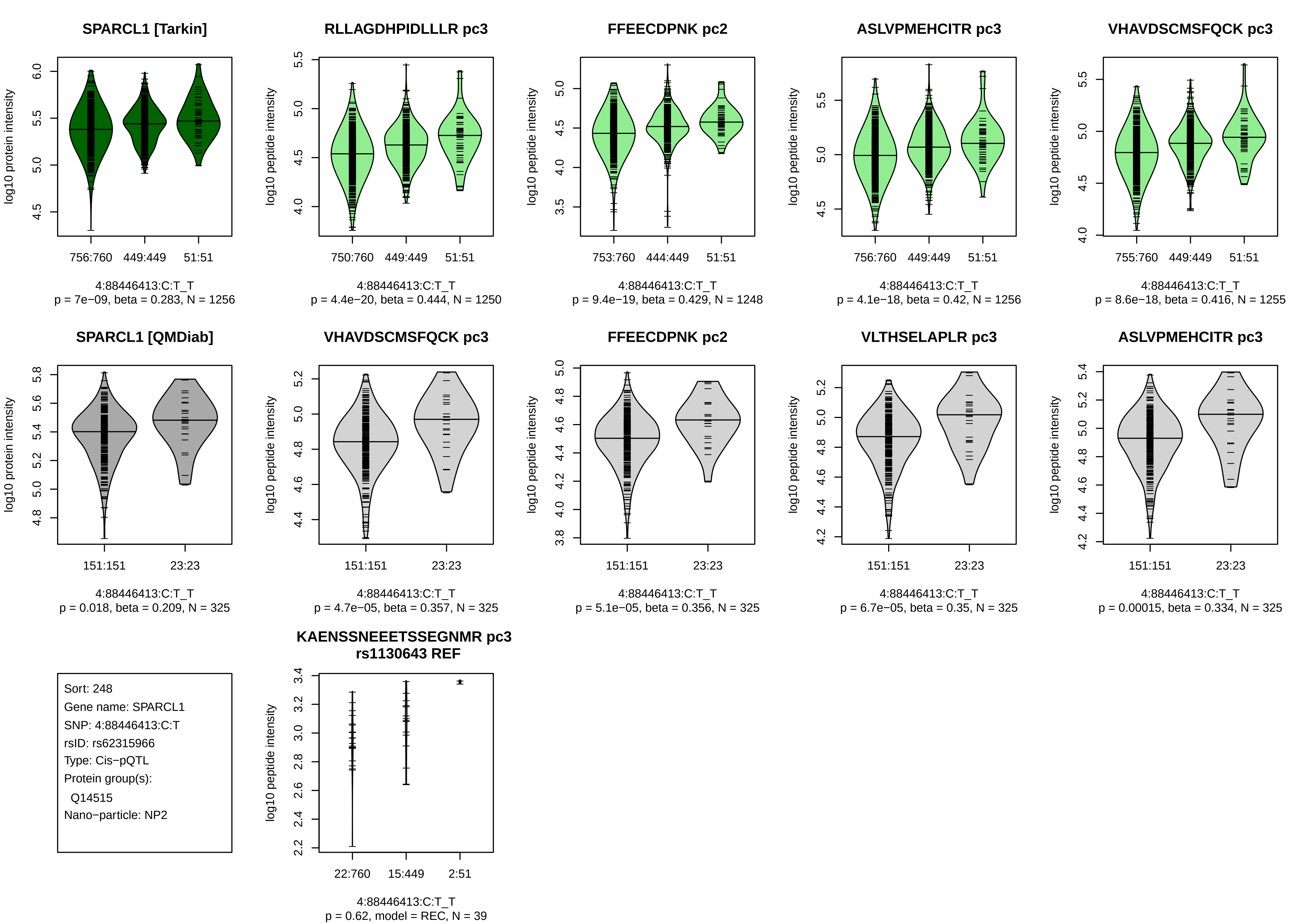
SPARCL1 [Tarkin]
log10 protein intensity
756:760
449:449
51:51
4:88446413:C:T_T
p = 7e−09, beta = 0.283, N = 1256
RLLAGDHPIDLLLR pc3
log10 peptide intensity
750:760
449:449
51:51
4:88446413:C:T_T
p = 4.4e−20, beta = 0.444, N = 1250
FFEECDPNK pc2
753:760
444:449
51:51
4:88446413:C:T_T
p = 9.4e−19, beta = 0.429, N = 1248
ASLVPMEHCITR pc3
756:760
449:449
51:51
4:88446413:C:T_T
p = 4.1e−18, beta = 0.42, N = 1256
VHAVDSCMSFQCK pc3
755:760
449:449
51:51
4:88446413:C:T_T
p = 8.6e−18, beta = 0.416, N = 1255
SPARCL1 [QMDiab]
151:151
23:23
4:88446413:C:T_T
p = 0.018, beta = 0.209, N = 325
VHAVDSCMSFQCK pc3
4:88446413:C:T_T
p = 4.7e−05, beta = 0.357, N = 325
FFEECDPNK pc2
4:88446413:C:T_T
p = 5.1e−05, beta = 0.356, N = 325
VLTHSELAPLR pc3
4:88446413:C:T_T
p = 6.7e−05, beta = 0.35, N = 325
ASLVPMEHCITR pc3
4:88446413:C:T_T
p = 0.00015, beta = 0.334, N = 325
Sort: 248
Gene name: SPARCL1
SNP: 4:88446413:C:T
rsID: rs62315966
Type: Cis−pQTL
Protein group(s):
Q14515
Nano−particle: NP2
KAENSSNEEETSSEGNMR pc3
rs1130643 REF
22:760
15:449
2:51
4:88446413:C:T_T
p = 0.62, model = REC, N = 39

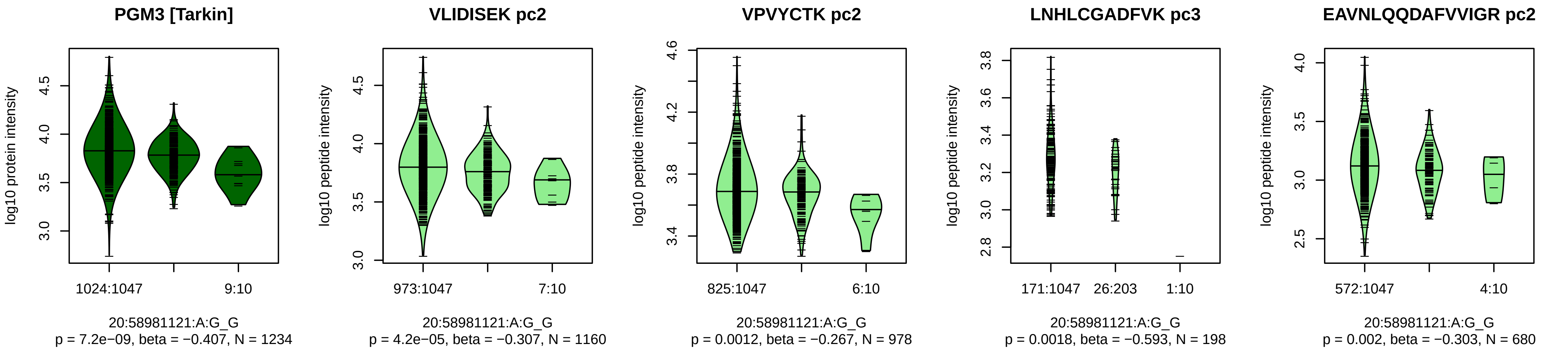

No protein data available
for replication in QMDiab

Sort: 249
Gene name: PGM3
SNP: 20:58981121:A:G
rsID: rs78912946
Type: Trans−pQTL
Protein group(s):
H0Y987
Nano−particle: NP5

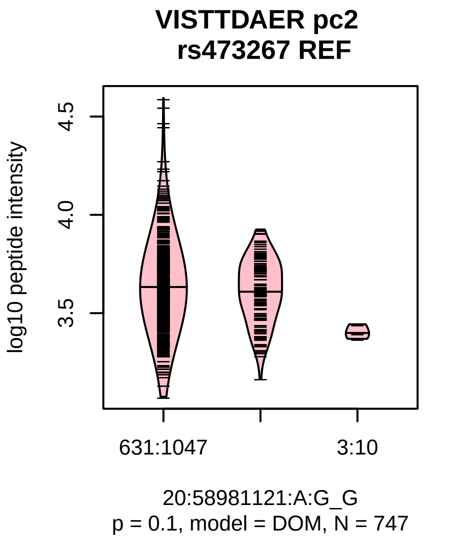

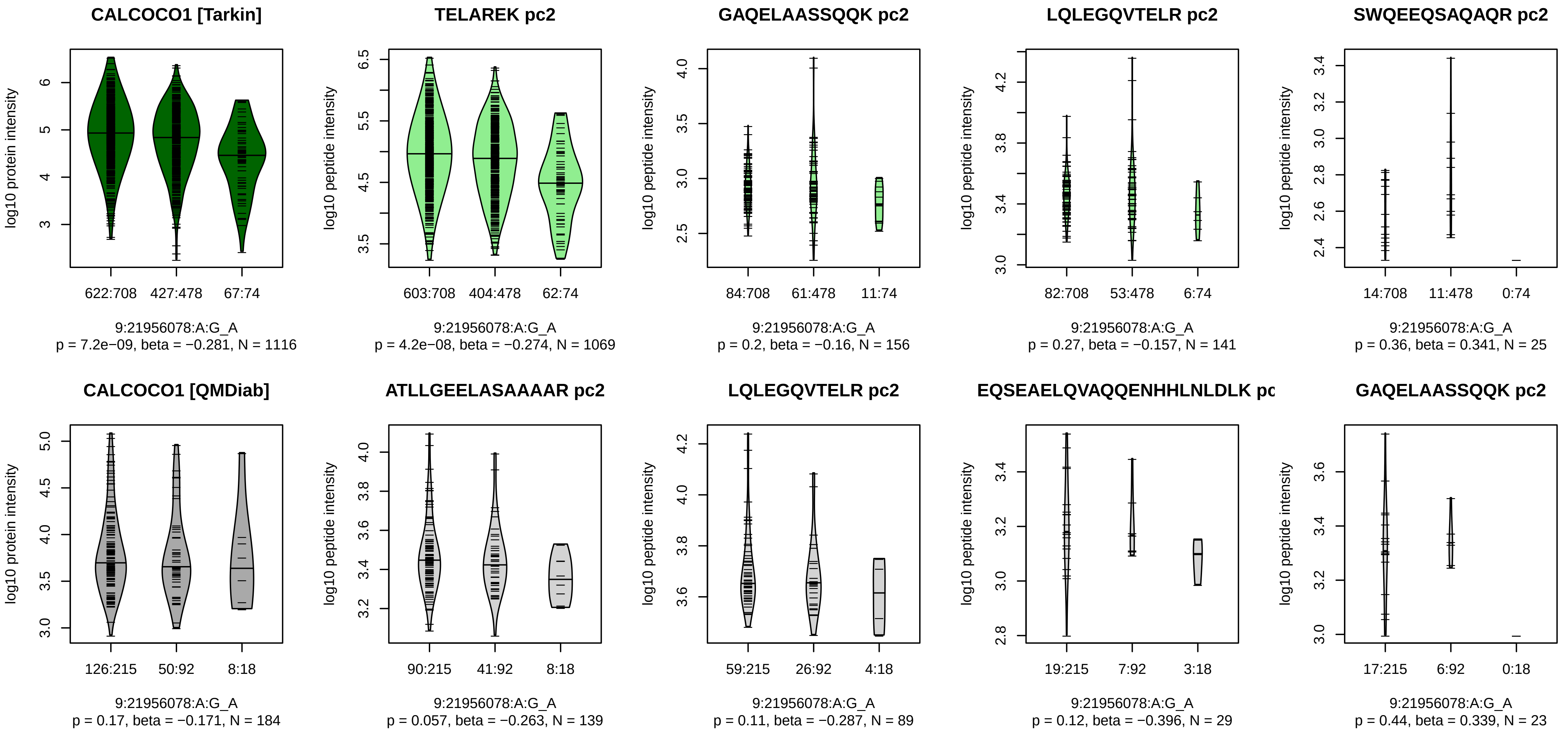

Sort: 250
Gene name: CALCOCO1
SNP: 9:21956078:A:G
rsID: rs2027938
Type: Trans−pQTL
Protein group(s):
Q9P1Z2;Q9P1Z2−2;Q9P1Z2−
Nano−particle: NP5

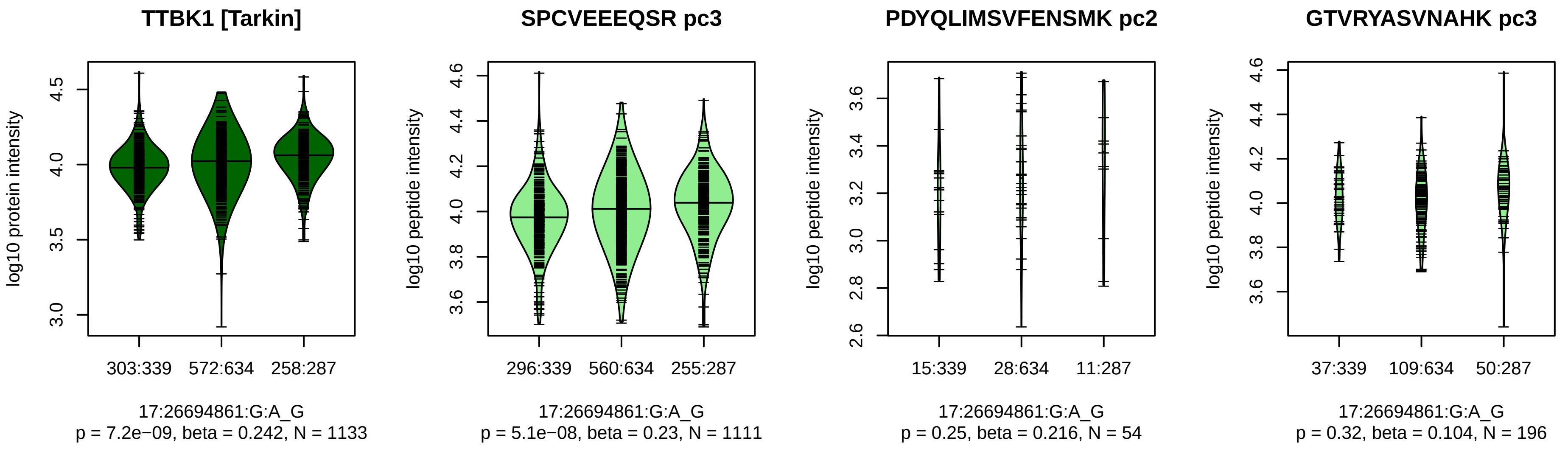

No protein data available
for replication in QMDiab

Sort: 251
Gene name: TTBK1
SNP: 17:26694861:G:A
rsID: rs704
Type: Trans−pQTL
Protein group(s):
X6R456
Nano−particle: NP2

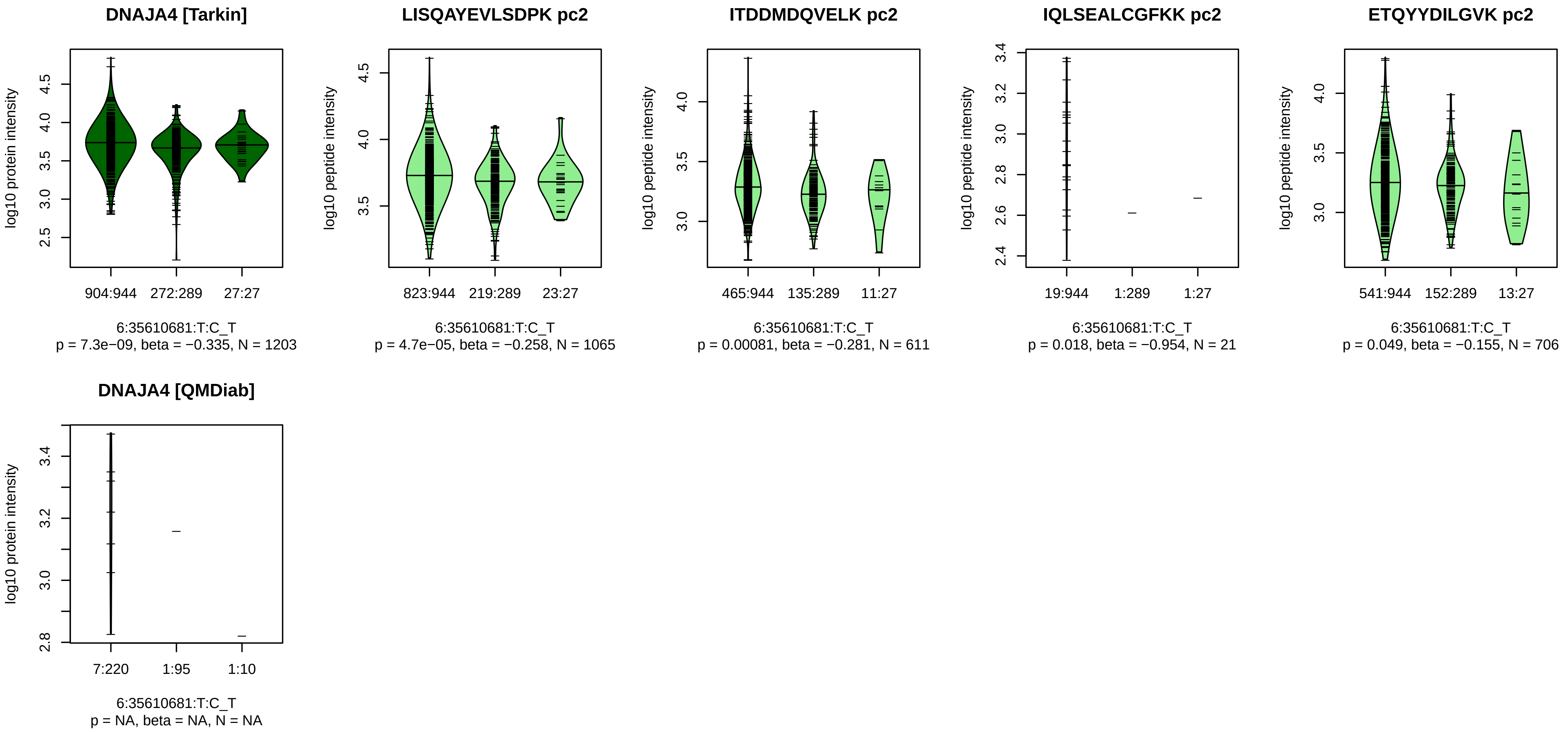

Sort: 252
Gene name: DNAJA4
SNP: 6:35610681:T:C
rsID: rs2143404
Type: Trans−pQTL
Protein group(s):
T: Q8WW22−2
Q: Q8WW22;Q8WW22−2
Nano−particle: NP1

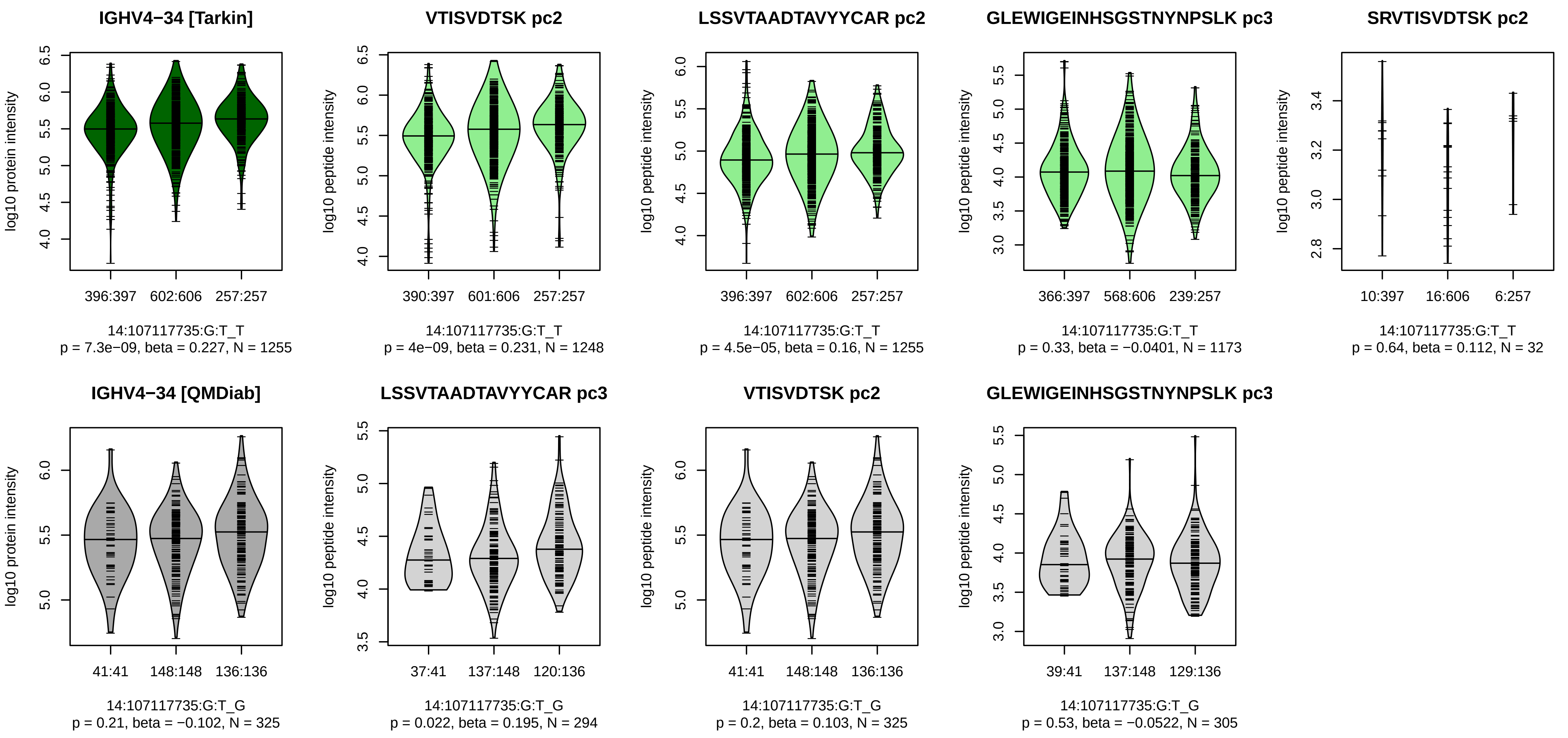

Sort: 253
Gene name: IGHV4−34
SNP: 14:107117735:G:T
rsID: rs1858687
Type: Cis−pQTL
Protein group(s):
P06331
Nano−particle: NP3

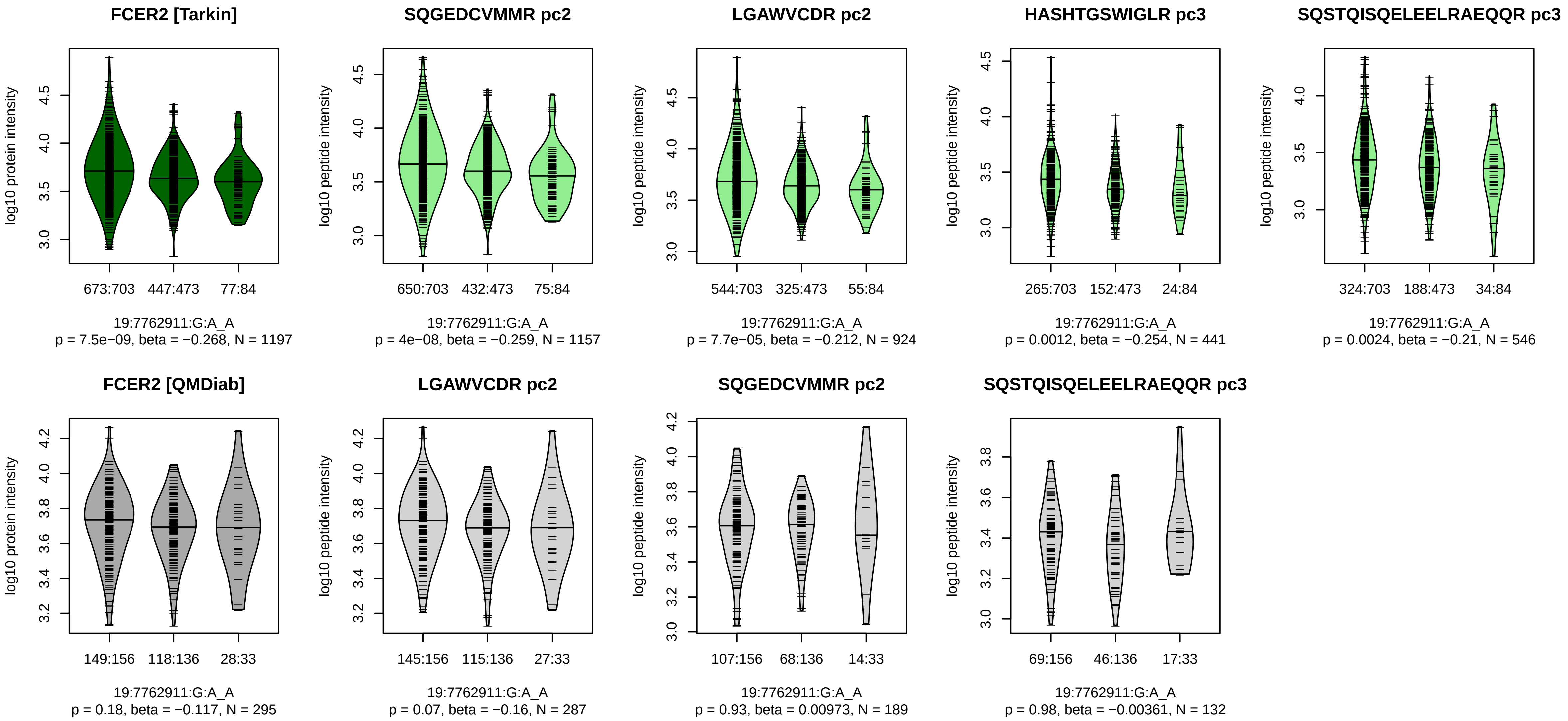

Sort: 254
Gene name: FCER2
SNP: 19:7762911:G:A
rsID: rs7249360
Type: Cis−pQTL
Protein group(s):
P06734
Nano−particle: NP2

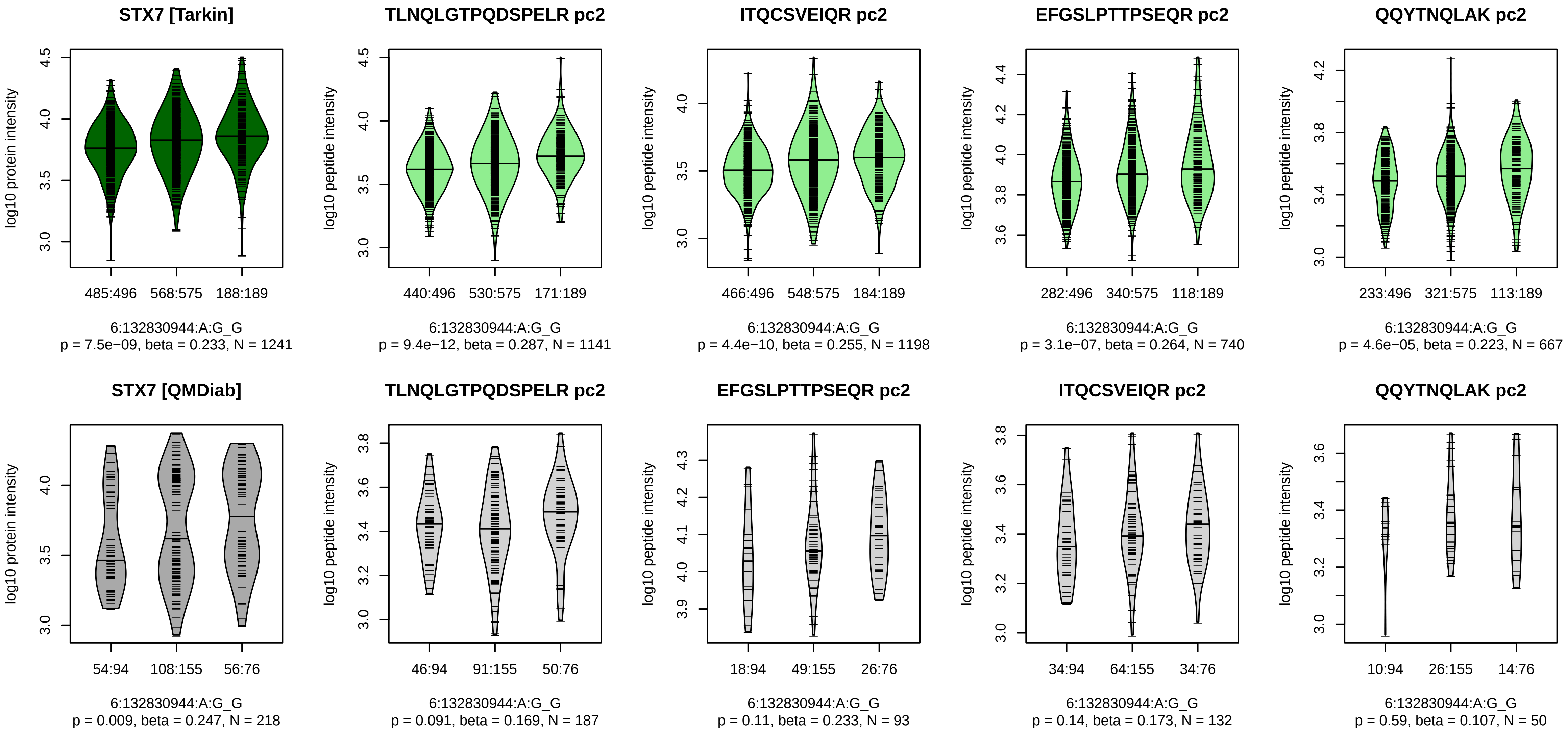

Sort: 255
Gene name: STX7
SNP: 6:132830944:A:G
rsID: rs9493341
Type: Cis−pQTL
Protein group(s):
O15400;O15400−2
Nano−particle: NP1

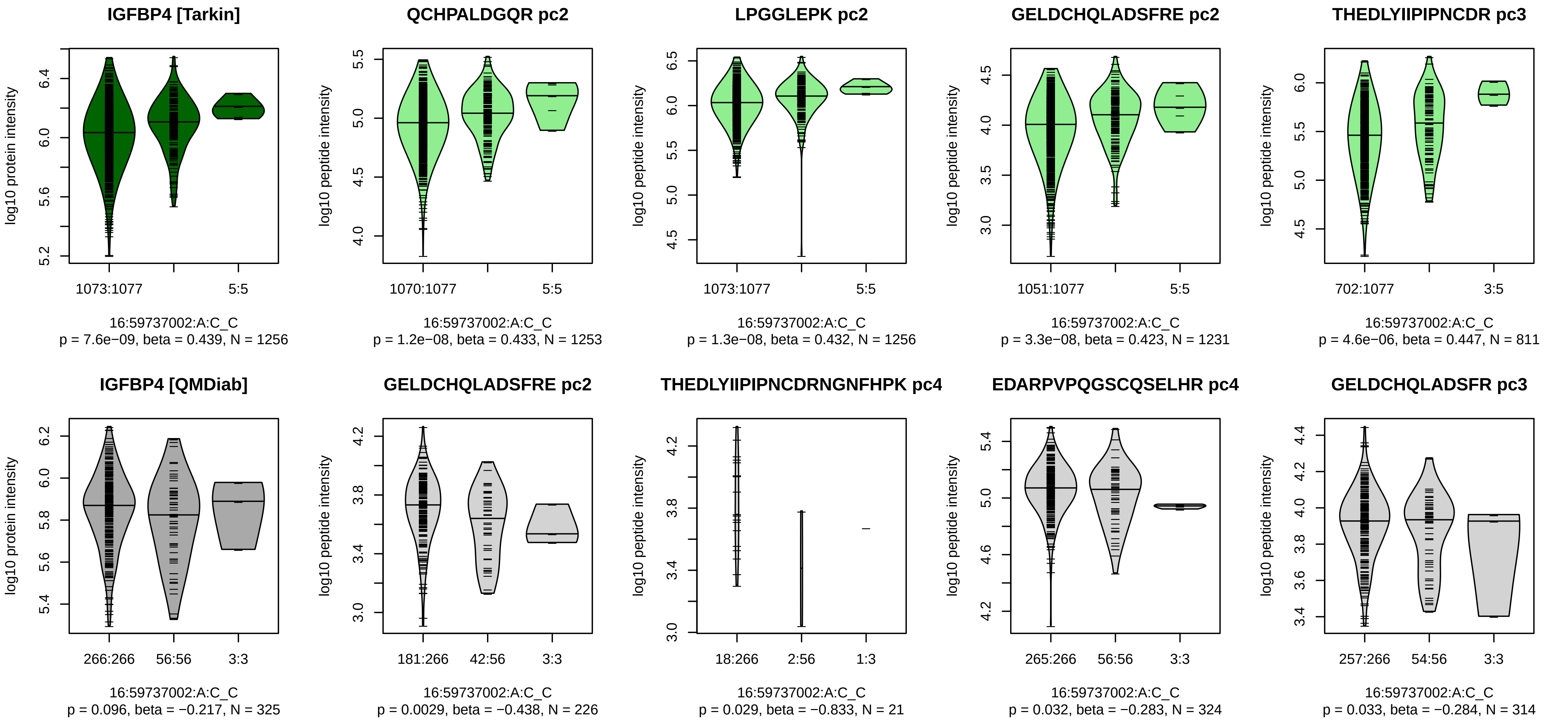

Sort: 256
Gene name: IGFBP4
SNP: 16:59737002:A:C
rsID: rs58794056
Type: Trans−pQTL
Protein group(s):
P22692
Nano−particle: NP1

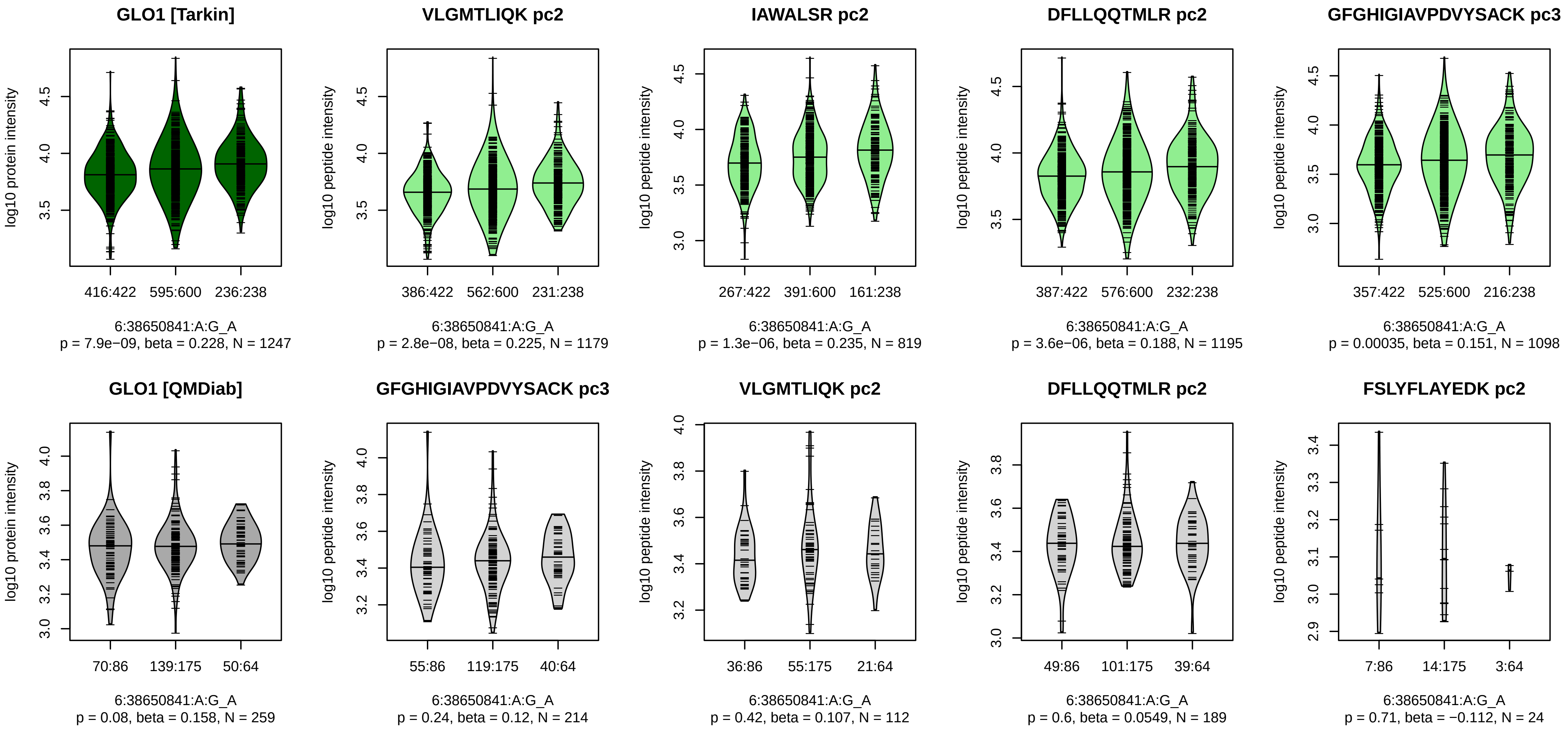

Sort: 257
Gene name: GLO1
SNP: 6:38650841:A:G
rsID: rs4714175
Type: Cis−pQTL
Protein group(s):
Q04760
Nano−particle: NP2

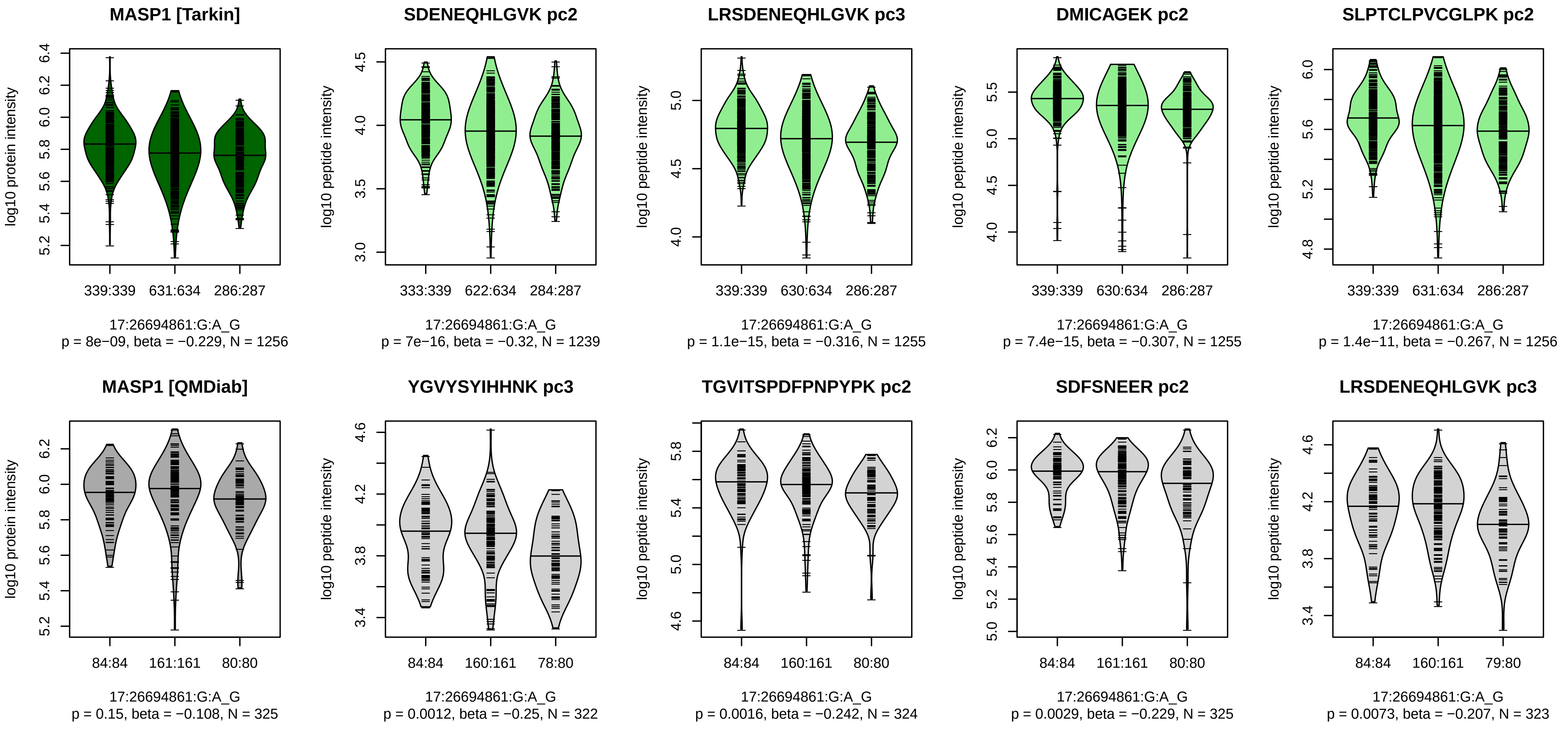

Sort: 258
Gene name: MASP1
SNP: 17:26694861:G:A
rsID: rs704
Type: Trans−pQTL
Protein group(s):
P48740
Nano−particle: NP2

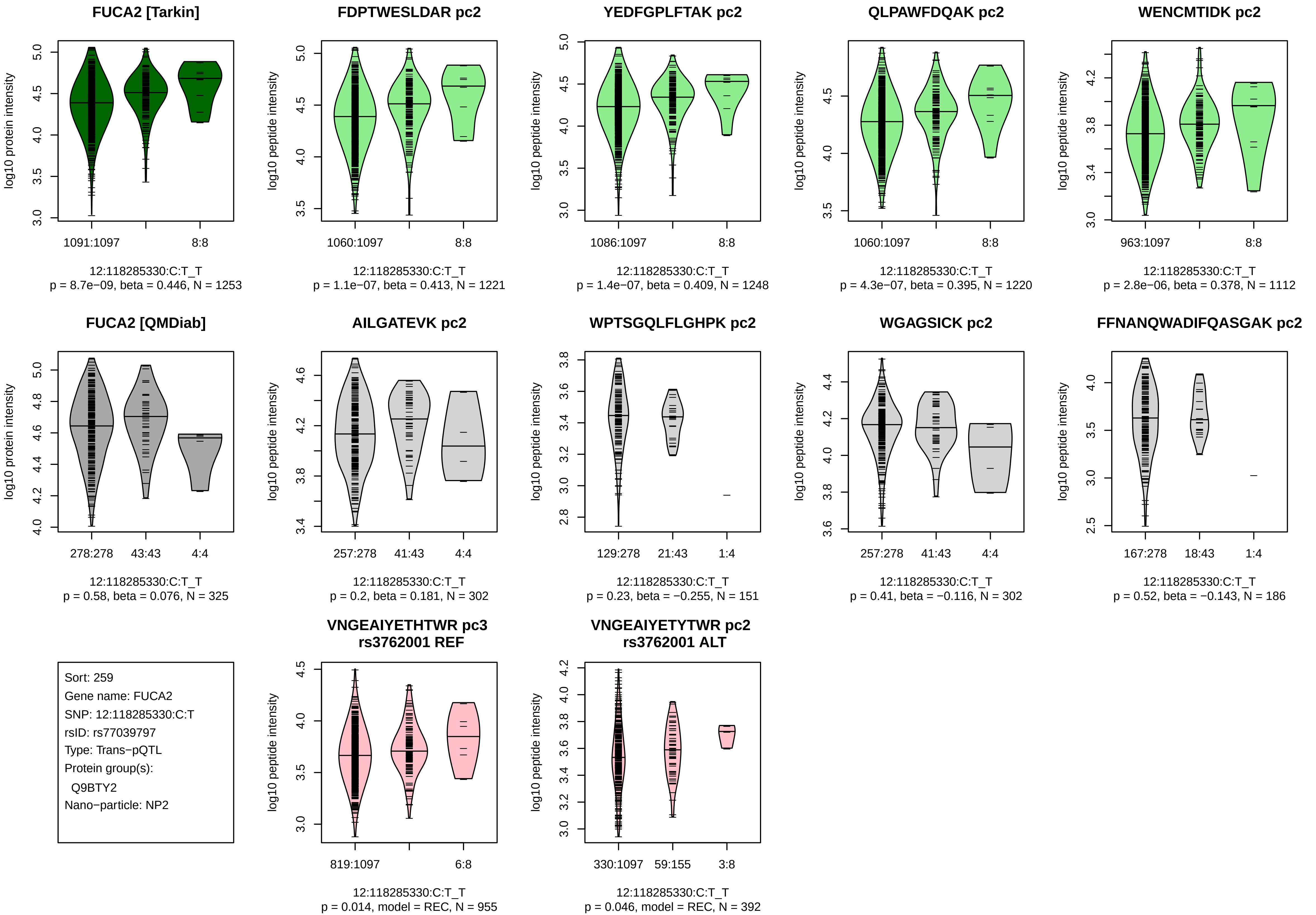
FUCA2 [Tarkin]
log10 protein intensity
1091:1097
8:8
12:118285330:C:T_T
p = 8.7e−09, beta = 0.446, N = 1253
FDPTWESLDAR pc2
log10 peptide intensity
1060:1097
8:8
12:118285330:C:T_T
p = 1.1e−07, beta = 0.413, N = 1221
YEDFGPLFTAK pc2
log10 peptide intensity
1086:1097
8:8
12:118285330:C:T_T
p = 1.4e−07, beta = 0.409, N = 1248
QLPAWFDQAK pc2
log10 peptide intensity
1060:1097
8:8
12:118285330:C:T_T
p = 4.3e−07, beta = 0.395, N = 1220
WENCMTIDK pc2
log10 peptide intensity
963:1097
8:8
12:118285330:C:T_T
p = 2.8e−06, beta = 0.378, N = 1112
FUCA2 [QMDiab]
log10 protein intensity
278:278
43:43
4:4
12:118285330:C:T_T
p = 0.58, beta = 0.076, N = 325
AILGATEVK pc2
log10 peptide intensity
257:278
41:43
4:4
12:118285330:C:T_T
p = 0.2, beta = 0.181, N = 302
WPTSGQLFLGHPK pc2
log10 peptide intensity
129:278
21:43
1:4
12:118285330:C:T_T
p = 0.23, beta = −0.255, N = 151
WGAGSICK pc2
log10 peptide intensity
257:278
41:43
4:4
12:118285330:C:T_T
p = 0.41, beta = −0.116, N = 302
FFNANQWADIFQASGAK pc2
log10 peptide intensity
167:278
18:43
1:4
12:118285330:C:T_T
p = 0.52, beta = −0.143, N = 186
Sort: 259
Gene name: FUCA2
SNP: 12:118285330:C:T
rsID: rs77039797
Type: Trans−pQTL
Protein group(s):
Q9BTY2
Nano−particle: NP2
VNGEAIYETHTWR pc3
rs3762001 REF
log10 peptide intensity
819:1097
6:8
12:118285330:C:T_T
p = 0.014, model = REC, N = 955
VNGEAIYETYTWR pc2
rs3762001 ALT
log10 peptide intensity
330:1097
59:155
3:8
12:118285330:C:T_T
p = 0.046, model = REC, N = 392

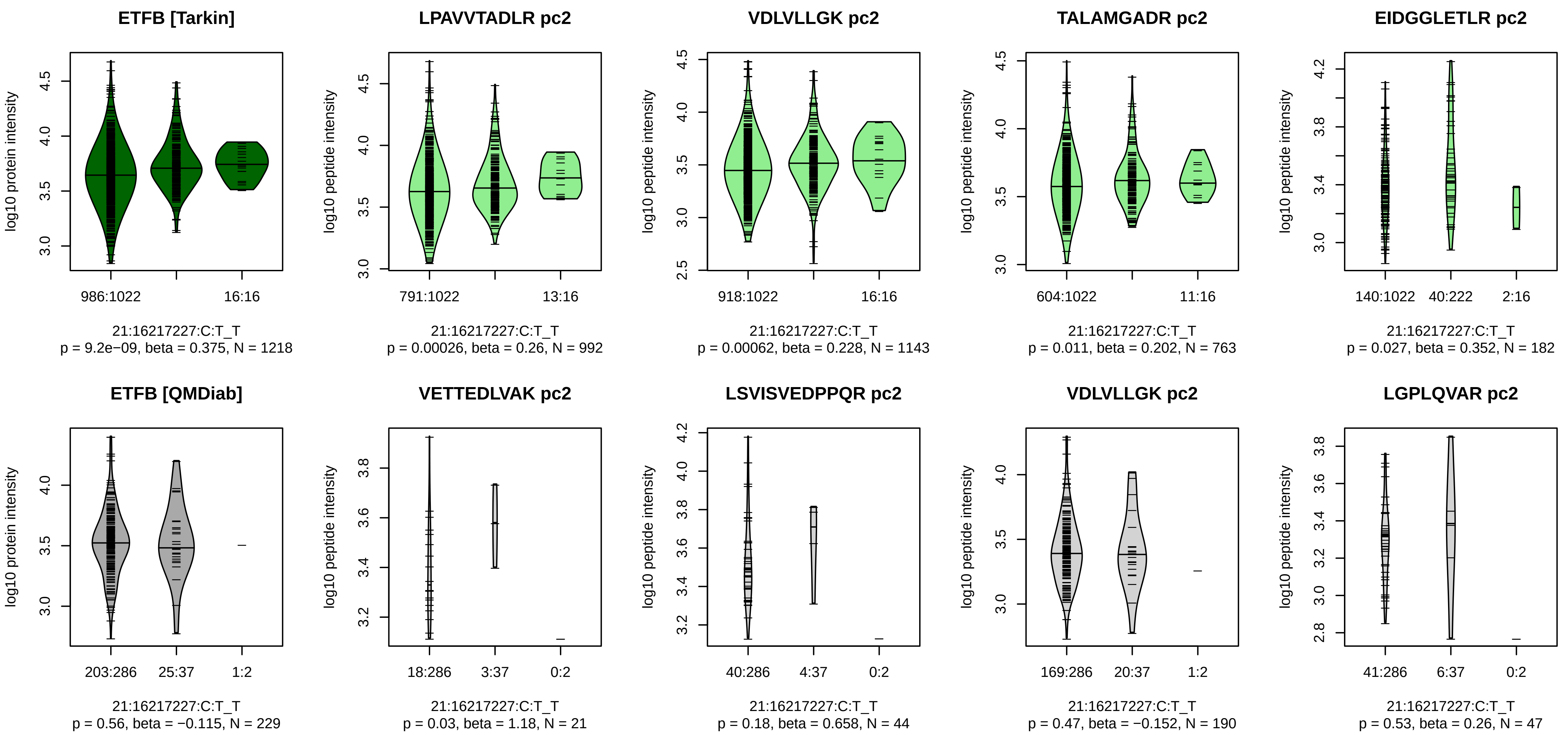

Sort: 260
Gene name: ETFB
SNP: 21:16217227:C:T
rsID: rs75418177
Type: Trans−pQTL
Protein group(s):
T: P38117−2
Q: P38117;P38117−2
Nano−particle: NP1

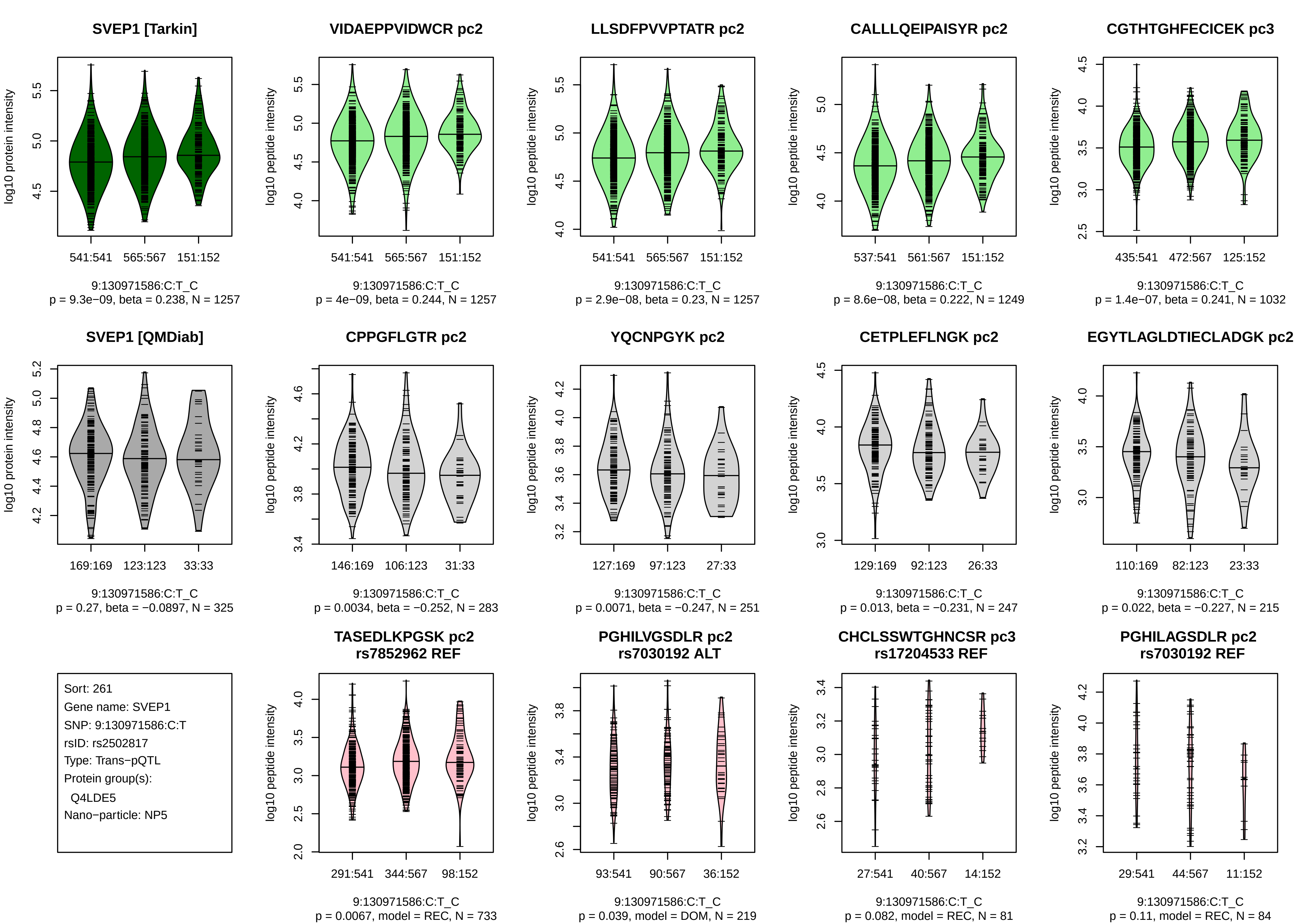
SVEP1 [Tarkin]
log10 protein intensity
541:541 565:567 151:152
9:130971586:C:T_C
p = 9.3e−09, beta = 0.238, N = 1257
VIDAEPPVIDWCR pc2
log10 peptide intensity
541:541 565:567 151:152
9:130971586:C:T_C
p = 4e−09, beta = 0.244, N = 1257
LLSDFPVVPTATR pc2
541:541 565:567 151:152
9:130971586:C:T_C
p = 2.9e−08, beta = 0.23, N = 1257
CALLLQEIPAISYR pc2
537:541 561:567 151:152
9:130971586:C:T_C
p = 8.6e−08, beta = 0.222, N = 1249
CGTHTGHFECICEK pc3
435:541 472:567 125:152
9:130971586:C:T_C
p = 1.4e−07, beta = 0.241, N = 1032
SVEP1 [QMDiab]
169:169 123:123 33:33
9:130971586:C:T_C
p = 0.27, beta = −0.0897, N = 325
CPPGFLGTR pc2
146:169 106:123 31:33
9:130971586:C:T_C
p = 0.0034, beta = −0.252, N = 283
YQCNPGYK pc2
127:169 97:123 27:33
9:130971586:C:T_C
p = 0.0071, beta = −0.247, N = 251
CETPLEFLNGK pc2
129:169 92:123 26:33
9:130971586:C:T_C
p = 0.013, beta = −0.231, N = 247
EGYTLAGLDTIECLADGK pc2
110:169 82:123 23:33
9:130971586:C:T_C
p = 0.022, beta = −0.227, N = 215
Sort: 261
Gene name: SVEP1
SNP: 9:130971586:C:T
rsID: rs2502817
Type: Trans−pQTL
Protein group(s):
Q4LDE5
Nano−particle: NP5
TASEDLKPGSK pc2
rs7852962 REF
291:541 344:567 98:152
9:130971586:C:T_C
p = 0.0067, model = REC, N = 733
PGHILVGSDLR pc2
rs7030192 ALT
93:541 90:567 36:152
9:130971586:C:T_C
p = 0.039, model = DOM, N = 219
CHCLSSWTGHNCSR pc3
rs17204533 REF
27:541 40:567 14:152
9:130971586:C:T_C
p = 0.082, model = REC, N = 81
PGHILAGSDLR pc2
rs7030192 REF
29:541 44:567 11:152
9:130971586:C:T_C
p = 0.11, model = REC, N = 84

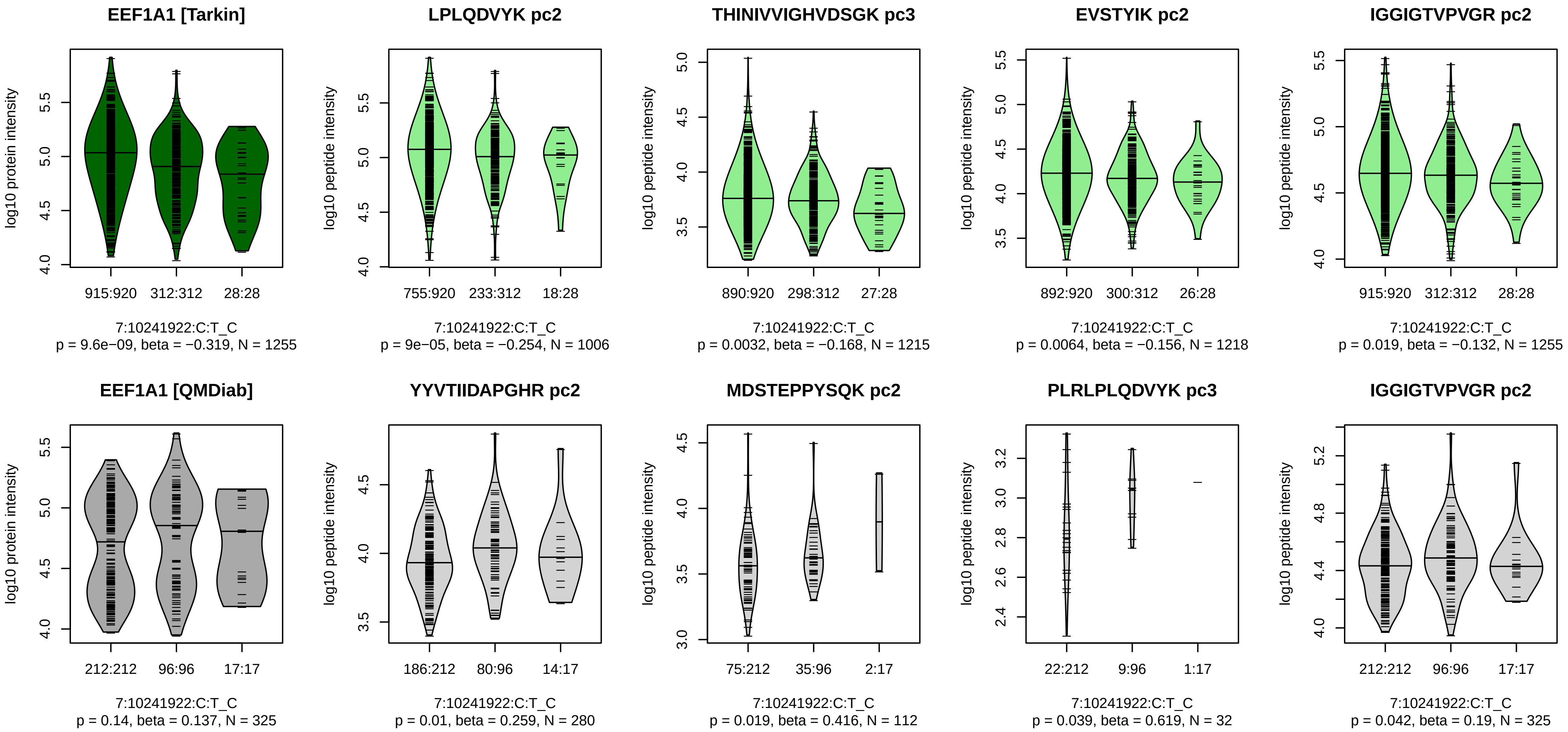

Sort: 262
Gene name: EEF1A1
SNP: 7:10241922:C:T
rsID: rs222328
Type: Trans−pQTL
Protein group(s):
T: A0A7I2V659
Q: A0A087WVQ9;A0A7I2V659;I
Nano−particle: NP3

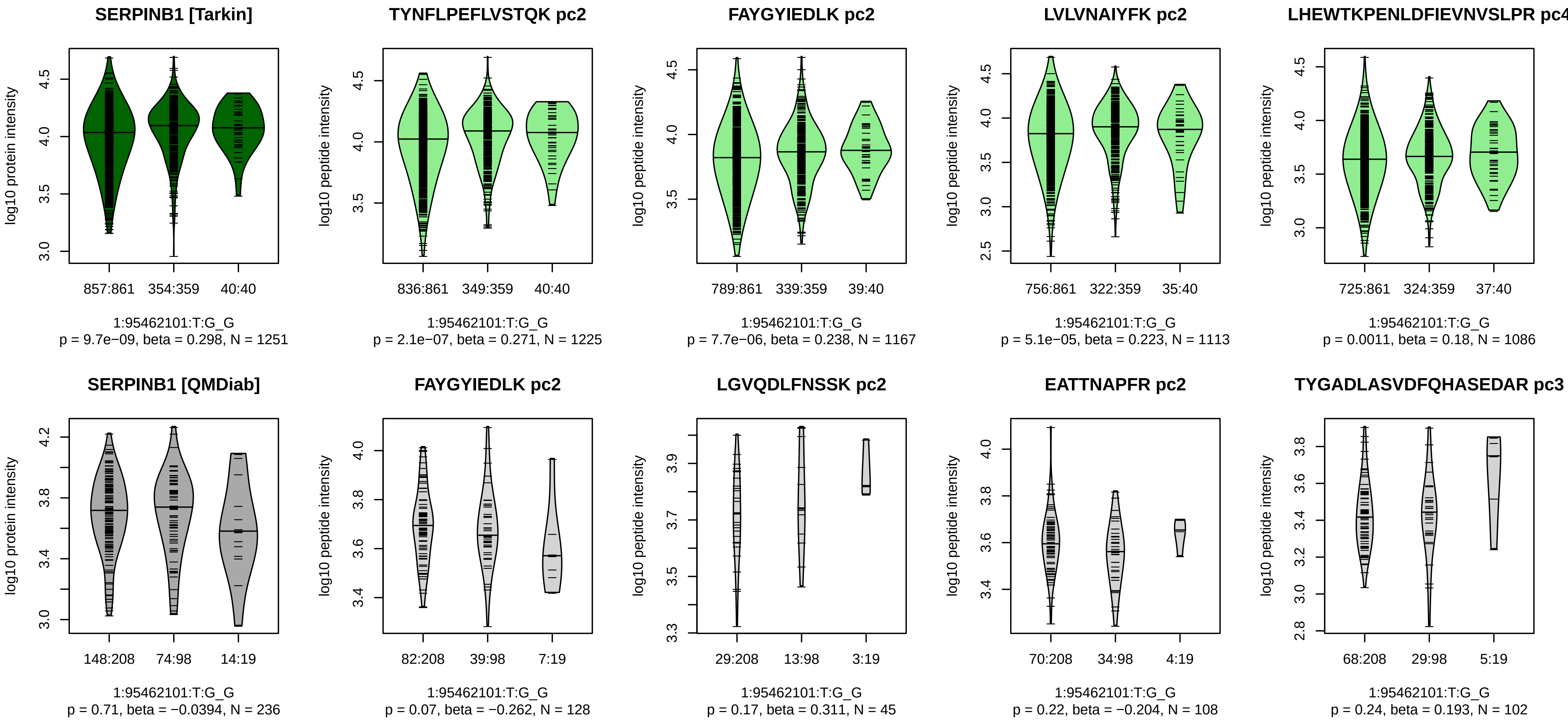

Sort: 263
Gene name: SERPINB1
SNP: 1:95462101:T:G
rsID: rs12036045
Type: Trans−pQTL
Protein group(s):
P30740
Nano−particle: NP4

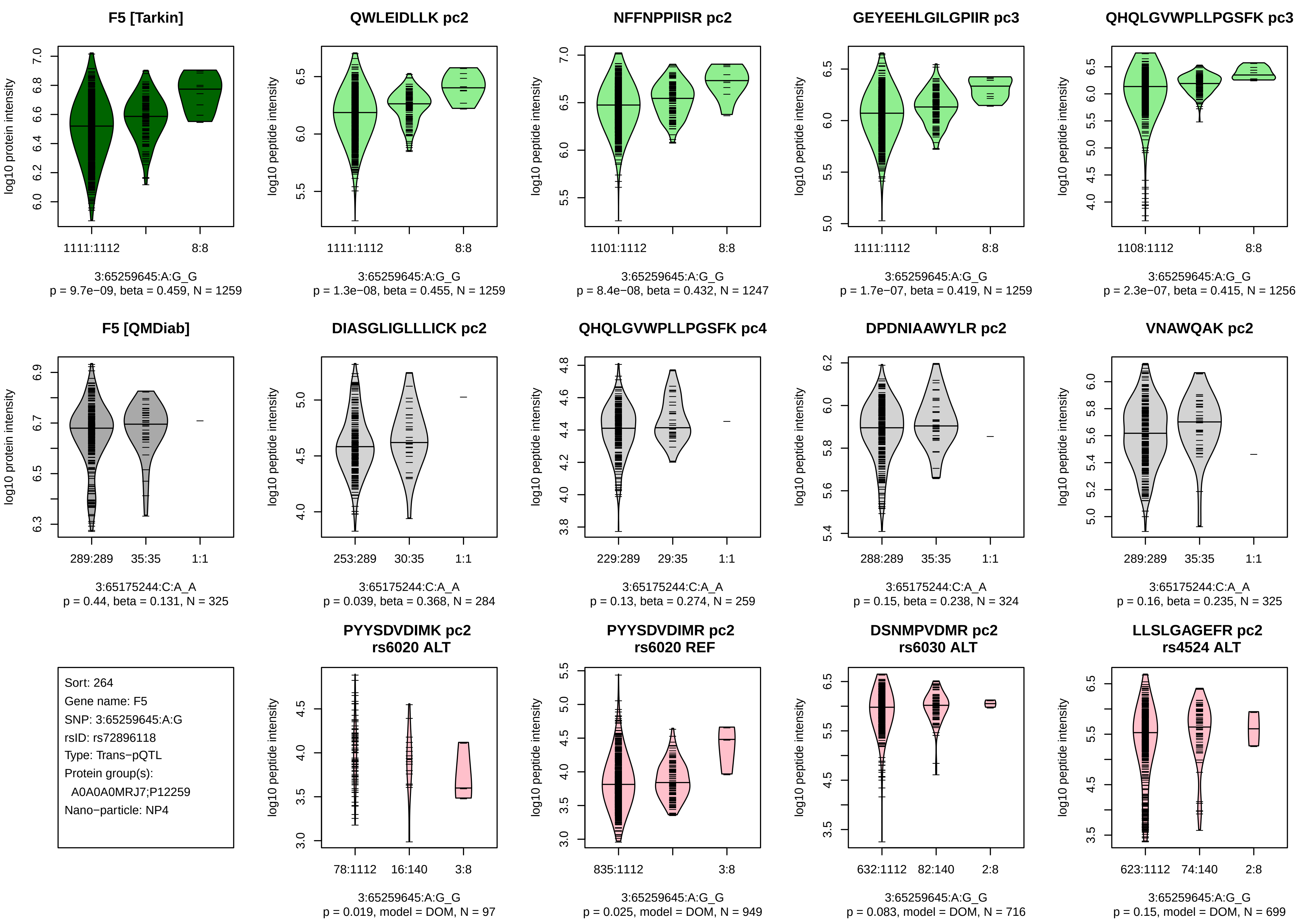
F5 [Tarkin]
log10 protein intensity
1111:1112
8:8
3:65259645:A:G_G
p = 9.7e−09, beta = 0.459, N = 1259
QWLEIDLLK pc2
log10 peptide intensity
1111:1112
8:8
3:65259645:A:G_G
p = 1.3e−08, beta = 0.455, N = 1259
NFFNPPIISR pc2
log10 peptide intensity
1101:1112
8:8
3:65259645:A:G_G
p = 8.4e−08, beta = 0.432, N = 1247
GEYEEHLGILGPIIR pc3
log10 peptide intensity
1111:1112
8:8
3:65259645:A:G_G
p = 1.7e−07, beta = 0.419, N = 1259
QHQLGVWPLLPGSFK pc3
log10 peptide intensity
1108:1112
8:8
3:65259645:A:G_G
p = 2.3e−07, beta = 0.415, N = 1256
F5 [QMDiab]
log10 protein intensity
289:289
35:35
1:1
3:65175244:C:A_A
p = 0.44, beta = 0.131, N = 325
DIASGLIGLLLICK pc2
log10 peptide intensity
253:289
30:35
1:1
3:65175244:C:A_A
p = 0.039, beta = 0.368, N = 284
QHQLGVWPLLPGSFK pc4
log10 peptide intensity
229:289
29:35
1:1
3:65175244:C:A_A
p = 0.13, beta = 0.274, N = 259
DPDNIAAWYLR pc2
log10 peptide intensity
288:289
35:35
1:1
3:65175244:C:A_A
p = 0.15, beta = 0.238, N = 324
VNAWQAK pc2
log10 peptide intensity
289:289
35:35
1:1
3:65175244:C:A_A
p = 0.16, beta = 0.235, N = 325
Sort: 264
Gene name: F5
SNP: 3:65259645:A:G
rsID: rs72896118
Type: Trans−pQTL
Protein group(s):
A0A0A0MRJ7;P12259
Nano−particle: NP4
PYYSDVDIMK pc2
rs6020 ALT
log10 peptide intensity
78:1112
16:140
3:8
3:65259645:A:G_G
p = 0.019, model = DOM, N = 97
PYYSDVDIMR pc2
rs6020 REF
log10 peptide intensity
835:1112
3:8
3:65259645:A:G_G
p = 0.025, model = DOM, N = 949
DSNMPVDMR pc2
rs6030 ALT
log10 peptide intensity
632:1112
82:140
2:8
3:65259645:A:G_G
p = 0.083, model = DOM, N = 716
LLSLGAGEFR pc2
rs4524 ALT
log10 peptide intensity
623:1112
74:140
2:8
3:65259645:A:G_G
p = 0.15, model = DOM, N = 699

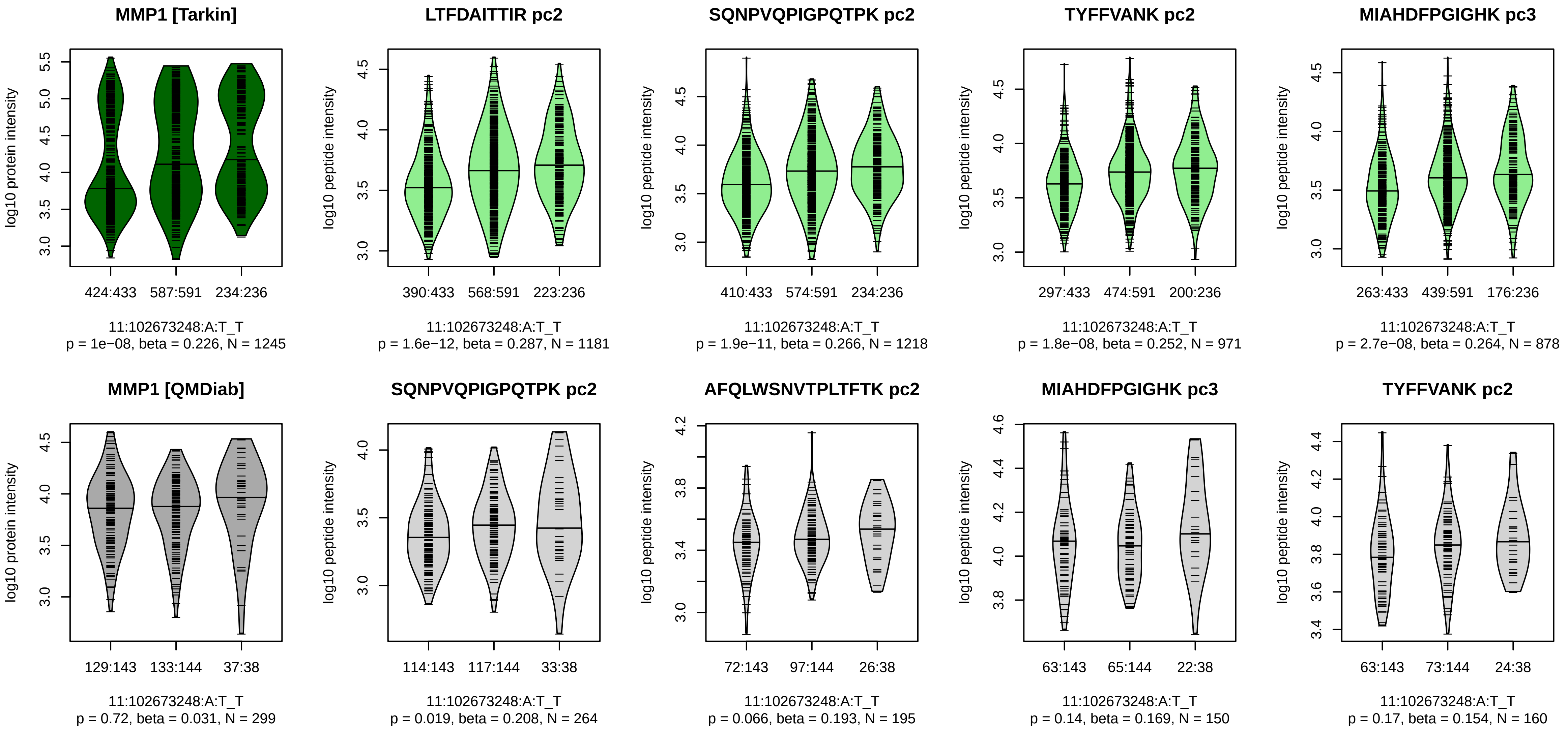

Sort: 265
Gene name: MMP1
SNP: 11:102673248:A:T
rsID: rs484915
Type: Cis−pQTL
Protein group(s):
P03956
Nano−particle: NP1

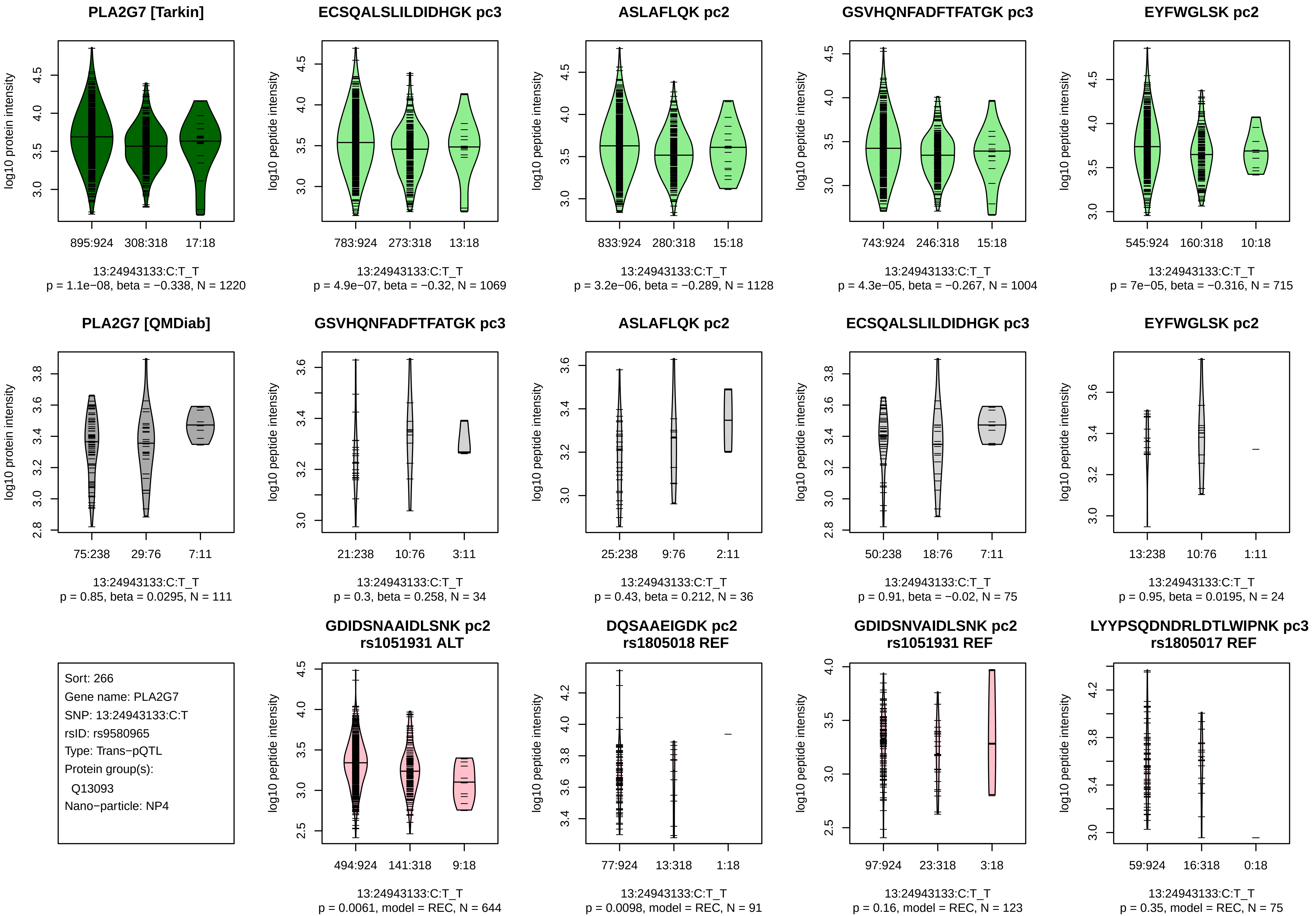

PLA2G7 [Tarkin]
log10 protein intensity
895:924
308:318
17:18
13:24943133:C:T_T
p = 1.1e−08, beta = −0.338, N = 1220
ECSQALSLILDIDHGK pc3
log10 peptide intensity
783:924
273:318
13:18
13:24943133:C:T_T
p = 4.9e−07, beta = −0.32, N = 1069
ASLAFLQK pc2
log10 peptide intensity
833:924
280:318
15:18
13:24943133:C:T_T
p = 3.2e−06, beta = −0.289, N = 1128
GSVHQNFADFTFATGK pc3
log10 peptide intensity
743:924
246:318
15:18
13:24943133:C:T_T
p = 4.3e−05, beta = −0.267, N = 1004
EYFWGLSK pc2
log10 peptide intensity
545:924
160:318
10:18
13:24943133:C:T_T
p = 7e−05, beta = −0.316, N = 715
PLA2G7 [QMDiab]
log10 protein intensity
75:238
29:76
7:11
13:24943133:C:T_T
p = 0.85, beta = 0.0295, N = 111
GSVHQNFADFTFATGK pc3
log10 peptide intensity
21:238
10:76
3:11
13:24943133:C:T_T
p = 0.3, beta = 0.258, N = 34
ASLAFLQK pc2
log10 peptide intensity
25:238
9:76
2:11
13:24943133:C:T_T
p = 0.43, beta = 0.212, N = 36
ECSQALSLILDIDHGK pc3
log10 peptide intensity
50:238
18:76
7:11
13:24943133:C:T_T
p = 0.91, beta = −0.02, N = 75
EYFWGLSK pc2
log10 peptide intensity
13:238
10:76
1:11
13:24943133:C:T_T
p = 0.95, beta = 0.0195, N = 24
Sort: 266
Gene name: PLA2G7
SNP: 13:24943133:C:T
rsID: rs9580965
Type: Trans−pQTL
Protein group(s):
Q13093
Nano−particle: NP4
GDIDSNAAIDLSNK pc2
rs1051931 ALT
log10 peptide intensity
494:924
141:318
9:18
13:24943133:C:T_T
p = 0.0061, model = REC, N = 644
DQSAAEIGDK pc2
rs1805018 REF
log10 peptide intensity
77:924
13:318
1:18
13:24943133:C:T_T
p = 0.0098, model = REC, N = 91
GDIDSNVAIDLSNK pc2
rs1051931 REF
log10 peptide intensity
97:924
23:318
3:18
13:24943133:C:T_T
p = 0.16, model = REC, N = 123
LYYPSQDNDRLDTLWIPNK pc3
rs1805017 REF
log10 peptide intensity
59:924
16:318
0:18
13:24943133:C:T_T
p = 0.35, model = REC, N = 75

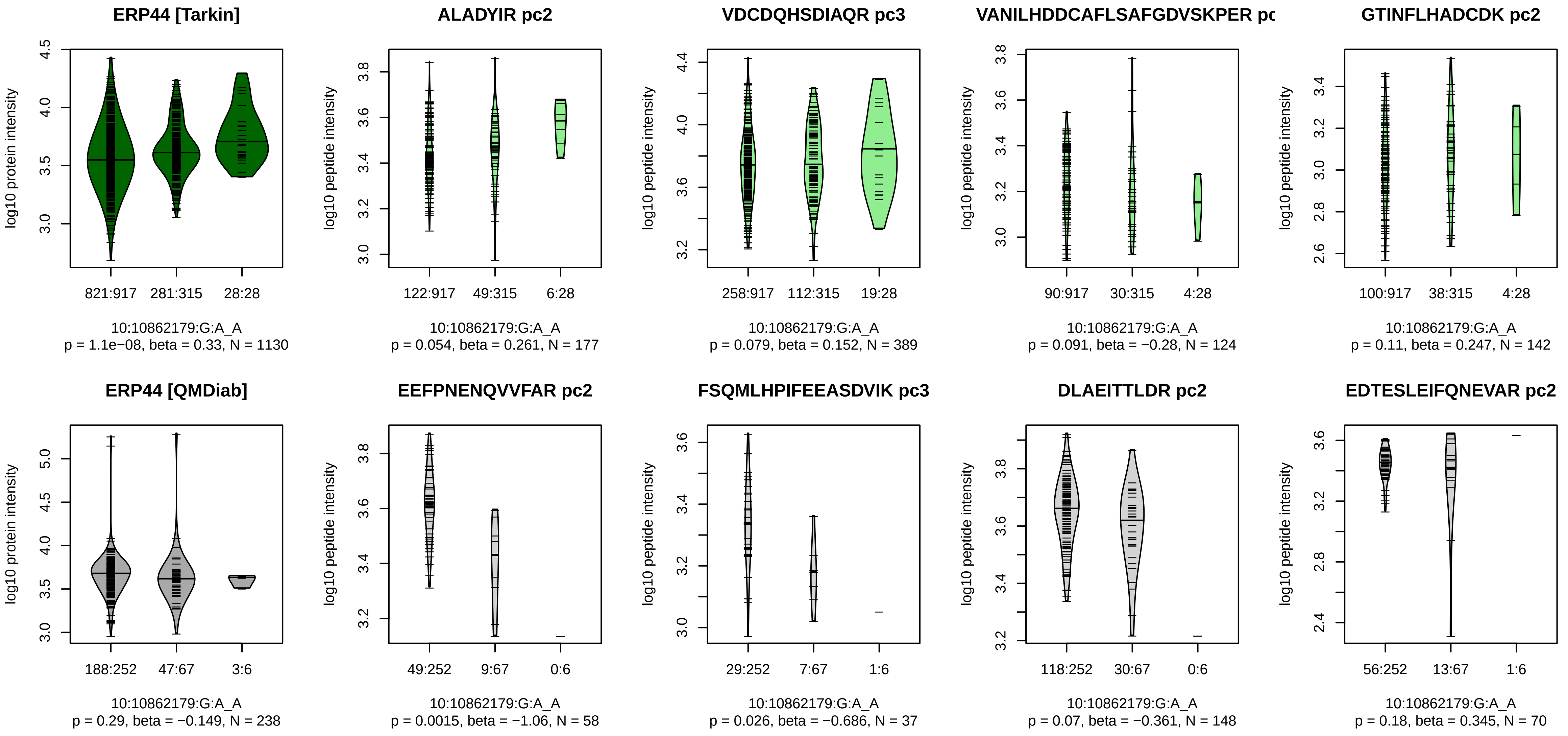

Sort: 267
Gene name: ERP44
SNP: 10:10862179:G:A
rsID: rs11256847
Type: Trans−pQTL
Protein group(s):
Q9BS26
Nano−particle: NP2

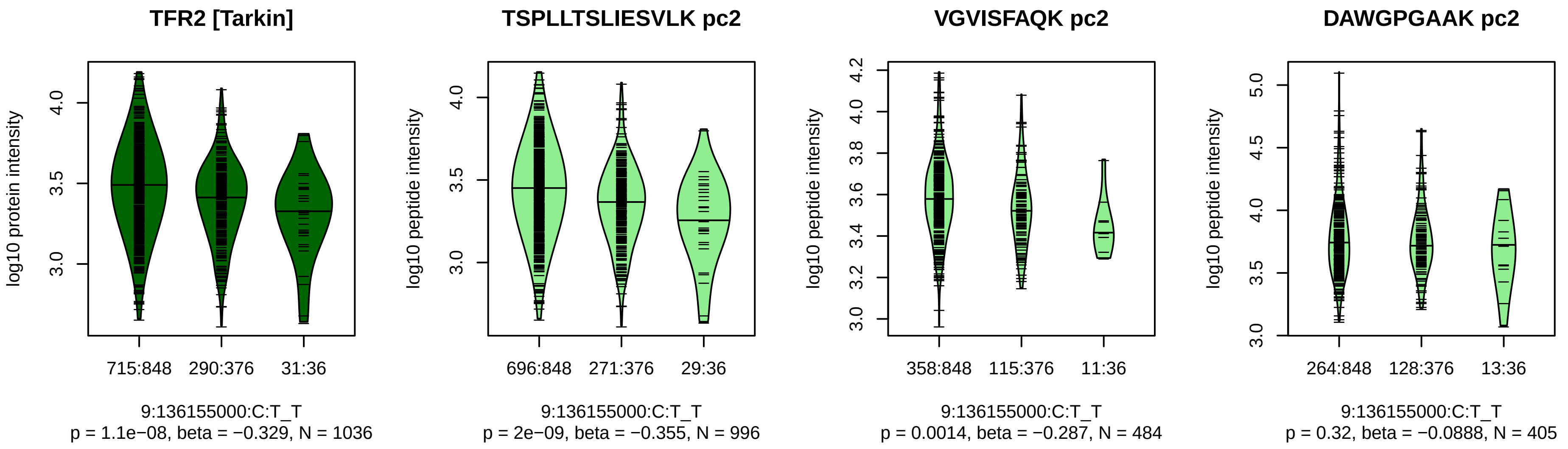

No protein data available
for replication in QMDiab

Sort: 268
Gene name: TFR2
SNP: 9:136155000:C:T
rsID: rs635634
Type: Trans−pQTL
Protein group(s):
Q9UP52
Nano−particle: NP5

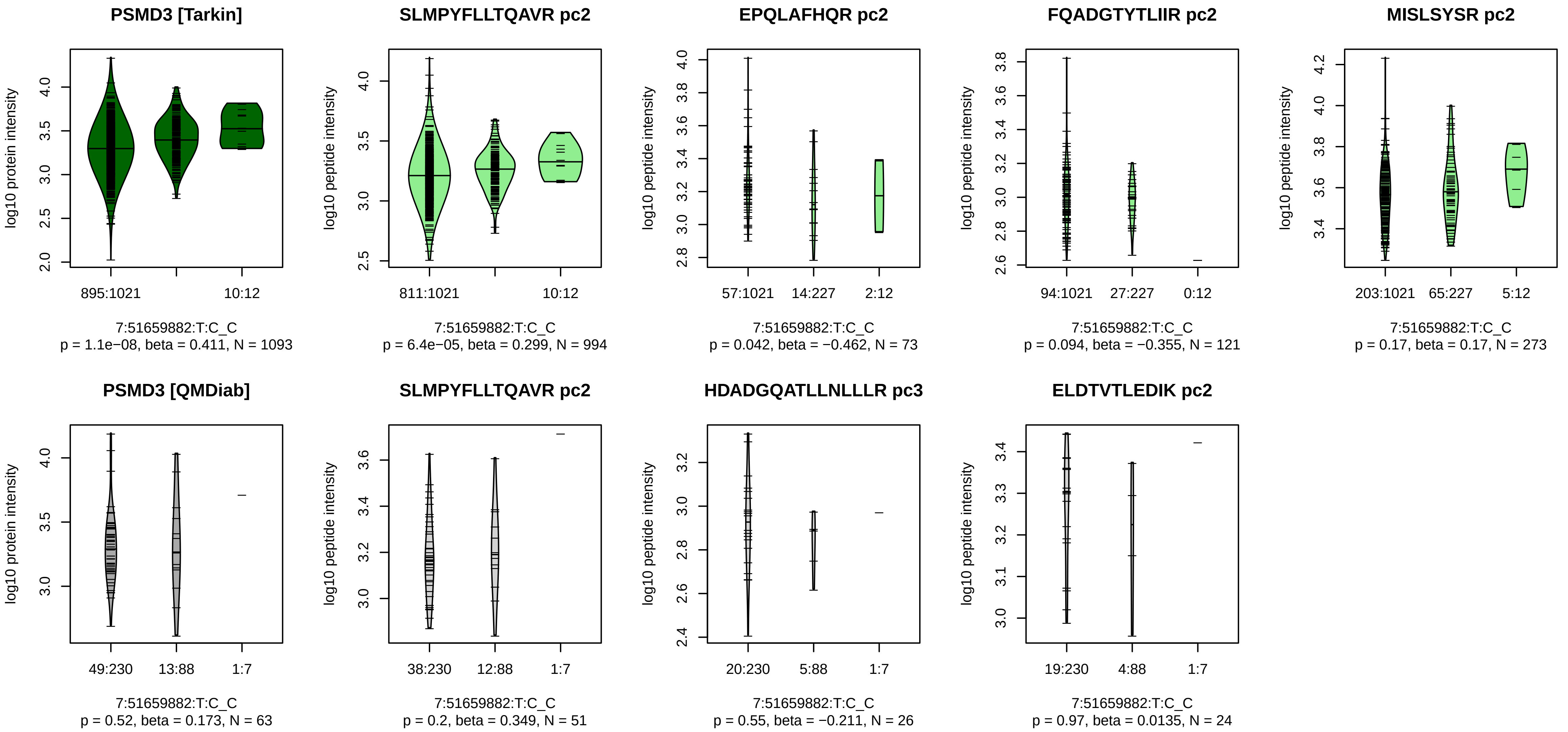

Sort: 269
Gene name: PSMD3
SNP: 7:51659882:T:C
rsID: rs1547952
Type: Trans−pQTL
Protein group(s):
O43242
Nano−particle: NP1

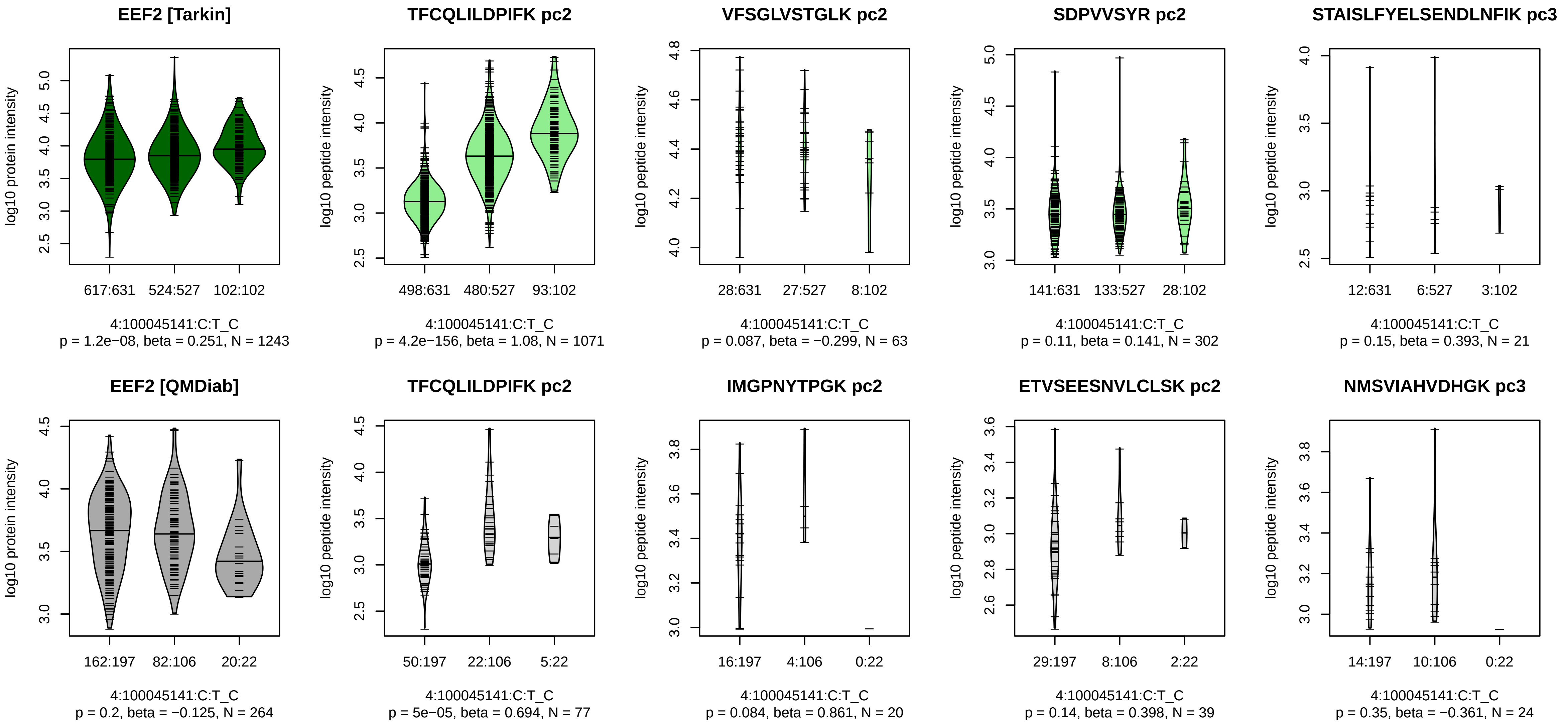

Sort: 270
Gene name: EEF2
SNP: 4:100045141:C:T
rsID: rs1139490
Type: Trans−pQTL
Protein group(s):
P13639
Nano−particle: NP1

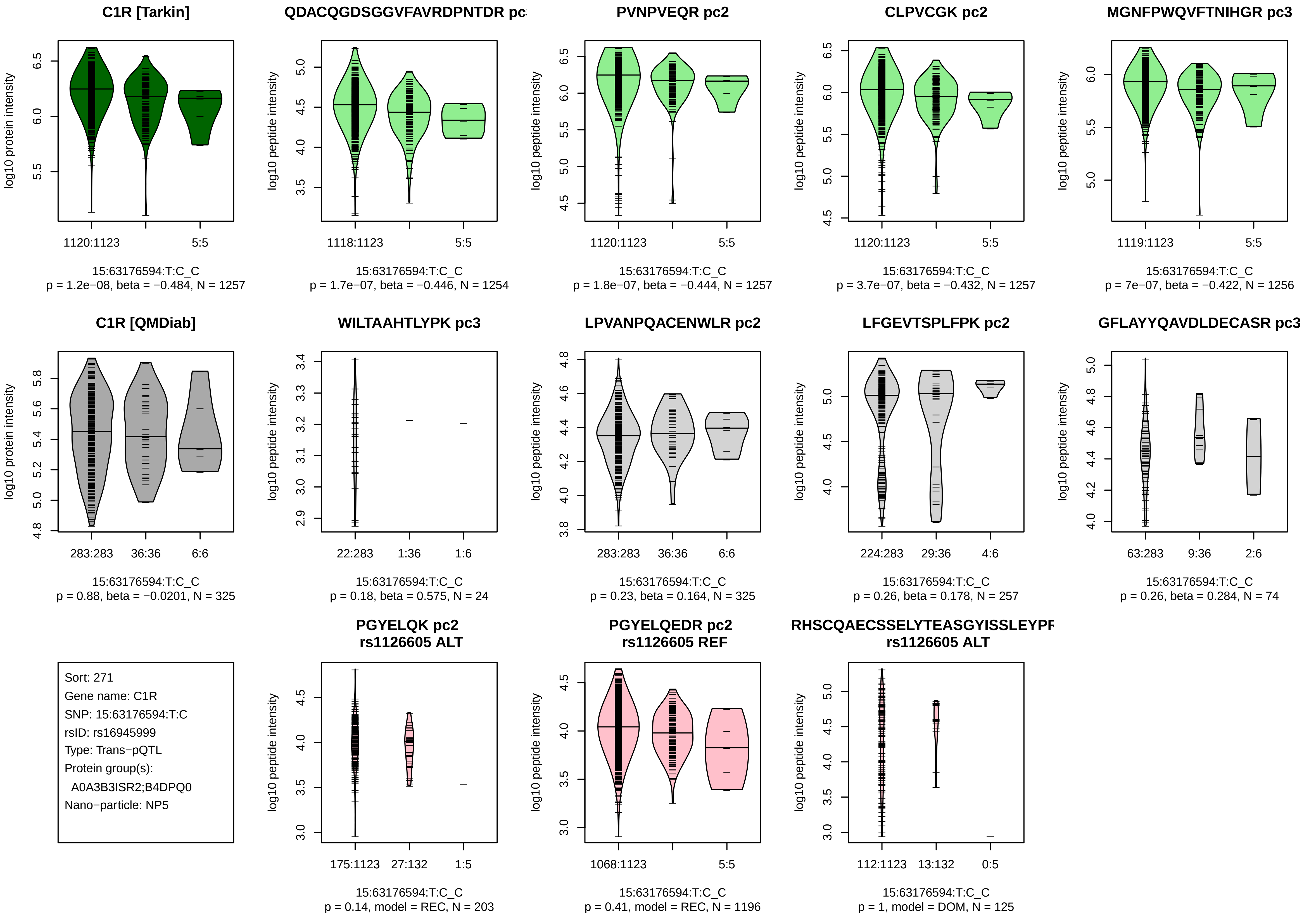

C1R [Tarkin]
log10 protein intensity
1120:1123
5:5
15:63176594:T:C_C
p = 1.2e−08, beta = −0.484, N = 1257
QDACQGDSGGVFAVRDPNTDR pc
log10 peptide intensity
1118:1123
5:5
15:63176594:T:C_C
p = 1.7e−07, beta = −0.446, N = 1254
PVNPVEQR pc2
log10 peptide intensity
1120:1123
5:5
15:63176594:T:C_C
p = 1.8e−07, beta = −0.444, N = 1257
CLPVCGK pc2
log10 peptide intensity
1120:1123
5:5
15:63176594:T:C_C
p = 3.7e−07, beta = −0.432, N = 1257
MGNFPWQVFTNIHGR pc3
log10 peptide intensity
1119:1123
5:5
15:63176594:T:C_C
p = 7e−07, beta = −0.422, N = 1256
C1R [QMDiab]
log10 protein intensity
283:283
36:36
6:6
15:63176594:T:C_C
p = 0.88, beta = −0.0201, N = 325
WILTAAHTLYPK pc3
log10 peptide intensity
22:283
1:36
1:6
15:63176594:T:C_C
p = 0.18, beta = 0.575, N = 24
LPVANPQACENWLR pc2
log10 peptide intensity
283:283
36:36
6:6
15:63176594:T:C_C
p = 0.23, beta = 0.164, N = 325
LFGEVTSPLFPK pc2
log10 peptide intensity
224:283
29:36
4:6
15:63176594:T:C_C
p = 0.26, beta = 0.178, N = 257
GFLAYYQAVDLDECASR pc3
log10 peptide intensity
63:283
9:36
2:6
15:63176594:T:C_C
p = 0.26, beta = 0.284, N = 74
Sort: 271
Gene name: C1R
SNP: 15:63176594:T:C
rsID: rs16945999
Type: Trans−pQTL
Protein group(s):
A0A3B3ISR2;B4DPQ0
Nano−particle: NP5
PGYELQK pc2
rs1126605 ALT
log10 peptide intensity
175:1123
27:132
1:5
15:63176594:T:C_C
p = 0.14, model = REC, N = 203
PGYELQEDR pc2
rs1126605 REF
log10 peptide intensity
1068:1123
5:5
15:63176594:T:C_C
p = 0.41, model = REC, N = 1196
RHSCQAECSSELYTEASGYISSLEYPF
rs1126605 ALT
log10 peptide intensity
112:1123
13:132
0:5
15:63176594:T:C_C
p = 1, model = DOM, N = 125

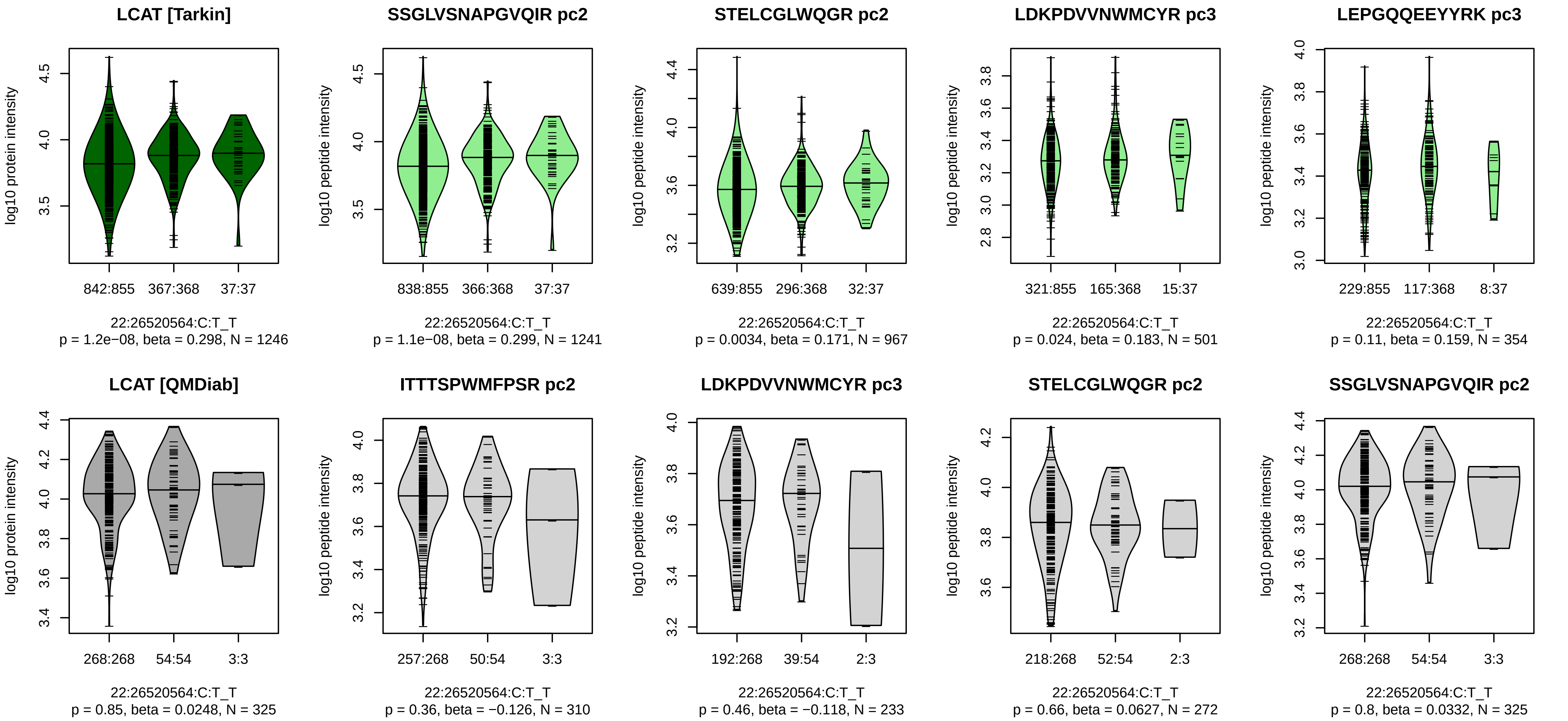

Sort: 272
Gene name: LCAT
SNP: 22:26520564:C:T
rsID: rs9613112
Type: Trans−pQTL
Protein group(s):
P04180
Nano−particle: NP2

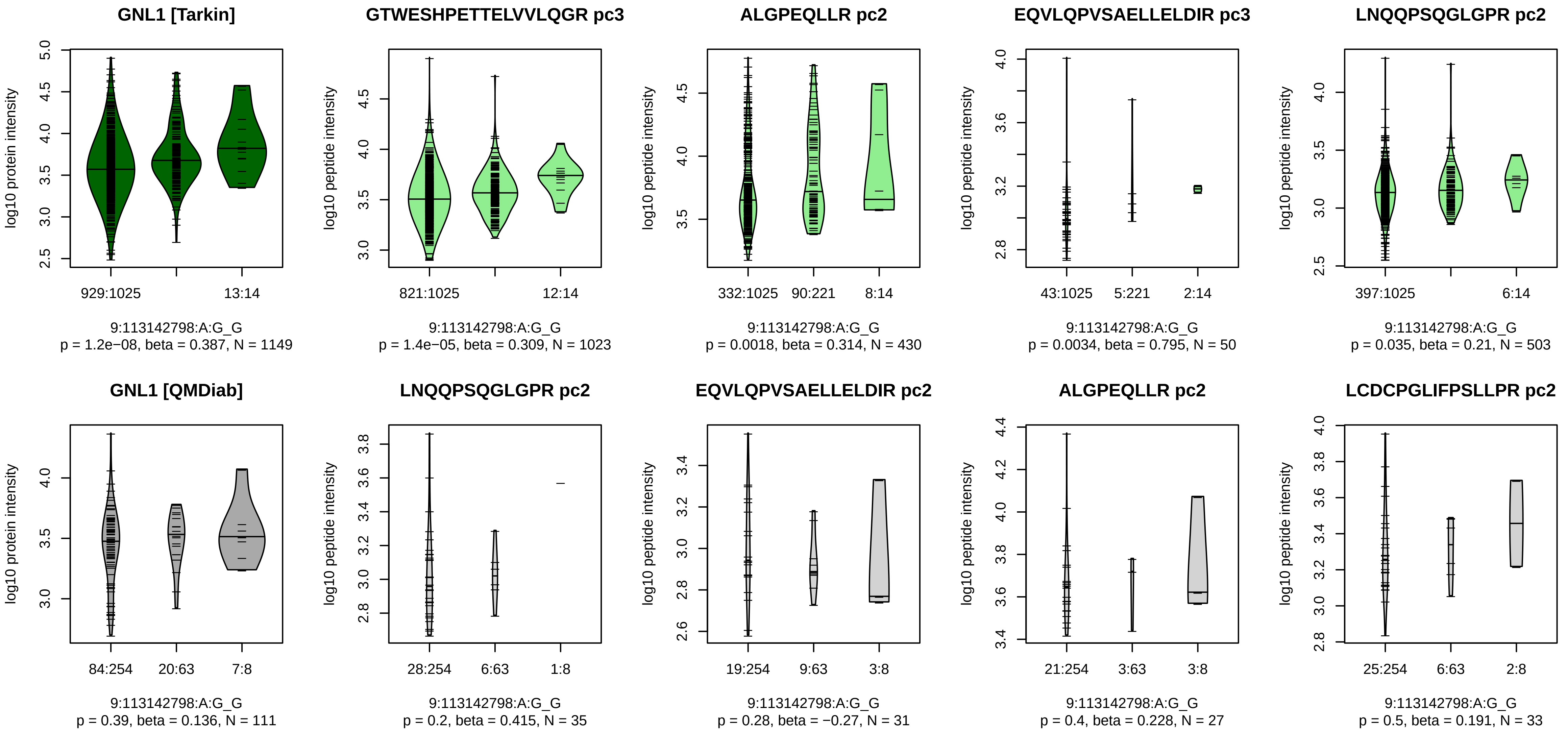

Sort: 273
Gene name: GNL1
SNP: 9:113142798:A:G
rsID: rs72762683
Type: Trans−pQTL
Protein group(s):
P36915
Nano−particle: NP5

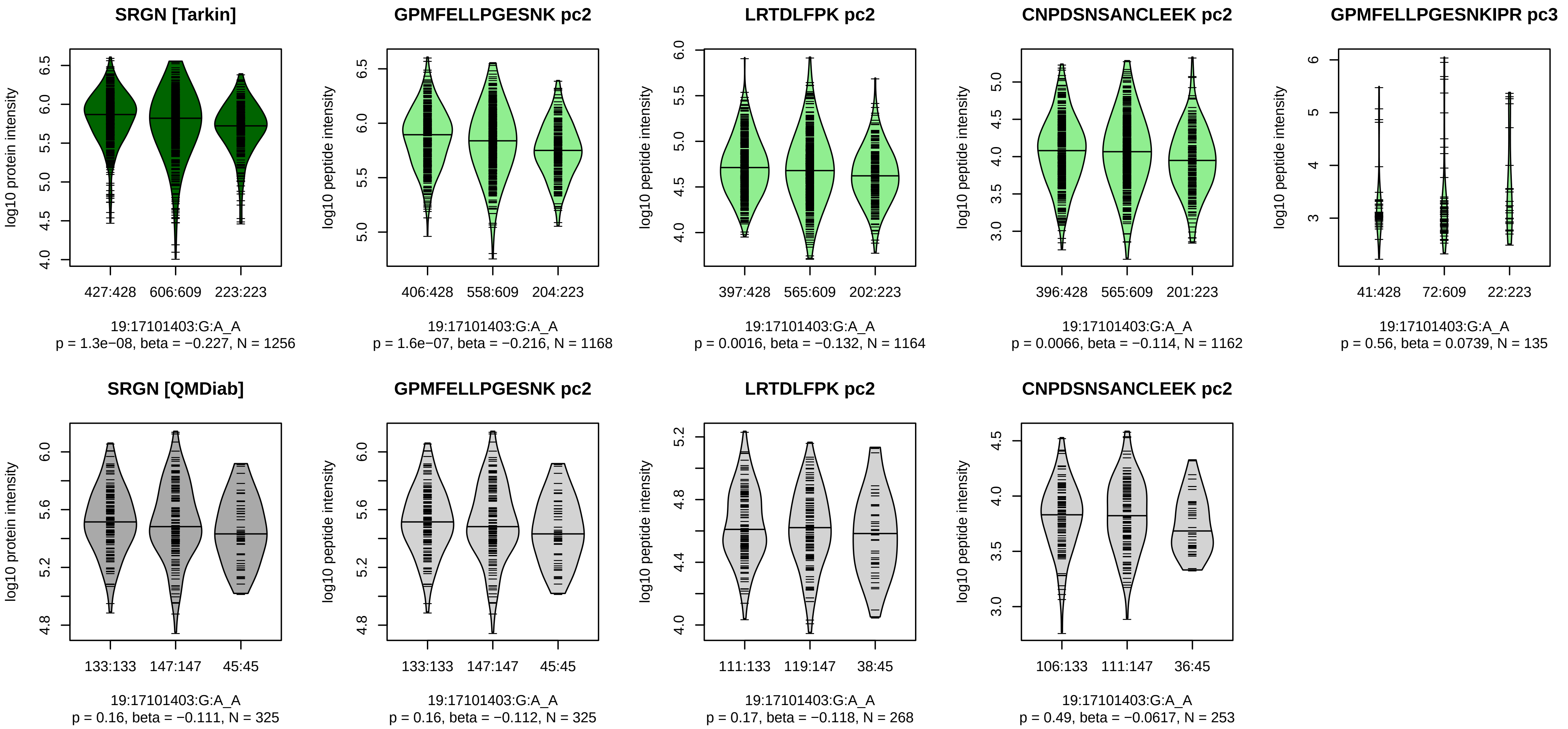

Sort: 274
Gene name: SRGN
SNP: 19:17101403:G:A
rsID: rs59665139
Type: Trans−pQTL
Protein group(s):
P10124
Nano−particle: NP2

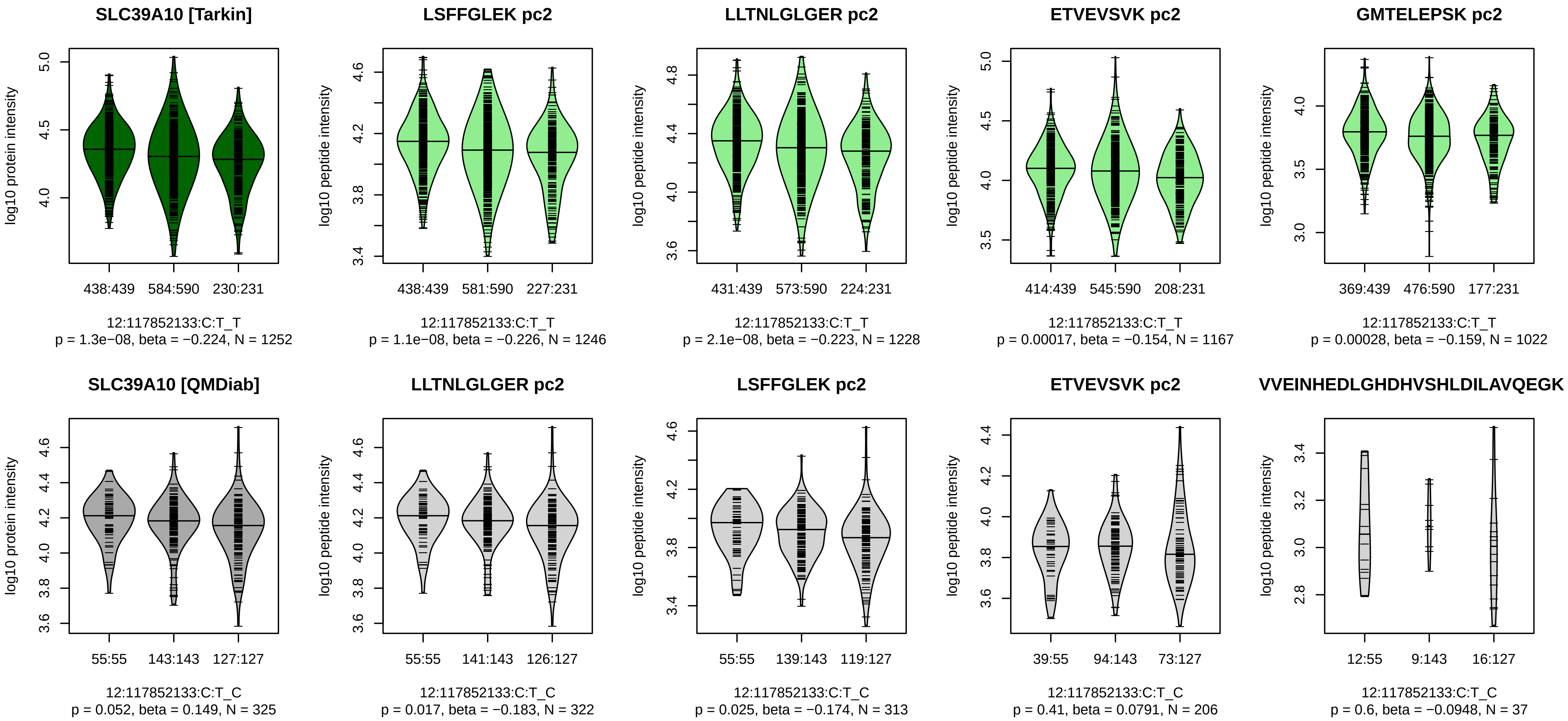

Sort: 275
Gene name: SLC39A10
SNP: 12:117852133:C:T
rsID: rs816273
Type: Trans−pQTL
Protein group(s):
Q9ULF5
Nano−particle: NP3

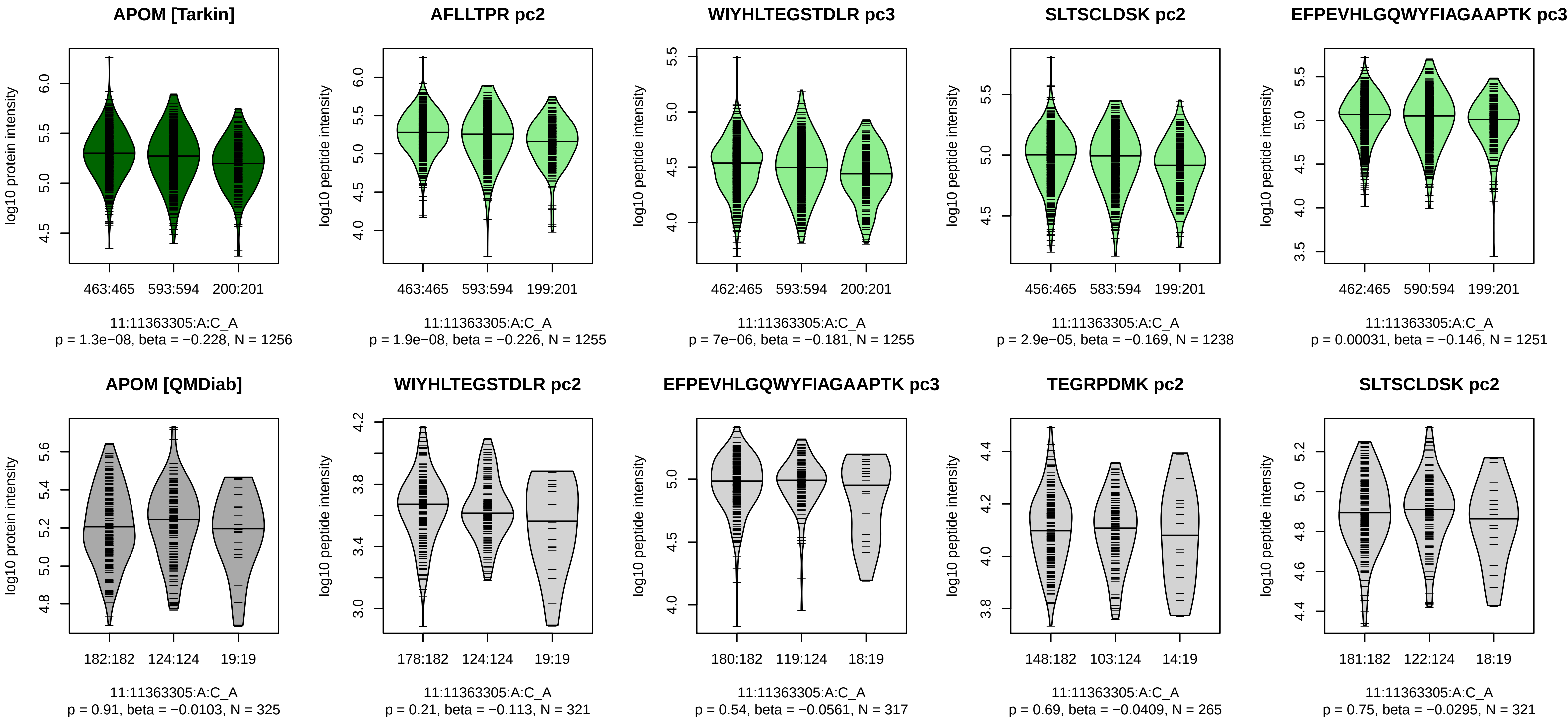

Sort: 276
Gene name: APOM
SNP: 11:11363305:A:C
rsID: rs9888138
Type: Trans−pQTL
Protein group(s):
O95445
Nano−particle: NP1

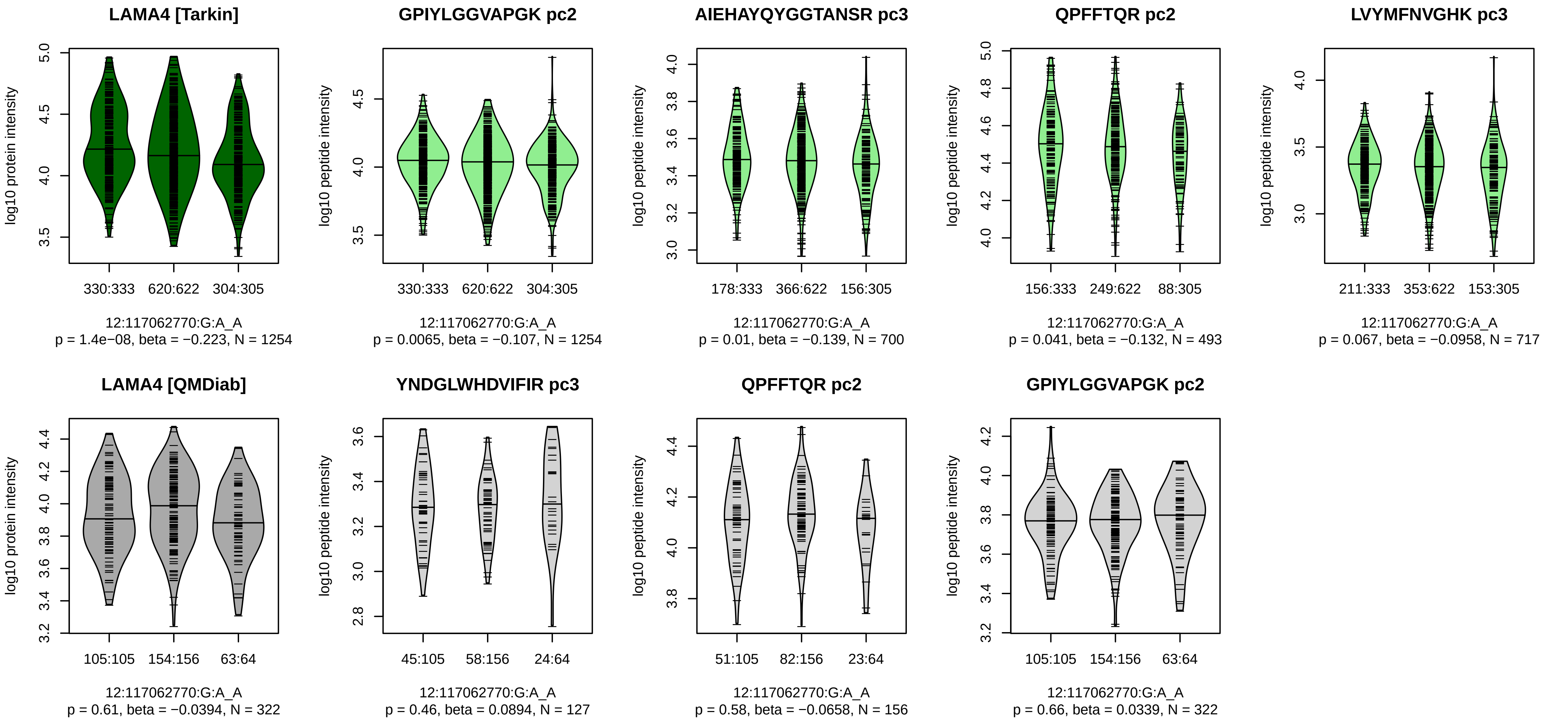

Sort: 277
Gene name: LAMA4
SNP: 12:117062770:G:A
rsID: rs10850683
Type: Trans−pQTL
Protein group(s):
A0A0A0MQS9;A0A0A0MTC7
Nano−particle: NP3

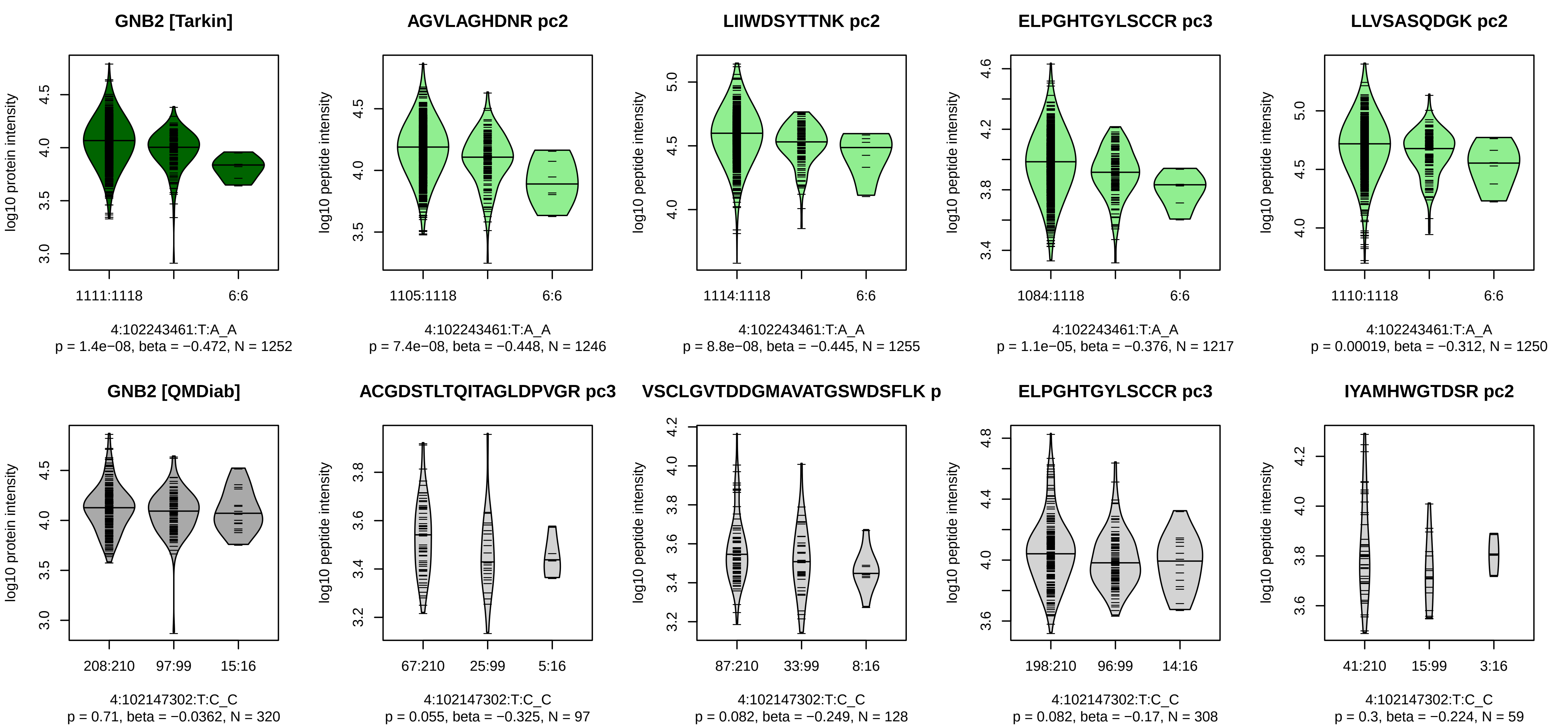

Sort: 278
Gene name: GNB2
SNP: 4:102243461:T:A
rsID: rs76455904
Type: Trans−pQTL
Protein group(s):
P62879
Nano−particle: NP1

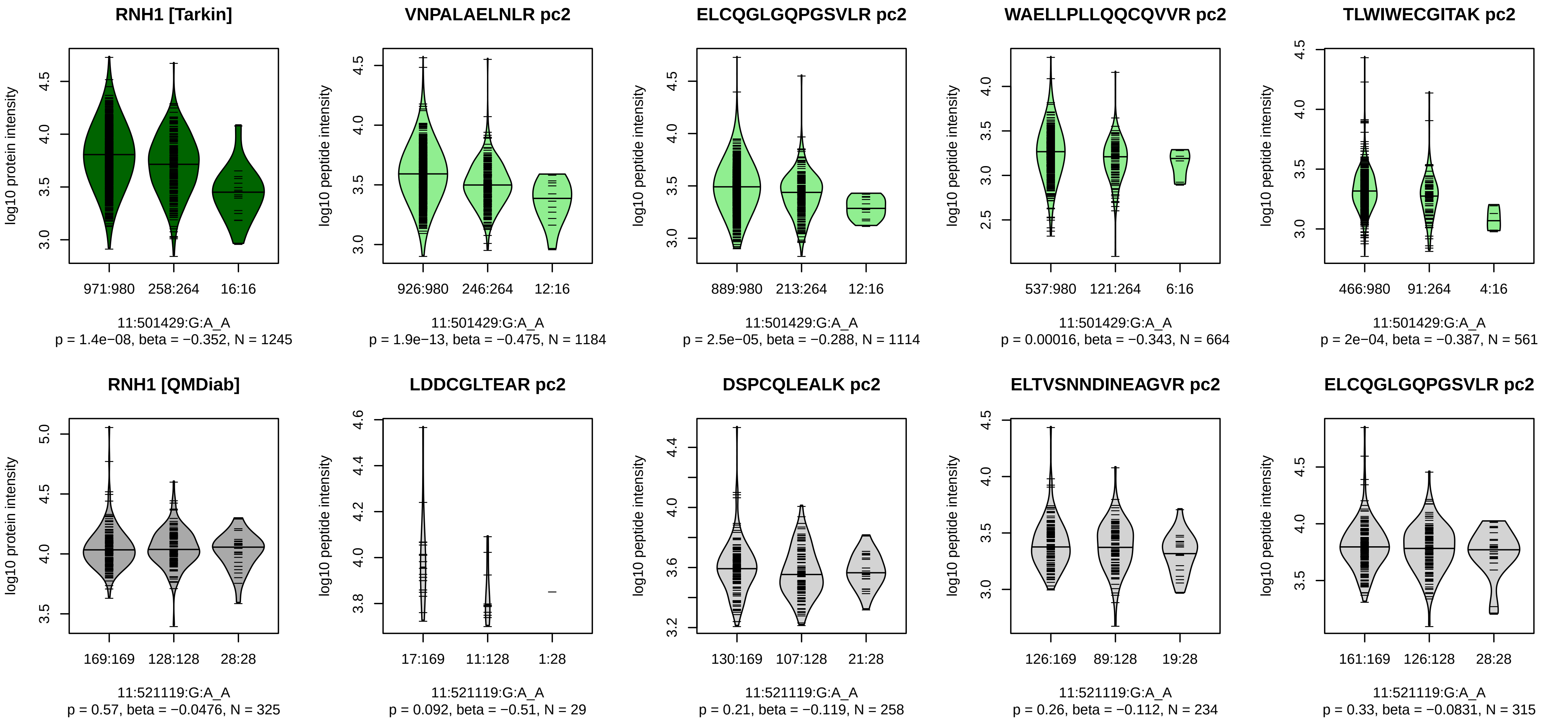

Sort: 279
Gene name: RNH1
SNP: 11:501429:G:A
rsID: rs12806062
Type: Cis−pQTL
Protein group(s):
P13489
Nano−particle: NP2

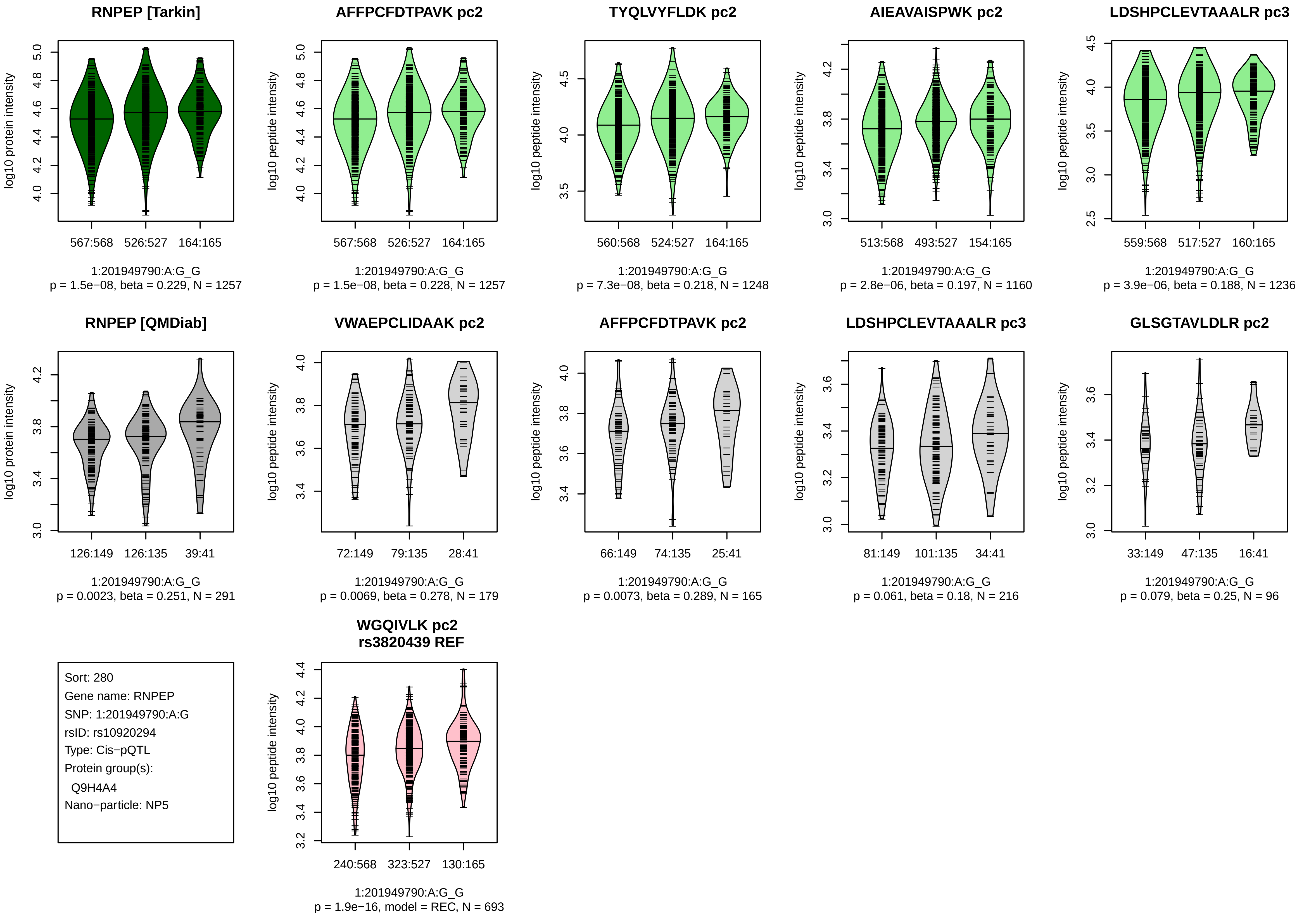
RNPEP [Tarkin]
log10 protein intensity
567:568 526:527 164:165
1:201949790:A:G_G
p = 1.5e−08, beta = 0.229, N = 1257
AFFPCFDTPAVK pc2
log10 peptide intensity
567:568 526:527 164:165
1:201949790:A:G_G
p = 1.5e−08, beta = 0.228, N = 1257
TYQLVYFLDK pc2
log10 peptide intensity
560:568 524:527 164:165
1:201949790:A:G_G
p = 7.3e−08, beta = 0.218, N = 1248
AIEAVAISPWK pc2
log10 peptide intensity
513:568 493:527 154:165
1:201949790:A:G_G
p = 2.8e−06, beta = 0.197, N = 1160
LDSHPCLEVTAAALR pc3
log10 peptide intensity
559:568 517:527 160:165
1:201949790:A:G_G
p = 3.9e−06, beta = 0.188, N = 1236
RNPEP [QMDiab]
log10 protein intensity
126:149 126:135 39:41
1:201949790:A:G_G
p = 0.0023, beta = 0.251, N = 291
VWAEPCLIDAAK pc2
log10 peptide intensity
72:149 79:135 28:41
1:201949790:A:G_G
p = 0.0069, beta = 0.278, N = 179
AFFPCFDTPAVK pc2
log10 peptide intensity
66:149 74:135 25:41
1:201949790:A:G_G
p = 0.0073, beta = 0.289, N = 165
LDSHPCLEVTAAALR pc3
log10 peptide intensity
81:149 101:135 34:41
1:201949790:A:G_G
p = 0.061, beta = 0.18, N = 216
GLSGTAVLDLR pc2
log10 peptide intensity
33:149 47:135 16:41
1:201949790:A:G_G
p = 0.079, beta = 0.25, N = 96
Sort: 280
Gene name: RNPEP
SNP: 1:201949790:A:G
rsID: rs10920294
Type: Cis−pQTL
Protein group(s):
Q9H4A4
Nano−particle: NP5
WGQIVLK pc2
rs3820439 REF
log10 peptide intensity
240:568 323:527 130:165
1:201949790:A:G_G
p = 1.9e−16, model = REC, N = 693

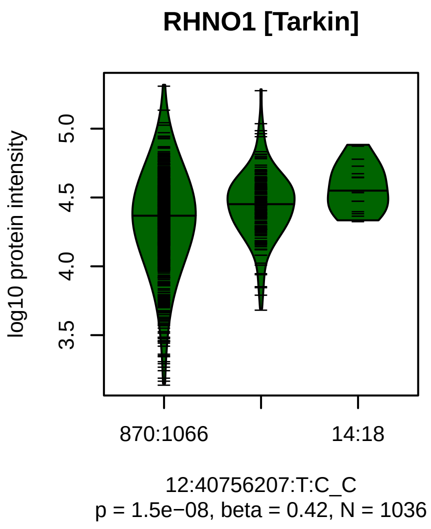

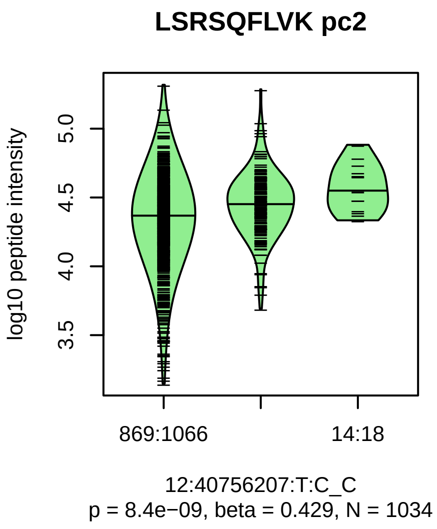

No protein data available
for replication in QMDiab

Sort: 281
Gene name: RHNO1
SNP: 12:40756207:T:C
rsID: rs11176195
Type: Trans−pQTL
Protein group(s):
Q9BSD3−2
Nano−particle: NP5

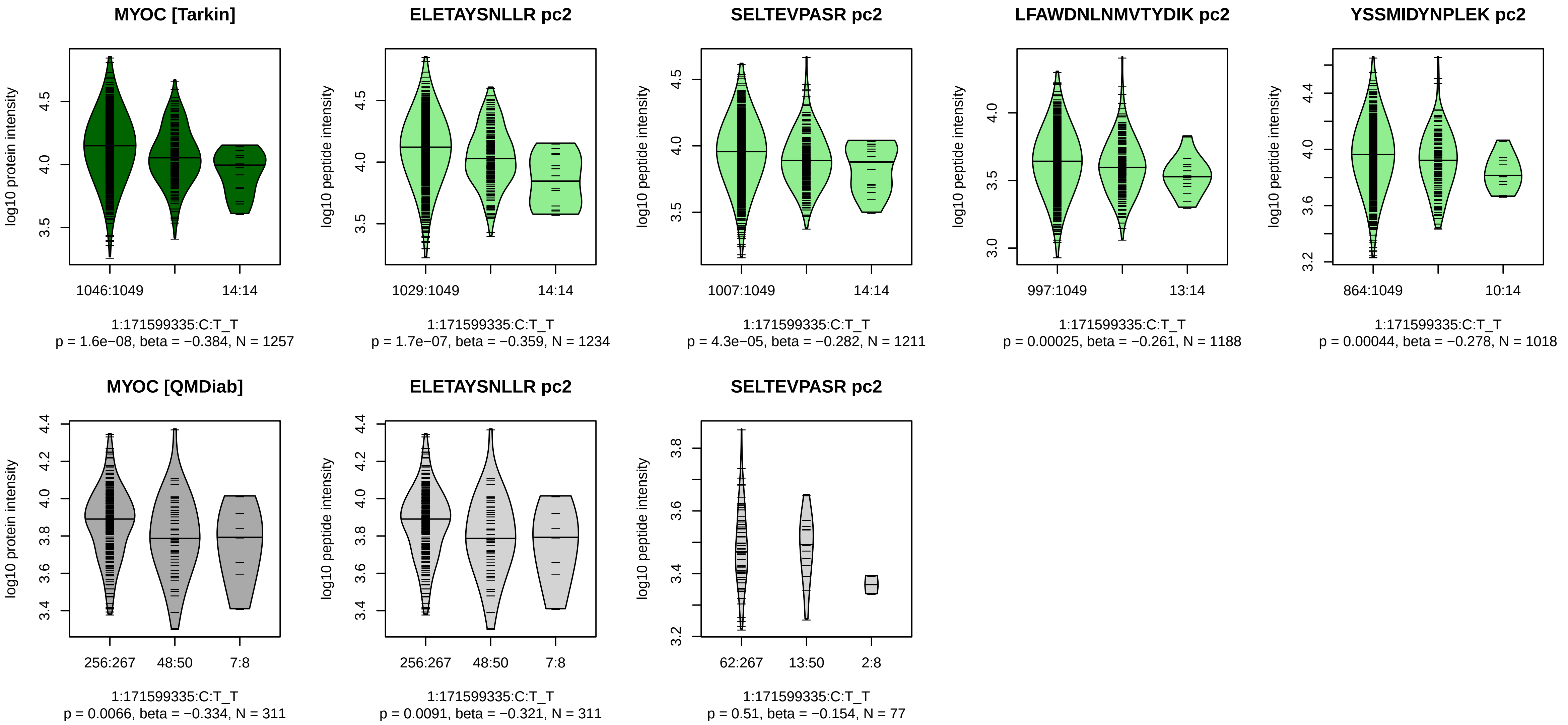

Sort: 282
Gene name: MYOC
SNP: 1:171599335:C:T
rsID: rs7530400
Type: Cis−pQTL
Protein group(s):
Q99972
Nano−particle: NP5

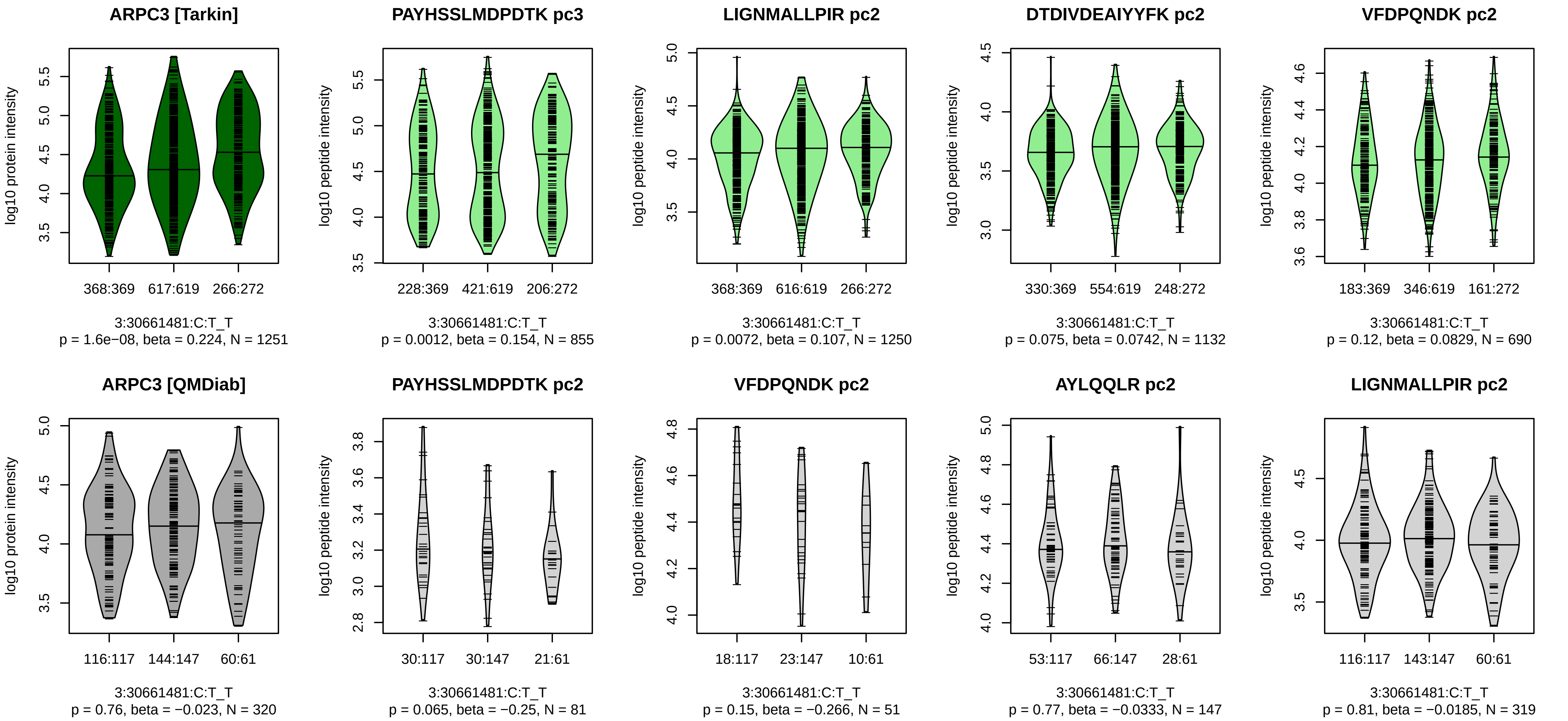

Sort: 283
Gene name: ARPC3
SNP: 3:30661481:C:T
rsID: rs17025741
Type: Trans−pQTL
Protein group(s):
O15145
Nano−particle: NP1

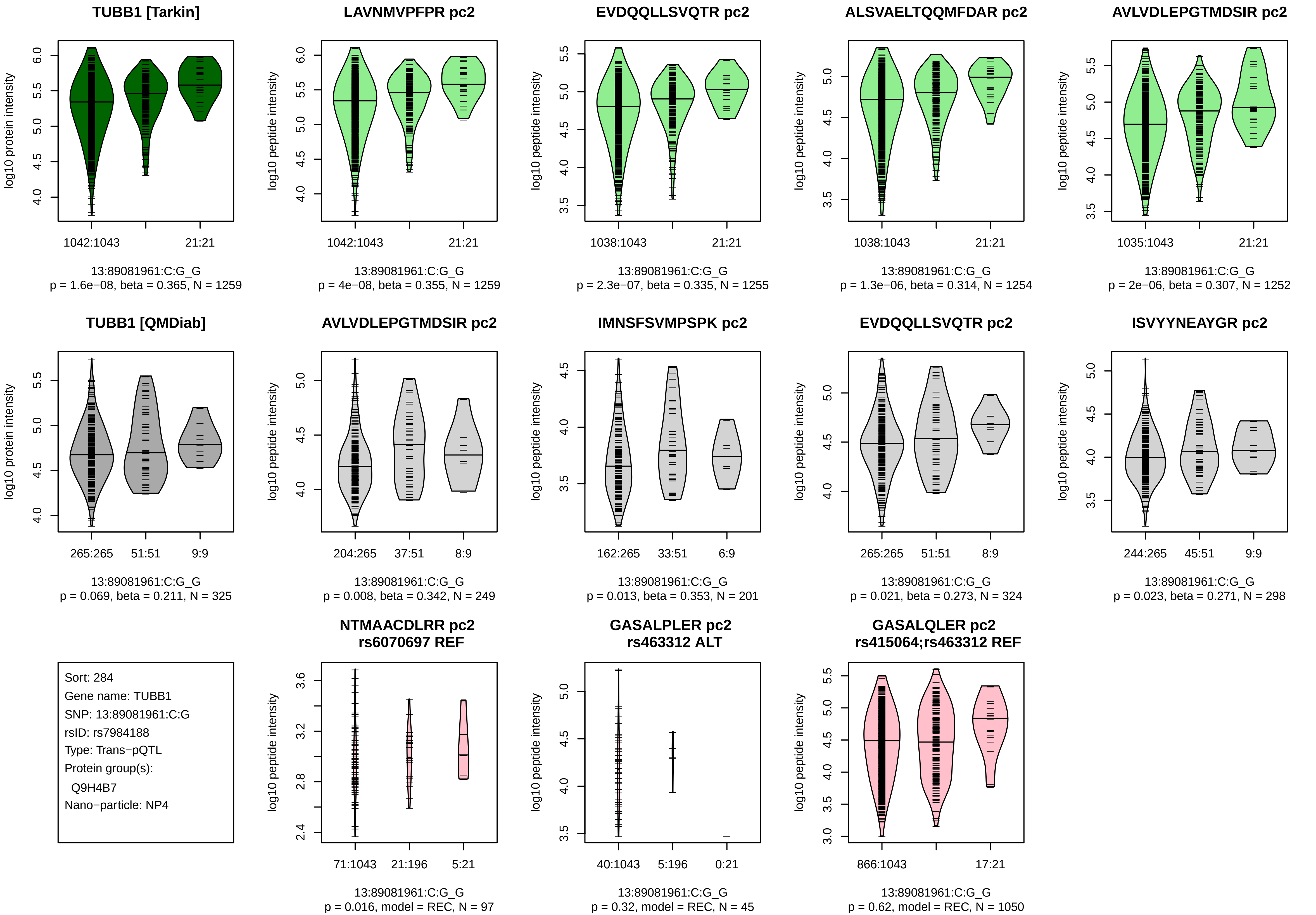
TUBB1 [Tarkin]
log10 protein intensity
1042:1043
21:21
13:89081961:C:G_G
p = 1.6e−08, beta = 0.365, N = 1259
LAVNMVPFPR pc2
log10 peptide intensity
1042:1043
21:21
13:89081961:C:G_G
p = 4e−08, beta = 0.355, N = 1259
EVDQQLLSVQTR pc2
log10 peptide intensity
1038:1043
21:21
13:89081961:C:G_G
p = 2.3e−07, beta = 0.335, N = 1255
ALSVAELTQQMFDAR pc2
log10 peptide intensity
1038:1043
21:21
13:89081961:C:G_G
p = 1.3e−06, beta = 0.314, N = 1254
AVLVDLEPGTMDSIR pc2
log10 peptide intensity
1035:1043
21:21
13:89081961:C:G_G
p = 2e−06, beta = 0.307, N = 1252
TUBB1 [QMDiab]
log10 protein intensity
265:265
51:51
9:9
13:89081961:C:G_G
p = 0.069, beta = 0.211, N = 325
AVLVDLEPGTMDSIR pc2
log10 peptide intensity
204:265
37:51
8:9
13:89081961:C:G_G
p = 0.008, beta = 0.342, N = 249
IMNSFSVMPSPK pc2
log10 peptide intensity
162:265
33:51
6:9
13:89081961:C:G_G
p = 0.013, beta = 0.353, N = 201
EVDQQLLSVQTR pc2
log10 peptide intensity
265:265
51:51
8:9
13:89081961:C:G_G
p = 0.021, beta = 0.273, N = 324
ISVYYNEAYGR pc2
log10 peptide intensity
244:265
45:51
9:9
13:89081961:C:G_G
p = 0.023, beta = 0.271, N = 298
Sort: 284
Gene name: TUBB1
SNP: 13:89081961:C:G
rsID: rs7984188
Type: Trans−pQTL
Protein group(s):
Q9H4B7
Nano−particle: NP4
NTMAACDLRR pc2
rs6070697 REF
log10 peptide intensity
71:1043
21:196
5:21
13:89081961:C:G_G
p = 0.016, model = REC, N = 97
GASALPLER pc2
rs463312 ALT
log10 peptide intensity
40:1043
5:196
0:21
13:89081961:C:G_G
p = 0.32, model = REC, N = 45
GASALQLER pc2
rs415064;rs463312 REF
log10 peptide intensity
866:1043
17:21
13:89081961:C:G_G
p = 0.62, model = REC, N = 1050

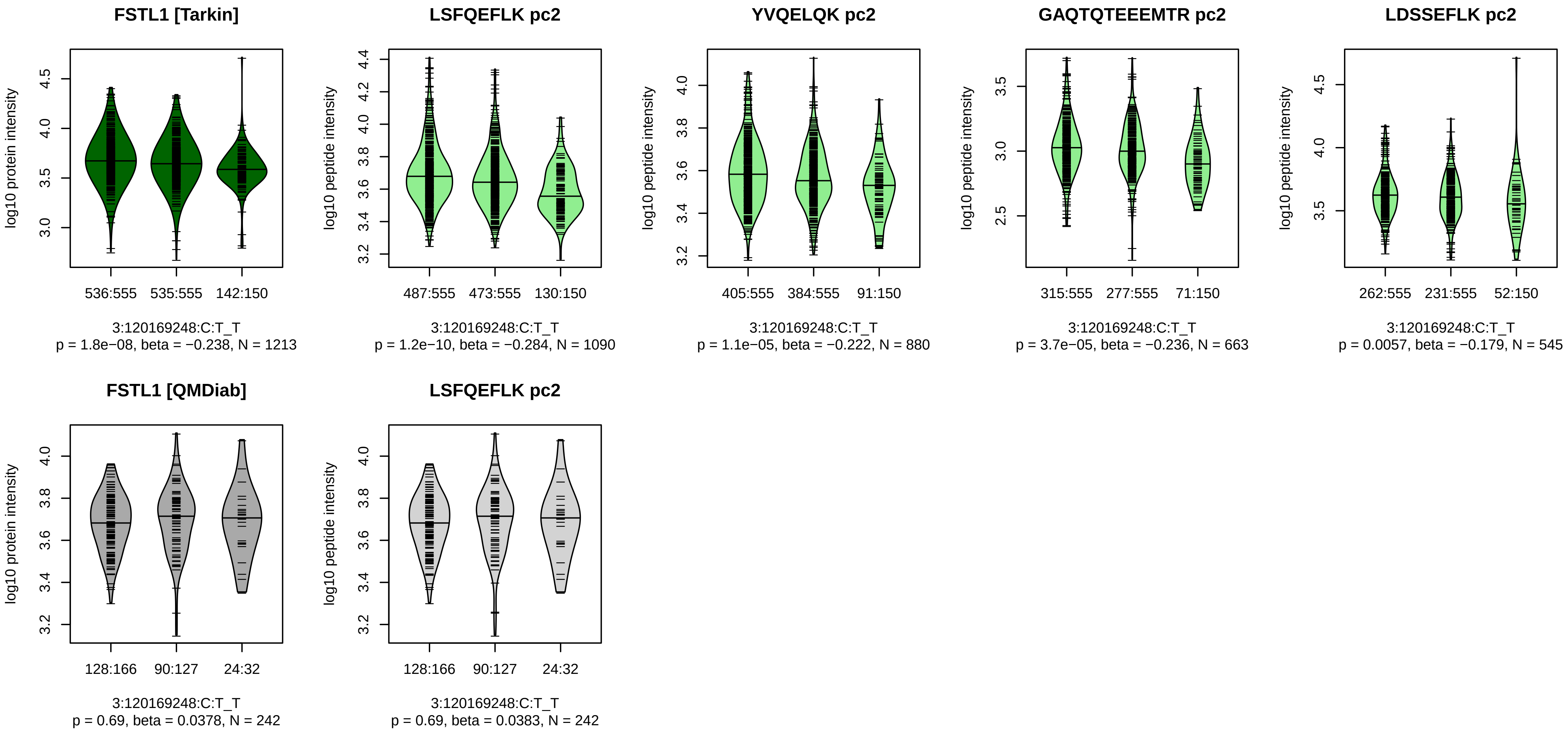

Sort: 285
Gene name: FSTL1
SNP: 3:120169248:C:T
rsID: rs1147707
Type: Cis−pQTL
Protein group(s):
Q12841
Nano−particle: NP2

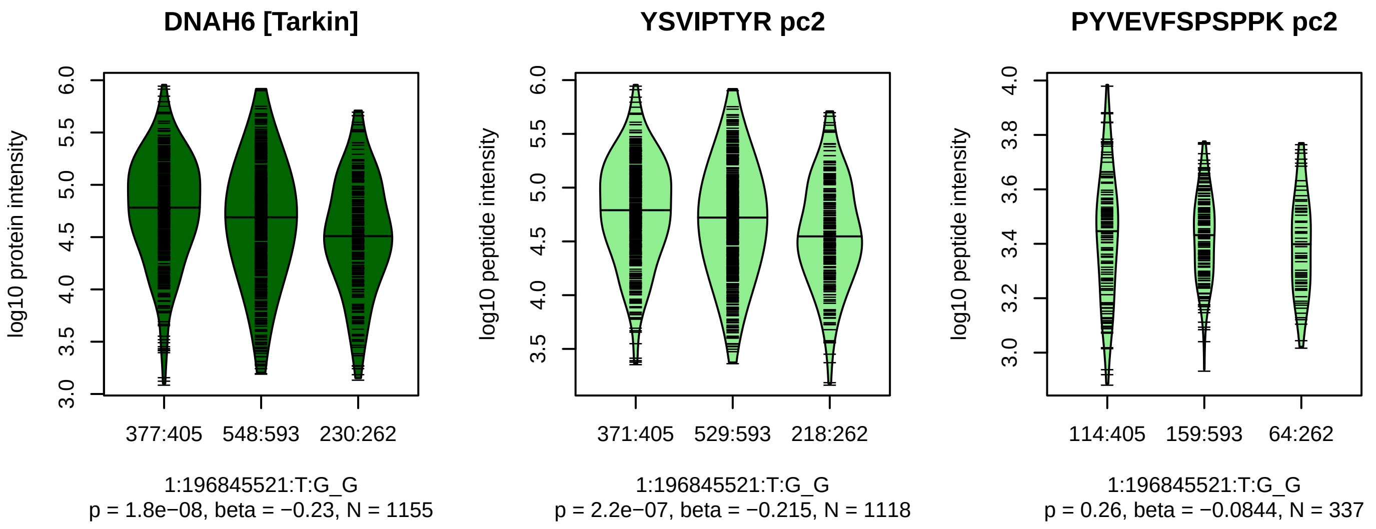

No protein data available
for replication in QMDiab

Sort: 286
Gene name: DNAH6
SNP: 1:196845521:T:G
rsID: rs6695525
Type: Trans−pQTL
Protein group(s):
Q9C0G6
Nano−particle: NP4

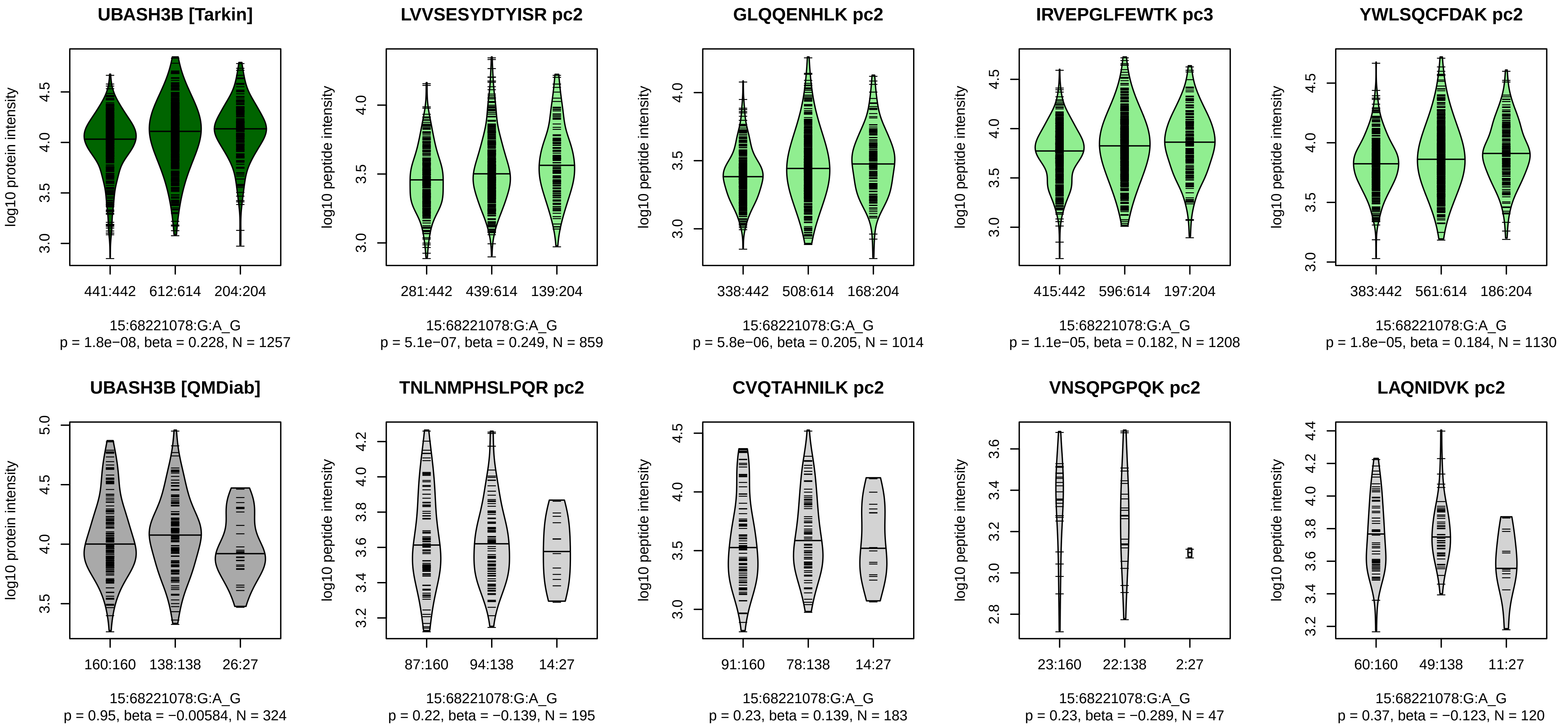

Sort: 287
Gene name: UBASH3B
SNP: 15:68221078:G:A
rsID: rs396174
Type: Trans−pQTL
Protein group(s):
Q8TF42
Nano−particle: NP4

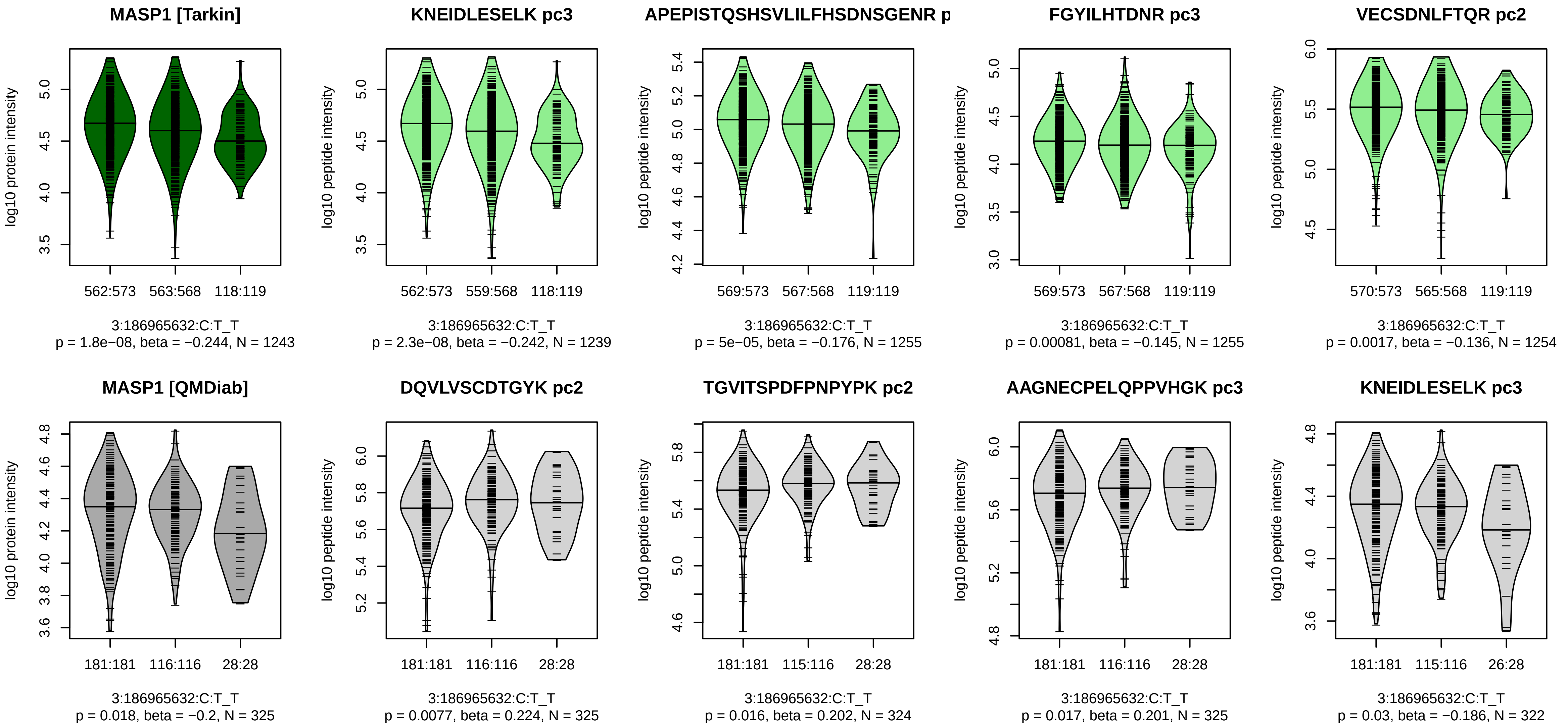

Sort: 288
Gene name: MASP1
SNP: 3:186965632:C:T
rsID: rs184750711
Type: Cis−pQTL
Protein group(s):
F8W876
Nano−particle: NP2

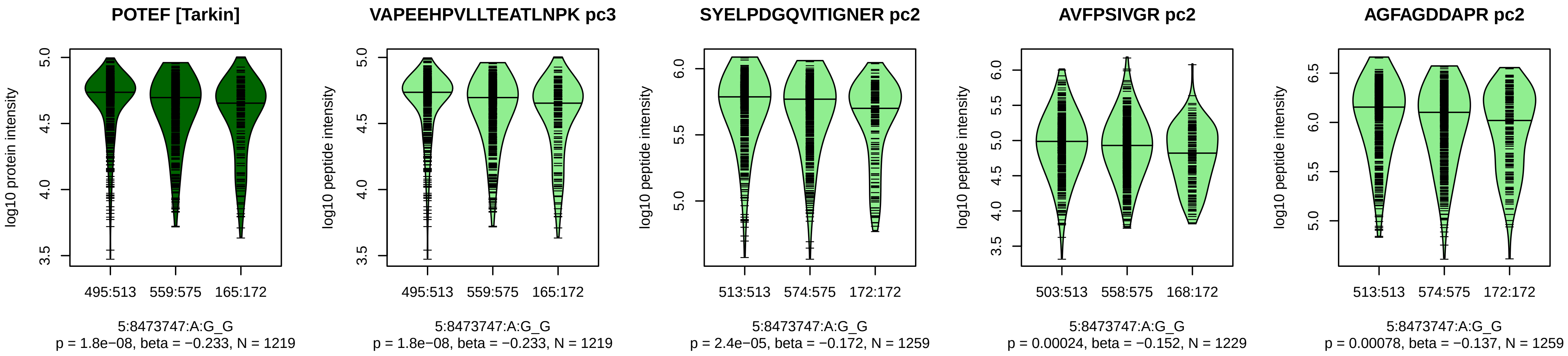

No protein data available
for replication in QMDiab

Sort: 289
Gene name: POTEF
SNP: 5:8473747:A:G
rsID: rs186893
Type: Trans−pQTL
Protein group(s):
A5A3E0
Nano−particle: NP4

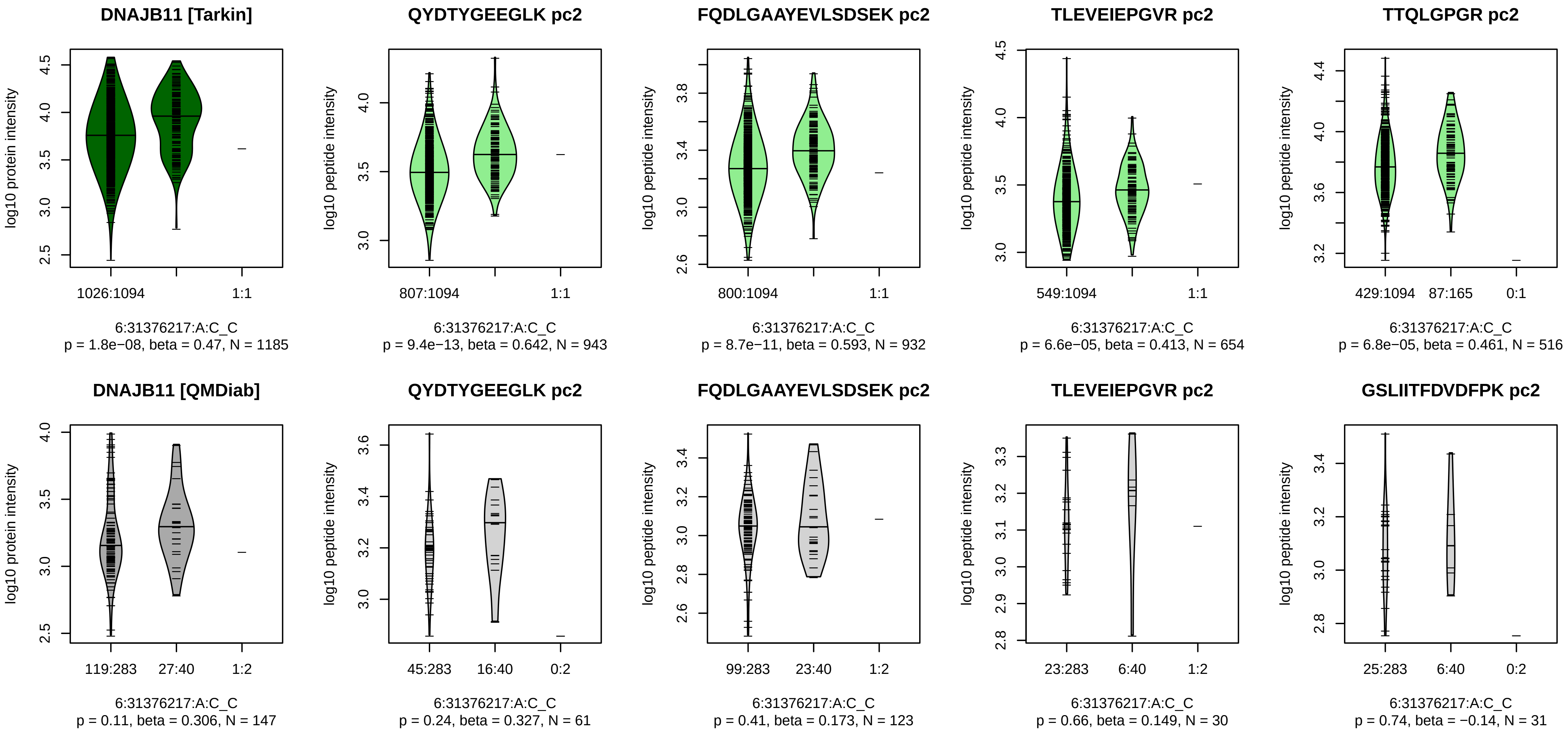

Sort: 290
Gene name: DNAJB11
SNP: 6:31376217:A:C
rsID: rs113708600
Type: Trans−pQTL
Protein group(s):
Q9UBS4
Nano−particle: NP3

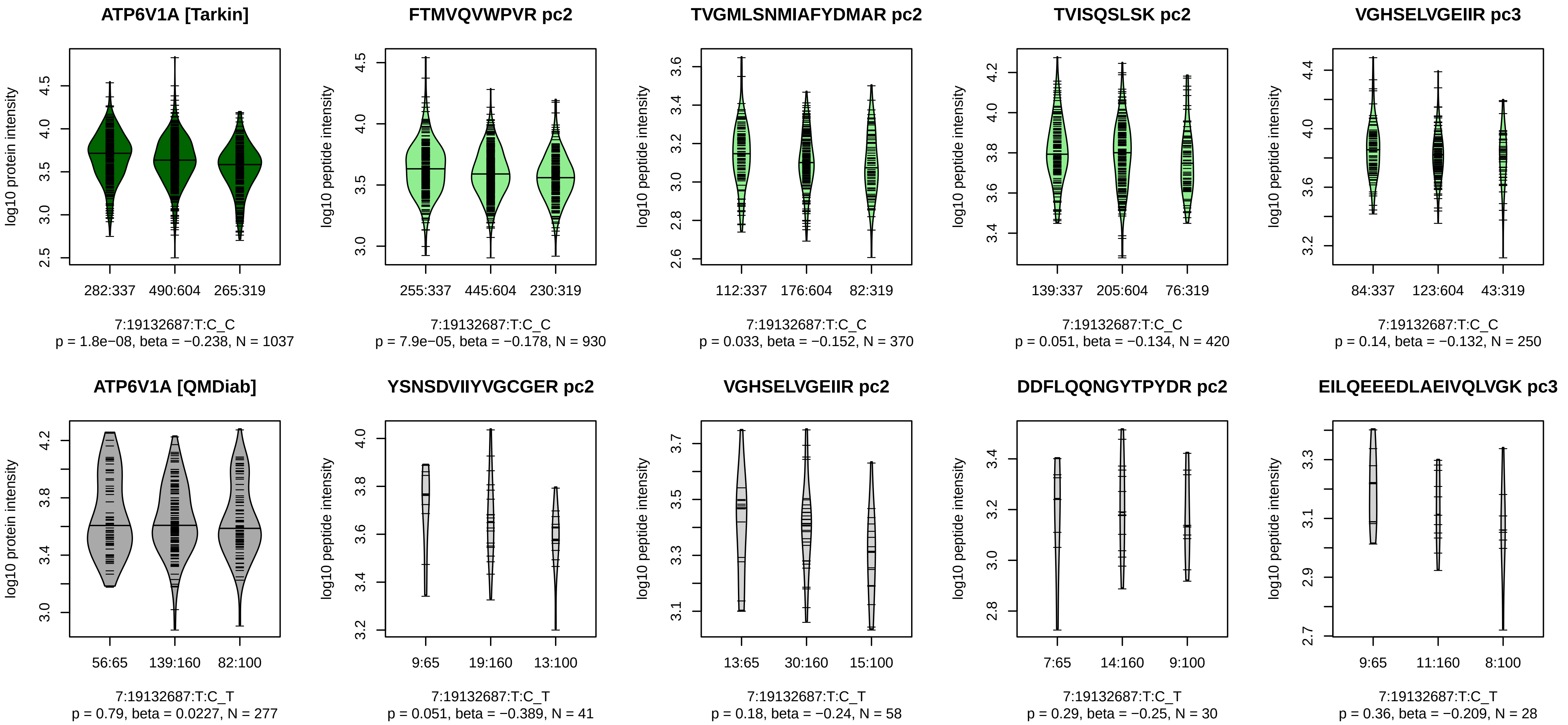

Sort: 291
Gene name: ATP6V1A
SNP: 7:19132687:T:C
rsID: rs10240058
Type: Trans−pQTL
Protein group(s):
P38606
Nano−particle: NP5

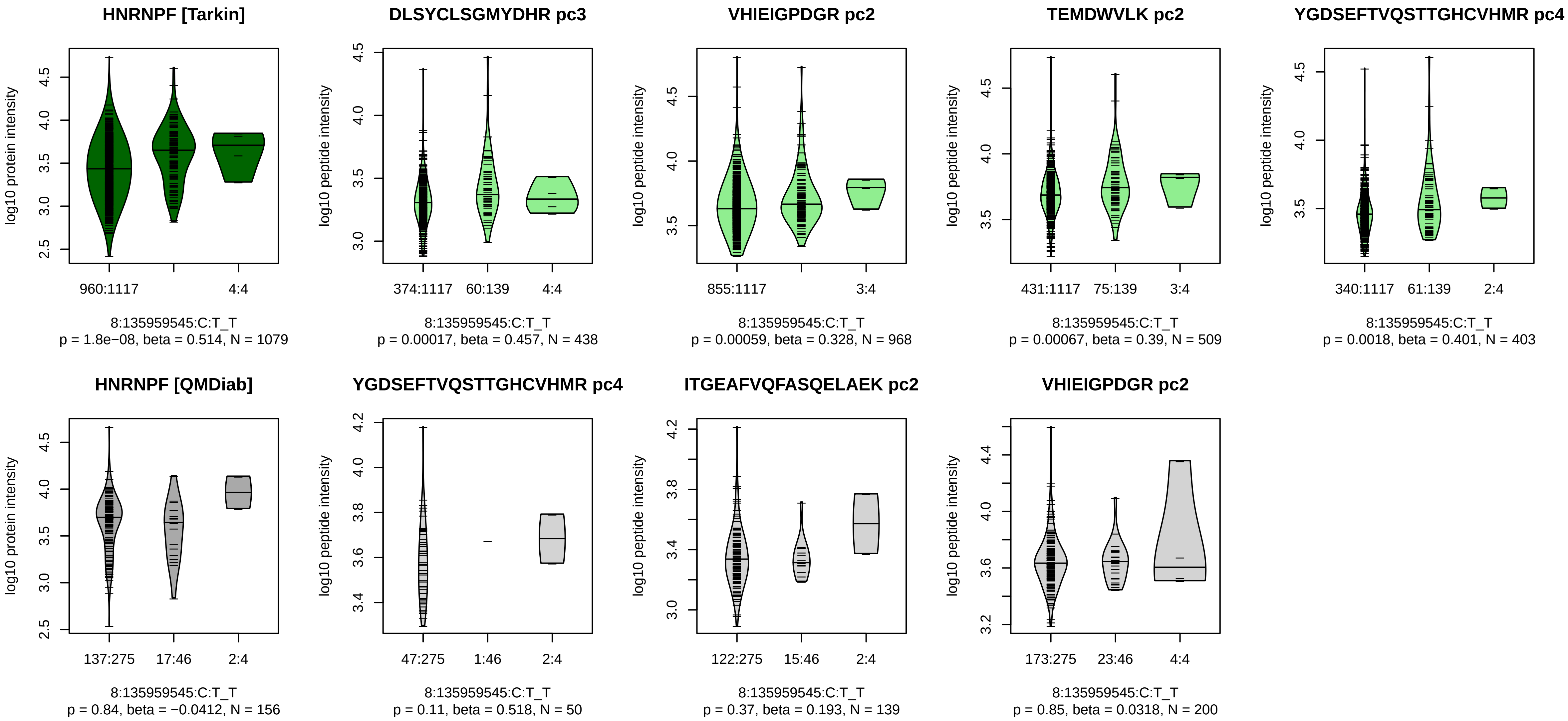

Sort: 292
Gene name: HNRNPF
SNP: 8:135959545:C:T
rsID: rs78923810
Type: Trans−pQTL
Protein group(s):
P52597
Nano−particle: NP2

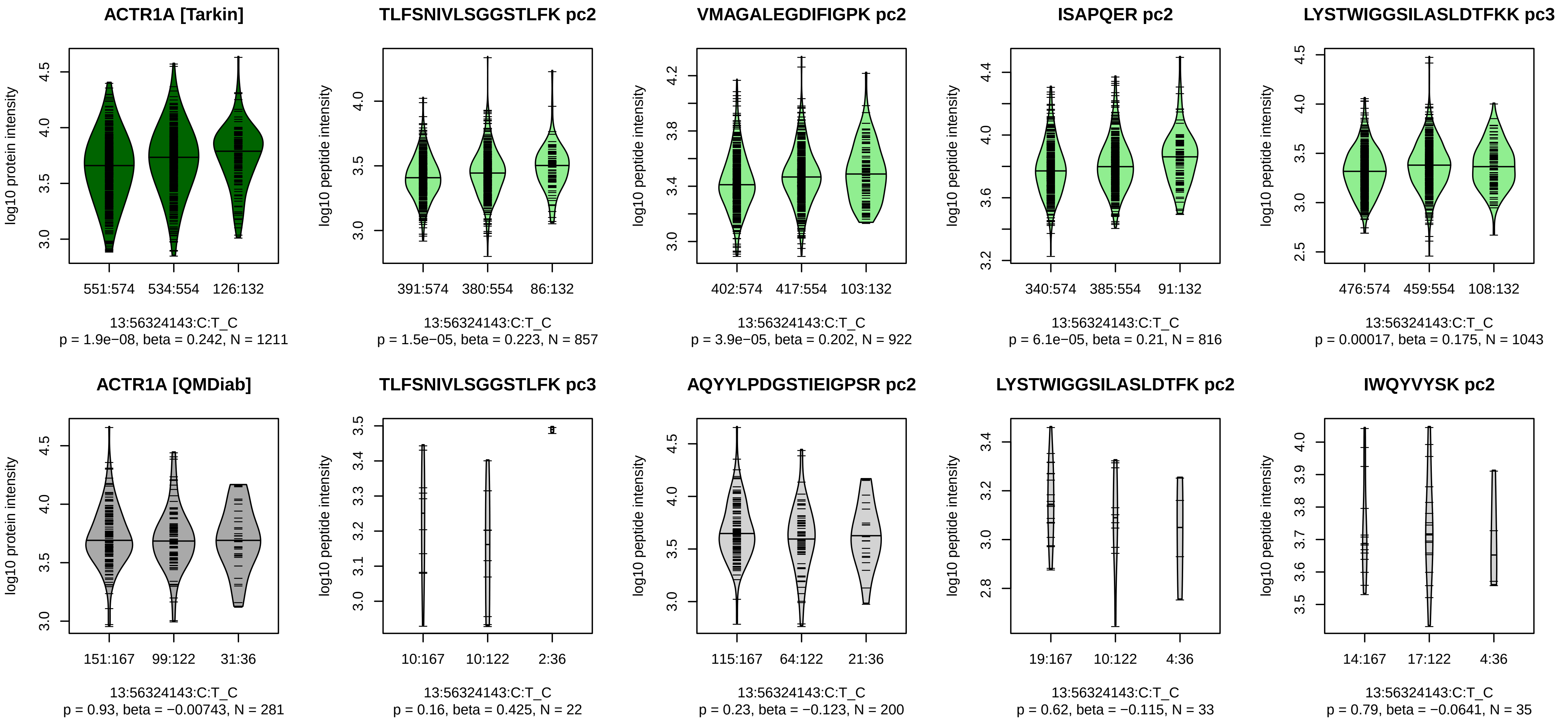

Sort: 293
Gene name: ACTR1A
SNP: 13:56324143:C:T
rsID: rs6561866
Type: Trans−pQTL
Protein group(s):
P61163
Nano−particle: NP5

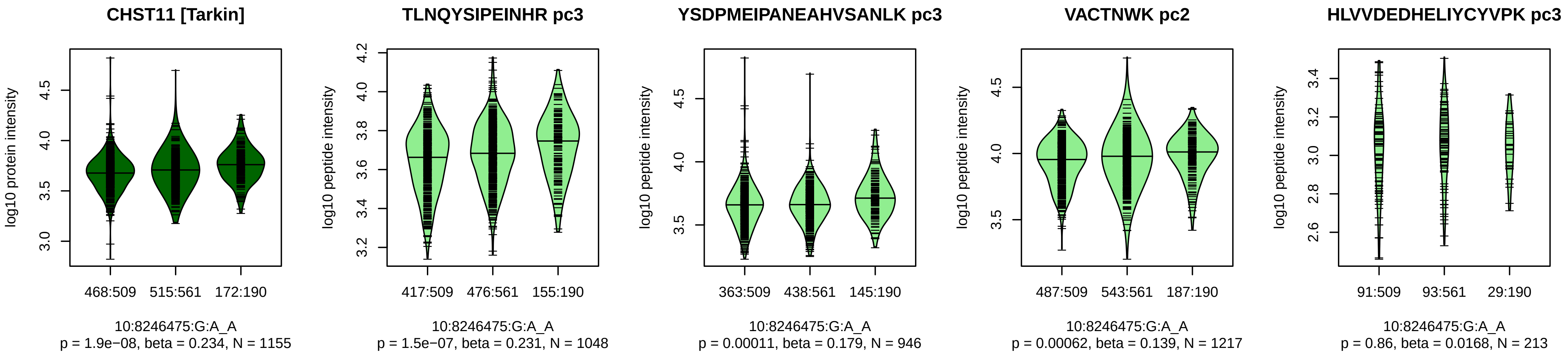

No protein data available
for replication in QMDiab

Sort: 294
Gene name: CHST11
SNP: 10:8246475:G:A
rsID: rs1629795
Type: Trans−pQTL
Protein group(s):
Q9NPF2;Q9NPF2−2
Nano−particle: NP4

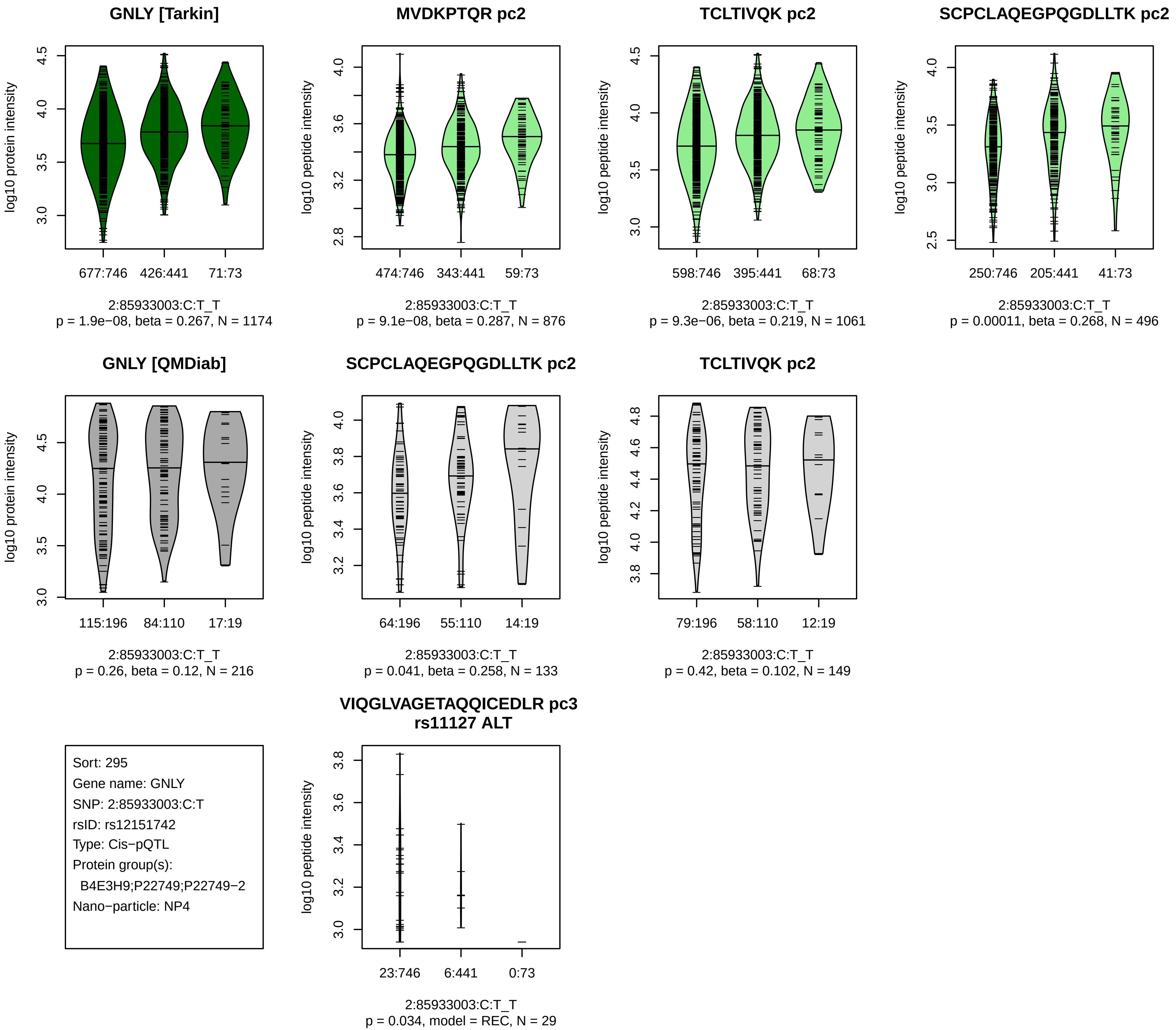
GNLY [Tarkin]
log10 protein intensity
677:746
426:441
71:73
2:85933003:C:T_T
p = 1.9e−08, beta = 0.267, N = 1174
MVDKPTQR pc2
log10 peptide intensity
474:746
343:441
59:73
2:85933003:C:T_T
p = 9.1e−08, beta = 0.287, N = 876
TCLTIVQK pc2
log10 peptide intensity
598:746
395:441
68:73
2:85933003:C:T_T
p = 9.3e−06, beta = 0.219, N = 1061
SCPCLAQEGPQGDLLTK pc2
log10 peptide intensity
250:746
205:441
41:73
2:85933003:C:T_T
p = 0.00011, beta = 0.268, N = 496
GNLY [QMDiab]
log10 protein intensity
115:196
84:110
17:19
2:85933003:C:T_T
p = 0.26, beta = 0.12, N = 216
SCPCLAQEGPQGDLLTK pc2
log10 peptide intensity
64:196
55:110
14:19
2:85933003:C:T_T
p = 0.041, beta = 0.258, N = 133
TCLTIVQK pc2
log10 peptide intensity
79:196
58:110
12:19
2:85933003:C:T_T
p = 0.42, beta = 0.102, N = 149
Sort: 295
Gene name: GNLY
SNP: 2:85933003:C:T
rsID: rs12151742
Type: Cis−pQTL
Protein group(s):
B4E3H9;P22749;P22749−2
Nano−particle: NP4
VIQGLVAGETAQQICEDLR pc3
rs11127 ALT
log10 peptide intensity
23:746
6:441
0:73
2:85933003:C:T_T
p = 0.034, model = REC, N = 29

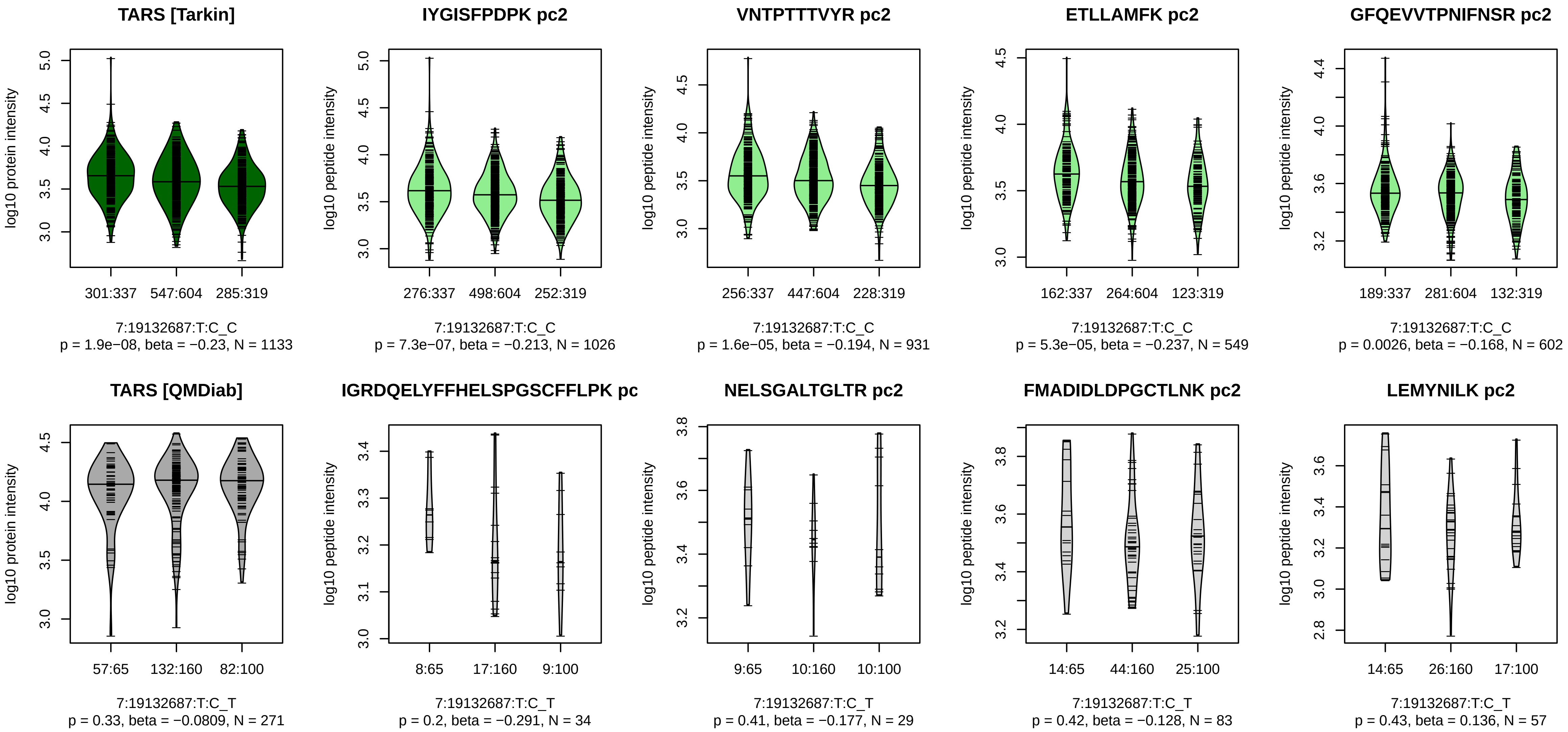

Sort: 296
Gene name: TARS
SNP: 7:19132687:T:C
rsID: rs10240058
Type: Trans−pQTL
Protein group(s):
T: P26639
Q: P26639;P26639−2
Nano−particle: NP5

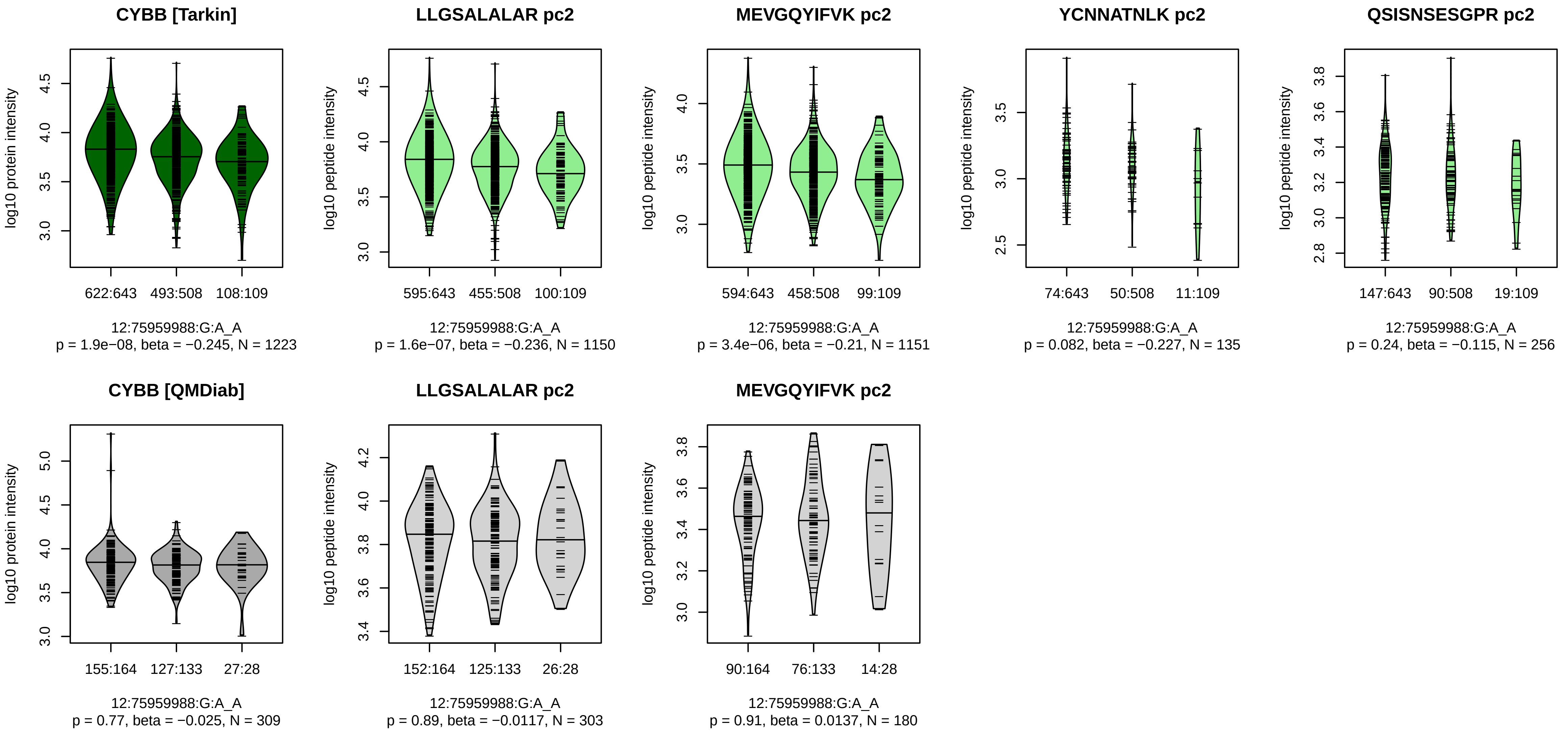

Sort: 297
Gene name: CYBB
SNP: 12:75959988:G:A
rsID: rs34170388
Type: Trans−pQTL
Protein group(s):
P04839
Nano−particle: NP1

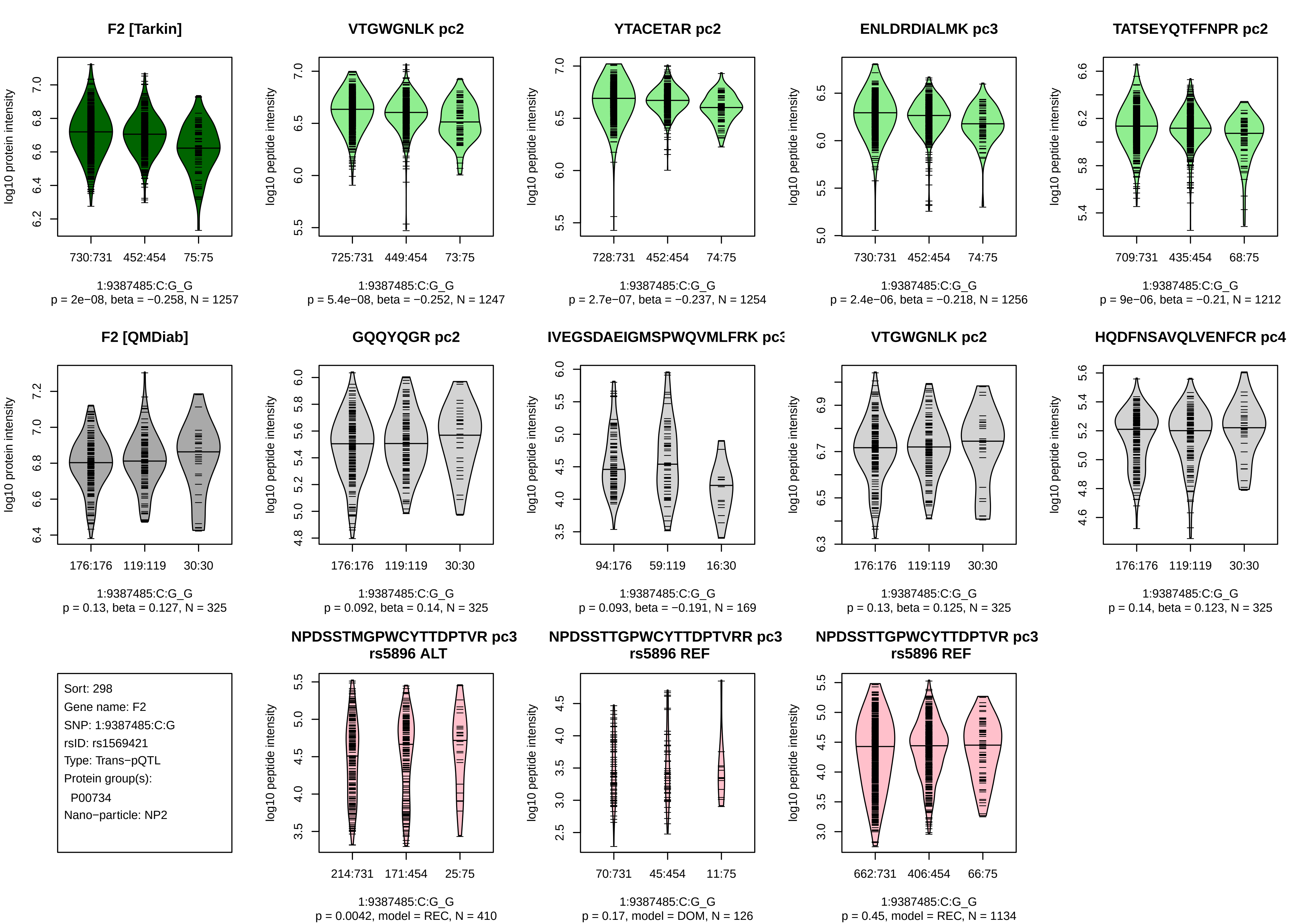

F2 [Tarkin]
log10 protein intensity
730:731
452:454
75:75
1:9387485:C:G_G
p = 2e−08, beta = −0.258, N = 1257
VTGWGNLK pc2
log10 peptide intensity
725:731
449:454
73:75
1:9387485:C:G_G
p = 5.4e−08, beta = −0.252, N = 1247
YTACETAR pc2
log10 peptide intensity
728:731
452:454
74:75
1:9387485:C:G_G
p = 2.7e−07, beta = −0.237, N = 1254
ENLDRDIALMK pc3
log10 peptide intensity
730:731
452:454
74:75
1:9387485:C:G_G
p = 2.4e−06, beta = −0.218, N = 1256
TATSEYQTFFNPR pc2
log10 peptide intensity
709:731
435:454
68:75
1:9387485:C:G_G
p = 9e−06, beta = −0.21, N = 1212
F2 [QMDiab]
log10 protein intensity
176:176
119:119
30:30
1:9387485:C:G_G
p = 0.13, beta = 0.127, N = 325
GQQYQGR pc2
log10 peptide intensity
176:176
119:119
30:30
1:9387485:C:G_G
p = 0.092, beta = 0.14, N = 325
IVEGSDAEIGMSPWQVMLFRK pc3
log10 peptide intensity
94:176
59:119
16:30
1:9387485:C:G_G
p = 0.093, beta = −0.191, N = 169
VTGWGNLK pc2
log10 peptide intensity
176:176
119:119
30:30
1:9387485:C:G_G
p = 0.13, beta = 0.125, N = 325
HQDFNSAVQLVENFCR pc4
log10 peptide intensity
176:176
119:119
30:30
1:9387485:C:G_G
p = 0.14, beta = 0.123, N = 325
Sort: 298
Gene name: F2
SNP: 1:9387485:C:G
rsID: rs1569421
Type: Trans−pQTL
Protein group(s):
P00734
Nano−particle: NP2
NPDSSTMGPWCYTTDPTVR pc3
rs5896 ALT
log10 peptide intensity
214:731
171:454
25:75
1:9387485:C:G_G
p = 0.0042, model = REC, N = 410
NPDSSTTGPWCYTTDPTVRR pc3
rs5896 REF
log10 peptide intensity
70:731
45:454
11:75
1:9387485:C:G_G
p = 0.17, model = DOM, N = 126
NPDSSTTGPWCYTTDPTVR pc3
rs5896 REF
log10 peptide intensity
662:731
406:454
66:75
1:9387485:C:G_G
p = 0.45, model = REC, N = 1134

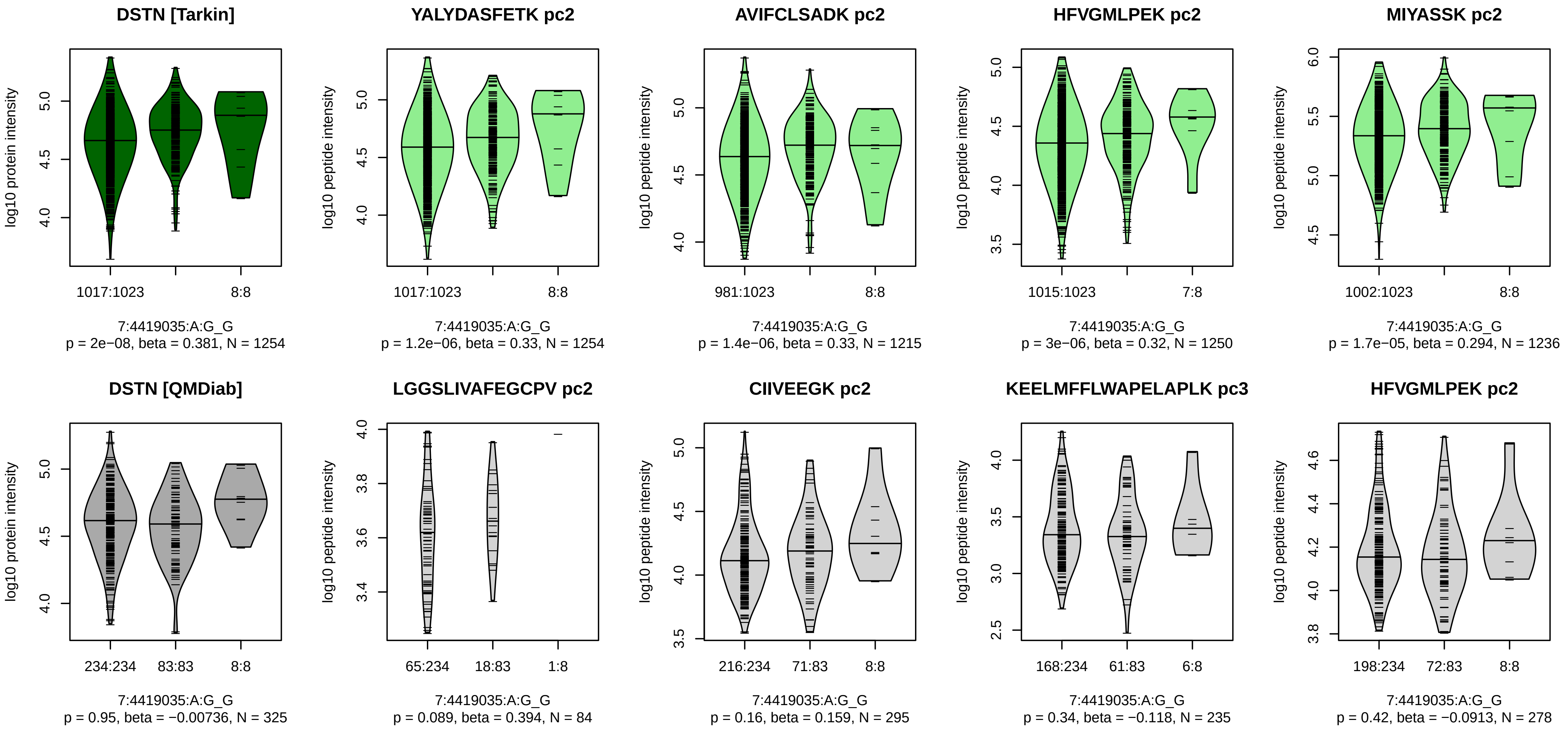

Sort: 299
Gene name: DSTN
SNP: 7:4419035:A:G
rsID: rs314625
Type: Trans−pQTL
Protein group(s):
P60981
Nano−particle: NP3

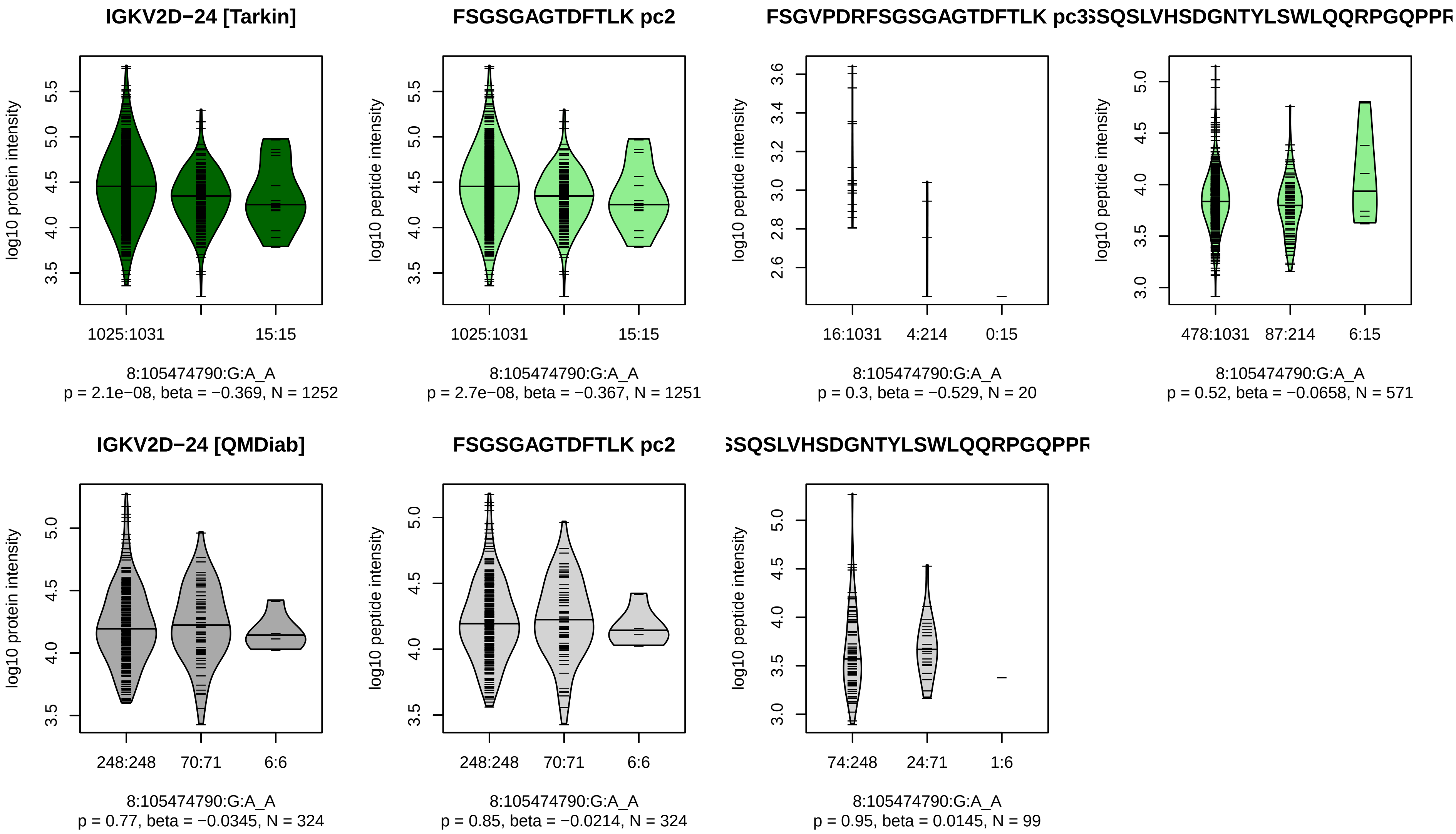

Sort: 300
Gene name: IGKV2D−24
SNP: 8:105474790:G:A
rsID: rs35787438
Type: Trans−pQTL
Protein group(s):
A0A075B6R9
Nano−particle: NP3

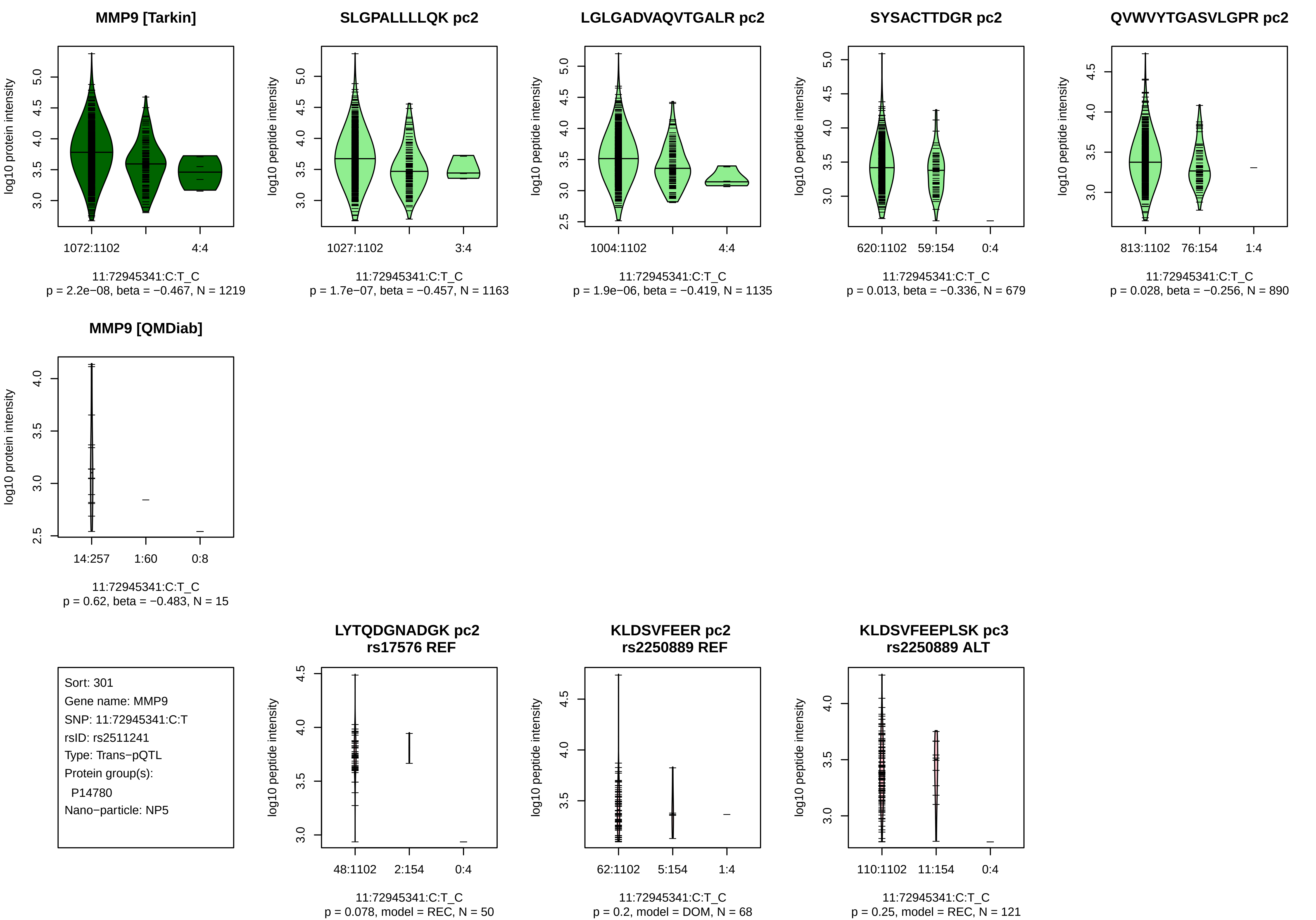

MMP9 [Tarkin]
log10 protein intensity
1072:1102
4:4
11:72945341:C:T_C
p = 2.2e−08, beta = −0.467, N = 1219
SLGPALLLLQK pc2
log10 peptide intensity
1027:1102
3:4
11:72945341:C:T_C
p = 1.7e−07, beta = −0.457, N = 1163
LGLGADVAQVTGALR pc2
log10 peptide intensity
1004:1102
4:4
11:72945341:C:T_C
p = 1.9e−06, beta = −0.419, N = 1135
SYSACTTDGR pc2
log10 peptide intensity
620:1102
59:154
0:4
11:72945341:C:T_C
p = 0.013, beta = −0.336, N = 679
QVWVYTGASVLGPR pc2
log10 peptide intensity
813:1102
76:154
1:4
11:72945341:C:T_C
p = 0.028, beta = −0.256, N = 890
MMP9 [QMDiab]
log10 protein intensity
14:257
1:60
0:8
11:72945341:C:T_C
p = 0.62, beta = −0.483, N = 15
Sort: 301
Gene name: MMP9
SNP: 11:72945341:C:T
rsID: rs2511241
Type: Trans−pQTL
Protein group(s):
P14780
Nano−particle: NP5
LYTQDGNADGK pc2
rs17576 REF
log10 peptide intensity
48:1102
2:154
0:4
11:72945341:C:T_C
p = 0.078, model = REC, N = 50
KLDSVFEER pc2
rs2250889 REF
log10 peptide intensity
62:1102
5:154
1:4
11:72945341:C:T_C
p = 0.2, model = DOM, N = 68
KLDSVFEEPLSK pc3
rs2250889 ALT
log10 peptide intensity
110:1102
11:154
0:4
11:72945341:C:T_C
p = 0.25, model = REC, N = 121

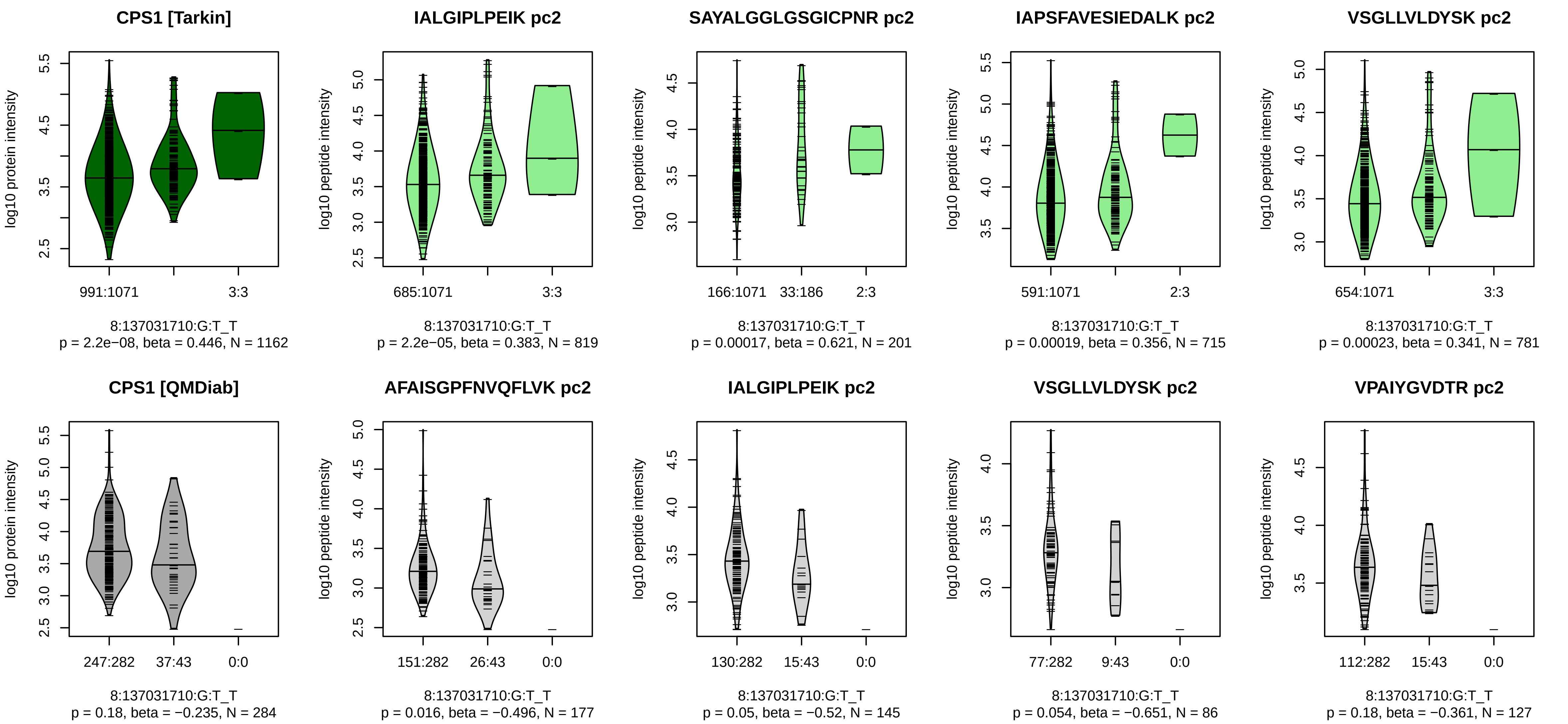

Sort: 302
Gene name: CPS1
SNP: 8:137031710:G:T
rsID: rs62528878
Type: Trans−pQTL
Protein group(s):
T: P31327−3
Q: P31327;P31327−3
Nano−particle: NP5

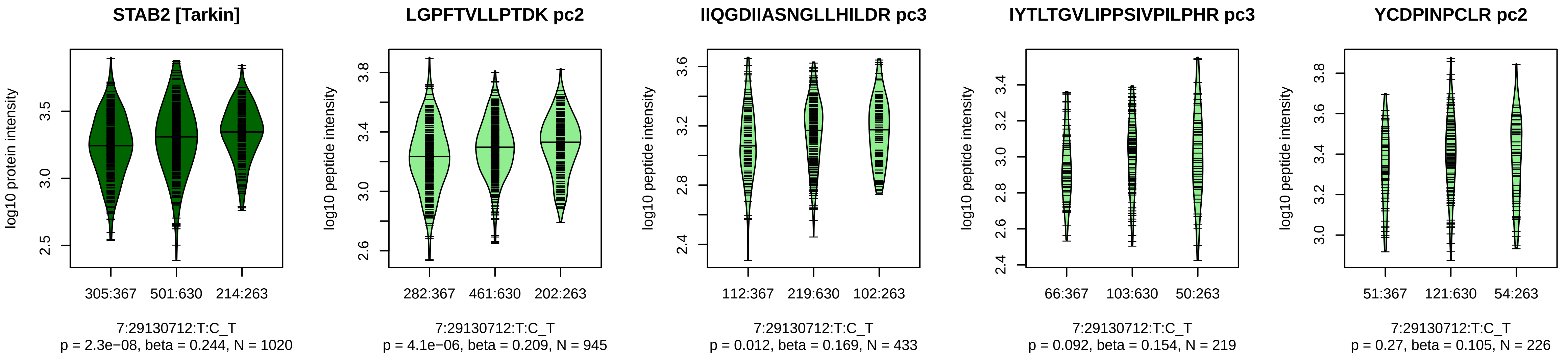

No protein data available
for replication in QMDiab

Sort: 303
Gene name: STAB2
SNP: 7:29130712:T:C
rsID: rs2075127
Type: Trans−pQTL
Protein group(s):
Q8WWQ8
Nano−particle: NP4

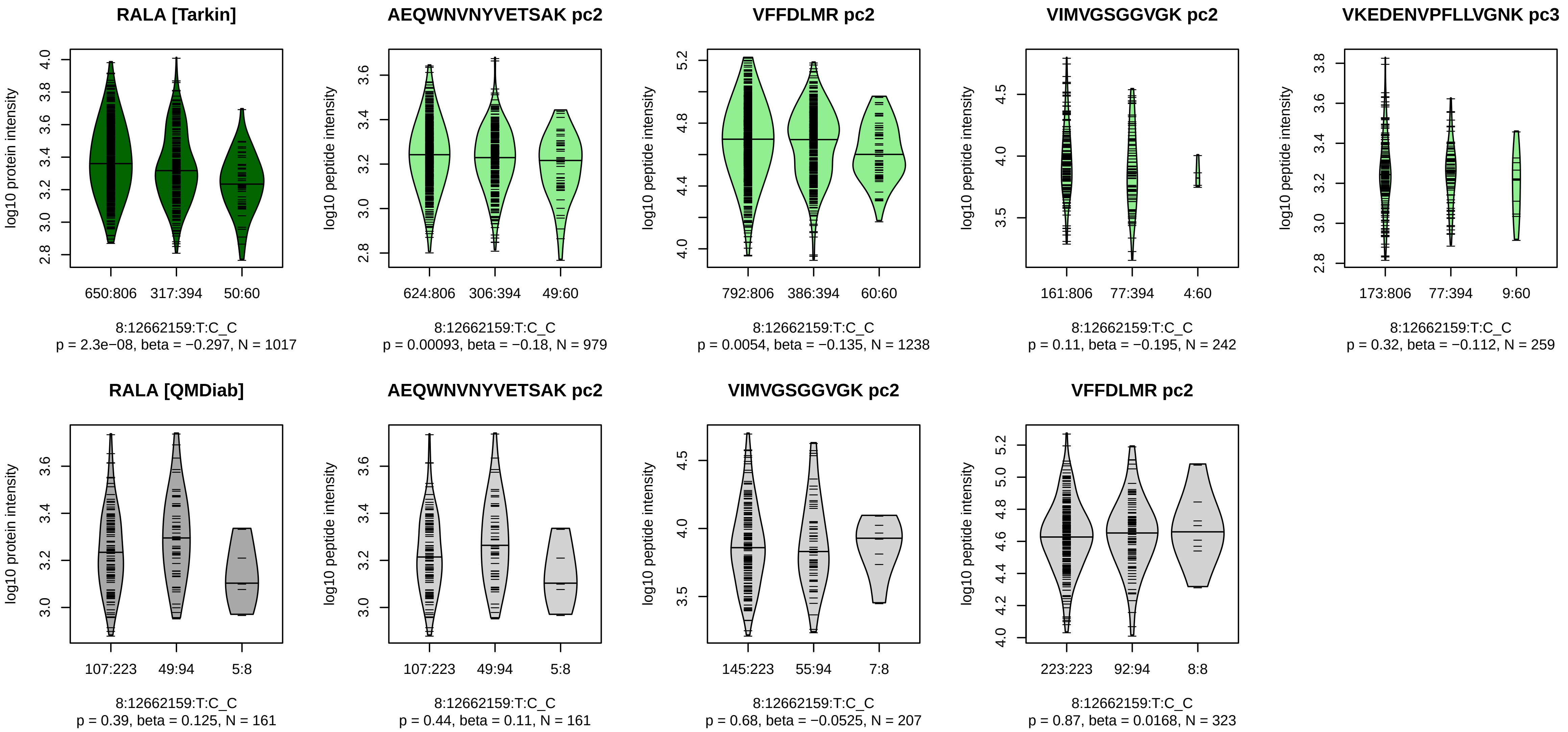

Sort: 304
Gene name: RALA
SNP: 8:12662159:T:C
rsID: rs4074557
Type: Trans−pQTL
Protein group(s):
P11233
Nano−particle: NP1

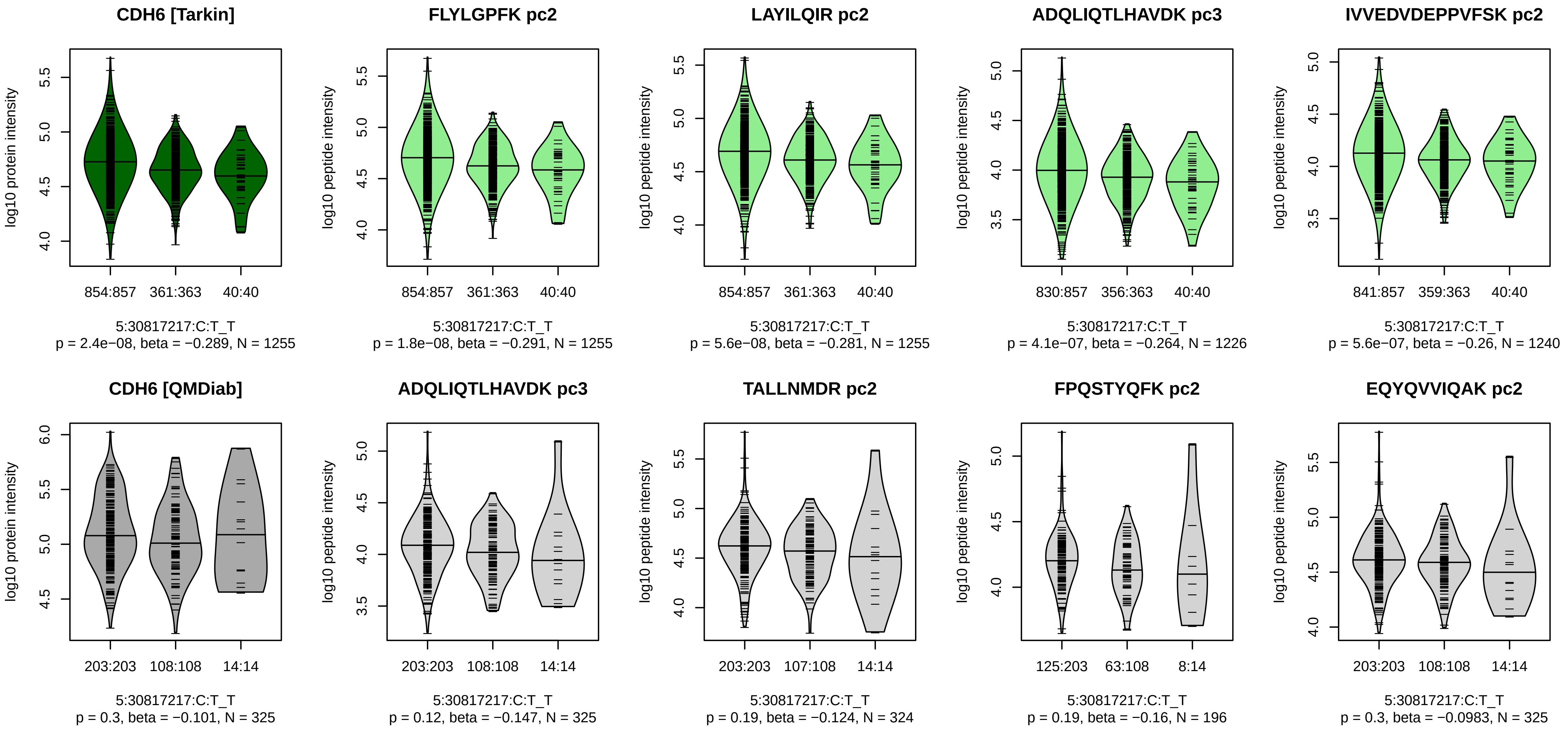

Sort: 305
Gene name: CDH6
SNP: 5:30817217:C:T
rsID: rs59682852
Type: Cis−pQTL
Protein group(s):
P55285
Nano−particle: NP2

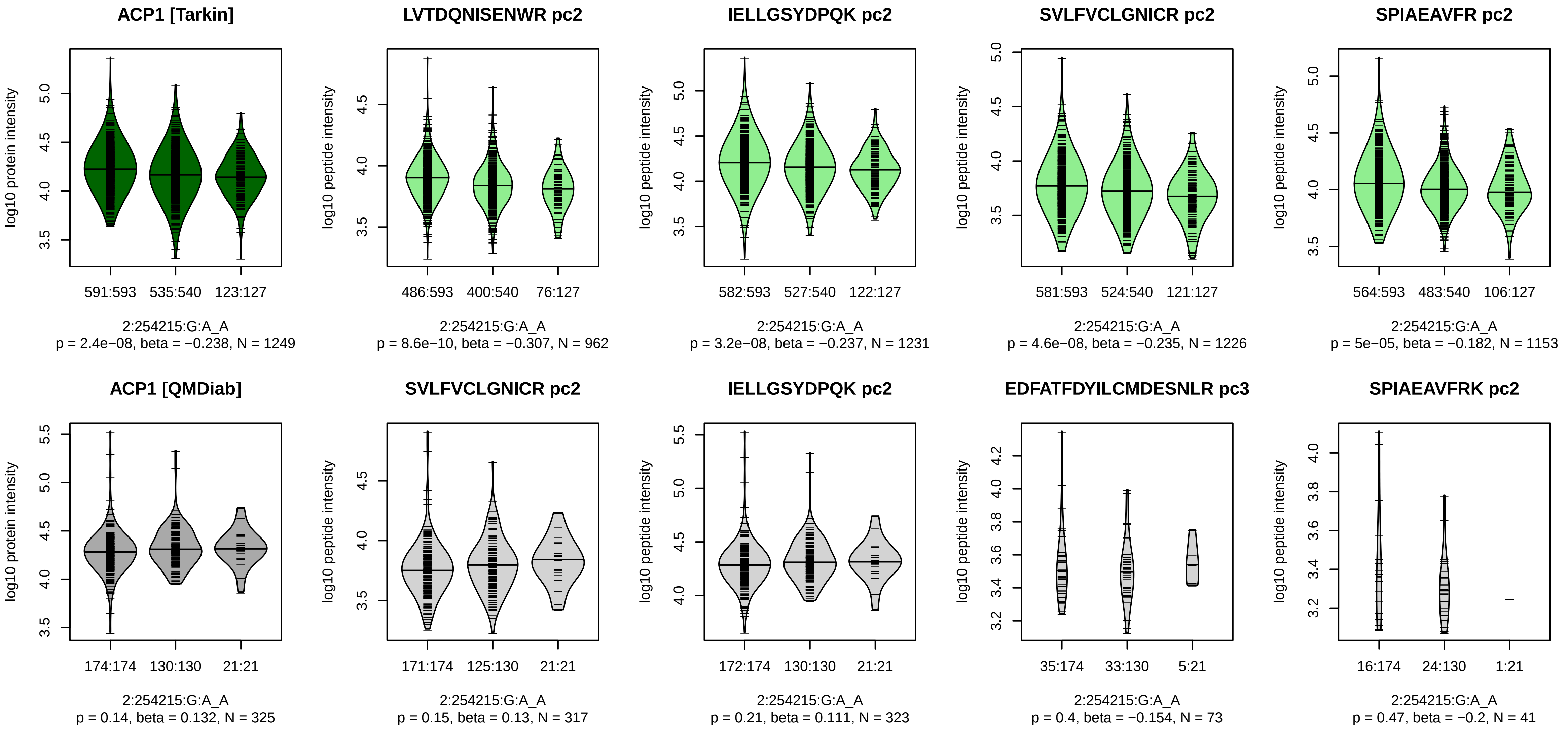

Sort: 306
Gene name: ACP1
SNP: 2:254215:G:A
rsID: rs17713879
Type: Cis−pQTL
Protein group(s):
P24666
Nano−particle: NP2

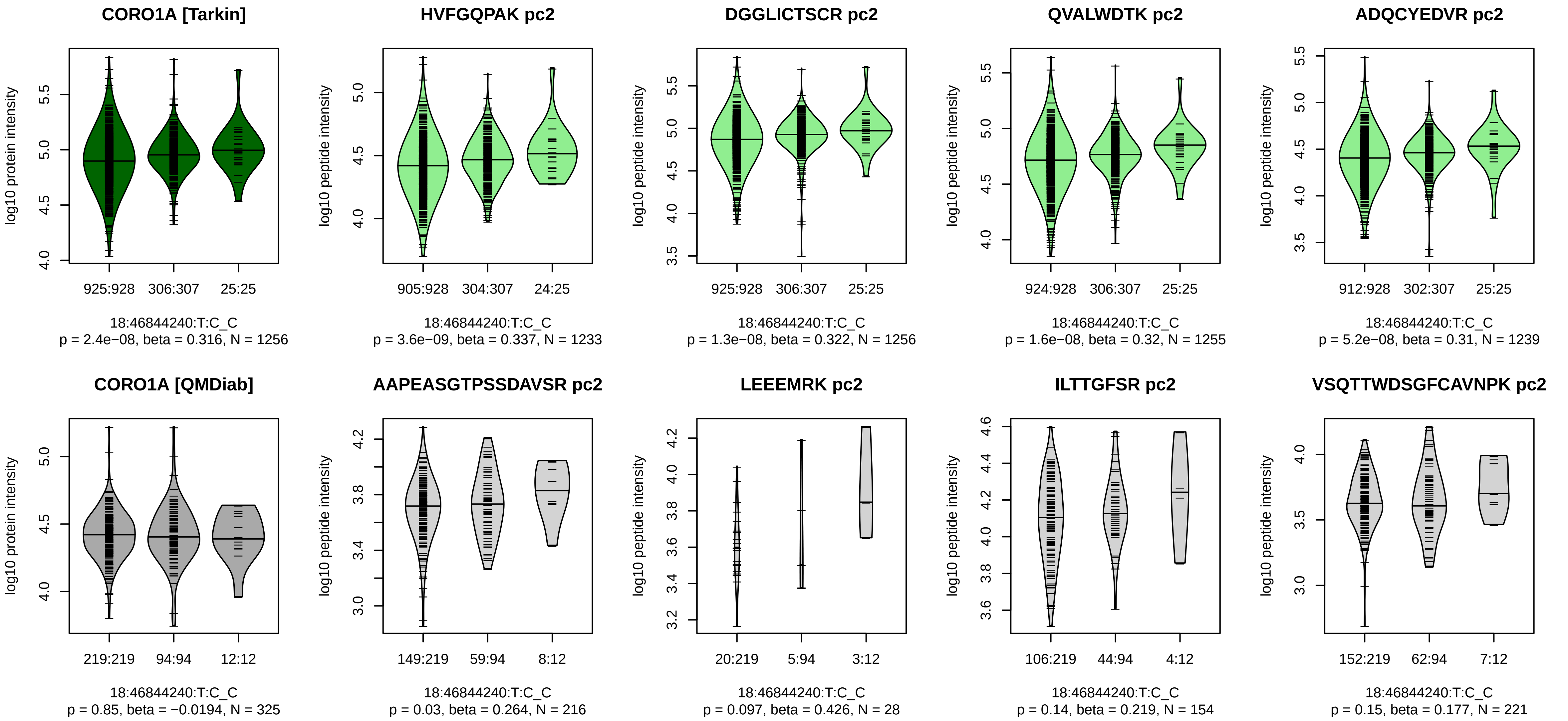

Sort: 307
Gene name: CORO1A
SNP: 18:46844240:T:C
rsID: rs77317783
Type: Trans−pQTL
Protein group(s):
P31146
Nano−particle: NP1

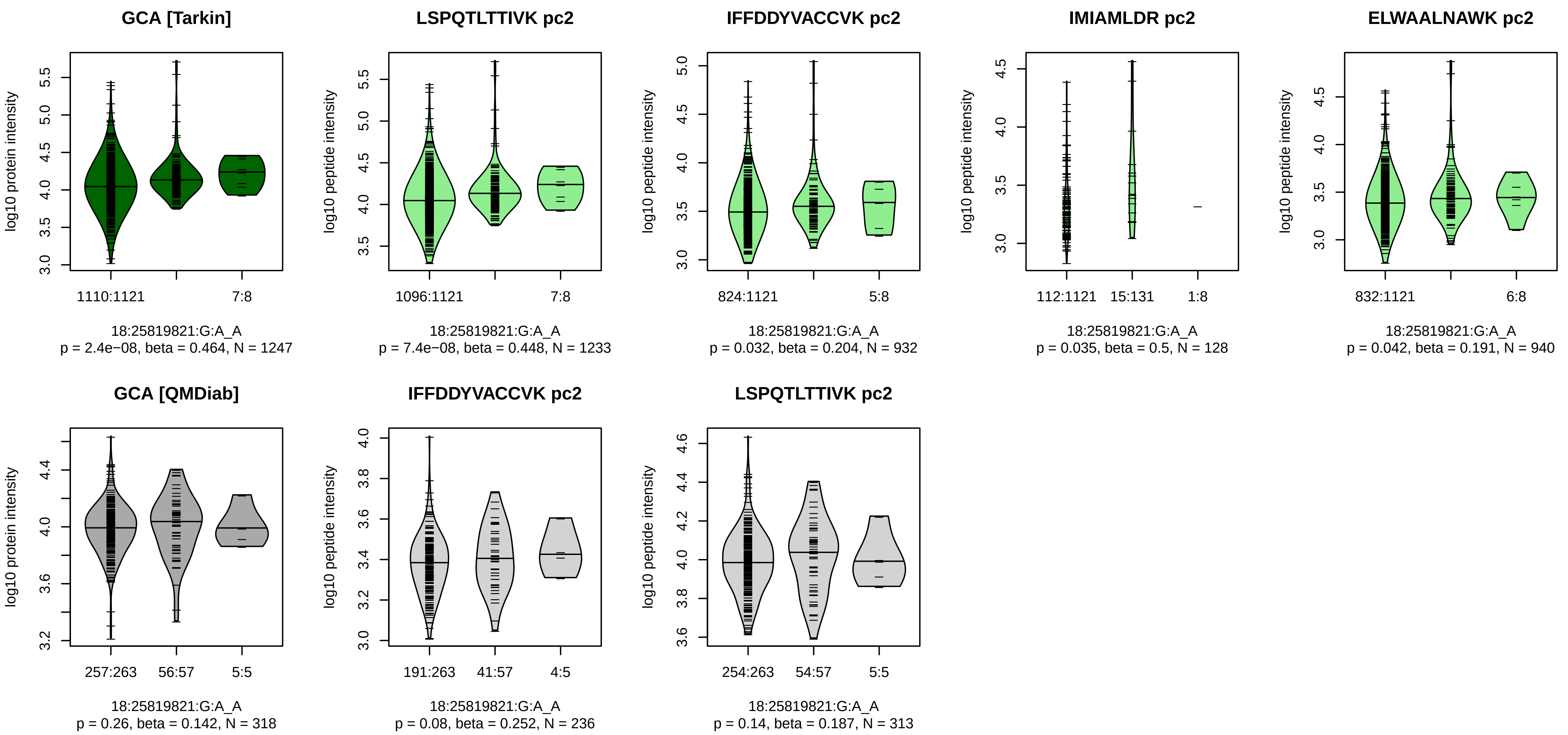

Sort: 308
Gene name: GCA
SNP: 18:25819821:G:A
rsID: rs12963915
Type: Trans−pQTL
Protein group(s):
H7BXD5;P28676
Nano−particle: NP2

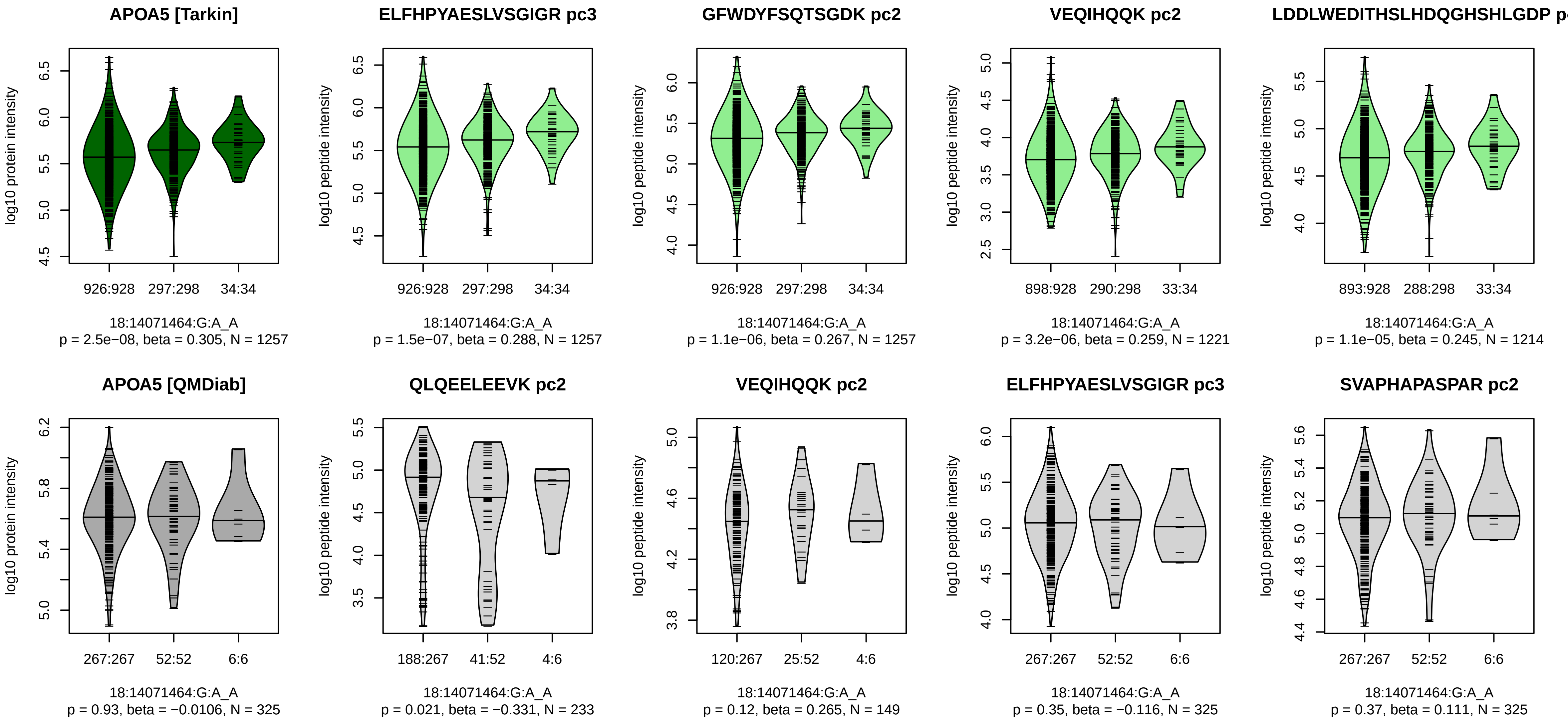

Sort: 309

Gene name: APOA5

SNP: 18:14071464:G:A

rsID: rs17619135

Type: Trans−pQTL

Protein group(s):

Q6Q788

Nano−particle: NP5

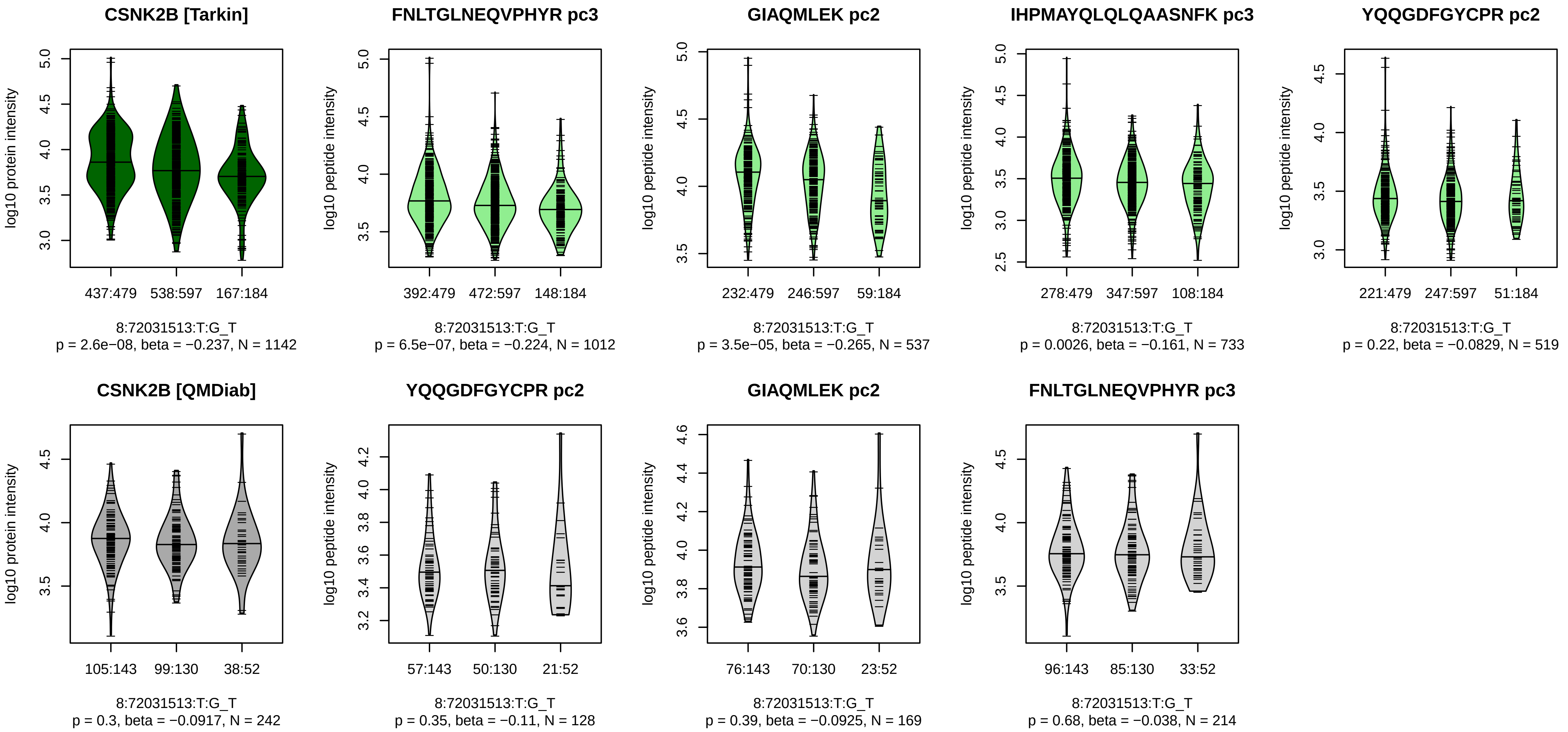

Sort: 310
Gene name: CSNK2B
SNP: 8:72031513:T:G
rsID: rs1481806
Type: Trans−pQTL
Protein group(s):
A0A7I2YQ78;P67870;Q5SRQ6
Nano−particle: NP2

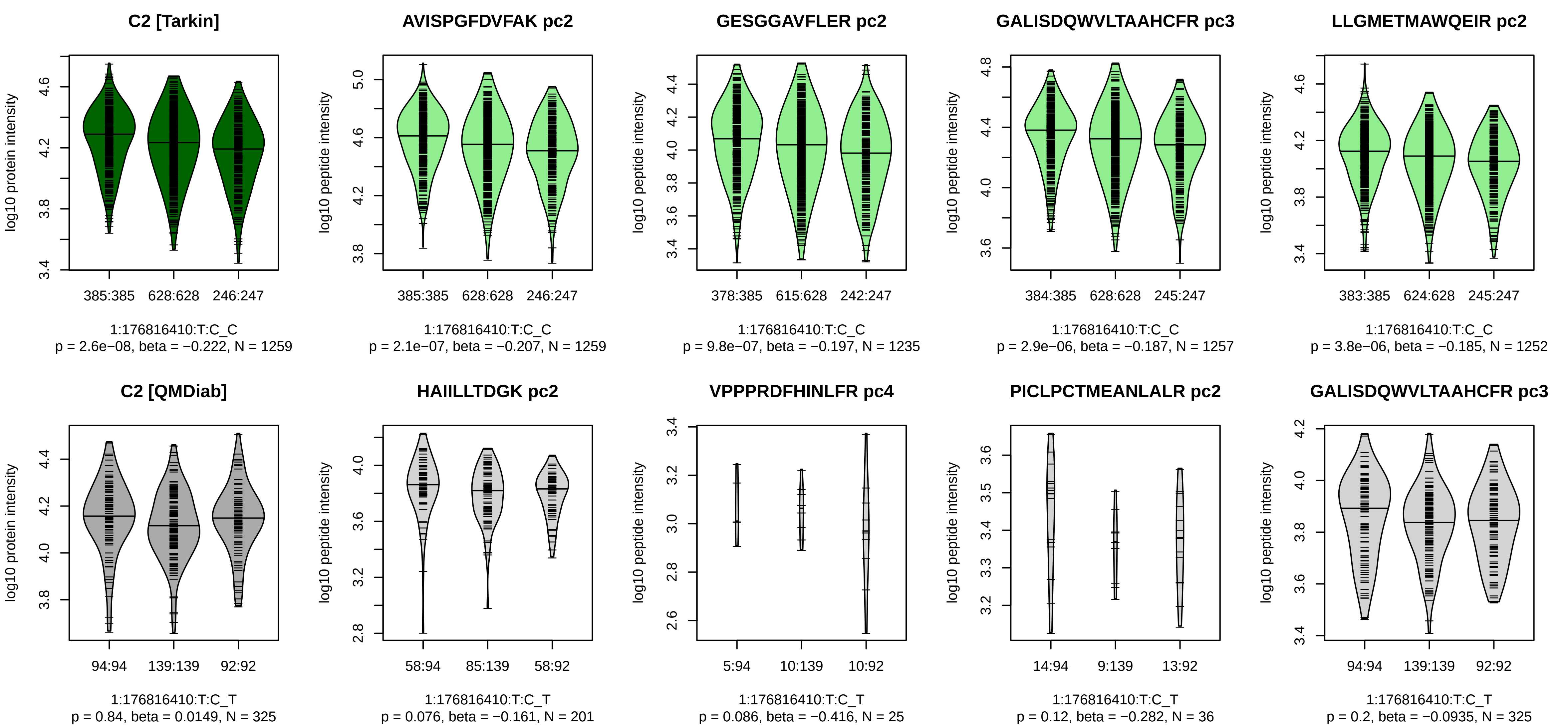

Sort: 311
Gene name: C2
SNP: 1:176816410:T:C
rsID: rs2038114
Type: Trans−pQTL
Protein group(s):
P06681
Nano−particle: NP4

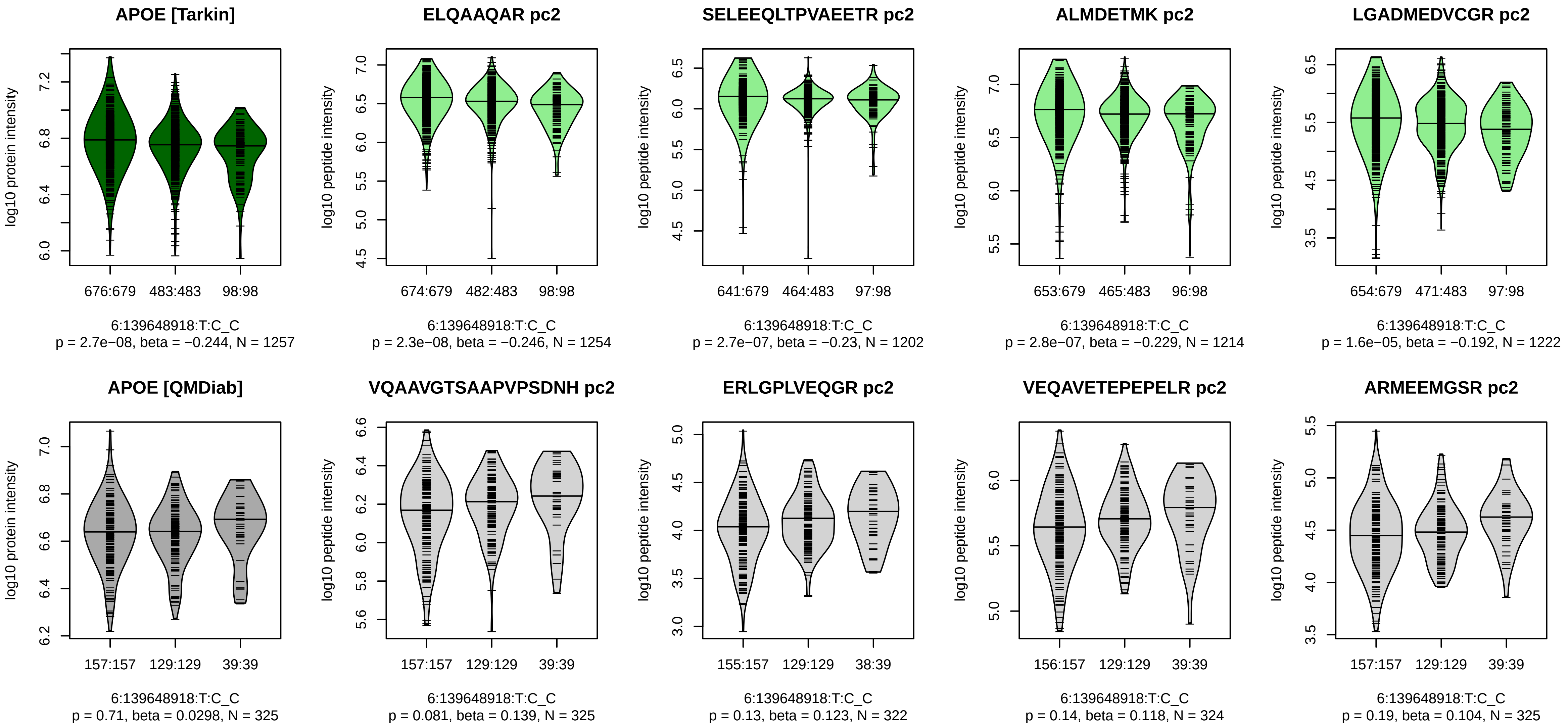

Sort: 312
Gene name: APOE
SNP: 6:139648918:T:C
rsID: rs4896448
Type: Trans−pQTL
Protein group(s):
P02649
Nano−particle: NP5

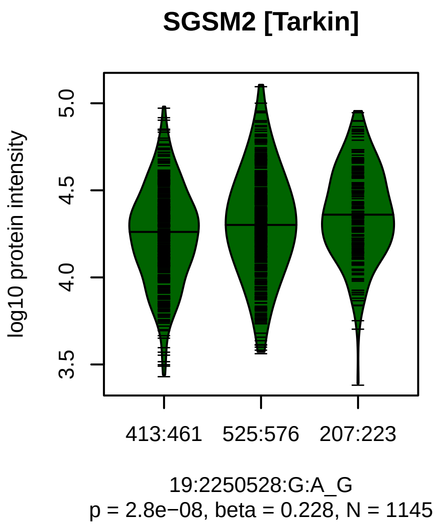

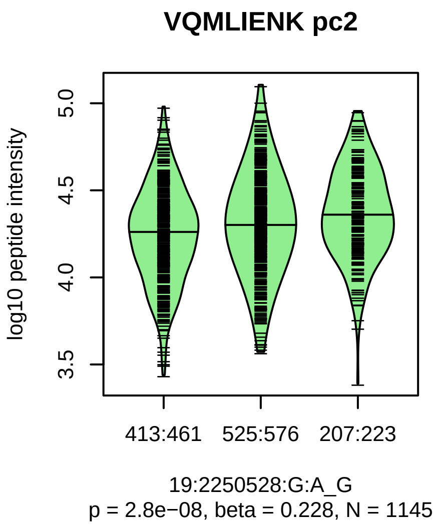

No protein data available
for replication in QMDiab

Sort: 313
Gene name: SGSM2
SNP: 19:2250528:G:A
rsID: rs8112524
Type: Trans−pQTL
Protein group(s):
O43147;O43147−2
Nano−particle: NP3

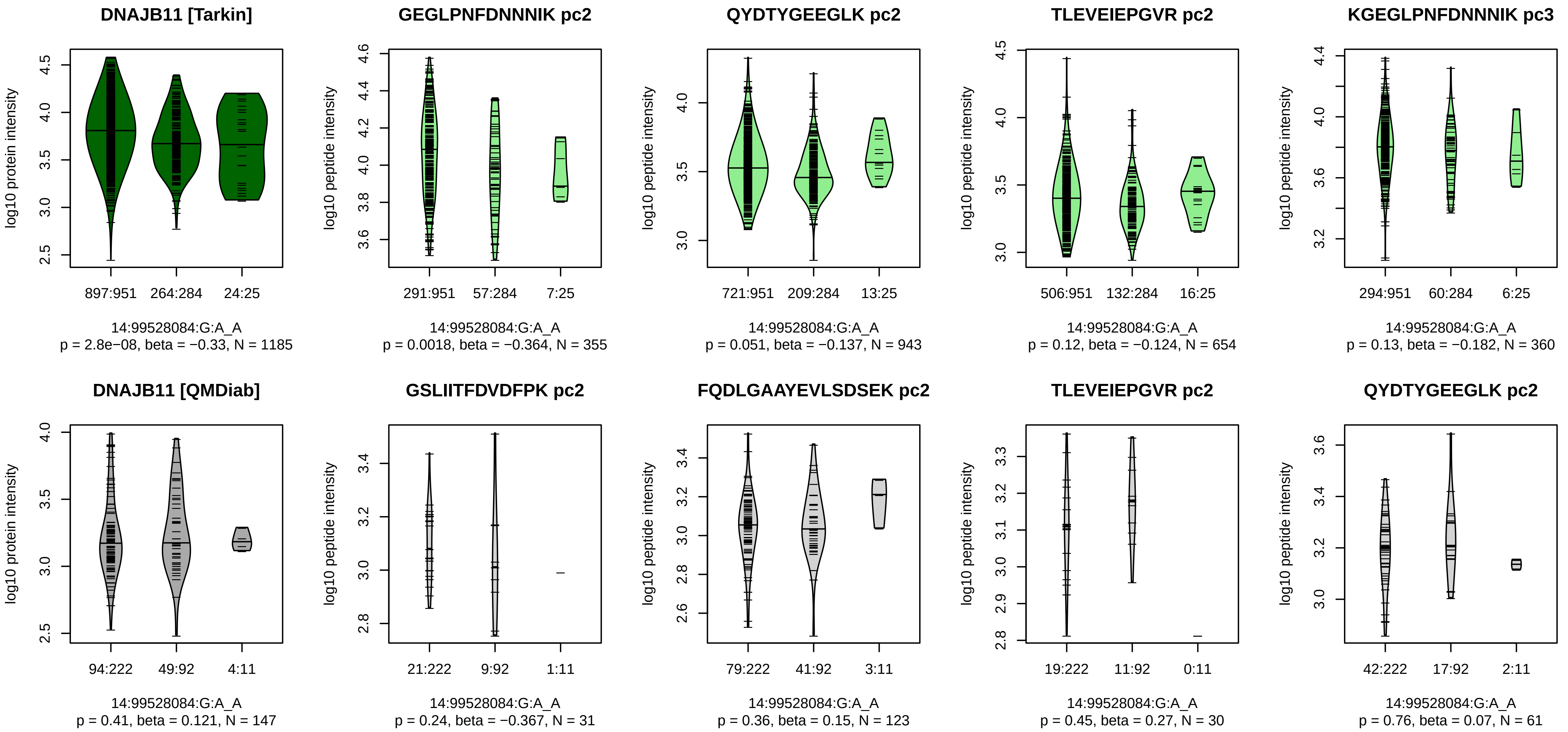

Sort: 314
Gene name: DNAJB11
SNP: 14:99528084:G:A
rsID: rs34232727
Type: Trans−pQTL
Protein group(s):
Q9UBS4
Nano−particle: NP3

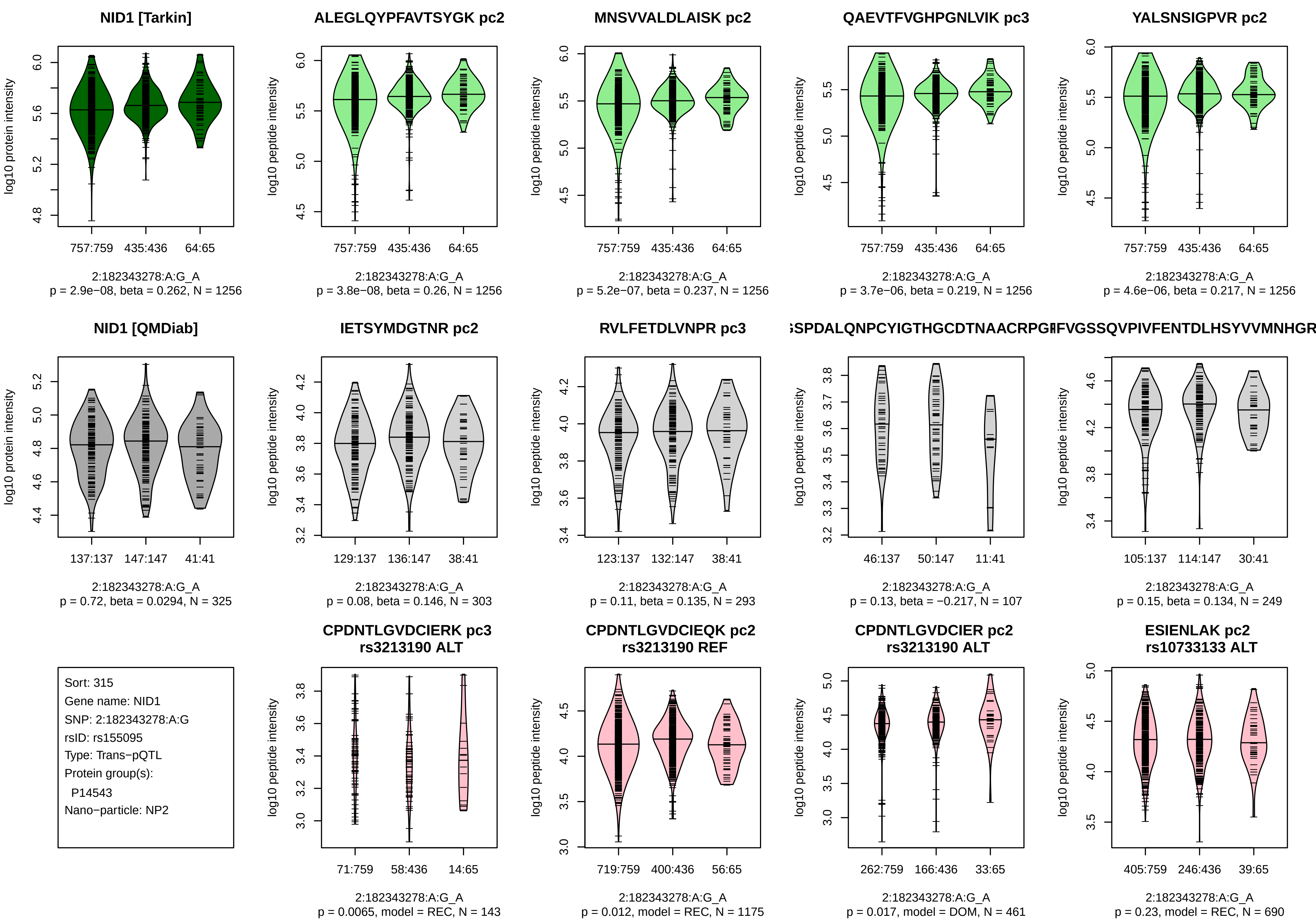
NID1 [Tarkin]
log10 protein intensity
757:759 435:436 64:65
2:182343278:A:G_A
p = 2.9e−08, beta = 0.262, N = 1256
ALEGLQYPFAVTSYGK pc2
log10 peptide intensity
757:759 435:436 64:65
2:182343278:A:G_A
p = 3.8e−08, beta = 0.26, N = 1256
MNSVVALDLAISK pc2
log10 peptide intensity
757:759 435:436 64:65
2:182343278:A:G_A
p = 5.2e−07, beta = 0.237, N = 1256
QAEVTFVGHPGNLVIK pc3
log10 peptide intensity
757:759 435:436 64:65
2:182343278:A:G_A
p = 3.7e−06, beta = 0.219, N = 1256
YALSNSIGPVR pc2
log10 peptide intensity
757:759 435:436 64:65
2:182343278:A:G_A
p = 4.6e−06, beta = 0.217, N = 1256
NID1 [QMDiab]
log10 protein intensity
137:137 147:147 41:41
2:182343278:A:G_A
p = 0.72, beta = 0.0294, N = 325
IETSYMDGTNR pc2
log10 peptide intensity
129:137 136:147 38:41
2:182343278:A:G_A
p = 0.08, beta = 0.146, N = 303
RVLFETDLVNPR pc3
log10 peptide intensity
123:137 132:147 38:41
2:182343278:A:G_A
p = 0.11, beta = 0.135, N = 293
SPDALQNPCYIGTHGCDTNAACRPGIIFVGSSQVPIVFENTDLHSYVVMNHGR
log10 peptide intensity
46:137 50:147 11:41
2:182343278:A:G_A
p = 0.13, beta = −0.217, N = 107
log10 peptide intensity
105:137 114:147 30:41
2:182343278:A:G_A
p = 0.15, beta = 0.134, N = 249
Sort: 315
Gene name: NID1
SNP: 2:182343278:A:G
rsID: rs155095
Type: Trans−pQTL
Protein group(s):
P14543
Nano−particle: NP2
CPDNTLGVDCIERK pc3
rs3213190 ALT
log10 peptide intensity
71:759 58:436 14:65
2:182343278:A:G_A
p = 0.0065, model = REC, N = 143
CPDNTLGVDCIEQK pc2
rs3213190 REF
log10 peptide intensity
719:759 400:436 56:65
2:182343278:A:G_A
p = 0.012, model = REC, N = 1175
CPDNTLGVDCIER pc2
rs3213190 ALT
log10 peptide intensity
262:759 166:436 33:65
2:182343278:A:G_A
p = 0.017, model = DOM, N = 461
ESIENLAK pc2
rs10733133 ALT
log10 peptide intensity
405:759 246:436 39:65
2:182343278:A:G_A
p = 0.23, model = REC, N = 690

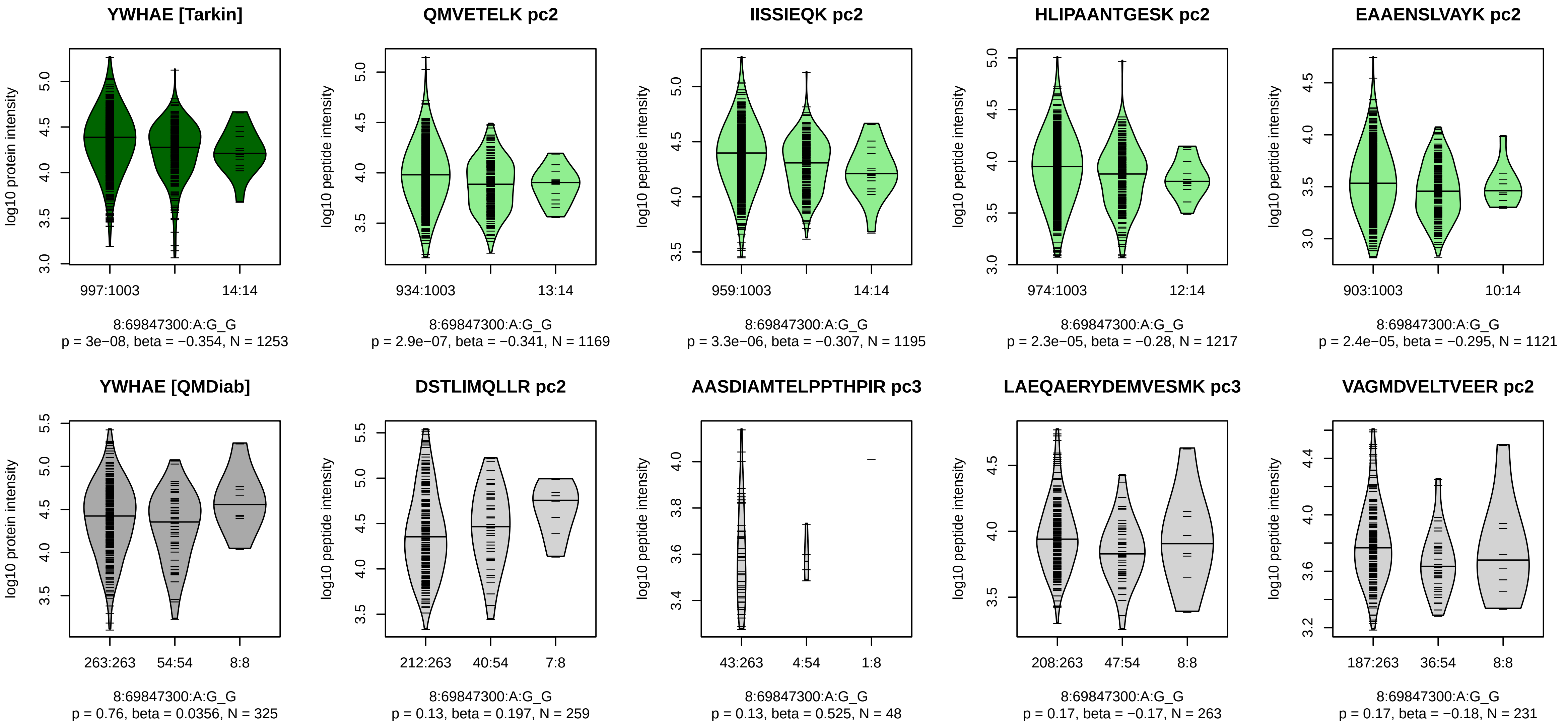

Sort: 316
Gene name: YWHAE
SNP: 8:69847300:A:G
rsID: rs71517217
Type: Trans−pQTL
Protein group(s):
P62258
Nano−particle: NP1

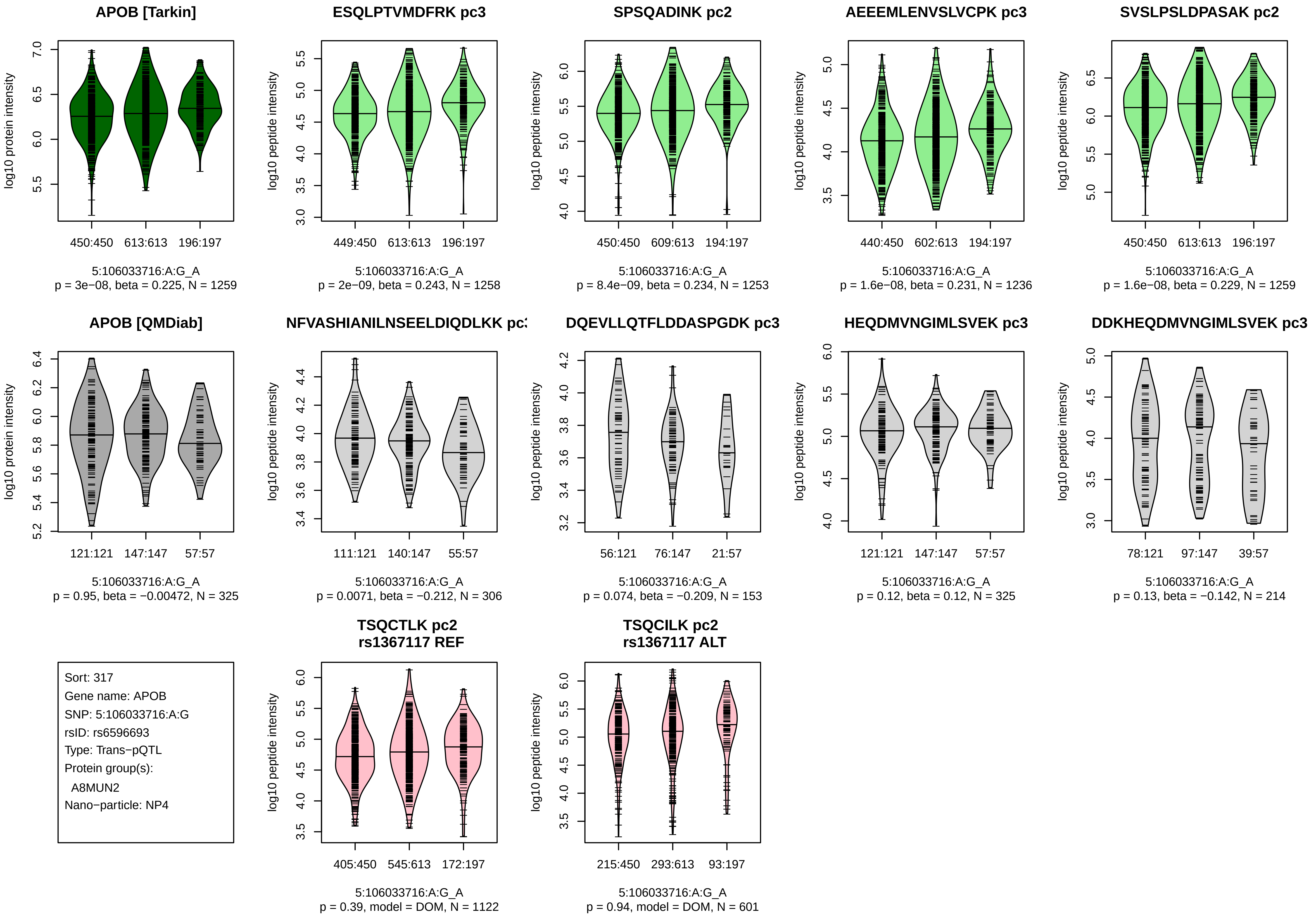

APOB [Tarkin]
log10 protein intensity
450:450 613:613 196:197
5:106033716:A:G_A
p = 3e−08, beta = 0.225, N = 1259
ESQLPTVMDFRK pc3
log10 peptide intensity
449:450 613:613 196:197
5:106033716:A:G_A
p = 2e−09, beta = 0.243, N = 1258
SPSQADINK pc2
log10 peptide intensity
450:450 609:613 194:197
5:106033716:A:G_A
p = 8.4e−09, beta = 0.234, N = 1253
AEEEMLENVSLVCPK pc3
log10 peptide intensity
440:450 602:613 194:197
5:106033716:A:G_A
p = 1.6e−08, beta = 0.231, N = 1236
SVSLPSLDPASAK pc2
log10 peptide intensity
450:450 613:613 196:197
5:106033716:A:G_A
p = 1.6e−08, beta = 0.229, N = 1259
APOB [QMDiab]
log10 protein intensity
121:121 147:147 57:57
5:106033716:A:G_A
p = 0.95, beta = −0.00472, N = 325
NFVASHIANILNSEELDIQDLKK pc3
log10 peptide intensity
111:121 140:147 55:57
5:106033716:A:G_A
p = 0.0071, beta = −0.212, N = 306
DQEVLLQTFLDDASPGDK pc3
log10 peptide intensity
56:121 76:147 21:57
5:106033716:A:G_A
p = 0.074, beta = −0.209, N = 153
HEQDMVNGIMLSVEK pc3
log10 peptide intensity
121:121 147:147 57:57
5:106033716:A:G_A
p = 0.12, beta = 0.12, N = 325
DDKHEQDMVNGIMLSVEK pc3
log10 peptide intensity
78:121 97:147 39:57
5:106033716:A:G_A
p = 0.13, beta = −0.142, N = 214
Sort: 317
Gene name: APOB
SNP: 5:106033716:A:G
rsID: rs6596693
Type: Trans−pQTL
Protein group(s):
A8MUN2
Nano−particle: NP4
TSQCTLK pc2
rs1367117 REF
log10 peptide intensity
405:450 545:613 172:197
5:106033716:A:G_A
p = 0.39, model = DOM, N = 1122
TSQCILK pc2
rs1367117 ALT
log10 peptide intensity
215:450 293:613 93:197
5:106033716:A:G_A
p = 0.94, model = DOM, N = 601

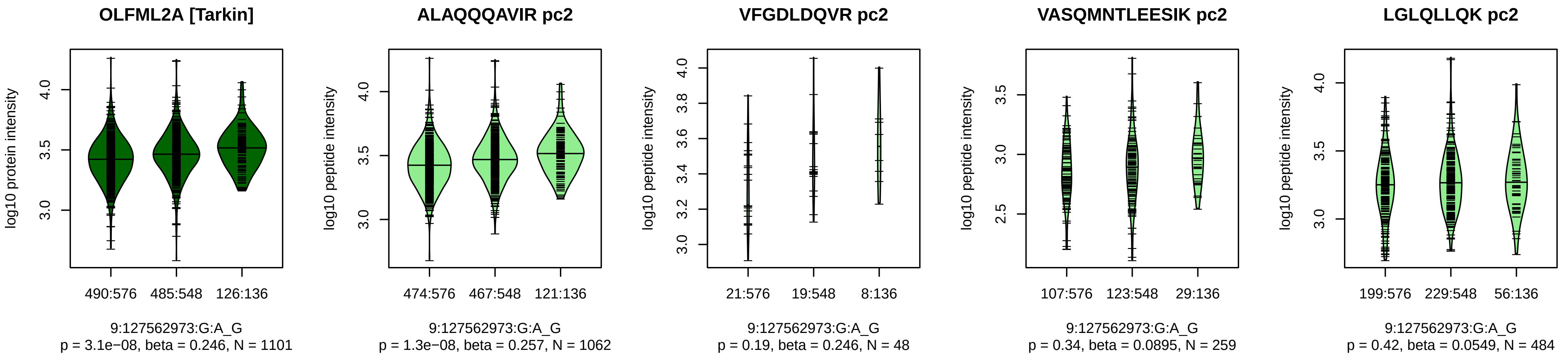

No protein data available
for replication in QMDiab

Sort: 318
Gene name: OLFML2A
SNP: 9:127562973:G:A
rsID: rs913232
Type: Cis−pQTL
Protein group(s):
Q68BL7
Nano−particle: NP3

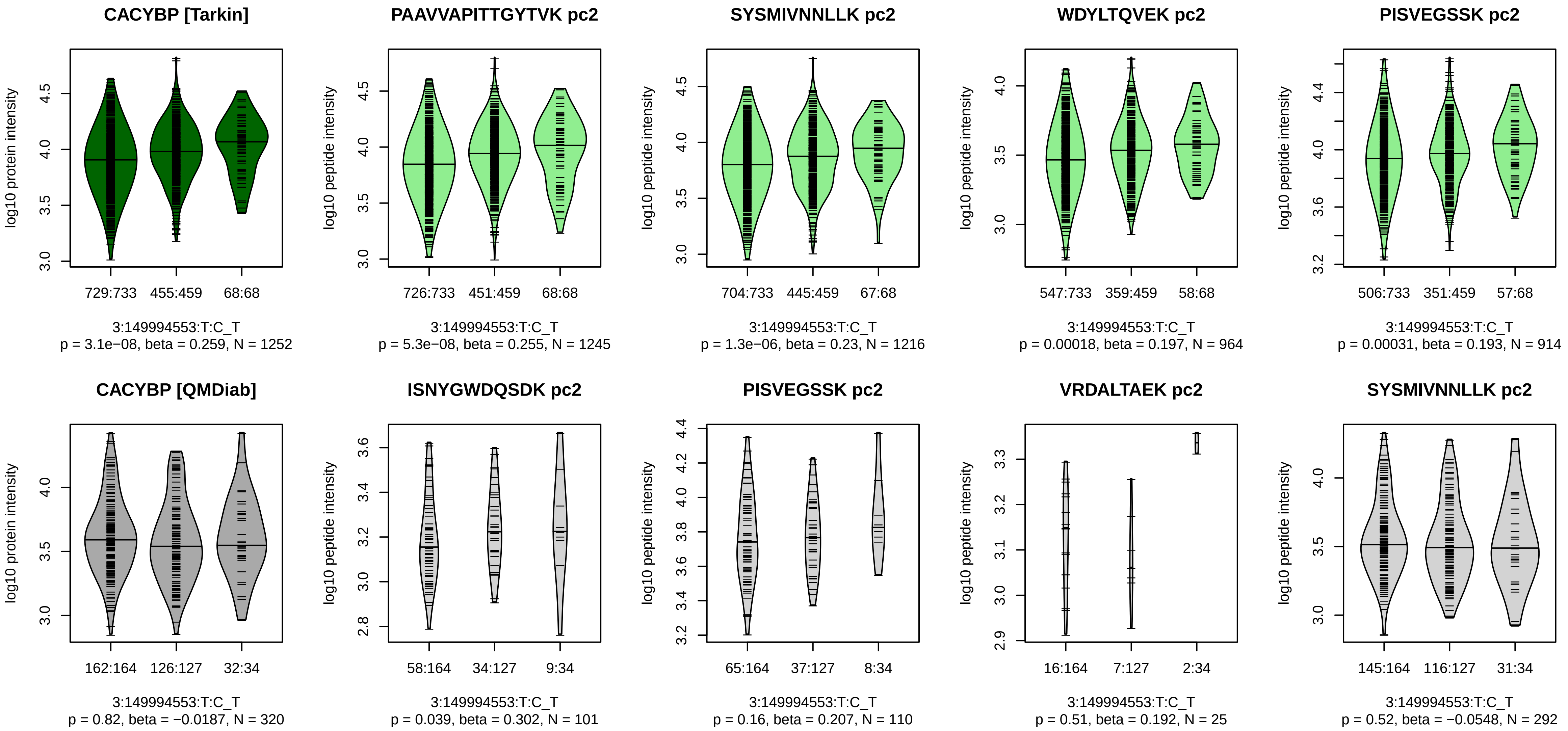

Sort: 319
Gene name: CACYBP
SNP: 3:149994553:T:C
rsID: rs4681600
Type: Trans−pQTL
Protein group(s):
Q9HB71
Nano−particle: NP3

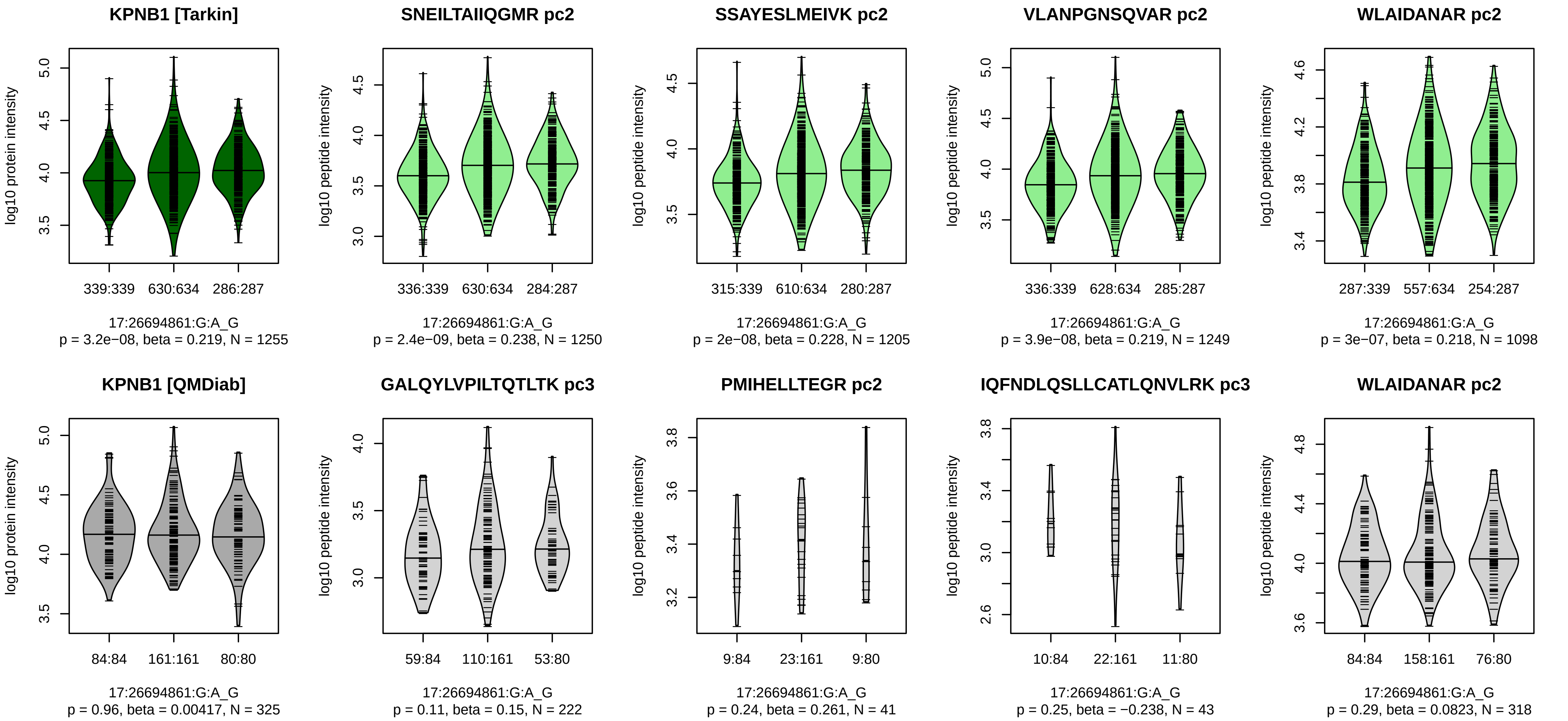

Sort: 320
Gene name: KPNB1
SNP: 17:26694861:G:A
rsID: rs704
Type: Trans−pQTL
Protein group(s):
Q14974
Nano−particle: NP2

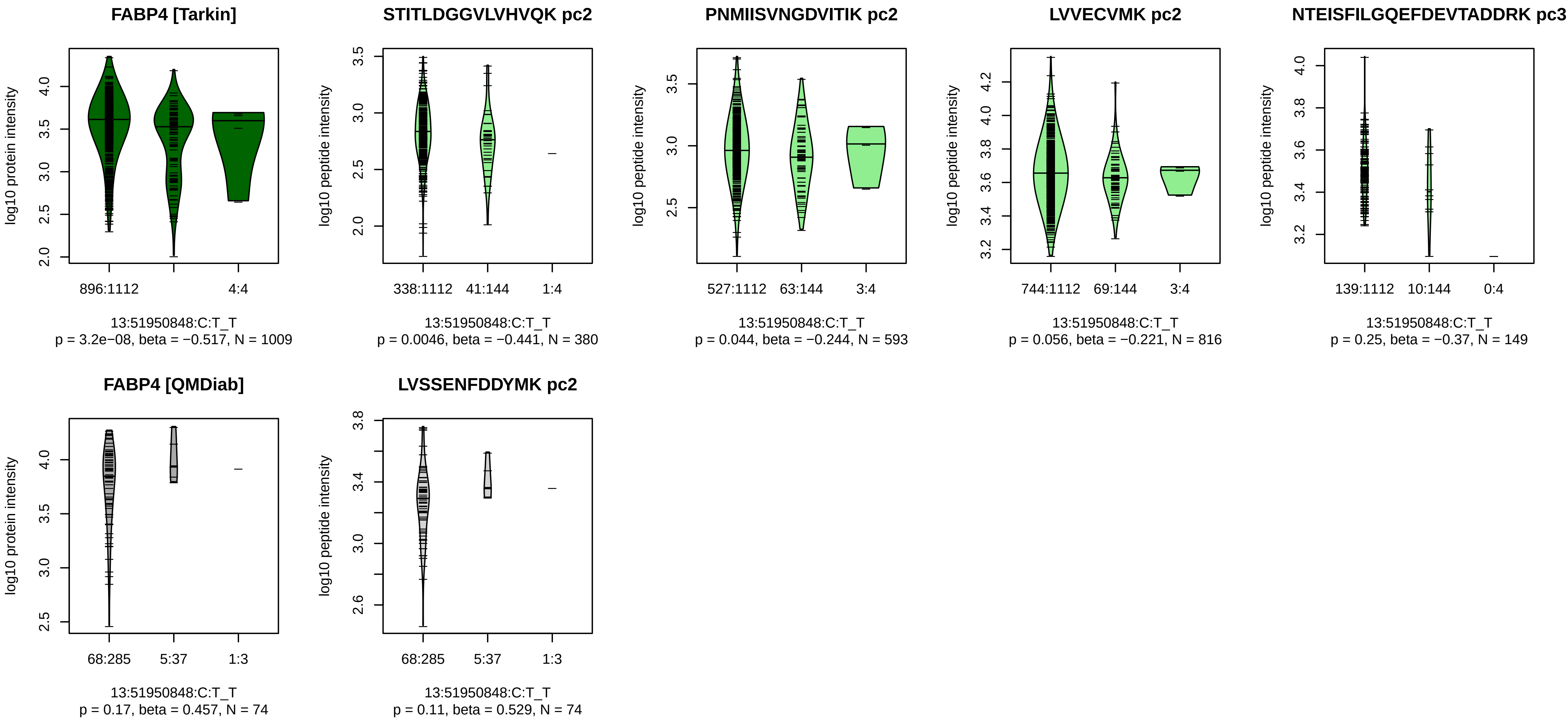

Sort: 321
Gene name: FABP4
SNP: 13:51950848:C:T
rsID: rs79789954
Type: Trans−pQTL
Protein group(s):
P15090
Nano−particle: NP2

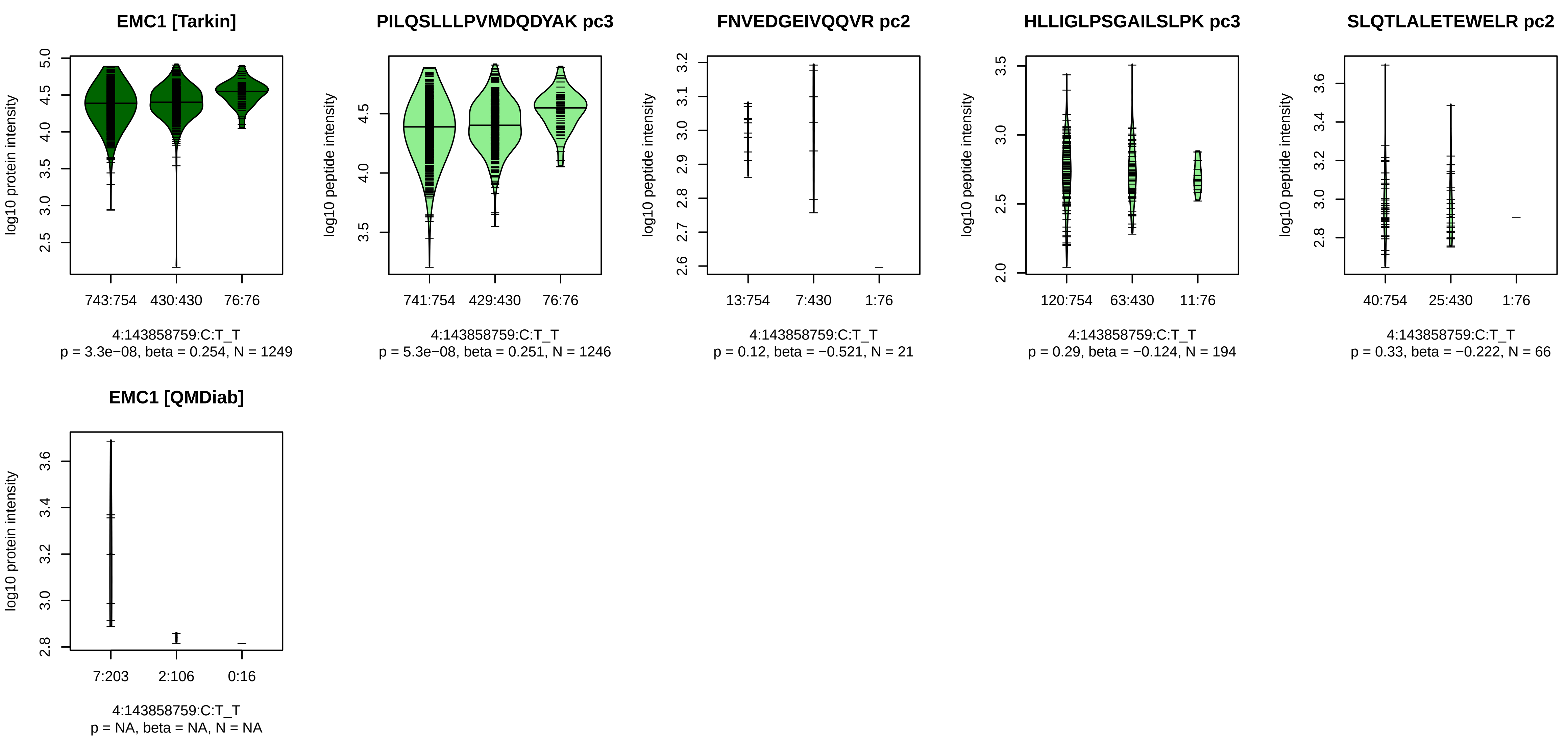

Sort: 322
Gene name: EMC1
SNP: 4:143858759:C:T
rsID: rs10011128
Type: Trans−pQTL
Protein group(s):
T: A0A8I5KW55;Q8N766;Q8N7[illegible]
Q: A0A8I5KW55;H7C5A2;Q8N7[illegible]
Nano−particle: NP4

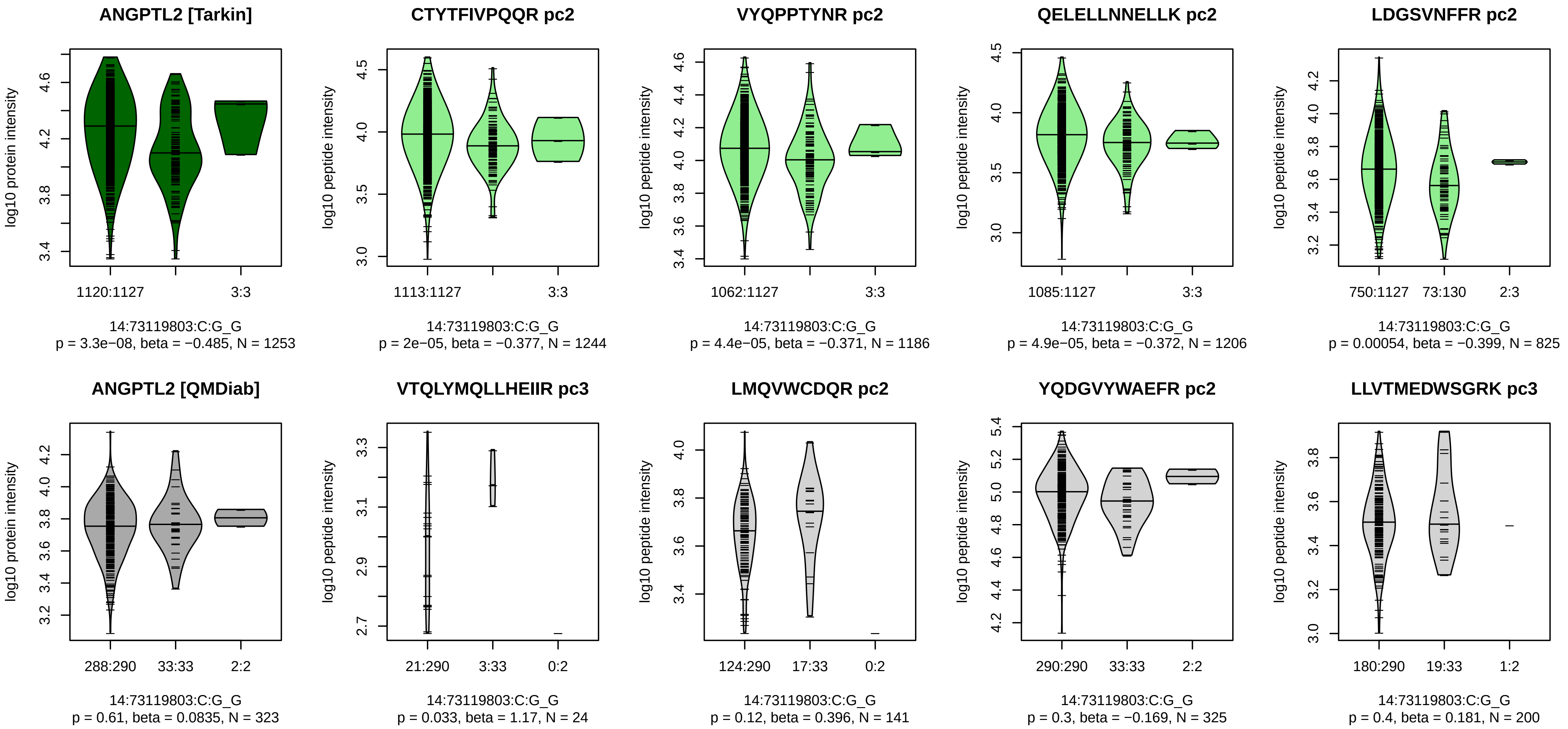

Sort: 323
Gene name: ANGPTL2
SNP: 14:73119803:C:G
rsID: rs115153943
Type: Trans−pQTL
Protein group(s):
Q9UKU9
Nano−particle: NP5

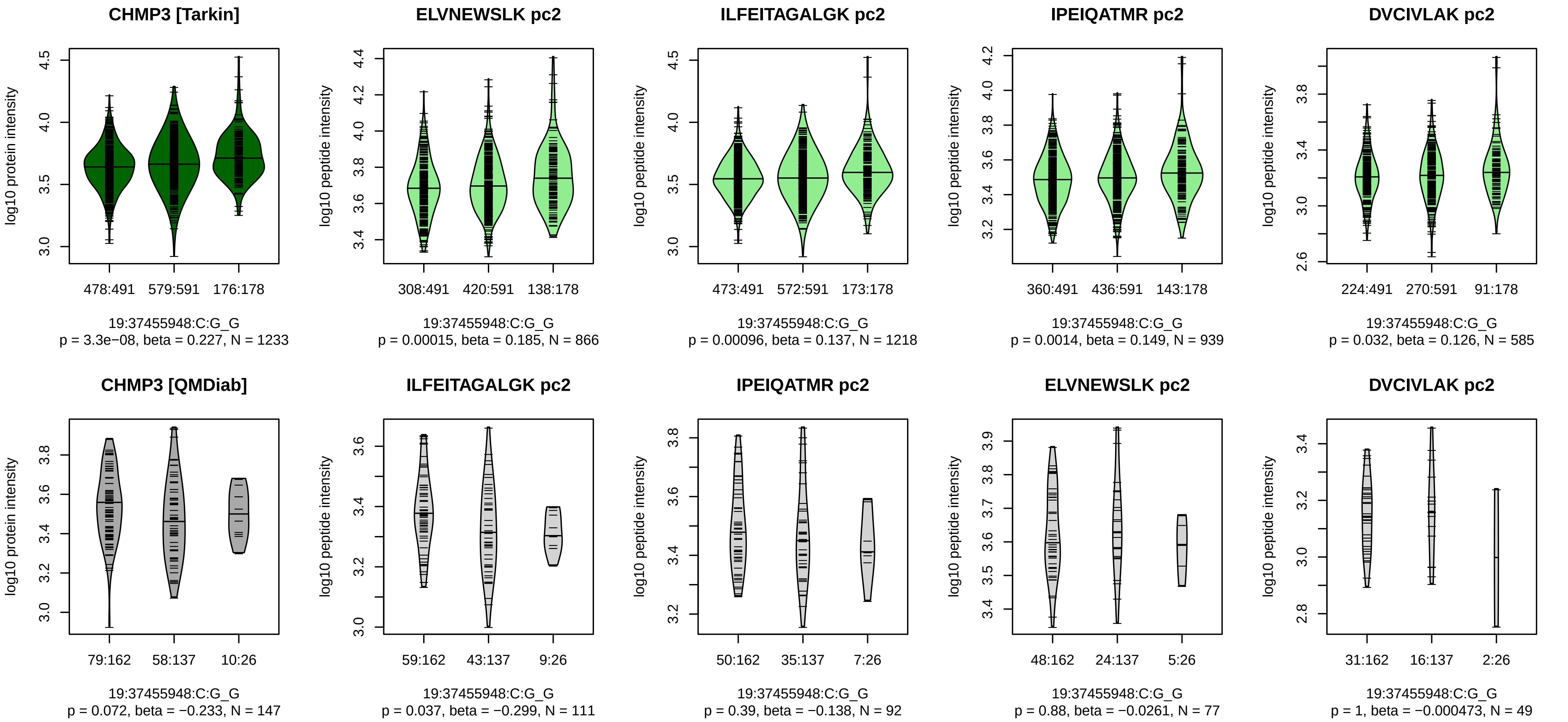

Sort: 324
Gene name: CHMP3
SNP: 19:37455948:C:G
rsID: rs519551
Type: Trans−pQTL
Protein group(s):
Q9Y3E7
Nano−particle: NP1

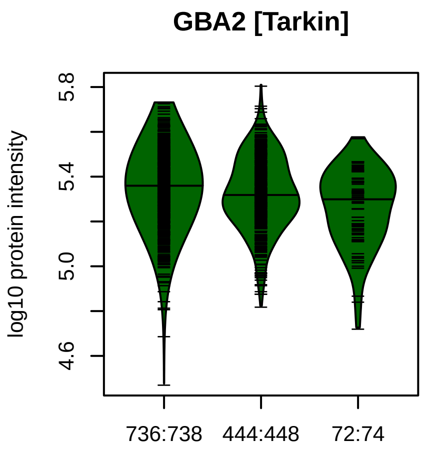

1:9387506:G:A_A
p = 3.3e−08, beta = −0.256, N = 1252

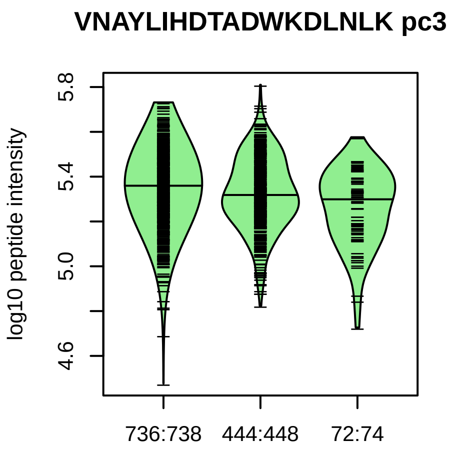

1:9387506:G:A_A
p = 3.3e−08, beta = −0.256, N = 1252

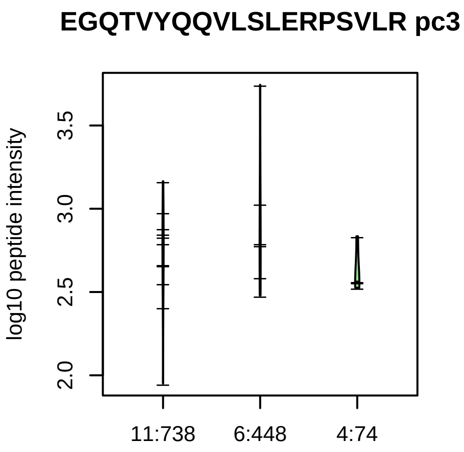

1:9387506:G:A_A
p = 0.58, beta = 0.144, N = 21

No protein data available
for replication in QMDiab

Sort: 325
Gene name: GBA2
SNP: 1:9387506:G:A
rsID: rs3944982
Type: Trans−pQTL
Protein group(s):
Q9HCG7
Nano−particle: NP2

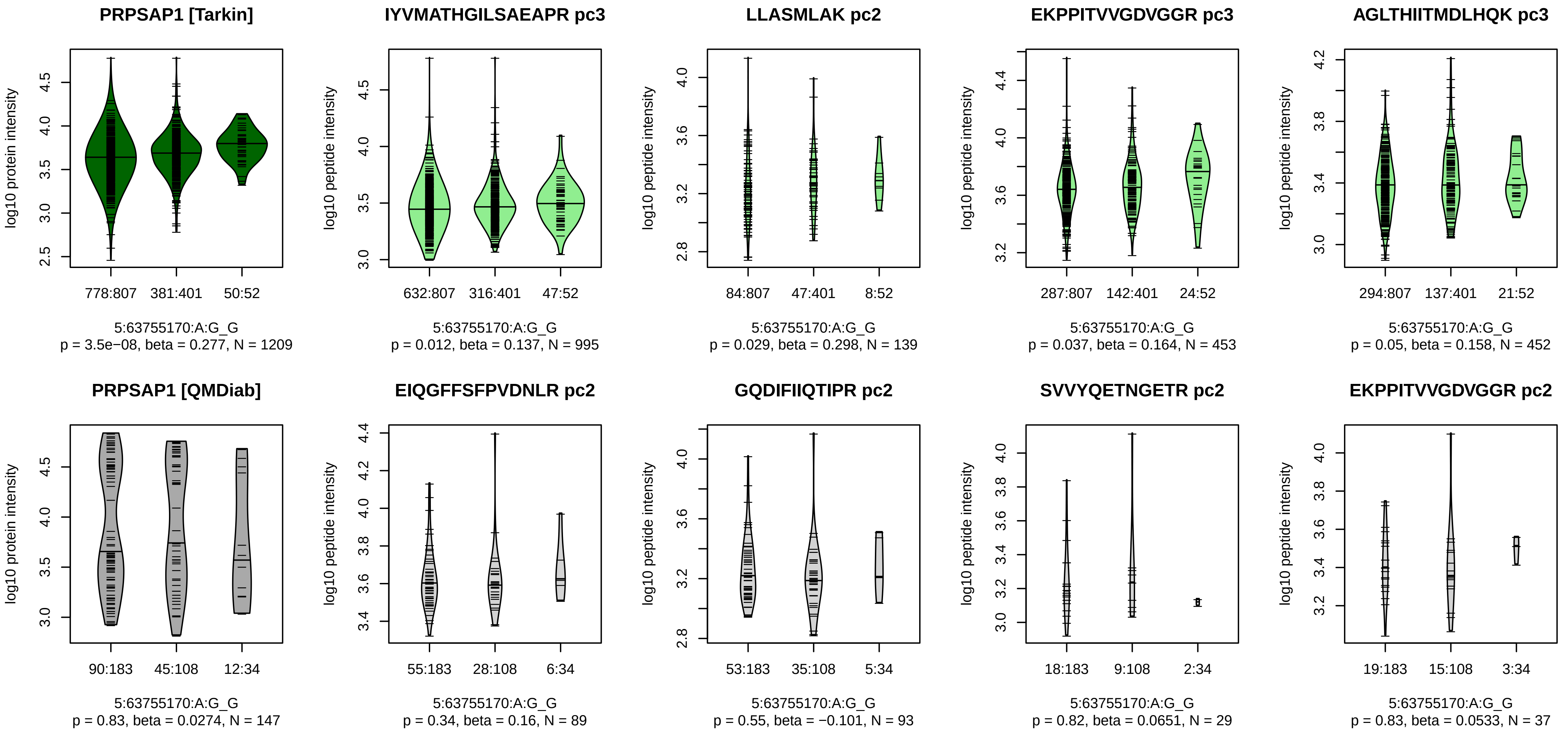

Sort: 326
Gene name: PRPSAP1
SNP: 5:63755170:A:G
rsID: rs271725
Type: Trans−pQTL
Protein group(s):
Q14558−2
Nano−particle: NP4

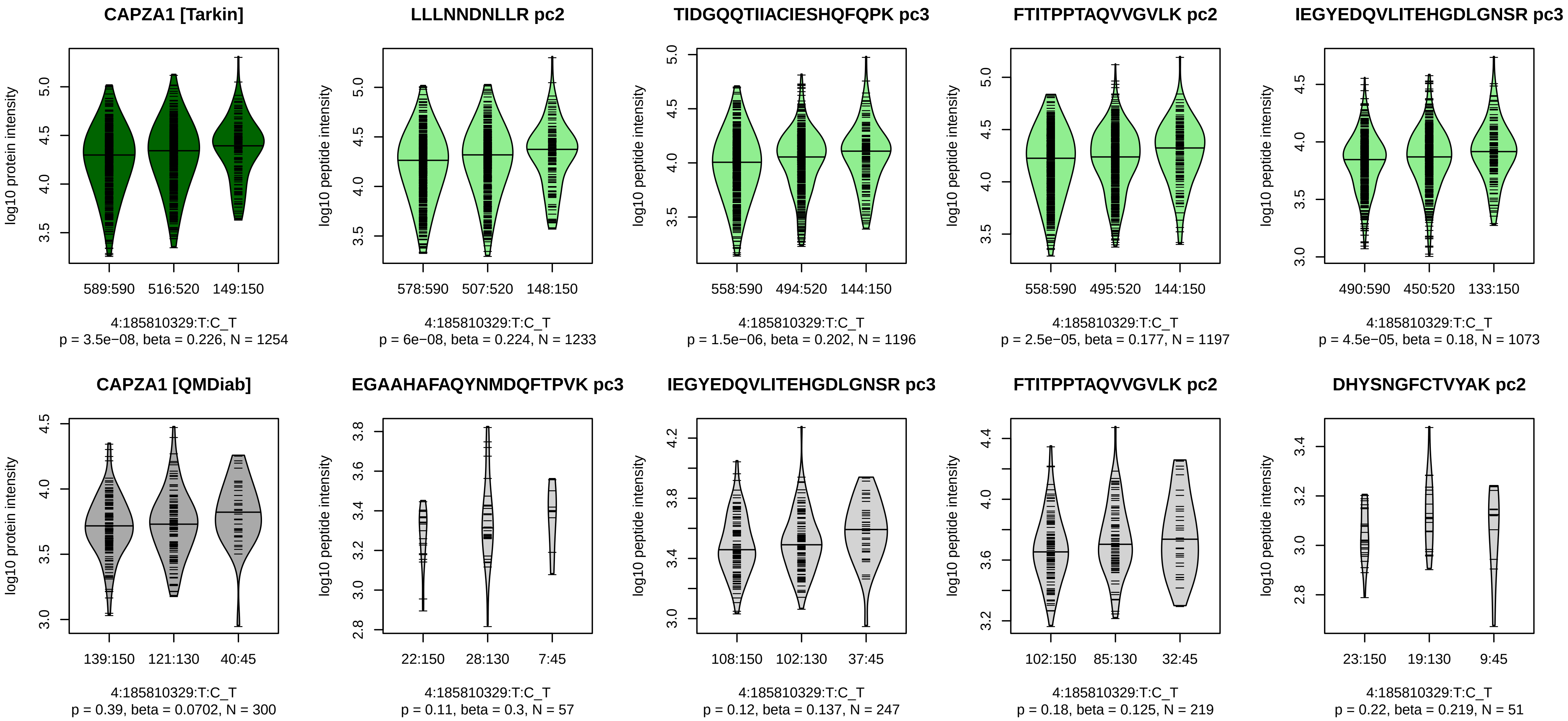

Sort: 327
Gene name: CAPZA1
SNP: 4:185810329:T:C
rsID: rs6856223
Type: Trans−pQTL
Protein group(s):
P52907
Nano−particle: NP4

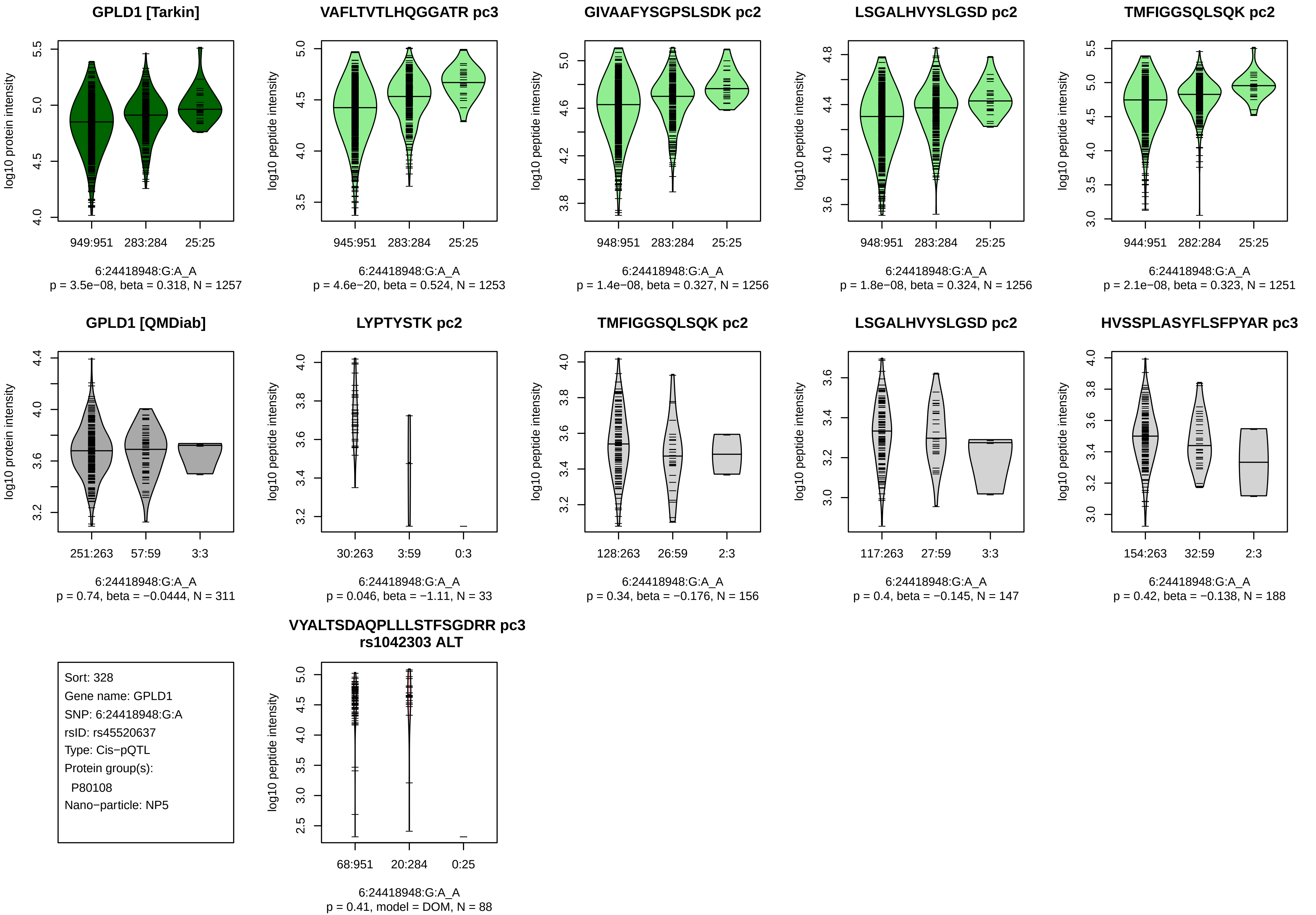

GPLD1 [Tarkin]
log10 protein intensity
949:951
283:284
25:25
6:24418948:G:A_A
p = 3.5e−08, beta = 0.318, N = 1257
VAFLTVTLHQGGATR pc3
log10 peptide intensity
945:951
283:284
25:25
6:24418948:G:A_A
p = 4.6e−20, beta = 0.524, N = 1253
GIVAAFYSGPSLSDK pc2
log10 peptide intensity
948:951
283:284
25:25
6:24418948:G:A_A
p = 1.4e−08, beta = 0.327, N = 1256
LSGALHVYSLGSD pc2
log10 peptide intensity
948:951
283:284
25:25
6:24418948:G:A_A
p = 1.8e−08, beta = 0.324, N = 1256
TMFIGGSQLSQK pc2
log10 peptide intensity
944:951
282:284
25:25
6:24418948:G:A_A
p = 2.1e−08, beta = 0.323, N = 1251
GPLD1 [QMDiab]
log10 protein intensity
251:263
57:59
3:3
6:24418948:G:A_A
p = 0.74, beta = −0.0444, N = 311
LYPTYSTK pc2
log10 peptide intensity
30:263
3:59
0:3
6:24418948:G:A_A
p = 0.046, beta = −1.11, N = 33
TMFIGGSQLSQK pc2
log10 peptide intensity
128:263
26:59
2:3
6:24418948:G:A_A
p = 0.34, beta = −0.176, N = 156
LSGALHVYSLGSD pc2
log10 peptide intensity
117:263
27:59
3:3
6:24418948:G:A_A
p = 0.4, beta = −0.145, N = 147
HVSSPLASYFLSFPYAR pc3
log10 peptide intensity
154:263
32:59
2:3
6:24418948:G:A_A
p = 0.42, beta = −0.138, N = 188
Sort: 328
Gene name: GPLD1
SNP: 6:24418948:G:A
rsID: rs45520637
Type: Cis−pQTL
Protein group(s):
P80108
Nano−particle: NP5
VYALTSDAQPLLLSTFSGDRR pc3
rs1042303 ALT
log10 peptide intensity
68:951
20:284
0:25
6:24418948:G:A_A
p = 0.41, model = DOM, N = 88

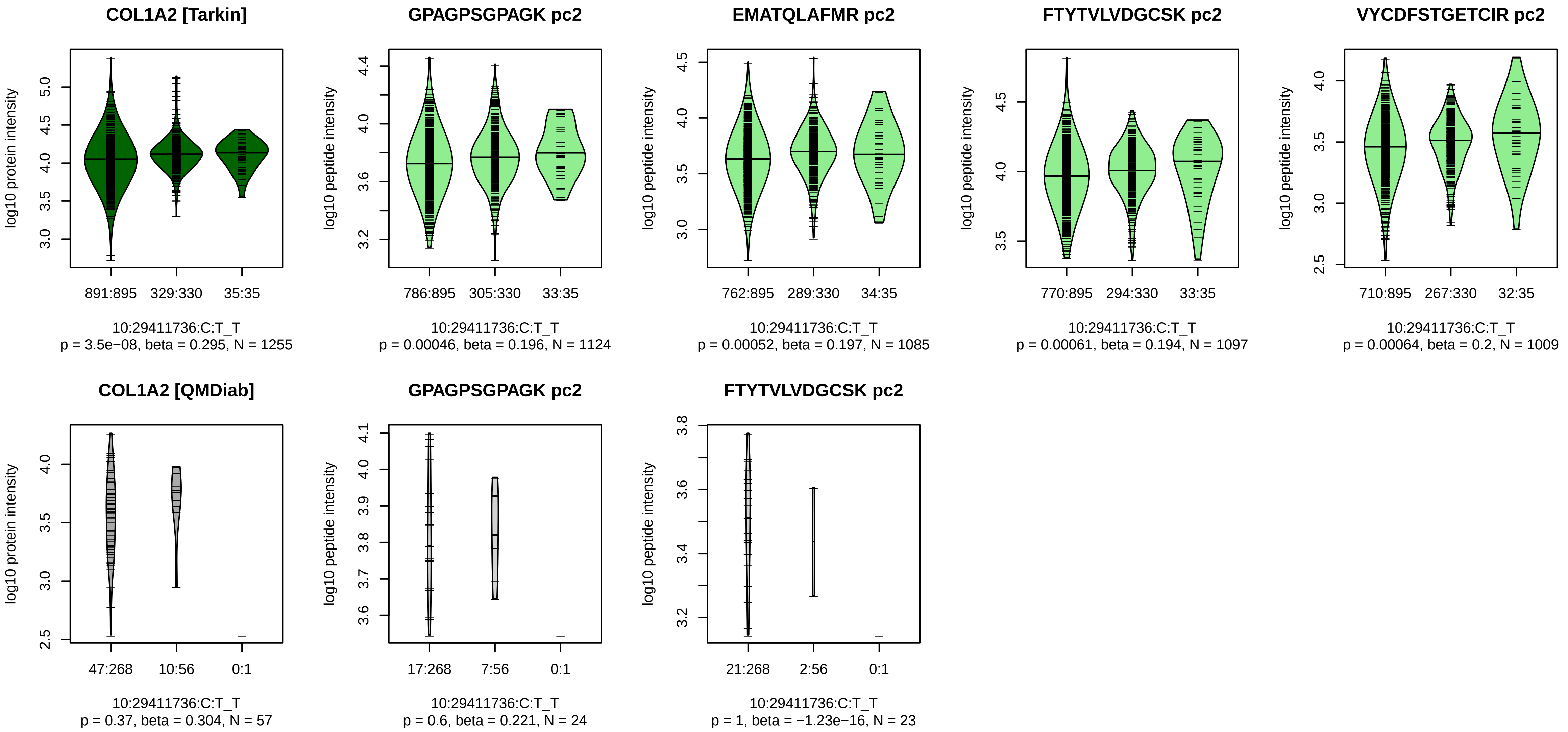

Sort: 329
Gene name: COL1A2
SNP: 10:29411736:C:T
rsID: rs11598329
Type: Trans−pQTL
Protein group(s):
P08123
Nano−particle: NP3

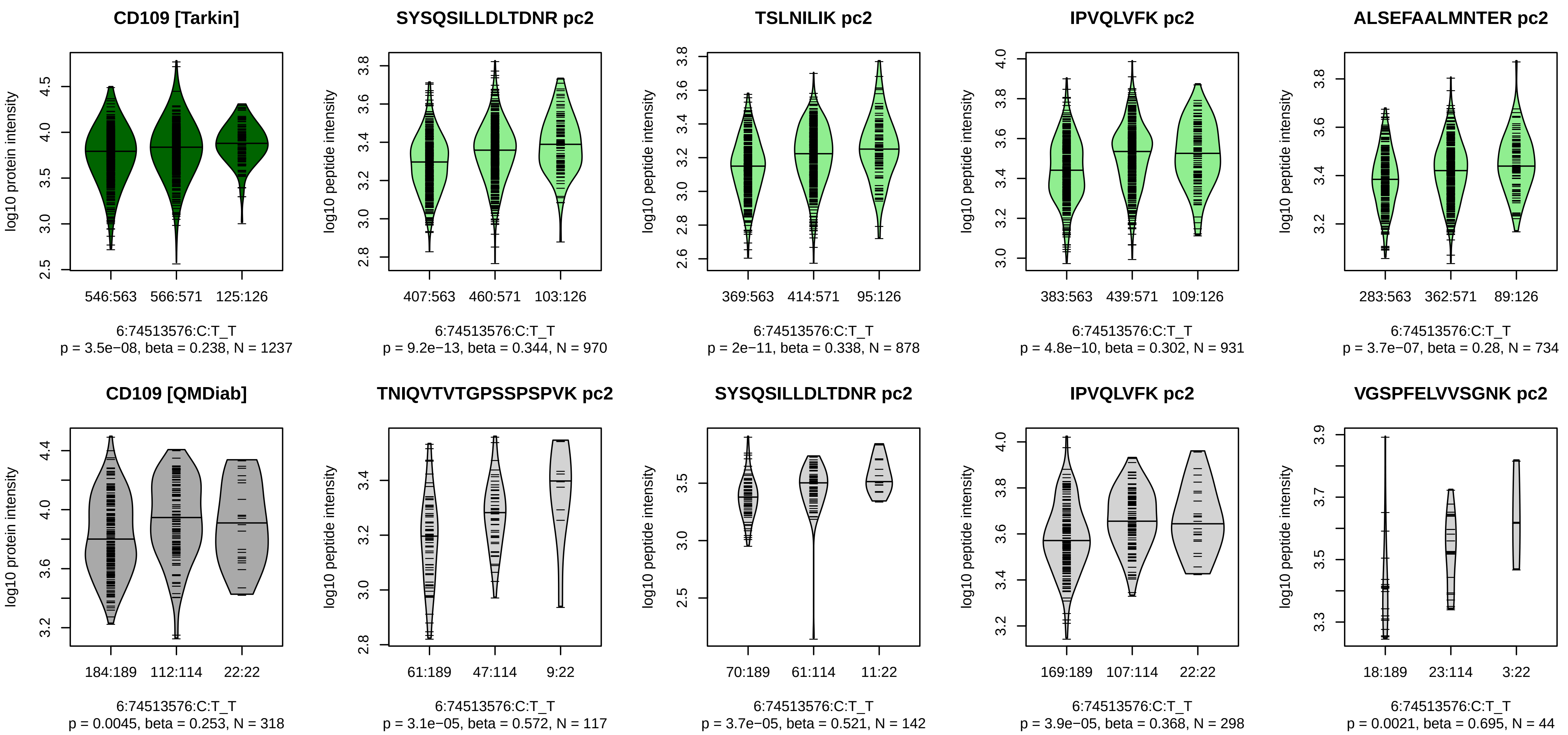

Sort: 330
Gene name: CD109
SNP: 6:74513576:C:T
rsID: rs9442970
Type: Cis−pQTL
Protein group(s):
Q6YHK3
Nano−particle: NP2

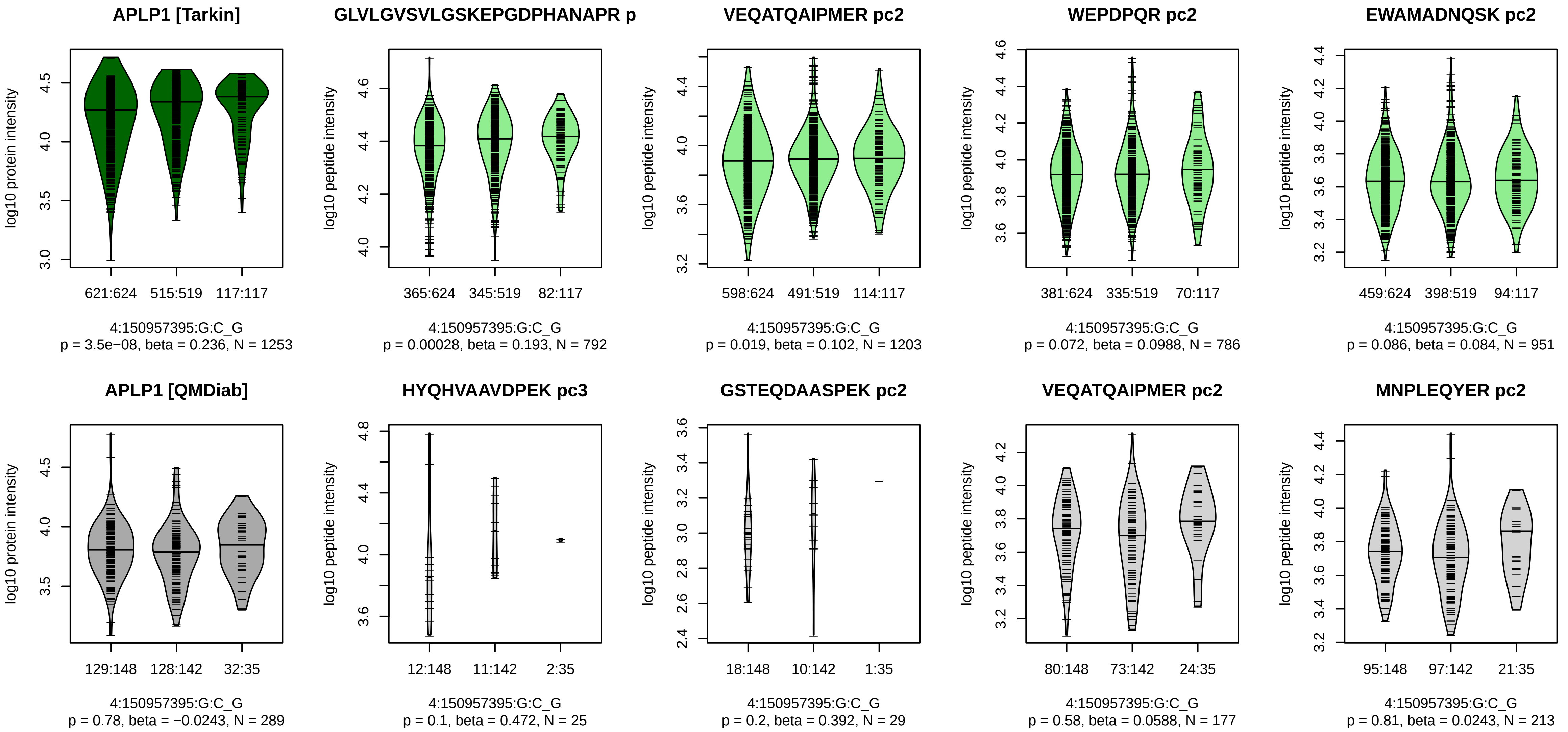

Sort: 331
Gene name: APLP1
SNP: 4:150957395:G:C
rsID: rs4835171
Type: Trans−pQTL
Protein group(s):
B7Z4G8
Nano−particle: NP2

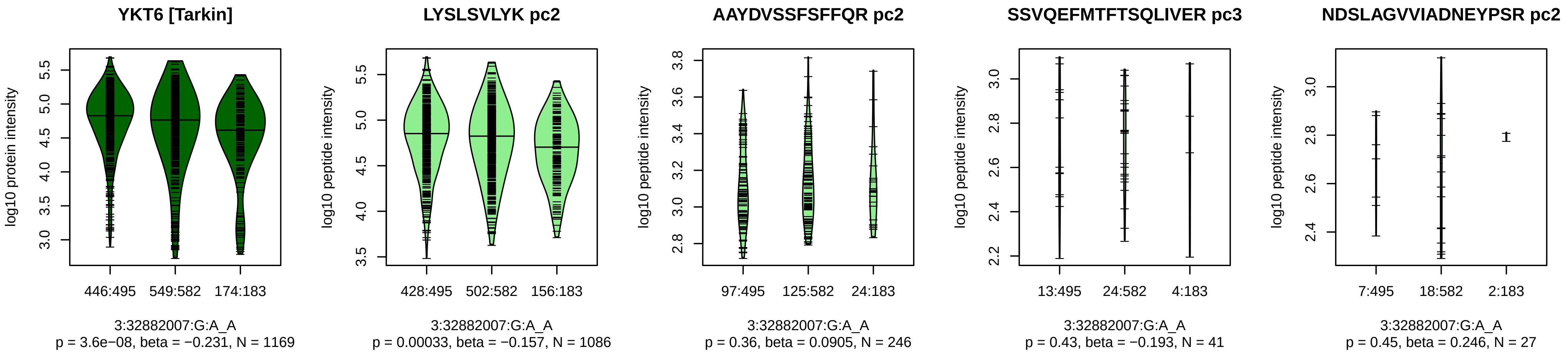

No protein data available
for replication in QMDiab

Sort: 332
Gene name: YKT6
SNP: 3:32882007:G:A
rsID: rs13075951
Type: Trans−pQTL
Protein group(s):
A0A7I2V4L6;O15498
Nano−particle: NP1

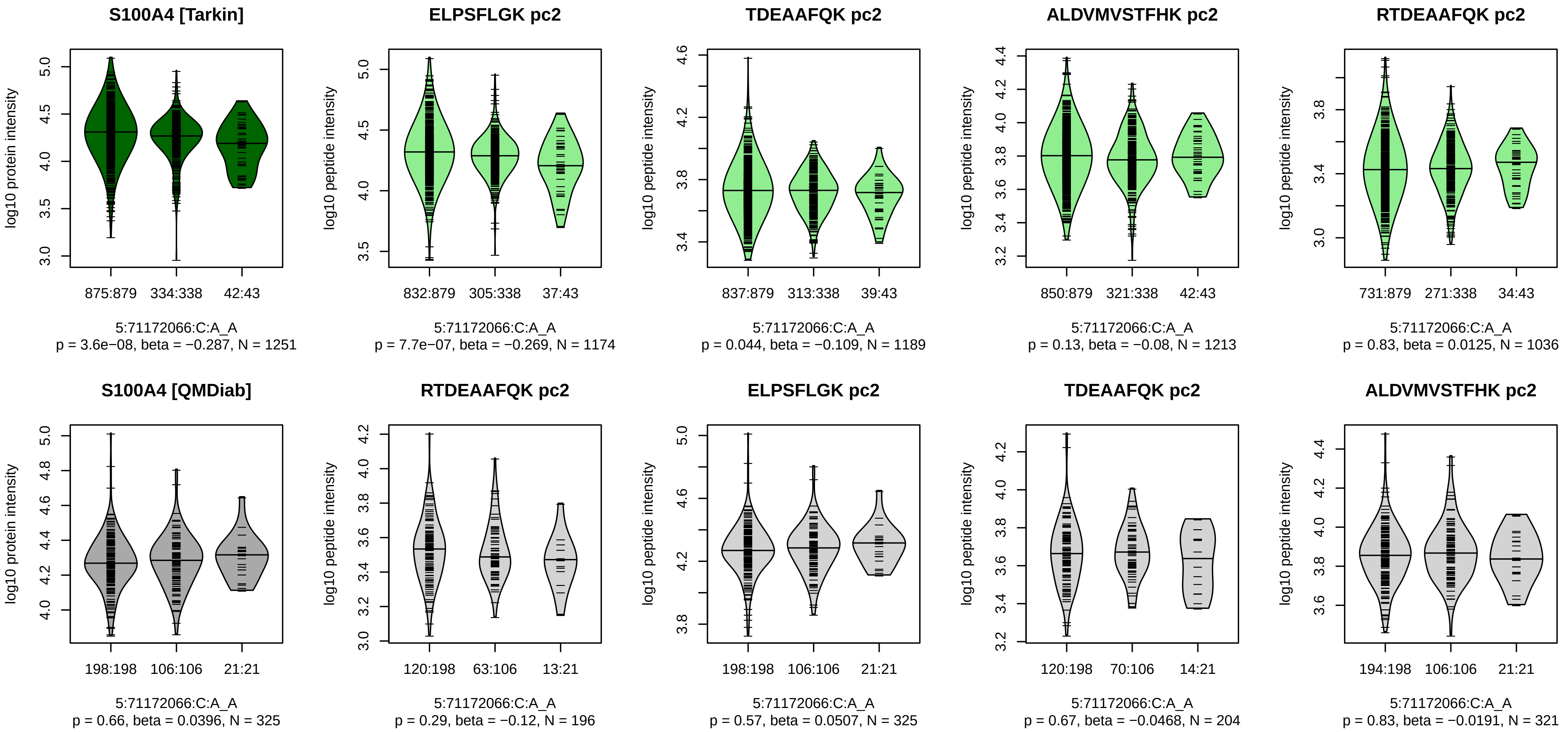

Sort: 333
Gene name: S100A4
SNP: 5:71172066:C:A
rsID: rs13160167
Type: Trans−pQTL
Protein group(s):
P26447
Nano−particle: NP2

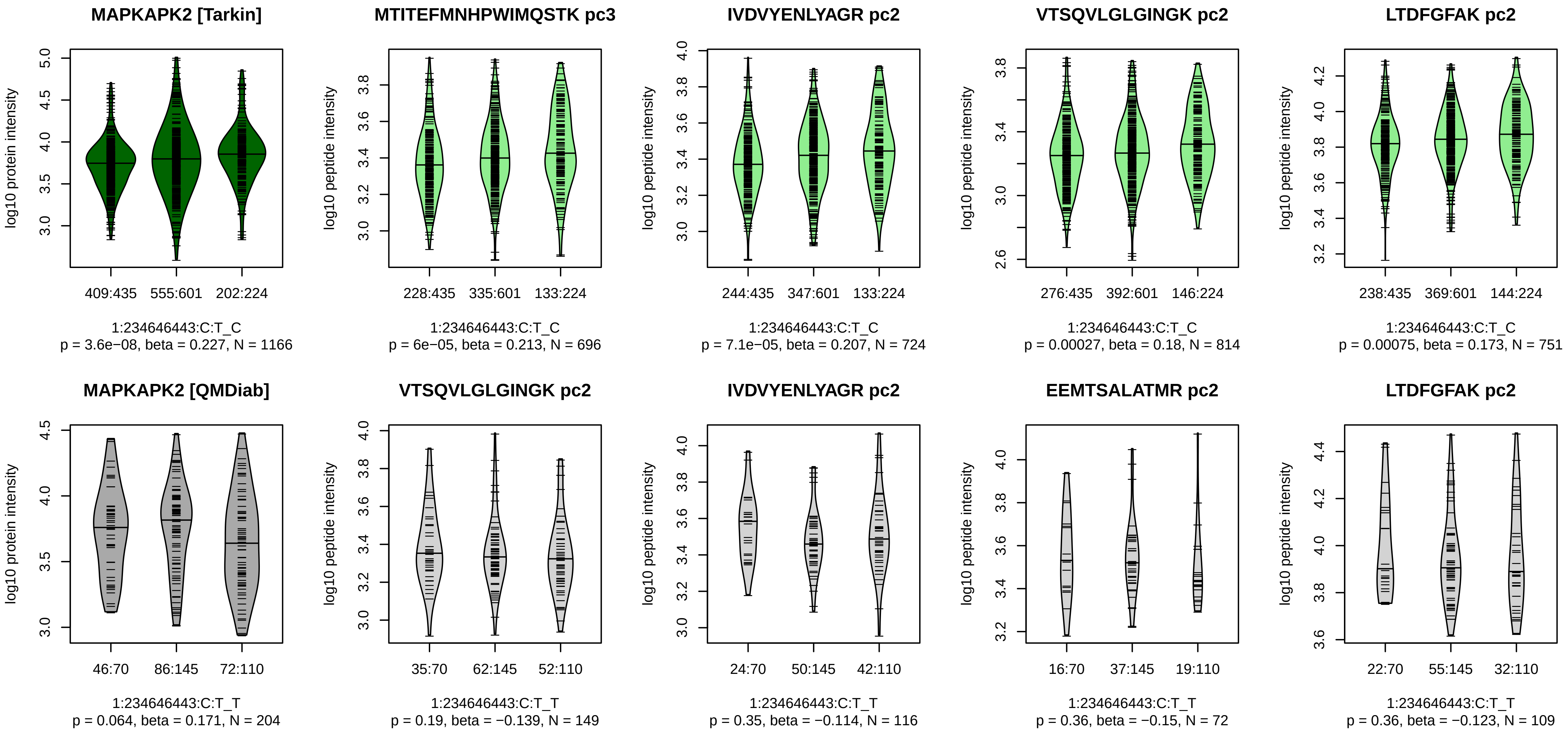

Sort: 334
Gene name: MAPKAPK2
SNP: 1:234646443:C:T
rsID: rs12754270
Type: Trans−pQTL
Protein group(s):
P49137
Nano−particle: NP1

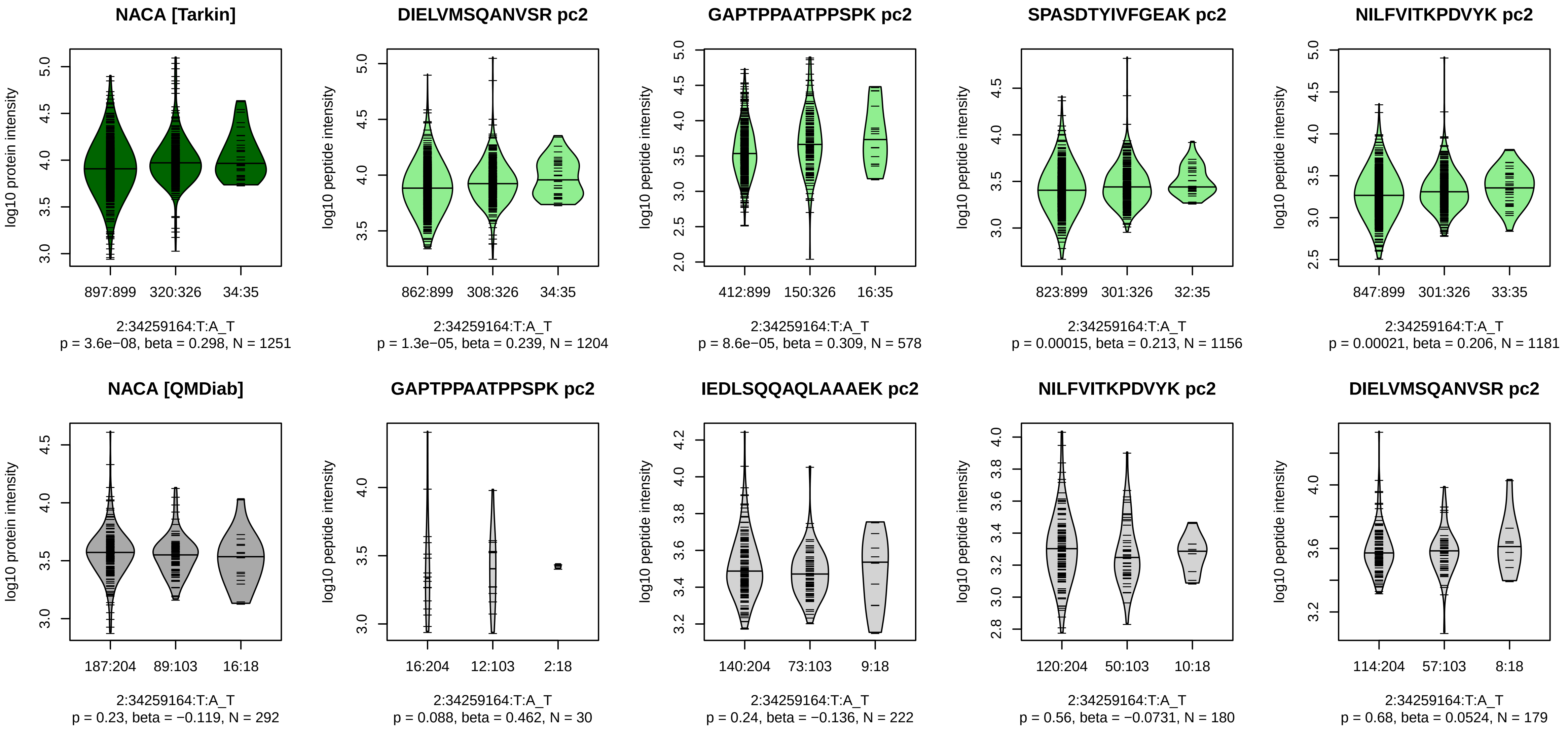

Sort: 335
Gene name: NACA
SNP: 2:34259164:T:A
rsID: rs12712368
Type: Trans−pQTL
Protein group(s):
E9PAV3
Nano−particle: NP4

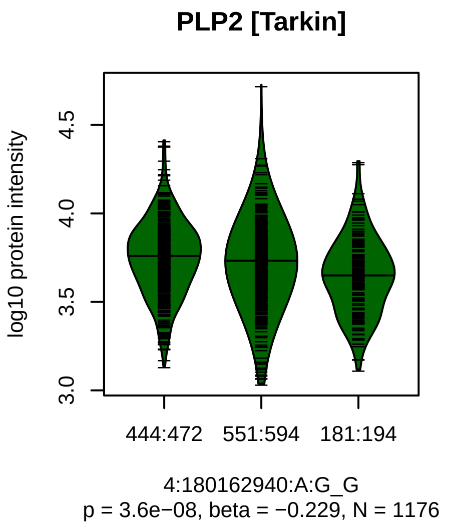

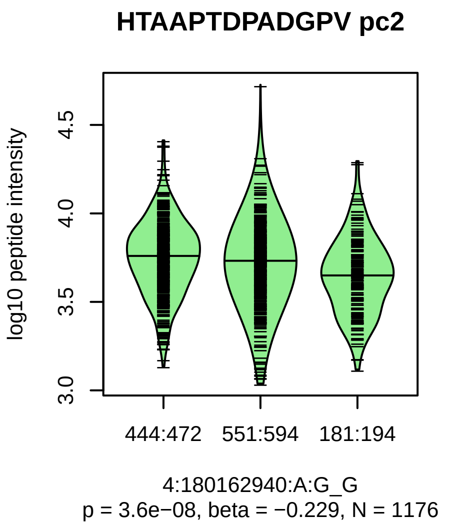

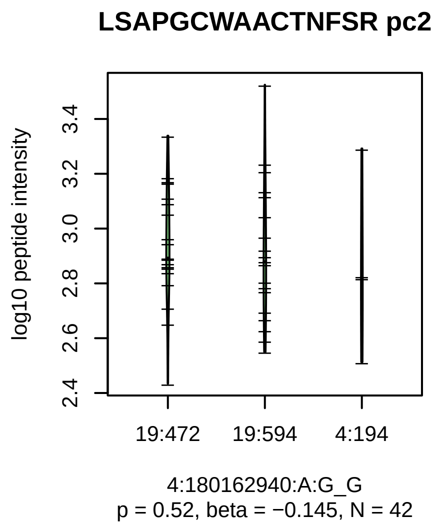

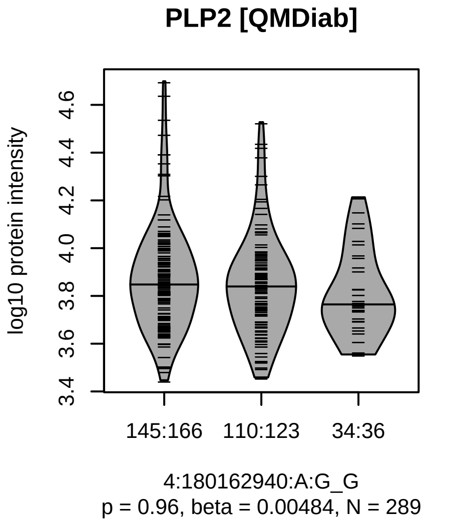

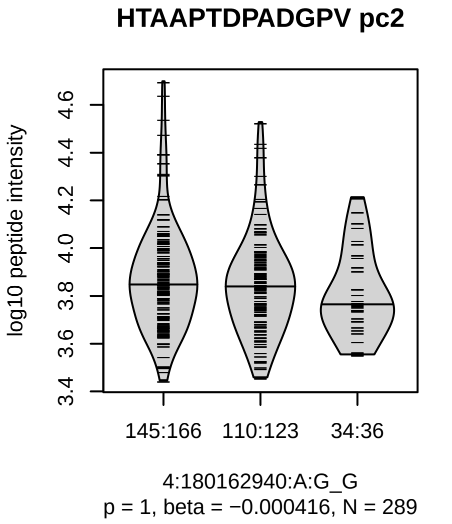

Sort: 336
Gene name: PLP2
SNP: 4:180162940:A:G
rsID: rs7696819
Type: Trans−pQTL
Protein group(s):
Q04941
Nano−particle: NP1

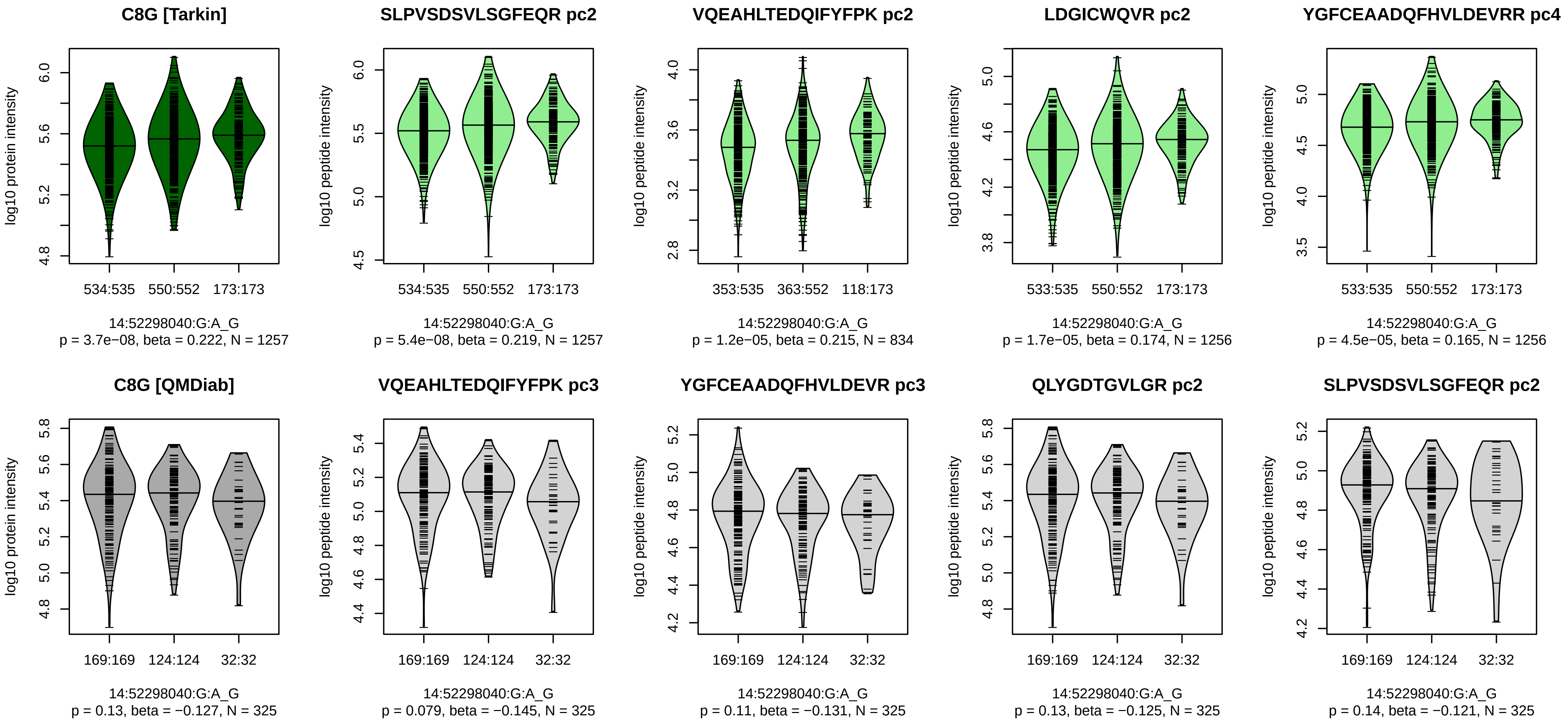

Sort: 337
Gene name: C8G
SNP: 14:52298040:G:A
rsID: rs4898719
Type: Trans−pQTL
Protein group(s):
P07360
Nano−particle: NP5

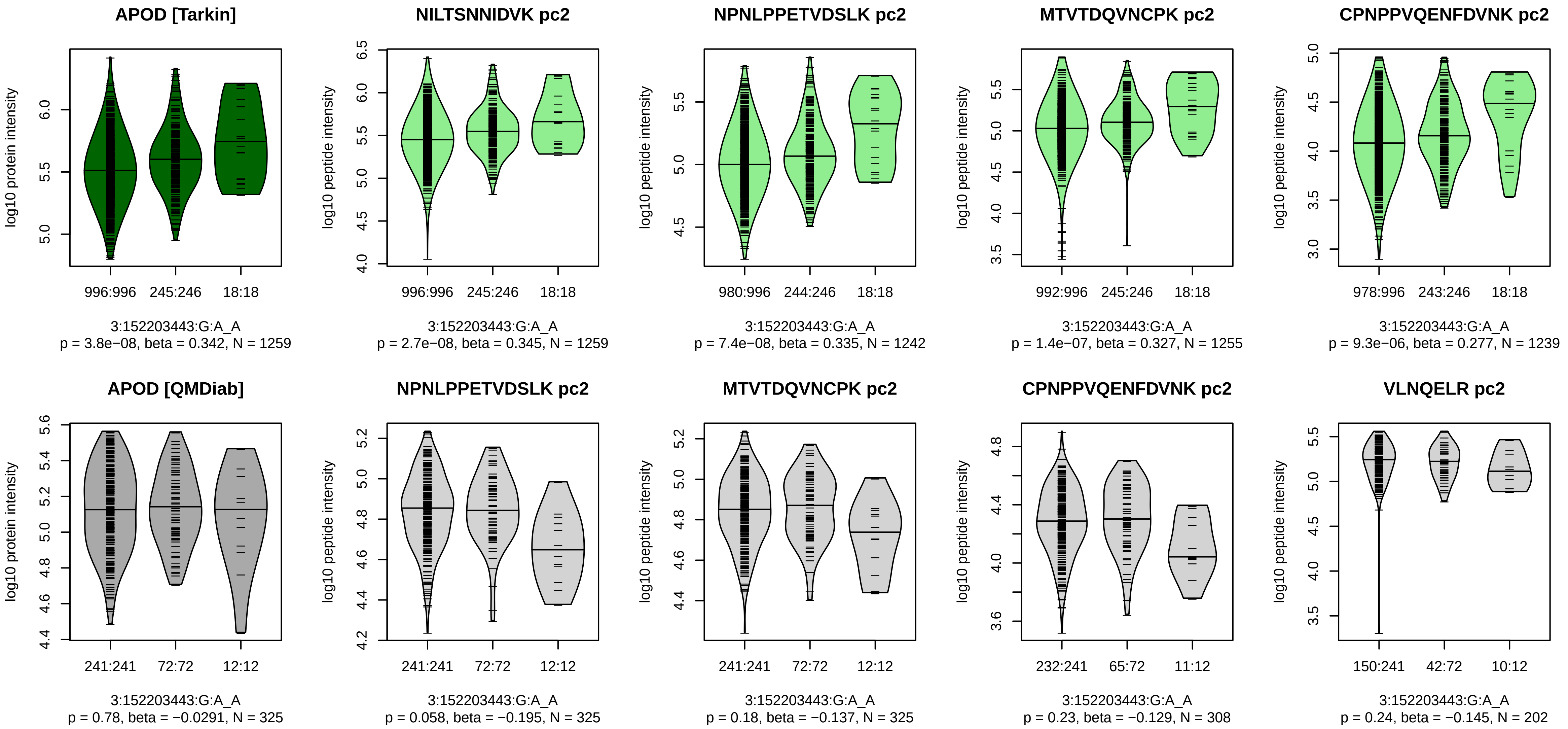

Sort: 338
Gene name: APOD
SNP: 3:152203443:G:A
rsID: rs28716728
Type: Trans−pQTL
Protein group(s):
P05090
Nano−particle: NP4

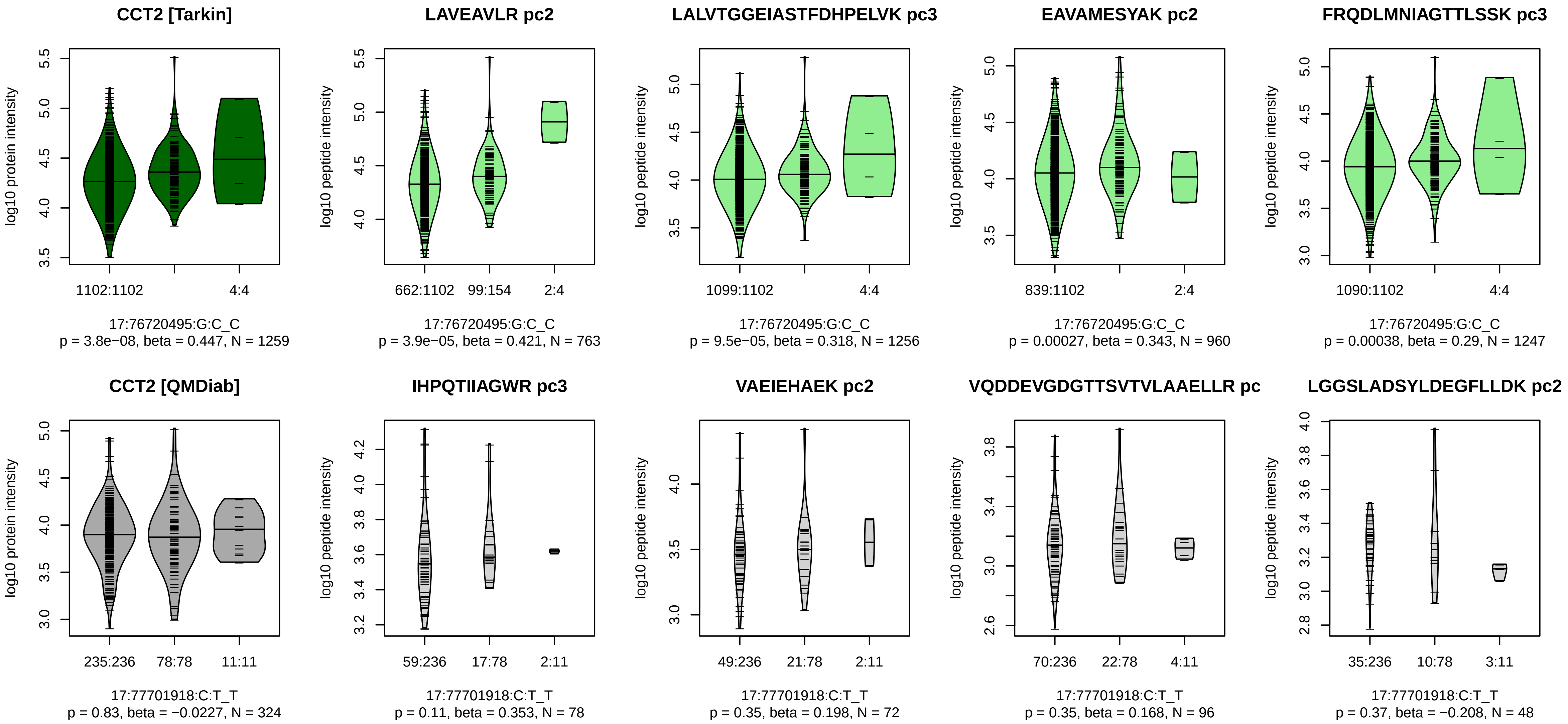

Sort: 339
Gene name: CCT2
SNP: 17:76720495:G:C
rsID: rs55666909
Type: Trans−pQTL
Protein group(s):
P78371
Nano−particle: NP4

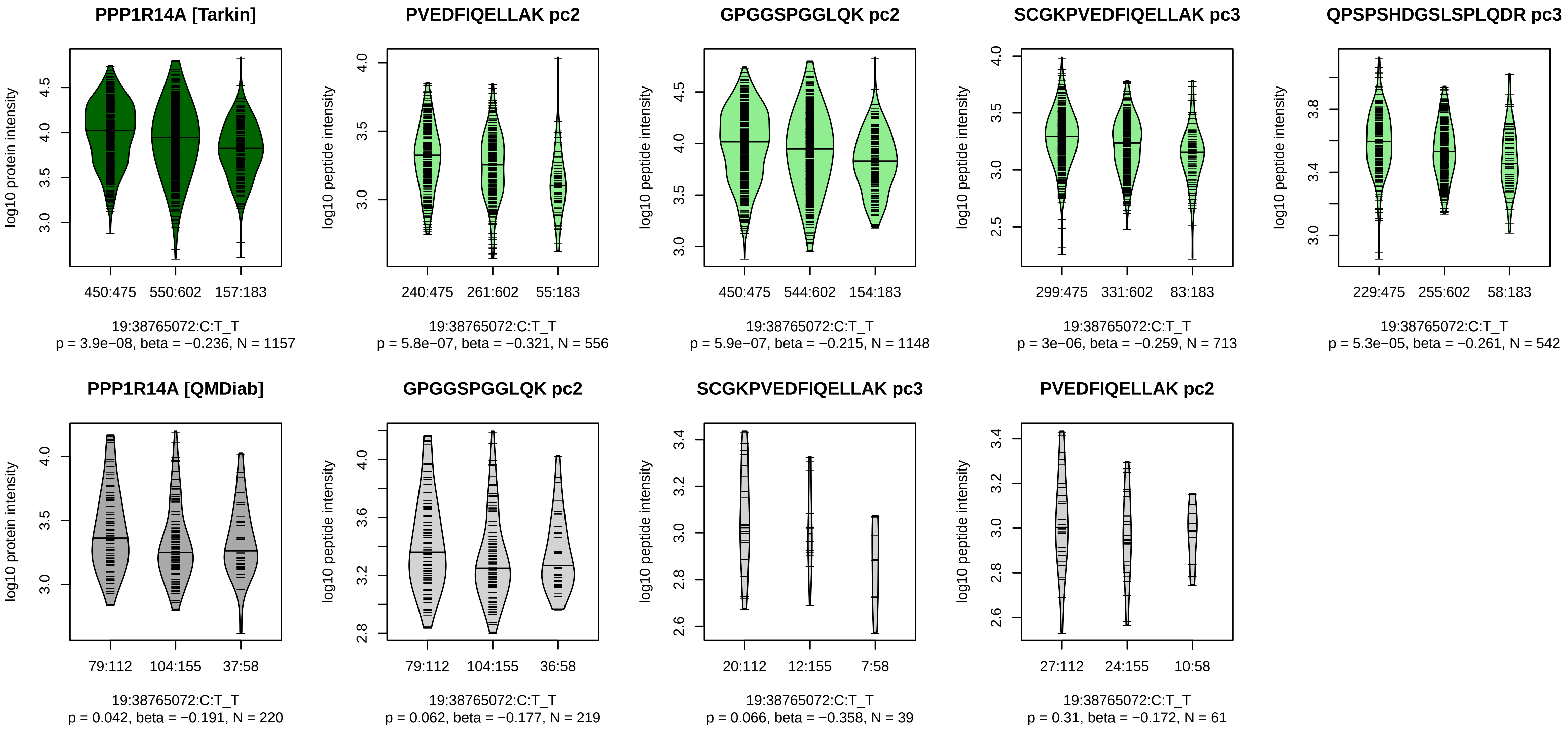

Sort: 340
Gene name: PPP1R14A
SNP: 19:38765072:C:T
rsID: rs8101725
Type: Cis−pQTL
Protein group(s):
T: Q96A00
Q: Q96A00;Q96A00−2
Nano−particle: NP3

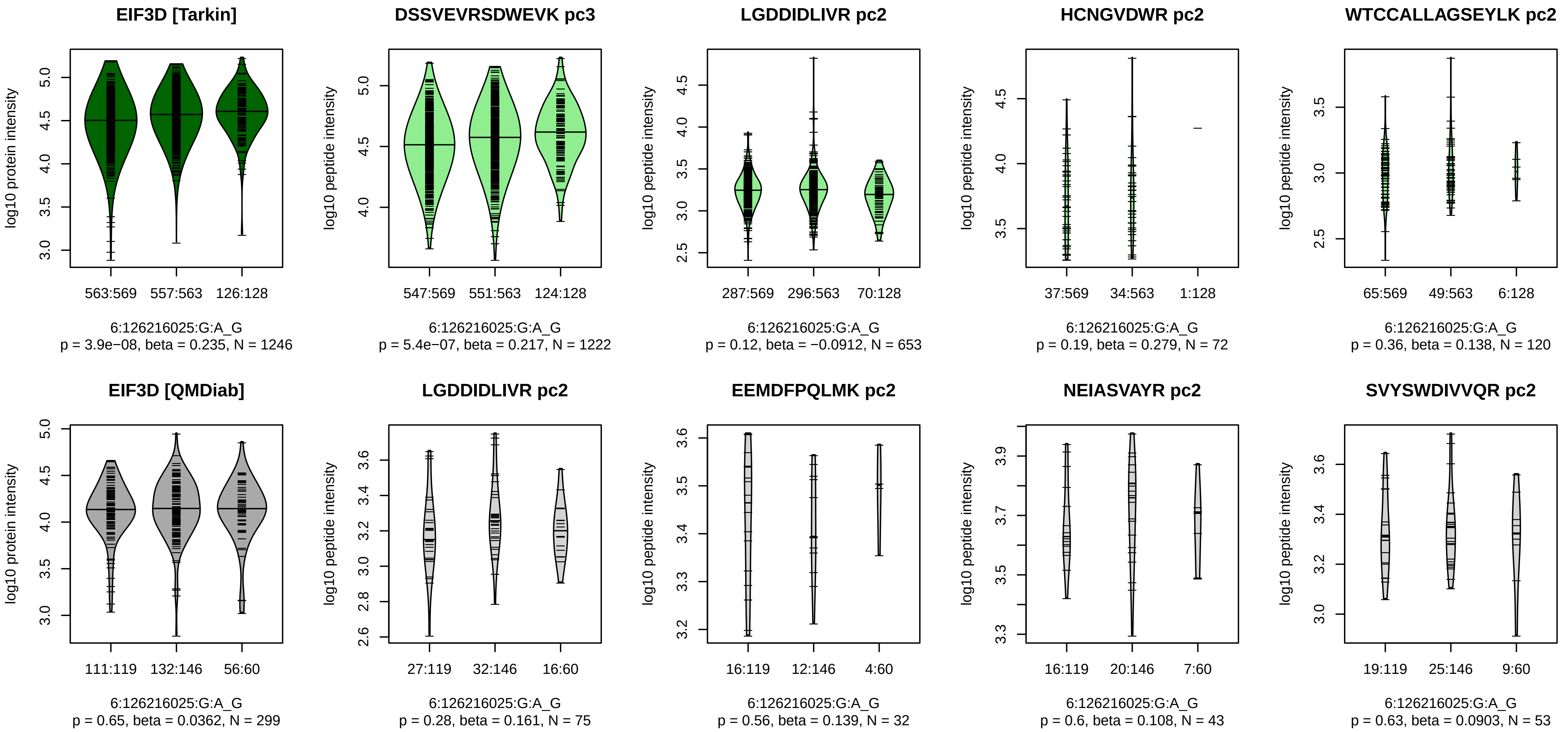

Sort: 341
Gene name: EIF3D
SNP: 6:126216025:G:A
rsID: rs1322757
Type: Trans−pQTL
Protein group(s):
O15371
Nano−particle: NP1

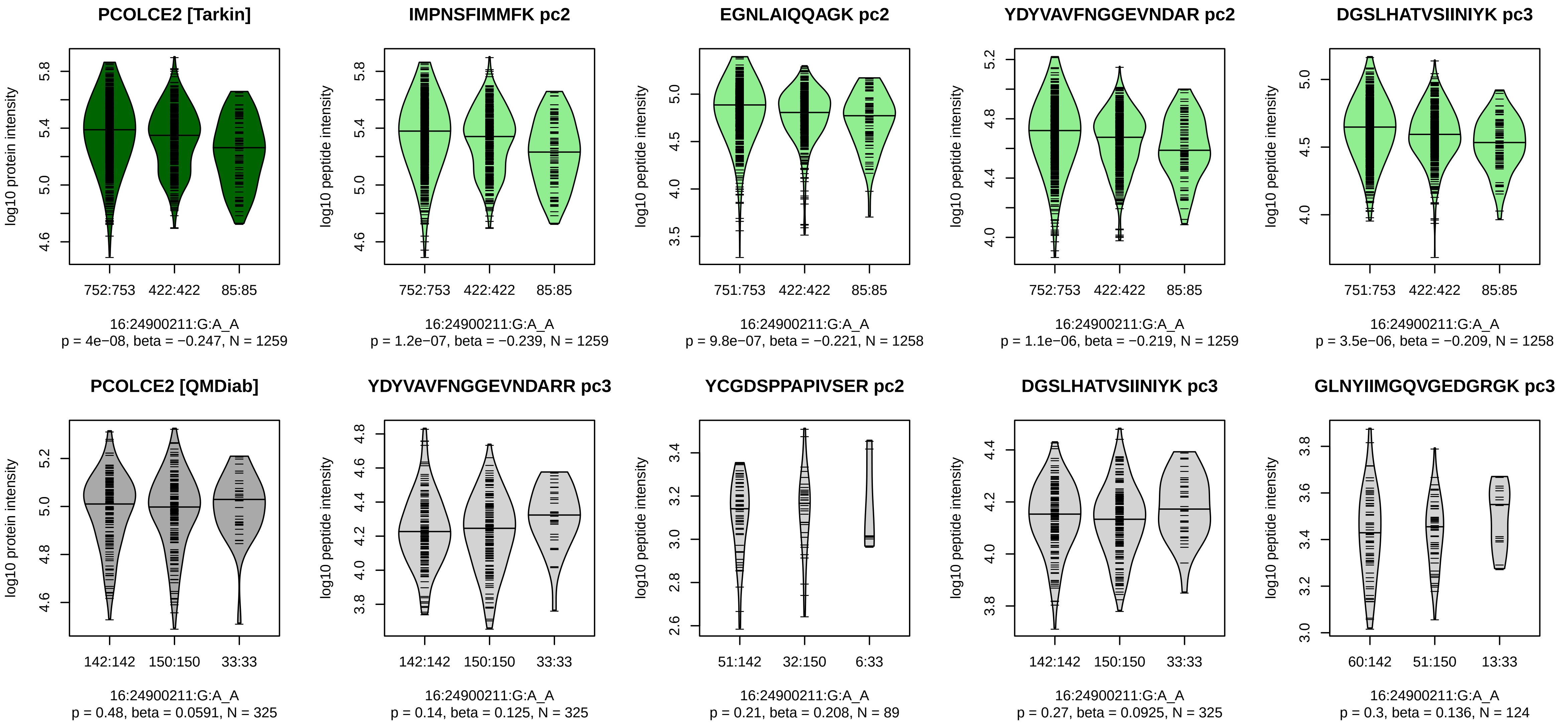

Sort: 342
Gene name: PCOLCE2
SNP: 16:24900211:G:A
rsID: rs274071
Type: Trans−pQTL
Protein group(s):
Q9UKZ9
Nano−particle: NP4

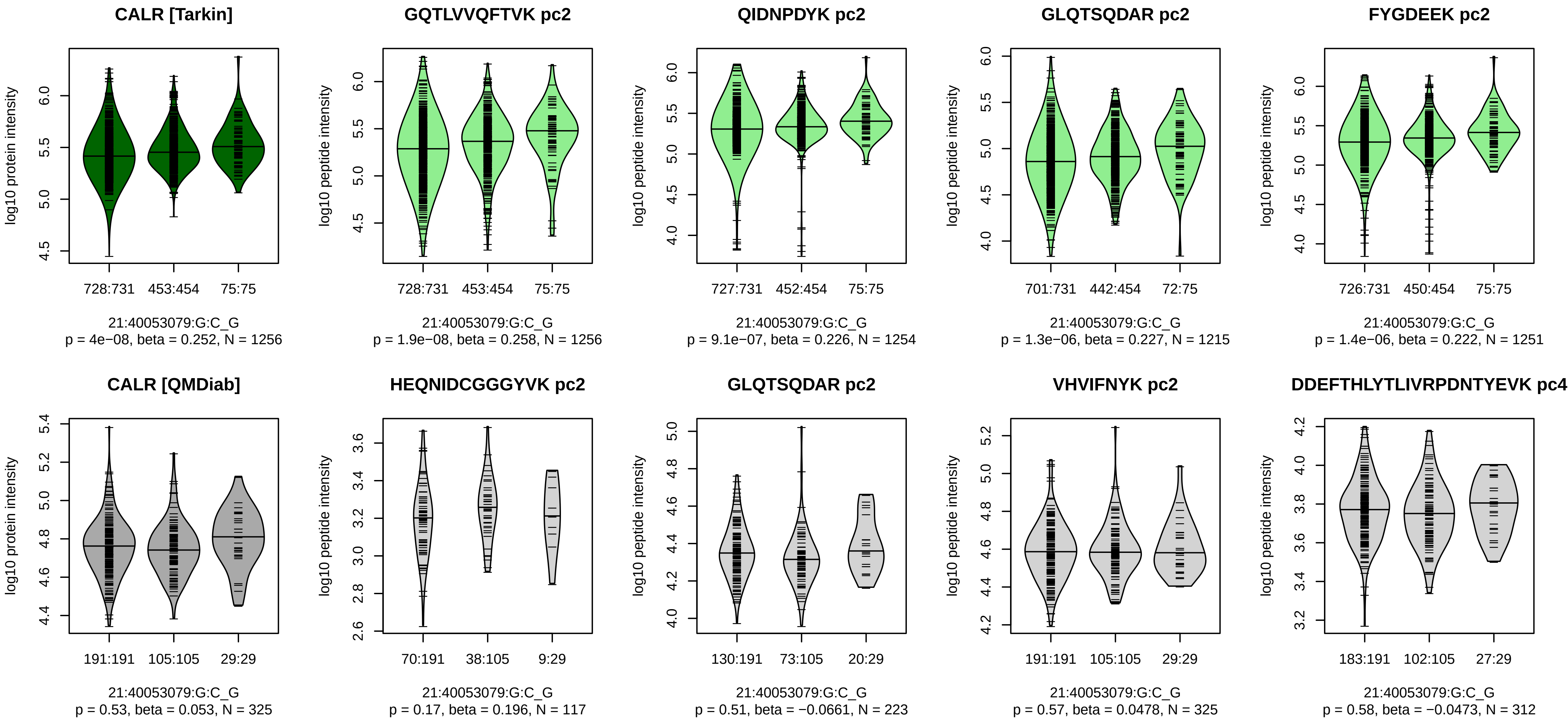

Sort: 343
Gene name: CALR
SNP: 21:40053079:G:C
rsID: rs2836620
Type: Trans−pQTL
Protein group(s):
P27797
Nano−particle: NP2

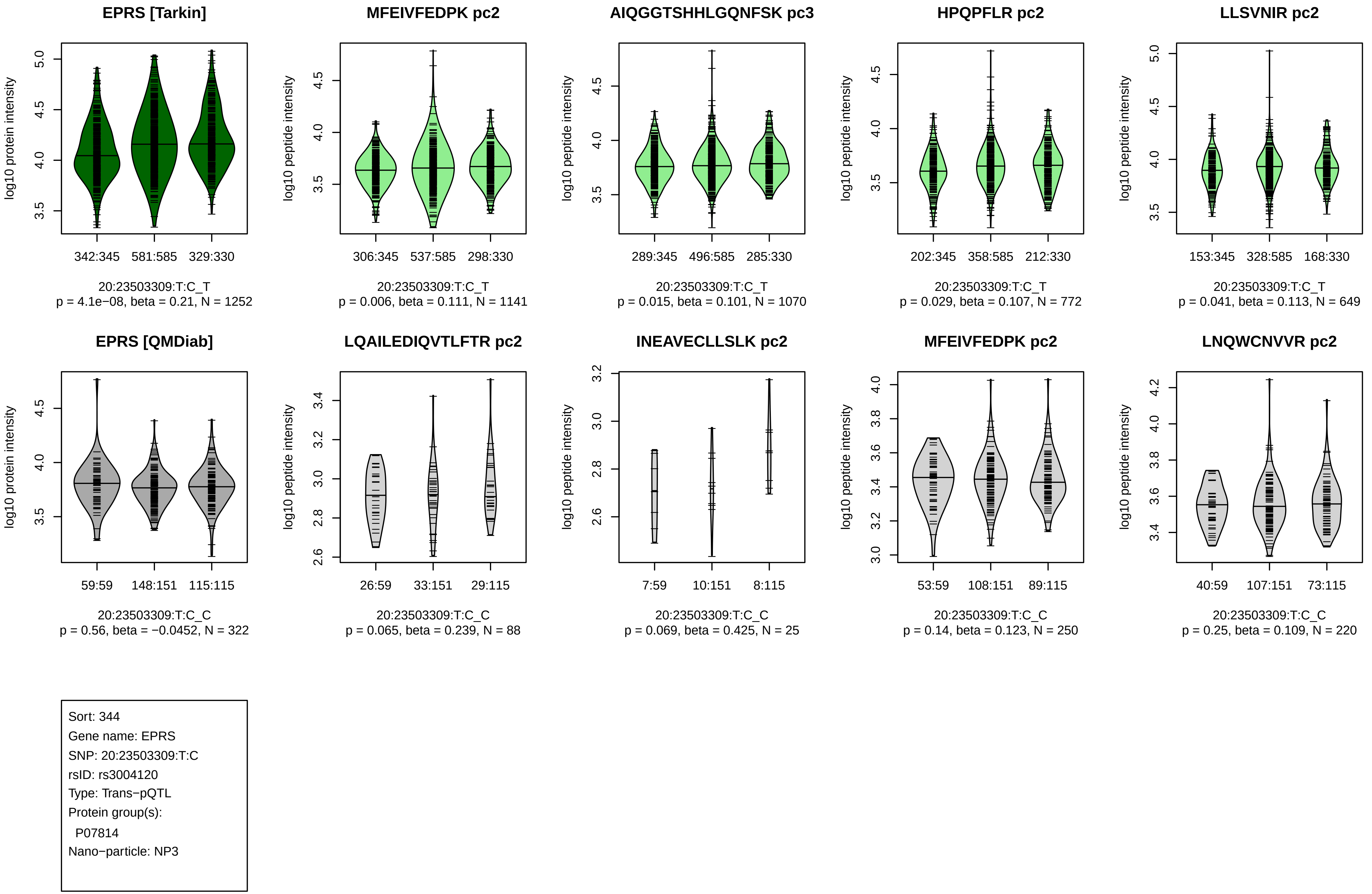

EPRS [Tarkin]
log10 protein intensity
342:345
581:585
329:330
20:23503309:T:C_T
p = 4.1e−08, beta = 0.21, N = 1252
MFEIVFEDPK pc2
log10 peptide intensity
306:345
537:585
298:330
20:23503309:T:C_T
p = 0.006, beta = 0.111, N = 1141
AIQGGTSHHLGQNFSK pc3
log10 peptide intensity
289:345
496:585
285:330
20:23503309:T:C_T
p = 0.015, beta = 0.101, N = 1070
HPQPFLR pc2
log10 peptide intensity
202:345
358:585
212:330
20:23503309:T:C_T
p = 0.029, beta = 0.107, N = 772
LLSVNIR pc2
log10 peptide intensity
153:345
328:585
168:330
20:23503309:T:C_T
p = 0.041, beta = 0.113, N = 649
EPRS [QMDiab]
log10 protein intensity
59:59
148:151
115:115
20:23503309:T:C_C
p = 0.56, beta = −0.0452, N = 322
LQAILEDIQVTLFTR pc2
log10 peptide intensity
26:59
33:151
29:115
20:23503309:T:C_C
p = 0.065, beta = 0.239, N = 88
INEAVECLLSLK pc2
log10 peptide intensity
7:59
10:151
8:115
20:23503309:T:C_C
p = 0.069, beta = 0.425, N = 25
MFEIVFEDPK pc2
log10 peptide intensity
53:59
108:151
89:115
20:23503309:T:C_C
p = 0.14, beta = 0.123, N = 250
LNQWCNVVR pc2
log10 peptide intensity
40:59
107:151
73:115
20:23503309:T:C_C
p = 0.25, beta = 0.109, N = 220
Sort: 344
Gene name: EPRS
SNP: 20:23503309:T:C
rsID: rs3004120
Type: Trans−pQTL
Protein group(s):
P07814
Nano−particle: NP3

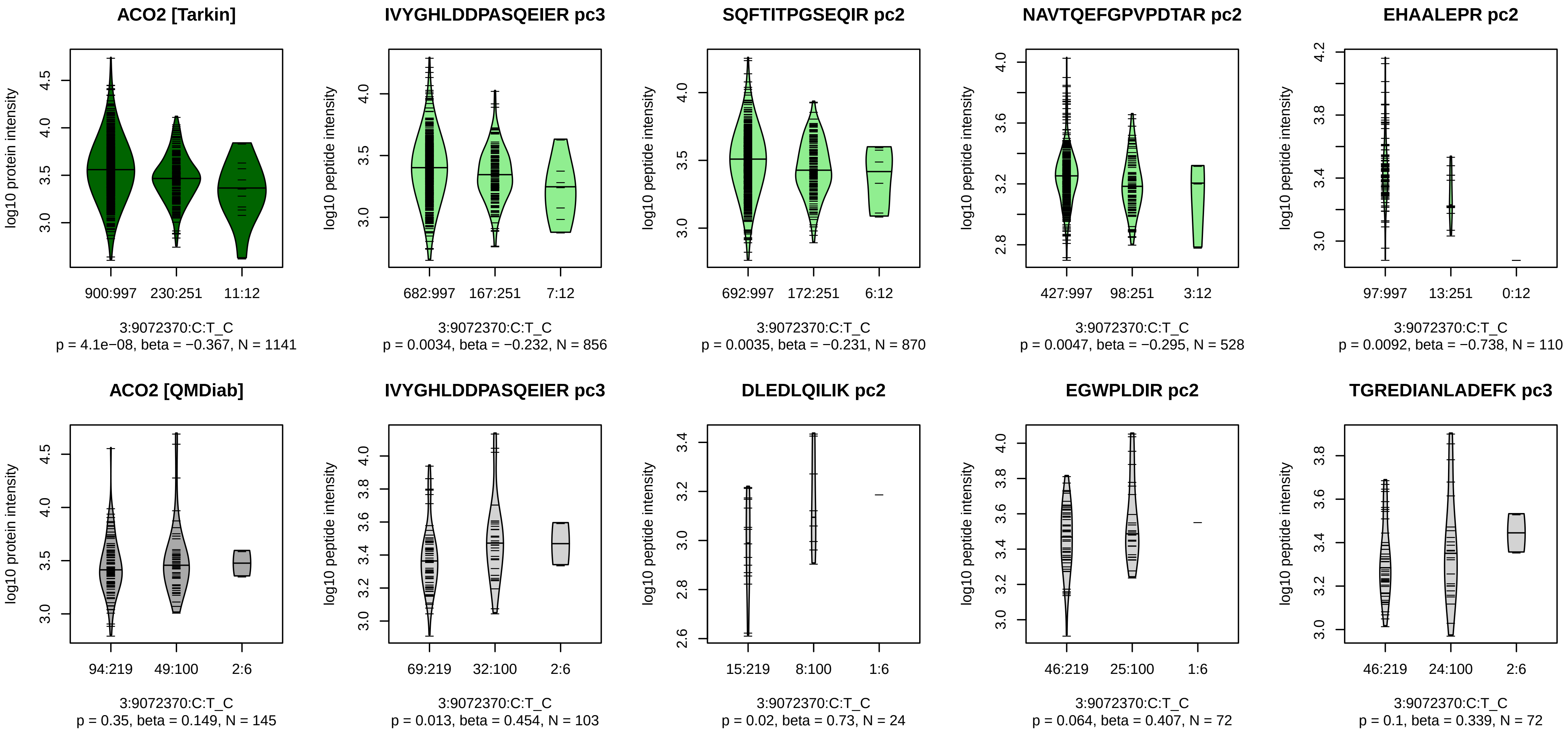

Sort: 345
Gene name: ACO2
SNP: 3:9072370:C:T
rsID: rs549413
Type: Trans−pQTL
Protein group(s):
A2A274;Q99798
Nano−particle: NP1

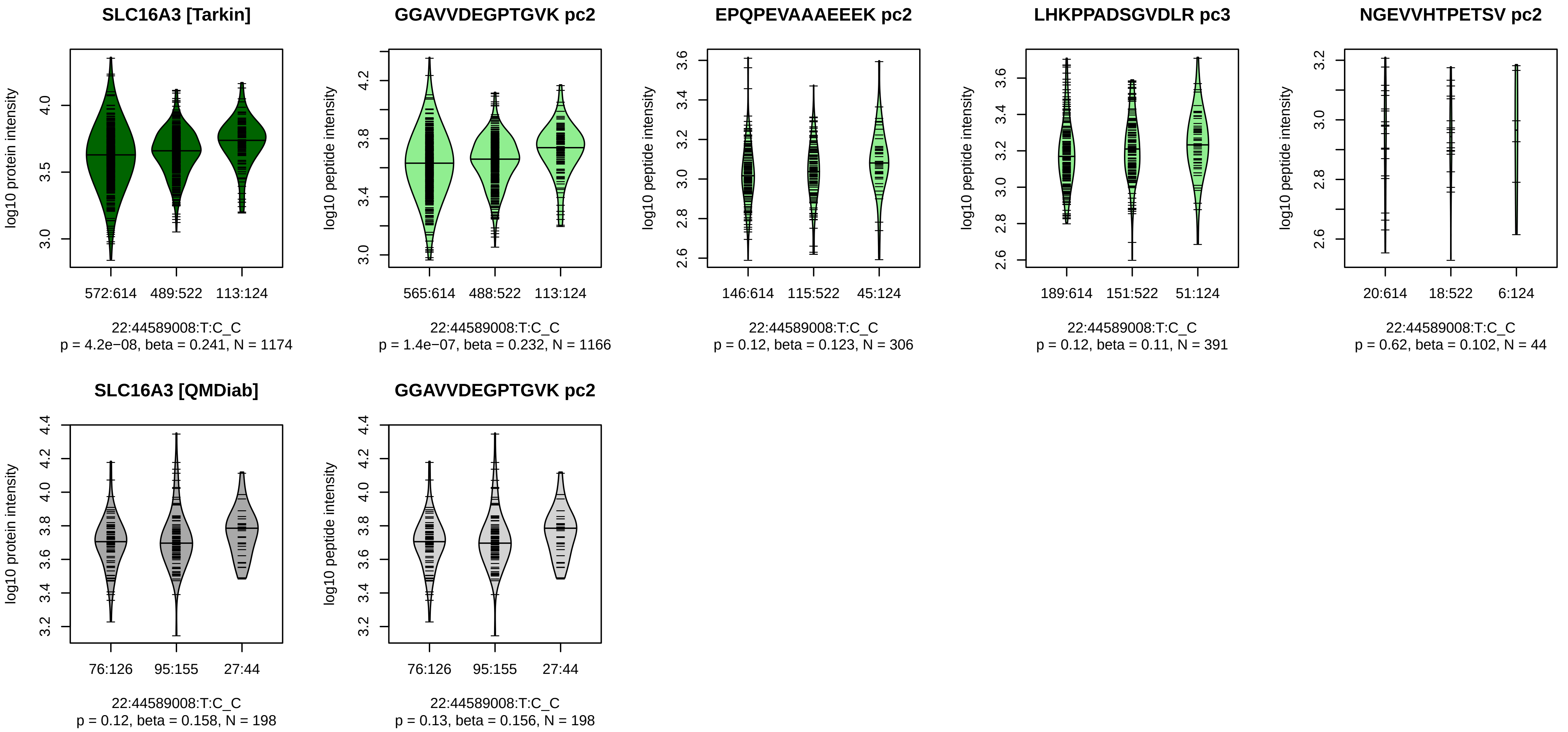

Sort: 346
Gene name: SLC16A3
SNP: 22:44589008:T:C
rsID: rs932381
Type: Trans−pQTL
Protein group(s):
J3KTM6;J3QQV2;O15427
Nano−particle: NP1

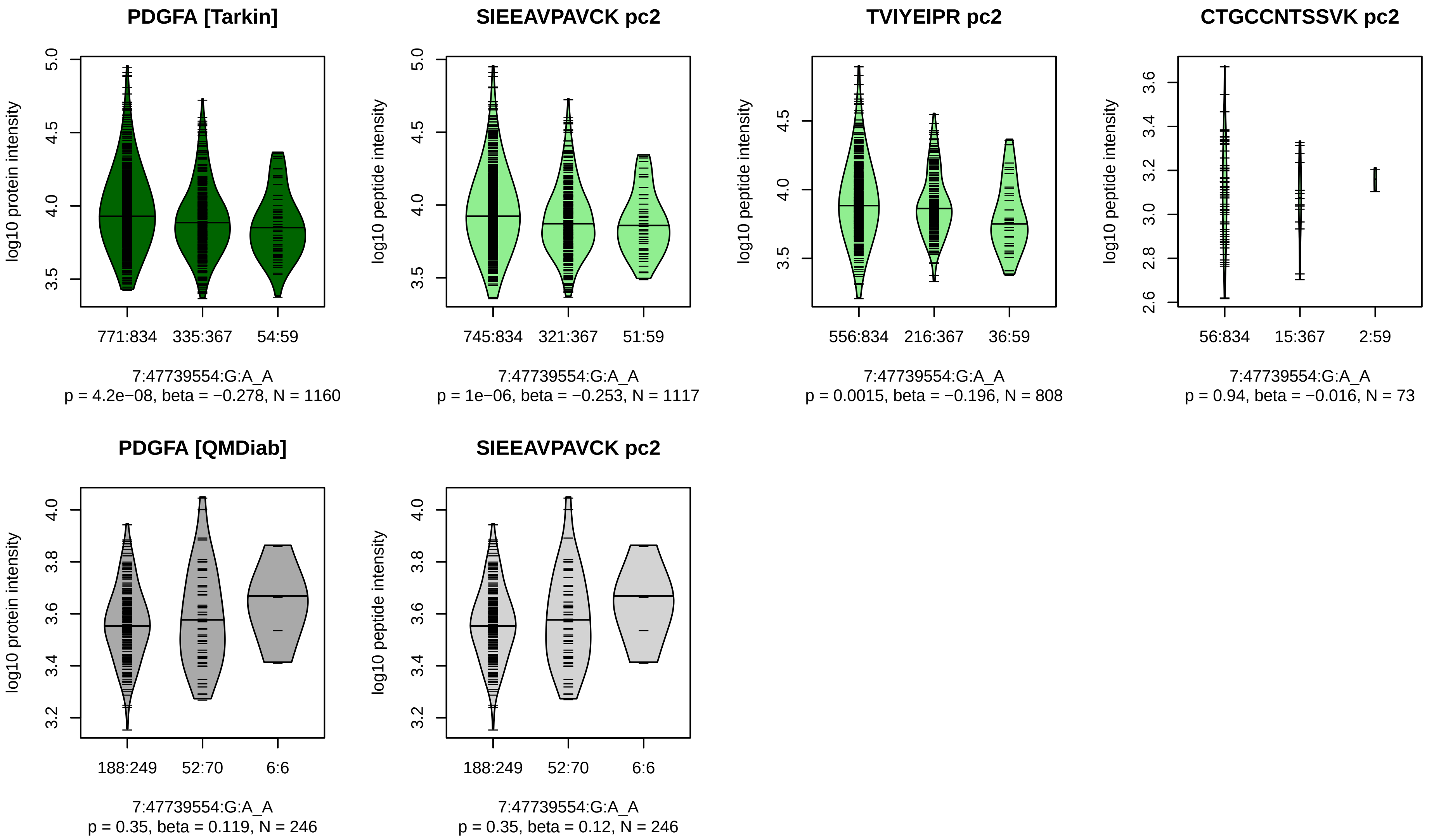

Sort: 347
Gene name: PDGFA
SNP: 7:47739554:G:A
rsID: rs67043063
Type: Trans−pQTL
Protein group(s):
T: P04085
Q: P04085;P04085−2
Nano−particle: NP3

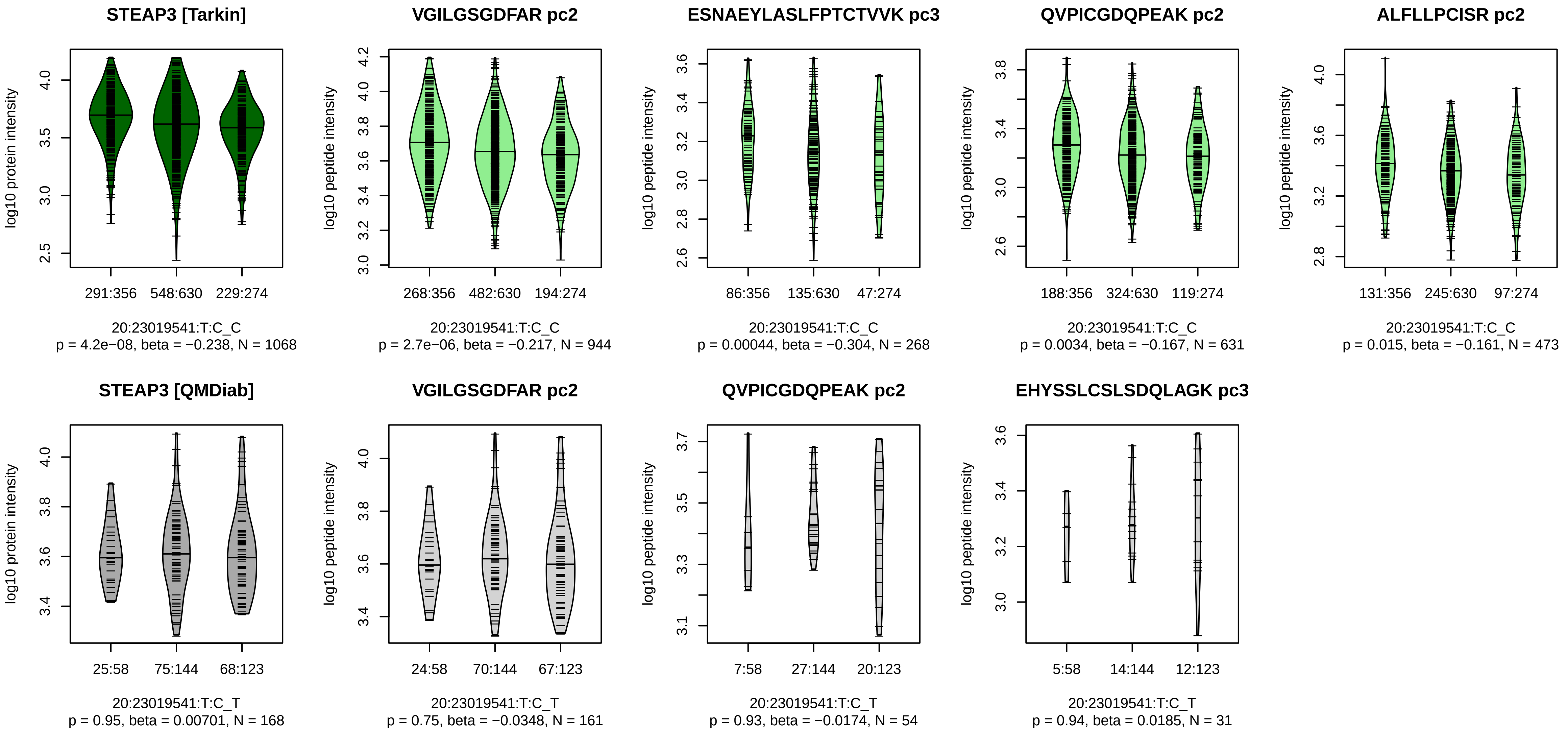

Sort: 348
Gene name: STEAP3
SNP: 20:23019541:T:C
rsID: rs2235951
Type: Trans−pQTL
Protein group(s):
Q658P3;Q658P3−2
Nano−particle: NP1

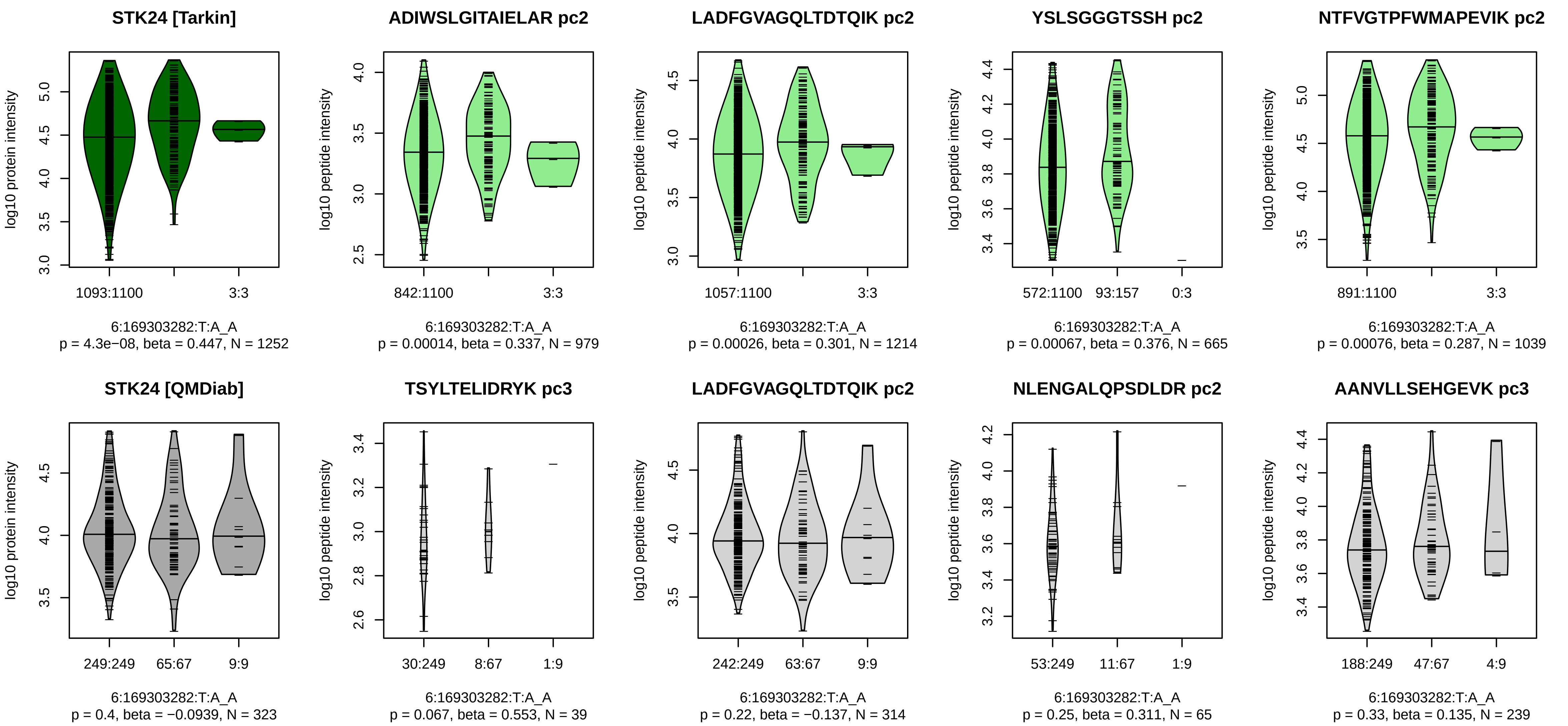

Sort: 349
Gene name: STK24
SNP: 6:169303282:T:A
rsID: rs58373438
Type: Trans−pQTL
Protein group(s):
T: B4DR80;Q9Y6E0−2
Q: B4DR80;Q9Y6E0;Q9Y6E0−2
Nano−particle: NP5

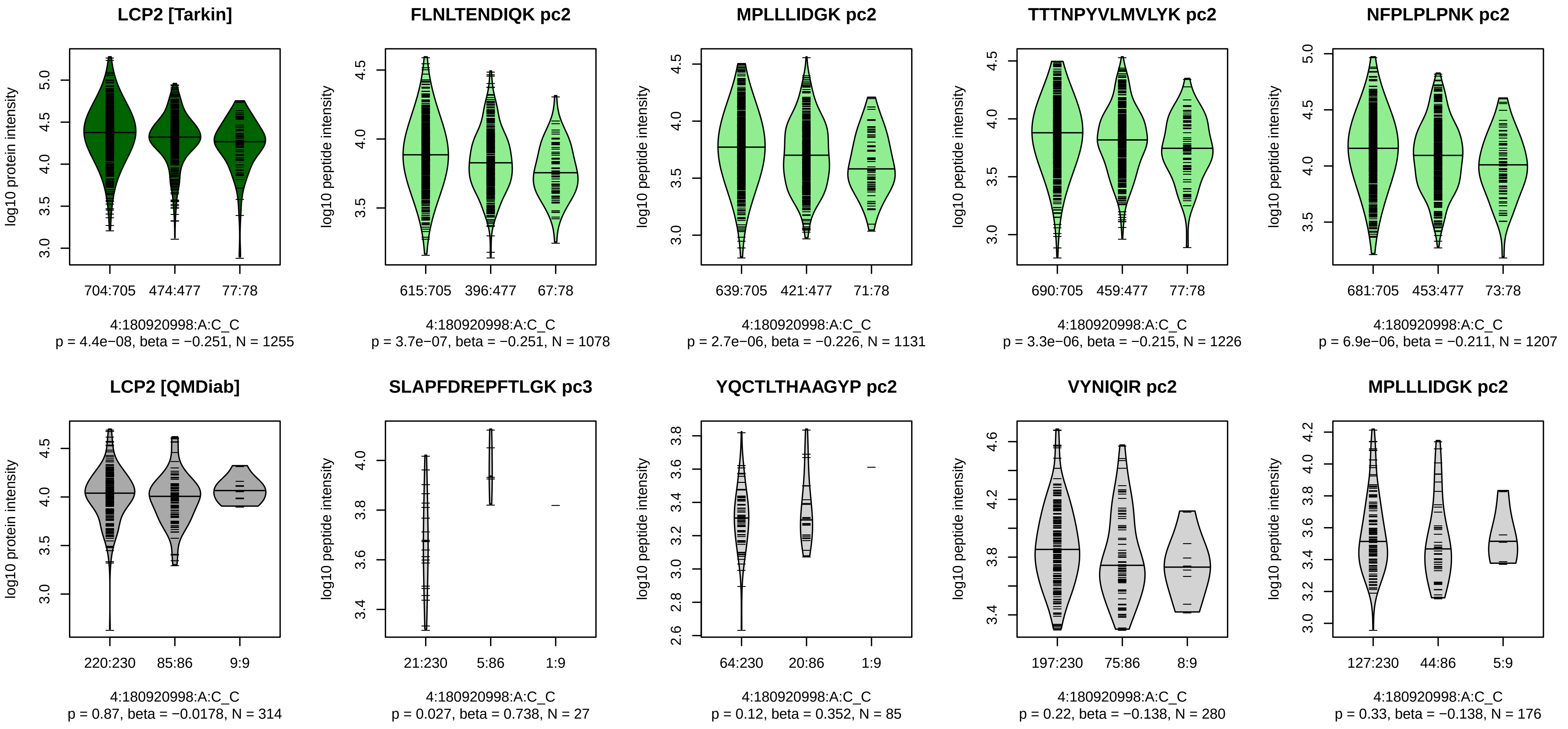

Sort: 350
Gene name: LCP2
SNP: 4:180920998:A:C
rsID: rs4861826
Type: Trans−pQTL
Protein group(s):
Q13094
Nano−particle: NP1

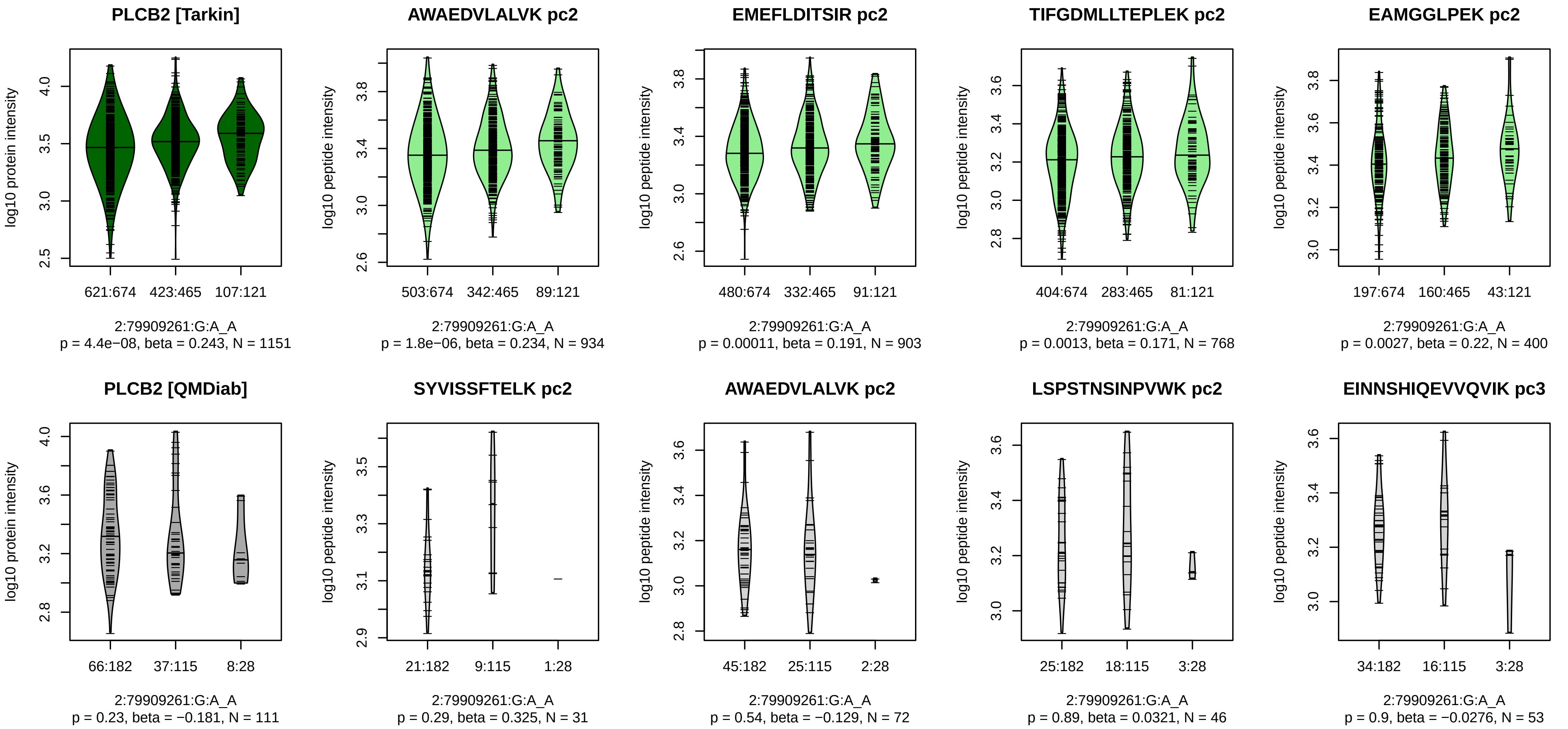

Sort: 351
Gene name: PLCB2
SNP: 2:79909261:G:A
rsID: rs7568201
Type: Trans−pQTL
Protein group(s):
T: Q00722;Q00722−2;Q00722−
Q: Q00722;Q00722−2
Nano−particle: NP1

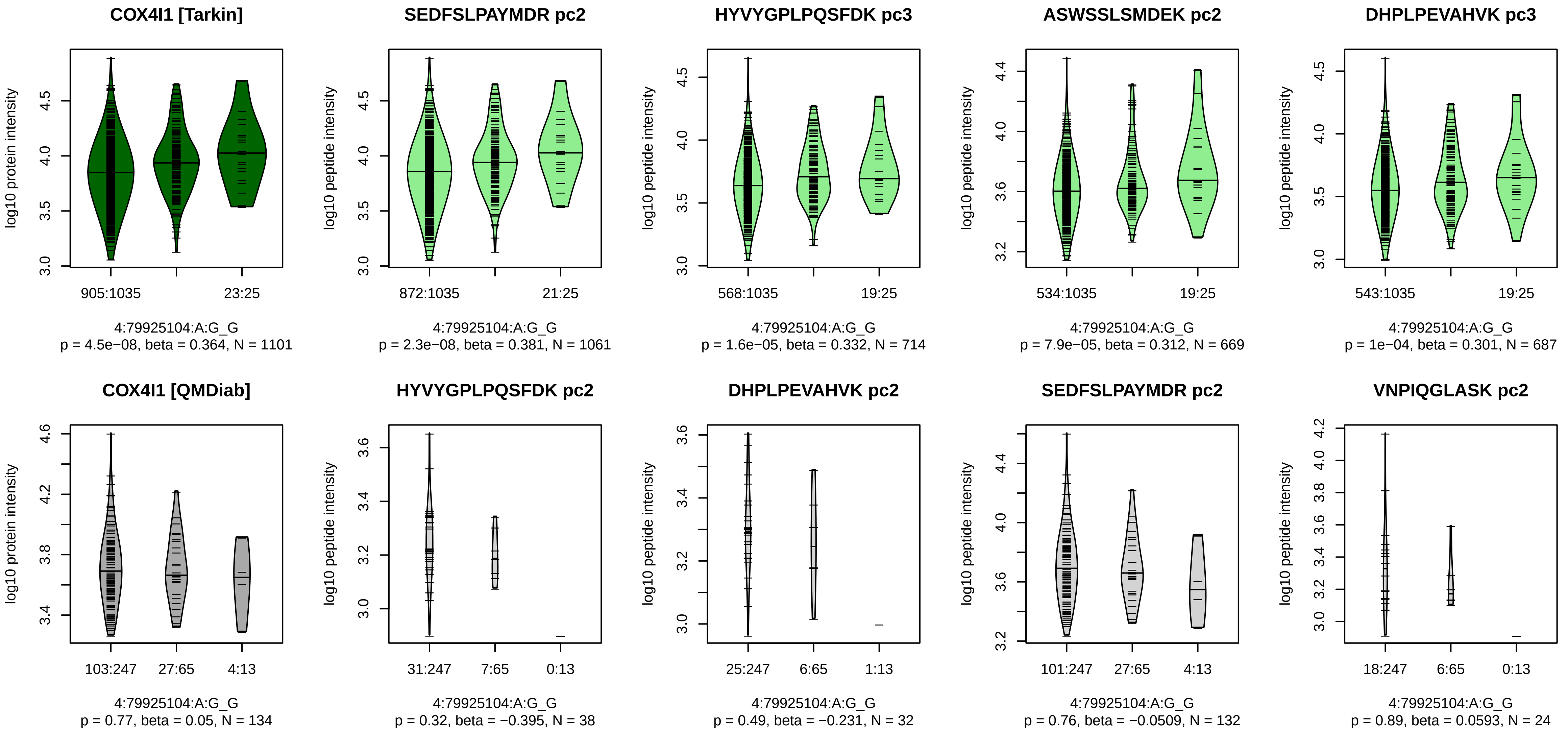

Sort: 352
Gene name: COX4I1
SNP: 4:79925104:A:G
rsID: rs1031936
Type: Trans−pQTL
Protein group(s):
P13073
Nano−particle: NP4

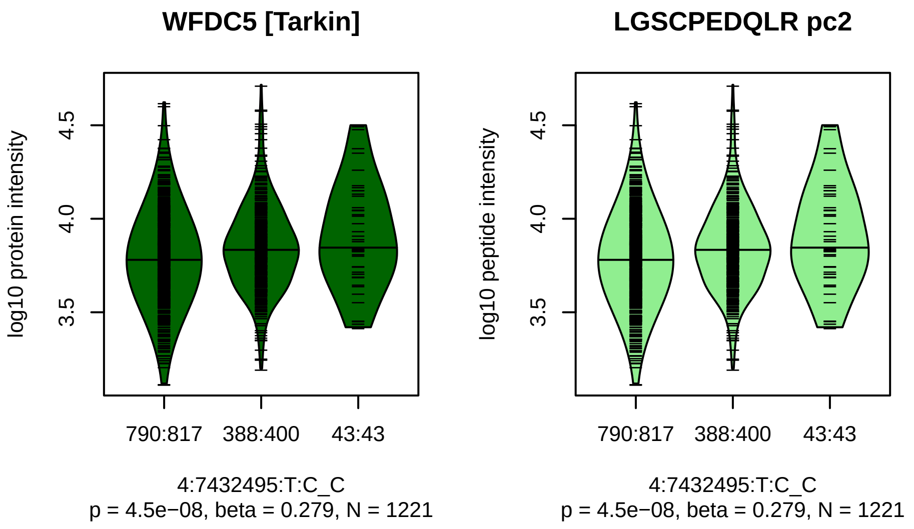

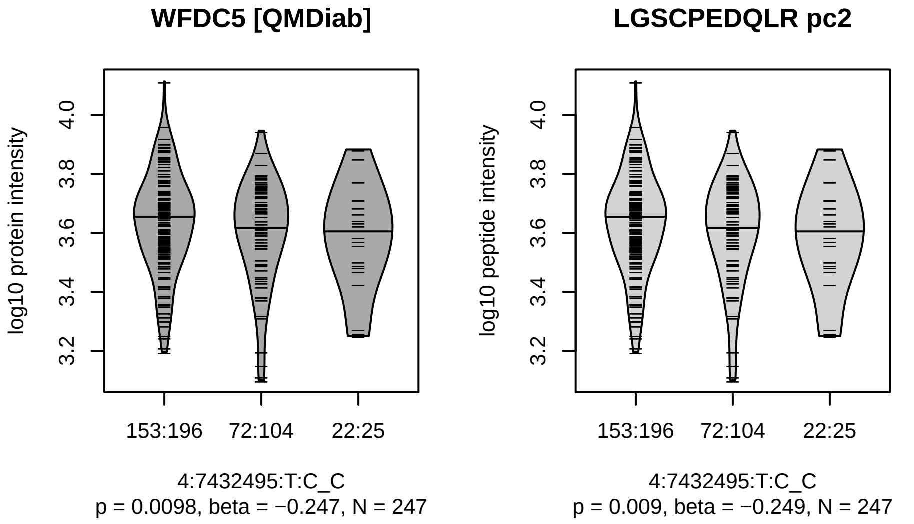

Sort: 353
Gene name: WFDC5
SNP: 4:7432495:T:C
rsID: rs58448418
Type: Trans−pQTL
Protein group(s):
Q8TCV5;Q8TCV5−2
Nano−particle: NP1

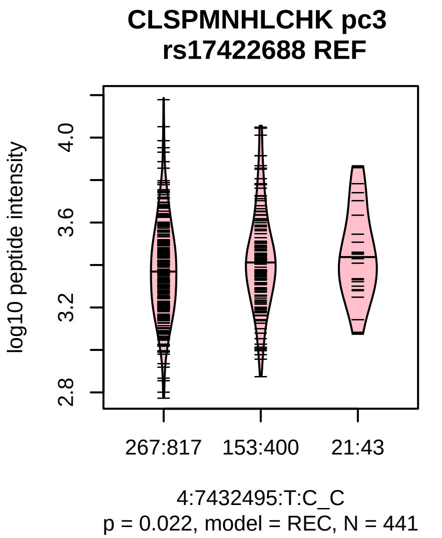

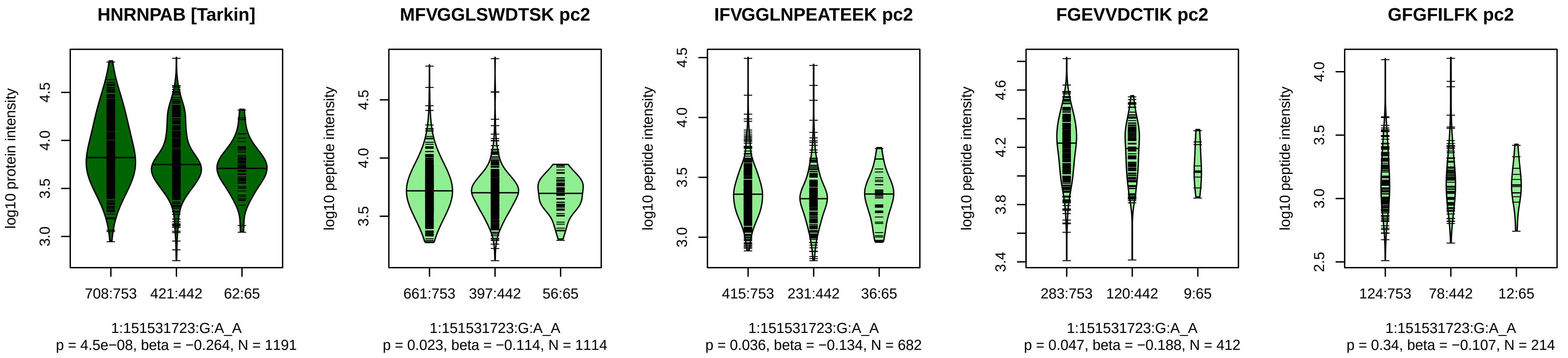

No protein data available
for replication in QMDiab

Sort: 354
Gene name: HNRNPAB
SNP: 1:151531723:G:A
rsID: rs72692719
Type: Trans−pQTL
Protein group(s):
D6RD18
Nano−particle: NP1

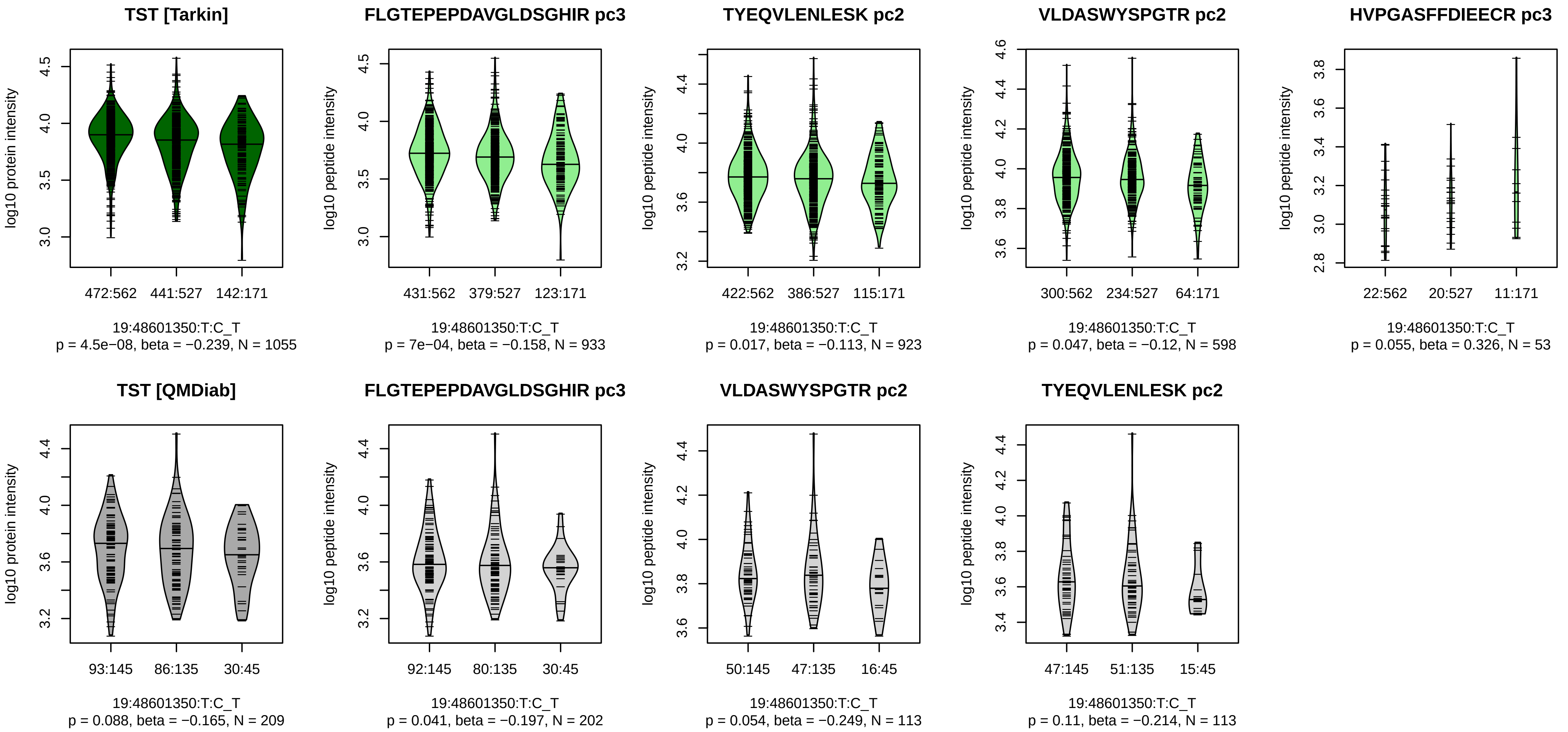

Sort: 355
Gene name: TST
SNP: 19:48601350:T:C
rsID: rs251685
Type: Trans−pQTL
Protein group(s):
Q16762
Nano−particle: NP4

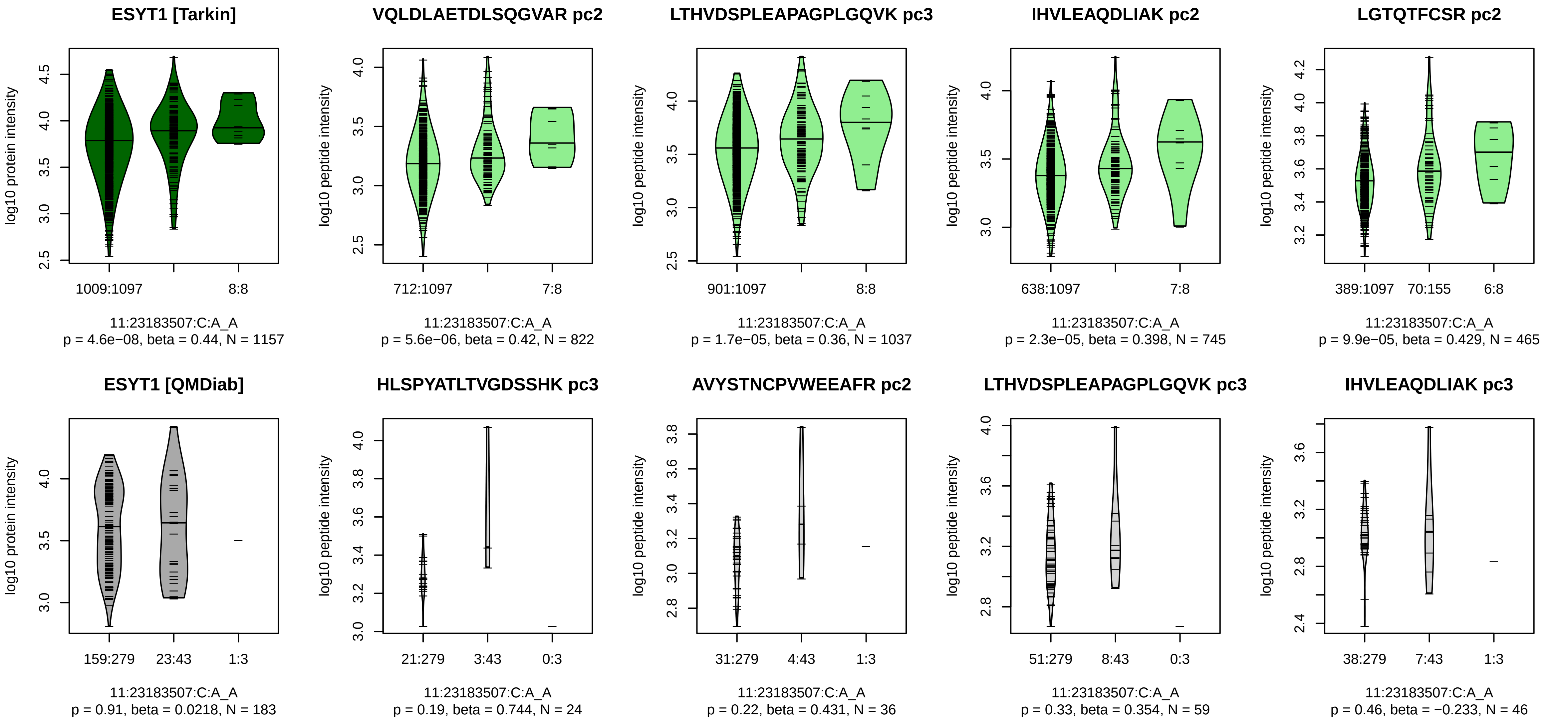

Sort: 356
Gene name: ESYT1
SNP: 11:23183507:C:A
rsID: rs72866988
Type: Trans−pQTL
Protein group(s):
Q9BSJ8;Q9BSJ8−2
Nano−particle: NP4

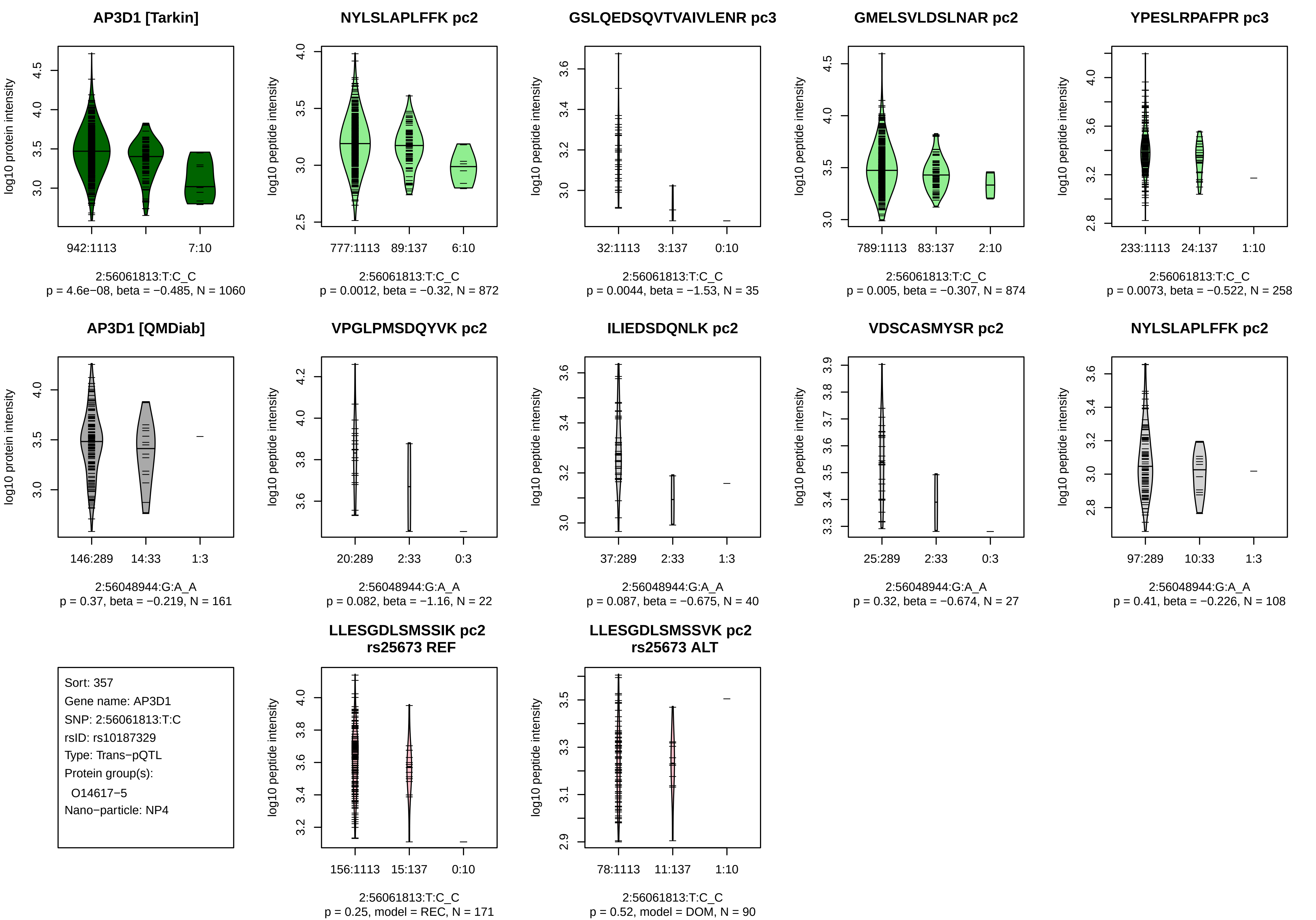
AP3D1 [Tarkin]
log10 protein intensity
942:1113
7:10
2:56061813:T:C_C
p = 4.6e−08, beta = −0.485, N = 1060
NYLSLAPLFFK pc2
log10 peptide intensity
777:1113 89:137 6:10
2:56061813:T:C_C
p = 0.0012, beta = −0.32, N = 872
GSLQEDSQVTVAIVLENR pc3
log10 peptide intensity
32:1113 3:137 0:10
2:56061813:T:C_C
p = 0.0044, beta = −1.53, N = 35
GMELSVLDSLNAR pc2
log10 peptide intensity
789:1113 83:137 2:10
2:56061813:T:C_C
p = 0.005, beta = −0.307, N = 874
YPESLRPAFPR pc3
log10 peptide intensity
233:1113 24:137 1:10
2:56061813:T:C_C
p = 0.0073, beta = −0.522, N = 258
AP3D1 [QMDiab]
log10 protein intensity
146:289 14:33 1:3
2:56048944:G:A_A
p = 0.37, beta = −0.219, N = 161
VPGLPMSDQYVK pc2
log10 peptide intensity
20:289 2:33 0:3
2:56048944:G:A_A
p = 0.082, beta = −1.16, N = 22
ILIEDSDQNLK pc2
log10 peptide intensity
37:289 2:33 1:3
2:56048944:G:A_A
p = 0.087, beta = −0.675, N = 40
VDSCASMYSR pc2
log10 peptide intensity
25:289 2:33 0:3
2:56048944:G:A_A
p = 0.32, beta = −0.674, N = 27
NYLSLAPLFFK pc2
log10 peptide intensity
97:289 10:33 1:3
2:56048944:G:A_A
p = 0.41, beta = −0.226, N = 108
Sort: 357
Gene name: AP3D1
SNP: 2:56061813:T:C
rsID: rs10187329
Type: Trans−pQTL
Protein group(s):
O14617−5
Nano−particle: NP4
LLESGDLSMSSIK pc2
rs25673 REF
log10 peptide intensity
156:1113 15:137 0:10
2:56061813:T:C_C
p = 0.25, model = REC, N = 171
LLESGDLSMSSVK pc2
rs25673 ALT
log10 peptide intensity
78:1113 11:137 1:10
2:56061813:T:C_C
p = 0.52, model = DOM, N = 90

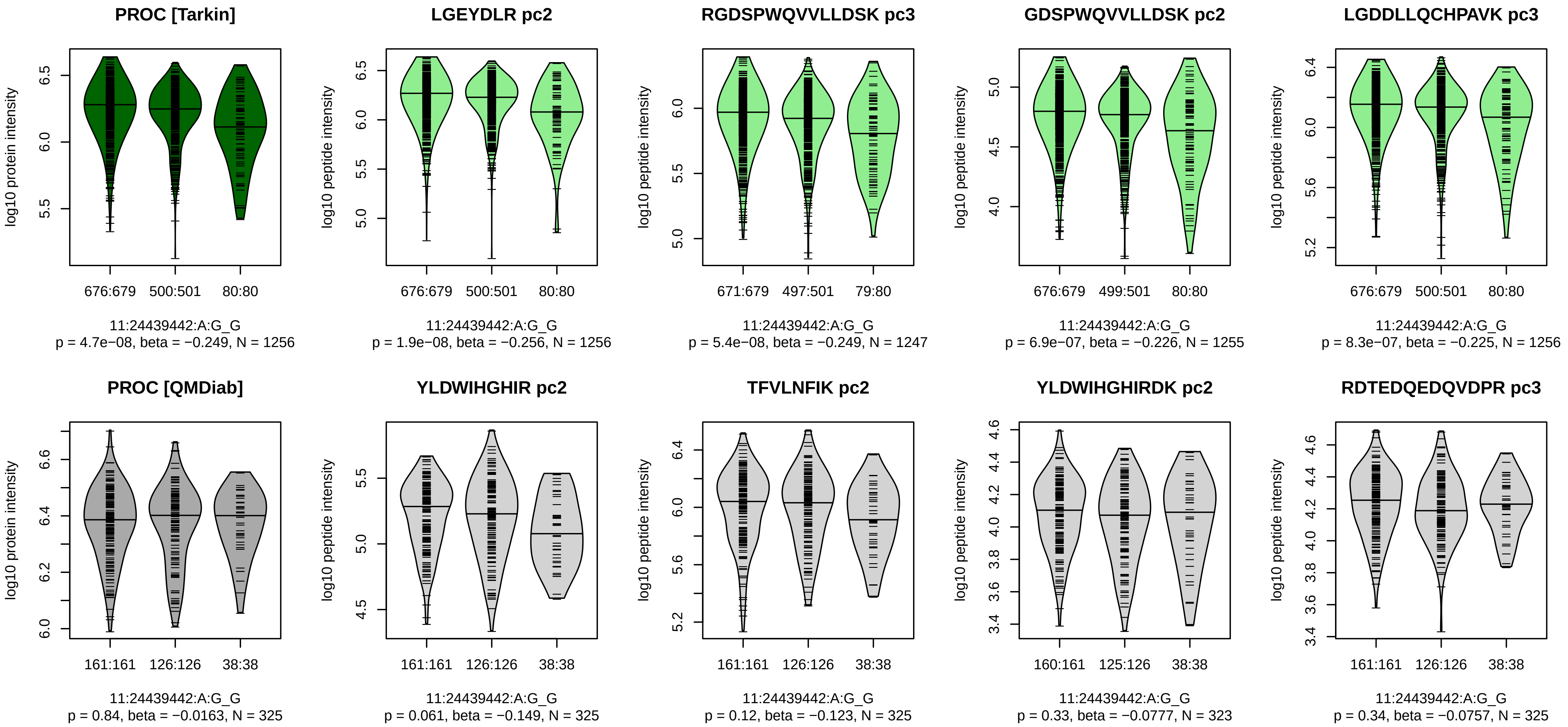

Sort: 358
Gene name: PROC
SNP: 11:24439442:A:G
rsID: rs7930800
Type: Trans−pQTL
Protein group(s):
E7END6
Nano−particle: NP2

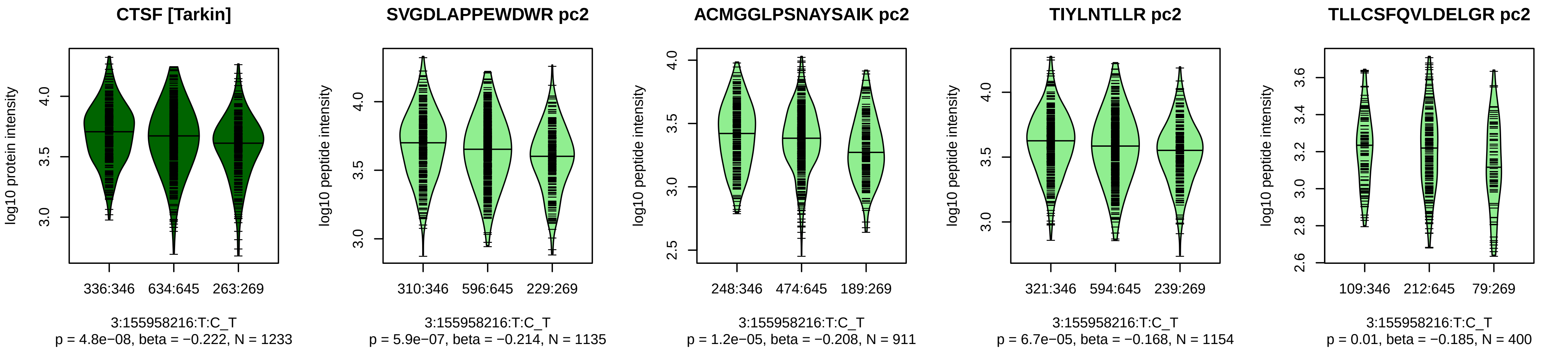

No protein data available
for replication in QMDiab

Sort: 359
Gene name: CTSF
SNP: 3:155958216:T:C
rsID: rs6764861
Type: Trans−pQTL
Protein group(s):
A0A7I2V313
Nano−particle: NP4

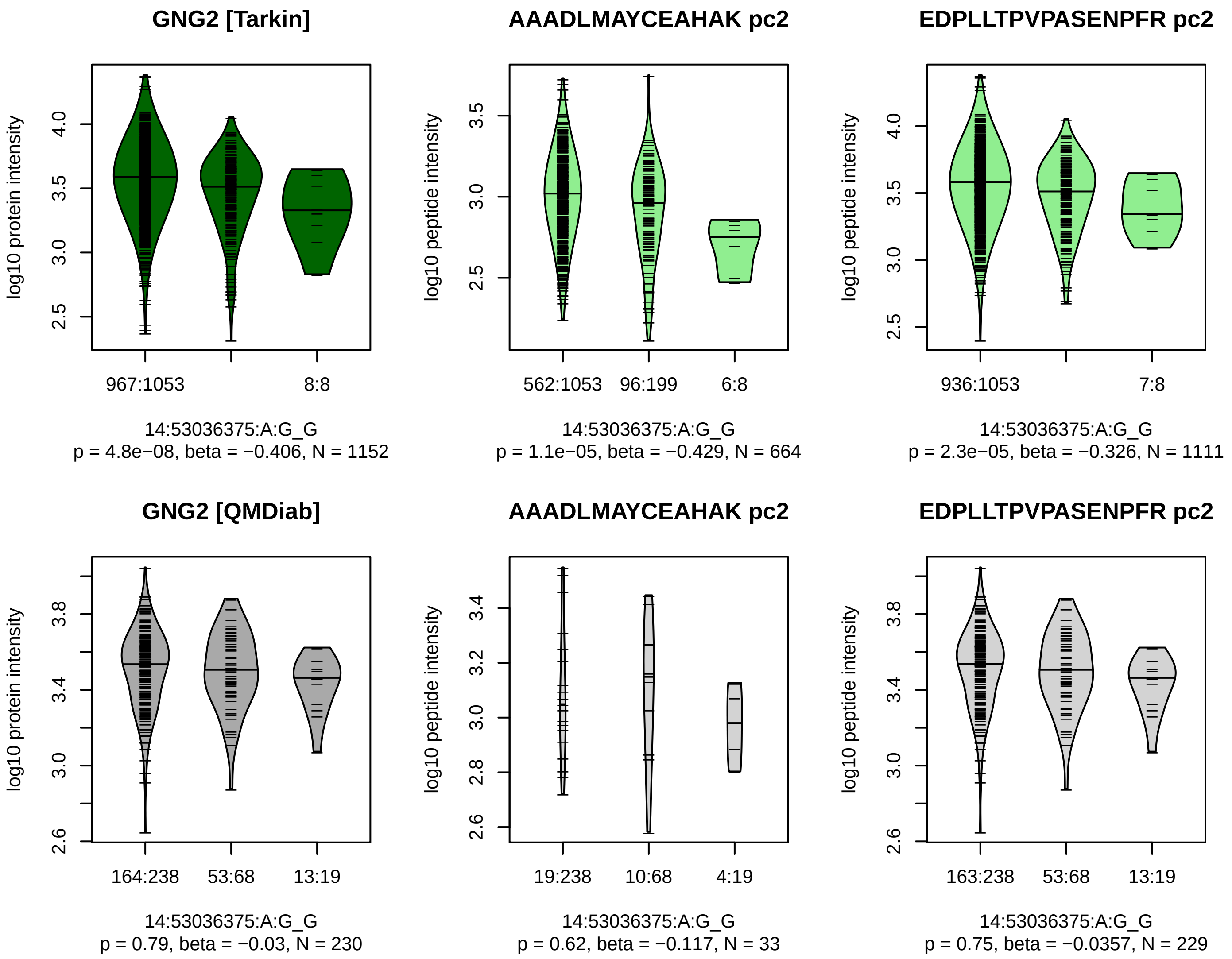

Sort: 360
Gene name: GNG2
SNP: 14:53036375:A:G
rsID: rs11157921
Type: Cis−pQTL
Protein group(s):
T: G3V3J9
Q: G3V2N0;G3V3J9;P59768
Nano−particle: NP1

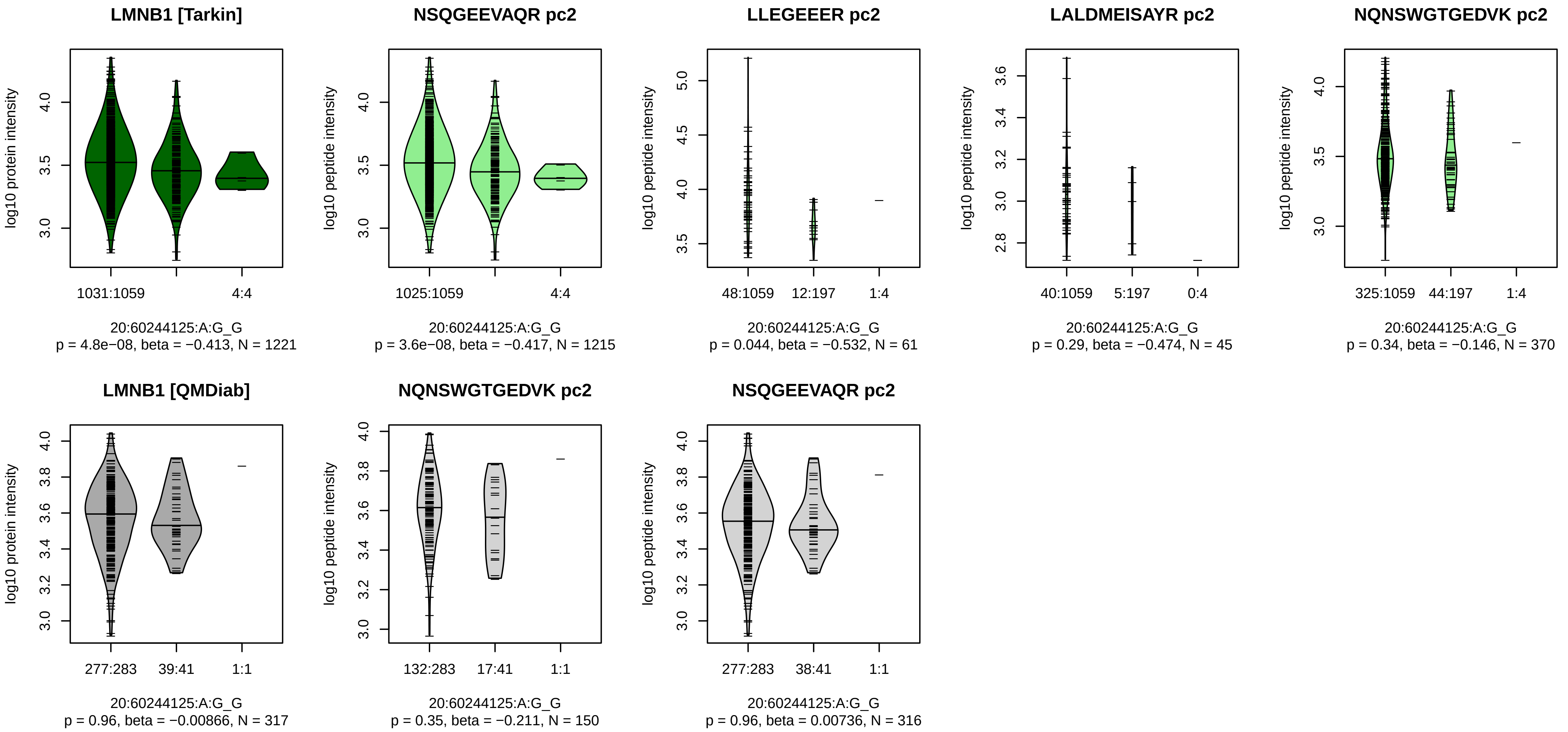

Sort: 361
Gene name: LMNB1
SNP: 20:60244125:A:G
rsID: rs113003549
Type: Trans−pQTL
Protein group(s):
P20700
Nano−particle: NP2

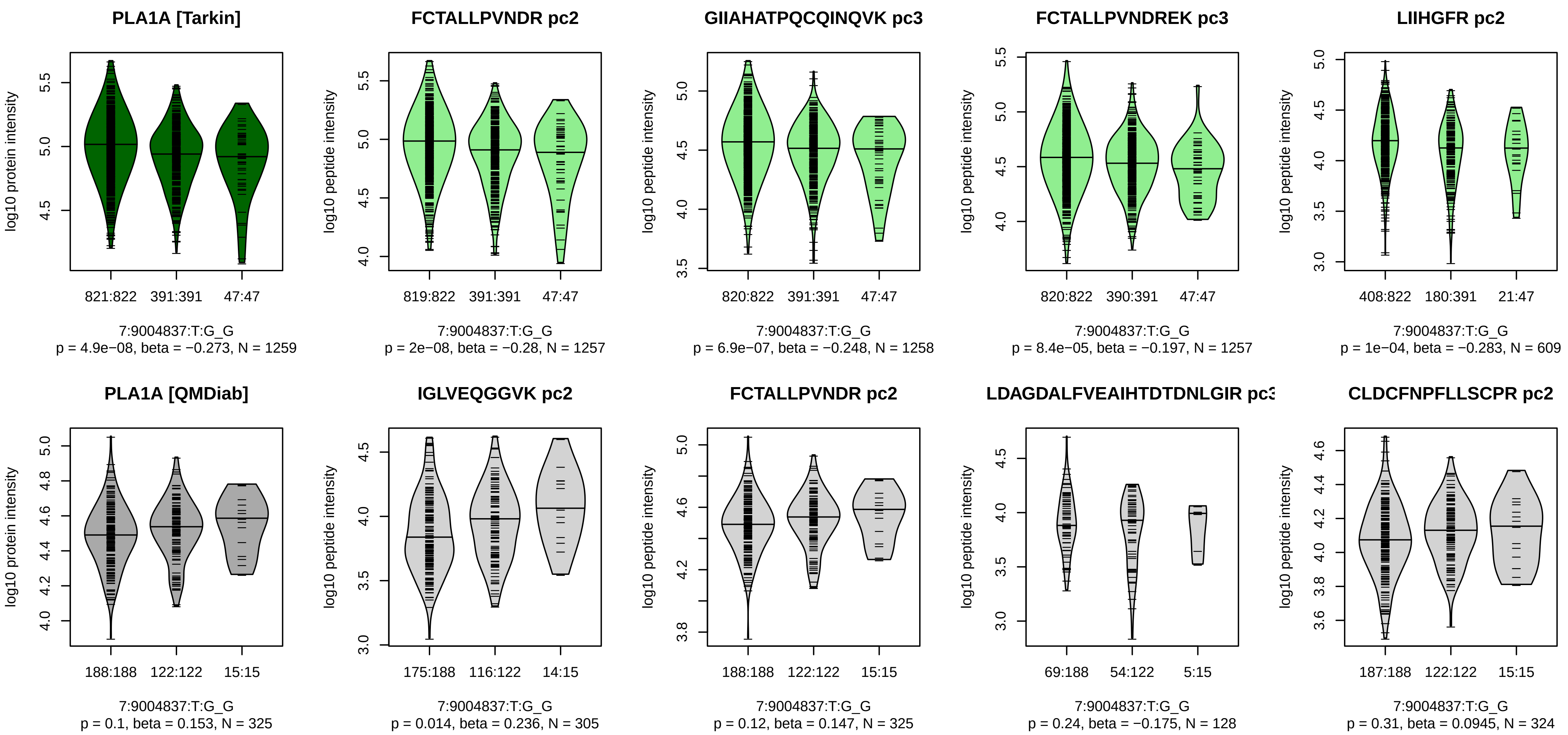

Sort: 362
Gene name: PLA1A
SNP: 7:9004837:T:G
rsID: rs55814095
Type: Trans−pQTL
Protein group(s):
G5E9W0;Q53H76
Nano−particle: NP4

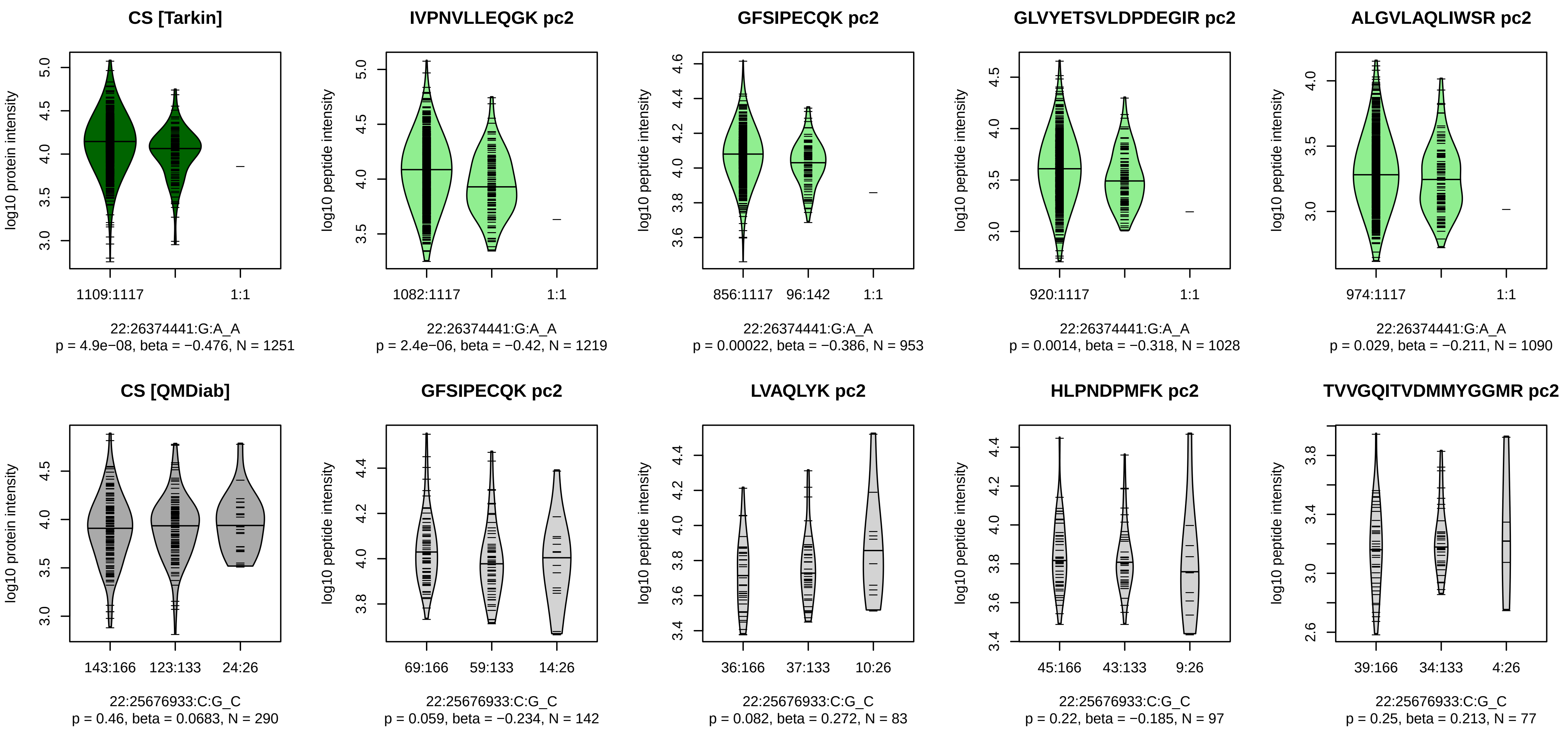

Sort: 363
Gene name: CS
SNP: 22:26374441:G:A
rsID: rs62224169
Type: Trans−pQTL
Protein group(s):
T: O75390
Q: B4DJV2;O75390
Nano−particle: NP1

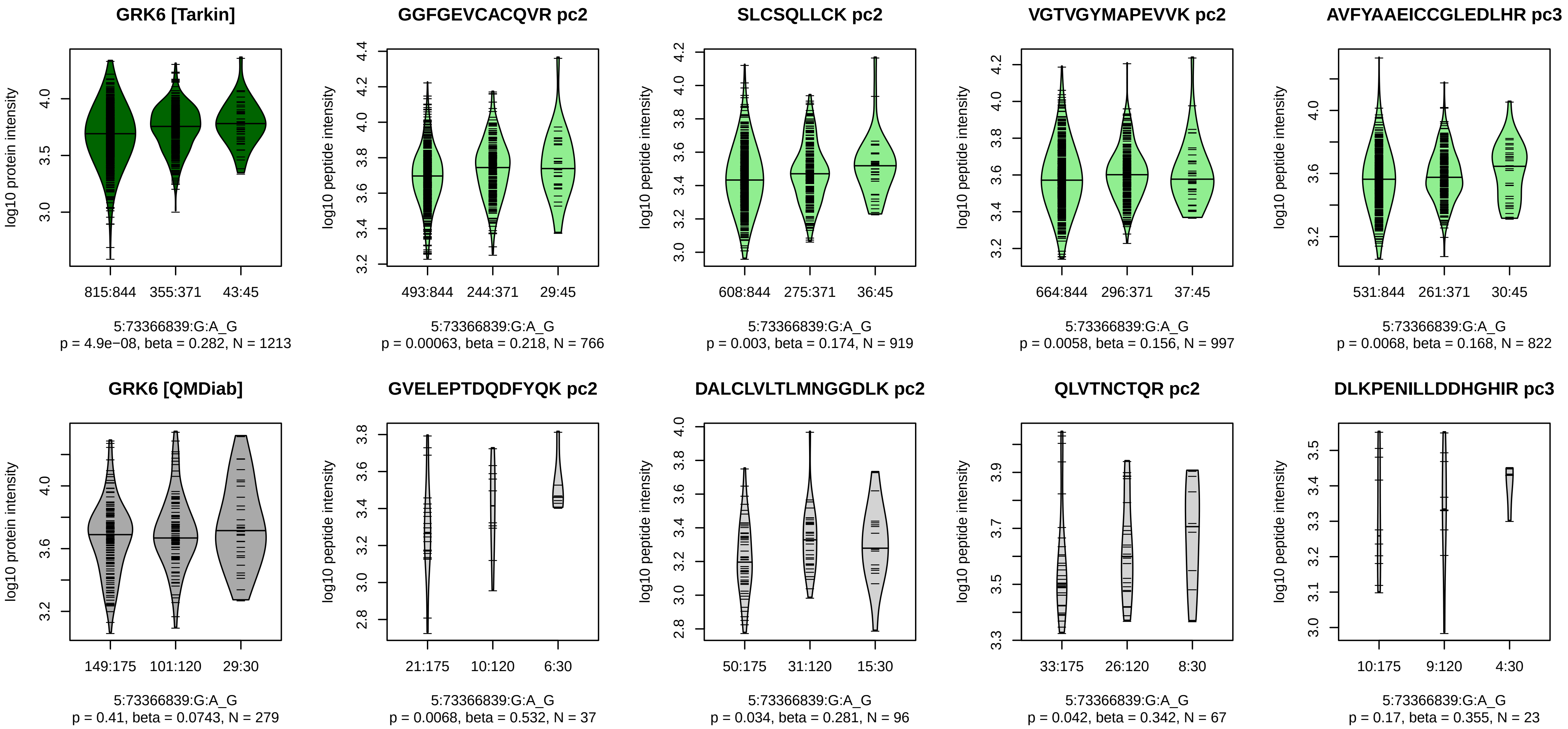

Sort: 364
Gene name: GRK6
SNP: 5:73366839:G:A
rsID: rs286644
Type: Trans−pQTL
Protein group(s):
T: P43250−2
Q: P43250;P43250−2;P43250−3
Nano−particle: NP1
