# Supplementary material for: A genome-wide association study of mass spectrometry proteomics using a nanoparticle enrichment platform
Source: Nat Genet. 2025 Nov 27;57(12):2987–96. doi: 10.1038/s41588-025-02413-w (PMC12695657; doi:10.1038/s41588-025-02413-w)
Supplement: Supplementary file 6 — Meta-analysis Forest plots and violin plots of protein and up to 19 peptide levels by genotype for 364 pQTLs identified in this study. [file 41588_2025_2413_MOESM6_ESM.pdf]

1. GALC (P54803)
Tarkin MSPA=0.981

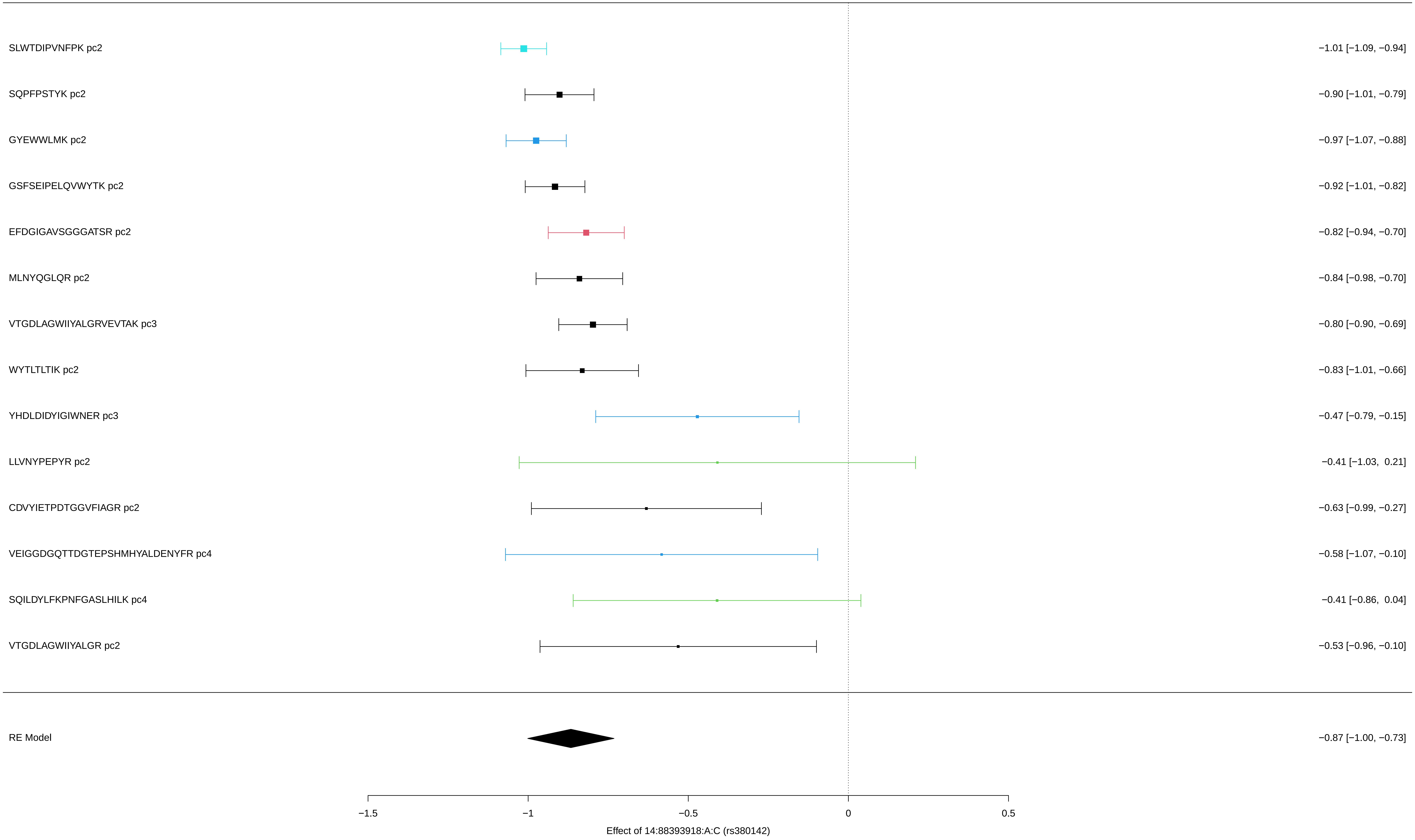

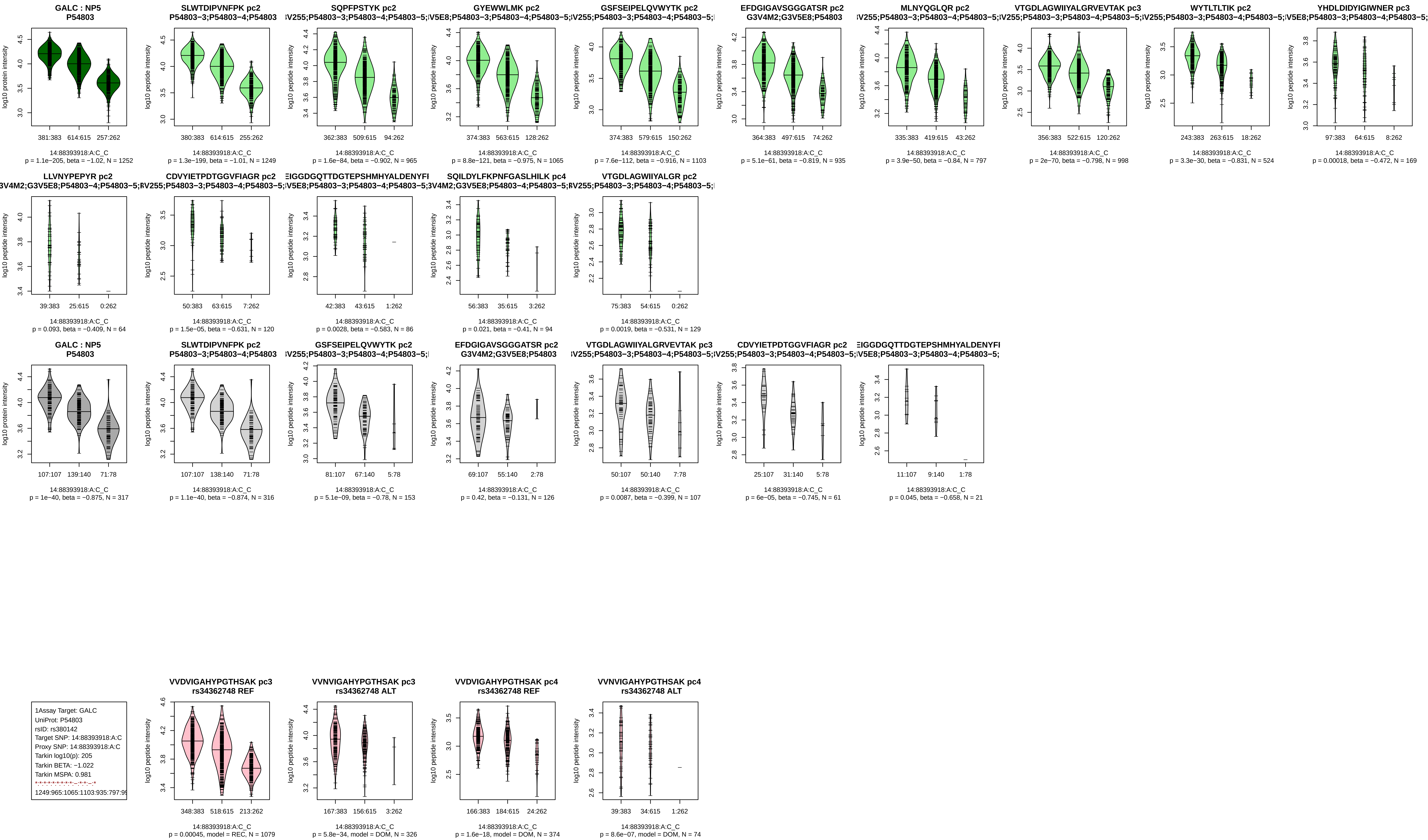
GALC : NP5
P54803
log10 protein intensity
381:383 614:615 257:262
14:88393918:A:C_C
p = 1.1e−205, beta = −1.02, N = 1252
SLWTDIPVNFPK pc2
P54803−3;P54803−4;P54803
log10 peptide intensity
380:383 614:615 255:262
14:88393918:A:C_C
p = 1.3e−199, beta = −1.01, N = 1249
SQPFPSTYK pc2
362:383 509:615 94:262
14:88393918:A:C_C
p = 1.6e−84, beta = −0.902, N = 965
GYEWWLMK pc2
374:383 563:615 128:262
14:88393918:A:C_C
p = 8.8e−121, beta = −0.975, N = 1065
GSFSEIPELQVWYTK pc2
374:383 579:615 150:262
14:88393918:A:C_C
p = 7.6e−112, beta = −0.916, N = 1103
EFDGIGAVSGGGATSR pc2
G3V4M2;G3V5E8;P54803
364:383 497:615 74:262
14:88393918:A:C_C
p = 5.1e−61, beta = −0.819, N = 935
MLNYQGLQR pc2
335:383 419:615 43:262
14:88393918:A:C_C
p = 3.9e−50, beta = −0.84, N = 797
VTGDLAGWIIYALGRVEVTAK pc3
356:383 522:615 120:262
14:88393918:A:C_C
p = 2e−70, beta = −0.798, N = 998
WYTLTLTIK pc2
243:383 263:615 18:262
14:88393918:A:C_C
p = 3.3e−30, beta = −0.831, N = 524
YHDLDIDYIGIWNER pc3
97:383 64:615 8:262
14:88393918:A:C_C
p = 0.00018, beta = −0.472, N = 169
LLVNYPEPYR pc2
39:383 25:615 0:262
14:88393918:A:C_C
p = 0.093, beta = −0.409, N = 64
CDVYIETPDTGGVFIAGR pc2
50:383 63:615 7:262
14:88393918:A:C_C
p = 1.5e−05, beta = −0.631, N = 120
42:383 43:615 1:262
14:88393918:A:C_C
p = 0.0028, beta = −0.583, N = 86
SQILDYLFKPNFGASLHILK pc4
56:383 35:615 3:262
14:88393918:A:C_C
p = 0.021, beta = −0.41, N = 94
VTGDLAGWIIYALGR pc2
75:383 54:615 0:262
14:88393918:A:C_C
p = 0.0019, beta = −0.531, N = 129
GALC : NP5
P54803
107:107 139:140 71:78
14:88393918:A:C_C
p = 1e−40, beta = −0.875, N = 317
SLWTDIPVNFPK pc2
P54803−3;P54803−4;P54803
107:107 138:140 71:78
14:88393918:A:C_C
p = 1.1e−40, beta = −0.874, N = 316
GSFSEIPELQVWYTK pc2
81:107 67:140 5:78
14:88393918:A:C_C
p = 5.1e−09, beta = −0.78, N = 153
EFDGIGAVSGGGATSR pc2
G3V4M2;G3V5E8;P54803
69:107 55:140 2:78
14:88393918:A:C_C
p = 0.42, beta = −0.131, N = 126
VTGDLAGWIIYALGRVEVTAK pc3
50:107 50:140 7:78
14:88393918:A:C_C
p = 0.0087, beta = −0.399, N = 107
CDVYIETPDTGGVFIAGR pc2
25:107 31:140 5:78
14:88393918:A:C_C
p = 6e−05, beta = −0.745, N = 61
11:107 9:140 1:78
14:88393918:A:C_C
p = 0.045, beta = −0.658, N = 21
1Assay Target: GALC
UniProt: P54803
rsID: rs380142
Target SNP: 14:88393918:A:C
Proxy SNP: 14:88393918:A:C
Tarkin log10(p): 205
Tarkin BETA: −1.022
Tarkin MSPA: 0.981
VVDVIGAHYPGTHSAK pc3
rs34362748 REF
348:383 518:615 213:262
14:88393918:A:C_C
p = 0.00045, model = REC, N = 1079
VVNVIGAHYPGTHSAK pc3
rs34362748 ALT
167:383 156:615 3:262
14:88393918:A:C_C
p = 5.8e−34, model = DOM, N = 326
VVDVIGAHYPGTHSAK pc4
rs34362748 REF
166:383 184:615 24:262
14:88393918:A:C_C
p = 1.6e−18, model = DOM, N = 374
VVNVIGAHYPGTHSAK pc4
rs34362748 ALT
39:383 34:615 1:262
14:88393918:A:C_C
p = 8.6e−07, model = DOM, N = 74

2. HEG1 (Q9ULI3)
Tarkin MSPA=0.65

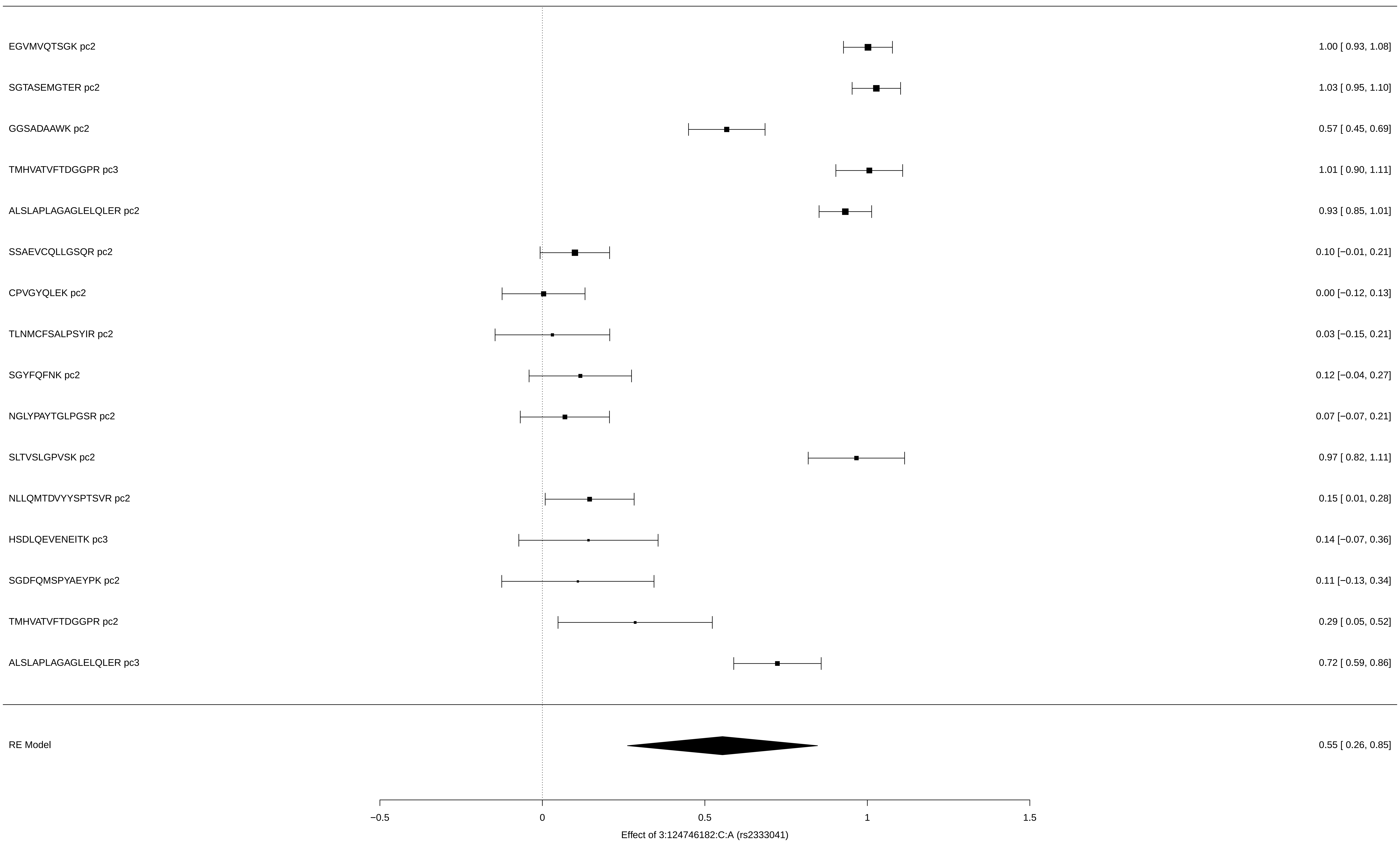

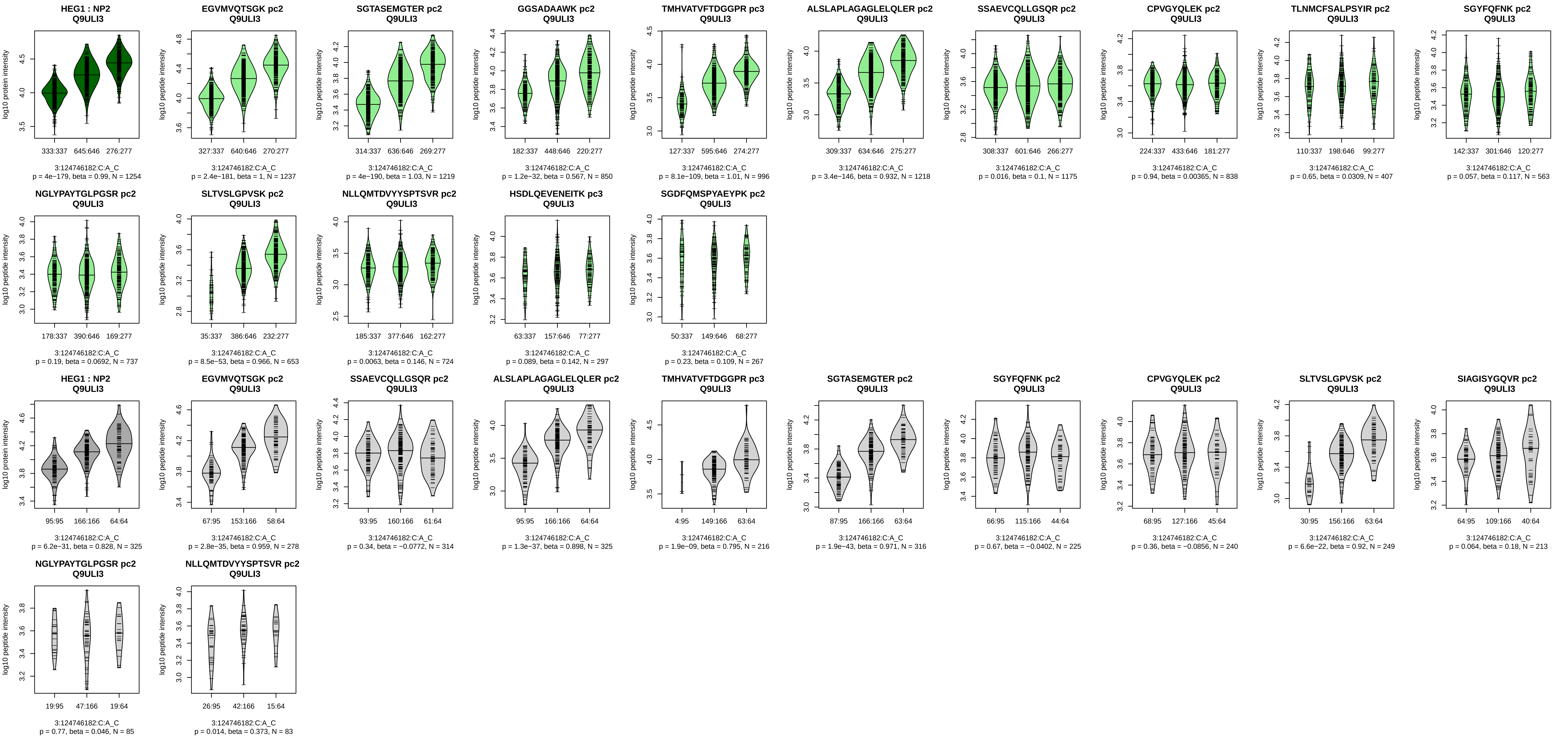

2Assay Target: HEG1
UniProt: Q9ULI3
rsID: rs2333041
Target SNP: 3:124746182:C:A
Proxy SNP: 3:124746182:C:A
Tarkin log10(p): 178.4
Tarkin BETA: 0.99
Tarkin MSPA: 0.65
::*:*:*:−:−:−:−:−:*:*:−:−:*:*:NA
1237:1219:850:996:1218:1175:8

3. HSP90B1 (P14625)
Tarkin MSPA=0.973

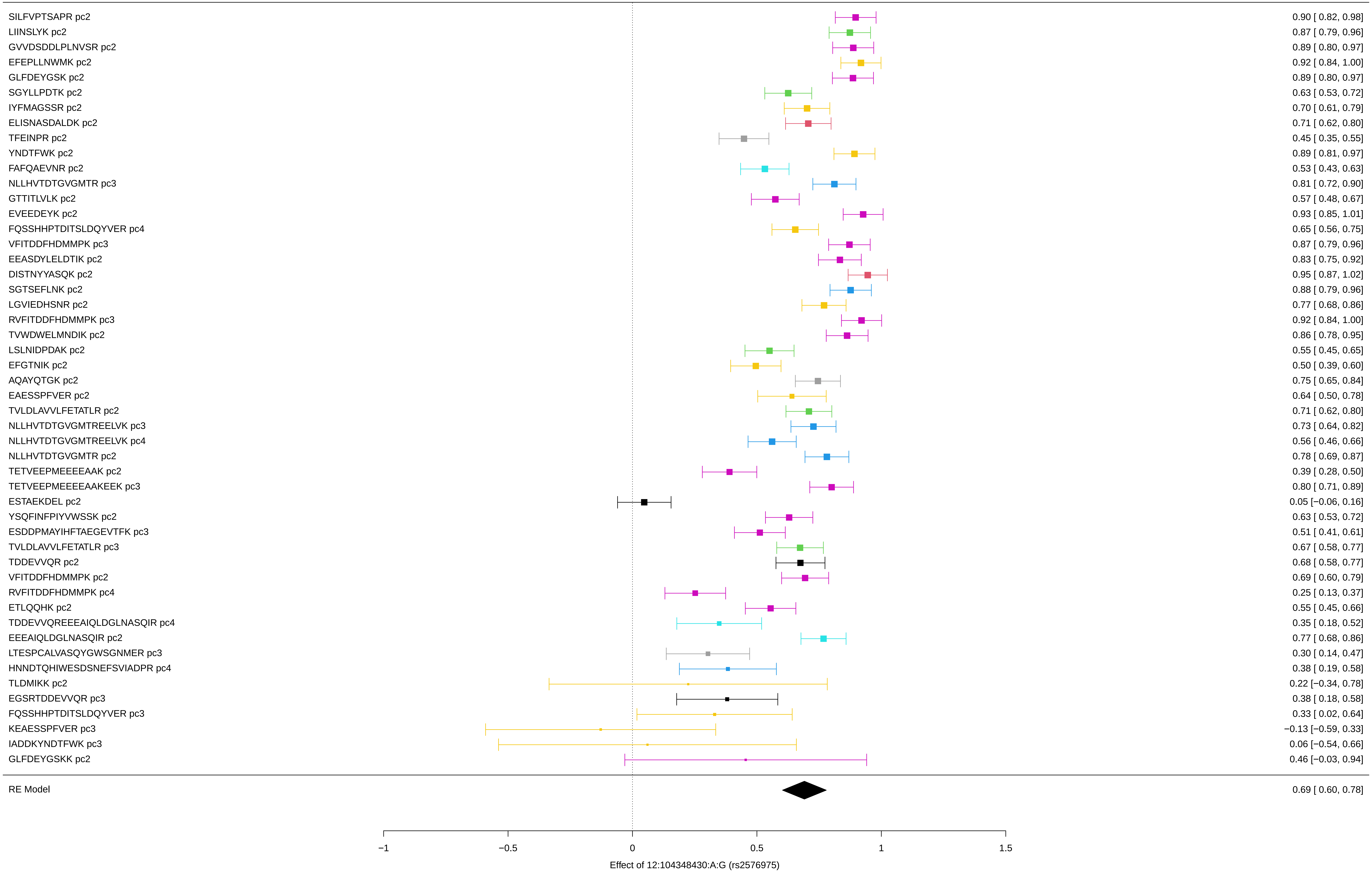

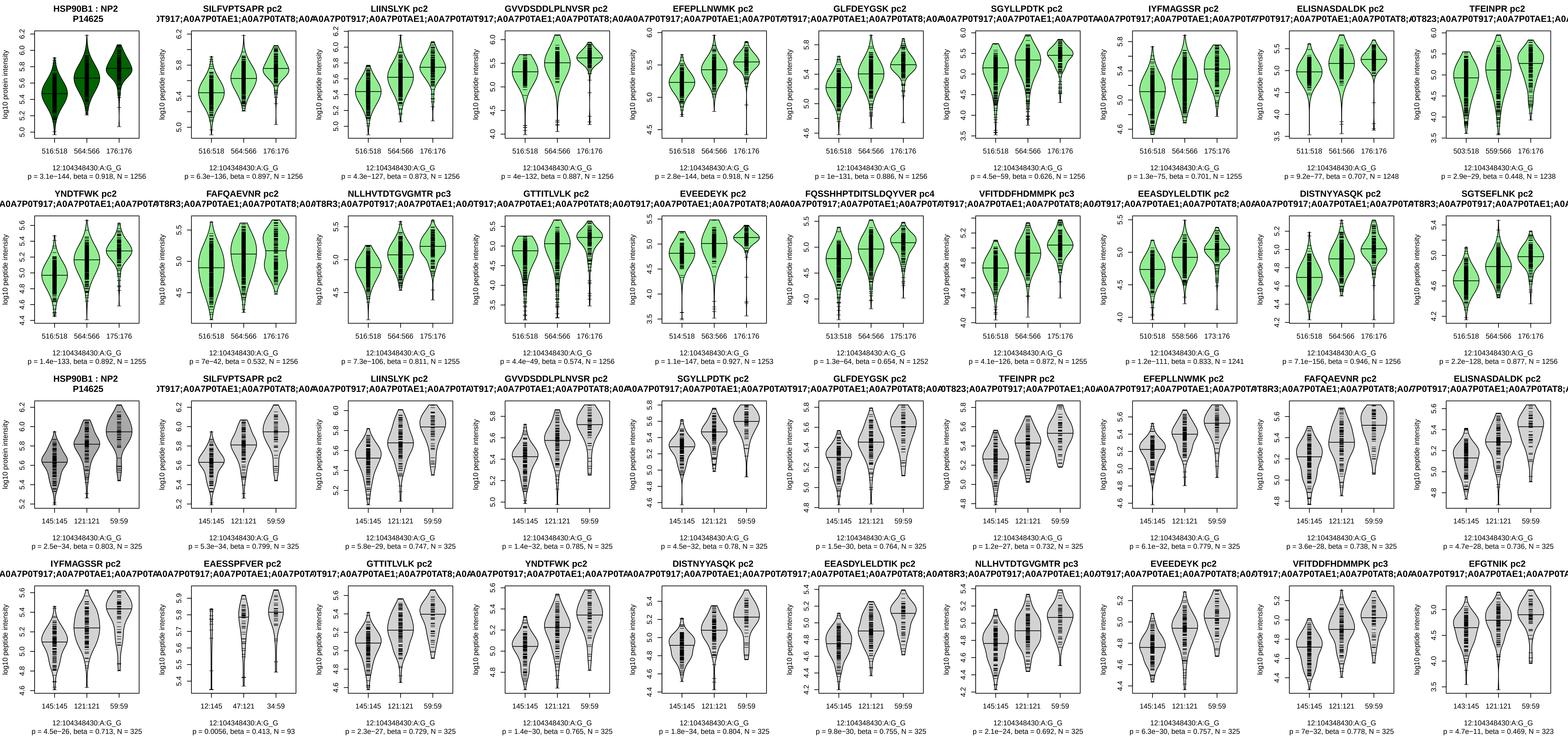

3Assay Target: HSP90B1
UniProt: P14625
rsID: rs2576975
Target SNP: 12:104348430:A:G
Proxy SNP: 12:104348430:A:G
Tarkin log10(p): 143.5
Tarkin BETA: 0.918
Tarkin MSPA: 0.973
1256:1256:1256:1256:1256:125

4. HP (P00738)
Tarkin MSPA=0.376

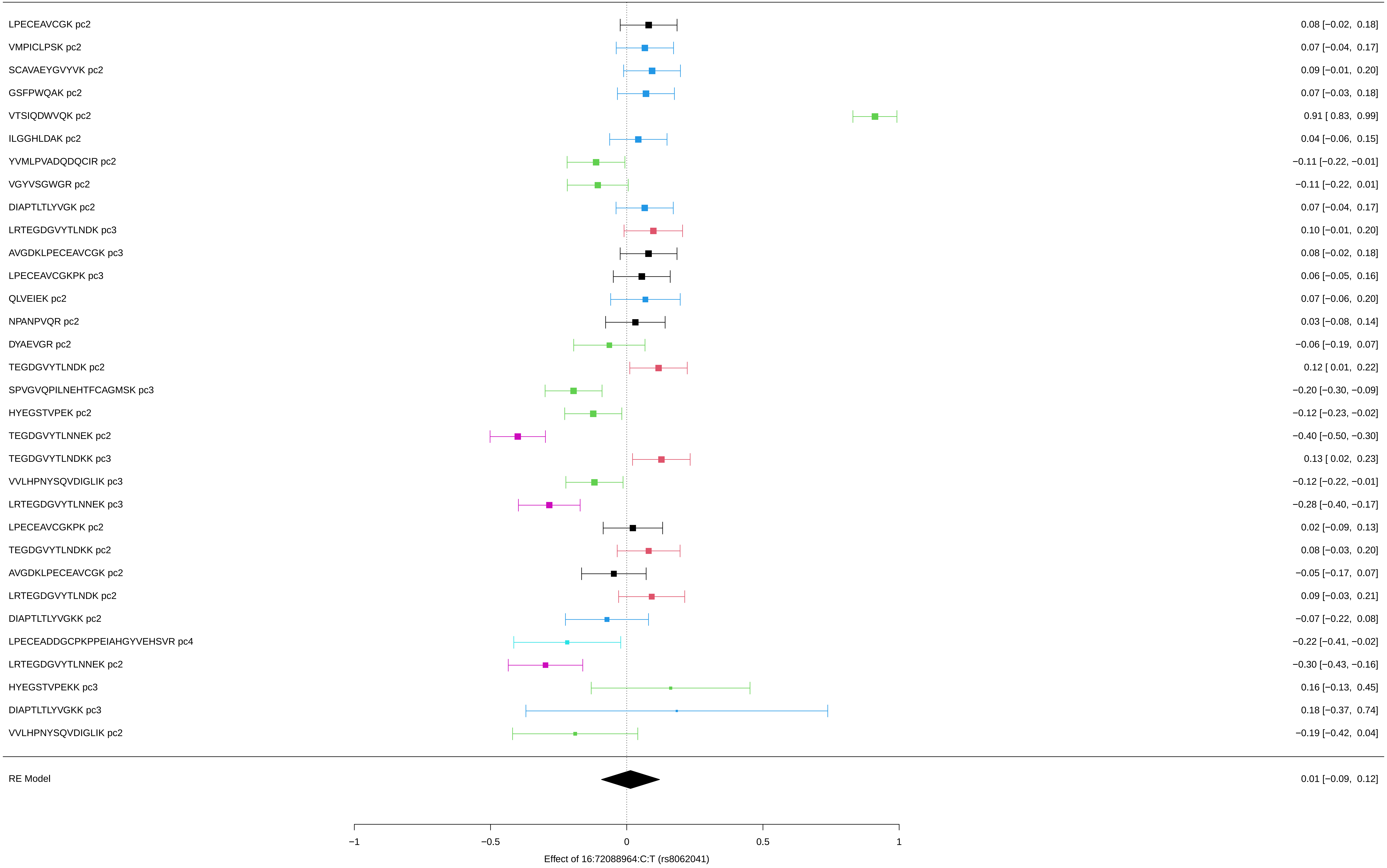

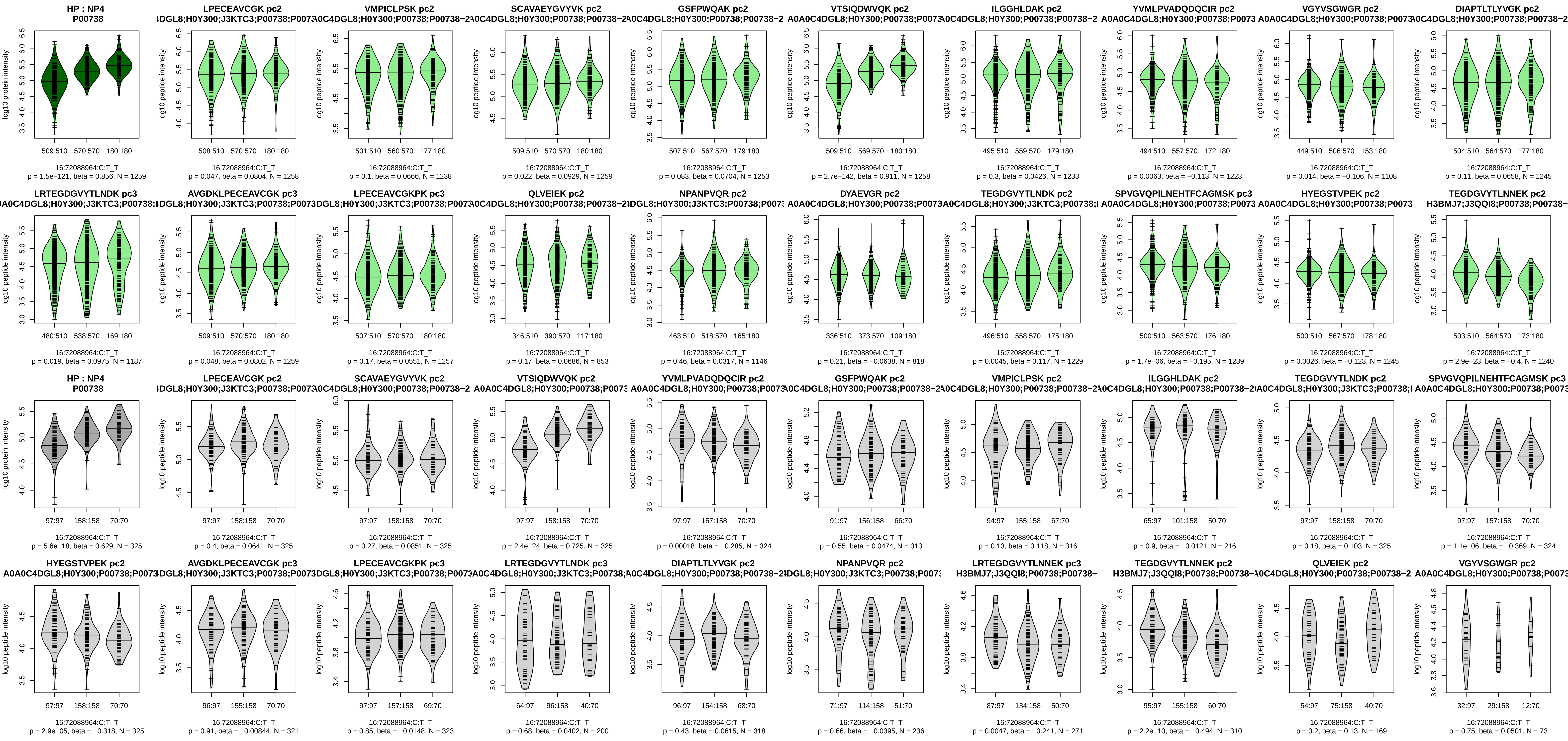

4Assay Target: HP
UniProt: P00738
rsID: rs8062041
Target SNP: 16:72088964:C:T
Proxy SNP: 16:72088964:C:T
Tarkin log10(p): 120.8
Tarkin BETA: 0.856
Tarkin MSPA: 0.376
1258:1238:1259:1253:1258:123

5. SFTPD (P35247)
Tarkin MSPA=1

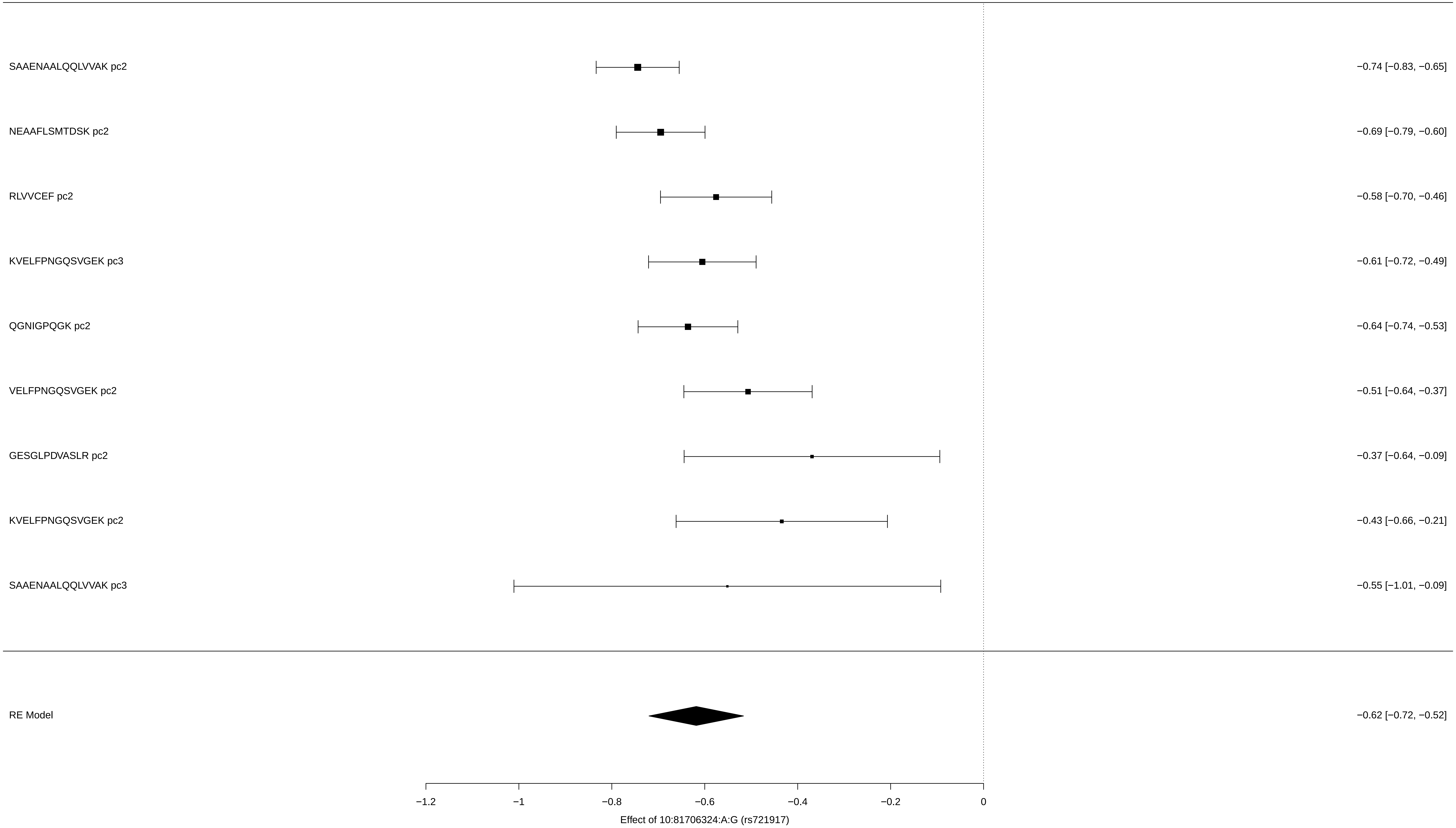

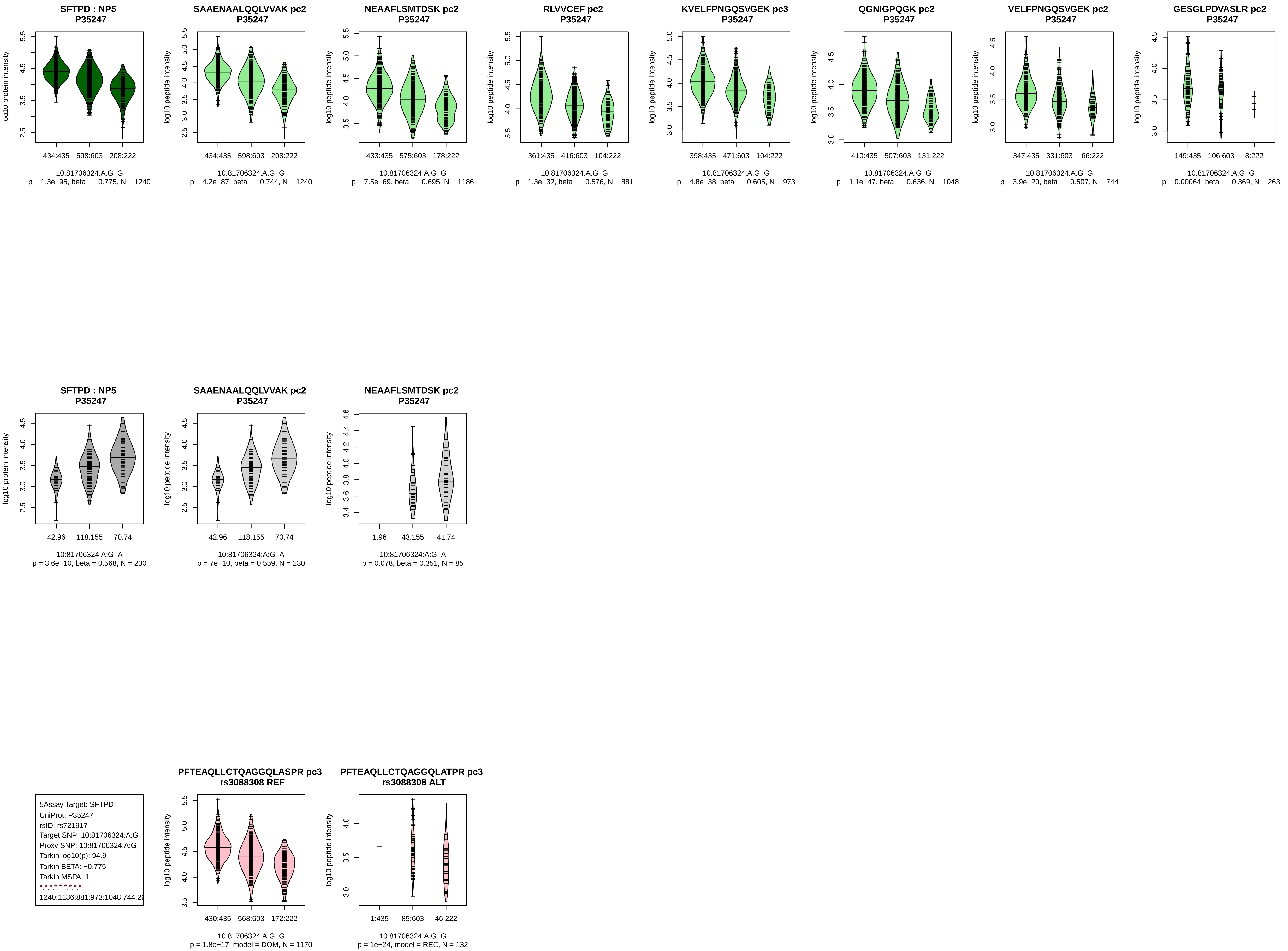

SFTPD : NP5
P35247
log10 protein intensity
434:435 598:603 208:222
10:81706324:A:G_G
p = 1.3e−95, beta = −0.775, N = 1240
SAAENAALQQLVVAK pc2
P35247
log10 peptide intensity
434:435 598:603 208:222
10:81706324:A:G_G
p = 4.2e−87, beta = −0.744, N = 1240
NEAAFLSMTDSK pc2
P35247
433:435 575:603 178:222
10:81706324:A:G_G
p = 7.5e−69, beta = −0.695, N = 1186
RLVVCEF pc2
P35247
361:435 416:603 104:222
10:81706324:A:G_G
p = 1.3e−32, beta = −0.576, N = 881
KVELFPNGQSVGEK pc3
P35247
398:435 471:603 104:222
10:81706324:A:G_G
p = 4.8e−38, beta = −0.605, N = 973
QGNIGPQGK pc2
P35247
410:435 507:603 131:222
10:81706324:A:G_G
p = 1.1e−47, beta = −0.636, N = 1048
VELFPNGQSVGEK pc2
P35247
347:435 331:603 66:222
10:81706324:A:G_G
p = 3.9e−20, beta = −0.507, N = 744
GESGLPDVASLR pc2
P35247
149:435 106:603 8:222
10:81706324:A:G_G
p = 0.00064, beta = −0.369, N = 263
SFTPD : NP5
P35247
42:96 118:155 70:74
10:81706324:A:G_A
p = 3.6e−10, beta = 0.568, N = 230
SAAENAALQQLVVAK pc2
P35247
42:96 118:155 70:74
10:81706324:A:G_A
p = 7e−10, beta = 0.559, N = 230
NEAAFLSMTDSK pc2
P35247
1:96 43:155 41:74
10:81706324:A:G_A
p = 0.078, beta = 0.351, N = 85
5Assay Target: SFTPD
UniProt: P35247
rsID: rs721917
Target SNP: 10:81706324:A:G
Proxy SNP: 10:81706324:A:G
Tarkin log10(p): 94.9
Tarkin BETA: −0.775
Tarkin MSPA: 1
1240:1186:881:973:1048:744:26
PFTEAQLLCTQAGGQLASPR pc3
rs3088308 REF
430:435 568:603 172:222
10:81706324:A:G_G
p = 1.8e−17, model = DOM, N = 1170
PFTEAQLLCTQAGGQLATPR pc3
rs3088308 ALT
1:435 85:603 46:222
10:81706324:A:G_G
p = 1e−24, model = REC, N = 132

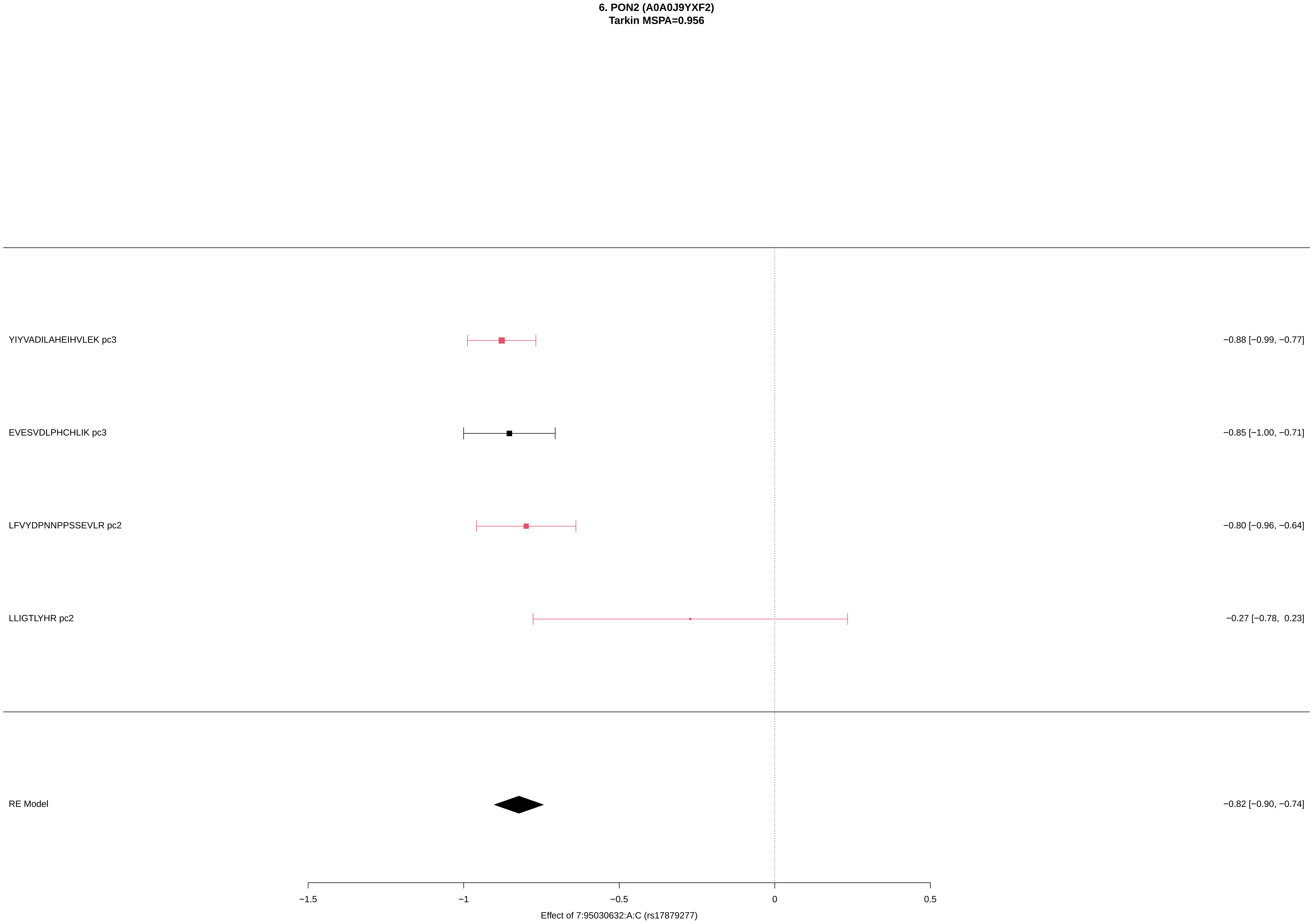
6. PON2 (A0A0J9YXF2)
Tarkin MSPA=0.956
YIYVADILAHEIHVLEK pc3
−0.88 [−0.99, −0.77]
EVESVDLPHCHLIK pc3
−0.85 [−1.00, −0.71]
LFVYDPNNPPSSEVLR pc2
−0.80 [−0.96, −0.64]
LLIGTLYHR pc2
−0.27 [−0.78, 0.23]
RE Model
−0.82 [−0.90, −0.74]
−1.5
−1
−0.5
0
0.5
Effect of 7:95030632:A:C (rs17879277)

PON2 : NP5
A0A0J9YXF2

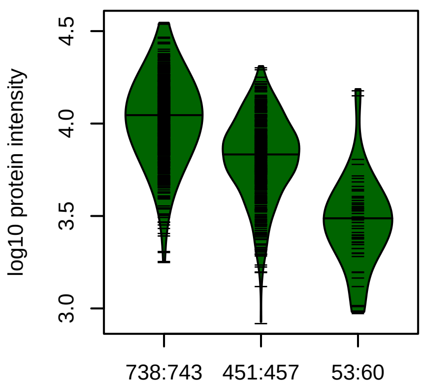

7:95030632:A:C_C
p = 2.3e−91, beta = −0.916, N = 1242

YIYVADILAHEIHVLEK pc3
A0A0J9YXF2;Q15165;Q15165−3

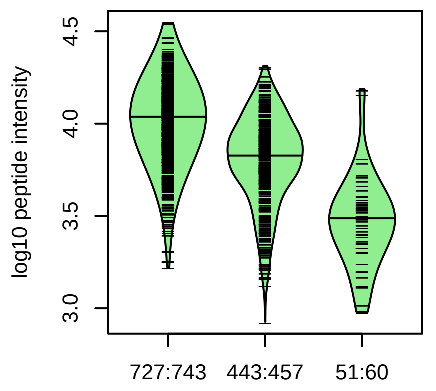

7:95030632:A:C_C
p = 7.1e−81, beta = −0.878, N = 1221

EVESVDLPHCHLIK pc3
A0A0J9YXM1;A0A2Q2SAK4;G3XAK4;

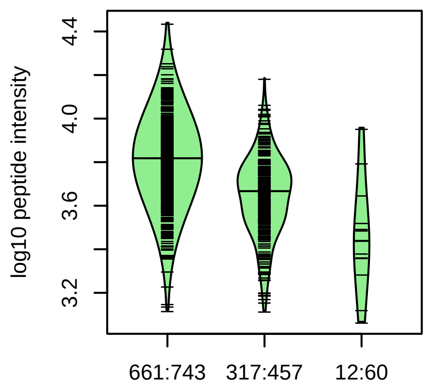

7:95030632:A:C_C
p = 1.2e−45, beta = −0.853, N = 990

LFVYDPNNPPSSEVLR pc2
A0A0J9YXF2;Q15165;Q15165−3

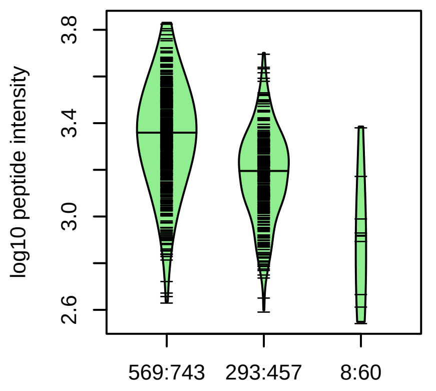

7:95030632:A:C_C
p = 8.2e−35, beta = −0.799, N = 870

LLIGTLYHR pc2
A0A0J9YXF2;Q15165;Q15165−3

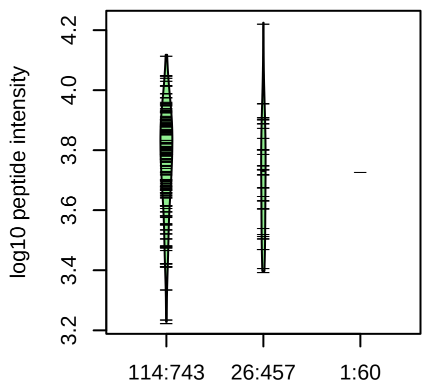

7:95030632:A:C_C
p = 0.17, beta = −0.271, N = 141

PON2 : NP5
A0A0J9YXF2

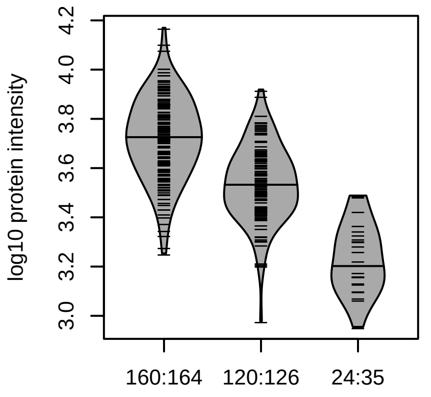

7:95030632:A:C_C
p = 7.2e−36, beta = −0.983, N = 304

YIYVADILAHEIHVLEK pc3
A0A0J9YXF2;Q15165;Q15165−3

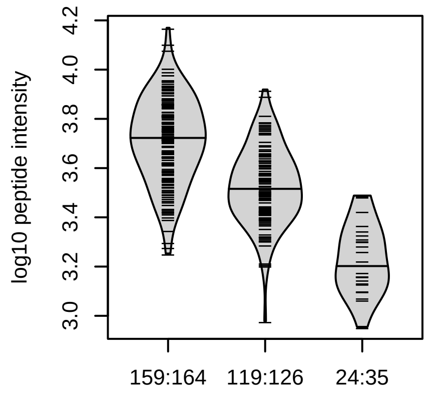

7:95030632:A:C_C
p = 3.3e−34, beta = −0.966, N = 302

LFVYDPNNPPSSEVLR pc2
A0A0J9YXF2;Q15165;Q15165−3

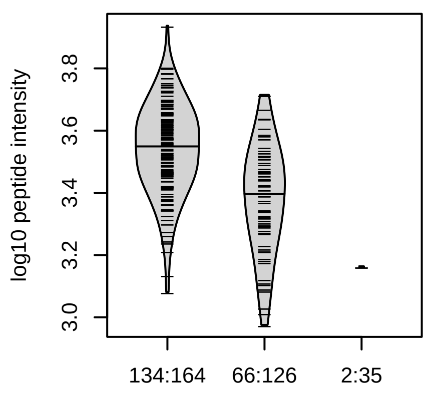

7:95030632:A:C_C
p = 1.5e−12, beta = −0.927, N = 202

6Assay Target: PON2
UniProt: A0A0J9YXF2
rsID: rs17879277
Target SNP: 7:95030632:A:C
Proxy SNP: 7:95030632:A:C
Tarkin log10(p): 90.6
Tarkin BETA: −0.916
Tarkin MSPA: 0.956
::*:−
1221:990:870:141

FEEAENSLLHLK pc3
rs12026 REF

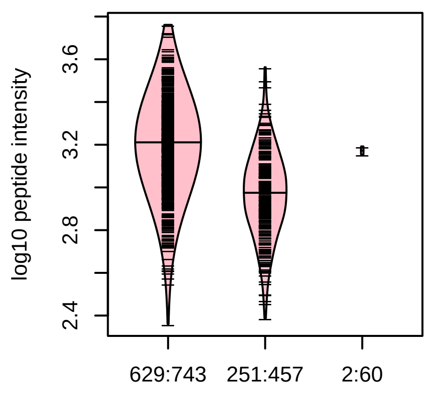

7:95030632:A:C_C
p = 4.9e−42, model = REC, N = 882

IQNILSEK pc2
rs7493 REF

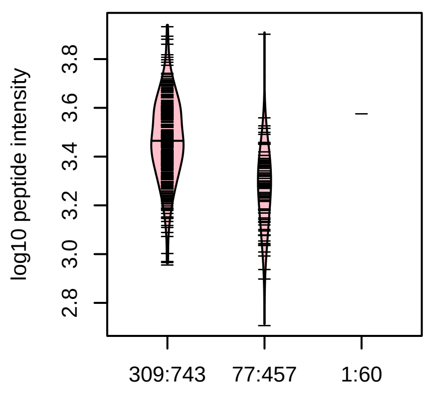

7:95030632:A:C_C
p = 6.8e−25, model = REC, N = 387

FEEAENSLLHLK pc2
rs12026 REF

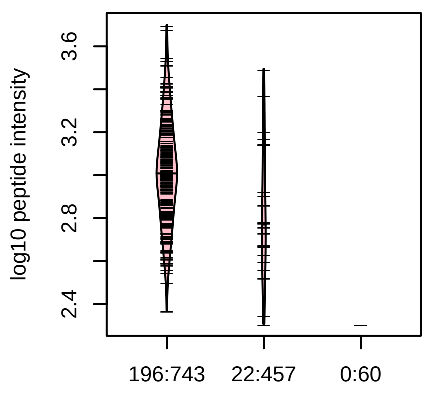

7:95030632:A:C_C
p = 5.6e−28, model = REC, N = 218

7. MST1 (G3XAK1)
Tarkin MSPA=0.996

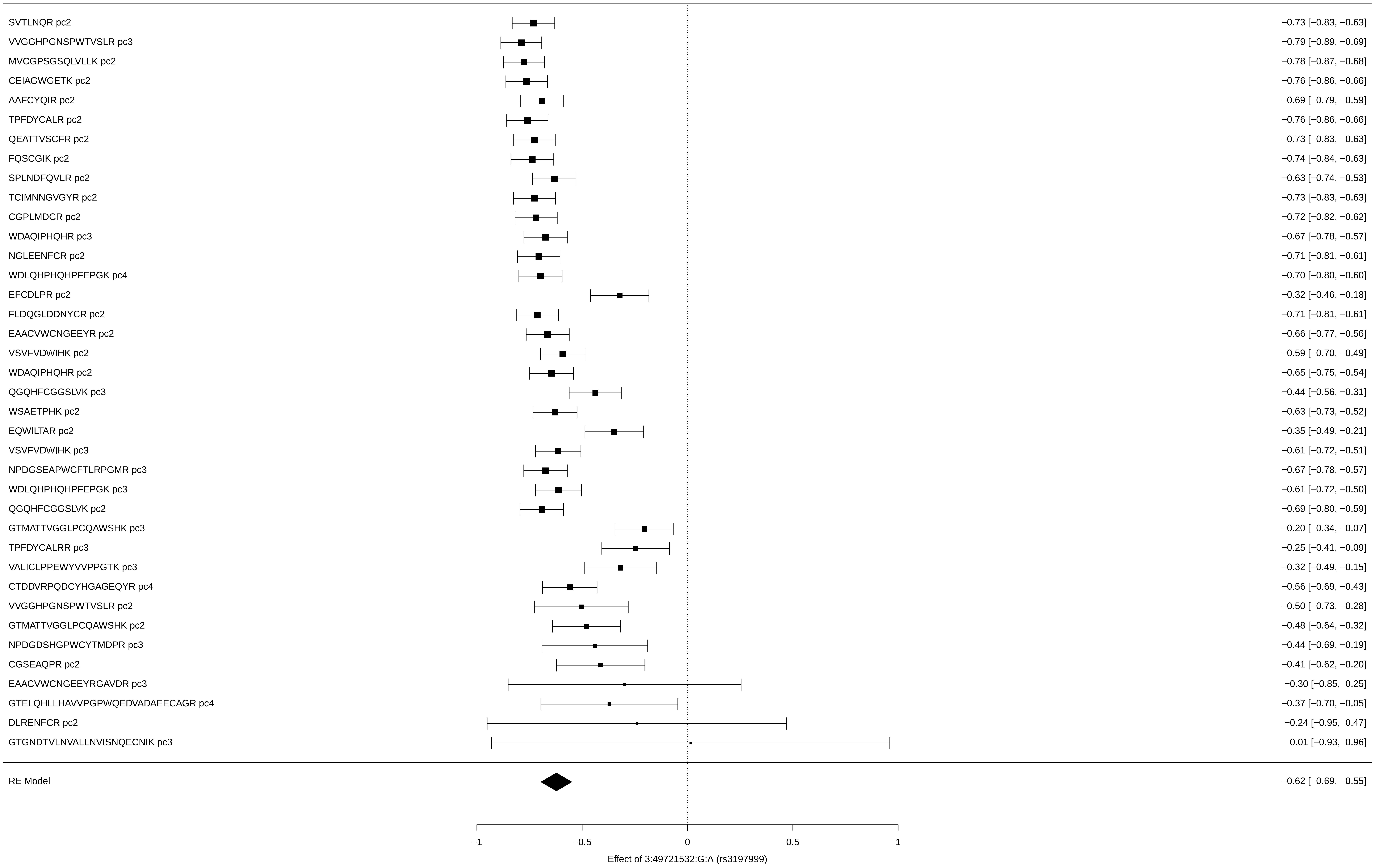

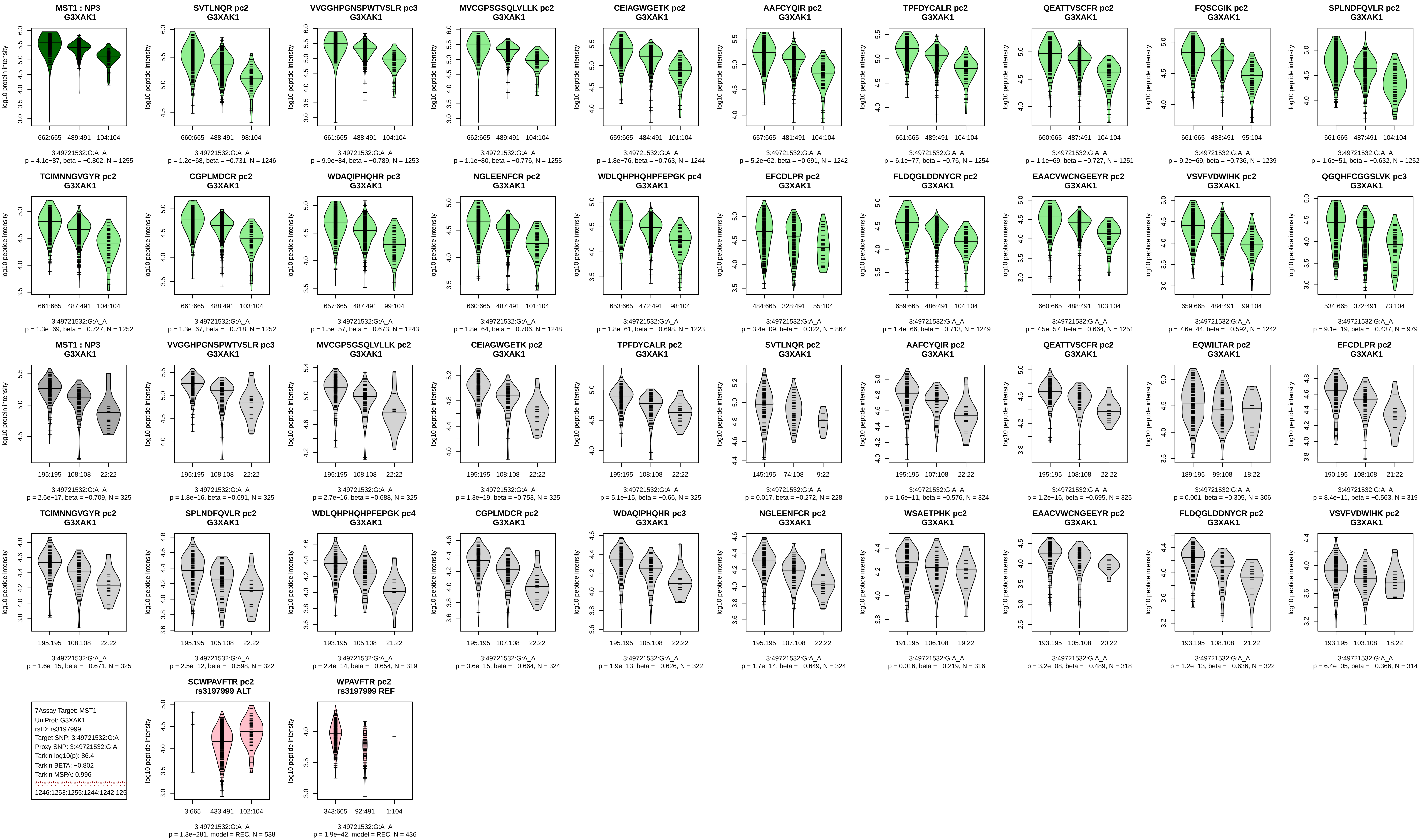
MST1 : NP3
G3XAK1
log10 protein intensity
662:665 489:491 104:104
3:49721532:G:A_A
p = 4.1e−87, beta = −0.802, N = 1255
SVTLNQR pc2
G3XAK1
log10 peptide intensity
660:665 488:491 98:104
3:49721532:G:A_A
p = 1.2e−68, beta = −0.731, N = 1246
VVGGHPGNSPWTVSLR pc3
G3XAK1
661:665 488:491 104:104
3:49721532:G:A_A
p = 9.9e−84, beta = −0.789, N = 1253
MVCGPSGSGQLVLLK pc2
G3XAK1
662:665 489:491 104:104
3:49721532:G:A_A
p = 1.1e−80, beta = −0.776, N = 1255
CEIAGWGETK pc2
G3XAK1
659:665 484:491 101:104
3:49721532:G:A_A
p = 1.8e−76, beta = −0.763, N = 1244
AAFCYQIR pc2
G3XAK1
657:665 481:491 104:104
3:49721532:G:A_A
p = 5.2e−62, beta = −0.691, N = 1242
TPFDYCALR pc2
G3XAK1
661:665 489:491 104:104
3:49721532:G:A_A
p = 6.1e−77, beta = −0.76, N = 1254
QEATTVSCFR pc2
G3XAK1
660:665 487:491 104:104
3:49721532:G:A_A
p = 1.1e−69, beta = −0.727, N = 1251
FQSCGIK pc2
G3XAK1
661:665 483:491 95:104
3:49721532:G:A_A
p = 9.2e−69, beta = −0.736, N = 1239
SPLNDFQVLR pc2
G3XAK1
661:665 487:491 104:104
3:49721532:G:A_A
p = 1.6e−51, beta = −0.632, N = 1252
TCIMNNGVGYR pc2
G3XAK1
661:665 487:491 104:104
3:49721532:G:A_A
p = 1.3e−69, beta = −0.727, N = 1252
CGPLMDCR pc2
G3XAK1
661:665 488:491 103:104
3:49721532:G:A_A
p = 1.3e−67, beta = −0.718, N = 1252
WDAQIPHQHR pc3
G3XAK1
657:665 487:491 99:104
3:49721532:G:A_A
p = 1.5e−57, beta = −0.673, N = 1243
NGLEENFCR pc2
G3XAK1
660:665 487:491 101:104
3:49721532:G:A_A
p = 1.8e−64, beta = −0.706, N = 1248
WDLQHPHQHPFEPGK pc4
G3XAK1
653:665 472:491 98:104
3:49721532:G:A_A
p = 1.8e−61, beta = −0.698, N = 1223
EFCDLPR pc2
G3XAK1
484:665 328:491 55:104
3:49721532:G:A_A
p = 3.4e−09, beta = −0.322, N = 867
FLDQGLDDNYCR pc2
G3XAK1
659:665 486:491 104:104
3:49721532:G:A_A
p = 1.4e−66, beta = −0.713, N = 1249
EAACVWCNGEEYR pc2
G3XAK1
660:665 488:491 103:104
3:49721532:G:A_A
p = 7.5e−57, beta = −0.664, N = 1251
VSVFVDWIHK pc2
G3XAK1
659:665 484:491 99:104
3:49721532:G:A_A
p = 7.6e−44, beta = −0.592, N = 1242
QGQHFCGGSLVK pc3
G3XAK1
534:665 372:491 73:104
3:49721532:G:A_A
p = 9.1e−19, beta = −0.437, N = 979
MST1 : NP3
G3XAK1
195:195 108:108 22:22
3:49721532:G:A_A
p = 2.6e−17, beta = −0.709, N = 325
VVGGHPGNSPWTVSLR pc3
G3XAK1
195:195 108:108 22:22
3:49721532:G:A_A
p = 1.8e−16, beta = −0.691, N = 325
MVCGPSGSGQLVLLK pc2
G3XAK1
195:195 108:108 22:22
3:49721532:G:A_A
p = 2.7e−16, beta = −0.688, N = 325
CEIAGWGETK pc2
G3XAK1
195:195 108:108 22:22
3:49721532:G:A_A
p = 1.3e−19, beta = −0.753, N = 325
TPFDYCALR pc2
G3XAK1
195:195 108:108 22:22
3:49721532:G:A_A
p = 5.1e−15, beta = −0.66, N = 325
SVTLNQR pc2
G3XAK1
145:195 74:108 9:22
3:49721532:G:A_A
p = 0.017, beta = −0.272, N = 228
AAFCYQIR pc2
G3XAK1
195:195 107:108 22:22
3:49721532:G:A_A
p = 1.6e−11, beta = −0.576, N = 324
QEATTVSCFR pc2
G3XAK1
195:195 108:108 22:22
3:49721532:G:A_A
p = 1.2e−16, beta = −0.695, N = 325
EQWILTAR pc2
G3XAK1
189:195 99:108 18:22
3:49721532:G:A_A
p = 0.001, beta = −0.305, N = 306
EFCDLPR pc2
G3XAK1
190:195 108:108 21:22
3:49721532:G:A_A
p = 8.4e−11, beta = −0.563, N = 319
TCIMNNGVGYR pc2
G3XAK1
195:195 108:108 22:22
3:49721532:G:A_A
p = 1.6e−15, beta = −0.671, N = 325
SPLNDFQVLR pc2
G3XAK1
195:195 105:108 22:22
3:49721532:G:A_A
p = 2.5e−12, beta = −0.598, N = 322
WDLQHPHQHPFEPGK pc4
G3XAK1
193:195 105:108 21:22
3:49721532:G:A_A
p = 2.4e−14, beta = −0.654, N = 319
CGPLMDCR pc2
G3XAK1
195:195 107:108 22:22
3:49721532:G:A_A
p = 3.6e−15, beta = −0.664, N = 324
WDAQIPHQHR pc3
G3XAK1
195:195 105:108 22:22
3:49721532:G:A_A
p = 1.9e−13, beta = −0.626, N = 322
NGLEENFCR pc2
G3XAK1
195:195 107:108 22:22
3:49721532:G:A_A
p = 1.7e−14, beta = −0.649, N = 324
WSAETPHK pc2
G3XAK1
191:195 106:108 19:22
3:49721532:G:A_A
p = 0.016, beta = −0.219, N = 316
EAACVWCNGEEYR pc2
G3XAK1
193:195 105:108 20:22
3:49721532:G:A_A
p = 3.2e−08, beta = −0.489, N = 318
FLDQGLDDNYCR pc2
G3XAK1
193:195 108:108 21:22
3:49721532:G:A_A
p = 1.2e−13, beta = −0.636, N = 322
VSVFVDWIHK pc2
G3XAK1
193:195 103:108 18:22
3:49721532:G:A_A
p = 6.4e−05, beta = −0.366, N = 314
7Assay Target: MST1
UniProt: G3XAK1
rsID: rs3197999
Target SNP: 3:49721532:G:A
Proxy SNP: 3:49721532:G:A
Tarkin log10(p): 86.4
Tarkin BETA: −0.802
Tarkin MSPA: 0.996
1246:1253:1255:1244:1242:125
SCWPAVFTR pc2
rs3197999 ALT
3:665 433:491 102:104
3:49721532:G:A_A
p = 1.3e−281, model = REC, N = 538
WPAVFTR pc2
rs3197999 REF
343:665 92:491 1:104
3:49721532:G:A_A
p = 1.9e−42, model = REC, N = 436

8. VMO1 (Q7Z5L0)
Tarkin MSPA=0.878

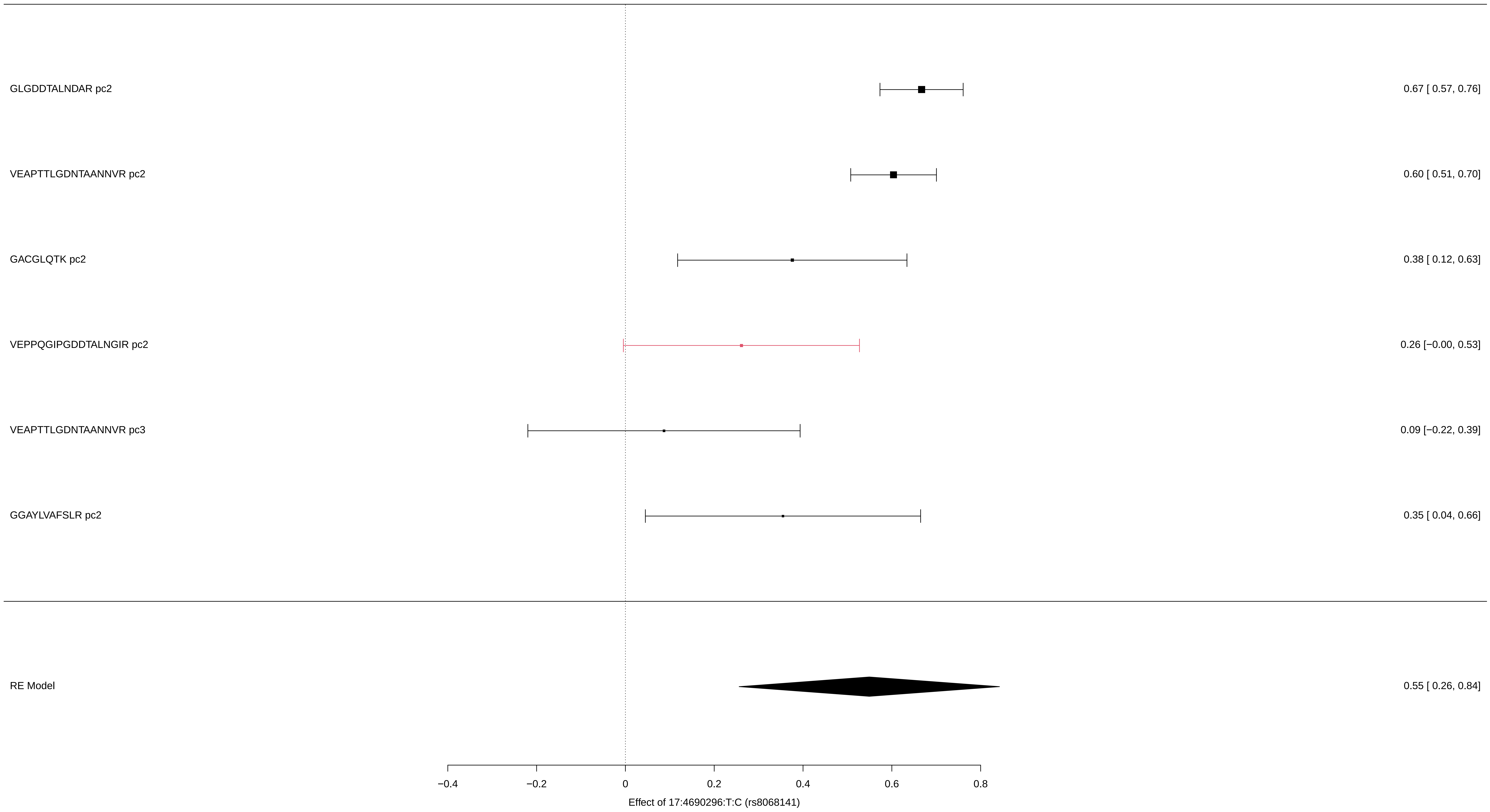

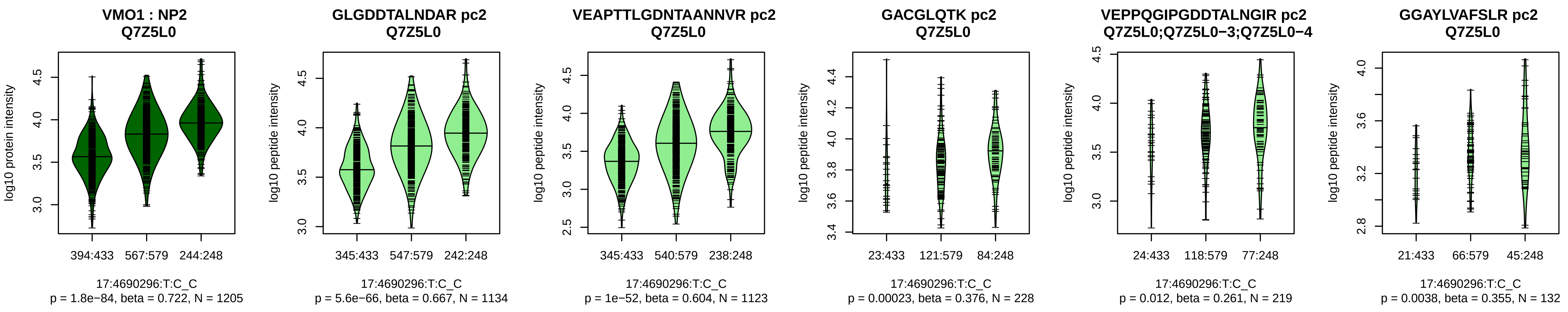

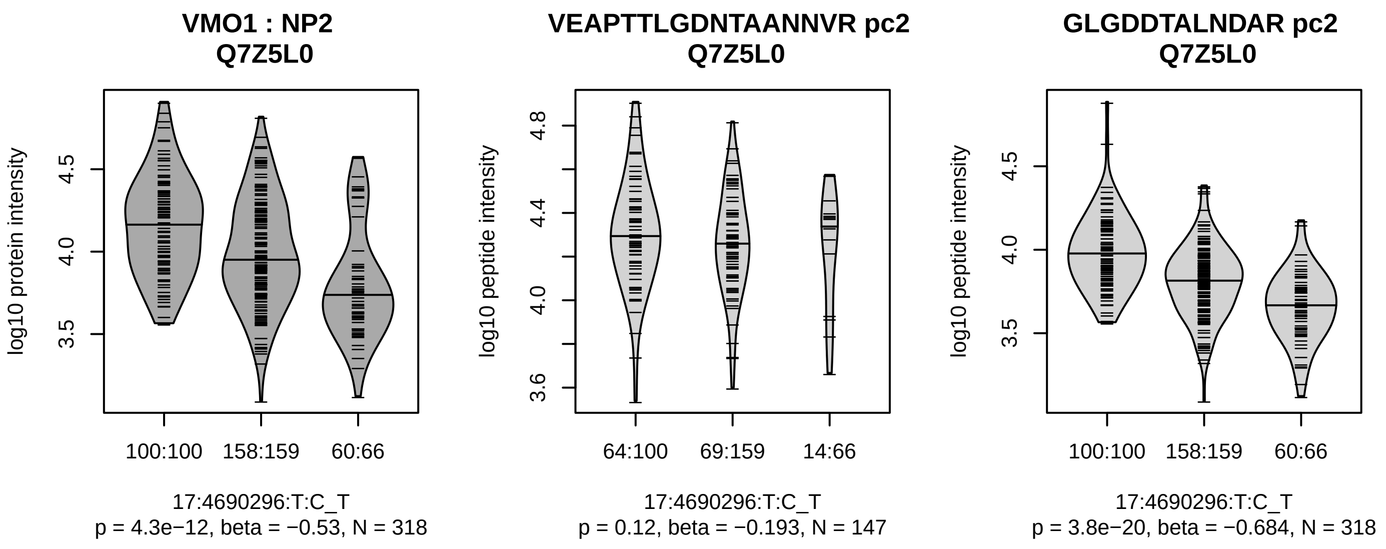

8Assay Target: VMO1
UniProt: Q7Z5L0
rsID: rs8068141
Target SNP: 17:4690296:T:C
Proxy SNP: 17:4690296:T:C
Tarkin log10(p): 83.7
Tarkin BETA: 0.722
Tarkin MSPA: 0.878
::*:–:–:*
1134:1123:228:219:144:132

9. MBL2 (P11226)
Tarkin MSPA=1

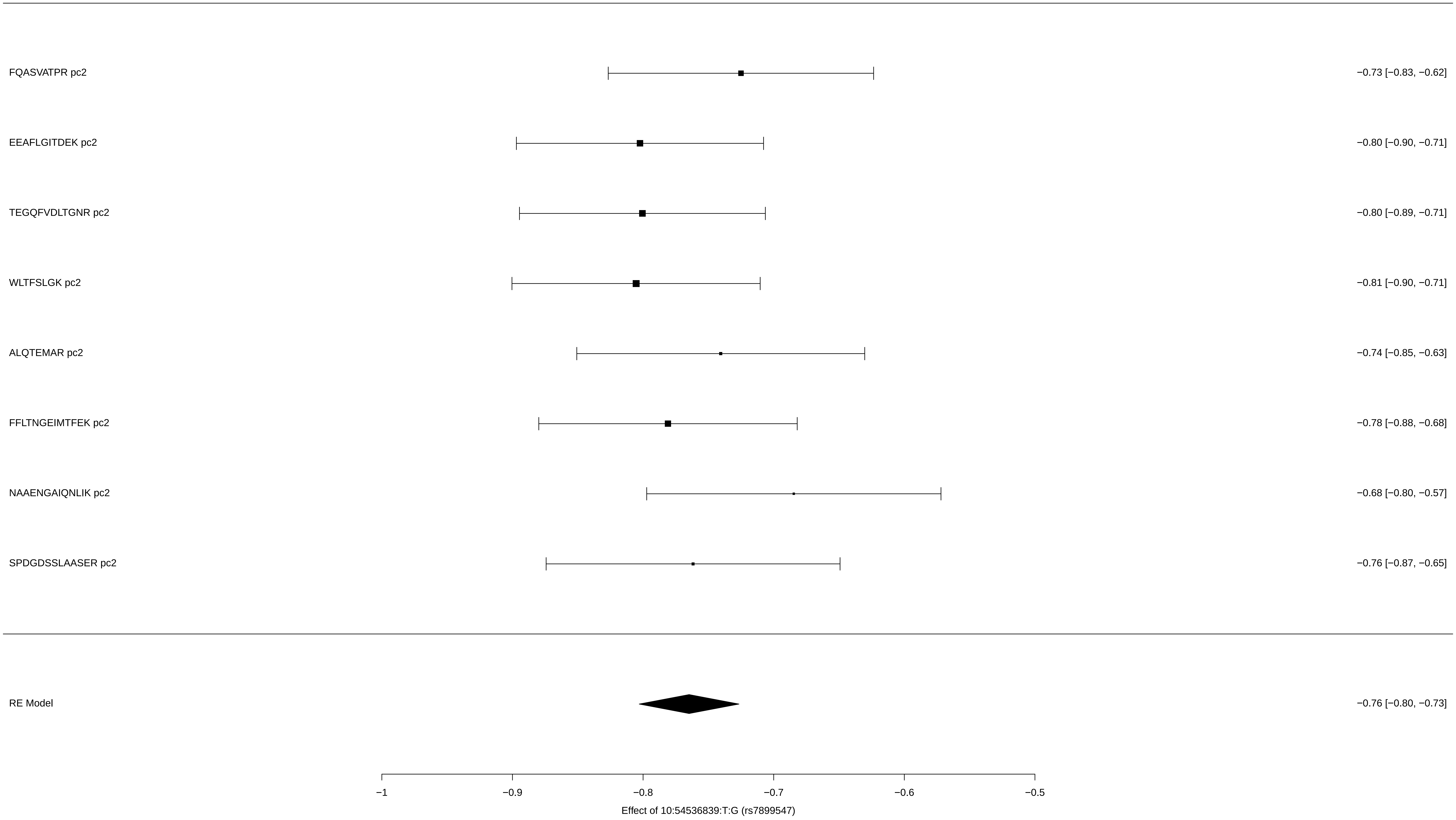

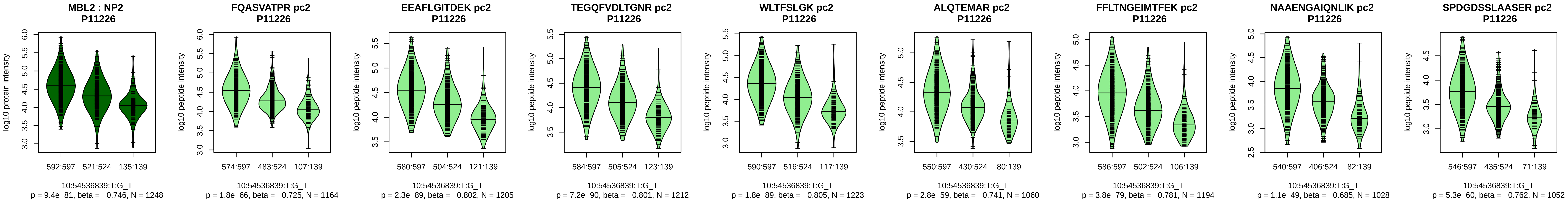

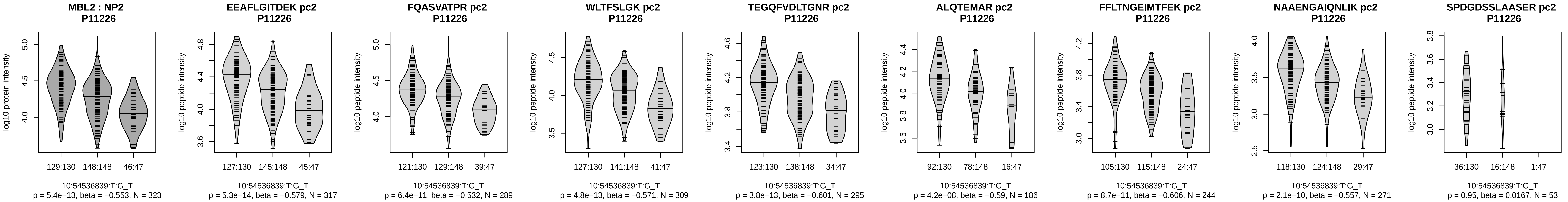

9Assay Target: MBL2
UniProt: P11226
rsID: rs7899547
Target SNP: 10:54536839:T:G
Proxy SNP: 10:54536839:T:G
Tarkin log10(p): 80
Tarkin BETA: −0.746
Tarkin MSPA: 1
::*:*:*:*:*:*:NA
1164:1205:1212:1223:1060:119

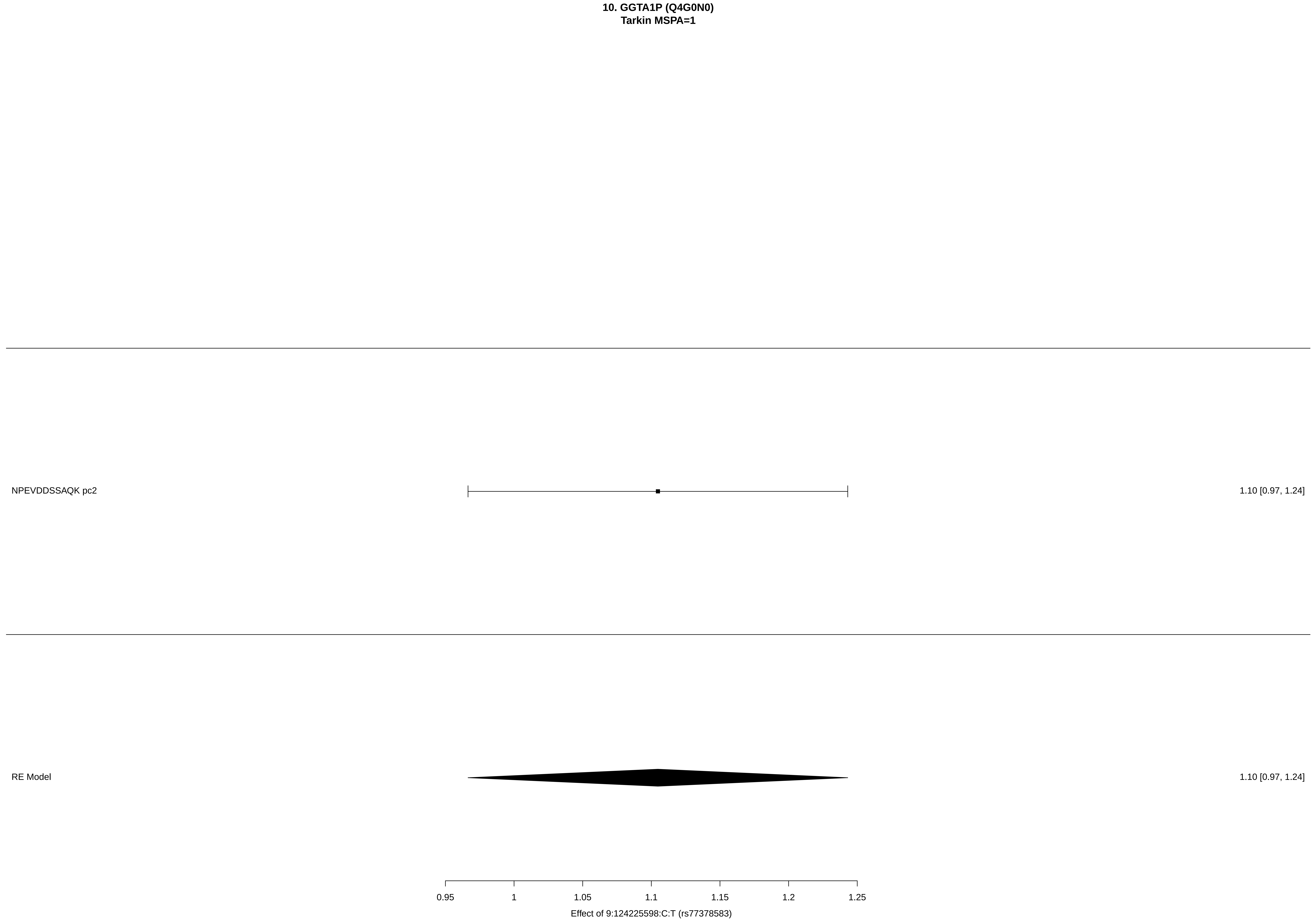
10. GGTA1P (Q4G0N0)
Tarkin MSPA=1
NPEVDDSSAQK pc2
1.10 [0.97, 1.24]
RE Model
1.10 [0.97, 1.24]
0.95
1
1.05
1.1
1.15
1.2
1.25
Effect of 9:124225598:C:T (rs77378583)

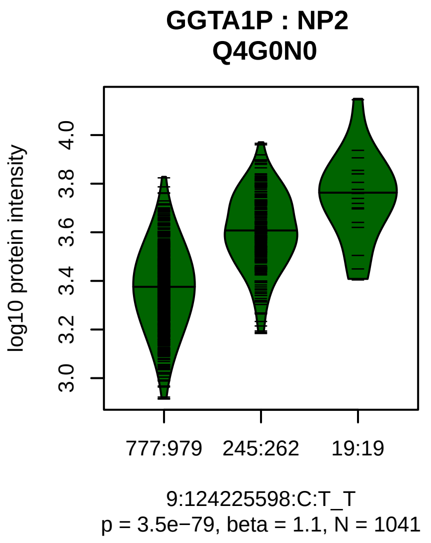

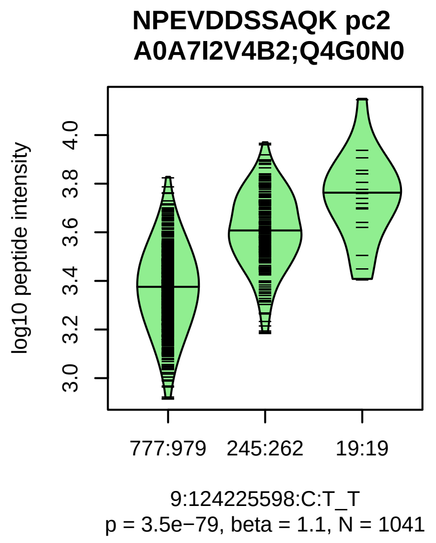

10Assay Target: GGTA1P
UniProt: Q4G0N0
rsID: rs77378583
Target SNP: 9:124225598:C:T
Proxy SNP: 9:124225598:C:T
Tarkin log10(p): 78.5
Tarkin BETA: 1.105
Tarkin MSPA: 1
*
1041

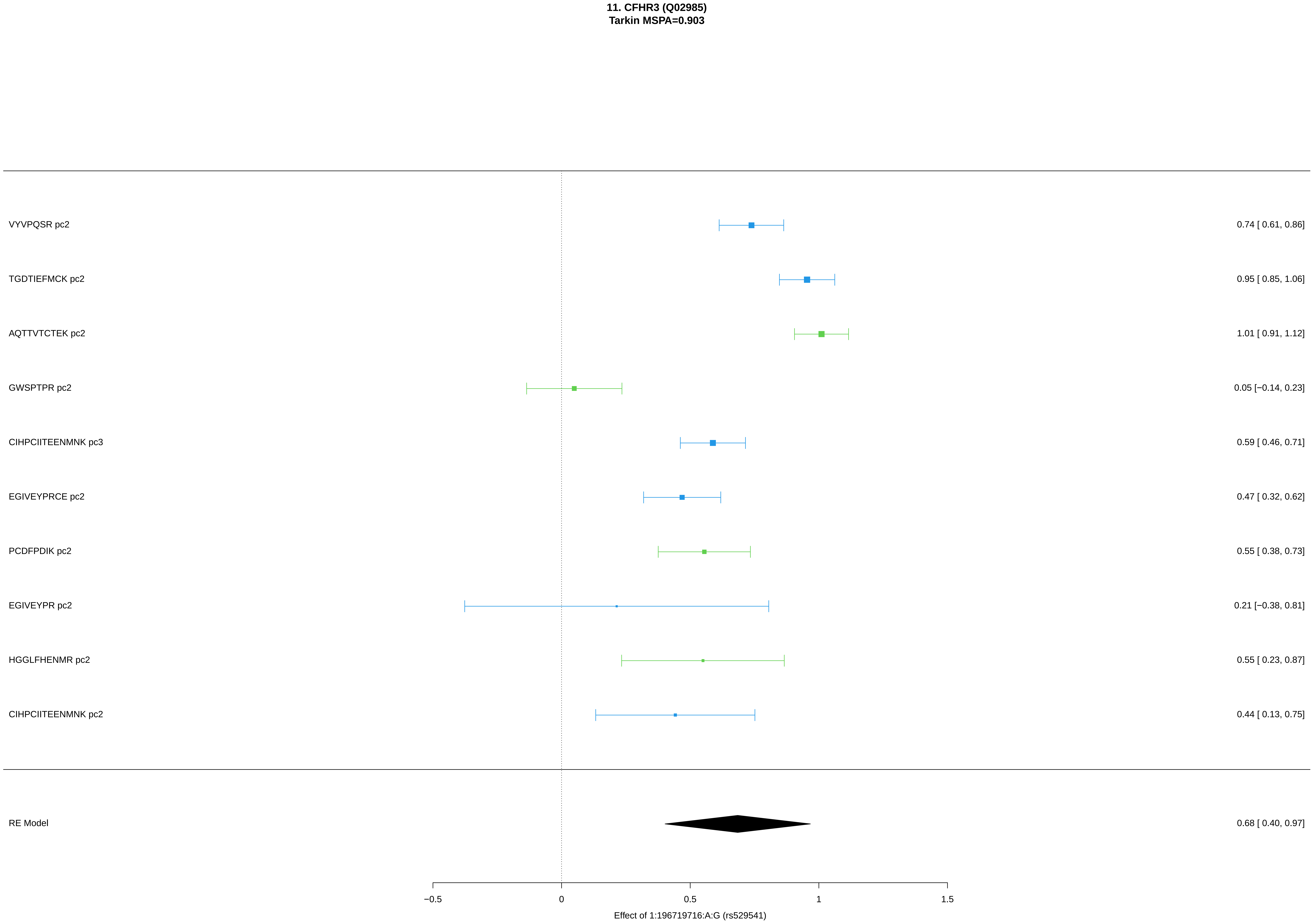
11. CFHR3 (Q02985)
Tarkin MSPA=0.903
VYVPQSR pc2
0.74 [0.61, 0.86]
TGDTIEFMCK pc2
0.95 [0.85, 1.06]
AQTTVTCTEK pc2
1.01 [0.91, 1.12]
GWSPTPR pc2
0.05 [−0.14, 0.23]
CIHPCIITEENMNK pc3
0.59 [0.46, 0.71]
EGIVEYPRCE pc2
0.47 [0.32, 0.62]
PCDFPDIK pc2
0.55 [0.38, 0.73]
EGIVEYPR pc2
0.21 [−0.38, 0.81]
HGGLFHENMR pc2
0.55 [0.23, 0.87]
CIHPCIITEENMNK pc2
0.44 [0.13, 0.75]
RE Model
0.68 [0.40, 0.97]
−0.5
0
0.5
1
1.5
Effect of 1:196719716:A:G (rs529541)

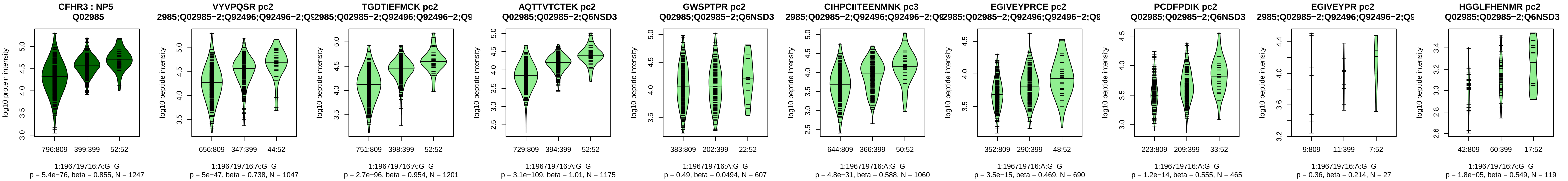

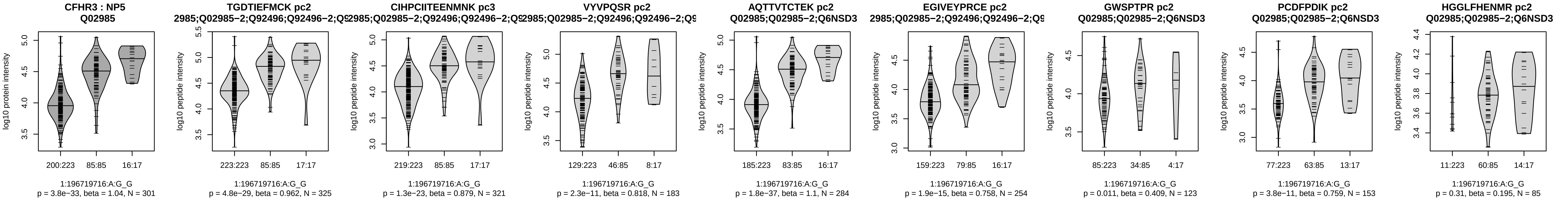

11Assay Target: CFHR3
UniProt: Q02985
rsID: rs529541
Target SNP: 1:196719716:A:G
Proxy SNP: 1:196719716:A:G
Tarkin log10(p): 75.3
Tarkin BETA: 0.855
Tarkin MSPA: 0.903
::*:−:*:*:*:−:*:*:NA:NA
1047:1201:1175:607:1060:690:4

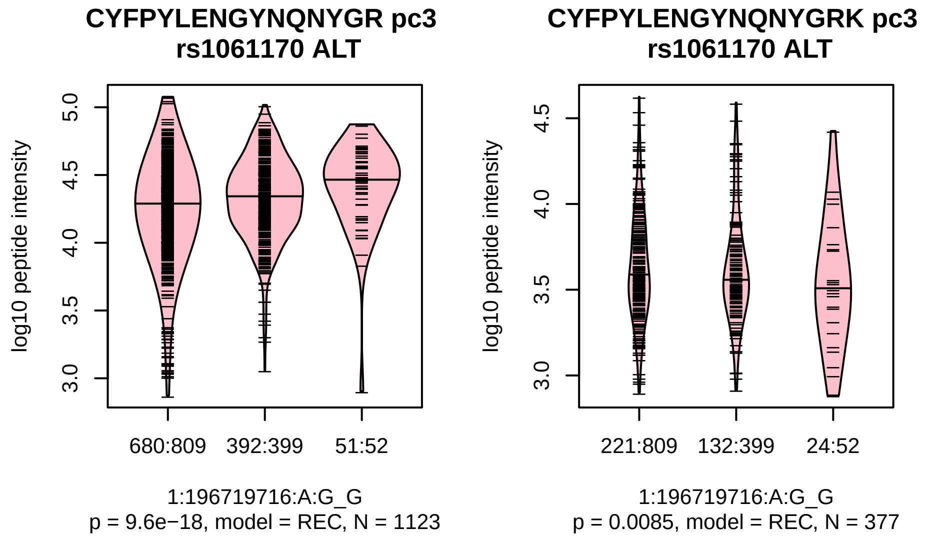

12. FN3K (Q9H479)
Tarkin MSPA=0.963

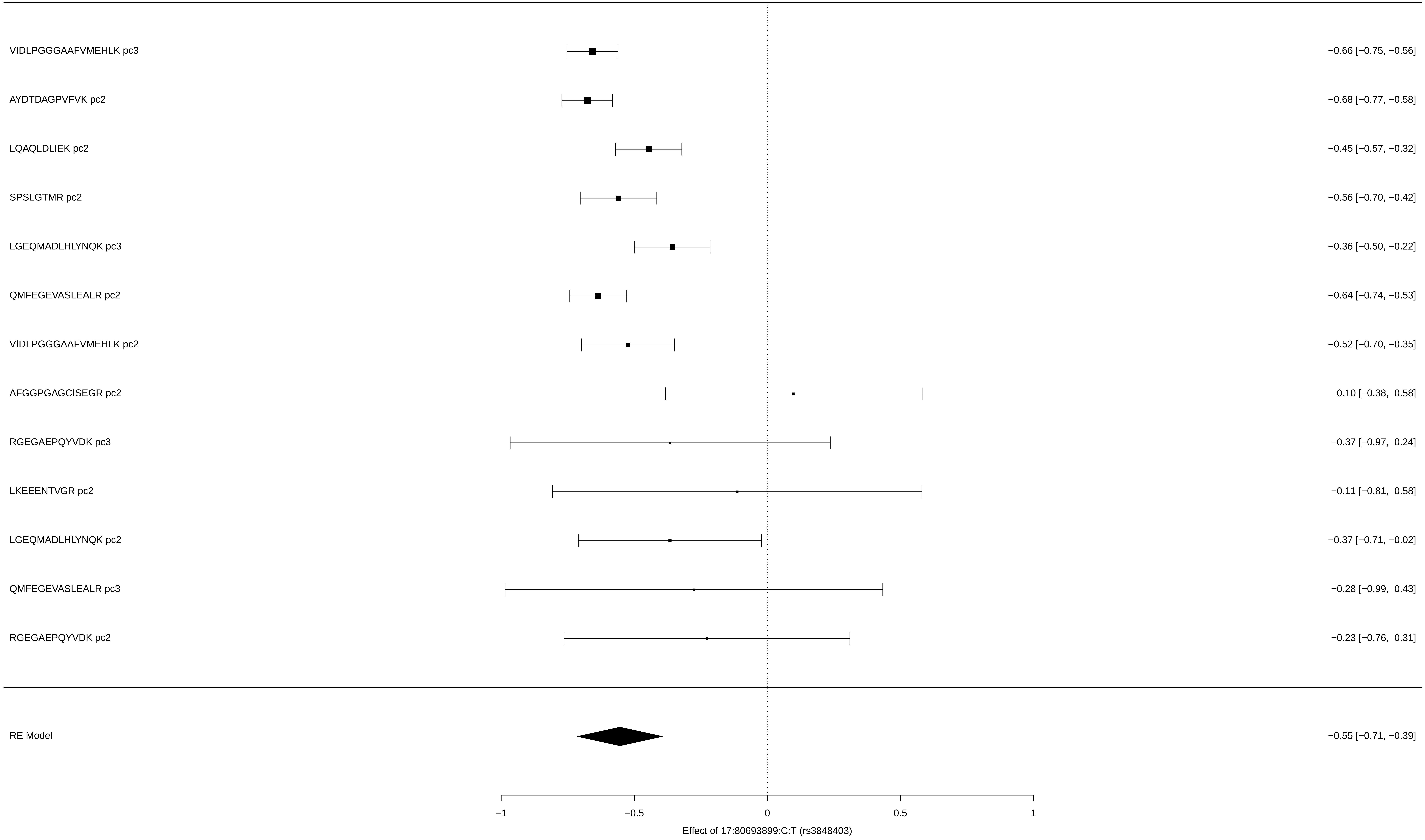

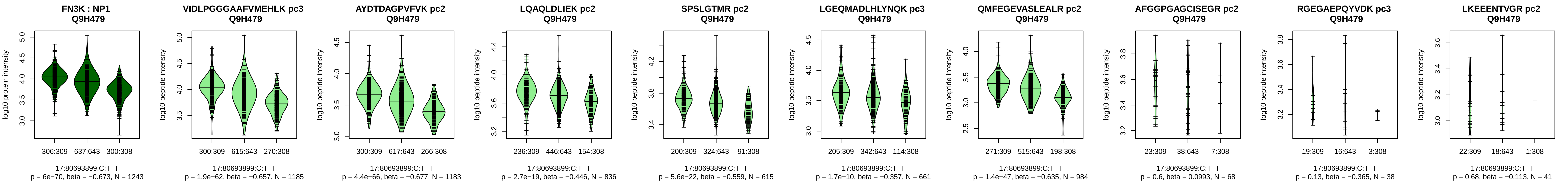

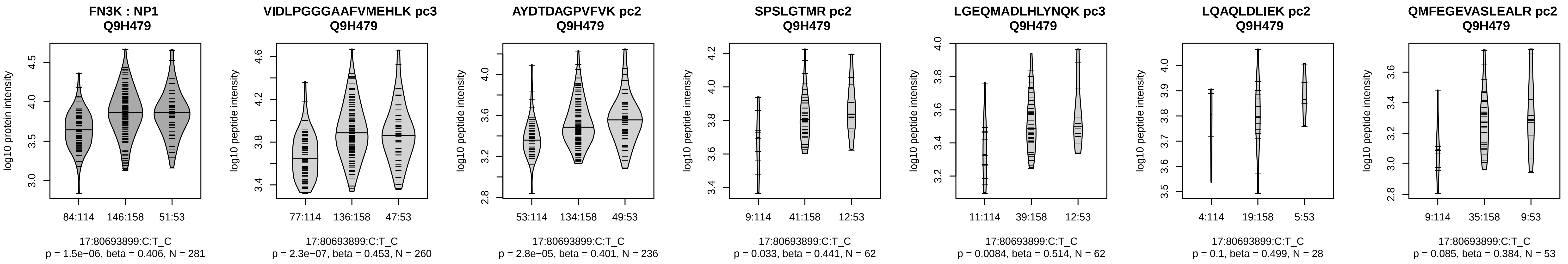

12Assay Target: FN3K
UniProt: Q9H479
rsID: rs3848403
Target SNP: 17:80693899:C:T
Proxy SNP: 17:80693899:C:T
Tarkin log10(p): 69.2
Tarkin BETA: −0.673
Tarkin MSPA: 0.963
::*:*:*:*:*:–:–:–:*:–:–:NA
1185:1183:836:615:661:984:415

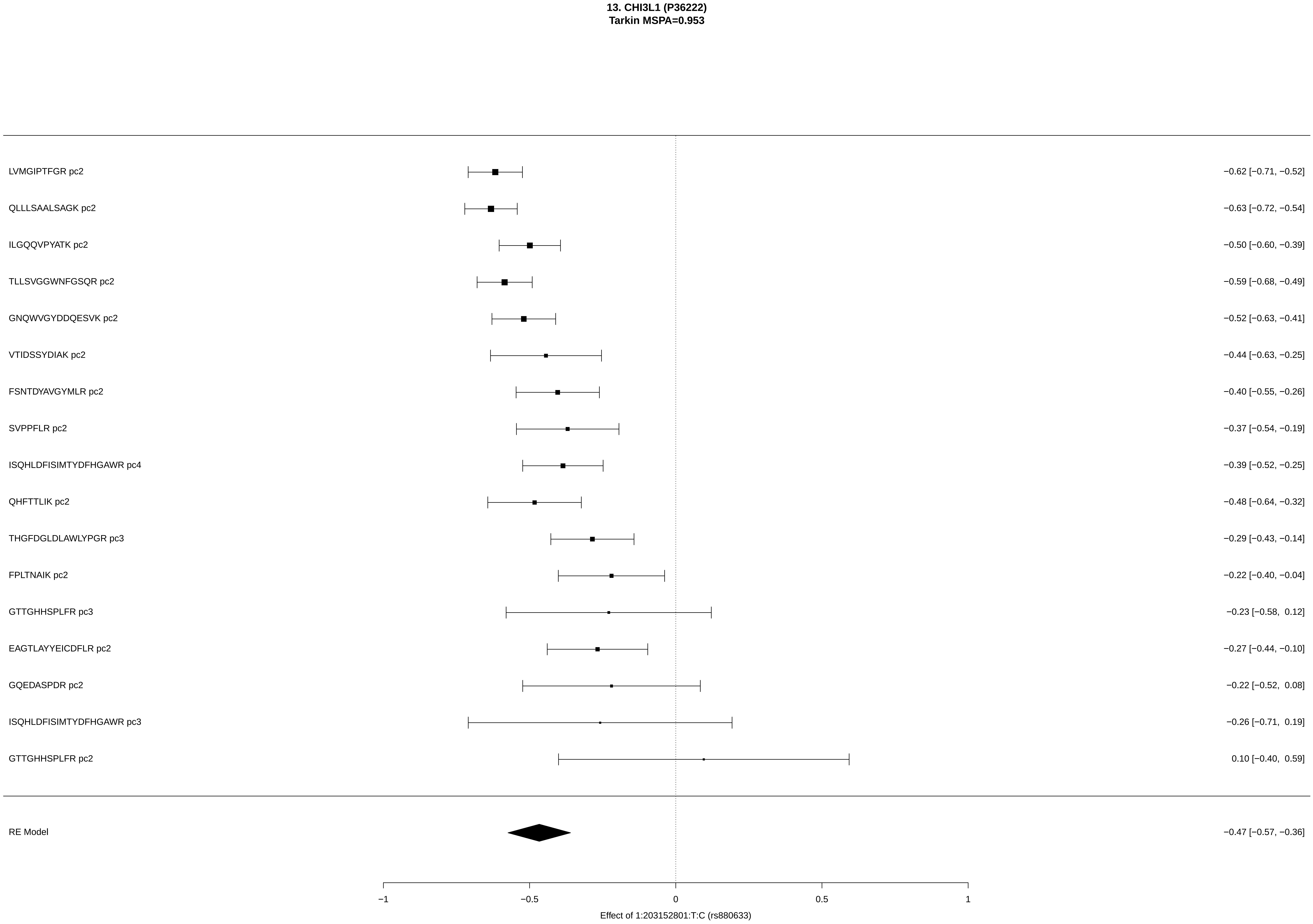

13. CHI3L1 (P36222)
Tarkin MSPA=0.953
LVMGIPTFGR pc2
−0.62 [−0.71, −0.52]
QLLLSAALSAGK pc2
−0.63 [−0.72, −0.54]
ILGQQVPYATK pc2
−0.50 [−0.60, −0.39]
TLLSVGGWNFGSQR pc2
−0.59 [−0.68, −0.49]
GNQWVGYDDQESVK pc2
−0.52 [−0.63, −0.41]
VTIDSSYDIAK pc2
−0.44 [−0.63, −0.25]
FSNTDYAVGYMLR pc2
−0.40 [−0.55, −0.26]
SVPPFLR pc2
−0.37 [−0.54, −0.19]
ISQHLDFISIMTYDFHGAWR pc4
−0.39 [−0.52, −0.25]
QHFTTLIK pc2
−0.48 [−0.64, −0.32]
THGFDGLDLAWLYPGR pc3
−0.29 [−0.43, −0.14]
FPLTNAIK pc2
−0.22 [−0.40, −0.04]
GTTGHHSPLFR pc3
−0.23 [−0.58, 0.12]
EAGTLAYYEICDFLR pc2
−0.27 [−0.44, −0.10]
GQEDASPDR pc2
−0.22 [−0.52, 0.08]
ISQHLDFISIMTYDFHGAWR pc3
−0.26 [−0.71, 0.19]
GTTGHHSPLFR pc2
0.10 [−0.40, 0.59]
RE Model
−0.47 [−0.57, −0.36]
−1
−0.5
0
0.5
1
Effect of 1:203152801:T:C (rs880633)

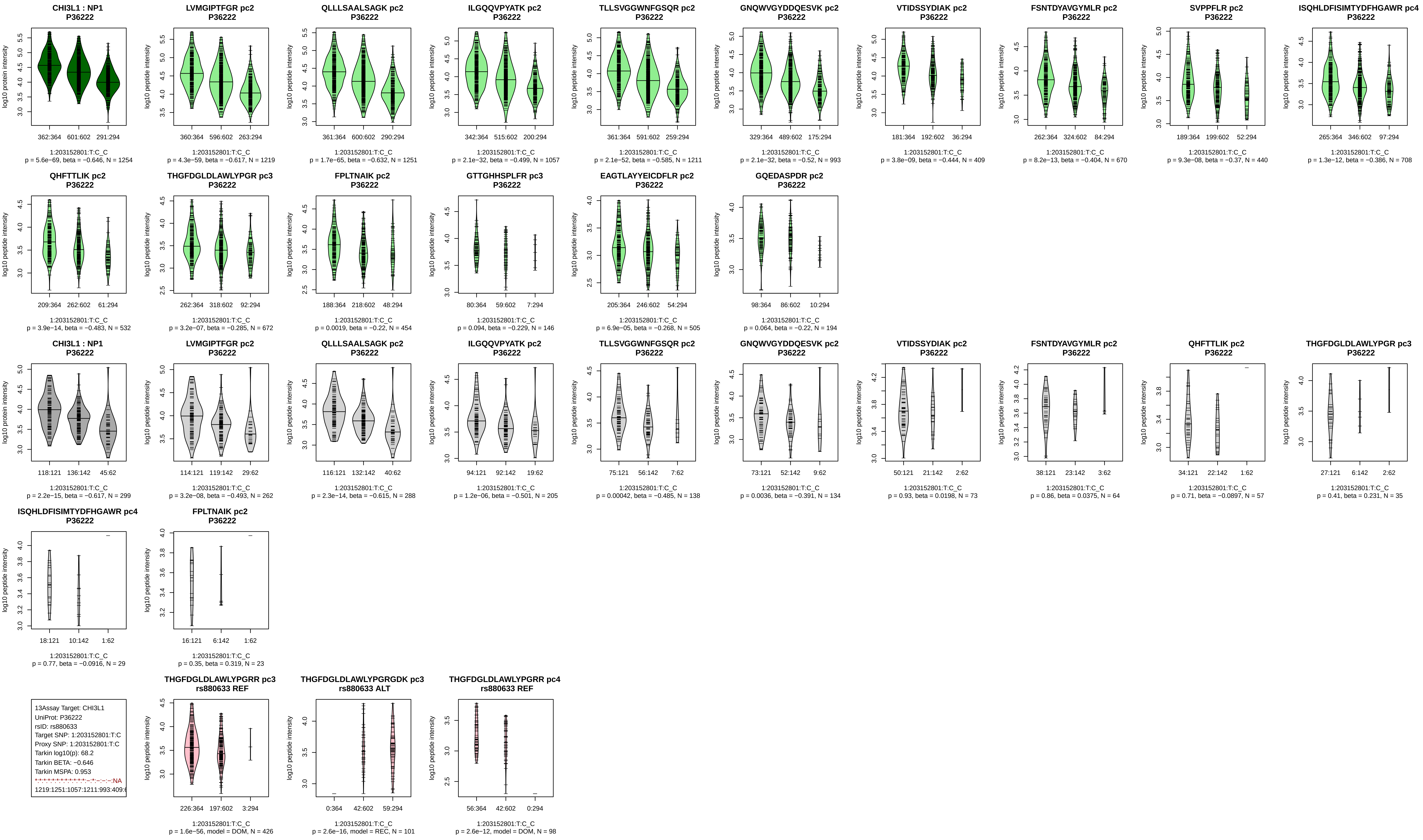

CHI3L1 : NP1
P36222
log10 protein intensity
362:364 601:602 291:294
1:203152801:T:C_C
p = 5.6e−69, beta = −0.646, N = 1254
LVMGIPTFGR pc2
P36222
log10 peptide intensity
360:364 596:602 263:294
1:203152801:T:C_C
p = 4.3e−59, beta = −0.617, N = 1219
QLLLSAALSAGK pc2
P36222
361:364 600:602 290:294
1:203152801:T:C_C
p = 1.7e−65, beta = −0.632, N = 1251
ILGQQVPYATK pc2
P36222
342:364 515:602 200:294
1:203152801:T:C_C
p = 2.1e−32, beta = −0.499, N = 1057
TLLSVGGWNFGSQR pc2
P36222
361:364 591:602 259:294
1:203152801:T:C_C
p = 2.1e−52, beta = −0.585, N = 1211
GNQWVGYDDQESVK pc2
P36222
329:364 489:602 175:294
1:203152801:T:C_C
p = 2.1e−32, beta = −0.52, N = 993
VTIDSSYDIAK pc2
P36222
181:364 192:602 36:294
1:203152801:T:C_C
p = 3.8e−09, beta = −0.444, N = 409
FSNTDYAVGYMLR pc2
P36222
262:364 324:602 84:294
1:203152801:T:C_C
p = 8.2e−13, beta = −0.404, N = 670
SVPPFLR pc2
P36222
189:364 199:602 52:294
1:203152801:T:C_C
p = 9.3e−08, beta = −0.37, N = 440
ISQHLDFISIMTYDFHGAWR pc4
P36222
265:364 346:602 97:294
1:203152801:T:C_C
p = 1.3e−12, beta = −0.386, N = 708
QHFTTLIK pc2
P36222
209:364 262:602 61:294
1:203152801:T:C_C
p = 3.9e−14, beta = −0.483, N = 532
THGFDGLDLAWLYPGR pc3
P36222
262:364 318:602 92:294
1:203152801:T:C_C
p = 3.2e−07, beta = −0.285, N = 672
FPLTNAIK pc2
P36222
188:364 218:602 48:294
1:203152801:T:C_C
p = 0.0019, beta = −0.22, N = 454
GTTGHHSPLFR pc3
P36222
80:364 59:602 7:294
1:203152801:T:C_C
p = 0.094, beta = −0.229, N = 146
EAGTLAYYEICDFLR pc2
P36222
205:364 246:602 54:294
1:203152801:T:C_C
p = 6.9e−05, beta = −0.268, N = 505
GQEDASPDR pc2
P36222
98:364 86:602 10:294
1:203152801:T:C_C
p = 0.064, beta = −0.22, N = 194
CHI3L1 : NP1
P36222
118:121 136:142 45:62
1:203152801:T:C_C
p = 2.2e−15, beta = −0.617, N = 299
LVMGIPTFGR pc2
P36222
114:121 119:142 29:62
1:203152801:T:C_C
p = 3.2e−08, beta = −0.493, N = 262
QLLLSAALSAGK pc2
P36222
116:121 132:142 40:62
1:203152801:T:C_C
p = 2.3e−14, beta = −0.615, N = 288
ILGQQVPYATK pc2
P36222
94:121 92:142 19:62
1:203152801:T:C_C
p = 1.2e−06, beta = −0.501, N = 205
TLLSVGGWNFGSQR pc2
P36222
75:121 56:142 7:62
1:203152801:T:C_C
p = 0.00042, beta = −0.485, N = 138
GNQWVGYDDQESVK pc2
P36222
73:121 52:142 9:62
1:203152801:T:C_C
p = 0.0036, beta = −0.391, N = 134
VTIDSSYDIAK pc2
P36222
50:121 21:142 2:62
1:203152801:T:C_C
p = 0.93, beta = 0.0198, N = 73
FSNTDYAVGYMLR pc2
P36222
38:121 23:142 3:62
1:203152801:T:C_C
p = 0.86, beta = 0.0375, N = 64
QHFTTLIK pc2
P36222
34:121 22:142 1:62
1:203152801:T:C_C
p = 0.71, beta = −0.0897, N = 57
THGFDGLDLAWLYPGR pc3
P36222
27:121 6:142 2:62
1:203152801:T:C_C
p = 0.41, beta = 0.231, N = 35
ISQHLDFISIMTYDFHGAWR pc4
P36222
18:121 10:142 1:62
1:203152801:T:C_C
p = 0.77, beta = −0.0916, N = 29
FPLTNAIK pc2
P36222
16:121 6:142 1:62
1:203152801:T:C_C
p = 0.35, beta = 0.319, N = 23
13Assay Target: CHI3L1
UniProt: P36222
rsID: rs880633
Target SNP: 1:203152801:T:C
Proxy SNP: 1:203152801:T:C
Tarkin log10(p): 68.2
Tarkin BETA: −0.646
Tarkin MSPA: 0.953
1219:1251:1057:1211:993:409:6
THGFDGLDLAWLYPGRR pc3
rs880633 REF
226:364 197:602 3:294
1:203152801:T:C_C
p = 1.6e−56, model = DOM, N = 426
THGFDGLDLAWLYPGRGDK pc3
rs880633 ALT
0:364 42:602 59:294
1:203152801:T:C_C
p = 2.6e−16, model = REC, N = 101
THGFDGLDLAWLYPGRR pc4
rs880633 REF
56:364 42:602 0:294
1:203152801:T:C_C
p = 2.6e−12, model = DOM, N = 98

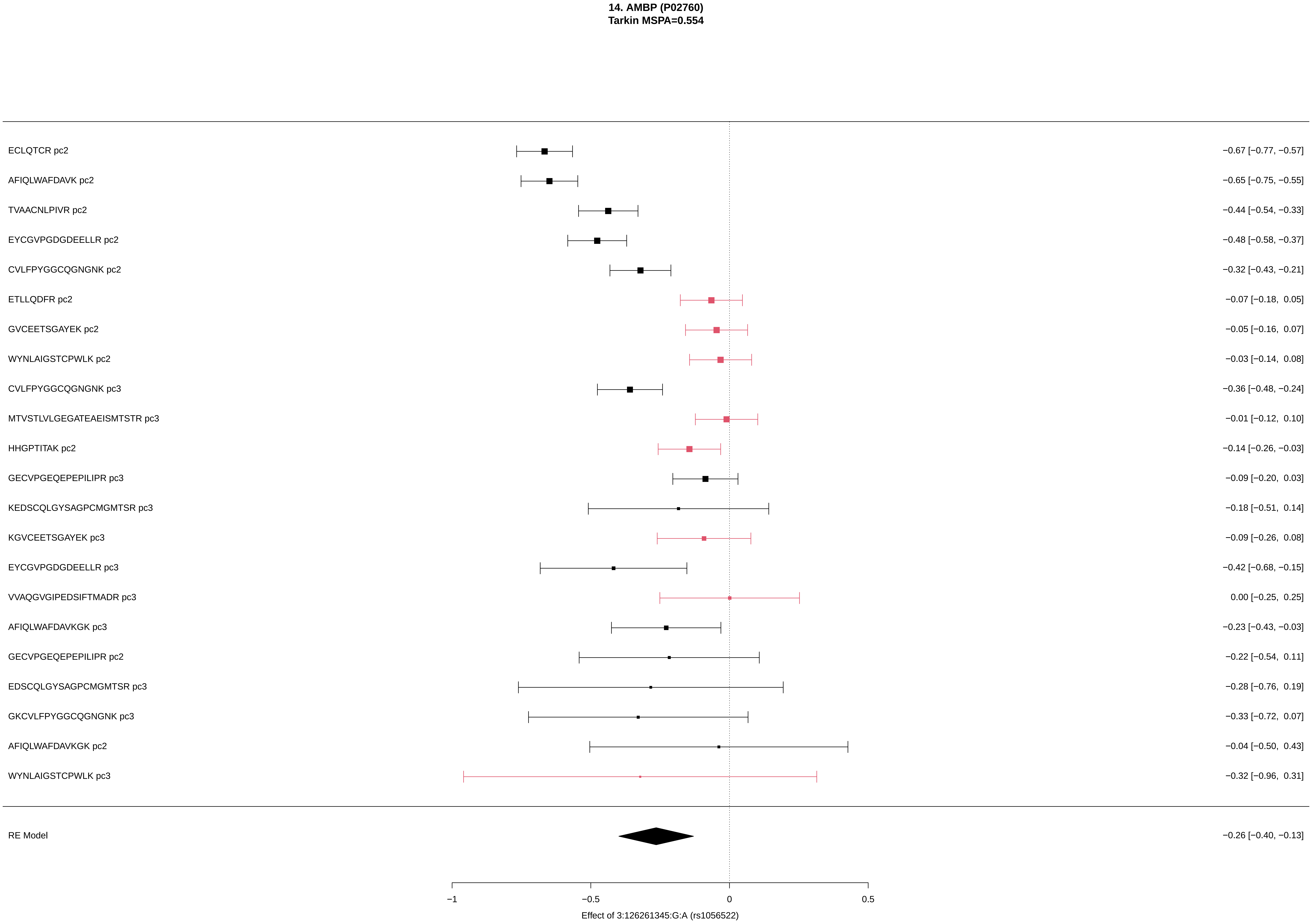
14. AMBP (P02760)
Tarkin MSPA=0.554
ECLQTCR pc2
AFIQLWAFDAVK pc2
TVAACNLPIVR pc2
EYCGVPGDGDEELLR pc2
CVLFPYGGCQGNGNK pc2
ETLLQDFR pc2
GVCEETSGAYEK pc2
WYNLAIGSTCPWLK pc2
CVLFPYGGCQGNGNK pc3
MTVSTLVLGEGATEAEISMTSTR pc3
HHGPTITAK pc2
GECVPGEQEPEPILIPR pc3
KEDSCQLGYSAGPCMGMTSR pc3
KGVCEETSGAYEK pc3
EYCGVPGDGDEELLR pc3
VVAQGVGIPEDSIFTMADR pc3
AFIQLWAFDAVKGK pc3
GECVPGEQEPEPILIPR pc2
EDSCQLGYSAGPCMGMTSR pc3
GKCVLFPYGGCQGNGNK pc3
AFIQLWAFDAVKGK pc2
WYNLAIGSTCPWLK pc3
RE Model
−0.67 [−0.77, −0.57]
−0.65 [−0.75, −0.55]
−0.44 [−0.54, −0.33]
−0.48 [−0.58, −0.37]
−0.32 [−0.43, −0.21]
−0.07 [−0.18, 0.05]
−0.05 [−0.16, 0.07]
−0.03 [−0.14, 0.08]
−0.36 [−0.48, −0.24]
−0.01 [−0.12, 0.10]
−0.14 [−0.26, −0.03]
−0.09 [−0.20, 0.03]
−0.18 [−0.51, 0.14]
−0.09 [−0.26, 0.08]
−0.42 [−0.68, −0.15]
0.00 [−0.25, 0.25]
−0.23 [−0.43, −0.03]
−0.22 [−0.54, 0.11]
−0.28 [−0.76, 0.19]
−0.33 [−0.72, 0.07]
−0.04 [−0.50, 0.43]
−0.32 [−0.96, 0.31]
−0.26 [−0.40, −0.13]
−1
−0.5
0
0.5
Effect of 3:126261345:G:A (rs1056522)

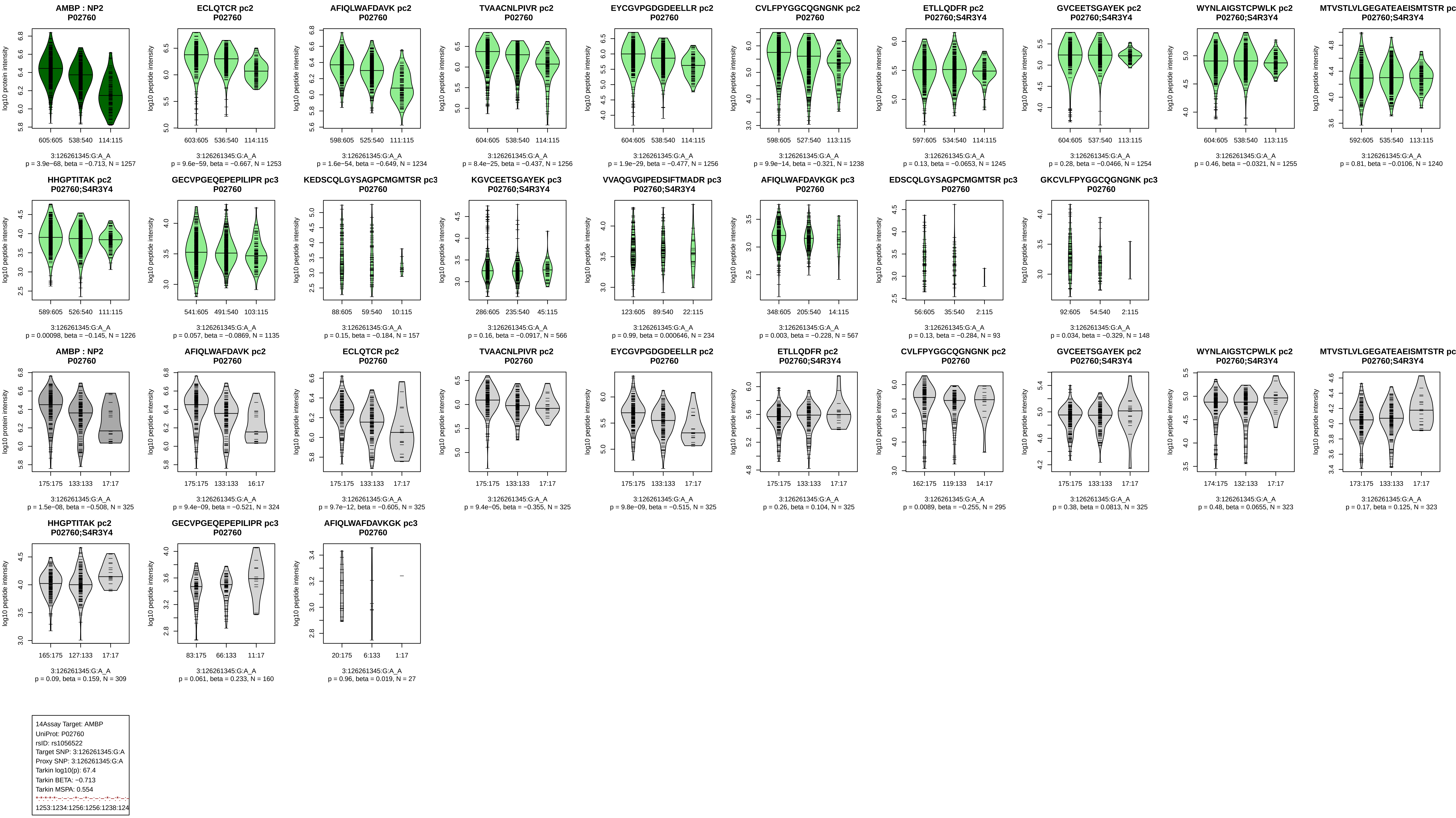

AMBP : NP2 P02760
log10 protein intensity
605:605 538:540 114:115
3:126261345:G:A_A
p = 3.9e−68, beta = −0.713, N = 1257
ECLQTCR pc2 P02760
log10 peptide intensity
603:605 536:540 114:115
3:126261345:G:A_A
p = 9.6e−59, beta = −0.667, N = 1253
AFIQLWAFDAVK pc2 P02760
598:605 525:540 111:115
3:126261345:G:A_A
p = 1.6e−54, beta = −0.649, N = 1234
TVAACNLPIVR pc2 P02760
604:605 538:540 114:115
3:126261345:G:A_A
p = 8.4e−25, beta = −0.437, N = 1256
EYCGVPGDGDEELLR pc2 P02760
604:605 538:540 114:115
3:126261345:G:A_A
p = 1.9e−29, beta = −0.477, N = 1256
CVLFPYGGCQGNGNK pc2 P02760
598:605 527:540 113:115
3:126261345:G:A_A
p = 9.9e−14, beta = −0.321, N = 1238
ETLLQDFR pc2 P02760;S4R3Y4
597:605 534:540 114:115
3:126261345:G:A_A
p = 0.13, beta = −0.0653, N = 1245
GVCEETSGAYEK pc2 P02760;S4R3Y4
604:605 537:540 113:115
3:126261345:G:A_A
p = 0.28, beta = −0.0466, N = 1254
WYNLAIGSTCPWLK pc2 P02760;S4R3Y4
604:605 538:540 113:115
3:126261345:G:A_A
p = 0.46, beta = −0.0321, N = 1255
MTVSTLVLGEGATEAEISMTSTR pc2 P02760;S4R3Y4
592:605 535:540 113:115
3:126261345:G:A_A
p = 0.81, beta = −0.0106, N = 1240
HHGPTITAK pc2 P02760;S4R3Y4
589:605 526:540 111:115
3:126261345:G:A_A
p = 0.00098, beta = −0.145, N = 1226
GECVPGEQEPEPILIPR pc3 P02760
541:605 491:540 103:115
3:126261345:G:A_A
p = 0.057, beta = −0.0869, N = 1135
KEDSCQLGYSAGPCMGMTSR pc3 P02760
88:605 59:540 10:115
3:126261345:G:A_A
p = 0.15, beta = −0.184, N = 157
KGVCEETSGAYEK pc3 P02760;S4R3Y4
286:605 235:540 45:115
3:126261345:G:A_A
p = 0.16, beta = −0.0917, N = 566
VVAQGVGIPEDSIFTMADR pc3 P02760;S4R3Y4
123:605 89:540 22:115
3:126261345:G:A_A
p = 0.99, beta = 0.000646, N = 234
AFIQLWAFDAVKGK pc3 P02760
348:605 205:540 14:115
3:126261345:G:A_A
p = 0.003, beta = −0.228, N = 567
EDSCQLGYSAGPCMGMTSR pc3 P02760
56:605 35:540 2:115
3:126261345:G:A_A
p = 0.13, beta = −0.284, N = 93
GKCVLFPYGGCQGNGNK pc3 P02760
92:605 54:540 2:115
3:126261345:G:A_A
p = 0.034, beta = −0.329, N = 148
AMBP : NP2 P02760
175:175 133:133 17:17
3:126261345:G:A_A
p = 1.5e−08, beta = −0.508, N = 325
AFIQLWAFDAVK pc2 P02760
175:175 133:133 16:17
3:126261345:G:A_A
p = 9.4e−09, beta = −0.521, N = 324
ECLQTCR pc2 P02760
175:175 133:133 17:17
3:126261345:G:A_A
p = 9.7e−12, beta = −0.605, N = 325
TVAACNLPIVR pc2 P02760
175:175 133:133 17:17
3:126261345:G:A_A
p = 9.4e−05, beta = −0.355, N = 325
EYCGVPGDGDEELLR pc2 P02760
175:175 133:133 17:17
3:126261345:G:A_A
p = 9.8e−09, beta = −0.515, N = 325
ETLLQDFR pc2 P02760;S4R3Y4
175:175 133:133 17:17
3:126261345:G:A_A
p = 0.26, beta = 0.104, N = 325
CVLFPYGGCQGNGNK pc2 P02760
162:175 119:133 14:17
3:126261345:G:A_A
p = 0.0089, beta = −0.255, N = 295
GVCEETSGAYEK pc2 P02760;S4R3Y4
175:175 133:133 17:17
3:126261345:G:A_A
p = 0.38, beta = 0.0813, N = 325
WYNLAIGSTCPWLK pc2 P02760;S4R3Y4
174:175 132:133 17:17
3:126261345:G:A_A
p = 0.48, beta = 0.0655, N = 323
MTVSTLVLGEGATEAEISMTSTR pc P02760;S4R3Y4
173:175 133:133 17:17
3:126261345:G:A_A
p = 0.17, beta = 0.125, N = 323
HHGPTITAK pc2 P02760;S4R3Y4
165:175 127:133 17:17
3:126261345:G:A_A
p = 0.09, beta = 0.159, N = 309
GECVPGEQEPEPILIPR pc3 P02760
83:175 66:133 11:17
3:126261345:G:A_A
p = 0.061, beta = 0.233, N = 160
AFIQLWAFDAVKGK pc3 P02760
20:175 6:133 1:17
3:126261345:G:A_A
p = 0.96, beta = 0.019, N = 27
14Assay Target: AMBP
UniProt: P02760
rsID: rs1056522
Target SNP: 3:126261345:G:A
Proxy SNP: 3:126261345:G:A
Tarkin log10(p): 67.4
Tarkin BETA: −0.713
Tarkin MSPA: 0.554
1253:1234:1256:1256:1238:124

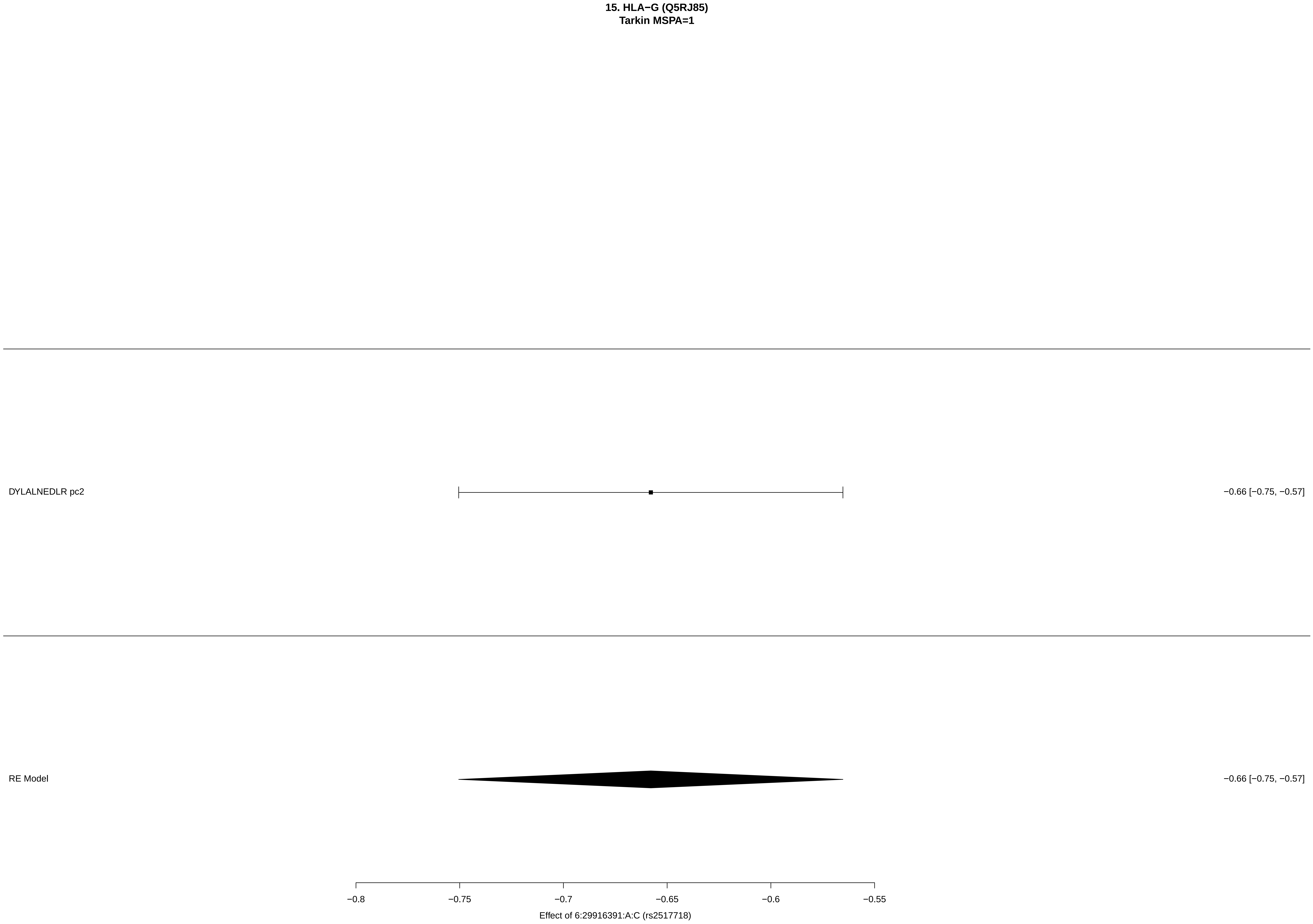

15. HLA−G (Q5RJ85)
Tarkin MSPA=1
DYLALNEDLR pc2
−0.66 [−0.75, −0.57]
RE Model
−0.66 [−0.75, −0.57]
−0.8
−0.75
−0.7
−0.65
−0.6
−0.55
Effect of 6:29916391:A:C (rs2517718)

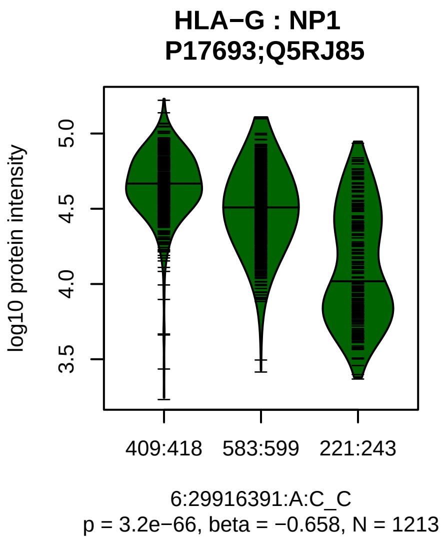

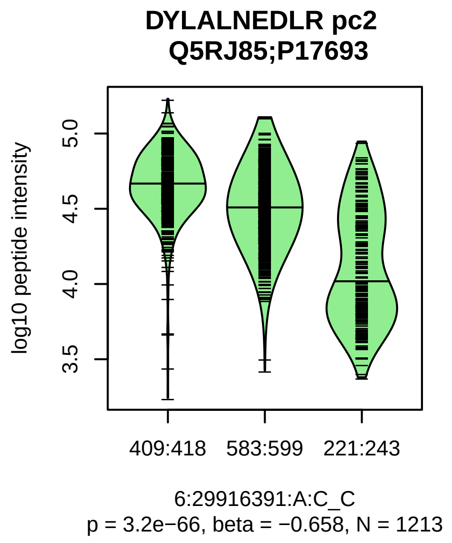

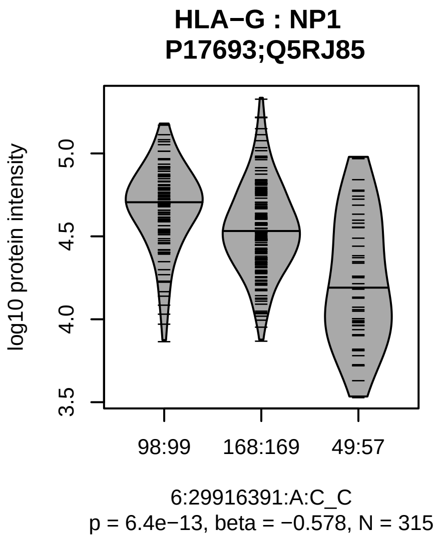

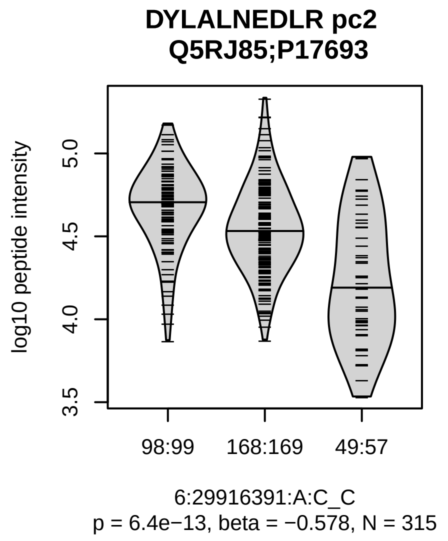

15Assay Target: HLA−G
UniProt: Q5RJ85
rsID: rs2517718
Target SNP: 6:29916391:A:C
Proxy SNP: 6:29916391:A:C
Tarkin log10(p): 65.5
Tarkin BETA: −0.658
Tarkin MSPA: 1
*
1213

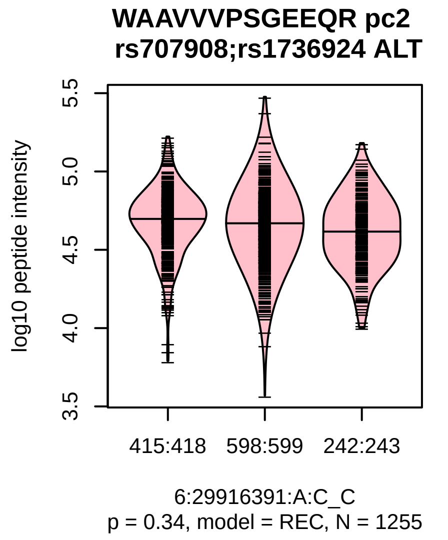

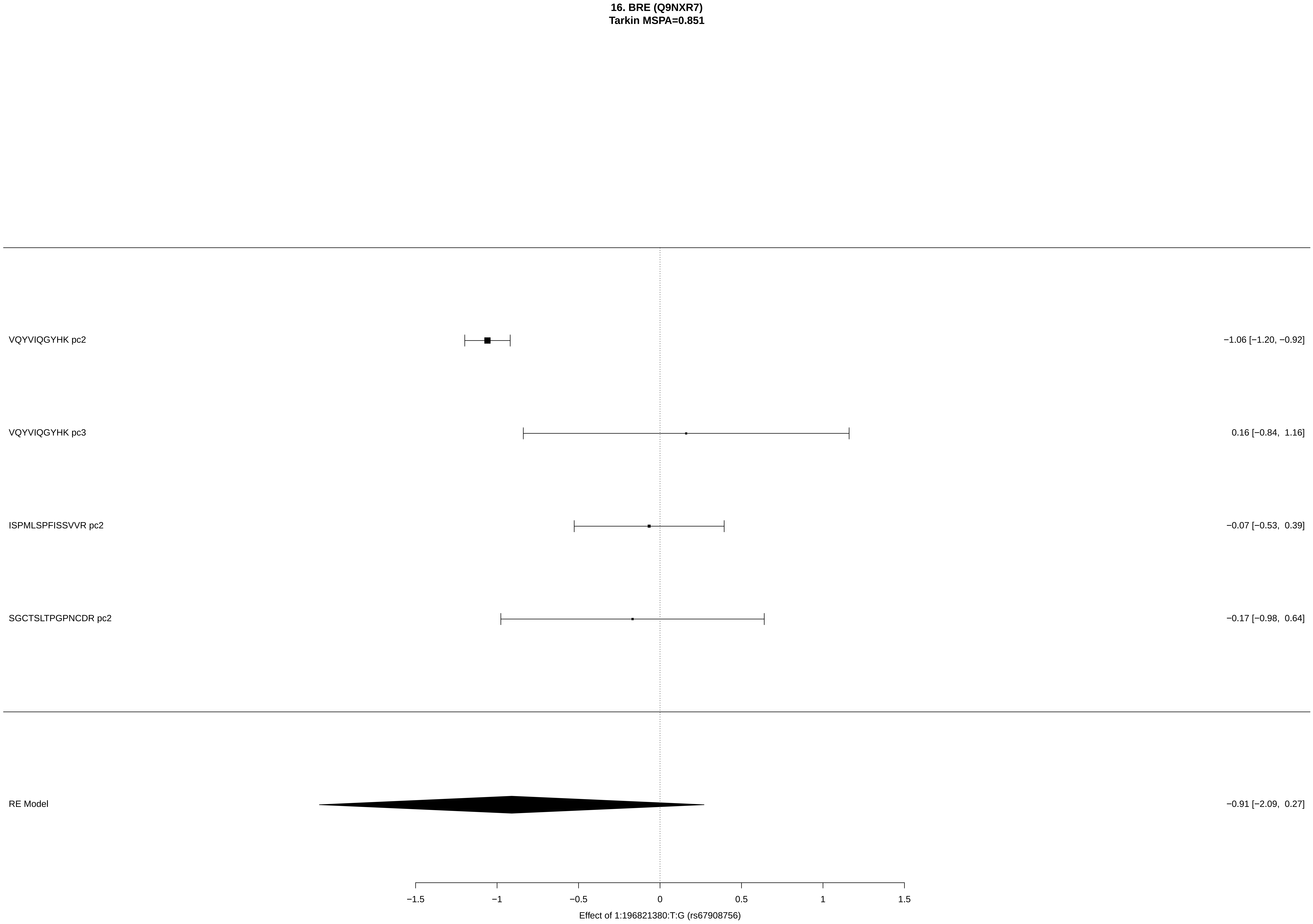
16. BRE (Q9NXR7)
Tarkin MSPA=0.851
VQYVIQGYHK pc2
−1.06 [−1.20, −0.92]
VQYVIQGYHK pc3
0.16 [−0.84, 1.16]
ISPMLSPFISSVVR pc2
−0.07 [−0.53, 0.39]
SGCTSLTPGPNCDR pc2
−0.17 [−0.98, 0.64]
RE Model
−0.91 [−2.09, 0.27]
−1.5
−1
−0.5
0
0.5
1
1.5
Effect of 1:196821380:T:G (rs67908756)

BRE : NP3
Q9NXR7

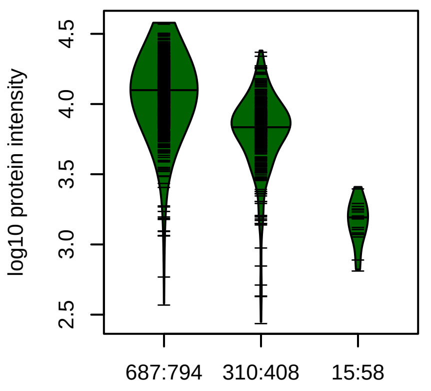

1:196821380:T:G_G
p = 1.9e−65, beta = −0.991, N = 1012

VQYVIQGYHK pc2
Q9NXR7;Q9NXR7−1;Q9NXR7−4

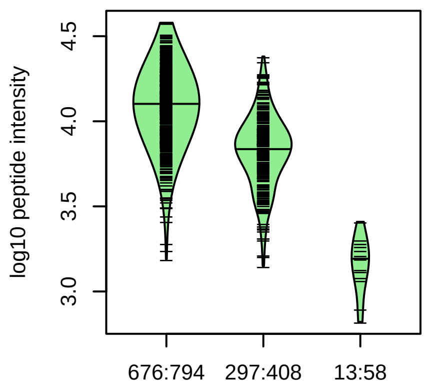

1:196821380:T:G_G
p = 3.3e−72, beta = −1.06, N = 986

ISPMLSPFISSVVR pc2
Q9NXR7;Q9NXR7−1;Q9NXR7−4

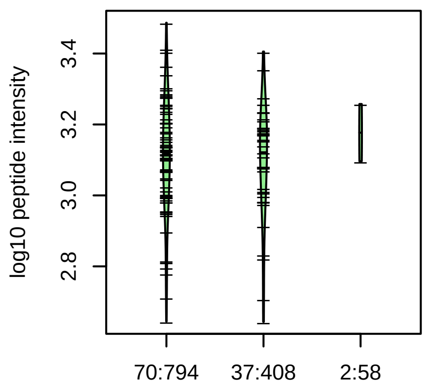

1:196821380:T:G_G
p = 0.71, beta = −0.0665, N = 109

SGCTSLTPGPNCDR pc2
Q9NXR7;Q9NXR7−1;Q9NXR7−4

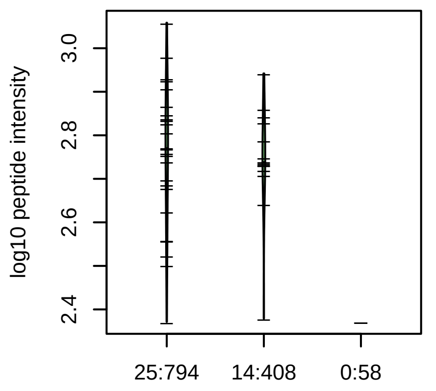

1:196821380:T:G_G
p = 0.59, beta = −0.169, N = 39

BRE : NP3
Q9NXR7

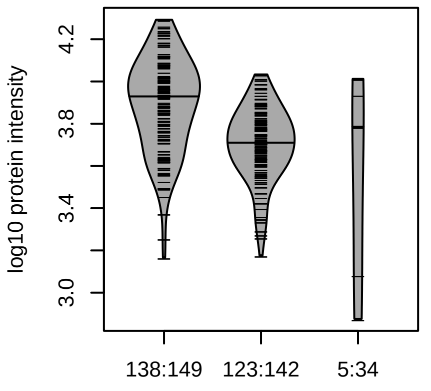

1:196821380:T:G_G
p = 1.7e−11, beta = −0.728, N = 266

VQYVIQGYHK pc2
Q9NXR7;Q9NXR7−1;Q9NXR7−4

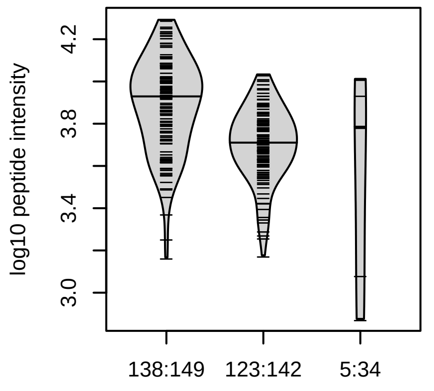

1:196821380:T:G_G
p = 1.7e−11, beta = −0.728, N = 266

16Assay Target: BRE
UniProt: Q9NXR7
rsID: rs67908756
Target SNP: 1:196821380:T:G
Proxy SNP: 1:196821380:T:G
Tarkin log10(p): 64.7
Tarkin BETA: −0.991
Tarkin MSPA: 0.851
*:−:−:−:NA:NA
986:24:109:39:7:19

17. CFH (A0A024R962)
Tarkin MSPA=0.878

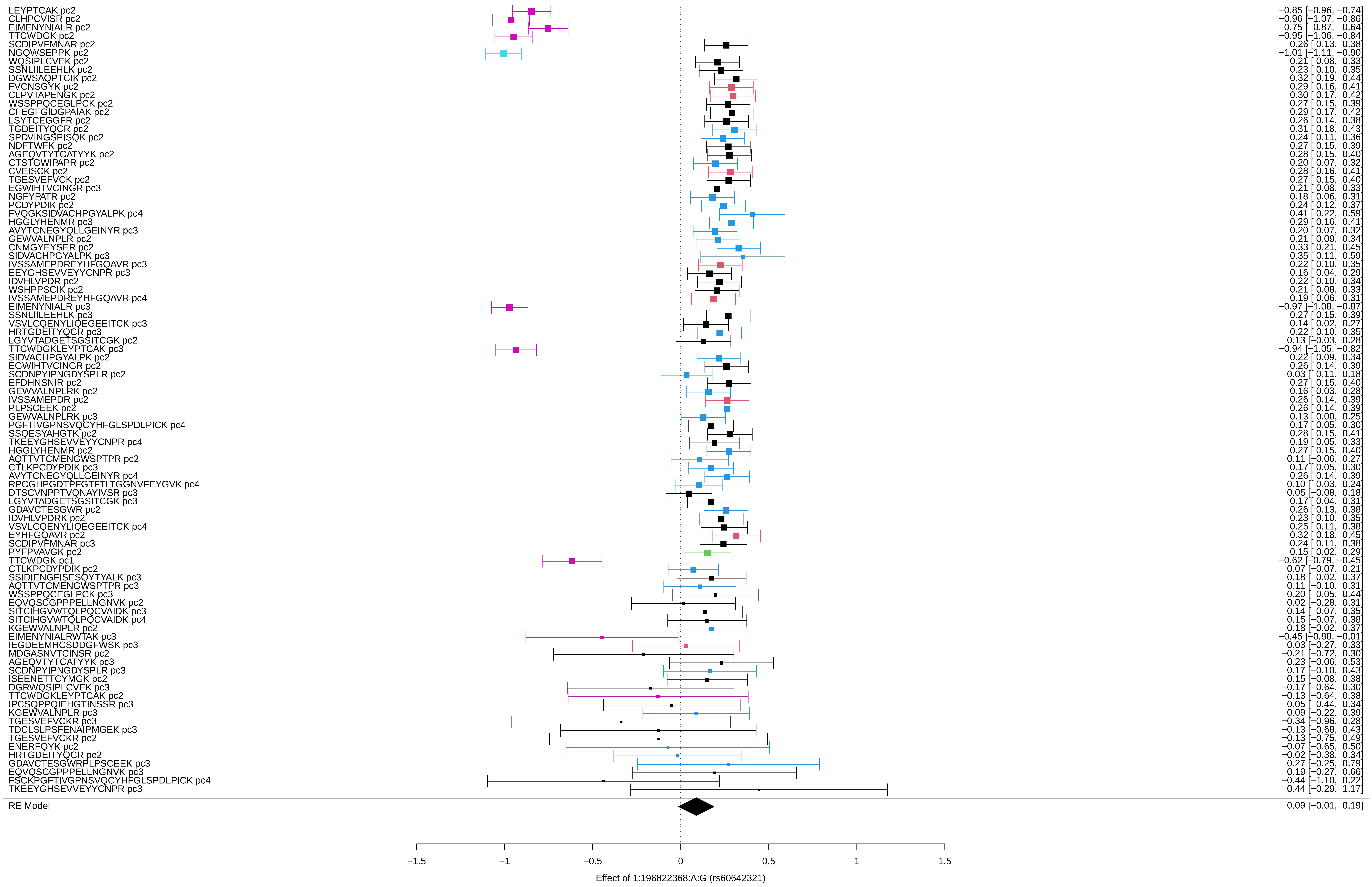

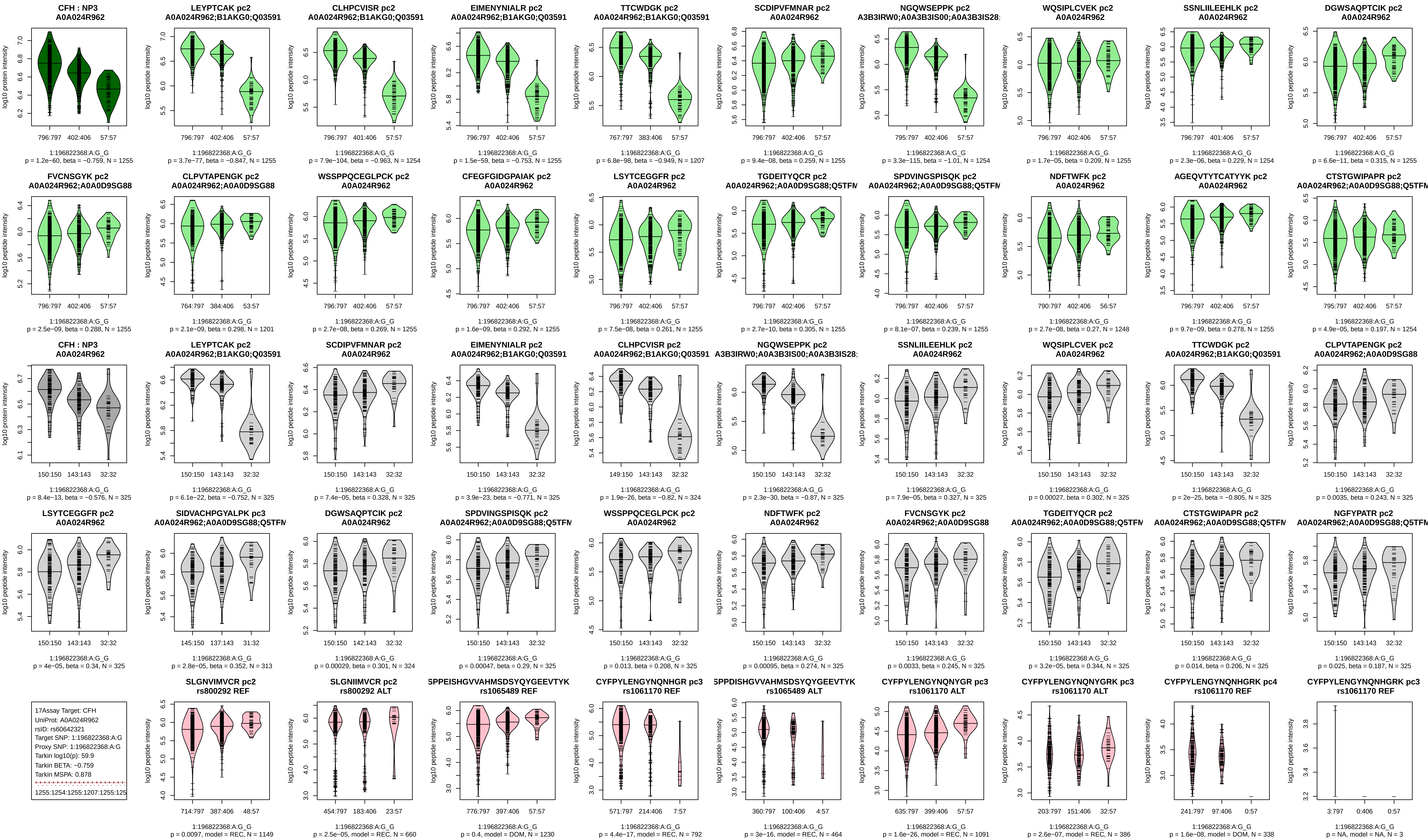
CFH : NP3 A0A024R962
796:797 402:406 57:57
1:196822368:A:G_G
p = 1.2e−60, beta = −0.759, N = 1255
LEYPTCAK pc2 A0A024R962;B1AKG0;Q03591
p = 3.7e−77, beta = −0.847, N = 1255
CLHPCVISR pc2 A0A024R962;B1AKG0;Q03591
p = 7.9e−104, beta = −0.963, N = 1254
EIMENYNIALR pc2 A0A024R962;B1AKG0;Q03591
p = 1.5e−59, beta = −0.753, N = 1255
TTCWDGK pc2 A0A024R962;B1AKG0;Q03591
p = 6.8e−98, beta = −0.949, N = 1207
SCDIPVFMNAR pc2 A0A024R962
p = 9.4e−08, beta = 0.259, N = 1255
NGQWSEPPK pc2 A3B3IRW0;A0A3B3IS00;A0A3B3IS28
p = 3.3e−115, beta = −1.01, N = 1254
WQSIPLCVEK pc2 A0A024R962
p = 1.7e−05, beta = 0.209, N = 1255
SSNLIILEEHLK pc2 A0A024R962
p = 2.3e−06, beta = 0.229, N = 1254
DGWSAQPTCIK pc2 A0A024R962
p = 6.6e−11, beta = 0.315, N = 1255
FVCNSGYK pc2 A0A024R962;A0A0D9SG88
p = 2.5e−09, beta = 0.288, N = 1255
CLPVTAPENGK pc2 A0A024R962;A0A0D9SG88
p = 2.1e−09, beta = 0.298, N = 1201
WSSPPQCEGLPCK pc2 A0A024R962
p = 2.7e−08, beta = 0.269, N = 1255
CFEGFGIDGPAIAK pc2 A0A024R962
p = 1.6e−09, beta = 0.292, N = 1255
LSYTCEGGFR pc2 A0A024R962
p = 7.5e−08, beta = 0.261, N = 1255
TGDEITYQCR pc2 A0A024R962;A0A0D9SG88;Q5TFM
p = 2.7e−10, beta = 0.305, N = 1255
SPDVINGSPISQK pc2 A0A024R962;A0A0D9SG88;Q5TFM
p = 8.1e−07, beta = 0.239, N = 1255
NDFTWFK pc2 A0A024R962
p = 2.7e−08, beta = 0.27, N = 1248
AGEQVTYTCATYYK pc2 A0A024R962
p = 9.7e−09, beta = 0.278, N = 1255
CTSTGWIPAPR pc2 A0A024R962;A0A0D9SG88;Q5TFM
p = 4.9e−05, beta = 0.197, N = 1254
CFH : NP3 A0A024R962
p = 8.4e−13, beta = −0.576, N = 325
LEYPTCAK pc2 A0A024R962;B1AKG0;Q03591
p = 6.1e−22, beta = −0.752, N = 325
SCDIPVFMNAR pc2 A0A024R962
p = 7.4e−05, beta = 0.328, N = 325
EIMENYNIALR pc2 A0A024R962;B1AKG0;Q03591
p = 3.9e−23, beta = −0.771, N = 325
CLHPCVISR pc2 A0A024R962;B1AKG0;Q03591
p = 1.9e−26, beta = −0.82, N = 324
NGQWSEPPK pc2 A3B3IRW0;A0A3B3IS00;A0A3B3IS28
p = 2.3e−30, beta = −0.87, N = 325
SSNLIILEEHLK pc2 A0A024R962
p = 7.9e−05, beta = 0.327, N = 325
WQSIPLCVEK pc2 A0A024R962
p = 0.00027, beta = 0.302, N = 325
TTCWDGK pc2 A0A024R962;B1AKG0;Q03591
p = 2e−25, beta = −0.805, N = 325
CLPVTAPENGK pc2 A0A024R962;A0A0D9SG88
p = 0.0035, beta = 0.243, N = 325
LSYTCEGGFR pc2 A0A024R962
p = 4e−05, beta = 0.34, N = 325
SIDVACHPGYALPK pc3 A0A024R962;A0A0D9SG88;Q5TFM
p = 2.8e−05, beta = 0.352, N = 313
DGWSAQPTCIK pc2 A0A024R962
p = 0.00029, beta = 0.301, N = 324
SPDVINGSPISQK pc2 A0A024R962;A0A0D9SG88;Q5TFM
p = 0.00047, beta = 0.29, N = 325
WSSPPQCEGLPCK pc2 A0A024R962
p = 0.013, beta = 0.208, N = 325
NDFTWFK pc2 A0A024R962
p = 0.00095, beta = 0.274, N = 325
FVCNSGYK pc2 A0A024R962;A0A0D9SG88
p = 0.0033, beta = 0.245, N = 325
TGDEITYQCR pc2 A0A024R962;A0A0D9SG88;Q5TFM
p = 3.2e−05, beta = 0.344, N = 325
CTSTGWIPAPR pc2 A0A024R962;A0A0D9SG88;Q5TFM
p = 0.014, beta = 0.206, N = 325
NGFYPATR pc2 A0A024R962;A0A0D9SG88;Q5TFM
p = 0.025, beta = 0.187, N = 325
17Assay Target: CFH
UniProt: A0A024R962
rsID: rs60642321
Target SNP: 1:196822368:A:G
Proxy SNP: 1:196822368:A:G
Tarkin log10(p): 59.9
Tarkin BETA: −0.759
Tarkin MSPA: 0.878
1255:1254:1255:1207:1255:125
SLGNVIMVCR pc2 rs800292 REF
p = 0.0097, model = REC, N = 1149
SLGNIIMVCR pc2 rs800292 ALT
p = 2.5e−05, model = REC, N = 660
SPPEISHGVVAHMSDSYQYGEEVTYK rs1065489 REF
p = 0.4, model = DOM, N = 1230
CYFPYLENGYNQNHGR pc3 rs1061170 REF
p = 4.4e−17, model = REC, N = 792
SPPDISHGVVAHMSDSYQYGEEVTYK rs1065489 ALT
p = 3e−16, model = REC, N = 464
CYFPYLENGYNQNYGR pc2 rs1061170 ALT
p = 1.6e−26, model = REC, N = 1091
CYFPYLENGYNQNYGRK pc3 rs1061170 ALT
p = 2.6e−07, model = REC, N = 386
CYFPYLENGYNQNHGRK pc4 rs1061170 REF
p = 1.6e−08, model = DOM, N = 338
CYFPYLENGYNQNHGRK pc3 rs1061170 REF
p = NA, model = NA, N = 3

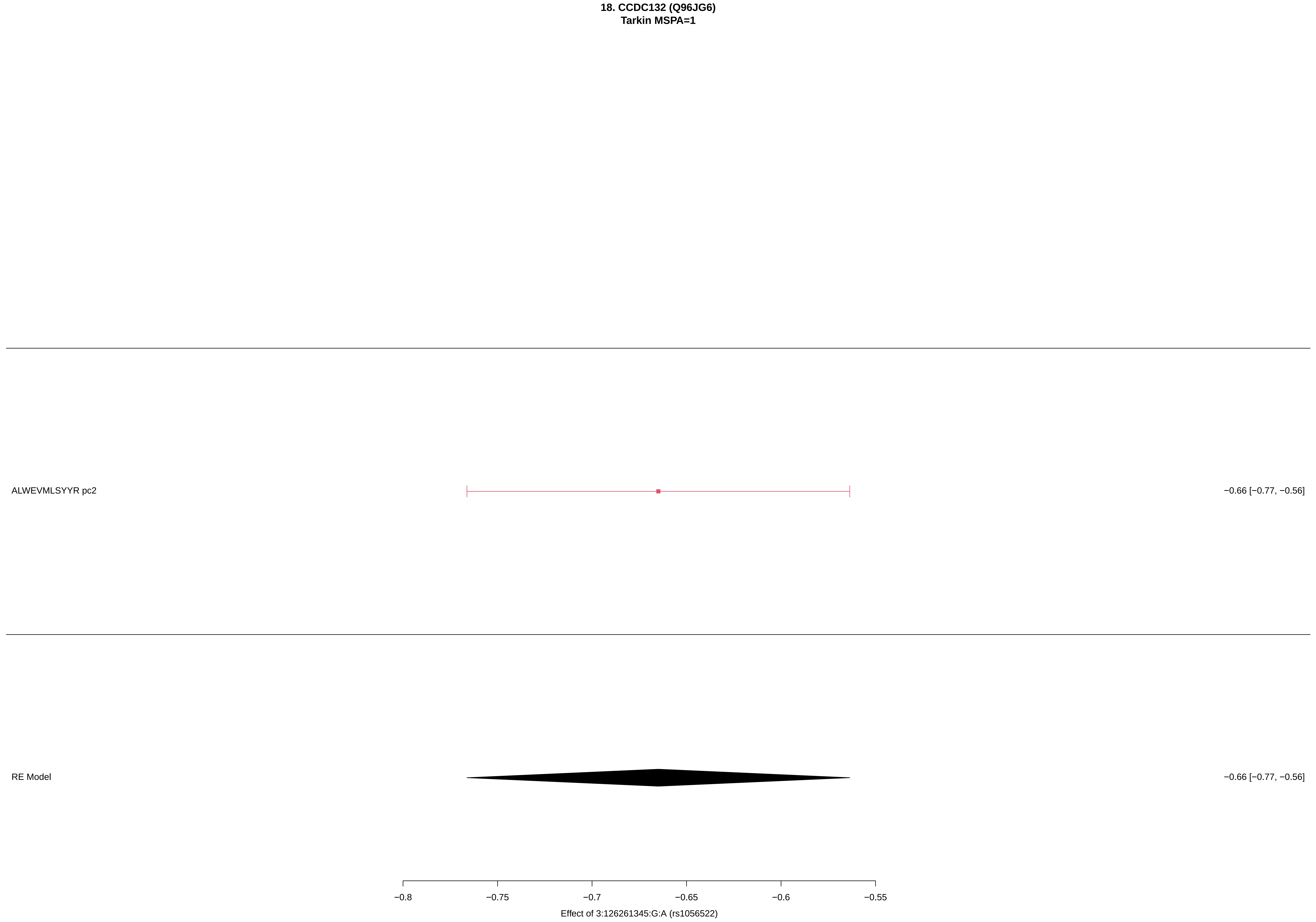
18. CCDC132 (Q96JG6)
Tarkin MSPA=1
ALWEVMLSYYR pc2
−0.66 [−0.77, −0.56]
RE Model
−0.66 [−0.77, −0.56]
−0.8
−0.75
−0.7
−0.65
−0.6
−0.55
Effect of 3:126261345:G:A (rs1056522)

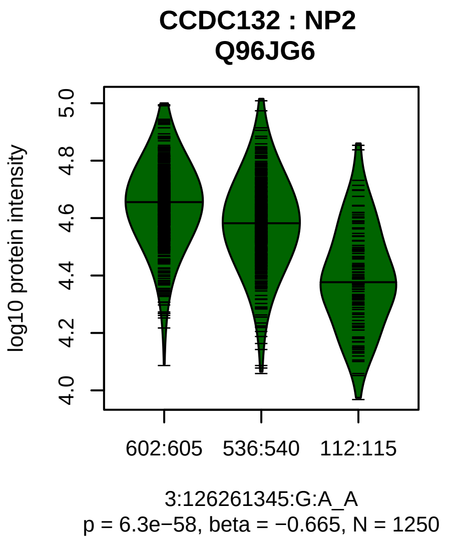

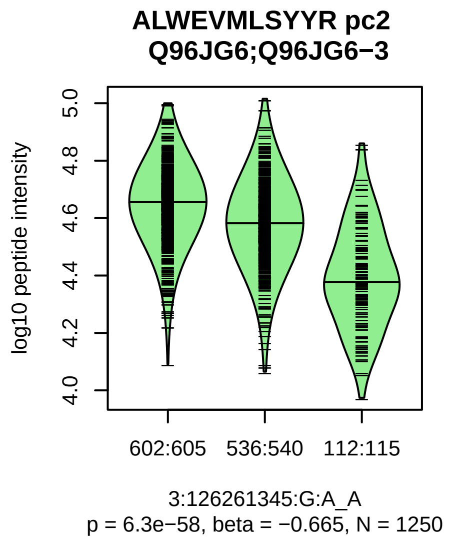

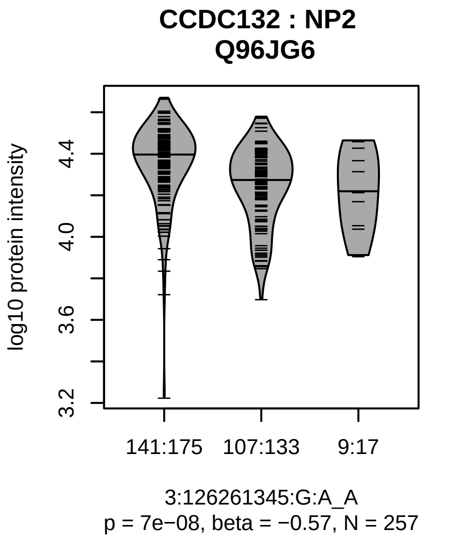

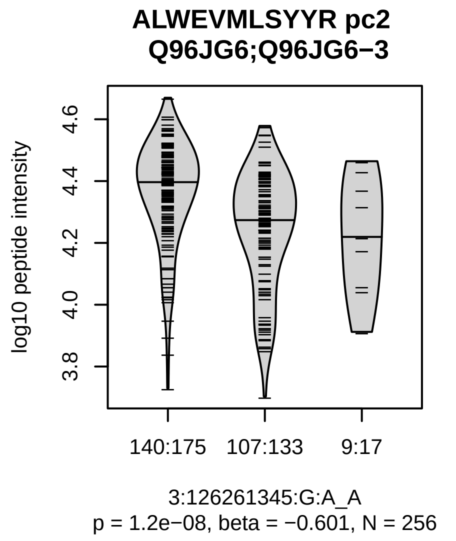

18Assay Target: CCDC132
UniProt: Q96JG6
rsID: rs1056522
Target SNP: 3:126261345:G:A
Proxy SNP: 3:126261345:G:A
Tarkin log10(p): 57.2
Tarkin BETA: −0.665
Tarkin MSPA: 1
*:NA
1250:10

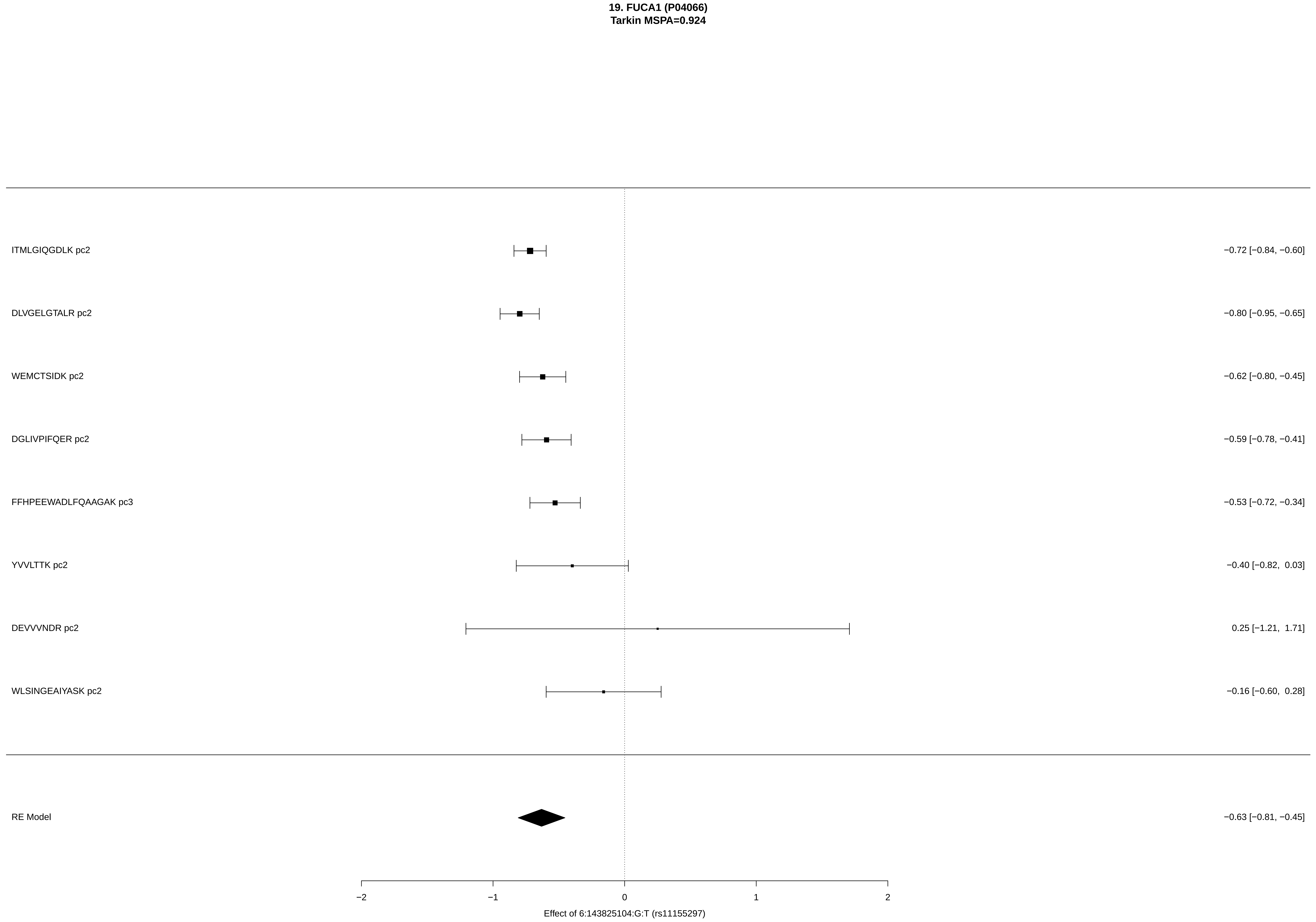

19. FUCA1 (P04066)
Tarkin MSPA=0.924
ITMLGIQGDLK pc2
−0.72 [−0.84, −0.60]
DLVGELGTALR pc2
−0.80 [−0.95, −0.65]
WEMCTSIDK pc2
−0.62 [−0.80, −0.45]
DGLIVPIFQER pc2
−0.59 [−0.78, −0.41]
FFHPEEWADLFQAAGAK pc3
−0.53 [−0.72, −0.34]
YVVLTTK pc2
−0.40 [−0.82, 0.03]
DEVVVNDR pc2
0.25 [−1.21, 1.71]
WLSINGEAIYASK pc2
−0.16 [−0.60, 0.28]
RE Model
−0.63 [−0.81, −0.45]
−2
−1
0
1
2
Effect of 6:143825104:G:T (rs11155297)

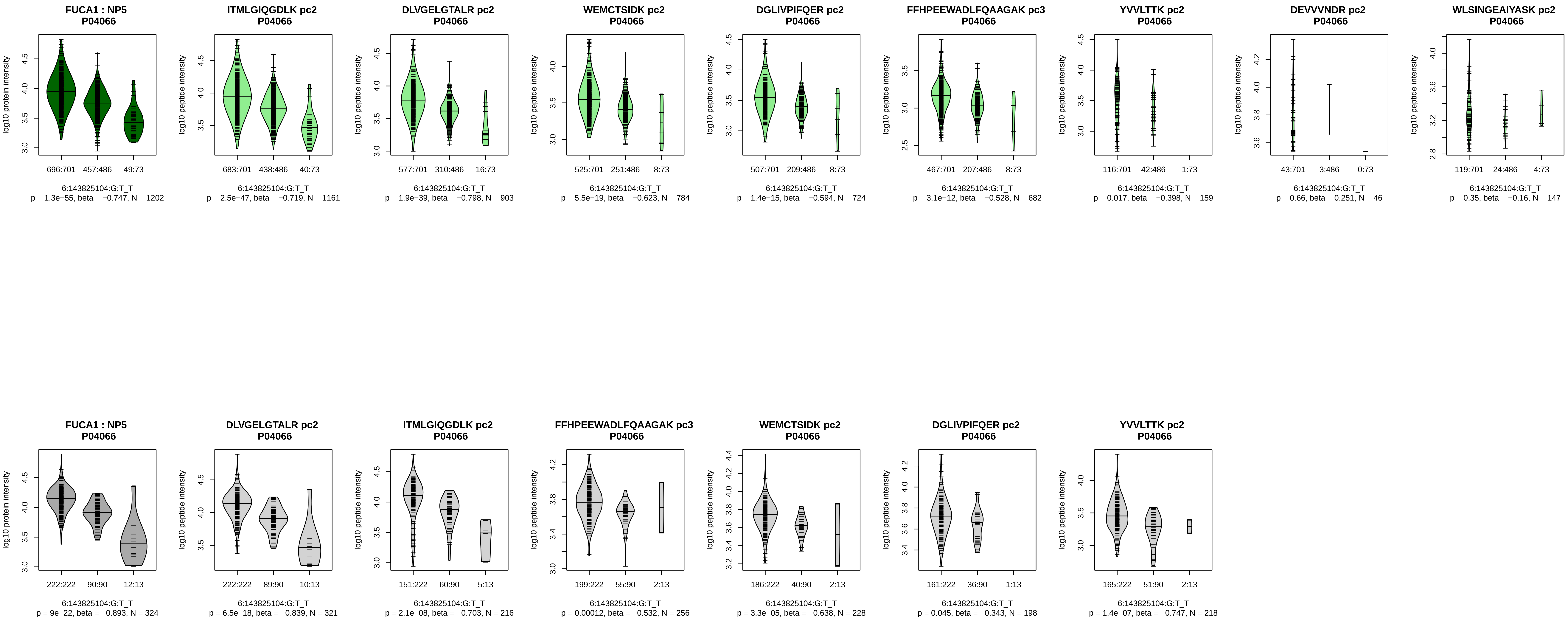

19Assay Target: FUCA1
UniProt: P04066
rsID: rs11155297
Target SNP: 6:143825104:G:T
Proxy SNP: 6:143825104:G:T
Tarkin log10(p): 54.9
Tarkin BETA: −0.747
Tarkin MSPA: 0.924
::*:*:*:–:–:–
1161:903:784:724:682:159:46:1

20. CTSH (A0A087X0D5)
Tarkin MSPA=0.97

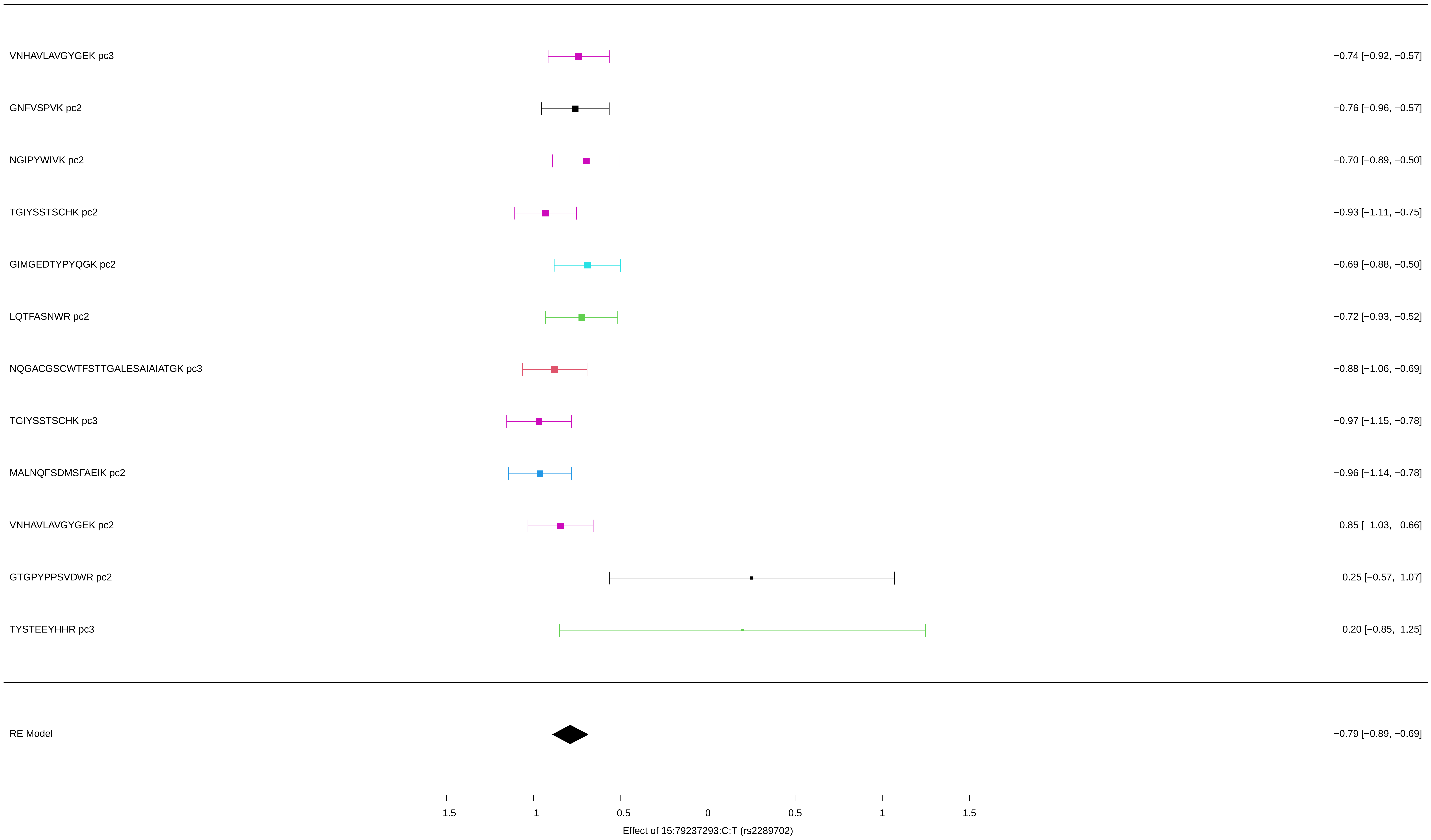

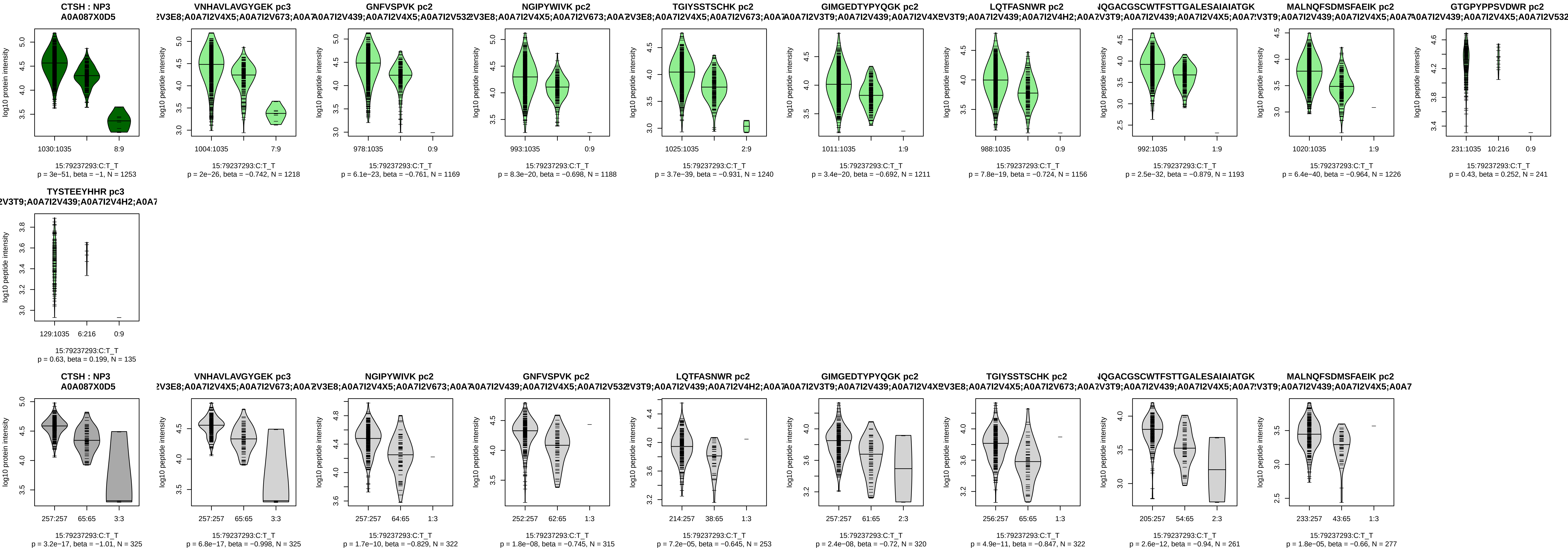

20Assay Target: CTSH
UniProt: A0A087X0D5
rsID: rs2289702
Target SNP: 15:79237293:C:T
Proxy SNP: 15:79237293:C:T
Tarkin log10(p): 50.5
Tarkin BETA: −1.003
Tarkin MSPA: 0.97
1218:1169:1188:1240:1211:115

21. HLA−G (Q5RJ85)
Tarkin MSPA=1

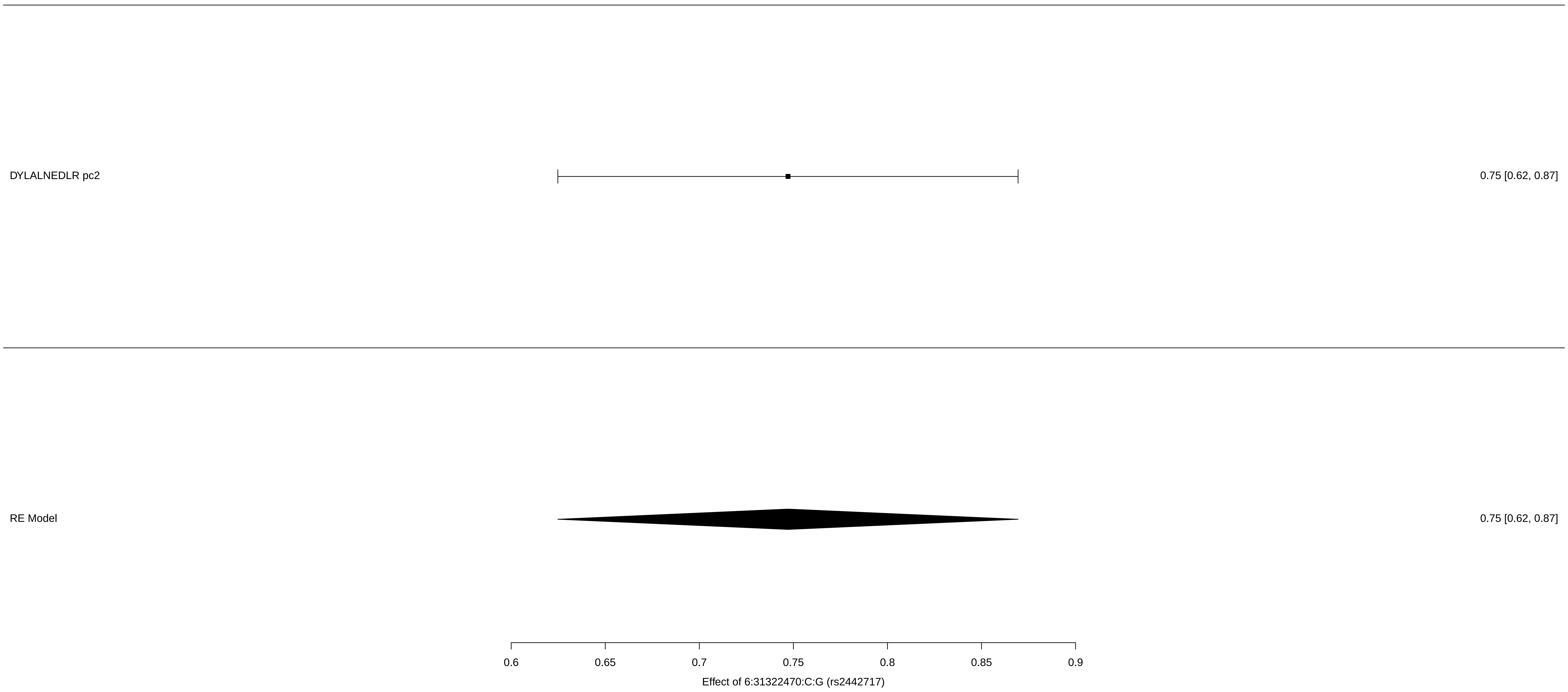

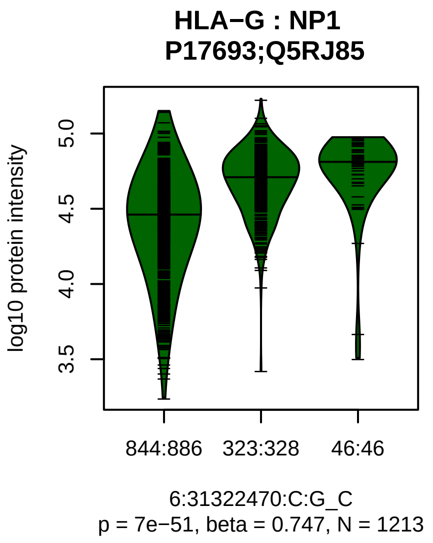

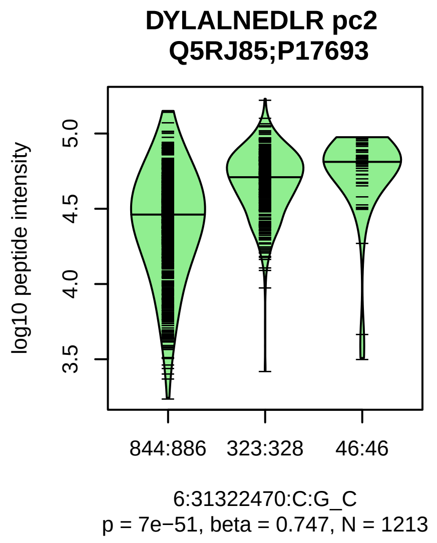

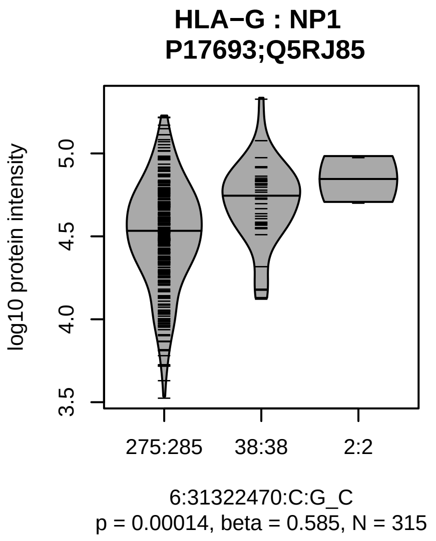

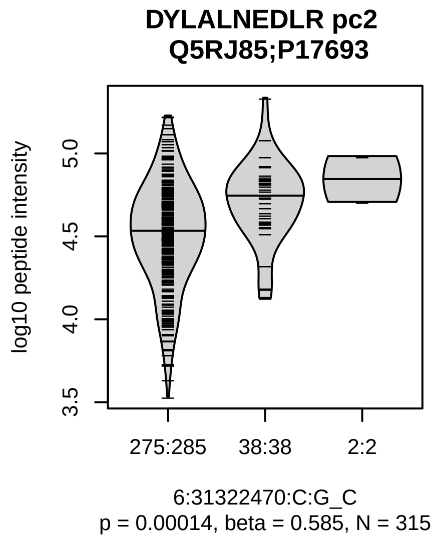

21Assay Target: HLA−G
UniProt: Q5RJ85
rsID: rs2442717
Target SNP: 6:31322470:C:G
Proxy SNP: 6:31322470:C:G
Tarkin log10(p): 50.2
Tarkin BETA: 0.747
Tarkin MSPA: 1
*
1213

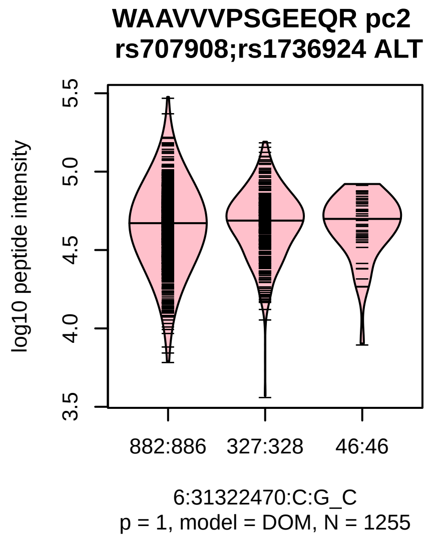

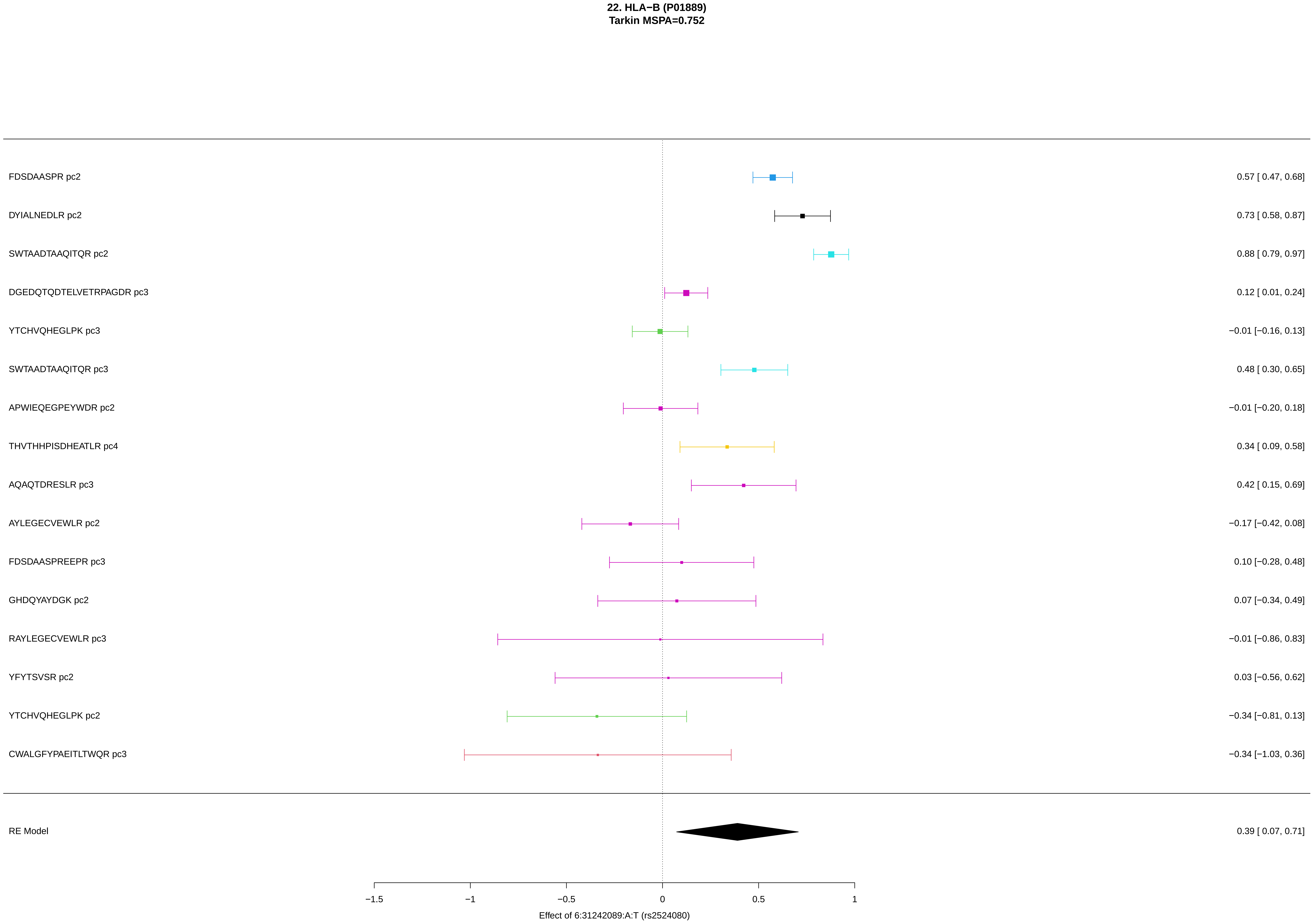
22. HLA−B (P01889)
Tarkin MSPA=0.752
FDSDAASPR pc2
0.57 [0.47, 0.68]
DYIALNEDLR pc2
0.73 [0.58, 0.87]
SWTAADTAAQITQR pc2
0.88 [0.79, 0.97]
DGEDQTQDTELVETRPAGDR pc3
0.12 [0.01, 0.24]
YTCHVQHEGLPK pc3
−0.01 [−0.16, 0.13]
SWTAADTAAQITQR pc3
0.48 [0.30, 0.65]
APWIEQEGPEYWDR pc2
−0.01 [−0.20, 0.18]
THVTHHPISDHEATLR pc4
0.34 [0.09, 0.58]
AQAQTDRESLR pc3
0.42 [0.15, 0.69]
AYLEGECVEWLR pc2
−0.17 [−0.42, 0.08]
FDSDAASPREEPR pc3
0.10 [−0.28, 0.48]
GHDQYAYDGK pc2
0.07 [−0.34, 0.49]
RAYLEGECVEWLR pc3
−0.01 [−0.86, 0.83]
YFYTSVSR pc2
0.03 [−0.56, 0.62]
YTCHVQHEGLPK pc2
−0.34 [−0.81, 0.13]
CWALGFYPAEITLTWQR pc3
−0.34 [−1.03, 0.36]
RE Model
0.39 [0.07, 0.71]
−1.5
−1
−0.5
0
0.5
1
Effect of 6:31242089:A:T (rs2524080)

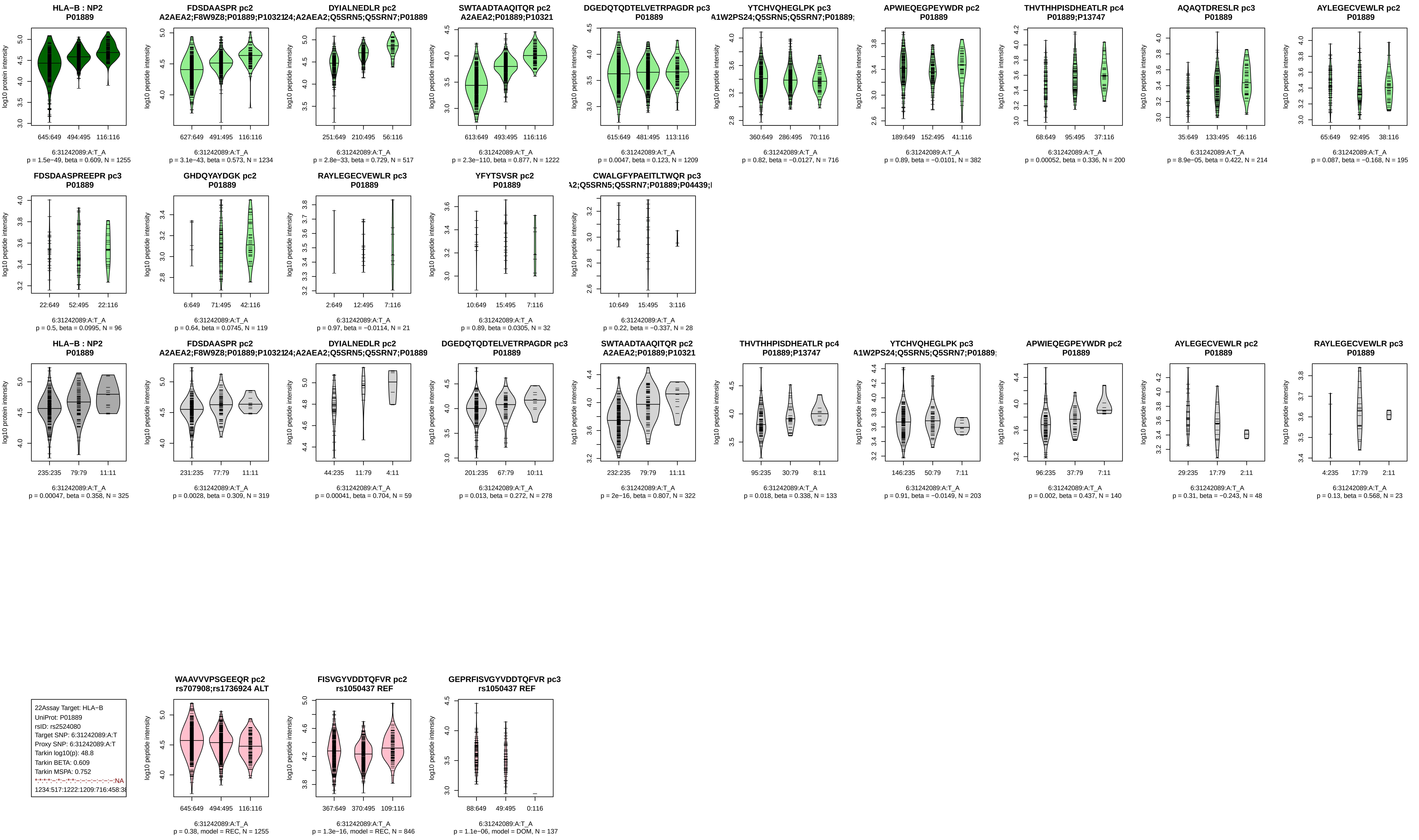
HLA−B : NP2
P01889
645:649 494:495 116:116
6:31242089:A:T_A
p = 1.5e−49, beta = 0.609, N = 1255
FDSDAASPR pc2
A2AEA2;F8W9Z8;P01889;P10321
627:649 491:495 116:116
6:31242089:A:T_A
p = 3.1e−43, beta = 0.573, N = 1234
DYIALNEDLR pc2
24;A2AEA2;Q5SRN5;Q5SRN7;P01889
251:649 210:495 56:116
6:31242089:A:T_A
p = 2.8e−33, beta = 0.729, N = 517
SWTAADTAAQITQR pc2
A2AEA2;P01889;P10321
613:649 493:495 116:116
6:31242089:A:T_A
p = 2.3e−110, beta = 0.877, N = 1222
DGEDQTQDTELVETRPAGDR pc3
P01889
615:649 481:495 113:116
6:31242089:A:T_A
p = 0.0047, beta = 0.123, N = 1209
YTCHVQHEGLPK pc3
A1W2PS24;Q5SRN5;Q5SRN7;P01889;
360:649 286:495 70:116
6:31242089:A:T_A
p = 0.82, beta = −0.0127, N = 716
APWIEQEGPEYWDR pc2
P01889
189:649 152:495 41:116
6:31242089:A:T_A
p = 0.89, beta = −0.0101, N = 382
THVTHHPISDHEATLR pc4
P01889;P13747
68:649 95:495 37:116
6:31242089:A:T_A
p = 0.00052, beta = 0.336, N = 200
AQAQTDRESLR pc3
P01889
35:649 133:495 46:116
6:31242089:A:T_A
p = 8.9e−05, beta = 0.422, N = 214
AYLEGECVEWLR pc2
P01889
65:649 92:495 38:116
6:31242089:A:T_A
p = 0.087, beta = −0.168, N = 195
FDSDAASPREEPR pc3
P01889
22:649 52:495 22:116
6:31242089:A:T_A
p = 0.5, beta = 0.0995, N = 96
GHDQYAYDGK pc2
P01889
6:649 71:495 42:116
6:31242089:A:T_A
p = 0.64, beta = 0.0745, N = 119
RAYLEGECVEWLR pc3
P01889
2:649 12:495 7:116
6:31242089:A:T_A
p = 0.97, beta = −0.0114, N = 21
YFYTSVSR pc2
P01889
10:649 15:495 7:116
6:31242089:A:T_A
p = 0.89, beta = 0.0305, N = 32
CWALGFYPAEITLTWQR pc3
A2;Q5SRN5;Q5SRN7;P01889;P04439;
10:649 15:495 3:116
6:31242089:A:T_A
p = 0.22, beta = −0.337, N = 28
HLA−B : NP2
P01889
235:235 79:79 11:11
6:31242089:A:T_A
p = 0.00047, beta = 0.358, N = 325
FDSDAASPR pc2
A2AEA2;F8W9Z8;P01889;P10321
231:235 77:79 11:11
6:31242089:A:T_A
p = 0.0028, beta = 0.309, N = 319
DYIALNEDLR pc2
24;A2AEA2;Q5SRN5;Q5SRN7;P01889
44:235 11:79 4:11
6:31242089:A:T_A
p = 0.00041, beta = 0.704, N = 59
DGEDQTQDTELVETRPAGDR pc3
P01889
201:235 67:79 10:11
6:31242089:A:T_A
p = 0.013, beta = 0.272, N = 278
SWTAADTAAQITQR pc2
A2AEA2;P01889;P10321
232:235 79:79 11:11
6:31242089:A:T_A
p = 2e−16, beta = 0.807, N = 322
THVTHHPISDHEATLR pc4
P01889;P13747
95:235 30:79 8:11
6:31242089:A:T_A
p = 0.018, beta = 0.338, N = 133
YTCHVQHEGLPK pc3
A1W2PS24;Q5SRN5;Q5SRN7;P01889;
146:235 50:79 7:11
6:31242089:A:T_A
p = 0.91, beta = −0.0149, N = 203
APWIEQEGPEYWDR pc2
P01889
96:235 37:79 7:11
6:31242089:A:T_A
p = 0.002, beta = 0.437, N = 140
AYLEGECVEWLR pc2
P01889
29:235 17:79 2:11
6:31242089:A:T_A
p = 0.31, beta = −0.243, N = 48
RAYLEGECVEWLR pc3
P01889
4:235 17:79 2:11
6:31242089:A:T_A
p = 0.13, beta = 0.568, N = 23
22Assay Target: HLA−B
UniProt: P01889
rsID: rs2524080
Target SNP: 6:31242089:A:T
Proxy SNP: 6:31242089:A:T
Tarkin log10(p): 48.8
Tarkin BETA: 0.609
Tarkin MSPA: 0.752
1234:517:1222:1209:716:458:38
WAAVVVPSGEEQR pc2
rs707908;rs1736924 ALT
645:649 494:495 116:116
6:31242089:A:T_A
p = 0.38, model = REC, N = 1255
FISVGYVDDTQFVR pc2
rs1050437 REF
367:649 370:495 109:116
6:31242089:A:T_A
p = 1.3e−16, model = REC, N = 846
GEPRFISVGYVDDTQFVR pc3
rs1050437 REF
88:649 49:495 0:116
6:31242089:A:T_A
p = 1.1e−06, model = DOM, N = 137

23. PEBP4 (Q96S96)
Tarkin MSPA=1

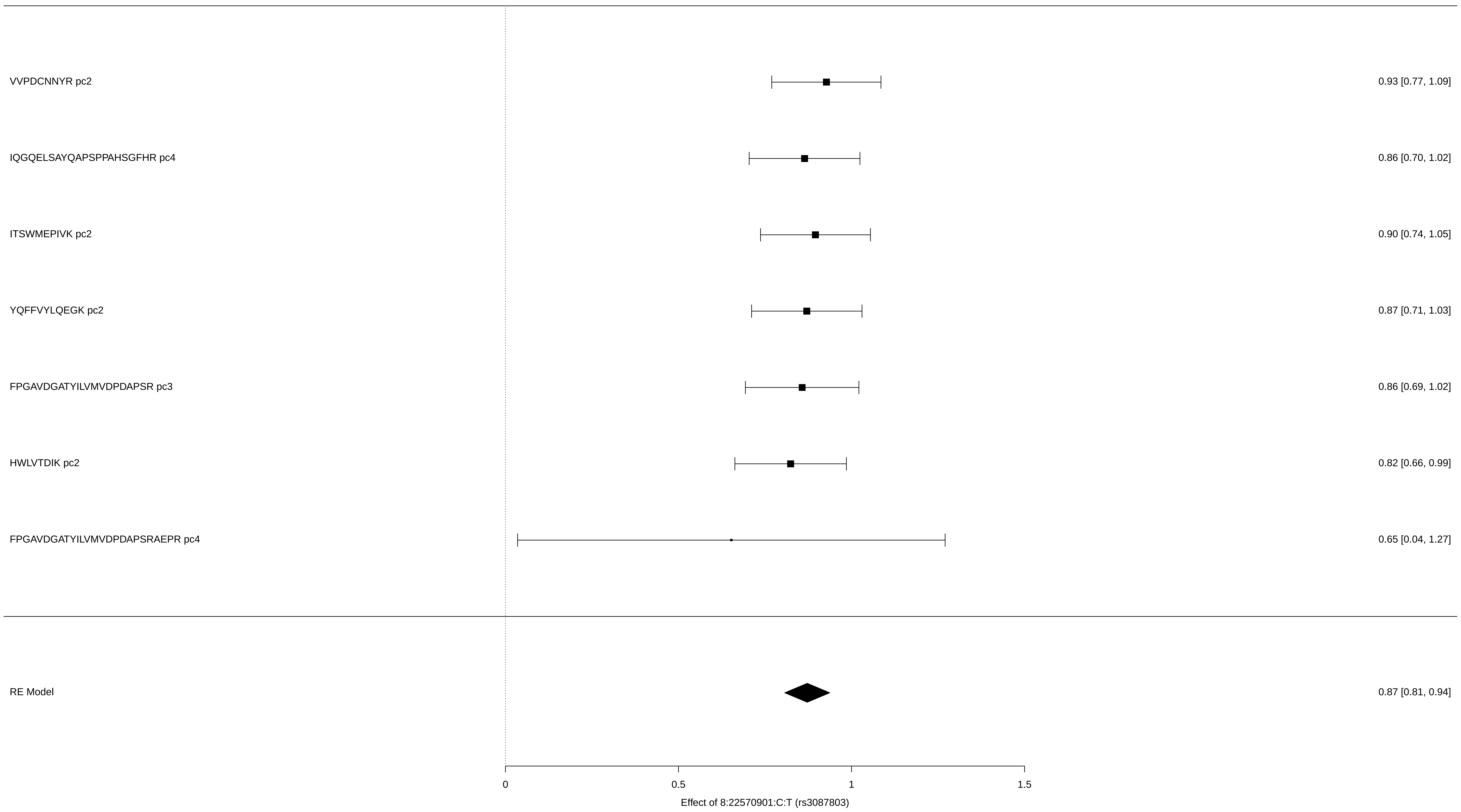

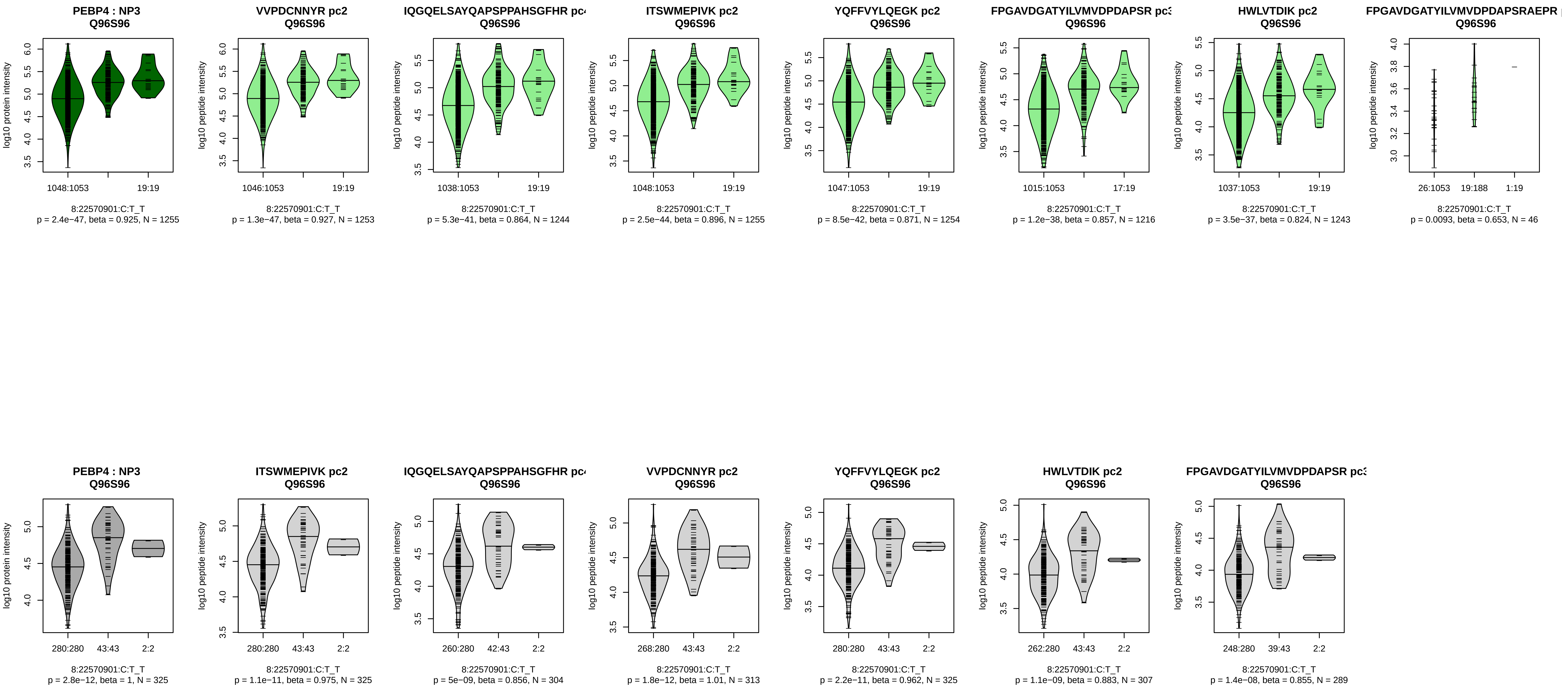

23Assay Target: PEBP4
UniProt: Q96S96
rsID: rs3087803
Target SNP: 8:22570901:C:T
Proxy SNP: 8:22570901:C:T
Tarkin log10(p): 46.6
Tarkin BETA: 0.925
Tarkin MSPA: 1
::*:*:*:*:*:NA:NA
1253:1244:1255:1254:1216:124

24. CCDC7 (Q96M83)
Tarkin MSPA=0.767

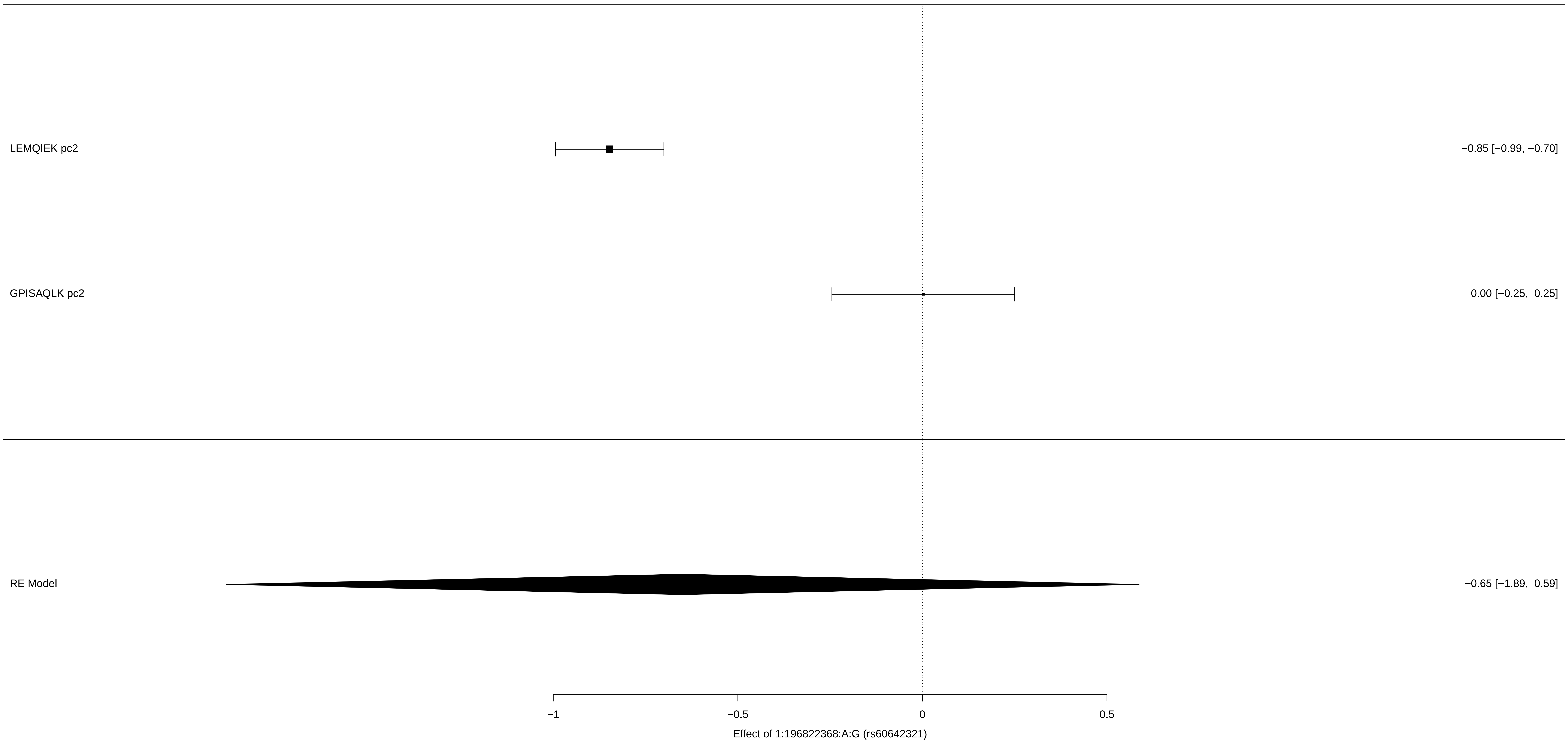

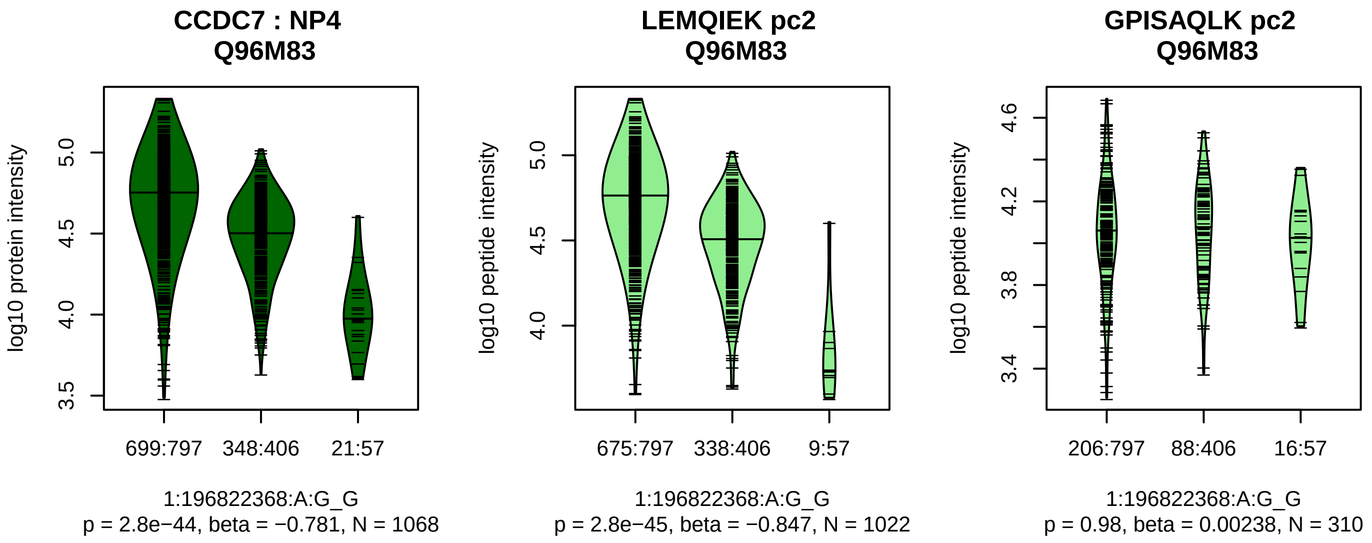

24Assay Target: CCDC7
UniProt: Q96M83
rsID: rs60642321
Target SNP: 1:196822368:A:G
Proxy SNP: 1:196822368:A:G
Tarkin log10(p): 43.6
Tarkin BETA: −0.781
Tarkin MSPA: 0.767
*:–
1022:310

25. CFB (B4E1Z4)
Tarkin MSPA=0.627

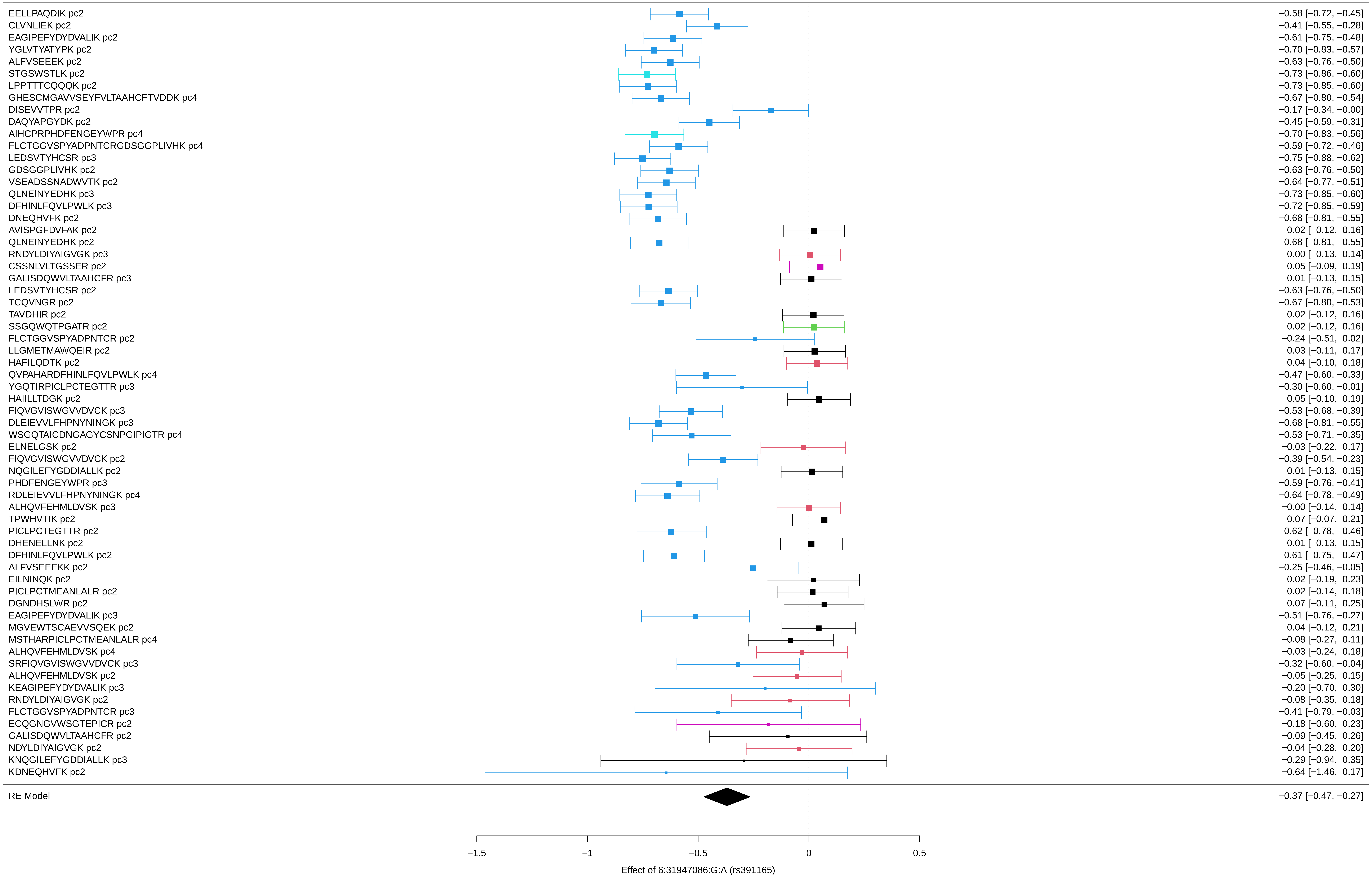

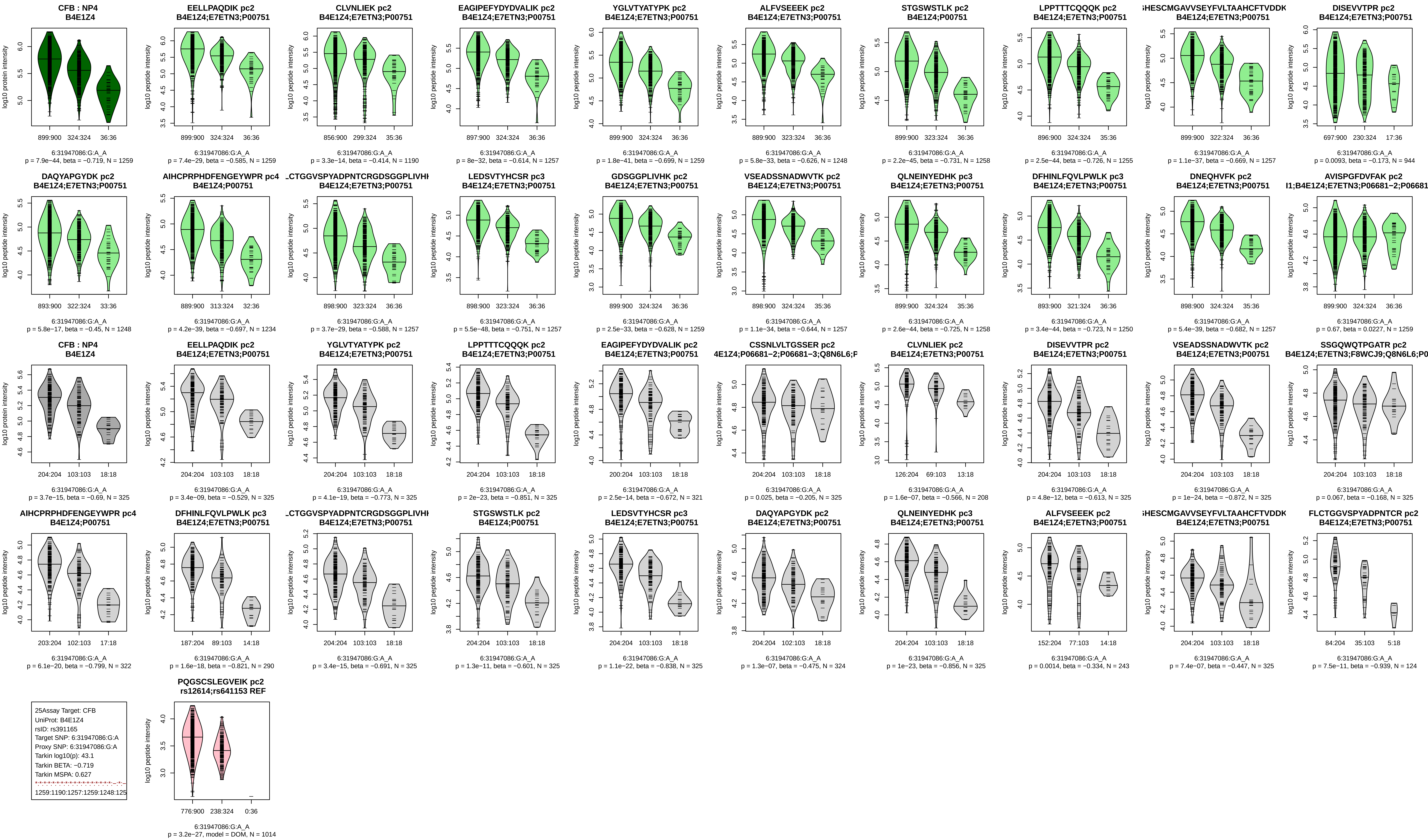
CFB : NP4
B4E1Z4
6:31947086:G:A_A
p = 7.9e−44, beta = −0.719, N = 1259
EELLPAQDIK pc2
B4E1Z4;E7ETN3;P00751
6:31947086:G:A_A
p = 7.4e−29, beta = −0.585, N = 1259
CLVNLIEK pc2
B4E1Z4;E7ETN3;P00751
6:31947086:G:A_A
p = 3.3e−14, beta = −0.414, N = 1190
EAGIPEFYDYDVALIK pc2
B4E1Z4;E7ETN3;P00751
6:31947086:G:A_A
p = 8e−32, beta = −0.614, N = 1257
YGLVTYATYPK pc2
B4E1Z4;E7ETN3;P00751
6:31947086:G:A_A
p = 1.8e−41, beta = −0.699, N = 1259
ALFVSEEEK pc2
B4E1Z4;E7ETN3;P00751
6:31947086:G:A_A
p = 5.8e−33, beta = −0.626, N = 1248
STGSWSTLK pc2
B4E1Z4;P00751
6:31947086:G:A_A
p = 2.2e−45, beta = −0.731, N = 1258
LPPTTTCQQQK pc2
B4E1Z4;E7ETN3;P00751
6:31947086:G:A_A
p = 2.5e−44, beta = −0.726, N = 1255
HESCMGAVVSEYFVLTAAHCFTVDDK
B4E1Z4;E7ETN3;P00751
6:31947086:G:A_A
p = 1.1e−37, beta = −0.669, N = 1257
DISEVVTPR pc2
B4E1Z4;E7ETN3;P00751
6:31947086:G:A_A
p = 0.0093, beta = −0.173, N = 944
DAQYAPGYDK pc2
B4E1Z4;E7ETN3;P00751
6:31947086:G:A_A
p = 5.8e−17, beta = −0.45, N = 1248
AIHCPRPHDFENGEYWPR pc4
B4E1Z4;P00751
6:31947086:G:A_A
p = 4.2e−39, beta = −0.697, N = 1234
CTGGVSPYADPNTCRGDSGGPLIVH
B4E1Z4;E7ETN3;P00751
6:31947086:G:A_A
p = 3.7e−29, beta = −0.588, N = 1257
LEDSVTYHCSR pc3
B4E1Z4;E7ETN3;P00751
6:31947086:G:A_A
p = 5.5e−48, beta = −0.751, N = 1257
GDSGGPLIVHK pc2
B4E1Z4;E7ETN3;P00751
6:31947086:G:A_A
p = 2.5e−33, beta = −0.628, N = 1259
VSEADSSNADWVTK pc2
B4E1Z4;E7ETN3;P00751
6:31947086:G:A_A
p = 1.1e−34, beta = −0.644, N = 1257
QLNEINYEDHK pc3
B4E1Z4;E7ETN3;P00751
6:31947086:G:A_A
p = 2.6e−44, beta = −0.725, N = 1258
DFHINLFQVLPWLK pc3
B4E1Z4;E7ETN3;P00751
6:31947086:G:A_A
p = 3.4e−44, beta = −0.723, N = 1250
DNEQHVFK pc2
B4E1Z4;E7ETN3;P00751
6:31947086:G:A_A
p = 5.4e−39, beta = −0.682, N = 1257
AVISPGFDVFAK pc2
I1;B4E1Z4;E7ETN3;P06681−2;P06681
6:31947086:G:A_A
p = 0.67, beta = 0.0227, N = 1259
CFB : NP4
B4E1Z4
6:31947086:G:A_A
p = 3.7e−15, beta = −0.69, N = 325
EELLPAQDIK pc2
B4E1Z4;E7ETN3;P00751
6:31947086:G:A_A
p = 3.4e−09, beta = −0.529, N = 325
YGLVTYATYPK pc2
B4E1Z4;E7ETN3;P00751
6:31947086:G:A_A
p = 4.1e−19, beta = −0.773, N = 325
LPPTTTCQQQK pc2
B4E1Z4;E7ETN3;P00751
6:31947086:G:A_A
p = 2e−23, beta = −0.851, N = 325
EAGIPEFYDYDVALIK pc2
B4E1Z4;E7ETN3;P00751
6:31947086:G:A_A
p = 2.5e−14, beta = −0.672, N = 321
CSSNLVLTGSSER pc2
4E1Z4;P06681−2;P06681−3;Q8N6L6;P
6:31947086:G:A_A
p = 0.025, beta = −0.205, N = 325
CLVNLIEK pc2
B4E1Z4;E7ETN3;P00751
6:31947086:G:A_A
p = 1.6e−07, beta = −0.566, N = 208
DISEVVTPR pc2
B4E1Z4;E7ETN3;P00751
6:31947086:G:A_A
p = 4.8e−12, beta = −0.613, N = 325
VSEADSSNADWVTK pc2
B4E1Z4;E7ETN3;P00751
6:31947086:G:A_A
p = 1e−24, beta = −0.872, N = 325
SSGQWQTPGATR pc2
B4E1Z4;E7ETN3;F8WCJ9;Q8N6L6;P0
6:31947086:G:A_A
p = 0.067, beta = −0.168, N = 325
AIHCPRPHDFENGEYWPR pc4
B4E1Z4;P00751
6:31947086:G:A_A
p = 6.1e−20, beta = −0.799, N = 322
DFHINLFQVLPWLK pc3
B4E1Z4;E7ETN3;P00751
6:31947086:G:A_A
p = 1.6e−18, beta = −0.821, N = 290
CTGGVSPYADPNTCRGDSGGPLIVH
B4E1Z4;E7ETN3;P00751
6:31947086:G:A_A
p = 3.4e−15, beta = −0.691, N = 325
STGSWSTLK pc2
B4E1Z4;P00751
6:31947086:G:A_A
p = 1.3e−11, beta = −0.601, N = 325
LEDSVTYHCSR pc3
B4E1Z4;E7ETN3;P00751
6:31947086:G:A_A
p = 1.1e−22, beta = −0.838, N = 325
DAQYAPGYDK pc2
B4E1Z4;E7ETN3;P00751
6:31947086:G:A_A
p = 1.3e−07, beta = −0.475, N = 324
QLNEINYEDHK pc3
B4E1Z4;E7ETN3;P00751
6:31947086:G:A_A
p = 1e−23, beta = −0.856, N = 325
ALFVSEEEK pc2
B4E1Z4;E7ETN3;P00751
6:31947086:G:A_A
p = 0.0014, beta = −0.334, N = 243
HESCMGAVVSEYFVLTAAHCFTVDDK
B4E1Z4;E7ETN3;P00751
6:31947086:G:A_A
p = 7.4e−07, beta = −0.447, N = 325
FLCTGGVSPYADPNTCR pc2
B4E1Z4;E7ETN3;P00751
6:31947086:G:A_A
p = 7.5e−11, beta = −0.939, N = 124
25Assay Target: CFB
UniProt: B4E1Z4
rsID: rs391165
Target SNP: 6:31947086:G:A
Proxy SNP: 6:31947086:G:A
Tarkin log10(p): 43.1
Tarkin BETA: −0.719
Tarkin MSPA: 0.627
1259:1190:1257:1259:1248:125
PQGSCSLEGVEIK pc2
rs12614;rs641153 REF
6:31947086:G:A_A
p = 3.2e−27, model = DOM, N = 1014

26. EBI3 (Q14213)
Tarkin MSPA=1

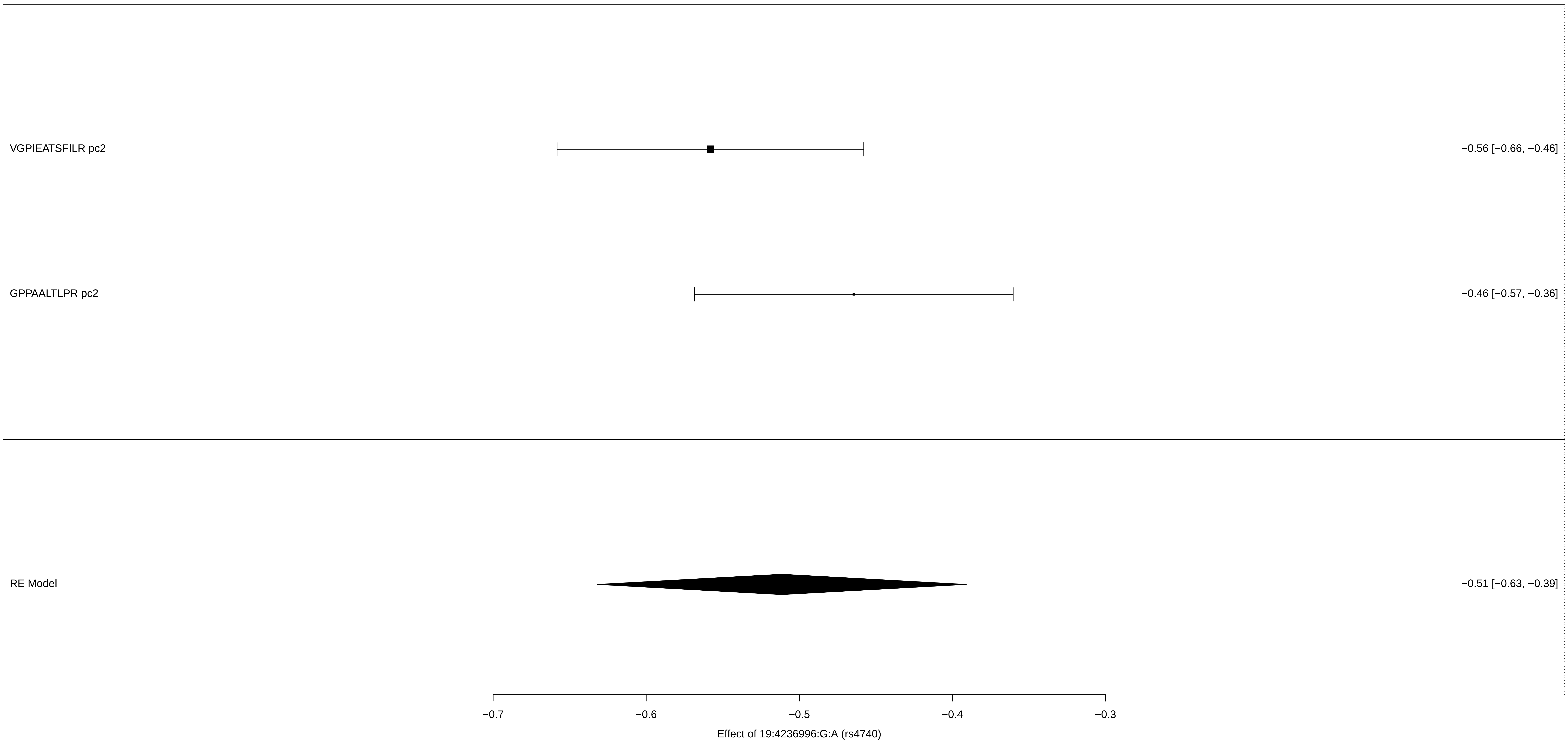

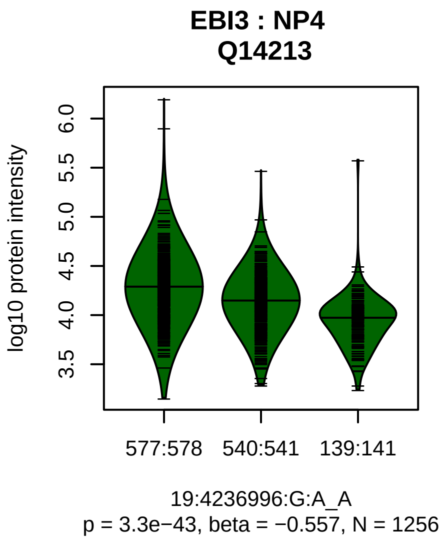

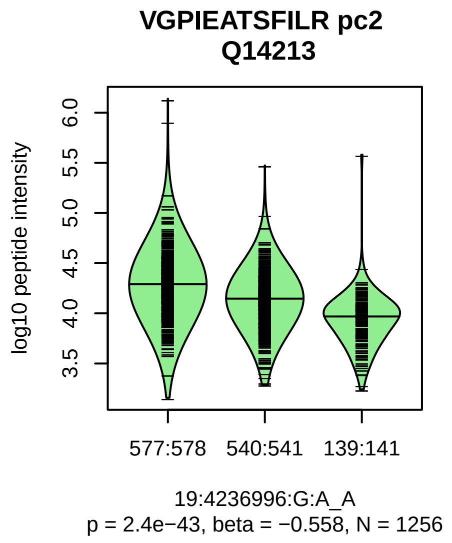

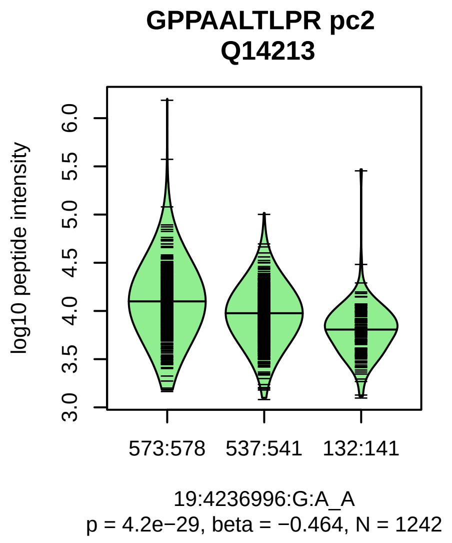

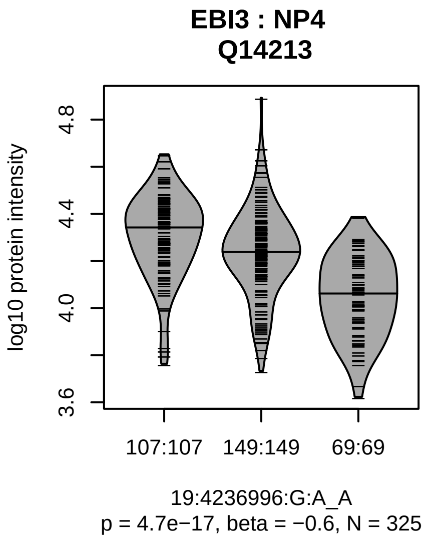

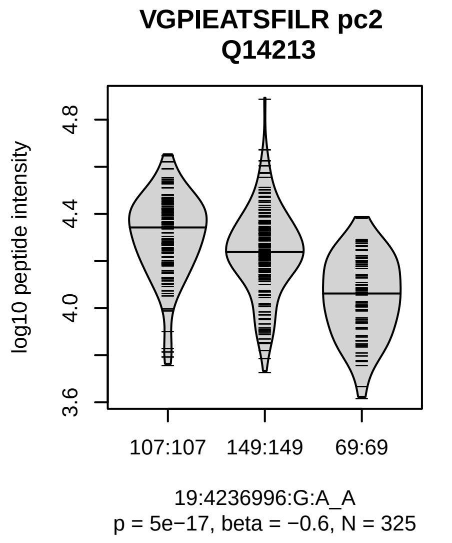

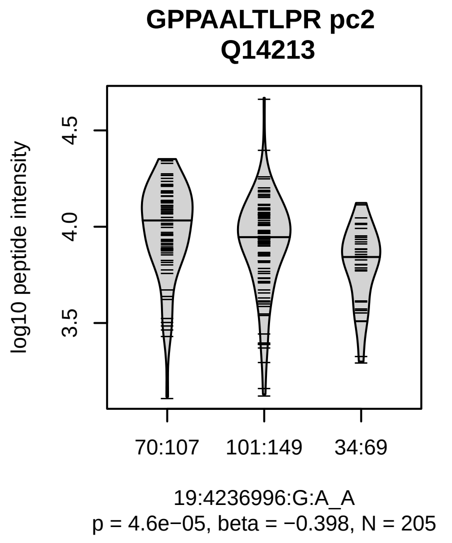

26Assay Target: EBI3
UniProt: Q14213
rsID: rs4740
Target SNP: 19:4236996:G:A
Proxy SNP: 19:4236996:G:A
Tarkin log10(p): 42.5
Tarkin BETA: −0.557
Tarkin MSPA: 1
:
1256:1242

27. ERAP1 (Q9NZ08)
Tarkin MSPA=0.877

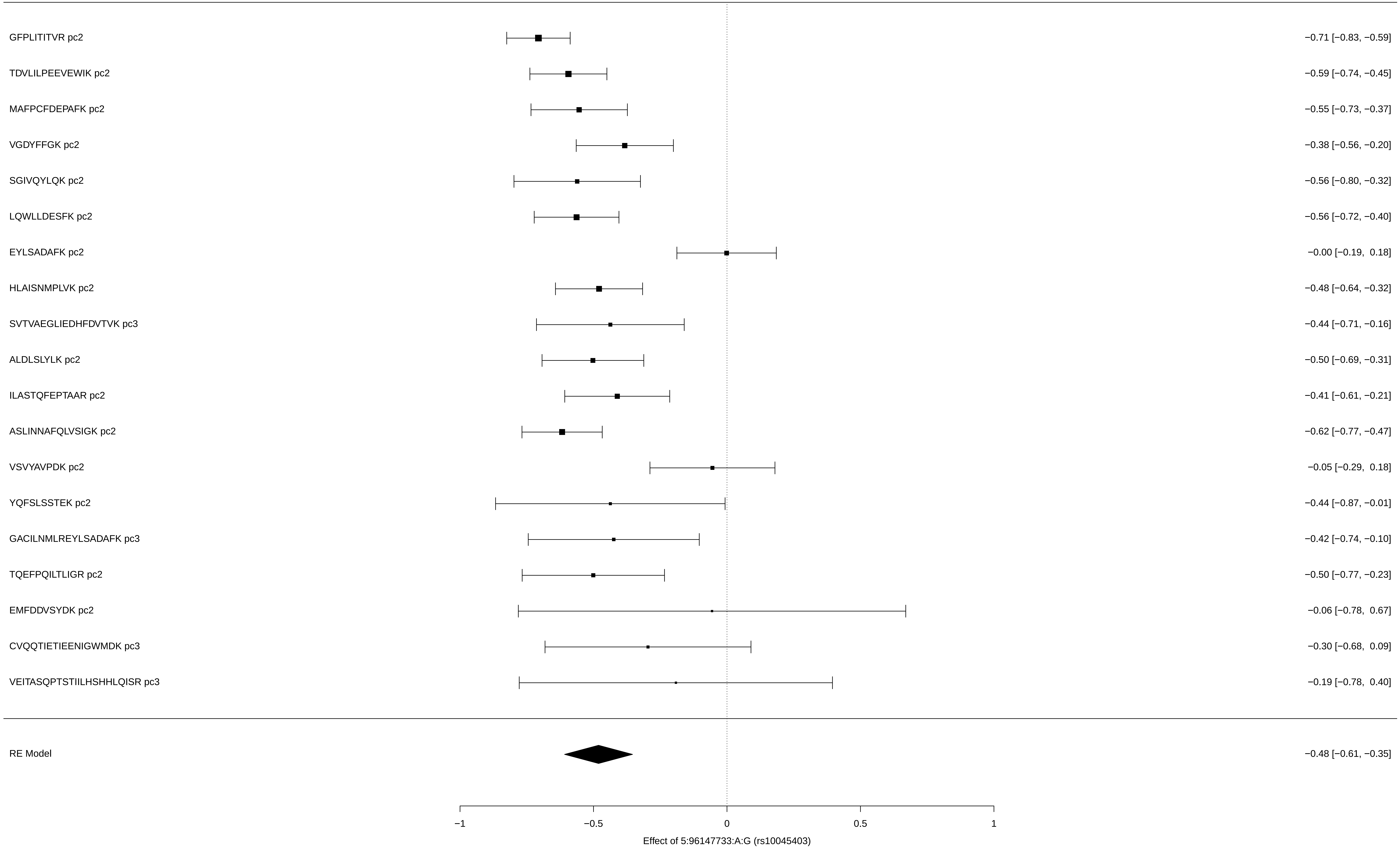

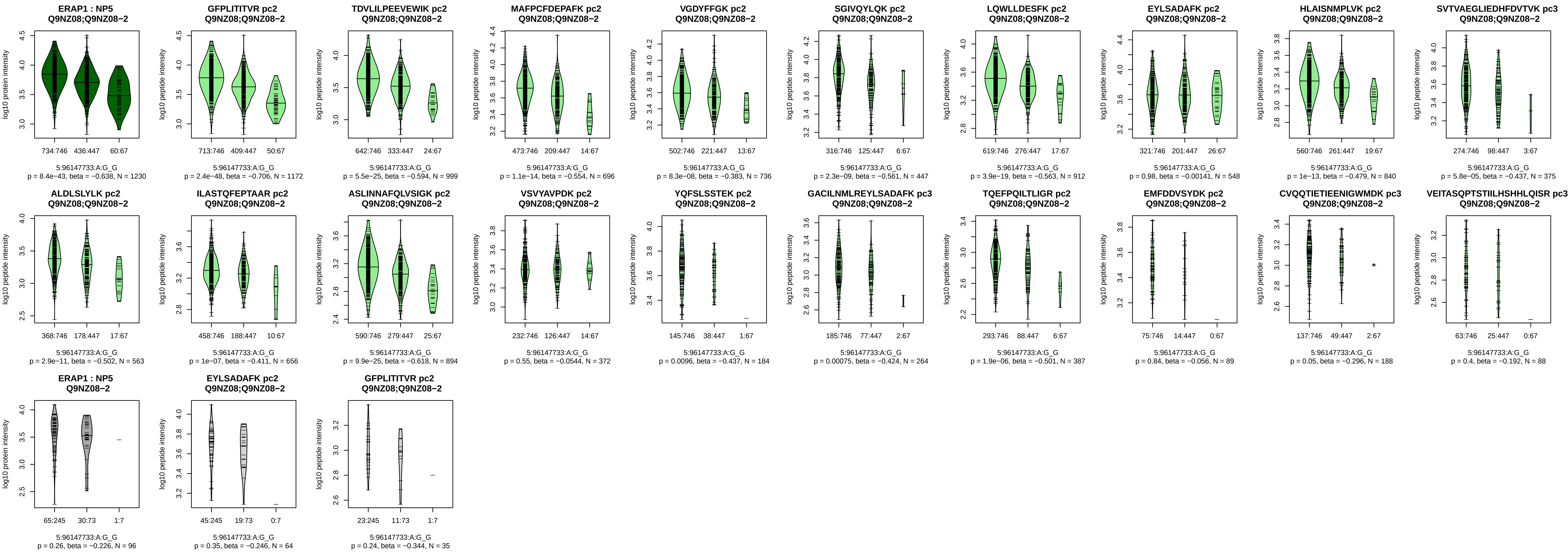

27Assay Target: ERAP1
UniProt: Q9NZ08
rsID: rs10045403
Target SNP: 5:96147733:A:G
Proxy SNP: 5:96147733:A:G
Tarkin log10(p): 42.1
Tarkin BETA: −0.638
Tarkin MSPA: 0.877
[illegible]
1172:999:696:736:447:912:548

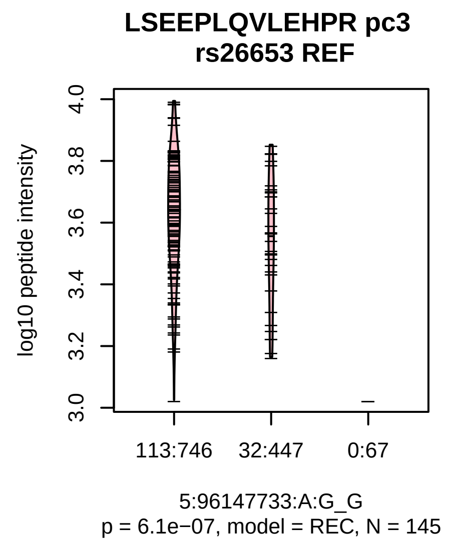

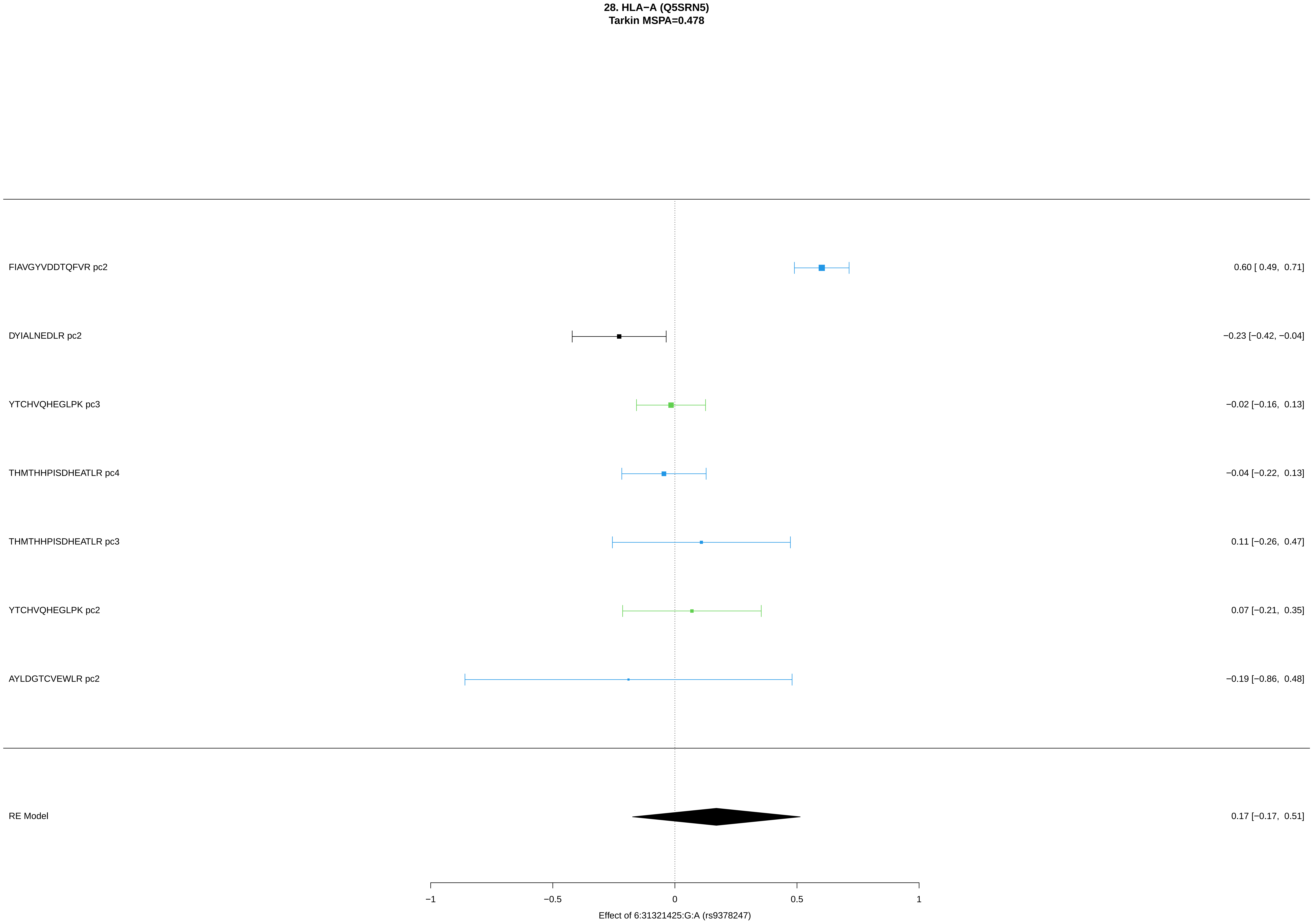
28. HLA−A (Q5SRN5)
Tarkin MSPA=0.478
FIAVGYVDDTQFVR pc2
0.60 [0.49, 0.71]
DYIALNEDLR pc2
−0.23 [−0.42, −0.04]
YTCHVQHEGLPK pc3
−0.02 [−0.16, 0.13]
THMTHHPISDHEATLR pc4
−0.04 [−0.22, 0.13]
THMTHHPISDHEATLR pc3
0.11 [−0.26, 0.47]
YTCHVQHEGLPK pc2
0.07 [−0.21, 0.35]
AYLDGTCVEWLR pc2
−0.19 [−0.86, 0.48]
RE Model
0.17 [−0.17, 0.51]
−1
−0.5
0
0.5
1
Effect of 6:31321425:G:A (rs9378247)

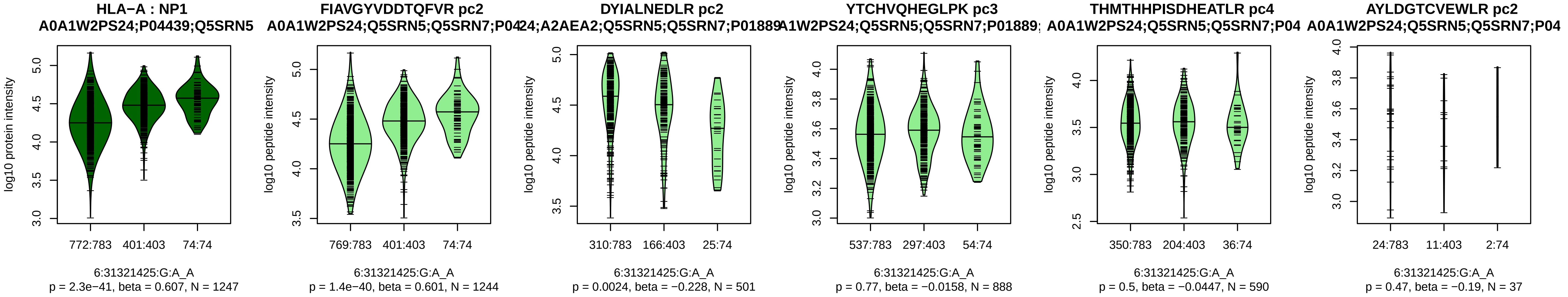

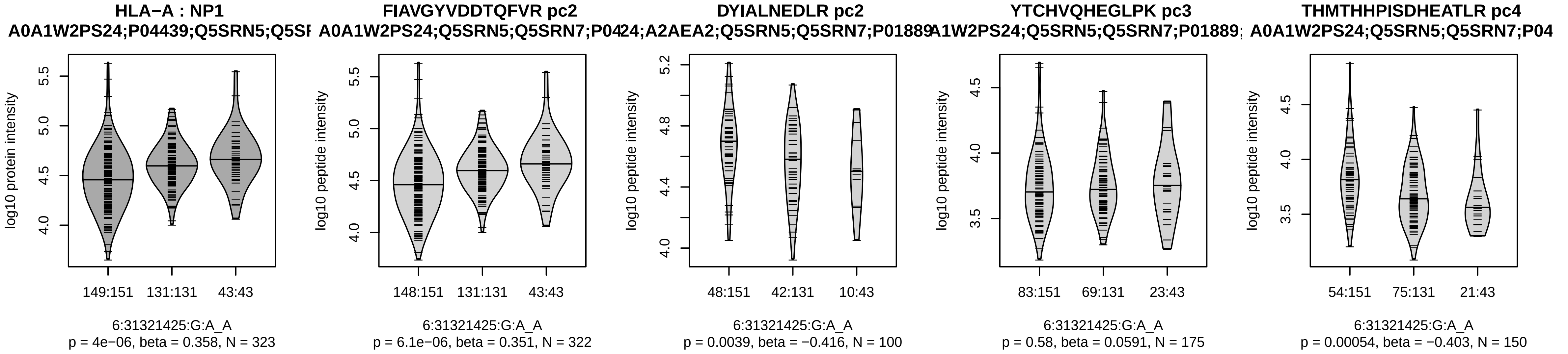

28Assay Target: HLA−A
UniProt: Q5SRN5
rsID: rs9378247
Target SNP: 6:31321425:G:A
Proxy SNP: 6:31321425:G:A
Tarkin log10(p): 40.6
Tarkin BETA: 0.607
Tarkin MSPA: 0.478
::−:−:−:−:−:NA
1244:501:888:590:166:223:37:1

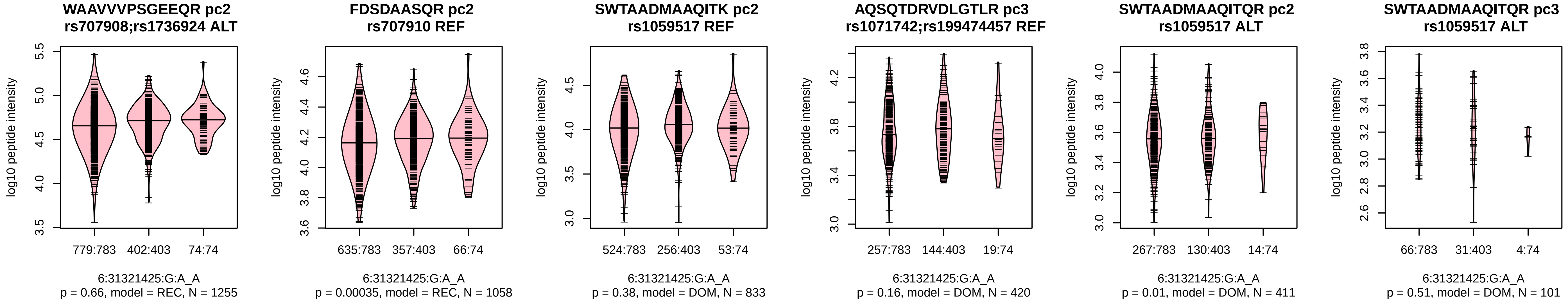

29. PGLYRP2 (Q96PD5)
Tarkin MSPA=0.964

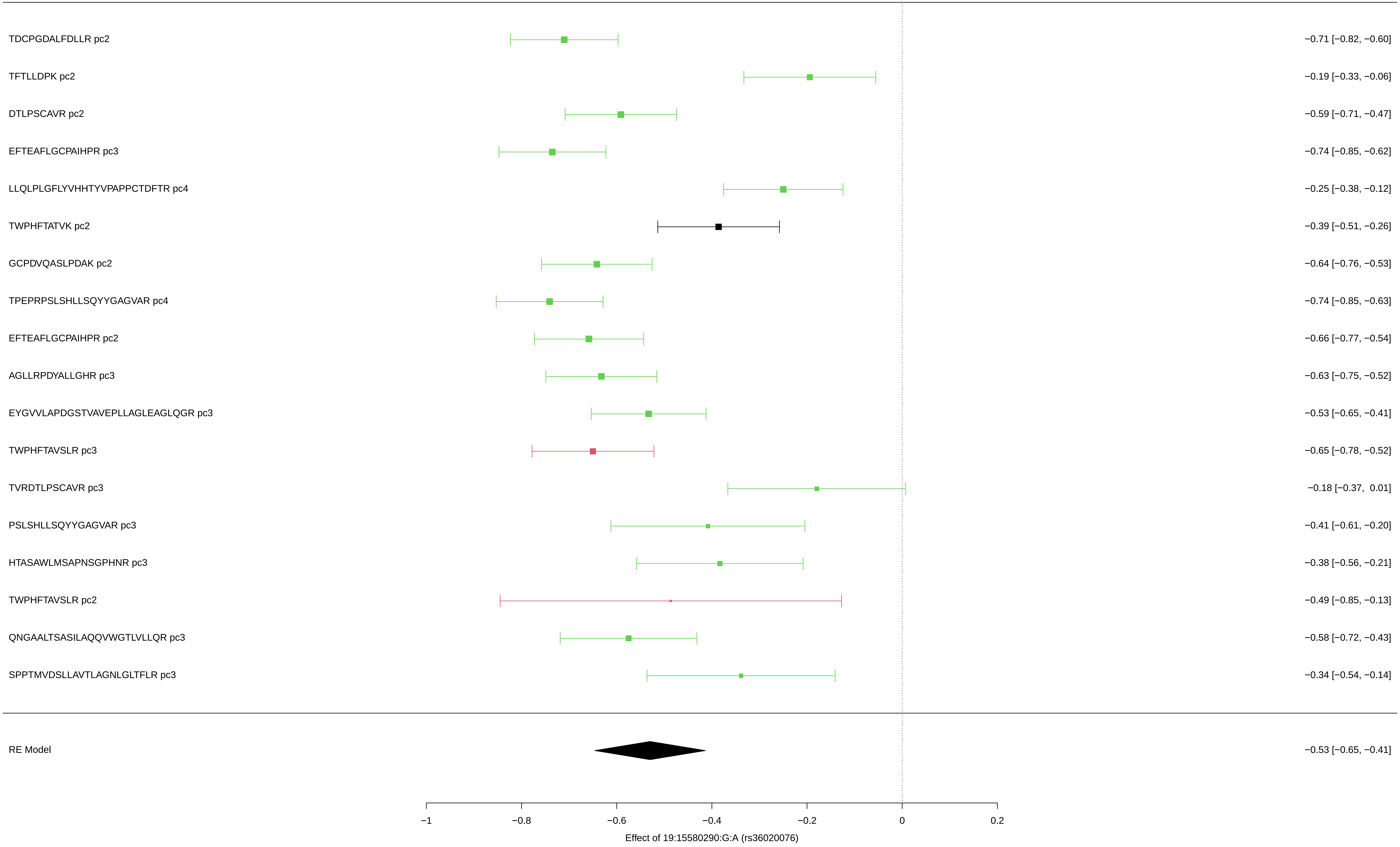

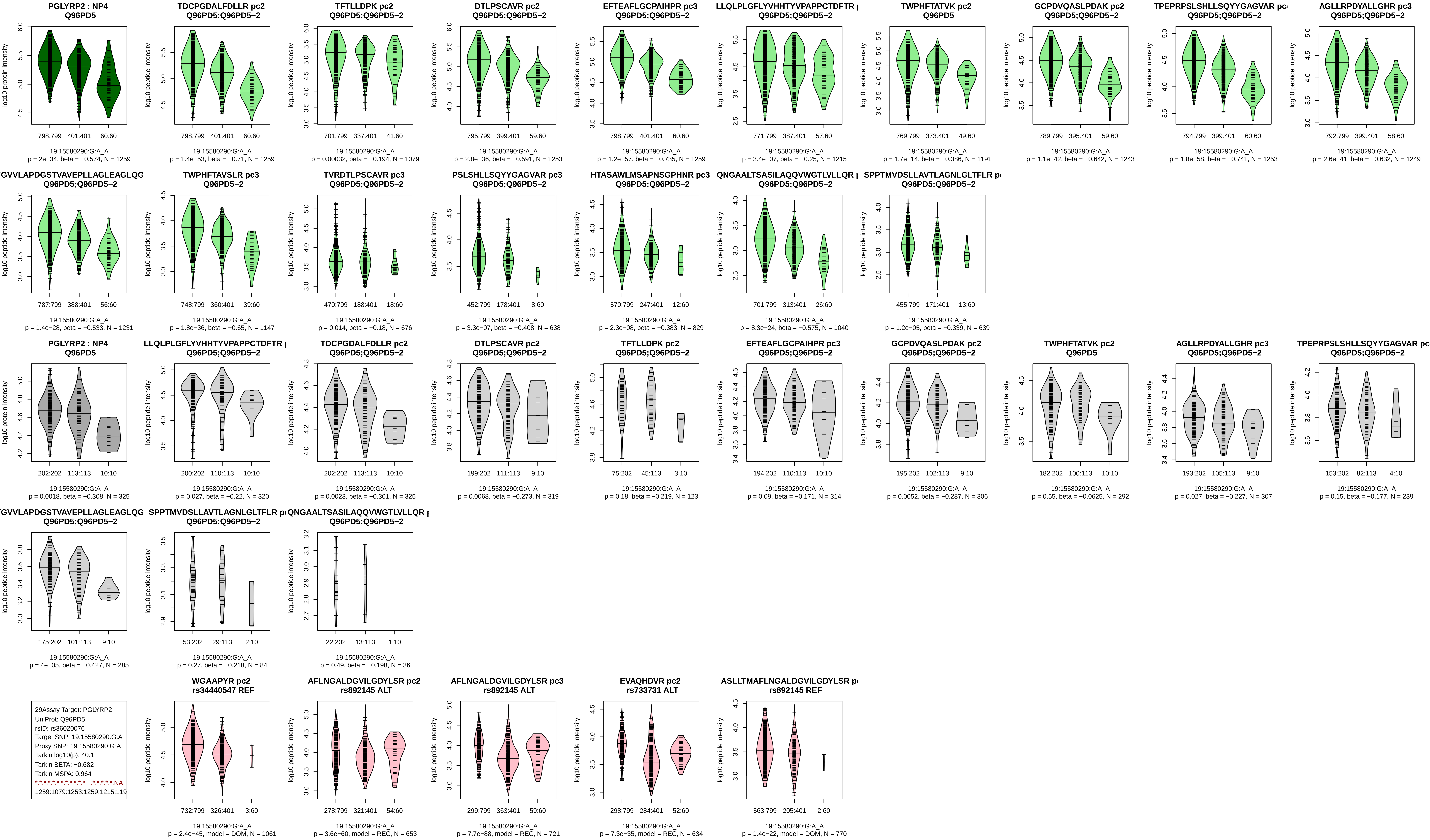

PGLYRP2 : NP4
Q96PD5
798:799 401:401 60:60
19:15580290:G:A_A
p = 2e−34, beta = −0.574, N = 1259
TDCPGDALFDLLR pc2
Q96PD5;Q96PD5−2
798:799 401:401 60:60
19:15580290:G:A_A
p = 1.4e−53, beta = −0.71, N = 1259
TFTLLDPK pc2
Q96PD5;Q96PD5−2
701:799 337:401 41:60
19:15580290:G:A_A
p = 0.00032, beta = −0.194, N = 1079
DTLPSCAVR pc2
Q96PD5;Q96PD5−2
795:799 399:401 59:60
19:15580290:G:A_A
p = 2.8e−36, beta = −0.591, N = 1253
EFTEAFLGCPAIHPR pc3
Q96PD5;Q96PD5−2
798:799 401:401 60:60
19:15580290:G:A_A
p = 1.2e−57, beta = −0.735, N = 1259
LLQLPLGFLYVHHTYVPAPPCTDFTR
Q96PD5;Q96PD5−2
771:799 387:401 57:60
19:15580290:G:A_A
p = 3.4e−07, beta = −0.25, N = 1215
TWPHFTATVK pc2
Q96PD5
769:799 373:401 49:60
19:15580290:G:A_A
p = 1.7e−14, beta = −0.386, N = 1191
GCPDVQASLPDAK pc2
Q96PD5;Q96PD5−2
789:799 395:401 59:60
19:15580290:G:A_A
p = 1.1e−42, beta = −0.642, N = 1243
TPEPRPSLSHLLSQYYGAGVAR pc
Q96PD5;Q96PD5−2
794:799 399:401 60:60
19:15580290:G:A_A
p = 1.8e−58, beta = −0.741, N = 1253
AGLLRPDYALLGHR pc3
Q96PD5;Q96PD5−2
792:799 399:401 58:60
19:15580290:G:A_A
p = 2.6e−41, beta = −0.632, N = 1249
GVVLAPDGSTVAVEPLLAGLEAGLQG
Q96PD5;Q96PD5−2
787:799 388:401 56:60
19:15580290:G:A_A
p = 1.4e−28, beta = −0.533, N = 1231
TWPHFTAVSLR pc3
Q96PD5−2
748:799 360:401 39:60
19:15580290:G:A_A
p = 1.8e−36, beta = −0.65, N = 1147
TVRDTLPSCAVR pc3
Q96PD5;Q96PD5−2
470:799 188:401 18:60
19:15580290:G:A_A
p = 0.014, beta = −0.18, N = 676
PSLSHLLSQYYGAGVAR pc3
Q96PD5;Q96PD5−2
452:799 178:401 8:60
19:15580290:G:A_A
p = 3.3e−07, beta = −0.408, N = 638
HTASAWLMSAPNSGPHNR pc3
Q96PD5;Q96PD5−2
570:799 247:401 12:60
19:15580290:G:A_A
p = 2.3e−08, beta = −0.383, N = 829
QNGAALTSASILAQQVWGTLVLLQR
Q96PD5;Q96PD5−2
701:799 313:401 26:60
19:15580290:G:A_A
p = 8.3e−24, beta = −0.575, N = 1040
SPPTMVDSLLAVTLAGNLGLTFLR p
Q96PD5;Q96PD5−2
455:799 171:401 13:60
19:15580290:G:A_A
p = 1.2e−05, beta = −0.339, N = 639
PGLYRP2 : NP4
Q96PD5
202:202 113:113 10:10
19:15580290:G:A_A
p = 0.0018, beta = −0.308, N = 325
LLQLPLGFLYVHHTYVPAPPCTDFTR
Q96PD5;Q96PD5−2
200:202 110:113 10:10
19:15580290:G:A_A
p = 0.027, beta = −0.22, N = 320
TDCPGDALFDLLR pc2
Q96PD5;Q96PD5−2
202:202 113:113 10:10
19:15580290:G:A_A
p = 0.0023, beta = −0.301, N = 325
DTLPSCAVR pc2
Q96PD5;Q96PD5−2
199:202 111:113 9:10
19:15580290:G:A_A
p = 0.0068, beta = −0.273, N = 319
TFTLLDPK pc2
Q96PD5;Q96PD5−2
75:202 45:113 3:10
19:15580290:G:A_A
p = 0.18, beta = −0.219, N = 123
EFTEAFLGCPAIHPR pc3
Q96PD5;Q96PD5−2
194:202 110:113 10:10
19:15580290:G:A_A
p = 0.09, beta = −0.171, N = 314
GCPDVQASLPDAK pc2
Q96PD5;Q96PD5−2
195:202 102:113 9:10
19:15580290:G:A_A
p = 0.0052, beta = −0.287, N = 306
TWPHFTATVK pc2
Q96PD5
182:202 100:113 10:10
19:15580290:G:A_A
p = 0.55, beta = −0.0625, N = 292
AGLLRPDYALLGHR pc3
Q96PD5;Q96PD5−2
193:202 105:113 9:10
19:15580290:G:A_A
p = 0.027, beta = −0.227, N = 307
TPEPRPSLSHLLSQYYGAGVAR pc
Q96PD5;Q96PD5−2
153:202 82:113 4:10
19:15580290:G:A_A
p = 0.15, beta = −0.177, N = 239
GVVLAPDGSTVAVEPLLAGLEAGLQG
Q96PD5;Q96PD5−2
175:202 101:113 9:10
19:15580290:G:A_A
p = 4e−05, beta = −0.427, N = 285
SPPTMVDSLLAVTLAGNLGLTFLR p
Q96PD5;Q96PD5−2
53:202 29:113 2:10
19:15580290:G:A_A
p = 0.27, beta = −0.218, N = 84
QNGAALTSASILAQQVWGTLVLLQR
Q96PD5;Q96PD5−2
22:202 13:113 1:10
19:15580290:G:A_A
p = 0.49, beta = −0.198, N = 36
29Assay Target: PGLYRP2
UniProt: Q96PD5
rsID: rs36020076
Target SNP: 19:15580290:G:A
Proxy SNP: 19:15580290:G:A
Tarkin log10(p): 40.1
Tarkin BETA: −0.682
Tarkin MSPA: 0.964
1259:1079:1253:1259:1215:119
WGAAPYR pc2
rs34440547 REF
732:799 326:401 3:60
19:15580290:G:A_A
p = 2.4e−45, model = DOM, N = 1061
AFLNGALDGVILGDYLSR pc2
rs892145 ALT
278:799 321:401 54:60
19:15580290:G:A_A
p = 3.6e−60, model = REC, N = 653
AFLNGALDGVILGDYLSR pc3
rs892145 ALT
299:799 363:401 59:60
19:15580290:G:A_A
p = 7.7e−88, model = REC, N = 721
EVAQHDVR pc2
rs733731 ALT
298:799 284:401 52:60
19:15580290:G:A_A
p = 7.3e−35, model = REC, N = 634
ASLLTMAFLNGALDGVILGDYLSR p
rs892145 REF
563:799 205:401 2:60
19:15580290:G:A_A
p = 1.4e−22, model = DOM, N = 770

30. SERPINF2 (P08697)
Tarkin MSPA=0.947

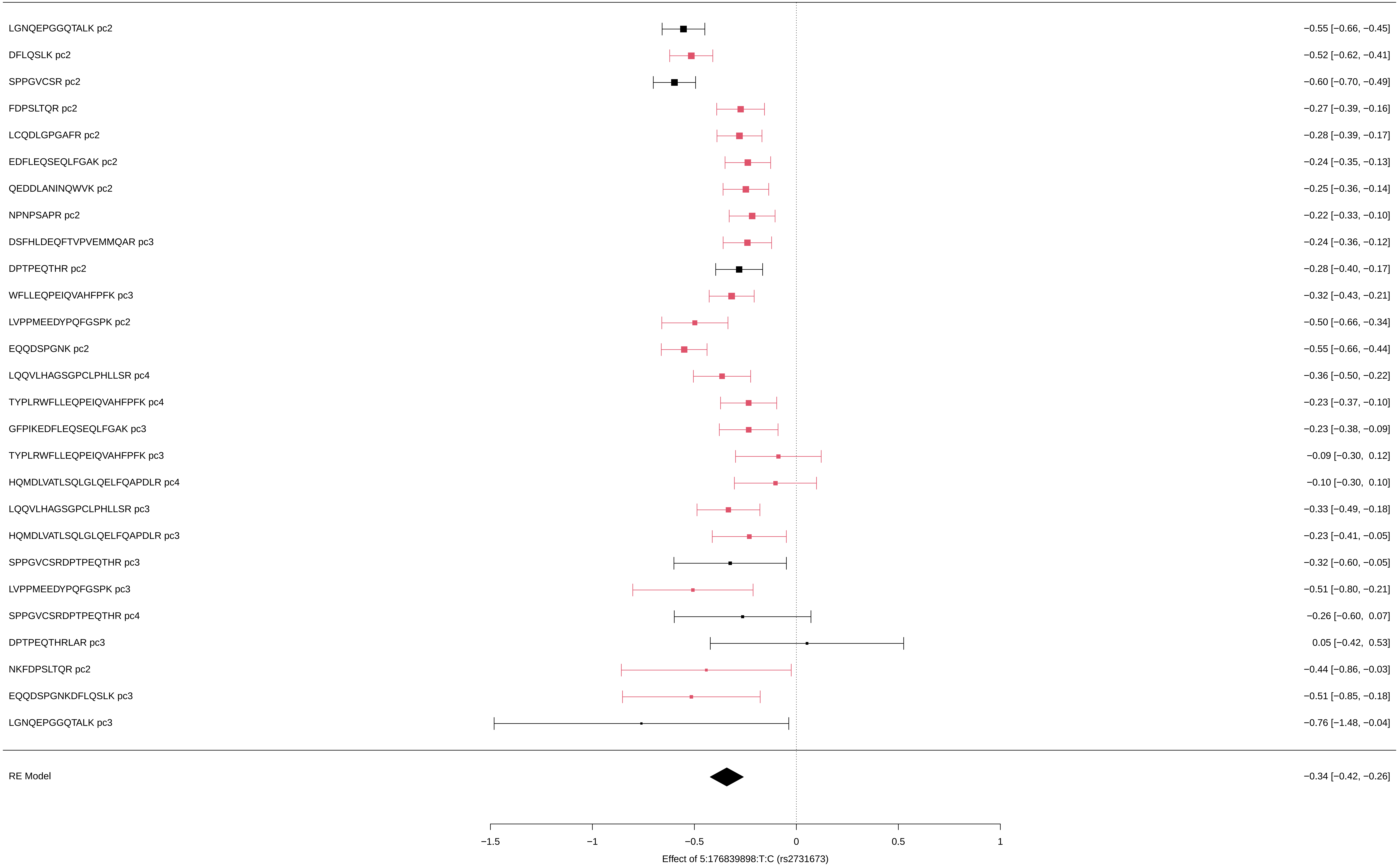

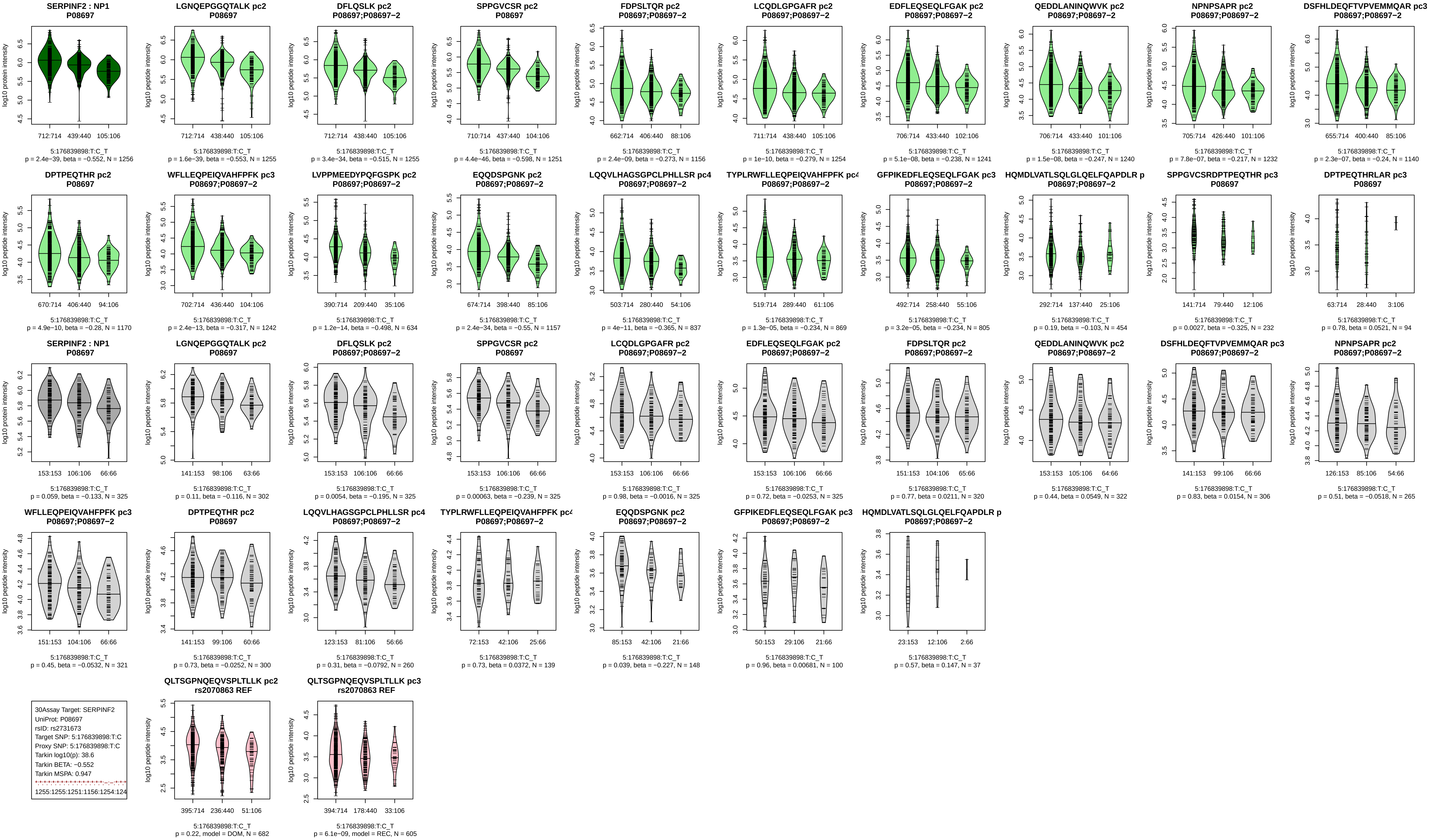
SERPINF2 : NP1
P08697
log10 protein intensity
712:714 439:440 105:106
5:176839898:T:C_T
p = 2.4e−39, beta = −0.552, N = 1256
LGNQEPGGQTALK pc2
P08697
log10 peptide intensity
712:714 438:440 105:106
5:176839898:T:C_T
p = 1.6e−39, beta = −0.553, N = 1255
DFLQSLK pc2
P08697;P08697−2
log10 peptide intensity
712:714 438:440 105:106
5:176839898:T:C_T
p = 3.4e−34, beta = −0.515, N = 1255
SPPGVCSR pc2
P08697
log10 peptide intensity
710:714 437:440 104:106
5:176839898:T:C_T
p = 4.4e−46, beta = −0.598, N = 1251
FDPSLTQR pc2
P08697;P08697−2
log10 peptide intensity
662:714 406:440 88:106
5:176839898:T:C_T
p = 2.4e−09, beta = −0.273, N = 1156
LCQDLGPGAFR pc2
P08697;P08697−2
log10 peptide intensity
711:714 438:440 105:106
5:176839898:T:C_T
p = 1e−10, beta = −0.279, N = 1254
EDFLEQSEQLFGAK pc2
P08697;P08697−2
log10 peptide intensity
706:714 433:440 102:106
5:176839898:T:C_T
p = 5.1e−08, beta = −0.238, N = 1241
QEDDLANINQWVK pc2
P08697;P08697−2
log10 peptide intensity
706:714 433:440 101:106
5:176839898:T:C_T
p = 1.5e−08, beta = −0.247, N = 1240
NPNPSAPR pc2
P08697;P08697−2
log10 peptide intensity
705:714 426:440 101:106
5:176839898:T:C_T
p = 7.8e−07, beta = −0.217, N = 1232
DSFHLDEQFTVPVEMMQAR pc3
P08697;P08697−2
log10 peptide intensity
655:714 400:440 85:106
5:176839898:T:C_T
p = 2.3e−07, beta = −0.24, N = 1140
DPTPEQTHR pc2
P08697
log10 peptide intensity
670:714 406:440 94:106
5:176839898:T:C_T
p = 4.9e−10, beta = −0.28, N = 1170
WFLLEQPEIQVAHFPFK pc3
P08697;P08697−2
log10 peptide intensity
702:714 436:440 104:106
5:176839898:T:C_T
p = 2.4e−13, beta = −0.317, N = 1242
LVPPMEEDYPQFGSPK pc2
P08697;P08697−2
log10 peptide intensity
390:714 209:440 35:106
5:176839898:T:C_T
p = 1.2e−14, beta = −0.498, N = 634
EQQDSPGNK pc2
P08697;P08697−2
log10 peptide intensity
674:714 398:440 85:106
5:176839898:T:C_T
p = 2.4e−34, beta = −0.55, N = 1157
LQQVLHAGSGPCLPHLLSR pc4
P08697;P08697−2
log10 peptide intensity
503:714 280:440 54:106
5:176839898:T:C_T
p = 4e−11, beta = −0.365, N = 837
TYPLRWFLLEQPEIQVAHFPFK pc4
P08697;P08697−2
log10 peptide intensity
519:714 289:440 61:106
5:176839898:T:C_T
p = 1.3e−05, beta = −0.234, N = 869
GFPIKEDFLEQSEQLFGAK pc3
P08697;P08697−2
log10 peptide intensity
492:714 258:440 55:106
5:176839898:T:C_T
p = 3.2e−05, beta = −0.234, N = 805
HQMDLVATLSQLGLQELFQAPDLR p
P08697;P08697−2
log10 peptide intensity
292:714 137:440 25:106
5:176839898:T:C_T
p = 0.19, beta = −0.103, N = 454
SPPGVCSRDPTPEQTHR pc3
P08697
log10 peptide intensity
141:714 79:440 12:106
5:176839898:T:C_T
p = 0.0027, beta = −0.325, N = 232
DPTPEQTHRLAR pc3
P08697
log10 peptide intensity
63:714 28:440 3:106
5:176839898:T:C_T
p = 0.78, beta = 0.0521, N = 94
SERPINF2 : NP1
P08697
log10 protein intensity
153:153 106:106 66:66
5:176839898:T:C_T
p = 0.059, beta = −0.133, N = 325
LGNQEPGGQTALK pc2
P08697
log10 peptide intensity
141:153 98:106 63:66
5:176839898:T:C_T
p = 0.11, beta = −0.116, N = 302
DFLQSLK pc2
P08697;P08697−2
log10 peptide intensity
153:153 106:106 66:66
5:176839898:T:C_T
p = 0.0054, beta = −0.195, N = 325
SPPGVCSR pc2
P08697
log10 peptide intensity
153:153 106:106 66:66
5:176839898:T:C_T
p = 0.00063, beta = −0.239, N = 325
LCQDLGPGAFR pc2
P08697;P08697−2
log10 peptide intensity
153:153 106:106 66:66
5:176839898:T:C_T
p = 0.98, beta = −0.0016, N = 325
EDFLEQSEQLFGAK pc2
P08697;P08697−2
log10 peptide intensity
153:153 106:106 66:66
5:176839898:T:C_T
p = 0.72, beta = −0.0253, N = 325
FDPSLTQR pc2
P08697;P08697−2
log10 peptide intensity
151:153 104:106 65:66
5:176839898:T:C_T
p = 0.77, beta = 0.0211, N = 320
QEDDLANINQWVK pc2
P08697;P08697−2
log10 peptide intensity
153:153 105:106 64:66
5:176839898:T:C_T
p = 0.44, beta = 0.0549, N = 322
DSFHLDEQFTVPVEMMQAR pc3
P08697;P08697−2
log10 peptide intensity
141:153 99:106 66:66
5:176839898:T:C_T
p = 0.83, beta = 0.0154, N = 306
NPNPSAPR pc2
P08697;P08697−2
log10 peptide intensity
126:153 85:106 54:66
5:176839898:T:C_T
p = 0.51, beta = −0.0518, N = 265
WFLLEQPEIQVAHFPFK pc3
P08697;P08697−2
log10 peptide intensity
151:153 104:106 66:66
5:176839898:T:C_T
p = 0.45, beta = −0.0532, N = 321
DPTPEQTHR pc2
P08697
log10 peptide intensity
141:153 99:106 60:66
5:176839898:T:C_T
p = 0.73, beta = −0.0252, N = 300
LQQVLHAGSGPCLPHLLSR pc4
P08697;P08697−2
log10 peptide intensity
123:153 81:106 56:66
5:176839898:T:C_T
p = 0.31, beta = −0.0792, N = 260
TYPLRWFLLEQPEIQVAHFPFK pc4
P08697;P08697−2
log10 peptide intensity
72:153 42:106 25:66
5:176839898:T:C_T
p = 0.73, beta = 0.0372, N = 139
EQQDSPGNK pc2
P08697;P08697−2
log10 peptide intensity
85:153 42:106 21:66
5:176839898:T:C_T
p = 0.039, beta = −0.227, N = 148
GFPIKEDFLEQSEQLFGAK pc3
P08697;P08697−2
log10 peptide intensity
50:153 29:106 21:66
5:176839898:T:C_T
p = 0.96, beta = 0.00681, N = 100
HQMDLVATLSQLGLQELFQAPDLR p
P08697;P08697−2
log10 peptide intensity
23:153 12:106 2:66
5:176839898:T:C_T
p = 0.57, beta = 0.147, N = 37
30Assay Target: SERPINF2
UniProt: P08697
rsID: rs2731673
Target SNP: 5:176839898:T:C
Proxy SNP: 5:176839898:T:C
Tarkin log10(p): 38.6
Tarkin BETA: −0.552
Tarkin MSPA: 0.947
1255:1255:1251:1156:1254:124
QLTSGPNQEQVSPLTLLK pc2
rs2070863 REF
log10 peptide intensity
395:714 236:440 51:106
5:176839898:T:C_T
p = 0.22, model = DOM, N = 682
QLTSGPNQEQVSPLTLLK pc3
rs2070863 REF
log10 peptide intensity
394:714 178:440 33:106
5:176839898:T:C_T
p = 6.1e−09, model = REC, N = 605

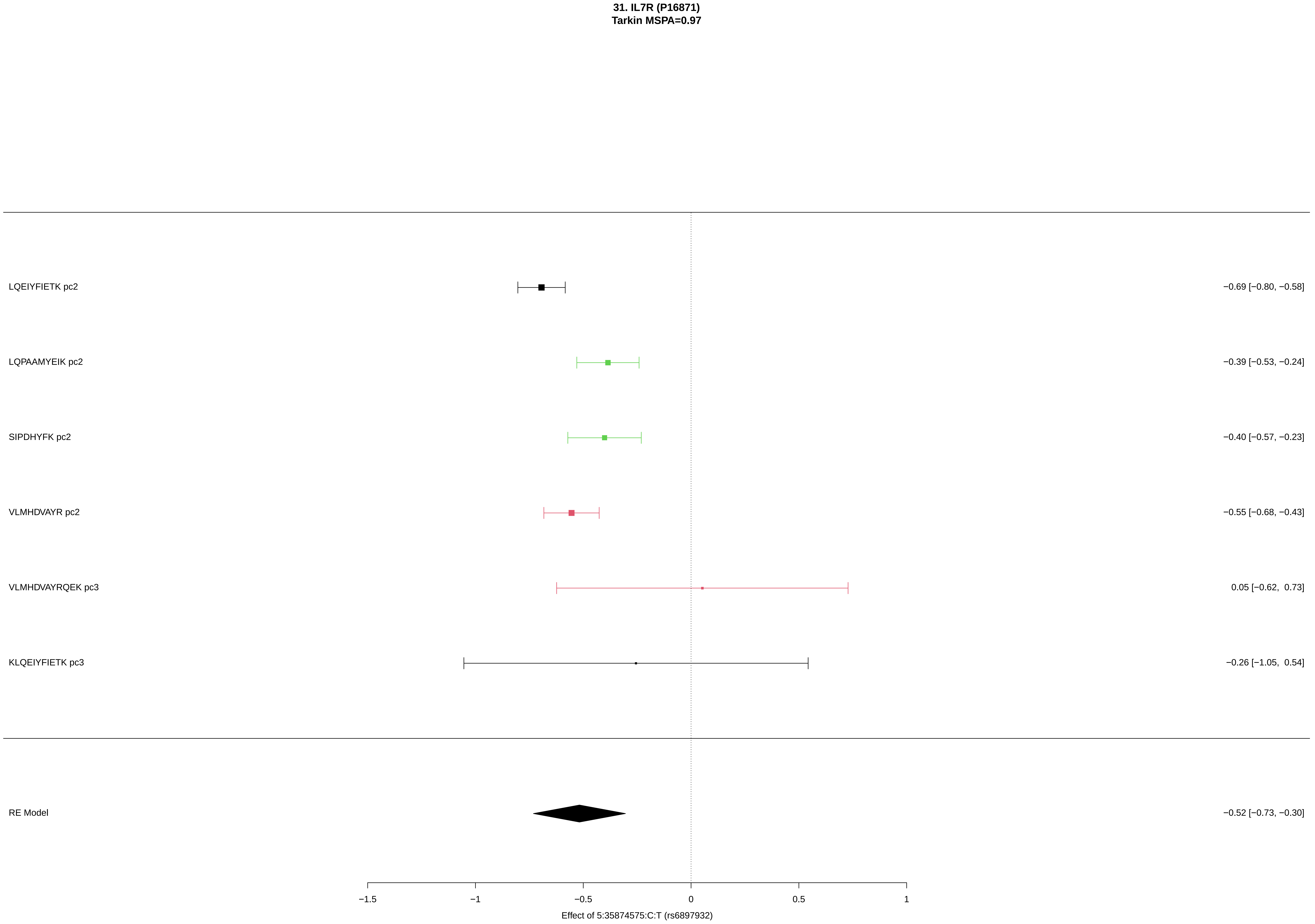
31. IL7R (P16871)
Tarkin MSPA=0.97
LQEIYFIETK pc2
−0.69 [−0.80, −0.58]
LQPAAMYEIK pc2
−0.39 [−0.53, −0.24]
SIPDHYFK pc2
−0.40 [−0.57, −0.23]
VLMHDVAYR pc2
−0.55 [−0.68, −0.43]
VLMHDVAYRQEK pc3
0.05 [−0.62, 0.73]
KLQEIYFIETK pc3
−0.26 [−1.05, 0.54]
RE Model
−0.52 [−0.73, −0.30]
−1.5
−1
−0.5
0
0.5
1
Effect of 5:35874575:C:T (rs6897932)

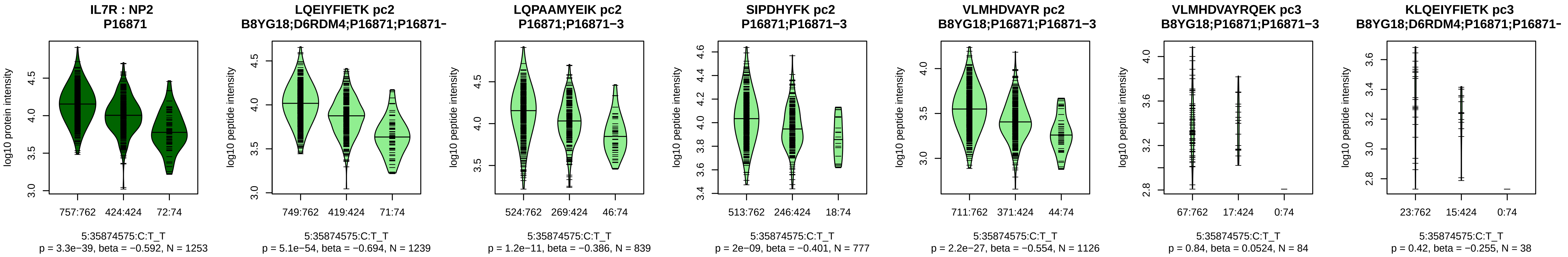

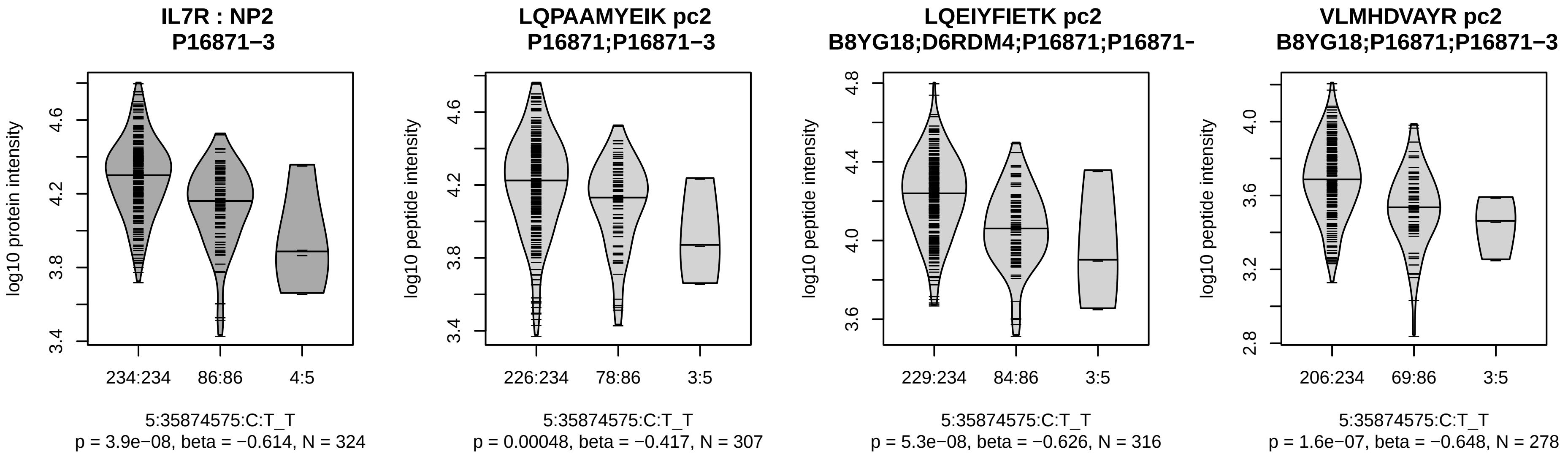

31Assay Target: IL7R
UniProt: P16871
rsID: rs6897932
Target SNP: 5:35874575:C:T
Proxy SNP: 5:35874575:C:T
Tarkin log10(p): 38.5
Tarkin BETA: −0.592
Tarkin MSPA: 0.97
::*:*:–:–
1239:839:777:1126:84:38

32. TIMP3 (P35625)
Tarkin MSPA=1

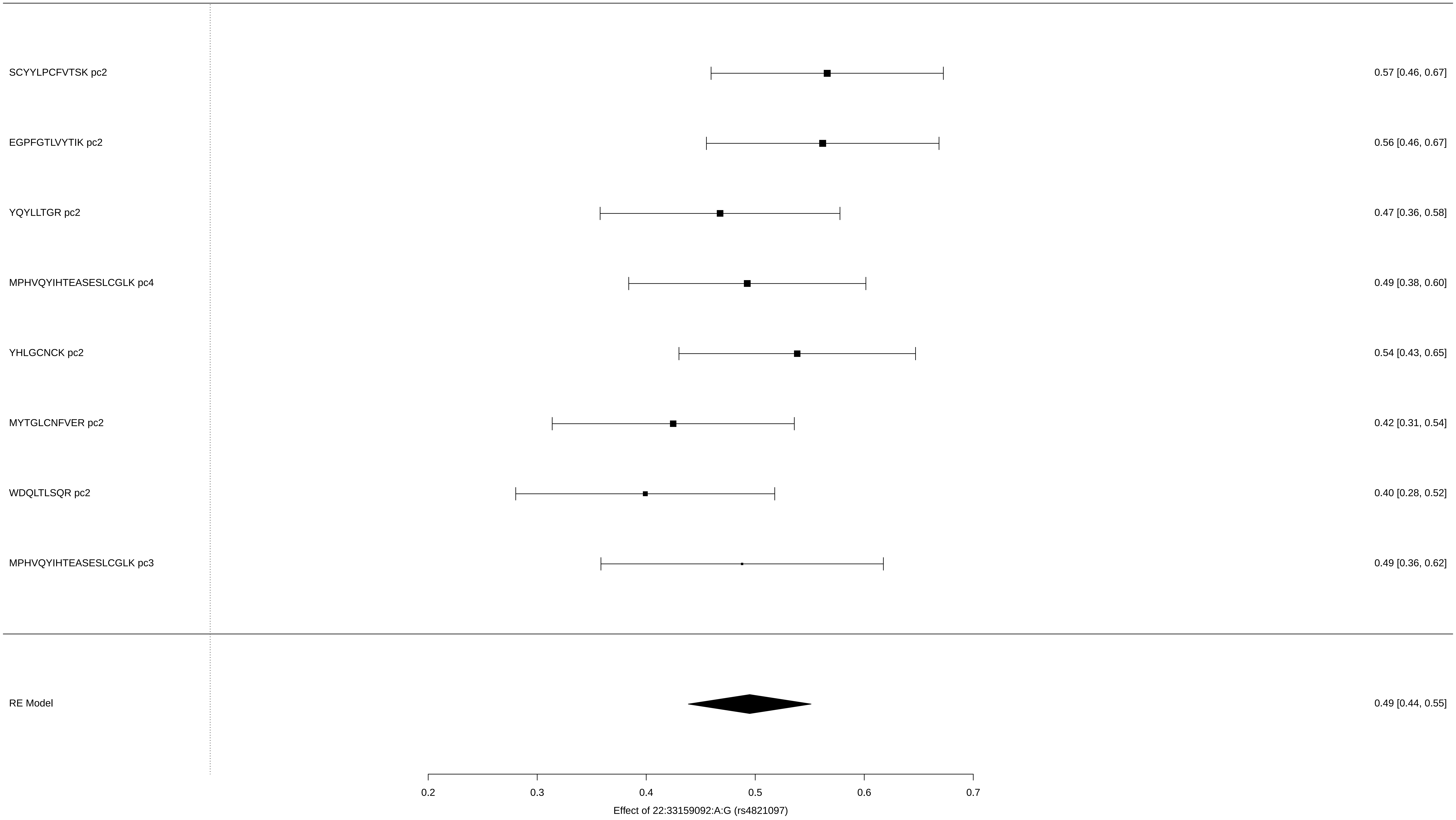

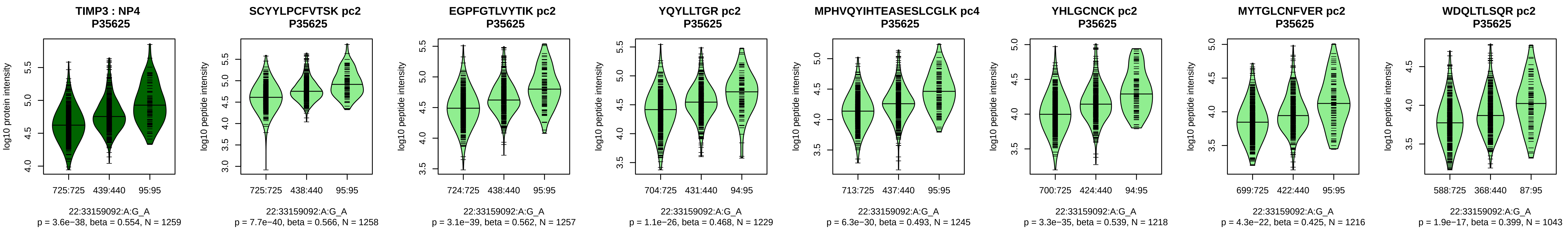

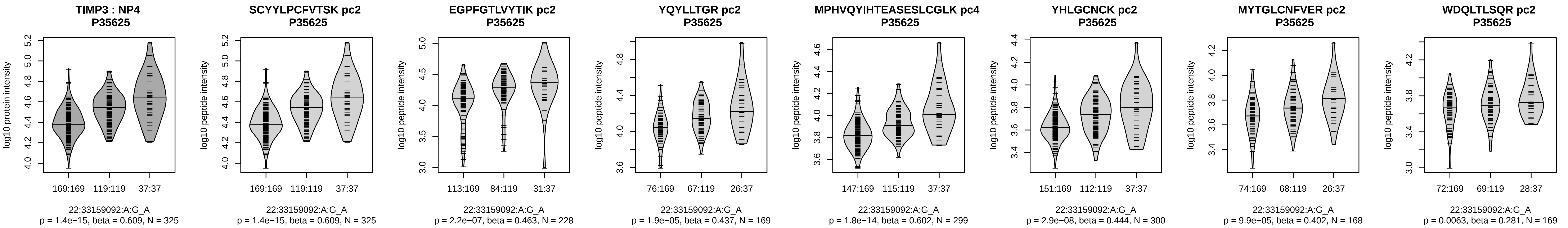

32Assay Target: TIMP3
UniProt: P35625
rsID: rs4821097
Target SNP: 22:33159092:A:G
Proxy SNP: 22:33159092:A:G
Tarkin log10(p): 37.4
Tarkin BETA: 0.554
Tarkin MSPA: 1
1258:1257:1229:1245:1218:121

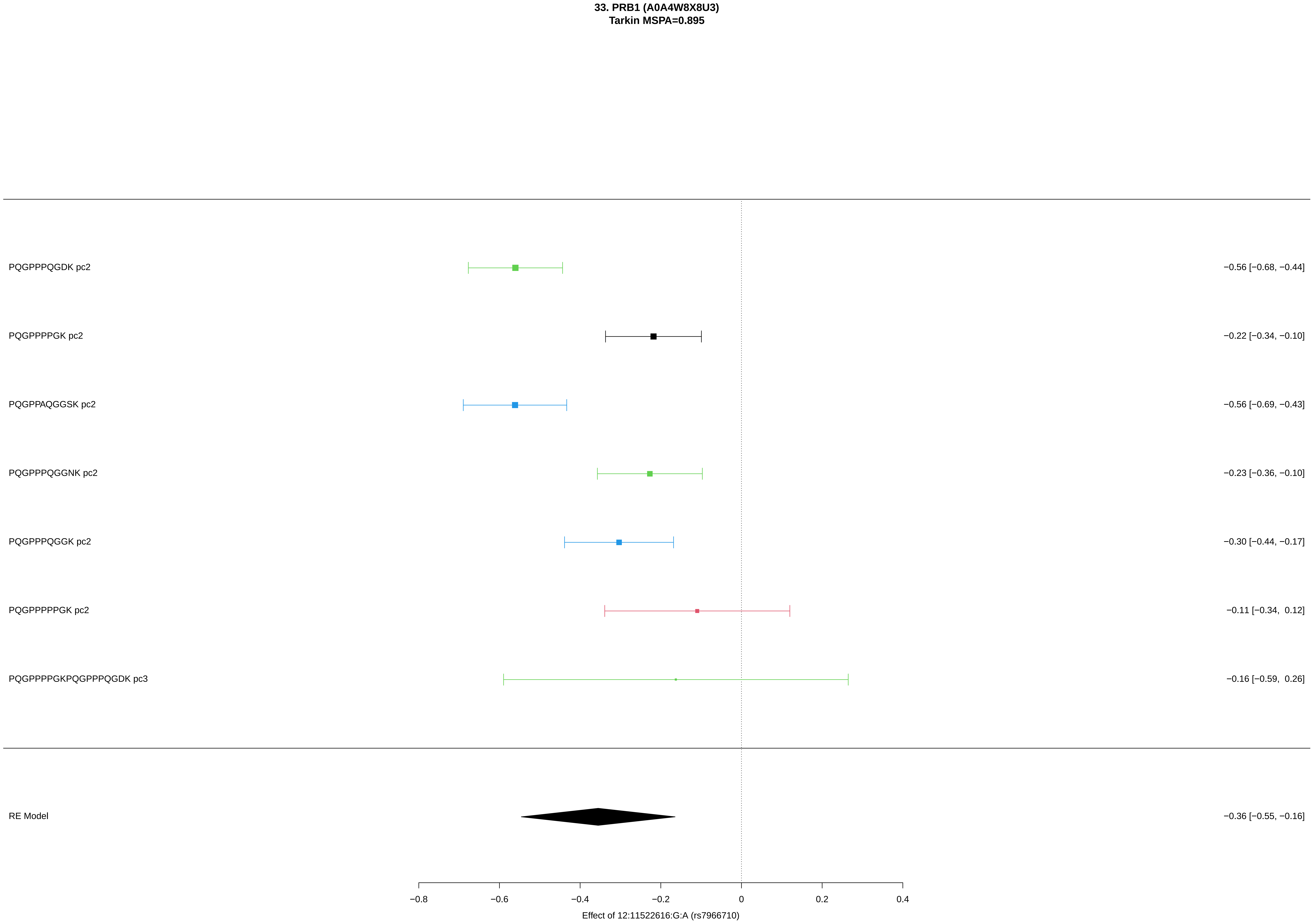
33. PRB1 (A0A4W8X8U3)
Tarkin MSPA=0.895
PQGPPPQGDK pc2
−0.56 [−0.68, −0.44]
PQGPPPPGK pc2
−0.22 [−0.34, −0.10]
PQGPPAQGGSK pc2
−0.56 [−0.69, −0.43]
PQGPPPQGGNK pc2
−0.23 [−0.36, −0.10]
PQGPPPQGGK pc2
−0.30 [−0.44, −0.17]
PQGPPPPPGK pc2
−0.11 [−0.34, 0.12]
PQGPPPPGKPQGPPPQGDK pc3
−0.16 [−0.59, 0.26]
RE Model
−0.36 [−0.55, −0.16]
−0.8
−0.6
−0.4
−0.2
0
0.2
0.4
Effect of 12:11522616:G:A (rs7966710)

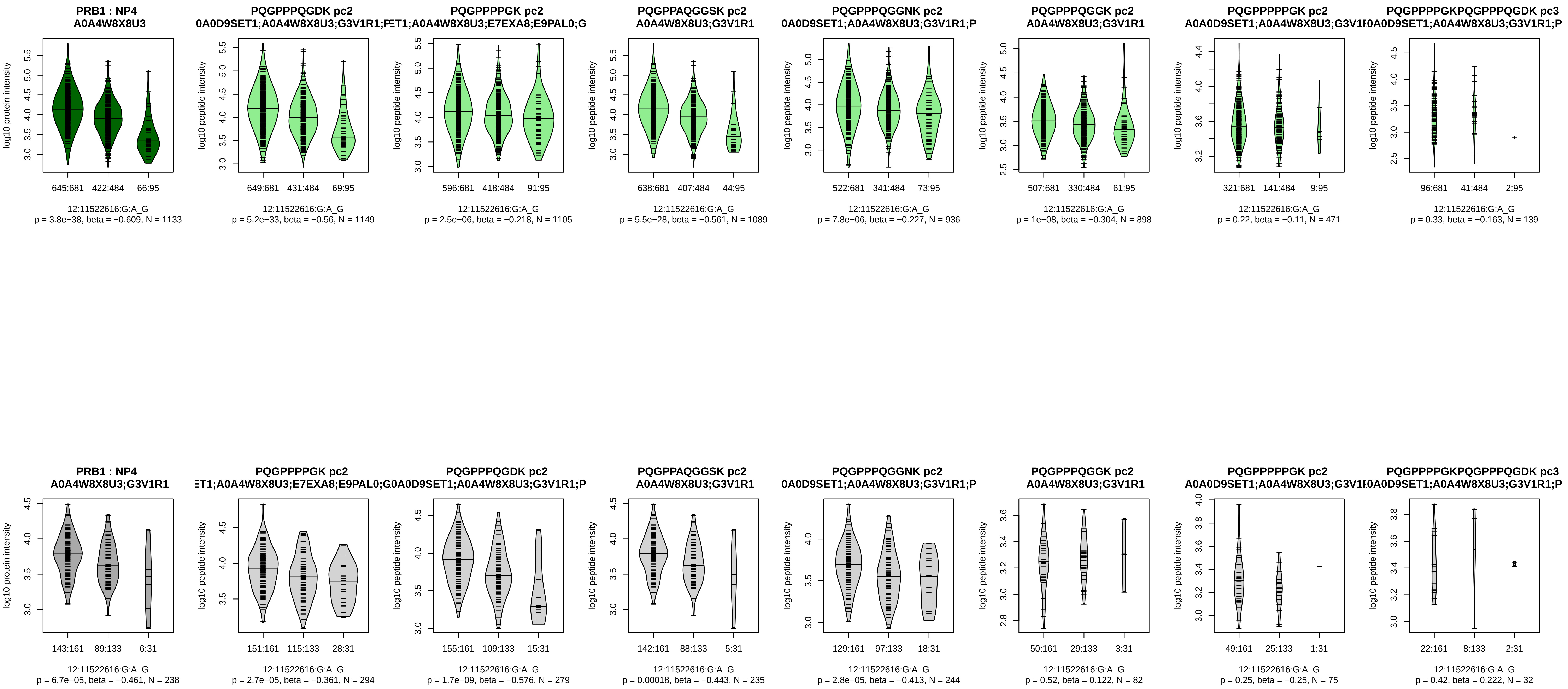

33Assay Target: PRB1
UniProt: A0A4W8X8U3
rsID: rs7966710
Target SNP: 12:11522616:G:A
Proxy SNP: 12:11522616:G:A
Tarkin log10(p): 37.4
Tarkin BETA: −0.609
Tarkin MSPA: 0.895
[illegible]
1149:1105:1089:936:898:471:1[illegible]

34. C2orf40 (Q9H1Z8)
Tarkin MSPA=0.975

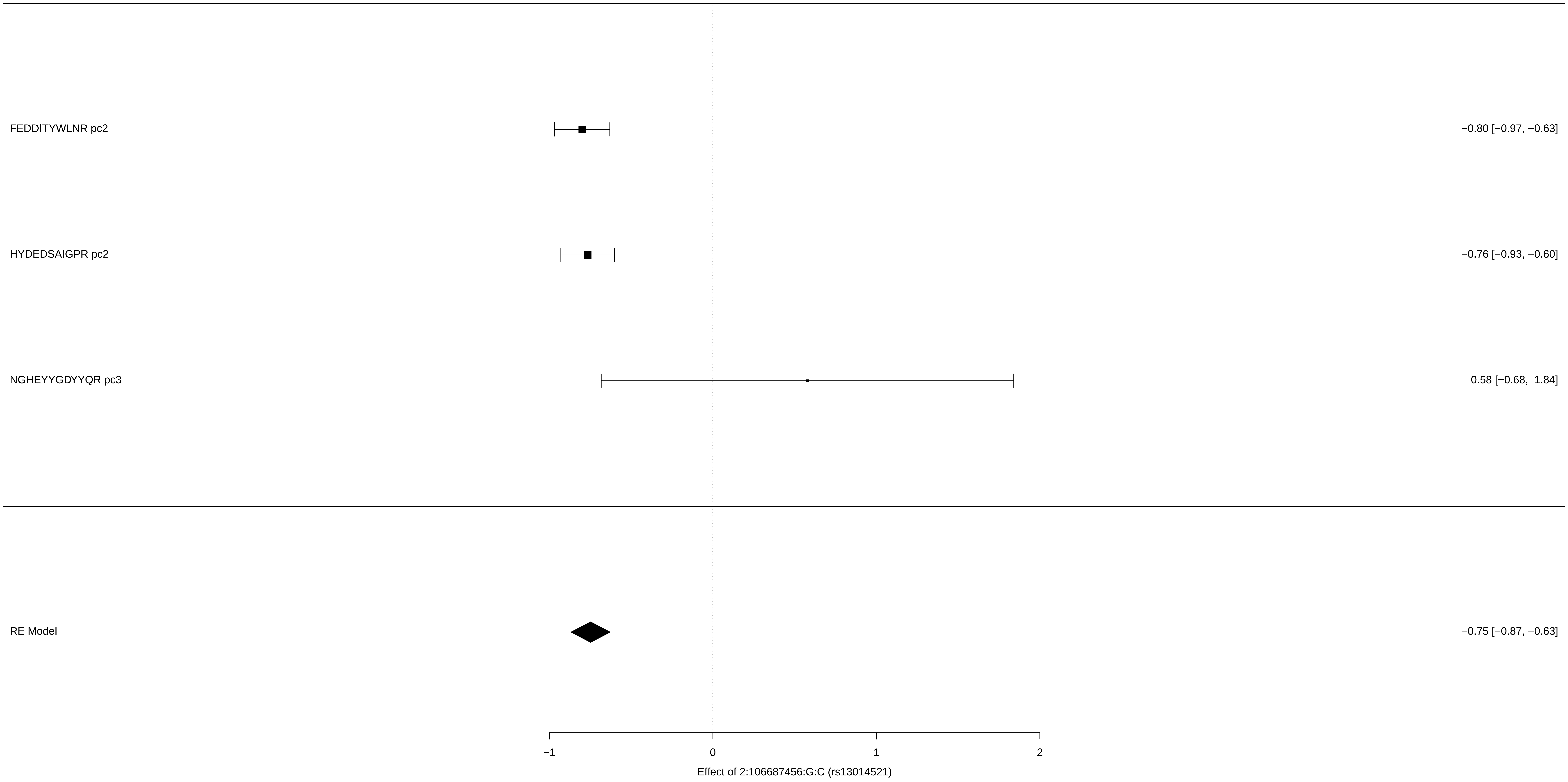

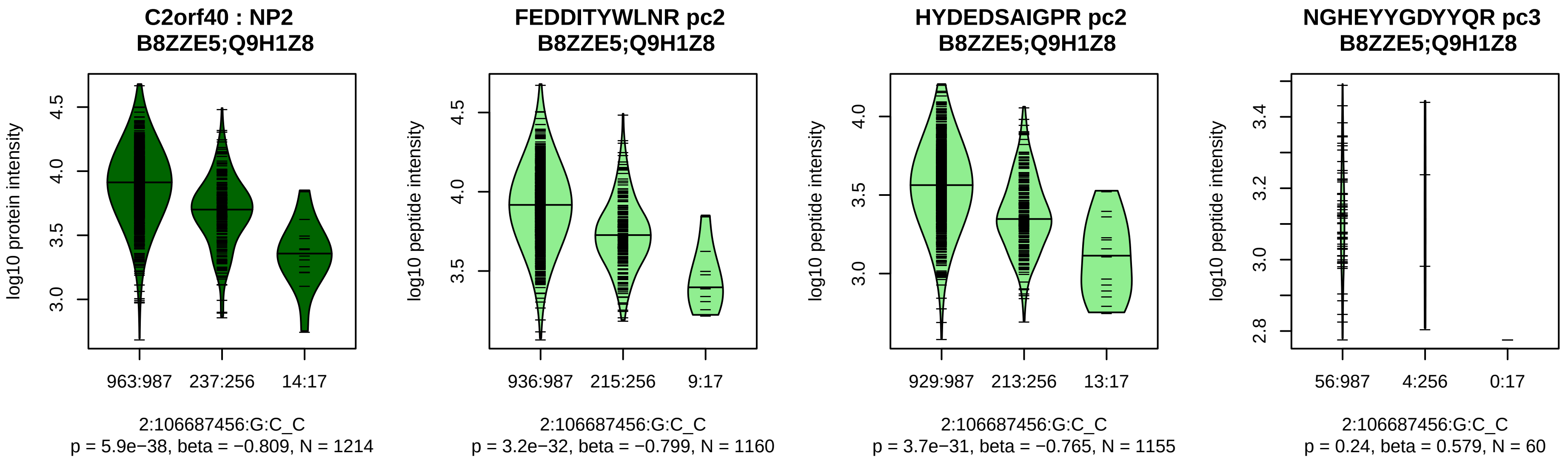

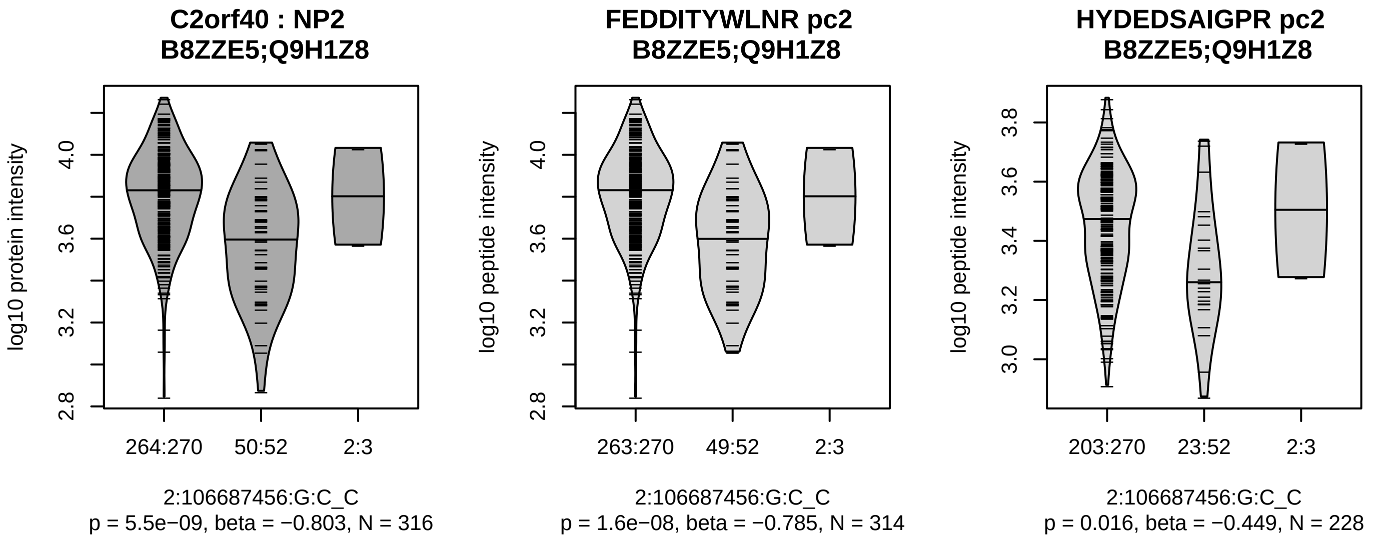

34Assay Target: C2orf40
UniProt: Q9H1Z8
rsID: rs13014521
Target SNP: 2:106687456:G:C
Proxy SNP: 2:106687456:G:C
Tarkin log10(p): 37.2
Tarkin BETA: −0.809
Tarkin MSPA: 0.975
::–
1160:1155:60

35. CPB2 (Q96IY4)
Tarkin MSPA=0.981

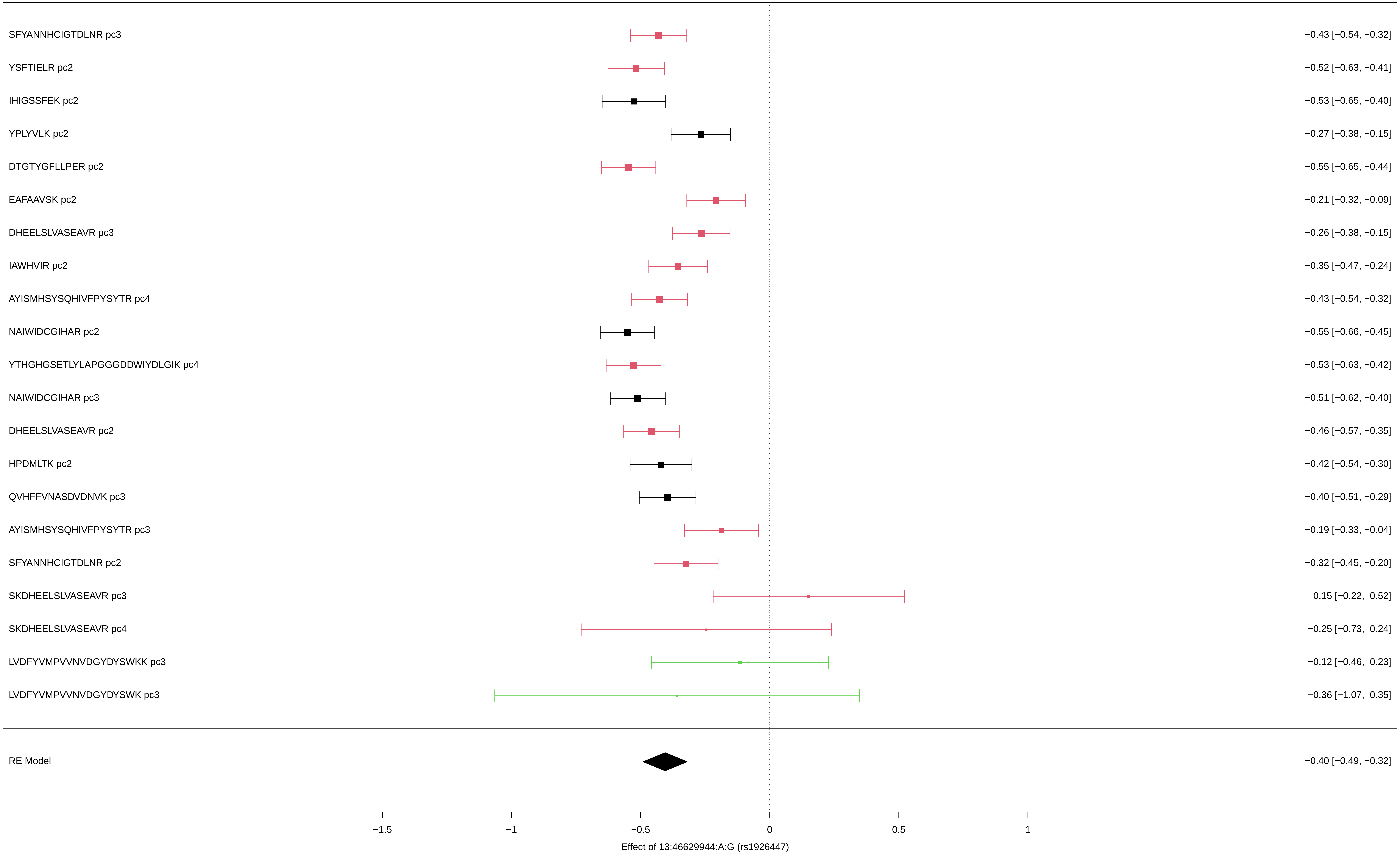

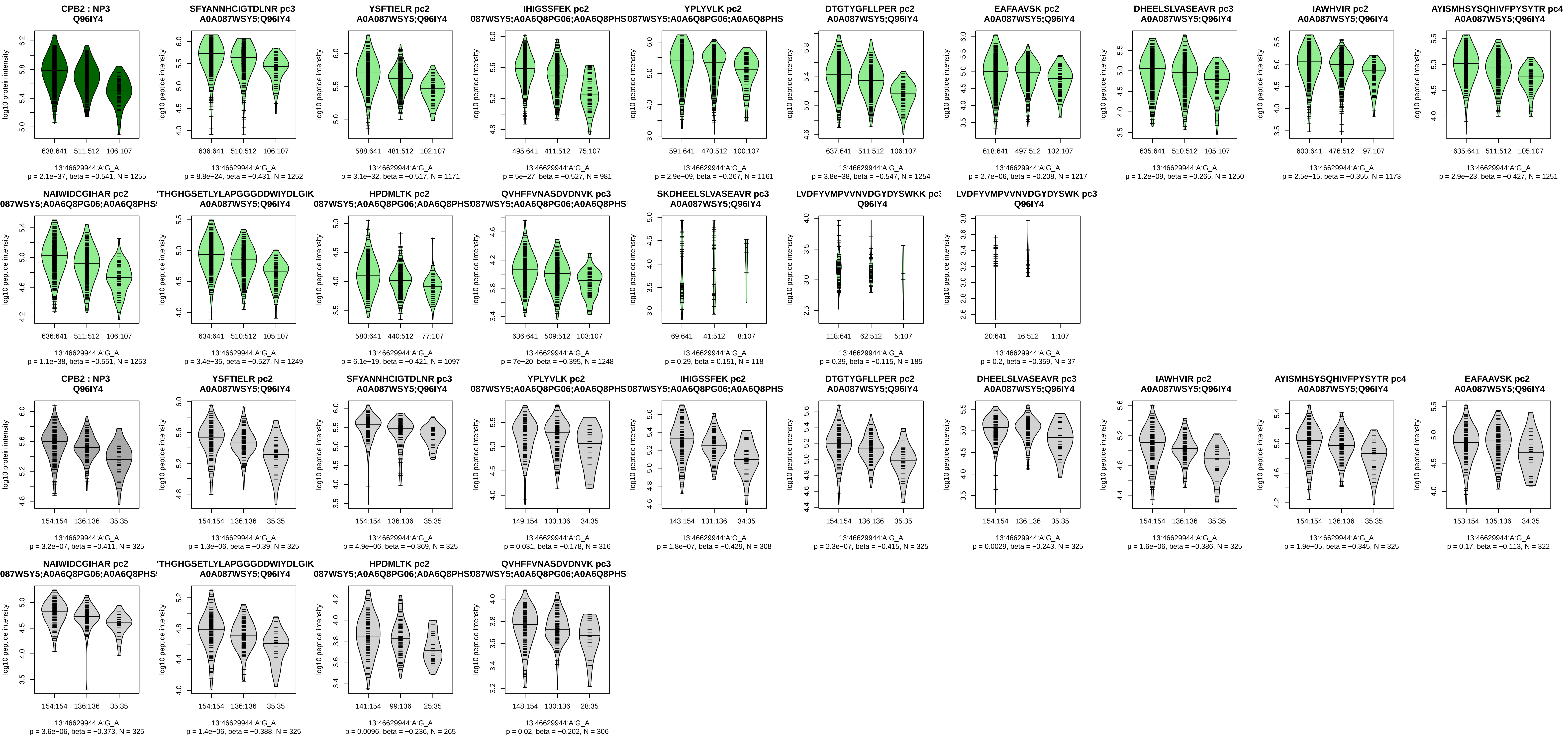

35Assay Target: CPB2
UniProt: Q96IY4
rsID: rs1926447
Target SNP: 13:46629944:A:G
Proxy SNP: 13:46629944:A:G
Tarkin log10(p): 36.7
Tarkin BETA: −0.541
Tarkin MSPA: 0.981
1252:1171:981:1161:1254:1217

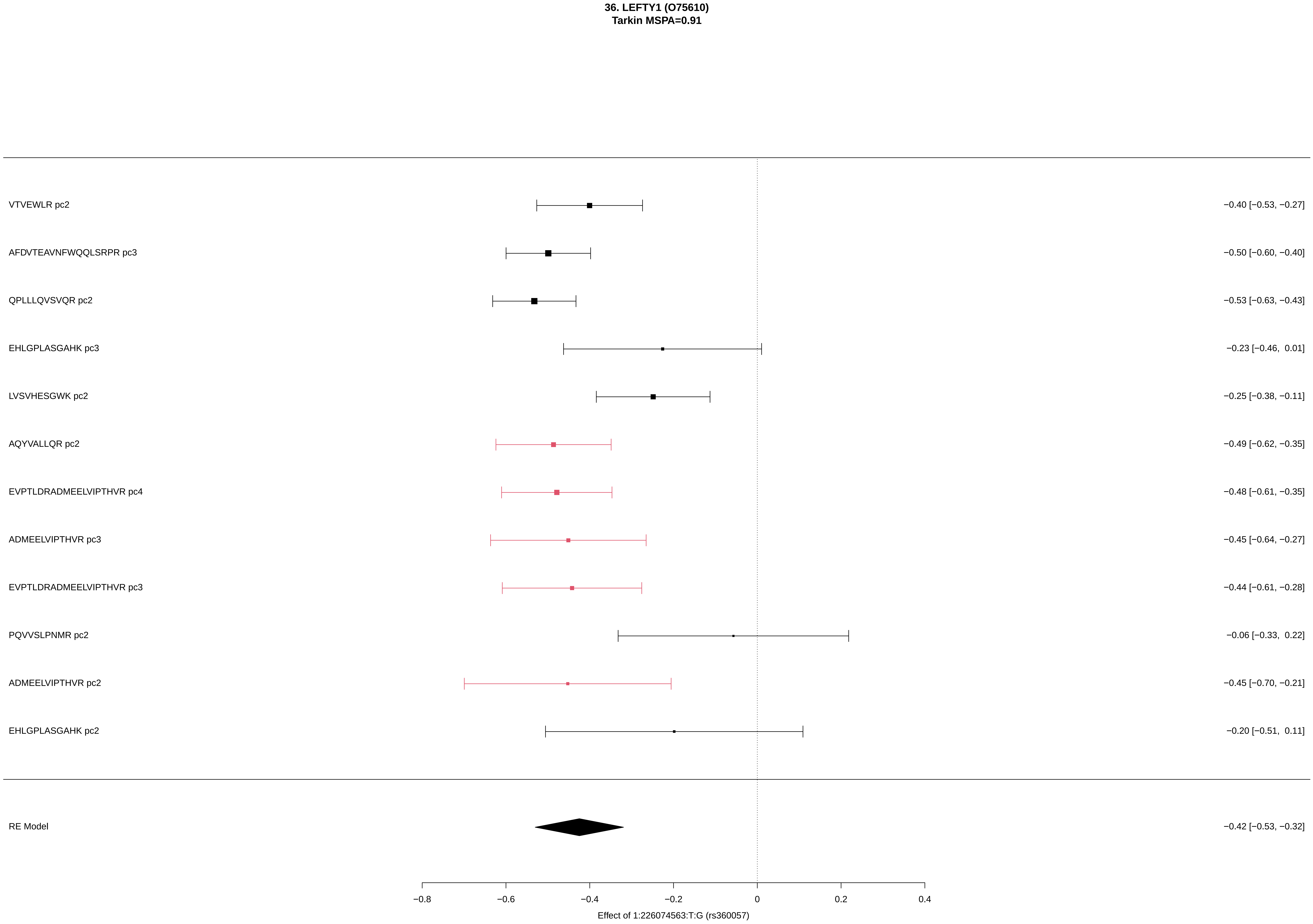
36. LEFTY1 (O75610)
Tarkin MSPA=0.91
VTVEWLR pc2
−0.40 [−0.53, −0.27]
AFDVTEAVNFWQQLSRPR pc3
−0.50 [−0.60, −0.40]
QPLLLQVSVQR pc2
−0.53 [−0.63, −0.43]
EHLGPLASGAHK pc3
−0.23 [−0.46, 0.01]
LVSVHESGWK pc2
−0.25 [−0.38, −0.11]
AQYVALLQR pc2
−0.49 [−0.62, −0.35]
EVPTLDRADMEELVIPTHVR pc4
−0.48 [−0.61, −0.35]
ADMEELVIPTHVR pc3
−0.45 [−0.64, −0.27]
EVPTLDRADMEELVIPTHVR pc3
−0.44 [−0.61, −0.28]
PQVVSLPNMR pc2
−0.06 [−0.33, 0.22]
ADMEELVIPTHVR pc2
−0.45 [−0.70, −0.21]
EHLGPLASGAHK pc2
−0.20 [−0.51, 0.11]
RE Model
−0.42 [−0.53, −0.32]
−0.8
−0.6
−0.4
−0.2
0
0.2
0.4
Effect of 1:226074563:T:G (rs360057)

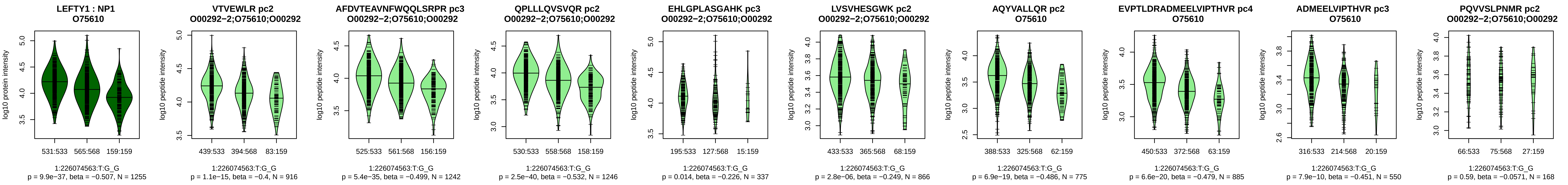

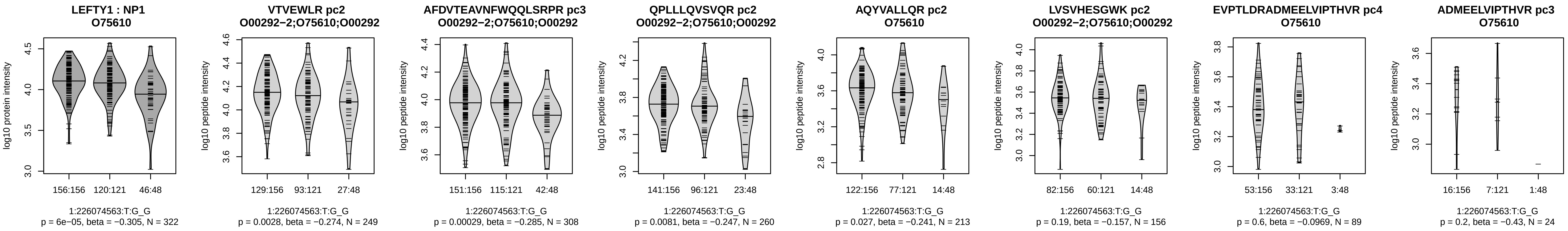

36Assay Target: LEFTY1
UniProt: O75610
rsID: rs360057
Target SNP: 1:226074563:T:G
Proxy SNP: 1:226074563:T:G
Tarkin log10(p): 36
Tarkin BETA: −0.507
Tarkin MSPA: 0.91
::*:–:*:*:*:*:*:–:*:–
916:1242:1246:337:866:775:885

37. RCN3 (Q96D15)
Tarkin MSPA=0.992

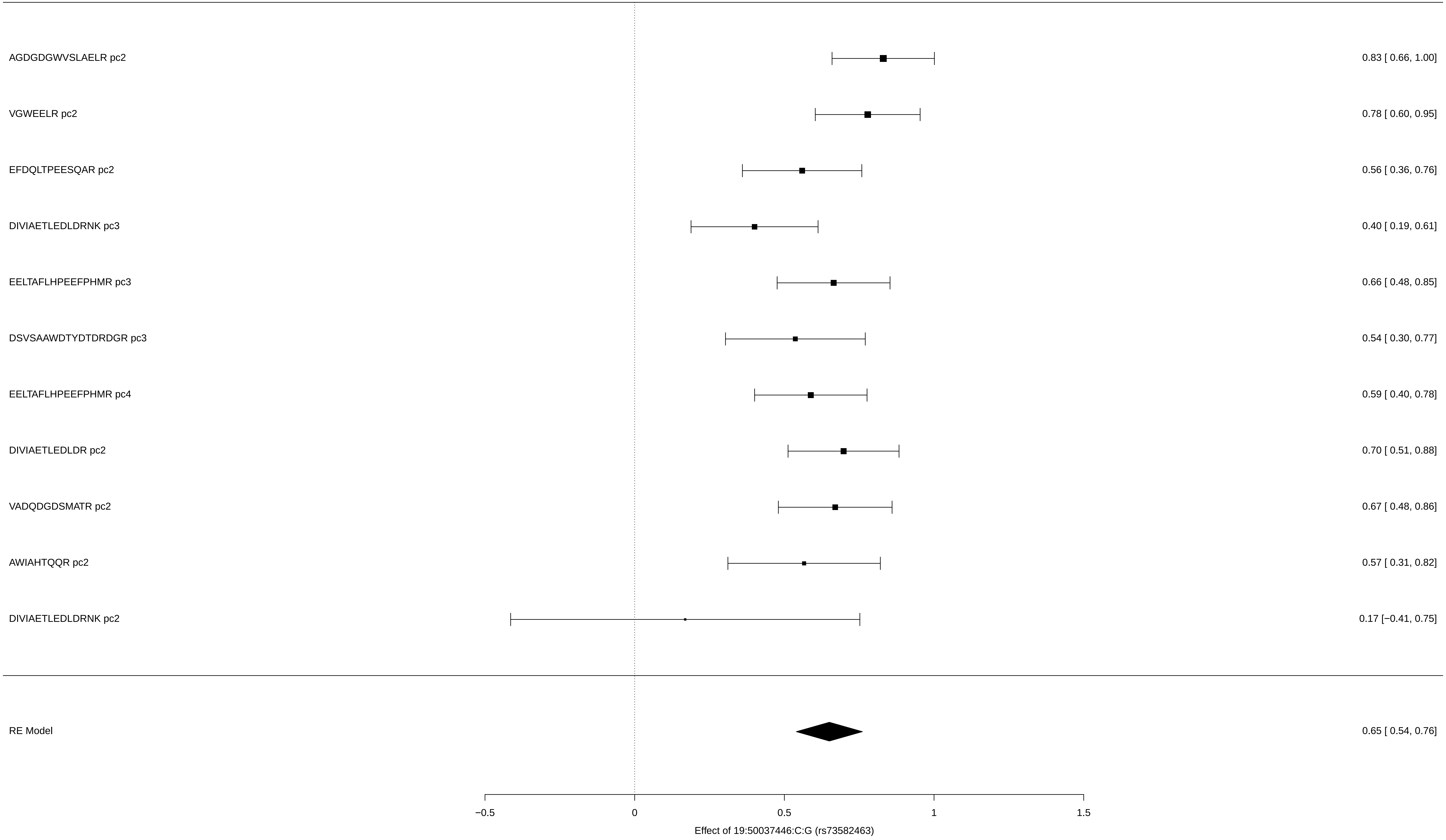

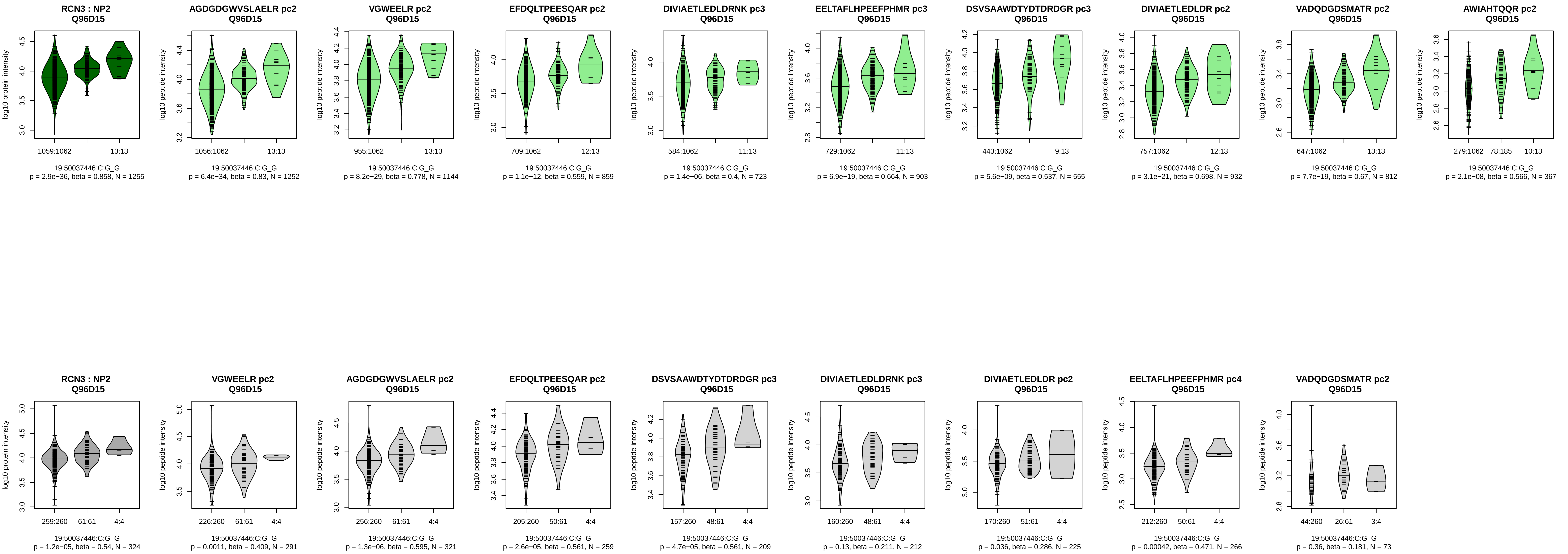

37Assay Target: RCN3
UniProt: Q96D15
rsID: rs73582463
Target SNP: 19:50037446:C:G
Proxy SNP: 19:50037446:C:G
Tarkin log10(p): 35.5
Tarkin BETA: 0.858
Tarkin MSPA: 0.992
1252:1144:859:723:903:555:928

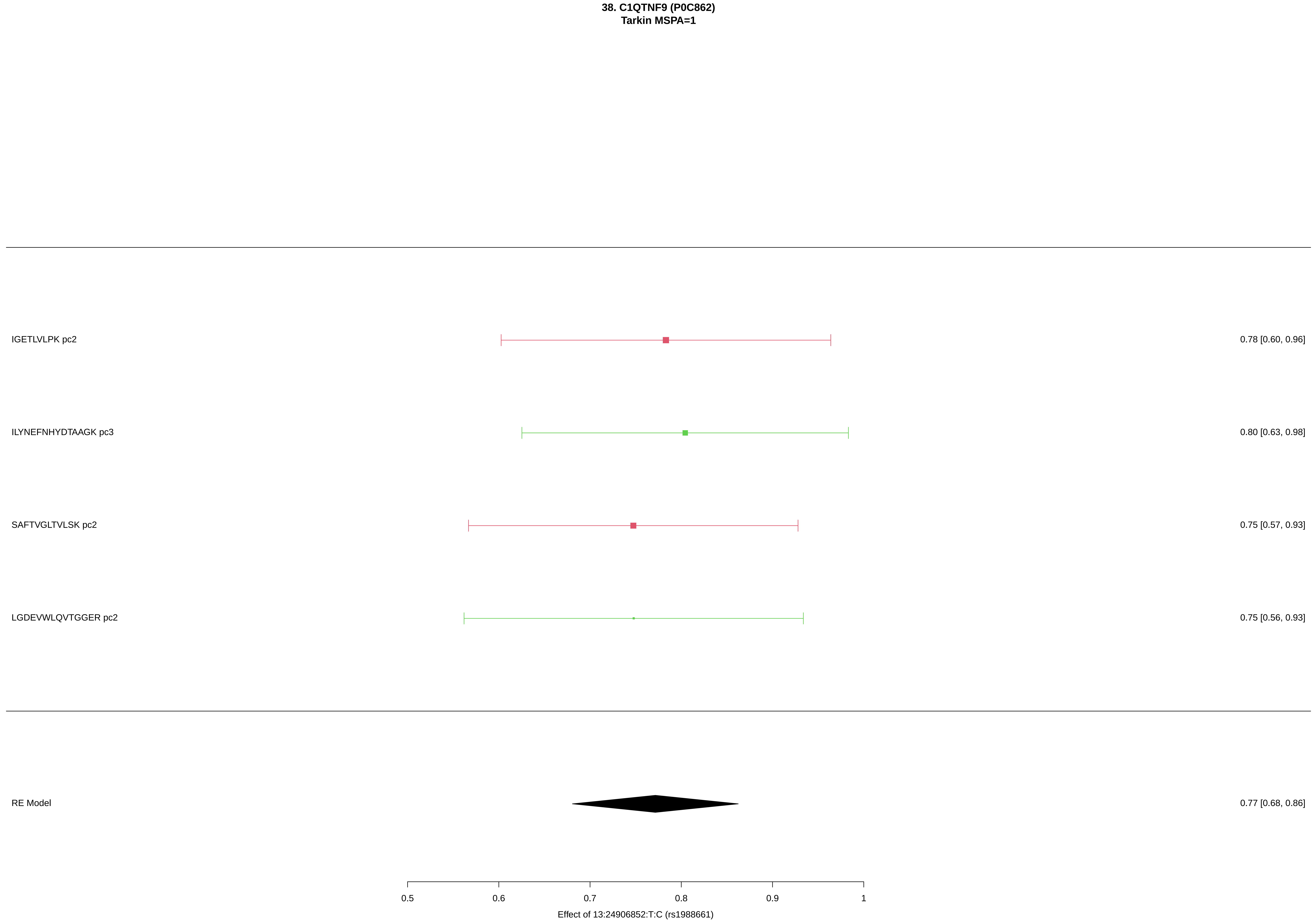

38. C1QTNF9 (P0C862)
Tarkin MSPA=1
IGETLVLPK pc2
0.78 [0.60, 0.96]
ILYNEFNHYDTAAGK pc3
0.80 [0.63, 0.98]
SAFTVGLTVLSK pc2
0.75 [0.57, 0.93]
LGDEVWLQVTGGER pc2
0.75 [0.56, 0.93]
RE Model
0.77 [0.68, 0.86]
0.5
0.6
0.7
0.8
0.9
1
Effect of 13:24906852:T:C (rs1988661)

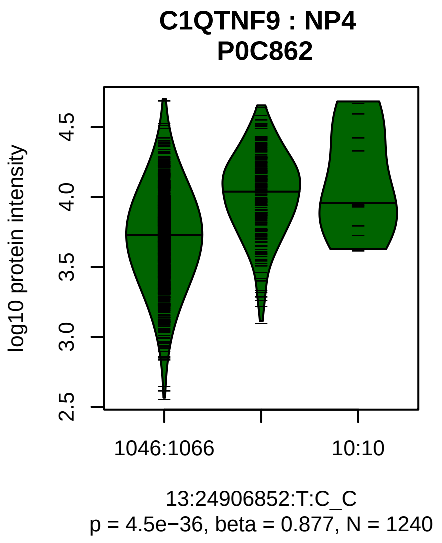

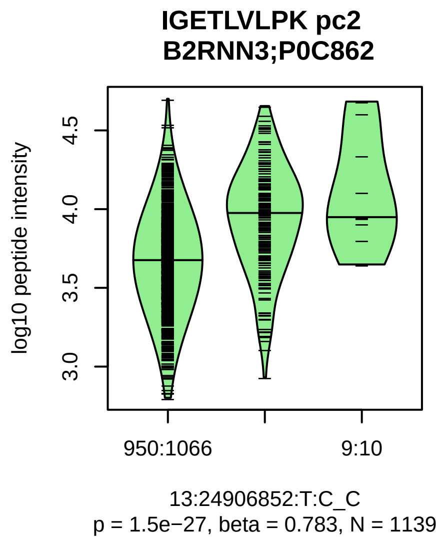

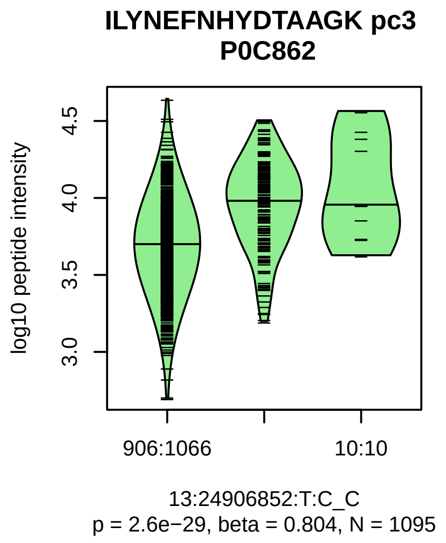

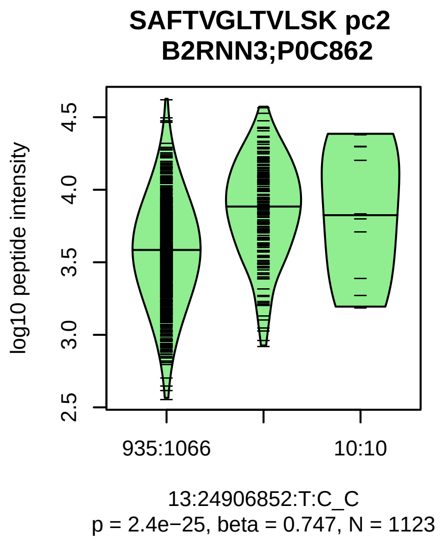

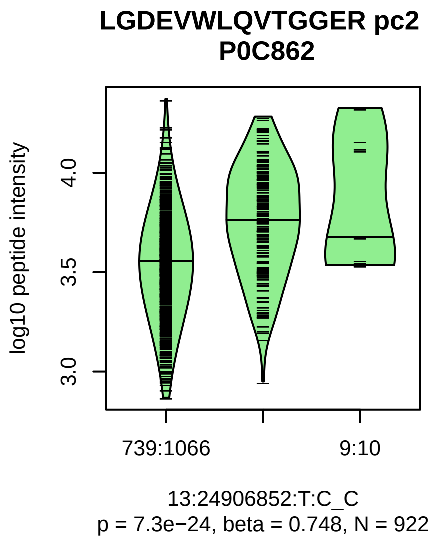

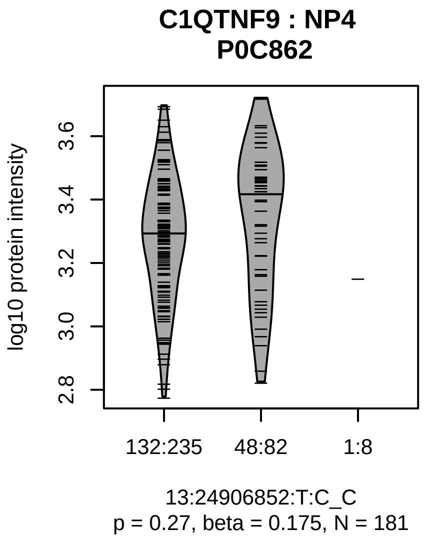

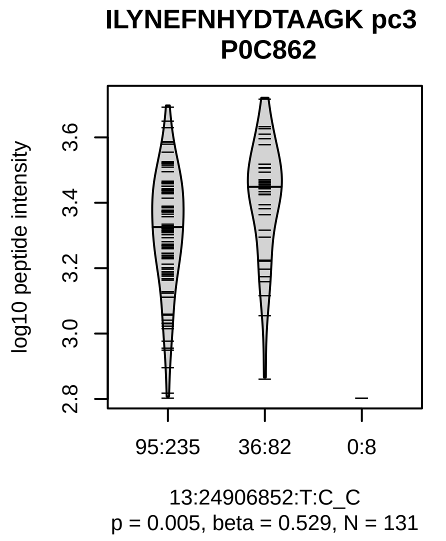

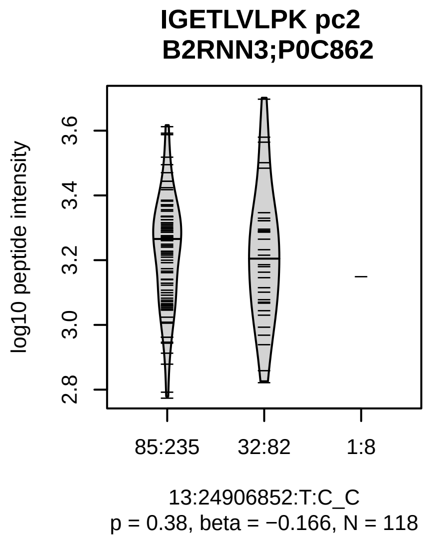

38Assay Target: C1QTNF9
UniProt: P0C862
rsID: rs1988661
Target SNP: 13:24906852:T:C
Proxy SNP: 13:24906852:T:C
Tarkin log10(p): 35.3
Tarkin BETA: 0.877
Tarkin MSPA: 1
::*:*:NA
1139:1095:1123:922:3

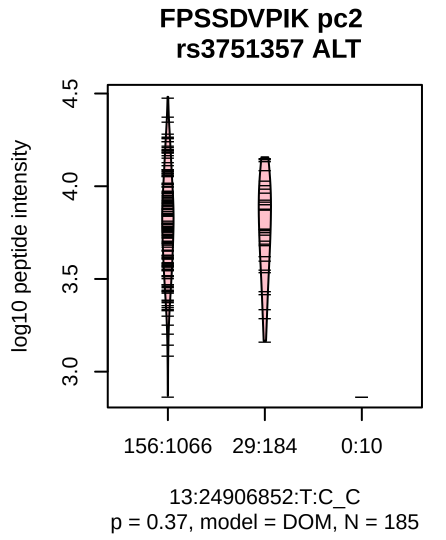

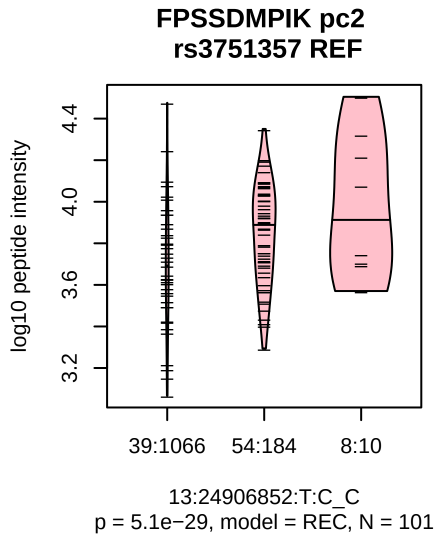

39. ITIH1 (P19827)
Tarkin MSPA=0.863

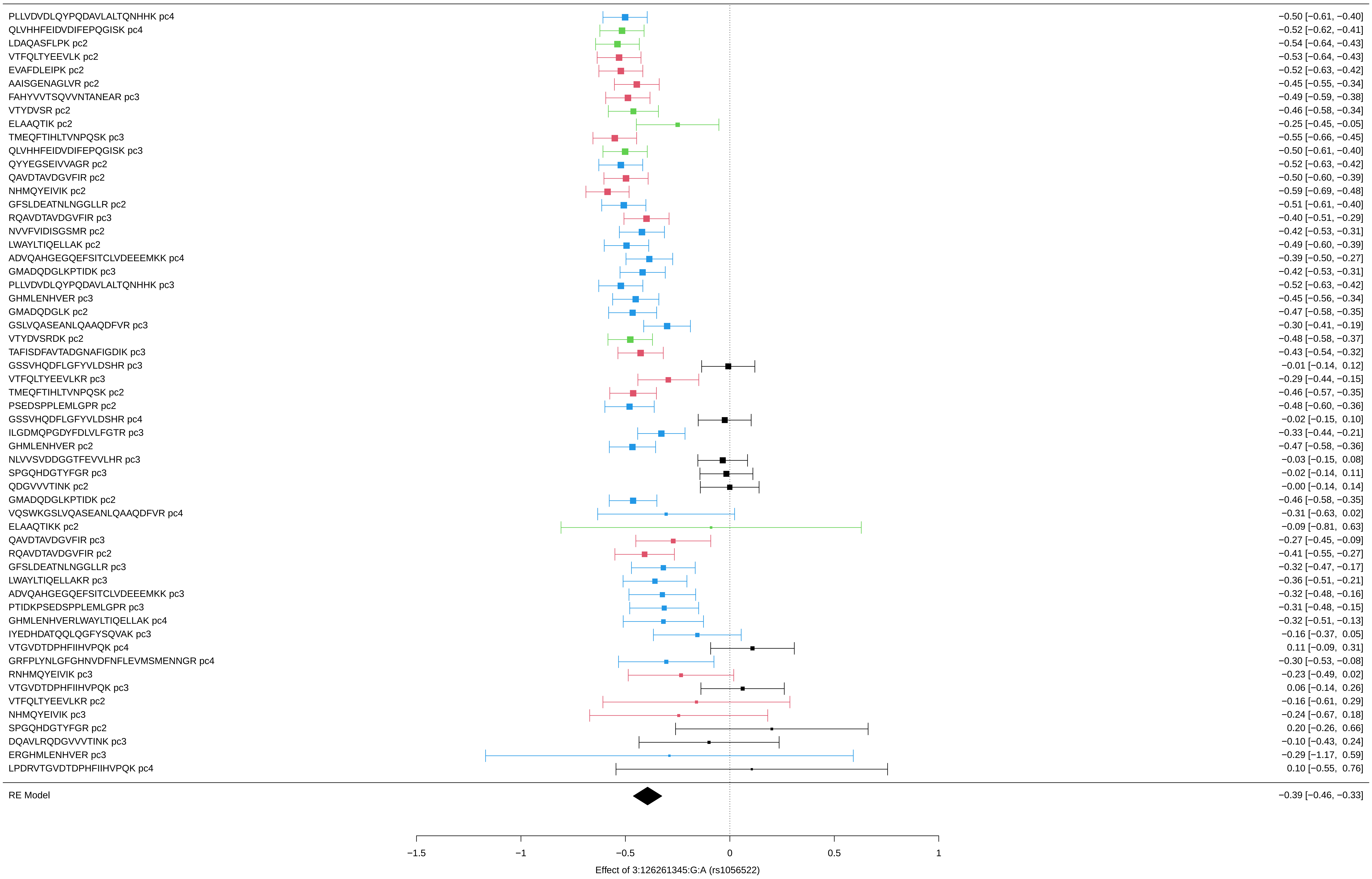

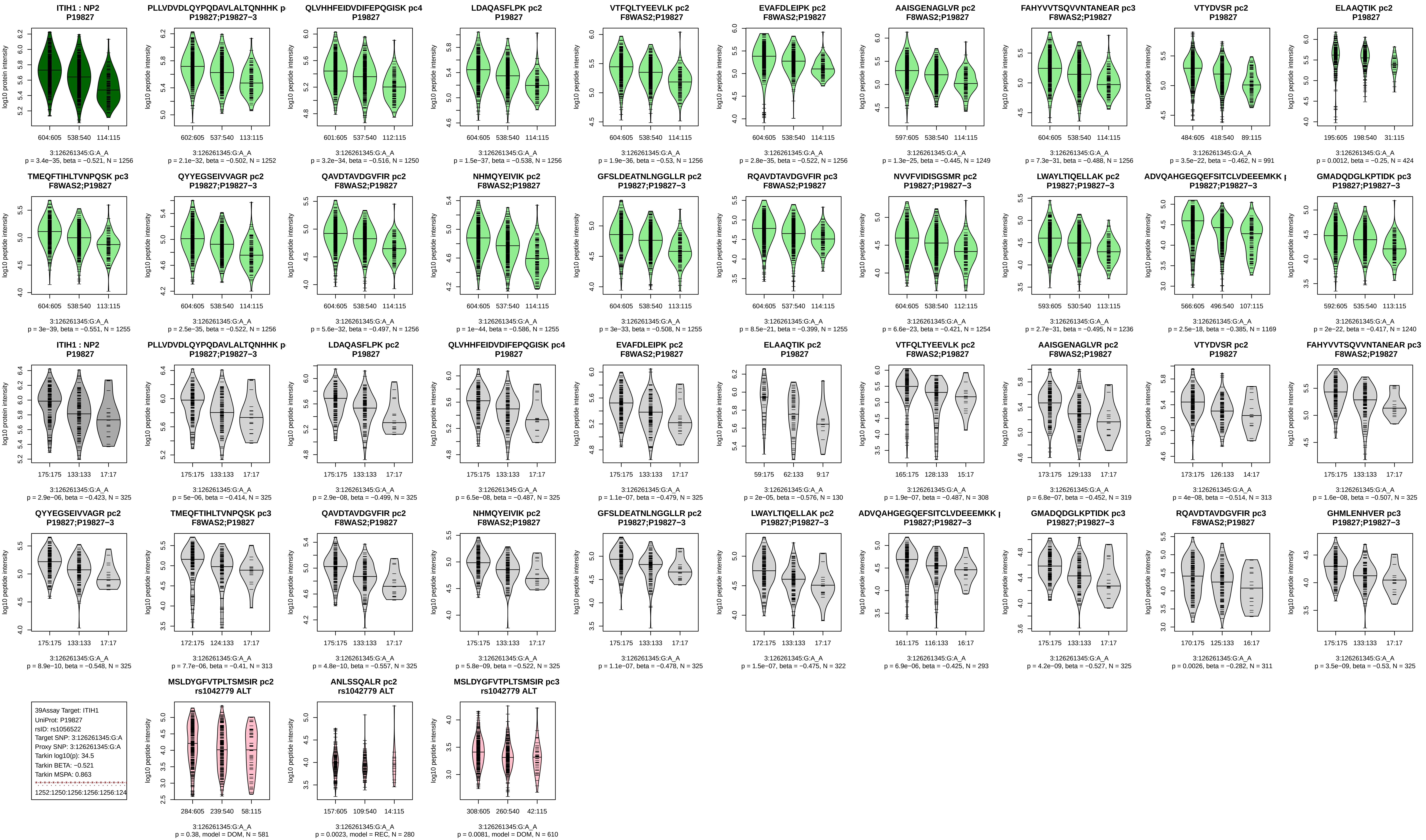
ITIH1 : NP2
P19827
604:605 538:540 114:115
3:126261345:G:A_A
p = 3.4e−35, beta = −0.521, N = 1256
PLLVDVDLQYPQDAVLALTQNHHK p
P19827;P19827−3
602:605 537:540 113:115
3:126261345:G:A_A
p = 2.1e−32, beta = −0.502, N = 1252
QLVHHFEIDVDIFEPQGISK pc4
P19827
601:605 537:540 112:115
3:126261345:G:A_A
p = 3.2e−34, beta = −0.516, N = 1250
LDAQASFLPK pc2
P19827
604:605 538:540 114:115
3:126261345:G:A_A
p = 1.5e−37, beta = −0.538, N = 1256
VTFQLTYEEVLK pc2
F8WAS2;P19827
604:605 538:540 114:115
3:126261345:G:A_A
p = 1.9e−36, beta = −0.53, N = 1256
EVAFDLEIPK pc2
F8WAS2;P19827
604:605 538:540 114:115
3:126261345:G:A_A
p = 2.8e−35, beta = −0.522, N = 1256
AAISGENAGLVR pc2
F8WAS2;P19827
597:605 538:540 114:115
3:126261345:G:A_A
p = 1.3e−25, beta = −0.445, N = 1249
FAHYVVTSQVVNTANEAR pc3
F8WAS2;P19827
604:605 538:540 114:115
3:126261345:G:A_A
p = 7.3e−31, beta = −0.488, N = 1256
VTYDVSR pc2
P19827
484:605 418:540 89:115
3:126261345:G:A_A
p = 3.5e−22, beta = −0.462, N = 991
ELAAQTIK pc2
P19827
195:605 198:540 31:115
3:126261345:G:A_A
p = 0.0012, beta = −0.25, N = 424
TMEQFTIHLTVNPQSK pc3
F8WAS2;P19827
604:605 538:540 113:115
3:126261345:G:A_A
p = 3e−39, beta = −0.551, N = 1255
QYYEGSEIVVAGR pc2
P19827;P19827−3
604:605 538:540 114:115
3:126261345:G:A_A
p = 2.5e−35, beta = −0.522, N = 1256
QAVDTAVDGVFIR pc2
F8WAS2;P19827
604:605 538:540 114:115
3:126261345:G:A_A
p = 5.6e−32, beta = −0.497, N = 1256
NHMQYEIVIK pc2
F8WAS2;P19827
604:605 537:540 114:115
3:126261345:G:A_A
p = 1e−44, beta = −0.586, N = 1255
GFSLDEATNLNGGLLR pc2
P19827;P19827−3
604:605 538:540 113:115
3:126261345:G:A_A
p = 3e−33, beta = −0.508, N = 1255
RQAVDTAVDGVFIR pc3
F8WAS2;P19827
604:605 537:540 114:115
3:126261345:G:A_A
p = 8.5e−21, beta = −0.399, N = 1255
NVVFVIDISGSMR pc2
P19827;P19827−3
604:605 538:540 112:115
3:126261345:G:A_A
p = 6.6e−23, beta = −0.421, N = 1254
LWAYLTIQELLAK pc2
P19827;P19827−3
593:605 530:540 113:115
3:126261345:G:A_A
p = 2.7e−31, beta = −0.495, N = 1236
ADVQAHGEGQEFSITCLVDEEEMKK p
P19827;P19827−3
566:605 496:540 107:115
3:126261345:G:A_A
p = 2.5e−18, beta = −0.385, N = 1169
GMADQDGLKPTIDK pc3
P19827;P19827−3
592:605 535:540 113:115
3:126261345:G:A_A
p = 2e−22, beta = −0.417, N = 1240
ITIH1 : NP2
P19827
175:175 133:133 17:17
3:126261345:G:A_A
p = 2.9e−06, beta = −0.423, N = 325
PLLVDVDLQYPQDAVLALTQNHHK p
P19827;P19827−3
175:175 133:133 17:17
3:126261345:G:A_A
p = 5e−06, beta = −0.414, N = 325
LDAQASFLPK pc2
P19827
175:175 133:133 17:17
3:126261345:G:A_A
p = 2.9e−08, beta = −0.499, N = 325
QLVHHFEIDVDIFEPQGISK pc4
P19827
175:175 133:133 17:17
3:126261345:G:A_A
p = 6.5e−08, beta = −0.487, N = 325
EVAFDLEIPK pc2
F8WAS2;P19827
175:175 133:133 17:17
3:126261345:G:A_A
p = 1.1e−07, beta = −0.479, N = 325
ELAAQTIK pc2
P19827
59:175 62:133 9:17
3:126261345:G:A_A
p = 2e−05, beta = −0.576, N = 130
VTFQLTYEEVLK pc2
F8WAS2;P19827
165:175 128:133 15:17
3:126261345:G:A_A
p = 1.9e−07, beta = −0.487, N = 308
AAISGENAGLVR pc2
F8WAS2;P19827
173:175 129:133 17:17
3:126261345:G:A_A
p = 6.8e−07, beta = −0.452, N = 319
VTYDVSR pc2
P19827
173:175 126:133 14:17
3:126261345:G:A_A
p = 4e−08, beta = −0.514, N = 313
FAHYVVTSQVVNTANEAR pc3
F8WAS2;P19827
175:175 133:133 17:17
3:126261345:G:A_A
p = 1.6e−08, beta = −0.507, N = 325
QYYEGSEIVVAGR pc2
P19827;P19827−3
175:175 133:133 17:17
3:126261345:G:A_A
p = 8.9e−10, beta = −0.548, N = 325
TMEQFTIHLTVNPQSK pc3
F8WAS2;P19827
172:175 124:133 17:17
3:126261345:G:A_A
p = 7.7e−06, beta = −0.41, N = 313
QAVDTAVDGVFIR pc2
F8WAS2;P19827
175:175 133:133 17:17
3:126261345:G:A_A
p = 4.8e−10, beta = −0.557, N = 325
NHMQYEIVIK pc2
F8WAS2;P19827
175:175 133:133 17:17
3:126261345:G:A_A
p = 5.8e−09, beta = −0.522, N = 325
GFSLDEATNLNGGLLR pc2
P19827;P19827−3
175:175 133:133 17:17
3:126261345:G:A_A
p = 1.1e−07, beta = −0.478, N = 325
LWAYLTIQELLAK pc2
P19827;P19827−3
172:175 133:133 17:17
3:126261345:G:A_A
p = 1.5e−07, beta = −0.475, N = 322
ADVQAHGEGQEFSITCLVDEEEMKK p
P19827;P19827−3
161:175 116:133 16:17
3:126261345:G:A_A
p = 6.9e−06, beta = −0.425, N = 293
GMADQDGLKPTIDK pc3
P19827;P19827−3
175:175 133:133 17:17
3:126261345:G:A_A
p = 4.2e−09, beta = −0.527, N = 325
RQAVDTAVDGVFIR pc3
F8WAS2;P19827
170:175 125:133 16:17
3:126261345:G:A_A
p = 0.0026, beta = −0.282, N = 311
GHMLENHVER pc3
P19827;P19827−3
175:175 133:133 17:17
3:126261345:G:A_A
p = 3.5e−09, beta = −0.53, N = 325
39Assay Target: ITIH1
UniProt: P19827
rsID: rs1056522
Target SNP: 3:126261345:G:A
Proxy SNP: 3:126261345:G:A
Tarkin log10(p): 34.5
Tarkin BETA: −0.521
Tarkin MSPA: 0.863
1252:1250:1256:1256:1256:124
MSLDYGFVTPLTSMSIR pc2
rs1042779 ALT
284:605 239:540 58:115
3:126261345:G:A_A
p = 0.38, model = DOM, N = 581
ANLSSQALR pc2
rs1042779 ALT
157:605 109:540 14:115
3:126261345:G:A_A
p = 0.0023, model = REC, N = 280
MSLDYGFVTPLTSMSIR pc3
rs1042779 ALT
308:605 260:540 42:115
3:126261345:G:A_A
p = 0.0081, model = DOM, N = 610
log10 protein intensity
log10 peptide intensity

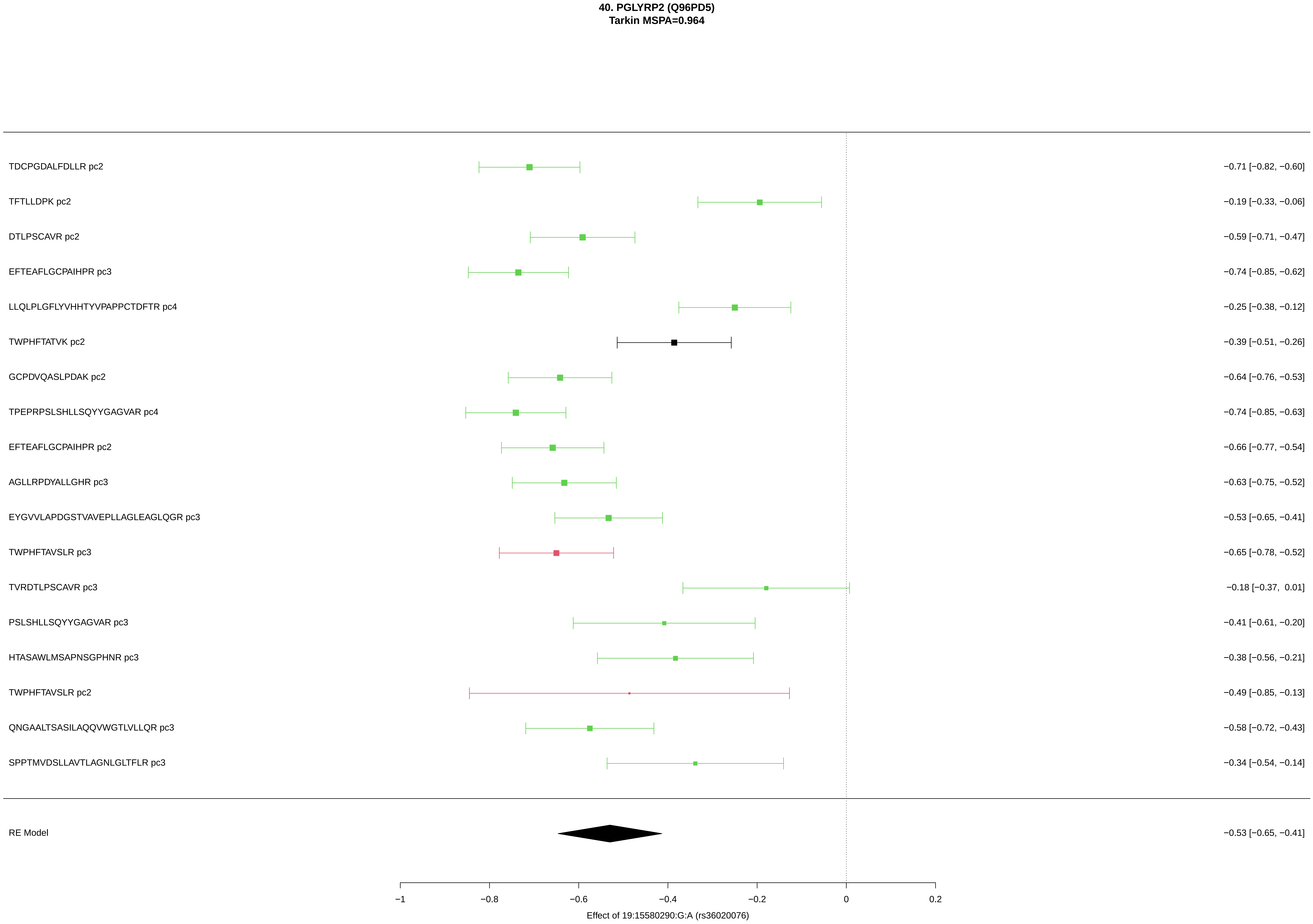

40. PGLYRP2 (Q96PD5)
Tarkin MSPA=0.964
TDCPGDALFDLLR pc2
−0.71 [−0.82, −0.60]
TFTLLDPK pc2
−0.19 [−0.33, −0.06]
DTLPSCAVR pc2
−0.59 [−0.71, −0.47]
EFTEAFLGCPAIHPR pc3
−0.74 [−0.85, −0.62]
LLQLPLGFLYVHHTYVPAPPCTDFTR pc4
−0.25 [−0.38, −0.12]
TWPHFTATVK pc2
−0.39 [−0.51, −0.26]
GCPDVQASLPDAK pc2
−0.64 [−0.76, −0.53]
TPEPRPSLSHLLSQYYGAGVAR pc4
−0.74 [−0.85, −0.63]
EFTEAFLGCPAIHPR pc2
−0.66 [−0.77, −0.54]
AGLLRPDYALLGHR pc3
−0.63 [−0.75, −0.52]
EYGVVLAPDGSTVAVEPLLAGLEAGLQGR pc3
−0.53 [−0.65, −0.41]
TWPHFTAVSLR pc3
−0.65 [−0.78, −0.52]
TVRDTLPSCAVR pc3
−0.18 [−0.37, 0.01]
PSLSHLLSQYYGAGVAR pc3
−0.41 [−0.61, −0.20]
HTASAWLMSAPNSGPHNR pc3
−0.38 [−0.56, −0.21]
TWPHFTAVSLR pc2
−0.49 [−0.85, −0.13]
QNGAALTSASILAQQVWGTLVLLQR pc3
−0.58 [−0.72, −0.43]
SPPTMVDSLLAVTLAGNLGLTFLR pc3
−0.34 [−0.54, −0.14]
RE Model
−0.53 [−0.65, −0.41]
−1
−0.8
−0.6
−0.4
−0.2
0
0.2
Effect of 19:15580290:G:A (rs36020076)

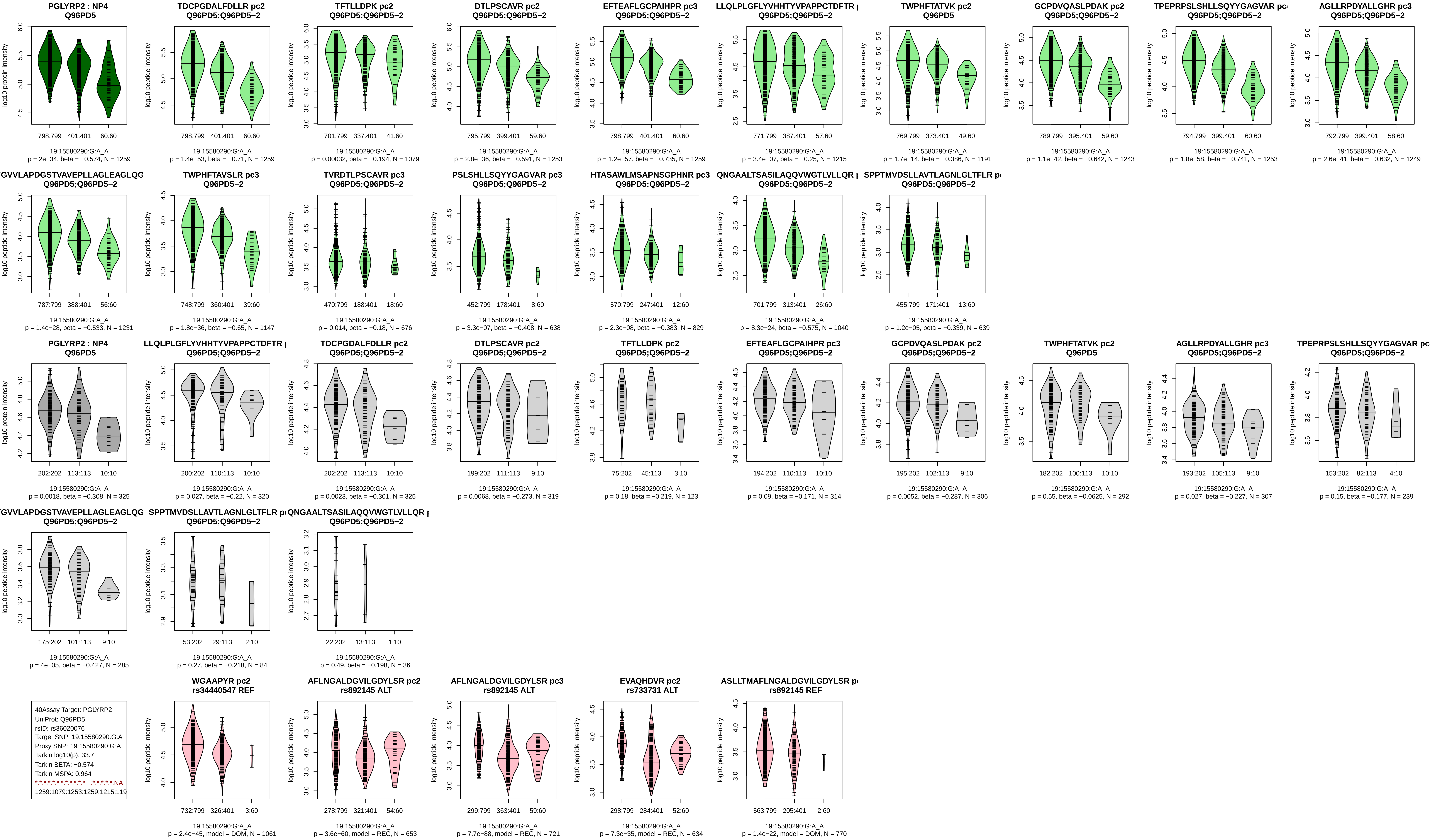
PGLYRP2 : NP4
Q96PD5
798:799 401:401 60:60
19:15580290:G:A_A
p = 2e−34, beta = −0.574, N = 1259
TDCPGDALFDLLR pc2
Q96PD5;Q96PD5−2
798:799 401:401 60:60
19:15580290:G:A_A
p = 1.4e−53, beta = −0.71, N = 1259
TFTLLDPK pc2
Q96PD5;Q96PD5−2
701:799 337:401 41:60
19:15580290:G:A_A
p = 0.00032, beta = −0.194, N = 1079
DTLPSCAVR pc2
Q96PD5;Q96PD5−2
795:799 399:401 59:60
19:15580290:G:A_A
p = 2.8e−36, beta = −0.591, N = 1253
EFTEAFLGCPAIHPR pc3
Q96PD5;Q96PD5−2
798:799 401:401 60:60
19:15580290:G:A_A
p = 1.2e−57, beta = −0.735, N = 1259
Q96PD5;Q96PD5−2
771:799 387:401 57:60
19:15580290:G:A_A
p = 3.4e−07, beta = −0.25, N = 1215
TWPHFTATVK pc2
Q96PD5
769:799 373:401 49:60
19:15580290:G:A_A
p = 1.7e−14, beta = −0.386, N = 1191
GCPDVQASLPDAK pc2
Q96PD5;Q96PD5−2
789:799 395:401 59:60
19:15580290:G:A_A
p = 1.1e−42, beta = −0.642, N = 1243
Q96PD5;Q96PD5−2
794:799 399:401 60:60
19:15580290:G:A_A
p = 1.8e−58, beta = −0.741, N = 1253
AGLLRPDYALLGHR pc3
Q96PD5;Q96PD5−2
792:799 399:401 58:60
19:15580290:G:A_A
p = 2.6e−41, beta = −0.632, N = 1249
Q96PD5;Q96PD5−2
787:799 388:401 56:60
19:15580290:G:A_A
p = 1.4e−28, beta = −0.533, N = 1231
TWPHFTAVSLR pc3
Q96PD5−2
748:799 360:401 39:60
19:15580290:G:A_A
p = 1.8e−36, beta = −0.65, N = 1147
TVRDTLPSCAVR pc3
Q96PD5;Q96PD5−2
470:799 188:401 18:60
19:15580290:G:A_A
p = 0.014, beta = −0.18, N = 676
PSLSHLLSQYYGAGVAR pc3
Q96PD5;Q96PD5−2
452:799 178:401 8:60
19:15580290:G:A_A
p = 3.3e−07, beta = −0.408, N = 638
HTASAWLMSAPNSGPHNR pc3
Q96PD5;Q96PD5−2
570:799 247:401 12:60
19:15580290:G:A_A
p = 2.3e−08, beta = −0.383, N = 829
Q96PD5;Q96PD5−2
701:799 313:401 26:60
19:15580290:G:A_A
p = 8.3e−24, beta = −0.575, N = 1040
Q96PD5;Q96PD5−2
455:799 171:401 13:60
19:15580290:G:A_A
p = 1.2e−05, beta = −0.339, N = 639
PGLYRP2 : NP4
Q96PD5
202:202 113:113 10:10
19:15580290:G:A_A
p = 0.0018, beta = −0.308, N = 325
Q96PD5;Q96PD5−2
200:202 110:113 10:10
19:15580290:G:A_A
p = 0.027, beta = −0.22, N = 320
TDCPGDALFDLLR pc2
Q96PD5;Q96PD5−2
202:202 113:113 10:10
19:15580290:G:A_A
p = 0.0023, beta = −0.301, N = 325
DTLPSCAVR pc2
Q96PD5;Q96PD5−2
199:202 111:113 9:10
19:15580290:G:A_A
p = 0.0068, beta = −0.273, N = 319
TFTLLDPK pc2
Q96PD5;Q96PD5−2
75:202 45:113 3:10
19:15580290:G:A_A
p = 0.18, beta = −0.219, N = 123
EFTEAFLGCPAIHPR pc3
Q96PD5;Q96PD5−2
194:202 110:113 10:10
19:15580290:G:A_A
p = 0.09, beta = −0.171, N = 314
GCPDVQASLPDAK pc2
Q96PD5;Q96PD5−2
195:202 102:113 9:10
19:15580290:G:A_A
p = 0.0052, beta = −0.287, N = 306
TWPHFTATVK pc2
Q96PD5
182:202 100:113 10:10
19:15580290:G:A_A
p = 0.55, beta = −0.0625, N = 292
AGLLRPDYALLGHR pc3
Q96PD5;Q96PD5−2
193:202 105:113 9:10
19:15580290:G:A_A
p = 0.027, beta = −0.227, N = 307
Q96PD5;Q96PD5−2
153:202 82:113 4:10
19:15580290:G:A_A
p = 0.15, beta = −0.177, N = 239
Q96PD5;Q96PD5−2
175:202 101:113 9:10
19:15580290:G:A_A
p = 4e−05, beta = −0.427, N = 285
Q96PD5;Q96PD5−2
53:202 29:113 2:10
19:15580290:G:A_A
p = 0.27, beta = −0.218, N = 84
Q96PD5;Q96PD5−2
22:202 13:113 1:10
19:15580290:G:A_A
p = 0.49, beta = −0.198, N = 36
40Assay Target: PGLYRP2
UniProt: Q96PD5
rsID: rs36020076
Target SNP: 19:15580290:G:A
Proxy SNP: 19:15580290:G:A
Tarkin log10(p): 33.7
Tarkin BETA: −0.574
Tarkin MSPA: 0.964
1259:1079:1253:1259:1215:119
WGAAPYR pc2
rs34440547 REF
732:799 326:401 3:60
19:15580290:G:A_A
p = 2.4e−45, model = DOM, N = 1061
AFLNGALDGVILGDYLSR pc2
rs892145 ALT
278:799 321:401 54:60
19:15580290:G:A_A
p = 3.6e−60, model = REC, N = 653
AFLNGALDGVILGDYLSR pc3
rs892145 ALT
299:799 363:401 59:60
19:15580290:G:A_A
p = 7.7e−88, model = REC, N = 721
EVAQHDVR pc2
rs733731 ALT
298:799 284:401 52:60
19:15580290:G:A_A
p = 7.3e−35, model = REC, N = 634
rs892145 REF
563:799 205:401 2:60
19:15580290:G:A_A
p = 1.4e−22, model = DOM, N = 770
log10 protein intensity
log10 peptide intensity

41. RNASE6 (Q93091)
Tarkin MSPA=0.947

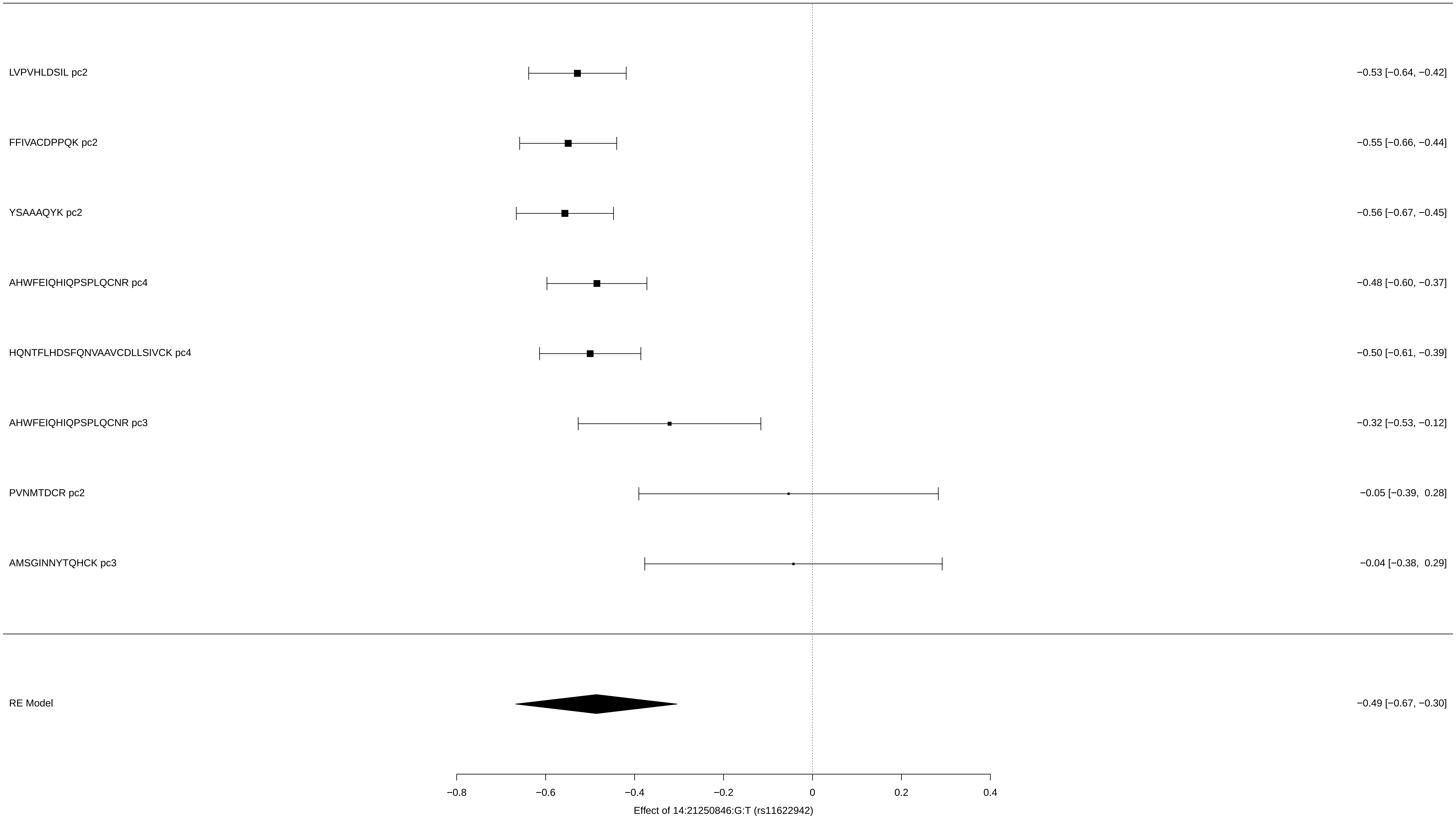

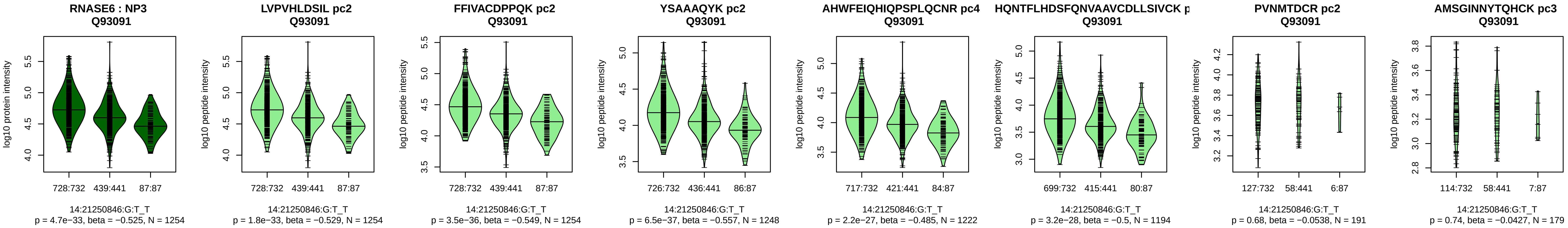

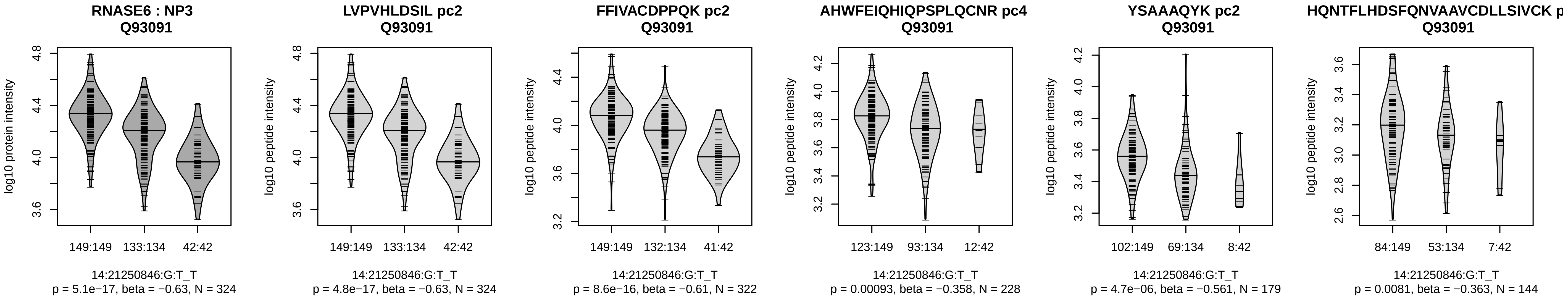

41Assay Target: RNASE6
UniProt: Q93091
rsID: rs11622942
Target SNP: 14:21250846:G:T
Proxy SNP: 14:21250846:G:T
Tarkin log10(p): 32.3
Tarkin BETA: −0.525
Tarkin MSPA: 0.947
::*:*:*:*:−:−:NA
1254:1254:1248:1222:1194:448

42. SPON2 (Q9BUD6)
Tarkin MSPA=0.965

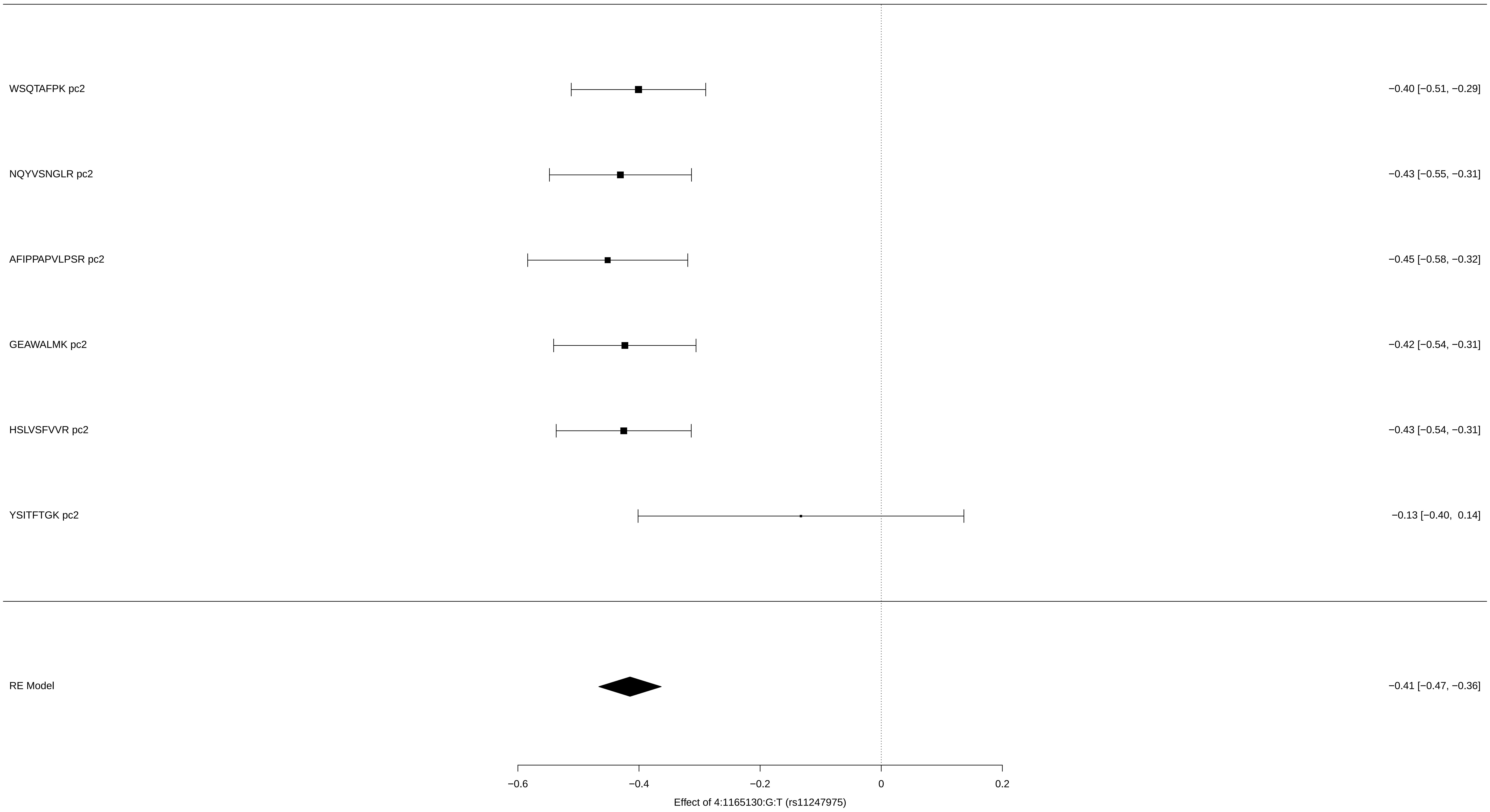

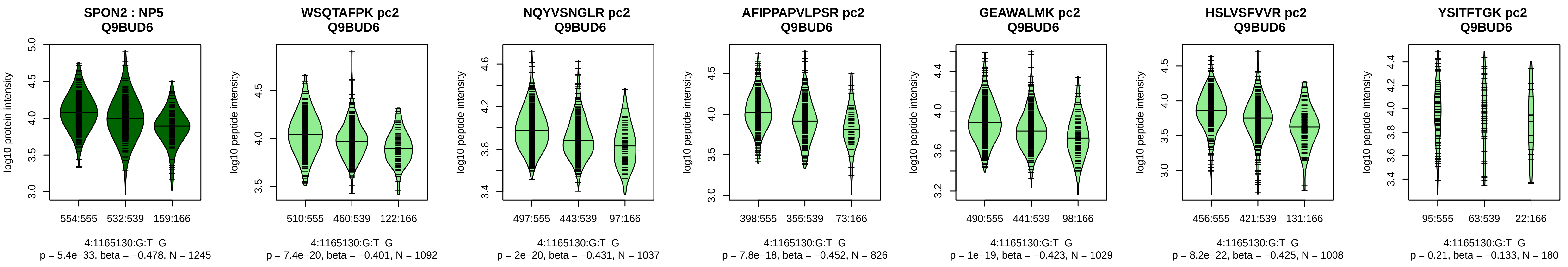

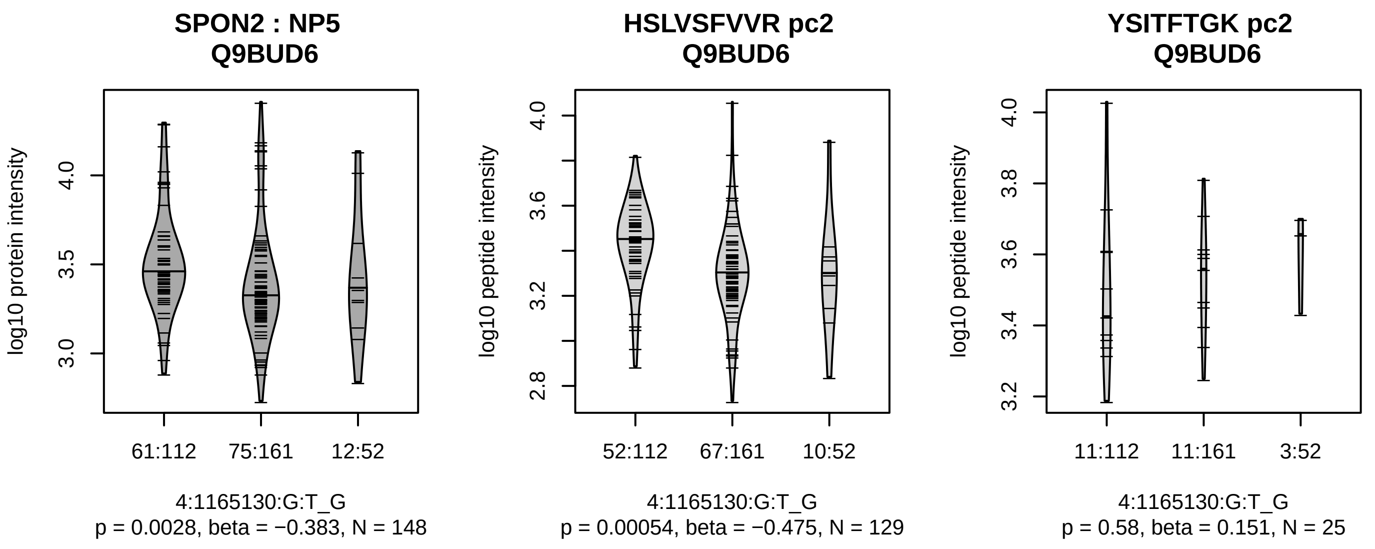

42Assay Target: SPON2
UniProt: Q9BUD6
rsID: rs11247975
Target SNP: 4:1165130:G:T
Proxy SNP: 4:1165130:G:T
Tarkin log10(p): 32.3
Tarkin BETA: −0.478
Tarkin MSPA: 0.965
··*·*·*·–
1092:1037:826:1029:1008:180

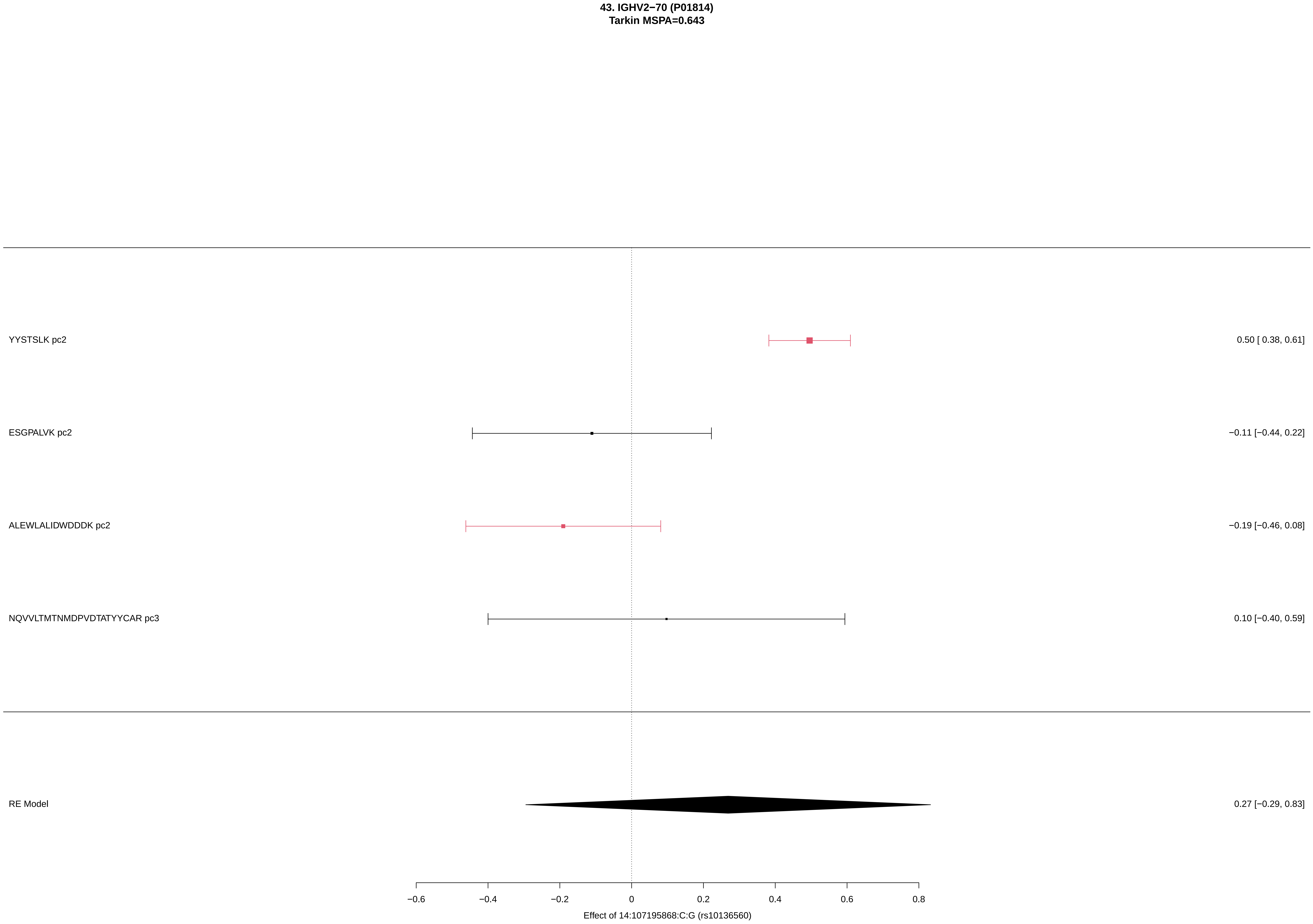

43. IGHV2−70 (P01814)
Tarkin MSPA=0.643
YYSTSLK pc2
0.50 [0.38, 0.61]
ESGPALVK pc2
−0.11 [−0.44, 0.22]
ALEWLALIDWDDDK pc2
−0.19 [−0.46, 0.08]
NQVVLTMTNMDPVDTATYYCAR pc3
0.10 [−0.40, 0.59]
RE Model
0.27 [−0.29, 0.83]
−0.6
−0.4
−0.2
0
0.2
0.4
0.6
0.8
Effect of 14:107195868:C:G (rs10136560)

IGHV2−70 : NP3
P01814

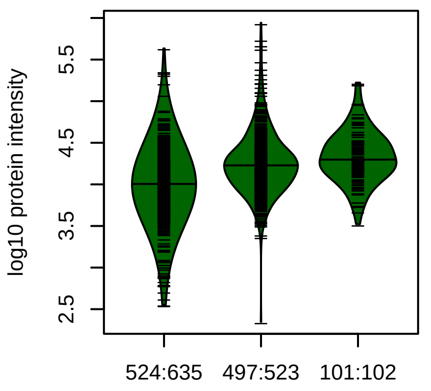

14:107195868:C:G_G
p = 1.6e−31, beta = 0.523, N = 1122

YYSTSLK pc2
P01814

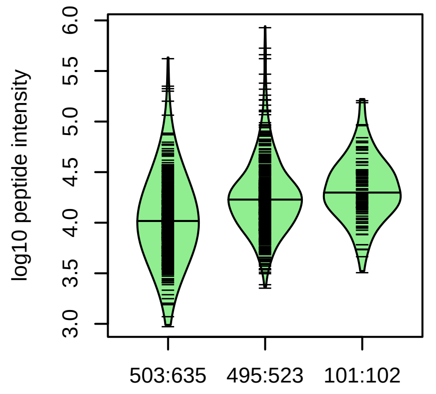

14:107195868:C:G_G
p = 7.7e−28, beta = 0.496, N = 1099

ESGPALVK pc2
A0A0C4DH43;P01814

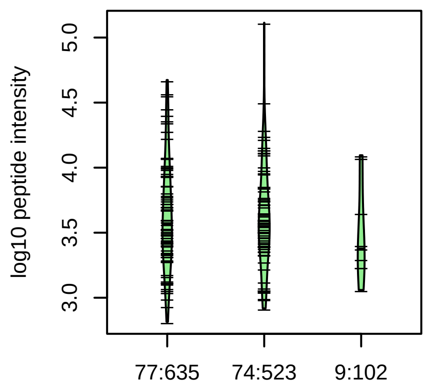

14:107195868:C:G_G
p = 0.39, beta = −0.111, N = 160

ALEWLALIDWDDDK pc2
P01814

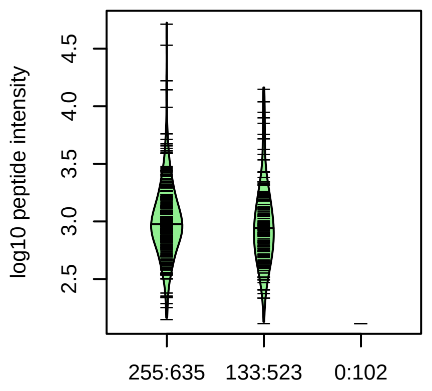

14:107195868:C:G_G
p = 0.072, beta = −0.19, N = 388

NQVVLTMTNMDPVDTATYYCAR pc
A0A0C4DH43;P01814

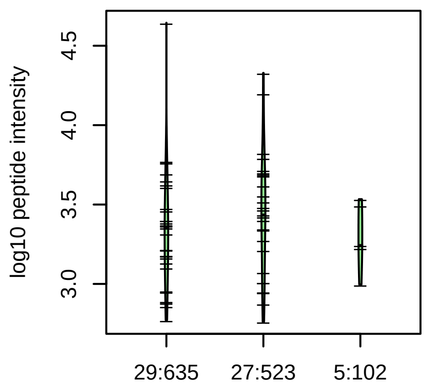

14:107195868:C:G_G
p = 0.62, beta = 0.097, N = 61

IGHV2−70 : NP3
P01814

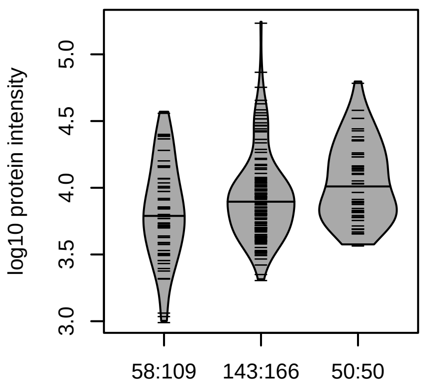

14:107195868:C:G_G
p = 0.001, beta = 0.309, N = 251

YYSTSLK pc2
P01814

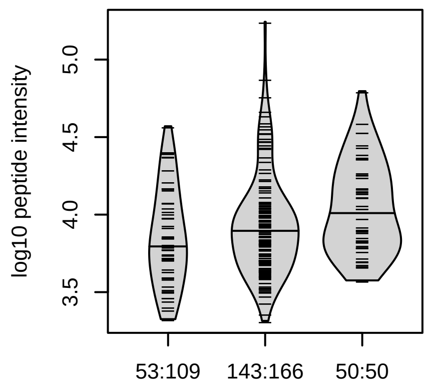

14:107195868:C:G_G
p = 0.0069, beta = 0.261, N = 246

ESGPALVK pc2
A0A0C4DH43;P01814

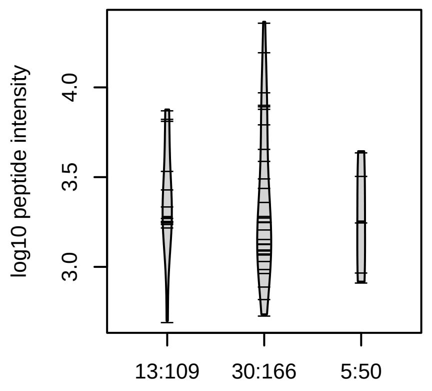

14:107195868:C:G_G
p = 0.56, beta = −0.135, N = 48

ALEWLALIDWDDDK pc2
P01814

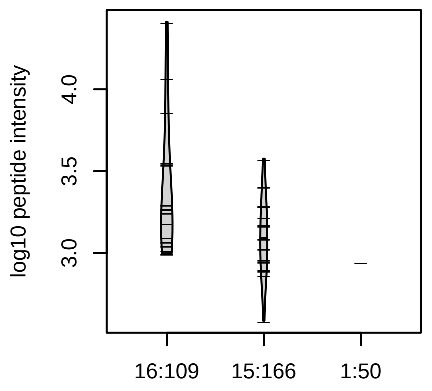

14:107195868:C:G_G
p = 0.6, beta = −0.155, N = 32

43Assay Target: IGHV2−70
UniProt: P01814
rsID: rs10136560
Target SNP: 14:107195868:C:G
Proxy SNP: 14:107195868:C:G
Tarkin log10(p): 30.8
Tarkin BETA: 0.523
Tarkin MSPA: 0.643
*:−:−:−
1099:160:388:61

44. FUCA2 (Q9BTY2)
Tarkin MSPA=0.993

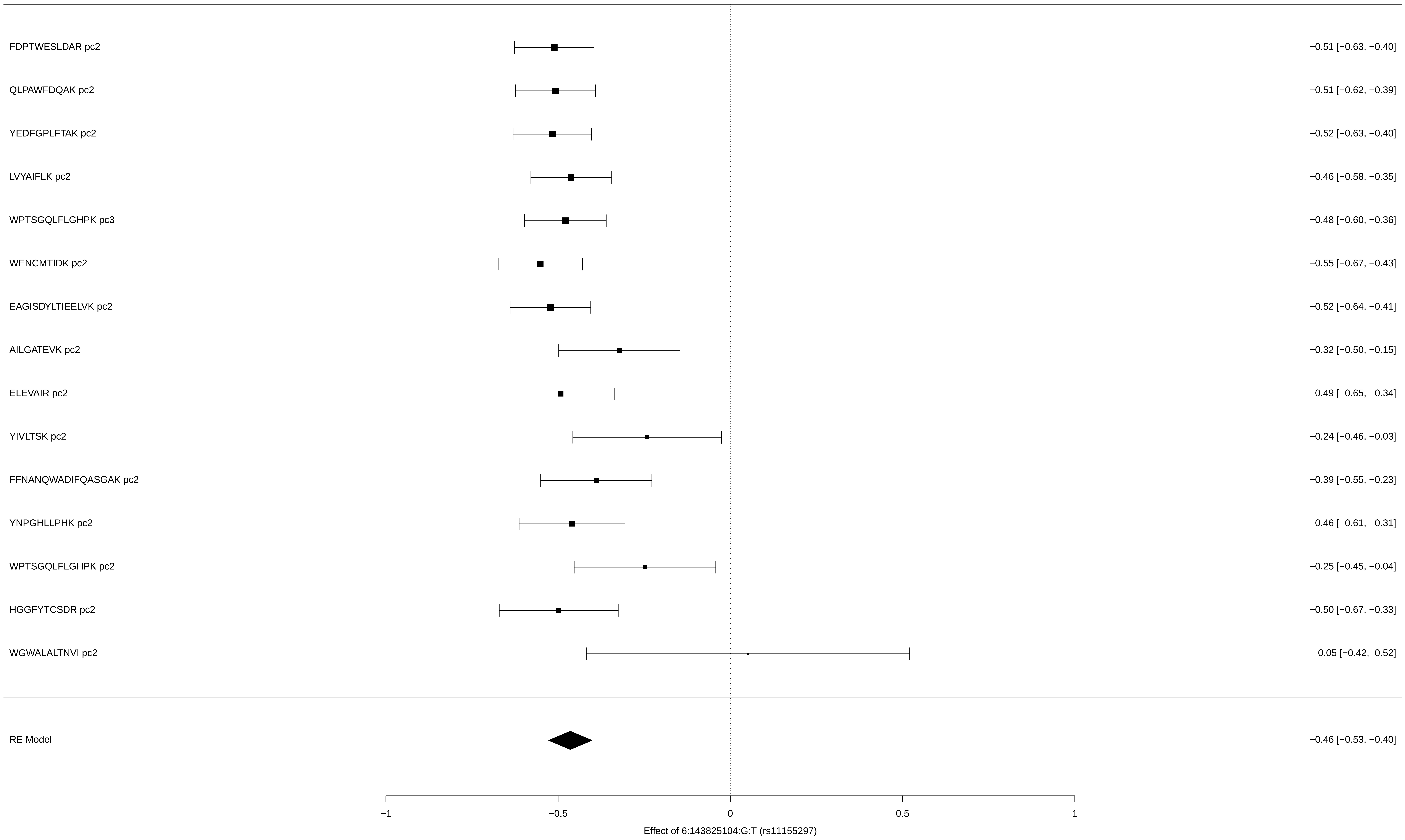

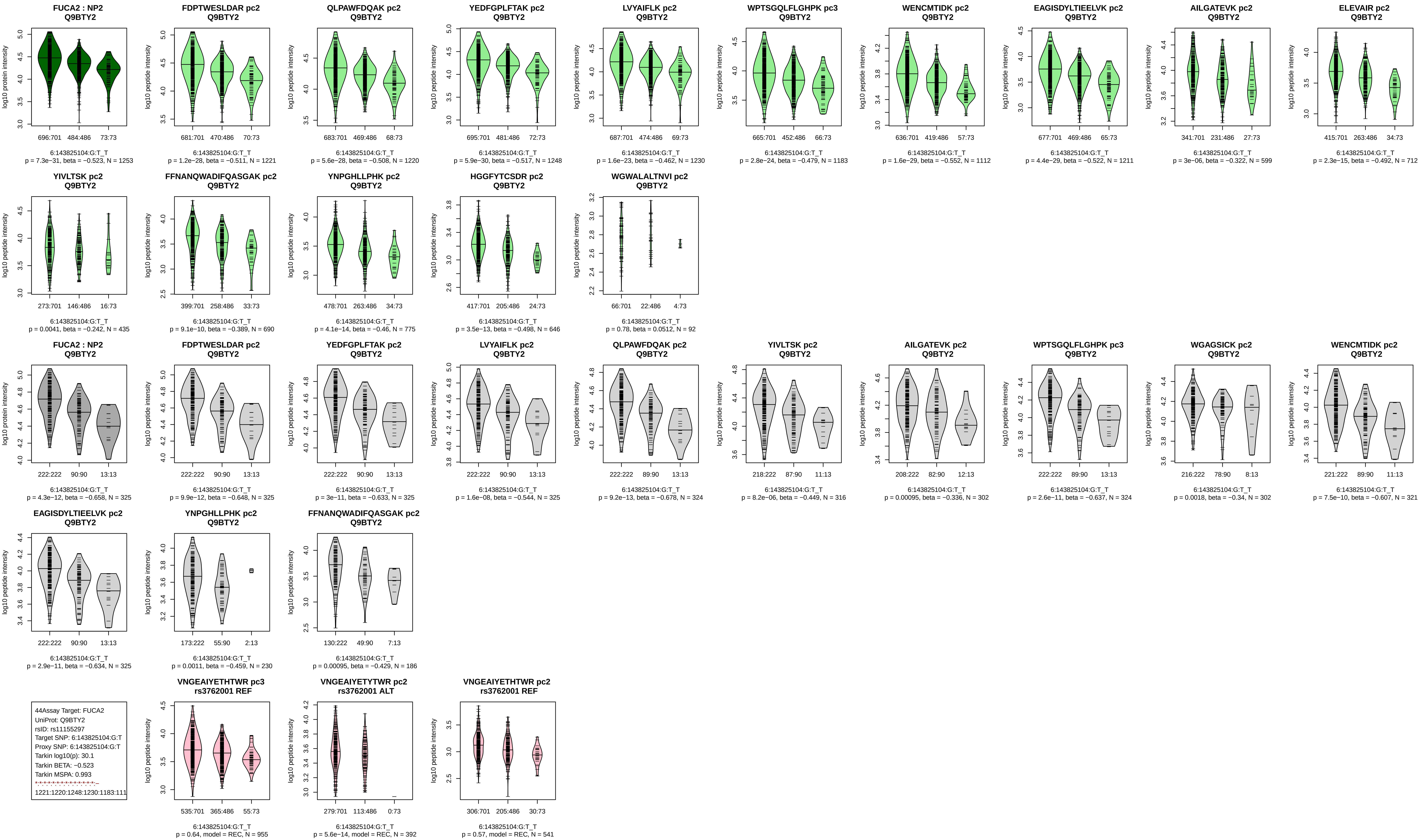
FUCA2 : NP2
Q9BTY2
log10 protein intensity
696:701 484:486 73:73
6:143825104:G:T_T
p = 7.3e−31, beta = −0.523, N = 1253
FDPTWESLDAR pc2
Q9BTY2
log10 peptide intensity
681:701 470:486 70:73
6:143825104:G:T_T
p = 1.2e−28, beta = −0.511, N = 1221
QLPAWFDQAK pc2
Q9BTY2
683:701 469:486 68:73
6:143825104:G:T_T
p = 5.6e−28, beta = −0.508, N = 1220
YEDFGPLFTAK pc2
Q9BTY2
695:701 481:486 72:73
6:143825104:G:T_T
p = 5.9e−30, beta = −0.517, N = 1248
LVYAIFLK pc2
Q9BTY2
687:701 474:486 69:73
6:143825104:G:T_T
p = 1.6e−23, beta = −0.462, N = 1230
WPTSGQLFLGHPK pc3
Q9BTY2
665:701 452:486 66:73
6:143825104:G:T_T
p = 2.8e−24, beta = −0.479, N = 1183
WENCMTIDK pc2
Q9BTY2
636:701 419:486 57:73
6:143825104:G:T_T
p = 1.6e−29, beta = −0.552, N = 1112
EAGISDYLTIEELVK pc2
Q9BTY2
677:701 469:486 65:73
6:143825104:G:T_T
p = 4.4e−29, beta = −0.522, N = 1211
AILGATEVK pc2
Q9BTY2
341:701 231:486 27:73
6:143825104:G:T_T
p = 3e−06, beta = −0.322, N = 599
ELEVAIR pc2
Q9BTY2
415:701 263:486 34:73
6:143825104:G:T_T
p = 2.3e−15, beta = −0.492, N = 712
YIVLTSK pc2
Q9BTY2
273:701 146:486 16:73
6:143825104:G:T_T
p = 0.0041, beta = −0.242, N = 435
FFNANQWADIFQASGAK pc2
Q9BTY2
399:701 258:486 33:73
6:143825104:G:T_T
p = 9.1e−10, beta = −0.389, N = 690
YNPGHLLPHK pc2
Q9BTY2
478:701 263:486 34:73
6:143825104:G:T_T
p = 4.1e−14, beta = −0.46, N = 775
HGGFYTCSDR pc2
Q9BTY2
417:701 205:486 24:73
6:143825104:G:T_T
p = 3.5e−13, beta = −0.498, N = 646
WGWALALTNVI pc2
Q9BTY2
66:701 22:486 4:73
6:143825104:G:T_T
p = 0.78, beta = 0.0512, N = 92
FUCA2 : NP2
Q9BTY2
222:222 90:90 13:13
6:143825104:G:T_T
p = 4.3e−12, beta = −0.658, N = 325
FDPTWESLDAR pc2
Q9BTY2
222:222 90:90 13:13
6:143825104:G:T_T
p = 9.9e−12, beta = −0.648, N = 325
YEDFGPLFTAK pc2
Q9BTY2
222:222 90:90 13:13
6:143825104:G:T_T
p = 3e−11, beta = −0.633, N = 325
LVYAIFLK pc2
Q9BTY2
222:222 90:90 13:13
6:143825104:G:T_T
p = 1.6e−08, beta = −0.544, N = 325
QLPAWFDQAK pc2
Q9BTY2
222:222 89:90 13:13
6:143825104:G:T_T
p = 9.2e−13, beta = −0.678, N = 324
YIVLTSK pc2
Q9BTY2
218:222 87:90 11:13
6:143825104:G:T_T
p = 8.2e−06, beta = −0.449, N = 316
AILGATEVK pc2
Q9BTY2
208:222 82:90 12:13
6:143825104:G:T_T
p = 0.00095, beta = −0.336, N = 302
WPTSGQLFLGHPK pc3
Q9BTY2
222:222 89:90 13:13
6:143825104:G:T_T
p = 2.6e−11, beta = −0.637, N = 324
WGAGSICK pc2
Q9BTY2
216:222 78:90 8:13
6:143825104:G:T_T
p = 0.0018, beta = −0.34, N = 302
WENCMTIDK pc2
Q9BTY2
221:222 89:90 11:13
6:143825104:G:T_T
p = 7.5e−10, beta = −0.607, N = 321
EAGISDYLTIEELVK pc2
Q9BTY2
222:222 90:90 13:13
6:143825104:G:T_T
p = 2.9e−11, beta = −0.634, N = 325
YNPGHLLPHK pc2
Q9BTY2
173:222 55:90 2:13
6:143825104:G:T_T
p = 0.0011, beta = −0.459, N = 230
FFNANQWADIFQASGAK pc2
Q9BTY2
130:222 49:90 7:13
6:143825104:G:T_T
p = 0.00095, beta = −0.429, N = 186
44Assay Target: FUCA2
UniProt: Q9BTY2
rsID: rs11155297
Target SNP: 6:143825104:G:T
Proxy SNP: 6:143825104:G:T
Tarkin log10(p): 30.1
Tarkin BETA: −0.523
Tarkin MSPA: 0.993
1221:1220:1248:1230:1183:111
VNGEAIYETHTWR pc3
rs3762001 REF
535:701 365:486 55:73
6:143825104:G:T_T
p = 0.64, model = REC, N = 955
VNGEAIYETYTWR pc2
rs3762001 ALT
279:701 113:486 0:73
6:143825104:G:T_T
p = 5.6e−14, model = REC, N = 392
VNGEAIYETHTWR pc2
rs3762001 REF
306:701 205:486 30:73
6:143825104:G:T_T
p = 0.57, model = REC, N = 541

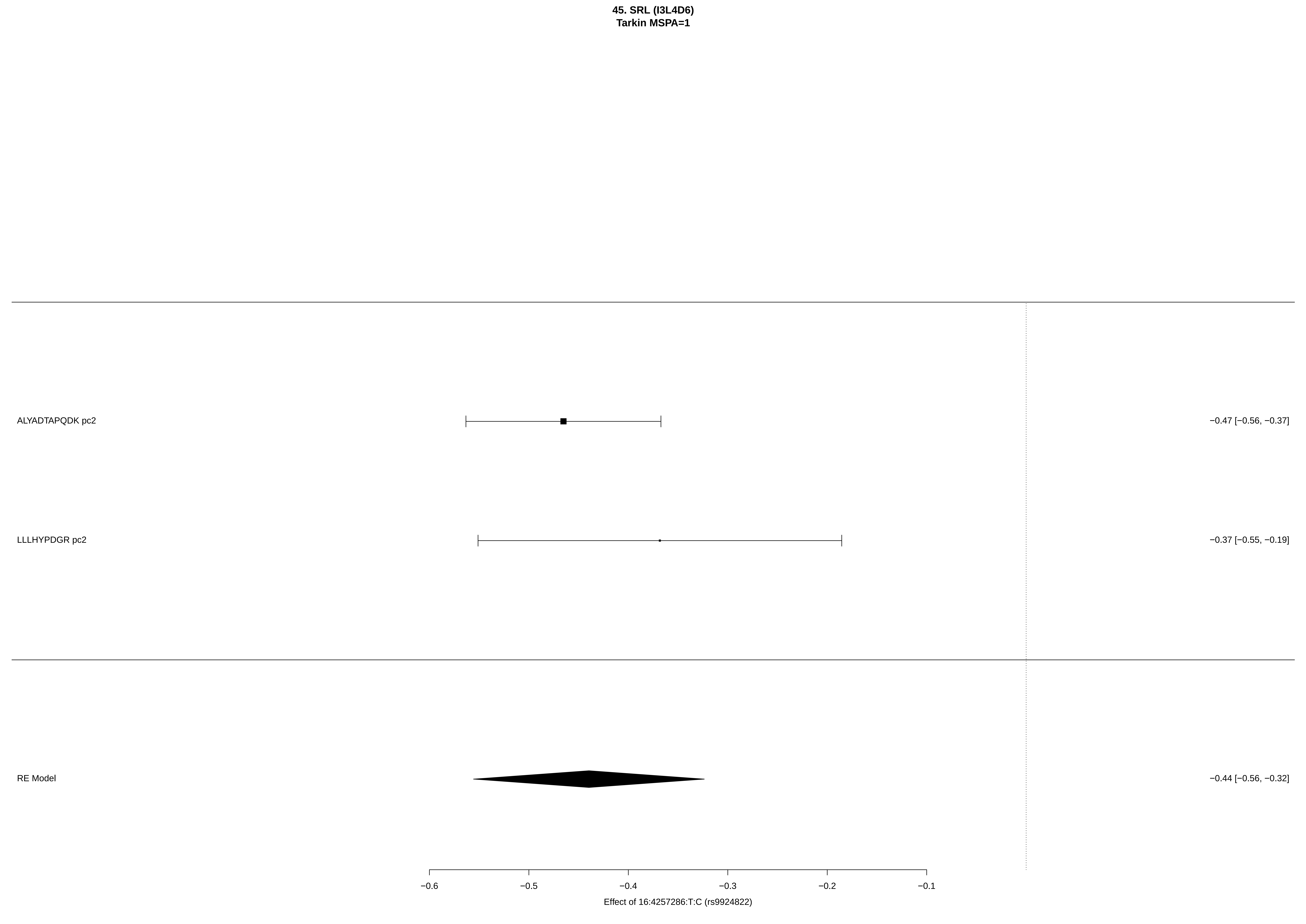
45. SRL (I3L4D6)
Tarkin MSPA=1
ALYADTAPQDK pc2
−0.47 [−0.56, −0.37]
LLLHYPDGR pc2
−0.37 [−0.55, −0.19]
RE Model
−0.44 [−0.56, −0.32]
−0.6
−0.5
−0.4
−0.3
−0.2
−0.1
Effect of 16:4257286:T:C (rs9924822)

SRL : NP2
I3L4D6

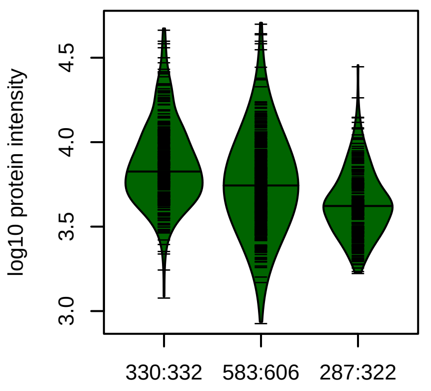

16:4257286:T:C_T
p = 9.5e−31, beta = −0.45, N = 1200

ALYADTAPQDK pc2
I3L4D6

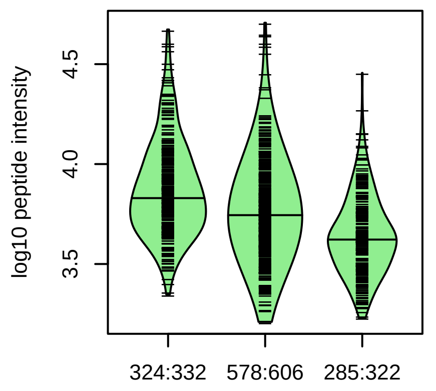

16:4257286:T:C_T
p = 1.9e−32, beta = −0.465, N = 1187

LLLHYPDGR pc2
I3L4D6

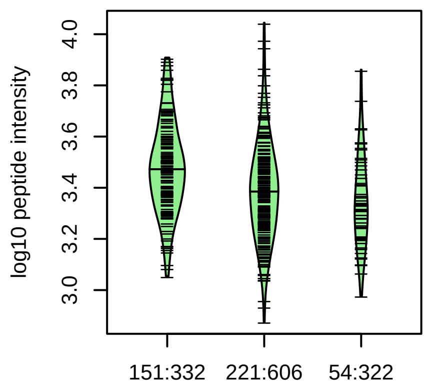

16:4257286:T:C_T
p = 3.3e−07, beta = −0.368, N = 426

SRL : NP2
I3L4D6

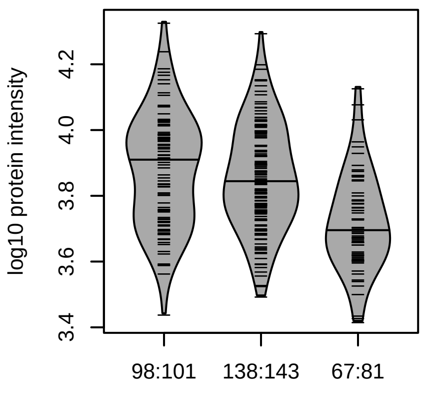

16:4257286:T:C_T
p = 5.2e−07, beta = −0.382, N = 303

ALYADTAPQDK pc2
I3L4D6

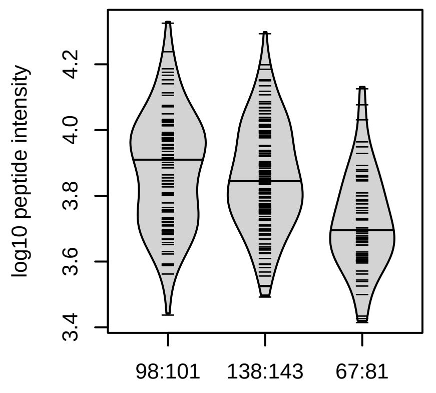

16:4257286:T:C_T
p = 5.2e−07, beta = −0.382, N = 303

LLLHYPDGR pc2
I3L4D6

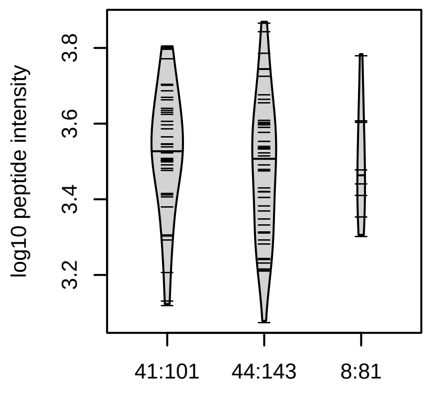

16:4257286:T:C_T
p = 0.56, beta = −0.0919, N = 93

45Assay Target: SRL
UniProt: I3L4D6
rsID: rs9924822
Target SNP: 16:4257286:T:C
Proxy SNP: 16:4257286:T:C
Tarkin log10(p): 30
Tarkin BETA: −0.45
Tarkin MSPA: 1
::NA
1187:426:12

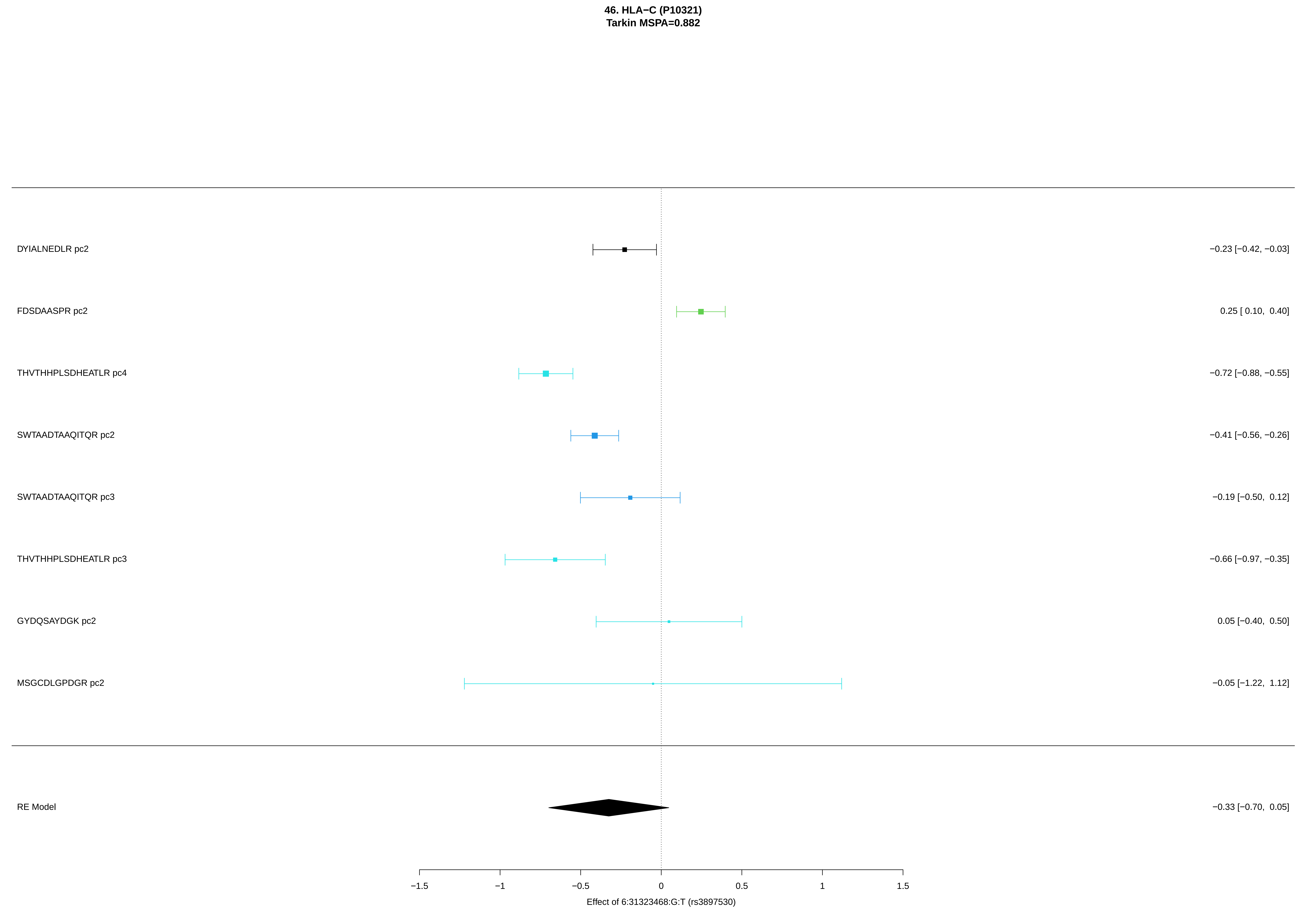

46. HLA−C (P10321)
Tarkin MSPA=0.882
DYIALNEDLR pc2
FDSDAASPR pc2
THVTHHPLSDHEATLR pc4
SWTAADTAAQITQR pc2
SWTAADTAAQITQR pc3
THVTHHPLSDHEATLR pc3
GYDQSAYDGK pc2
MSGCDLGPDGR pc2
RE Model
−0.23 [−0.42, −0.03]
0.25 [0.10, 0.40]
−0.72 [−0.88, −0.55]
−0.41 [−0.56, −0.26]
−0.19 [−0.50, 0.12]
−0.66 [−0.97, −0.35]
0.05 [−0.40, 0.50]
−0.05 [−1.22, 1.12]
−0.33 [−0.70, 0.05]
−1.5
−1
−0.5
0
0.5
1
1.5
Effect of 6:31323468:G:T (rs3897530)

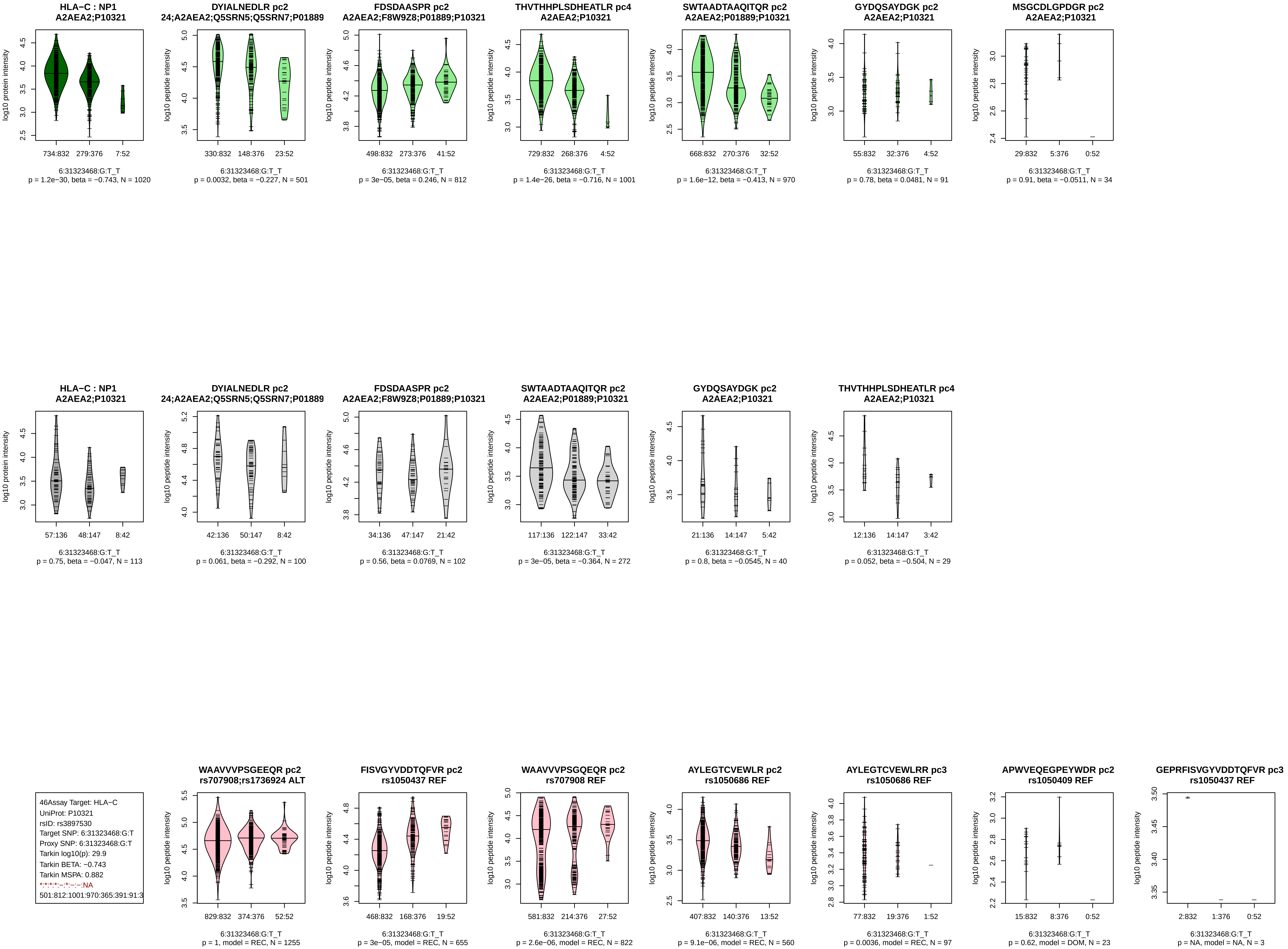
HLA−C : NP1
A2AEA2;P10321
log10 protein intensity
734:832 279:376 7:52
6:31323468:G:T_T
p = 1.2e−30, beta = −0.743, N = 1020
DYIALNEDLR pc2
24;A2AEA2;Q5SRN5;Q5SRN7;P01889
log10 peptide intensity
330:832 148:376 23:52
6:31323468:G:T_T
p = 0.0032, beta = −0.227, N = 501
FDSDAASPR pc2
A2AEA2;F8W9Z8;P01889;P10321
498:832 273:376 41:52
6:31323468:G:T_T
p = 3e−05, beta = 0.246, N = 812
THVTHHPLSDHEATLR pc4
A2AEA2;P10321
729:832 268:376 4:52
6:31323468:G:T_T
p = 1.4e−26, beta = −0.716, N = 1001
SWTAADTAAQITQR pc2
A2AEA2;P01889;P10321
668:832 270:376 32:52
6:31323468:G:T_T
p = 1.6e−12, beta = −0.413, N = 970
GYDQSAYDGK pc2
A2AEA2;P10321
55:832 32:376 4:52
6:31323468:G:T_T
p = 0.78, beta = 0.0481, N = 91
MSGCDLGPDGR pc2
A2AEA2;P10321
29:832 5:376 0:52
6:31323468:G:T_T
p = 0.91, beta = −0.0511, N = 34
HLA−C : NP1
A2AEA2;P10321
57:136 48:147 8:42
6:31323468:G:T_T
p = 0.75, beta = −0.047, N = 113
DYIALNEDLR pc2
24;A2AEA2;Q5SRN5;Q5SRN7;P01889
42:136 50:147 8:42
6:31323468:G:T_T
p = 0.061, beta = −0.292, N = 100
FDSDAASPR pc2
A2AEA2;F8W9Z8;P01889;P10321
34:136 47:147 21:42
6:31323468:G:T_T
p = 0.56, beta = 0.0769, N = 102
SWTAADTAAQITQR pc2
A2AEA2;P01889;P10321
117:136 122:147 33:42
6:31323468:G:T_T
p = 3e−05, beta = −0.364, N = 272
GYDQSAYDGK pc2
A2AEA2;P10321
21:136 14:147 5:42
6:31323468:G:T_T
p = 0.8, beta = −0.0545, N = 40
THVTHHPLSDHEATLR pc4
A2AEA2;P10321
12:136 14:147 3:42
6:31323468:G:T_T
p = 0.052, beta = −0.504, N = 29
46Assay Target: HLA−C
UniProt: P10321
rsID: rs3897530
Target SNP: 6:31323468:G:T
Proxy SNP: 6:31323468:G:T
Tarkin log10(p): 29.9
Tarkin BETA: −0.743
Tarkin MSPA: 0.882
::*:*:−:*:−:−:NA
501:812:1001:970:365:391:91:3
WAAVVVPSGEEQR pc2
rs707908;rs1736924 ALT
829:832 374:376 52:52
6:31323468:G:T_T
p = 1, model = REC, N = 1255
FISVGYVDDTQFVR pc2
rs1050437 REF
468:832 168:376 19:52
6:31323468:G:T_T
p = 3e−05, model = REC, N = 655
WAAVVVPSGQEQR pc2
rs707908 REF
581:832 214:376 27:52
6:31323468:G:T_T
p = 2.6e−06, model = REC, N = 822
AYLEGTCVEWLR pc2
rs1050686 REF
407:832 140:376 13:52
6:31323468:G:T_T
p = 9.1e−06, model = REC, N = 560
AYLEGTCVEWLRR pc3
rs1050686 REF
77:832 19:376 1:52
6:31323468:G:T_T
p = 0.0036, model = REC, N = 97
APWVEQEGPEYWDR pc2
rs1050409 REF
15:832 8:376 0:52
6:31323468:G:T_T
p = 0.62, model = DOM, N = 23
GEPRFISVGYVDDTQFVR pc3
rs1050437 REF
2:832 1:376 0:52
6:31323468:G:T_T
p = NA, model = NA, N = 3

47. FGL1 (Q08830)
Tarkin MSPA=0.982

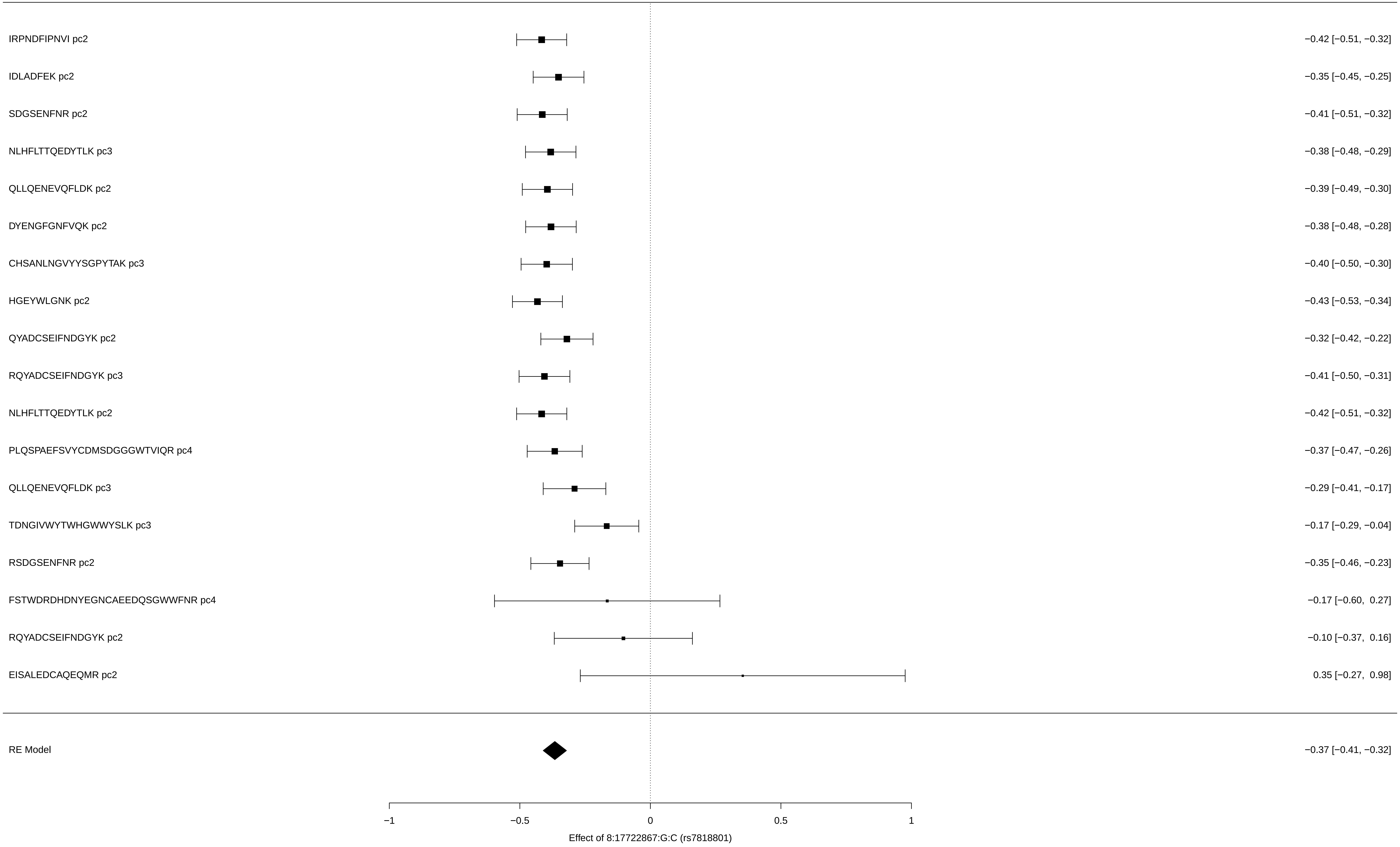

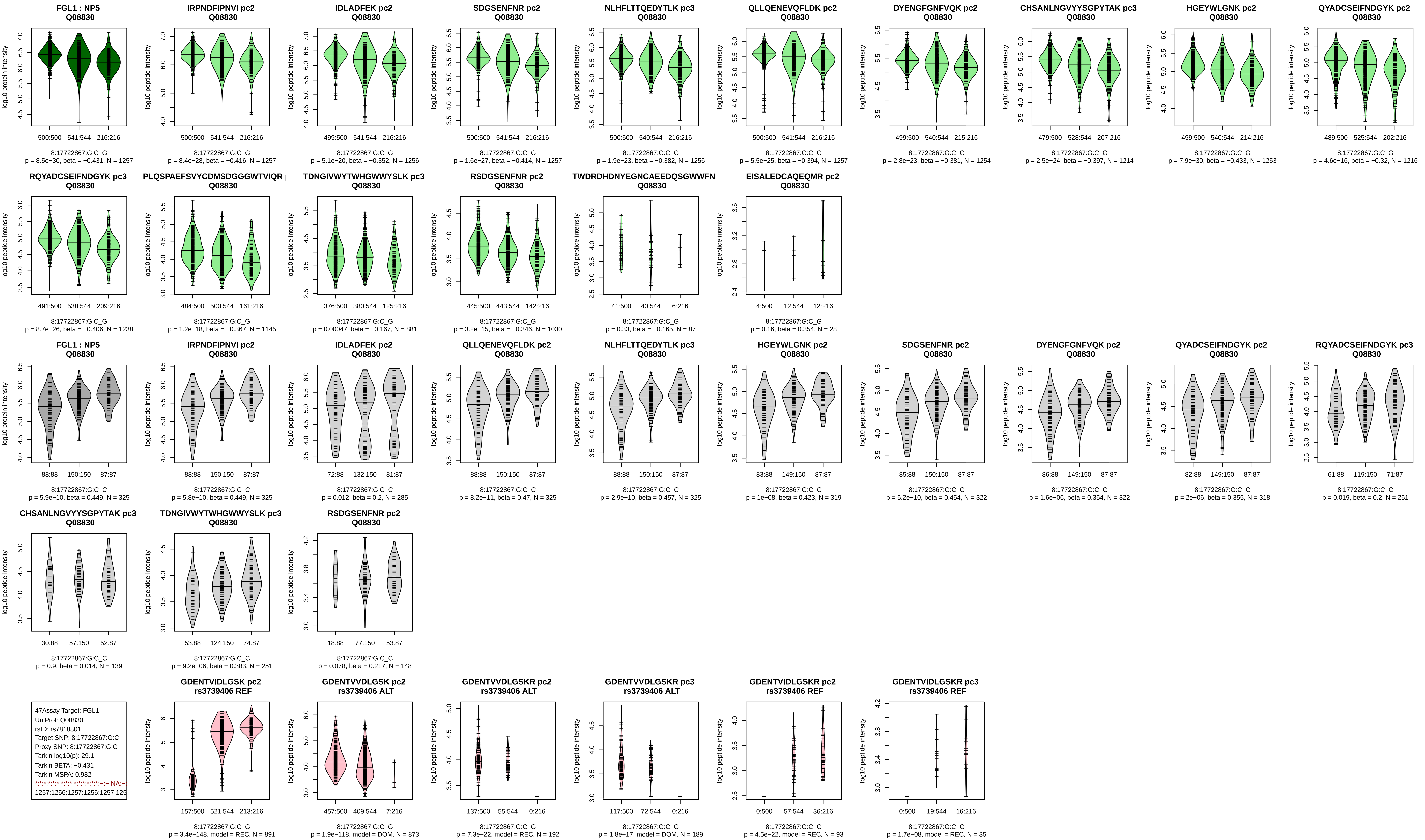

FGL1 : NP5
Q08830
log10 protein intensity
500:500 541:544 216:216
8:17722867:G:C_G
p = 8.5e−30, beta = −0.431, N = 1257
IRPNDFIPNVI pc2
Q08830
log10 peptide intensity
500:500 541:544 216:216
8:17722867:G:C_G
p = 8.4e−28, beta = −0.416, N = 1257
IDLADFEK pc2
Q08830
499:500 541:544 216:216
8:17722867:G:C_G
p = 5.1e−20, beta = −0.352, N = 1256
SDGSENFNR pc2
Q08830
500:500 541:544 216:216
8:17722867:G:C_G
p = 1.6e−27, beta = −0.414, N = 1257
NLHFLTTQEDYTLK pc3
Q08830
500:500 540:544 216:216
8:17722867:G:C_G
p = 1.9e−23, beta = −0.382, N = 1256
QLLQENEVQFLDK pc2
Q08830
500:500 541:544 216:216
8:17722867:G:C_G
p = 5.5e−25, beta = −0.394, N = 1257
DYENGFGNFVQK pc2
Q08830
499:500 540:544 215:216
8:17722867:G:C_G
p = 2.8e−23, beta = −0.381, N = 1254
CHSANLNGVYYSGPYTAK pc3
Q08830
479:500 528:544 207:216
8:17722867:G:C_G
p = 2.5e−24, beta = −0.397, N = 1214
HGEYWLGNK pc2
Q08830
499:500 540:544 214:216
8:17722867:G:C_G
p = 7.9e−30, beta = −0.433, N = 1253
QYADCSEIFNDGYK pc2
Q08830
489:500 525:544 202:216
8:17722867:G:C_G
p = 4.6e−16, beta = −0.32, N = 1216
RQYADCSEIFNDGYK pc3
Q08830
491:500 538:544 209:216
8:17722867:G:C_G
p = 8.7e−26, beta = −0.406, N = 1238
PLQSPAEFSVYCDMSDGGGWTVIQR
Q08830
484:500 500:544 161:216
8:17722867:G:C_G
p = 1.2e−18, beta = −0.367, N = 1145
TDNGIVWYTWHGWWYSLK pc3
Q08830
376:500 380:544 125:216
8:17722867:G:C_G
p = 0.00047, beta = −0.167, N = 881
RSDGSENFNR pc2
Q08830
445:500 443:544 142:216
8:17722867:G:C_G
p = 3.2e−15, beta = −0.346, N = 1030
TWDRDHDNYEGNCAEEDQSGWWFN
Q08830
41:500 40:544 6:216
8:17722867:G:C_G
p = 0.33, beta = −0.165, N = 87
EISALEDCAQEQMR pc2
Q08830
4:500 12:544 12:216
8:17722867:G:C_G
p = 0.16, beta = 0.354, N = 28
FGL1 : NP5
Q08830
88:88 150:150 87:87
8:17722867:G:C_C
p = 5.9e−10, beta = 0.449, N = 325
IRPNDFIPNVI pc2
Q08830
88:88 150:150 87:87
8:17722867:G:C_C
p = 5.8e−10, beta = 0.449, N = 325
IDLADFEK pc2
Q08830
72:88 132:150 81:87
8:17722867:G:C_C
p = 0.012, beta = 0.2, N = 285
QLLQENEVQFLDK pc2
Q08830
88:88 150:150 87:87
8:17722867:G:C_C
p = 8.2e−11, beta = 0.47, N = 325
NLHFLTTQEDYTLK pc3
Q08830
88:88 150:150 87:87
8:17722867:G:C_C
p = 2.9e−10, beta = 0.457, N = 325
HGEYWLGNK pc2
Q08830
83:88 149:150 87:87
8:17722867:G:C_C
p = 1e−08, beta = 0.423, N = 319
SDGSENFNR pc2
Q08830
85:88 150:150 87:87
8:17722867:G:C_C
p = 5.2e−10, beta = 0.454, N = 322
DYENGFGNFVQK pc2
Q08830
86:88 149:150 87:87
8:17722867:G:C_C
p = 1.6e−06, beta = 0.354, N = 322
QYADCSEIFNDGYK pc2
Q08830
82:88 149:150 87:87
8:17722867:G:C_C
p = 2e−06, beta = 0.355, N = 318
RQYADCSEIFNDGYK pc3
Q08830
61:88 119:150 71:87
8:17722867:G:C_C
p = 0.019, beta = 0.2, N = 251
CHSANLNGVYYSGPYTAK pc3
Q08830
30:88 57:150 52:87
8:17722867:G:C_C
p = 0.9, beta = 0.014, N = 139
TDNGIVWYTWHGWWYSLK pc3
Q08830
53:88 124:150 74:87
8:17722867:G:C_C
p = 9.2e−06, beta = 0.383, N = 251
RSDGSENFNR pc2
Q08830
18:88 77:150 53:87
8:17722867:G:C_C
p = 0.078, beta = 0.217, N = 148
47Assay Target: FGL1
UniProt: Q08830
rsID: rs7818801
Target SNP: 8:17722867:G:C
Proxy SNP: 8:17722867:G:C
Tarkin log10(p): 29.1
Tarkin BETA: −0.431
Tarkin MSPA: 0.982
1257:1256:1257:1256:1257:125
GDENTVIDLGSK pc2
rs3739406 REF
157:500 521:544 213:216
8:17722867:G:C_G
p = 3.4e−148, model = REC, N = 891
GDENTVVDLGSK pc2
rs3739406 ALT
457:500 409:544 7:216
8:17722867:G:C_G
p = 1.9e−118, model = DOM, N = 873
GDENTVVDLGSKR pc2
rs3739406 ALT
137:500 55:544 0:216
8:17722867:G:C_G
p = 7.3e−22, model = REC, N = 192
GDENTVVDLGSKR pc3
rs3739406 ALT
117:500 72:544 0:216
8:17722867:G:C_G
p = 1.8e−17, model = DOM, N = 189
GDENTVIDLGSKR pc2
rs3739406 REF
0:500 57:544 36:216
8:17722867:G:C_G
p = 4.5e−22, model = REC, N = 93
GDENTVIDLGSKR pc3
rs3739406 REF
0:500 19:544 16:216
8:17722867:G:C_G
p = 1.7e−08, model = REC, N = 35

48. PLA2G2A (P14555)
Tarkin MSPA=1

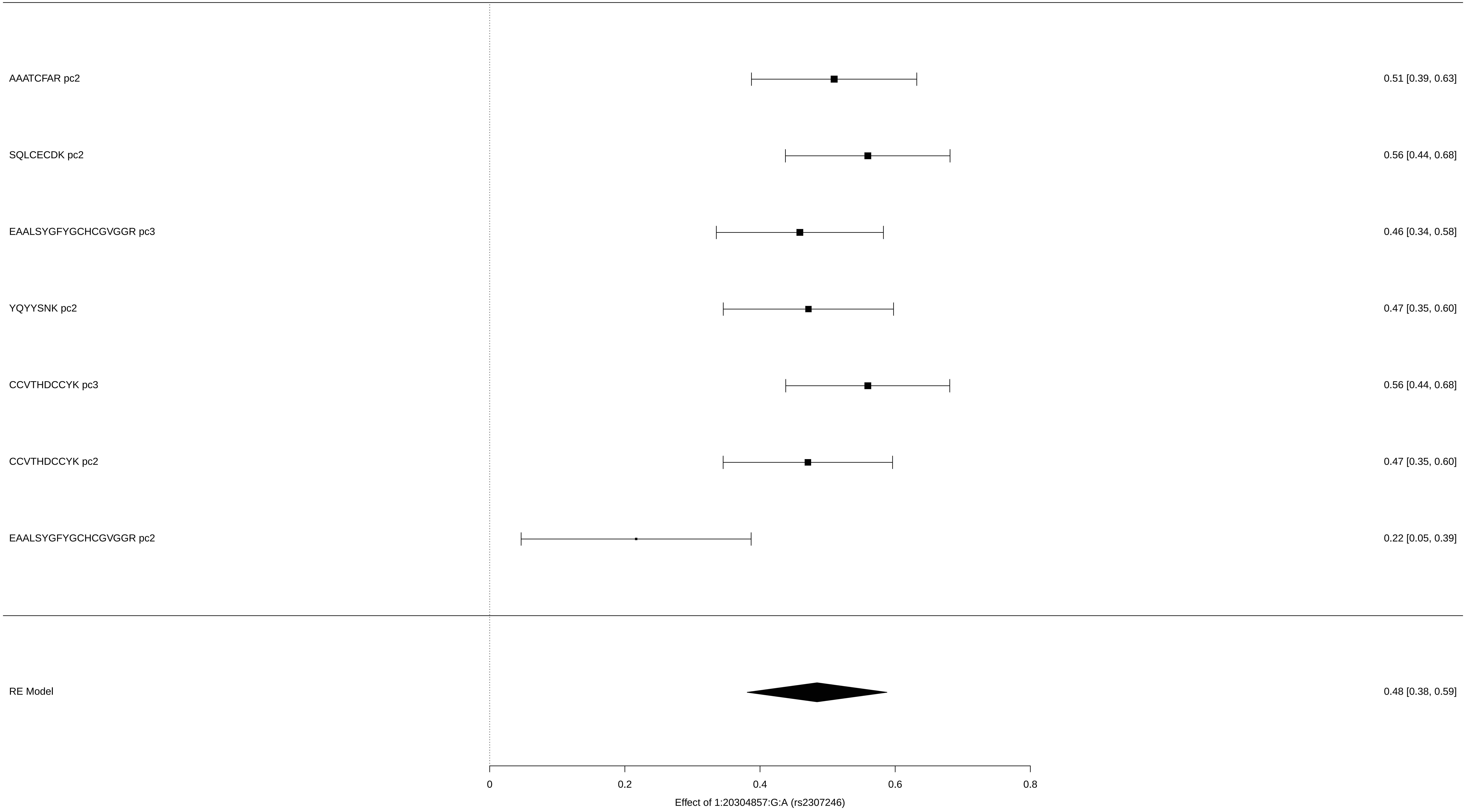

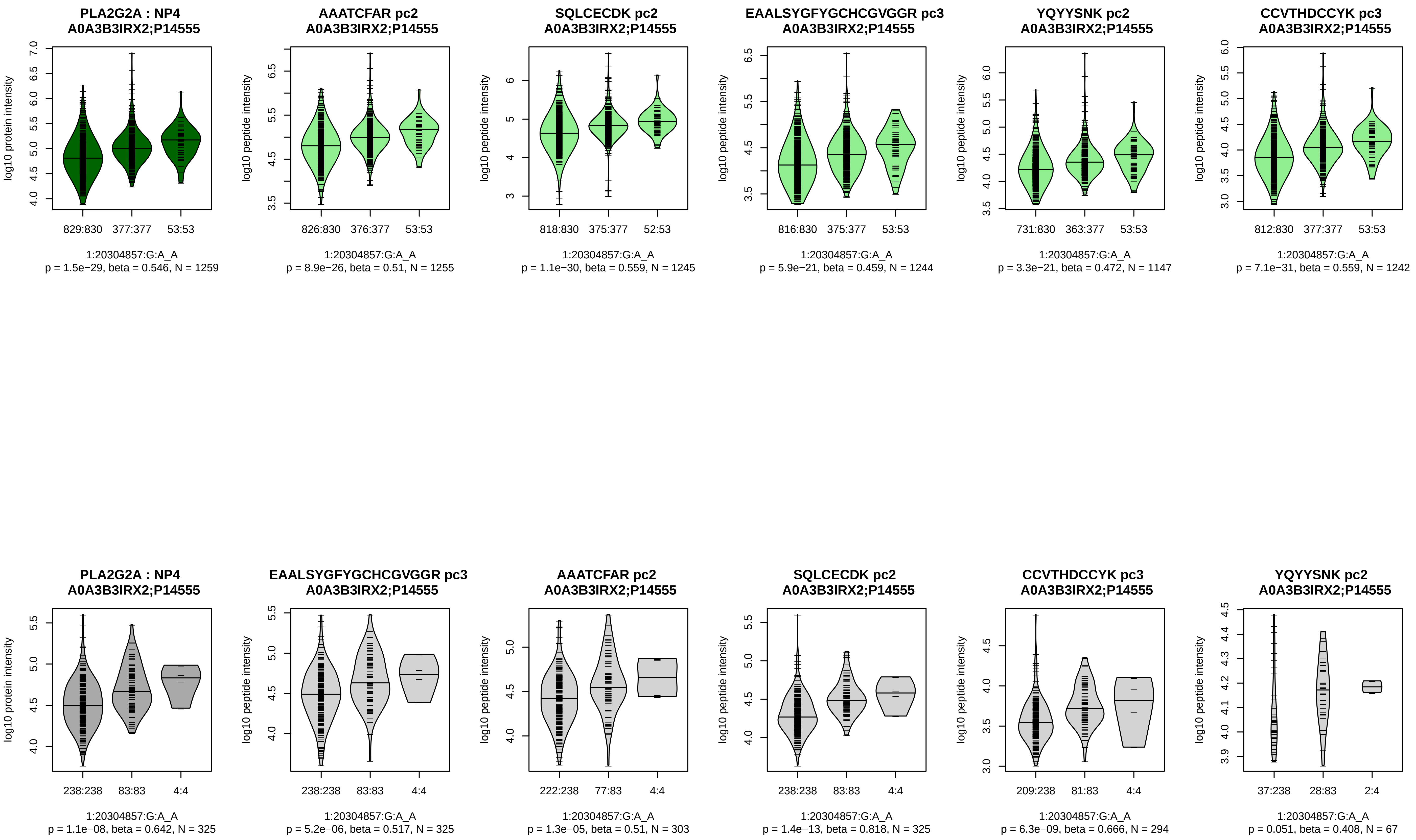

48Assay Target: PLA2G2A
UniProt: P14555
rsID: rs2307246
Target SNP: 1:20304857:G:A
Proxy SNP: 1:20304857:G:A
Tarkin log10(p): 28.8
Tarkin BETA: 0.546
Tarkin MSPA: 1
::*:*:*:*:*
1255:1245:1244:1147:1242:118

49. COL15A1 (P39059)
Tarkin MSPA=0.87

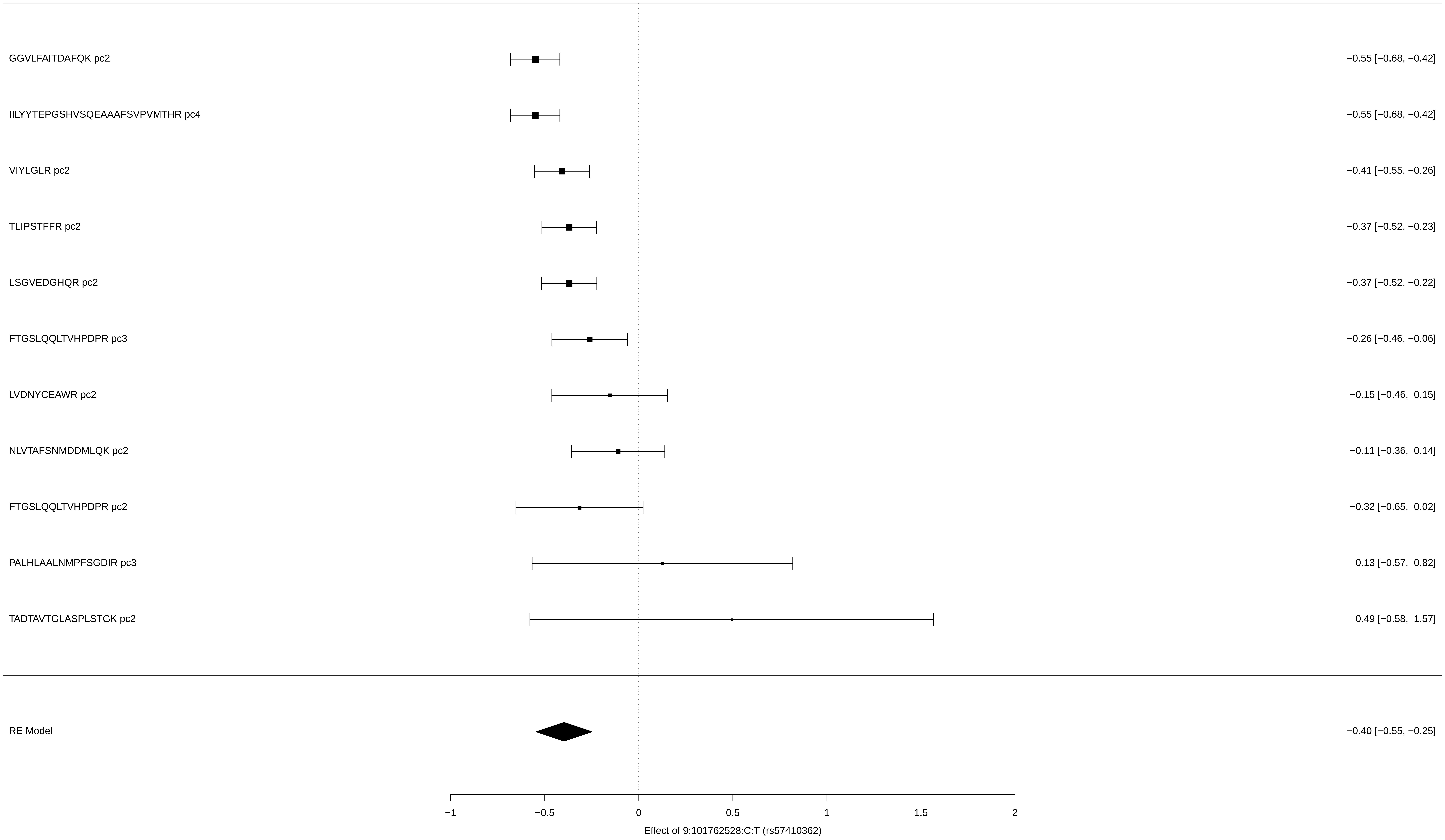

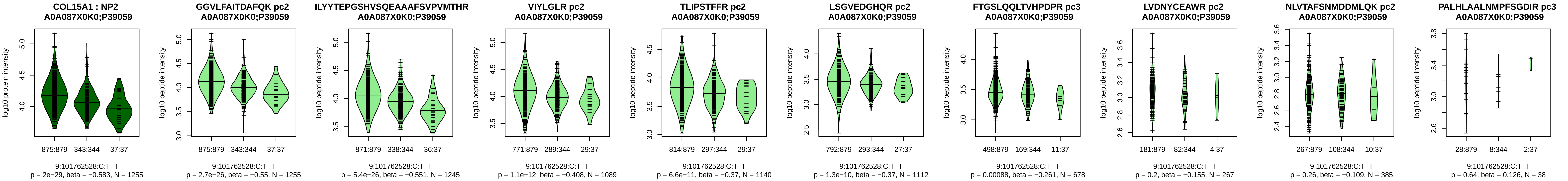

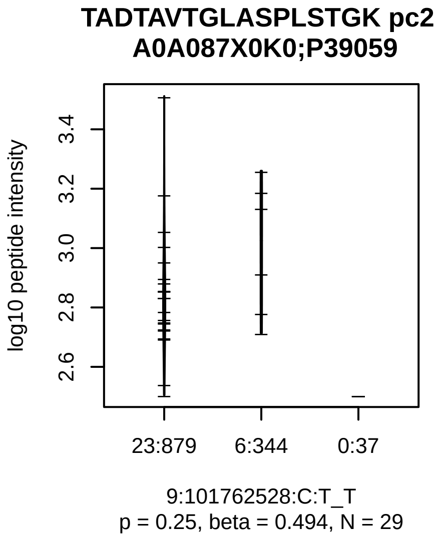

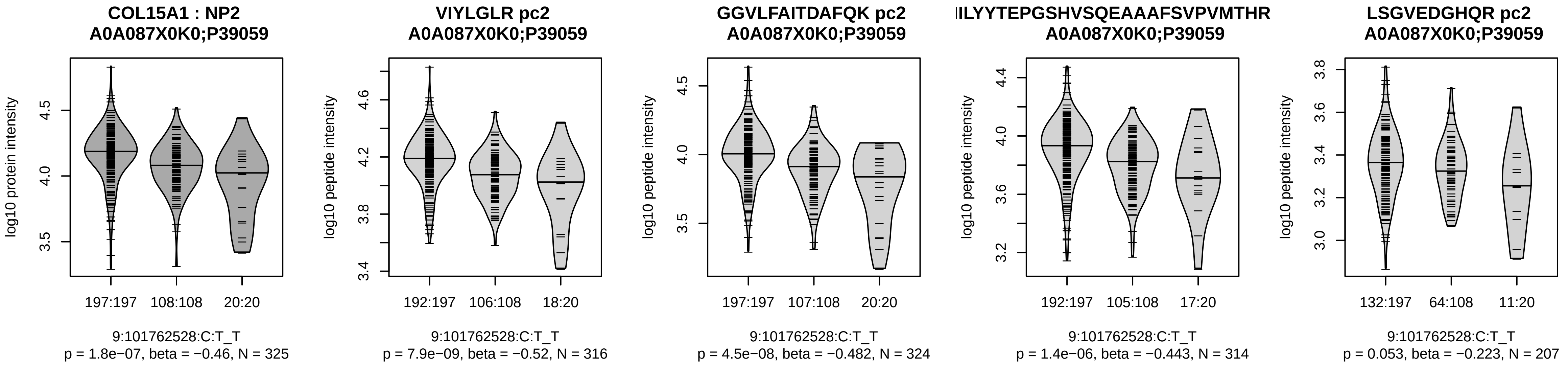

49Assay Target: COL15A1
UniProt: P39059
rsID: rs57410362
Target SNP: 9:101762528:C:T
Proxy SNP: 9:101762528:C:T
Tarkin log10(p): 28.7
Tarkin BETA: −0.583
Tarkin MSPA: 0.87
1255:1245:1089:1140:1112:678

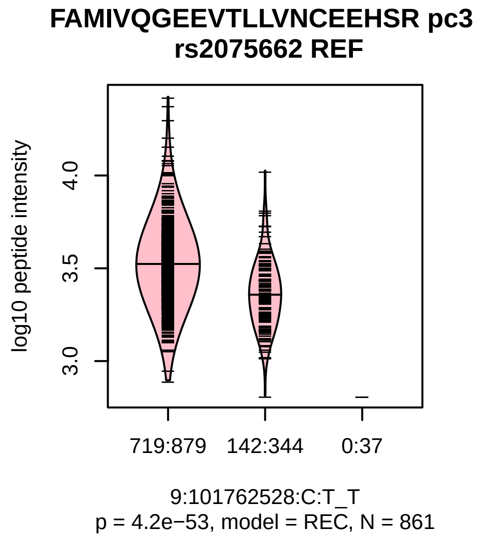

50. COLEC12 (Q5KU26)
Tarkin MSPA=0.989

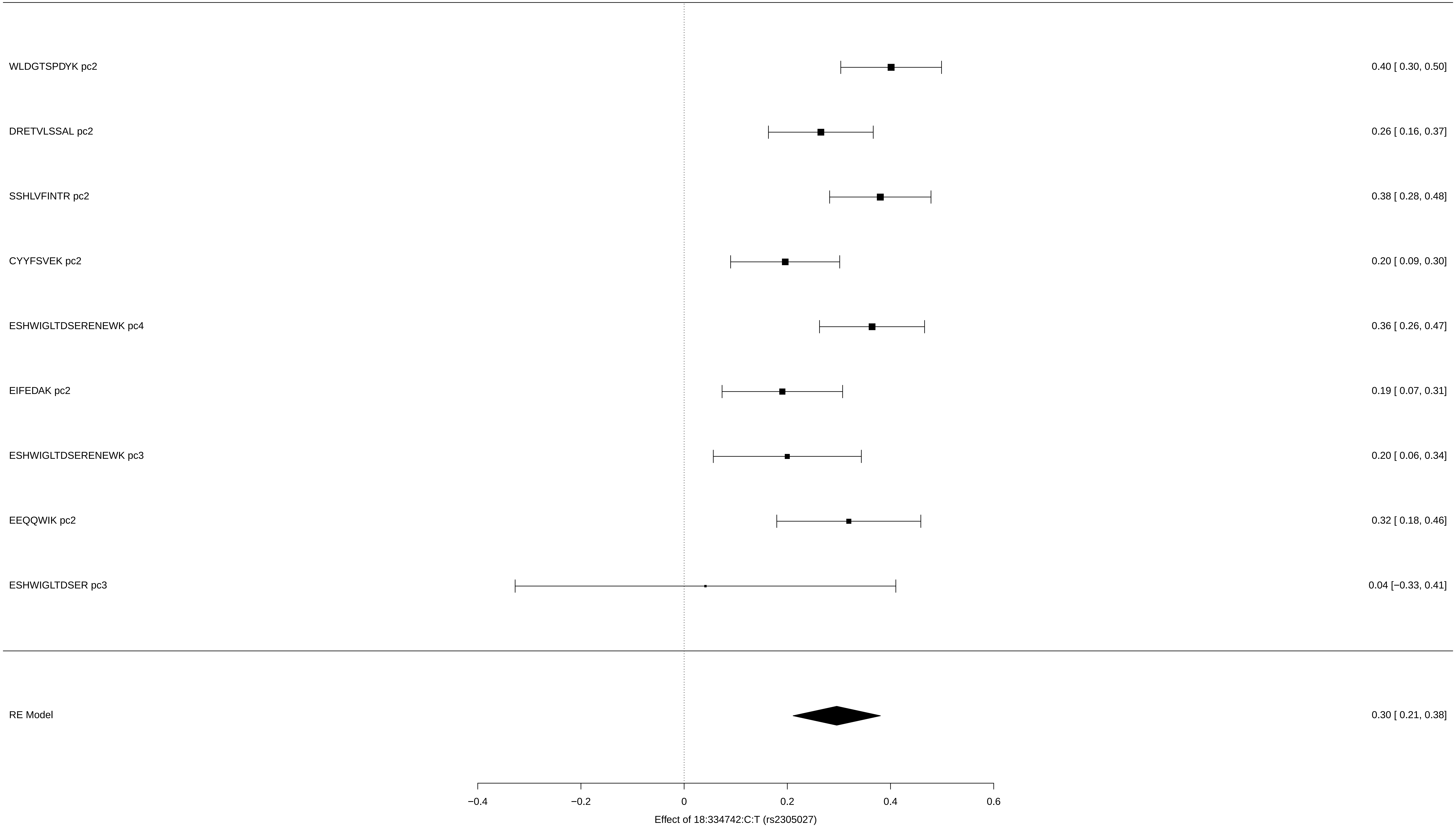

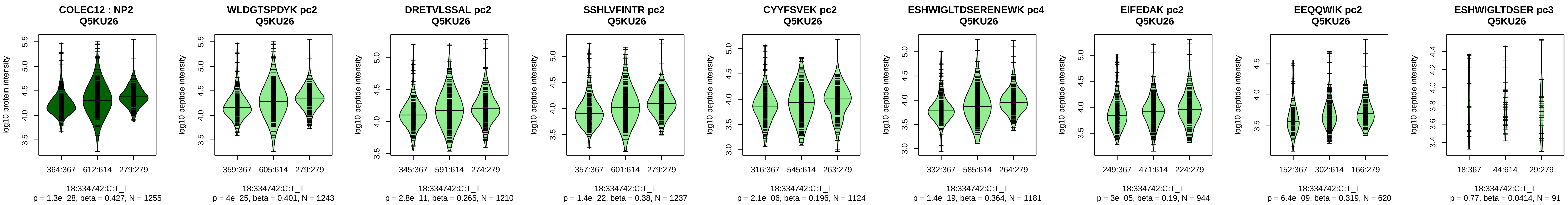

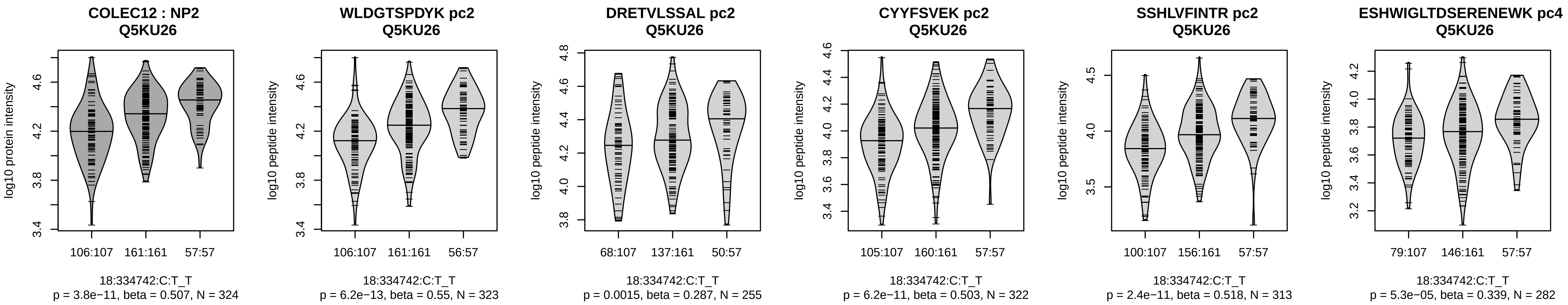

50Assay Target: COLEC12
UniProt: Q5KU26
rsID: rs2305027
Target SNP: 18:334742:C:T
Proxy SNP: 18:334742:C:T
Tarkin log10(p): 27.9
Tarkin BETA: 0.427
Tarkin MSPA: 0.989
1243:1210:1237:1124:1181:944

51. IGHV2−70 (A0A0C4DH43)
Tarkin MSPA=0.46

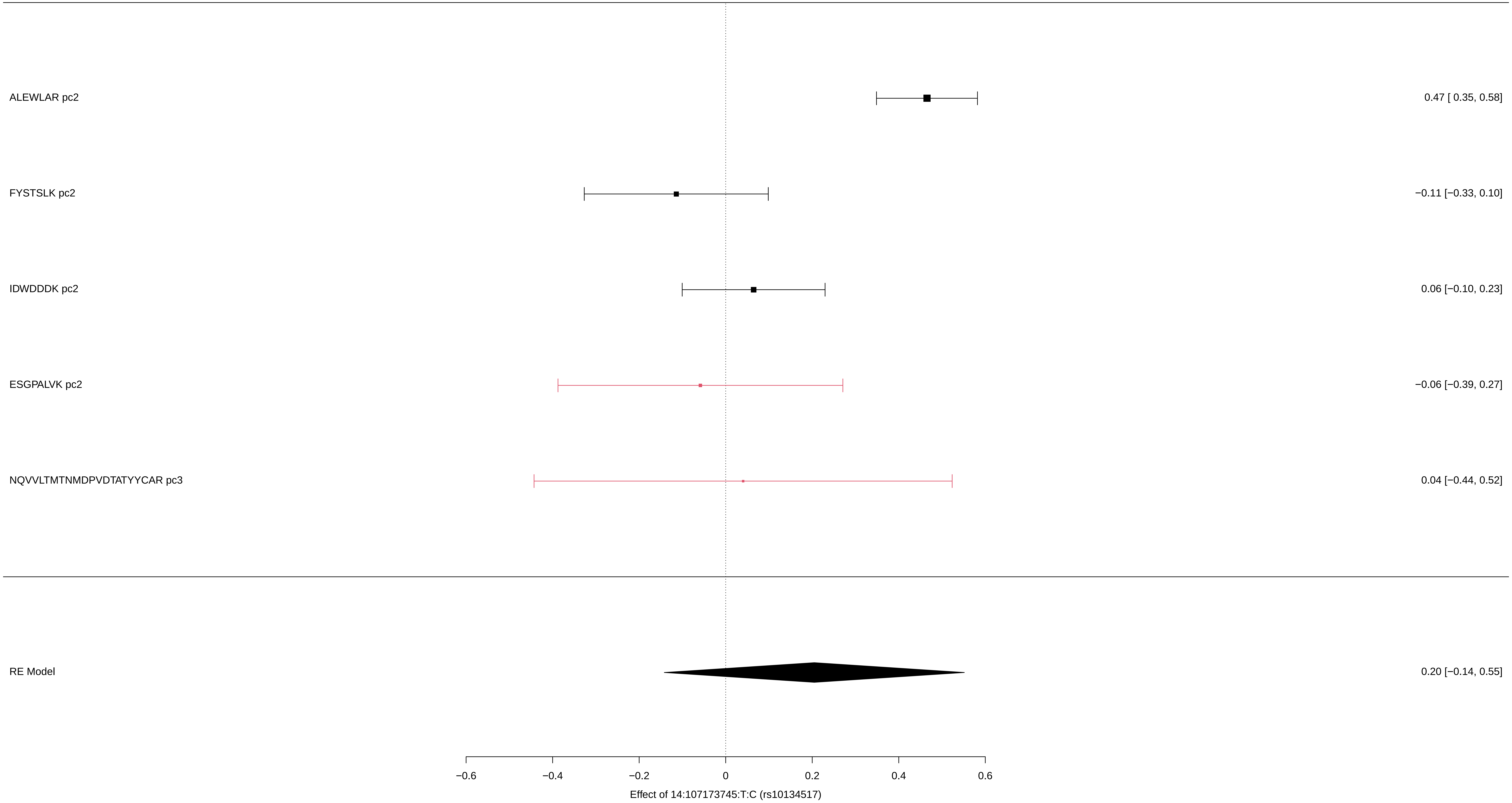

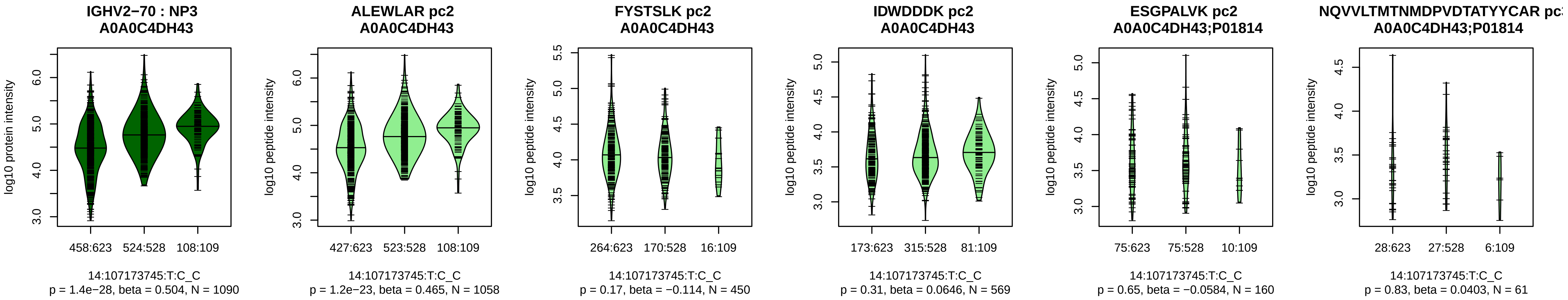

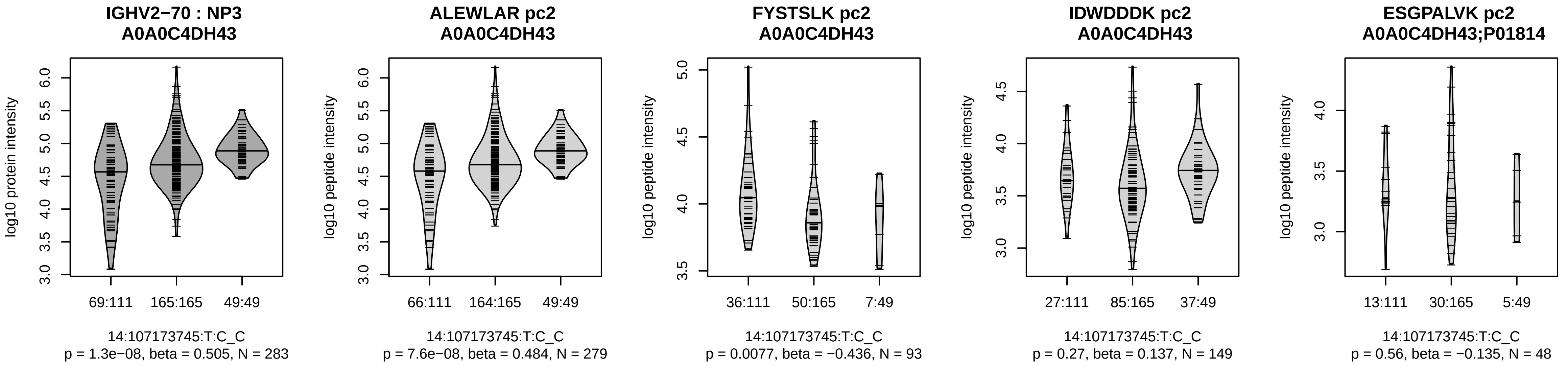

51Assay Target: IGHV2−70
UniProt: A0A0C4DH43
rsID: rs10134517
Target SNP: 14:107173745:T:C
Proxy SNP: 14:107173745:T:C
Tarkin log10(p): 27.9
Tarkin BETA: 0.504
Tarkin MSPA: 0.46
*:−:−:−:−:NA
1058:450:569:160:61:1

52. ITIH3 (Q06033)
Tarkin MSPA=0.741

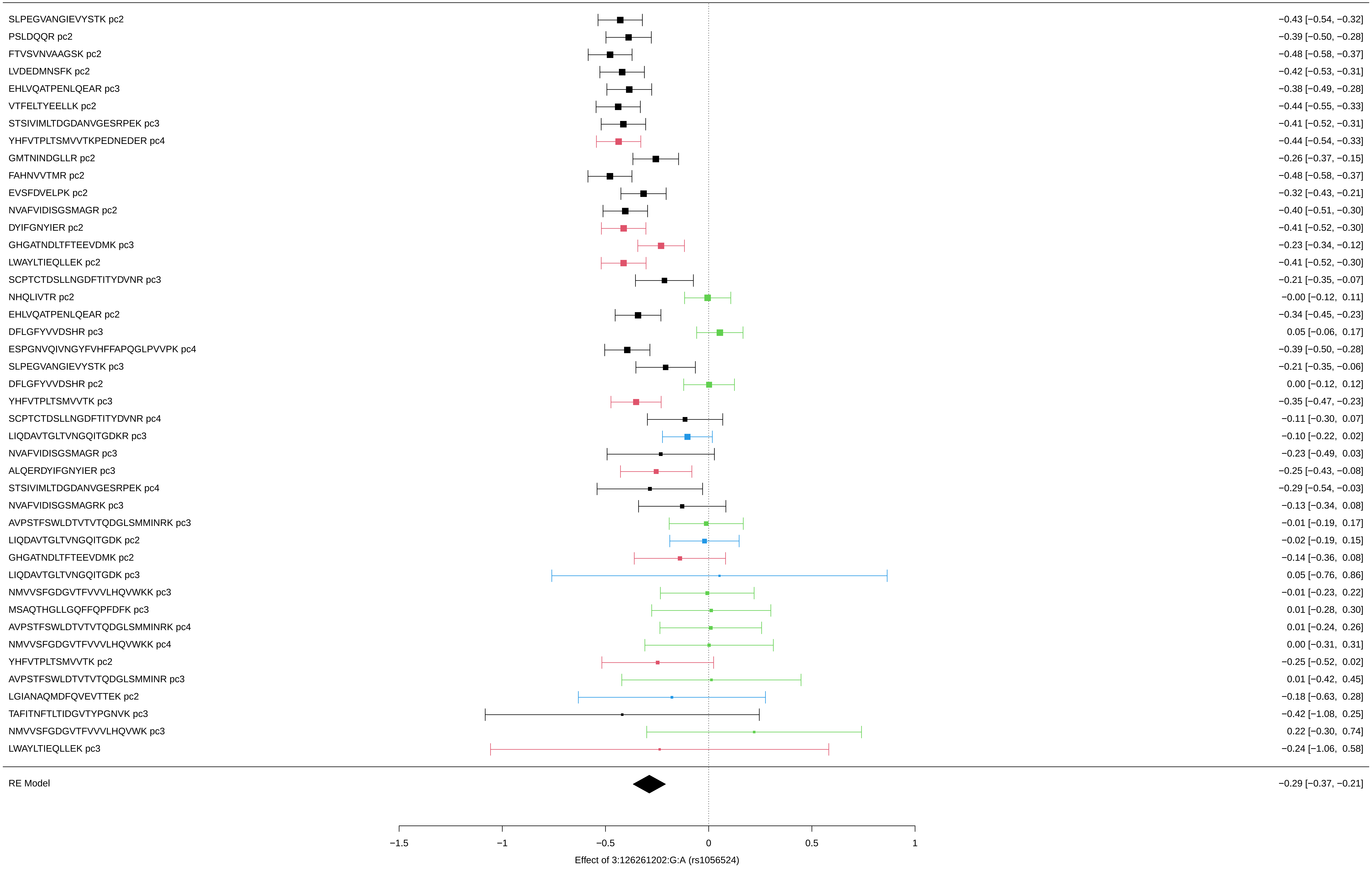

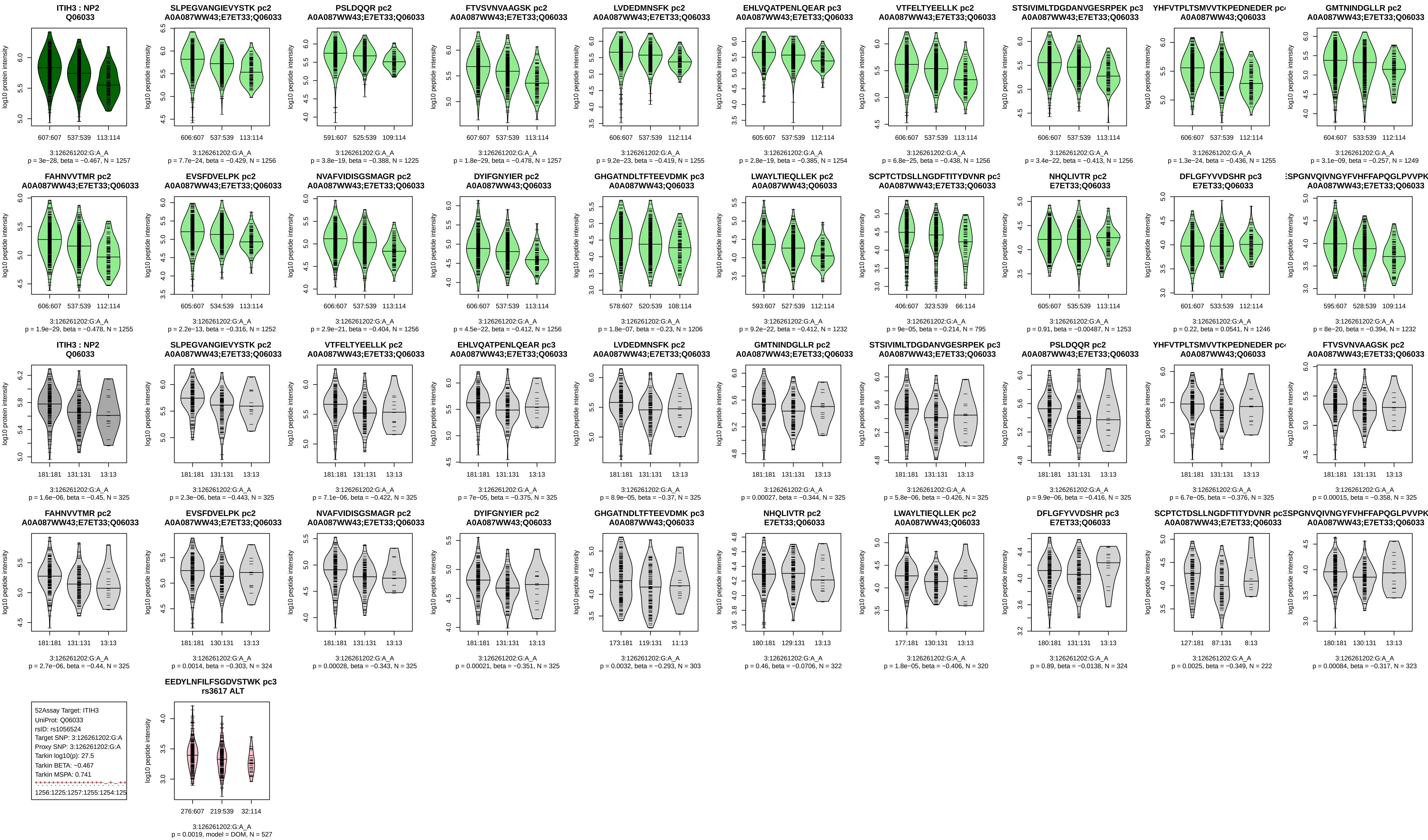
ITIH3 : NP2
Q06033
607:607 537:539 113:114
3:126261202:G:A_A
p = 3e−28, beta = −0.467, N = 1257
SLPEGVANGIEVYSTK pc2
A0A087WW43;E7ET33;Q06033
606:607 537:539 113:114
3:126261202:G:A_A
p = 7.7e−24, beta = −0.429, N = 1256
PSLDQQR pc2
A0A087WW43;E7ET33;Q06033
591:607 525:539 109:114
3:126261202:G:A_A
p = 3.8e−19, beta = −0.388, N = 1225
FTVSVNVAAGSK pc2
A0A087WW43;E7ET33;Q06033
607:607 537:539 113:114
3:126261202:G:A_A
p = 1.8e−29, beta = −0.478, N = 1257
LVDEDMNSFK pc2
A0A087WW43;E7ET33;Q06033
606:607 537:539 112:114
3:126261202:G:A_A
p = 9.2e−23, beta = −0.419, N = 1255
EHLVQATPENLQEAR pc3
A0A087WW43;E7ET33;Q06033
605:607 537:539 112:114
3:126261202:G:A_A
p = 2.8e−19, beta = −0.385, N = 1254
VTFELTYEELLK pc2
A0A087WW43;E7ET33;Q06033
606:607 537:539 113:114
3:126261202:G:A_A
p = 6.8e−25, beta = −0.438, N = 1256
STSIVIMLTDGDANVGESRPEK pc3
A0A087WW43;E7ET33;Q06033
606:607 537:539 113:114
3:126261202:G:A_A
p = 3.4e−22, beta = −0.413, N = 1256
YHFVTPLTSMVVTKPEDNEDER pc
A0A087WW43;Q06033
606:607 537:539 112:114
3:126261202:G:A_A
p = 1.3e−24, beta = −0.436, N = 1255
GMTNINDGLLR pc2
A0A087WW43;E7ET33;Q06033
604:607 533:539 112:114
3:126261202:G:A_A
p = 3.1e−09, beta = −0.257, N = 1249
FAHNVVTMR pc2
A0A087WW43;E7ET33;Q06033
606:607 537:539 112:114
3:126261202:G:A_A
p = 1.9e−29, beta = −0.478, N = 1255
EVSFDVELPK pc2
A0A087WW43;E7ET33;Q06033
605:607 534:539 113:114
3:126261202:G:A_A
p = 2.2e−13, beta = −0.316, N = 1252
NVAFVIDISGSMAGR pc2
A0A087WW43;E7ET33;Q06033
606:607 537:539 113:114
3:126261202:G:A_A
p = 2.9e−21, beta = −0.404, N = 1256
DYIFGNYIER pc2
A0A087WW43;Q06033
606:607 537:539 113:114
3:126261202:G:A_A
p = 4.5e−22, beta = −0.412, N = 1256
GHGATNDLTFTEEVDMK pc3
A0A087WW43;Q06033
578:607 520:539 108:114
3:126261202:G:A_A
p = 1.8e−07, beta = −0.23, N = 1206
LWAYLTIEQLLEK pc2
A0A087WW43;Q06033
593:607 527:539 112:114
3:126261202:G:A_A
p = 9.2e−22, beta = −0.412, N = 1232
SCPTCTDSLLNGDFTITYDVNR pc3
A0A087WW43;E7ET33;Q06033
406:607 323:539 66:114
3:126261202:G:A_A
p = 9e−05, beta = −0.214, N = 795
NHQLIVTR pc2
E7ET33;Q06033
605:607 535:539 113:114
3:126261202:G:A_A
p = 0.91, beta = −0.00487, N = 1253
DFLGFYVVDSHR pc3
E7ET33;Q06033
601:607 533:539 112:114
3:126261202:G:A_A
p = 0.22, beta = 0.0541, N = 1246
SPGNVQIVNGYFVHFFAPQGLPVVPK
A0A087WW43;E7ET33;Q06033
595:607 528:539 109:114
3:126261202:G:A_A
p = 8e−20, beta = −0.394, N = 1232
ITIH3 : NP2
Q06033
181:181 131:131 13:13
3:126261202:G:A_A
p = 1.6e−06, beta = −0.45, N = 325
SLPEGVANGIEVYSTK pc2
A0A087WW43;E7ET33;Q06033
181:181 131:131 13:13
3:126261202:G:A_A
p = 2.3e−06, beta = −0.443, N = 325
VTFELTYEELLK pc2
A0A087WW43;E7ET33;Q06033
181:181 131:131 13:13
3:126261202:G:A_A
p = 7.1e−06, beta = −0.422, N = 325
EHLVQATPENLQEAR pc3
A0A087WW43;E7ET33;Q06033
181:181 131:131 13:13
3:126261202:G:A_A
p = 7e−05, beta = −0.375, N = 325
LVDEDMNSFK pc2
A0A087WW43;E7ET33;Q06033
181:181 131:131 13:13
3:126261202:G:A_A
p = 8.9e−05, beta = −0.37, N = 325
GMTNINDGLLR pc2
A0A087WW43;E7ET33;Q06033
181:181 131:131 13:13
3:126261202:G:A_A
p = 0.00027, beta = −0.344, N = 325
STSIVIMLTDGDANVGESRPEK pc3
A0A087WW43;E7ET33;Q06033
181:181 131:131 13:13
3:126261202:G:A_A
p = 5.8e−06, beta = −0.426, N = 325
PSLDQQR pc2
A0A087WW43;E7ET33;Q06033
181:181 131:131 13:13
3:126261202:G:A_A
p = 9.9e−06, beta = −0.416, N = 325
YHFVTPLTSMVVTKPEDNEDER pc
A0A087WW43;Q06033
181:181 131:131 13:13
3:126261202:G:A_A
p = 6.7e−05, beta = −0.376, N = 325
FTVSVNVAAGSK pc2
A0A087WW43;E7ET33;Q06033
181:181 131:131 13:13
3:126261202:G:A_A
p = 0.00015, beta = −0.358, N = 325
FAHNVVTMR pc2
A0A087WW43;E7ET33;Q06033
181:181 131:131 13:13
3:126261202:G:A_A
p = 2.7e−06, beta = −0.44, N = 325
EVSFDVELPK pc2
A0A087WW43;E7ET33;Q06033
181:181 130:131 13:13
3:126261202:G:A_A
p = 0.0014, beta = −0.303, N = 324
NVAFVIDISGSMAGR pc2
A0A087WW43;E7ET33;Q06033
181:181 131:131 13:13
3:126261202:G:A_A
p = 0.00028, beta = −0.343, N = 325
DYIFGNYIER pc2
A0A087WW43;Q06033
181:181 131:131 13:13
3:126261202:G:A_A
p = 0.00021, beta = −0.351, N = 325
GHGATNDLTFTEEVDMK pc3
A0A087WW43;Q06033
173:181 119:131 11:13
3:126261202:G:A_A
p = 0.0032, beta = −0.293, N = 303
NHQLIVTR pc2
E7ET33;Q06033
180:181 129:131 13:13
3:126261202:G:A_A
p = 0.46, beta = −0.0706, N = 322
LWAYLTIEQLLEK pc2
A0A087WW43;Q06033
177:181 130:131 13:13
3:126261202:G:A_A
p = 1.8e−05, beta = −0.406, N = 320
DFLGFYVVDSHR pc3
E7ET33;Q06033
180:181 131:131 13:13
3:126261202:G:A_A
p = 0.89, beta = −0.0138, N = 324
SCPTCTDSLLNGDFTITYDVNR pc3
A0A087WW43;E7ET33;Q06033
127:181 87:131 8:13
3:126261202:G:A_A
p = 0.0025, beta = −0.349, N = 222
SPGNVQIVNGYFVHFFAPQGLPVVPK
A0A087WW43;E7ET33;Q06033
180:181 130:131 13:13
3:126261202:G:A_A
p = 0.00084, beta = −0.317, N = 323
52Assay Target: ITIH3
UniProt: Q06033
rsID: rs1056524
Target SNP: 3:126261202:G:A
Proxy SNP: 3:126261202:G:A
Tarkin log10(p): 27.5
Tarkin BETA: −0.467
Tarkin MSPA: 0.741
1256:1225:1257:1255:1254:125
EEDYLNFILFSGDVSTWK pc3
rs3617 ALT
276:607 219:539 32:114
3:126261202:G:A_A
p = 0.0019, model = DOM, N = 527

53. PDIA5 (Q14554)
Tarkin MSPA=0.989

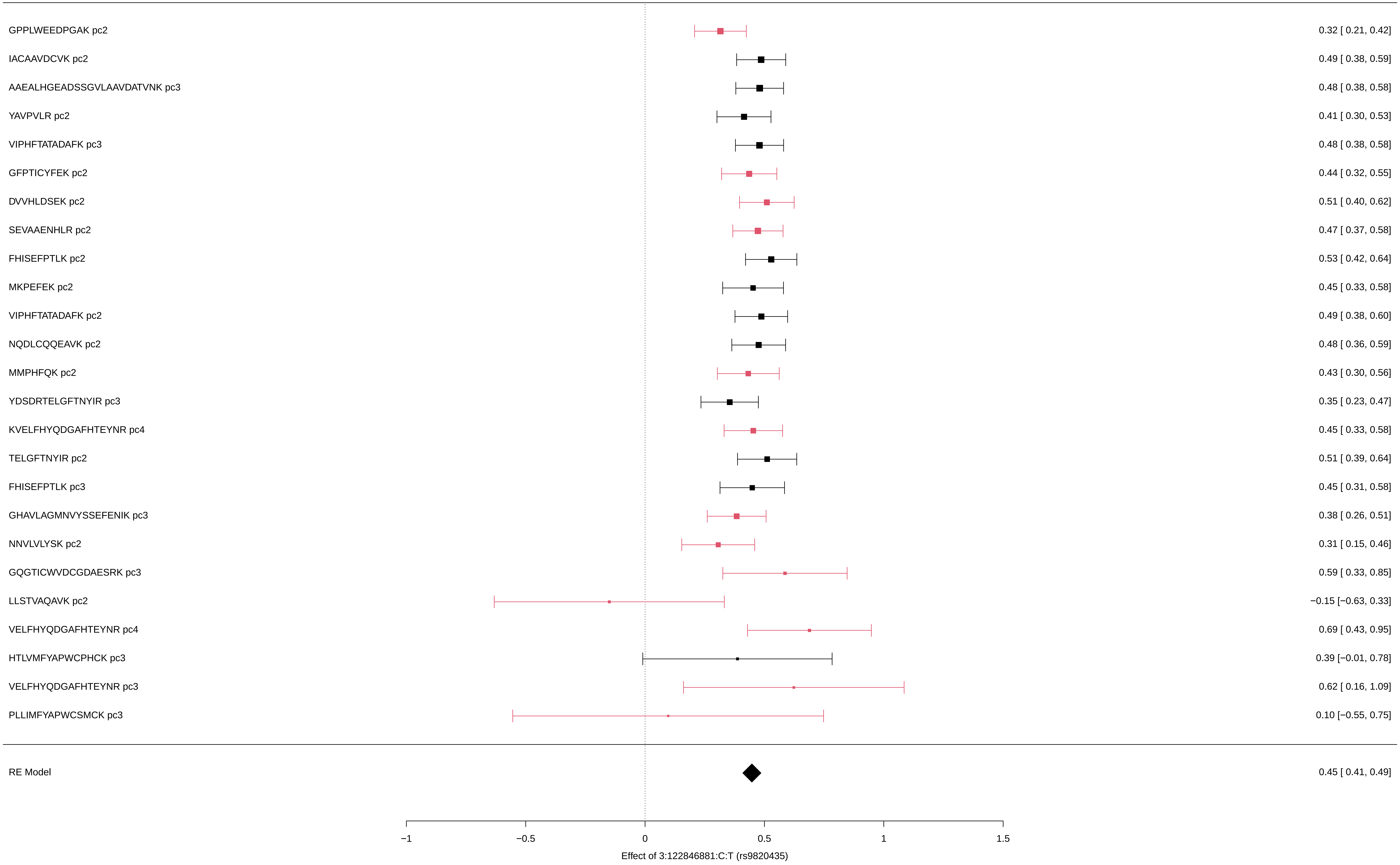

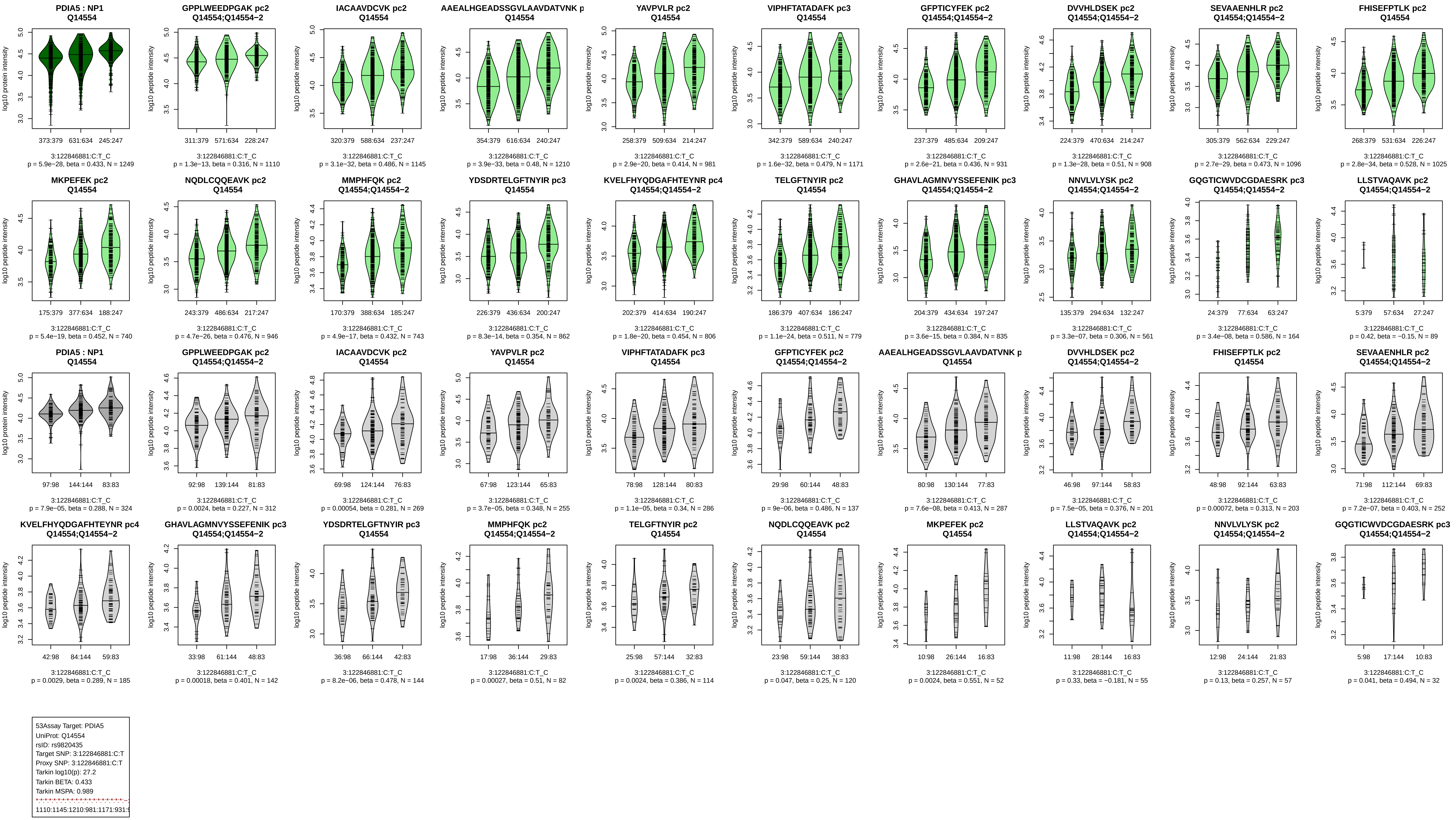
PDIA5 : NP1
Q14554
3:122846881:C:T_C
p = 5.9e−28, beta = 0.433, N = 1249
GPPLWEEDPGAK pc2
Q14554;Q14554−2
3:122846881:C:T_C
p = 1.3e−13, beta = 0.316, N = 1110
IACAAVDCVK pc2
Q14554
3:122846881:C:T_C
p = 3.1e−32, beta = 0.486, N = 1145
AAEALHGEADSSGVLAAVDATVNK p
Q14554
3:122846881:C:T_C
p = 3.9e−33, beta = 0.48, N = 1210
YAVPVLR pc2
Q14554
3:122846881:C:T_C
p = 2.9e−20, beta = 0.414, N = 981
VIPHFTATADAFK pc3
Q14554
3:122846881:C:T_C
p = 1.6e−32, beta = 0.479, N = 1171
GFPTICYFEK pc2
Q14554;Q14554−2
3:122846881:C:T_C
p = 2.6e−21, beta = 0.436, N = 931
DVVHLDSEK pc2
Q14554;Q14554−2
3:122846881:C:T_C
p = 1.3e−28, beta = 0.51, N = 908
SEVAAENHLR pc2
Q14554;Q14554−2
3:122846881:C:T_C
p = 2.7e−29, beta = 0.473, N = 1096
FHISEFPTLK pc2
Q14554
3:122846881:C:T_C
p = 2.8e−34, beta = 0.528, N = 1025
MKPEFEK pc2
Q14554
3:122846881:C:T_C
p = 5.4e−19, beta = 0.452, N = 740
NQDLCQQEAVK pc2
Q14554
3:122846881:C:T_C
p = 4.7e−26, beta = 0.476, N = 946
MMPHFQK pc2
Q14554;Q14554−2
3:122846881:C:T_C
p = 4.9e−17, beta = 0.432, N = 743
YDSDRTELGFTNYIR pc3
Q14554
3:122846881:C:T_C
p = 8.3e−14, beta = 0.354, N = 862
KVELFHYQDGAFHTEYNR pc4
Q14554;Q14554−2
3:122846881:C:T_C
p = 1.8e−20, beta = 0.454, N = 806
TELGFTNYIR pc2
Q14554
3:122846881:C:T_C
p = 1.1e−24, beta = 0.511, N = 779
GHAVLAGMNVYSSEFENIK pc3
Q14554;Q14554−2
3:122846881:C:T_C
p = 3.6e−15, beta = 0.384, N = 835
NNVLVLYSK pc2
Q14554;Q14554−2
3:122846881:C:T_C
p = 3.3e−07, beta = 0.306, N = 561
GQGTICWVDCGDAESRK pc3
Q14554;Q14554−2
3:122846881:C:T_C
p = 3.4e−08, beta = 0.586, N = 164
LLSTVAQAVK pc2
Q14554;Q14554−2
3:122846881:C:T_C
p = 0.42, beta = −0.15, N = 89
PDIA5 : NP1
Q14554
3:122846881:C:T_C
p = 7.9e−05, beta = 0.288, N = 324
GPPLWEEDPGAK pc2
Q14554;Q14554−2
3:122846881:C:T_C
p = 0.0024, beta = 0.227, N = 312
IACAAVDCVK pc2
Q14554
3:122846881:C:T_C
p = 0.00054, beta = 0.281, N = 269
YAVPVLR pc2
Q14554
3:122846881:C:T_C
p = 3.7e−05, beta = 0.348, N = 255
VIPHFTATADAFK pc3
Q14554
3:122846881:C:T_C
p = 1.1e−05, beta = 0.34, N = 286
GFPTICYFEK pc2
Q14554;Q14554−2
3:122846881:C:T_C
p = 9e−06, beta = 0.486, N = 137
AAEALHGEADSSGVLAAVDATVNK p
Q14554
3:122846881:C:T_C
p = 7.6e−08, beta = 0.413, N = 287
DVVHLDSEK pc2
Q14554;Q14554−2
3:122846881:C:T_C
p = 7.5e−05, beta = 0.376, N = 201
FHISEFPTLK pc2
Q14554
3:122846881:C:T_C
p = 0.00072, beta = 0.313, N = 203
SEVAAENHLR pc2
Q14554;Q14554−2
3:122846881:C:T_C
p = 7.2e−07, beta = 0.403, N = 252
KVELFHYQDGAFHTEYNR pc4
Q14554;Q14554−2
3:122846881:C:T_C
p = 0.0029, beta = 0.289, N = 185
GHAVLAGMNVYSSEFENIK pc3
Q14554;Q14554−2
3:122846881:C:T_C
p = 0.00018, beta = 0.401, N = 142
YDSDRTELGFTNYIR pc3
Q14554
3:122846881:C:T_C
p = 8.2e−06, beta = 0.478, N = 144
MMPHFQK pc2
Q14554;Q14554−2
3:122846881:C:T_C
p = 0.00027, beta = 0.51, N = 82
TELGFTNYIR pc2
Q14554
3:122846881:C:T_C
p = 0.0024, beta = 0.386, N = 114
NQDLCQQEAVK pc2
Q14554
3:122846881:C:T_C
p = 0.047, beta = 0.25, N = 120
MKPEFEK pc2
Q14554
3:122846881:C:T_C
p = 0.0024, beta = 0.551, N = 52
LLSTVAQAVK pc2
Q14554;Q14554−2
3:122846881:C:T_C
p = 0.33, beta = −0.181, N = 55
NNVLVLYSK pc2
Q14554;Q14554−2
3:122846881:C:T_C
p = 0.13, beta = 0.257, N = 57
GQGTICWVDCGDAESRK pc3
Q14554;Q14554−2
3:122846881:C:T_C
p = 0.041, beta = 0.494, N = 32
53Assay Target: PDIA5
UniProt: Q14554
rsID: rs9820435
Target SNP: 3:122846881:C:T
Proxy SNP: 3:122846881:C:T
Tarkin log10(p): 27.2
Tarkin BETA: 0.433
Tarkin MSPA: 0.989
1110:1145:1210:981:1171:931:9

54. SERPINE2 (P07093)
Tarkin MSPA=0.944

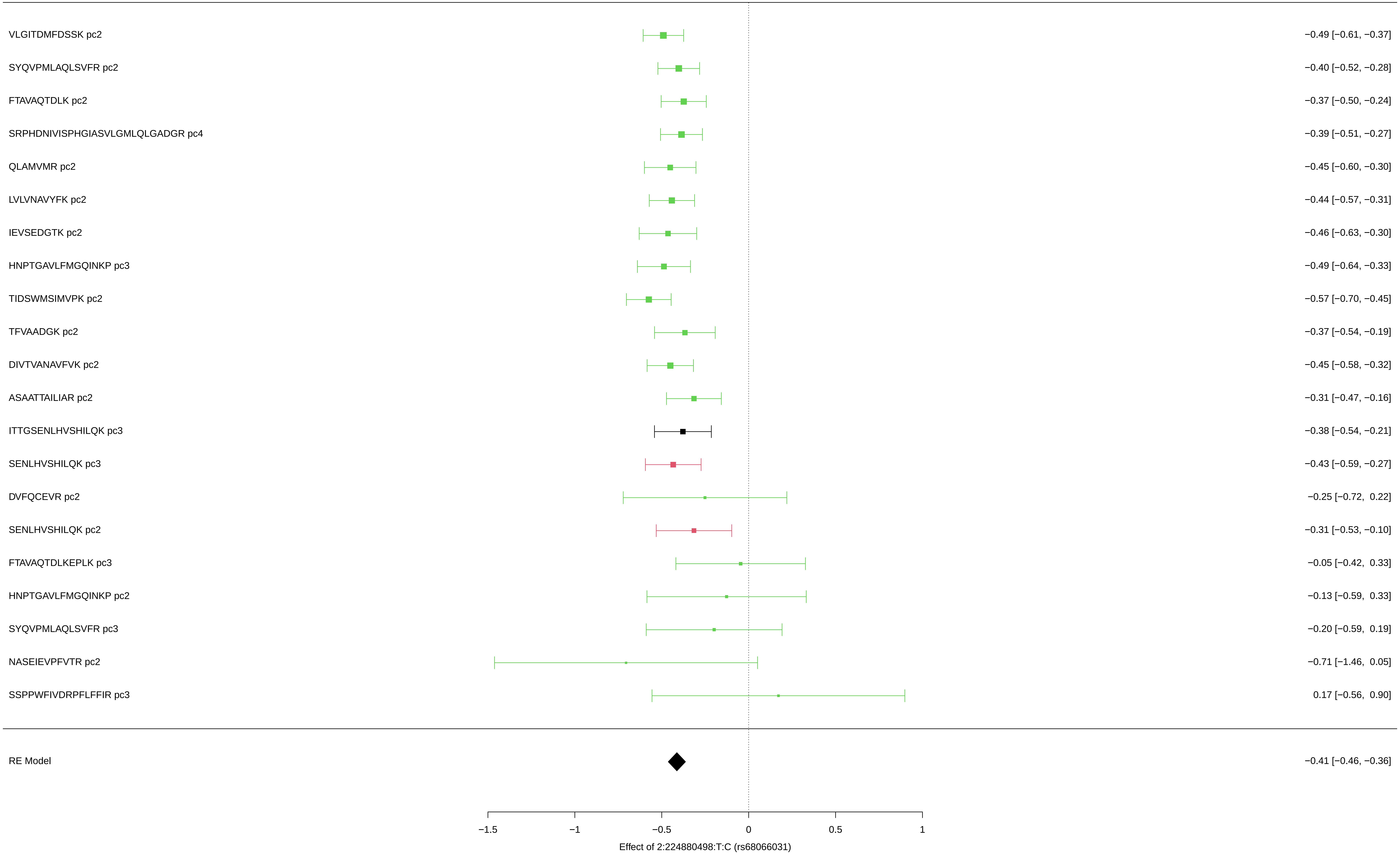

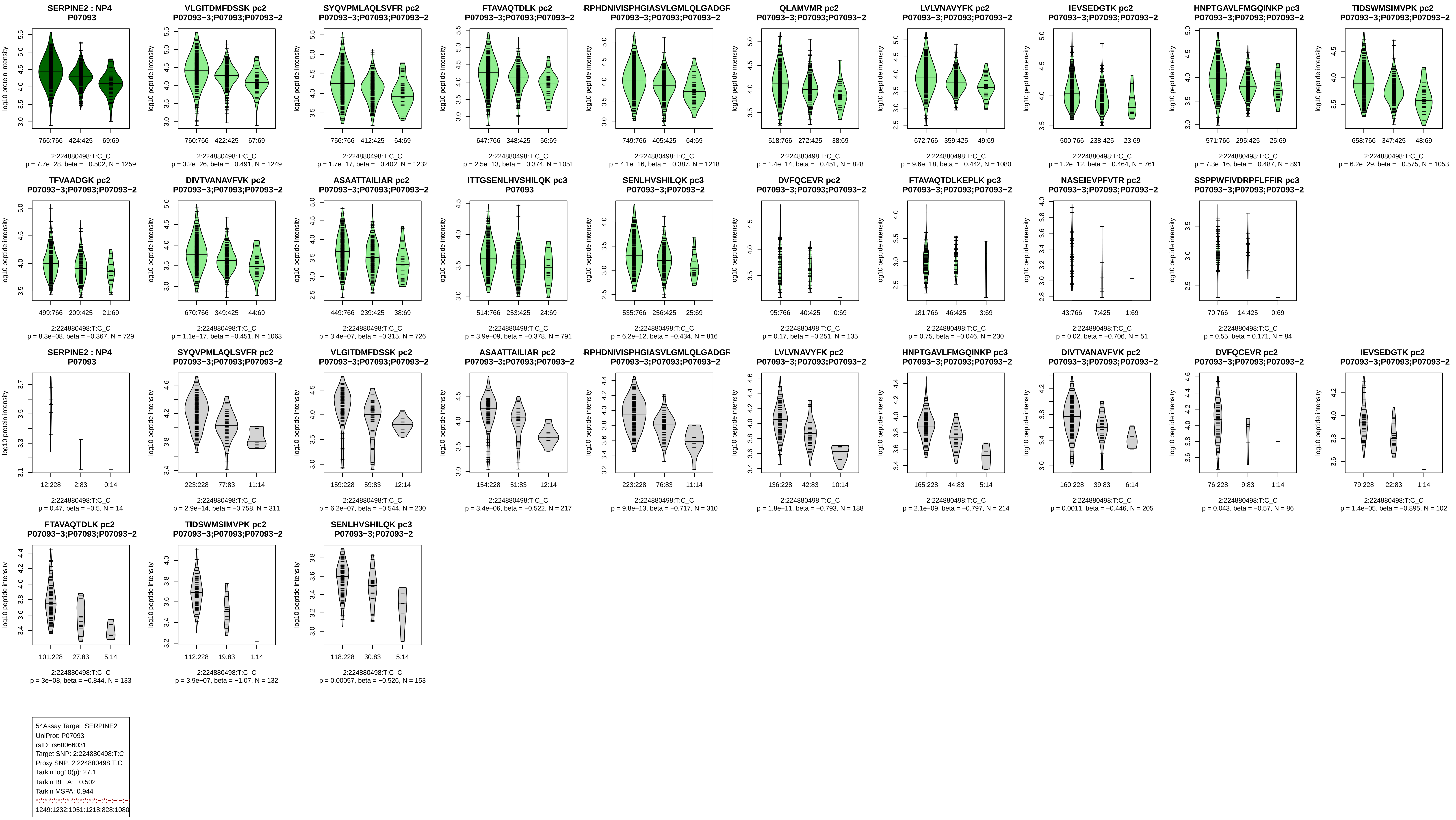

SERPINE2 : NP4
P07093
log10 protein intensity
766:766 424:425 69:69
2:224880498:T:C_C
p = 7.7e−28, beta = −0.502, N = 1259
VLGITDMFDSSK pc2
P07093−3;P07093;P07093−2
log10 peptide intensity
760:766 422:425 67:69
2:224880498:T:C_C
p = 3.2e−26, beta = −0.491, N = 1249
SYQVPMLAQLSVFR pc2
P07093−3;P07093;P07093−2
756:766 412:425 64:69
2:224880498:T:C_C
p = 1.7e−17, beta = −0.402, N = 1232
FTAVAQTDLK pc2
P07093−3;P07093;P07093−2
647:766 348:425 56:69
2:224880498:T:C_C
p = 2.5e−13, beta = −0.374, N = 1051
RPHDNIVISPHGIASVLGMLQLGADGF
P07093−3;P07093;P07093−2
749:766 405:425 64:69
2:224880498:T:C_C
p = 4.1e−16, beta = −0.387, N = 1218
QLAMVMR pc2
P07093−3;P07093;P07093−2
518:766 272:425 38:69
2:224880498:T:C_C
p = 1.4e−14, beta = −0.451, N = 828
LVLVNAVYFK pc2
P07093−3;P07093;P07093−2
672:766 359:425 49:69
2:224880498:T:C_C
p = 9.6e−18, beta = −0.442, N = 1080
IEVSEDGTK pc2
P07093−3;P07093;P07093−2
500:766 238:425 23:69
2:224880498:T:C_C
p = 1.2e−12, beta = −0.464, N = 761
HNPTGAVLFMGQINKP pc3
P07093−3;P07093;P07093−2
571:766 295:425 25:69
2:224880498:T:C_C
p = 7.3e−16, beta = −0.487, N = 891
TIDSWMSIMVPK pc2
P07093−3;P07093;P07093−2
658:766 347:425 48:69
2:224880498:T:C_C
p = 6.2e−29, beta = −0.575, N = 1053
TFVAADGK pc2
P07093−3;P07093;P07093−2
499:766 209:425 21:69
2:224880498:T:C_C
p = 8.3e−08, beta = −0.367, N = 729
DIVTVANAVFVK pc2
P07093−3;P07093;P07093−2
670:766 349:425 44:69
2:224880498:T:C_C
p = 1.1e−17, beta = −0.451, N = 1063
ASAATTAILIAR pc2
P07093−3;P07093;P07093−2
449:766 239:425 38:69
2:224880498:T:C_C
p = 3.4e−07, beta = −0.315, N = 726
ITTGSENLHVSHILQK pc3
P07093
514:766 253:425 24:69
2:224880498:T:C_C
p = 3.9e−09, beta = −0.378, N = 791
SENLHVSHILQK pc3
P07093−3;P07093;P07093−2
535:766 256:425 25:69
2:224880498:T:C_C
p = 6.2e−12, beta = −0.434, N = 816
DVFQCEVR pc2
P07093−3;P07093;P07093−2
95:766 40:425 0:69
2:224880498:T:C_C
p = 0.17, beta = −0.251, N = 135
FTAVAQTDLKEPLK pc3
P07093−3;P07093;P07093−2
181:766 46:425 3:69
2:224880498:T:C_C
p = 0.75, beta = −0.046, N = 230
NASEIEVPFVTR pc2
P07093−3;P07093;P07093−2
43:766 7:425 1:69
2:224880498:T:C_C
p = 0.02, beta = −0.706, N = 51
SSPPWFIVDRPFLFFIR pc3
P07093−3;P07093;P07093−2
70:766 14:425 0:69
2:224880498:T:C_C
p = 0.55, beta = 0.171, N = 84
SERPINE2 : NP4
P07093
12:228 2:83 0:14
2:224880498:T:C_C
p = 0.47, beta = −0.5, N = 14
SYQVPMLAQLSVFR pc2
P07093−3;P07093;P07093−2
223:228 77:83 11:14
2:224880498:T:C_C
p = 2.9e−14, beta = −0.758, N = 311
VLGITDMFDSSK pc2
P07093−3;P07093;P07093−2
159:228 59:83 12:14
2:224880498:T:C_C
p = 6.2e−07, beta = −0.544, N = 230
ASAATTAILIAR pc2
P07093−3;P07093;P07093−2
154:228 51:83 12:14
2:224880498:T:C_C
p = 3.4e−06, beta = −0.522, N = 217
RPHDNIVISPHGIASVLGMLQLGADGF
P07093−3;P07093;P07093−2
223:228 76:83 11:14
2:224880498:T:C_C
p = 9.8e−13, beta = −0.717, N = 310
LVLVNAVYFK pc2
P07093−3;P07093;P07093−2
136:228 42:83 10:14
2:224880498:T:C_C
p = 1.8e−11, beta = −0.793, N = 188
HNPTGAVLFMGQINKP pc3
P07093−3;P07093;P07093−2
165:228 44:83 5:14
2:224880498:T:C_C
p = 2.1e−09, beta = −0.797, N = 214
DIVTVANAVFVK pc2
P07093−3;P07093;P07093−2
160:228 39:83 6:14
2:224880498:T:C_C
p = 0.0011, beta = −0.446, N = 205
DVFQCEVR pc2
P07093−3;P07093;P07093−2
76:228 9:83 1:14
2:224880498:T:C_C
p = 0.043, beta = −0.57, N = 86
IEVSEDGTK pc2
P07093−3;P07093;P07093−2
79:228 22:83 1:14
2:224880498:T:C_C
p = 1.4e−05, beta = −0.895, N = 102
FTAVAQTDLK pc2
P07093−3;P07093;P07093−2
101:228 27:83 5:14
2:224880498:T:C_C
p = 3e−08, beta = −0.844, N = 133
TIDSWMSIMVPK pc2
P07093−3;P07093;P07093−2
112:228 19:83 1:14
2:224880498:T:C_C
p = 3.9e−07, beta = −1.07, N = 132
SENLHVSHILQK pc3
P07093−3;P07093−2
118:228 30:83 5:14
2:224880498:T:C_C
p = 0.00057, beta = −0.526, N = 153
54Assay Target: SERPINE2
UniProt: P07093
rsID: rs68066031
Target SNP: 2:224880498:T:C
Proxy SNP: 2:224880498:T:C
Tarkin log10(p): 27.1
Tarkin BETA: −0.502
Tarkin MSPA: 0.944
1249:1232:1051:1218:828:1080

55. TCN2 (F8WE86)
Tarkin MSPA=0.957

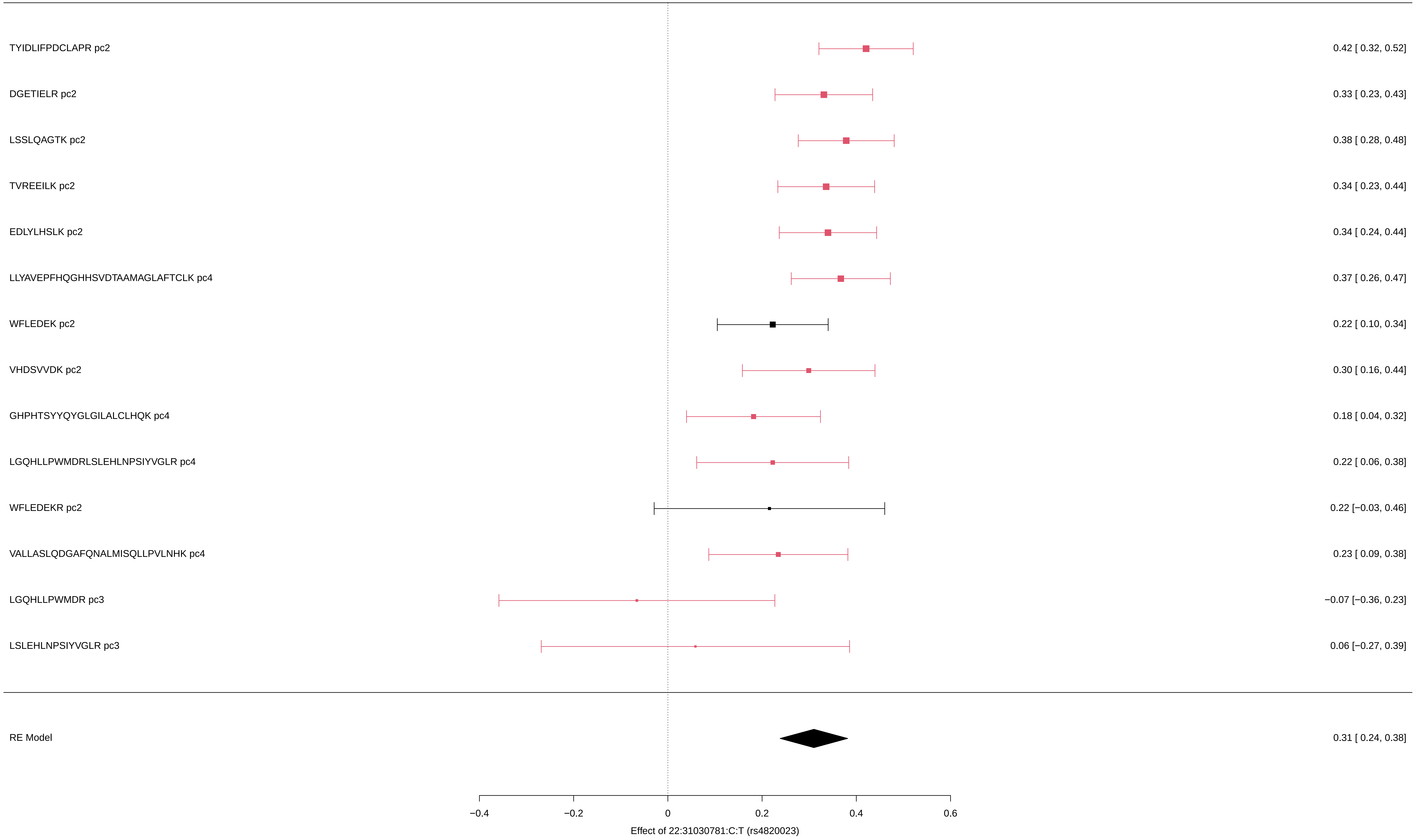

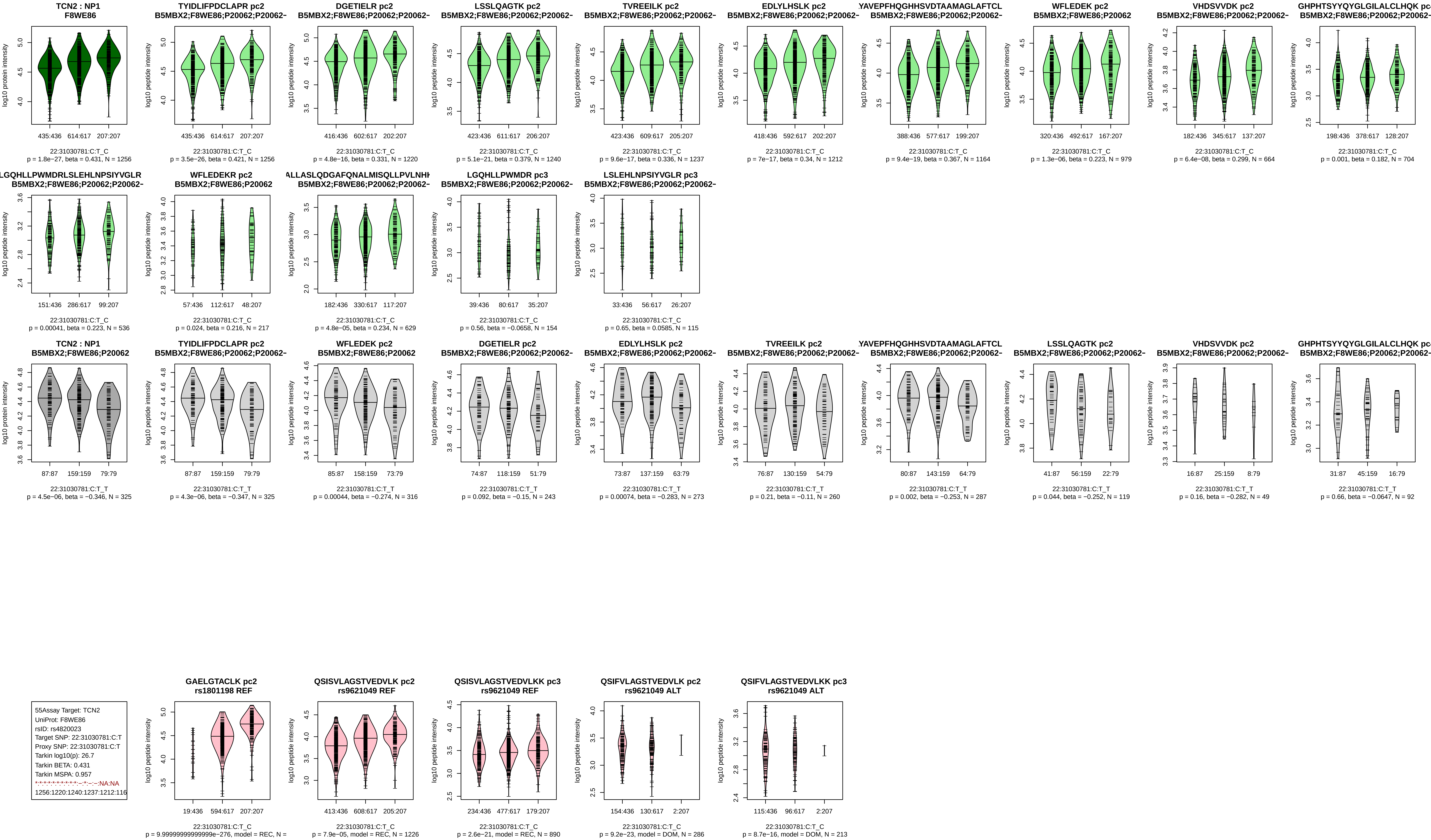

TCN2 : NP1
F8WE86
log10 protein intensity
435:436 614:617 207:207
22:31030781:C:T_C
p = 1.8e−27, beta = 0.431, N = 1256
TYIDLIFPDCLAPR pc2
B5MBX2;F8WE86;P20062;P20062−
log10 peptide intensity
435:436 614:617 207:207
22:31030781:C:T_C
p = 3.5e−26, beta = 0.421, N = 1256
DGETIELR pc2
B5MBX2;F8WE86;P20062;P20062−
416:436 602:617 202:207
22:31030781:C:T_C
p = 4.8e−16, beta = 0.331, N = 1220
LSSLQAGTK pc2
B5MBX2;F8WE86;P20062;P20062−
423:436 611:617 206:207
22:31030781:C:T_C
p = 5.1e−21, beta = 0.379, N = 1240
TVREEILK pc2
B5MBX2;F8WE86;P20062;P20062−
423:436 609:617 205:207
22:31030781:C:T_C
p = 9.6e−17, beta = 0.336, N = 1237
EDLYLHSLK pc2
B5MBX2;F8WE86;P20062;P20062−
418:436 592:617 202:207
22:31030781:C:T_C
p = 7e−17, beta = 0.34, N = 1212
YAVEPFHQGHHSVDTAAMAGLAFTCL
B5MBX2;F8WE86;P20062;P20062−
388:436 577:617 199:207
22:31030781:C:T_C
p = 9.4e−19, beta = 0.367, N = 1164
WFLEDEK pc2
B5MBX2;F8WE86;P20062
320:436 492:617 167:207
22:31030781:C:T_C
p = 1.3e−06, beta = 0.223, N = 979
VHDSVVDK pc2
B5MBX2;F8WE86;P20062;P20062−
182:436 345:617 137:207
22:31030781:C:T_C
p = 6.4e−08, beta = 0.299, N = 664
GHPHTSYYQYGLGILALCLHQK pc
B5MBX2;F8WE86;P20062;P20062−
198:436 378:617 128:207
22:31030781:C:T_C
p = 0.001, beta = 0.182, N = 704
LGQHLLPWMDRLSLEHLNPSIYVGLR
B5MBX2;F8WE86;P20062;P20062−
151:436 286:617 99:207
22:31030781:C:T_C
p = 0.00041, beta = 0.223, N = 536
WFLEDEKR pc2
B5MBX2;F8WE86;P20062
57:436 112:617 48:207
22:31030781:C:T_C
p = 0.024, beta = 0.216, N = 217
ALLASLQDGAFQNALMISQLLPVLNH
B5MBX2;F8WE86;P20062;P20062−
182:436 330:617 117:207
22:31030781:C:T_C
p = 4.8e−05, beta = 0.234, N = 629
LGQHLLPWMDR pc3
B5MBX2;F8WE86;P20062;P20062−
39:436 80:617 35:207
22:31030781:C:T_C
p = 0.56, beta = −0.0658, N = 154
LSLEHLNPSIYVGLR pc3
B5MBX2;F8WE86;P20062;P20062−
33:436 56:617 26:207
22:31030781:C:T_C
p = 0.65, beta = 0.0585, N = 115
TCN2 : NP1
B5MBX2;F8WE86;P20062
87:87 159:159 79:79
22:31030781:C:T_T
p = 4.5e−06, beta = −0.346, N = 325
TYIDLIFPDCLAPR pc2
B5MBX2;F8WE86;P20062;P20062−
87:87 159:159 79:79
22:31030781:C:T_T
p = 4.3e−06, beta = −0.347, N = 325
WFLEDEK pc2
B5MBX2;F8WE86;P20062
85:87 158:159 73:79
22:31030781:C:T_T
p = 0.00044, beta = −0.274, N = 316
DGETIELR pc2
B5MBX2;F8WE86;P20062;P20062−
74:87 118:159 51:79
22:31030781:C:T_T
p = 0.092, beta = −0.15, N = 243
EDLYLHSLK pc2
B5MBX2;F8WE86;P20062;P20062−
73:87 137:159 63:79
22:31030781:C:T_T
p = 0.00074, beta = −0.283, N = 273
TVREEILK pc2
B5MBX2;F8WE86;P20062;P20062−
76:87 130:159 54:79
22:31030781:C:T_T
p = 0.21, beta = −0.11, N = 260
YAVEPFHQGHHSVDTAAMAGLAFTCL
B5MBX2;F8WE86;P20062;P20062−
80:87 143:159 64:79
22:31030781:C:T_T
p = 0.002, beta = −0.253, N = 287
LSSLQAGTK pc2
B5MBX2;F8WE86;P20062;P20062−
41:87 56:159 22:79
22:31030781:C:T_T
p = 0.044, beta = −0.252, N = 119
VHDSVVDK pc2
B5MBX2;F8WE86;P20062;P20062−
16:87 25:159 8:79
22:31030781:C:T_T
p = 0.16, beta = −0.282, N = 49
GHPHTSYYQYGLGILALCLHQK pc
B5MBX2;F8WE86;P20062;P20062−
31:87 45:159 16:79
22:31030781:C:T_T
p = 0.66, beta = −0.0647, N = 92
55Assay Target: TCN2
UniProt: F8WE86
rsID: rs4820023
Target SNP: 22:31030781:C:T
Proxy SNP: 22:31030781:C:T
Tarkin log10(p): 26.7
Tarkin BETA: 0.431
Tarkin MSPA: 0.957
1256:1220:1240:1237:1212:116
GAELGTACLK pc2
rs1801198 REF
19:436 594:617 207:207
22:31030781:C:T_C
p = 9.99999999999999e−276, model = REC, N =
QSISVLAGSTVEDVLK pc2
rs9621049 REF
413:436 608:617 205:207
22:31030781:C:T_C
p = 7.9e−05, model = REC, N = 1226
QSISVLAGSTVEDVLKK pc3
rs9621049 REF
234:436 477:617 179:207
22:31030781:C:T_C
p = 2.6e−21, model = REC, N = 890
QSIFVLAGSTVEDVLK pc2
rs9621049 ALT
154:436 130:617 2:207
22:31030781:C:T_C
p = 9.2e−23, model = DOM, N = 286
QSIFVLAGSTVEDVLKK pc3
rs9621049 ALT
115:436 96:617 2:207
22:31030781:C:T_C
p = 8.7e−16, model = DOM, N = 213

56. CCL23 (P55773)
Tarkin MSPA=0.239

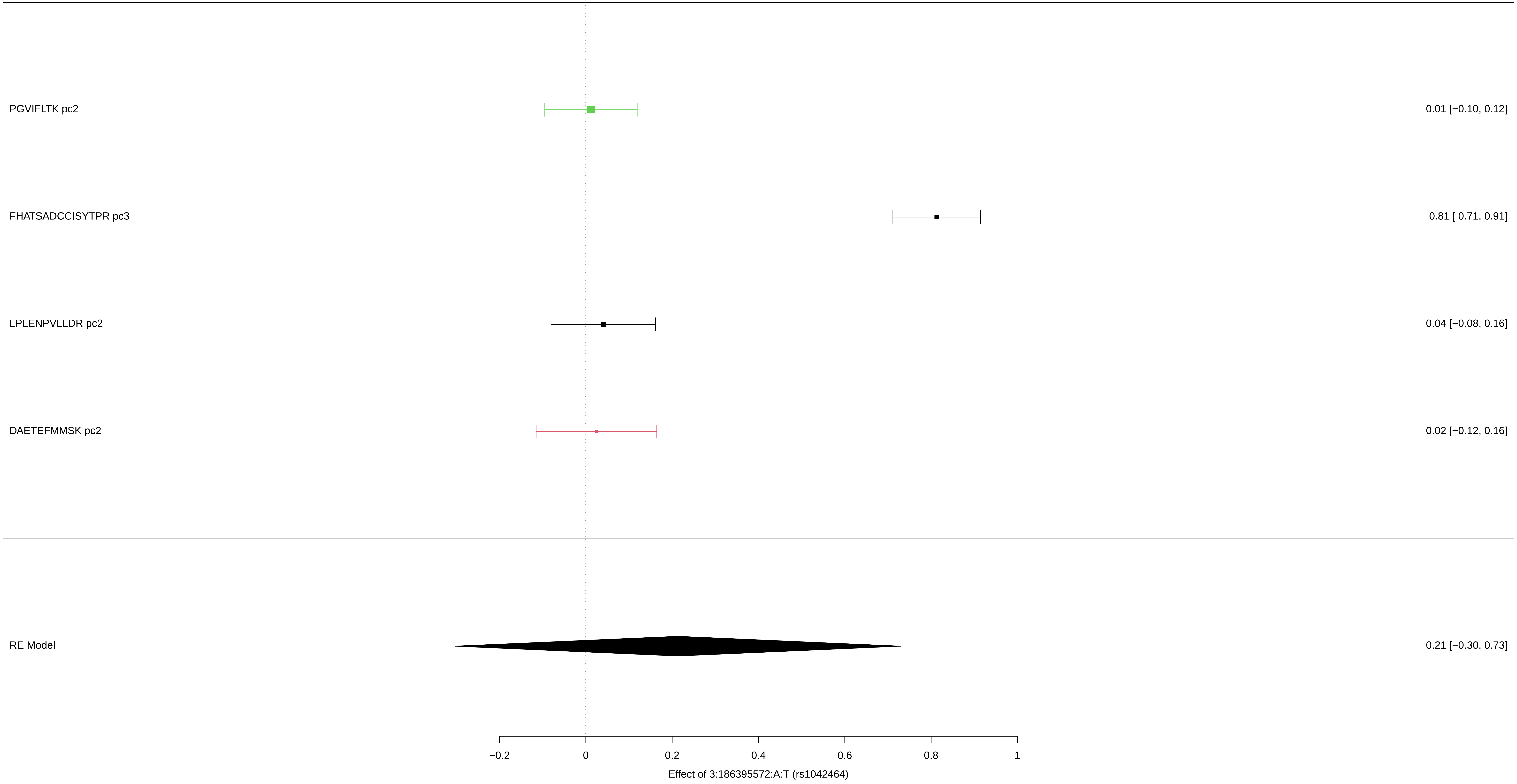

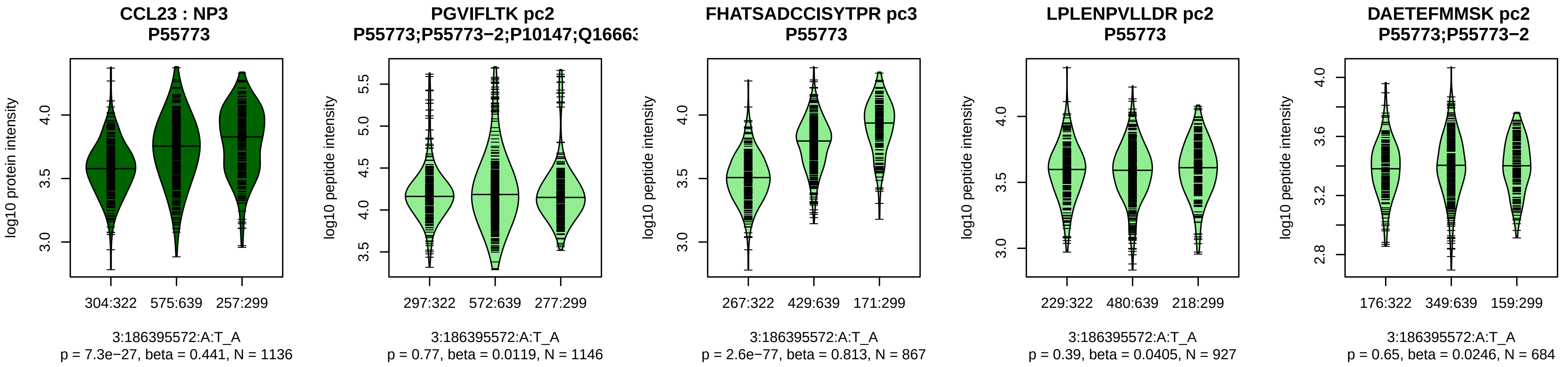

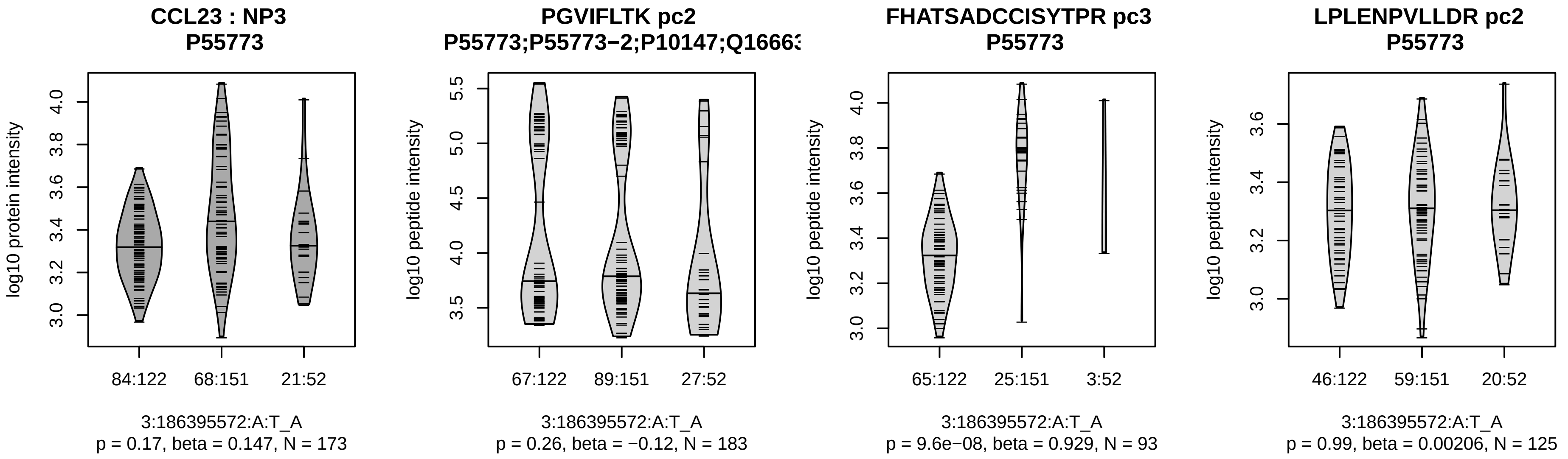

56Assay Target: CCL23
UniProt: P55773
rsID: rs1042464
Target SNP: 3:186395572:A:T
Proxy SNP: 3:186395572:A:T
Tarkin log10(p): 26.1
Tarkin BETA: 0.441
Tarkin MSPA: 0.239
–:*:–:–
1146:867:927:684

57. CCL16 (O15467)
Tarkin MSPA=0.995

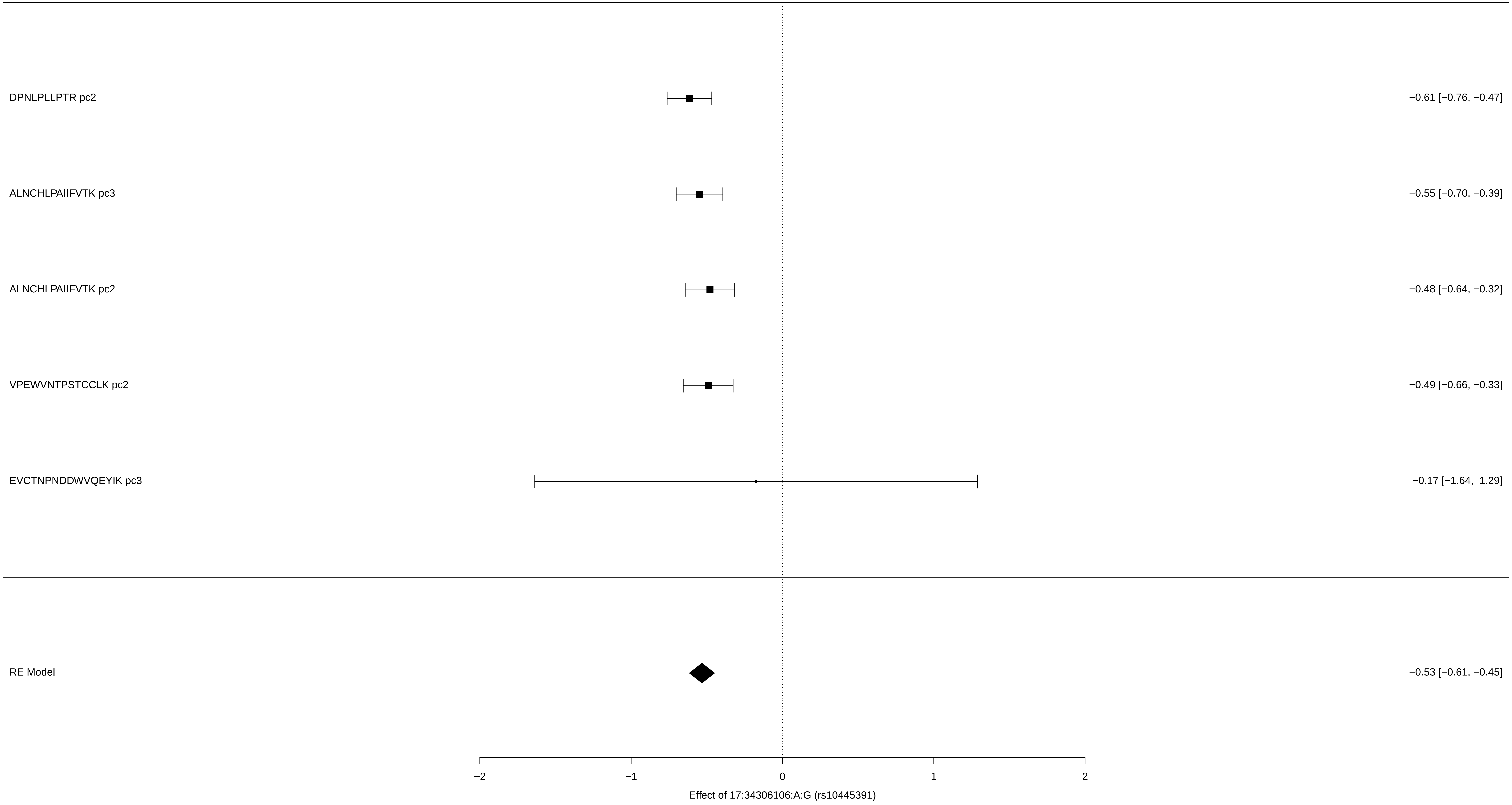

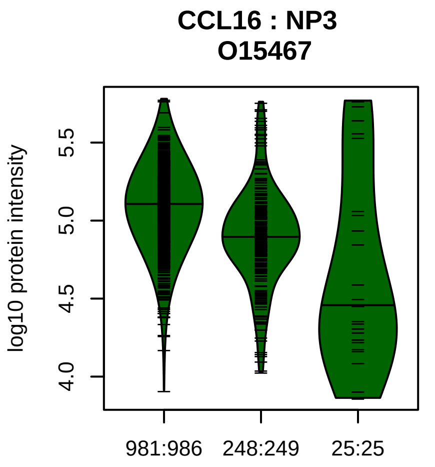

17:34306106:A:G_G
p = 5.6e−26, beta = −0.617, N = 1254

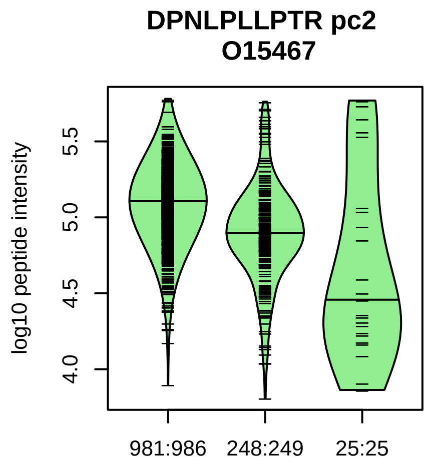

17:34306106:A:G_G
p = 8.6e−26, beta = −0.615, N = 1254

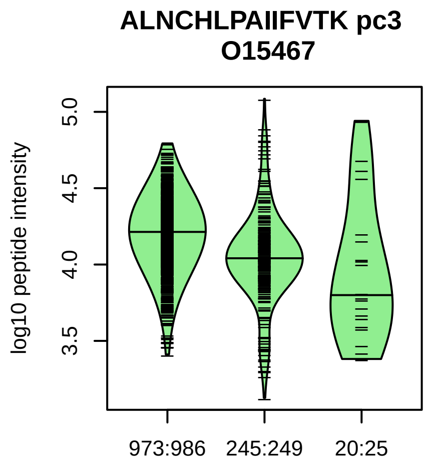

17:34306106:A:G_G
p = 2e−19, beta = −0.548, N = 1238

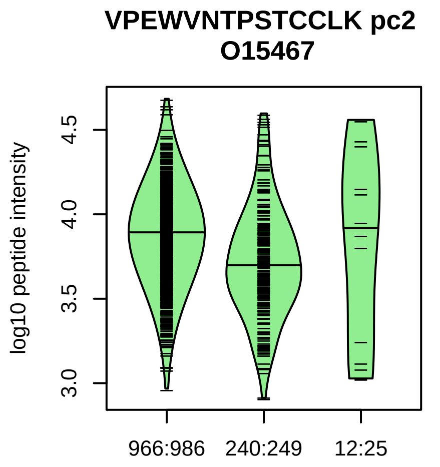

17:34306106:A:G_G
p = 3.5e−14, beta = −0.491, N = 1218

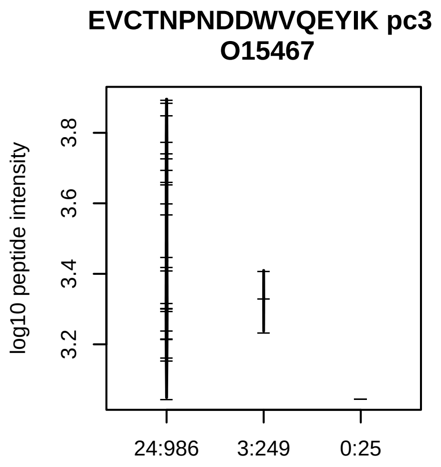

17:34306106:A:G_G
p = 0.76, beta = −0.174, N = 27

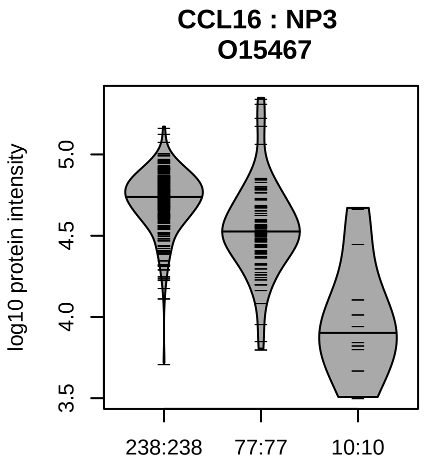

17:34306106:A:G_G
p = 6.8e−17, beta = −0.834, N = 325

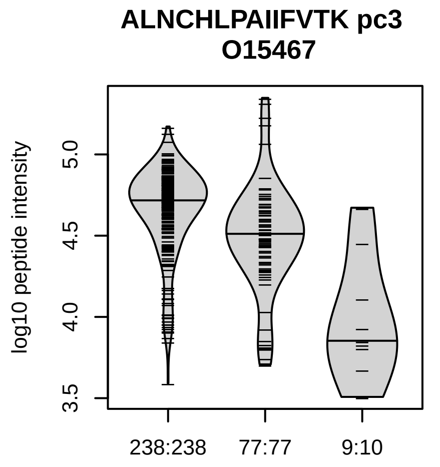

17:34306106:A:G_G
p = 6.6e−12, beta = −0.709, N = 324

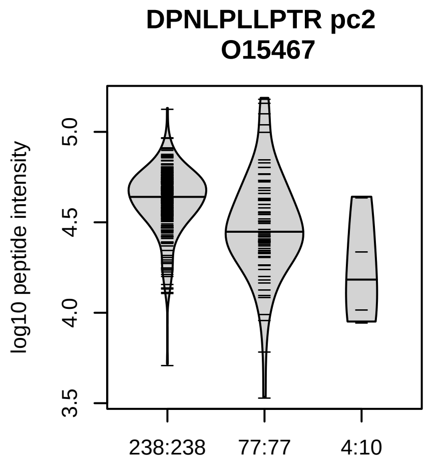

17:34306106:A:G_G
p = 2e−08, beta = −0.645, N = 319

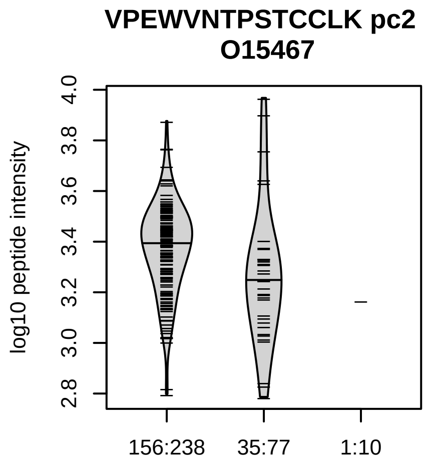

17:34306106:A:G_G
p = 0.00069, beta = −0.582, N = 192

57Assay Target: CCL16
UniProt: O15467
rsID: rs10445391
Target SNP: 17:34306106:A:G
Proxy SNP: 17:34306106:A:G
Tarkin log10(p): 25.2
Tarkin BETA: −0.617
Tarkin MSPA: 0.995
::*:*:–
1254:1238:1219:1218:27

58. SERPINA10 (G3V2W1)
Tarkin MSPA=0.944

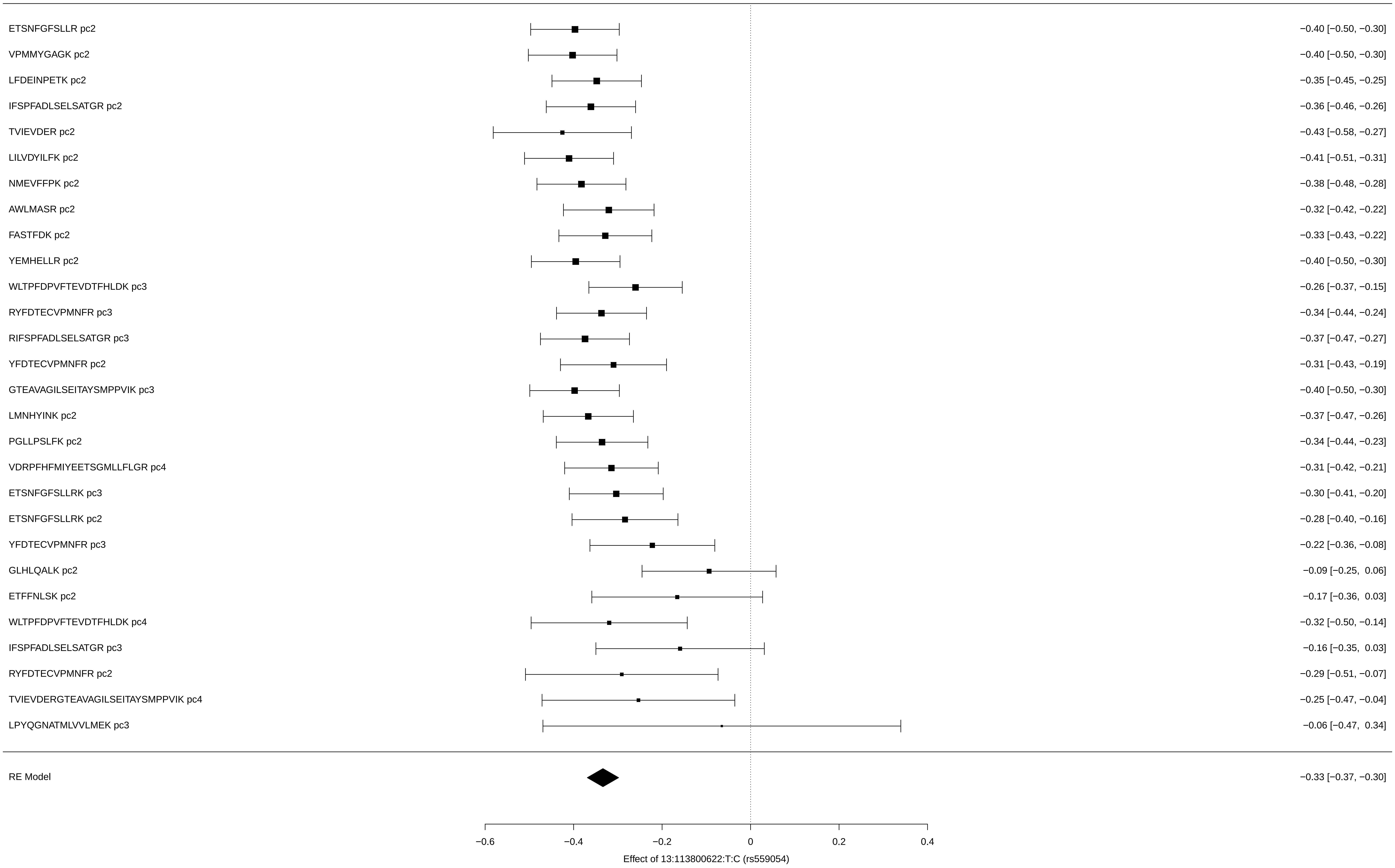

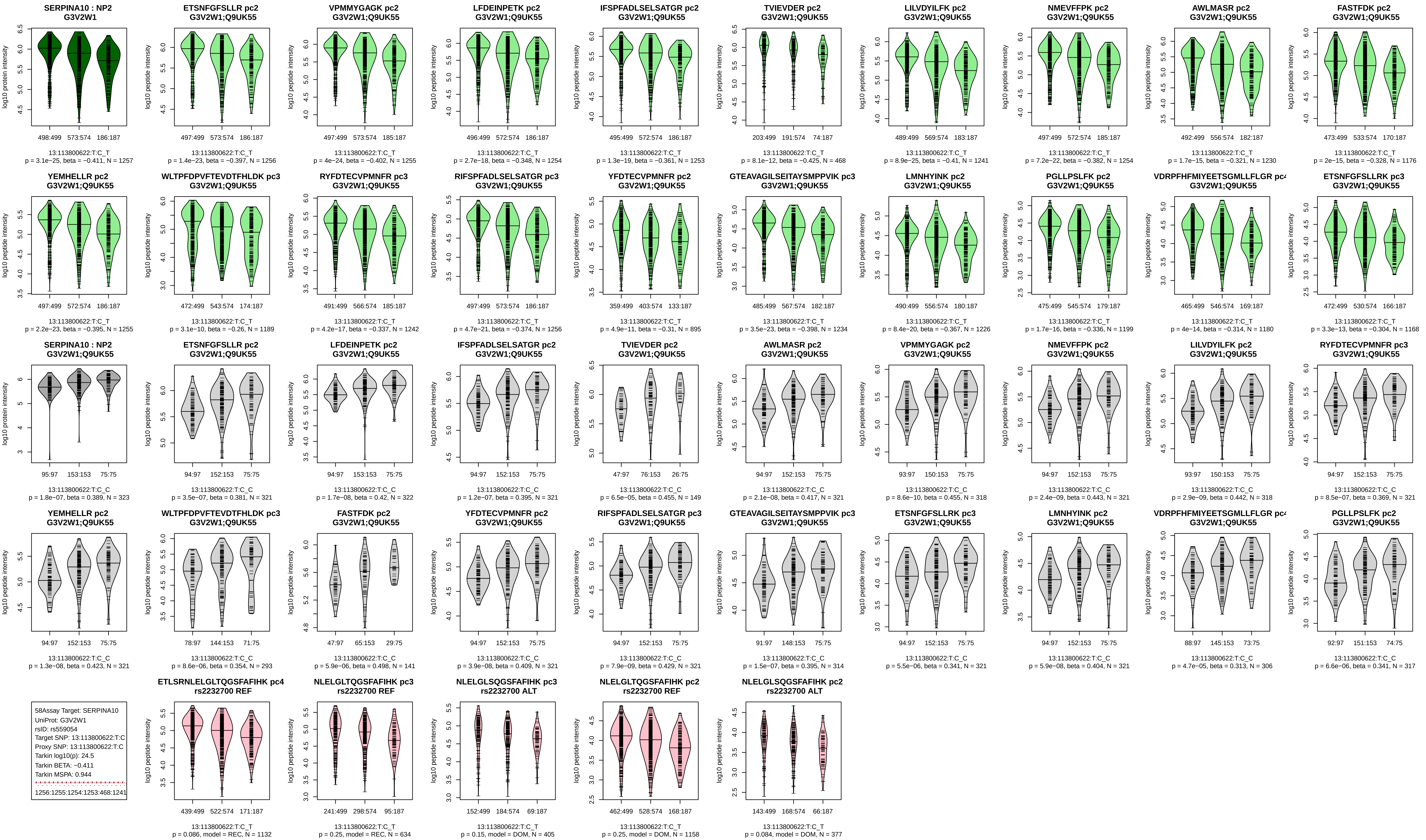
SERPINA10 : NP2
G3V2W1
log10 protein intensity
498:499 573:574 186:187
13:113800622:T:C_T
p = 3.1e−25, beta = −0.411, N = 1257
ETSNFGFSLLR pc2
G3V2W1;Q9UK55
log10 peptide intensity
497:499 573:574 186:187
13:113800622:T:C_T
p = 1.4e−23, beta = −0.397, N = 1256
VPMMYGAGK pc2
G3V2W1;Q9UK55
497:499 573:574 185:187
13:113800622:T:C_T
p = 4e−24, beta = −0.402, N = 1255
LFDEINPETK pc2
G3V2W1;Q9UK55
496:499 572:574 186:187
13:113800622:T:C_T
p = 2.7e−18, beta = −0.348, N = 1254
IFSPFADLSELSATGR pc2
G3V2W1;Q9UK55
495:499 572:574 186:187
13:113800622:T:C_T
p = 1.3e−19, beta = −0.361, N = 1253
TVIEVDER pc2
G3V2W1;Q9UK55
203:499 191:574 74:187
13:113800622:T:C_T
p = 8.1e−12, beta = −0.425, N = 468
LILVDYILFK pc2
G3V2W1;Q9UK55
489:499 569:574 183:187
13:113800622:T:C_T
p = 8.9e−25, beta = −0.41, N = 1241
NMEVFFPK pc2
G3V2W1;Q9UK55
497:499 572:574 185:187
13:113800622:T:C_T
p = 7.2e−22, beta = −0.382, N = 1254
AWLMASR pc2
G3V2W1;Q9UK55
492:499 556:574 182:187
13:113800622:T:C_T
p = 1.7e−15, beta = −0.321, N = 1230
FASTFDK pc2
G3V2W1;Q9UK55
473:499 533:574 170:187
13:113800622:T:C_T
p = 2e−15, beta = −0.328, N = 1176
YEMHELLR pc2
G3V2W1;Q9UK55
497:499 572:574 186:187
13:113800622:T:C_T
p = 2.2e−23, beta = −0.395, N = 1255
WLTPFDPVFTEVDTFHLDK pc3
G3V2W1;Q9UK55
472:499 543:574 174:187
13:113800622:T:C_T
p = 3.1e−10, beta = −0.26, N = 1189
RYFDTECVPMNFR pc3
G3V2W1;Q9UK55
491:499 566:574 185:187
13:113800622:T:C_T
p = 4.2e−17, beta = −0.337, N = 1242
RIFSPFADLSELSATGR pc3
G3V2W1;Q9UK55
497:499 573:574 186:187
13:113800622:T:C_T
p = 4.7e−21, beta = −0.374, N = 1256
YFDTECVPMNFR pc2
G3V2W1;Q9UK55
359:499 403:574 133:187
13:113800622:T:C_T
p = 4.9e−11, beta = −0.31, N = 895
GTEAVAGILSEITAYSMPPVIK pc3
G3V2W1;Q9UK55
485:499 567:574 182:187
13:113800622:T:C_T
p = 3.5e−23, beta = −0.398, N = 1234
LMNHYINK pc2
G3V2W1;Q9UK55
490:499 556:574 180:187
13:113800622:T:C_T
p = 8.4e−20, beta = −0.367, N = 1226
PGLLPSLFK pc2
G3V2W1;Q9UK55
475:499 545:574 179:187
13:113800622:T:C_T
p = 1.7e−16, beta = −0.336, N = 1199
VDRPFHFMIYEETSGMLLFLGR pc
G3V2W1;Q9UK55
465:499 546:574 169:187
13:113800622:T:C_T
p = 4e−14, beta = −0.314, N = 1180
ETSNFGFSLLRK pc3
G3V2W1;Q9UK55
472:499 530:574 166:187
13:113800622:T:C_T
p = 3.3e−13, beta = −0.304, N = 1168
SERPINA10 : NP2
G3V2W1;Q9UK55
95:97 153:153 75:75
13:113800622:T:C_C
p = 1.8e−07, beta = 0.389, N = 323
ETSNFGFSLLR pc2
G3V2W1;Q9UK55
94:97 152:153 75:75
13:113800622:T:C_C
p = 3.5e−07, beta = 0.381, N = 321
LFDEINPETK pc2
G3V2W1;Q9UK55
94:97 153:153 75:75
13:113800622:T:C_C
p = 1.7e−08, beta = 0.42, N = 322
IFSPFADLSELSATGR pc2
G3V2W1;Q9UK55
94:97 152:153 75:75
13:113800622:T:C_C
p = 1.2e−07, beta = 0.395, N = 321
TVIEVDER pc2
G3V2W1;Q9UK55
47:97 76:153 26:75
13:113800622:T:C_C
p = 6.5e−05, beta = 0.455, N = 149
AWLMASR pc2
G3V2W1;Q9UK55
94:97 152:153 75:75
13:113800622:T:C_C
p = 2.1e−08, beta = 0.417, N = 321
VPMMYGAGK pc2
G3V2W1;Q9UK55
93:97 150:153 75:75
13:113800622:T:C_C
p = 8.6e−10, beta = 0.455, N = 318
NMEVFFPK pc2
G3V2W1;Q9UK55
94:97 152:153 75:75
13:113800622:T:C_C
p = 2.4e−09, beta = 0.443, N = 321
LILVDYILFK pc2
G3V2W1;Q9UK55
93:97 150:153 75:75
13:113800622:T:C_C
p = 2.9e−09, beta = 0.442, N = 318
RYFDTECVPMNFR pc3
G3V2W1;Q9UK55
94:97 152:153 75:75
13:113800622:T:C_C
p = 8.5e−07, beta = 0.369, N = 321
YEMHELLR pc2
G3V2W1;Q9UK55
94:97 152:153 75:75
13:113800622:T:C_C
p = 1.3e−08, beta = 0.423, N = 321
WLTPFDPVFTEVDTFHLDK pc3
G3V2W1;Q9UK55
78:97 144:153 71:75
13:113800622:T:C_C
p = 8.6e−06, beta = 0.354, N = 293
FASTFDK pc2
G3V2W1;Q9UK55
47:97 65:153 29:75
13:113800622:T:C_C
p = 5.9e−06, beta = 0.498, N = 141
YFDTECVPMNFR pc2
G3V2W1;Q9UK55
94:97 152:153 75:75
13:113800622:T:C_C
p = 3.9e−08, beta = 0.409, N = 321
RIFSPFADLSELSATGR pc3
G3V2W1;Q9UK55
94:97 152:153 75:75
13:113800622:T:C_C
p = 7.9e−09, beta = 0.429, N = 321
GTEAVAGILSEITAYSMPPVIK pc3
G3V2W1;Q9UK55
91:97 148:153 75:75
13:113800622:T:C_C
p = 1.5e−07, beta = 0.395, N = 314
ETSNFGFSLLRK pc3
G3V2W1;Q9UK55
94:97 152:153 75:75
13:113800622:T:C_C
p = 5.5e−06, beta = 0.341, N = 321
LMNHYINK pc2
G3V2W1;Q9UK55
94:97 152:153 75:75
13:113800622:T:C_C
p = 5.9e−08, beta = 0.404, N = 321
VDRPFHFMIYEETSGMLLFLGR pc
G3V2W1;Q9UK55
88:97 145:153 73:75
13:113800622:T:C_C
p = 4.7e−05, beta = 0.313, N = 306
PGLLPSLFK pc2
G3V2W1;Q9UK55
92:97 151:153 74:75
13:113800622:T:C_C
p = 6.6e−06, beta = 0.341, N = 317
58Assay Target: SERPINA10
UniProt: G3V2W1
rsID: rs559054
Target SNP: 13:113800622:T:C
Proxy SNP: 13:113800622:T:C
Tarkin log10(p): 24.5
Tarkin BETA: −0.411
Tarkin MSPA: 0.944
1256:1255:1254:1253:468:1241
ETLSRNLELGLTQGSFAFIHK pc4
rs2232700 REF
439:499 522:574 171:187
13:113800622:T:C_T
p = 0.086, model = REC, N = 1132
NLELGLTQGSFAFIHK pc3
rs2232700 REF
241:499 298:574 95:187
13:113800622:T:C_T
p = 0.25, model = REC, N = 634
NLELGLSQGSFAFIHK pc3
rs2232700 ALT
152:499 184:574 69:187
13:113800622:T:C_T
p = 0.15, model = DOM, N = 405
NLELGLTQGSFAFIHK pc2
rs2232700 REF
462:499 528:574 168:187
13:113800622:T:C_T
p = 0.25, model = DOM, N = 1158
NLELGLSQGSFAFIHK pc2
rs2232700 ALT
143:499 168:574 66:187
13:113800622:T:C_T
p = 0.084, model = DOM, N = 377

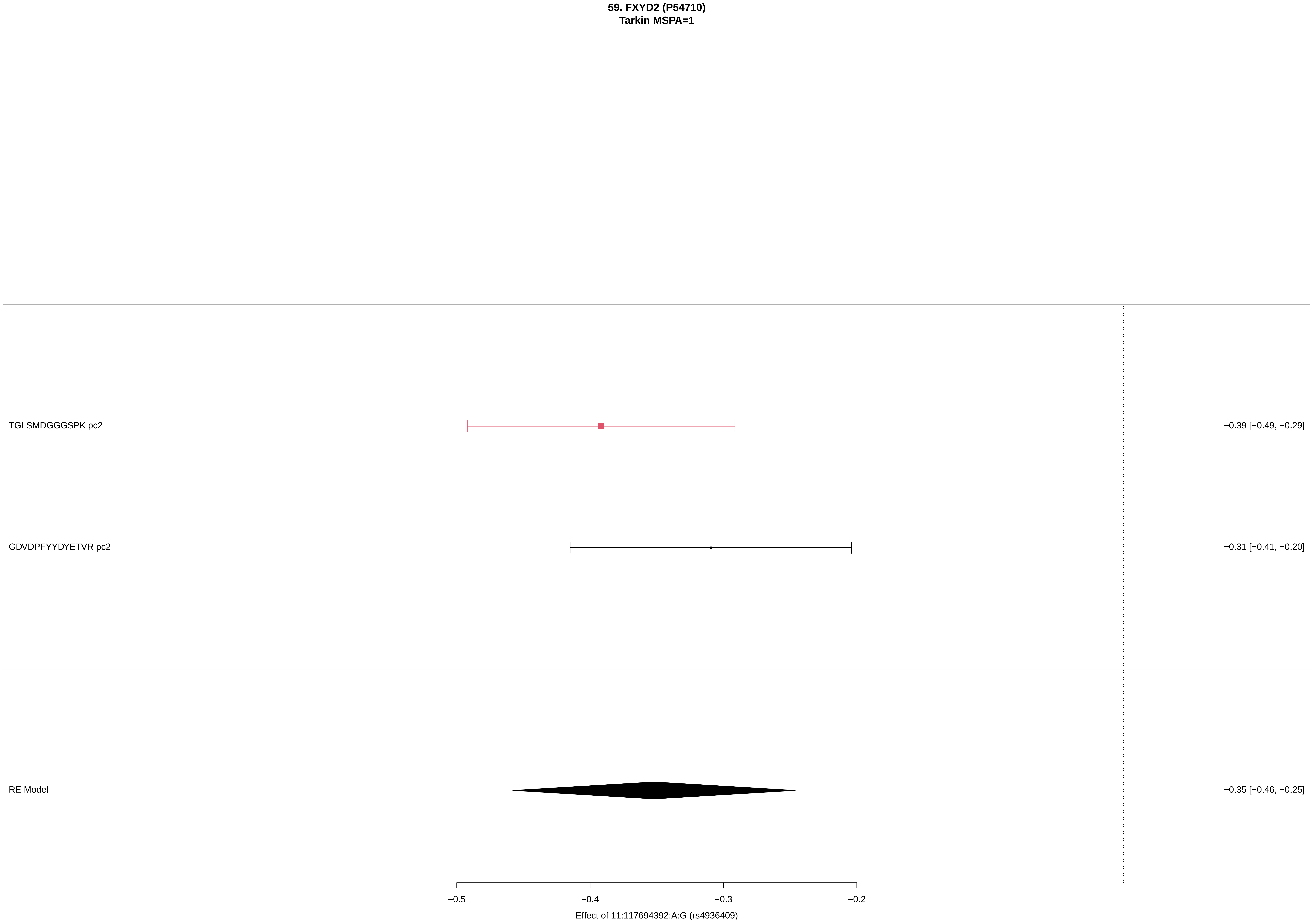
59. FXYD2 (P54710)
Tarkin MSPA=1
TGLSMDGGGSPK pc2
−0.39 [−0.49, −0.29]
GDVDPFYYDYETVR pc2
−0.31 [−0.41, −0.20]
RE Model
−0.35 [−0.46, −0.25]
−0.5
−0.4
−0.3
−0.2
Effect of 11:117694392:A:G (rs4936409)

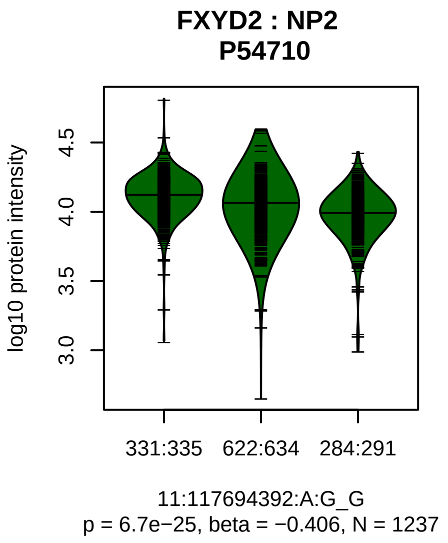

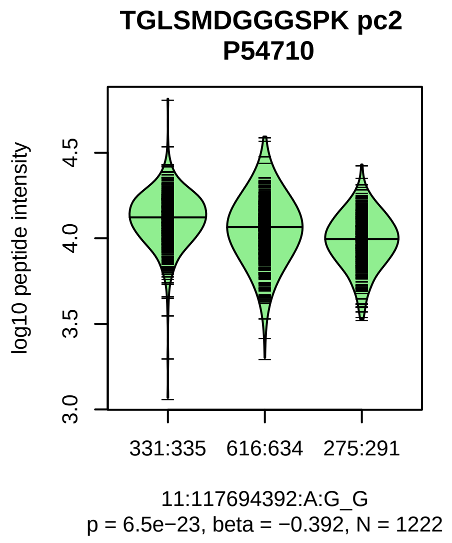

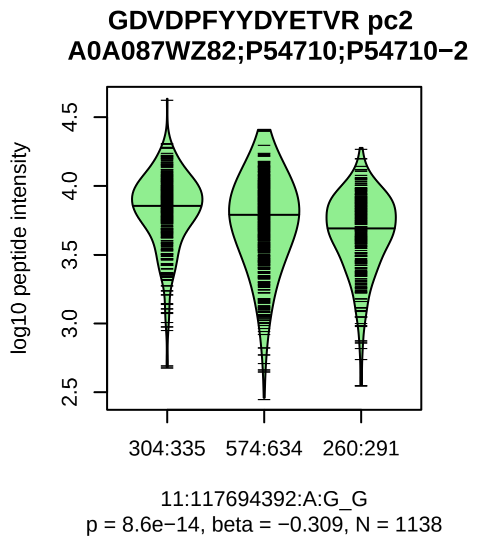

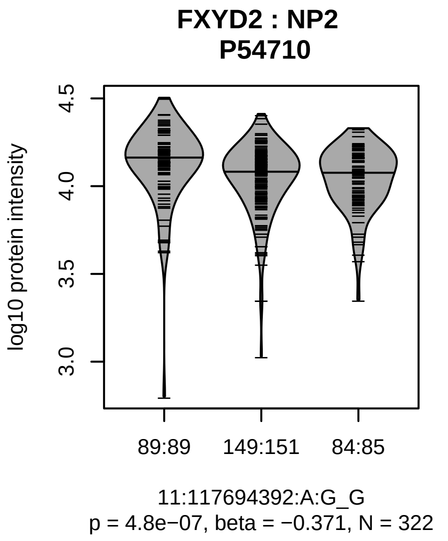

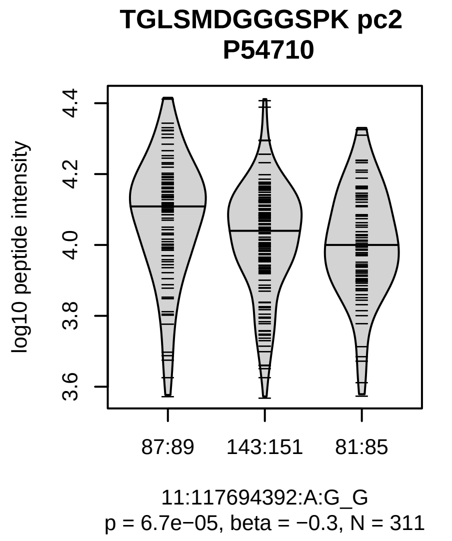

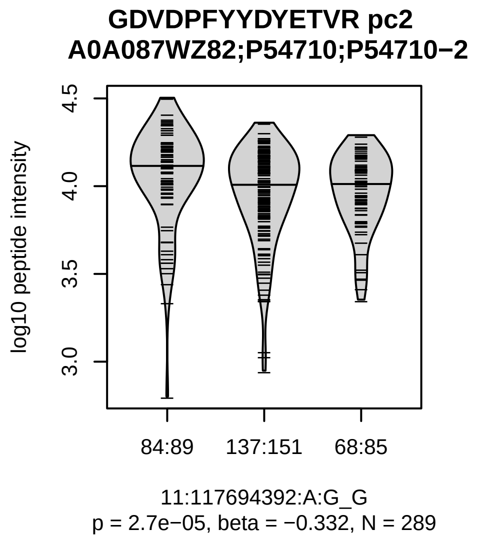

59Assay Target: FXYD2
UniProt: P54710
rsID: rs4936409
Target SNP: 11:117694392:A:G
Proxy SNP: 11:117694392:A:G
Tarkin log10(p): 24.2
Tarkin BETA: −0.406
Tarkin MSPA: 1
:
1222:1138

60. BPIFA1 (Q9NP55)
Tarkin MSPA=0.955

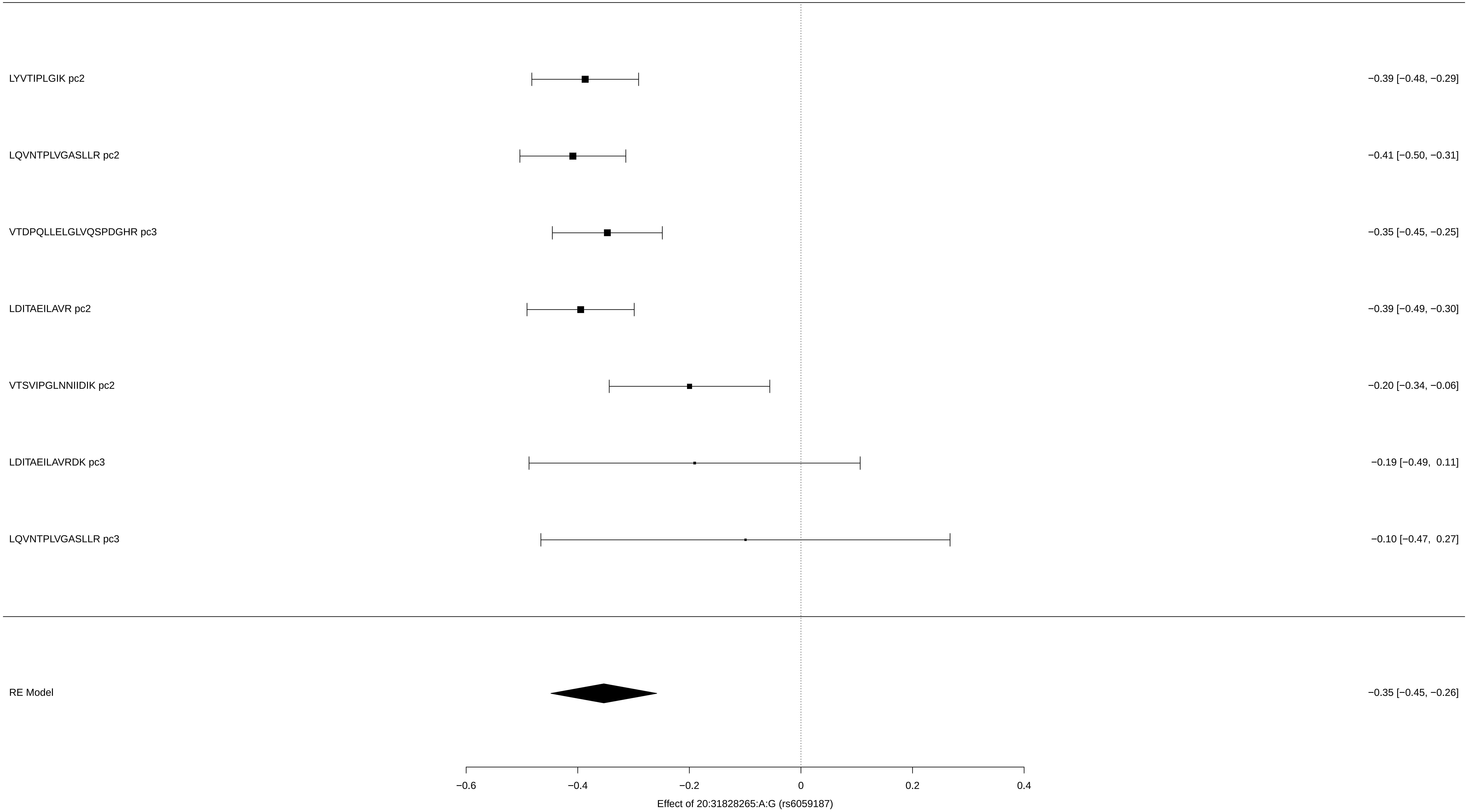

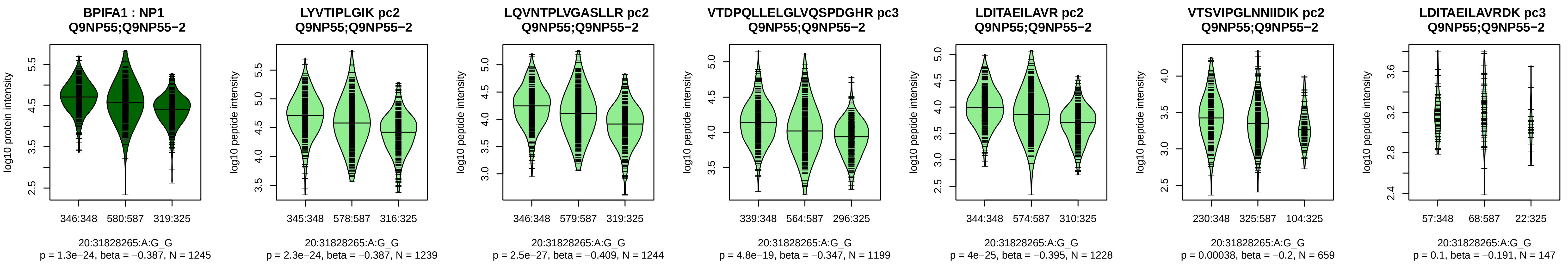

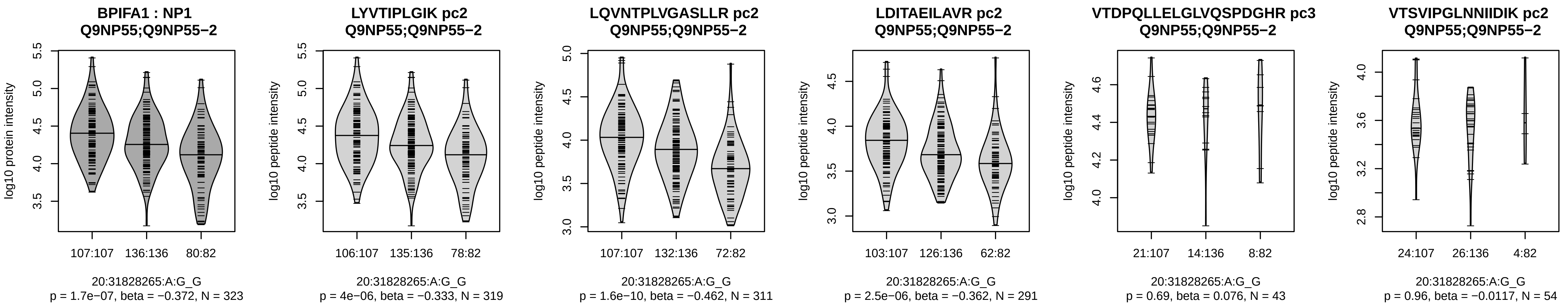

60Assay Target: BPIFA1
UniProt: Q9NP55
rsID: rs6059187
Target SNP: 20:31828265:A:G
Proxy SNP: 20:31828265:A:G
Tarkin log10(p): 23.9
Tarkin BETA: −0.388
Tarkin MSPA: 0.955
::*:*:*:−:−:NA
1239:1244:1199:1228:659:147:1

61. ZNF618 (Q5T7W0)
Tarkin MSPA=0.911

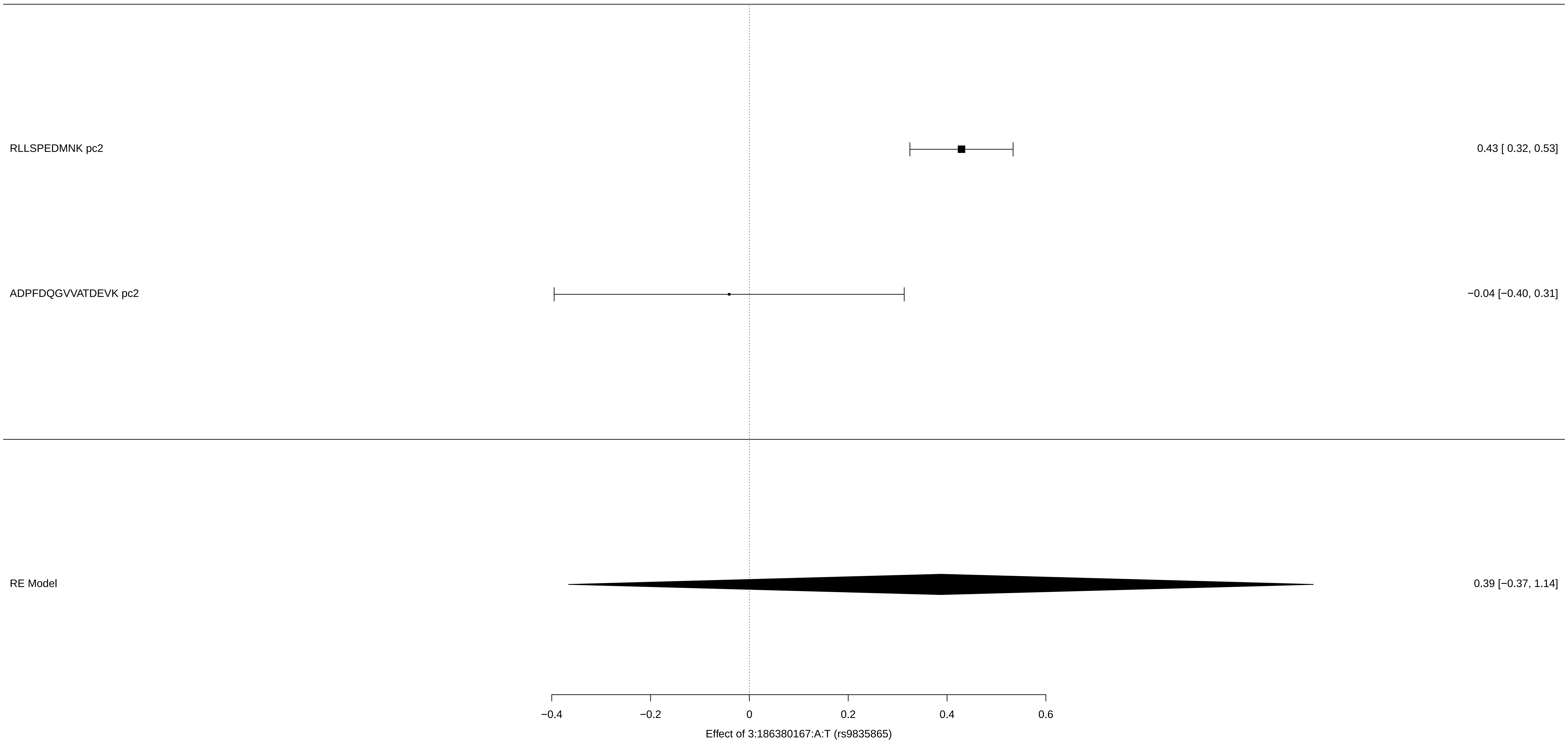

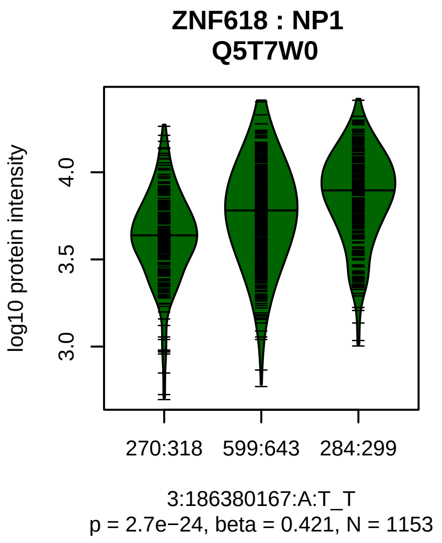

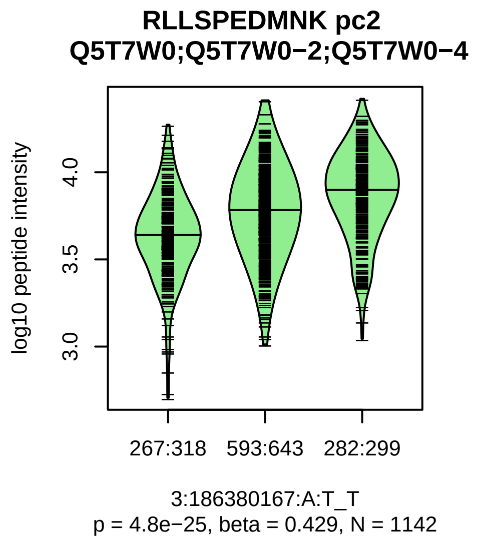

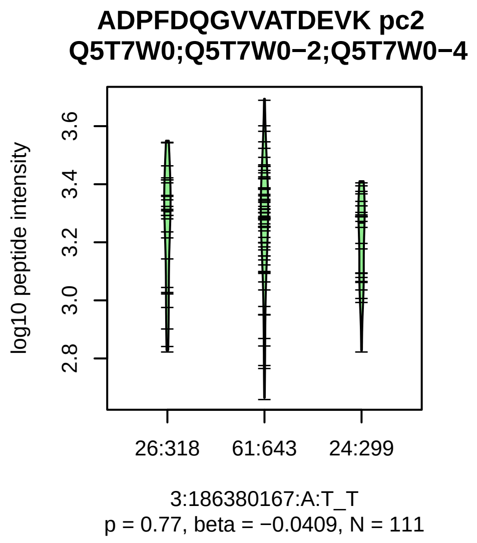

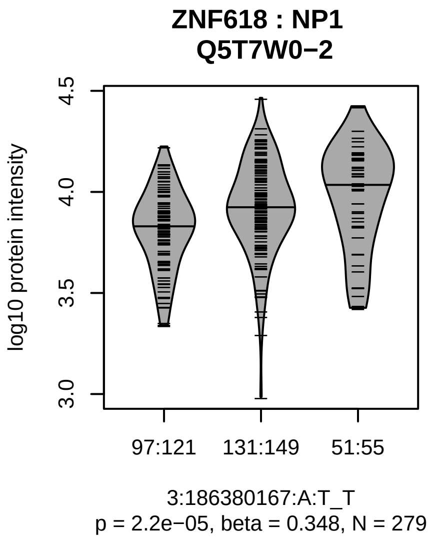

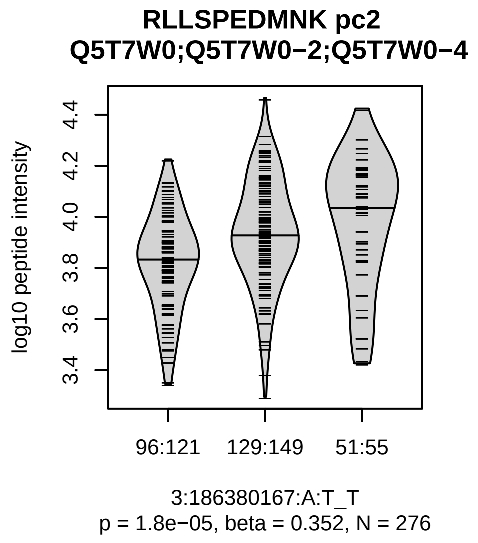

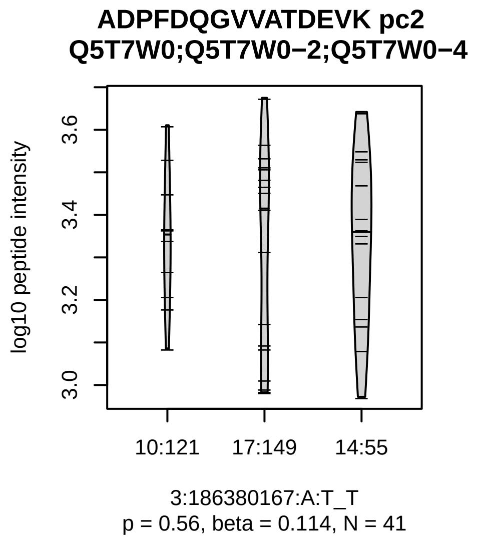

61Assay Target: ZNF618
UniProt: Q5T7W0
rsID: rs9835865
Target SNP: 3:186380167:A:T
Proxy SNP: 3:186380167:A:T
Tarkin log10(p): 23.6
Tarkin BETA: 0.421
Tarkin MSPA: 0.911
*:−:NA:NA
1142:111:5:1

62. SERPINE2 (P07093)
Tarkin MSPA=0.982

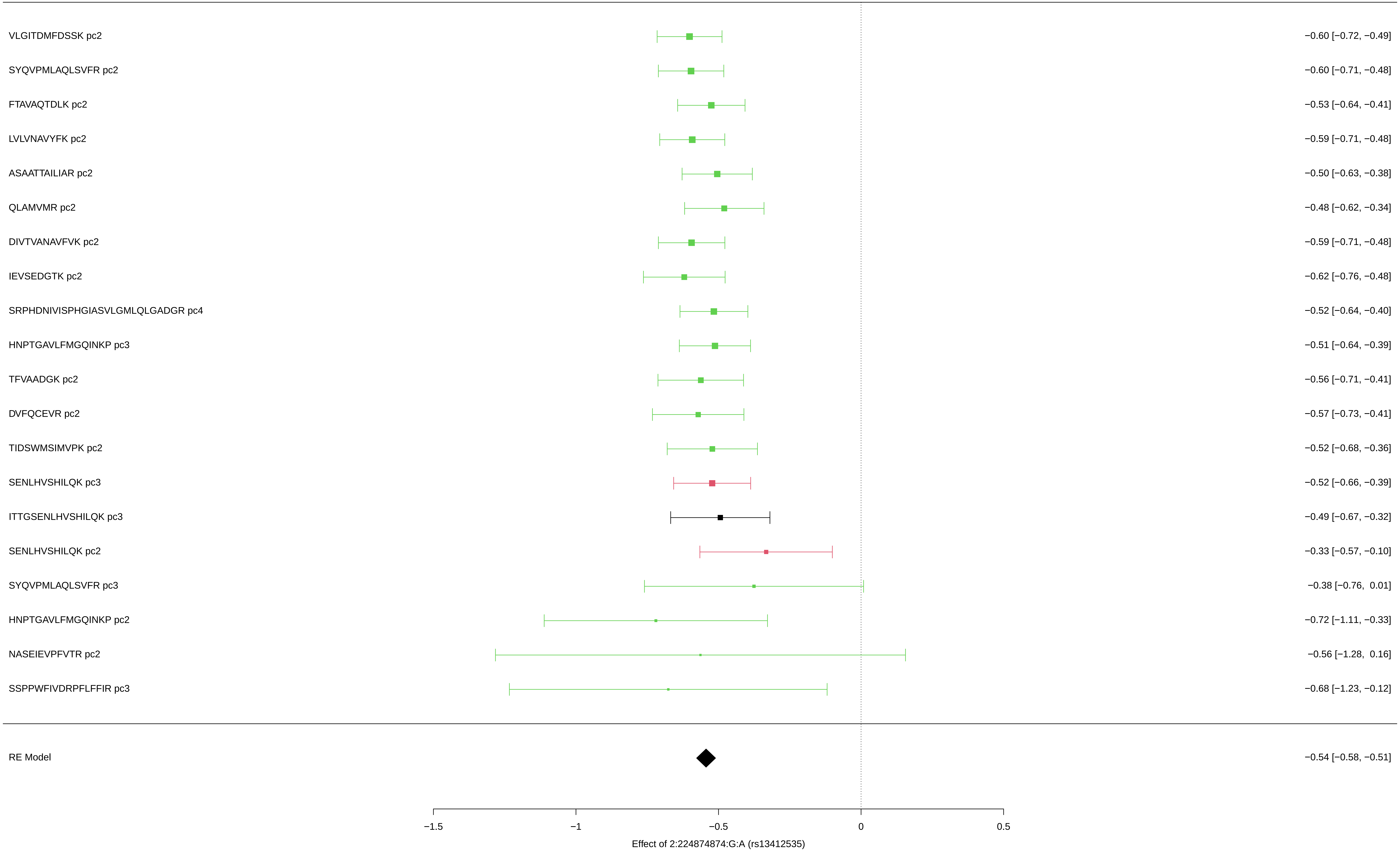

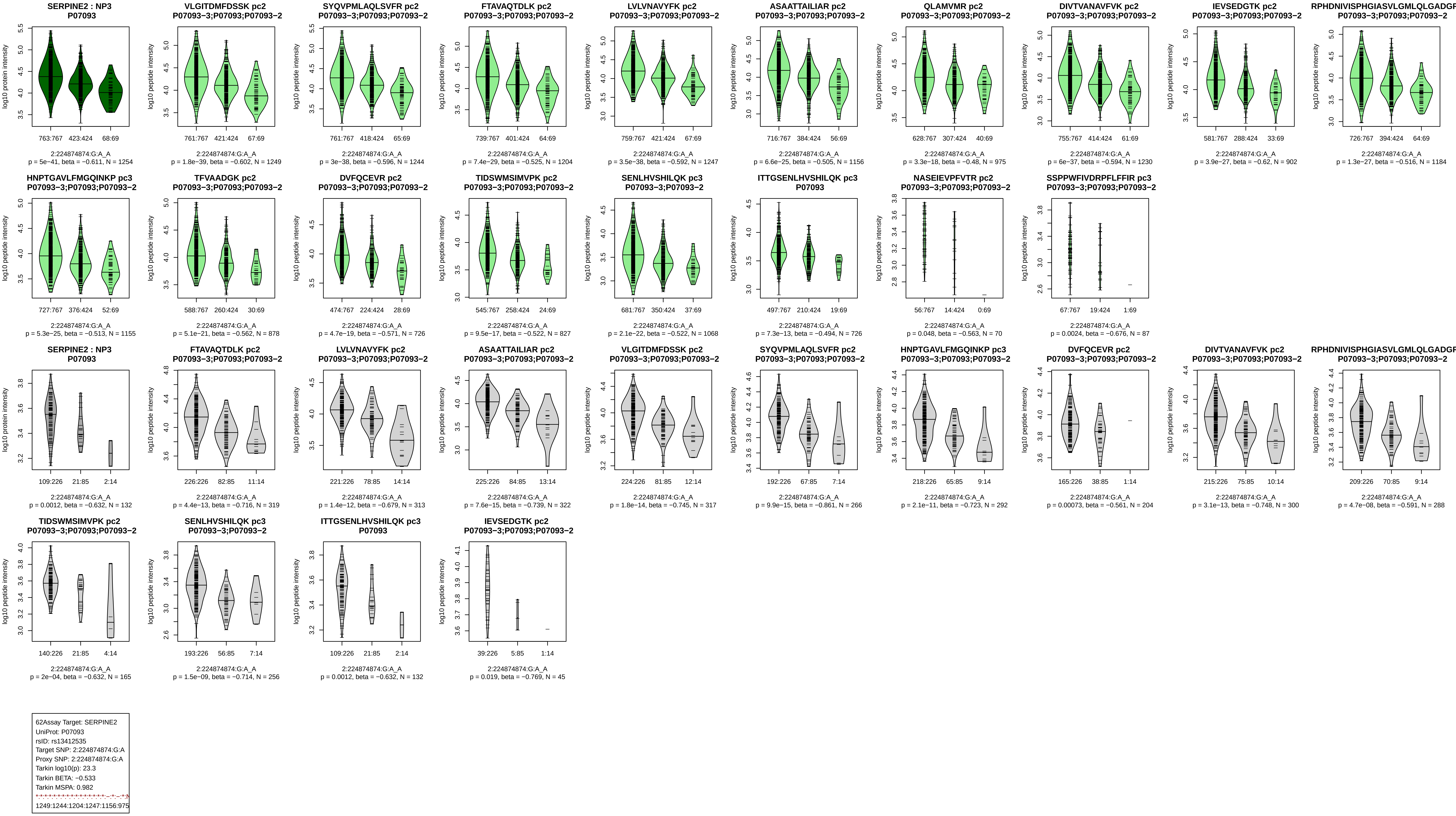
SERPINE2 : NP3
P07093
log10 protein intensity
763:767 423:424 68:69
2:224874874:G:A_A
p = 5e−41, beta = −0.611, N = 1254
VLGITDMFDSSK pc2
P07093−3;P07093;P07093−2
log10 peptide intensity
761:767 421:424 67:69
2:224874874:G:A_A
p = 1.8e−39, beta = −0.602, N = 1249
SYQVPMLAQLSVFR pc2
P07093−3;P07093;P07093−2
log10 peptide intensity
761:767 418:424 65:69
2:224874874:G:A_A
p = 3e−38, beta = −0.596, N = 1244
FTAVAQTDLK pc2
P07093−3;P07093;P07093−2
log10 peptide intensity
739:767 401:424 64:69
2:224874874:G:A_A
p = 7.4e−29, beta = −0.525, N = 1204
LVLVNAVYFK pc2
P07093−3;P07093;P07093−2
log10 peptide intensity
759:767 421:424 67:69
2:224874874:G:A_A
p = 3.5e−38, beta = −0.592, N = 1247
ASAATTAILIAR pc2
P07093−3;P07093;P07093−2
log10 peptide intensity
716:767 384:424 56:69
2:224874874:G:A_A
p = 6.6e−25, beta = −0.505, N = 1156
QLAMVMR pc2
P07093−3;P07093;P07093−2
log10 peptide intensity
628:767 307:424 40:69
2:224874874:G:A_A
p = 3.3e−18, beta = −0.48, N = 975
DIVTVANAVFVK pc2
P07093−3;P07093;P07093−2
log10 peptide intensity
755:767 414:424 61:69
2:224874874:G:A_A
p = 6e−37, beta = −0.594, N = 1230
IEVSEDGTK pc2
P07093−3;P07093;P07093−2
log10 peptide intensity
581:767 288:424 33:69
2:224874874:G:A_A
p = 3.9e−27, beta = −0.62, N = 902
RPHDNIVISPHGIASVLGMLQLGADGF
P07093−3;P07093;P07093−2
log10 peptide intensity
726:767 394:424 64:69
2:224874874:G:A_A
p = 1.3e−27, beta = −0.516, N = 1184
HNPTGAVLFMGQINKP pc3
P07093−3;P07093;P07093−2
log10 peptide intensity
727:767 376:424 52:69
2:224874874:G:A_A
p = 5.3e−25, beta = −0.513, N = 1155
TFVAADGK pc2
P07093−3;P07093;P07093−2
log10 peptide intensity
588:767 260:424 30:69
2:224874874:G:A_A
p = 5.1e−21, beta = −0.562, N = 878
DVFQCEVR pc2
P07093−3;P07093;P07093−2
log10 peptide intensity
474:767 224:424 28:69
2:224874874:G:A_A
p = 4.7e−19, beta = −0.571, N = 726
TIDSWMSIMVPK pc2
P07093−3;P07093;P07093−2
log10 peptide intensity
545:767 258:424 24:69
2:224874874:G:A_A
p = 9.5e−17, beta = −0.522, N = 827
SENLHVSHILQK pc3
P07093−3;P07093−2
log10 peptide intensity
681:767 350:424 37:69
2:224874874:G:A_A
p = 2.1e−22, beta = −0.522, N = 1068
ITTGSENLHVSHILQK pc3
P07093
log10 peptide intensity
497:767 210:424 19:69
2:224874874:G:A_A
p = 7.3e−13, beta = −0.494, N = 726
NASEIEVPFVTR pc2
P07093−3;P07093;P07093−2
log10 peptide intensity
56:767 14:424 0:69
2:224874874:G:A_A
p = 0.048, beta = −0.563, N = 70
SSPPWFIVDRPFLFFIR pc3
P07093−3;P07093;P07093−2
log10 peptide intensity
67:767 19:424 1:69
2:224874874:G:A_A
p = 0.0024, beta = −0.676, N = 87
SERPINE2 : NP3
P07093
log10 protein intensity
109:226 21:85 2:14
2:224874874:G:A_A
p = 0.0012, beta = −0.632, N = 132
FTAVAQTDLK pc2
P07093−3;P07093;P07093−2
log10 peptide intensity
226:226 82:85 11:14
2:224874874:G:A_A
p = 4.4e−13, beta = −0.716, N = 319
LVLVNAVYFK pc2
P07093−3;P07093;P07093−2
log10 peptide intensity
221:226 78:85 14:14
2:224874874:G:A_A
p = 1.4e−12, beta = −0.679, N = 313
ASAATTAILIAR pc2
P07093−3;P07093;P07093−2
log10 peptide intensity
225:226 84:85 13:14
2:224874874:G:A_A
p = 7.6e−15, beta = −0.739, N = 322
VLGITDMFDSSK pc2
P07093−3;P07093;P07093−2
log10 peptide intensity
224:226 81:85 12:14
2:224874874:G:A_A
p = 1.8e−14, beta = −0.745, N = 317
SYQVPMLAQLSVFR pc2
P07093−3;P07093;P07093−2
log10 peptide intensity
192:226 67:85 7:14
2:224874874:G:A_A
p = 9.9e−15, beta = −0.861, N = 266
HNPTGAVLFMGQINKP pc3
P07093−3;P07093;P07093−2
log10 peptide intensity
218:226 65:85 9:14
2:224874874:G:A_A
p = 2.1e−11, beta = −0.723, N = 292
DVFQCEVR pc2
P07093−3;P07093;P07093−2
log10 peptide intensity
165:226 38:85 1:14
2:224874874:G:A_A
p = 0.00073, beta = −0.561, N = 204
DIVTVANAVFVK pc2
P07093−3;P07093;P07093−2
log10 peptide intensity
215:226 75:85 10:14
2:224874874:G:A_A
p = 3.1e−13, beta = −0.748, N = 300
RPHDNIVISPHGIASVLGMLQLGADGF
P07093−3;P07093;P07093−2
log10 peptide intensity
209:226 70:85 9:14
2:224874874:G:A_A
p = 4.7e−08, beta = −0.591, N = 288
TIDSWMSIMVPK pc2
P07093−3;P07093;P07093−2
log10 peptide intensity
140:226 21:85 4:14
2:224874874:G:A_A
p = 2e−04, beta = −0.632, N = 165
SENLHVSHILQK pc3
P07093−3;P07093−2
log10 peptide intensity
193:226 56:85 7:14
2:224874874:G:A_A
p = 1.5e−09, beta = −0.714, N = 256
ITTGSENLHVSHILQK pc3
P07093
log10 peptide intensity
109:226 21:85 2:14
2:224874874:G:A_A
p = 0.0012, beta = −0.632, N = 132
IEVSEDGTK pc2
P07093−3;P07093;P07093−2
log10 peptide intensity
39:226 5:85 1:14
2:224874874:G:A_A
p = 0.019, beta = −0.769, N = 45
62Assay Target: SERPINE2
UniProt: P07093
rsID: rs13412535
Target SNP: 2:224874874:G:A
Proxy SNP: 2:224874874:G:A
Tarkin log10(p): 23.3
Tarkin BETA: −0.533
Tarkin MSPA: 0.982
1249:1244:1204:1247:1156:975

63. MSN (P26038)
Tarkin MSPA=0.791

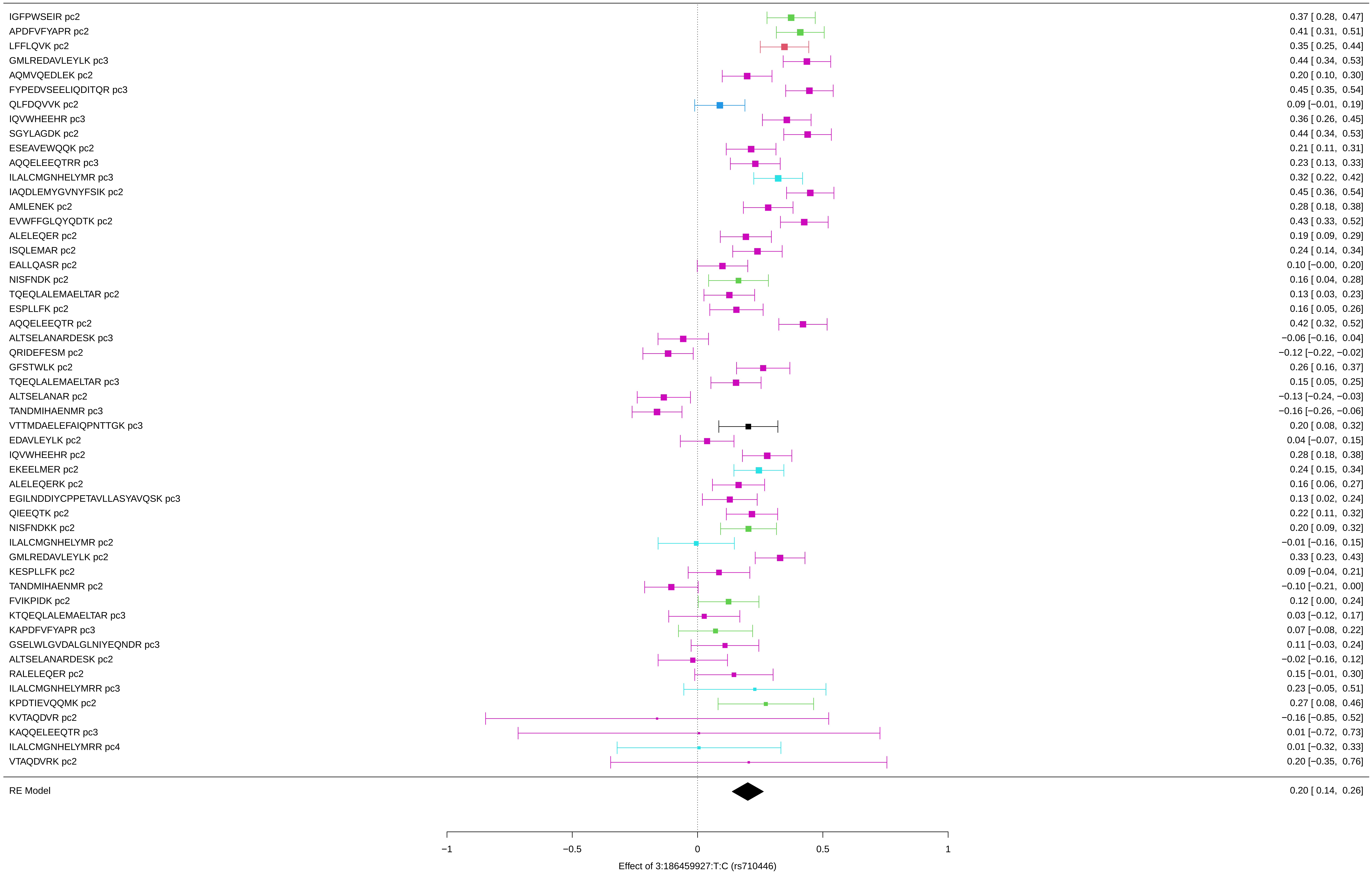

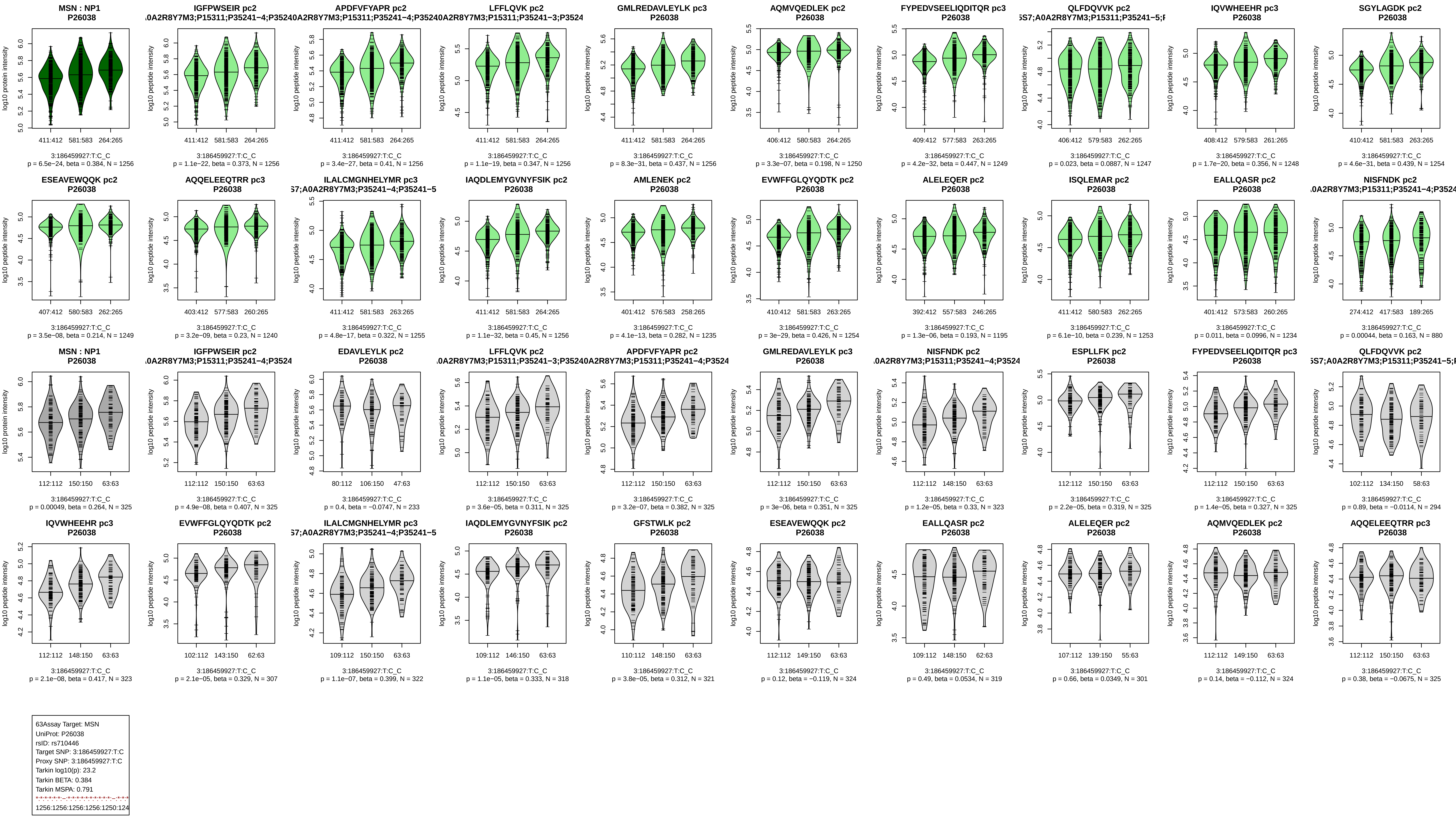

MSN : NP1
P26038
3:186459927:T:C_C
p = 6.5e−24, beta = 0.384, N = 1256
IGFPWSEIR pc2
p = 1.1e−22, beta = 0.373, N = 1256
APDFVFYAPR pc2
p = 3.4e−27, beta = 0.41, N = 1256
LFFLQVK pc2
p = 1.1e−19, beta = 0.347, N = 1256
GMLREDAVLEYLK pc3
p = 8.3e−31, beta = 0.437, N = 1256
AQMVQEDLEK pc2
p = 3.3e−07, beta = 0.198, N = 1250
FYPEDVSEELIQDITQR pc3
p = 4.2e−32, beta = 0.447, N = 1249
QLFDQVVK pc2
p = 0.023, beta = 0.0887, N = 1247
IQVWHEEHR pc3
p = 1.7e−20, beta = 0.356, N = 1248
SGYLAGDK pc2
p = 4.6e−31, beta = 0.439, N = 1254
ESEAVEWQQK pc2
p = 3.5e−08, beta = 0.214, N = 1249
AQQELEEQTRR pc3
p = 3.2e−09, beta = 0.23, N = 1240
ILALCMGNHELYMR pc3
p = 4.8e−17, beta = 0.322, N = 1255
IAQDLEMYGVNYFSIK pc2
p = 1.1e−32, beta = 0.45, N = 1256
AMLENEK pc2
p = 4.1e−13, beta = 0.282, N = 1235
EVWFFGLQYQDTK pc2
p = 3e−29, beta = 0.426, N = 1254
ALELEQER pc2
p = 1.3e−06, beta = 0.193, N = 1195
ISQLEMAR pc2
p = 6.1e−10, beta = 0.239, N = 1253
EALLQASR pc2
p = 0.011, beta = 0.0996, N = 1234
NISFNDK pc2
p = 0.00044, beta = 0.163, N = 880
MSN : NP1
p = 0.00049, beta = 0.264, N = 325
IGFPWSEIR pc2
p = 4.9e−08, beta = 0.407, N = 325
EDAVLEYLK pc2
p = 0.4, beta = −0.0747, N = 233
LFFLQVK pc2
p = 3.6e−05, beta = 0.311, N = 325
APDFVFYAPR pc2
p = 3.2e−07, beta = 0.382, N = 325
GMLREDAVLEYLK pc3
p = 3e−06, beta = 0.351, N = 325
NISFNDK pc2
p = 1.2e−05, beta = 0.33, N = 323
ESPLLFK pc2
p = 2.2e−05, beta = 0.319, N = 325
FYPEDVSEELIQDITQR pc3
p = 1.4e−05, beta = 0.327, N = 325
QLFDQVVK pc2
p = 0.89, beta = −0.0114, N = 294
IQVWHEEHR pc3
p = 2.1e−08, beta = 0.417, N = 323
EVWFFGLQYQDTK pc2
p = 2.1e−05, beta = 0.329, N = 307
ILALCMGNHELYMR pc3
p = 1.1e−07, beta = 0.399, N = 322
IAQDLEMYGVNYFSIK pc2
p = 1.1e−05, beta = 0.333, N = 318
GFSTWLK pc2
p = 3.8e−05, beta = 0.312, N = 321
ESEAVEWQQK pc2
p = 0.12, beta = −0.119, N = 324
EALLQASR pc2
p = 0.49, beta = 0.0534, N = 319
ALELEQER pc2
p = 0.66, beta = 0.0349, N = 301
AQMVQEDLEK pc2
p = 0.14, beta = −0.112, N = 324
AQQELEEQTRR pc3
p = 0.38, beta = −0.0675, N = 325
63Assay Target: MSN
UniProt: P26038
rsID: rs710446
Target SNP: 3:186459927:T:C
Proxy SNP: 3:186459927:T:C
Tarkin log10(p): 23.2
Tarkin BETA: 0.384
Tarkin MSPA: 0.791
1256:1256:1256:1256:1250:124

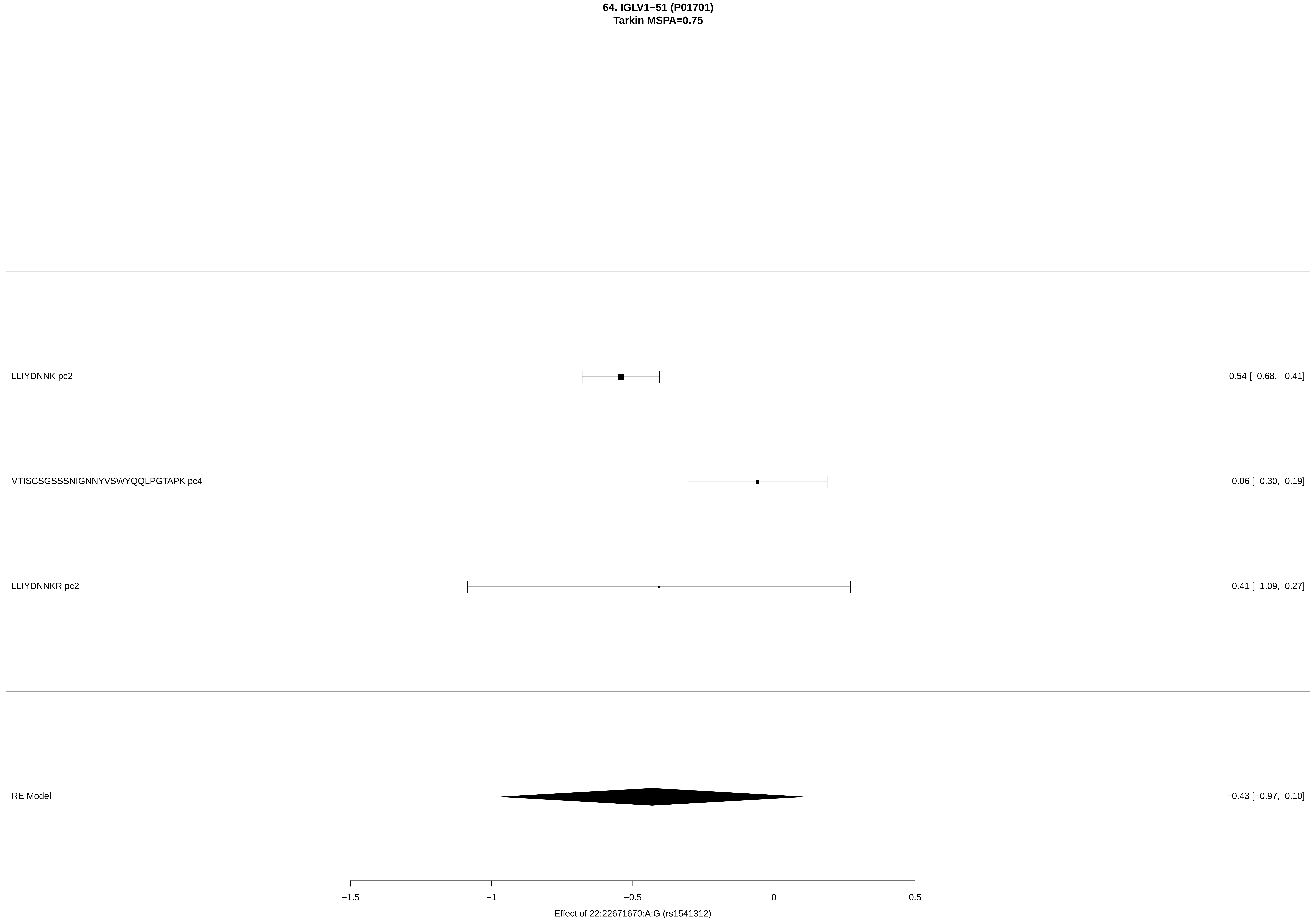

64. IGLV1−51 (P01701)
Tarkin MSPA=0.75
LLIYDNNK pc2
−0.54 [−0.68, −0.41]
VTISCSGSSSNIGNNYVSWYQQLPGTAPK pc4
−0.06 [−0.30, 0.19]
LLIYDNNKR pc2
−0.41 [−1.09, 0.27]
RE Model
−0.43 [−0.97, 0.10]
−1.5
−1
−0.5
0
0.5
Effect of 22:22671670:A:G (rs1541312)

IGLV1−51 : NP3
P01701

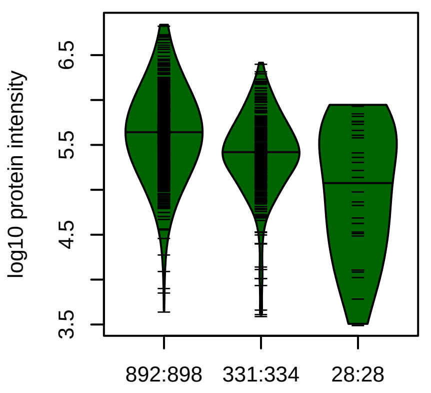

22:22670081:G:A_A
p = 1.7e−23, beta = −0.543, N = 1251

LLIYDNNK pc2
P01701

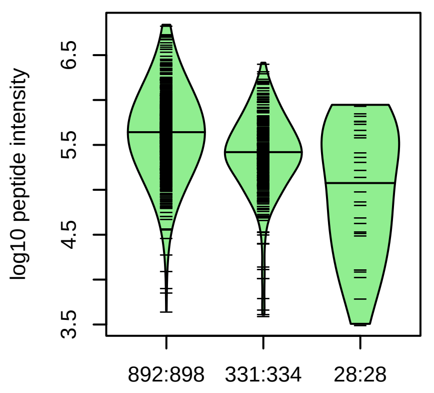

22:22670081:G:A_A
p = 1.7e−23, beta = −0.542, N = 1251

TISCSGSSSNIGNNYVSWYQQLPGTAP
P01701

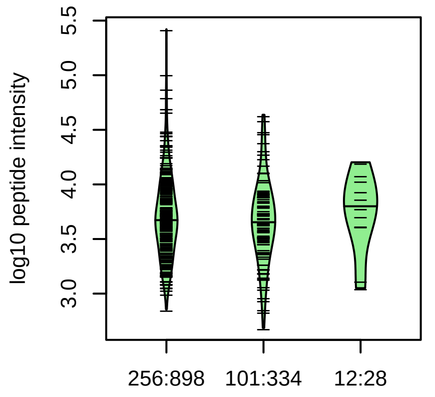

22:22670081:G:A_A
p = 0.54, beta = −0.0581, N = 369

LLIYDNNKR pc2
P01701

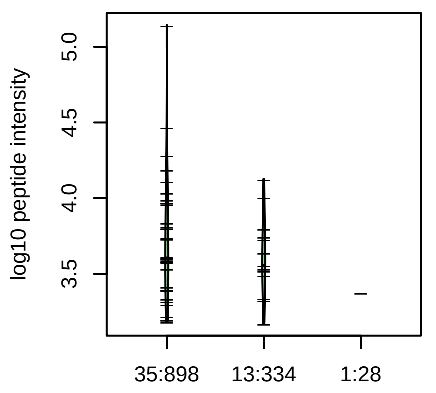

22:22670081:G:A_A
p = 0.13, beta = −0.407, N = 49

IGLV1−51 : NP3
P01701

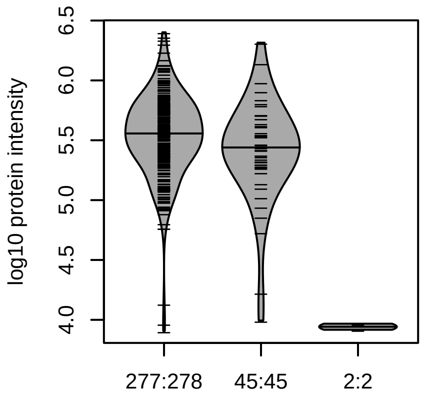

22:22670081:G:A_A
p = 0.0013, beta = −0.468, N = 324

LLIYDNNK pc2
P01701

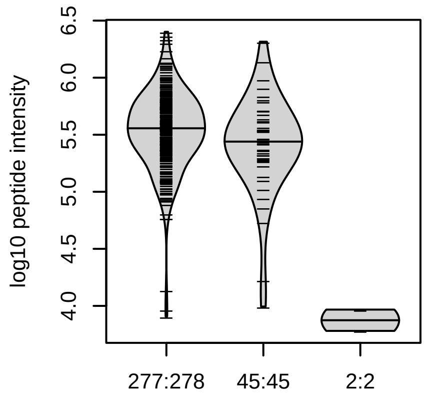

22:22670081:G:A_A
p = 0.0012, beta = −0.469, N = 324

TISCSGSSSNIGNNYVSWYQQLPGTAP
P01701

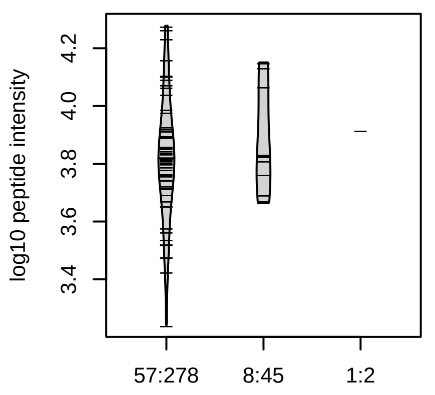

22:22670081:G:A_A
p = 0.48, beta = 0.21, N = 66

64Assay Target: IGLV1−51
UniProt: P01701
rsID: rs1541312
Target SNP: 22:22671670:A:G
Proxy SNP: 22:22670081:G:A
Tarkin log10(p): 23
Tarkin BETA: −0.589
Tarkin MSPA: 0.75
*:−:−
1251:369:49

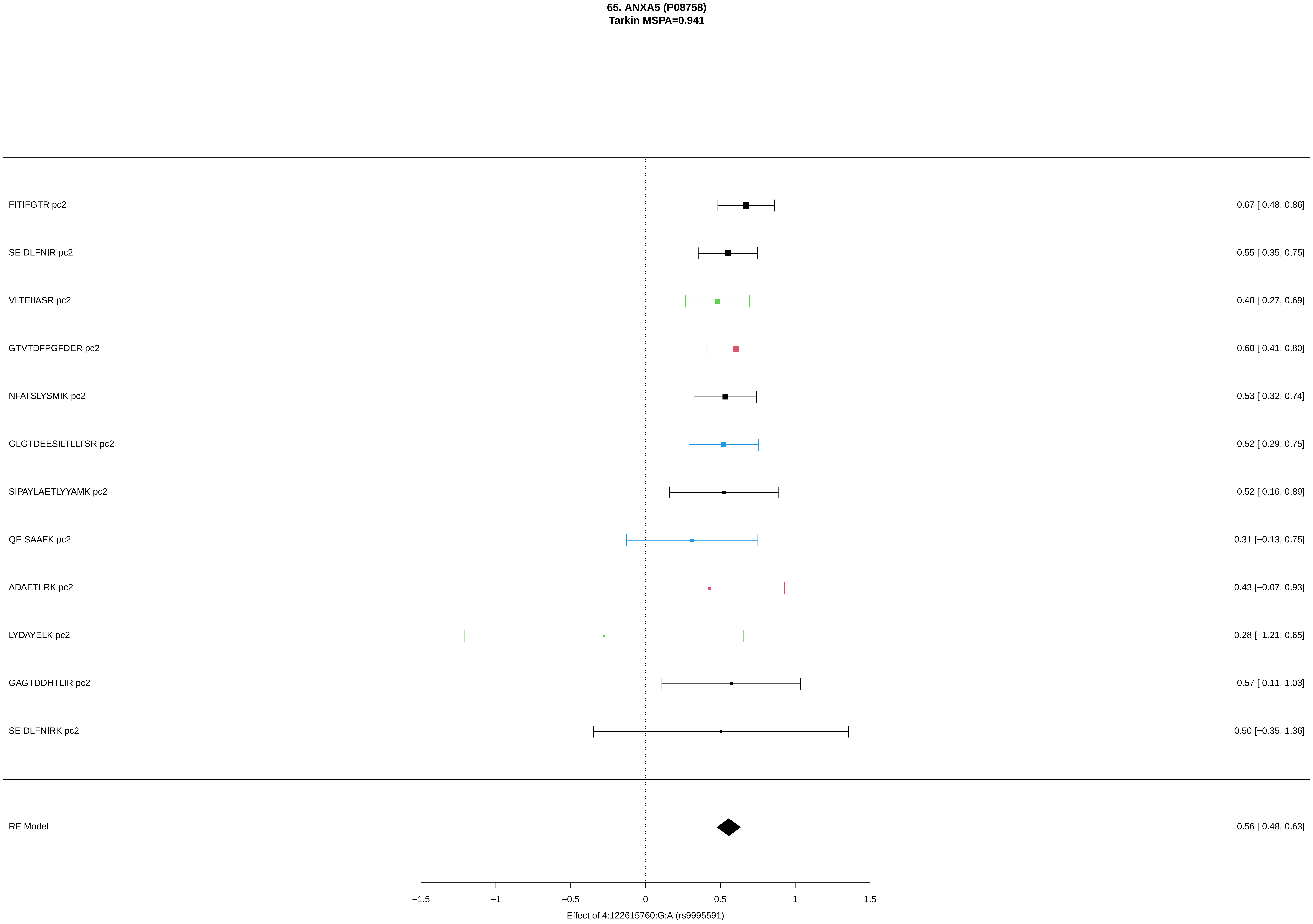
65. ANXA5 (P08758)
Tarkin MSPA=0.941
FITIFGTR pc2
0.67 [0.48, 0.86]
SEIDLFNIR pc2
0.55 [0.35, 0.75]
VLTEIIASR pc2
0.48 [0.27, 0.69]
GTVTDFPGFDER pc2
0.60 [0.41, 0.80]
NFATSLYSMIK pc2
0.53 [0.32, 0.74]
GLGTDEESILTLLTSR pc2
0.52 [0.29, 0.75]
SIPAYLAETLYYAMK pc2
0.52 [0.16, 0.89]
QEISAAFK pc2
0.31 [−0.13, 0.75]
ADAETLRK pc2
0.43 [−0.07, 0.93]
LYDAYELK pc2
−0.28 [−1.21, 0.65]
GAGTDDHTLIR pc2
0.57 [0.11, 1.03]
SEIDLFNIRK pc2
0.50 [−0.35, 1.36]
RE Model
0.56 [0.48, 0.63]
−1.5
−1
−0.5
0
0.5
1
1.5
Effect of 4:122615760:G:A (rs9995591)

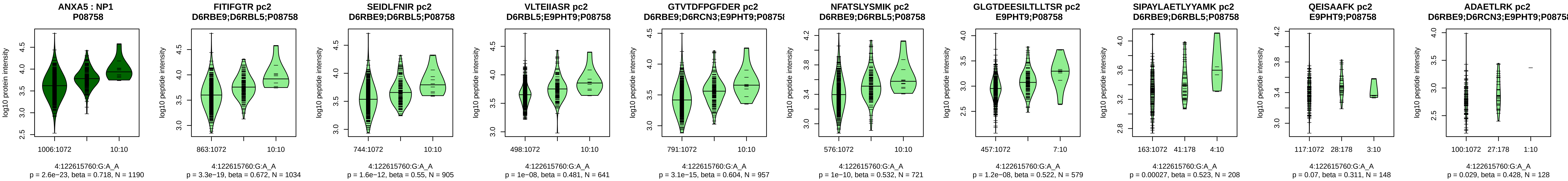

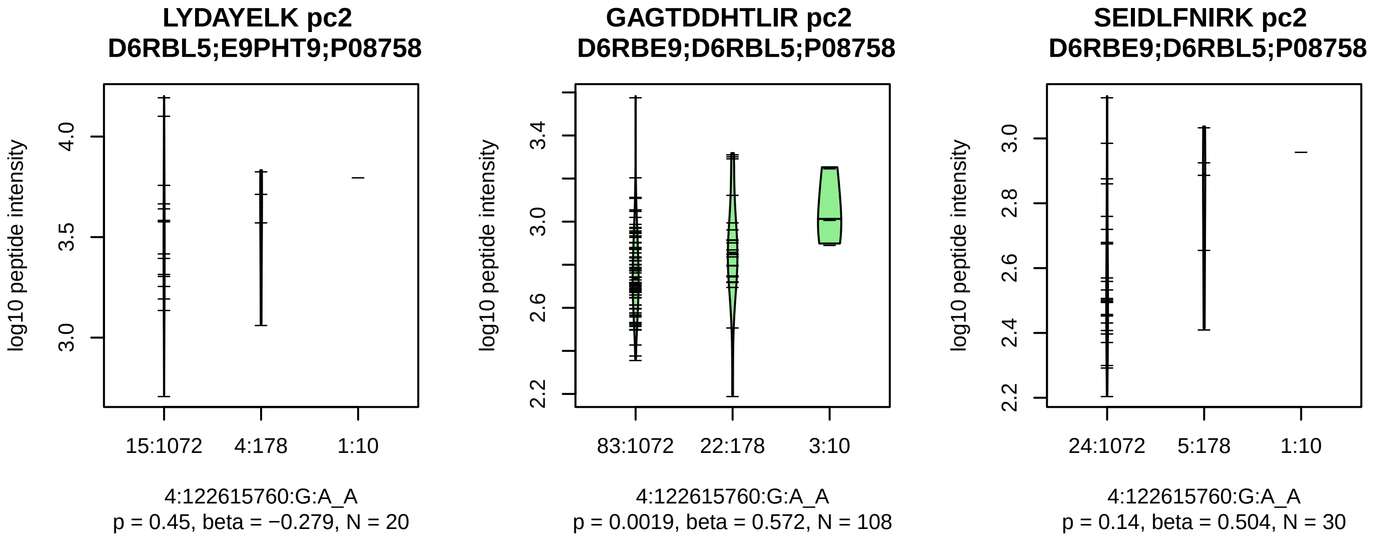

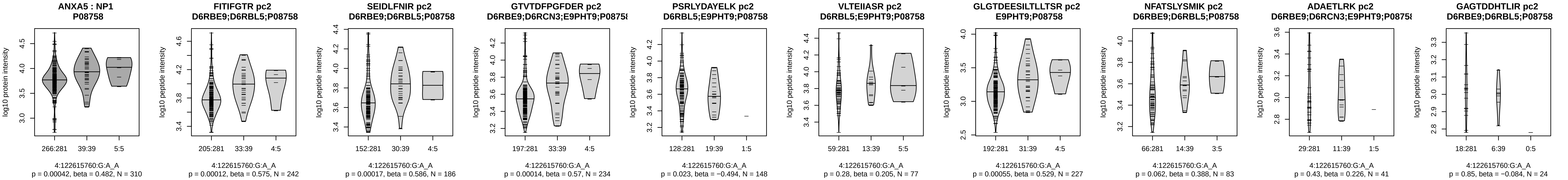

65Assay Target: ANXA5
UniProt: P08758
rsID: rs9995591
Target SNP: 4:122615760:G:A
Proxy SNP: 4:122615760:G:A
Tarkin log10(p): 22.6
Tarkin BETA: 0.718
Tarkin MSPA: 0.941
::*:*:*:*:*:-:-:-:*:-:NA
1034:905:641:957:721:579:208:

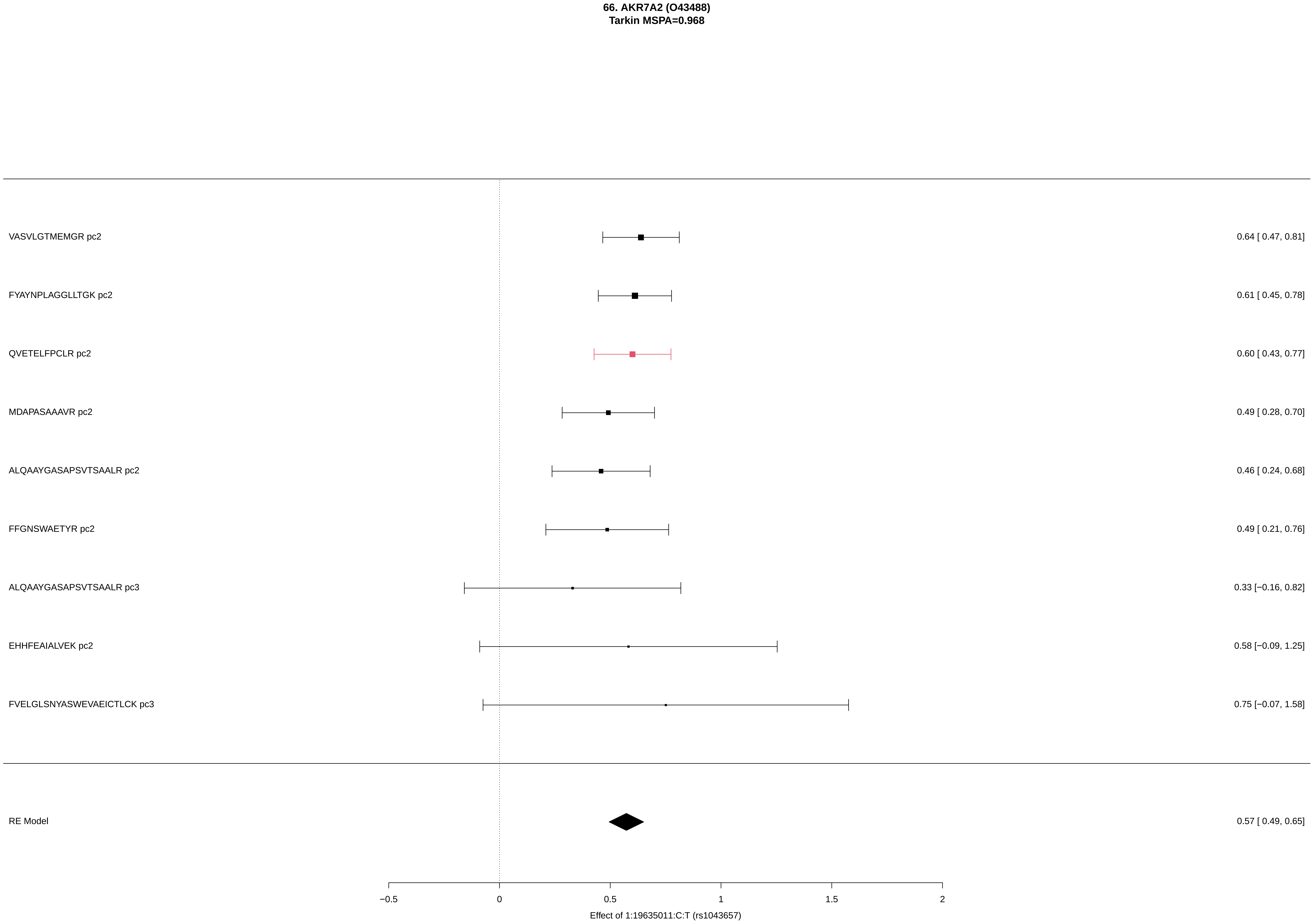
66. AKR7A2 (O43488)
Tarkin MSPA=0.968
VASVLGTMEMGR pc2
0.64 [0.47, 0.81]
FYAYNPLAGGLLTGK pc2
0.61 [0.45, 0.78]
QVETELFPCLR pc2
0.60 [0.43, 0.77]
MDAPASAAAVR pc2
0.49 [0.28, 0.70]
ALQAAYGASAPSVTSAALR pc2
0.46 [0.24, 0.68]
FFGNSWAETYR pc2
0.49 [0.21, 0.76]
ALQAAYGASAPSVTSAALR pc3
0.33 [−0.16, 0.82]
EHHFEAIALVEK pc2
0.58 [−0.09, 1.25]
FVELGLSNYASWEVAEICTLCK pc3
0.75 [−0.07, 1.58]
RE Model
0.57 [0.49, 0.65]
−0.5
0
0.5
1
1.5
2
Effect of 1:19635011:C:T (rs1043657)

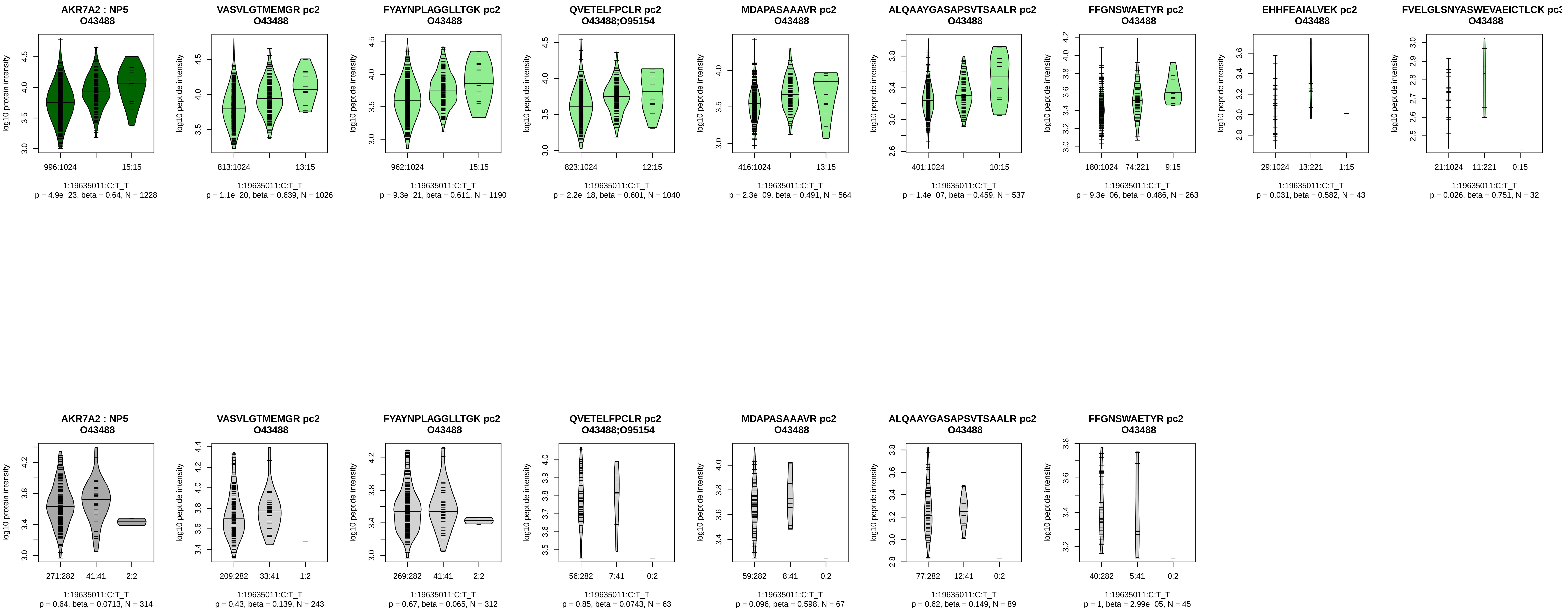

66Assay Target: AKR7A2
UniProt: O43488
rsID: rs1043657
Target SNP: 1:19635011:C:T
Proxy SNP: 1:19635011:C:T
Tarkin log10(p): 22.3
Tarkin BETA: 0.64
Tarkin MSPA: 0.968
::*:*:*:*:–:–:–
1026:1190:1040:564:537:263:78

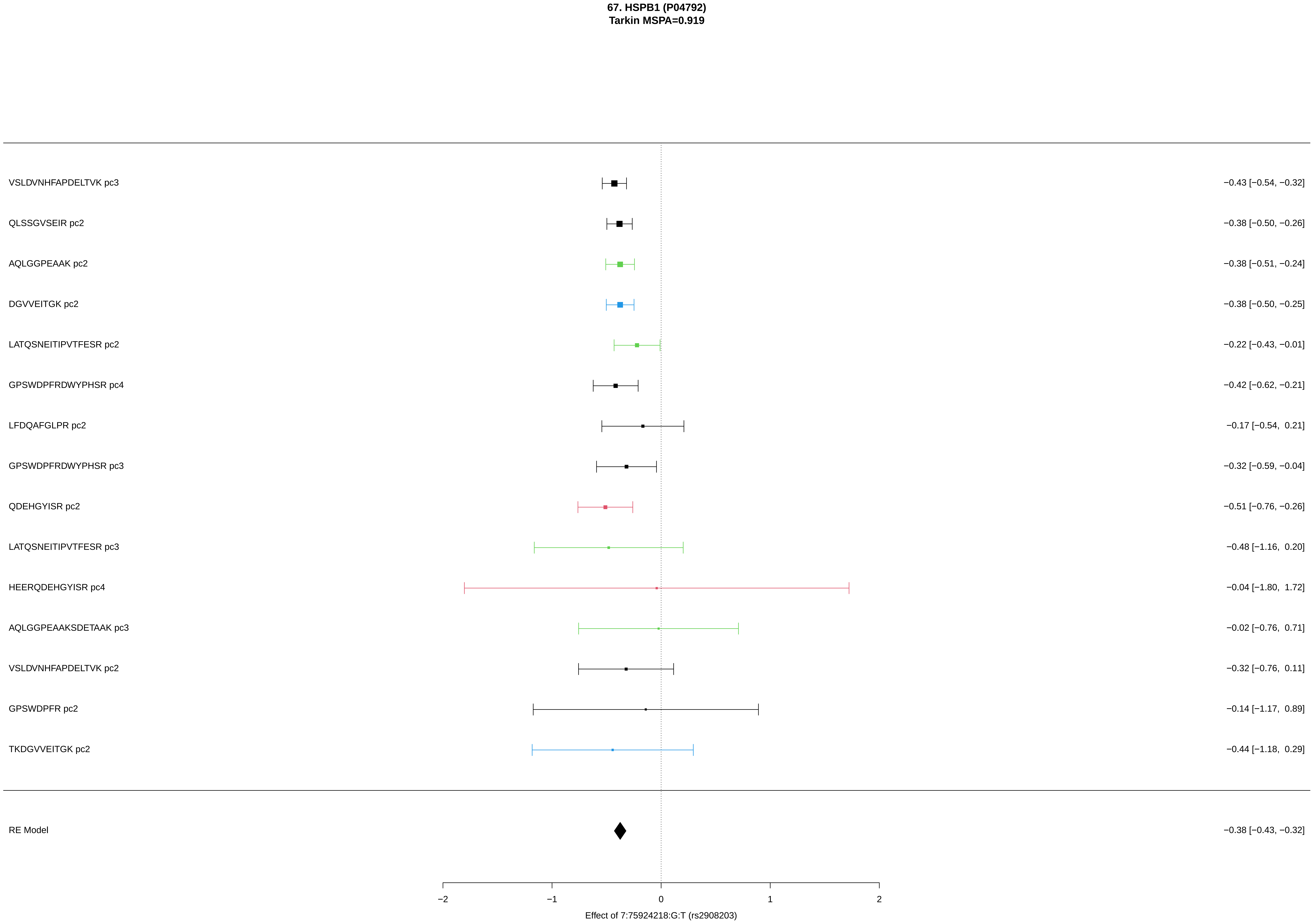
67. HSPB1 (P04792)
Tarkin MSPA=0.919
VSLDVNHFAPDELTVK pc3
−0.43 [−0.54, −0.32]
QLSSGVSEIR pc2
−0.38 [−0.50, −0.26]
AQLGGPEAAK pc2
−0.38 [−0.51, −0.24]
DGVVEITGK pc2
−0.38 [−0.50, −0.25]
LATQSNEITIPVTFESR pc2
−0.22 [−0.43, −0.01]
GPSWDPFRDWYPHSR pc4
−0.42 [−0.62, −0.21]
LFDQAFGLPR pc2
−0.17 [−0.54, 0.21]
GPSWDPFRDWYPHSR pc3
−0.32 [−0.59, −0.04]
QDEHGYISR pc2
−0.51 [−0.76, −0.26]
LATQSNEITIPVTFESR pc3
−0.48 [−1.16, 0.20]
HEERQDEHGYISR pc4
−0.04 [−1.80, 1.72]
AQLGGPEAAKSDETAAK pc3
−0.02 [−0.76, 0.71]
VSLDVNHFAPDELTVK pc2
−0.32 [−0.76, 0.11]
GPSWDPFR pc2
−0.14 [−1.17, 0.89]
TKDGVVEITGK pc2
−0.44 [−1.18, 0.29]
RE Model
−0.38 [−0.43, −0.32]
−2
−1
0
1
2
Effect of 7:75924218:G:T (rs2908203)

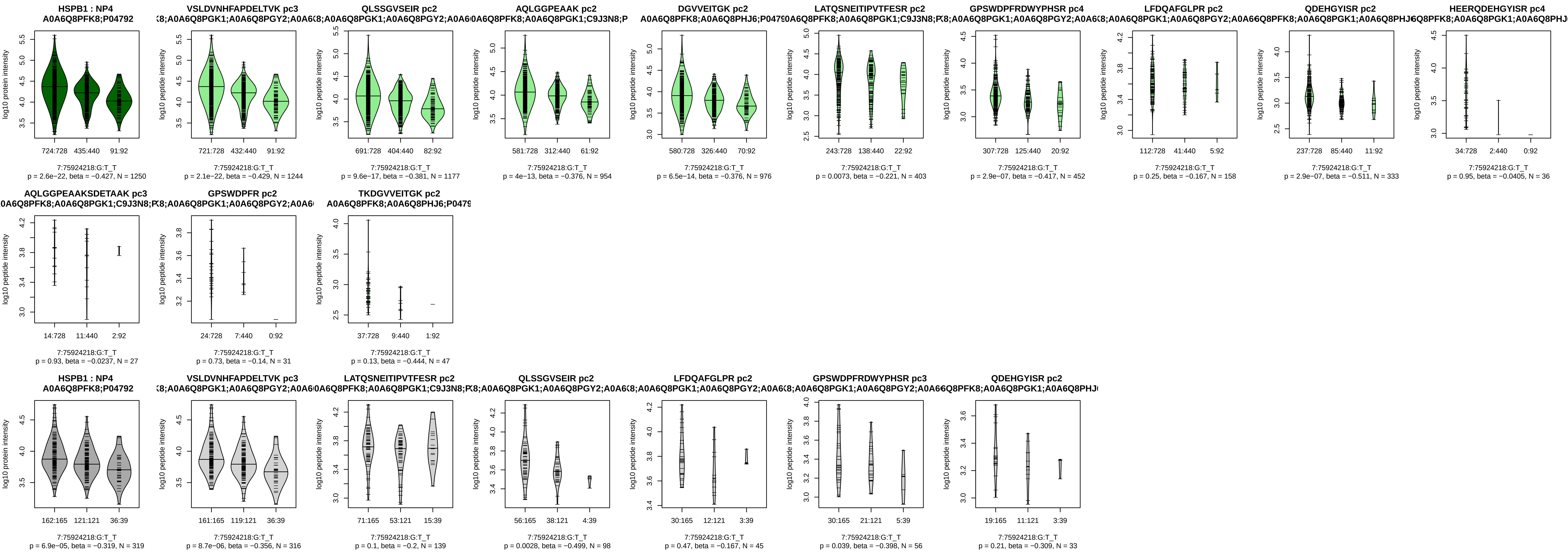

67Assay Target: HSPB1
UniProt: P04792
rsID: rs2908203
Target SNP: 7:75924218:G:T
Proxy SNP: 7:75924218:G:T
Tarkin log10(p): 21.6
Tarkin BETA: −0.427
Tarkin MSPA: 0.919
::*:*:*:*:−:*:*:−:−:−:−:−:−:NA:NA
1244:1177:954:976:403:452:158

68. ITIH2 (P19823)
Tarkin MSPA=0.871

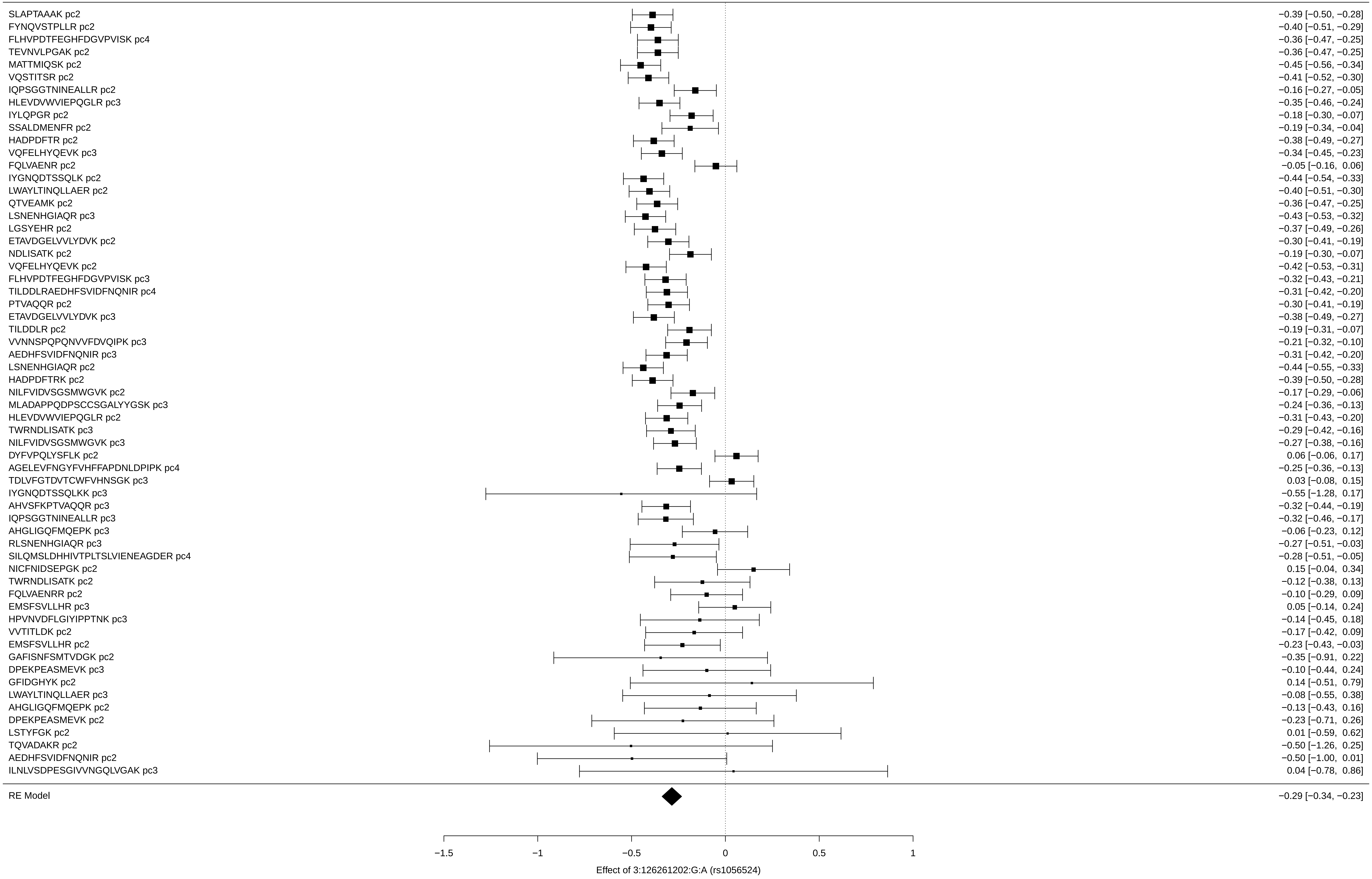

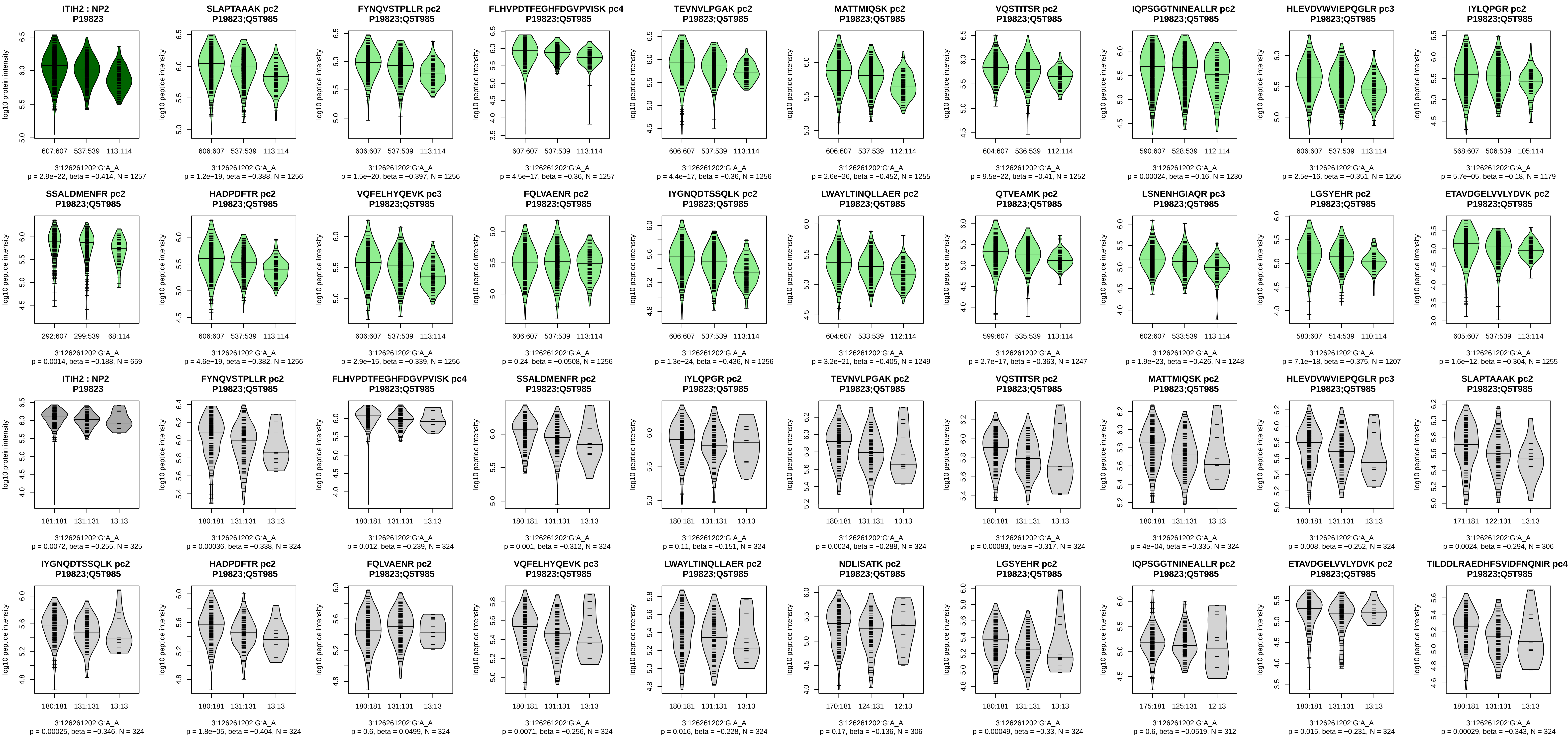

68Assay Target: ITIH2
UniProt: P19823
rsID: rs1056524
Target SNP: 3:126261202:G:A
Proxy SNP: 3:126261202:G:A
Tarkin log10(p): 21.5
Tarkin BETA: −0.414
Tarkin MSPA: 0.871
1256:1256:1257:1256:1255:125

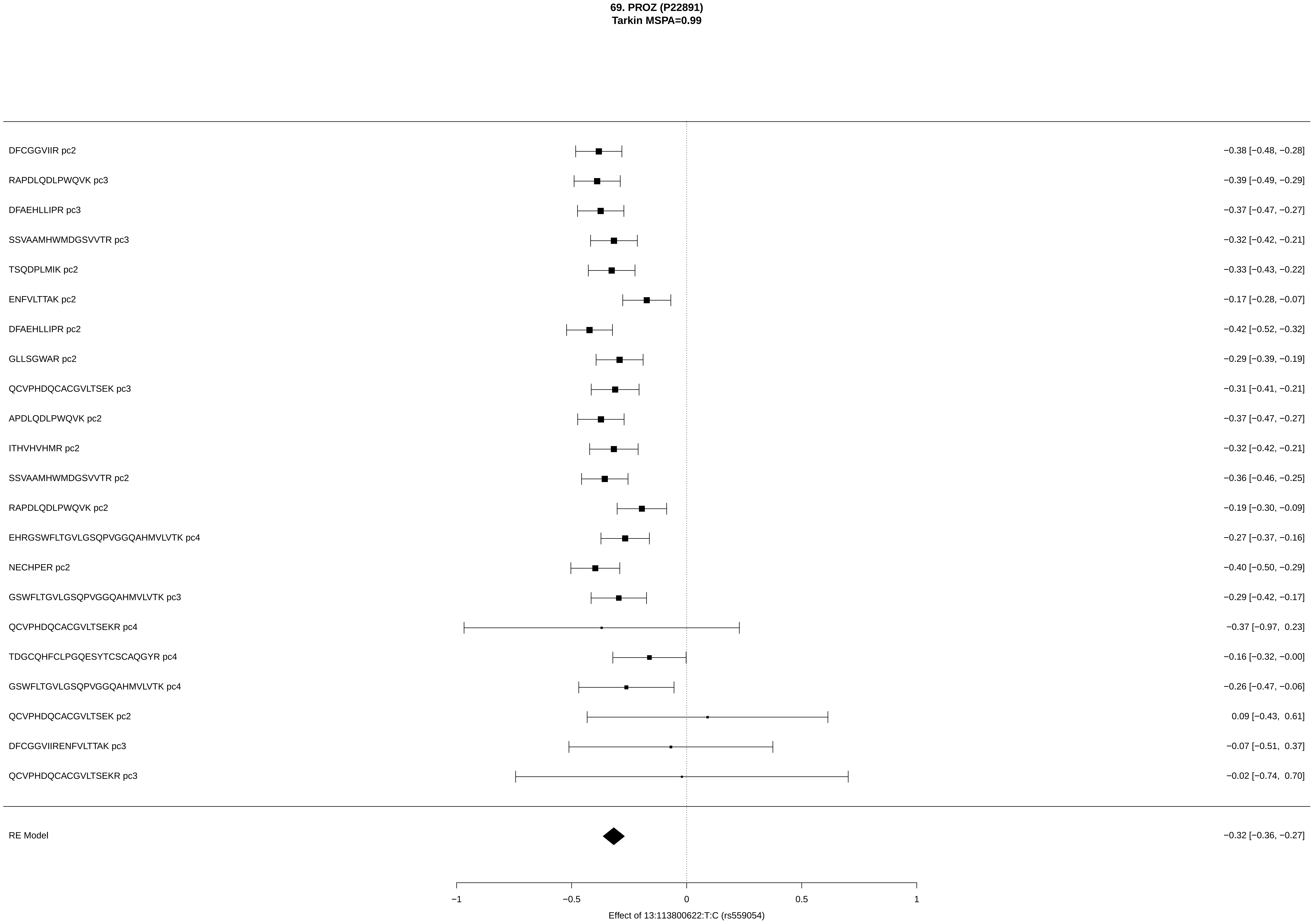

69. PROZ (P22891)
Tarkin MSPA=0.99
DFCGGVIIR pc2
RAPDLQDLPWQVK pc3
DFAEHLLIPR pc3
SSVAAMHWMDGSVVTR pc3
TSQDPLMIK pc2
ENFVLTTAK pc2
DFAEHLLIPR pc2
GLLSGWAR pc2
QCVPHDQCACGVLTSEK pc3
APDLQDLPWQVK pc2
ITHVHVHMR pc2
SSVAAMHWMDGSVVTR pc2
RAPDLQDLPWQVK pc2
EHRGSWFLTGVLGSQPVGGQAHMVLVTK pc4
NECHPER pc2
GSWFLTGVLGSQPVGGQAHMVLVTK pc3
QCVPHDQCACGVLTSEKR pc4
TDGCQHFCLPGQESYTCSCAQGYR pc4
GSWFLTGVLGSQPVGGQAHMVLVTK pc4
QCVPHDQCACGVLTSEK pc2
DFCGGVIIRENFVLTTAK pc3
QCVPHDQCACGVLTSEKR pc3
RE Model
−0.38 [−0.48, −0.28]
−0.39 [−0.49, −0.29]
−0.37 [−0.47, −0.27]
−0.32 [−0.42, −0.21]
−0.33 [−0.43, −0.22]
−0.17 [−0.28, −0.07]
−0.42 [−0.52, −0.32]
−0.29 [−0.39, −0.19]
−0.31 [−0.41, −0.21]
−0.37 [−0.47, −0.27]
−0.32 [−0.42, −0.21]
−0.36 [−0.46, −0.25]
−0.19 [−0.30, −0.09]
−0.27 [−0.37, −0.16]
−0.40 [−0.50, −0.29]
−0.29 [−0.42, −0.17]
−0.37 [−0.97, 0.23]
−0.16 [−0.32, −0.00]
−0.26 [−0.47, −0.06]
0.09 [−0.43, 0.61]
−0.07 [−0.51, 0.37]
−0.02 [−0.74, 0.70]
−0.32 [−0.36, −0.27]
−1
−0.5
0
0.5
1
Effect of 13:113800622:T:C (rs559054)

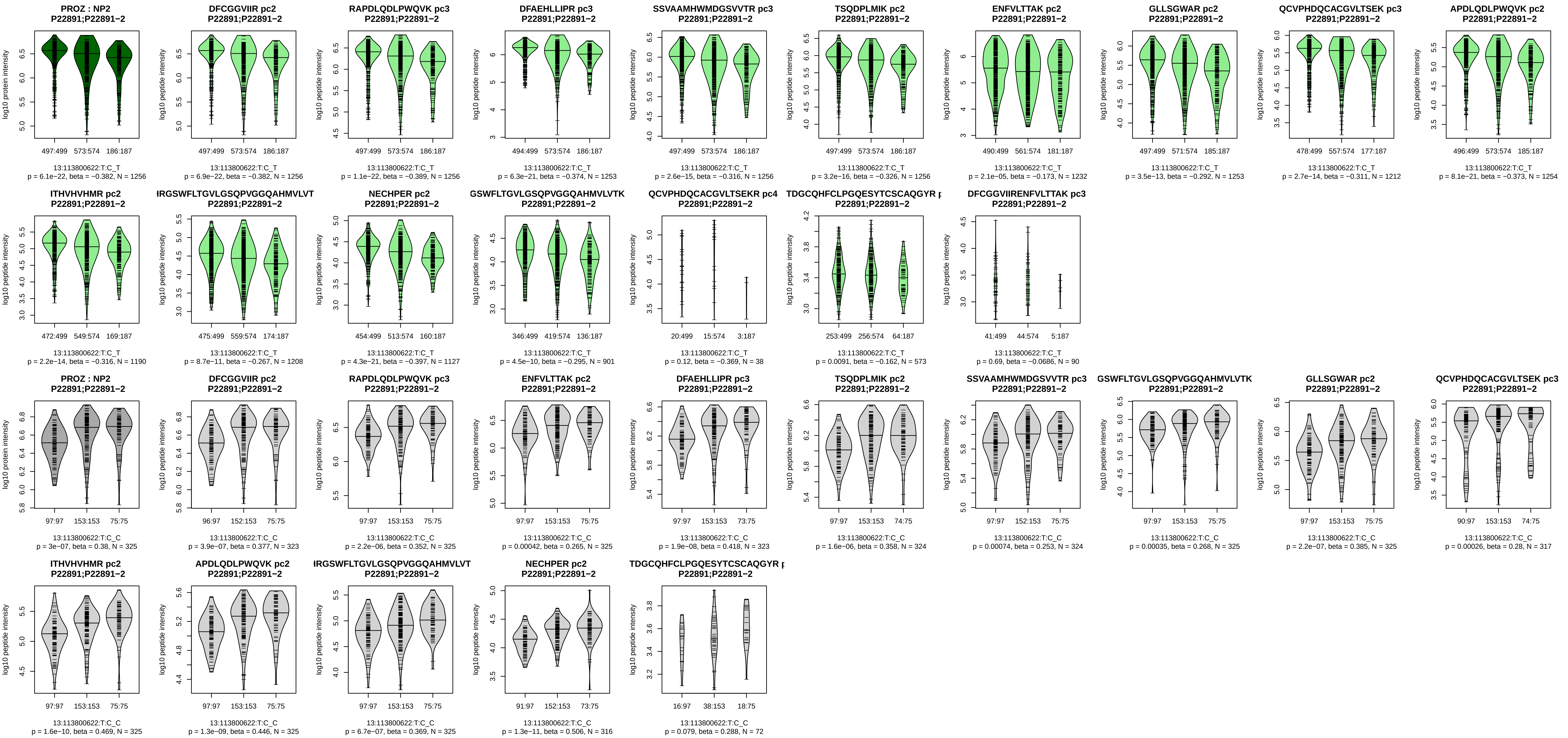

69Assay Target: PROZ
UniProt: P22891
rsID: rs559054
Target SNP: 13:113800622:T:C
Proxy SNP: 13:113800622:T:C
Tarkin log10(p): 21.2
Tarkin BETA: −0.382
Tarkin MSPA: 0.99
1256:1256:1253:1256:1256:123

70. CSTF3 (Q12996)
Tarkin MSPA=1

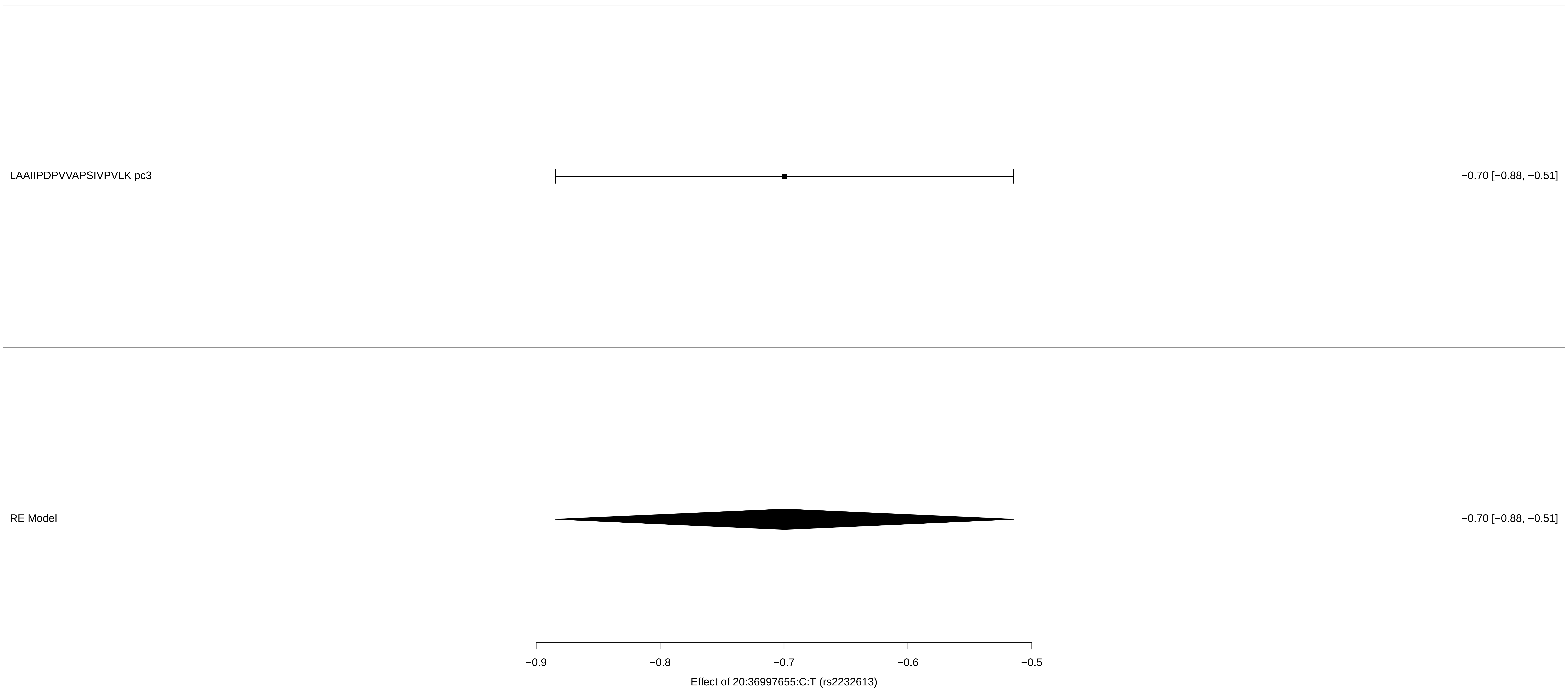

CSTF3 : NP4
Q12996

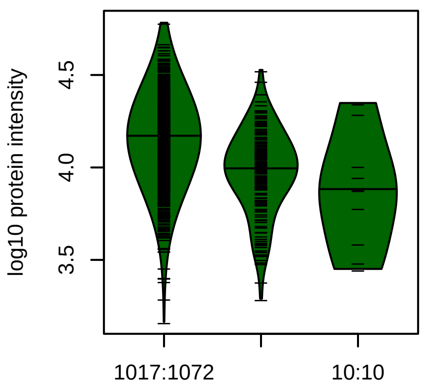

20:36997655:C:T_T
p = 1.1e−21, beta = −0.7, N = 1195

LAAIIPDPVVAPSIVPVLK pc3
E9PLP8;Q12996

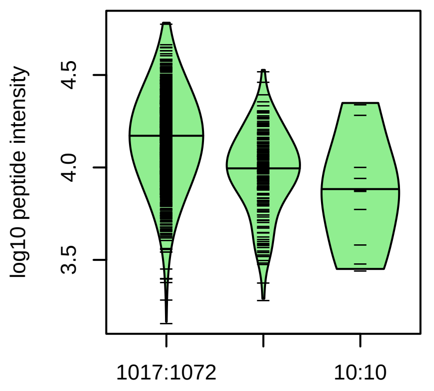

20:36997655:C:T_T
p = 1.1e−21, beta = −0.7, N = 1195

70Assay Target: CSTF3
UniProt: Q12996
rsID: rs2232613
Target SNP: 20:36997655:C:T
Proxy SNP: 20:36997655:C:T
Tarkin log10(p): 20.9
Tarkin BETA: −0.7
Tarkin MSPA: 1
*
1195

71. SERPING1 (P05155)
Tarkin MSPA=0.878

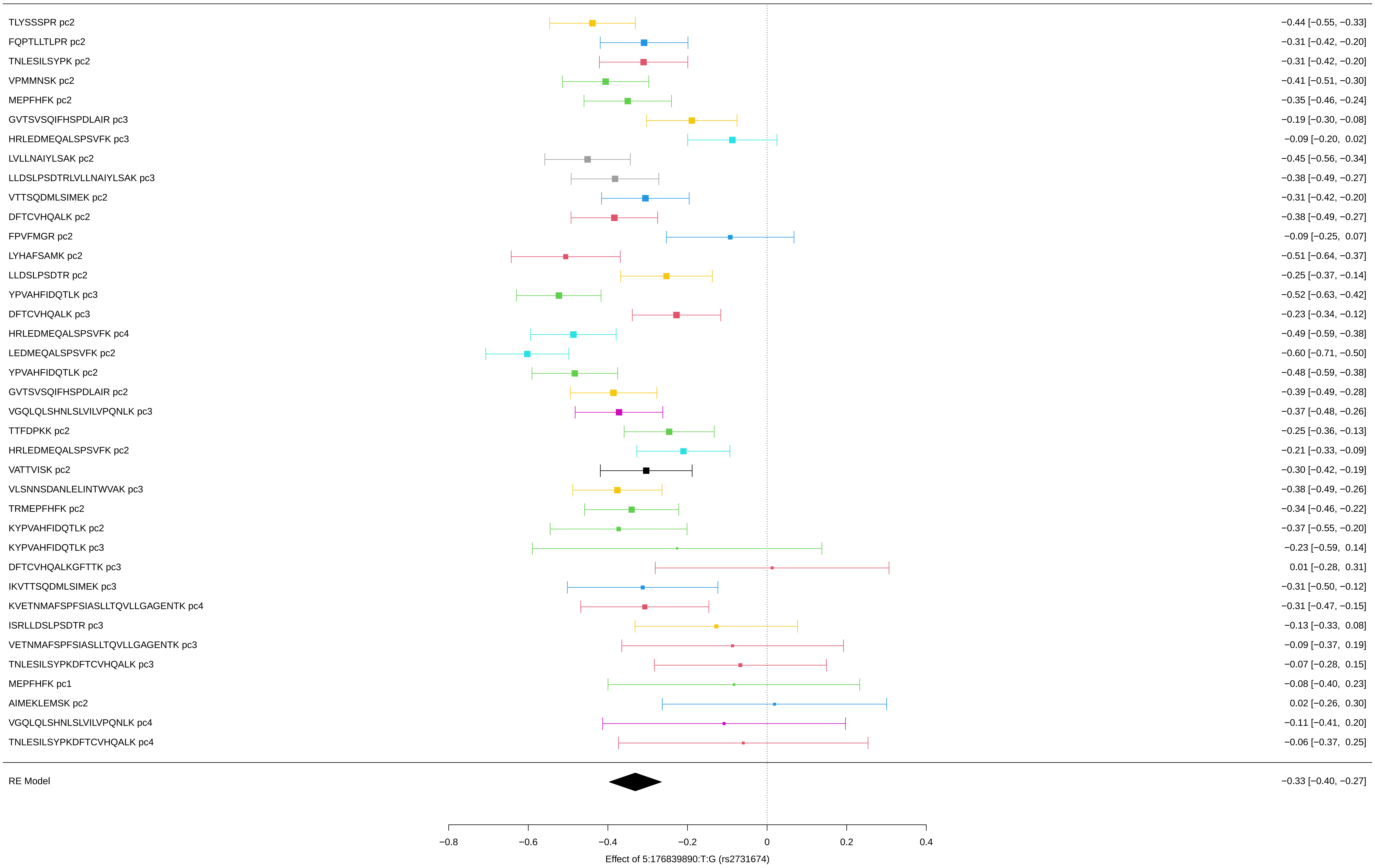

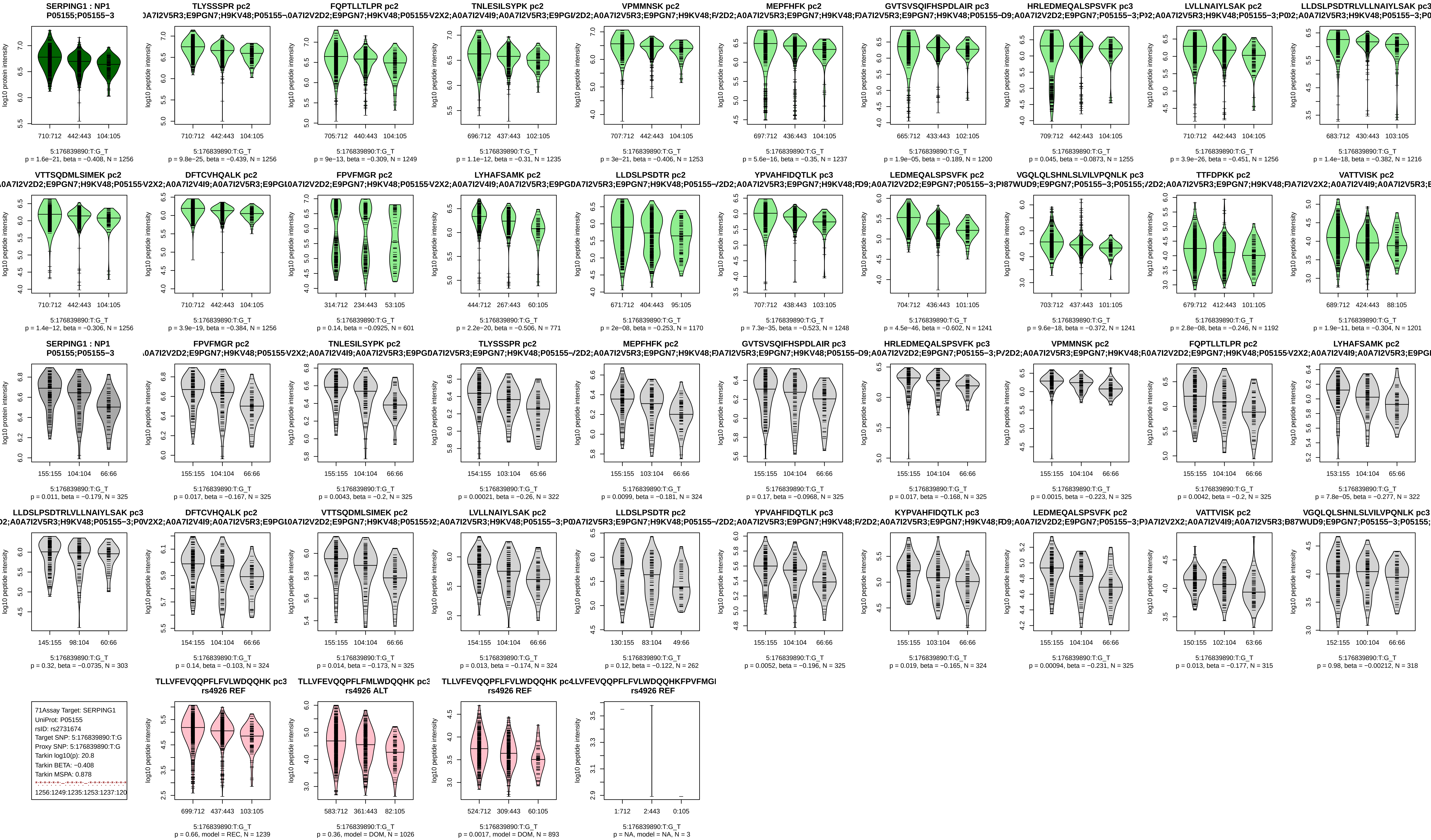
71Assay Target: SERPING1
UniProt: P05155
rsID: rs2731674
Target SNP: 5:176839890:T:G
Proxy SNP: 5:176839890:T:G
Tarkin log10(p): 20.8
Tarkin BETA: −0.408
Tarkin MSPA: 0.878

72. S100B (P04271)
Tarkin MSPA=1

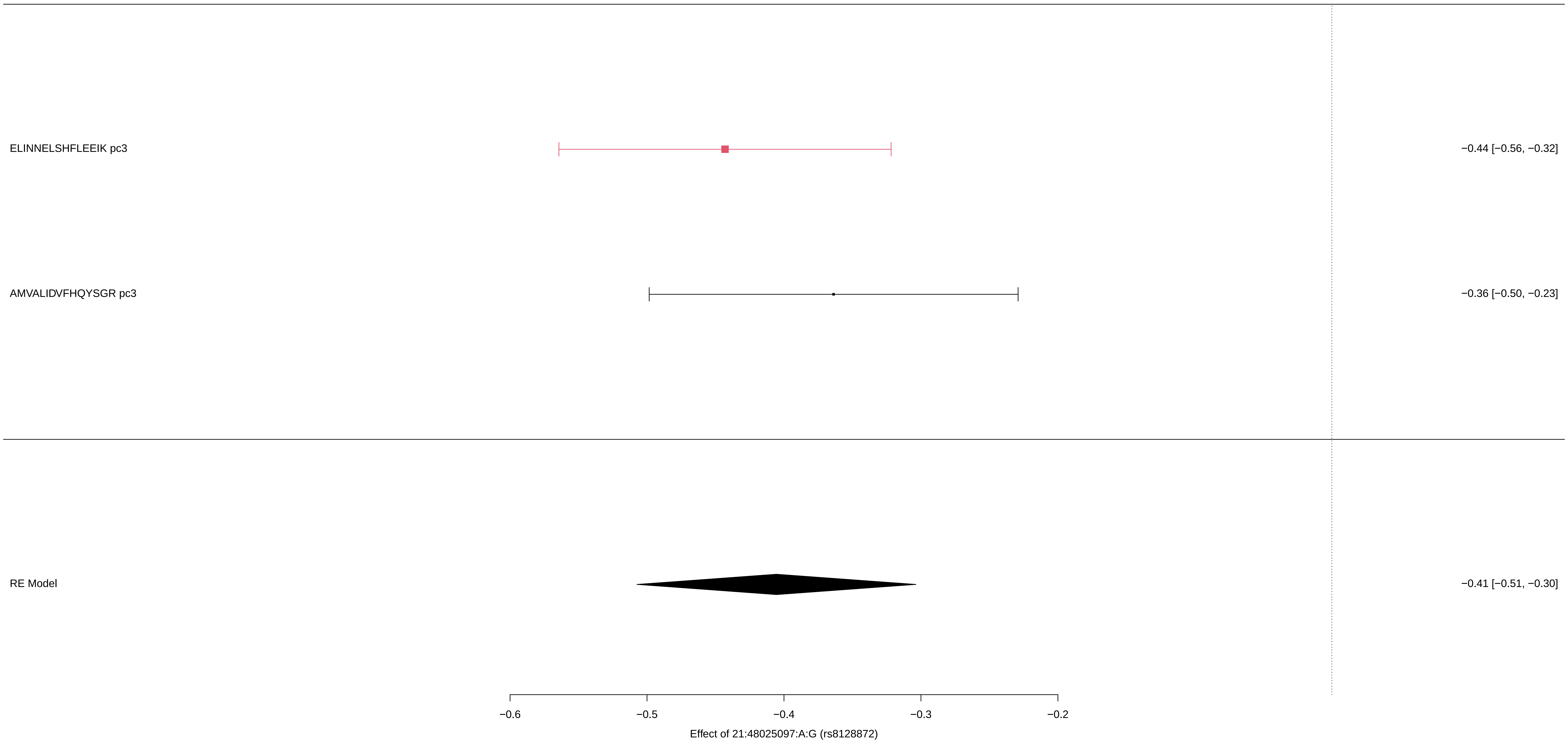

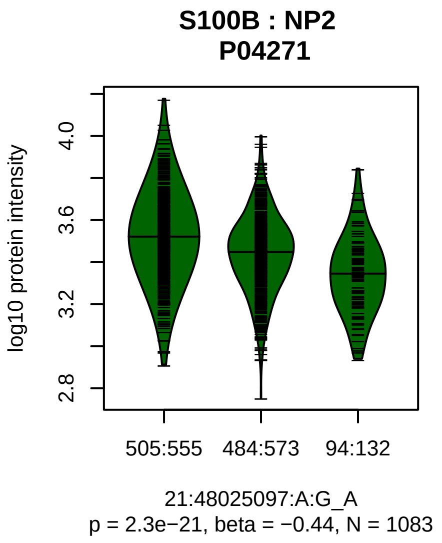

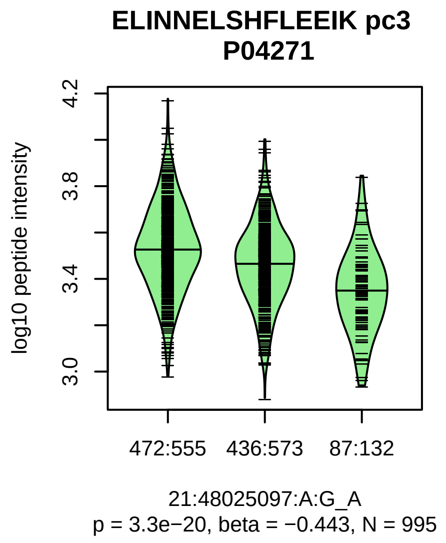

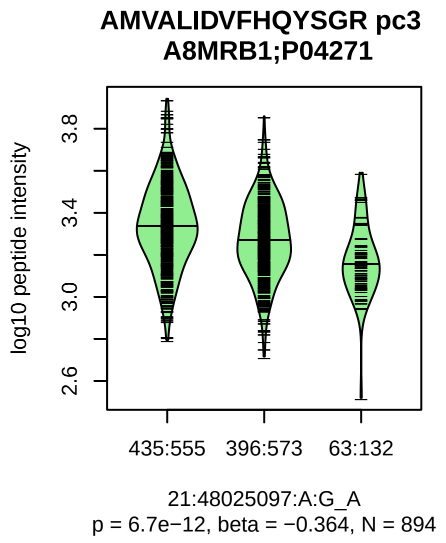

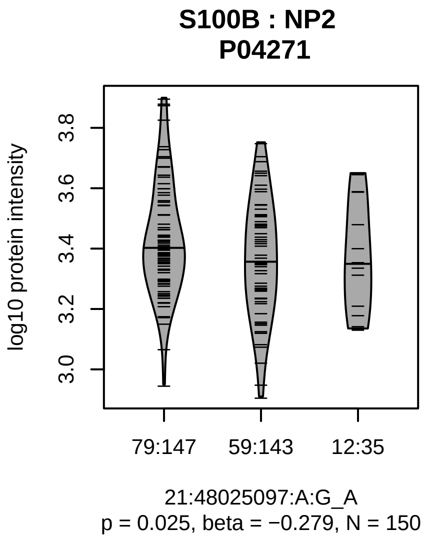

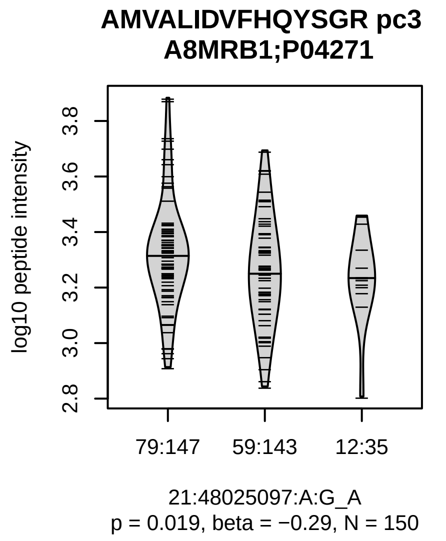

72Assay Target: S100B
UniProt: P04271
rsID: rs8128872
Target SNP: 21:48025097:A:G
Proxy SNP: 21:48025097:A:G
Tarkin log10(p): 20.6
Tarkin BETA: −0.44
Tarkin MSPA: 1
:
995:894

73. LPA (P08519)
Tarkin MSPA=0.859

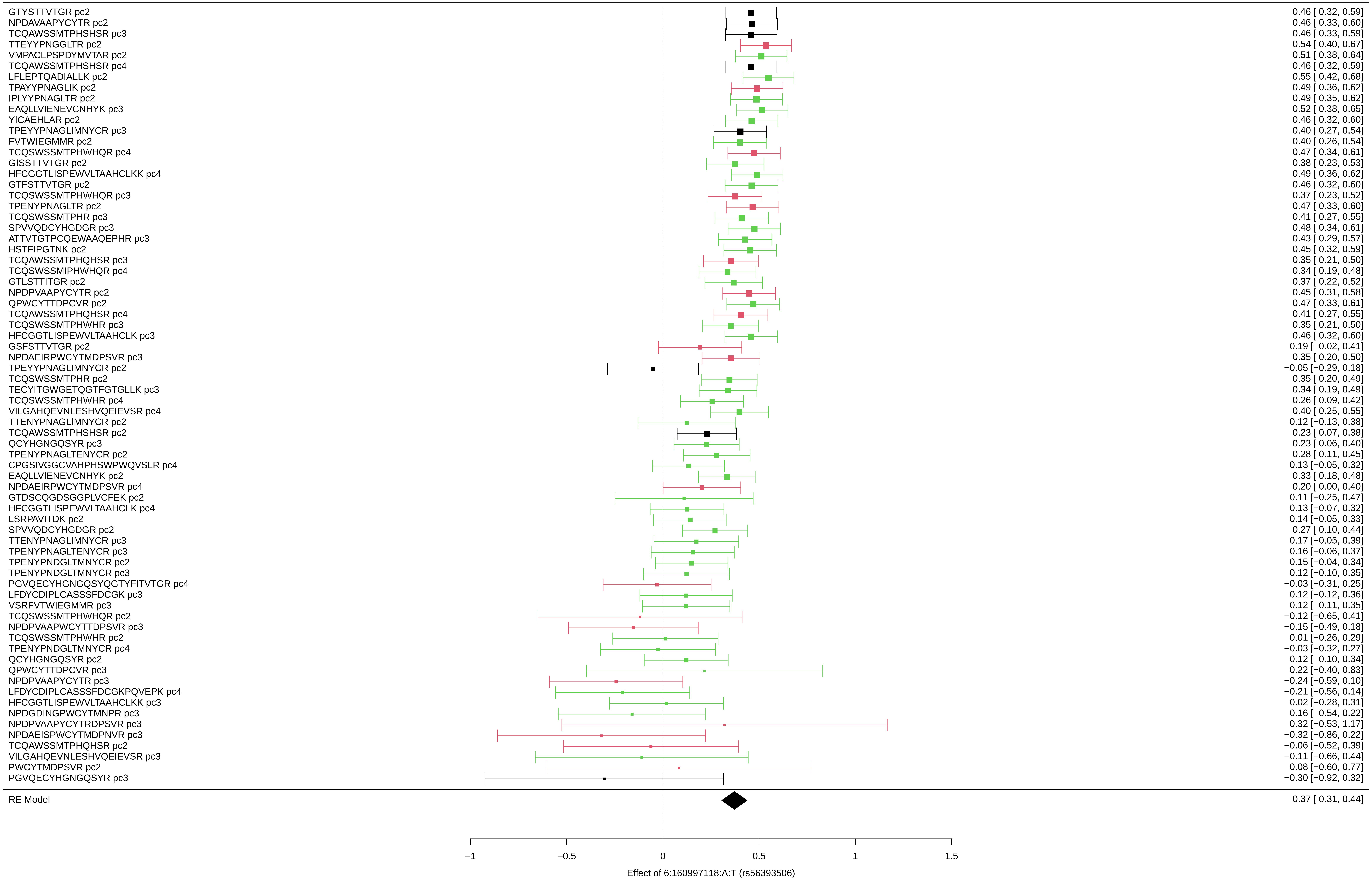

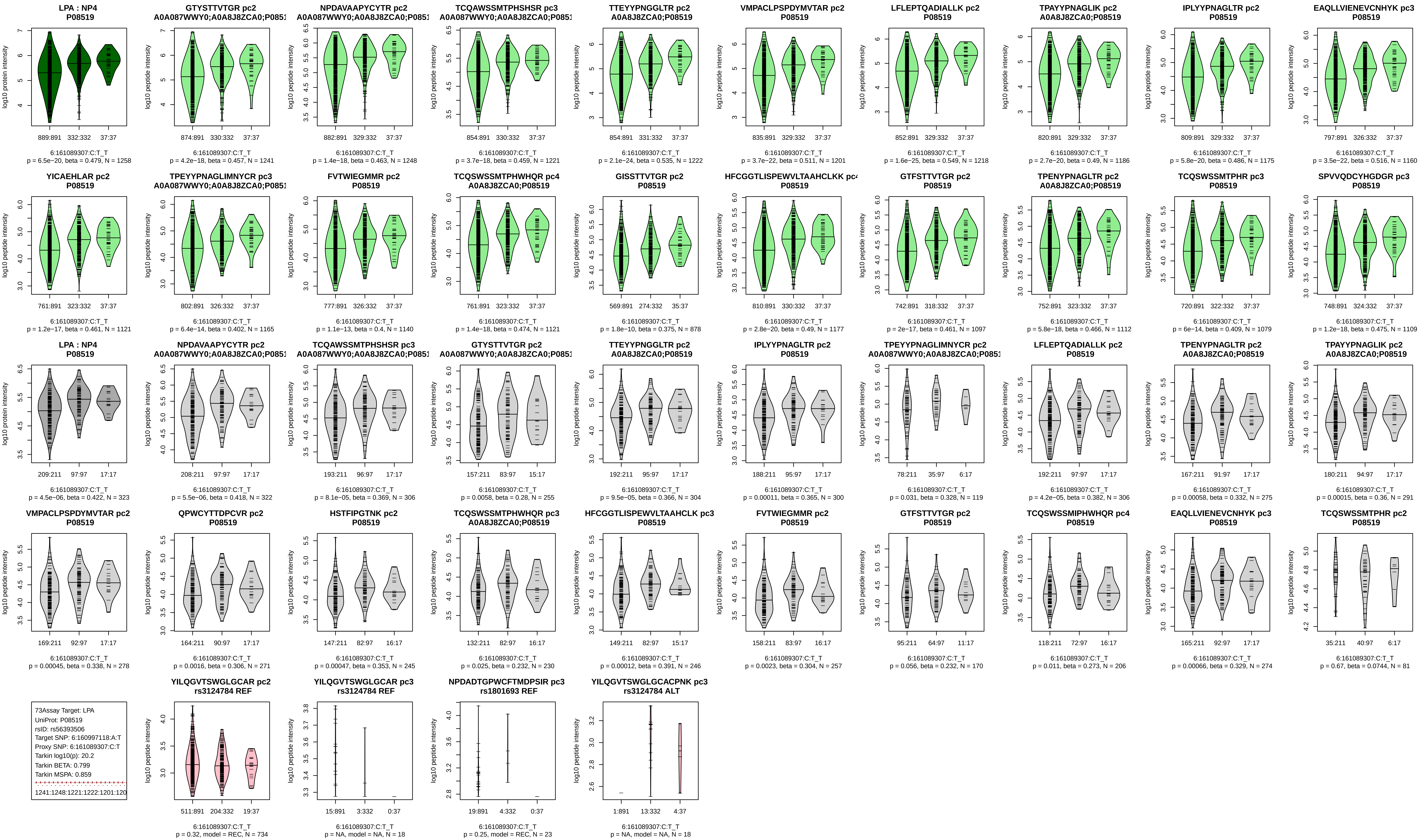

LPA : NP4
P08519
889:891 332:332 37:37
6:161089307:C:T_T
p = 6.5e−20, beta = 0.479, N = 1258
GTYSTTVTGR pc2
A0A087WWY0;A0A8J8ZCA0;P0851
874:891 330:332 37:37
6:161089307:C:T_T
p = 4.2e−18, beta = 0.457, N = 1241
NPDAVAAPYCYTR pc2
A0A087WWY0;A0A8J8ZCA0;P0851
882:891 329:332 37:37
6:161089307:C:T_T
p = 1.4e−18, beta = 0.463, N = 1248
TCQAWSSMTPHSHSR pc3
A0A087WWY0;A0A8J8ZCA0;P0851
854:891 330:332 37:37
6:161089307:C:T_T
p = 3.7e−18, beta = 0.459, N = 1221
TTEYYPNGGLTR pc2
A0A8J8ZCA0;P08519
854:891 331:332 37:37
6:161089307:C:T_T
p = 2.1e−24, beta = 0.535, N = 1222
VMPACLPSPDYMVTAR pc2
P08519
835:891 329:332 37:37
6:161089307:C:T_T
p = 3.7e−22, beta = 0.511, N = 1201
LFLEPTQADIALLK pc2
P08519
852:891 329:332 37:37
6:161089307:C:T_T
p = 1.6e−25, beta = 0.549, N = 1218
TPAYYPNAGLIK pc2
A0A8J8ZCA0;P08519
820:891 329:332 37:37
6:161089307:C:T_T
p = 2.7e−20, beta = 0.49, N = 1186
IPLYYPNAGLTR pc2
P08519
809:891 329:332 37:37
6:161089307:C:T_T
p = 5.8e−20, beta = 0.486, N = 1175
EAQLLVIENEVCNHYK pc3
P08519
797:891 326:332 37:37
6:161089307:C:T_T
p = 3.5e−22, beta = 0.516, N = 1160
YICAEHLAR pc2
P08519
761:891 323:332 37:37
6:161089307:C:T_T
p = 1.2e−17, beta = 0.461, N = 1121
TPEYYPNAGLIMNYCR pc3
A0A087WWY0;A0A8J8ZCA0;P0851
802:891 326:332 37:37
6:161089307:C:T_T
p = 6.4e−14, beta = 0.402, N = 1165
FVTWIEGMMR pc2
P08519
777:891 326:332 37:37
6:161089307:C:T_T
p = 1.1e−13, beta = 0.4, N = 1140
TCQSWSSMTPHWHQR pc4
A0A8J8ZCA0;P08519
761:891 323:332 37:37
6:161089307:C:T_T
p = 1.4e−18, beta = 0.474, N = 1121
GISSTTVTGR pc2
P08519
569:891 274:332 35:37
6:161089307:C:T_T
p = 1.8e−10, beta = 0.375, N = 878
HFCGGTLISPEWVLTAAHCLKK pc4
P08519
810:891 330:332 37:37
6:161089307:C:T_T
p = 2.8e−20, beta = 0.49, N = 1177
GTFSTTVTGR pc2
P08519
742:891 318:332 37:37
6:161089307:C:T_T
p = 2e−17, beta = 0.461, N = 1097
TPENYPNAGLTR pc2
A0A8J8ZCA0;P08519
752:891 323:332 37:37
6:161089307:C:T_T
p = 5.8e−18, beta = 0.466, N = 1112
TCQSWSSMTPHR pc3
P08519
720:891 322:332 37:37
6:161089307:C:T_T
p = 6e−14, beta = 0.409, N = 1079
SPVVQDCYHGDGR pc3
P08519
748:891 324:332 37:37
6:161089307:C:T_T
p = 1.2e−18, beta = 0.475, N = 1109
LPA : NP4
P08519
209:211 97:97 17:17
6:161089307:C:T_T
p = 4.5e−06, beta = 0.422, N = 323
NPDAVAAPYCYTR pc2
A0A087WWY0;A0A8J8ZCA0;P0851
208:211 97:97 17:17
6:161089307:C:T_T
p = 5.5e−06, beta = 0.418, N = 322
TCQAWSSMTPHSHSR pc3
A0A087WWY0;A0A8J8ZCA0;P0851
193:211 96:97 17:17
6:161089307:C:T_T
p = 8.1e−05, beta = 0.369, N = 306
GTYSTTVTGR pc2
A0A087WWY0;A0A8J8ZCA0;P0851
157:211 83:97 15:17
6:161089307:C:T_T
p = 0.0058, beta = 0.28, N = 255
TTEYYPNGGLTR pc2
A0A8J8ZCA0;P08519
192:211 95:97 17:17
6:161089307:C:T_T
p = 9.5e−05, beta = 0.366, N = 304
IPLYYPNAGLTR pc2
P08519
188:211 95:97 17:17
6:161089307:C:T_T
p = 0.00011, beta = 0.365, N = 300
TPEYYPNAGLIMNYCR pc2
A0A087WWY0;A0A8J8ZCA0;P0851
78:211 35:97 6:17
6:161089307:C:T_T
p = 0.031, beta = 0.328, N = 119
LFLEPTQADIALLK pc2
P08519
192:211 97:97 17:17
6:161089307:C:T_T
p = 4.2e−05, beta = 0.382, N = 306
TPENYPNAGLTR pc2
A0A8J8ZCA0;P08519
167:211 91:97 17:17
6:161089307:C:T_T
p = 0.00058, beta = 0.332, N = 275
TPAYYPNAGLIK pc2
A0A8J8ZCA0;P08519
180:211 94:97 17:17
6:161089307:C:T_T
p = 0.00015, beta = 0.36, N = 291
VMPACLPSPDYMVTAR pc2
P08519
169:211 92:97 17:17
6:161089307:C:T_T
p = 0.00045, beta = 0.338, N = 278
QPWCYTTDPCVR pc2
P08519
164:211 90:97 17:17
6:161089307:C:T_T
p = 0.0016, beta = 0.306, N = 271
HSTFIPGTNK pc2
P08519
147:211 82:97 16:17
6:161089307:C:T_T
p = 0.00047, beta = 0.353, N = 245
TCQSWSSMTPHWHQR pc3
A0A8J8ZCA0;P08519
132:211 82:97 16:17
6:161089307:C:T_T
p = 0.025, beta = 0.232, N = 230
HFCGGTLISPEWVLTAAHCLK pc3
P08519
149:211 82:97 15:17
6:161089307:C:T_T
p = 0.00012, beta = 0.391, N = 246
FVTWIEGMMR pc2
P08519
158:211 83:97 16:17
6:161089307:C:T_T
p = 0.0023, beta = 0.304, N = 257
GTFSTTVTGR pc2
P08519
95:211 64:97 11:17
6:161089307:C:T_T
p = 0.056, beta = 0.232, N = 170
TCQSWSSMIPHWHQR pc4
P08519
118:211 72:97 16:17
6:161089307:C:T_T
p = 0.011, beta = 0.273, N = 206
EAQLLVIENEVCNHYK pc3
P08519
165:211 92:97 17:17
6:161089307:C:T_T
p = 0.00066, beta = 0.329, N = 274
TCQSWSSMTPHR pc2
P08519
35:211 40:97 6:17
6:161089307:C:T_T
p = 0.67, beta = 0.0744, N = 81
73Assay Target: LPA
UniProt: P08519
rsID: rs56393506
Target SNP: 6:160997118:A:T
Proxy SNP: 6:161089307:C:T
Tarkin log10(p): 20.2
Tarkin BETA: 0.799
Tarkin MSPA: 0.859
1241:1248:1221:1222:1201:120
YILQGVTSWGLGCAR pc2
rs3124784 REF
511:891 204:332 19:37
6:161089307:C:T_T
p = 0.32, model = REC, N = 734
YILQGVTSWGLGCAR pc3
rs3124784 REF
15:891 3:332 0:37
6:161089307:C:T_T
p = NA, model = NA, N = 18
NPDADTGPWCFTMDPSIR pc3
rs1801693 REF
19:891 4:332 0:37
6:161089307:C:T_T
p = 0.25, model = REC, N = 23
YILQGVTSWGLGCACPNK pc3
rs3124784 ALT
1:891 13:332 4:37
6:161089307:C:T_T
p = NA, model = NA, N = 18
log10 protein intensity
log10 peptide intensity

74. OAF (Q86UD1)
Tarkin MSPA=0.977

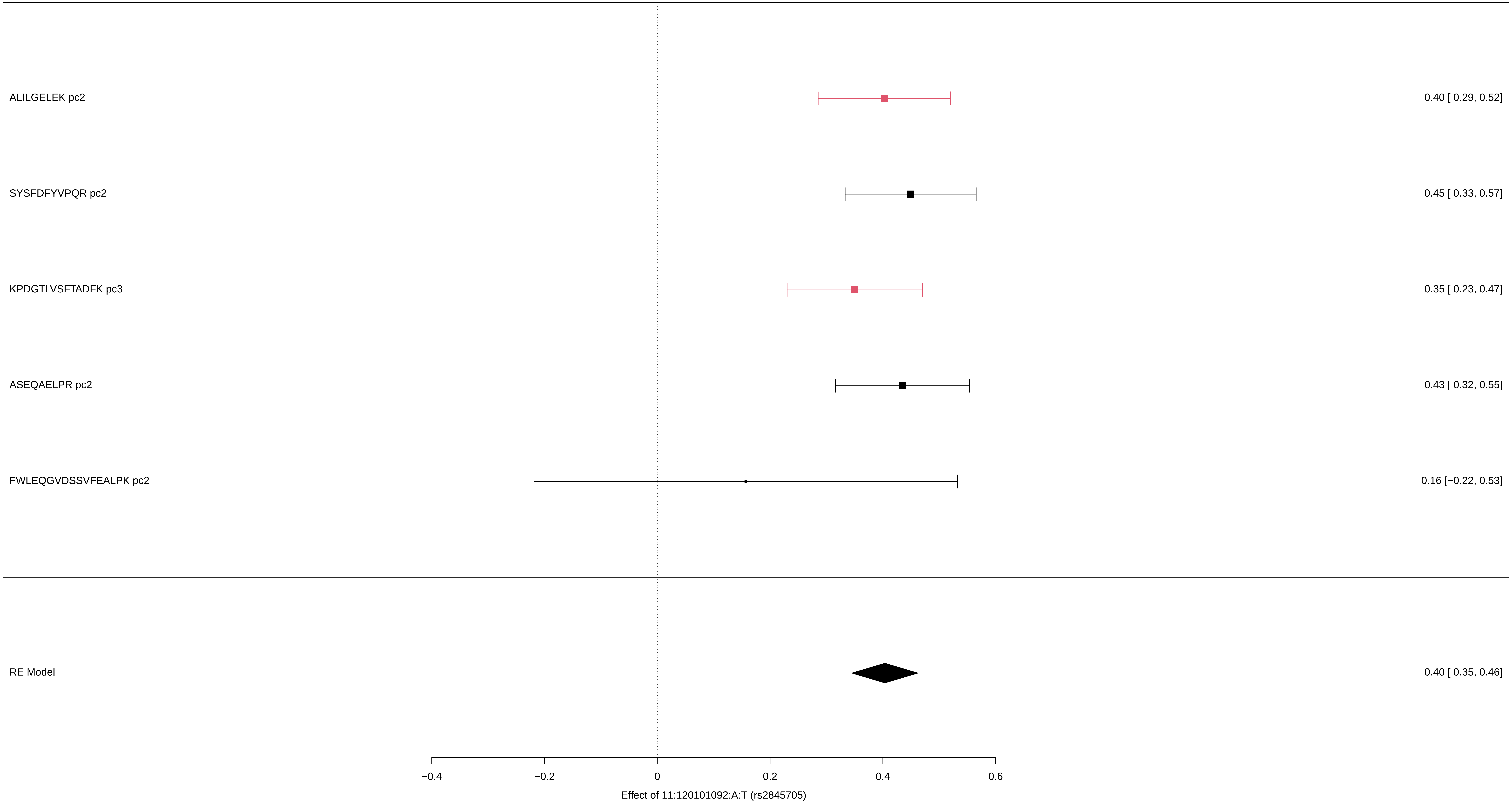

OAF : NP5
Q86UD1

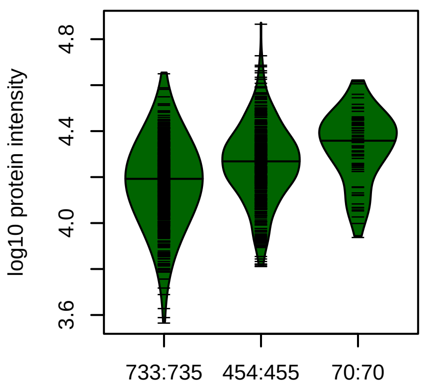

11:120101092:A:T_A
p = 6.9e−21, beta = 0.431, N = 1257

ALILGELEK pc2
Q86UD1

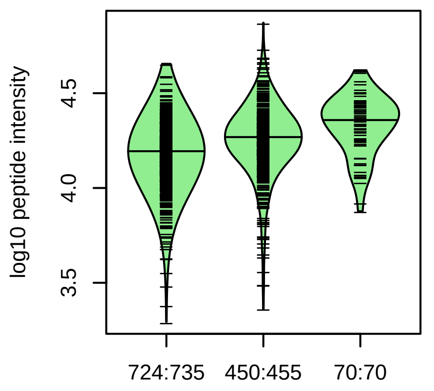

11:120101092:A:T_A
p = 3.2e−18, beta = 0.403, N = 1244

SYSFDFYVPQR pc2
E9PJ29;Q86UD1

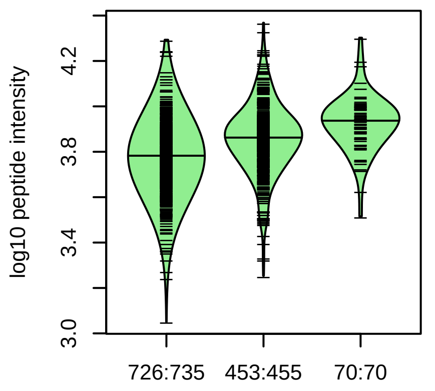

11:120101092:A:T_A
p = 1.5e−22, beta = 0.449, N = 1249

KPDGTLVSFTADFK pc3
Q86UD1

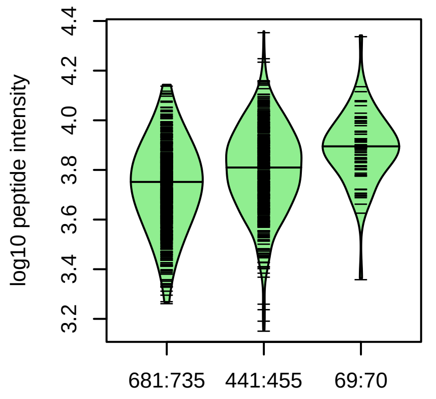

11:120101092:A:T_A
p = 1.1e−13, beta = 0.35, N = 1191

ASEQAELPR pc2
E9PJ29;Q86UD1

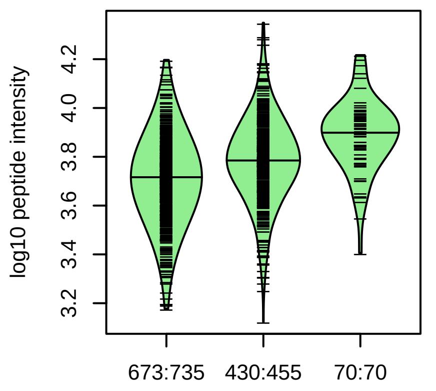

11:120101092:A:T_A
p = 2.3e−20, beta = 0.435, N = 1173

FWLEQGVDSSVFEALPK pc2
E9PJ29;Q86UD1

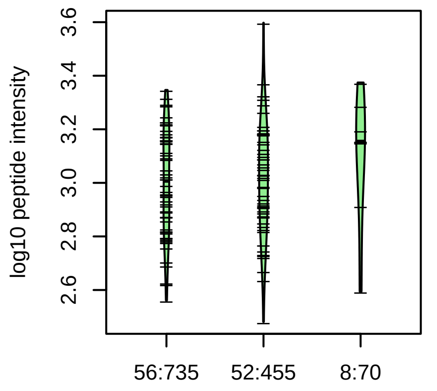

11:120101092:A:T_A
p = 0.28, beta = 0.157, N = 116

OAF : NP5
Q86UD1

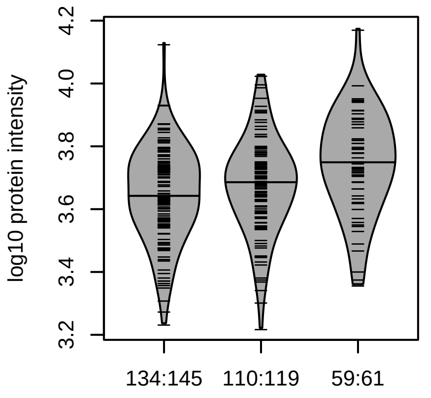

11:120101092:A:T_A
p = 0.00055, beta = 0.256, N = 303

SYSFDFYVPQR pc2
E9PJ29;Q86UD1

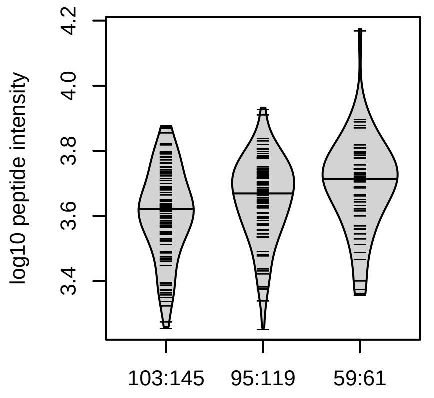

11:120101092:A:T_A
p = 0.012, beta = 0.198, N = 257

ALILGELEK pc2
Q86UD1

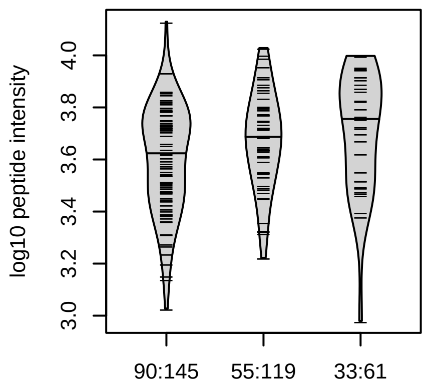

11:120101092:A:T_A
p = 0.011, beta = 0.242, N = 178

KPDGTLVSFTADFK pc3
Q86UD1

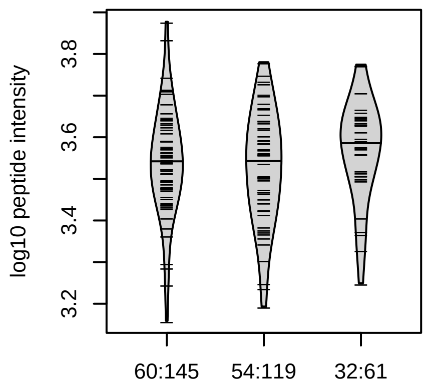

11:120101092:A:T_A
p = 0.61, beta = 0.053, N = 146

ASEQAELPR pc2
E9PJ29;Q86UD1

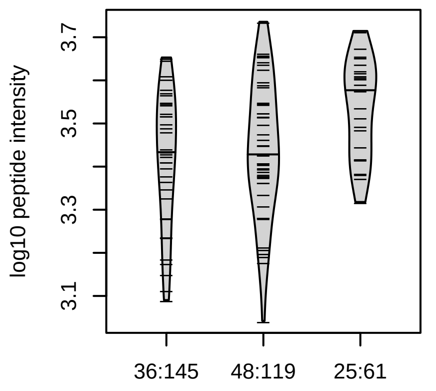

11:120101092:A:T_A
p = 0.024, beta = 0.28, N = 109

74Assay Target: OAF
UniProt: Q86UD1
rsID: rs2845705
Target SNP: 11:120101092:A:T
Proxy SNP: 11:120101092:A:T
Tarkin log10(p): 20.2
Tarkin BETA: 0.431
Tarkin MSPA: 0.977
::*:*:–
1244:1249:1191:1173:116

YGLSLAWYPCMLK pc2
rs2508490 REF

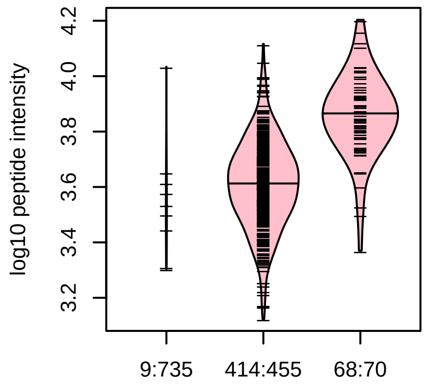

11:120101092:A:T_A
p = 2.5e−281, model = REC, N = 491

75. PLEC (Q15149)
Tarkin MSPA=0.666

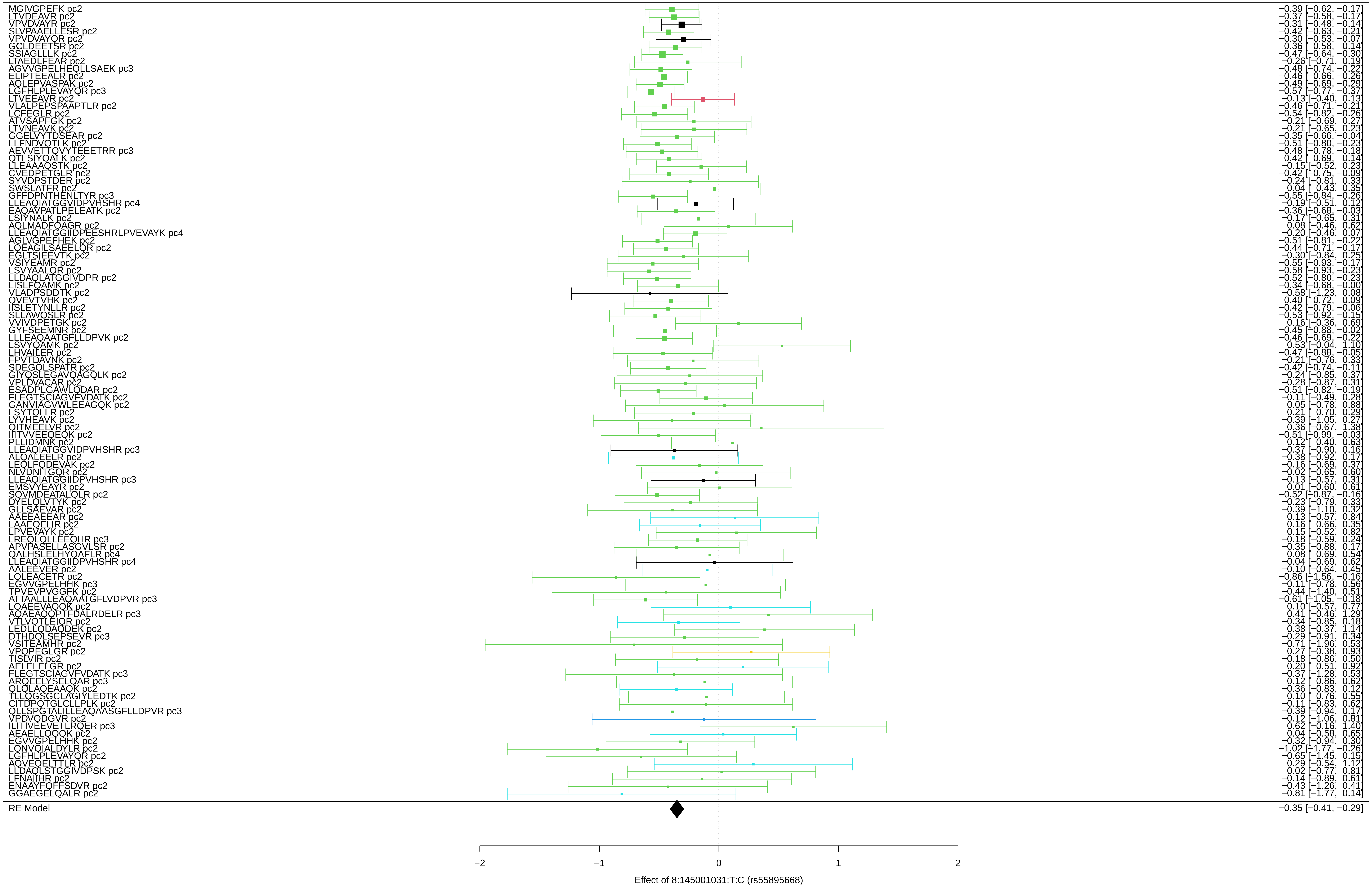

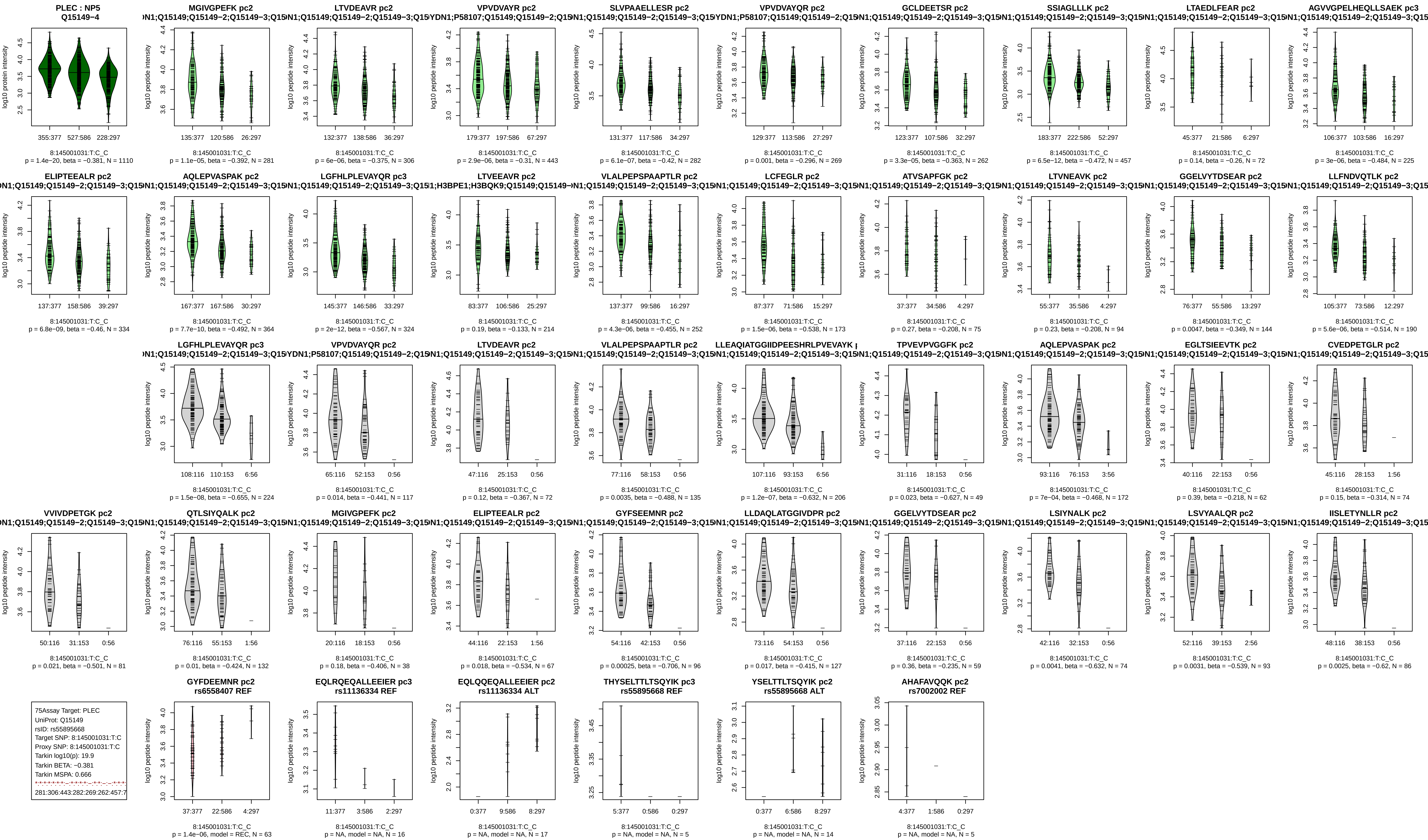
PLEC : NP5
Q15149−4
355:377 527:586 228:297
8:145001031:T:C_C
p = 1.4e−20, beta = −0.381, N = 1110
MGIVGPEFK pc2
135:377 120:586 26:297
8:145001031:T:C_C
p = 1.1e−05, beta = −0.392, N = 281
LTVDEAVR pc2
132:377 138:586 36:297
8:145001031:T:C_C
p = 6e−06, beta = −0.375, N = 306
VPVDVAYR pc2
179:377 197:586 67:297
8:145001031:T:C_C
p = 2.9e−06, beta = −0.31, N = 443
SLVPAAELLESR pc2
131:377 117:586 34:297
8:145001031:T:C_C
p = 6.1e−07, beta = −0.42, N = 282
VPVDVAYQR pc2
129:377 113:586 27:297
8:145001031:T:C_C
p = 0.001, beta = −0.296, N = 269
GCLDEETSR pc2
123:377 107:586 32:297
8:145001031:T:C_C
p = 3.3e−05, beta = −0.363, N = 262
SSIAGLLLK pc2
183:377 222:586 52:297
8:145001031:T:C_C
p = 6.5e−12, beta = −0.472, N = 457
LTAEDLFEAR pc2
45:377 21:586 6:297
8:145001031:T:C_C
p = 0.14, beta = −0.26, N = 72
AGVVGPELHEQLLSAEK pc3
106:377 103:586 16:297
8:145001031:T:C_C
p = 3e−06, beta = −0.484, N = 225
ELIPTEEALR pc2
137:377 158:586 39:297
8:145001031:T:C_C
p = 6.8e−09, beta = −0.46, N = 334
AQLEPVASPAK pc2
167:377 167:586 30:297
8:145001031:T:C_C
p = 7.7e−10, beta = −0.492, N = 364
LGFHLPLEVAYQR pc3
145:377 146:586 33:297
8:145001031:T:C_C
p = 2e−12, beta = −0.567, N = 324
LTVEEAVR pc2
83:377 106:586 25:297
8:145001031:T:C_C
p = 0.19, beta = −0.133, N = 214
VLALPEPSPAAPTLR pc2
137:377 99:586 16:297
8:145001031:T:C_C
p = 4.3e−06, beta = −0.455, N = 252
LCFEGLR pc2
87:377 71:586 15:297
8:145001031:T:C_C
p = 1.5e−06, beta = −0.538, N = 173
ATVSAPFGK pc2
37:377 34:586 4:297
8:145001031:T:C_C
p = 0.27, beta = −0.208, N = 75
LTVNEAVK pc2
55:377 35:586 4:297
8:145001031:T:C_C
p = 0.23, beta = −0.208, N = 94
GGELVYTDSEAR pc2
76:377 55:586 13:297
8:145001031:T:C_C
p = 0.0047, beta = −0.349, N = 144
LLFNDVQTLK pc2
105:377 73:586 12:297
8:145001031:T:C_C
p = 5.6e−06, beta = −0.514, N = 190
LGFHLPLEVAYQR pc3
108:116 110:153 6:56
8:145001031:T:C_C
p = 1.5e−08, beta = −0.655, N = 224
VPVDVAYQR pc2
65:116 52:153 0:56
8:145001031:T:C_C
p = 0.014, beta = −0.441, N = 117
LTVDEAVR pc2
47:116 25:153 0:56
8:145001031:T:C_C
p = 0.12, beta = −0.367, N = 72
VLALPEPSPAAPTLR pc2
77:116 58:153 0:56
8:145001031:T:C_C
p = 0.0035, beta = −0.488, N = 135
107:116 93:153 6:56
8:145001031:T:C_C
p = 1.2e−07, beta = −0.632, N = 206
TPVEVPVGGFK pc2
31:116 18:153 0:56
8:145001031:T:C_C
p = 0.023, beta = −0.627, N = 49
AQLEPVASPAK pc2
93:116 76:153 3:56
8:145001031:T:C_C
p = 7e−04, beta = −0.468, N = 172
EGLTSIEEVTK pc2
40:116 22:153 0:56
8:145001031:T:C_C
p = 0.39, beta = −0.218, N = 62
CVEDPETGLR pc2
45:116 28:153 1:56
8:145001031:T:C_C
p = 0.15, beta = −0.314, N = 74
VVIVDPETGK pc2
50:116 31:153 0:56
8:145001031:T:C_C
p = 0.021, beta = −0.501, N = 81
QTLSIYQALK pc2
76:116 55:153 1:56
8:145001031:T:C_C
p = 0.01, beta = −0.424, N = 132
MGIVGPEFK pc2
20:116 18:153 0:56
8:145001031:T:C_C
p = 0.18, beta = −0.406, N = 38
ELIPTEEALR pc2
44:116 22:153 1:56
8:145001031:T:C_C
p = 0.018, beta = −0.534, N = 67
GYFSEEMNR pc2
54:116 42:153 0:56
8:145001031:T:C_C
p = 0.00025, beta = −0.706, N = 96
LLDAQLATGGIVDPR pc2
73:116 54:153 0:56
8:145001031:T:C_C
p = 0.017, beta = −0.415, N = 127
GGELVYTDSEAR pc2
37:116 22:153 0:56
8:145001031:T:C_C
p = 0.36, beta = −0.235, N = 59
LSIYNALK pc2
42:116 32:153 0:56
8:145001031:T:C_C
p = 0.0041, beta = −0.632, N = 74
LSVYAALQR pc2
52:116 39:153 2:56
8:145001031:T:C_C
p = 0.0031, beta = −0.539, N = 93
IISLETYNLLR pc2
48:116 38:153 0:56
8:145001031:T:C_C
p = 0.0025, beta = −0.62, N = 86
75Assay Target: PLEC
UniProt: Q15149
rsID: rs55895668
Target SNP: 8:145001031:T:C
Proxy SNP: 8:145001031:T:C
Tarkin log10(p): 19.9
Tarkin BETA: −0.381
Tarkin MSPA: 0.666
GYFDEEMNR pc2
rs6558407 REF
37:377 22:586 4:297
8:145001031:T:C_C
p = 1.4e−06, model = REC, N = 63
EQLRQEQALLEEIER pc3
rs11136334 REF
11:377 3:586 2:297
8:145001031:T:C_C
p = NA, model = NA, N = 16
EQLQQEQALLEEIER pc2
rs11136334 ALT
0:377 9:586 8:297
8:145001031:T:C_C
p = NA, model = NA, N = 17
THYSELTTLTSQYIK pc3
rs55895668 REF
5:377 0:586 0:297
8:145001031:T:C_C
p = NA, model = NA, N = 5
YSELTTLTSQYIK pc2
rs55895668 ALT
0:377 6:586 8:297
8:145001031:T:C_C
p = NA, model = NA, N = 14
AHAFAVQQK pc2
rs7002002 REF
4:377 1:586 0:297
8:145001031:T:C_C
p = NA, model = NA, N = 5

76. PON1 (P27169)
Tarkin MSPA=0.945

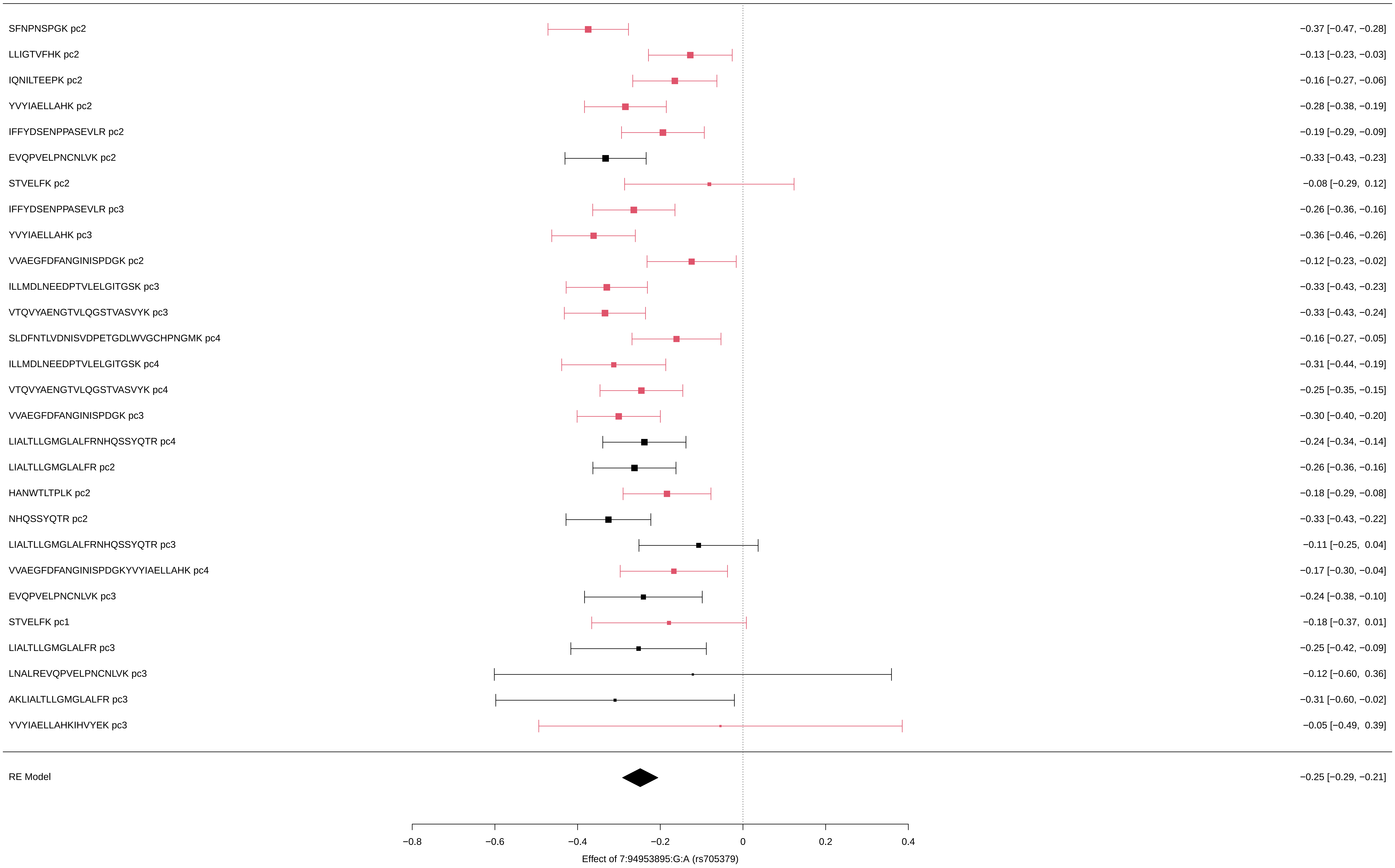

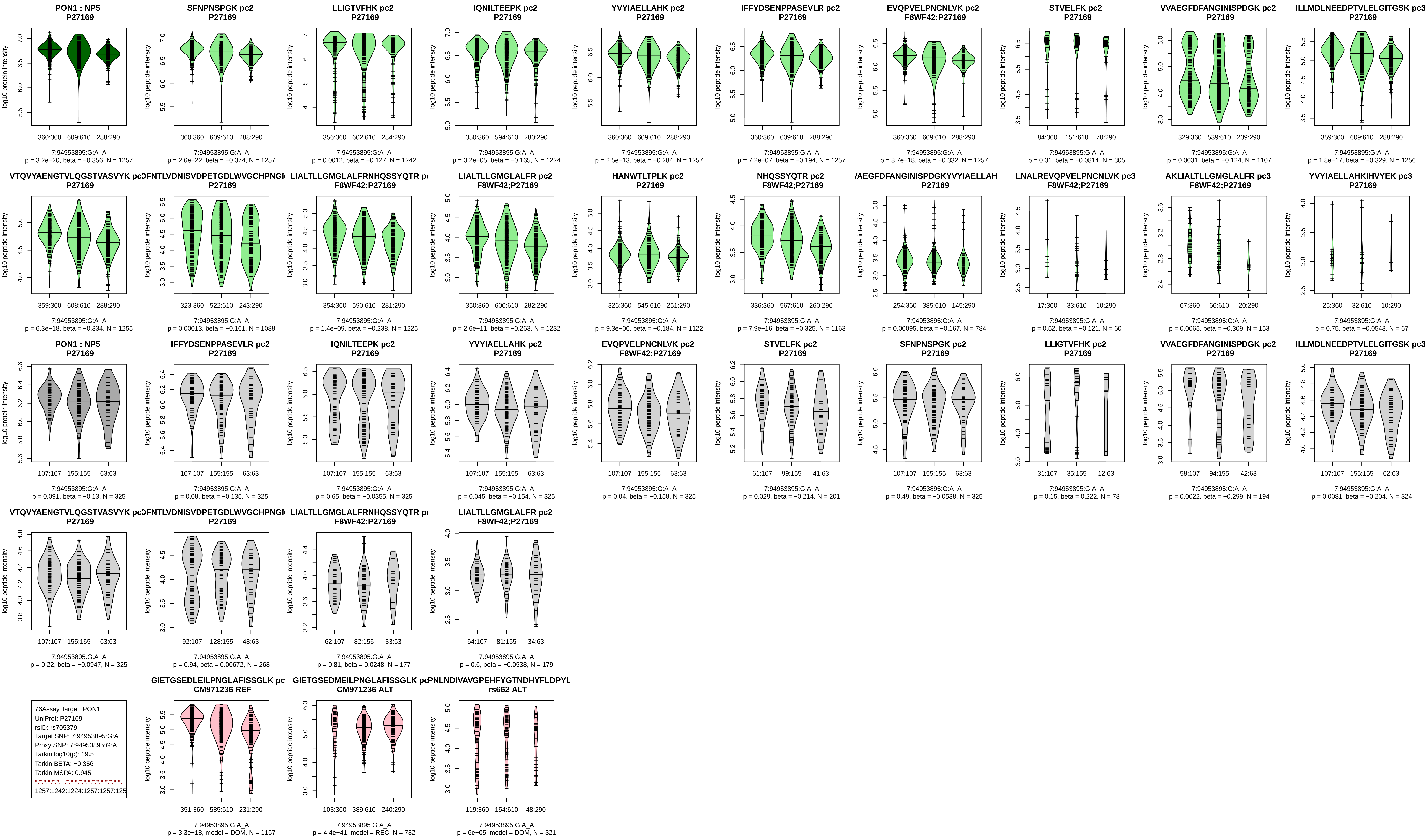
log10 protein intensity
log10 peptide intensity
PON1 : NP5
P27169
360:360 609:610 288:290
7:94953895:G:A_A
p = 3.2e−20, beta = −0.356, N = 1257
SFNPNSPGK pc2
P27169
360:360 609:610 288:290
7:94953895:G:A_A
p = 2.6e−22, beta = −0.374, N = 1257
LLIGTVFHK pc2
P27169
356:360 602:610 284:290
7:94953895:G:A_A
p = 0.0012, beta = −0.127, N = 1242
IQNILTEEPK pc2
P27169
350:360 594:610 280:290
7:94953895:G:A_A
p = 3.2e−05, beta = −0.165, N = 1224
YVYIAELLAHK pc2
P27169
360:360 609:610 288:290
7:94953895:G:A_A
p = 2.5e−13, beta = −0.284, N = 1257
IFFYDSENPPASEVLR pc2
P27169
360:360 609:610 288:290
7:94953895:G:A_A
p = 7.2e−07, beta = −0.194, N = 1257
EVQPVELPNCNLVK pc2
F8WF42;P27169
360:360 609:610 288:290
7:94953895:G:A_A
p = 8.7e−18, beta = −0.332, N = 1257
STVELFK pc2
P27169
84:360 151:610 70:290
7:94953895:G:A_A
p = 0.31, beta = −0.0814, N = 305
VVAEGFDFANGINISPDGK pc2
P27169
329:360 539:610 239:290
7:94953895:G:A_A
p = 0.0031, beta = −0.124, N = 1107
ILLMDLNEEDPTVLELGITGSK pc3
P27169
359:360 609:610 288:290
7:94953895:G:A_A
p = 1.8e−17, beta = −0.329, N = 1256
VTQVYAENGTVLQGSTVASVYK pc
P27169
359:360 608:610 288:290
7:94953895:G:A_A
p = 6.3e−18, beta = −0.334, N = 1255
FNTLVDNISVDPETGDLWVGCHPNGN
P27169
323:360 522:610 243:290
7:94953895:G:A_A
p = 0.00013, beta = −0.161, N = 1088
LIALTLLGMGLALFRNHQSSYQTR p
F8WF42;P27169
354:360 590:610 281:290
7:94953895:G:A_A
p = 1.4e−09, beta = −0.238, N = 1225
LIALTLLGMGLALFR pc2
F8WF42;P27169
350:360 600:610 282:290
7:94953895:G:A_A
p = 2.6e−11, beta = −0.263, N = 1232
HANWTLTPLK pc2
P27169
326:360 545:610 251:290
7:94953895:G:A_A
p = 9.3e−06, beta = −0.184, N = 1122
NHQSSYQTR pc2
F8WF42;P27169
336:360 567:610 260:290
7:94953895:G:A_A
p = 7.9e−16, beta = −0.325, N = 1163
AEGFDFANGINISPDGKYVYIAELLAH
P27169
254:360 385:610 145:290
7:94953895:G:A_A
p = 0.00095, beta = −0.167, N = 784
LNALREVQPVELPNCNLVK pc3
F8WF42;P27169
17:360 33:610 10:290
7:94953895:G:A_A
p = 0.52, beta = −0.121, N = 60
AKLIALTLLGMGLALFR pc3
F8WF42;P27169
67:360 66:610 20:290
7:94953895:G:A_A
p = 0.0065, beta = −0.309, N = 153
YVYIAELLAHKIHVYEK pc3
P27169
25:360 32:610 10:290
7:94953895:G:A_A
p = 0.75, beta = −0.0543, N = 67
PON1 : NP5
P27169
107:107 155:155 63:63
7:94953895:G:A_A
p = 0.091, beta = −0.13, N = 325
IFFYDSENPPASEVLR pc2
P27169
107:107 155:155 63:63
7:94953895:G:A_A
p = 0.08, beta = −0.135, N = 325
IQNILTEEPK pc2
P27169
107:107 155:155 63:63
7:94953895:G:A_A
p = 0.65, beta = −0.0355, N = 325
YVYIAELLAHK pc2
P27169
107:107 155:155 63:63
7:94953895:G:A_A
p = 0.045, beta = −0.154, N = 325
EVQPVELPNCNLVK pc2
F8WF42;P27169
107:107 155:155 63:63
7:94953895:G:A_A
p = 0.04, beta = −0.158, N = 325
STVELFK pc2
P27169
61:107 99:155 41:63
7:94953895:G:A_A
p = 0.029, beta = −0.214, N = 201
SFNPNSPGK pc2
P27169
107:107 155:155 63:63
7:94953895:G:A_A
p = 0.49, beta = −0.0538, N = 325
LLIGTVFHK pc2
P27169
31:107 35:155 12:63
7:94953895:G:A_A
p = 0.15, beta = 0.222, N = 78
VVAEGFDFANGINISPDGK pc2
P27169
58:107 94:155 42:63
7:94953895:G:A_A
p = 0.0022, beta = −0.299, N = 194
ILLMDLNEEDPTVLELGITGSK pc3
P27169
107:107 155:155 62:63
7:94953895:G:A_A
p = 0.0081, beta = −0.204, N = 324
VTQVYAENGTVLQGSTVASVYK pc
P27169
107:107 155:155 63:63
7:94953895:G:A_A
p = 0.22, beta = −0.0947, N = 325
FNTLVDNISVDPETGDLWVGCHPNGN
P27169
92:107 128:155 48:63
7:94953895:G:A_A
p = 0.94, beta = 0.00672, N = 268
LIALTLLGMGLALFRNHQSSYQTR p
F8WF42;P27169
62:107 82:155 33:63
7:94953895:G:A_A
p = 0.81, beta = 0.0248, N = 177
LIALTLLGMGLALFR pc2
F8WF42;P27169
64:107 81:155 34:63
7:94953895:G:A_A
p = 0.6, beta = −0.0538, N = 179
76Assay Target: PON1
UniProt: P27169
rsID: rs705379
Target SNP: 7:94953895:G:A
Proxy SNP: 7:94953895:G:A
Tarkin log10(p): 19.5
Tarkin BETA: −0.356
Tarkin MSPA: 0.945
1257:1242:1224:1257:1257:125
GIETGSEDLEILPNGLAFISSGLK pc
CM971236 REF
351:360 585:610 231:290
7:94953895:G:A_A
p = 3.3e−18, model = DOM, N = 1167
GIETGSEDMEILPNGLAFISSGLK pc
CM971236 ALT
103:360 389:610 240:290
7:94953895:G:A_A
p = 4.4e−41, model = REC, N = 732
PNLNDIVAVGPEHFYGTNDHYFLDPYL
rs662 ALT
119:360 154:610 48:290
7:94953895:G:A_A
p = 6e−05, model = DOM, N = 321

77. HEATR6 (Q6AI08)
Tarkin MSPA=1

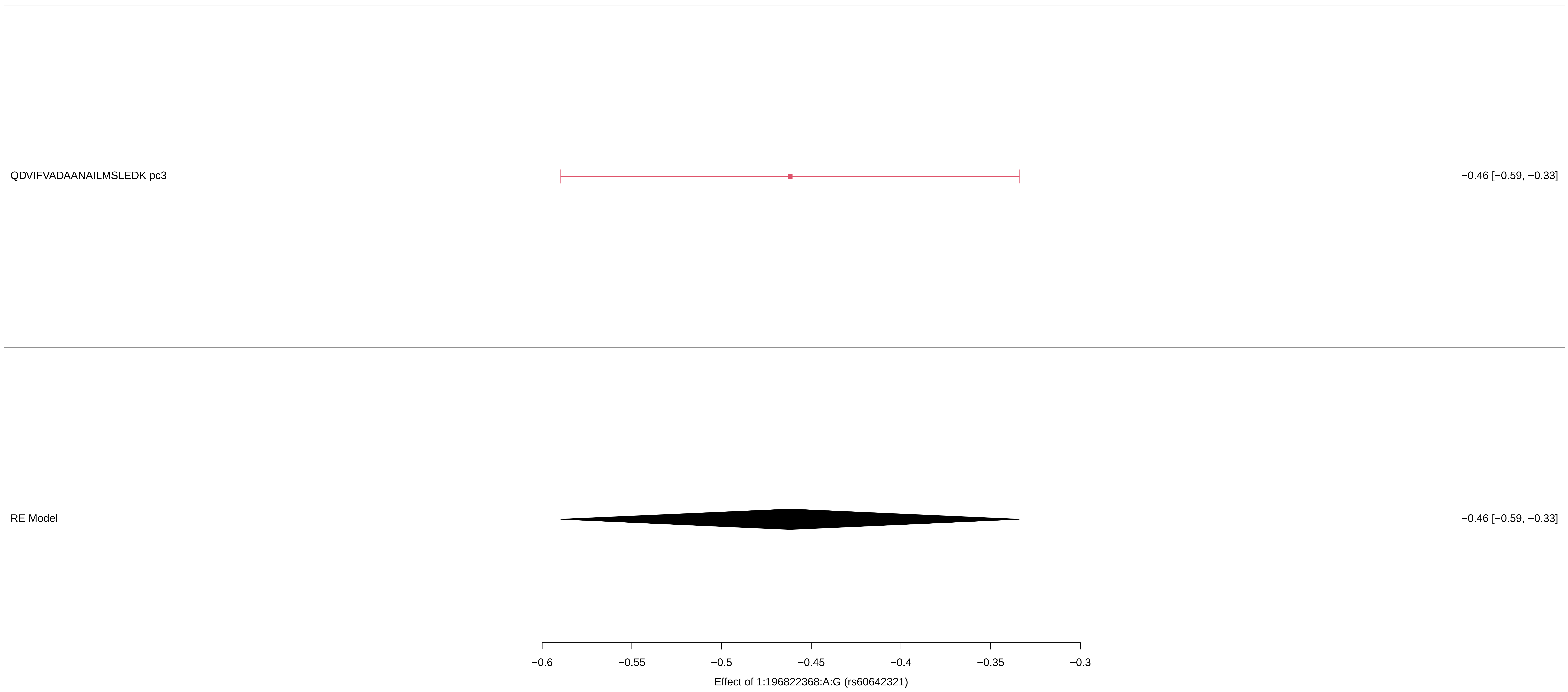

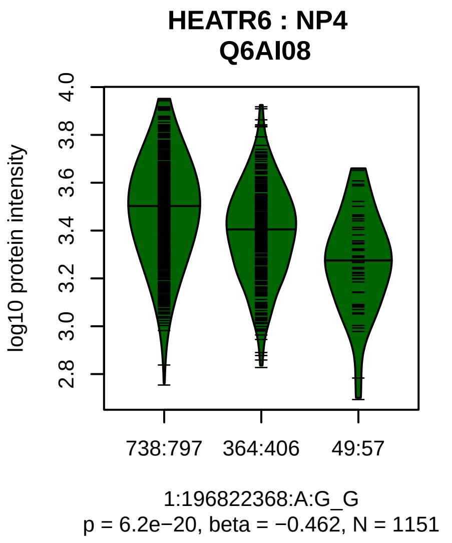

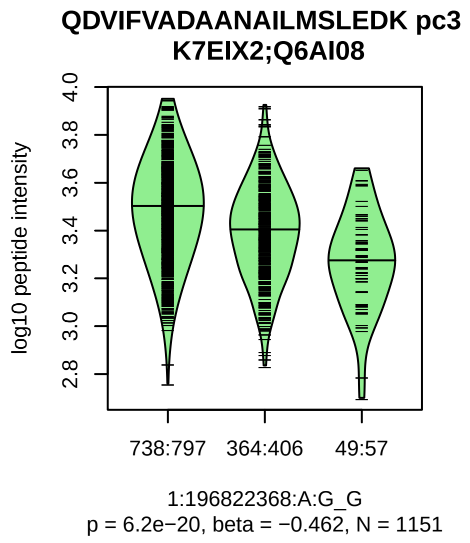

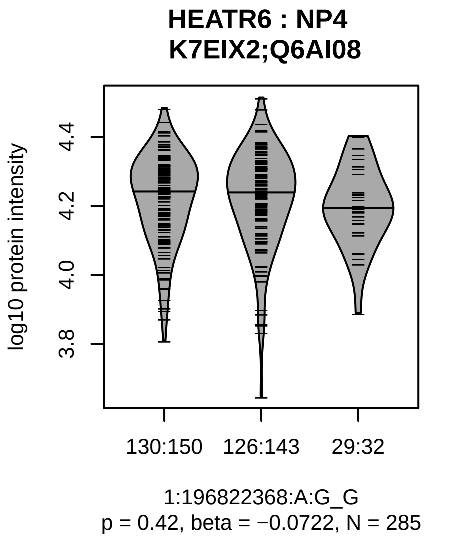

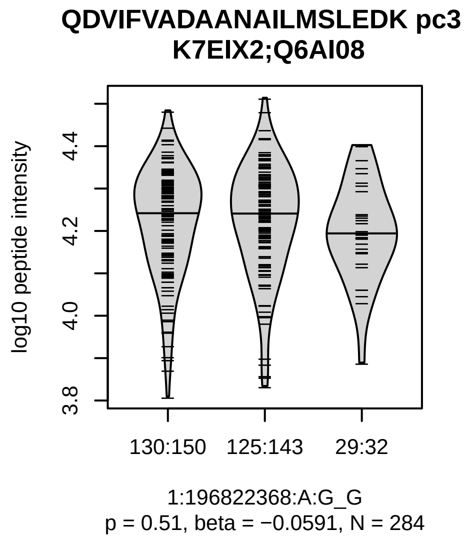

77Assay Target: HEATR6
UniProt: Q6AI08
rsID: rs60642321
Target SNP: 1:196822368:A:G
Proxy SNP: 1:196822368:A:G
Tarkin log10(p): 19.2
Tarkin BETA: −0.462
Tarkin MSPA: 1
*:NA
1151:6

78. DYNC1H1 (Q14204)
Tarkin MSPA=0.915

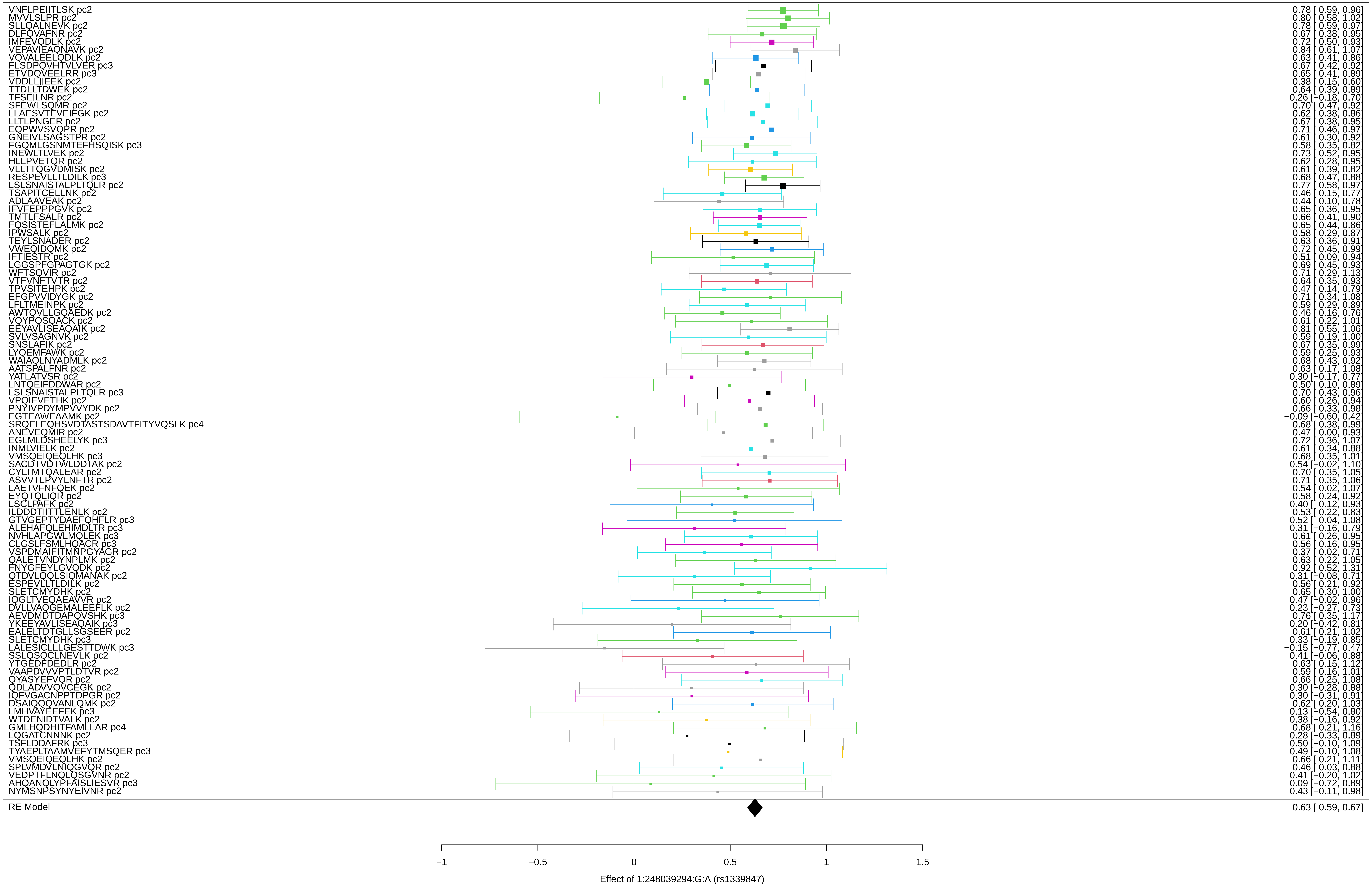

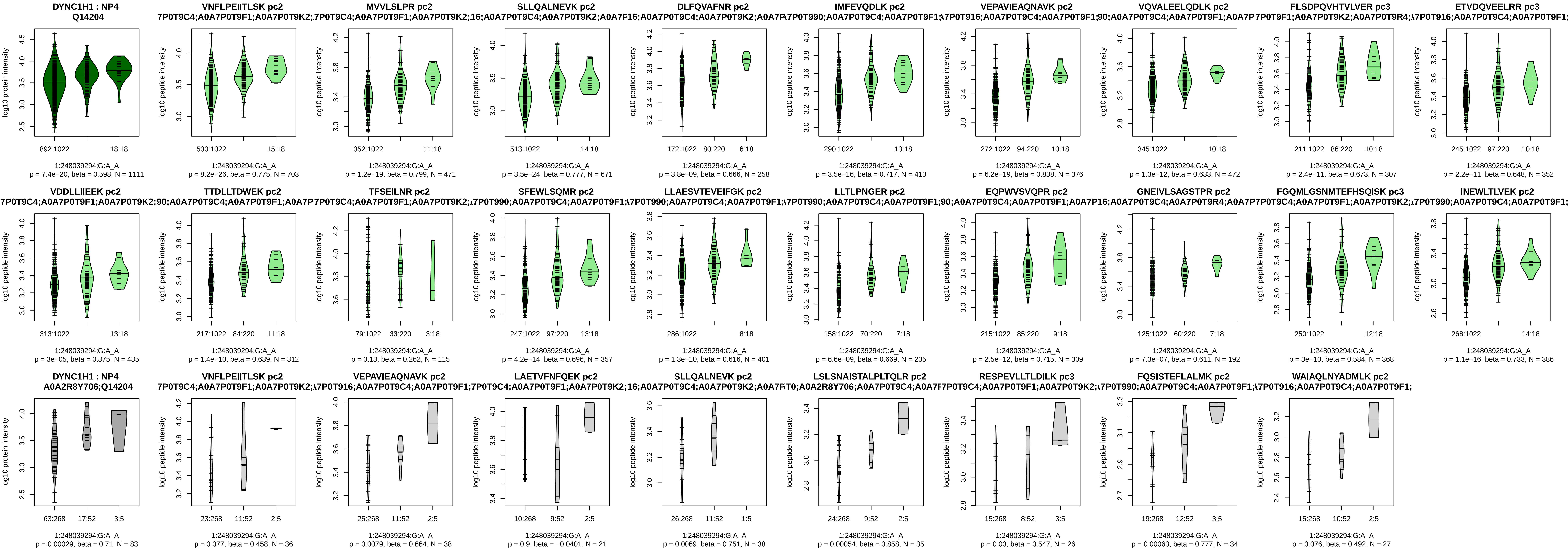

78Assay Target: DYNC1H1
UniProt: Q14204
rsID: rs1339847
Target SNP: 1:248039294:G:A
Proxy SNP: 1:248039294:G:A
Tarkin log10(p): 19.1
Tarkin BETA: 0.598
Tarkin MSPA: 0.915
703:471:671:258:413:376:472:3

79. C6 (P13671)
Tarkin MSPA=0.983

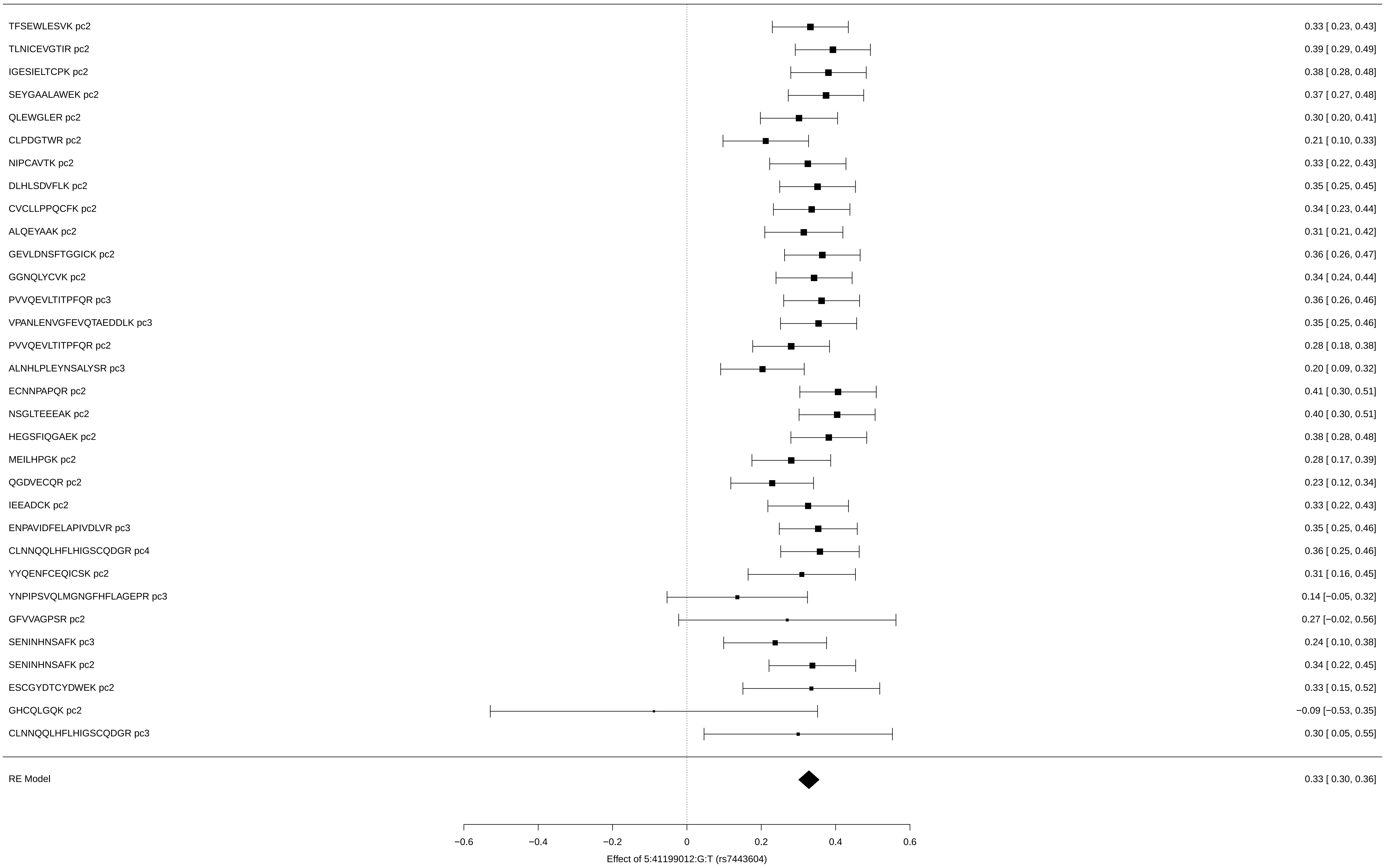

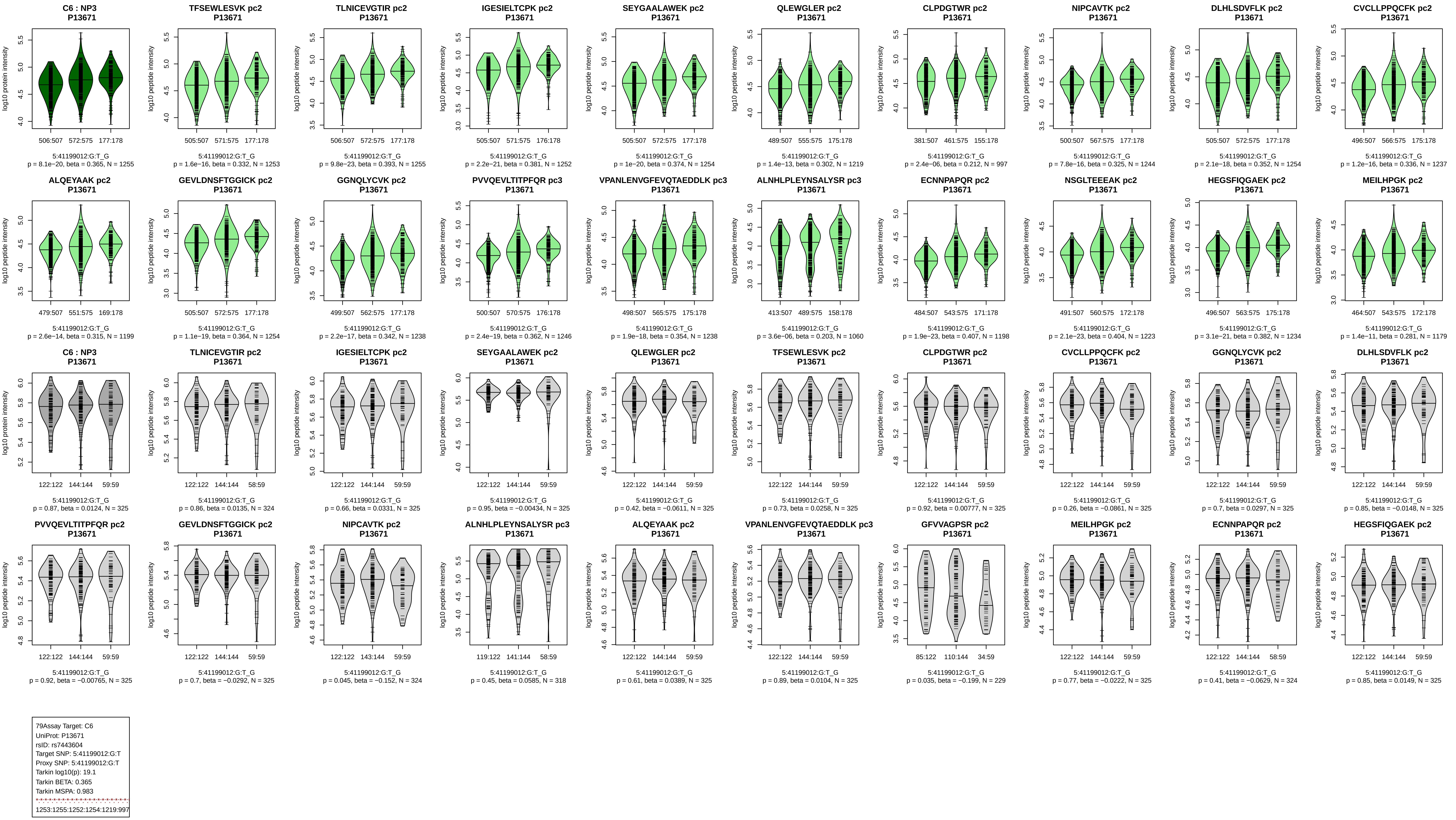
C6 : NP3 P13671
5:41199012:G:T_G
p = 8.1e−20, beta = 0.365, N = 1255
TFSEWLESVK pc2 P13671
5:41199012:G:T_G
p = 1.6e−16, beta = 0.332, N = 1253
TLNICEVGTIR pc2 P13671
5:41199012:G:T_G
p = 9.8e−23, beta = 0.393, N = 1255
IGESIELTCPK pc2 P13671
5:41199012:G:T_G
p = 2.2e−21, beta = 0.381, N = 1252
SEYGAALAWEK pc2 P13671
5:41199012:G:T_G
p = 1e−20, beta = 0.374, N = 1254
QLEWGLER pc2 P13671
5:41199012:G:T_G
p = 1.4e−13, beta = 0.302, N = 1219
CLPDGTWR pc2 P13671
5:41199012:G:T_G
p = 2.4e−06, beta = 0.212, N = 997
NIPCAVTK pc2 P13671
5:41199012:G:T_G
p = 7.8e−16, beta = 0.325, N = 1244
DLHLSDVFLK pc2 P13671
5:41199012:G:T_G
p = 2.1e−18, beta = 0.352, N = 1254
CVCLLPPQCFK pc2 P13671
5:41199012:G:T_G
p = 1.2e−16, beta = 0.336, N = 1237
ALQEYAAK pc2 P13671
5:41199012:G:T_G
p = 2.6e−14, beta = 0.315, N = 1199
GEVLDNSFTGGICK pc2 P13671
5:41199012:G:T_G
p = 1.1e−19, beta = 0.364, N = 1254
GGNQLYCVK pc2 P13671
5:41199012:G:T_G
p = 2.2e−17, beta = 0.342, N = 1238
PVVQEVLTITPFQR pc3 P13671
5:41199012:G:T_G
p = 2.4e−19, beta = 0.362, N = 1246
VPANLENVGFEVQTAEDDLK pc3 P13671
5:41199012:G:T_G
p = 1.9e−18, beta = 0.354, N = 1238
ALNHLPLEYNSALYSR pc3 P13671
5:41199012:G:T_G
p = 3.6e−06, beta = 0.203, N = 1060
ECNNPAPQR pc2 P13671
5:41199012:G:T_G
p = 1.9e−23, beta = 0.407, N = 1198
NSGLTEEEAK pc2 P13671
5:41199012:G:T_G
p = 2.1e−23, beta = 0.404, N = 1223
HEGSFIQGAEK pc2 P13671
5:41199012:G:T_G
p = 3.1e−21, beta = 0.382, N = 1234
MEILHPGK pc2 P13671
5:41199012:G:T_G
p = 1.4e−11, beta = 0.281, N = 1179
C6 : NP3 P13671
5:41199012:G:T_G
p = 0.87, beta = 0.0124, N = 325
TLNICEVGTIR pc2 P13671
5:41199012:G:T_G
p = 0.86, beta = 0.0135, N = 324
IGESIELTCPK pc2 P13671
5:41199012:G:T_G
p = 0.66, beta = 0.0331, N = 325
SEYGAALAWEK pc2 P13671
5:41199012:G:T_G
p = 0.95, beta = −0.00434, N = 325
QLEWGLER pc2 P13671
5:41199012:G:T_G
p = 0.42, beta = −0.0611, N = 325
TFSEWLESVK pc2 P13671
5:41199012:G:T_G
p = 0.73, beta = 0.0258, N = 325
CLPDGTWR pc2 P13671
5:41199012:G:T_G
p = 0.92, beta = 0.00777, N = 325
CVCLLPPQCFK pc2 P13671
5:41199012:G:T_G
p = 0.26, beta = −0.0861, N = 325
GGNQLYCVK pc2 P13671
5:41199012:G:T_G
p = 0.7, beta = 0.0297, N = 325
DLHLSDVFLK pc2 P13671
5:41199012:G:T_G
p = 0.85, beta = −0.0148, N = 325
PVVQEVLTITPFQR pc2 P13671
5:41199012:G:T_G
p = 0.92, beta = −0.00765, N = 325
GEVLDNSFTGGICK pc2 P13671
5:41199012:G:T_G
p = 0.7, beta = −0.0292, N = 325
NIPCAVTK pc2 P13671
5:41199012:G:T_G
p = 0.045, beta = −0.152, N = 324
ALNHLPLEYNSALYSR pc3 P13671
5:41199012:G:T_G
p = 0.45, beta = 0.0585, N = 318
ALQEYAAK pc2 P13671
5:41199012:G:T_G
p = 0.61, beta = 0.0389, N = 325
VPANLENVGFEVQTAEDDLK pc3 P13671
5:41199012:G:T_G
p = 0.89, beta = 0.0104, N = 325
GFVVAGPSR pc2 P13671
5:41199012:G:T_G
p = 0.035, beta = −0.199, N = 229
MEILHPGK pc2 P13671
5:41199012:G:T_G
p = 0.77, beta = −0.0222, N = 325
ECNNPAPQR pc2 P13671
5:41199012:G:T_G
p = 0.41, beta = −0.0629, N = 324
HEGSFIQGAEK pc2 P13671
5:41199012:G:T_G
p = 0.85, beta = 0.0149, N = 325
79Assay Target: C6
UniProt: P13671
rsID: rs7443604
Target SNP: 5:41199012:G:T
Proxy SNP: 5:41199012:G:T
Tarkin log10(p): 19.1
Tarkin BETA: 0.365
Tarkin MSPA: 0.983
1253:1255:1252:1254:1219:997

80. LBP (P18428)
Tarkin MSPA=0.826

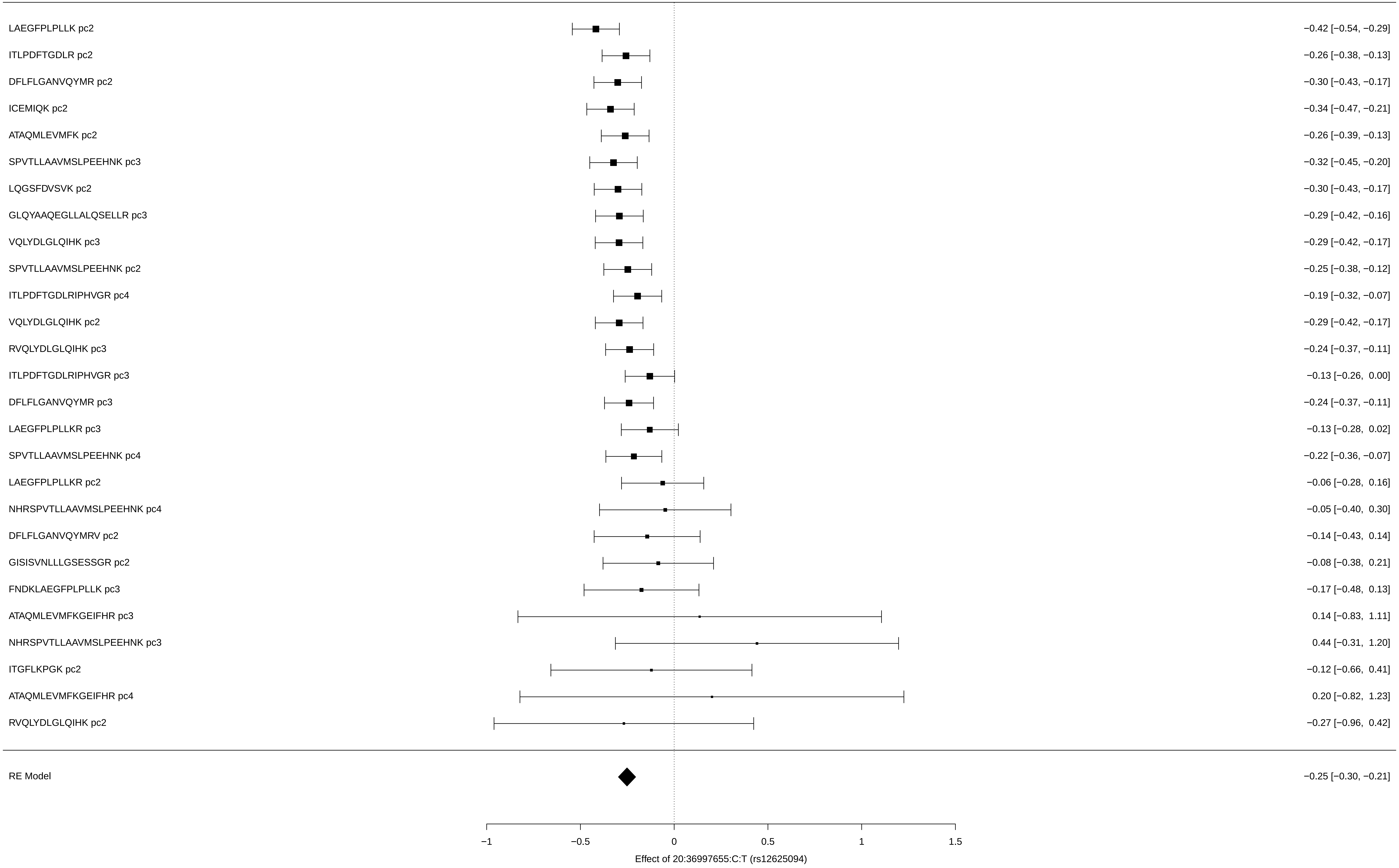

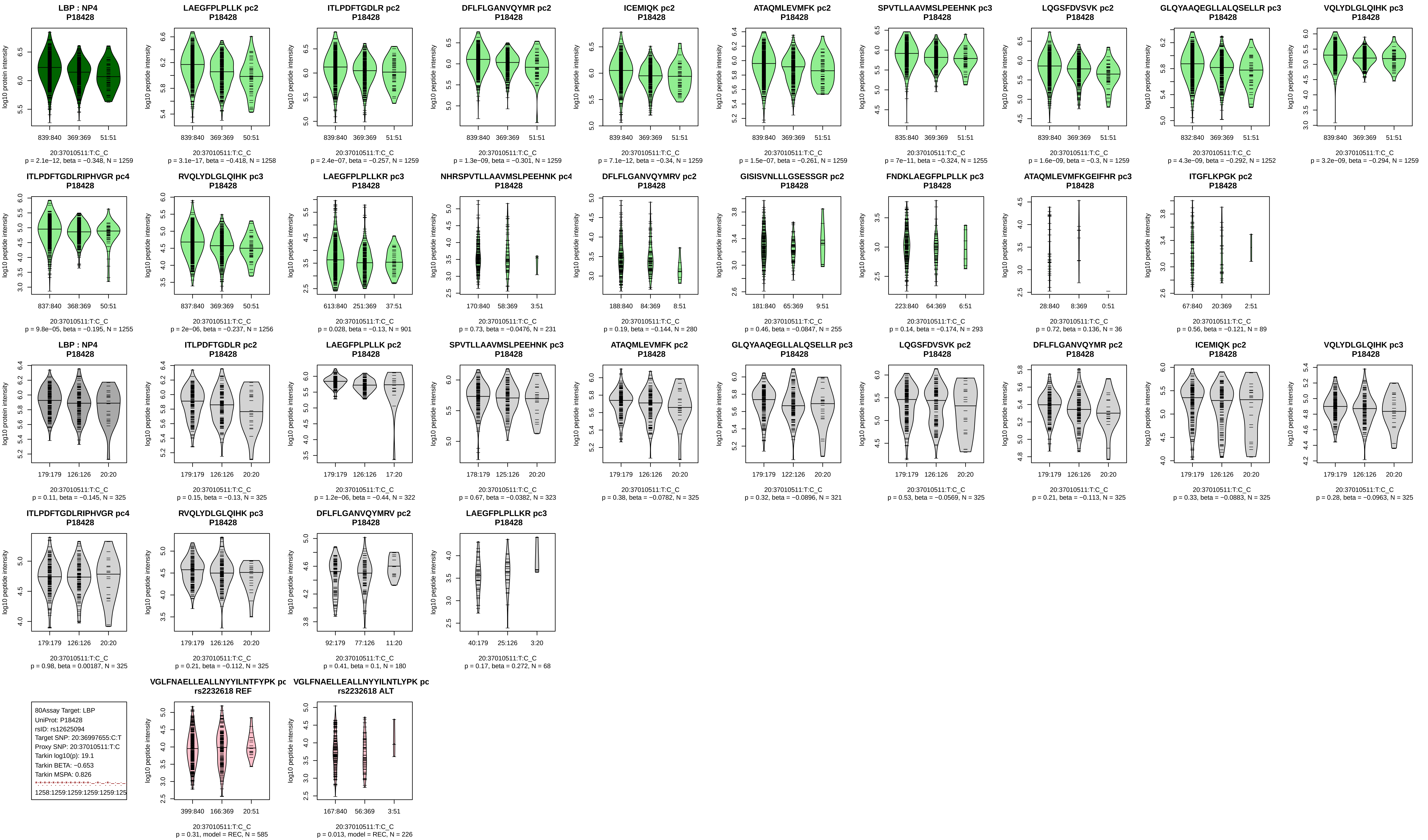
LBP : NP4
P18428
log10 protein intensity
839:840 369:369 51:51
20:37010511:T:C_C
p = 2.1e−12, beta = −0.348, N = 1259
LAEGFPLPLLK pc2
P18428
log10 peptide intensity
839:840 369:369 50:51
20:37010511:T:C_C
p = 3.1e−17, beta = −0.418, N = 1258
ITLPDFTGDLR pc2
P18428
839:840 369:369 51:51
20:37010511:T:C_C
p = 2.4e−07, beta = −0.257, N = 1259
DFLFLGANVQYMR pc2
P18428
839:840 369:369 51:51
20:37010511:T:C_C
p = 1.3e−09, beta = −0.301, N = 1259
ICEMIQK pc2
P18428
839:840 369:369 51:51
20:37010511:T:C_C
p = 7.1e−12, beta = −0.34, N = 1259
ATAQMLEVMFK pc2
P18428
839:840 369:369 51:51
20:37010511:T:C_C
p = 1.5e−07, beta = −0.261, N = 1259
SPVTLLAAVMSLPEEHNK pc3
P18428
835:840 369:369 51:51
20:37010511:T:C_C
p = 7e−11, beta = −0.324, N = 1255
LQGSFDVSVK pc2
P18428
839:840 369:369 51:51
20:37010511:T:C_C
p = 1.6e−09, beta = −0.3, N = 1259
GLQYAAQEGLLALQSELLR pc3
P18428
832:840 369:369 51:51
20:37010511:T:C_C
p = 4.3e−09, beta = −0.292, N = 1252
VQLYDLGLQIHK pc3
P18428
839:840 369:369 51:51
20:37010511:T:C_C
p = 3.2e−09, beta = −0.294, N = 1259
ITLPDFTGDLRIPHVGR pc4
P18428
837:840 368:369 50:51
20:37010511:T:C_C
p = 9.8e−05, beta = −0.195, N = 1255
RVQLYDLGLQIHK pc3
P18428
837:840 369:369 50:51
20:37010511:T:C_C
p = 2e−06, beta = −0.237, N = 1256
LAEGFPLPLLKR pc3
P18428
613:840 251:369 37:51
20:37010511:T:C_C
p = 0.028, beta = −0.13, N = 901
NHRSPVTLLAAVMSLPEEHNK pc4
P18428
170:840 58:369 3:51
20:37010511:T:C_C
p = 0.73, beta = −0.0476, N = 231
DFLFLGANVQYMRV pc2
P18428
188:840 84:369 8:51
20:37010511:T:C_C
p = 0.19, beta = −0.144, N = 280
GISISVNLLLGSESSGR pc2
P18428
181:840 65:369 9:51
20:37010511:T:C_C
p = 0.46, beta = −0.0847, N = 255
FNDKLAEGFPLPLLK pc3
P18428
223:840 64:369 6:51
20:37010511:T:C_C
p = 0.14, beta = −0.174, N = 293
ATAQMLEVMFKGEIFHR pc3
P18428
28:840 8:369 0:51
20:37010511:T:C_C
p = 0.72, beta = 0.136, N = 36
ITGFLKPGK pc2
P18428
67:840 20:369 2:51
20:37010511:T:C_C
p = 0.56, beta = −0.121, N = 89
LBP : NP4
P18428
179:179 126:126 20:20
20:37010511:T:C_C
p = 0.11, beta = −0.145, N = 325
ITLPDFTGDLR pc2
P18428
179:179 126:126 20:20
20:37010511:T:C_C
p = 0.15, beta = −0.13, N = 325
LAEGFPLPLLK pc2
P18428
179:179 126:126 17:20
20:37010511:T:C_C
p = 1.2e−06, beta = −0.44, N = 322
SPVTLLAAVMSLPEEHNK pc3
P18428
178:179 125:126 20:20
20:37010511:T:C_C
p = 0.67, beta = −0.0382, N = 323
ATAQMLEVMFK pc2
P18428
179:179 126:126 20:20
20:37010511:T:C_C
p = 0.38, beta = −0.0782, N = 325
GLQYAAQEGLLALQSELLR pc3
P18428
179:179 122:126 20:20
20:37010511:T:C_C
p = 0.32, beta = −0.0896, N = 321
LQGSFDVSVK pc2
P18428
179:179 126:126 20:20
20:37010511:T:C_C
p = 0.53, beta = −0.0569, N = 325
DFLFLGANVQYMR pc2
P18428
179:179 126:126 20:20
20:37010511:T:C_C
p = 0.21, beta = −0.113, N = 325
ICEMIQK pc2
P18428
179:179 126:126 20:20
20:37010511:T:C_C
p = 0.33, beta = −0.0883, N = 325
VQLYDLGLQIHK pc3
P18428
179:179 126:126 20:20
20:37010511:T:C_C
p = 0.28, beta = −0.0963, N = 325
ITLPDFTGDLRIPHVGR pc4
P18428
179:179 126:126 20:20
20:37010511:T:C_C
p = 0.98, beta = 0.00187, N = 325
RVQLYDLGLQIHK pc3
P18428
179:179 126:126 20:20
20:37010511:T:C_C
p = 0.21, beta = −0.112, N = 325
DFLFLGANVQYMRV pc2
P18428
92:179 77:126 11:20
20:37010511:T:C_C
p = 0.41, beta = 0.1, N = 180
LAEGFPLPLLKR pc3
P18428
40:179 25:126 3:20
20:37010511:T:C_C
p = 0.17, beta = 0.272, N = 68
80Assay Target: LBP
UniProt: P18428
rsID: rs12625094
Target SNP: 20:36997655:C:T
Proxy SNP: 20:37010511:T:C
Tarkin log10(p): 19.1
Tarkin BETA: −0.653
Tarkin MSPA: 0.826
1258:1259:1259:1259:1259:125
VGLFNAELLEALLNYYILNTFYPK pc
rs2232618 REF
399:840 166:369 20:51
20:37010511:T:C_C
p = 0.31, model = REC, N = 585
VGLFNAELLEALLNYYILNTLYPK pc
rs2232618 ALT
167:840 56:369 3:51
20:37010511:T:C_C
p = 0.013, model = REC, N = 226

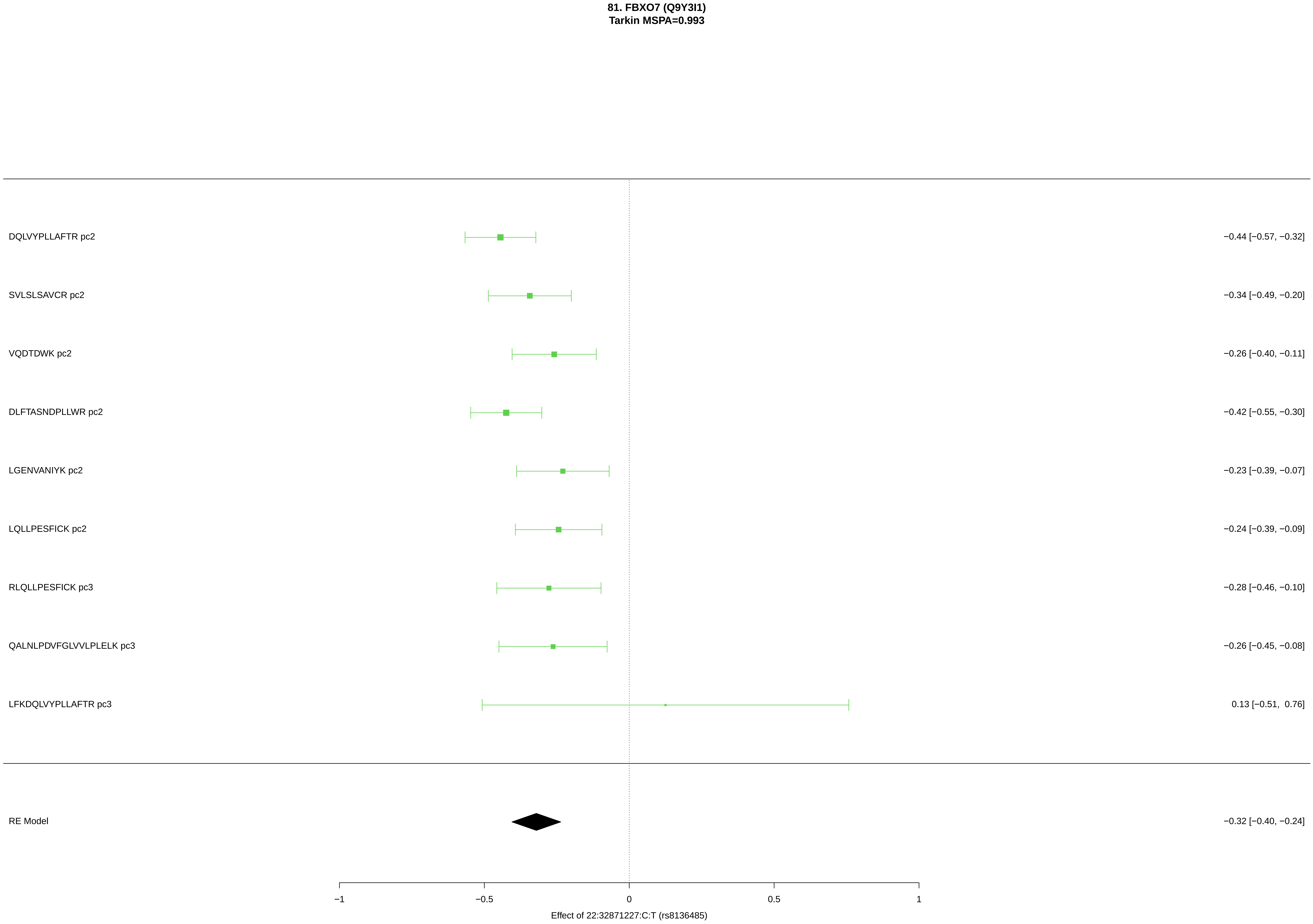
81. FBXO7 (Q9Y3I1)
Tarkin MSPA=0.993
DQLVYPLLAFTR pc2
−0.44 [−0.57, −0.32]
SVLSLSAVCR pc2
−0.34 [−0.49, −0.20]
VQDTDWK pc2
−0.26 [−0.40, −0.11]
DLFTASNDPLLWR pc2
−0.42 [−0.55, −0.30]
LGENVANIYK pc2
−0.23 [−0.39, −0.07]
LQLLPESFICK pc2
−0.24 [−0.39, −0.09]
RLQLLPESFICK pc3
−0.28 [−0.46, −0.10]
QALNLPDVFGLVVLPLELK pc3
−0.26 [−0.45, −0.08]
LFKDQLVYPLLAFTR pc3
0.13 [−0.51, 0.76]
RE Model
−0.32 [−0.40, −0.24]
−1
−0.5
0
0.5
1
Effect of 22:32871227:C:T (rs8136485)

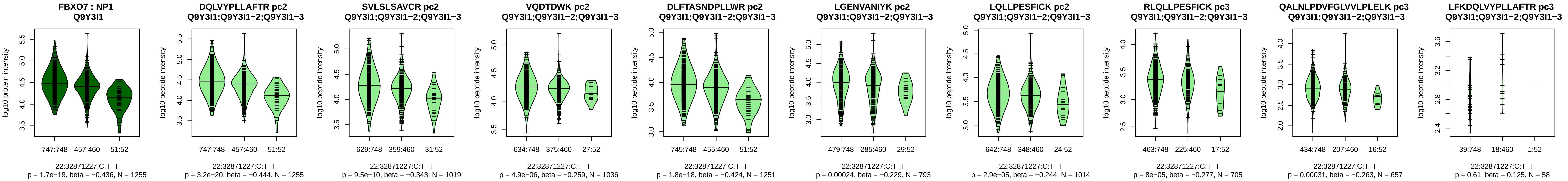

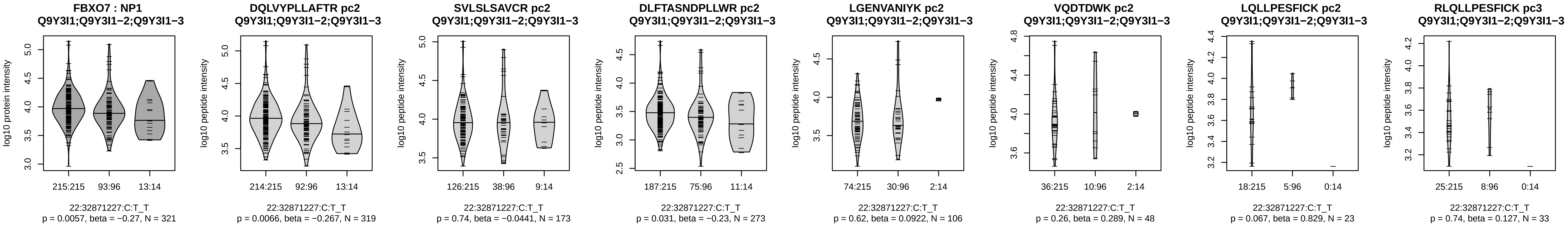

81Assay Target: FBXO7
UniProt: Q9Y3I1
rsID: rs8136485
Target SNP: 22:32871227:C:T
Proxy SNP: 22:32871227:C:T
Tarkin log10(p): 18.8
Tarkin BETA: −0.436
Tarkin MSPA: 0.993
::*:*:*:*:*:−:NA:NA:NA
1255:1019:1036:1251:793:1014

82. MASP2 (O00187)
Tarkin MSPA=0.806

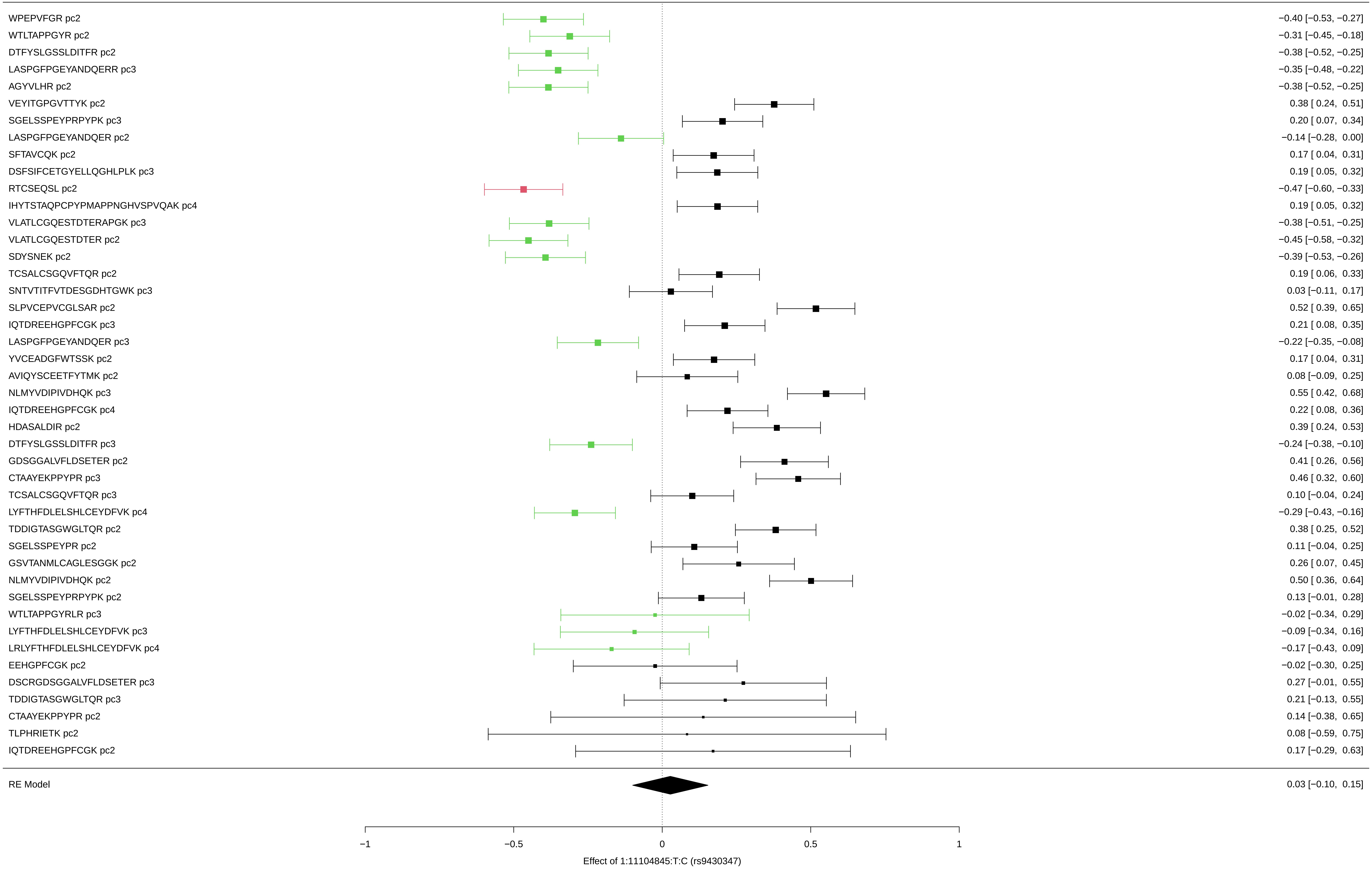

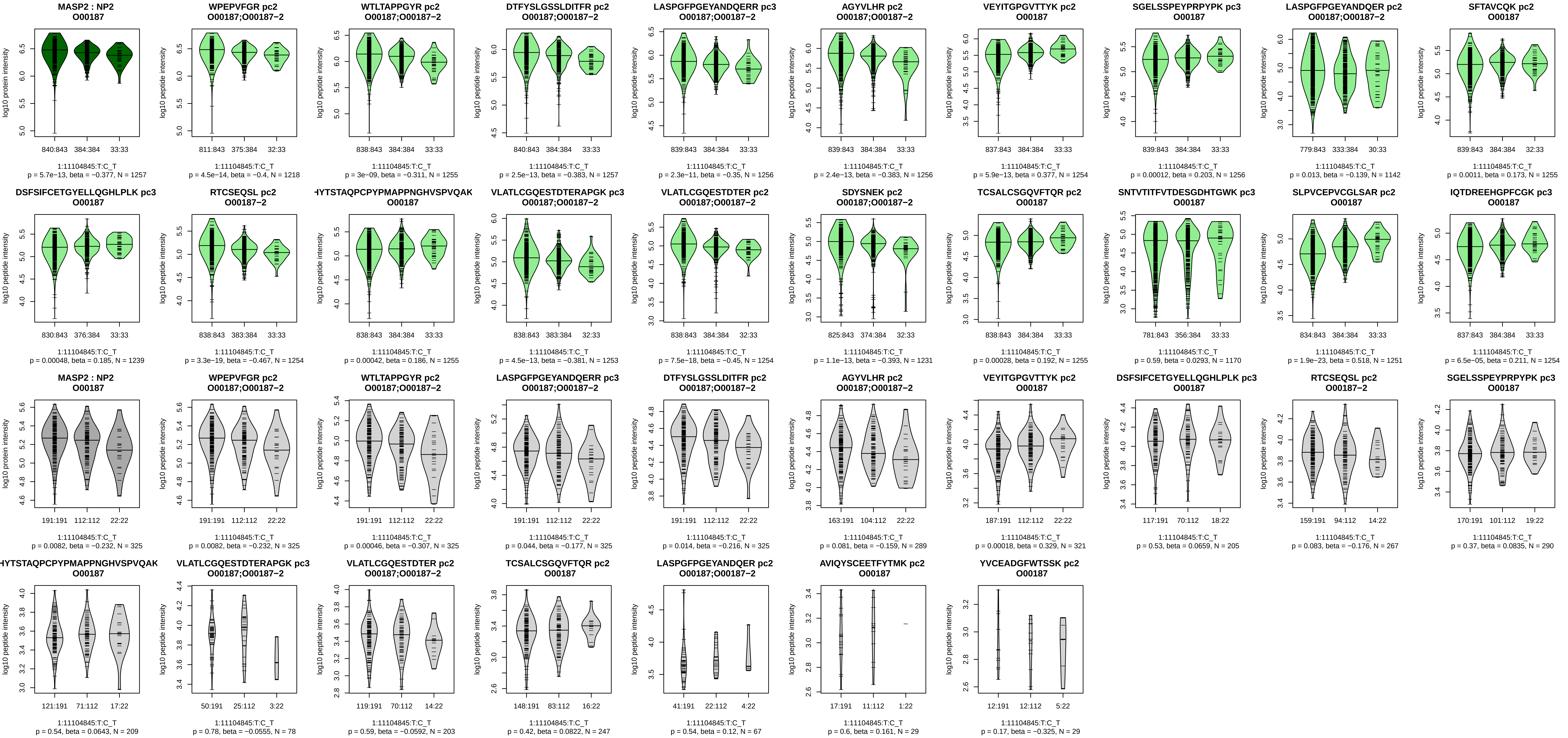

82Assay Target: MASP2
UniProt: O00187
rsID: rs9430347
Target SNP: 1:11104845:T:C
Proxy SNP: 1:11104845:T:C
Tarkin log10(p): 18.5
Tarkin BETA: −0.467
Tarkin MSPA: 0.806
[illegible]
1218:1255:1257:1256:1256:125

83. CPXM2 (Q8N436)
Tarkin MSPA=0.812

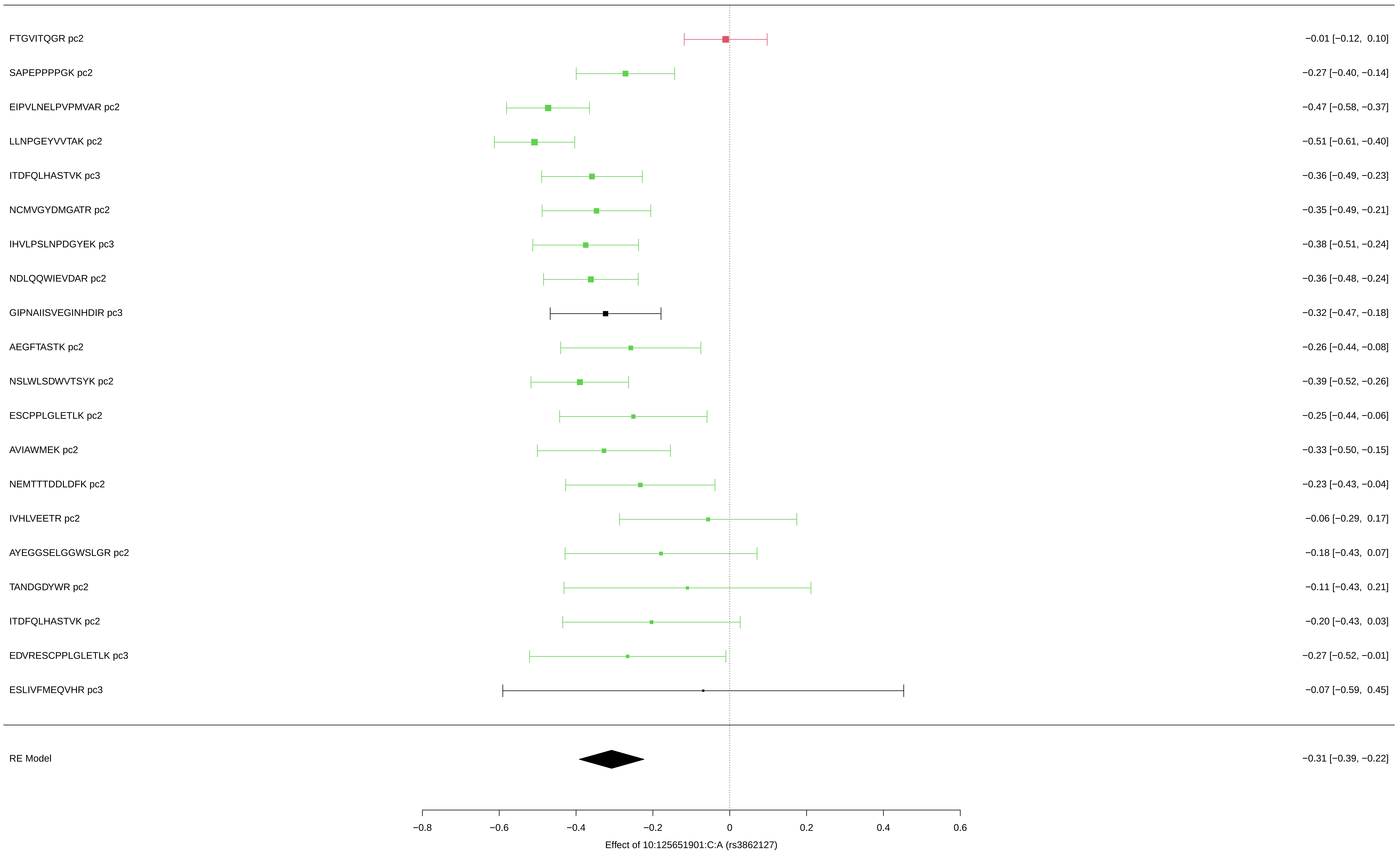

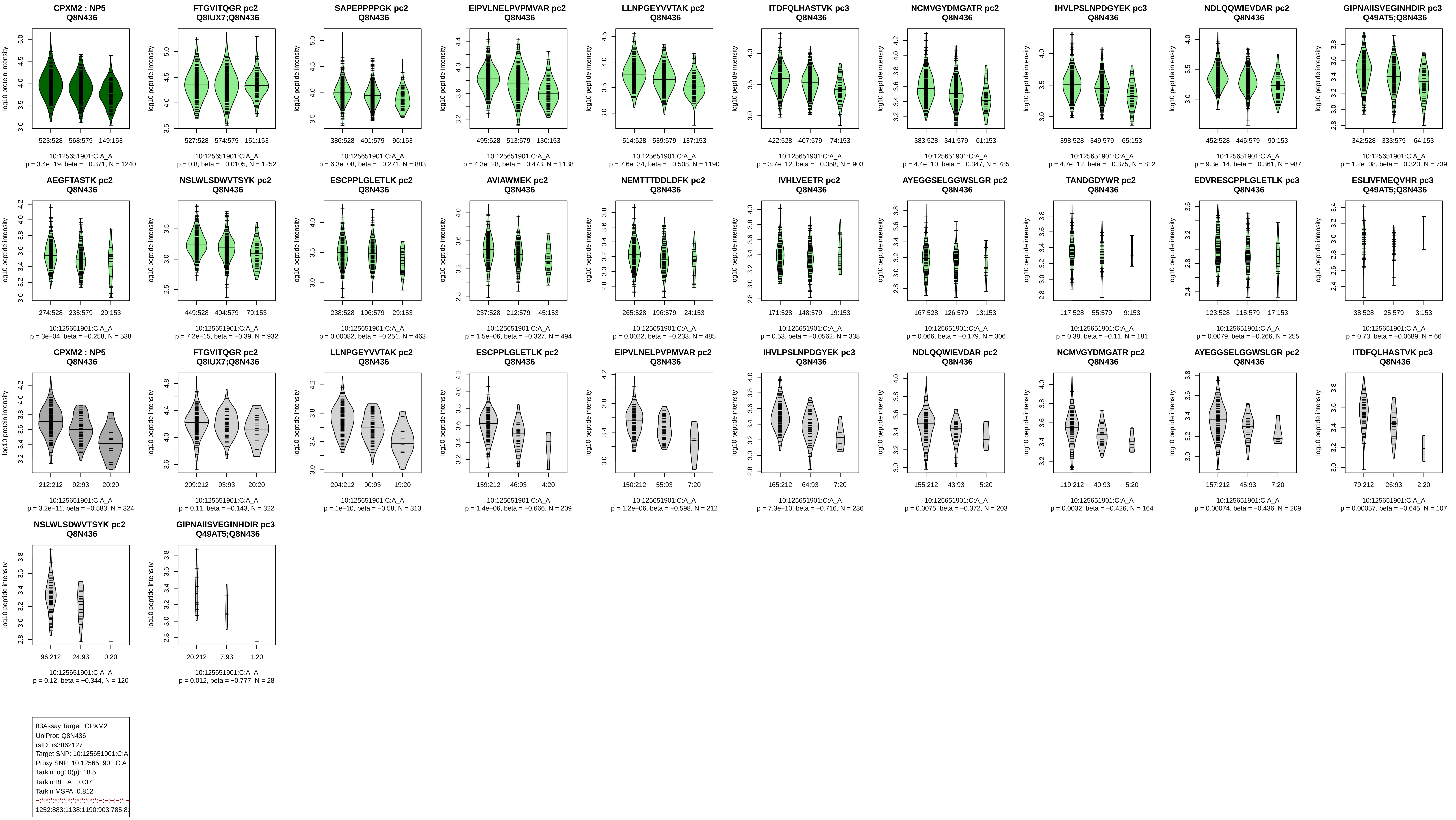
CPXM2 : NP5
Q8N436
log10 protein intensity
523:528 568:579 149:153
10:125651901:C:A_A
p = 3.4e−19, beta = −0.371, N = 1240
FTGVITQGR pc2
Q8IUX7;Q8N436
log10 peptide intensity
527:528 574:579 151:153
10:125651901:C:A_A
p = 0.8, beta = −0.0105, N = 1252
SAPEPPPPGK pc2
Q8N436
386:528 401:579 96:153
10:125651901:C:A_A
p = 6.3e−08, beta = −0.271, N = 883
EIPVLNELPVPMVAR pc2
Q8N436
495:528 513:579 130:153
10:125651901:C:A_A
p = 4.3e−28, beta = −0.473, N = 1138
LLNPGEYVVTAK pc2
Q8N436
514:528 539:579 137:153
10:125651901:C:A_A
p = 7.6e−34, beta = −0.508, N = 1190
ITDFQLHASTVK pc3
Q8N436
422:528 407:579 74:153
10:125651901:C:A_A
p = 3.7e−12, beta = −0.358, N = 903
NCMVGYDMGATR pc2
Q8N436
383:528 341:579 61:153
10:125651901:C:A_A
p = 4.4e−10, beta = −0.347, N = 785
IHVLPSLNPDGYEK pc3
Q8N436
398:528 349:579 65:153
10:125651901:C:A_A
p = 4.7e−12, beta = −0.375, N = 812
NDLQQWIEVDAR pc2
Q8N436
452:528 445:579 90:153
10:125651901:C:A_A
p = 9.3e−14, beta = −0.361, N = 987
GIPNAIISVEGINHDIR pc3
Q49AT5;Q8N436
342:528 333:579 64:153
10:125651901:C:A_A
p = 1.2e−08, beta = −0.323, N = 739
AEGFTASTK pc2
Q8N436
274:528 235:579 29:153
10:125651901:C:A_A
p = 3e−04, beta = −0.258, N = 538
NSLWLSDWVTSYK pc2
Q8N436
449:528 404:579 79:153
10:125651901:C:A_A
p = 7.2e−15, beta = −0.39, N = 932
ESCPPLGLETLK pc2
Q8N436
238:528 196:579 29:153
10:125651901:C:A_A
p = 0.00082, beta = −0.251, N = 463
AVIAWMEK pc2
Q8N436
237:528 212:579 45:153
10:125651901:C:A_A
p = 1.5e−06, beta = −0.327, N = 494
NEMTTTDDLDFK pc2
Q8N436
265:528 196:579 24:153
10:125651901:C:A_A
p = 0.0022, beta = −0.233, N = 485
IVHLVEETR pc2
Q8N436
171:528 148:579 19:153
10:125651901:C:A_A
p = 0.53, beta = −0.0562, N = 338
AYEGGSELGGWSLGR pc2
Q8N436
167:528 126:579 13:153
10:125651901:C:A_A
p = 0.066, beta = −0.179, N = 306
TANDGDYWR pc2
Q8N436
117:528 55:579 9:153
10:125651901:C:A_A
p = 0.38, beta = −0.11, N = 181
EDVRESCPPLGLETLK pc3
Q8N436
123:528 115:579 17:153
10:125651901:C:A_A
p = 0.0079, beta = −0.266, N = 255
ESLIVFMEQVHR pc3
Q49AT5;Q8N436
38:528 25:579 3:153
10:125651901:C:A_A
p = 0.73, beta = −0.0689, N = 66
CPXM2 : NP5
Q8N436
log10 protein intensity
212:212 92:93 20:20
10:125651901:C:A_A
p = 3.2e−11, beta = −0.583, N = 324
FTGVITQGR pc2
Q8IUX7;Q8N436
209:212 93:93 20:20
10:125651901:C:A_A
p = 0.11, beta = −0.143, N = 322
LLNPGEYVVTAK pc2
Q8N436
204:212 90:93 19:20
10:125651901:C:A_A
p = 1e−10, beta = −0.58, N = 313
ESCPPLGLETLK pc2
Q8N436
159:212 46:93 4:20
10:125651901:C:A_A
p = 1.4e−06, beta = −0.666, N = 209
EIPVLNELPVPMVAR pc2
Q8N436
150:212 55:93 7:20
10:125651901:C:A_A
p = 1.2e−06, beta = −0.598, N = 212
IHVLPSLNPDGYEK pc3
Q8N436
165:212 64:93 7:20
10:125651901:C:A_A
p = 7.3e−10, beta = −0.716, N = 236
NDLQQWIEVDAR pc2
Q8N436
155:212 43:93 5:20
10:125651901:C:A_A
p = 0.0075, beta = −0.372, N = 203
NCMVGYDMGATR pc2
Q8N436
119:212 40:93 5:20
10:125651901:C:A_A
p = 0.0032, beta = −0.426, N = 164
AYEGGSELGGWSLGR pc2
Q8N436
157:212 45:93 7:20
10:125651901:C:A_A
p = 0.00074, beta = −0.436, N = 209
ITDFQLHASTVK pc3
Q8N436
79:212 26:93 2:20
10:125651901:C:A_A
p = 0.00057, beta = −0.645, N = 107
NSLWLSDWVTSYK pc2
Q8N436
96:212 24:93 0:20
10:125651901:C:A_A
p = 0.12, beta = −0.344, N = 120
GIPNAIISVEGINHDIR pc3
Q49AT5;Q8N436
20:212 7:93 1:20
10:125651901:C:A_A
p = 0.012, beta = −0.777, N = 28
83Assay Target: CPXM2
UniProt: Q8N436
rsID: rs3862127
Target SNP: 10:125651901:C:A
Proxy SNP: 10:125651901:C:A
Tarkin log10(p): 18.5
Tarkin BETA: −0.371
Tarkin MSPA: 0.812
1252:883:1138:1190:903:785:8

84. MMP3 (P08254)
Tarkin MSPA=0.964

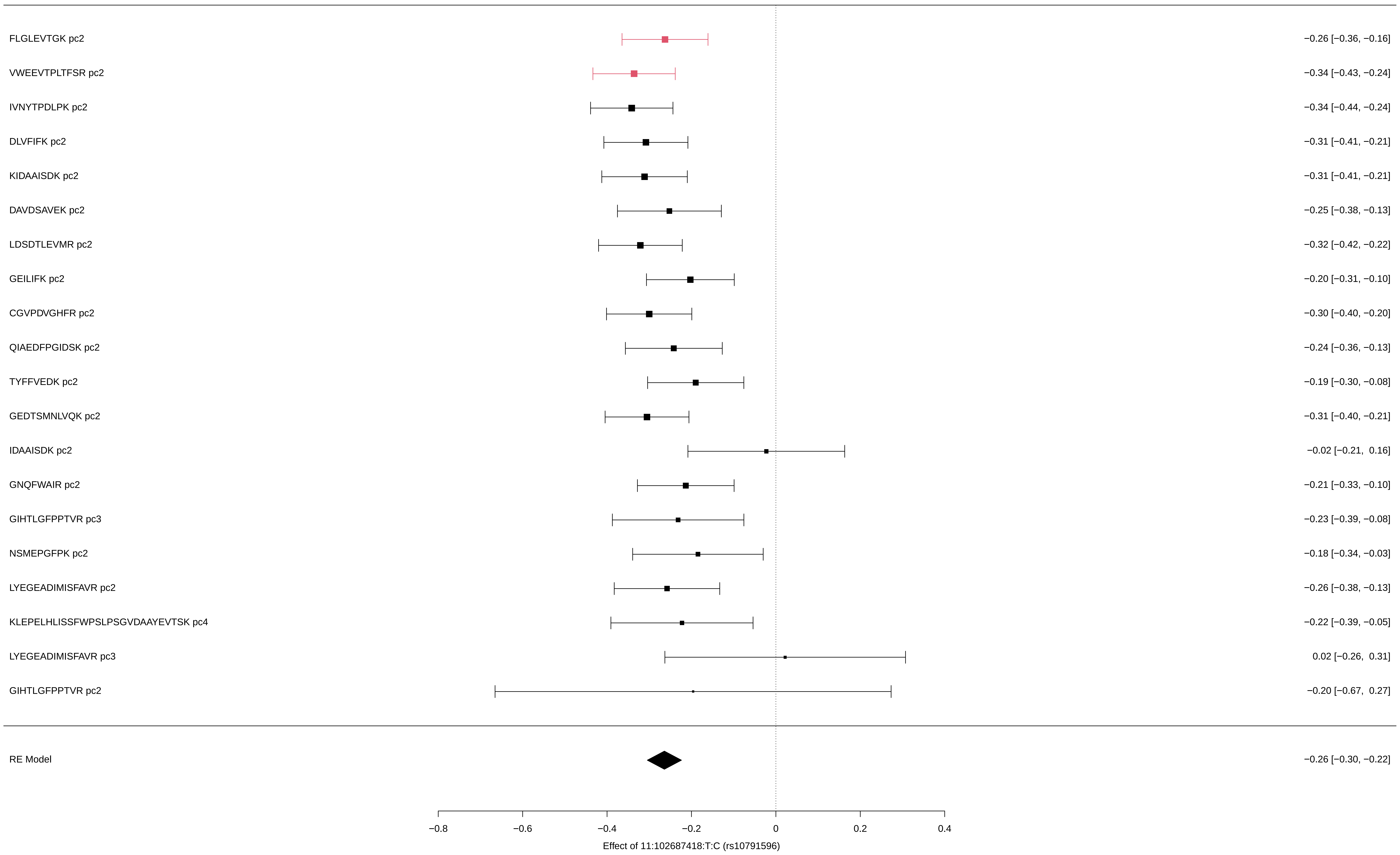

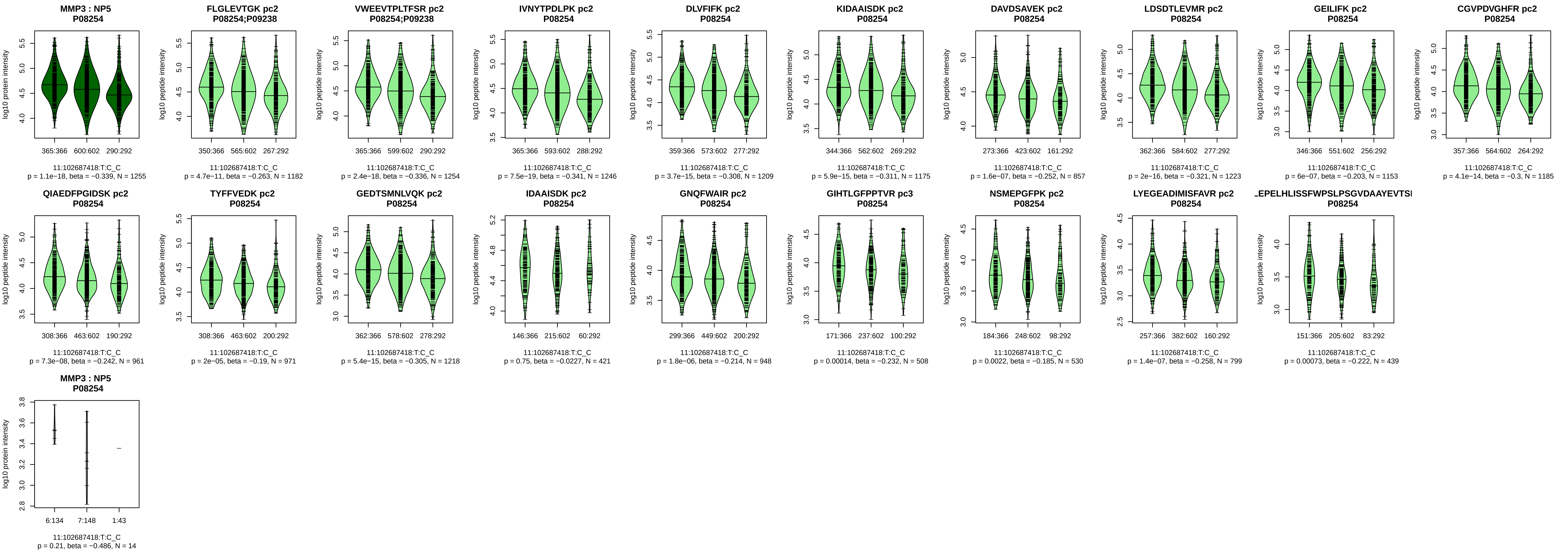

84Assay Target: MMP3
UniProt: P08254
rsID: rs10791596
Target SNP: 11:102687418:T:C
Proxy SNP: 11:102687418:T:C
Tarkin log10(p): 18
Tarkin BETA: −0.339
Tarkin MSPA: 0.964
1182:1254:1246:1209:1175:857

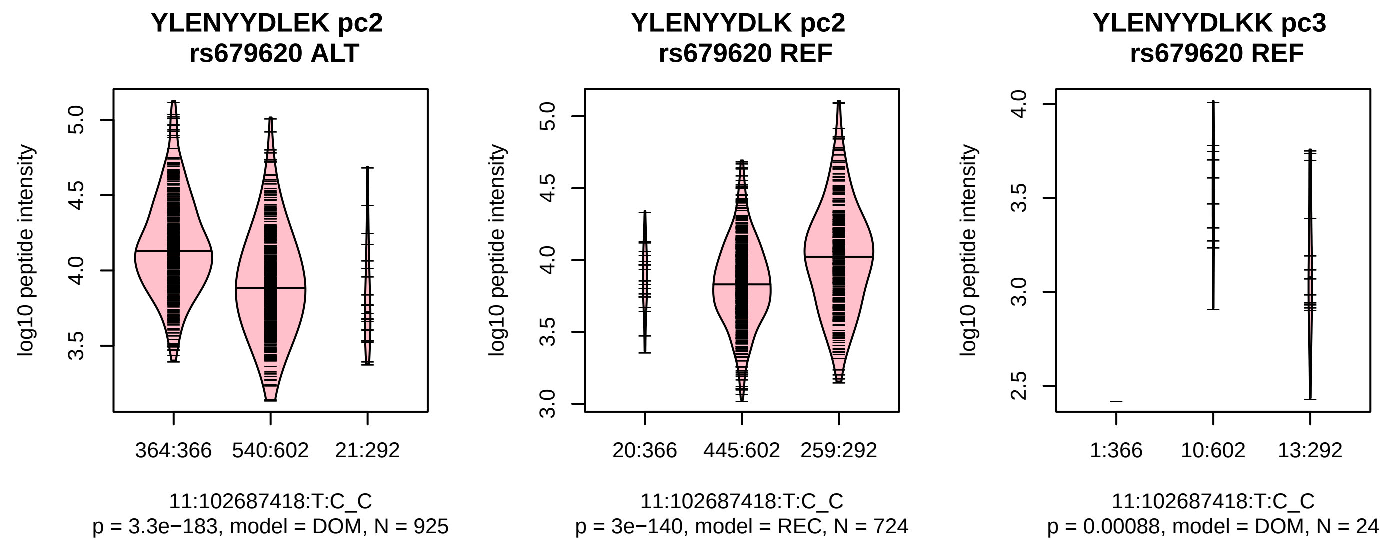

85. COLEC10 (Q9Y6Z7)
Tarkin MSPA=0.748

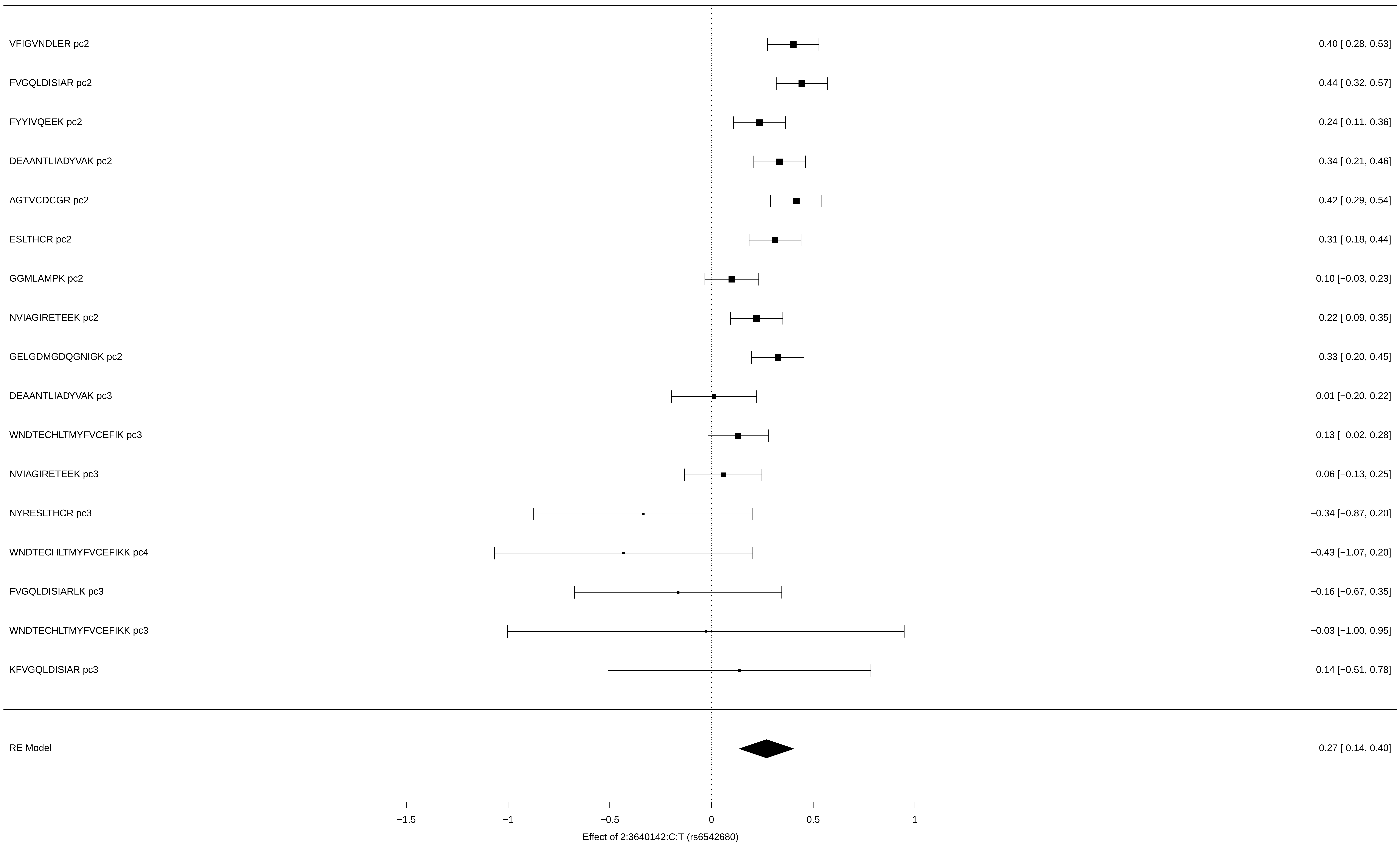

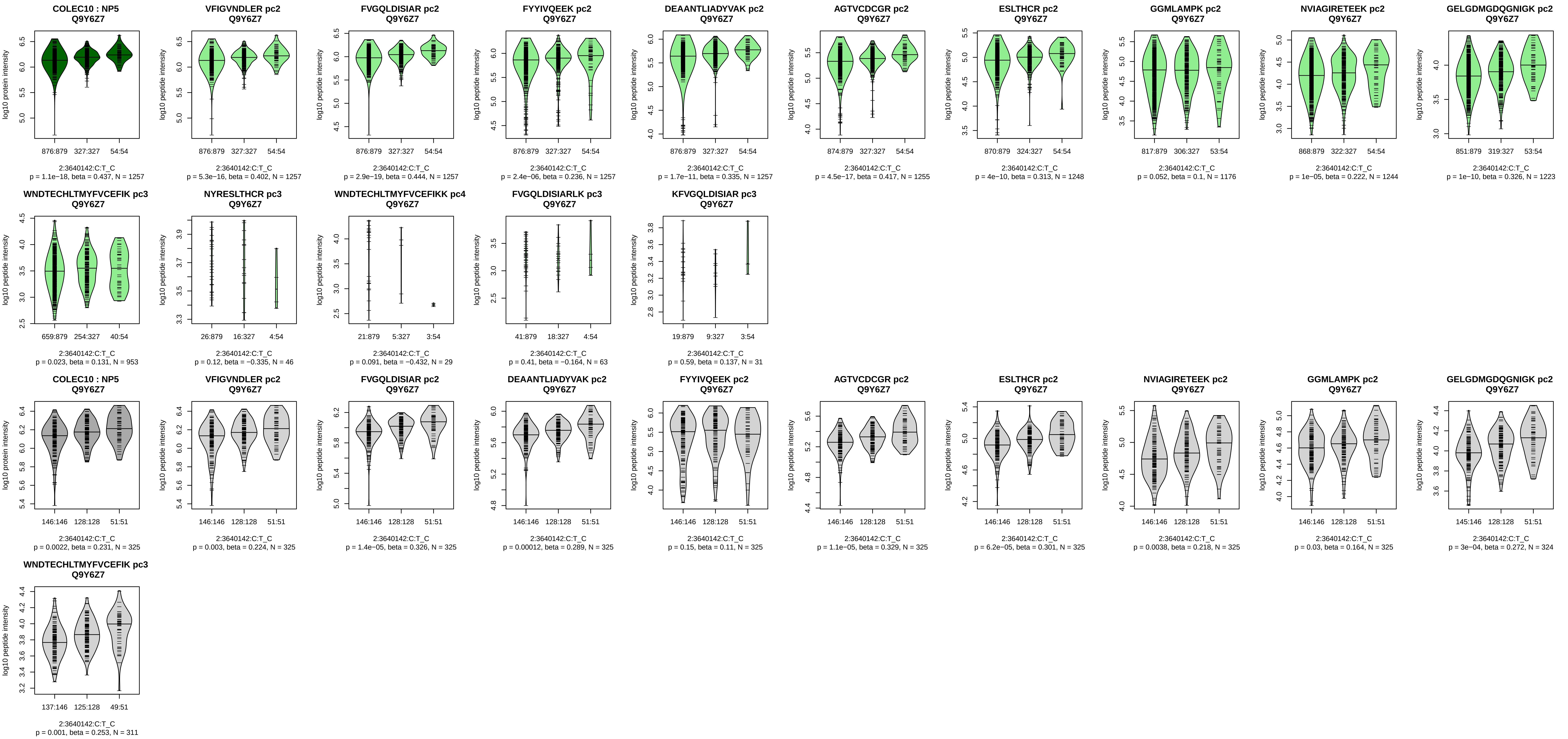

85Assay Target: COLEC10
UniProt: Q9Y6Z7
rsID: rs6542680
Target SNP: 2:3640142:C:T
Proxy SNP: 2:3640142:C:T
Tarkin log10(p): 17.9
Tarkin BETA: 0.437
Tarkin MSPA: 0.748
1257:1257:1257:1257:1255:124

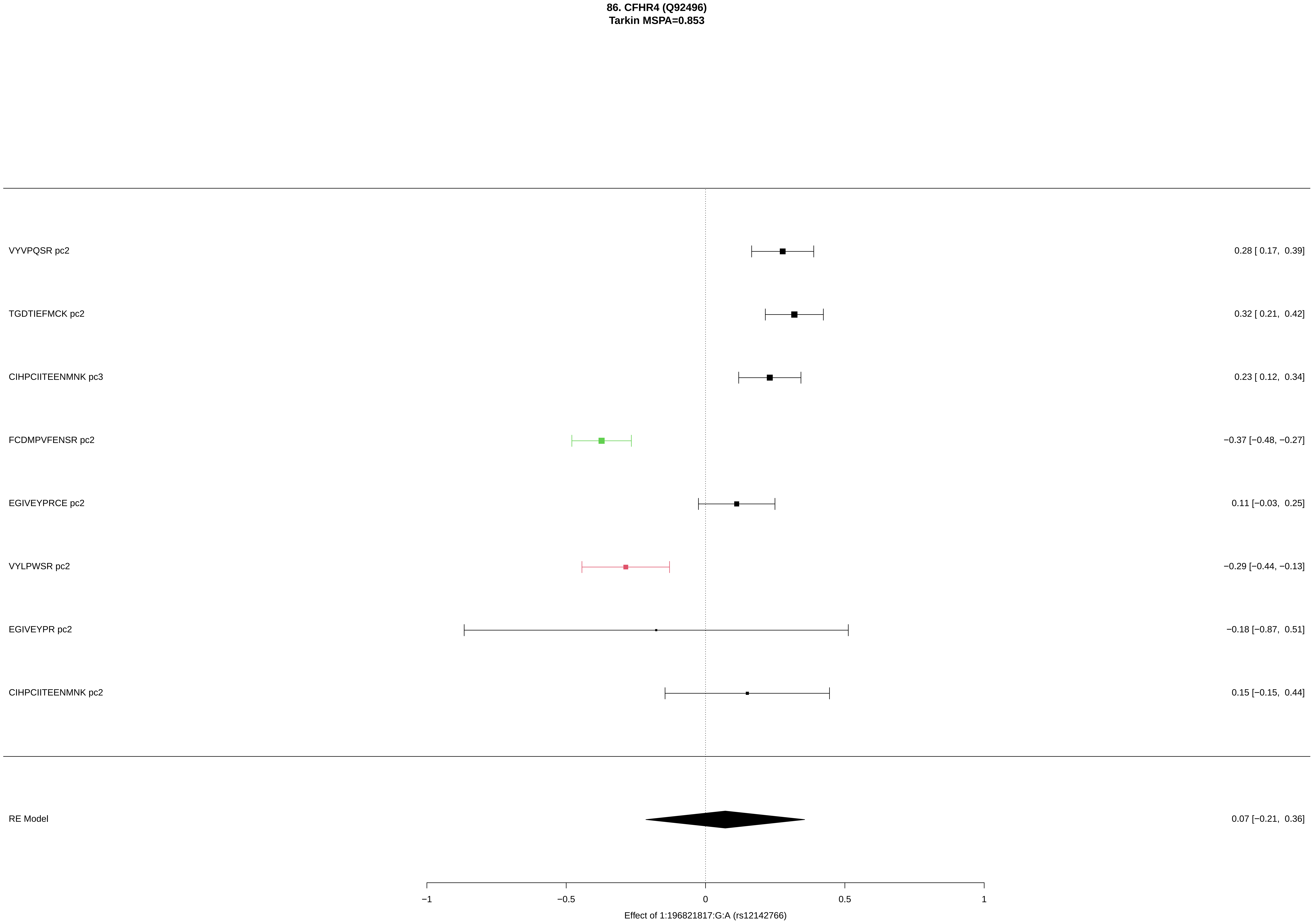
86. CFHR4 (Q92496)
Tarkin MSPA=0.853
VYVPQSR pc2
0.28 [0.17, 0.39]
TGDTIEFMCK pc2
0.32 [0.21, 0.42]
CIHPCIITEENMNK pc3
0.23 [0.12, 0.34]
FCDMPVFENSR pc2
−0.37 [−0.48, −0.27]
EGIVEYPRCE pc2
0.11 [−0.03, 0.25]
VYLPWSR pc2
−0.29 [−0.44, −0.13]
EGIVEYPR pc2
−0.18 [−0.87, 0.51]
CIHPCIITEENMNK pc2
0.15 [−0.15, 0.44]
RE Model
0.07 [−0.21, 0.36]
−1
−0.5
0
0.5
1
Effect of 1:196821817:G:A (rs12142766)

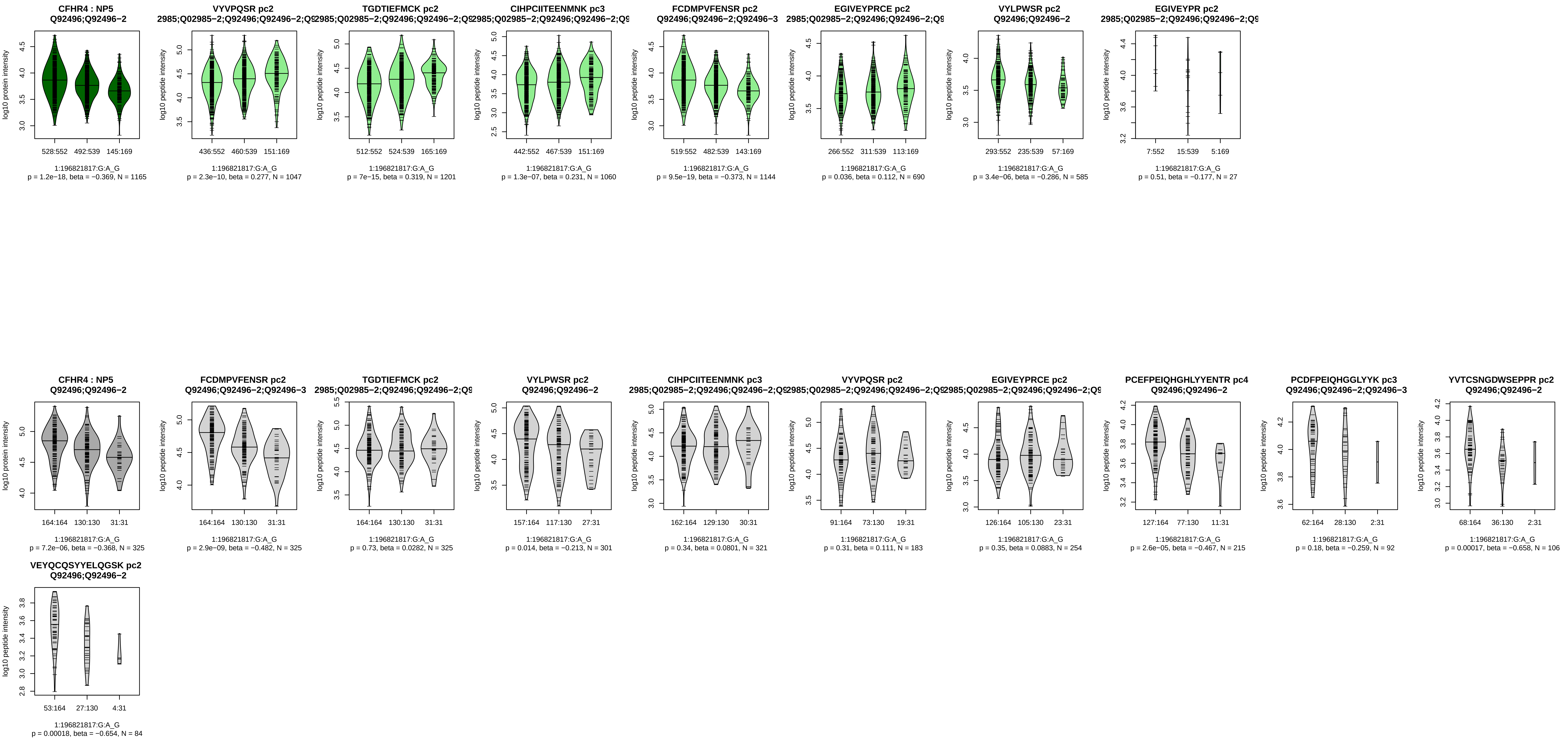

86Assay Target: CFHR4
UniProt: Q92496
rsID: rs12142766
Target SNP: 1:196821817:G:A
Proxy SNP: 1:196821817:G:A
Tarkin log10(p): 17.9
Tarkin BETA: −0.369
Tarkin MSPA: 0.853
1047:1201:1060:1144:690:585:2

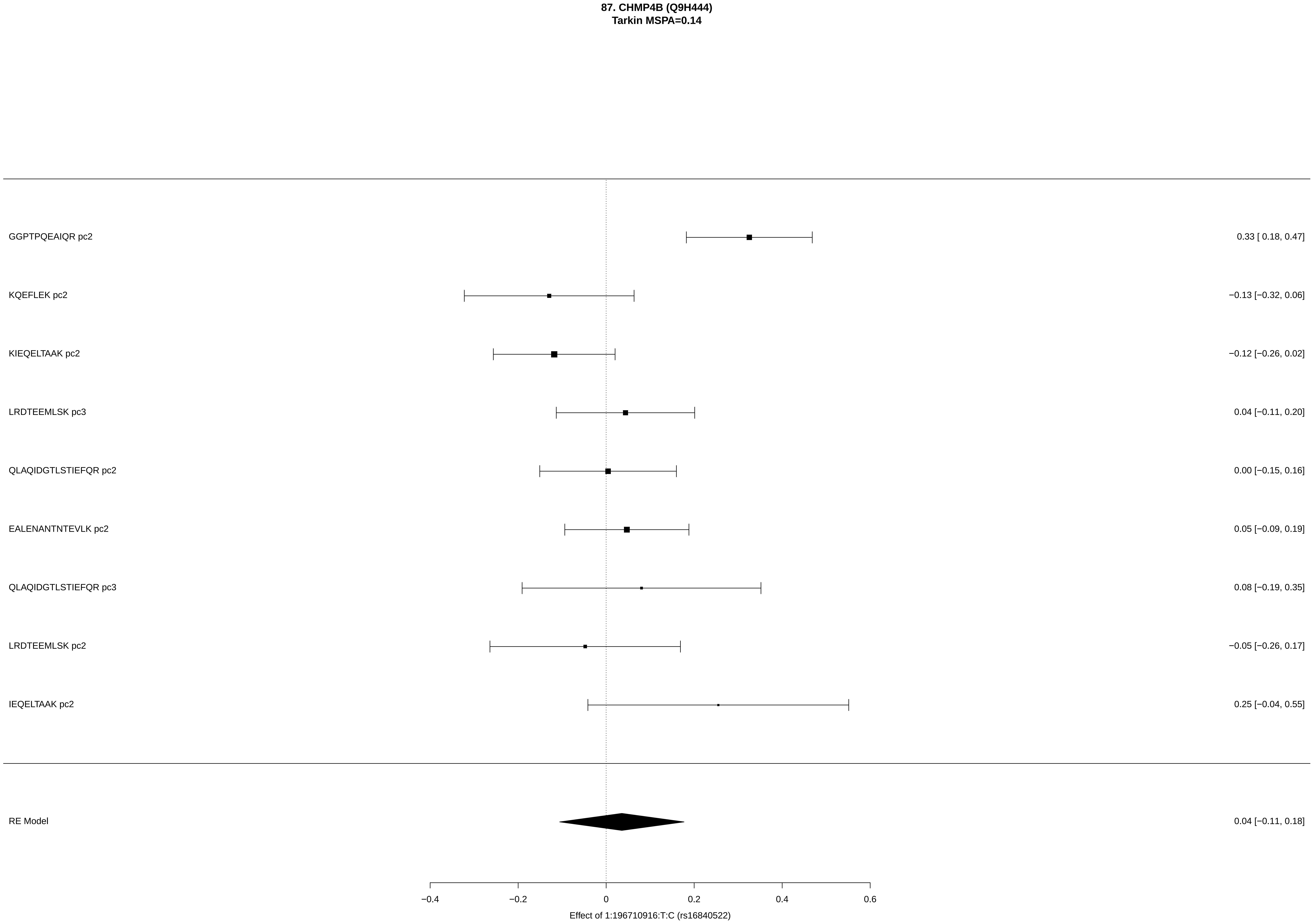
87. CHMP4B (Q9H444)
Tarkin MSPA=0.14
GGPTPQEAIQR pc2
0.33 [0.18, 0.47]
KQEFLEK pc2
−0.13 [−0.32, 0.06]
KIEQELTAAK pc2
−0.12 [−0.26, 0.02]
LRDTEEMLSK pc3
0.04 [−0.11, 0.20]
QLAQIDGTLSTIEFQR pc2
0.00 [−0.15, 0.16]
EALENANTNTEVLK pc2
0.05 [−0.09, 0.19]
QLAQIDGTLSTIEFQR pc3
0.08 [−0.19, 0.35]
LRDTEEMLSK pc2
−0.05 [−0.26, 0.17]
IEQELTAAK pc2
0.25 [−0.04, 0.55]
RE Model
0.04 [−0.11, 0.18]
−0.4
−0.2
0
0.2
0.4
0.6
Effect of 1:196710916:T:C (rs16840522)

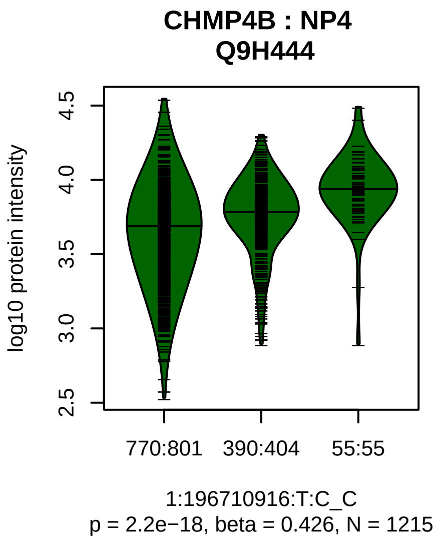

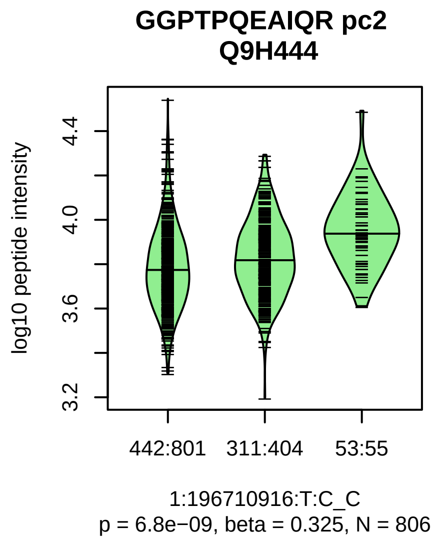

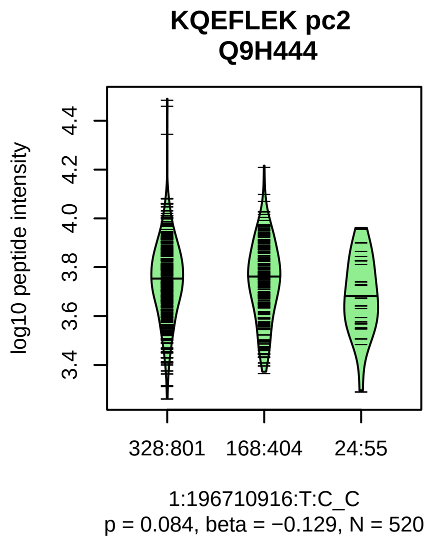

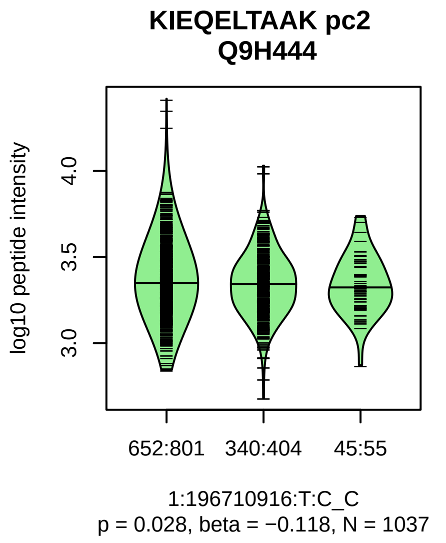

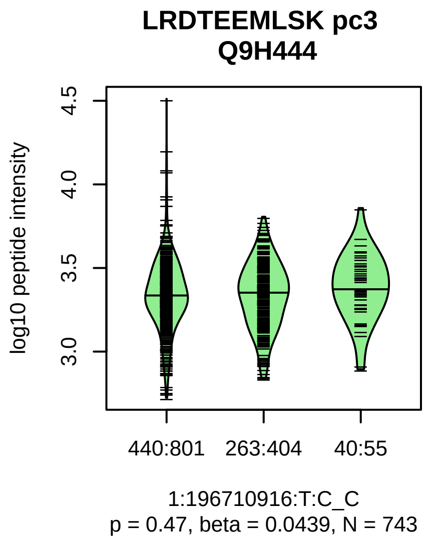

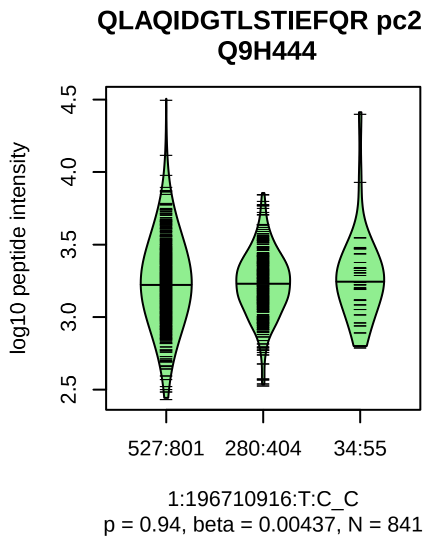

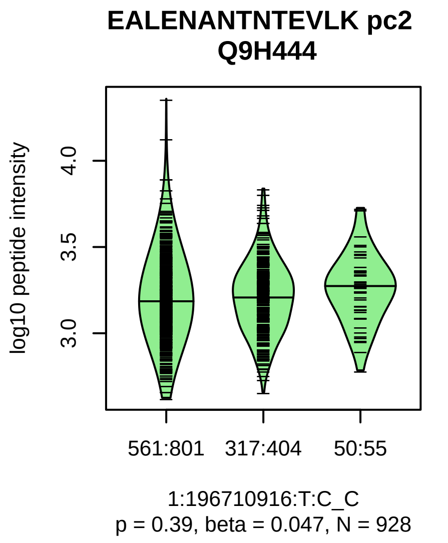

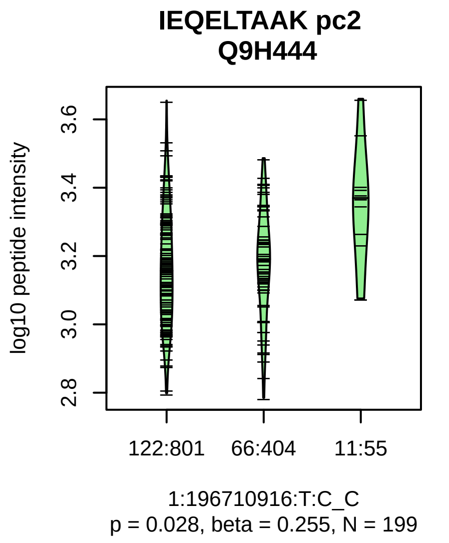

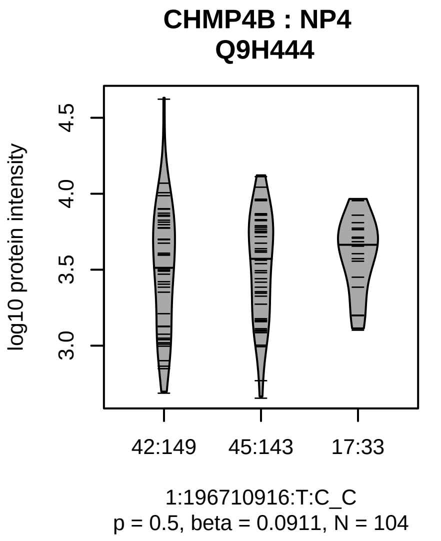

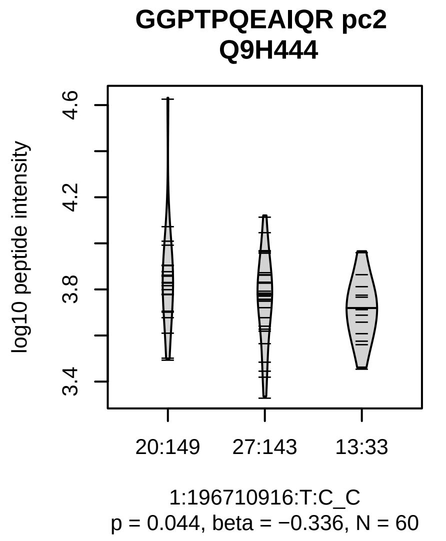

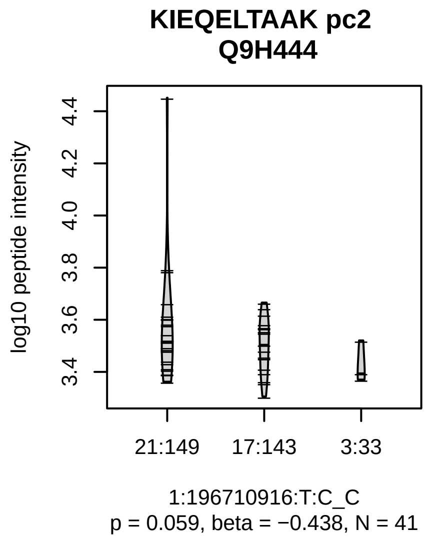

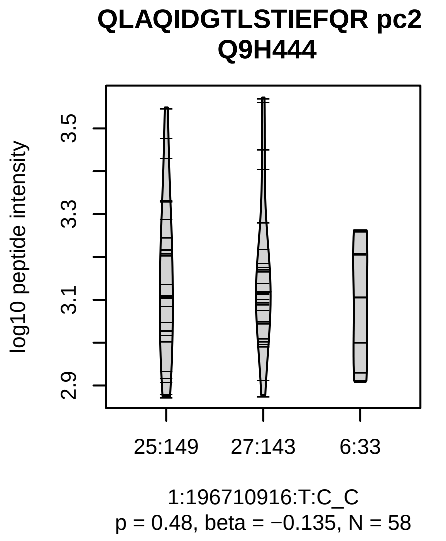

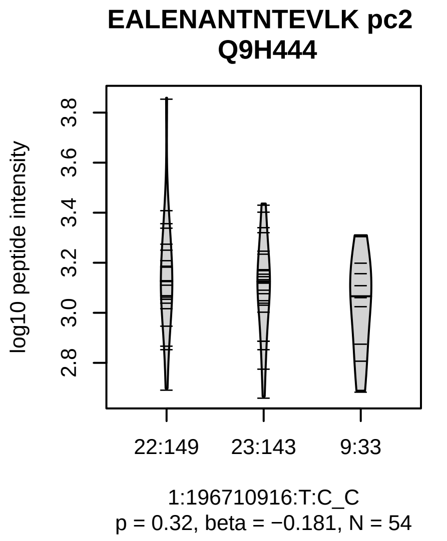

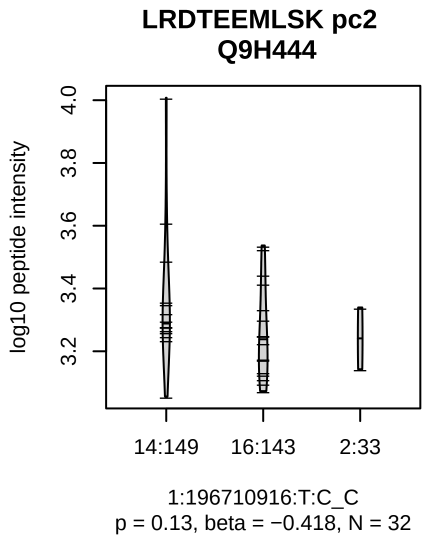

87Assay Target: CHMP4B
UniProt: Q9H444
rsID: rs16840522
Target SNP: 1:196710916:T:C
Proxy SNP: 1:196710916:T:C
Tarkin log10(p): 17.7
Tarkin BETA: 0.426
Tarkin MSPA: 0.14
*:–:–:–:–:–:–:–:–
806:520:1037:743:841:928:283:

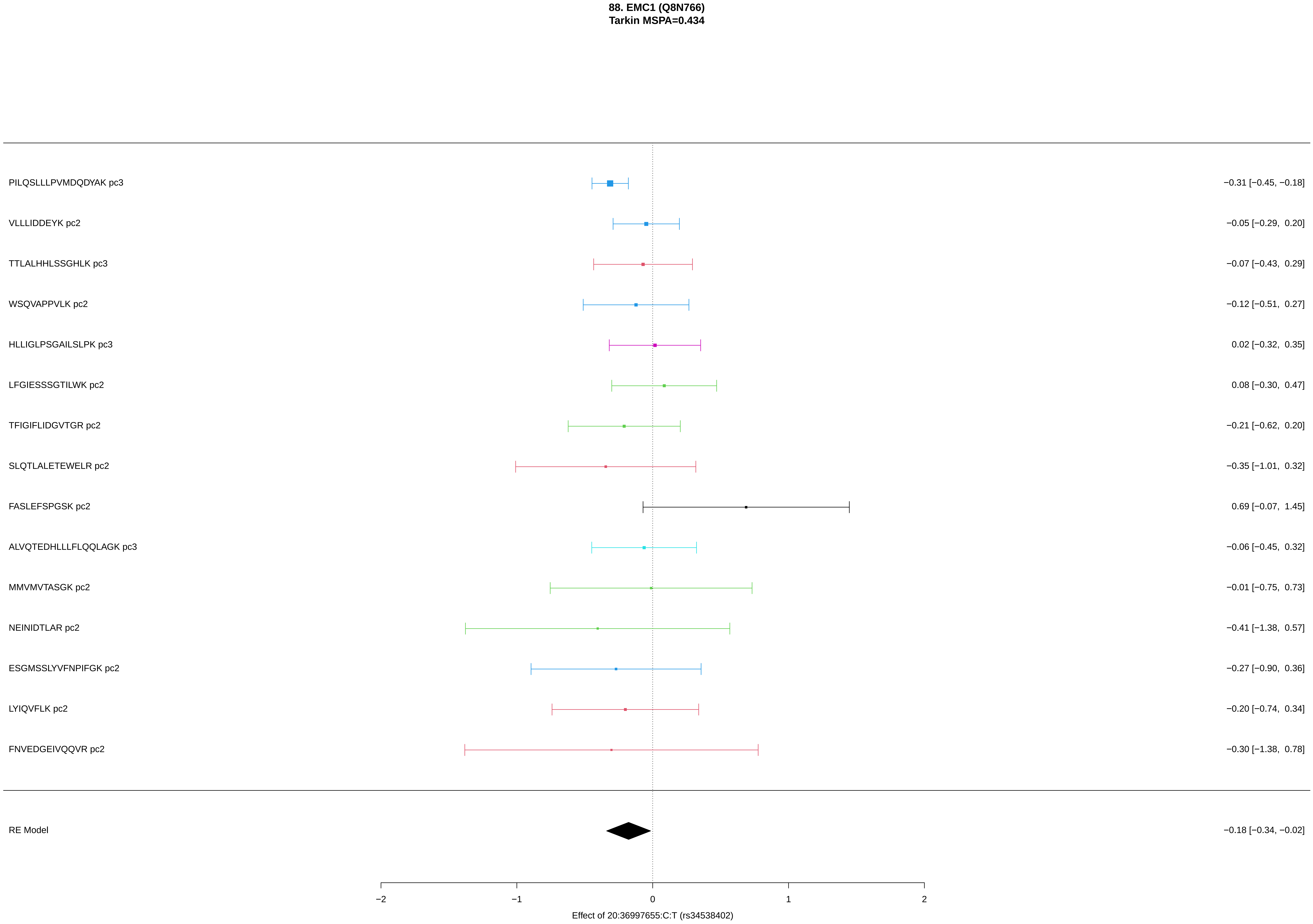

88. EMC1 (Q8N766)
Tarkin MSPA=0.434
PILQSLLLPVMDQDYAK pc3
−0.31 [−0.45, −0.18]
VLLLIDDEYK pc2
−0.05 [−0.29, 0.20]
TTLALHHLSSGHLK pc3
−0.07 [−0.43, 0.29]
WSQVAPPVLK pc2
−0.12 [−0.51, 0.27]
HLLIGLPSGAILSLPK pc3
0.02 [−0.32, 0.35]
LFGIESSSGTILWK pc2
0.08 [−0.30, 0.47]
TFIGIFLIDGVTGR pc2
−0.21 [−0.62, 0.20]
SLQTLALETEWELR pc2
−0.35 [−1.01, 0.32]
FASLEFSPGSK pc2
0.69 [−0.07, 1.45]
ALVQTEDHLLLFLQQLAGK pc3
−0.06 [−0.45, 0.32]
MMVMVTASGK pc2
−0.01 [−0.75, 0.73]
NEINIDTLAR pc2
−0.41 [−1.38, 0.57]
ESGMSSLYVFNPIFGK pc2
−0.27 [−0.90, 0.36]
LYIQVFLK pc2
−0.20 [−0.74, 0.34]
FNVEDGEIVQQVR pc2
−0.30 [−1.38, 0.78]
RE Model
−0.18 [−0.34, −0.02]
−2
−1
0
1
2
Effect of 20:36997655:C:T (rs34538402)

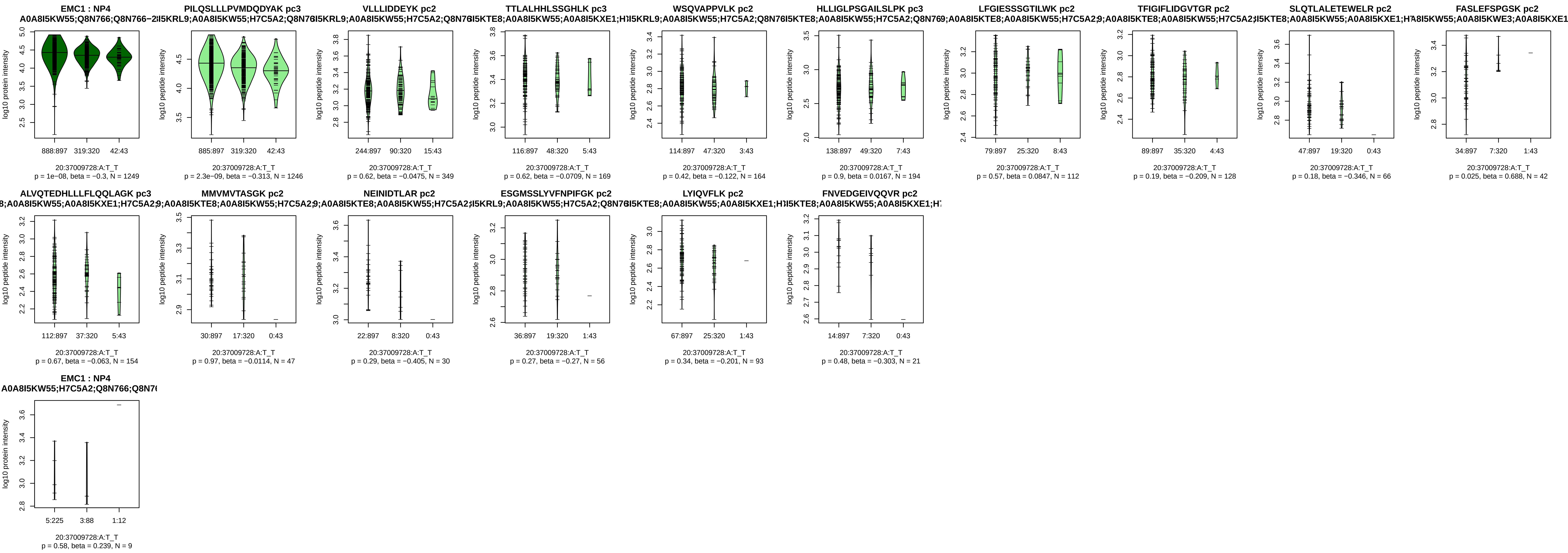

88Assay Target: EMC1
UniProt: Q8N766
rsID: rs34538402
Target SNP: 20:36997655:C:T
Proxy SNP: 20:37009728:A:T
Tarkin log10(p): 17.6
Tarkin BETA: −0.633
Tarkin MSPA: 0.434
*-:-:-:-:-:-:-:-:-:-:-:-:-:-:-:-:NA
1246:349:169:164:194:112:128:

89. ADAMDEC1 (O15204)
Tarkin MSPA=0.848

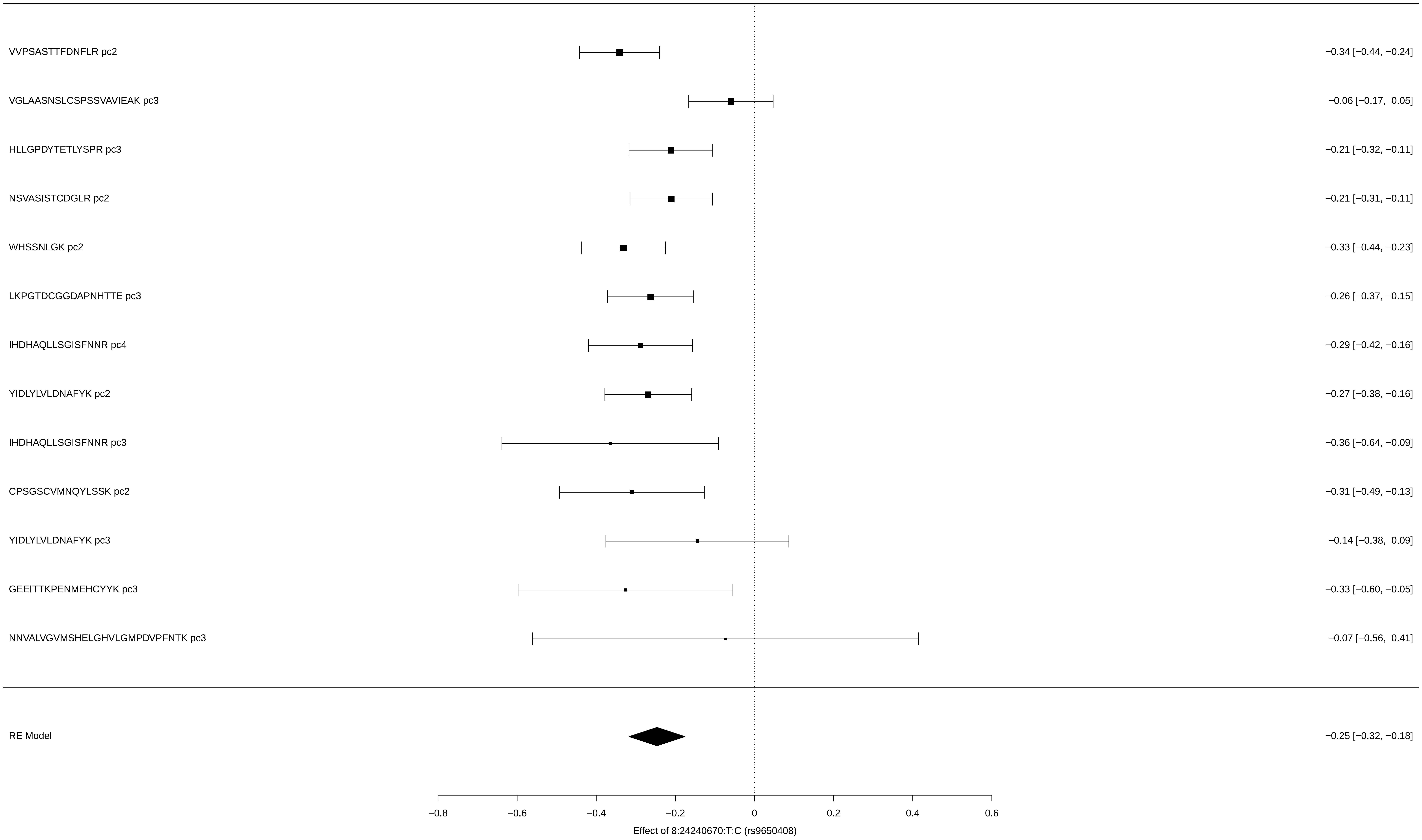

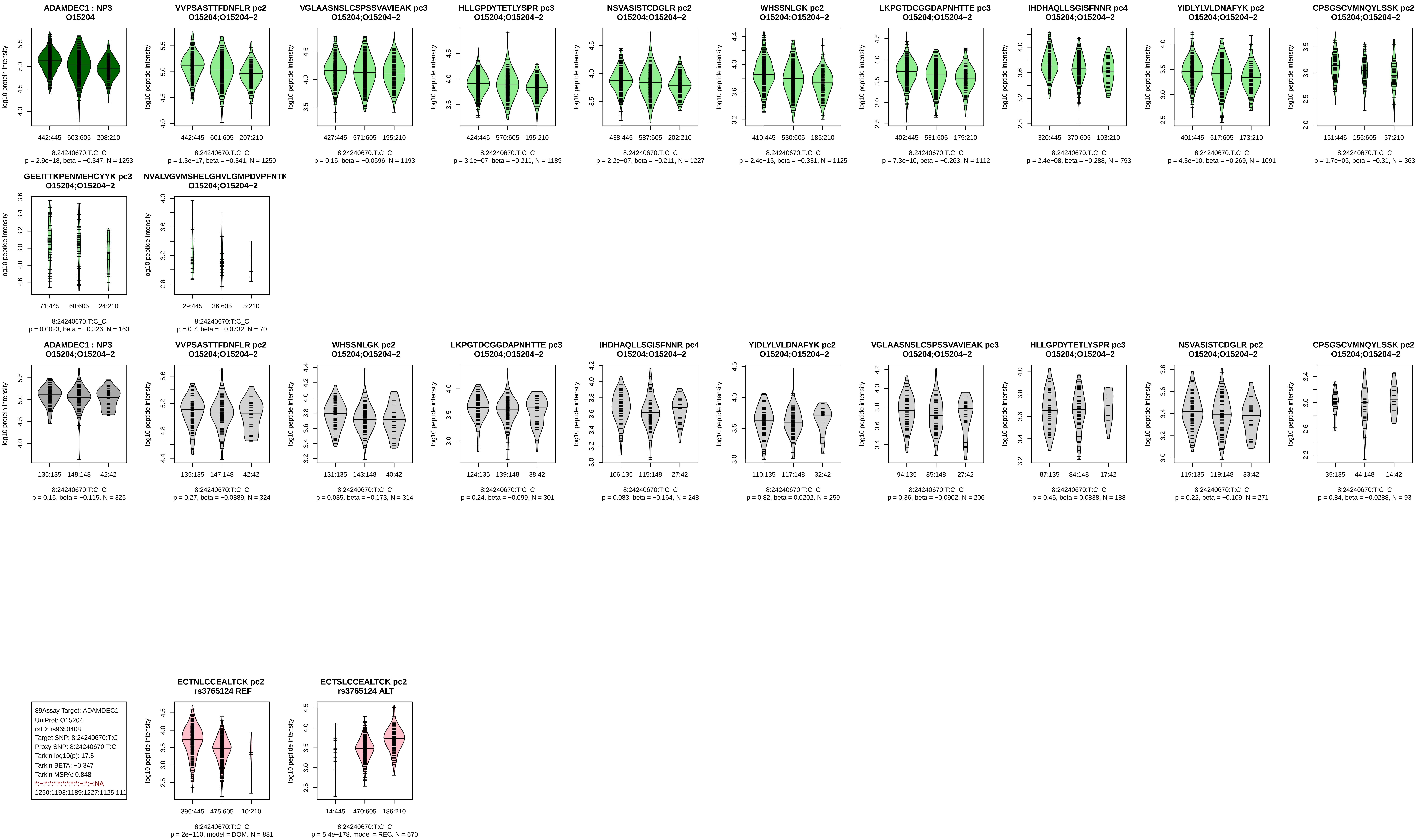
ADAMDEC1 : NP3
O15204
log10 protein intensity
442:445 603:605 208:210
8:24240670:T:C_C
p = 2.9e−18, beta = −0.347, N = 1253
VVPSASTTFDNFLR pc2
O15204;O15204−2
log10 peptide intensity
442:445 601:605 207:210
8:24240670:T:C_C
p = 1.3e−17, beta = −0.341, N = 1250
VGLAASNSLCSPSSVAVIEAK pc3
O15204;O15204−2
427:445 571:605 195:210
8:24240670:T:C_C
p = 0.15, beta = −0.0596, N = 1193
HLLGPDYTETLYSPR pc3
O15204;O15204−2
424:445 570:605 195:210
8:24240670:T:C_C
p = 3.1e−07, beta = −0.211, N = 1189
NSVASISTCDGLR pc2
O15204;O15204−2
438:445 587:605 202:210
8:24240670:T:C_C
p = 2.2e−07, beta = −0.211, N = 1227
WHSSNLGK pc2
O15204;O15204−2
410:445 530:605 185:210
8:24240670:T:C_C
p = 2.4e−15, beta = −0.331, N = 1125
LKPGTDCGGDAPNHTTE pc3
O15204;O15204−2
402:445 531:605 179:210
8:24240670:T:C_C
p = 7.3e−10, beta = −0.263, N = 1112
IHDHAQLLSGISFNNR pc4
O15204;O15204−2
320:445 370:605 103:210
8:24240670:T:C_C
p = 2.4e−08, beta = −0.288, N = 793
YIDLYLVLDNAFYK pc2
O15204;O15204−2
401:445 517:605 173:210
8:24240670:T:C_C
p = 4.3e−10, beta = −0.269, N = 1091
CPSGSCVMNQYLSSK pc2
O15204;O15204−2
151:445 155:605 57:210
8:24240670:T:C_C
p = 1.7e−05, beta = −0.31, N = 363
GEEITTKPENMEHCYYK pc3
O15204;O15204−2
71:445 68:605 24:210
8:24240670:T:C_C
p = 0.0023, beta = −0.326, N = 163
INVALVGVMSHELGHVLGMPDVPFNTK
O15204;O15204−2
29:445 36:605 5:210
8:24240670:T:C_C
p = 0.7, beta = −0.0732, N = 70
ADAMDEC1 : NP3
O15204;O15204−2
135:135 148:148 42:42
8:24240670:T:C_C
p = 0.15, beta = −0.115, N = 325
VVPSASTTFDNFLR pc2
O15204;O15204−2
135:135 147:148 42:42
8:24240670:T:C_C
p = 0.27, beta = −0.0889, N = 324
WHSSNLGK pc2
O15204;O15204−2
131:135 143:148 40:42
8:24240670:T:C_C
p = 0.035, beta = −0.173, N = 314
LKPGTDCGGDAPNHTTE pc3
O15204;O15204−2
124:135 139:148 38:42
8:24240670:T:C_C
p = 0.24, beta = −0.099, N = 301
IHDHAQLLSGISFNNR pc4
O15204;O15204−2
106:135 115:148 27:42
8:24240670:T:C_C
p = 0.083, beta = −0.164, N = 248
YIDLYLVLDNAFYK pc2
O15204;O15204−2
110:135 117:148 32:42
8:24240670:T:C_C
p = 0.82, beta = 0.0202, N = 259
VGLAASNSLCSPSSVAVIEAK pc3
O15204;O15204−2
94:135 85:148 27:42
8:24240670:T:C_C
p = 0.36, beta = −0.0902, N = 206
HLLGPDYTETLYSPR pc3
O15204;O15204−2
87:135 84:148 17:42
8:24240670:T:C_C
p = 0.45, beta = 0.0838, N = 188
NSVASISTCDGLR pc2
O15204;O15204−2
119:135 119:148 33:42
8:24240670:T:C_C
p = 0.22, beta = −0.109, N = 271
CPSGSCVMNQYLSSK pc2
O15204;O15204−2
35:135 44:148 14:42
8:24240670:T:C_C
p = 0.84, beta = −0.0288, N = 93
89Assay Target: ADAMDEC1
UniProt: O15204
rsID: rs9650408
Target SNP: 8:24240670:T:C
Proxy SNP: 8:24240670:T:C
Tarkin log10(p): 17.5
Tarkin BETA: −0.347
Tarkin MSPA: 0.848
:−::*:*:*:*:*:*:−:*:−:NA
1250:1193:1189:1227:1125:111
ECTNLCCEALTCK pc2
rs3765124 REF
396:445 475:605 10:210
8:24240670:T:C_C
p = 2e−110, model = DOM, N = 881
ECTSLCCEALTCK pc2
rs3765124 ALT
14:445 470:605 186:210
8:24240670:T:C_C
p = 5.4e−178, model = REC, N = 670

90. BPIFB1 (Q8TDL5)
Tarkin MSPA=0.834

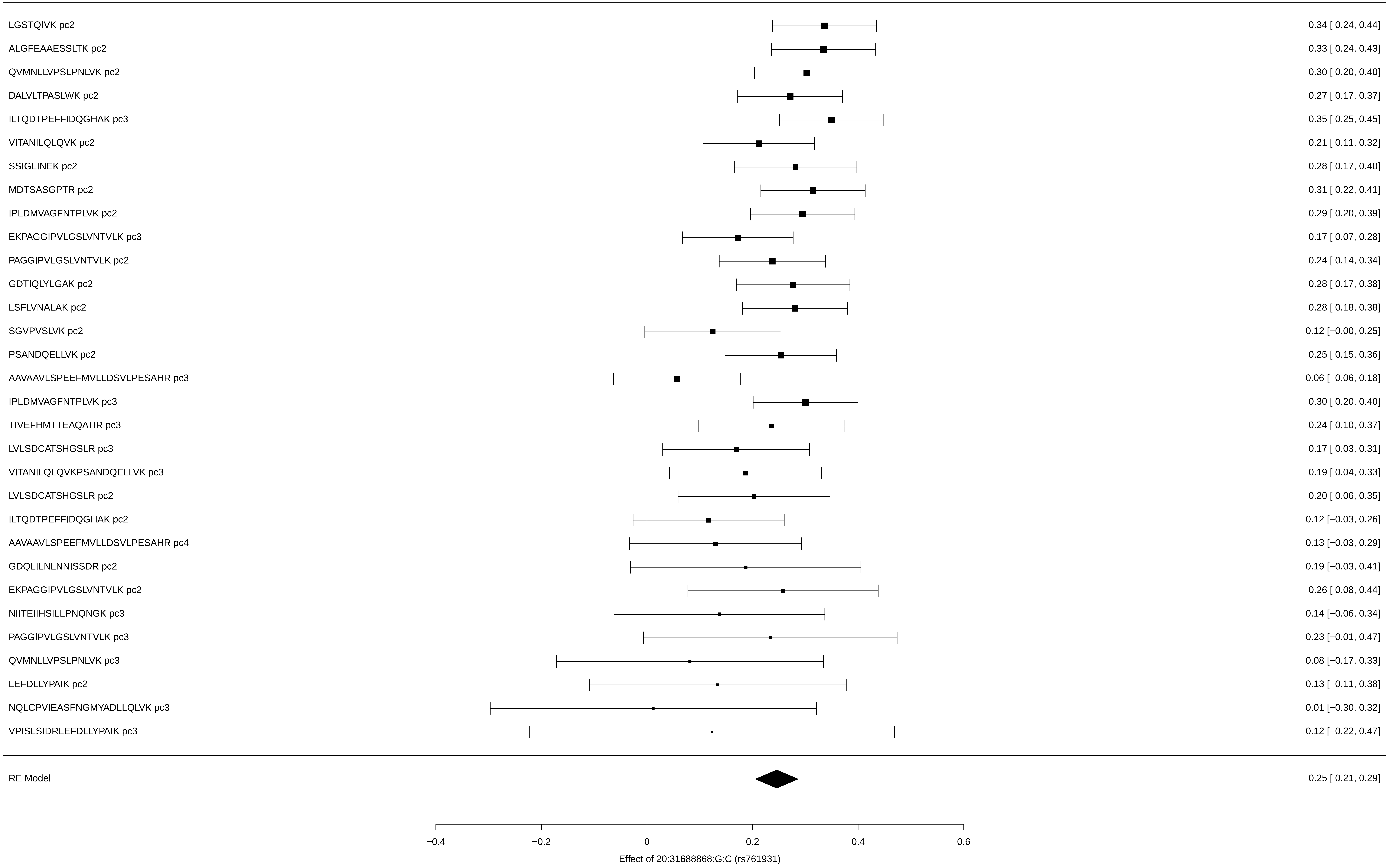

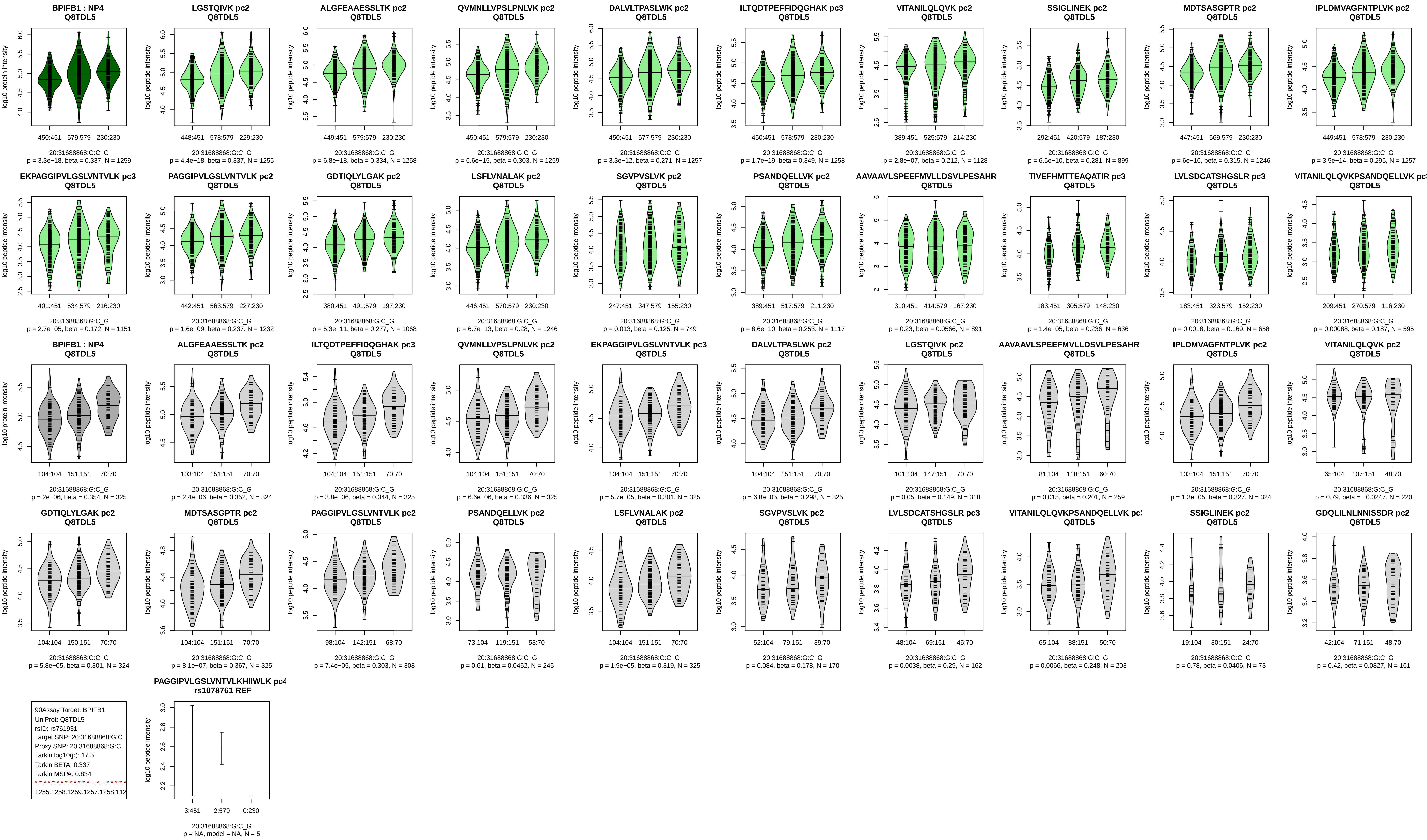

BPIFB1 : NP4
Q8TDL5
log10 protein intensity
450:451 579:579 230:230
20:31688868:G:C_G
p = 3.3e−18, beta = 0.337, N = 1259
LGSTQIVK pc2
Q8TDL5
log10 peptide intensity
448:451 578:579 229:230
20:31688868:G:C_G
p = 4.4e−18, beta = 0.337, N = 1255
ALGFEAAESSLTK pc2
Q8TDL5
449:451 579:579 230:230
20:31688868:G:C_G
p = 6.8e−18, beta = 0.334, N = 1258
QVMNLLVPSLPNLVK pc2
Q8TDL5
450:451 579:579 230:230
20:31688868:G:C_G
p = 6.6e−15, beta = 0.303, N = 1259
DALVLTPASLWK pc2
Q8TDL5
450:451 577:579 230:230
20:31688868:G:C_G
p = 3.3e−12, beta = 0.271, N = 1257
ILTQDTPEFFIDQGHAK pc3
Q8TDL5
450:451 578:579 230:230
20:31688868:G:C_G
p = 1.7e−19, beta = 0.349, N = 1258
VITANILQLQVK pc2
Q8TDL5
389:451 525:579 214:230
20:31688868:G:C_G
p = 2.8e−07, beta = 0.212, N = 1128
SSIGLINEK pc2
Q8TDL5
292:451 420:579 187:230
20:31688868:G:C_G
p = 6.5e−10, beta = 0.281, N = 899
MDTSASGPTR pc2
Q8TDL5
447:451 569:579 230:230
20:31688868:G:C_G
p = 6e−16, beta = 0.315, N = 1246
IPLDMVAGFNTPLVK pc2
Q8TDL5
449:451 578:579 230:230
20:31688868:G:C_G
p = 3.5e−14, beta = 0.295, N = 1257
EKPAGGIPVLGSLVNTVLK pc3
Q8TDL5
401:451 534:579 216:230
20:31688868:G:C_G
p = 2.7e−05, beta = 0.172, N = 1151
PAGGIPVLGSLVNTVLK pc2
Q8TDL5
442:451 563:579 227:230
20:31688868:G:C_G
p = 1.6e−09, beta = 0.237, N = 1232
GDTIQLYLGAK pc2
Q8TDL5
380:451 491:579 197:230
20:31688868:G:C_G
p = 5.3e−11, beta = 0.277, N = 1068
LSFLVNALAK pc2
Q8TDL5
446:451 570:579 230:230
20:31688868:G:C_G
p = 6.7e−13, beta = 0.28, N = 1246
SGVPVSLVK pc2
Q8TDL5
247:451 347:579 155:230
20:31688868:G:C_G
p = 0.013, beta = 0.125, N = 749
PSANDQELLVK pc2
Q8TDL5
389:451 517:579 211:230
20:31688868:G:C_G
p = 8.6e−10, beta = 0.253, N = 1117
AAVAAVLSPEEFMVLLDSVLPESAHR
Q8TDL5
310:451 414:579 167:230
20:31688868:G:C_G
p = 0.23, beta = 0.0566, N = 891
TIVEFHMTTEAQATIR pc3
Q8TDL5
183:451 305:579 148:230
20:31688868:G:C_G
p = 1.4e−05, beta = 0.236, N = 636
LVLSDCATSHGSLR pc3
Q8TDL5
183:451 323:579 152:230
20:31688868:G:C_G
p = 0.0018, beta = 0.169, N = 658
VITANILQLQVKPSANDQELLVK pc
Q8TDL5
209:451 270:579 116:230
20:31688868:G:C_G
p = 0.00088, beta = 0.187, N = 595
BPIFB1 : NP4
Q8TDL5
104:104 151:151 70:70
20:31688868:G:C_G
p = 2e−06, beta = 0.354, N = 325
ALGFEAAESSLTK pc2
Q8TDL5
103:104 151:151 70:70
20:31688868:G:C_G
p = 2.4e−06, beta = 0.352, N = 324
ILTQDTPEFFIDQGHAK pc3
Q8TDL5
104:104 151:151 70:70
20:31688868:G:C_G
p = 3.8e−06, beta = 0.344, N = 325
QVMNLLVPSLPNLVK pc2
Q8TDL5
104:104 151:151 70:70
20:31688868:G:C_G
p = 6.6e−06, beta = 0.336, N = 325
EKPAGGIPVLGSLVNTVLK pc3
Q8TDL5
104:104 151:151 70:70
20:31688868:G:C_G
p = 5.7e−05, beta = 0.301, N = 325
DALVLTPASLWK pc2
Q8TDL5
104:104 151:151 70:70
20:31688868:G:C_G
p = 6.8e−05, beta = 0.298, N = 325
LGSTQIVK pc2
Q8TDL5
101:104 147:151 70:70
20:31688868:G:C_G
p = 0.05, beta = 0.149, N = 318
AAVAAVLSPEEFMVLLDSVLPESAHR
Q8TDL5
81:104 118:151 60:70
20:31688868:G:C_G
p = 0.015, beta = 0.201, N = 259
IPLDMVAGFNTPLVK pc2
Q8TDL5
103:104 151:151 70:70
20:31688868:G:C_G
p = 1.3e−05, beta = 0.327, N = 324
VITANILQLQVK pc2
Q8TDL5
65:104 107:151 48:70
20:31688868:G:C_G
p = 0.79, beta = −0.0247, N = 220
GDTIQLYLGAK pc2
Q8TDL5
104:104 150:151 70:70
20:31688868:G:C_G
p = 5.8e−05, beta = 0.301, N = 324
MDTSASGPTR pc2
Q8TDL5
104:104 151:151 70:70
20:31688868:G:C_G
p = 8.1e−07, beta = 0.367, N = 325
PAGGIPVLGSLVNTVLK pc2
Q8TDL5
98:104 142:151 68:70
20:31688868:G:C_G
p = 7.4e−05, beta = 0.303, N = 308
PSANDQELLVK pc2
Q8TDL5
73:104 119:151 53:70
20:31688868:G:C_G
p = 0.61, beta = 0.0452, N = 245
LSFLVNALAK pc2
Q8TDL5
104:104 151:151 70:70
20:31688868:G:C_G
p = 1.9e−05, beta = 0.319, N = 325
SGVPVSLVK pc2
Q8TDL5
52:104 79:151 39:70
20:31688868:G:C_G
p = 0.084, beta = 0.178, N = 170
LVLSDCATSHGSLR pc3
Q8TDL5
48:104 69:151 45:70
20:31688868:G:C_G
p = 0.0038, beta = 0.29, N = 162
VITANILQLQVKPSANDQELLVK pc
Q8TDL5
65:104 88:151 50:70
20:31688868:G:C_G
p = 0.0066, beta = 0.248, N = 203
SSIGLINEK pc2
Q8TDL5
19:104 30:151 24:70
20:31688868:G:C_G
p = 0.78, beta = 0.0406, N = 73
GDQLILNLNNISSDR pc2
Q8TDL5
42:104 71:151 48:70
20:31688868:G:C_G
p = 0.42, beta = 0.0827, N = 161
90Assay Target: BPIFB1
UniProt: Q8TDL5
rsID: rs761931
Target SNP: 20:31688868:G:C
Proxy SNP: 20:31688868:G:C
Tarkin log10(p): 17.5
Tarkin BETA: 0.337
Tarkin MSPA: 0.834
1255:1258:1259:1257:1258:112
PAGGIPVLGSLVNTVLKHIIWLK pc4
rs1078761 REF
3:451 2:579 0:230
20:31688868:G:C_G
p = NA, model = NA, N = 5

91. APMAP (Q9HDC9)
Tarkin MSPA=0.87

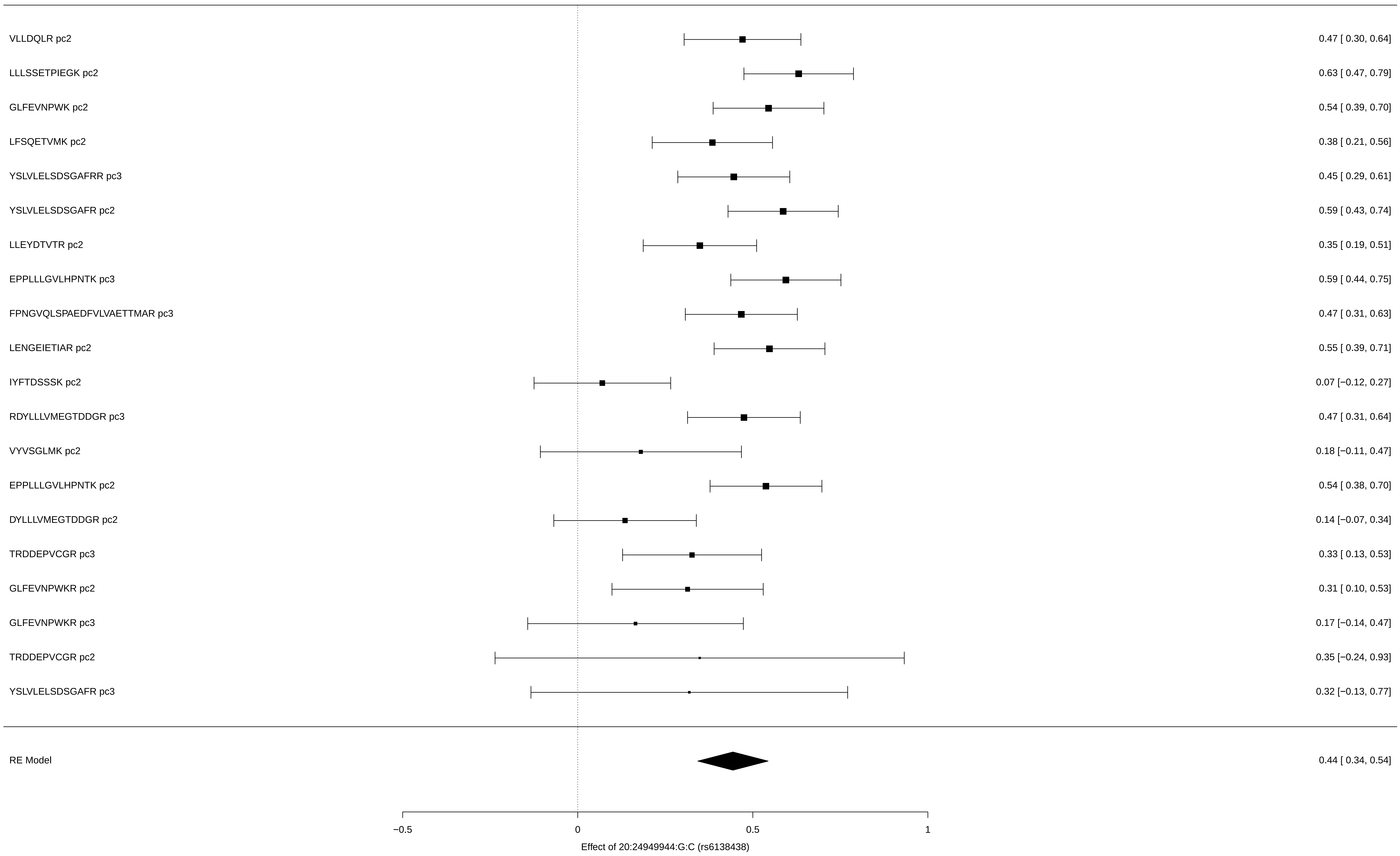

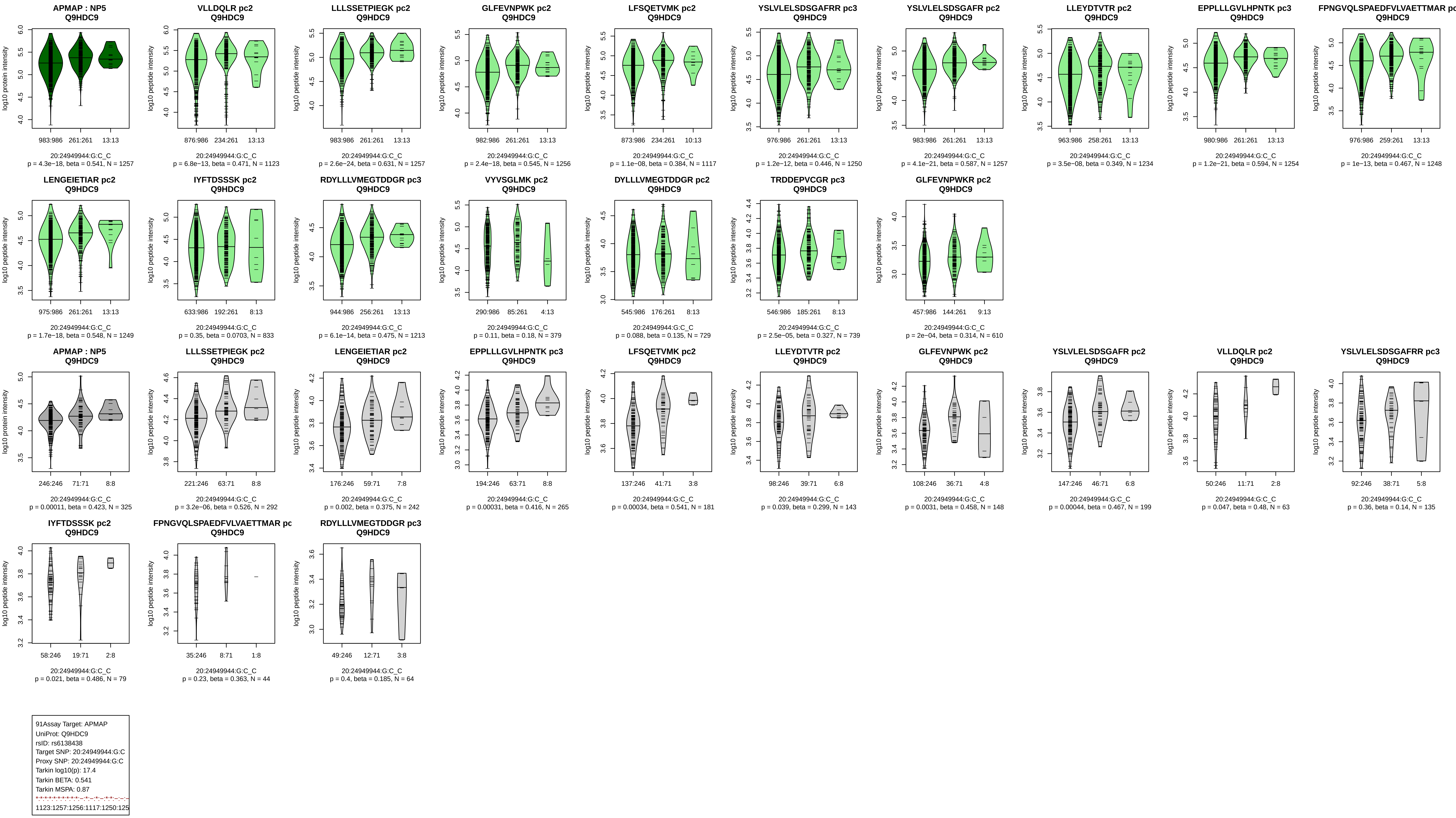

APMAP : NP5 Q9HDC9
983:986 261:261 13:13
20:24949944:G:C_C
p = 4.3e−18, beta = 0.541, N = 1257
VLLDQLR pc2 Q9HDC9
876:986 234:261 13:13
20:24949944:G:C_C
p = 6.8e−13, beta = 0.471, N = 1123
LLLSSETPIEGK pc2 Q9HDC9
983:986 261:261 13:13
20:24949944:G:C_C
p = 2.6e−24, beta = 0.631, N = 1257
GLFEVNPWK pc2 Q9HDC9
982:986 261:261 13:13
20:24949944:G:C_C
p = 2.4e−18, beta = 0.545, N = 1256
LFSQETVMK pc2 Q9HDC9
873:986 234:261 10:13
20:24949944:G:C_C
p = 1.1e−08, beta = 0.384, N = 1117
YSLVLELSDSGAFRR pc3 Q9HDC9
976:986 261:261 13:13
20:24949944:G:C_C
p = 1.2e−12, beta = 0.446, N = 1250
YSLVLELSDSGAFR pc2 Q9HDC9
983:986 261:261 13:13
20:24949944:G:C_C
p = 4.1e−21, beta = 0.587, N = 1257
LLEYDTVTR pc2 Q9HDC9
963:986 258:261 13:13
20:24949944:G:C_C
p = 3.5e−08, beta = 0.349, N = 1234
EPPLLLGVLHPNTK pc3 Q9HDC9
980:986 261:261 13:13
20:24949944:G:C_C
p = 1.2e−21, beta = 0.594, N = 1254
FPNGVQLSPAEDFVLVAETTMAR pc Q9HDC9
976:986 259:261 13:13
20:24949944:G:C_C
p = 1e−13, beta = 0.467, N = 1248
LENGEIETIAR pc2 Q9HDC9
975:986 261:261 13:13
20:24949944:G:C_C
p = 1.7e−18, beta = 0.548, N = 1249
IYFTDSSSK pc2 Q9HDC9
633:986 192:261 8:13
20:24949944:G:C_C
p = 0.35, beta = 0.0703, N = 833
RDYLLLVMEGTDDGR pc3 Q9HDC9
944:986 256:261 13:13
20:24949944:G:C_C
p = 6.1e−14, beta = 0.475, N = 1213
VYVSGLMK pc2 Q9HDC9
290:986 85:261 4:13
20:24949944:G:C_C
p = 0.11, beta = 0.18, N = 379
DYLLLVMEGTDDGR pc2 Q9HDC9
545:986 176:261 8:13
20:24949944:G:C_C
p = 0.088, beta = 0.135, N = 729
TRDDEPVCGR pc3 Q9HDC9
546:986 185:261 8:13
20:24949944:G:C_C
p = 2.5e−05, beta = 0.327, N = 739
GLFEVNPWKR pc2 Q9HDC9
457:986 144:261 9:13
20:24949944:G:C_C
p = 2e−04, beta = 0.314, N = 610
APMAP : NP5 Q9HDC9
246:246 71:71 8:8
20:24949944:G:C_C
p = 0.00011, beta = 0.423, N = 325
LLLSSETPIEGK pc2 Q9HDC9
221:246 63:71 8:8
20:24949944:G:C_C
p = 3.2e−06, beta = 0.526, N = 292
LENGEIETIAR pc2 Q9HDC9
176:246 59:71 7:8
20:24949944:G:C_C
p = 0.002, beta = 0.375, N = 242
EPPLLLGVLHPNTK pc3 Q9HDC9
194:246 63:71 8:8
20:24949944:G:C_C
p = 0.00031, beta = 0.416, N = 265
LFSQETVMK pc2 Q9HDC9
137:246 41:71 3:8
20:24949944:G:C_C
p = 0.00034, beta = 0.541, N = 181
LLEYDTVTR pc2 Q9HDC9
98:246 39:71 6:8
20:24949944:G:C_C
p = 0.039, beta = 0.299, N = 143
GLFEVNPWK pc2 Q9HDC9
108:246 36:71 4:8
20:24949944:G:C_C
p = 0.0031, beta = 0.458, N = 148
YSLVLELSDSGAFR pc2 Q9HDC9
147:246 46:71 6:8
20:24949944:G:C_C
p = 0.00044, beta = 0.467, N = 199
VLLDQLR pc2 Q9HDC9
50:246 11:71 2:8
20:24949944:G:C_C
p = 0.047, beta = 0.48, N = 63
YSLVLELSDSGAFRR pc3 Q9HDC9
92:246 38:71 5:8
20:24949944:G:C_C
p = 0.36, beta = 0.14, N = 135
IYFTDSSSK pc2 Q9HDC9
58:246 19:71 2:8
20:24949944:G:C_C
p = 0.021, beta = 0.486, N = 79
FPNGVQLSPAEDFVLVAETTMAR pc Q9HDC9
35:246 8:71 1:8
20:24949944:G:C_C
p = 0.23, beta = 0.363, N = 44
RDYLLLVMEGTDDGR pc3 Q9HDC9
49:246 12:71 3:8
20:24949944:G:C_C
p = 0.4, beta = 0.185, N = 64
log10 protein intensity
log10 peptide intensity
91Assay Target: APMAP
UniProt: Q9HDC9
rsID: rs6138438
Target SNP: 20:24949944:G:C
Proxy SNP: 20:24949944:G:C
Tarkin log10(p): 17.4
Tarkin BETA: 0.541
Tarkin MSPA: 0.87
1123:1257:1256:1117:1250:125

92. C19orf33 (Q9GZP8)
Tarkin MSPA=1

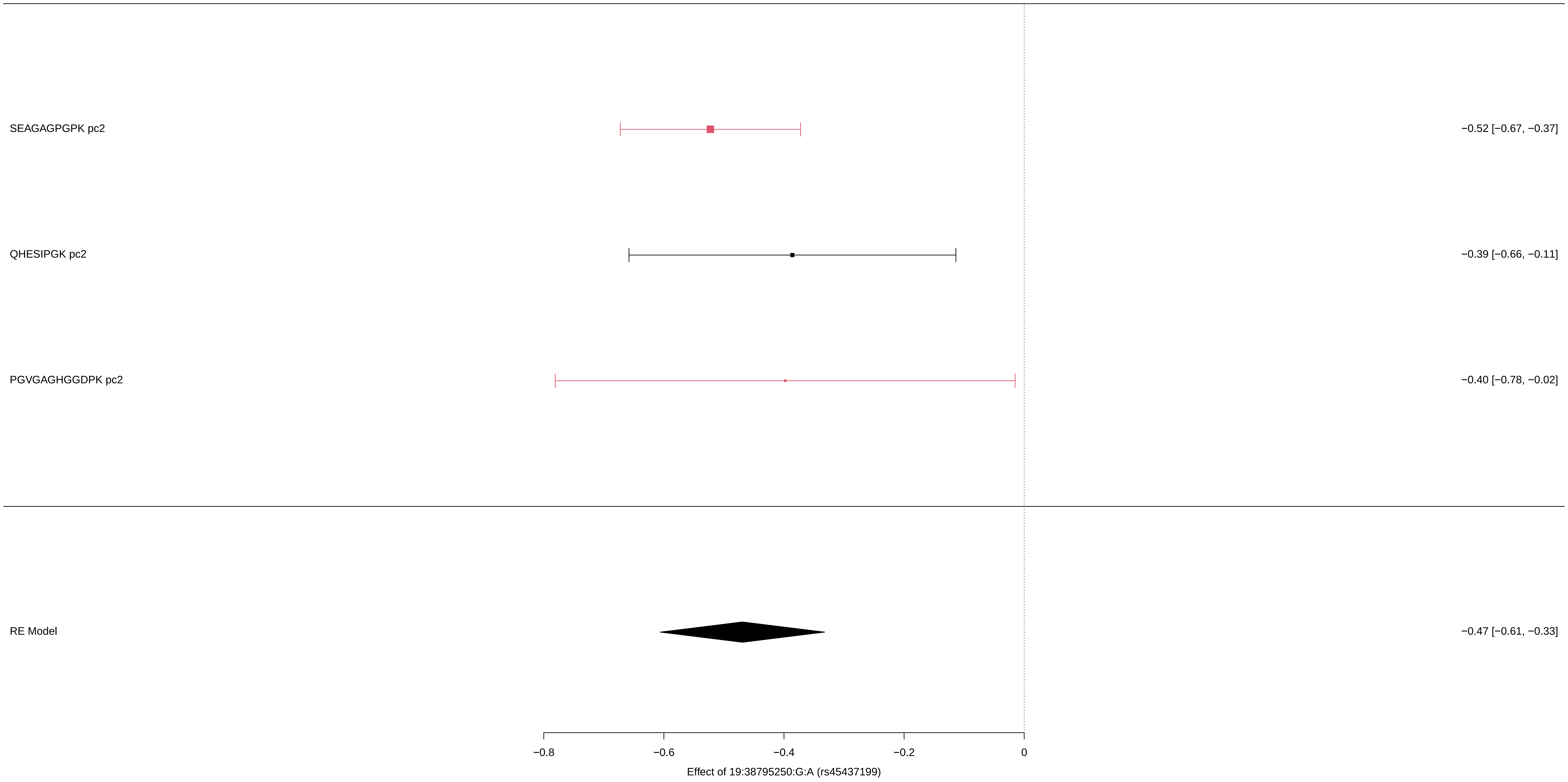

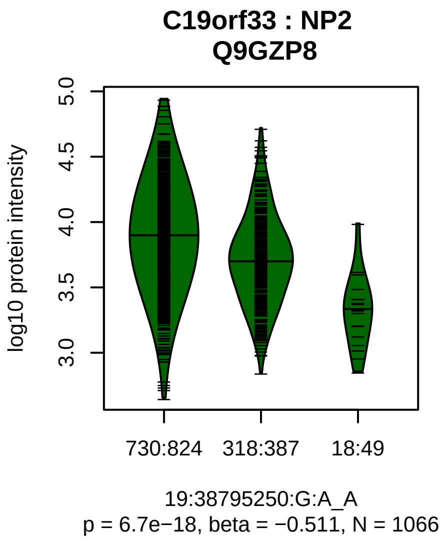

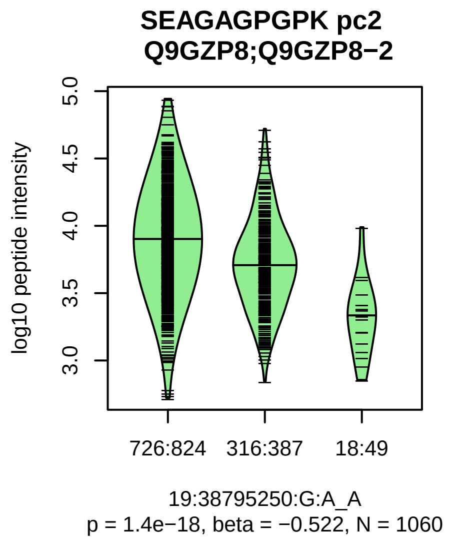

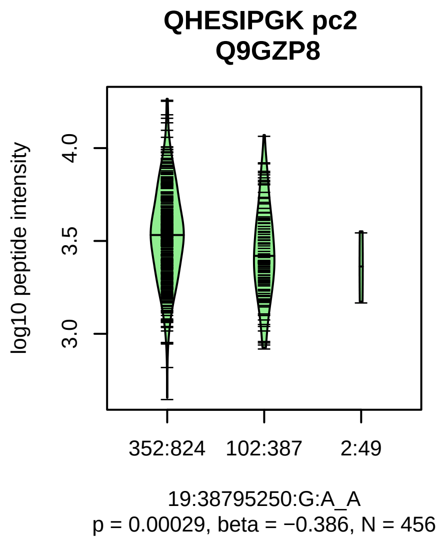

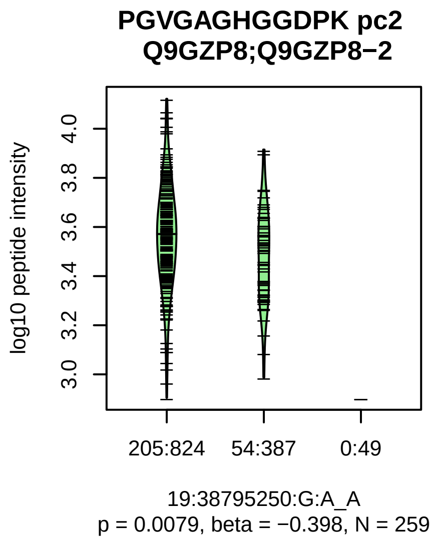

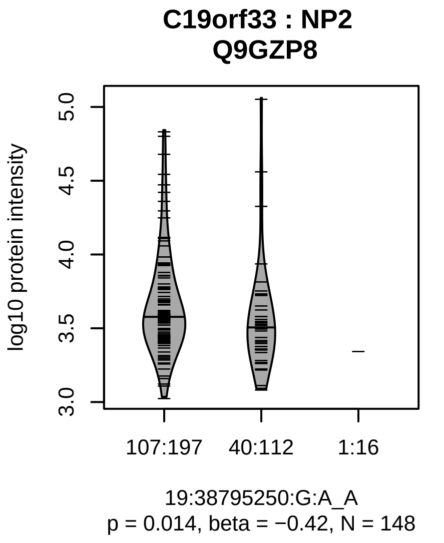

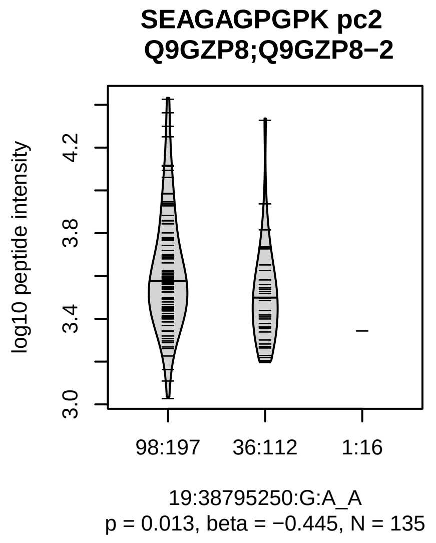

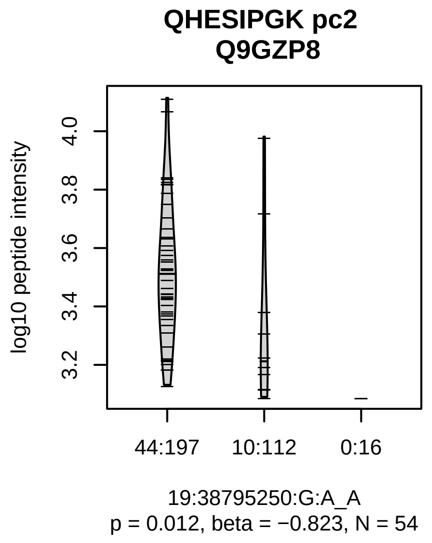

92Assay Target: C19orf33
UniProt: Q9GZP8
rsID: rs45437199
Target SNP: 19:38795250:G:A
Proxy SNP: 19:38795250:G:A
Tarkin log10(p): 17.2
Tarkin BETA: −0.511
Tarkin MSPA: 1
::*
1060:456:259

93. ITGA2 (P17301)
Tarkin MSPA=0.917

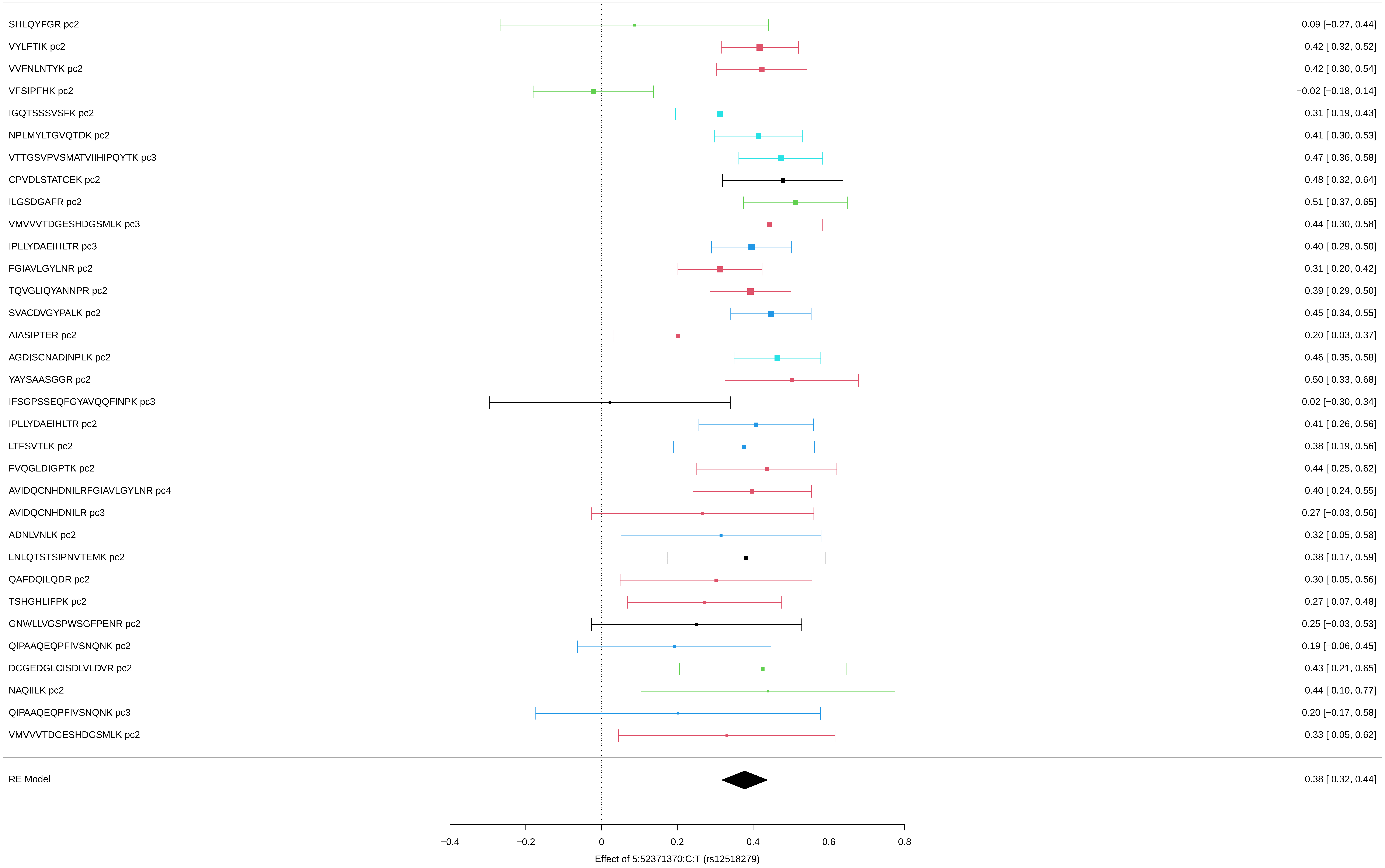

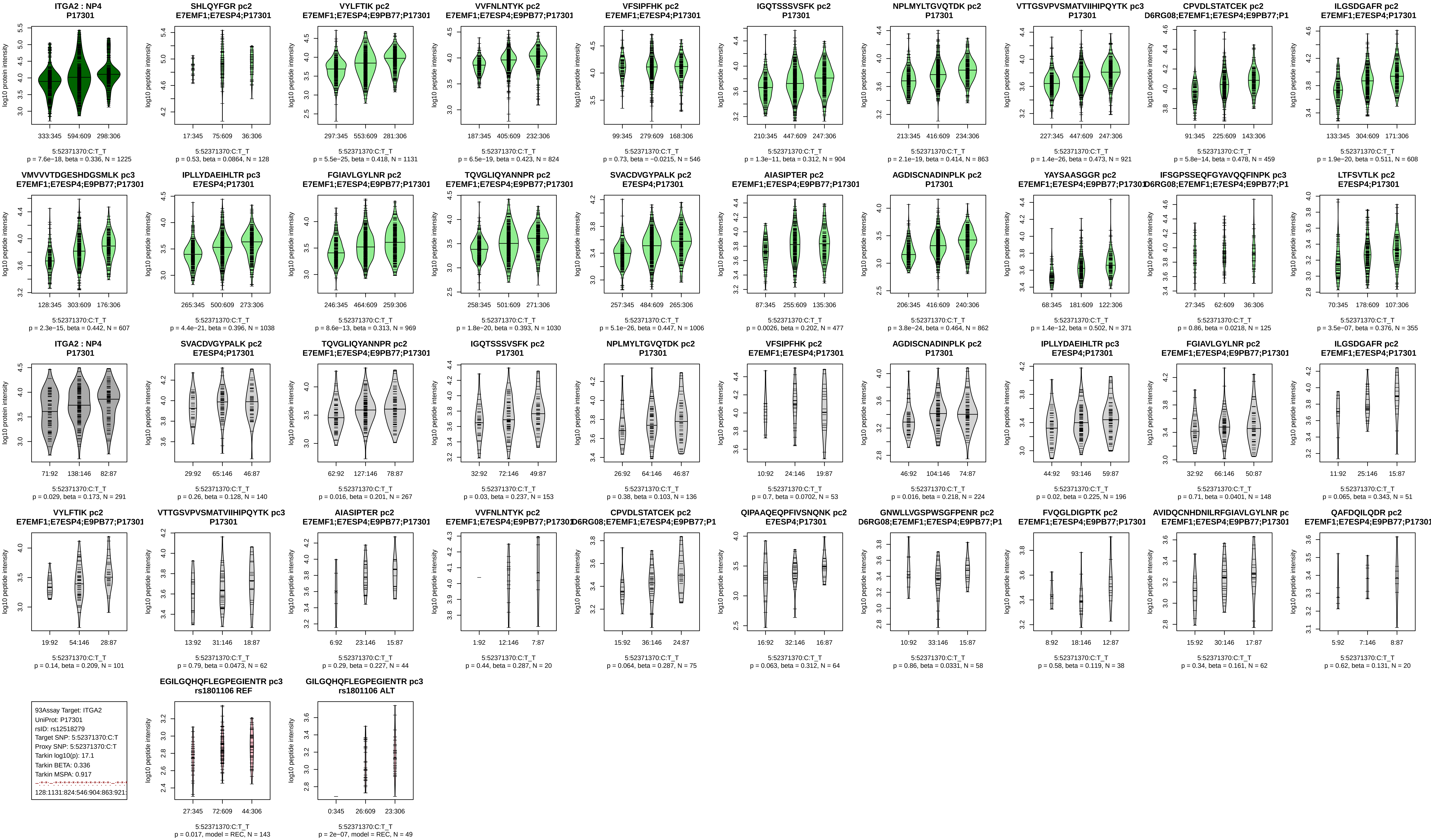

ITGA2 : NP4
P17301
log10 protein intensity
333:345 594:609 298:306
5:52371370:C:T_T
p = 7.6e−18, beta = 0.336, N = 1225
SHLQYFGR pc2
E7EMF1;E7ESP4;P17301
log10 peptide intensity
17:345 75:609 36:306
5:52371370:C:T_T
p = 0.53, beta = 0.0864, N = 128
VYLFTIK pc2
E7EMF1;E7ESP4;E9PB77;P17301
297:345 553:609 281:306
5:52371370:C:T_T
p = 5.5e−25, beta = 0.418, N = 1131
VVFNLNTYK pc2
E7EMF1;E7ESP4;E9PB77;P17301
187:345 405:609 232:306
5:52371370:C:T_T
p = 6.5e−19, beta = 0.423, N = 824
VFSIPFHK pc2
E7EMF1;E7ESP4;P17301
99:345 279:609 168:306
5:52371370:C:T_T
p = 0.73, beta = −0.0215, N = 546
IGQTSSSVSFK pc2
P17301
210:345 447:609 247:306
5:52371370:C:T_T
p = 1.3e−11, beta = 0.312, N = 904
NPLMYLTGVQTDK pc2
P17301
213:345 416:609 234:306
5:52371370:C:T_T
p = 2.1e−19, beta = 0.414, N = 863
VTTGSVPVSMATVIIHIPQYTK pc3
P17301
227:345 447:609 247:306
5:52371370:C:T_T
p = 1.4e−26, beta = 0.473, N = 921
CPVDLSTATCEK pc2
D6RG08;E7EMF1;E7ESP4;E9PB77;P1
91:345 225:609 143:306
5:52371370:C:T_T
p = 5.8e−14, beta = 0.478, N = 459
ILGSDGAFR pc2
E7EMF1;E7ESP4;P17301
133:345 304:609 171:306
5:52371370:C:T_T
p = 1.9e−20, beta = 0.511, N = 608
VMVVVTDGESHDGSMLK pc3
E7EMF1;E7ESP4;E9PB77;P17301
128:345 303:609 176:306
5:52371370:C:T_T
p = 2.3e−15, beta = 0.442, N = 607
IPLLYDAEIHLTR pc3
E7ESP4;P17301
265:345 500:609 273:306
5:52371370:C:T_T
p = 4.4e−21, beta = 0.396, N = 1038
FGIAVLGYLNR pc2
E7EMF1;E7ESP4;E9PB77;P17301
246:345 464:609 259:306
5:52371370:C:T_T
p = 8.6e−13, beta = 0.313, N = 969
TQVGLIQYANNPR pc2
E7EMF1;E7ESP4;E9PB77;P17301
258:345 501:609 271:306
5:52371370:C:T_T
p = 1.8e−20, beta = 0.393, N = 1030
SVACDVGYPALK pc2
E7ESP4;P17301
257:345 484:609 265:306
5:52371370:C:T_T
p = 5.1e−26, beta = 0.447, N = 1006
AIASIPTER pc2
E7EMF1;E7ESP4;E9PB77;P17301
87:345 255:609 135:306
5:52371370:C:T_T
p = 0.0026, beta = 0.202, N = 477
AGDISCNADINPLK pc2
P17301
206:345 416:609 240:306
5:52371370:C:T_T
p = 3.8e−24, beta = 0.464, N = 862
YAYSAASGGR pc2
E7EMF1;E7ESP4;E9PB77;P17301
68:345 181:609 122:306
5:52371370:C:T_T
p = 1.4e−12, beta = 0.502, N = 371
IFSGPSSEQFGYAVQQFINPK pc3
D6RG08;E7EMF1;E7ESP4;E9PB77;P1
27:345 62:609 36:306
5:52371370:C:T_T
p = 0.86, beta = 0.0218, N = 125
LTFSVTLK pc2
E7ESP4;P17301
70:345 178:609 107:306
5:52371370:C:T_T
p = 3.5e−07, beta = 0.376, N = 355
ITGA2 : NP4
P17301
71:92 138:146 82:87
5:52371370:C:T_T
p = 0.029, beta = 0.173, N = 291
SVACDVGYPALK pc2
E7ESP4;P17301
29:92 65:146 46:87
5:52371370:C:T_T
p = 0.26, beta = 0.128, N = 140
TQVGLIQYANNPR pc2
E7EMF1;E7ESP4;E9PB77;P17301
62:92 127:146 78:87
5:52371370:C:T_T
p = 0.016, beta = 0.201, N = 267
IGQTSSSVSFK pc2
P17301
32:92 72:146 49:87
5:52371370:C:T_T
p = 0.03, beta = 0.237, N = 153
NPLMYLTGVQTDK pc2
P17301
26:92 64:146 46:87
5:52371370:C:T_T
p = 0.38, beta = 0.103, N = 136
VFSIPFHK pc2
E7EMF1;E7ESP4;P17301
10:92 24:146 19:87
5:52371370:C:T_T
p = 0.7, beta = 0.0702, N = 53
AGDISCNADINPLK pc2
P17301
46:92 104:146 74:87
5:52371370:C:T_T
p = 0.016, beta = 0.218, N = 224
IPLLYDAEIHLTR pc3
E7ESP4;P17301
44:92 93:146 59:87
5:52371370:C:T_T
p = 0.02, beta = 0.225, N = 196
FGIAVLGYLNR pc2
E7EMF1;E7ESP4;E9PB77;P17301
32:92 66:146 50:87
5:52371370:C:T_T
p = 0.71, beta = 0.0401, N = 148
ILGSDGAFR pc2
E7EMF1;E7ESP4;P17301
11:92 25:146 15:87
5:52371370:C:T_T
p = 0.065, beta = 0.343, N = 51
VYLFTIK pc2
E7EMF1;E7ESP4;E9PB77;P17301
19:92 54:146 28:87
5:52371370:C:T_T
p = 0.14, beta = 0.209, N = 101
VTTGSVPVSMATVIIHIPQYTK pc3
P17301
13:92 31:146 18:87
5:52371370:C:T_T
p = 0.79, beta = 0.0473, N = 62
AIASIPTER pc2
E7EMF1;E7ESP4;E9PB77;P17301
6:92 23:146 15:87
5:52371370:C:T_T
p = 0.29, beta = 0.227, N = 44
VVFNLNTYK pc2
E7EMF1;E7ESP4;E9PB77;P17301
1:92 12:146 7:87
5:52371370:C:T_T
p = 0.44, beta = 0.287, N = 20
CPVDLSTATCEK pc2
D6RG08;E7EMF1;E7ESP4;E9PB77;P1
15:92 36:146 24:87
5:52371370:C:T_T
p = 0.064, beta = 0.287, N = 75
QIPAAQEQPFIVSNQNK pc2
E7ESP4;P17301
16:92 32:146 16:87
5:52371370:C:T_T
p = 0.063, beta = 0.312, N = 64
GNWLLVGSPWSGFPENR pc2
D6RG08;E7EMF1;E7ESP4;E9PB77;P1
10:92 33:146 15:87
5:52371370:C:T_T
p = 0.86, beta = 0.0331, N = 58
FVQGLDIGPTK pc2
E7EMF1;E7ESP4;E9PB77;P17301
8:92 18:146 12:87
5:52371370:C:T_T
p = 0.58, beta = 0.119, N = 38
AVIDQCNHDNILRFGIAVLGYLNR pc
E7EMF1;E7ESP4;E9PB77;P17301
15:92 30:146 17:87
5:52371370:C:T_T
p = 0.34, beta = 0.161, N = 62
QAFDQILQDR pc2
E7EMF1;E7ESP4;E9PB77;P17301
5:92 7:146 8:87
5:52371370:C:T_T
p = 0.62, beta = 0.131, N = 20
93Assay Target: ITGA2
UniProt: P17301
rsID: rs12518279
Target SNP: 5:52371370:C:T
Proxy SNP: 5:52371370:C:T
Tarkin log10(p): 17.1
Tarkin BETA: 0.336
Tarkin MSPA: 0.917
128:1131:824:546:904:863:921:
EGILGQHQFLEGPEGIENTR pc3
rs1801106 REF
27:345 72:609 44:306
5:52371370:C:T_T
p = 0.017, model = REC, N = 143
GILGQHQFLEGPEGIENTR pc3
rs1801106 ALT
0:345 26:609 23:306
5:52371370:C:T_T
p = 2e−07, model = REC, N = 49

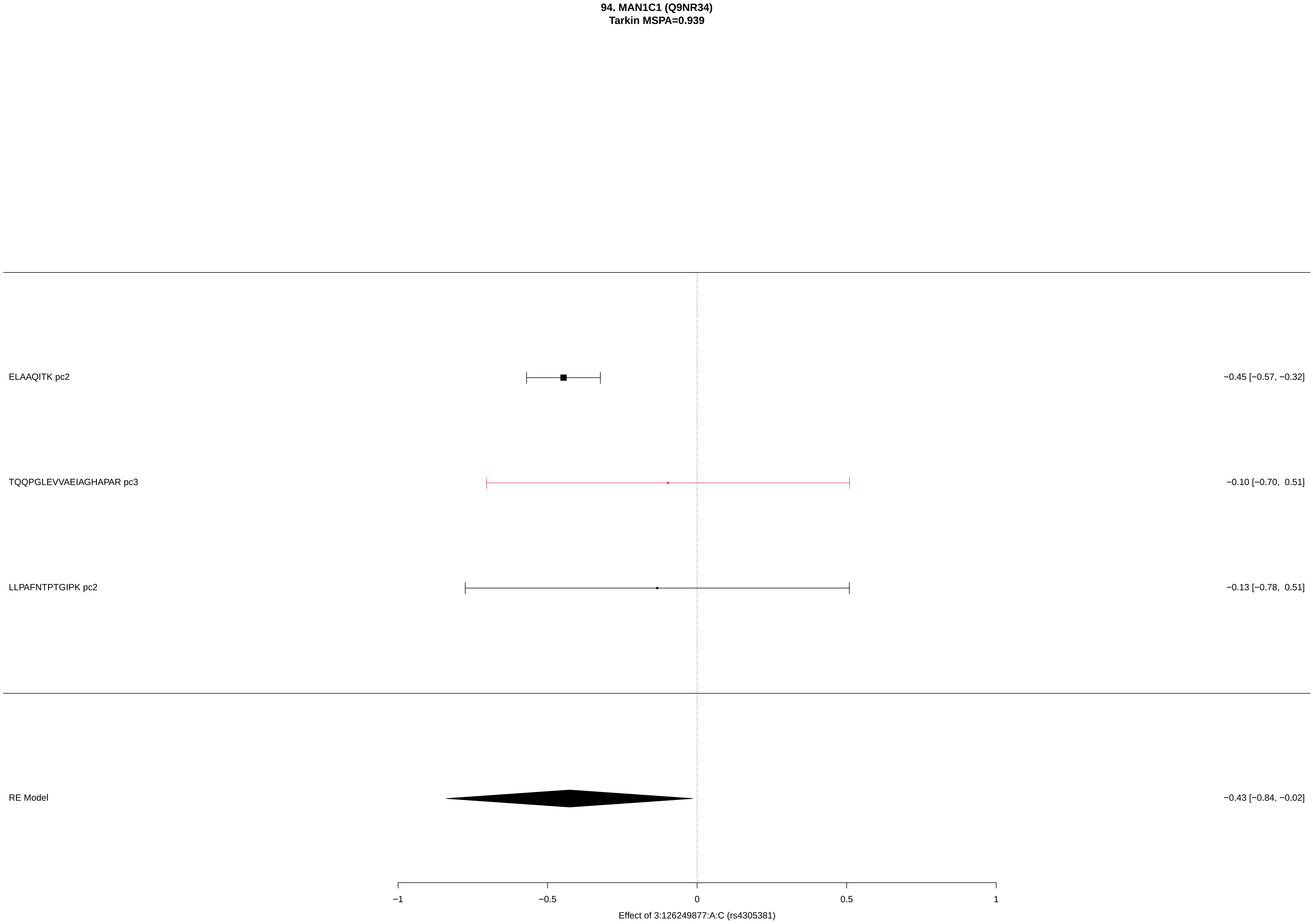
94. MAN1C1 (Q9NR34)
Tarkin MSPA=0.939
ELAAQITK pc2
−0.45 [−0.57, −0.32]
TQQPGLEVVAEIAGHAPAR pc3
−0.10 [−0.70, 0.51]
LLPAFNTPTGIPK pc2
−0.13 [−0.78, 0.51]
RE Model
−0.43 [−0.84, −0.02]
−1
−0.5
0
0.5
1
Effect of 3:126249877:A:C (rs4305381)

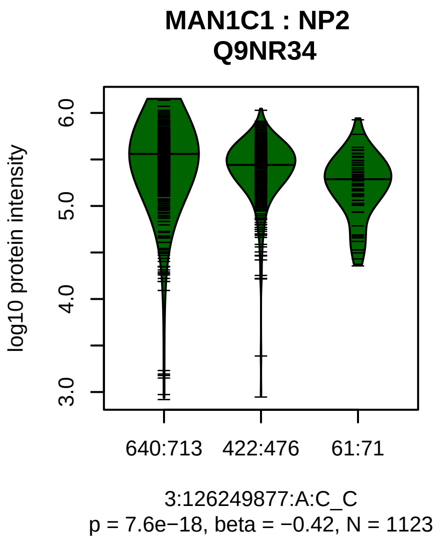

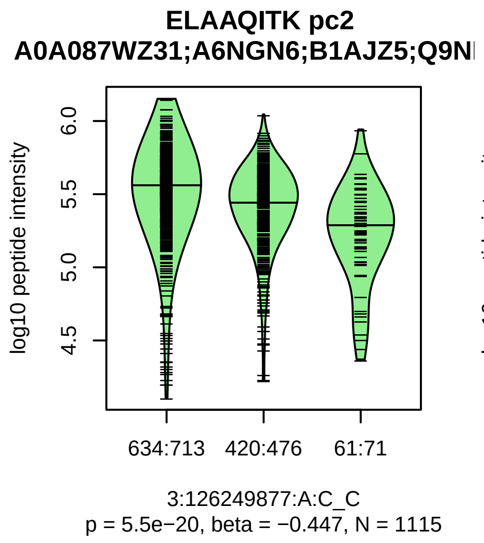

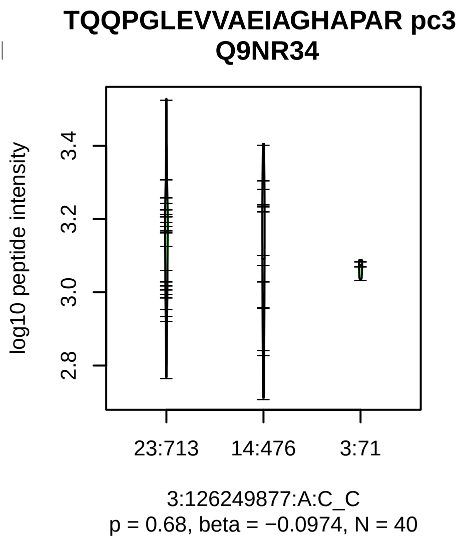

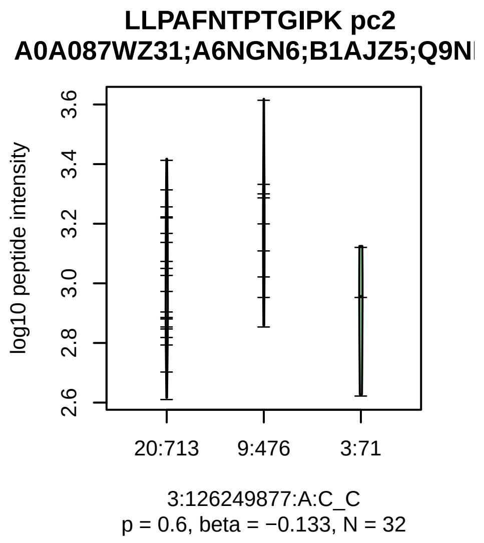

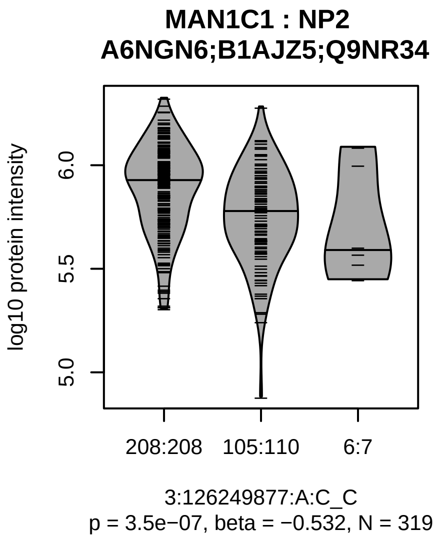

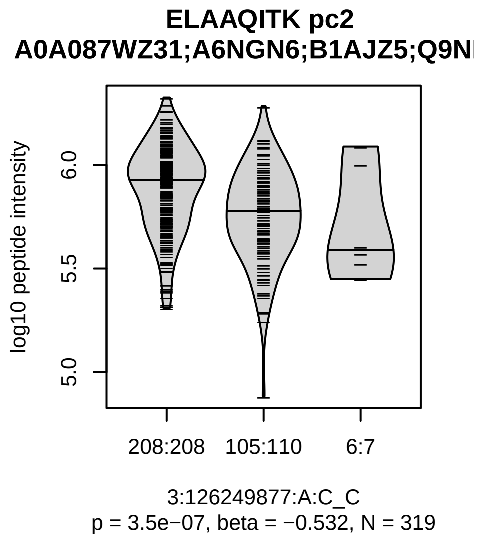

94Assay Target: MAN1C1
UniProt: Q9NR34
rsID: rs4305381
Target SNP: 3:126249877:A:C
Proxy SNP: 3:126249877:A:C
Tarkin log10(p): 17.1
Tarkin BETA: −0.42
Tarkin MSPA: 0.939
*:−:−
1115:40:32

95. PZP (P20742)
Tarkin MSPA=0.459

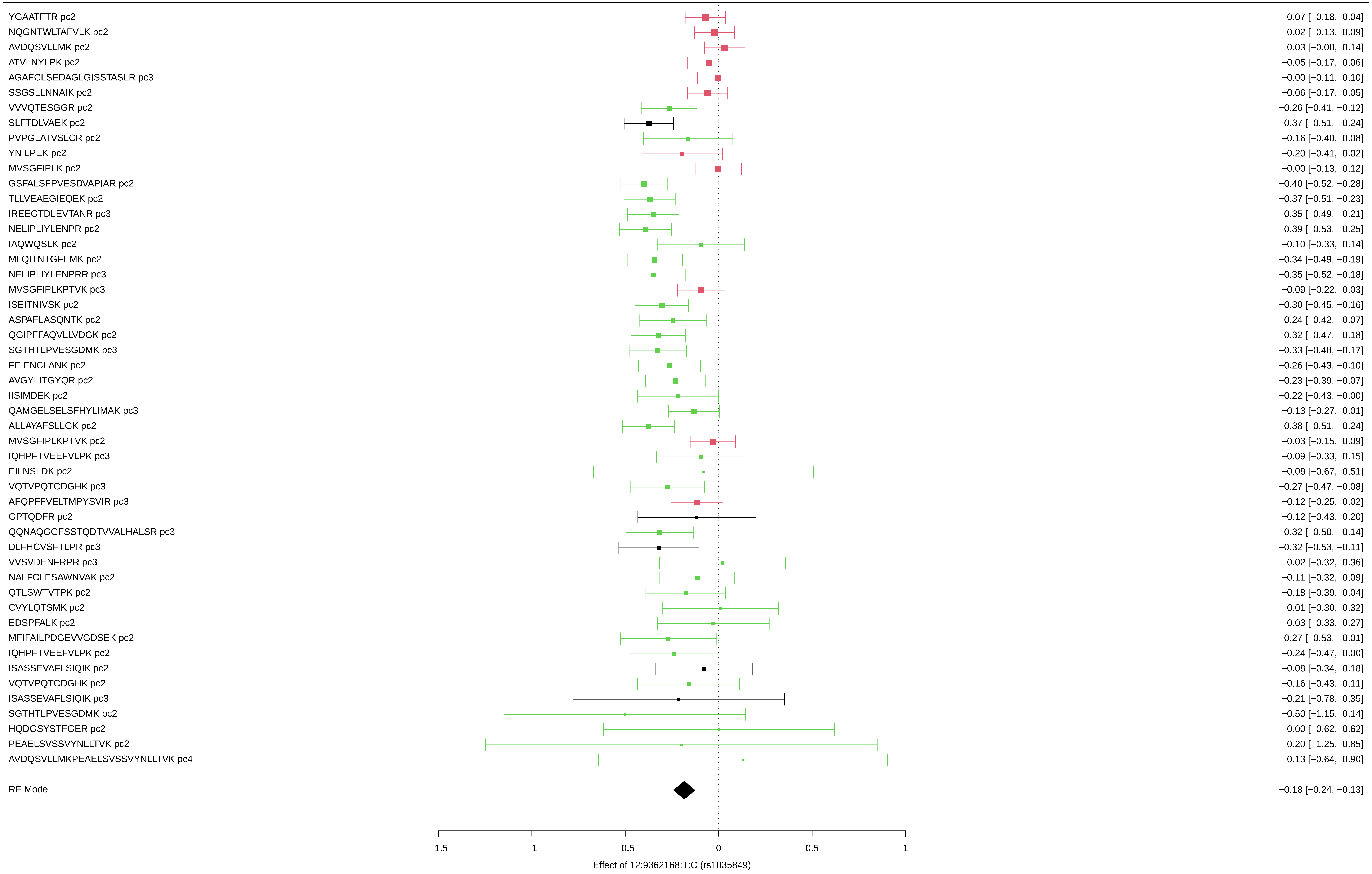

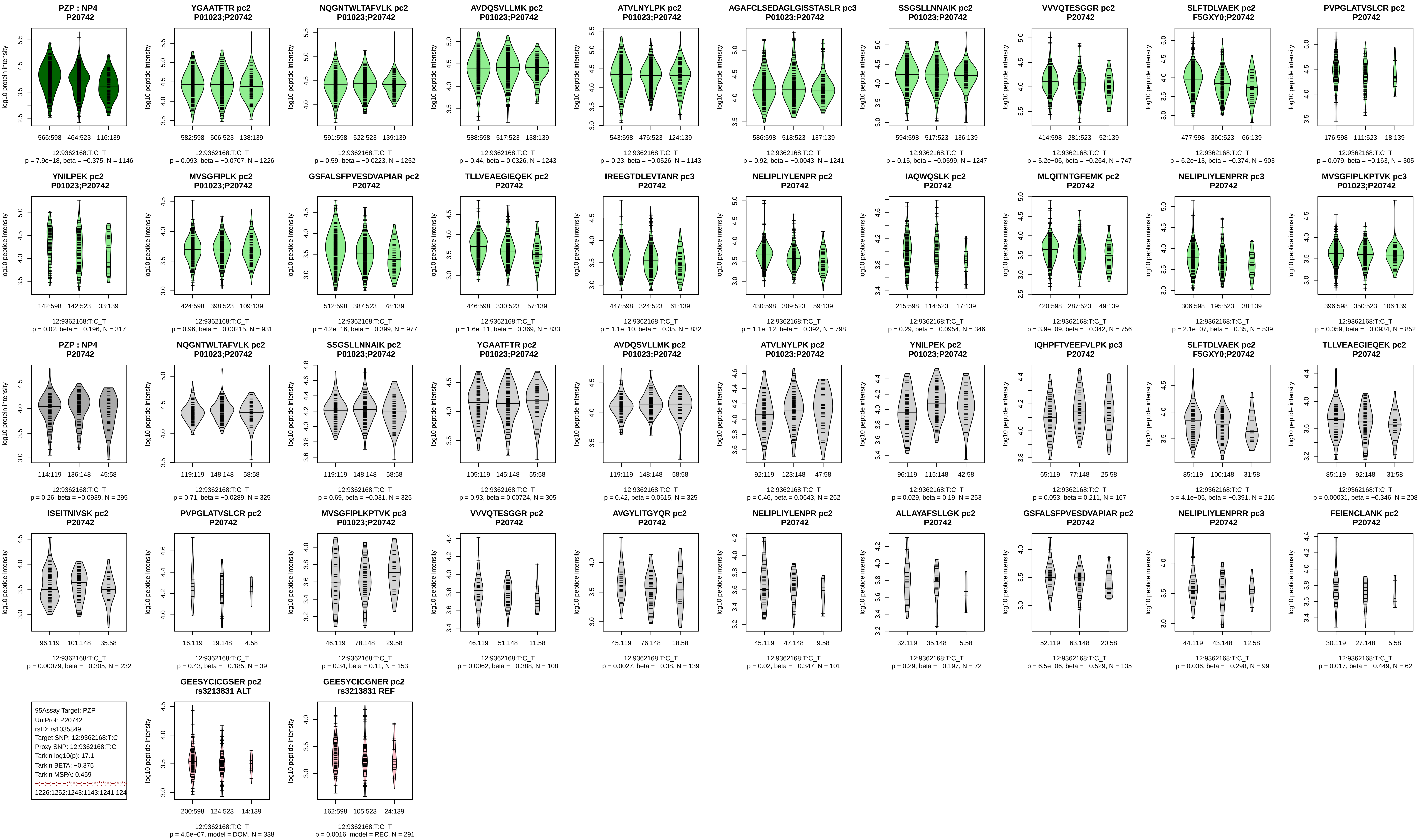
PZP : NP4
P20742
log10 protein intensity
566:598 464:523 116:139
12:9362168:T:C_T
p = 7.9e−18, beta = −0.375, N = 1146
YGAATFTR pc2
P01023;P20742
log10 peptide intensity
582:598 506:523 138:139
12:9362168:T:C_T
p = 0.093, beta = −0.0707, N = 1226
NQGNTWLTAFVLK pc2
P01023;P20742
591:598 522:523 139:139
12:9362168:T:C_T
p = 0.59, beta = −0.0223, N = 1252
AVDQSVLLMK pc2
P01023;P20742
588:598 517:523 138:139
12:9362168:T:C_T
p = 0.44, beta = 0.0326, N = 1243
ATVLNYLPK pc2
P01023;P20742
543:598 476:523 124:139
12:9362168:T:C_T
p = 0.23, beta = −0.0526, N = 1143
AGAFCLSEDAGLGISSTASLR pc3
P01023;P20742
586:598 518:523 137:139
12:9362168:T:C_T
p = 0.92, beta = −0.0043, N = 1241
SSGSLLNNAIK pc2
P01023;P20742
594:598 517:523 136:139
12:9362168:T:C_T
p = 0.15, beta = −0.0599, N = 1247
VVVQTESGGR pc2
P20742
414:598 281:523 52:139
12:9362168:T:C_T
p = 5.2e−06, beta = −0.264, N = 747
SLFTDLVAEK pc2
F5GXY0;P20742
477:598 360:523 66:139
12:9362168:T:C_T
p = 6.2e−13, beta = −0.374, N = 903
PVPGLATVSLCR pc2
P20742
176:598 111:523 18:139
12:9362168:T:C_T
p = 0.079, beta = −0.163, N = 305
YNILPEK pc2
P01023;P20742
142:598 142:523 33:139
12:9362168:T:C_T
p = 0.02, beta = −0.196, N = 317
MVSGFIPLK pc2
P01023;P20742
424:598 398:523 109:139
12:9362168:T:C_T
p = 0.96, beta = −0.00215, N = 931
GSFALSFPVESDVAPIAR pc2
P20742
512:598 387:523 78:139
12:9362168:T:C_T
p = 4.2e−16, beta = −0.399, N = 977
TLLVEAEGIEQEK pc2
P20742
446:598 330:523 57:139
12:9362168:T:C_T
p = 1.6e−11, beta = −0.369, N = 833
IREEGTDLEVTANR pc3
P20742
447:598 324:523 61:139
12:9362168:T:C_T
p = 1.1e−10, beta = −0.35, N = 832
NELIPLIYLENPR pc2
P20742
430:598 309:523 59:139
12:9362168:T:C_T
p = 1.1e−12, beta = −0.392, N = 798
IAQWQSLK pc2
P20742
215:598 114:523 17:139
12:9362168:T:C_T
p = 0.29, beta = −0.0954, N = 346
MLQITNTGFEMK pc2
P20742
420:598 287:523 49:139
12:9362168:T:C_T
p = 3.9e−09, beta = −0.342, N = 756
NELIPLIYLENPRR pc3
P20742
306:598 195:523 38:139
12:9362168:T:C_T
p = 2.1e−07, beta = −0.35, N = 539
MVSGFIPLKPTVK pc3
P01023;P20742
396:598 350:523 106:139
12:9362168:T:C_T
p = 0.059, beta = −0.0934, N = 852
PZP : NP4
P20742
114:119 136:148 45:58
12:9362168:T:C_T
p = 0.26, beta = −0.0939, N = 295
NQGNTWLTAFVLK pc2
P01023;P20742
119:119 148:148 58:58
12:9362168:T:C_T
p = 0.71, beta = −0.0289, N = 325
SSGSLLNNAIK pc2
P01023;P20742
119:119 148:148 58:58
12:9362168:T:C_T
p = 0.69, beta = −0.031, N = 325
YGAATFTR pc2
P01023;P20742
105:119 145:148 55:58
12:9362168:T:C_T
p = 0.93, beta = 0.00724, N = 305
AVDQSVLLMK pc2
P01023;P20742
119:119 148:148 58:58
12:9362168:T:C_T
p = 0.42, beta = 0.0615, N = 325
ATVLNYLPK pc2
P01023;P20742
92:119 123:148 47:58
12:9362168:T:C_T
p = 0.46, beta = 0.0643, N = 262
YNILPEK pc2
P01023;P20742
96:119 115:148 42:58
12:9362168:T:C_T
p = 0.029, beta = 0.19, N = 253
IQHPFTVEEFVLPK pc3
P20742
65:119 77:148 25:58
12:9362168:T:C_T
p = 0.053, beta = 0.211, N = 167
SLFTDLVAEK pc2
F5GXY0;P20742
85:119 100:148 31:58
12:9362168:T:C_T
p = 4.1e−05, beta = −0.391, N = 216
TLLVEAEGIEQEK pc2
P20742
85:119 92:148 31:58
12:9362168:T:C_T
p = 0.00031, beta = −0.346, N = 208
ISEITNIVSK pc2
P20742
96:119 101:148 35:58
12:9362168:T:C_T
p = 0.00079, beta = −0.305, N = 232
PVPGLATVSLCR pc2
P20742
16:119 19:148 4:58
12:9362168:T:C_T
p = 0.43, beta = −0.185, N = 39
MVSGFIPLKPTVK pc3
P01023;P20742
46:119 78:148 29:58
12:9362168:T:C_T
p = 0.34, beta = 0.11, N = 153
VVVQTESGGR pc2
P20742
46:119 51:148 11:58
12:9362168:T:C_T
p = 0.0062, beta = −0.388, N = 108
AVGYLITGYQR pc2
P20742
45:119 76:148 18:58
12:9362168:T:C_T
p = 0.0027, beta = −0.38, N = 139
NELIPLIYLENPR pc2
P20742
45:119 47:148 9:58
12:9362168:T:C_T
p = 0.02, beta = −0.347, N = 101
ALLAYAFSLLGK pc2
P20742
32:119 35:148 5:58
12:9362168:T:C_T
p = 0.29, beta = −0.197, N = 72
GSFALSFPVESDVAPIAR pc2
P20742
52:119 63:148 20:58
12:9362168:T:C_T
p = 6.5e−06, beta = −0.529, N = 135
NELIPLIYLENPRR pc3
P20742
44:119 43:148 12:58
12:9362168:T:C_T
p = 0.036, beta = −0.298, N = 99
FEIENCLANK pc2
P20742
30:119 27:148 5:58
12:9362168:T:C_T
p = 0.017, beta = −0.449, N = 62
95Assay Target: PZP
UniProt: P20742
rsID: rs1035849
Target SNP: 12:9362168:T:C
Proxy SNP: 12:9362168:T:C
Tarkin log10(p): 17.1
Tarkin BETA: −0.375
Tarkin MSPA: 0.459
1226:1252:1243:1143:1241:124
GEESYCICGSER pc2
rs3213831 ALT
200:598 124:523 14:139
12:9362168:T:C_T
p = 4.5e−07, model = DOM, N = 338
GEESYCICGNER pc2
rs3213831 REF
162:598 105:523 24:139
12:9362168:T:C_T
p = 0.0016, model = REC, N = 291

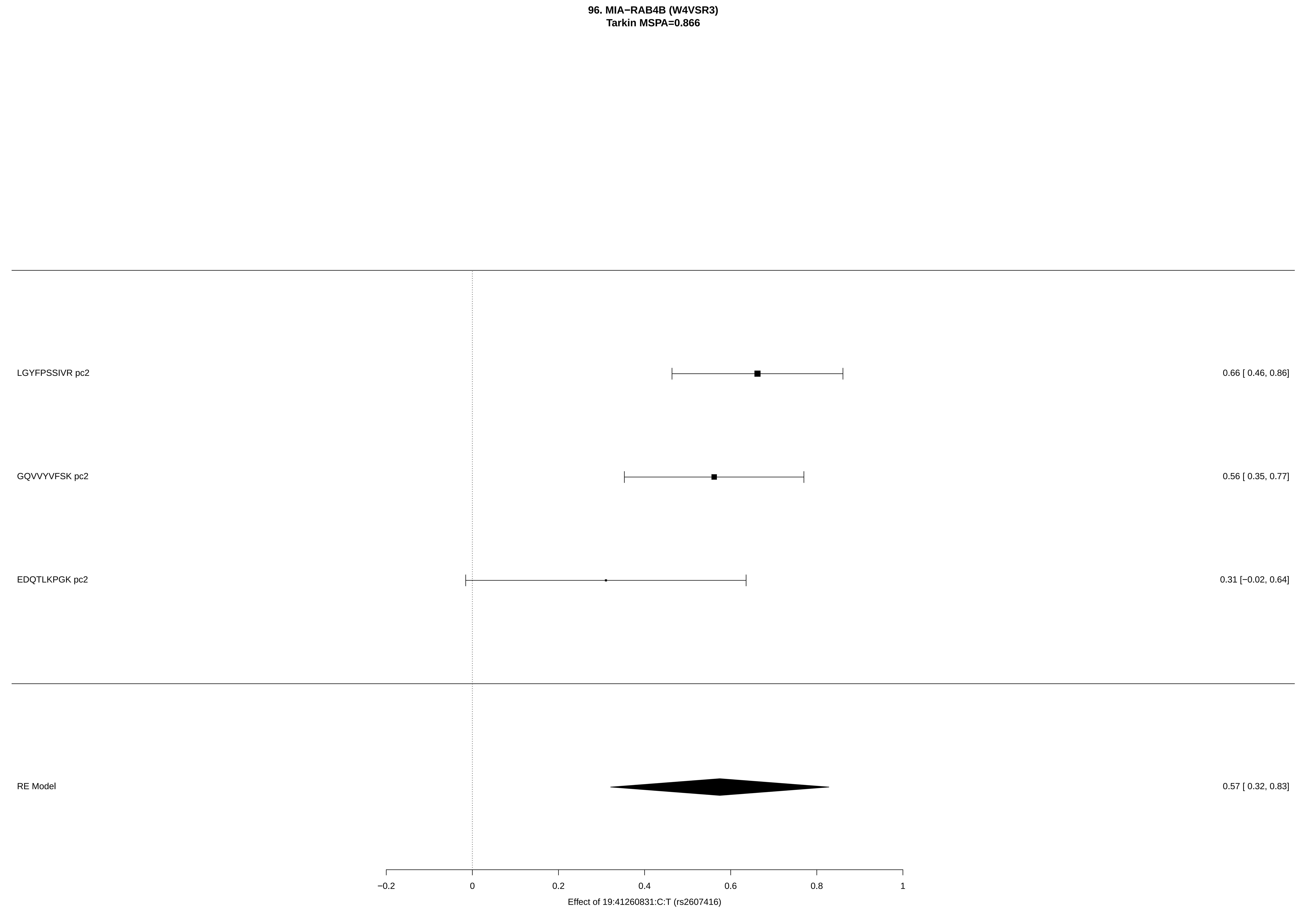
96. MIA−RAB4B (W4VSR3)
Tarkin MSPA=0.866
LGYFPSSIVR pc2
0.66 [0.46, 0.86]
GQVVYVFSK pc2
0.56 [0.35, 0.77]
EDQTLKPGK pc2
0.31 [−0.02, 0.64]
RE Model
0.57 [0.32, 0.83]
−0.2
0
0.2
0.4
0.6
0.8
1
Effect of 19:41260831:C:T (rs2607416)

**MIA−RAB4B : NP3
W4VSR3**

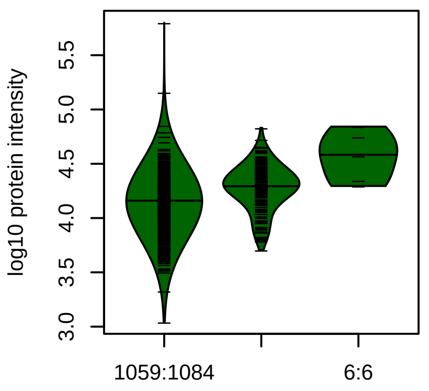

19:41260831:C:T_T
p = 9.2e−18, beta = 0.656, N = 1232

**LGYFPSSIVR pc2
W4VSR3;Q16674**

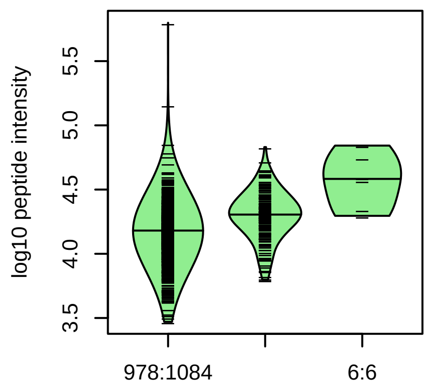

19:41260831:C:T_T
p = 2.8e−17, beta = 0.662, N = 1142

**GQVVYVFSK pc2
W4VSR3;Q16674**

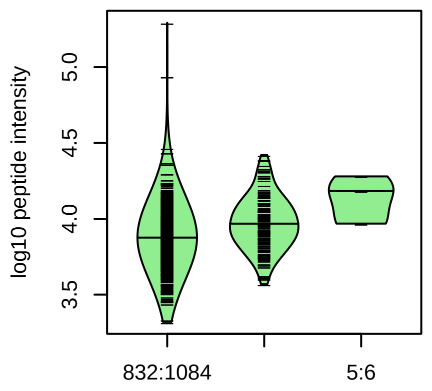

19:41260831:C:T_T
p = 7.2e−12, beta = 0.562, N = 987

**EDQTLKPGK pc2
W4VSR3;Q16674**

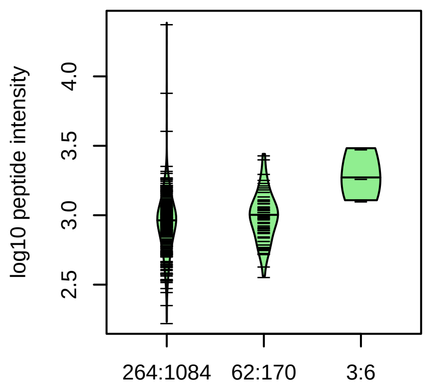

19:41260831:C:T_T
p = 0.015, beta = 0.31, N = 329

**LGYFPSSIVR pc2
W4VSR3;Q16674**

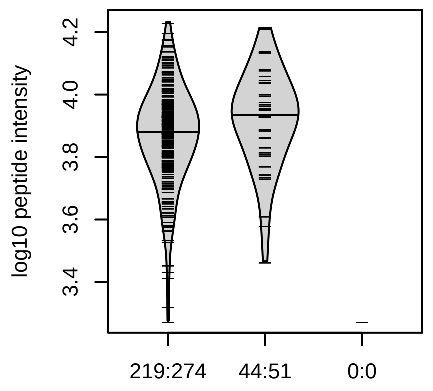

19:41260831:C:T_T
p = 0.42, beta = 0.132, N = 263

**GQVVYVFSK pc2
W4VSR3;Q16674**

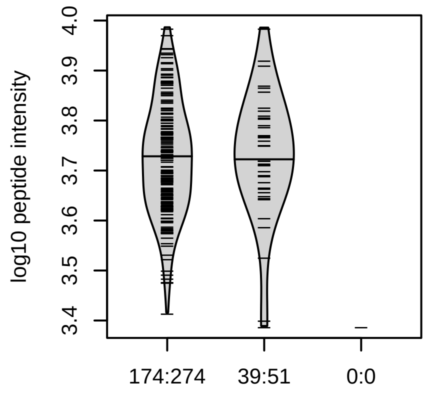

19:41260831:C:T_T
p = 0.9, beta = 0.0215, N = 213

96Assay Target: MIA−RAB4B
UniProt: W4VSR3
rsID: rs2607416
Target SNP: 19:41260831:C:T
Proxy SNP: 19:41260831:C:T
Tarkin log10(p): 17
Tarkin BETA: 0.656
Tarkin MSPA: 0.866
::−
1142:987:329

97. FUT8 (Q9BYC5)
Tarkin MSPA=0.894

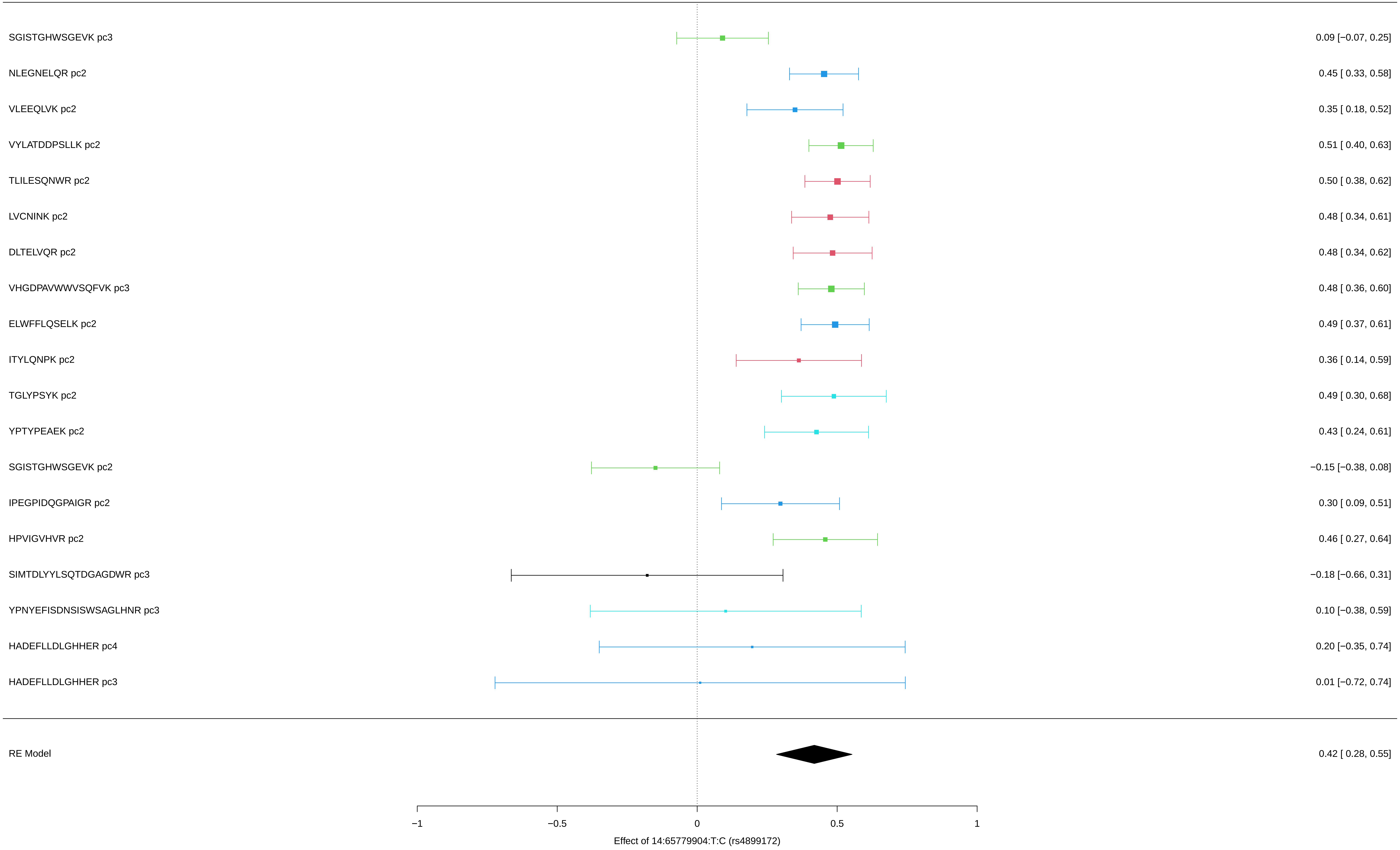

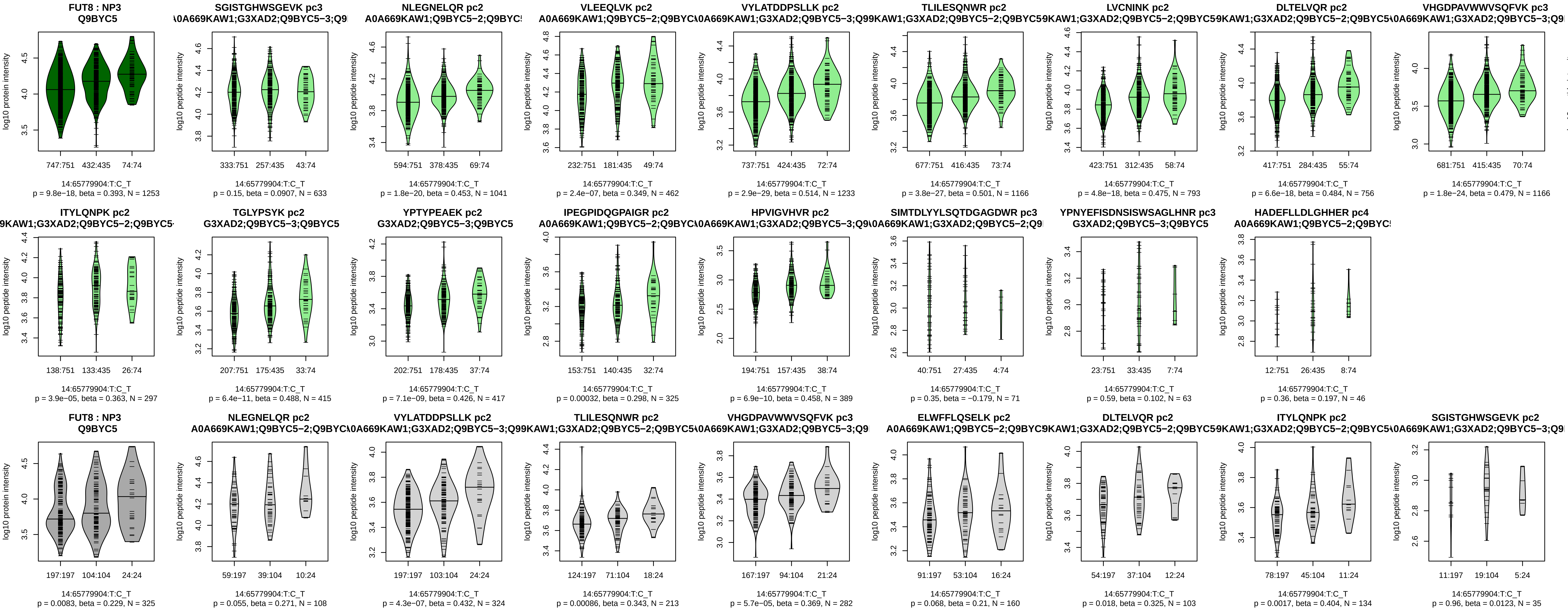

97Assay Target: FUT8
UniProt: Q9BYC5
rsID: rs4899172
Target SNP: 14:65779904:T:C
Proxy SNP: 14:65779904:T:C
Tarkin log10(p): 17
Tarkin BETA: 0.393
Tarkin MSPA: 0.894
–:*:*:*:*:*:*:*:*:*:*:*:–:*:*:–:–:–:–
633:1041:462:1233:1166:793:75

98. FETUB (Q9UGM5)
Tarkin MSPA=0.306

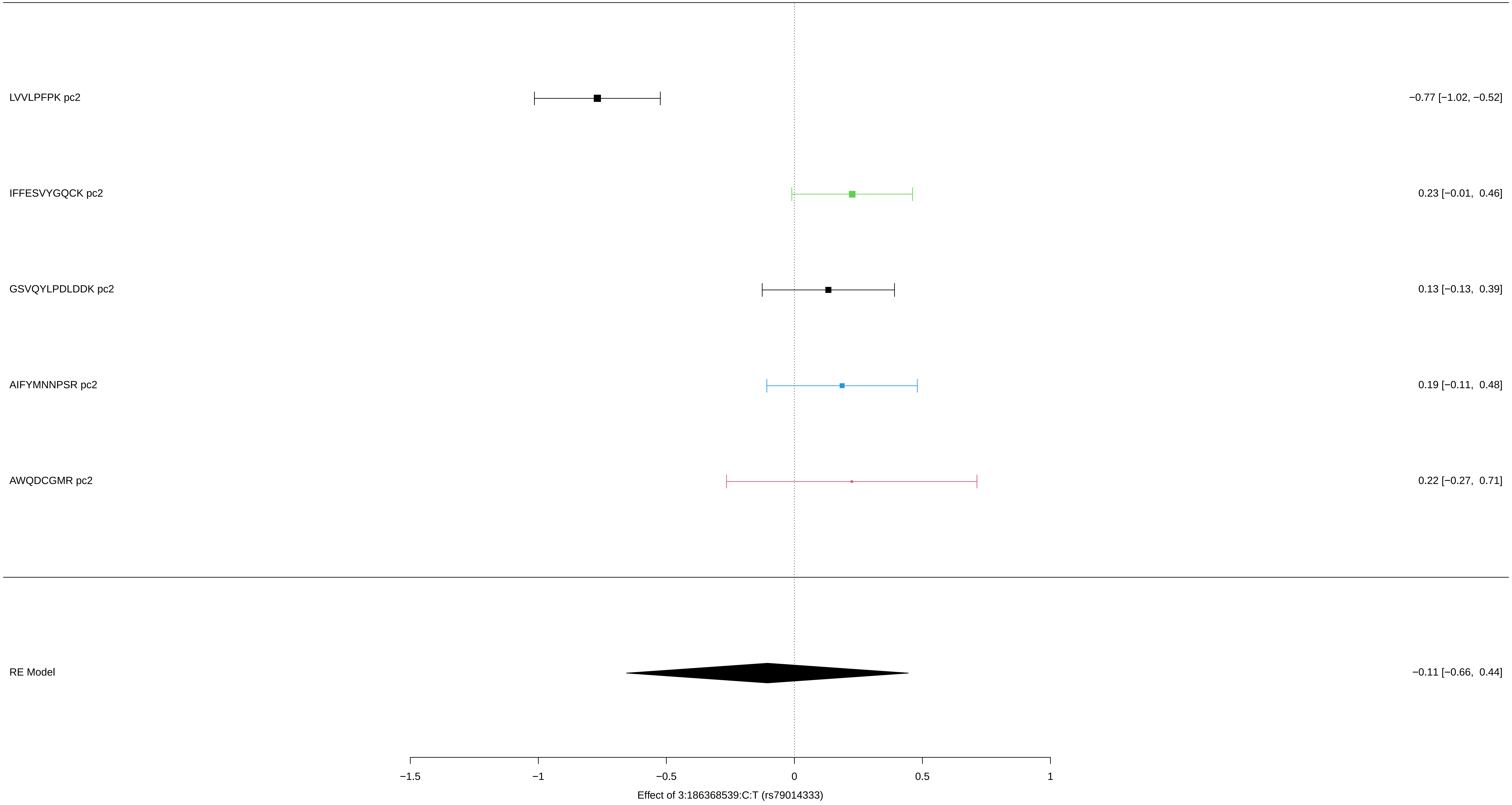

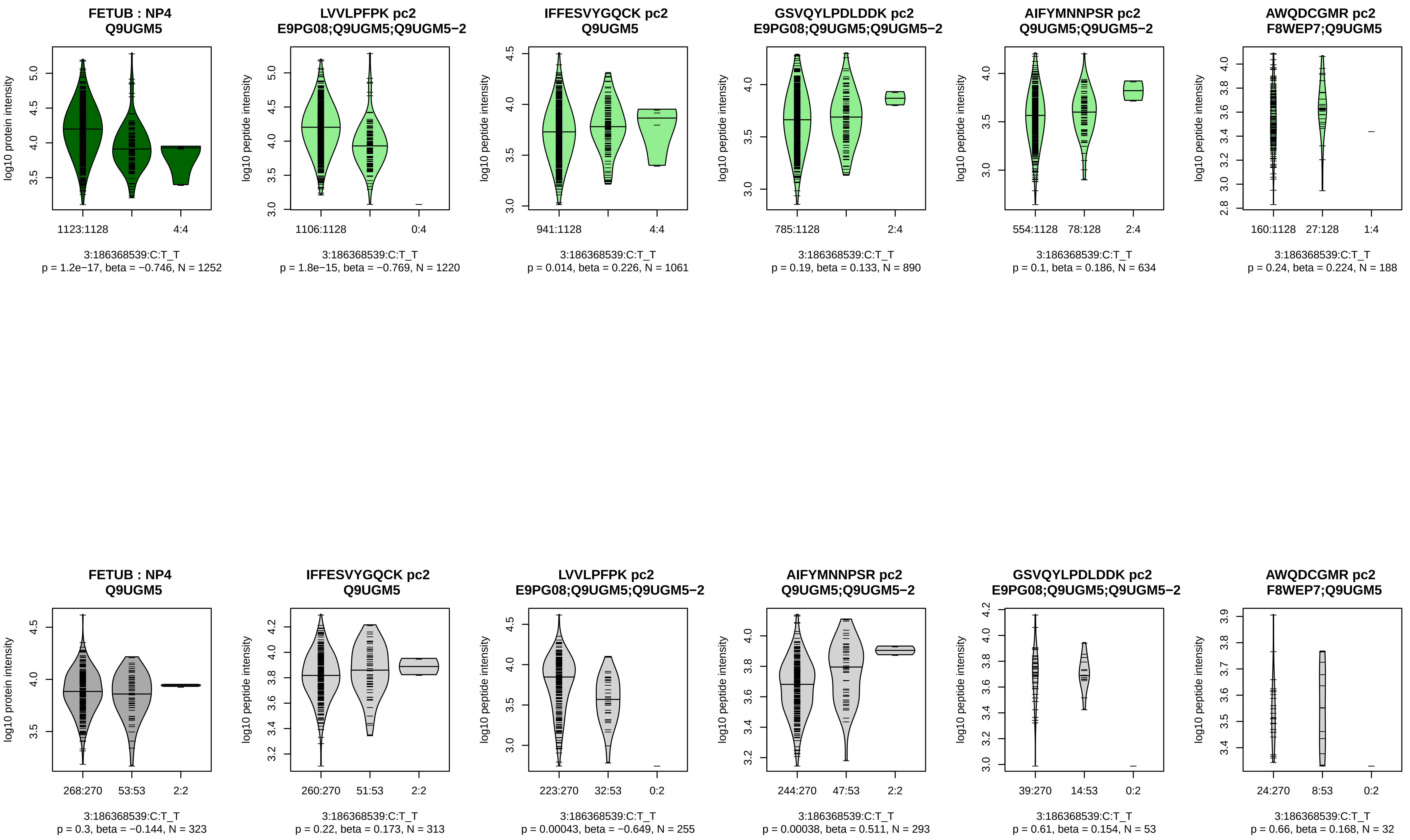

98Assay Target: FETUB
UniProt: Q9UGM5
rsID: rs79014333
Target SNP: 3:186368539:C:T
Proxy SNP: 3:186368539:C:T
Tarkin log10(p): 16.9
Tarkin BETA: −0.746
Tarkin MSPA: 0.306
*:−:−:−:−
1220:1061:890:634:188

99. HNRNPA3 (A0A7I2V4G0)
Tarkin MSPA=0.092

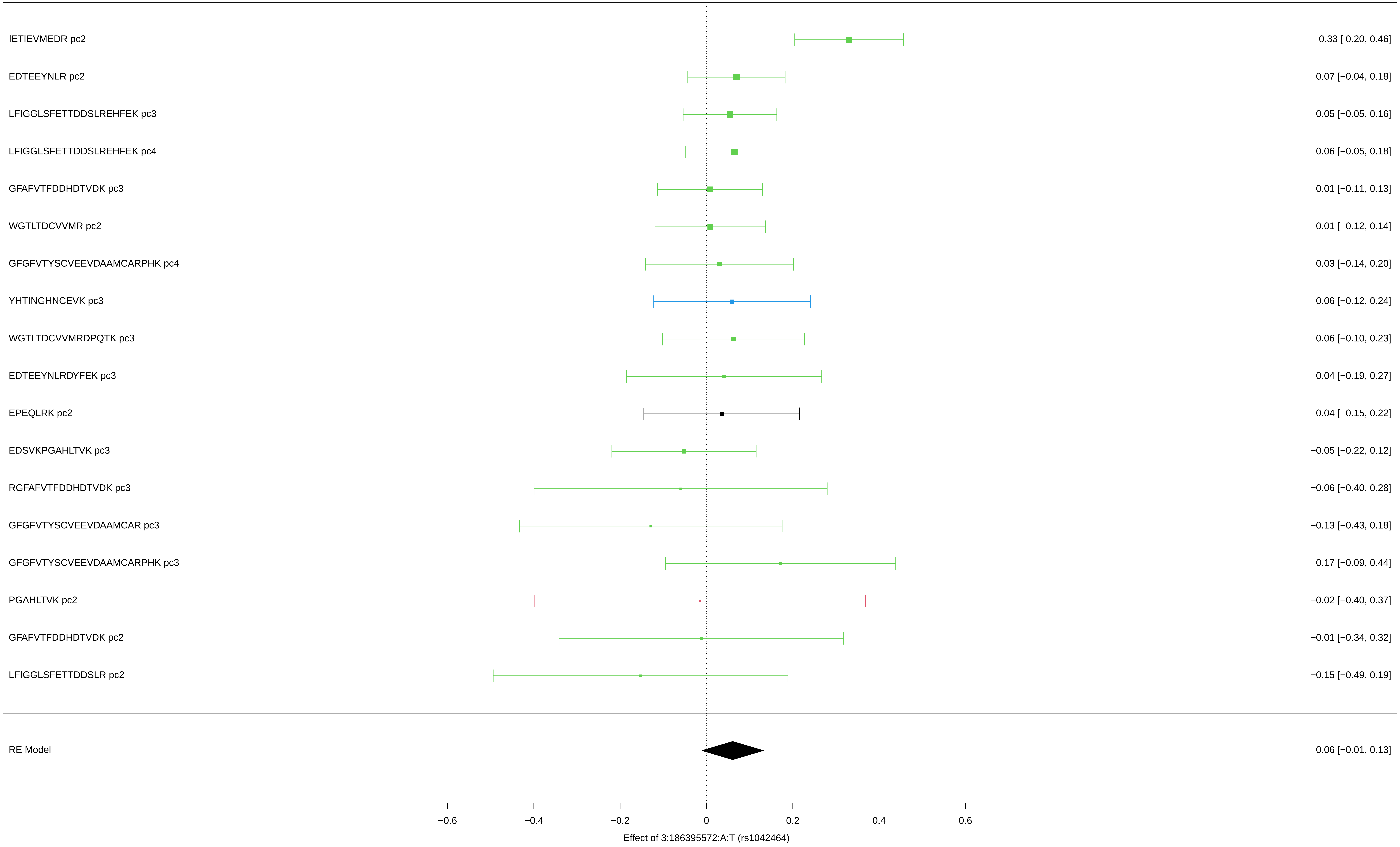

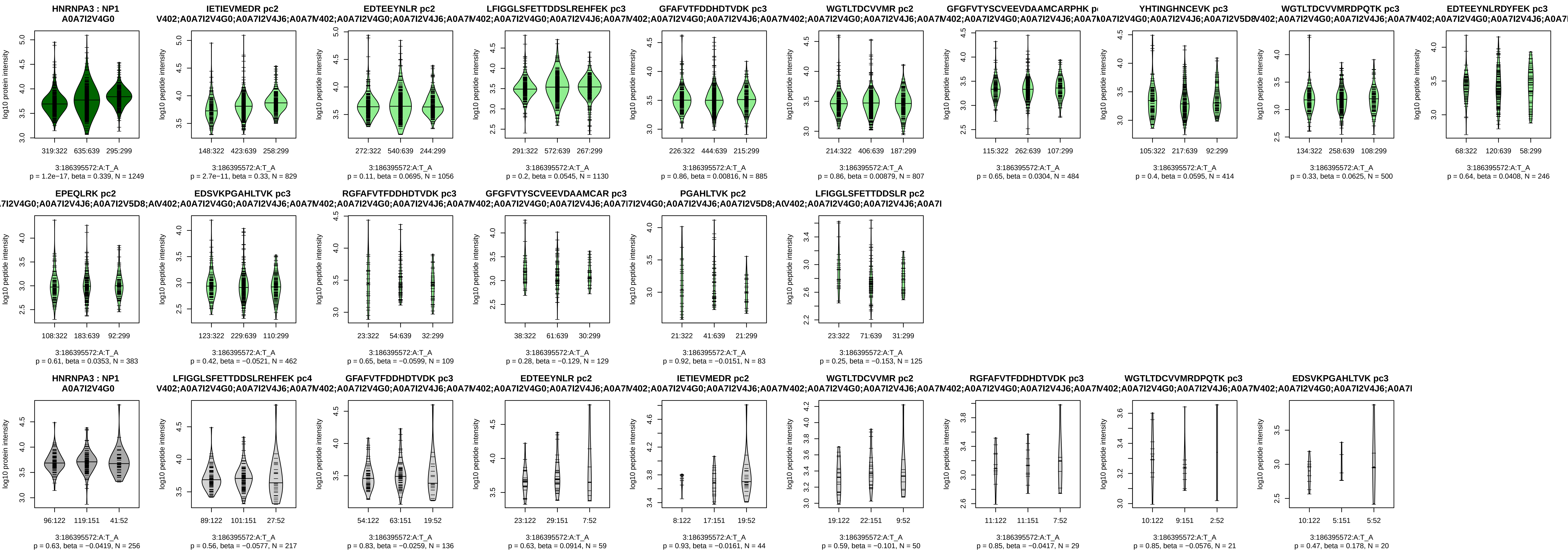

99Assay Target: HNRNPA3
UniProt: A0A7I2V4G0
rsID: rs1042464
Target SNP: 3:186395572:A:T
Proxy SNP: 3:186395572:A:T
Tarkin log10(p): 16.9
Tarkin BETA: 0.339
Tarkin MSPA: 0.092
829:1056:1130:1062:885:807:48

100. GALNT12 (Q8IXK2)
Tarkin MSPA=0.572

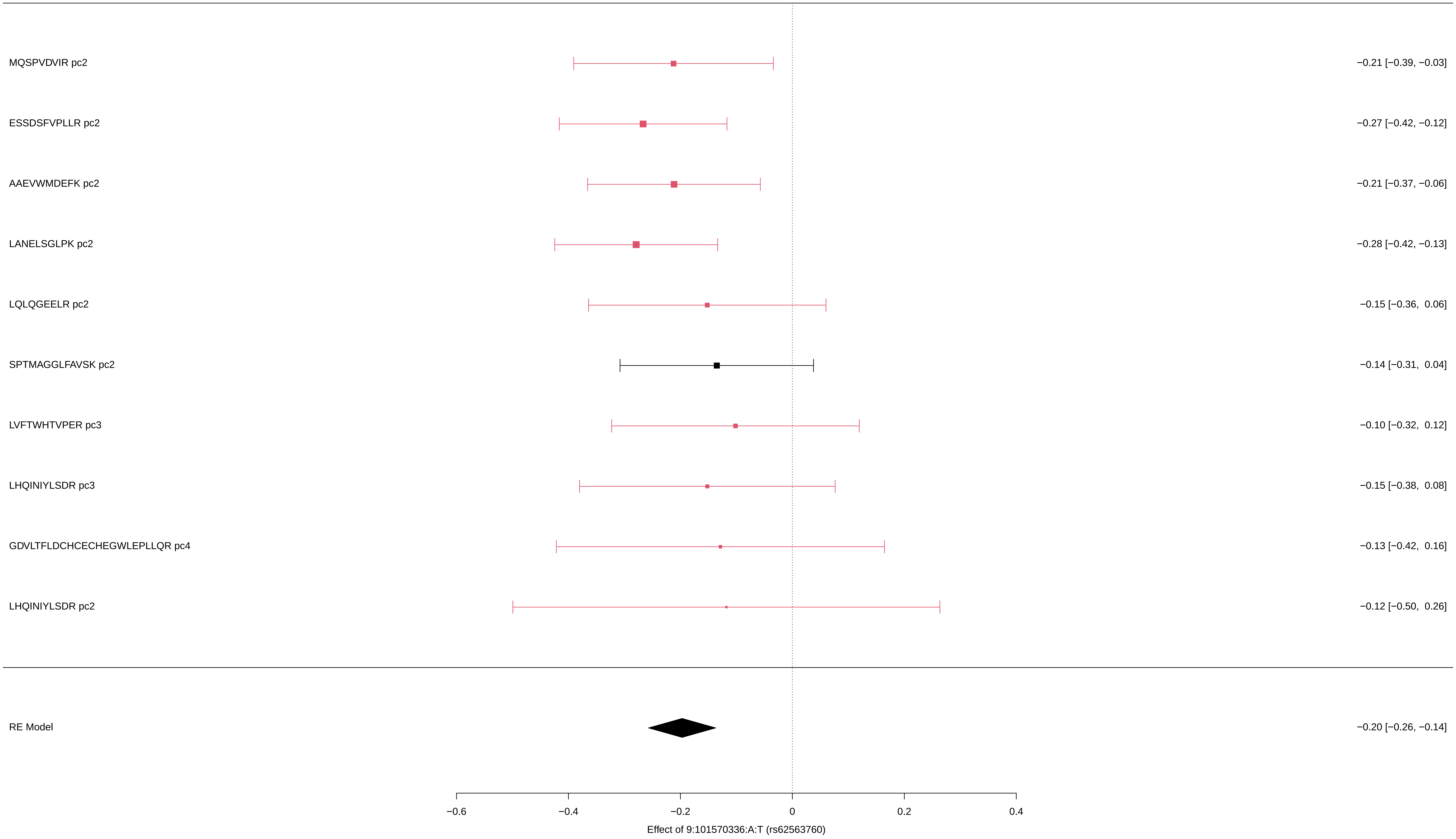

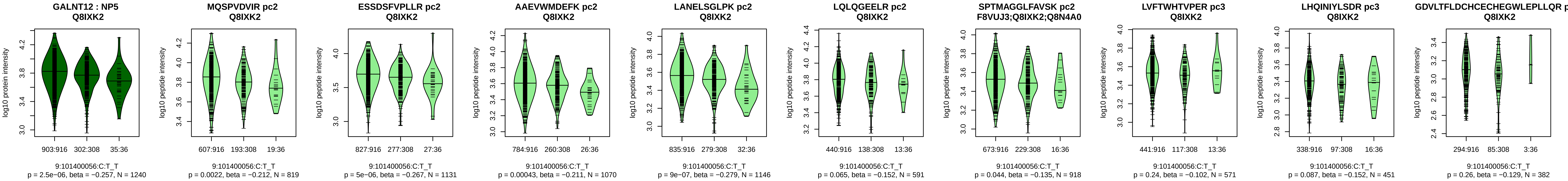

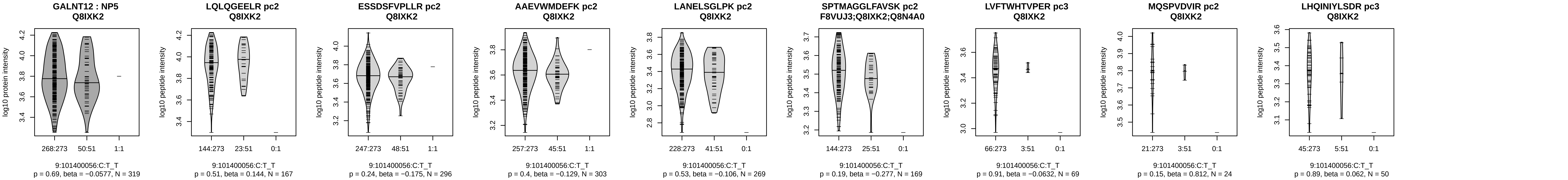

100Assay Target: GALNT12
UniProt: Q8IXK2
rsID: rs62563760
Target SNP: 9:101570336:A:T
Proxy SNP: 9:101400056:C:T
Tarkin log10(p): 16.7
Tarkin BETA: −0.616
Tarkin MSPA: 0.572
::*:*:–:–:–:–:–:–
819:1131:1070:1146:591:918:57

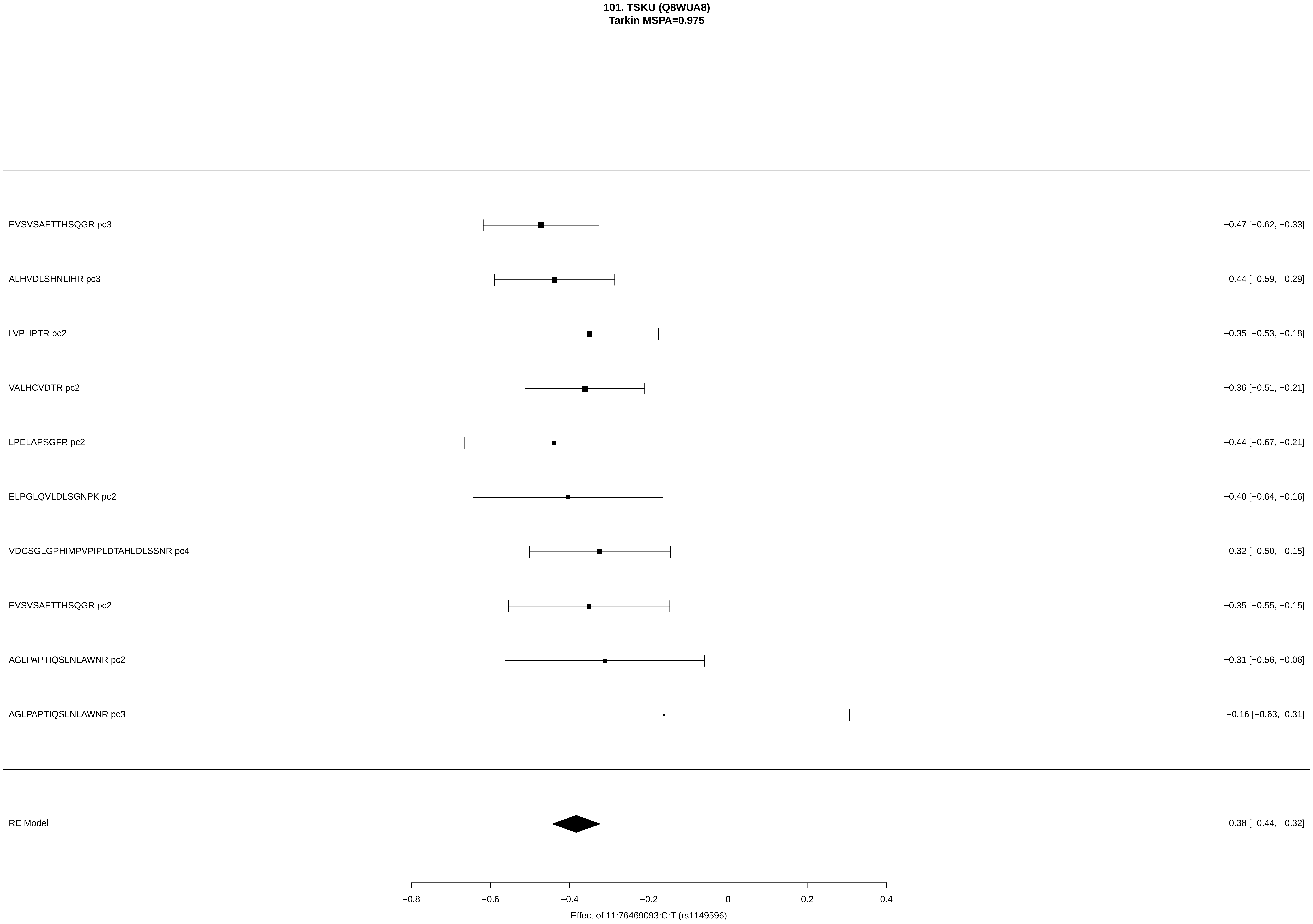

101. TSKU (Q8WUA8)
Tarkin MSPA=0.975
EVSVSAFTTHSQGR pc3
−0.47 [−0.62, −0.33]
ALHVDLSHNLIHR pc3
−0.44 [−0.59, −0.29]
LVPHPTR pc2
−0.35 [−0.53, −0.18]
VALHCVDTR pc2
−0.36 [−0.51, −0.21]
LPELAPSGFR pc2
−0.44 [−0.67, −0.21]
ELPGLQVLDLSGNPK pc2
−0.40 [−0.64, −0.16]
VDCSGLGPHIMPVPIPLDTAHLDLSSNR pc4
−0.32 [−0.50, −0.15]
EVSVSAFTTHSQGR pc2
−0.35 [−0.55, −0.15]
AGLPAPTIQSLNLAWNR pc2
−0.31 [−0.56, −0.06]
AGLPAPTIQSLNLAWNR pc3
−0.16 [−0.63, 0.31]
RE Model
−0.38 [−0.44, −0.32]
−0.8
−0.6
−0.4
−0.2
0
0.2
0.4
Effect of 11:76469093:C:T (rs1149596)

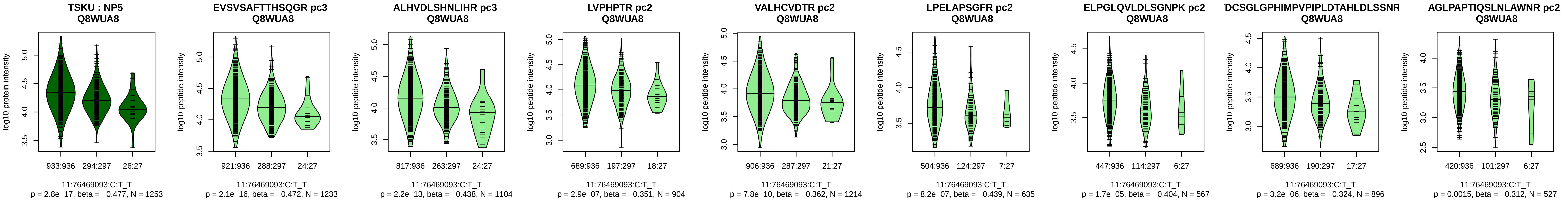

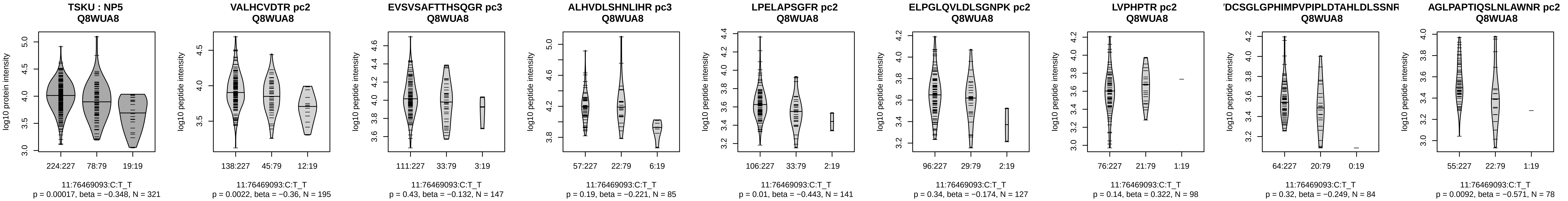

101Assay Target: TSKU
UniProt: Q8WUA8
rsID: rs1149596
Target SNP: 11:76469093:C:T
Proxy SNP: 11:76469093:C:T
Tarkin log10(p): 16.5
Tarkin BETA: −0.477
Tarkin MSPA: 0.975
::*:*:*:*:*:*:*:−:NA
1233:1104:904:1214:635:567:89

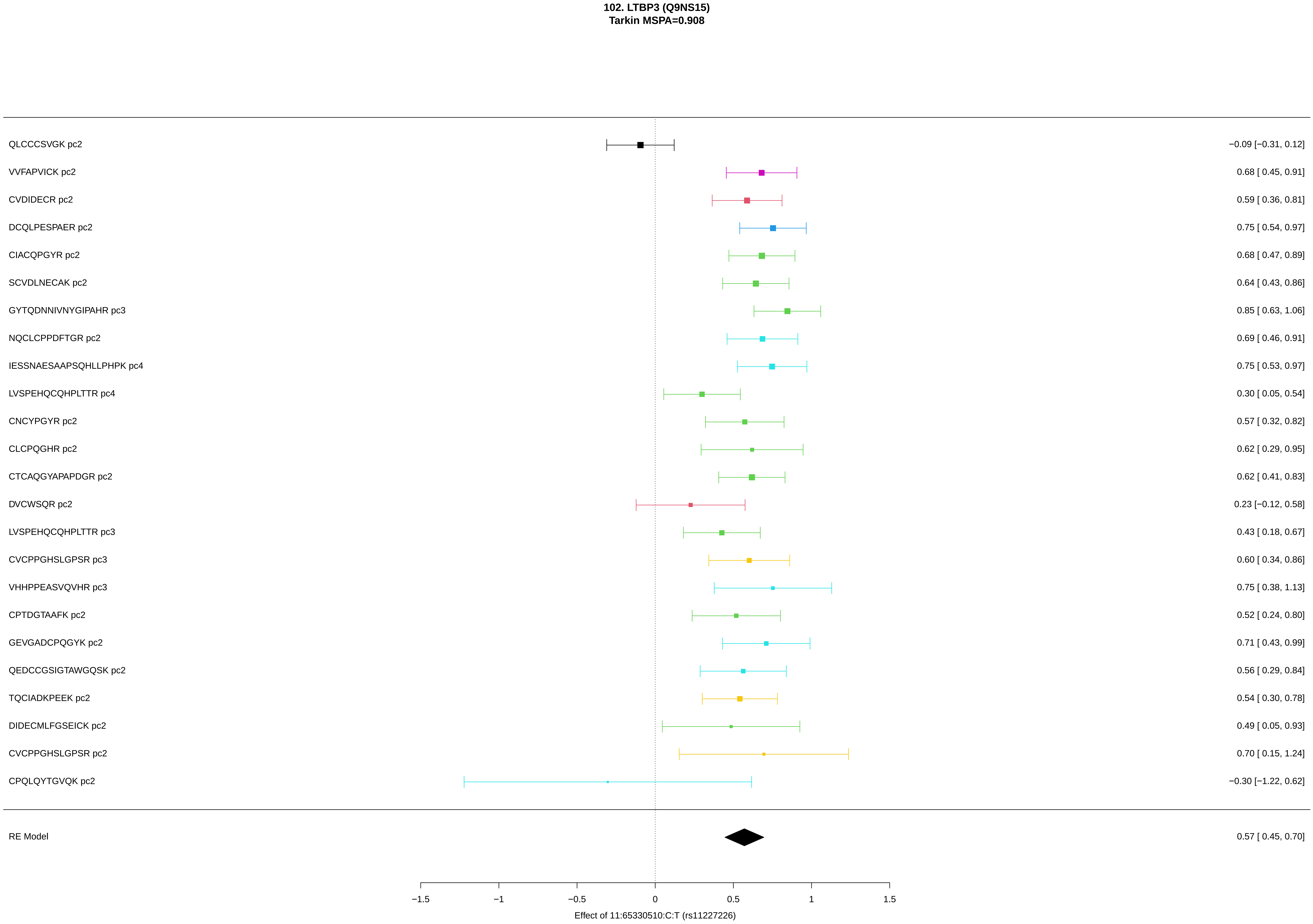
102. LTBP3 (Q9NS15)
Tarkin MSPA=0.908
QLCCCSVGK pc2 −0.09 [−0.31, 0.12]
VVFAPVICK pc2 0.68 [0.45, 0.91]
CVDIDECR pc2 0.59 [0.36, 0.81]
DCQLPESPAER pc2 0.75 [0.54, 0.97]
CIACQPGYR pc2 0.68 [0.47, 0.89]
SCVDLNECAK pc2 0.64 [0.43, 0.86]
GYTQDNNIVNYGIPAHR pc3 0.85 [0.63, 1.06]
NQCLCPPDFTGR pc2 0.69 [0.46, 0.91]
IESSNAESAAPSQHLLPHPK pc4 0.75 [0.53, 0.97]
LVSPEHQCQHPLTTR pc4 0.30 [0.05, 0.54]
CNCYPGYR pc2 0.57 [0.32, 0.82]
CLCPQGHR pc2 0.62 [0.29, 0.95]
CTCAQGYAPAPDGR pc2 0.62 [0.41, 0.83]
DVCWSQR pc2 0.23 [−0.12, 0.58]
LVSPEHQCQHPLTTR pc3 0.43 [0.18, 0.67]
CVCPPGHSLGPSR pc3 0.60 [0.34, 0.86]
VHHPPEASVQVHR pc3 0.75 [0.38, 1.13]
CPTDGTAAFK pc2 0.52 [0.24, 0.80]
GEVGADCPQGYK pc2 0.71 [0.43, 0.99]
QEDCCGSIGTAWGQSK pc2 0.56 [0.29, 0.84]
TQCIADKPEEK pc2 0.54 [0.30, 0.78]
DIDECMLFGSEICK pc2 0.49 [0.05, 0.93]
CVCPPGHSLGPSR pc2 0.70 [0.15, 1.24]
CPQLQYTGVQK pc2 −0.30 [−1.22, 0.62]
RE Model 0.57 [0.45, 0.70]
−1.5 −1 −0.5 0 0.5 1 1.5
Effect of 11:65330510:C:T (rs11227226)

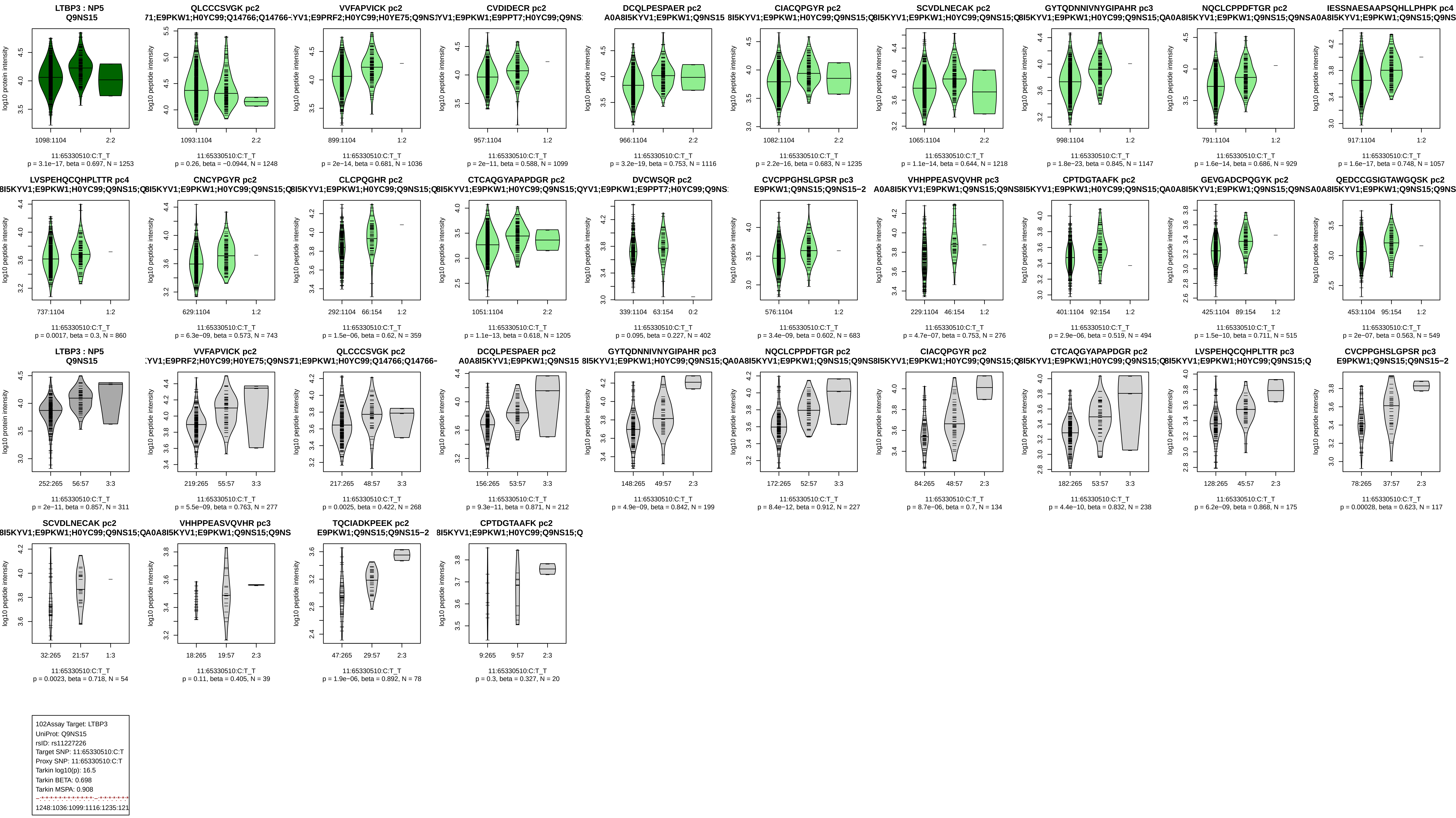

LTBP3 : NP5
Q9NS15
11:65330510:C:T_T
p = 3.1e−17, beta = 0.697, N = 1253
QLCCCSVGK pc2
p = 0.26, beta = −0.0944, N = 1248
VVFAPVICK pc2
p = 2e−14, beta = 0.681, N = 1036
CVDIDECR pc2
p = 2e−11, beta = 0.588, N = 1099
DCQLPESPAER pc2
p = 3.2e−19, beta = 0.753, N = 1116
CIACQPGYR pc2
p = 2.2e−16, beta = 0.683, N = 1235
SCVDLNECAK pc2
p = 1.1e−14, beta = 0.644, N = 1218
GYTQDNNIVNYGIPAHR pc3
p = 1.8e−23, beta = 0.845, N = 1147
NQCLCPPDFTGR pc2
p = 1.6e−14, beta = 0.686, N = 929
IESSNAESAAPSQHLLPHPK pc4
p = 1.6e−17, beta = 0.748, N = 1057
LVSPEHQCQHPLTTR pc4
p = 0.0017, beta = 0.3, N = 860
CNCYPGYR pc2
p = 6.3e−09, beta = 0.573, N = 743
CLCPQGHR pc2
p = 1.5e−06, beta = 0.62, N = 359
CTCAQGYAPAPDGR pc2
p = 1.1e−13, beta = 0.618, N = 1205
DVCWSQR pc2
p = 0.095, beta = 0.227, N = 402
CVCPPGHSLGPSR pc3
p = 3.4e−09, beta = 0.602, N = 683
VHHPPEASVQVHR pc3
p = 4.7e−07, beta = 0.753, N = 276
CPTDGTAAFK pc2
p = 2.9e−06, beta = 0.519, N = 494
GEVGADCPQGYK pc2
p = 1.5e−10, beta = 0.711, N = 515
QEDCCGSIGTAWGQSK pc2
p = 2e−07, beta = 0.563, N = 549
LTBP3 : NP5
Q9NS15
p = 2e−11, beta = 0.857, N = 311
VVFAPVICK pc2
p = 5.5e−09, beta = 0.763, N = 277
QLCCCSVGK pc2
p = 0.0025, beta = 0.422, N = 268
DCQLPESPAER pc2
p = 9.3e−11, beta = 0.871, N = 212
GYTQDNNIVNYGIPAHR pc3
p = 4.9e−09, beta = 0.842, N = 199
NQCLCPPDFTGR pc2
p = 8.4e−12, beta = 0.912, N = 227
CIACQPGYR pc2
p = 8.7e−06, beta = 0.7, N = 134
CTCAQGYAPAPDGR pc2
p = 4.4e−10, beta = 0.832, N = 238
LVSPEHQCQHPLTTR pc3
p = 6.2e−09, beta = 0.868, N = 175
CVCPPGHSLGPSR pc3
p = 0.00028, beta = 0.623, N = 117
SCVDLNECAK pc2
p = 0.0023, beta = 0.718, N = 54
VHHPPEASVQVHR pc3
p = 0.11, beta = 0.405, N = 39
TQCIADKPEEK pc2
p = 1.9e−06, beta = 0.892, N = 78
CPTDGTAAFK pc2
p = 0.3, beta = 0.327, N = 20
102Assay Target: LTBP3
UniProt: Q9NS15
rsID: rs11227226
Target SNP: 11:65330510:C:T
Proxy SNP: 11:65330510:C:T
Tarkin log10(p): 16.5
Tarkin BETA: 0.698
Tarkin MSPA: 0.908
1248:1036:1099:1116:1235:121

103. COLEC11 (Q9BWP8)
Tarkin MSPA=0.813

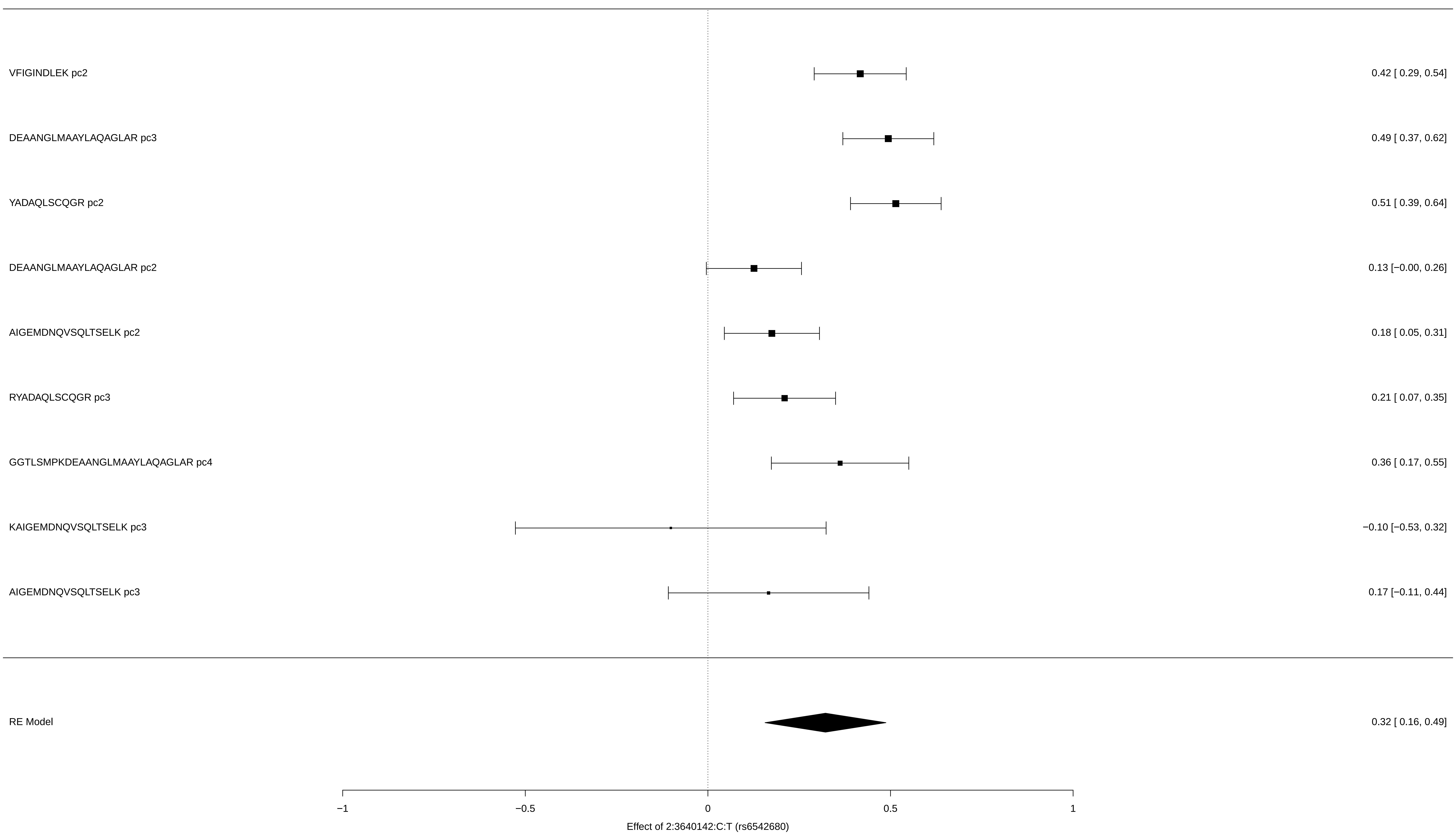

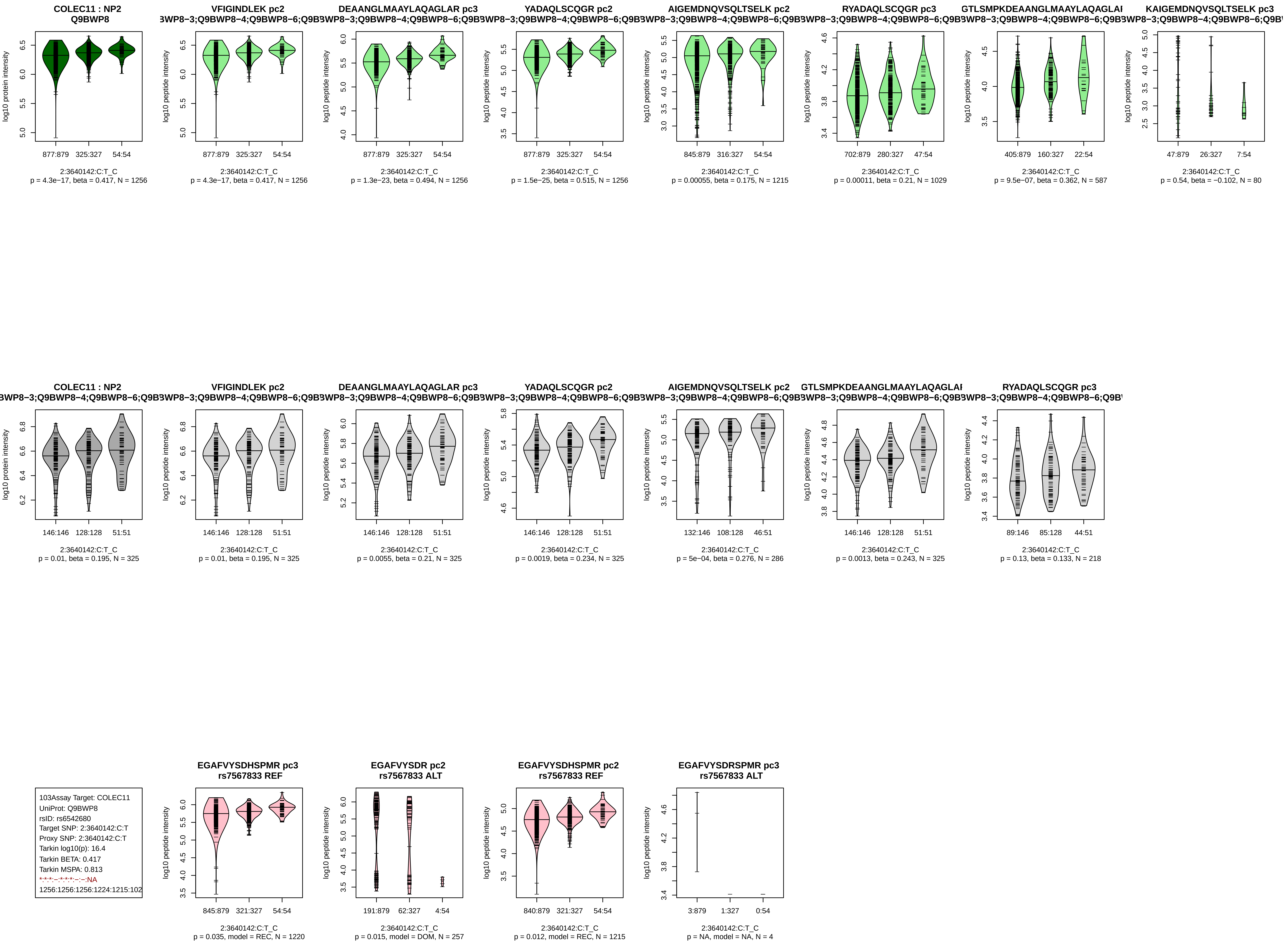
COLEC11 : NP2
Q9BWP8
VFIGINDLEK pc2
DEAANGLMAAYLAQAGLAR pc3
YADAQLSCQGR pc2
AIGEMDNQVSQLTSELK pc2
RYADAQLSCQGR pc3
GTLSMPKDEAANGLMAAYLAQAGLAI
KAIGEMDNQVSQLTSELK pc3
log10 protein intensity
log10 peptide intensity
877:879 325:327 54:54
845:879 316:327 54:54
702:879 280:327 47:54
405:879 160:327 22:54
47:879 26:327 7:54
2:3640142:C:T_C
p = 4.3e−17, beta = 0.417, N = 1256
p = 4.3e−17, beta = 0.417, N = 1256
p = 1.3e−23, beta = 0.494, N = 1256
p = 1.5e−25, beta = 0.515, N = 1256
p = 0.00055, beta = 0.175, N = 1215
p = 0.00011, beta = 0.21, N = 1029
p = 9.5e−07, beta = 0.362, N = 587
p = 0.54, beta = −0.102, N = 80
COLEC11 : NP2
VFIGINDLEK pc2
DEAANGLMAAYLAQAGLAR pc3
YADAQLSCQGR pc2
AIGEMDNQVSQLTSELK pc2
GTLSMPKDEAANGLMAAYLAQAGLAI
RYADAQLSCQGR pc3
146:146 128:128 51:51
132:146 108:128 46:51
89:146 85:128 44:51
2:3640142:C:T_C
p = 0.01, beta = 0.195, N = 325
p = 0.01, beta = 0.195, N = 325
p = 0.0055, beta = 0.21, N = 325
p = 0.0019, beta = 0.234, N = 325
p = 5e−04, beta = 0.276, N = 286
p = 0.0013, beta = 0.243, N = 325
p = 0.13, beta = 0.133, N = 218
103Assay Target: COLEC11
UniProt: Q9BWP8
rsID: rs6542680
Target SNP: 2:3640142:C:T
Proxy SNP: 2:3640142:C:T
Tarkin log10(p): 16.4
Tarkin BETA: 0.417
Tarkin MSPA: 0.813
::*:−:*:*:*:−:−:NA
1256:1256:1256:1224:1215:102
EGAFVYSDHSPMR pc3
rs7567833 REF
EGAFVYSDR pc2
rs7567833 ALT
EGAFVYSDHSPMR pc2
rs7567833 REF
EGAFVYSDRSPMR pc3
rs7567833 ALT
845:879 321:327 54:54
191:879 62:327 4:54
840:879 321:327 54:54
3:879 1:327 0:54
2:3640142:C:T_C
p = 0.035, model = REC, N = 1220
p = 0.015, model = DOM, N = 257
p = 0.012, model = REC, N = 1215
p = NA, model = NA, N = 4

104. CCL15 (Q16663)
Tarkin MSPA=1

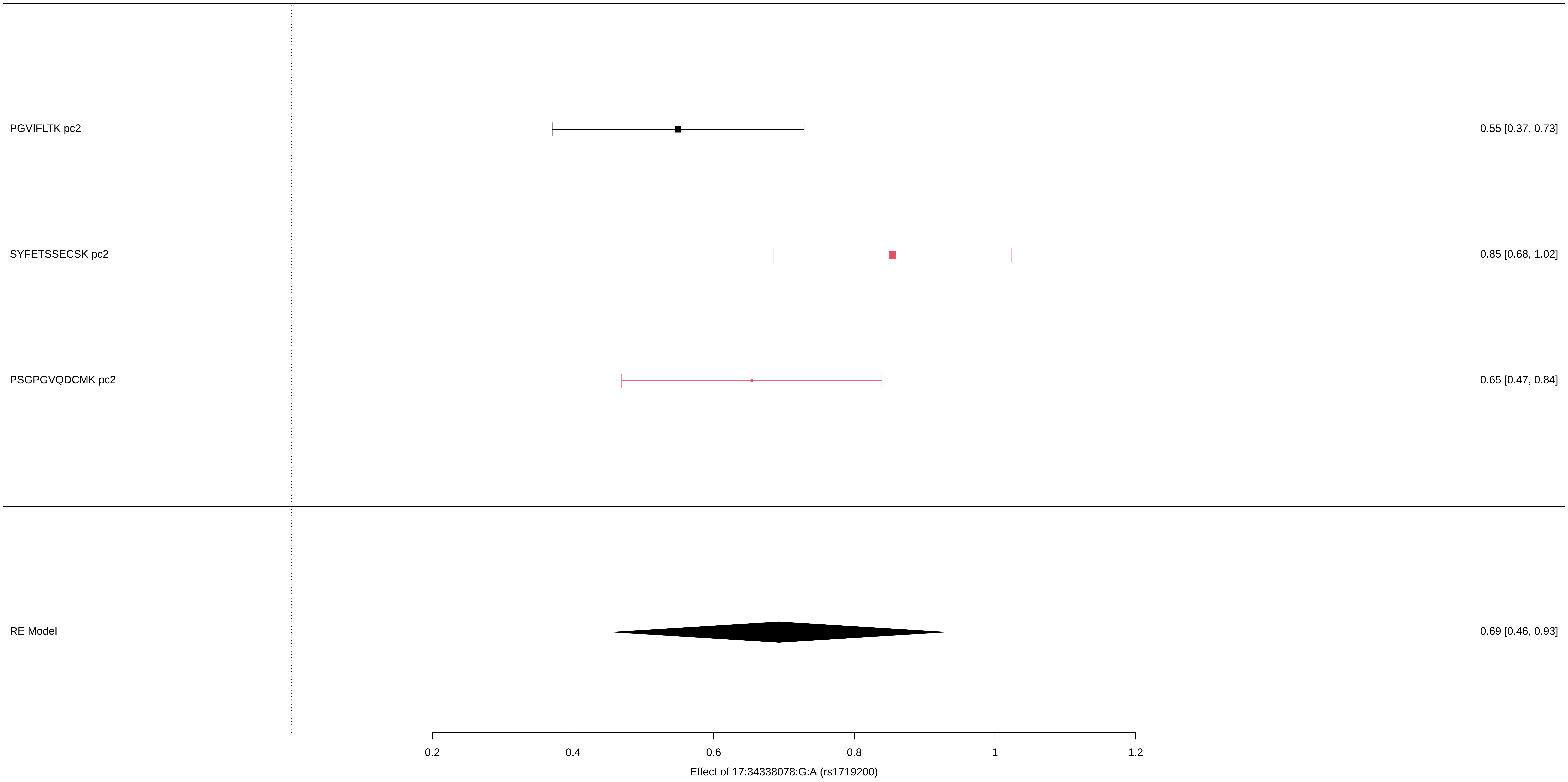

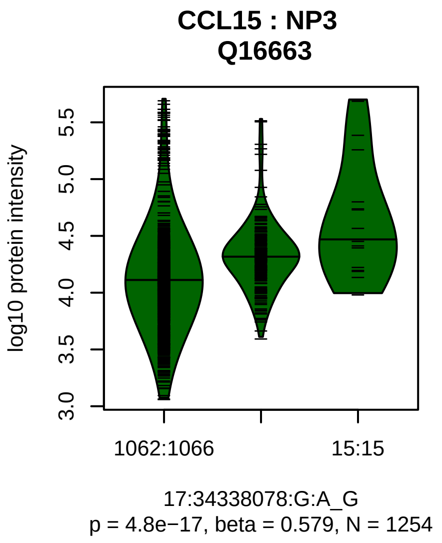

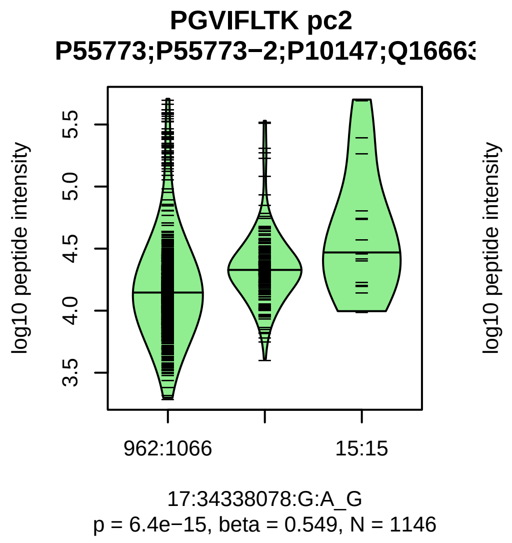

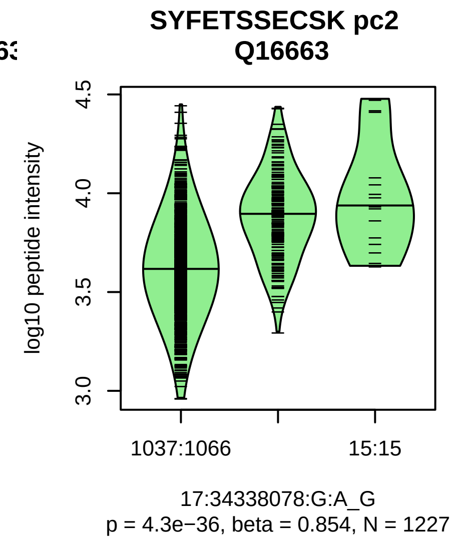

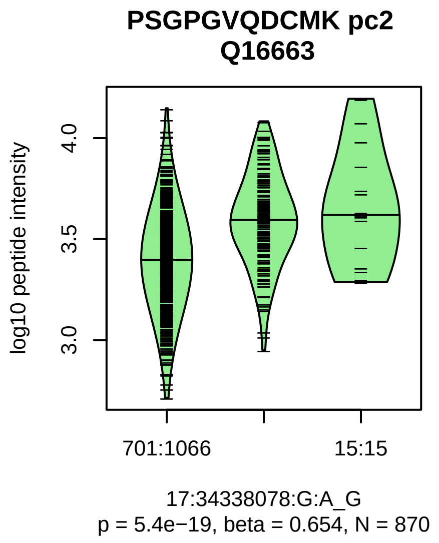

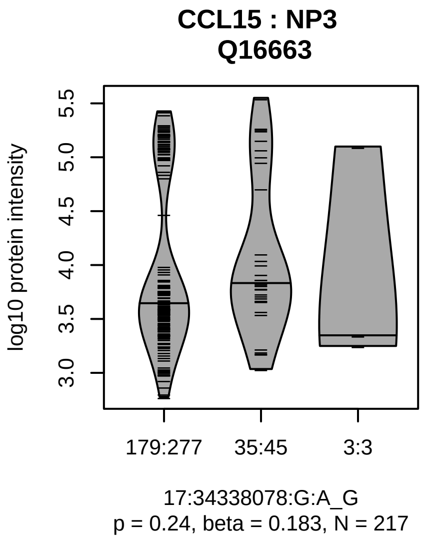

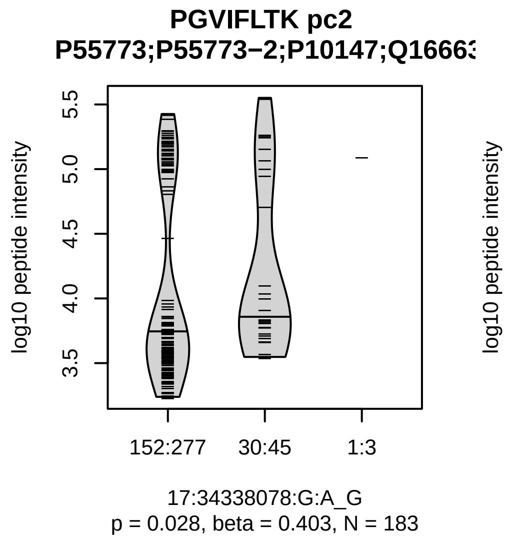

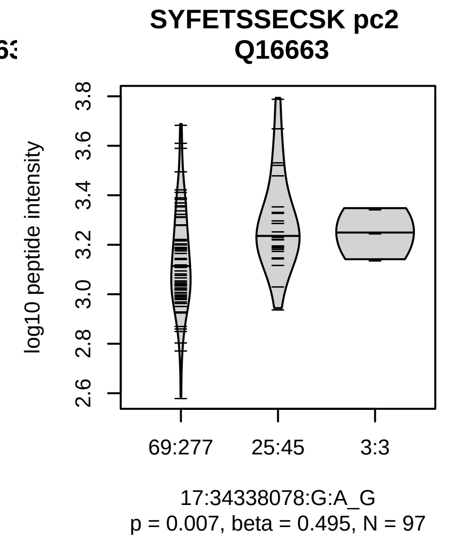

104Assay Target: CCL15
UniProt: Q16663
rsID: rs1719200
Target SNP: 17:34338078:G:A
Proxy SNP: 17:34338078:G:A
Tarkin log10(p): 16.3
Tarkin BETA: 0.579
Tarkin MSPA: 1
::*:NA
1146:1227:870:16

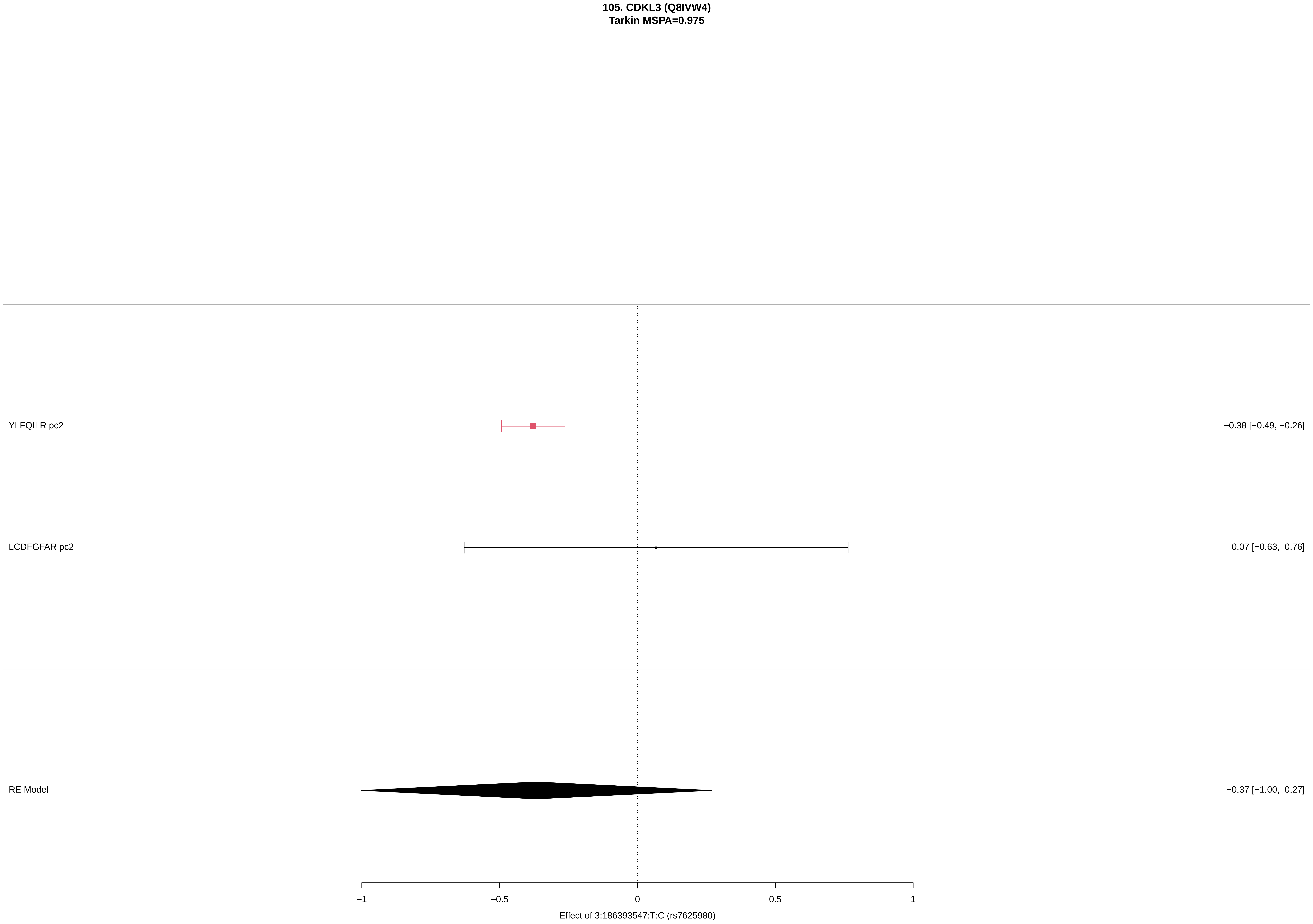
105. CDKL3 (Q8IVW4)
Tarkin MSPA=0.975
YLFQILR pc2
−0.38 [−0.49, −0.26]
LCDFGFAR pc2
0.07 [−0.63, 0.76]
RE Model
−0.37 [−1.00, 0.27]
−1
−0.5
0
0.5
1
Effect of 3:186393547:T:C (rs7625980)

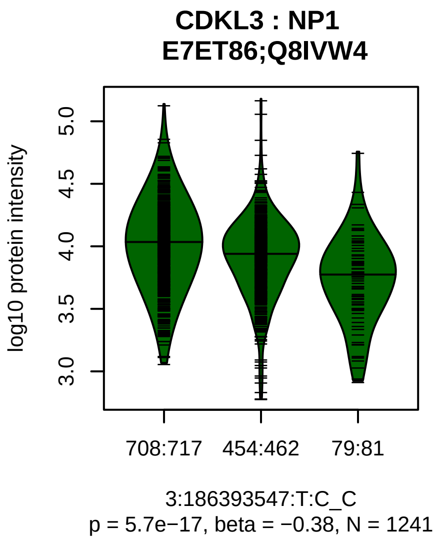

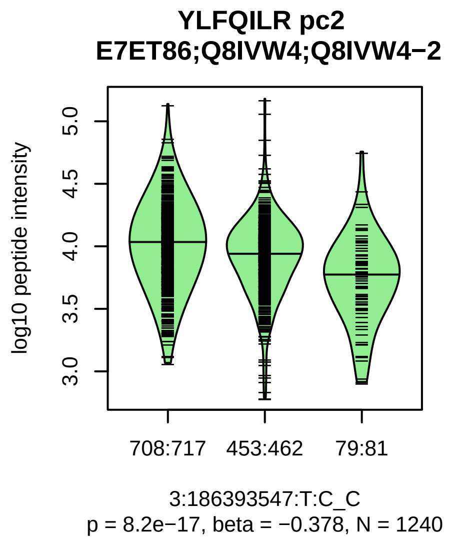

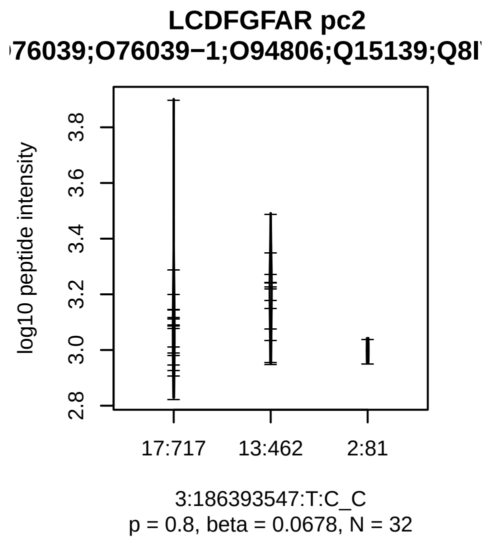

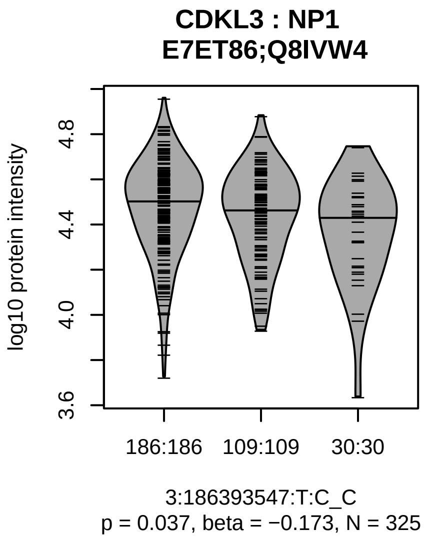

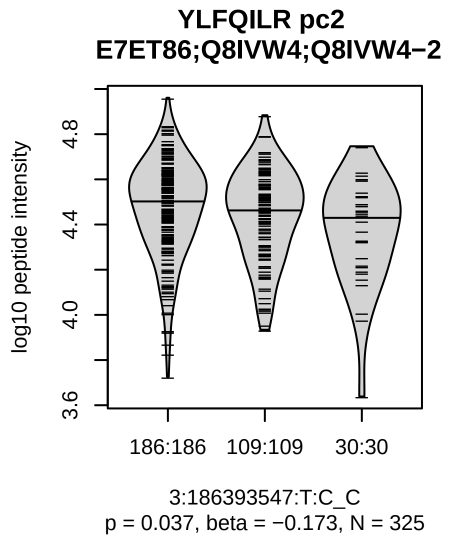

105Assay Target: CDKL3
UniProt: Q8IVW4
rsID: rs7625980
Target SNP: 3:186393547:T:C
Proxy SNP: 3:186393547:T:C
Tarkin log10(p): 16.2
Tarkin BETA: −0.38
Tarkin MSPA: 0.975
*:–
1240:32

106. LGALS4 (P56470)
Tarkin MSPA=0.992

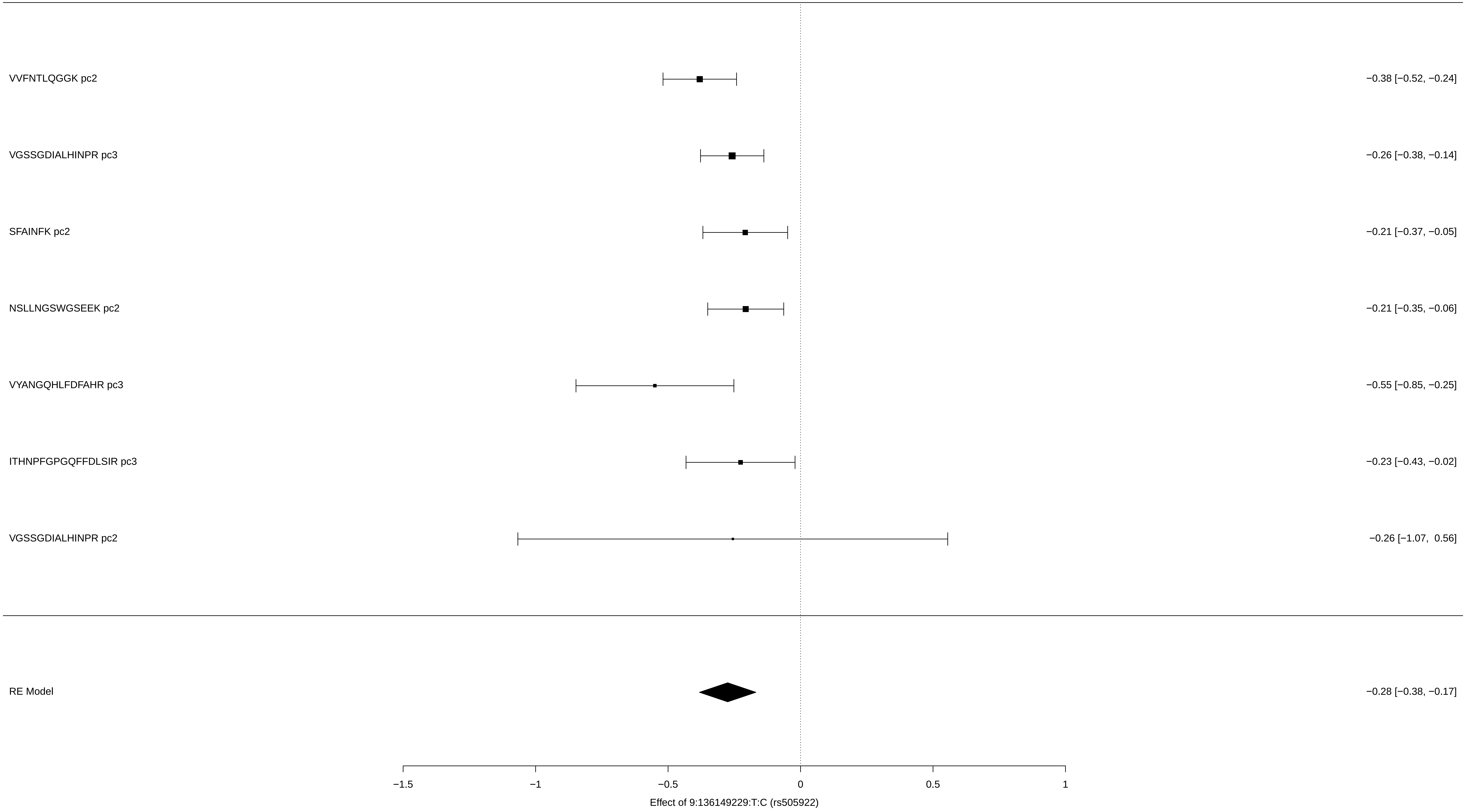

LGALS4 : NP3
P56470

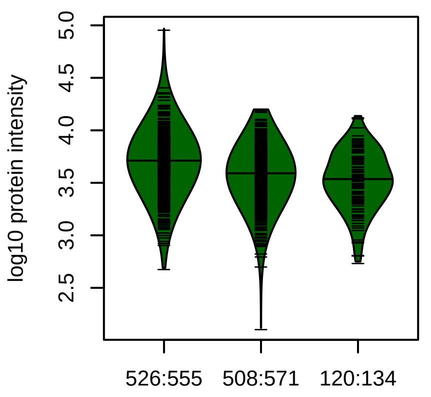

9:136149229:T:C_C
p = 6.4e−17, beta = −0.365, N = 1154

VVFNTLQGGK pc2
P56470

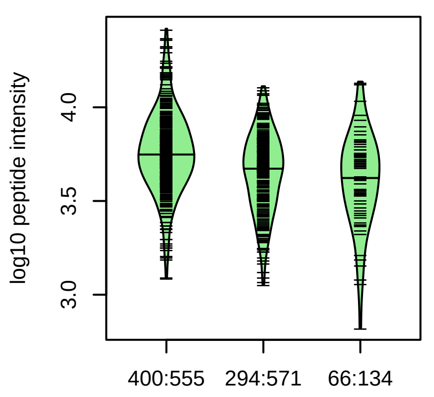

9:136149229:T:C_C
p = 3.8e−12, beta = −0.38, N = 760

VGSSGDIALHINPR pc3
P56470

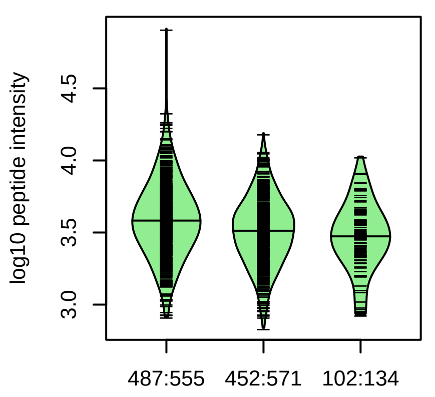

9:136149229:T:C_C
p = 3.4e−08, beta = −0.258, N = 1041

SFAINFK pc2
P56470

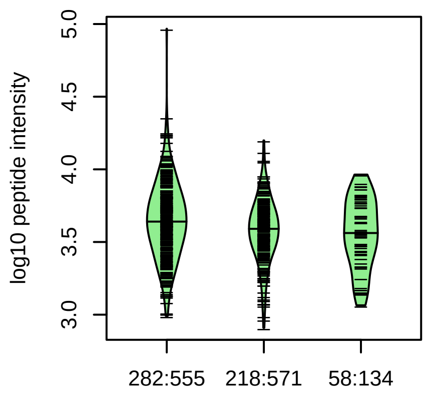

9:136149229:T:C_C
p = 0.00083, beta = −0.209, N = 558

NSLLNGSWGSEEK pc2
P56470

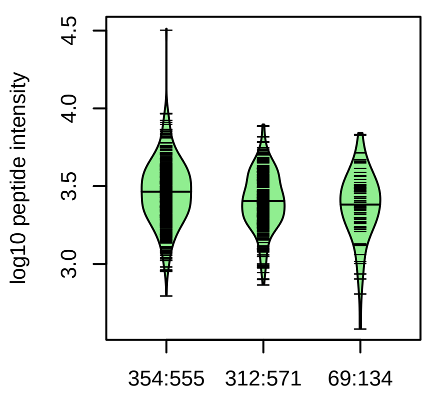

9:136149229:T:C_C
p = 0.00022, beta = −0.207, N = 735

VYANGQHLFDFAHR pc3
P56470

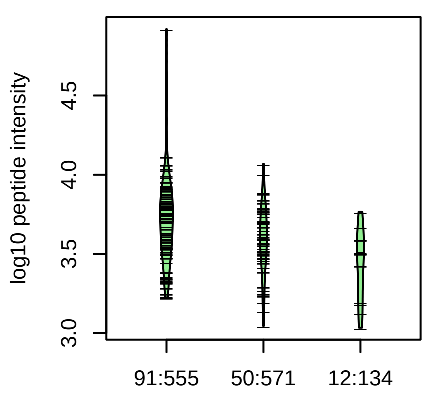

9:136149229:T:C_C
p = 4.7e−06, beta = −0.55, N = 153

ITHNPFGPGQFFDLSIR pc3
P56470

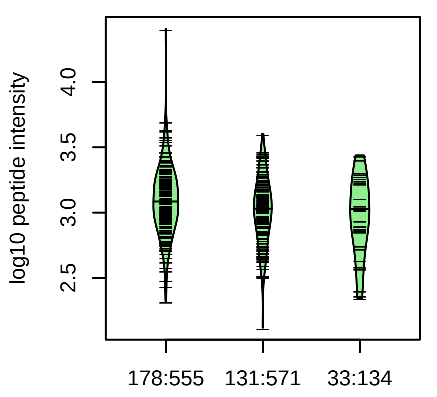

9:136149229:T:C_C
p = 0.0048, beta = −0.226, N = 342

LGALS4 : NP3
P56470

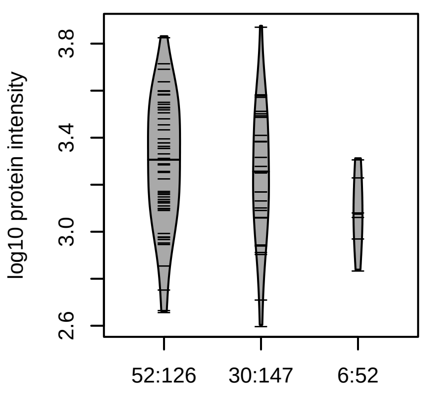

9:136149229:T:C_C
p = 0.042, beta = −0.334, N = 88

VVFNTLQGGK pc2
P56470

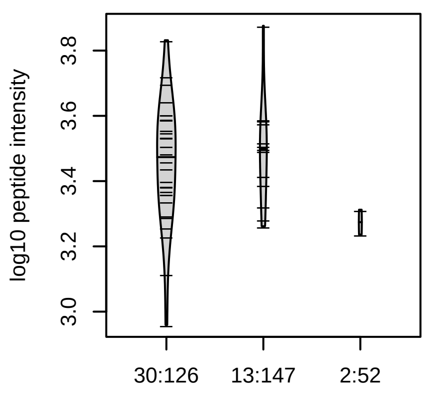

9:136149229:T:C_C
p = 0.7, beta = −0.0978, N = 45

VGSSGDIALHINPR pc3
P56470

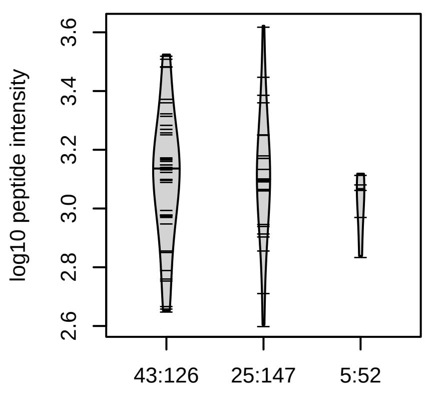

9:136149229:T:C_C
p = 0.3, beta = −0.187, N = 73

106Assay Target: LGALS4
UniProt: P56470
rsID: rs505922
Target SNP: 9:136149229:T:C
Proxy SNP: 9:136149229:T:C
Tarkin log10(p): 16.2
Tarkin BETA: −0.366
Tarkin MSPA: 0.992
::*:*:*:*:–
760:1041:558:735:153:342:29

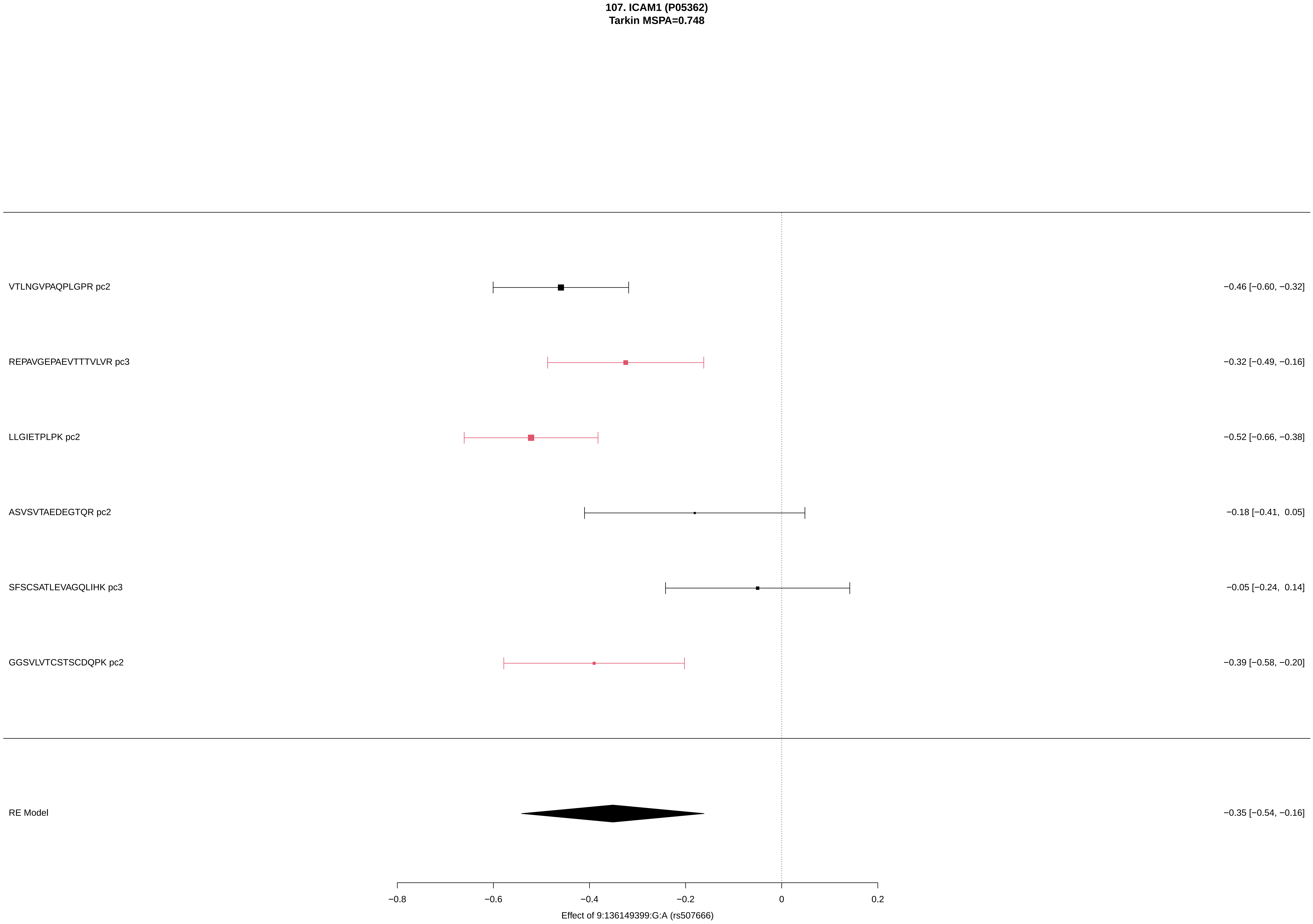

107. ICAM1 (P05362)
Tarkin MSPA=0.748
VTLNGVPAQPLGPR pc2
−0.46 [−0.60, −0.32]
REPAVGEPAEVTTTVLVR pc3
−0.32 [−0.49, −0.16]
LLGIETPLPK pc2
−0.52 [−0.66, −0.38]
ASVSVTAEDEGTQR pc2
−0.18 [−0.41, 0.05]
SFSCSATLEVAGQLIHK pc3
−0.05 [−0.24, 0.14]
GGSVLVTCSTSCDQPK pc2
−0.39 [−0.58, −0.20]
RE Model
−0.35 [−0.54, −0.16]
−0.8
−0.6
−0.4
−0.2
0
0.2
Effect of 9:136149399:G:A (rs507666)

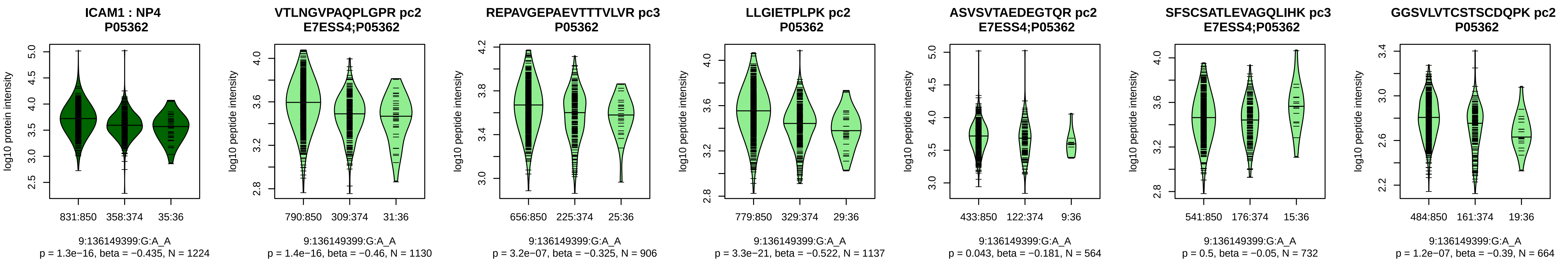

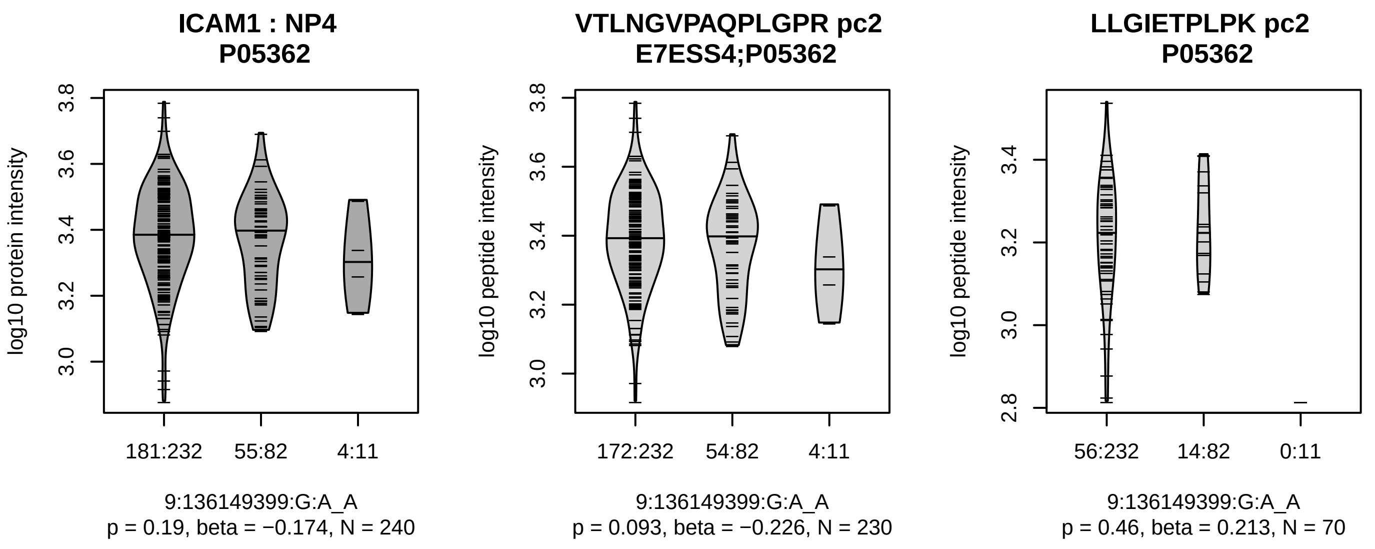

107Assay Target: ICAM1
UniProt: P05362
rsID: rs507666
Target SNP: 9:136149399:G:A
Proxy SNP: 9:136149399:G:A
Tarkin log10(p): 15.9
Tarkin BETA: −0.436
Tarkin MSPA: 0.748
::*:–:–:*
1130:906:1137:564:732:664

108. CDH19 (Q9H159)
Tarkin MSPA=0.805

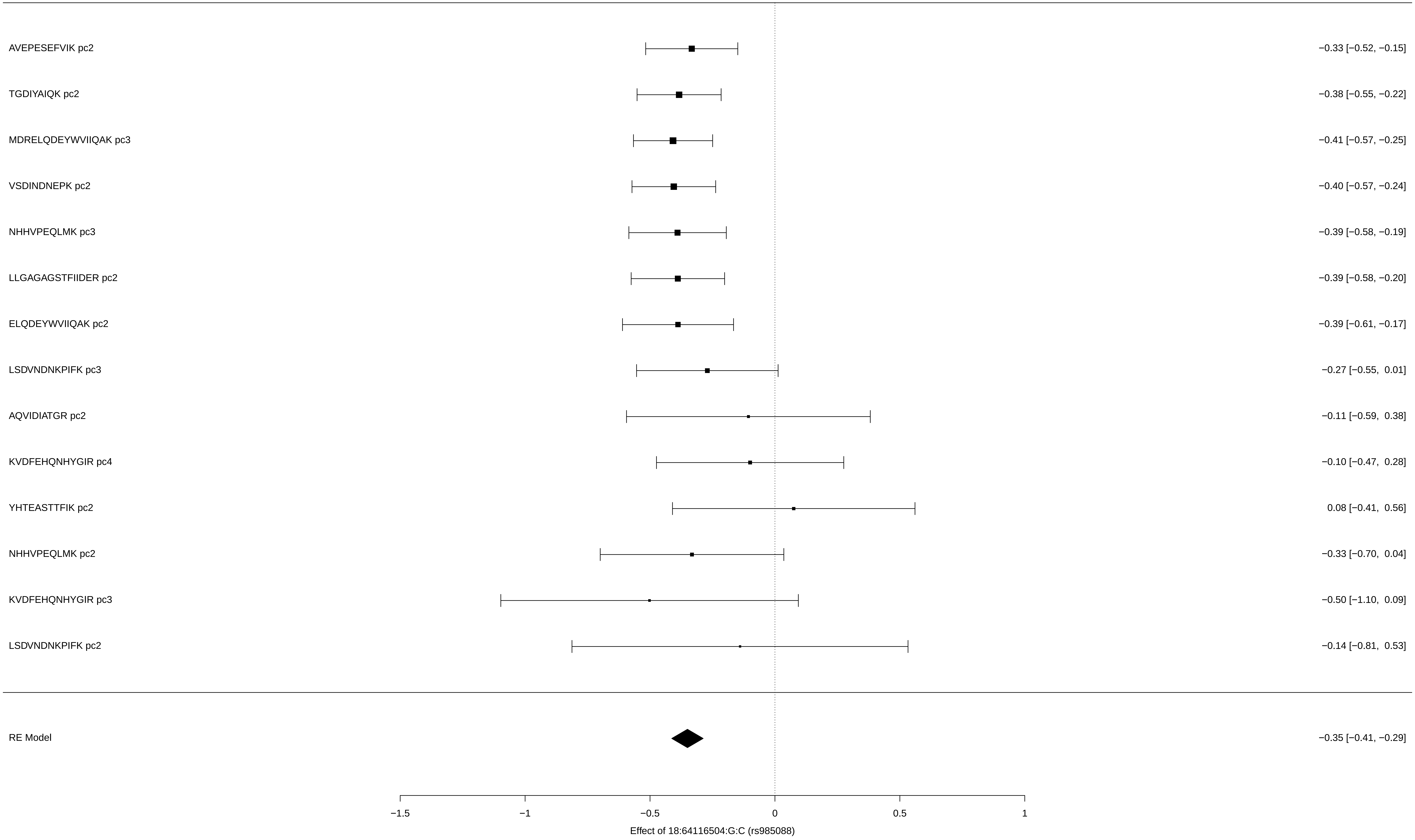

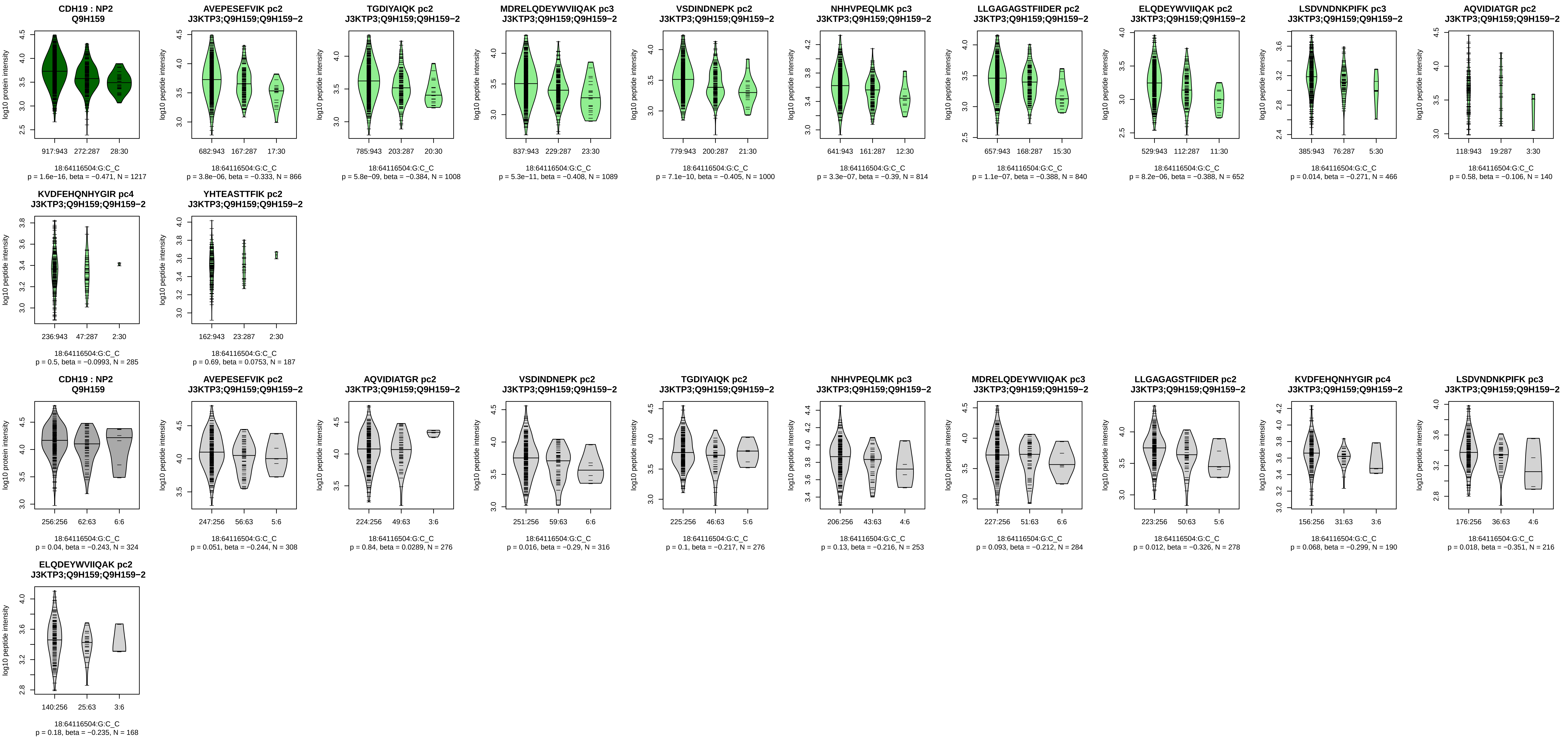

108Assay Target: CDH19
UniProt: Q9H159
rsID: rs985088
Target SNP: 18:64116504:G:C
Proxy SNP: 18:64116504:G:C
Tarkin log10(p): 15.8
Tarkin BETA: −0.471
Tarkin MSPA: 0.805
866:1008:1089:1000:814:840:65

109. SAA1 (E9PQD6)
Tarkin MSPA=0.912

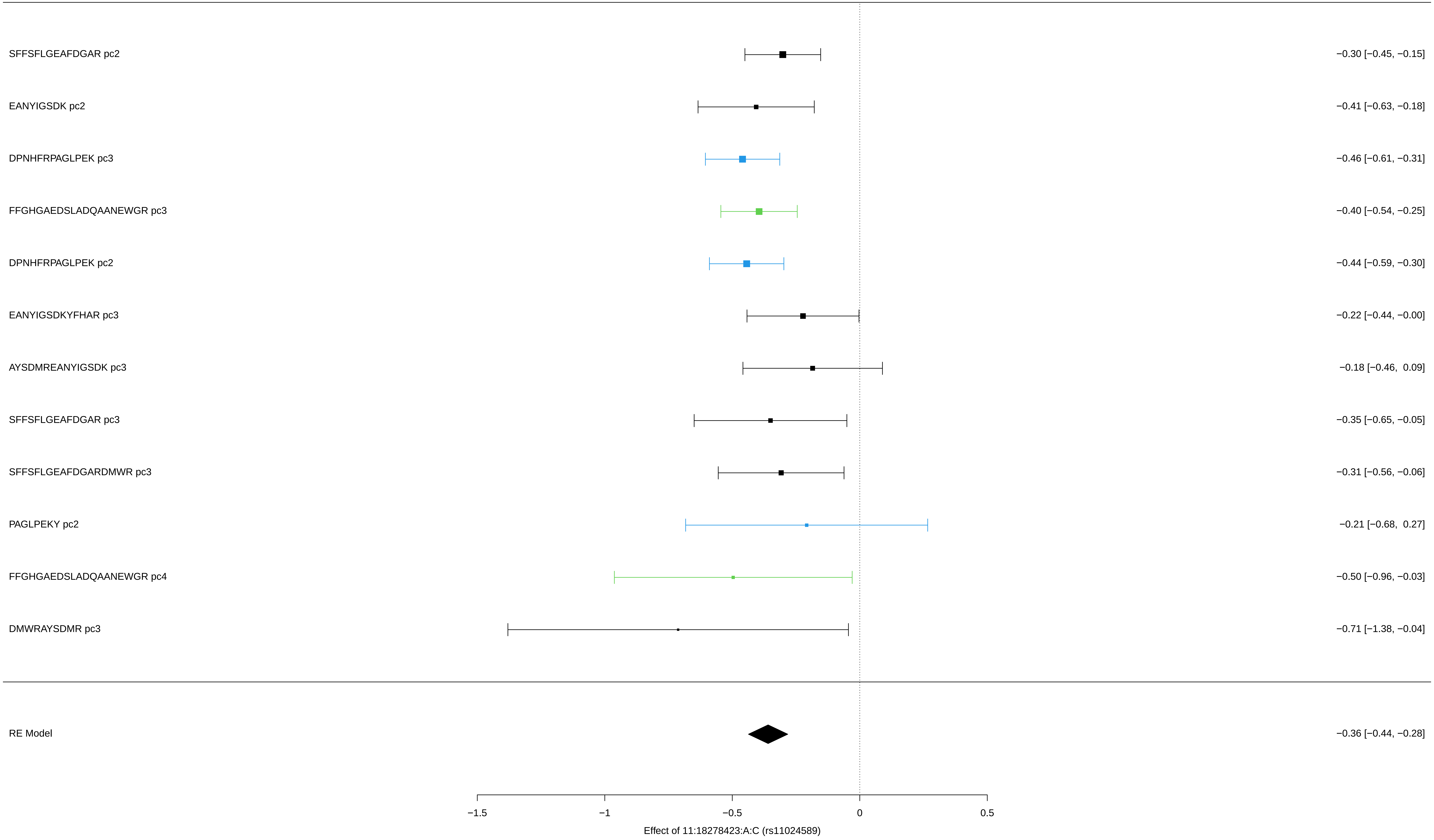

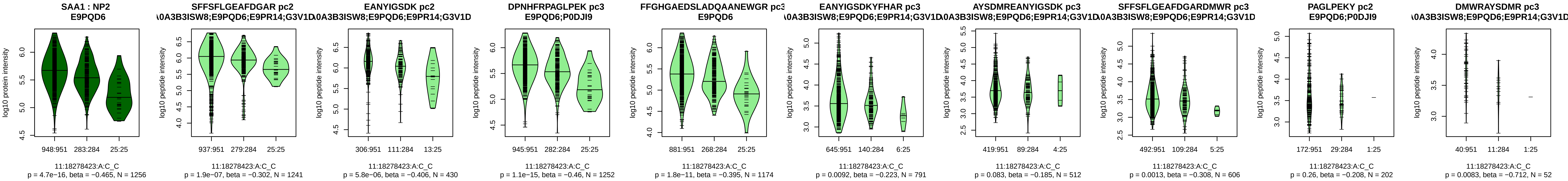

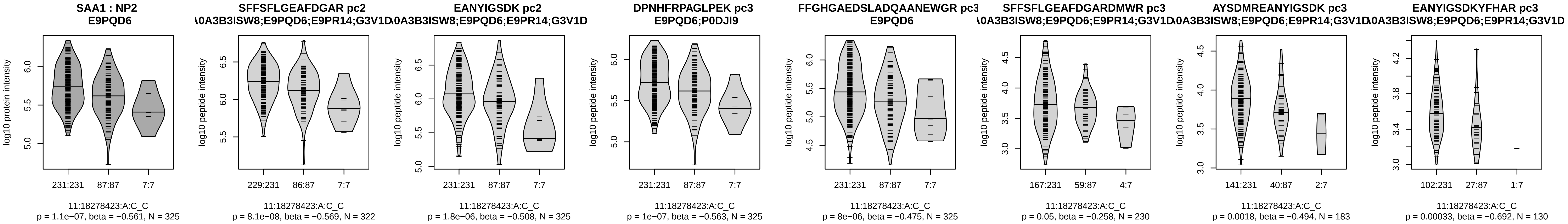

109Assay Target: SAA1
UniProt: E9PQD6
rsID: rs11024589
Target SNP: 11:18278423:A:C
Proxy SNP: 11:18278423:A:C
Tarkin log10(p): 15.3
Tarkin BETA: −0.465
Tarkin MSPA: 0.912
::*:*:*:*:–:*:*:–:*:*:NA:NA:NA
1241:430:1252:1174:1253:791:5

110. C1RL (Q9NZP8)
Tarkin MSPA=0.66

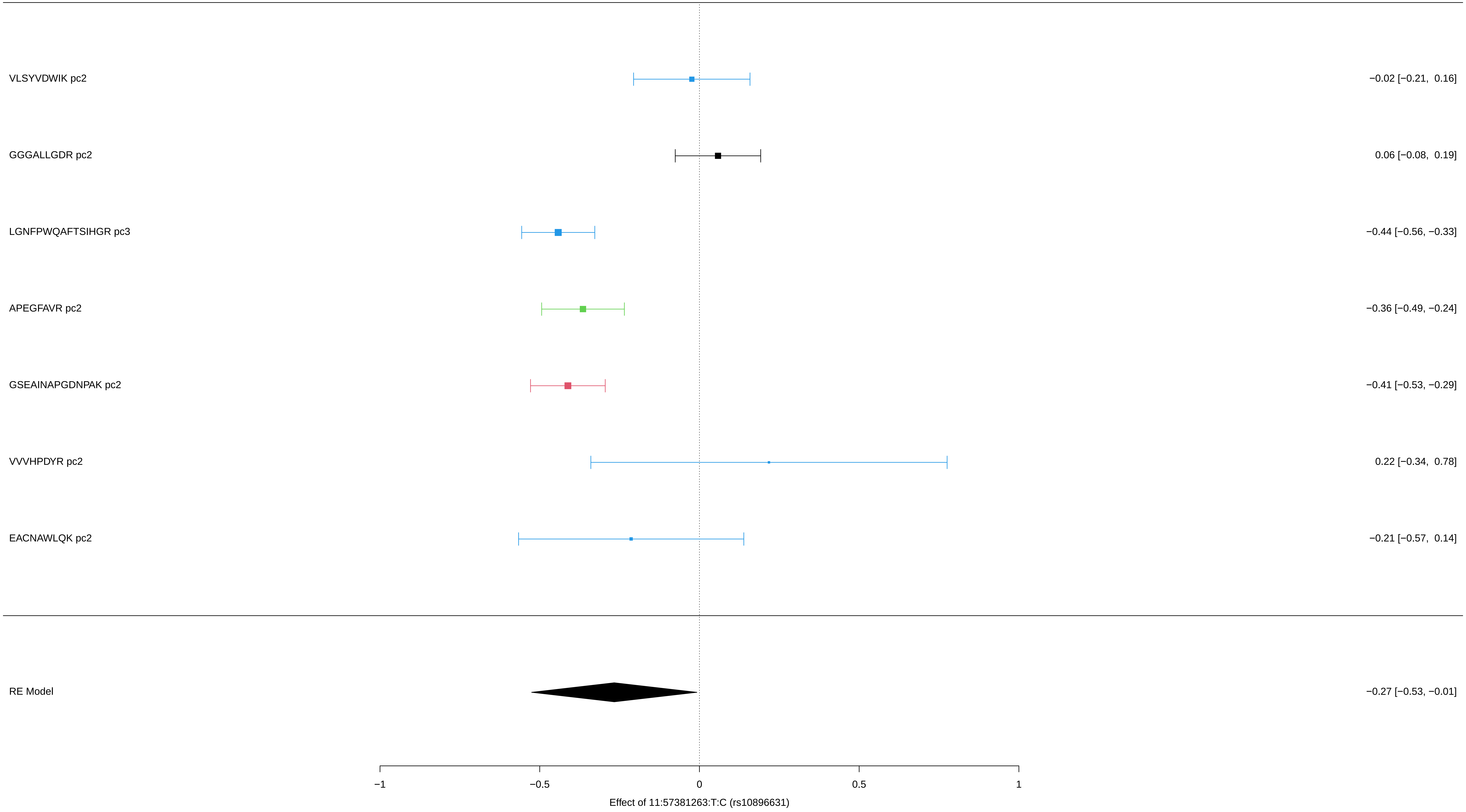

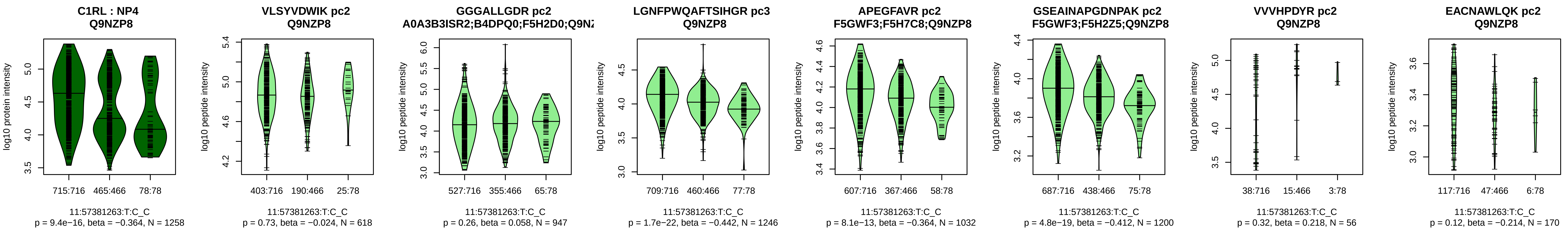

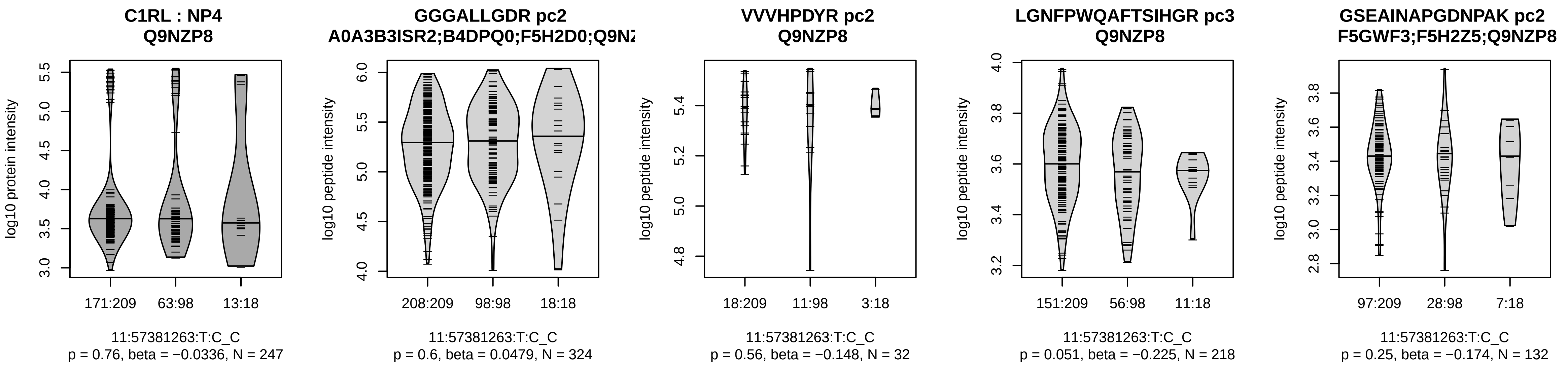

110Assay Target: C1RL
UniProt: Q9NZP8
rsID: rs10896631
Target SNP: 11:57381263:T:C
Proxy SNP: 11:57381263:T:C
Tarkin log10(p): 15
Tarkin BETA: −0.364
Tarkin MSPA: 0.66
−:−:*:*:*:−:−
618:947:1246:1032:1200:56:170

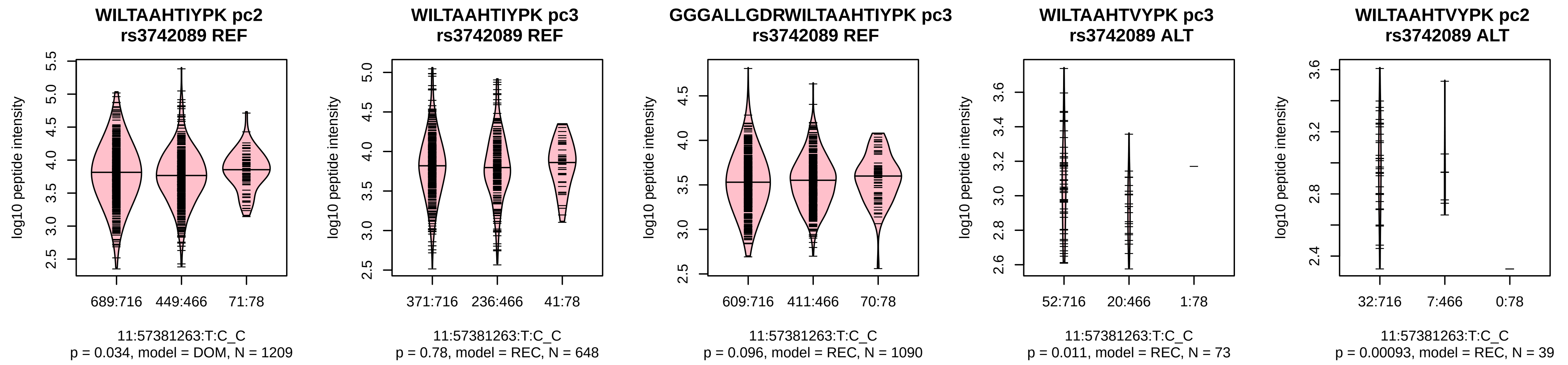

111. FAM177A1 (Q8N128)
Tarkin MSPA=0.985

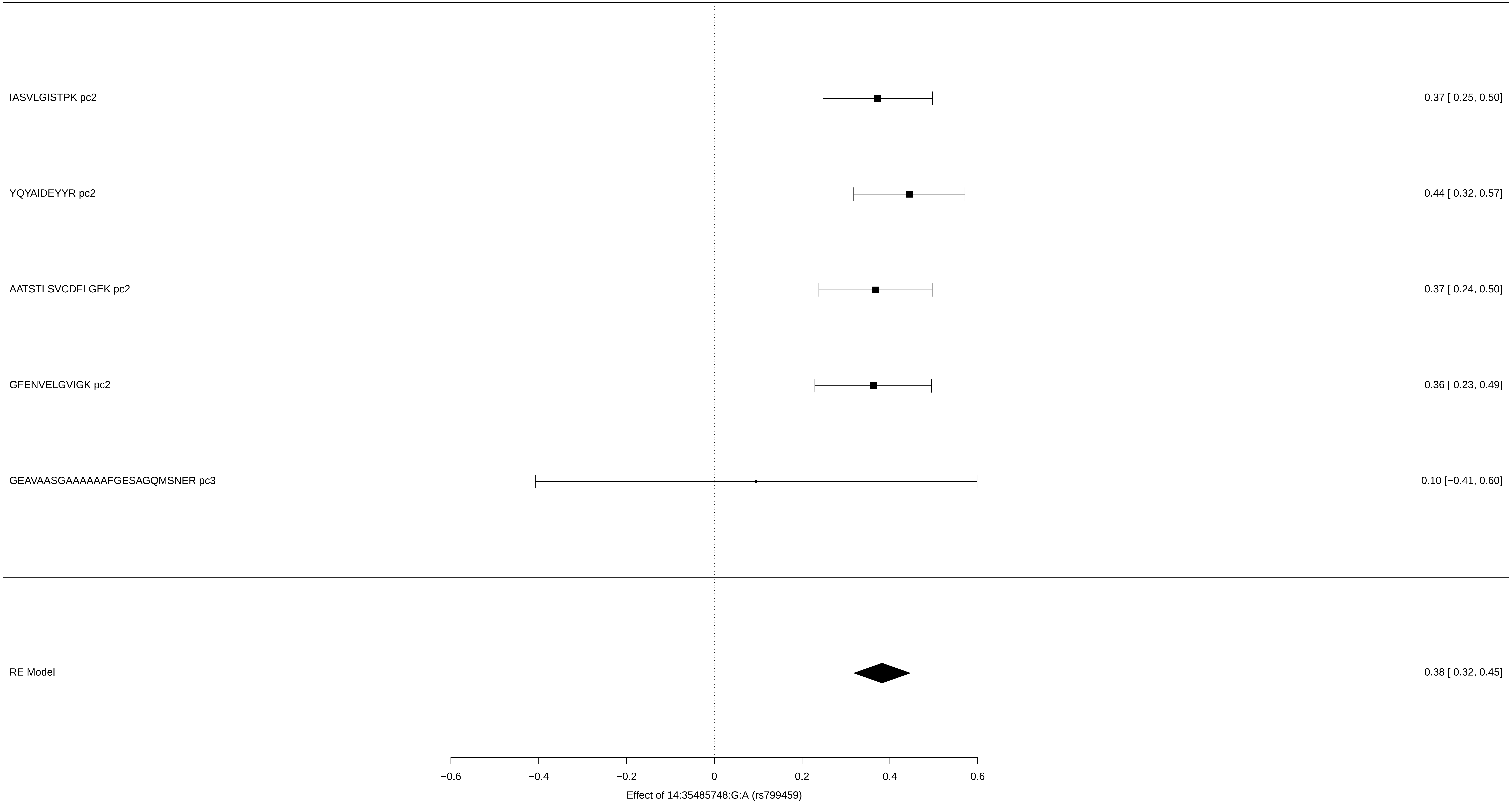

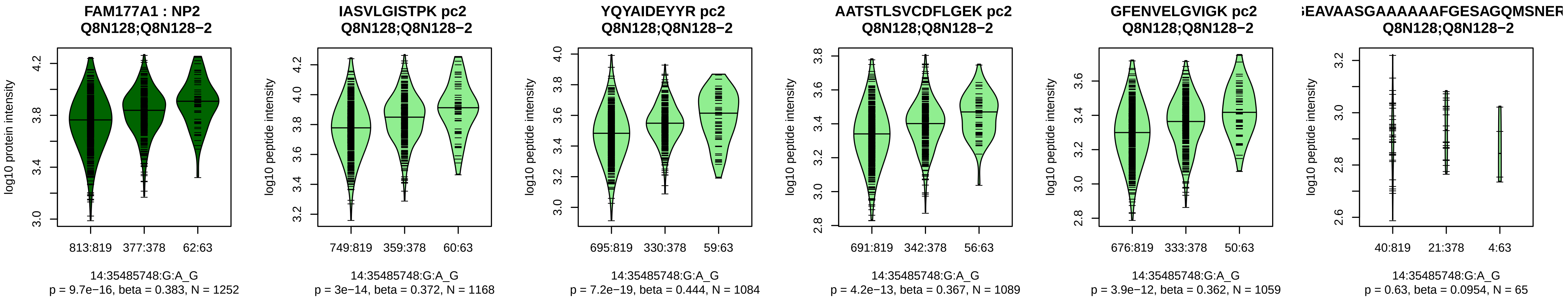

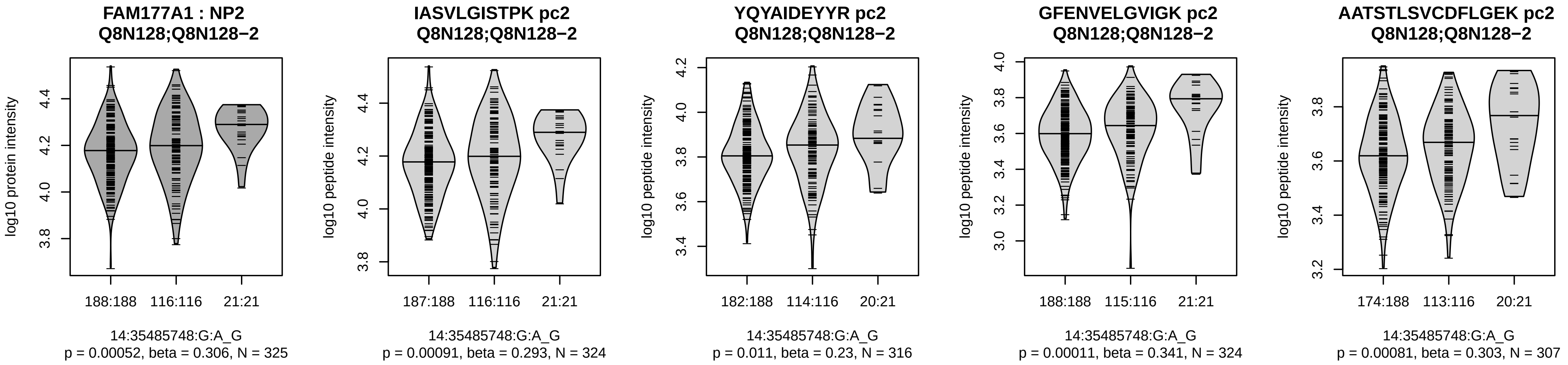

111Assay Target: FAM177A1
UniProt: Q8N128
rsID: rs799459
Target SNP: 14:35485748:G:A
Proxy SNP: 14:35485748:G:A
Tarkin log10(p): 15
Tarkin BETA: 0.383
Tarkin MSPA: 0.985
::*:*:–
1168:1084:1089:1059:65

112. FCGR3A (P08637)
Tarkin MSPA=0.482

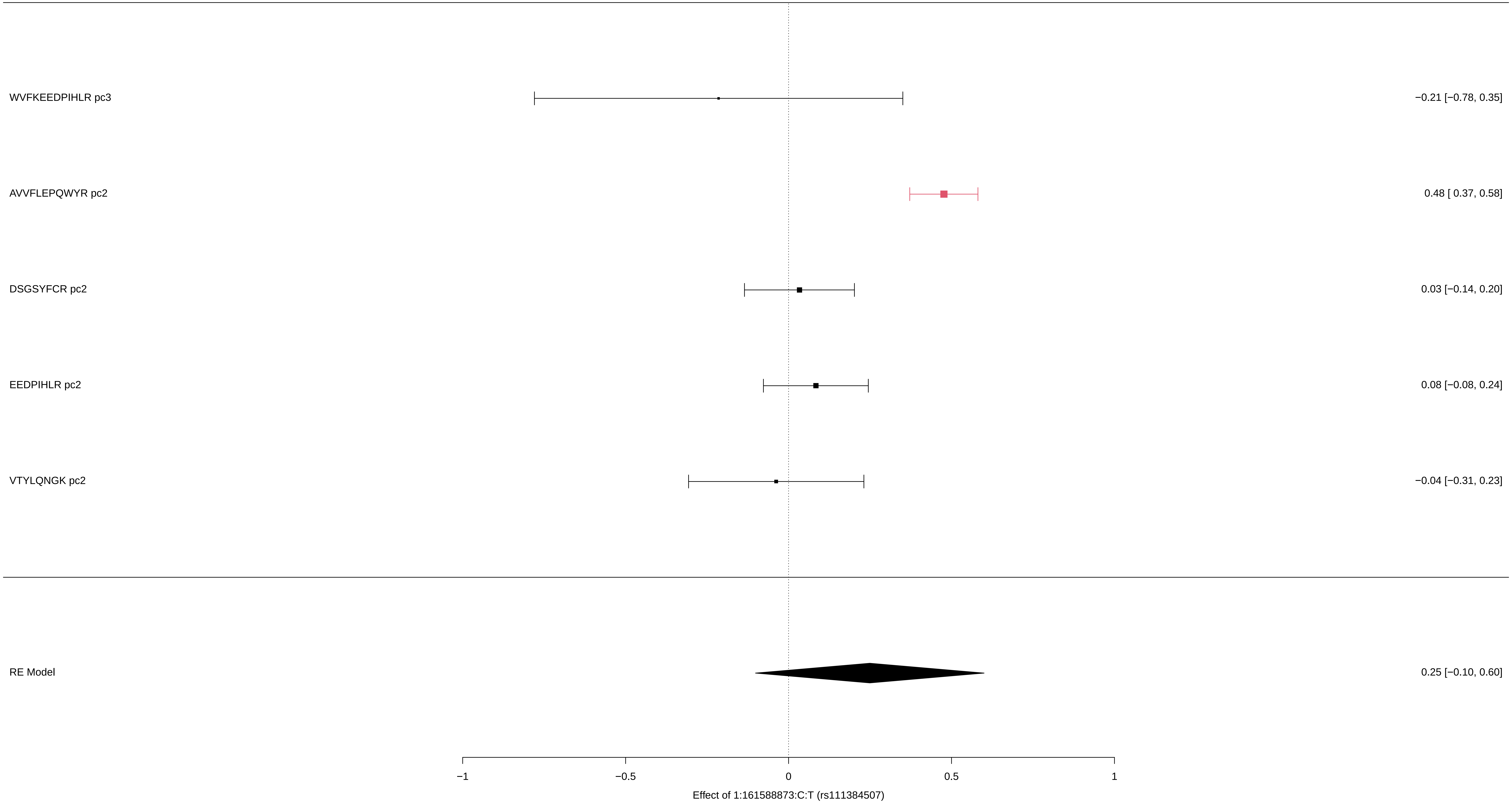

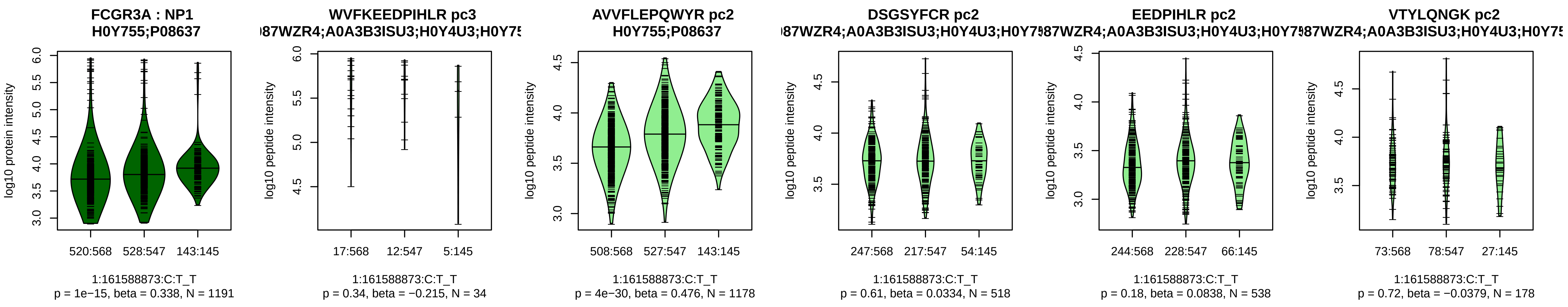

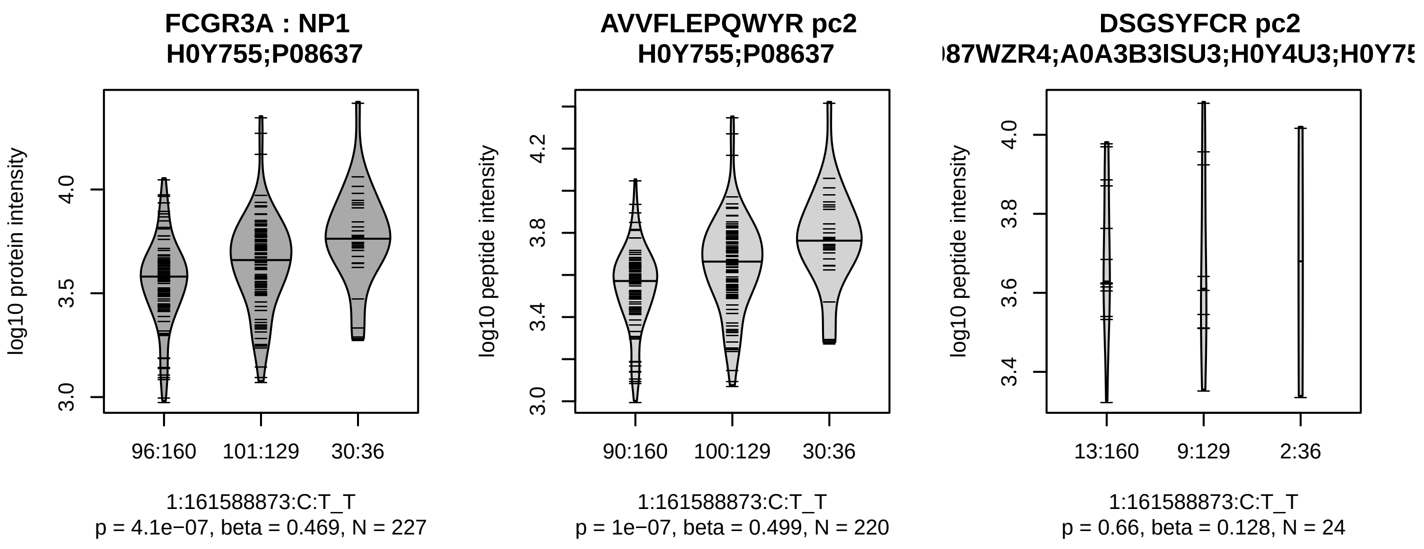

112Assay Target: FCGR3A
UniProt: P08637
rsID: rs111384507
Target SNP: 1:161588873:C:T
Proxy SNP: 1:161588873:C:T
Tarkin log10(p): 15
Tarkin BETA: 0.338
Tarkin MSPA: 0.482
–:*:–:–:–
34:1178:518:538:178

113. C3 (P01024)
Tarkin MSPA=0.939

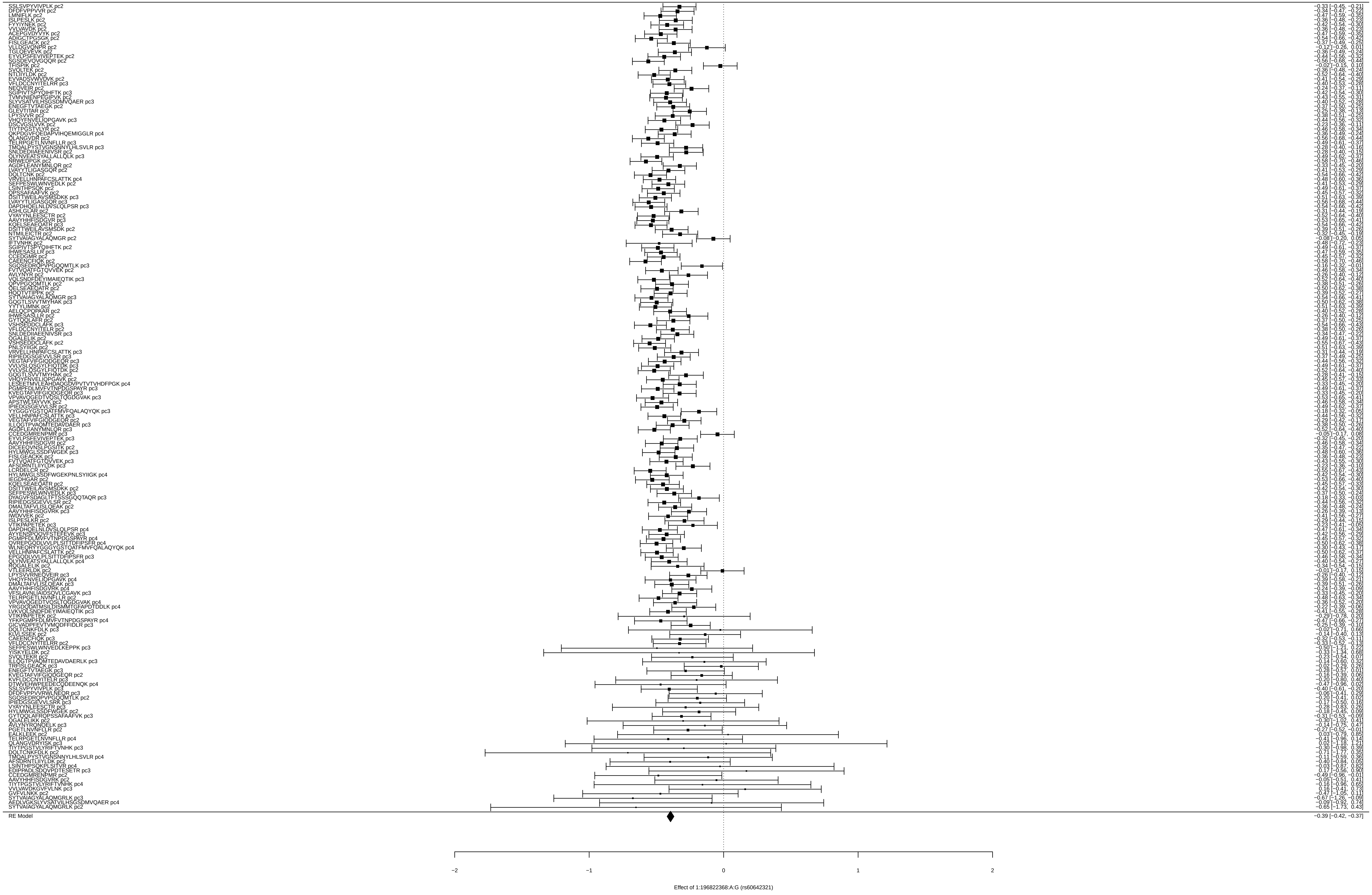

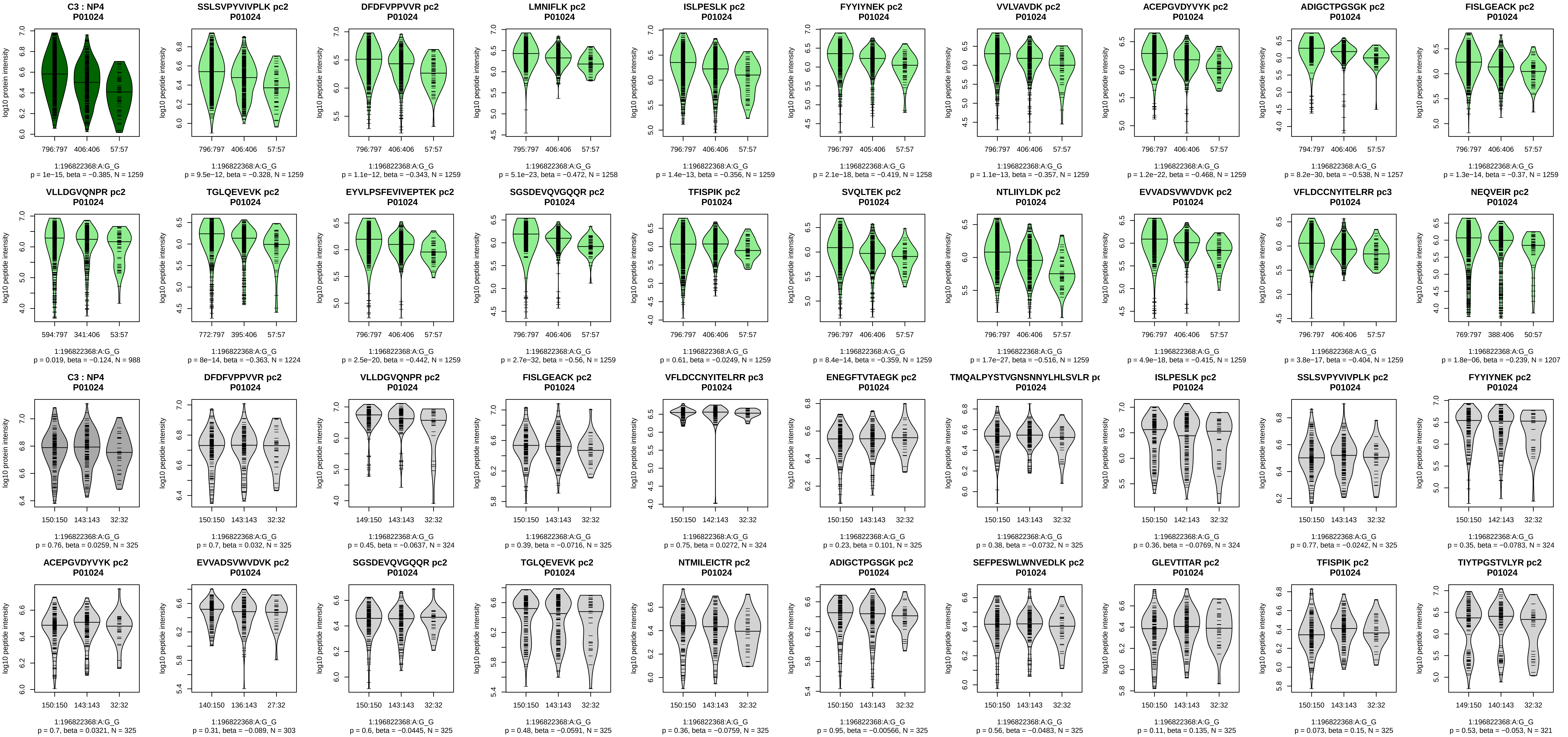

113Assay Target: C3
UniProt: P01024
rsID: rs60642321
Target SNP: 1:196822368:A:G
Proxy SNP: 1:196822368:A:G
Tarkin log10(p): 15
Tarkin BETA: −0.385
Tarkin MSPA: 0.939
1259:1259:1258:1259:1258:125

114. PLG (P00747)
Tarkin MSPA=0.804

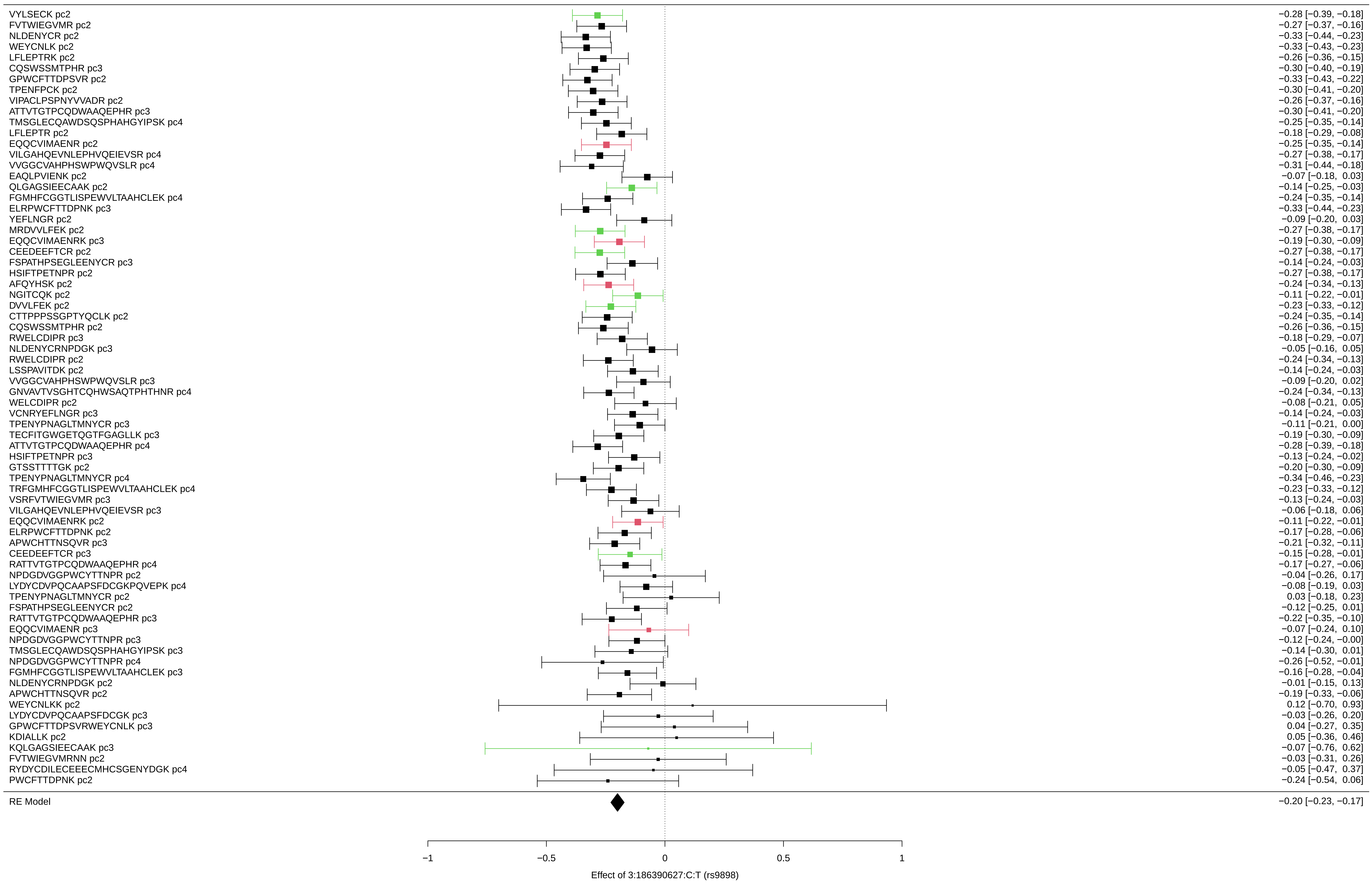

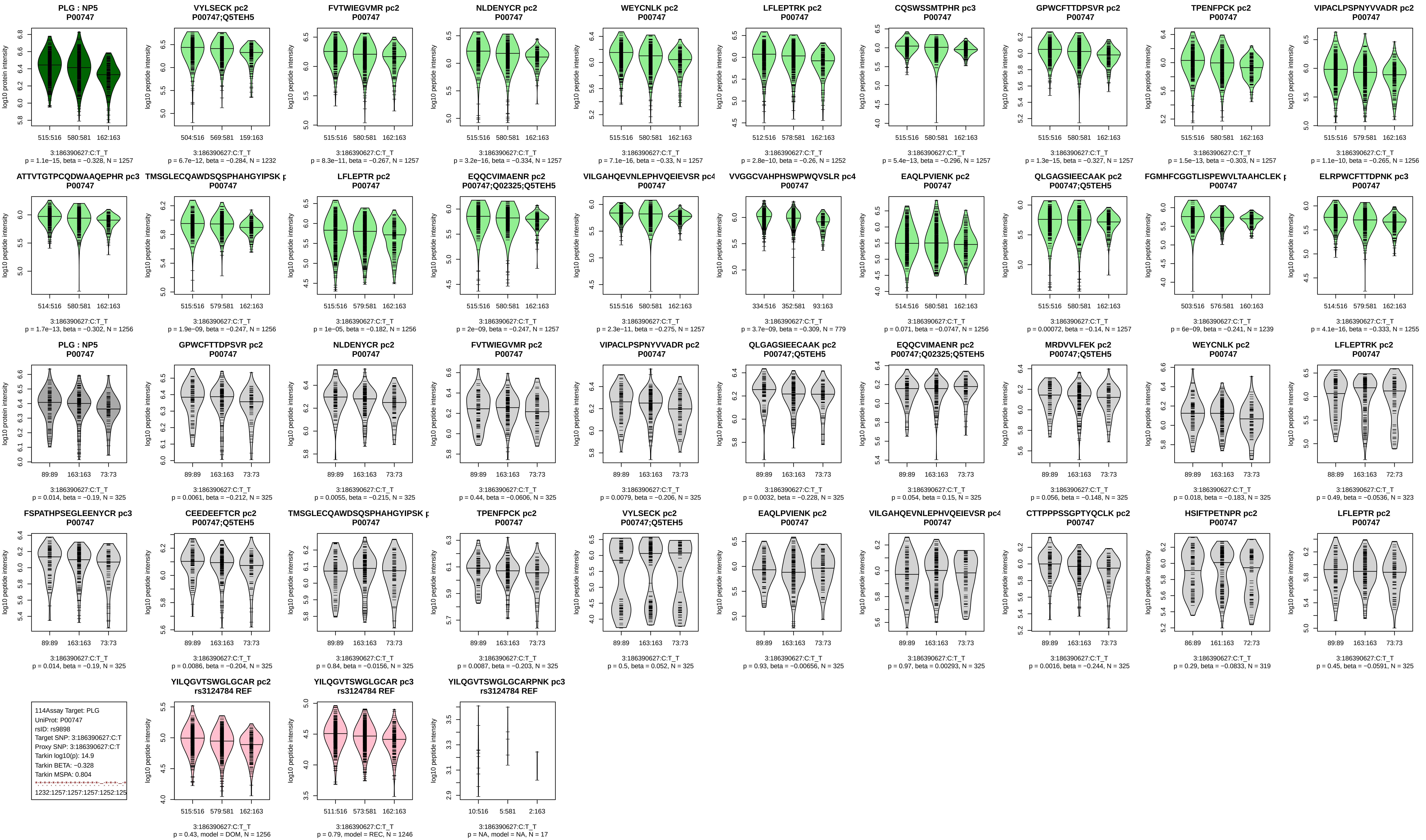

PLG : NP5
P00747
log10 protein intensity
515:516 580:581 162:163
3:186390627:C:T_T
p = 1.1e−15, beta = −0.328, N = 1257
VYLSECK pc2
P00747;Q5TEH5
log10 peptide intensity
504:516 569:581 159:163
3:186390627:C:T_T
p = 6.7e−12, beta = −0.284, N = 1232
FVTWIEGVMR pc2
P00747
515:516 580:581 162:163
3:186390627:C:T_T
p = 8.3e−11, beta = −0.267, N = 1257
NLDENYCR pc2
P00747
515:516 580:581 162:163
3:186390627:C:T_T
p = 3.2e−16, beta = −0.334, N = 1257
WEYCNLK pc2
P00747
515:516 580:581 162:163
3:186390627:C:T_T
p = 7.1e−16, beta = −0.33, N = 1257
LFLEPTRK pc2
P00747
512:516 578:581 162:163
3:186390627:C:T_T
p = 2.8e−10, beta = −0.26, N = 1252
CQSWSSMTPHR pc3
P00747
515:516 580:581 162:163
3:186390627:C:T_T
p = 5.4e−13, beta = −0.296, N = 1257
GPWCFTTDPSVR pc2
P00747
515:516 580:581 162:163
3:186390627:C:T_T
p = 1.3e−15, beta = −0.327, N = 1257
TPENFPCK pc2
P00747
515:516 580:581 162:163
3:186390627:C:T_T
p = 1.5e−13, beta = −0.303, N = 1257
VIPACLPSPNYVVADR pc2
P00747
515:516 579:581 162:163
3:186390627:C:T_T
p = 1.1e−10, beta = −0.265, N = 1256
ATTVTGTPCQDWAAQEPHR pc3
P00747
514:516 580:581 162:163
3:186390627:C:T_T
p = 1.7e−13, beta = −0.302, N = 1256
TMSGLECQAWDSQSPHAHGYIPSK p
P00747
515:516 579:581 162:163
3:186390627:C:T_T
p = 1.9e−09, beta = −0.247, N = 1256
LFLEPTR pc2
P00747
515:516 579:581 162:163
3:186390627:C:T_T
p = 1e−05, beta = −0.182, N = 1256
EQQCVIMAENR pc2
P00747;Q02325;Q5TEH5
515:516 580:581 162:163
3:186390627:C:T_T
p = 2e−09, beta = −0.247, N = 1257
VILGAHQEVNLEPHVQEIEVSR pc4
P00747
515:516 580:581 162:163
3:186390627:C:T_T
p = 2.3e−11, beta = −0.275, N = 1257
VVGGCVAHPHSWPWQVSLR pc4
P00747
334:516 352:581 93:163
3:186390627:C:T_T
p = 3.7e−09, beta = −0.309, N = 779
EAQLPVIENK pc2
P00747
514:516 580:581 162:163
3:186390627:C:T_T
p = 0.071, beta = −0.0747, N = 1256
QLGAGSIEECAAK pc2
P00747;Q5TEH5
515:516 580:581 162:163
3:186390627:C:T_T
p = 0.00072, beta = −0.14, N = 1257
FGMHFCGGTLISPEWVLTAAHCLEK p
P00747
503:516 576:581 160:163
3:186390627:C:T_T
p = 6e−09, beta = −0.241, N = 1239
ELRPWCFTTDPNK pc3
P00747
514:516 579:581 162:163
3:186390627:C:T_T
p = 4.1e−16, beta = −0.333, N = 1255
PLG : NP5
P00747
89:89 163:163 73:73
3:186390627:C:T_T
p = 0.014, beta = −0.19, N = 325
GPWCFTTDPSVR pc2
P00747
89:89 163:163 73:73
3:186390627:C:T_T
p = 0.0061, beta = −0.212, N = 325
NLDENYCR pc2
P00747
89:89 163:163 73:73
3:186390627:C:T_T
p = 0.0055, beta = −0.215, N = 325
FVTWIEGVMR pc2
P00747
89:89 163:163 73:73
3:186390627:C:T_T
p = 0.44, beta = −0.0606, N = 325
VIPACLPSPNYVVADR pc2
P00747
89:89 163:163 73:73
3:186390627:C:T_T
p = 0.0079, beta = −0.206, N = 325
QLGAGSIEECAAK pc2
P00747;Q5TEH5
89:89 163:163 73:73
3:186390627:C:T_T
p = 0.0032, beta = −0.228, N = 325
EQQCVIMAENR pc2
P00747;Q02325;Q5TEH5
89:89 163:163 73:73
3:186390627:C:T_T
p = 0.054, beta = 0.15, N = 325
MRDVVLFEK pc2
P00747;Q5TEH5
89:89 163:163 73:73
3:186390627:C:T_T
p = 0.056, beta = −0.148, N = 325
WEYCNLK pc2
P00747
89:89 163:163 73:73
3:186390627:C:T_T
p = 0.018, beta = −0.183, N = 325
LFLEPTRK pc2
P00747
88:89 163:163 72:73
3:186390627:C:T_T
p = 0.49, beta = −0.0536, N = 323
FSPATHPSEGLEENYCR pc3
P00747
89:89 163:163 73:73
3:186390627:C:T_T
p = 0.014, beta = −0.19, N = 325
CEEDEEFTCR pc2
P00747;Q5TEH5
89:89 163:163 73:73
3:186390627:C:T_T
p = 0.0086, beta = −0.204, N = 325
TMSGLECQAWDSQSPHAHGYIPSK p
P00747
89:89 163:163 73:73
3:186390627:C:T_T
p = 0.84, beta = −0.0156, N = 325
TPENFPCK pc2
P00747
89:89 163:163 73:73
3:186390627:C:T_T
p = 0.0087, beta = −0.203, N = 325
VYLSECK pc2
P00747;Q5TEH5
89:89 163:163 73:73
3:186390627:C:T_T
p = 0.5, beta = 0.052, N = 325
EAQLPVIENK pc2
P00747
89:89 163:163 73:73
3:186390627:C:T_T
p = 0.93, beta = −0.00656, N = 325
VILGAHQEVNLEPHVQEIEVSR pc4
P00747
89:89 163:163 73:73
3:186390627:C:T_T
p = 0.97, beta = 0.00293, N = 325
CTTPPPSSGPTYQCLK pc2
P00747
89:89 163:163 73:73
3:186390627:C:T_T
p = 0.0016, beta = −0.244, N = 325
HSIFTPETNPR pc2
P00747
86:89 161:163 72:73
3:186390627:C:T_T
p = 0.29, beta = −0.0833, N = 319
LFLEPTR pc2
P00747
89:89 163:163 73:73
3:186390627:C:T_T
p = 0.45, beta = −0.0591, N = 325
114Assay Target: PLG
UniProt: P00747
rsID: rs9898
Target SNP: 3:186390627:C:T
Proxy SNP: 3:186390627:C:T
Tarkin log10(p): 14.9
Tarkin BETA: −0.328
Tarkin MSPA: 0.804
1232:1257:1257:1257:1252:125
YILQGVTSWGLGCAR pc2
rs3124784 REF
515:516 579:581 162:163
3:186390627:C:T_T
p = 0.43, model = DOM, N = 1256
YILQGVTSWGLGCAR pc3
rs3124784 REF
511:516 573:581 162:163
3:186390627:C:T_T
p = 0.79, model = REC, N = 1246
YILQGVTSWGLGCARPNK pc3
rs3124784 REF
10:516 5:581 2:163
3:186390627:C:T_T
p = NA, model = NA, N = 17

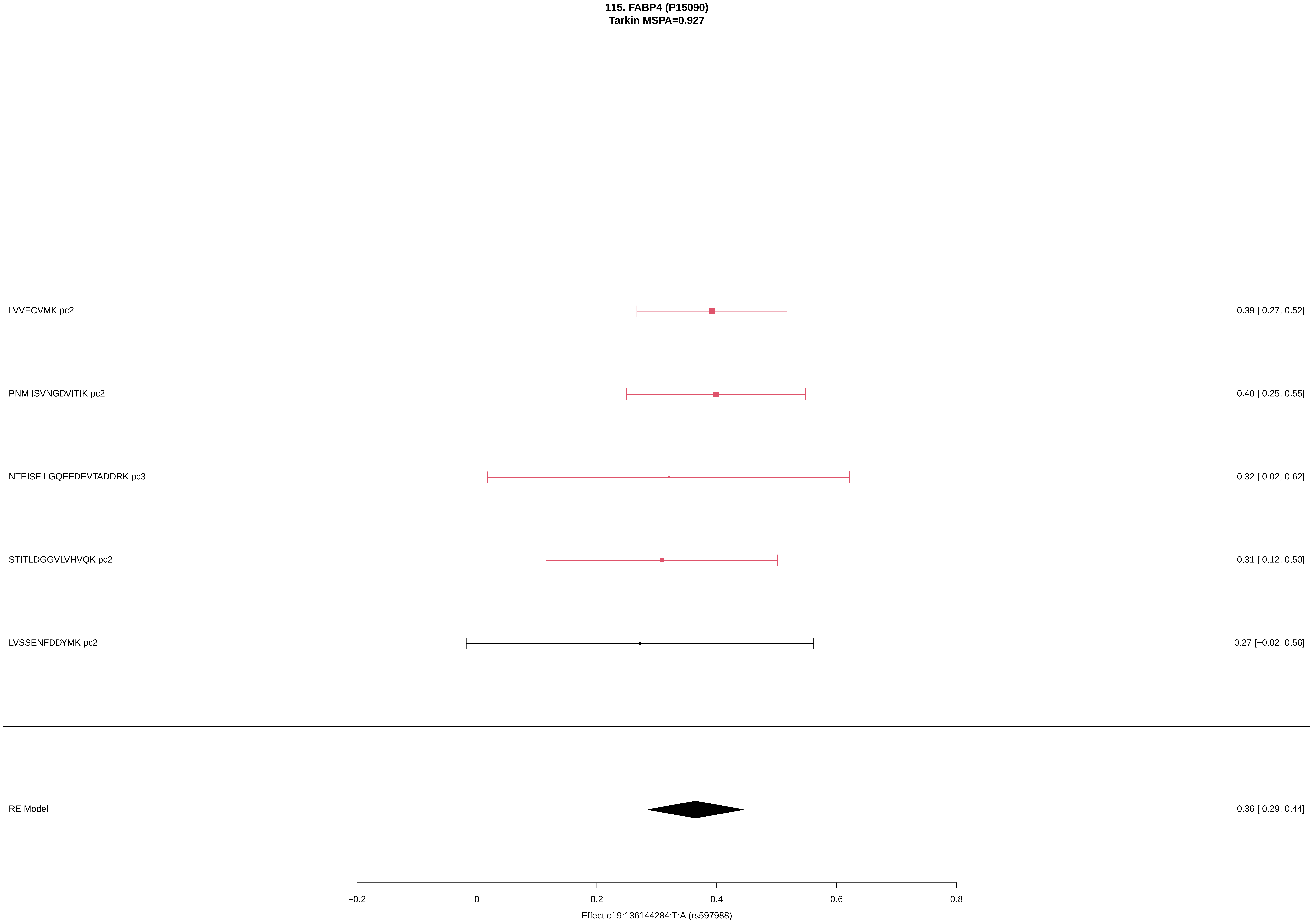
115. FABP4 (P15090)
Tarkin MSPA=0.927
LVVECVMK pc2
0.39 [0.27, 0.52]
PNMIISVNGDVITIK pc2
0.40 [0.25, 0.55]
NTEISFILGQEFDEVTADDRK pc3
0.32 [0.02, 0.62]
STITLDGGVLVHVQK pc2
0.31 [0.12, 0.50]
LVSSENFDDYMK pc2
0.27 [−0.02, 0.56]
RE Model
0.36 [0.29, 0.44]
−0.2
0
0.2
0.4
0.6
0.8
Effect of 9:136144284:T:A (rs597988)

FABP4 : NP2
P15090

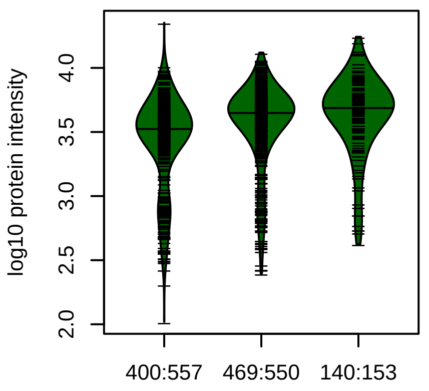

9:136144284:T:A_A
p = 1.2e−15, beta = 0.361, N = 1009

LVVECVMK pc2
P15090

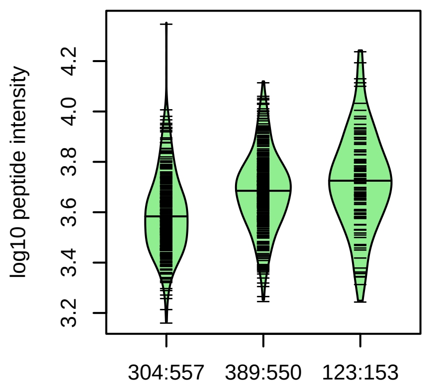

9:136144284:T:A_A
p = 2.7e−15, beta = 0.392, N = 816

PNMIISVNGDVITIK pc2
P15090

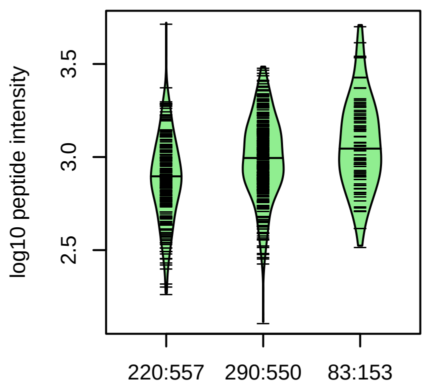

9:136144284:T:A_A
p = 1.5e−11, beta = 0.399, N = 593

NTEISFILGQEFDEVTADDRK pc3
P15090

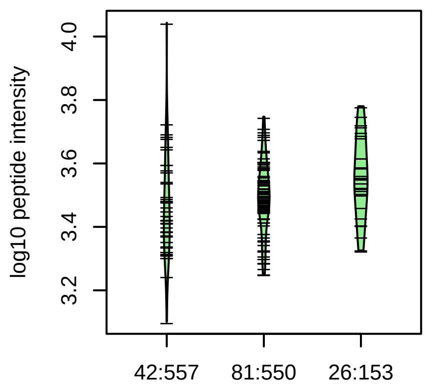

9:136144284:T:A_A
p = 0.0071, beta = 0.32, N = 149

STITLDGGVLVHVQK pc2
P15090

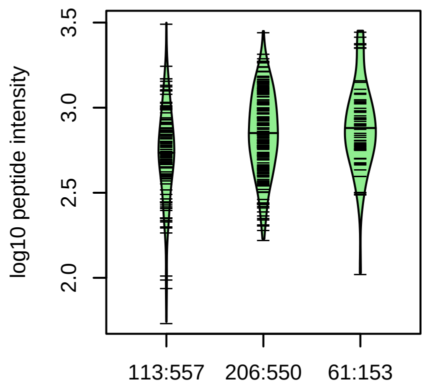

9:136144284:T:A_A
p = 4.8e−05, beta = 0.308, N = 380

LVSSENFDDYMK pc2
E5RH45;E5RIR0;P02689;P15090

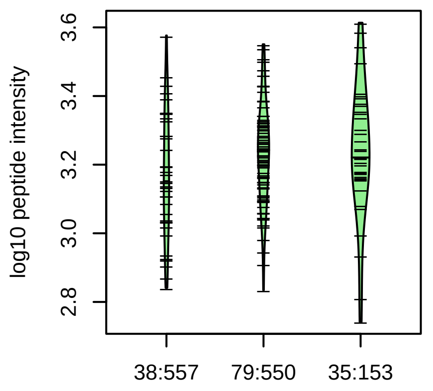

9:136144284:T:A_A
p = 0.017, beta = 0.272, N = 152

FABP4 : NP2
P15090

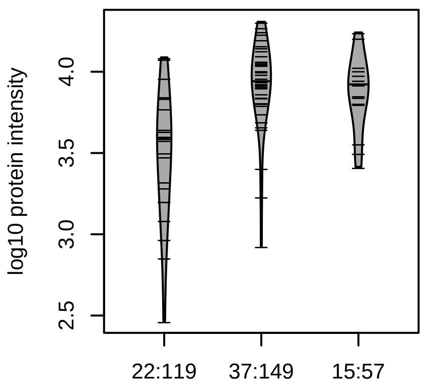

9:136144284:T:A_A
p = 7e−04, beta = 0.522, N = 74

LVSSENFDDYMK pc2
E5RH45;E5RIR0;P02689;P15090

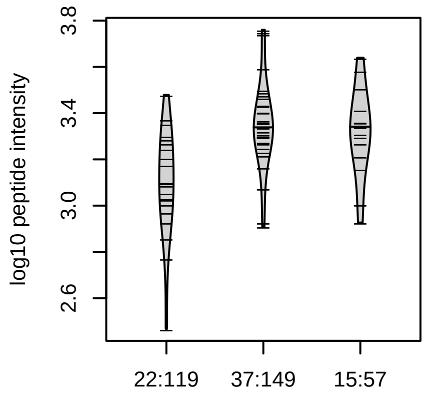

9:136144284:T:A_A
p = 7.9e−05, beta = 0.6, N = 74

115Assay Target: FABP4
UniProt: P15090
rsID: rs597988
Target SNP: 9:136144284:T:A
Proxy SNP: 9:136144284:T:A
Tarkin log10(p): 14.9
Tarkin BETA: 0.36
Tarkin MSPA: 0.927
::*:*:–
816:593:149:380:152

116. GPR116 (Q8IZF2)
Tarkin MSPA=0.99

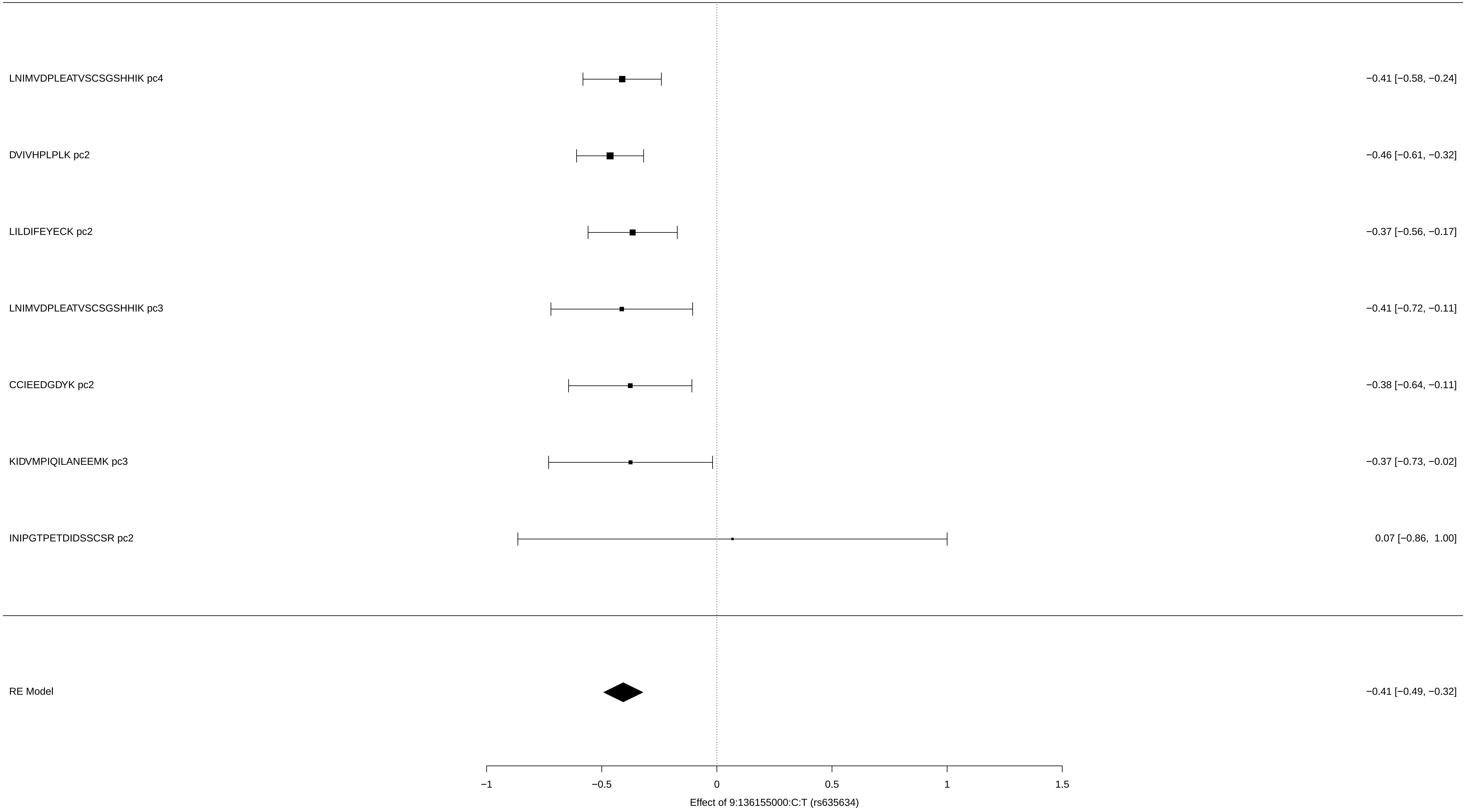

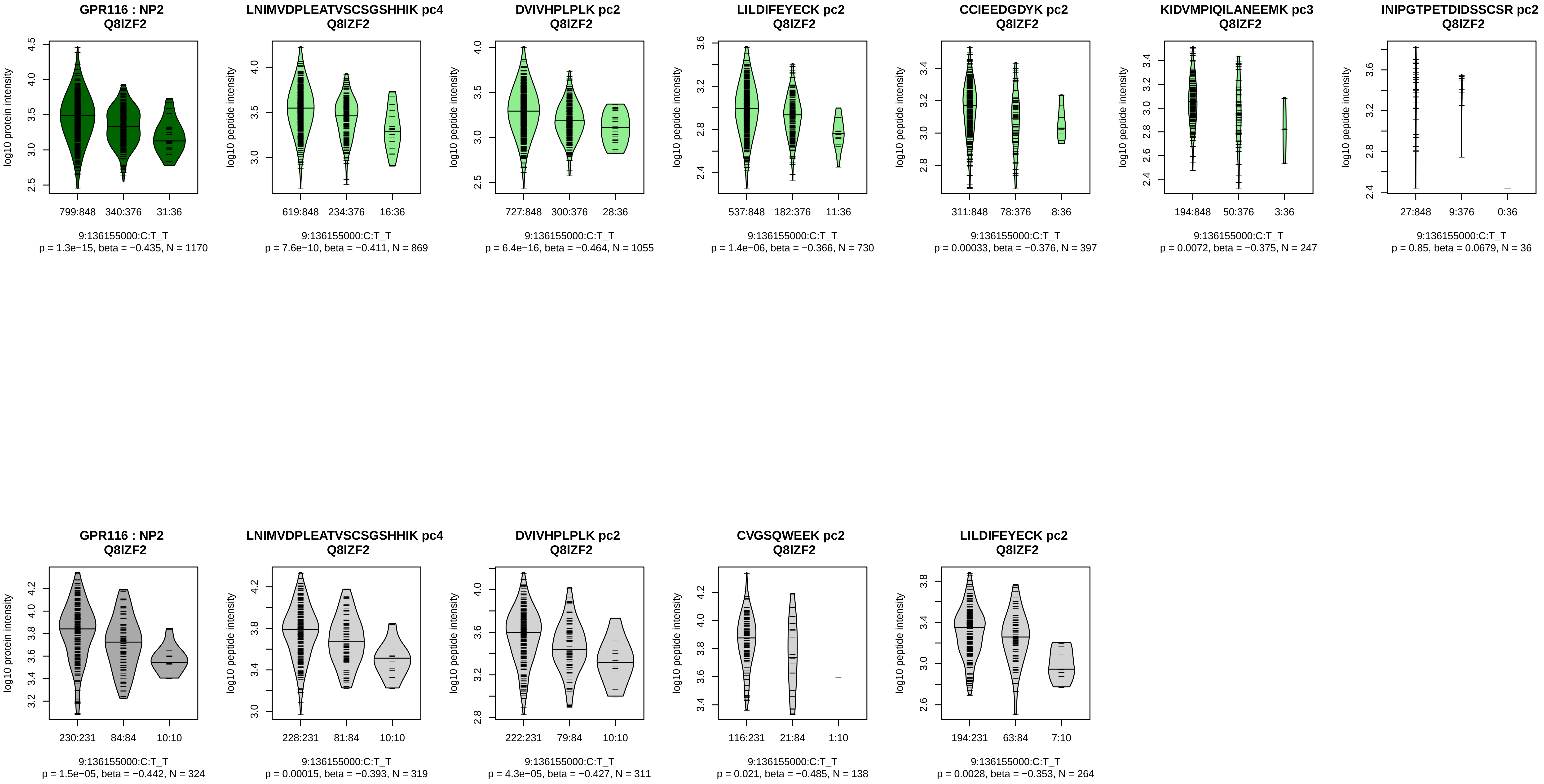

116Assay Target: GPR116
UniProt: Q8IZF2
rsID: rs635634
Target SNP: 9:136155000:C:T
Proxy SNP: 9:136155000:C:T
Tarkin log10(p): 14.9
Tarkin BETA: −0.435
Tarkin MSPA: 0.99
::*:*:*:*:−:NA
869:1055:730:331:397:247:36:2

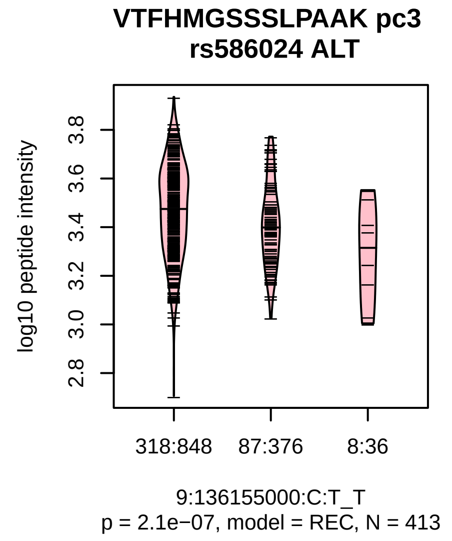

117. CD34 (P28906)
Tarkin MSPA=1

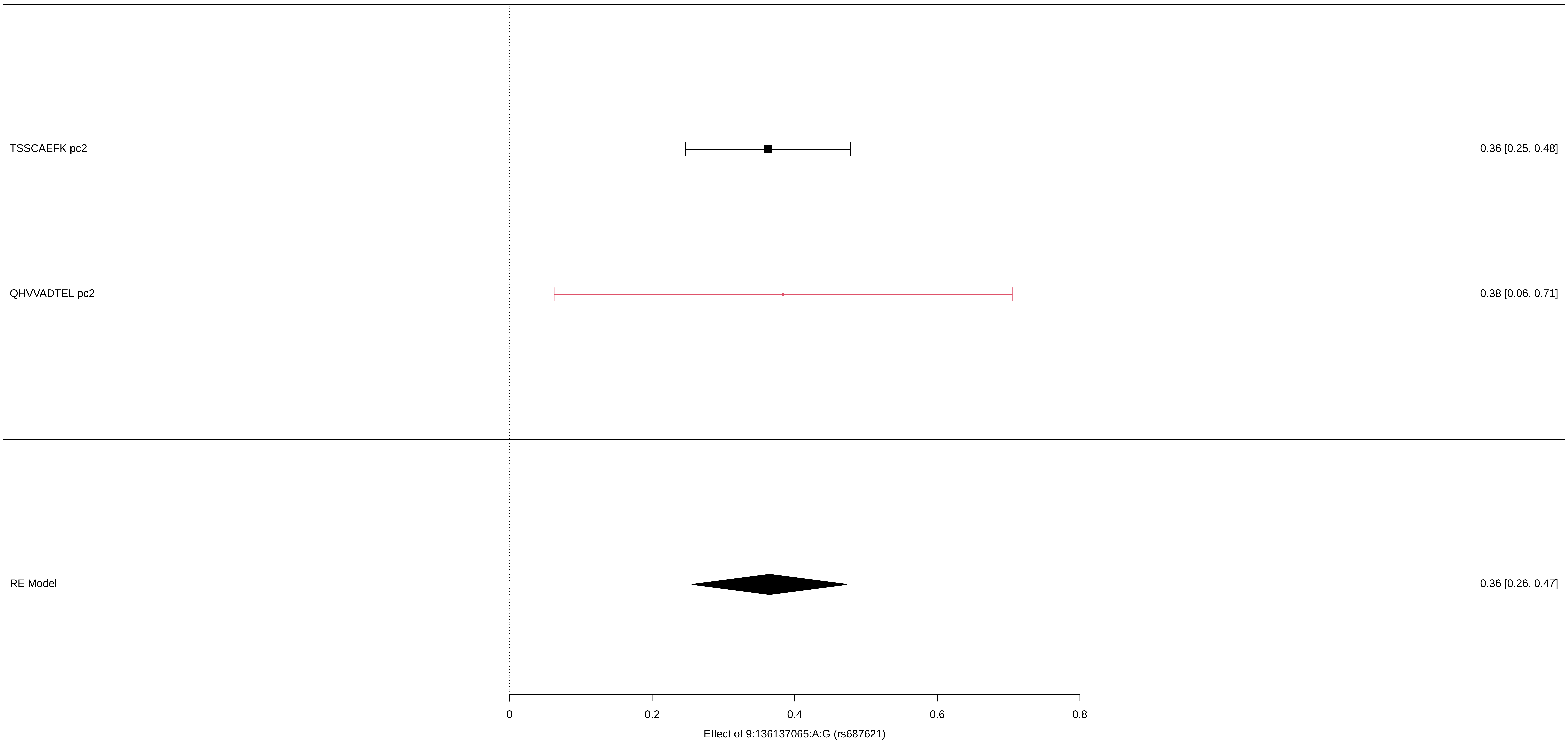

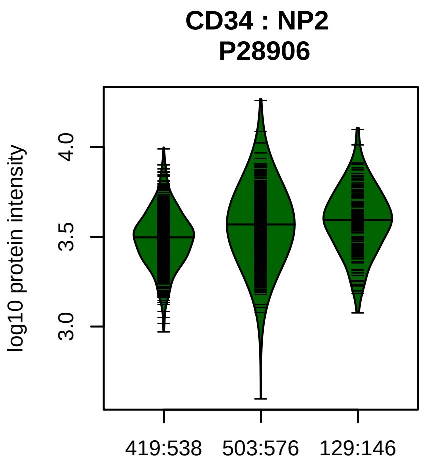

9:136137065:A:G_G
p = 3.2e−15, beta = 0.357, N = 1051

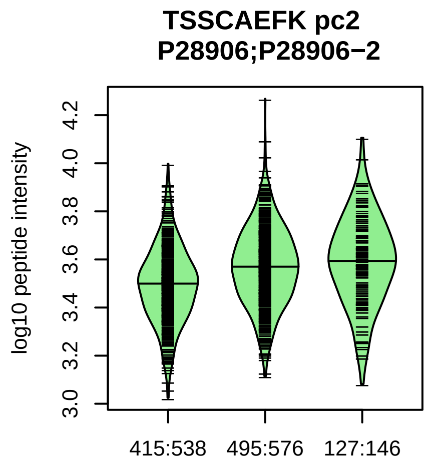

9:136137065:A:G_G
p = 2e−15, beta = 0.362, N = 1037

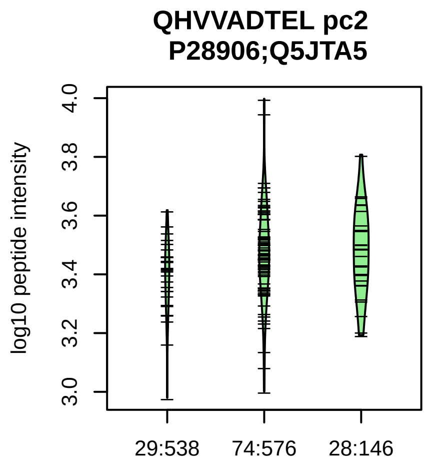

9:136137065:A:G_G
p = 0.0026, beta = 0.384, N = 131

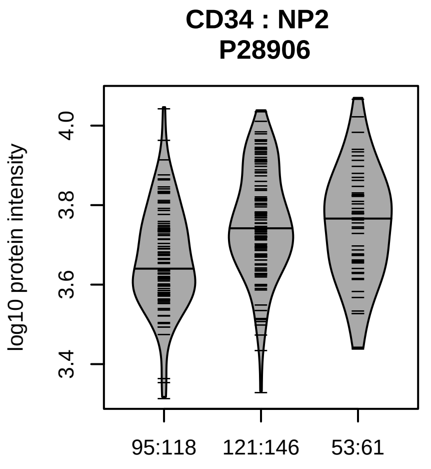

9:136137065:A:G_G
p = 3.7e−06, beta = 0.376, N = 269

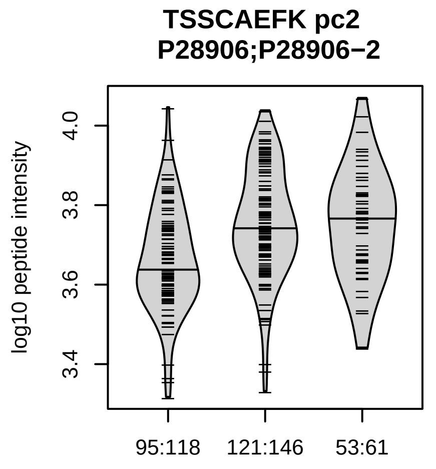

9:136137065:A:G_G
p = 1.7e−06, beta = 0.389, N = 269

117Assay Target: CD34
UniProt: P28906
rsID: rs687621
Target SNP: 9:136137065:A:G
Proxy SNP: 9:136137065:A:G
Tarkin log10(p): 14.5
Tarkin BETA: 0.357
Tarkin MSPA: 1
::NA
1037:131:9

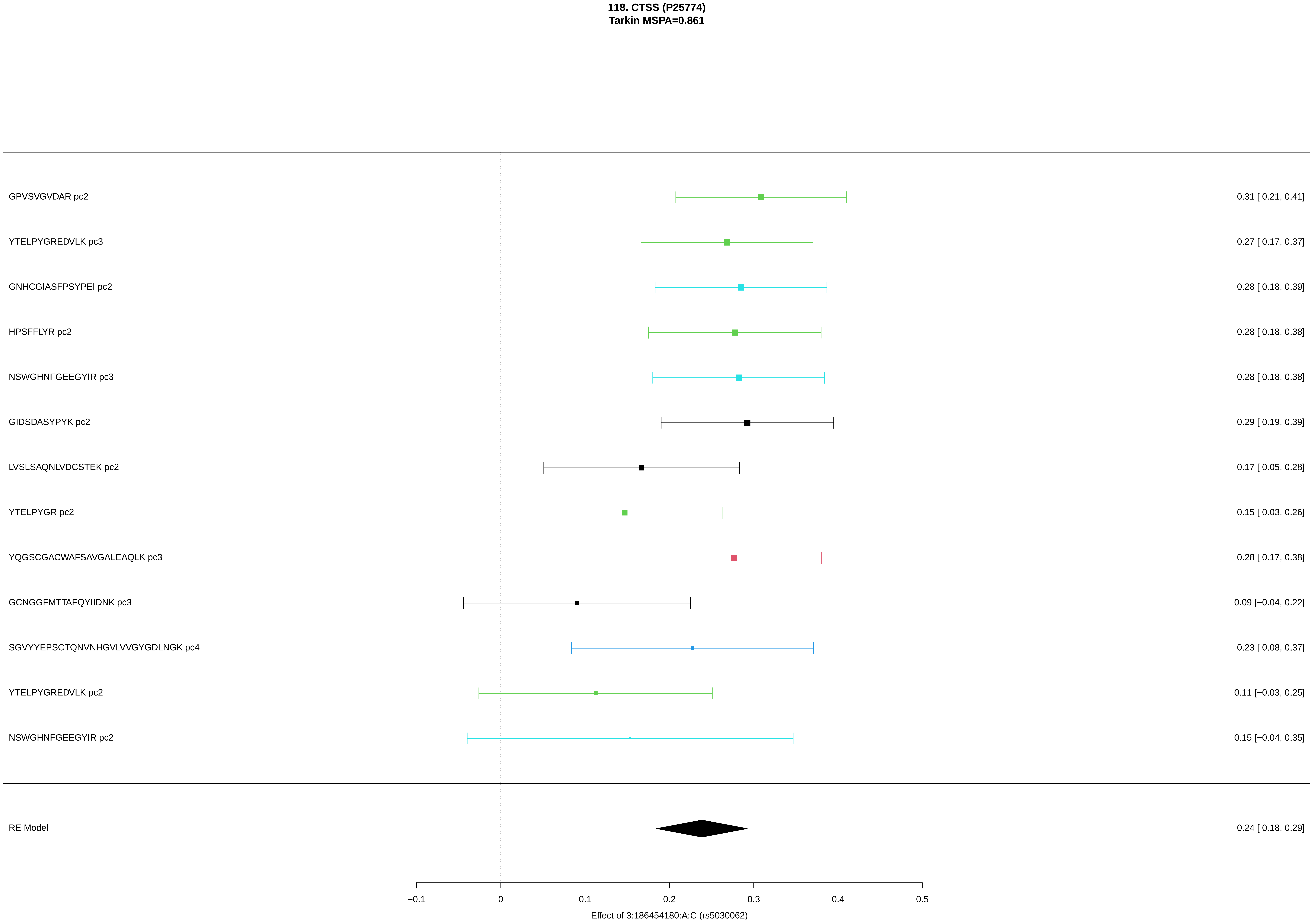
118. CTSS (P25774)
Tarkin MSPA=0.861
GPVSVGVDAR pc2
0.31 [0.21, 0.41]
YTELPYGREDVLK pc3
0.27 [0.17, 0.37]
GNHCGIASFPSYPEI pc2
0.28 [0.18, 0.39]
HPSFFLYR pc2
0.28 [0.18, 0.38]
NSWGHNFGEEGYIR pc3
0.28 [0.18, 0.38]
GIDSDASYPYK pc2
0.29 [0.19, 0.39]
LVSLSAQNLVDCSTEK pc2
0.17 [0.05, 0.28]
YTELPYGR pc2
0.15 [0.03, 0.26]
YQGSCGACWAFSAVGALEAQLK pc3
0.28 [0.17, 0.38]
GCNGGFMTTAFQYIIDNK pc3
0.09 [−0.04, 0.22]
SGVYYEPSCTQNVNHGVLVVGYGDLNGK pc4
0.23 [0.08, 0.37]
YTELPYGREDVLK pc2
0.11 [−0.03, 0.25]
NSWGHNFGEEGYIR pc2
0.15 [−0.04, 0.35]
RE Model
0.24 [0.18, 0.29]
−0.1
0
0.1
0.2
0.3
0.4
0.5
Effect of 3:186454180:A:C (rs5030062)

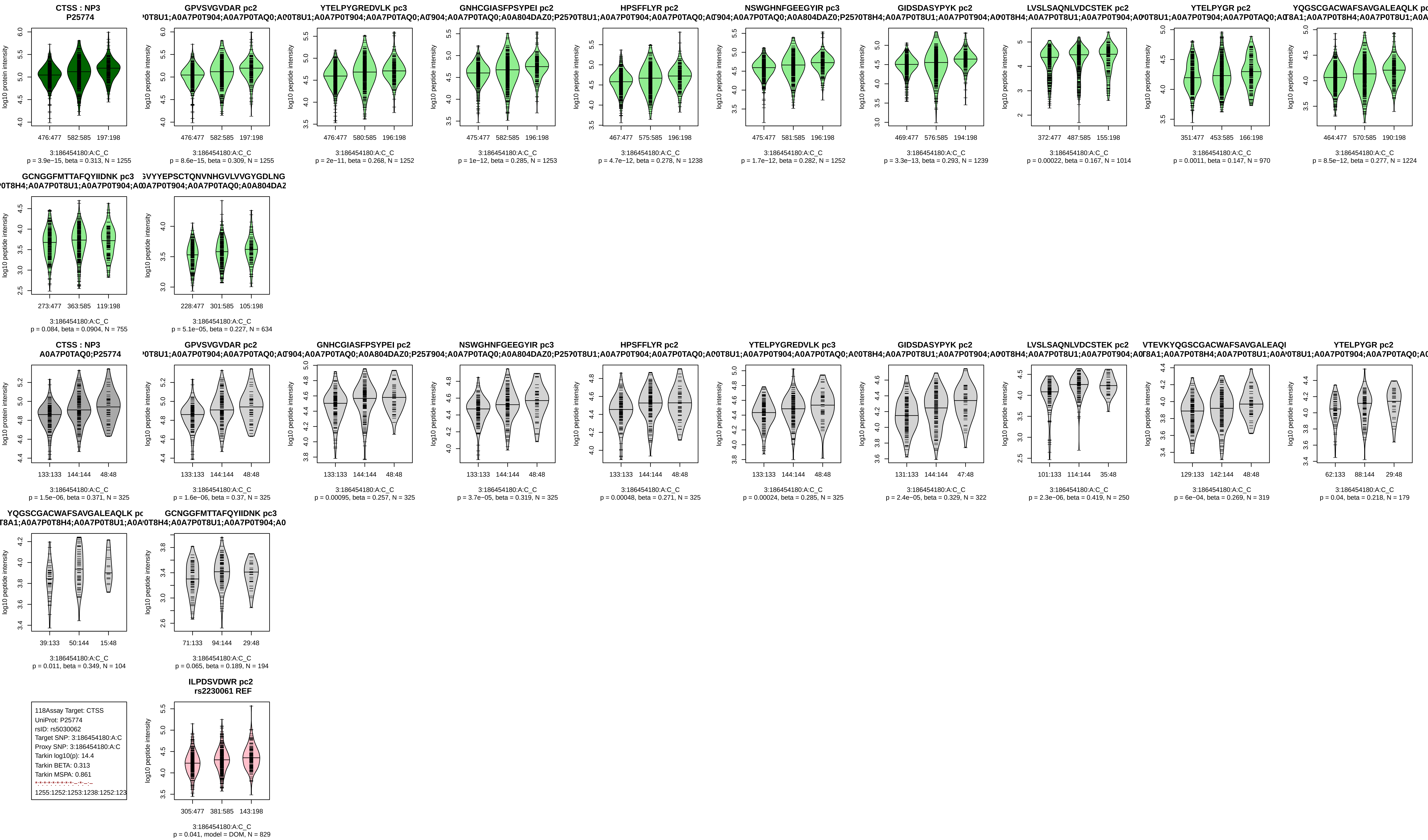
CTSS : NP3
P25774
GPVSVGVDAR pc2
YTELPYGREDVLK pc3
GNHCGIASFPSYPEI pc2
HPSFFLYR pc2
NSWGHNFGEEGYIR pc3
GIDSDASYPYK pc2
LVSLSAQNLVDCSTEK pc2
YTELPYGR pc2
log10 protein intensity
log10 peptide intensity
476:477
582:585
197:198
3:186454180:A:C_C
p = 3.9e−15, beta = 0.313, N = 1255
p = 8.6e−15, beta = 0.309, N = 1255
p = 2e−11, beta = 0.268, N = 1252
p = 1e−12, beta = 0.285, N = 1253
p = 4.7e−12, beta = 0.278, N = 1238
p = 1.7e−12, beta = 0.282, N = 1252
p = 3.3e−13, beta = 0.293, N = 1239
p = 0.00022, beta = 0.167, N = 1014
p = 0.0011, beta = 0.147, N = 970
p = 8.5e−12, beta = 0.277, N = 1224
GCNGGFMTTAFQYIIDNK pc3
p = 0.084, beta = 0.0904, N = 755
p = 5.1e−05, beta = 0.227, N = 634
CTSS : NP3
A0A7P0TAQ0;P25774
p = 1.5e−06, beta = 0.371, N = 325
p = 1.6e−06, beta = 0.37, N = 325
p = 0.00095, beta = 0.257, N = 325
p = 3.7e−05, beta = 0.319, N = 325
p = 0.00048, beta = 0.271, N = 325
p = 0.00024, beta = 0.285, N = 325
p = 2.4e−05, beta = 0.329, N = 322
p = 2.3e−06, beta = 0.419, N = 250
p = 6e−04, beta = 0.269, N = 319
p = 0.04, beta = 0.218, N = 179
p = 0.011, beta = 0.349, N = 104
p = 0.065, beta = 0.189, N = 194
118Assay Target: CTSS
UniProt: P25774
rsID: rs5030062
Target SNP: 3:186454180:A:C
Proxy SNP: 3:186454180:A:C
Tarkin log10(p): 14.4
Tarkin BETA: 0.313
Tarkin MSPA: 0.861
1255:1252:1253:1238:1252:123
ILPDSVDWR pc2
rs2230061 REF
p = 0.041, model = DOM, N = 829

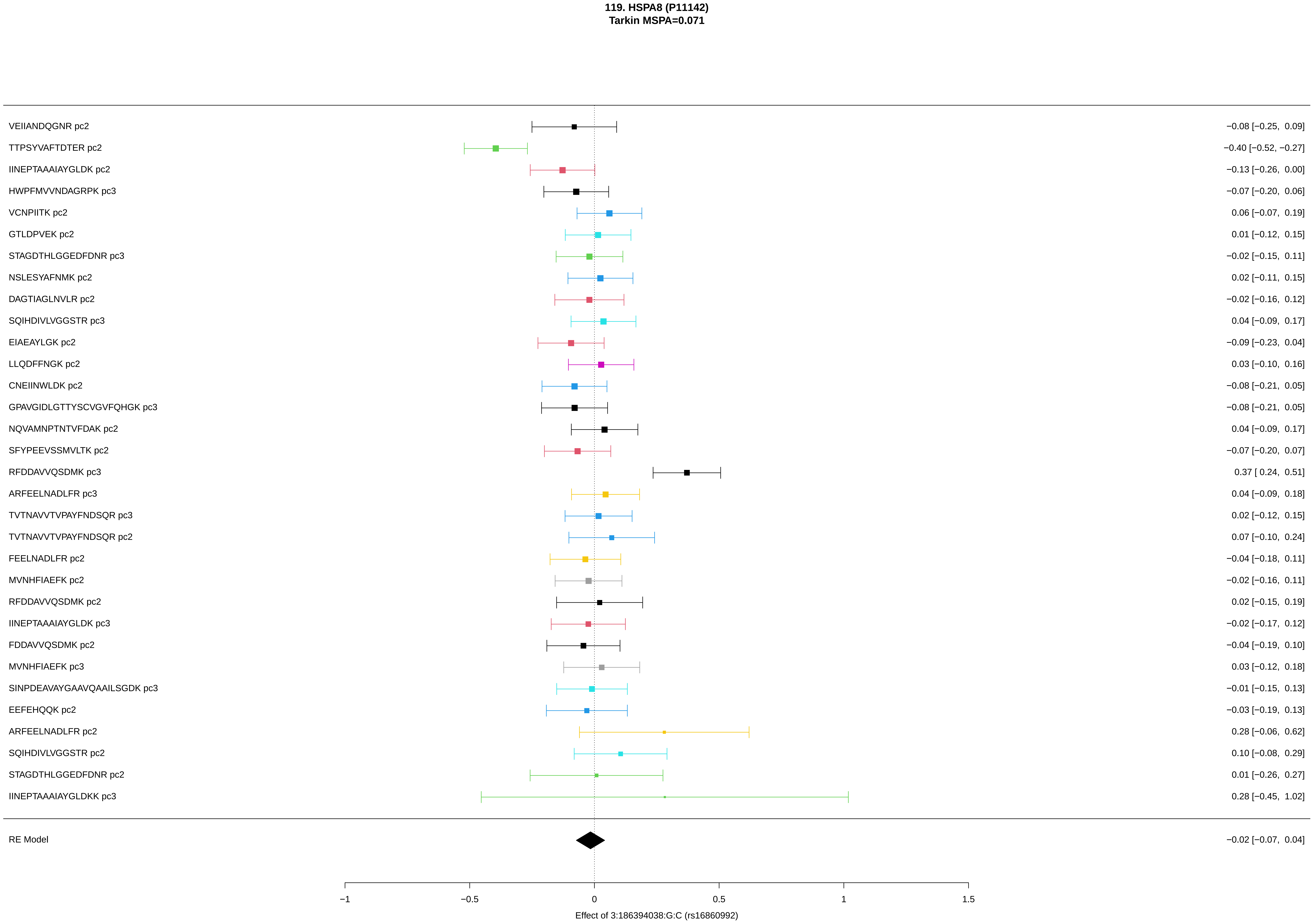
119. HSPA8 (P11142)
Tarkin MSPA=0.071
VEIIANDQGNR pc2
−0.08 [−0.25, 0.09]
TTPSYVAFTDTER pc2
−0.40 [−0.52, −0.27]
IINEPTAAAIAYGLDK pc2
−0.13 [−0.26, 0.00]
HWPFMVVNDAGRPK pc3
−0.07 [−0.20, 0.06]
VCNPIITK pc2
0.06 [−0.07, 0.19]
GTLDPVEK pc2
0.01 [−0.12, 0.15]
STAGDTHLGGEDFDNR pc3
−0.02 [−0.15, 0.11]
NSLESYAFNMK pc2
0.02 [−0.11, 0.15]
DAGTIAGLNVLR pc2
−0.02 [−0.16, 0.12]
SQIHDIVLVGGSTR pc3
0.04 [−0.09, 0.17]
EIAEAYLGK pc2
−0.09 [−0.23, 0.04]
LLQDFFNGK pc2
0.03 [−0.10, 0.16]
CNEIINWLDK pc2
−0.08 [−0.21, 0.05]
GPAVGIDLGTTYSCVGVFQHGK pc3
−0.08 [−0.21, 0.05]
NQVAMNPTNTVFDAK pc2
0.04 [−0.09, 0.17]
SFYPEEVSSMVLTK pc2
−0.07 [−0.20, 0.07]
RFDDAVVQSDMK pc3
0.37 [0.24, 0.51]
ARFEELNADLFR pc3
0.04 [−0.09, 0.18]
TVTNAVVTVPAYFNDSQR pc3
0.02 [−0.12, 0.15]
TVTNAVVTVPAYFNDSQR pc2
0.07 [−0.10, 0.24]
FEELNADLFR pc2
−0.04 [−0.18, 0.11]
MVNHFIAEFK pc2
−0.02 [−0.16, 0.11]
RFDDAVVQSDMK pc2
0.02 [−0.15, 0.19]
IINEPTAAAIAYGLDK pc3
−0.02 [−0.17, 0.12]
FDDAVVQSDMK pc2
−0.04 [−0.19, 0.10]
MVNHFIAEFK pc3
0.03 [−0.12, 0.18]
SINPDEAVAYGAAVQAAILSGDK pc3
−0.01 [−0.15, 0.13]
EEFEHQQK pc2
−0.03 [−0.19, 0.13]
ARFEELNADLFR pc2
0.28 [−0.06, 0.62]
SQIHDIVLVGGSTR pc2
0.10 [−0.08, 0.29]
STAGDTHLGGEDFDNR pc2
0.01 [−0.26, 0.27]
IINEPTAAAIAYGLDKK pc3
0.28 [−0.45, 1.02]
RE Model
−0.02 [−0.07, 0.04]
−1
−0.5
0
0.5
1
1.5
Effect of 3:186394038:G:C (rs16860992)

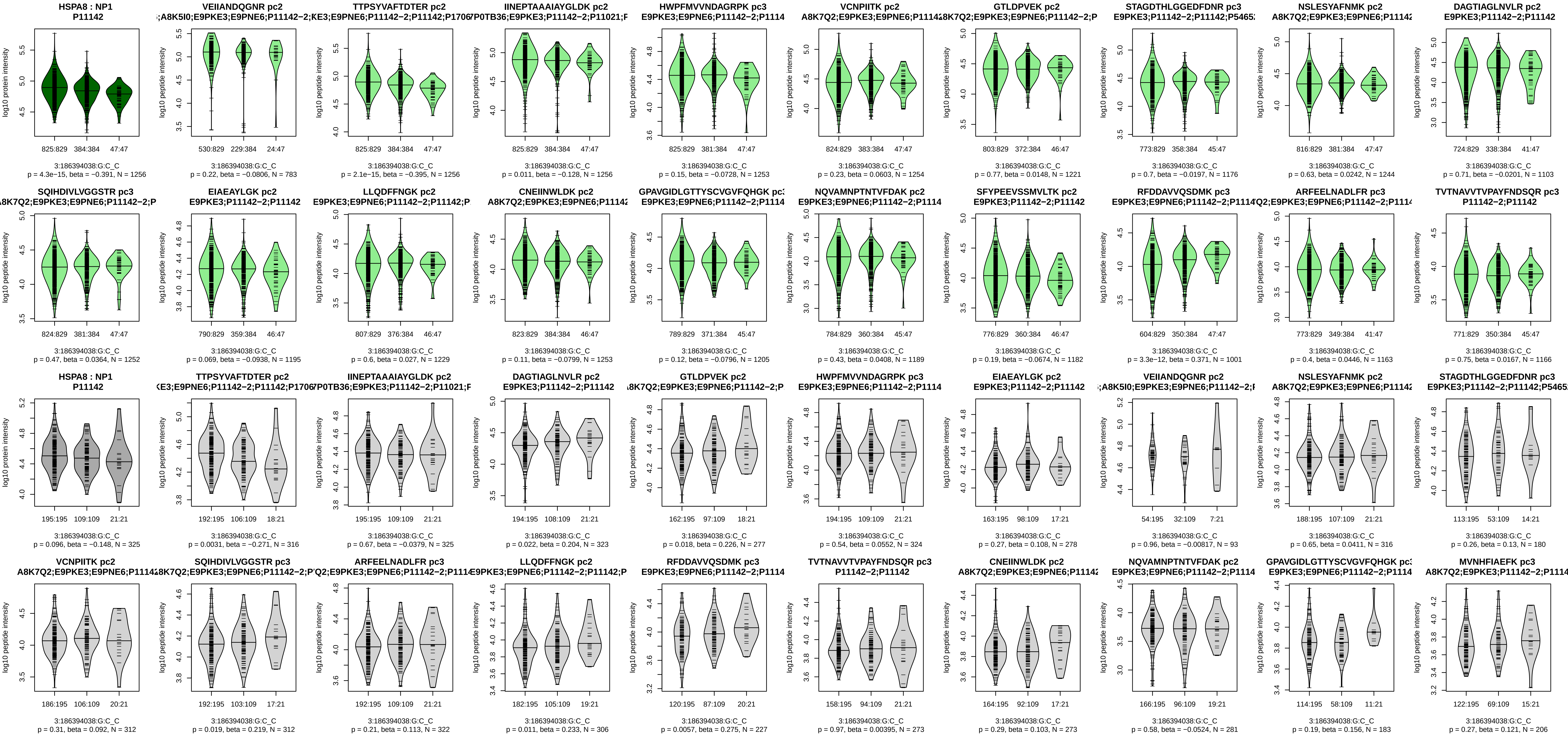

119Assay Target: HSPA8
UniProt: P11142
rsID: rs16860992
Target SNP: 3:186394038:G:C
Proxy SNP: 3:186394038:G:C
Tarkin log10(p): 14.4
Tarkin BETA: −0.391
Tarkin MSPA: 0.071
783:1256:1256:1253:1254:1221

120. C8G (P07360)
Tarkin MSPA=0.787

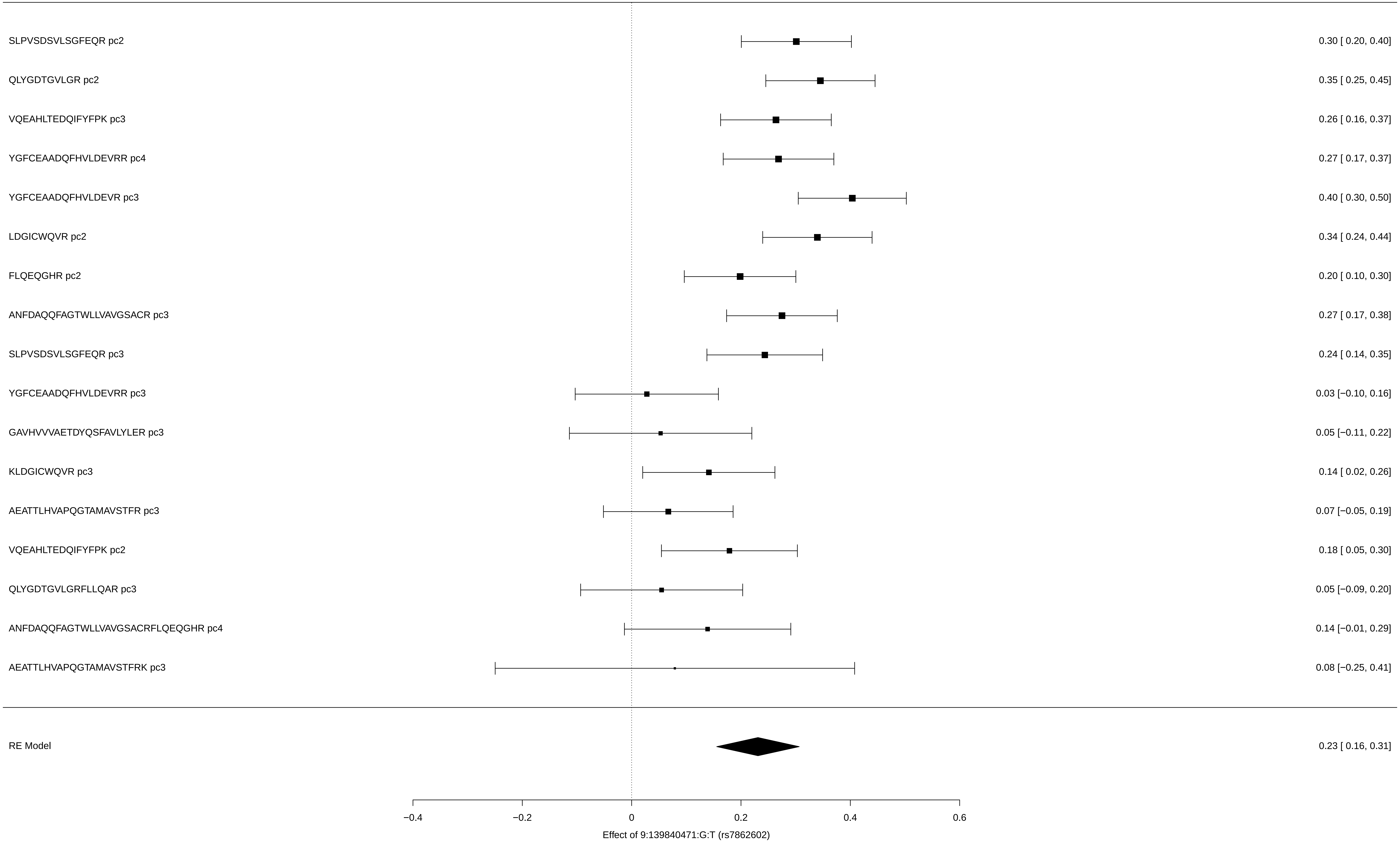

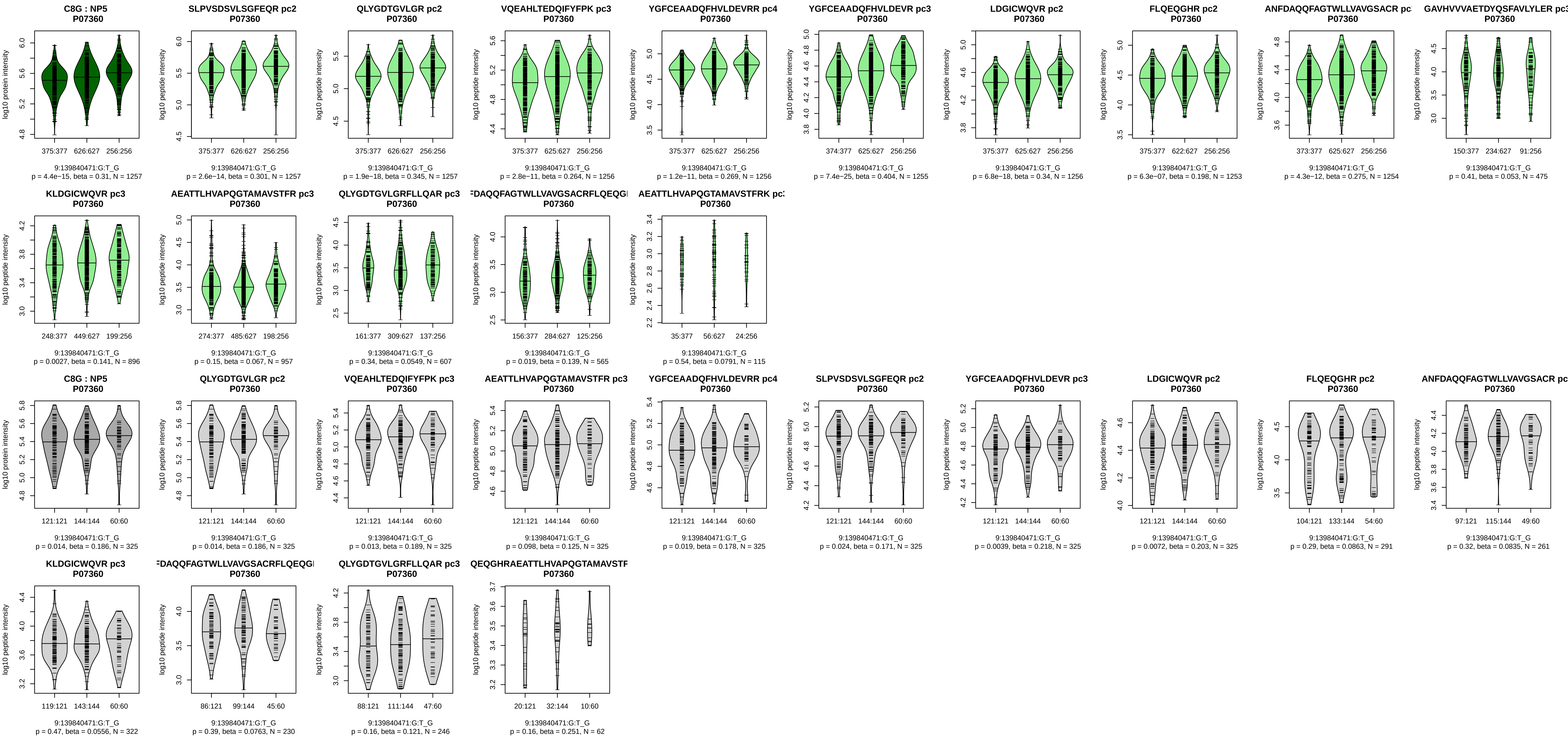

120Assay Target: C8G
UniProt: P07360
rsID: rs7862602
Target SNP: 9:139840471:G:T
Proxy SNP: 9:139840471:G:T
Tarkin log10(p): 14.4
Tarkin BETA: 0.31
Tarkin MSPA: 0.787
[illegible]
1257:1257:1256:1256:1255:125

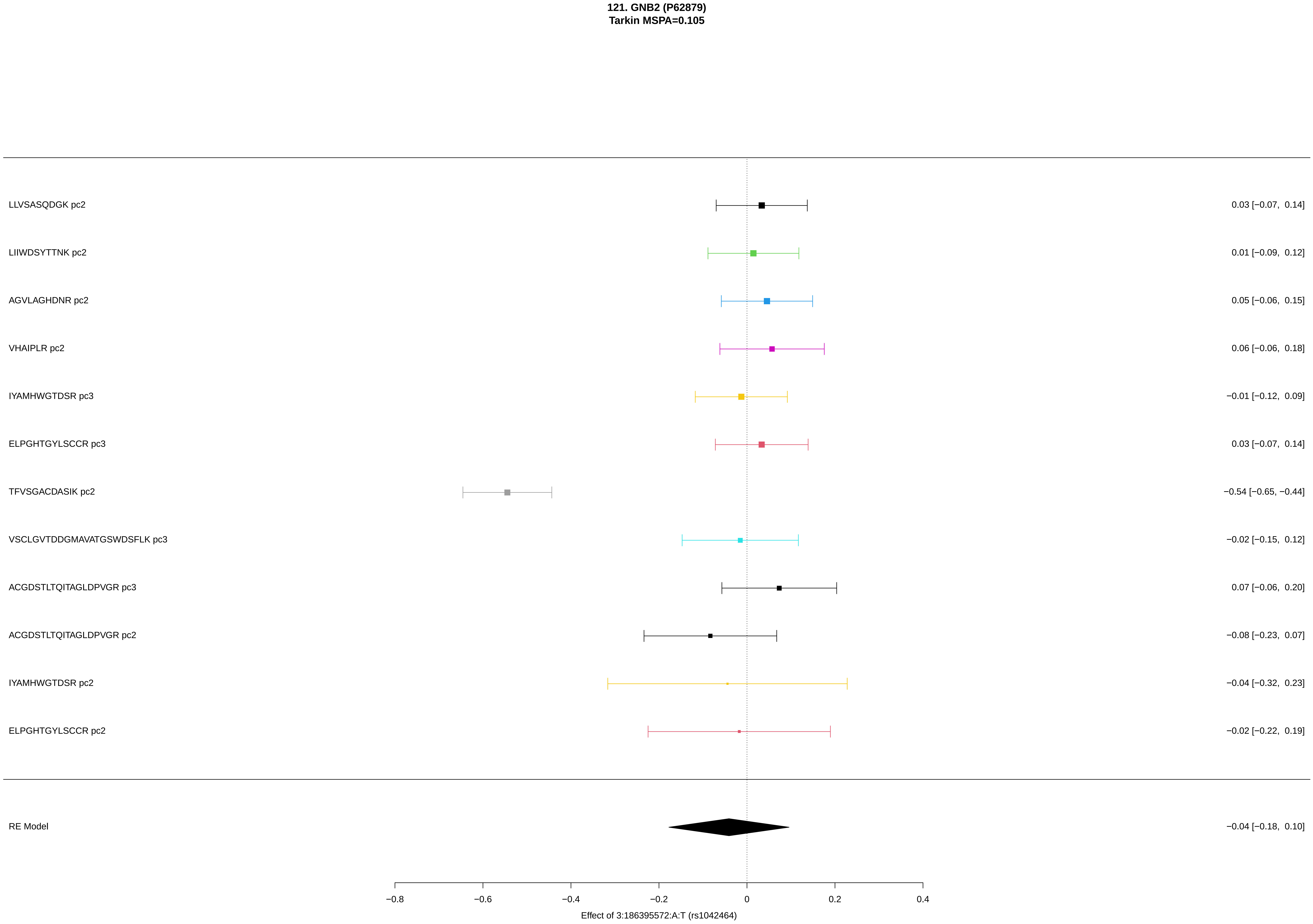

121. GNB2 (P62879)
Tarkin MSPA=0.105
LLVSASQDGK pc2
LIIWDSYTTNK pc2
AGVLAGHDNR pc2
VHAIPLR pc2
IYAMHWGTDSR pc3
ELPGHTGYLSCCR pc3
TFVSGACDASIK pc2
VSCLGVTDDGMAVATGSWDSFLK pc3
ACGDSTLTQITAGLDPVGR pc3
ACGDSTLTQITAGLDPVGR pc2
IYAMHWGTDSR pc2
ELPGHTGYLSCCR pc2
RE Model
0.03 [−0.07, 0.14]
0.01 [−0.09, 0.12]
0.05 [−0.06, 0.15]
0.06 [−0.06, 0.18]
−0.01 [−0.12, 0.09]
0.03 [−0.07, 0.14]
−0.54 [−0.65, −0.44]
−0.02 [−0.15, 0.12]
0.07 [−0.06, 0.20]
−0.08 [−0.23, 0.07]
−0.04 [−0.32, 0.23]
−0.02 [−0.22, 0.19]
−0.04 [−0.18, 0.10]
−0.8
−0.6
−0.4
−0.2
0
0.2
0.4
Effect of 3:186395572:A:T (rs1042464)

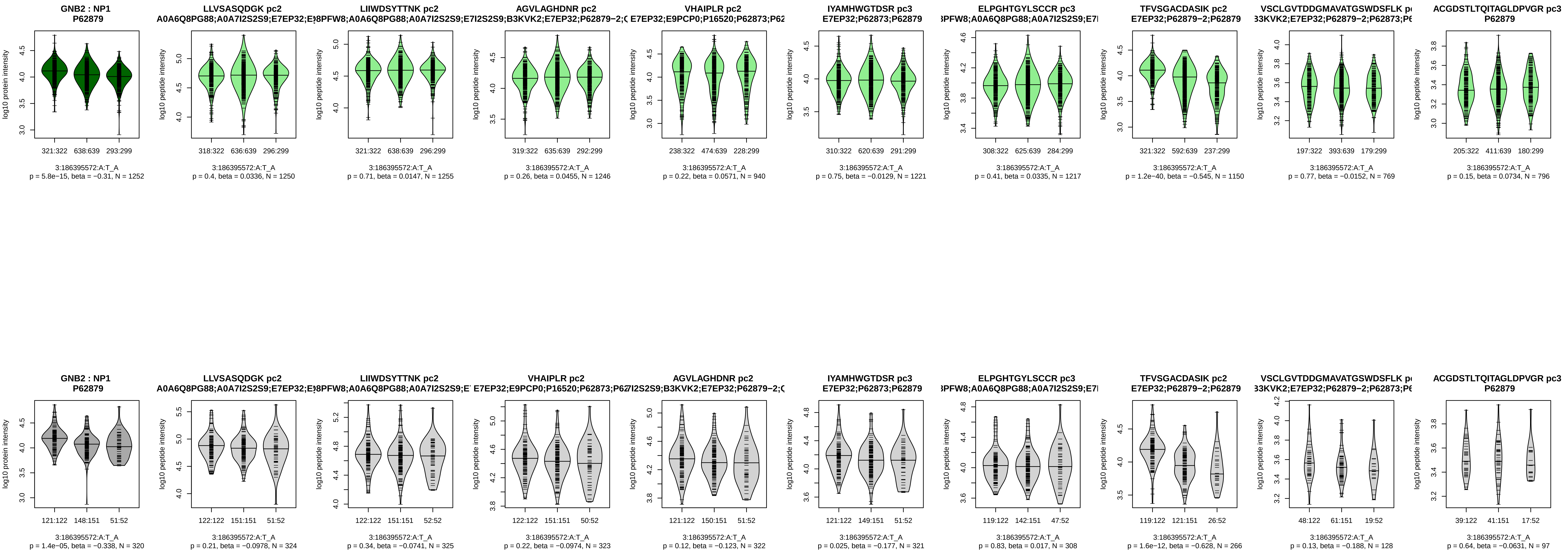

121Assay Target: GNB2
UniProt: P62879
rsID: rs1042464
Target SNP: 3:186395572:A:T
Proxy SNP: 3:186395572:A:T
Tarkin log10(p): 14.2
Tarkin BETA: −0.31
Tarkin MSPA: 0.105
−:−:−:−:−:−:*:−:−:−:−:−:NA
1250:1255:1246:940:1221:1217

122. DIAPH1 (O60610)
Tarkin MSPA=0.958

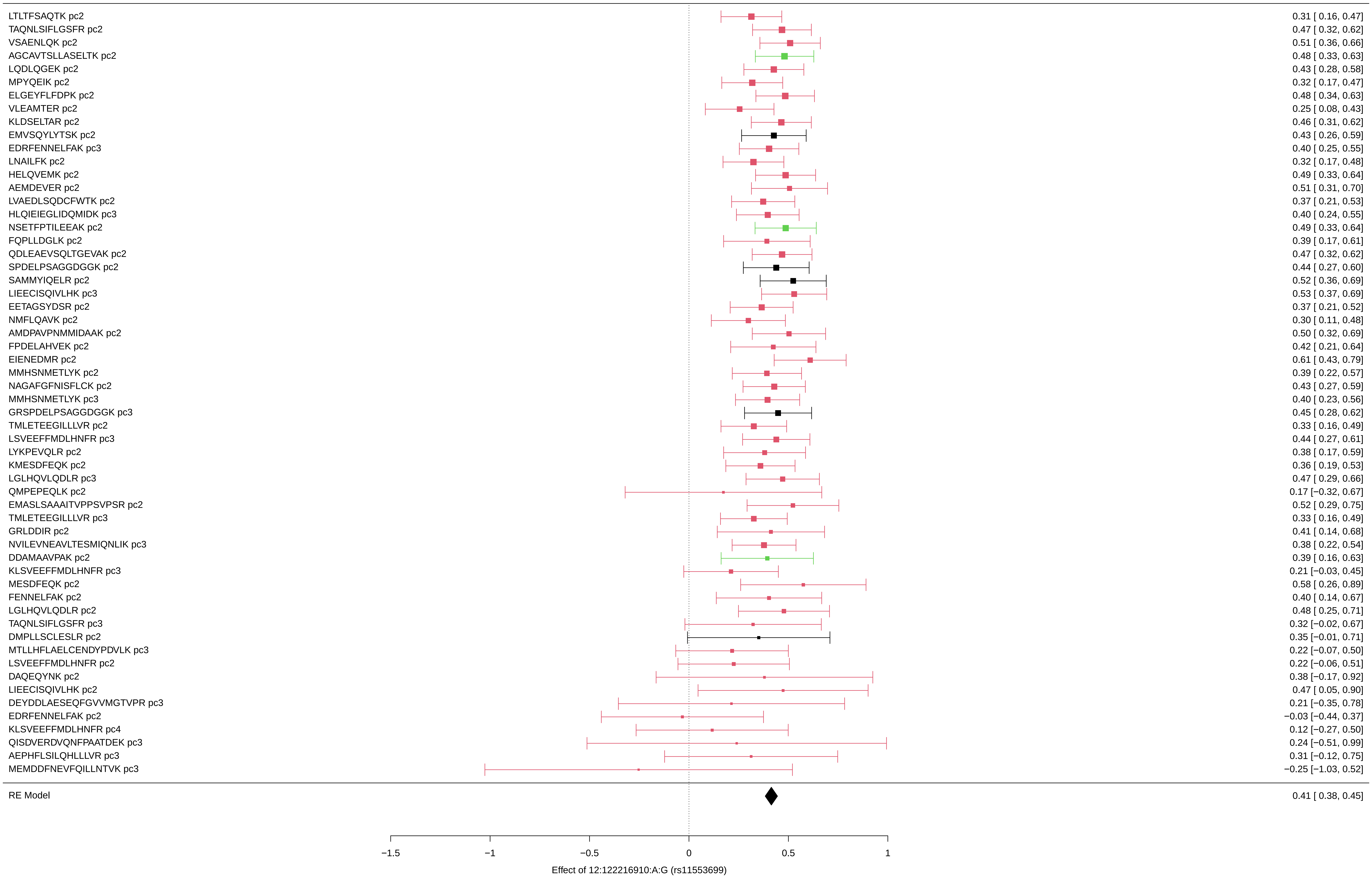

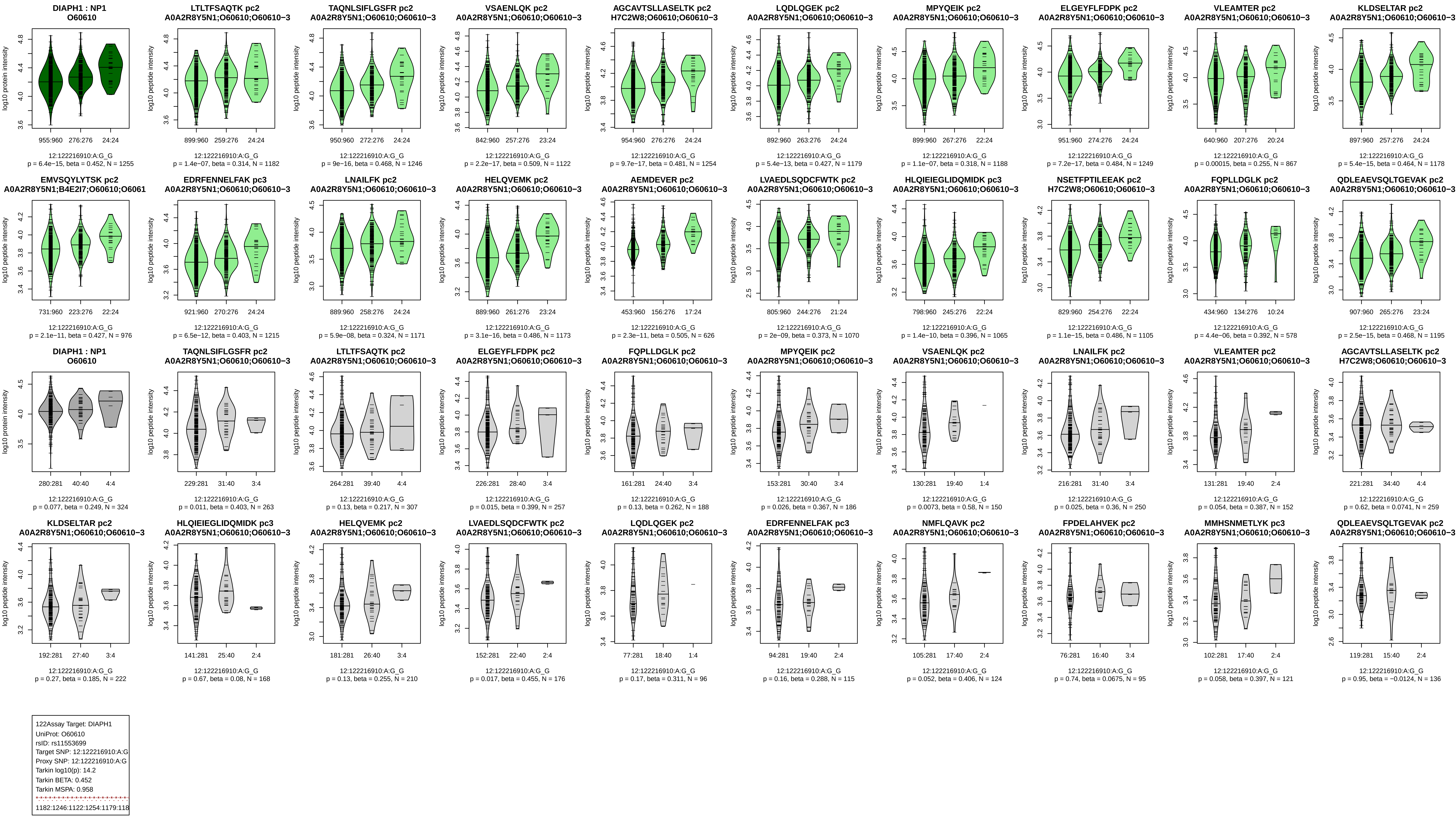
DIAPH1 : NP1
O60610
log10 protein intensity
955:960 276:276 24:24
12:122216910:A:G_G
p = 6.4e−15, beta = 0.452, N = 1255
LTLTFSAQTK pc2
A0A2R8Y5N1;O60610;O60610−3
log10 peptide intensity
899:960 259:276 24:24
12:122216910:A:G_G
p = 1.4e−07, beta = 0.314, N = 1182
TAQNLSIFLGSFR pc2
A0A2R8Y5N1;O60610;O60610−3
950:960 272:276 24:24
12:122216910:A:G_G
p = 9e−16, beta = 0.468, N = 1246
VSAENLQK pc2
A0A2R8Y5N1;O60610;O60610−3
842:960 257:276 23:24
12:122216910:A:G_G
p = 2.2e−17, beta = 0.509, N = 1122
AGCAVTSLLASELTK pc2
H7C2W8;O60610;O60610−3
954:960 276:276 24:24
12:122216910:A:G_G
p = 9.7e−17, beta = 0.481, N = 1254
LQDLQGEK pc2
A0A2R8Y5N1;O60610;O60610−3
892:960 263:276 24:24
12:122216910:A:G_G
p = 5.4e−13, beta = 0.427, N = 1179
MPYQEIK pc2
A0A2R8Y5N1;O60610;O60610−3
899:960 267:276 22:24
12:122216910:A:G_G
p = 1.1e−07, beta = 0.318, N = 1188
ELGEYFLFDPK pc2
A0A2R8Y5N1;O60610;O60610−3
951:960 274:276 24:24
12:122216910:A:G_G
p = 7.2e−17, beta = 0.484, N = 1249
VLEAMTER pc2
A0A2R8Y5N1;O60610;O60610−3
640:960 207:276 20:24
12:122216910:A:G_G
p = 0.00015, beta = 0.255, N = 867
KLDSELTAR pc2
A0A2R8Y5N1;O60610;O60610−3
897:960 257:276 24:24
12:122216910:A:G_G
p = 5.4e−15, beta = 0.464, N = 1178
EMVSQYLYTSK pc2
A0A2R8Y5N1;B4E2I7;O60610;O6061
log10 peptide intensity
731:960 223:276 22:24
12:122216910:A:G_G
p = 2.1e−11, beta = 0.427, N = 976
EDRFENNELFAK pc3
A0A2R8Y5N1;O60610;O60610−3
921:960 270:276 24:24
12:122216910:A:G_G
p = 6.5e−12, beta = 0.403, N = 1215
LNAILFK pc2
A0A2R8Y5N1;O60610;O60610−3
889:960 258:276 24:24
12:122216910:A:G_G
p = 5.9e−08, beta = 0.324, N = 1171
HELQVEMK pc2
A0A2R8Y5N1;O60610;O60610−3
889:960 261:276 23:24
12:122216910:A:G_G
p = 3.1e−16, beta = 0.486, N = 1173
AEMDEVER pc2
A0A2R8Y5N1;O60610;O60610−3
453:960 156:276 17:24
12:122216910:A:G_G
p = 2.3e−11, beta = 0.505, N = 626
LVAEDLSQDCFWTK pc2
A0A2R8Y5N1;O60610;O60610−3
805:960 244:276 21:24
12:122216910:A:G_G
p = 2e−09, beta = 0.373, N = 1070
HLQIEIEGLIDQMIDK pc3
A0A2R8Y5N1;O60610;O60610−3
798:960 245:276 22:24
12:122216910:A:G_G
p = 1.4e−10, beta = 0.396, N = 1065
NSETFPTILEEAK pc2
H7C2W8;O60610;O60610−3
829:960 254:276 22:24
12:122216910:A:G_G
p = 1.1e−15, beta = 0.486, N = 1105
FQPLLDGLK pc2
A0A2R8Y5N1;O60610;O60610−3
434:960 134:276 10:24
12:122216910:A:G_G
p = 4.4e−06, beta = 0.392, N = 578
QDLEAEVSQLTGEVAK pc2
A0A2R8Y5N1;O60610;O60610−3
907:960 265:276 23:24
12:122216910:A:G_G
p = 2.5e−15, beta = 0.468, N = 1195
DIAPH1 : NP1
O60610
log10 protein intensity
280:281 40:40 4:4
12:122216910:A:G_G
p = 0.077, beta = 0.249, N = 324
TAQNLSIFLGSFR pc2
A0A2R8Y5N1;O60610;O60610−3
log10 peptide intensity
229:281 31:40 3:4
12:122216910:A:G_G
p = 0.011, beta = 0.403, N = 263
LTLTFSAQTK pc2
A0A2R8Y5N1;O60610;O60610−3
264:281 39:40 4:4
12:122216910:A:G_G
p = 0.13, beta = 0.217, N = 307
ELGEYFLFDPK pc2
A0A2R8Y5N1;O60610;O60610−3
226:281 28:40 3:4
12:122216910:A:G_G
p = 0.015, beta = 0.399, N = 257
FQPLLDGLK pc2
A0A2R8Y5N1;O60610;O60610−3
161:281 24:40 3:4
12:122216910:A:G_G
p = 0.13, beta = 0.262, N = 188
MPYQEIK pc2
A0A2R8Y5N1;O60610;O60610−3
153:281 30:40 3:4
12:122216910:A:G_G
p = 0.026, beta = 0.367, N = 186
VSAENLQK pc2
A0A2R8Y5N1;O60610;O60610−3
130:281 19:40 1:4
12:122216910:A:G_G
p = 0.0073, beta = 0.58, N = 150
LNAILFK pc2
A0A2R8Y5N1;O60610;O60610−3
216:281 31:40 3:4
12:122216910:A:G_G
p = 0.025, beta = 0.36, N = 250
VLEAMTER pc2
A0A2R8Y5N1;O60610;O60610−3
131:281 19:40 2:4
12:122216910:A:G_G
p = 0.054, beta = 0.387, N = 152
AGCAVTSLLASELTK pc2
H7C2W8;O60610;O60610−3
221:281 34:40 4:4
12:122216910:A:G_G
p = 0.62, beta = 0.0741, N = 259
KLDSELTAR pc2
A0A2R8Y5N1;O60610;O60610−3
log10 peptide intensity
192:281 27:40 3:4
12:122216910:A:G_G
p = 0.27, beta = 0.185, N = 222
HLQIEIEGLIDQMIDK pc3
A0A2R8Y5N1;O60610;O60610−3
141:281 25:40 2:4
12:122216910:A:G_G
p = 0.67, beta = 0.08, N = 168
HELQVEMK pc2
A0A2R8Y5N1;O60610;O60610−3
181:281 26:40 3:4
12:122216910:A:G_G
p = 0.13, beta = 0.255, N = 210
LVAEDLSQDCFWTK pc2
A0A2R8Y5N1;O60610;O60610−3
152:281 22:40 2:4
12:122216910:A:G_G
p = 0.017, beta = 0.455, N = 176
LQDLQGEK pc2
A0A2R8Y5N1;O60610;O60610−3
77:281 18:40 1:4
12:122216910:A:G_G
p = 0.17, beta = 0.311, N = 96
EDRFENNELFAK pc3
A0A2R8Y5N1;O60610;O60610−3
94:281 19:40 2:4
12:122216910:A:G_G
p = 0.16, beta = 0.288, N = 115
NMFLQAVK pc2
A0A2R8Y5N1;O60610;O60610−3
105:281 17:40 2:4
12:122216910:A:G_G
p = 0.052, beta = 0.406, N = 124
FPDELAHVEK pc2
A0A2R8Y5N1;O60610;O60610−3
76:281 16:40 3:4
12:122216910:A:G_G
p = 0.74, beta = 0.0675, N = 95
MMHSNMETLYK pc3
A0A2R8Y5N1;O60610;O60610−3
102:281 17:40 2:4
12:122216910:A:G_G
p = 0.058, beta = 0.397, N = 121
QDLEAEVSQLTGEVAK pc2
A0A2R8Y5N1;O60610;O60610−3
119:281 15:40 2:4
12:122216910:A:G_G
p = 0.95, beta = −0.0124, N = 136
122Assay Target: DIAPH1
UniProt: O60610
rsID: rs11553699
Target SNP: 12:122216910:A:G
Proxy SNP: 12:122216910:A:G
Tarkin log10(p): 14.2
Tarkin BETA: 0.452
Tarkin MSPA: 0.958
1182:1246:1122:1254:1179:118

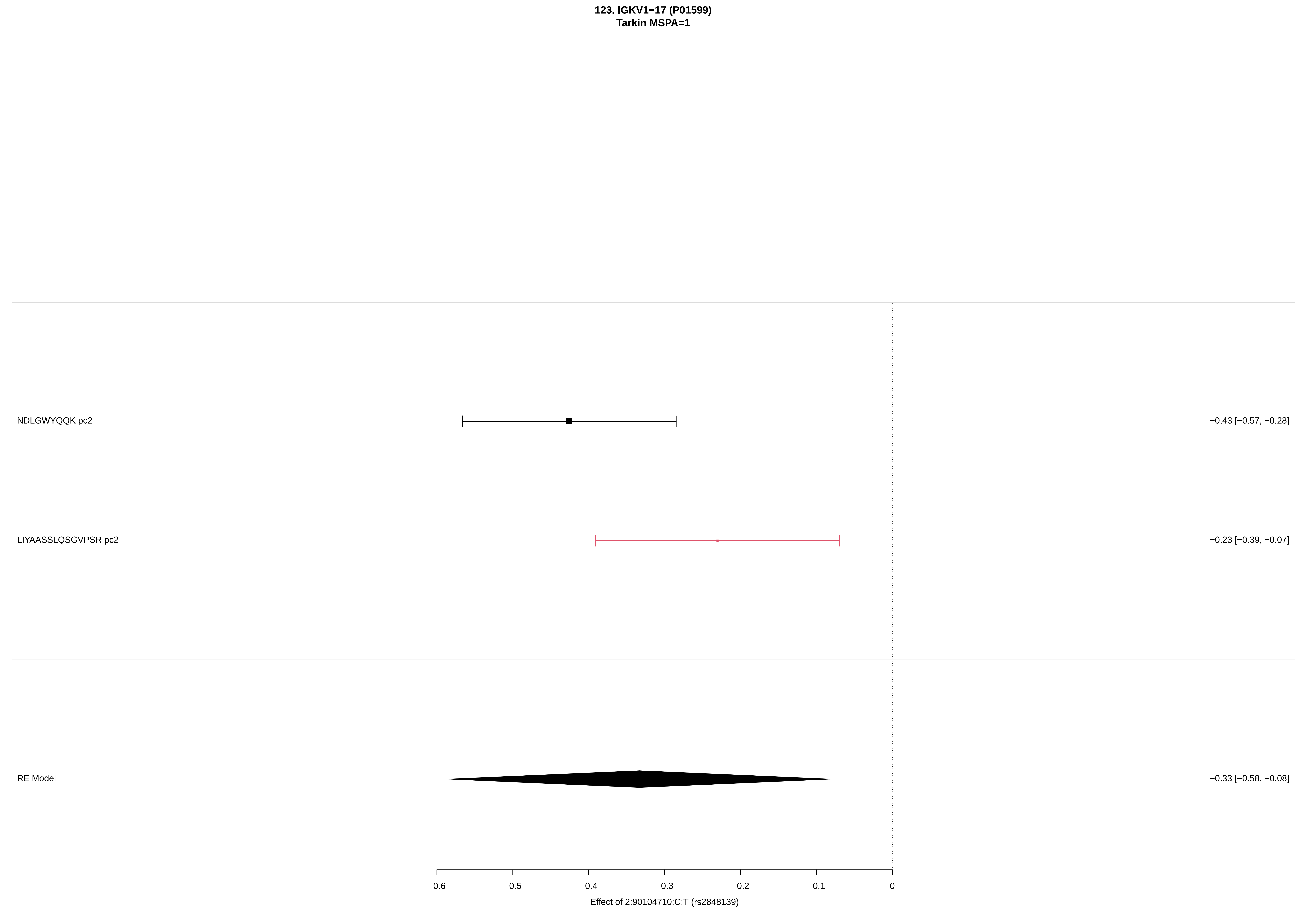
123. IGKV1−17 (P01599)
Tarkin MSPA=1
NDLGWYQQK pc2
−0.43 [−0.57, −0.28]
LIYAASSLQSGVPSR pc2
−0.23 [−0.39, −0.07]
RE Model
−0.33 [−0.58, −0.08]
−0.6
−0.5
−0.4
−0.3
−0.2
−0.1
0
Effect of 2:90104710:C:T (rs2848139)

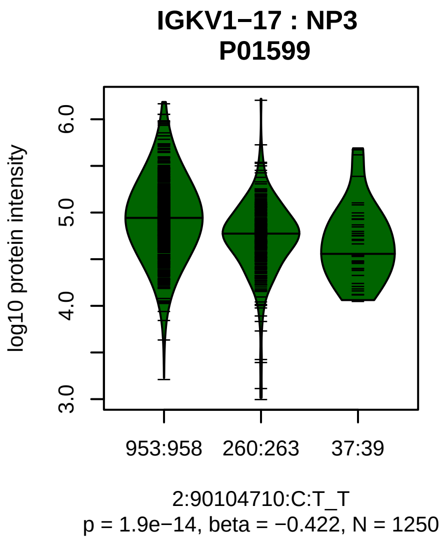

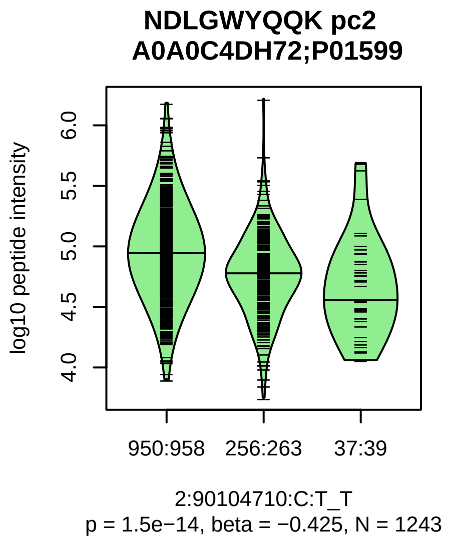

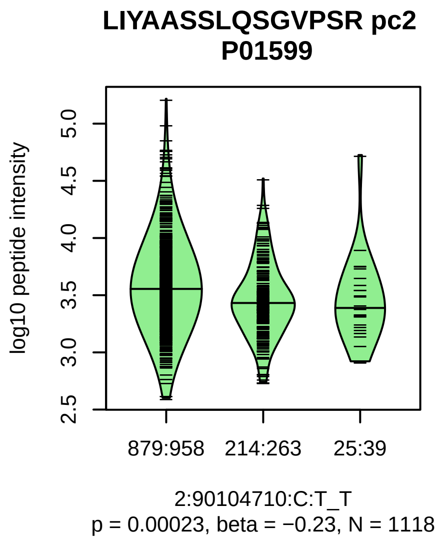

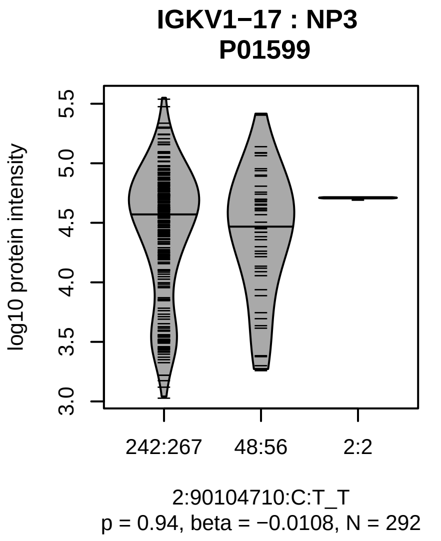

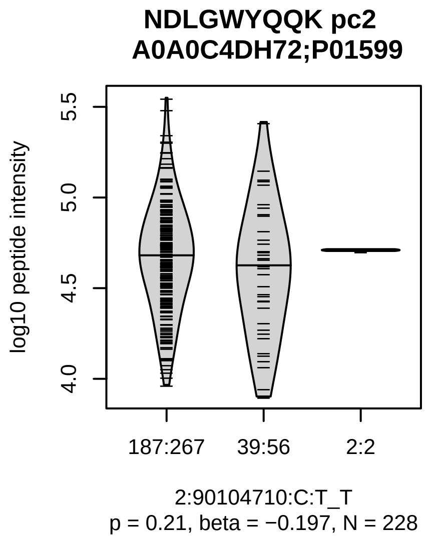

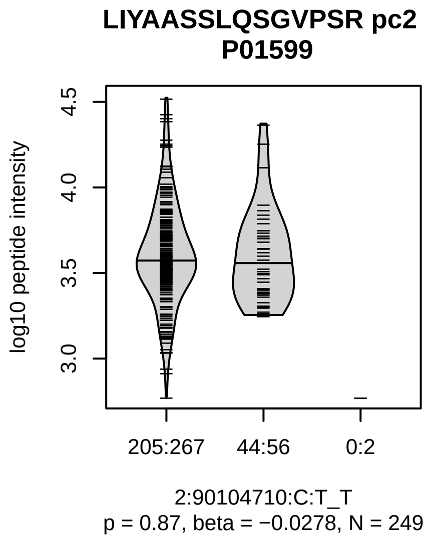

123Assay Target: IGKV1−17
UniProt: P01599
rsID: rs2848139
Target SNP: 2:90104710:C:T
Proxy SNP: 2:90104710:C:T
Tarkin log10(p): 13.7
Tarkin BETA: −0.422
Tarkin MSPA: 1
::NA:NA
1243:1118:4:2

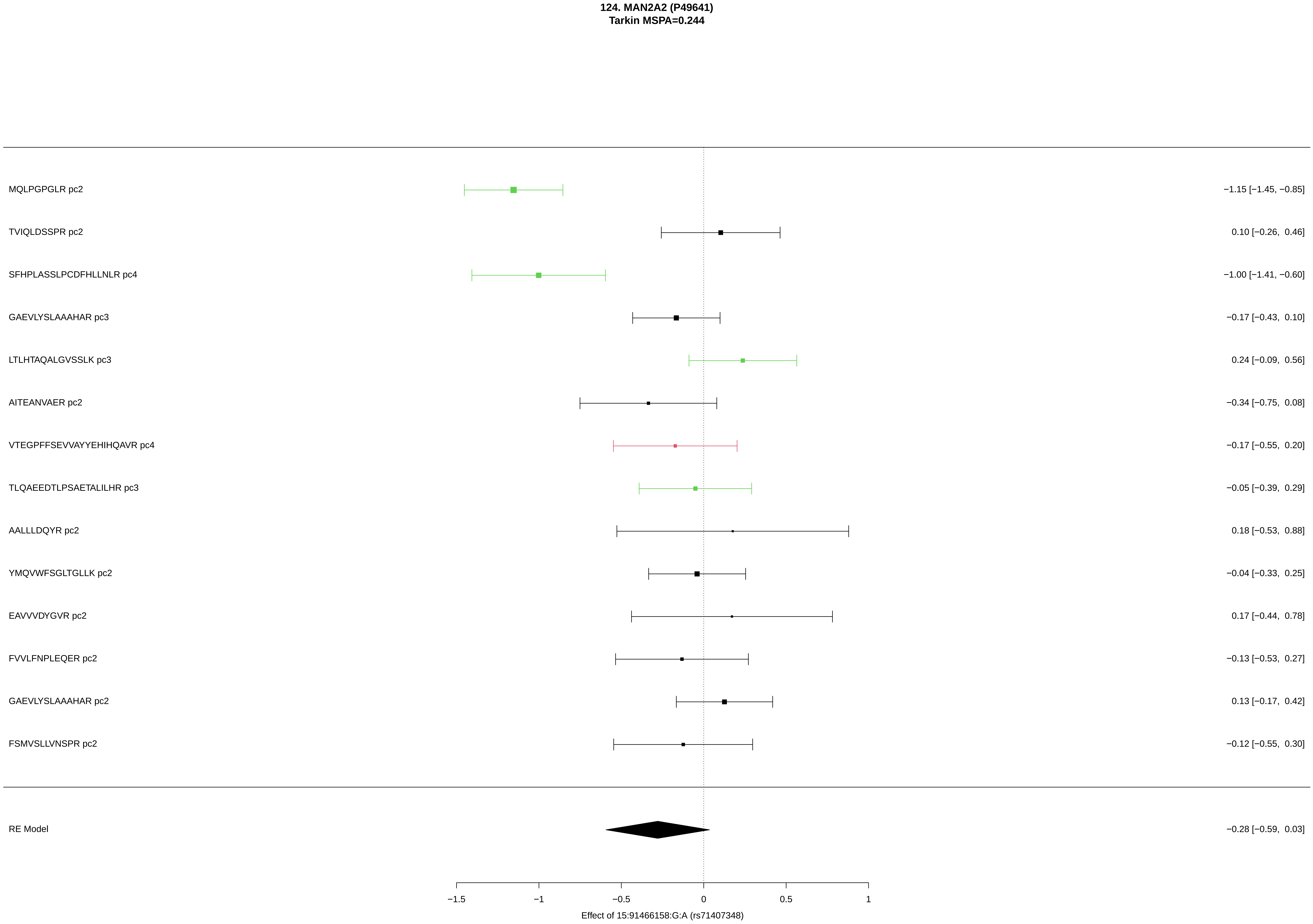

124. MAN2A2 (P49641)
Tarkin MSPA=0.244
MQLPGPGLR pc2
−1.15 [−1.45, −0.85]
TVIQLDSSPR pc2
0.10 [−0.26, 0.46]
SFHPLASSLPCDFHLLNLR pc4
−1.00 [−1.41, −0.60]
GAEVLYSLAAAHAR pc3
−0.17 [−0.43, 0.10]
LTLHTAQALGVSSLK pc3
0.24 [−0.09, 0.56]
AITEANVAER pc2
−0.34 [−0.75, 0.08]
VTEGPFFSEVVAYYEHIHQAVR pc4
−0.17 [−0.55, 0.20]
TLQAEEDTLPSAETALILHR pc3
−0.05 [−0.39, 0.29]
AALLLDQYR pc2
0.18 [−0.53, 0.88]
YMQVWFSGLTGLLK pc2
−0.04 [−0.33, 0.25]
EAVVVDYGVR pc2
0.17 [−0.44, 0.78]
FVVLFNPLEQER pc2
−0.13 [−0.53, 0.27]
GAEVLYSLAAAHAR pc2
0.13 [−0.17, 0.42]
FSMVSLLVNSPR pc2
−0.12 [−0.55, 0.30]
RE Model
−0.28 [−0.59, 0.03]
−1.5
−1
−0.5
0
0.5
1
Effect of 15:91466158:G:A (rs71407348)

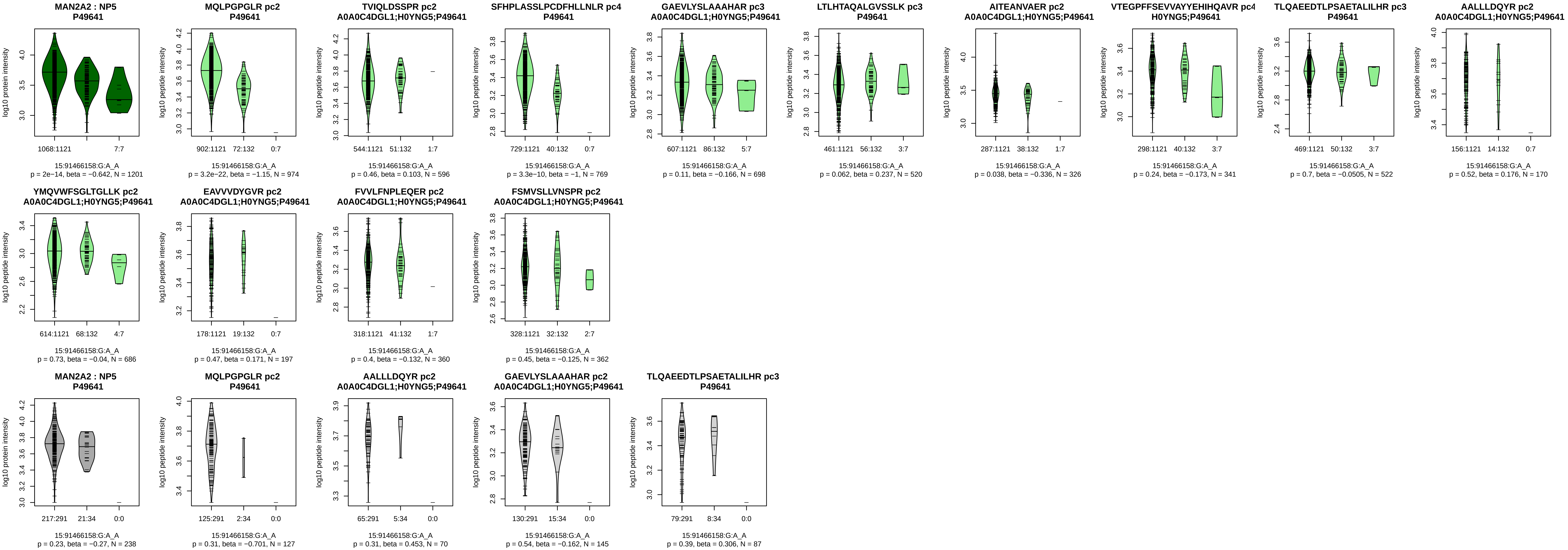

124Assay Target: MAN2A2
UniProt: P49641
rsID: rs71407348
Target SNP: 15:91466158:G:A
Proxy SNP: 15:91466158:G:A
Tarkin log10(p): 13.7
Tarkin BETA: −0.642
Tarkin MSPA: 0.244
:−::−:−:−:−:−:−:−:−:−:−:−
974:596:769:698:520:326:341:5

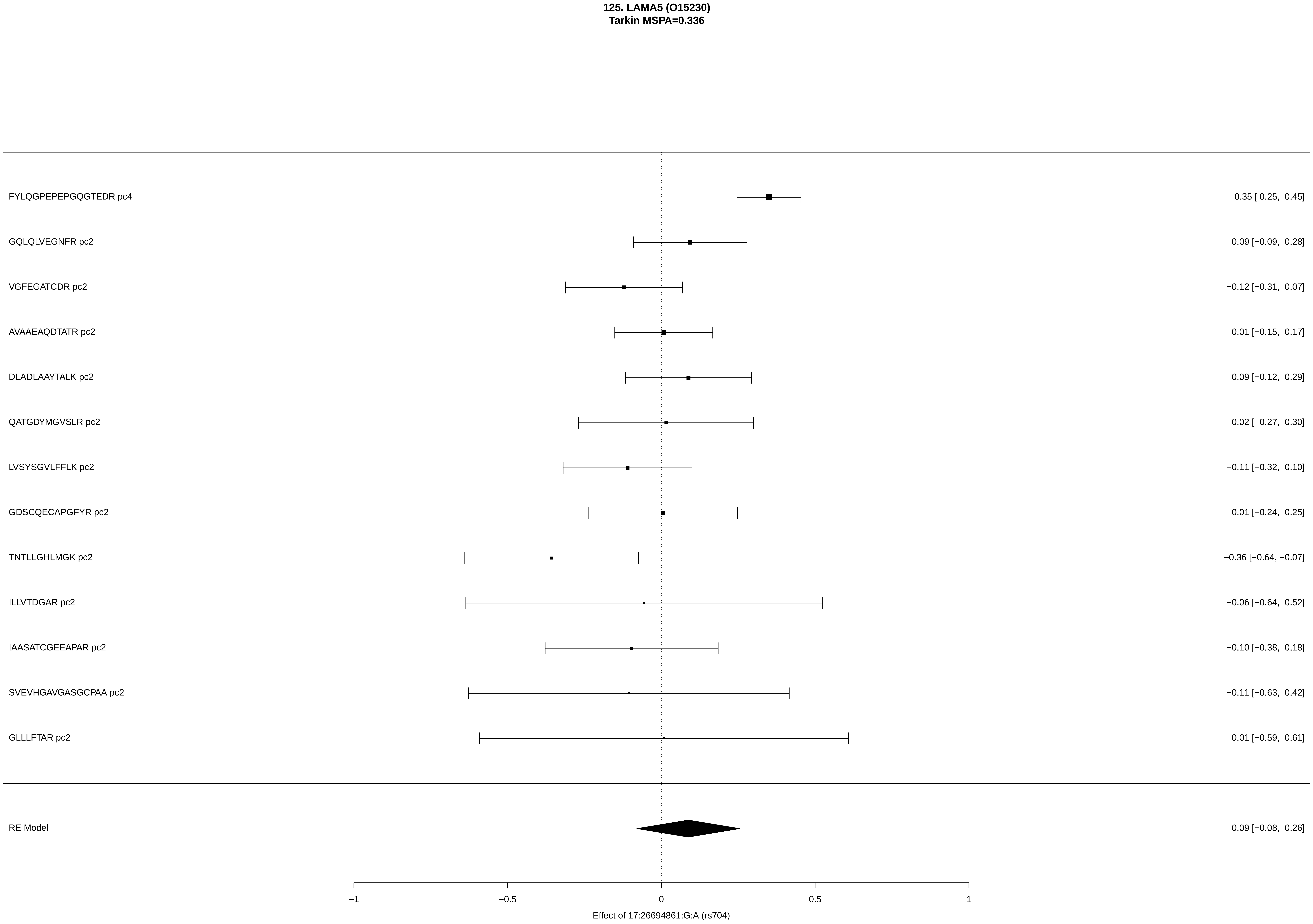

125. LAMA5 (O15230)
Tarkin MSPA=0.336
FYLQGPEPEPGQGTEDR pc4
0.35 [0.25, 0.45]
GQLQLVEGNFR pc2
0.09 [−0.09, 0.28]
VGFEGATCDR pc2
−0.12 [−0.31, 0.07]
AVAAEAQDTATR pc2
0.01 [−0.15, 0.17]
DLADLAAYTALK pc2
0.09 [−0.12, 0.29]
QATGDYMGVSLR pc2
0.02 [−0.27, 0.30]
LVSYSGVLFFLK pc2
−0.11 [−0.32, 0.10]
GDSCQECAPGFYR pc2
0.01 [−0.24, 0.25]
TNTLLGHLMGK pc2
−0.36 [−0.64, −0.07]
ILLVTDGAR pc2
−0.06 [−0.64, 0.52]
IAASATCGEEAPAR pc2
−0.10 [−0.38, 0.18]
SVEVHGAVGASGCPAA pc2
−0.11 [−0.63, 0.42]
GLLLFTAR pc2
0.01 [−0.59, 0.61]
RE Model
0.09 [−0.08, 0.26]
−1
−0.5
0
0.5
1
Effect of 17:26694861:G:A (rs704)

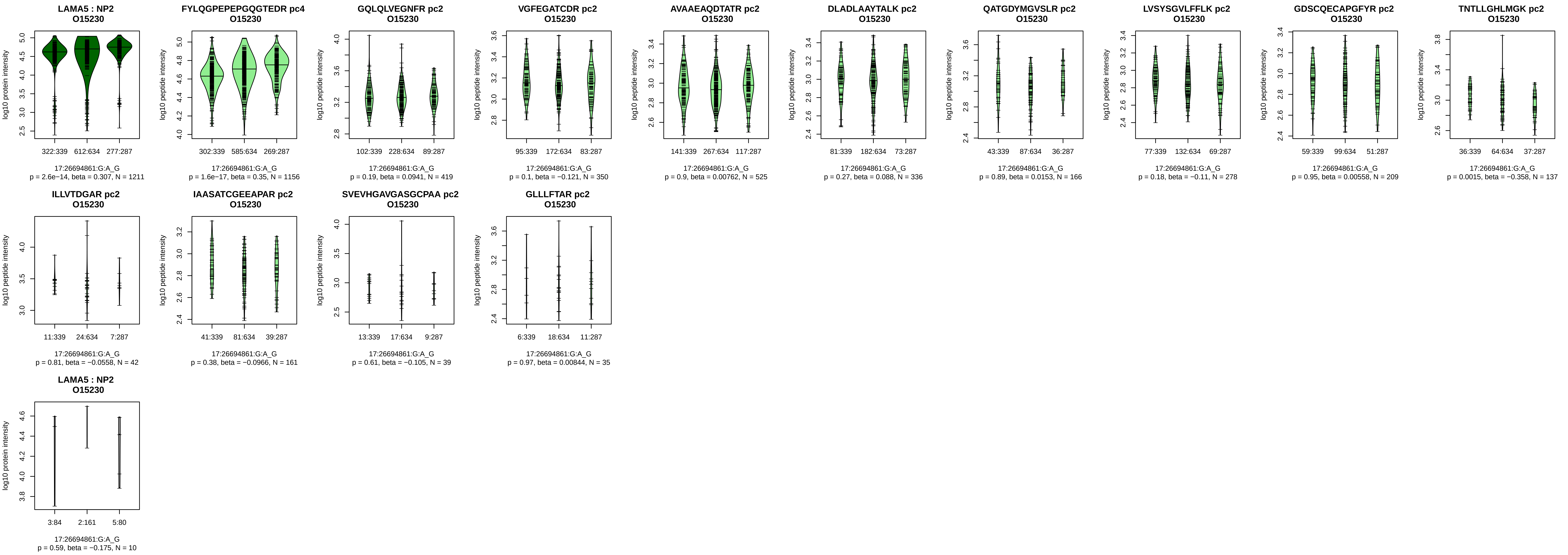

125Assay Target: LAMA5
UniProt: O15230
rsID: rs704
Target SNP: 17:26694861:G:A
Proxy SNP: 17:26694861:G:A
Tarkin log10(p): 13.6
Tarkin BETA: 0.307
Tarkin MSPA: 0.336
:−:−:−:−:−:−::−:−:−:−:NA:NA:
1156:419:350:525:336:166:278:

126. SERPINA11 (Q86U17)
Tarkin MSPA=1

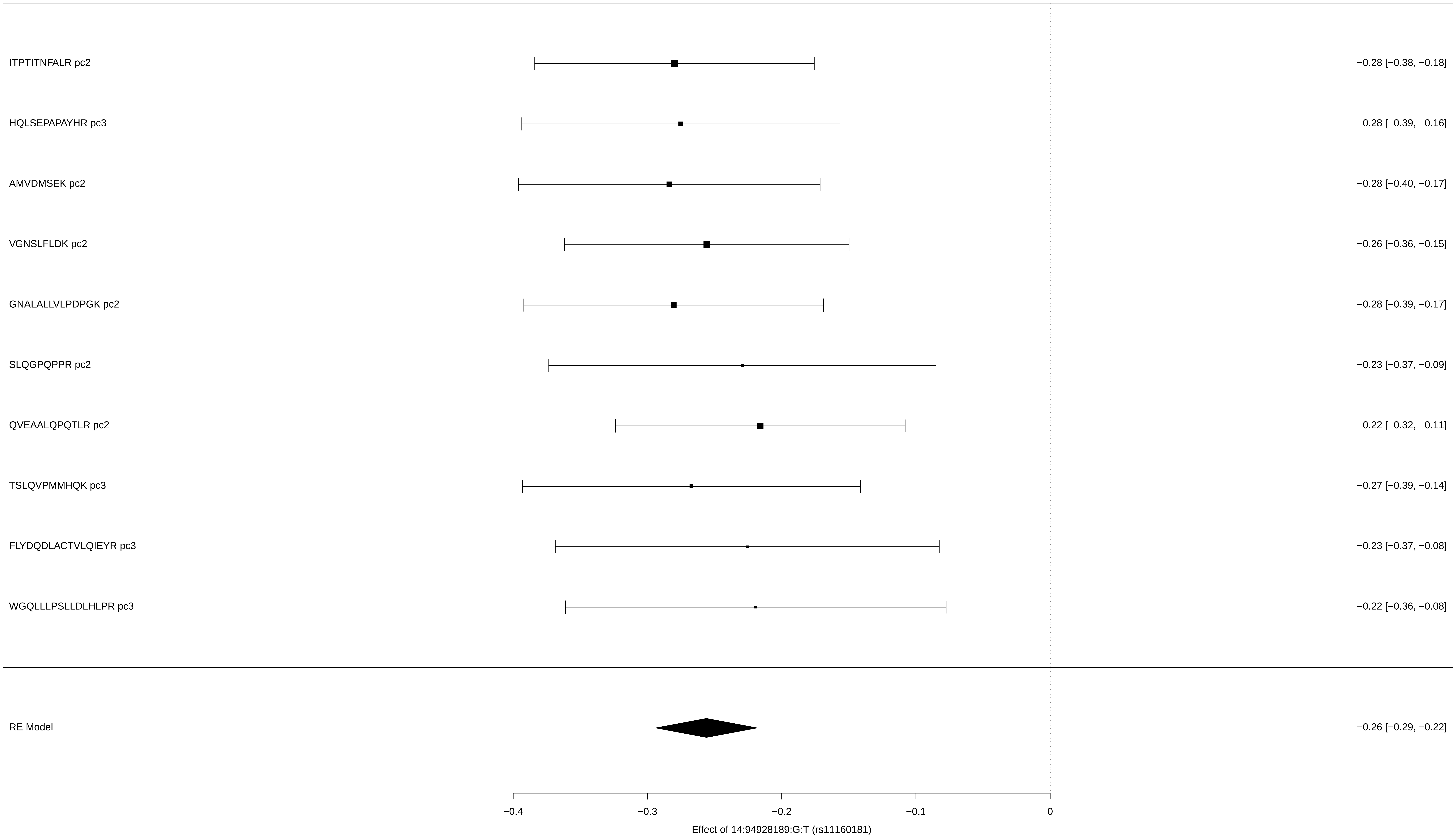

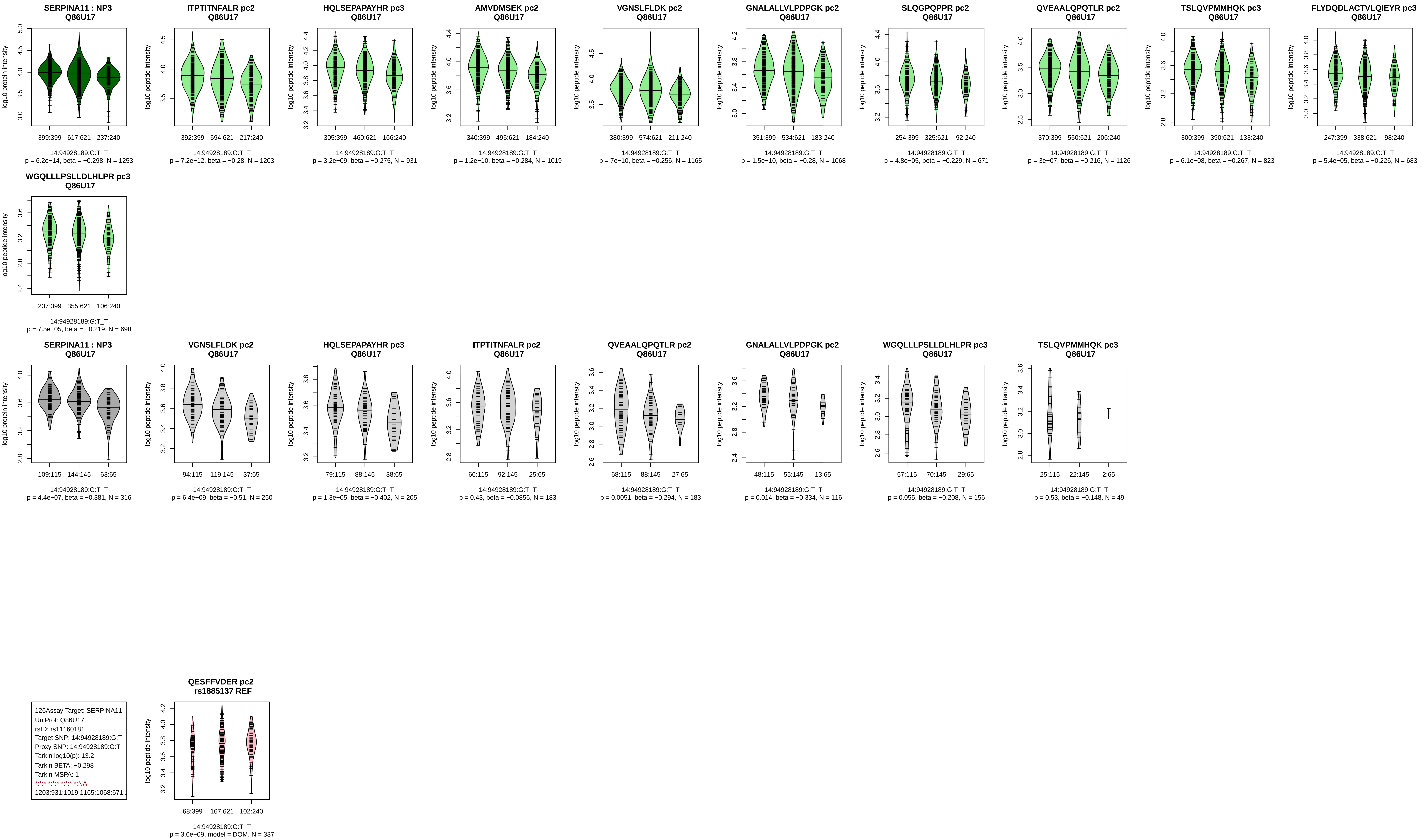
SERPINA11 : NP3 Q86U17
14:94928189:G:T_T
p = 6.2e−14, beta = −0.298, N = 1253
ITPTITNFALR pc2 Q86U17
14:94928189:G:T_T
p = 7.2e−12, beta = −0.28, N = 1203
HQLSEPAPAYHR pc3 Q86U17
14:94928189:G:T_T
p = 3.2e−09, beta = −0.275, N = 931
AMVDMSEK pc2 Q86U17
14:94928189:G:T_T
p = 1.2e−10, beta = −0.284, N = 1019
VGNSLFLDK pc2 Q86U17
14:94928189:G:T_T
p = 7e−10, beta = −0.256, N = 1165
GNALALLVLPDPGK pc2 Q86U17
14:94928189:G:T_T
p = 1.5e−10, beta = −0.28, N = 1068
SLQGPQPPR pc2 Q86U17
14:94928189:G:T_T
p = 4.8e−05, beta = −0.229, N = 671
QVEAALQPQTLR pc2 Q86U17
14:94928189:G:T_T
p = 3e−07, beta = −0.216, N = 1126
TSLQVPMMHQK pc3 Q86U17
14:94928189:G:T_T
p = 6.1e−08, beta = −0.267, N = 823
FLYDQDLACTVLQIEYR pc3 Q86U17
14:94928189:G:T_T
p = 5.4e−05, beta = −0.226, N = 683
WGQLLLPSLLDLHLPR pc3 Q86U17
14:94928189:G:T_T
p = 7.5e−05, beta = −0.219, N = 698
SERPINA11 : NP3 Q86U17
14:94928189:G:T_T
p = 4.4e−07, beta = −0.381, N = 316
VGNSLFLDK pc2 Q86U17
14:94928189:G:T_T
p = 6.4e−09, beta = −0.51, N = 250
HQLSEPAPAYHR pc3 Q86U17
14:94928189:G:T_T
p = 1.3e−05, beta = −0.402, N = 205
ITPTITNFALR pc2 Q86U17
14:94928189:G:T_T
p = 0.43, beta = −0.0856, N = 183
QVEAALQPQTLR pc2 Q86U17
14:94928189:G:T_T
p = 0.0051, beta = −0.294, N = 183
GNALALLVLPDPGK pc2 Q86U17
14:94928189:G:T_T
p = 0.014, beta = −0.334, N = 116
WGQLLLPSLLDLHLPR pc3 Q86U17
14:94928189:G:T_T
p = 0.055, beta = −0.208, N = 156
TSLQVPMMHQK pc3 Q86U17
14:94928189:G:T_T
p = 0.53, beta = −0.148, N = 49
126Assay Target: SERPINA11
UniProt: Q86U17
rsID: rs11160181
Target SNP: 14:94928189:G:T
Proxy SNP: 14:94928189:G:T
Tarkin log10(p): 13.2
Tarkin BETA: −0.298
Tarkin MSPA: 1
::*:*:*:*:*:*:*:*:NA
1203:931:1019:1165:1068:671:
QESFFVDER pc2 rs1885137 REF
14:94928189:G:T_T
p = 3.6e−09, model = DOM, N = 337

127. ORM2 (P19652)
Tarkin MSPA=0.721

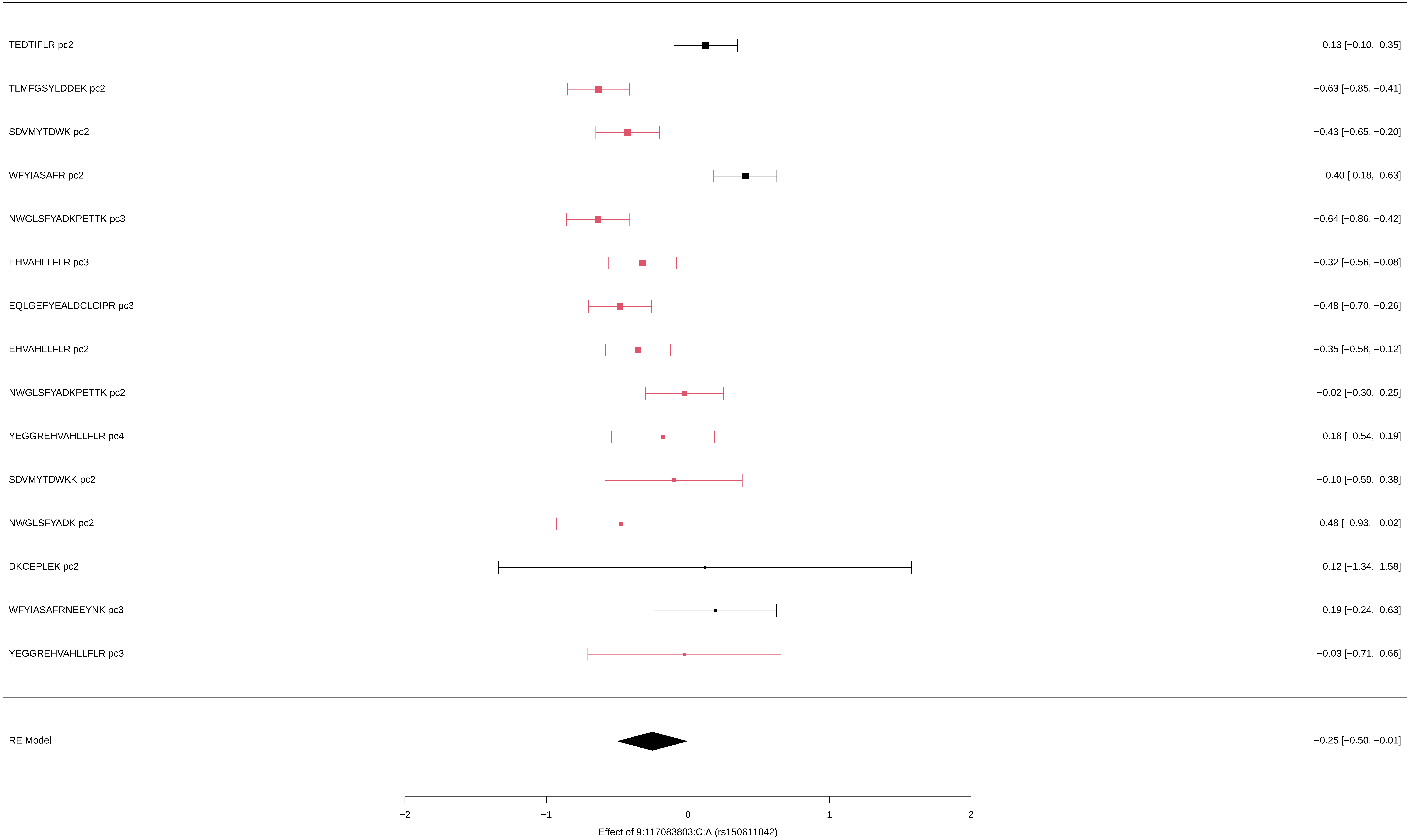

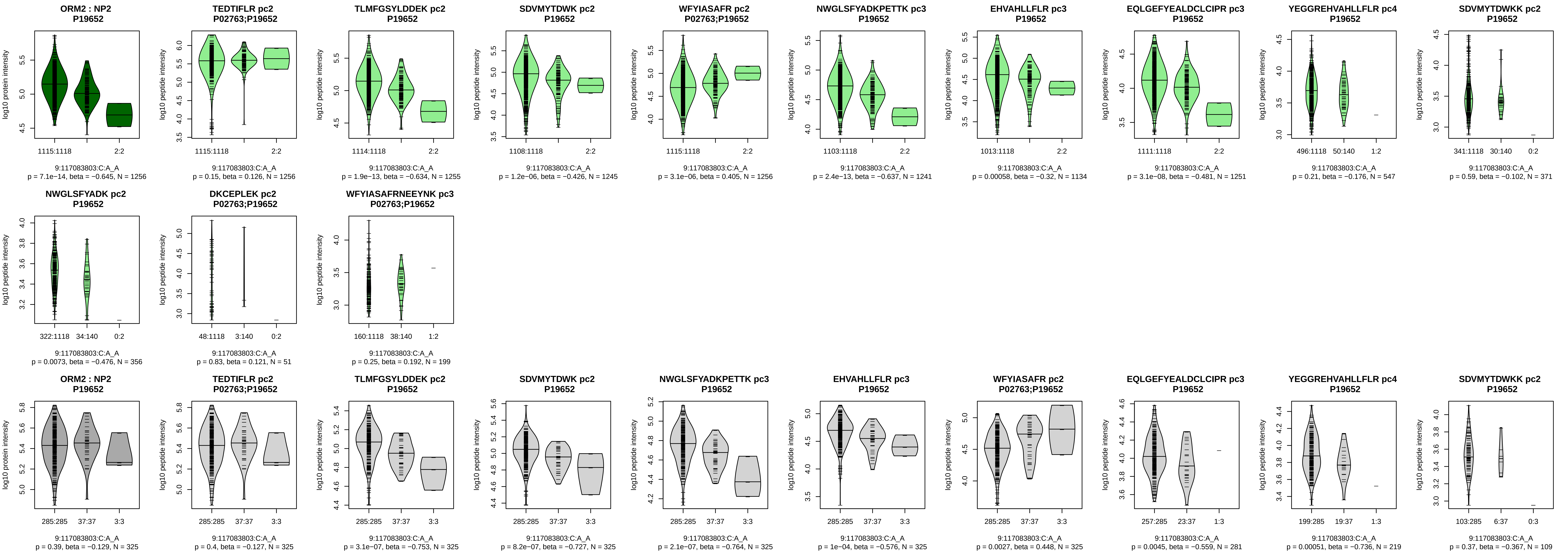

127Assay Target: ORM2
UniProt: P19652
rsID: rs150611042
Target SNP: 9:117083803:C:A
Proxy SNP: 9:117083803:C:A
Tarkin log10(p): 13.1
Tarkin BETA: −0.645
Tarkin MSPA: 0.721
−:*:*:*:*:*:*:−:−:−:*:−:−:−:NA:NA
1256:1255:1245:1256:1241:113

128. TNFAIP6 (P98066)
Tarkin MSPA=0.821

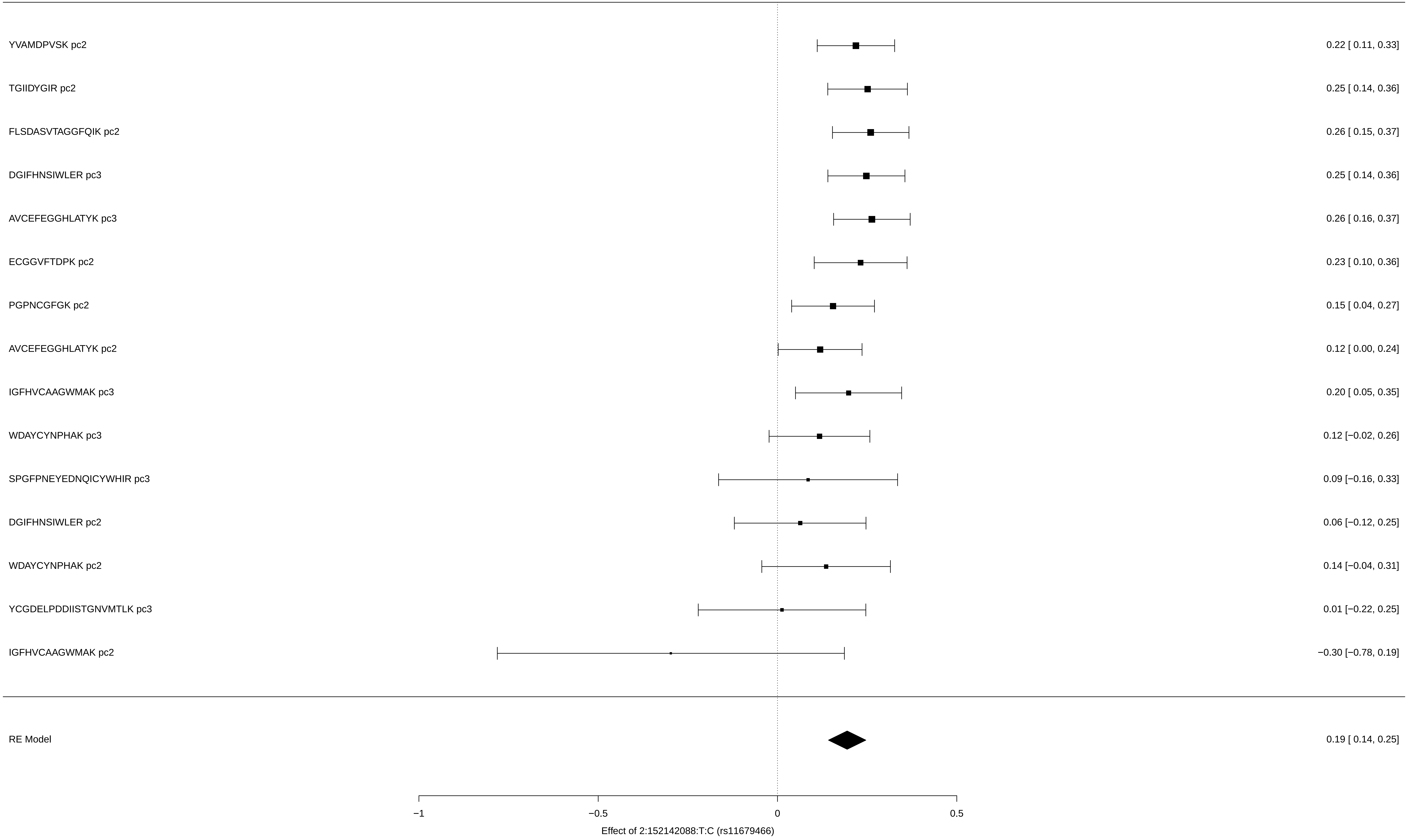

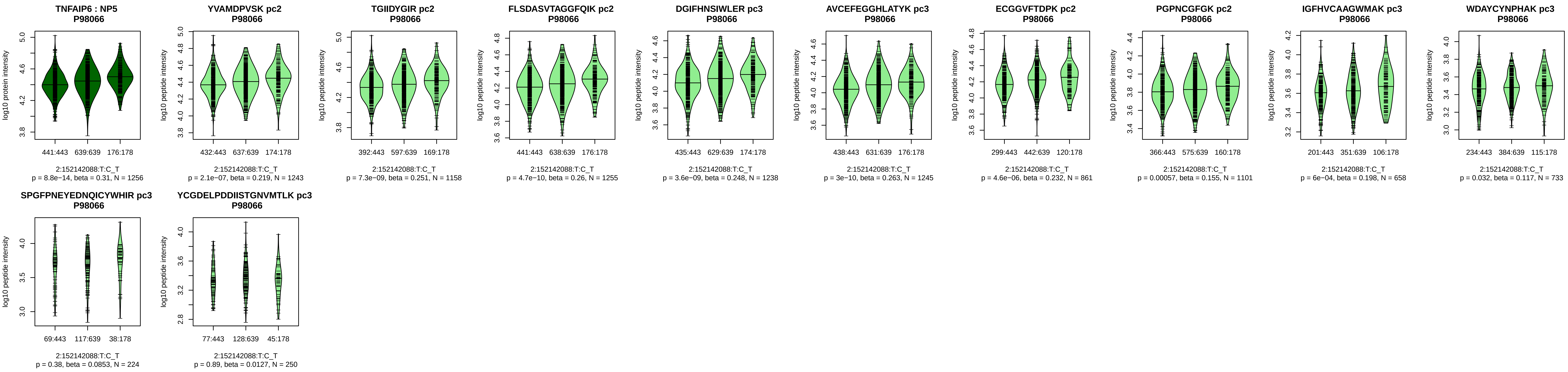

128Assay Target: TNFAIP6
UniProt: P98066
rsID: rs11679466
Target SNP: 2:152142088:T:C
Proxy SNP: 2:152142088:T:C
Tarkin log10(p): 13.1
Tarkin BETA: 0.31
Tarkin MSPA: 0.821
1243:1158:1255:1238:1245:861

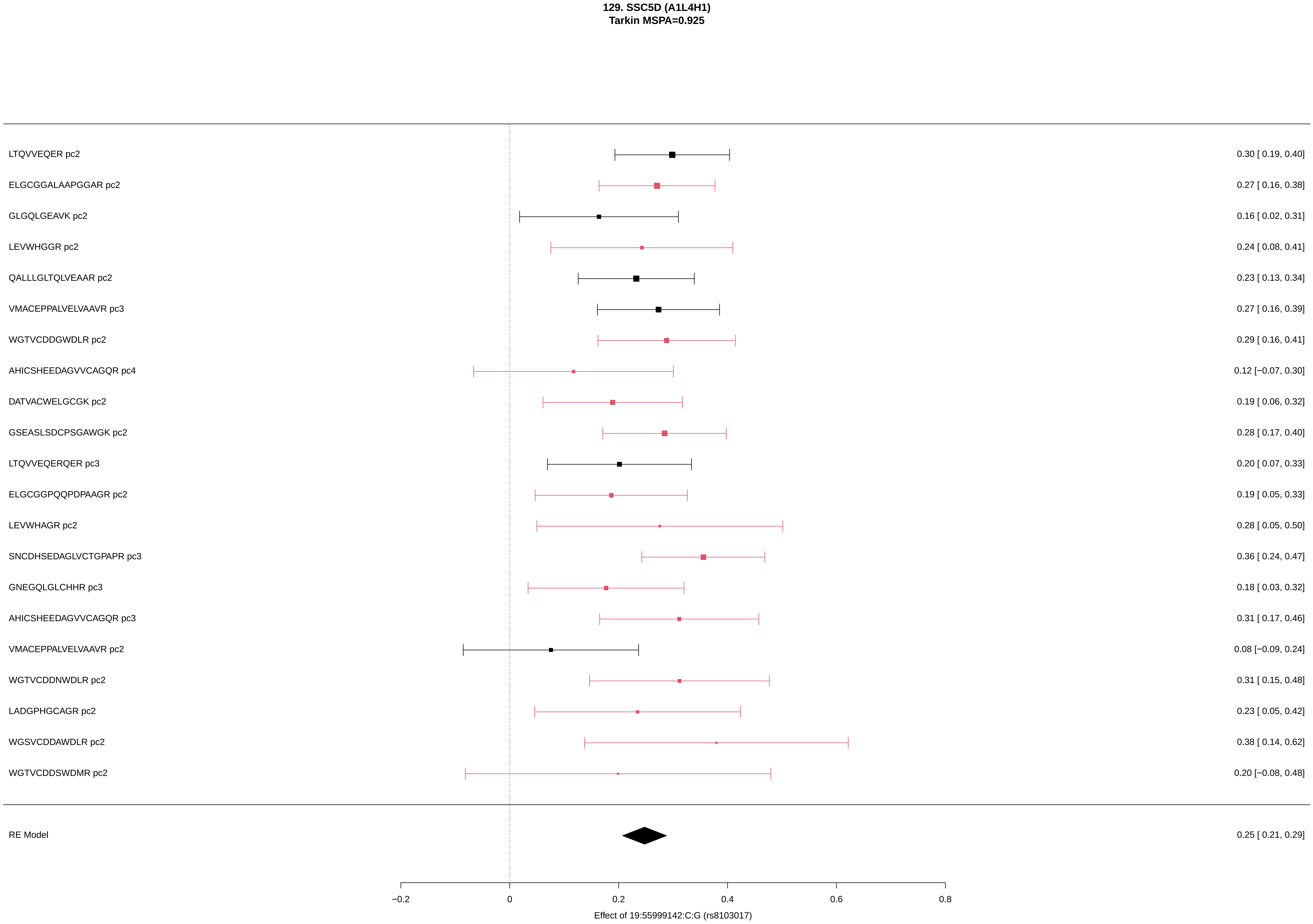
129. SSC5D (A1L4H1)
Tarkin MSPA=0.925
LTQVVEQER pc2
ELGCGGALAAPGGAR pc2
GLGQLGEAVK pc2
LEVWHGGR pc2
QALLLGLTQLVEAAR pc2
VMACEPPALVELVAAVR pc3
WGTVCDDGWDLR pc2
AHICSHEEDAGVVCAGQR pc4
DATVACWELGCGK pc2
GSEASLSDCPSGAWGK pc2
LTQVVEQERQER pc3
ELGCGGPQQPDPAAGR pc2
LEVWHAGR pc2
SNCDHSEDAGLVCTGPAPR pc3
GNEGQLGLCHHR pc3
AHICSHEEDAGVVCAGQR pc3
VMACEPPALVELVAAVR pc2
WGTVCDDNWDLR pc2
LADGPHGCAGR pc2
WGSVCDDAWDLR pc2
WGTVCDDSWDMR pc2
RE Model
0.30 [0.19, 0.40]
0.27 [0.16, 0.38]
0.16 [0.02, 0.31]
0.24 [0.08, 0.41]
0.23 [0.13, 0.34]
0.27 [0.16, 0.39]
0.29 [0.16, 0.41]
0.12 [−0.07, 0.30]
0.19 [0.06, 0.32]
0.28 [0.17, 0.40]
0.20 [0.07, 0.33]
0.19 [0.05, 0.33]
0.28 [0.05, 0.50]
0.36 [0.24, 0.47]
0.18 [0.03, 0.32]
0.31 [0.17, 0.46]
0.08 [−0.09, 0.24]
0.31 [0.15, 0.48]
0.23 [0.05, 0.42]
0.38 [0.14, 0.62]
0.20 [−0.08, 0.48]
0.25 [0.21, 0.29]
−0.2
0
0.2
0.4
0.6
0.8
Effect of 19:55999142:C:G (rs8103017)

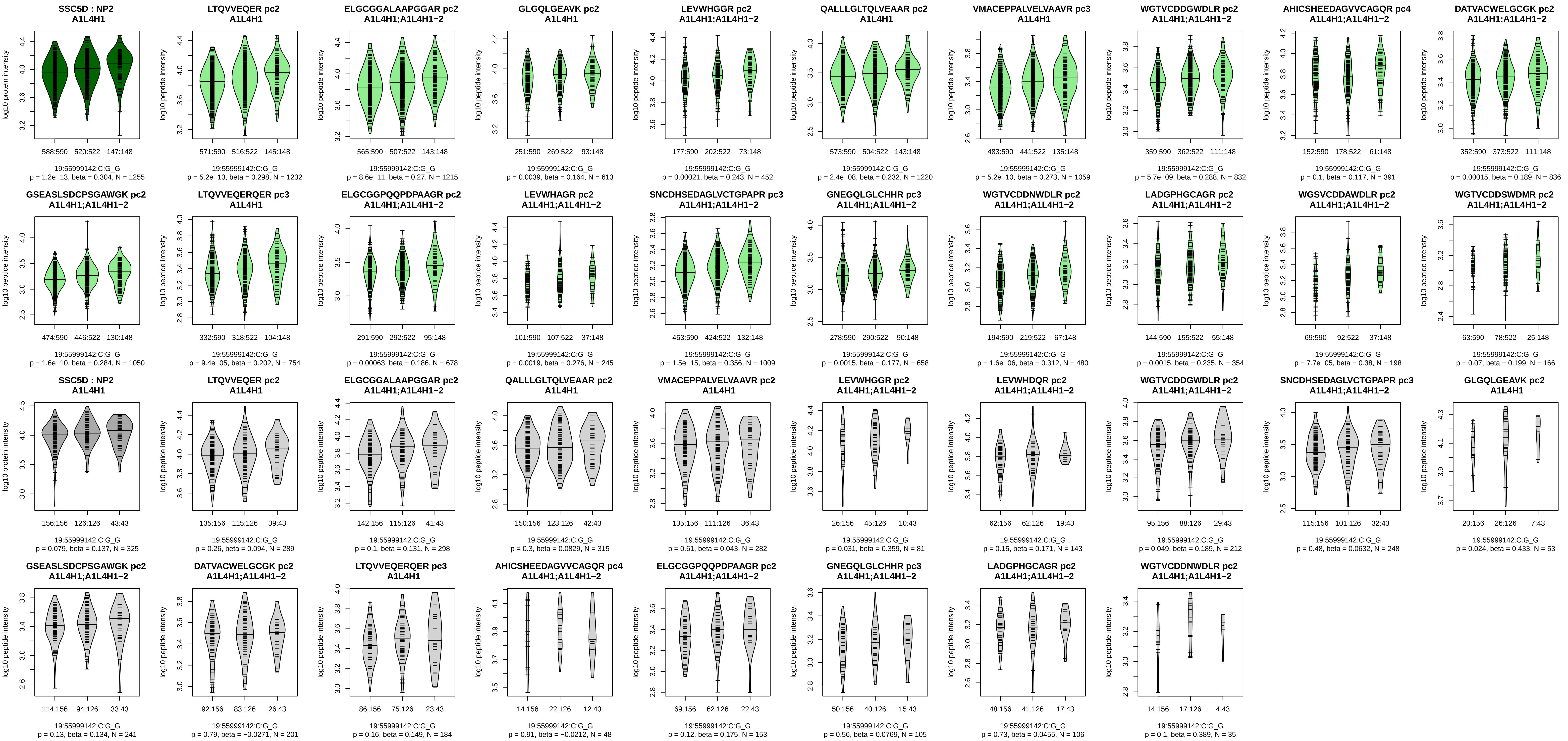

129Assay Target: SSC5D
UniProt: A1L4H1
rsID: rs8103017
Target SNP: 19:55999142:C:G
Proxy SNP: 19:55999142:C:G
Tarkin log10(p): 12.9
Tarkin BETA: 0.304
Tarkin MSPA: 0.925
1232:1215:613:452:1220:1059:8

130. TPSAB1 (Q15661)
Tarkin MSPA=1

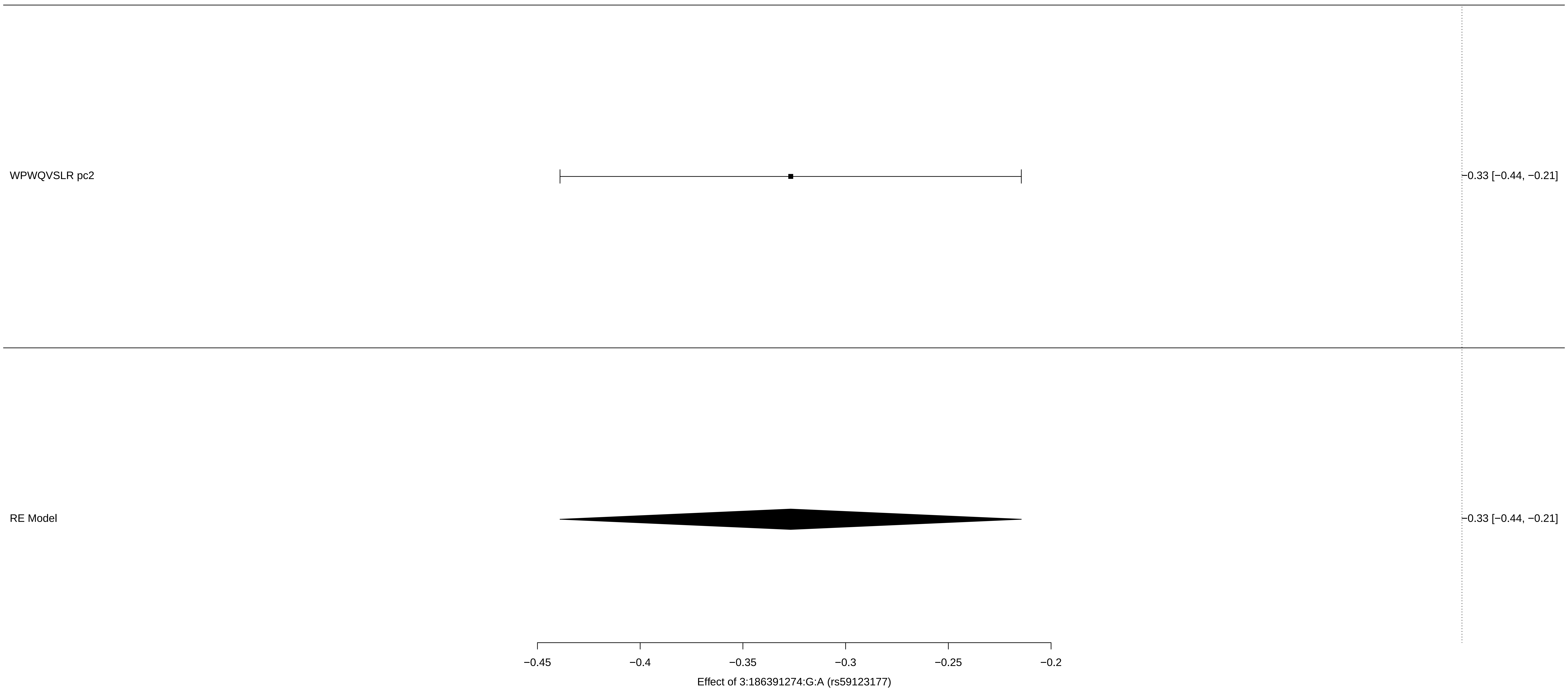

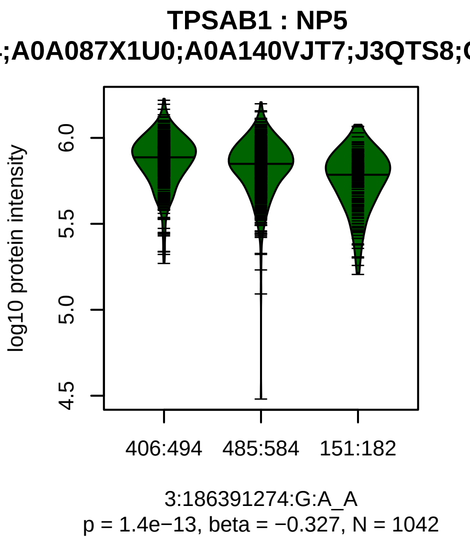

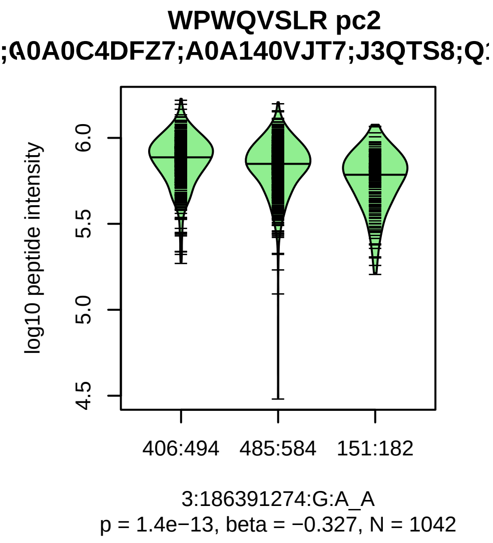

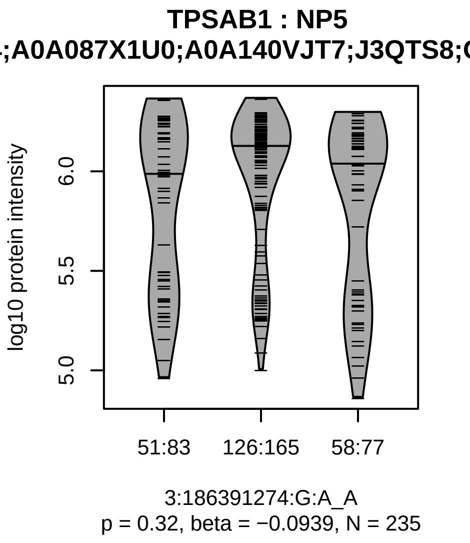

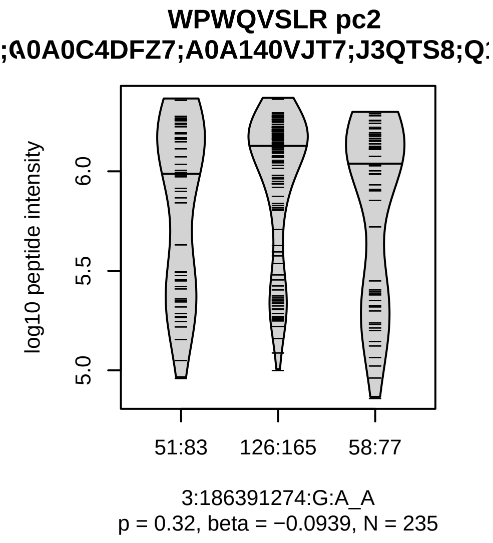

130Assay Target: TPSAB1
UniProt: Q15661
rsID: rs59123177
Target SNP: 3:186391274:G:A
Proxy SNP: 3:186391274:G:A
Tarkin log10(p): 12.9
Tarkin BETA: −0.327
Tarkin MSPA: 1
*
1042

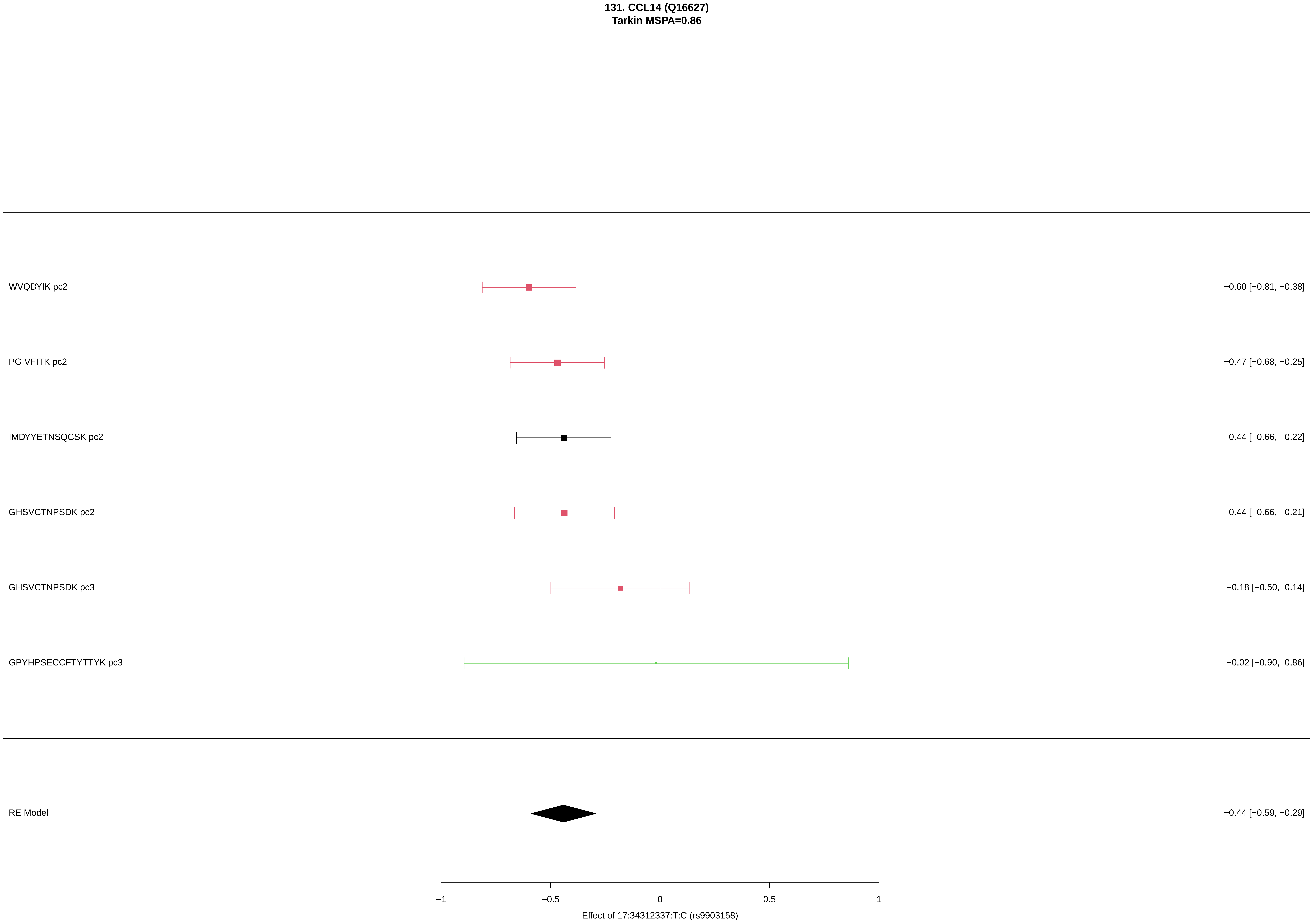
131. CCL14 (Q16627)
Tarkin MSPA=0.86
WVQDYIK pc2
−0.60 [−0.81, −0.38]
PGIVFITK pc2
−0.47 [−0.68, −0.25]
IMDYYETNSQCSK pc2
−0.44 [−0.66, −0.22]
GHSVCTNPSDK pc2
−0.44 [−0.66, −0.21]
GHSVCTNPSDK pc3
−0.18 [−0.50, 0.14]
GPYHPSECCFTYTTYK pc3
−0.02 [−0.90, 0.86]
RE Model
−0.44 [−0.59, −0.29]
−1
−0.5
0
0.5
1
Effect of 17:34312337:T:C (rs9903158)

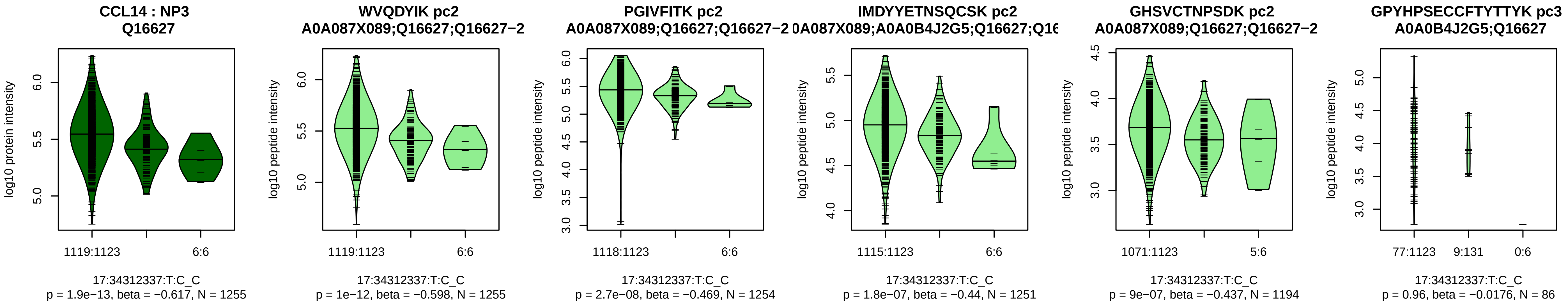

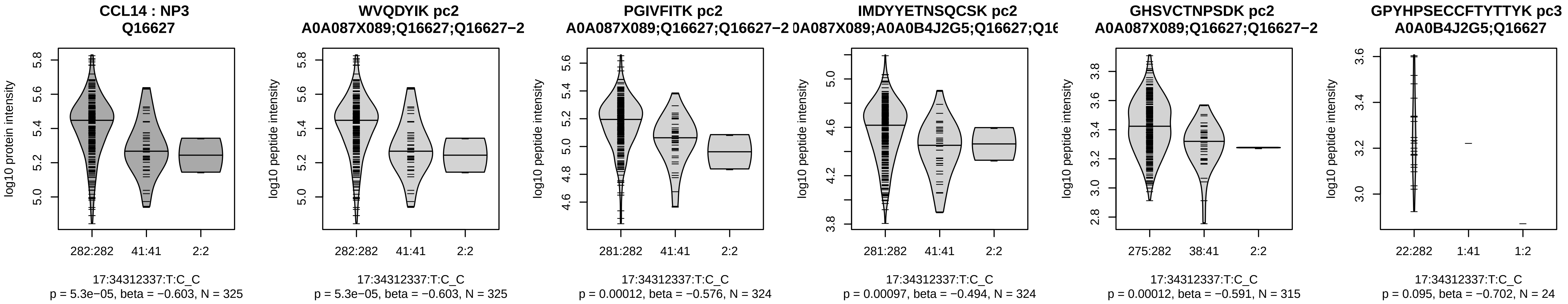

131Assay Target: CCL14
UniProt: Q16627
rsID: rs9903158
Target SNP: 17:34312337:T:C
Proxy SNP: 17:34312337:T:C
Tarkin log10(p): 12.7
Tarkin BETA: −0.617
Tarkin MSPA: 0.86
::*:*:–:–
1255:1254:1251:1194:722:86

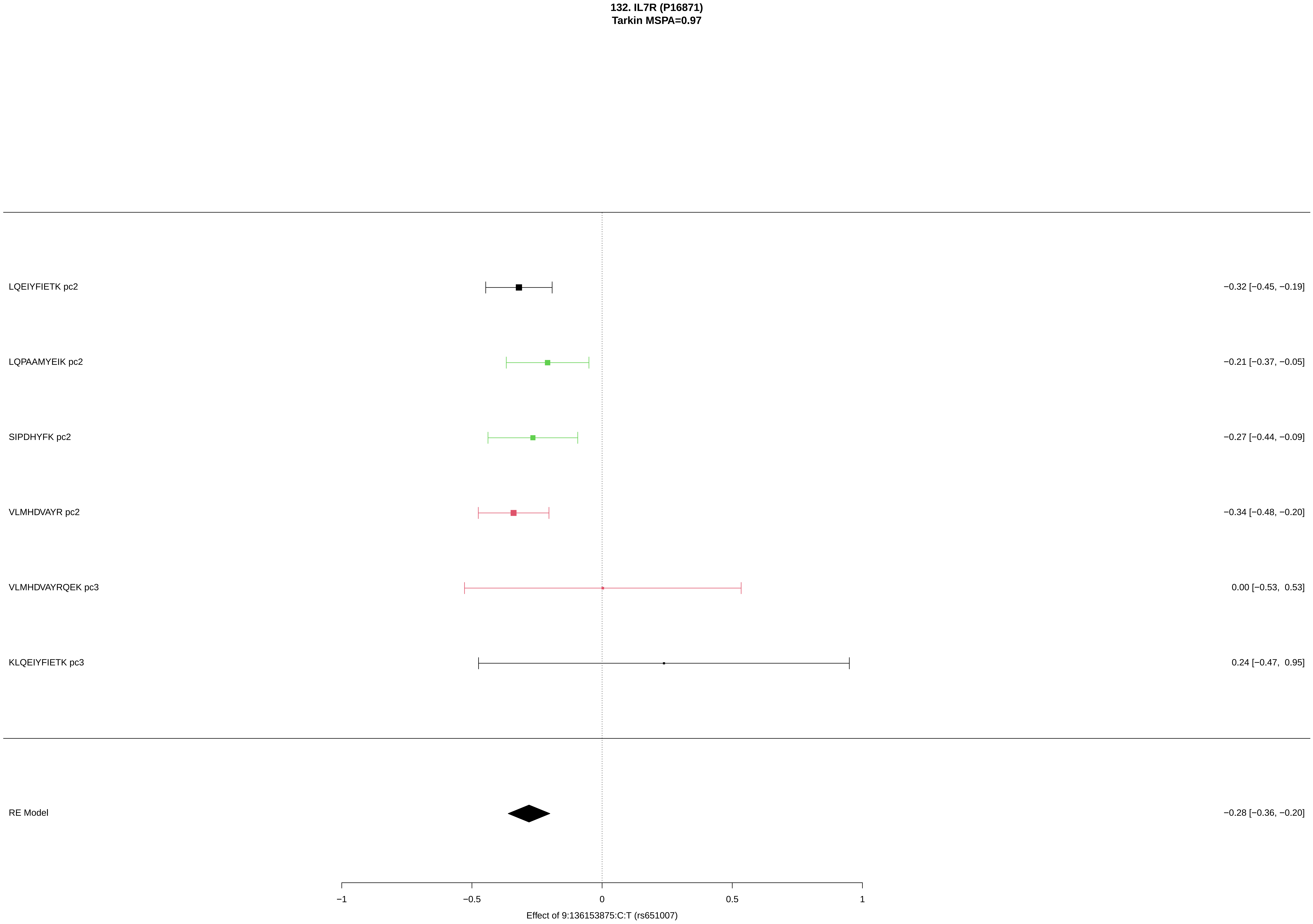
132. IL7R (P16871)
Tarkin MSPA=0.97
LQEIYFIETK pc2
−0.32 [−0.45, −0.19]
LQPAAMYEIK pc2
−0.21 [−0.37, −0.05]
SIPDHYFK pc2
−0.27 [−0.44, −0.09]
VLMHDVAYR pc2
−0.34 [−0.48, −0.20]
VLMHDVAYRQEK pc3
0.00 [−0.53, 0.53]
KLQEIYFIETK pc3
0.24 [−0.47, 0.95]
RE Model
−0.28 [−0.36, −0.20]
−1
−0.5
0
0.5
1
Effect of 9:136153875:C:T (rs651007)

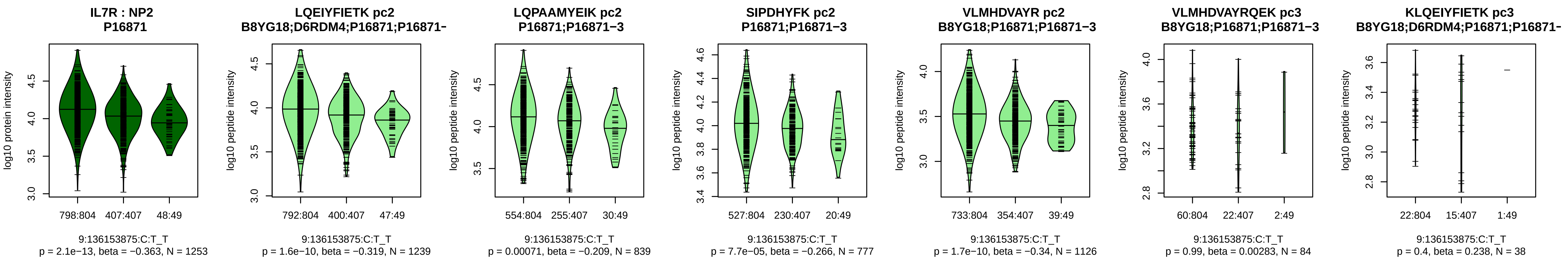

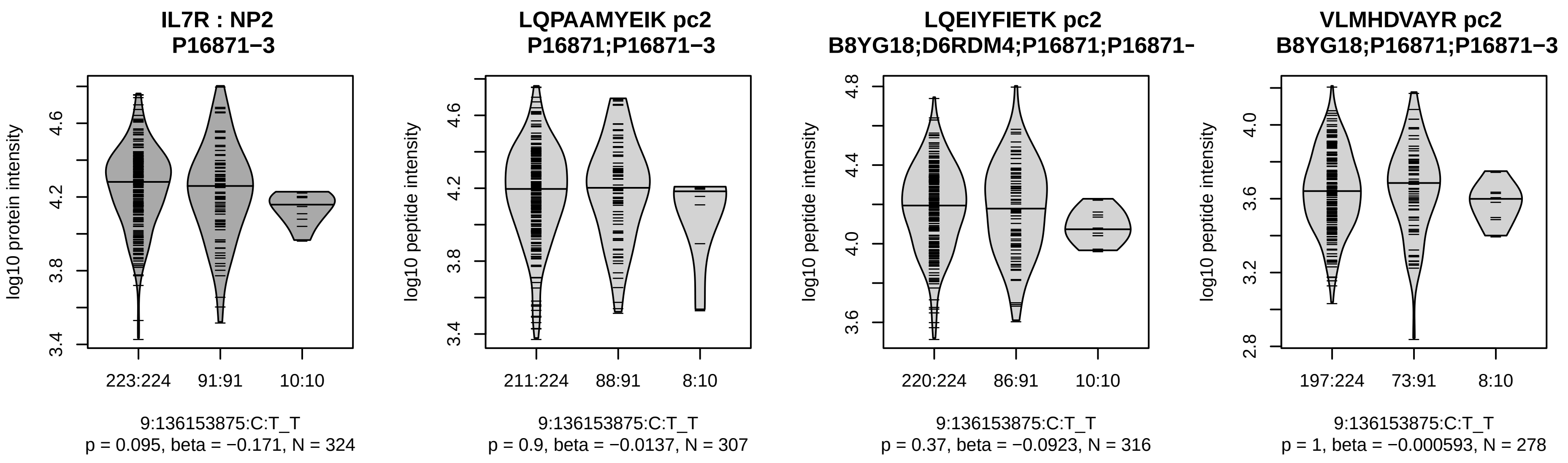

132Assay Target: IL7R
UniProt: P16871
rsID: rs651007
Target SNP: 9:136153875:C:T
Proxy SNP: 9:136153875:C:T
Tarkin log10(p): 12.7
Tarkin BETA: −0.363
Tarkin MSPA: 0.97
::*:*:–:–
1239:839:777:1126:84:38

133. TAC3 (Q9UHF0)
Tarkin MSPA=1

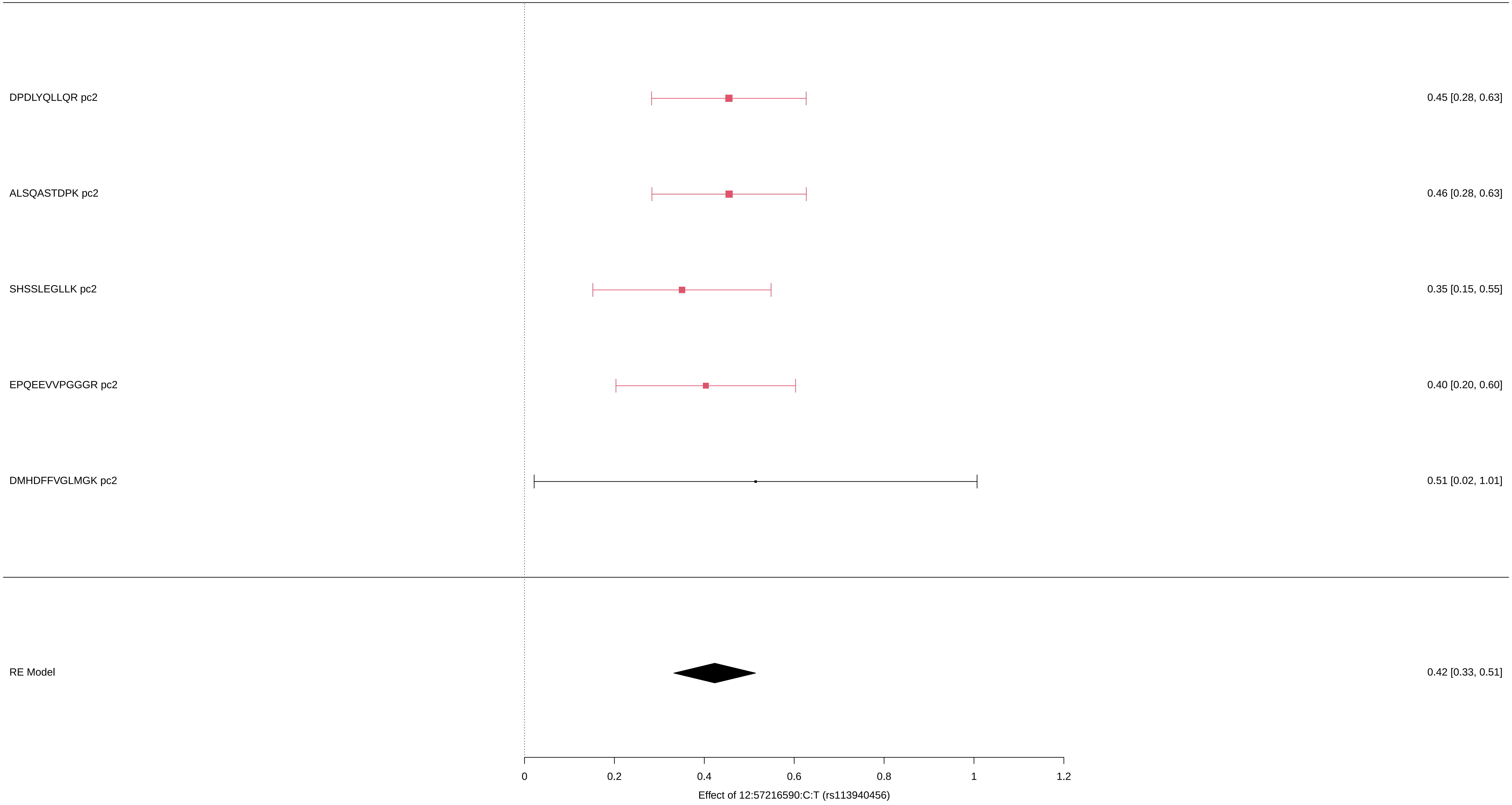

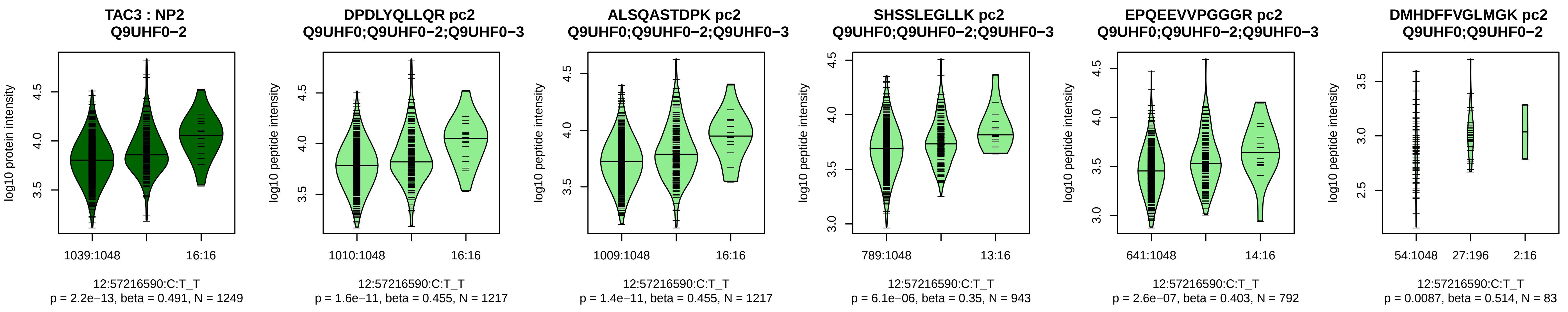

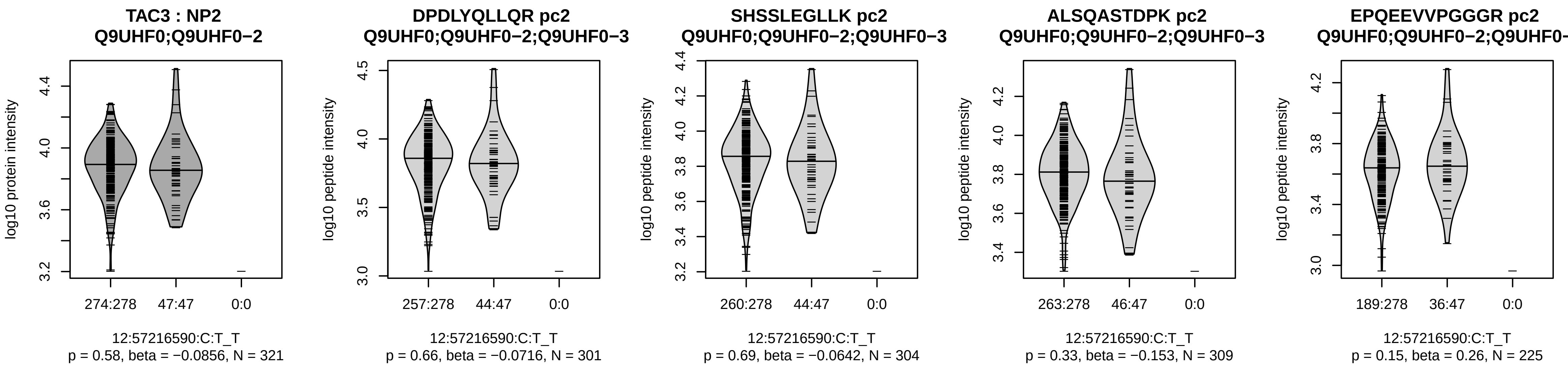

133Assay Target: TAC3
UniProt: Q9UHF0
rsID: rs113940456
Target SNP: 12:57216590:C:T
Proxy SNP: 12:57216590:C:T
Tarkin log10(p): 12.7
Tarkin BETA: 0.491
Tarkin MSPA: 1
::*:*:*
1217:1217:943:792:83

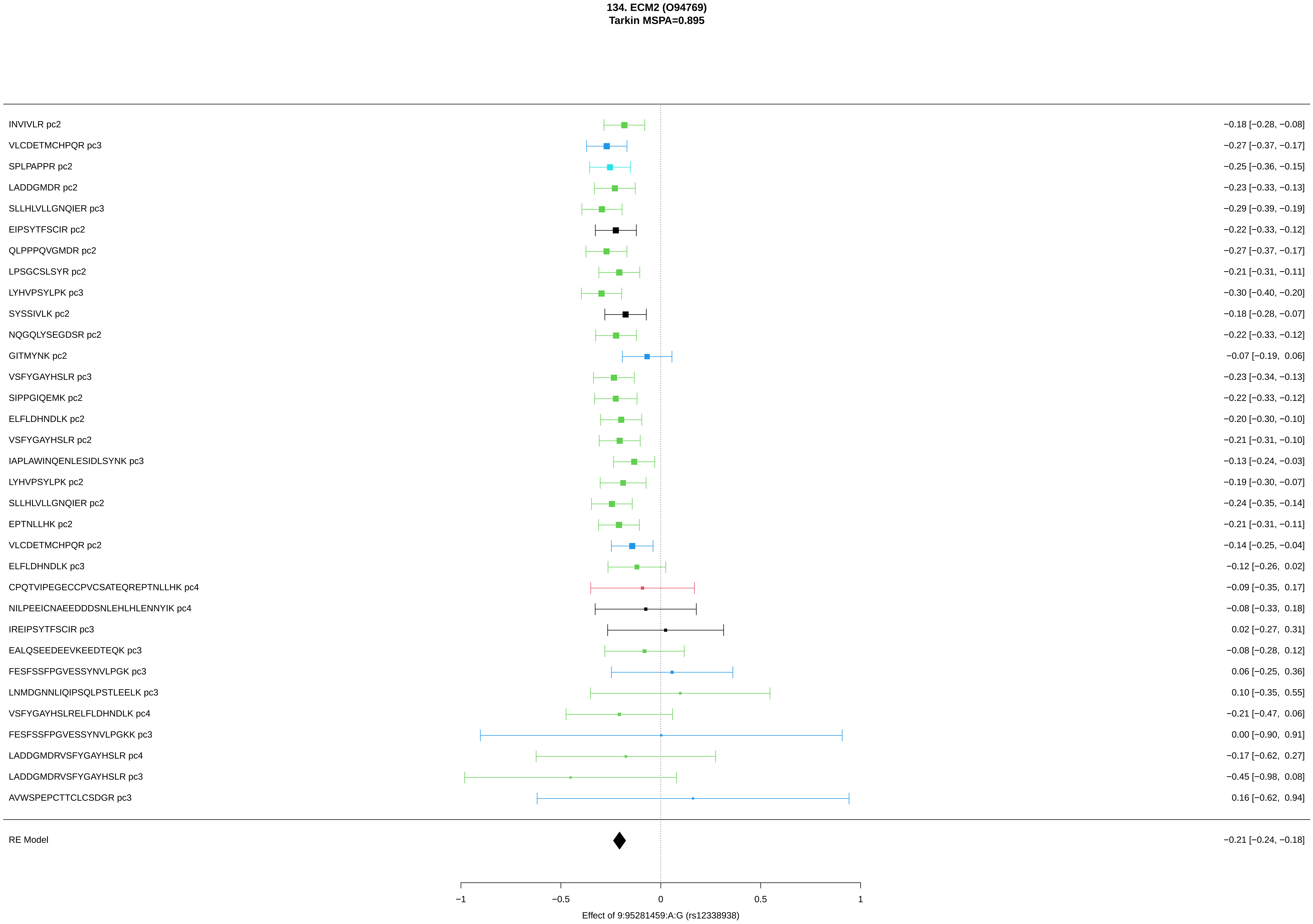
134. ECM2 (O94769)
Tarkin MSPA=0.895
INVIVLR pc2 −0.18 [−0.28, −0.08]
VLCDETMCHPQR pc3 −0.27 [−0.37, −0.17]
SPLPAPPR pc2 −0.25 [−0.36, −0.15]
LADDGMDR pc2 −0.23 [−0.33, −0.13]
SLLHLVLLGNQIER pc3 −0.29 [−0.39, −0.19]
EIPSYTFSCIR pc2 −0.22 [−0.33, −0.12]
QLPPPQVGMDR pc2 −0.27 [−0.37, −0.17]
LPSGCSLSYR pc2 −0.21 [−0.31, −0.11]
LYHVPSYLPK pc3 −0.30 [−0.40, −0.20]
SYSSIVLK pc2 −0.18 [−0.28, −0.07]
NQGQLYSEGDSR pc2 −0.22 [−0.33, −0.12]
GITMYNK pc2 −0.07 [−0.19, 0.06]
VSFYGAYHSLR pc3 −0.23 [−0.34, −0.13]
SIPPGIQEMK pc2 −0.22 [−0.33, −0.12]
ELFLDHNDLK pc2 −0.20 [−0.30, −0.10]
VSFYGAYHSLR pc2 −0.21 [−0.31, −0.10]
IAPLAWINQENLESIDLSYNK pc3 −0.13 [−0.24, −0.03]
LYHVPSYLPK pc2 −0.19 [−0.30, −0.07]
SLLHLVLLGNQIER pc2 −0.24 [−0.35, −0.14]
EPTNLLHK pc2 −0.21 [−0.31, −0.11]
VLCDETMCHPQR pc2 −0.14 [−0.25, −0.04]
ELFLDHNDLK pc3 −0.12 [−0.26, 0.02]
CPQTVIPEGECCPVCSATEQREPTNLLHK pc4 −0.09 [−0.35, 0.17]
NILPEEICNAEEDDDSNLEHLHLENNYIK pc4 −0.08 [−0.33, 0.18]
IREIPSYTFSCIR pc3 0.02 [−0.27, 0.31]
EALQSEEDEEVKEEDTEQK pc3 −0.08 [−0.28, 0.12]
FESFSSFPGVESSYNVLPGK pc3 0.06 [−0.25, 0.36]
LNMDGNNLIQIPSQLPSTLEELK pc3 0.10 [−0.35, 0.55]
VSFYGAYHSLRELFLDHNDLK pc4 −0.21 [−0.47, 0.06]
FESFSSFPGVESSYNVLPGKK pc3 0.00 [−0.90, 0.91]
LADDGMDRVSFYGAYHSLR pc4 −0.17 [−0.62, 0.27]
LADDGMDRVSFYGAYHSLR pc3 −0.45 [−0.98, 0.08]
AVWSPEPCTTCLCSDGR pc3 0.16 [−0.62, 0.94]
RE Model −0.21 [−0.24, −0.18]
−1
−0.5
0
0.5
1
Effect of 9:95281459:A:G (rs12338938)

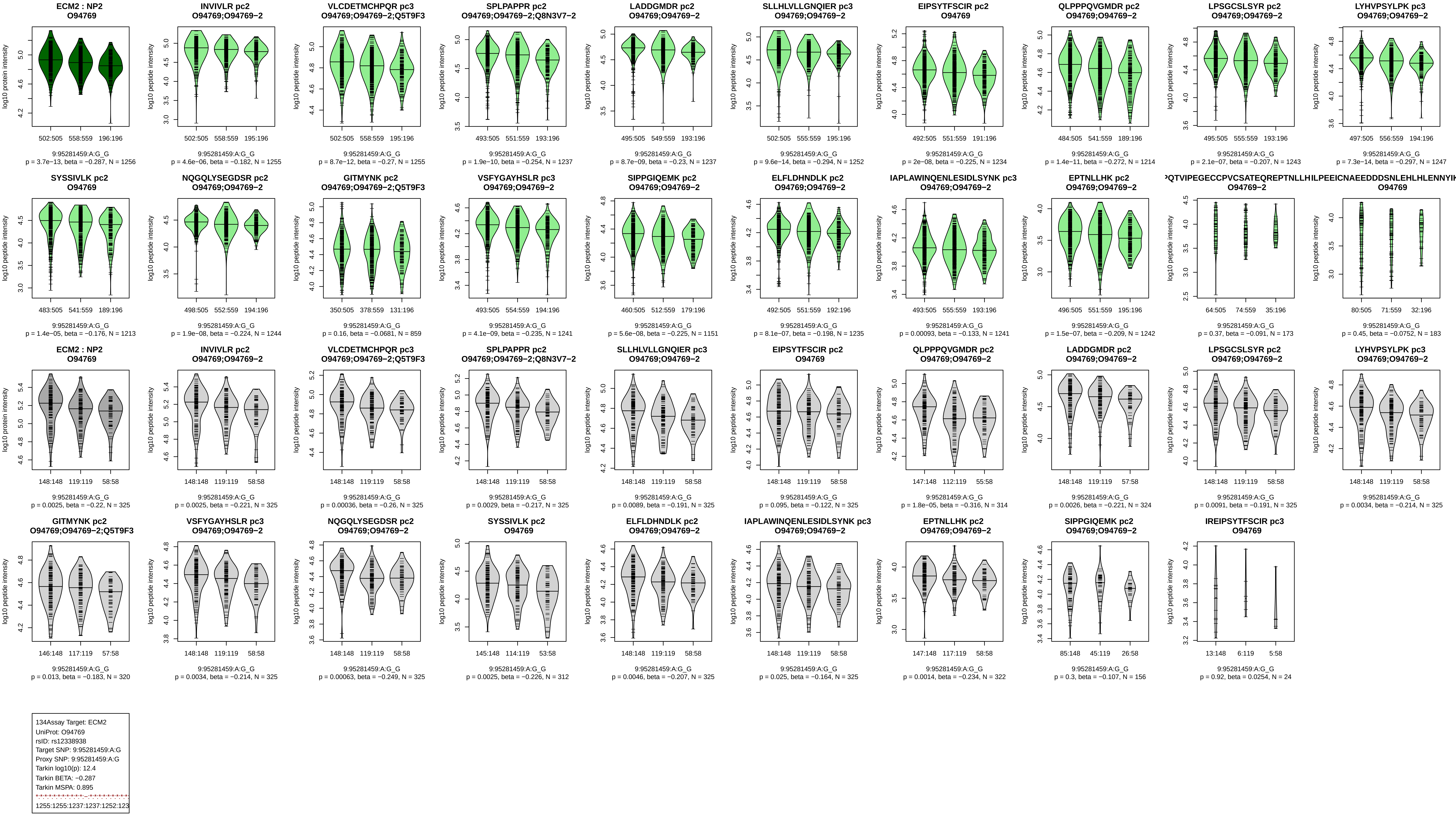
ECM2 : NP2 O94769
p = 3.7e-13, beta = −0.287, N = 1256
INVIVLR pc2 O94769;O94769−2
p = 4.6e−06, beta = −0.182, N = 1255
VLCDETMCHPQR pc3 O94769;O94769−2;Q5T9F3
p = 8.7e−12, beta = −0.27, N = 1255
SPLPAPPR pc2 O94769;O94769−2;Q8N3V7−2
p = 1.9e−10, beta = −0.254, N = 1237
LADDGMDR pc2 O94769;O94769−2
p = 8.7e−09, beta = −0.23, N = 1237
SLLHLVLLGNQIER pc3 O94769;O94769−2
p = 9.6e−14, beta = −0.294, N = 1252
EIPSYTFSCIR pc2 O94769
p = 2e−08, beta = −0.225, N = 1234
QLPPPQVGMDR pc2 O94769;O94769−2
p = 1.4e−11, beta = −0.272, N = 1214
LPSGCSLSYR pc2 O94769;O94769−2
p = 2.1e−07, beta = −0.207, N = 1243
LYHVPSYLPK pc3 O94769;O94769−2
p = 7.3e−14, beta = −0.297, N = 1247
SYSSIVLK pc2 O94769
p = 1.4e−05, beta = −0.176, N = 1213
NQGQLYSEGDSR pc2 O94769;O94769−2
p = 1.9e−08, beta = −0.224, N = 1244
GITMYNK pc2 O94769;O94769−2;Q5T9F3
p = 0.16, beta = −0.0681, N = 859
VSFYGAYHSLR pc3 O94769;O94769−2
p = 4.1e−09, beta = −0.235, N = 1241
SIPPGIQEMK pc2 O94769;O94769−2
p = 5.6e−08, beta = −0.225, N = 1151
ELFLDHNDLK pc2 O94769;O94769−2
p = 8.1e−07, beta = −0.198, N = 1235
IAPLAWINQENLESIDLSYNK pc3 O94769;O94769−2
p = 0.00093, beta = −0.133, N = 1241
EPTNLLHK pc2 O94769;O94769−2
p = 1.5e−07, beta = −0.209, N = 1242
QTVIPEGECCPVCSATEQREPTNLLH O94769−2
p = 0.37, beta = −0.091, N = 173
ILPEEICNAEEDDSNLEHLHLENNYIK O94769
p = 0.45, beta = −0.0752, N = 183
ECM2 : NP2 O94769
p = 0.0025, beta = −0.22, N = 325
INVIVLR pc2 O94769;O94769−2
p = 0.0025, beta = −0.221, N = 325
VLCDETMCHPQR pc3 O94769;O94769−2;Q5T9F3
p = 0.00036, beta = −0.26, N = 325
SPLPAPPR pc2 O94769;O94769−2;Q8N3V7−2
p = 0.0029, beta = −0.217, N = 325
SLLHLVLLGNQIER pc3 O94769;O94769−2
p = 0.0089, beta = −0.191, N = 325
EIPSYTFSCIR pc2 O94769
p = 0.095, beta = −0.122, N = 325
QLPPPQVGMDR pc2 O94769;O94769−2
p = 1.8e−05, beta = −0.316, N = 314
LADDGMDR pc2 O94769;O94769−2
p = 0.0026, beta = −0.221, N = 324
LPSGCSLSYR pc2 O94769;O94769−2
p = 0.0091, beta = −0.191, N = 325
LYHVPSYLPK pc3 O94769;O94769−2
p = 0.0034, beta = −0.214, N = 325
GITMYNK pc2 O94769;O94769−2;Q5T9F3
p = 0.013, beta = −0.183, N = 320
VSFYGAYHSLR pc3 O94769;O94769−2
p = 0.0034, beta = −0.214, N = 325
NQGQLYSEGDSR pc2 O94769;O94769−2
p = 0.00063, beta = −0.249, N = 325
SYSSIVLK pc2 O94769
p = 0.0025, beta = −0.226, N = 312
ELFLDHNDLK pc2 O94769;O94769−2
p = 0.0046, beta = −0.207, N = 325
IAPLAWINQENLESIDLSYNK pc3 O94769;O94769−2
p = 0.025, beta = −0.164, N = 325
EPTNLLHK pc2 O94769;O94769−2
p = 0.0014, beta = −0.234, N = 322
SIPPGIQEMK pc2 O94769;O94769−2
p = 0.3, beta = −0.107, N = 156
IREIPSYTFSCIR pc3 O94769
p = 0.92, beta = 0.0254, N = 24
9:95281459:A:G_G
134Assay Target: ECM2
UniProt: O94769
rsID: rs12338938
Target SNP: 9:95281459:A:G
Proxy SNP: 9:95281459:A:G
Tarkin log10(p): 12.4
Tarkin BETA: −0.287
Tarkin MSPA: 0.895
1255:1255:1237:1237:1252:123

135. ITIH4 (Q14624)
Tarkin MSPA=0.142

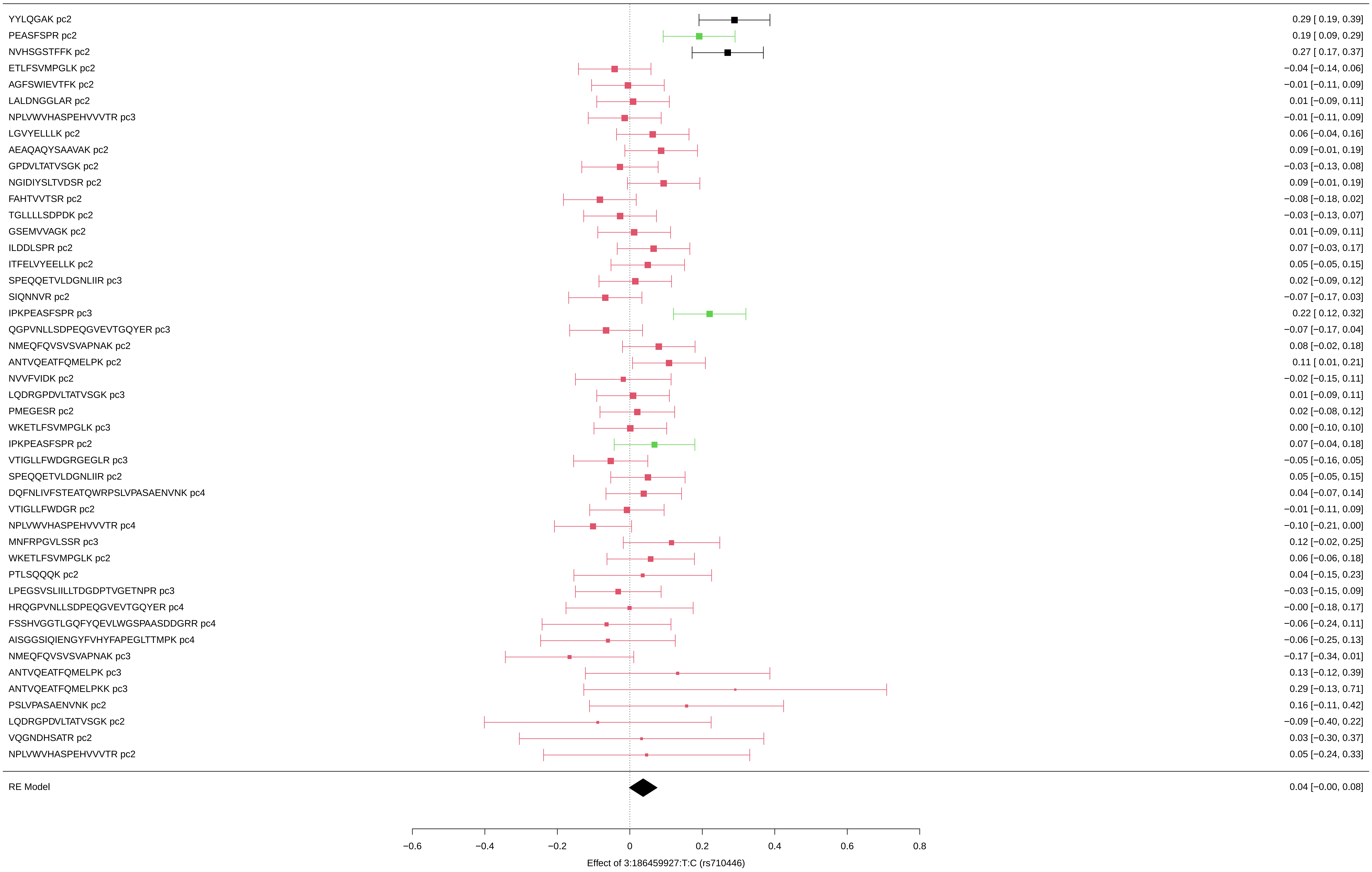

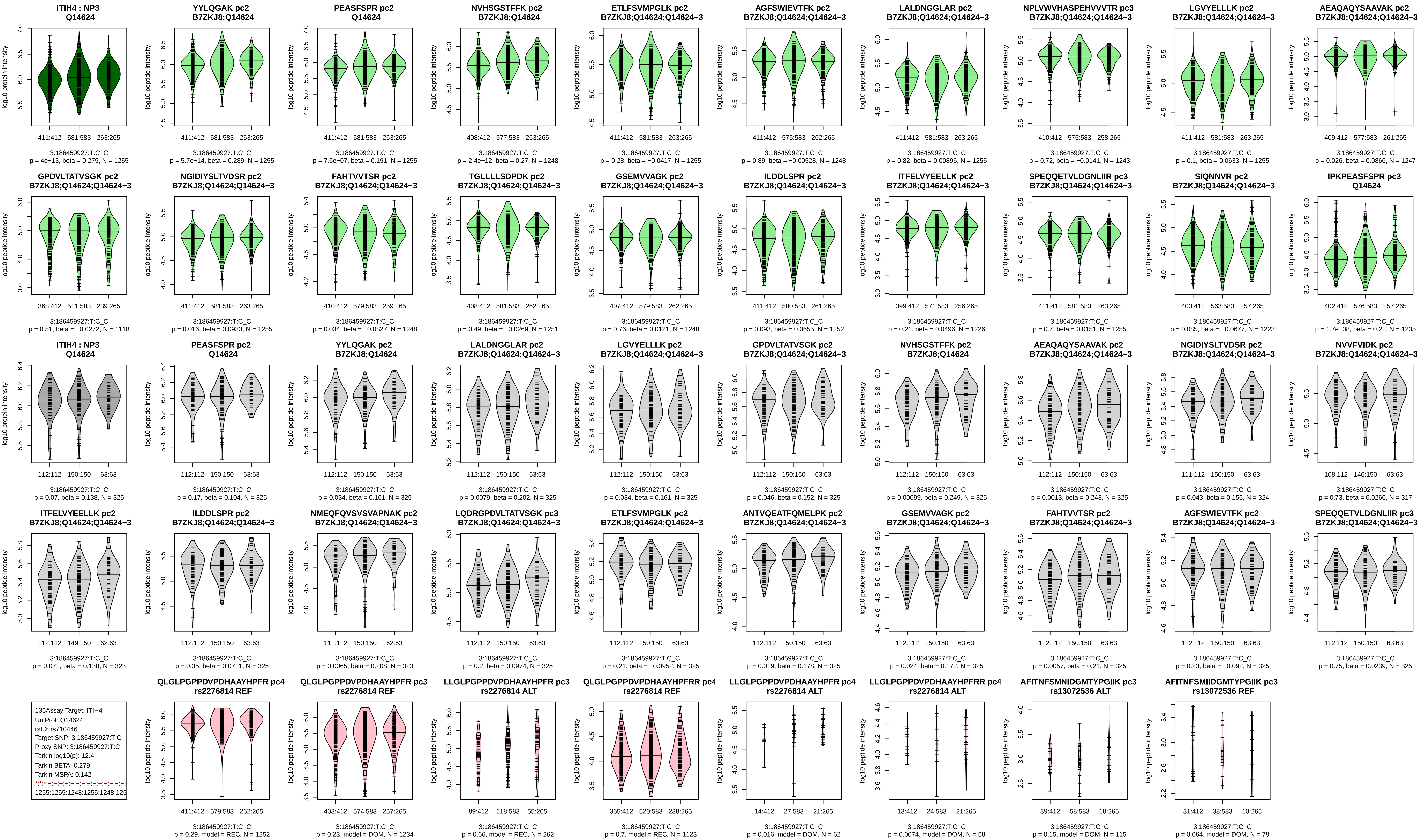

ITIH4 : NP3 Q14624
log10 protein intensity
411:412 581:583 263:265
3:186459927:T:C_C
p = 4e−13, beta = 0.279, N = 1255
YYLQGAK pc2 B7ZKJ8;Q14624
log10 peptide intensity
411:412 581:583 263:265
3:186459927:T:C_C
p = 5.7e−14, beta = 0.289, N = 1255
PEASFSPR pc2 Q14624
411:412 581:583 263:265
3:186459927:T:C_C
p = 7.6e−07, beta = 0.191, N = 1255
NVHSGSTFFK pc2 B7ZKJ8;Q14624
408:412 577:583 263:265
3:186459927:T:C_C
p = 2.4e−12, beta = 0.27, N = 1248
ETLFSVMPGLK pc2 B7ZKJ8;Q14624;Q14624−3
411:412 581:583 263:265
3:186459927:T:C_C
p = 0.28, beta = −0.0417, N = 1255
AGFSWIEVTFK pc2 B7ZKJ8;Q14624;Q14624−3
411:412 575:583 262:265
3:186459927:T:C_C
p = 0.89, beta = −0.00528, N = 1248
LALDNGGLAR pc2 B7ZKJ8;Q14624;Q14624−3
411:412 581:583 263:265
3:186459927:T:C_C
p = 0.82, beta = 0.00896, N = 1255
NPLVWVHASPEHVVVTR pc3 B7ZKJ8;Q14624;Q14624−3
410:412 575:583 258:265
3:186459927:T:C_C
p = 0.72, beta = −0.0141, N = 1243
LGVYELLLK pc2 B7ZKJ8;Q14624;Q14624−3
411:412 581:583 263:265
3:186459927:T:C_C
p = 0.1, beta = 0.0633, N = 1255
AEAQAQYSAAVAK pc2 B7ZKJ8;Q14624;Q14624−3
409:412 577:583 261:265
3:186459927:T:C_C
p = 0.026, beta = 0.0866, N = 1247
GPDVLTATVSGK pc2 B7ZKJ8;Q14624;Q14624−3
368:412 511:583 239:265
3:186459927:T:C_C
p = 0.51, beta = −0.0272, N = 1118
NGIDIYSLTVDSR pc2 B7ZKJ8;Q14624;Q14624−3
411:412 581:583 263:265
3:186459927:T:C_C
p = 0.016, beta = 0.0933, N = 1255
FAHTVVTSR pc2 B7ZKJ8;Q14624;Q14624−3
410:412 579:583 259:265
3:186459927:T:C_C
p = 0.034, beta = −0.0827, N = 1248
TGLLLLSDPDK pc2 B7ZKJ8;Q14624;Q14624−3
408:412 581:583 262:265
3:186459927:T:C_C
p = 0.49, beta = −0.0269, N = 1251
GSEMVVAGK pc2 B7ZKJ8;Q14624;Q14624−3
407:412 579:583 262:265
3:186459927:T:C_C
p = 0.76, beta = 0.0121, N = 1248
ILDDLSPR pc2 B7ZKJ8;Q14624;Q14624−3
411:412 580:583 261:265
3:186459927:T:C_C
p = 0.093, beta = 0.0655, N = 1252
ITFELVYEELLK pc2 B7ZKJ8;Q14624;Q14624−3
399:412 571:583 256:265
3:186459927:T:C_C
p = 0.21, beta = 0.0496, N = 1226
SPEQQETVLDGNLIIR pc3 B7ZKJ8;Q14624;Q14624−3
411:412 581:583 263:265
3:186459927:T:C_C
p = 0.7, beta = 0.0151, N = 1255
SIQNNVR pc2 B7ZKJ8;Q14624;Q14624−3
403:412 563:583 257:265
3:186459927:T:C_C
p = 0.085, beta = −0.0677, N = 1223
IPKPEASFSPR pc3 Q14624
402:412 576:583 257:265
3:186459927:T:C_C
p = 1.7e−08, beta = 0.22, N = 1235
ITIH4 : NP3 Q14624
112:112 150:150 63:63
3:186459927:T:C_C
p = 0.07, beta = 0.138, N = 325
PEASFSPR pc2 Q14624
112:112 150:150 63:63
3:186459927:T:C_C
p = 0.17, beta = 0.104, N = 325
YYLQGAK pc2 B7ZKJ8;Q14624
112:112 150:150 63:63
3:186459927:T:C_C
p = 0.034, beta = 0.161, N = 325
LALDNGGLAR pc2 B7ZKJ8;Q14624;Q14624−3
112:112 150:150 63:63
3:186459927:T:C_C
p = 0.0079, beta = 0.202, N = 325
LGVYELLLK pc2 B7ZKJ8;Q14624;Q14624−3
112:112 150:150 63:63
3:186459927:T:C_C
p = 0.034, beta = 0.161, N = 325
GPDVLTATVSGK pc2 B7ZKJ8;Q14624;Q14624−3
112:112 150:150 63:63
3:186459927:T:C_C
p = 0.046, beta = 0.152, N = 325
NVHSGSTFFK pc2 B7ZKJ8;Q14624
112:112 150:150 63:63
3:186459927:T:C_C
p = 0.00099, beta = 0.249, N = 325
AEAQAQYSAAVAK pc2 B7ZKJ8;Q14624;Q14624−3
112:112 150:150 63:63
3:186459927:T:C_C
p = 0.0013, beta = 0.243, N = 325
NGIDIYSLTVDSR pc2 B7ZKJ8;Q14624;Q14624−3
111:112 150:150 63:63
3:186459927:T:C_C
p = 0.043, beta = 0.155, N = 324
NVVFVIDK pc2 B7ZKJ8;Q14624;Q14624−3
108:112 146:150 63:63
3:186459927:T:C_C
p = 0.73, beta = 0.0266, N = 317
ITFELVYEELLK pc2 B7ZKJ8;Q14624;Q14624−3
112:112 149:150 62:63
3:186459927:T:C_C
p = 0.071, beta = 0.138, N = 323
ILDDLSPR pc2 B7ZKJ8;Q14624;Q14624−3
112:112 150:150 63:63
3:186459927:T:C_C
p = 0.35, beta = 0.0711, N = 325
NMEQFQVSVSVAPNAK pc2 B7ZKJ8;Q14624;Q14624−3
111:112 150:150 62:63
3:186459927:T:C_C
p = 0.0065, beta = 0.208, N = 323
LQDRGPDVLTATVSGK pc3 B7ZKJ8;Q14624;Q14624−3
112:112 150:150 63:63
3:186459927:T:C_C
p = 0.2, beta = 0.0974, N = 325
ETLFSVMPGLK pc2 B7ZKJ8;Q14624;Q14624−3
112:112 150:150 63:63
3:186459927:T:C_C
p = 0.21, beta = −0.0952, N = 325
ANTVQEATFQMELPK pc2 B7ZKJ8;Q14624;Q14624−3
112:112 150:150 63:63
3:186459927:T:C_C
p = 0.019, beta = 0.178, N = 325
GSEMVVAGK pc2 B7ZKJ8;Q14624;Q14624−3
112:112 150:150 63:63
3:186459927:T:C_C
p = 0.024, beta = 0.172, N = 325
FAHTVVTSR pc2 B7ZKJ8;Q14624;Q14624−3
112:112 150:150 63:63
3:186459927:T:C_C
p = 0.0057, beta = 0.21, N = 325
AGFSWIEVTFK pc2 B7ZKJ8;Q14624;Q14624−3
112:112 150:150 63:63
3:186459927:T:C_C
p = 0.23, beta = −0.092, N = 325
SPEQQETVLDGNLIIR pc3 B7ZKJ8;Q14624;Q14624−3
112:112 150:150 63:63
3:186459927:T:C_C
p = 0.75, beta = 0.0239, N = 325
135Assay Target: ITIH4
UniProt: Q14624
rsID: rs710446
Target SNP: 3:186459927:T:C
Proxy SNP: 3:186459927:T:C
Tarkin log10(p): 12.4
Tarkin BETA: 0.279
Tarkin MSPA: 0.142
1255:1255:1248:1255:1248:125
QLGLPGPPDVPDHAAYHPFR pc4 rs2276814 REF
411:412 579:583 262:265
3:186459927:T:C_C
p = 0.29, model = REC, N = 1252
QLGLPGPPDVPDHAAYHPFR pc3 rs2276814 REF
403:412 574:583 257:265
3:186459927:T:C_C
p = 0.23, model = DOM, N = 1234
LLGLPGPPDVPDHAAYHPFR pc3 rs2276814 ALT
89:412 118:583 55:265
3:186459927:T:C_C
p = 0.66, model = REC, N = 262
QLGLPGPPDVPDHAAYHPFRR pc4 rs2276814 REF
365:412 520:583 238:265
3:186459927:T:C_C
p = 0.7, model = REC, N = 1123
LLGLPGPPDVPDHAAYHPFR pc4 rs2276814 ALT
14:412 27:583 21:265
3:186459927:T:C_C
p = 0.016, model = DOM, N = 62
LLGLPGPPDVPDHAAYHPFRR pc4 rs2276814 ALT
13:412 24:583 21:265
3:186459927:T:C_C
p = 0.0074, model = DOM, N = 58
AFITNFSMNIDGMTYPGIIK pc3 rs13072536 ALT
39:412 58:583 18:265
3:186459927:T:C_C
p = 0.15, model = DOM, N = 115
AFITNFSMIIDGMTYPGIIK pc3 rs13072536 REF
31:412 38:583 10:265
3:186459927:T:C_C
p = 0.064, model = DOM, N = 79

136. CNDP2 (Q96KP4)
Tarkin MSPA=0.813

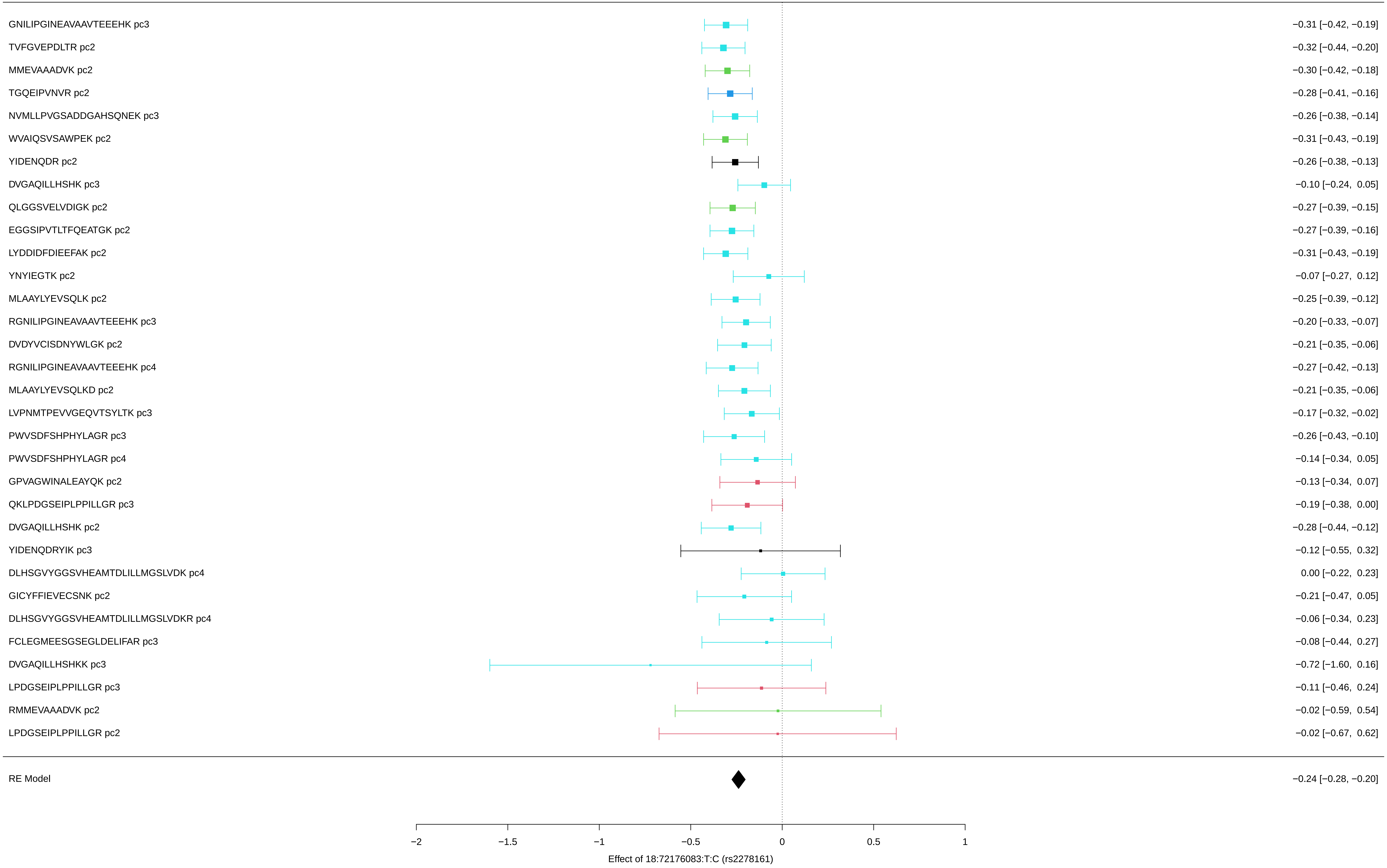

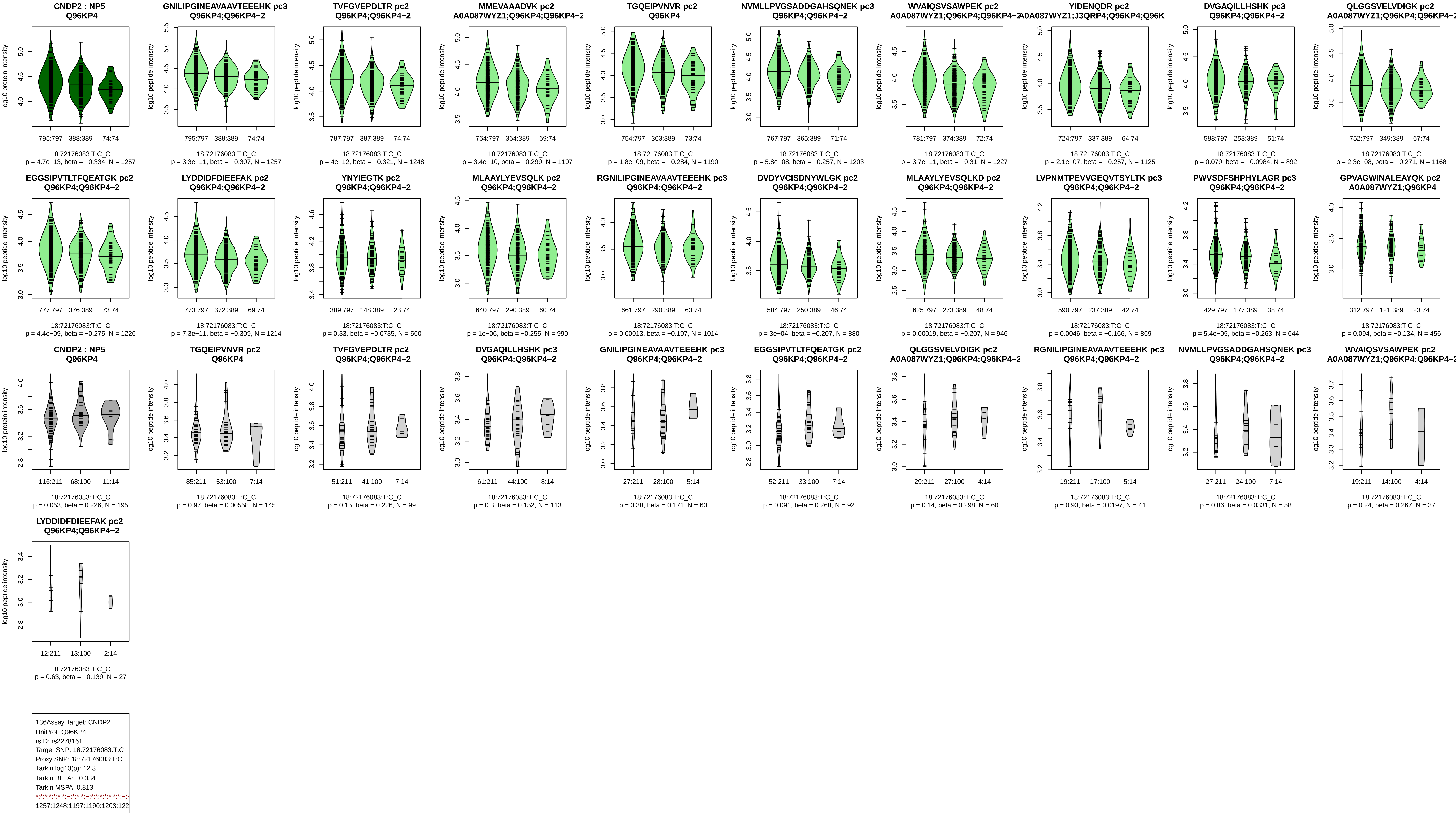
CNDP2 : NP5
Q96KP4
795:797 388:389 74:74
18:72176083:T:C_C
p = 4.7e−13, beta = −0.334, N = 1257
GNILIPGINEAVAAVTEEEHK pc3
Q96KP4;Q96KP4−2
795:797 388:389 74:74
18:72176083:T:C_C
p = 3.3e−11, beta = −0.307, N = 1257
TVFGVEPDLTR pc2
Q96KP4;Q96KP4−2
787:797 387:389 74:74
18:72176083:T:C_C
p = 4e−12, beta = −0.321, N = 1248
MMEVAAADVK pc2
A0A087WYZ1;Q96KP4;Q96KP4−2
764:797 364:389 69:74
18:72176083:T:C_C
p = 3.4e−10, beta = −0.299, N = 1197
TGQEIPVNVR pc2
Q96KP4
754:797 363:389 73:74
18:72176083:T:C_C
p = 1.8e−09, beta = −0.284, N = 1190
NVMLLPVGSADDGAHSQNEK pc3
Q96KP4;Q96KP4−2
767:797 365:389 71:74
18:72176083:T:C_C
p = 5.8e−08, beta = −0.257, N = 1203
WVAIQSVSAWPEK pc2
A0A087WYZ1;Q96KP4;Q96KP4−2
781:797 374:389 72:74
18:72176083:T:C_C
p = 3.7e−11, beta = −0.31, N = 1227
YIDENQDR pc2
A0A087WYZ1;J3QRP4;Q96KP4;Q96K
724:797 337:389 64:74
18:72176083:T:C_C
p = 2.1e−07, beta = −0.257, N = 1125
DVGAQILLHSHK pc3
Q96KP4;Q96KP4−2
588:797 253:389 51:74
18:72176083:T:C_C
p = 0.079, beta = −0.0984, N = 892
QLGGSVELVDIGK pc2
A0A087WYZ1;Q96KP4;Q96KP4−2
752:797 349:389 67:74
18:72176083:T:C_C
p = 2.3e−08, beta = −0.271, N = 1168
EGGSIPVTLTFQEATGK pc2
Q96KP4;Q96KP4−2
777:797 376:389 73:74
18:72176083:T:C_C
p = 4.4e−09, beta = −0.277, N = 1226
LYDDIDFDIEEFAK pc2
Q96KP4;Q96KP4−2
773:797 372:389 69:74
18:72176083:T:C_C
p = 7.3e−11, beta = −0.309, N = 1214
YNYIEGTK pc2
Q96KP4;Q96KP4−2
389:797 148:389 23:74
18:72176083:T:C_C
p = 0.33, beta = −0.0735, N = 560
MLAAYLYEVSQLK pc2
Q96KP4;Q96KP4−2
640:797 290:389 60:74
18:72176083:T:C_C
p = 1e−06, beta = −0.255, N = 990
RGNILIPGINEAVAAVTEEEHK pc3
Q96KP4;Q96KP4−2
661:797 290:389 63:74
18:72176083:T:C_C
p = 0.00013, beta = −0.197, N = 1014
DVDYVCISDNYWLGK pc2
Q96KP4;Q96KP4−2
584:797 250:389 46:74
18:72176083:T:C_C
p = 3e−04, beta = −0.207, N = 880
MLAAYLYEVSQLKD pc2
Q96KP4;Q96KP4−2
625:797 273:389 48:74
18:72176083:T:C_C
p = 0.00019, beta = −0.207, N = 946
LVPNMTPEVVGEQVTSYLTK pc3
Q96KP4;Q96KP4−2
590:797 237:389 42:74
18:72176083:T:C_C
p = 0.0046, beta = −0.166, N = 869
PWVSDFSHPHYLAGR pc3
Q96KP4;Q96KP4−2
429:797 177:389 38:74
18:72176083:T:C_C
p = 5.4e−05, beta = −0.263, N = 644
GPVAGWINALEAYQK pc2
A0A087WYZ1;Q96KP4
312:797 121:389 23:74
18:72176083:T:C_C
p = 0.094, beta = −0.134, N = 456
CNDP2 : NP5
Q96KP4
116:211 68:100 11:14
18:72176083:T:C_C
p = 0.053, beta = 0.226, N = 195
TGQEIPVNVR pc2
Q96KP4
85:211 53:100 7:14
18:72176083:T:C_C
p = 0.97, beta = 0.00558, N = 145
TVFGVEPDLTR pc2
Q96KP4;Q96KP4−2
51:211 41:100 7:14
18:72176083:T:C_C
p = 0.15, beta = 0.226, N = 99
DVGAQILLHSHK pc3
Q96KP4;Q96KP4−2
61:211 44:100 8:14
18:72176083:T:C_C
p = 0.3, beta = 0.152, N = 113
GNILIPGINEAVAAVTEEEHK pc3
Q96KP4;Q96KP4−2
27:211 28:100 5:14
18:72176083:T:C_C
p = 0.38, beta = 0.171, N = 60
EGGSIPVTLTFQEATGK pc2
Q96KP4;Q96KP4−2
52:211 33:100 7:14
18:72176083:T:C_C
p = 0.091, beta = 0.268, N = 92
QLGGSVELVDIGK pc2
A0A087WYZ1;Q96KP4;Q96KP4−2
29:211 27:100 4:14
18:72176083:T:C_C
p = 0.14, beta = 0.298, N = 60
RGNILIPGINEAVAAVTEEEHK pc3
Q96KP4;Q96KP4−2
19:211 17:100 5:14
18:72176083:T:C_C
p = 0.93, beta = 0.0197, N = 41
NVMLLPVGSADDGAHSQNEK pc3
Q96KP4;Q96KP4−2
27:211 24:100 7:14
18:72176083:T:C_C
p = 0.86, beta = 0.0331, N = 58
WVAIQSVSAWPEK pc2
A0A087WYZ1;Q96KP4;Q96KP4−2
19:211 14:100 4:14
18:72176083:T:C_C
p = 0.24, beta = 0.267, N = 37
LYDDIDFDIEEFAK pc2
Q96KP4;Q96KP4−2
12:211 13:100 2:14
18:72176083:T:C_C
p = 0.63, beta = −0.139, N = 27
log10 protein intensity
log10 peptide intensity
136Assay Target: CNDP2
UniProt: Q96KP4
rsID: rs2278161
Target SNP: 18:72176083:T:C
Proxy SNP: 18:72176083:T:C
Tarkin log10(p): 12.3
Tarkin BETA: −0.334
Tarkin MSPA: 0.813
1257:1248:1197:1190:1203:122

137. IGKV2D−29 (A0A075B6S2)
Tarkin MSPA=0.841

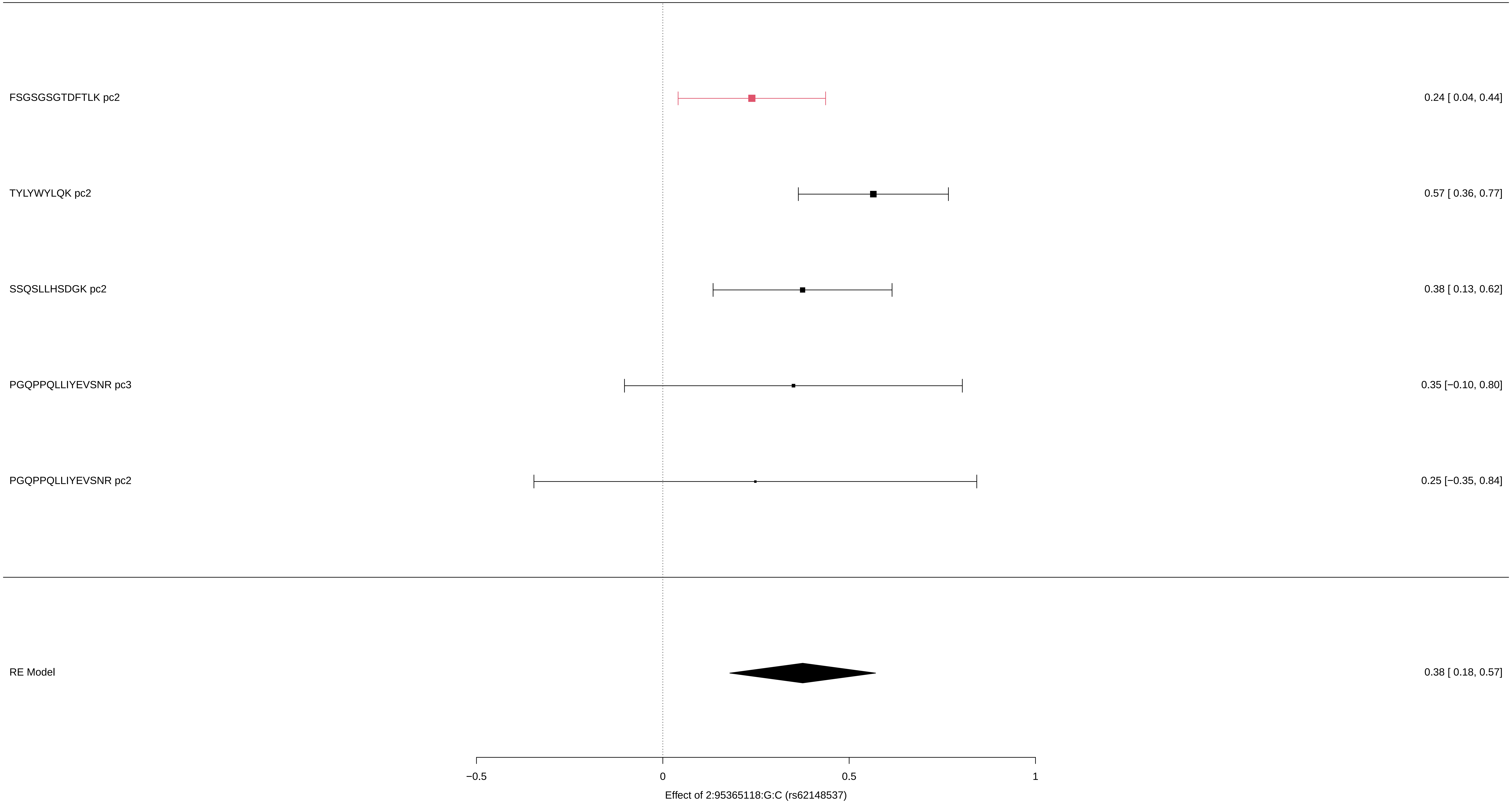

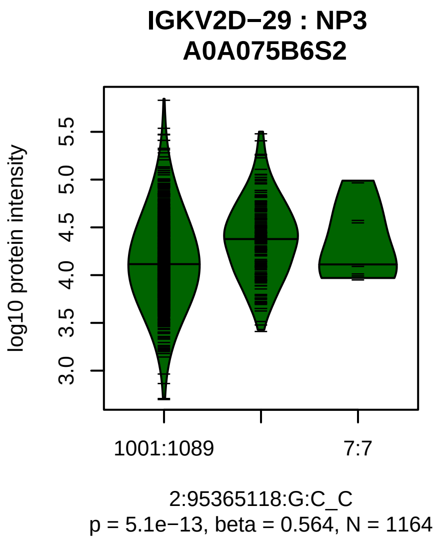

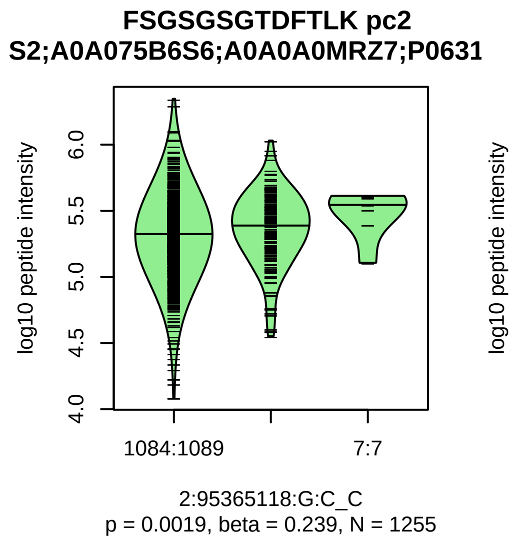

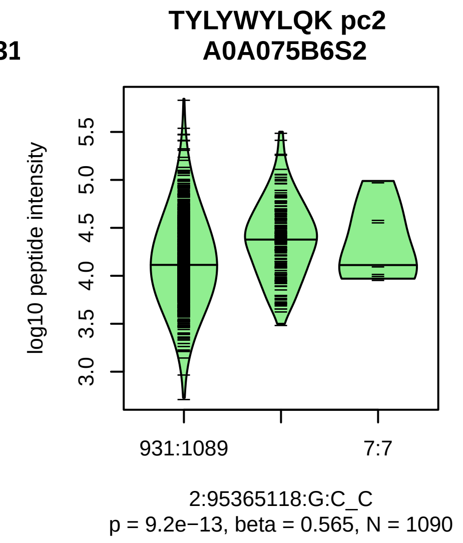

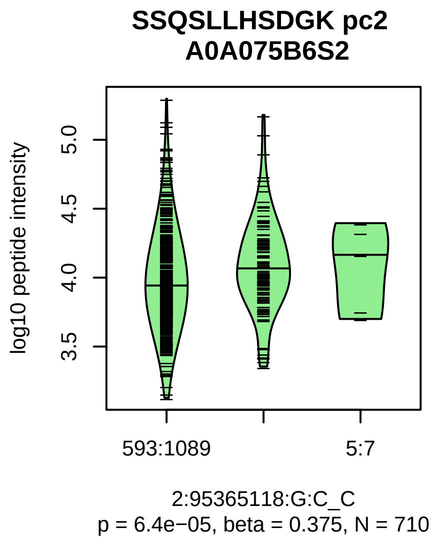

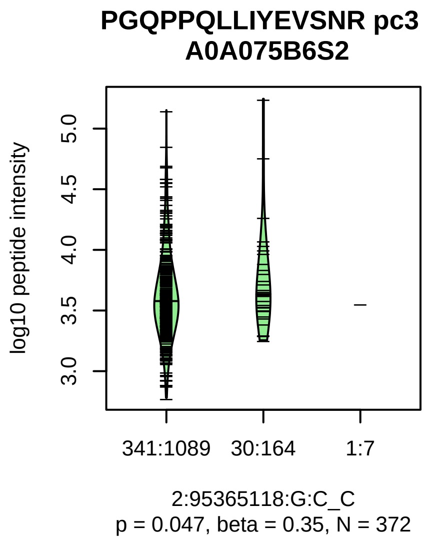

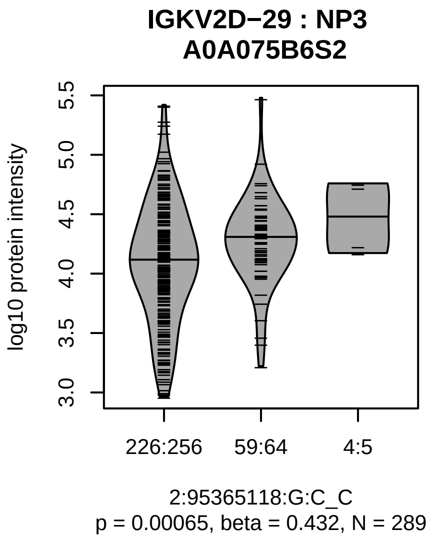

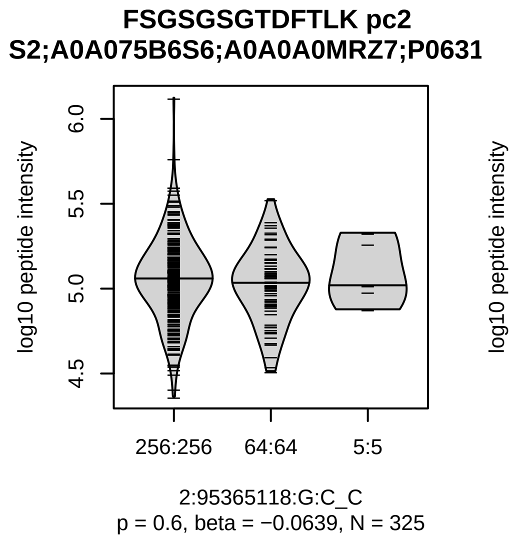

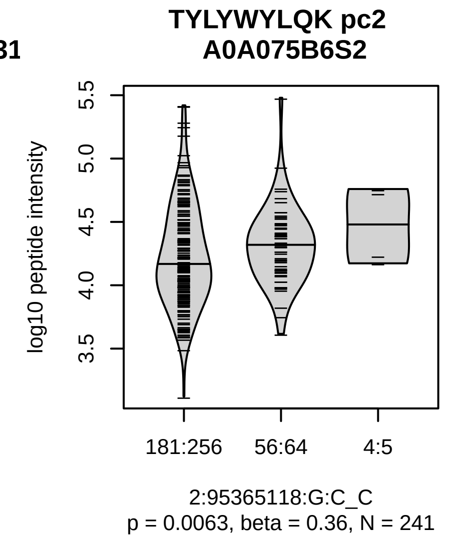

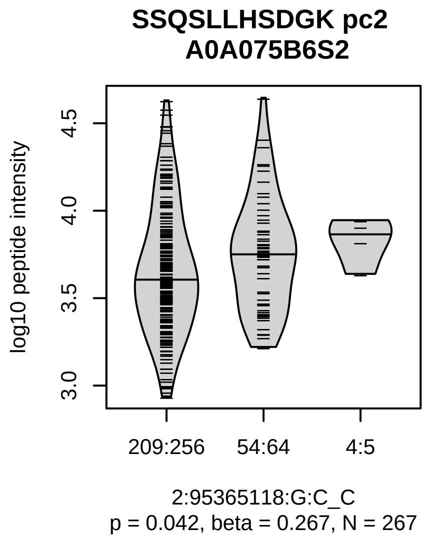

137Assay Target: IGKV2D−29
UniProt: A0A075B6S2
rsID: rs62148537
Target SNP: 2:95365118:G:C
Proxy SNP: 2:95365118:G:C
Tarkin log10(p): 12.3
Tarkin BETA: 0.564
Tarkin MSPA: 0.841
::*:−:−:NA
1255:1090:710:372:204:7

138. AEBP1 (Q8IUX7)
Tarkin MSPA=0.87

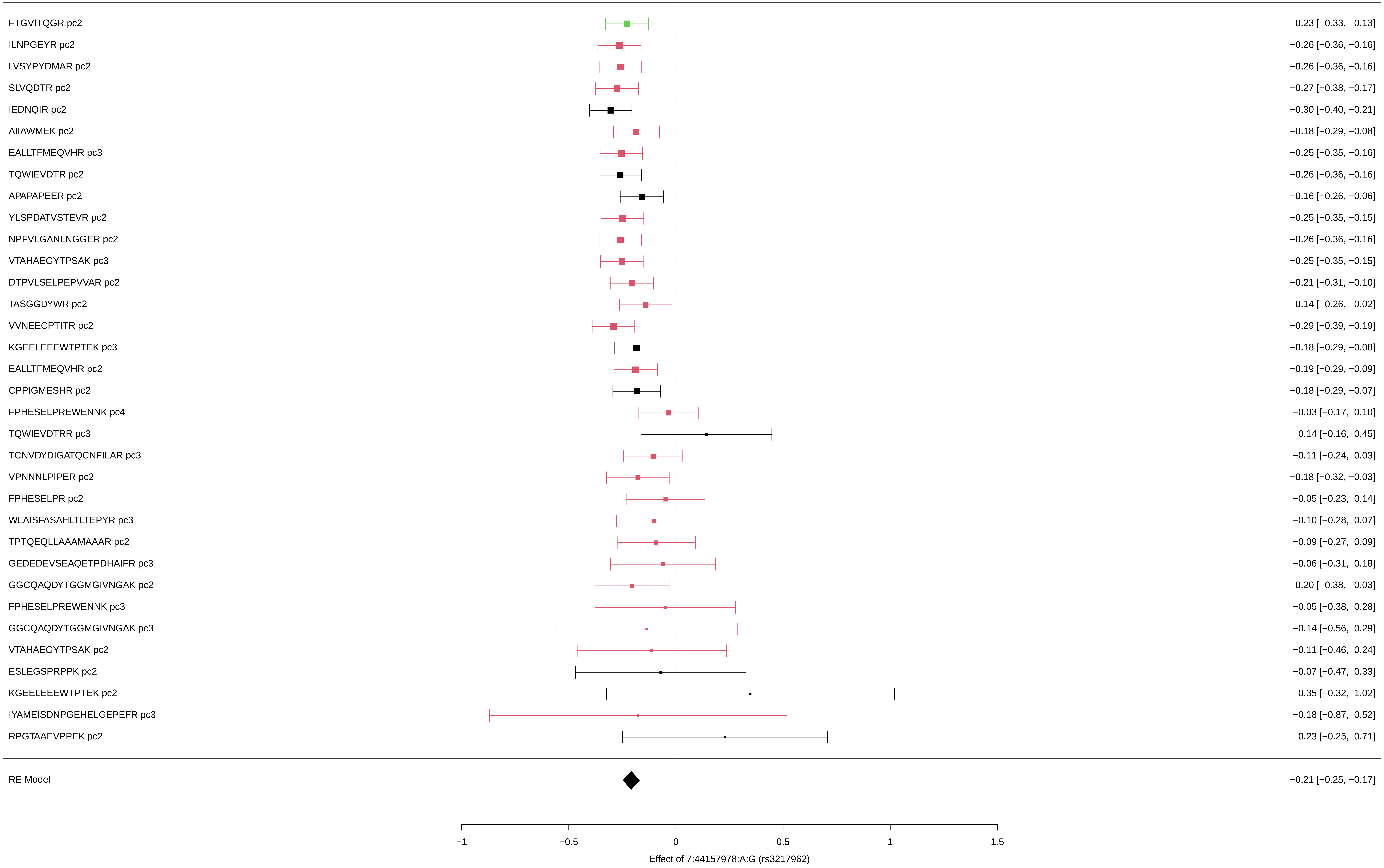

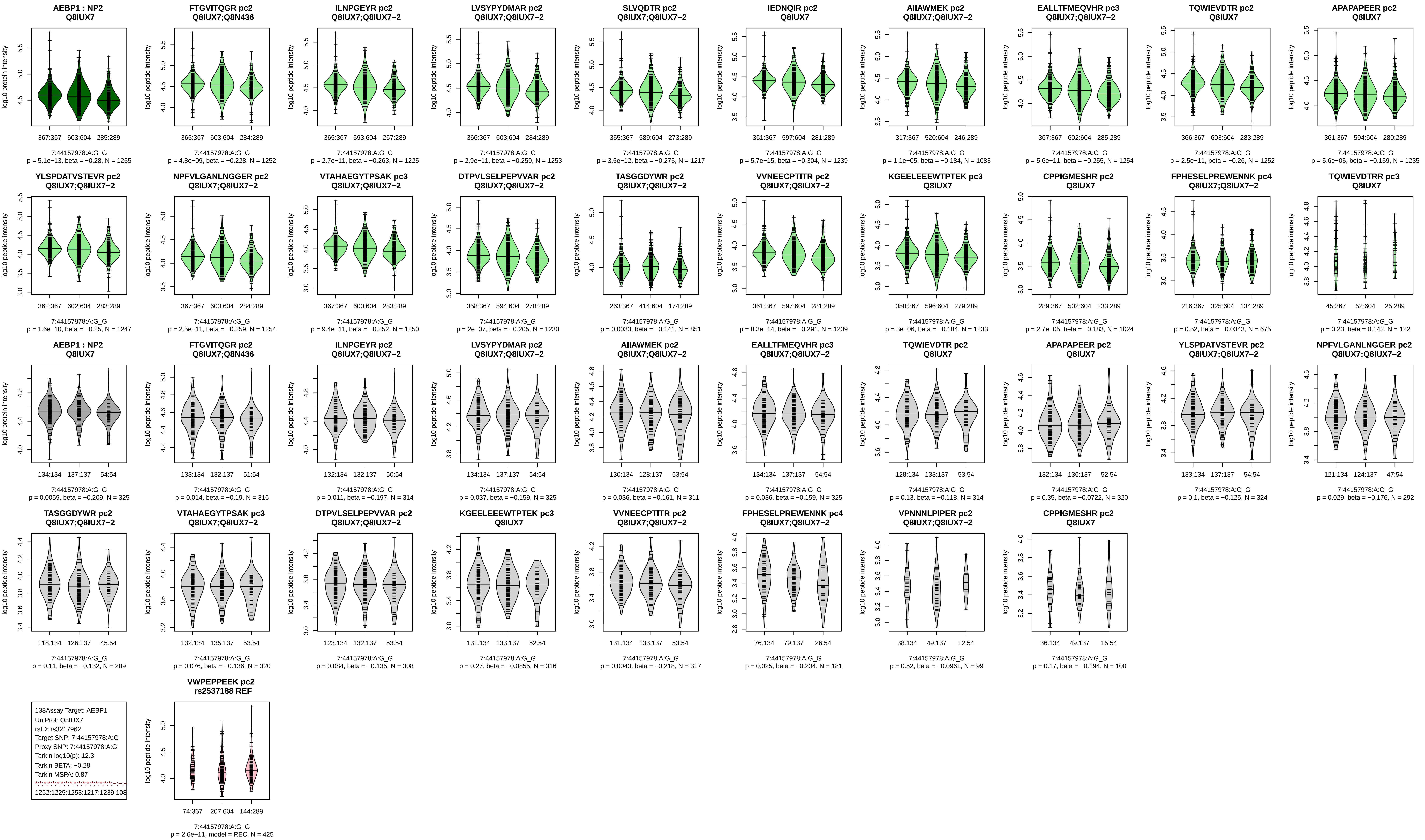
AEBP1 : NP2 Q8IUX7
7:44157978:A:G_G p = 5.1e−13, beta = −0.28, N = 1255
FTGVITQGR pc2 Q8IUX7;Q8N436
7:44157978:A:G_G p = 4.8e−09, beta = −0.228, N = 1252
ILNPGEYR pc2 Q8IUX7;Q8IUX7−2
7:44157978:A:G_G p = 2.7e−11, beta = −0.263, N = 1225
LVSYPYDMAR pc2 Q8IUX7;Q8IUX7−2
7:44157978:A:G_G p = 2.9e−11, beta = −0.259, N = 1253
SLVQDTR pc2 Q8IUX7;Q8IUX7−2
7:44157978:A:G_G p = 3.5e−12, beta = −0.275, N = 1217
IEDNQIR pc2 Q8IUX7
7:44157978:A:G_G p = 5.7e−15, beta = −0.304, N = 1239
AIIAWMEK pc2 Q8IUX7;Q8IUX7−2
7:44157978:A:G_G p = 1.1e−05, beta = −0.184, N = 1083
EALLTFMEQVHR pc3 Q8IUX7;Q8IUX7−2
7:44157978:A:G_G p = 5.6e−11, beta = −0.255, N = 1254
TQWIEVDTR pc2 Q8IUX7
7:44157978:A:G_G p = 2.5e−11, beta = −0.26, N = 1252
APAPAPEER pc2 Q8IUX7
7:44157978:A:G_G p = 5.6e−05, beta = −0.159, N = 1235
YLSPDATVSTEVR pc2 Q8IUX7;Q8IUX7−2
7:44157978:A:G_G p = 1.6e−10, beta = −0.25, N = 1247
NPFVLGANLNGGER pc2 Q8IUX7;Q8IUX7−2
7:44157978:A:G_G p = 2.5e−11, beta = −0.259, N = 1254
VTAHAEGYTPSAK pc3 Q8IUX7;Q8IUX7−2
7:44157978:A:G_G p = 9.4e−11, beta = −0.252, N = 1250
DTPVLSELPEPVVAR pc2 Q8IUX7;Q8IUX7−2
7:44157978:A:G_G p = 2e−07, beta = −0.205, N = 1230
TASGGDYWR pc2 Q8IUX7;Q8IUX7−2
7:44157978:A:G_G p = 0.0033, beta = −0.141, N = 851
VVNEECPTITR pc2 Q8IUX7;Q8IUX7−2
7:44157978:A:G_G p = 8.3e−14, beta = −0.291, N = 1239
KGEELEEEWTPTEK pc3 Q8IUX7
7:44157978:A:G_G p = 3e−06, beta = −0.184, N = 1233
CPPIGMESHR pc2 Q8IUX7
7:44157978:A:G_G p = 2.7e−05, beta = −0.183, N = 1024
FPHESELPREWENNK pc4 Q8IUX7;Q8IUX7−2
7:44157978:A:G_G p = 0.52, beta = −0.0343, N = 675
TQWIEVDTRR pc3 Q8IUX7
7:44157978:A:G_G p = 0.23, beta = 0.142, N = 122
AEBP1 : NP2 Q8IUX7
7:44157978:A:G_G p = 0.0059, beta = −0.209, N = 325
FTGVITQGR pc2 Q8IUX7;Q8N436
7:44157978:A:G_G p = 0.014, beta = −0.19, N = 316
ILNPGEYR pc2 Q8IUX7;Q8IUX7−2
7:44157978:A:G_G p = 0.011, beta = −0.197, N = 314
LVSYPYDMAR pc2 Q8IUX7;Q8IUX7−2
7:44157978:A:G_G p = 0.037, beta = −0.159, N = 325
AIIAWMEK pc2 Q8IUX7;Q8IUX7−2
7:44157978:A:G_G p = 0.036, beta = −0.161, N = 311
EALLTFMEQVHR pc3 Q8IUX7;Q8IUX7−2
7:44157978:A:G_G p = 0.036, beta = −0.159, N = 325
TQWIEVDTR pc2 Q8IUX7
7:44157978:A:G_G p = 0.13, beta = −0.118, N = 314
APAPAPEER pc2 Q8IUX7
7:44157978:A:G_G p = 0.35, beta = −0.0722, N = 320
YLSPDATVSTEVR pc2 Q8IUX7;Q8IUX7−2
7:44157978:A:G_G p = 0.1, beta = −0.125, N = 324
NPFVLGANLNGGER pc2 Q8IUX7;Q8IUX7−2
7:44157978:A:G_G p = 0.029, beta = −0.176, N = 292
TASGGDYWR pc2 Q8IUX7;Q8IUX7−2
7:44157978:A:G_G p = 0.11, beta = −0.132, N = 289
VTAHAEGYTPSAK pc3 Q8IUX7;Q8IUX7−2
7:44157978:A:G_G p = 0.076, beta = −0.136, N = 320
DTPVLSELPEPVVAR pc2 Q8IUX7;Q8IUX7−2
7:44157978:A:G_G p = 0.084, beta = −0.135, N = 308
KGEELEEEWTPTEK pc3 Q8IUX7
7:44157978:A:G_G p = 0.27, beta = −0.0855, N = 316
VVNEECPTITR pc2 Q8IUX7;Q8IUX7−2
7:44157978:A:G_G p = 0.0043, beta = −0.218, N = 317
FPHESELPREWENNK pc4 Q8IUX7;Q8IUX7−2
7:44157978:A:G_G p = 0.025, beta = −0.234, N = 181
VPNNNLPIPER pc2 Q8IUX7;Q8IUX7−2
7:44157978:A:G_G p = 0.52, beta = −0.0961, N = 99
CPPIGMESHR pc2 Q8IUX7
7:44157978:A:G_G p = 0.17, beta = −0.194, N = 100
138Assay Target: AEBP1
UniProt: Q8IUX7
rsID: rs3217962
Target SNP: 7:44157978:A:G
Proxy SNP: 7:44157978:A:G
Tarkin log10(p): 12.3
Tarkin BETA: −0.28
Tarkin MSPA: 0.87
1252:1225:1253:1217:1239:108
VWPEPPEEK pc2 rs2537188 REF
7:44157978:A:G_G p = 2.6e−11, model = REC, N = 425

139. MASP2 (O00187)
Tarkin MSPA=0.806

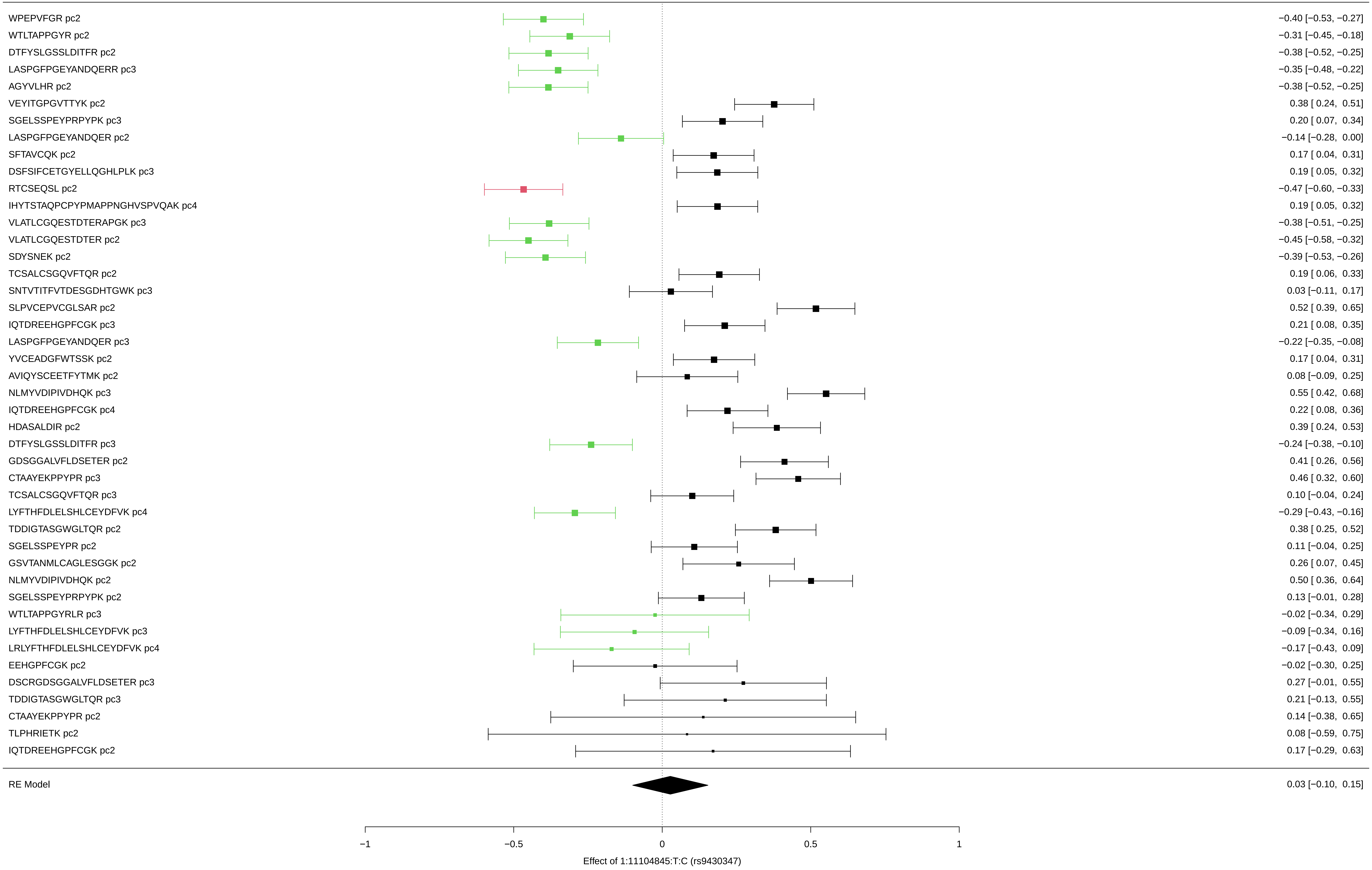

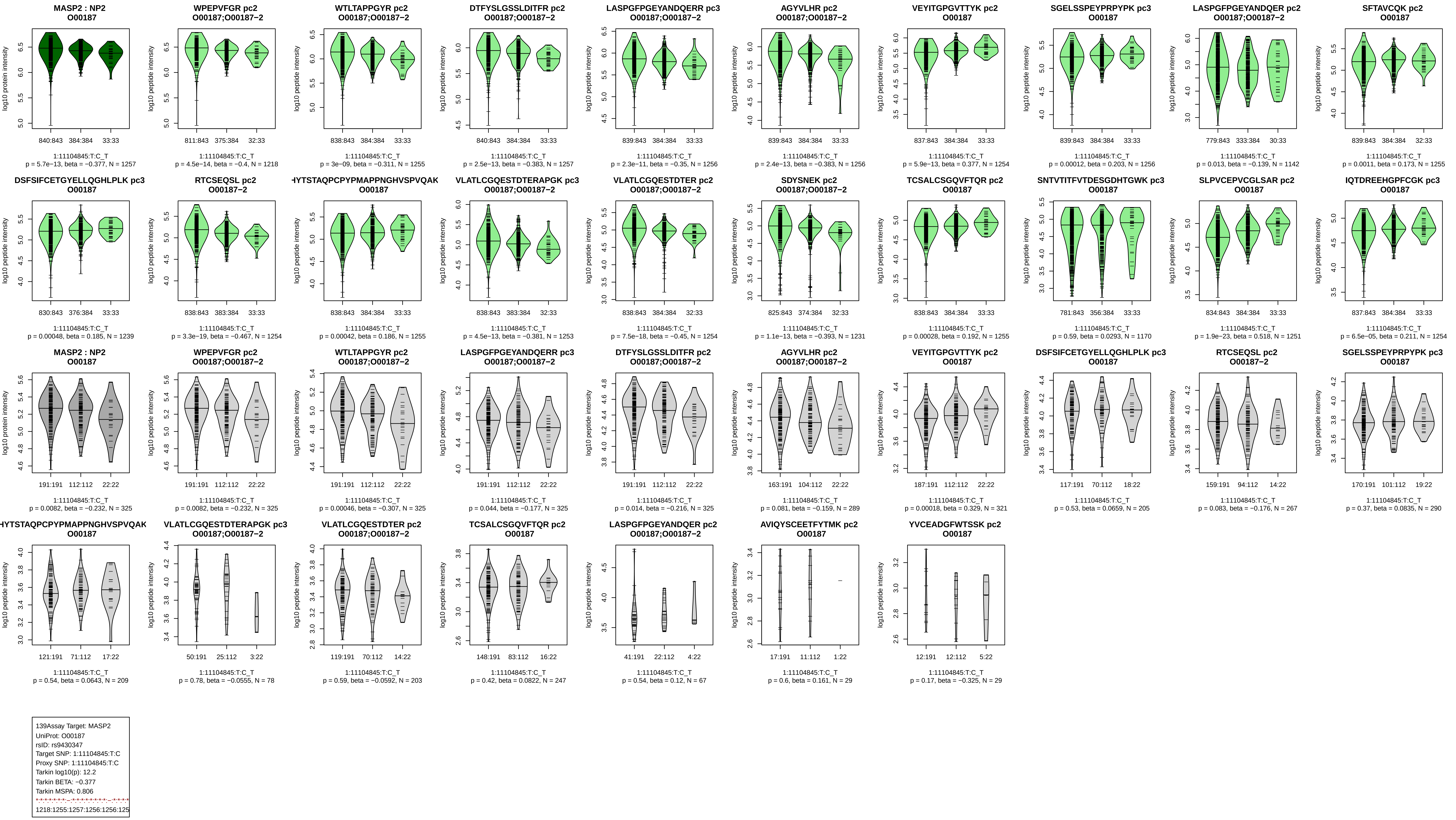
MASP2 : NP2
O00187
log10 protein intensity
840:843 384:384 33:33
1:11104845:T:C_T
p = 5.7e−13, beta = −0.377, N = 1257
WPEPVFGR pc2
O00187;O00187−2
log10 peptide intensity
811:843 375:384 32:33
1:11104845:T:C_T
p = 4.5e−14, beta = −0.4, N = 1218
WTLTAPPGYR pc2
O00187;O00187−2
838:843 384:384 33:33
1:11104845:T:C_T
p = 3e−09, beta = −0.311, N = 1255
DTFYSLGSSLDITFR pc2
O00187;O00187−2
840:843 384:384 33:33
1:11104845:T:C_T
p = 2.5e−13, beta = −0.383, N = 1257
LASPGFPGEYANDQERR pc3
O00187;O00187−2
839:843 384:384 33:33
1:11104845:T:C_T
p = 2.3e−11, beta = −0.35, N = 1256
AGYVLHR pc2
O00187;O00187−2
839:843 384:384 33:33
1:11104845:T:C_T
p = 2.4e−13, beta = −0.383, N = 1256
VEYITGPGVTTYK pc2
O00187
837:843 384:384 33:33
1:11104845:T:C_T
p = 5.9e−13, beta = 0.377, N = 1254
SGELSSPEYPRPYPK pc3
O00187
839:843 384:384 33:33
1:11104845:T:C_T
p = 0.00012, beta = 0.203, N = 1256
LASPGFPGEYANDQER pc2
O00187;O00187−2
779:843 333:384 30:33
1:11104845:T:C_T
p = 0.013, beta = −0.139, N = 1142
SFTAVCQK pc2
O00187
839:843 384:384 32:33
1:11104845:T:C_T
p = 0.0011, beta = 0.173, N = 1255
DSFSIFCETGYELLQGHLPLK pc3
O00187
830:843 376:384 33:33
1:11104845:T:C_T
p = 0.00048, beta = 0.185, N = 1239
RTCSEQSL pc2
O00187−2
838:843 383:384 33:33
1:11104845:T:C_T
p = 3.3e−19, beta = −0.467, N = 1254
HYTSTAQPCPYPMAPPNGHVSPVQAK
O00187
838:843 384:384 33:33
1:11104845:T:C_T
p = 0.00042, beta = 0.186, N = 1255
VLATLCGQESTDTERAPGK pc3
O00187;O00187−2
838:843 383:384 32:33
1:11104845:T:C_T
p = 4.5e−13, beta = −0.381, N = 1253
VLATLCGQESTDTER pc2
O00187;O00187−2
838:843 384:384 32:33
1:11104845:T:C_T
p = 7.5e−18, beta = −0.45, N = 1254
SDYSNEK pc2
O00187;O00187−2
825:843 374:384 32:33
1:11104845:T:C_T
p = 1.1e−13, beta = −0.393, N = 1231
TCSALCSGQVFTQR pc2
O00187
838:843 384:384 33:33
1:11104845:T:C_T
p = 0.00028, beta = 0.192, N = 1255
SNTVTITFVTDESGDHTGWK pc3
O00187
781:843 356:384 33:33
1:11104845:T:C_T
p = 0.59, beta = 0.0293, N = 1170
SLPVCEPVCGLSAR pc2
O00187
834:843 384:384 33:33
1:11104845:T:C_T
p = 1.9e−23, beta = 0.518, N = 1251
IQTDREEHGPFCGK pc3
O00187
837:843 384:384 33:33
1:11104845:T:C_T
p = 6.5e−05, beta = 0.211, N = 1254
MASP2 : NP2
O00187
191:191 112:112 22:22
1:11104845:T:C_T
p = 0.0082, beta = −0.232, N = 325
WPEPVFGR pc2
O00187;O00187−2
191:191 112:112 22:22
1:11104845:T:C_T
p = 0.0082, beta = −0.232, N = 325
WTLTAPPGYR pc2
O00187;O00187−2
191:191 112:112 22:22
1:11104845:T:C_T
p = 0.00046, beta = −0.307, N = 325
LASPGFPGEYANDQERR pc3
O00187;O00187−2
191:191 112:112 22:22
1:11104845:T:C_T
p = 0.044, beta = −0.177, N = 325
DTFYSLGSSLDITFR pc2
O00187;O00187−2
191:191 112:112 22:22
1:11104845:T:C_T
p = 0.014, beta = −0.216, N = 325
AGYVLHR pc2
O00187;O00187−2
163:191 104:112 22:22
1:11104845:T:C_T
p = 0.081, beta = −0.159, N = 289
VEYITGPGVTTYK pc2
O00187
187:191 112:112 22:22
1:11104845:T:C_T
p = 0.00018, beta = 0.329, N = 321
DSFSIFCETGYELLQGHLPLK pc3
O00187
117:191 70:112 18:22
1:11104845:T:C_T
p = 0.53, beta = 0.0659, N = 205
RTCSEQSL pc2
O00187−2
159:191 94:112 14:22
1:11104845:T:C_T
p = 0.083, beta = −0.176, N = 267
SGELSSPEYPRPYPK pc3
O00187
170:191 101:112 19:22
1:11104845:T:C_T
p = 0.37, beta = 0.0835, N = 290
HYTSTAQPCPYPMAPPNGHVSPVQAK
O00187
121:191 71:112 17:22
1:11104845:T:C_T
p = 0.54, beta = 0.0643, N = 209
VLATLCGQESTDTERAPGK pc3
O00187;O00187−2
50:191 25:112 3:22
1:11104845:T:C_T
p = 0.78, beta = −0.0555, N = 78
VLATLCGQESTDTER pc2
O00187;O00187−2
119:191 70:112 14:22
1:11104845:T:C_T
p = 0.59, beta = −0.0592, N = 203
TCSALCSGQVFTQR pc2
O00187
148:191 83:112 16:22
1:11104845:T:C_T
p = 0.42, beta = 0.0822, N = 247
LASPGFPGEYANDQER pc2
O00187;O00187−2
41:191 22:112 4:22
1:11104845:T:C_T
p = 0.54, beta = 0.12, N = 67
AVIQYSCEETFYTMK pc2
O00187
17:191 11:112 1:22
1:11104845:T:C_T
p = 0.6, beta = 0.161, N = 29
YVCEADGFWTSSK pc2
O00187
12:191 12:112 5:22
1:11104845:T:C_T
p = 0.17, beta = −0.325, N = 29
139Assay Target: MASP2
UniProt: O00187
rsID: rs9430347
Target SNP: 1:11104845:T:C
Proxy SNP: 1:11104845:T:C
Tarkin log10(p): 12.2
Tarkin BETA: −0.377
Tarkin MSPA: 0.806
1218:1255:1257:1256:1256:125

140. FCN1 (O00602)
Tarkin MSPA=0.973

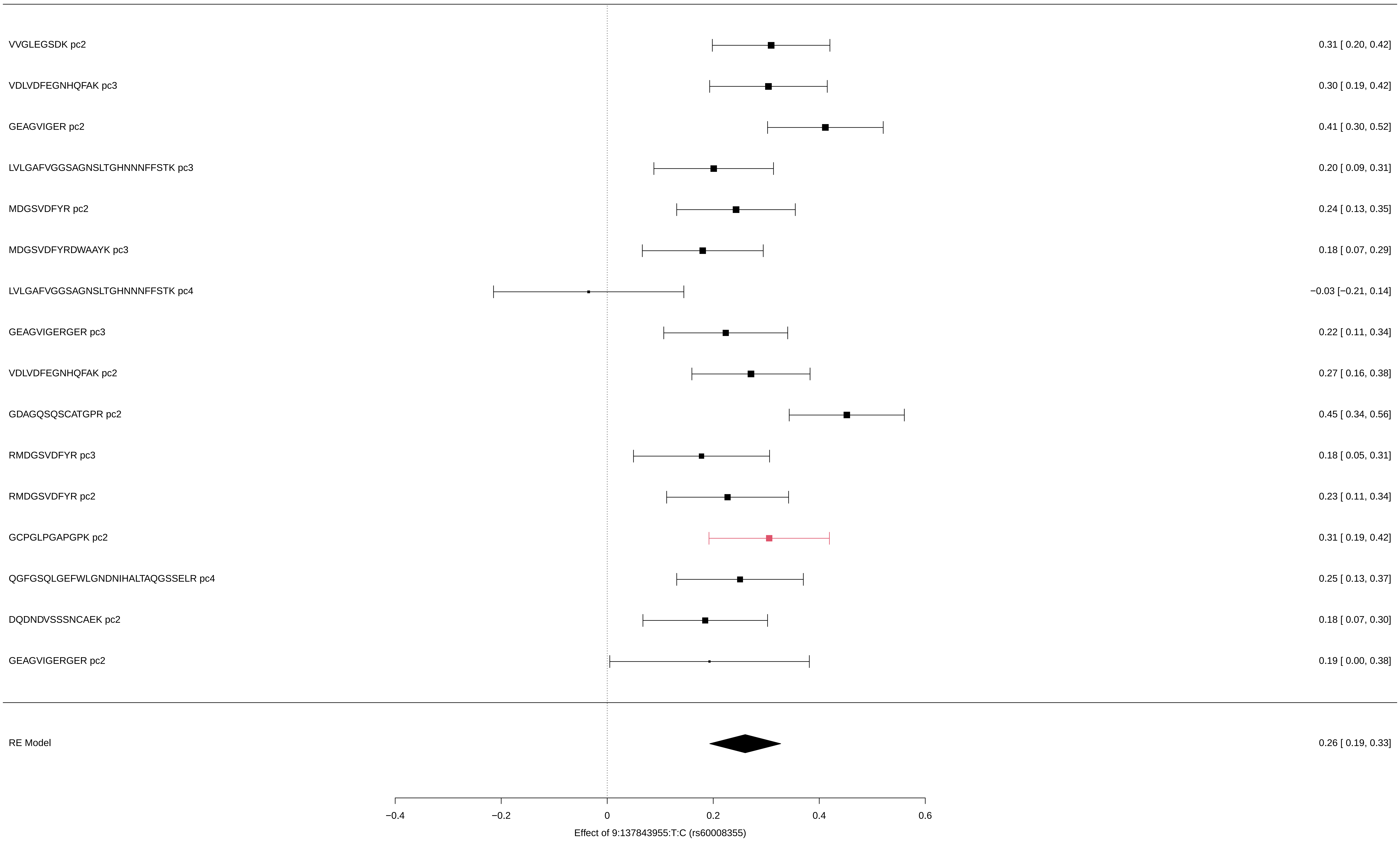

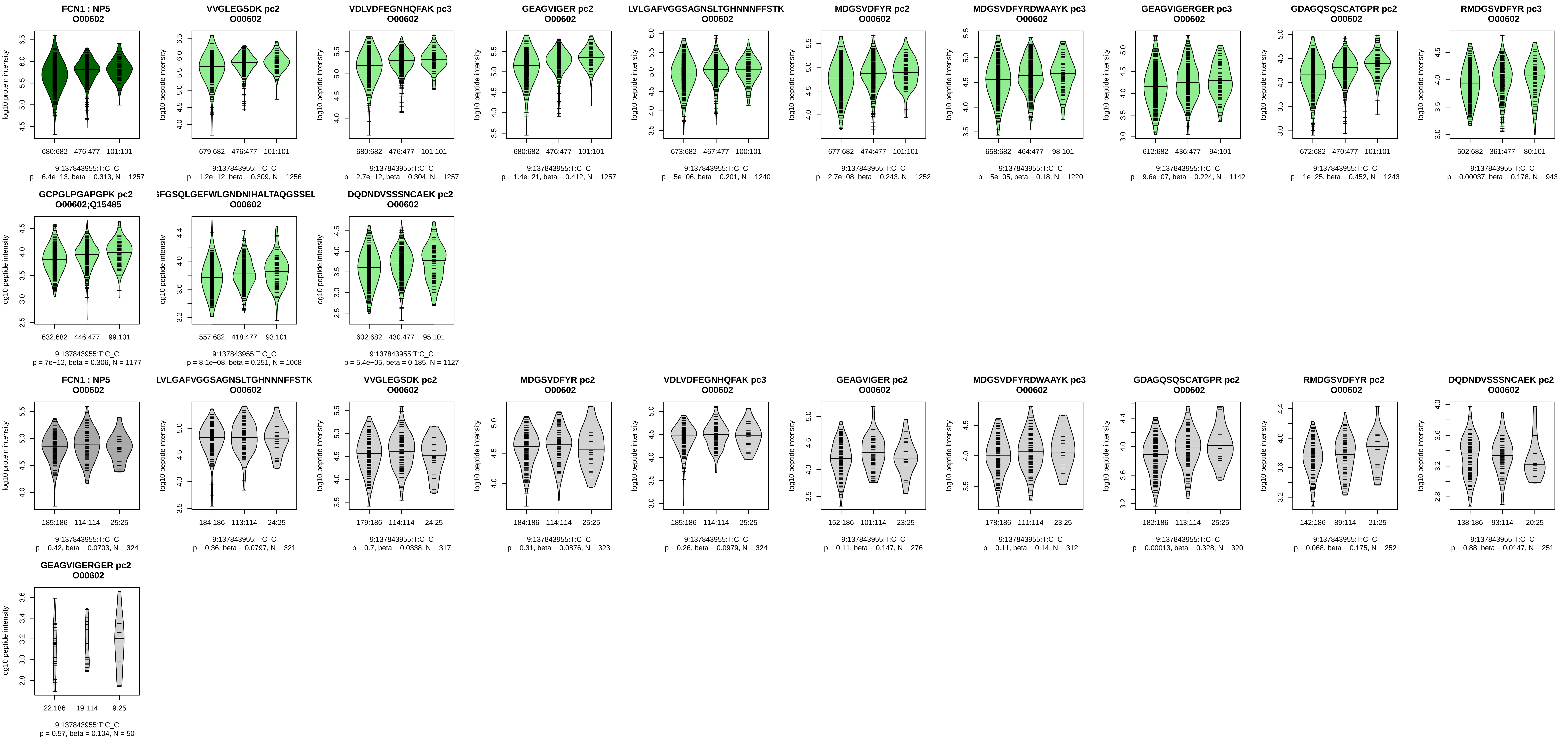

140Assay Target: FCN1
UniProt: O00602
rsID: rs60008355
Target SNP: 9:137843955:T:C
Proxy SNP: 9:137843955:T:C
Tarkin log10(p): 12.2
Tarkin BETA: 0.313
Tarkin MSPA: 0.973
::*:*:*:*:–:*:*:*:*:*:*:*:*:*:NA:NA
1256:1257:1257:1240:1252:122

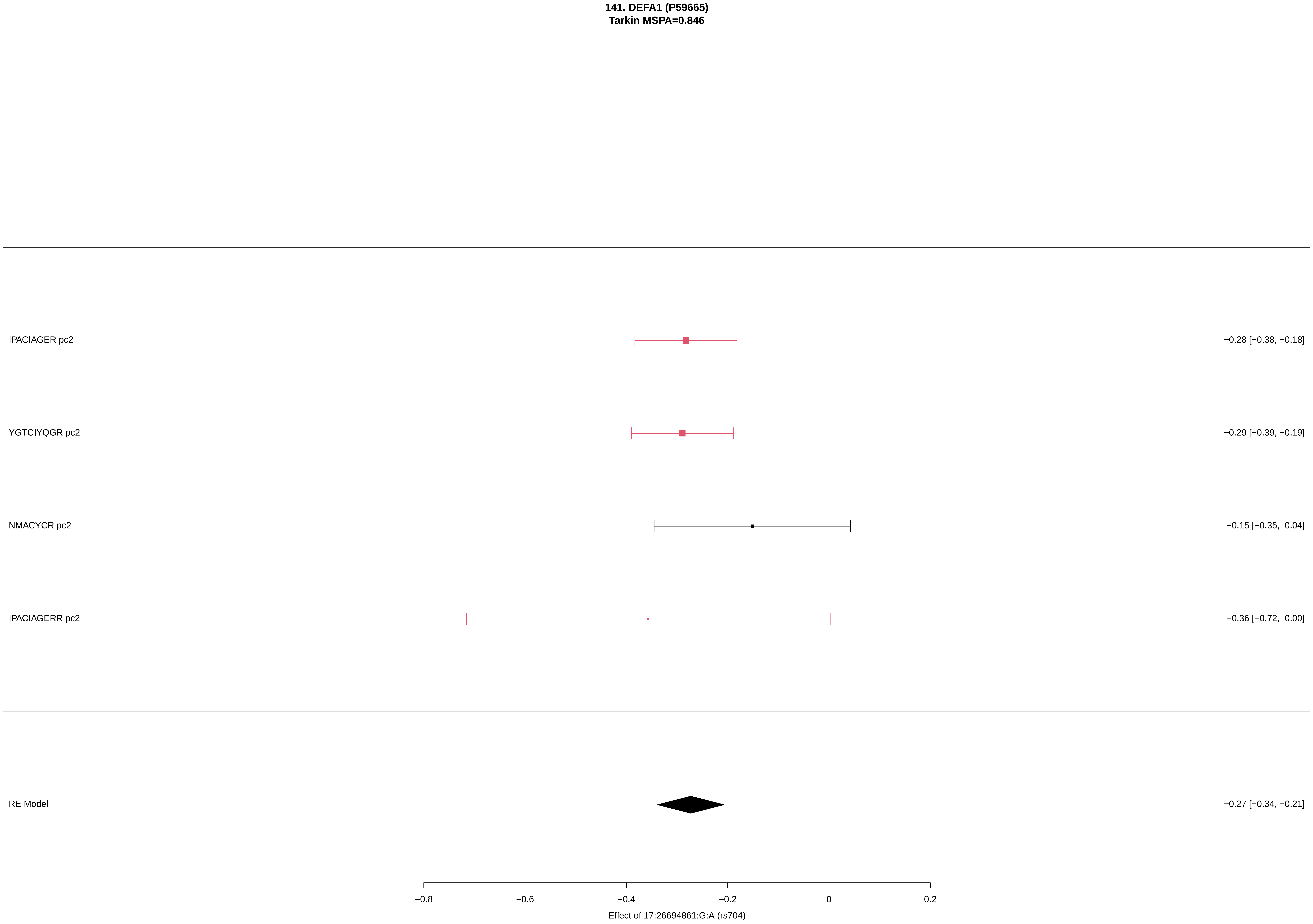
141. DEFA1 (P59665)
Tarkin MSPA=0.846
IPACIAGER pc2
−0.28 [−0.38, −0.18]
YGTCIYQGR pc2
−0.29 [−0.39, −0.19]
NMACYCR pc2
−0.15 [−0.35, 0.04]
IPACIAGERR pc2
−0.36 [−0.72, 0.00]
RE Model
−0.27 [−0.34, −0.21]
−0.8
−0.6
−0.4
−0.2
0
0.2
Effect of 17:26694861:G:A (rs704)

DEFA1 : NP4
P59665

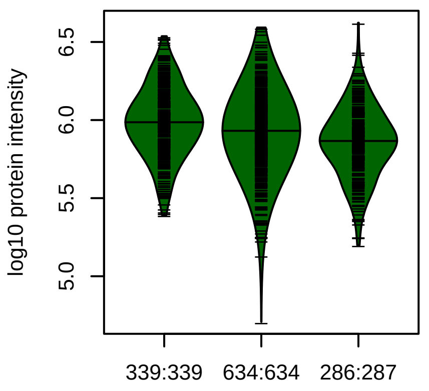

17:26694861:G:A_G
p = 9.2e−13, beta = −0.282, N = 1259

IPACIAGER pc2
P59665;P59666

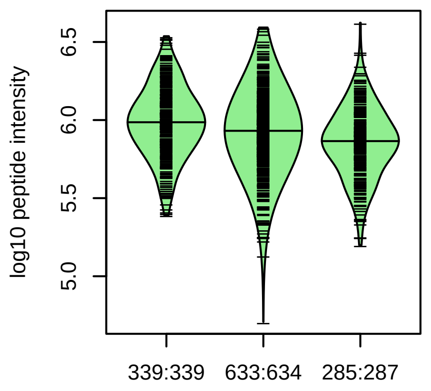

17:26694861:G:A_G
p = 9.2e−13, beta = −0.282, N = 1257

YGTCIYQGR pc2
P59665;P59666

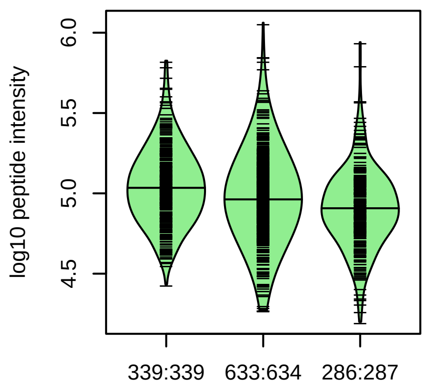

17:26694861:G:A_G
p = 2.3e−13, beta = −0.289, N = 1258

NMACYCR pc2
P59665

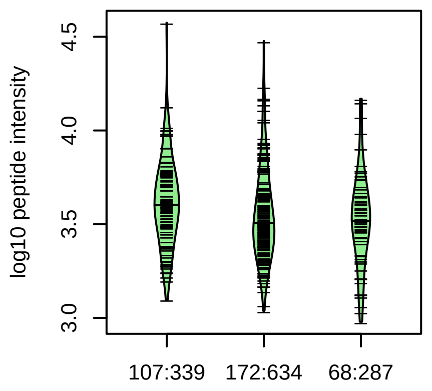

17:26694861:G:A_G
p = 0.045, beta = −0.151, N = 347

IPACIAGERR pc2
P59665;P59666

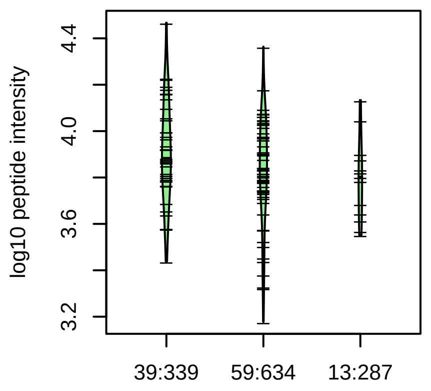

17:26694861:G:A_G
p = 0.012, beta = −0.357, N = 111

IPACIAGER pc2
P59665;P59666

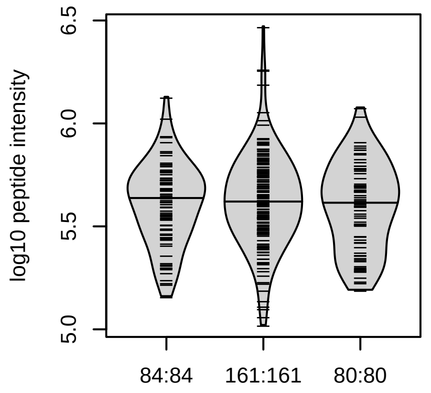

17:26694861:G:A_G
p = 0.81, beta = −0.0185, N = 325

YGTCIYQGR pc2
P59665;P59666

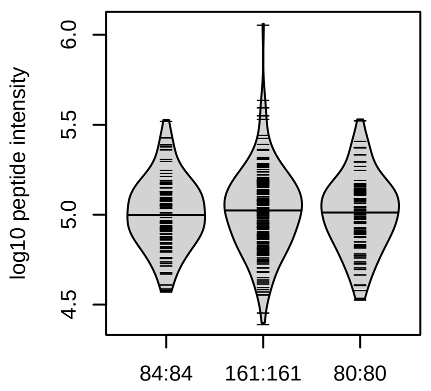

17:26694861:G:A_G
p = 0.95, beta = −0.00506, N = 325

IPACIAGERR pc2
P59665;P59666

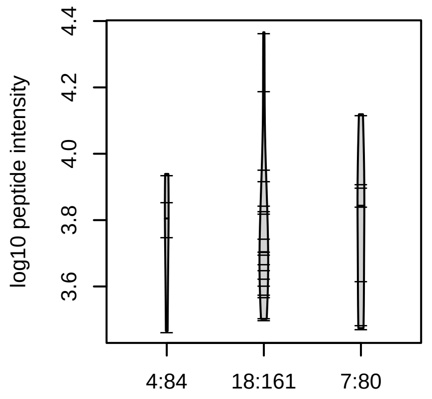

17:26694861:G:A_G
p = 0.38, beta = 0.252, N = 29

141Assay Target: DEFA1
UniProt: P59665
rsID: rs704
Target SNP: 17:26694861:G:A
Proxy SNP: 17:26694861:G:A
Tarkin log10(p): 12
Tarkin BETA: −0.282
Tarkin MSPA: 0.846
::–:–
1257:1258:347:111

142. F12 (P00748)
Tarkin MSPA=0.925

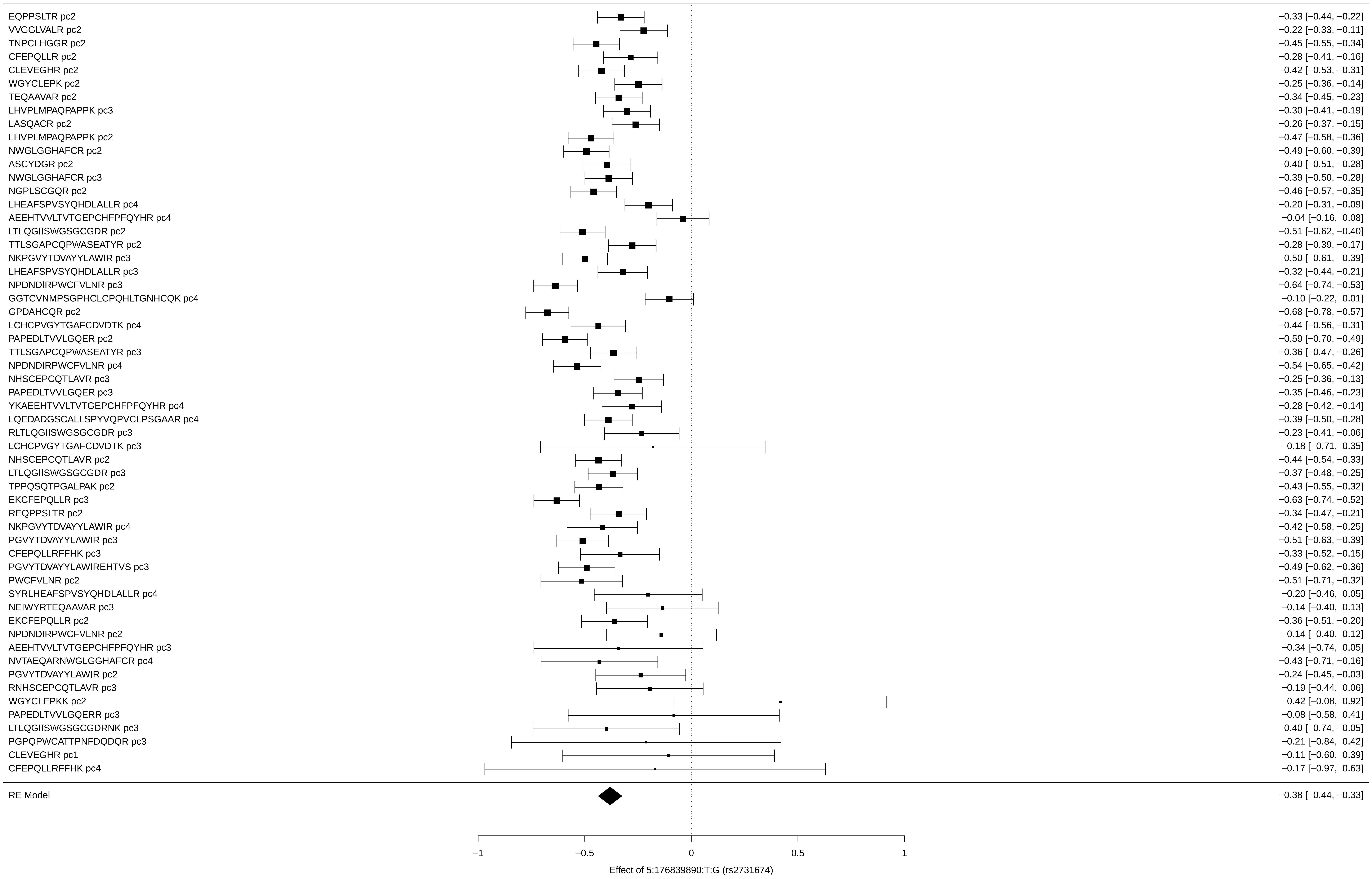

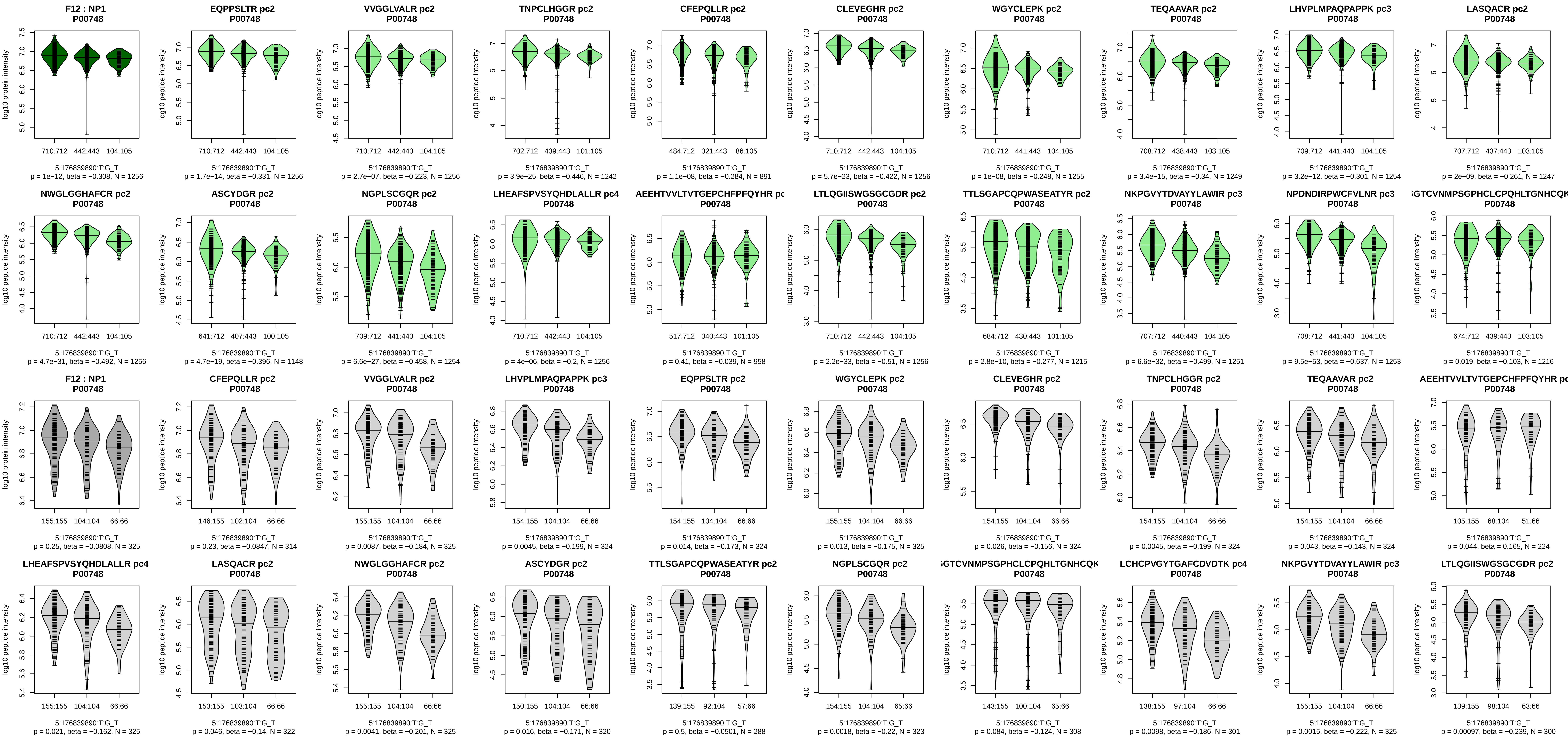

142Assay Target: F12
UniProt: P00748
rsID: rs2731674
Target SNP: 5:176839890:T:G
Proxy SNP: 5:176839890:T:G
Tarkin log10(p): 12
Tarkin BETA: −0.308
Tarkin MSPA: 0.925
1256:1256:1242:891:1256:1255

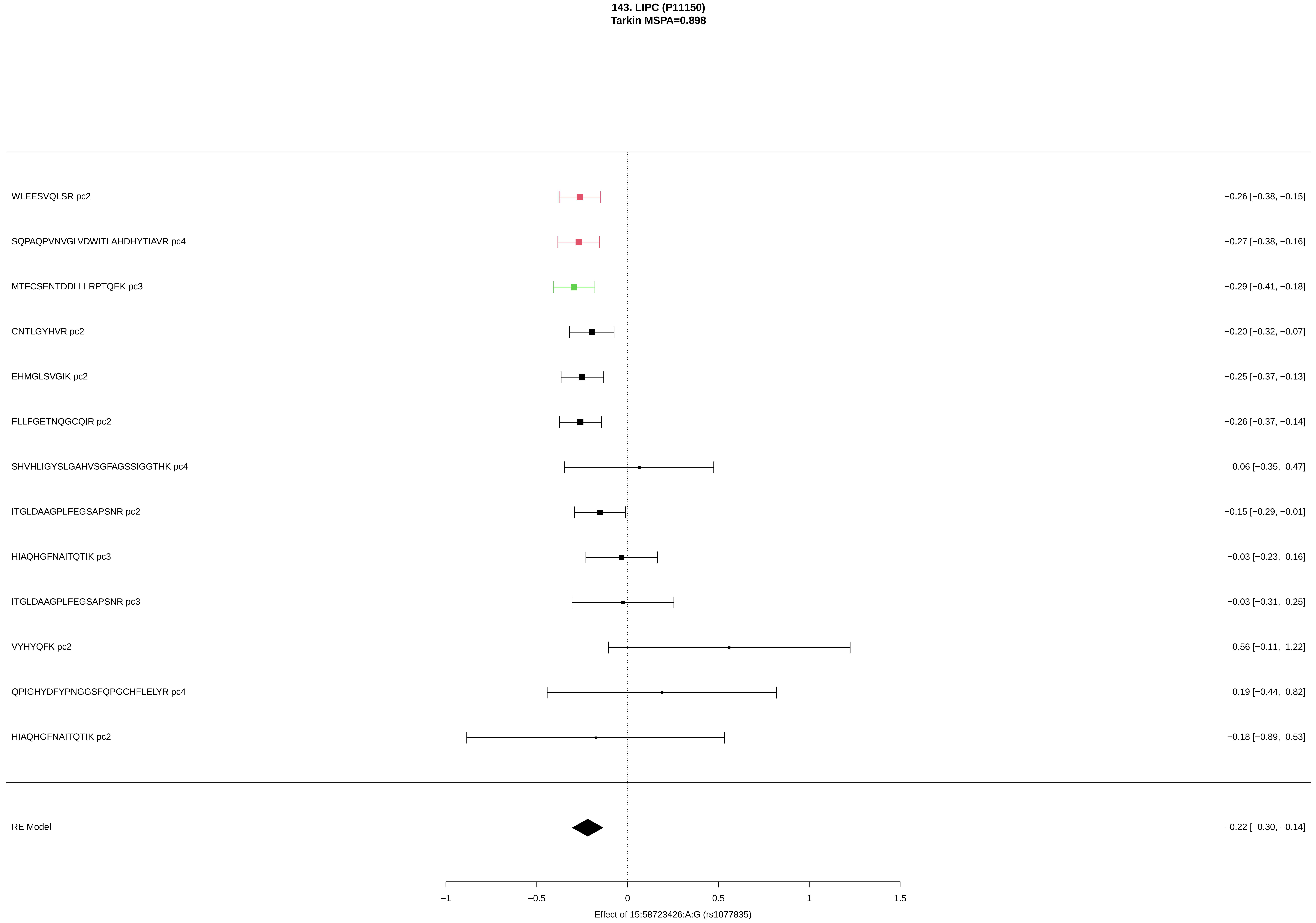
143. LIPC (P11150)
Tarkin MSPA=0.898
WLEESVQLSR pc2
−0.26 [−0.38, −0.15]
SQPAQPVNVGLVDWITLAHDHYTIAVR pc4
−0.27 [−0.38, −0.16]
MTFCSENTDDLLLRPTQEK pc3
−0.29 [−0.41, −0.18]
CNTLGYHVR pc2
−0.20 [−0.32, −0.07]
EHMGLSVGIK pc2
−0.25 [−0.37, −0.13]
FLLFGETNQGCQIR pc2
−0.26 [−0.37, −0.14]
SHVHLIGYSLGAHVSGFAGSSIGGTHK pc4
0.06 [−0.35, 0.47]
ITGLDAAGPLFEGSAPSNR pc2
−0.15 [−0.29, −0.01]
HIAQHGFNAITQTIK pc3
−0.03 [−0.23, 0.16]
ITGLDAAGPLFEGSAPSNR pc3
−0.03 [−0.31, 0.25]
VYHYQFK pc2
0.56 [−0.11, 1.22]
QPIGHYDFYPNGGSFQPGCHFLELYR pc4
0.19 [−0.44, 0.82]
HIAQHGFNAITQTIK pc2
−0.18 [−0.89, 0.53]
RE Model
−0.22 [−0.30, −0.14]
−1
−0.5
0
0.5
1
1.5
Effect of 15:58723426:A:G (rs1077835)

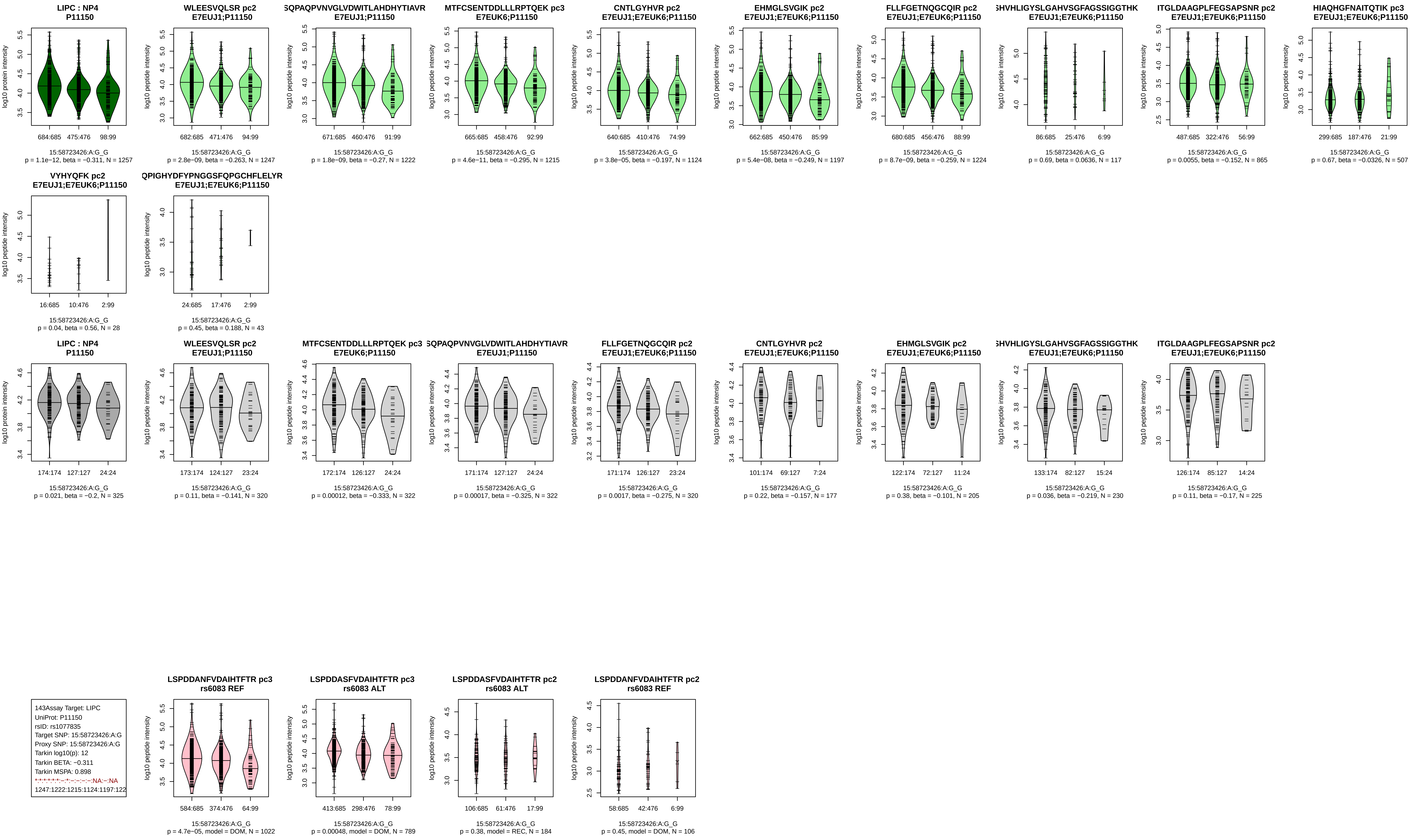
LIPC : NP4
P11150
log10 protein intensity
684:685 475:476 98:99
15:58723426:A:G_G
p = 1.1e−12, beta = −0.311, N = 1257
WLEESVQLSR pc2
E7EUJ1;P11150
log10 peptide intensity
682:685 471:476 94:99
15:58723426:A:G_G
p = 2.8e−09, beta = −0.263, N = 1247
SQPAQPVNVGLVDWITLAHDHYTIAVR
E7EUJ1;P11150
671:685 460:476 91:99
15:58723426:A:G_G
p = 1.8e−09, beta = −0.27, N = 1222
MTFCSENTDDLLLRPTQEK pc3
E7EUK6;P11150
665:685 458:476 92:99
15:58723426:A:G_G
p = 4.6e−11, beta = −0.295, N = 1215
CNTLGYHVR pc2
E7EUJ1;E7EUK6;P11150
640:685 410:476 74:99
15:58723426:A:G_G
p = 3.8e−05, beta = −0.197, N = 1124
EHMGLSVGIK pc2
E7EUJ1;E7EUK6;P11150
662:685 450:476 85:99
15:58723426:A:G_G
p = 5.4e−08, beta = −0.249, N = 1197
FLLFGETNQGCQIR pc2
E7EUJ1;E7EUK6;P11150
680:685 456:476 88:99
15:58723426:A:G_G
p = 8.7e−09, beta = −0.259, N = 1224
SHVHLIGYSLGAHVSGFAGSSIGGTHK
E7EUJ1;E7EUK6;P11150
86:685 25:476 6:99
15:58723426:A:G_G
p = 0.69, beta = 0.0636, N = 117
ITGLDAAGPLFEGSAPSNR pc2
E7EUJ1;E7EUK6;P11150
487:685 322:476 56:99
15:58723426:A:G_G
p = 0.0055, beta = −0.152, N = 865
HIAQHGFNAITQTIK pc3
E7EUJ1;E7EUK6;P11150
299:685 187:476 21:99
15:58723426:A:G_G
p = 0.67, beta = −0.0326, N = 507
VYHYQFK pc2
E7EUJ1;E7EUK6;P11150
16:685 10:476 2:99
15:58723426:A:G_G
p = 0.04, beta = 0.56, N = 28
QPIGHYDFYPNGGSFQPGCHFLELYR
E7EUJ1;E7EUK6;P11150
24:685 17:476 2:99
15:58723426:A:G_G
p = 0.45, beta = 0.188, N = 43
LIPC : NP4
P11150
174:174 127:127 24:24
15:58723426:A:G_G
p = 0.021, beta = −0.2, N = 325
WLEESVQLSR pc2
E7EUJ1;P11150
173:174 124:127 23:24
15:58723426:A:G_G
p = 0.11, beta = −0.141, N = 320
MTFCSENTDDLLLRPTQEK pc3
E7EUK6;P11150
172:174 126:127 24:24
15:58723426:A:G_G
p = 0.00012, beta = −0.333, N = 322
SQPAQPVNVGLVDWITLAHDHYTIAVR
E7EUJ1;P11150
171:174 127:127 24:24
15:58723426:A:G_G
p = 0.00017, beta = −0.325, N = 322
FLLFGETNQGCQIR pc2
E7EUJ1;E7EUK6;P11150
171:174 126:127 23:24
15:58723426:A:G_G
p = 0.0017, beta = −0.275, N = 320
CNTLGYHVR pc2
E7EUJ1;E7EUK6;P11150
101:174 69:127 7:24
15:58723426:A:G_G
p = 0.22, beta = −0.157, N = 177
EHMGLSVGIK pc2
E7EUJ1;E7EUK6;P11150
122:174 72:127 11:24
15:58723426:A:G_G
p = 0.38, beta = −0.101, N = 205
SHVHLIGYSLGAHVSGFAGSSIGGTHK
E7EUJ1;E7EUK6;P11150
133:174 82:127 15:24
15:58723426:A:G_G
p = 0.036, beta = −0.219, N = 230
ITGLDAAGPLFEGSAPSNR pc2
E7EUJ1;E7EUK6;P11150
126:174 85:127 14:24
15:58723426:A:G_G
p = 0.11, beta = −0.17, N = 225
143Assay Target: LIPC
UniProt: P11150
rsID: rs1077835
Target SNP: 15:58723426:A:G
Proxy SNP: 15:58723426:A:G
Tarkin log10(p): 12
Tarkin BETA: −0.311
Tarkin MSPA: 0.898
::*:*:*:*:−:*:−:−:−:−:−:NA:−:NA
1247:1222:1215:1124:1197:122
LSPDDANFVDAIHTFTR pc3
rs6083 REF
584:685 374:476 64:99
15:58723426:A:G_G
p = 4.7e−05, model = DOM, N = 1022
LSPDDASFVDAIHTFTR pc3
rs6083 ALT
413:685 298:476 78:99
15:58723426:A:G_G
p = 0.00048, model = DOM, N = 789
LSPDDASFVDAIHTFTR pc2
rs6083 ALT
106:685 61:476 17:99
15:58723426:A:G_G
p = 0.38, model = REC, N = 184
LSPDDANFVDAIHTFTR pc2
rs6083 REF
58:685 42:476 6:99
15:58723426:A:G_G
p = 0.45, model = DOM, N = 106

144. NUCB2 (P80303)
Tarkin MSPA=0.773

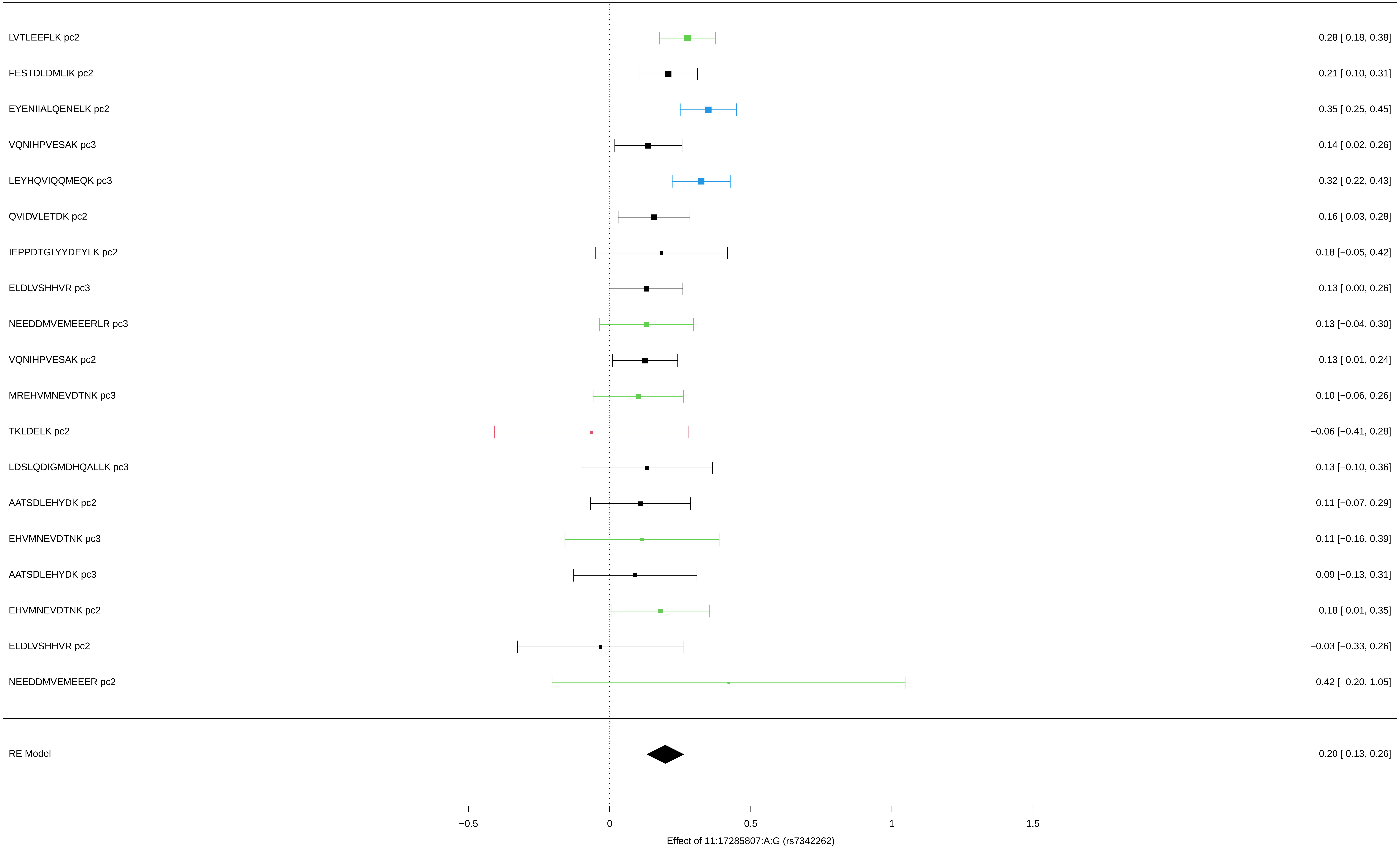

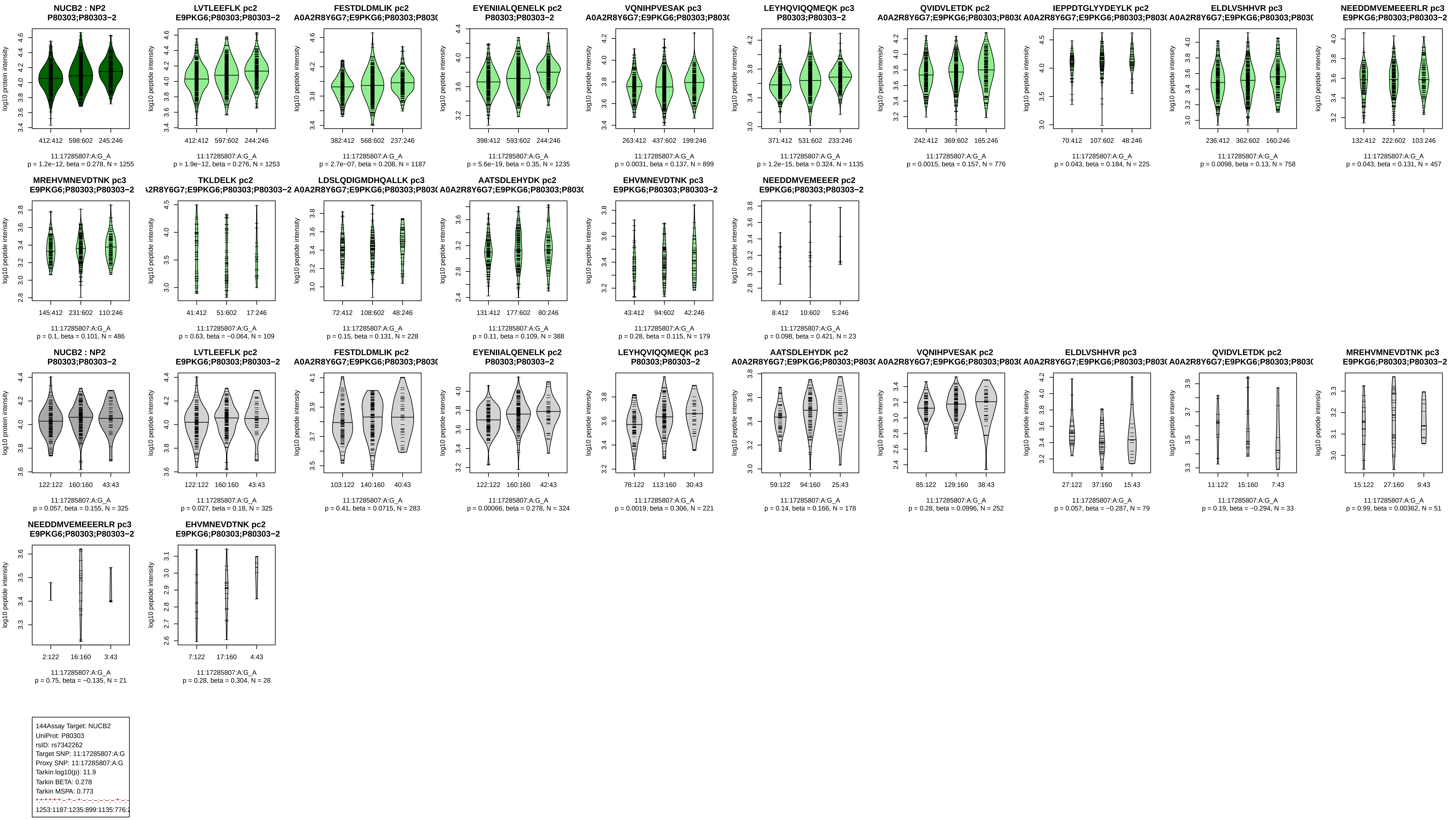
NUCB2 : NP2
P80303;P80303−2
412:412 598:602 245:246
11:17285807:A:G_A
p = 1.2e−12, beta = 0.278, N = 1255
LVTLEEFLK pc2
E9PKG6;P80303;P80303−2
412:412 597:602 244:246
11:17285807:A:G_A
p = 1.9e−12, beta = 0.276, N = 1253
FESTDLDMLIK pc2
A0A2R8Y6G7;E9PKG6;P80303;P8030
382:412 568:602 237:246
11:17285807:A:G_A
p = 2.7e−07, beta = 0.208, N = 1187
EYENIIALQENELK pc2
P80303;P80303−2
398:412 593:602 244:246
11:17285807:A:G_A
p = 5.6e−19, beta = 0.35, N = 1235
VQNIHPVESAK pc3
A0A2R8Y6G7;E9PKG6;P80303;P8030
263:412 437:602 199:246
11:17285807:A:G_A
p = 0.0031, beta = 0.137, N = 899
LEYHQVIQQMEQK pc3
P80303;P80303−2
371:412 531:602 233:246
11:17285807:A:G_A
p = 1.2e−15, beta = 0.324, N = 1135
QVIDVLETDK pc2
A0A2R8Y6G7;E9PKG6;P80303;P8030
242:412 369:602 165:246
11:17285807:A:G_A
p = 0.0015, beta = 0.157, N = 776
IEPPDTGLYYDEYLK pc2
A0A2R8Y6G7;E9PKG6;P80303;P8030
70:412 107:602 48:246
11:17285807:A:G_A
p = 0.043, beta = 0.184, N = 225
ELDLVSHHVR pc3
A0A2R8Y6G7;E9PKG6;P80303;P8030
236:412 362:602 160:246
11:17285807:A:G_A
p = 0.0098, beta = 0.13, N = 758
NEEDDMVEMEEERLR pc3
E9PKG6;P80303;P80303−2
132:412 222:602 103:246
11:17285807:A:G_A
p = 0.043, beta = 0.131, N = 457
MREHVMNEVDTNK pc3
E9PKG6;P80303;P80303−2
145:412 231:602 110:246
11:17285807:A:G_A
p = 0.1, beta = 0.101, N = 486
TKLDELK pc2
A2R8Y6G7;E9PKG6;P80303;P80303−2
41:412 51:602 17:246
11:17285807:A:G_A
p = 0.63, beta = −0.064, N = 109
LDSLQDIGMDHQALLK pc3
A0A2R8Y6G7;E9PKG6;P80303;P8030
72:412 108:602 48:246
11:17285807:A:G_A
p = 0.15, beta = 0.131, N = 228
AATSDLEHYDK pc2
A0A2R8Y6G7;E9PKG6;P80303;P8030
131:412 177:602 80:246
11:17285807:A:G_A
p = 0.11, beta = 0.109, N = 388
EHVMNEVDTNK pc3
E9PKG6;P80303;P80303−2
43:412 94:602 42:246
11:17285807:A:G_A
p = 0.28, beta = 0.115, N = 179
NEEDDMVEMEEER pc2
E9PKG6;P80303;P80303−2
8:412 10:602 5:246
11:17285807:A:G_A
p = 0.098, beta = 0.421, N = 23
NUCB2 : NP2
P80303;P80303−2
122:122 160:160 43:43
11:17285807:A:G_A
p = 0.057, beta = 0.155, N = 325
LVTLEEFLK pc2
E9PKG6;P80303;P80303−2
122:122 160:160 43:43
11:17285807:A:G_A
p = 0.027, beta = 0.18, N = 325
FESTDLDMLIK pc2
A0A2R8Y6G7;E9PKG6;P80303;P8030
103:122 140:160 40:43
11:17285807:A:G_A
p = 0.41, beta = 0.0715, N = 283
EYENIIALQENELK pc2
P80303;P80303−2
122:122 160:160 42:43
11:17285807:A:G_A
p = 0.00066, beta = 0.278, N = 324
LEYHQVIQQMEQK pc3
P80303;P80303−2
78:122 113:160 30:43
11:17285807:A:G_A
p = 0.0019, beta = 0.306, N = 221
AATSDLEHYDK pc2
A0A2R8Y6G7;E9PKG6;P80303;P8030
59:122 94:160 25:43
11:17285807:A:G_A
p = 0.14, beta = 0.166, N = 178
VQNIHPVESAK pc2
A0A2R8Y6G7;E9PKG6;P80303;P8030
85:122 129:160 38:43
11:17285807:A:G_A
p = 0.28, beta = 0.0996, N = 252
ELDLVSHHVR pc3
A0A2R8Y6G7;E9PKG6;P80303;P8030
27:122 37:160 15:43
11:17285807:A:G_A
p = 0.057, beta = −0.287, N = 79
QVIDVLETDK pc2
A0A2R8Y6G7;E9PKG6;P80303;P8030
11:122 15:160 7:43
11:17285807:A:G_A
p = 0.19, beta = −0.294, N = 33
MREHVMNEVDTNK pc3
E9PKG6;P80303;P80303−2
15:122 27:160 9:43
11:17285807:A:G_A
p = 0.99, beta = 0.00362, N = 51
NEEDDMVEMEEERLR pc3
E9PKG6;P80303;P80303−2
2:122 16:160 3:43
11:17285807:A:G_A
p = 0.75, beta = −0.135, N = 21
EHVMNEVDTNK pc2
E9PKG6;P80303;P80303−2
7:122 17:160 4:43
11:17285807:A:G_A
p = 0.28, beta = 0.304, N = 28
log10 protein intensity
log10 peptide intensity
144Assay Target: NUCB2
UniProt: P80303
rsID: rs7342262
Target SNP: 11:17285807:A:G
Proxy SNP: 11:17285807:A:G
Tarkin log10(p): 11.9
Tarkin BETA: 0.278
Tarkin MSPA: 0.773
1253:1187:1235:899:1135:776:2

145. ITIH4 (Q14624)
Tarkin MSPA=0.325

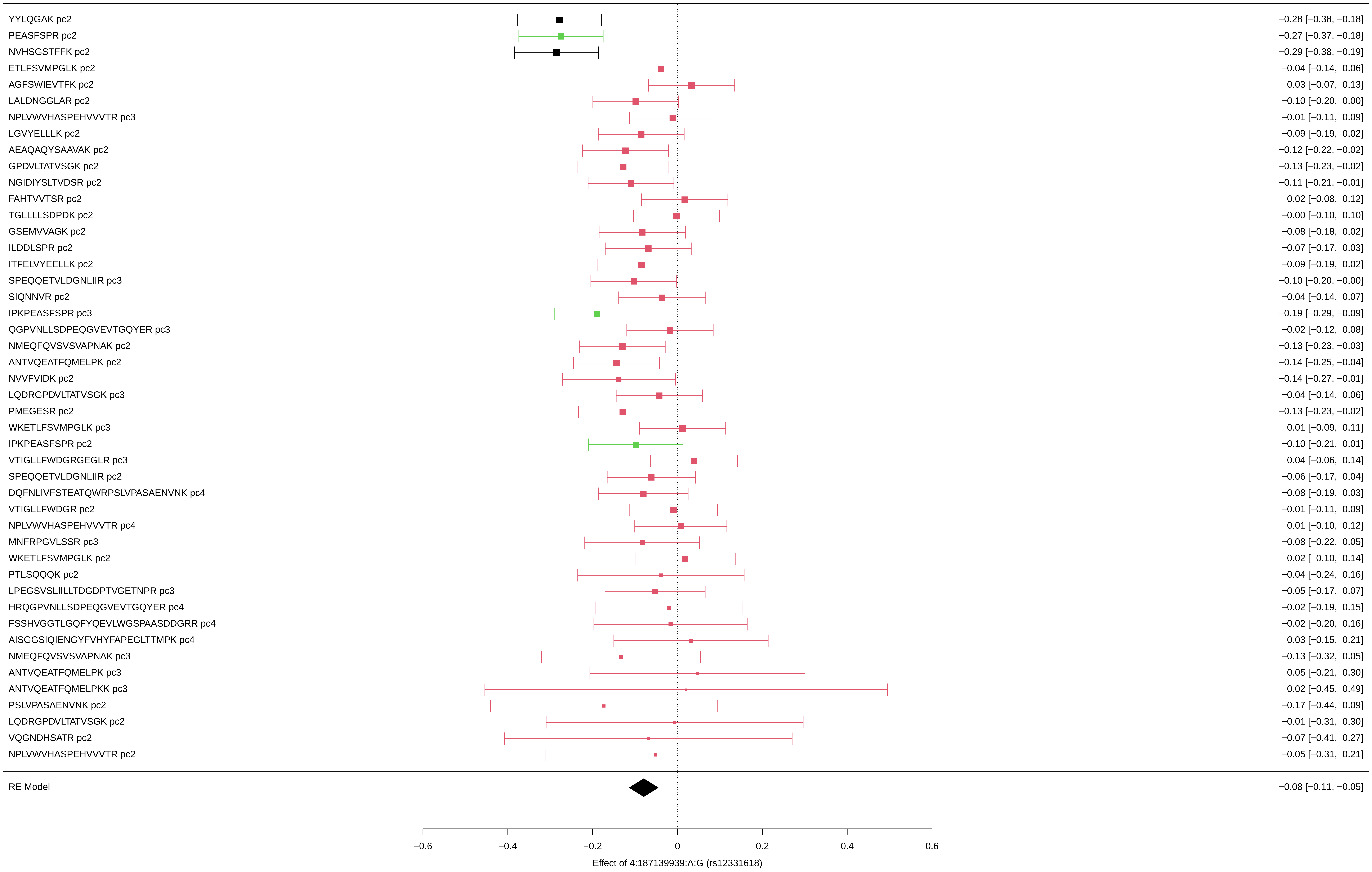

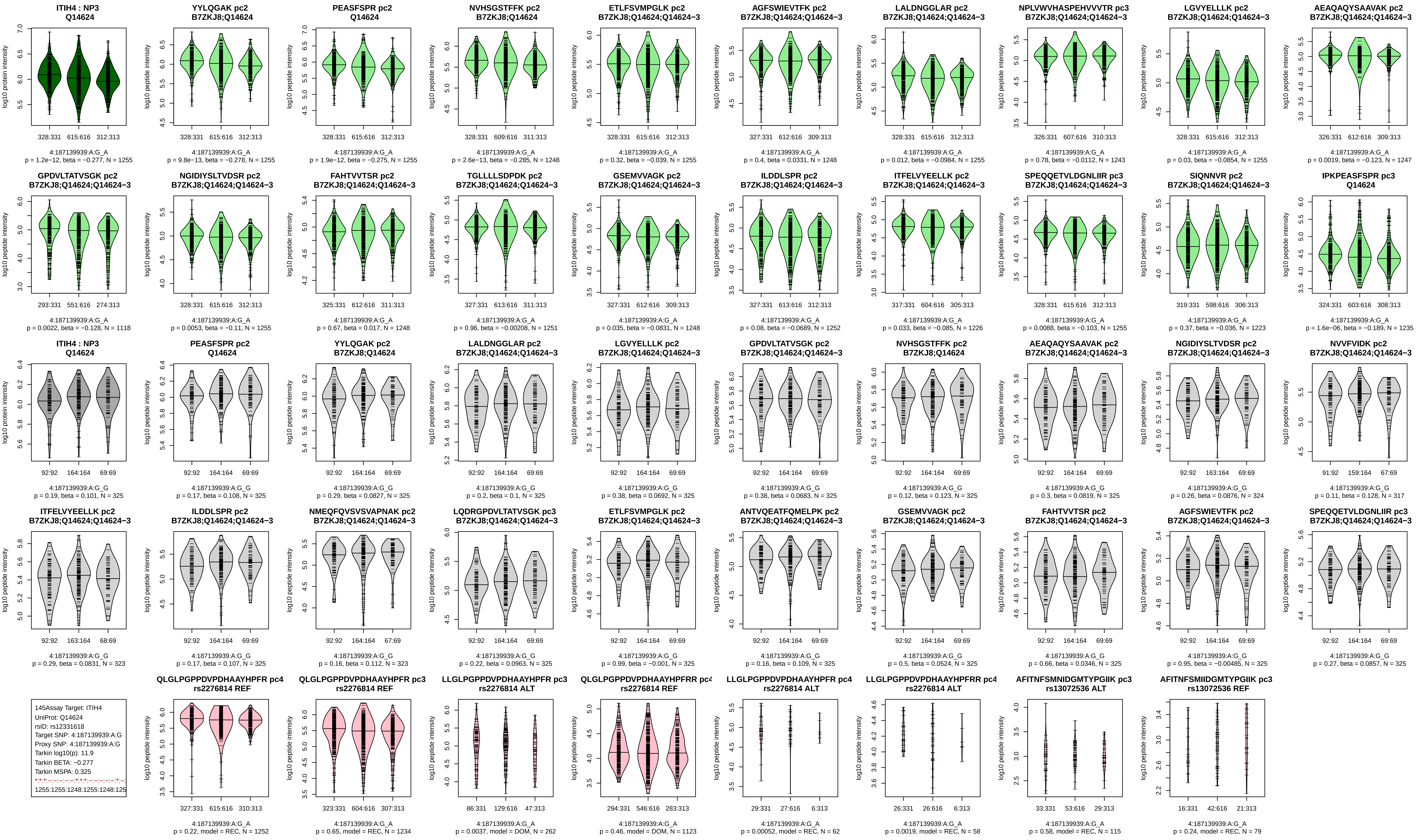

ITIH4 : NP3 Q14624
log10 protein intensity
328:331 615:616 312:313
4:187139939:A:G_A
p = 1.2e−12, beta = −0.277, N = 1255
YYLQGAK pc2 B7ZKJ8;Q14624
log10 peptide intensity
328:331 615:616 312:313
4:187139939:A:G_A
p = 9.8e−13, beta = −0.278, N = 1255
PEASFSPR pc2 Q14624
328:331 615:616 312:313
4:187139939:A:G_A
p = 1.9e−12, beta = −0.275, N = 1255
NVHSGSTFFK pc2 B7ZKJ8;Q14624
328:331 609:616 311:313
4:187139939:A:G_A
p = 2.6e−13, beta = −0.285, N = 1248
ETLFSVMPGLK pc2 B7ZKJ8;Q14624;Q14624−3
328:331 615:616 312:313
4:187139939:A:G_A
p = 0.32, beta = −0.039, N = 1255
AGFSWIEVTFK pc2 B7ZKJ8;Q14624;Q14624−3
327:331 612:616 309:313
4:187139939:A:G_A
p = 0.4, beta = 0.0331, N = 1248
LALDNGGLAR pc2 B7ZKJ8;Q14624;Q14624−3
328:331 615:616 312:313
4:187139939:A:G_A
p = 0.012, beta = −0.0984, N = 1255
NPLVWVHASPEHVVVTR pc3 B7ZKJ8;Q14624;Q14624−3
326:331 607:616 310:313
4:187139939:A:G_A
p = 0.78, beta = −0.0112, N = 1243
LGVYELLLK pc2 B7ZKJ8;Q14624;Q14624−3
328:331 615:616 312:313
4:187139939:A:G_A
p = 0.03, beta = −0.0854, N = 1255
AEAQAQYSAAVAK pc2 B7ZKJ8;Q14624;Q14624−3
326:331 612:616 309:313
4:187139939:A:G_A
p = 0.0019, beta = −0.123, N = 1247
GPDVLTATVSGK pc2 B7ZKJ8;Q14624;Q14624−3
293:331 551:616 274:313
4:187139939:A:G_A
p = 0.0022, beta = −0.128, N = 1118
NGIDIYSLTVDSR pc2 B7ZKJ8;Q14624;Q14624−3
328:331 615:616 312:313
4:187139939:A:G_A
p = 0.0053, beta = −0.11, N = 1255
FAHTVVTSR pc2 B7ZKJ8;Q14624;Q14624−3
325:331 612:616 311:313
4:187139939:A:G_A
p = 0.67, beta = 0.017, N = 1248
TGLLLLSDPDK pc2 B7ZKJ8;Q14624;Q14624−3
327:331 613:616 311:313
4:187139939:A:G_A
p = 0.96, beta = −0.00208, N = 1251
GSEMVVAGK pc2 B7ZKJ8;Q14624;Q14624−3
327:331 612:616 309:313
4:187139939:A:G_A
p = 0.035, beta = −0.0831, N = 1248
ILDDLSPR pc2 B7ZKJ8;Q14624;Q14624−3
327:331 613:616 312:313
4:187139939:A:G_A
p = 0.08, beta = −0.0689, N = 1252
ITFELVYEELLK pc2 B7ZKJ8;Q14624;Q14624−3
317:331 604:616 305:313
4:187139939:A:G_A
p = 0.033, beta = −0.085, N = 1226
SPEQQETVLDGNLIIR pc3 B7ZKJ8;Q14624;Q14624−3
328:331 615:616 312:313
4:187139939:A:G_A
p = 0.0088, beta = −0.103, N = 1255
SIQNNVR pc2 B7ZKJ8;Q14624;Q14624−3
319:331 598:616 306:313
4:187139939:A:G_A
p = 0.37, beta = −0.036, N = 1223
IPKPEASFSPR pc3 Q14624
324:331 603:616 308:313
4:187139939:A:G_A
p = 1.6e−06, beta = −0.189, N = 1235
ITIH4 : NP3 Q14624
92:92 164:164 69:69
4:187139939:A:G_G
p = 0.19, beta = 0.101, N = 325
PEASFSPR pc2 Q14624
92:92 164:164 69:69
4:187139939:A:G_G
p = 0.17, beta = 0.108, N = 325
YYLQGAK pc2 B7ZKJ8;Q14624
92:92 164:164 69:69
4:187139939:A:G_G
p = 0.29, beta = 0.0827, N = 325
LALDNGGLAR pc2 B7ZKJ8;Q14624;Q14624−3
92:92 164:164 69:69
4:187139939:A:G_G
p = 0.2, beta = 0.1, N = 325
LGVYELLLK pc2 B7ZKJ8;Q14624;Q14624−3
92:92 164:164 69:69
4:187139939:A:G_G
p = 0.38, beta = 0.0692, N = 325
GPDVLTATVSGK pc2 B7ZKJ8;Q14624;Q14624−3
92:92 164:164 69:69
4:187139939:A:G_G
p = 0.38, beta = 0.0683, N = 325
NVHSGSTFFK pc2 B7ZKJ8;Q14624
92:92 164:164 69:69
4:187139939:A:G_G
p = 0.12, beta = 0.123, N = 325
AEAQAQYSAAVAK pc2 B7ZKJ8;Q14624;Q14624−3
92:92 164:164 69:69
4:187139939:A:G_G
p = 0.3, beta = 0.0819, N = 325
NGIDIYSLTVDSR pc2 B7ZKJ8;Q14624;Q14624−3
92:92 163:164 69:69
4:187139939:A:G_G
p = 0.26, beta = 0.0876, N = 324
NVVFVIDK pc2 B7ZKJ8;Q14624;Q14624−3
91:92 159:164 67:69
4:187139939:A:G_G
p = 0.11, beta = 0.128, N = 317
ITFELVYEELLK pc2 B7ZKJ8;Q14624;Q14624−3
92:92 163:164 68:69
4:187139939:A:G_G
p = 0.29, beta = 0.0831, N = 323
ILDDLSPR pc2 B7ZKJ8;Q14624;Q14624−3
92:92 164:164 69:69
4:187139939:A:G_G
p = 0.17, beta = 0.107, N = 325
NMEQFQVSVSVAPNAK pc2 B7ZKJ8;Q14624;Q14624−3
92:92 164:164 67:69
4:187139939:A:G_G
p = 0.16, beta = 0.112, N = 323
LQDRGPDVLTATVSGK pc3 B7ZKJ8;Q14624;Q14624−3
92:92 164:164 69:69
4:187139939:A:G_G
p = 0.22, beta = 0.0963, N = 325
ETLFSVMPGLK pc2 B7ZKJ8;Q14624;Q14624−3
92:92 164:164 69:69
4:187139939:A:G_G
p = 0.99, beta = −0.001, N = 325
ANTVQEATFQMELPK pc2 B7ZKJ8;Q14624;Q14624−3
92:92 164:164 69:69
4:187139939:A:G_G
p = 0.16, beta = 0.109, N = 325
GSEMVVAGK pc2 B7ZKJ8;Q14624;Q14624−3
92:92 164:164 69:69
4:187139939:A:G_G
p = 0.5, beta = 0.0524, N = 325
FAHTVVTSR pc2 B7ZKJ8;Q14624;Q14624−3
92:92 164:164 69:69
4:187139939:A:G_G
p = 0.66, beta = 0.0346, N = 325
AGFSWIEVTFK pc2 B7ZKJ8;Q14624;Q14624−3
92:92 164:164 69:69
4:187139939:A:G_G
p = 0.95, beta = −0.00485, N = 325
SPEQQETVLDGNLIIR pc3 B7ZKJ8;Q14624;Q14624−3
92:92 164:164 69:69
4:187139939:A:G_G
p = 0.27, beta = 0.0857, N = 325
145Assay Target: ITIH4
UniProt: Q14624
rsID: rs12331618
Target SNP: 4:187139939:A:G
Proxy SNP: 4:187139939:A:G
Tarkin log10(p): 11.9
Tarkin BETA: −0.277
Tarkin MSPA: 0.325
1255:1255:1248:1255:1248:125
QLGLPGPPDVPDHAAYHPFR pc4 rs2276814 REF
327:331 615:616 310:313
4:187139939:A:G_A
p = 0.22, model = REC, N = 1252
QLGLPGPPDVPDHAAYHPFR pc3 rs2276814 REF
323:331 604:616 307:313
4:187139939:A:G_A
p = 0.65, model = REC, N = 1234
LLGLPGPPDVPDHAAYHPFR pc3 rs2276814 ALT
86:331 129:616 47:313
4:187139939:A:G_A
p = 0.0037, model = DOM, N = 262
QLGLPGPPDVPDHAAYHPFRR pc4 rs2276814 REF
294:331 546:616 283:313
4:187139939:A:G_A
p = 0.46, model = DOM, N = 1123
LLGLPGPPDVPDHAAYHPFR pc4 rs2276814 ALT
29:331 27:616 6:313
4:187139939:A:G_A
p = 0.00052, model = REC, N = 62
LLGLPGPPDVPDHAAYHPFRR pc4 rs2276814 ALT
26:331 26:616 6:313
4:187139939:A:G_A
p = 0.0019, model = REC, N = 58
AFITNFSMNIDGMTYPGIIK pc3 rs13072536 ALT
33:331 53:616 29:313
4:187139939:A:G_A
p = 0.58, model = REC, N = 115
AFITNFSMIIDGMTYPGIIK pc3 rs13072536 REF
16:331 42:616 21:313
4:187139939:A:G_A
p = 0.24, model = REC, N = 79

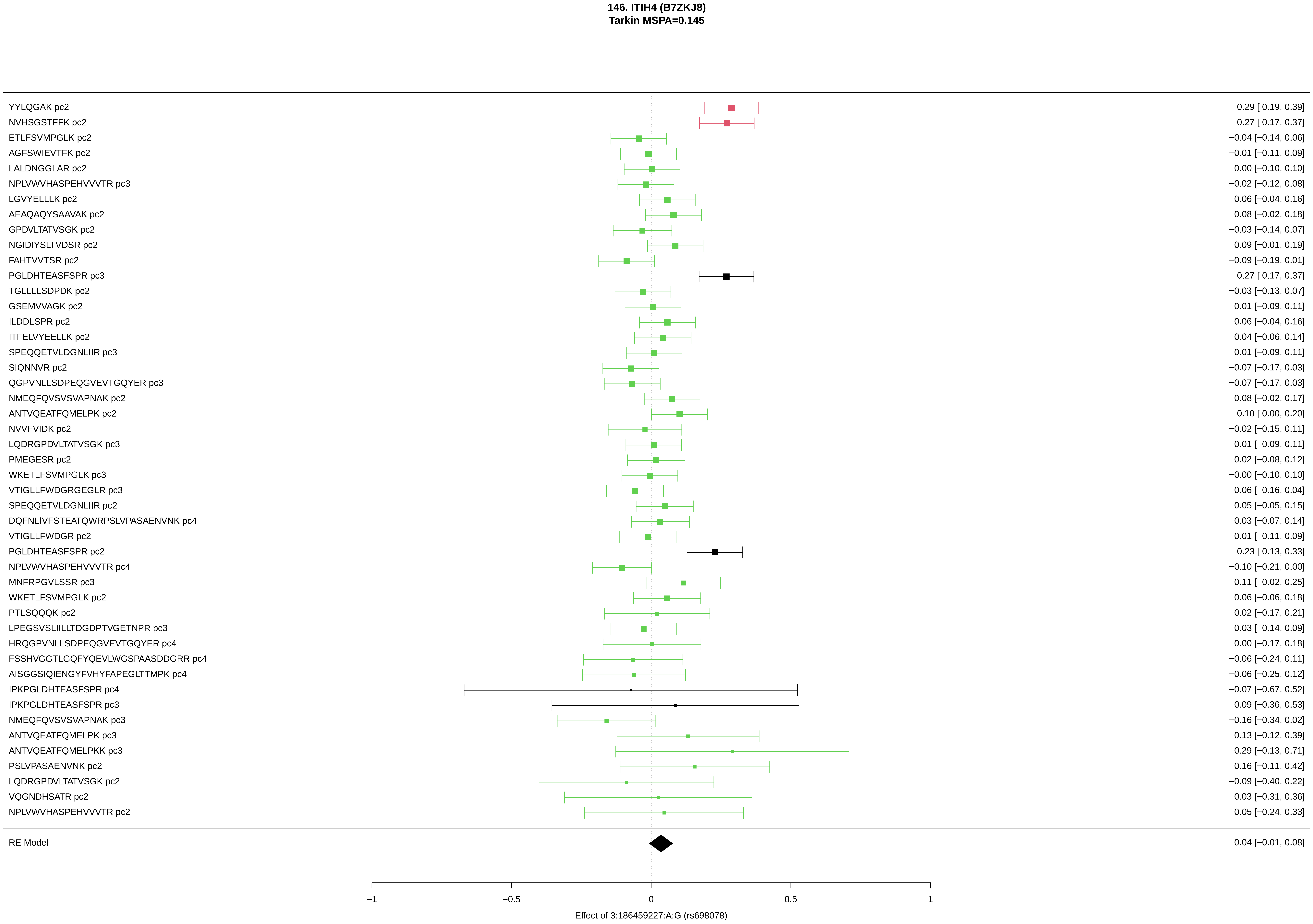
146. ITIH4 (B7ZKJ8)
Tarkin MSPA=0.145
YYLQGAK pc2
NVHSGSTFFK pc2
ETLFSVMPGLK pc2
AGFSWIEVTFK pc2
LALDNGGLAR pc2
NPLVWVHASPEHVVVTR pc3
LGVYELLLK pc2
AEAQAQYSAAVAK pc2
GPDVLTATVSGK pc2
NGIDIYSLTVDSR pc2
FAHTVVTSR pc2
PGLDHTEASFSPR pc3
TGLLLLSDPDK pc2
GSEMVVAGK pc2
ILDDLSPR pc2
ITFELVYEELLK pc2
SPEQQETVLDGNLIIR pc3
SIQNNVR pc2
QGPVNLLSDPEQGVEVTGQYER pc3
NMEQFQVSVSVAPNAK pc2
ANTVQEATFQMELPK pc2
NVVFVIDK pc2
LQDRGPDVLTATVSGK pc3
PMEGESR pc2
WKETLFSVMPGLK pc3
VTIGLLFWDGRGEGLR pc3
SPEQQETVLDGNLIIR pc2
DQFNLIVFSTEATQWRPSLVPASAENVNK pc4
VTIGLLFWDGR pc2
PGLDHTEASFSPR pc2
NPLVWVHASPEHVVVTR pc4
MNFRPGVLSSR pc3
WKETLFSVMPGLK pc2
PTLSQQQK pc2
LPEGSVSLIILLTDGDPTVGETNPR pc3
HRQGPVNLLSDPEQGVEVTGQYER pc4
FSSHVGGTLGQFYQEVLWGSPAASDDGRR pc4
AISGGSIQIENGYFVHYFAPEGLTTMPK pc4
IPKPGLDHTEASFSPR pc4
IPKPGLDHTEASFSPR pc3
NMEQFQVSVSVAPNAK pc3
ANTVQEATFQMELPK pc3
ANTVQEATFQMELPKK pc3
PSLVPASAENVNK pc2
LQDRGPDVLTATVSGK pc2
VQGNDHSATR pc2
NPLVWVHASPEHVVVTR pc2
RE Model
0.29 [0.19, 0.39]
0.27 [0.17, 0.37]
−0.04 [−0.14, 0.06]
−0.01 [−0.11, 0.09]
0.00 [−0.10, 0.10]
−0.02 [−0.12, 0.08]
0.06 [−0.04, 0.16]
0.08 [−0.02, 0.18]
−0.03 [−0.14, 0.07]
0.09 [−0.01, 0.19]
−0.09 [−0.19, 0.01]
0.27 [0.17, 0.37]
−0.03 [−0.13, 0.07]
0.01 [−0.09, 0.11]
0.06 [−0.04, 0.16]
0.04 [−0.06, 0.14]
0.01 [−0.09, 0.11]
−0.07 [−0.17, 0.03]
−0.07 [−0.17, 0.03]
0.08 [−0.02, 0.17]
0.10 [0.00, 0.20]
−0.02 [−0.15, 0.11]
0.01 [−0.09, 0.11]
0.02 [−0.08, 0.12]
−0.00 [−0.10, 0.10]
−0.06 [−0.16, 0.04]
0.05 [−0.05, 0.15]
0.03 [−0.07, 0.14]
−0.01 [−0.11, 0.09]
0.23 [0.13, 0.33]
−0.10 [−0.21, 0.00]
0.11 [−0.02, 0.25]
0.06 [−0.06, 0.18]
0.02 [−0.17, 0.21]
−0.03 [−0.14, 0.09]
0.00 [−0.17, 0.18]
−0.06 [−0.24, 0.11]
−0.06 [−0.25, 0.12]
−0.07 [−0.67, 0.52]
0.09 [−0.36, 0.53]
−0.16 [−0.34, 0.02]
0.13 [−0.12, 0.39]
0.29 [−0.13, 0.71]
0.16 [−0.11, 0.42]
−0.09 [−0.40, 0.22]
0.03 [−0.31, 0.36]
0.05 [−0.24, 0.33]
0.04 [−0.01, 0.08]
−1
−0.5
0
0.5
1
Effect of 3:186459227:A:G (rs698078)

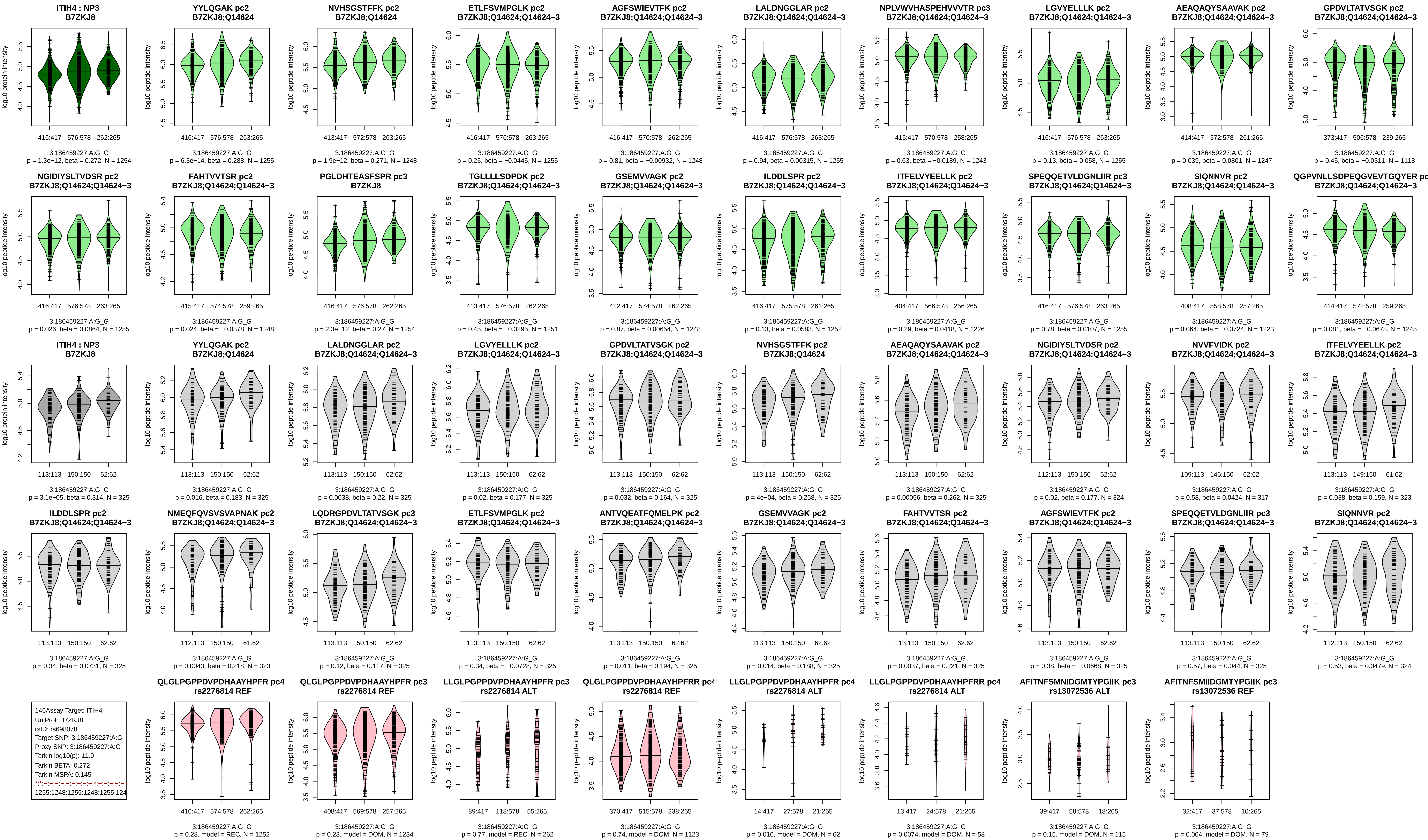
ITIH4 : NP3
B7ZKJ8
416:417 576:578 262:265
3:186459227:A:G_G
p = 1.3e−12, beta = 0.272, N = 1254
YYLQGAK pc2
B7ZKJ8;Q14624
416:417 576:578 263:265
3:186459227:A:G_G
p = 6.3e−14, beta = 0.288, N = 1255
NVHSGSTFFK pc2
B7ZKJ8;Q14624
413:417 572:578 263:265
3:186459227:A:G_G
p = 1.9e−12, beta = 0.271, N = 1248
ETLFSVMPGLK pc2
B7ZKJ8;Q14624;Q14624−3
416:417 576:578 263:265
3:186459227:A:G_G
p = 0.25, beta = −0.0445, N = 1255
AGFSWIEVTFK pc2
B7ZKJ8;Q14624;Q14624−3
416:417 570:578 262:265
3:186459227:A:G_G
p = 0.81, beta = −0.00932, N = 1248
LALDNGGLAR pc2
B7ZKJ8;Q14624;Q14624−3
416:417 576:578 263:265
3:186459227:A:G_G
p = 0.94, beta = 0.00315, N = 1255
NPLVWVHASPEHVVVTR pc3
B7ZKJ8;Q14624;Q14624−3
415:417 570:578 258:265
3:186459227:A:G_G
p = 0.63, beta = −0.0189, N = 1243
LGVYELLLK pc2
B7ZKJ8;Q14624;Q14624−3
416:417 576:578 263:265
3:186459227:A:G_G
p = 0.13, beta = 0.058, N = 1255
AEAQAQYSAAVAK pc2
B7ZKJ8;Q14624;Q14624−3
414:417 572:578 261:265
3:186459227:A:G_G
p = 0.039, beta = 0.0801, N = 1247
GPDVLTATVSGK pc2
B7ZKJ8;Q14624;Q14624−3
373:417 506:578 239:265
3:186459227:A:G_G
p = 0.45, beta = −0.0311, N = 1118
NGIDIYSLTVDSR pc2
B7ZKJ8;Q14624;Q14624−3
416:417 576:578 263:265
3:186459227:A:G_G
p = 0.026, beta = 0.0864, N = 1255
FAHTVVTSR pc2
B7ZKJ8;Q14624;Q14624−3
415:417 574:578 259:265
3:186459227:A:G_G
p = 0.024, beta = −0.0878, N = 1248
PGLDHTEASFSPR pc3
B7ZKJ8
416:417 576:578 262:265
3:186459227:A:G_G
p = 2.3e−12, beta = 0.27, N = 1254
TGLLLLSDPDK pc2
B7ZKJ8;Q14624;Q14624−3
413:417 576:578 262:265
3:186459227:A:G_G
p = 0.45, beta = −0.0295, N = 1251
GSEMVVAGK pc2
B7ZKJ8;Q14624;Q14624−3
412:417 574:578 262:265
3:186459227:A:G_G
p = 0.87, beta = 0.00654, N = 1248
ILDDLSPR pc2
B7ZKJ8;Q14624;Q14624−3
416:417 575:578 261:265
3:186459227:A:G_G
p = 0.13, beta = 0.0583, N = 1252
ITFELVYEELLK pc2
B7ZKJ8;Q14624;Q14624−3
404:417 566:578 256:265
3:186459227:A:G_G
p = 0.29, beta = 0.0418, N = 1226
SPEQQETVLDGNLIIR pc3
B7ZKJ8;Q14624;Q14624−3
416:417 576:578 263:265
3:186459227:A:G_G
p = 0.78, beta = 0.0107, N = 1255
SIQNNVR pc2
B7ZKJ8;Q14624;Q14624−3
408:417 558:578 257:265
3:186459227:A:G_G
p = 0.064, beta = −0.0724, N = 1223
QGPVNLLSDPEQGVEVTGQYER pc
B7ZKJ8;Q14624;Q14624−3
414:417 572:578 259:265
3:186459227:A:G_G
p = 0.081, beta = −0.0678, N = 1245
ITIH4 : NP3
B7ZKJ8
113:113 150:150 62:62
3:186459227:A:G_G
p = 3.1e−05, beta = 0.314, N = 325
YYLQGAK pc2
B7ZKJ8;Q14624
113:113 150:150 62:62
3:186459227:A:G_G
p = 0.016, beta = 0.183, N = 325
LALDNGGLAR pc2
B7ZKJ8;Q14624;Q14624−3
113:113 150:150 62:62
3:186459227:A:G_G
p = 0.0038, beta = 0.22, N = 325
LGVYELLLK pc2
B7ZKJ8;Q14624;Q14624−3
113:113 150:150 62:62
3:186459227:A:G_G
p = 0.02, beta = 0.177, N = 325
GPDVLTATVSGK pc2
B7ZKJ8;Q14624;Q14624−3
113:113 150:150 62:62
3:186459227:A:G_G
p = 0.032, beta = 0.164, N = 325
NVHSGSTFFK pc2
B7ZKJ8;Q14624
113:113 150:150 62:62
3:186459227:A:G_G
p = 4e−04, beta = 0.268, N = 325
AEAQAQYSAAVAK pc2
B7ZKJ8;Q14624;Q14624−3
113:113 150:150 62:62
3:186459227:A:G_G
p = 0.00056, beta = 0.262, N = 325
NGIDIYSLTVDSR pc2
B7ZKJ8;Q14624;Q14624−3
112:113 150:150 62:62
3:186459227:A:G_G
p = 0.02, beta = 0.177, N = 324
NVVFVIDK pc2
B7ZKJ8;Q14624;Q14624−3
109:113 146:150 62:62
3:186459227:A:G_G
p = 0.58, beta = 0.0424, N = 317
ITFELVYEELLK pc2
B7ZKJ8;Q14624;Q14624−3
113:113 149:150 61:62
3:186459227:A:G_G
p = 0.038, beta = 0.159, N = 323
ILDDLSPR pc2
B7ZKJ8;Q14624;Q14624−3
113:113 150:150 62:62
3:186459227:A:G_G
p = 0.34, beta = 0.0731, N = 325
NMEQFQVSVSVAPNAK pc2
B7ZKJ8;Q14624;Q14624−3
112:113 150:150 61:62
3:186459227:A:G_G
p = 0.0043, beta = 0.218, N = 323
LQDRGPDVLTATVSGK pc3
B7ZKJ8;Q14624;Q14624−3
113:113 150:150 62:62
3:186459227:A:G_G
p = 0.12, beta = 0.117, N = 325
ETLFSVMPGLK pc2
B7ZKJ8;Q14624;Q14624−3
113:113 150:150 62:62
3:186459227:A:G_G
p = 0.34, beta = −0.0728, N = 325
ANTVQEATFQMELPK pc2
B7ZKJ8;Q14624;Q14624−3
113:113 150:150 62:62
3:186459227:A:G_G
p = 0.011, beta = 0.194, N = 325
GSEMVVAGK pc2
B7ZKJ8;Q14624;Q14624−3
113:113 150:150 62:62
3:186459227:A:G_G
p = 0.014, beta = 0.188, N = 325
FAHTVVTSR pc2
B7ZKJ8;Q14624;Q14624−3
113:113 150:150 62:62
3:186459227:A:G_G
p = 0.0037, beta = 0.221, N = 325
AGFSWIEVTFK pc2
B7ZKJ8;Q14624;Q14624−3
113:113 150:150 62:62
3:186459227:A:G_G
p = 0.38, beta = −0.0668, N = 325
SPEQQETVLDGNLIIR pc3
B7ZKJ8;Q14624;Q14624−3
113:113 150:150 62:62
3:186459227:A:G_G
p = 0.57, beta = 0.044, N = 325
SIQNNVR pc2
B7ZKJ8;Q14624;Q14624−3
112:113 150:150 62:62
3:186459227:A:G_G
p = 0.53, beta = 0.0479, N = 324
146Assay Target: ITIH4
UniProt: B7ZKJ8
rsID: rs698078
Target SNP: 3:186459227:A:G
Proxy SNP: 3:186459227:A:G
Tarkin log10(p): 11.9
Tarkin BETA: 0.272
Tarkin MSPA: 0.145
1255:1248:1255:1248:1255:124
QLGLPGPPDVPDHAAYHPFR pc4
rs2276814 REF
416:417 574:578 262:265
3:186459227:A:G_G
p = 0.28, model = REC, N = 1252
QLGLPGPPDVPDHAAYHPFR pc3
rs2276814 REF
408:417 569:578 257:265
3:186459227:A:G_G
p = 0.23, model = DOM, N = 1234
LLGLPGPPDVPDHAAYHPFR pc3
rs2276814 ALT
89:417 118:578 55:265
3:186459227:A:G_G
p = 0.77, model = REC, N = 262
QLGLPGPPDVPDHAAYHPFRR pc4
rs2276814 REF
370:417 515:578 238:265
3:186459227:A:G_G
p = 0.74, model = DOM, N = 1123
LLGLPGPPDVPDHAAYHPFR pc4
rs2276814 ALT
14:417 27:578 21:265
3:186459227:A:G_G
p = 0.016, model = DOM, N = 62
LLGLPGPPDVPDHAAYHPFRR pc4
rs2276814 ALT
13:417 24:578 21:265
3:186459227:A:G_G
p = 0.0074, model = DOM, N = 58
AFITNFSMNIDGMTYPGIIK pc3
rs13072536 ALT
39:417 58:578 18:265
3:186459227:A:G_G
p = 0.15, model = DOM, N = 115
AFITNFSMIIDGMTYPGIIK pc3
rs13072536 REF
32:417 37:578 10:265
3:186459227:A:G_G
p = 0.064, model = DOM, N = 79
log10 protein intensity
log10 peptide intensity

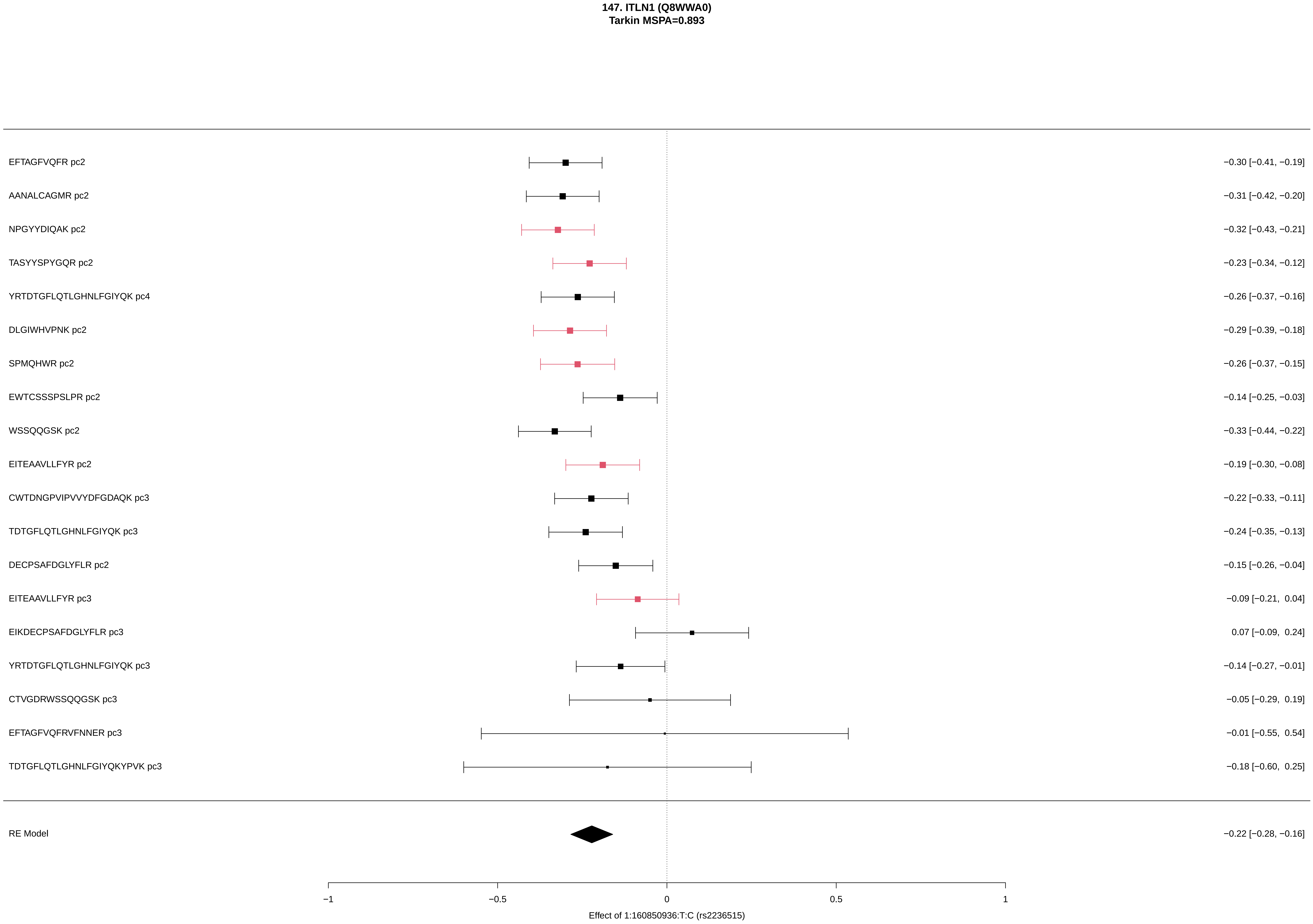
147. ITLN1 (Q8WWA0)
Tarkin MSPA=0.893
EFTAGFVQFR pc2
−0.30 [−0.41, −0.19]
AANALCAGMR pc2
−0.31 [−0.42, −0.20]
NPGYYDIQAK pc2
−0.32 [−0.43, −0.21]
TASYYSPYGQR pc2
−0.23 [−0.34, −0.12]
YRTDTGFLQTLGHNLFGIYQK pc4
−0.26 [−0.37, −0.16]
DLGIWHVPNK pc2
−0.29 [−0.39, −0.18]
SPMQHWR pc2
−0.26 [−0.37, −0.15]
EWTCSSSPSLPR pc2
−0.14 [−0.25, −0.03]
WSSQQGSK pc2
−0.33 [−0.44, −0.22]
EITEAAVLLFYR pc2
−0.19 [−0.30, −0.08]
CWTDNGPVIPVVYDFGDAQK pc3
−0.22 [−0.33, −0.11]
TDTGFLQTLGHNLFGIYQK pc3
−0.24 [−0.35, −0.13]
DECPSAFDGLYFLR pc2
−0.15 [−0.26, −0.04]
EITEAAVLLFYR pc3
−0.09 [−0.21, 0.04]
EIKDECPSAFDGLYFLR pc3
0.07 [−0.09, 0.24]
YRTDTGFLQTLGHNLFGIYQK pc3
−0.14 [−0.27, −0.01]
CTVGDRWSSQQGSK pc3
−0.05 [−0.29, 0.19]
EFTAGFVQFRVFNNER pc3
−0.01 [−0.55, 0.54]
TDTGFLQTLGHNLFGIYQKYPVK pc3
−0.18 [−0.60, 0.25]
RE Model
−0.22 [−0.28, −0.16]
−1
−0.5
0
0.5
1
Effect of 1:160850936:T:C (rs2236515)

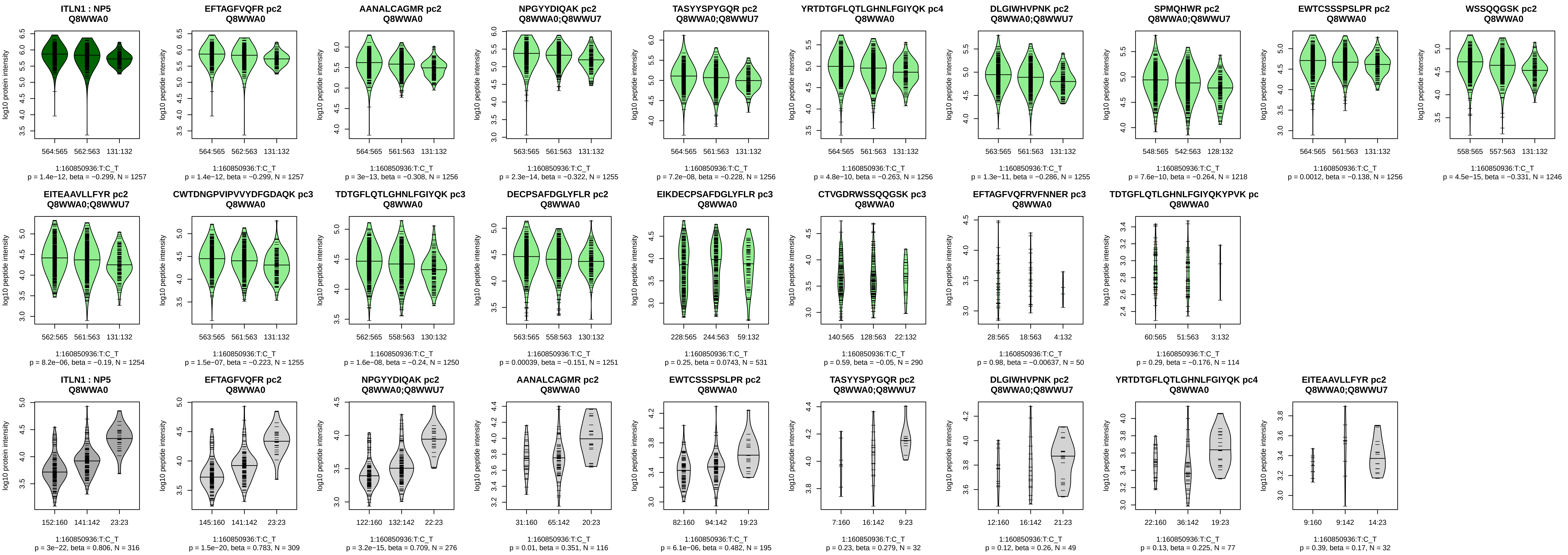

147Assay Target: ITLN1
UniProt: Q8WWA0
rsID: rs2236515
Target SNP: 1:160850936:T:C
Proxy SNP: 1:160850936:T:C
Tarkin log10(p): 11.9
Tarkin BETA: −0.299
Tarkin MSPA: 0.893
::*:*:*:*:*:*:*:*:*:*:*:−:−:*:−:−:−:N
1257:1256:1255:1256:1256:125

148. BPI (P17213)
Tarkin MSPA=0.839

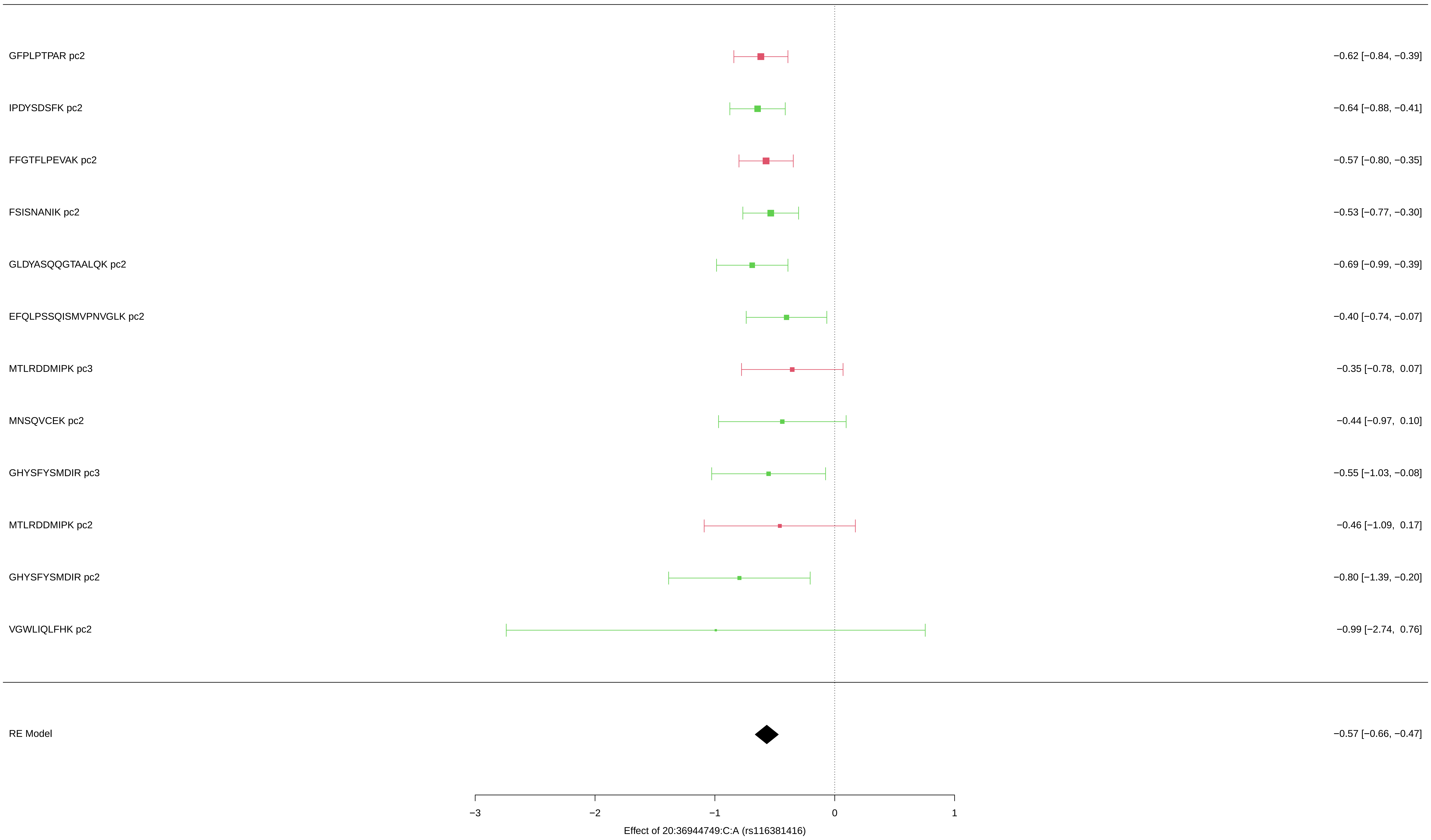

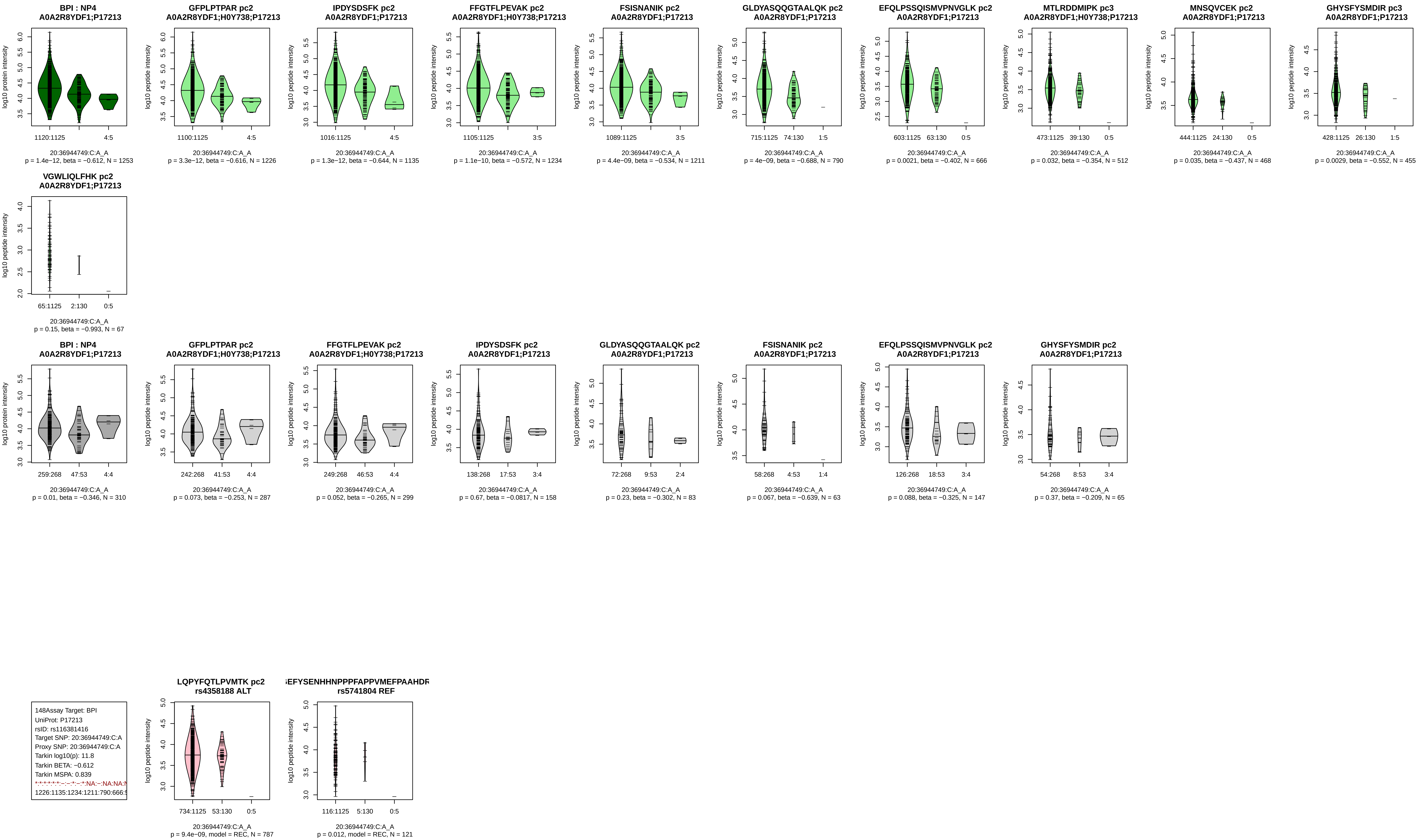
BPI : NP4
A0A2R8YDF1;P17213
20:36944749:C:A_A
p = 1.4e−12, beta = −0.612, N = 1253
GFPLPTPAR pc2
A0A2R8YDF1;H0Y738;P17213
p = 3.3e−12, beta = −0.616, N = 1226
IPDYSDSFK pc2
A0A2R8YDF1;P17213
p = 1.3e−12, beta = −0.644, N = 1135
FFGTFLPEVAK pc2
A0A2R8YDF1;H0Y738;P17213
p = 1.1e−10, beta = −0.572, N = 1234
FSISNANIK pc2
A0A2R8YDF1;P17213
p = 4.4e−09, beta = −0.534, N = 1211
GLDYASQQGTAALQK pc2
A0A2R8YDF1;P17213
p = 4e−09, beta = −0.688, N = 790
EFQLPSSQISMVPNVGLK pc2
A0A2R8YDF1;P17213
p = 0.0021, beta = −0.402, N = 666
MTLRDDMIPK pc3
A0A2R8YDF1;H0Y738;P17213
p = 0.032, beta = −0.354, N = 512
MNSQVCEK pc2
A0A2R8YDF1;P17213
p = 0.035, beta = −0.437, N = 468
GHYSFYSMDIR pc3
A0A2R8YDF1;P17213
p = 0.0029, beta = −0.552, N = 455
VGWLIQLFHK pc2
A0A2R8YDF1;P17213
p = 0.15, beta = −0.993, N = 67
BPI : NP4
A0A2R8YDF1;P17213
p = 0.01, beta = −0.346, N = 310
GFPLPTPAR pc2
A0A2R8YDF1;H0Y738;P17213
p = 0.073, beta = −0.253, N = 287
FFGTFLPEVAK pc2
A0A2R8YDF1;H0Y738;P17213
p = 0.052, beta = −0.265, N = 299
IPDYSDSFK pc2
A0A2R8YDF1;P17213
p = 0.67, beta = −0.0817, N = 158
GLDYASQQGTAALQK pc2
A0A2R8YDF1;P17213
p = 0.23, beta = −0.302, N = 83
FSISNANIK pc2
A0A2R8YDF1;P17213
p = 0.067, beta = −0.639, N = 63
EFQLPSSQISMVPNVGLK pc2
A0A2R8YDF1;P17213
p = 0.088, beta = −0.325, N = 147
GHYSFYSMDIR pc2
A0A2R8YDF1;P17213
p = 0.37, beta = −0.209, N = 65
148Assay Target: BPI
UniProt: P17213
rsID: rs116381416
Target SNP: 20:36944749:C:A
Proxy SNP: 20:36944749:C:A
Tarkin log10(p): 11.8
Tarkin BETA: −0.612
Tarkin MSPA: 0.839
LQPYFQTLPVMTK pc2
rs4358188 ALT
p = 9.4e−09, model = REC, N = 787
rs5741804 REF
p = 0.012, model = REC, N = 121

149. ITIH4 (B7ZKJ8)
Tarkin MSPA=0.315

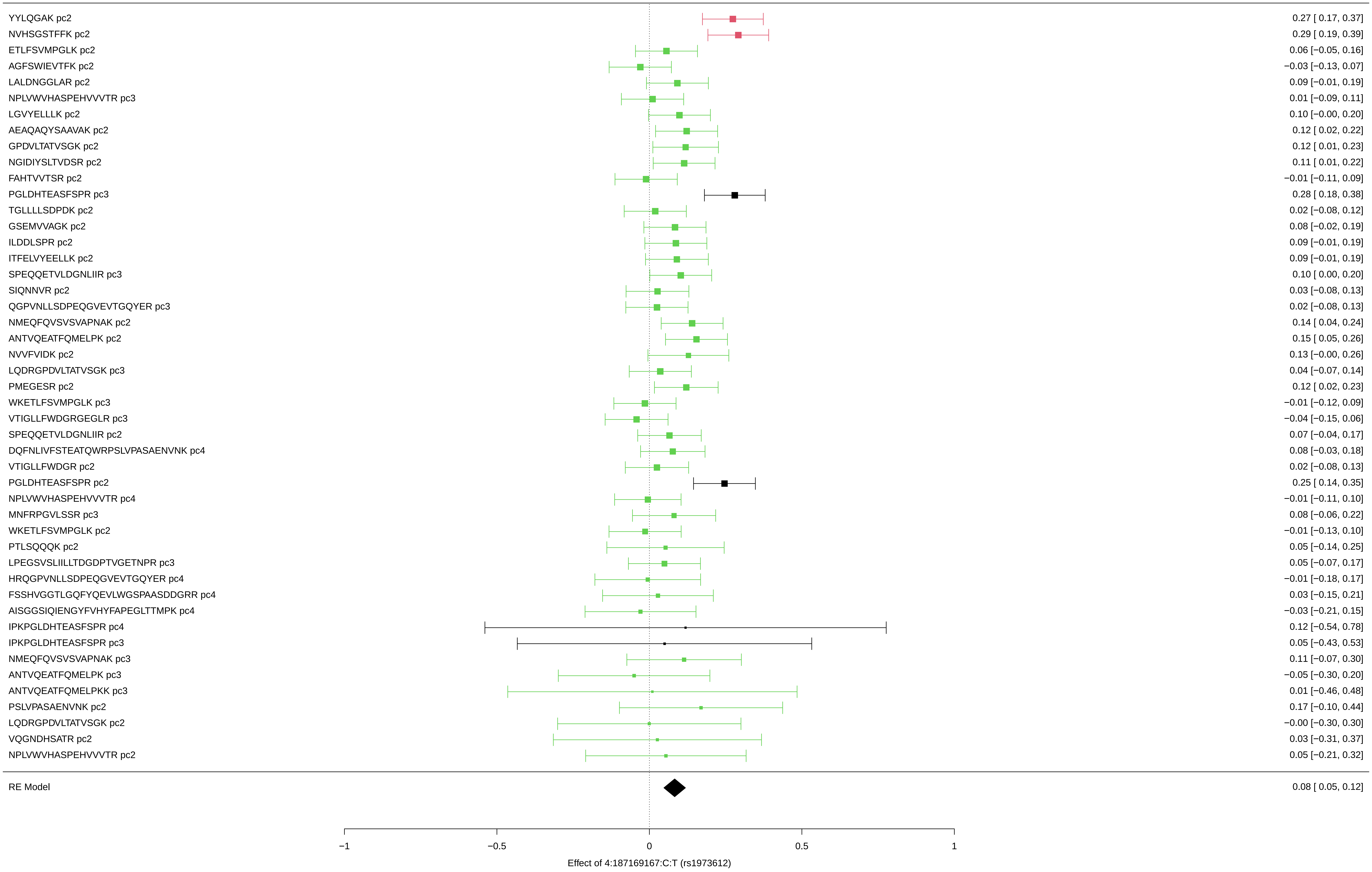

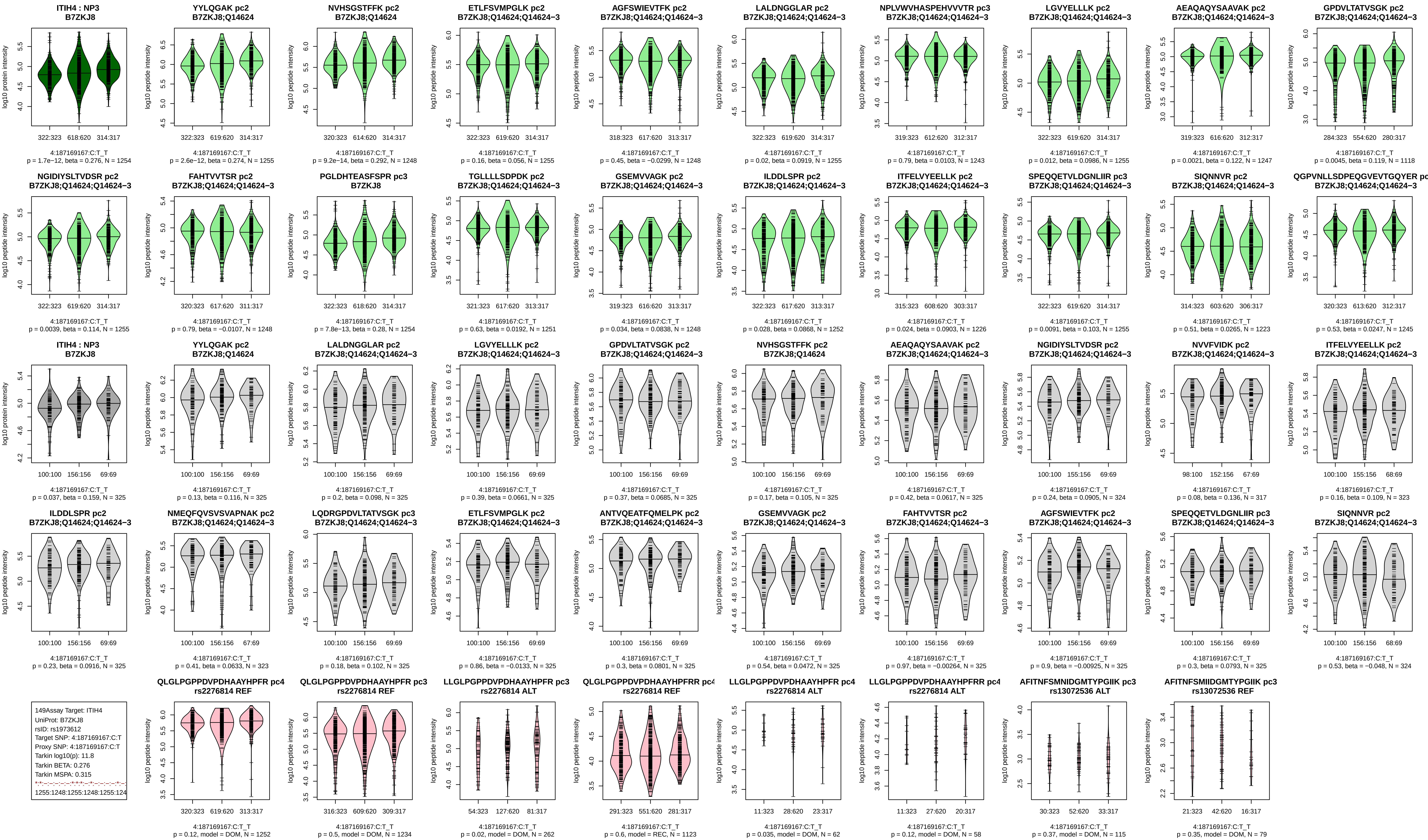
ITIH4 : NP3
B7ZKJ8
log10 protein intensity
322:323 618:620 314:317
4:187169167:C:T_T
p = 1.7e−12, beta = 0.276, N = 1254
YYLQGAK pc2
B7ZKJ8;Q14624
log10 peptide intensity
322:323 619:620 314:317
4:187169167:C:T_T
p = 2.6e−12, beta = 0.274, N = 1255
NVHSGSTFFK pc2
B7ZKJ8;Q14624
320:323 614:620 314:317
4:187169167:C:T_T
p = 9.2e−14, beta = 0.292, N = 1248
ETLFSVMPGLK pc2
B7ZKJ8;Q14624;Q14624−3
322:323 619:620 314:317
4:187169167:C:T_T
p = 0.16, beta = 0.056, N = 1255
AGFSWIEVTFK pc2
B7ZKJ8;Q14624;Q14624−3
318:323 617:620 313:317
4:187169167:C:T_T
p = 0.45, beta = −0.0299, N = 1248
LALDNGGLAR pc2
B7ZKJ8;Q14624;Q14624−3
322:323 619:620 314:317
4:187169167:C:T_T
p = 0.02, beta = 0.0919, N = 1255
NPLVWVHASPEHVVVTR pc3
B7ZKJ8;Q14624;Q14624−3
319:323 612:620 312:317
4:187169167:C:T_T
p = 0.79, beta = 0.0103, N = 1243
LGVYELLLK pc2
B7ZKJ8;Q14624;Q14624−3
322:323 619:620 314:317
4:187169167:C:T_T
p = 0.012, beta = 0.0986, N = 1255
AEAQAQYSAAVAK pc2
B7ZKJ8;Q14624;Q14624−3
319:323 616:620 312:317
4:187169167:C:T_T
p = 0.0021, beta = 0.122, N = 1247
GPDVLTATVSGK pc2
B7ZKJ8;Q14624;Q14624−3
284:323 554:620 280:317
4:187169167:C:T_T
p = 0.0045, beta = 0.119, N = 1118
NGIDIYSLTVDSR pc2
B7ZKJ8;Q14624;Q14624−3
322:323 619:620 314:317
4:187169167:C:T_T
p = 0.0039, beta = 0.114, N = 1255
FAHTVVTSR pc2
B7ZKJ8;Q14624;Q14624−3
320:323 617:620 311:317
4:187169167:C:T_T
p = 0.79, beta = −0.0107, N = 1248
PGLDHTEASFSPR pc3
B7ZKJ8
322:323 618:620 314:317
4:187169167:C:T_T
p = 7.8e−13, beta = 0.28, N = 1254
TGLLLLSDPDK pc2
B7ZKJ8;Q14624;Q14624−3
321:323 617:620 313:317
4:187169167:C:T_T
p = 0.63, beta = 0.0192, N = 1251
GSEMVVAGK pc2
B7ZKJ8;Q14624;Q14624−3
319:323 616:620 313:317
4:187169167:C:T_T
p = 0.034, beta = 0.0838, N = 1248
ILDDLSPR pc2
B7ZKJ8;Q14624;Q14624−3
322:323 617:620 313:317
4:187169167:C:T_T
p = 0.028, beta = 0.0868, N = 1252
ITFELVYEELLK pc2
B7ZKJ8;Q14624;Q14624−3
315:323 608:620 303:317
4:187169167:C:T_T
p = 0.024, beta = 0.0903, N = 1226
SPEQQETVLDGNLIIR pc3
B7ZKJ8;Q14624;Q14624−3
322:323 619:620 314:317
4:187169167:C:T_T
p = 0.0091, beta = 0.103, N = 1255
SIQNNVR pc2
B7ZKJ8;Q14624;Q14624−3
314:323 603:620 306:317
4:187169167:C:T_T
p = 0.51, beta = 0.0265, N = 1223
QGPVNLLSDPEQGVEVTGQYER pc
B7ZKJ8;Q14624;Q14624−3
320:323 613:620 312:317
4:187169167:C:T_T
p = 0.53, beta = 0.0247, N = 1245
ITIH4 : NP3
B7ZKJ8
100:100 156:156 69:69
4:187169167:C:T_T
p = 0.037, beta = 0.159, N = 325
YYLQGAK pc2
B7ZKJ8;Q14624
100:100 156:156 69:69
4:187169167:C:T_T
p = 0.13, beta = 0.116, N = 325
LALDNGGLAR pc2
B7ZKJ8;Q14624;Q14624−3
100:100 156:156 69:69
4:187169167:C:T_T
p = 0.2, beta = 0.098, N = 325
LGVYELLLK pc2
B7ZKJ8;Q14624;Q14624−3
100:100 156:156 69:69
4:187169167:C:T_T
p = 0.39, beta = 0.0661, N = 325
GPDVLTATVSGK pc2
B7ZKJ8;Q14624;Q14624−3
100:100 156:156 69:69
4:187169167:C:T_T
p = 0.37, beta = 0.0685, N = 325
NVHSGSTFFK pc2
B7ZKJ8;Q14624
100:100 156:156 69:69
4:187169167:C:T_T
p = 0.17, beta = 0.105, N = 325
AEAQAQYSAAVAK pc2
B7ZKJ8;Q14624;Q14624−3
100:100 156:156 69:69
4:187169167:C:T_T
p = 0.42, beta = 0.0617, N = 325
NGIDIYSLTVDSR pc2
B7ZKJ8;Q14624;Q14624−3
100:100 155:156 69:69
4:187169167:C:T_T
p = 0.24, beta = 0.0905, N = 324
NVVFVIDK pc2
B7ZKJ8;Q14624;Q14624−3
98:100 152:156 67:69
4:187169167:C:T_T
p = 0.08, beta = 0.136, N = 317
ITFELVYEELLK pc2
B7ZKJ8;Q14624;Q14624−3
100:100 155:156 68:69
4:187169167:C:T_T
p = 0.16, beta = 0.109, N = 323
ILDDLSPR pc2
B7ZKJ8;Q14624;Q14624−3
100:100 156:156 69:69
4:187169167:C:T_T
p = 0.23, beta = 0.0916, N = 325
NMEQFQVSVSVAPNAK pc2
B7ZKJ8;Q14624;Q14624−3
100:100 156:156 67:69
4:187169167:C:T_T
p = 0.41, beta = 0.0633, N = 323
LQDRGPDVLTATVSGK pc3
B7ZKJ8;Q14624;Q14624−3
100:100 156:156 69:69
4:187169167:C:T_T
p = 0.18, beta = 0.102, N = 325
ETLFSVMPGLK pc2
B7ZKJ8;Q14624;Q14624−3
100:100 156:156 69:69
4:187169167:C:T_T
p = 0.86, beta = −0.0133, N = 325
ANTVQEATFQMELPK pc2
B7ZKJ8;Q14624;Q14624−3
100:100 156:156 69:69
4:187169167:C:T_T
p = 0.3, beta = 0.0801, N = 325
GSEMVVAGK pc2
B7ZKJ8;Q14624;Q14624−3
100:100 156:156 69:69
4:187169167:C:T_T
p = 0.54, beta = 0.0472, N = 325
FAHTVVTSR pc2
B7ZKJ8;Q14624;Q14624−3
100:100 156:156 69:69
4:187169167:C:T_T
p = 0.97, beta = −0.00264, N = 325
AGFSWIEVTFK pc2
B7ZKJ8;Q14624;Q14624−3
100:100 156:156 69:69
4:187169167:C:T_T
p = 0.9, beta = −0.00925, N = 325
SPEQQETVLDGNLIIR pc3
B7ZKJ8;Q14624;Q14624−3
100:100 156:156 69:69
4:187169167:C:T_T
p = 0.3, beta = 0.0793, N = 325
SIQNNVR pc2
B7ZKJ8;Q14624;Q14624−3
100:100 156:156 68:69
4:187169167:C:T_T
p = 0.53, beta = −0.048, N = 324
149Assay Target: ITIH4
UniProt: B7ZKJ8
rsID: rs1973612
Target SNP: 4:187169167:C:T
Proxy SNP: 4:187169167:C:T
Tarkin log10(p): 11.8
Tarkin BETA: 0.276
Tarkin MSPA: 0.315
1255:1248:1255:1248:1255:124
QLGLPGPPDVPDHAAYHPFR pc4
rs2276814 REF
320:323 619:620 313:317
4:187169167:C:T_T
p = 0.12, model = DOM, N = 1252
QLGLPGPPDVPDHAAYHPFR pc3
rs2276814 REF
316:323 609:620 309:317
4:187169167:C:T_T
p = 0.5, model = DOM, N = 1234
LLGLPGPPDVPDHAAYHPFR pc3
rs2276814 ALT
54:323 127:620 81:317
4:187169167:C:T_T
p = 0.02, model = DOM, N = 262
QLGLPGPPDVPDHAAYHPFRR pc4
rs2276814 REF
291:323 551:620 281:317
4:187169167:C:T_T
p = 0.6, model = REC, N = 1123
LLGLPGPPDVPDHAAYHPFR pc4
rs2276814 ALT
11:323 28:620 23:317
4:187169167:C:T_T
p = 0.035, model = DOM, N = 62
LLGLPGPPDVPDHAAYHPFRR pc4
rs2276814 ALT
11:323 27:620 20:317
4:187169167:C:T_T
p = 0.12, model = DOM, N = 58
AFITNFSMNIDGMTYPGIIK pc3
rs13072536 ALT
30:323 52:620 33:317
4:187169167:C:T_T
p = 0.37, model = DOM, N = 115
AFITNFSMIIDGMTYPGIIK pc3
rs13072536 REF
21:323 42:620 16:317
4:187169167:C:T_T
p = 0.35, model = DOM, N = 79

150. FCN2 (Q15485)
Tarkin MSPA=0.88

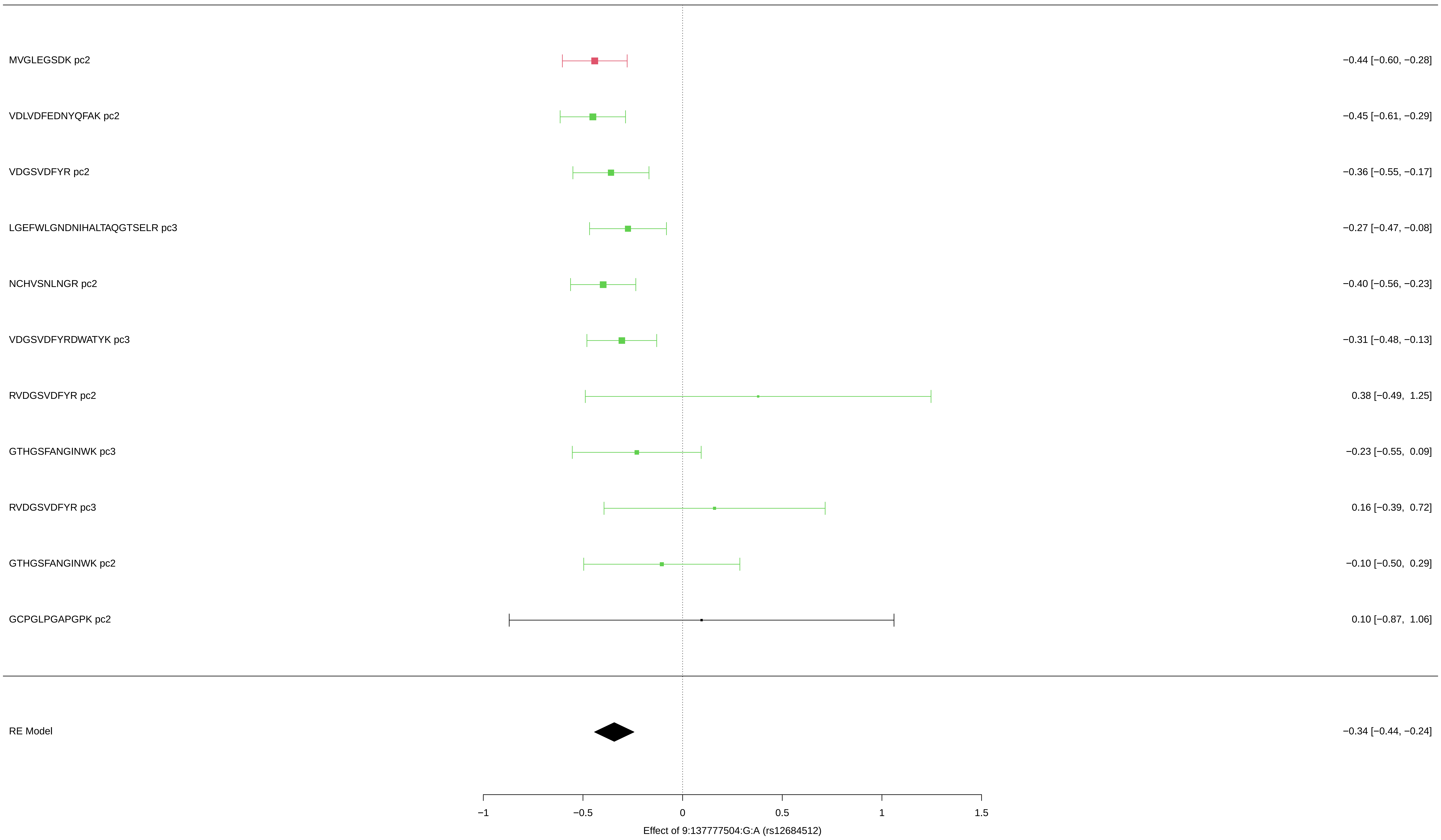

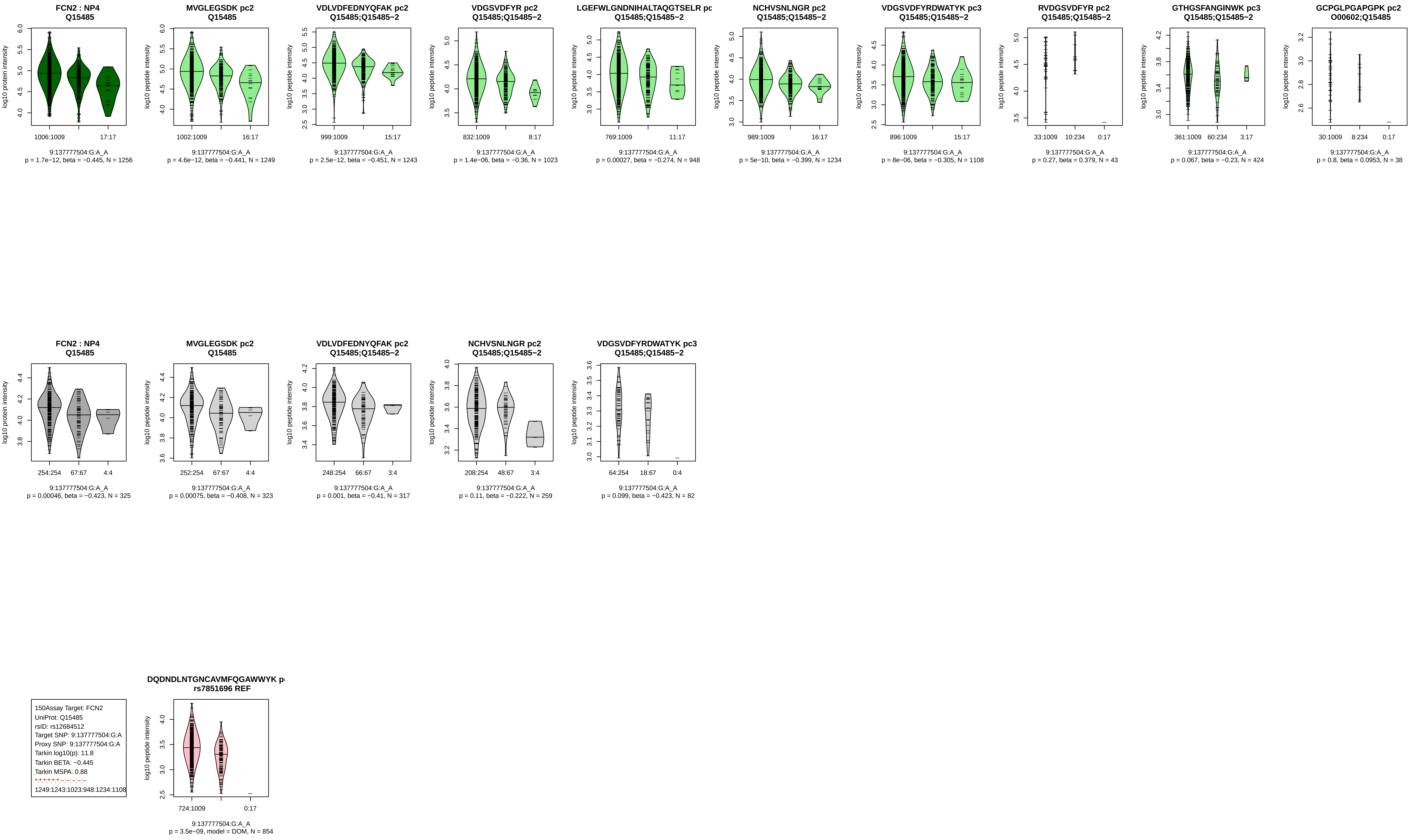
FCN2 : NP4
Q15485
log10 protein intensity
1006:1009
17:17
9:137777504:G:A_A
p = 1.7e−12, beta = −0.445, N = 1256
MVGLEGSDK pc2
Q15485
log10 peptide intensity
1002:1009
16:17
9:137777504:G:A_A
p = 4.6e−12, beta = −0.441, N = 1249
VDLVDFEDNYQFAK pc2
Q15485;Q15485−2
999:1009
15:17
9:137777504:G:A_A
p = 2.5e−12, beta = −0.451, N = 1243
VDGSVDFYR pc2
Q15485;Q15485−2
832:1009
8:17
9:137777504:G:A_A
p = 1.4e−06, beta = −0.36, N = 1023
LGEFWLGNDNIHALTAQGTSELR pc
Q15485;Q15485−2
769:1009
11:17
9:137777504:G:A_A
p = 0.00027, beta = −0.274, N = 948
NCHVSNLNGR pc2
Q15485;Q15485−2
989:1009
16:17
9:137777504:G:A_A
p = 5e−10, beta = −0.399, N = 1234
VDGSVDFYRDWATYK pc3
Q15485;Q15485−2
896:1009
15:17
9:137777504:G:A_A
p = 8e−06, beta = −0.305, N = 1108
RVDGSVDFYR pc2
Q15485;Q15485−2
33:1009
10:234
0:17
9:137777504:G:A_A
p = 0.27, beta = 0.379, N = 43
GTHGSFANGINWK pc3
Q15485;Q15485−2
361:1009
60:234
3:17
9:137777504:G:A_A
p = 0.067, beta = −0.23, N = 424
GCPGLPGAPGPK pc2
O00602;Q15485
30:1009
8:234
0:17
9:137777504:G:A_A
p = 0.8, beta = 0.0953, N = 38
FCN2 : NP4
Q15485
254:254
67:67
4:4
9:137777504:G:A_A
p = 0.00046, beta = −0.423, N = 325
MVGLEGSDK pc2
Q15485
252:254
67:67
4:4
9:137777504:G:A_A
p = 0.00075, beta = −0.408, N = 323
VDLVDFEDNYQFAK pc2
Q15485;Q15485−2
248:254
66:67
3:4
9:137777504:G:A_A
p = 0.001, beta = −0.41, N = 317
NCHVSNLNGR pc2
Q15485;Q15485−2
208:254
48:67
3:4
9:137777504:G:A_A
p = 0.11, beta = −0.222, N = 259
VDGSVDFYRDWATYK pc3
Q15485;Q15485−2
64:254
18:67
0:4
9:137777504:G:A_A
p = 0.099, beta = −0.423, N = 82
150Assay Target: FCN2
UniProt: Q15485
rsID: rs12684512
Target SNP: 9:137777504:G:A
Proxy SNP: 9:137777504:G:A
Tarkin log10(p): 11.8
Tarkin BETA: −0.445
Tarkin MSPA: 0.88
1249:1243:1023:948:1234:1108
DQDNDLNTGNCAVMFQGAWWYK p
rs7851696 REF
724:1009
0:17
9:137777504:G:A_A
p = 3.5e−09, model = DOM, N = 854

151. PDCD5 (O14737)
Tarkin MSPA=0.866

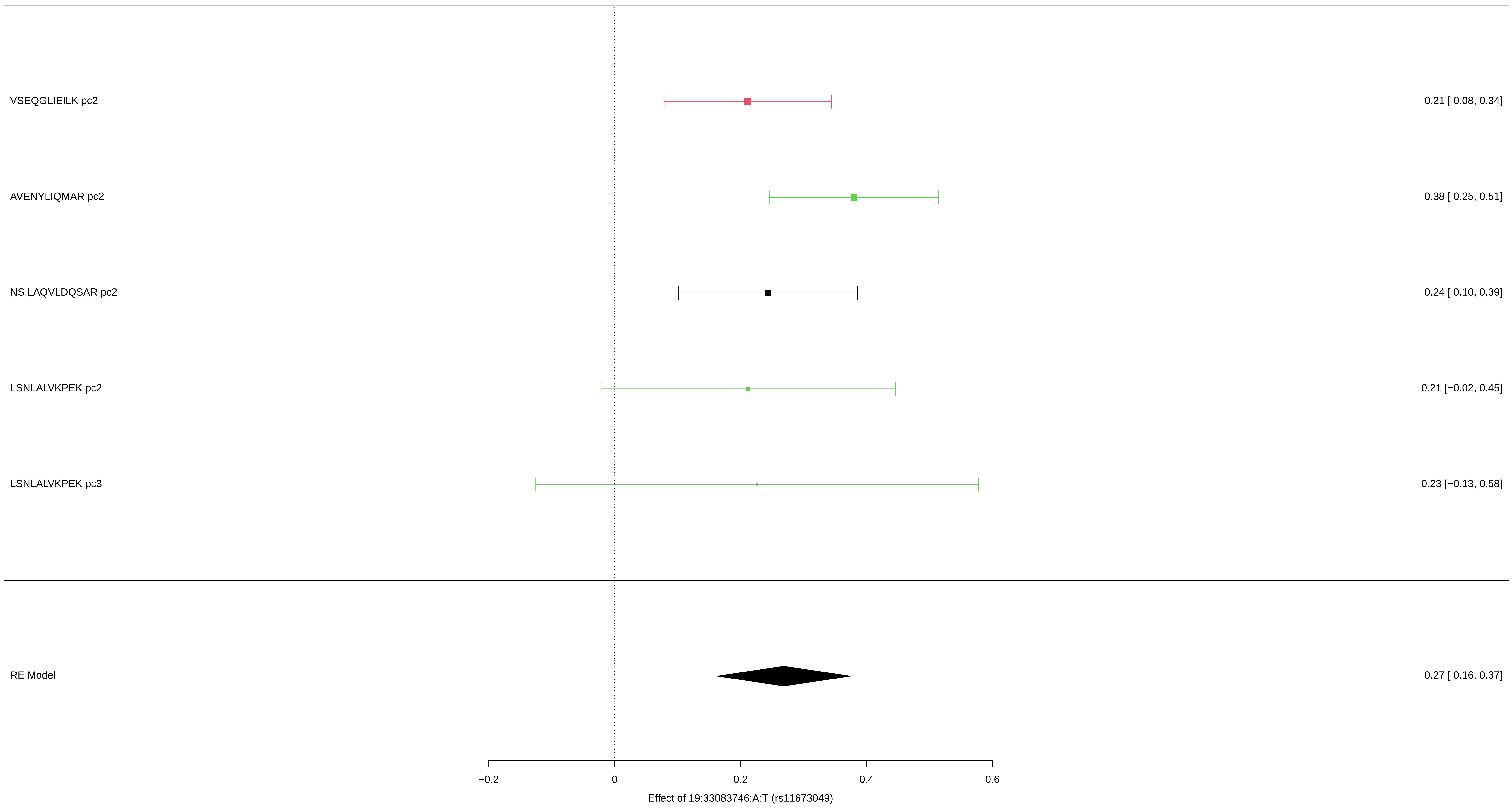

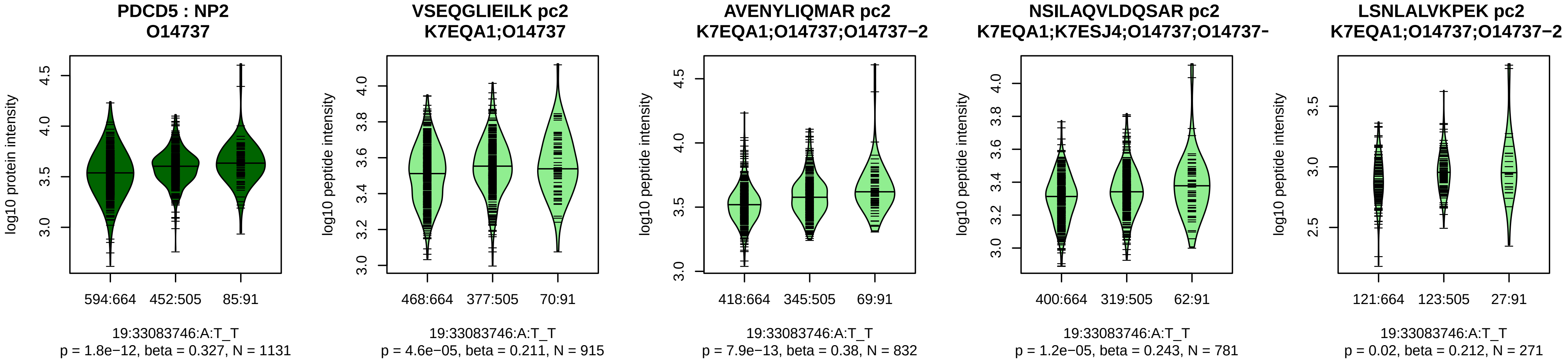

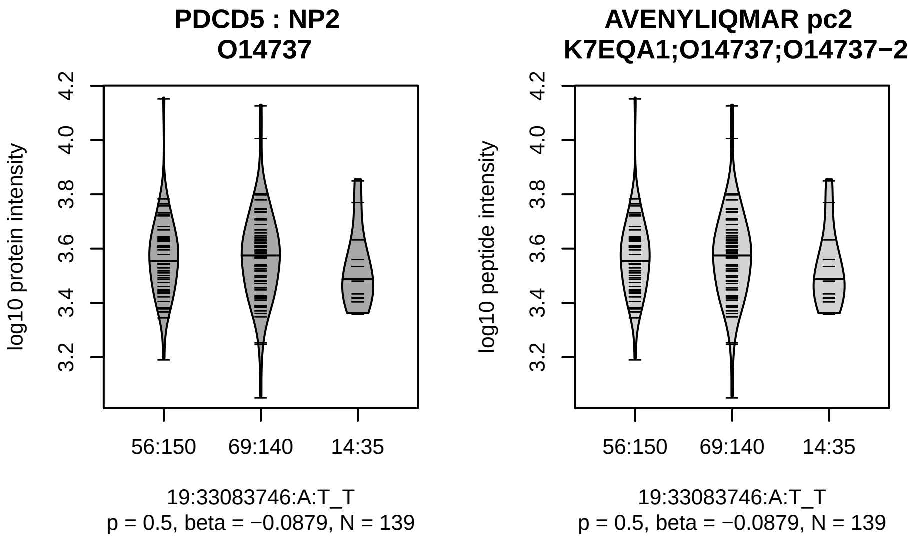

151Assay Target: PDCD5
UniProt: O14737
rsID: rs11673049
Target SNP: 19:33083746:A:T
Proxy SNP: 19:33083746:A:T
Tarkin log10(p): 11.7
Tarkin BETA: 0.327
Tarkin MSPA: 0.866
::*:−:−:NA:NA
915:832:781:271:121:2:1

152. FREM2 (Q5SZK8)
Tarkin MSPA=0.602

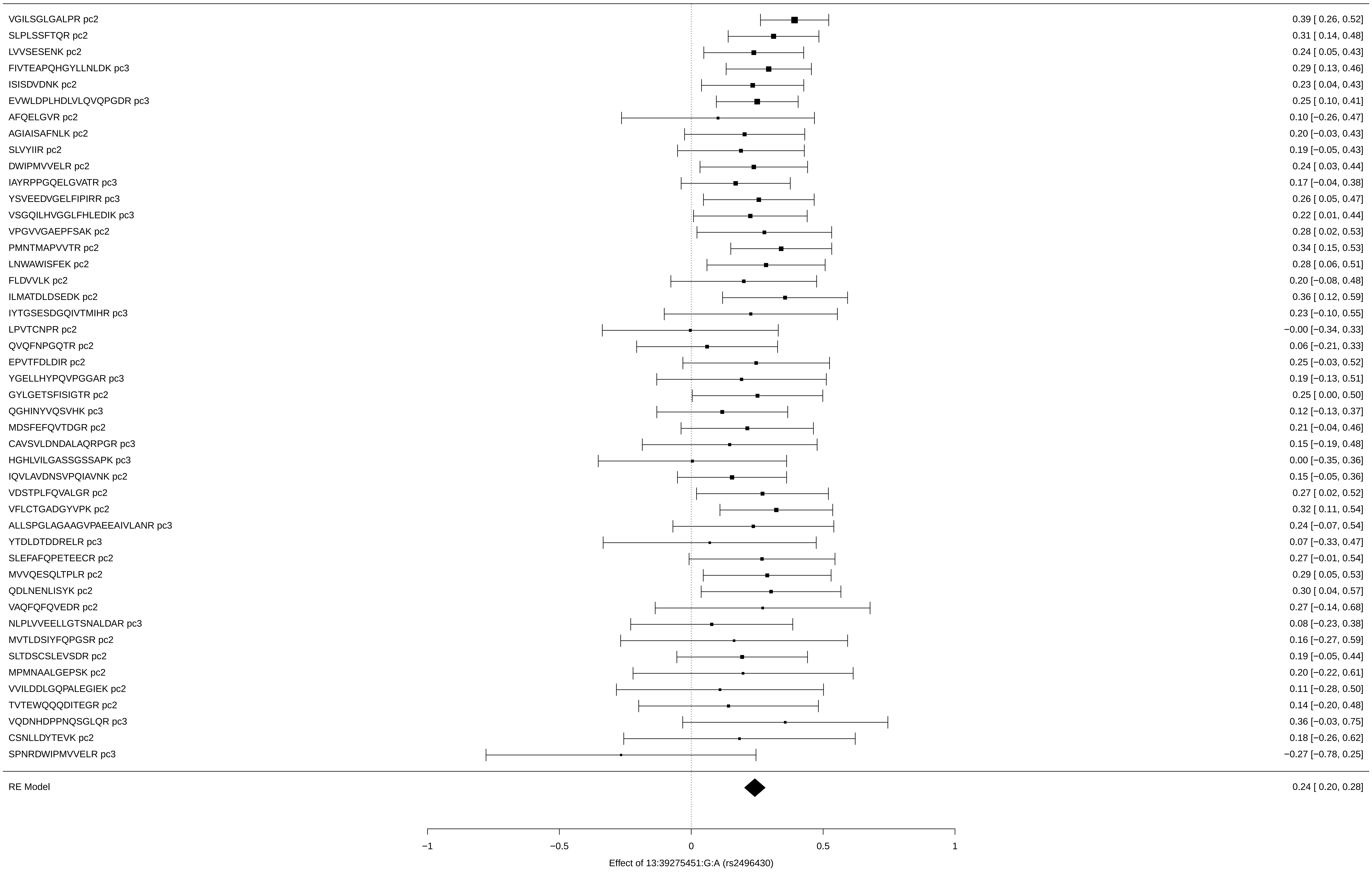

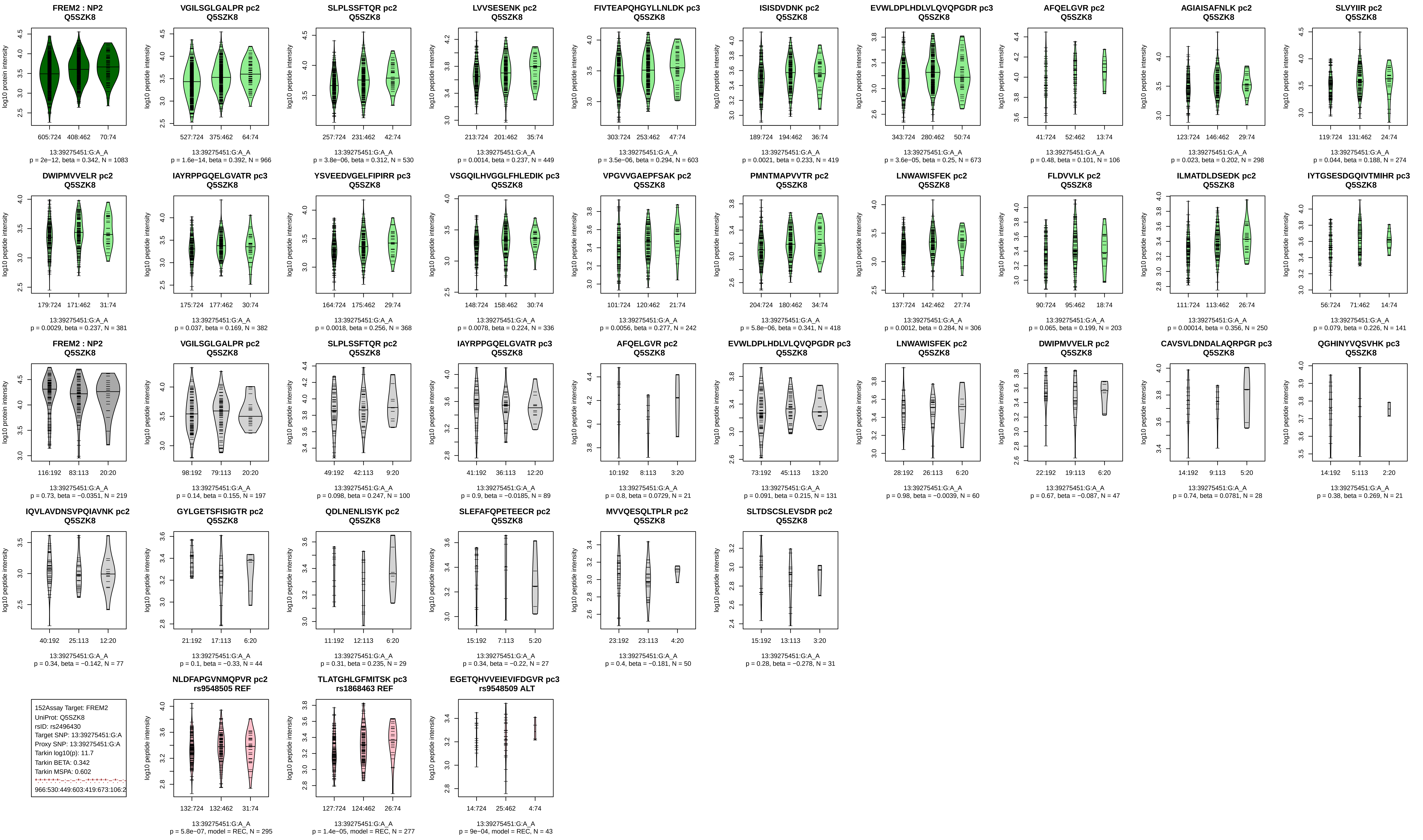

FREM2 : NP2
Q5SZK8
605:724 408:462 70:74
13:39275451:G:A_A
p = 2e−12, beta = 0.342, N = 1083
VGILSGLGALPR pc2
Q5SZK8
527:724 375:462 64:74
13:39275451:G:A_A
p = 1.6e−14, beta = 0.392, N = 966
SLPLSSFTQR pc2
Q5SZK8
257:724 231:462 42:74
13:39275451:G:A_A
p = 3.8e−06, beta = 0.312, N = 530
LVVSESENK pc2
Q5SZK8
213:724 201:462 35:74
13:39275451:G:A_A
p = 0.0014, beta = 0.237, N = 449
FIVTEAPQHGYLLNLDK pc3
Q5SZK8
303:724 253:462 47:74
13:39275451:G:A_A
p = 3.5e−06, beta = 0.294, N = 603
ISISDVDNK pc2
Q5SZK8
189:724 194:462 36:74
13:39275451:G:A_A
p = 0.0021, beta = 0.233, N = 419
EVWLDPLHDLVLQVQPGDR pc3
Q5SZK8
343:724 280:462 50:74
13:39275451:G:A_A
p = 3.6e−05, beta = 0.25, N = 673
AFQELGVR pc2
Q5SZK8
41:724 52:462 13:74
13:39275451:G:A_A
p = 0.48, beta = 0.101, N = 106
AGIAISAFNLK pc2
Q5SZK8
123:724 146:462 29:74
13:39275451:G:A_A
p = 0.023, beta = 0.202, N = 298
SLVYIIR pc2
Q5SZK8
119:724 131:462 24:74
13:39275451:G:A_A
p = 0.044, beta = 0.188, N = 274
DWIPMVVELR pc2
Q5SZK8
179:724 171:462 31:74
13:39275451:G:A_A
p = 0.0029, beta = 0.237, N = 381
IAYRPPGQELGVATR pc3
Q5SZK8
175:724 177:462 30:74
13:39275451:G:A_A
p = 0.037, beta = 0.169, N = 382
YSVEEDVGELFIPIRR pc3
Q5SZK8
164:724 175:462 29:74
13:39275451:G:A_A
p = 0.0018, beta = 0.256, N = 368
VSGQILHVGGLFHLEDIK pc3
Q5SZK8
148:724 158:462 30:74
13:39275451:G:A_A
p = 0.0078, beta = 0.224, N = 336
VPGVVGAEPFSAK pc2
Q5SZK8
101:724 120:462 21:74
13:39275451:G:A_A
p = 0.0056, beta = 0.277, N = 242
PMNTMAPVVTR pc2
Q5SZK8
204:724 180:462 34:74
13:39275451:G:A_A
p = 5.8e−06, beta = 0.341, N = 418
LNWAWISFEK pc2
Q5SZK8
137:724 142:462 27:74
13:39275451:G:A_A
p = 0.0012, beta = 0.284, N = 306
FLDVVLK pc2
Q5SZK8
90:724 95:462 18:74
13:39275451:G:A_A
p = 0.065, beta = 0.199, N = 203
ILMATDLDSEDK pc2
Q5SZK8
111:724 113:462 26:74
13:39275451:G:A_A
p = 0.00014, beta = 0.356, N = 250
IYTGSESDGQIVTMIHR pc3
Q5SZK8
56:724 71:462 14:74
13:39275451:G:A_A
p = 0.079, beta = 0.226, N = 141
FREM2 : NP2
Q5SZK8
116:192 83:113 20:20
13:39275451:G:A_A
p = 0.73, beta = −0.0351, N = 219
VGILSGLGALPR pc2
Q5SZK8
98:192 79:113 20:20
13:39275451:G:A_A
p = 0.14, beta = 0.155, N = 197
SLPLSSFTQR pc2
Q5SZK8
49:192 42:113 9:20
13:39275451:G:A_A
p = 0.098, beta = 0.247, N = 100
IAYRPPGQELGVATR pc3
Q5SZK8
41:192 36:113 12:20
13:39275451:G:A_A
p = 0.9, beta = −0.0185, N = 89
AFQELGVR pc2
Q5SZK8
10:192 8:113 3:20
13:39275451:G:A_A
p = 0.8, beta = 0.0729, N = 21
EVWLDPLHDLVLQVQPGDR pc3
Q5SZK8
73:192 45:113 13:20
13:39275451:G:A_A
p = 0.091, beta = 0.215, N = 131
LNWAWISFEK pc2
Q5SZK8
28:192 26:113 6:20
13:39275451:G:A_A
p = 0.98, beta = −0.0039, N = 60
DWIPMVVELR pc2
Q5SZK8
22:192 19:113 6:20
13:39275451:G:A_A
p = 0.67, beta = −0.087, N = 47
CAVSVLDNDALAQRPGR pc3
Q5SZK8
14:192 9:113 5:20
13:39275451:G:A_A
p = 0.74, beta = 0.0781, N = 28
QGHINYVQSVHK pc3
Q5SZK8
14:192 5:113 2:20
13:39275451:G:A_A
p = 0.38, beta = 0.269, N = 21
IQVLAVDNSVPQIAVNK pc2
Q5SZK8
40:192 25:113 12:20
13:39275451:G:A_A
p = 0.34, beta = −0.142, N = 77
GYLGETSFISIGTR pc2
Q5SZK8
21:192 17:113 6:20
13:39275451:G:A_A
p = 0.1, beta = −0.33, N = 44
QDLNENLISYK pc2
Q5SZK8
11:192 12:113 6:20
13:39275451:G:A_A
p = 0.31, beta = 0.235, N = 29
SLEFAFQPETEECR pc2
Q5SZK8
15:192 7:113 5:20
13:39275451:G:A_A
p = 0.34, beta = −0.22, N = 27
MVVQESQLTPLR pc2
Q5SZK8
23:192 23:113 4:20
13:39275451:G:A_A
p = 0.4, beta = −0.181, N = 50
SLTDSCSLEVSDR pc2
Q5SZK8
15:192 13:113 3:20
13:39275451:G:A_A
p = 0.28, beta = −0.278, N = 31
152Assay Target: FREM2
UniProt: Q5SZK8
rsID: rs2496430
Target SNP: 13:39275451:G:A
Proxy SNP: 13:39275451:G:A
Tarkin log10(p): 11.7
Tarkin BETA: 0.342
Tarkin MSPA: 0.602
966:530:449:603:419:673:106:2
NLDFAPGVNMQPVR pc2
rs9548505 REF
132:724 132:462 31:74
13:39275451:G:A_A
p = 5.8e−07, model = REC, N = 295
TLATGHLGFMITSK pc3
rs1868463 REF
127:724 124:462 26:74
13:39275451:G:A_A
p = 1.4e−05, model = REC, N = 277
EGETQHVVEIEVIFDGVR pc3
rs9548509 ALT
14:724 25:462 4:74
13:39275451:G:A_A
p = 9e−04, model = REC, N = 43

153. MMRN2 (Q9H8L6)
Tarkin MSPA=0.789

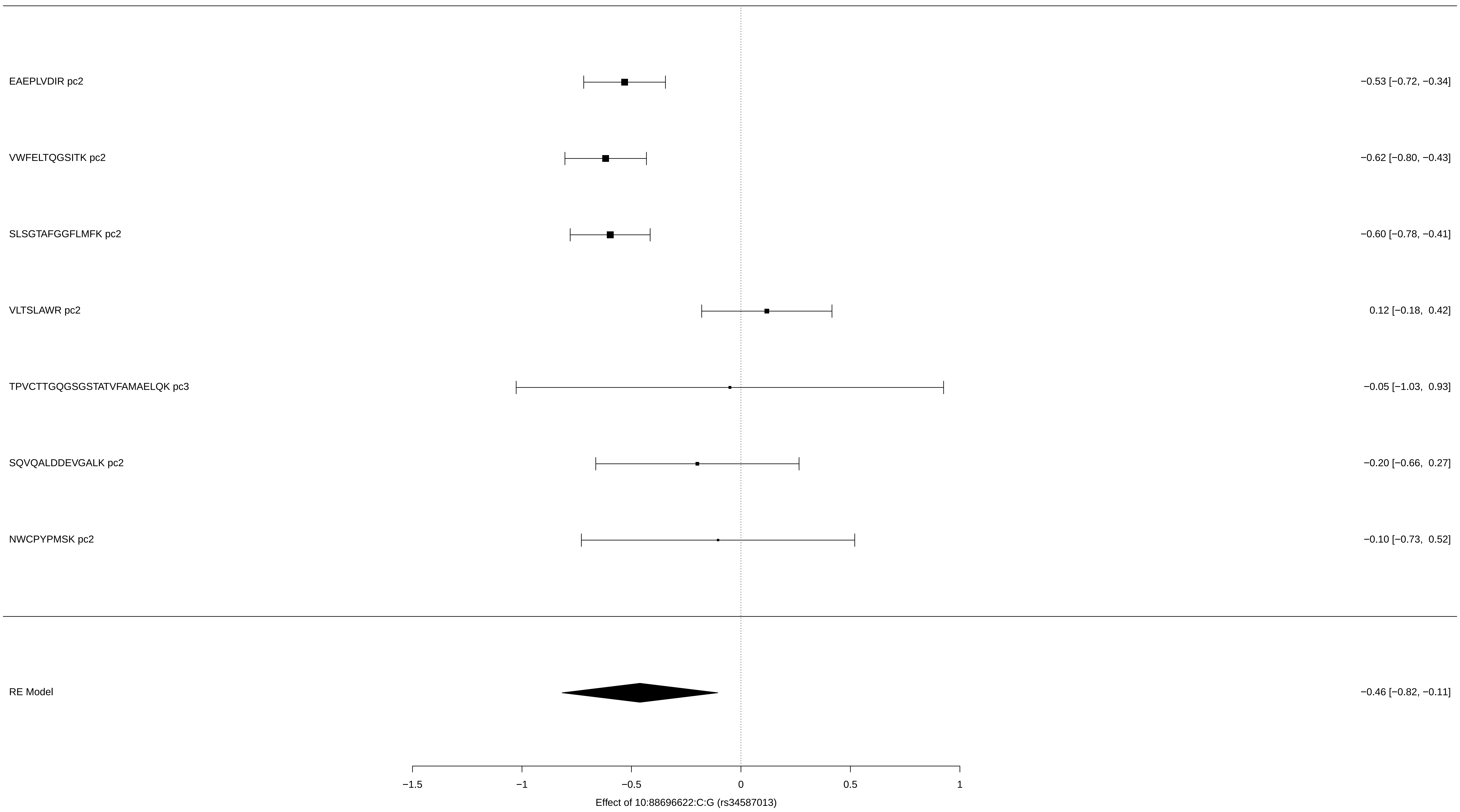

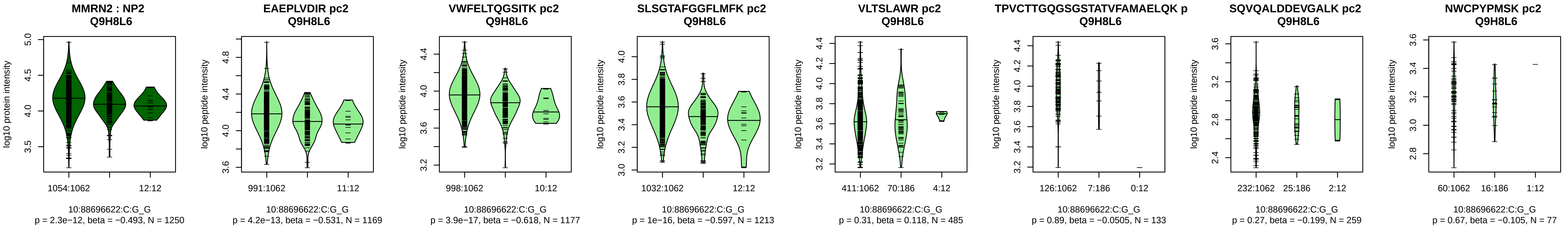

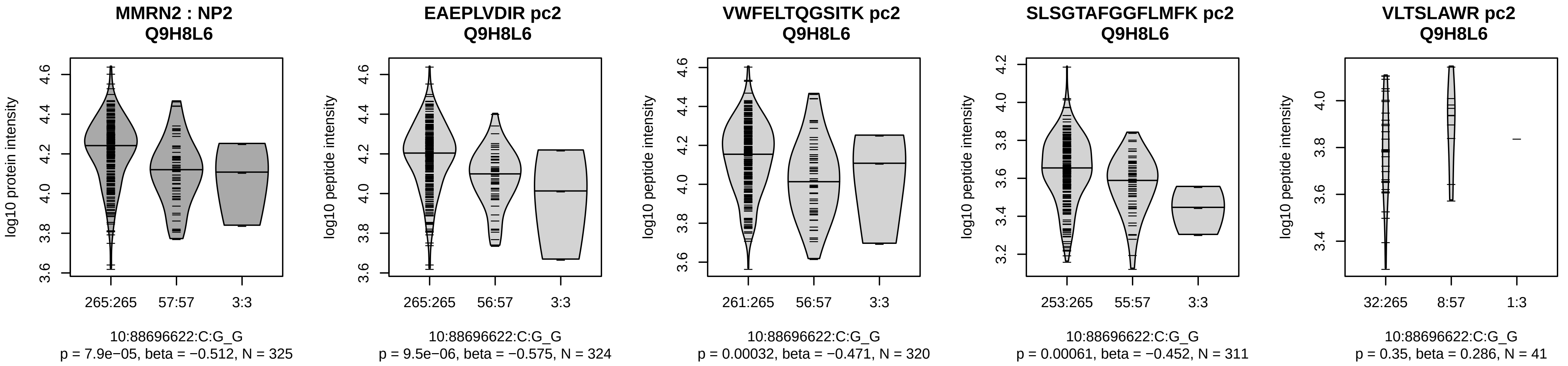

153Assay Target: MMRN2
UniProt: Q9H8L6
rsID: rs34587013
Target SNP: 10:88696622:C:G
Proxy SNP: 10:88696622:C:G
Tarkin log10(p): 11.6
Tarkin BETA: −0.492
Tarkin MSPA: 0.789
::*:−:−:−:−:NA
1169:1177:1213:485:133:259:77

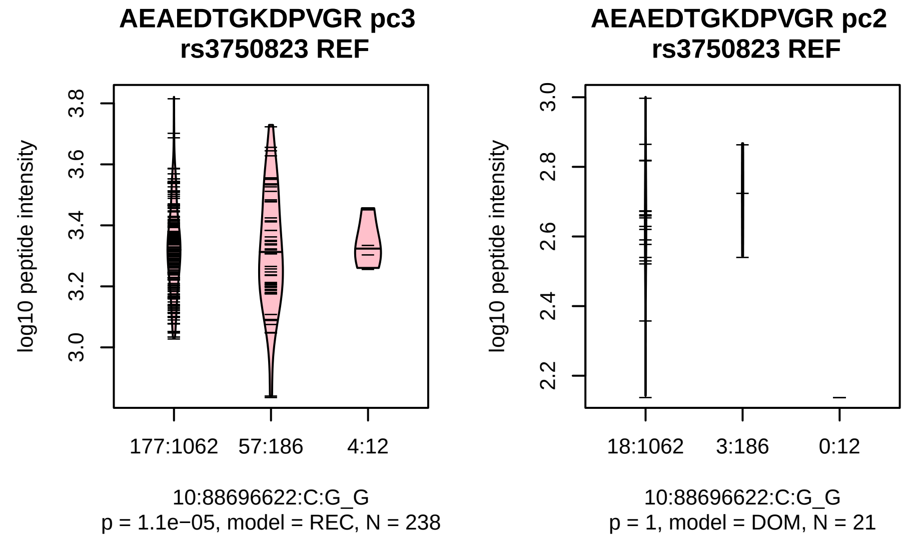

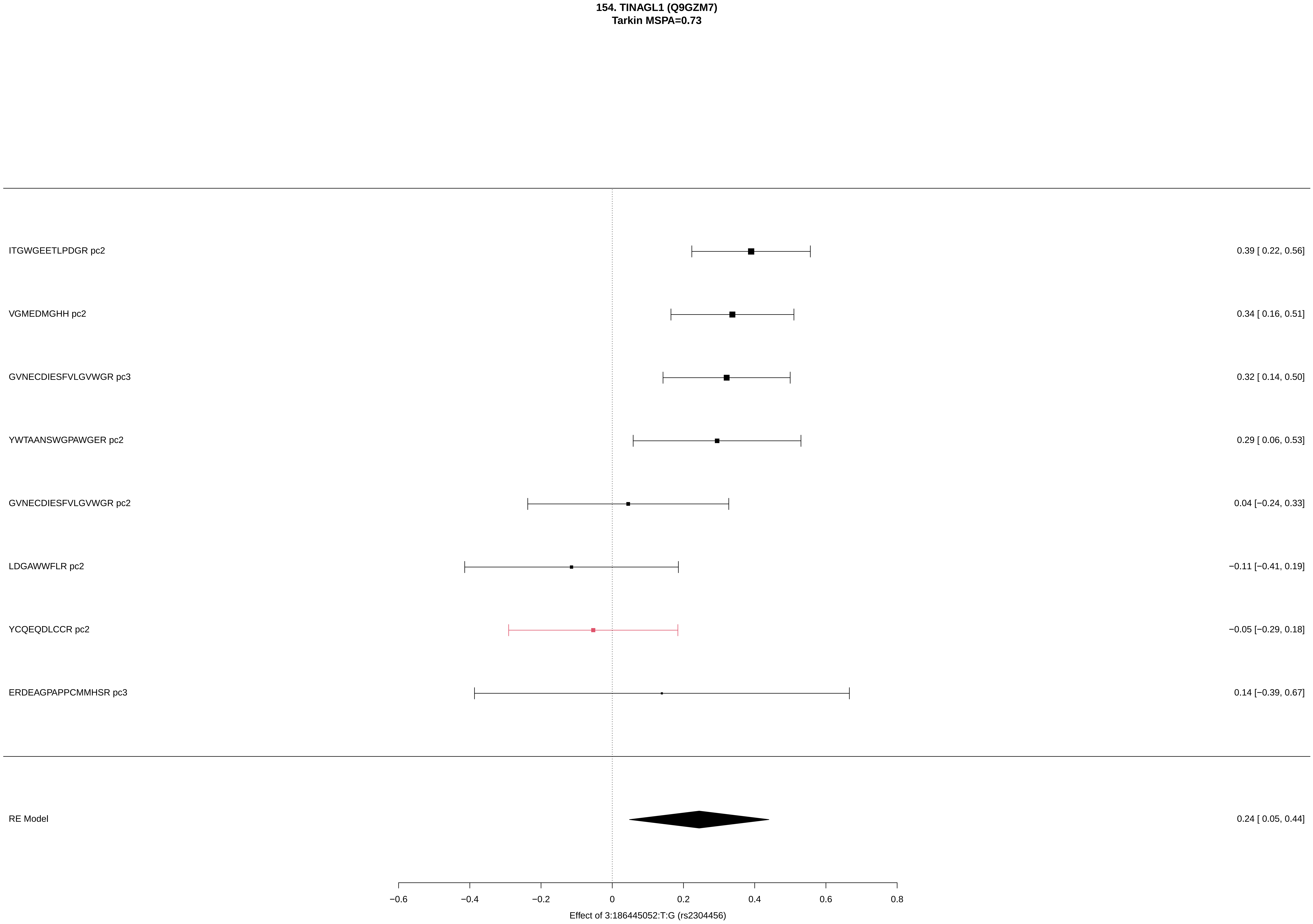
154. TINAGL1 (Q9GZM7)
Tarkin MSPA=0.73
ITGWGEETLPDGR pc2
0.39 [0.22, 0.56]
VGMEDMGHH pc2
0.34 [0.16, 0.51]
GVNECDIESFVLGVWGR pc3
0.32 [0.14, 0.50]
YWTAANSWGPAWGER pc2
0.29 [0.06, 0.53]
GVNECDIESFVLGVWGR pc2
0.04 [−0.24, 0.33]
LDGAWWFLR pc2
−0.11 [−0.41, 0.19]
YCQEQDLCCR pc2
−0.05 [−0.29, 0.18]
ERDEAGPAPPCMMHSR pc3
0.14 [−0.39, 0.67]
RE Model
0.24 [0.05, 0.44]
−0.6
−0.4
−0.2
0
0.2
0.4
0.6
0.8
Effect of 3:186445052:T:G (rs2304456)

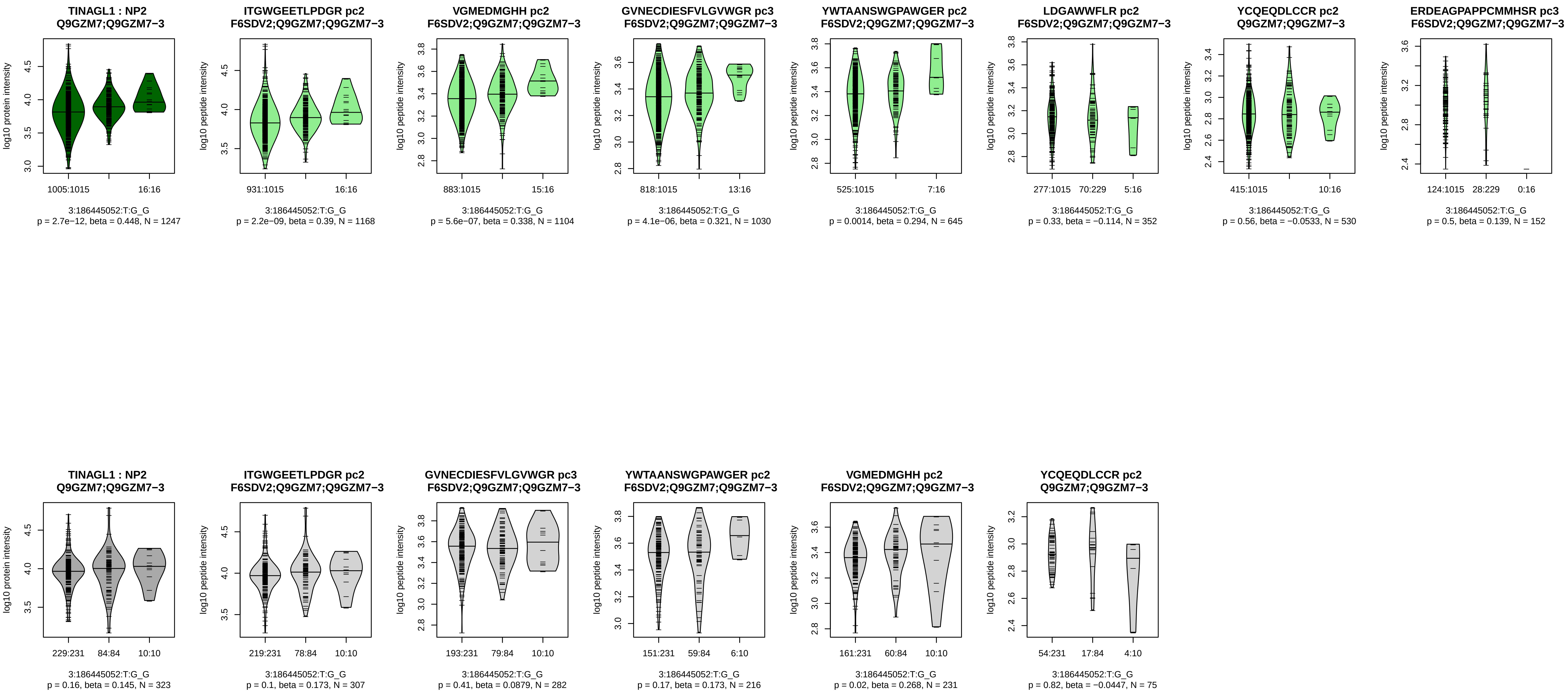

154Assay Target: TINAGL1
UniProt: Q9GZM7
rsID: rs2304456
Target SNP: 3:186445052:T:G
Proxy SNP: 3:186445052:T:G
Tarkin log10(p): 11.6
Tarkin BETA: 0.448
Tarkin MSPA: 0.73
::*:*:–:–:–:–
1168:1104:1030:645:427:352:53

155. CDH15 (P55291)
Tarkin MSPA=0.9

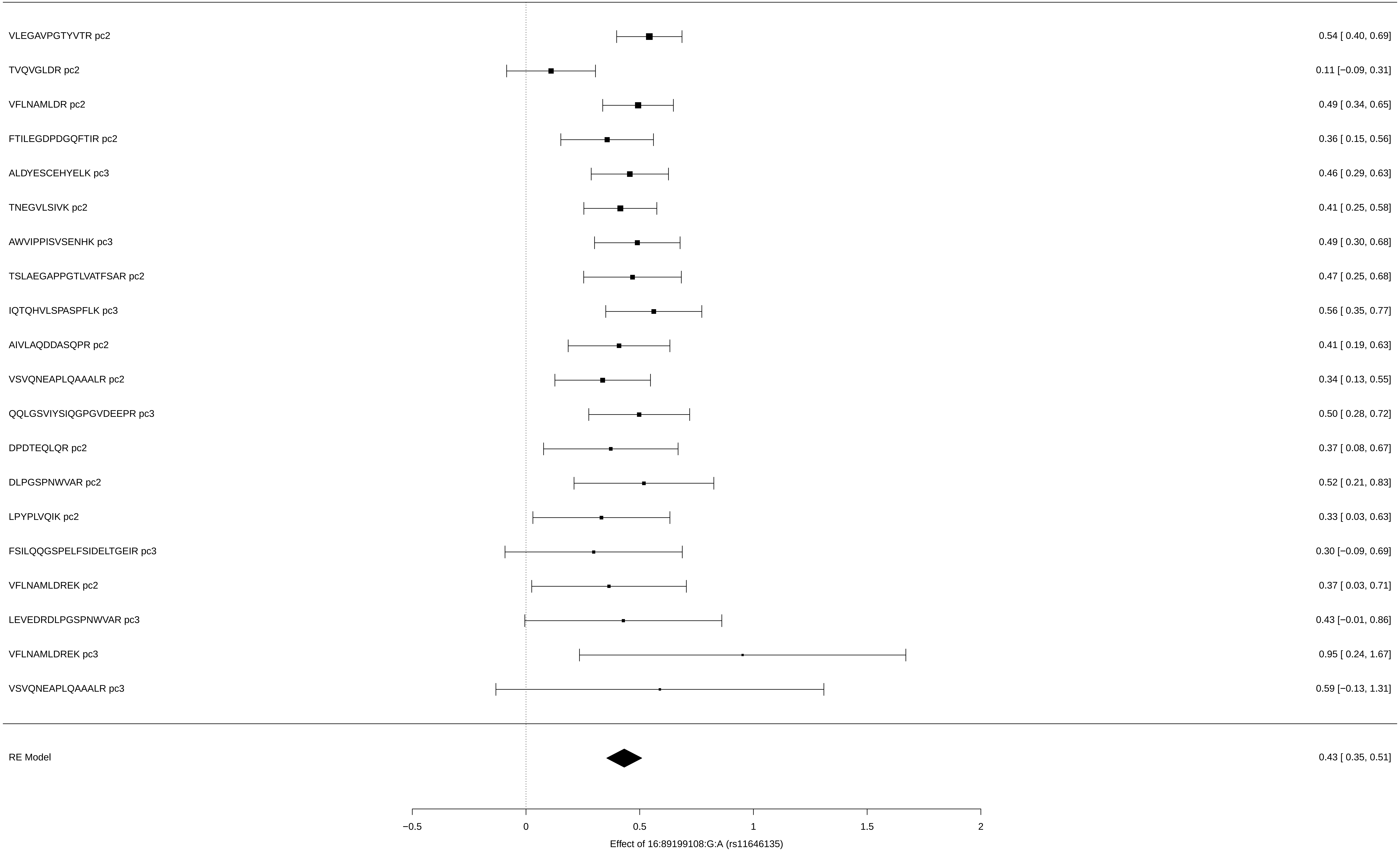

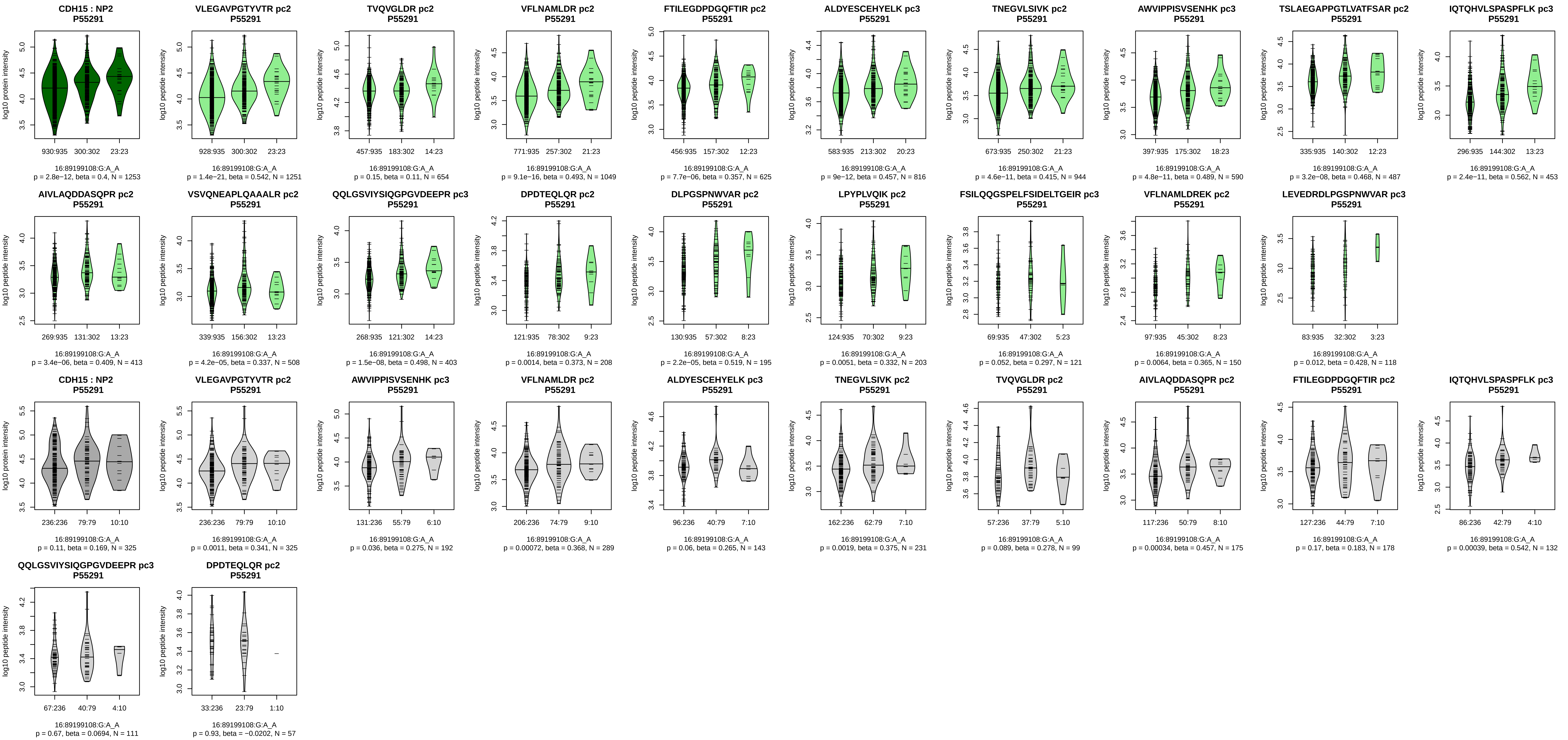

155Assay Target: CDH15
UniProt: P55291
rsID: rs11646135
Target SNP: 16:89199108:G:A
Proxy SNP: 16:89199108:G:A
Tarkin log10(p): 11.5
Tarkin BETA: 0.4
Tarkin MSPA: 0.9
1251:654:1049:625:816:944:59[illegible]

156. IGKV1−17 (P01599)
Tarkin MSPA=1

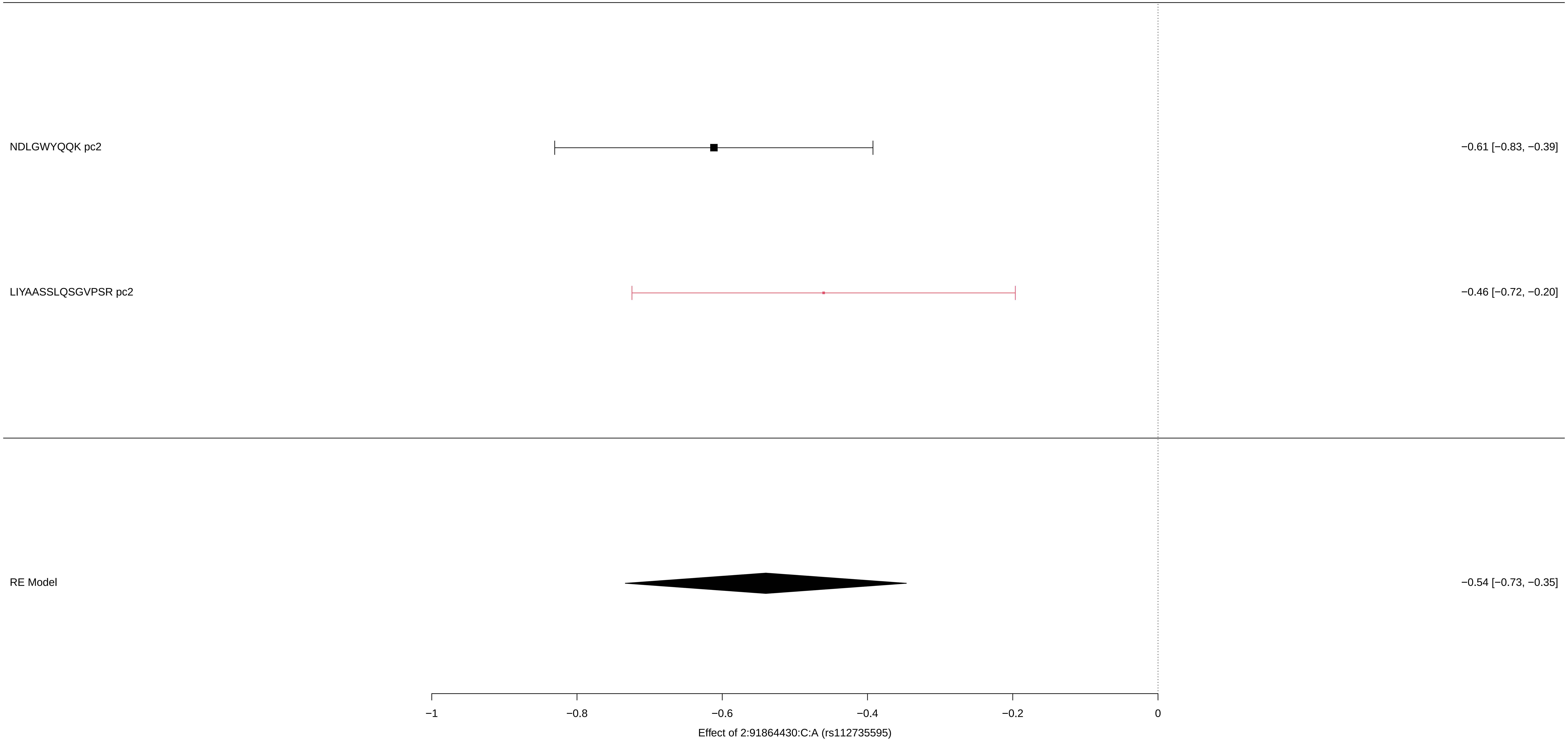

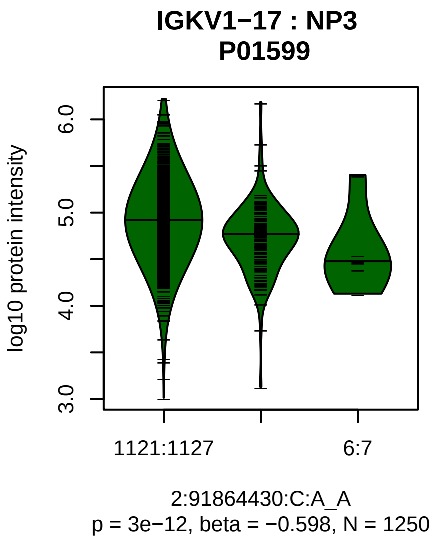

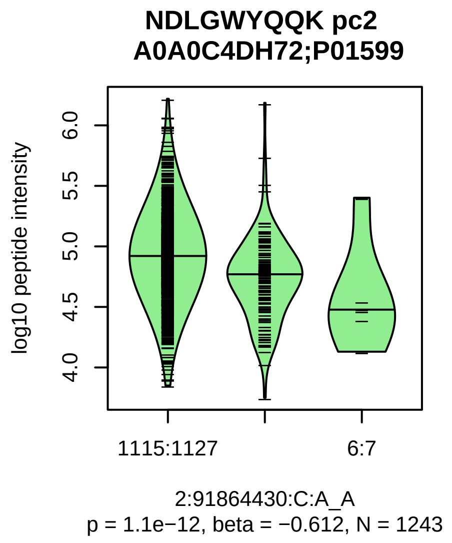

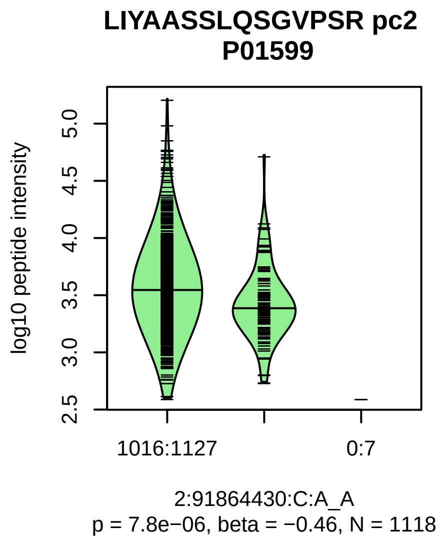

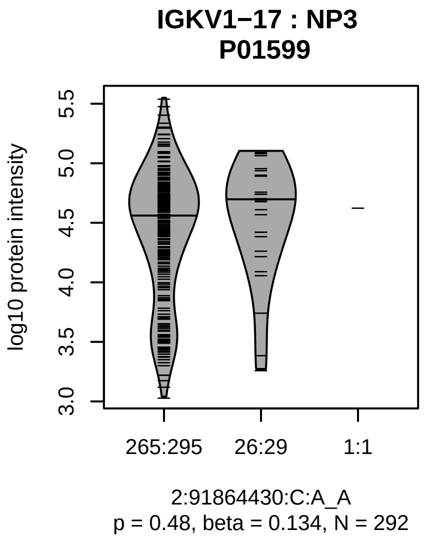

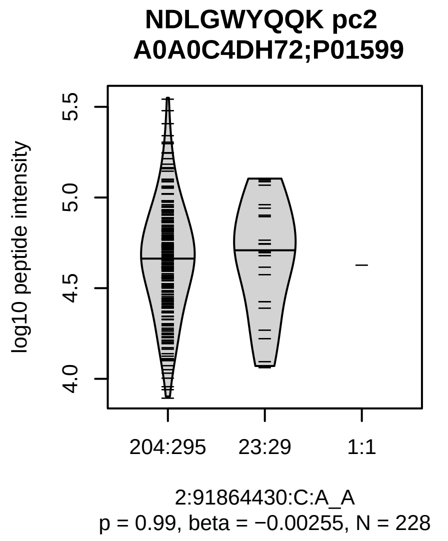

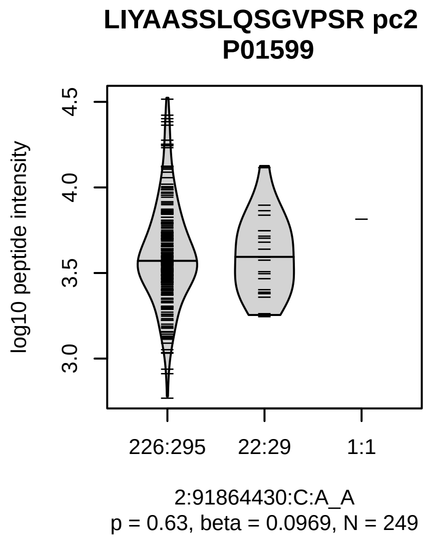

156Assay Target: IGKV1−17
UniProt: P01599
rsID: rs112735595
Target SNP: 2:91864430:C:A
Proxy SNP: 2:91864430:C:A
Tarkin log10(p): 11.5
Tarkin BETA: −0.598
Tarkin MSPA: 1
::NA:NA
1243:1118:4:2

157. FCN3 (O75636)
Tarkin MSPA=0.512

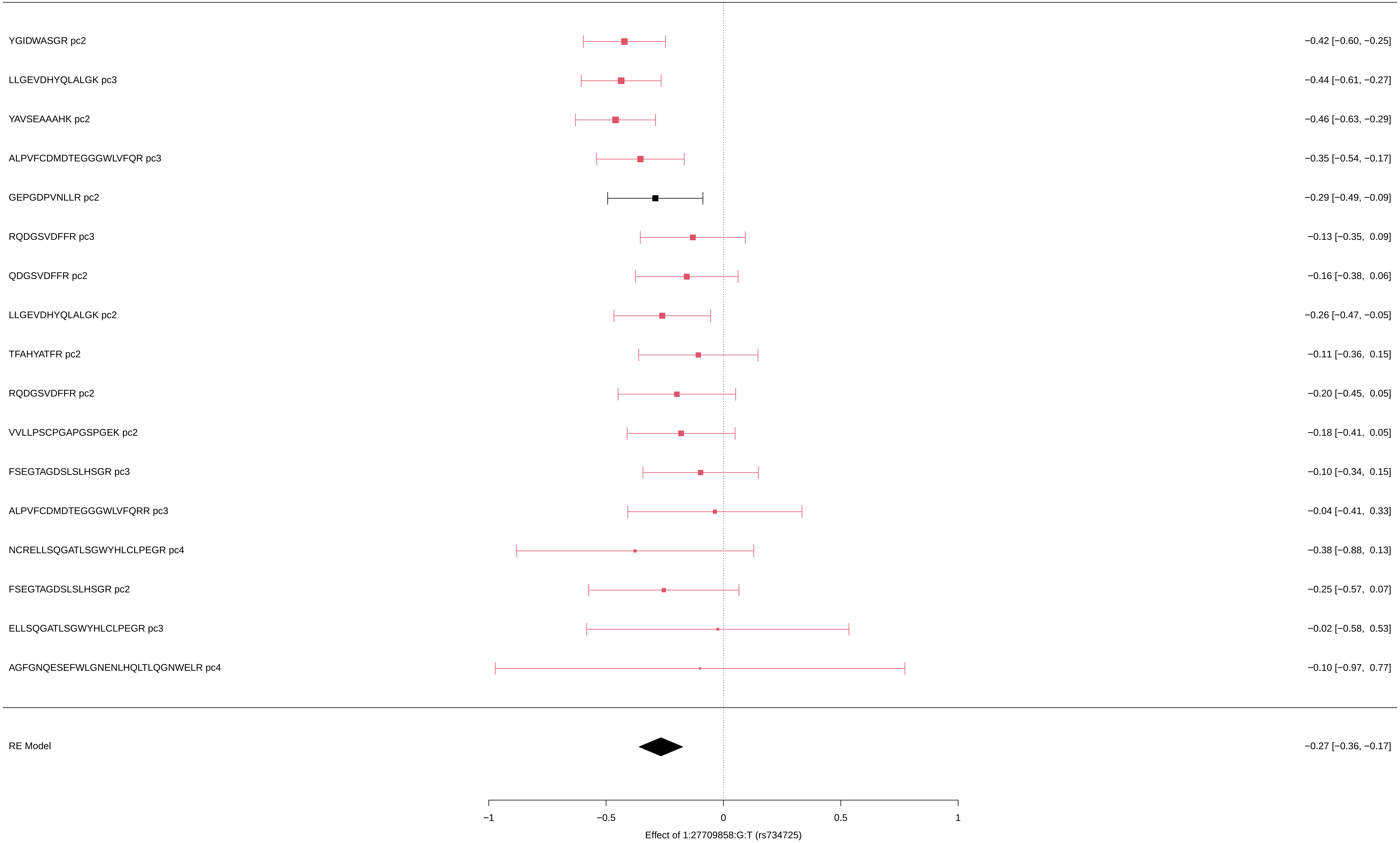

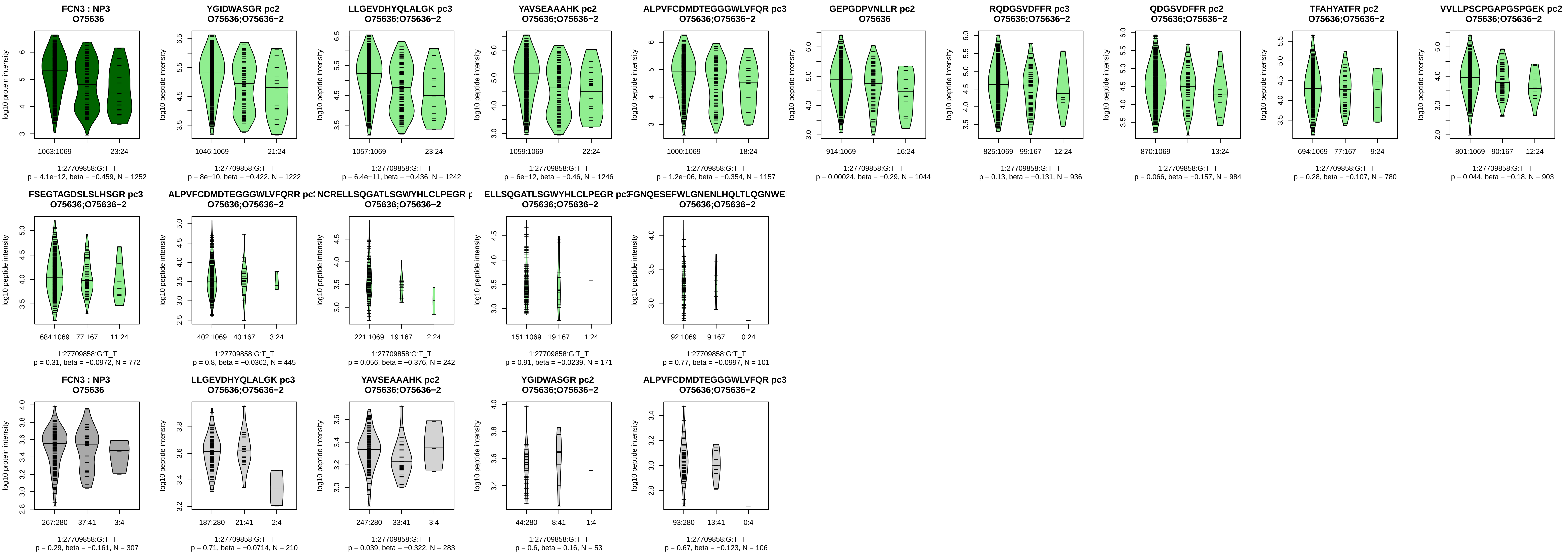

157Assay Target: FCN3
UniProt: O75636
rsID: rs734725
Target SNP: 1:27709858:G:T
Proxy SNP: 1:27709858:G:T
Tarkin log10(p): 11.4
Tarkin BETA: −0.459
Tarkin MSPA: 0.512
1222:1242:1246:1157:1044:936

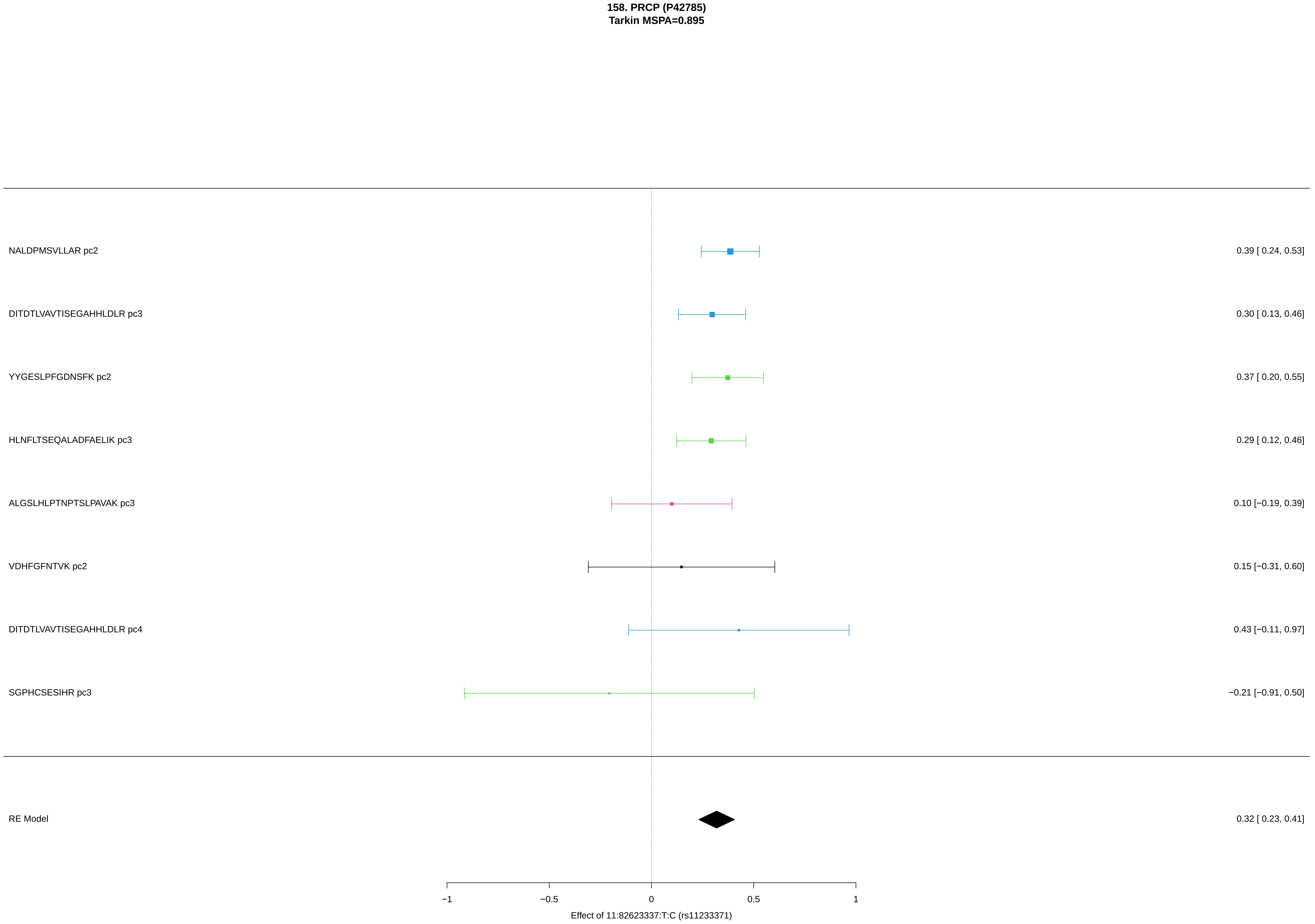

158. PRCP (P42785)
Tarkin MSPA=0.895
NALDPMSVLLAR pc2
0.39 [0.24, 0.53]
DITDTLVAVTISEGAHHLDLR pc3
0.30 [0.13, 0.46]
YYGESLPFGDNSFK pc2
0.37 [0.20, 0.55]
HLNFLTSEQALADFAELIK pc3
0.29 [0.12, 0.46]
ALGSLHLPTNPTSLPAVAK pc3
0.10 [−0.19, 0.39]
VDHFGFNTVK pc2
0.15 [−0.31, 0.60]
DITDTLVAVTISEGAHHLDLR pc4
0.43 [−0.11, 0.97]
SGPHCSESIHR pc3
−0.21 [−0.91, 0.50]
RE Model
0.32 [0.23, 0.41]
−1
−0.5
0
0.5
1
Effect of 11:82623337:T:C (rs11233371)

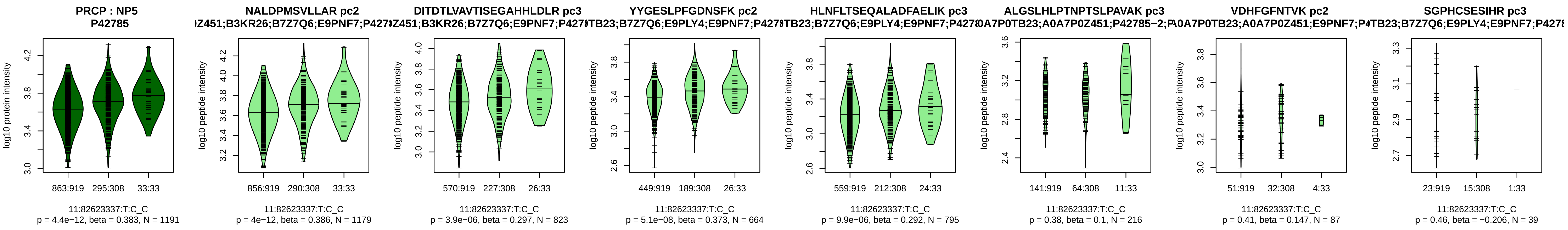

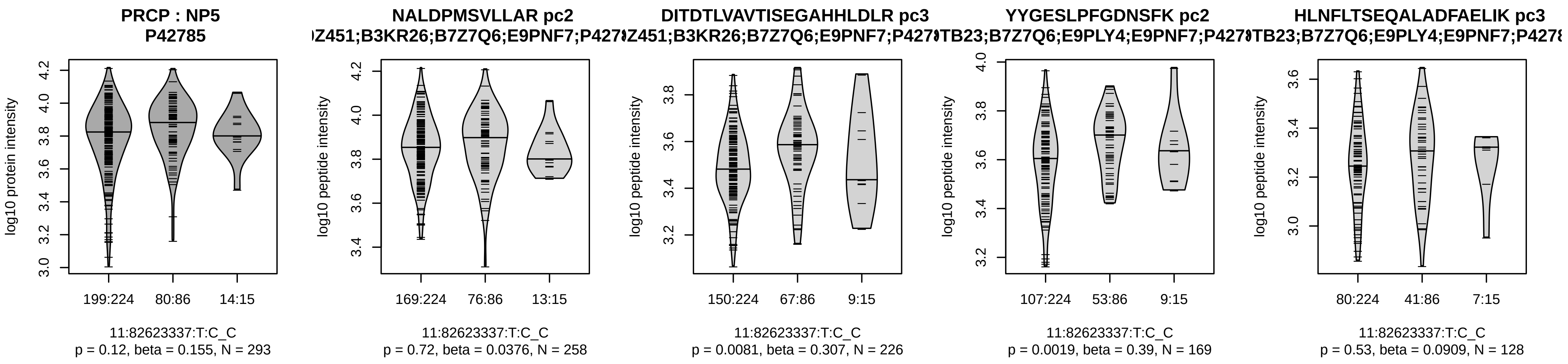

158Assay Target: PRCP
UniProt: P42785
rsID: rs11233371
Target SNP: 11:82623337:T:C
Proxy SNP: 11:82623337:T:C
Tarkin log10(p): 11.4
Tarkin BETA: 0.383
Tarkin MSPA: 0.895
::*:*:–:–:–:–
1179:823:664:795:216:87:63:39

159. IGKV1−5 (P01602)
Tarkin MSPA=0.47

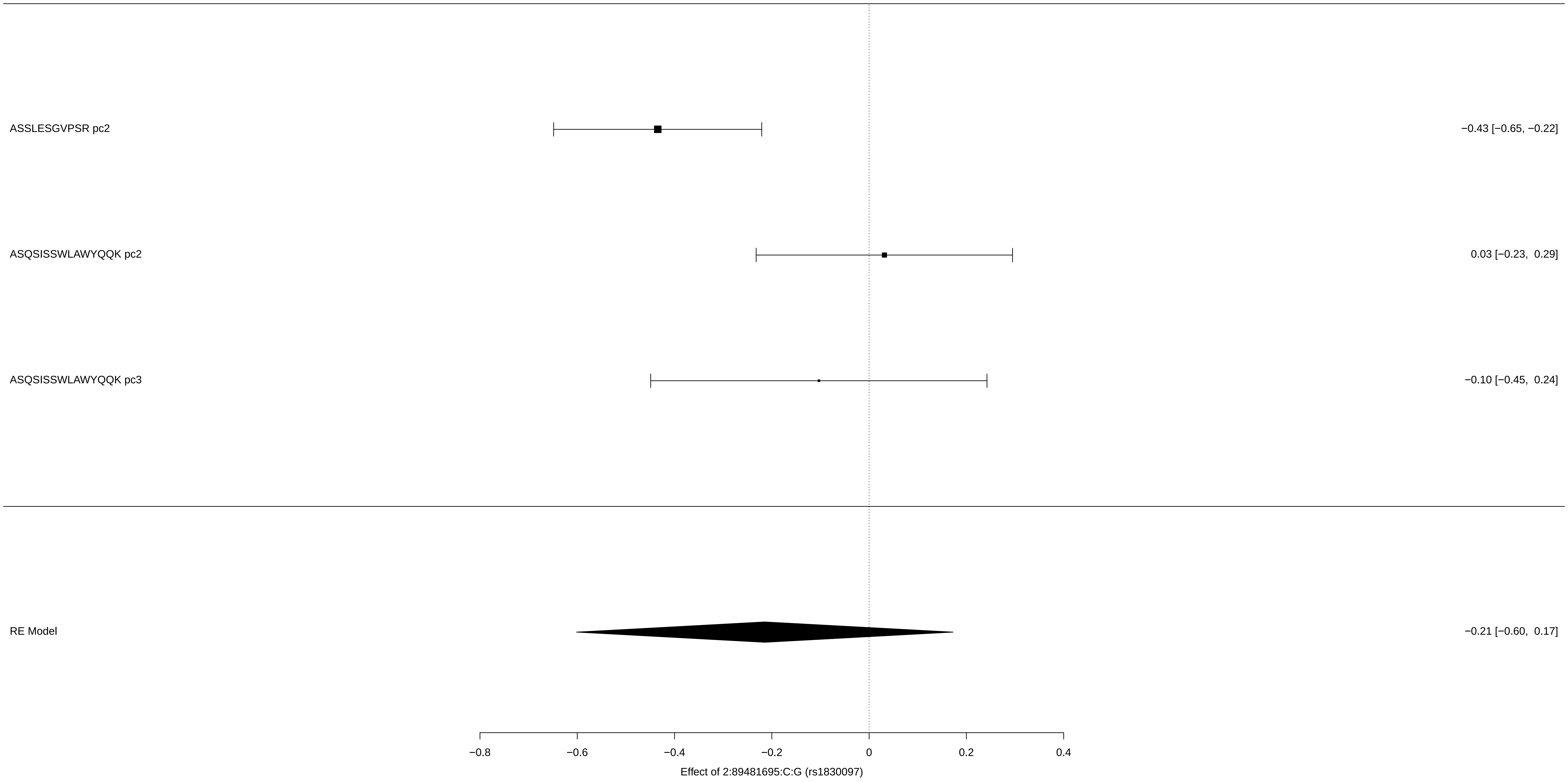

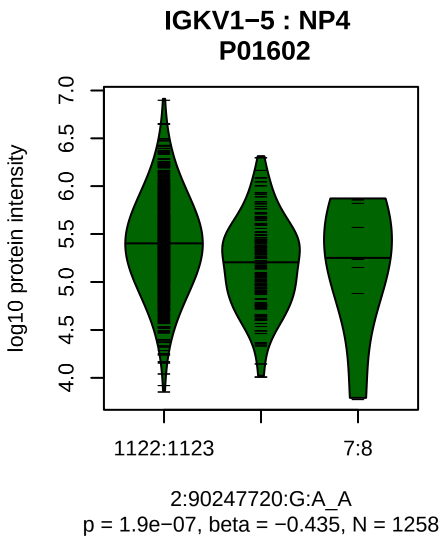

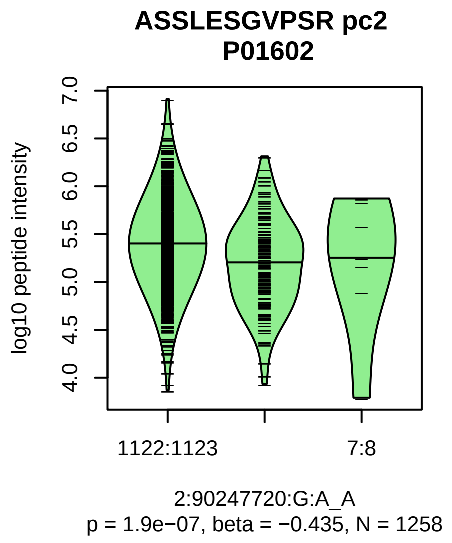

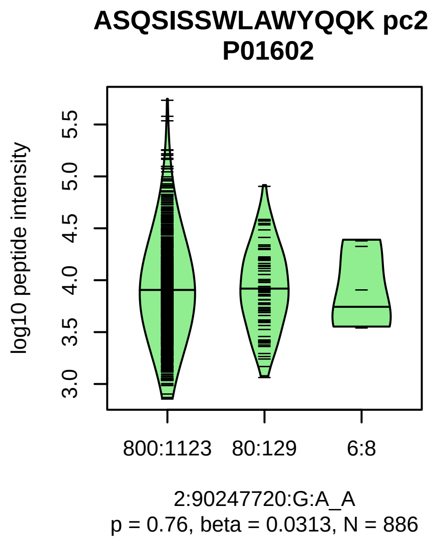

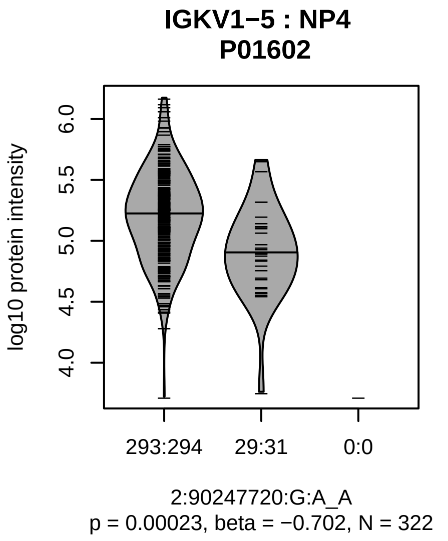

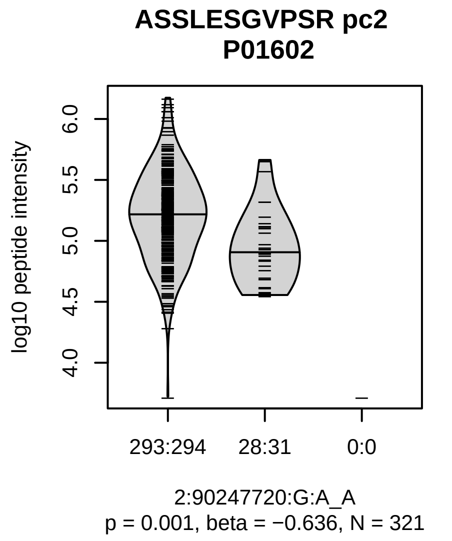

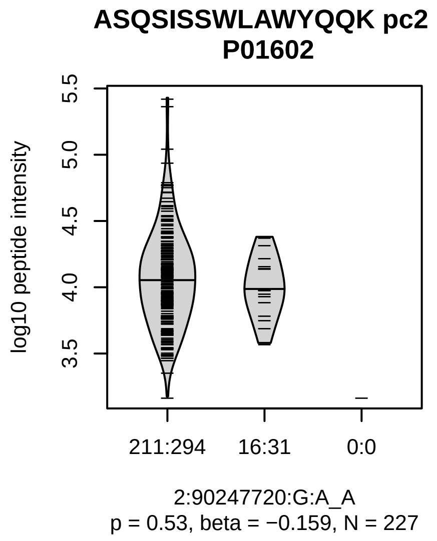

159Assay Target: IGKV1−5
UniProt: P01602
rsID: rs1830097
Target SNP: 2:89481695:C:G
Proxy SNP: 2:90247720:G:A
Tarkin log10(p): 11.3
Tarkin BETA: −0.547
Tarkin MSPA: 0.47
*:−:−
1258:886:534

160. ANGPTL6 (Q8NI99)
Tarkin MSPA=0.876

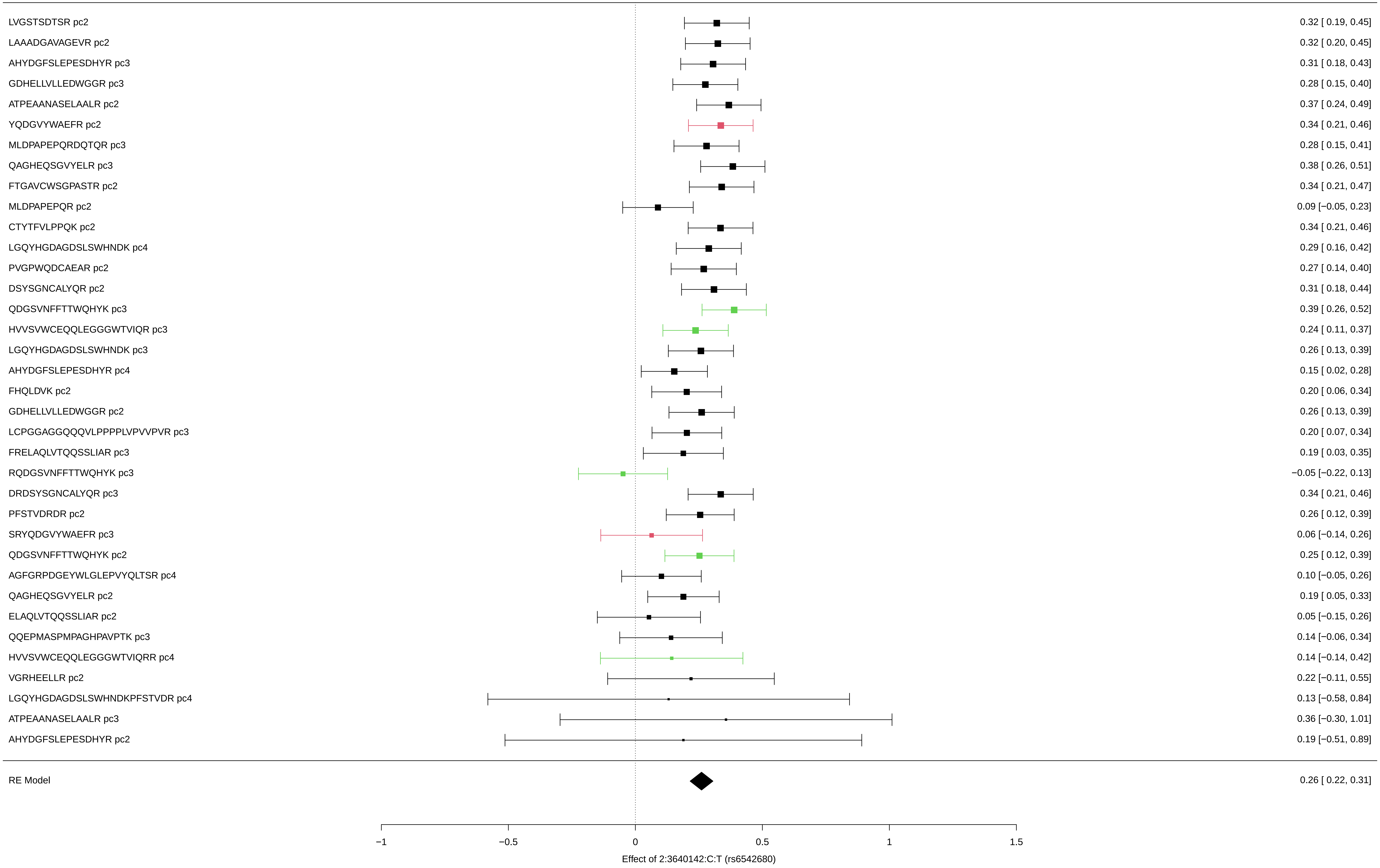

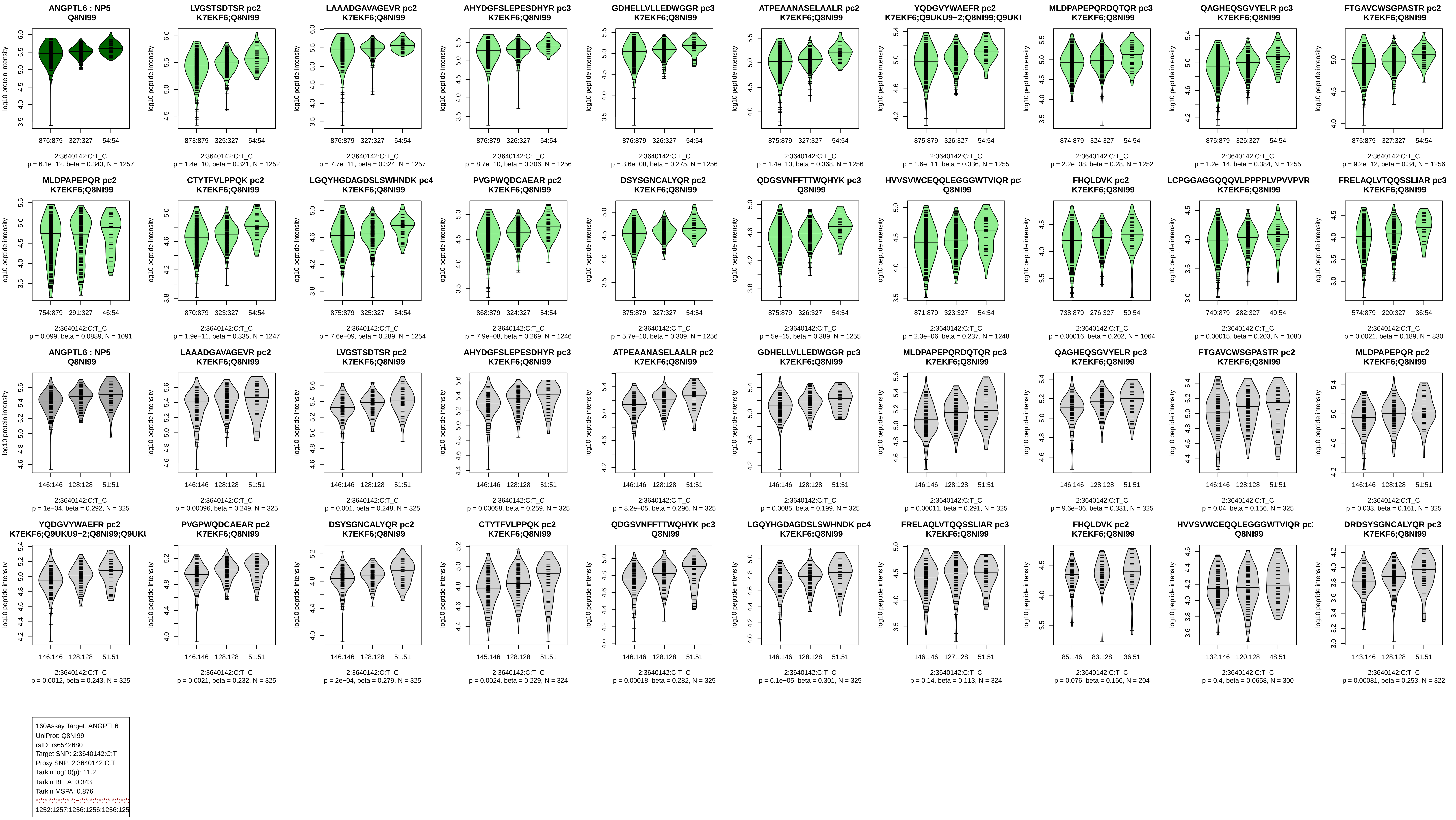
ANGPTL6 : NP5
Q8NI99
log10 protein intensity
876:879 327:327 54:54
2:3640142:C:T_C
p = 6.1e-12, beta = 0.343, N = 1257
LVGSTSDTSR pc2
K7EKF6;Q8NI99
log10 peptide intensity
873:879 325:327 54:54
2:3640142:C:T_C
p = 1.4e-10, beta = 0.321, N = 1252
LAAADGAVAGEVR pc2
K7EKF6;Q8NI99
876:879 327:327 54:54
2:3640142:C:T_C
p = 7.7e-11, beta = 0.324, N = 1257
AHYDGFSLEPESDHYR pc3
K7EKF6;Q8NI99
876:879 326:327 54:54
2:3640142:C:T_C
p = 8.7e-10, beta = 0.306, N = 1256
GDHELLVLLEDWGGR pc3
K7EKF6;Q8NI99
876:879 326:327 54:54
2:3640142:C:T_C
p = 3.6e-08, beta = 0.275, N = 1256
ATPEAANASELAALR pc2
K7EKF6;Q8NI99
875:879 327:327 54:54
2:3640142:C:T_C
p = 1.4e-13, beta = 0.368, N = 1256
YQDGVYWAEFR pc2
K7EKF6;Q9UKU9−2;Q8NI99;Q9UKU
875:879 326:327 54:54
2:3640142:C:T_C
p = 1.6e-11, beta = 0.336, N = 1255
MLDPAPEPQRDQTQR pc3
K7EKF6;Q8NI99
874:879 324:327 54:54
2:3640142:C:T_C
p = 2.2e−08, beta = 0.28, N = 1252
QAGHEQSGVYELR pc3
K7EKF6;Q8NI99
875:879 326:327 54:54
2:3640142:C:T_C
p = 1.2e-14, beta = 0.384, N = 1255
FTGAVCWSGPASTR pc2
K7EKF6;Q8NI99
875:879 327:327 54:54
2:3640142:C:T_C
p = 9.2e-12, beta = 0.34, N = 1256
MLDPAPEPQR pc2
K7EKF6;Q8NI99
754:879 291:327 46:54
2:3640142:C:T_C
p = 0.099, beta = 0.0889, N = 1091
CTYTFVLPPQK pc2
K7EKF6;Q8NI99
870:879 323:327 54:54
2:3640142:C:T_C
p = 1.9e-11, beta = 0.335, N = 1247
LGQYHGDAGDSLSWHNDK pc4
K7EKF6;Q8NI99
875:879 325:327 54:54
2:3640142:C:T_C
p = 7.6e-09, beta = 0.289, N = 1254
PVGPWQDCAEAR pc2
K7EKF6;Q8NI99
868:879 324:327 54:54
2:3640142:C:T_C
p = 7.9e-08, beta = 0.269, N = 1246
DSYSGNCALYQR pc2
K7EKF6;Q8NI99
875:879 327:327 54:54
2:3640142:C:T_C
p = 5.7e-10, beta = 0.309, N = 1256
QDGSVNFFTTWQHYK pc3
Q8NI99
875:879 326:327 54:54
2:3640142:C:T_C
p = 5e-15, beta = 0.389, N = 1255
HVVSVWCEQQLEGGGWTVIQR pc
Q8NI99
871:879 323:327 54:54
2:3640142:C:T_C
p = 2.3e-06, beta = 0.237, N = 1248
FHQLDVK pc2
K7EKF6;Q8NI99
738:879 276:327 50:54
2:3640142:C:T_C
p = 0.00016, beta = 0.202, N = 1064
LCPGGAGGQQQVLPPPPLVPVVPVR
K7EKF6;Q8NI99
749:879 282:327 49:54
2:3640142:C:T_C
p = 0.00015, beta = 0.203, N = 1080
FRELAQLVTQQSSLIAR pc3
K7EKF6;Q8NI99
574:879 220:327 36:54
2:3640142:C:T_C
p = 0.0021, beta = 0.189, N = 830
ANGPTL6 : NP5
Q8NI99
146:146 128:128 51:51
2:3640142:C:T_C
p = 1e-04, beta = 0.292, N = 325
LAAADGAVAGEVR pc2
K7EKF6;Q8NI99
146:146 128:128 51:51
2:3640142:C:T_C
p = 0.00096, beta = 0.249, N = 325
LVGSTSDTSR pc2
K7EKF6;Q8NI99
146:146 128:128 51:51
2:3640142:C:T_C
p = 0.001, beta = 0.248, N = 325
AHYDGFSLEPESDHYR pc3
K7EKF6;Q8NI99
146:146 128:128 51:51
2:3640142:C:T_C
p = 0.00058, beta = 0.259, N = 325
ATPEAANASELAALR pc2
K7EKF6;Q8NI99
146:146 128:128 51:51
2:3640142:C:T_C
p = 8.2e-05, beta = 0.296, N = 325
GDHELLVLLEDWGGR pc3
K7EKF6;Q8NI99
146:146 128:128 51:51
2:3640142:C:T_C
p = 0.0085, beta = 0.199, N = 325
MLDPAPEPQRDQTQR pc3
K7EKF6;Q8NI99
146:146 128:128 51:51
2:3640142:C:T_C
p = 0.00011, beta = 0.291, N = 325
QAGHEQSGVYELR pc3
K7EKF6;Q8NI99
146:146 128:128 51:51
2:3640142:C:T_C
p = 9.6e-06, beta = 0.331, N = 325
FTGAVCWSGPASTR pc2
K7EKF6;Q8NI99
146:146 128:128 51:51
2:3640142:C:T_C
p = 0.04, beta = 0.156, N = 325
MLDPAPEPQR pc2
K7EKF6;Q8NI99
146:146 128:128 51:51
2:3640142:C:T_C
p = 0.033, beta = 0.161, N = 325
YQDGVYWAEFR pc2
K7EKF6;Q9UKU9−2;Q8NI99;Q9UKU
146:146 128:128 51:51
2:3640142:C:T_C
p = 0.0012, beta = 0.243, N = 325
PVGPWQDCAEAR pc2
K7EKF6;Q8NI99
146:146 128:128 51:51
2:3640142:C:T_C
p = 0.0021, beta = 0.232, N = 325
DSYSGNCALYQR pc2
K7EKF6;Q8NI99
146:146 128:128 51:51
2:3640142:C:T_C
p = 2e-04, beta = 0.279, N = 325
CTYTFVLPPQK pc2
K7EKF6;Q8NI99
145:146 128:128 51:51
2:3640142:C:T_C
p = 0.0024, beta = 0.229, N = 324
QDGSVNFFTTWQHYK pc3
Q8NI99
146:146 128:128 51:51
2:3640142:C:T_C
p = 0.00018, beta = 0.282, N = 325
LGQYHGDAGDSLSWHNDK pc4
K7EKF6;Q8NI99
146:146 128:128 51:51
2:3640142:C:T_C
p = 6.1e-05, beta = 0.301, N = 325
FRELAQLVTQQSSLIAR pc3
K7EKF6;Q8NI99
146:146 127:128 51:51
2:3640142:C:T_C
p = 0.14, beta = 0.113, N = 324
FHQLDVK pc2
K7EKF6;Q8NI99
85:146 83:128 36:51
2:3640142:C:T_C
p = 0.076, beta = 0.166, N = 204
HVVSVWCEQQLEGGGWTVIQR pc
Q8NI99
132:146 120:128 48:51
2:3640142:C:T_C
p = 0.4, beta = 0.0658, N = 300
DRDSYSGNCALYQR pc3
K7EKF6;Q8NI99
143:146 128:128 51:51
2:3640142:C:T_C
p = 0.00081, beta = 0.253, N = 322
160Assay Target: ANGPTL6
UniProt: Q8NI99
rsID: rs6542680
Target SNP: 2:3640142:C:T
Proxy SNP: 2:3640142:C:T
Tarkin log10(p): 11.2
Tarkin BETA: 0.343
Tarkin MSPA: 0.876
1252:1257:1256:1256:1256:125

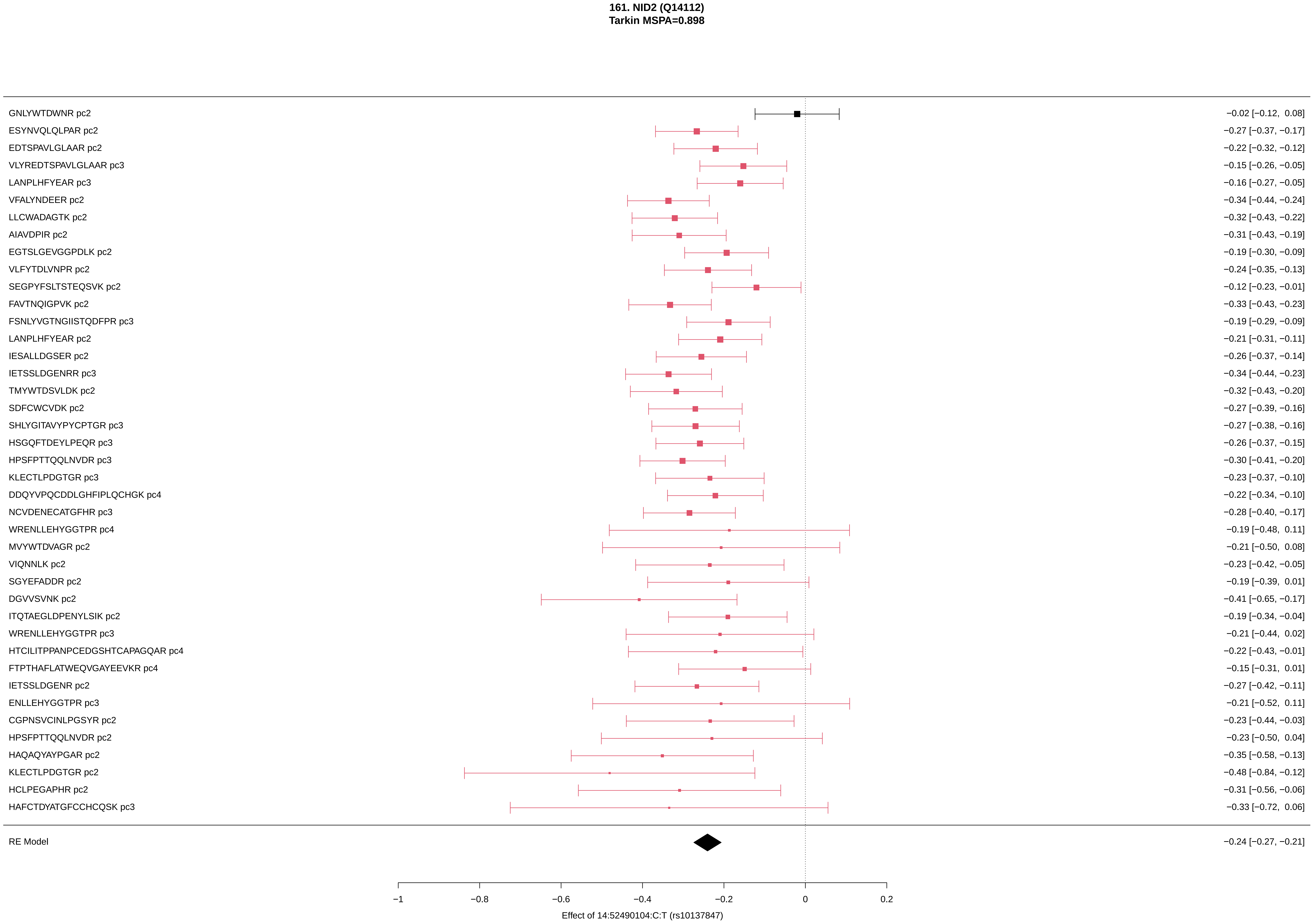
161. NID2 (Q14112)
Tarkin MSPA=0.898
GNLYWTDWNR pc2 −0.02 [−0.12, 0.08]
ESYNVQLQLPAR pc2 −0.27 [−0.37, −0.17]
EDTSPAVLGLAAR pc2 −0.22 [−0.32, −0.12]
VLYREDTSPAVLGLAAR pc3 −0.15 [−0.26, −0.05]
LANPLHFYEAR pc3 −0.16 [−0.27, −0.05]
VFALYNDEER pc2 −0.34 [−0.44, −0.24]
LLCWADAGTK pc2 −0.32 [−0.43, −0.22]
AIAVDPIR pc2 −0.31 [−0.43, −0.19]
EGTSLGEVGGPDLK pc2 −0.19 [−0.30, −0.09]
VLFYTDLVNPR pc2 −0.24 [−0.35, −0.13]
SEGPYFSLTSTEQSVK pc2 −0.12 [−0.23, −0.01]
FAVTNQIGPVK pc2 −0.33 [−0.43, −0.23]
FSNLYVGTNGIISTQDFPR pc3 −0.19 [−0.29, −0.09]
LANPLHFYEAR pc2 −0.21 [−0.31, −0.11]
IESALLDGSER pc2 −0.26 [−0.37, −0.14]
IETSSLDGENRR pc3 −0.34 [−0.44, −0.23]
TMYWTDSVLDK pc2 −0.32 [−0.43, −0.20]
SDFCWCVDK pc2 −0.27 [−0.39, −0.16]
SHLYGITAVYPYCPTGR pc3 −0.27 [−0.38, −0.16]
HSGQFTDEYLPEQR pc3 −0.26 [−0.37, −0.15]
HPSFPTTQQLNVDR pc3 −0.30 [−0.41, −0.20]
KLECTLPDGTGR pc3 −0.23 [−0.37, −0.10]
DDQYVPQCDDLGHFIPLQCHGK pc4 −0.22 [−0.34, −0.10]
NCVDENECATGFHR pc3 −0.28 [−0.40, −0.17]
WRENLLEHYGGTPR pc4 −0.19 [−0.48, 0.11]
MVYWTDVAGR pc2 −0.21 [−0.50, 0.08]
VIQNNLK pc2 −0.23 [−0.42, −0.05]
SGYEFADDR pc2 −0.19 [−0.39, 0.01]
DGVVSVNK pc2 −0.41 [−0.65, −0.17]
ITQTAEGLDPENYLSIK pc2 −0.19 [−0.34, −0.04]
WRENLLEHYGGTPR pc3 −0.21 [−0.44, 0.02]
HTCILITPPANPCEDGSHTCAPAGQAR pc4 −0.22 [−0.43, −0.01]
FTPTHAFLATWEQVGAYEEVKR pc4 −0.15 [−0.31, 0.01]
IETSSLDGENR pc2 −0.27 [−0.42, −0.11]
ENLLEHYGGTPR pc3 −0.21 [−0.52, 0.11]
CGPNSVCINLPGSYR pc2 −0.23 [−0.44, −0.03]
HPSFPTTQQLNVDR pc2 −0.23 [−0.50, 0.04]
HAQAQYAYPGAR pc2 −0.35 [−0.58, −0.13]
KLECTLPDGTGR pc2 −0.48 [−0.84, −0.12]
HCLPEGAPHR pc2 −0.31 [−0.56, −0.06]
HAFCTDYATGFCCHCQSK pc3 −0.33 [−0.72, 0.06]
RE Model −0.24 [−0.27, −0.21]
−1
−0.8
−0.6
−0.4
−0.2
0
0.2
Effect of 14:52490104:C:T (rs10137847)

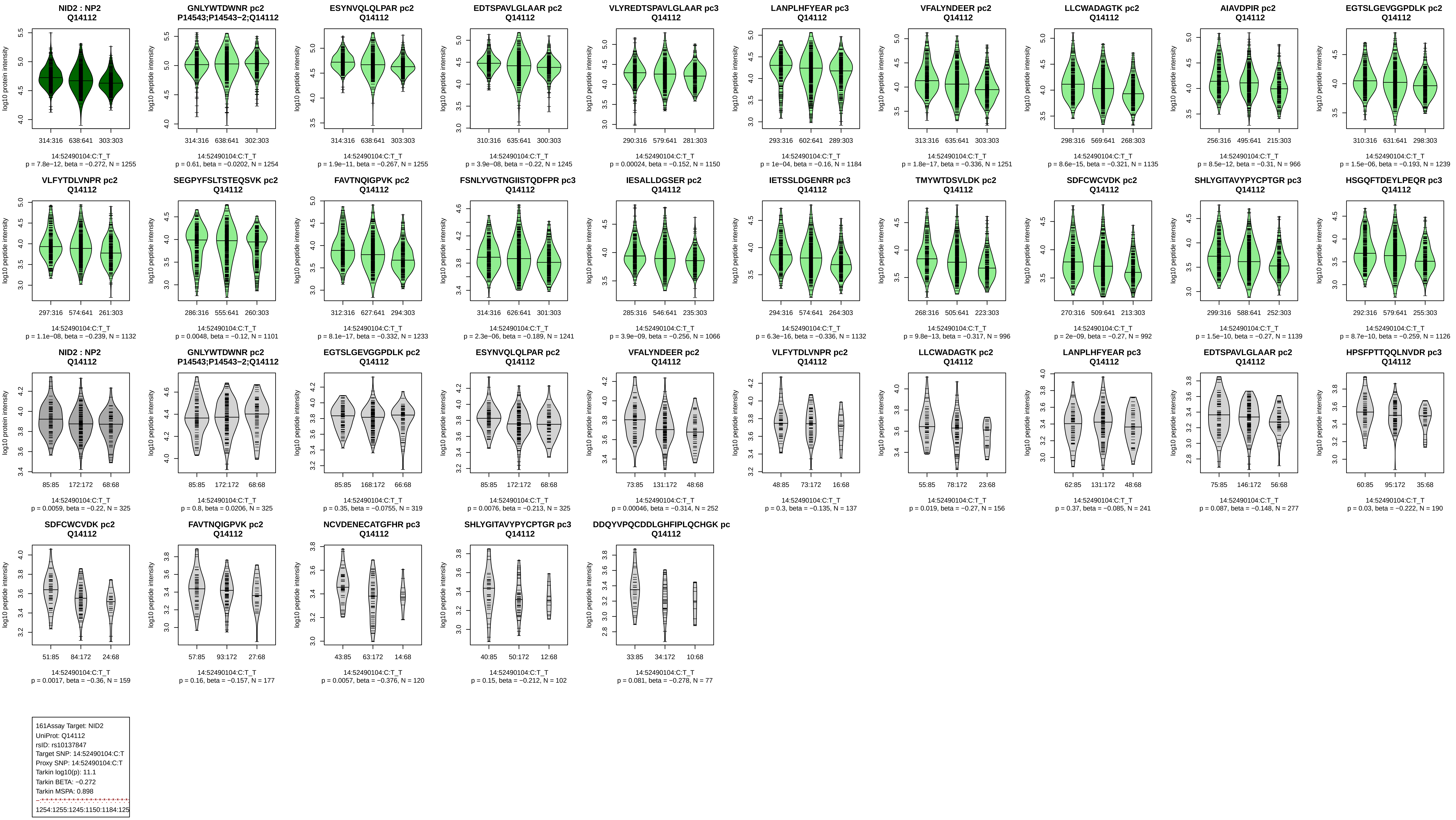
NID2 : NP2
Q14112
log10 protein intensity
314:316 638:641 303:303
14:52490104:C:T_T
p = 7.8e−12, beta = −0.272, N = 1255
GNLYWTDWNR pc2
P14543;P14543−2;Q14112
log10 peptide intensity
314:316 638:641 302:303
14:52490104:C:T_T
p = 0.61, beta = −0.0202, N = 1254
ESYNVQLQLPAR pc2
Q14112
314:316 638:641 303:303
14:52490104:C:T_T
p = 1.9e−11, beta = −0.267, N = 1255
EDTSPAVLGLAAR pc2
Q14112
310:316 635:641 300:303
14:52490104:C:T_T
p = 3.9e−08, beta = −0.22, N = 1245
VLYREDTSPAVLGLAAR pc3
Q14112
290:316 579:641 281:303
14:52490104:C:T_T
p = 0.00024, beta = −0.152, N = 1150
LANPLHFYEAR pc3
Q14112
293:316 602:641 289:303
14:52490104:C:T_T
p = 1e−04, beta = −0.16, N = 1184
VFALYNDEER pc2
Q14112
313:316 635:641 303:303
14:52490104:C:T_T
p = 1.8e−17, beta = −0.336, N = 1251
LLCWADAGTK pc2
Q14112
298:316 569:641 268:303
14:52490104:C:T_T
p = 8.6e−15, beta = −0.321, N = 1135
AIAVDPIR pc2
Q14112
256:316 495:641 215:303
14:52490104:C:T_T
p = 8.5e−12, beta = −0.31, N = 966
EGTSLGEVGGPDLK pc2
Q14112
310:316 631:641 298:303
14:52490104:C:T_T
p = 1.5e−06, beta = −0.193, N = 1239
VLFYTDLVNPR pc2
Q14112
297:316 574:641 261:303
14:52490104:C:T_T
p = 1.1e−08, beta = −0.239, N = 1132
SEGPYFSLTSTEQSVK pc2
Q14112
286:316 555:641 260:303
14:52490104:C:T_T
p = 0.0048, beta = −0.12, N = 1101
FAVTNQIGPVK pc2
Q14112
312:316 627:641 294:303
14:52490104:C:T_T
p = 8.1e−17, beta = −0.332, N = 1233
FSNLYVGTNGIISTQDFPR pc3
Q14112
314:316 626:641 301:303
14:52490104:C:T_T
p = 2.3e−06, beta = −0.189, N = 1241
IESALLDGSER pc2
Q14112
285:316 546:641 235:303
14:52490104:C:T_T
p = 3.9e−09, beta = −0.256, N = 1066
IETSSLDGENRR pc3
Q14112
294:316 574:641 264:303
14:52490104:C:T_T
p = 6.3e−16, beta = −0.336, N = 1132
TMYWTDSVLDK pc2
Q14112
268:316 505:641 223:303
14:52490104:C:T_T
p = 9.8e−13, beta = −0.317, N = 996
SDFCWCVDK pc2
Q14112
270:316 509:641 213:303
14:52490104:C:T_T
p = 2e−09, beta = −0.27, N = 992
SHLYGITAVYPYCPTGR pc3
Q14112
299:316 588:641 252:303
14:52490104:C:T_T
p = 1.5e−10, beta = −0.27, N = 1139
HSGQFTDEYLPEQR pc3
Q14112
292:316 579:641 255:303
14:52490104:C:T_T
p = 8.7e−10, beta = −0.259, N = 1126
NID2 : NP2
Q14112
85:85 172:172 68:68
14:52490104:C:T_T
p = 0.0059, beta = −0.22, N = 325
GNLYWTDWNR pc2
P14543;P14543−2;Q14112
85:85 172:172 68:68
14:52490104:C:T_T
p = 0.8, beta = 0.0206, N = 325
EGTSLGEVGGPDLK pc2
Q14112
85:85 168:172 66:68
14:52490104:C:T_T
p = 0.35, beta = −0.0755, N = 319
ESYNVQLQLPAR pc2
Q14112
85:85 172:172 68:68
14:52490104:C:T_T
p = 0.0076, beta = −0.213, N = 325
VFALYNDEER pc2
Q14112
73:85 131:172 48:68
14:52490104:C:T_T
p = 0.00046, beta = −0.314, N = 252
VLFYTDLVNPR pc2
Q14112
48:85 73:172 16:68
14:52490104:C:T_T
p = 0.3, beta = −0.135, N = 137
LLCWADAGTK pc2
Q14112
55:85 78:172 23:68
14:52490104:C:T_T
p = 0.019, beta = −0.27, N = 156
LANPLHFYEAR pc3
Q14112
62:85 131:172 48:68
14:52490104:C:T_T
p = 0.37, beta = −0.085, N = 241
EDTSPAVLGLAAR pc2
Q14112
75:85 146:172 56:68
14:52490104:C:T_T
p = 0.087, beta = −0.148, N = 277
HPSFPTTQQLNVDR pc3
Q14112
60:85 95:172 35:68
14:52490104:C:T_T
p = 0.03, beta = −0.222, N = 190
SDFCWCVDK pc2
Q14112
51:85 84:172 24:68
14:52490104:C:T_T
p = 0.0017, beta = −0.36, N = 159
FAVTNQIGPVK pc2
Q14112
57:85 93:172 27:68
14:52490104:C:T_T
p = 0.16, beta = −0.157, N = 177
NCVDENECATGFHR pc3
Q14112
43:85 63:172 14:68
14:52490104:C:T_T
p = 0.0057, beta = −0.376, N = 120
SHLYGITAVYPYCPTGR pc3
Q14112
40:85 50:172 12:68
14:52490104:C:T_T
p = 0.15, beta = −0.212, N = 102
DDQYVPQCDDLGHFIPLQCHGK pc
Q14112
33:85 34:172 10:68
14:52490104:C:T_T
p = 0.081, beta = −0.278, N = 77
161Assay Target: NID2
UniProt: Q14112
rsID: rs10137847
Target SNP: 14:52490104:C:T
Proxy SNP: 14:52490104:C:T
Tarkin log10(p): 11.1
Tarkin BETA: −0.272
Tarkin MSPA: 0.898
1254:1255:1245:1150:1184:125

162. SRCRB4D (Q8WTU2)
Tarkin MSPA=0.989

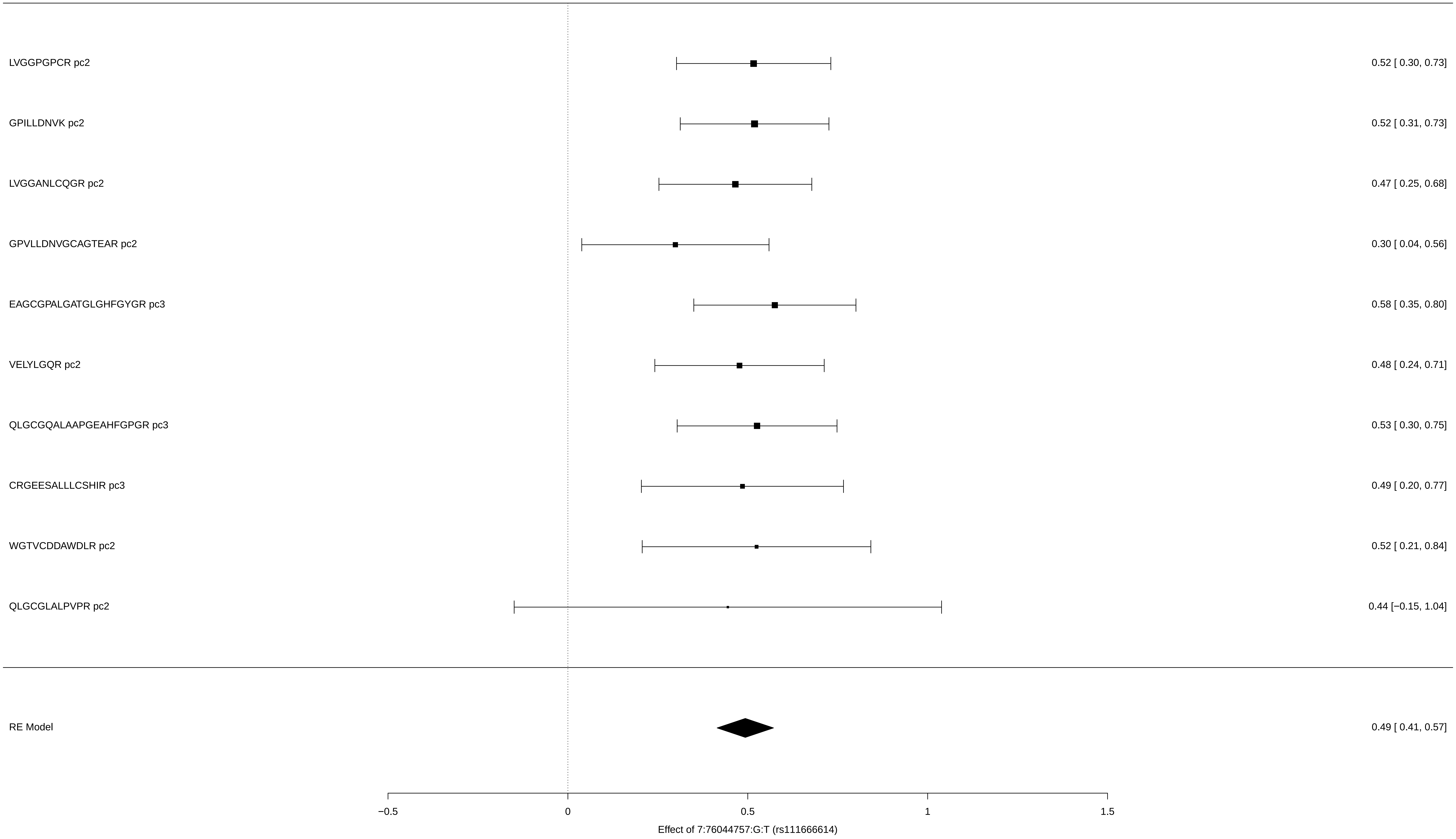

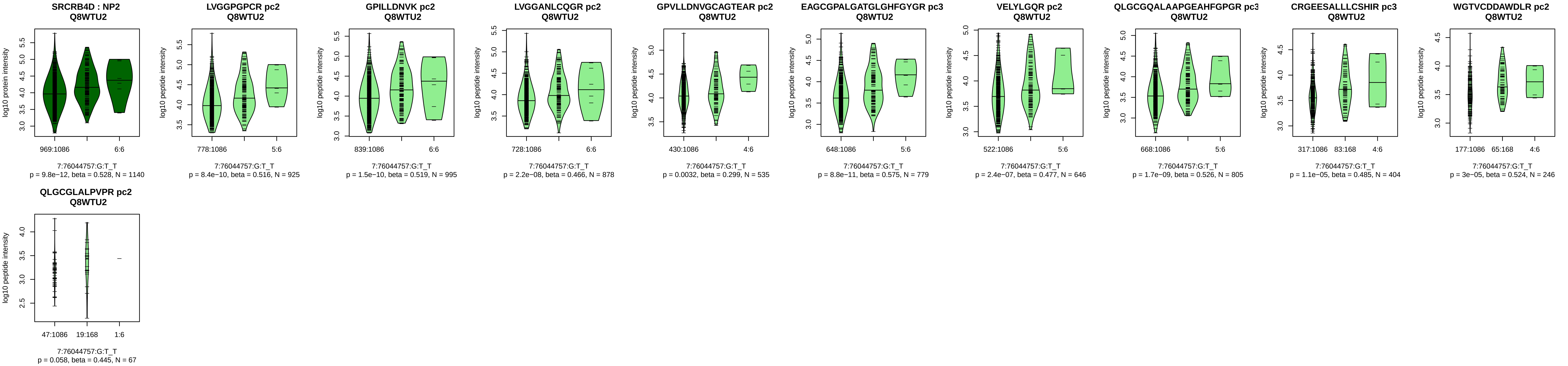
SRCRB4D : NP2 Q8WTU2
969:1086 6:6
7:76044757:G:T_T
p = 9.8e−12, beta = 0.528, N = 1140
LVGGPGPCR pc2 Q8WTU2
778:1086 5:6
p = 8.4e−10, beta = 0.516, N = 925
GPILLDNVK pc2 Q8WTU2
839:1086 6:6
p = 1.5e−10, beta = 0.519, N = 995
LVGGANLCQGR pc2 Q8WTU2
728:1086 6:6
p = 2.2e−08, beta = 0.466, N = 878
GPVLLDNVGCAGTEAR pc2 Q8WTU2
430:1086 4:6
p = 0.0032, beta = 0.299, N = 535
EAGCGPALGATGLGHFGYGR pc3 Q8WTU2
648:1086 5:6
p = 8.8e−11, beta = 0.575, N = 779
VELYLGQR pc2 Q8WTU2
522:1086 5:6
p = 2.4e−07, beta = 0.477, N = 646
QLGCGQALAAPGEAHFGPGR pc3 Q8WTU2
668:1086 5:6
p = 1.7e−09, beta = 0.526, N = 805
CRGEESALLLCSHIR pc3 Q8WTU2
317:1086 83:168 4:6
p = 1.1e−05, beta = 0.485, N = 404
WGTVCDDAWDLR pc2 Q8WTU2
177:1086 65:168 4:6
p = 3e−05, beta = 0.524, N = 246
QLGCGLALPVPR pc2 Q8WTU2
47:1086 19:168 1:6
p = 0.058, beta = 0.445, N = 67
log10 protein intensity
log10 peptide intensity

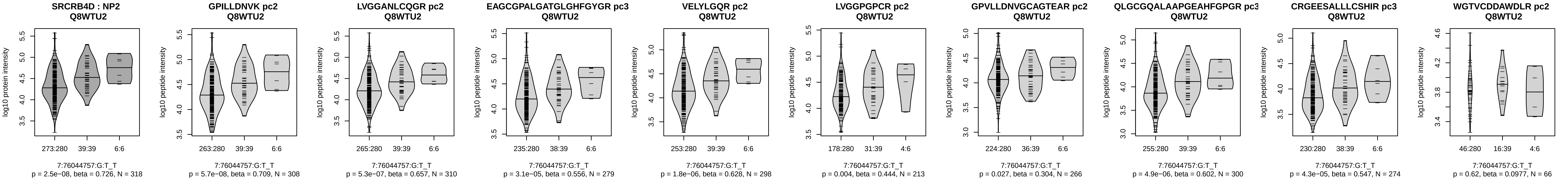
SRCRB4D : NP2 Q8WTU2
273:280 39:39 6:6
p = 2.5e−08, beta = 0.726, N = 318
GPILLDNVK pc2 Q8WTU2
263:280 39:39 6:6
p = 5.7e−08, beta = 0.709, N = 308
LVGGANLCQGR pc2 Q8WTU2
265:280 39:39 6:6
p = 5.3e−07, beta = 0.657, N = 310
EAGCGPALGATGLGHFGYGR pc3 Q8WTU2
235:280 38:39 6:6
p = 3.1e−05, beta = 0.556, N = 279
VELYLGQR pc2 Q8WTU2
253:280 39:39 6:6
p = 1.8e−06, beta = 0.628, N = 298
LVGGPGPCR pc2 Q8WTU2
178:280 31:39 4:6
p = 0.004, beta = 0.444, N = 213
GPVLLDNVGCAGTEAR pc2 Q8WTU2
224:280 36:39 6:6
p = 0.027, beta = 0.304, N = 266
QLGCGQALAAPGEAHFGPGR pc3 Q8WTU2
255:280 39:39 6:6
p = 4.9e−06, beta = 0.602, N = 300
CRGEESALLLCSHIR pc3 Q8WTU2
230:280 38:39 6:6
p = 4.3e−05, beta = 0.547, N = 274
WGTVCDDAWDLR pc2 Q8WTU2
46:280 16:39 4:6
p = 0.62, beta = 0.0977, N = 66
7:76044757:G:T_T

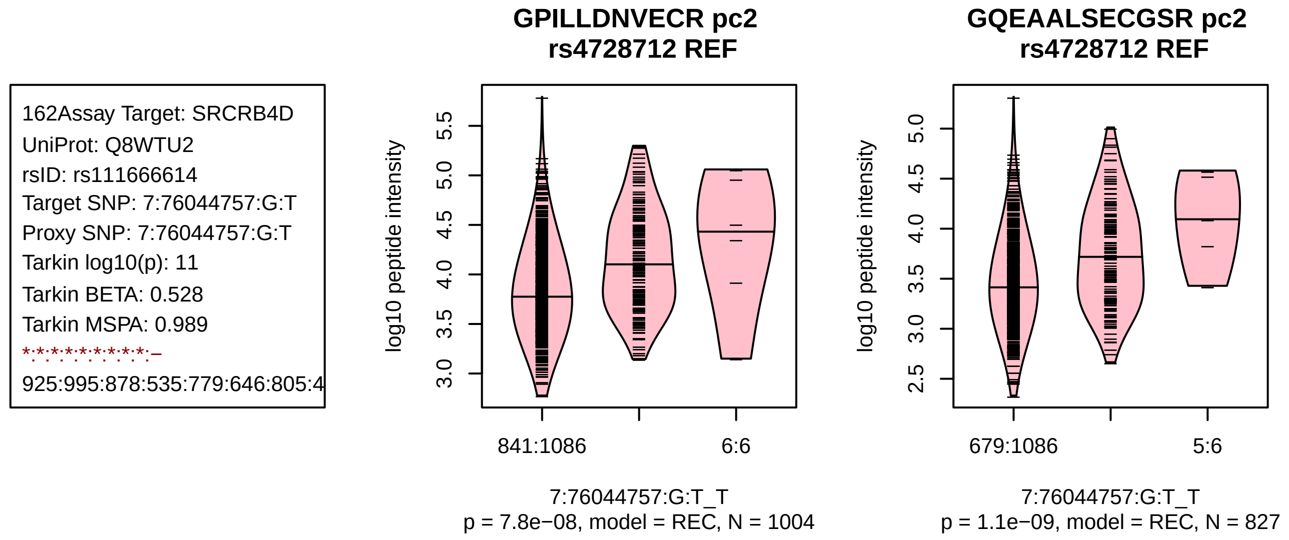
162Assay Target: SRCRB4D
UniProt: Q8WTU2
rsID: rs111666614
Target SNP: 7:76044757:G:T
Proxy SNP: 7:76044757:G:T
Tarkin log10(p): 11
Tarkin BETA: 0.528
Tarkin MSPA: 0.989
925:995:878:535:779:646:805:4
GPILLDNVECR pc2 rs4728712 REF
841:1086 6:6
7:76044757:G:T_T
p = 7.8e−08, model = REC, N = 1004
GQEAALSECGSR pc2 rs4728712 REF
679:1086 5:6
7:76044757:G:T_T
p = 1.1e−09, model = REC, N = 827

163. PADI4 (Q9UM07)
Tarkin MSPA=0.503

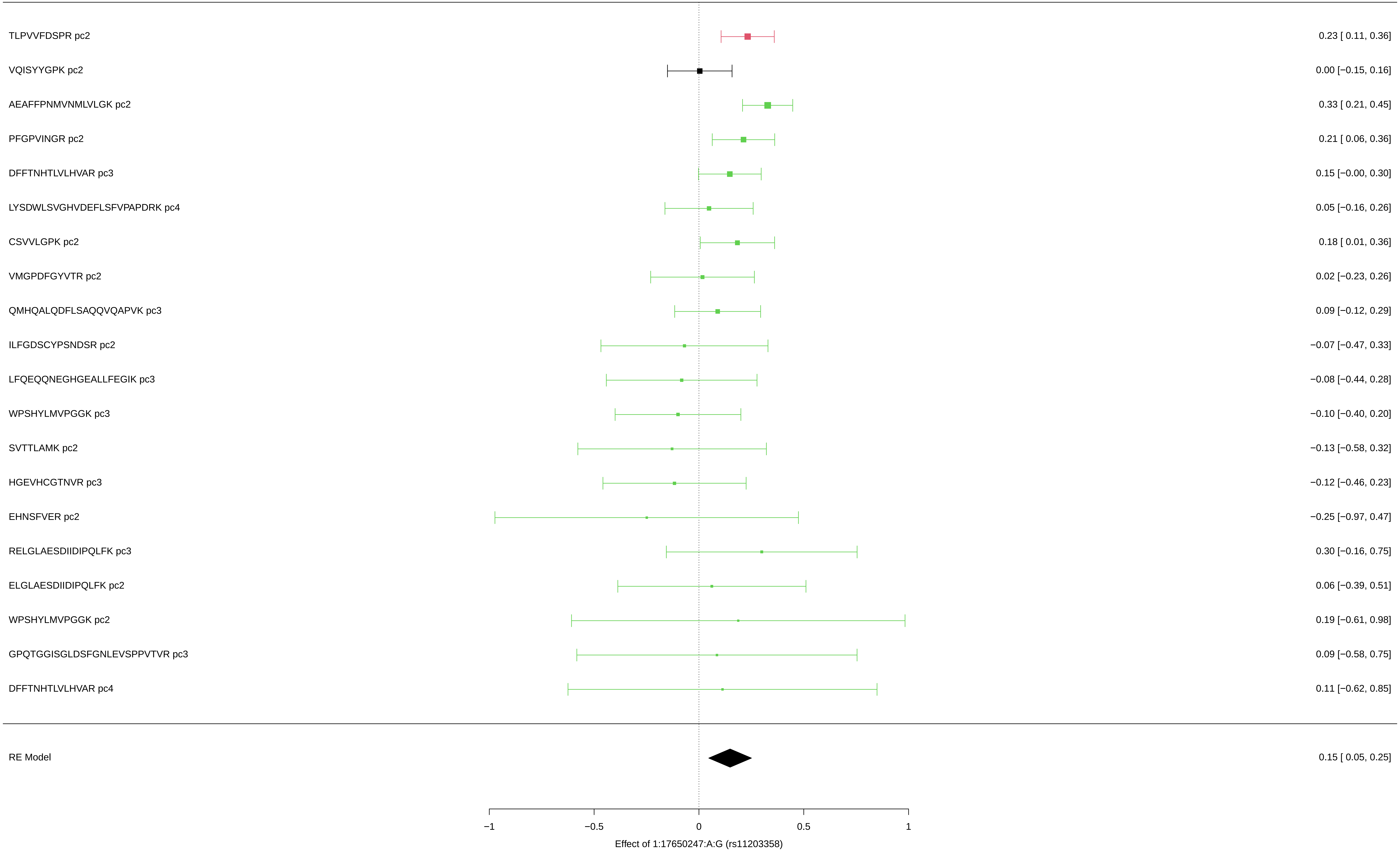

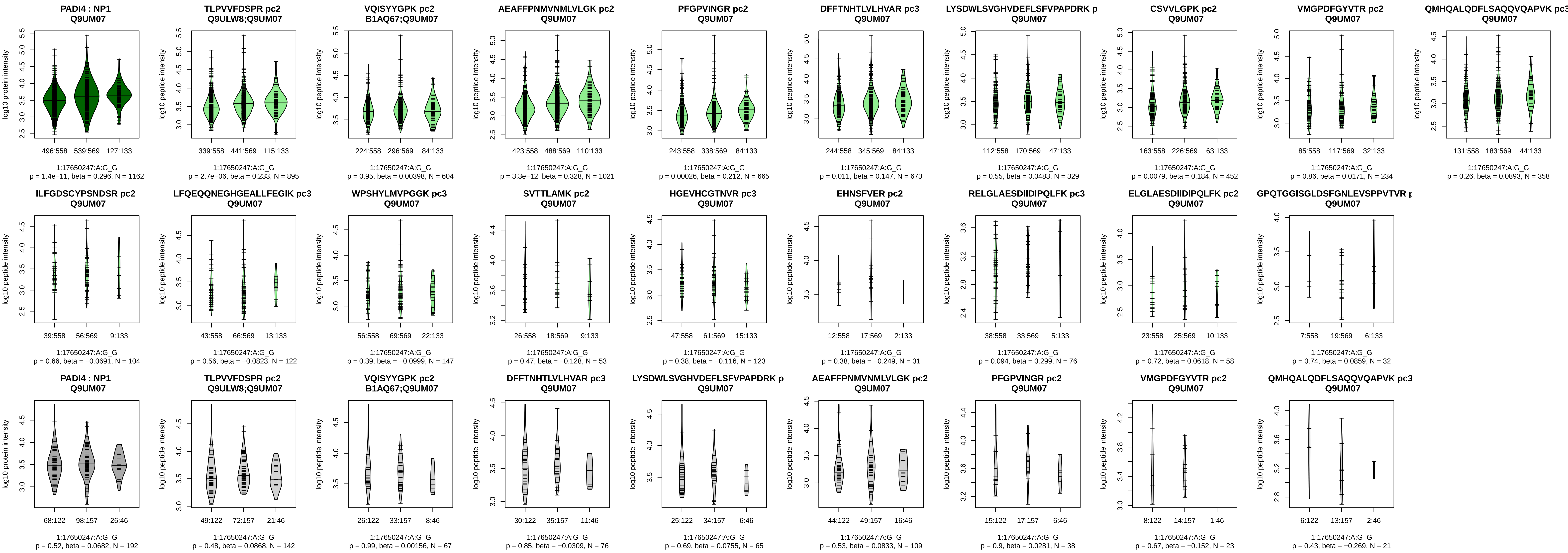

163Assay Target: PADI4
UniProt: Q9UM07
rsID: rs11203358
Target SNP: 1:17650247:A:G
Proxy SNP: 1:17650247:A:G
Tarkin log10(p): 10.9
Tarkin BETA: 0.296
Tarkin MSPA: 0.503
:–::*:–:–:*:–:–:–:–:–:–:–:–:–:–:–
895:604:1021:665:673:329:452:

164. LAMC1 (P11047)
Tarkin MSPA=0.864

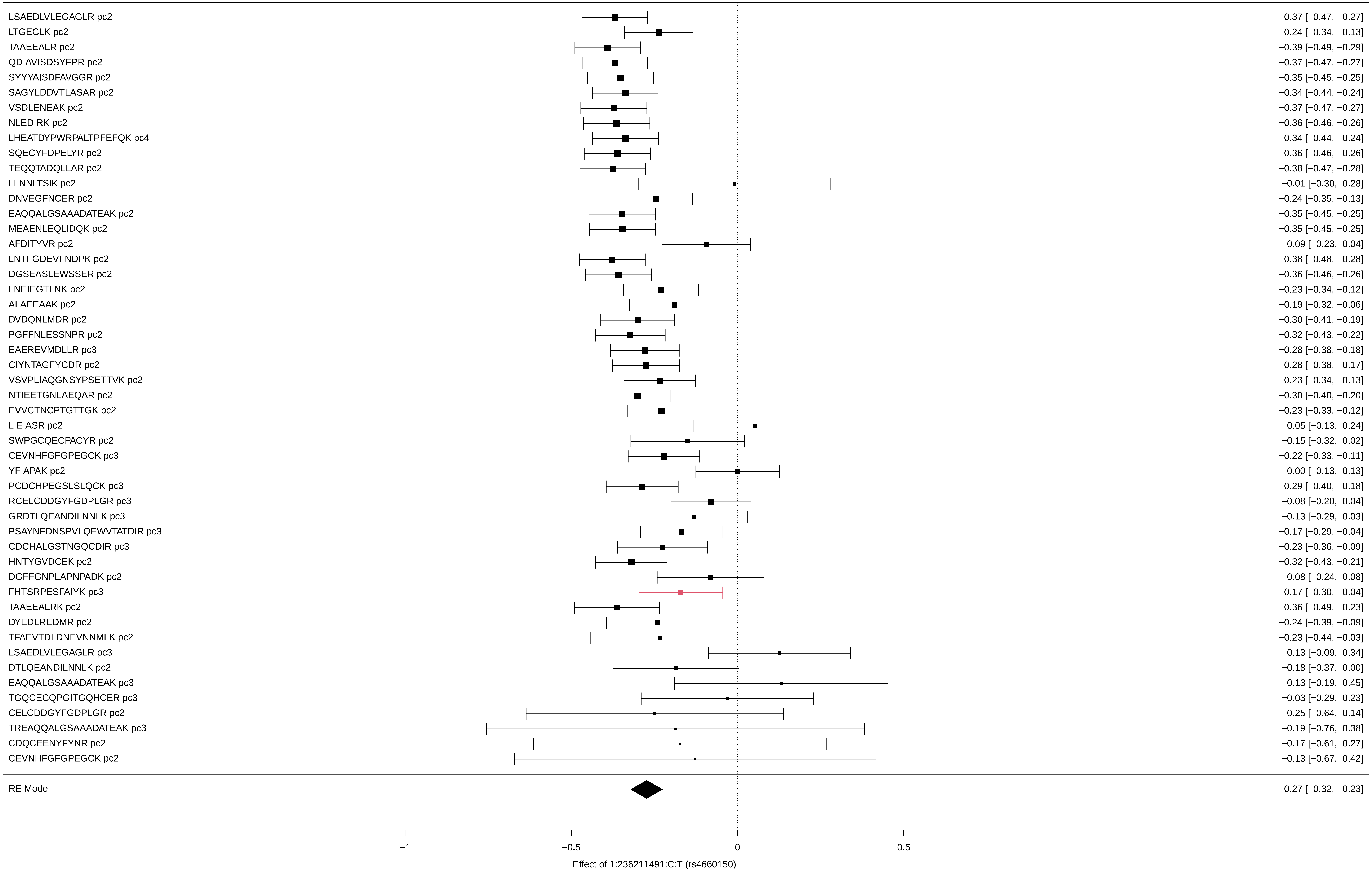

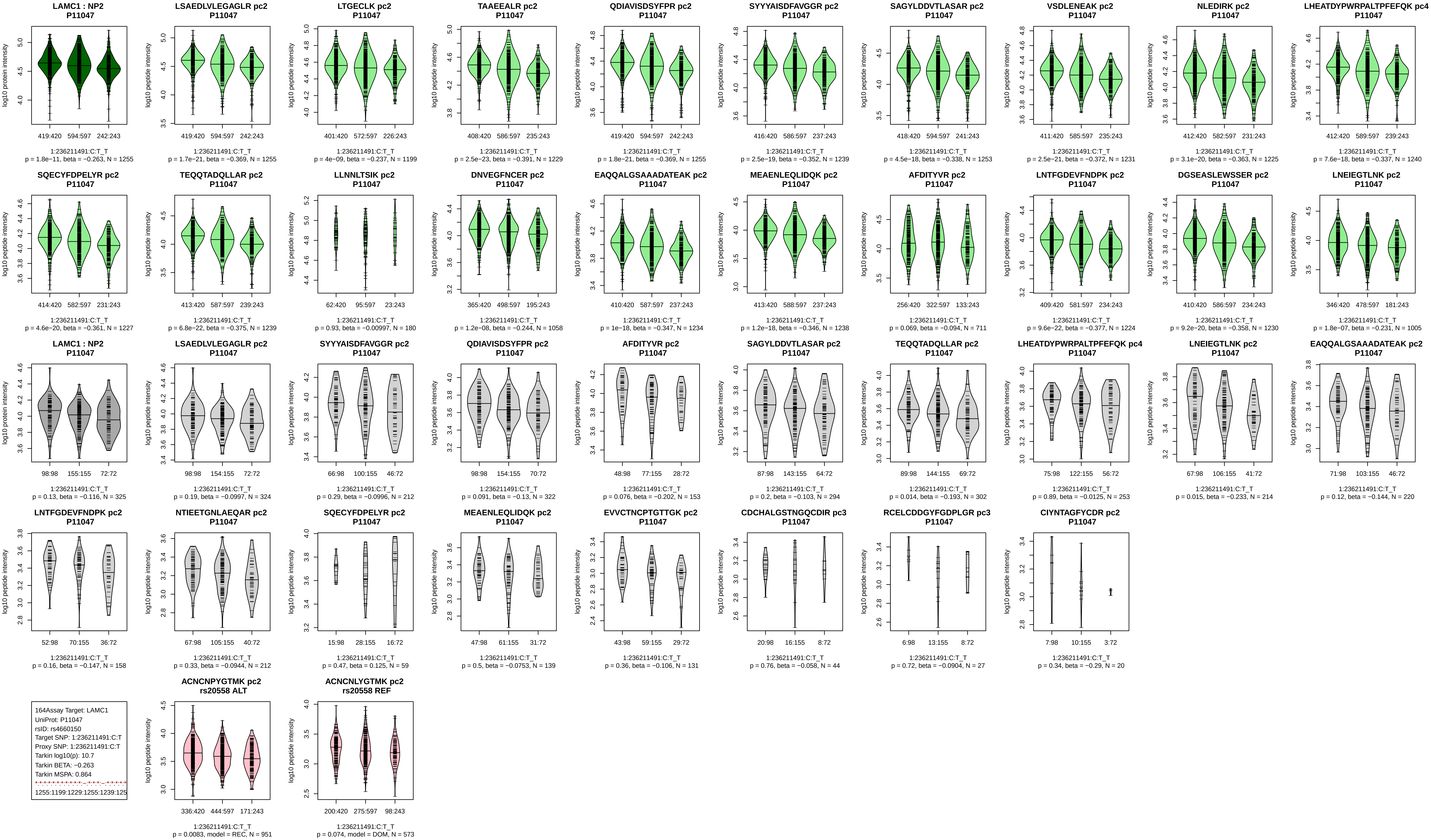

LAMC1 : NP2
P11047
log10 protein intensity
419:420 594:597 242:243
1:236211491:C:T_T
p = 1.8e−11, beta = −0.263, N = 1255
LSAEDLVLEGAGLR pc2
P11047
log10 peptide intensity
419:420 594:597 242:243
1:236211491:C:T_T
p = 1.7e−21, beta = −0.369, N = 1255
LTGECLK pc2
P11047
401:420 572:597 226:243
1:236211491:C:T_T
p = 4e−09, beta = −0.237, N = 1199
TAAEEALR pc2
P11047
408:420 586:597 235:243
1:236211491:C:T_T
p = 2.5e−23, beta = −0.391, N = 1229
QDIAVISDSYFPR pc2
P11047
419:420 594:597 242:243
1:236211491:C:T_T
p = 1.8e−21, beta = −0.369, N = 1255
SYYYAISDFAVGGR pc2
P11047
416:420 586:597 237:243
1:236211491:C:T_T
p = 2.5e−19, beta = −0.352, N = 1239
SAGYLDDVTLASAR pc2
P11047
418:420 594:597 241:243
1:236211491:C:T_T
p = 4.5e−18, beta = −0.338, N = 1253
VSDLENEAK pc2
P11047
411:420 585:597 235:243
1:236211491:C:T_T
p = 2.5e−21, beta = −0.372, N = 1231
NLEDIRK pc2
P11047
412:420 582:597 231:243
1:236211491:C:T_T
p = 3.1e−20, beta = −0.363, N = 1225
LHEATDYPWRPALTPFEFQK pc4
P11047
412:420 589:597 239:243
1:236211491:C:T_T
p = 7.6e−18, beta = −0.337, N = 1240
SQECYFDPELYR pc2
P11047
414:420 582:597 231:243
1:236211491:C:T_T
p = 4.6e−20, beta = −0.361, N = 1227
TEQQTADQLLAR pc2
P11047
413:420 587:597 239:243
1:236211491:C:T_T
p = 6.8e−22, beta = −0.375, N = 1239
LLNNLTSIK pc2
P11047
62:420 95:597 23:243
1:236211491:C:T_T
p = 0.93, beta = −0.00997, N = 180
DNVEGFNCER pc2
P11047
365:420 498:597 195:243
1:236211491:C:T_T
p = 1.2e−08, beta = −0.244, N = 1058
EAQQALGSAAADATEAK pc2
P11047
410:420 587:597 237:243
1:236211491:C:T_T
p = 1e−18, beta = −0.347, N = 1234
MEAENLEQLIDQK pc2
P11047
413:420 588:597 237:243
1:236211491:C:T_T
p = 1.2e−18, beta = −0.346, N = 1238
AFDITYVR pc2
P11047
256:420 322:597 133:243
1:236211491:C:T_T
p = 0.069, beta = −0.094, N = 711
LNTFGDEVFNDPK pc2
P11047
409:420 581:597 234:243
1:236211491:C:T_T
p = 9.6e−22, beta = −0.377, N = 1224
DGSEASLEWSSER pc2
P11047
410:420 586:597 234:243
1:236211491:C:T_T
p = 9.2e−20, beta = −0.358, N = 1230
LNEIEGTLNK pc2
P11047
346:420 478:597 181:243
1:236211491:C:T_T
p = 1.8e−07, beta = −0.231, N = 1005
LAMC1 : NP2
P11047
98:98 155:155 72:72
1:236211491:C:T_T
p = 0.13, beta = −0.116, N = 325
LSAEDLVLEGAGLR pc2
P11047
98:98 154:155 72:72
1:236211491:C:T_T
p = 0.19, beta = −0.0997, N = 324
SYYYAISDFAVGGR pc2
P11047
66:98 100:155 46:72
1:236211491:C:T_T
p = 0.29, beta = −0.0996, N = 212
QDIAVISDSYFPR pc2
P11047
98:98 154:155 70:72
1:236211491:C:T_T
p = 0.091, beta = −0.13, N = 322
AFDITYVR pc2
P11047
48:98 77:155 28:72
1:236211491:C:T_T
p = 0.076, beta = −0.202, N = 153
SAGYLDDVTLASAR pc2
P11047
87:98 143:155 64:72
1:236211491:C:T_T
p = 0.2, beta = −0.103, N = 294
TEQQTADQLLAR pc2
P11047
89:98 144:155 69:72
1:236211491:C:T_T
p = 0.014, beta = −0.193, N = 302
LHEATDYPWRPALTPFEFQK pc4
P11047
75:98 122:155 56:72
1:236211491:C:T_T
p = 0.89, beta = −0.0125, N = 253
LNEIEGTLNK pc2
P11047
67:98 106:155 41:72
1:236211491:C:T_T
p = 0.015, beta = −0.233, N = 214
EAQQALGSAAADATEAK pc2
P11047
71:98 103:155 46:72
1:236211491:C:T_T
p = 0.12, beta = −0.144, N = 220
LNTFGDEVFNDPK pc2
P11047
52:98 70:155 36:72
1:236211491:C:T_T
p = 0.16, beta = −0.147, N = 158
NTIEETGNLAEQAR pc2
P11047
67:98 105:155 40:72
1:236211491:C:T_T
p = 0.33, beta = −0.0944, N = 212
SQECYFDPELYR pc2
P11047
15:98 28:155 16:72
1:236211491:C:T_T
p = 0.47, beta = 0.125, N = 59
MEAENLEQLIDQK pc2
P11047
47:98 61:155 31:72
1:236211491:C:T_T
p = 0.5, beta = −0.0753, N = 139
EVVCTNCPTGTTGK pc2
P11047
43:98 59:155 29:72
1:236211491:C:T_T
p = 0.36, beta = −0.106, N = 131
CDCHALGSTNGQCDIR pc3
P11047
20:98 16:155 8:72
1:236211491:C:T_T
p = 0.76, beta = −0.058, N = 44
RCELCDDGYFGDPLGR pc3
P11047
6:98 13:155 8:72
1:236211491:C:T_T
p = 0.72, beta = −0.0904, N = 27
CIYNTAGFYCDR pc2
P11047
7:98 10:155 3:72
1:236211491:C:T_T
p = 0.34, beta = −0.29, N = 20
164Assay Target: LAMC1
UniProt: P11047
rsID: rs4660150
Target SNP: 1:236211491:C:T
Proxy SNP: 1:236211491:C:T
Tarkin log10(p): 10.7
Tarkin BETA: −0.263
Tarkin MSPA: 0.864
1255:1199:1229:1255:1239:125
ACNCNPYGTMK pc2
rs20558 ALT
336:420 444:597 171:243
1:236211491:C:T_T
p = 0.0083, model = REC, N = 951
ACNCNLYGTMK pc2
rs20558 REF
200:420 275:597 98:243
1:236211491:C:T_T
p = 0.074, model = DOM, N = 573

165. RNASE3 (P12724)
Tarkin MSPA=0.786

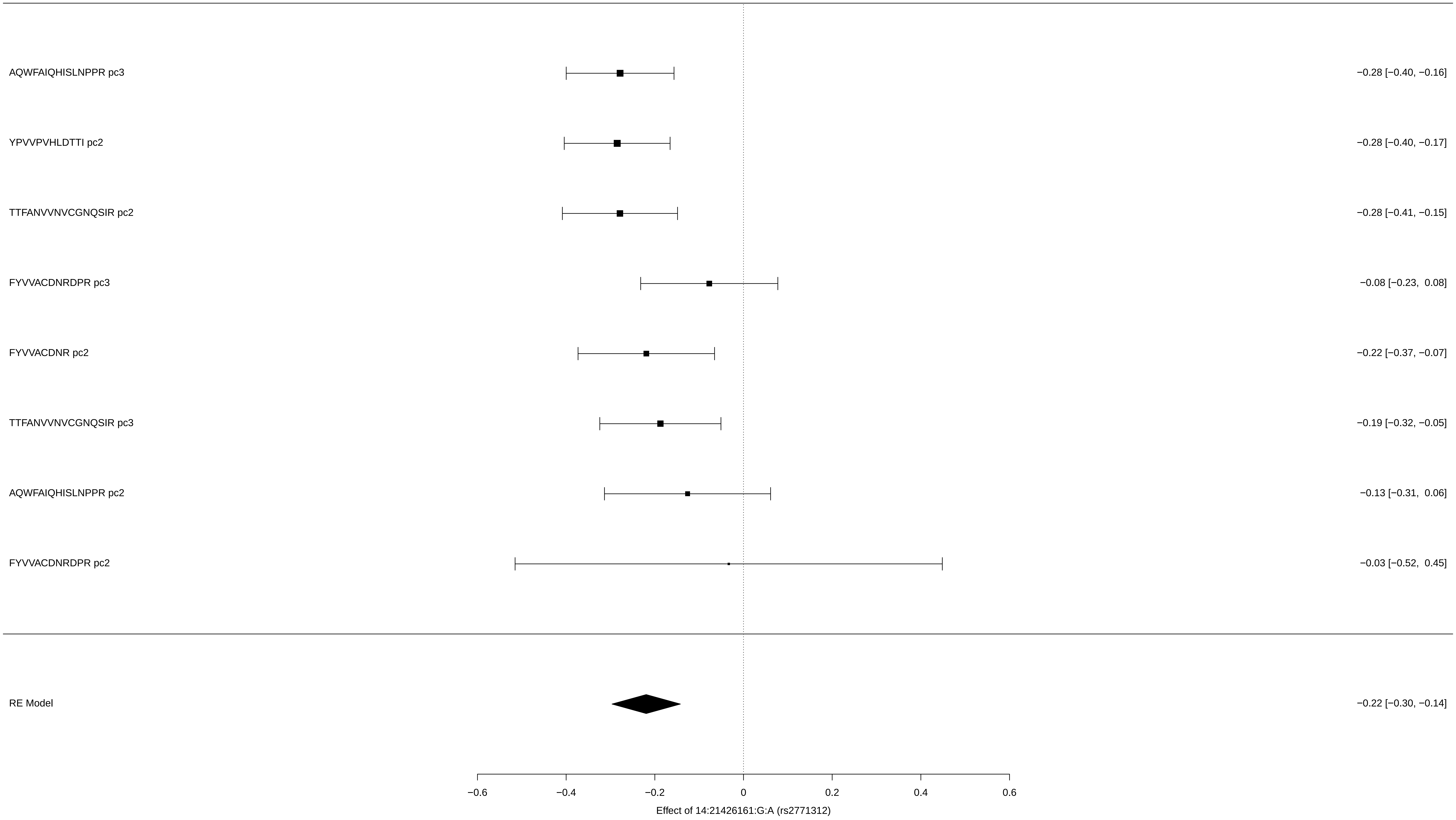

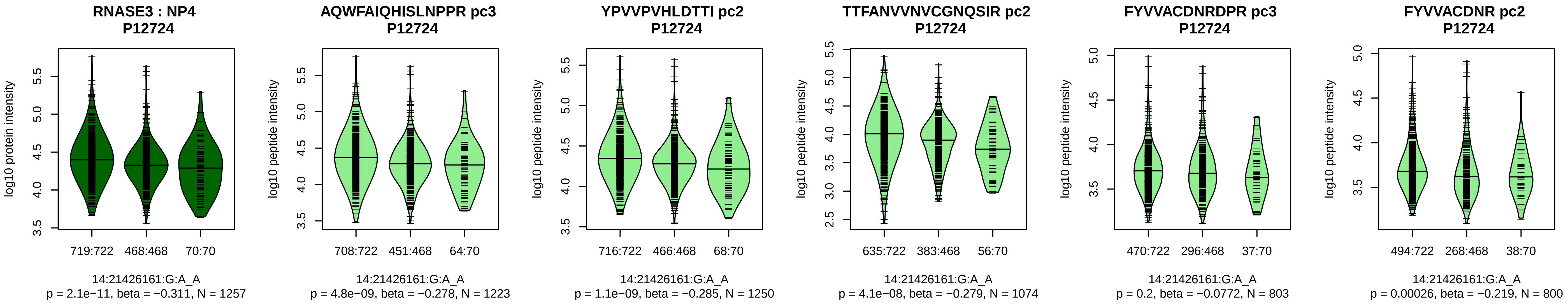

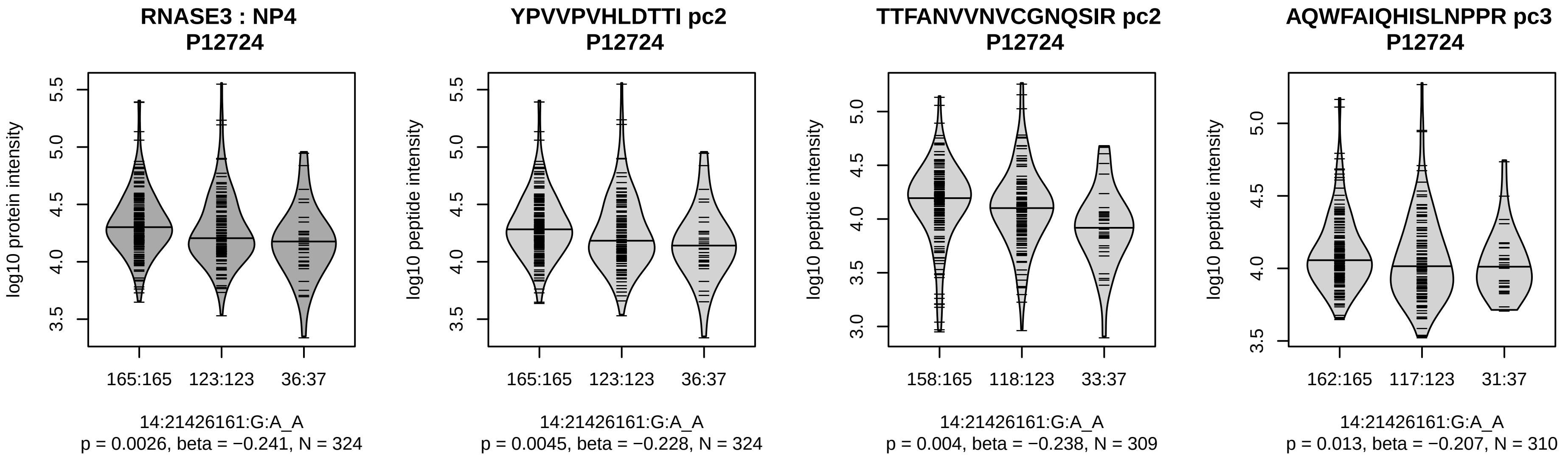

165Assay Target: RNASE3
UniProt: P12724
rsID: rs2771312
Target SNP: 14:21426161:G:A
Proxy SNP: 14:21426161:G:A
Tarkin log10(p): 10.7
Tarkin BETA: −0.311
Tarkin MSPA: 0.786
::*:–:*:*:–:–
1223:1250:1074:803:800:1037:5

166. CCL18 (P55774)
Tarkin MSPA=0.937

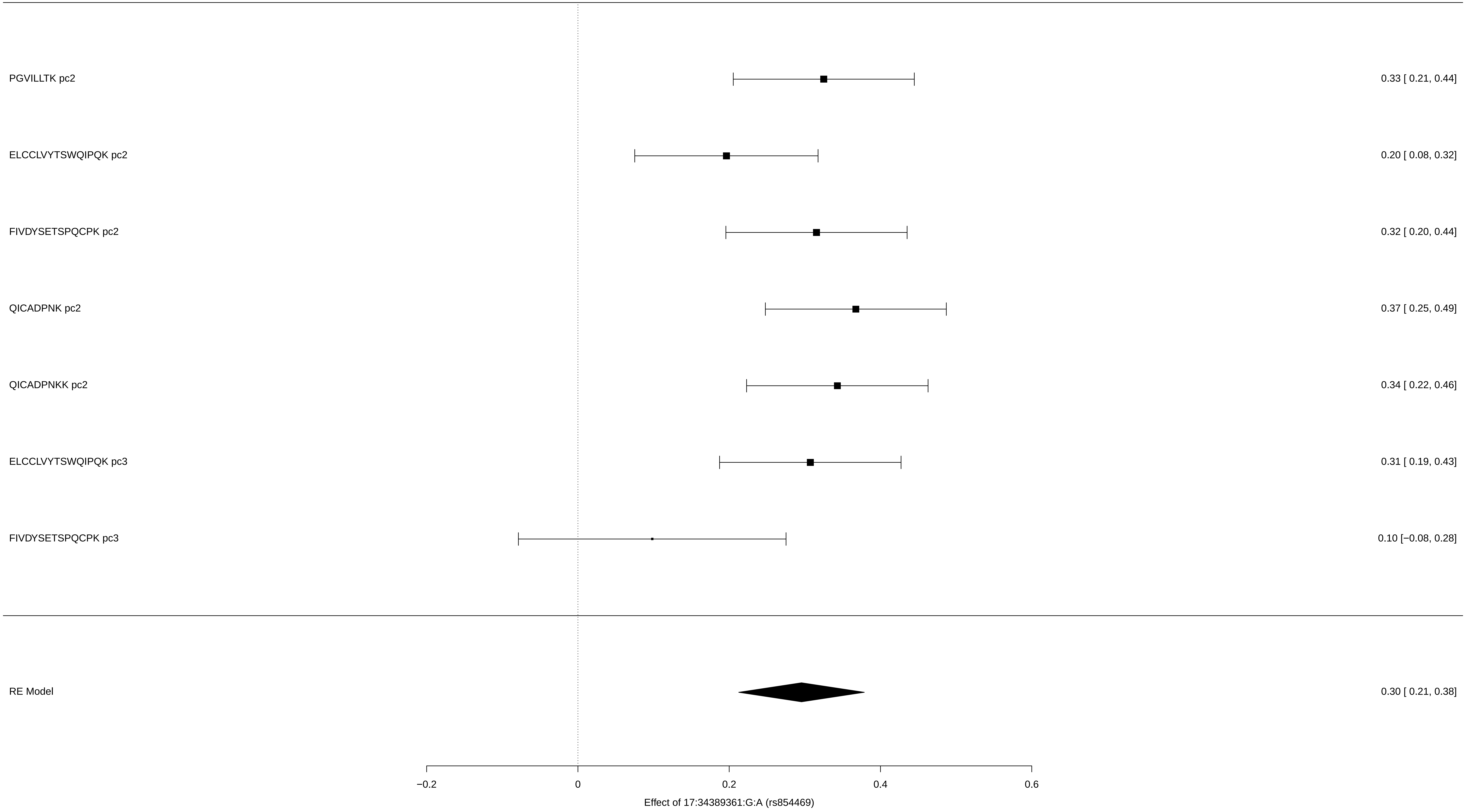

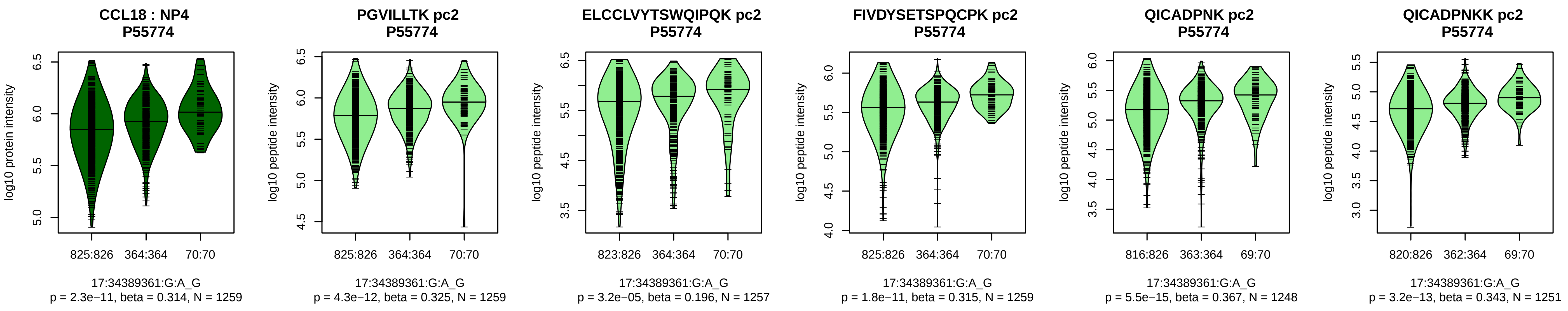

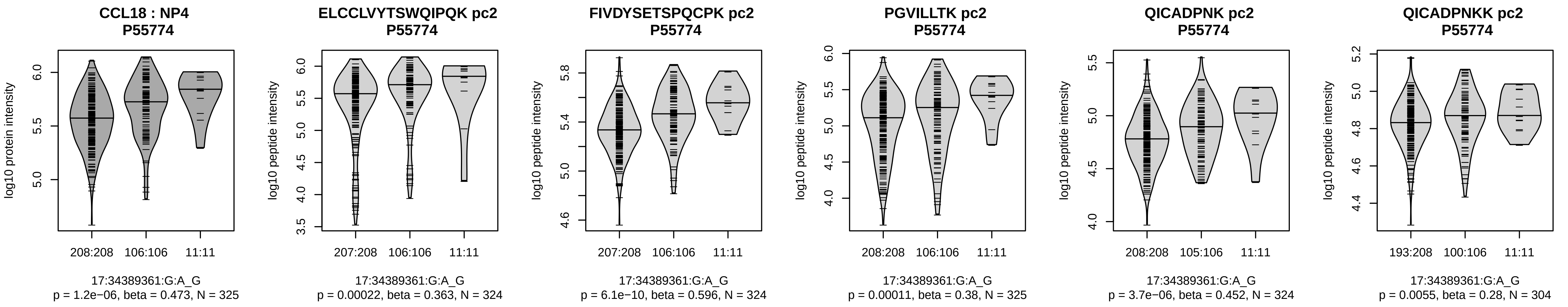

166Assay Target: CCL18
UniProt: P55774
rsID: rs854469
Target SNP: 17:34389361:G:A
Proxy SNP: 17:34389361:G:A
Tarkin log10(p): 10.6
Tarkin BETA: 0.314
Tarkin MSPA: 0.937
::*:*:*:*:–
1259:1257:1259:1248:1251:125

167. KNG1 (P01042)
Tarkin MSPA=0.393

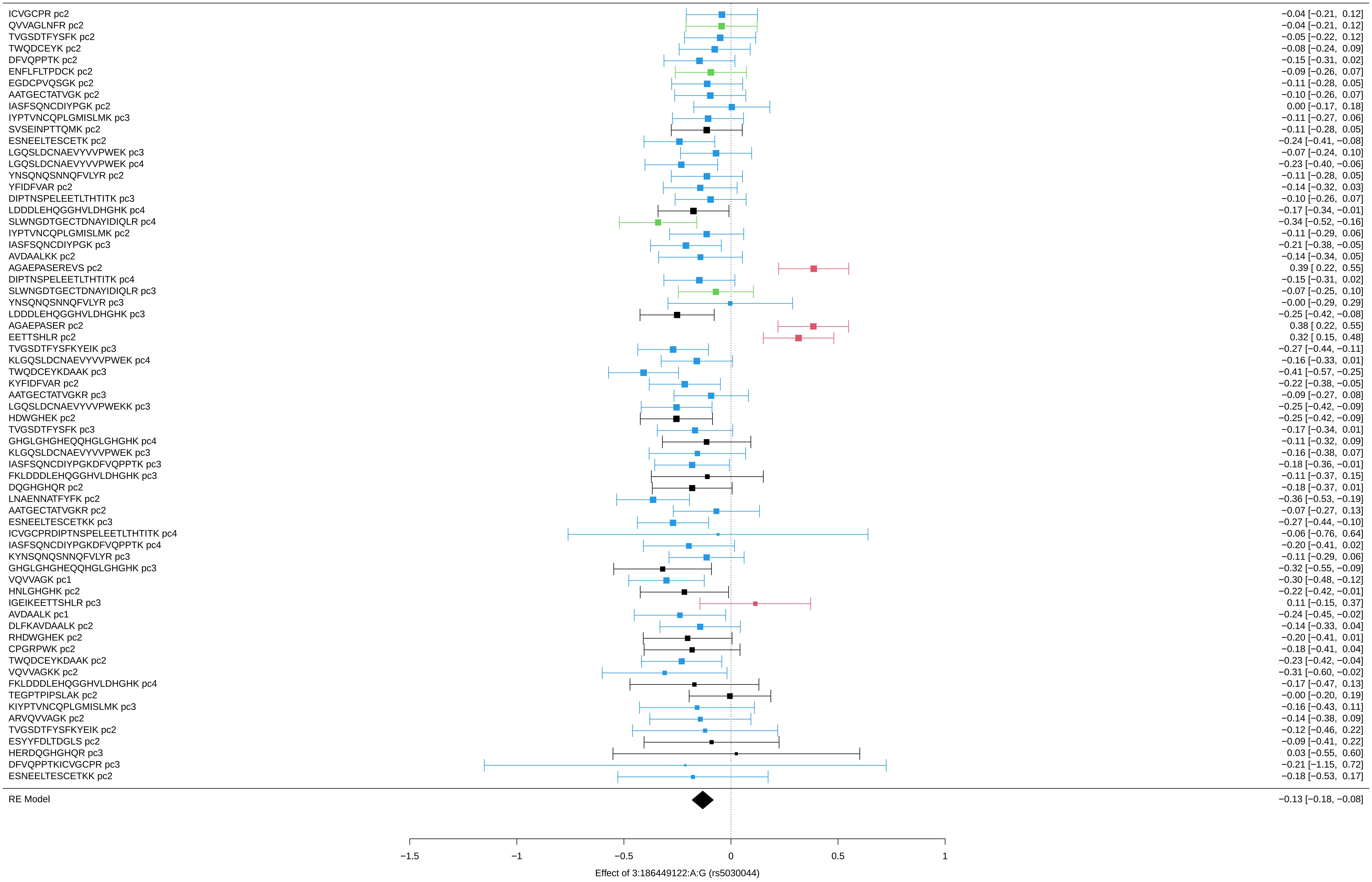

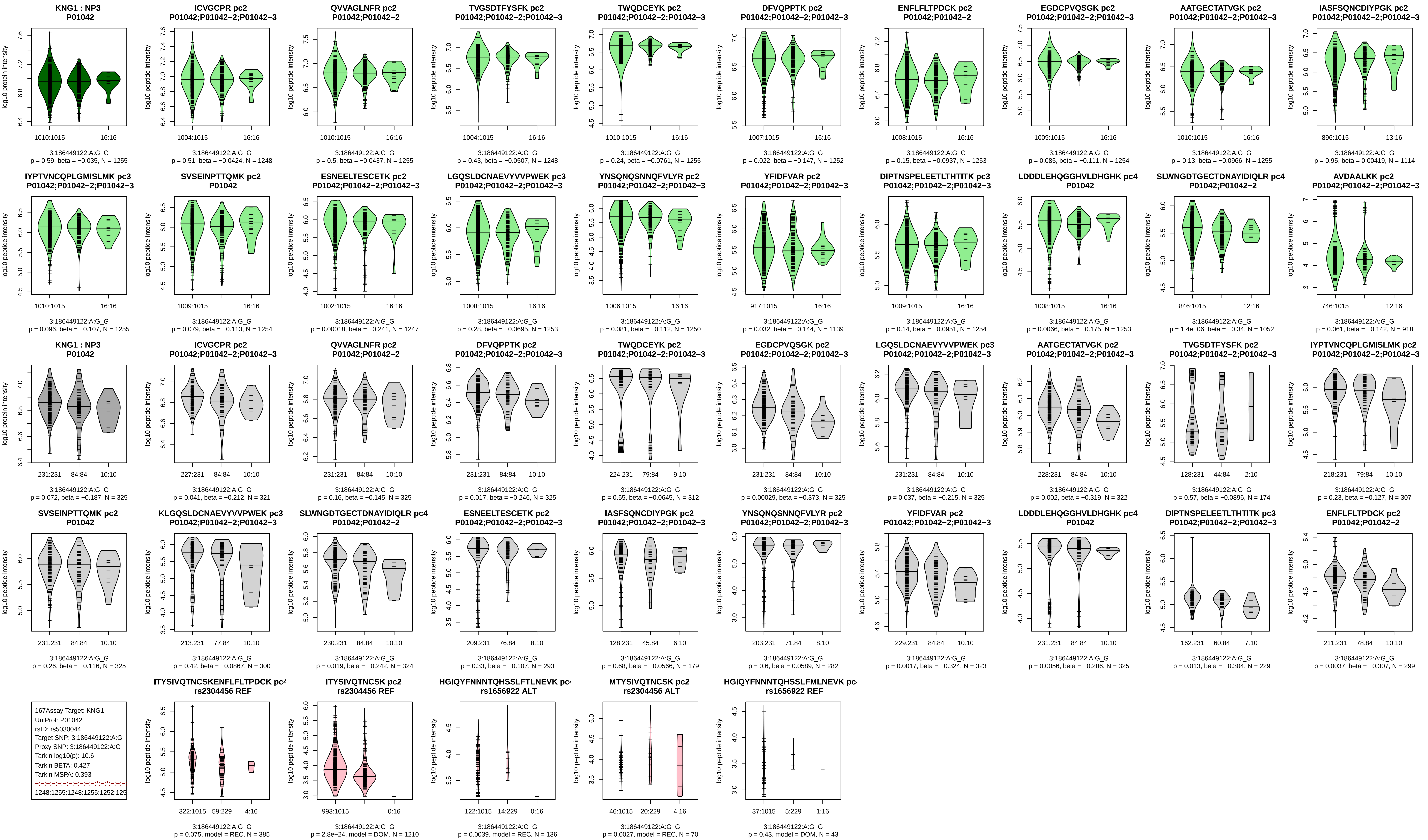
log10 protein intensity
log10 peptide intensity
KNG1 : NP3
P01042
1010:1015
16:16
3:186449122:A:G_G
p = 0.59, beta = −0.035, N = 1255
ICVGCPR pc2
P01042;P01042−2;P01042−3
1004:1015
16:16
3:186449122:A:G_G
p = 0.51, beta = −0.0424, N = 1248
QVVAGLNFR pc2
P01042;P01042−2
1010:1015
16:16
3:186449122:A:G_G
p = 0.5, beta = −0.0437, N = 1255
TVGSDTFYSFK pc2
P01042;P01042−2;P01042−3
1004:1015
16:16
3:186449122:A:G_G
p = 0.43, beta = −0.0507, N = 1248
TWQDCEYK pc2
P01042;P01042−2;P01042−3
1010:1015
16:16
3:186449122:A:G_G
p = 0.24, beta = −0.0761, N = 1255
DFVQPPTK pc2
P01042;P01042−2;P01042−3
1007:1015
16:16
3:186449122:A:G_G
p = 0.022, beta = −0.147, N = 1252
ENFLFLTPDCK pc2
P01042;P01042−2
1008:1015
16:16
3:186449122:A:G_G
p = 0.15, beta = −0.0937, N = 1253
EGDCPVQSGK pc2
P01042;P01042−2;P01042−3
1009:1015
16:16
3:186449122:A:G_G
p = 0.085, beta = −0.111, N = 1254
AATGECTATVGK pc2
P01042;P01042−2;P01042−3
1010:1015
16:16
3:186449122:A:G_G
p = 0.13, beta = −0.0966, N = 1255
IASFSQNCDIYPGK pc2
P01042;P01042−2;P01042−3
896:1015
13:16
3:186449122:A:G_G
p = 0.95, beta = 0.00419, N = 1114
IYPTVNCQPLGMISLMK pc3
P01042;P01042−2;P01042−3
1010:1015
16:16
3:186449122:A:G_G
p = 0.096, beta = −0.107, N = 1255
SVSEINPTTQMK pc2
P01042
1009:1015
16:16
3:186449122:A:G_G
p = 0.079, beta = −0.113, N = 1254
ESNEELTESCETK pc2
P01042;P01042−2;P01042−3
1002:1015
16:16
3:186449122:A:G_G
p = 0.00018, beta = −0.241, N = 1247
LGQSLDCNAEVYVVPWEK pc3
P01042;P01042−2;P01042−3
1008:1015
16:16
3:186449122:A:G_G
p = 0.28, beta = −0.0695, N = 1253
YNSQNQSNNQFVLYR pc2
P01042;P01042−2;P01042−3
1006:1015
16:16
3:186449122:A:G_G
p = 0.081, beta = −0.112, N = 1250
YFIDFVAR pc2
P01042;P01042−2;P01042−3
917:1015
16:16
3:186449122:A:G_G
p = 0.032, beta = −0.144, N = 1139
DIPTNSPELEETLTHTITK pc3
P01042;P01042−2;P01042−3
1009:1015
16:16
3:186449122:A:G_G
p = 0.14, beta = −0.0951, N = 1254
LDDDLEHQGGHVLDHGHK pc4
P01042
1008:1015
16:16
3:186449122:A:G_G
p = 0.0066, beta = −0.175, N = 1253
SLWNGDTGECTDNAYIDIQLR pc4
P01042;P01042−2
846:1015
12:16
3:186449122:A:G_G
p = 1.4e−06, beta = −0.34, N = 1052
AVDAALKK pc2
P01042;P01042−2;P01042−3
746:1015
12:16
3:186449122:A:G_G
p = 0.061, beta = −0.142, N = 918
KNG1 : NP3
P01042
231:231
84:84
10:10
3:186449122:A:G_G
p = 0.072, beta = −0.187, N = 325
ICVGCPR pc2
P01042;P01042−2;P01042−3
227:231
84:84
10:10
3:186449122:A:G_G
p = 0.041, beta = −0.212, N = 321
QVVAGLNFR pc2
P01042;P01042−2
231:231
84:84
10:10
3:186449122:A:G_G
p = 0.16, beta = −0.145, N = 325
DFVQPPTK pc2
P01042;P01042−2;P01042−3
231:231
84:84
10:10
3:186449122:A:G_G
p = 0.017, beta = −0.246, N = 325
TWQDCEYK pc2
P01042;P01042−2;P01042−3
224:231
79:84
9:10
3:186449122:A:G_G
p = 0.55, beta = −0.0645, N = 312
EGDCPVQSGK pc2
P01042;P01042−2;P01042−3
231:231
84:84
10:10
3:186449122:A:G_G
p = 0.00029, beta = −0.373, N = 325
LGQSLDCNAEVYVVPWEK pc3
P01042;P01042−2;P01042−3
231:231
84:84
10:10
3:186449122:A:G_G
p = 0.037, beta = −0.215, N = 325
AATGECTATVGK pc2
P01042;P01042−2;P01042−3
228:231
84:84
10:10
3:186449122:A:G_G
p = 0.002, beta = −0.319, N = 322
TVGSDTFYSFK pc2
P01042;P01042−2;P01042−3
128:231
44:84
2:10
3:186449122:A:G_G
p = 0.57, beta = −0.0896, N = 174
IYPTVNCQPLGMISLMK pc2
P01042;P01042−2;P01042−3
218:231
79:84
10:10
3:186449122:A:G_G
p = 0.23, beta = −0.127, N = 307
SVSEINPTTQMK pc2
P01042
231:231
84:84
10:10
3:186449122:A:G_G
p = 0.26, beta = −0.116, N = 325
KLGQSLDCNAEVYVVPWEK pc3
P01042;P01042−2;P01042−3
213:231
77:84
10:10
3:186449122:A:G_G
p = 0.42, beta = −0.0867, N = 300
SLWNGDTGECTDNAYIDIQLR pc4
P01042;P01042−2
230:231
84:84
10:10
3:186449122:A:G_G
p = 0.019, beta = −0.242, N = 324
ESNEELTESCETK pc2
P01042;P01042−2;P01042−3
209:231
76:84
8:10
3:186449122:A:G_G
p = 0.33, beta = −0.107, N = 293
IASFSQNCDIYPGK pc2
P01042;P01042−2;P01042−3
128:231
45:84
6:10
3:186449122:A:G_G
p = 0.68, beta = −0.0566, N = 179
YNSQNQSNNQFVLYR pc2
P01042;P01042−2;P01042−3
203:231
71:84
8:10
3:186449122:A:G_G
p = 0.6, beta = 0.0589, N = 282
YFIDFVAR pc2
P01042;P01042−2;P01042−3
229:231
84:84
10:10
3:186449122:A:G_G
p = 0.0017, beta = −0.324, N = 323
LDDDLEHQGGHVLDHGHK pc4
P01042
231:231
84:84
10:10
3:186449122:A:G_G
p = 0.0056, beta = −0.286, N = 325
DIPTNSPELEETLTHTITK pc3
P01042;P01042−2;P01042−3
162:231
60:84
7:10
3:186449122:A:G_G
p = 0.013, beta = −0.304, N = 229
ENFLFLTPDCK pc2
P01042;P01042−2
211:231
78:84
10:10
3:186449122:A:G_G
p = 0.0037, beta = −0.307, N = 299
167Assay Target: KNG1
UniProt: P01042
rsID: rs5030044
Target SNP: 3:186449122:A:G
Proxy SNP: 3:186449122:A:G
Tarkin log10(p): 10.6
Tarkin BETA: 0.427
Tarkin MSPA: 0.393
1248:1255:1248:1255:1252:125
ITYSIVQTNCSKENFLFLTPDCK pc
rs2304456 REF
322:1015
59:229
4:16
3:186449122:A:G_G
p = 0.075, model = REC, N = 385
ITYSIVQTNCSK pc2
rs2304456 REF
993:1015
0:16
3:186449122:A:G_G
p = 2.8e−24, model = DOM, N = 1210
HGIQYFNNNTQHSSLFTLNEVK pc
rs1656922 ALT
122:1015
14:229
0:16
3:186449122:A:G_G
p = 0.0039, model = REC, N = 136
MTYSIVQTNCSK pc2
rs2304456 ALT
46:1015
20:229
4:16
3:186449122:A:G_G
p = 0.0027, model = REC, N = 70
HGIQYFNNNTQHSSLFMLNEVK pc
rs1656922 REF
37:1015
5:229
1:16
3:186449122:A:G_G
p = 0.43, model = DOM, N = 43

168. TGFBI (Q15582)
Tarkin MSPA=0.917

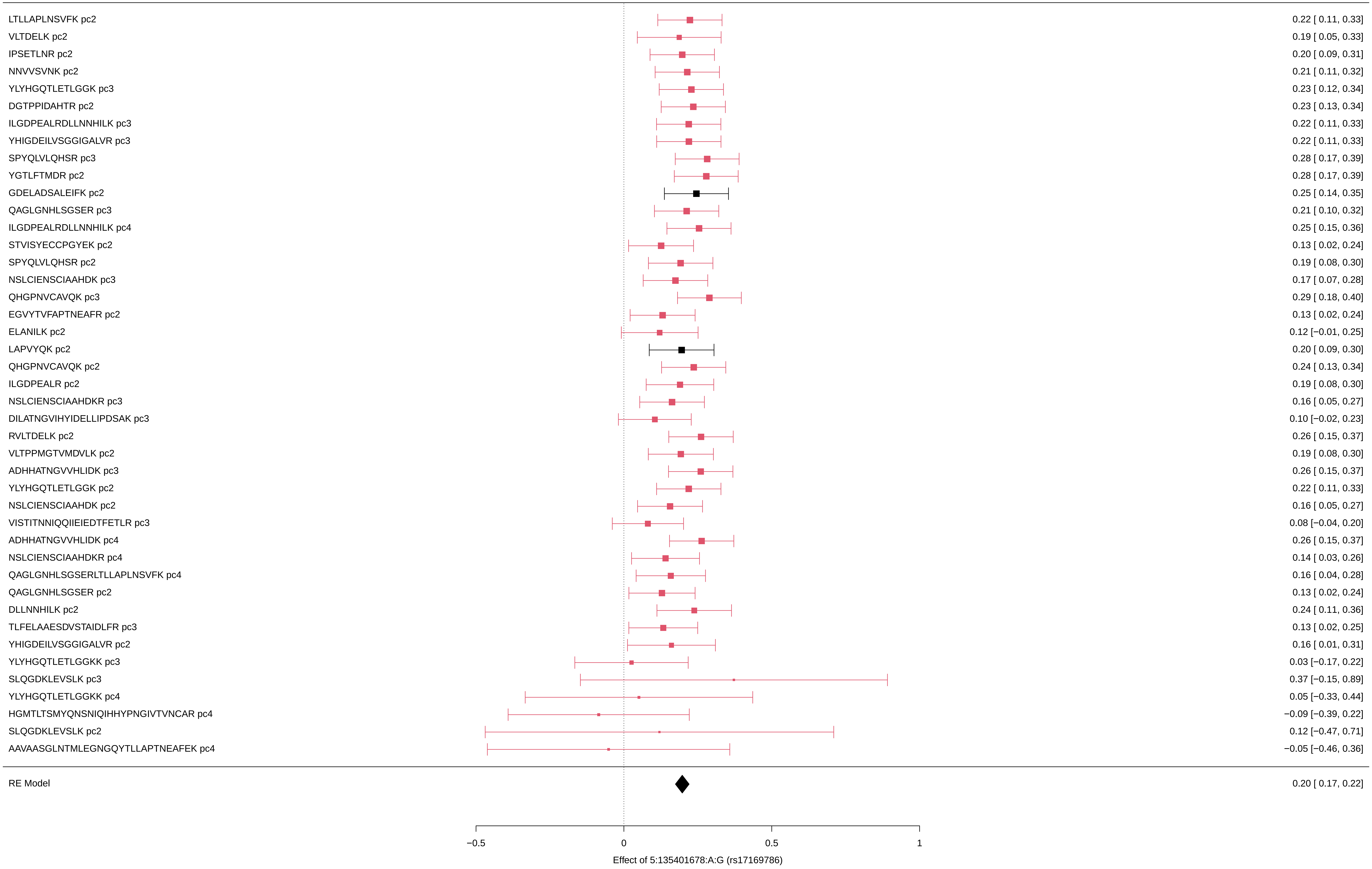

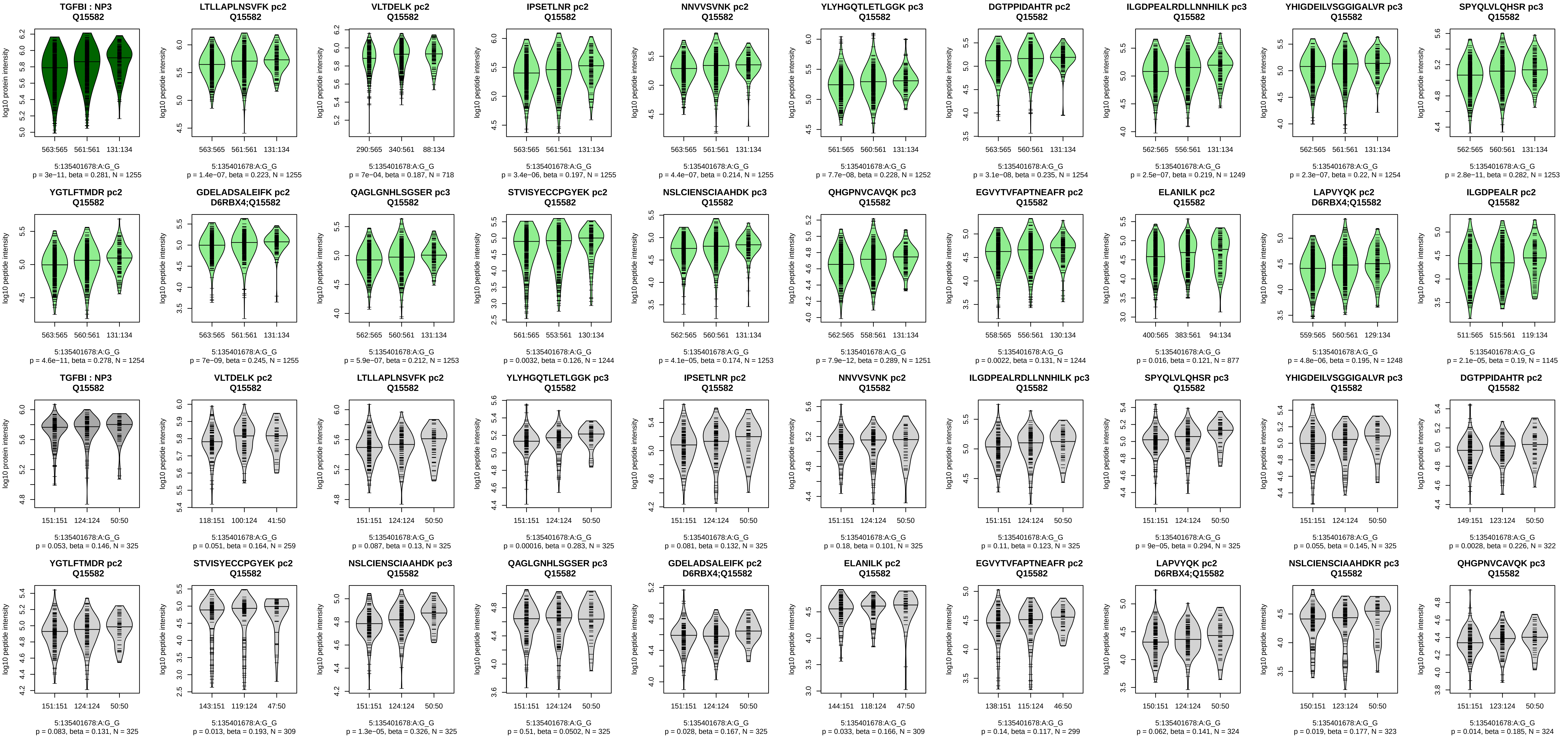

168Assay Target: TGFBI
UniProt: Q15582
rsID: rs17169786
Target SNP: 5:135401678:A:G
Proxy SNP: 5:135401678:A:G
Tarkin log10(p): 10.5
Tarkin BETA: 0.281
Tarkin MSPA: 0.917
1255:718:1255:1255:1252:1254

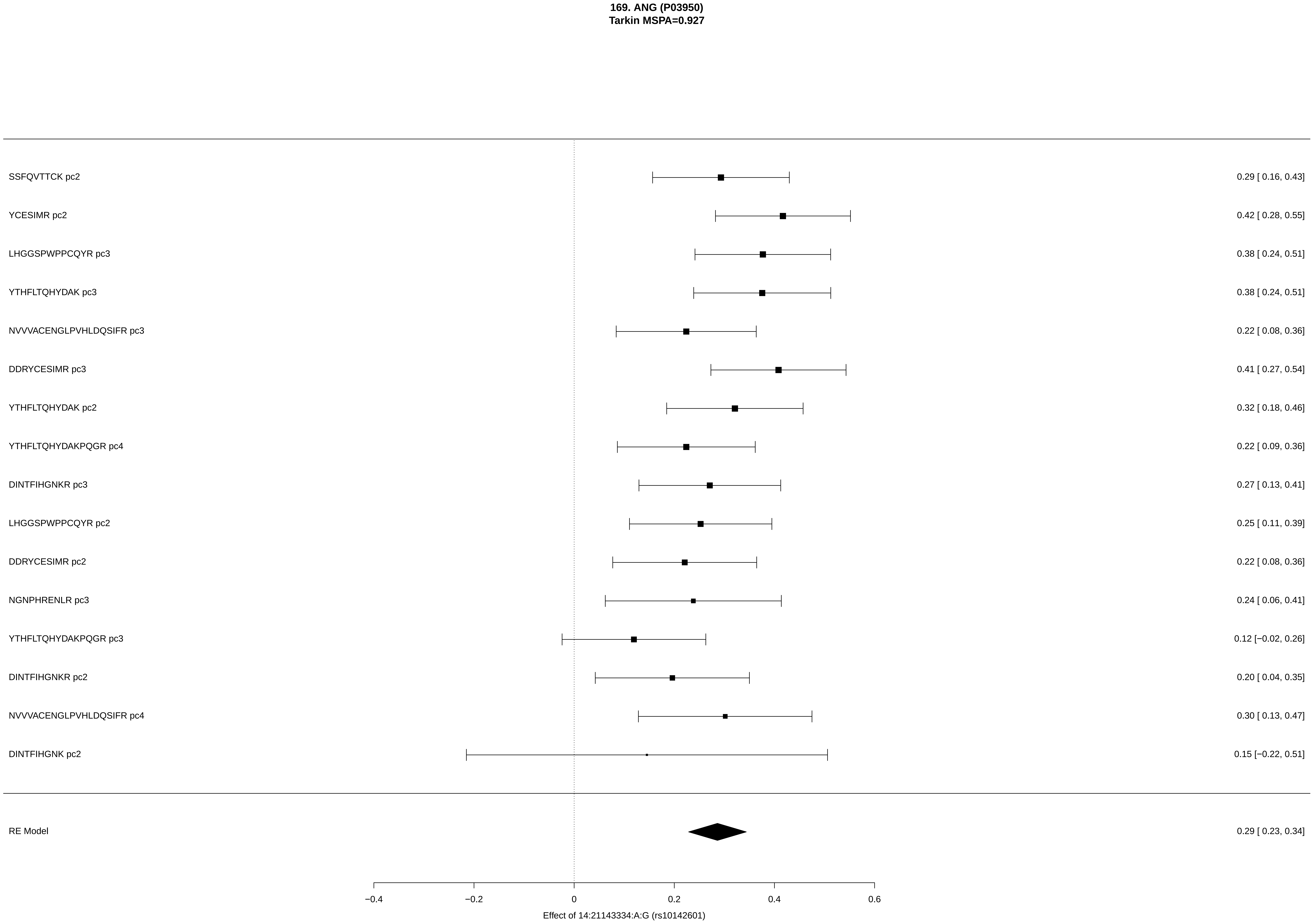
169. ANG (P03950)
Tarkin MSPA=0.927
SSFQVTTCK pc2
0.29 [0.16, 0.43]
YCESIMR pc2
0.42 [0.28, 0.55]
LHGGSPWPPCQYR pc3
0.38 [0.24, 0.51]
YTHFLTQHYDAK pc3
0.38 [0.24, 0.51]
NVVVACENGLPVHLDQSIFR pc3
0.22 [0.08, 0.36]
DDRYCESIMR pc3
0.41 [0.27, 0.54]
YTHFLTQHYDAK pc2
0.32 [0.18, 0.46]
YTHFLTQHYDAKPQGR pc4
0.22 [0.09, 0.36]
DINTFIHGNKR pc3
0.27 [0.13, 0.41]
LHGGSPWPPCQYR pc2
0.25 [0.11, 0.39]
DDRYCESIMR pc2
0.22 [0.08, 0.36]
NGNPHRENLR pc3
0.24 [0.06, 0.41]
YTHFLTQHYDAKPQGR pc3
0.12 [−0.02, 0.26]
DINTFIHGNKR pc2
0.20 [0.04, 0.35]
NVVVACENGLPVHLDQSIFR pc4
0.30 [0.13, 0.47]
DINTFIHGNK pc2
0.15 [−0.22, 0.51]
RE Model
0.29 [0.23, 0.34]
−0.4
−0.2
0
0.2
0.4
0.6
Effect of 14:21143334:A:G (rs10142601)

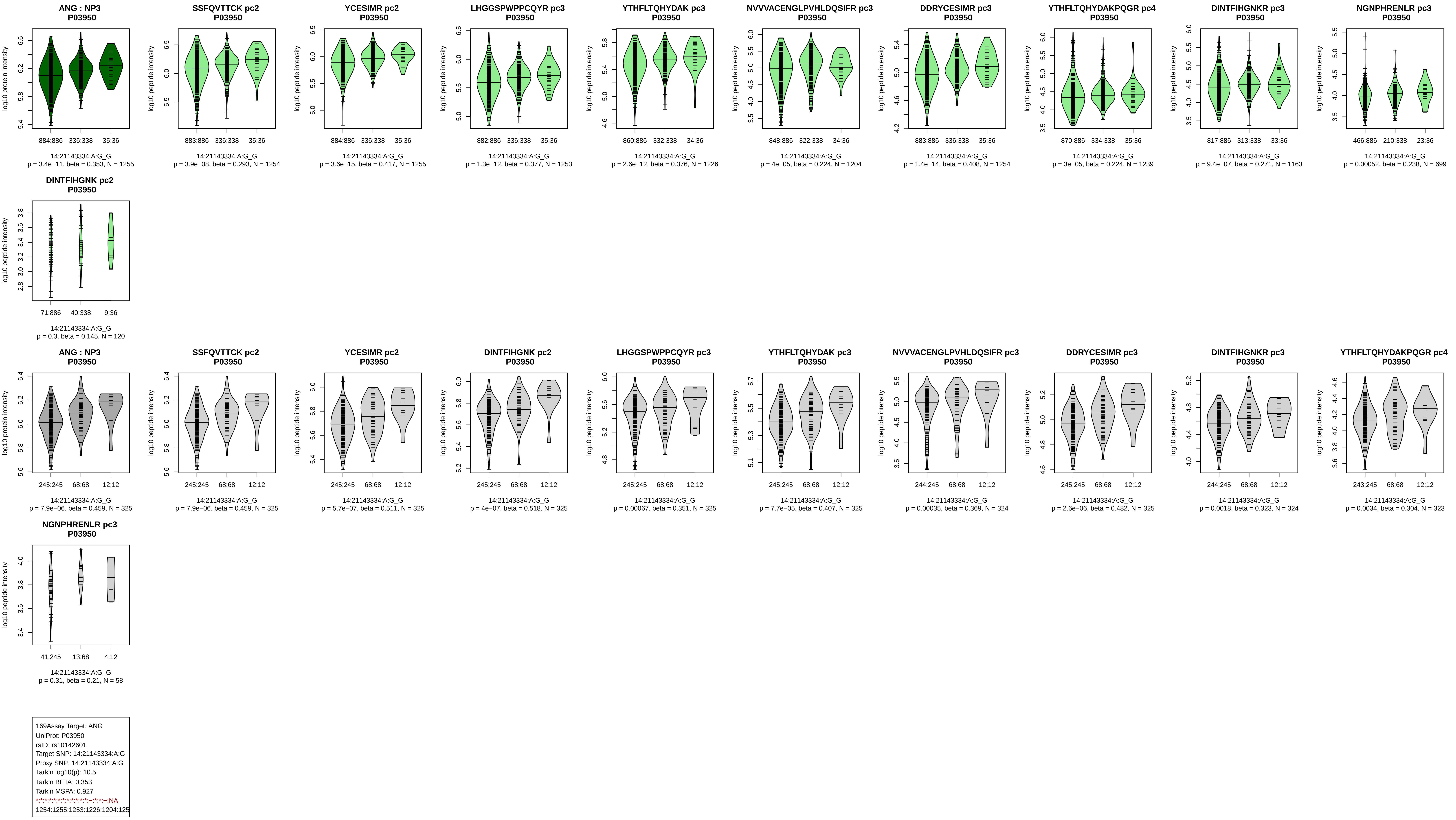

ANG : NP3 P03950
884:886 336:338 35:36
14:21143334:A:G_G
p = 3.4e−11, beta = 0.353, N = 1255
SSFQVTTCK pc2 P03950
883:886 336:338 35:36
14:21143334:A:G_G
p = 3.9e−08, beta = 0.293, N = 1254
YCESIMR pc2 P03950
884:886 336:338 35:36
14:21143334:A:G_G
p = 3.6e−15, beta = 0.417, N = 1255
LHGGSPWPPCQYR pc3 P03950
882:886 336:338 35:36
14:21143334:A:G_G
p = 1.3e−12, beta = 0.377, N = 1253
YTHFLTQHYDAK pc3 P03950
860:886 332:338 34:36
14:21143334:A:G_G
p = 2.6e−12, beta = 0.376, N = 1226
NVVVACENGLPVHLDQSIFR pc3 P03950
848:886 322:338 34:36
14:21143334:A:G_G
p = 4e−05, beta = 0.224, N = 1204
DDRYCESIMR pc3 P03950
883:886 336:338 35:36
14:21143334:A:G_G
p = 1.4e−14, beta = 0.408, N = 1254
YTHFLTQHYDAKPQGR pc4 P03950
870:886 334:338 35:36
14:21143334:A:G_G
p = 3e−05, beta = 0.224, N = 1239
DINTFIHGNKR pc3 P03950
817:886 313:338 33:36
14:21143334:A:G_G
p = 9.4e−07, beta = 0.271, N = 1163
NGNPHRENLR pc3 P03950
466:886 210:338 23:36
14:21143334:A:G_G
p = 0.00052, beta = 0.238, N = 699
DINTFIHGNK pc2 P03950
71:886 40:338 9:36
14:21143334:A:G_G
p = 0.3, beta = 0.145, N = 120
ANG : NP3 P03950
245:245 68:68 12:12
14:21143334:A:G_G
p = 7.9e−06, beta = 0.459, N = 325
SSFQVTTCK pc2 P03950
245:245 68:68 12:12
14:21143334:A:G_G
p = 7.9e−06, beta = 0.459, N = 325
YCESIMR pc2 P03950
245:245 68:68 12:12
14:21143334:A:G_G
p = 5.7e−07, beta = 0.511, N = 325
DINTFIHGNK pc2 P03950
245:245 68:68 12:12
14:21143334:A:G_G
p = 4e−07, beta = 0.518, N = 325
LHGGSPWPPCQYR pc3 P03950
245:245 68:68 12:12
14:21143334:A:G_G
p = 0.00067, beta = 0.351, N = 325
YTHFLTQHYDAK pc3 P03950
245:245 68:68 12:12
14:21143334:A:G_G
p = 7.7e−05, beta = 0.407, N = 325
NVVVACENGLPVHLDQSIFR pc3 P03950
244:245 68:68 12:12
14:21143334:A:G_G
p = 0.00035, beta = 0.369, N = 324
DDRYCESIMR pc3 P03950
245:245 68:68 12:12
14:21143334:A:G_G
p = 2.6e−06, beta = 0.482, N = 325
DINTFIHGNKR pc3 P03950
244:245 68:68 12:12
14:21143334:A:G_G
p = 0.0018, beta = 0.323, N = 324
YTHFLTQHYDAKPQGR pc4 P03950
243:245 68:68 12:12
14:21143334:A:G_G
p = 0.0034, beta = 0.304, N = 323
NGNPHRENLR pc3 P03950
41:245 13:68 4:12
14:21143334:A:G_G
p = 0.31, beta = 0.21, N = 58
log10 protein intensity
log10 peptide intensity
169Assay Target: ANG
UniProt: P03950
rsID: rs10142601
Target SNP: 14:21143334:A:G
Proxy SNP: 14:21143334:A:G
Tarkin log10(p): 10.5
Tarkin BETA: 0.353
Tarkin MSPA: 0.927
1254:1255:1253:1226:1204:125

170. RHOF (Q9HBH0)
Tarkin MSPA=0.975

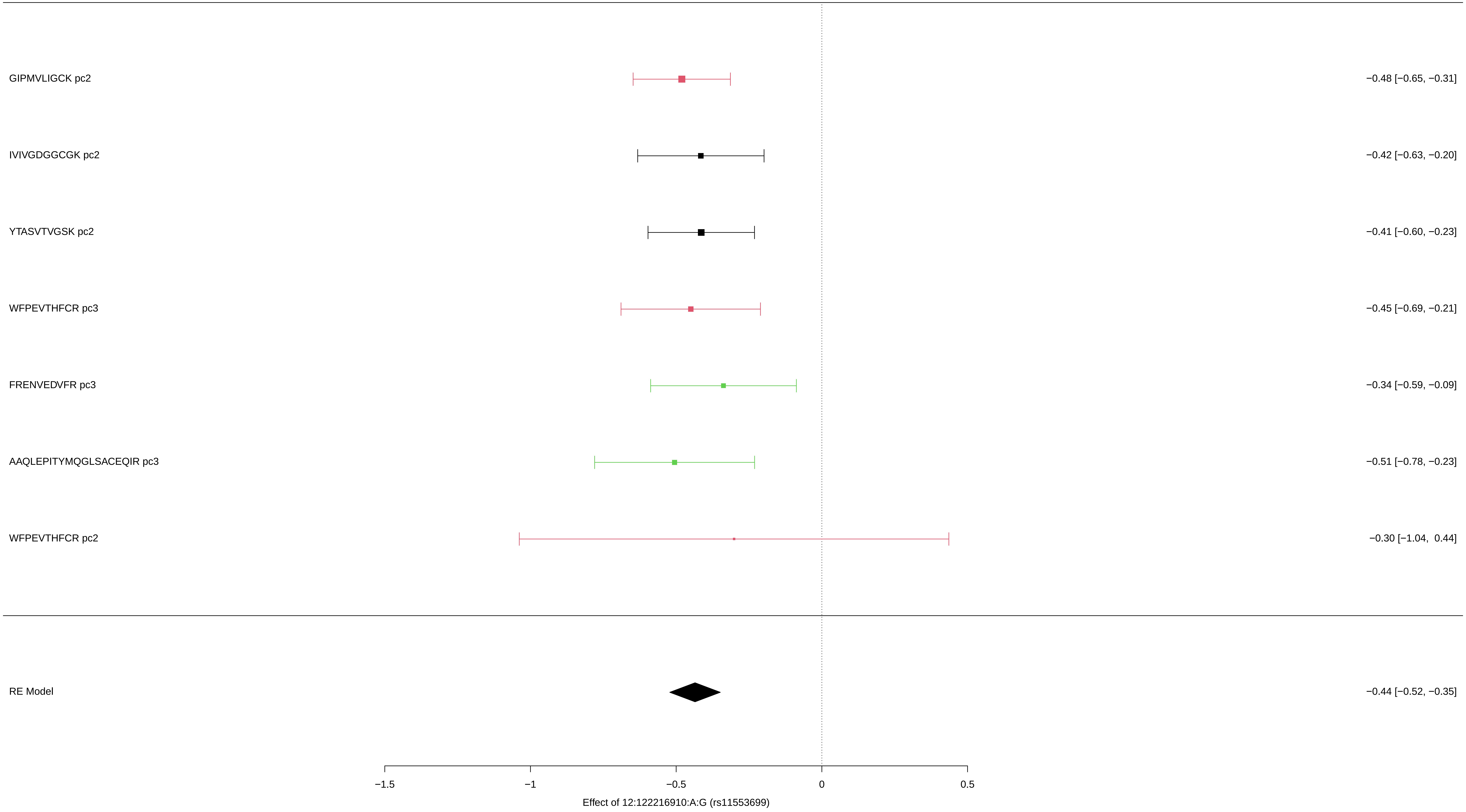

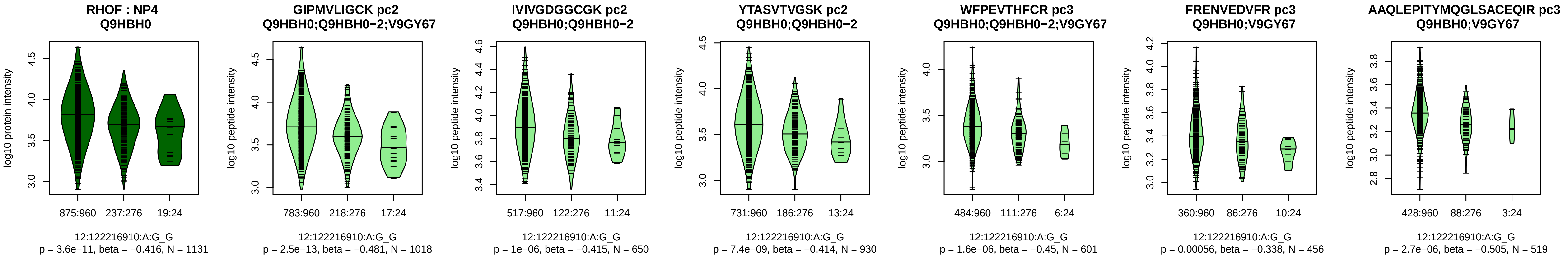

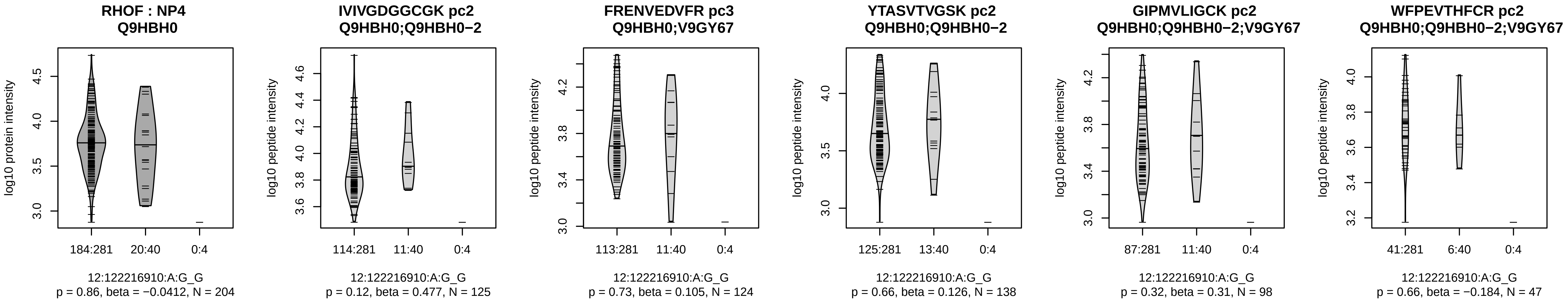

170Assay Target: RHOF
UniProt: Q9HBH0
rsID: rs11553699
Target SNP: 12:122216910:A:G
Proxy SNP: 12:122216910:A:G
Tarkin log10(p): 10.4
Tarkin BETA: −0.416
Tarkin MSPA: 0.975
::*:*:*:*:–
1018:650:930:601:456:519:106

171. KLHDC1 (Q8N7A1)
Tarkin MSPA=1

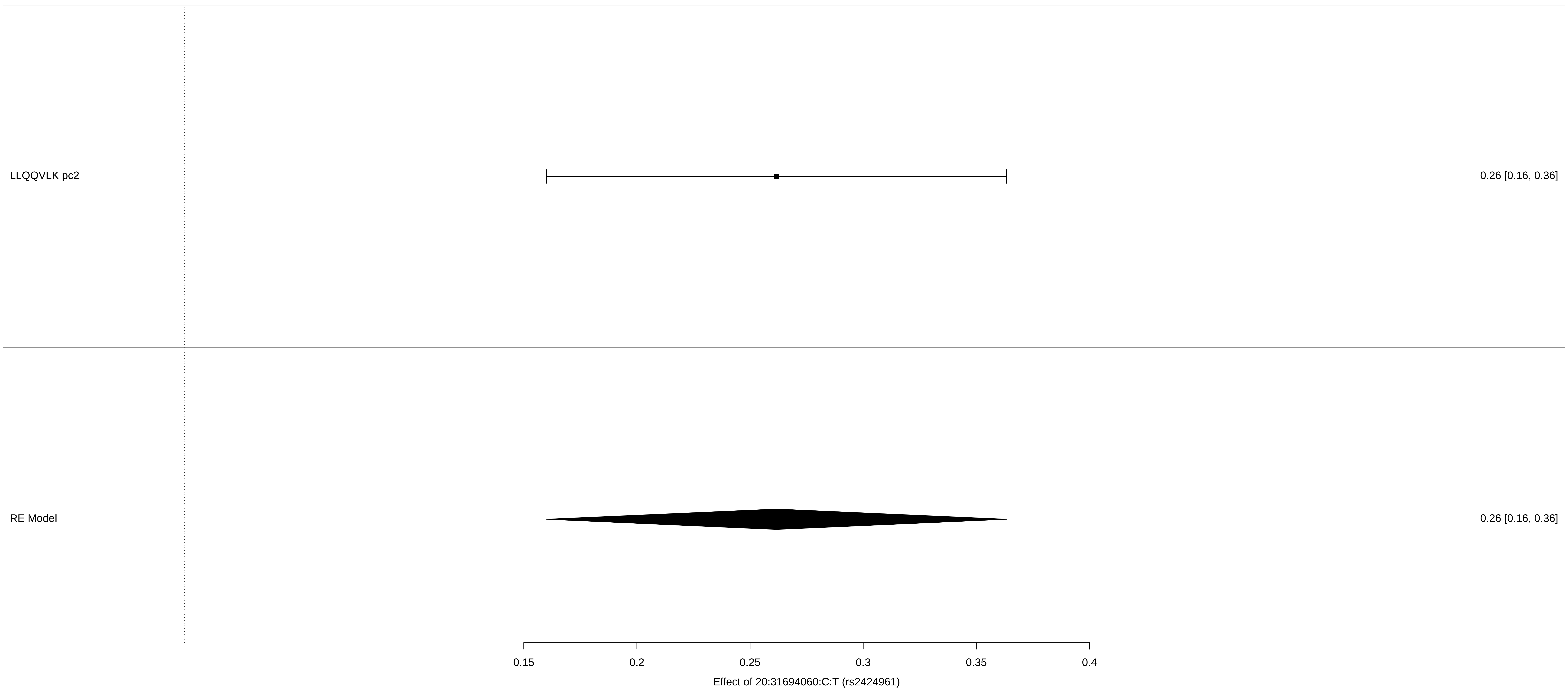

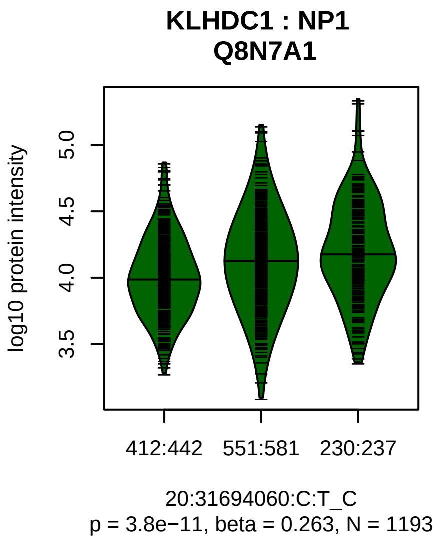

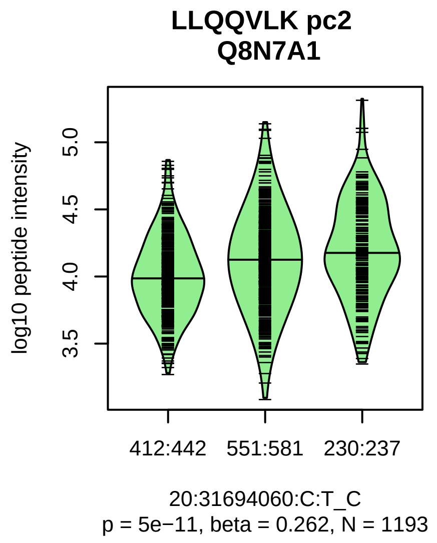

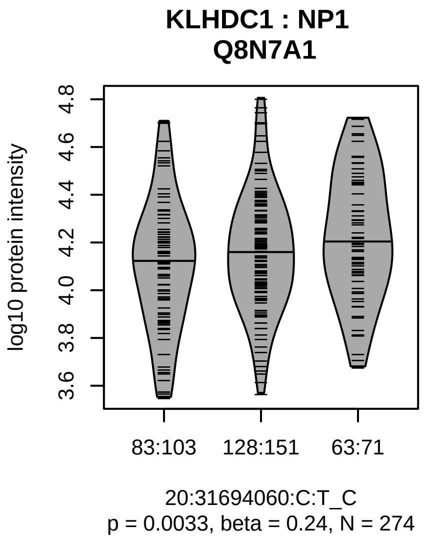

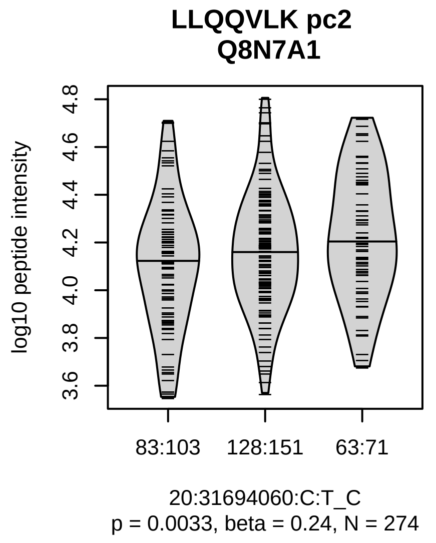

171Assay Target: KLHDC1
UniProt: Q8N7A1
rsID: rs2424961
Target SNP: 20:31694060:C:T
Proxy SNP: 20:31694060:C:T
Tarkin log10(p): 10.4
Tarkin BETA: 0.263
Tarkin MSPA: 1
*:NA
1193:8

172. SPTBN1 (Q01082)
Tarkin MSPA=0.554

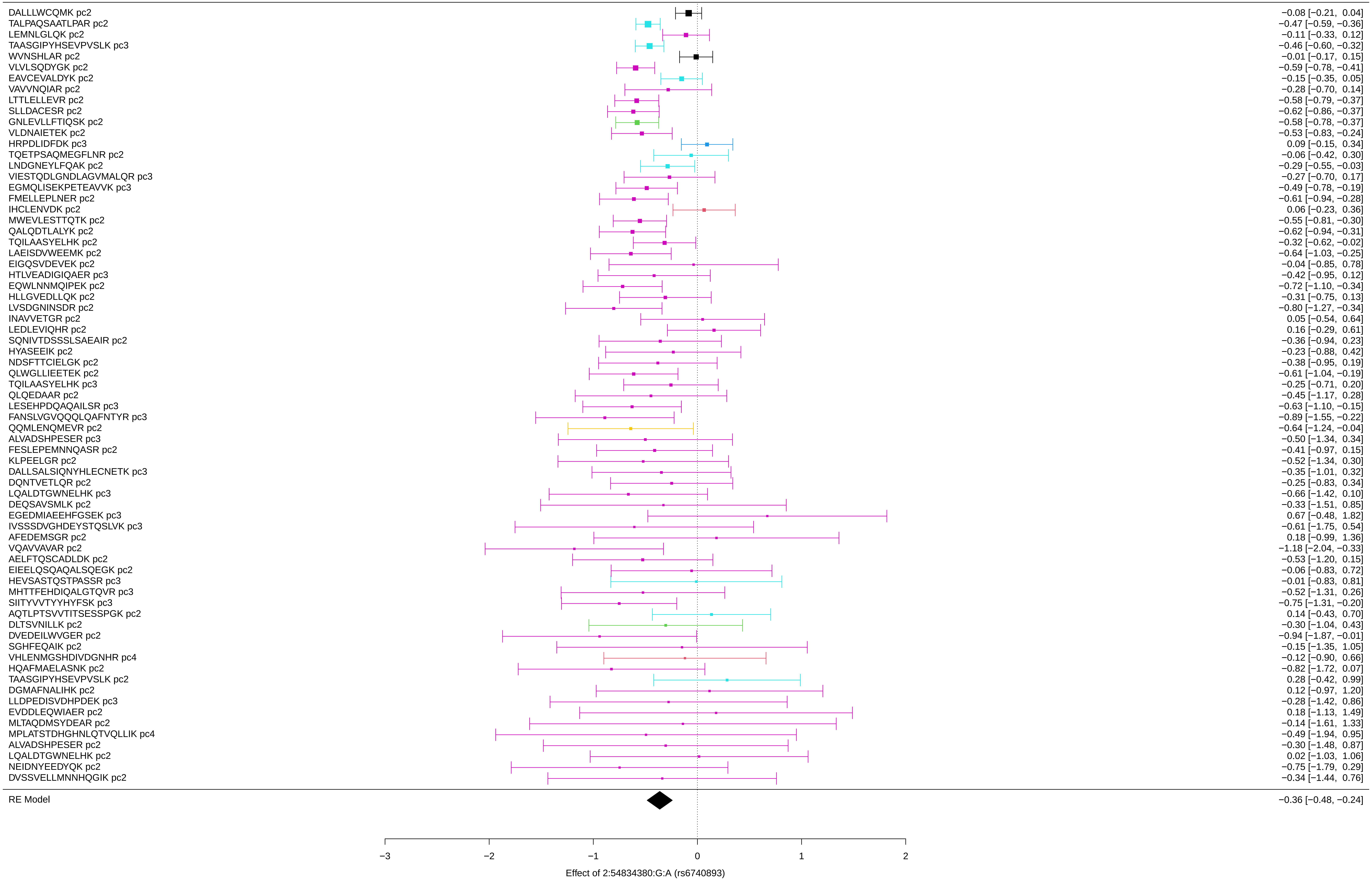

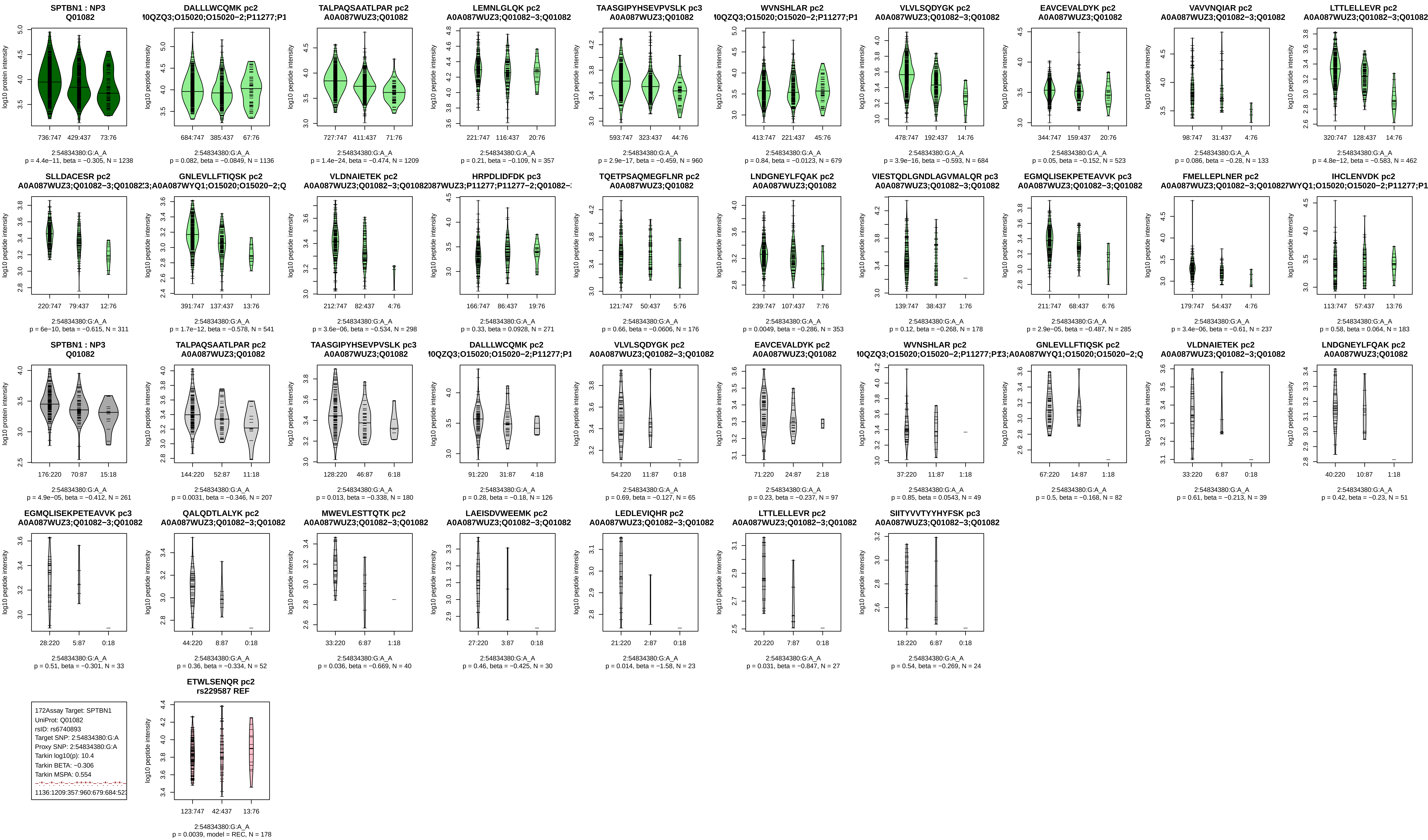
SPTBN1 : NP3
Q01082
log10 protein intensity
736:747 429:437 73:76
2:54834380:G:A_A
p = 4.4e−11, beta = −0.305, N = 1238
DALLLWCQMK pc2
I0QZQ3;O15020;O15020−2;P11277;P1
log10 peptide intensity
684:747 385:437 67:76
2:54834380:G:A_A
p = 0.082, beta = −0.0849, N = 1136
TALPAQSAATLPAR pc2
A0A087WUZ3;Q01082
727:747 411:437 71:76
2:54834380:G:A_A
p = 1.4e−24, beta = −0.474, N = 1209
LEMNLGLQK pc2
A0A087WUZ3;Q01082−3;Q01082
221:747 116:437 20:76
2:54834380:G:A_A
p = 0.21, beta = −0.109, N = 357
TAASGIPYHSEVPVSLK pc3
A0A087WUZ3;Q01082
593:747 323:437 44:76
2:54834380:G:A_A
p = 2.9e−17, beta = −0.459, N = 960
WVNSHLAR pc2
I0QZQ3;O15020;O15020−2;P11277;P1
413:747 221:437 45:76
2:54834380:G:A_A
p = 0.84, beta = −0.0123, N = 679
VLVLSQDYGK pc2
A0A087WUZ3;Q01082−3;Q01082
478:747 192:437 14:76
2:54834380:G:A_A
p = 3.9e−16, beta = −0.593, N = 684
EAVCEVALDYK pc2
A0A087WUZ3;Q01082
344:747 159:437 20:76
2:54834380:G:A_A
p = 0.05, beta = −0.152, N = 523
VAVVNQIAR pc2
A0A087WUZ3;Q01082−3;Q01082
98:747 31:437 4:76
2:54834380:G:A_A
p = 0.086, beta = −0.28, N = 133
LTTLELLEVR pc2
A0A087WUZ3;Q01082−3;Q01082
320:747 128:437 14:76
2:54834380:G:A_A
p = 4.8e−12, beta = −0.583, N = 462
SLLDACESR pc2
A0A087WUZ3;Q01082−3;Q01082
220:747 79:437 12:76
2:54834380:G:A_A
p = 6e−10, beta = −0.615, N = 311
GNLEVLLFTIQSK pc2
3;A0A087WYQ1;O15020;O15020−2;Q
391:747 137:437 13:76
2:54834380:G:A_A
p = 1.7e−12, beta = −0.578, N = 541
VLDNAIETEK pc2
A0A087WUZ3;Q01082−3;Q01082
212:747 82:437 4:76
2:54834380:G:A_A
p = 3.6e−06, beta = −0.534, N = 298
HRPDLIDFDK pc3
87WUZ3;P11277;P11277−2;Q01082−
166:747 86:437 19:76
2:54834380:G:A_A
p = 0.33, beta = 0.0928, N = 271
TQETPSAQMEGFLNR pc2
A0A087WUZ3;Q01082
121:747 50:437 5:76
2:54834380:G:A_A
p = 0.66, beta = −0.0606, N = 176
LNDGNEYLFQAK pc2
A0A087WUZ3;Q01082
239:747 107:437 7:76
2:54834380:G:A_A
p = 0.0049, beta = −0.286, N = 353
VIESTQDLGNDLAGVMALQR pc3
A0A087WUZ3;Q01082−3;Q01082
139:747 38:437 1:76
2:54834380:G:A_A
p = 0.12, beta = −0.268, N = 178
EGMQLISEKPETEAVVK pc3
A0A087WUZ3;Q01082−3;Q01082
211:747 68:437 6:76
2:54834380:G:A_A
p = 2.9e−05, beta = −0.487, N = 285
FMELLEPLNER pc2
A0A087WUZ3;Q01082−3;Q01082
179:747 54:437 4:76
2:54834380:G:A_A
p = 3.4e−06, beta = −0.61, N = 237
IHCLENVDK pc2
7WYQ1;O15020;O15020−2;P11277;P1
113:747 57:437 13:76
2:54834380:G:A_A
p = 0.58, beta = 0.064, N = 183
SPTBN1 : NP3
Q01082
log10 protein intensity
176:220 70:87 15:18
2:54834380:G:A_A
p = 4.9e−05, beta = −0.412, N = 261
TALPAQSAATLPAR pc2
A0A087WUZ3;Q01082
144:220 52:87 11:18
2:54834380:G:A_A
p = 0.0031, beta = −0.346, N = 207
TAASGIPYHSEVPVSLK pc3
A0A087WUZ3;Q01082
128:220 46:87 6:18
2:54834380:G:A_A
p = 0.013, beta = −0.338, N = 180
DALLLWCQMK pc2
I0QZQ3;O15020;O15020−2;P11277;P1
91:220 31:87 4:18
2:54834380:G:A_A
p = 0.28, beta = −0.18, N = 126
VLVLSQDYGK pc2
A0A087WUZ3;Q01082−3;Q01082
54:220 11:87 0:18
2:54834380:G:A_A
p = 0.69, beta = −0.127, N = 65
EAVCEVALDYK pc2
A0A087WUZ3;Q01082
71:220 24:87 2:18
2:54834380:G:A_A
p = 0.23, beta = −0.237, N = 97
WVNSHLAR pc2
I0QZQ3;O15020;O15020−2;P11277;P1
37:220 11:87 1:18
2:54834380:G:A_A
p = 0.85, beta = 0.0543, N = 49
GNLEVLLFTIQSK pc2
3;A0A087WYQ1;O15020;O15020−2;Q
67:220 14:87 1:18
2:54834380:G:A_A
p = 0.5, beta = −0.168, N = 82
VLDNAIETEK pc2
A0A087WUZ3;Q01082−3;Q01082
33:220 6:87 0:18
2:54834380:G:A_A
p = 0.61, beta = −0.213, N = 39
LNDGNEYLFQAK pc2
A0A087WUZ3;Q01082
40:220 10:87 1:18
2:54834380:G:A_A
p = 0.42, beta = −0.23, N = 51
EGMQLISEKPETEAVVK pc3
A0A087WUZ3;Q01082−3;Q01082
28:220 5:87 0:18
2:54834380:G:A_A
p = 0.51, beta = −0.301, N = 33
QALQDTLALYK pc2
A0A087WUZ3;Q01082−3;Q01082
44:220 8:87 0:18
2:54834380:G:A_A
p = 0.36, beta = −0.334, N = 52
MWEVLESTTQTK pc2
A0A087WUZ3;Q01082−3;Q01082
33:220 6:87 1:18
2:54834380:G:A_A
p = 0.036, beta = −0.669, N = 40
LAEISDVWEEMK pc2
A0A087WUZ3;Q01082−3;Q01082
27:220 3:87 0:18
2:54834380:G:A_A
p = 0.46, beta = −0.425, N = 30
LEDLEVIQHR pc2
A0A087WUZ3;Q01082−3;Q01082
21:220 2:87 0:18
2:54834380:G:A_A
p = 0.014, beta = −1.58, N = 23
LTTLELLEVR pc2
A0A087WUZ3;Q01082−3;Q01082
20:220 7:87 0:18
2:54834380:G:A_A
p = 0.031, beta = −0.847, N = 27
SIITYVVTYYHYFSK pc3
A0A087WUZ3;Q01082−3;Q01082
18:220 6:87 0:18
2:54834380:G:A_A
p = 0.54, beta = −0.269, N = 24
172Assay Target: SPTBN1
UniProt: Q01082
rsID: rs6740893
Target SNP: 2:54834380:G:A
Proxy SNP: 2:54834380:G:A
Tarkin log10(p): 10.4
Tarkin BETA: −0.306
Tarkin MSPA: 0.554
1136:1209:357:960:679:684:523
ETWLSENQR pc2
rs229587 REF
123:747 42:437 13:76
2:54834380:G:A_A
p = 0.0039, model = REC, N = 178

173. COL6A2 (P12110)
Tarkin MSPA=0.096

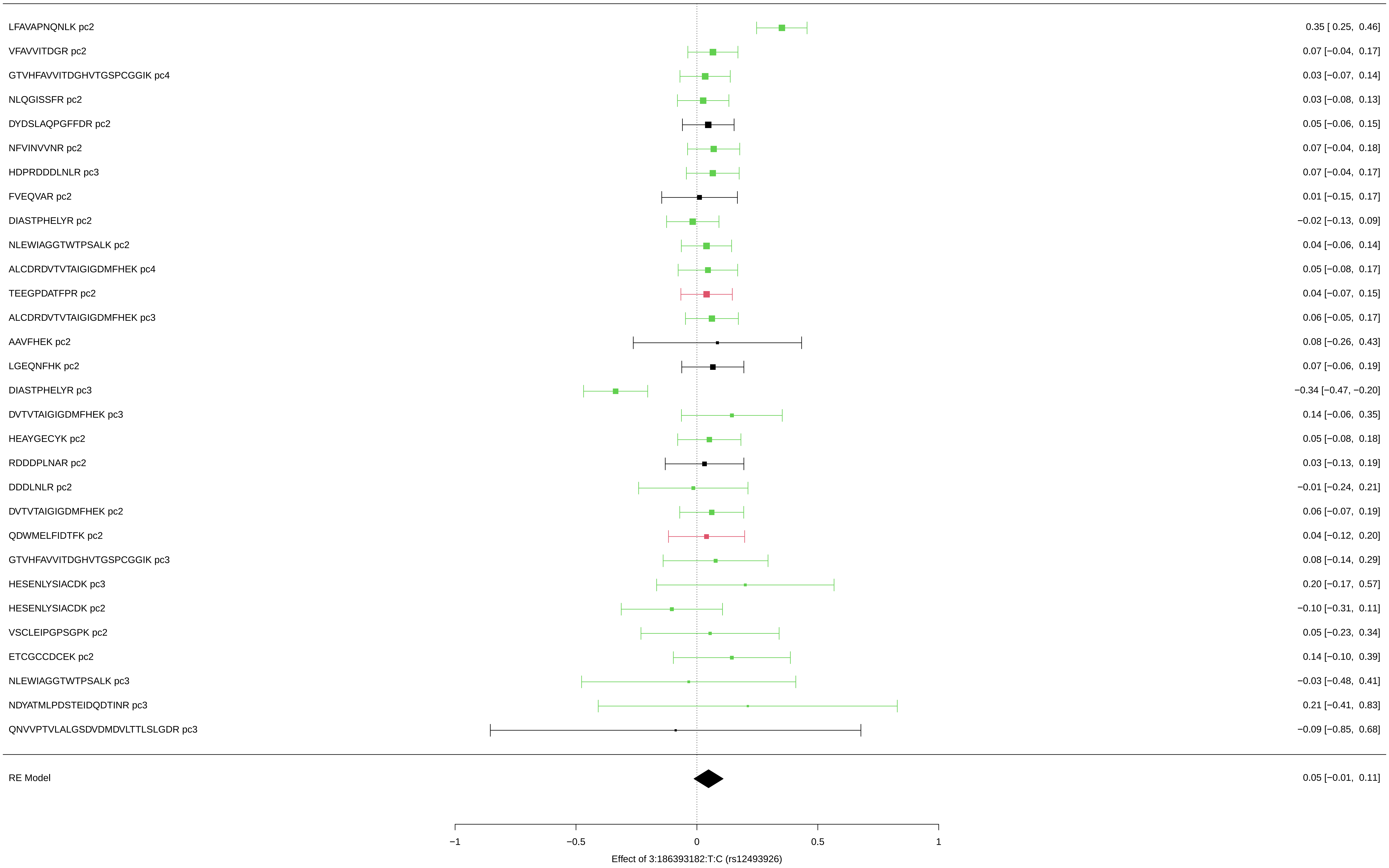

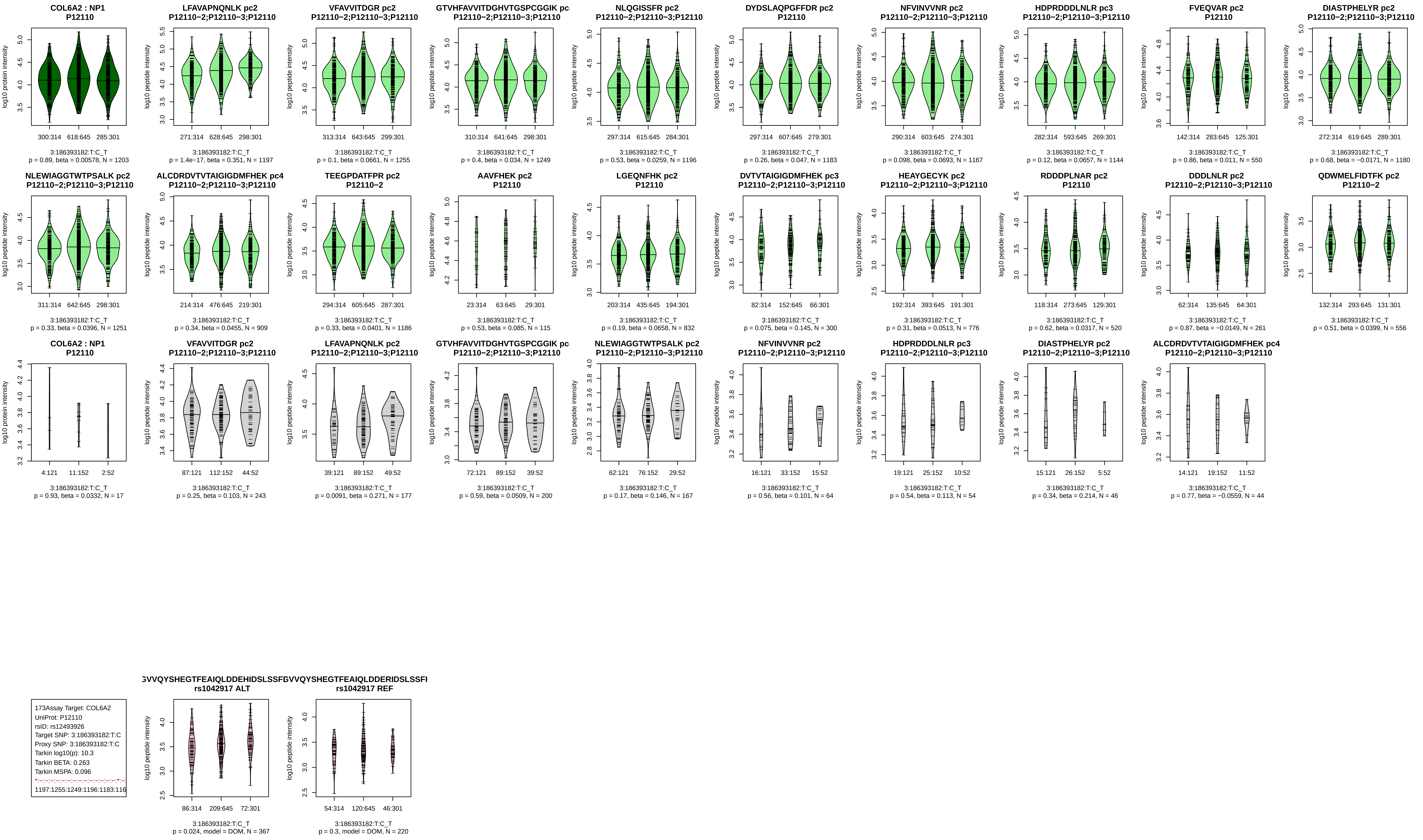

COL6A2 : NP1
P12110
log10 protein intensity
300:314 618:645 285:301
3:186393182:T:C_T
p = 0.89, beta = 0.00578, N = 1203
LFAVAPNQNLK pc2
P12110−2;P12110−3;P12110
271:314 628:645 298:301
p = 1.4e−17, beta = 0.351, N = 1197
VFAVVITDGR pc2
313:314 643:645 299:301
p = 0.1, beta = 0.0661, N = 1255
GTVHFAVVITDGHVTGSPCGGIK pc
310:314 641:645 298:301
p = 0.4, beta = 0.034, N = 1249
NLQGISSFR pc2
297:314 615:645 284:301
p = 0.53, beta = 0.0259, N = 1196
DYDSLAQPGFFDR pc2
P12110
297:314 607:645 279:301
p = 0.26, beta = 0.047, N = 1183
NFVINVVNR pc2
290:314 603:645 274:301
p = 0.098, beta = 0.0693, N = 1167
HDPRDDDLNLR pc3
282:314 593:645 269:301
p = 0.12, beta = 0.0657, N = 1144
FVEQVAR pc2
142:314 283:645 125:301
p = 0.86, beta = 0.011, N = 550
DIASTPHELYR pc2
272:314 619:645 289:301
p = 0.68, beta = −0.0171, N = 1180
NLEWIAGGTWTPSALK pc2
311:314 642:645 298:301
p = 0.33, beta = 0.0396, N = 1251
ALCDRDVTVTAIGIGDMFHEK pc4
214:314 476:645 219:301
p = 0.34, beta = 0.0455, N = 909
TEEGPDATFPR pc2
P12110−2
294:314 605:645 287:301
p = 0.33, beta = 0.0401, N = 1186
AAVFHEK pc2
23:314 63:645 29:301
p = 0.53, beta = 0.085, N = 115
LGEQNFHK pc2
203:314 435:645 194:301
p = 0.19, beta = 0.0658, N = 832
DVTVTAIGIGDMFHEK pc3
82:314 152:645 66:301
p = 0.075, beta = 0.145, N = 300
HEAYGECYK pc2
192:314 393:645 191:301
p = 0.31, beta = 0.0513, N = 776
RDDDPLNAR pc2
118:314 273:645 129:301
p = 0.62, beta = 0.0317, N = 520
DDDLNLR pc2
62:314 135:645 64:301
p = 0.87, beta = −0.0149, N = 261
QDWMELFIDTFK pc2
132:314 293:645 131:301
p = 0.51, beta = 0.0399, N = 556
COL6A2 : NP1
4:121 11:152 2:52
p = 0.93, beta = 0.0332, N = 17
VFAVVITDGR pc2
87:121 112:152 44:52
p = 0.25, beta = 0.103, N = 243
LFAVAPNQNLK pc2
39:121 89:152 49:52
p = 0.0091, beta = 0.271, N = 177
GTVHFAVVITDGHVTGSPCGGIK pc
72:121 89:152 39:52
p = 0.59, beta = 0.0509, N = 200
NLEWIAGGTWTPSALK pc2
62:121 76:152 29:52
p = 0.17, beta = 0.146, N = 167
NFVINVVNR pc2
16:121 33:152 15:52
p = 0.56, beta = 0.101, N = 64
HDPRDDDLNLR pc3
19:121 25:152 10:52
p = 0.54, beta = 0.113, N = 54
DIASTPHELYR pc2
15:121 26:152 5:52
p = 0.34, beta = 0.214, N = 46
ALCDRDVTVTAIGIGDMFHEK pc4
14:121 19:152 11:52
p = 0.77, beta = −0.0559, N = 44
173Assay Target: COL6A2
UniProt: P12110
rsID: rs12493926
Target SNP: 3:186393182:T:C
Proxy SNP: 3:186393182:T:C
Tarkin log10(p): 10.3
Tarkin BETA: 0.263
Tarkin MSPA: 0.096
1197:1255:1249:1196:1183:116
GVVQYSHEGTFEAIQLDDEHIDSLSSF
rs1042917 ALT
86:314 209:645 72:301
p = 0.024, model = DOM, N = 367
GVVQYSHEGTFEAIQLDDERIDSLSSFI
rs1042917 REF
54:314 120:645 46:301
p = 0.3, model = DOM, N = 220

174. HHIPL1 (Q96JK4)
Tarkin MSPA=0.675

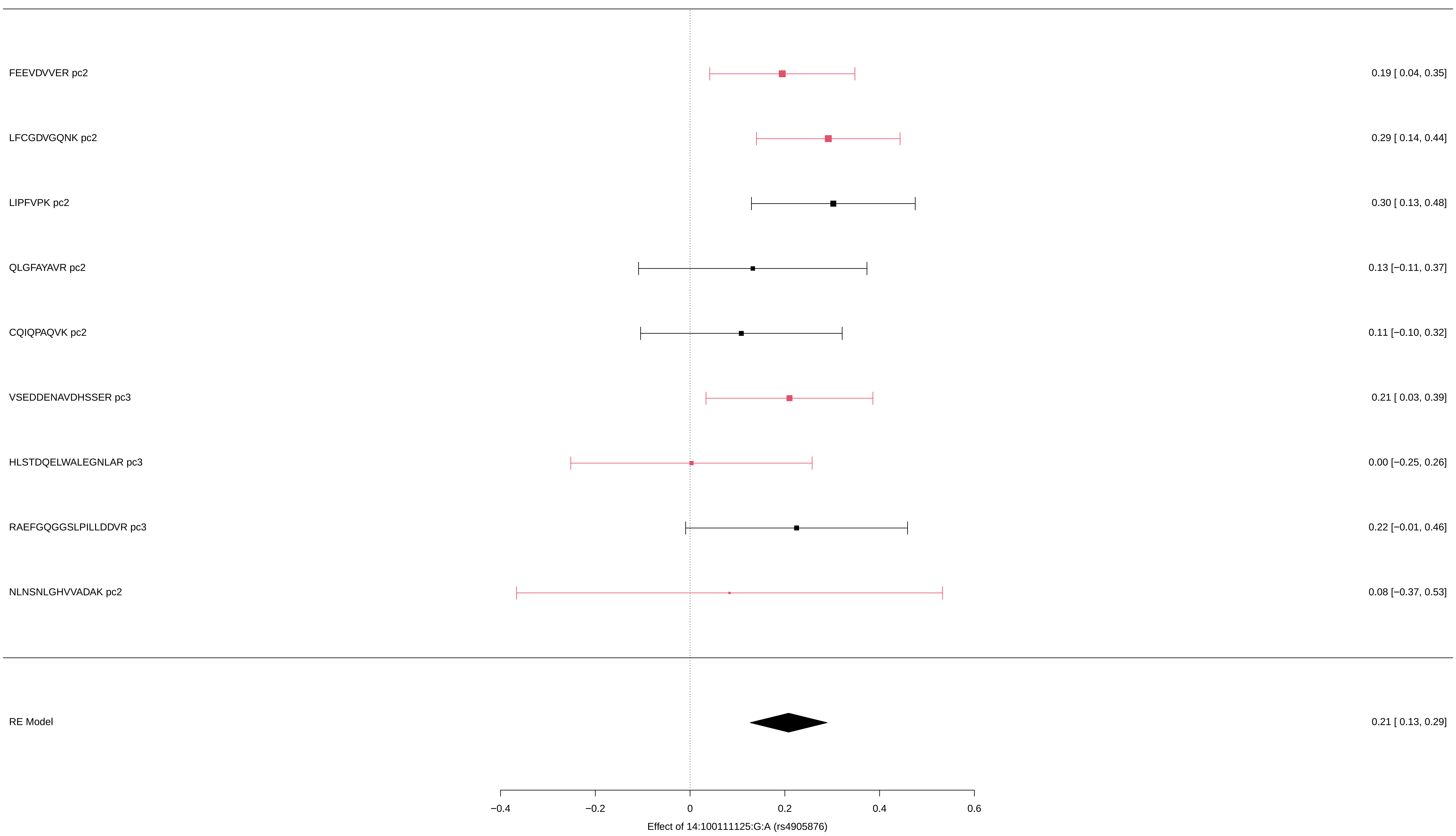

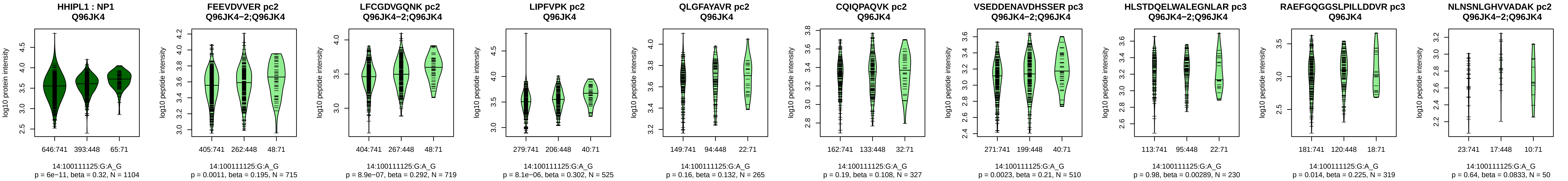

174Assay Target: HHIPL1
UniProt: Q96JK4
rsID: rs4905876
Target SNP: 14:100111125:G:A
Proxy SNP: 14:100111125:G:A
Tarkin log10(p): 10.2
Tarkin BETA: 0.32
Tarkin MSPA: 0.675
::*:–:–:*:–:–:–
715:719:525:265:327:510:230:3

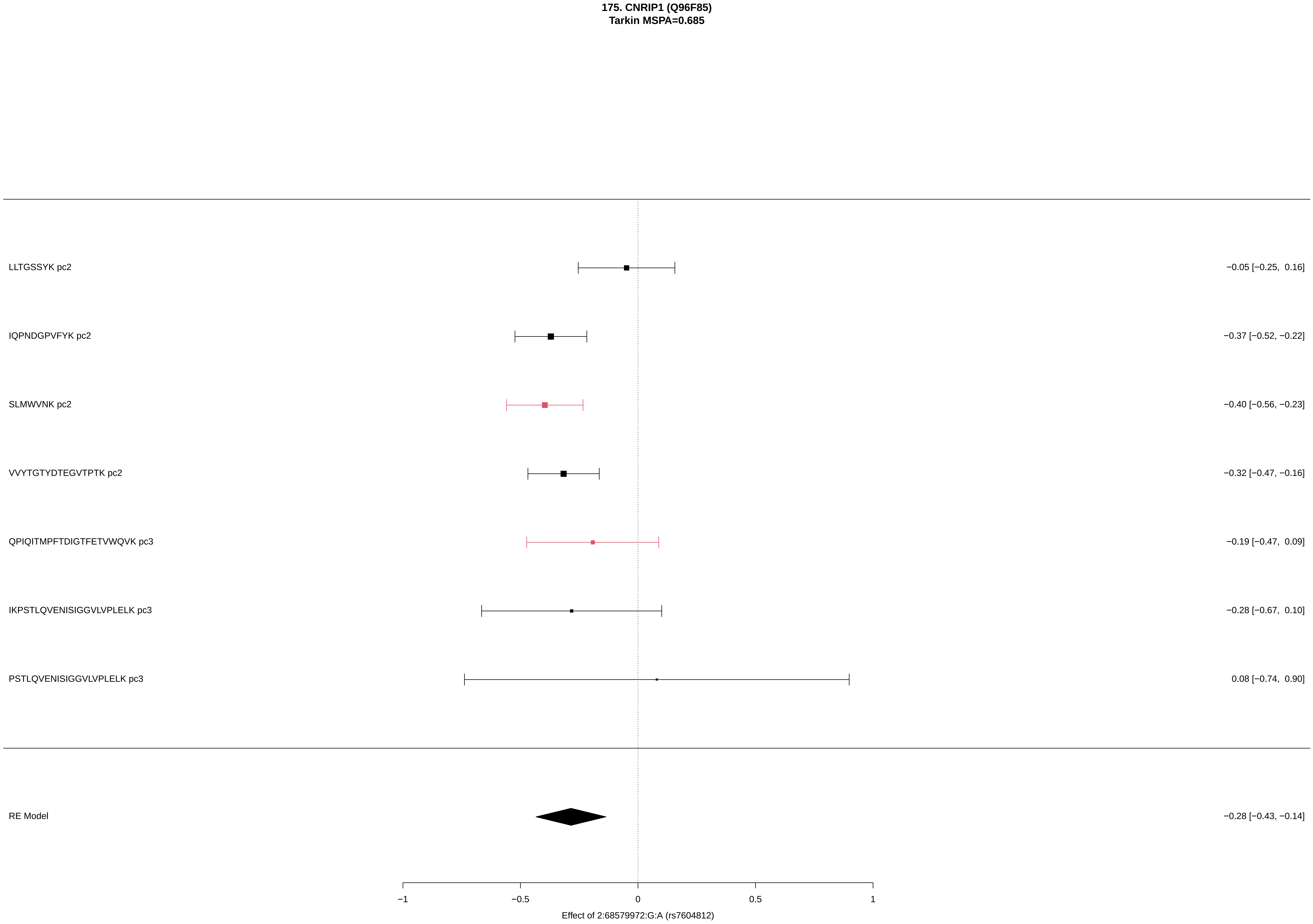

175. CNRIP1 (Q96F85)
Tarkin MSPA=0.685
LLTGSSYK pc2
−0.05 [−0.25, 0.16]
IQPNDGPVFYK pc2
−0.37 [−0.52, −0.22]
SLMWVNK pc2
−0.40 [−0.56, −0.23]
VVYTGTYDTEGVTPTK pc2
−0.32 [−0.47, −0.16]
QPIQITMPFTDIGTFETVWQVK pc3
−0.19 [−0.47, 0.09]
IKPSTLQVENISIGGVLVPLELK pc3
−0.28 [−0.67, 0.10]
PSTLQVENISIGGVLVPLELK pc3
0.08 [−0.74, 0.90]
RE Model
−0.28 [−0.43, −0.14]
−1
−0.5
0
0.5
1
Effect of 2:68579972:G:A (rs7604812)

CNRIP1 : NP3
Q96F85

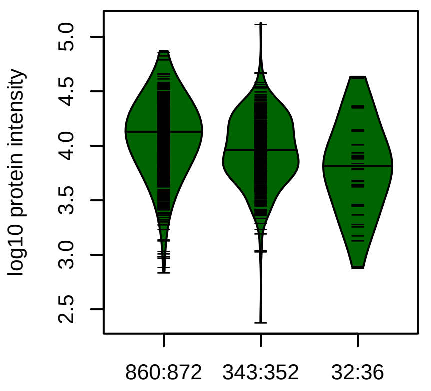

2:68579972:G:A_A
p = 6.6e−11, beta = −0.351, N = 1235

LLTGSSYK pc2
B8ZZB8;Q96F85;Q96F85−2

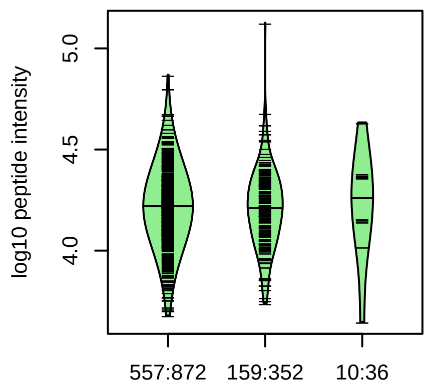

2:68579972:G:A_A
p = 0.54, beta = −0.0486, N = 726

IQPNDGPVFYK pc2
B8ZZB8;Q96F85;Q96F85−2

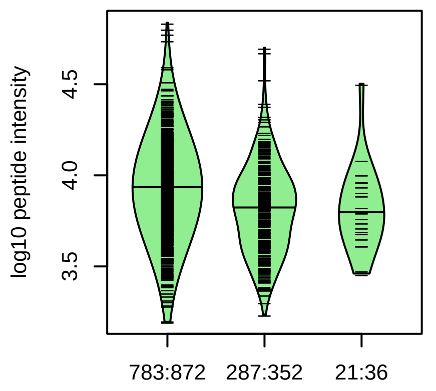

2:68579972:G:A_A
p = 6.1e−10, beta = −0.37, N = 1091

SLMWVNK pc2
Q96F85

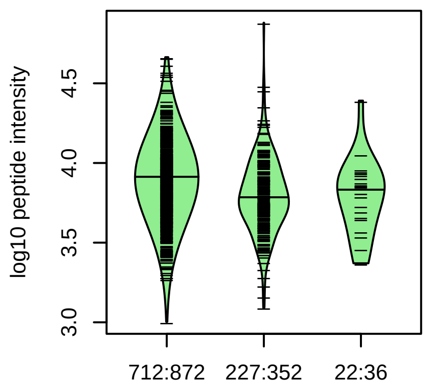

2:68579972:G:A_A
p = 5.2e−10, beta = −0.396, N = 961

VVYTGTYDTEGVTPTK pc2
B8ZZB8;Q96F85;Q96F85−2

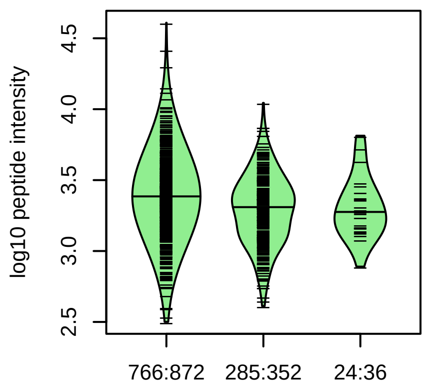

2:68579972:G:A_A
p = 9.7e−08, beta = −0.316, N = 1075

QPIQITMPFTDIGTFETVWQVK pc3
Q96F85

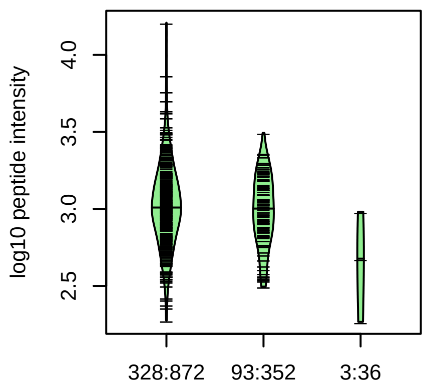

2:68579972:G:A_A
p = 0.078, beta = −0.192, N = 424

IKPSTLQVENISIGGVLVPLELK pc3
B8ZZB8;Q96F85;Q96F85−2

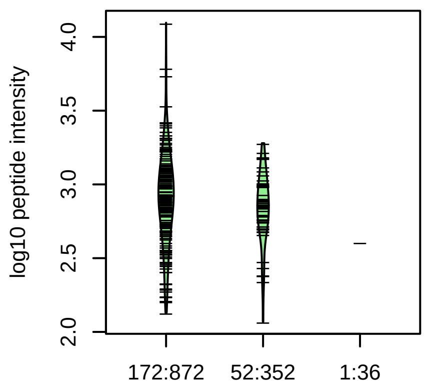

2:68579972:G:A_A
p = 0.059, beta = −0.282, N = 225

PSTLQVENISIGGVLVPLELK pc3
B8ZZB8;Q96F85;Q96F85−2

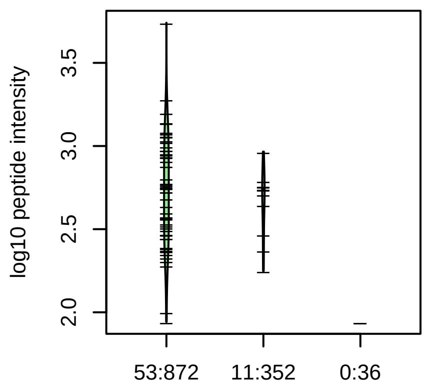

2:68579972:G:A_A
p = 0.8, beta = 0.0802, N = 64

CNRIP1 : NP3
Q96F85

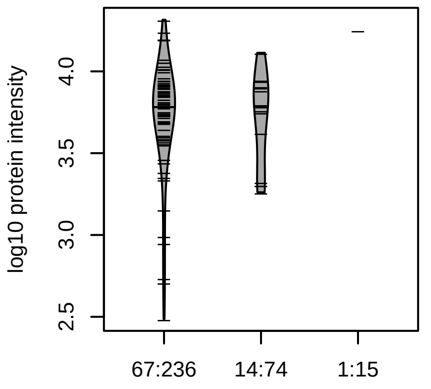

2:68579972:G:A_A
p = 0.34, beta = 0.238, N = 82

IQPNDGPVFYK pc2
B8ZZB8;Q96F85;Q96F85−2

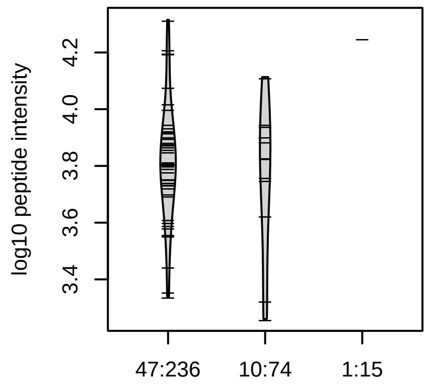

2:68579972:G:A_A
p = 0.7, beta = 0.11, N = 58

IKPSTLQVENISIGGVLVPLELK pc3
B8ZZB8;Q96F85;Q96F85−2

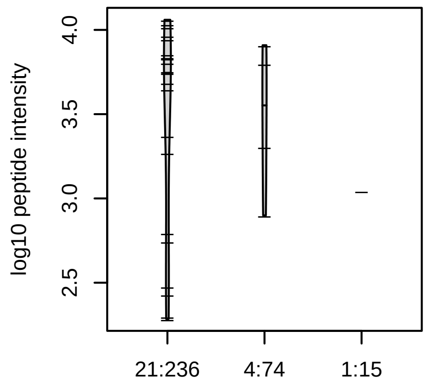

2:68579972:G:A_A
p = 0.48, beta = −0.257, N = 26

175Assay Target: CNRIP1
UniProt: Q96F85
rsID: rs7604812
Target SNP: 2:68579972:G:A
Proxy SNP: 2:68579972:G:A
Tarkin log10(p): 10.2
Tarkin BETA: −0.351
Tarkin MSPA: 0.685
−:*:*:*:−:−:−:NA
726:1091:961:1075:424:225:64:

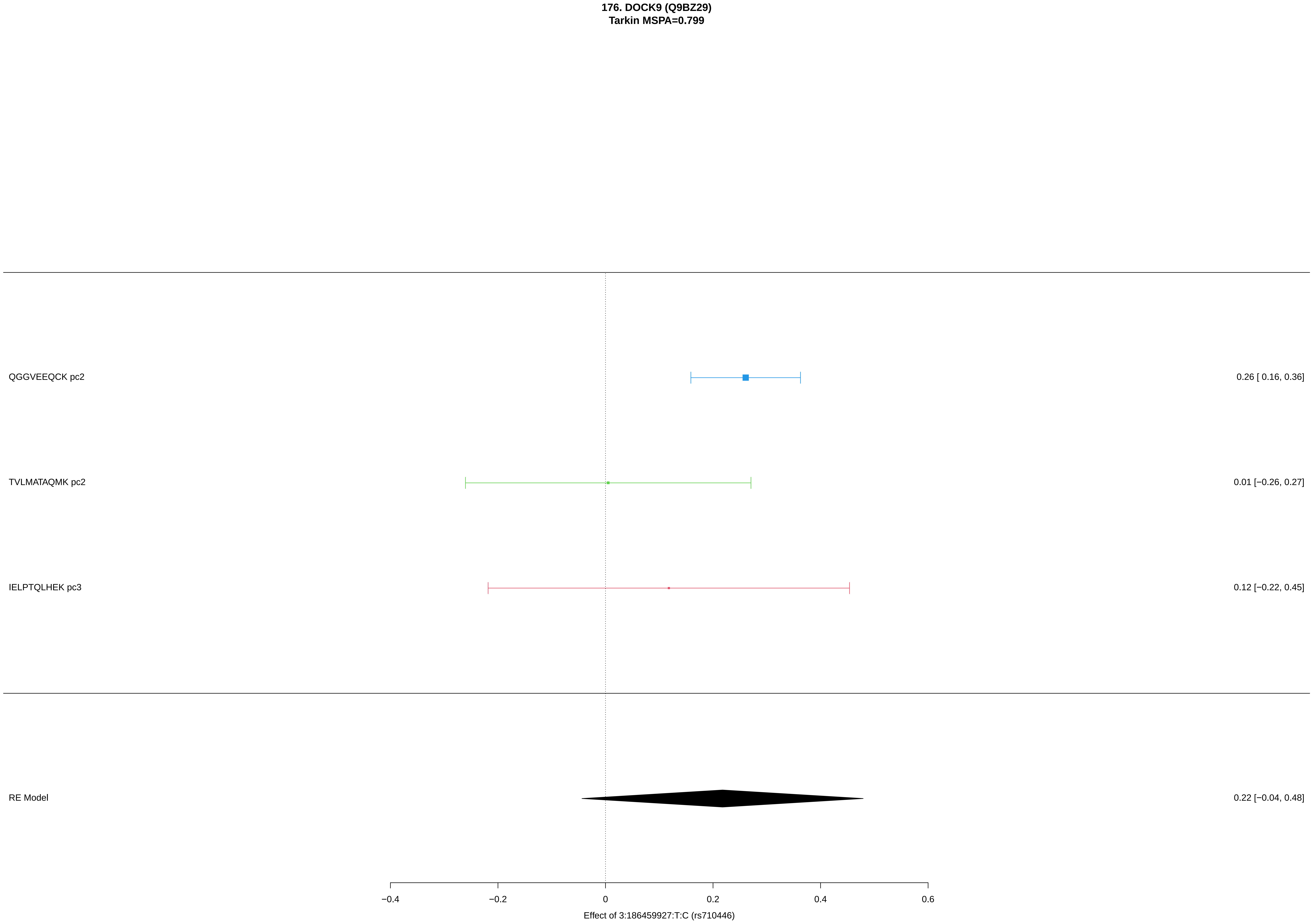

176. DOCK9 (Q9BZ29)
Tarkin MSPA=0.799
QGGVEEQCK pc2
0.26 [0.16, 0.36]
TVLMATAQMK pc2
0.01 [−0.26, 0.27]
IELPTQLHEK pc3
0.12 [−0.22, 0.45]
RE Model
0.22 [−0.04, 0.48]
−0.4
−0.2
0
0.2
0.4
0.6
Effect of 3:186459927:T:C (rs710446)

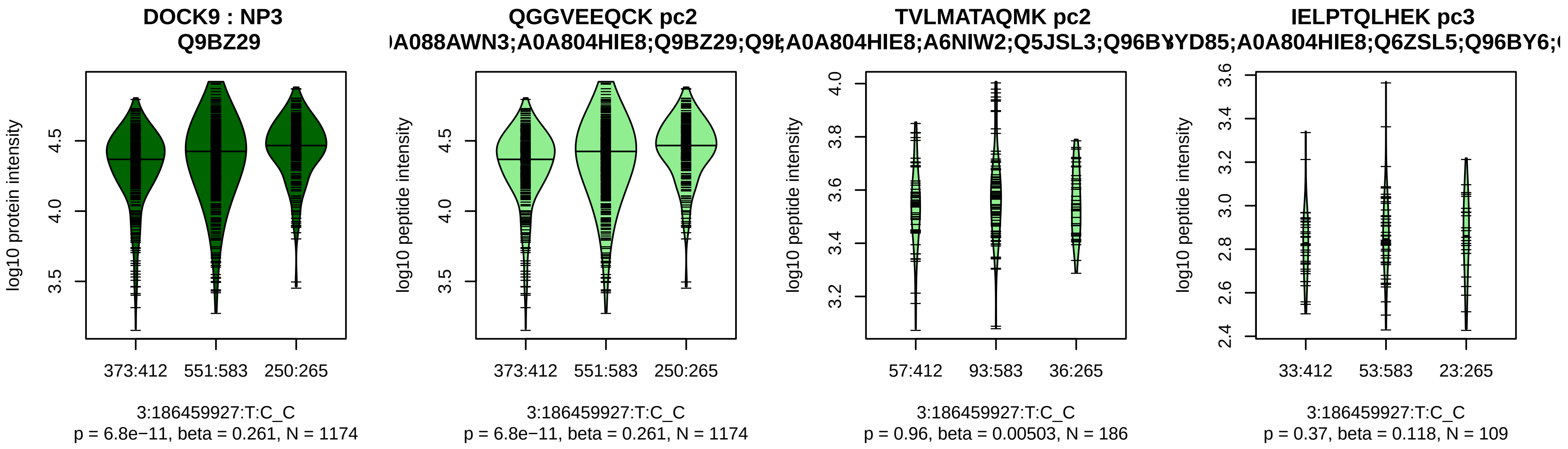

176Assay Target: DOCK9
UniProt: Q9BZ29
rsID: rs710446
Target SNP: 3:186459927:T:C
Proxy SNP: 3:186459927:T:C
Tarkin log10(p): 10.2
Tarkin BETA: 0.261
Tarkin MSPA: 0.799
*:−:−:NA:NA
1174:186:109:19:1

177. CD151 (P48509)
Tarkin MSPA=0.92

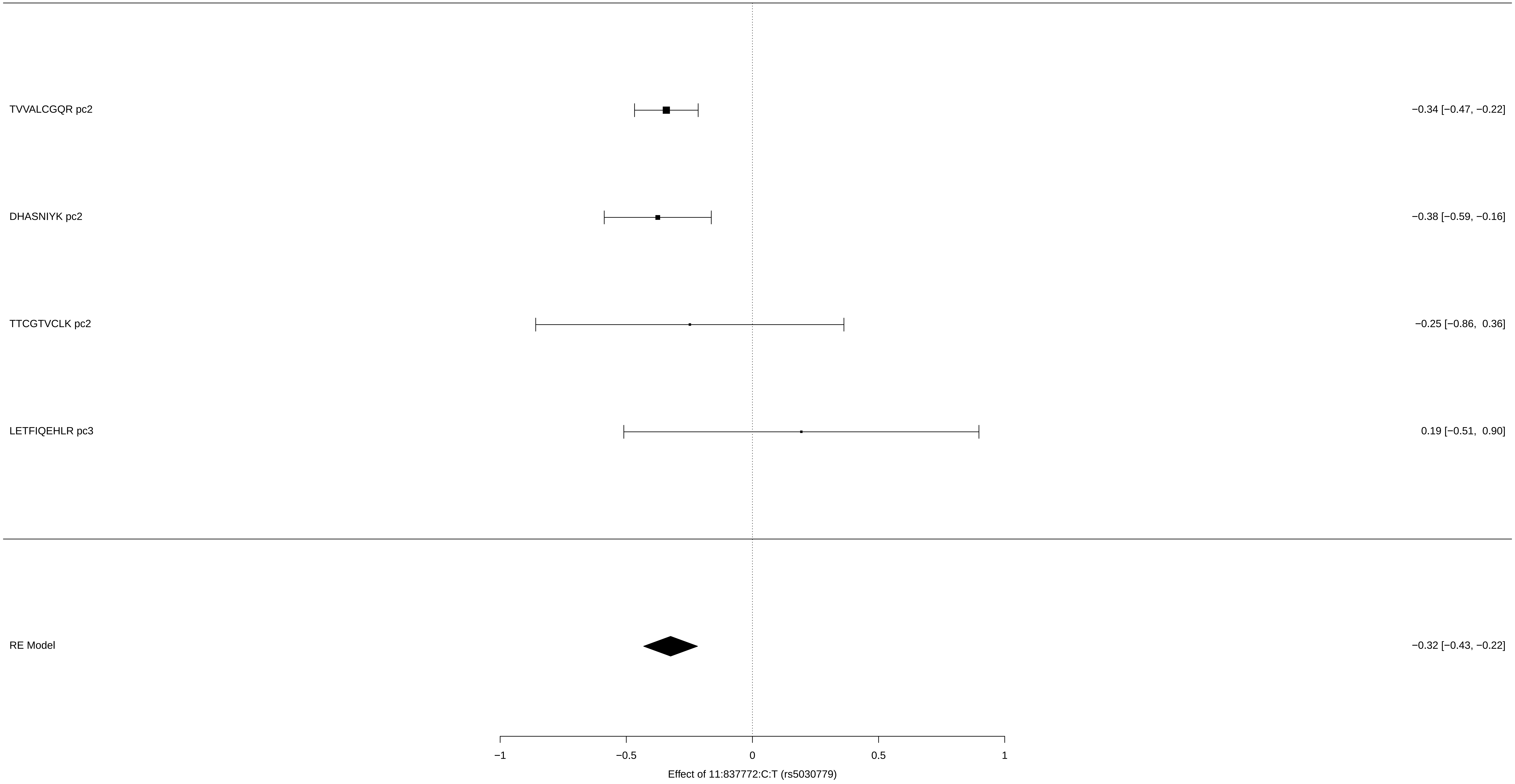

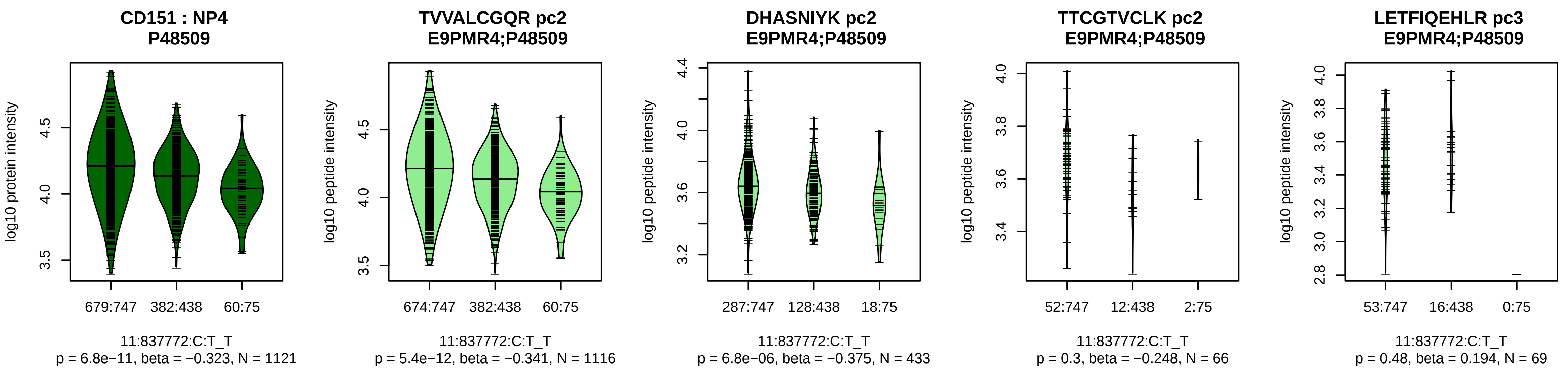

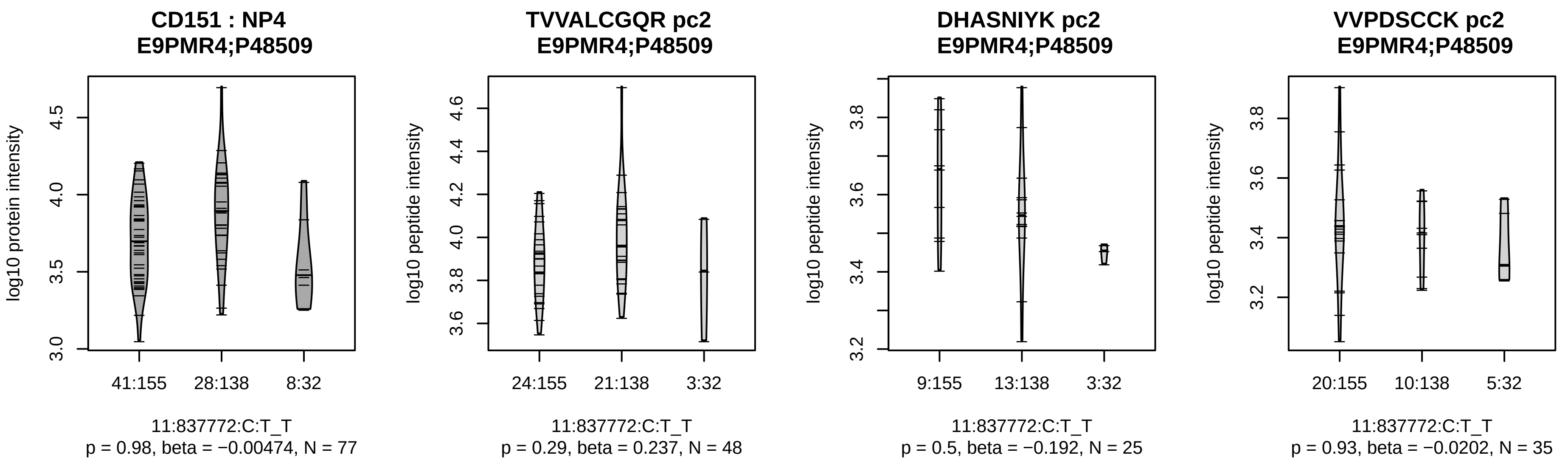

177Assay Target: CD151
UniProt: P48509
rsID: rs5030779
Target SNP: 11:837772:C:T
Proxy SNP: 11:837772:C:T
Tarkin log10(p): 10.2
Tarkin BETA: −0.323
Tarkin MSPA: 0.92
::–:–
1116:433:66:69

178. PON3 (Q15166)
Tarkin MSPA=0.597

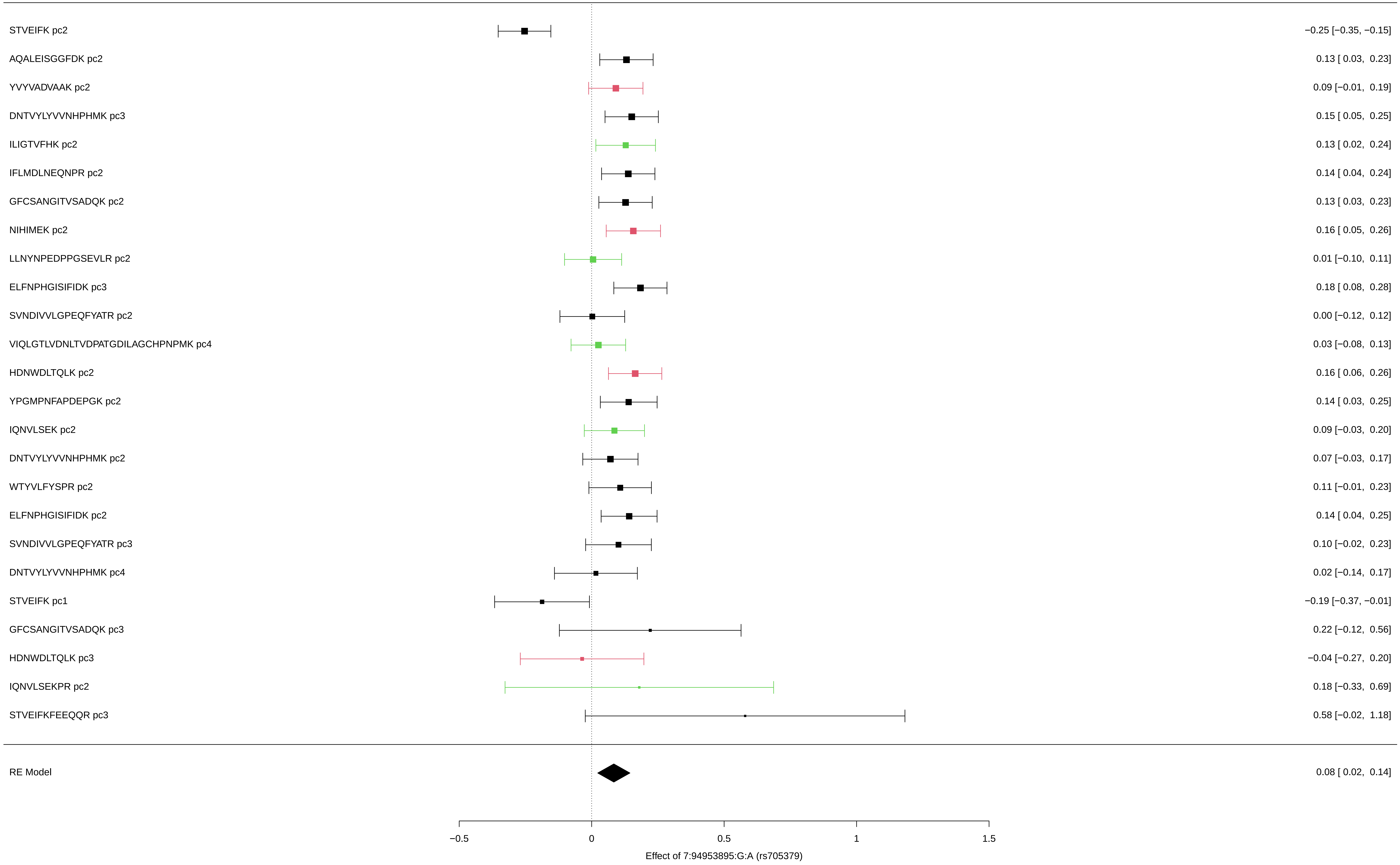

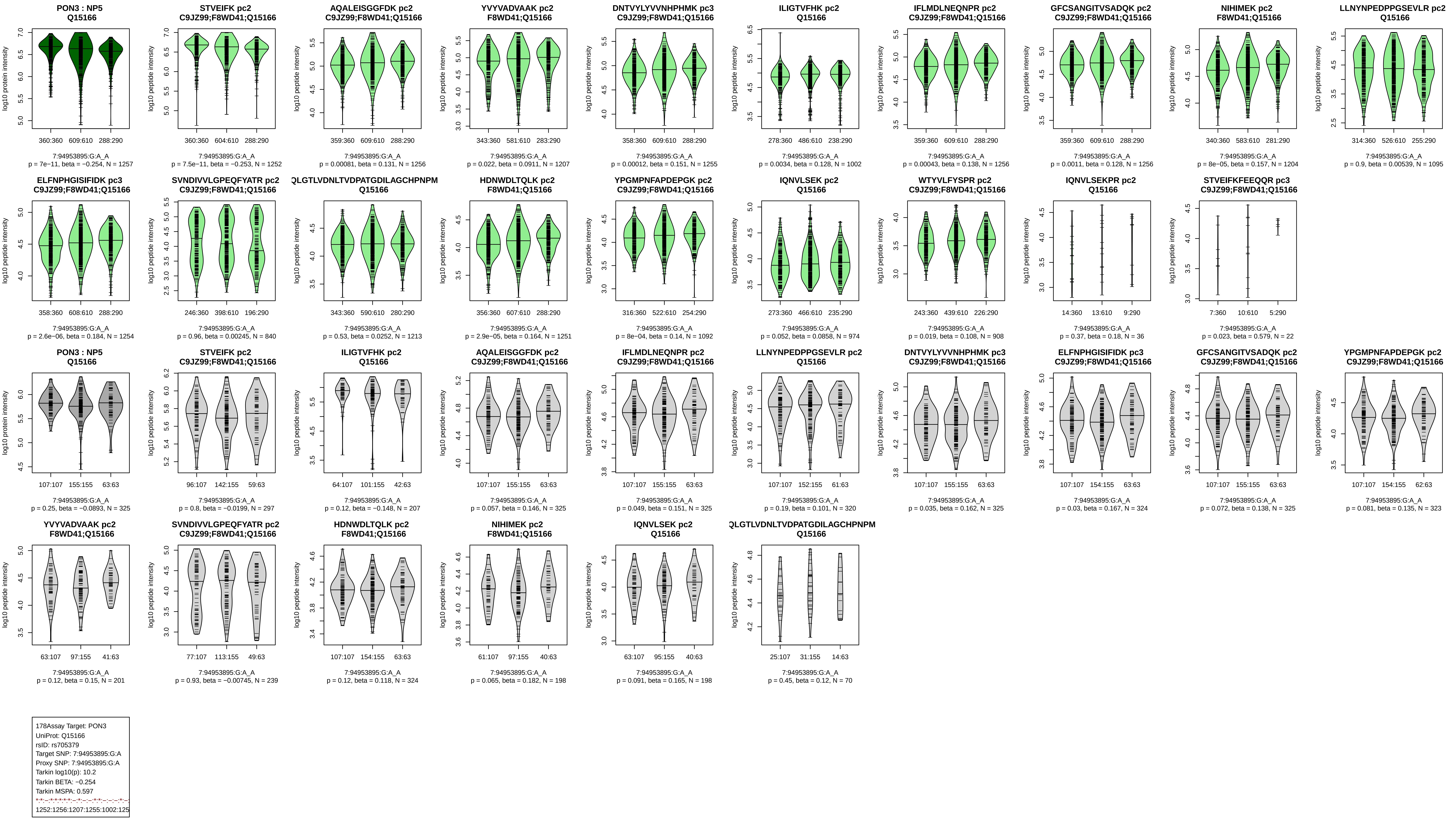
PON3 : NP5
Q15166
360:360 609:610 288:290
7:94953895:G:A_A
p = 7e−11, beta = −0.254, N = 1257
STVEIFK pc2
C9JZ99;F8WD41;Q15166
360:360 604:610 288:290
7:94953895:G:A_A
p = 7.5e−11, beta = −0.253, N = 1252
AQALEISGGFDK pc2
C9JZ99;F8WD41;Q15166
359:360 609:610 288:290
7:94953895:G:A_A
p = 0.00081, beta = 0.131, N = 1256
YVYVADVAAK pc2
F8WD41;Q15166
343:360 581:610 283:290
7:94953895:G:A_A
p = 0.022, beta = 0.0911, N = 1207
DNTVYLYVVNHPHMK pc3
C9JZ99;F8WD41;Q15166
358:360 609:610 288:290
7:94953895:G:A_A
p = 0.00012, beta = 0.151, N = 1255
ILIGTVFHK pc2
Q15166
278:360 486:610 238:290
7:94953895:G:A_A
p = 0.0034, beta = 0.128, N = 1002
IFLMDLNEQNPR pc2
C9JZ99;F8WD41;Q15166
359:360 609:610 288:290
7:94953895:G:A_A
p = 0.00043, beta = 0.138, N = 1256
GFCSANGITVSADQK pc2
C9JZ99;F8WD41;Q15166
359:360 609:610 288:290
7:94953895:G:A_A
p = 0.0011, beta = 0.128, N = 1256
NIHIMEK pc2
F8WD41;Q15166
340:360 583:610 281:290
7:94953895:G:A_A
p = 8e−05, beta = 0.157, N = 1204
LLNYNPEDPPGSEVLR pc2
Q15166
314:360 526:610 255:290
7:94953895:G:A_A
p = 0.9, beta = 0.00539, N = 1095
ELFNPHGISIFIDK pc3
C9JZ99;F8WD41;Q15166
358:360 608:610 288:290
7:94953895:G:A_A
p = 2.6e−06, beta = 0.184, N = 1254
SVNDIVVLGPEQFYATR pc2
C9JZ99;F8WD41;Q15166
246:360 398:610 196:290
7:94953895:G:A_A
p = 0.96, beta = 0.00245, N = 840
QLGTLVDNLTVDPATGDILAGCHPNPM
Q15166
343:360 590:610 280:290
7:94953895:G:A_A
p = 0.53, beta = 0.0252, N = 1213
HDNWDLTQLK pc2
C9JZ99;F8WD41;Q15166
356:360 607:610 288:290
7:94953895:G:A_A
p = 2.9e−05, beta = 0.164, N = 1251
YPGMPNFAPDEPGK pc2
C9JZ99;F8WD41;Q15166
316:360 522:610 254:290
7:94953895:G:A_A
p = 8e−04, beta = 0.14, N = 1092
IQNVLSEK pc2
Q15166
273:360 466:610 235:290
7:94953895:G:A_A
p = 0.052, beta = 0.0858, N = 974
WTYVLFYSPR pc2
C9JZ99;F8WD41;Q15166
243:360 439:610 226:290
7:94953895:G:A_A
p = 0.019, beta = 0.108, N = 908
IQNVLSEKPR pc2
Q15166
14:360 13:610 9:290
7:94953895:G:A_A
p = 0.37, beta = 0.18, N = 36
STVEIFKFEEQR pc3
C9JZ99;F8WD41;Q15166
7:360 10:610 5:290
7:94953895:G:A_A
p = 0.023, beta = 0.579, N = 22
PON3 : NP5
Q15166
107:107 155:155 63:63
7:94953895:G:A_A
p = 0.25, beta = −0.0893, N = 325
STVEIFK pc2
C9JZ99;F8WD41;Q15166
96:107 142:155 59:63
7:94953895:G:A_A
p = 0.8, beta = −0.0199, N = 297
ILIGTVFHK pc2
Q15166
64:107 101:155 42:63
7:94953895:G:A_A
p = 0.12, beta = −0.148, N = 207
AQALEISGGFDK pc2
C9JZ99;F8WD41;Q15166
107:107 155:155 63:63
7:94953895:G:A_A
p = 0.057, beta = 0.146, N = 325
IFLMDLNEQNPR pc2
C9JZ99;F8WD41;Q15166
107:107 155:155 63:63
7:94953895:G:A_A
p = 0.049, beta = 0.151, N = 325
LLNYNPEDPPGSEVLR pc2
Q15166
107:107 152:155 61:63
7:94953895:G:A_A
p = 0.19, beta = 0.101, N = 320
DNTVYLYVVNHPHMK pc3
C9JZ99;F8WD41;Q15166
107:107 155:155 63:63
7:94953895:G:A_A
p = 0.035, beta = 0.162, N = 325
ELFNPHGISIFIDK pc3
C9JZ99;F8WD41;Q15166
107:107 154:155 63:63
7:94953895:G:A_A
p = 0.03, beta = 0.167, N = 324
GFCSANGITVSADQK pc2
C9JZ99;F8WD41;Q15166
107:107 155:155 63:63
7:94953895:G:A_A
p = 0.072, beta = 0.138, N = 325
YPGMPNFAPDEPGK pc2
C9JZ99;F8WD41;Q15166
107:107 154:155 62:63
7:94953895:G:A_A
p = 0.081, beta = 0.135, N = 323
YVYVADVAAK pc2
F8WD41;Q15166
63:107 97:155 41:63
7:94953895:G:A_A
p = 0.12, beta = 0.15, N = 201
SVNDIVVLGPEQFYATR pc2
C9JZ99;F8WD41;Q15166
77:107 113:155 49:63
7:94953895:G:A_A
p = 0.93, beta = −0.00745, N = 239
HDNWDLTQLK pc2
F8WD41;Q15166
107:107 154:155 63:63
7:94953895:G:A_A
p = 0.12, beta = 0.118, N = 324
NIHIMEK pc2
F8WD41;Q15166
61:107 97:155 40:63
7:94953895:G:A_A
p = 0.065, beta = 0.182, N = 198
IQNVLSEK pc2
Q15166
63:107 95:155 40:63
7:94953895:G:A_A
p = 0.091, beta = 0.165, N = 198
QLGTLVDNLTVDPATGDILAGCHPNPM
Q15166
25:107 31:155 14:63
7:94953895:G:A_A
p = 0.45, beta = 0.12, N = 70
log10 protein intensity
log10 peptide intensity
178Assay Target: PON3
UniProt: Q15166
rsID: rs705379
Target SNP: 7:94953895:G:A
Proxy SNP: 7:94953895:G:A
Tarkin log10(p): 10.2
Tarkin BETA: −0.254
Tarkin MSPA: 0.597
1252:1256:1207:1255:1002:125

179. FABP5 (Q01469)
Tarkin MSPA=0.802

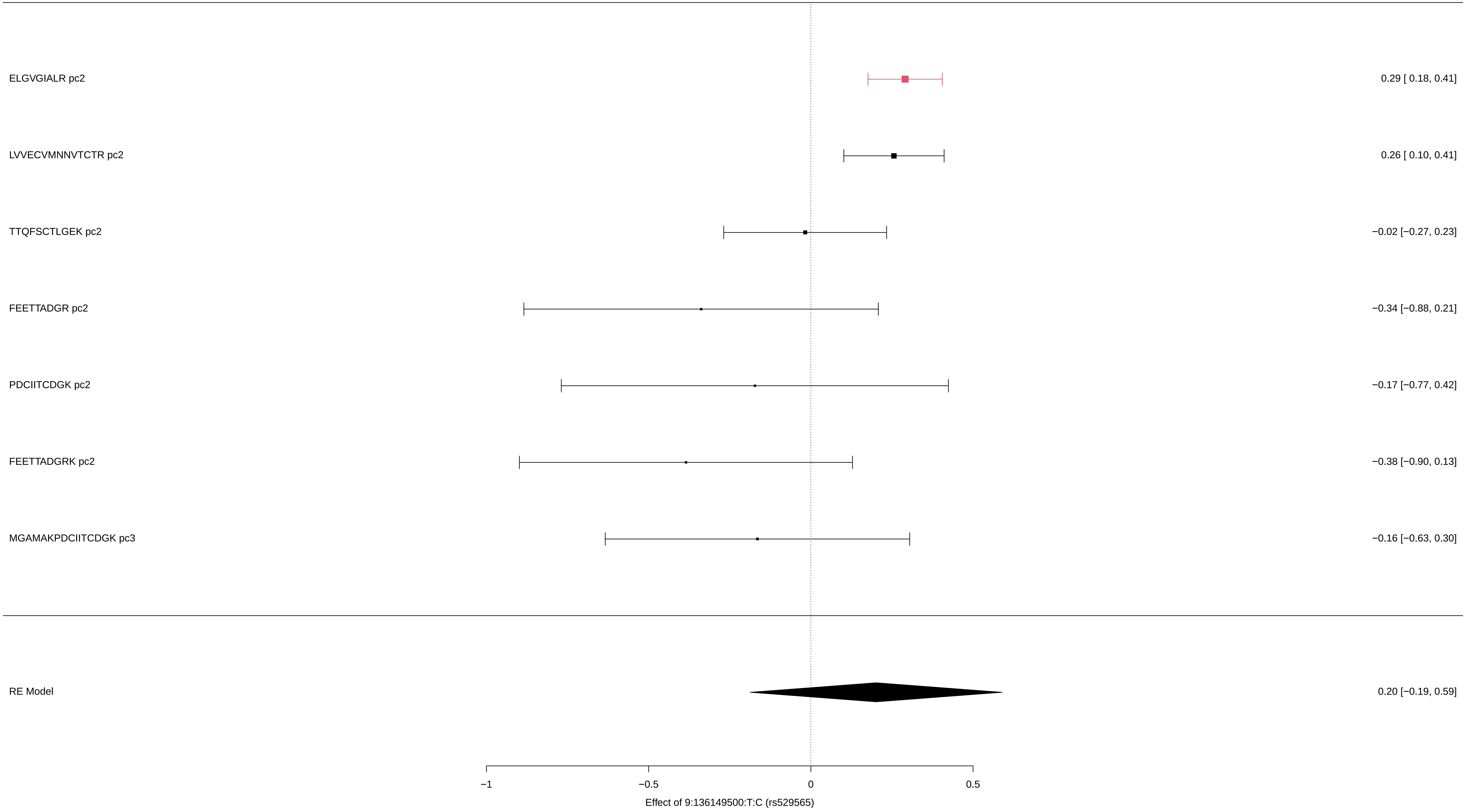

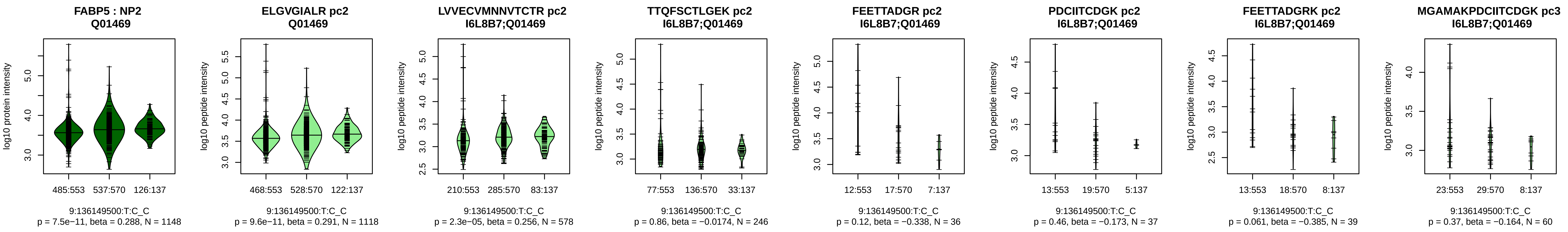

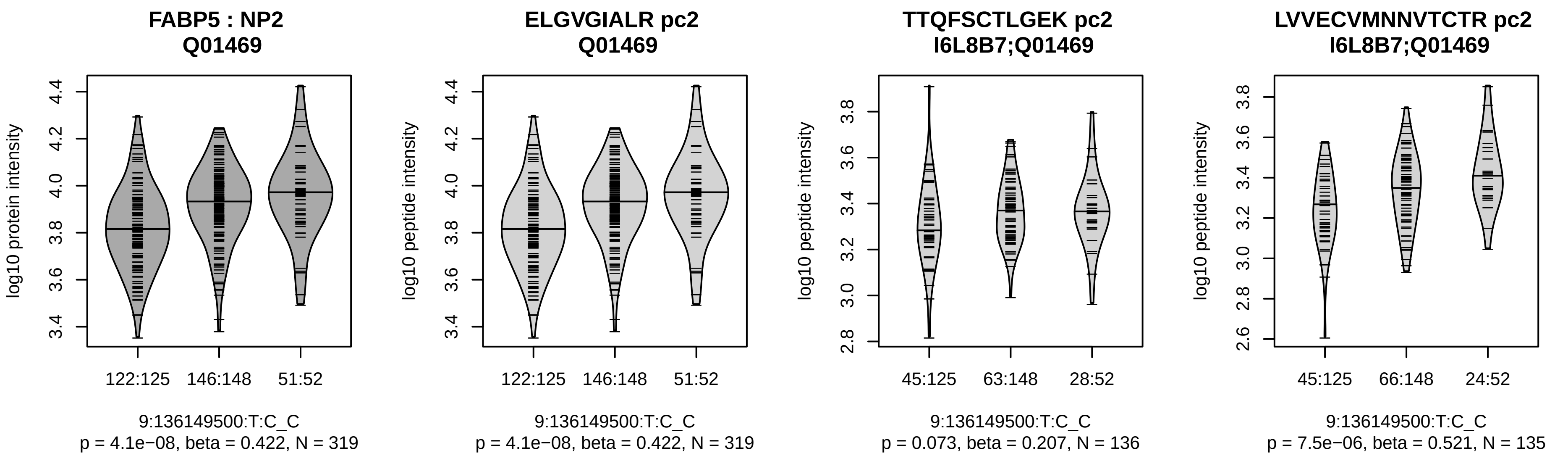

179Assay Target: FABP5
UniProt: Q01469
rsID: rs529565
Target SNP: 9:136149500:T:C
Proxy SNP: 9:136149500:T:C
Tarkin log10(p): 10.1
Tarkin BETA: 0.288
Tarkin MSPA: 0.802
::−:−:−:−:−:NA:NA
1118:578:246:36:37:39:60:4:2

180. COCH (O43405)
Tarkin MSPA=0.987

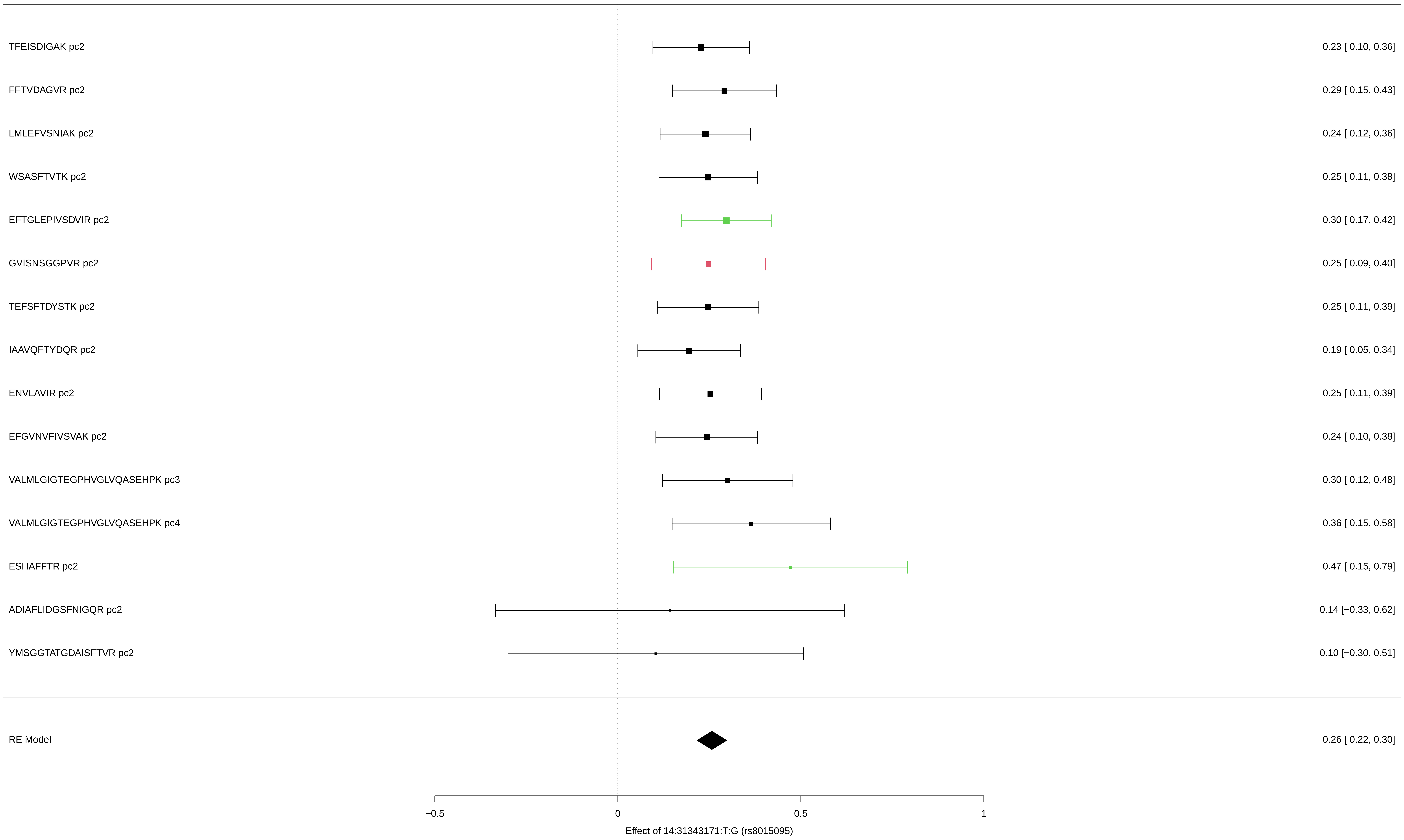

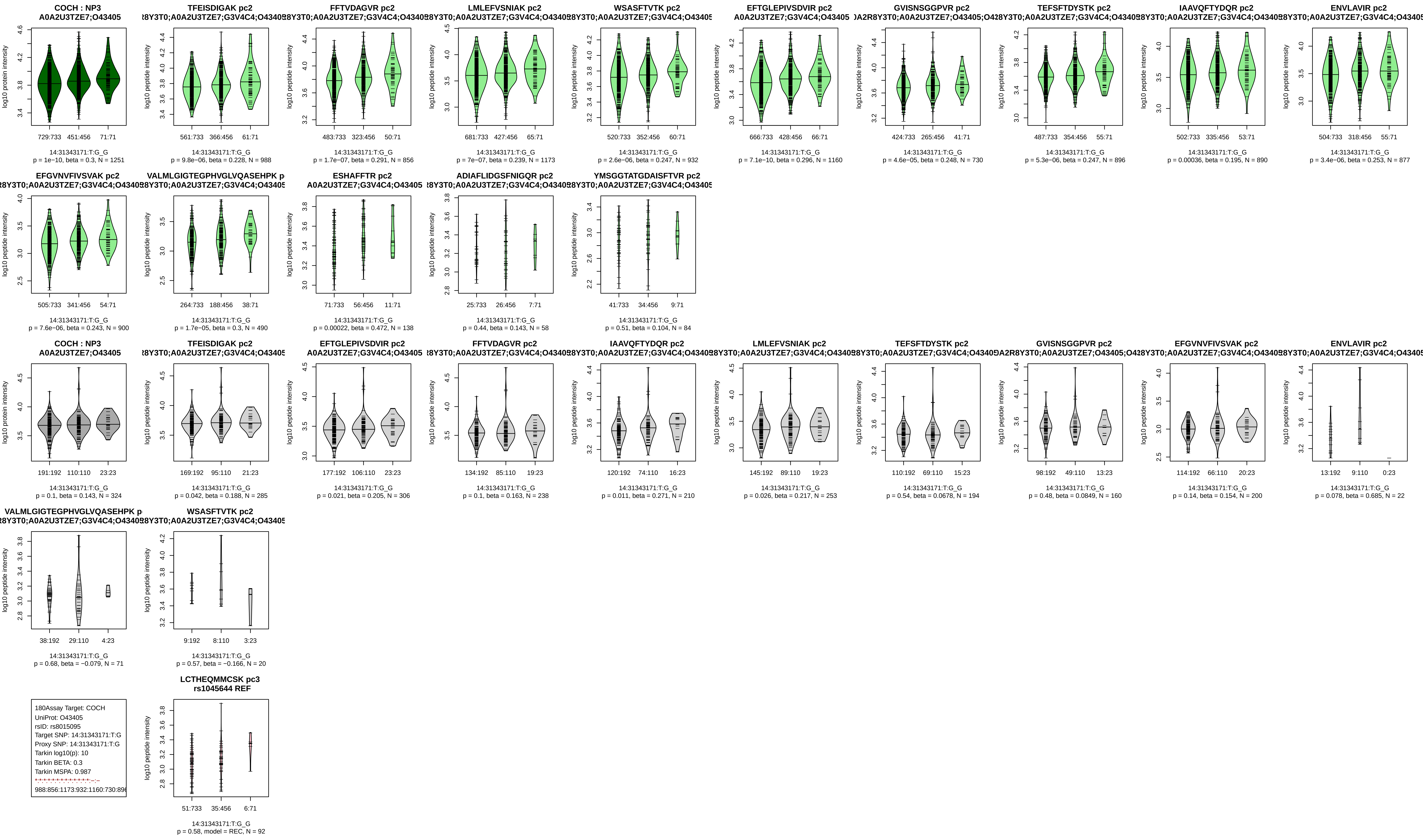
COCH : NP3
A0A2U3TZE7;O43405
729:733 451:456 71:71
14:31343171:T:G_G
p = 1e−10, beta = 0.3, N = 1251
TFEISDIGAK pc2
561:733 366:456 61:71
p = 9.8e−06, beta = 0.228, N = 988
FFTVDAGVR pc2
483:733 323:456 50:71
p = 1.7e−07, beta = 0.291, N = 856
LMLEFVSNIAK pc2
681:733 427:456 65:71
p = 7e−07, beta = 0.239, N = 1173
WSASFTVTK pc2
520:733 352:456 60:71
p = 2.6e−06, beta = 0.247, N = 932
EFTGLEPIVSDVIR pc2
A0A2U3TZE7;G3V4C4;O43405
666:733 428:456 66:71
p = 7.1e−10, beta = 0.296, N = 1160
GVISNSGGPVR pc2
424:733 265:456 41:71
p = 4.6e−05, beta = 0.248, N = 730
TEFSFTDYSTK pc2
487:733 354:456 55:71
p = 5.3e−06, beta = 0.247, N = 896
IAAVQFTYDQR pc2
502:733 335:456 53:71
p = 0.00036, beta = 0.195, N = 890
ENVLAVIR pc2
504:733 318:456 55:71
p = 3.4e−06, beta = 0.253, N = 877
EFGVNVFIVSVAK pc2
505:733 341:456 54:71
p = 7.6e−06, beta = 0.243, N = 900
VALMLGIGTEGPHVGLVQASEHPK pc2
264:733 188:456 38:71
p = 1.7e−05, beta = 0.3, N = 490
ESHAFFTR pc2
A0A2U3TZE7;G3V4C4;O43405
71:733 56:456 11:71
p = 0.00022, beta = 0.472, N = 138
ADIAFLIDGSFNIGQR pc2
25:733 26:456 7:71
p = 0.44, beta = 0.143, N = 58
YMSGGTATGDAISFTVR pc2
41:733 34:456 9:71
p = 0.51, beta = 0.104, N = 84
COCH : NP3
A0A2U3TZE7;O43405
191:192 110:110 23:23
p = 0.1, beta = 0.143, N = 324
TFEISDIGAK pc2
169:192 95:110 21:23
p = 0.042, beta = 0.188, N = 285
EFTGLEPIVSDVIR pc2
A0A2U3TZE7;G3V4C4;O43405
177:192 106:110 23:23
p = 0.021, beta = 0.205, N = 306
FFTVDAGVR pc2
134:192 85:110 19:23
p = 0.1, beta = 0.163, N = 238
IAAVQFTYDQR pc2
120:192 74:110 16:23
p = 0.011, beta = 0.271, N = 210
LMLEFVSNIAK pc2
145:192 89:110 19:23
p = 0.026, beta = 0.217, N = 253
TEFSFTDYSTK pc2
110:192 69:110 15:23
p = 0.54, beta = 0.0678, N = 194
GVISNSGGPVR pc2
98:192 49:110 13:23
p = 0.48, beta = 0.0849, N = 160
EFGVNVFIVSVAK pc2
114:192 66:110 20:23
p = 0.14, beta = 0.154, N = 200
ENVLAVIR pc2
13:192 9:110 0:23
p = 0.078, beta = 0.685, N = 22
VALMLGIGTEGPHVGLVQASEHPK p
38:192 29:110 4:23
p = 0.68, beta = −0.079, N = 71
WSASFTVTK pc2
9:192 8:110 3:23
p = 0.57, beta = −0.166, N = 20
180Assay Target: COCH
UniProt: O43405
rsID: rs8015095
Target SNP: 14:31343171:T:G
Proxy SNP: 14:31343171:T:G
Tarkin log10(p): 10
Tarkin BETA: 0.3
Tarkin MSPA: 0.987
988:856:1173:932:1160:730:89
LCTHEQMMCSK pc3
rs1045644 REF
51:733 35:456 6:71
14:31343171:T:G_G
p = 0.58, model = REC, N = 92
log10 protein intensity
log10 peptide intensity

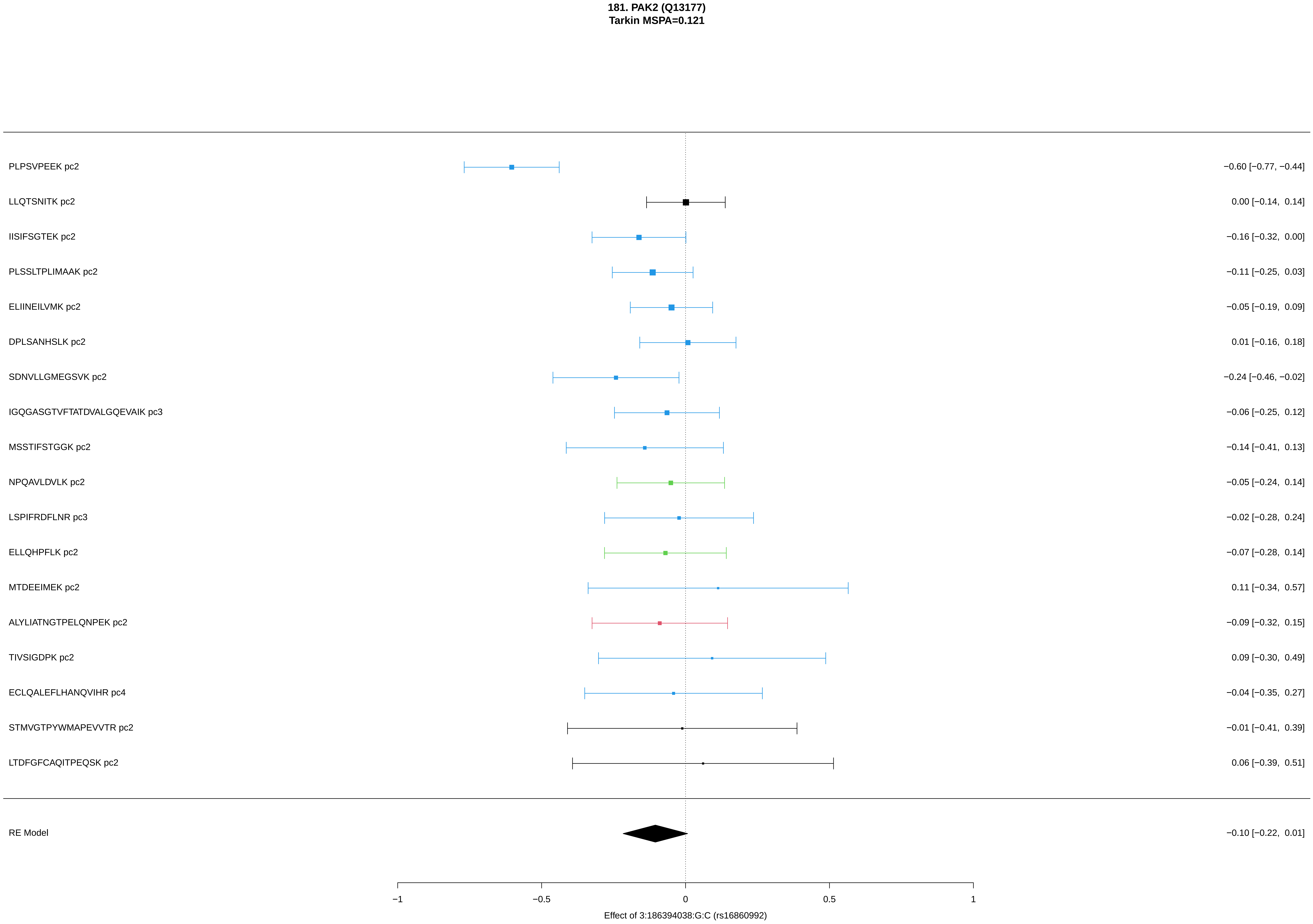
181. PAK2 (Q13177)
Tarkin MSPA=0.121
PLPSVPEEK pc2
LLQTSNITK pc2
IISIFSGTEK pc2
PLSSLTPLIMAAK pc2
ELIINEILVMK pc2
DPLSANHSLK pc2
SDNVLLGMEGSVK pc2
IGQGASGTVFTATDVALGQEVAIK pc3
MSSTIFSTGGK pc2
NPQAVLDVLK pc2
LSPIFRDFLNR pc3
ELLQHPFLK pc2
MTDEEIMEK pc2
ALYLIATNGTPELQNPEK pc2
TIVSIGDPK pc2
ECLQALEFLHANQVIHR pc4
STMVGTPYWMAPEVVTR pc2
LTDFGFCAQITPEQSK pc2
RE Model
−0.60 [−0.77, −0.44]
0.00 [−0.14, 0.14]
−0.16 [−0.32, 0.00]
−0.11 [−0.25, 0.03]
−0.05 [−0.19, 0.09]
0.01 [−0.16, 0.18]
−0.24 [−0.46, −0.02]
−0.06 [−0.25, 0.12]
−0.14 [−0.41, 0.13]
−0.05 [−0.24, 0.14]
−0.02 [−0.28, 0.24]
−0.07 [−0.28, 0.14]
0.11 [−0.34, 0.57]
−0.09 [−0.32, 0.15]
0.09 [−0.30, 0.49]
−0.04 [−0.35, 0.27]
−0.01 [−0.41, 0.39]
0.06 [−0.39, 0.51]
−0.10 [−0.22, 0.01]
−1
−0.5
0
0.5
1
Effect of 3:186394038:G:C (rs16860992)

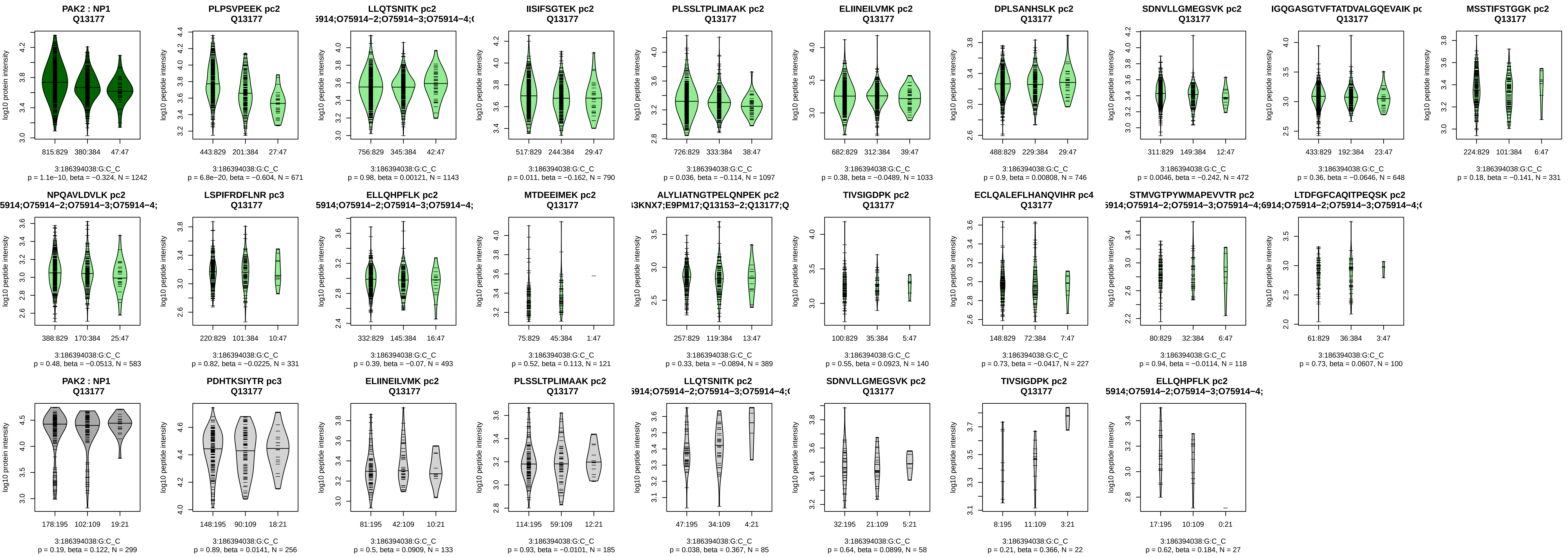

181Assay Target: PAK2
UniProt: Q13177
rsID: rs16860992
Target SNP: 3:186394038:G:C
Proxy SNP: 3:186394038:G:C
Tarkin log10(p): 10
Tarkin BETA: −0.324
Tarkin MSPA: 0.121
-:-:-:-:-:-:-:-:-:-:-:-:-:-:-:-:-
671:1143:790:1097:1033:746:4

182. PGD (P52209)
Tarkin MSPA=0.724

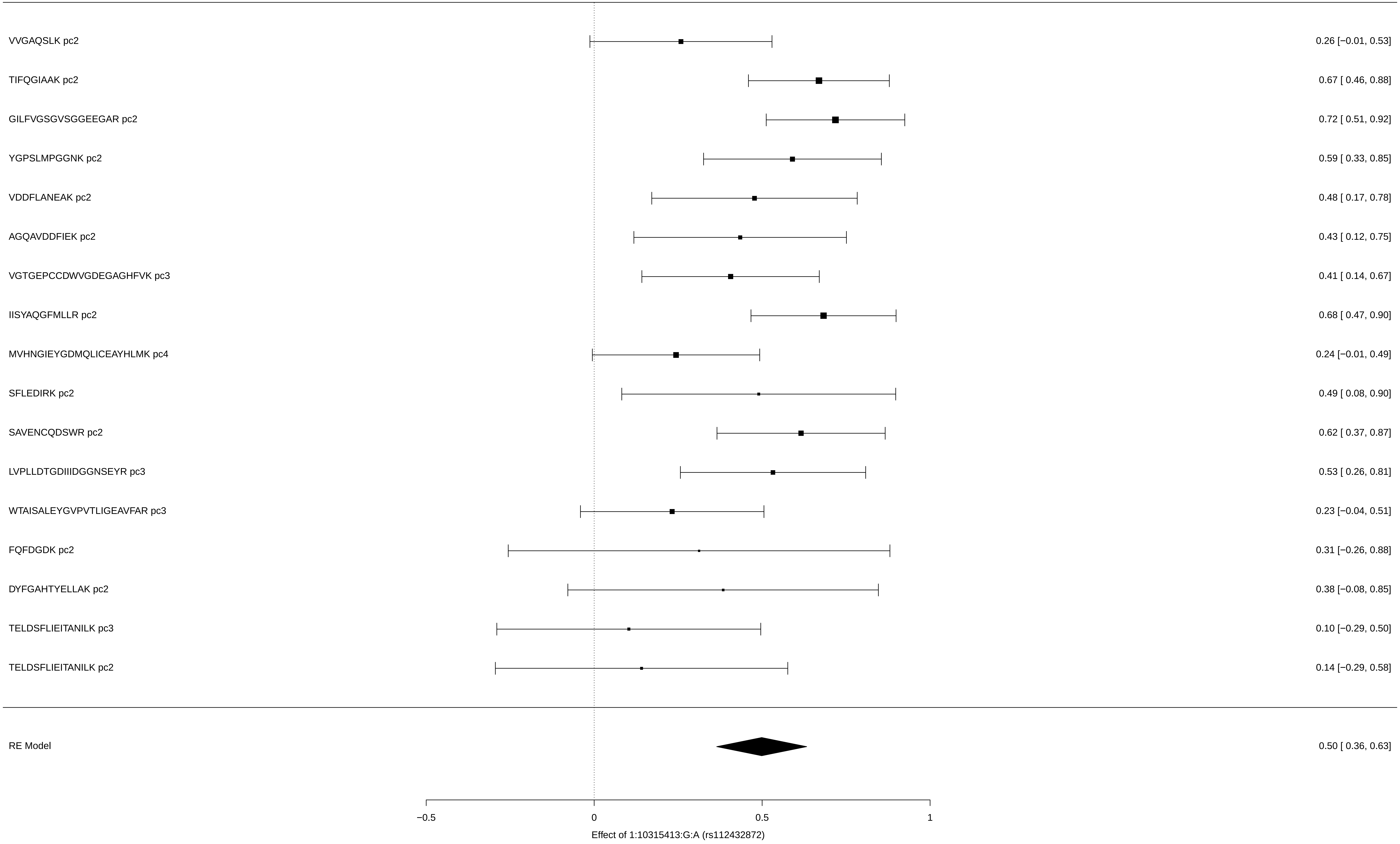

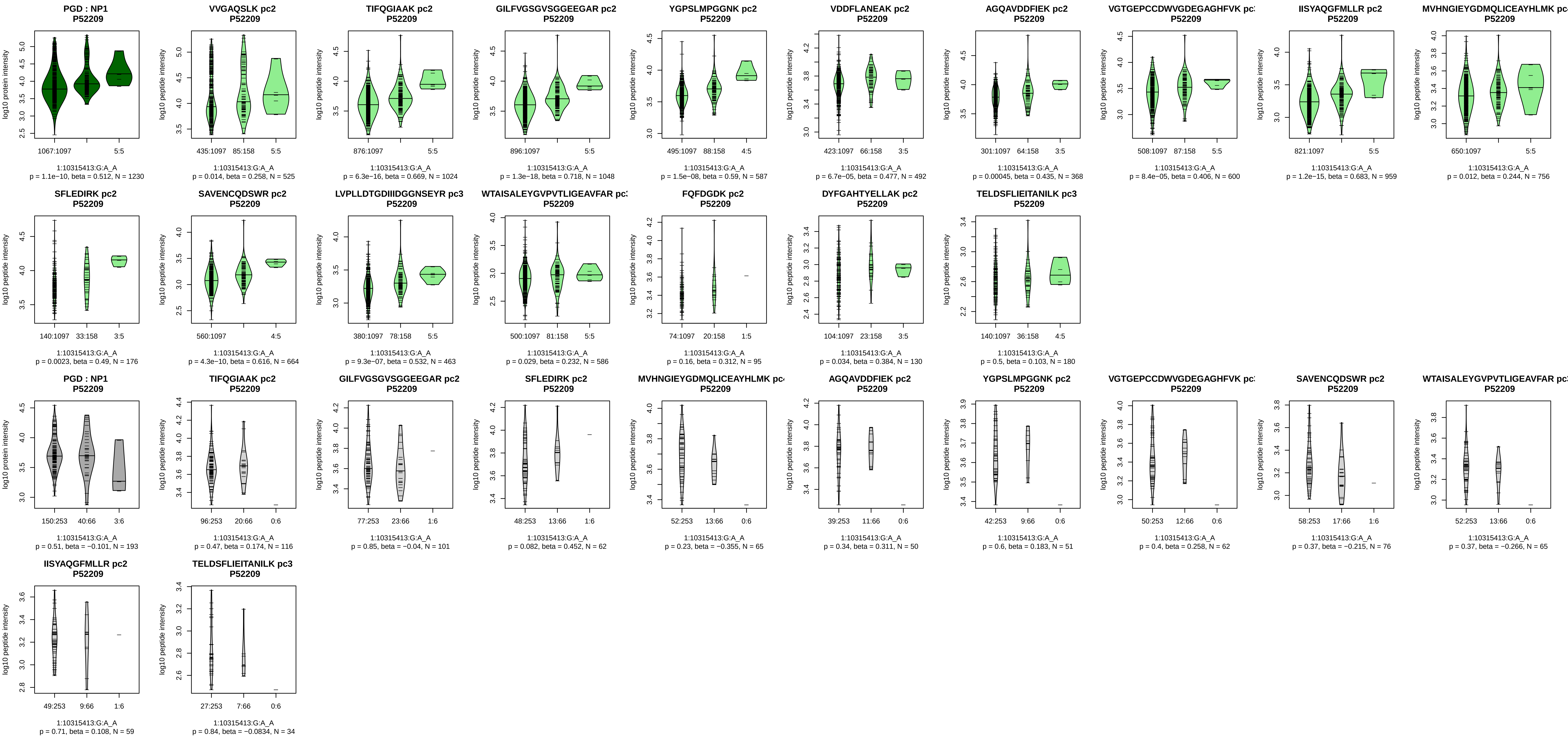

182Assay Target: PGD
UniProt: P52209
rsID: rs112432872
Target SNP: 1:10315413:G:A
Proxy SNP: 1:10315413:G:A
Tarkin log10(p): 10
Tarkin BETA: 0.512
Tarkin MSPA: 0.724
–:*:*:*:*:*:*:*:–:*:*:*:–:–:–:–:–:NA
525:1024:1048:587:492:368:600

183. SPINK5 (Q9NQ38)
Tarkin MSPA=0.447

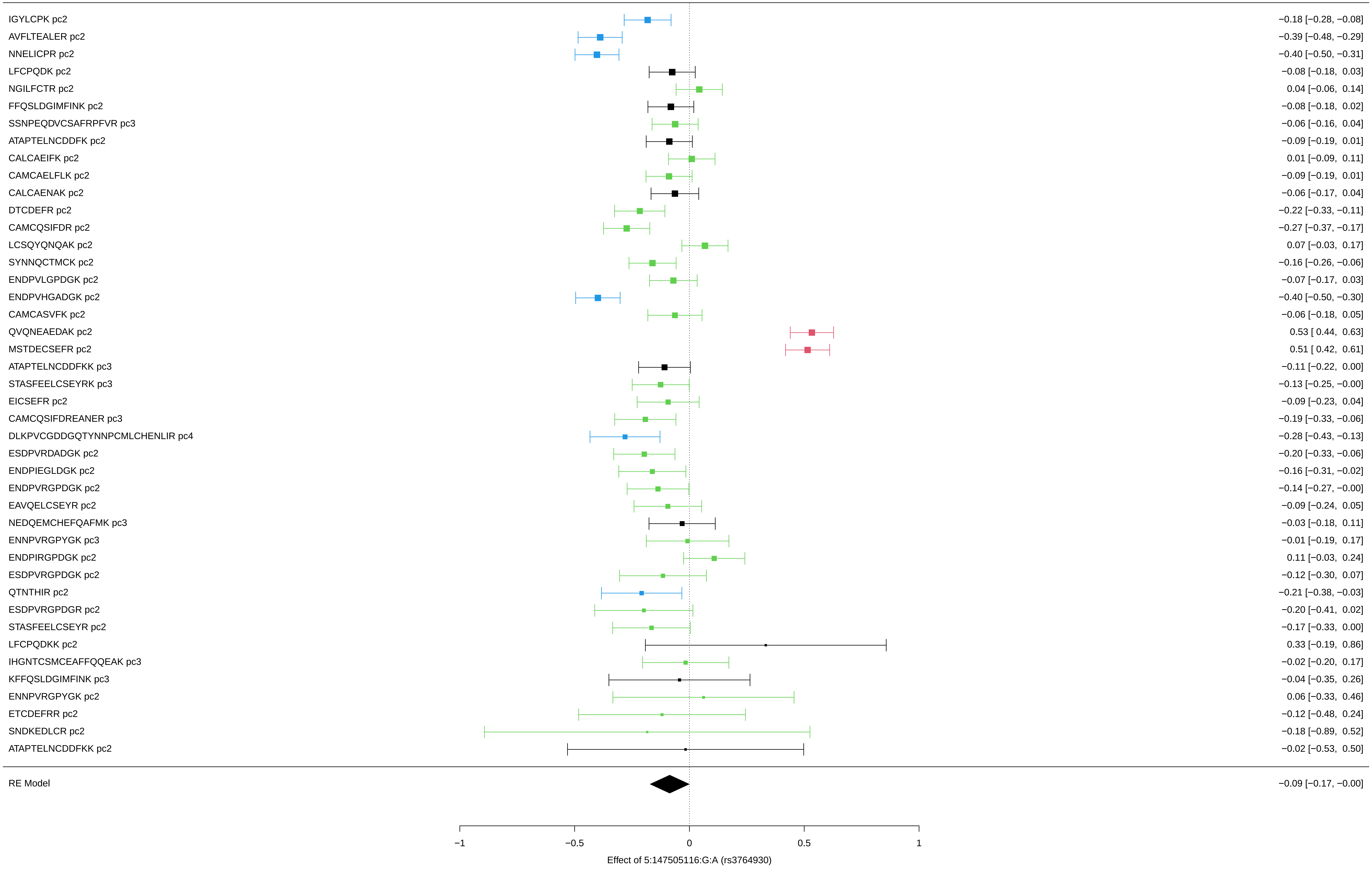

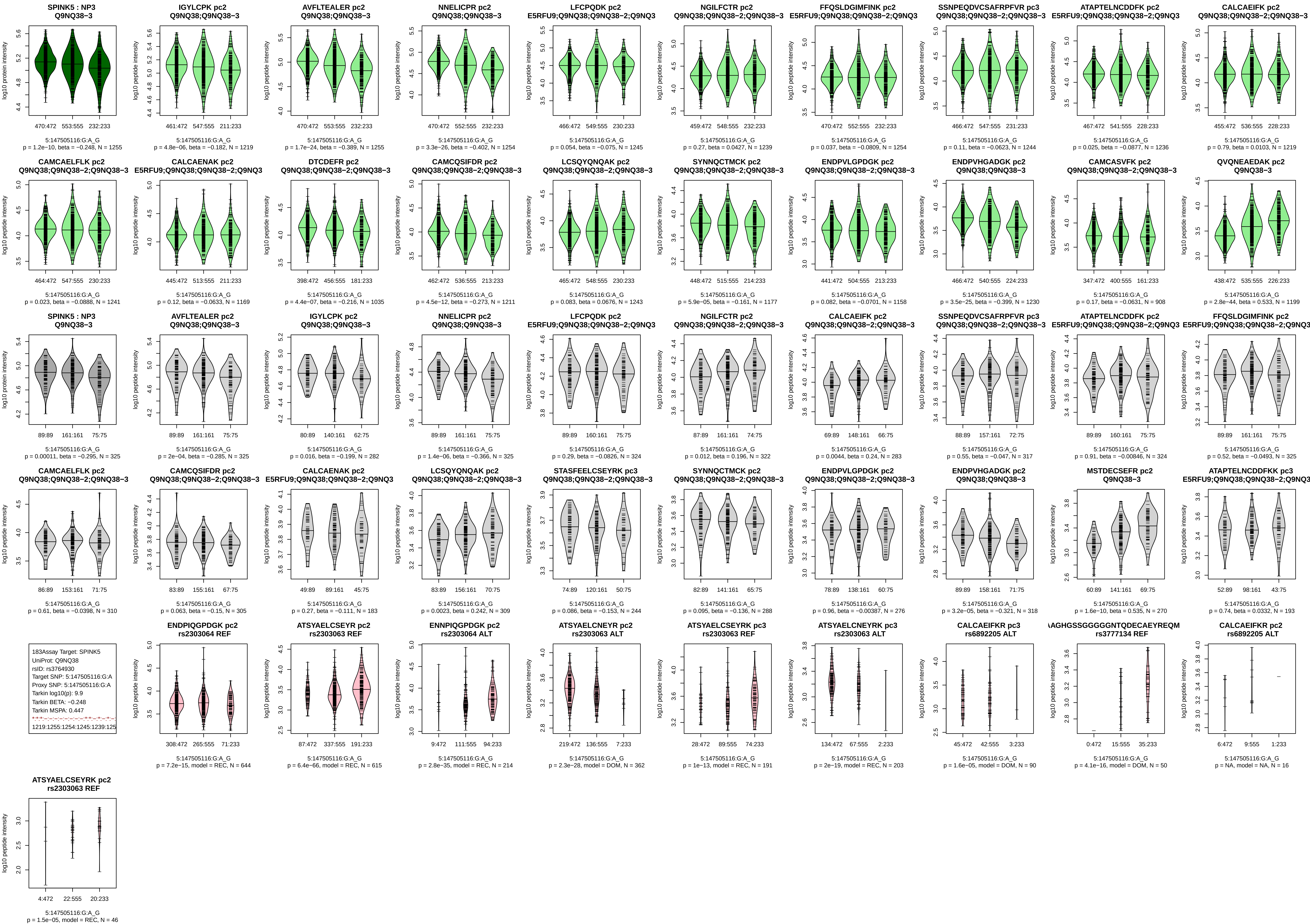
SPINK5 : NP3
Q9NQ38−3
log10 protein intensity
470:472 553:555 232:233
5:147505116:G:A_G
p = 1.2e−10, beta = −0.248, N = 1255
IGYLCPK pc2
Q9NQ38;Q9NQ38−3
461:472 547:555 211:233
5:147505116:G:A_G
p = 4.8e−06, beta = −0.182, N = 1219
AVFLTEALER pc2
Q9NQ38;Q9NQ38−3
470:472 553:555 232:233
5:147505116:G:A_G
p = 1.7e−24, beta = −0.389, N = 1255
NNELICPR pc2
Q9NQ38;Q9NQ38−3
470:472 552:555 232:233
5:147505116:G:A_G
p = 3.3e−26, beta = −0.402, N = 1254
LFCPQDK pc2
E5RFU9;Q9NQ38;Q9NQ38−2;Q9NQ3
466:472 549:555 230:233
5:147505116:G:A_G
p = 0.054, beta = −0.075, N = 1245
NGILFCTR pc2
Q9NQ38;Q9NQ38−2;Q9NQ38−3
459:472 548:555 232:233
5:147505116:G:A_G
p = 0.27, beta = 0.0427, N = 1239
FFQSLDGIMFINK pc2
E5RFU9;Q9NQ38;Q9NQ38−2;Q9NQ3
470:472 552:555 232:233
5:147505116:G:A_G
p = 0.037, beta = −0.0809, N = 1254
SSNPEQDVCSAFRPFVR pc3
Q9NQ38;Q9NQ38−2;Q9NQ38−3
466:472 547:555 231:233
5:147505116:G:A_G
p = 0.11, beta = −0.0623, N = 1244
ATAPTELNCDDFK pc2
E5RFU9;Q9NQ38;Q9NQ38−2;Q9NQ3
467:472 541:555 228:233
5:147505116:G:A_G
p = 0.025, beta = −0.0877, N = 1236
CALCAEIFK pc2
Q9NQ38;Q9NQ38−2;Q9NQ38−3
455:472 536:555 228:233
5:147505116:G:A_G
p = 0.79, beta = 0.0103, N = 1219
CAMCAELFLK pc2
Q9NQ38;Q9NQ38−2;Q9NQ38−3
464:472 547:555 230:233
5:147505116:G:A_G
p = 0.023, beta = −0.0888, N = 1241
CALCAENAK pc2
E5RFU9;Q9NQ38;Q9NQ38−2;Q9NQ3
445:472 513:555 211:233
5:147505116:G:A_G
p = 0.12, beta = −0.0633, N = 1169
DTCDEFR pc2
Q9NQ38;Q9NQ38−2;Q9NQ38−3
398:472 456:555 181:233
5:147505116:G:A_G
p = 4.4e−07, beta = −0.216, N = 1035
CAMCQSIFDR pc2
Q9NQ38;Q9NQ38−2;Q9NQ38−3
462:472 536:555 213:233
5:147505116:G:A_G
p = 4.5e−12, beta = −0.273, N = 1211
LCSQYQNQAK pc2
Q9NQ38;Q9NQ38−2;Q9NQ38−3
465:472 548:555 230:233
5:147505116:G:A_G
p = 0.083, beta = 0.0676, N = 1243
SYNNQCTMCK pc2
Q9NQ38;Q9NQ38−2;Q9NQ38−3
448:472 515:555 214:233
5:147505116:G:A_G
p = 5.9e−05, beta = −0.161, N = 1177
ENDPVLGPDGK pc2
Q9NQ38;Q9NQ38−2;Q9NQ38−3
441:472 504:555 213:233
5:147505116:G:A_G
p = 0.082, beta = −0.0701, N = 1158
ENDPVHGADGK pc2
Q9NQ38;Q9NQ38−3
466:472 540:555 224:233
5:147505116:G:A_G
p = 3.5e−25, beta = −0.399, N = 1230
CAMCASVFK pc2
Q9NQ38;Q9NQ38−2;Q9NQ38−3
347:472 400:555 161:233
5:147505116:G:A_G
p = 0.17, beta = −0.0631, N = 908
QVQNEAEDAK pc2
Q9NQ38−3
438:472 535:555 226:233
5:147505116:G:A_G
p = 2.8e−44, beta = 0.533, N = 1199
SPINK5 : NP3
Q9NQ38−3
89:89 161:161 75:75
5:147505116:G:A_G
p = 0.00011, beta = −0.295, N = 325
AVFLTEALER pc2
Q9NQ38;Q9NQ38−3
89:89 161:161 75:75
5:147505116:G:A_G
p = 2e−04, beta = −0.285, N = 325
IGYLCPK pc2
Q9NQ38;Q9NQ38−3
80:89 140:161 62:75
5:147505116:G:A_G
p = 0.016, beta = −0.199, N = 282
NNELICPR pc2
Q9NQ38;Q9NQ38−3
89:89 161:161 75:75
5:147505116:G:A_G
p = 1.4e−06, beta = −0.366, N = 325
LFCPQDK pc2
E5RFU9;Q9NQ38;Q9NQ38−2;Q9NQ3
89:89 160:161 75:75
5:147505116:G:A_G
p = 0.29, beta = −0.0826, N = 324
NGILFCTR pc2
Q9NQ38;Q9NQ38−2;Q9NQ38−3
87:89 161:161 74:75
5:147505116:G:A_G
p = 0.012, beta = 0.196, N = 322
CALCAEIFK pc2
Q9NQ38;Q9NQ38−2;Q9NQ38−3
69:89 148:161 66:75
5:147505116:G:A_G
p = 0.0044, beta = 0.24, N = 283
SSNPEQDVCSAFRPFVR pc3
Q9NQ38;Q9NQ38−2;Q9NQ38−3
88:89 157:161 72:75
5:147505116:G:A_G
p = 0.55, beta = −0.047, N = 317
ATAPTELNCDDFK pc2
E5RFU9;Q9NQ38;Q9NQ38−2;Q9NQ3
89:89 160:161 75:75
5:147505116:G:A_G
p = 0.91, beta = −0.00846, N = 324
FFQSLDGIMFINK pc2
E5RFU9;Q9NQ38;Q9NQ38−2;Q9NQ3
89:89 161:161 75:75
5:147505116:G:A_G
p = 0.52, beta = −0.0493, N = 325
CAMCAELFLK pc2
Q9NQ38;Q9NQ38−2;Q9NQ38−3
86:89 153:161 71:75
5:147505116:G:A_G
p = 0.61, beta = −0.0398, N = 310
CAMCQSIFDR pc2
Q9NQ38;Q9NQ38−2;Q9NQ38−3
83:89 155:161 67:75
5:147505116:G:A_G
p = 0.063, beta = −0.15, N = 305
CALCAENAK pc2
E5RFU9;Q9NQ38;Q9NQ38−2;Q9NQ3
49:89 89:161 45:75
5:147505116:G:A_G
p = 0.27, beta = −0.111, N = 183
LCSQYQNQAK pc2
Q9NQ38;Q9NQ38−2;Q9NQ38−3
83:89 156:161 70:75
5:147505116:G:A_G
p = 0.0023, beta = 0.242, N = 309
STASFEELCSEYRK pc3
Q9NQ38;Q9NQ38−2;Q9NQ38−3
74:89 120:161 50:75
5:147505116:G:A_G
p = 0.086, beta = −0.153, N = 244
SYNNQCTMCK pc2
Q9NQ38;Q9NQ38−2;Q9NQ38−3
82:89 141:161 65:75
5:147505116:G:A_G
p = 0.095, beta = −0.136, N = 288
ENDPVLGPDGK pc2
Q9NQ38;Q9NQ38−2;Q9NQ38−3
78:89 138:161 60:75
5:147505116:G:A_G
p = 0.96, beta = −0.00387, N = 276
ENDPVHGADGK pc2
Q9NQ38;Q9NQ38−3
89:89 158:161 71:75
5:147505116:G:A_G
p = 3.2e−05, beta = −0.321, N = 318
MSTDECSEFR pc2
Q9NQ38−3
60:89 141:161 69:75
5:147505116:G:A_G
p = 1.6e−10, beta = 0.535, N = 270
ATAPTELNCDDFKK pc3
E5RFU9;Q9NQ38;Q9NQ38−2;Q9NQ3
52:89 98:161 43:75
5:147505116:G:A_G
p = 0.74, beta = 0.0332, N = 193
183Assay Target: SPINK5
UniProt: Q9NQ38
rsID: rs3764930
Target SNP: 5:147505116:G:A
Proxy SNP: 5:147505116:G:A
Tarkin log10(p): 9.9
Tarkin BETA: −0.248
Tarkin MSPA: 0.447
1219:1255:1254:1245:1239:125
ENDPIQGPDGK pc2
rs2303064 REF
308:472 265:555 71:233
5:147505116:G:A_G
p = 7.2e−15, model = REC, N = 644
ATSYAELCSEYR pc2
rs2303063 REF
87:472 337:555 191:233
5:147505116:G:A_G
p = 6.4e−66, model = REC, N = 615
ENNPIQGPDGK pc2
rs2303064 ALT
9:472 111:555 94:233
5:147505116:G:A_G
p = 2.8e−35, model = REC, N = 214
ATSYAELCNEYR pc2
rs2303063 ALT
219:472 136:555 7:233
5:147505116:G:A_G
p = 2.3e−28, model = DOM, N = 362
ATSYAELCSEYRK pc3
rs2303063 REF
28:472 89:555 74:233
5:147505116:G:A_G
p = 1e−13, model = REC, N = 191
ATSYAELCNEYRK pc3
rs2303063 ALT
134:472 67:555 2:233
5:147505116:G:A_G
p = 2e−19, model = REC, N = 203
CALCAEIFKR pc3
rs6892205 ALT
45:472 42:555 3:233
5:147505116:G:A_G
p = 1.6e−05, model = DOM, N = 90
AAGHGSSGGGGGNTQDECAEYREQM
rs3777134 REF
0:472 15:555 35:233
5:147505116:G:A_G
p = 4.1e−16, model = DOM, N = 50
CALCAEIFKR pc2
rs6892205 ALT
6:472 9:555 1:233
5:147505116:G:A_G
p = NA, model = NA, N = 16
ATSYAELCSEYRK pc2
rs2303063 REF
4:472 22:555 20:233
5:147505116:G:A_G
p = 1.5e−05, model = REC, N = 46
log10 peptide intensity

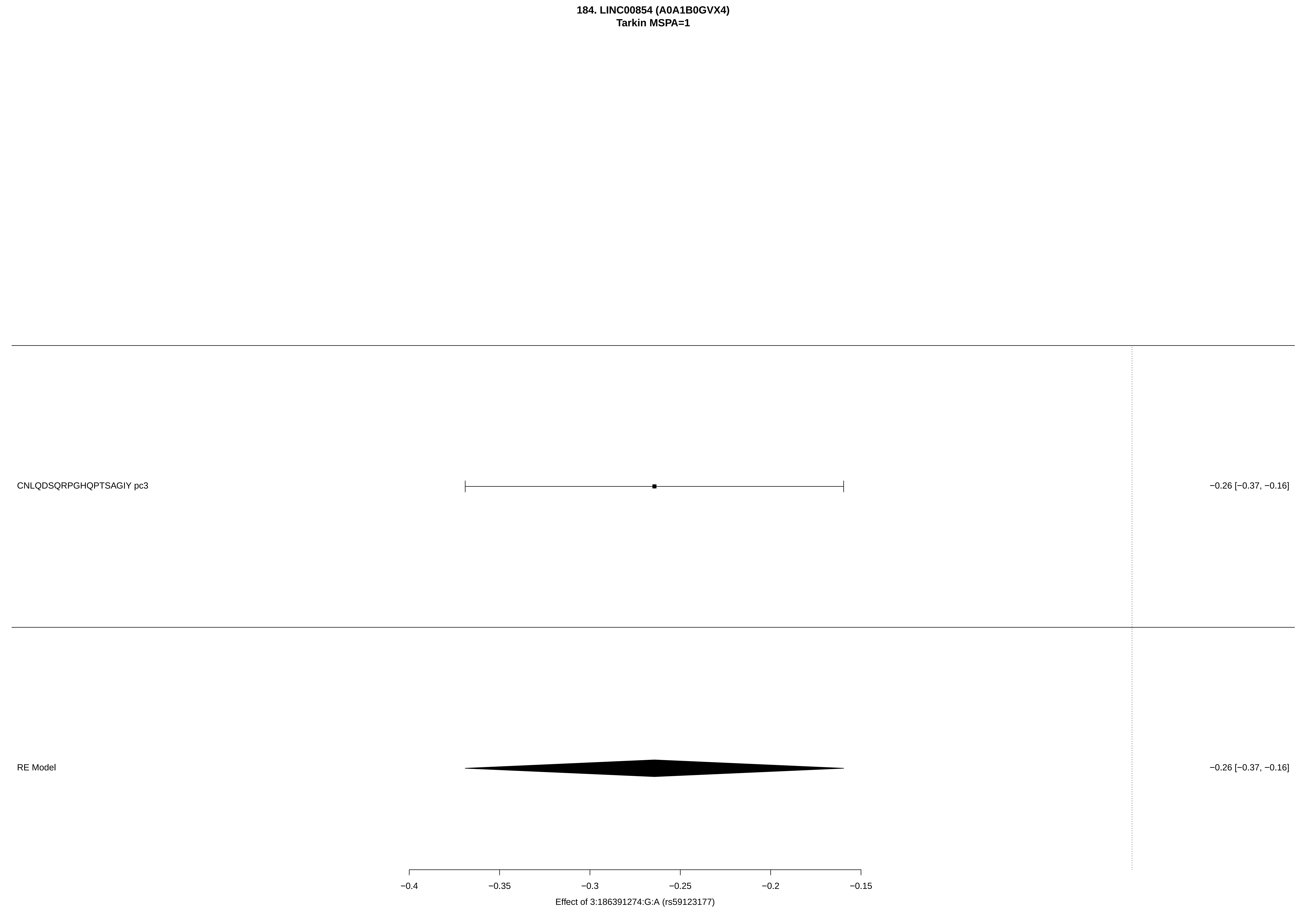
184. LINC00854 (A0A1B0GVX4)
Tarkin MSPA=1
CNLQDSQRPGHQPTSAGIY pc3
−0.26 [−0.37, −0.16]
RE Model
−0.26 [−0.37, −0.16]
−0.4
−0.35
−0.3
−0.25
−0.2
−0.15
Effect of 3:186391274:G:A (rs59123177)

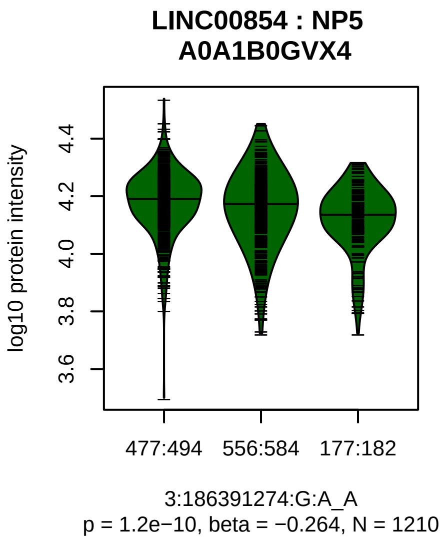

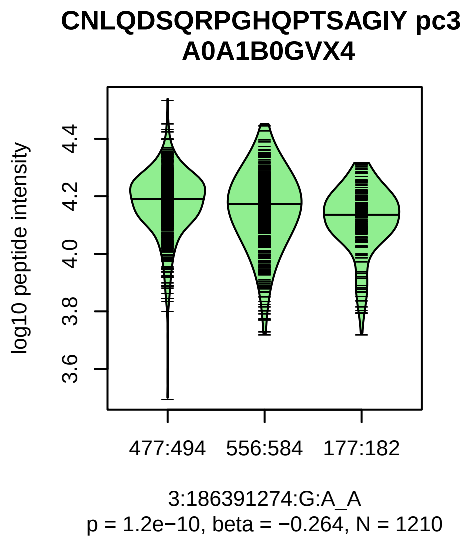

184Assay Target: LINC00854
UniProt: A0A1B0GVX4
rsID: rs59123177
Target SNP: 3:186391274:G:A
Proxy SNP: 3:186391274:G:A
Tarkin log10(p): 9.9
Tarkin BETA: −0.264
Tarkin MSPA: 1
*
1210

185. QSOX1 (O00391)
Tarkin MSPA=0.722

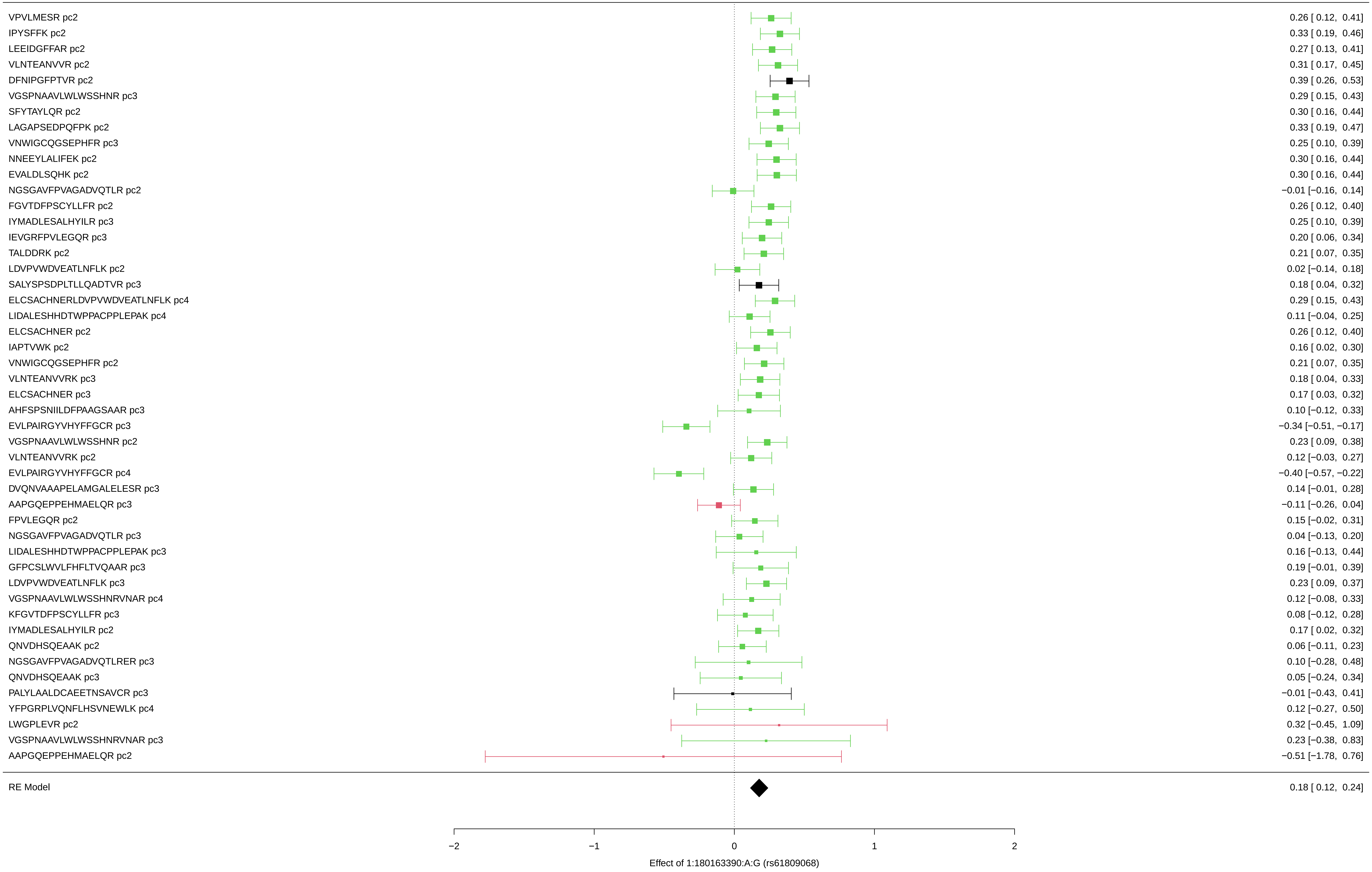

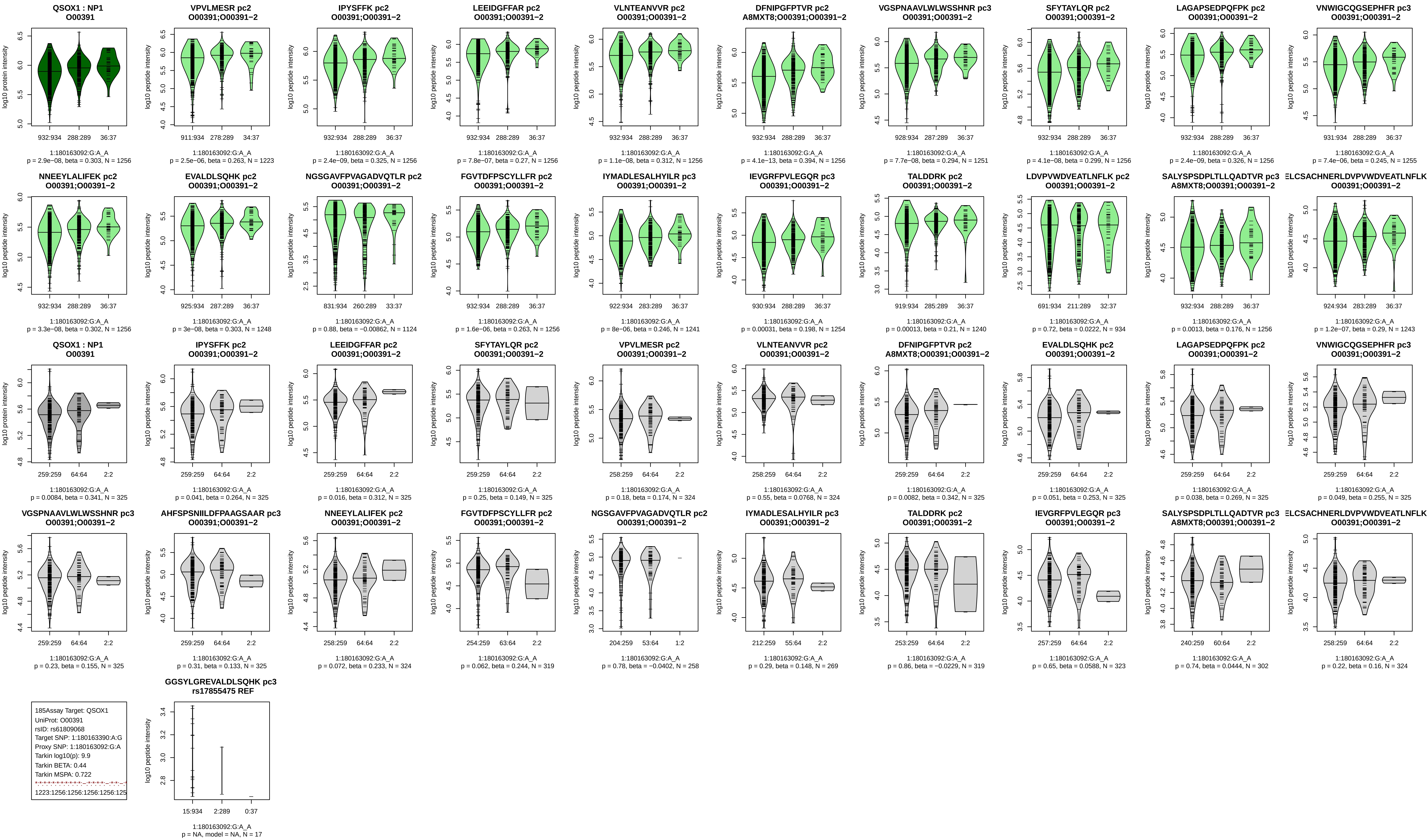

QSOX1 : NP1
O00391
log10 protein intensity
932:934 288:289 36:37
1:180163092:G:A_A
p = 2.9e−08, beta = 0.303, N = 1256
VPVLMESR pc2
O00391;O00391−2
log10 peptide intensity
911:934 278:289 34:37
1:180163092:G:A_A
p = 2.5e−06, beta = 0.263, N = 1223
IPYSFFK pc2
O00391;O00391−2
932:934 288:289 36:37
1:180163092:G:A_A
p = 2.4e−09, beta = 0.325, N = 1256
LEEIDGFFAR pc2
O00391;O00391−2
932:934 288:289 36:37
1:180163092:G:A_A
p = 7.8e−07, beta = 0.27, N = 1256
VLNTEANVVR pc2
O00391;O00391−2
932:934 288:289 36:37
1:180163092:G:A_A
p = 1.1e−08, beta = 0.312, N = 1256
DFNIPGFPTVR pc2
A8MXT8;O00391;O00391−2
932:934 288:289 36:37
1:180163092:G:A_A
p = 4.1e−13, beta = 0.394, N = 1256
VGSPNAAVLWLWSSHNR pc3
O00391;O00391−2
928:934 287:289 36:37
1:180163092:G:A_A
p = 7.7e−08, beta = 0.294, N = 1251
SFYTAYLQR pc2
O00391;O00391−2
932:934 288:289 36:37
1:180163092:G:A_A
p = 4.1e−08, beta = 0.299, N = 1256
LAGAPSEDPQFPK pc2
O00391;O00391−2
932:934 288:289 36:37
1:180163092:G:A_A
p = 2.4e−09, beta = 0.326, N = 1256
VNWIGCQGSEPHFR pc3
O00391;O00391−2
931:934 288:289 36:37
1:180163092:G:A_A
p = 7.4e−06, beta = 0.245, N = 1255
NNEEYLALIFEK pc2
O00391;O00391−2
932:934 288:289 36:37
1:180163092:G:A_A
p = 3.3e−08, beta = 0.302, N = 1256
EVALDLSQHK pc2
O00391;O00391−2
925:934 287:289 36:37
1:180163092:G:A_A
p = 3e−08, beta = 0.303, N = 1248
NGSGAVFPVAGADVQTLR pc2
O00391;O00391−2
831:934 260:289 33:37
1:180163092:G:A_A
p = 0.88, beta = −0.00862, N = 1124
FGVTDFPSCYLLFR pc2
O00391;O00391−2
932:934 288:289 36:37
1:180163092:G:A_A
p = 1.6e−06, beta = 0.263, N = 1256
IYMADLESALHYILR pc3
O00391;O00391−2
922:934 283:289 36:37
1:180163092:G:A_A
p = 8e−06, beta = 0.246, N = 1241
IEVGRFPVLEGQR pc3
O00391;O00391−2
930:934 288:289 36:37
1:180163092:G:A_A
p = 0.00031, beta = 0.198, N = 1254
TALDDRK pc2
O00391;O00391−2
919:934 285:289 36:37
1:180163092:G:A_A
p = 0.00013, beta = 0.21, N = 1240
LDVPVWDVEATLNFLK pc2
O00391;O00391−2
691:934 211:289 32:37
1:180163092:G:A_A
p = 0.72, beta = 0.0222, N = 934
SALYSPSDPLTLLQADTVR pc3
A8MXT8;O00391;O00391−2
932:934 288:289 36:37
1:180163092:G:A_A
p = 0.0013, beta = 0.176, N = 1256
ELCSACHNERLDVPVWDVEATLNFLK
O00391;O00391−2
924:934 283:289 36:37
1:180163092:G:A_A
p = 1.2e−07, beta = 0.29, N = 1243
QSOX1 : NP1
O00391
259:259 64:64 2:2
1:180163092:G:A_A
p = 0.0084, beta = 0.341, N = 325
IPYSFFK pc2
O00391;O00391−2
259:259 64:64 2:2
1:180163092:G:A_A
p = 0.041, beta = 0.264, N = 325
LEEIDGFFAR pc2
O00391;O00391−2
259:259 64:64 2:2
1:180163092:G:A_A
p = 0.016, beta = 0.312, N = 325
SFYTAYLQR pc2
O00391;O00391−2
259:259 64:64 2:2
1:180163092:G:A_A
p = 0.25, beta = 0.149, N = 325
VPVLMESR pc2
O00391;O00391−2
258:259 64:64 2:2
1:180163092:G:A_A
p = 0.18, beta = 0.174, N = 324
VLNTEANVVR pc2
O00391;O00391−2
258:259 64:64 2:2
1:180163092:G:A_A
p = 0.55, beta = 0.0768, N = 324
DFNIPGFPTVR pc2
A8MXT8;O00391;O00391−2
259:259 64:64 2:2
1:180163092:G:A_A
p = 0.0082, beta = 0.342, N = 325
EVALDLSQHK pc2
O00391;O00391−2
259:259 64:64 2:2
1:180163092:G:A_A
p = 0.051, beta = 0.253, N = 325
LAGAPSEDPQFPK pc2
O00391;O00391−2
259:259 64:64 2:2
1:180163092:G:A_A
p = 0.038, beta = 0.269, N = 325
VNWIGCQGSEPHFR pc3
O00391;O00391−2
259:259 64:64 2:2
1:180163092:G:A_A
p = 0.049, beta = 0.255, N = 325
VGSPNAAVLWLWSSHNR pc3
O00391;O00391−2
259:259 64:64 2:2
1:180163092:G:A_A
p = 0.23, beta = 0.155, N = 325
AHFSPSNIILDFPAAGSAAR pc3
O00391;O00391−2
259:259 64:64 2:2
1:180163092:G:A_A
p = 0.31, beta = 0.133, N = 325
NNEEYLALIFEK pc2
O00391;O00391−2
258:259 64:64 2:2
1:180163092:G:A_A
p = 0.072, beta = 0.233, N = 324
FGVTDFPSCYLLFR pc2
O00391;O00391−2
254:259 63:64 2:2
1:180163092:G:A_A
p = 0.062, beta = 0.244, N = 319
NGSGAVFPVAGADVQTLR pc2
O00391;O00391−2
204:259 53:64 1:2
1:180163092:G:A_A
p = 0.78, beta = −0.0402, N = 258
IYMADLESALHYILR pc3
O00391;O00391−2
212:259 55:64 2:2
1:180163092:G:A_A
p = 0.29, beta = 0.148, N = 269
TALDDRK pc2
O00391;O00391−2
253:259 64:64 2:2
1:180163092:G:A_A
p = 0.86, beta = −0.0229, N = 319
IEVGRFPVLEGQR pc3
O00391;O00391−2
257:259 64:64 2:2
1:180163092:G:A_A
p = 0.65, beta = 0.0588, N = 323
SALYSPSDPLTLLQADTVR pc3
A8MXT8;O00391;O00391−2
240:259 60:64 2:2
1:180163092:G:A_A
p = 0.74, beta = 0.0444, N = 302
ELCSACHNERLDVPVWDVEATLNFLK
O00391;O00391−2
258:259 64:64 2:2
1:180163092:G:A_A
p = 0.22, beta = 0.16, N = 324
185Assay Target: QSOX1
UniProt: O00391
rsID: rs61809068
Target SNP: 1:180163390:A:G
Proxy SNP: 1:180163092:G:A
Tarkin log10(p): 9.9
Tarkin BETA: 0.44
Tarkin MSPA: 0.722
1223:1256:1256:1256:1256:125
GGSYLGREVALDLSQHK pc3
rs17855475 REF
15:934 2:289 0:37
1:180163092:G:A_A
p = NA, model = NA, N = 17

186. IGKV1−17 (P01599)
Tarkin MSPA=1

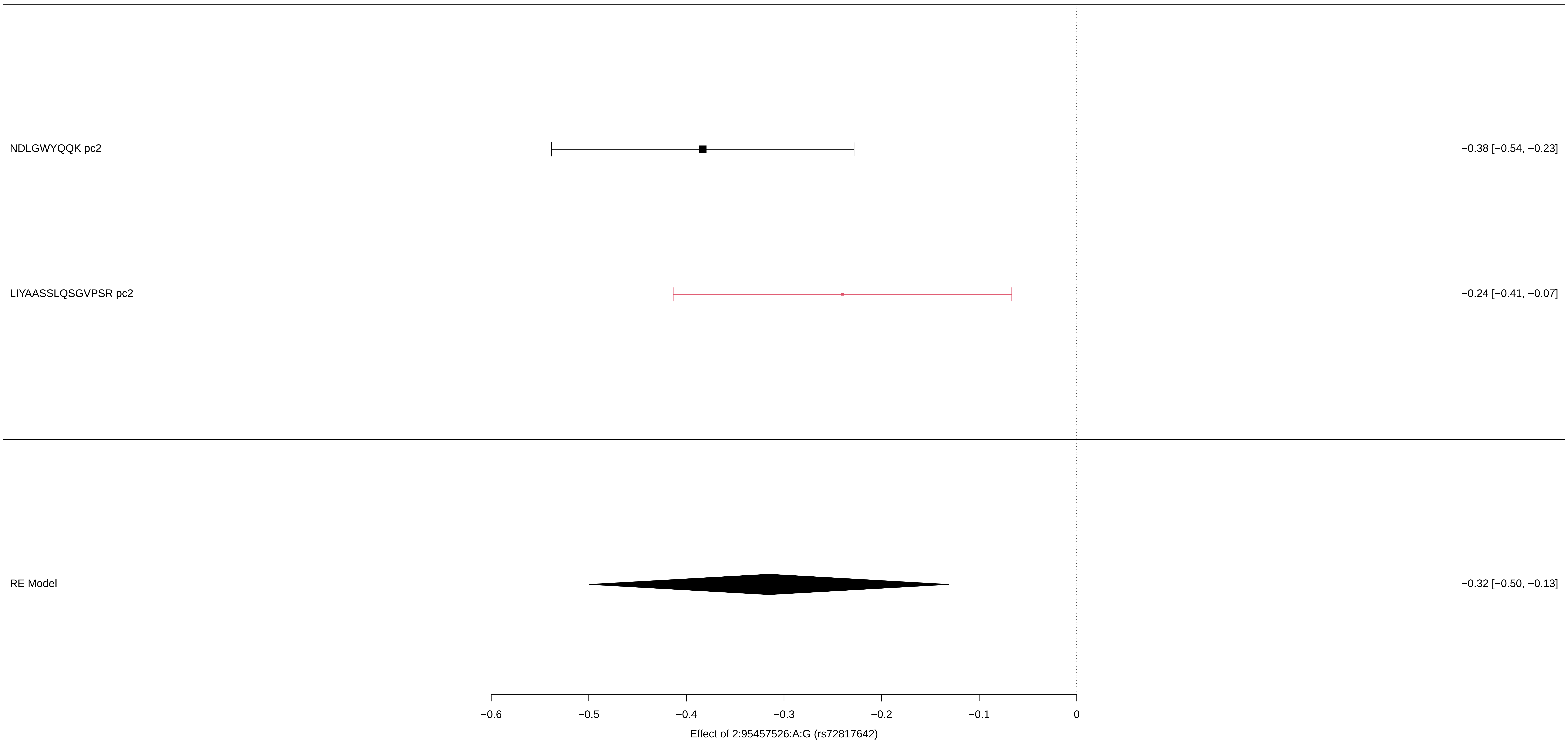

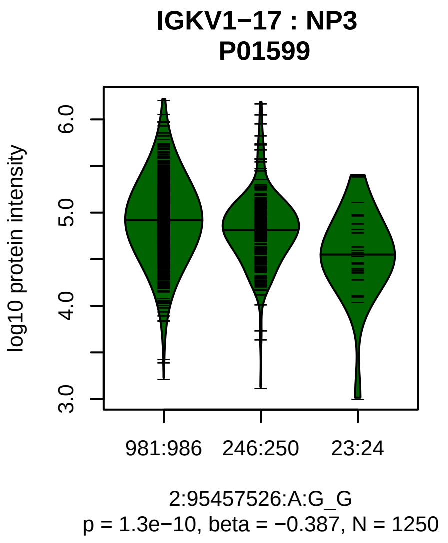

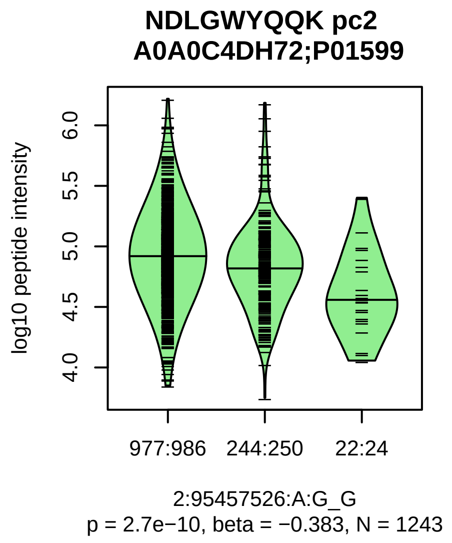

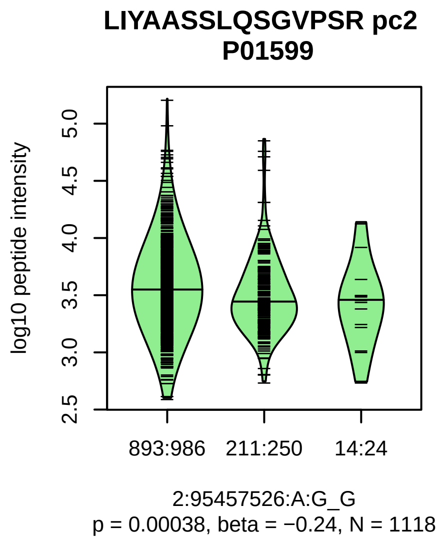

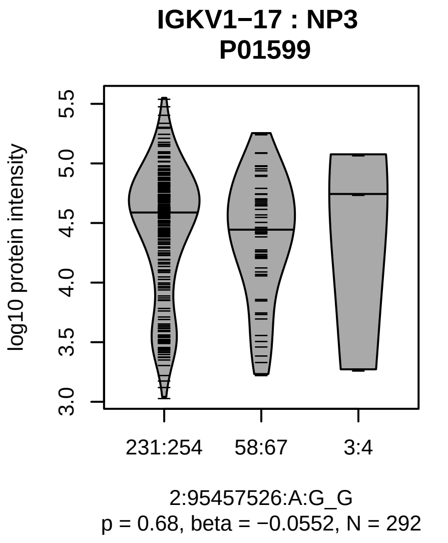

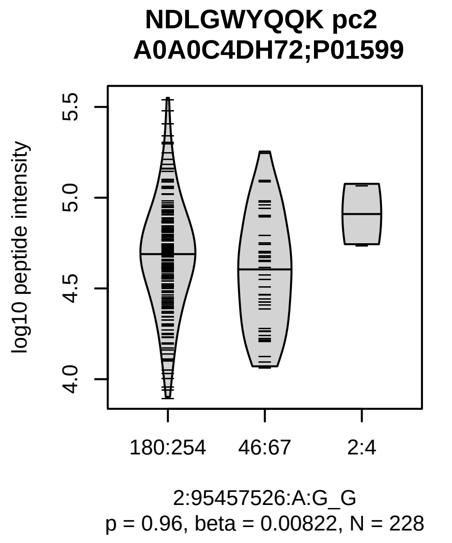

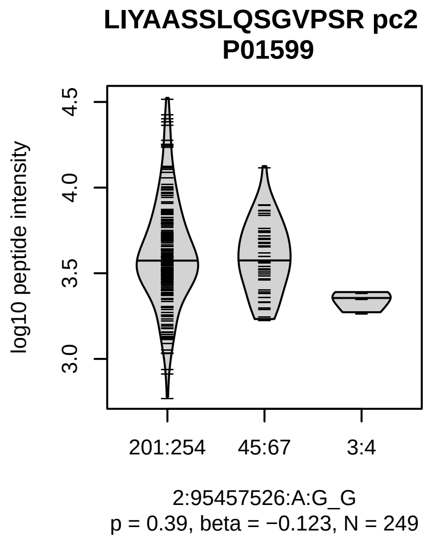

186Assay Target: IGKV1−17
UniProt: P01599
rsID: rs72817642
Target SNP: 2:95457526:A:G
Proxy SNP: 2:95457526:A:G
Tarkin log10(p): 9.9
Tarkin BETA: −0.387
Tarkin MSPA: 1
::NA:NA
1243:1118:4:2

187. PGM3 (H0Y987)
Tarkin MSPA=0.806

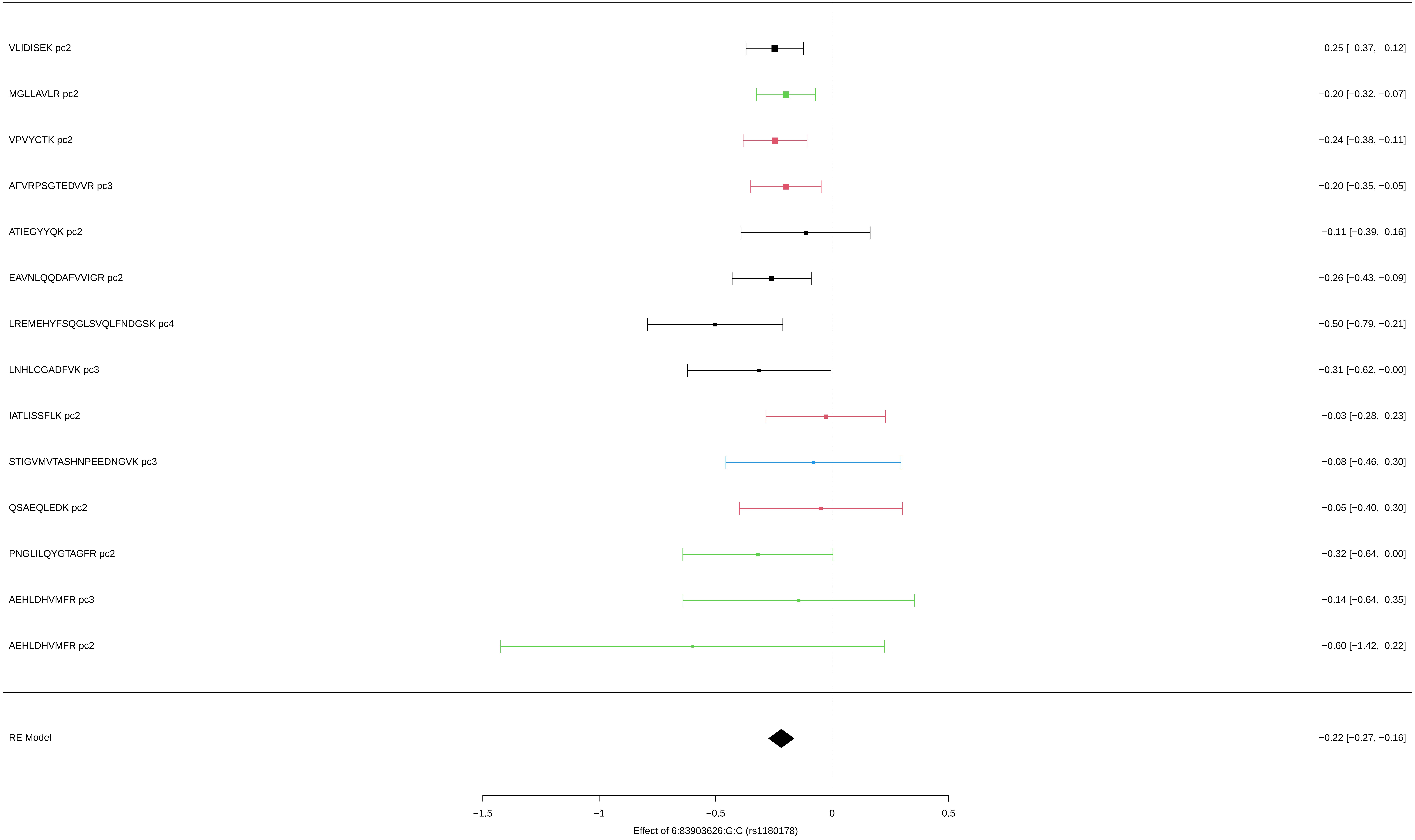

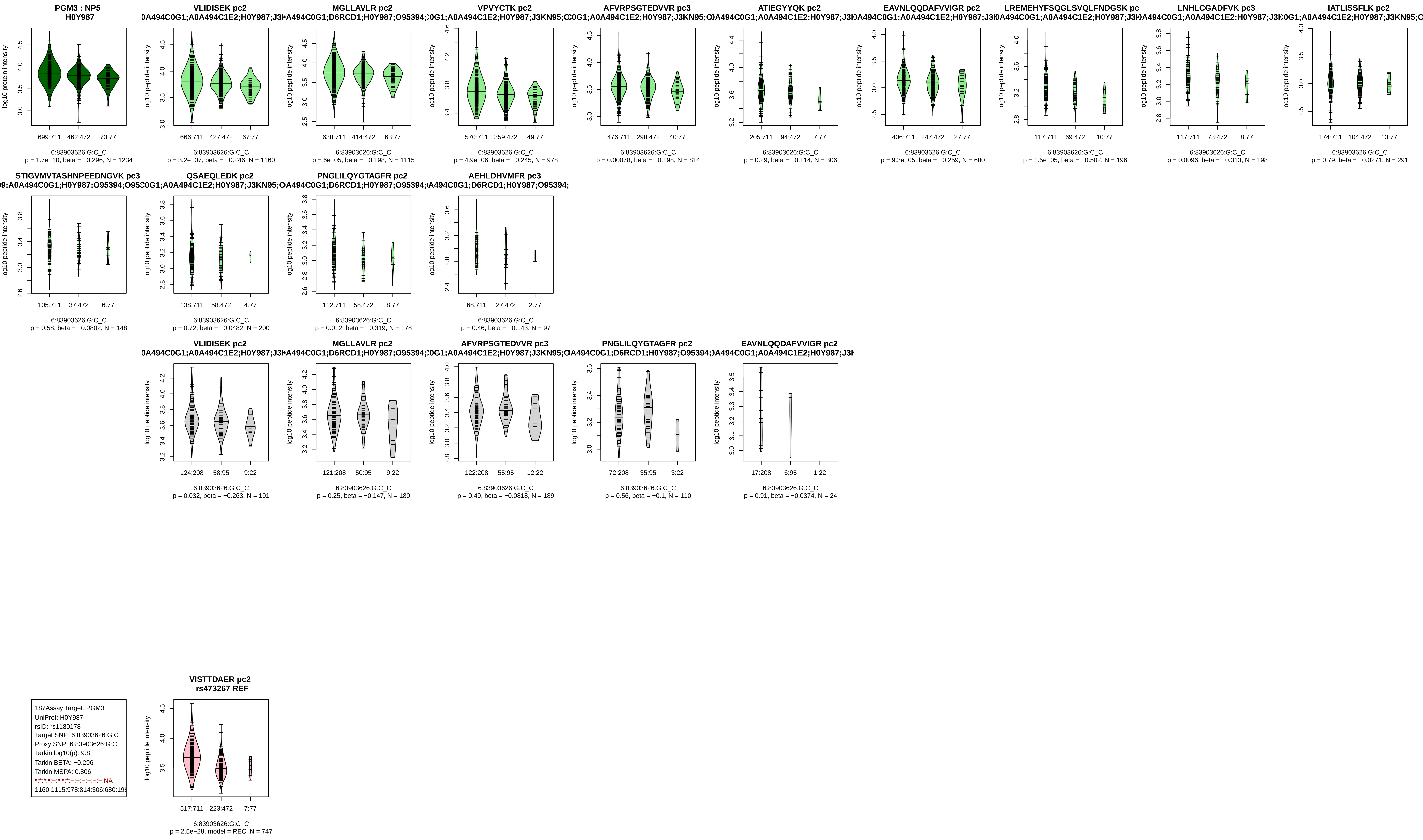
PGM3 : NP5
H0Y987
log10 protein intensity
699:711 462:472 73:77
6:83903626:G:C_C
p = 1.7e−10, beta = −0.296, N = 1234
VLIDISEK pc2
log10 peptide intensity
666:711 427:472 67:77
6:83903626:G:C_C
p = 3.2e−07, beta = −0.246, N = 1160
MGLLAVLR pc2
638:711 414:472 63:77
6:83903626:G:C_C
p = 6e−05, beta = −0.198, N = 1115
VPVYCTK pc2
570:711 359:472 49:77
6:83903626:G:C_C
p = 4.9e−06, beta = −0.245, N = 978
AFVRPSGTEDVVR pc3
476:711 298:472 40:77
6:83903626:G:C_C
p = 0.00078, beta = −0.198, N = 814
ATIEGYYQK pc2
205:711 94:472 7:77
6:83903626:G:C_C
p = 0.29, beta = −0.114, N = 306
EAVNLQQDAFVVIGR pc2
406:711 247:472 27:77
6:83903626:G:C_C
p = 9.3e−05, beta = −0.259, N = 680
LREMEHYFSQGLSVQLFNDGSK pc
117:711 69:472 10:77
6:83903626:G:C_C
p = 1.5e−05, beta = −0.502, N = 196
LNHLCGADFVK pc3
117:711 73:472 8:77
6:83903626:G:C_C
p = 0.0096, beta = −0.313, N = 198
IATLISSFLK pc2
174:711 104:472 13:77
6:83903626:G:C_C
p = 0.79, beta = −0.0271, N = 291
STIGVMVTASHNPEEDNGVK pc3
105:711 37:472 6:77
6:83903626:G:C_C
p = 0.58, beta = −0.0802, N = 148
QSAEQLEDK pc2
138:711 58:472 4:77
6:83903626:G:C_C
p = 0.72, beta = −0.0482, N = 200
PNGLILQYGTAGFR pc2
112:711 58:472 8:77
6:83903626:G:C_C
p = 0.012, beta = −0.319, N = 178
AEHLDHVMFR pc3
68:711 27:472 2:77
6:83903626:G:C_C
p = 0.46, beta = −0.143, N = 97
VLIDISEK pc2
124:208 58:95 9:22
6:83903626:G:C_C
p = 0.032, beta = −0.263, N = 191
MGLLAVLR pc2
121:208 50:95 9:22
6:83903626:G:C_C
p = 0.25, beta = −0.147, N = 180
AFVRPSGTEDVVR pc3
122:208 55:95 12:22
6:83903626:G:C_C
p = 0.49, beta = −0.0818, N = 189
PNGLILQYGTAGFR pc2
72:208 35:95 3:22
6:83903626:G:C_C
p = 0.56, beta = −0.1, N = 110
EAVNLQQDAFVVIGR pc2
17:208 6:95 1:22
6:83903626:G:C_C
p = 0.91, beta = −0.0374, N = 24
187Assay Target: PGM3
UniProt: H0Y987
rsID: rs1180178
Target SNP: 6:83903626:G:C
Proxy SNP: 6:83903626:G:C
Tarkin log10(p): 9.8
Tarkin BETA: −0.296
Tarkin MSPA: 0.806
VISTTDAER pc2
rs473267 REF
517:711 223:472 7:77
6:83903626:G:C_C
p = 2.5e−28, model = REC, N = 747

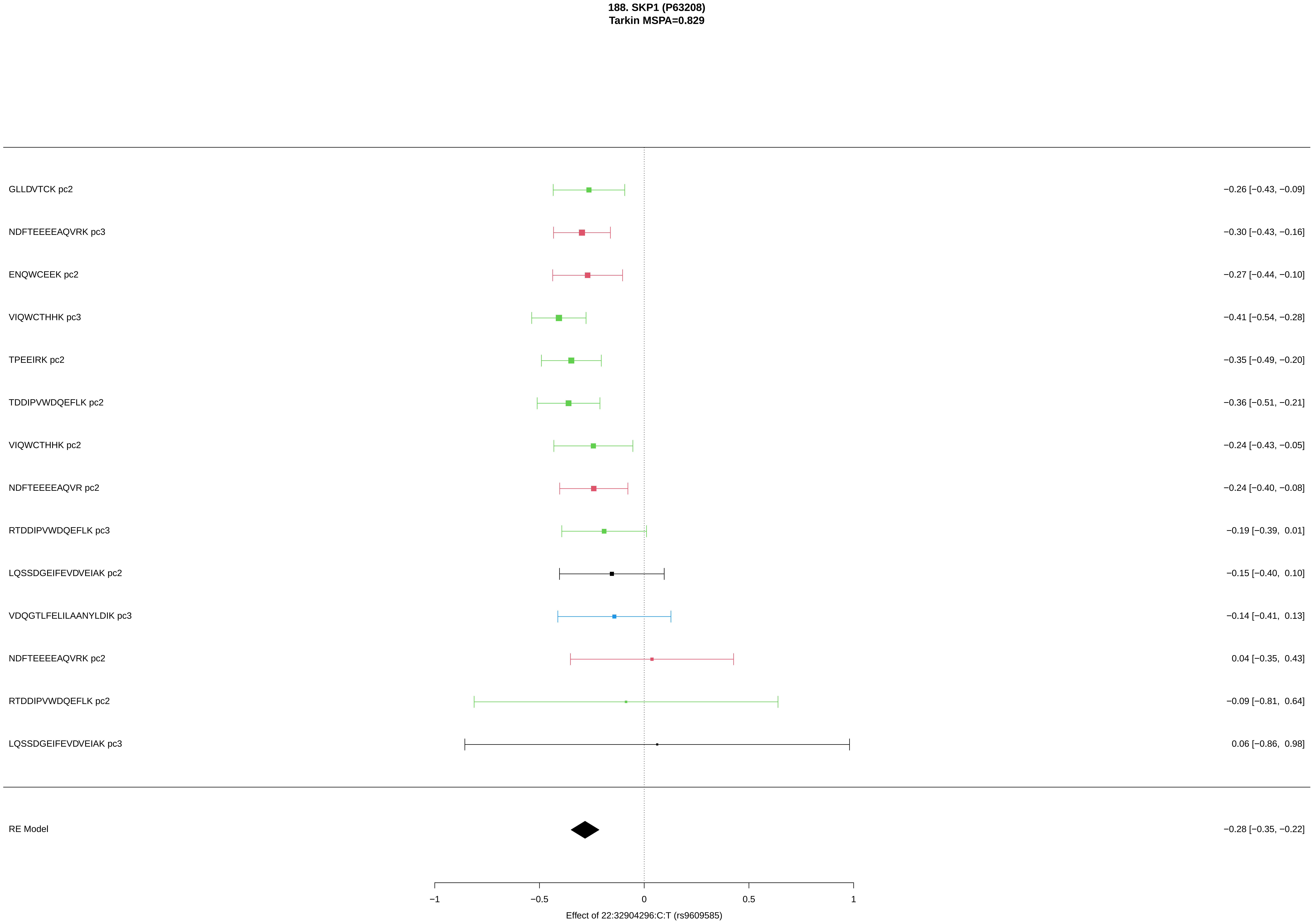

188. SKP1 (P63208)
Tarkin MSPA=0.829
GLLDVTCK pc2
−0.26 [−0.43, −0.09]
NDFTEEEEAQVRK pc3
−0.30 [−0.43, −0.16]
ENQWCEEK pc2
−0.27 [−0.44, −0.10]
VIQWCTHHK pc3
−0.41 [−0.54, −0.28]
TPEEIRK pc2
−0.35 [−0.49, −0.20]
TDDIPVWDQEFLK pc2
−0.36 [−0.51, −0.21]
VIQWCTHHK pc2
−0.24 [−0.43, −0.05]
NDFTEEEEAQVR pc2
−0.24 [−0.40, −0.08]
RTDDIPVWDQEFLK pc3
−0.19 [−0.39, 0.01]
LQSSDGEIFEVDVEIAK pc2
−0.15 [−0.40, 0.10]
VDQGTLFELILAANYLDIK pc3
−0.14 [−0.41, 0.13]
NDFTEEEEAQVRK pc2
0.04 [−0.35, 0.43]
RTDDIPVWDQEFLK pc2
−0.09 [−0.81, 0.64]
LQSSDGEIFEVDVEIAK pc3
0.06 [−0.86, 0.98]
RE Model
−0.28 [−0.35, −0.22]
−1
−0.5
0
0.5
1
Effect of 22:32904296:C:T (rs9609585)

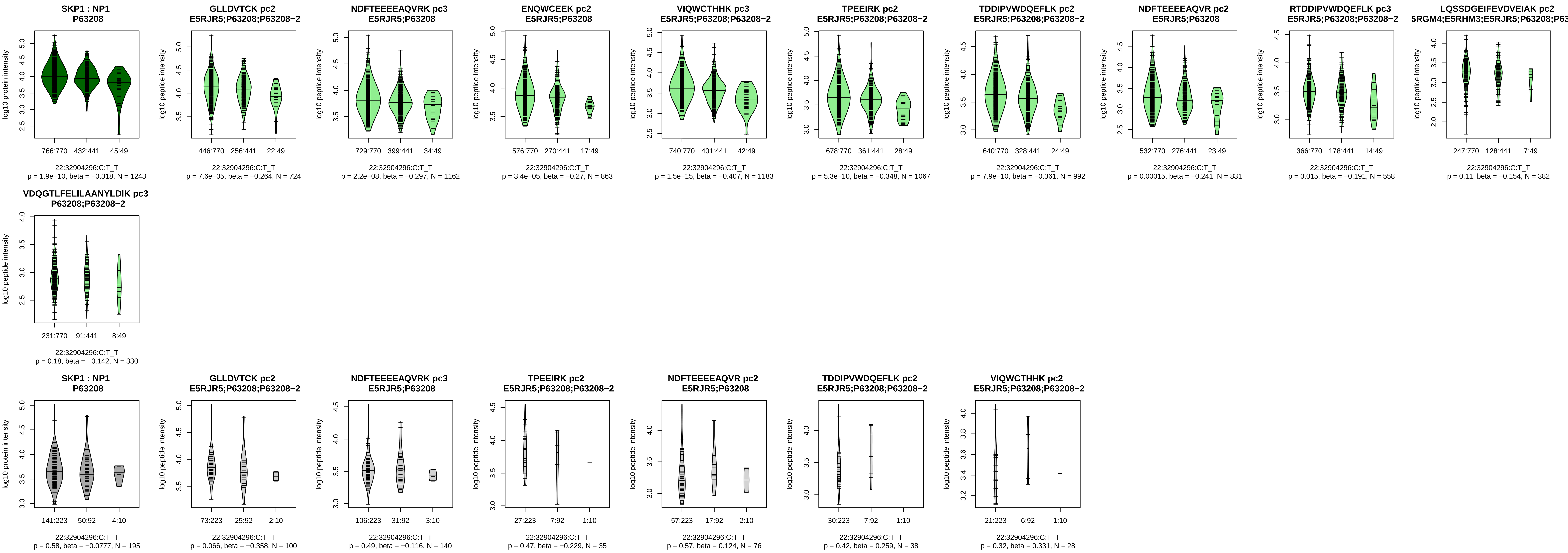

188Assay Target: SKP1
UniProt: P63208
rsID: rs9609585
Target SNP: 22:32904296:C:T
Proxy SNP: 22:32904296:C:T
Tarkin log10(p): 9.7
Tarkin BETA: −0.318
Tarkin MSPA: 0.829
724:1162:863:1183:1067:992:6

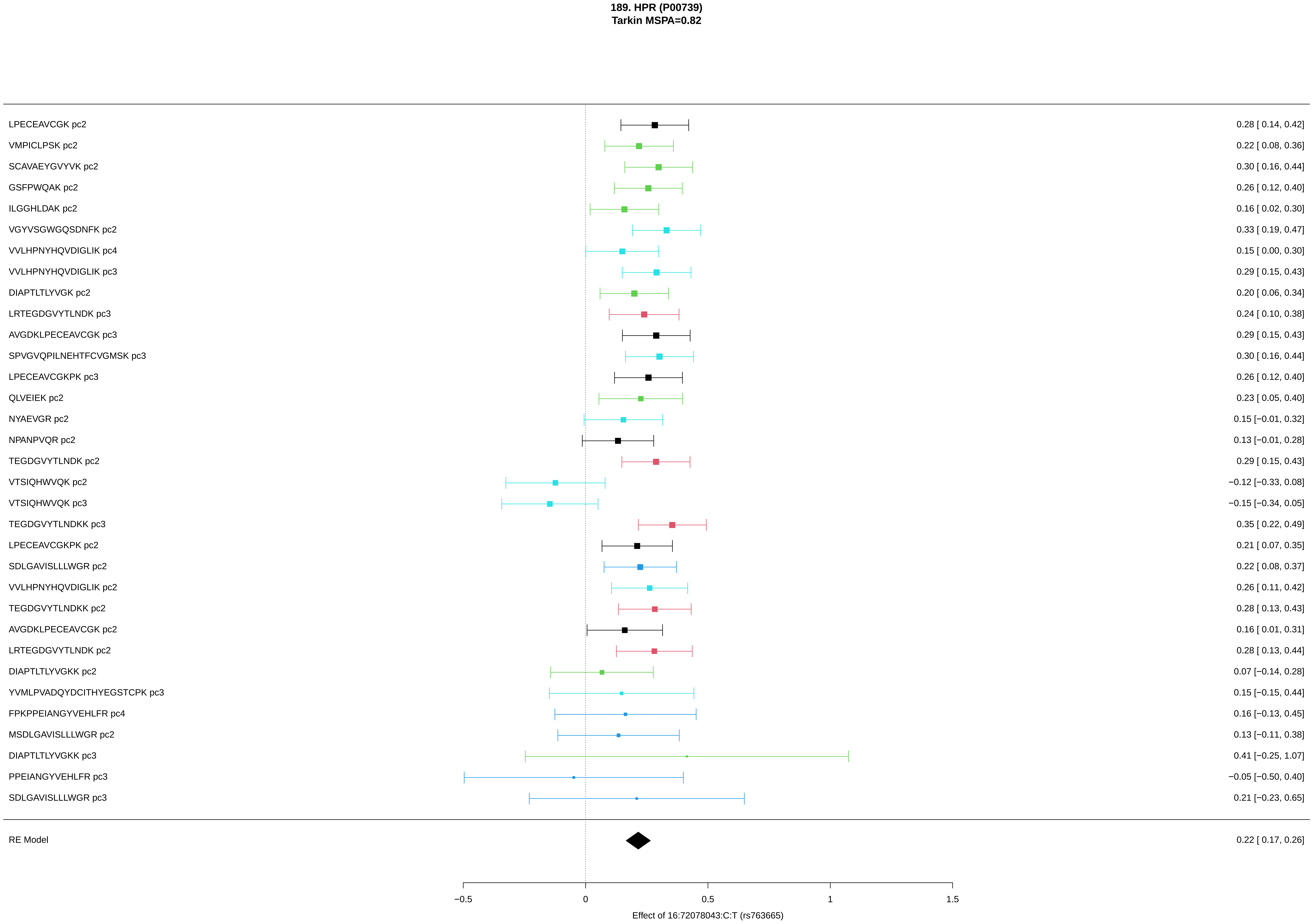
189. HPR (P00739)
Tarkin MSPA=0.82
LPECEAVCGK pc2 0.28 [0.14, 0.42]
VMPICLPSK pc2 0.22 [0.08, 0.36]
SCAVAEYGVYVK pc2 0.30 [0.16, 0.44]
GSFPWQAK pc2 0.26 [0.12, 0.40]
ILGGHLDAK pc2 0.16 [0.02, 0.30]
VGYVSGWGQSDNFK pc2 0.33 [0.19, 0.47]
VVLHPNYHQVDIGLIK pc4 0.15 [0.00, 0.30]
VVLHPNYHQVDIGLIK pc3 0.29 [0.15, 0.43]
DIAPTLTLYVGK pc2 0.20 [0.06, 0.34]
LRTEGDGVYTLNDK pc3 0.24 [0.10, 0.38]
AVGDKLPECEAVCGK pc3 0.29 [0.15, 0.43]
SPVGVQPILNEHTFCVGMSK pc3 0.30 [0.16, 0.44]
LPECEAVCGKPK pc3 0.26 [0.12, 0.40]
QLVEIEK pc2 0.23 [0.05, 0.40]
NYAEVGR pc2 0.15 [−0.01, 0.32]
NPANPVQR pc2 0.13 [−0.01, 0.28]
TEGDGVYTLNDK pc2 0.29 [0.15, 0.43]
VTSIQHWVQK pc2 −0.12 [−0.33, 0.08]
VTSIQHWVQK pc3 −0.15 [−0.34, 0.05]
TEGDGVYTLNDKK pc3 0.35 [0.22, 0.49]
LPECEAVCGKPK pc2 0.21 [0.07, 0.35]
SDLGAVISLLLWGR pc2 0.22 [0.08, 0.37]
VVLHPNYHQVDIGLIK pc2 0.26 [0.11, 0.42]
TEGDGVYTLNDKK pc2 0.28 [0.13, 0.43]
AVGDKLPECEAVCGK pc2 0.16 [0.01, 0.31]
LRTEGDGVYTLNDK pc2 0.28 [0.13, 0.44]
DIAPTLTLYVGKK pc2 0.07 [−0.14, 0.28]
YVMLPVADQYDCITHYEGSTCPK pc3 0.15 [−0.15, 0.44]
FPKPPEIANGYVEHLFR pc4 0.16 [−0.13, 0.45]
MSDLGAVISLLLWGR pc2 0.13 [−0.11, 0.38]
DIAPTLTLYVGKK pc3 0.41 [−0.25, 1.07]
PPEIANGYVEHLFR pc3 −0.05 [−0.50, 0.40]
SDLGAVISLLLWGR pc3 0.21 [−0.23, 0.65]
RE Model 0.22 [0.17, 0.26]
−0.5
0
0.5
1
1.5
Effect of 16:72078043:C:T (rs763665)

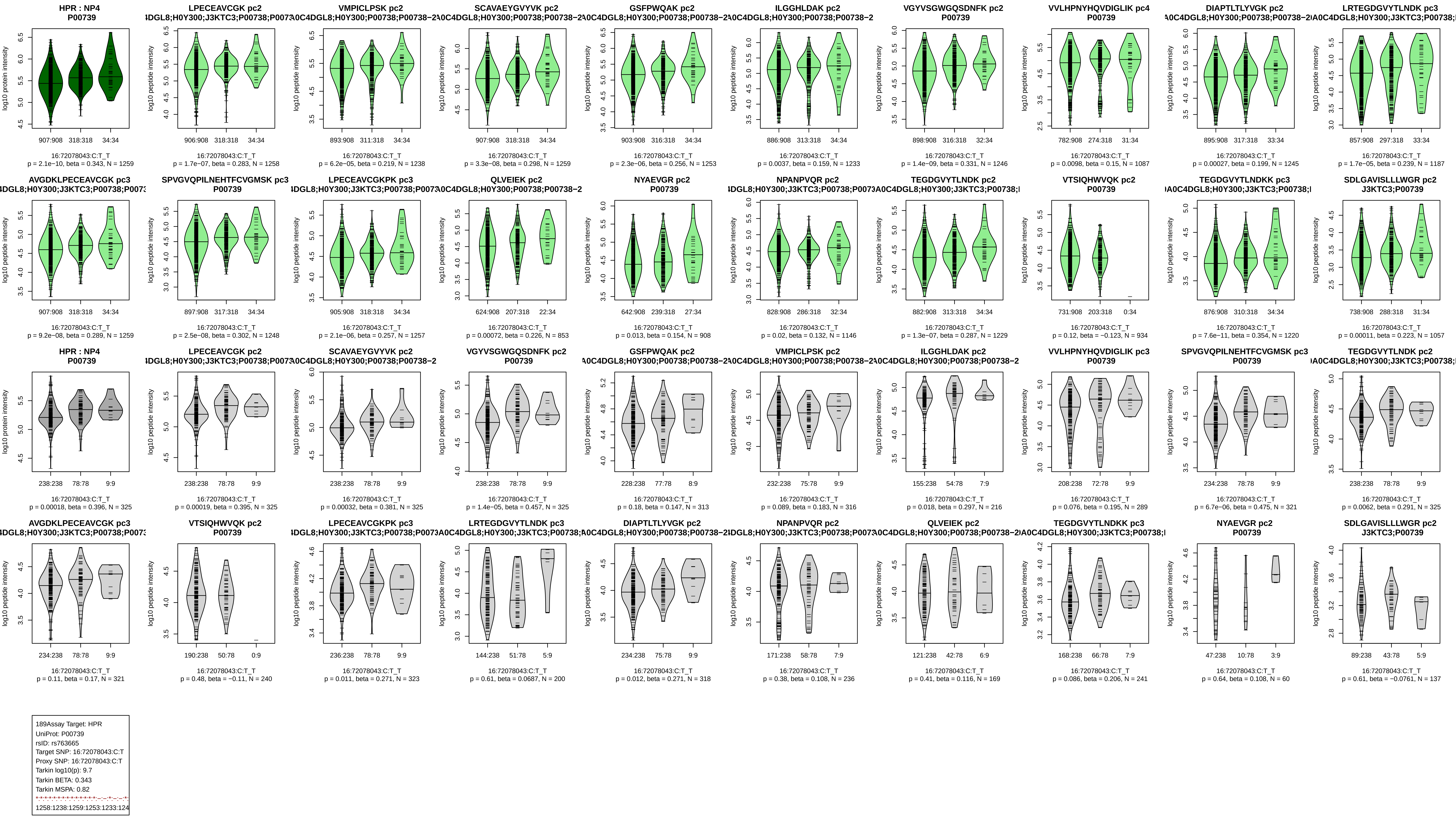

HPR : NP4
P00739
16:72078043:C:T_T
p = 2.1e−10, beta = 0.343, N = 1259
LPECEAVCGK pc2
p = 1.7e−07, beta = 0.283, N = 1258
VMPICLPSK pc2
p = 6.2e−05, beta = 0.219, N = 1238
SCAVAEYGVYVK pc2
p = 3.3e−08, beta = 0.298, N = 1259
GSFPWQAK pc2
p = 2.3e−06, beta = 0.256, N = 1253
ILGGHLDAK pc2
p = 0.0037, beta = 0.159, N = 1233
VGYVSGWGQSDNFK pc2
P00739
p = 1.4e−09, beta = 0.331, N = 1246
VVLHPNYHQVDIGLIK pc4
P00739
p = 0.0098, beta = 0.15, N = 1087
DIAPTLTLYVGK pc2
p = 0.00027, beta = 0.199, N = 1245
LRTEGDGVYTLNDK pc3
p = 1.7e−05, beta = 0.239, N = 1187
AVGDKLPECEAVCGK pc3
p = 9.2e−08, beta = 0.289, N = 1259
SPVGVQPILNEHTFCVGMSK pc3
P00739
p = 2.5e−08, beta = 0.302, N = 1248
LPECEAVCGKPK pc3
p = 2.1e−06, beta = 0.257, N = 1257
QLVEIEK pc2
p = 0.00072, beta = 0.226, N = 853
NYAEVGR pc2
P00739
p = 0.013, beta = 0.154, N = 908
NPANPVQR pc2
p = 0.02, beta = 0.132, N = 1146
TEGDGVYTLNDK pc2
p = 1.3e−07, beta = 0.287, N = 1229
VTSIQHWVQK pc2
P00739
p = 0.12, beta = −0.123, N = 934
TEGDGVYTLNDKK pc3
p = 7.6e−11, beta = 0.354, N = 1220
SDLGAVISLLLWGR pc2
J3KTC3;P00739
p = 0.00011, beta = 0.223, N = 1057
HPR : NP4
P00739
p = 0.00018, beta = 0.396, N = 325
LPECEAVCGK pc2
p = 0.00019, beta = 0.395, N = 325
SCAVAEYGVYVK pc2
p = 0.00032, beta = 0.381, N = 325
VGYVSGWGQSDNFK pc2
P00739
p = 1.4e−05, beta = 0.457, N = 325
GSFPWQAK pc2
p = 0.18, beta = 0.147, N = 313
VMPICLPSK pc2
p = 0.089, beta = 0.183, N = 316
ILGGHLDAK pc2
p = 0.018, beta = 0.297, N = 216
VVLHPNYHQVDIGLIK pc3
P00739
p = 0.076, beta = 0.195, N = 289
SPVGVQPILNEHTFCVGMSK pc3
P00739
p = 6.7e−06, beta = 0.475, N = 321
TEGDGVYTLNDK pc2
p = 0.0062, beta = 0.291, N = 325
AVGDKLPECEAVCGK pc3
p = 0.11, beta = 0.17, N = 321
VTSIQHWVQK pc2
P00739
p = 0.48, beta = −0.11, N = 240
LPECEAVCGKPK pc3
p = 0.011, beta = 0.271, N = 323
LRTEGDGVYTLNDK pc3
p = 0.61, beta = 0.0687, N = 200
DIAPTLTLYVGK pc2
p = 0.012, beta = 0.271, N = 318
NPANPVQR pc2
p = 0.38, beta = 0.108, N = 236
QLVEIEK pc2
p = 0.41, beta = 0.116, N = 169
TEGDGVYTLNDKK pc3
p = 0.086, beta = 0.206, N = 241
NYAEVGR pc2
P00739
p = 0.64, beta = 0.108, N = 60
SDLGAVISLLLWGR pc2
J3KTC3;P00739
p = 0.61, beta = −0.0761, N = 137
189Assay Target: HPR
UniProt: P00739
rsID: rs763665
Target SNP: 16:72078043:C:T
Proxy SNP: 16:72078043:C:T
Tarkin log10(p): 9.7
Tarkin BETA: 0.343
Tarkin MSPA: 0.82

190. IGKV1−33 (P01594)
Tarkin MSPA=1

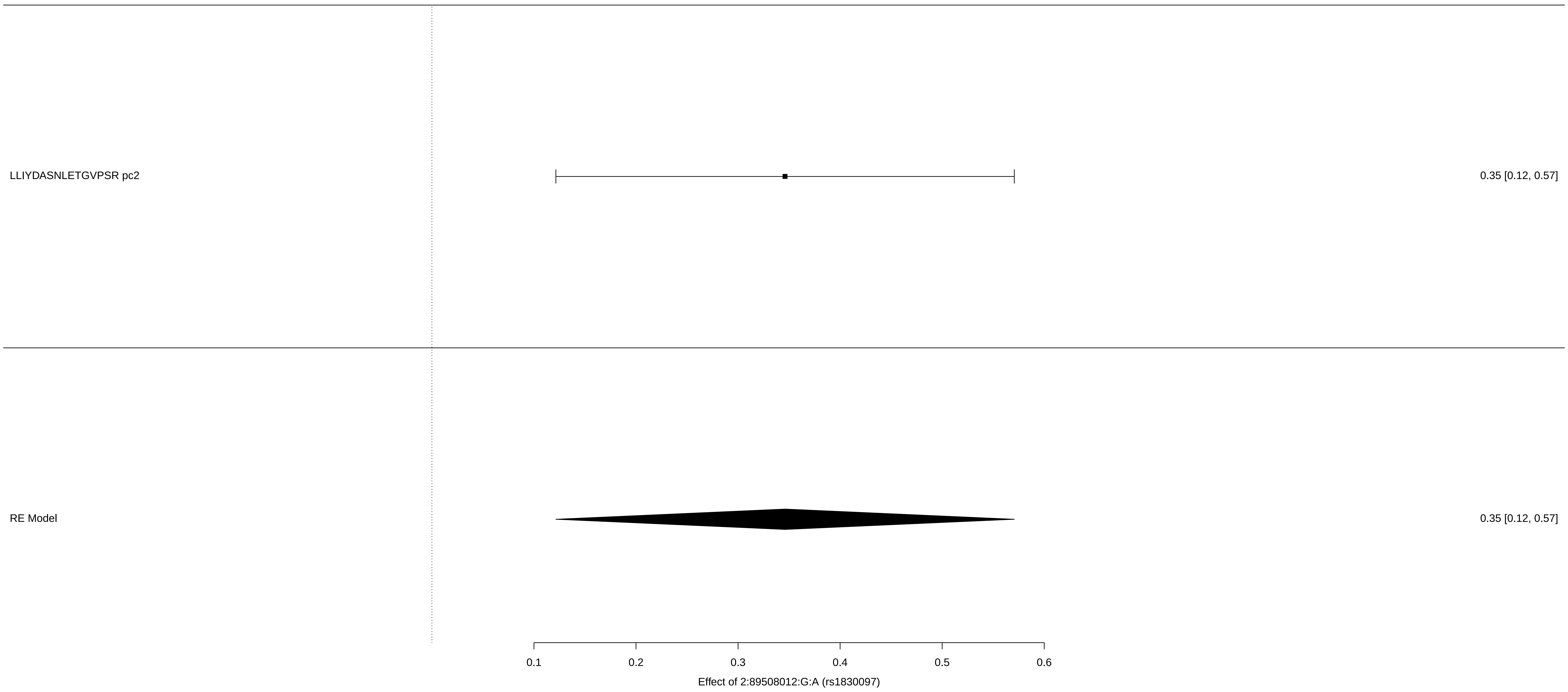

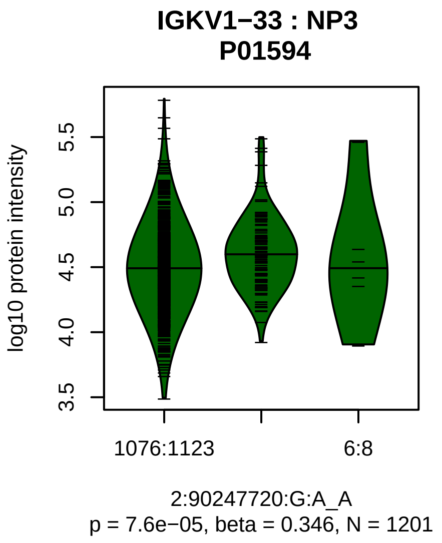

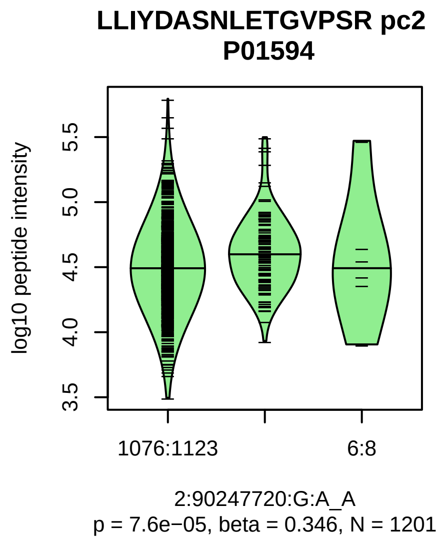

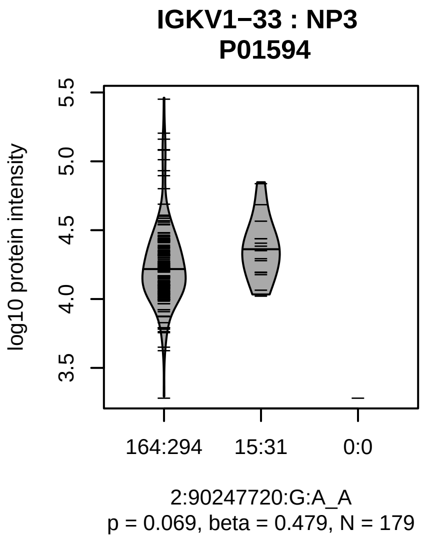

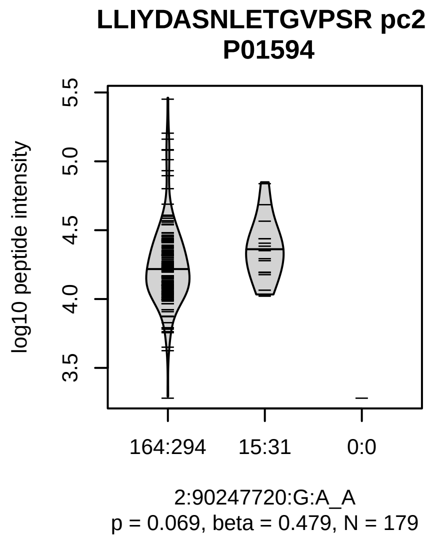

190Assay Target: IGKV1−33
UniProt: P01594
rsID: rs1830097
Target SNP: 2:89508012:G:A
Proxy SNP: 2:90247720:G:A
Tarkin log10(p): 9.6
Tarkin BETA: 0.445
Tarkin MSPA: 1
*:NA:NA
1201:12:1

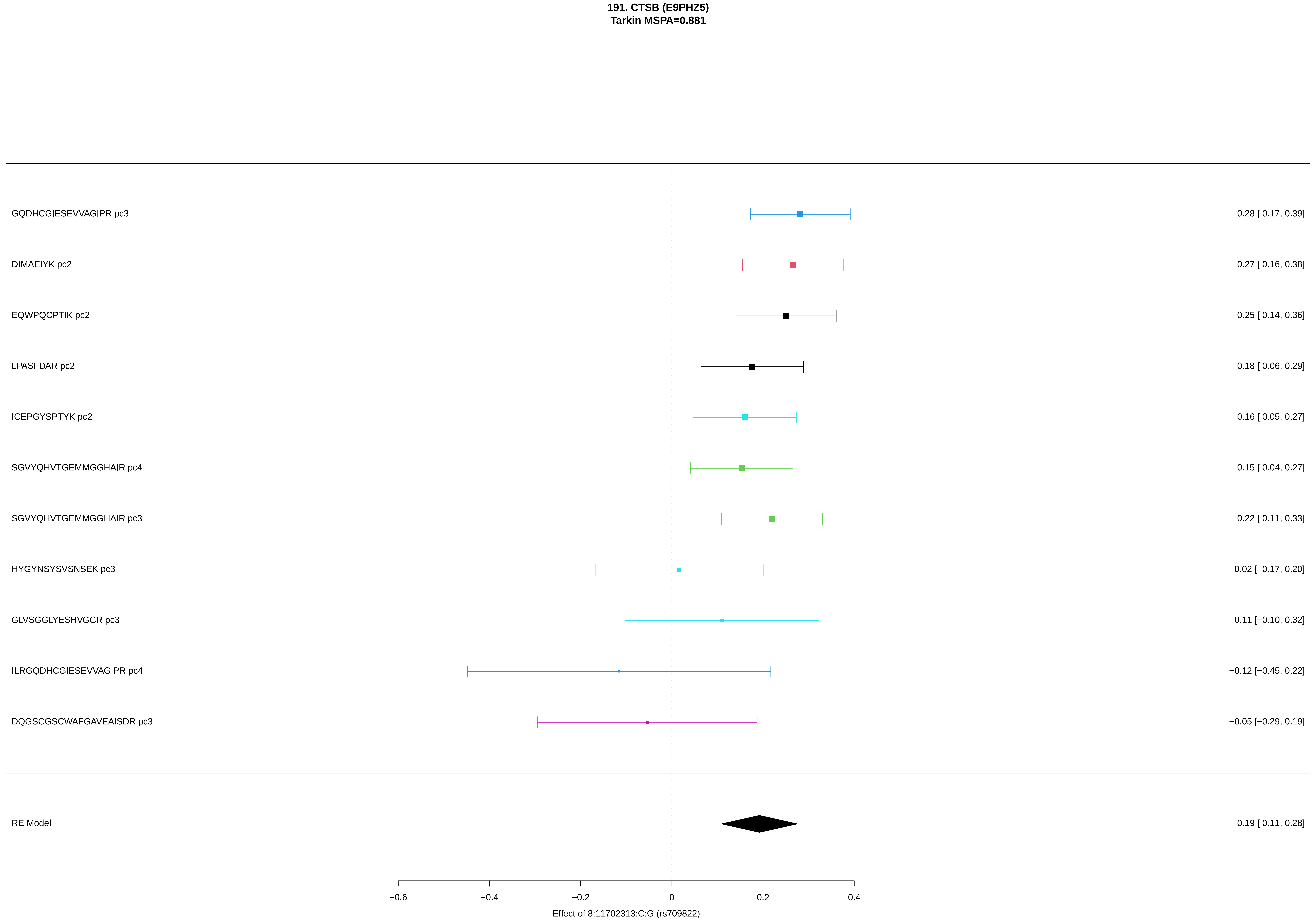

191. CTSB (E9PHZ5)
Tarkin MSPA=0.881
GQDHCGIESEVVAGIPR pc3
0.28 [0.17, 0.39]
DIMAEIYK pc2
0.27 [0.16, 0.38]
EQWPQCPTIK pc2
0.25 [0.14, 0.36]
LPASFDAR pc2
0.18 [0.06, 0.29]
ICEPGYSPTYK pc2
0.16 [0.05, 0.27]
SGVYQHVTGEMMGGHAIR pc4
0.15 [0.04, 0.27]
SGVYQHVTGEMMGGHAIR pc3
0.22 [0.11, 0.33]
HYGYNSYSVSNSEK pc3
0.02 [−0.17, 0.20]
GLVSGGLYESHVGCR pc3
0.11 [−0.10, 0.32]
ILRGQDHCGIESEVVAGIPR pc4
−0.12 [−0.45, 0.22]
DQGSCGSCWAFGAVEAISDR pc3
−0.05 [−0.29, 0.19]
RE Model
0.19 [0.11, 0.28]
−0.6
−0.4
−0.2
0
0.2
0.4
Effect of 8:11702313:C:G (rs709822)

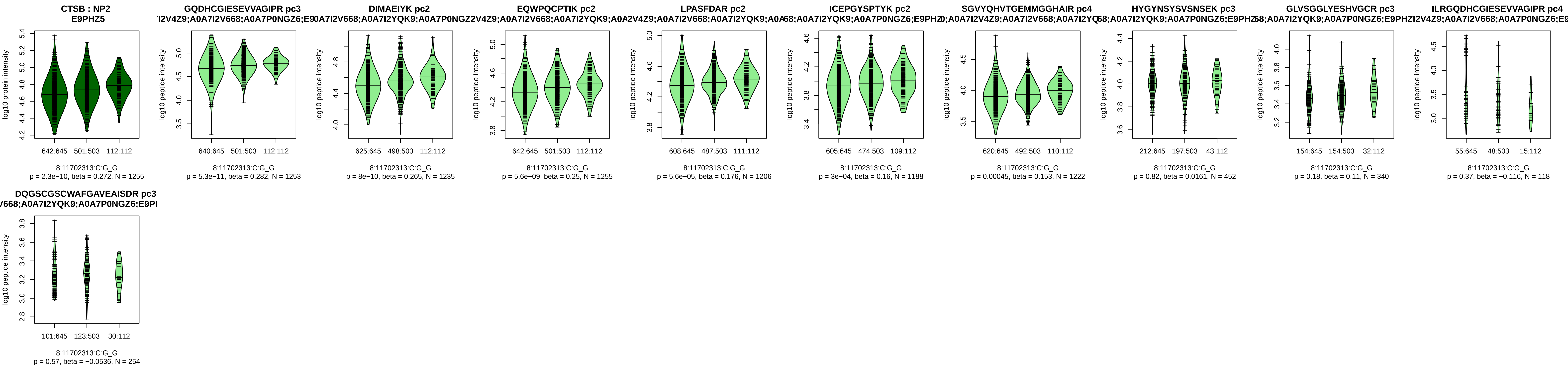

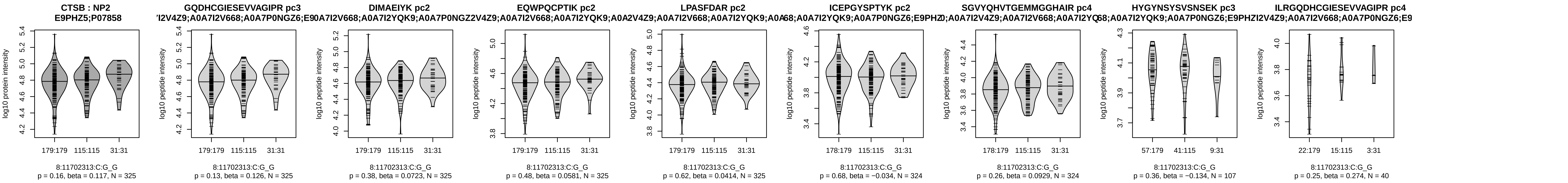

191Assay Target: CTSB
UniProt: E9PHZ5
rsID: rs709822
Target SNP: 8:11702313:C:G
Proxy SNP: 8:11702313:C:G
Tarkin log10(p): 9.6
Tarkin BETA: 0.272
Tarkin MSPA: 0.881
::*:*:*:*:–:–:–:–:NA
1253:1235:1255:1206:1188:122

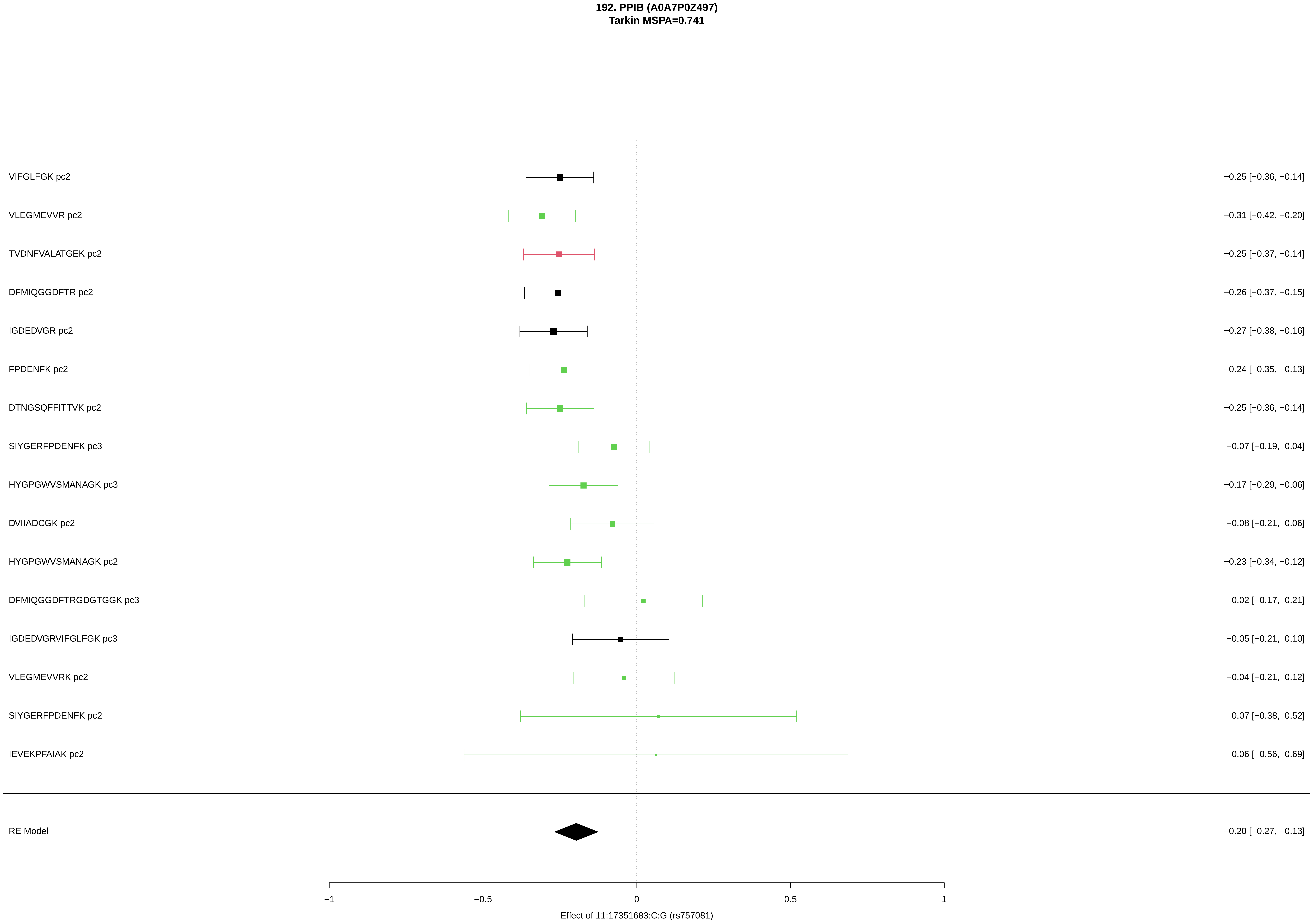

192. PPIB (A0A7P0Z497)
Tarkin MSPA=0.741
VIFGLFGK pc2
VLEGMEVVR pc2
TVDNFVALATGEK pc2
DFMIQGGDFTR pc2
IGDEDVGR pc2
FPDENFK pc2
DTNGSQFFITTVK pc2
SIYGERFPDENFK pc3
HYGPGWVSMANAGK pc3
DVIIADCGK pc2
HYGPGWVSMANAGK pc2
DFMIQGGDFTRGDGTGGK pc3
IGDEDVGRVIFGLFGK pc3
VLEGMEVVRK pc2
SIYGERFPDENFK pc2
IEVEKPFAIAK pc2
RE Model
−0.25 [−0.36, −0.14]
−0.31 [−0.42, −0.20]
−0.25 [−0.37, −0.14]
−0.26 [−0.37, −0.15]
−0.27 [−0.38, −0.16]
−0.24 [−0.35, −0.13]
−0.25 [−0.36, −0.14]
−0.07 [−0.19, 0.04]
−0.17 [−0.29, −0.06]
−0.08 [−0.21, 0.06]
−0.23 [−0.34, −0.12]
0.02 [−0.17, 0.21]
−0.05 [−0.21, 0.10]
−0.04 [−0.21, 0.12]
0.07 [−0.38, 0.52]
0.06 [−0.56, 0.69]
−0.20 [−0.27, −0.13]
−1
−0.5
0
0.5
1
Effect of 11:17351683:C:G (rs757081)

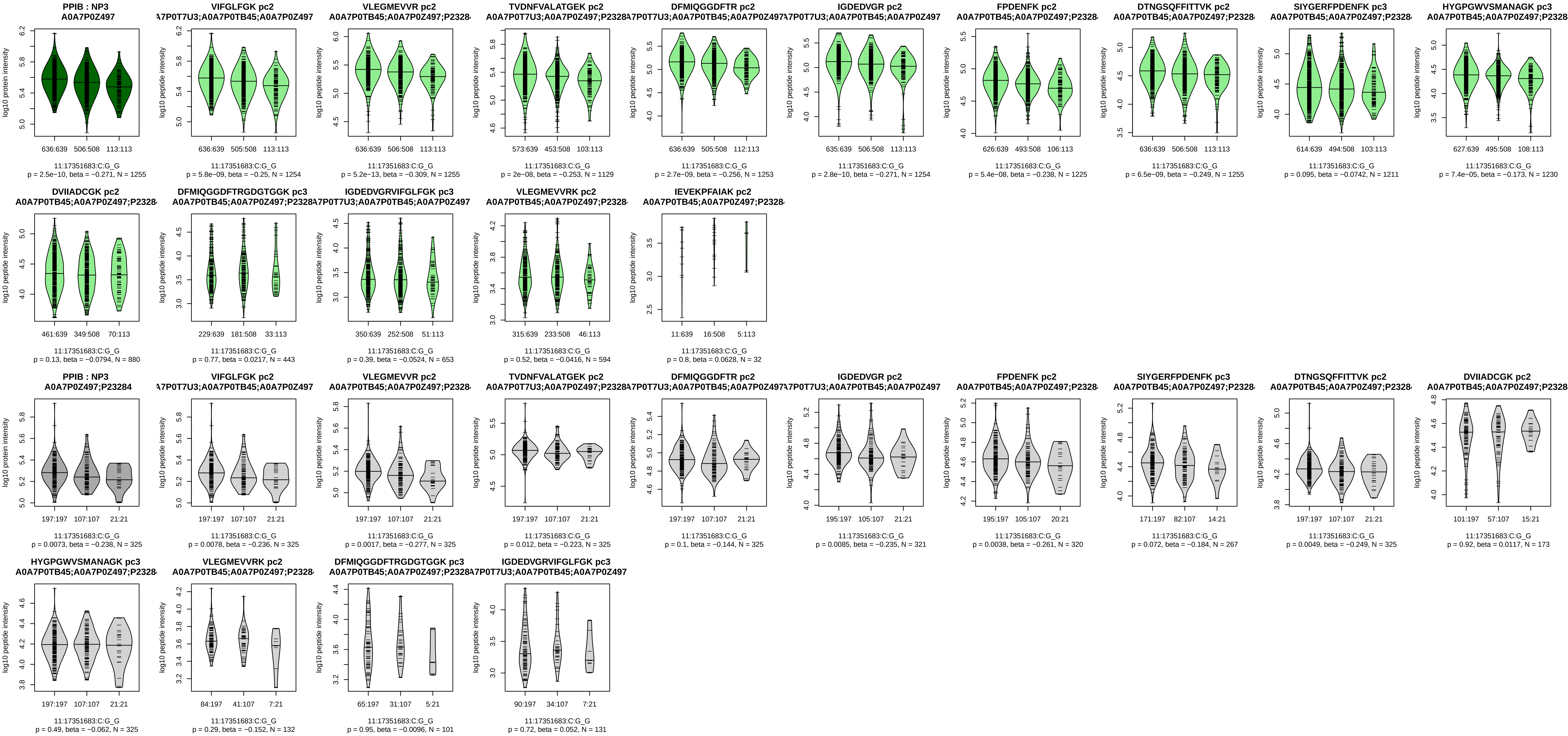

192Assay Target: PPIB
UniProt: A0A7P0Z497
rsID: rs757081
Target SNP: 11:17351683:C:G
Proxy SNP: 11:17351683:C:G
Tarkin log10(p): 9.6
Tarkin BETA: −0.271
Tarkin MSPA: 0.741
1254:1255:1129:1253:1254:122

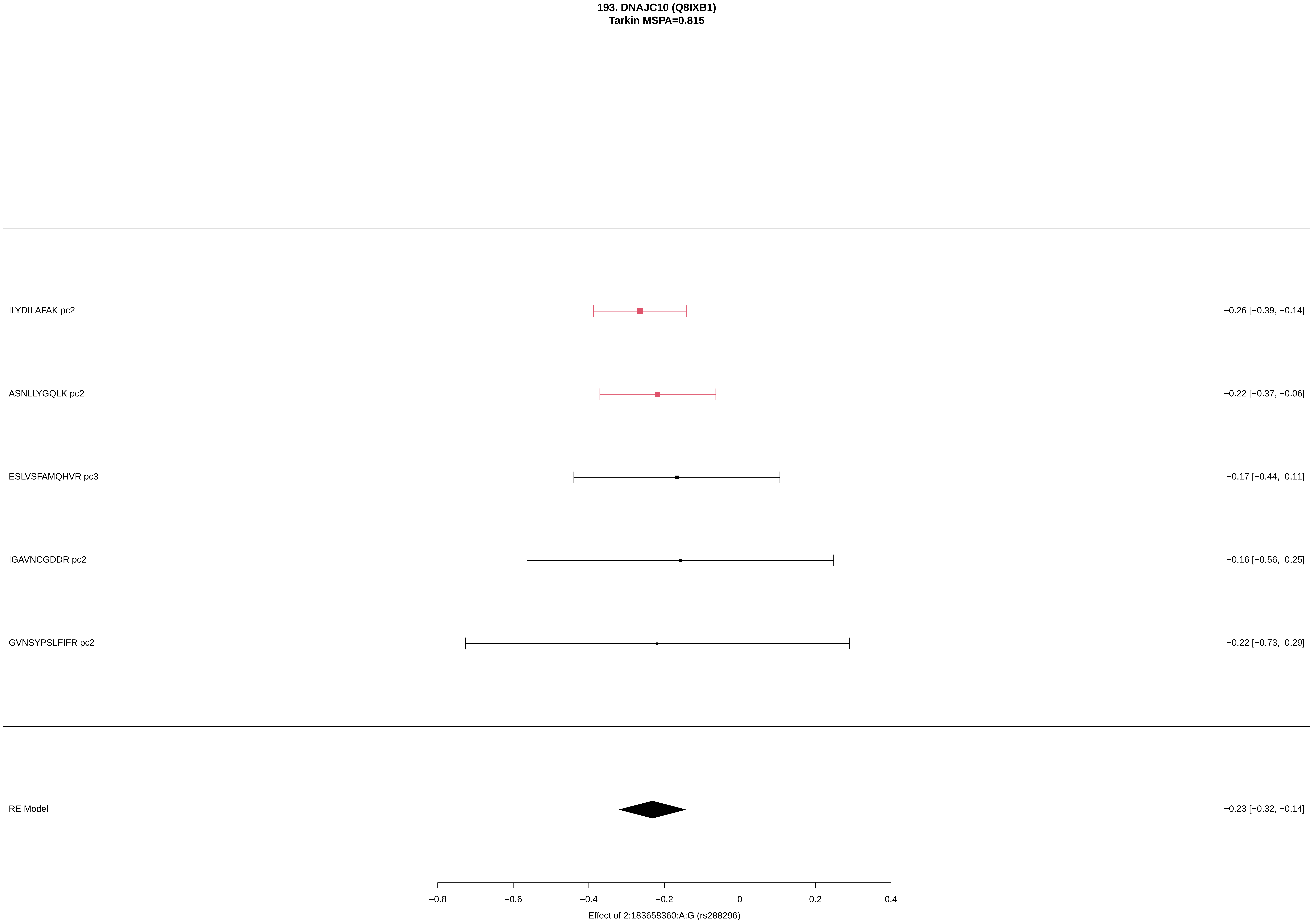

193. DNAJC10 (Q8IXB1)
Tarkin MSPA=0.815
ILYDILAFAK pc2
−0.26 [−0.39, −0.14]
ASNLLYGQLK pc2
−0.22 [−0.37, −0.06]
ESLVSFAMQHVR pc3
−0.17 [−0.44, 0.11]
IGAVNCGDDR pc2
−0.16 [−0.56, 0.25]
GVNSYPSLFIFR pc2
−0.22 [−0.73, 0.29]
RE Model
−0.23 [−0.32, −0.14]
−0.8
−0.6
−0.4
−0.2
0
0.2
0.4
Effect of 2:183658360:A:G (rs288296)

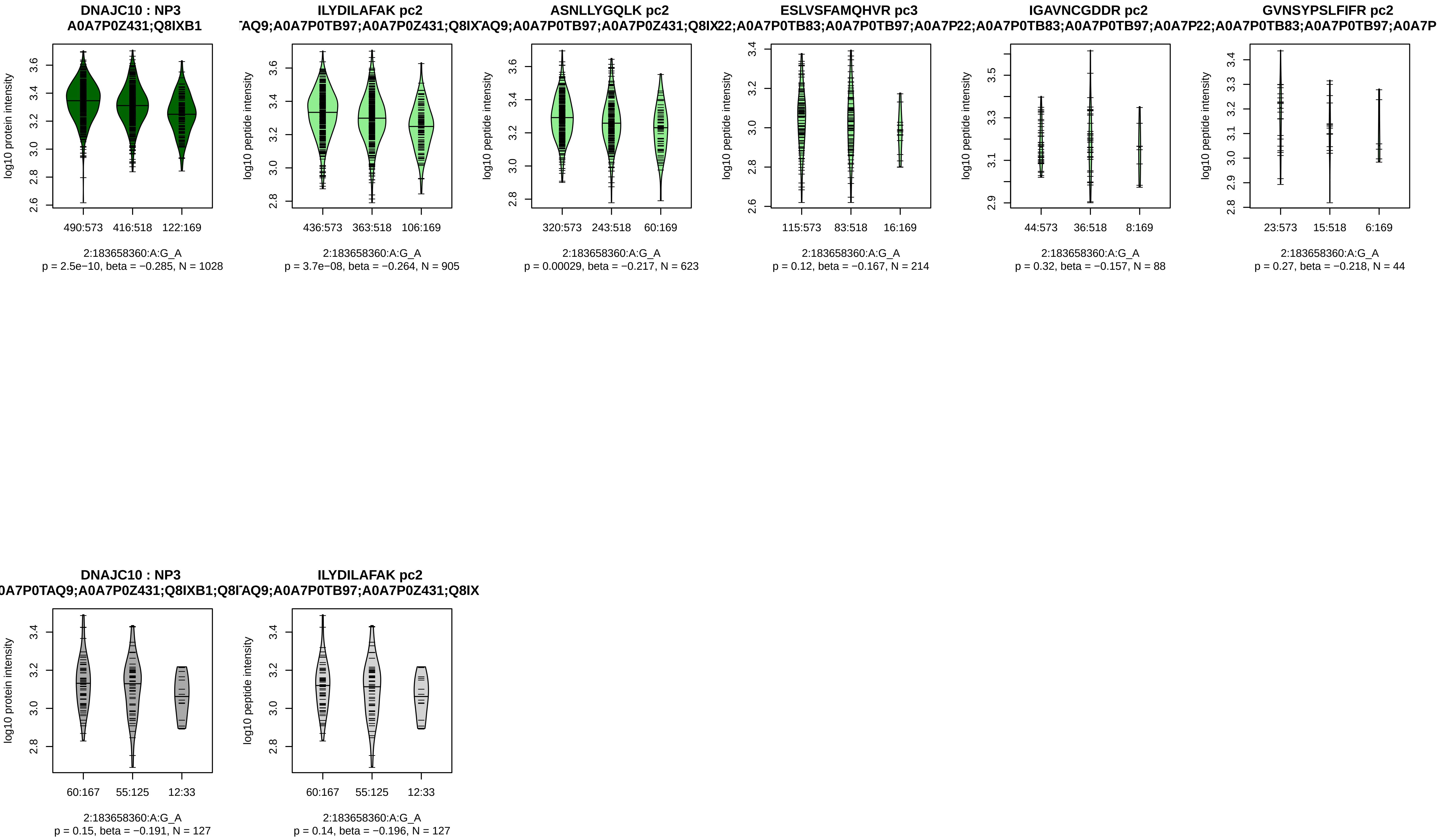

193Assay Target: DNAJC10
UniProt: Q8IXB1
rsID: rs288296
Target SNP: 2:183658360:A:G
Proxy SNP: 2:183658360:A:G
Tarkin log10(p): 9.6
Tarkin BETA: −0.285
Tarkin MSPA: 0.815
::–:–:–
905:623:214:88:44

194. ST14 (Q9Y5Y6)
Tarkin MSPA=0.429

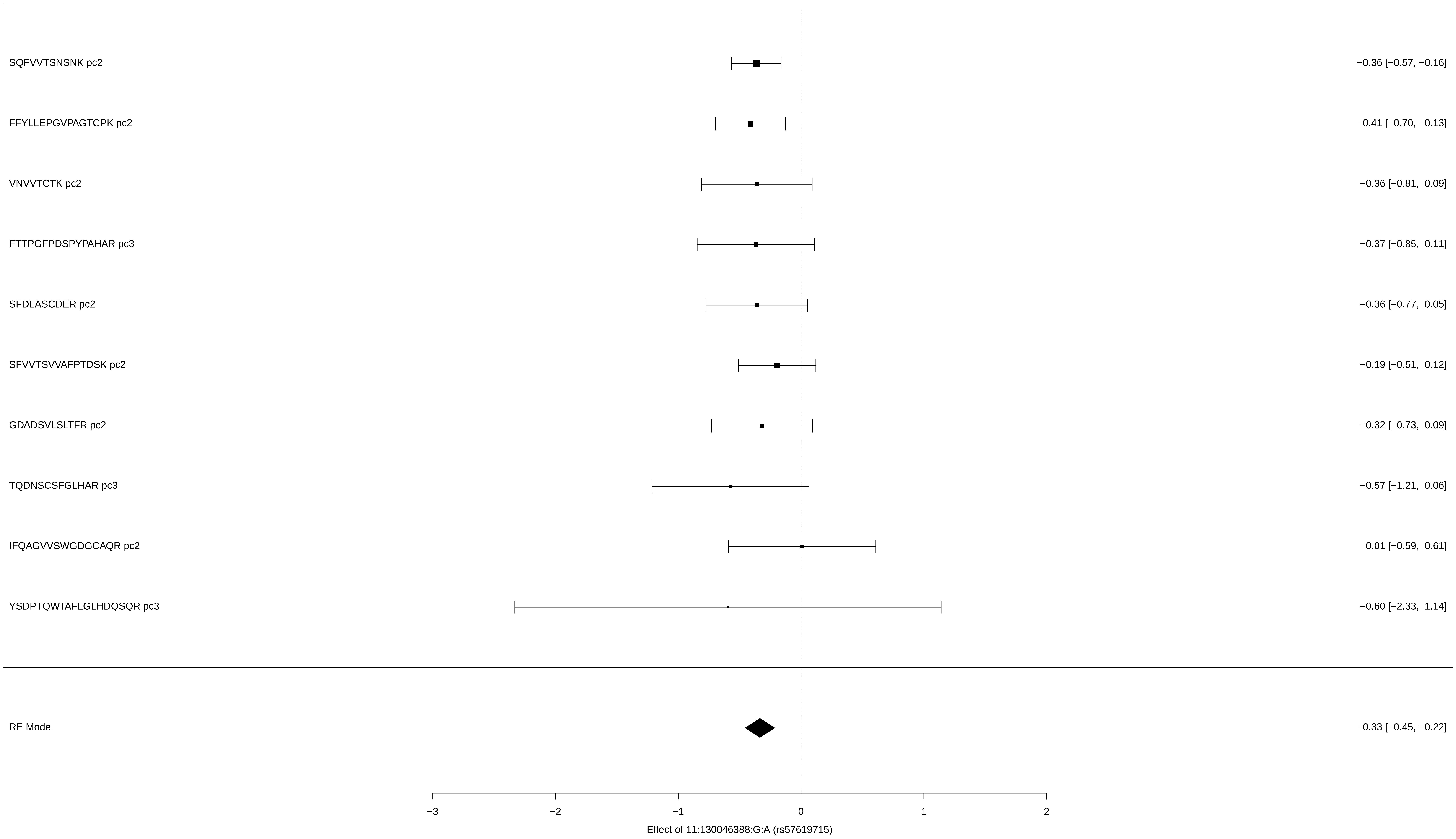

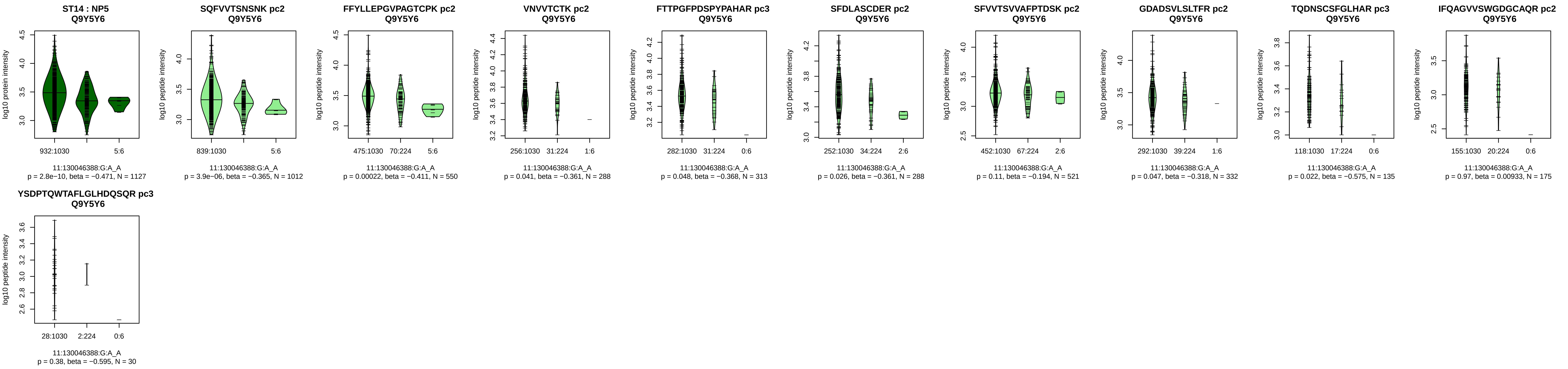

194Assay Target: ST14
UniProt: Q9Y5Y6
rsID: rs57619715
Target SNP: 11:130046388:G:A
Proxy SNP: 11:130046388:G:A
Tarkin log10(p): 9.6
Tarkin BETA: −0.472
Tarkin MSPA: 0.429
::–:–:–:–:–:–:–:–
1012:550:288:313:288:521:332:

195. PLA2G7 (Q13093)
Tarkin MSPA=0.948

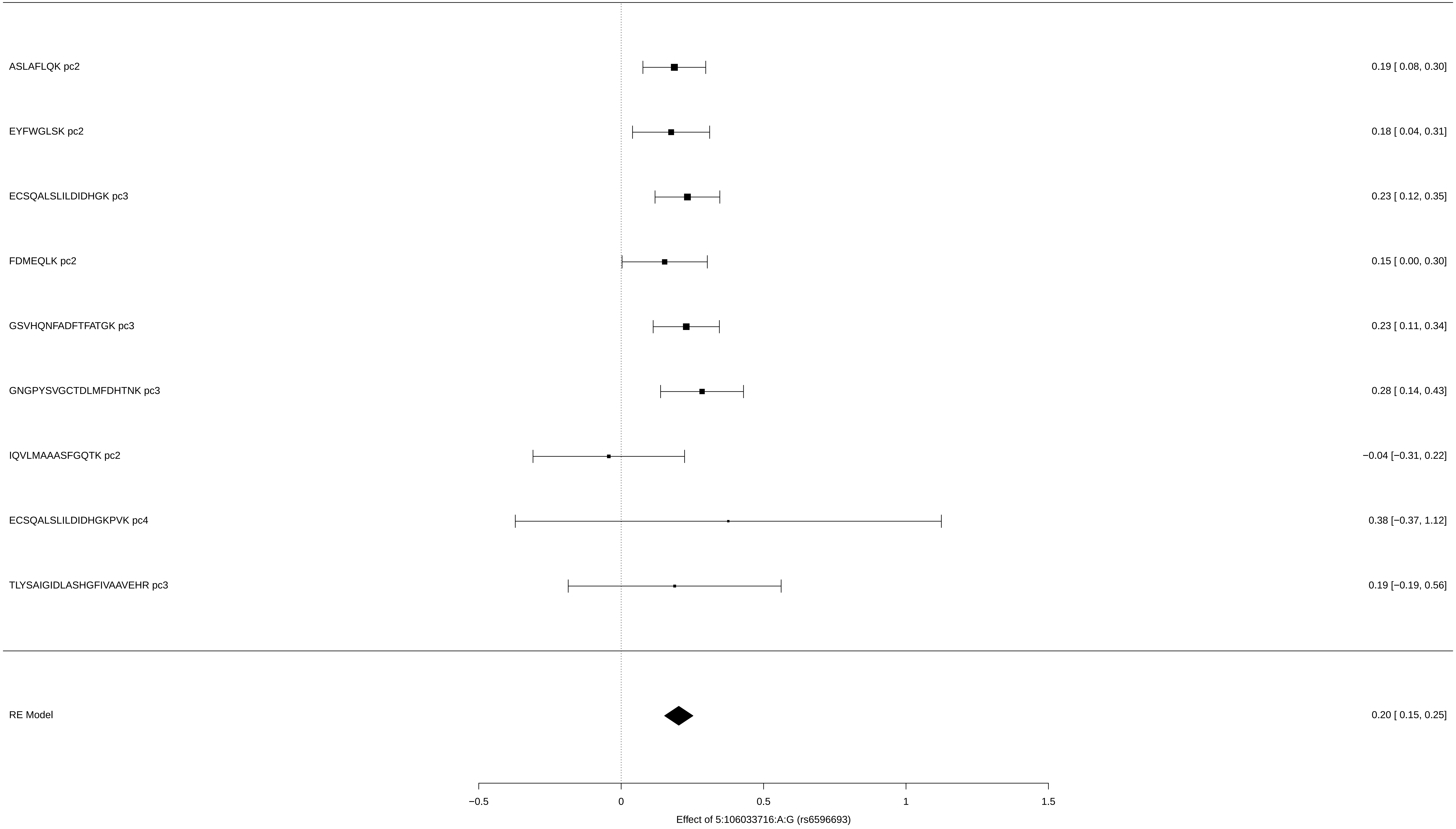

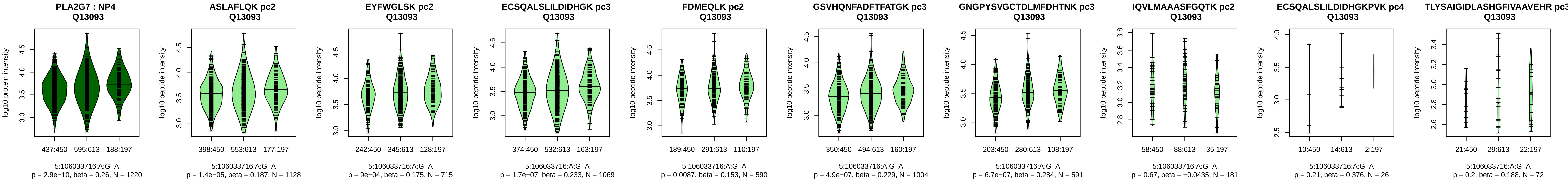

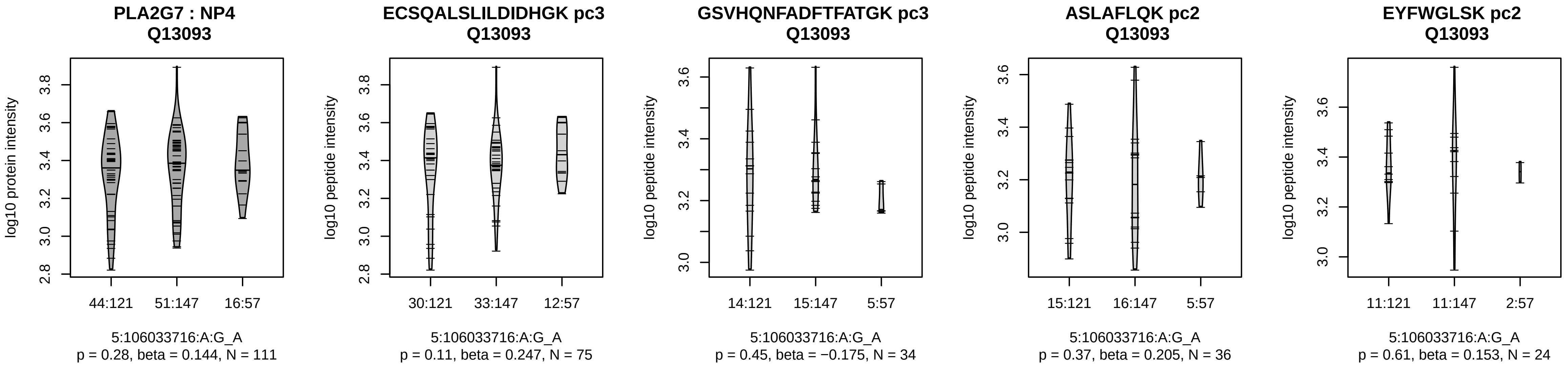

195Assay Target: PLA2G7
UniProt: Q13093
rsID: rs6596693
Target SNP: 5:106033716:A:G
Proxy SNP: 5:106033716:A:G
Tarkin log10(p): 9.5
Tarkin BETA: 0.26
Tarkin MSPA: 0.948
::*:*:*:*:−:−:−:NA
1128:715:1069:590:1004:591:18

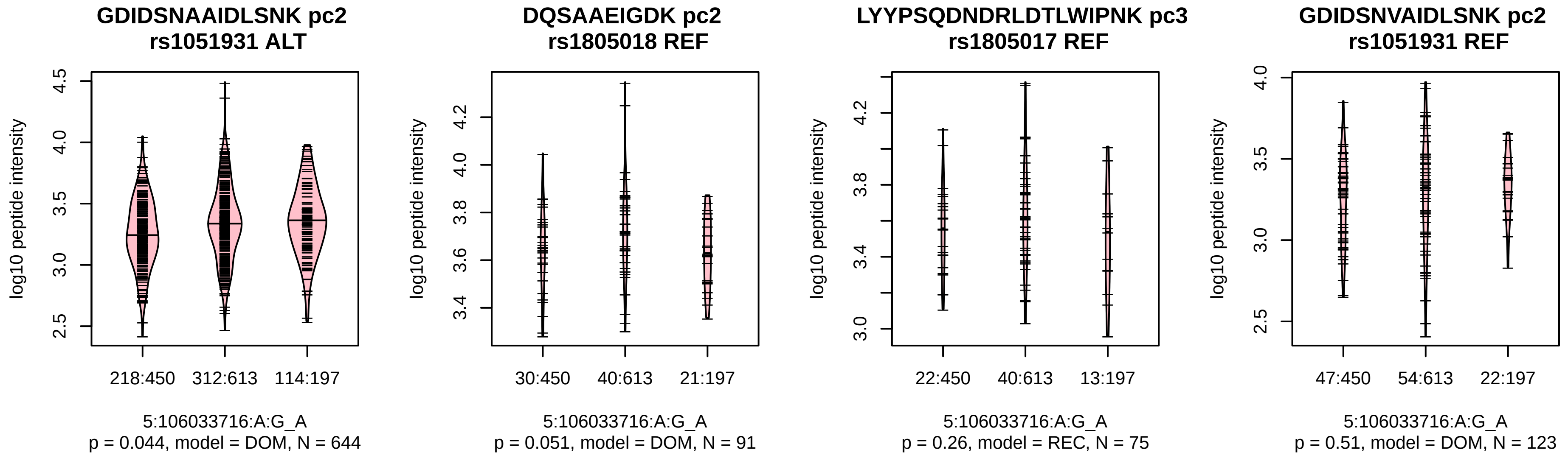

196. SERPINA4 (P29622)
Tarkin MSPA=0.9

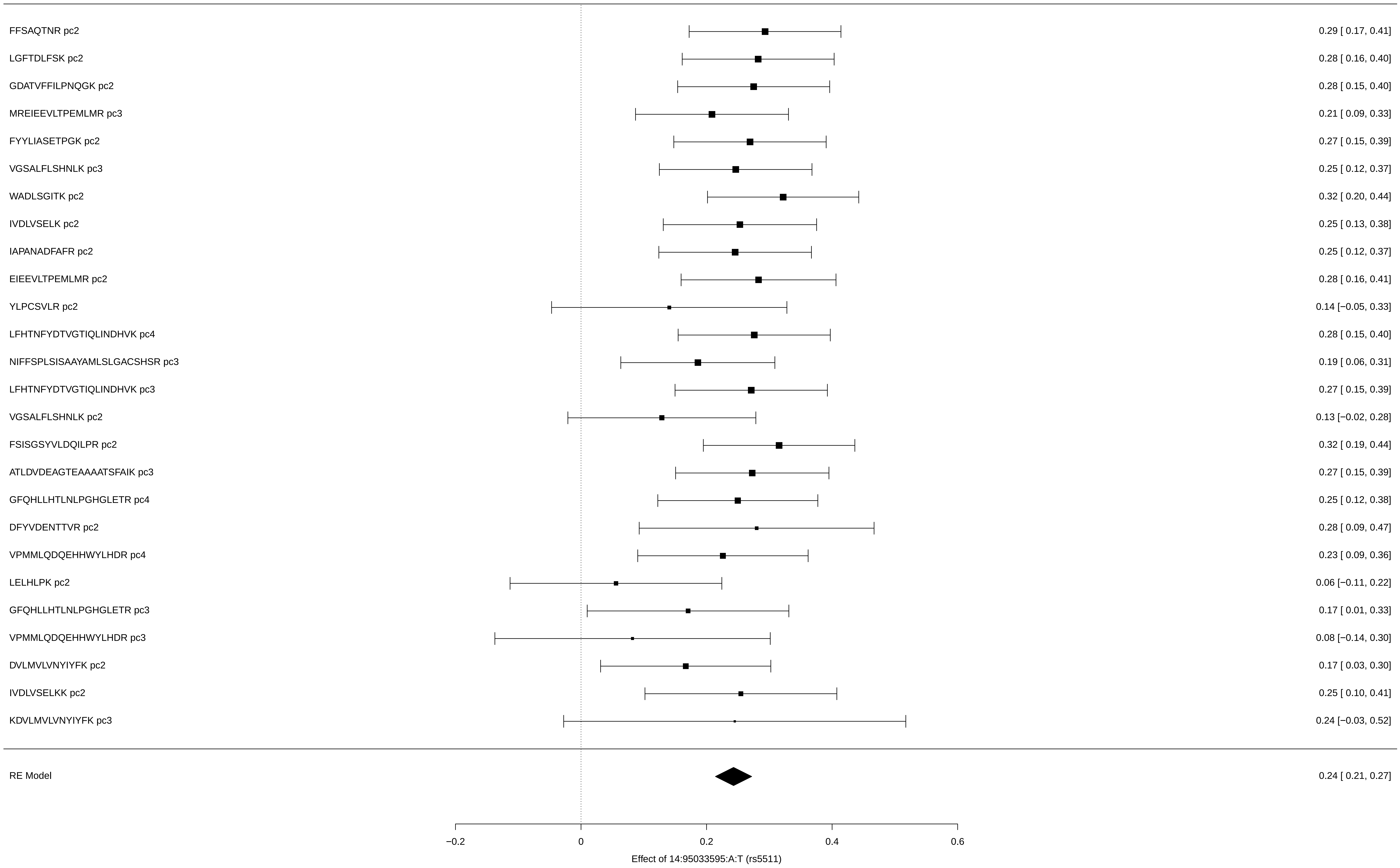

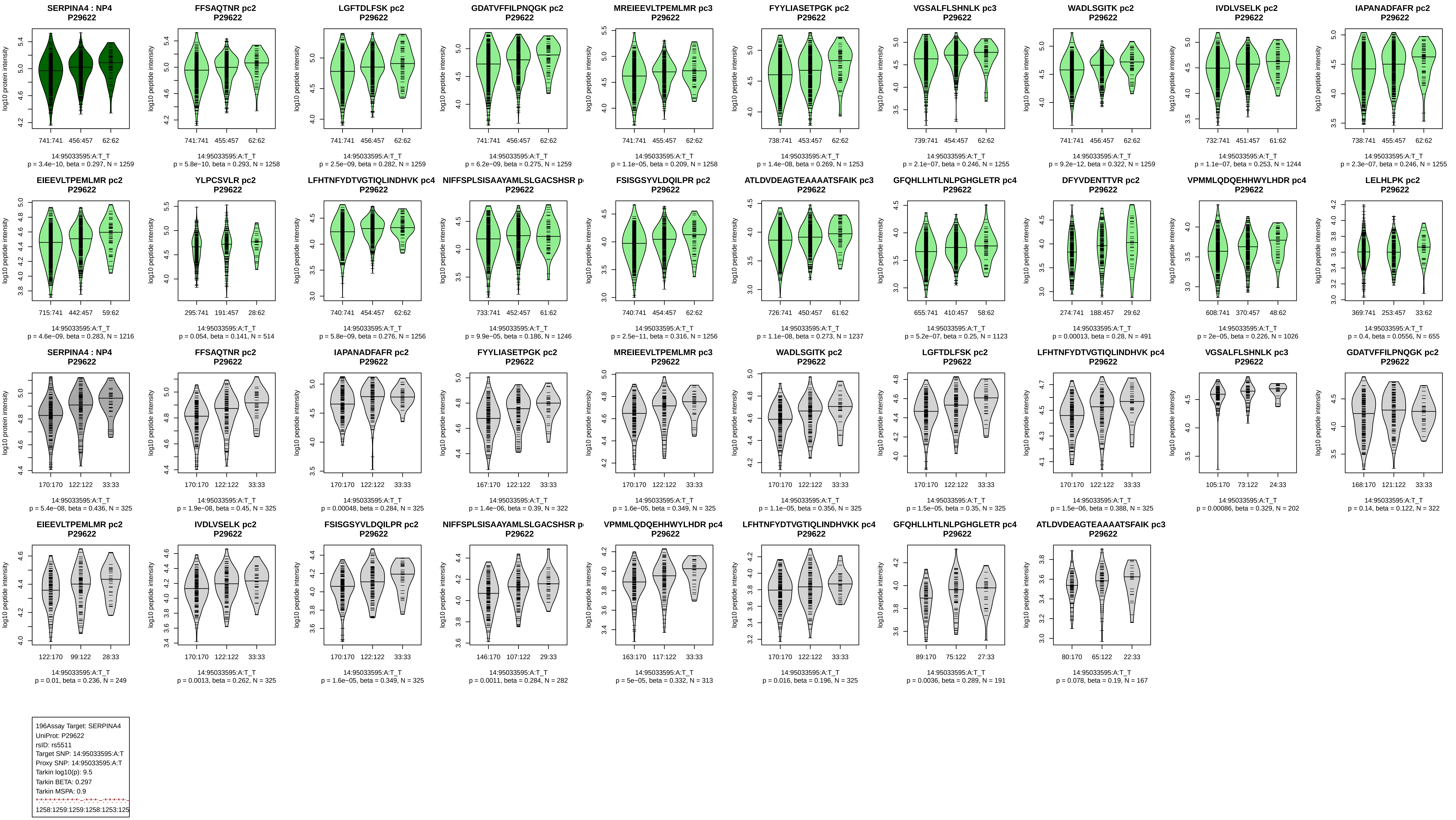
SERPINA4 : NP4 P29622
p = 3.4e−10, beta = 0.297, N = 1259
FFSAQTNR pc2 P29622
p = 5.8e−10, beta = 0.293, N = 1258
LGFTDLFSK pc2 P29622
p = 2.5e−09, beta = 0.282, N = 1259
GDATVFFILPNQGK pc2 P29622
p = 6.2e−09, beta = 0.275, N = 1259
MREIEEVLTPEMLMR pc3 P29622
p = 1.1e−05, beta = 0.209, N = 1258
FYYLIASETPGK pc2 P29622
p = 1.4e−08, beta = 0.269, N = 1253
VGSALFLSHNLK pc3 P29622
p = 2.1e−07, beta = 0.246, N = 1255
WADLSGITK pc2 P29622
p = 9.2e−12, beta = 0.322, N = 1259
IVDLVSELK pc2 P29622
p = 1.1e−07, beta = 0.253, N = 1244
IAPANADFAFR pc2 P29622
p = 2.3e−07, beta = 0.246, N = 1255
EIEEVLTPEMLMR pc2 P29622
p = 4.6e−09, beta = 0.283, N = 1216
YLPCSVLR pc2 P29622
p = 0.054, beta = 0.141, N = 514
LFHTNFYDTVGTIQLINDHVK pc4 P29622
p = 5.8e−09, beta = 0.276, N = 1256
NIFFSPLSISAAYAMLSLGACSHSR pc P29622
p = 9.9e−05, beta = 0.186, N = 1246
FSISGSYVLDQILPR pc2 P29622
p = 2.5e−11, beta = 0.316, N = 1256
ATLDVDEAGTEAAAATSFAIK pc3 P29622
p = 1.1e−08, beta = 0.273, N = 1237
GFQHLLHTLNLPGHGLETR pc4 P29622
p = 5.2e−07, beta = 0.25, N = 1123
DFYVDENTTVR pc2 P29622
p = 0.00013, beta = 0.28, N = 491
VPMMLQDQEHHWYLHDR pc4 P29622
p = 2e−05, beta = 0.226, N = 1026
LELHLPK pc2 P29622
p = 0.4, beta = 0.0556, N = 655
SERPINA4 : NP4 P29622
p = 5.4e−08, beta = 0.436, N = 325
FFSAQTNR pc2 P29622
p = 1.9e−08, beta = 0.45, N = 325
IAPANADFAFR pc2 P29622
p = 0.00048, beta = 0.284, N = 325
FYYLIASETPGK pc2 P29622
p = 1.4e−06, beta = 0.39, N = 322
MREIEEVLTPEMLMR pc3 P29622
p = 1.6e−05, beta = 0.349, N = 325
WADLSGITK pc2 P29622
p = 1.1e−05, beta = 0.356, N = 325
LGFTDLFSK pc2 P29622
p = 1.5e−05, beta = 0.35, N = 325
LFHTNFYDTVGTIQLINDHVK pc4 P29622
p = 1.5e−06, beta = 0.388, N = 325
VGSALFLSHNLK pc3 P29622
p = 0.00086, beta = 0.329, N = 202
GDATVFFILPNQGK pc2 P29622
p = 0.14, beta = 0.122, N = 322
EIEEVLTPEMLMR pc2 P29622
p = 0.01, beta = 0.236, N = 249
IVDLVSELK pc2 P29622
p = 0.0013, beta = 0.262, N = 325
FSISGSYVLDQILPR pc2 P29622
p = 1.6e−05, beta = 0.349, N = 325
NIFFSPLSISAAYAMLSLGACSHSR pc P29622
p = 0.0011, beta = 0.284, N = 282
VPMMLQDQEHHWYLHDR pc4 P29622
p = 5e−05, beta = 0.332, N = 313
LFHTNFYDTVGTIQLINDHVKK pc4 P29622
p = 0.016, beta = 0.196, N = 325
GFQHLLHTLNLPGHGLETR pc4 P29622
p = 0.0036, beta = 0.289, N = 191
ATLDVDEAGTEAAAATSFAIK pc3 P29622
p = 0.078, beta = 0.19, N = 167
14:95033595:A:T_T
196Assay Target: SERPINA4
UniProt: P29622
rsID: rs5511
Target SNP: 14:95033595:A:T
Proxy SNP: 14:95033595:A:T
Tarkin log10(p): 9.5
Tarkin BETA: 0.297
Tarkin MSPA: 0.9
1258:1259:1259:1258:1253:125

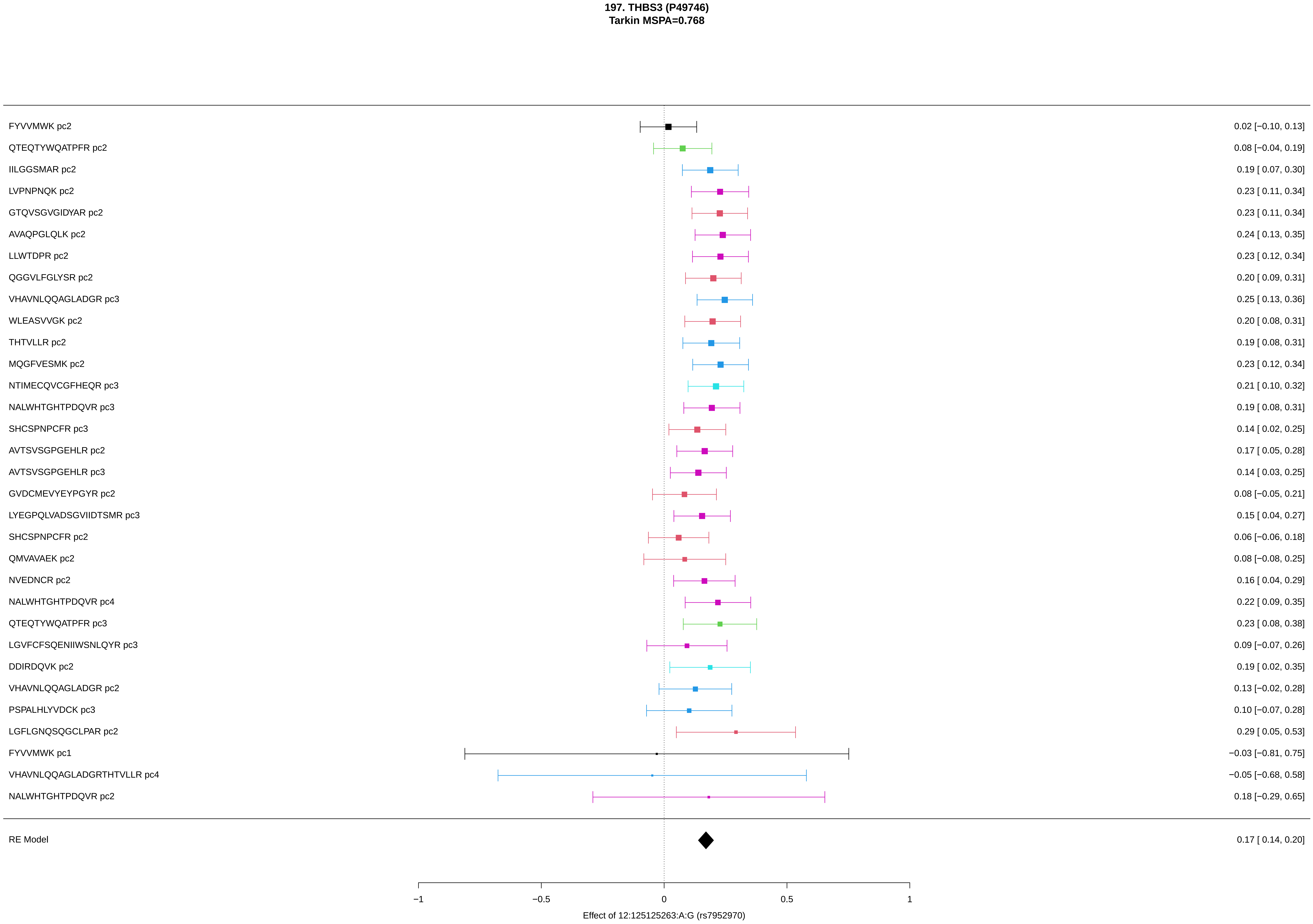
197. THBS3 (P49746)
Tarkin MSPA=0.768
FYVVMWK pc2 0.02 [−0.10, 0.13]
QTEQTYWQATPFR pc2 0.08 [−0.04, 0.19]
IILGGSMAR pc2 0.19 [0.07, 0.30]
LVPNPNQK pc2 0.23 [0.11, 0.34]
GTQVSGVGIDYAR pc2 0.23 [0.11, 0.34]
AVAQPGLQLK pc2 0.24 [0.13, 0.35]
LLWTDPR pc2 0.23 [0.12, 0.34]
QGGVLFGLYSR pc2 0.20 [0.09, 0.31]
VHAVNLQQAGLADGR pc3 0.25 [0.13, 0.36]
WLEASVVGK pc2 0.20 [0.08, 0.31]
THTVLLR pc2 0.19 [0.08, 0.31]
MQGFVESMK pc2 0.23 [0.12, 0.34]
NTIMECQVCGFHEQR pc3 0.21 [0.10, 0.32]
NALWHTGHTPDQVR pc3 0.19 [0.08, 0.31]
SHCSPNPCFR pc3 0.14 [0.02, 0.25]
AVTSVSGPGEHLR pc2 0.17 [0.05, 0.28]
AVTSVSGPGEHLR pc3 0.14 [0.03, 0.25]
GVDCMEVYEYPGYR pc2 0.08 [−0.05, 0.21]
LYEGPQLVADSGVIIDTSMR pc3 0.15 [0.04, 0.27]
SHCSPNPCFR pc2 0.06 [−0.06, 0.18]
QMVAVAEK pc2 0.08 [−0.08, 0.25]
NVEDNCR pc2 0.16 [0.04, 0.29]
NALWHTGHTPDQVR pc4 0.22 [0.09, 0.35]
QTEQTYWQATPFR pc3 0.23 [0.08, 0.38]
LGVFCFSQENIIWSNLQYR pc3 0.09 [−0.07, 0.26]
DDIRDQVK pc2 0.19 [0.02, 0.35]
VHAVNLQQAGLADGR pc2 0.13 [−0.02, 0.28]
PSPALHLYVDCK pc3 0.10 [−0.07, 0.28]
LGFLGNQSQGCLPAR pc2 0.29 [0.05, 0.53]
FYVVMWK pc1 −0.03 [−0.81, 0.75]
VHAVNLQQAGLADGRTHTVLLR pc4 −0.05 [−0.68, 0.58]
NALWHTGHTPDQVR pc2 0.18 [−0.29, 0.65]
RE Model 0.17 [0.14, 0.20]
−1
−0.5
0
0.5
1
Effect of 12:125125263:A:G (rs7952970)

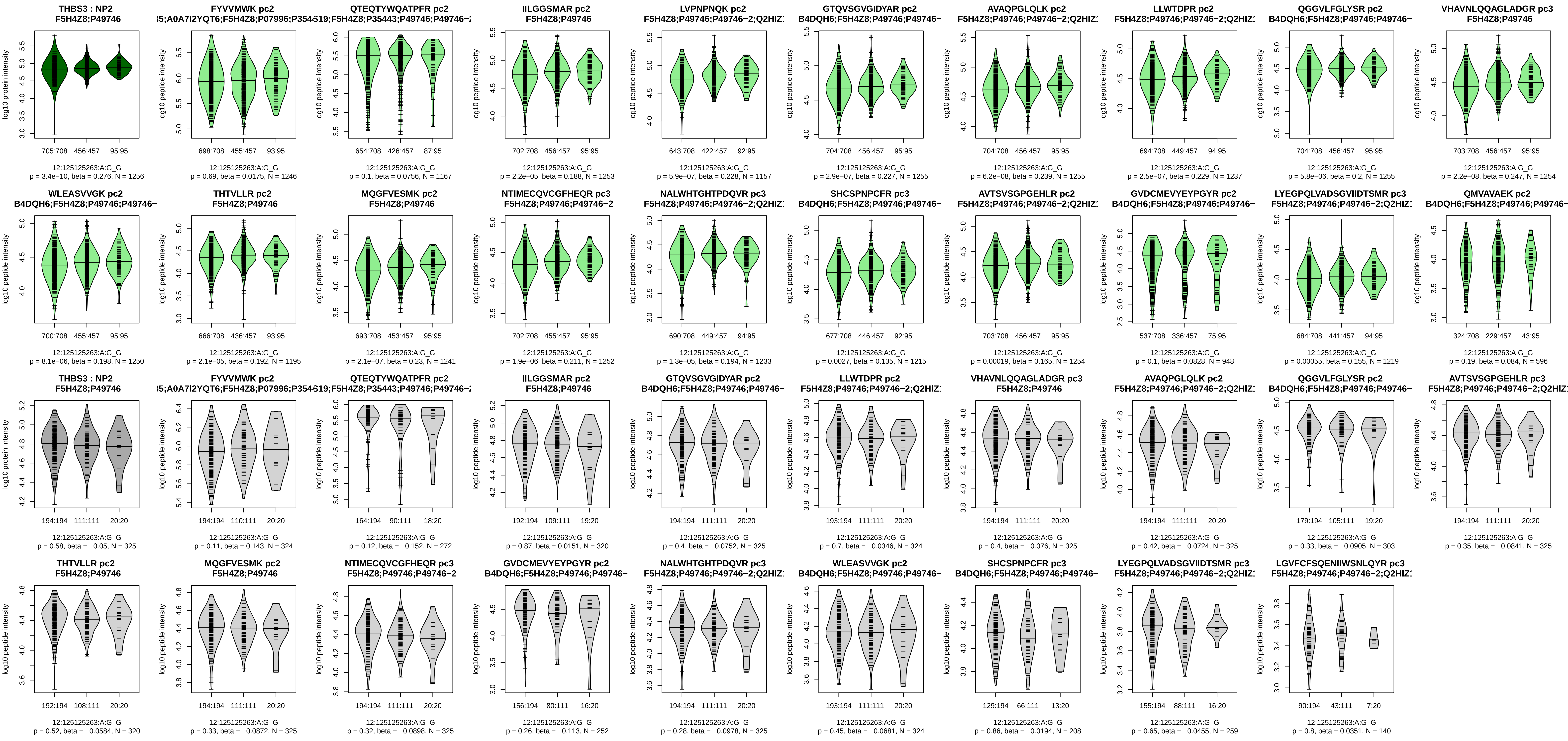

197Assay Target: THBS3
UniProt: P49746
rsID: rs7952970
Target SNP: 12:125125263:A:G
Proxy SNP: 12:125125263:A:G
Tarkin log10(p): 9.5
Tarkin BETA: 0.276
Tarkin MSPA: 0.768
1246:1167:1253:1157:1255:125

198. FCN2 (Q15485)
Tarkin MSPA=0.736

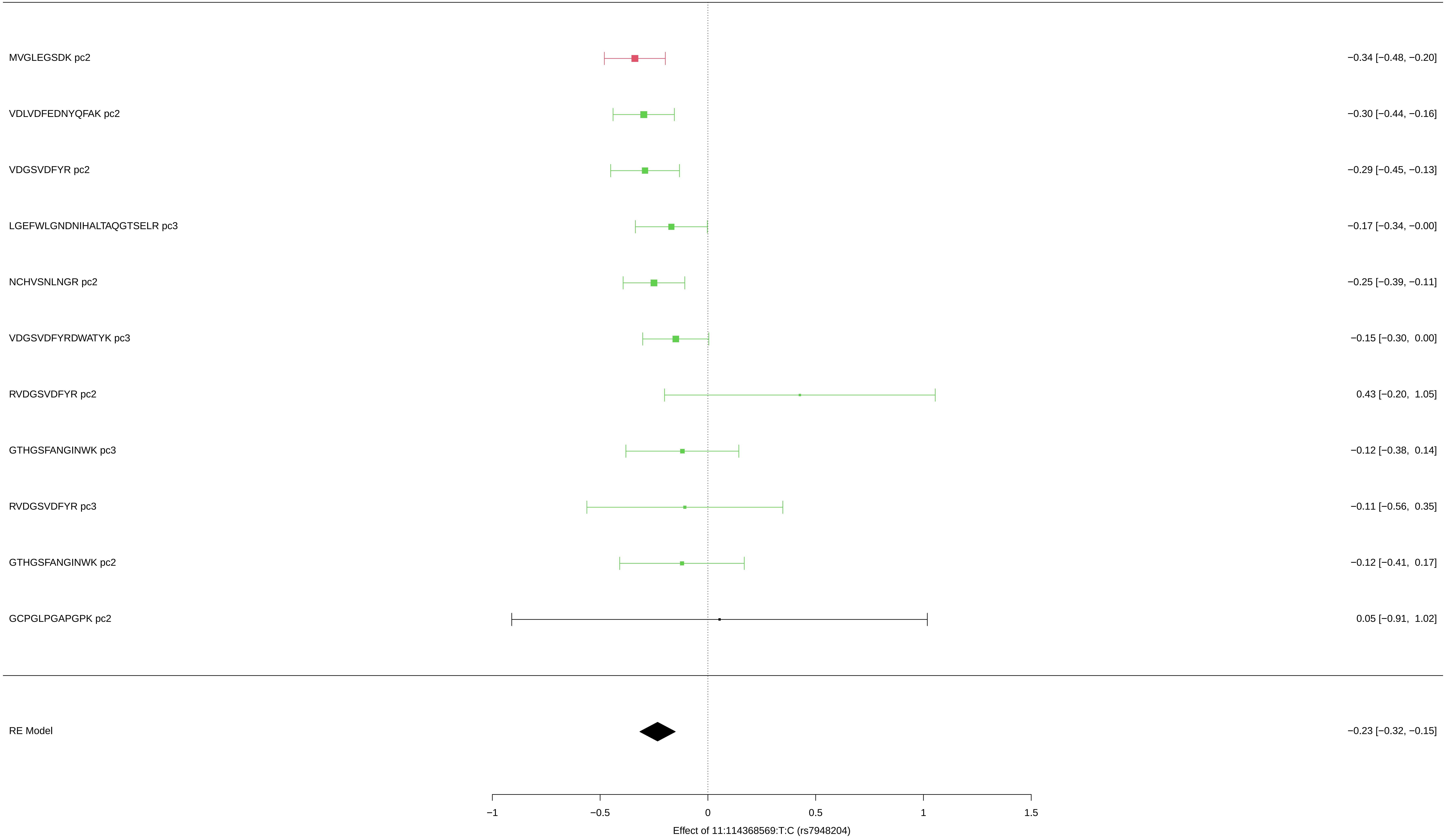

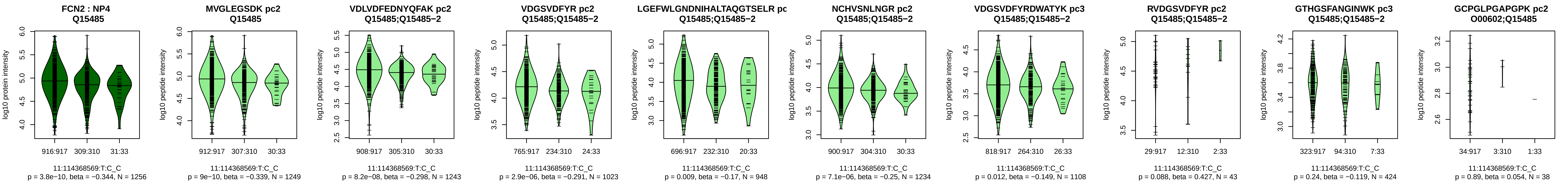

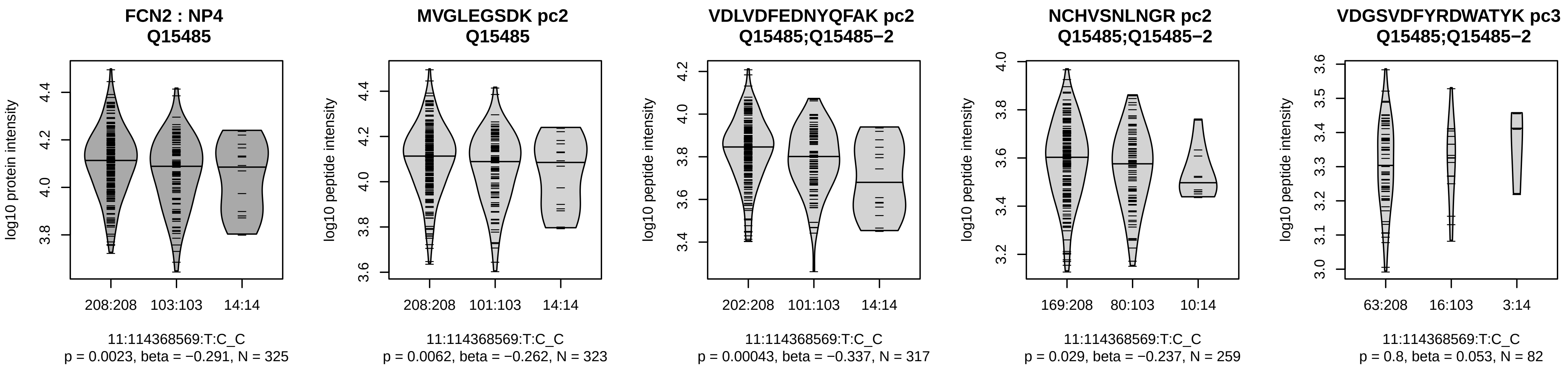

198Assay Target: FCN2
UniProt: Q15485
rsID: rs7948204
Target SNP: 11:114368569:T:C
Proxy SNP: 11:114368569:T:C
Tarkin log10(p): 9.4
Tarkin BETA: −0.344
Tarkin MSPA: 0.736
1249:1243:1023:948:1234:1108

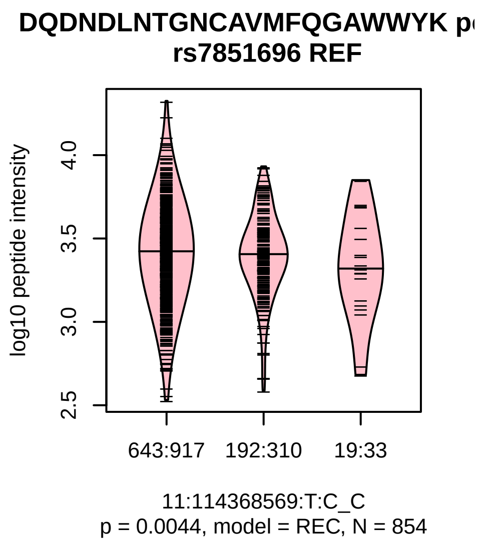

199. C4A (P0C0L4)
Tarkin MSPA=0.249

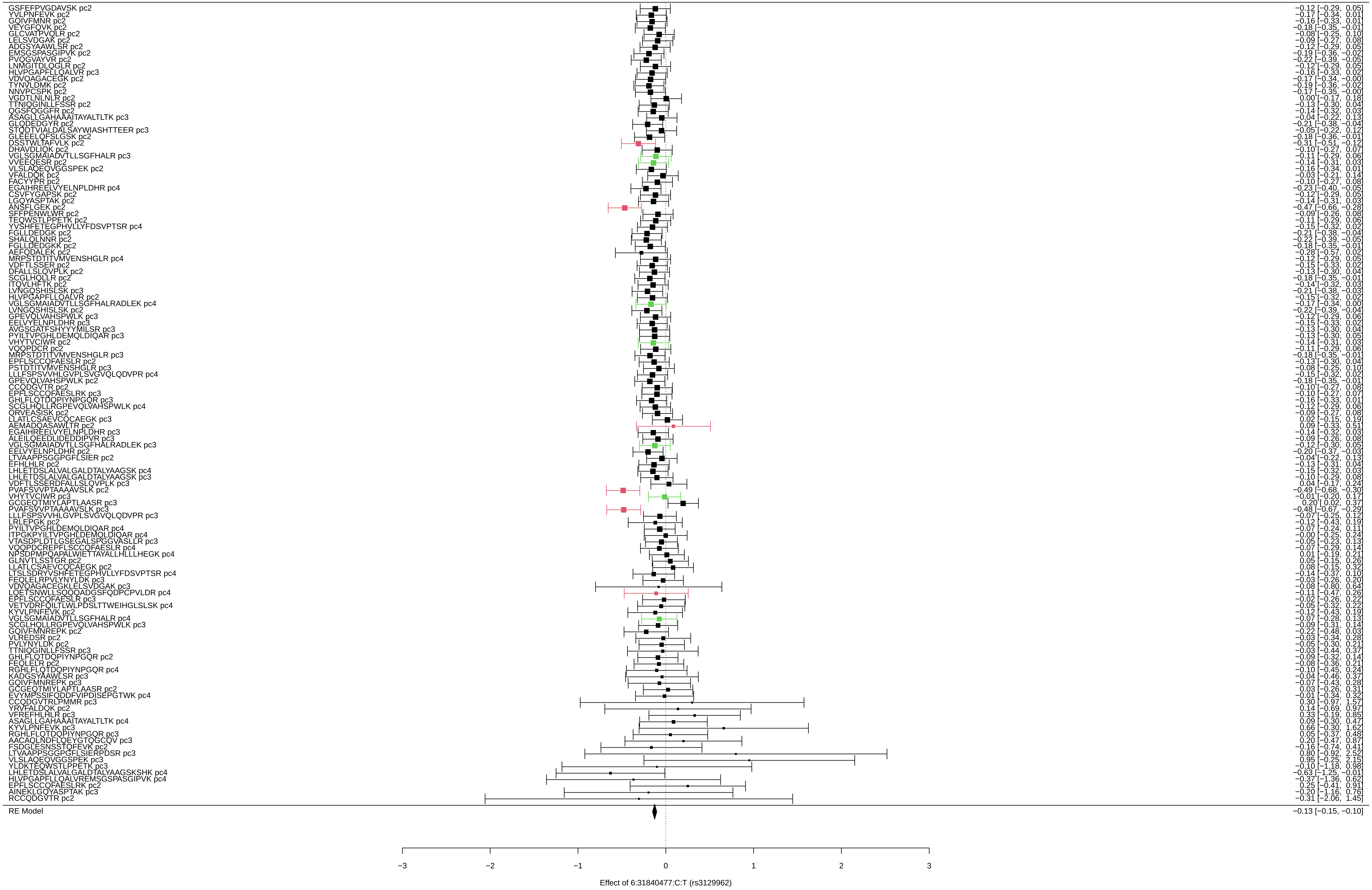

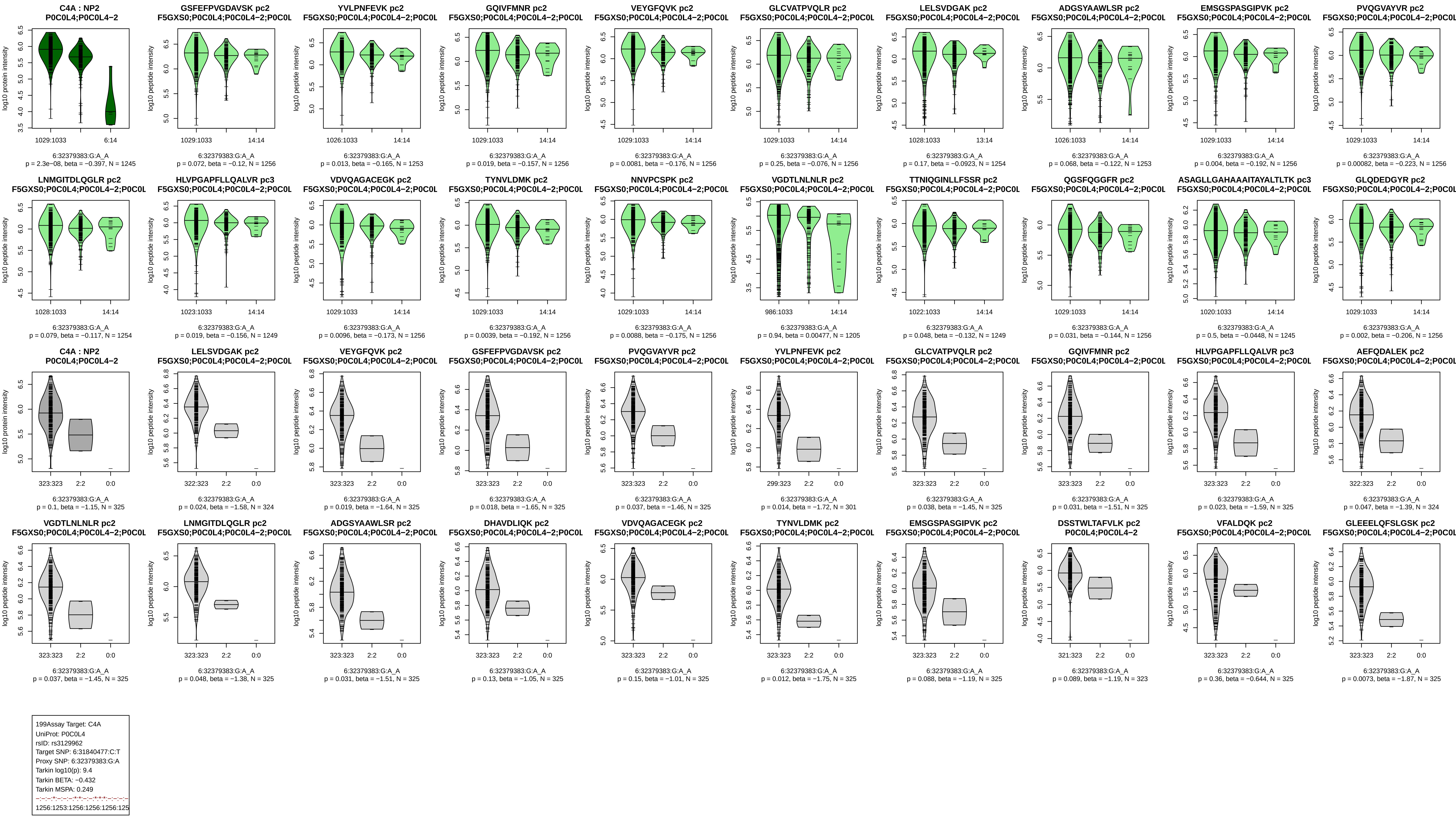
C4A : NP2
P0C0L4;P0C0L4−2
6:32379383:G:A_A
p = 2.3e−08, beta = −0.397, N = 1245
GSFEFPVGDAVSK pc2
F5GXS0;P0C0L4;P0C0L4−2;P0C0L
p = 0.072, beta = −0.12, N = 1256
YVLPNFEVK pc2
p = 0.013, beta = −0.165, N = 1253
GQIVFMNR pc2
p = 0.019, beta = −0.157, N = 1256
VEYGFQVK pc2
p = 0.0081, beta = −0.176, N = 1256
GLCVATPVQLR pc2
p = 0.25, beta = −0.076, N = 1256
LELSVDGAK pc2
p = 0.17, beta = −0.0923, N = 1254
ADGSYAAWLSR pc2
p = 0.068, beta = −0.122, N = 1253
EMSGSPASGIPVK pc2
p = 0.004, beta = −0.192, N = 1256
PVQGVAYVR pc2
p = 0.00082, beta = −0.223, N = 1256
LNMGITDLQGLR pc2
p = 0.079, beta = −0.117, N = 1254
HLVPGAPFLLQALVR pc3
p = 0.019, beta = −0.156, N = 1249
VDVQAGACEGK pc2
p = 0.0096, beta = −0.173, N = 1256
TYNVLDMK pc2
p = 0.0039, beta = −0.192, N = 1256
NNVPCSPK pc2
p = 0.0088, beta = −0.175, N = 1256
VGDTLNLNLR pc2
p = 0.94, beta = 0.00477, N = 1205
TTNIQGINLLFSSR pc2
p = 0.048, beta = −0.132, N = 1249
QGSFQGGFR pc2
p = 0.031, beta = −0.144, N = 1256
ASAGLLGAHAAAITAYALTLTK pc3
p = 0.5, beta = −0.0448, N = 1245
GLQDEDGYR pc2
p = 0.002, beta = −0.206, N = 1256
C4A : NP2
p = 0.1, beta = −1.15, N = 325
LELSVDGAK pc2
p = 0.024, beta = −1.58, N = 324
VEYGFQVK pc2
p = 0.019, beta = −1.64, N = 325
GSFEFPVGDAVSK pc2
p = 0.018, beta = −1.65, N = 325
PVQGVAYVR pc2
p = 0.037, beta = −1.46, N = 325
YVLPNFEVK pc2
p = 0.014, beta = −1.72, N = 301
GLCVATPVQLR pc2
p = 0.038, beta = −1.45, N = 325
GQIVFMNR pc2
p = 0.031, beta = −1.51, N = 325
HLVPGAPFLLQALVR pc3
p = 0.023, beta = −1.59, N = 325
AEFQDALEK pc2
p = 0.047, beta = −1.39, N = 324
VGDTLNLNLR pc2
p = 0.037, beta = −1.45, N = 325
LNMGITDLQGLR pc2
p = 0.048, beta = −1.38, N = 325
ADGSYAAWLSR pc2
p = 0.031, beta = −1.51, N = 325
DHAVDLIQK pc2
p = 0.13, beta = −1.05, N = 325
VDVQAGACEGK pc2
p = 0.15, beta = −1.01, N = 325
TYNVLDMK pc2
p = 0.012, beta = −1.75, N = 325
EMSGSPASGIPVK pc2
p = 0.088, beta = −1.19, N = 325
DSSTWLTAFVLK pc2
P0C0L4;P0C0L4−2
p = 0.089, beta = −1.19, N = 323
VFALDQK pc2
p = 0.36, beta = −0.644, N = 325
GLEEELQFSLGSK pc2
p = 0.0073, beta = −1.87, N = 325
199Assay Target: C4A
UniProt: P0C0L4
rsID: rs3129962
Target SNP: 6:31840477:C:T
Proxy SNP: 6:32379383:G:A
Tarkin log10(p): 9.4
Tarkin BETA: −0.432
Tarkin MSPA: 0.249
1256:1253:1256:1256:1256:125

200. LAMB1 (P07942)
Tarkin MSPA=0.89

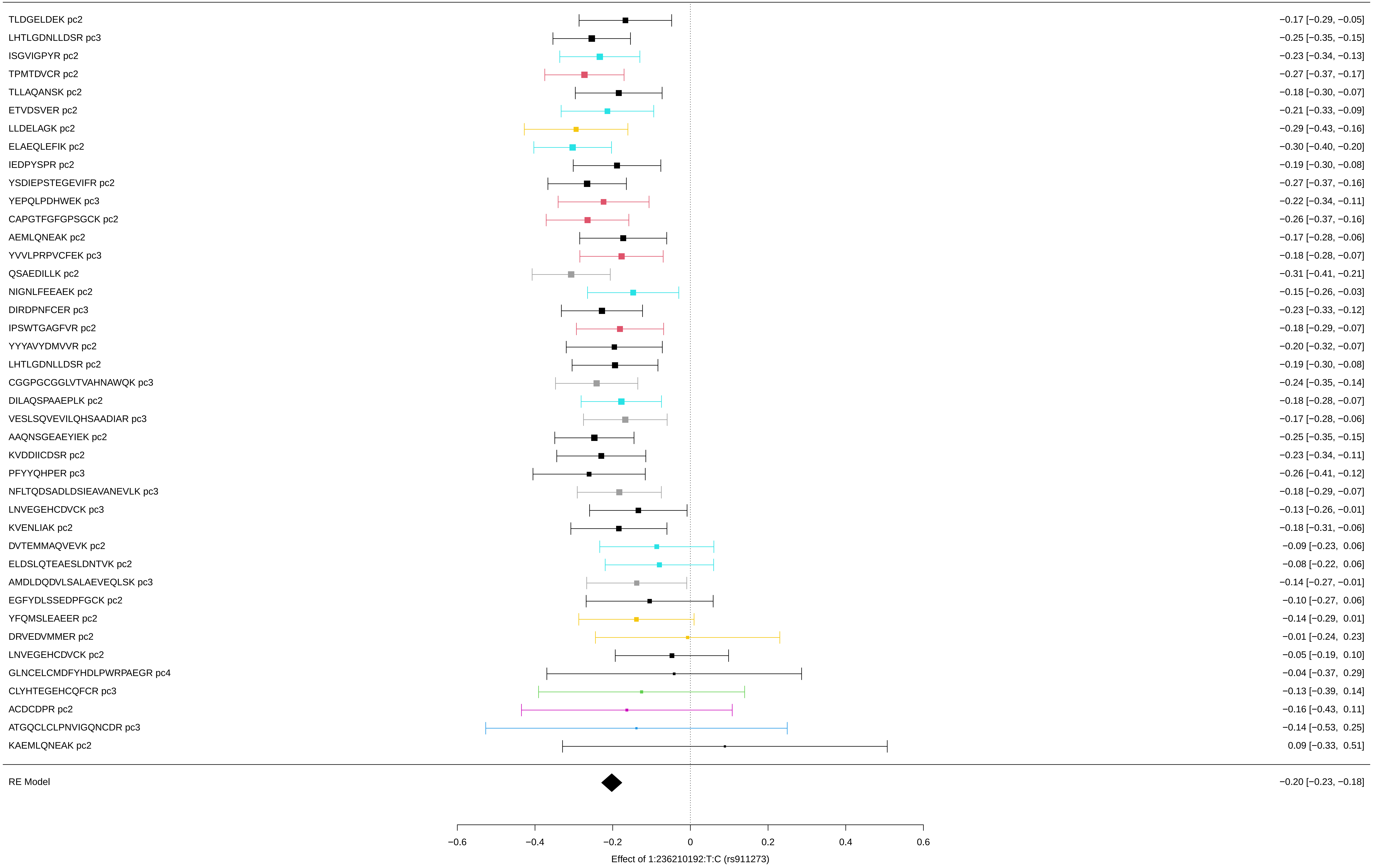

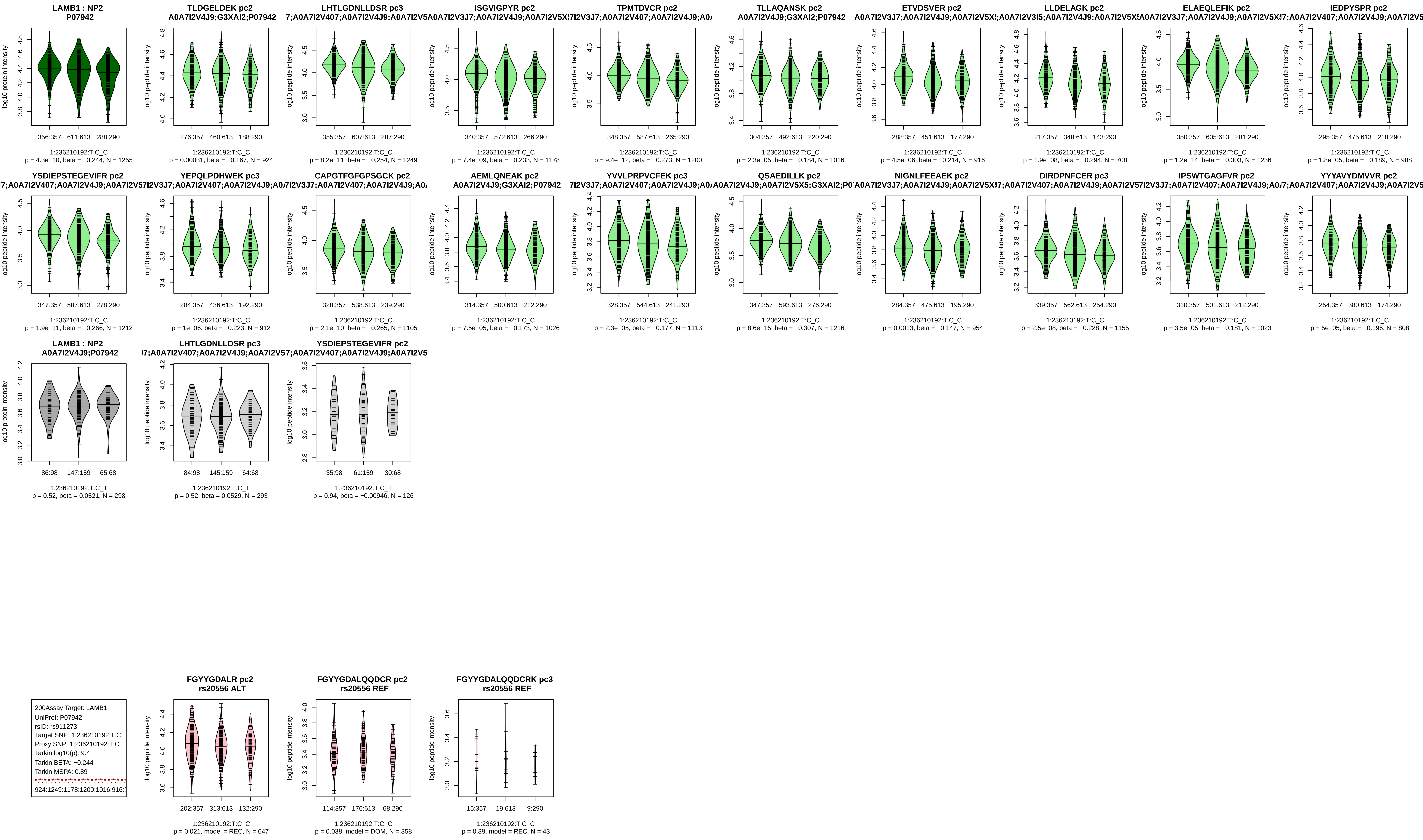
LAMB1 : NP2
P07942
1:236210192:T:C_C
p = 4.3e−10, beta = −0.244, N = 1255
TLDGELDEK pc2
A0A7I2V4J9;G3XAI2;P07942
1:236210192:T:C_C
p = 0.00031, beta = −0.167, N = 924
LHTLGDNLLDSR pc3
1:236210192:T:C_C
p = 8.2e−11, beta = −0.254, N = 1249
ISGVIGPYR pc2
1:236210192:T:C_C
p = 7.4e−09, beta = −0.233, N = 1178
TPMTDVCR pc2
1:236210192:T:C_C
p = 9.4e−12, beta = −0.273, N = 1200
TLLAQANSK pc2
A0A7I2V4J9;G3XAI2;P07942
1:236210192:T:C_C
p = 2.3e−05, beta = −0.184, N = 1016
ETVDSVER pc2
1:236210192:T:C_C
p = 4.5e−06, beta = −0.214, N = 916
LLDELAGK pc2
1:236210192:T:C_C
p = 1.9e−08, beta = −0.294, N = 708
ELAEQLEFIK pc2
1:236210192:T:C_C
p = 1.2e−14, beta = −0.303, N = 1236
IEDPYSPR pc2
1:236210192:T:C_C
p = 1.8e−05, beta = −0.189, N = 988
YSDIEPSTEGEVIFR pc2
1:236210192:T:C_C
p = 1.9e−11, beta = −0.266, N = 1212
YEPQLPDHWEK pc3
1:236210192:T:C_C
p = 1e−06, beta = −0.223, N = 912
CAPGTFGFGPSGCK pc2
1:236210192:T:C_C
p = 2.1e−10, beta = −0.265, N = 1105
AEMLQNEAK pc2
A0A7I2V4J9;G3XAI2;P07942
1:236210192:T:C_C
p = 7.5e−05, beta = −0.173, N = 1026
YVVLPRPVCFEK pc3
1:236210192:T:C_C
p = 2.3e−05, beta = −0.177, N = 1113
QSAEDILLK pc2
1:236210192:T:C_C
p = 8.6e−15, beta = −0.307, N = 1216
NIGNLFEEAEK pc2
1:236210192:T:C_C
p = 0.0013, beta = −0.147, N = 954
DIRDPNFCER pc3
1:236210192:T:C_C
p = 2.5e−08, beta = −0.228, N = 1155
IPSWTGAGFVR pc2
1:236210192:T:C_C
p = 3.5e−05, beta = −0.181, N = 1023
YYYAVYDMVVR pc2
1:236210192:T:C_C
p = 5e−05, beta = −0.196, N = 808
LAMB1 : NP2
A0A7I2V4J9;P07942
1:236210192:T:C_T
p = 0.52, beta = 0.0521, N = 298
LHTLGDNLLDSR pc3
1:236210192:T:C_T
p = 0.52, beta = 0.0529, N = 293
YSDIEPSTEGEVIFR pc2
1:236210192:T:C_T
p = 0.94, beta = −0.00946, N = 126
200Assay Target: LAMB1
UniProt: P07942
rsID: rs911273
Target SNP: 1:236210192:T:C
Proxy SNP: 1:236210192:T:C
Tarkin log10(p): 9.4
Tarkin BETA: −0.244
Tarkin MSPA: 0.89
FGYYGDALR pc2
rs20556 ALT
1:236210192:T:C_C
p = 0.021, model = REC, N = 647
FGYYGDALQQDCR pc2
rs20556 REF
1:236210192:T:C_C
p = 0.038, model = DOM, N = 358
FGYYGDALQQDCRK pc3
rs20556 REF
1:236210192:T:C_C
p = 0.39, model = REC, N = 43

201. LTBP1 (Q14766)
Tarkin MSPA=0.023

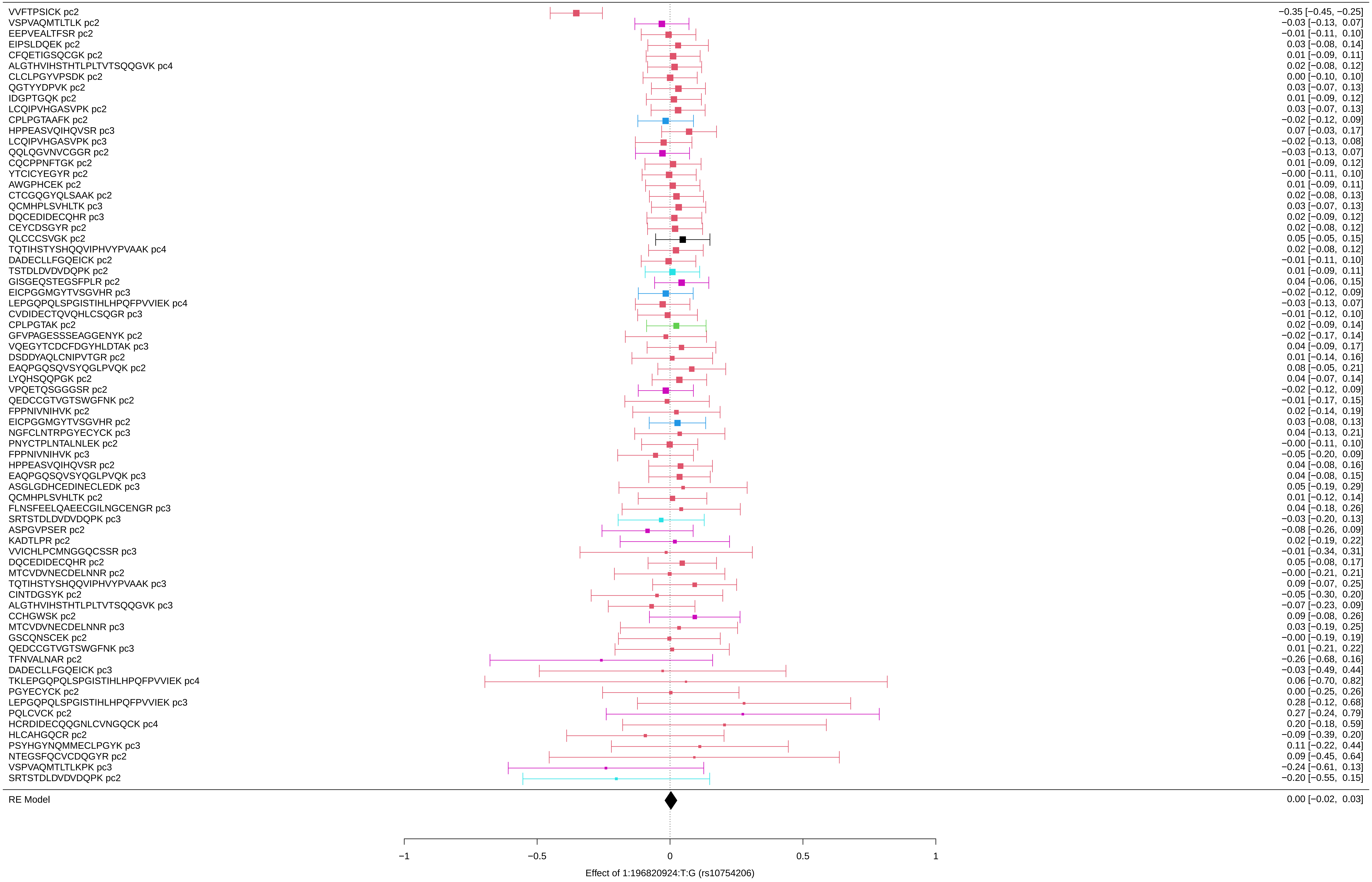

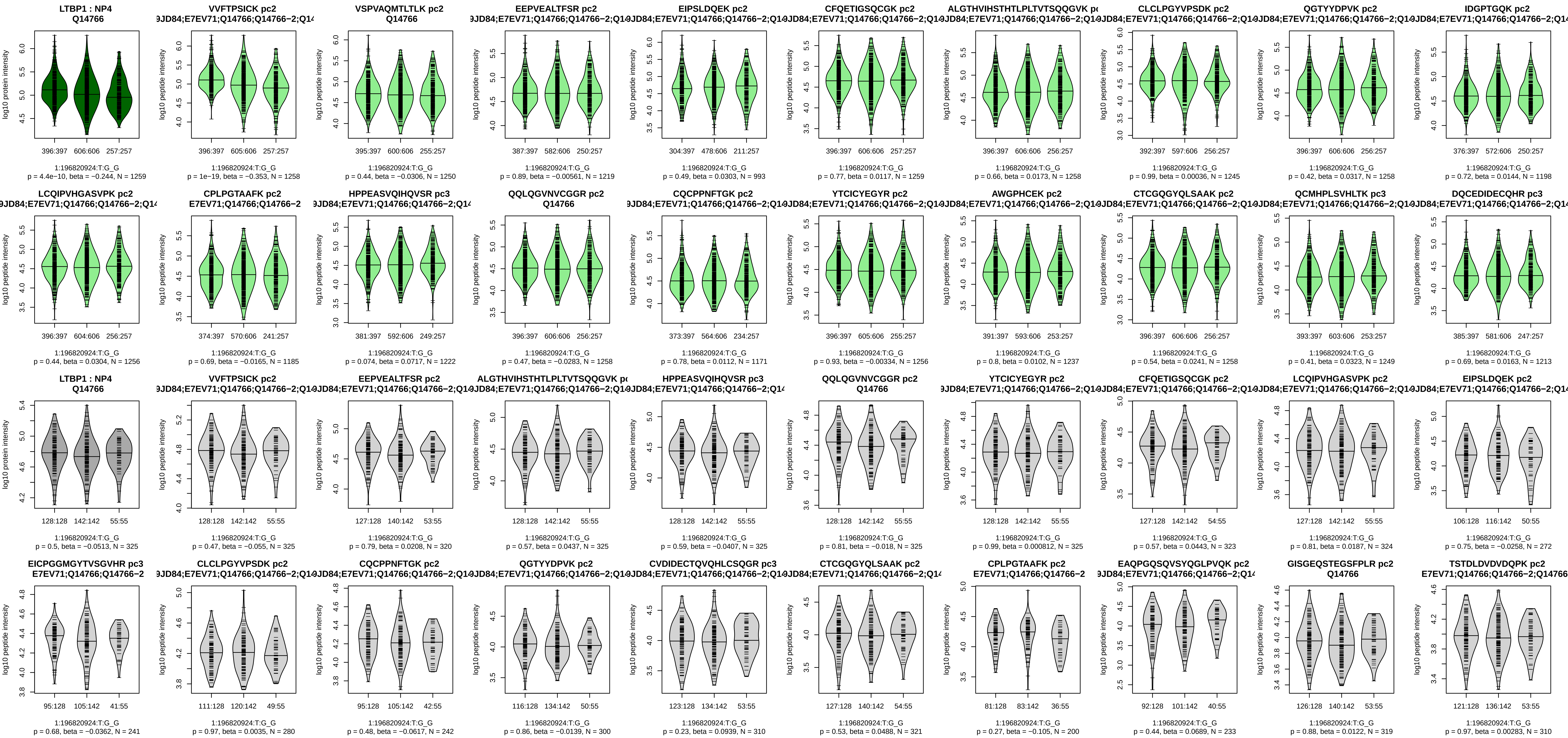

201Assay Target: LTBP1
UniProt: Q14766
rsID: rs10754206
Target SNP: 1:196820924:T:G
Proxy SNP: 1:196820924:T:G
Tarkin log10(p): 9.4
Tarkin BETA: −0.244
Tarkin MSPA: 0.023
1258:1250:1219:993:1259:1258

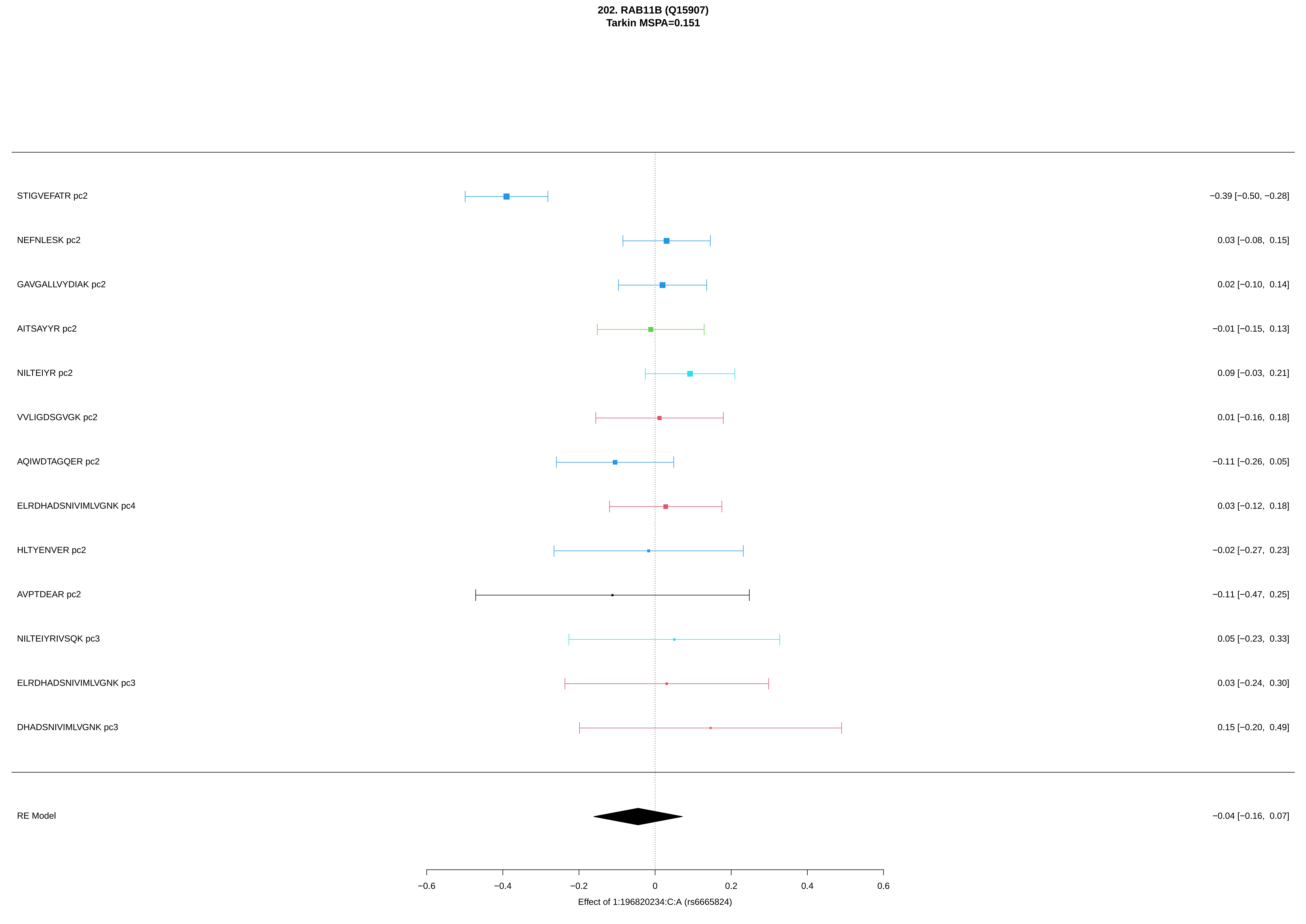
202. RAB11B (Q15907)
Tarkin MSPA=0.151
STIGVEFATR pc2
−0.39 [−0.50, −0.28]
NEFNLESK pc2
0.03 [−0.08, 0.15]
GAVGALLVYDIAK pc2
0.02 [−0.10, 0.14]
AITSAYYR pc2
−0.01 [−0.15, 0.13]
NILTEIYR pc2
0.09 [−0.03, 0.21]
VVLIGDSGVGK pc2
0.01 [−0.16, 0.18]
AQIWDTAGQER pc2
−0.11 [−0.26, 0.05]
ELRDHADSNIVIMLVGNK pc4
0.03 [−0.12, 0.18]
HLTYENVER pc2
−0.02 [−0.27, 0.23]
AVPTDEAR pc2
−0.11 [−0.47, 0.25]
NILTEIYRIVSQK pc3
0.05 [−0.23, 0.33]
ELRDHADSNIVIMLVGNK pc3
0.03 [−0.24, 0.30]
DHADSNIVIMLVGNK pc3
0.15 [−0.20, 0.49]
RE Model
−0.04 [−0.16, 0.07]
−0.6
−0.4
−0.2
0
0.2
0.4
0.6
Effect of 1:196820234:C:A (rs6665824)

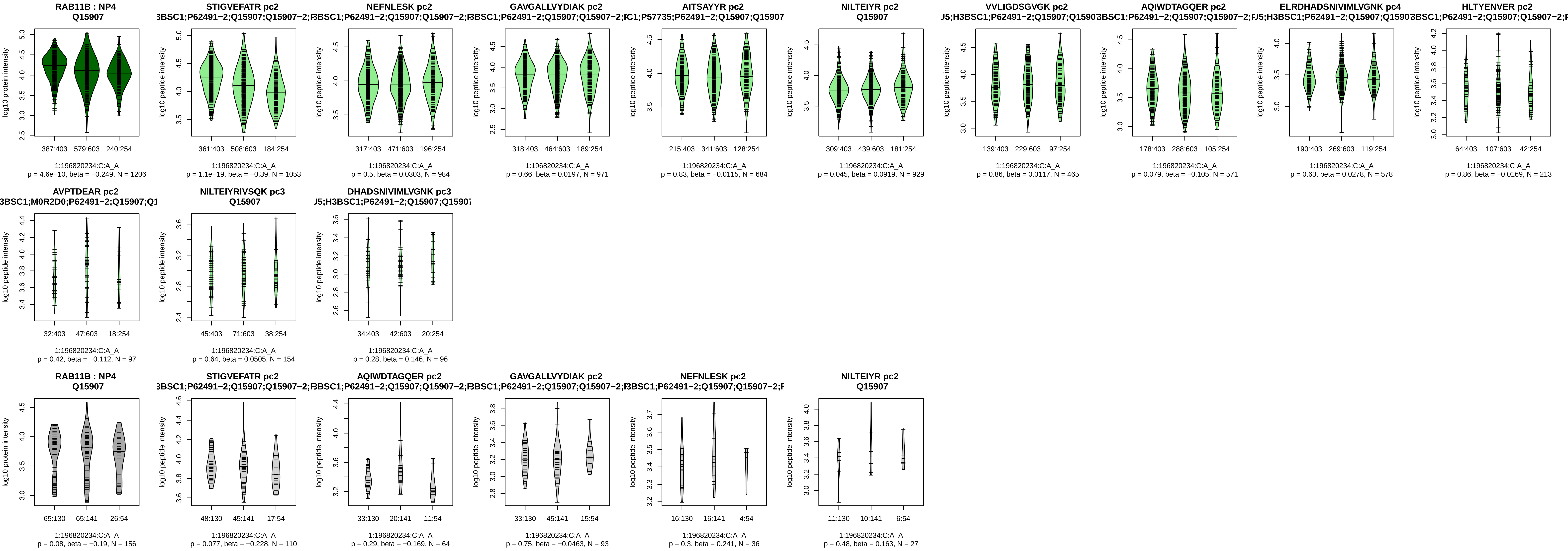

202Assay Target: RAB11B
UniProt: Q15907
rsID: rs6665824
Target SNP: 1:196820234:C:A
Proxy SNP: 1:196820234:C:A
Tarkin log10(p): 9.3
Tarkin BETA: −0.249
Tarkin MSPA: 0.151
*:−:−:−:−:−:−:−:−:−:−:NA
1053:984:971:684:929:465:571:

203. APOE (P02649)
Tarkin MSPA=0.814

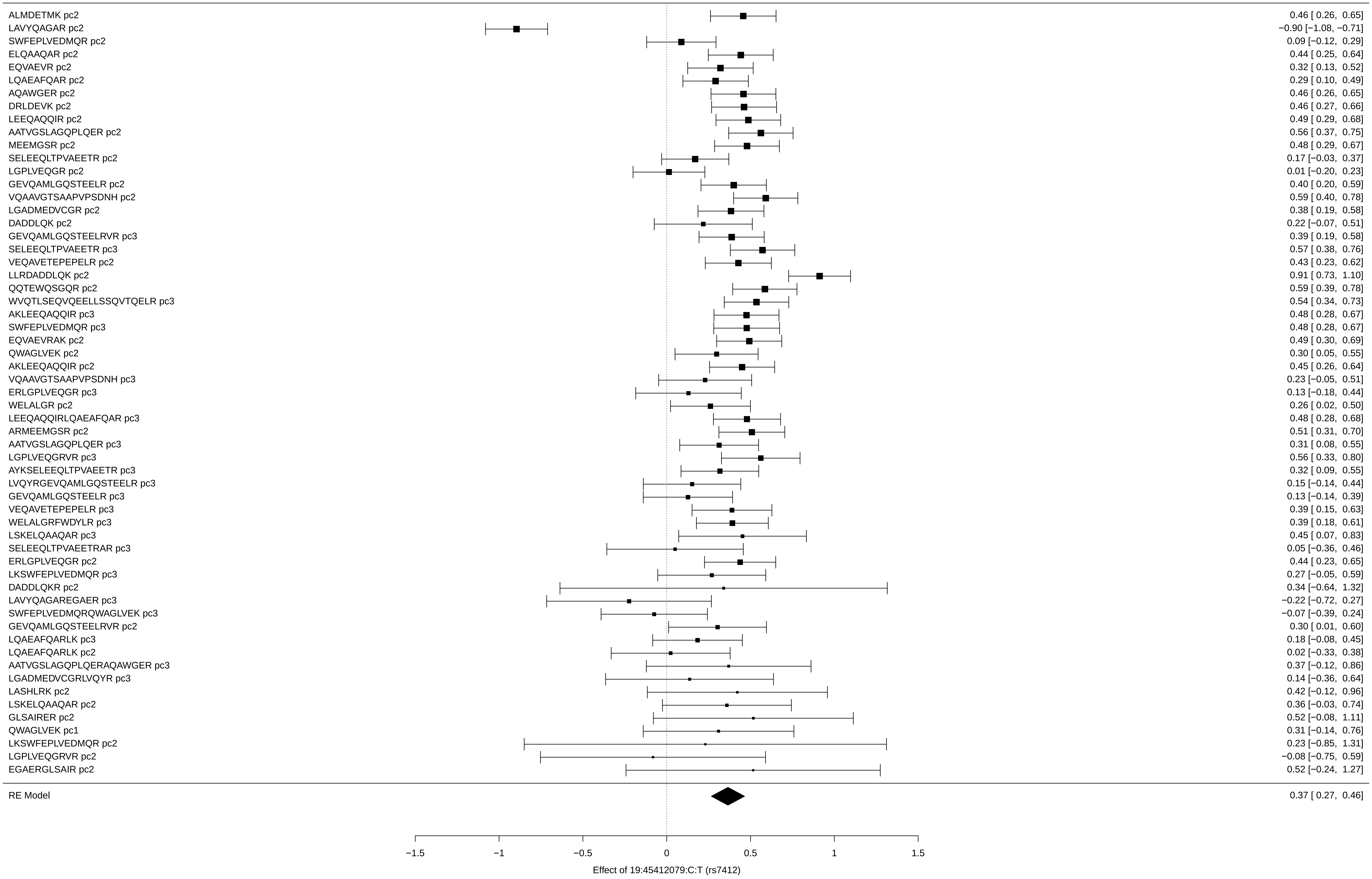

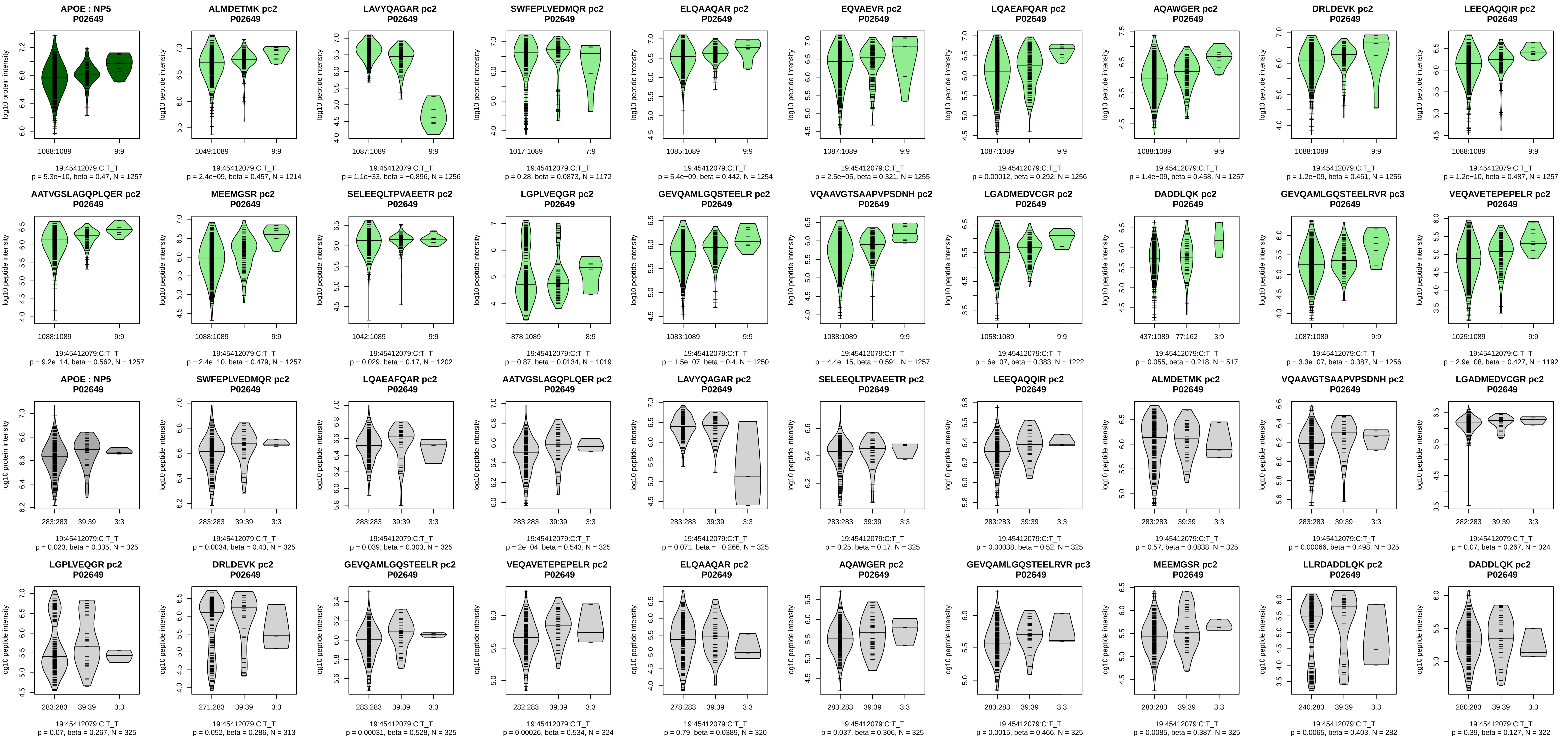

203Assay Target: APOE
UniProt: P02649
rsID: rs7412
Target SNP: 19:45412079:C:T
Proxy SNP: 19:45412079:C:T
Tarkin log10(p): 9.3
Tarkin BETA: 0.47
Tarkin MSPA: 0.814
::_:*:*:*:*:*:*:*:*:_:_:*:*:*:_:*:*:*:
1214:1256:1172:1254:1255:125

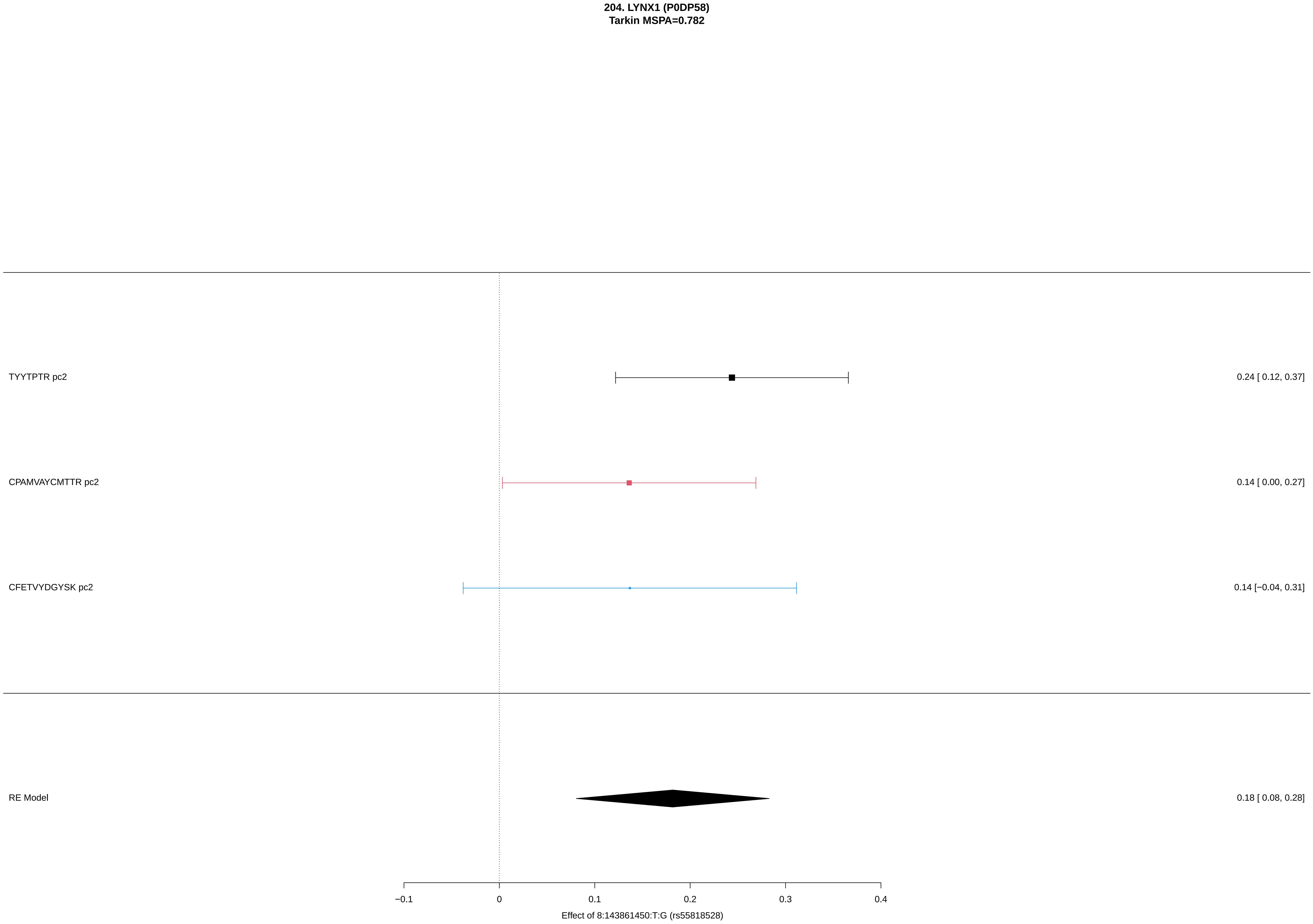
204. LYNX1 (P0DP58)
Tarkin MSPA=0.782
TYYTPTR pc2
0.24 [0.12, 0.37]
CPAMVAYCMTTR pc2
0.14 [0.00, 0.27]
CFETVYDGYSK pc2
0.14 [−0.04, 0.31]
RE Model
0.18 [0.08, 0.28]
−0.1
0
0.1
0.2
0.3
0.4
Effect of 8:143861450:T:G (rs55818528)

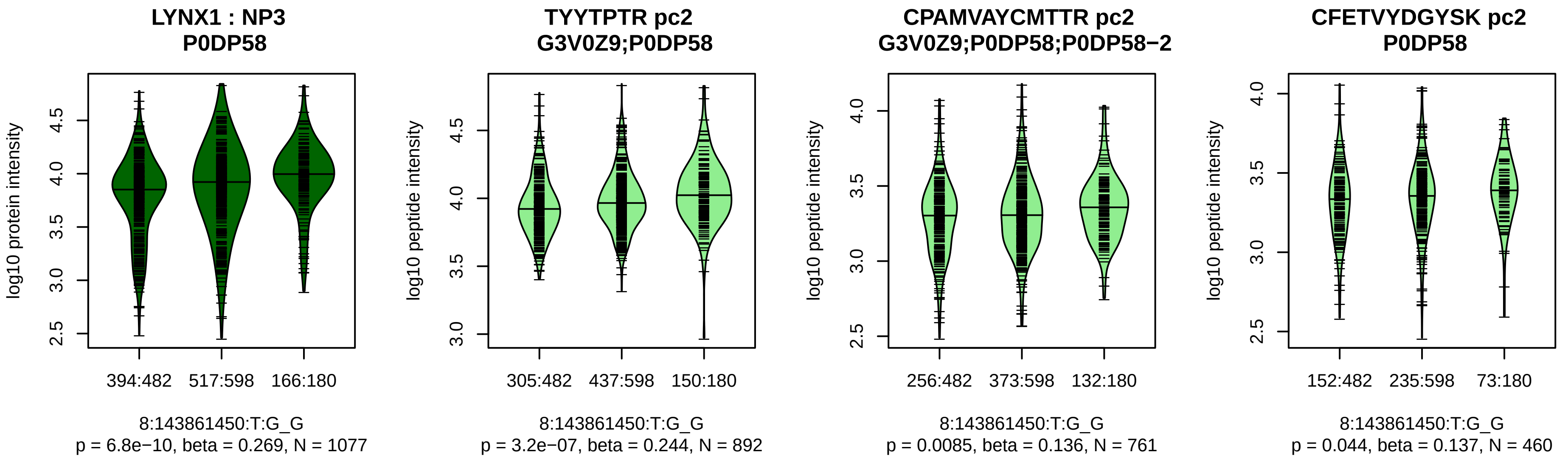

204Assay Target: LYNX1
UniProt: P0DP58
rsID: rs55818528
Target SNP: 8:143861450:T:G
Proxy SNP: 8:143861450:T:G
Tarkin log10(p): 9.2
Tarkin BETA: 0.269
Tarkin MSPA: 0.782
::−:NA
892:761:460:1

205. CTSS (P25774)
Tarkin MSPA=0.915

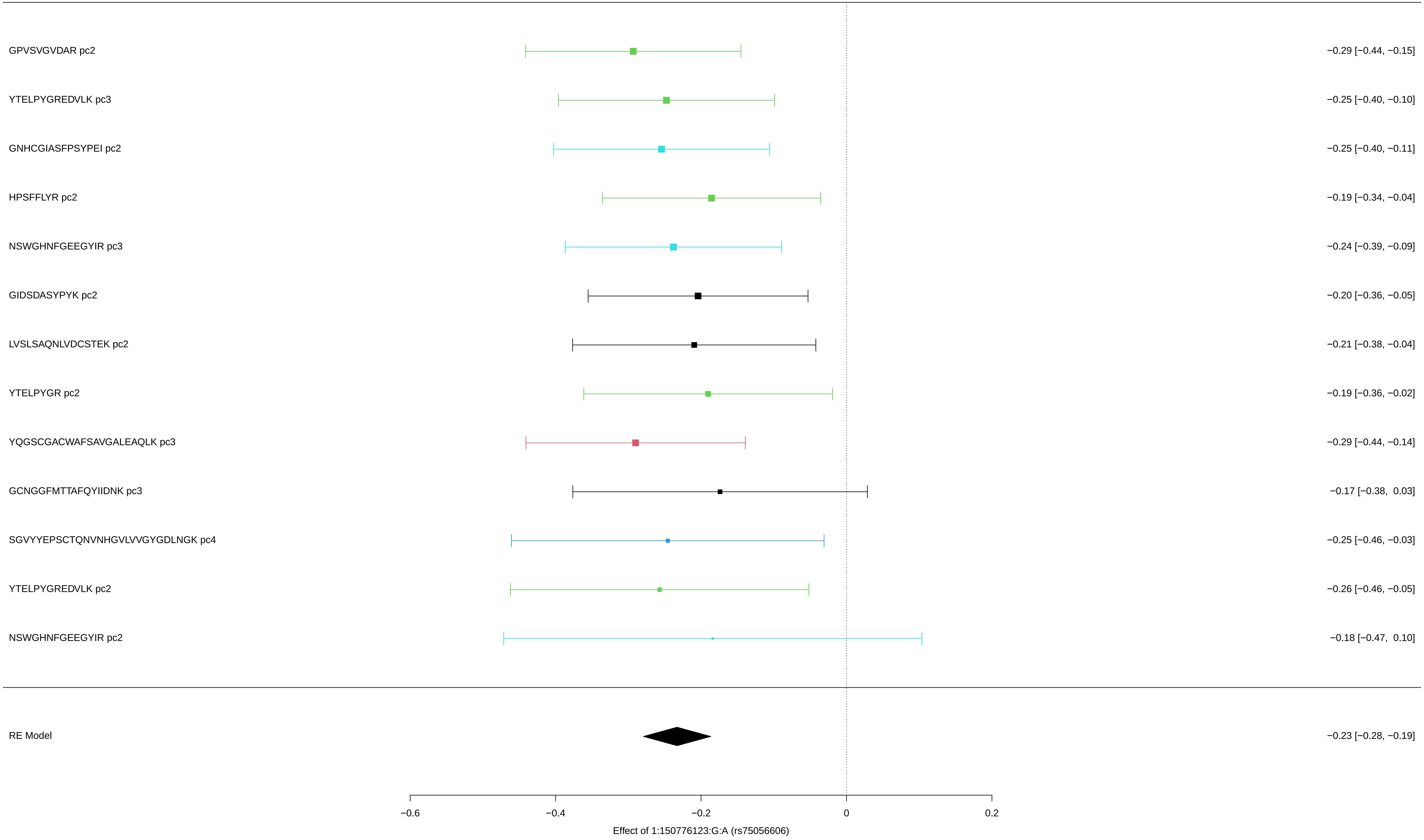

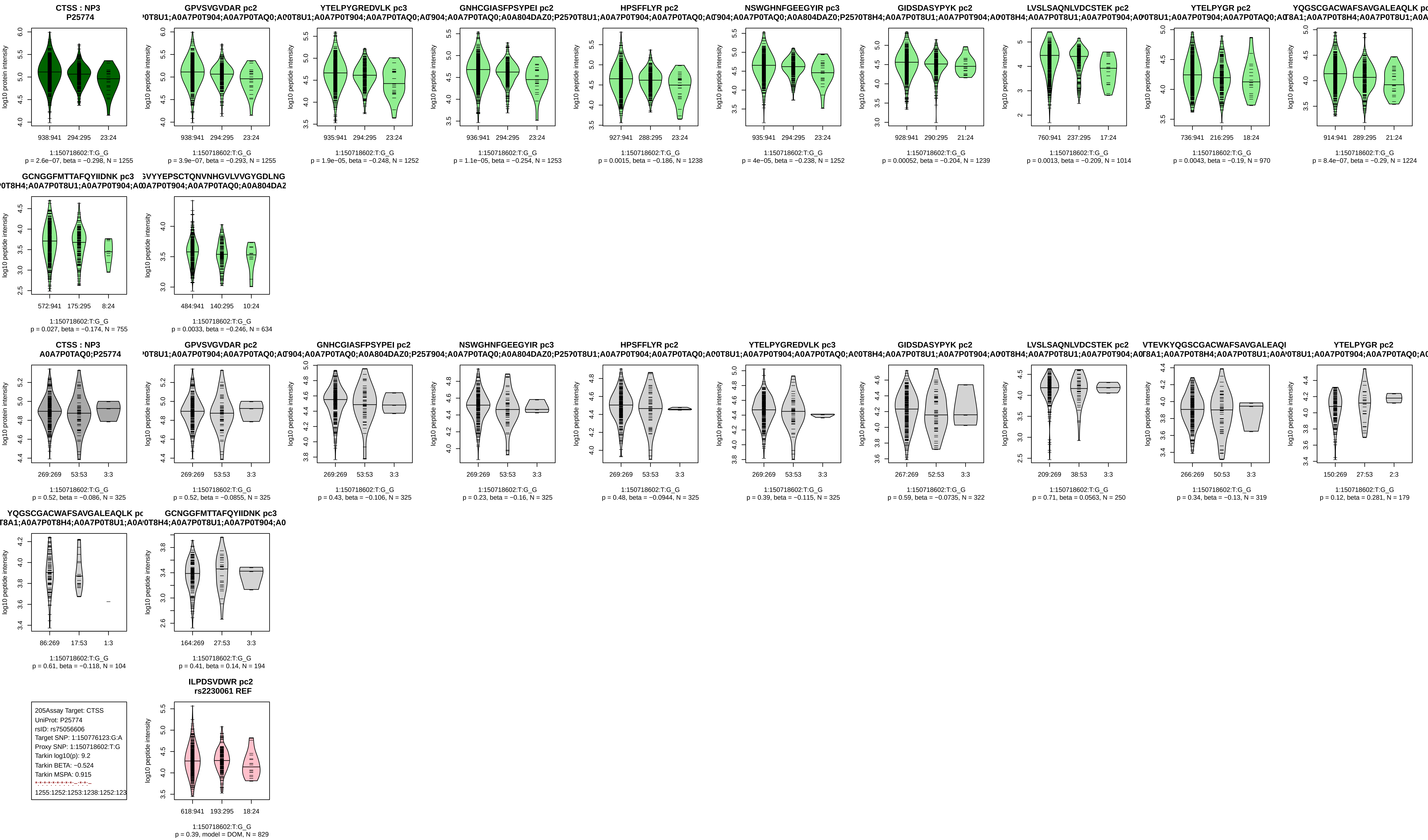
CTSS : NP3
P25774
log10 protein intensity
938:941 294:295 23:24
1:150718602:T:G_G
p = 2.6e−07, beta = −0.298, N = 1255
GPVSVGVDAR pc2
log10 peptide intensity
938:941 294:295 23:24
1:150718602:T:G_G
p = 3.9e−07, beta = −0.293, N = 1255
YTELPYGREDVLK pc3
935:941 294:295 23:24
1:150718602:T:G_G
p = 1.9e−05, beta = −0.248, N = 1252
GNHCGIASFPSYPEI pc2
936:941 294:295 23:24
1:150718602:T:G_G
p = 1.1e−05, beta = −0.254, N = 1253
HPSFFLYR pc2
927:941 288:295 23:24
1:150718602:T:G_G
p = 0.0015, beta = −0.186, N = 1238
NSWGHNFGEEGYIR pc3
935:941 294:295 23:24
1:150718602:T:G_G
p = 4e−05, beta = −0.238, N = 1252
GIDSDASYPYK pc2
928:941 290:295 21:24
1:150718602:T:G_G
p = 0.00052, beta = −0.204, N = 1239
LVSLSAQNLVDCSTEK pc2
760:941 237:295 17:24
1:150718602:T:G_G
p = 0.0013, beta = −0.209, N = 1014
YTELPYGR pc2
736:941 216:295 18:24
1:150718602:T:G_G
p = 0.0043, beta = −0.19, N = 970
YQGSCGACWAFSAVGALEAQLK pc
914:941 289:295 21:24
1:150718602:T:G_G
p = 8.4e−07, beta = −0.29, N = 1224
GCNGGFMTTAFQYIIDNK pc3
572:941 175:295 8:24
1:150718602:T:G_G
p = 0.027, beta = −0.174, N = 755
GVYYEPSCTQNVNHGVLVVGYGDLNG
484:941 140:295 10:24
1:150718602:T:G_G
p = 0.0033, beta = −0.246, N = 634
CTSS : NP3
A0A7P0TAQ0;P25774
269:269 53:53 3:3
1:150718602:T:G_G
p = 0.52, beta = −0.086, N = 325
GPVSVGVDAR pc2
269:269 53:53 3:3
1:150718602:T:G_G
p = 0.52, beta = −0.0855, N = 325
GNHCGIASFPSYPEI pc2
269:269 53:53 3:3
1:150718602:T:G_G
p = 0.43, beta = −0.106, N = 325
NSWGHNFGEEGYIR pc3
269:269 53:53 3:3
1:150718602:T:G_G
p = 0.23, beta = −0.16, N = 325
HPSFFLYR pc2
269:269 53:53 3:3
1:150718602:T:G_G
p = 0.48, beta = −0.0944, N = 325
YTELPYGREDVLK pc3
269:269 53:53 3:3
1:150718602:T:G_G
p = 0.39, beta = −0.115, N = 325
GIDSDASYPYK pc2
267:269 52:53 3:3
1:150718602:T:G_G
p = 0.59, beta = −0.0735, N = 322
LVSLSAQNLVDCSTEK pc2
209:269 38:53 3:3
1:150718602:T:G_G
p = 0.71, beta = 0.0563, N = 250
VTEVKYQGSCGACWAFSAVGALEAQI
266:269 50:53 3:3
1:150718602:T:G_G
p = 0.34, beta = −0.13, N = 319
YTELPYGR pc2
150:269 27:53 2:3
1:150718602:T:G_G
p = 0.12, beta = 0.281, N = 179
YQGSCGACWAFSAVGALEAQLK pc
86:269 17:53 1:3
1:150718602:T:G_G
p = 0.61, beta = −0.118, N = 104
GCNGGFMTTAFQYIIDNK pc3
164:269 27:53 3:3
1:150718602:T:G_G
p = 0.41, beta = 0.14, N = 194
205Assay Target: CTSS
UniProt: P25774
rsID: rs75056606
Target SNP: 1:150776123:G:A
Proxy SNP: 1:150718602:T:G
Tarkin log10(p): 9.2
Tarkin BETA: −0.524
Tarkin MSPA: 0.915
1255:1252:1253:1238:1252:123
ILPDSVDWR pc2
rs2230061 REF
618:941 193:295 18:24
1:150718602:T:G_G
p = 0.39, model = DOM, N = 829

206. MAN2A1 (Q16706)
Tarkin MSPA=0.769

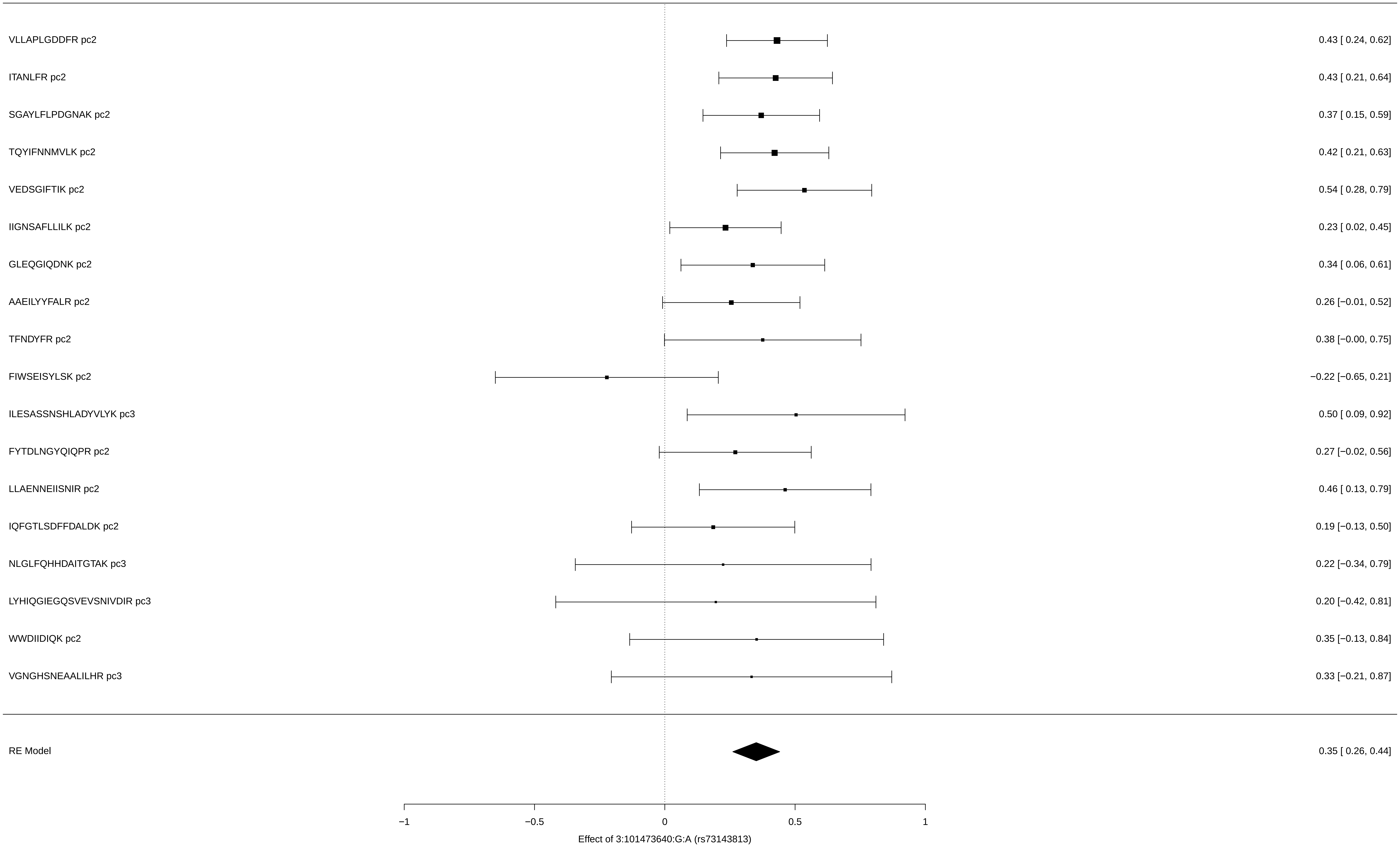

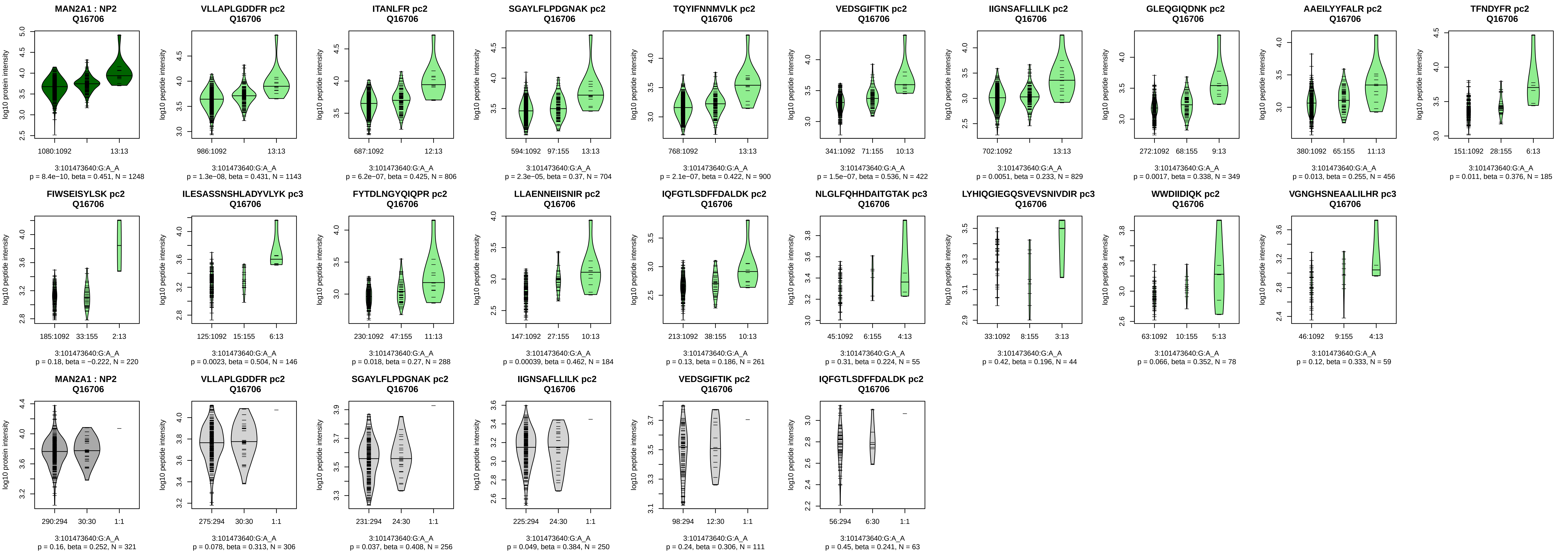

206Assay Target: MAN2A1
UniProt: Q16706
rsID: rs73143813
Target SNP: 3:101473640:G:A
Proxy SNP: 3:101473640:G:A
Tarkin log10(p): 9.1
Tarkin BETA: 0.451
Tarkin MSPA: 0.769
::*:*:*:*:*:–:–:–:*:–:*:–:–:–:–:–:N
1143:806:704:900:422:829:349:

207. UBR1 (Q8IWV7)
Tarkin MSPA=0.465

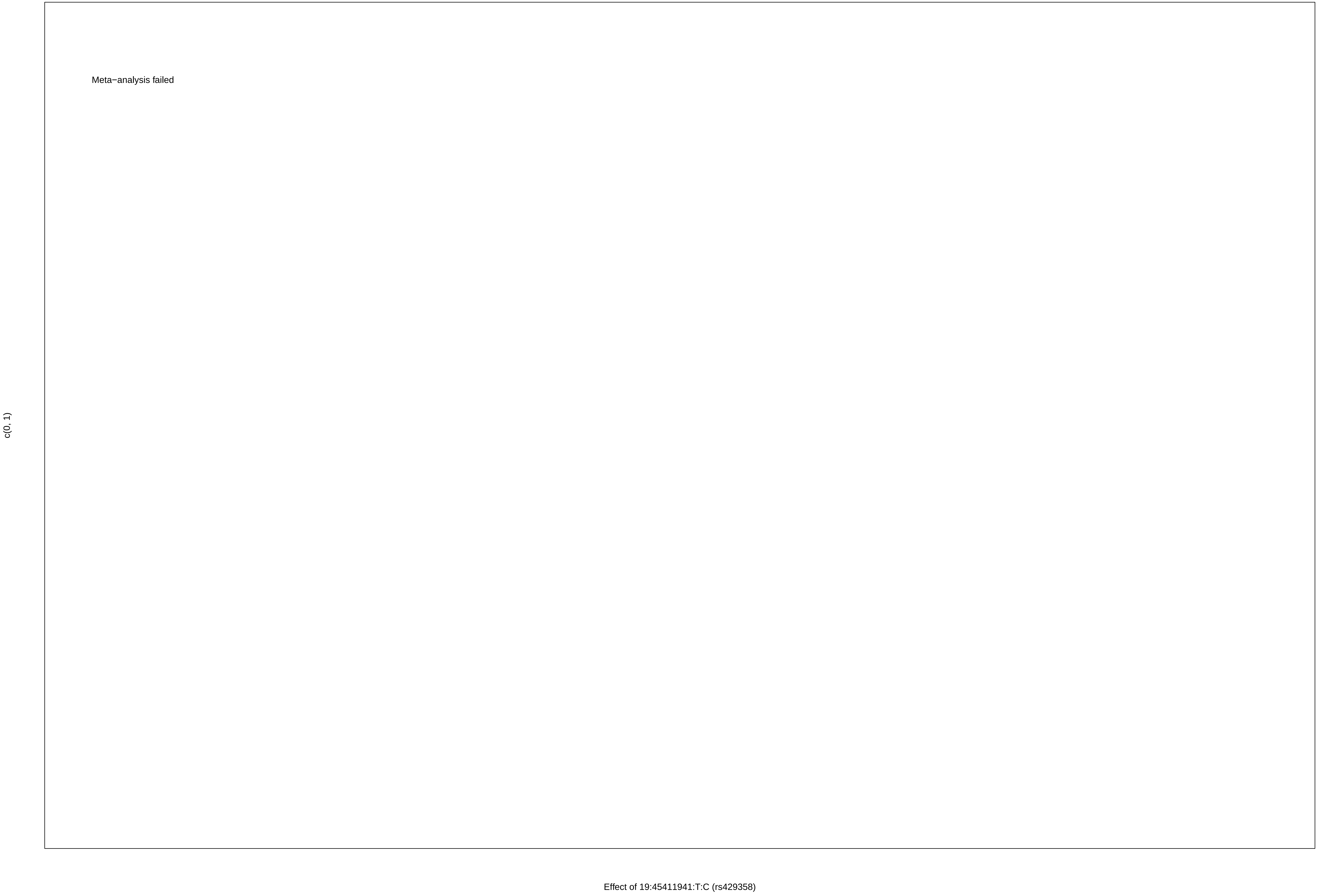

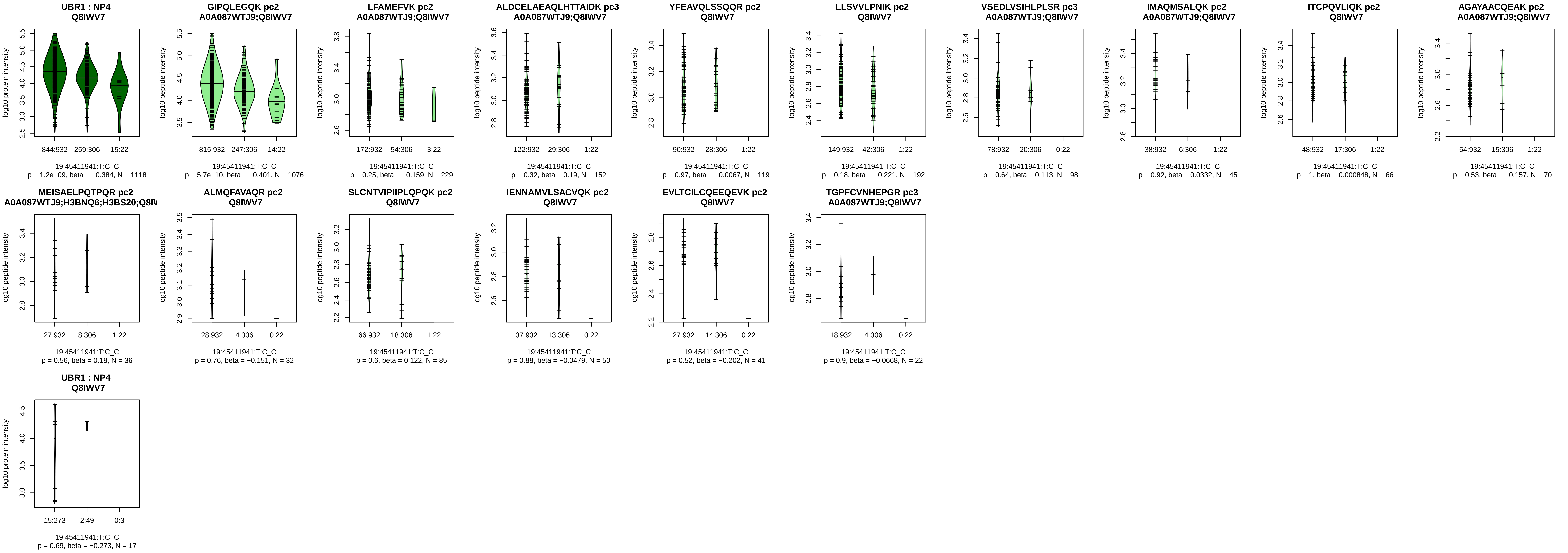

207Assay Target: UBR1
UniProt: Q8IWV7
rsID: rs429358
Target SNP: 19:45411941:T:C
Proxy SNP: 19:45411941:T:C
Tarkin log10(p): 8.9
Tarkin BETA: −0.384
Tarkin MSPA: 0.465
*:−:−:−:−:−:−:−:−:−:−:−:−:−:NA
1076:229:152:119:192:98:45:66

208. PAIP1 (Q9H074)
Tarkin MSPA=0.868

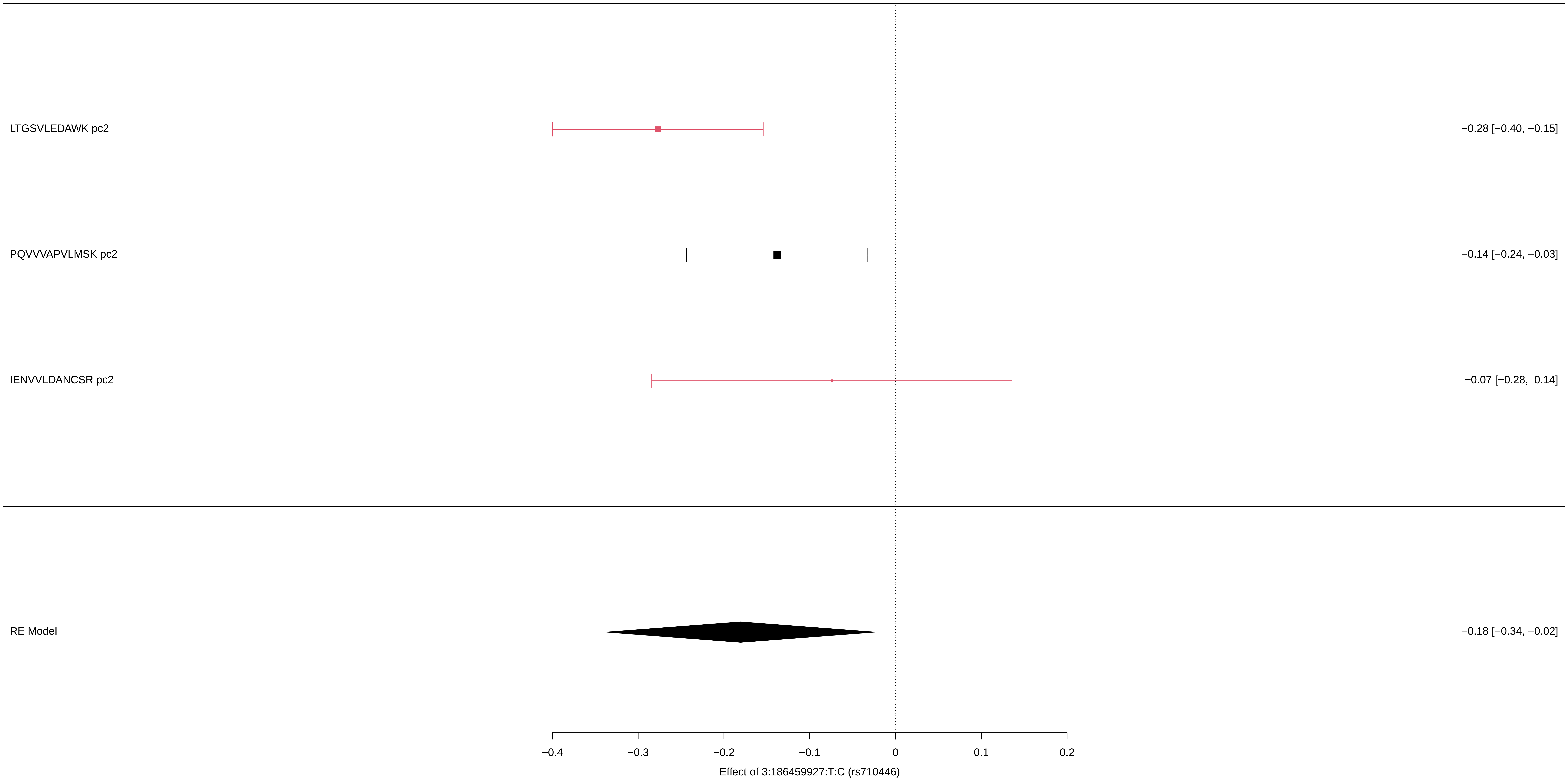

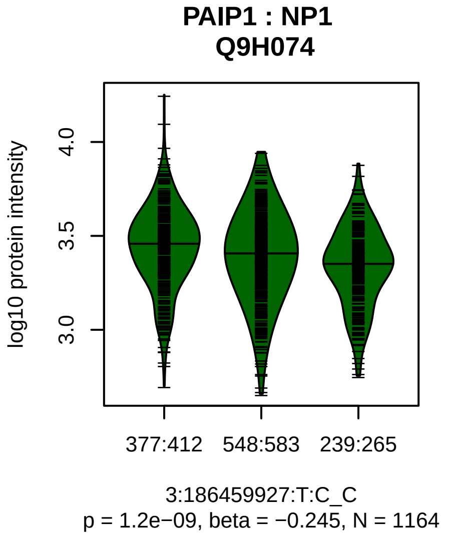

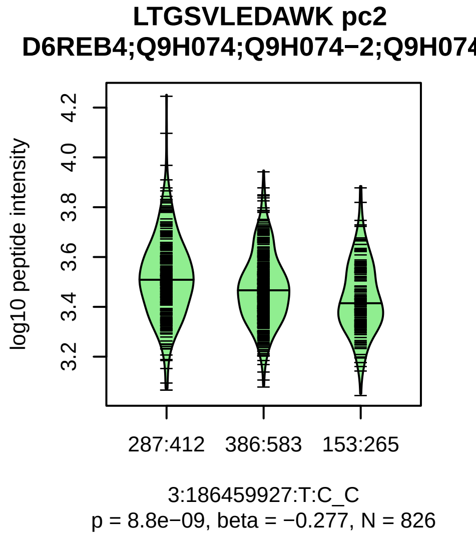

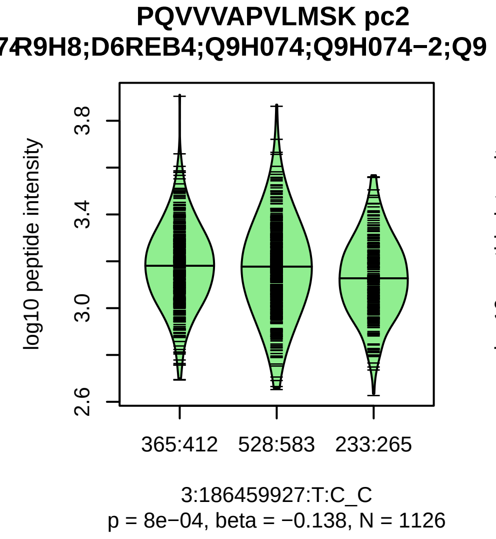

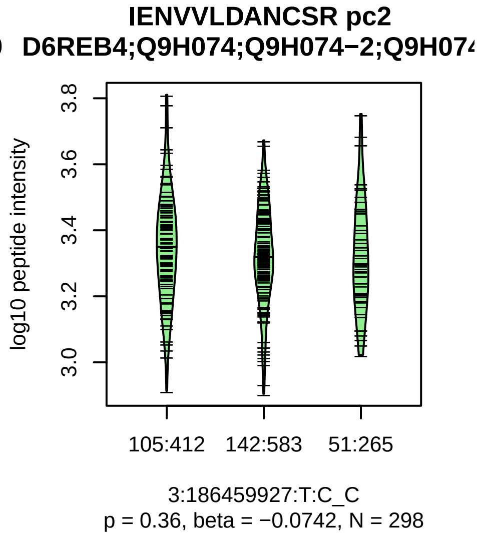

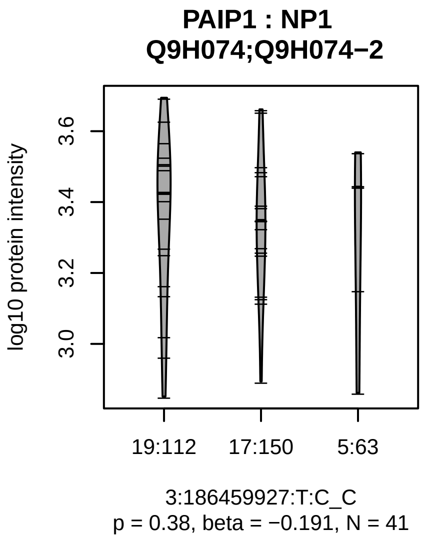

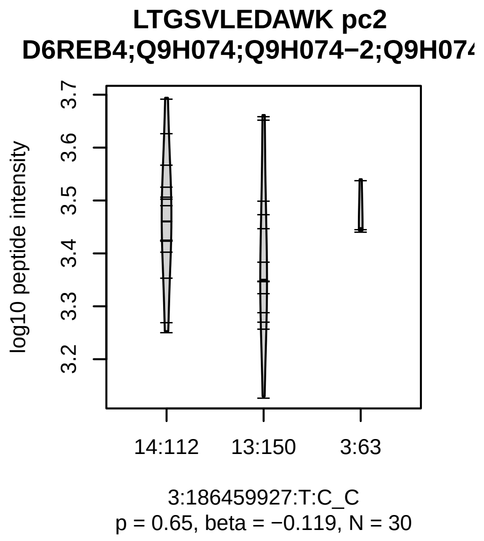

208Assay Target: PAIP1
UniProt: Q9H074
rsID: rs710446
Target SNP: 3:186459927:T:C
Proxy SNP: 3:186459927:T:C
Tarkin log10(p): 8.9
Tarkin BETA: −0.245
Tarkin MSPA: 0.868
::−
826:1126:298

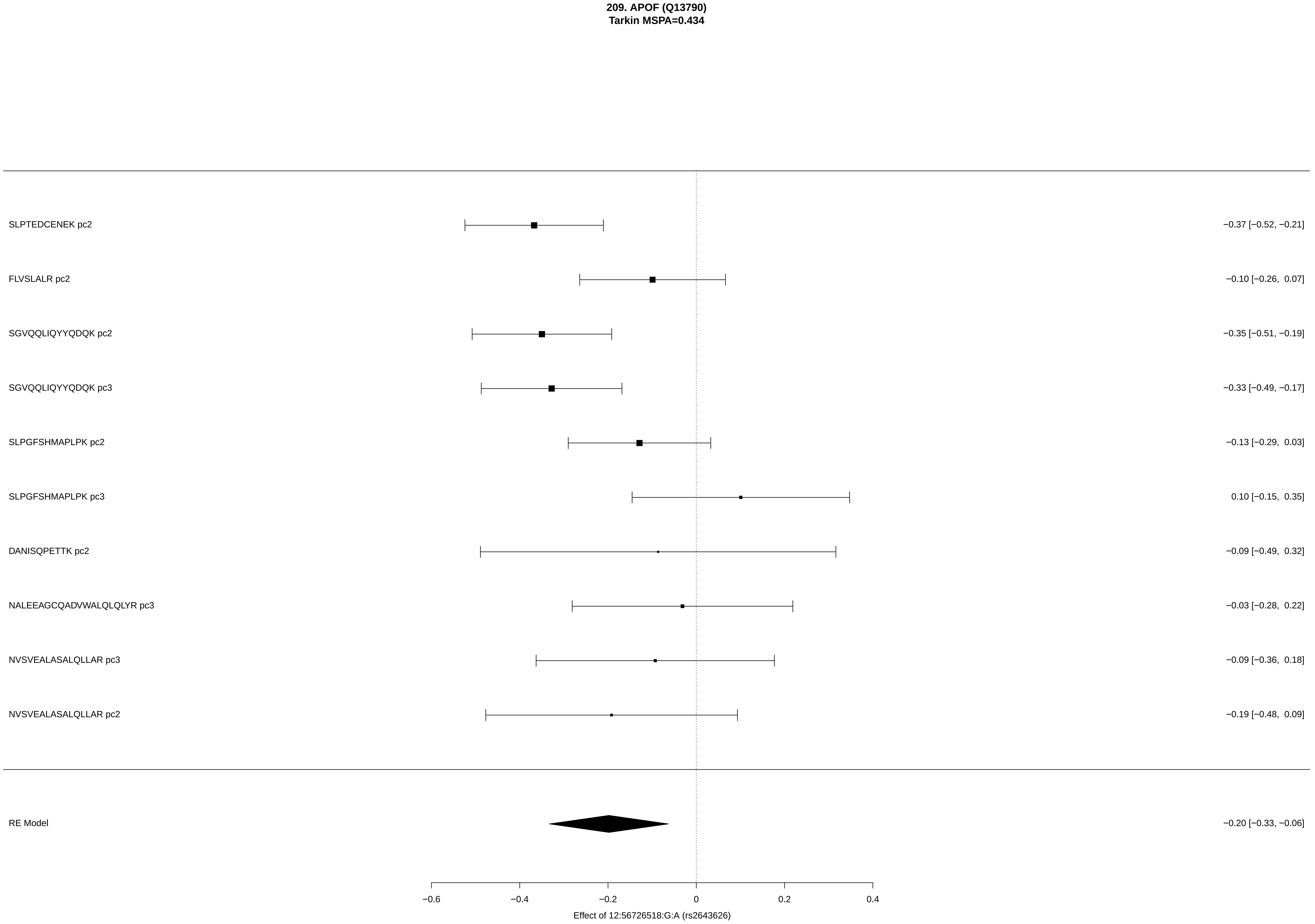

209. APOF (Q13790)
Tarkin MSPA=0.434
SLPTEDCENEK pc2
−0.37 [−0.52, −0.21]
FLVSLALR pc2
−0.10 [−0.26, 0.07]
SGVQQLIQYYQDQK pc2
−0.35 [−0.51, −0.19]
SGVQQLIQYYQDQK pc3
−0.33 [−0.49, −0.17]
SLPGFSHMAPLPK pc2
−0.13 [−0.29, 0.03]
SLPGFSHMAPLPK pc3
0.10 [−0.15, 0.35]
DANISQPETTK pc2
−0.09 [−0.49, 0.32]
NALEEAGCQADVWALQLQLYR pc3
−0.03 [−0.28, 0.22]
NVSVEALASALQLLAR pc3
−0.09 [−0.36, 0.18]
NVSVEALASALQLLAR pc2
−0.19 [−0.48, 0.09]
RE Model
−0.20 [−0.33, −0.06]
−0.6
−0.4
−0.2
0
0.2
0.4
Effect of 12:56726518:G:A (rs2643626)

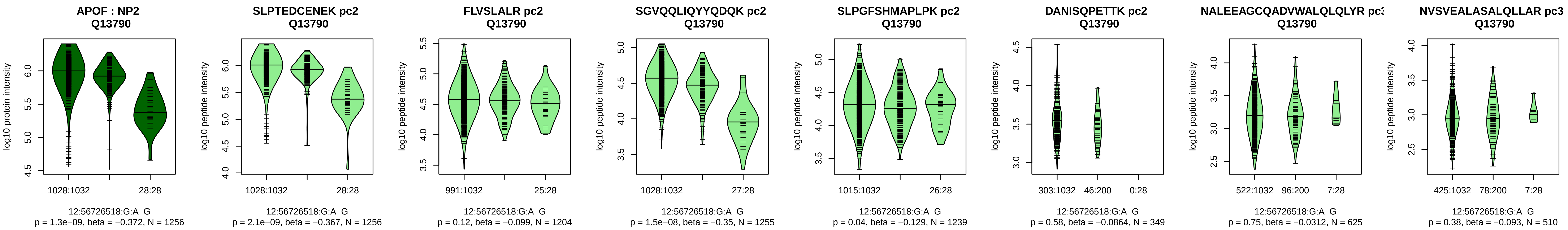

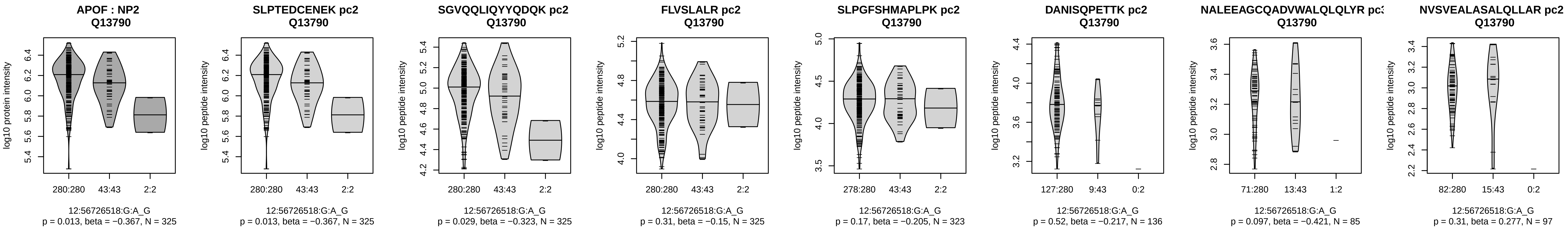

209Assay Target: APOF
UniProt: Q13790
rsID: rs2643626
Target SNP: 12:56726518:G:A
Proxy SNP: 12:56726518:G:A
Tarkin log10(p): 8.9
Tarkin BETA: −0.372
Tarkin MSPA: 0.434
:–::*:–:–:–:–:–
1256:1204:1255:1254:1239:538

210. LECT2 (O14960)
Tarkin MSPA=0.728

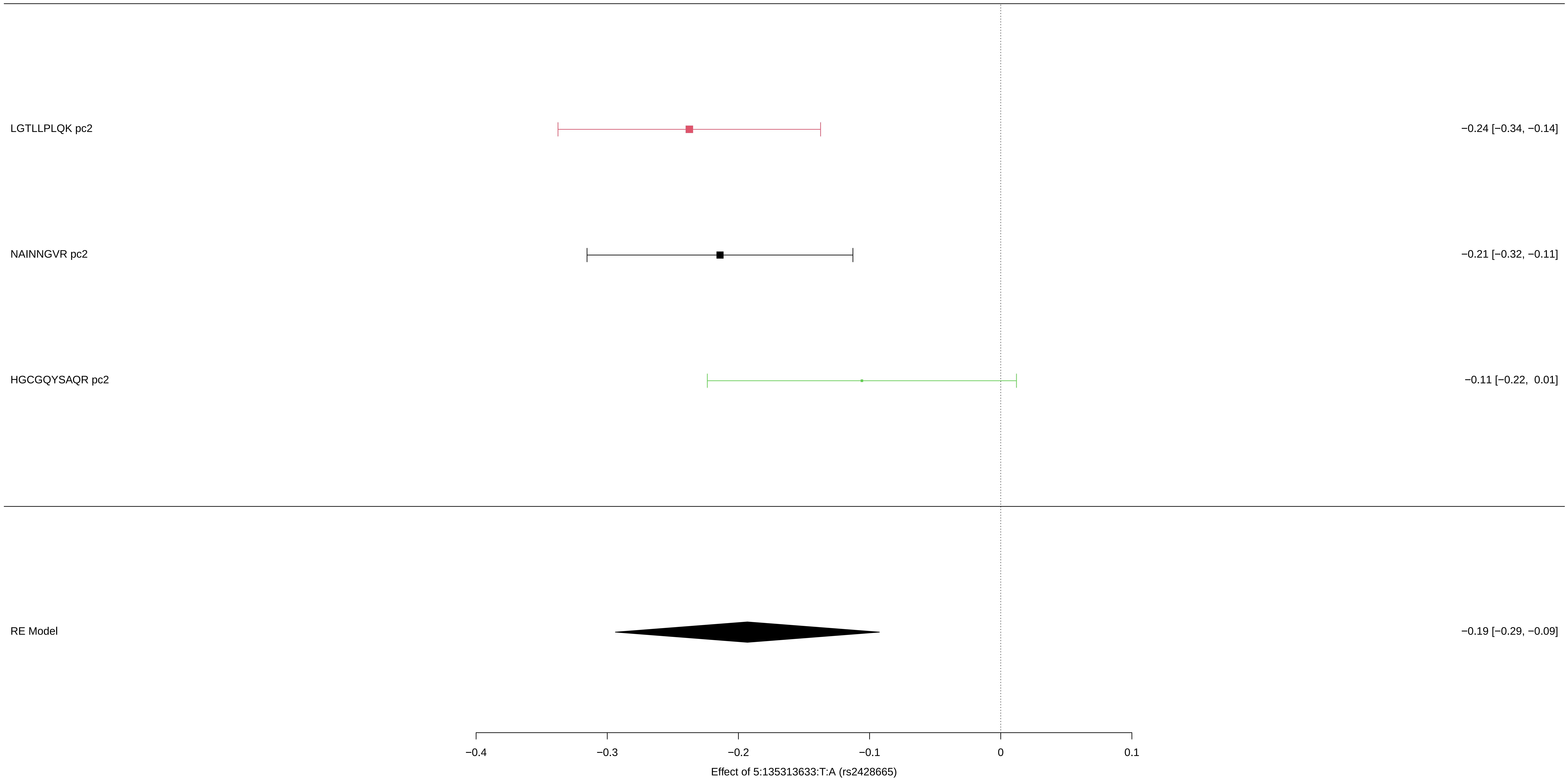

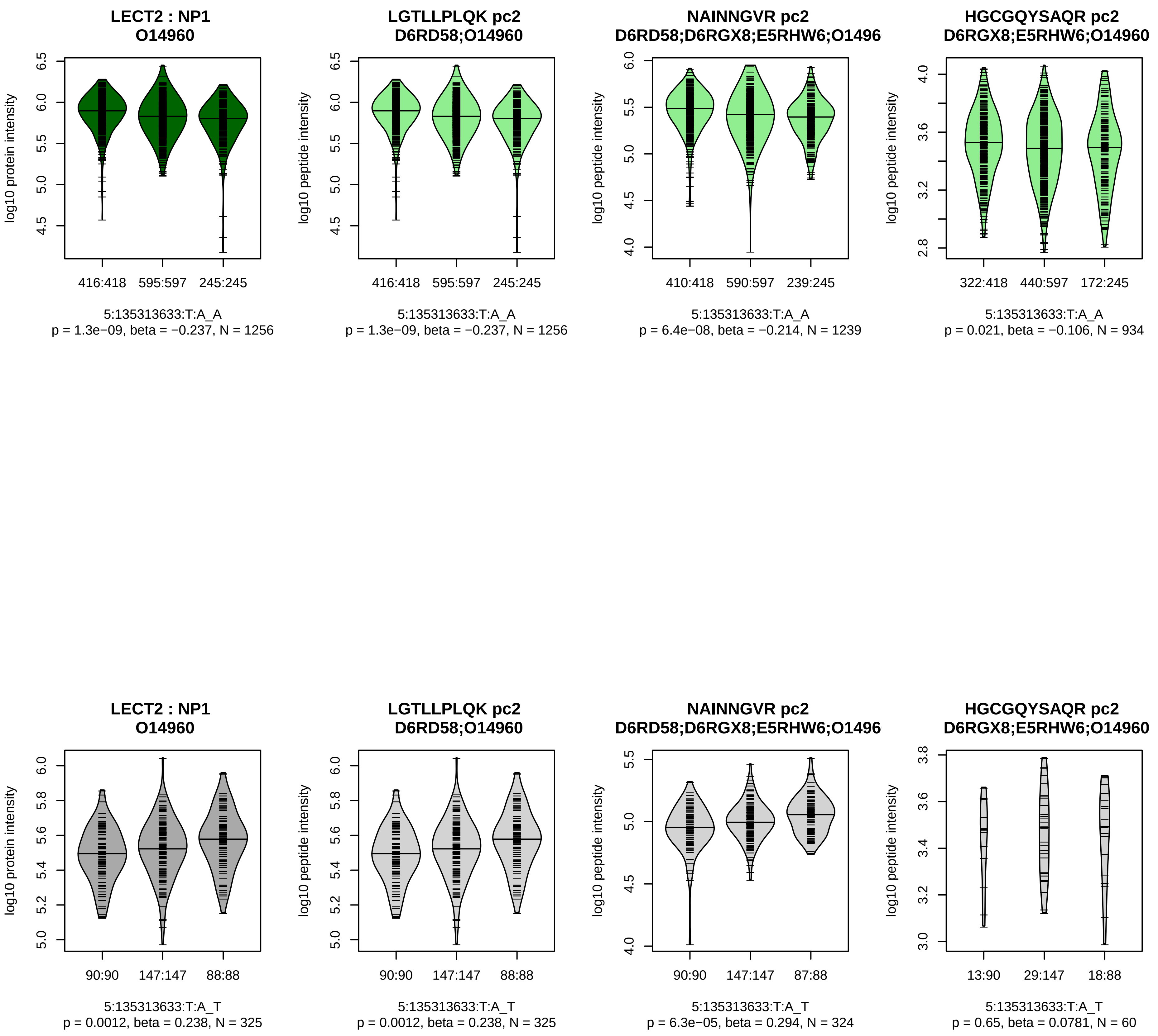

210Assay Target: LECT2
UniProt: O14960
rsID: rs2428665
Target SNP: 5:135313633:T:A
Proxy SNP: 5:135313633:T:A
Tarkin log10(p): 8.9
Tarkin BETA: −0.238
Tarkin MSPA: 0.728
::–
1256:1239:934

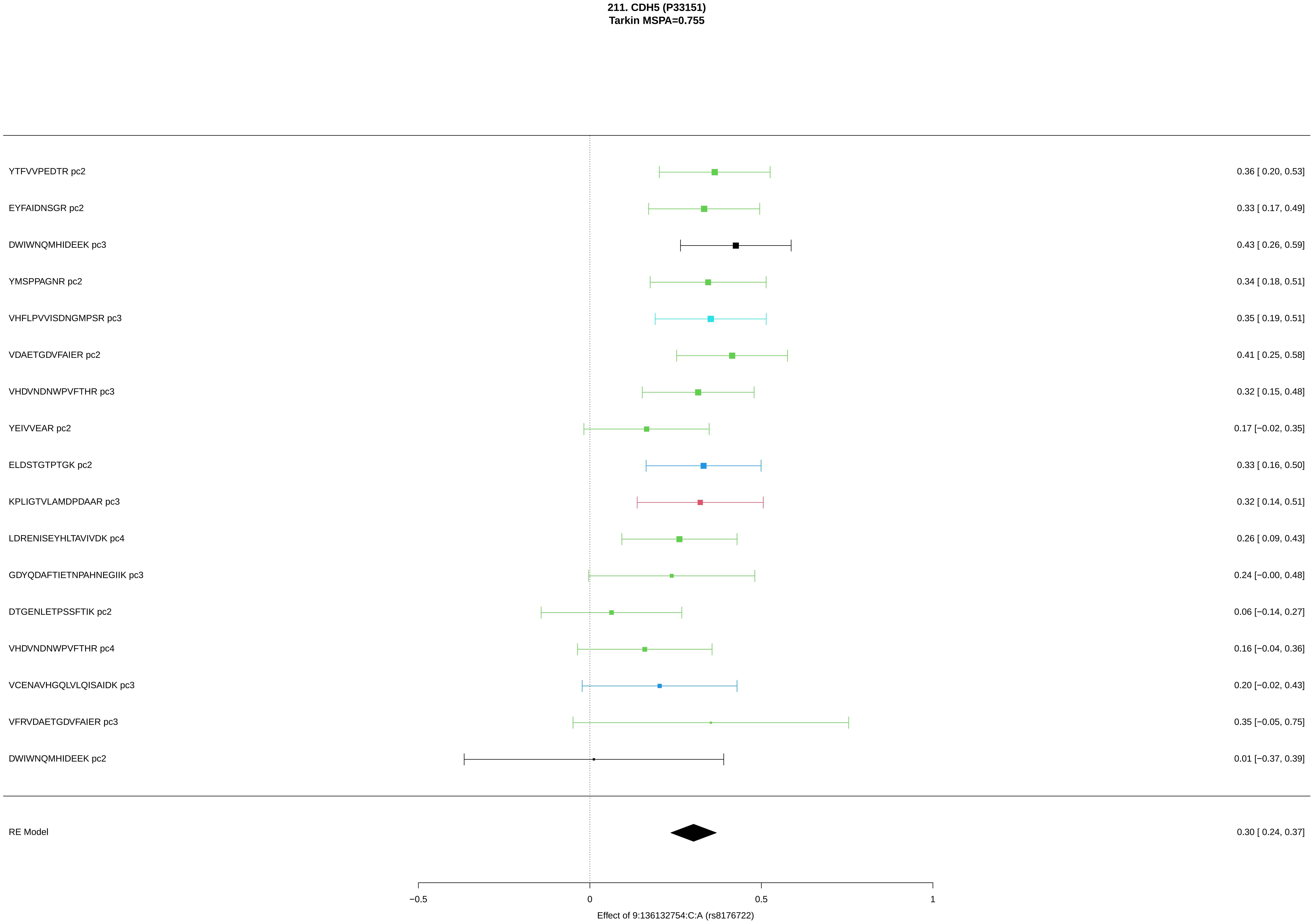

211. CDH5 (P33151)
Tarkin MSPA=0.755
YTFVVPEDTR pc2
0.36 [0.20, 0.53]
EYFAIDNSGR pc2
0.33 [0.17, 0.49]
DWIWNQMHIDEEK pc3
0.43 [0.26, 0.59]
YMSPPAGNR pc2
0.34 [0.18, 0.51]
VHFLPVVISDNGMPSR pc3
0.35 [0.19, 0.51]
VDAETGDVFAIER pc2
0.41 [0.25, 0.58]
VHDVNDNWPVFTHR pc3
0.32 [0.15, 0.48]
YEIVVEAR pc2
0.17 [−0.02, 0.35]
ELDSTGTPTGK pc2
0.33 [0.16, 0.50]
KPLIGTVLAMDPDAAR pc3
0.32 [0.14, 0.51]
LDRENISEYHLTAVIVDK pc4
0.26 [0.09, 0.43]
GDYQDAFTIETNPAHNEGIIK pc3
0.24 [−0.00, 0.48]
DTGENLETPSSFTIK pc2
0.06 [−0.14, 0.27]
VHDVNDNWPVFTHR pc4
0.16 [−0.04, 0.36]
VCENAVHGQLVLQISAIDK pc3
0.20 [−0.02, 0.43]
VFRVDAETGDVFAIER pc3
0.35 [−0.05, 0.75]
DWIWNQMHIDEEK pc2
0.01 [−0.37, 0.39]
RE Model
0.30 [0.24, 0.37]
−0.5
0
0.5
1
Effect of 9:136132754:C:A (rs8176722)

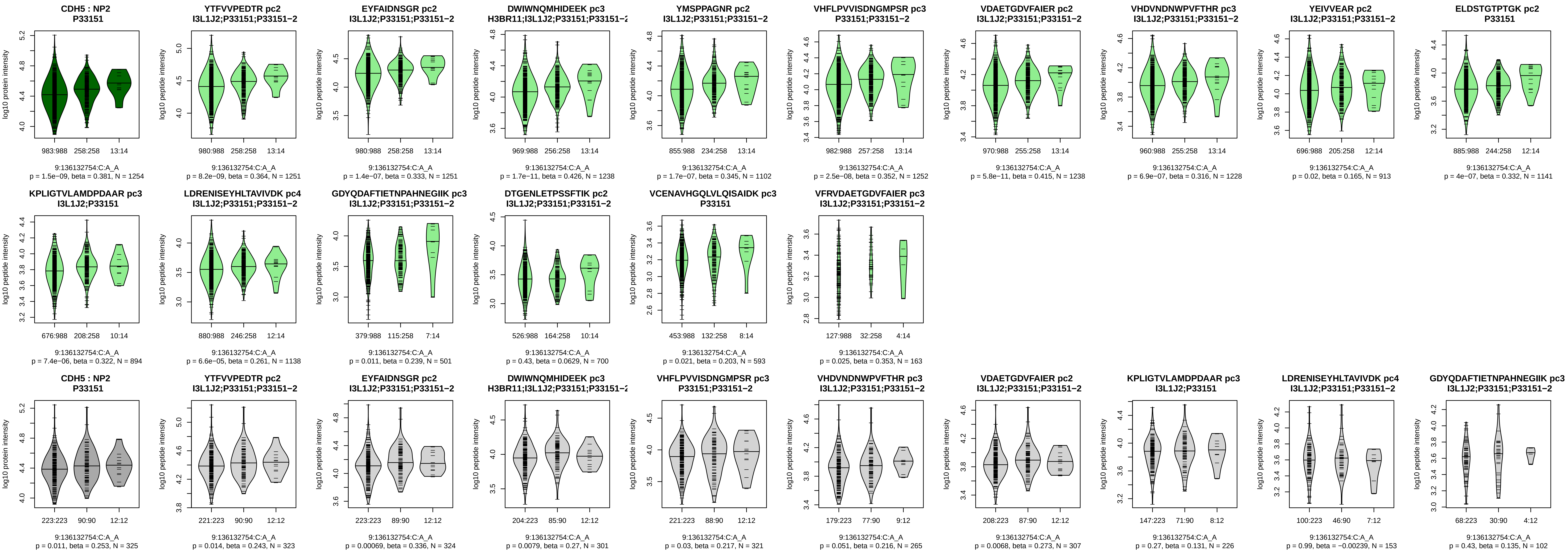

211Assay Target: CDH5
UniProt: P33151
rsID: rs8176722
Target SNP: 9:136132754:C:A
Proxy SNP: 9:136132754:C:A
Tarkin log10(p): 8.8
Tarkin BETA: 0.381
Tarkin MSPA: 0.755
1251:1251:1238:1102:1252:123

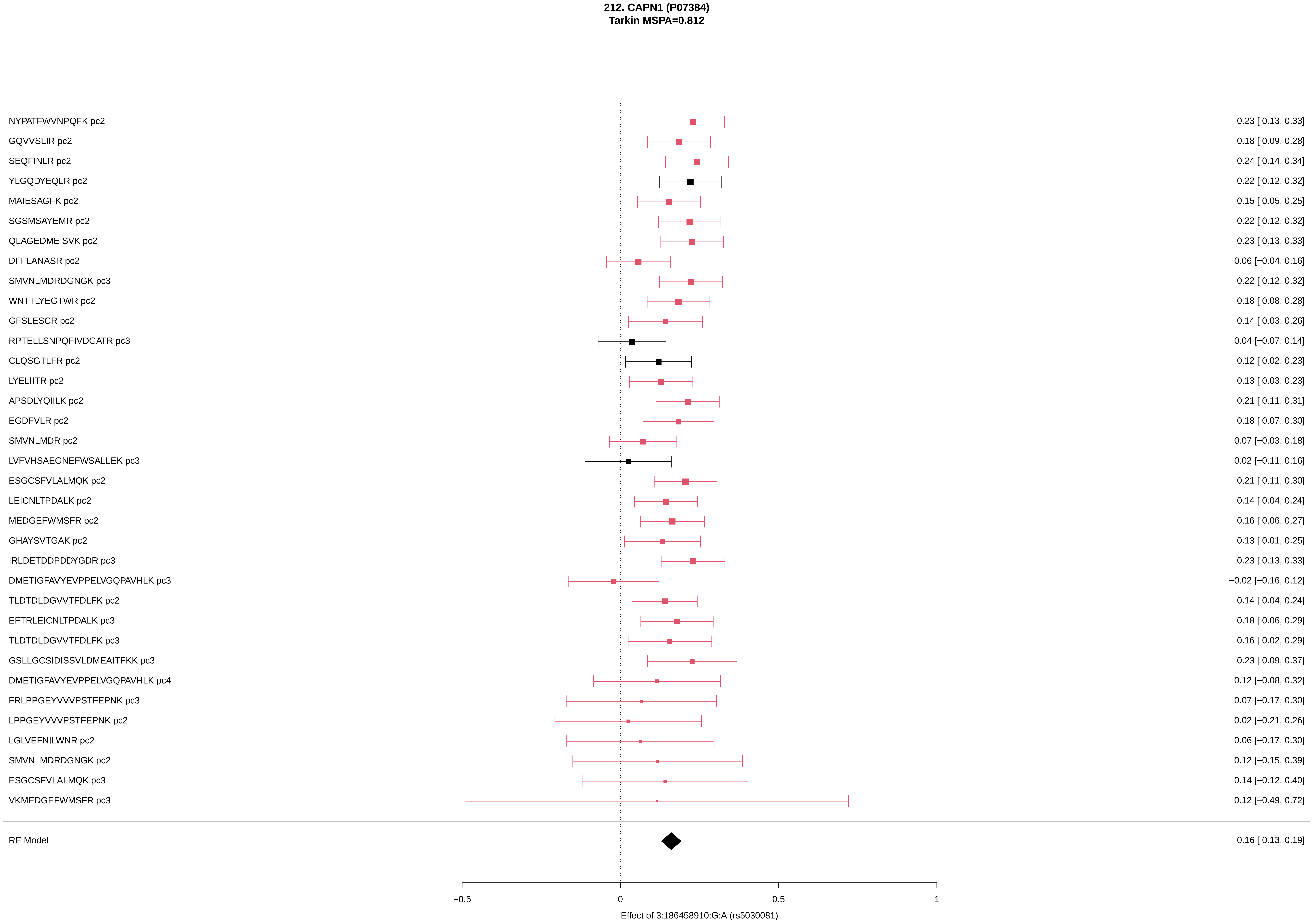

212. CAPN1 (P07384)
Tarkin MSPA=0.812
NYPATFWVNPQFK pc2
0.23 [0.13, 0.33]
GQVVSLIR pc2
0.18 [0.09, 0.28]
SEQFINLR pc2
0.24 [0.14, 0.34]
YLGQDYEQLR pc2
0.22 [0.12, 0.32]
MAIESAGFK pc2
0.15 [0.05, 0.25]
SGSMSAYEMR pc2
0.22 [0.12, 0.32]
QLAGEDMEISVK pc2
0.23 [0.13, 0.33]
DFFLANASR pc2
0.06 [−0.04, 0.16]
SMVNLMDRDGNGK pc3
0.22 [0.12, 0.32]
WNTTLYEGTWR pc2
0.18 [0.08, 0.28]
GFSLESCR pc2
0.14 [0.03, 0.26]
RPTELLSNPQFIVDGATR pc3
0.04 [−0.07, 0.14]
CLQSGTLFR pc2
0.12 [0.02, 0.23]
LYELIITR pc2
0.13 [0.03, 0.23]
APSDLYQIILK pc2
0.21 [0.11, 0.31]
EGDFVLR pc2
0.18 [0.07, 0.30]
SMVNLMDR pc2
0.07 [−0.03, 0.18]
LVFVHSAEGNEFWSALLEK pc3
0.02 [−0.11, 0.16]
ESGCSFVLALMQK pc2
0.21 [0.11, 0.30]
LEICNLTPDALK pc2
0.14 [0.04, 0.24]
MEDGEFWMSFR pc2
0.16 [0.06, 0.27]
GHAYSVTGAK pc2
0.13 [0.01, 0.25]
IRLDETDDPDDYGDR pc3
0.23 [0.13, 0.33]
DMETIGFAVYEVPPELVGQPAVHLK pc3
−0.02 [−0.16, 0.12]
TLDTDLDGVVTFDLFK pc2
0.14 [0.04, 0.24]
EFTRLEICNLTPDALK pc3
0.18 [0.06, 0.29]
TLDTDLDGVVTFDLFK pc3
0.16 [0.02, 0.29]
GSLLGCSIDISSVLDMEAITFKK pc3
0.23 [0.09, 0.37]
DMETIGFAVYEVPPELVGQPAVHLK pc4
0.12 [−0.08, 0.32]
FRLPPGEYVVVPSTFEPNK pc3
0.07 [−0.17, 0.30]
LPPGEYVVVPSTFEPNK pc2
0.02 [−0.21, 0.26]
LGLVEFNILWNR pc2
0.06 [−0.17, 0.30]
SMVNLMDRDGNGK pc2
0.12 [−0.15, 0.39]
ESGCSFVLALMQK pc3
0.14 [−0.12, 0.40]
VKMEDGEFWMSFR pc3
0.12 [−0.49, 0.72]
RE Model
0.16 [0.13, 0.19]
−0.5
0
0.5
1
Effect of 3:186458910:G:A (rs5030081)

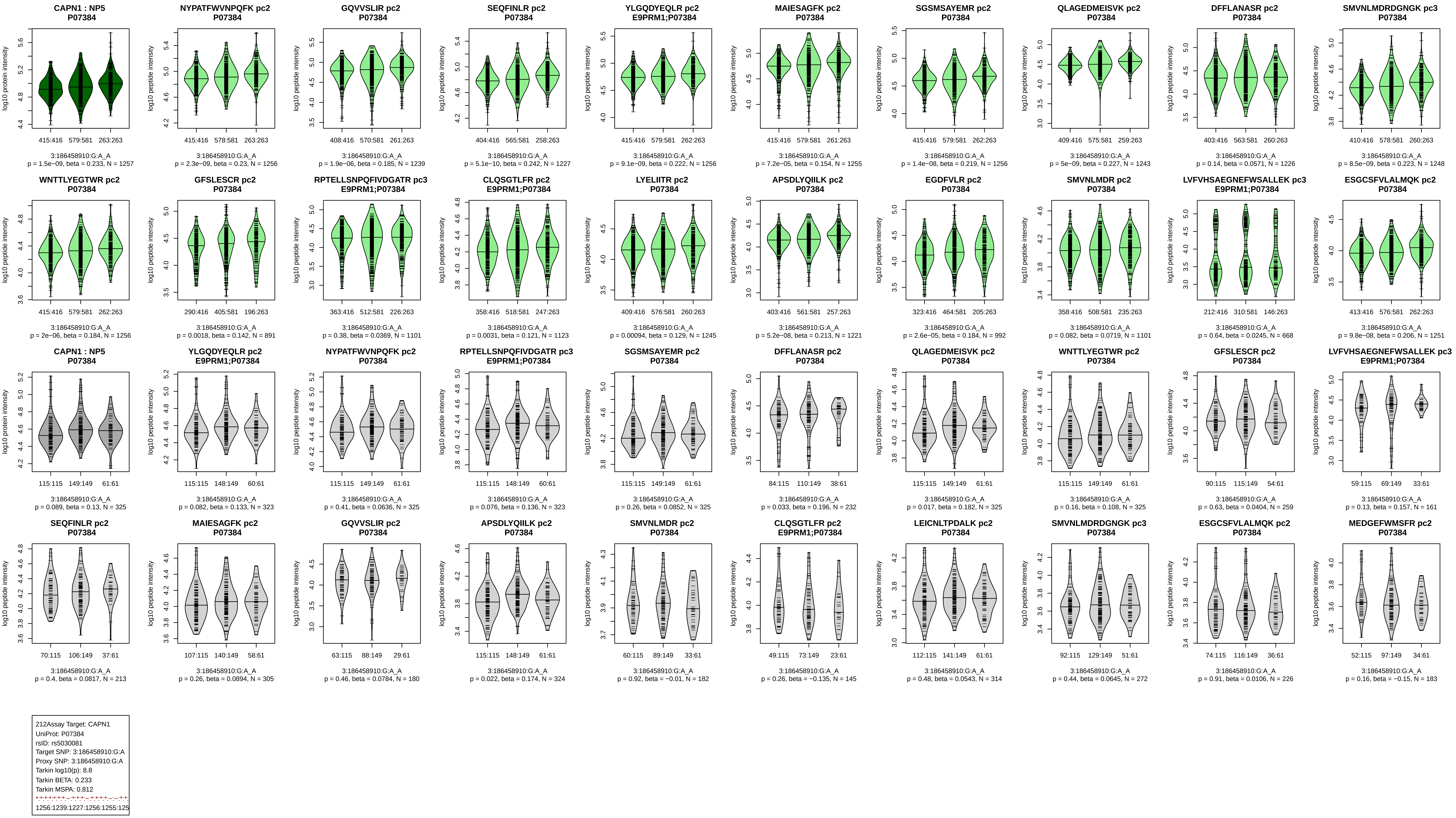
CAPN1 : NP5
P07384
log10 protein intensity
415:416 579:581 263:263
3:186458910:G:A_A
p = 1.5e−09, beta = 0.233, N = 1257
NYPATFWVNPQFK pc2
P07384
log10 peptide intensity
415:416 578:581 263:263
3:186458910:G:A_A
p = 2.3e−09, beta = 0.23, N = 1256
GQVVSLIR pc2
P07384
log10 peptide intensity
408:416 570:581 261:263
3:186458910:G:A_A
p = 1.9e−06, beta = 0.185, N = 1239
SEQFINLR pc2
P07384
log10 peptide intensity
404:416 565:581 258:263
3:186458910:G:A_A
p = 5.1e−10, beta = 0.242, N = 1227
YLGQDYEQLR pc2
E9PRM1;P07384
log10 peptide intensity
415:416 579:581 262:263
3:186458910:G:A_A
p = 9.1e−09, beta = 0.222, N = 1256
MAIESAGFK pc2
P07384
log10 peptide intensity
415:416 579:581 261:263
3:186458910:G:A_A
p = 7.2e−05, beta = 0.154, N = 1255
SGSMSAYEMR pc2
P07384
log10 peptide intensity
415:416 579:581 262:263
3:186458910:G:A_A
p = 1.4e−08, beta = 0.219, N = 1256
QLAGEDMEISVK pc2
P07384
log10 peptide intensity
409:416 575:581 259:263
3:186458910:G:A_A
p = 5e−09, beta = 0.227, N = 1243
DFFLANASR pc2
P07384
log10 peptide intensity
403:416 563:581 260:263
3:186458910:G:A_A
p = 0.14, beta = 0.0571, N = 1226
SMVNLMDRDGNGK pc3
P07384
log10 peptide intensity
410:416 578:581 260:263
3:186458910:G:A_A
p = 8.5e−09, beta = 0.223, N = 1248
WNTTLYEGTWR pc2
P07384
log10 peptide intensity
415:416 579:581 262:263
3:186458910:G:A_A
p = 2e−06, beta = 0.184, N = 1256
GFSLESCR pc2
P07384
log10 peptide intensity
290:416 405:581 196:263
3:186458910:G:A_A
p = 0.0018, beta = 0.142, N = 891
RPTELLSNPQFIVDGATR pc3
E9PRM1;P07384
log10 peptide intensity
363:416 512:581 226:263
3:186458910:G:A_A
p = 0.38, beta = 0.0369, N = 1101
CLQSGTLFR pc2
E9PRM1;P07384
log10 peptide intensity
358:416 518:581 247:263
3:186458910:G:A_A
p = 0.0031, beta = 0.121, N = 1123
LYELIITR pc2
P07384
log10 peptide intensity
409:416 576:581 260:263
3:186458910:G:A_A
p = 0.00094, beta = 0.129, N = 1245
APSDLYQIILK pc2
P07384
log10 peptide intensity
403:416 561:581 257:263
3:186458910:G:A_A
p = 5.2e−08, beta = 0.213, N = 1221
EGDFVLR pc2
P07384
log10 peptide intensity
323:416 464:581 205:263
3:186458910:G:A_A
p = 2.6e−05, beta = 0.184, N = 992
SMVNLMDR pc2
P07384
log10 peptide intensity
358:416 508:581 235:263
3:186458910:G:A_A
p = 0.082, beta = 0.0719, N = 1101
LVFVHSAEGNEFWSALLEK pc3
E9PRM1;P07384
log10 peptide intensity
212:416 310:581 146:263
3:186458910:G:A_A
p = 0.64, beta = 0.0245, N = 668
ESGCSFVLALMQK pc2
P07384
log10 peptide intensity
413:416 576:581 262:263
3:186458910:G:A_A
p = 9.8e−08, beta = 0.206, N = 1251
CAPN1 : NP5
P07384
log10 protein intensity
115:115 149:149 61:61
3:186458910:G:A_A
p = 0.089, beta = 0.13, N = 325
YLGQDYEQLR pc2
E9PRM1;P07384
log10 peptide intensity
115:115 148:149 60:61
3:186458910:G:A_A
p = 0.082, beta = 0.133, N = 323
NYPATFWVNPQFK pc2
P07384
log10 peptide intensity
115:115 149:149 61:61
3:186458910:G:A_A
p = 0.41, beta = 0.0636, N = 325
RPTELLSNPQFIVDGATR pc3
E9PRM1;P07384
log10 peptide intensity
115:115 148:149 60:61
3:186458910:G:A_A
p = 0.076, beta = 0.136, N = 323
SGSMSAYEMR pc2
P07384
log10 peptide intensity
115:115 149:149 61:61
3:186458910:G:A_A
p = 0.26, beta = 0.0852, N = 325
DFFLANASR pc2
P07384
log10 peptide intensity
84:115 110:149 38:61
3:186458910:G:A_A
p = 0.033, beta = 0.196, N = 232
QLAGEDMEISVK pc2
P07384
log10 peptide intensity
115:115 149:149 61:61
3:186458910:G:A_A
p = 0.017, beta = 0.182, N = 325
WNTTLYEGTWR pc2
P07384
log10 peptide intensity
115:115 149:149 61:61
3:186458910:G:A_A
p = 0.16, beta = 0.108, N = 325
GFSLESCR pc2
P07384
log10 peptide intensity
90:115 115:149 54:61
3:186458910:G:A_A
p = 0.63, beta = 0.0404, N = 259
LVFVHSAEGNEFWSALLEK pc3
E9PRM1;P07384
log10 peptide intensity
59:115 69:149 33:61
3:186458910:G:A_A
p = 0.13, beta = 0.157, N = 161
SEQFINLR pc2
P07384
log10 peptide intensity
70:115 106:149 37:61
3:186458910:G:A_A
p = 0.4, beta = 0.0817, N = 213
MAIESAGFK pc2
P07384
log10 peptide intensity
107:115 140:149 58:61
3:186458910:G:A_A
p = 0.26, beta = 0.0894, N = 305
GQVVSLIR pc2
P07384
log10 peptide intensity
63:115 88:149 29:61
3:186458910:G:A_A
p = 0.46, beta = 0.0784, N = 180
APSDLYQIILK pc2
P07384
log10 peptide intensity
115:115 148:149 61:61
3:186458910:G:A_A
p = 0.022, beta = 0.174, N = 324
SMVNLMDR pc2
P07384
log10 peptide intensity
60:115 89:149 33:61
3:186458910:G:A_A
p = 0.92, beta = −0.01, N = 182
CLQSGTLFR pc2
E9PRM1;P07384
log10 peptide intensity
49:115 73:149 23:61
3:186458910:G:A_A
p = 0.26, beta = −0.135, N = 145
LEICNLTPDALK pc2
P07384
log10 peptide intensity
112:115 141:149 61:61
3:186458910:G:A_A
p = 0.48, beta = 0.0543, N = 314
SMVNLMDRDGNGK pc3
P07384
log10 peptide intensity
92:115 129:149 51:61
3:186458910:G:A_A
p = 0.44, beta = 0.0645, N = 272
ESGCSFVLALMQK pc2
P07384
log10 peptide intensity
74:115 116:149 36:61
3:186458910:G:A_A
p = 0.91, beta = 0.0106, N = 226
MEDGEFWMSFR pc2
P07384
log10 peptide intensity
52:115 97:149 34:61
3:186458910:G:A_A
p = 0.16, beta = −0.15, N = 183
212Assay Target: CAPN1
UniProt: P07384
rsID: rs5030081
Target SNP: 3:186458910:G:A
Proxy SNP: 3:186458910:G:A
Tarkin log10(p): 8.8
Tarkin BETA: 0.233
Tarkin MSPA: 0.812
1256:1239:1227:1256:1255:125

213. F8 (P00451)
Tarkin MSPA=0.769

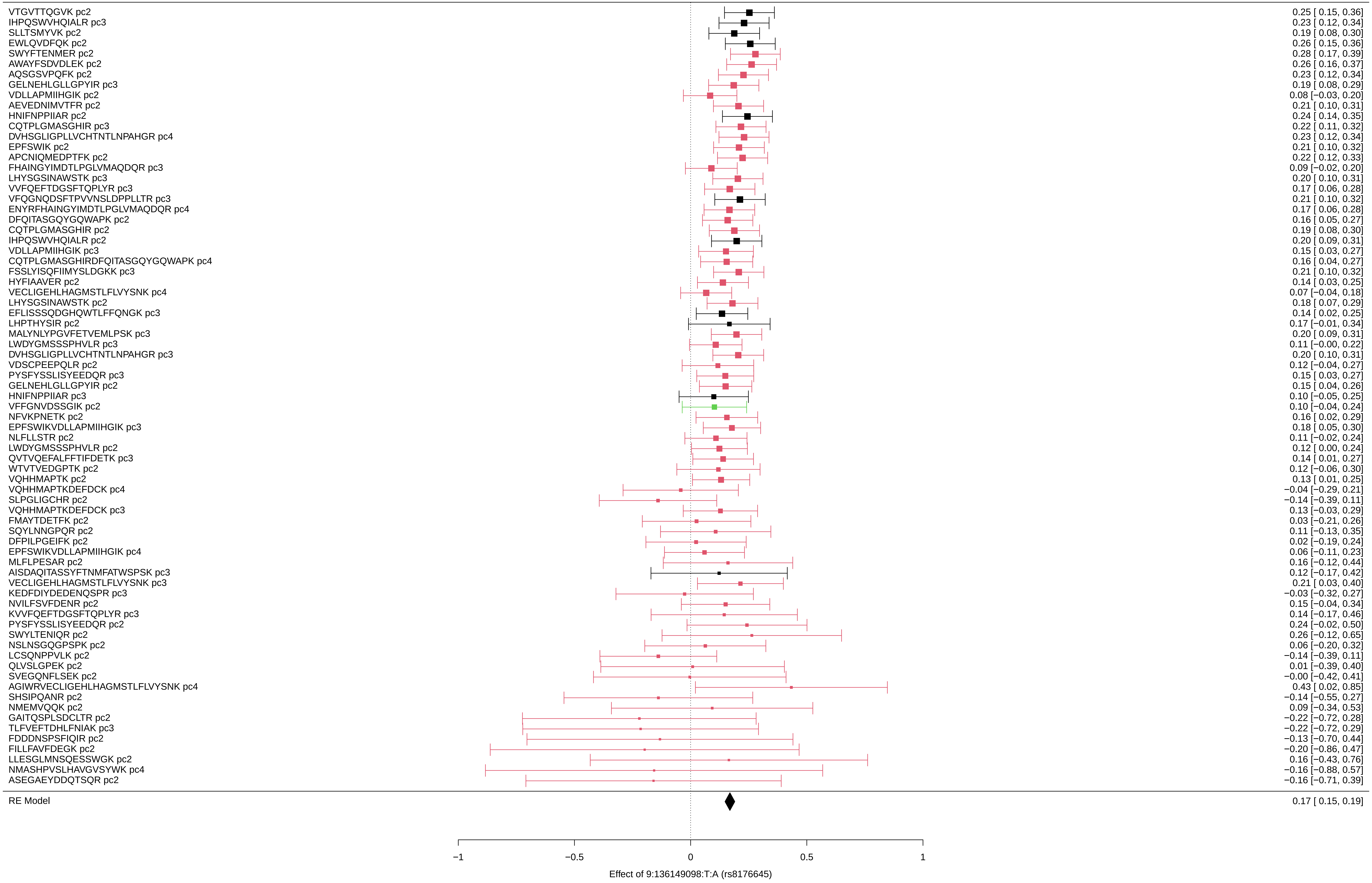

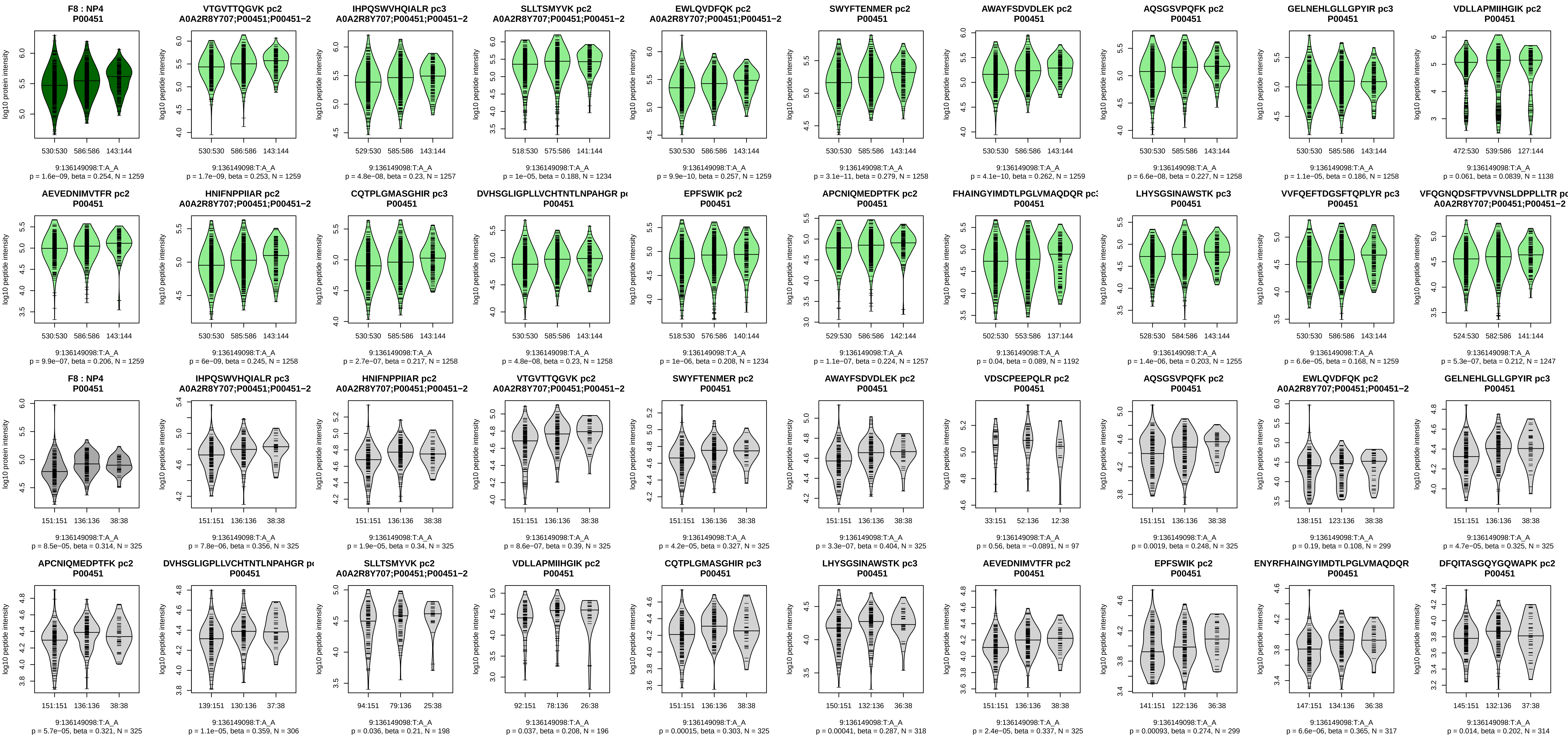

213Assay Target: F8
UniProt: P00451
rsID: rs8176645
Target SNP: 9:136149098:T:A
Proxy SNP: 9:136149098:T:A
Tarkin log10(p): 8.8
Tarkin BETA: 0.254
Tarkin MSPA: 0.769
1259:1257:1234:1259:1258:125

214. ALDH9A1 (P49189)
Tarkin MSPA=0.777

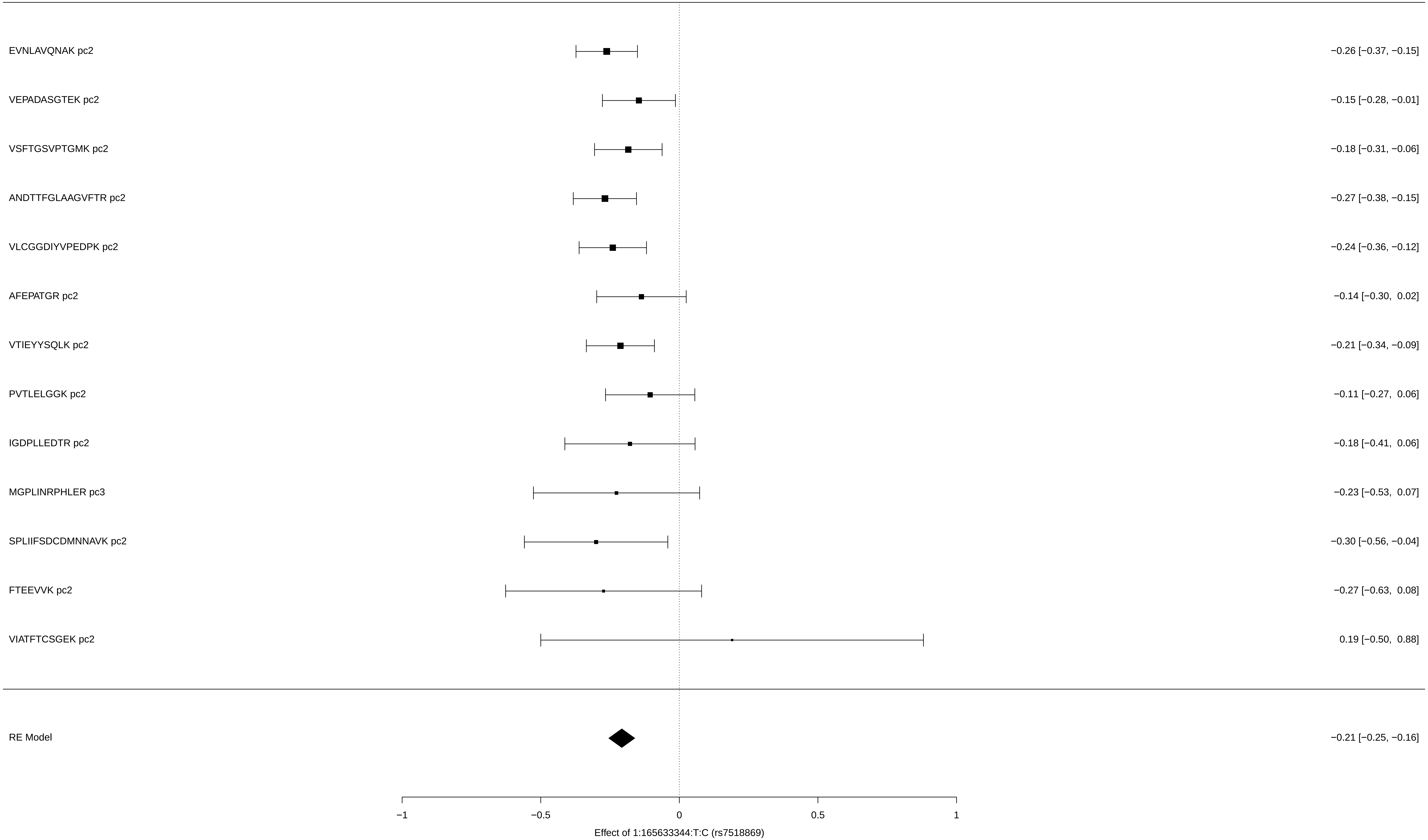

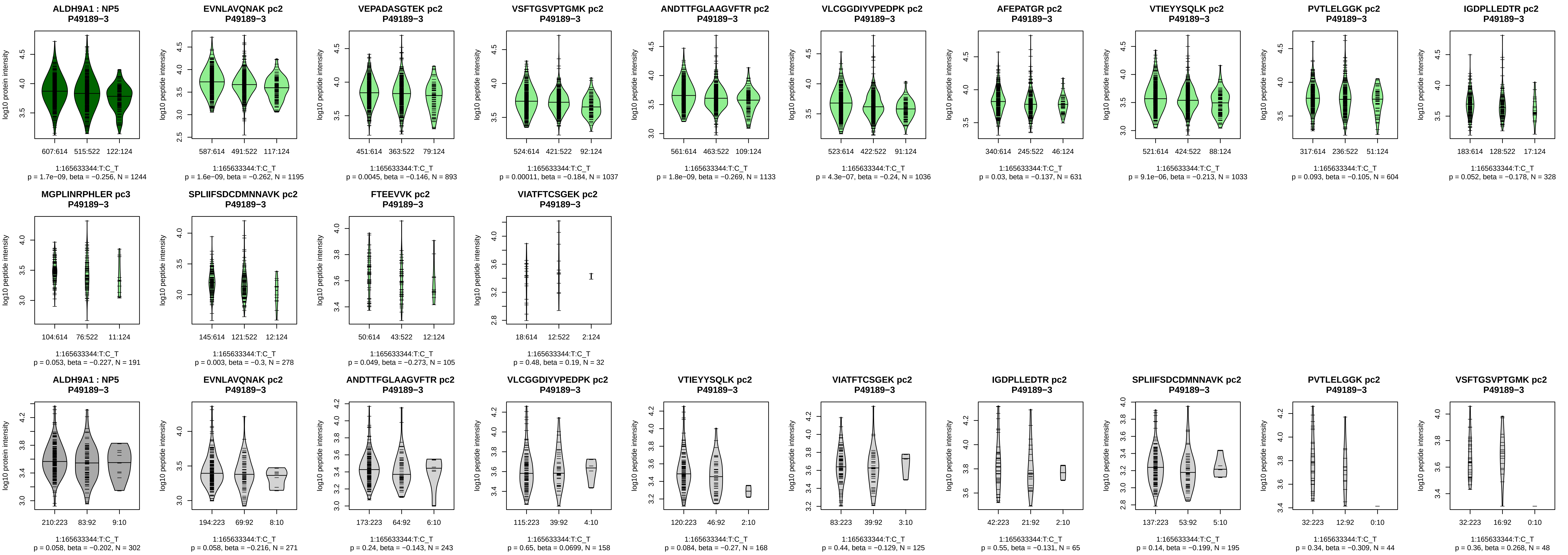

214Assay Target: ALDH9A1
UniProt: P49189
rsID: rs7518869
Target SNP: 1:165633344:T:C
Proxy SNP: 1:165633344:T:C
Tarkin log10(p): 8.8
Tarkin BETA: −0.256
Tarkin MSPA: 0.777
[illegible]
1195:893:1037:1133:1036:631:1

215. CTRB2 (Q6GPI1)
Tarkin MSPA=0.884

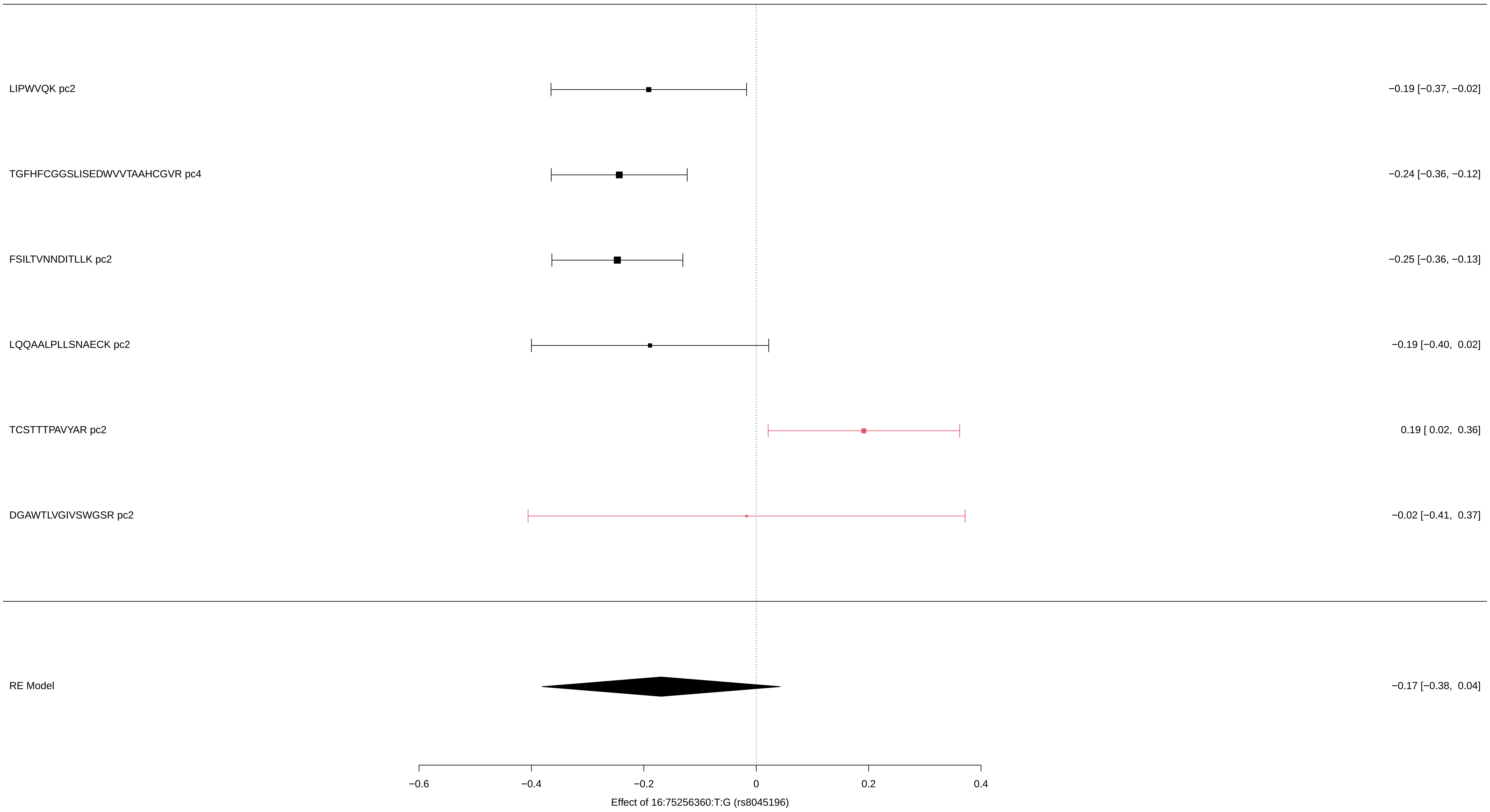

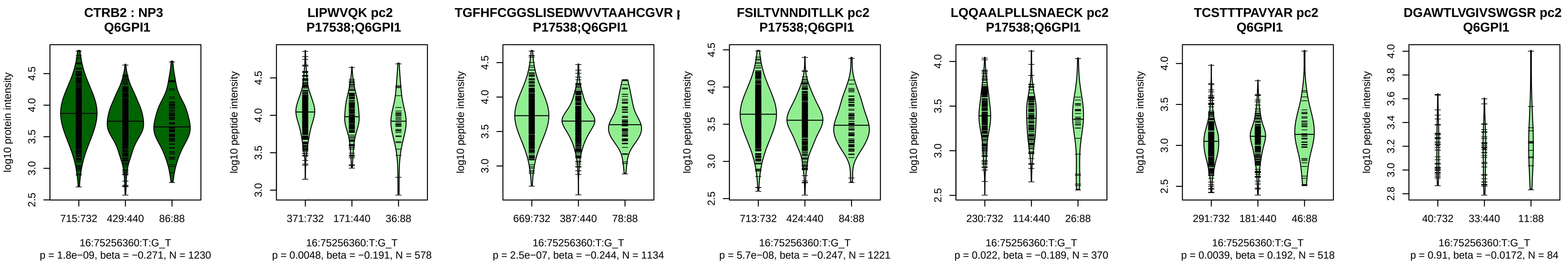

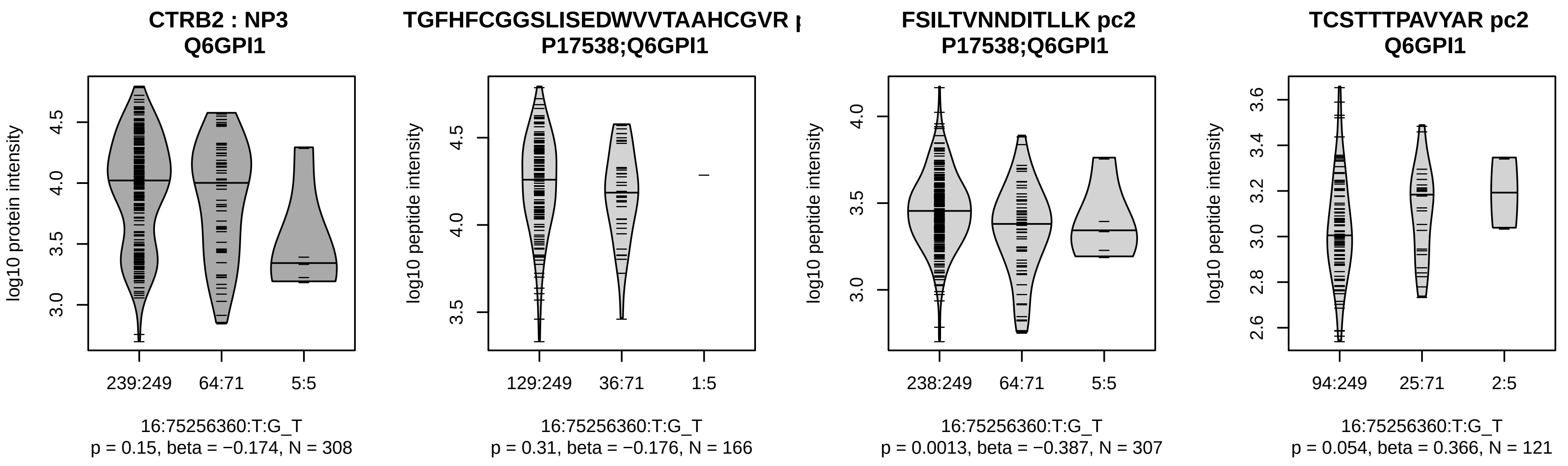

215Assay Target: CTRB2
UniProt: Q6GPI1
rsID: rs8045196
Target SNP: 16:75256360:T:G
Proxy SNP: 16:75256360:T:G
Tarkin log10(p): 8.7
Tarkin BETA: −0.272
Tarkin MSPA: 0.884
::*:–:*:–
578:1134:1221:370:518:84

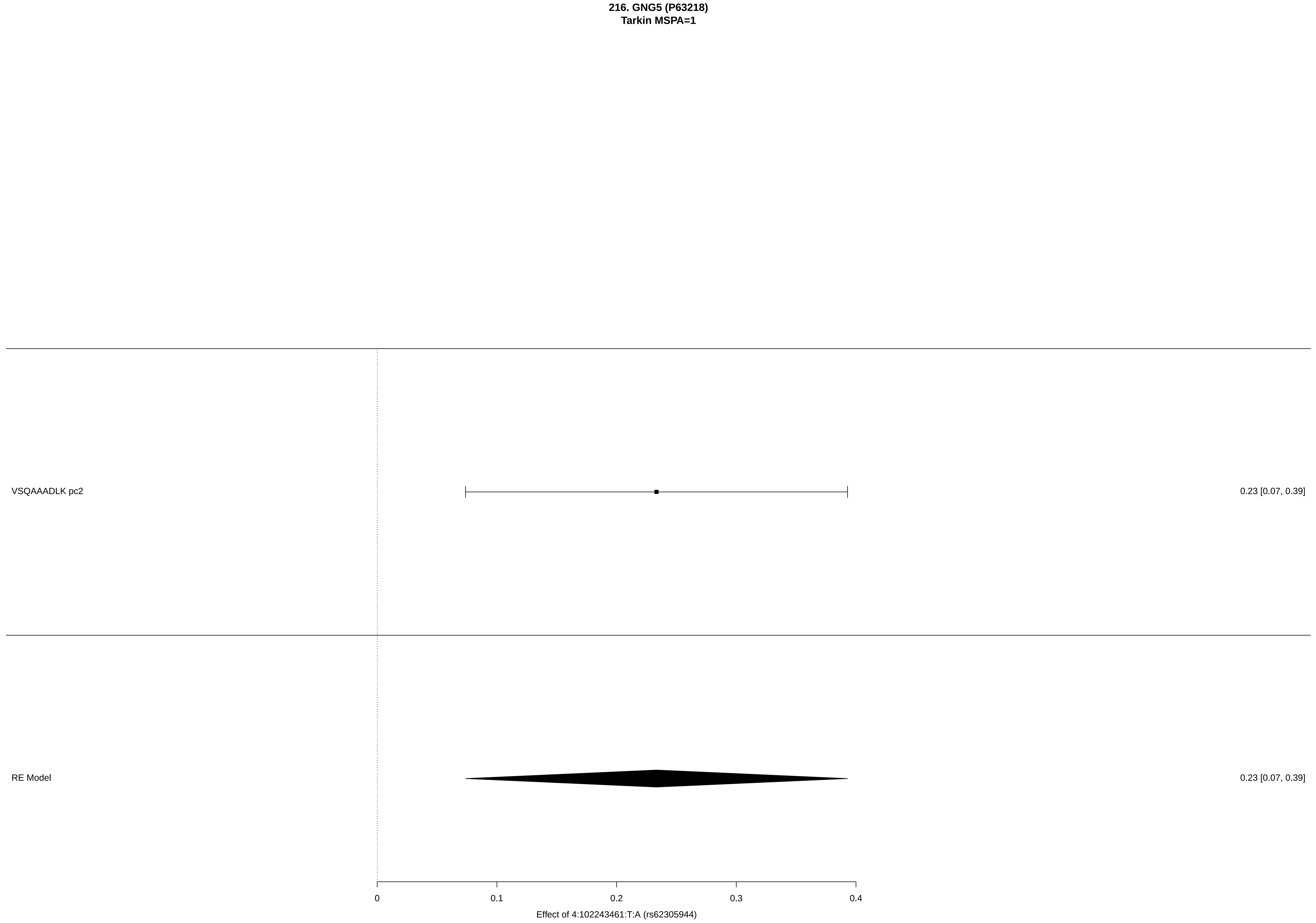
216. GNG5 (P63218)
Tarkin MSPA=1
VSQAAADLK pc2
0.23 [0.07, 0.39]
RE Model
0.23 [0.07, 0.39]
0
0.1
0.2
0.3
0.4
Effect of 4:102243461:T:A (rs62305944)

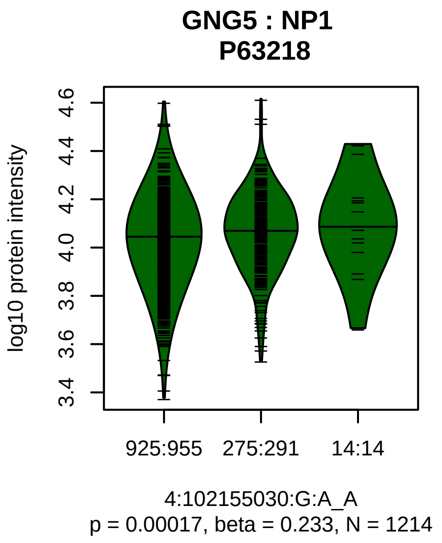

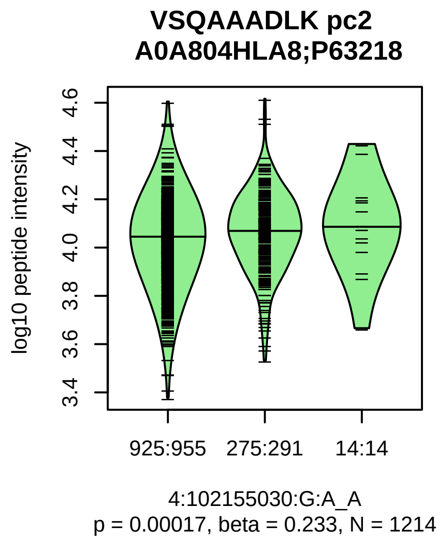

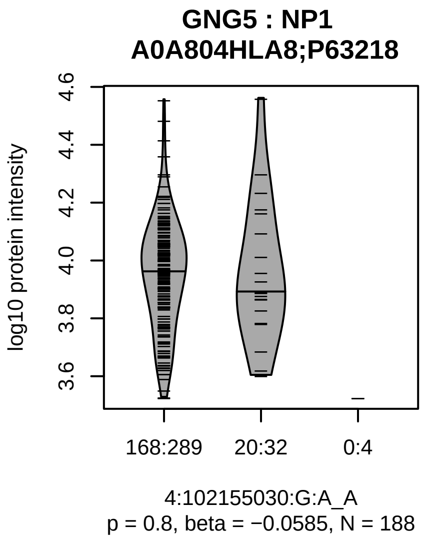

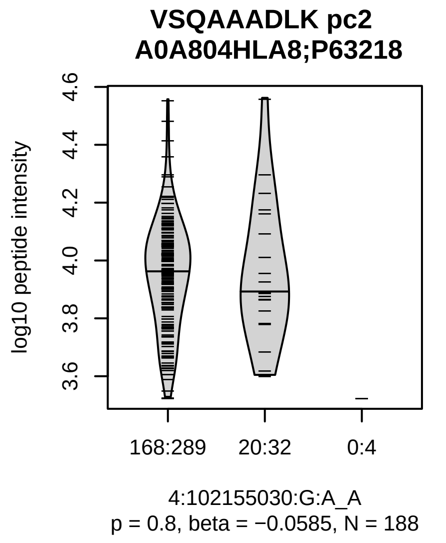

216Assay Target: GNG5
UniProt: P63218
rsID: rs62305944
Target SNP: 4:102243461:T:A
Proxy SNP: 4:102155030:G:A
Tarkin log10(p): 8.7
Tarkin BETA: −0.504
Tarkin MSPA: 1
*
1214

217. PTPRO (Q16827)
Tarkin MSPA=0.724

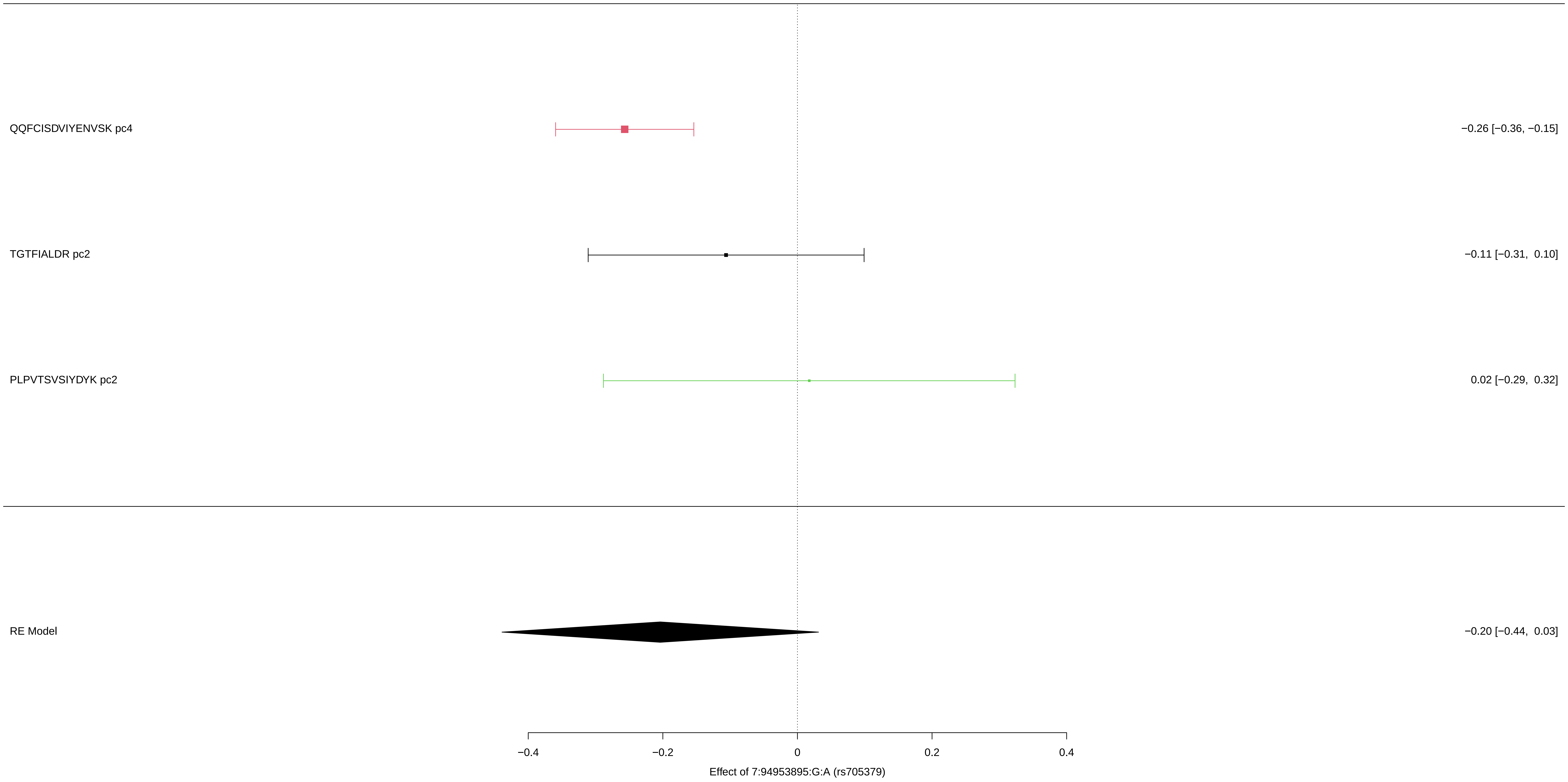

PTPRO : NP2
Q16827

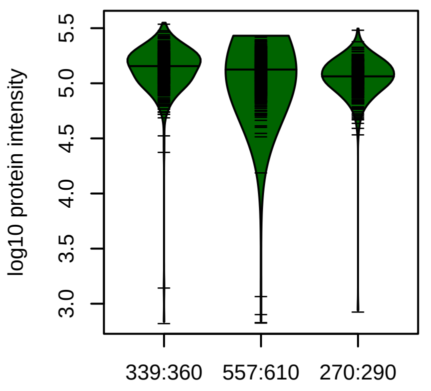

7:94953895:G:A_A
p = 2e−09, beta = −0.241, N = 1166

QQFCISDVIYENVSK pc4
I8PRB8;A0A6I8PRG8;Q16827;Q16827-

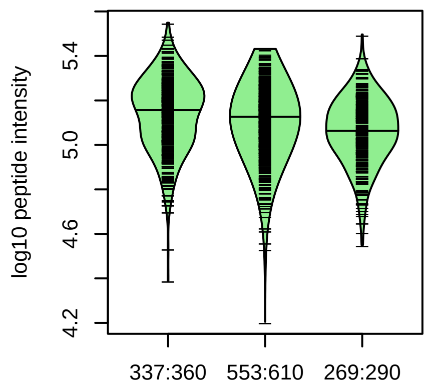

7:94953895:G:A_A
p = 1.8e−10, beta = −0.257, N = 1159

TGTFIALDR pc2
VU56;P23467;P23467−2;P23467−3;P2

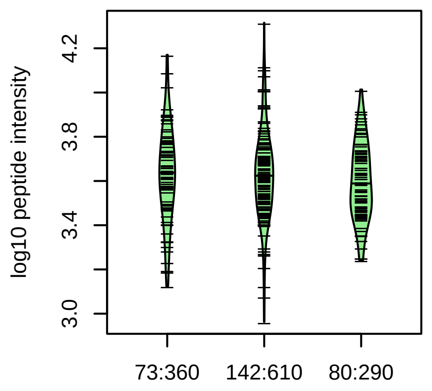

7:94953895:G:A_A
p = 0.18, beta = −0.106, N = 295

PLPVTSVSIYDYK pc2
PL17;A0A6I8PRZ5;Q16827;Q16827−2

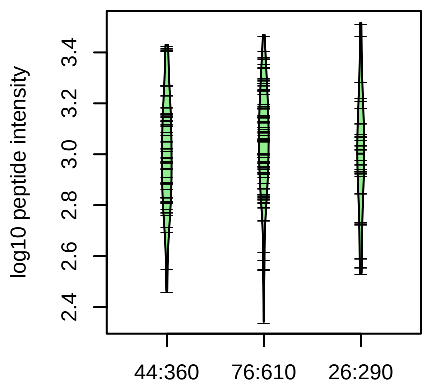

7:94953895:G:A_A
p = 0.88, beta = 0.0175, N = 146

PTPRO : NP2
Q16827−2

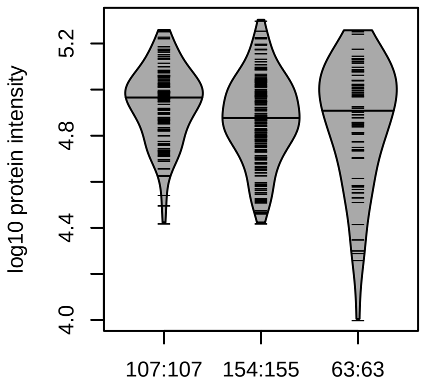

7:94953895:G:A_A
p = 0.028, beta = −0.169, N = 324

QQFCISDVIYENVSK pc4
I8PRB8;A0A6I8PRG8;Q16827;Q16827-

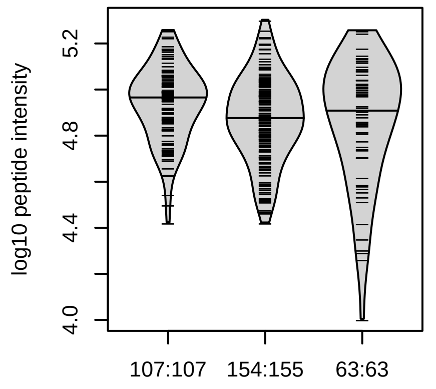

7:94953895:G:A_A
p = 0.028, beta = −0.169, N = 324

217Assay Target: PTPRO
UniProt: Q16827
rsID: rs705379
Target SNP: 7:94953895:G:A
Proxy SNP: 7:94953895:G:A
Tarkin log10(p): 8.7
Tarkin BETA: −0.241
Tarkin MSPA: 0.724
*:−:−
1159:295:146

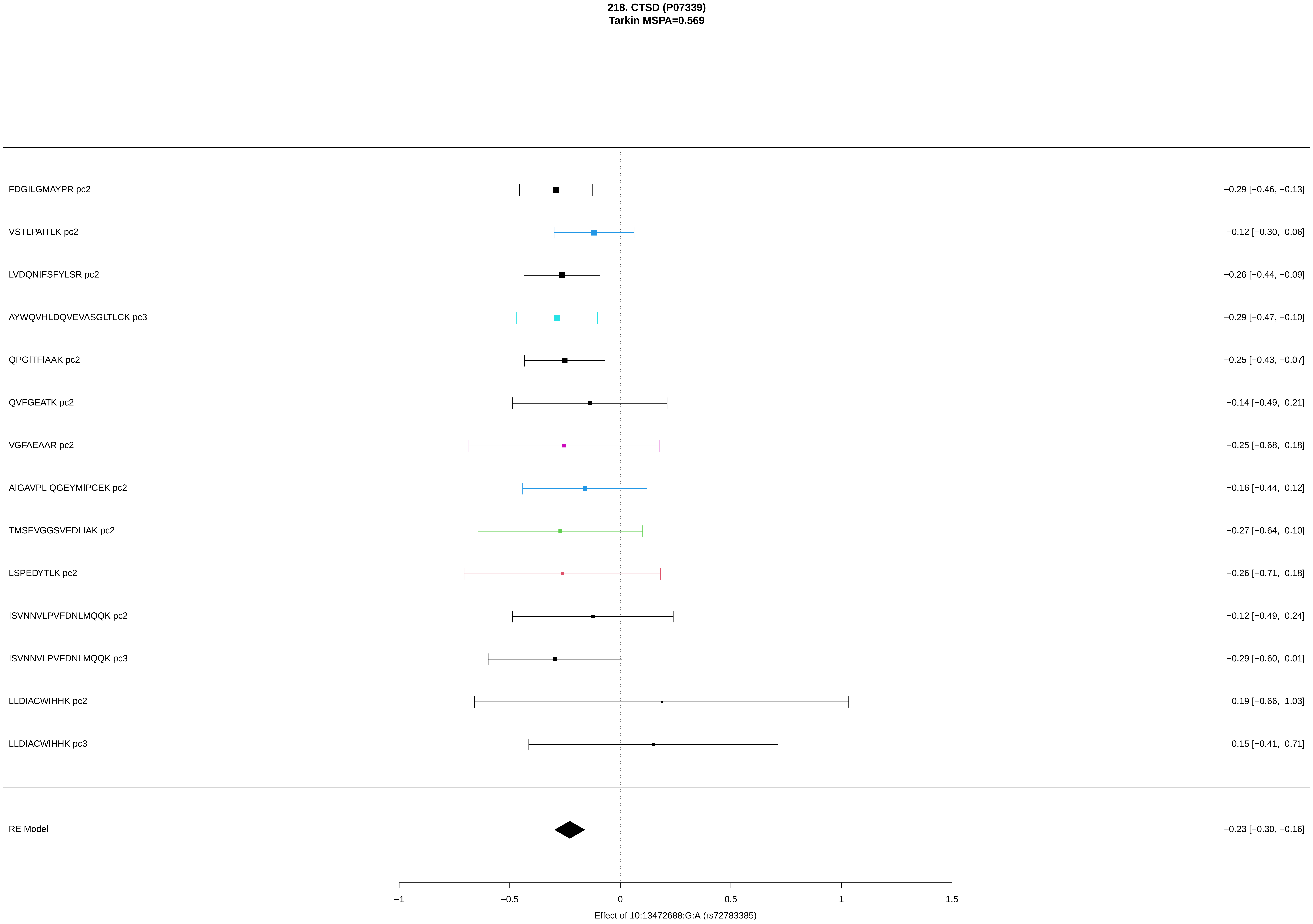

218. CTSD (P07339)
Tarkin MSPA=0.569
FDGILGMAYPR pc2
−0.29 [−0.46, −0.13]
VSTLPAITLK pc2
−0.12 [−0.30, 0.06]
LVDQNIFSFYLSR pc2
−0.26 [−0.44, −0.09]
AYWQVHLDQVEVASGLTLCK pc3
−0.29 [−0.47, −0.10]
QPGITFIAAK pc2
−0.25 [−0.43, −0.07]
QVFGEATK pc2
−0.14 [−0.49, 0.21]
VGFAEAAR pc2
−0.25 [−0.68, 0.18]
AIGAVPLIQGEYMIPCEK pc2
−0.16 [−0.44, 0.12]
TMSEVGGSVEDLIAK pc2
−0.27 [−0.64, 0.10]
LSPEDYTLK pc2
−0.26 [−0.71, 0.18]
ISVNNVLPVFDNLMQQK pc2
−0.12 [−0.49, 0.24]
ISVNNVLPVFDNLMQQK pc3
−0.29 [−0.60, 0.01]
LLDIACWIHHK pc2
0.19 [−0.66, 1.03]
LLDIACWIHHK pc3
0.15 [−0.41, 0.71]
RE Model
−0.23 [−0.30, −0.16]
−1
−0.5
0
0.5
1
1.5
Effect of 10:13472688:G:A (rs72783385)

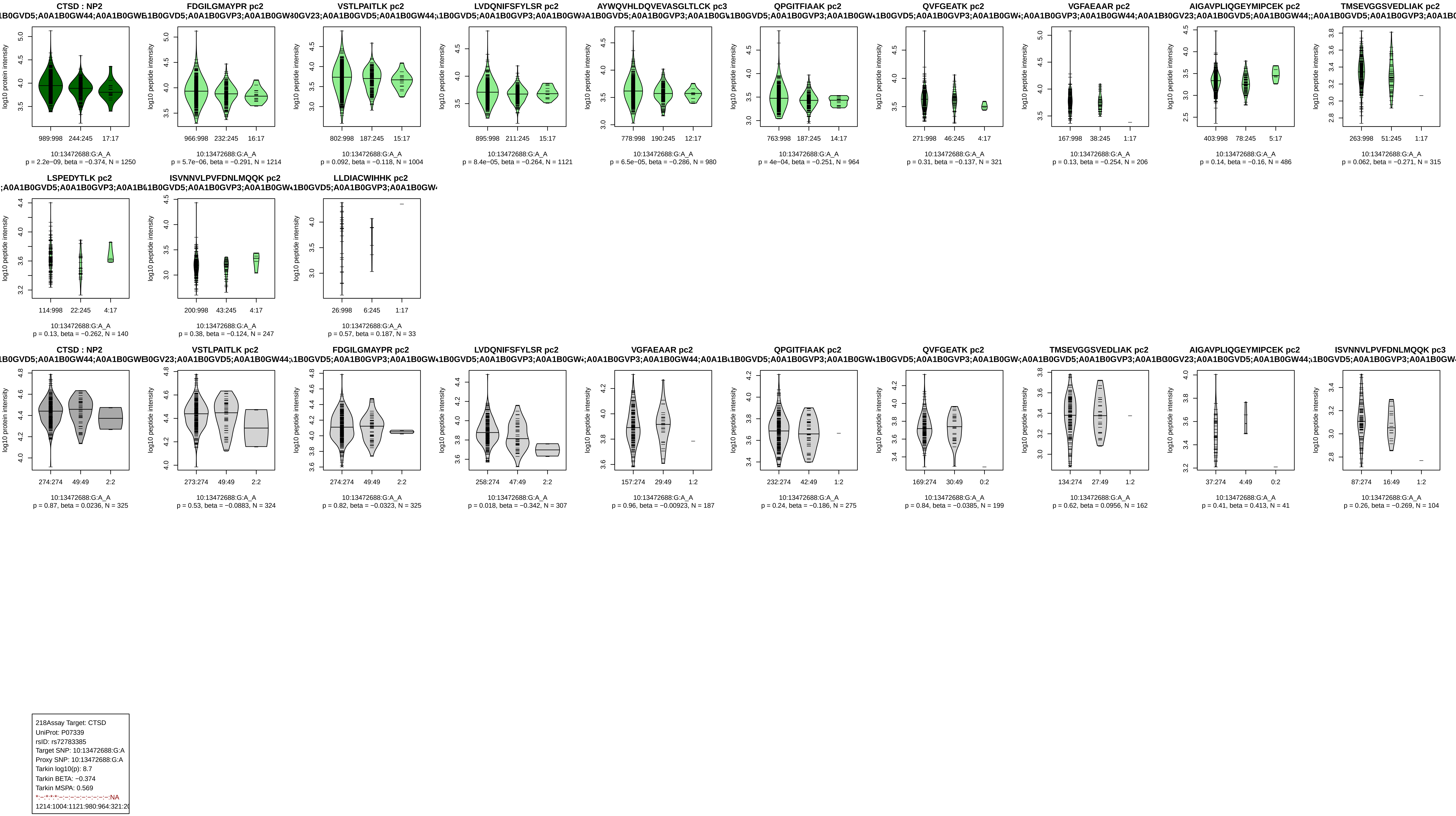

CTSD : NP2
FDGILGMAYPR pc2
VSTLPAITLK pc2
LVDQNIFSFYLSR pc2
AYWQVHLDQVEVASGLTLCK pc3
QPGITFIAAK pc2
QVFGEATK pc2
VGFAEAAR pc2
AIGAVPLIQGEYMIPCEK pc2
TMSEVGGSVEDLIAK pc2
log10 protein intensity
log10 peptide intensity
989:998 244:245 17:17
966:998 232:245 16:17
802:998 187:245 15:17
895:998 211:245 15:17
778:998 190:245 12:17
763:998 187:245 14:17
271:998 46:245 4:17
167:998 38:245 1:17
403:998 78:245 5:17
263:998 51:245 1:17
10:13472688:G:A_A
p = 2.2e−09, beta = −0.374, N = 1250
p = 5.7e−06, beta = −0.291, N = 1214
p = 0.092, beta = −0.118, N = 1004
p = 8.4e−05, beta = −0.264, N = 1121
p = 6.5e−05, beta = −0.286, N = 980
p = 4e−04, beta = −0.251, N = 964
p = 0.31, beta = −0.137, N = 321
p = 0.13, beta = −0.254, N = 206
p = 0.14, beta = −0.16, N = 486
p = 0.062, beta = −0.271, N = 315
LSPEDYTLK pc2
ISVNNVLPVFDNLMQQK pc2
LLDIACWIHHK pc2
114:998 22:245 4:17
200:998 43:245 4:17
26:998 6:245 1:17
p = 0.13, beta = −0.262, N = 140
p = 0.38, beta = −0.124, N = 247
p = 0.57, beta = 0.187, N = 33
CTSD : NP2
VSTLPAITLK pc2
FDGILGMAYPR pc2
LVDQNIFSFYLSR pc2
VGFAEAAR pc2
QPGITFIAAK pc2
QVFGEATK pc2
TMSEVGGSVEDLIAK pc2
AIGAVPLIQGEYMIPCEK pc2
ISVNNVLPVFDNLMQQK pc3
274:274 49:49 2:2
273:274 49:49 2:2
274:274 49:49 2:2
258:274 47:49 2:2
157:274 29:49 1:2
232:274 42:49 1:2
169:274 30:49 0:2
134:274 27:49 1:2
37:274 4:49 0:2
87:274 16:49 1:2
p = 0.87, beta = 0.0236, N = 325
p = 0.53, beta = −0.0883, N = 324
p = 0.82, beta = −0.0323, N = 325
p = 0.018, beta = −0.342, N = 307
p = 0.96, beta = −0.00923, N = 187
p = 0.24, beta = −0.186, N = 275
p = 0.84, beta = −0.0385, N = 199
p = 0.62, beta = 0.0956, N = 162
p = 0.41, beta = 0.413, N = 41
p = 0.26, beta = −0.269, N = 104
218Assay Target: CTSD
UniProt: P07339
rsID: rs72783385
Target SNP: 10:13472688:G:A
Proxy SNP: 10:13472688:G:A
Tarkin log10(p): 8.7
Tarkin BETA: −0.374
Tarkin MSPA: 0.569

219. DKK3 (Q9UBP4)
Tarkin MSPA=0.813

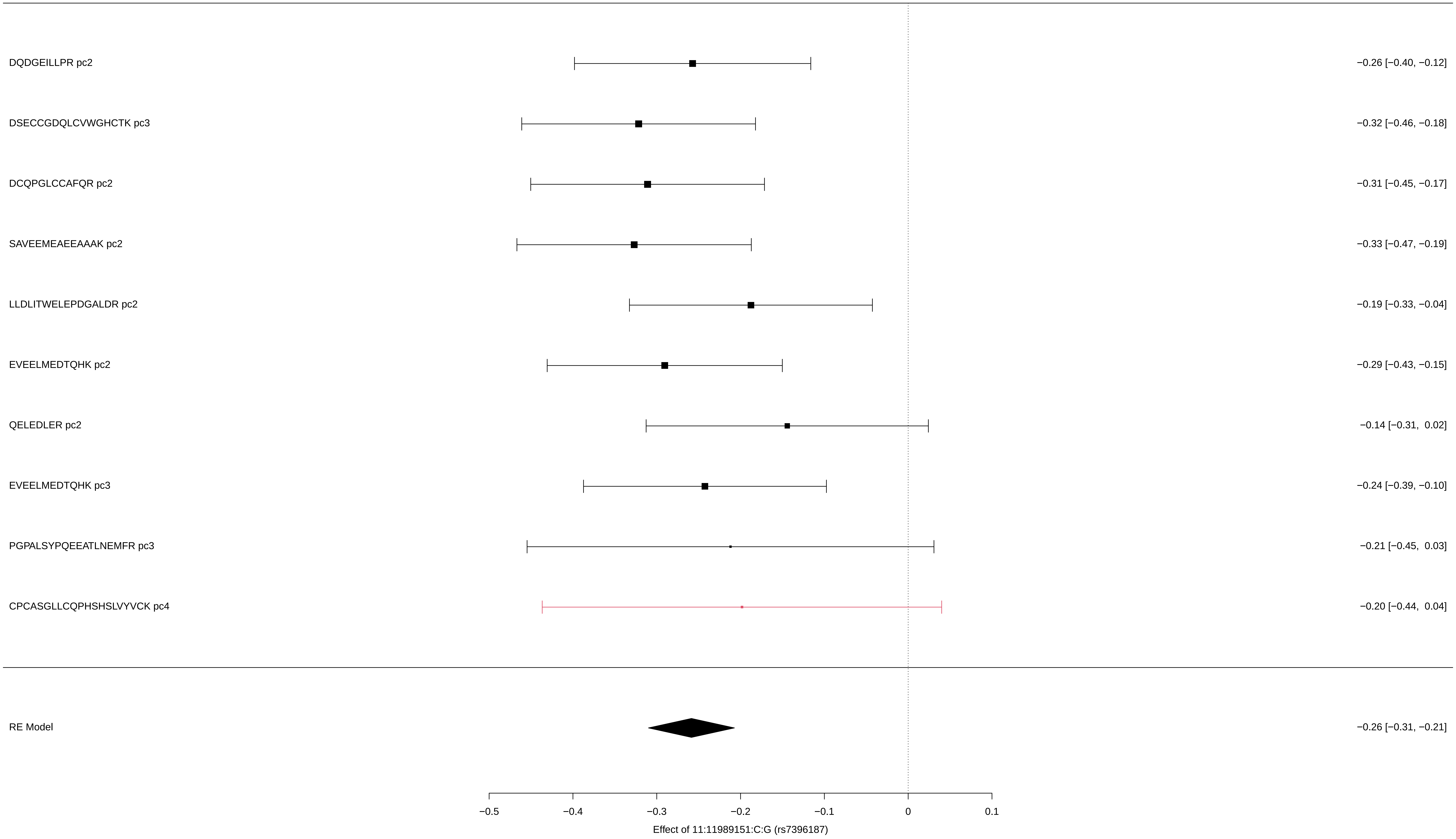

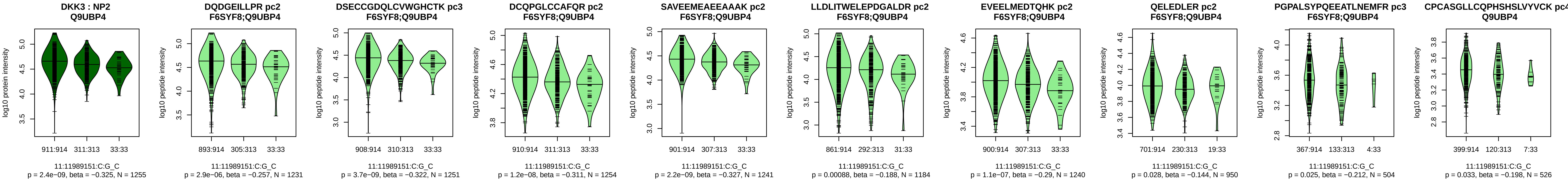

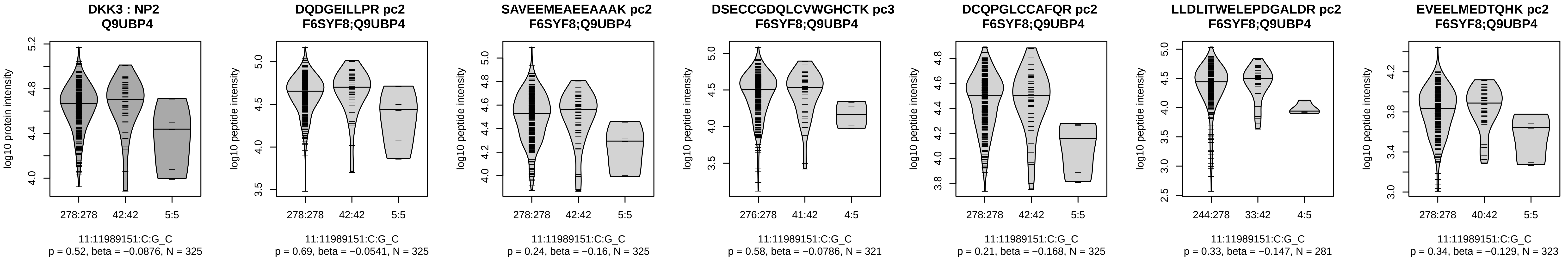

219Assay Target: DKK3
UniProt: Q9UBP4
rsID: rs7396187
Target SNP: 11:11989151:C:G
Proxy SNP: 11:11989151:C:G
Tarkin log10(p): 8.6
Tarkin BETA: −0.325
Tarkin MSPA: 0.813
1231:1251:1254:1241:1184:124

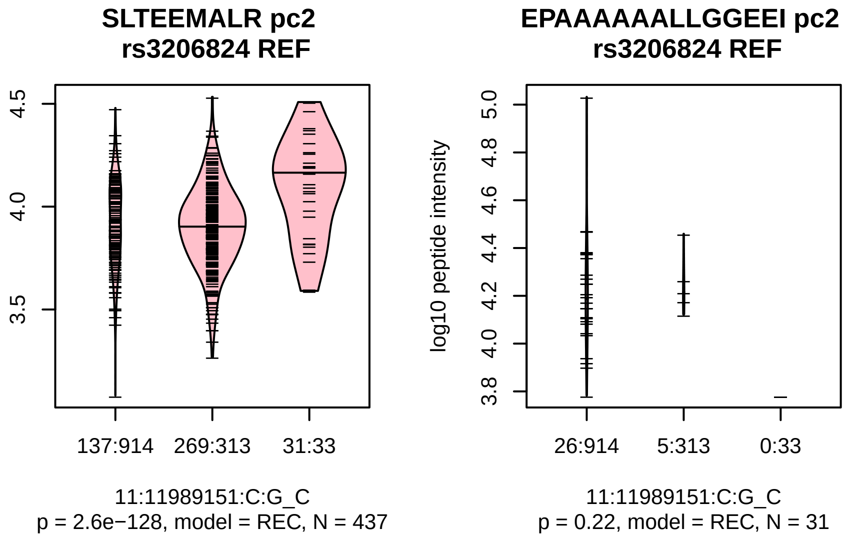

220. COL18A1 (P39060)
Tarkin MSPA=0.434

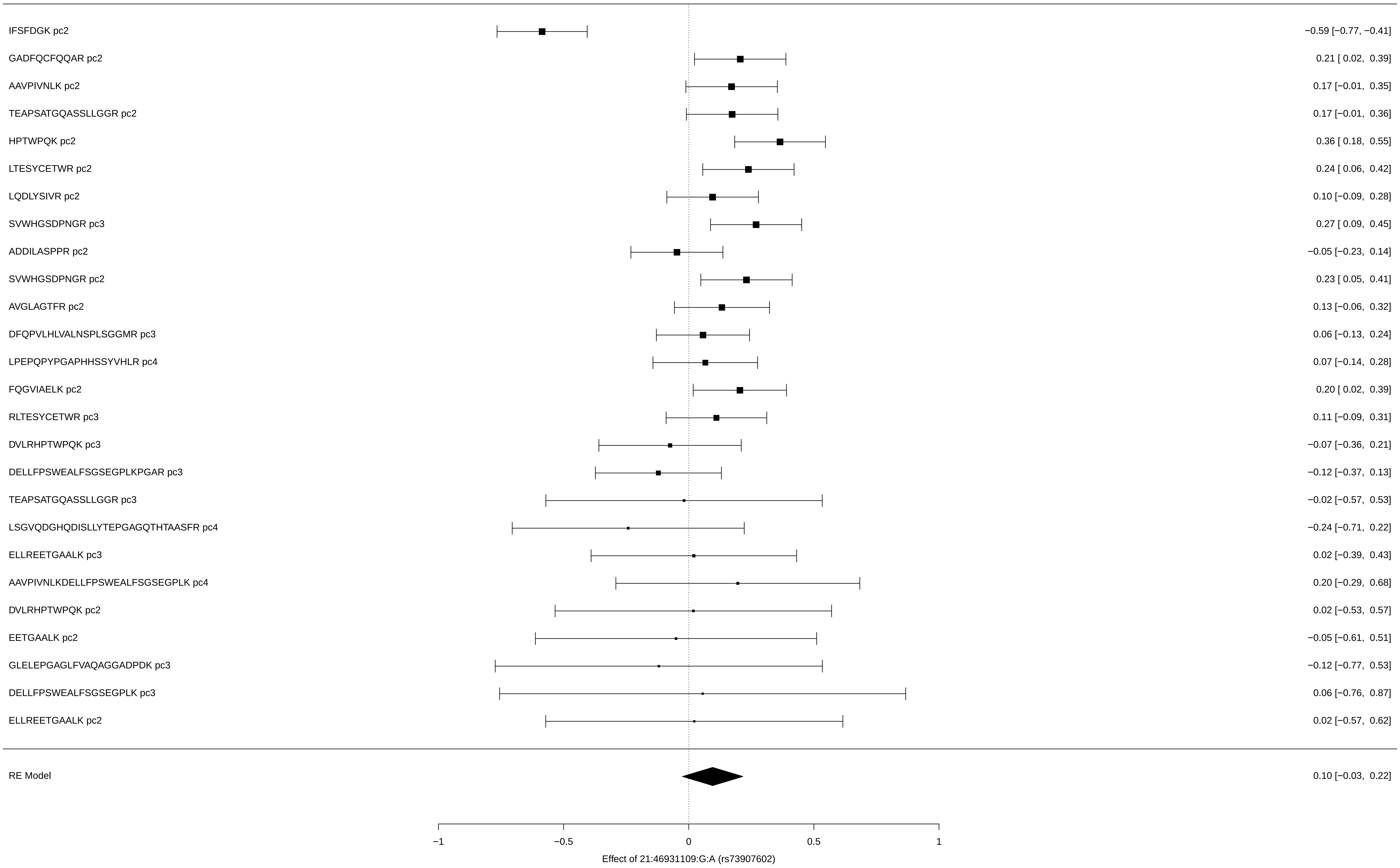

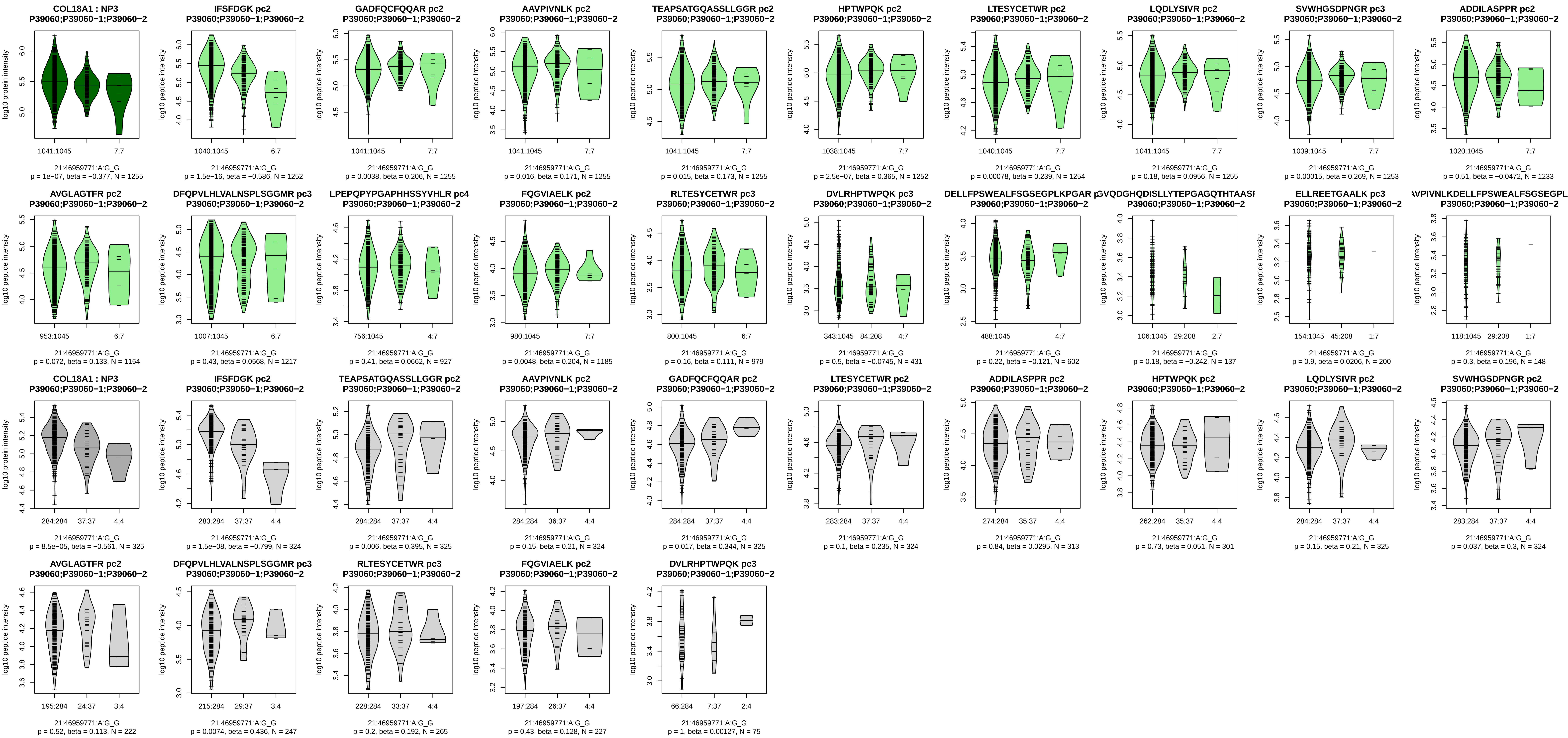

220Assay Target: COL18A1
UniProt: P39060
rsID: rs73907602
Target SNP: 21:46931109:G:A
Proxy SNP: 21:46959771:A:G
Tarkin log10(p): 8.6
Tarkin BETA: −0.453
Tarkin MSPA: 0.434
[illegible]
1252:1255:1255:1255:1252:125

221. PAM (P19021)
Tarkin MSPA=0.859

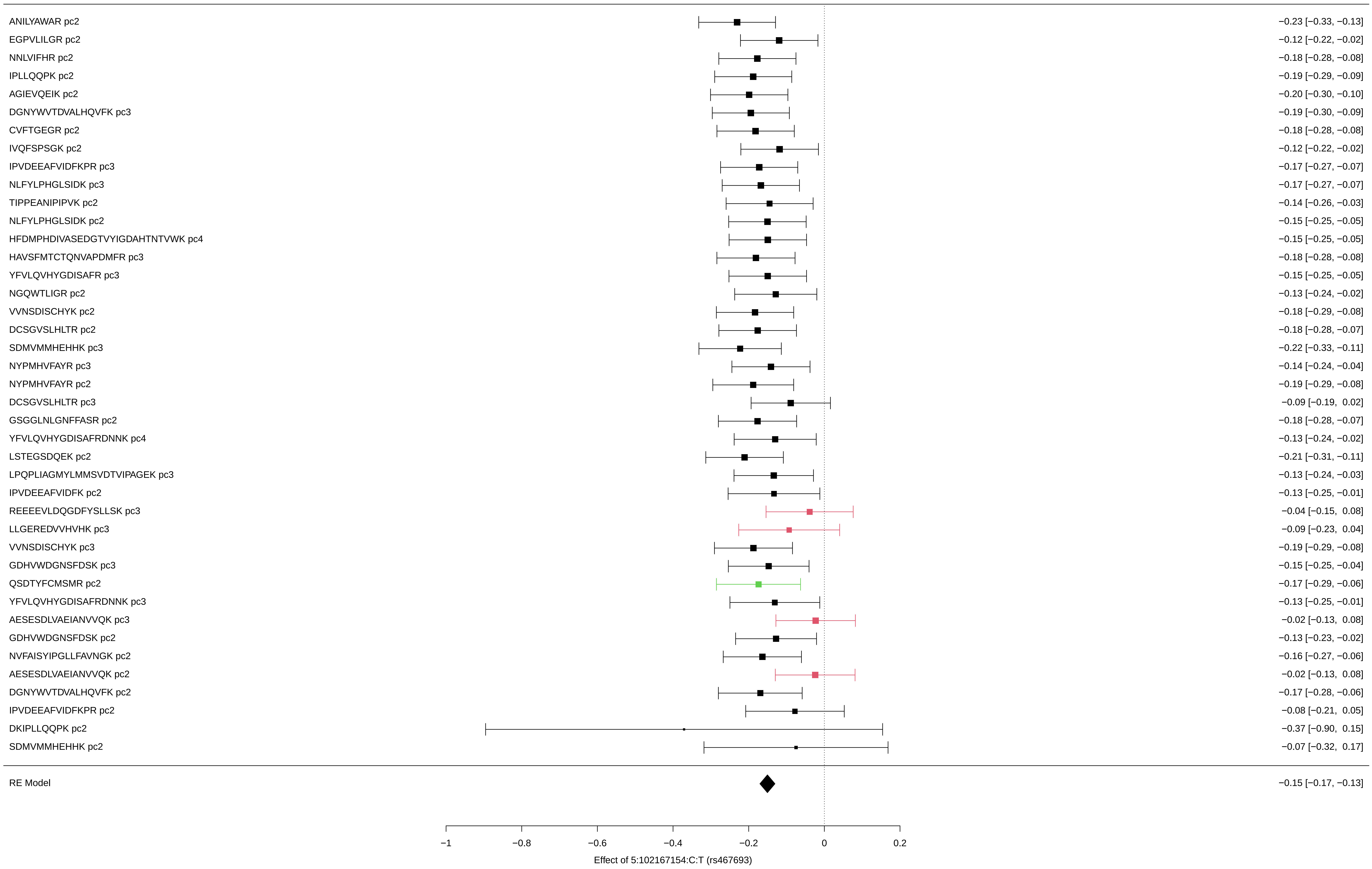

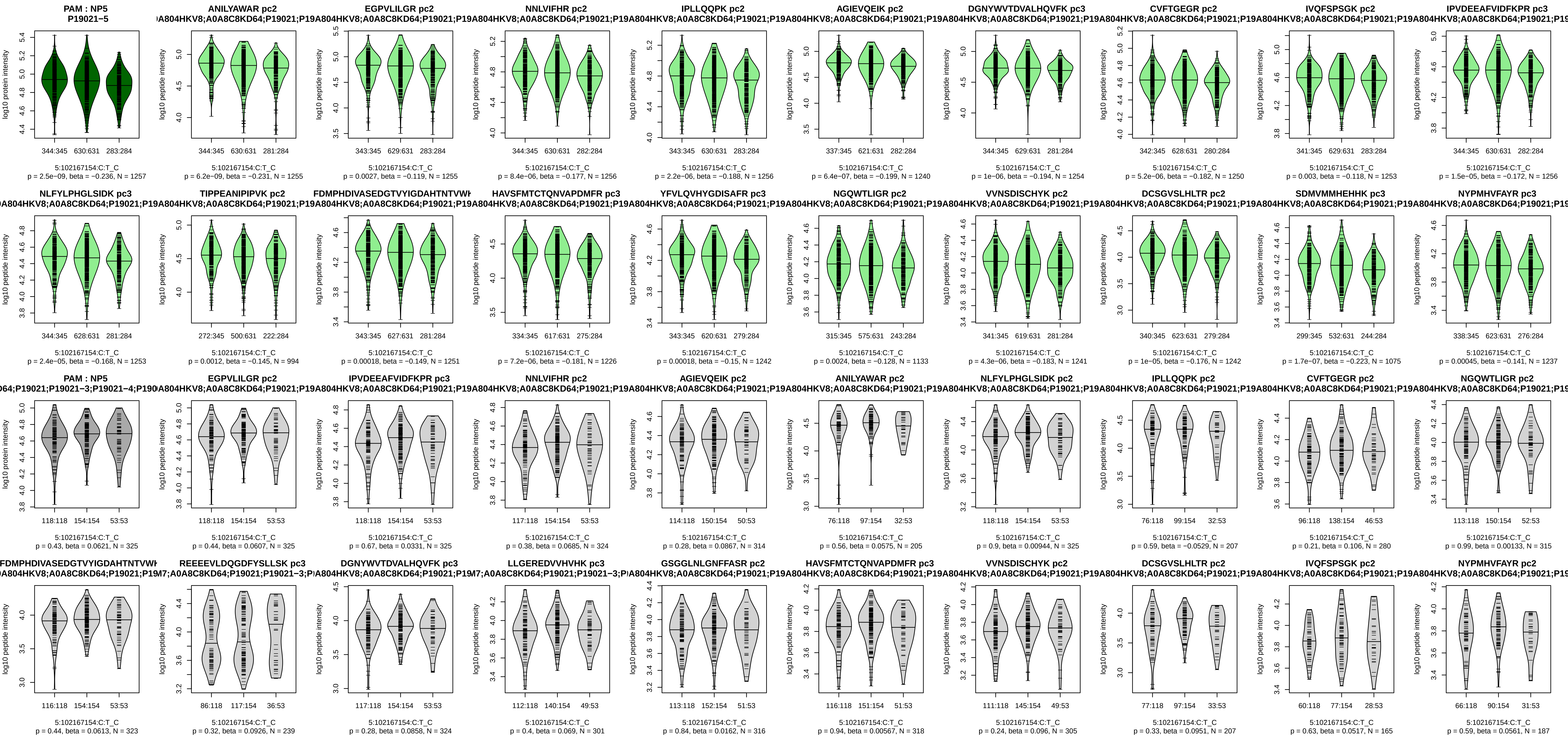

221Assay Target: PAM
UniProt: P19021
rsID: rs467693
Target SNP: 5:102167154:C:T
Proxy SNP: 5:102167154:C:T
Tarkin log10(p): 8.6
Tarkin BETA: −0.236
Tarkin MSPA: 0.859
1255:1255:1256:1256:1240:125

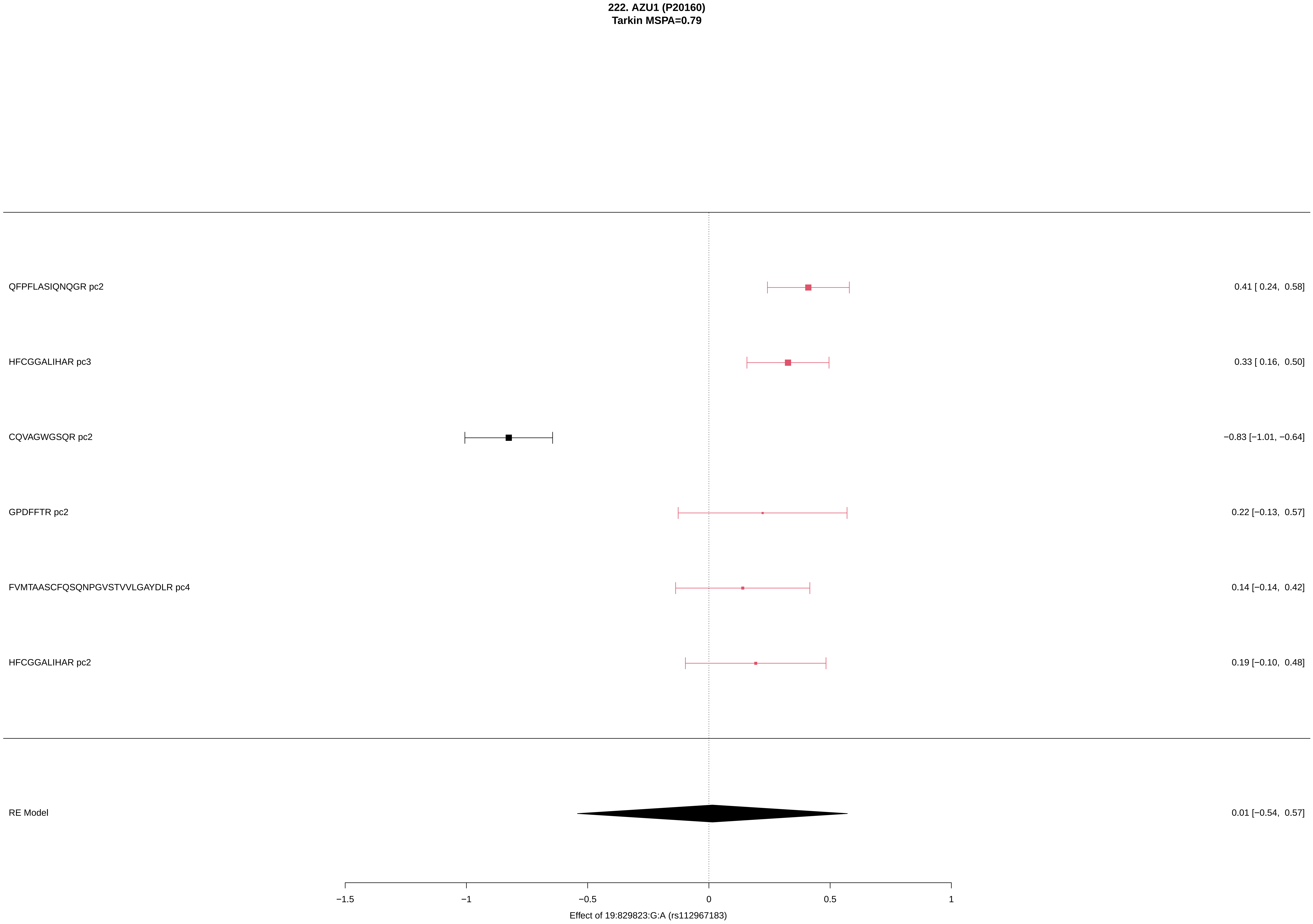

222. AZU1 (P20160)
Tarkin MSPA=0.79
QFPFLASIQNQGR pc2
0.41 [0.24, 0.58]
HFCGGALIHAR pc3
0.33 [0.16, 0.50]
CQVAGWGSQR pc2
−0.83 [−1.01, −0.64]
GPDFFTR pc2
0.22 [−0.13, 0.57]
FVMTAASCFQSQNPGVSTVVLGAYDLR pc4
0.14 [−0.14, 0.42]
HFCGGALIHAR pc2
0.19 [−0.10, 0.48]
RE Model
0.01 [−0.54, 0.57]
−1.5
−1
−0.5
0
0.5
1
Effect of 19:829823:G:A (rs112967183)

AZU1 : NP4 P20160 | **QFPFLASIQNQGR pc2 P20160** | **HFCGGALIHAR pc3 P20160** | **CQVAGWGSQR pc2 A0A087WXP0;P20160** | **GPDFFTR pc2 P20160** | **VMTAASCFQSQNPGVSTVVLGAYDLR P20160**

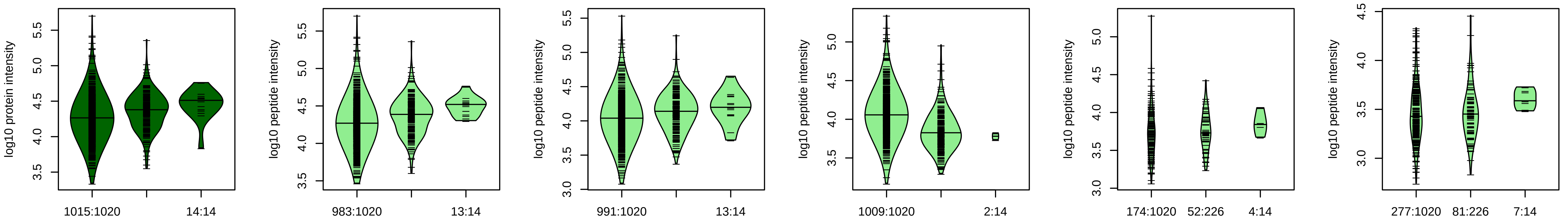

19:829823:G:A_A
p = 2.5e−09, beta = 0.389, N = 1254

19:829823:G:A_A
p = 5.4e−10, beta = 0.41, N = 1218

19:829823:G:A_A
p = 7.9e−07, beta = 0.326, N = 1228

19:829823:G:A_A
p = 2.8e−30, beta = −0.826, N = 1221

19:829823:G:A_A
p = 0.1, beta = 0.222, N = 230

19:829823:G:A_A
p = 0.19, beta = 0.14, N = 365

AZU1 : NP4 P20160 | **QFPFLASIQNQGR pc2 P20160** | **CQVAGWGSQR pc2 A0A087WXP0;P20160** | **HFCGGALIHAR pc3 P20160**

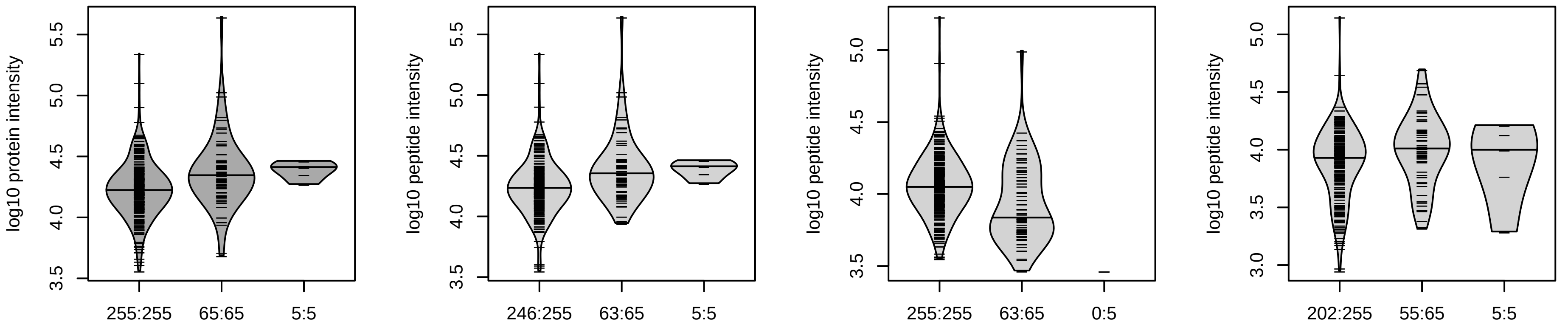

19:829823:G:A_A
p = 3.4e−05, beta = 0.492, N = 325

19:829823:G:A_A
p = 1.4e−05, beta = 0.52, N = 314

19:829823:G:A_A
p = 8.5e−06, beta = −0.609, N = 318

19:829823:G:A_A
p = 0.03, beta = 0.278, N = 262

222Assay Target: AZU1
UniProt: P20160
rsID: rs112967183
Target SNP: 19:829823:G:A
Proxy SNP: 19:829823:G:A
Tarkin log10(p): 8.6
Tarkin BETA: 0.389
Tarkin MSPA: 0.79
::*:−:−:−:NA
1218:1228:1221:230:365:379:1

223. PFKP (Q01813)
Tarkin MSPA=0.85

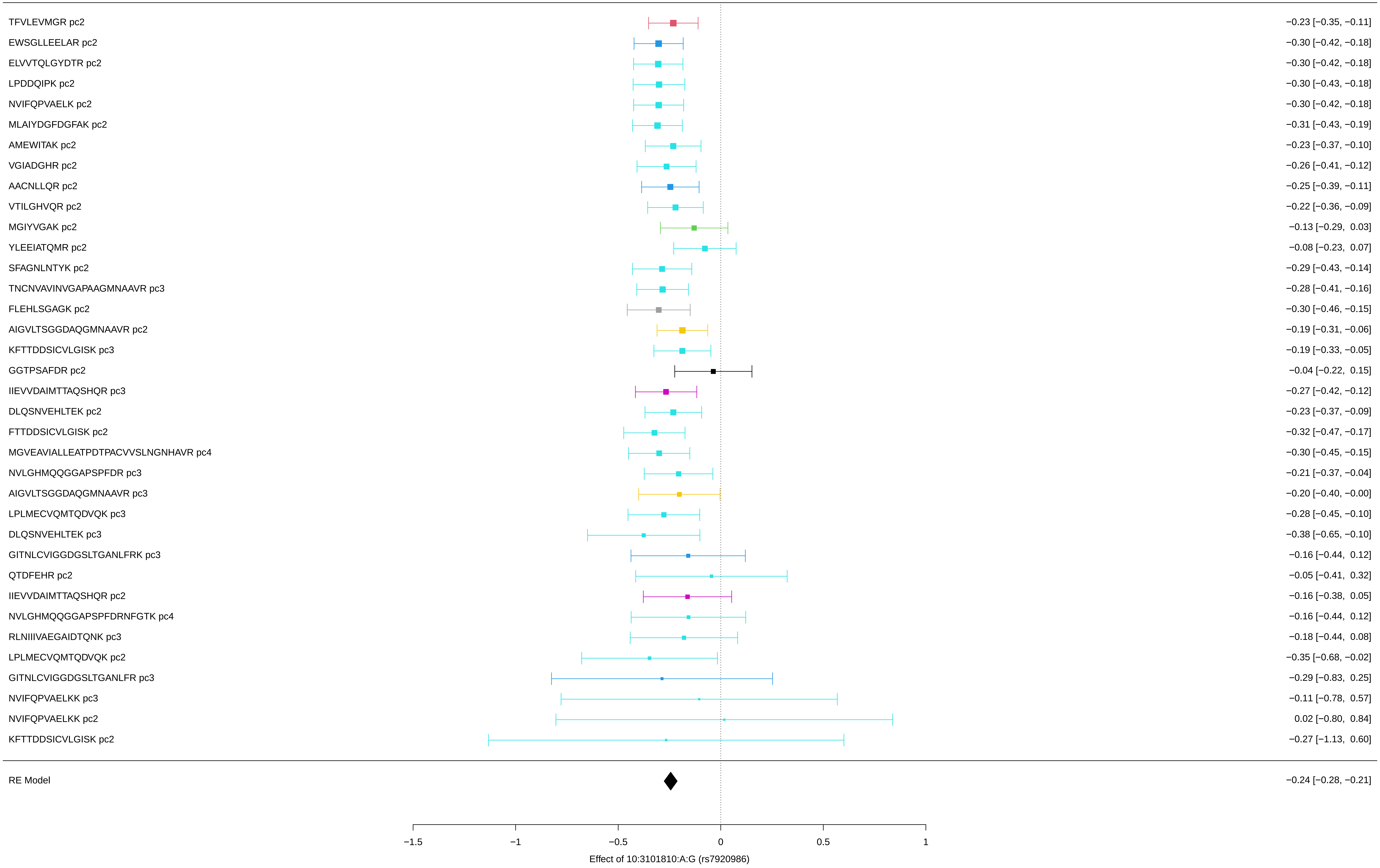

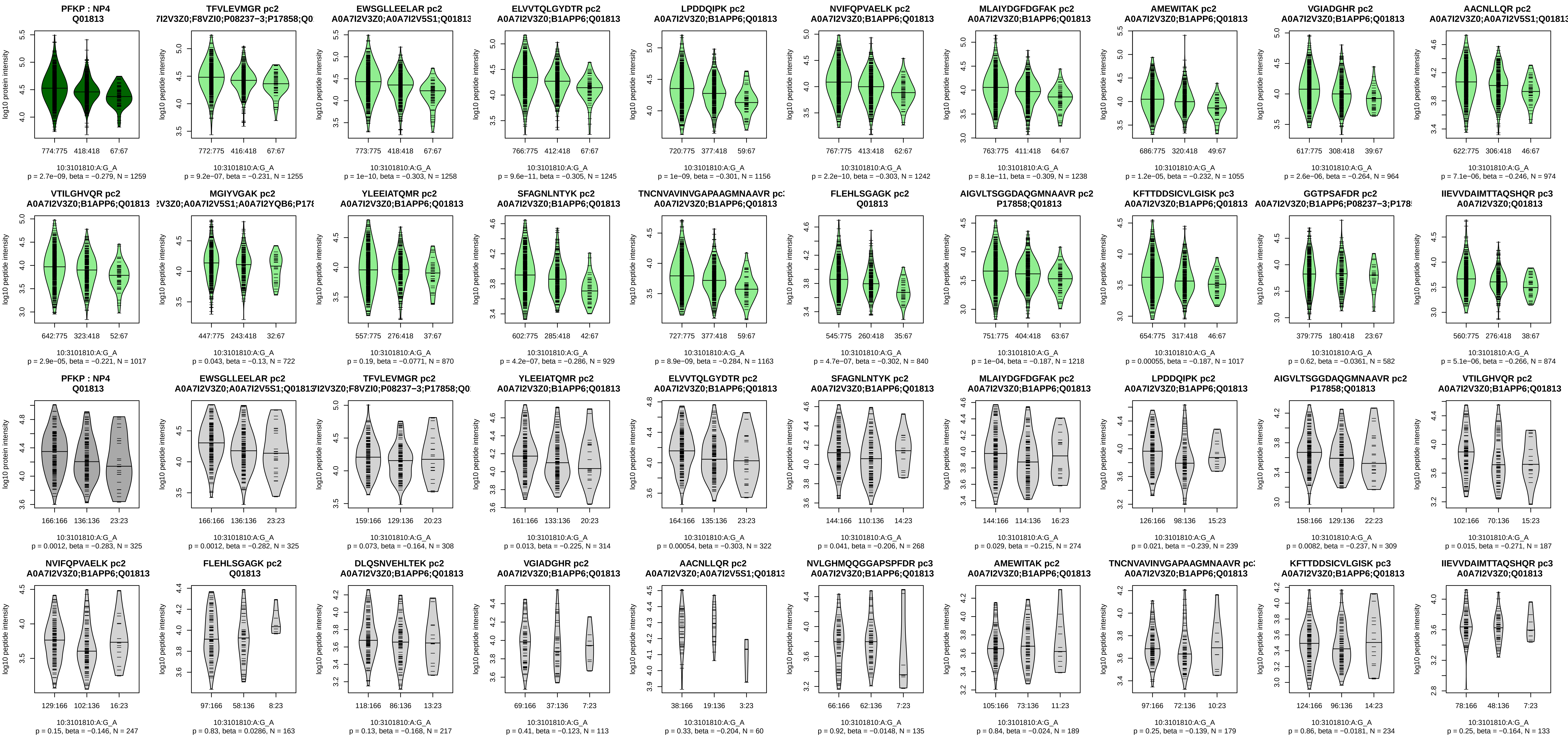

223Assay Target: PFKP
UniProt: Q01813
rsID: rs7920986
Target SNP: 10:3101810:A:G
Proxy SNP: 10:3101810:A:G
Tarkin log10(p): 8.6
Tarkin BETA: −0.279
Tarkin MSPA: 0.85
1255:1258:1245:1156:1242:123

224. SCP2 (P22307)
Tarkin MSPA=0.458

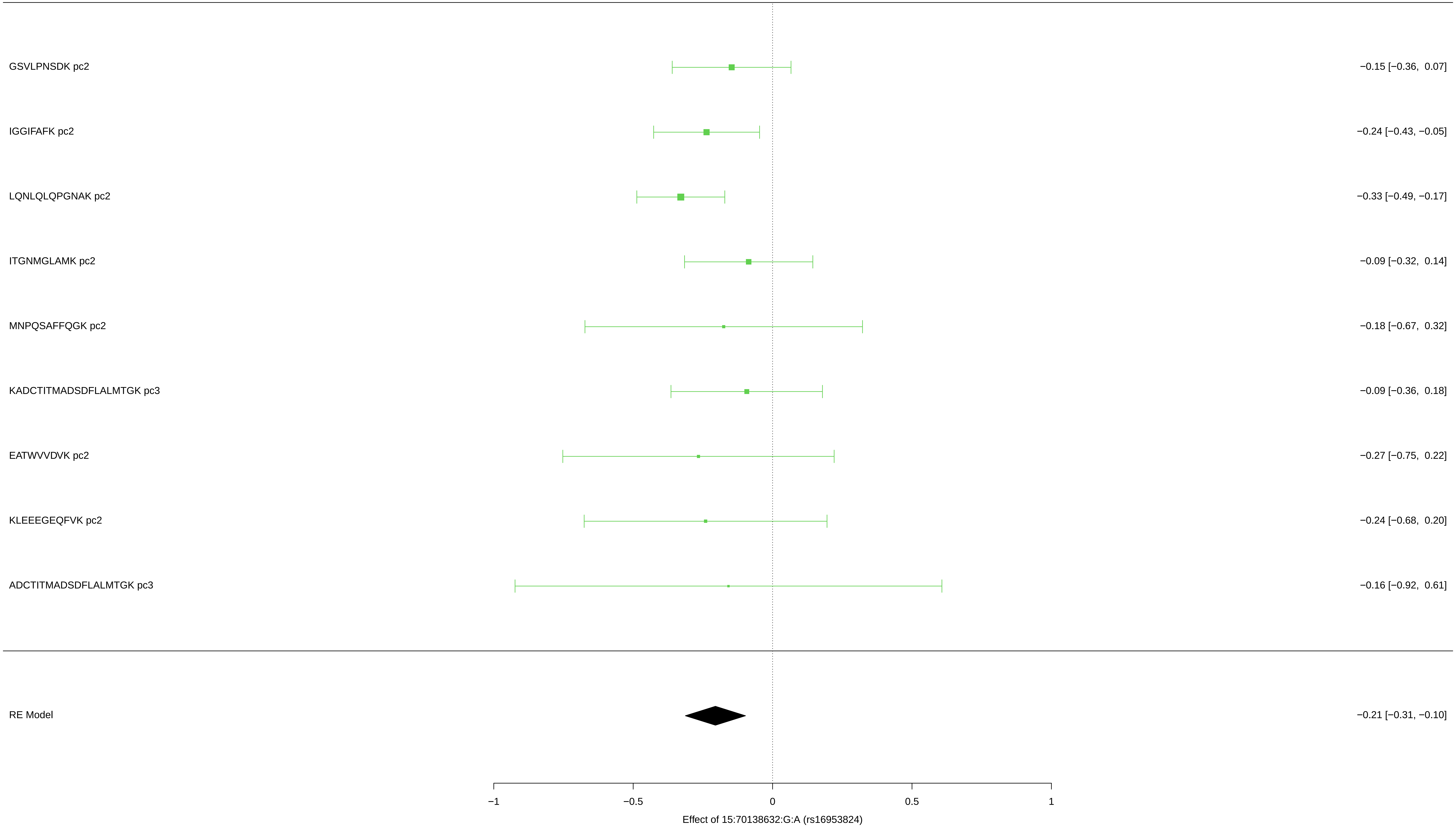

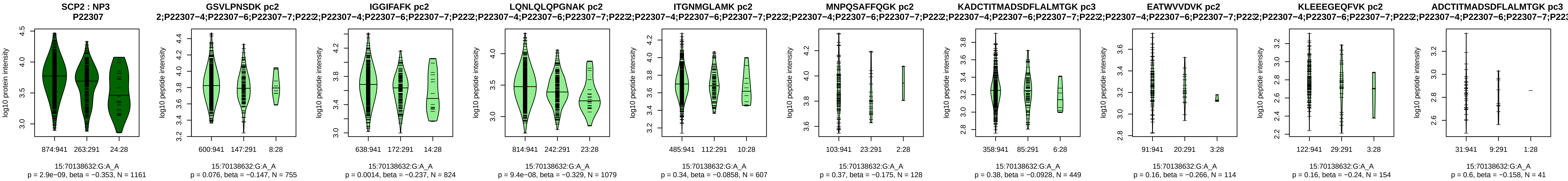

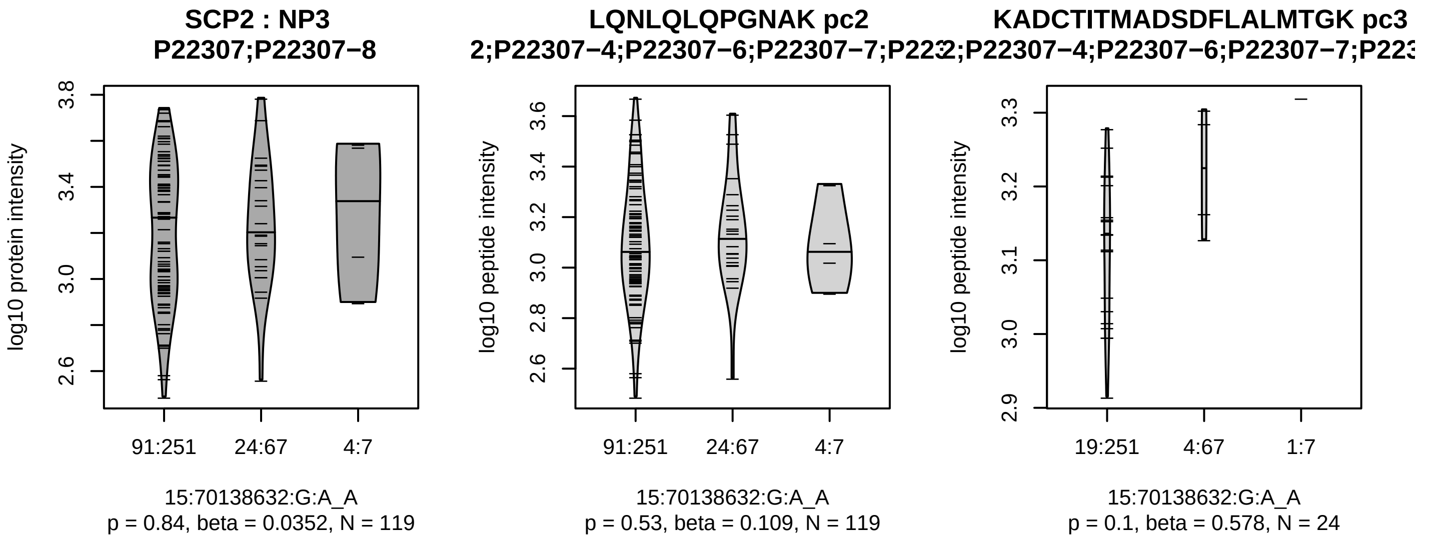

224Assay Target: SCP2
UniProt: P22307
rsID: rs16953824
Target SNP: 15:70138632:G:A
Proxy SNP: 15:70138632:G:A
Tarkin log10(p): 8.5
Tarkin BETA: −0.353
Tarkin MSPA: 0.458
−:*:*:−:−:−:−:−:NA:−:NA:NA:NA
755:824:1079:607:128:449:114:

225. NCF1 (P14598)
Tarkin MSPA=0.781

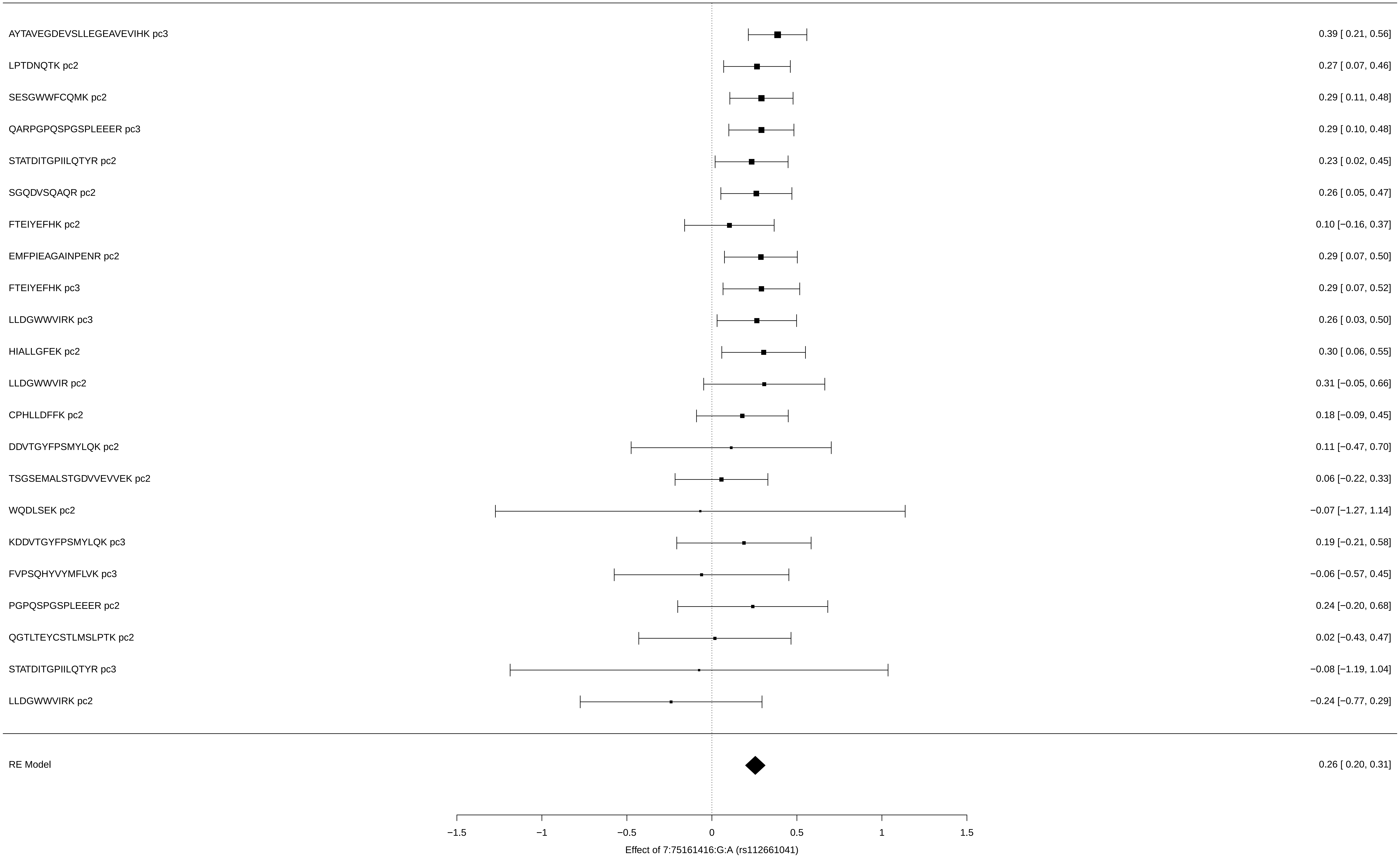

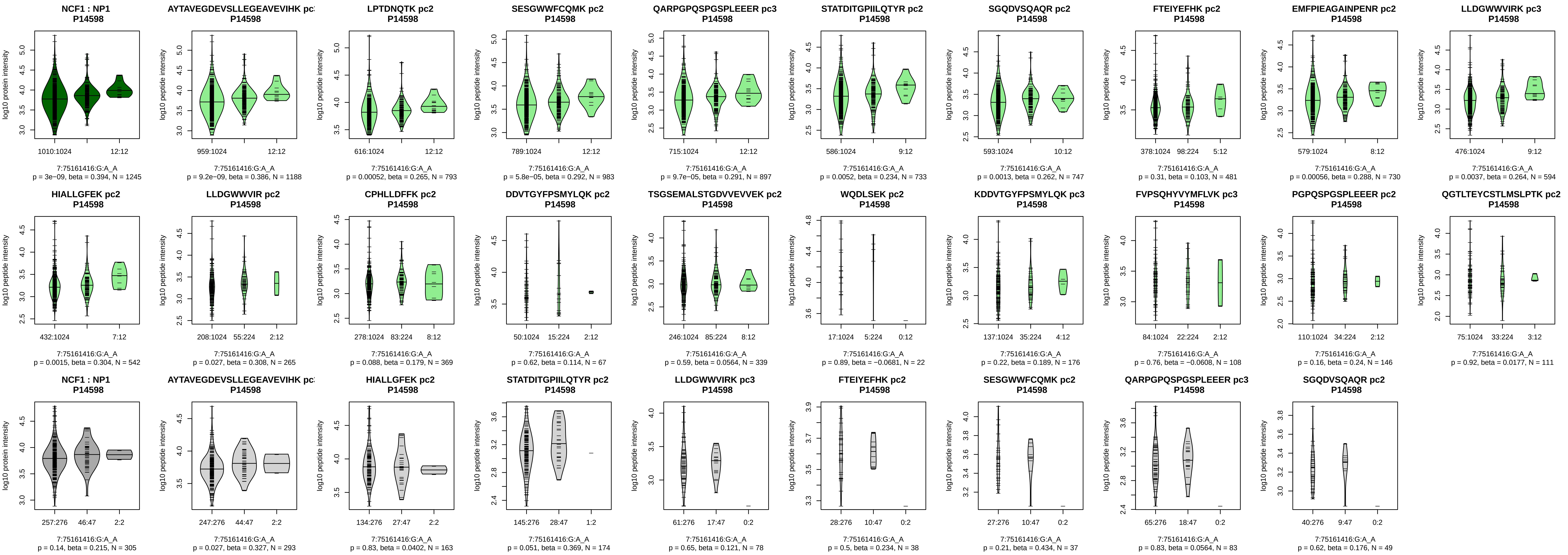

225Assay Target: NCF1
UniProt: P14598
rsID: rs112661041
Target SNP: 7:75161416:G:A
Proxy SNP: 7:75161416:G:A
Tarkin log10(p): 8.5
Tarkin BETA: 0.394
Tarkin MSPA: 0.781
::*:*:*:*:–:*:*:*:*:–:–:–:–:–:–:–:N
1188:793:983:897:733:747:481:

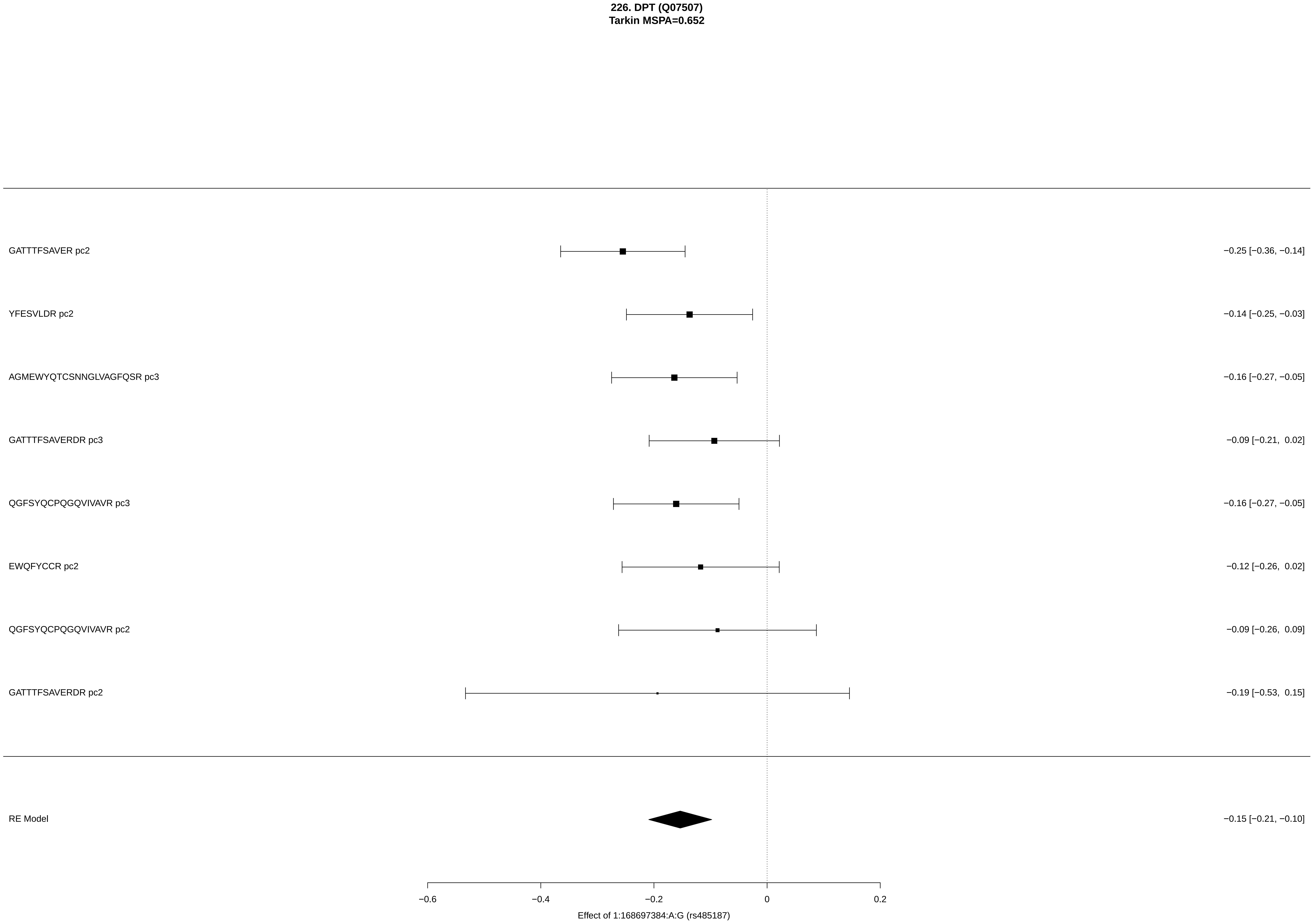
226. DPT (Q07507)
Tarkin MSPA=0.652
GATTTFSAVER pc2
−0.25 [−0.36, −0.14]
YFESVLDR pc2
−0.14 [−0.25, −0.03]
AGMEWYQTCSNNGLVAGFQSR pc3
−0.16 [−0.27, −0.05]
GATTTFSAVERDR pc3
−0.09 [−0.21, 0.02]
QGFSYQCPQGQVIVAVR pc3
−0.16 [−0.27, −0.05]
EWQFYCCR pc2
−0.12 [−0.26, 0.02]
QGFSYQCPQGQVIVAVR pc2
−0.09 [−0.26, 0.09]
GATTTFSAVERDR pc2
−0.19 [−0.53, 0.15]
RE Model
−0.15 [−0.21, −0.10]
−0.6
−0.4
−0.2
0
0.2
Effect of 1:168697384:A:G (rs485187)

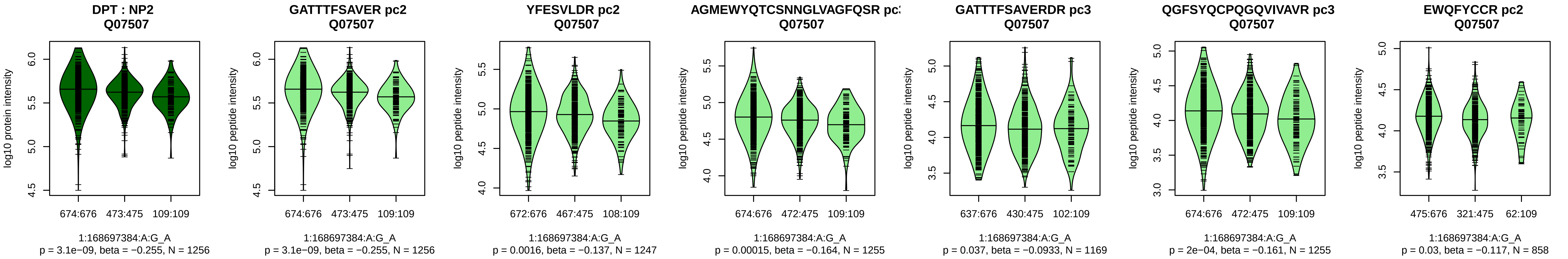

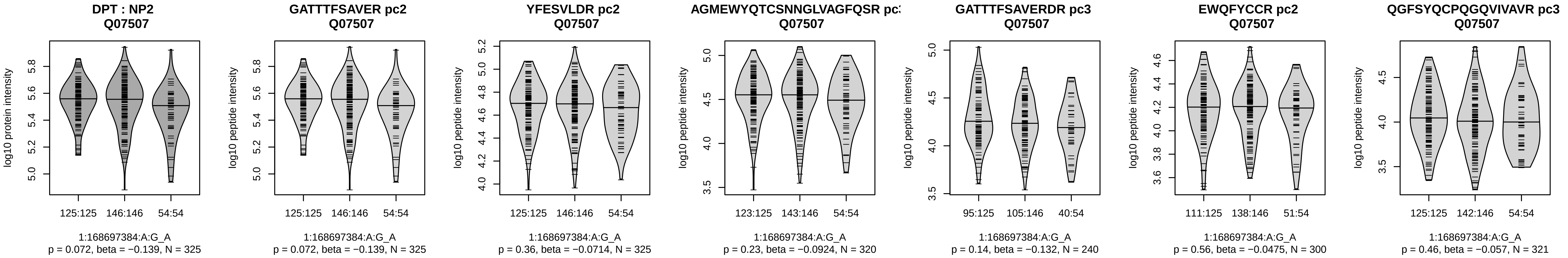

226Assay Target: DPT
UniProt: Q07507
rsID: rs485187
Target SNP: 1:168697384:A:G
Proxy SNP: 1:168697384:A:G
Tarkin log10(p): 8.5
Tarkin BETA: −0.255
Tarkin MSPA: 0.652
::*:–:*:–:–:–
1256:1247:1255:1169:1255:858

227. FCN2 (Q15485)
Tarkin MSPA=0.161

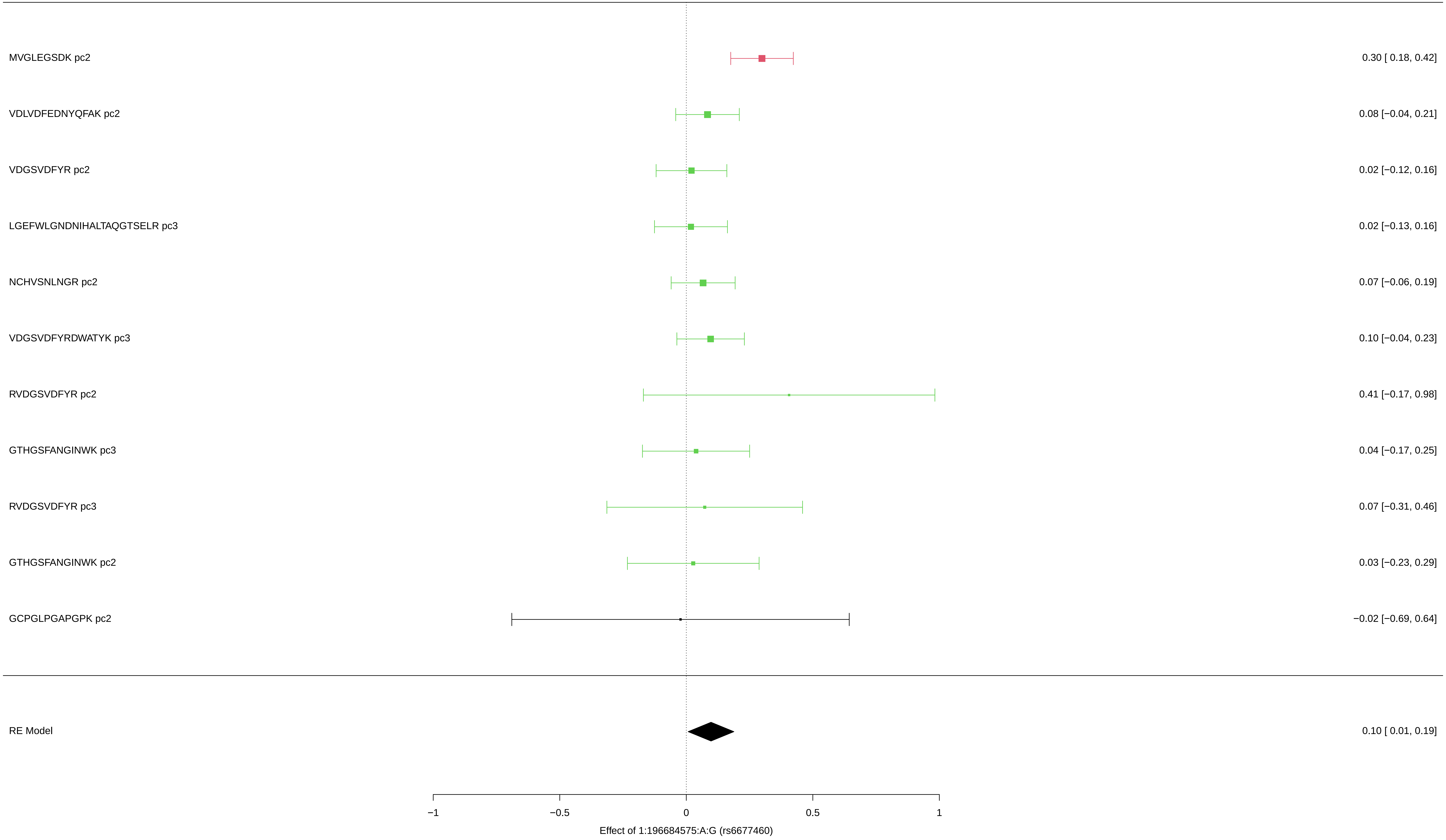

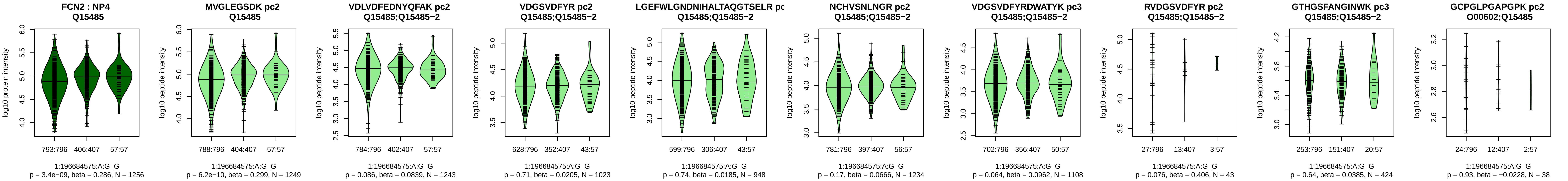

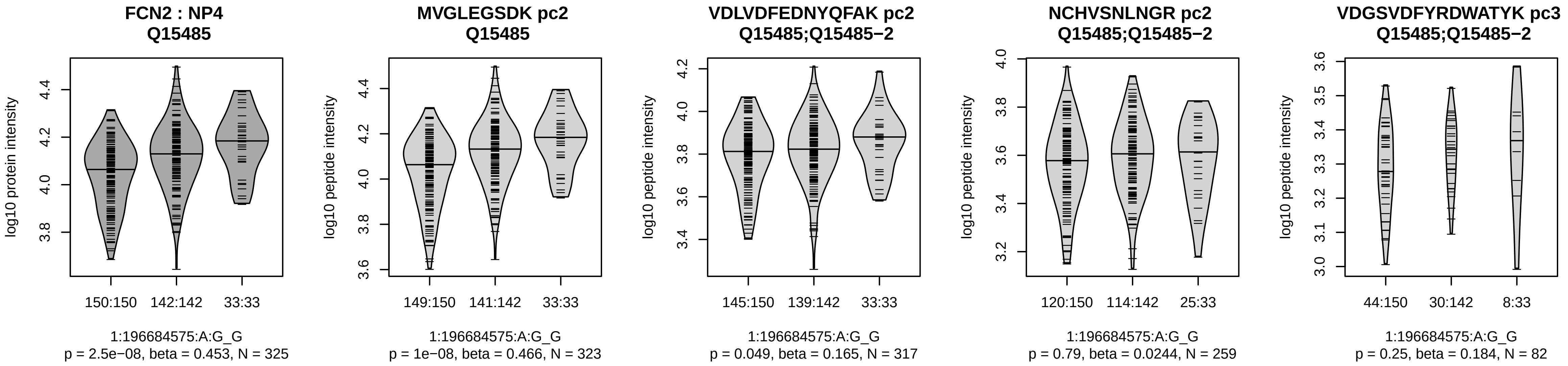

227Assay Target: FCN2
UniProt: Q15485
rsID: rs6677460
Target SNP: 1:196684575:A:G
Proxy SNP: 1:196684575:A:G
Tarkin log10(p): 8.5
Tarkin BETA: 0.286
Tarkin MSPA: 0.161
*:–:–:–:–:–:–:–
1249:1243:1023:948:1234:1108

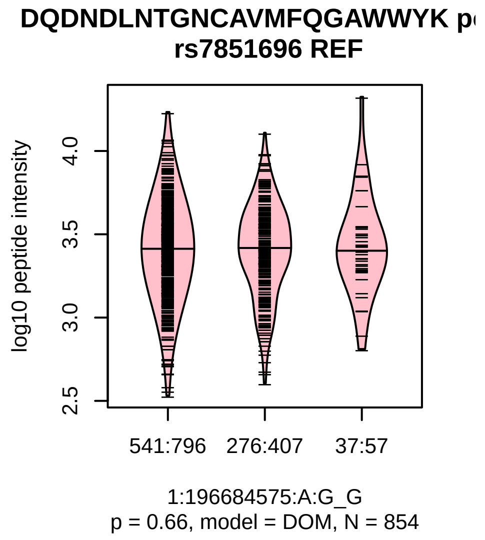

228. CYBB (P04839)
Tarkin MSPA=0.628

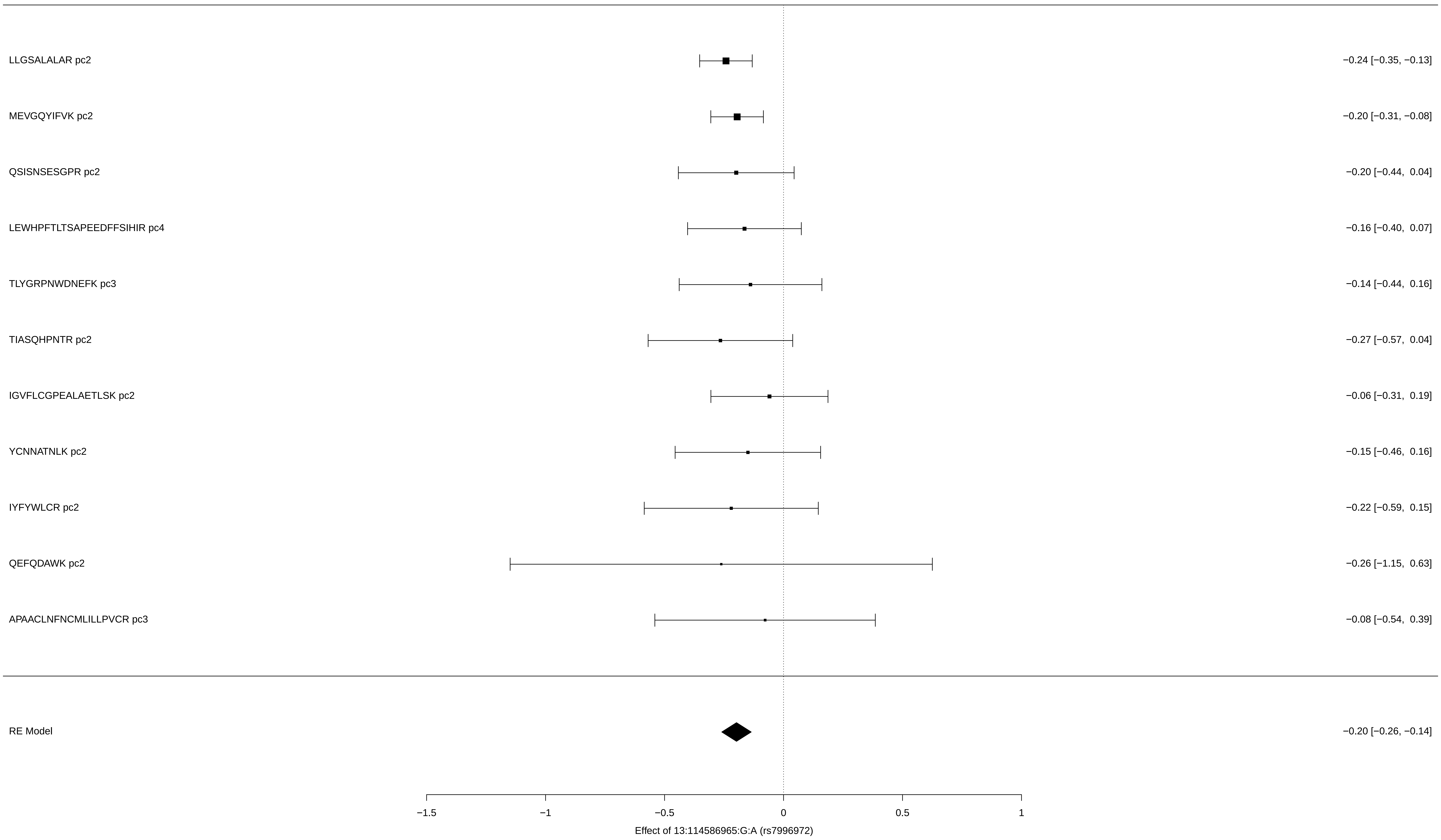

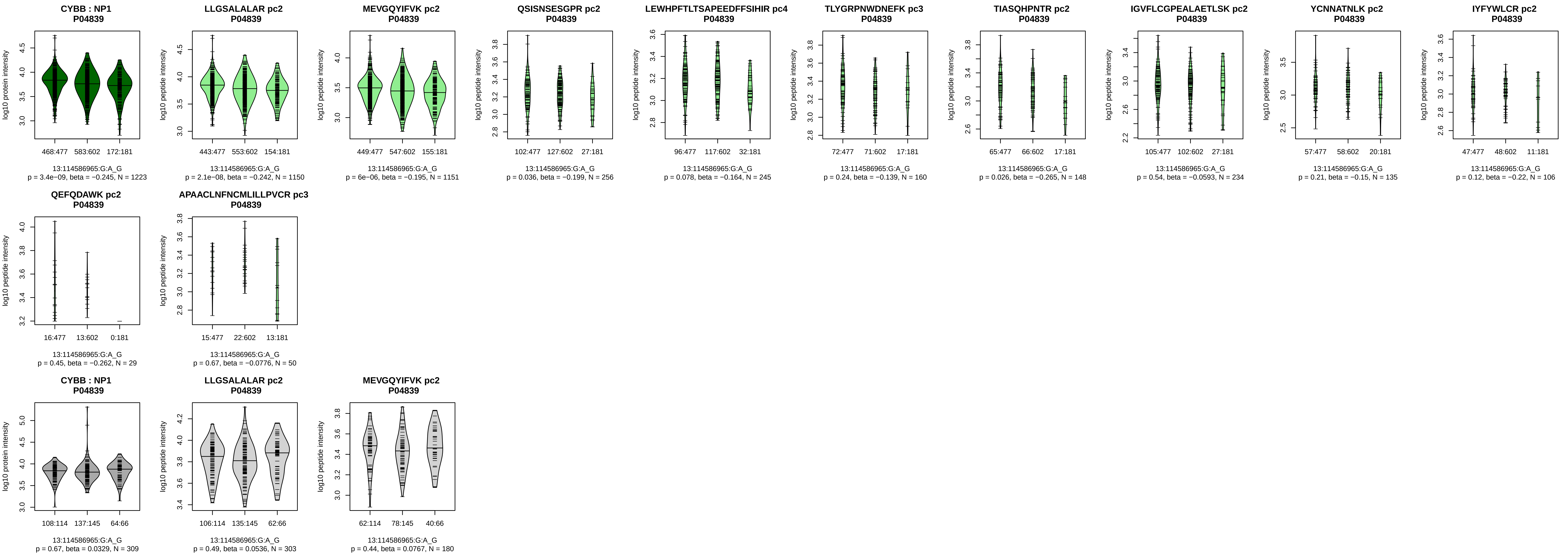

228Assay Target: CYBB
UniProt: P04839
rsID: rs7996972
Target SNP: 13:114586965:G:A
Proxy SNP: 13:114586965:G:A
Tarkin log10(p): 8.5
Tarkin BETA: −0.245
Tarkin MSPA: 0.628
::−:−:−:−:−:−:−:−:−:NA
1150:1151:256:245:160:148:234

229. SLC25A3 (Q00325)
Tarkin MSPA=0.317

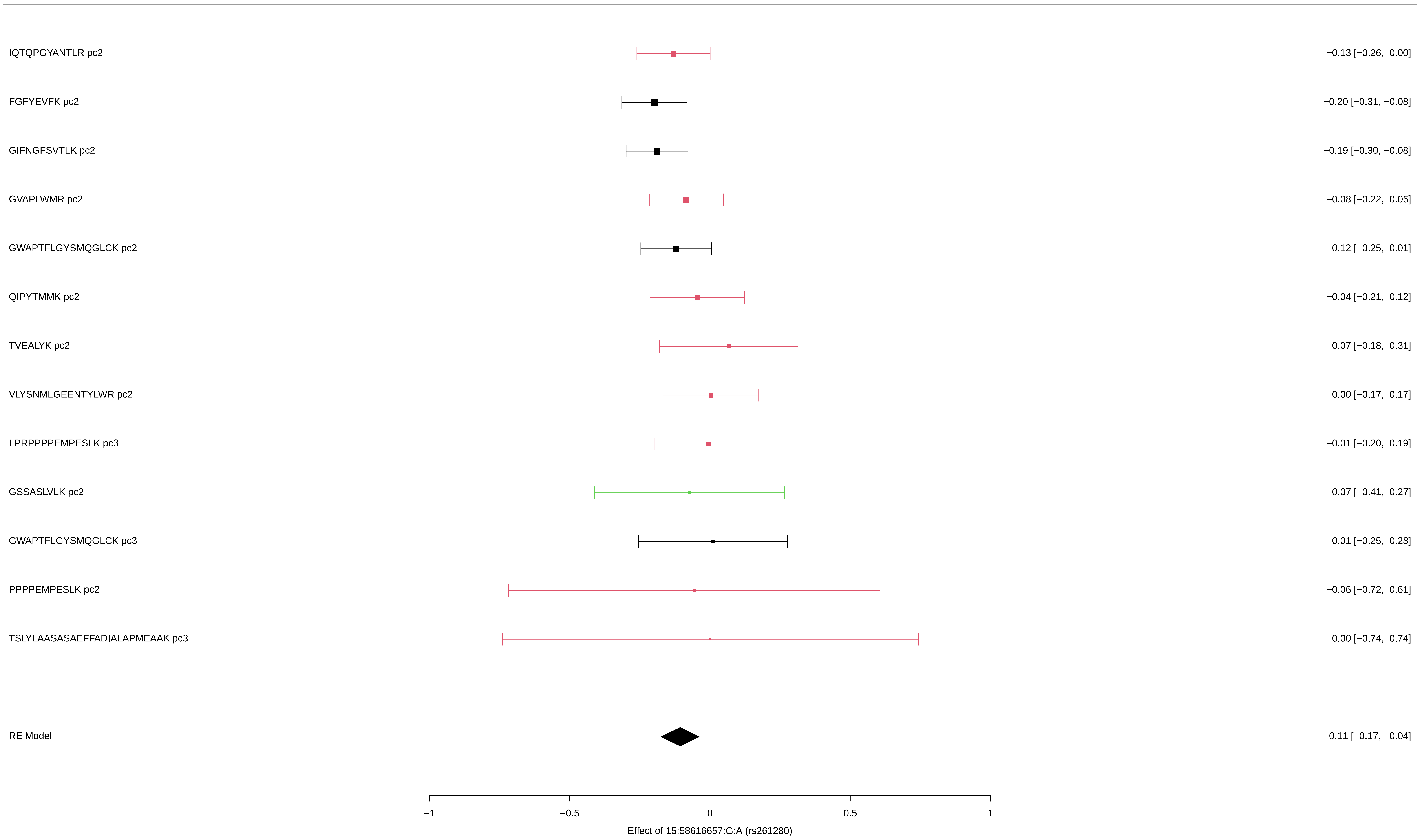

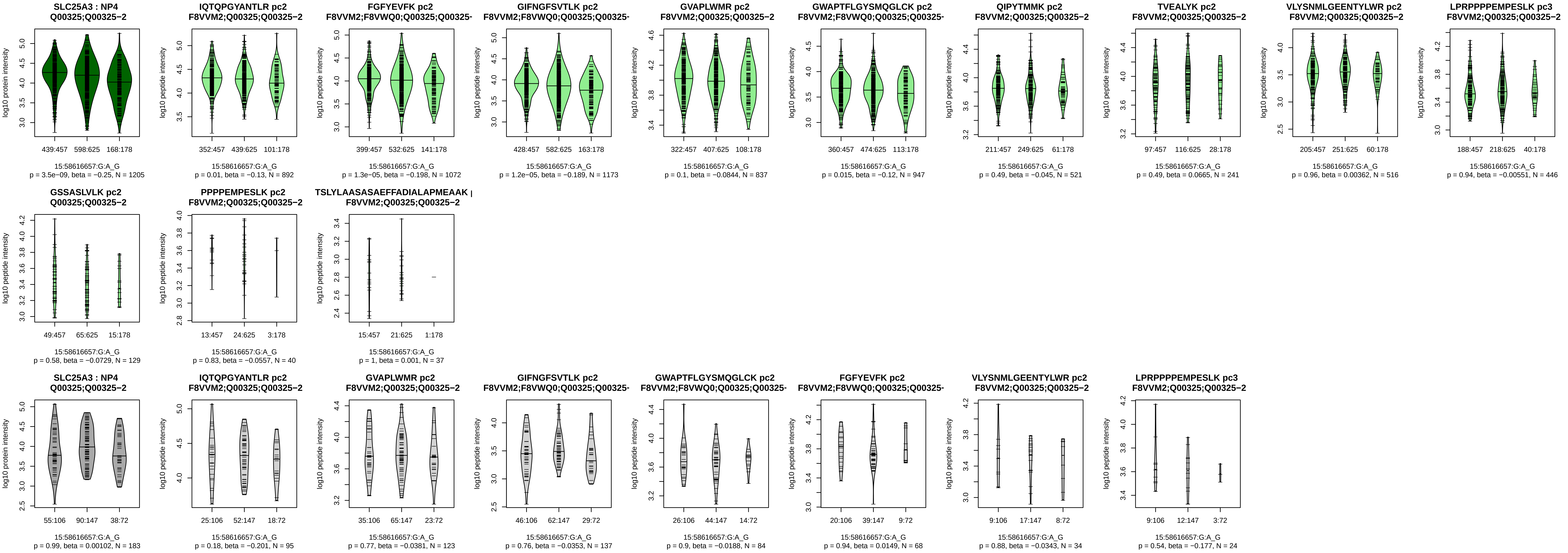

229Assay Target: SLC25A3
UniProt: Q00325
rsID: rs261280
Target SNP: 15:58616657:G:A
Proxy SNP: 15:58616657:G:A
Tarkin log10(p): 8.5
Tarkin BETA: −0.25
Tarkin MSPA: 0.317
–:*:*:–:–:–:–:–:–:–:–
892:1072:1173:837:947:521:24

230. DDX6 (P26196)
Tarkin MSPA=0.364

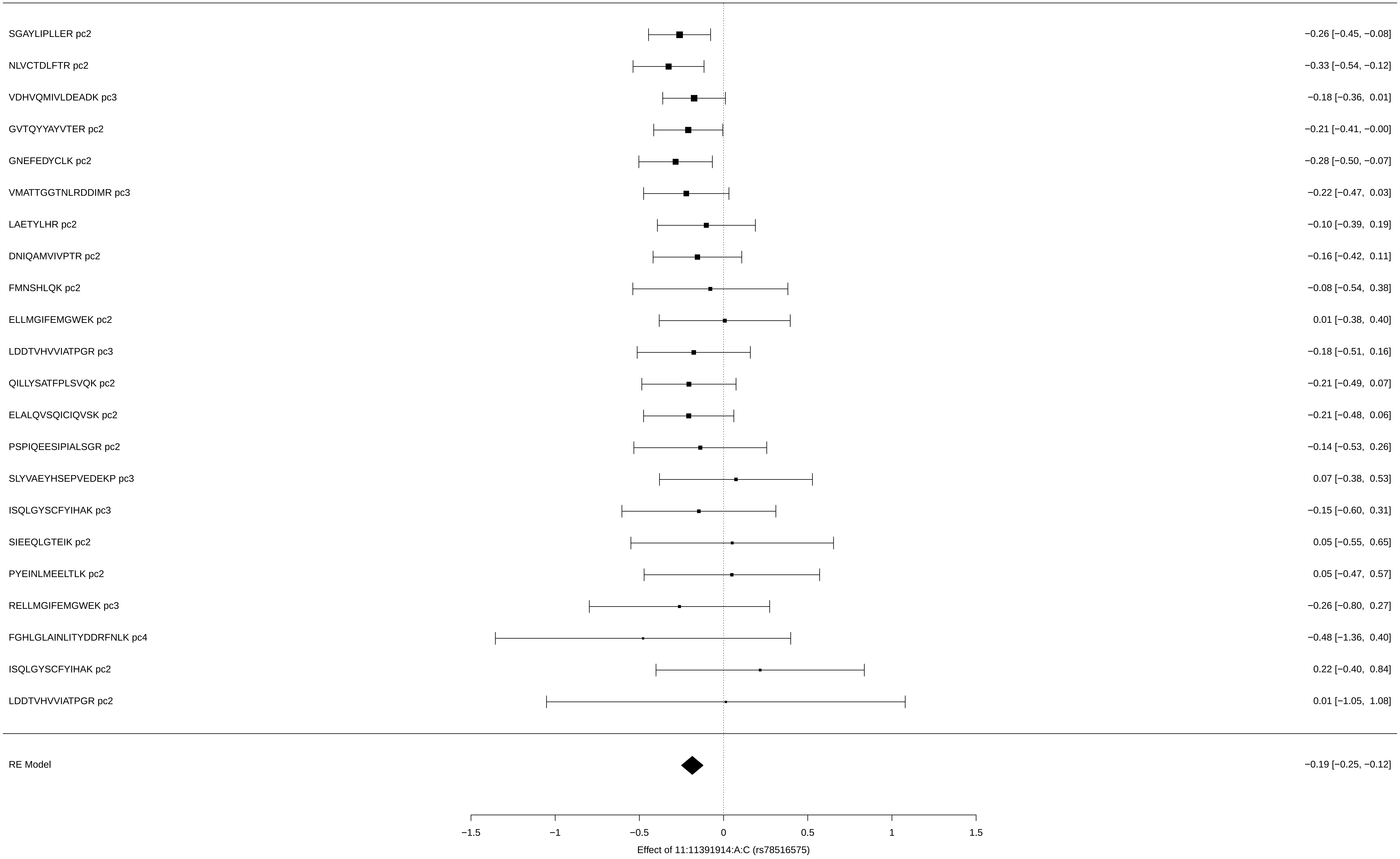

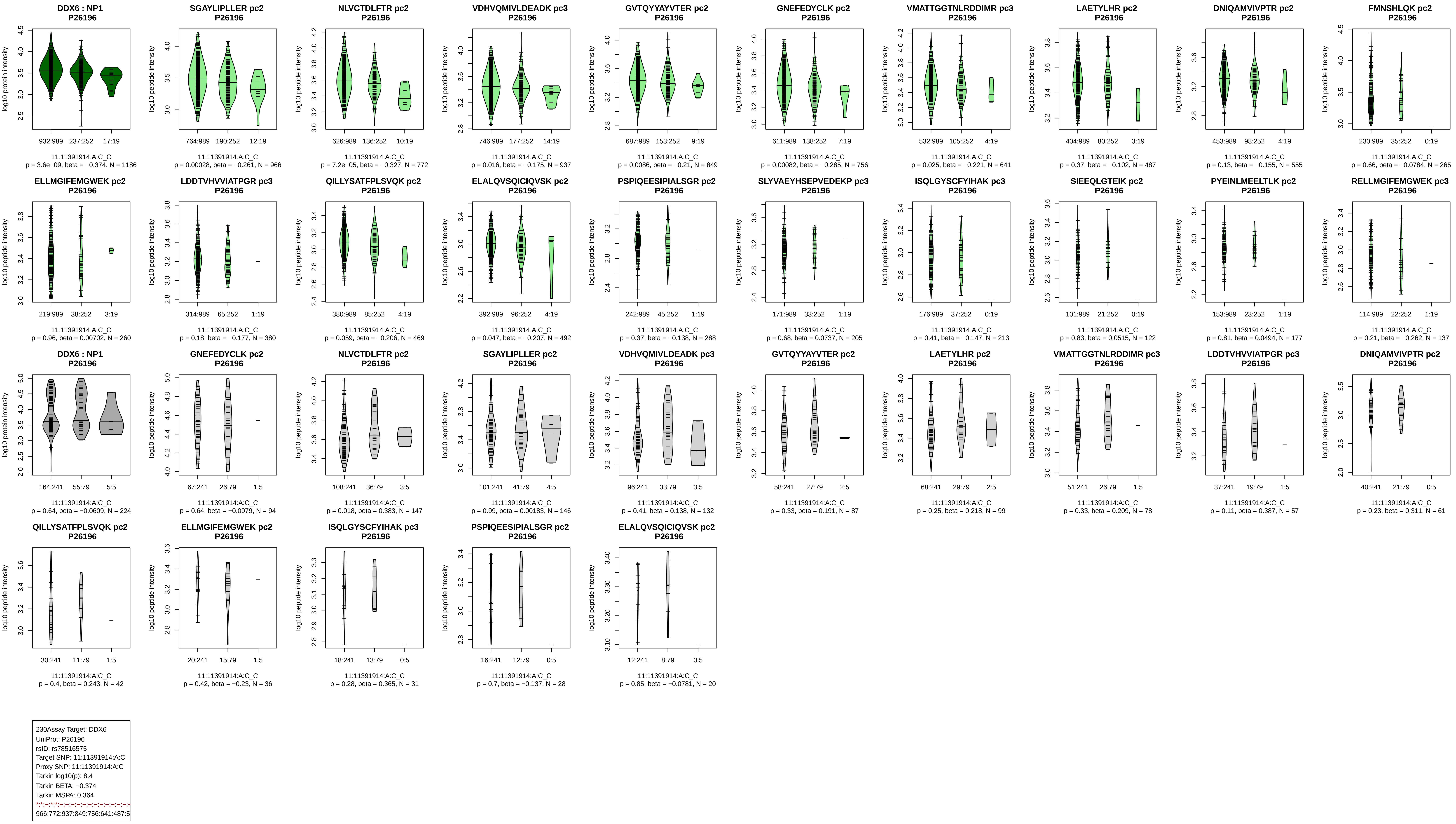
DDX6 : NP1 P26196
p = 3.6e−09, beta = −0.374, N = 1186
SGAYLIPLLER pc2 P26196
p = 0.00028, beta = −0.261, N = 966
NLVCTDLFTR pc2 P26196
p = 7.2e−05, beta = −0.327, N = 772
VDHVQMIVLDEADK pc3 P26196
p = 0.016, beta = −0.175, N = 937
GVTQYYAYVTER pc2 P26196
p = 0.0086, beta = −0.21, N = 849
GNEFEDYCLK pc2 P26196
p = 0.00082, beta = −0.285, N = 756
VMATTGGTNLRDDIMR pc3 P26196
p = 0.025, beta = −0.221, N = 641
LAETYLHR pc2 P26196
p = 0.37, beta = −0.102, N = 487
DNIQAMVIVPTR pc2 P26196
p = 0.13, beta = −0.155, N = 555
FMNSHLQK pc2 P26196
p = 0.66, beta = −0.0784, N = 265
ELLMGIFEMGWEK pc2 P26196
p = 0.96, beta = 0.00702, N = 260
LDDTVHVVIATPGR pc3 P26196
p = 0.18, beta = −0.177, N = 380
QILLYSATFPLSVQK pc2 P26196
p = 0.059, beta = −0.206, N = 469
ELALQVSQICIQVSK pc2 P26196
p = 0.047, beta = −0.207, N = 492
PSPIQEESIPIALSGR pc2 P26196
p = 0.37, beta = −0.138, N = 288
SLYVAEYHSEPVEDEKP pc3 P26196
p = 0.68, beta = 0.0737, N = 205
ISQLGYSCFYIHAK pc3 P26196
p = 0.41, beta = −0.147, N = 213
SIEEQLGTEIK pc2 P26196
p = 0.83, beta = 0.0515, N = 122
PYEINLMEELTLK pc2 P26196
p = 0.81, beta = 0.0494, N = 177
RELLMGIFEMGWEK pc3 P26196
p = 0.21, beta = −0.262, N = 137
DDX6 : NP1 P26196
p = 0.64, beta = −0.0609, N = 224
GNEFEDYCLK pc2 P26196
p = 0.64, beta = −0.0979, N = 94
NLVCTDLFTR pc2 P26196
p = 0.018, beta = 0.383, N = 147
SGAYLIPLLER pc2 P26196
p = 0.99, beta = 0.00183, N = 146
VDHVQMIVLDEADK pc3 P26196
p = 0.41, beta = 0.138, N = 132
GVTQYYAYVTER pc2 P26196
p = 0.33, beta = 0.191, N = 87
LAETYLHR pc2 P26196
p = 0.25, beta = 0.218, N = 99
VMATTGGTNLRDDIMR pc3 P26196
p = 0.33, beta = 0.209, N = 78
LDDTVHVVIATPGR pc3 P26196
p = 0.11, beta = 0.387, N = 57
DNIQAMVIVPTR pc2 P26196
p = 0.23, beta = 0.311, N = 61
QILLYSATFPLSVQK pc2 P26196
p = 0.4, beta = 0.243, N = 42
ELLMGIFEMGWEK pc2 P26196
p = 0.42, beta = −0.23, N = 36
ISQLGYSCFYIHAK pc3 P26196
p = 0.28, beta = 0.365, N = 31
PSPIQEESIPIALSGR pc2 P26196
p = 0.7, beta = −0.137, N = 28
ELALQVSQICIQVSK pc2 P26196
p = 0.85, beta = −0.0781, N = 20
11:11391914:A:C_C
230Assay Target: DDX6
UniProt: P26196
rsID: rs78516575
Target SNP: 11:11391914:A:C
Proxy SNP: 11:11391914:A:C
Tarkin log10(p): 8.4
Tarkin BETA: −0.374
Tarkin MSPA: 0.364
966:772:937:849:756:641:487:5

231. ADAMTS13 (Q76LX8)
Tarkin MSPA=0.606

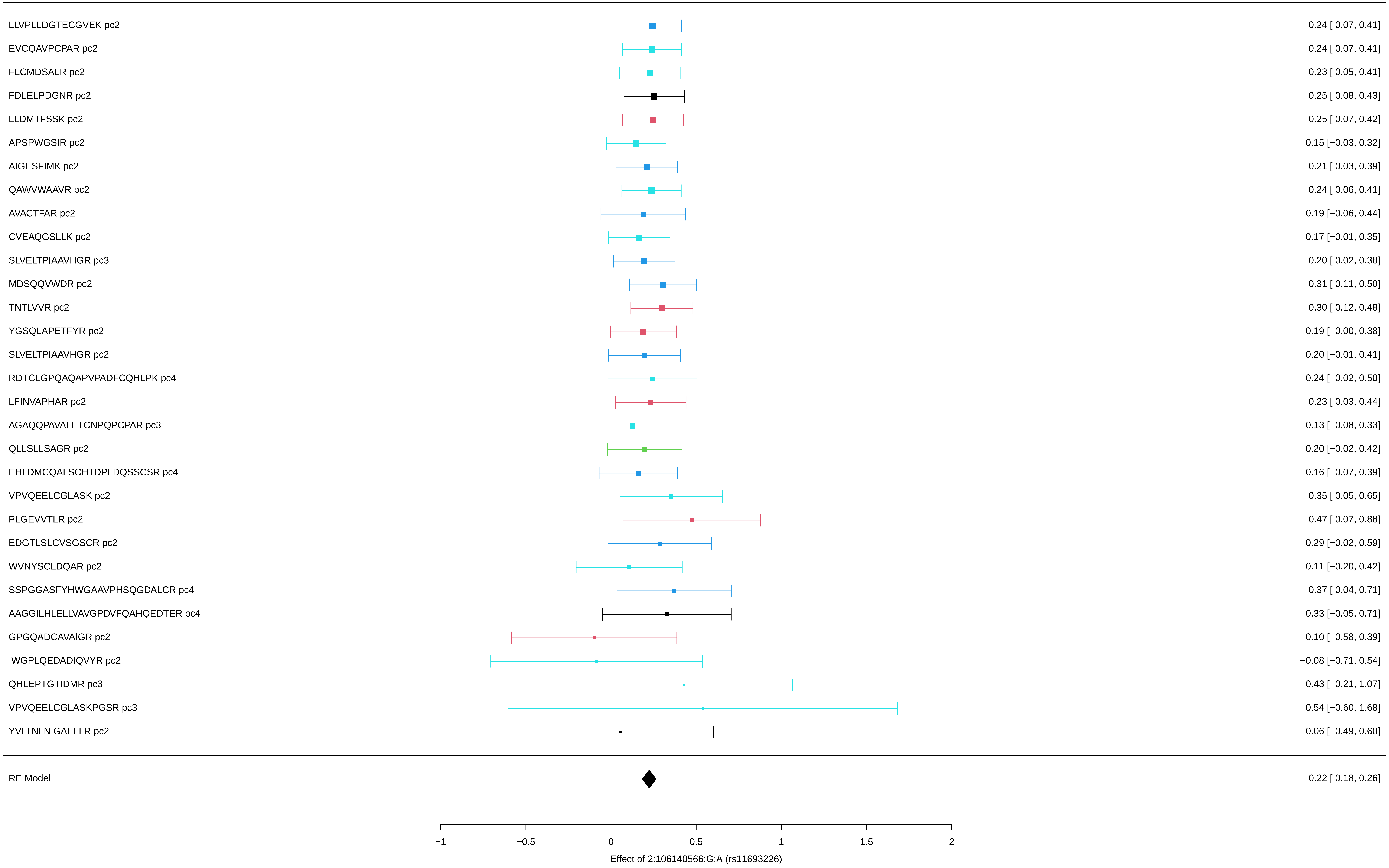

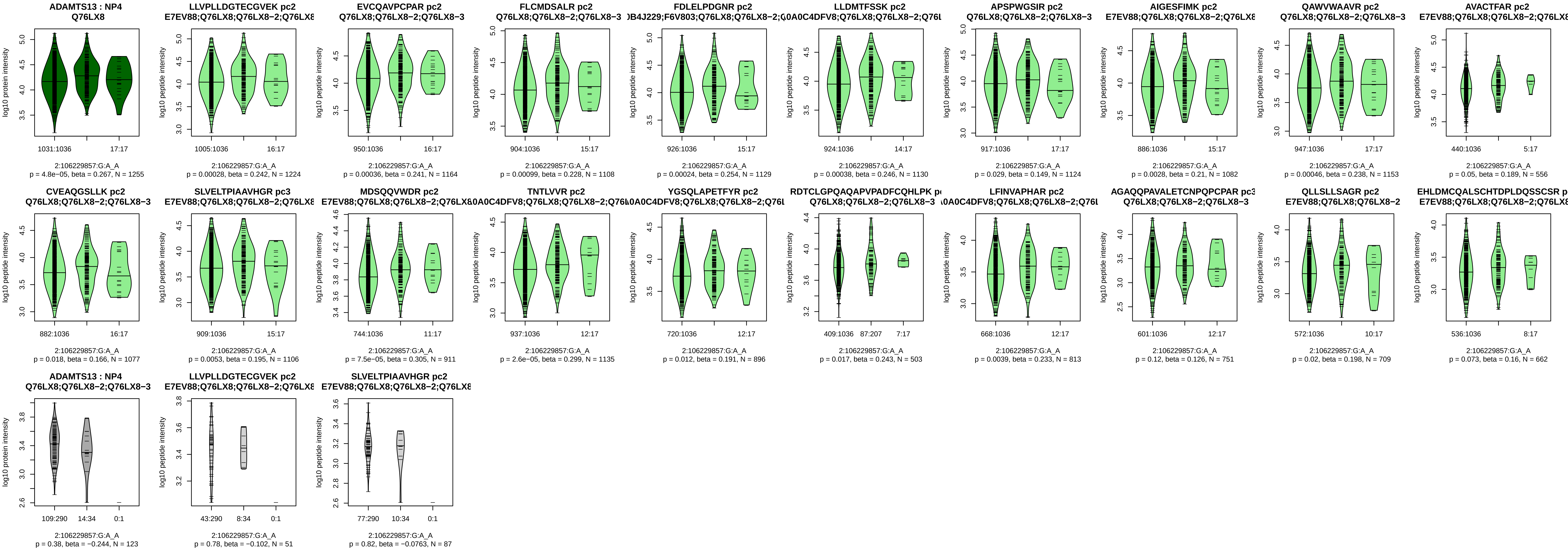
ADAMTS13 : NP4
Q76LX8
log10 protein intensity
1031:1036
17:17
2:106229857:G:A_A
p = 4.8e−05, beta = 0.267, N = 1255
LLVPLLDGTECGVEK pc2
log10 peptide intensity
1005:1036
16:17
p = 0.00028, beta = 0.242, N = 1224
EVCQAVPCPAR pc2
Q76LX8;Q76LX8−2;Q76LX8−3
950:1036
16:17
p = 0.00036, beta = 0.241, N = 1164
FLCMDSALR pc2
Q76LX8;Q76LX8−2;Q76LX8−3
904:1036
15:17
p = 0.00099, beta = 0.228, N = 1108
FDLELPDGNR pc2
926:1036
15:17
p = 0.00024, beta = 0.254, N = 1129
LLDMTFSSK pc2
924:1036
14:17
p = 0.00038, beta = 0.246, N = 1130
APSPWGSIR pc2
Q76LX8;Q76LX8−2;Q76LX8−3
917:1036
17:17
p = 0.029, beta = 0.149, N = 1124
AIGESFIMK pc2
886:1036
15:17
p = 0.0028, beta = 0.21, N = 1082
QAWVWAAVR pc2
Q76LX8;Q76LX8−2;Q76LX8−3
947:1036
17:17
p = 0.00046, beta = 0.238, N = 1153
AVACTFAR pc2
440:1036
5:17
p = 0.05, beta = 0.189, N = 556
CVEAQGSLLK pc2
Q76LX8;Q76LX8−2;Q76LX8−3
882:1036
16:17
p = 0.018, beta = 0.166, N = 1077
SLVELTPIAAVHGR pc3
909:1036
15:17
p = 0.0053, beta = 0.195, N = 1106
MDSQQVWDR pc2
744:1036
11:17
p = 7.5e−05, beta = 0.305, N = 911
TNTLVVR pc2
937:1036
12:17
p = 2.6e−05, beta = 0.299, N = 1135
YGSQLAPETFYR pc2
720:1036
12:17
p = 0.012, beta = 0.191, N = 896
Q76LX8;Q76LX8−2;Q76LX8−3
409:1036
87:207
7:17
p = 0.017, beta = 0.243, N = 503
LFINVAPHAR pc2
668:1036
12:17
p = 0.0039, beta = 0.233, N = 813
Q76LX8;Q76LX8−2;Q76LX8−3
601:1036
12:17
p = 0.12, beta = 0.126, N = 751
QLLSLLSAGR pc2
E7EV88;Q76LX8;Q76LX8−2
572:1036
10:17
p = 0.02, beta = 0.198, N = 709
536:1036
8:17
p = 0.073, beta = 0.16, N = 662
ADAMTS13 : NP4
Q76LX8;Q76LX8−2;Q76LX8−3
109:290
14:34
0:1
p = 0.38, beta = −0.244, N = 123
LLVPLLDGTECGVEK pc2
43:290
8:34
0:1
p = 0.78, beta = −0.102, N = 51
SLVELTPIAAVHGR pc2
77:290
10:34
0:1
p = 0.82, beta = −0.0763, N = 87

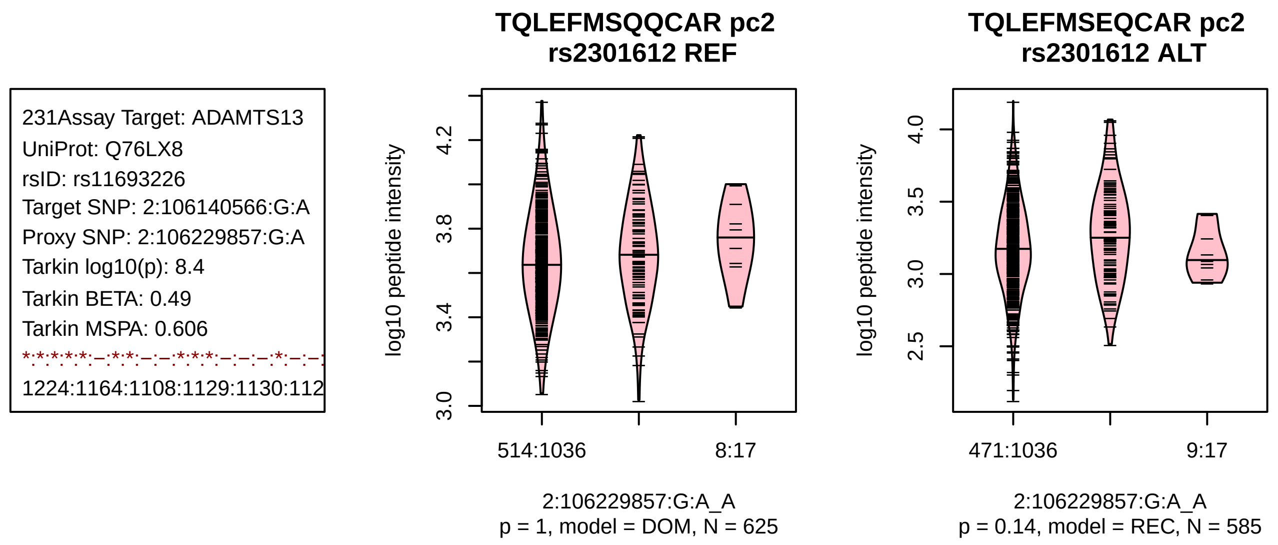
231Assay Target: ADAMTS13
UniProt: Q76LX8
rsID: rs11693226
Target SNP: 2:106140566:G:A
Proxy SNP: 2:106229857:G:A
Tarkin log10(p): 8.4
Tarkin BETA: 0.49
Tarkin MSPA: 0.606
TQLEFMSQQCAR pc2
rs2301612 REF
log10 peptide intensity
514:1036
8:17
2:106229857:G:A_A
p = 1, model = DOM, N = 625
TQLEFMSEQCAR pc2
rs2301612 ALT
471:1036
9:17
p = 0.14, model = REC, N = 585

232. ENDOV (Q8N8Q3)
Tarkin MSPA=1

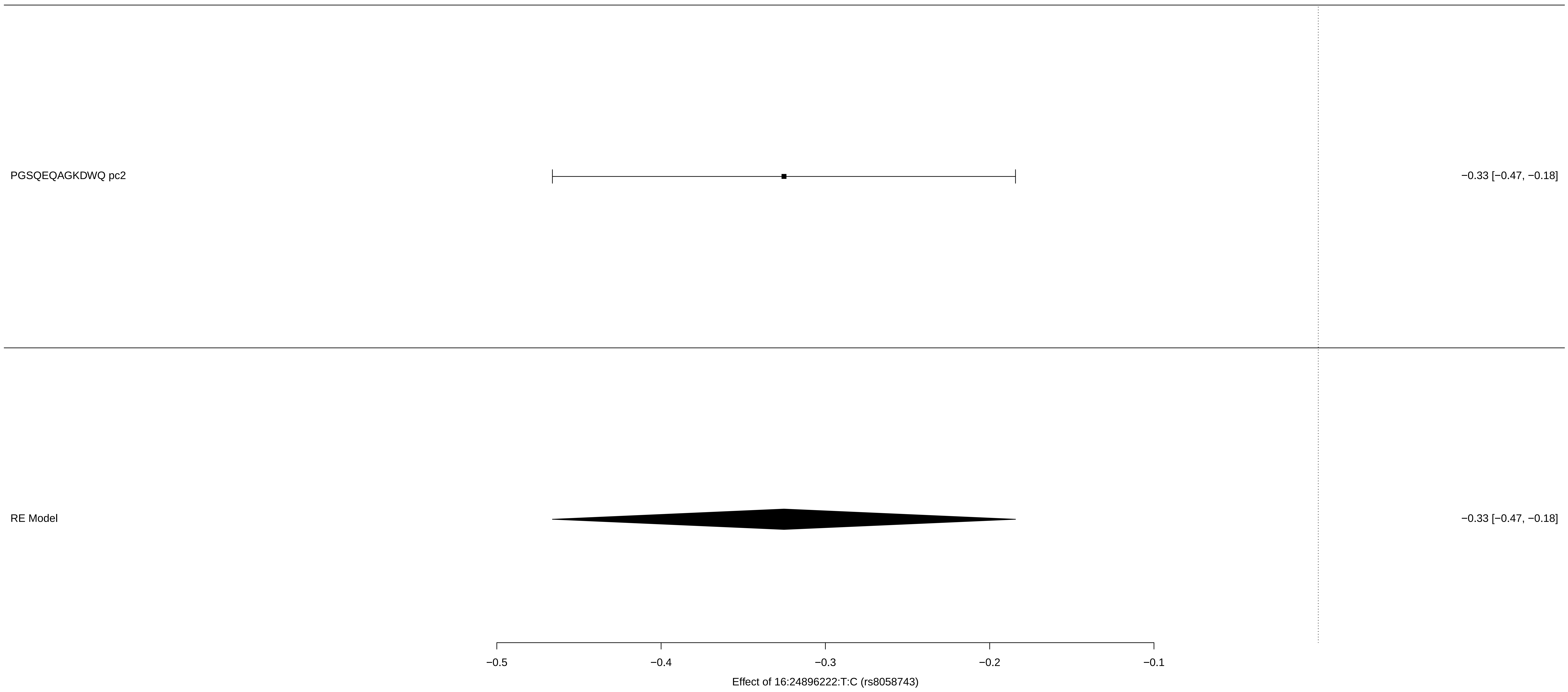

ENDOV : NP2
Q8N8Q3−3

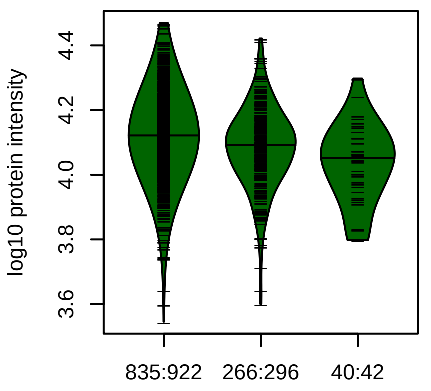

16:24896222:T:C_T
p = 3.7e−09, beta = −0.325, N = 1141

PGSQEQAGKDWQ pc2
I3L0L4;Q8N8Q3−3

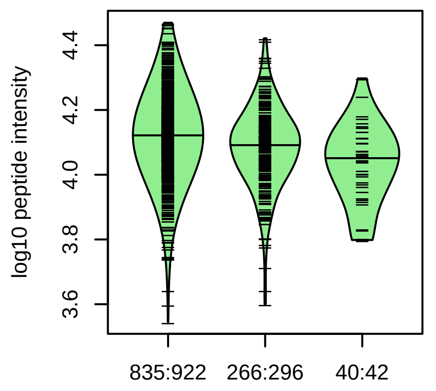

16:24896222:T:C_T
p = 3.7e−09, beta = −0.325, N = 1141

ENDOV : NP2
Q8N8Q3−3

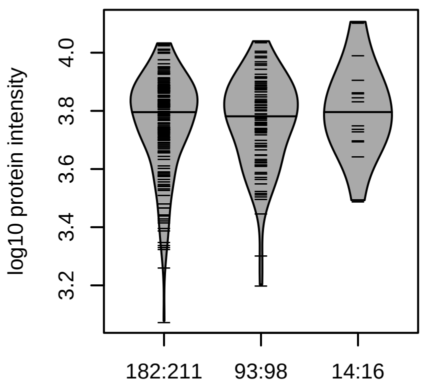

16:24896222:T:C_T
p = 0.84, beta = 0.0205, N = 289

PGSQEQAGKDWQ pc2
I3L0L4;Q8N8Q3−3

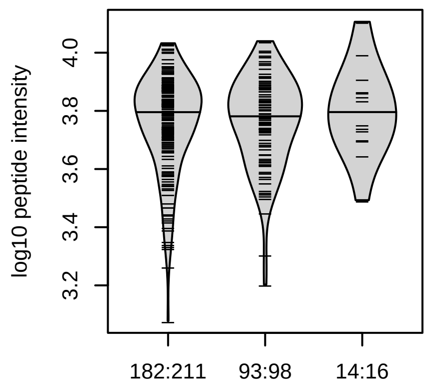

16:24896222:T:C_T
p = 0.84, beta = 0.0205, N = 289

232Assay Target: ENDOV
UniProt: Q8N8Q3
rsID: rs8058743
Target SNP: 16:24896222:T:C
Proxy SNP: 16:24896222:T:C
Tarkin log10(p): 8.4
Tarkin BETA: −0.325
Tarkin MSPA: 1
*
1141

233. GPC4 (O75487)
Tarkin MSPA=0.475

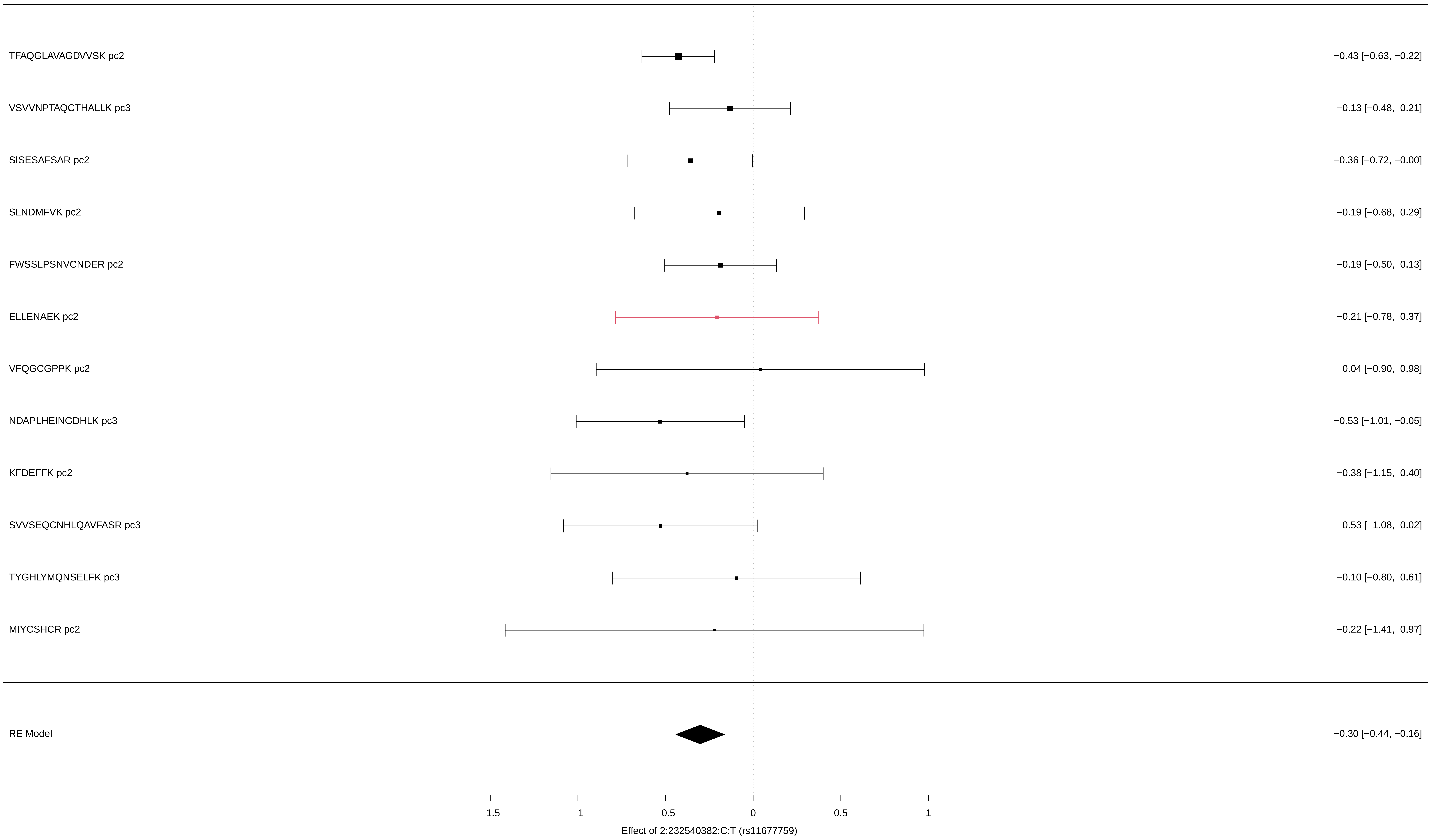

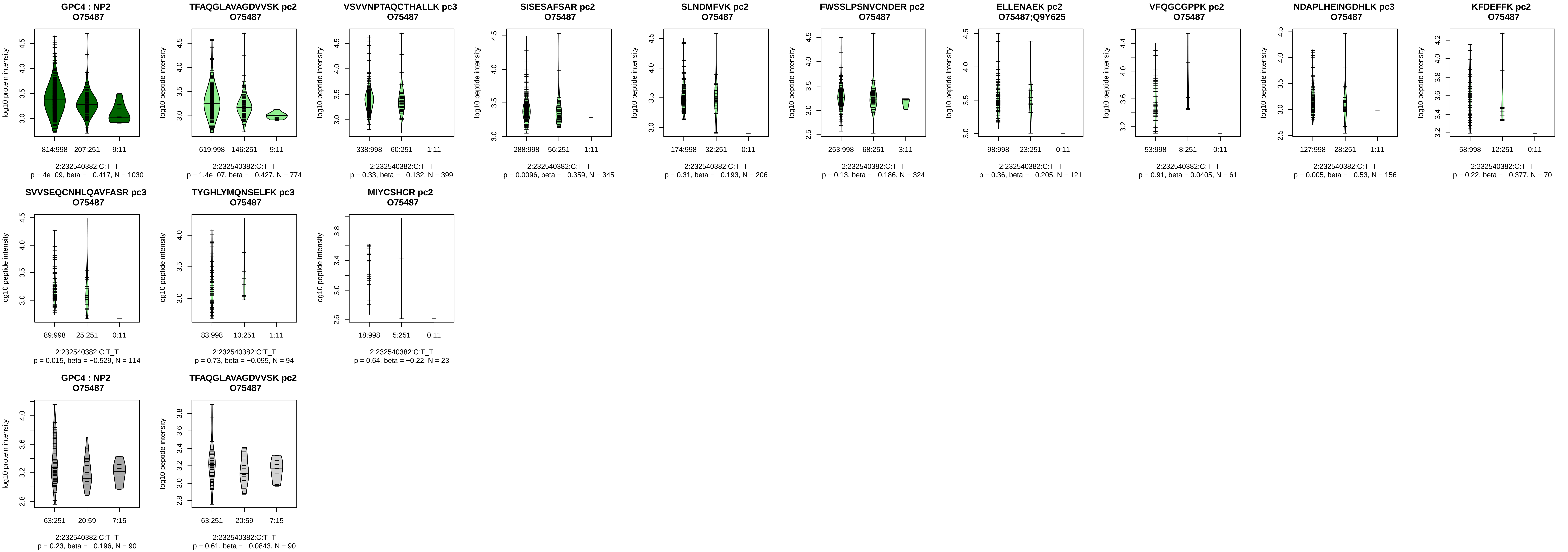

233Assay Target: GPC4
UniProt: O75487
rsID: rs11677759
Target SNP: 2:232540382:C:T
Proxy SNP: 2:232540382:C:T
Tarkin log10(p): 8.4
Tarkin BETA: −0.417
Tarkin MSPA: 0.475
:−::−:−:−:−:*:−:−:−:−:NA
774:399:345:206:324:121:61:156:70:114:94:23:90:90:1030

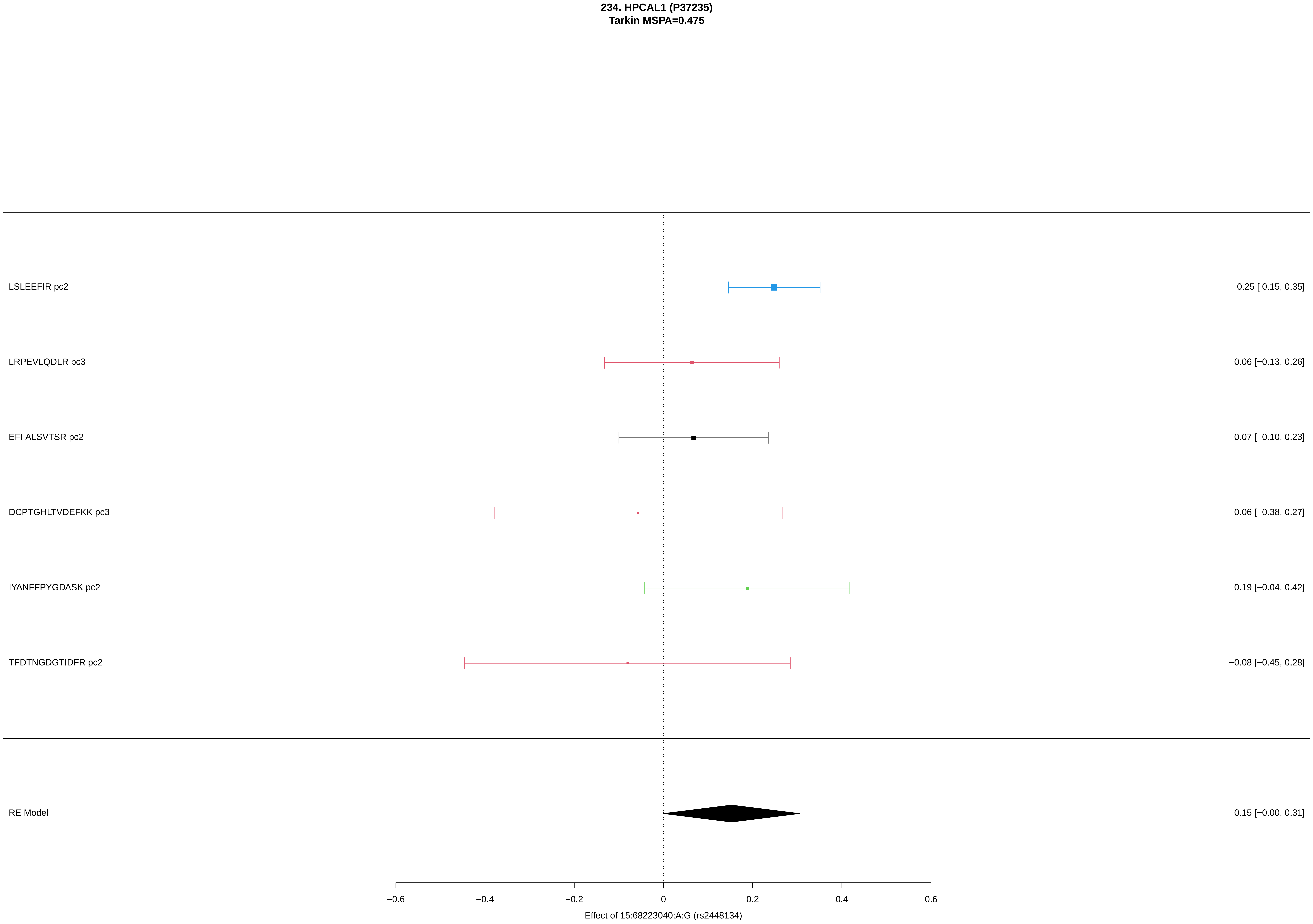
234. HPCAL1 (P37235)
Tarkin MSPA=0.475
LSLEEFIR pc2
0.25 [0.15, 0.35]
LRPEVLQDLR pc3
0.06 [−0.13, 0.26]
EFIIALSVTSR pc2
0.07 [−0.10, 0.23]
DCPTGHLTVDEFKK pc3
−0.06 [−0.38, 0.27]
IYANFFPYGDASK pc2
0.19 [−0.04, 0.42]
TFDTNGDGTIDFR pc2
−0.08 [−0.45, 0.28]
RE Model
0.15 [−0.00, 0.31]
−0.6
−0.4
−0.2
0
0.2
0.4
0.6
Effect of 15:68223040:A:G (rs2448134)

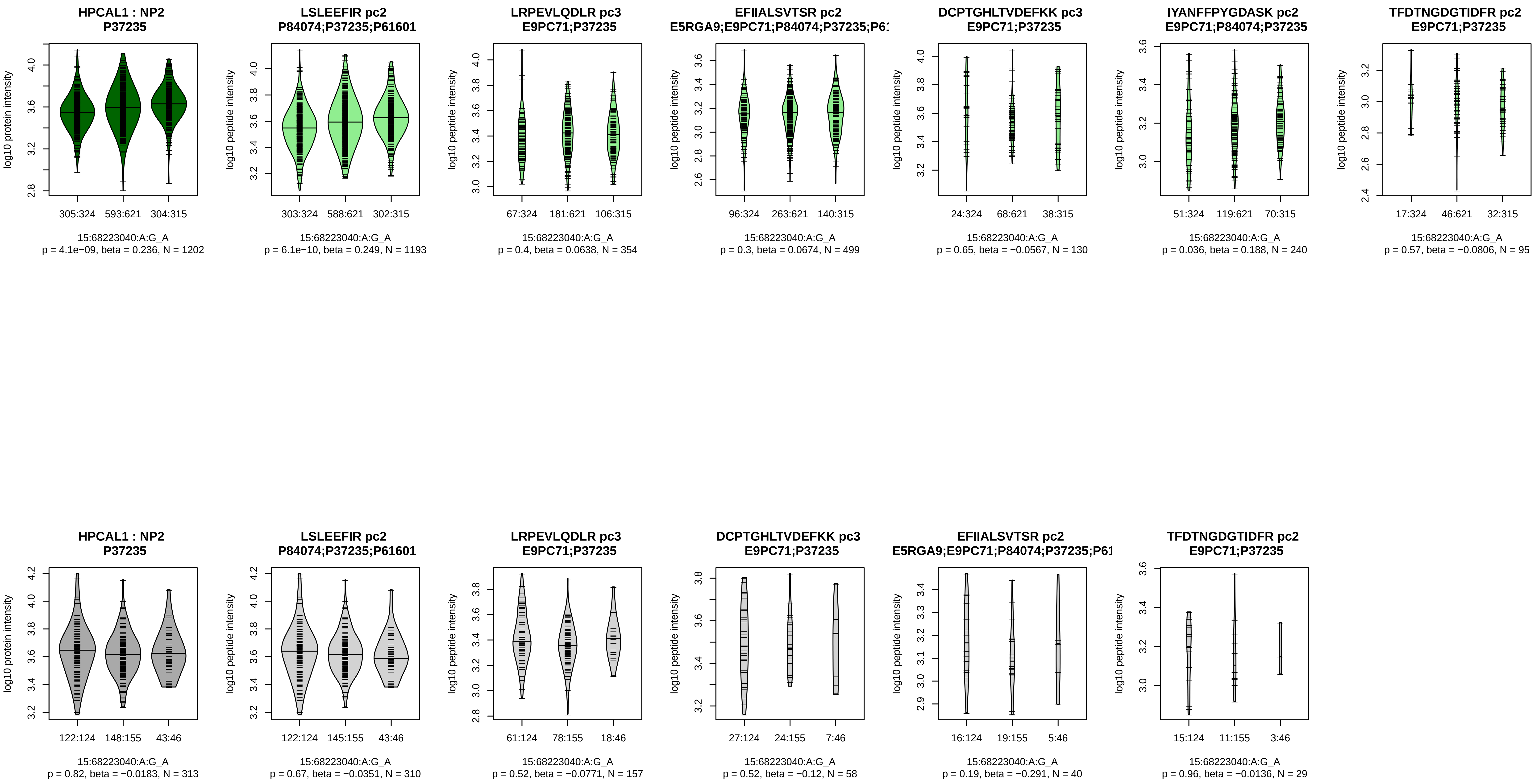

234Assay Target: HPCAL1
UniProt: P37235
rsID: rs2448134
Target SNP: 15:68223040:A:G
Proxy SNP: 15:68223040:A:G
Tarkin log10(p): 8.4
Tarkin BETA: 0.236
Tarkin MSPA: 0.475
*:−:−:−:−:−:NA
1193:354:499:130:240:95:2

235. KIF2A (O00139)
Tarkin MSPA=0.126

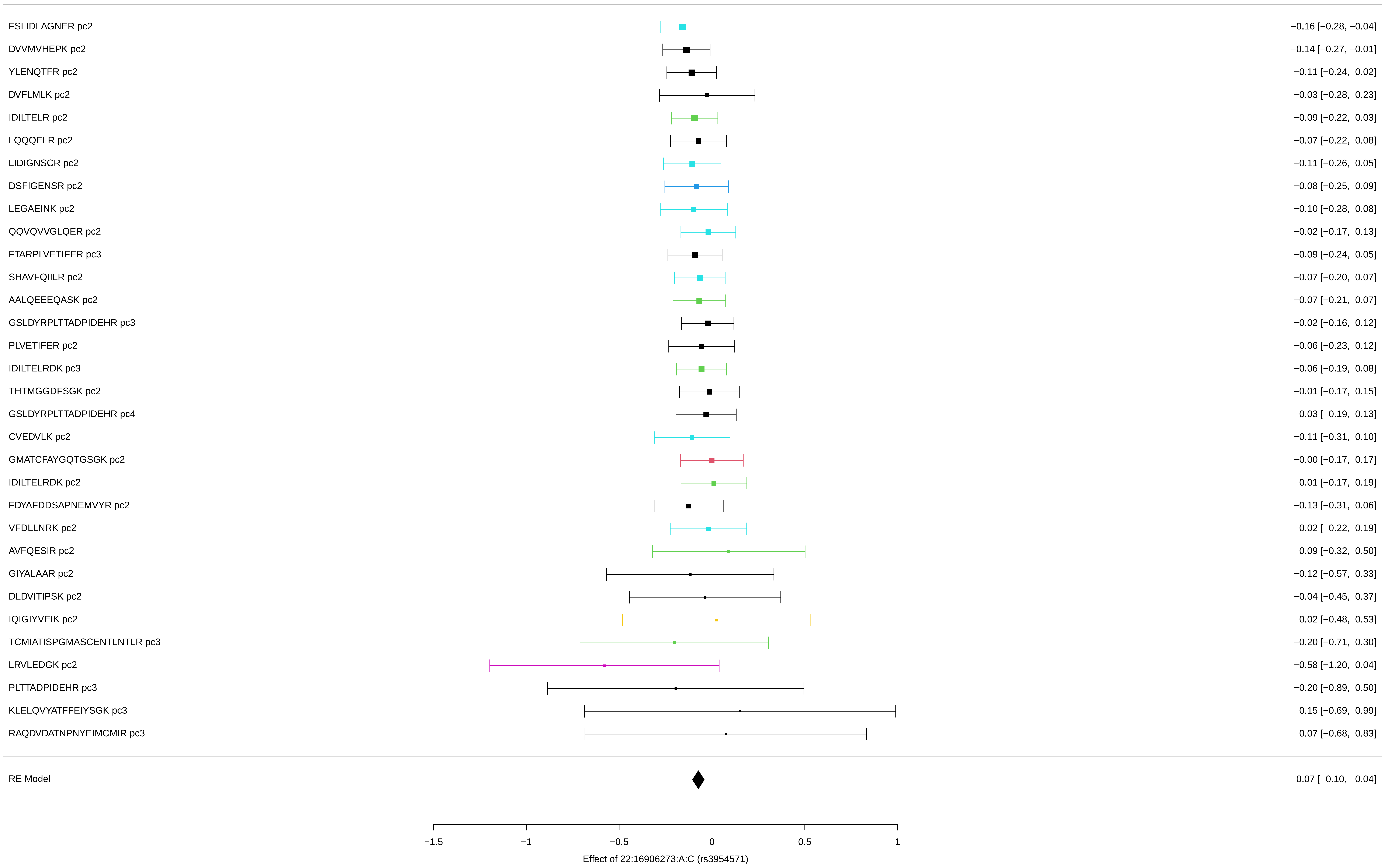

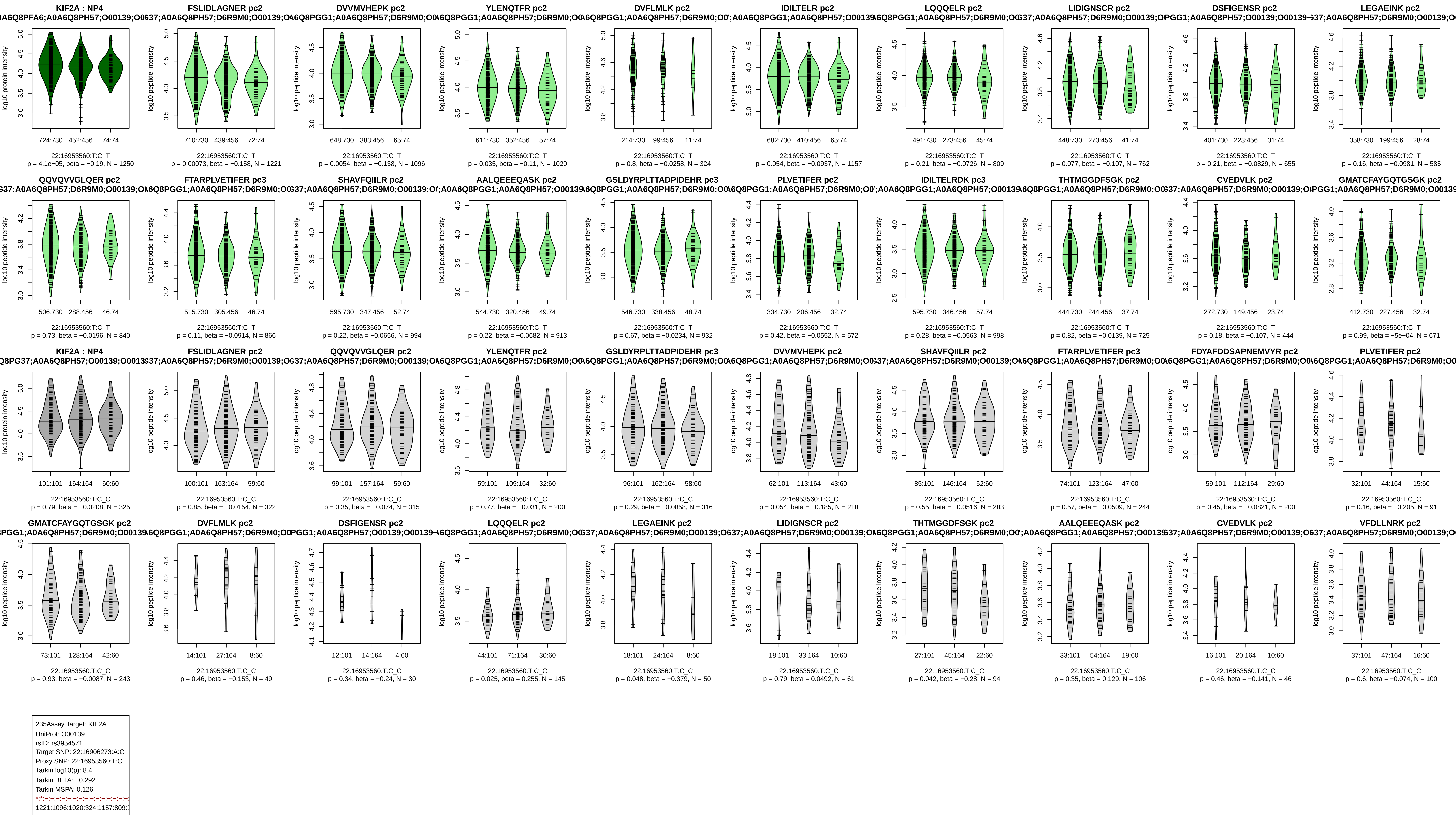

KIF2A : NP4
724:730 452:456 74:74
22:16953560:T:C_T
p = 4.1e−05, beta = −0.19, N = 1250
FSLIDLAGNER pc2
710:730 439:456 72:74
22:16953560:T:C_T
p = 0.00073, beta = −0.158, N = 1221
DVVMVHEPK pc2
648:730 383:456 65:74
22:16953560:T:C_T
p = 0.0054, beta = −0.138, N = 1096
YLENQTFR pc2
611:730 352:456 57:74
22:16953560:T:C_T
p = 0.035, beta = −0.11, N = 1020
DVFLMLK pc2
214:730 99:456 11:74
22:16953560:T:C_T
p = 0.8, beta = −0.0258, N = 324
IDILTELR pc2
682:730 410:456 65:74
22:16953560:T:C_T
p = 0.054, beta = −0.0937, N = 1157
LQQQELR pc2
491:730 273:456 45:74
22:16953560:T:C_T
p = 0.21, beta = −0.0726, N = 809
LIDIGNSCR pc2
448:730 273:456 41:74
22:16953560:T:C_T
p = 0.077, beta = −0.107, N = 762
DSFIGENSR pc2
401:730 223:456 31:74
22:16953560:T:C_T
p = 0.21, beta = −0.0829, N = 655
LEGAEINK pc2
358:730 199:456 28:74
22:16953560:T:C_T
p = 0.16, beta = −0.0981, N = 585
QQVQVVGLQER pc2
506:730 288:456 46:74
22:16953560:T:C_T
p = 0.73, beta = −0.0196, N = 840
FTARPLVETIFER pc3
515:730 305:456 46:74
22:16953560:T:C_T
p = 0.11, beta = −0.0914, N = 866
SHAVFQIILR pc2
595:730 347:456 52:74
22:16953560:T:C_T
p = 0.22, beta = −0.0656, N = 994
AALQEEEQASK pc2
544:730 320:456 49:74
22:16953560:T:C_T
p = 0.22, beta = −0.0682, N = 913
GSLDYRPLTTADPIDEHR pc3
546:730 338:456 48:74
22:16953560:T:C_T
p = 0.67, beta = −0.0234, N = 932
PLVETIFER pc2
334:730 206:456 32:74
22:16953560:T:C_T
p = 0.42, beta = −0.0552, N = 572
IDILTELRDK pc2
595:730 346:456 57:74
22:16953560:T:C_T
p = 0.28, beta = −0.0563, N = 998
THTMGGDFSGK pc2
444:730 244:456 37:74
22:16953560:T:C_T
p = 0.82, beta = −0.0139, N = 725
CVEDVLK pc2
272:730 149:456 23:74
22:16953560:T:C_T
p = 0.18, beta = −0.107, N = 444
GMATCFAYGQTGSGK pc2
412:730 227:456 32:74
22:16953560:T:C_T
p = 0.99, beta = −5e−04, N = 671
KIF2A : NP4
101:101 164:164 60:60
22:16953560:T:C_C
p = 0.79, beta = −0.0208, N = 325
FSLIDLAGNER pc2
100:101 163:164 59:60
22:16953560:T:C_C
p = 0.85, beta = −0.0154, N = 322
QQVQVVGLQER pc2
99:101 157:164 59:60
22:16953560:T:C_C
p = 0.35, beta = −0.074, N = 315
YLENQTFR pc2
59:101 109:164 32:60
22:16953560:T:C_C
p = 0.77, beta = −0.031, N = 200
GSLDYRPLTTADPIDEHR pc3
96:101 162:164 58:60
22:16953560:T:C_C
p = 0.29, beta = −0.0858, N = 316
DVVMVHEPK pc2
62:101 113:164 43:60
22:16953560:T:C_C
p = 0.054, beta = −0.185, N = 218
SHAVFQIILR pc2
85:101 146:164 52:60
22:16953560:T:C_C
p = 0.55, beta = −0.0516, N = 283
FTARPLVETIFER pc3
74:101 123:164 47:60
22:16953560:T:C_C
p = 0.57, beta = −0.0509, N = 244
FDYAFDDSAPNEMVYR pc2
59:101 112:164 29:60
22:16953560:T:C_C
p = 0.45, beta = −0.0821, N = 200
PLVETIFER pc2
32:101 44:164 15:60
22:16953560:T:C_C
p = 0.16, beta = −0.205, N = 91
GMATCFAYGQTGSGK pc2
73:101 128:164 42:60
22:16953560:T:C_C
p = 0.93, beta = −0.0087, N = 243
DVFLMLK pc2
14:101 27:164 8:60
22:16953560:T:C_C
p = 0.46, beta = −0.153, N = 49
DSFIGENSR pc2
12:101 14:164 4:60
22:16953560:T:C_C
p = 0.34, beta = −0.24, N = 30
LQQQELR pc2
44:101 71:164 30:60
22:16953560:T:C_C
p = 0.025, beta = 0.255, N = 145
LEGAEINK pc2
18:101 24:164 8:60
22:16953560:T:C_C
p = 0.048, beta = −0.379, N = 50
LIDIGNSCR pc2
18:101 33:164 10:60
22:16953560:T:C_C
p = 0.79, beta = 0.0492, N = 61
THTMGGDFSGK pc2
27:101 45:164 22:60
22:16953560:T:C_C
p = 0.042, beta = −0.28, N = 94
AALQEEEQASK pc2
33:101 54:164 19:60
22:16953560:T:C_C
p = 0.35, beta = 0.129, N = 106
CVEDVLK pc2
16:101 20:164 10:60
22:16953560:T:C_C
p = 0.46, beta = −0.141, N = 46
VFDLLNRK pc2
37:101 47:164 16:60
22:16953560:T:C_C
p = 0.6, beta = −0.074, N = 100
235Assay Target: KIF2A
UniProt: O00139
rsID: rs3954571
Target SNP: 22:16906273:A:C
Proxy SNP: 22:16953560:T:C
Tarkin log10(p): 8.4
Tarkin BETA: −0.292
Tarkin MSPA: 0.126
1221:1096:1020:324:1157:809:

236. FUCA1 (P04066)
Tarkin MSPA=0.457

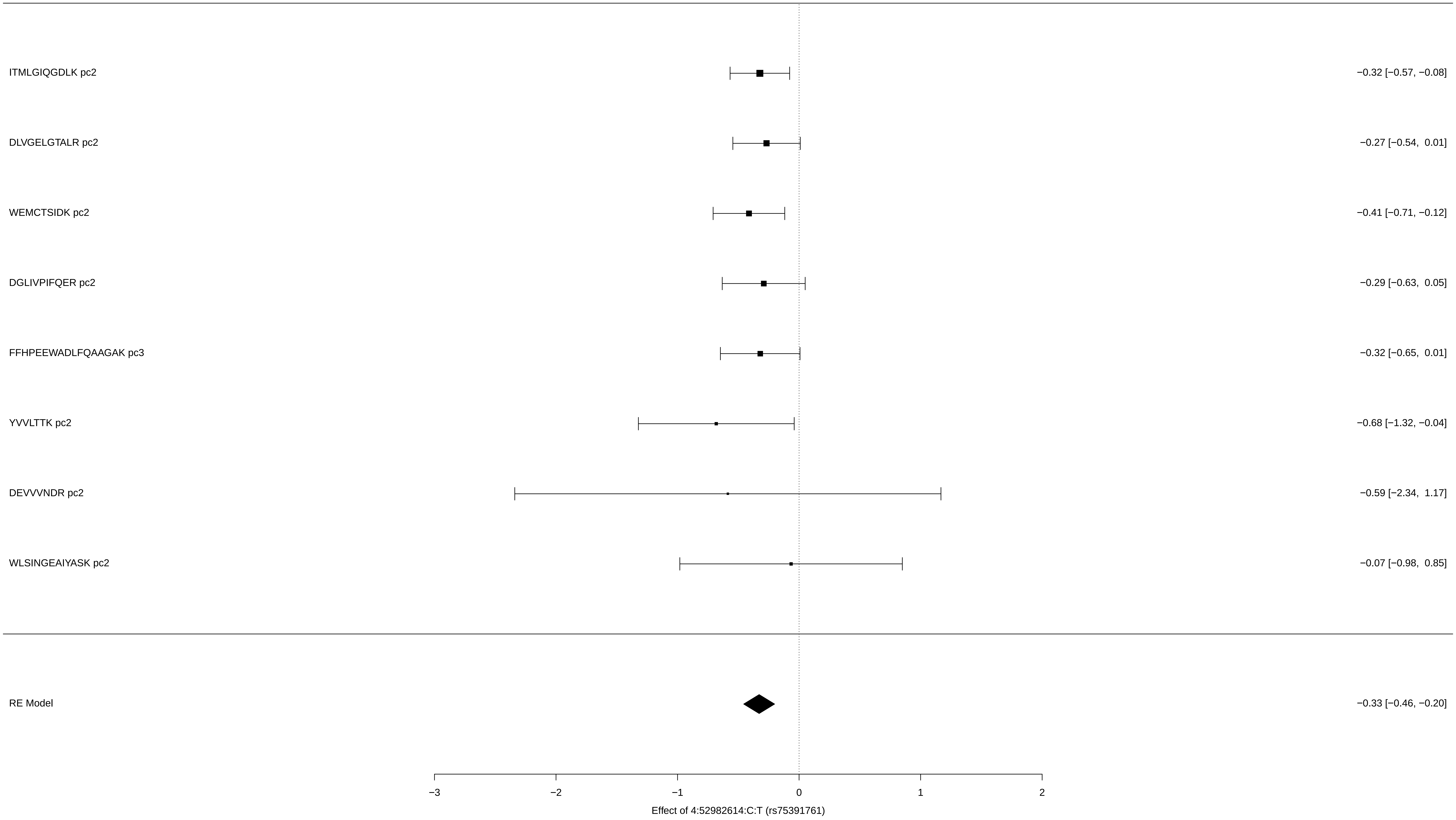

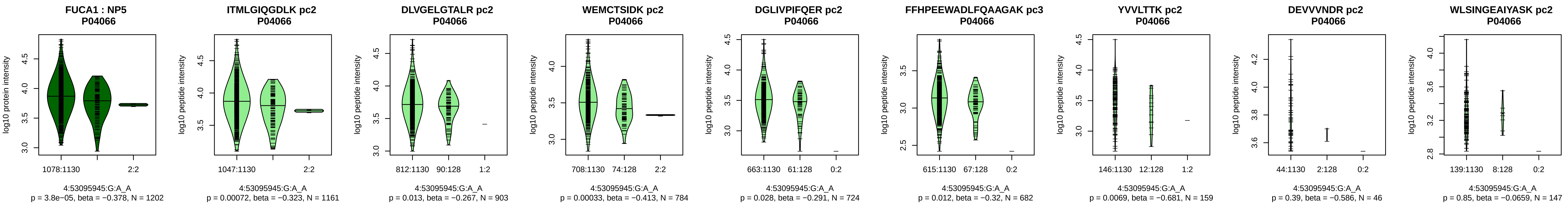

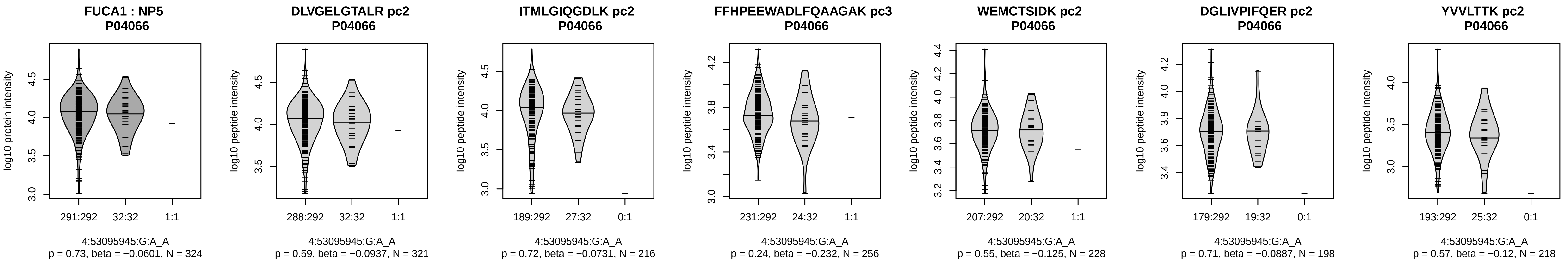

236Assay Target: FUCA1
UniProt: P04066
rsID: rs75391761
Target SNP: 4:52982614:C:T
Proxy SNP: 4:53095945:G:A
Tarkin log10(p): 8.3
Tarkin BETA: −0.475
Tarkin MSPA: 0.457
:–::–:–:*:–:–
1161:903:784:724:682:159:46:1

237. CDH6 (P55285)
Tarkin MSPA=0.85

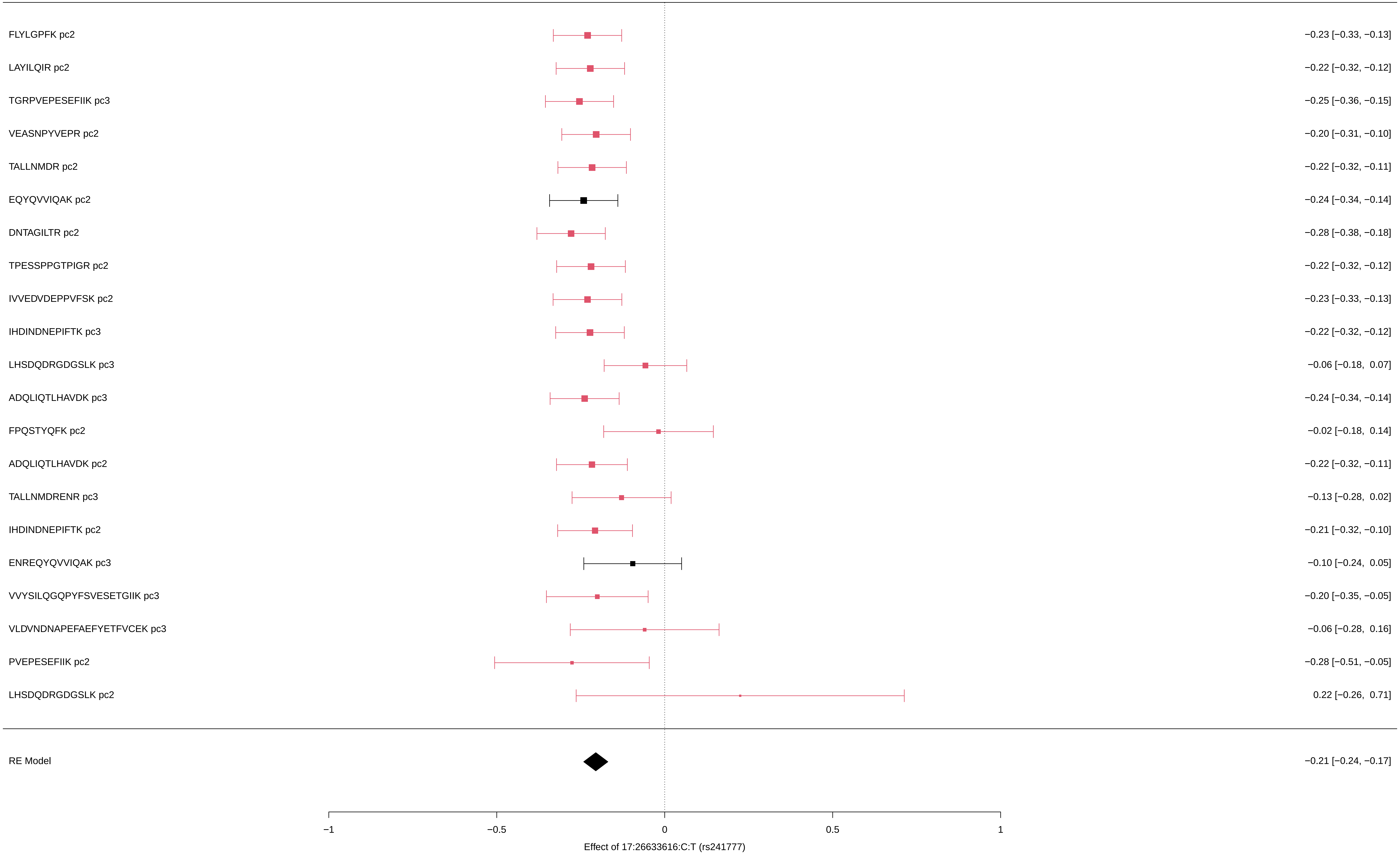

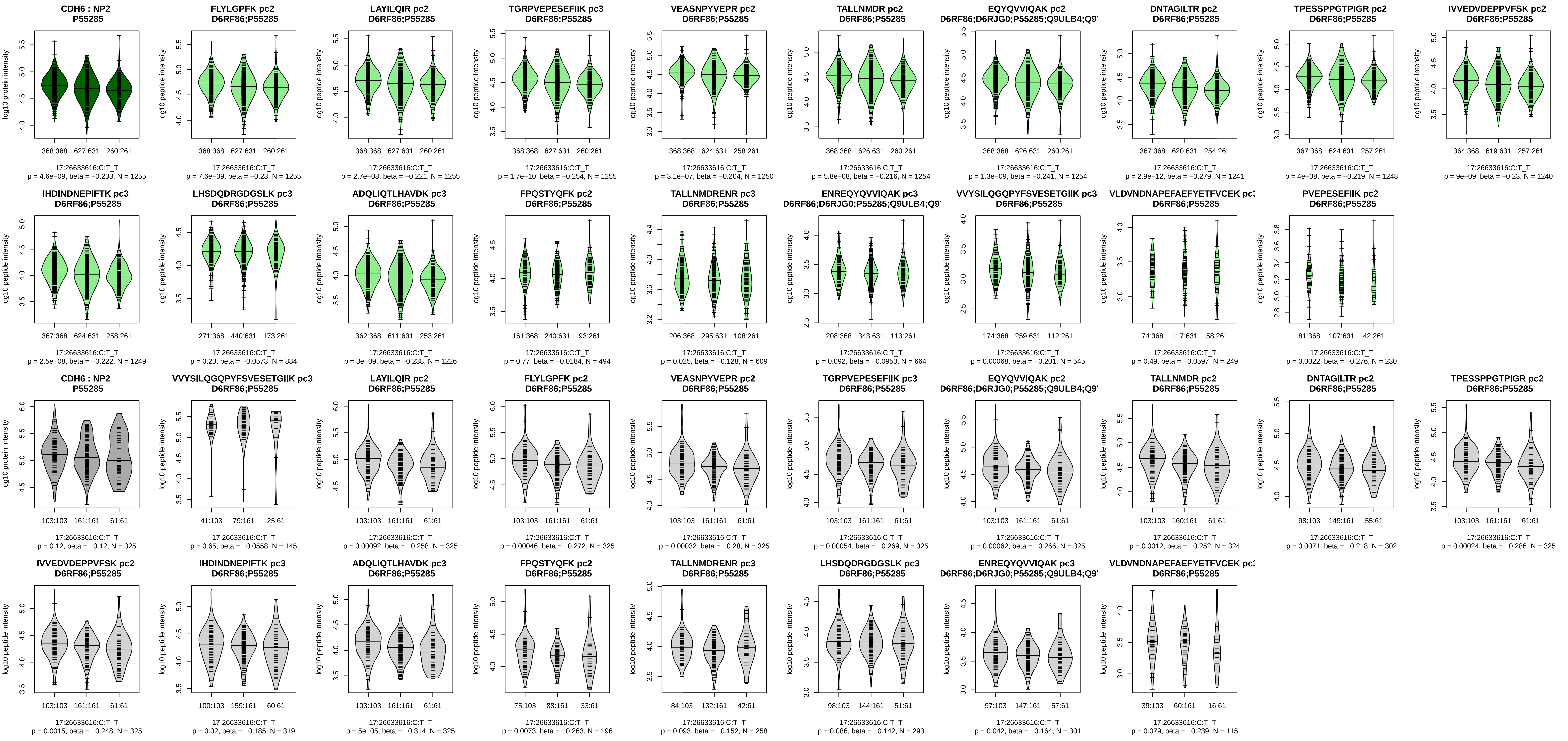

237Assay Target: CDH6
UniProt: P55285
rsID: rs241777
Target SNP: 17:26633616:C:T
Proxy SNP: 17:26633616:C:T
Tarkin log10(p): 8.3
Tarkin BETA: −0.233
Tarkin MSPA: 0.85
1255:1255:1255:1250:1254:125

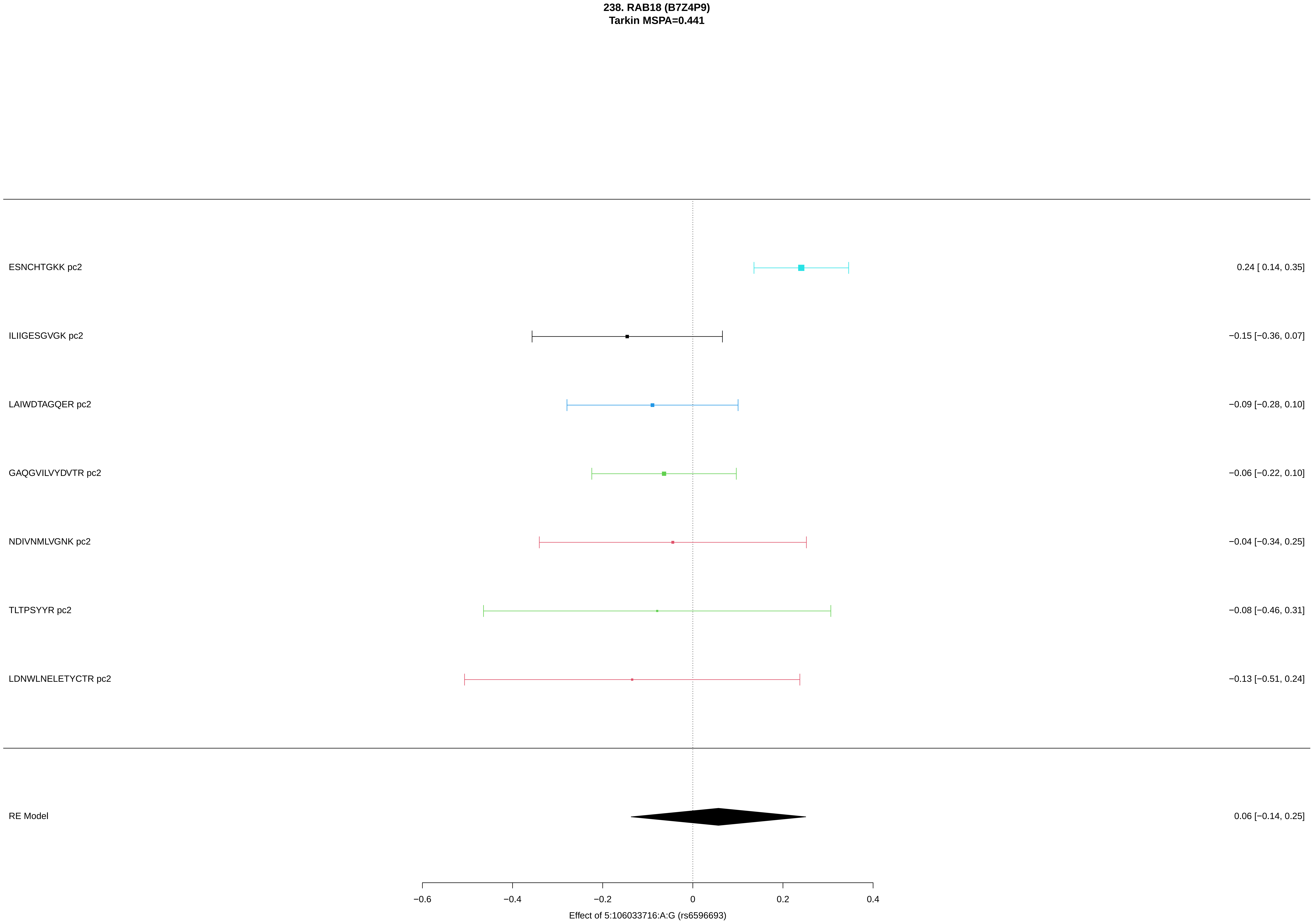
238. RAB18 (B7Z4P9)
Tarkin MSPA=0.441
ESNCHTGKK pc2
0.24 [0.14, 0.35]
ILIIGESGVGK pc2
−0.15 [−0.36, 0.07]
LAIWDTAGQER pc2
−0.09 [−0.28, 0.10]
GAQGVILVYDVTR pc2
−0.06 [−0.22, 0.10]
NDIVNMLVGNK pc2
−0.04 [−0.34, 0.25]
TLTPSYYR pc2
−0.08 [−0.46, 0.31]
LDNWLNELETYCTR pc2
−0.13 [−0.51, 0.24]
RE Model
0.06 [−0.14, 0.25]
−0.6
−0.4
−0.2
0
0.2
0.4
Effect of 5:106033716:A:G (rs6596693)

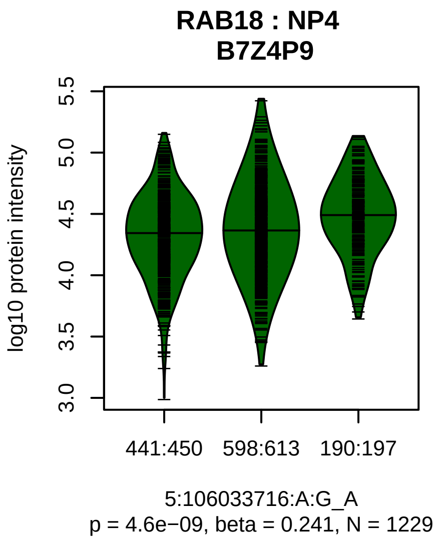

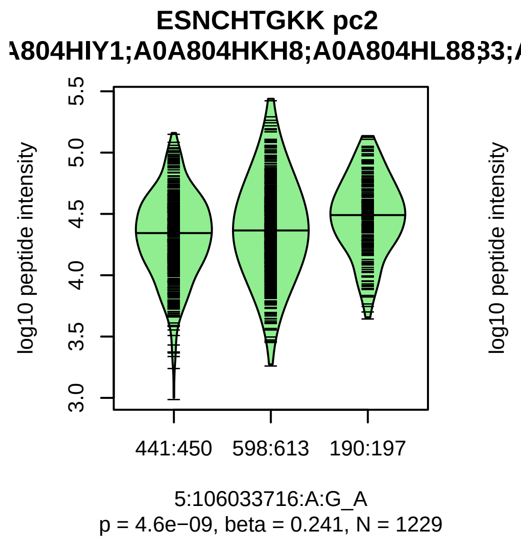

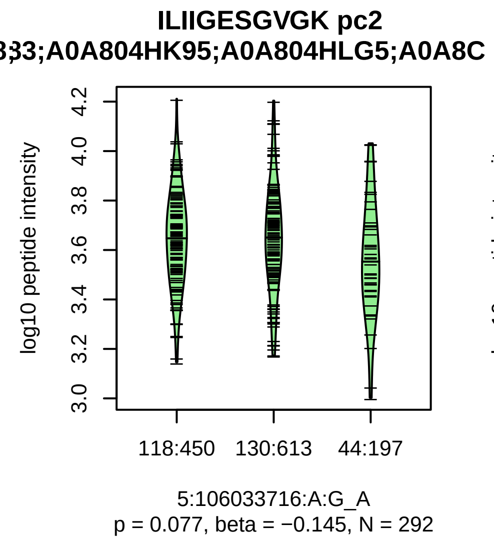

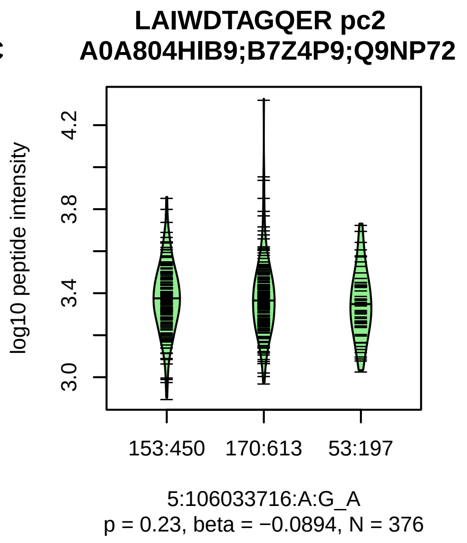

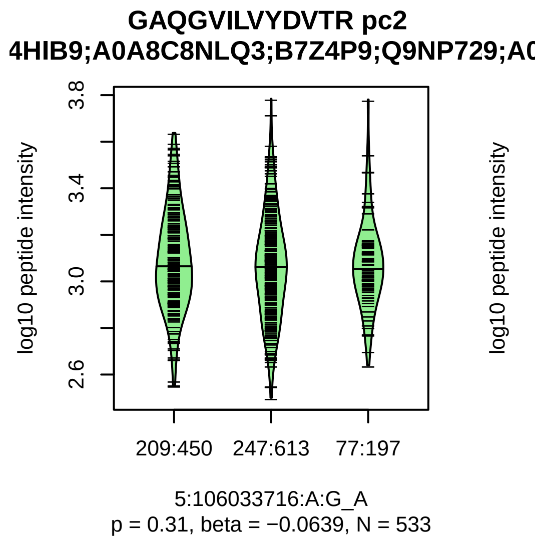

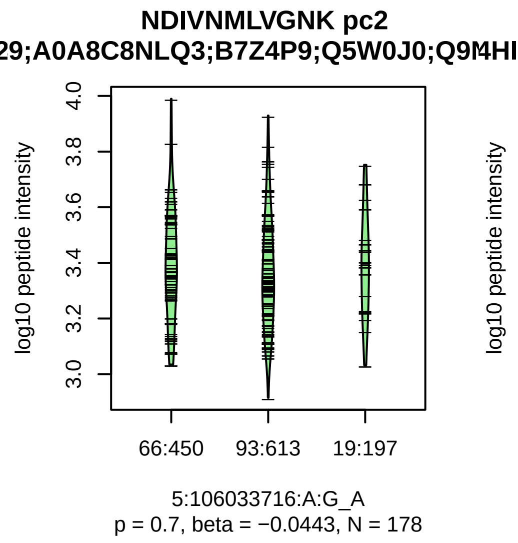

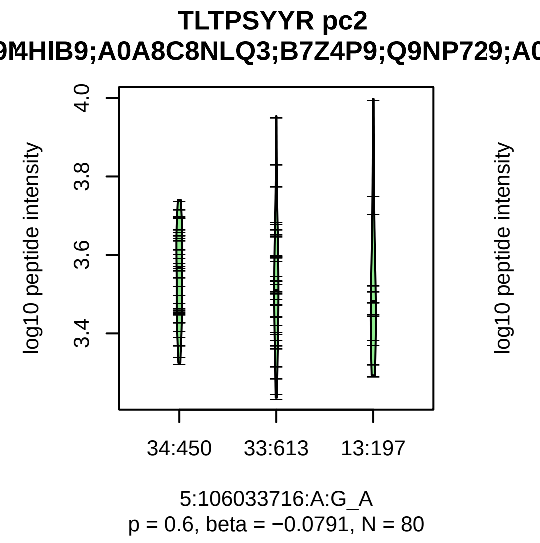

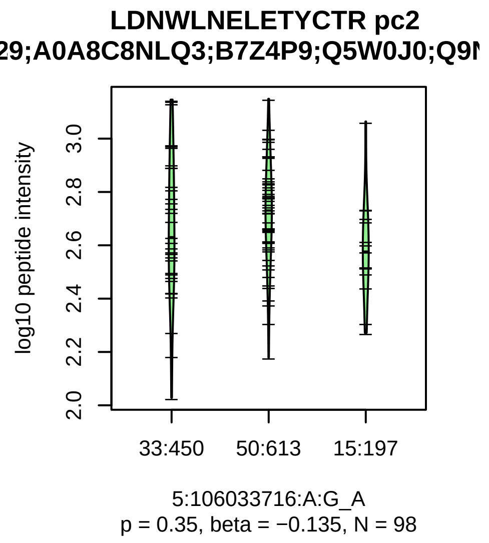

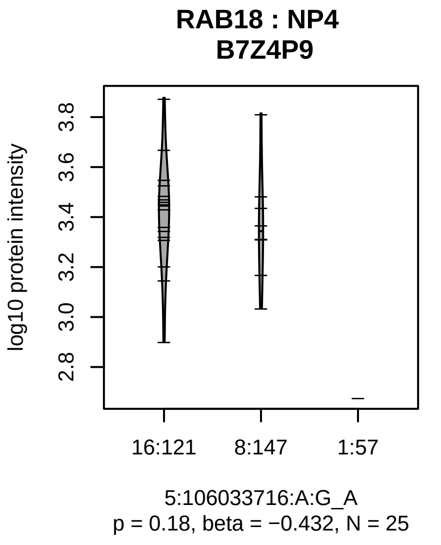

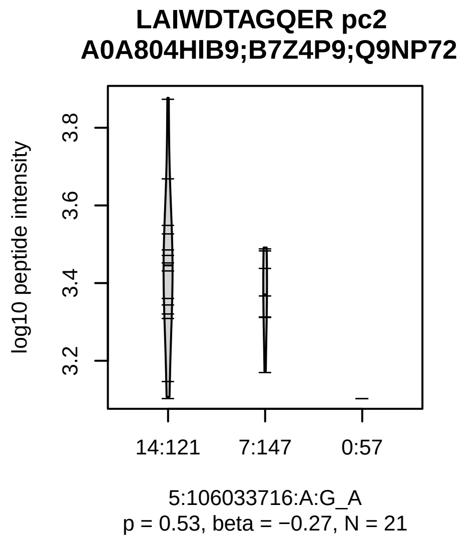

238Assay Target: RAB18
UniProt: B7Z4P9
rsID: rs6596693
Target SNP: 5:106033716:A:G
Proxy SNP: 5:106033716:A:G
Tarkin log10(p): 8.3
Tarkin BETA: 0.241
Tarkin MSPA: 0.441
*:–:–:–:–:–:–
1229:292:376:533:178:80:98

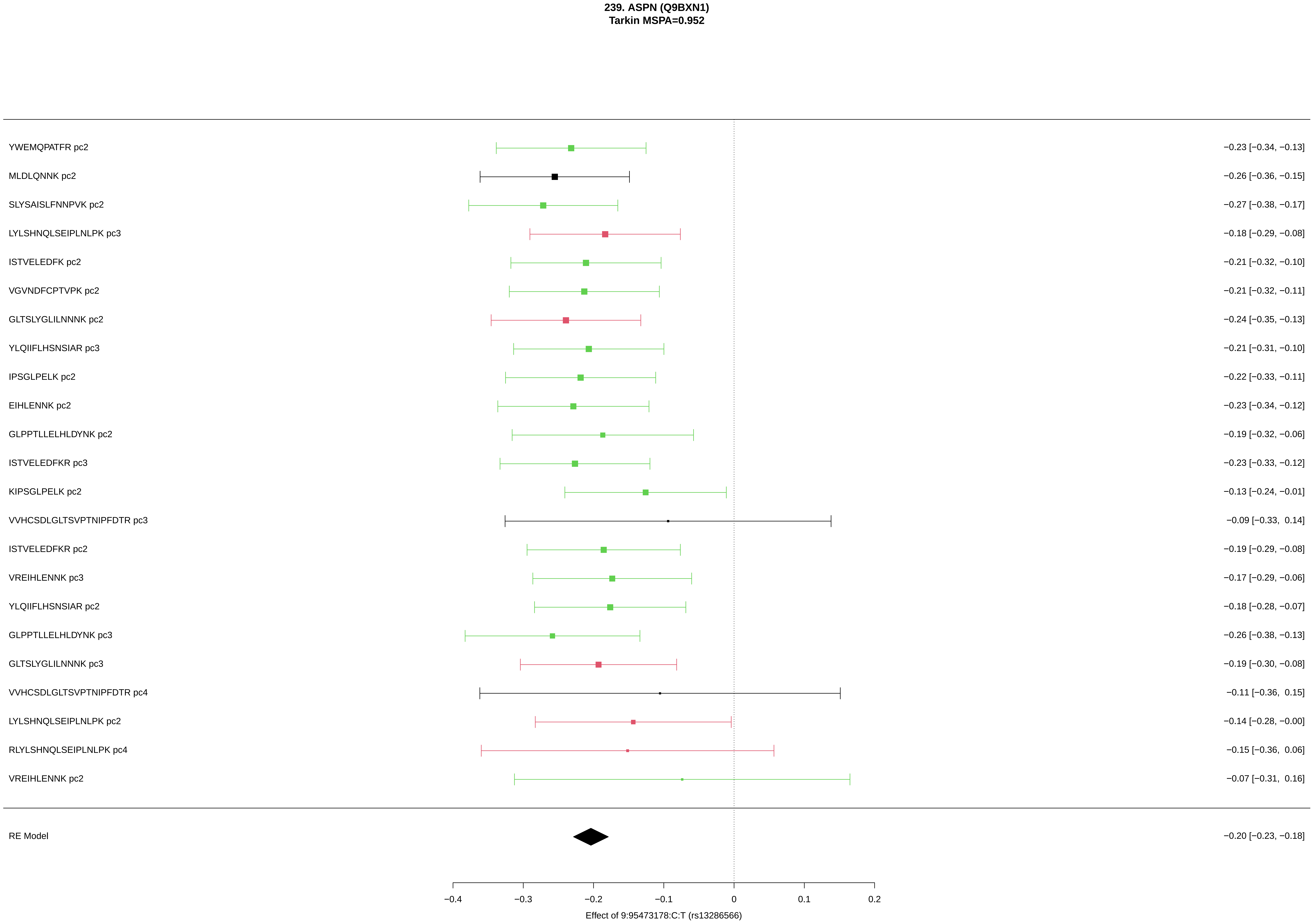
239. ASPN (Q9BXN1)
Tarkin MSPA=0.952
YWEMQPATFR pc2 −0.23 [−0.34, −0.13]
MLDLQNNK pc2 −0.26 [−0.36, −0.15]
SLYSAISLFNNPVK pc2 −0.27 [−0.38, −0.17]
LYLSHNQLSEIPLNLPK pc3 −0.18 [−0.29, −0.08]
ISTVELEDFK pc2 −0.21 [−0.32, −0.10]
VGVNDFCPTVPK pc2 −0.21 [−0.32, −0.11]
GLTSLYGLILNNNK pc2 −0.24 [−0.35, −0.13]
YLQIIFLHSNSIAR pc3 −0.21 [−0.31, −0.10]
IPSGLPELK pc2 −0.22 [−0.33, −0.11]
EIHLENNK pc2 −0.23 [−0.34, −0.12]
GLPPTLLELHLDYNK pc2 −0.19 [−0.32, −0.06]
ISTVELEDFKR pc3 −0.23 [−0.33, −0.12]
KIPSGLPELK pc2 −0.13 [−0.24, −0.01]
VVHCSDLGLTSVPTNIPFDTR pc3 −0.09 [−0.33, 0.14]
ISTVELEDFKR pc2 −0.19 [−0.29, −0.08]
VREIHLENNK pc3 −0.17 [−0.29, −0.06]
YLQIIFLHSNSIAR pc2 −0.18 [−0.28, −0.07]
GLPPTLLELHLDYNK pc3 −0.26 [−0.38, −0.13]
GLTSLYGLILNNNK pc3 −0.19 [−0.30, −0.08]
VVHCSDLGLTSVPTNIPFDTR pc4 −0.11 [−0.36, 0.15]
LYLSHNQLSEIPLNLPK pc2 −0.14 [−0.28, −0.00]
RLYLSHNQLSEIPLNLPK pc4 −0.15 [−0.36, 0.06]
VREIHLENNK pc2 −0.07 [−0.31, 0.16]
RE Model −0.20 [−0.23, −0.18]
−0.4
−0.3
−0.2
−0.1
0
0.1
0.2
Effect of 9:95473178:C:T (rs13286566)

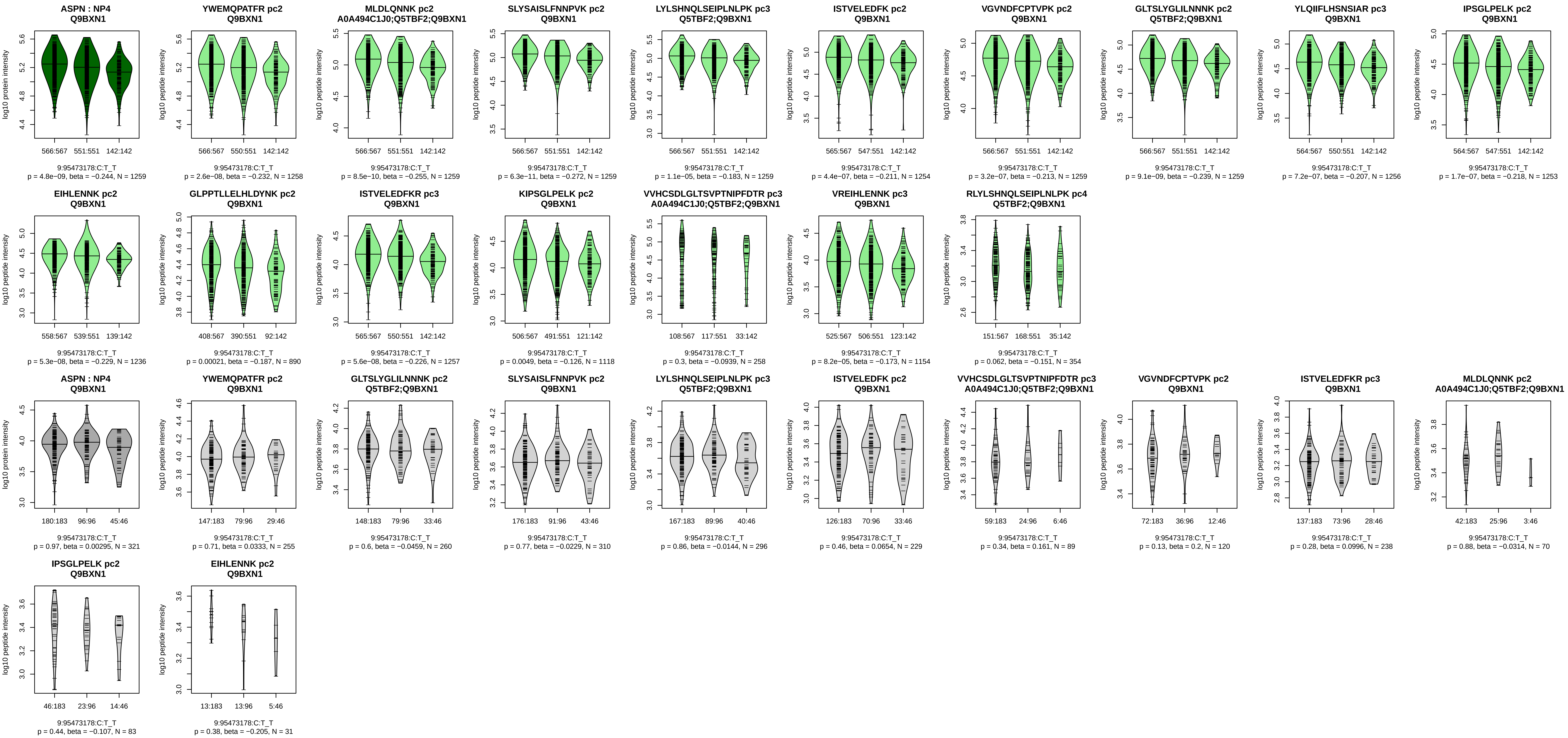

239Assay Target: ASPN
UniProt: Q9BXN1
rsID: rs13286566
Target SNP: 9:95473178:C:T
Proxy SNP: 9:95473178:C:T
Tarkin log10(p): 8.3
Tarkin BETA: −0.244
Tarkin MSPA: 0.952
1258:1259:1259:1259:1254:125

240. AIMP1 (Q12904)
Tarkin MSPA=0.486

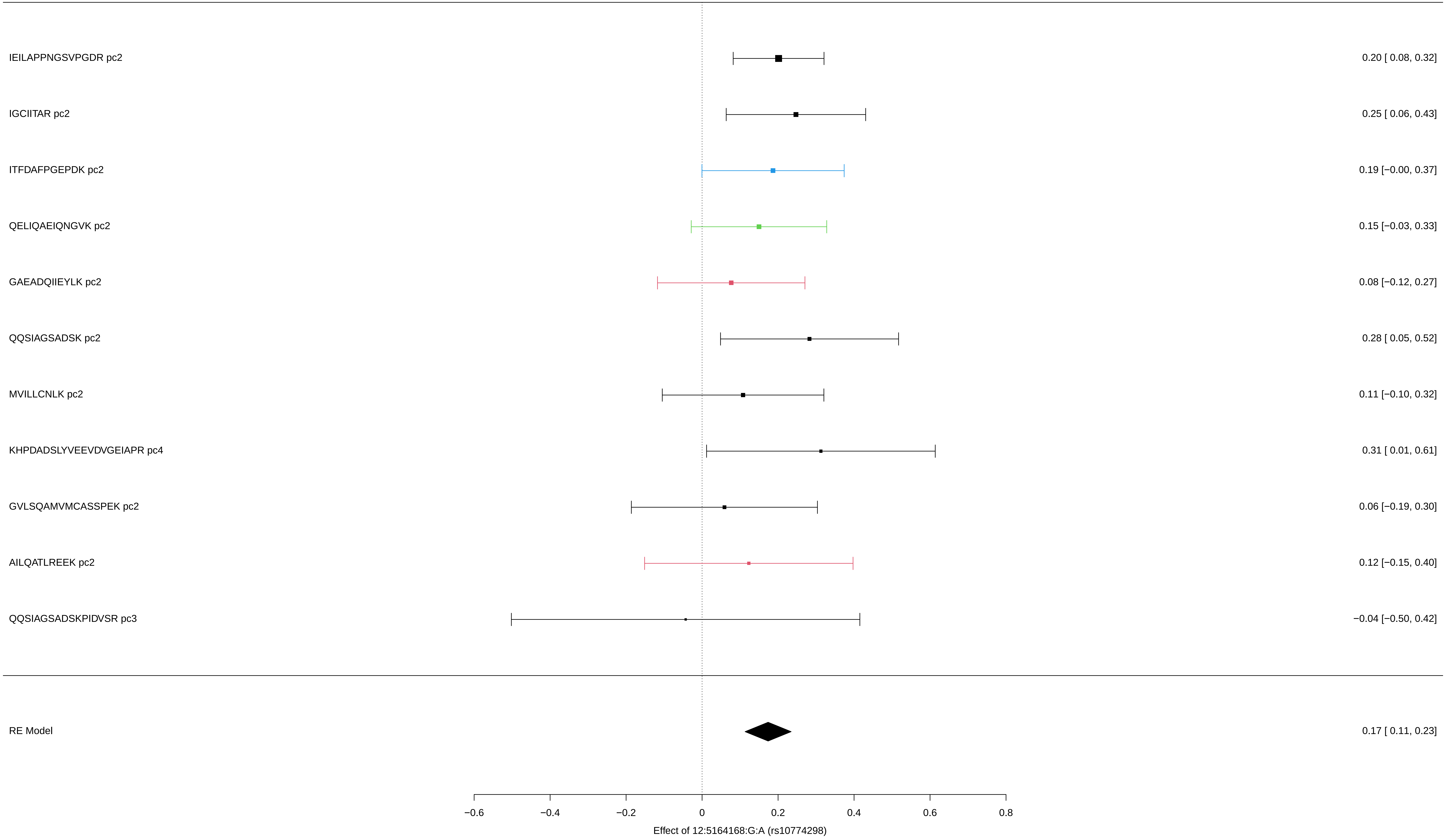

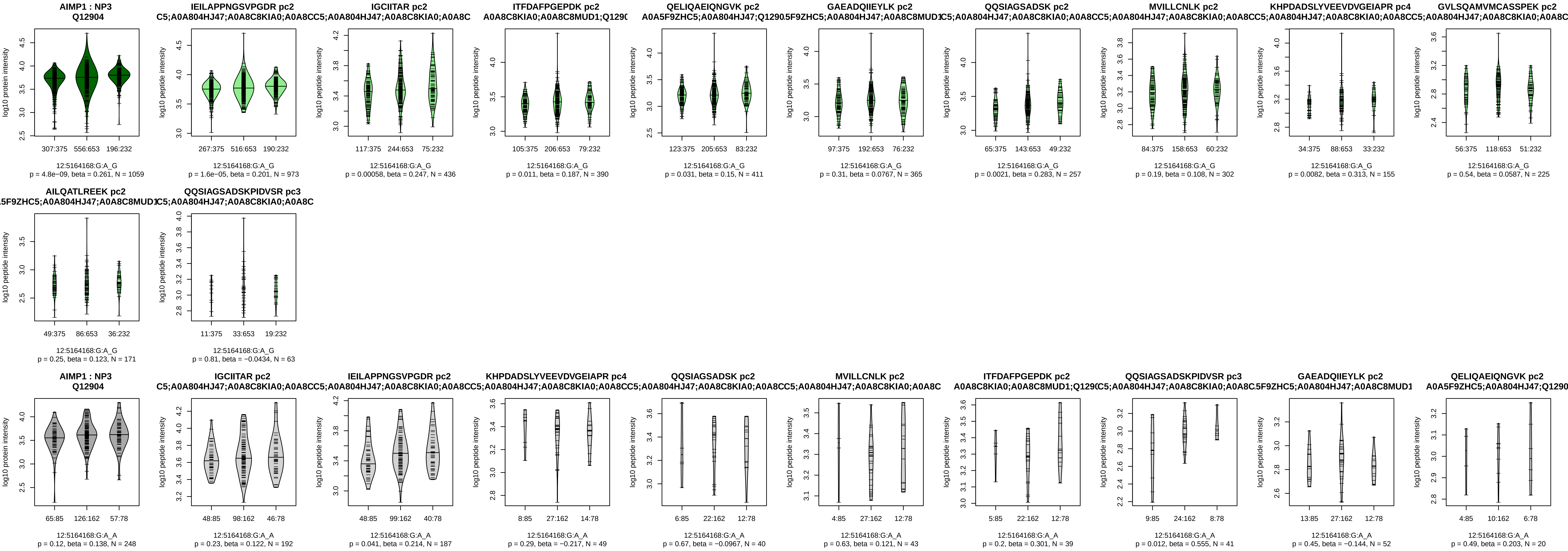

240Assay Target: AIMP1
UniProt: Q12904
rsID: rs10774298
Target SNP: 12:5164168:G:A
Proxy SNP: 12:5164168:G:A
Tarkin log10(p): 8.3
Tarkin BETA: 0.261
Tarkin MSPA: 0.486
::–:–:–:*:–:*:–:–:–:NA
973:436:390:411:365:257:302:1

241. BMP10 (O95393)
Tarkin MSPA=0.583

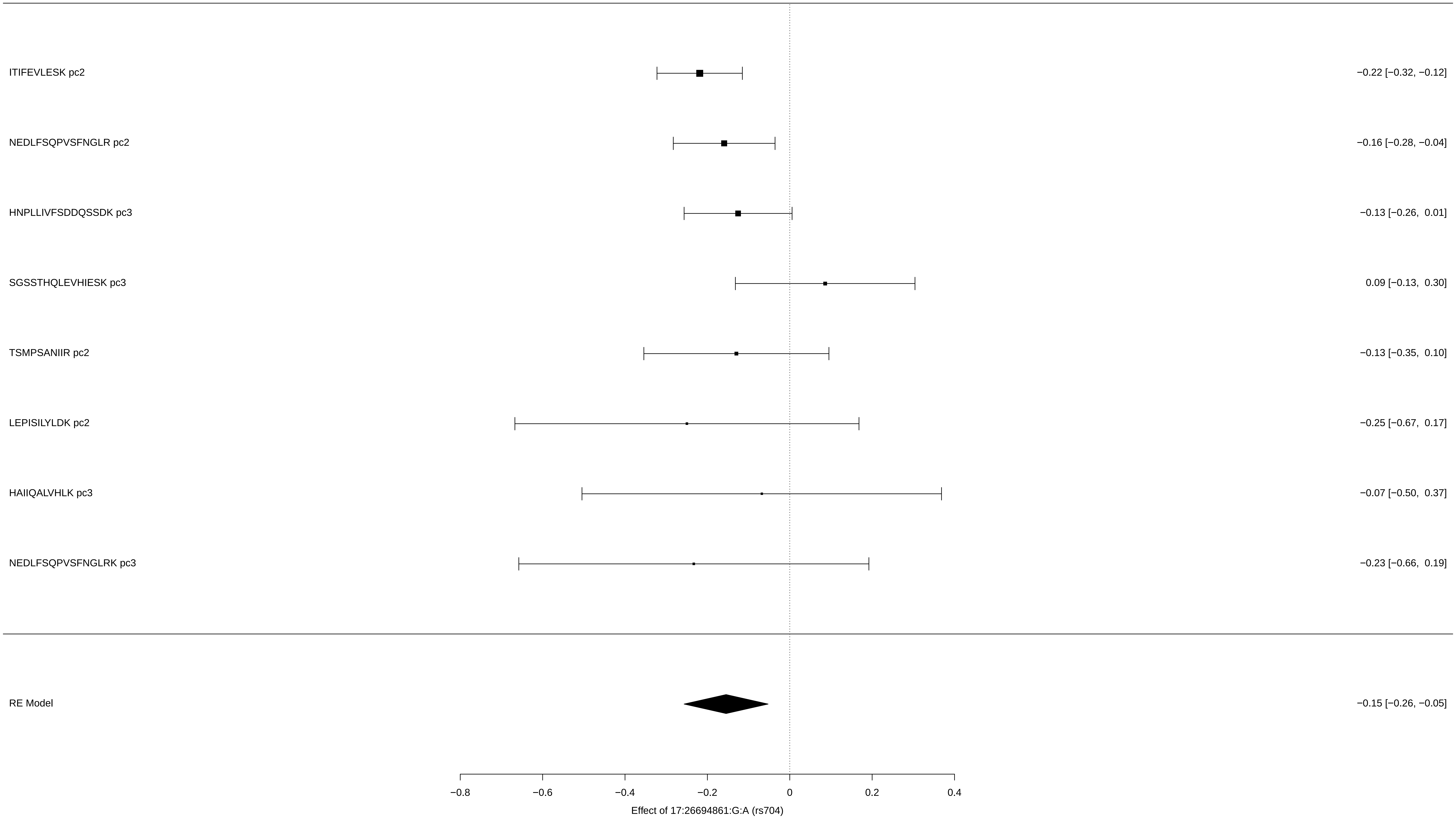

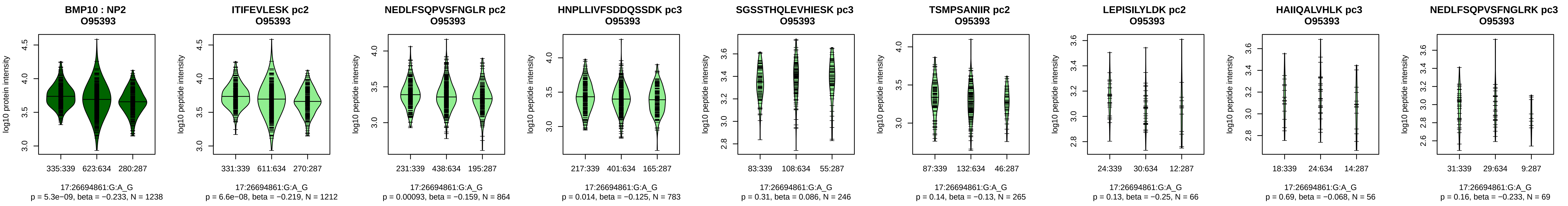

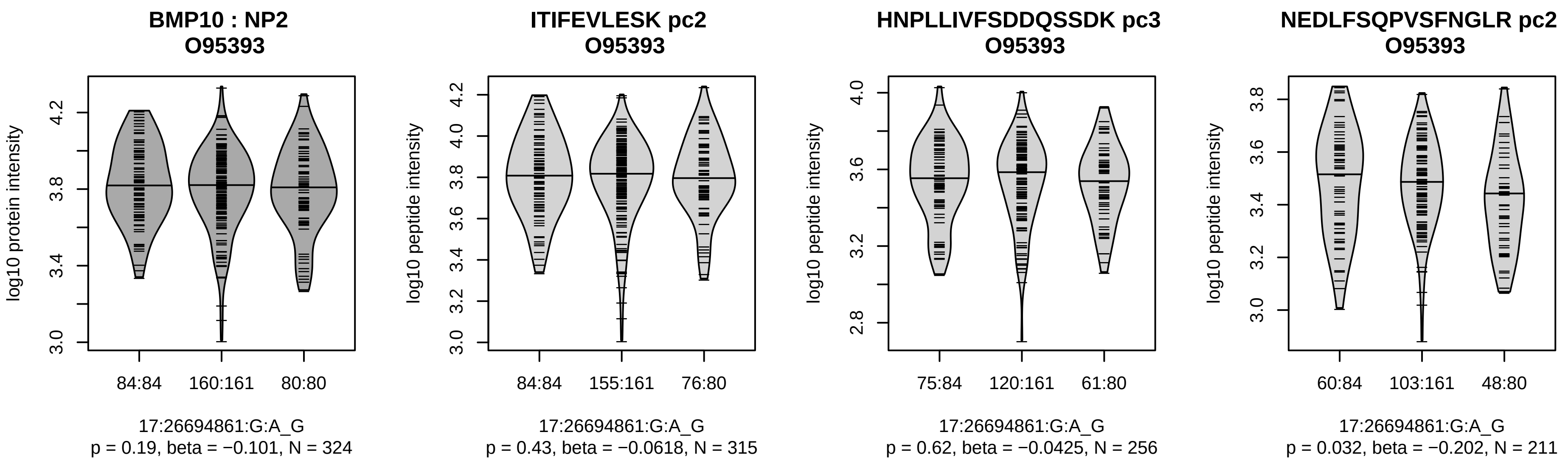

241Assay Target: BMP10
UniProt: O95393
rsID: rs704
Target SNP: 17:26694861:G:A
Proxy SNP: 17:26694861:G:A
Tarkin log10(p): 8.3
Tarkin BETA: −0.233
Tarkin MSPA: 0.583
::–:–:–:–:–:–
1212:864:783:246:265:66:56:69

242. C19orf80 (Q6UXH0)
Tarkin MSPA=0.926

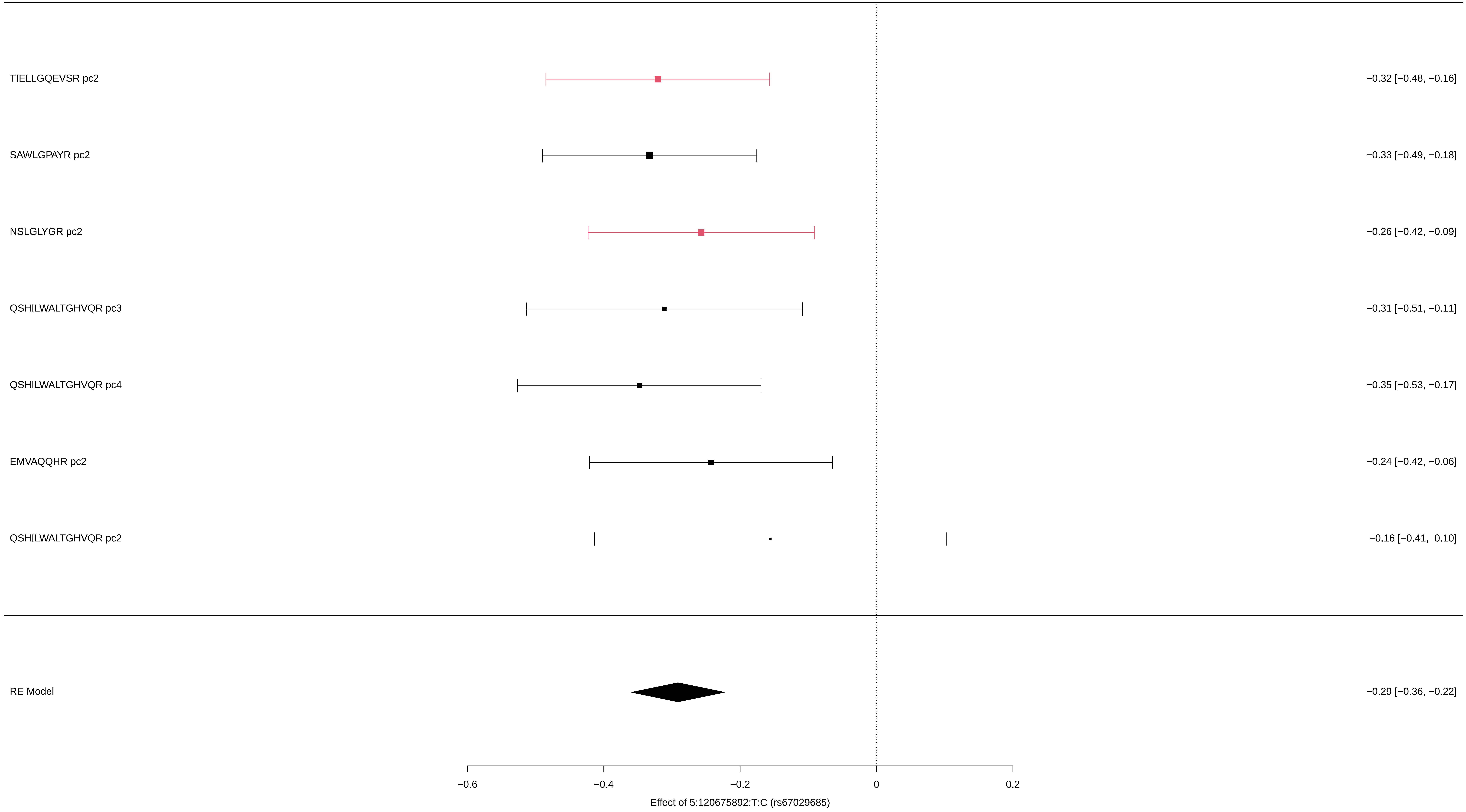

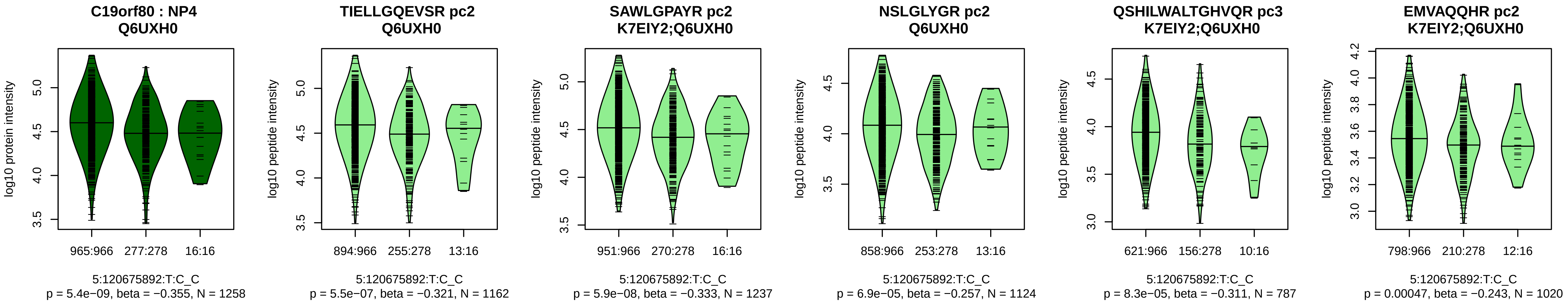

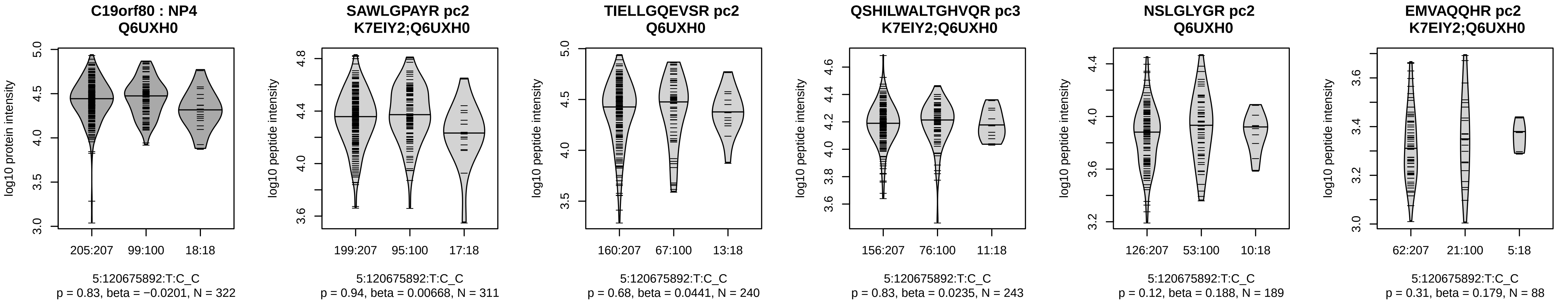

242Assay Target: C19orf80
UniProt: Q6UXH0
rsID: rs67029685
Target SNP: 5:120675892:T:C
Proxy SNP: 5:120675892:T:C
Tarkin log10(p): 8.3
Tarkin BETA: −0.354
Tarkin MSPA: 0.926
::*:*:*:–
1162:1237:1124:787:939:1020:4

243. PSMF1 (Q92530)
Tarkin MSPA=0.941

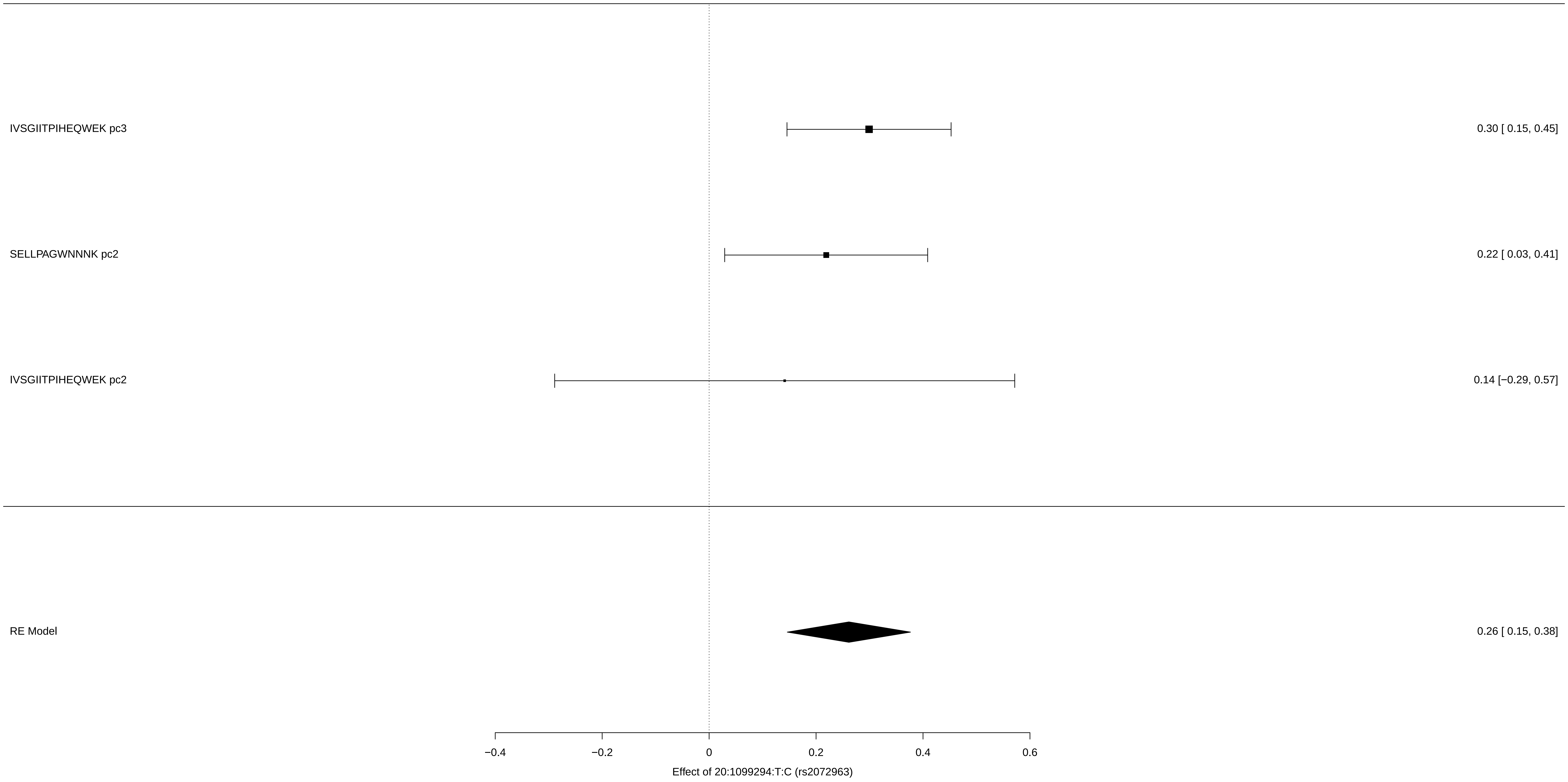

PSMF1 : NP1
Q5QPM7;Q92530

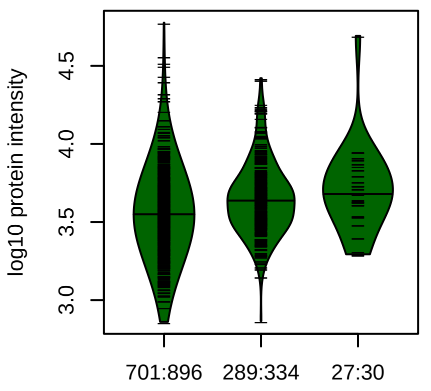

20:1099294:T:C_C
p = 5.5e−09, beta = 0.343, N = 1017

IVSGIITPIHEQWEK pc3
Q5QPM7;Q92530

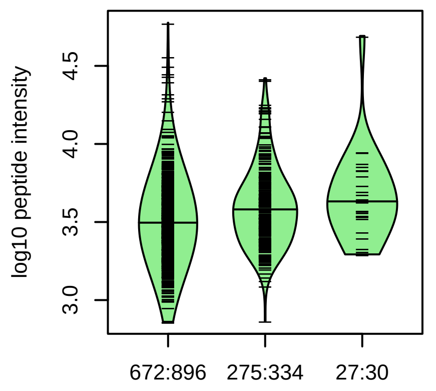

20:1099294:T:C_C
p = 6.1e−07, beta = 0.299, N = 974

SELLPAGWNNNK pc2
Q5QPM7;Q92530

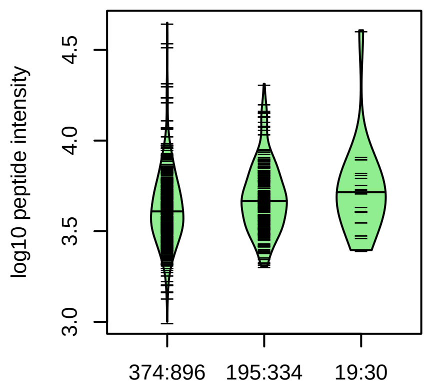

20:1099294:T:C_C
p = 0.0031, beta = 0.219, N = 588

IVSGIITPIHEQWEK pc3
Q5QPM7;Q92530

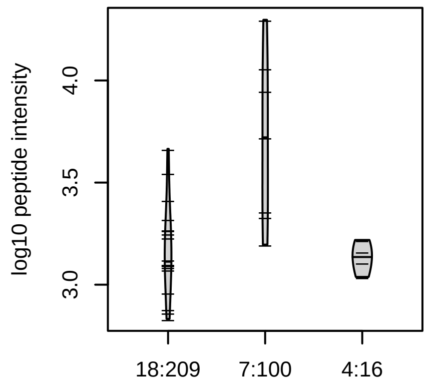

20:1099294:T:C_C
p = 0.13, beta = 0.36, N = 29

243Assay Target: PSMF1
UniProt: Q92530
rsID: rs2072963
Target SNP: 20:1099294:T:C
Proxy SNP: 20:1099294:T:C
Tarkin log10(p): 8.3
Tarkin BETA: 0.343
Tarkin MSPA: 0.941
::−:NA:NA
974:588:98:11:12

244. RPS18 (P62269)
Tarkin MSPA=0.854

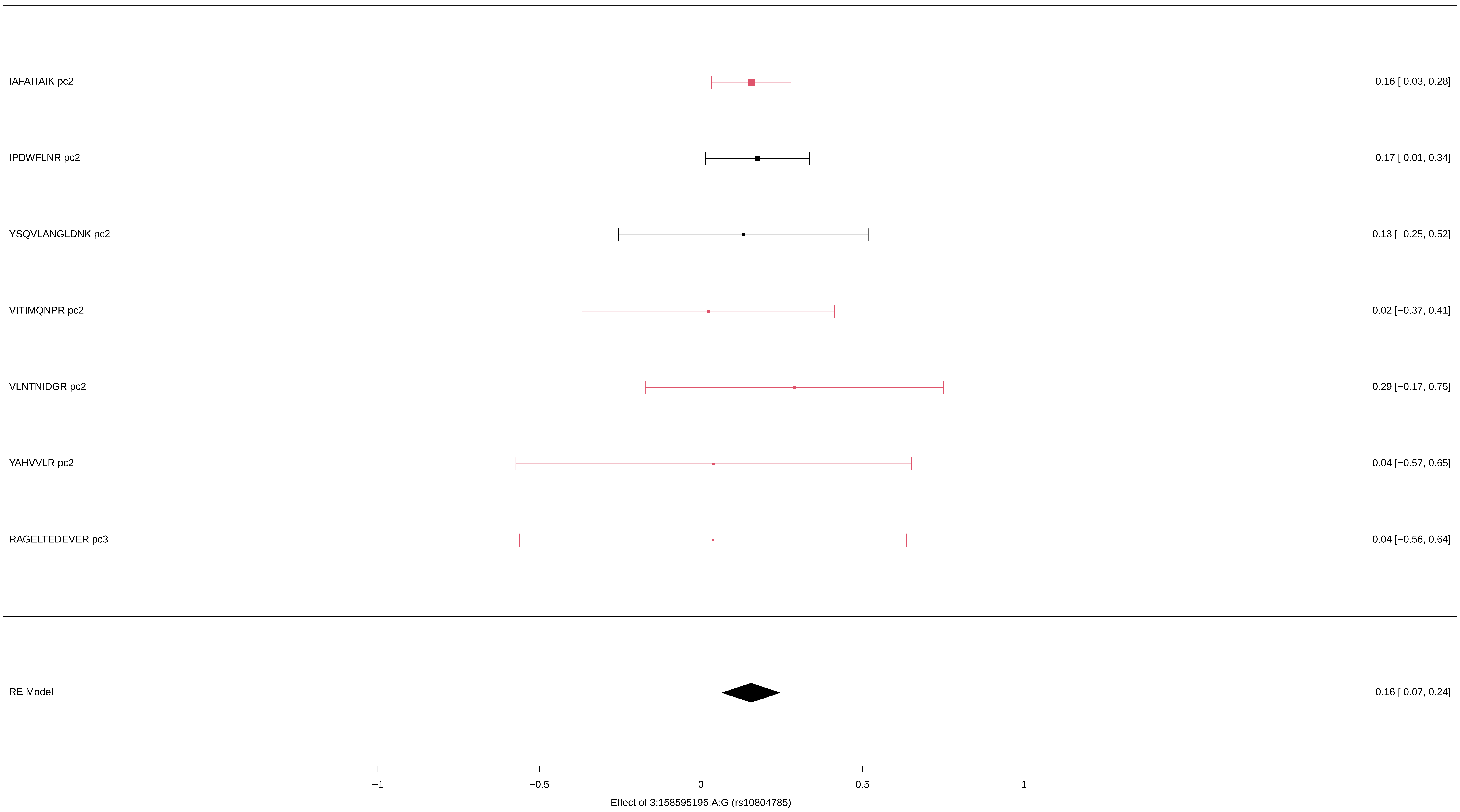

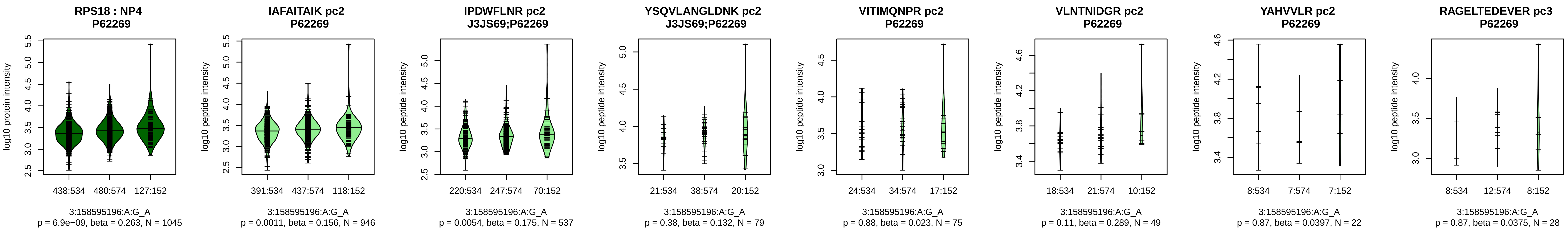

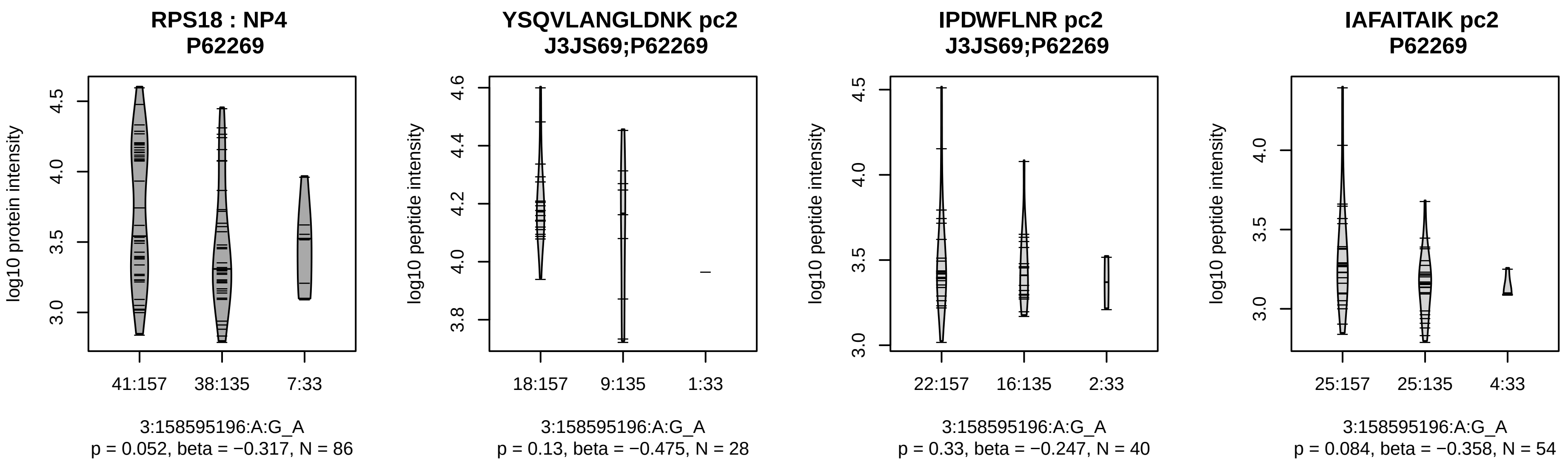

244Assay Target: RPS18
UniProt: P62269
rsID: rs10804785
Target SNP: 3:158595196:A:G
Proxy SNP: 3:158595196:A:G
Tarkin log10(p): 8.2
Tarkin BETA: 0.263
Tarkin MSPA: 0.854
::−:−:−:−:−:NA:NA:NA
946:537:79:75:49:22:28:12:4:5

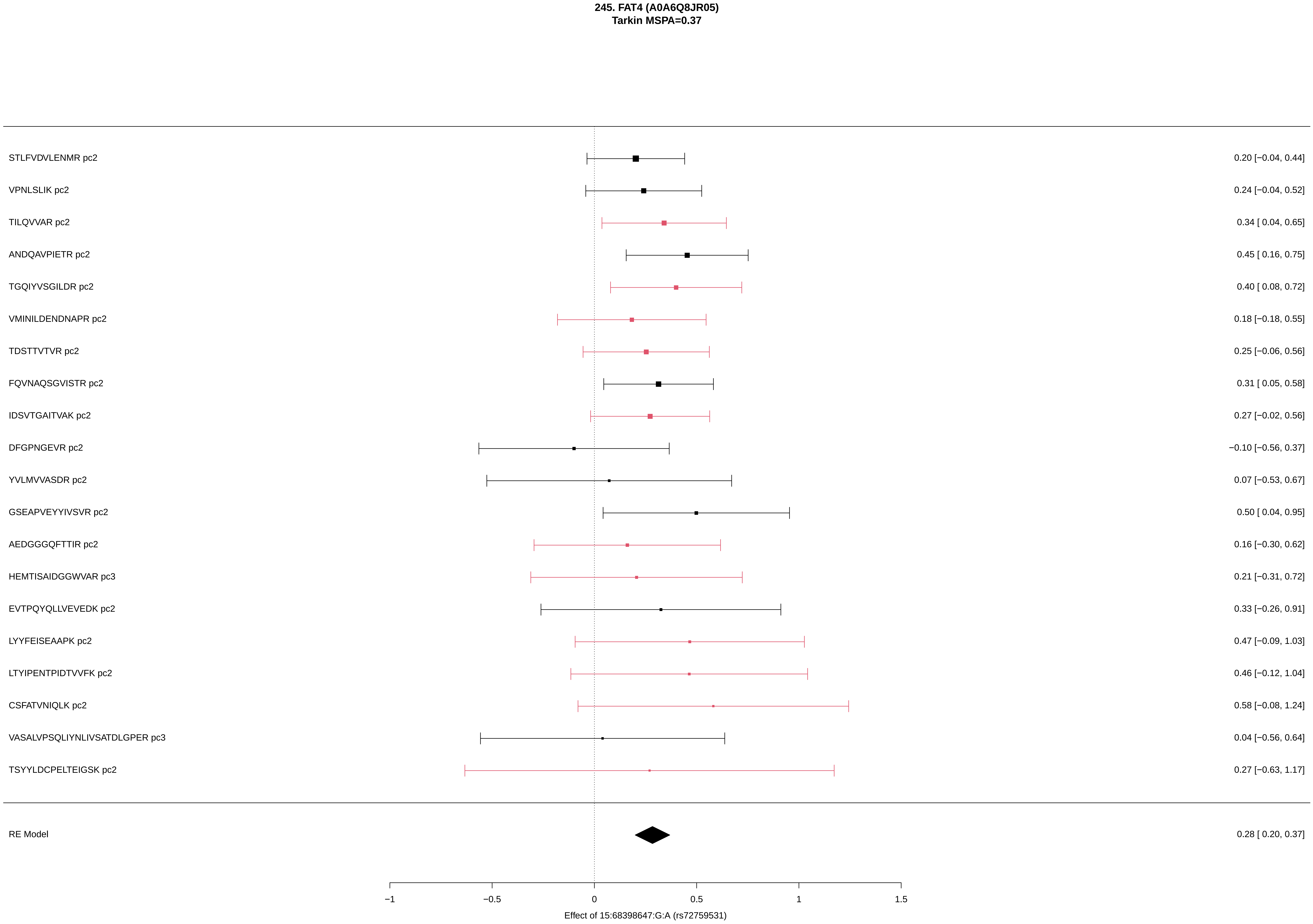
245. FAT4 (A0A6Q8JR05)
Tarkin MSPA=0.37
STLFVDVLENMR pc2
VPNLSLIK pc2
TILQVVAR pc2
ANDQAVPIETR pc2
TGQIYVSGILDR pc2
VMINILDENDNAPR pc2
TDSTTVTVR pc2
FQVNAQSGVISTR pc2
IDSVTGAITVAK pc2
DFGPNGEVR pc2
YVLMVVASDR pc2
GSEAPVEYYIVSVR pc2
AEDGGGQFTTIR pc2
HEMTISAIDGGWVAR pc3
EVTPQYQLLVEVEDK pc2
LYYFEISEAAPK pc2
LTYIPENTPIDTVVFK pc2
CSFATVNIQLK pc2
VASALVPSQLIYNLIVSATDLGPER pc3
TSYYLDCPELTEIGSK pc2
RE Model
0.20 [−0.04, 0.44]
0.24 [−0.04, 0.52]
0.34 [0.04, 0.65]
0.45 [0.16, 0.75]
0.40 [0.08, 0.72]
0.18 [−0.18, 0.55]
0.25 [−0.06, 0.56]
0.31 [0.05, 0.58]
0.27 [−0.02, 0.56]
−0.10 [−0.56, 0.37]
0.07 [−0.53, 0.67]
0.50 [0.04, 0.95]
0.16 [−0.30, 0.62]
0.21 [−0.31, 0.72]
0.33 [−0.26, 0.91]
0.47 [−0.09, 1.03]
0.46 [−0.12, 1.04]
0.58 [−0.08, 1.24]
0.04 [−0.56, 0.64]
0.27 [−0.63, 1.17]
0.28 [0.20, 0.37]
−1
−0.5
0
0.5
1
1.5
Effect of 15:68398647:G:A (rs72759531)

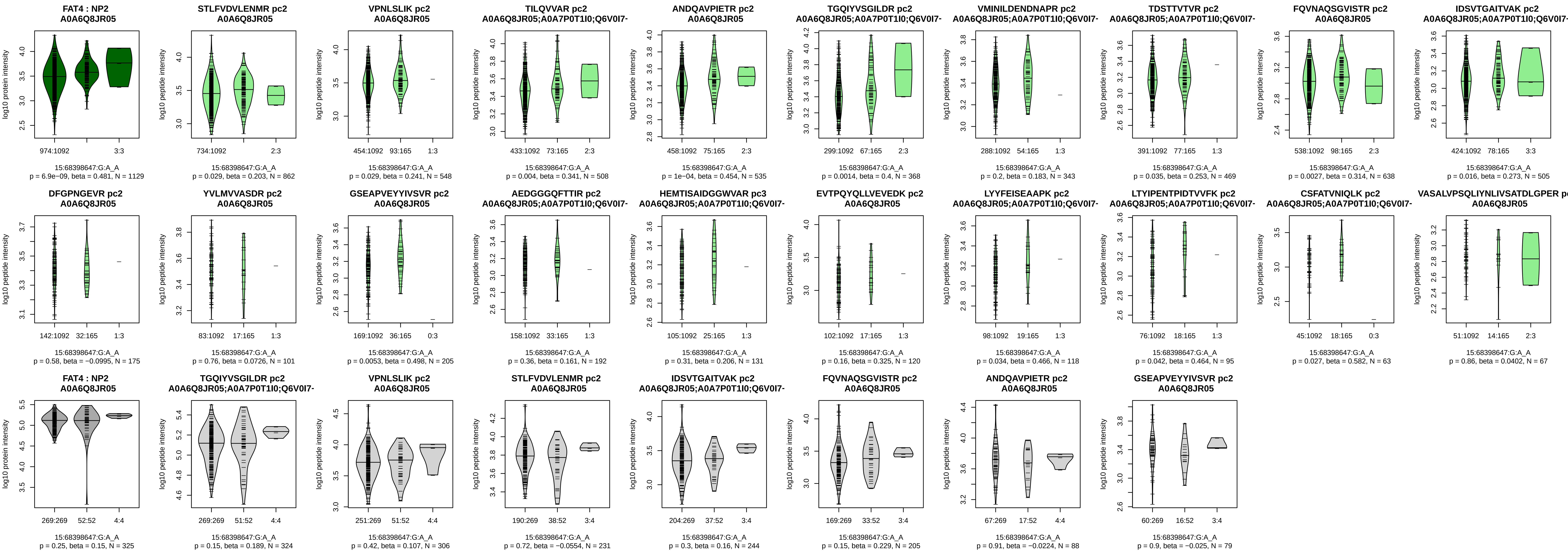
FAT4 : NP2
A0A6Q8JR05
log10 protein intensity
974:1092
3:3
15:68398647:G:A_A
p = 6.9e−09, beta = 0.481, N = 1129
STLFVDVLENMR pc2
A0A6Q8JR05
log10 peptide intensity
734:1092
2:3
15:68398647:G:A_A
p = 0.029, beta = 0.203, N = 862
VPNLSLIK pc2
A0A6Q8JR05
454:1092
93:165
1:3
15:68398647:G:A_A
p = 0.029, beta = 0.241, N = 548
TILQVVAR pc2
A0A6Q8JR05;A0A7P0T1I0;Q6V0I7-
433:1092
73:165
2:3
15:68398647:G:A_A
p = 0.004, beta = 0.341, N = 508
ANDQAVPIETR pc2
A0A6Q8JR05
458:1092
75:165
2:3
15:68398647:G:A_A
p = 1e−04, beta = 0.454, N = 535
TGQIYVSGILDR pc2
A0A6Q8JR05;A0A7P0T1I0;Q6V0I7-
299:1092
67:165
2:3
15:68398647:G:A_A
p = 0.0014, beta = 0.4, N = 368
VMINILDENDNAPR pc2
A0A6Q8JR05;A0A7P0T1I0;Q6V0I7-
288:1092
54:165
1:3
15:68398647:G:A_A
p = 0.2, beta = 0.183, N = 343
TDSTTVTVR pc2
A0A6Q8JR05;A0A7P0T1I0;Q6V0I7-
391:1092
77:165
1:3
15:68398647:G:A_A
p = 0.035, beta = 0.253, N = 469
FQVNAQSGVISTR pc2
A0A6Q8JR05
538:1092
98:165
2:3
15:68398647:G:A_A
p = 0.0027, beta = 0.314, N = 638
IDSVTGAITVAK pc2
A0A6Q8JR05;A0A7P0T1I0;Q6V0I7-
424:1092
78:165
3:3
15:68398647:G:A_A
p = 0.016, beta = 0.273, N = 505
DFGPNGEVR pc2
A0A6Q8JR05
142:1092
32:165
1:3
15:68398647:G:A_A
p = 0.58, beta = −0.0995, N = 175
YVLMVVASDR pc2
A0A6Q8JR05
83:1092
17:165
1:3
15:68398647:G:A_A
p = 0.76, beta = 0.0726, N = 101
GSEAPVEYYIVSVR pc2
A0A6Q8JR05
169:1092
36:165
0:3
15:68398647:G:A_A
p = 0.0053, beta = 0.498, N = 205
AEDGGGQFTTIR pc2
A0A6Q8JR05;A0A7P0T1I0;Q6V0I7-
158:1092
33:165
1:3
15:68398647:G:A_A
p = 0.36, beta = 0.161, N = 192
HEMTISAIDGGWVAR pc3
A0A6Q8JR05;A0A7P0T1I0;Q6V0I7-
105:1092
25:165
1:3
15:68398647:G:A_A
p = 0.31, beta = 0.206, N = 131
EVTPQYQLLVEVEDK pc2
A0A6Q8JR05
102:1092
17:165
1:3
15:68398647:G:A_A
p = 0.16, beta = 0.325, N = 120
LYYFEISEAAPK pc2
A0A6Q8JR05;A0A7P0T1I0;Q6V0I7-
98:1092
19:165
1:3
15:68398647:G:A_A
p = 0.034, beta = 0.466, N = 118
LTYIPENTPIDTVVFK pc2
A0A6Q8JR05;A0A7P0T1I0;Q6V0I7-
76:1092
18:165
1:3
15:68398647:G:A_A
p = 0.042, beta = 0.464, N = 95
CSFATVNIQLK pc2
A0A6Q8JR05;A0A7P0T1I0;Q6V0I7-
45:1092
18:165
0:3
15:68398647:G:A_A
p = 0.027, beta = 0.582, N = 63
VASALVPSQLIYNLIVSATDLGPER pc
A0A6Q8JR05
51:1092
14:165
2:3
15:68398647:G:A_A
p = 0.86, beta = 0.0402, N = 67
FAT4 : NP2
A0A6Q8JR05
269:269
52:52
4:4
15:68398647:G:A_A
p = 0.25, beta = 0.15, N = 325
TGQIYVSGILDR pc2
A0A6Q8JR05;A0A7P0T1I0;Q6V0I7-
269:269
51:52
4:4
15:68398647:G:A_A
p = 0.15, beta = 0.189, N = 324
VPNLSLIK pc2
A0A6Q8JR05
251:269
51:52
4:4
15:68398647:G:A_A
p = 0.42, beta = 0.107, N = 306
STLFVDVLENMR pc2
A0A6Q8JR05
190:269
38:52
3:4
15:68398647:G:A_A
p = 0.72, beta = −0.0554, N = 231
IDSVTGAITVAK pc2
A0A6Q8JR05;A0A7P0T1I0;Q6V0I7-
204:269
37:52
3:4
15:68398647:G:A_A
p = 0.3, beta = 0.16, N = 244
FQVNAQSGVISTR pc2
A0A6Q8JR05
169:269
33:52
3:4
15:68398647:G:A_A
p = 0.15, beta = 0.229, N = 205
ANDQAVPIETR pc2
A0A6Q8JR05
67:269
17:52
4:4
15:68398647:G:A_A
p = 0.91, beta = −0.0224, N = 88
GSEAPVEYYIVSVR pc2
A0A6Q8JR05
60:269
16:52
3:4
15:68398647:G:A_A
p = 0.9, beta = −0.025, N = 79

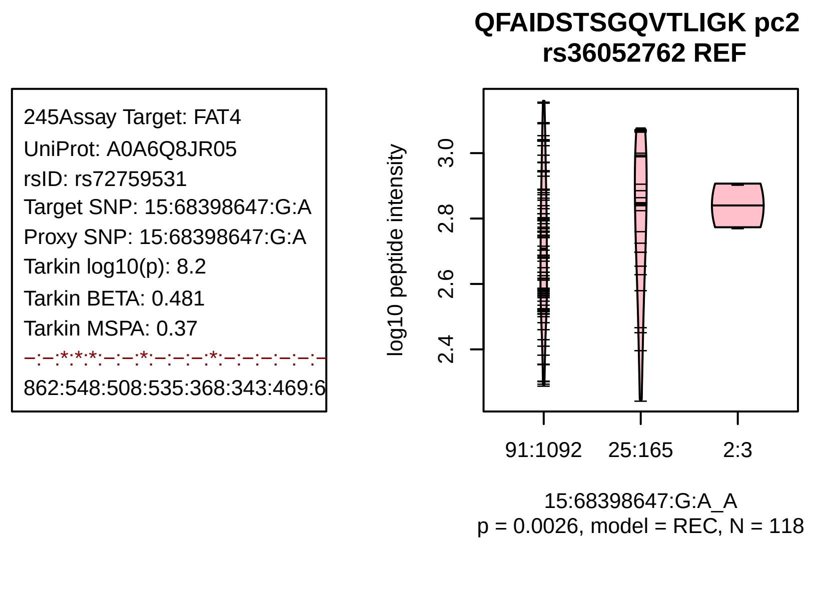
245Assay Target: FAT4
UniProt: A0A6Q8JR05
rsID: rs72759531
Target SNP: 15:68398647:G:A
Proxy SNP: 15:68398647:G:A
Tarkin log10(p): 8.2
Tarkin BETA: 0.481
Tarkin MSPA: 0.37
862:548:508:535:368:343:469:6
QFAIDSTSGQVTLIGK pc2
rs36052762 REF
log10 peptide intensity
91:1092
25:165
2:3
15:68398647:G:A_A
p = 0.0026, model = REC, N = 118

246. SPARCL1 (Q14515)
Tarkin MSPA=0.932

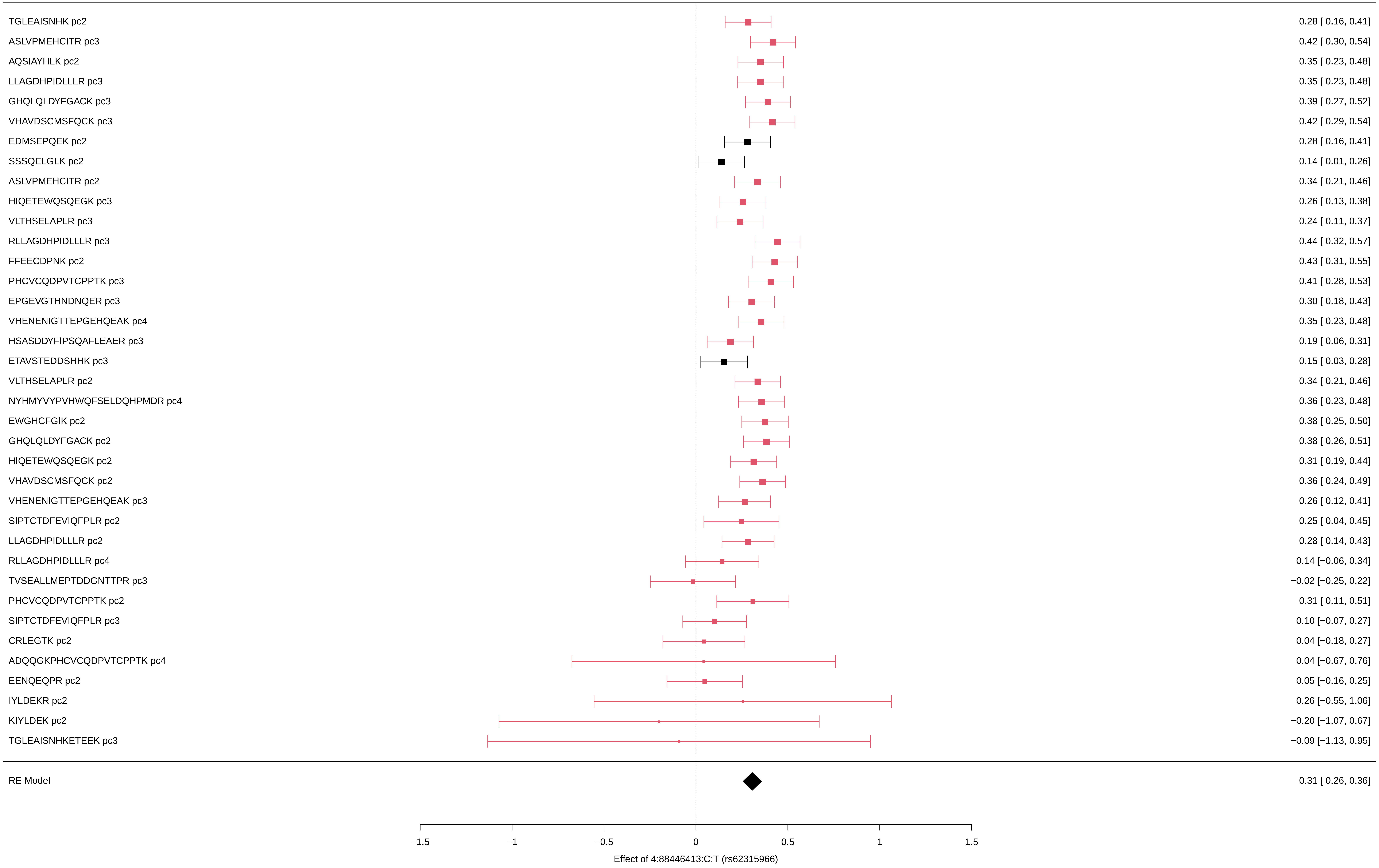

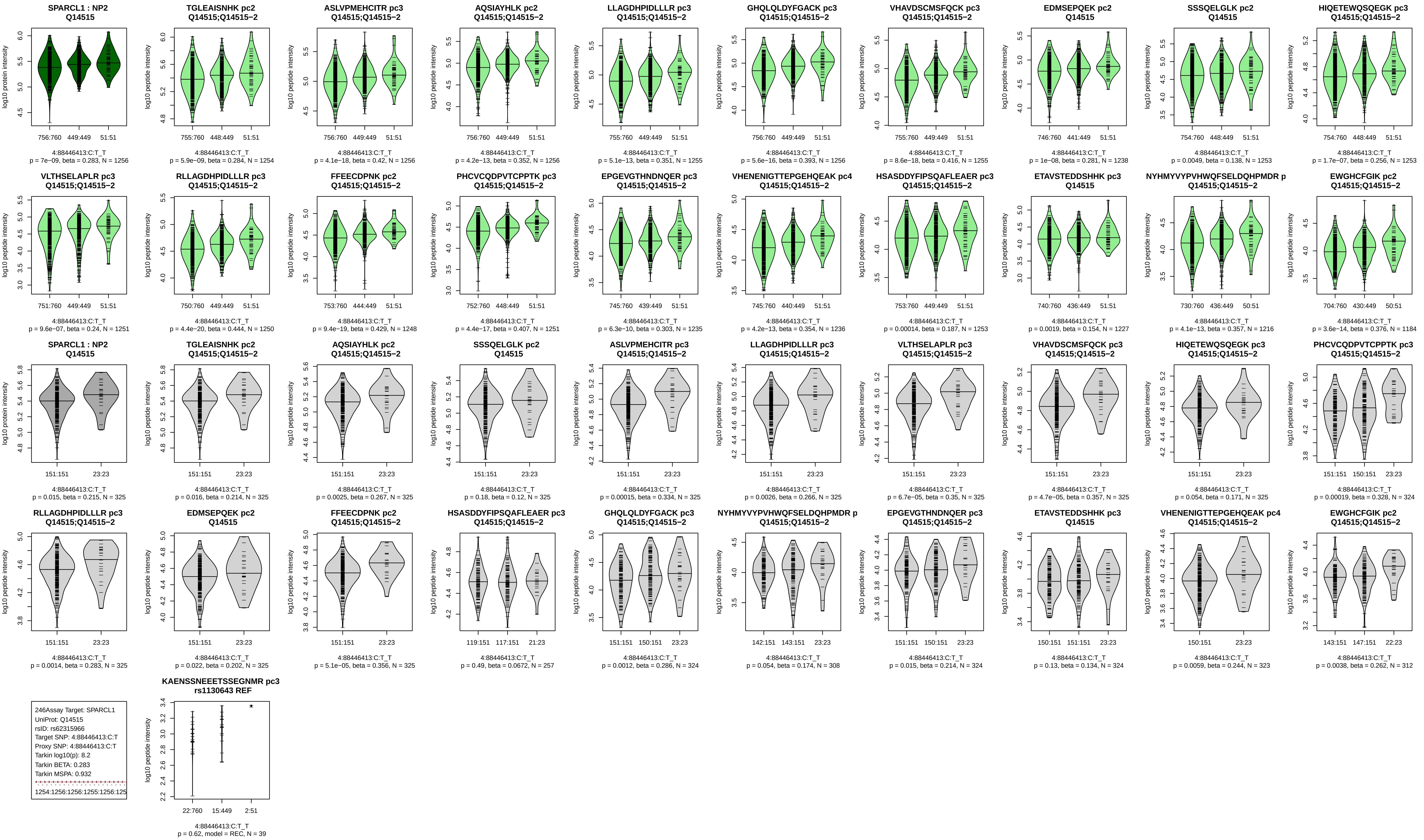
SPARCL1 : NP2
Q14515
756:760 449:449 51:51
4:88446413:C:T_T
p = 7e−09, beta = 0.283, N = 1256
TGLEAISNHK pc2
Q14515;Q14515−2
755:760 448:449 51:51
4:88446413:C:T_T
p = 5.9e−09, beta = 0.284, N = 1254
ASLVPMEHCITR pc3
Q14515;Q14515−2
756:760 449:449 51:51
4:88446413:C:T_T
p = 4.1e−18, beta = 0.42, N = 1256
AQSIAYHLK pc2
Q14515;Q14515−2
756:760 449:449 51:51
4:88446413:C:T_T
p = 4.2e−13, beta = 0.352, N = 1256
LLAGDHPIDLLLR pc3
Q14515;Q14515−2
755:760 449:449 51:51
4:88446413:C:T_T
p = 5.1e−13, beta = 0.351, N = 1255
GHQLQLDYFGACK pc3
Q14515;Q14515−2
756:760 449:449 51:51
4:88446413:C:T_T
p = 5.6e−16, beta = 0.393, N = 1256
VHAVDSCMSFQCK pc3
Q14515;Q14515−2
755:760 449:449 51:51
4:88446413:C:T_T
p = 8.6e−18, beta = 0.416, N = 1255
EDMSEPQEK pc2
Q14515
746:760 441:449 51:51
4:88446413:C:T_T
p = 1e−08, beta = 0.281, N = 1238
SSSQELGLK pc2
Q14515
754:760 448:449 51:51
4:88446413:C:T_T
p = 0.0049, beta = 0.138, N = 1253
HIQETEWQSQEGK pc3
Q14515;Q14515−2
754:760 448:449 51:51
4:88446413:C:T_T
p = 1.7e−07, beta = 0.256, N = 1253
VLTHSELAPLR pc3
Q14515;Q14515−2
751:760 449:449 51:51
4:88446413:C:T_T
p = 9.6e−07, beta = 0.24, N = 1251
RLLAGDHPIDLLLR pc3
Q14515;Q14515−2
750:760 449:449 51:51
4:88446413:C:T_T
p = 4.4e−20, beta = 0.444, N = 1250
FFEECDPNK pc2
Q14515;Q14515−2
753:760 444:449 51:51
4:88446413:C:T_T
p = 9.4e−19, beta = 0.429, N = 1248
PHCVCQDPVTCPPTK pc3
Q14515;Q14515−2
752:760 448:449 51:51
4:88446413:C:T_T
p = 4.4e−17, beta = 0.407, N = 1251
EPGEVGTHNDNQER pc3
Q14515;Q14515−2
745:760 439:449 51:51
4:88446413:C:T_T
p = 6.3e−10, beta = 0.303, N = 1235
VHENENIGTTEPGEHQEAK pc4
Q14515;Q14515−2
745:760 440:449 51:51
4:88446413:C:T_T
p = 4.2e−13, beta = 0.354, N = 1236
HSASDDYFIPSQAFLEAER pc3
Q14515;Q14515−2
753:760 449:449 51:51
4:88446413:C:T_T
p = 0.00014, beta = 0.187, N = 1253
ETAVSTEDDSHHK pc3
Q14515
740:760 436:449 51:51
4:88446413:C:T_T
p = 0.0019, beta = 0.154, N = 1227
NYHMYVYPVHWQFSELDQHPMDR p
Q14515;Q14515−2
730:760 436:449 50:51
4:88446413:C:T_T
p = 4.1e−13, beta = 0.357, N = 1216
EWGHCFGIK pc2
Q14515;Q14515−2
704:760 430:449 50:51
4:88446413:C:T_T
p = 3.6e−14, beta = 0.376, N = 1184
SPARCL1 : NP2
Q14515
151:151 23:23
4:88446413:C:T_T
p = 0.015, beta = 0.215, N = 325
TGLEAISNHK pc2
Q14515;Q14515−2
151:151 23:23
4:88446413:C:T_T
p = 0.016, beta = 0.214, N = 325
AQSIAYHLK pc2
Q14515;Q14515−2
151:151 23:23
4:88446413:C:T_T
p = 0.0025, beta = 0.267, N = 325
SSSQELGLK pc2
Q14515
151:151 23:23
4:88446413:C:T_T
p = 0.18, beta = 0.12, N = 325
ASLVPMEHCITR pc3
Q14515;Q14515−2
151:151 23:23
4:88446413:C:T_T
p = 0.00015, beta = 0.334, N = 325
LLAGDHPIDLLLR pc3
Q14515;Q14515−2
151:151 23:23
4:88446413:C:T_T
p = 0.0026, beta = 0.266, N = 325
VLTHSELAPLR pc3
Q14515;Q14515−2
151:151 23:23
4:88446413:C:T_T
p = 6.7e−05, beta = 0.35, N = 325
VHAVDSCMSFQCK pc3
Q14515;Q14515−2
151:151 23:23
4:88446413:C:T_T
p = 4.7e−05, beta = 0.357, N = 325
HIQETEWQSQEGK pc3
Q14515;Q14515−2
151:151 23:23
4:88446413:C:T_T
p = 0.054, beta = 0.171, N = 325
PHCVCQDPVTCPPTK pc3
Q14515;Q14515−2
151:151 150:151 23:23
4:88446413:C:T_T
p = 0.00019, beta = 0.328, N = 324
RLLAGDHPIDLLLR pc3
Q14515;Q14515−2
151:151 23:23
4:88446413:C:T_T
p = 0.0014, beta = 0.283, N = 325
EDMSEPQEK pc2
Q14515
151:151 23:23
4:88446413:C:T_T
p = 0.022, beta = 0.202, N = 325
FFEECDPNK pc2
Q14515;Q14515−2
151:151 23:23
4:88446413:C:T_T
p = 5.1e−05, beta = 0.356, N = 325
HSASDDYFIPSQAFLEAER pc3
Q14515;Q14515−2
119:151 117:151 21:23
4:88446413:C:T_T
p = 0.49, beta = 0.0672, N = 257
GHQLQLDYFGACK pc3
Q14515;Q14515−2
151:151 150:151 23:23
4:88446413:C:T_T
p = 0.0012, beta = 0.286, N = 324
NYHMYVYPVHWQFSELDQHPMDR p
Q14515;Q14515−2
142:151 143:151 23:23
4:88446413:C:T_T
p = 0.054, beta = 0.174, N = 308
EPGEVGTHNDNQER pc3
Q14515;Q14515−2
151:151 150:151 23:23
4:88446413:C:T_T
p = 0.015, beta = 0.214, N = 324
ETAVSTEDDSHHK pc3
Q14515
150:151 151:151 23:23
4:88446413:C:T_T
p = 0.13, beta = 0.134, N = 324
VHENENIGTTEPGEHQEAK pc4
Q14515;Q14515−2
150:151 23:23
4:88446413:C:T_T
p = 0.0059, beta = 0.244, N = 323
EWGHCFGIK pc2
Q14515;Q14515−2
143:151 147:151 22:23
4:88446413:C:T_T
p = 0.0038, beta = 0.262, N = 312
246Assay Target: SPARCL1
UniProt: Q14515
rsID: rs62315966
Target SNP: 4:88446413:C:T
Proxy SNP: 4:88446413:C:T
Tarkin log10(p): 8.2
Tarkin BETA: 0.283
Tarkin MSPA: 0.932
1254:1256:1256:1255:1256:125
KAENSSNEEETSSEGNMR pc3
rs1130643 REF
22:760 15:449 2:51
4:88446413:C:T_T
p = 0.62, model = REC, N = 39
log10 protein intensity
log10 peptide intensity

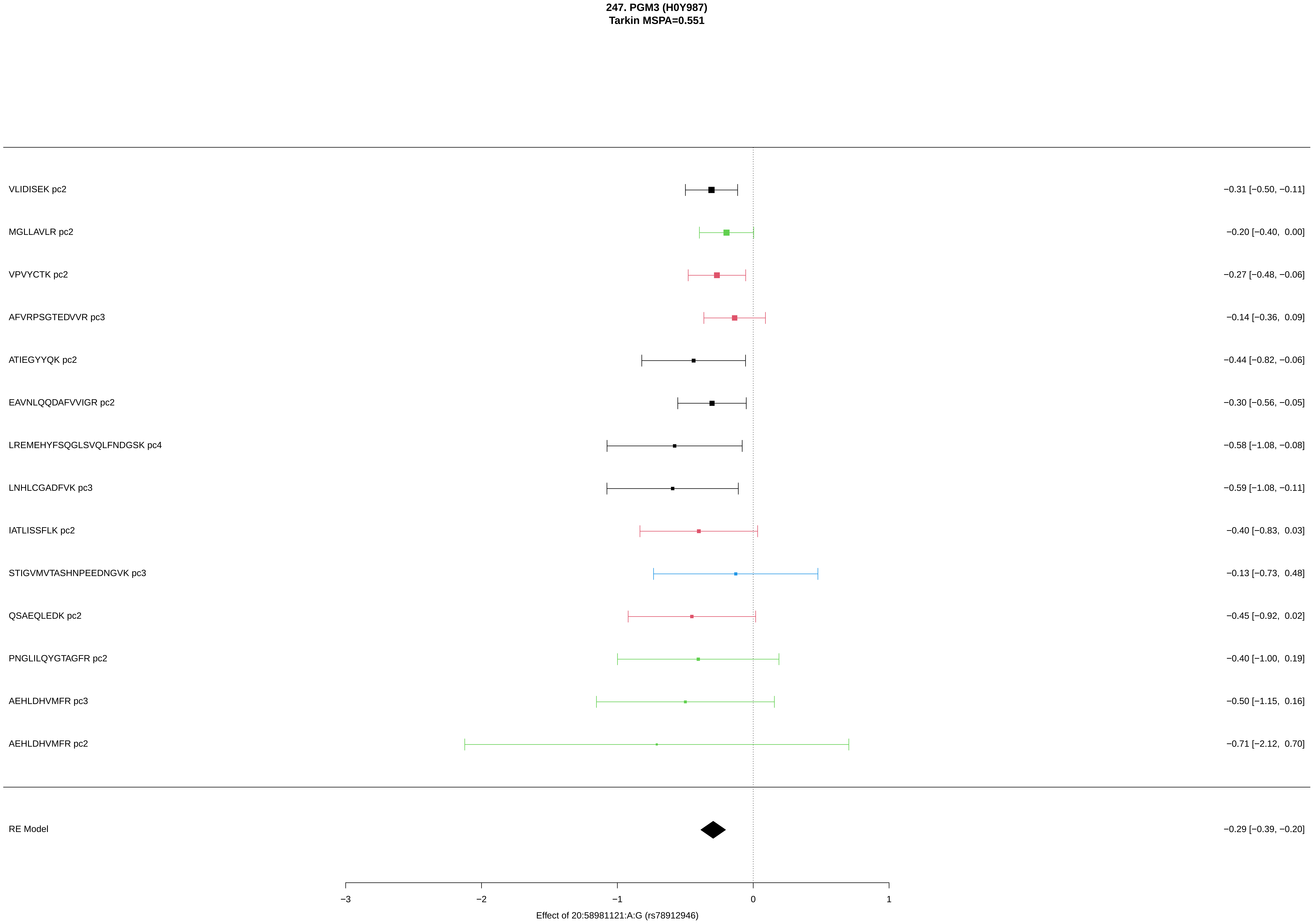
247. PGM3 (H0Y987)
Tarkin MSPA=0.551
VLIDISEK pc2
MGLLAVLR pc2
VPVYCTK pc2
AFVRPSGTEDVVR pc3
ATIEGYYQK pc2
EAVNLQQDAFVVIGR pc2
LREMEHYFSQGLSVQLFNDGSK pc4
LNHLCGADFVK pc3
IATLISSFLK pc2
STIGVMVTASHNPEEDNGVK pc3
QSAEQLEDK pc2
PNGLILQYGTAGFR pc2
AEHLDHVMFR pc3
AEHLDHVMFR pc2
RE Model
−0.31 [−0.50, −0.11]
−0.20 [−0.40, 0.00]
−0.27 [−0.48, −0.06]
−0.14 [−0.36, 0.09]
−0.44 [−0.82, −0.06]
−0.30 [−0.56, −0.05]
−0.58 [−1.08, −0.08]
−0.59 [−1.08, −0.11]
−0.40 [−0.83, 0.03]
−0.13 [−0.73, 0.48]
−0.45 [−0.92, 0.02]
−0.40 [−1.00, 0.19]
−0.50 [−1.15, 0.16]
−0.71 [−2.12, 0.70]
−0.29 [−0.39, −0.20]
−3
−2
−1
0
1
Effect of 20:58981121:A:G (rs78912946)

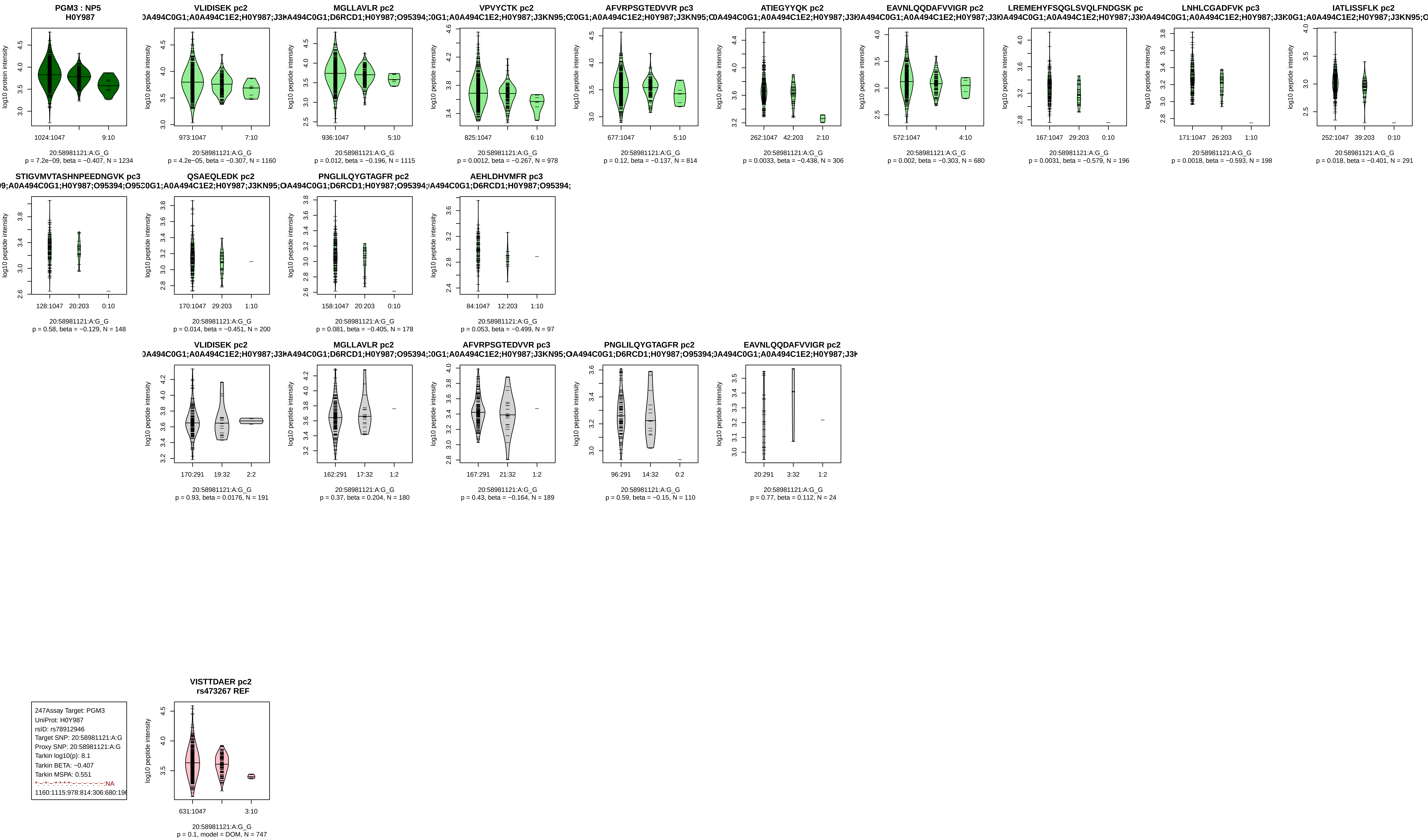
PGM3 : NP5
H0Y987
log10 protein intensity
1024:1047
9:10
20:58981121:A:G_G
p = 7.2e−09, beta = −0.407, N = 1234
VLIDISEK pc2
973:1047
7:10
p = 4.2e−05, beta = −0.307, N = 1160
MGLLAVLR pc2
936:1047
5:10
p = 0.012, beta = −0.196, N = 1115
VPVYCTK pc2
825:1047
6:10
p = 0.0012, beta = −0.267, N = 978
AFVRPSGTEDVVR pc3
677:1047
5:10
p = 0.12, beta = −0.137, N = 814
ATIEGYYQK pc2
262:1047
42:203
2:10
p = 0.0033, beta = −0.438, N = 306
EAVNLQQDAFVVIGR pc2
572:1047
4:10
p = 0.002, beta = −0.303, N = 680
LREMEHYFSQGLSVQLFNDGSK pc
167:1047
29:203
0:10
p = 0.0031, beta = −0.579, N = 196
LNHLCGADFVK pc3
171:1047
26:203
1:10
p = 0.0018, beta = −0.593, N = 198
IATLISSFLK pc2
252:1047
39:203
0:10
p = 0.018, beta = −0.401, N = 291
STIGVMVTASHNPEEDNGVK pc3
128:1047
20:203
0:10
p = 0.58, beta = −0.129, N = 148
QSAEQLEDK pc2
170:1047
29:203
1:10
p = 0.014, beta = −0.451, N = 200
PNGLILQYGTAGFR pc2
158:1047
20:203
0:10
p = 0.081, beta = −0.405, N = 178
AEHLDHVMFR pc3
84:1047
12:203
1:10
p = 0.053, beta = −0.499, N = 97
VLIDISEK pc2
170:291
19:32
2:2
p = 0.93, beta = 0.0176, N = 191
MGLLAVLR pc2
162:291
17:32
1:2
p = 0.37, beta = 0.204, N = 180
AFVRPSGTEDVVR pc3
167:291
21:32
1:2
p = 0.43, beta = −0.164, N = 189
PNGLILQYGTAGFR pc2
96:291
14:32
0:2
p = 0.59, beta = −0.15, N = 110
EAVNLQQDAFVVIGR pc2
20:291
3:32
1:2
p = 0.77, beta = 0.112, N = 24
log10 peptide intensity
247Assay Target: PGM3
UniProt: H0Y987
rsID: rs78912946
Target SNP: 20:58981121:A:G
Proxy SNP: 20:58981121:A:G
Tarkin log10(p): 8.1
Tarkin BETA: −0.407
Tarkin MSPA: 0.551
VISTTDAER pc2
rs473267 REF
631:1047
3:10
p = 0.1, model = DOM, N = 747

248. CALCOCO1 (Q9P1Z2)
Tarkin MSPA=0.553

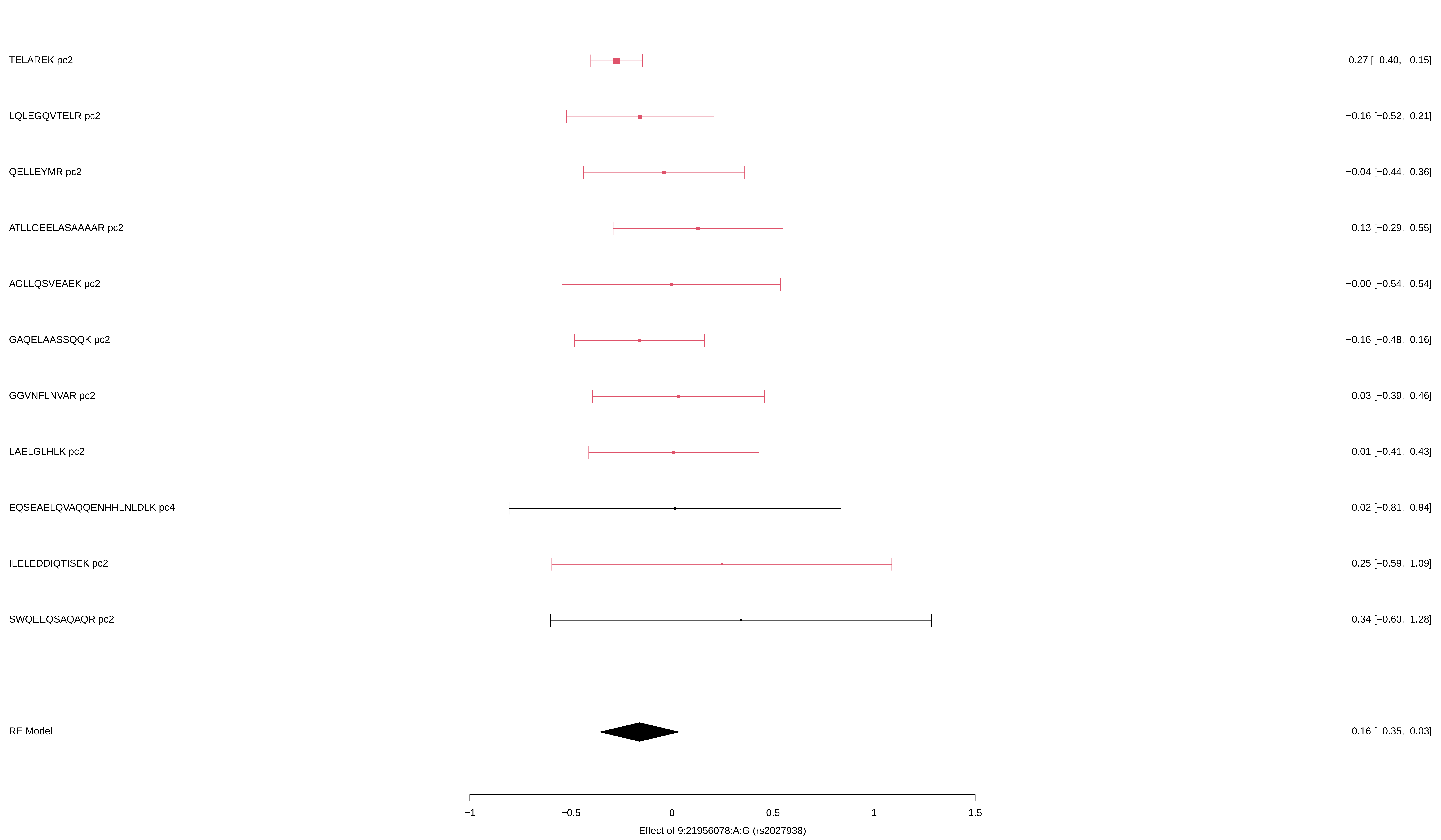

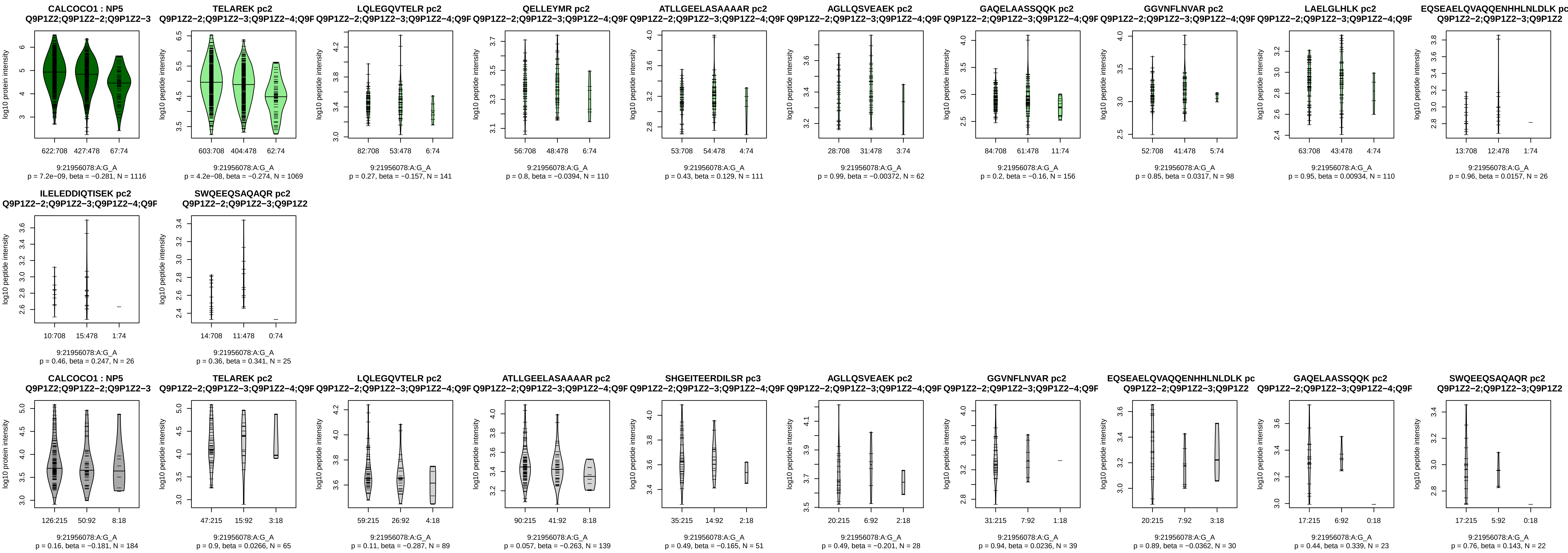

248Assay Target: CALCOCO1
UniProt: Q9P1Z2
rsID: rs2027938
Target SNP: 9:21956078:A:G
Proxy SNP: 9:21956078:A:G
Tarkin log10(p): 8.1
Tarkin BETA: −0.281
Tarkin MSPA: 0.553
*:−:−:−:−:−:−:−:−:−:NA:NA:NA
1069:141:110:111:62:156:98:11

249. TTBK1 (X6R456)
Tarkin MSPA=0.816

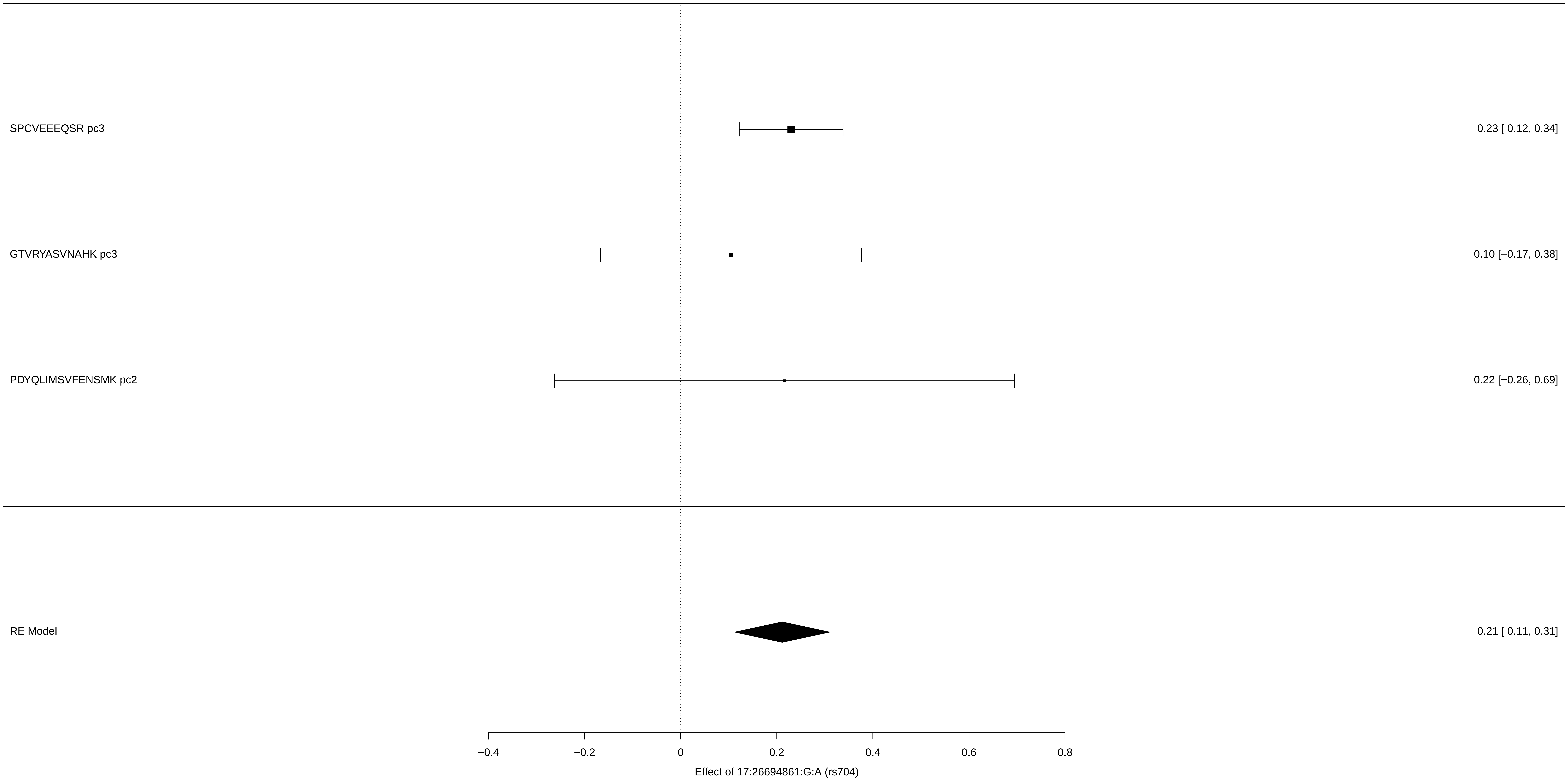

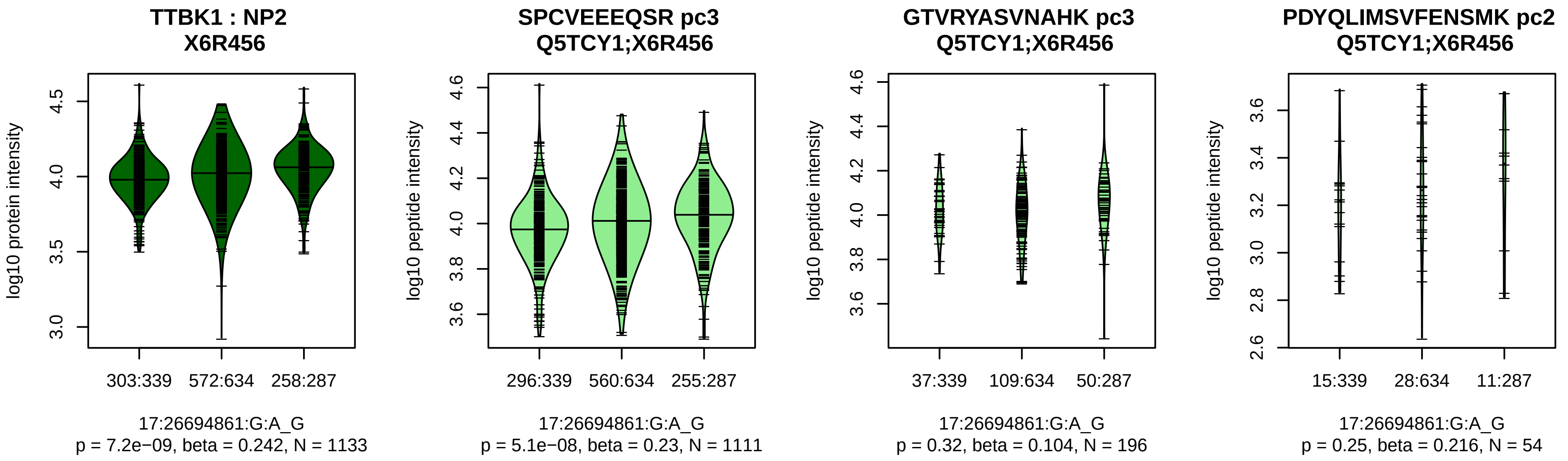

249Assay Target: TTBK1
UniProt: X6R456
rsID: rs704
Target SNP: 17:26694861:G:A
Proxy SNP: 17:26694861:G:A
Tarkin log10(p): 8.1
Tarkin BETA: 0.242
Tarkin MSPA: 0.816
*:−:−
1111:196:54

250. DNAJA4 (Q8WW22)
Tarkin MSPA=0.268

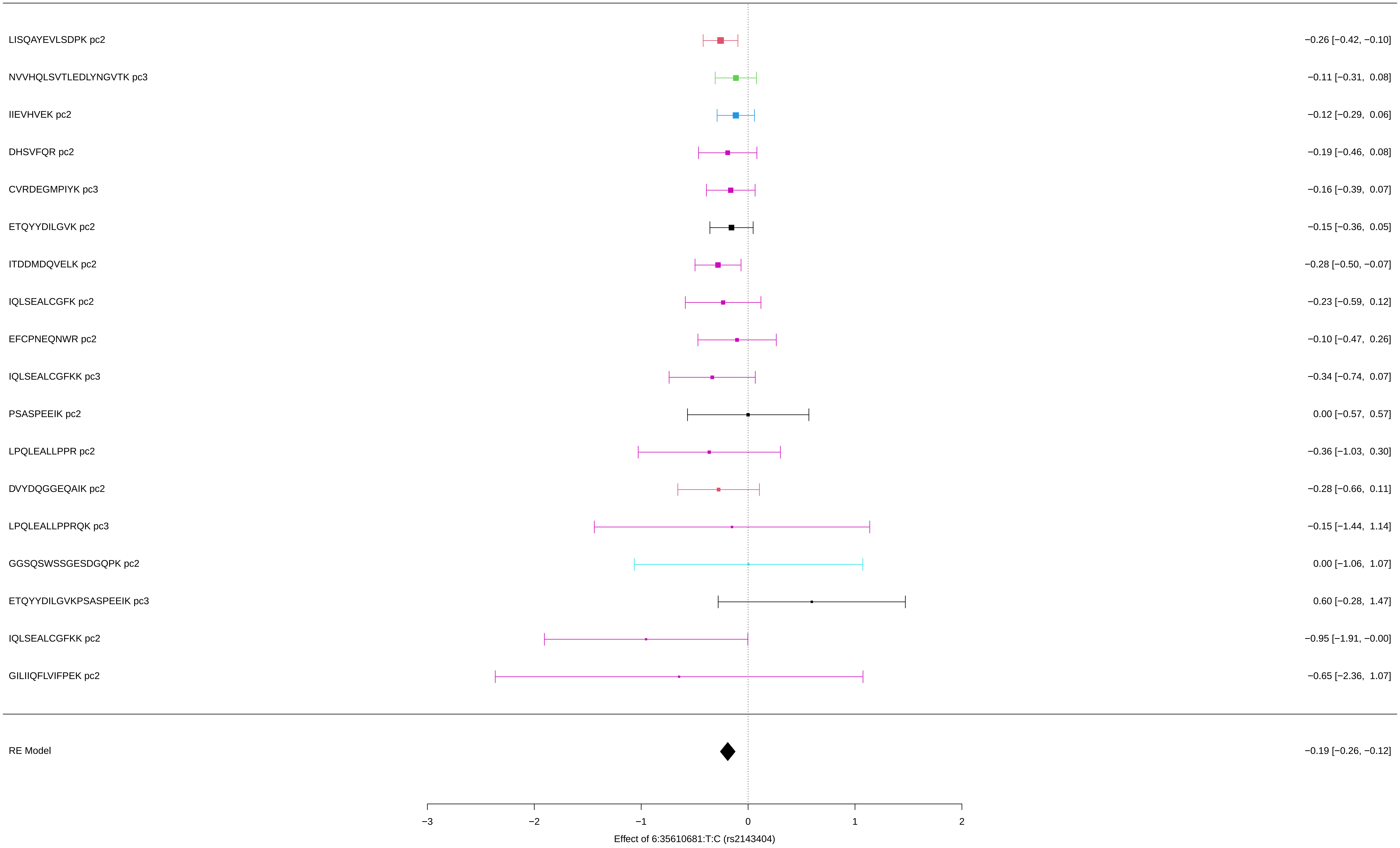

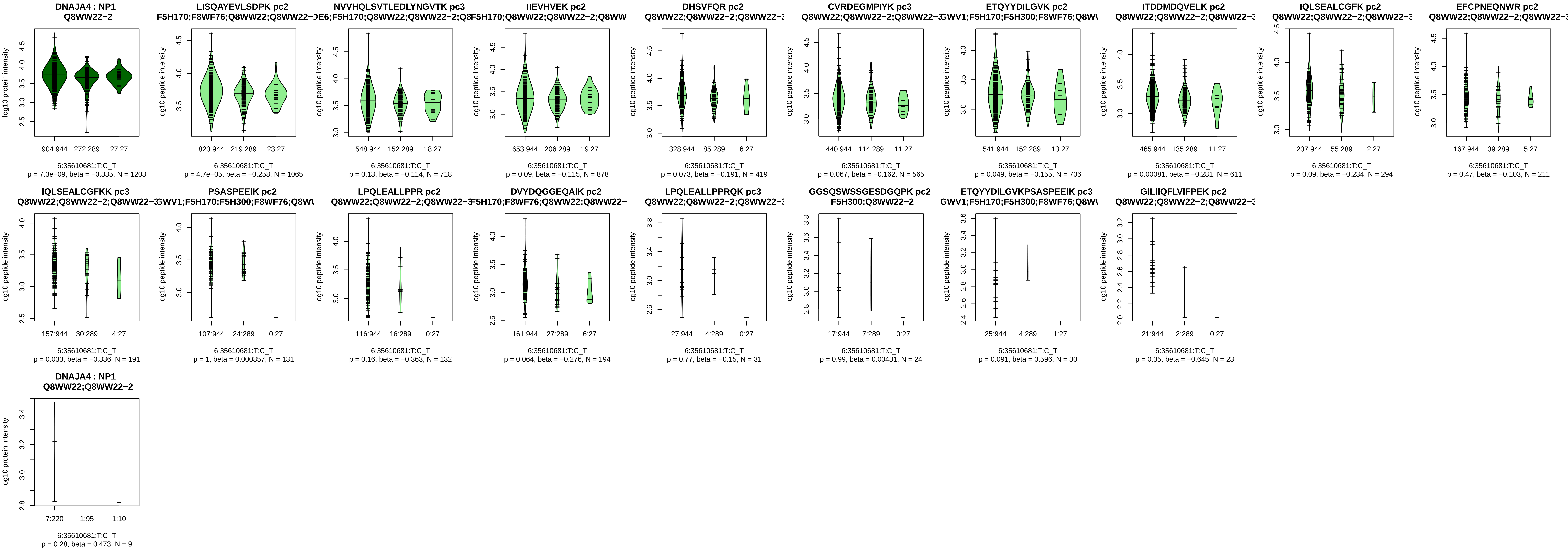

250Assay Target: DNAJA4
UniProt: Q8WW22
rsID: rs2143404
Target SNP: 6:35610681:T:C
Proxy SNP: 6:35610681:T:C
Tarkin log10(p): 8.1
Tarkin BETA: −0.335
Tarkin MSPA: 0.268
:−:−:−:−:−::−:−:−:−:−:−:−:−:−:−:
1065:718:878:419:565:706:611:

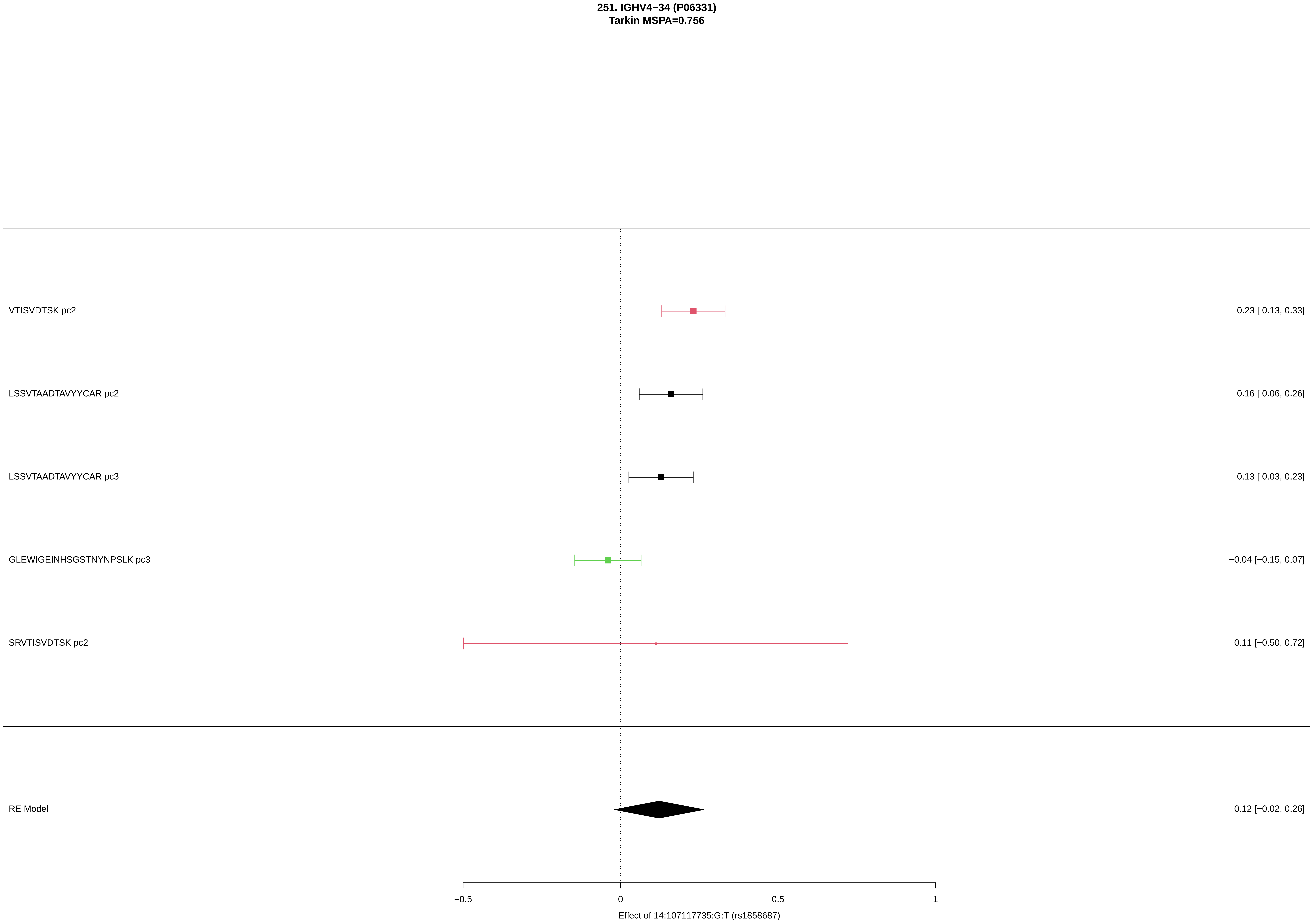
251. IGHV4−34 (P06331)
Tarkin MSPA=0.756
VTISVDTSK pc2
0.23 [0.13, 0.33]
LSSVTAADTAVYYCAR pc2
0.16 [0.06, 0.26]
LSSVTAADTAVYYCAR pc3
0.13 [0.03, 0.23]
GLEWIGEINHSGSTNYNPSLK pc3
−0.04 [−0.15, 0.07]
SRVTISVDTSK pc2
0.11 [−0.50, 0.72]
RE Model
0.12 [−0.02, 0.26]
−0.5
0
0.5
1
Effect of 14:107117735:G:T (rs1858687)

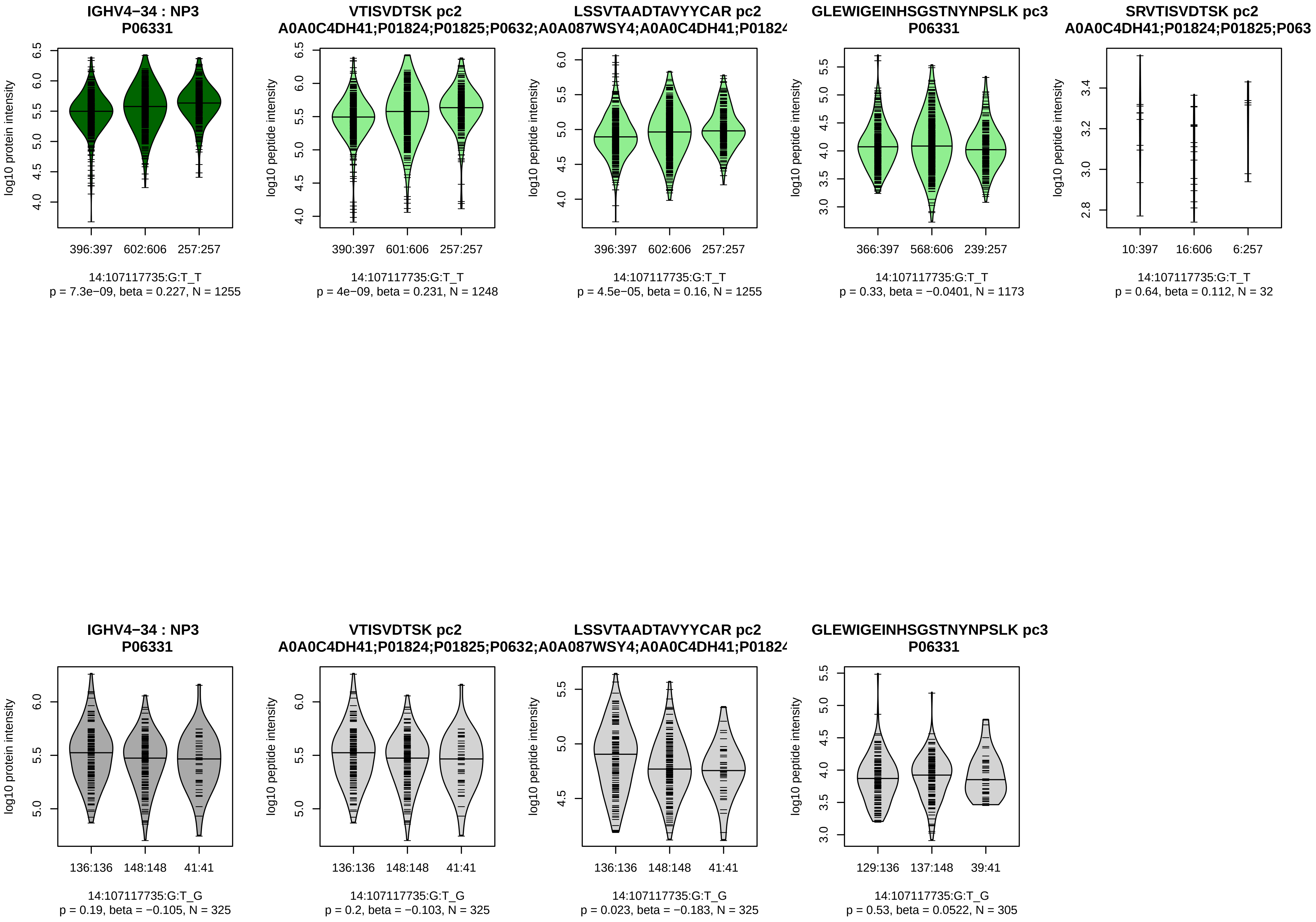

251Assay Target: IGHV4−34
UniProt: P06331
rsID: rs1858687
Target SNP: 14:107117735:G:T
Proxy SNP: 14:107117735:G:T
Tarkin log10(p): 8.1
Tarkin BETA: 0.227
Tarkin MSPA: 0.756
::*:−:NA:−
1248:1255:1227:1173:6:32

252. FCER2 (P06734)
Tarkin MSPA=0.937

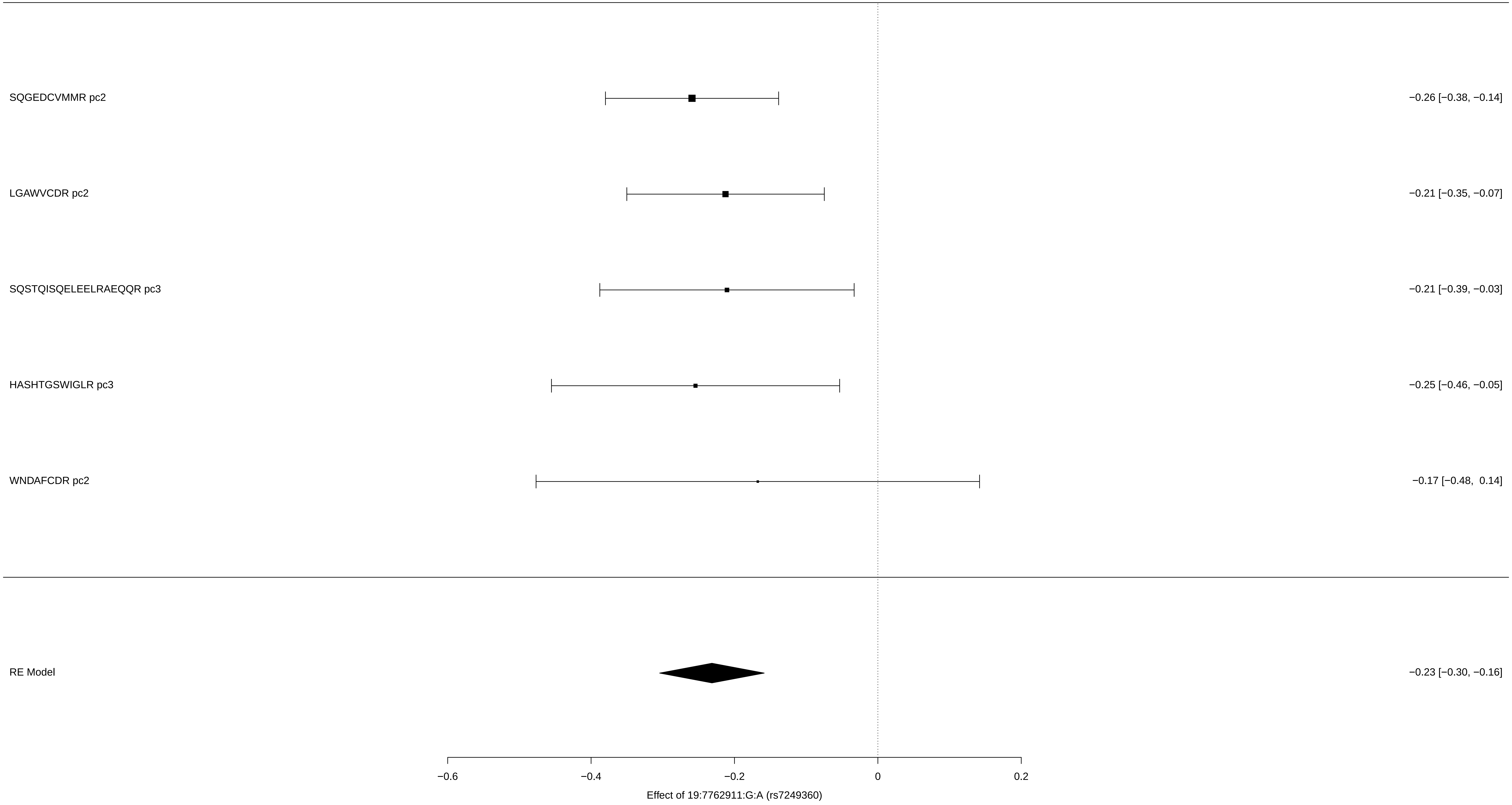

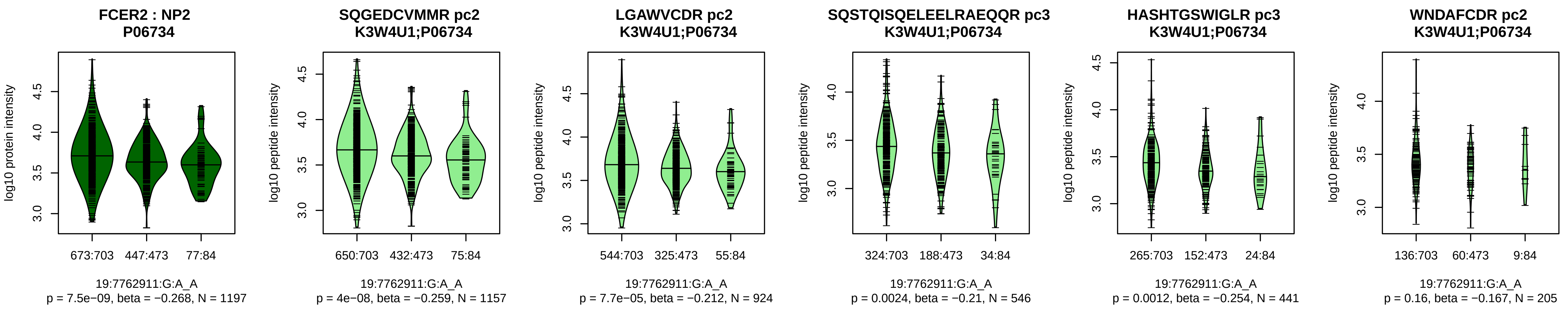

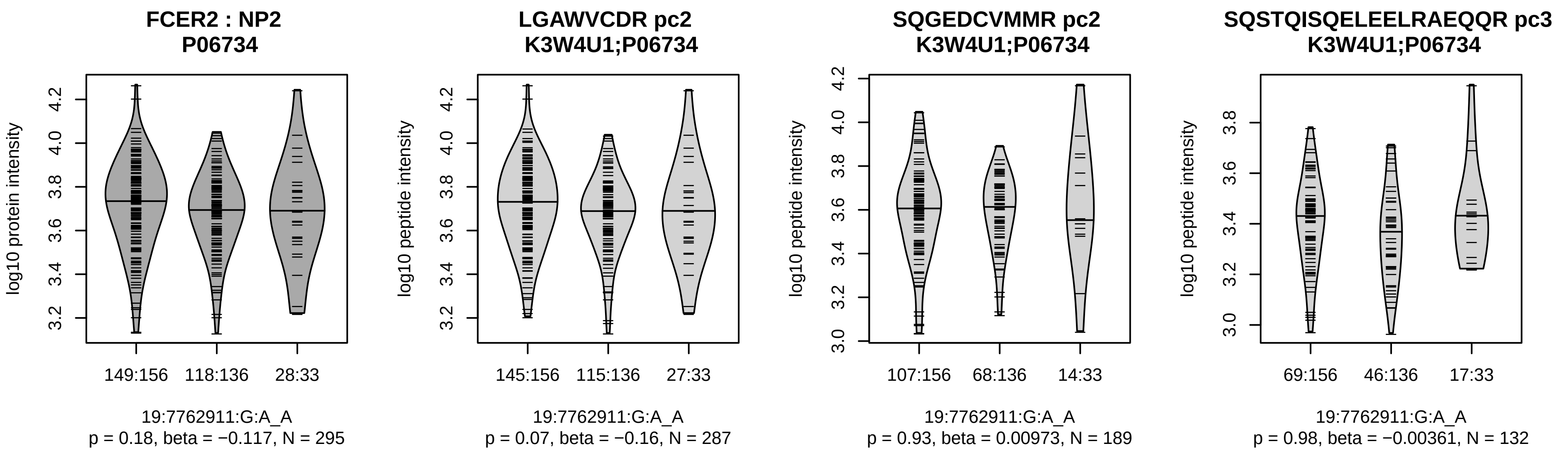

252Assay Target: FCER2
UniProt: P06734
rsID: rs7249360
Target SNP: 19:7762911:G:A
Proxy SNP: 19:7762911:G:A
Tarkin log10(p): 8.1
Tarkin BETA: −0.268
Tarkin MSPA: 0.937
::*:*:–
1157:924:546:441:205

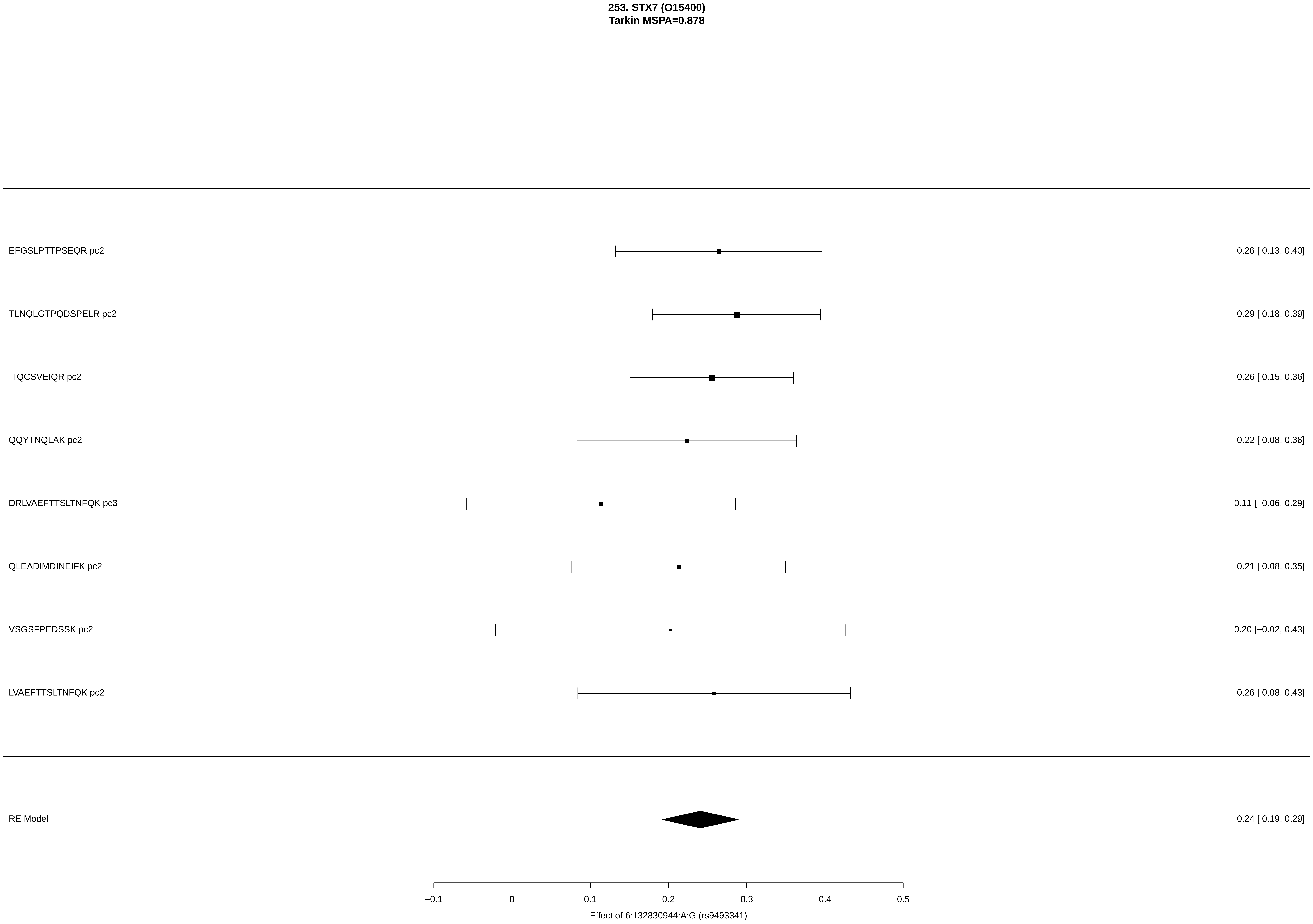
253. STX7 (O15400)
Tarkin MSPA=0.878
EFGSLPTTPSEQR pc2
0.26 [0.13, 0.40]
TLNQLGTPQDSPELR pc2
0.29 [0.18, 0.39]
ITQCSVEIQR pc2
0.26 [0.15, 0.36]
QQYTNQLAK pc2
0.22 [0.08, 0.36]
DRLVAEFTTSLTNFQK pc3
0.11 [−0.06, 0.29]
QLEADIMDINEIFK pc2
0.21 [0.08, 0.35]
VSGSFPEDSSK pc2
0.20 [−0.02, 0.43]
LVAEFTTSLTNFQK pc2
0.26 [0.08, 0.43]
RE Model
0.24 [0.19, 0.29]
−0.1
0
0.1
0.2
0.3
0.4
0.5
Effect of 6:132830944:A:G (rs9493341)

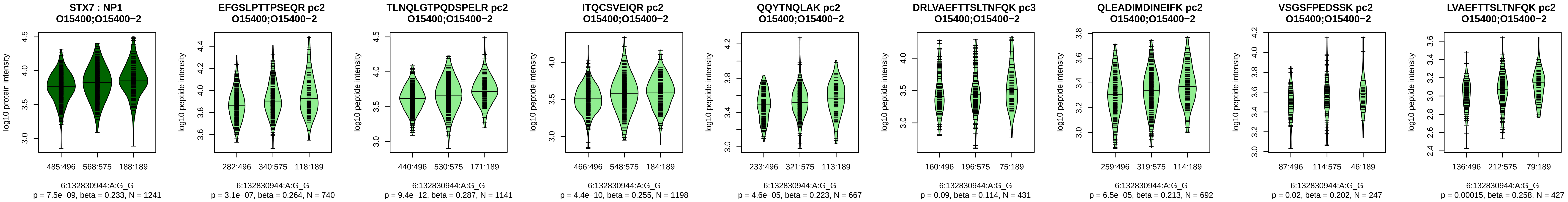

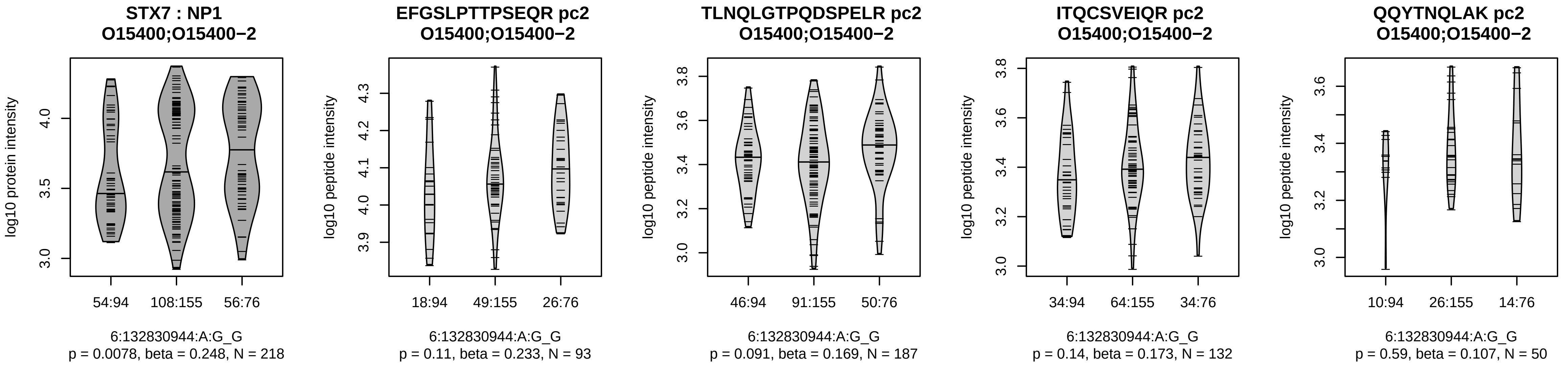

253Assay Target: STX7
UniProt: O15400
rsID: rs9493341
Target SNP: 6:132830944:A:G
Proxy SNP: 6:132830944:A:G
Tarkin log10(p): 8.1
Tarkin BETA: 0.233
Tarkin MSPA: 0.878
::*:*:–:*:–:*
740:1141:1198:667:431:692:247

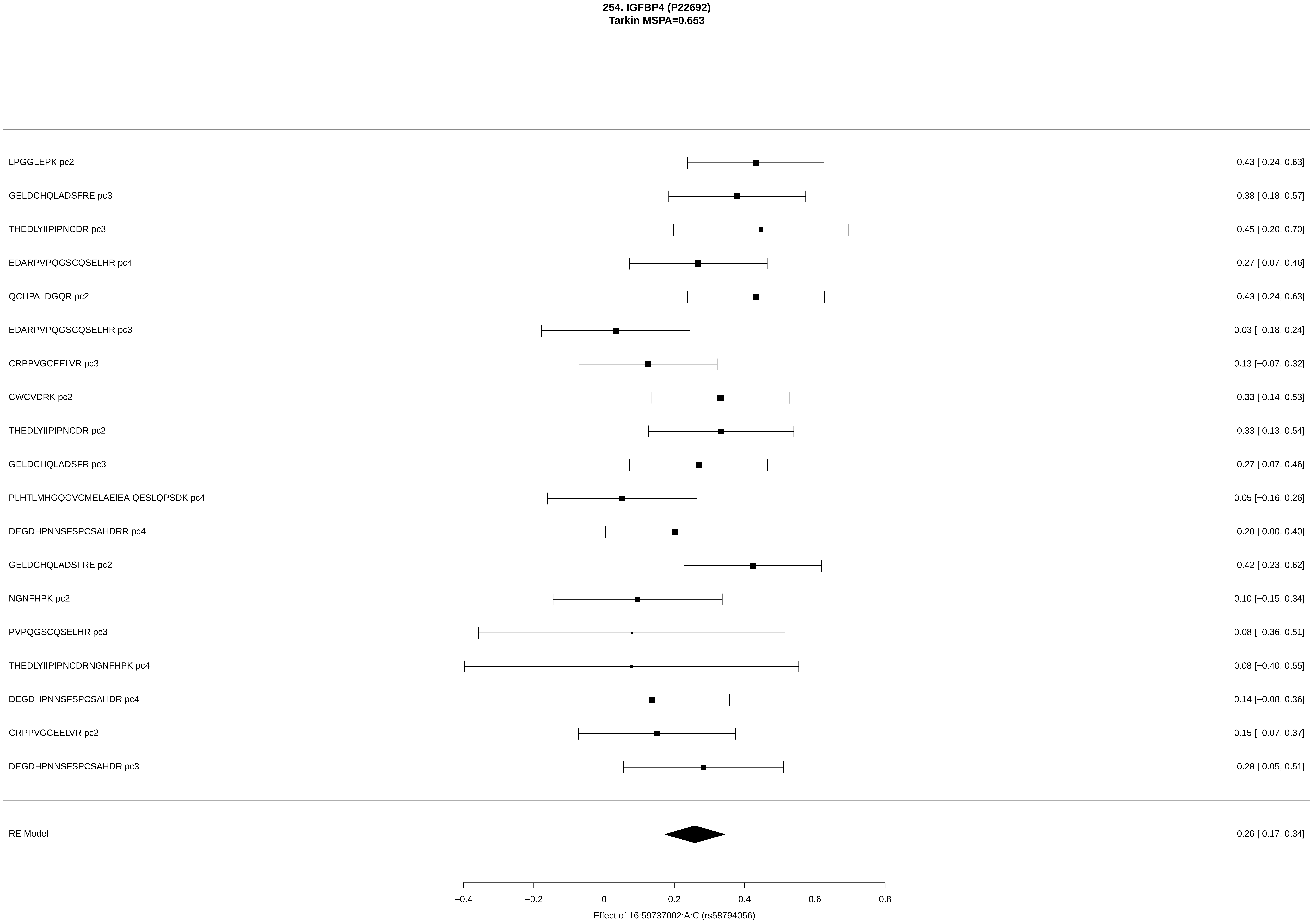
254. IGFBP4 (P22692)
Tarkin MSPA=0.653
LPGGLEPK pc2
0.43 [0.24, 0.63]
GELDCHQLADSFRE pc3
0.38 [0.18, 0.57]
THEDLYIIPIPNCDR pc3
0.45 [0.20, 0.70]
EDARPVPQGSCQSELHR pc4
0.27 [0.07, 0.46]
QCHPALDGQR pc2
0.43 [0.24, 0.63]
EDARPVPQGSCQSELHR pc3
0.03 [−0.18, 0.24]
CRPPVGCEELVR pc3
0.13 [−0.07, 0.32]
CWCVDRK pc2
0.33 [0.14, 0.53]
THEDLYIIPIPNCDR pc2
0.33 [0.13, 0.54]
GELDCHQLADSFR pc3
0.27 [0.07, 0.46]
PLHTLMHGQGVCMELAEIEAIQESLQPSDK pc4
0.05 [−0.16, 0.26]
DEGDHPNNSFSPCSAHDRR pc4
0.20 [0.00, 0.40]
GELDCHQLADSFRE pc2
0.42 [0.23, 0.62]
NGNFHPK pc2
0.10 [−0.15, 0.34]
PVPQGSCQSELHR pc3
0.08 [−0.36, 0.51]
THEDLYIIPIPNCDRNGNFHPK pc4
0.08 [−0.40, 0.55]
DEGDHPNNSFSPCSAHDR pc4
0.14 [−0.08, 0.36]
CRPPVGCEELVR pc2
0.15 [−0.07, 0.37]
DEGDHPNNSFSPCSAHDR pc3
0.28 [0.05, 0.51]
RE Model
0.26 [0.17, 0.34]
−0.4
−0.2
0
0.2
0.4
0.6
0.8
Effect of 16:59737002:A:C (rs58794056)

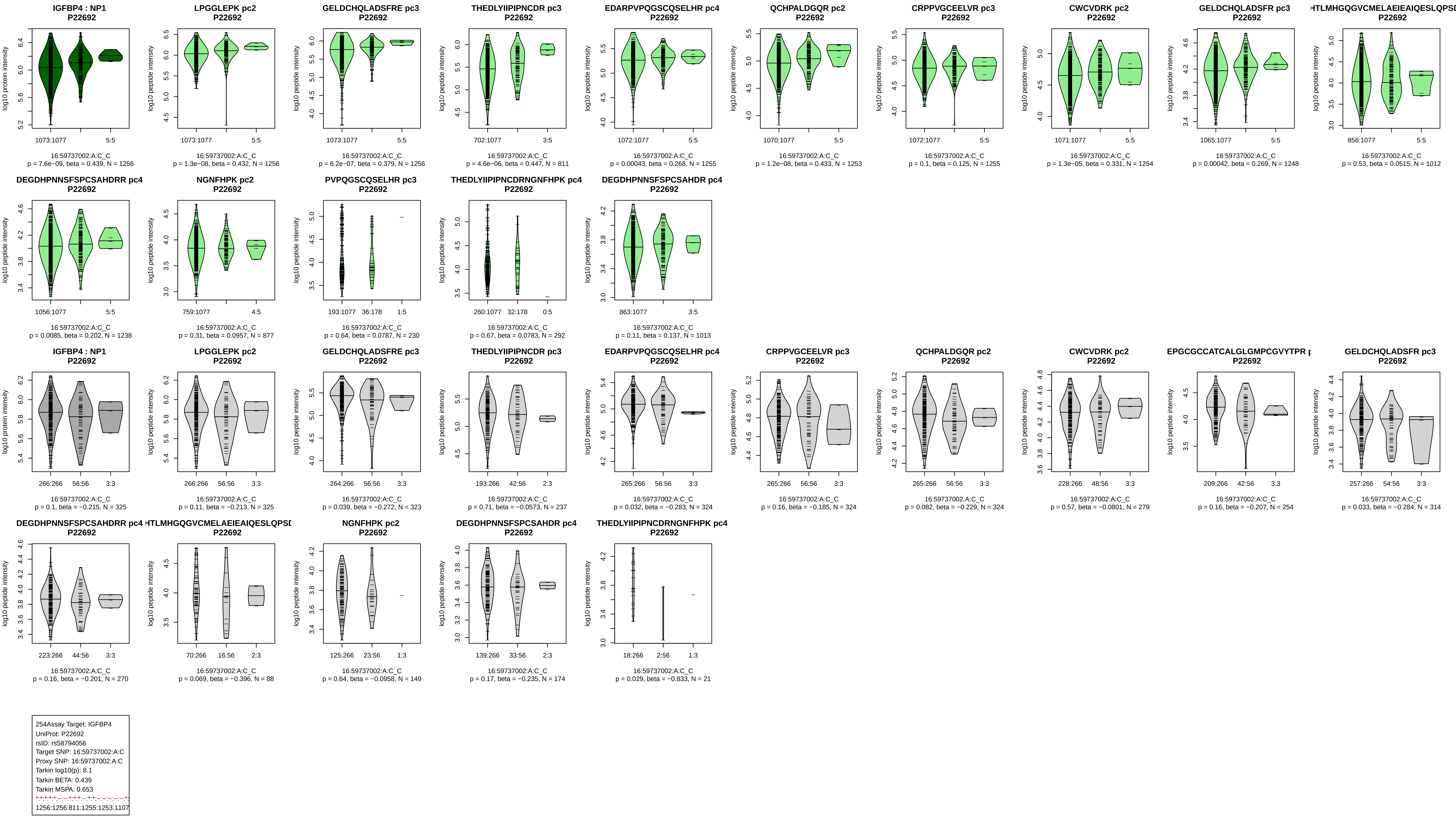
IGFBP4 : NP1
P22692
log10 protein intensity
1073:1077
5:5
16:59737002:A:C_C
p = 7.6e−09, beta = 0.439, N = 1256
LPGGLEPK pc2
P22692
log10 peptide intensity
1073:1077
5:5
16:59737002:A:C_C
p = 1.3e−08, beta = 0.432, N = 1256
GELDCHQLADSFRE pc3
P22692
1073:1077
5:5
16:59737002:A:C_C
p = 6.2e−07, beta = 0.379, N = 1256
THEDLYIIPIPNCDR pc3
P22692
702:1077
3:5
16:59737002:A:C_C
p = 4.6e−06, beta = 0.447, N = 811
EDARPVPQGSCQSELHR pc4
P22692
1072:1077
5:5
16:59737002:A:C_C
p = 0.00043, beta = 0.268, N = 1255
QCHPALDGQR pc2
P22692
1070:1077
5:5
16:59737002:A:C_C
p = 1.2e−08, beta = 0.433, N = 1253
CRPPVGCEELVR pc3
P22692
1072:1077
5:5
16:59737002:A:C_C
p = 0.1, beta = 0.125, N = 1255
CWCVDRK pc2
P22692
1071:1077
5:5
16:59737002:A:C_C
p = 1.3e−05, beta = 0.331, N = 1254
GELDCHQLADSFR pc3
P22692
1065:1077
5:5
16:59737002:A:C_C
p = 0.00042, beta = 0.269, N = 1248
HTLMHGQGVCMELAEIEAIQESLQPSD
P22692
856:1077
5:5
16:59737002:A:C_C
p = 0.53, beta = 0.0515, N = 1012
DEGDHPNNSFSPCSAHDRR pc4
P22692
1056:1077
5:5
16:59737002:A:C_C
p = 0.0085, beta = 0.202, N = 1238
NGNFHPK pc2
P22692
759:1077
4:5
16:59737002:A:C_C
p = 0.31, beta = 0.0957, N = 877
PVPQGSCQSELHR pc3
P22692
193:1077
36:178
1:5
16:59737002:A:C_C
p = 0.64, beta = 0.0787, N = 230
THEDLYIIPIPNCDRNGNFHPK pc4
P22692
260:1077
32:178
0:5
16:59737002:A:C_C
p = 0.67, beta = 0.0783, N = 292
DEGDHPNNSFSPCSAHDR pc4
P22692
863:1077
3:5
16:59737002:A:C_C
p = 0.11, beta = 0.137, N = 1013
IGFBP4 : NP1
P22692
266:266
56:56
3:3
16:59737002:A:C_C
p = 0.1, beta = −0.215, N = 325
LPGGLEPK pc2
P22692
266:266
56:56
3:3
16:59737002:A:C_C
p = 0.11, beta = −0.213, N = 325
GELDCHQLADSFRE pc3
P22692
264:266
56:56
3:3
16:59737002:A:C_C
p = 0.039, beta = −0.272, N = 323
THEDLYIIPIPNCDR pc3
P22692
193:266
42:56
2:3
16:59737002:A:C_C
p = 0.71, beta = −0.0573, N = 237
EDARPVPQGSCQSELHR pc4
P22692
265:266
56:56
3:3
16:59737002:A:C_C
p = 0.032, beta = −0.283, N = 324
CRPPVGCEELVR pc3
P22692
265:266
56:56
3:3
16:59737002:A:C_C
p = 0.16, beta = −0.185, N = 324
QCHPALDGQR pc2
P22692
265:266
56:56
3:3
16:59737002:A:C_C
p = 0.082, beta = −0.229, N = 324
CWCVDRK pc2
P22692
228:266
48:56
3:3
16:59737002:A:C_C
p = 0.57, beta = −0.0801, N = 279
EPGCGCCATCALGLGMPCGVYTPR p
P22692
209:266
42:56
3:3
16:59737002:A:C_C
p = 0.16, beta = −0.207, N = 254
GELDCHQLADSFR pc3
P22692
257:266
54:56
3:3
16:59737002:A:C_C
p = 0.033, beta = −0.284, N = 314
DEGDHPNNSFSPCSAHDRR pc4
P22692
223:266
44:56
3:3
16:59737002:A:C_C
p = 0.16, beta = −0.201, N = 270
HTLMHGQGVCMELAEIEAIQESLQPSD
P22692
70:266
16:56
2:3
16:59737002:A:C_C
p = 0.069, beta = −0.396, N = 88
NGNFHPK pc2
P22692
125:266
23:56
1:3
16:59737002:A:C_C
p = 0.64, beta = −0.0958, N = 149
DEGDHPNNSFSPCSAHDR pc4
P22692
139:266
33:56
2:3
16:59737002:A:C_C
p = 0.17, beta = −0.235, N = 174
THEDLYIIPIPNCDRNGNFHPK pc4
P22692
18:266
2:56
1:3
16:59737002:A:C_C
p = 0.029, beta = −0.833, N = 21
254Assay Target: IGFBP4
UniProt: P22692
rsID: rs58794056
Target SNP: 16:59737002:A:C
Proxy SNP: 16:59737002:A:C
Tarkin log10(p): 8.1
Tarkin BETA: 0.439
Tarkin MSPA: 0.653
1256:1256:811:1255:1253:1107

255. GLO1 (Q04760)
Tarkin MSPA=0.913

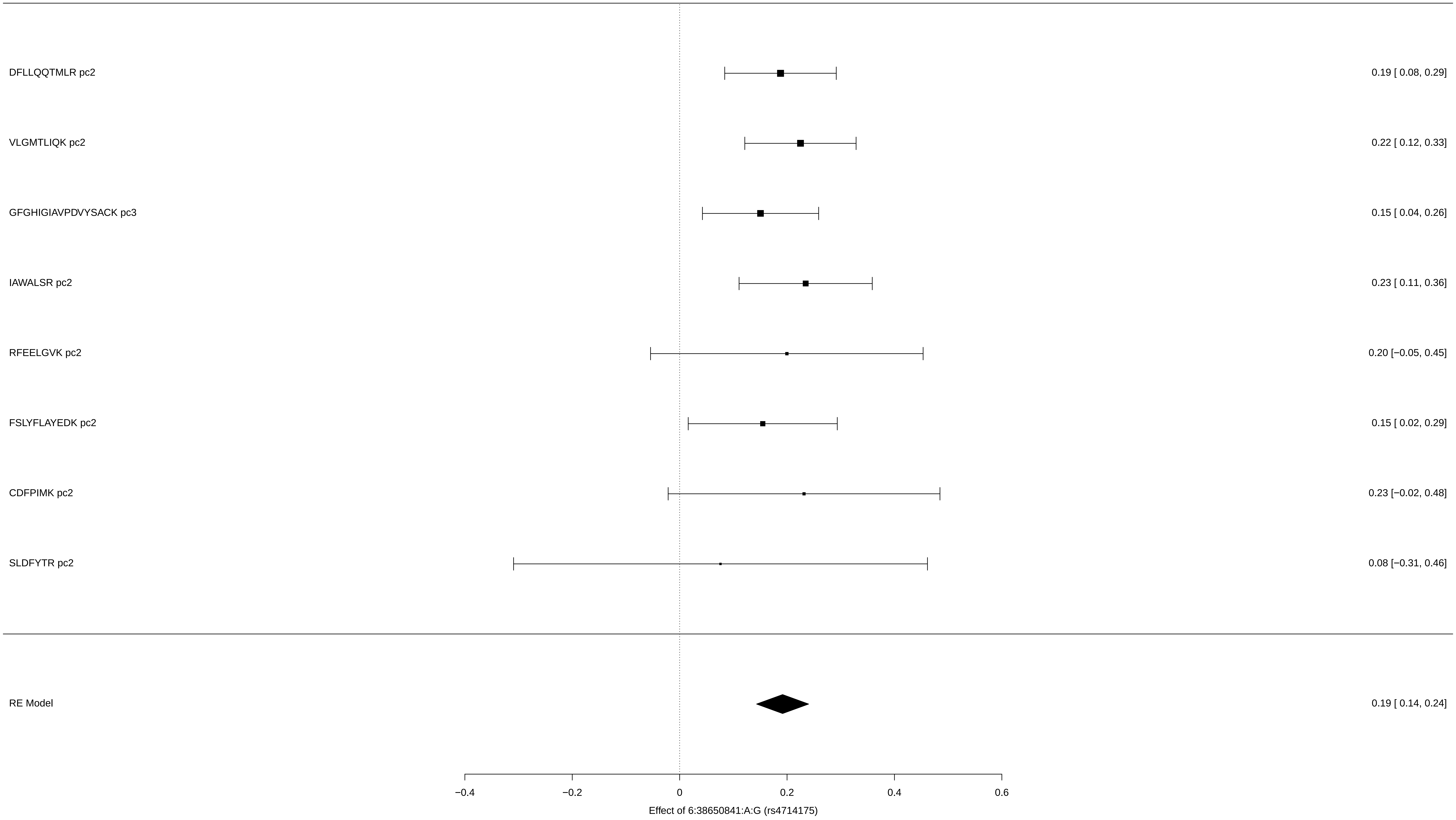

GLO1 : NP2
Q04760

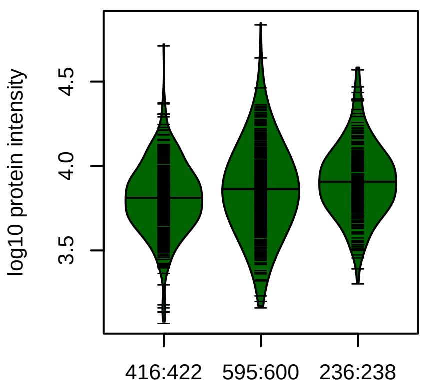

6:38650841:A:G_A
p = 7.9e−09, beta = 0.228, N = 1247

DFLLQQTMLR pc2
Q04760

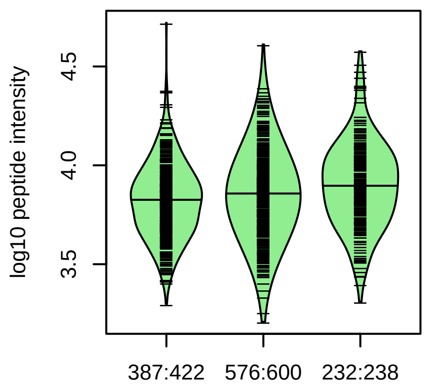

6:38650841:A:G_A
p = 3.6e−06, beta = 0.188, N = 1195

VLGMTLIQK pc2
Q04760

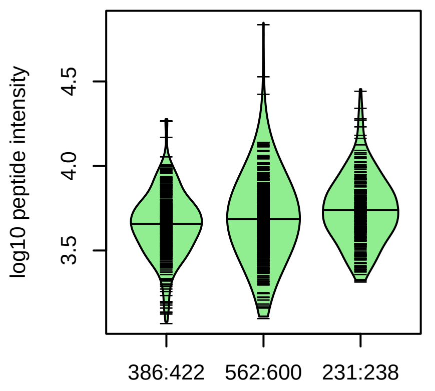

6:38650841:A:G_A
p = 2.8e−08, beta = 0.225, N = 1179

GFGHIGIAVPDVYSACK pc3
Q04760

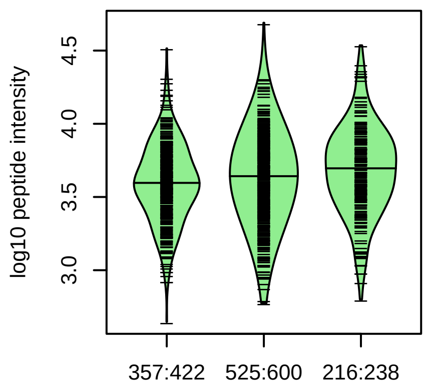

6:38650841:A:G_A
p = 0.00035, beta = 0.151, N = 1098

IAWALSR pc2
Q04760

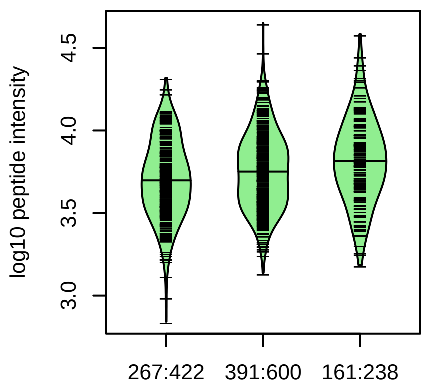

6:38650841:A:G_A
p = 1.3e−06, beta = 0.235, N = 819

RFEELGVK pc2
Q04760

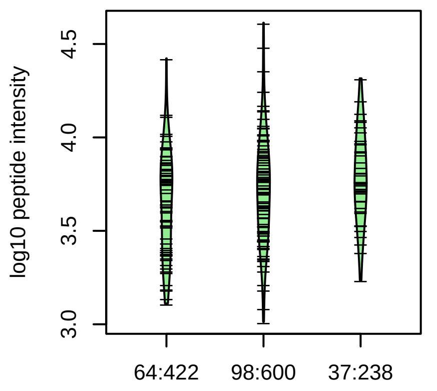

6:38650841:A:G_A
p = 0.044, beta = 0.2, N = 199

FSLYFLAYEDK pc2
Q04760

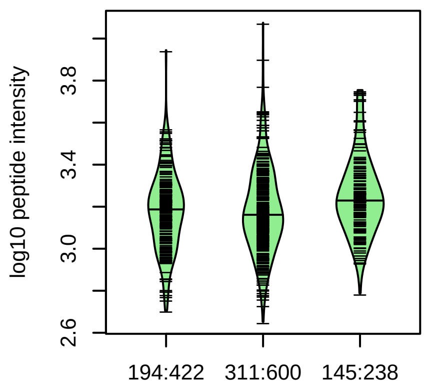

6:38650841:A:G_A
p = 0.0042, beta = 0.155, N = 650

CDFPIMK pc2
Q04760

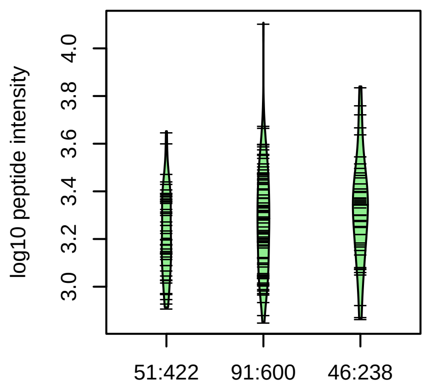

6:38650841:A:G_A
p = 0.019, beta = 0.232, N = 188

SLDFYTR pc2
Q04760

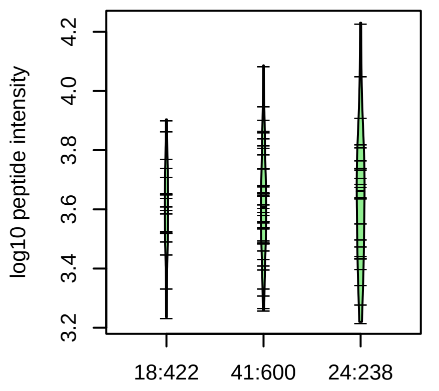

6:38650841:A:G_A
p = 0.61, beta = 0.0761, N = 83

GLO1 : NP2
Q04760

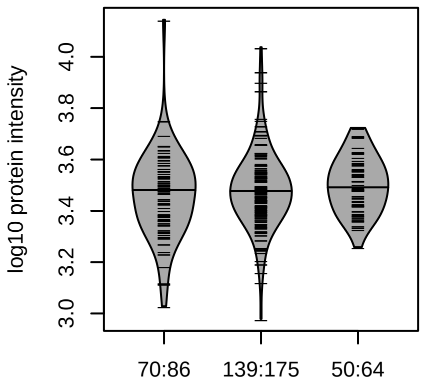

6:38650841:A:G_A
p = 0.08, beta = 0.158, N = 259

GFGHIGIAVPDVYSACK pc3
Q04760

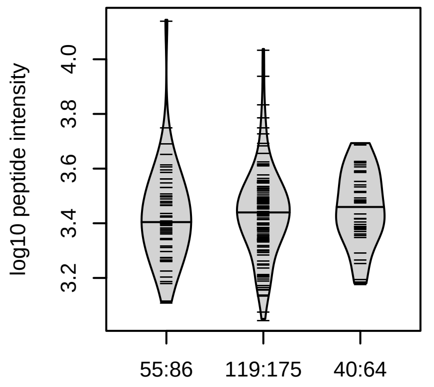

6:38650841:A:G_A
p = 0.24, beta = 0.12, N = 214

DFLLQQTMLR pc2
Q04760

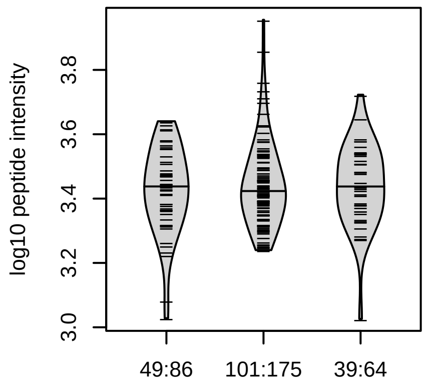

6:38650841:A:G_A
p = 0.6, beta = 0.0549, N = 189

VLGMTLIQK pc2
Q04760

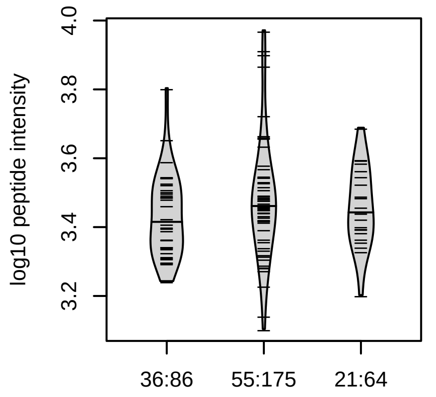

6:38650841:A:G_A
p = 0.42, beta = 0.107, N = 112

FSLYFLAYEDK pc2
Q04760

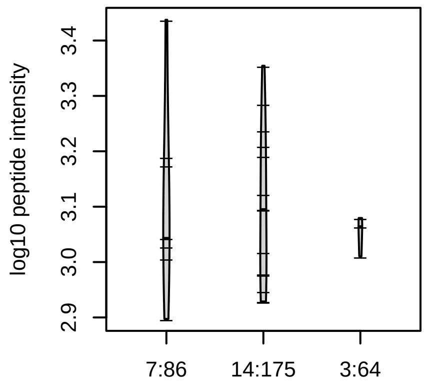

6:38650841:A:G_A
p = 0.71, beta = −0.112, N = 24

255Assay Target: GLO1
UniProt: Q04760
rsID: rs4714175
Target SNP: 6:38650841:A:G
Proxy SNP: 6:38650841:A:G
Tarkin log10(p): 8.1
Tarkin BETA: 0.228
Tarkin MSPA: 0.913
::*:*:–:*:–:–
1195:1179:1098:819:199:650:18

256. MASP1 (P48740)
Tarkin MSPA=0.654

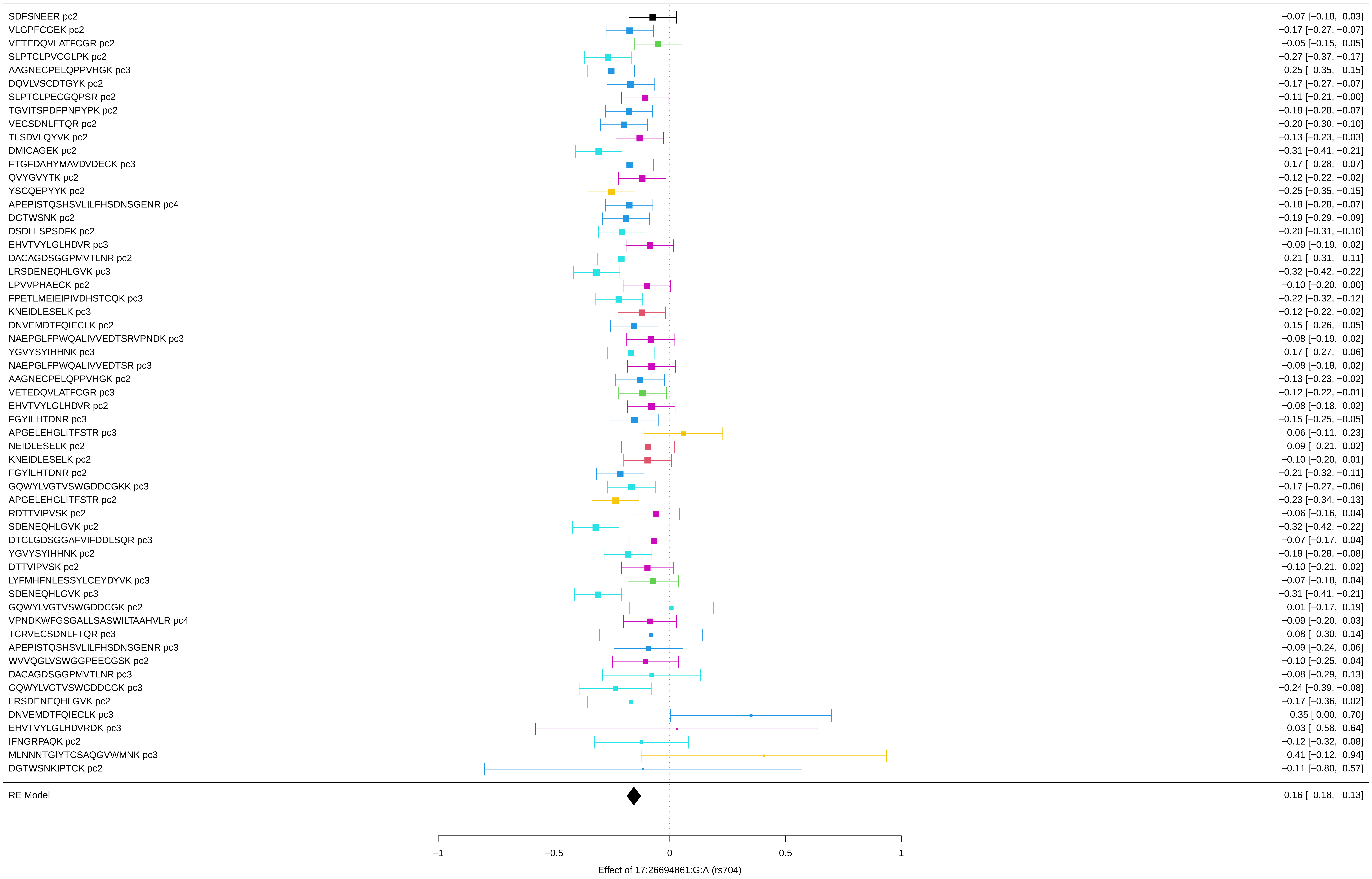

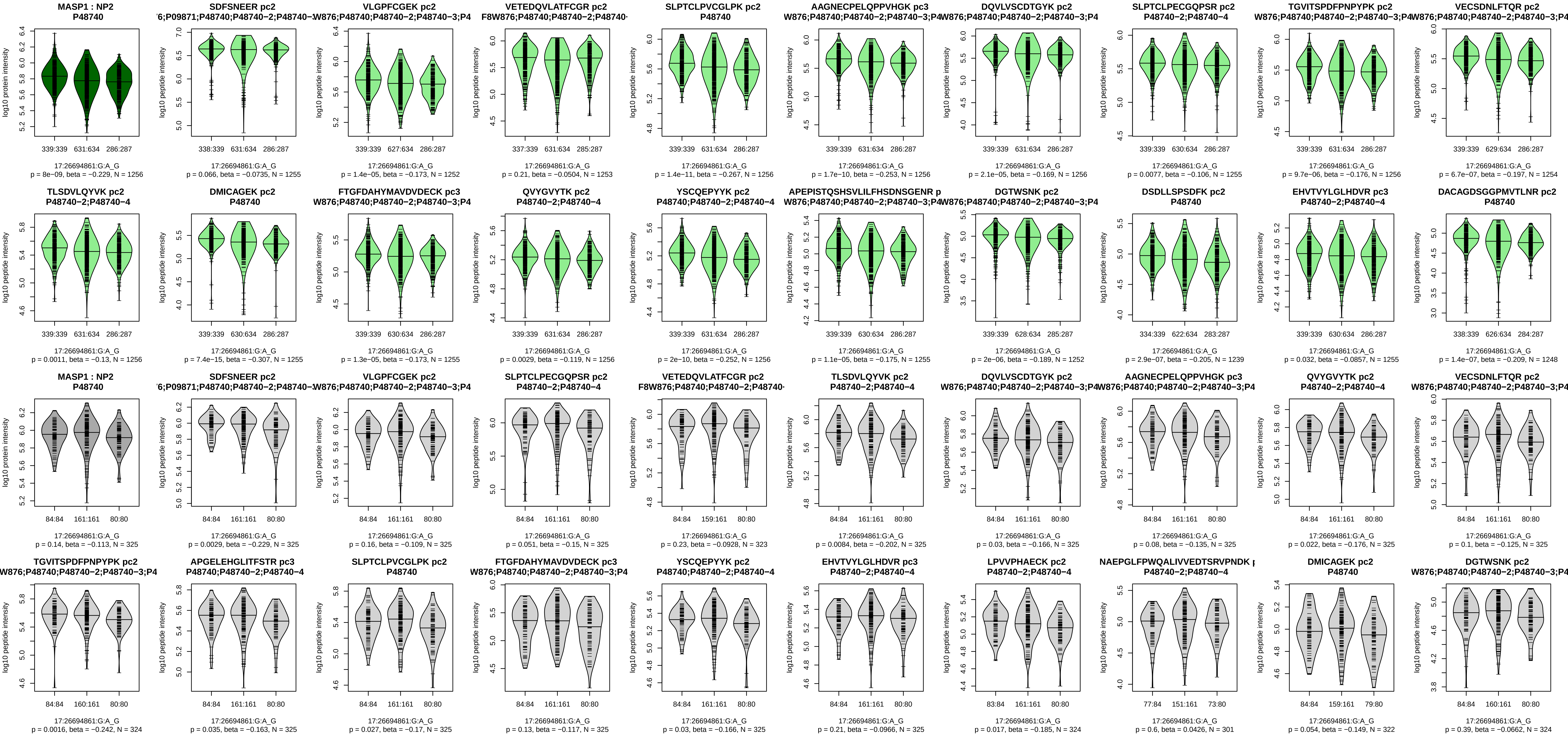

256Assay Target: MASP1
UniProt: P48740
rsID: rs704
Target SNP: 17:26694861:G:A
Proxy SNP: 17:26694861:G:A
Tarkin log10(p): 8.1
Tarkin BETA: −0.229
Tarkin MSPA: 0.654
1255:1252:1253:1256:1256:125

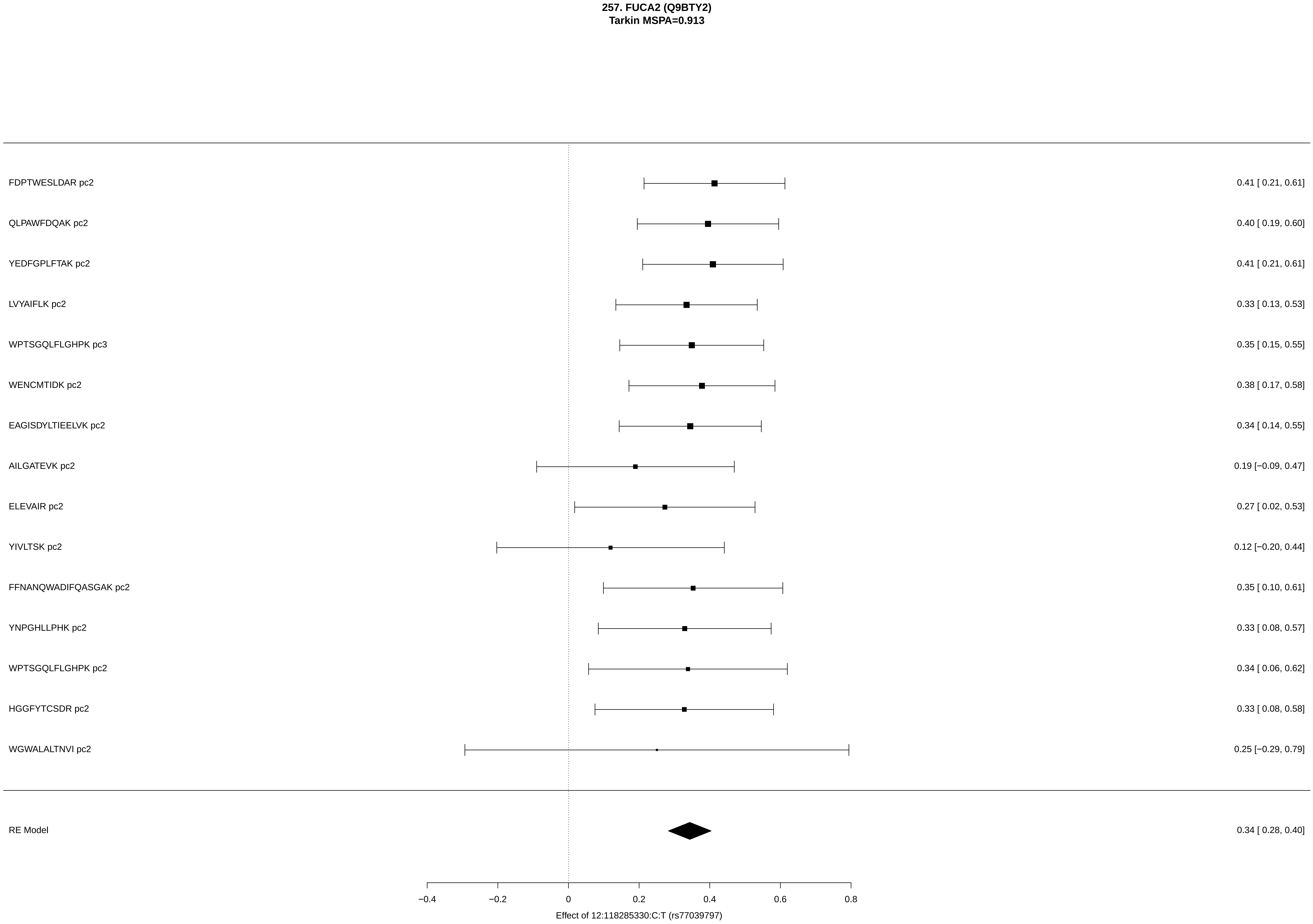
257. FUCA2 (Q9BTY2)
Tarkin MSPA=0.913
FDPTWESLDAR pc2
0.41 [0.21, 0.61]
QLPAWFDQAK pc2
0.40 [0.19, 0.60]
YEDFGPLFTAK pc2
0.41 [0.21, 0.61]
LVYAIFLK pc2
0.33 [0.13, 0.53]
WPTSGQLFLGHPK pc3
0.35 [0.15, 0.55]
WENCMTIDK pc2
0.38 [0.17, 0.58]
EAGISDYLTIEELVK pc2
0.34 [0.14, 0.55]
AILGATEVK pc2
0.19 [−0.09, 0.47]
ELEVAIR pc2
0.27 [0.02, 0.53]
YIVLTSK pc2
0.12 [−0.20, 0.44]
FFNANQWADIFQASGAK pc2
0.35 [0.10, 0.61]
YNPGHLLPHK pc2
0.33 [0.08, 0.57]
WPTSGQLFLGHPK pc2
0.34 [0.06, 0.62]
HGGFYTCSDR pc2
0.33 [0.08, 0.58]
WGWALALTNVI pc2
0.25 [−0.29, 0.79]
RE Model
0.34 [0.28, 0.40]
−0.4
−0.2
0
0.2
0.4
0.6
0.8
Effect of 12:118285330:C:T (rs77039797)

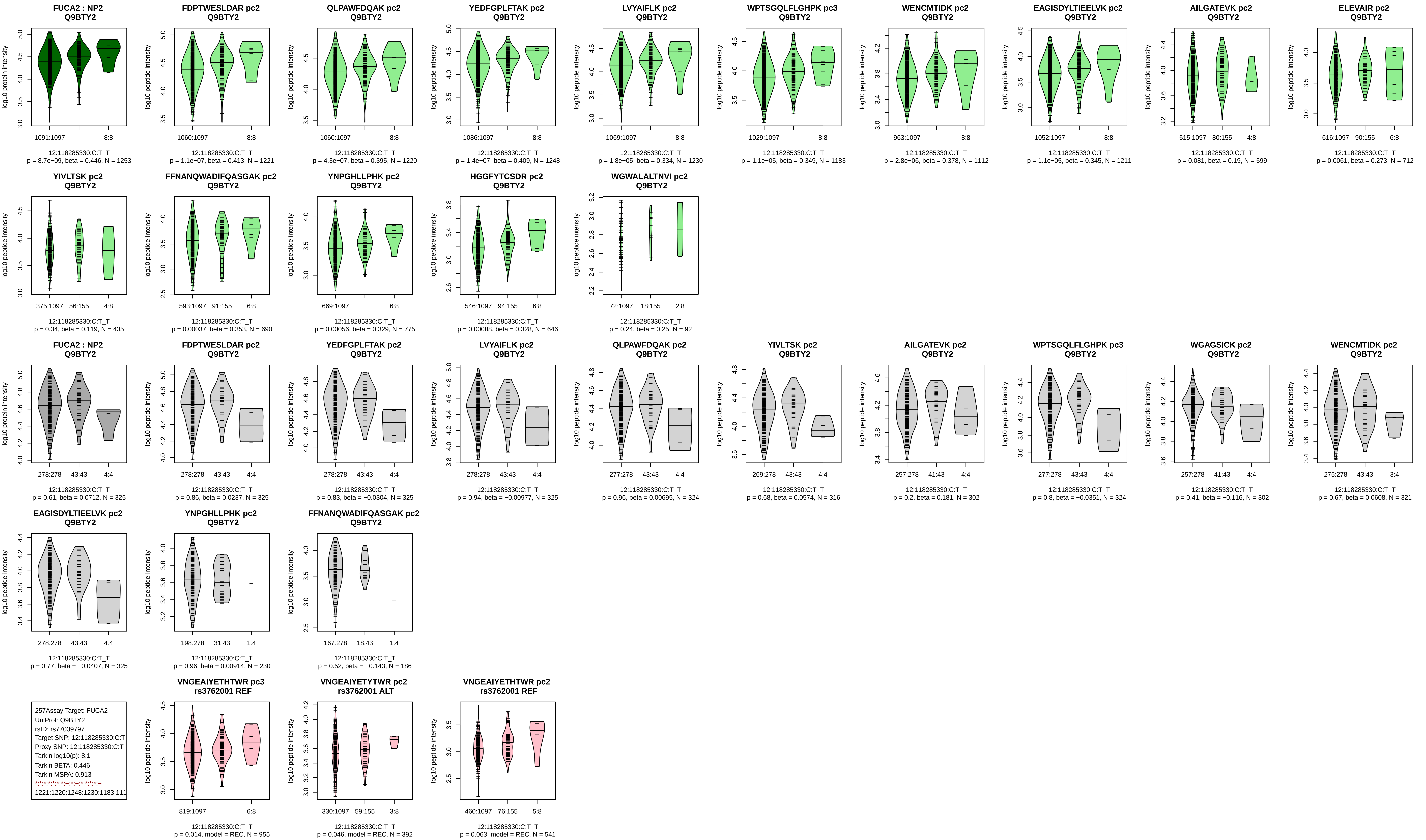
FUCA2 : NP2 Q9BTY2
1091:1097 8:8
12:118285330:C:T_T
p = 8.7e−09, beta = 0.446, N = 1253
FDPTWESLDAR pc2 Q9BTY2
1060:1097 8:8
12:118285330:C:T_T
p = 1.1e−07, beta = 0.413, N = 1221
QLPAWFDQAK pc2 Q9BTY2
1060:1097 8:8
12:118285330:C:T_T
p = 4.3e−07, beta = 0.395, N = 1220
YEDFGPLFTAK pc2 Q9BTY2
1086:1097 8:8
12:118285330:C:T_T
p = 1.4e−07, beta = 0.409, N = 1248
LVYAIFLK pc2 Q9BTY2
1069:1097 8:8
12:118285330:C:T_T
p = 1.8e−05, beta = 0.334, N = 1230
WPTSGQLFLGHPK pc3 Q9BTY2
1029:1097 8:8
12:118285330:C:T_T
p = 1.1e−05, beta = 0.349, N = 1183
WENCMTIDK pc2 Q9BTY2
963:1097 8:8
12:118285330:C:T_T
p = 2.8e−06, beta = 0.378, N = 1112
EAGISDYLTIEELVK pc2 Q9BTY2
1052:1097 8:8
12:118285330:C:T_T
p = 1.1e−05, beta = 0.345, N = 1211
AILGATEVK pc2 Q9BTY2
515:1097 80:155 4:8
12:118285330:C:T_T
p = 0.081, beta = 0.19, N = 599
ELEVAIR pc2 Q9BTY2
616:1097 90:155 6:8
12:118285330:C:T_T
p = 0.0061, beta = 0.273, N = 712
YIVLTSK pc2 Q9BTY2
375:1097 56:155 4:8
12:118285330:C:T_T
p = 0.34, beta = 0.119, N = 435
FFNANQWADIFQASGAK pc2 Q9BTY2
593:1097 91:155 6:8
12:118285330:C:T_T
p = 0.00037, beta = 0.353, N = 690
YNPGHLLPHK pc2 Q9BTY2
669:1097 6:8
12:118285330:C:T_T
p = 0.00056, beta = 0.329, N = 775
HGGFYTCSDR pc2 Q9BTY2
546:1097 94:155 6:8
12:118285330:C:T_T
p = 0.00088, beta = 0.328, N = 646
WGWALALTNVI pc2 Q9BTY2
72:1097 18:155 2:8
12:118285330:C:T_T
p = 0.24, beta = 0.25, N = 92
FUCA2 : NP2 Q9BTY2
278:278 43:43 4:4
12:118285330:C:T_T
p = 0.61, beta = 0.0712, N = 325
FDPTWESLDAR pc2 Q9BTY2
278:278 43:43 4:4
12:118285330:C:T_T
p = 0.86, beta = 0.0237, N = 325
YEDFGPLFTAK pc2 Q9BTY2
278:278 43:43 4:4
12:118285330:C:T_T
p = 0.83, beta = −0.0304, N = 325
LVYAIFLK pc2 Q9BTY2
278:278 43:43 4:4
12:118285330:C:T_T
p = 0.94, beta = −0.00977, N = 325
QLPAWFDQAK pc2 Q9BTY2
277:278 43:43 4:4
12:118285330:C:T_T
p = 0.96, beta = 0.00695, N = 324
YIVLTSK pc2 Q9BTY2
269:278 43:43 4:4
12:118285330:C:T_T
p = 0.68, beta = 0.0574, N = 316
AILGATEVK pc2 Q9BTY2
257:278 41:43 4:4
12:118285330:C:T_T
p = 0.2, beta = 0.181, N = 302
WPTSGQLFLGHPK pc3 Q9BTY2
277:278 43:43 4:4
12:118285330:C:T_T
p = 0.8, beta = −0.0351, N = 324
WGAGSICK pc2 Q9BTY2
257:278 41:43 4:4
12:118285330:C:T_T
p = 0.41, beta = −0.116, N = 302
WENCMTIDK pc2 Q9BTY2
275:278 43:43 3:4
12:118285330:C:T_T
p = 0.67, beta = 0.0608, N = 321
EAGISDYLTIEELVK pc2 Q9BTY2
278:278 43:43 4:4
12:118285330:C:T_T
p = 0.77, beta = −0.0407, N = 325
YNPGHLLPHK pc2 Q9BTY2
198:278 31:43 1:4
12:118285330:C:T_T
p = 0.96, beta = 0.00914, N = 230
FFNANQWADIFQASGAK pc2 Q9BTY2
167:278 18:43 1:4
12:118285330:C:T_T
p = 0.52, beta = −0.143, N = 186
257Assay Target: FUCA2
UniProt: Q9BTY2
rsID: rs77039797
Target SNP: 12:118285330:C:T
Proxy SNP: 12:118285330:C:T
Tarkin log10(p): 8.1
Tarkin BETA: 0.446
Tarkin MSPA: 0.913
1221:1220:1248:1230:1183:111
VNGEAIYETHTWR pc3 rs3762001 REF
819:1097 6:8
12:118285330:C:T_T
p = 0.014, model = REC, N = 955
VNGEAIYETYTWR pc2 rs3762001 ALT
330:1097 59:155 3:8
12:118285330:C:T_T
p = 0.046, model = REC, N = 392
VNGEAIYETHTWR pc2 rs3762001 REF
460:1097 76:155 5:8
12:118285330:C:T_T
p = 0.063, model = REC, N = 541

258. ETFB (P38117)
Tarkin MSPA=0.359

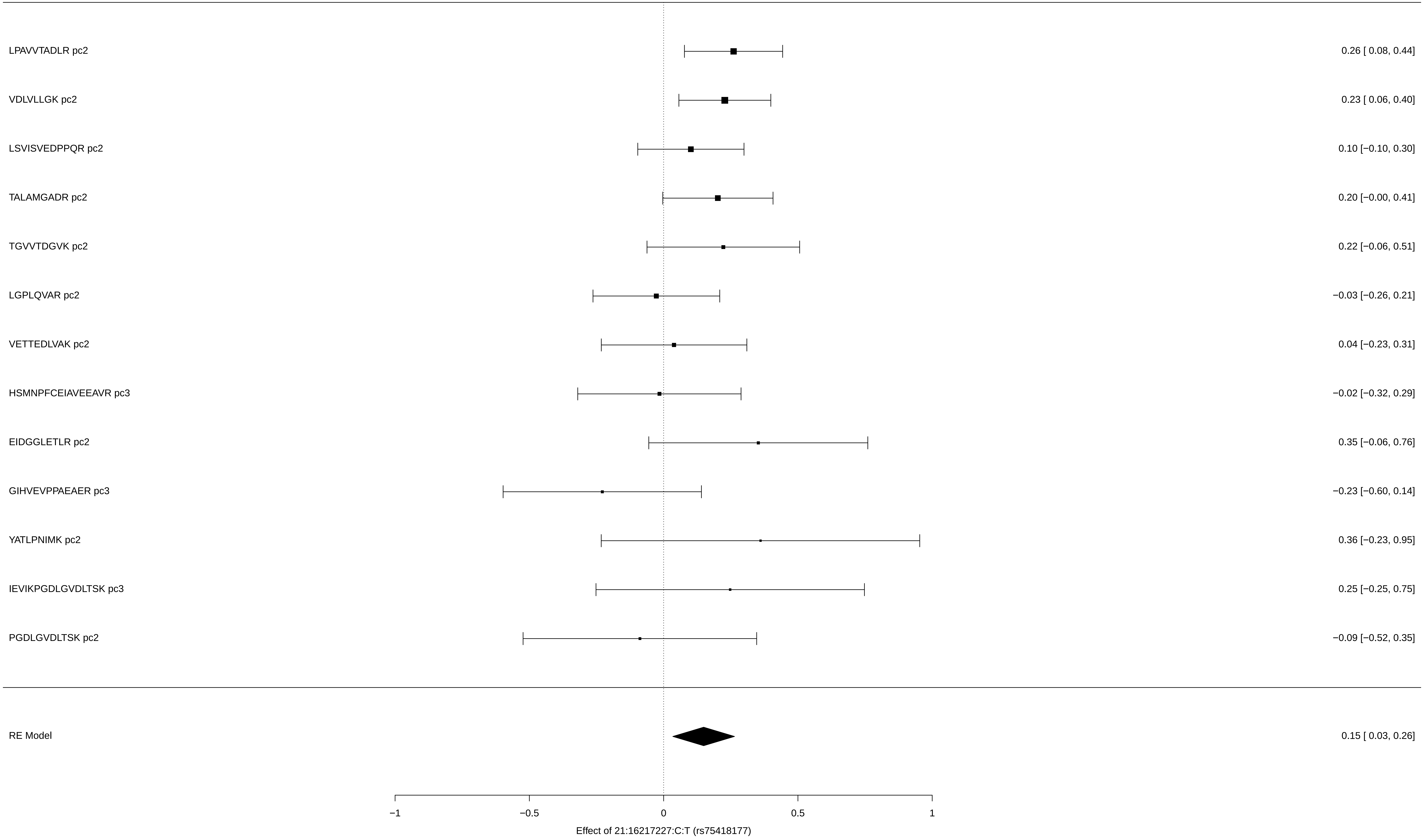

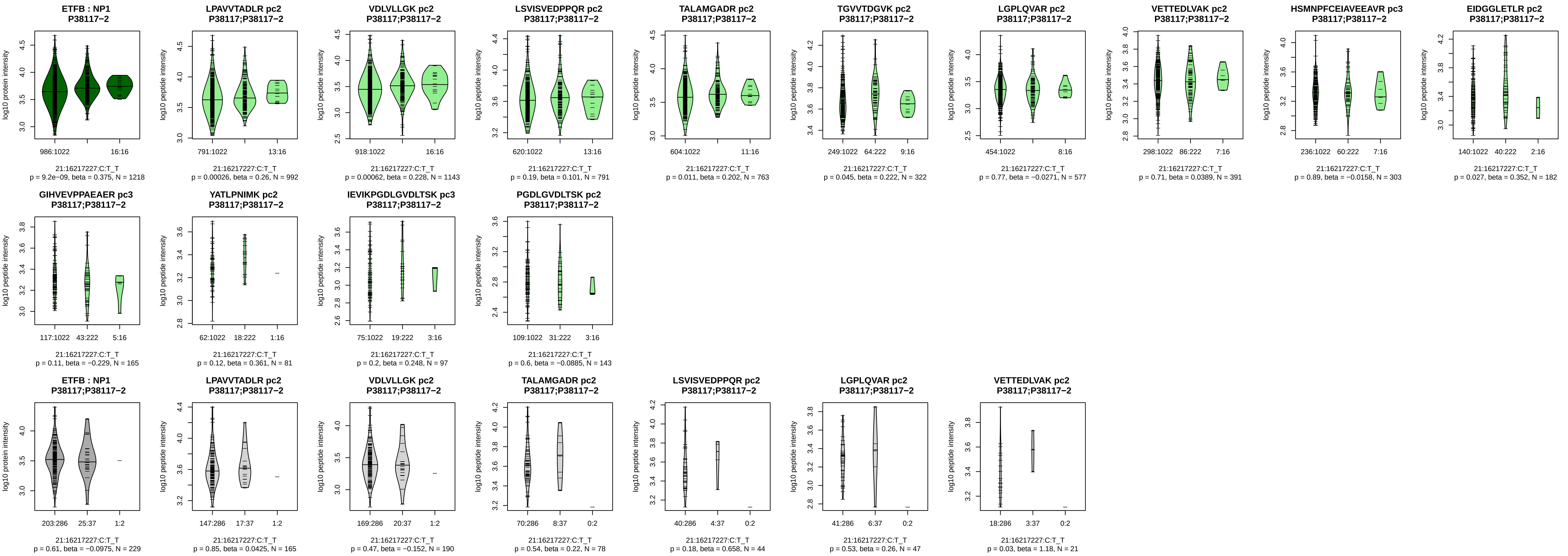

258Assay Target: ETFB
UniProt: P38117
rsID: rs75418177
Target SNP: 21:16217227:C:T
Proxy SNP: 21:16217227:C:T
Tarkin log10(p): 8
Tarkin BETA: 0.375
Tarkin MSPA: 0.359
::−:−:−:−:−:−:−:−:−:−:−:NA:NA
992:1143:791:763:322:577:391:

259. SVEP1 (Q4LDE5)
Tarkin MSPA=0.81

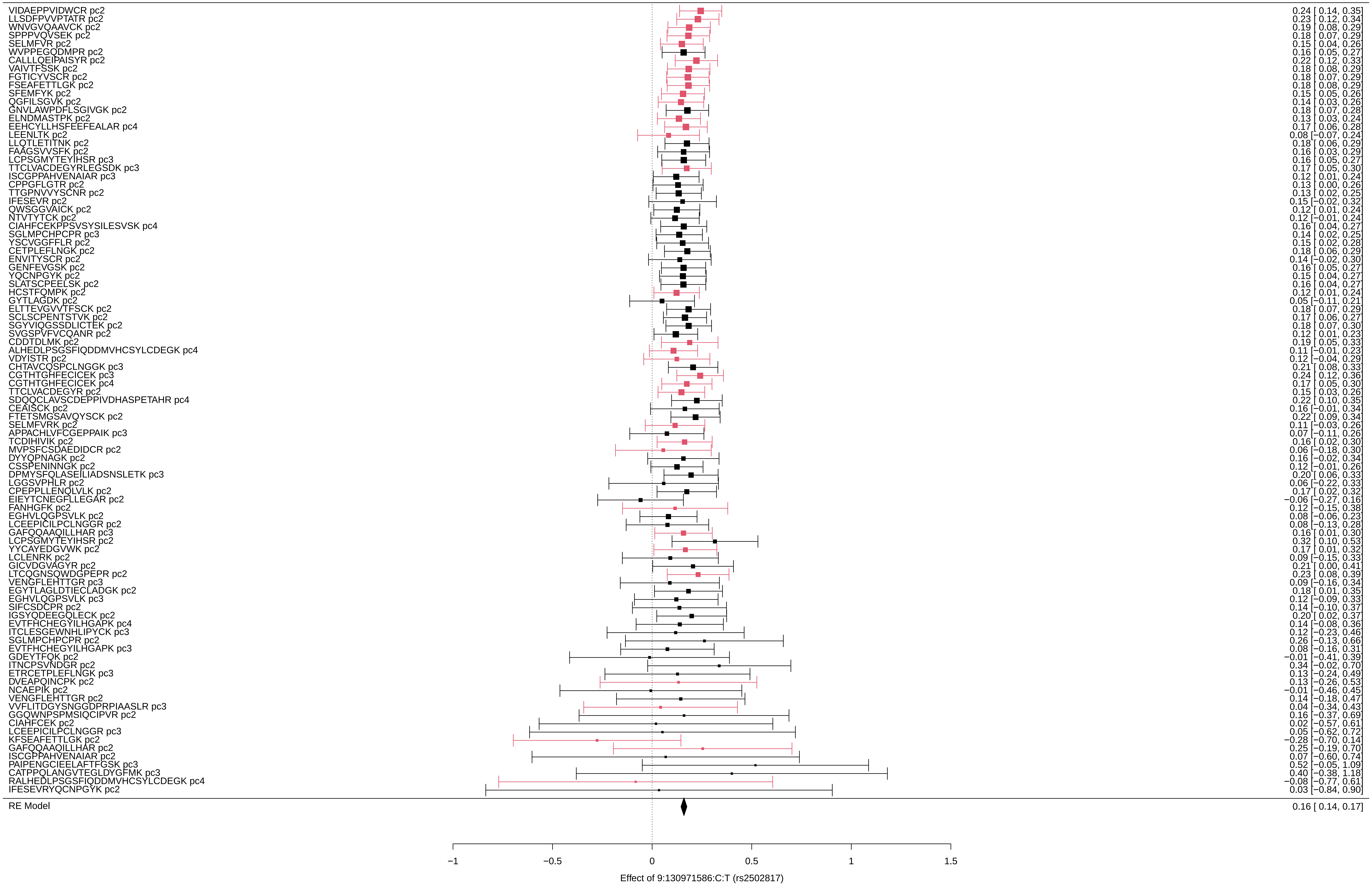

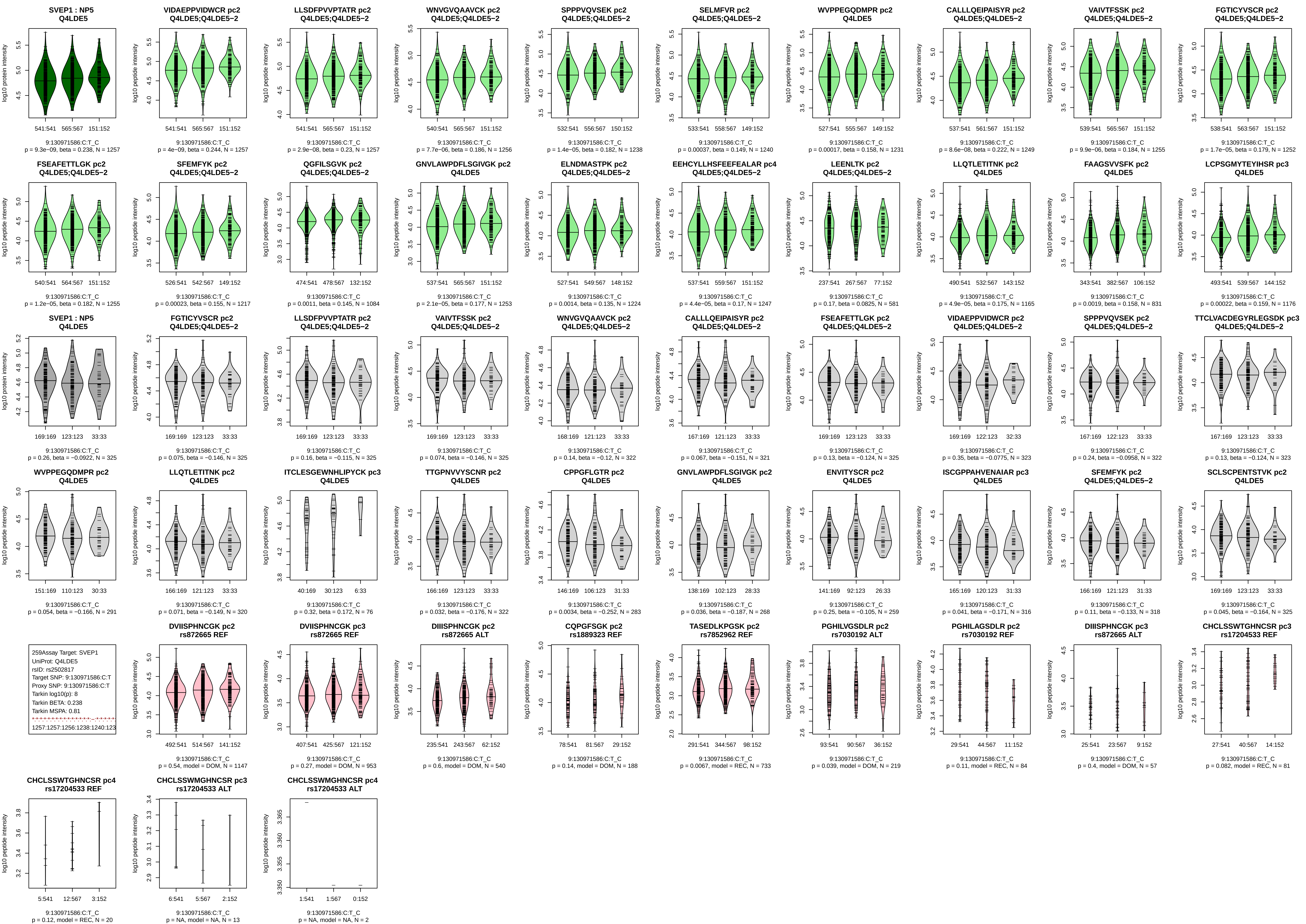
SVEP1 : NP5
Q4LDE5
log10 protein intensity
541:541 565:567 151:152
9:130971586:C:T_C
p = 9.3e−09, beta = 0.238, N = 1257
VIDAEPPVIDWCR pc2
Q4LDE5;Q4LDE5−2
log10 peptide intensity
541:541 565:567 151:152
9:130971586:C:T_C
p = 4e−09, beta = 0.244, N = 1257
LLSDFPVVPTATR pc2
Q4LDE5;Q4LDE5−2
541:541 565:567 151:152
9:130971586:C:T_C
p = 2.9e−08, beta = 0.23, N = 1257
WNVGVQAAVCK pc2
Q4LDE5;Q4LDE5−2
540:541 565:567 151:152
9:130971586:C:T_C
p = 7.7e−06, beta = 0.186, N = 1256
SPPPVQVSEK pc2
Q4LDE5;Q4LDE5−2
532:541 556:567 150:152
9:130971586:C:T_C
p = 1.4e−05, beta = 0.182, N = 1238
SELMFVR pc2
Q4LDE5;Q4LDE5−2
533:541 558:567 149:152
9:130971586:C:T_C
p = 0.00037, beta = 0.149, N = 1240
WVPPEGQDMPR pc2
Q4LDE5
527:541 555:567 149:152
9:130971586:C:T_C
p = 0.00017, beta = 0.158, N = 1231
CALLLQEIPAISYR pc2
Q4LDE5;Q4LDE5−2
537:541 561:567 151:152
9:130971586:C:T_C
p = 8.6e−08, beta = 0.222, N = 1249
VAIVTFSSK pc2
Q4LDE5;Q4LDE5−2
539:541 565:567 151:152
9:130971586:C:T_C
p = 9.9e−06, beta = 0.184, N = 1255
FGTICYVSCR pc2
Q4LDE5;Q4LDE5−2
538:541 563:567 151:152
9:130971586:C:T_C
p = 1.7e−05, beta = 0.179, N = 1252
FSEAFETTLGK pc2
Q4LDE5;Q4LDE5−2
540:541 564:567 151:152
9:130971586:C:T_C
p = 1.2e−05, beta = 0.182, N = 1255
SFEMFYK pc2
Q4LDE5;Q4LDE5−2
526:541 542:567 149:152
9:130971586:C:T_C
p = 0.00023, beta = 0.155, N = 1217
QGFILSGVK pc2
Q4LDE5;Q4LDE5−2
474:541 478:567 132:152
9:130971586:C:T_C
p = 0.0011, beta = 0.145, N = 1084
GNVLAWPDFLSGIVGK pc2
Q4LDE5
537:541 565:567 151:152
9:130971586:C:T_C
p = 2.1e−05, beta = 0.177, N = 1253
ELNDMASTPK pc2
Q4LDE5;Q4LDE5−2
527:541 549:567 148:152
9:130971586:C:T_C
p = 0.0014, beta = 0.135, N = 1224
EEHCYLLHSFEEFEALAR pc4
Q4LDE5;Q4LDE5−2
537:541 559:567 151:152
9:130971586:C:T_C
p = 4.4e−05, beta = 0.17, N = 1247
LEENLTK pc2
Q4LDE5;Q4LDE5−2
237:541 267:567 77:152
9:130971586:C:T_C
p = 0.17, beta = 0.0825, N = 581
LLQTLETITNK pc2
Q4LDE5
490:541 532:567 143:152
9:130971586:C:T_C
p = 4.9e−05, beta = 0.175, N = 1165
FAAGSVVSFK pc2
Q4LDE5
343:541 382:567 106:152
9:130971586:C:T_C
p = 0.0019, beta = 0.158, N = 831
LCPSGMYTEYIHSR pc3
Q4LDE5
493:541 539:567 144:152
9:130971586:C:T_C
p = 0.00022, beta = 0.159, N = 1176
SVEP1 : NP5
Q4LDE5
169:169 123:123 33:33
9:130971586:C:T_C
p = 0.26, beta = −0.0922, N = 325
FGTICYVSCR pc2
Q4LDE5;Q4LDE5−2
169:169 123:123 33:33
9:130971586:C:T_C
p = 0.075, beta = −0.146, N = 325
LLSDFPVVPTATR pc2
Q4LDE5;Q4LDE5−2
169:169 123:123 33:33
9:130971586:C:T_C
p = 0.16, beta = −0.115, N = 325
VAIVTFSSK pc2
Q4LDE5;Q4LDE5−2
169:169 123:123 33:33
9:130971586:C:T_C
p = 0.074, beta = −0.146, N = 325
WNVGVQAAVCK pc2
Q4LDE5;Q4LDE5−2
168:169 121:123 33:33
9:130971586:C:T_C
p = 0.14, beta = −0.12, N = 322
CALLLQEIPAISYR pc2
Q4LDE5;Q4LDE5−2
167:169 121:123 33:33
9:130971586:C:T_C
p = 0.067, beta = −0.151, N = 321
FSEAFETTLGK pc2
Q4LDE5;Q4LDE5−2
169:169 123:123 33:33
9:130971586:C:T_C
p = 0.13, beta = −0.124, N = 325
VIDAEPPVIDWCR pc2
Q4LDE5;Q4LDE5−2
169:169 122:123 32:33
9:130971586:C:T_C
p = 0.35, beta = −0.0775, N = 323
SPPPVQVSEK pc2
Q4LDE5;Q4LDE5−2
167:169 122:123 33:33
9:130971586:C:T_C
p = 0.24, beta = −0.0958, N = 322
TTCLVACDEGYRLEGSDK pc3
Q4LDE5;Q4LDE5−2
167:169 123:123 33:33
9:130971586:C:T_C
p = 0.13, beta = −0.124, N = 323
WVPPEGQDMPR pc2
Q4LDE5
151:169 110:123 30:33
9:130971586:C:T_C
p = 0.054, beta = −0.166, N = 291
LLQTLETITNK pc2
Q4LDE5
166:169 121:123 33:33
9:130971586:C:T_C
p = 0.071, beta = −0.149, N = 320
ITCLESGEWNHLIPYCK pc3
Q4LDE5
40:169 30:123 6:33
9:130971586:C:T_C
p = 0.32, beta = 0.172, N = 76
TTGPNVVYSCNR pc2
Q4LDE5
166:169 123:123 33:33
9:130971586:C:T_C
p = 0.032, beta = −0.176, N = 322
CPPGFLGTR pc2
Q4LDE5
146:169 106:123 31:33
9:130971586:C:T_C
p = 0.0034, beta = −0.252, N = 283
GNVLAWPDFLSGIVGK pc2
Q4LDE5
138:169 102:123 28:33
9:130971586:C:T_C
p = 0.036, beta = −0.187, N = 268
ENVITYSCR pc2
Q4LDE5
141:169 92:123 26:33
9:130971586:C:T_C
p = 0.25, beta = −0.105, N = 259
ISCGPPAHVENAIAR pc3
Q4LDE5
165:169 120:123 31:33
9:130971586:C:T_C
p = 0.041, beta = −0.171, N = 316
SFEMFYK pc2
Q4LDE5;Q4LDE5−2
166:169 121:123 31:33
9:130971586:C:T_C
p = 0.11, beta = −0.133, N = 318
SCLSCPENTSTVK pc2
Q4LDE5
169:169 123:123 33:33
9:130971586:C:T_C
p = 0.045, beta = −0.164, N = 325
259Assay Target: SVEP1
UniProt: Q4LDE5
rsID: rs2502817
Target SNP: 9:130971586:C:T
Proxy SNP: 9:130971586:C:T
Tarkin log10(p): 8
Tarkin BETA: 0.238
Tarkin MSPA: 0.81
1257:1257:1256:1238:1240:123
DVIISPHNCGK pc2
rs872665 REF
492:541 514:567 141:152
9:130971586:C:T_C
p = 0.54, model = DOM, N = 1147
DVIISPHNCGK pc3
rs872665 REF
407:541 425:567 121:152
9:130971586:C:T_C
p = 0.27, model = DOM, N = 953
DIIISPHNCGK pc2
rs872665 ALT
235:541 243:567 62:152
9:130971586:C:T_C
p = 0.6, model = DOM, N = 540
CQPGFSGK pc2
rs1889323 REF
78:541 81:567 29:152
9:130971586:C:T_C
p = 0.14, model = DOM, N = 188
TASEDLKPGSK pc2
rs7852962 REF
291:541 344:567 98:152
9:130971586:C:T_C
p = 0.0067, model = REC, N = 733
PGHILVGSDLR pc2
rs7030192 ALT
93:541 90:567 36:152
9:130971586:C:T_C
p = 0.039, model = DOM, N = 219
PGHILAGSDLR pc2
rs7030192 REF
29:541 44:567 11:152
9:130971586:C:T_C
p = 0.11, model = REC, N = 84
DIIISPHNCGK pc3
rs872665 ALT
25:541 23:567 9:152
9:130971586:C:T_C
p = 0.4, model = DOM, N = 57
CHCLSSWTGHNCSR pc3
rs17204533 REF
27:541 40:567 14:152
9:130971586:C:T_C
p = 0.082, model = REC, N = 81
CHCLSSWTGHNCSR pc4
rs17204533 REF
5:541 12:567 3:152
9:130971586:C:T_C
p = 0.12, model = REC, N = 20
CHCLSSWMGHNCSR pc3
rs17204533 ALT
6:541 5:567 2:152
9:130971586:C:T_C
p = NA, model = NA, N = 13
CHCLSSWMGHNCSR pc4
rs17204533 ALT
1:541 1:567 0:152
9:130971586:C:T_C
p = NA, model = NA, N = 2

260. EEF1A1 (A0A7I2V659)
Tarkin MSPA=0.178

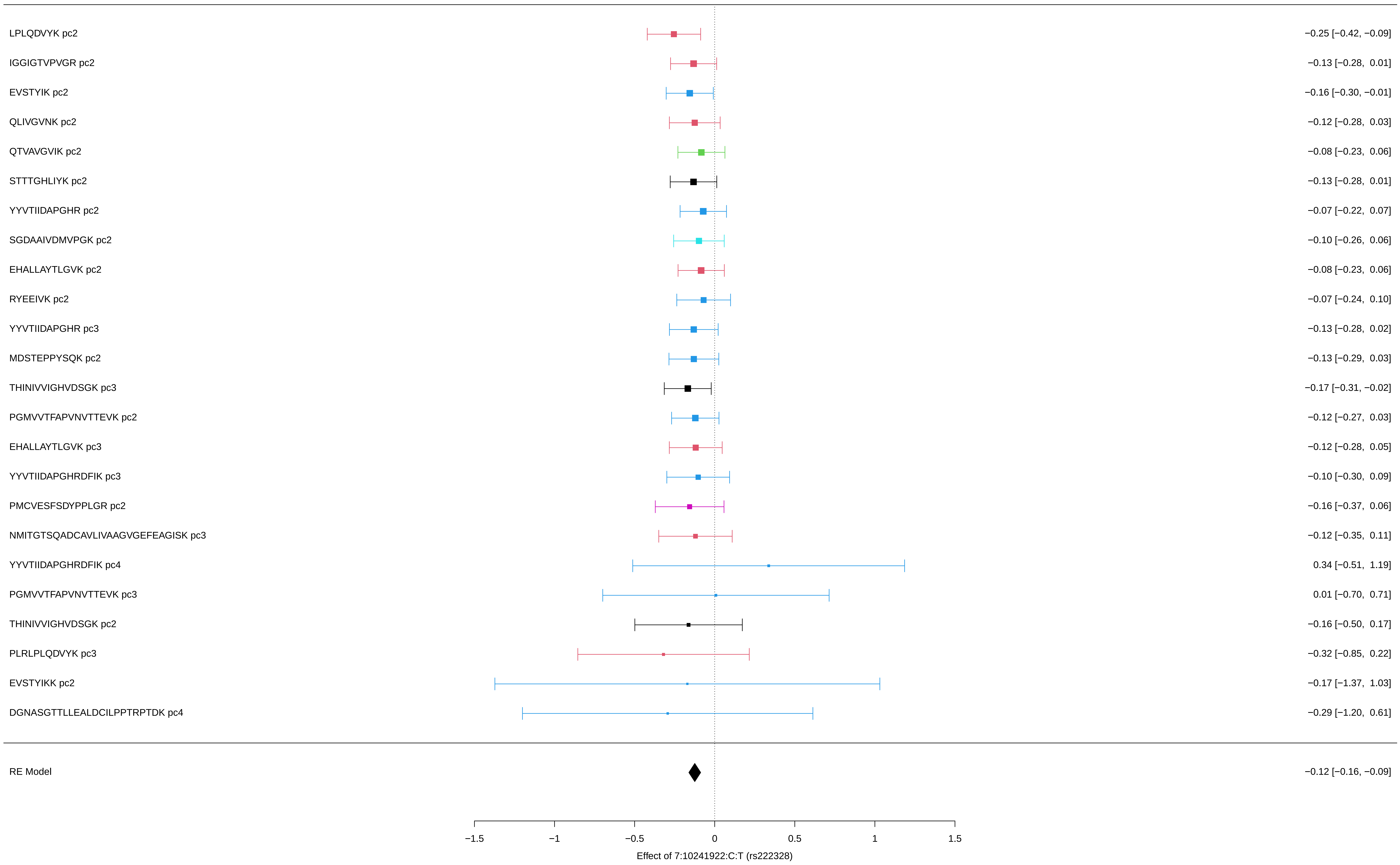

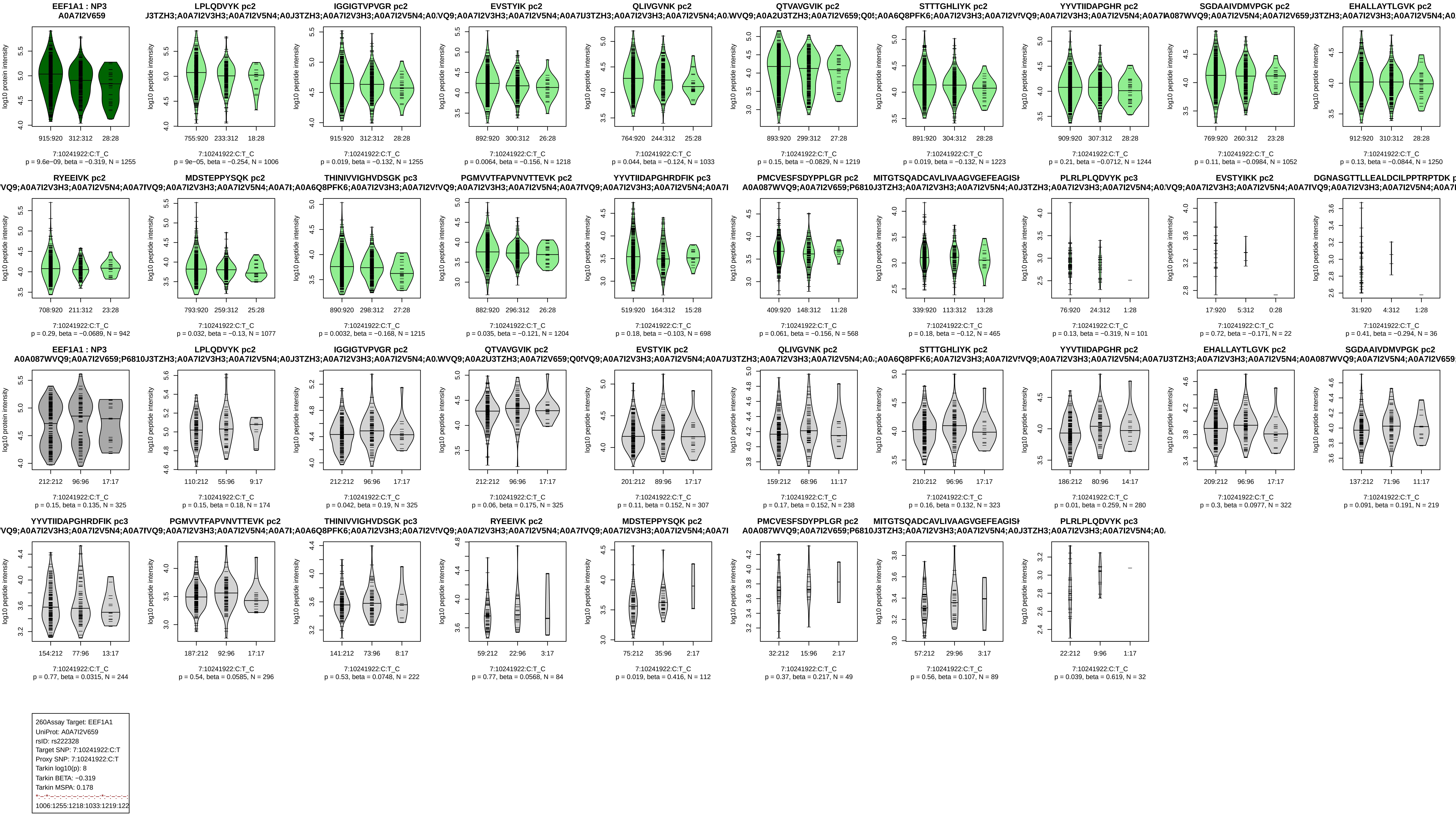
EEF1A1 : NP3
A0A7I2V659
LPLQDVYK pc2
IGGIGTVPVGR pc2
EVSTYIK pc2
QLIVGVNK pc2
QTVAVGVIK pc2
STTTGHLIYK pc2
YYVTIIDAPGHR pc2
SGDAAIVDMVPGK pc2
EHALLAYTLGVK pc2
log10 protein intensity
log10 peptide intensity
7:10241922:C:T_C
p = 9.6e−09, beta = −0.319, N = 1255
p = 9e−05, beta = −0.254, N = 1006
p = 0.019, beta = −0.132, N = 1255
p = 0.0064, beta = −0.156, N = 1218
p = 0.044, beta = −0.124, N = 1033
p = 0.15, beta = −0.0829, N = 1219
p = 0.019, beta = −0.132, N = 1223
p = 0.21, beta = −0.0712, N = 1244
p = 0.11, beta = −0.0984, N = 1052
p = 0.13, beta = −0.0844, N = 1250
RYEEIVK pc2
MDSTEPPYSQK pc2
THINIVVIGHVDSGK pc3
PGMVVTFAPVNVTTEVK pc2
YYVTIIDAPGHRDFIK pc3
PMCVESFSDYPPLGR pc2
PLRLPLQDVYK pc3
EVSTYIKK pc2
p = 0.29, beta = −0.0689, N = 942
p = 0.032, beta = −0.13, N = 1077
p = 0.0032, beta = −0.168, N = 1215
p = 0.035, beta = −0.121, N = 1204
p = 0.18, beta = −0.103, N = 698
p = 0.061, beta = −0.156, N = 568
p = 0.18, beta = −0.12, N = 465
p = 0.13, beta = −0.319, N = 101
p = 0.72, beta = −0.171, N = 22
p = 0.41, beta = −0.294, N = 36
EEF1A1 : NP3
p = 0.15, beta = 0.135, N = 325
p = 0.15, beta = 0.18, N = 174
p = 0.042, beta = 0.19, N = 325
p = 0.06, beta = 0.175, N = 325
p = 0.11, beta = 0.152, N = 307
p = 0.17, beta = 0.152, N = 238
p = 0.16, beta = 0.132, N = 323
p = 0.01, beta = 0.259, N = 280
p = 0.3, beta = 0.0977, N = 322
p = 0.091, beta = 0.191, N = 219
p = 0.77, beta = 0.0315, N = 244
p = 0.54, beta = 0.0585, N = 296
p = 0.53, beta = 0.0748, N = 222
p = 0.77, beta = 0.0568, N = 84
p = 0.019, beta = 0.416, N = 112
p = 0.37, beta = 0.217, N = 49
p = 0.56, beta = 0.107, N = 89
p = 0.039, beta = 0.619, N = 32
260Assay Target: EEF1A1
UniProt: A0A7I2V659
rsID: rs222328
Target SNP: 7:10241922:C:T
Proxy SNP: 7:10241922:C:T
Tarkin log10(p): 8
Tarkin BETA: −0.319
Tarkin MSPA: 0.178
1006:1255:1218:1033:1219:122

261. SERPINB1 (P30740)
Tarkin MSPA=0.358

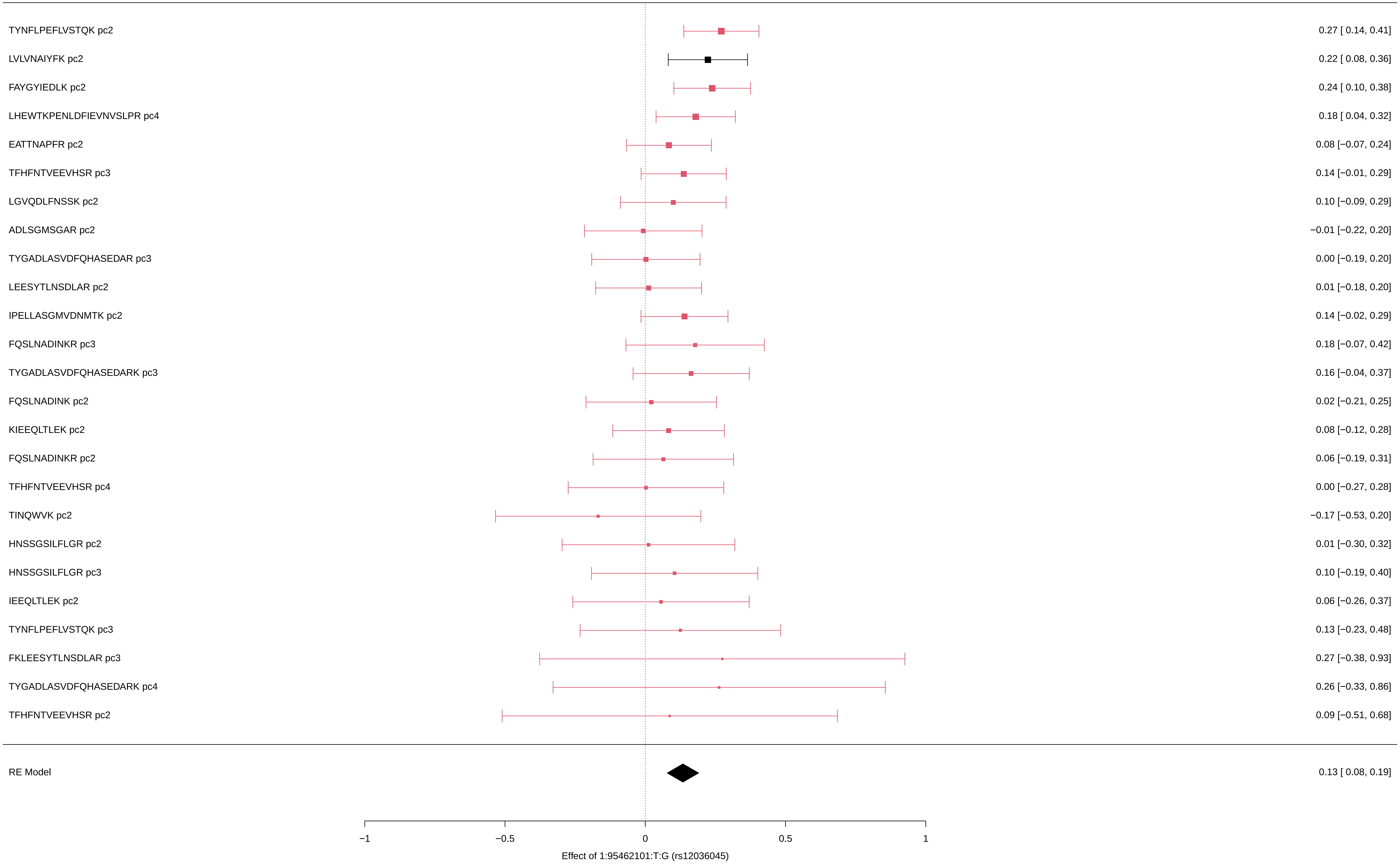

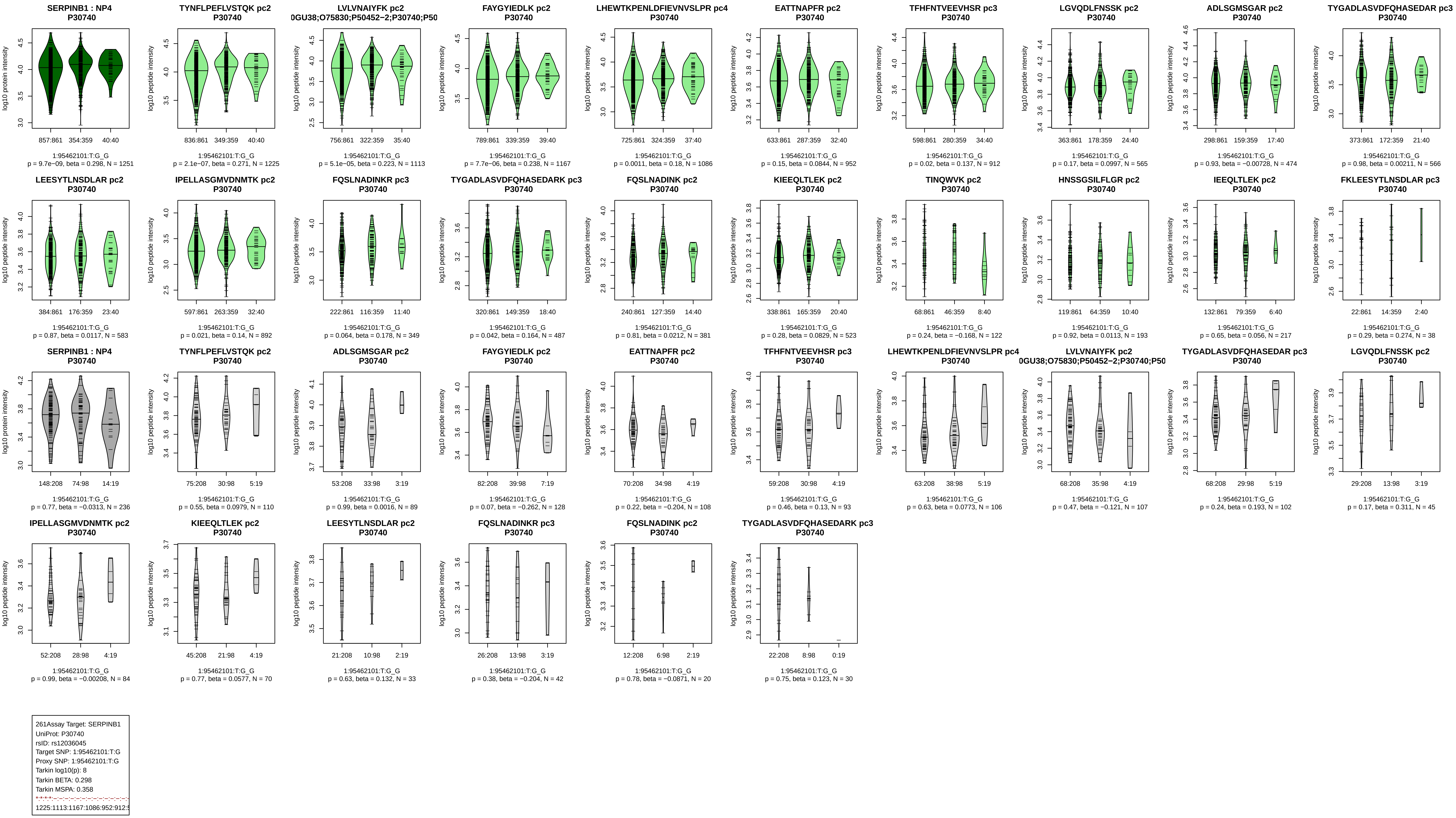
SERPINB1 : NP4
P30740
log10 protein intensity
857:861 354:359 40:40
1:95462101:T:G_G
p = 9.7e−09, beta = 0.298, N = 1251
TYNFLPEFLVSTQK pc2
P30740
log10 peptide intensity
836:861 349:359 40:40
1:95462101:T:G_G
p = 2.1e−07, beta = 0.271, N = 1225
LVLVNAIYFK pc2
0GU38;O75830;P50452−2;P30740;P50
756:861 322:359 35:40
1:95462101:T:G_G
p = 5.1e−05, beta = 0.223, N = 1113
FAYGYIEDLK pc2
P30740
789:861 339:359 39:40
1:95462101:T:G_G
p = 7.7e−06, beta = 0.238, N = 1167
LHEWTKPENLDFIEVNVSLPR pc4
P30740
725:861 324:359 37:40
1:95462101:T:G_G
p = 0.0011, beta = 0.18, N = 1086
EATTNAPFR pc2
P30740
633:861 287:359 32:40
1:95462101:T:G_G
p = 0.15, beta = 0.0844, N = 952
TFHFNTVEEVHSR pc3
P30740
598:861 280:359 34:40
1:95462101:T:G_G
p = 0.02, beta = 0.137, N = 912
LGVQDLFNSSK pc2
P30740
363:861 178:359 24:40
1:95462101:T:G_G
p = 0.17, beta = 0.0997, N = 565
ADLSGMSGAR pc2
P30740
298:861 159:359 17:40
1:95462101:T:G_G
p = 0.93, beta = −0.00728, N = 474
TYGADLASVDFQHASEDAR pc3
P30740
373:861 172:359 21:40
1:95462101:T:G_G
p = 0.98, beta = 0.00211, N = 566
LEESYTLNSDLAR pc2
P30740
384:861 176:359 23:40
1:95462101:T:G_G
p = 0.87, beta = 0.0117, N = 583
IPELLASGMVDNMTK pc2
P30740
597:861 263:359 32:40
1:95462101:T:G_G
p = 0.021, beta = 0.14, N = 892
FQSLNADINKR pc3
P30740
222:861 116:359 11:40
1:95462101:T:G_G
p = 0.064, beta = 0.178, N = 349
TYGADLASVDFQHASEDARK pc3
P30740
320:861 149:359 18:40
1:95462101:T:G_G
p = 0.042, beta = 0.164, N = 487
FQSLNADINK pc2
P30740
240:861 127:359 14:40
1:95462101:T:G_G
p = 0.81, beta = 0.0212, N = 381
KIEEQLTLEK pc2
P30740
338:861 165:359 20:40
1:95462101:T:G_G
p = 0.28, beta = 0.0829, N = 523
TINQWVK pc2
P30740
68:861 46:359 8:40
1:95462101:T:G_G
p = 0.24, beta = −0.168, N = 122
HNSSGSILFLGR pc2
P30740
119:861 64:359 10:40
1:95462101:T:G_G
p = 0.92, beta = 0.0113, N = 193
IEEQLTLEK pc2
P30740
132:861 79:359 6:40
1:95462101:T:G_G
p = 0.65, beta = 0.056, N = 217
FKLEESYTLNSDLAR pc3
P30740
22:861 14:359 2:40
1:95462101:T:G_G
p = 0.29, beta = 0.274, N = 38
SERPINB1 : NP4
P30740
148:208 74:98 14:19
1:95462101:T:G_G
p = 0.77, beta = −0.0313, N = 236
TYNFLPEFLVSTQK pc2
P30740
75:208 30:98 5:19
1:95462101:T:G_G
p = 0.55, beta = 0.0979, N = 110
ADLSGMSGAR pc2
P30740
53:208 33:98 3:19
1:95462101:T:G_G
p = 0.99, beta = 0.0016, N = 89
FAYGYIEDLK pc2
P30740
82:208 39:98 7:19
1:95462101:T:G_G
p = 0.07, beta = −0.262, N = 128
EATTNAPFR pc2
P30740
70:208 34:98 4:19
1:95462101:T:G_G
p = 0.22, beta = −0.204, N = 108
TFHFNTVEEVHSR pc3
P30740
59:208 30:98 4:19
1:95462101:T:G_G
p = 0.46, beta = 0.13, N = 93
LHEWTKPENLDFIEVNVSLPR pc4
P30740
63:208 38:98 5:19
1:95462101:T:G_G
p = 0.63, beta = 0.0773, N = 106
LVLVNAIYFK pc2
0GU38;O75830;P50452−2;P30740;P50
68:208 35:98 4:19
1:95462101:T:G_G
p = 0.47, beta = −0.121, N = 107
TYGADLASVDFQHASEDAR pc3
P30740
68:208 29:98 5:19
1:95462101:T:G_G
p = 0.24, beta = 0.193, N = 102
LGVQDLFNSSK pc2
P30740
29:208 13:98 3:19
1:95462101:T:G_G
p = 0.17, beta = 0.311, N = 45
IPELLASGMVDNMTK pc2
P30740
52:208 28:98 4:19
1:95462101:T:G_G
p = 0.99, beta = −0.00208, N = 84
KIEEQLTLEK pc2
P30740
45:208 21:98 4:19
1:95462101:T:G_G
p = 0.77, beta = 0.0577, N = 70
LEESYTLNSDLAR pc2
P30740
21:208 10:98 2:19
1:95462101:T:G_G
p = 0.63, beta = 0.132, N = 33
FQSLNADINKR pc3
P30740
26:208 13:98 3:19
1:95462101:T:G_G
p = 0.38, beta = −0.204, N = 42
FQSLNADINK pc2
P30740
12:208 6:98 2:19
1:95462101:T:G_G
p = 0.78, beta = −0.0871, N = 20
TYGADLASVDFQHASEDARK pc3
P30740
22:208 8:98 0:19
1:95462101:T:G_G
p = 0.75, beta = 0.123, N = 30
261Assay Target: SERPINB1
UniProt: P30740
rsID: rs12036045
Target SNP: 1:95462101:T:G
Proxy SNP: 1:95462101:T:G
Tarkin log10(p): 8
Tarkin BETA: 0.298
Tarkin MSPA: 0.358
1225:1113:1167:1086:952:912:5

262. F5 (P12259)
Tarkin MSPA=0.476

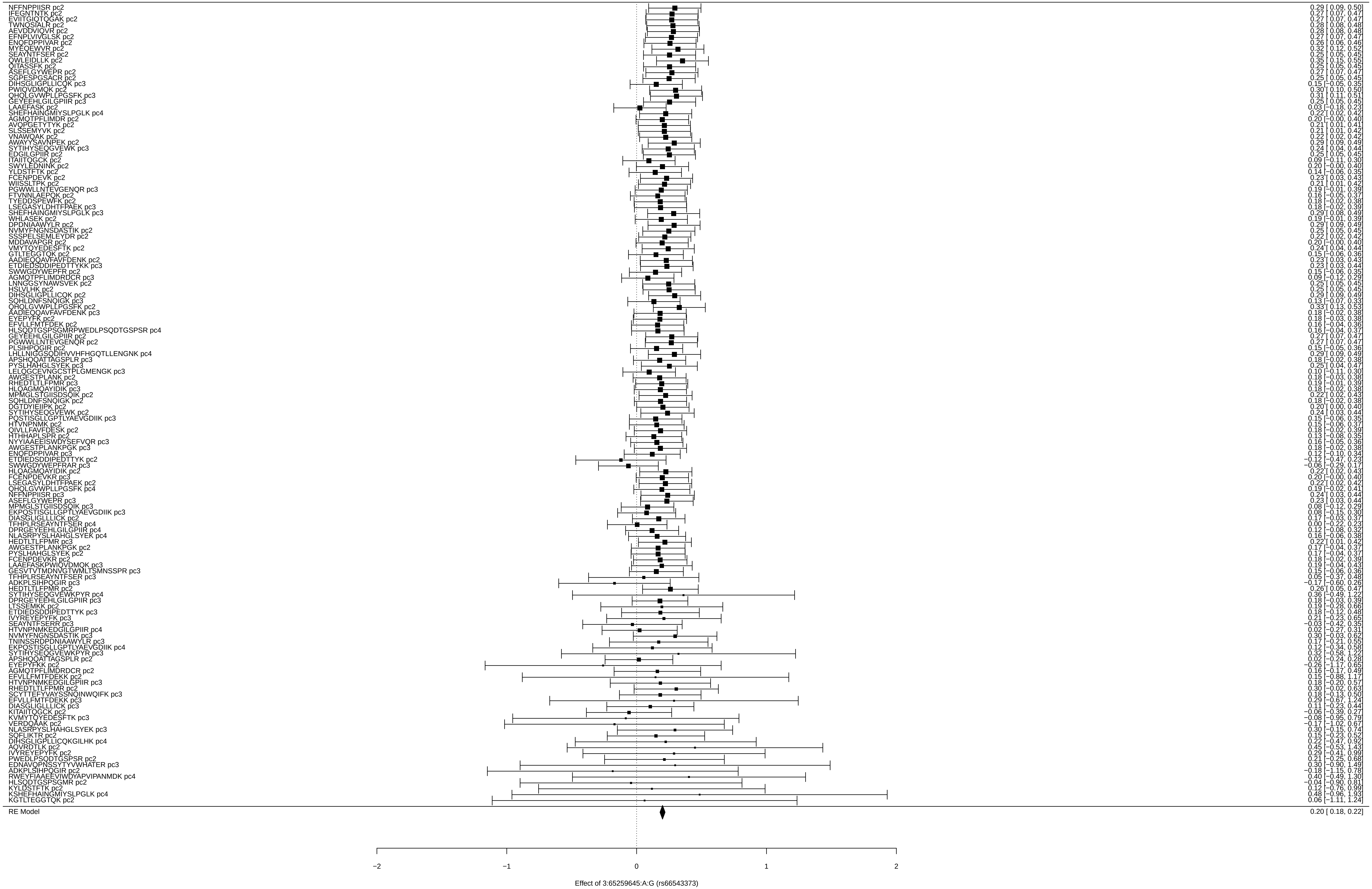

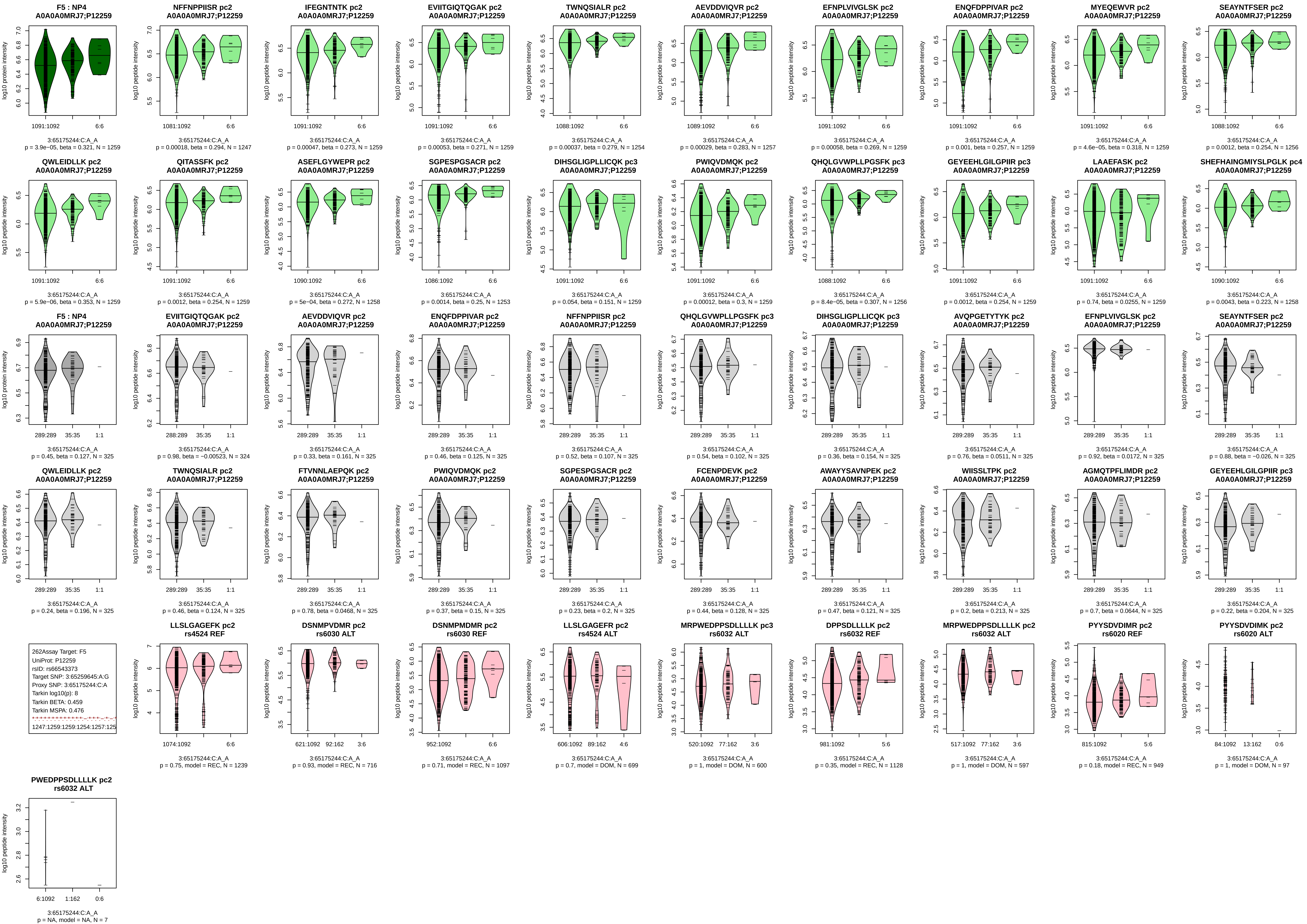
F5 : NP4 A0A0A0MRJ7;P12259
log10 protein intensity
1091:1092 6:6
3:65175244:C:A_A
p = 3.9e−05, beta = 0.321, N = 1259
NFFNPPIISR pc2 A0A0A0MRJ7;P12259
p = 0.00018, beta = 0.294, N = 1247
IFEGNTNTK pc2 A0A0A0MRJ7;P12259
p = 0.00047, beta = 0.273, N = 1259
EVIITGIQTQGAK pc2 A0A0A0MRJ7;P12259
p = 0.00053, beta = 0.271, N = 1259
TWNQSIALR pc2 A0A0A0MRJ7;P12259
p = 0.00037, beta = 0.279, N = 1254
AEVDDVIQVR pc2 A0A0A0MRJ7;P12259
p = 0.00029, beta = 0.283, N = 1257
EFNPLVIVGLSK pc2 A0A0A0MRJ7;P12259
p = 0.00058, beta = 0.269, N = 1259
ENQFDPPIVAR pc2 A0A0A0MRJ7;P12259
p = 0.001, beta = 0.257, N = 1259
MYEQEWVR pc2 A0A0A0MRJ7;P12259
p = 4.6e−05, beta = 0.318, N = 1259
SEAYNTFSER pc2 A0A0A0MRJ7;P12259
p = 0.0012, beta = 0.254, N = 1256
QWLEIDLLK pc2 A0A0A0MRJ7;P12259
p = 5.9e−06, beta = 0.353, N = 1259
QITASSFK pc2 A0A0A0MRJ7;P12259
p = 0.0012, beta = 0.254, N = 1259
ASEFLGYWEPR pc2 A0A0A0MRJ7;P12259
p = 5e−04, beta = 0.272, N = 1258
SGPESPGSACR pc2 A0A0A0MRJ7;P12259
p = 0.0014, beta = 0.25, N = 1253
DIHSGLIGPLLICQK pc3 A0A0A0MRJ7;P12259
p = 0.054, beta = 0.151, N = 1259
PWIQVDMQK pc2 A0A0A0MRJ7;P12259
p = 0.00012, beta = 0.3, N = 1259
QHQLGVWPLLPGSFK pc3 A0A0A0MRJ7;P12259
p = 8.4e−05, beta = 0.307, N = 1256
GEYEEHLGILGPIIR pc3 A0A0A0MRJ7;P12259
p = 0.0012, beta = 0.254, N = 1259
LAAEFASK pc2 A0A0A0MRJ7;P12259
p = 0.74, beta = 0.0255, N = 1259
SHEFHAINGMIYSLPGLK pc4 A0A0A0MRJ7;P12259
p = 0.0043, beta = 0.223, N = 1258
F5 : NP4 A0A0A0MRJ7;P12259
289:289 35:35 1:1
p = 0.45, beta = 0.127, N = 325
EVIITGIQTQGAK pc2 A0A0A0MRJ7;P12259
p = 0.98, beta = −0.00523, N = 324
AEVDDVIQVR pc2 A0A0A0MRJ7;P12259
p = 0.33, beta = 0.161, N = 325
ENQFDPPIVAR pc2 A0A0A0MRJ7;P12259
p = 0.46, beta = 0.125, N = 325
NFFNPPIISR pc2 A0A0A0MRJ7;P12259
p = 0.52, beta = 0.107, N = 325
QHQLGVWPLLPGSFK pc3 A0A0A0MRJ7;P12259
p = 0.54, beta = 0.102, N = 325
DIHSGLIGPLLICQK pc3 A0A0A0MRJ7;P12259
p = 0.36, beta = 0.154, N = 325
AVQPGETYTYK pc2 A0A0A0MRJ7;P12259
p = 0.76, beta = 0.0511, N = 325
EFNPLVIVGLSK pc2 A0A0A0MRJ7;P12259
p = 0.92, beta = 0.0172, N = 325
SEAYNTFSER pc2 A0A0A0MRJ7;P12259
p = 0.88, beta = −0.026, N = 325
QWLEIDLLK pc2 A0A0A0MRJ7;P12259
p = 0.24, beta = 0.196, N = 325
TWNQSIALR pc2 A0A0A0MRJ7;P12259
p = 0.46, beta = 0.124, N = 325
FTVNNLAEPQK pc2 A0A0A0MRJ7;P12259
p = 0.78, beta = 0.0468, N = 325
PWIQVDMQK pc2 A0A0A0MRJ7;P12259
p = 0.37, beta = 0.15, N = 325
SGPESPGSACR pc2 A0A0A0MRJ7;P12259
p = 0.23, beta = 0.2, N = 325
FCENPDEVK pc2 A0A0A0MRJ7;P12259
p = 0.44, beta = 0.128, N = 325
AWAYYSAVNPEK pc2 A0A0A0MRJ7;P12259
p = 0.47, beta = 0.121, N = 325
WIISSLTPK pc2 A0A0A0MRJ7;P12259
p = 0.2, beta = 0.213, N = 325
AGMQTPFLIMDR pc2 A0A0A0MRJ7;P12259
p = 0.7, beta = 0.0644, N = 325
GEYEEHLGILGPIIR pc3 A0A0A0MRJ7;P12259
p = 0.22, beta = 0.204, N = 325
262Assay Target: F5
UniProt: P12259
rsID: rs6543373
Target SNP: 3:65259645:A:G
Proxy SNP: 3:65175244:C:A
Tarkin log10(p): 8
Tarkin BETA: 0.459
Tarkin MSPA: 0.476
1247:1259:1259:1254:1257:125
LLSLGAGEFK pc2 rs4524 REF
1074:1092 6:6
p = 0.75, model = REC, N = 1239
DSNMPVDMR pc2 rs6030 ALT
621:1092 92:162 3:6
p = 0.93, model = REC, N = 716
DSNMPMDMR pc2 rs6030 REF
952:1092 6:6
p = 0.71, model = REC, N = 1097
LLSLGAGEFR pc2 rs4524 ALT
606:1092 89:162 4:6
p = 0.7, model = DOM, N = 699
MRPWEDPPSDLLLLK pc3 rs6032 ALT
520:1092 77:162 3:6
p = 1, model = DOM, N = 600
DPPSDLLLLK pc2 rs6032 REF
981:1092 5:6
p = 0.35, model = REC, N = 1128
MRPWEDPPSDLLLLK pc2 rs6032 ALT
517:1092 77:162 3:6
p = 1, model = DOM, N = 597
PYYSDVDIMR pc2 rs6020 REF
815:1092 5:6
p = 0.18, model = REC, N = 949
PYYSDVDIMK pc2 rs6020 ALT
84:1092 13:162 0:6
p = 1, model = DOM, N = 97
PWEDPPSDLLLLK pc2 rs6032 ALT
6:1092 1:162 0:6
p = NA, model = NA, N = 7

263. MMP1 (P03956)
Tarkin MSPA=0.714

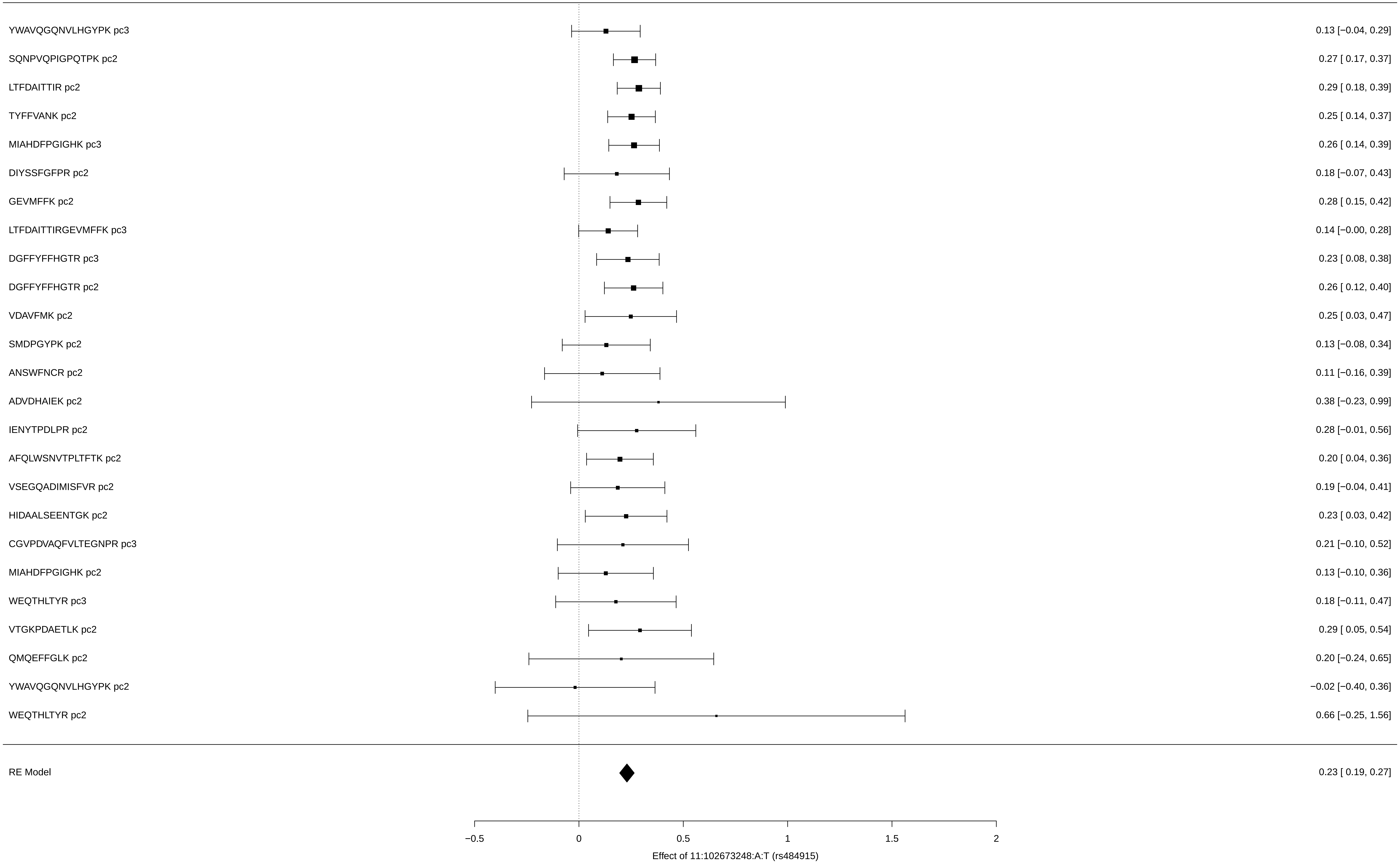

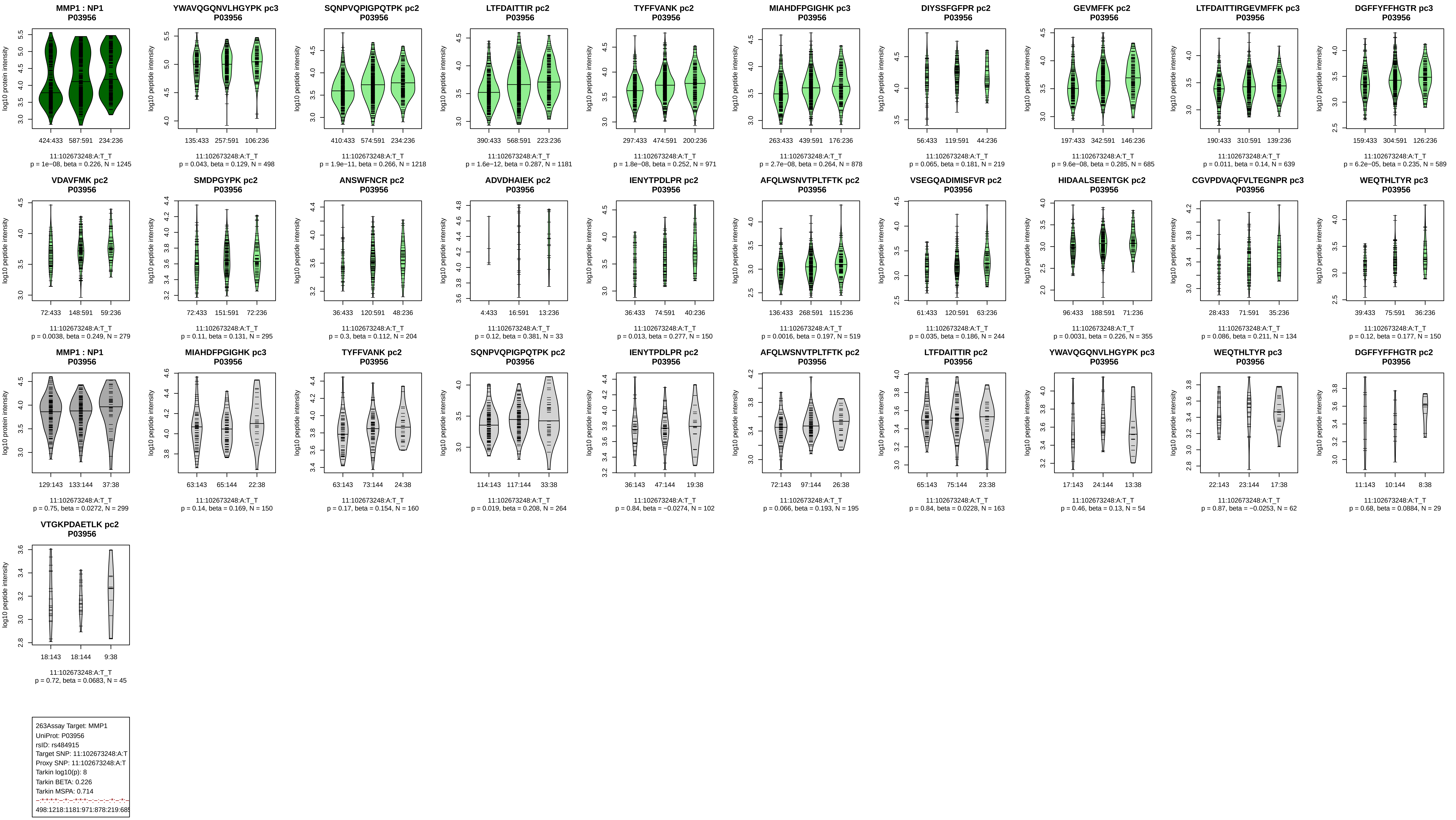
MMP1 : NP1
P03956
p = 1e−08, beta = 0.226, N = 1245
YWAVQGQNVLHGYPK pc3
P03956
p = 0.043, beta = 0.129, N = 498
SQNPVQPIGPQTPK pc2
P03956
p = 1.9e−11, beta = 0.266, N = 1218
LTFDAITTIR pc2
P03956
p = 1.6e−12, beta = 0.287, N = 1181
TYFFVANK pc2
P03956
p = 1.8e−08, beta = 0.252, N = 971
MIAHDFPGIGHK pc3
P03956
p = 2.7e−08, beta = 0.264, N = 878
DIYSSFGFPR pc2
P03956
p = 0.065, beta = 0.181, N = 219
GEVMFFK pc2
P03956
p = 9.6e−08, beta = 0.285, N = 685
LTFDAITTIRGEVMFFK pc3
P03956
p = 0.011, beta = 0.14, N = 639
DGFFYFFHGTR pc3
P03956
p = 6.2e−05, beta = 0.235, N = 589
VDAVFMK pc2
P03956
p = 0.0038, beta = 0.249, N = 279
SMDPGYPK pc2
P03956
p = 0.11, beta = 0.131, N = 295
ANSWFNCR pc2
P03956
p = 0.3, beta = 0.112, N = 204
ADVDHAIEK pc2
P03956
p = 0.12, beta = 0.381, N = 33
IENYTPDLPR pc2
P03956
p = 0.013, beta = 0.277, N = 150
AFQLWSNVTPLTFTK pc2
P03956
p = 0.0016, beta = 0.197, N = 519
VSEGQADIMISFVR pc2
P03956
p = 0.035, beta = 0.186, N = 244
HIDAALSEENTGK pc2
P03956
p = 0.0031, beta = 0.226, N = 355
CGVPDVAQFVLTEGNPR pc3
P03956
p = 0.086, beta = 0.211, N = 134
WEQTHLTYR pc3
P03956
p = 0.12, beta = 0.177, N = 150
MMP1 : NP1
P03956
p = 0.75, beta = 0.0272, N = 299
MIAHDFPGIGHK pc3
P03956
p = 0.14, beta = 0.169, N = 150
TYFFVANK pc2
P03956
p = 0.17, beta = 0.154, N = 160
SQNPVQPIGPQTPK pc2
P03956
p = 0.019, beta = 0.208, N = 264
IENYTPDLPR pc2
P03956
p = 0.84, beta = −0.0274, N = 102
AFQLWSNVTPLTFTK pc2
P03956
p = 0.066, beta = 0.193, N = 195
LTFDAITTIR pc2
P03956
p = 0.84, beta = 0.0228, N = 163
YWAVQGQNVLHGYPK pc3
P03956
p = 0.46, beta = 0.13, N = 54
WEQTHLTYR pc3
P03956
p = 0.87, beta = −0.0253, N = 62
DGFFYFFHGTR pc2
P03956
p = 0.68, beta = 0.0884, N = 29
VTGKPDAETLK pc2
P03956
p = 0.72, beta = 0.0683, N = 45
11:102673248:A:T_T
log10 protein intensity
log10 peptide intensity
263Assay Target: MMP1
UniProt: P03956
rsID: rs484915
Target SNP: 11:102673248:A:T
Proxy SNP: 11:102673248:A:T
Tarkin log10(p): 8
Tarkin BETA: 0.226
Tarkin MSPA: 0.714
498:1218:1181:971:878:219:685

264. PLA2G7 (Q13093)
Tarkin MSPA=0.961

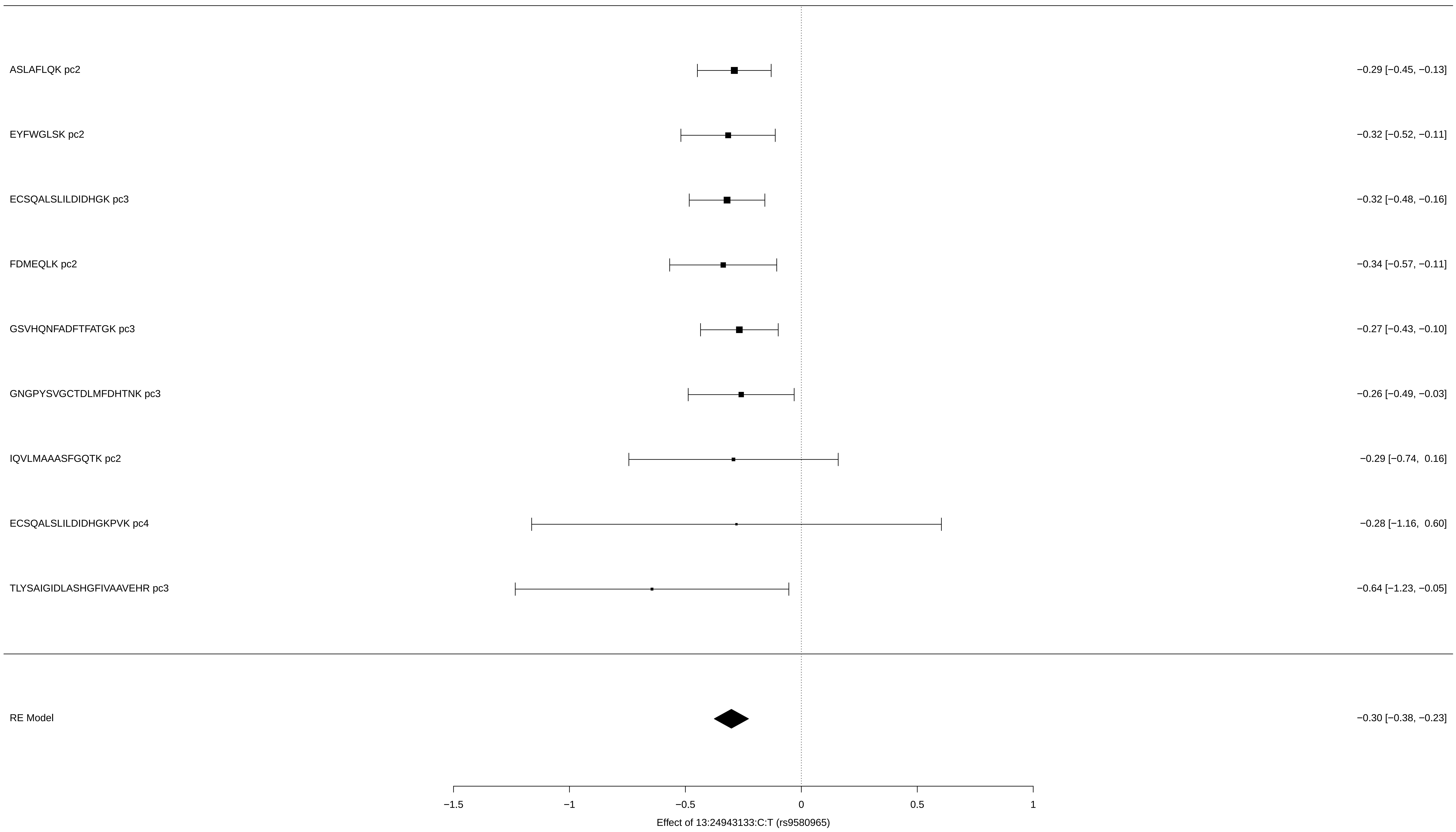

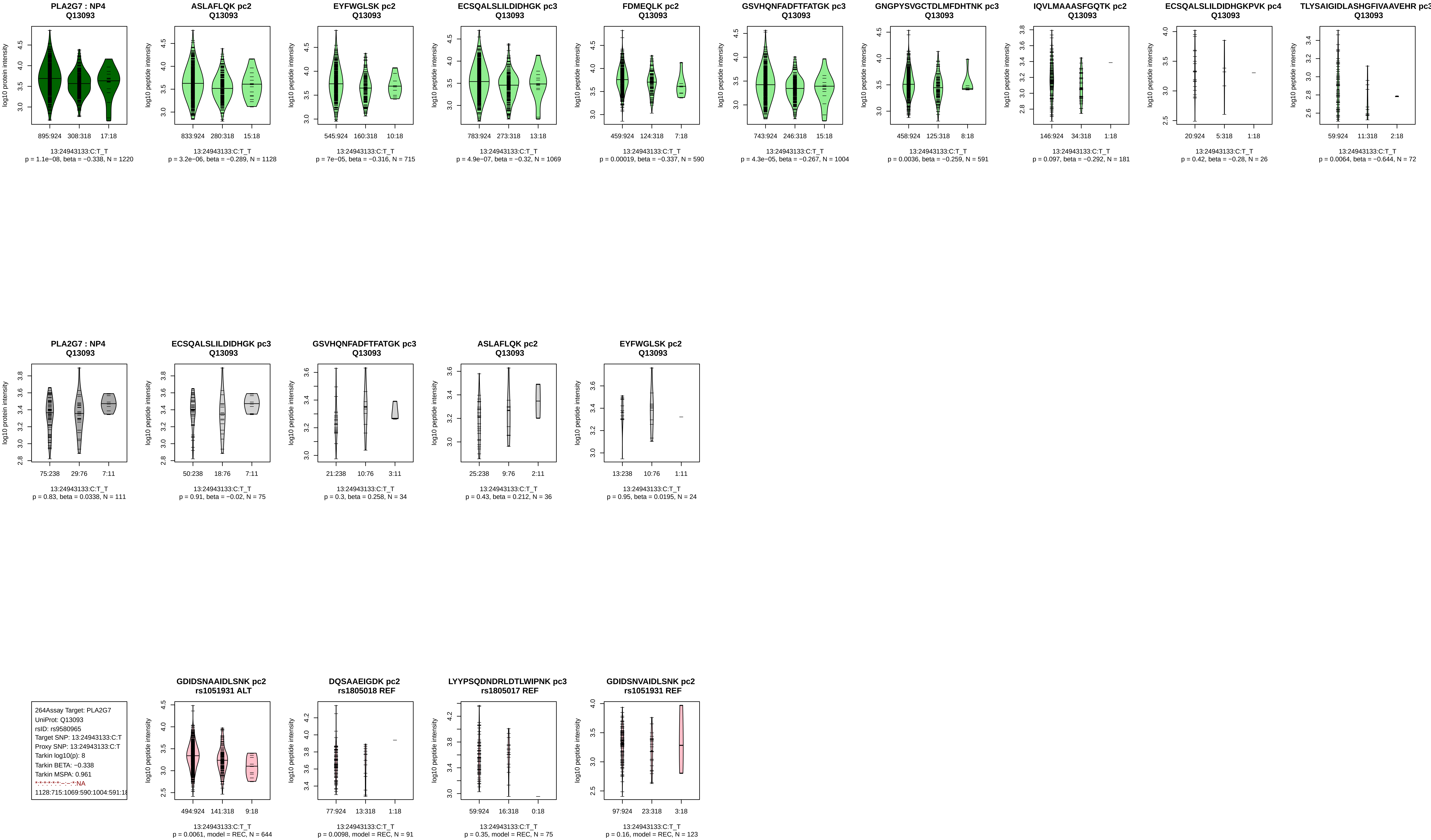
PLA2G7 : NP4
Q13093
log10 protein intensity
895:924 308:318 17:18
13:24943133:C:T_T
p = 1.1e−08, beta = −0.338, N = 1220
ASLAFLQK pc2
Q13093
log10 peptide intensity
833:924 280:318 15:18
13:24943133:C:T_T
p = 3.2e−06, beta = −0.289, N = 1128
EYFWGLSK pc2
Q13093
545:924 160:318 10:18
13:24943133:C:T_T
p = 7e−05, beta = −0.316, N = 715
ECSQALSLILDIDHGK pc3
Q13093
783:924 273:318 13:18
13:24943133:C:T_T
p = 4.9e−07, beta = −0.32, N = 1069
FDMEQLK pc2
Q13093
459:924 124:318 7:18
13:24943133:C:T_T
p = 0.00019, beta = −0.337, N = 590
GSVHQNFADFTFATGK pc3
Q13093
743:924 246:318 15:18
13:24943133:C:T_T
p = 4.3e−05, beta = −0.267, N = 1004
GNGPYSVGCTDLMFDHTNK pc3
Q13093
458:924 125:318 8:18
13:24943133:C:T_T
p = 0.0036, beta = −0.259, N = 591
IQVLMAAASFGQTK pc2
Q13093
146:924 34:318 1:18
13:24943133:C:T_T
p = 0.097, beta = −0.292, N = 181
ECSQALSLILDIDHGKPVK pc4
Q13093
20:924 5:318 1:18
13:24943133:C:T_T
p = 0.42, beta = −0.28, N = 26
TLYSAIGIDLASHGFIVAAVEHR pc3
Q13093
59:924 11:318 2:18
13:24943133:C:T_T
p = 0.0064, beta = −0.644, N = 72
PLA2G7 : NP4
Q13093
log10 protein intensity
75:238 29:76 7:11
13:24943133:C:T_T
p = 0.83, beta = 0.0338, N = 111
ECSQALSLILDIDHGK pc3
Q13093
50:238 18:76 7:11
13:24943133:C:T_T
p = 0.91, beta = −0.02, N = 75
GSVHQNFADFTFATGK pc3
Q13093
21:238 10:76 3:11
13:24943133:C:T_T
p = 0.3, beta = 0.258, N = 34
ASLAFLQK pc2
Q13093
25:238 9:76 2:11
13:24943133:C:T_T
p = 0.43, beta = 0.212, N = 36
EYFWGLSK pc2
Q13093
13:238 10:76 1:11
13:24943133:C:T_T
p = 0.95, beta = 0.0195, N = 24
264Assay Target: PLA2G7
UniProt: Q13093
rsID: rs9580965
Target SNP: 13:24943133:C:T
Proxy SNP: 13:24943133:C:T
Tarkin log10(p): 8
Tarkin BETA: −0.338
Tarkin MSPA: 0.961
::*:*:*:*:−:−:*:NA
1128:715:1069:590:1004:591:18
GDIDSNAAIDLSNK pc2
rs1051931 ALT
494:924 141:318 9:18
13:24943133:C:T_T
p = 0.0061, model = REC, N = 644
DQSAAEIGDK pc2
rs1805018 REF
77:924 13:318 1:18
13:24943133:C:T_T
p = 0.0098, model = REC, N = 91
LYYPSQDNDRLDTLWIPNK pc3
rs1805017 REF
59:924 16:318 0:18
13:24943133:C:T_T
p = 0.35, model = REC, N = 75
GDIDSNVAIDLSNK pc2
rs1051931 REF
97:924 23:318 3:18
13:24943133:C:T_T
p = 0.16, model = REC, N = 123

265. ERP44 (Q9BS26)
Tarkin MSPA=0

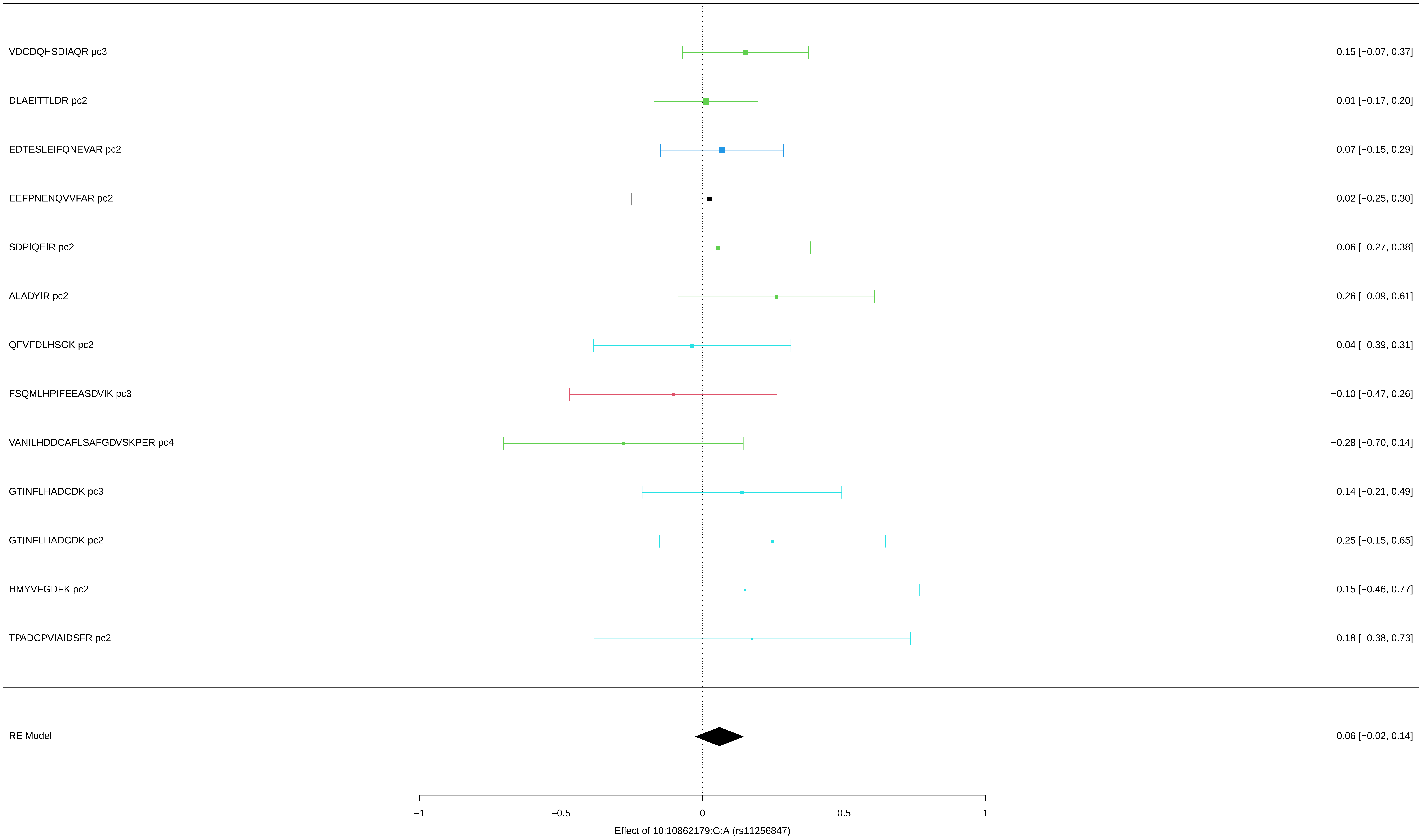

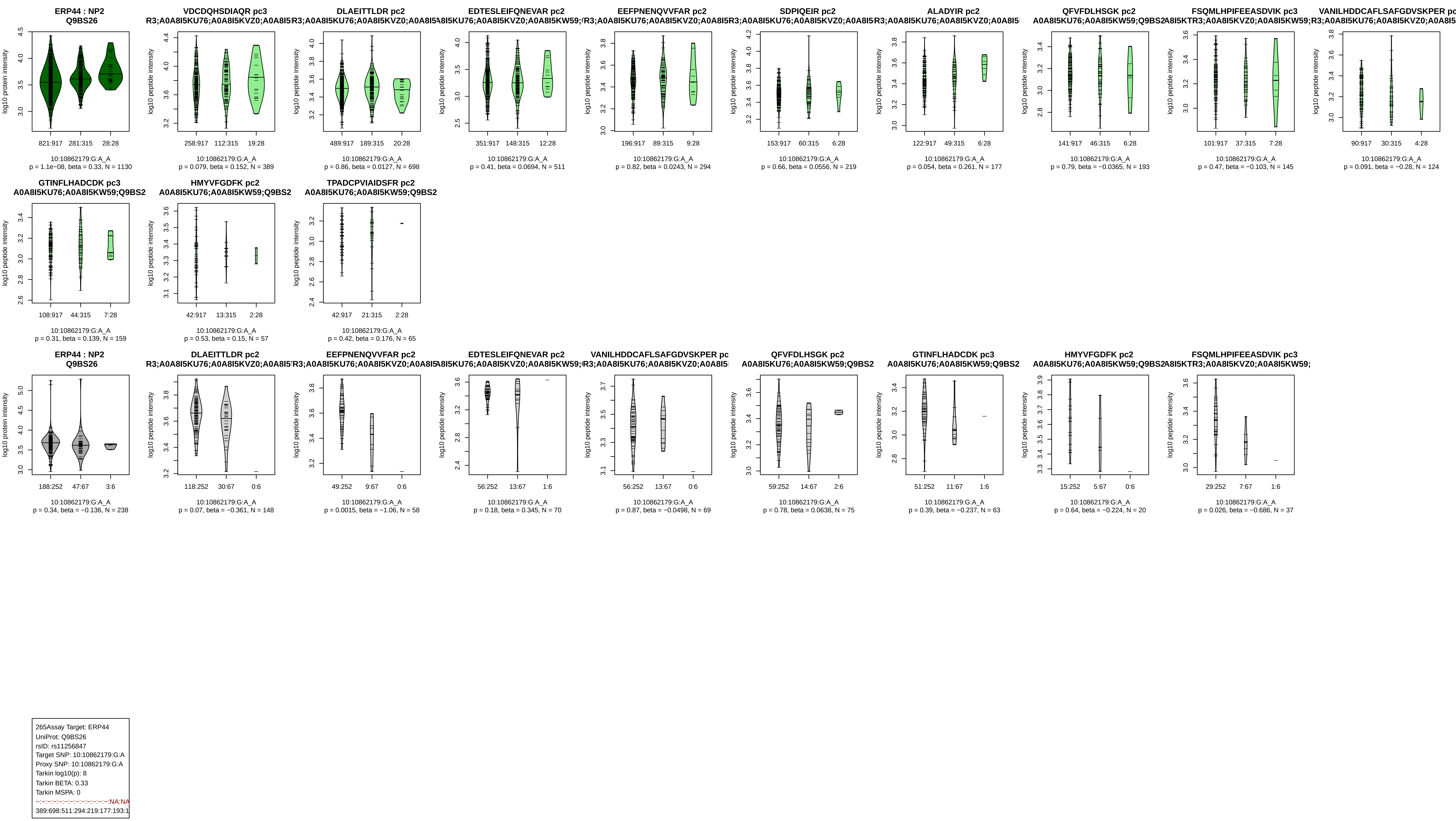
ERP44 : NP2
Q9BS26
10:10862179:G:A_A
p = 1.1e−08, beta = 0.33, N = 1130
VDCDQHSDIAQR pc3
p = 0.079, beta = 0.152, N = 389
DLAEITTLDR pc2
p = 0.86, beta = 0.0127, N = 698
EDTESLEIFQNEVAR pc2
p = 0.41, beta = 0.0694, N = 511
EEFPNENQVVFAR pc2
p = 0.82, beta = 0.0243, N = 294
SDPIQEIR pc2
p = 0.66, beta = 0.0556, N = 219
ALADYIR pc2
p = 0.054, beta = 0.261, N = 177
QFVFDLHSGK pc2
p = 0.79, beta = −0.0365, N = 193
FSQMLHPIFEEASDVIK pc3
p = 0.47, beta = −0.103, N = 145
VANILHDDCAFLSAFGDVSKPER pc2
p = 0.091, beta = −0.28, N = 124
GTINFLHADCDK pc3
p = 0.31, beta = 0.139, N = 159
HMYVFGDFK pc2
p = 0.53, beta = 0.15, N = 57
TPADCPVIAIDSFR pc2
p = 0.42, beta = 0.176, N = 65
ERP44 : NP2
Q9BS26
p = 0.34, beta = −0.136, N = 238
DLAEITTLDR pc2
p = 0.07, beta = −0.361, N = 148
EEFPNENQVVFAR pc2
p = 0.0015, beta = −1.06, N = 58
EDTESLEIFQNEVAR pc2
p = 0.18, beta = 0.345, N = 70
VANILHDDCAFLSAFGDVSKPER pc
p = 0.87, beta = −0.0498, N = 69
QFVFDLHSGK pc2
p = 0.78, beta = 0.0638, N = 75
GTINFLHADCDK pc3
p = 0.39, beta = −0.237, N = 63
HMYVFGDFK pc2
p = 0.64, beta = −0.224, N = 20
FSQMLHPIFEEASDVIK pc3
p = 0.026, beta = −0.686, N = 37
265Assay Target: ERP44
UniProt: Q9BS26
rsID: rs11256847
Target SNP: 10:10862179:G:A
Proxy SNP: 10:10862179:G:A
Tarkin log10(p): 8
Tarkin BETA: 0.33
Tarkin MSPA: 0
389:698:511:294:219:177:193:1

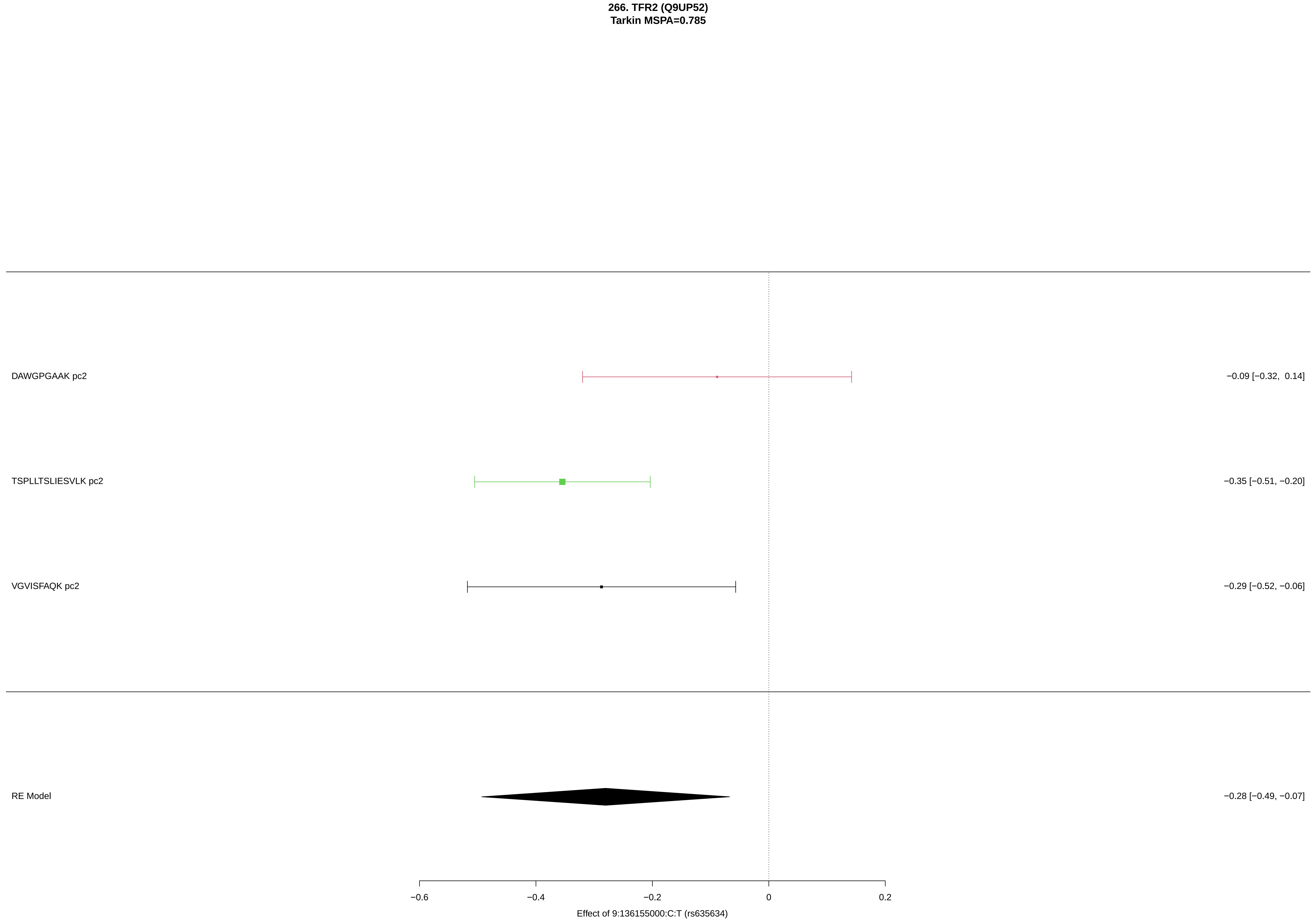
266. TFR2 (Q9UP52)
Tarkin MSPA=0.785
DAWGPGAAK pc2
−0.09 [−0.32, 0.14]
TSPLLTSLIESVLK pc2
−0.35 [−0.51, −0.20]
VGVISFAQK pc2
−0.29 [−0.52, −0.06]
RE Model
−0.28 [−0.49, −0.07]
−0.6
−0.4
−0.2
0
0.2
Effect of 9:136155000:C:T (rs635634)

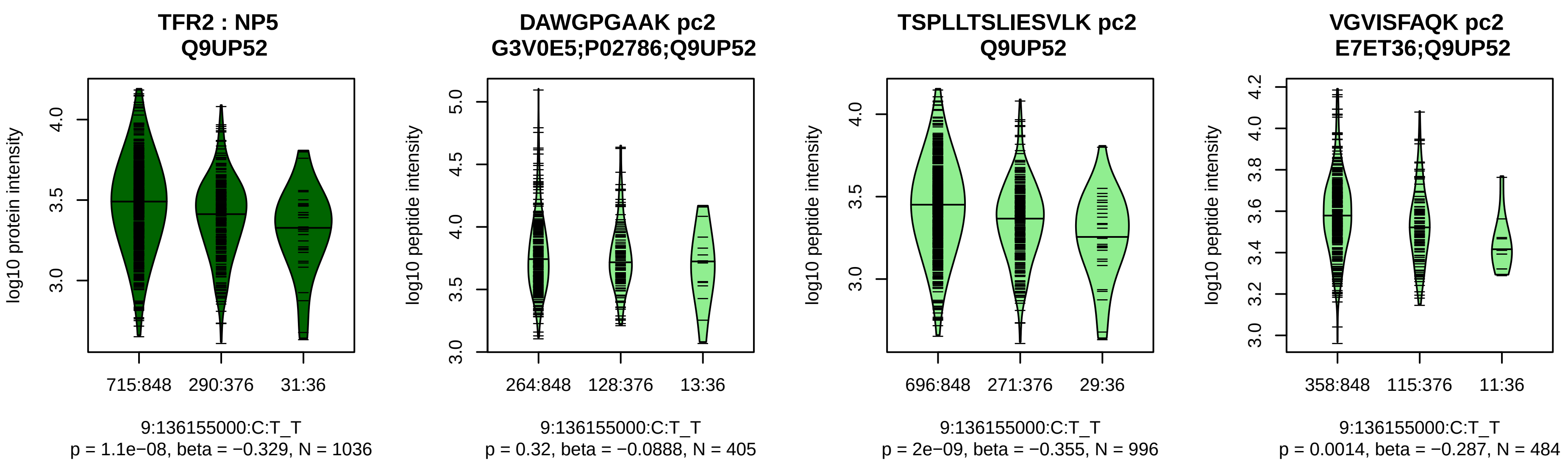

266Assay Target: TFR2
UniProt: Q9UP52
rsID: rs635634
Target SNP: 9:136155000:C:T
Proxy SNP: 9:136155000:C:T
Tarkin log10(p): 8
Tarkin BETA: −0.329
Tarkin MSPA: 0.785
–:*:*
405:996:484

267. PSMD3 (O43242)
Tarkin MSPA=0.262

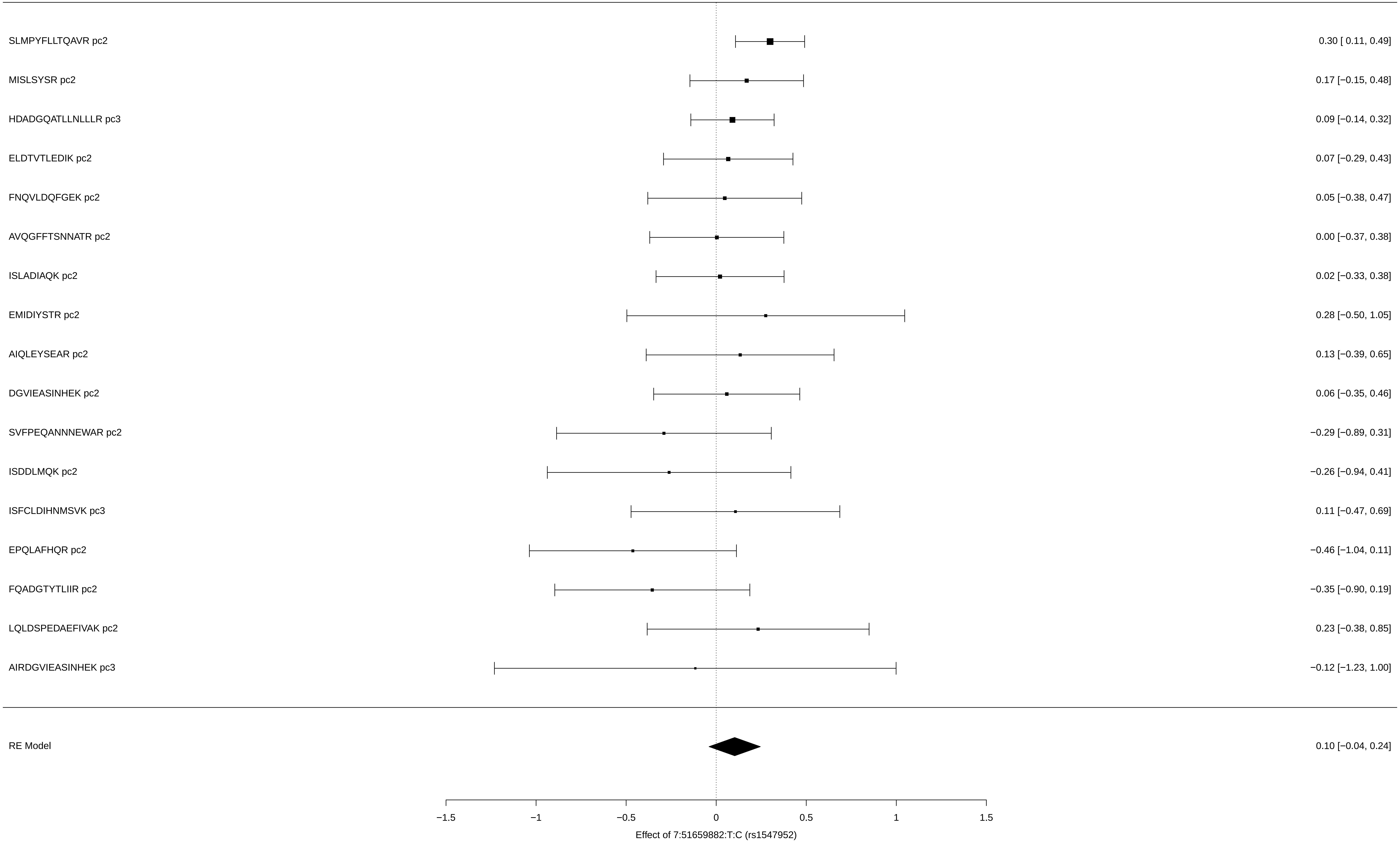

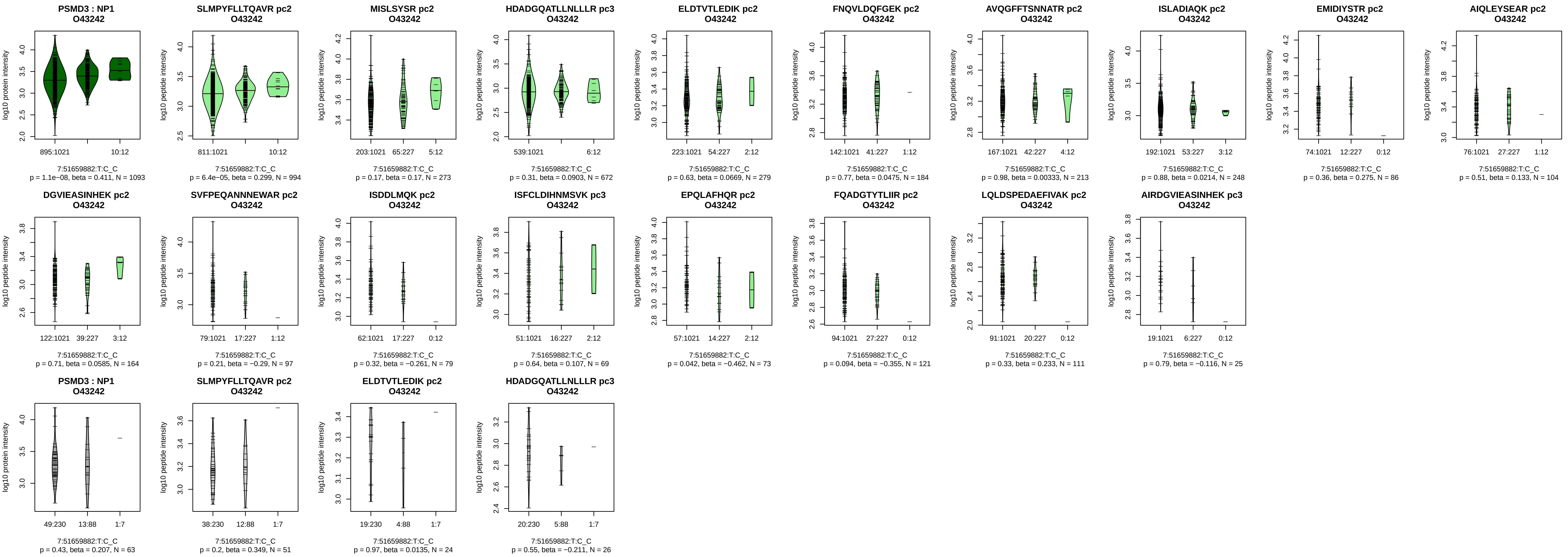

267Assay Target: PSMD3
UniProt: O43242
rsID: rs1547952
Target SNP: 7:51659882:T:C
Proxy SNP: 7:51659882:T:C
Tarkin log10(p): 8
Tarkin BETA: 0.411
Tarkin MSPA: 0.262
*-:-:-:-:-:-:-:-:-:-:-:-:-:-:-:-:-:-
994:273:672:279:184:213:248:8

268. EEF2 (P13639)
Tarkin MSPA=0.088

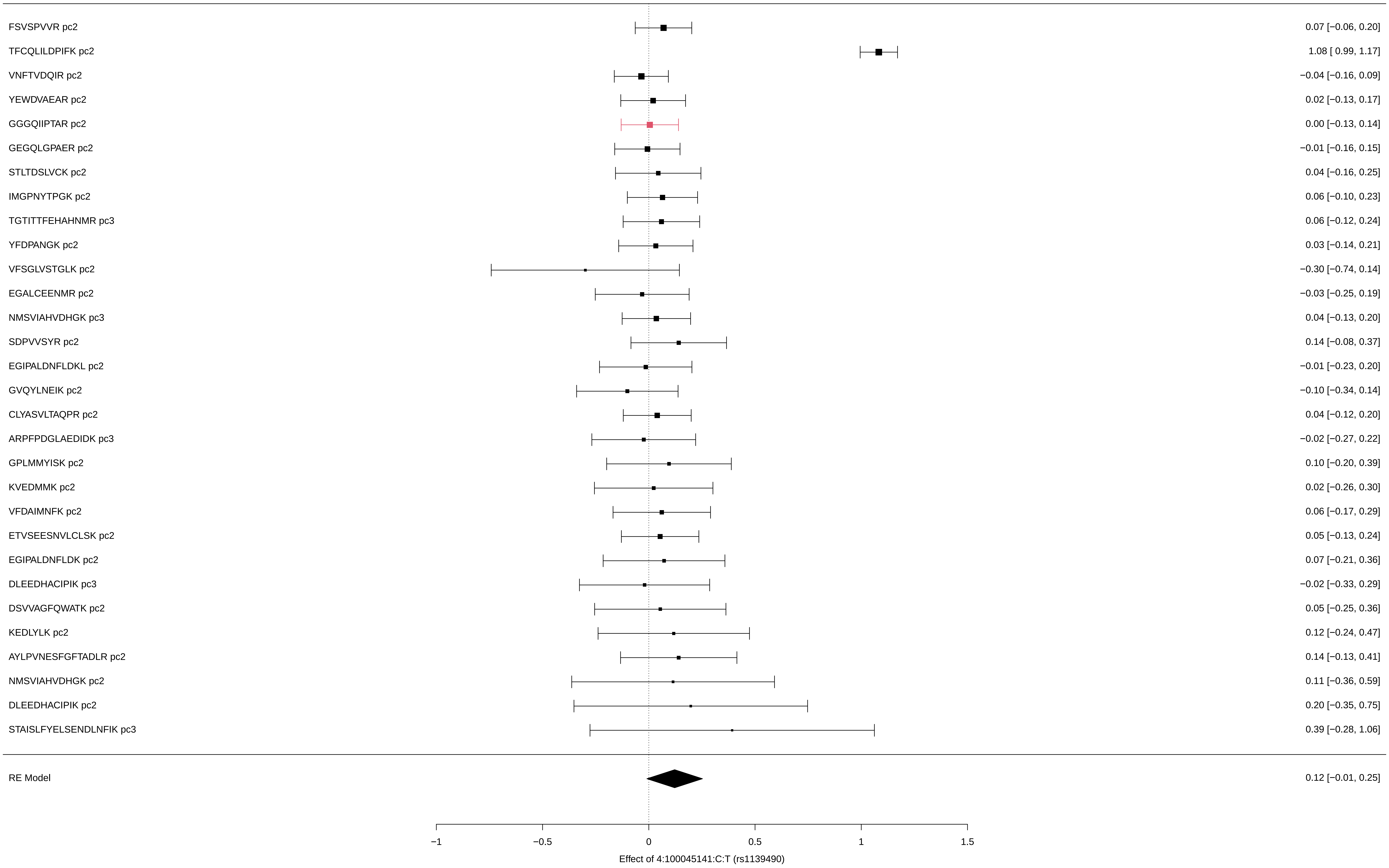

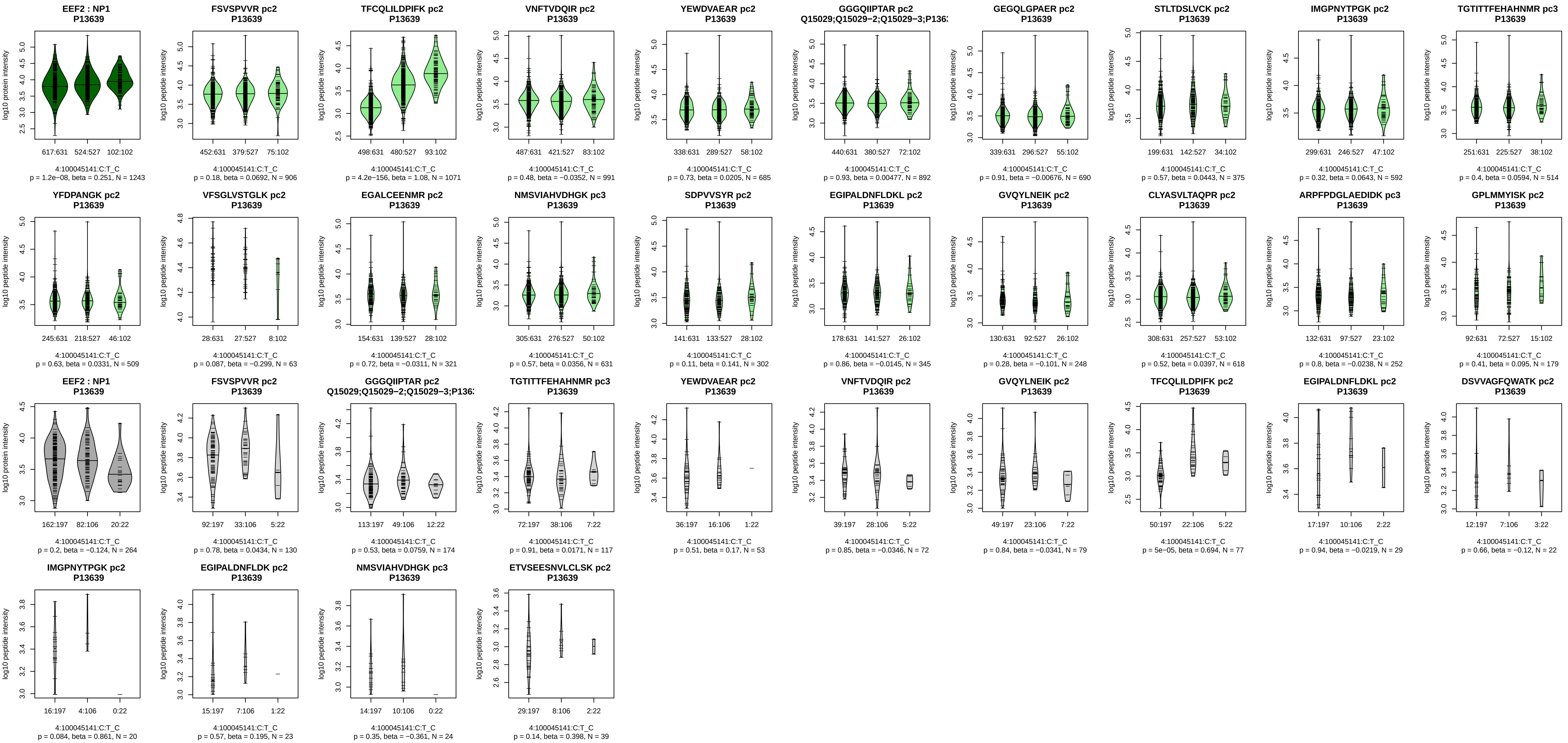

268Assay Target: EEF2
UniProt: P13639
rsID: rs1139490
Target SNP: 4:100045141:C:T
Proxy SNP: 4:100045141:C:T
Tarkin log10(p): 7.9
Tarkin BETA: 0.251
Tarkin MSPA: 0.088
906:1071:991:685:892:690:375

269. C1R (B4DPQ0)
Tarkin MSPA=0.607

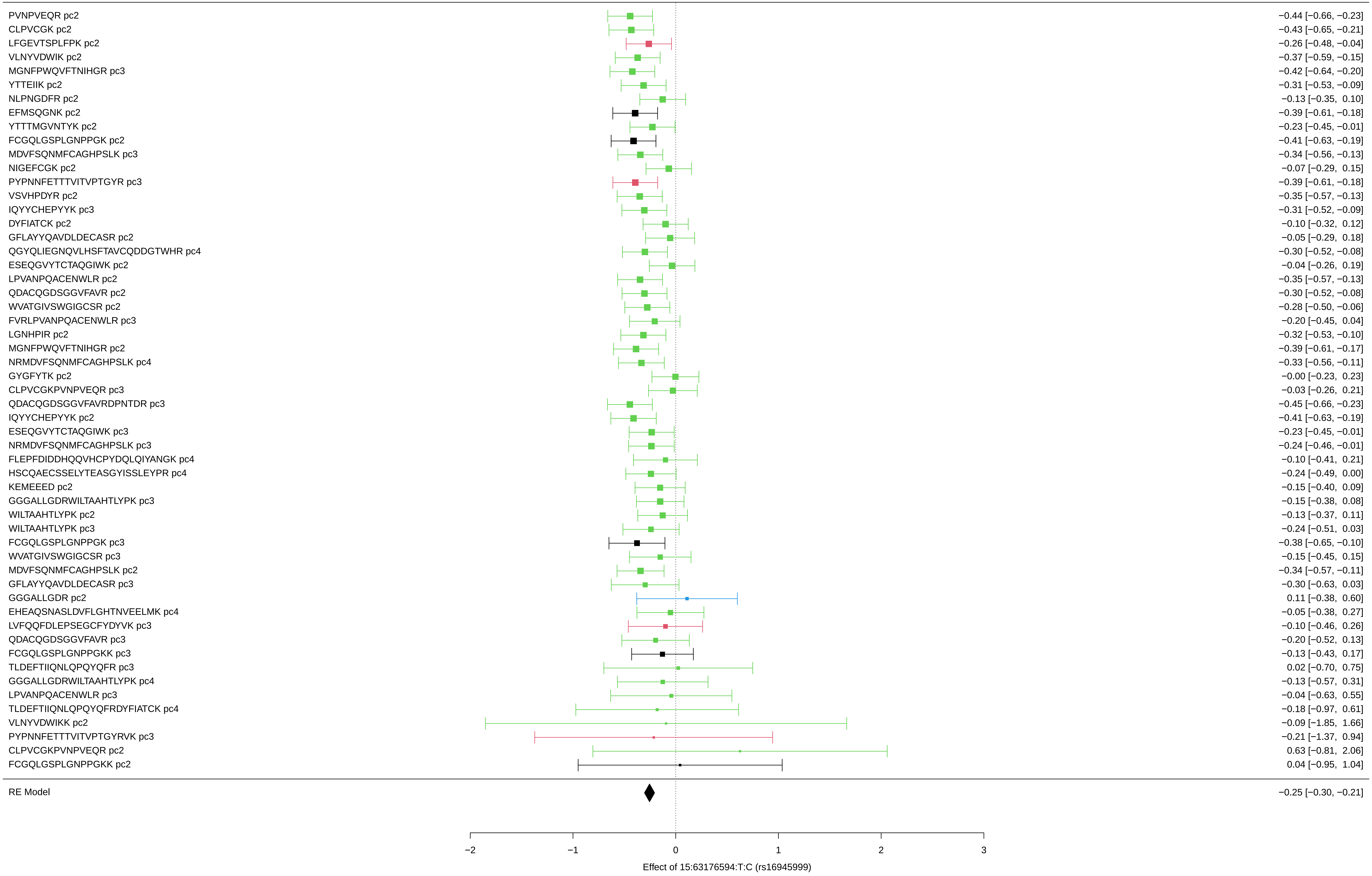

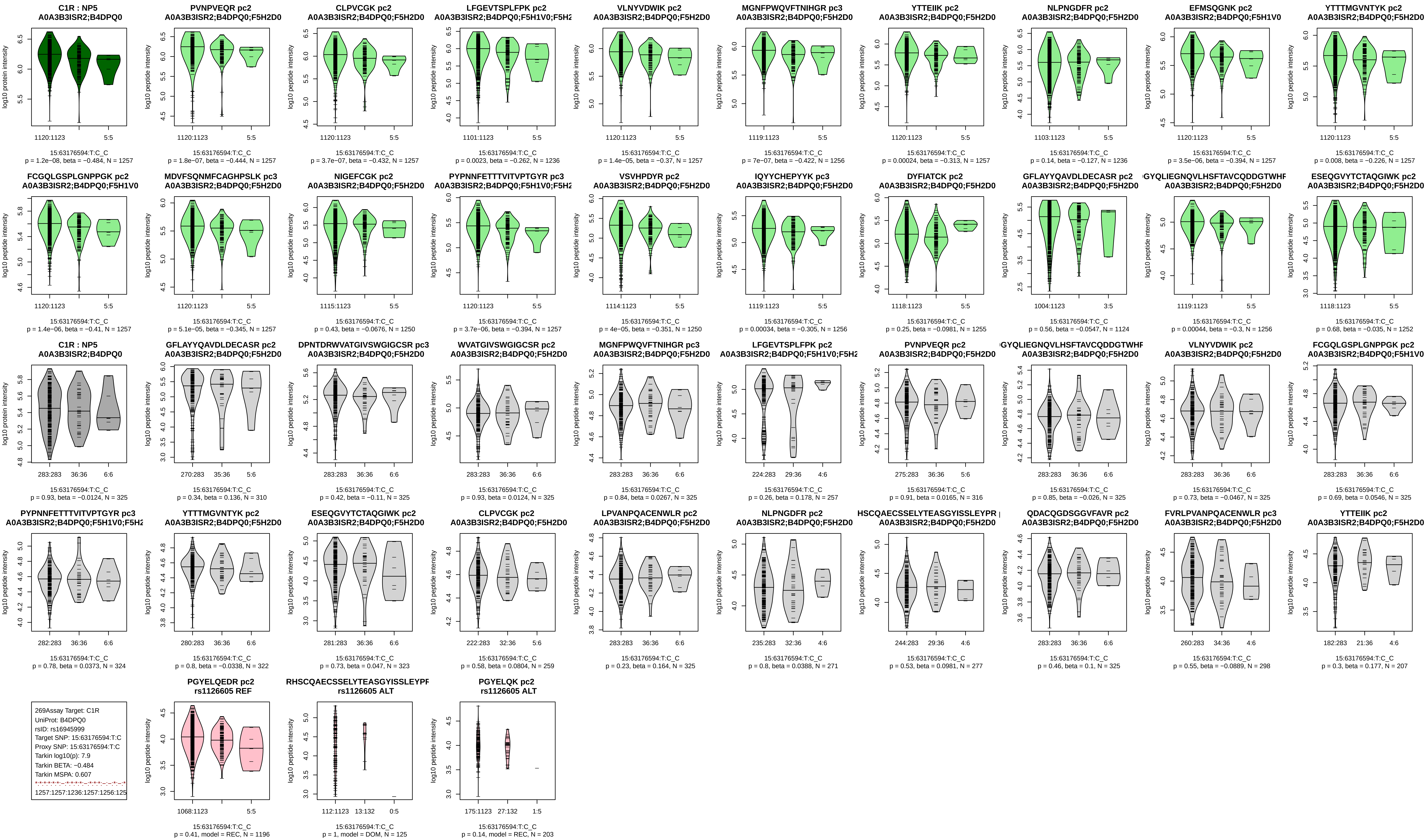

C1R : NP5
A0A3B3ISR2;B4DPQ0
log10 protein intensity
1120:1123
5:5
15:63176594:T:C_C
p = 1.2e−08, beta = −0.484, N = 1257
PVNPVEQR pc2
A0A3B3ISR2;B4DPQ0;F5H2D0
log10 peptide intensity
1120:1123
5:5
15:63176594:T:C_C
p = 1.8e−07, beta = −0.444, N = 1257
CLPVCGK pc2
A0A3B3ISR2;B4DPQ0;F5H2D0
1120:1123
5:5
15:63176594:T:C_C
p = 3.7e−07, beta = −0.432, N = 1257
LFGEVTSPLFPK pc2
A0A3B3ISR2;B4DPQ0;F5H1V0;F5H2
1101:1123
5:5
15:63176594:T:C_C
p = 0.0023, beta = −0.262, N = 1236
VLNYVDWIK pc2
A0A3B3ISR2;B4DPQ0;F5H2D0
1120:1123
5:5
15:63176594:T:C_C
p = 1.4e−05, beta = −0.37, N = 1257
MGNFPWQVFTNIHGR pc3
A0A3B3ISR2;B4DPQ0;F5H2D0
1119:1123
5:5
15:63176594:T:C_C
p = 7e−07, beta = −0.422, N = 1256
YTTEIIK pc2
A0A3B3ISR2;B4DPQ0;F5H2D0
1120:1123
5:5
15:63176594:T:C_C
p = 0.00024, beta = −0.313, N = 1257
NLPNGDFR pc2
A0A3B3ISR2;B4DPQ0;F5H2D0
1103:1123
5:5
15:63176594:T:C_C
p = 0.14, beta = −0.127, N = 1236
EFMSQGNK pc2
A0A3B3ISR2;B4DPQ0;F5H1V0
1120:1123
5:5
15:63176594:T:C_C
p = 3.5e−06, beta = −0.394, N = 1257
YTTTMGVNTYK pc2
A0A3B3ISR2;B4DPQ0;F5H2D0
1120:1123
5:5
15:63176594:T:C_C
p = 0.008, beta = −0.226, N = 1257
FCGQLGSPLGNPPGK pc2
A0A3B3ISR2;B4DPQ0;F5H1V0
1120:1123
5:5
15:63176594:T:C_C
p = 1.4e−06, beta = −0.41, N = 1257
MDVFSQNMFCAGHPSLK pc3
A0A3B3ISR2;B4DPQ0;F5H2D0
1120:1123
5:5
15:63176594:T:C_C
p = 5.1e−05, beta = −0.345, N = 1257
NIGEFCGK pc2
A0A3B3ISR2;B4DPQ0;F5H2D0
1115:1123
5:5
15:63176594:T:C_C
p = 0.43, beta = −0.0676, N = 1250
PYPNNFETTTVITVPTGYR pc3
A0A3B3ISR2;B4DPQ0;F5H1V0;F5H2
1120:1123
5:5
15:63176594:T:C_C
p = 3.7e−06, beta = −0.394, N = 1257
VSVHPDYR pc2
A0A3B3ISR2;B4DPQ0;F5H2D0
1114:1123
5:5
15:63176594:T:C_C
p = 4e−05, beta = −0.351, N = 1250
IQYYCHEPYYK pc3
A0A3B3ISR2;B4DPQ0;F5H2D0
1119:1123
5:5
15:63176594:T:C_C
p = 0.00034, beta = −0.305, N = 1256
DYFIATCK pc2
A0A3B3ISR2;B4DPQ0;F5H2D0
1118:1123
5:5
15:63176594:T:C_C
p = 0.25, beta = −0.0981, N = 1255
GFLAYYQAVDLDECASR pc2
A0A3B3ISR2;B4DPQ0;F5H2D0
1004:1123
3:5
15:63176594:T:C_C
p = 0.56, beta = −0.0547, N = 1124
GYQLIEGNQVLHSFTAVCQDDGTWHF
A0A3B3ISR2;B4DPQ0;F5H2D0
1119:1123
5:5
15:63176594:T:C_C
p = 0.00044, beta = −0.3, N = 1256
ESEQGVYTCTAQGIWK pc2
A0A3B3ISR2;B4DPQ0;F5H2D0
1118:1123
5:5
15:63176594:T:C_C
p = 0.68, beta = −0.035, N = 1252
C1R : NP5
A0A3B3ISR2;B4DPQ0
283:283
36:36
6:6
15:63176594:T:C_C
p = 0.93, beta = −0.0124, N = 325
GFLAYYQAVDLDECASR pc2
A0A3B3ISR2;B4DPQ0;F5H2D0
270:283
35:36
5:6
15:63176594:T:C_C
p = 0.34, beta = 0.136, N = 310
DPNTDRWVATGIVSWGIGCSR pc3
A0A3B3ISR2;B4DPQ0;F5H2D0
283:283
36:36
6:6
15:63176594:T:C_C
p = 0.42, beta = −0.11, N = 325
WVATGIVSWGIGCSR pc2
A0A3B3ISR2;B4DPQ0;F5H2D0
283:283
36:36
6:6
15:63176594:T:C_C
p = 0.93, beta = 0.0124, N = 325
MGNFPWQVFTNIHGR pc3
A0A3B3ISR2;B4DPQ0;F5H2D0
283:283
36:36
6:6
15:63176594:T:C_C
p = 0.84, beta = 0.0267, N = 325
LFGEVTSPLFPK pc2
A0A3B3ISR2;B4DPQ0;F5H1V0;F5H2
224:283
29:36
4:6
15:63176594:T:C_C
p = 0.26, beta = 0.178, N = 257
PVNPVEQR pc2
A0A3B3ISR2;B4DPQ0;F5H2D0
275:283
36:36
5:6
15:63176594:T:C_C
p = 0.91, beta = 0.0165, N = 316
GYQLIEGNQVLHSFTAVCQDDGTWHF
A0A3B3ISR2;B4DPQ0;F5H2D0
283:283
36:36
6:6
15:63176594:T:C_C
p = 0.85, beta = −0.026, N = 325
VLNYVDWIK pc2
A0A3B3ISR2;B4DPQ0;F5H2D0
283:283
36:36
6:6
15:63176594:T:C_C
p = 0.73, beta = −0.0467, N = 325
FCGQLGSPLGNPPGK pc2
A0A3B3ISR2;B4DPQ0;F5H1V0
283:283
36:36
6:6
15:63176594:T:C_C
p = 0.69, beta = 0.0546, N = 325
PYPNNFETTTVITVPTGYR pc3
A0A3B3ISR2;B4DPQ0;F5H1V0;F5H2
282:283
36:36
6:6
15:63176594:T:C_C
p = 0.78, beta = 0.0373, N = 324
YTTTMGVNTYK pc2
A0A3B3ISR2;B4DPQ0;F5H2D0
280:283
36:36
6:6
15:63176594:T:C_C
p = 0.8, beta = −0.0338, N = 322
ESEQGVYTCTAQGIWK pc2
A0A3B3ISR2;B4DPQ0;F5H2D0
281:283
36:36
6:6
15:63176594:T:C_C
p = 0.73, beta = 0.047, N = 323
CLPVCGK pc2
A0A3B3ISR2;B4DPQ0;F5H2D0
222:283
32:36
5:6
15:63176594:T:C_C
p = 0.58, beta = 0.0804, N = 259
LPVANPQACENWLR pc2
A0A3B3ISR2;B4DPQ0;F5H2D0
283:283
36:36
6:6
15:63176594:T:C_C
p = 0.23, beta = 0.164, N = 325
NLPNGDFR pc2
A0A3B3ISR2;B4DPQ0;F5H2D0
235:283
32:36
4:6
15:63176594:T:C_C
p = 0.8, beta = 0.0388, N = 271
HSCQAECSSELYTEASGYISSLEYPR
A0A3B3ISR2;B4DPQ0;F5H2D0
244:283
29:36
4:6
15:63176594:T:C_C
p = 0.53, beta = 0.0981, N = 277
QDACQGDSGGVFAVR pc2
A0A3B3ISR2;B4DPQ0;F5H2D0
283:283
36:36
6:6
15:63176594:T:C_C
p = 0.46, beta = 0.1, N = 325
FVRLPVANPQACENWLR pc3
A0A3B3ISR2;B4DPQ0;F5H2D0
260:283
34:36
4:6
15:63176594:T:C_C
p = 0.55, beta = −0.0889, N = 298
YTTEIIK pc2
A0A3B3ISR2;B4DPQ0;F5H2D0
182:283
21:36
4:6
15:63176594:T:C_C
p = 0.3, beta = 0.177, N = 207
269Assay Target: C1R
UniProt: B4DPQ0
rsID: rs16945999
Target SNP: 15:63176594:T:C
Proxy SNP: 15:63176594:T:C
Tarkin log10(p): 7.9
Tarkin BETA: −0.484
Tarkin MSPA: 0.607
1257:1257:1236:1257:1256:125
PGYELQEDR pc2
rs1126605 REF
1068:1123
5:5
15:63176594:T:C_C
p = 0.41, model = REC, N = 1196
RHSCQAECSSELYTEASGYISSLEYPF
rs1126605 ALT
112:1123
13:132
0:5
15:63176594:T:C_C
p = 1, model = DOM, N = 125
PGYELQK pc2
rs1126605 ALT
175:1123
27:132
1:5
15:63176594:T:C_C
p = 0.14, model = REC, N = 203

270. LCAT (P04180)
Tarkin MSPA=0.557

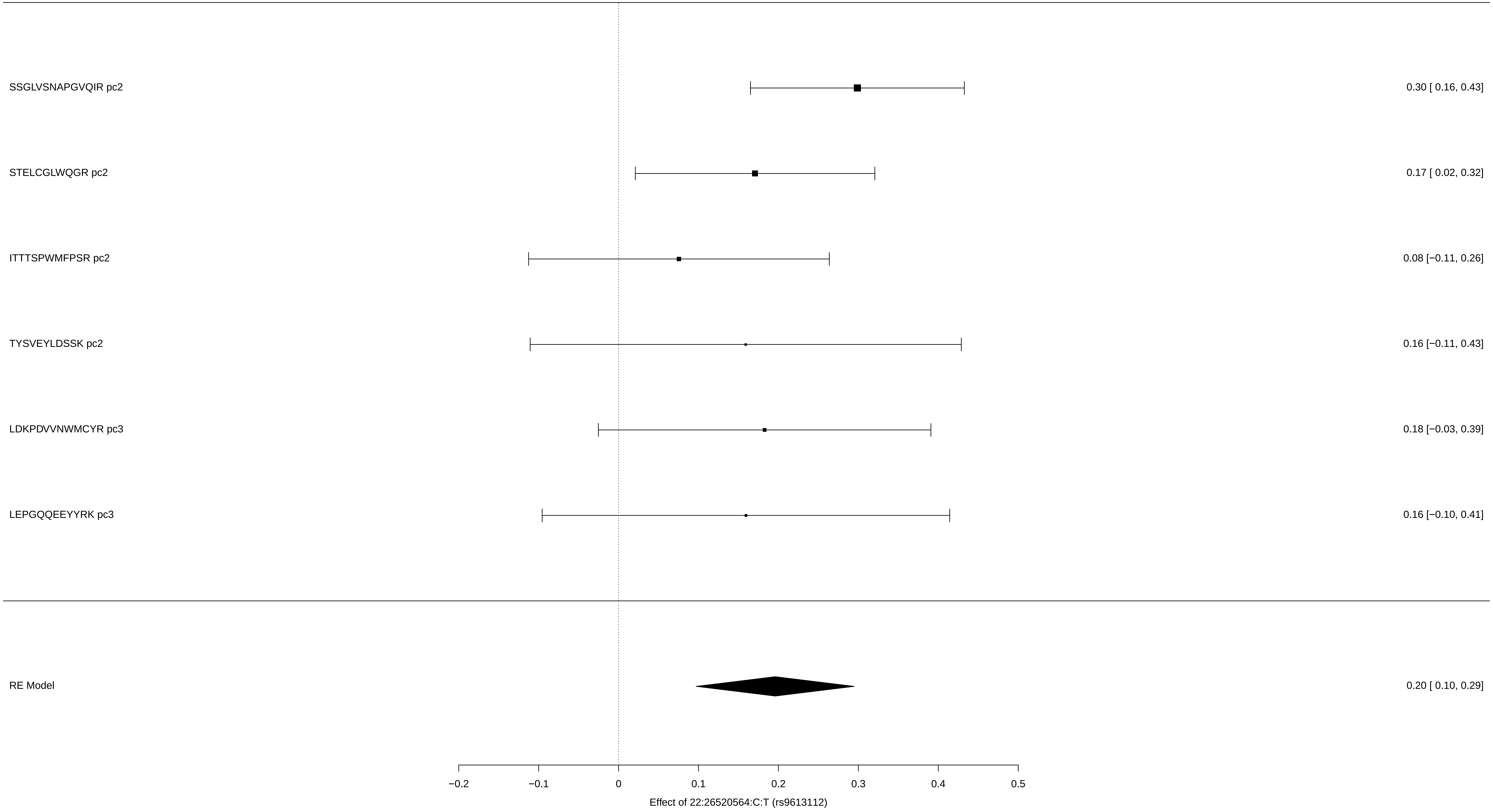

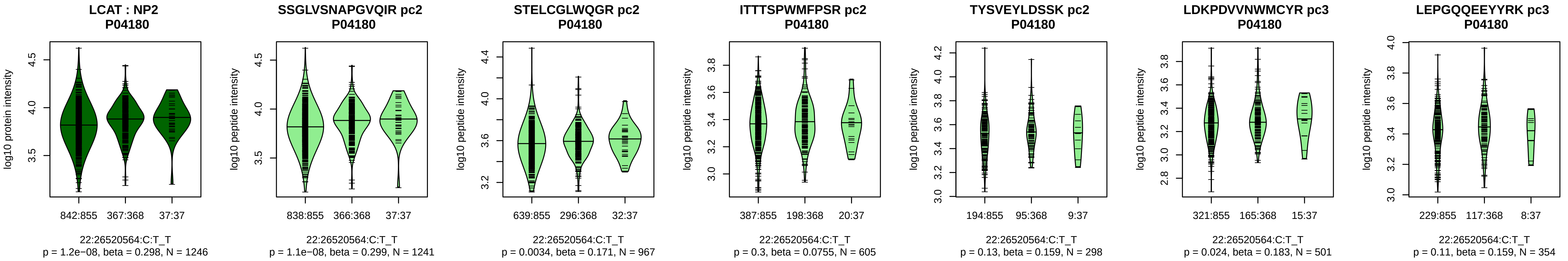

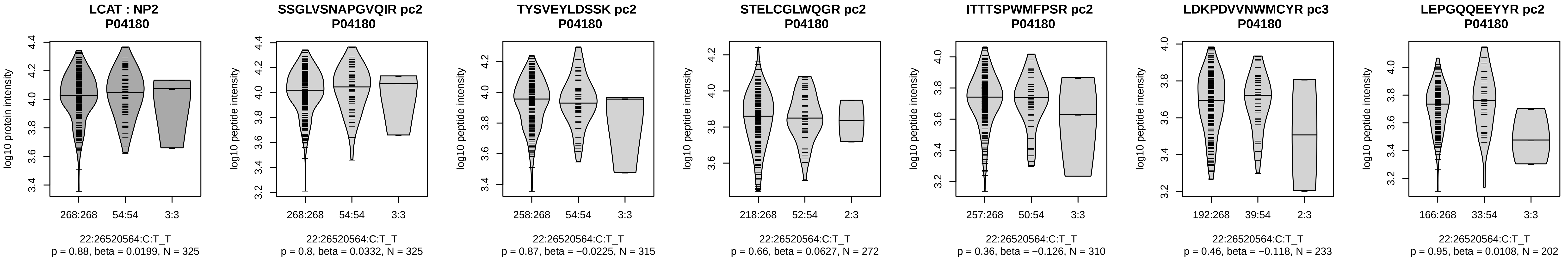

270Assay Target: LCAT
UniProt: P04180
rsID: rs9613112
Target SNP: 22:26520564:C:T
Proxy SNP: 22:26520564:C:T
Tarkin log10(p): 7.9
Tarkin BETA: 0.298
Tarkin MSPA: 0.557
::–:–:–:–
1241:967:605:298:501:354

271. GNL1 (P36915)
Tarkin MSPA=0.304

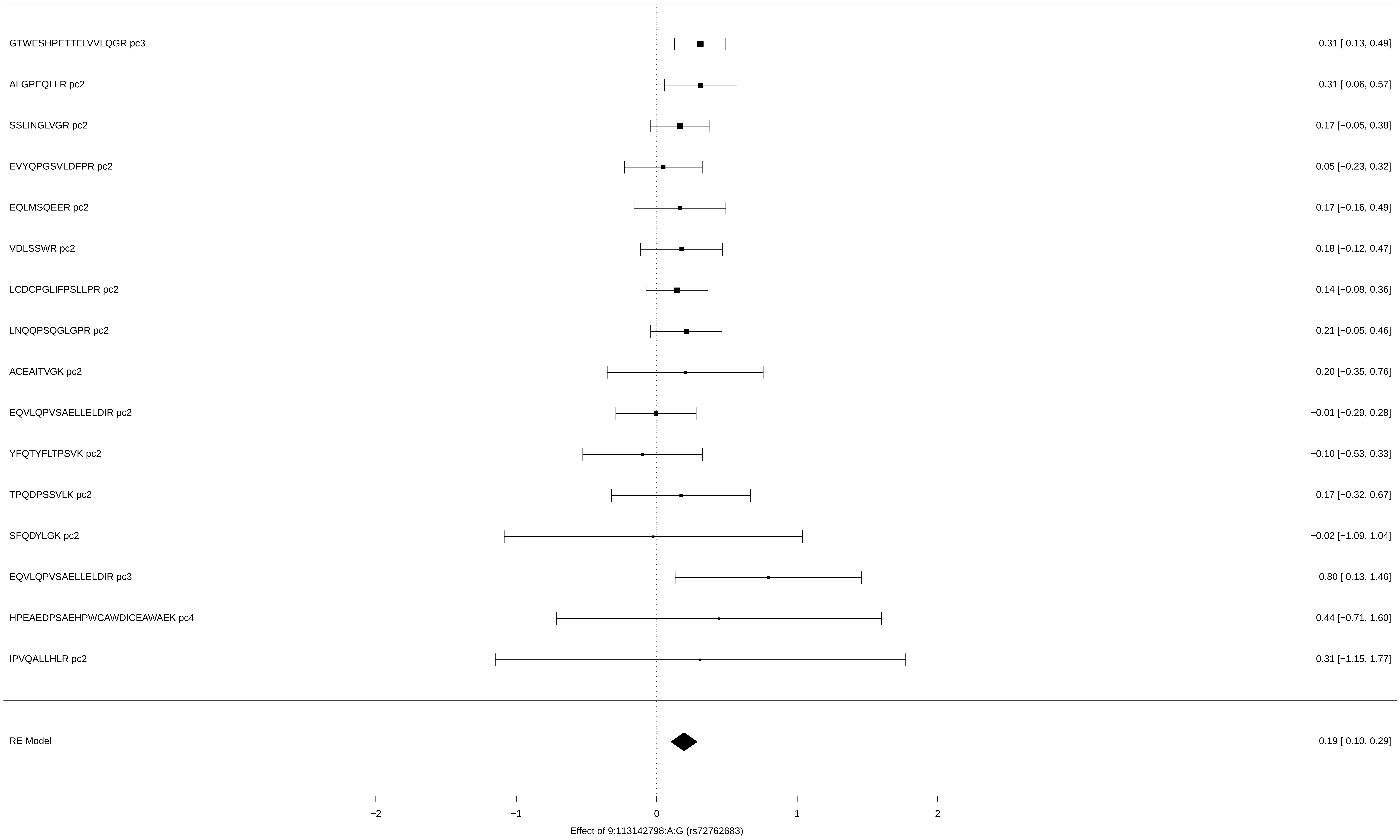

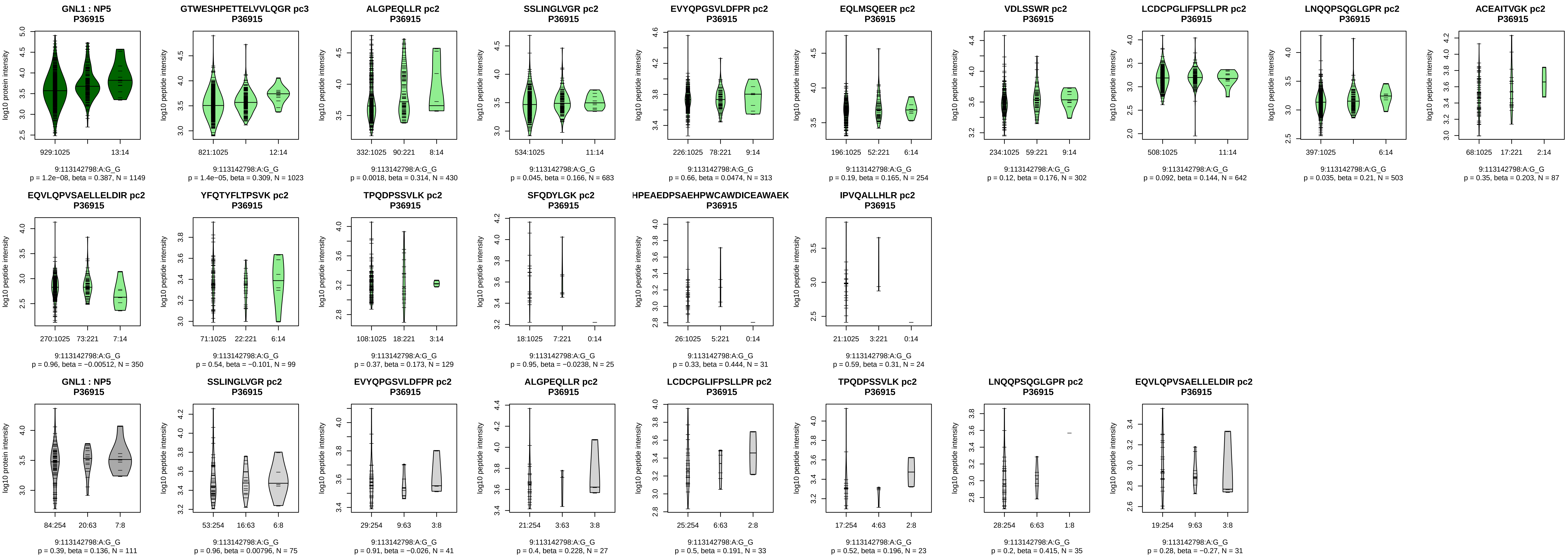

271Assay Target: GNL1
UniProt: P36915
rsID: rs72762683
Target SNP: 9:113142798:A:G
Proxy SNP: 9:113142798:A:G
Tarkin log10(p): 7.9
Tarkin BETA: 0.387
Tarkin MSPA: 0.304
::−:−:−:−:−:−:−:−:−:−:−:*:−:−:NA
1023:430:683:313:254:302:642:

272. SRGN (P10124)
Tarkin MSPA=0.957

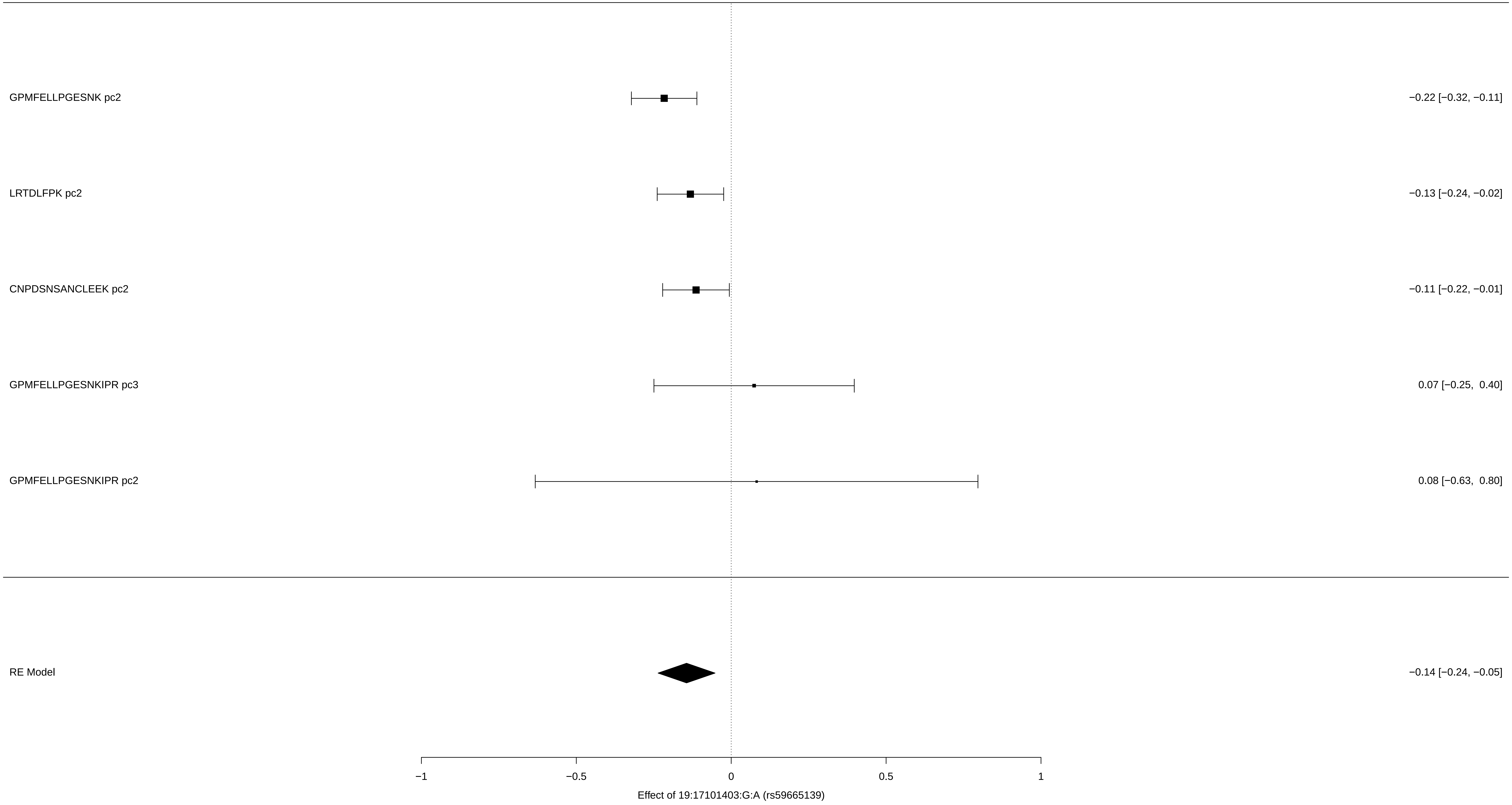

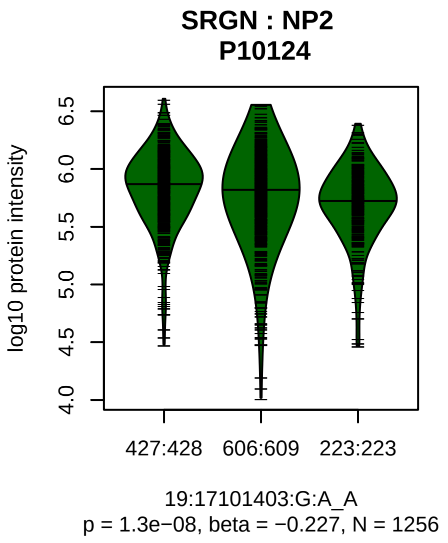

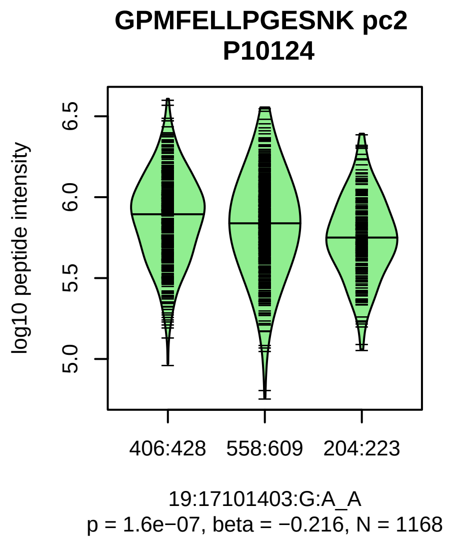

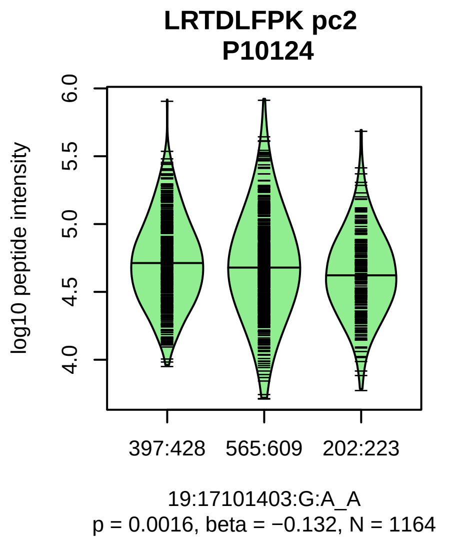

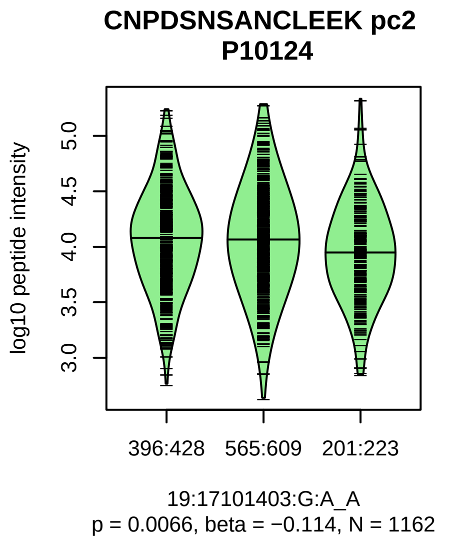

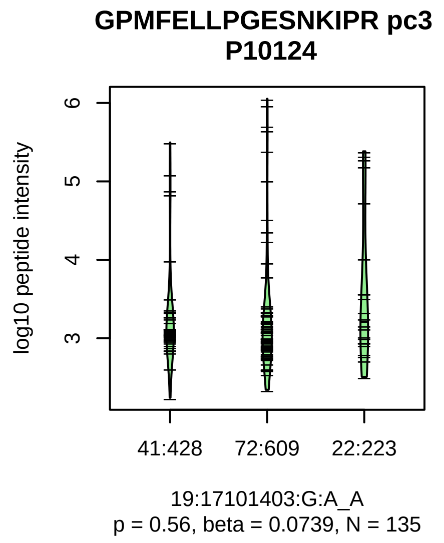

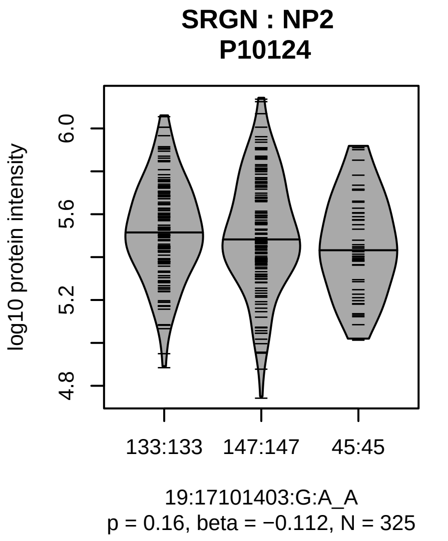

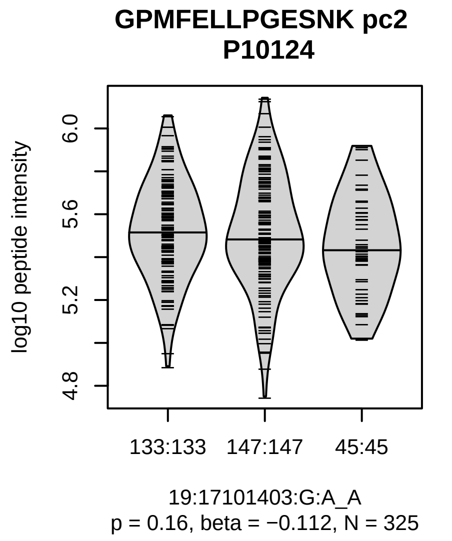

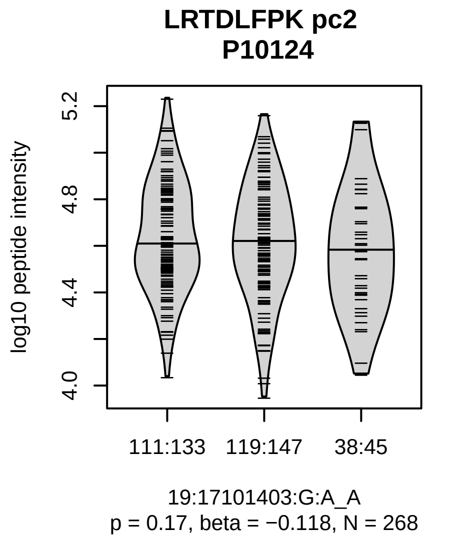

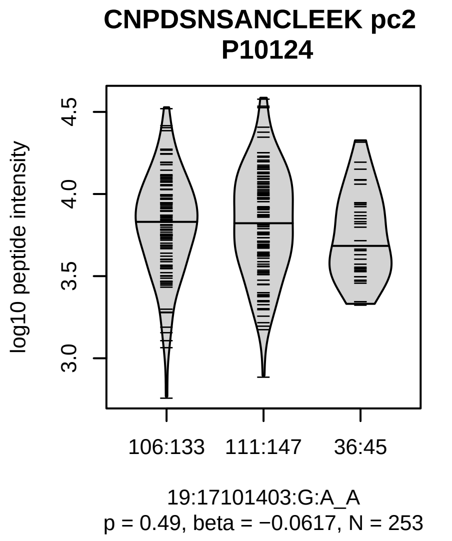

272Assay Target: SRGN
UniProt: P10124
rsID: rs59665139
Target SNP: 19:17101403:G:A
Proxy SNP: 19:17101403:G:A
Tarkin log10(p): 7.9
Tarkin BETA: −0.227
Tarkin MSPA: 0.957
::*:–:–
1168:1164:1162:135:22

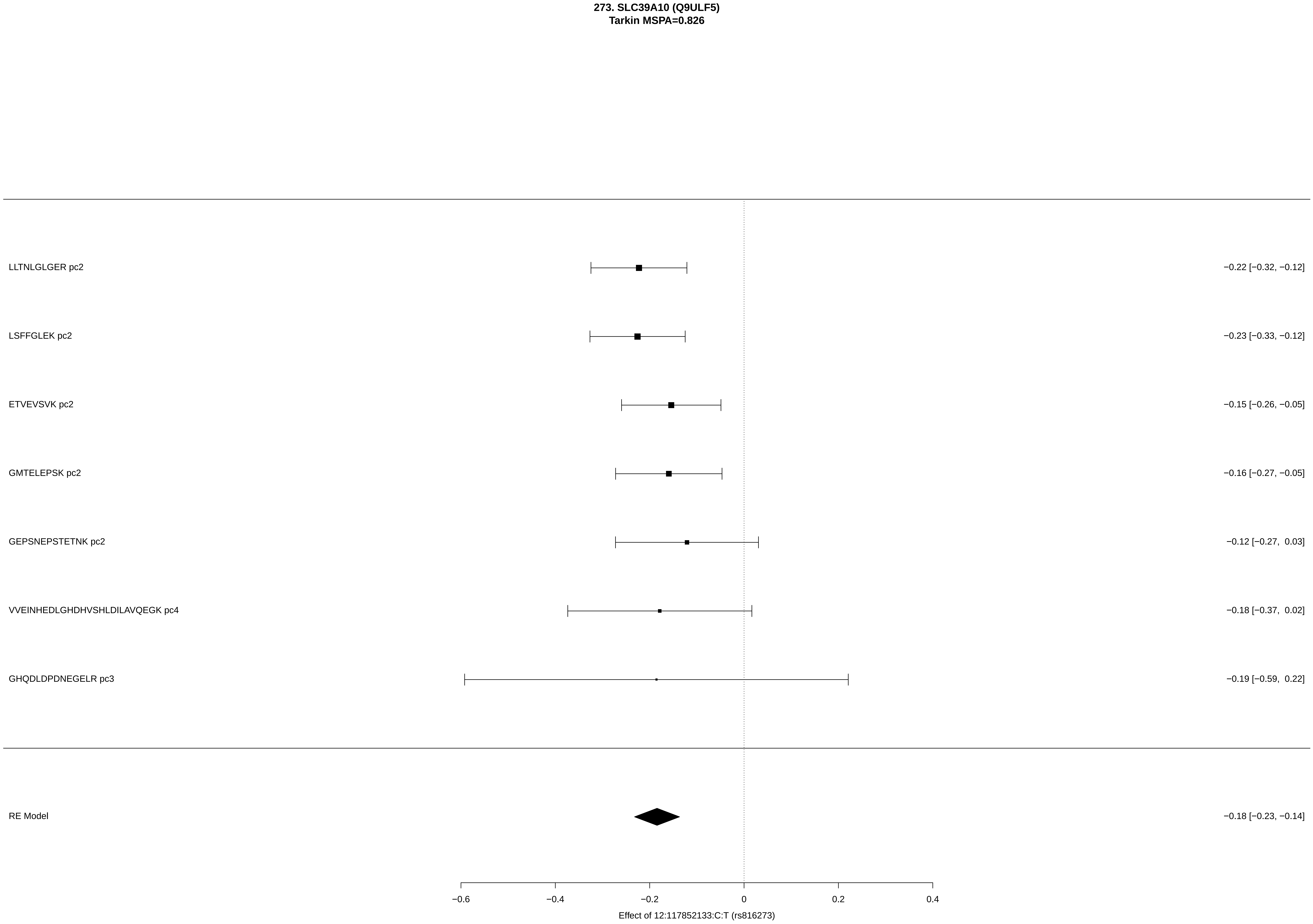
273. SLC39A10 (Q9ULF5)
Tarkin MSPA=0.826
LLTNLGLGER pc2
−0.22 [−0.32, −0.12]
LSFFGLEK pc2
−0.23 [−0.33, −0.12]
ETVEVSVK pc2
−0.15 [−0.26, −0.05]
GMTELEPSK pc2
−0.16 [−0.27, −0.05]
GEPSNEPSTETNK pc2
−0.12 [−0.27, 0.03]
VVEINHEDLGHDHVSHLDILAVQEGK pc4
−0.18 [−0.37, 0.02]
GHQDLDPDNEGELR pc3
−0.19 [−0.59, 0.22]
RE Model
−0.18 [−0.23, −0.14]
−0.6
−0.4
−0.2
0
0.2
0.4
Effect of 12:117852133:C:T (rs816273)

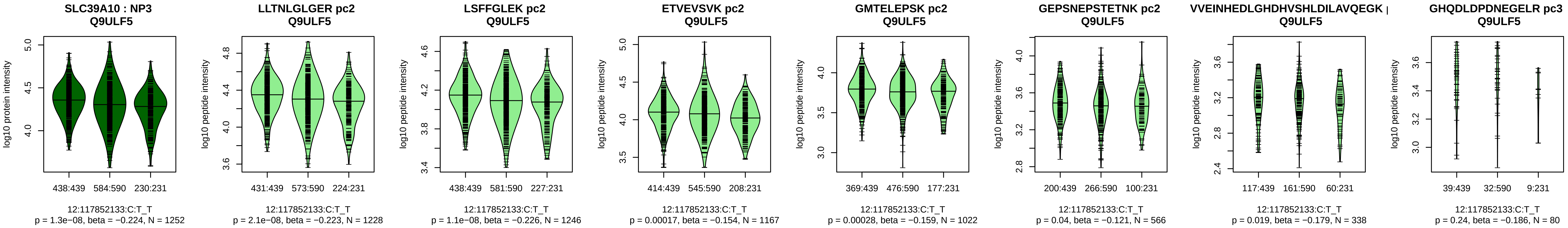

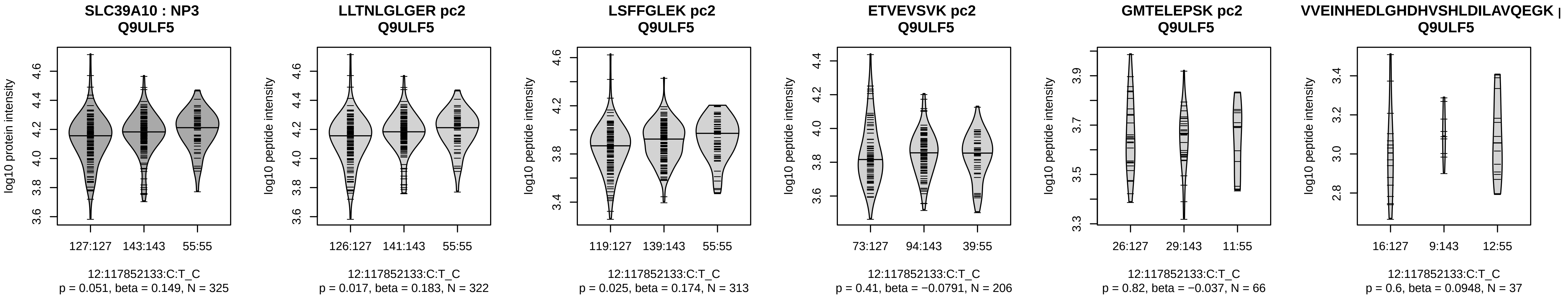

273Assay Target: SLC39A10
UniProt: Q9ULF5
rsID: rs816273
Target SNP: 12:117852133:C:T
Proxy SNP: 12:117852133:C:T
Tarkin log10(p): 7.9
Tarkin BETA: −0.224
Tarkin MSPA: 0.826
::*:*:–:–:–
1228:1246:1167:1022:566:338:8

274. APOM (O95445)
Tarkin MSPA=0.792

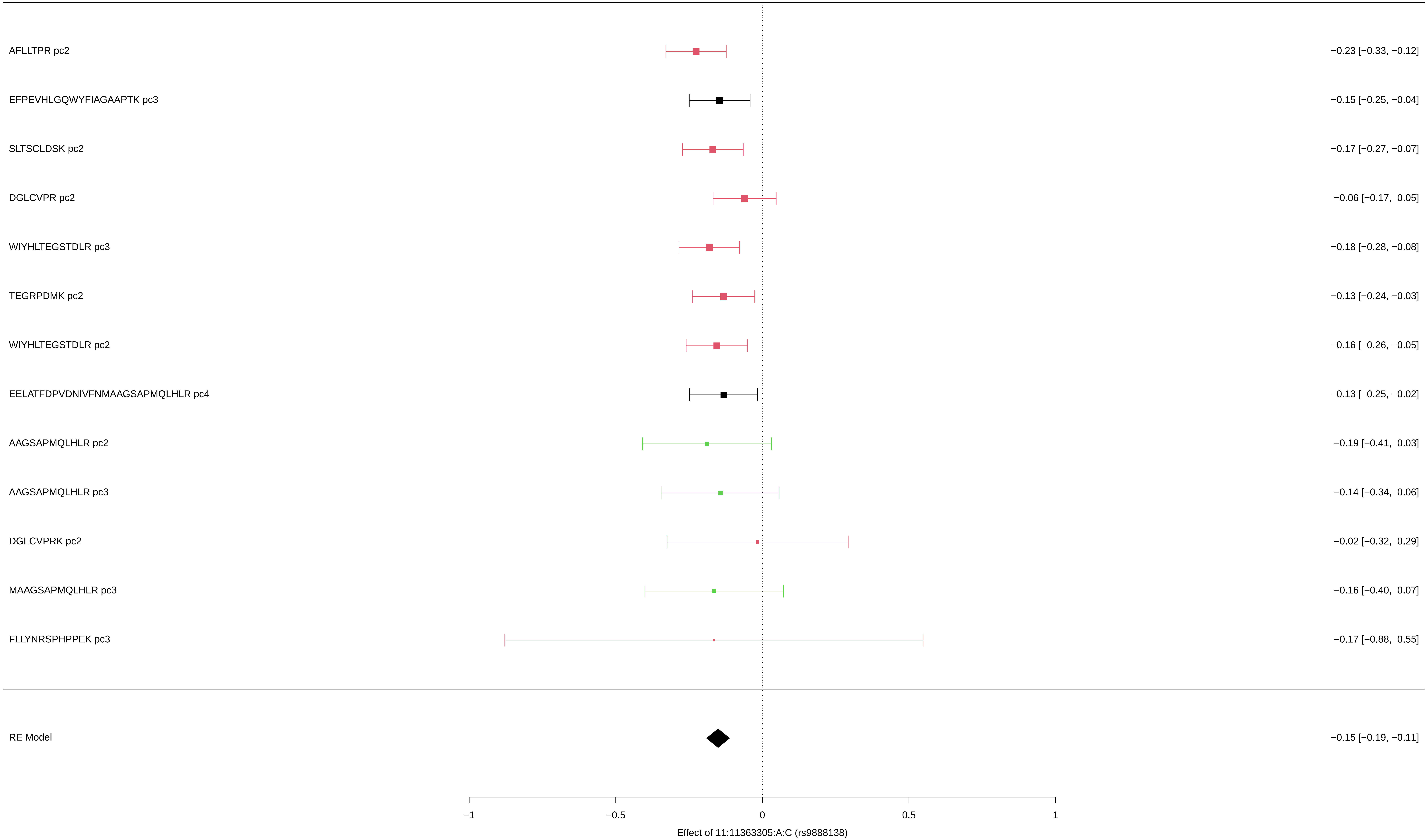

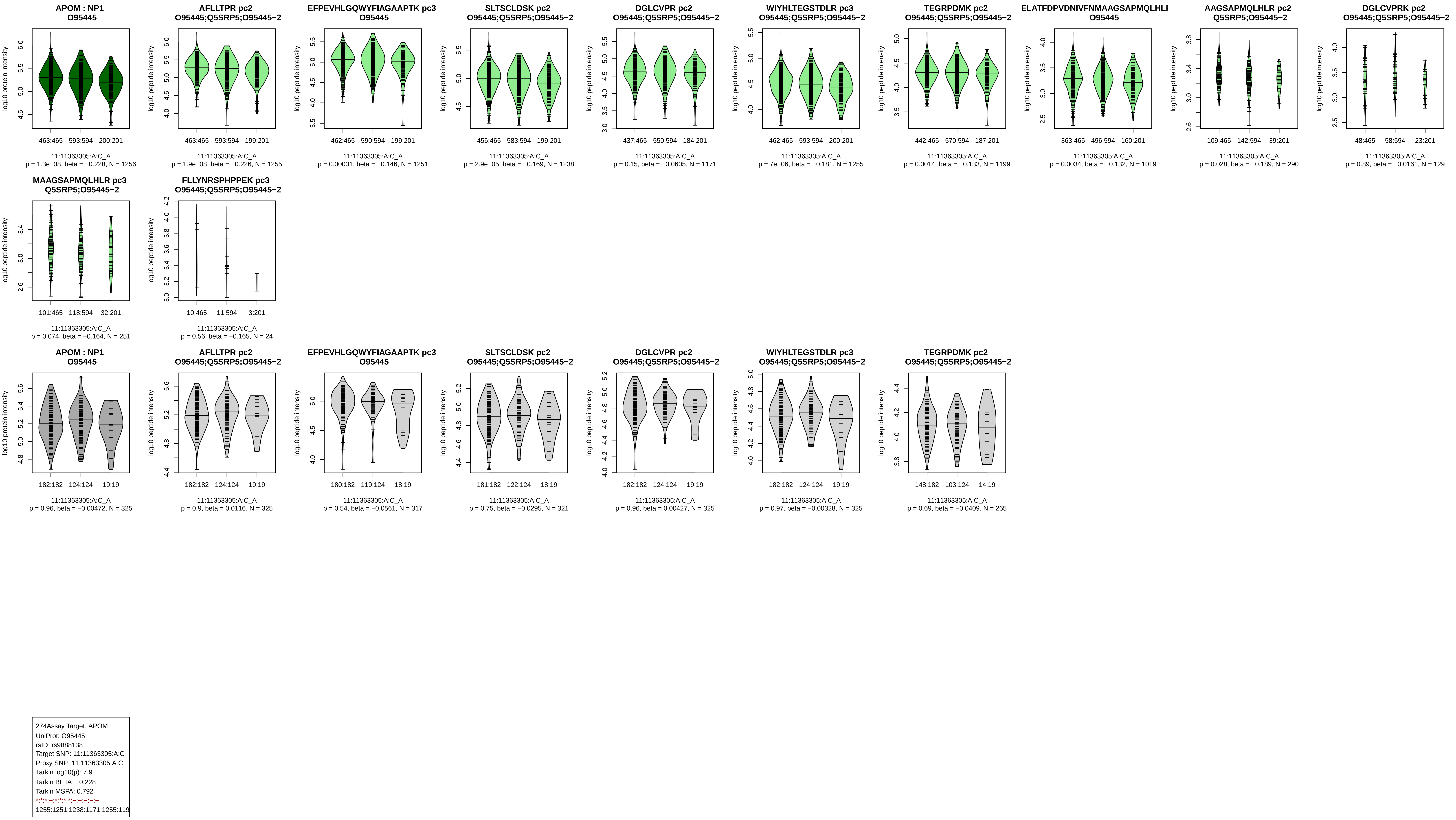

APOM : NP1
O95445
log10 protein intensity
463:465 593:594 200:201
11:11363305:A:C_A
p = 1.3e−08, beta = −0.228, N = 1256
AFLLTPR pc2
O95445;Q5SRP5;O95445−2
log10 peptide intensity
463:465 593:594 199:201
11:11363305:A:C_A
p = 1.9e−08, beta = −0.226, N = 1255
EFPEVHLGQWYFIAGAAPTK pc3
O95445
462:465 590:594 199:201
11:11363305:A:C_A
p = 0.00031, beta = −0.146, N = 1251
SLTSCLDSK pc2
O95445;Q5SRP5;O95445−2
456:465 583:594 199:201
11:11363305:A:C_A
p = 2.9e−05, beta = −0.169, N = 1238
DGLCVPR pc2
O95445;Q5SRP5;O95445−2
437:465 550:594 184:201
11:11363305:A:C_A
p = 0.15, beta = −0.0605, N = 1171
WIYHLTEGSTDLR pc3
O95445;Q5SRP5;O95445−2
462:465 593:594 200:201
11:11363305:A:C_A
p = 7e−06, beta = −0.181, N = 1255
TEGRPDMK pc2
O95445;Q5SRP5;O95445−2
442:465 570:594 187:201
11:11363305:A:C_A
p = 0.0014, beta = −0.133, N = 1199
ELATFDPVDNIVFNMAAGSAPMQLHL
O95445
363:465 496:594 160:201
11:11363305:A:C_A
p = 0.0034, beta = −0.132, N = 1019
AAGSAPMQLHLR pc2
Q5SRP5;O95445−2
109:465 142:594 39:201
11:11363305:A:C_A
p = 0.028, beta = −0.189, N = 290
DGLCVPRK pc2
O95445;Q5SRP5;O95445−2
48:465 58:594 23:201
11:11363305:A:C_A
p = 0.89, beta = −0.0161, N = 129
MAAGSAPMQLHLR pc3
Q5SRP5;O95445−2
101:465 118:594 32:201
11:11363305:A:C_A
p = 0.074, beta = −0.164, N = 251
FLLYNRSPHPPEK pc3
Q5SRP5;O95445−2
10:465 11:594 3:201
11:11363305:A:C_A
p = 0.56, beta = −0.165, N = 24
APOM : NP1
O95445
182:182 124:124 19:19
11:11363305:A:C_A
p = 0.96, beta = −0.00472, N = 325
AFLLTPR pc2
O95445;Q5SRP5;O95445−2
182:182 124:124 19:19
11:11363305:A:C_A
p = 0.9, beta = 0.0116, N = 325
EFPEVHLGQWYFIAGAAPTK pc3
O95445
180:182 119:124 18:19
11:11363305:A:C_A
p = 0.54, beta = −0.0561, N = 317
SLTSCLDSK pc2
O95445;Q5SRP5;O95445−2
181:182 122:124 18:19
11:11363305:A:C_A
p = 0.75, beta = −0.0295, N = 321
DGLCVPR pc2
O95445;Q5SRP5;O95445−2
182:182 124:124 19:19
11:11363305:A:C_A
p = 0.96, beta = 0.00427, N = 325
WIYHLTEGSTDLR pc3
O95445;Q5SRP5;O95445−2
182:182 124:124 19:19
11:11363305:A:C_A
p = 0.97, beta = −0.00328, N = 325
TEGRPDMK pc2
O95445;Q5SRP5;O95445−2
148:182 103:124 14:19
11:11363305:A:C_A
p = 0.69, beta = −0.0409, N = 265
274Assay Target: APOM
UniProt: O95445
rsID: rs9888138
Target SNP: 11:11363305:A:C
Proxy SNP: 11:11363305:A:C
Tarkin log10(p): 7.9
Tarkin BETA: −0.228
Tarkin MSPA: 0.792
1255:1251:1238:1171:1255:119

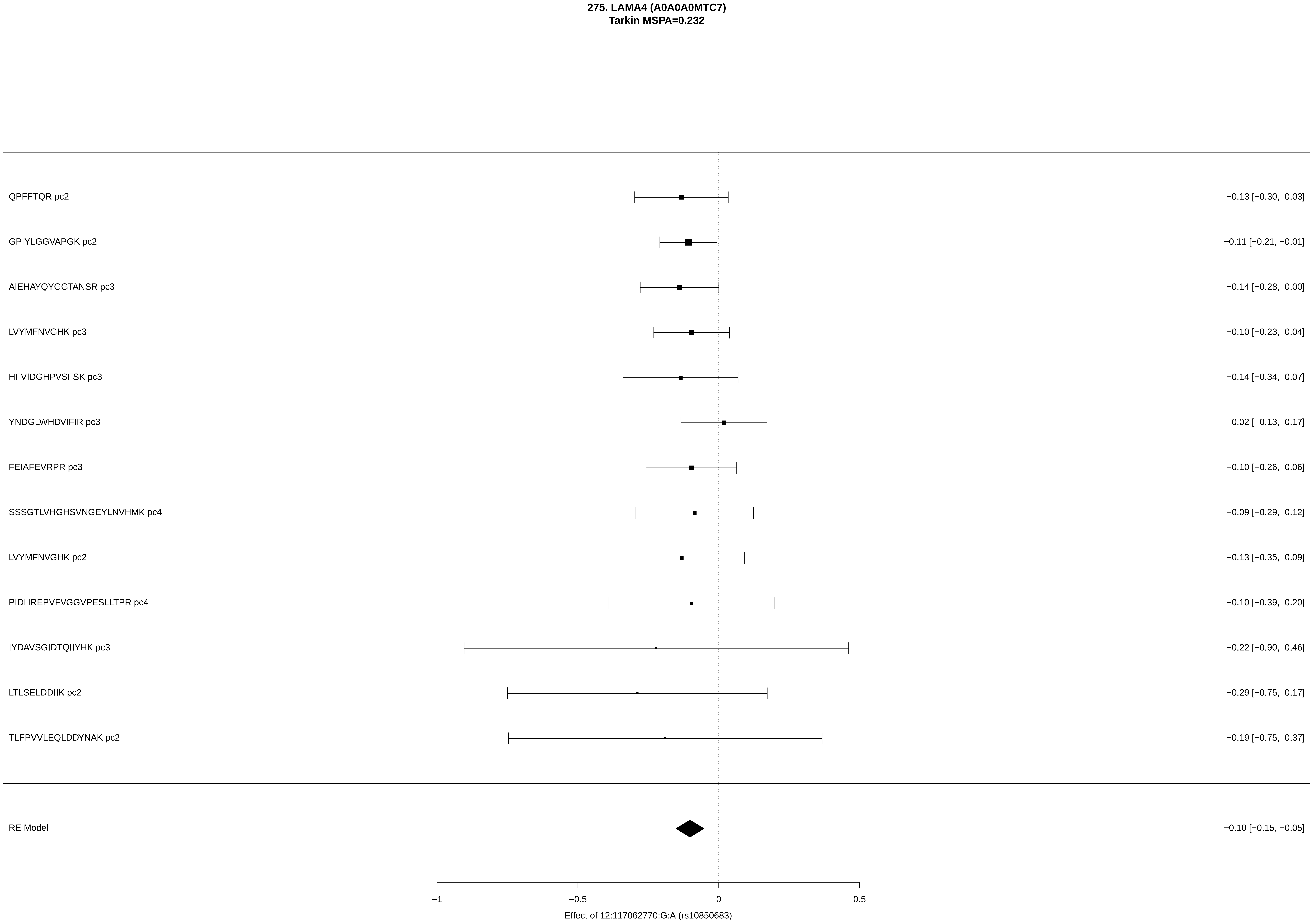

275. LAMA4 (A0A0A0MTC7)
Tarkin MSPA=0.232
QPFFTQR pc2
−0.13 [−0.30, 0.03]
GPIYLGGVAPGK pc2
−0.11 [−0.21, −0.01]
AIEHAYQYGGTANSR pc3
−0.14 [−0.28, 0.00]
LVYMFNVGHK pc3
−0.10 [−0.23, 0.04]
HFVIDGHPVSFSK pc3
−0.14 [−0.34, 0.07]
YNDGLWHDVIFIR pc3
0.02 [−0.13, 0.17]
FEIAFEVRPR pc3
−0.10 [−0.26, 0.06]
SSSGTLVHGHSVNGEYLNVHMK pc4
−0.09 [−0.29, 0.12]
LVYMFNVGHK pc2
−0.13 [−0.35, 0.09]
PIDHREPVFVGGVPESLLTPR pc4
−0.10 [−0.39, 0.20]
IYDAVSGIDTQIIYHK pc3
−0.22 [−0.90, 0.46]
LTLSELDDIIK pc2
−0.29 [−0.75, 0.17]
TLFPVVLEQLDDYNAK pc2
−0.19 [−0.75, 0.37]
RE Model
−0.10 [−0.15, −0.05]
−1
−0.5
0
0.5
Effect of 12:117062770:G:A (rs10850683)

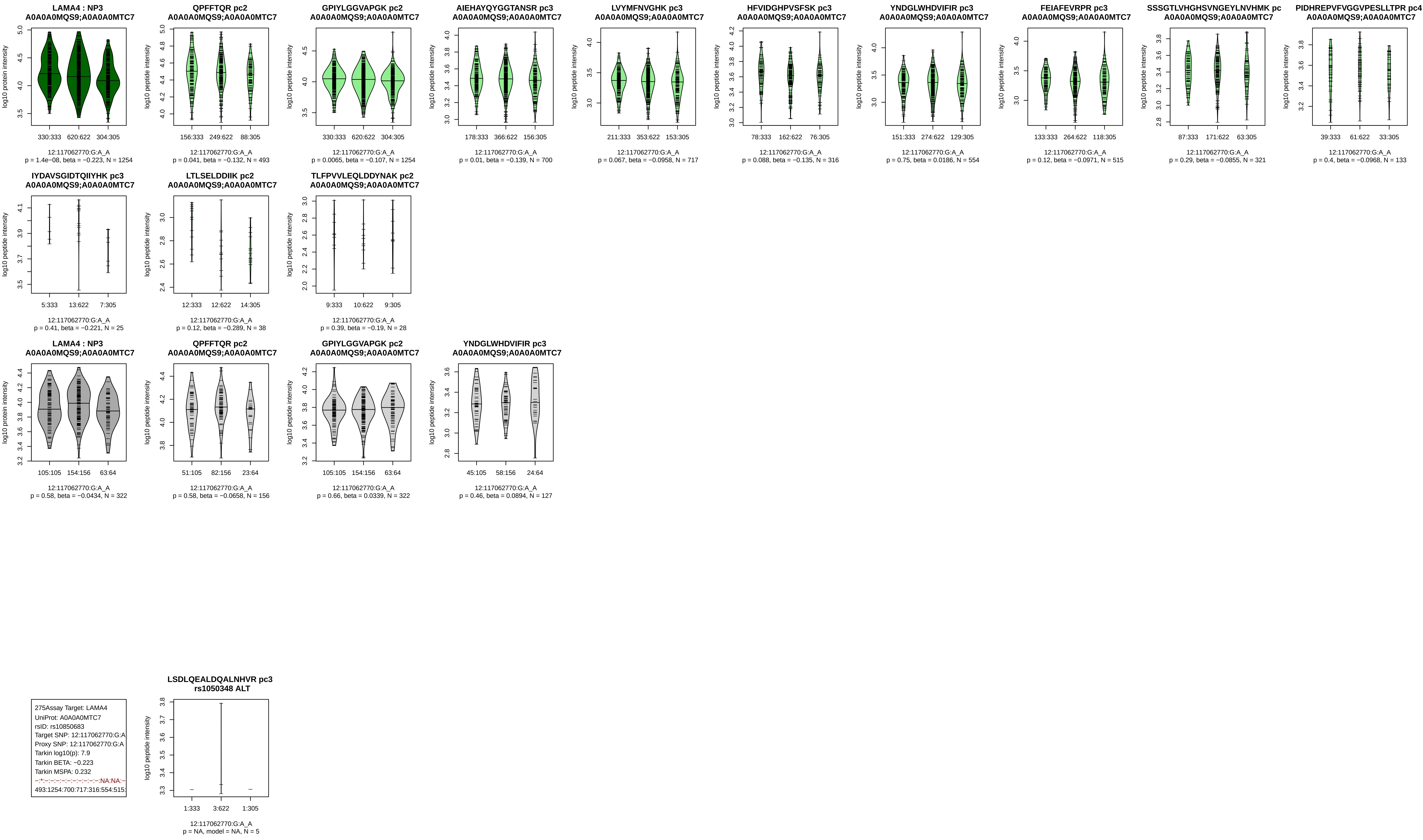
LAMA4 : NP3
A0A0A0MQS9;A0A0A0MTC7
log10 protein intensity
330:333 620:622 304:305
12:117062770:G:A_A
p = 1.4e−08, beta = −0.223, N = 1254
QPFFTQR pc2
A0A0A0MQS9;A0A0A0MTC7
log10 peptide intensity
156:333 249:622 88:305
12:117062770:G:A_A
p = 0.041, beta = −0.132, N = 493
GPIYLGGVAPGK pc2
A0A0A0MQS9;A0A0A0MTC7
330:333 620:622 304:305
12:117062770:G:A_A
p = 0.0065, beta = −0.107, N = 1254
AIEHAYQYGGTANSR pc3
A0A0A0MQS9;A0A0A0MTC7
178:333 366:622 156:305
12:117062770:G:A_A
p = 0.01, beta = −0.139, N = 700
LVYMFNVGHK pc3
A0A0A0MQS9;A0A0A0MTC7
211:333 353:622 153:305
12:117062770:G:A_A
p = 0.067, beta = −0.0958, N = 717
HFVIDGHPVSFSK pc3
A0A0A0MQS9;A0A0A0MTC7
78:333 162:622 76:305
12:117062770:G:A_A
p = 0.088, beta = −0.135, N = 316
YNDGLWHDVIFIR pc3
A0A0A0MQS9;A0A0A0MTC7
151:333 274:622 129:305
12:117062770:G:A_A
p = 0.75, beta = 0.0186, N = 554
FEIAFEVRPR pc3
A0A0A0MQS9;A0A0A0MTC7
133:333 264:622 118:305
12:117062770:G:A_A
p = 0.12, beta = −0.0971, N = 515
SSSGTLVHGHSVNGEYLNVHMK pc
A0A0A0MQS9;A0A0A0MTC7
87:333 171:622 63:305
12:117062770:G:A_A
p = 0.29, beta = −0.0855, N = 321
PIDHREPVFVGGVPESLLTPR pc4
A0A0A0MQS9;A0A0A0MTC7
39:333 61:622 33:305
12:117062770:G:A_A
p = 0.4, beta = −0.0968, N = 133
IYDAVSGIDTQIIYHK pc3
A0A0A0MQS9;A0A0A0MTC7
5:333 13:622 7:305
12:117062770:G:A_A
p = 0.41, beta = −0.221, N = 25
LTLSELDDIIK pc2
A0A0A0MQS9;A0A0A0MTC7
12:333 12:622 14:305
12:117062770:G:A_A
p = 0.12, beta = −0.289, N = 38
TLFPVVLEQLDDYNAK pc2
A0A0A0MQS9;A0A0A0MTC7
9:333 10:622 9:305
12:117062770:G:A_A
p = 0.39, beta = −0.19, N = 28
LAMA4 : NP3
A0A0A0MQS9;A0A0A0MTC7
105:105 154:156 63:64
12:117062770:G:A_A
p = 0.58, beta = −0.0434, N = 322
QPFFTQR pc2
A0A0A0MQS9;A0A0A0MTC7
51:105 82:156 23:64
12:117062770:G:A_A
p = 0.58, beta = −0.0658, N = 156
GPIYLGGVAPGK pc2
A0A0A0MQS9;A0A0A0MTC7
105:105 154:156 63:64
12:117062770:G:A_A
p = 0.66, beta = 0.0339, N = 322
YNDGLWHDVIFIR pc3
A0A0A0MQS9;A0A0A0MTC7
45:105 58:156 24:64
12:117062770:G:A_A
p = 0.46, beta = 0.0894, N = 127
275Assay Target: LAMA4
UniProt: A0A0A0MTC7
rsID: rs10850683
Target SNP: 12:117062770:G:A
Proxy SNP: 12:117062770:G:A
Tarkin log10(p): 7.9
Tarkin BETA: −0.223
Tarkin MSPA: 0.232
−:*:−:−:−:−:−:−:−:−:−:NA:NA:−
493:1254:700:717:316:554:515:
LSDLQEALDQALNHVR pc3
rs1050348 ALT
1:333 3:622 1:305
12:117062770:G:A_A
p = NA, model = NA, N = 5

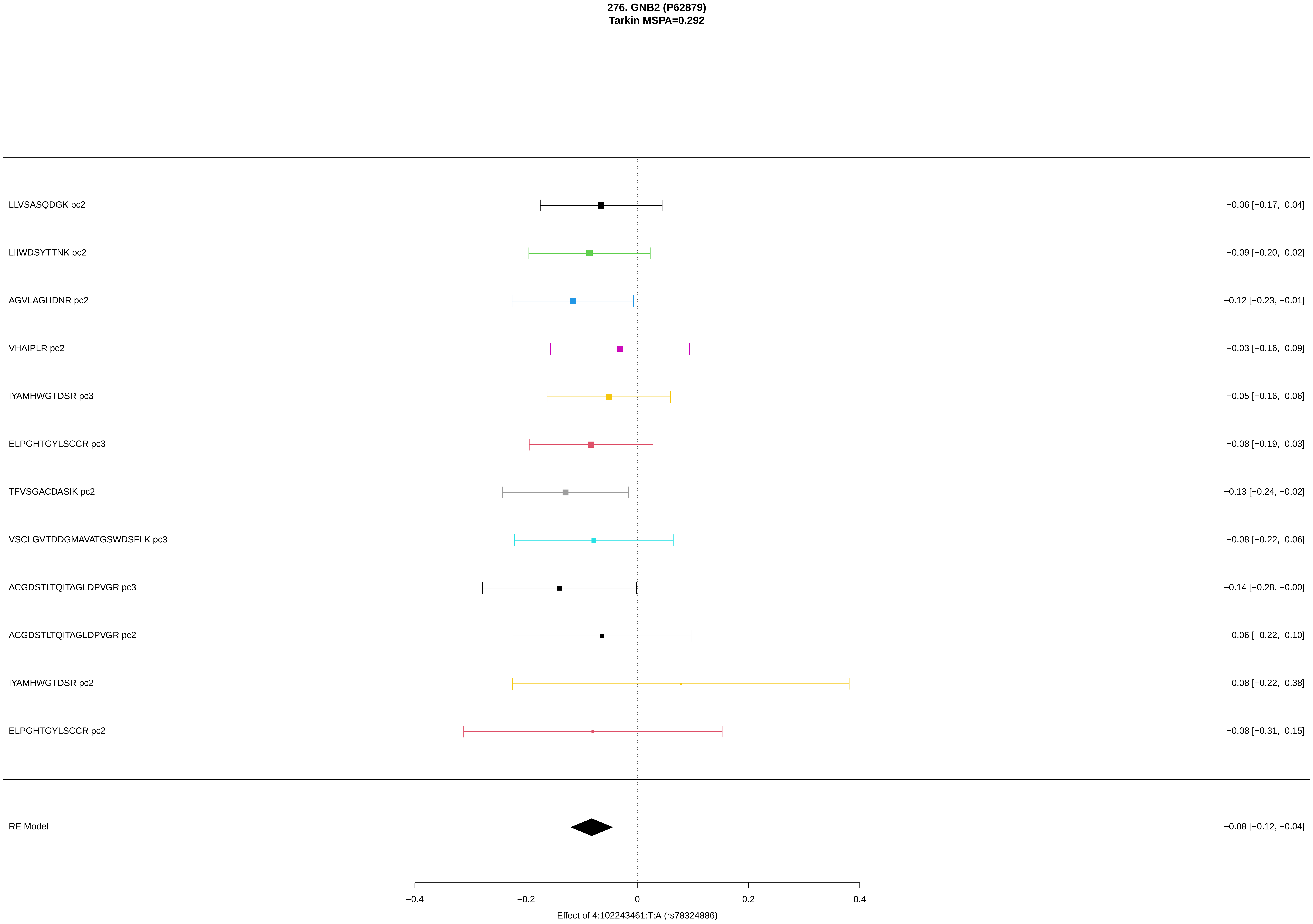
276. GNB2 (P62879)
Tarkin MSPA=0.292
LLVSASQDGK pc2
LIIWDSYTTNK pc2
AGVLAGHDNR pc2
VHAIPLR pc2
IYAMHWGTDSR pc3
ELPGHTGYLSCCR pc3
TFVSGACDASIK pc2
VSCLGVTDDGMAVATGSWDSFLK pc3
ACGDSTLTQITAGLDPVGR pc3
ACGDSTLTQITAGLDPVGR pc2
IYAMHWGTDSR pc2
ELPGHTGYLSCCR pc2
RE Model
−0.06 [−0.17, 0.04]
−0.09 [−0.20, 0.02]
−0.12 [−0.23, −0.01]
−0.03 [−0.16, 0.09]
−0.05 [−0.16, 0.06]
−0.08 [−0.19, 0.03]
−0.13 [−0.24, −0.02]
−0.08 [−0.22, 0.06]
−0.14 [−0.28, −0.00]
−0.06 [−0.22, 0.10]
0.08 [−0.22, 0.38]
−0.08 [−0.31, 0.15]
−0.08 [−0.12, −0.04]
−0.4
−0.2
0
0.2
0.4
Effect of 4:102243461:T:A (rs78324886)

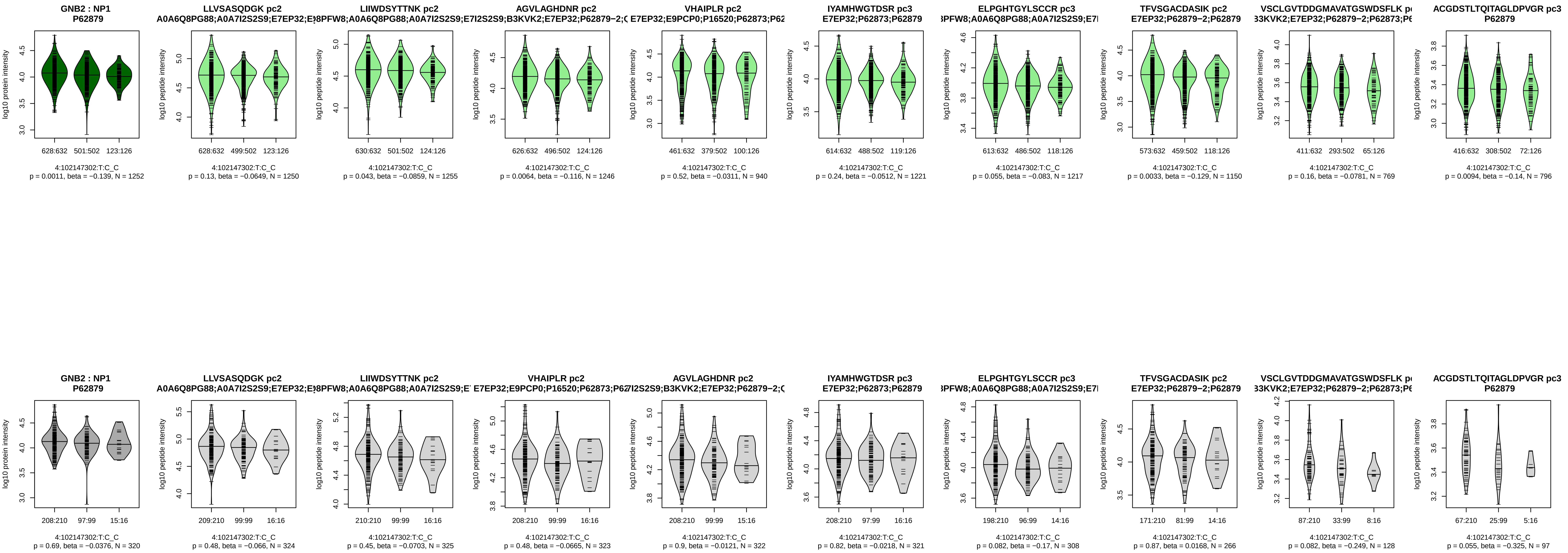

276Assay Target: GNB2
UniProt: P62879
rsID: rs78324886
Target SNP: 4:102243461:T:A
Proxy SNP: 4:102147302:T:C
Tarkin log10(p): 7.9
Tarkin BETA: −0.472
Tarkin MSPA: 0.292
−:−:*:−:−:−:*:−:*:−:−:−:−:NA
1250:1255:1246:940:1221:1217

277. RNH1 (P13489)
Tarkin MSPA=0.241

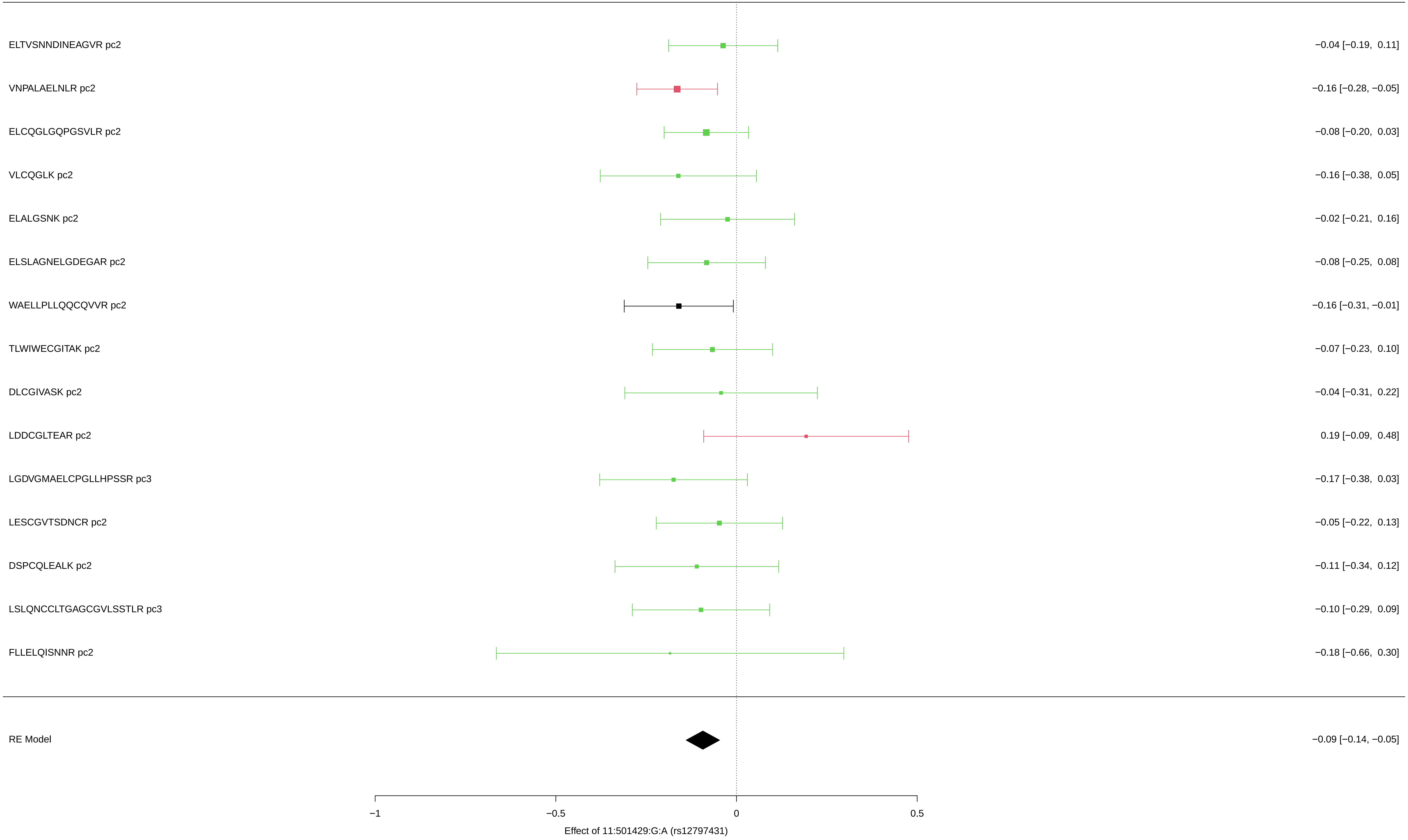

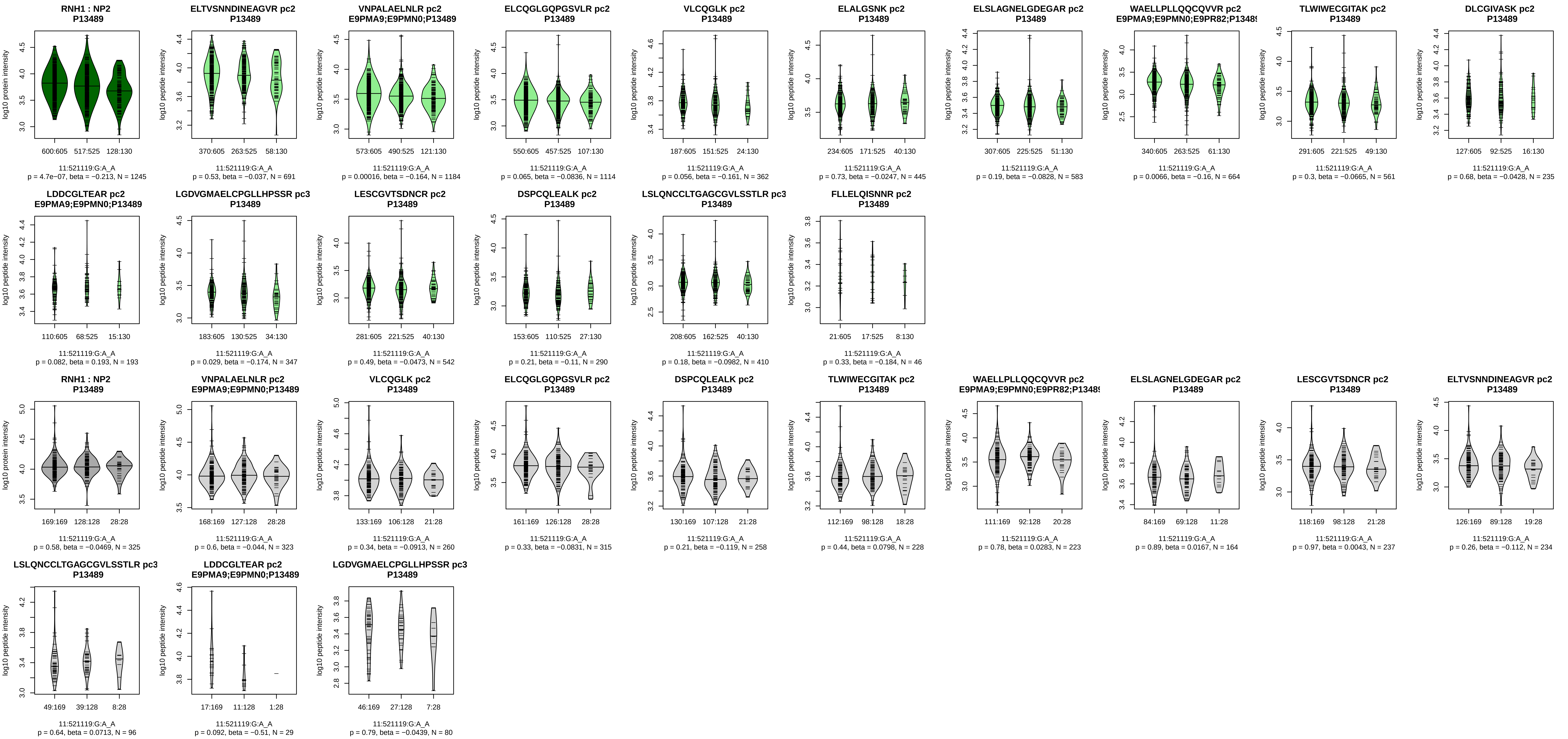

277Assay Target: RNH1
UniProt: P13489
rsID: rs12797431
Target SNP: 11:501429:G:A
Proxy SNP: 11:521119:G:A
Tarkin log10(p): 7.8
Tarkin BETA: −0.352
Tarkin MSPA: 0.241
−:*:−:−:−:−:*:−:−:−:−:−:−:−:−:NA
691:1184:1114:362:445:583:664

278. RNPEP (Q9H4A4)
Tarkin MSPA=0.847

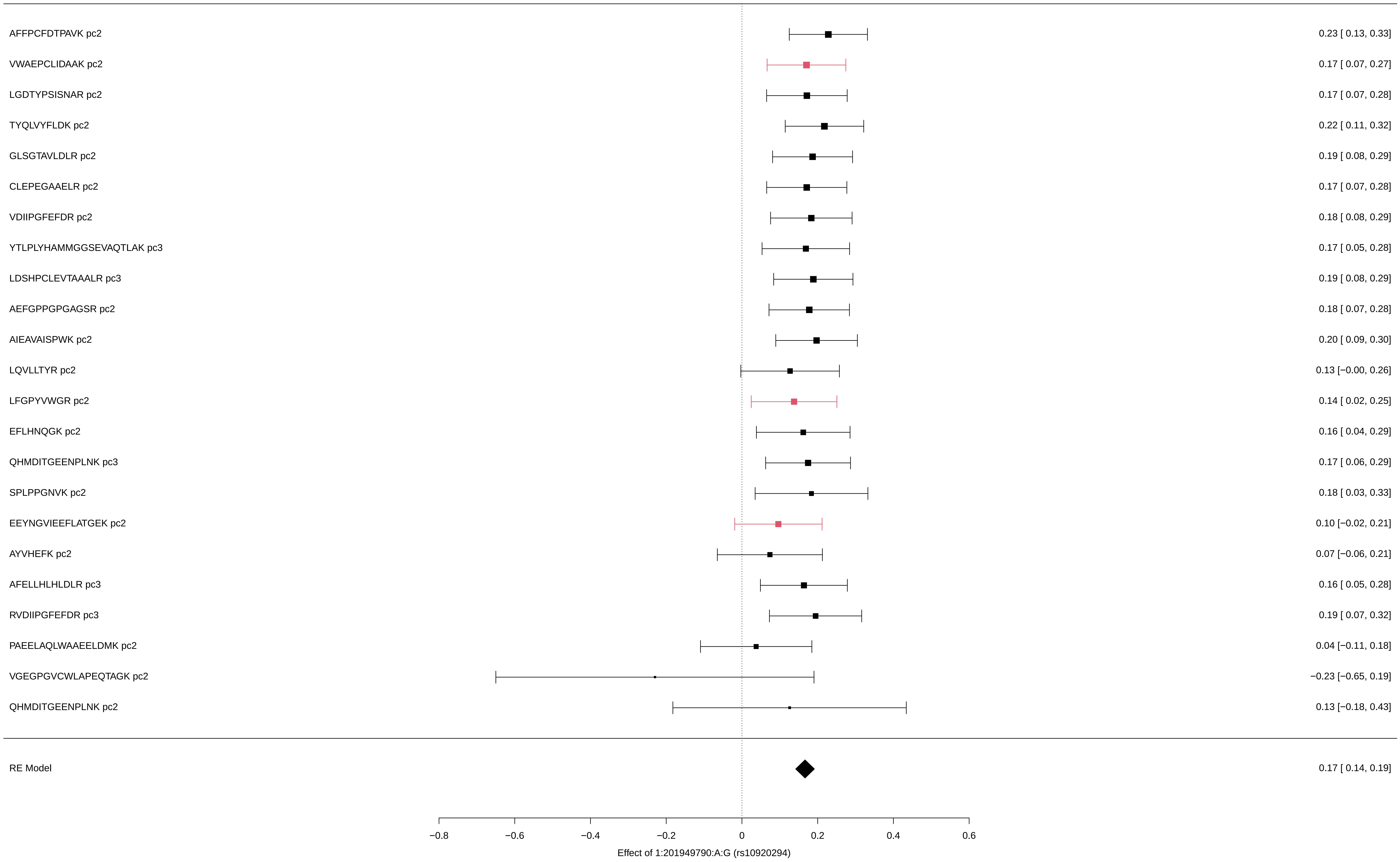

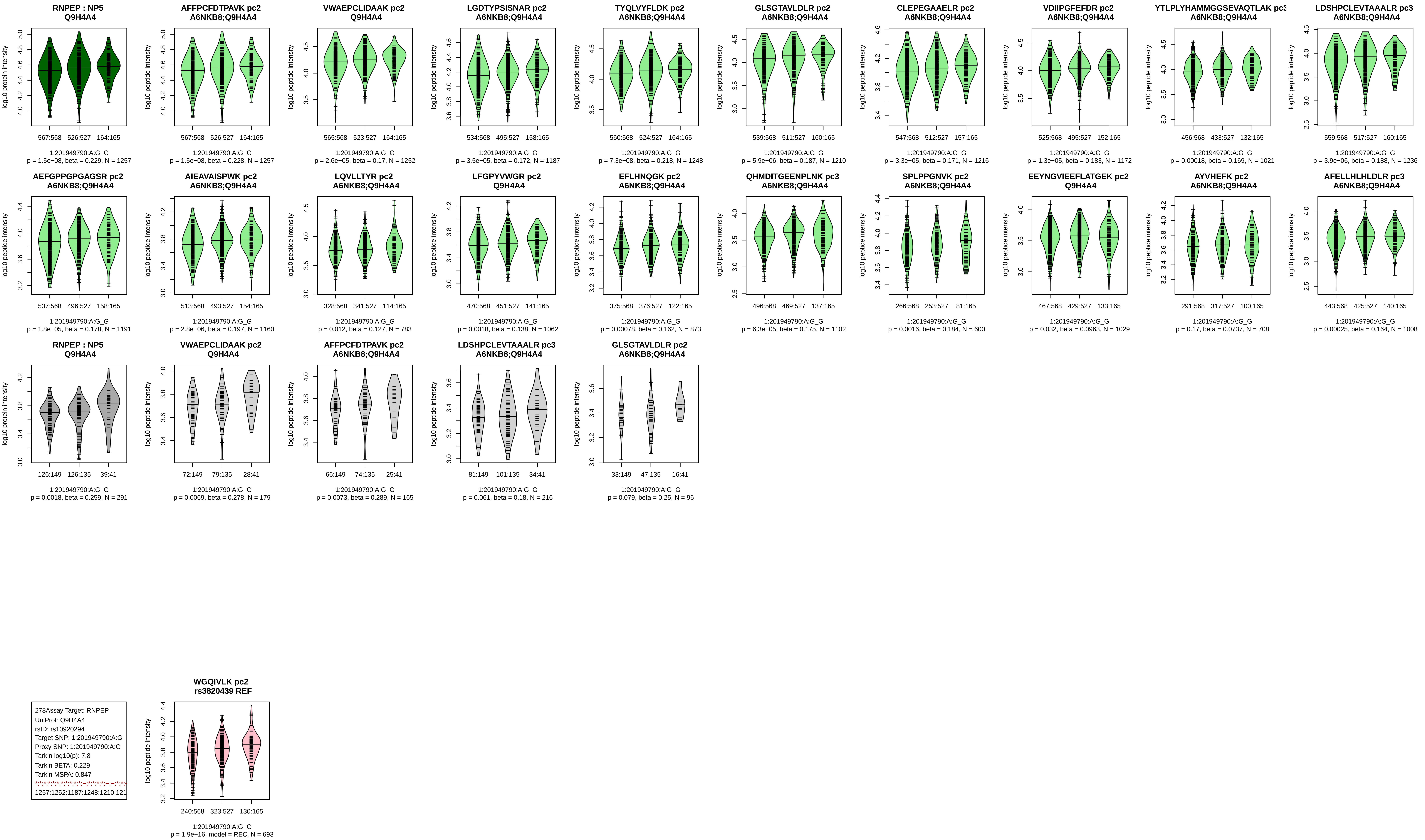

RNPEP : NP5
Q9H4A4
log10 protein intensity
567:568 526:527 164:165
1:201949790:A:G_G
p = 1.5e−08, beta = 0.229, N = 1257
AFFPCFDTPAVK pc2
A6NKB8;Q9H4A4
log10 peptide intensity
567:568 526:527 164:165
1:201949790:A:G_G
p = 1.5e−08, beta = 0.228, N = 1257
VWAEPCLIDAAK pc2
Q9H4A4
565:568 523:527 164:165
1:201949790:A:G_G
p = 2.6e−05, beta = 0.17, N = 1252
LGDTYPSISNAR pc2
A6NKB8;Q9H4A4
534:568 495:527 158:165
1:201949790:A:G_G
p = 3.5e−05, beta = 0.172, N = 1187
TYQLVYFLDK pc2
A6NKB8;Q9H4A4
560:568 524:527 164:165
1:201949790:A:G_G
p = 7.3e−08, beta = 0.218, N = 1248
GLSGTAVLDLR pc2
A6NKB8;Q9H4A4
539:568 511:527 160:165
1:201949790:A:G_G
p = 5.9e−06, beta = 0.187, N = 1210
CLEPEGAAELR pc2
A6NKB8;Q9H4A4
547:568 512:527 157:165
1:201949790:A:G_G
p = 3.3e−05, beta = 0.171, N = 1216
VDIIPGFEFDR pc2
A6NKB8;Q9H4A4
525:568 495:527 152:165
1:201949790:A:G_G
p = 1.3e−05, beta = 0.183, N = 1172
YTLPLYHAMMGGSEVAQTLAK pc3
A6NKB8;Q9H4A4
456:568 433:527 132:165
1:201949790:A:G_G
p = 0.00018, beta = 0.169, N = 1021
LDSHPCLEVTAAALR pc3
A6NKB8;Q9H4A4
559:568 517:527 160:165
1:201949790:A:G_G
p = 3.9e−06, beta = 0.188, N = 1236
AEFGPPGPGAGSR pc2
A6NKB8;Q9H4A4
537:568 496:527 158:165
1:201949790:A:G_G
p = 1.8e−05, beta = 0.178, N = 1191
AIEAVAISPWK pc2
A6NKB8;Q9H4A4
513:568 493:527 154:165
1:201949790:A:G_G
p = 2.8e−06, beta = 0.197, N = 1160
LQVLLTYR pc2
A6NKB8;Q9H4A4
328:568 341:527 114:165
1:201949790:A:G_G
p = 0.012, beta = 0.127, N = 783
LFGPYVWGR pc2
Q9H4A4
470:568 451:527 141:165
1:201949790:A:G_G
p = 0.0018, beta = 0.138, N = 1062
EFLHNQGK pc2
A6NKB8;Q9H4A4
375:568 376:527 122:165
1:201949790:A:G_G
p = 0.00078, beta = 0.162, N = 873
QHMDITGEENPLNK pc3
A6NKB8;Q9H4A4
496:568 469:527 137:165
1:201949790:A:G_G
p = 6.3e−05, beta = 0.175, N = 1102
SPLPPGNVK pc2
A6NKB8;Q9H4A4
266:568 253:527 81:165
1:201949790:A:G_G
p = 0.0016, beta = 0.184, N = 600
EEYNGVIEEFLATGEK pc2
Q9H4A4
467:568 429:527 133:165
1:201949790:A:G_G
p = 0.032, beta = 0.0963, N = 1029
AYVHEFK pc2
A6NKB8;Q9H4A4
291:568 317:527 100:165
1:201949790:A:G_G
p = 0.17, beta = 0.0737, N = 708
AFELLHLHLDLR pc3
A6NKB8;Q9H4A4
443:568 425:527 140:165
1:201949790:A:G_G
p = 0.00025, beta = 0.164, N = 1008
RNPEP : NP5
Q9H4A4
126:149 126:135 39:41
1:201949790:A:G_G
p = 0.0018, beta = 0.259, N = 291
VWAEPCLIDAAK pc2
Q9H4A4
72:149 79:135 28:41
1:201949790:A:G_G
p = 0.0069, beta = 0.278, N = 179
AFFPCFDTPAVK pc2
A6NKB8;Q9H4A4
66:149 74:135 25:41
1:201949790:A:G_G
p = 0.0073, beta = 0.289, N = 165
LDSHPCLEVTAAALR pc3
A6NKB8;Q9H4A4
81:149 101:135 34:41
1:201949790:A:G_G
p = 0.061, beta = 0.18, N = 216
GLSGTAVLDLR pc2
A6NKB8;Q9H4A4
33:149 47:135 16:41
1:201949790:A:G_G
p = 0.079, beta = 0.25, N = 96
278Assay Target: RNPEP
UniProt: Q9H4A4
rsID: rs10920294
Target SNP: 1:201949790:A:G
Proxy SNP: 1:201949790:A:G
Tarkin log10(p): 7.8
Tarkin BETA: 0.229
Tarkin MSPA: 0.847
1257:1252:1187:1248:1210:121
WGQIVLK pc2
rs3820439 REF
240:568 323:527 130:165
1:201949790:A:G_G
p = 1.9e−16, model = REC, N = 693

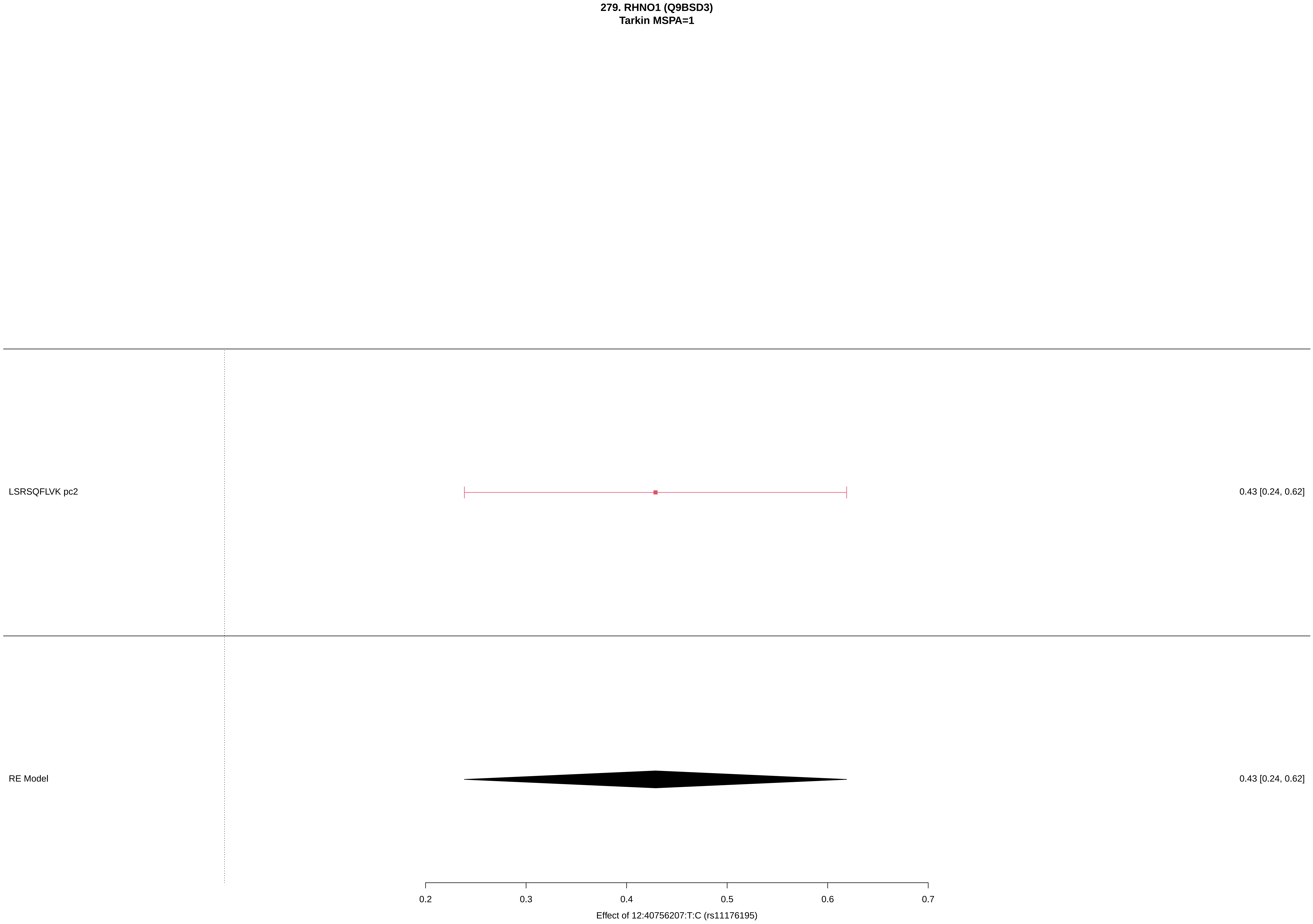

279. RHNO1 (Q9BSD3)
Tarkin MSPA=1
LSRSQFLVK pc2
0.43 [0.24, 0.62]
RE Model
0.43 [0.24, 0.62]
0.2
0.3
0.4
0.5
0.6
0.7
Effect of 12:40756207:T:C (rs11176195)

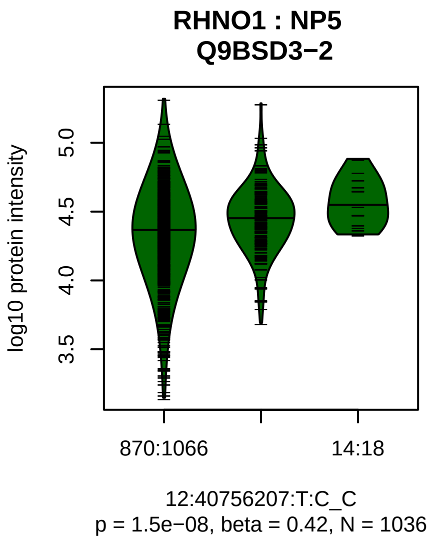

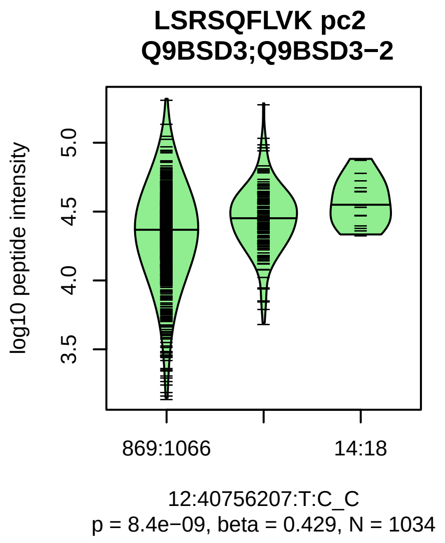

279Assay Target: RHNO1
UniProt: Q9BSD3
rsID: rs11176195
Target SNP: 12:40756207:T:C
Proxy SNP: 12:40756207:T:C
Tarkin log10(p): 7.8
Tarkin BETA: 0.42
Tarkin MSPA: 1
*:NA
1034:9

280. MYOC (Q99972)
Tarkin MSPA=0.428

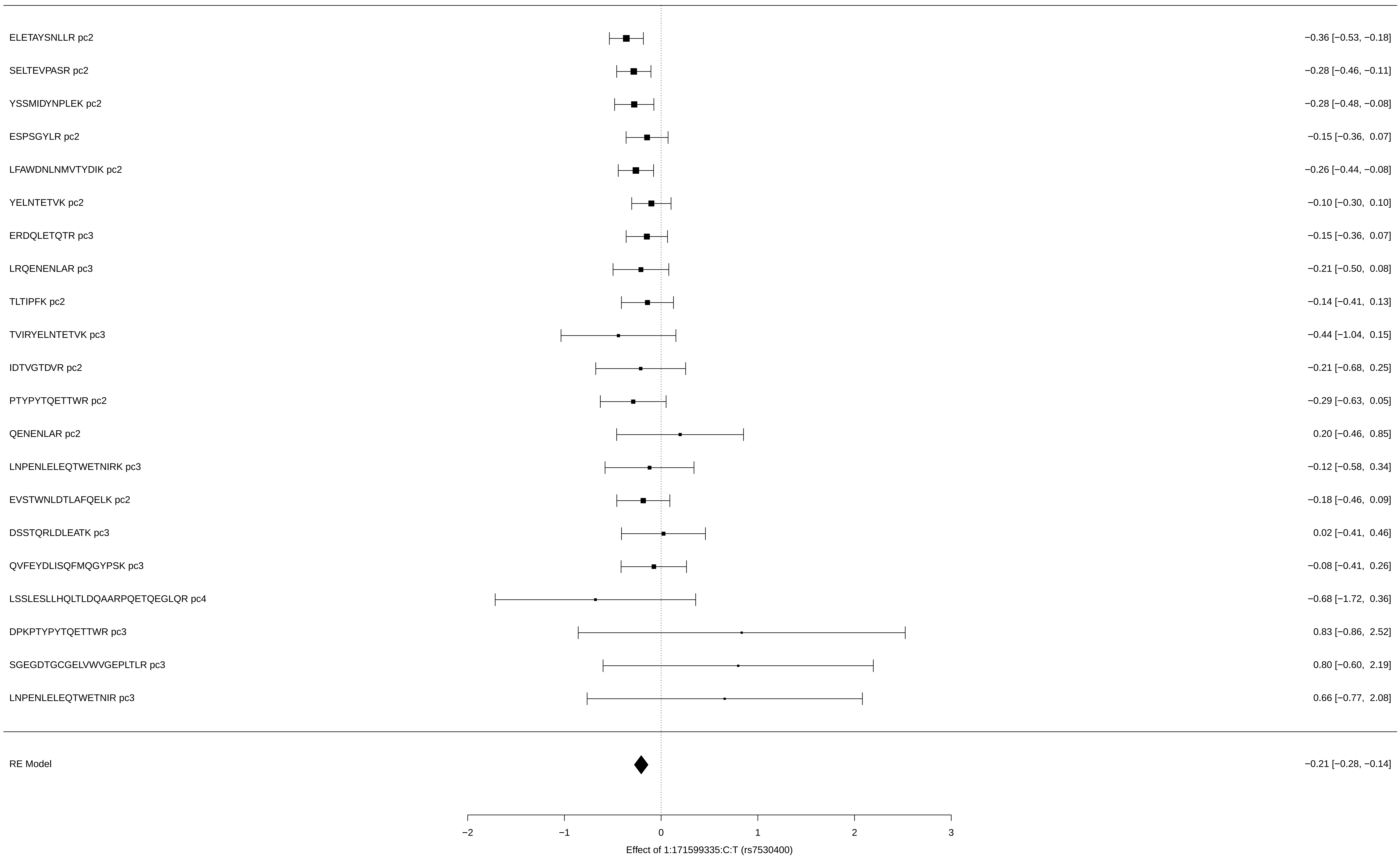

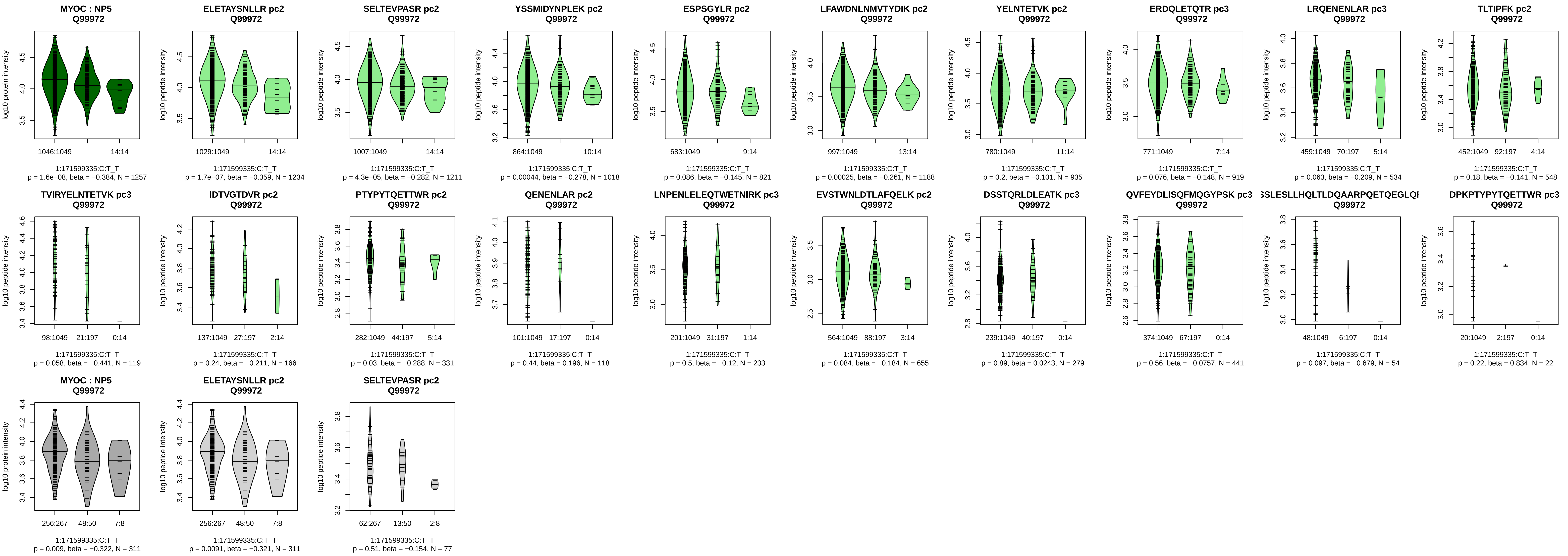

280Assay Target: MYOC
UniProt: Q99972
rsID: rs7530400
Target SNP: 1:171599335:C:T
Proxy SNP: 1:171599335:C:T
Tarkin log10(p): 7.8
Tarkin BETA: −0.384
Tarkin MSPA: 0.428
1234:1211:1018:821:1188:935:[illegible]

281. ARPC3 (O15145)
Tarkin MSPA=0.347

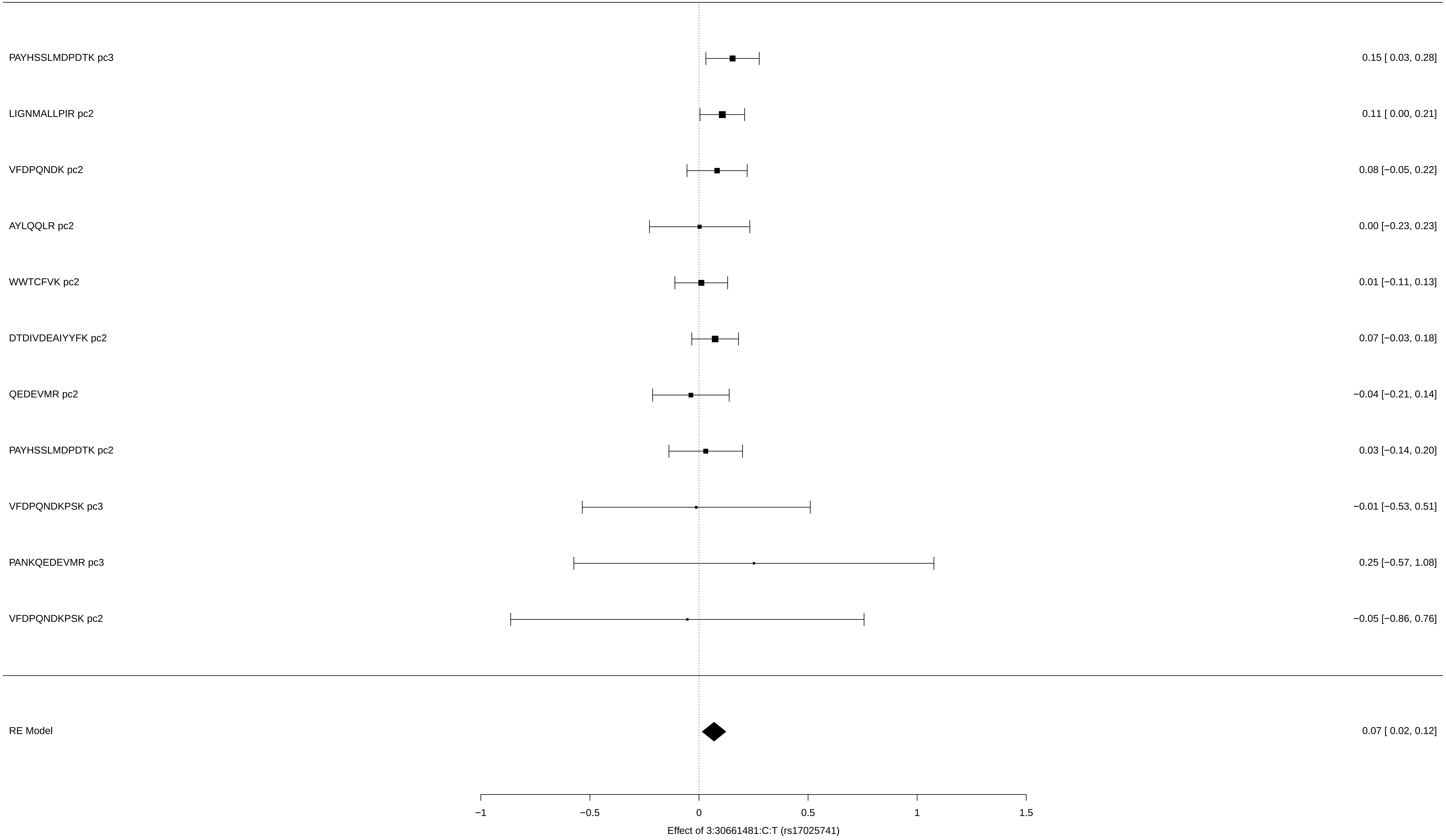

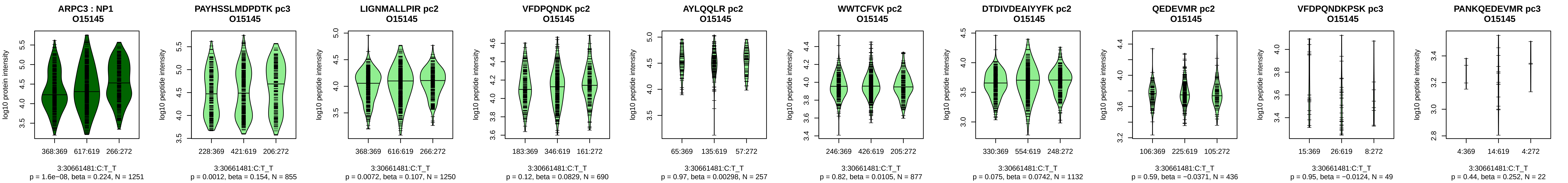

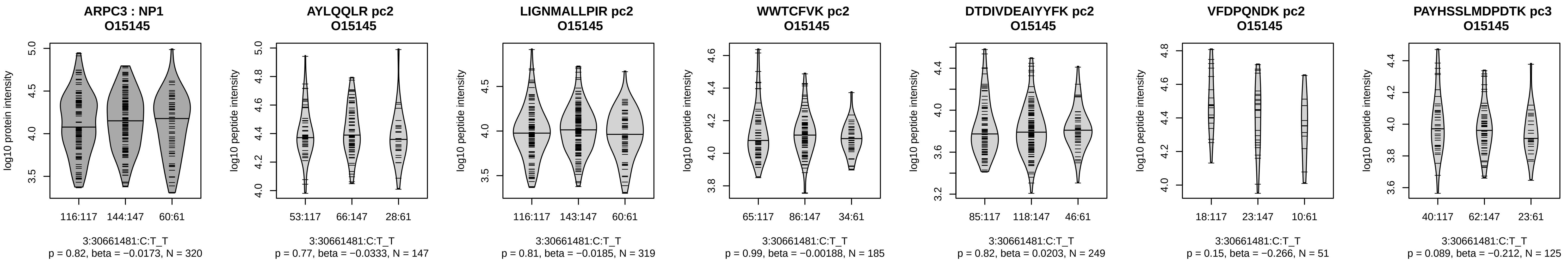

281Assay Target: ARPC3
UniProt: O15145
rsID: rs17025741
Target SNP: 3:30661481:C:T
Proxy SNP: 3:30661481:C:T
Tarkin log10(p): 7.8
Tarkin BETA: 0.224
Tarkin MSPA: 0.347
::–:–:–:–:–:–:–:–:NA:NA
855:1250:690:257:877:1132:43[illegible]

282. TUBB1 (Q9H4B7)
Tarkin MSPA=0.755

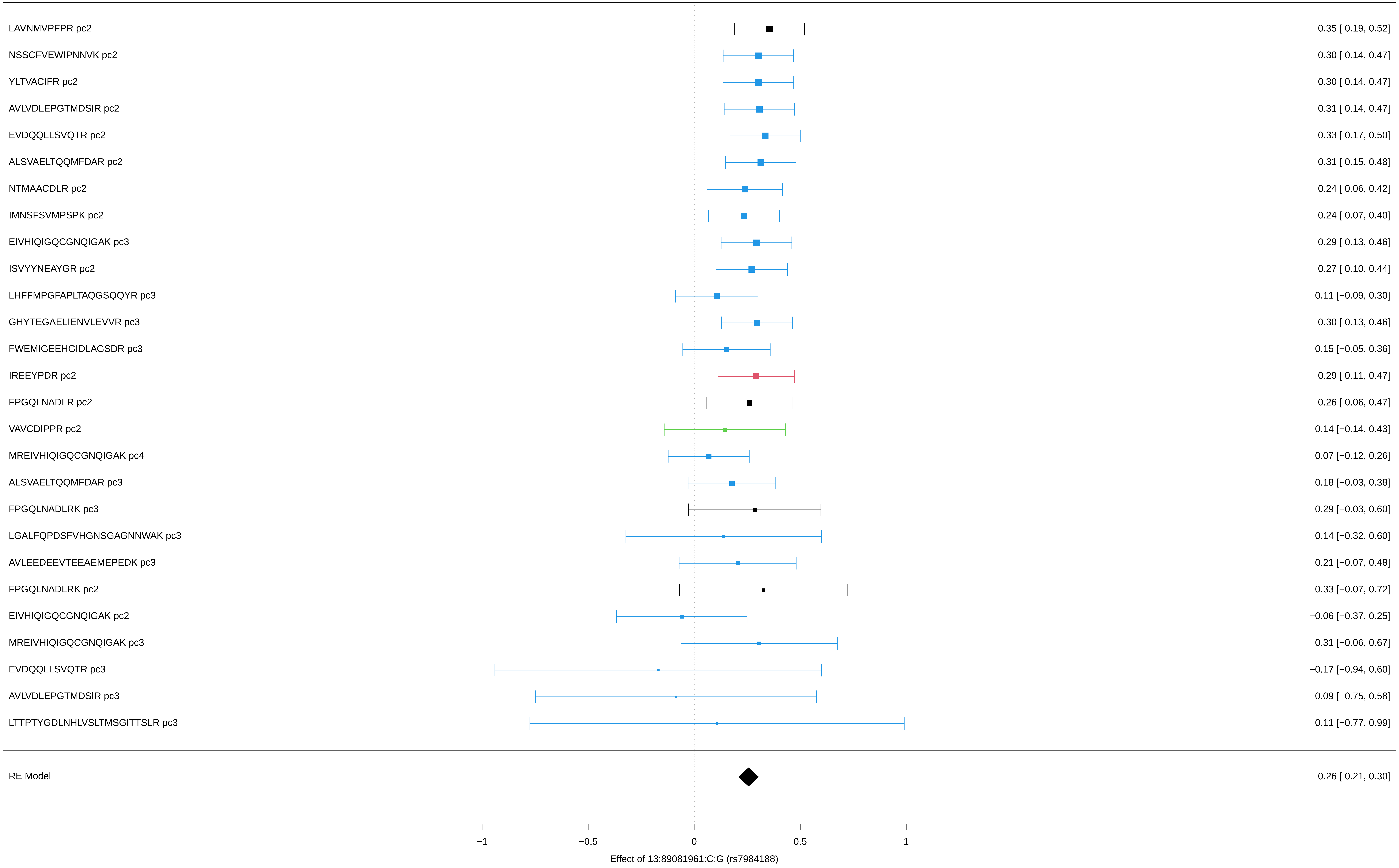

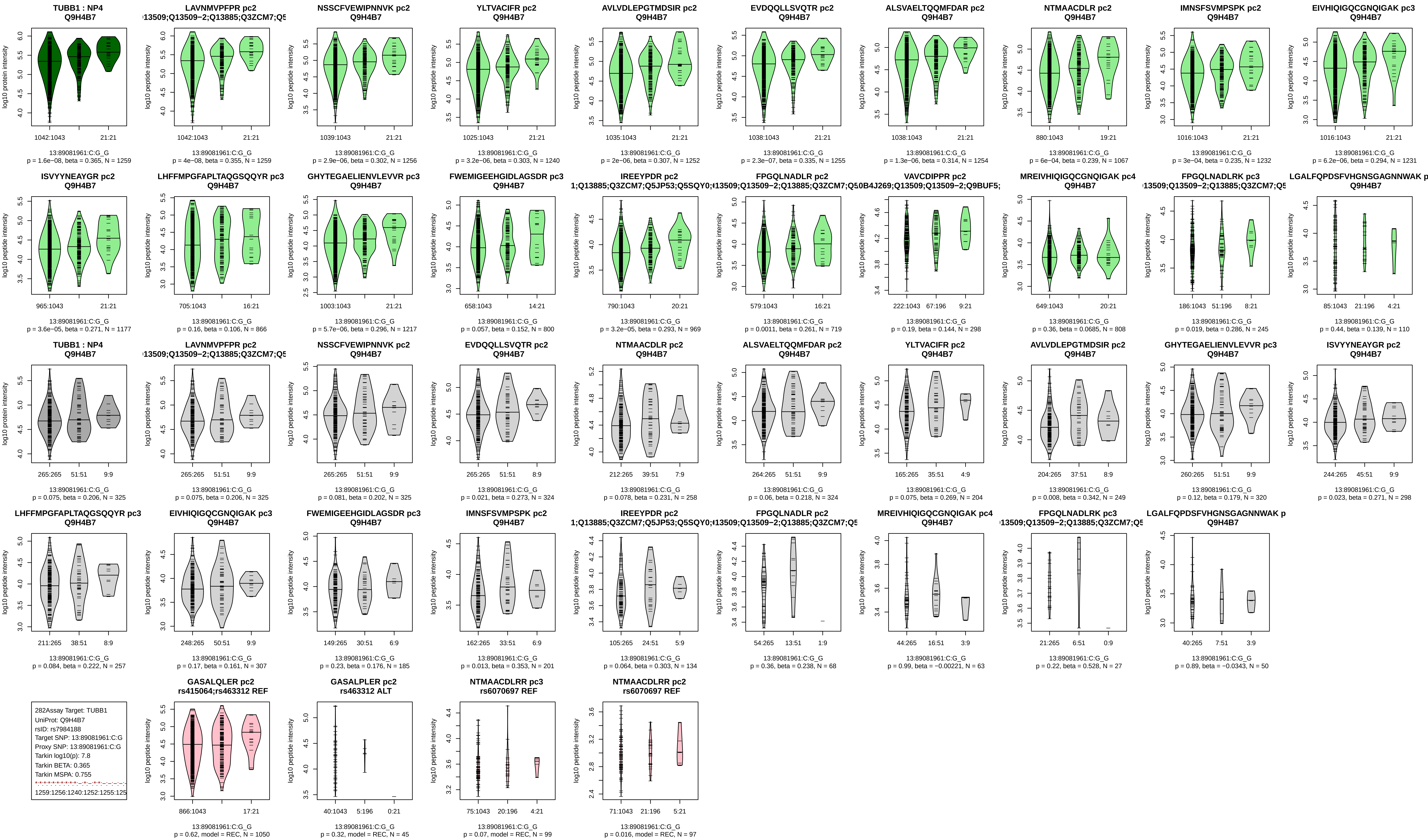
TUBB1 : NP4
Q9H4B7
log10 protein intensity
1042:1043
21:21
13:89081961:C:G_G
p = 1.6e−08, beta = 0.365, N = 1259
LAVNMVPFPR pc2
13509;Q13509−2;Q13885;Q3ZCM7;Q5
log10 peptide intensity
1042:1043
21:21
13:89081961:C:G_G
p = 4e−08, beta = 0.355, N = 1259
NSSCFVEWIPNNVK pc2
Q9H4B7
1039:1043
21:21
13:89081961:C:G_G
p = 2.9e−06, beta = 0.302, N = 1256
YLTVACIFR pc2
Q9H4B7
1025:1043
21:21
13:89081961:C:G_G
p = 3.2e−06, beta = 0.303, N = 1240
AVLVDLEPGTMDSIR pc2
Q9H4B7
1035:1043
21:21
13:89081961:C:G_G
p = 2e−06, beta = 0.307, N = 1252
EVDQQLLSVQTR pc2
Q9H4B7
1038:1043
21:21
13:89081961:C:G_G
p = 2.3e−07, beta = 0.335, N = 1255
ALSVAELTQQMFDAR pc2
Q9H4B7
1038:1043
21:21
13:89081961:C:G_G
p = 1.3e−06, beta = 0.314, N = 1254
NTMAACDLR pc2
Q9H4B7
880:1043
19:21
13:89081961:C:G_G
p = 6e−04, beta = 0.239, N = 1067
IMNSFSVMPSPK pc2
Q9H4B7
1016:1043
21:21
13:89081961:C:G_G
p = 3e−04, beta = 0.235, N = 1232
EIVHIQIGQCGNQIGAK pc3
Q9H4B7
1016:1043
21:21
13:89081961:C:G_G
p = 6.2e−06, beta = 0.294, N = 1231
ISVYYNEAYGR pc2
Q9H4B7
965:1043
21:21
13:89081961:C:G_G
p = 3.6e−05, beta = 0.271, N = 1177
LHFFMPGFAPLTAQGSQQYR pc3
Q9H4B7
705:1043
16:21
13:89081961:C:G_G
p = 0.16, beta = 0.106, N = 866
GHYTEGAELIENVLEVVR pc3
Q9H4B7
1003:1043
21:21
13:89081961:C:G_G
p = 5.7e−06, beta = 0.296, N = 1217
FWEMIGEEHGIDLAGSDR pc3
Q9H4B7
658:1043
14:21
13:89081961:C:G_G
p = 0.057, beta = 0.152, N = 800
IREEYPDR pc2
1;Q13885;Q3ZCM7;Q5JP53;Q5SQY0;
790:1043
20:21
13:89081961:C:G_G
p = 3.2e−05, beta = 0.293, N = 969
FPGQLNADLR pc2
13509;Q13509−2;Q13885;Q3ZCM7;Q5
579:1043
16:21
13:89081961:C:G_G
p = 0.0011, beta = 0.261, N = 719
VAVCDIPPR pc2
0B4J269;Q13509;Q13509−2;Q9BUF5;
222:1043
67:196
9:21
13:89081961:C:G_G
p = 0.19, beta = 0.144, N = 298
MREIVHIQIGQCGNQIGAK pc4
Q9H4B7
649:1043
20:21
13:89081961:C:G_G
p = 0.36, beta = 0.0685, N = 808
FPGQLNADLRK pc3
13509;Q13509−2;Q13885;Q3ZCM7;Q5
186:1043
51:196
8:21
13:89081961:C:G_G
p = 0.019, beta = 0.286, N = 245
LGALFQPDSFVHGNSGAGNNWAK p
Q9H4B7
85:1043
21:196
4:21
13:89081961:C:G_G
p = 0.44, beta = 0.139, N = 110
TUBB1 : NP4
Q9H4B7
265:265
51:51
9:9
13:89081961:C:G_G
p = 0.075, beta = 0.206, N = 325
LAVNMVPFPR pc2
13509;Q13509−2;Q13885;Q3ZCM7;Q5
265:265
51:51
9:9
13:89081961:C:G_G
p = 0.075, beta = 0.206, N = 325
NSSCFVEWIPNNVK pc2
Q9H4B7
265:265
51:51
9:9
13:89081961:C:G_G
p = 0.081, beta = 0.202, N = 325
EVDQQLLSVQTR pc2
Q9H4B7
265:265
51:51
8:9
13:89081961:C:G_G
p = 0.021, beta = 0.273, N = 324
NTMAACDLR pc2
Q9H4B7
212:265
39:51
7:9
13:89081961:C:G_G
p = 0.078, beta = 0.231, N = 258
ALSVAELTQQMFDAR pc2
Q9H4B7
264:265
51:51
9:9
13:89081961:C:G_G
p = 0.06, beta = 0.218, N = 324
YLTVACIFR pc2
Q9H4B7
165:265
35:51
4:9
13:89081961:C:G_G
p = 0.075, beta = 0.269, N = 204
AVLVDLEPGTMDSIR pc2
Q9H4B7
204:265
37:51
8:9
13:89081961:C:G_G
p = 0.008, beta = 0.342, N = 249
GHYTEGAELIENVLEVVR pc3
Q9H4B7
260:265
51:51
9:9
13:89081961:C:G_G
p = 0.12, beta = 0.179, N = 320
ISVYYNEAYGR pc2
Q9H4B7
244:265
45:51
9:9
13:89081961:C:G_G
p = 0.023, beta = 0.271, N = 298
LHFFMPGFAPLTAQGSQQYR pc3
Q9H4B7
211:265
38:51
8:9
13:89081961:C:G_G
p = 0.084, beta = 0.222, N = 257
EIVHIQIGQCGNQIGAK pc3
Q9H4B7
248:265
50:51
9:9
13:89081961:C:G_G
p = 0.17, beta = 0.161, N = 307
FWEMIGEEHGIDLAGSDR pc3
Q9H4B7
149:265
30:51
6:9
13:89081961:C:G_G
p = 0.23, beta = 0.176, N = 185
IMNSFSVMPSPK pc2
Q9H4B7
162:265
33:51
6:9
13:89081961:C:G_G
p = 0.013, beta = 0.353, N = 201
IREEYPDR pc2
1;Q13885;Q3ZCM7;Q5JP53;Q5SQY0;
105:265
24:51
5:9
13:89081961:C:G_G
p = 0.064, beta = 0.303, N = 134
FPGQLNADLR pc2
13509;Q13509−2;Q13885;Q3ZCM7;Q5
54:265
13:51
1:9
13:89081961:C:G_G
p = 0.36, beta = 0.238, N = 68
MREIVHIQIGQCGNQIGAK pc4
Q9H4B7
44:265
16:51
3:9
13:89081961:C:G_G
p = 0.99, beta = −0.00221, N = 63
FPGQLNADLRK pc3
13509;Q13509−2;Q13885;Q3ZCM7;Q5
21:265
6:51
0:9
13:89081961:C:G_G
p = 0.22, beta = 0.528, N = 27
LGALFQPDSFVHGNSGAGNNWAK p
Q9H4B7
40:265
7:51
3:9
13:89081961:C:G_G
p = 0.89, beta = −0.0343, N = 50
282Assay Target: TUBB1
UniProt: Q9H4B7
rsID: rs7984188
Target SNP: 13:89081961:C:G
Proxy SNP: 13:89081961:C:G
Tarkin log10(p): 7.8
Tarkin BETA: 0.365
Tarkin MSPA: 0.755
1259:1256:1240:1252:1255:125
GASALQLER pc2
rs415064;rs463312 REF
866:1043
17:21
13:89081961:C:G_G
p = 0.62, model = REC, N = 1050
GASALPLER pc2
rs463312 ALT
40:1043
5:196
0:21
13:89081961:C:G_G
p = 0.32, model = REC, N = 45
NTMAACDLRR pc3
rs6070697 REF
75:1043
20:196
4:21
13:89081961:C:G_G
p = 0.07, model = REC, N = 99
NTMAACDLRR pc2
rs6070697 REF
71:1043
21:196
5:21
13:89081961:C:G_G
p = 0.016, model = REC, N = 97

283. FSTL1 (Q12841)
Tarkin MSPA=0.741

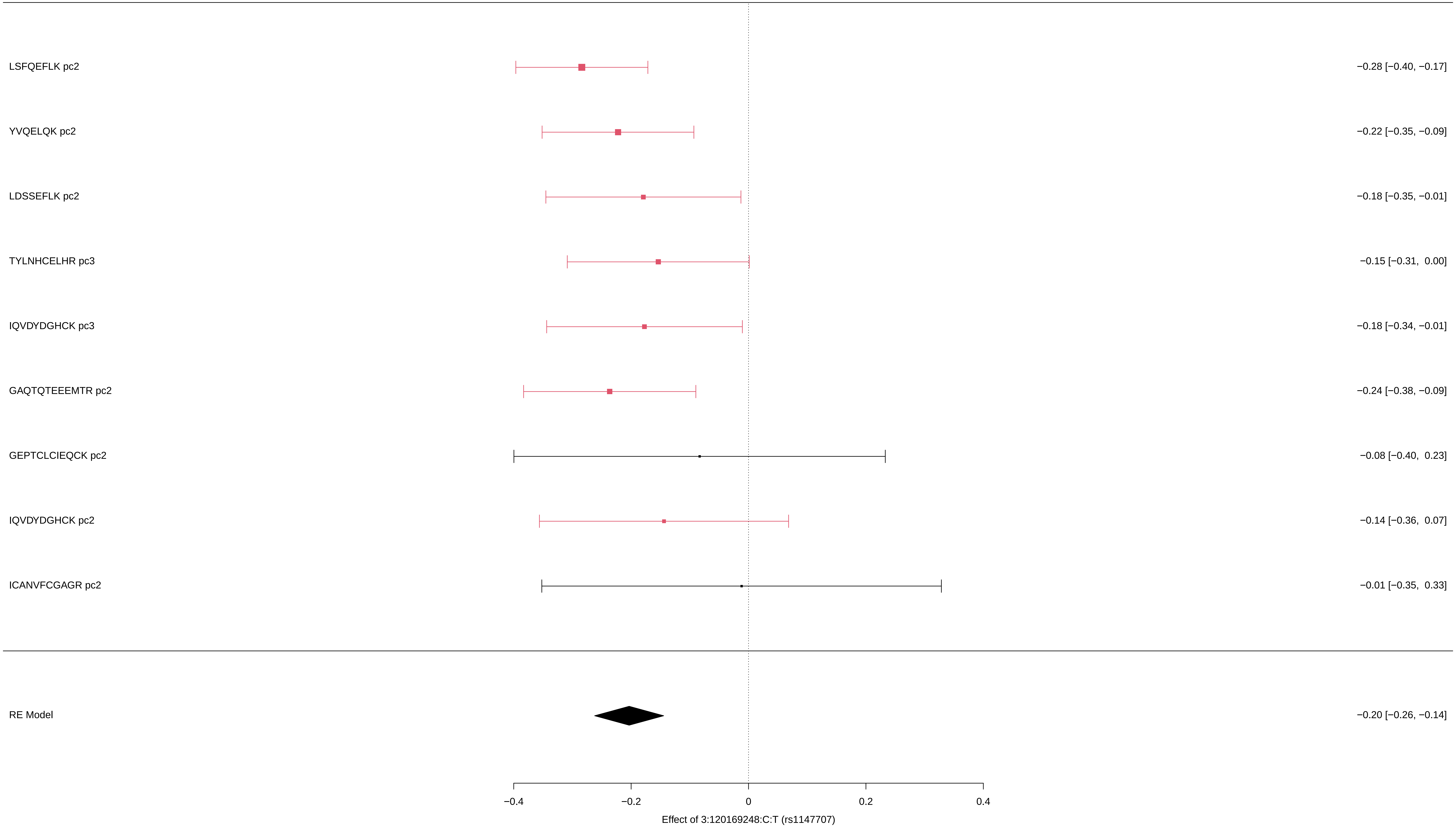

FSTL1 : NP2
Q12841

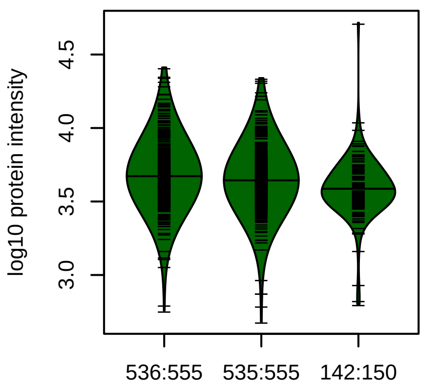

3:120169248:C:T_T
p = 1.8e−08, beta = −0.238, N = 1213

LSFQEFLK pc2
Q12841;Q12841−2

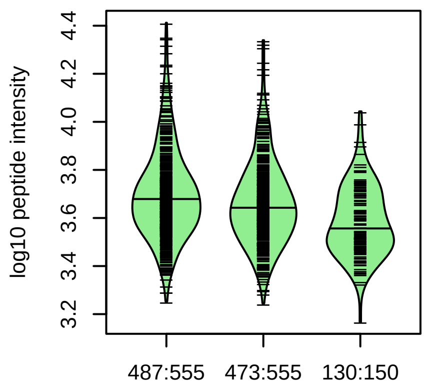

3:120169248:C:T_T
p = 1.2e−10, beta = −0.284, N = 1090

YVQELQK pc2
Q12841;Q12841−2

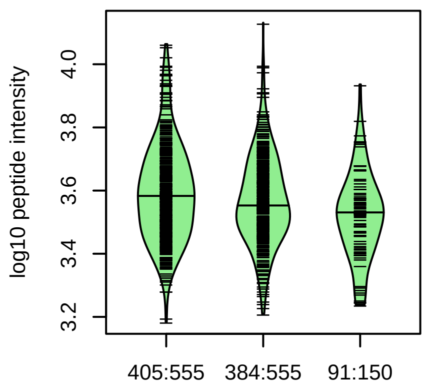

3:120169248:C:T_T
p = 1.1e−05, beta = −0.222, N = 880

LDSSEFLK pc2
Q12841;Q12841−2

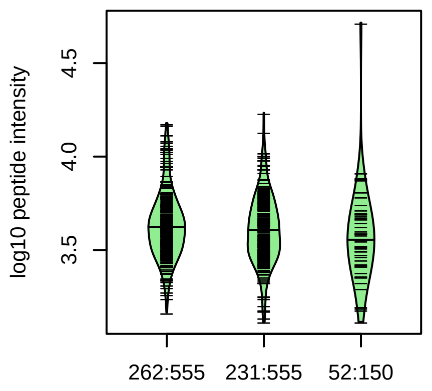

3:120169248:C:T_T
p = 0.0057, beta = −0.179, N = 545

TYLNHCELHR pc3
Q12841;Q12841−2

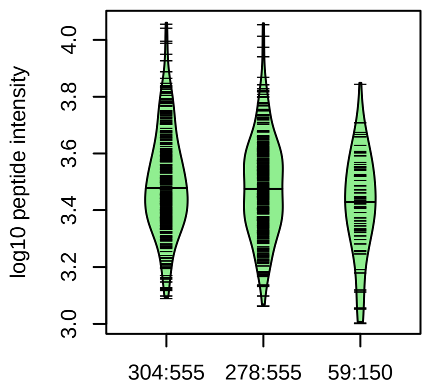

3:120169248:C:T_T
p = 0.011, beta = −0.154, N = 641

IQVDYDGHCK pc3
Q12841;Q12841−2

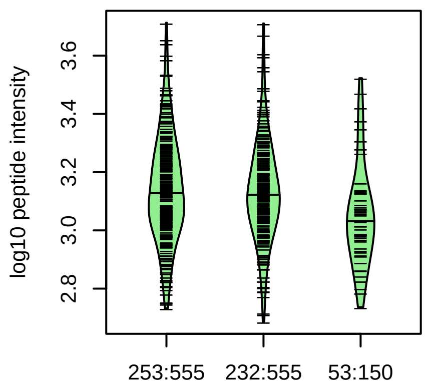

3:120169248:C:T_T
p = 0.0064, beta = −0.177, N = 538

GAQTQTEEEMTR pc2
Q12841;Q12841−2

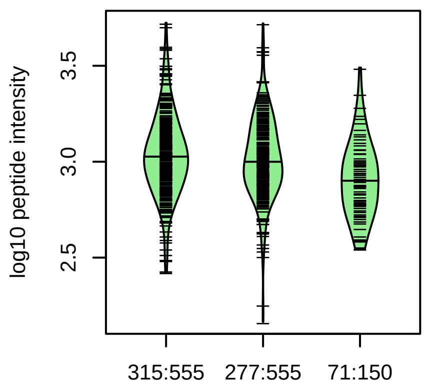

3:120169248:C:T_T
p = 3.7e−05, beta = −0.236, N = 663

GEPTCLCIEQCK pc2
Q12841

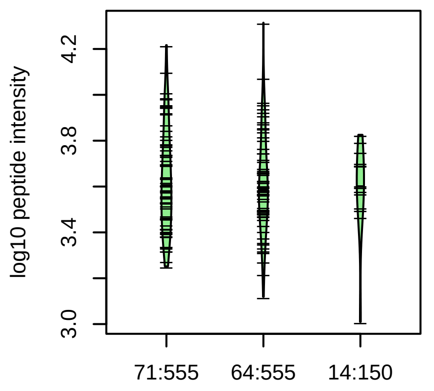

3:120169248:C:T_T
p = 0.5, beta = −0.0834, N = 149

ICANVFCGAGR pc2
Q12841

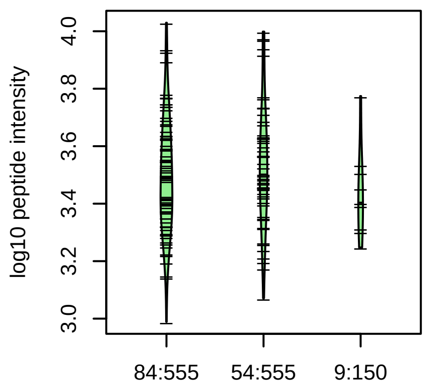

3:120169248:C:T_T
p = 0.93, beta = −0.0118, N = 147

FSTL1 : NP2
Q12841

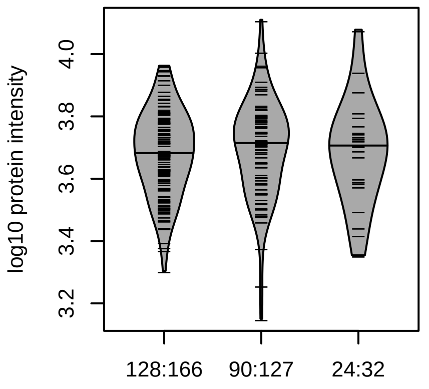

3:120169248:C:T_T
p = 0.66, beta = 0.0412, N = 242

LSFQEFLK pc2
Q12841;Q12841−2

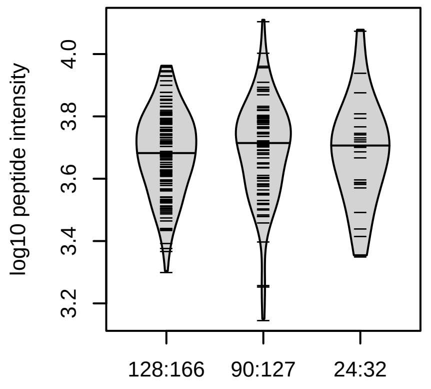

3:120169248:C:T_T
p = 0.69, beta = 0.0383, N = 242

283Assay Target: FSTL1
UniProt: Q12841
rsID: rs1147707
Target SNP: 3:120169248:C:T
Proxy SNP: 3:120169248:C:T
Tarkin log10(p): 7.8
Tarkin BETA: −0.238
Tarkin MSPA: 0.741
::*:–:*:*:–:–:–
1090:880:545:641:538:663:149:

284. DNAH6 (Q9C0G6)
Tarkin MSPA=0.768

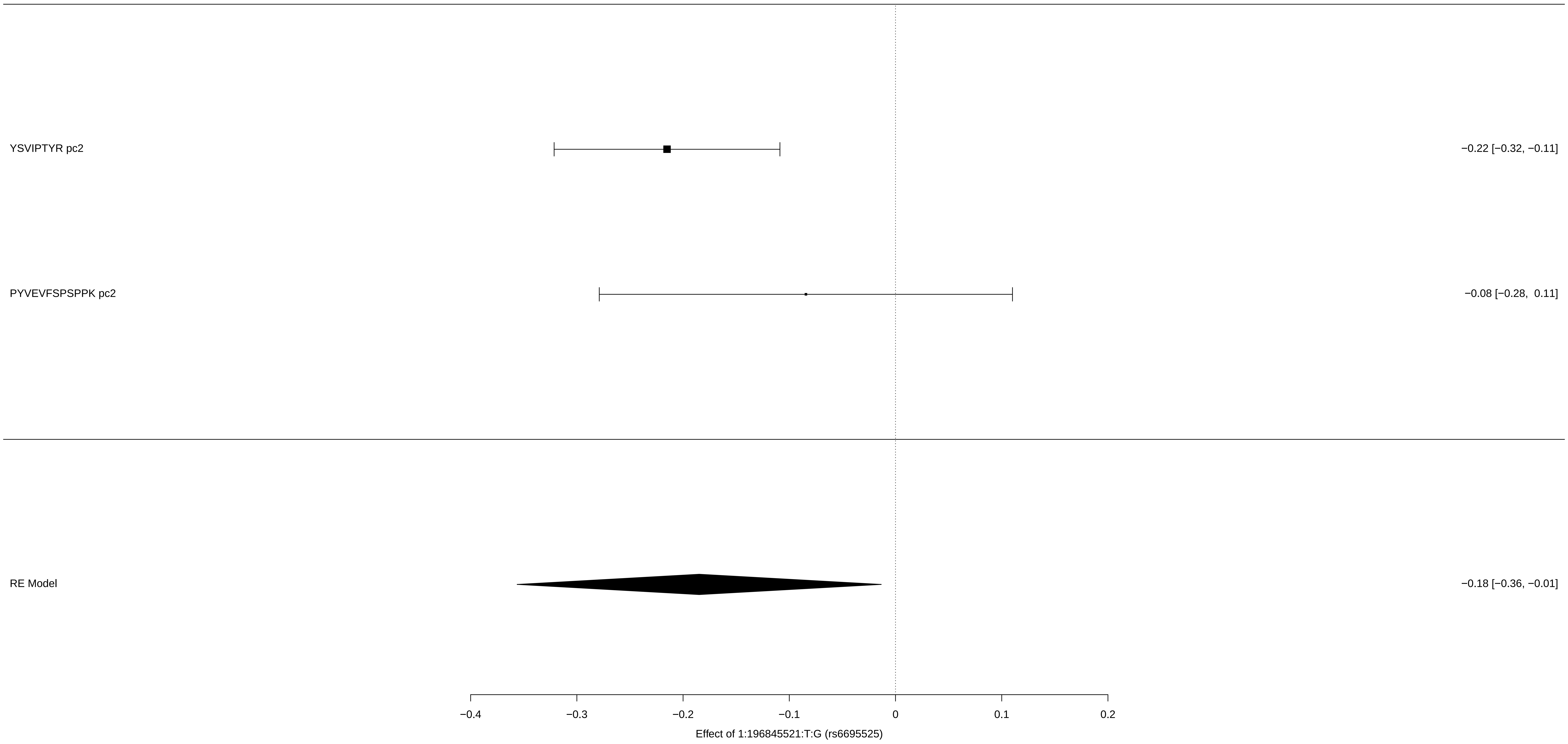

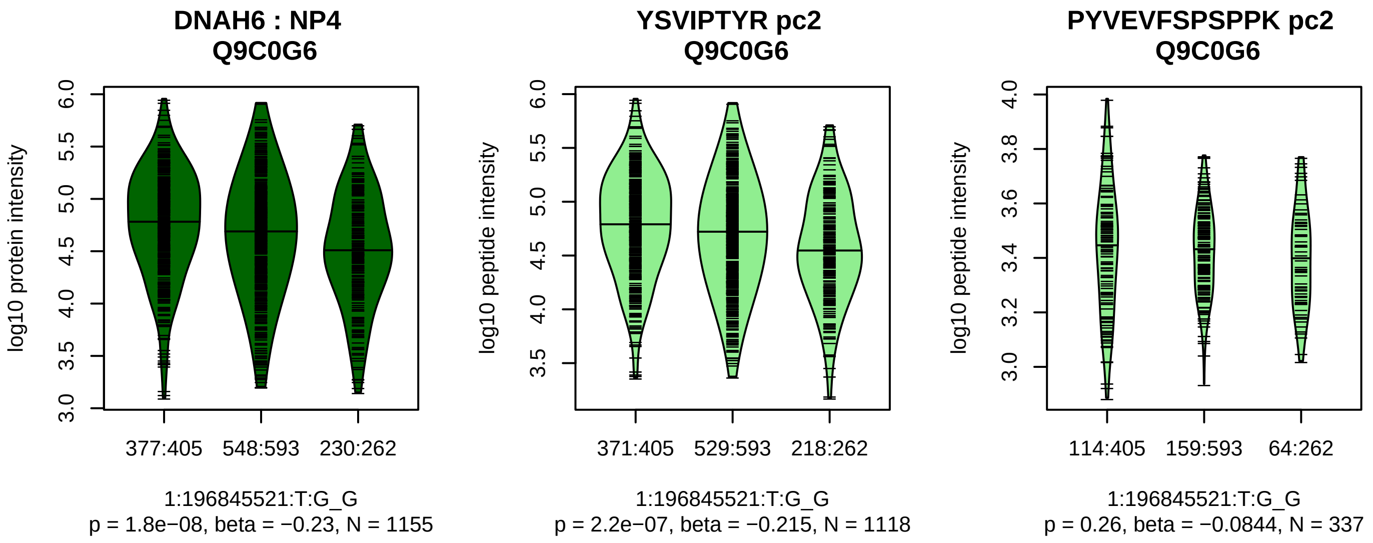

284Assay Target: DNAH6
UniProt: Q9C0G6
rsID: rs6695525
Target SNP: 1:196845521:T:G
Proxy SNP: 1:196845521:T:G
Tarkin log10(p): 7.8
Tarkin BETA: −0.23
Tarkin MSPA: 0.768
*:–
1118:337

285. UBASH3B (Q8TF42)
Tarkin MSPA=0.719

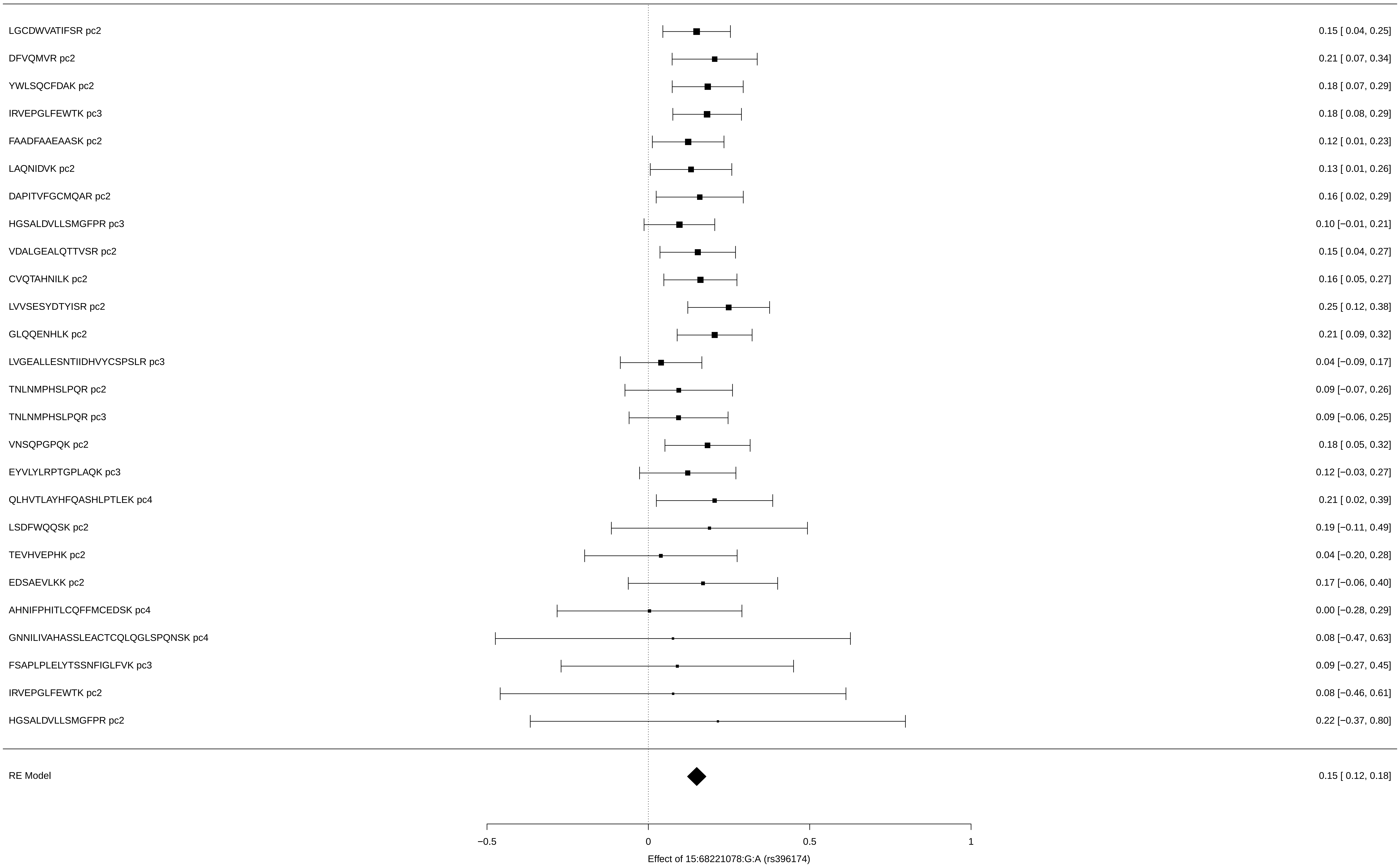

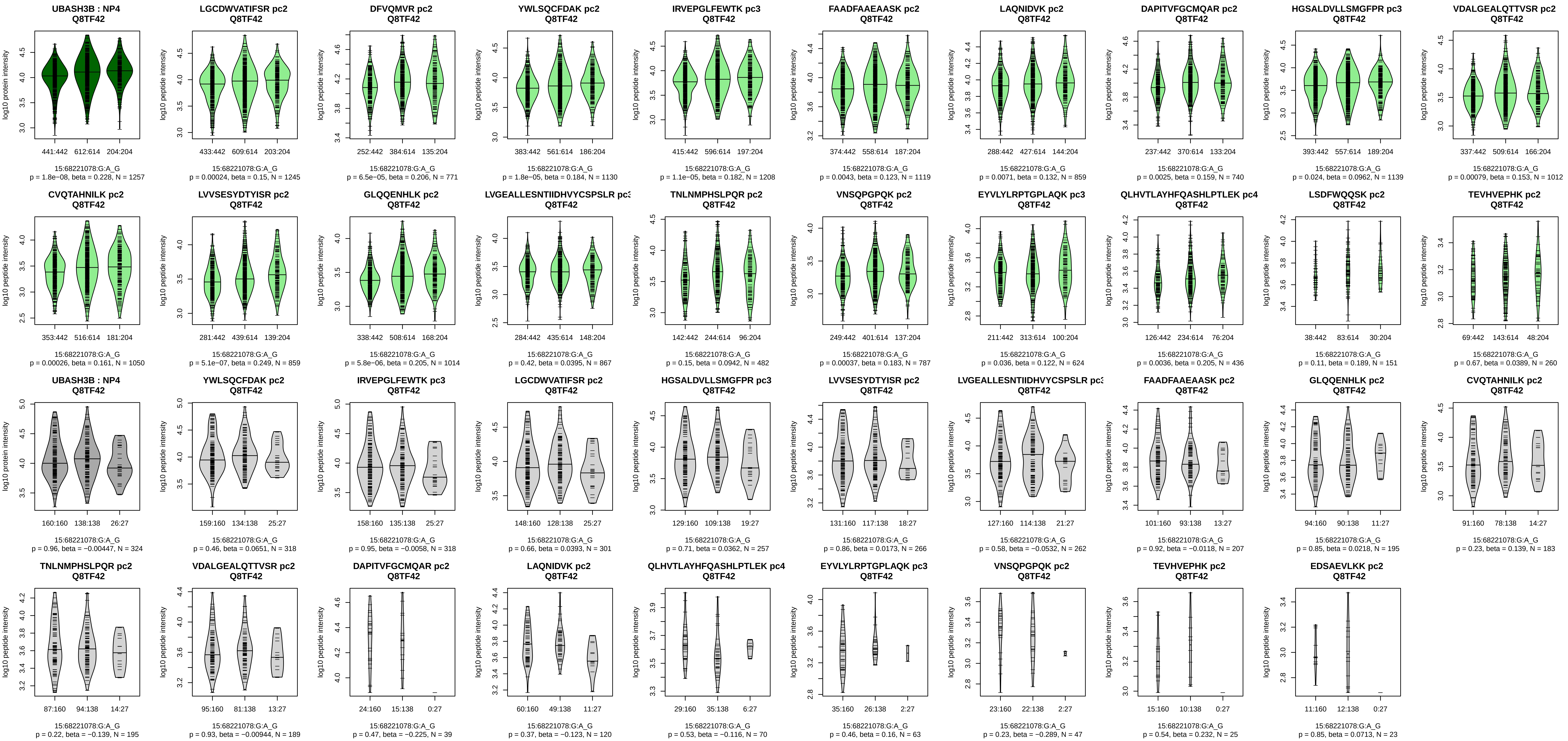

285Assay Target: UBASH3B
UniProt: Q8TF42
rsID: rs396174
Target SNP: 15:68221078:G:A
Proxy SNP: 15:68221078:G:A
Tarkin log10(p): 7.8
Tarkin BETA: 0.228
Tarkin MSPA: 0.719
1245:771:1130:1208:1119:859:[illegible]

286. MASP1 (F8W876)
Tarkin MSPA=0.403

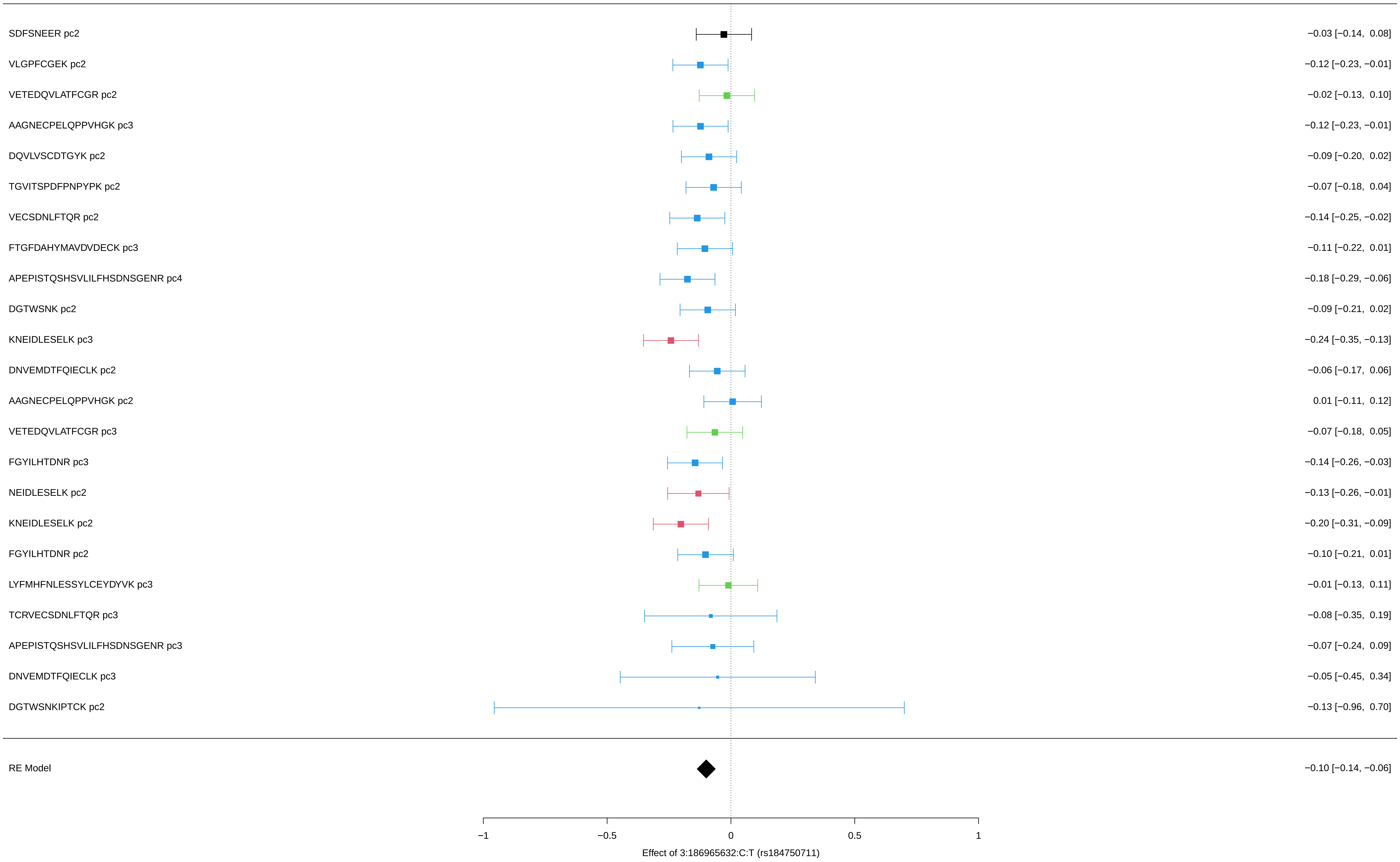

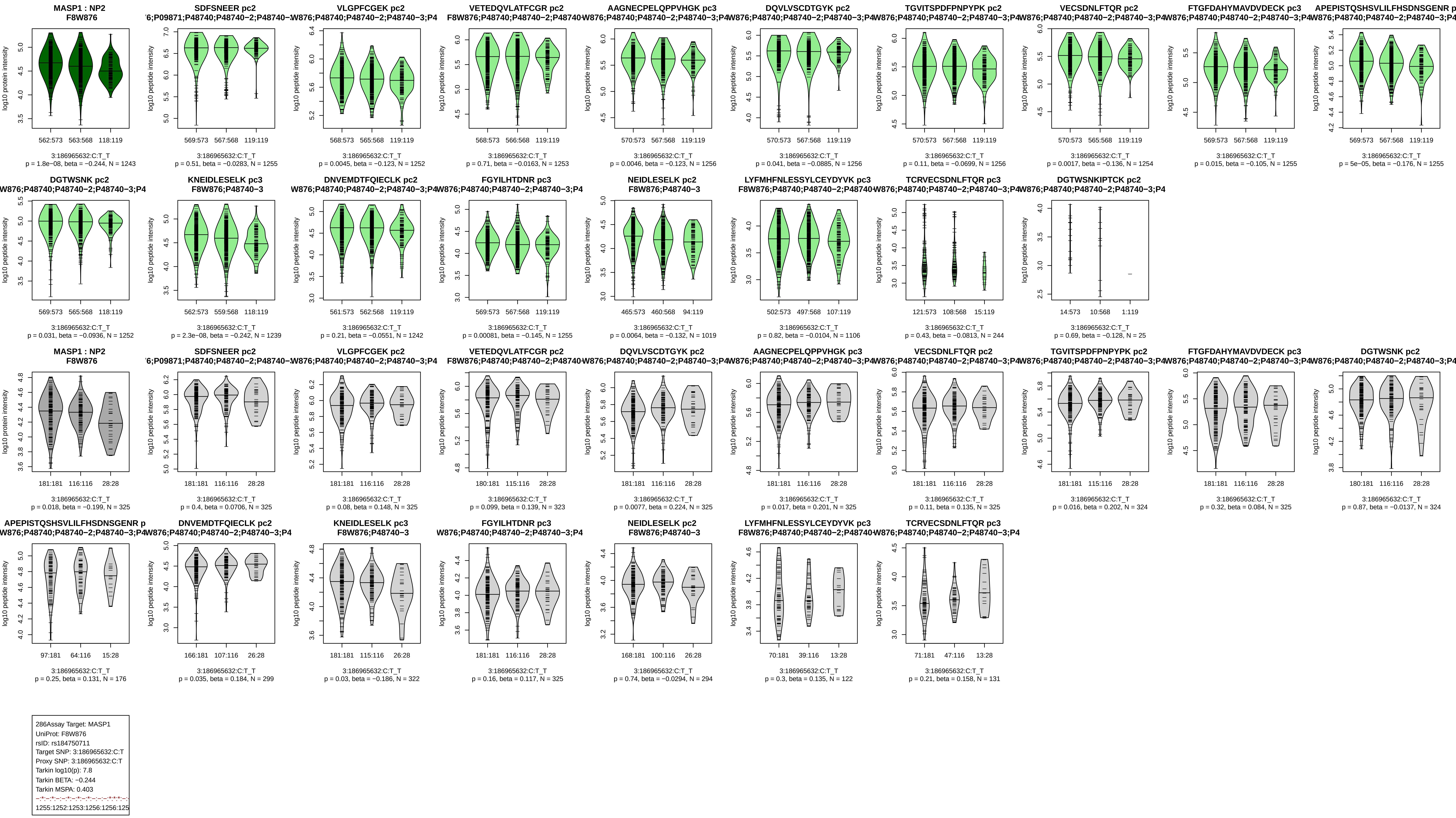
MASP1 : NP2
F8W876
log10 protein intensity
562:573 563:568 118:119
3:186965632:C:T_T
p = 1.8e−08, beta = −0.244, N = 1243
SDFSNEER pc2
'6;P09871;P48740;P48740−2;P48740−
log10 peptide intensity
569:573 567:568 119:119
3:186965632:C:T_T
p = 0.51, beta = −0.0283, N = 1255
VLGPFCGEK pc2
W876;P48740;P48740−2;P48740−3;P4
568:573 565:568 119:119
3:186965632:C:T_T
p = 0.0045, beta = −0.123, N = 1252
VETEDQVLATFCGR pc2
F8W876;P48740;P48740−2;P48740
568:573 566:568 119:119
3:186965632:C:T_T
p = 0.71, beta = −0.0163, N = 1253
AAGNECPELQPPVHGK pc3
W876;P48740;P48740−2;P48740−3;P4
570:573 567:568 119:119
3:186965632:C:T_T
p = 0.0046, beta = −0.123, N = 1256
DQVLVSCDTGYK pc2
W876;P48740;P48740−2;P48740−3;P4
570:573 567:568 119:119
3:186965632:C:T_T
p = 0.041, beta = −0.0885, N = 1256
TGVITSPDFPNPYPK pc2
W876;P48740;P48740−2;P48740−3;P4
570:573 567:568 119:119
3:186965632:C:T_T
p = 0.11, beta = −0.0699, N = 1256
VECSDNLFTQR pc2
W876;P48740;P48740−2;P48740−3;P4
570:573 565:568 119:119
3:186965632:C:T_T
p = 0.0017, beta = −0.136, N = 1254
FTGFDAHYMAVDVDECK pc3
W876;P48740;P48740−2;P48740−3;P4
569:573 567:568 119:119
3:186965632:C:T_T
p = 0.015, beta = −0.105, N = 1255
APEPISTQSHSVLILFHSDNSGENR p
W876;P48740;P48740−2;P48740−3;P4
569:573 567:568 119:119
3:186965632:C:T_T
p = 5e−05, beta = −0.176, N = 1255
DGTWSNK pc2
W876;P48740;P48740−2;P48740−3;P4
569:573 565:568 118:119
3:186965632:C:T_T
p = 0.031, beta = −0.0936, N = 1252
KNEIDLESELK pc3
F8W876;P48740−3
562:573 559:568 118:119
3:186965632:C:T_T
p = 2.3e−08, beta = −0.242, N = 1239
DNVEMDTFQIECLK pc2
W876;P48740;P48740−2;P48740−3;P4
561:573 562:568 119:119
3:186965632:C:T_T
p = 0.21, beta = −0.0551, N = 1242
FGYILHTDNR pc3
W876;P48740;P48740−2;P48740−3;P4
569:573 567:568 119:119
3:186965632:C:T_T
p = 0.00081, beta = −0.145, N = 1255
NEIDLESELK pc2
F8W876;P48740−3
465:573 460:568 94:119
3:186965632:C:T_T
p = 0.0064, beta = −0.132, N = 1019
LYFMHFNLESSYLCEYDYVK pc3
F8W876;P48740;P48740−2;P48740
502:573 497:568 107:119
3:186965632:C:T_T
p = 0.82, beta = −0.0104, N = 1106
TCRVECSDNLFTQR pc3
W876;P48740;P48740−2;P48740−3;P4
121:573 108:568 15:119
3:186965632:C:T_T
p = 0.43, beta = −0.0813, N = 244
DGTWSNKIPTCK pc2
W876;P48740;P48740−2;P48740−3;P4
14:573 10:568 1:119
3:186965632:C:T_T
p = 0.69, beta = −0.128, N = 25
MASP1 : NP2
F8W876
181:181 116:116 28:28
3:186965632:C:T_T
p = 0.018, beta = −0.199, N = 325
SDFSNEER pc2
'6;P09871;P48740;P48740−2;P48740−
181:181 116:116 28:28
3:186965632:C:T_T
p = 0.4, beta = 0.0706, N = 325
VLGPFCGEK pc2
W876;P48740;P48740−2;P48740−3;P4
181:181 116:116 28:28
3:186965632:C:T_T
p = 0.08, beta = 0.148, N = 325
VETEDQVLATFCGR pc2
F8W876;P48740;P48740−2;P48740
180:181 115:116 28:28
3:186965632:C:T_T
p = 0.099, beta = 0.139, N = 323
DQVLVSCDTGYK pc2
W876;P48740;P48740−2;P48740−3;P4
181:181 116:116 28:28
3:186965632:C:T_T
p = 0.0077, beta = 0.224, N = 325
AAGNECPELQPPVHGK pc3
W876;P48740;P48740−2;P48740−3;P4
181:181 116:116 28:28
3:186965632:C:T_T
p = 0.017, beta = 0.201, N = 325
VECSDNLFTQR pc2
W876;P48740;P48740−2;P48740−3;P4
181:181 116:116 28:28
3:186965632:C:T_T
p = 0.11, beta = 0.135, N = 325
TGVITSPDFPNPYPK pc2
W876;P48740;P48740−2;P48740−3;P4
181:181 115:116 28:28
3:186965632:C:T_T
p = 0.016, beta = 0.202, N = 324
FTGFDAHYMAVDVDECK pc3
W876;P48740;P48740−2;P48740−3;P4
181:181 116:116 28:28
3:186965632:C:T_T
p = 0.32, beta = 0.084, N = 325
DGTWSNK pc2
W876;P48740;P48740−2;P48740−3;P4
180:181 116:116 28:28
3:186965632:C:T_T
p = 0.87, beta = −0.0137, N = 324
APEPISTQSHSVLILFHSDNSGENR p
W876;P48740;P48740−2;P48740−3;P4
97:181 64:116 15:28
3:186965632:C:T_T
p = 0.25, beta = 0.131, N = 176
DNVEMDTFQIECLK pc2
W876;P48740;P48740−2;P48740−3;P4
166:181 107:116 26:28
3:186965632:C:T_T
p = 0.035, beta = 0.184, N = 299
KNEIDLESELK pc3
F8W876;P48740−3
181:181 115:116 26:28
3:186965632:C:T_T
p = 0.03, beta = −0.186, N = 322
FGYILHTDNR pc3
W876;P48740;P48740−2;P48740−3;P4
181:181 116:116 28:28
3:186965632:C:T_T
p = 0.16, beta = 0.117, N = 325
NEIDLESELK pc2
F8W876;P48740−3
168:181 100:116 26:28
3:186965632:C:T_T
p = 0.74, beta = −0.0294, N = 294
LYFMHFNLESSYLCEYDYVK pc3
F8W876;P48740;P48740−2;P48740
70:181 39:116 13:28
3:186965632:C:T_T
p = 0.3, beta = 0.135, N = 122
TCRVECSDNLFTQR pc3
W876;P48740;P48740−2;P48740−3;P4
71:181 47:116 13:28
3:186965632:C:T_T
p = 0.21, beta = 0.158, N = 131
286Assay Target: MASP1
UniProt: F8W876
rsID: rs184750711
Target SNP: 3:186965632:C:T
Proxy SNP: 3:186965632:C:T
Tarkin log10(p): 7.8
Tarkin BETA: −0.244
Tarkin MSPA: 0.403
1255:1252:1253:1256:1256:125

287. POTEF (A5A3E0)
Tarkin MSPA=0.529

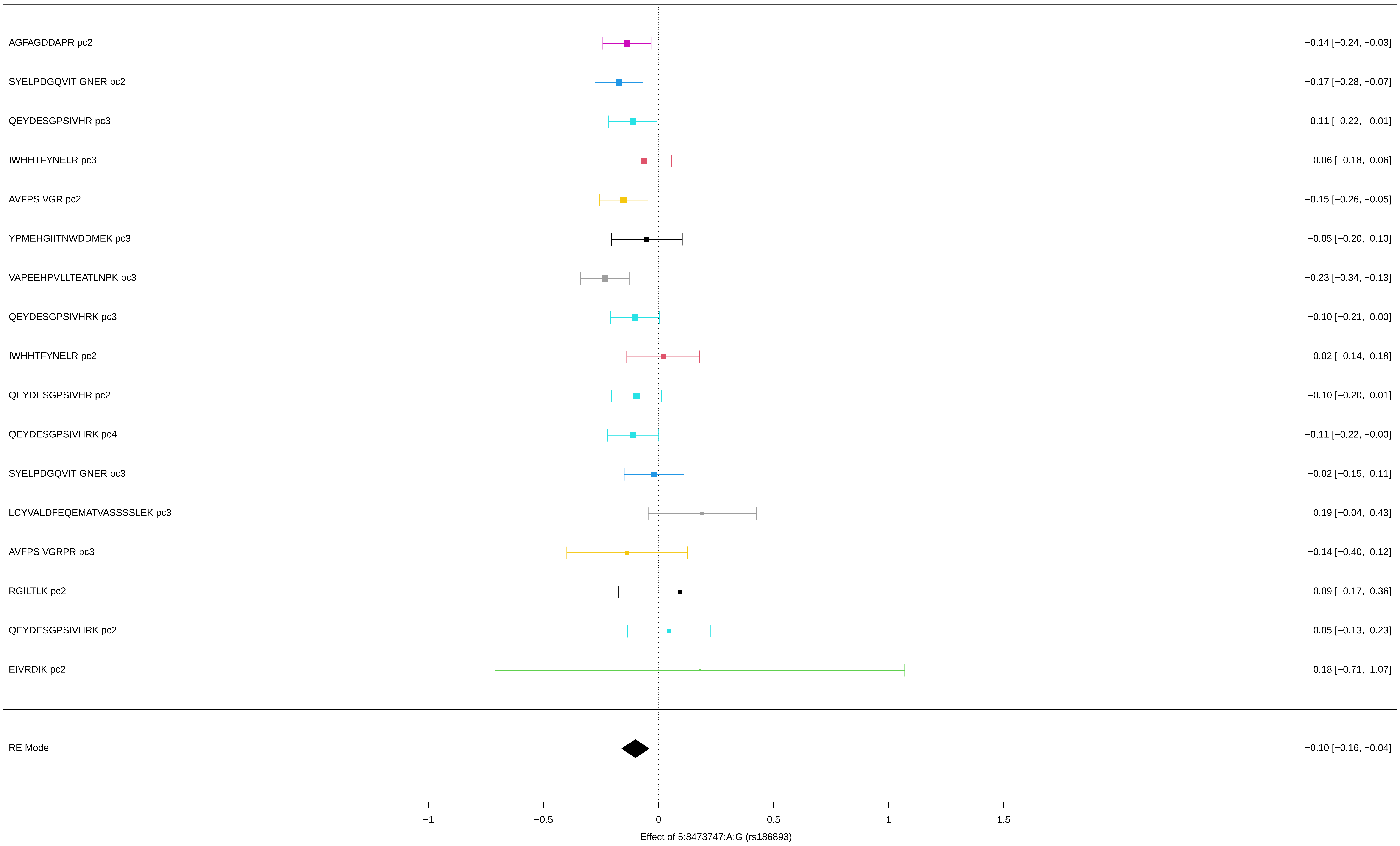

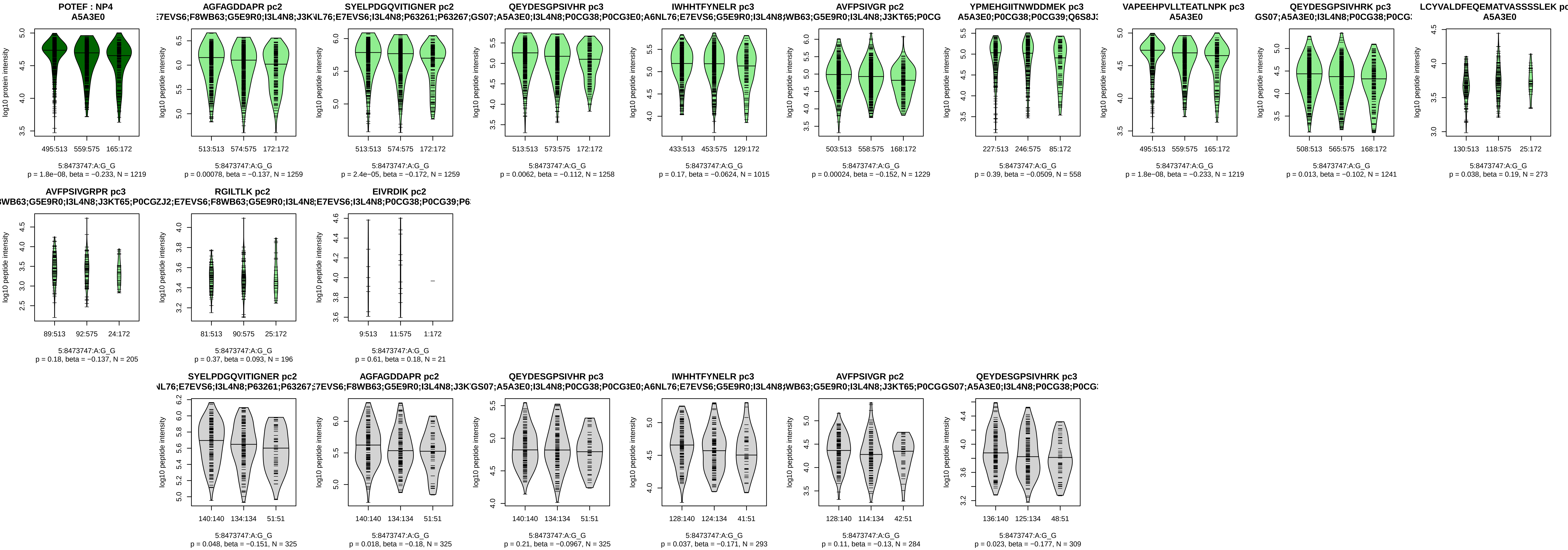

287Assay Target: POTEF
UniProt: A5A3E0
rsID: rs186893
Target SNP: 5:8473747:A:G
Proxy SNP: 5:8473747:A:G
Tarkin log10(p): 7.8
Tarkin BETA: −0.233
Tarkin MSPA: 0.529
··*·–·*·–·*·–·–·–·*·–·–·–·–·–·–
1259:1259:1258:1015:1229:558

288. DNAJB11 (Q9UBS4)
Tarkin MSPA=0.868

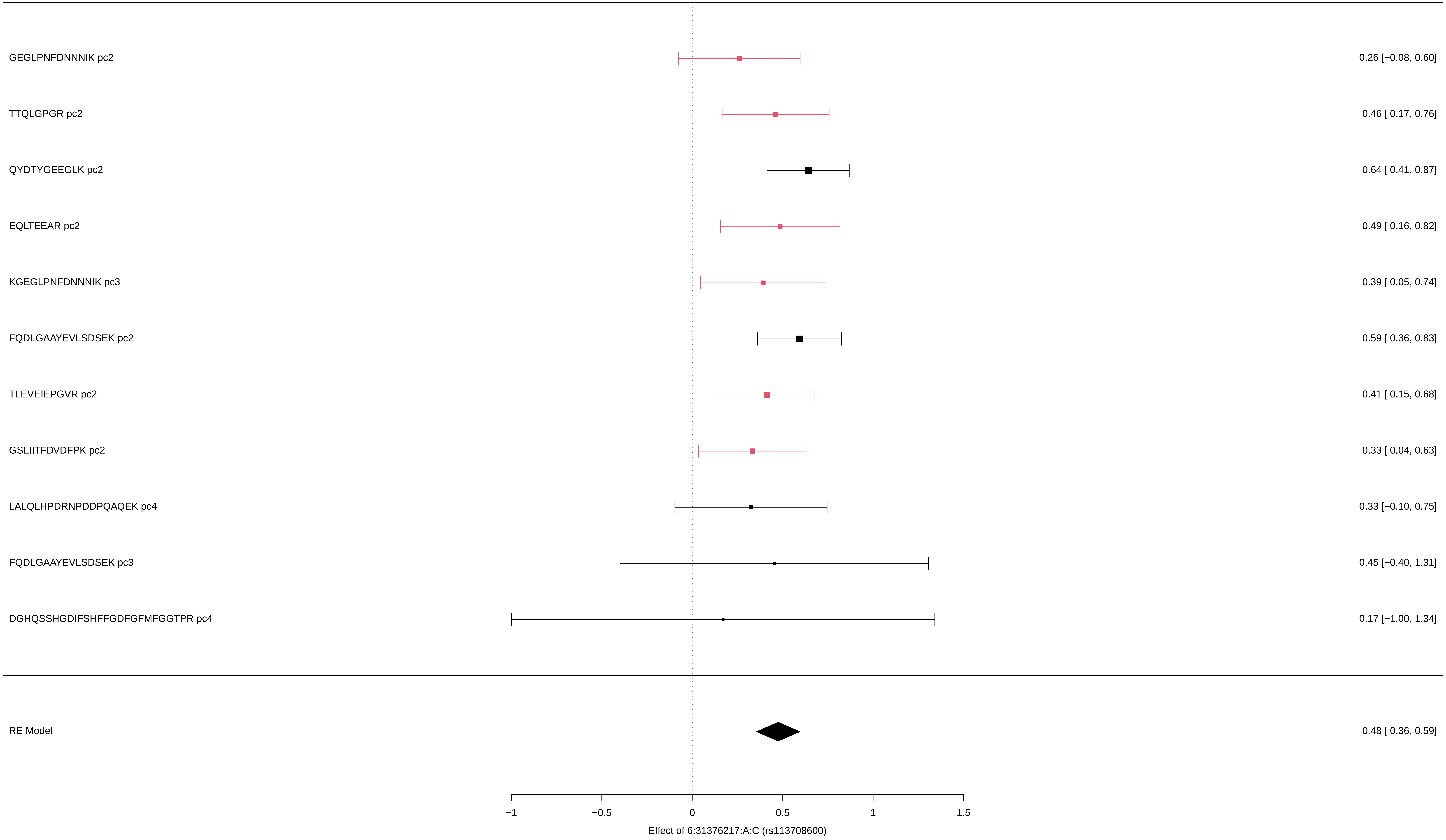

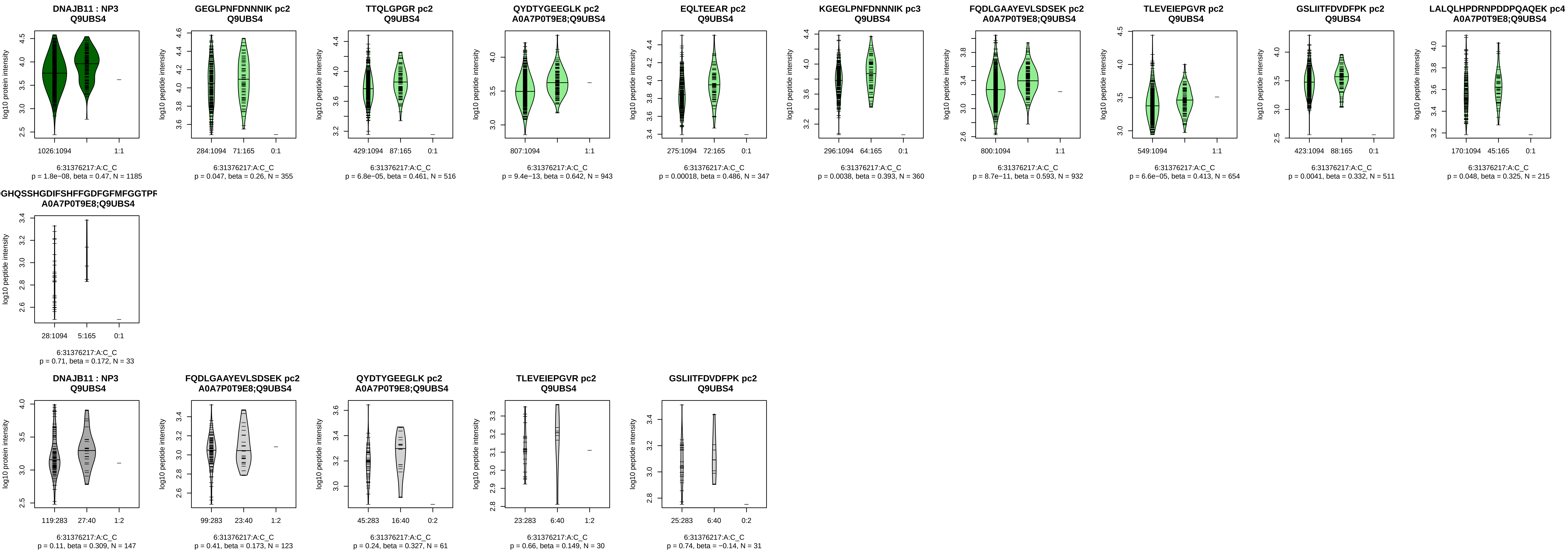

288Assay Target: DNAJB11
UniProt: Q9UBS4
rsID: rs113708600
Target SNP: 6:31376217:A:C
Proxy SNP: 6:31376217:A:C
Tarkin log10(p): 7.7
Tarkin BETA: 0.47
Tarkin MSPA: 0.868
−:*:*:*:*:*:*:−:−:−:NA:NA
355:516:943:347:360:932:654:5

289. ATP6V1A (P38606)
Tarkin MSPA=0.191

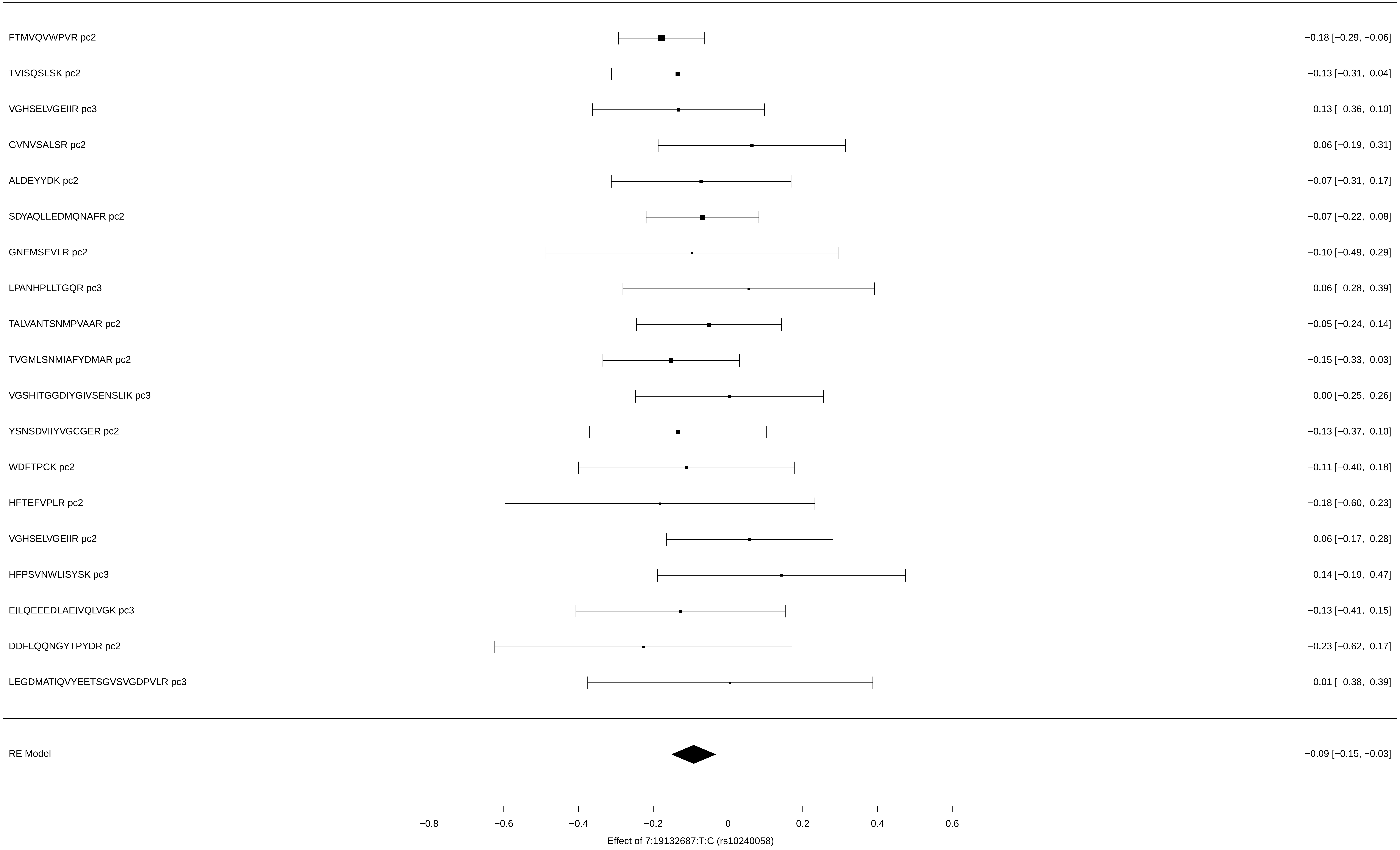

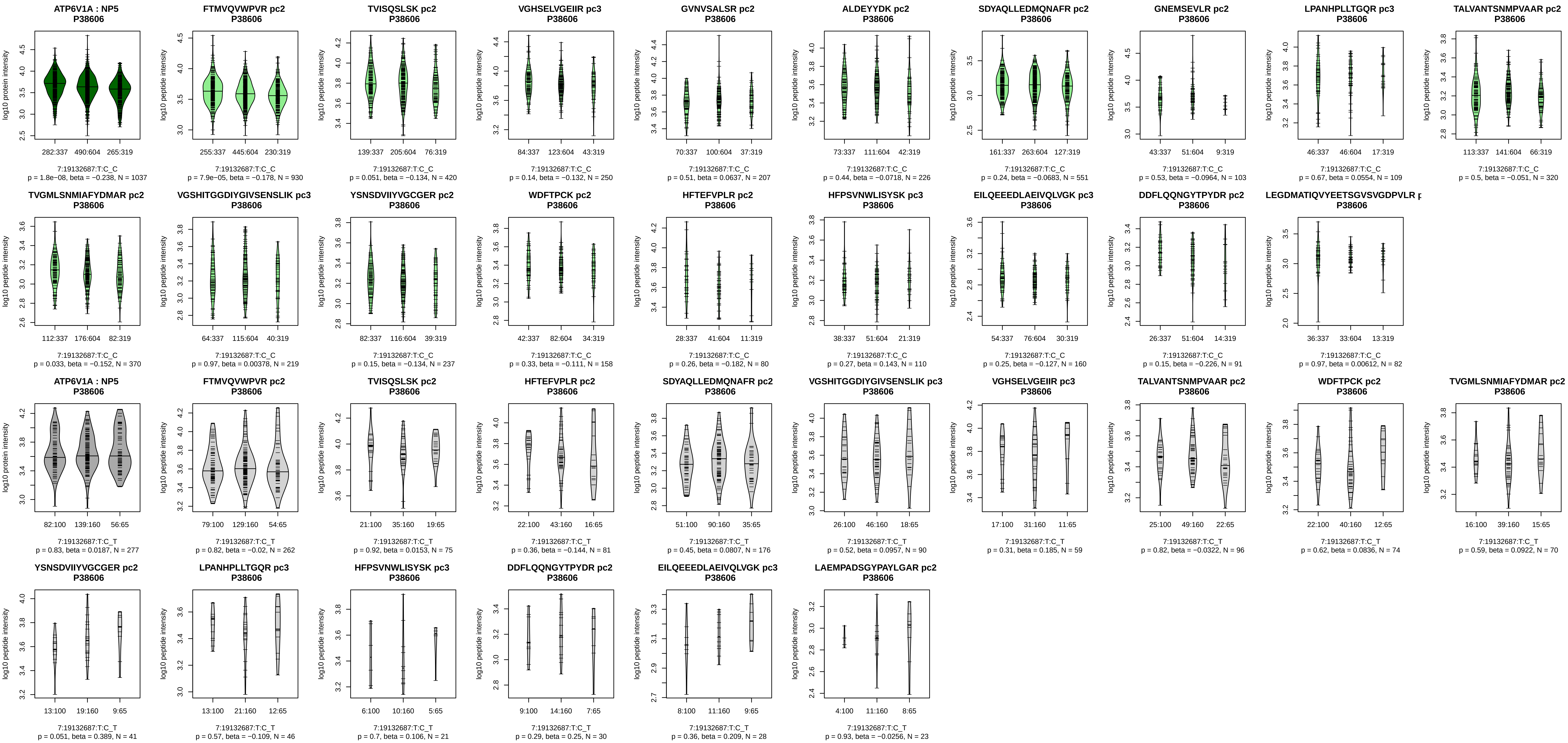

289Assay Target: ATP6V1A
UniProt: P38606
rsID: rs10240058
Target SNP: 7:19132687:T:C
Proxy SNP: 7:19132687:T:C
Tarkin log10(p): 7.7
Tarkin BETA: −0.238
Tarkin MSPA: 0.191
930:420:250:207:226:551:103:1

290. HNRNPF (P52597)
Tarkin MSPA=0.758

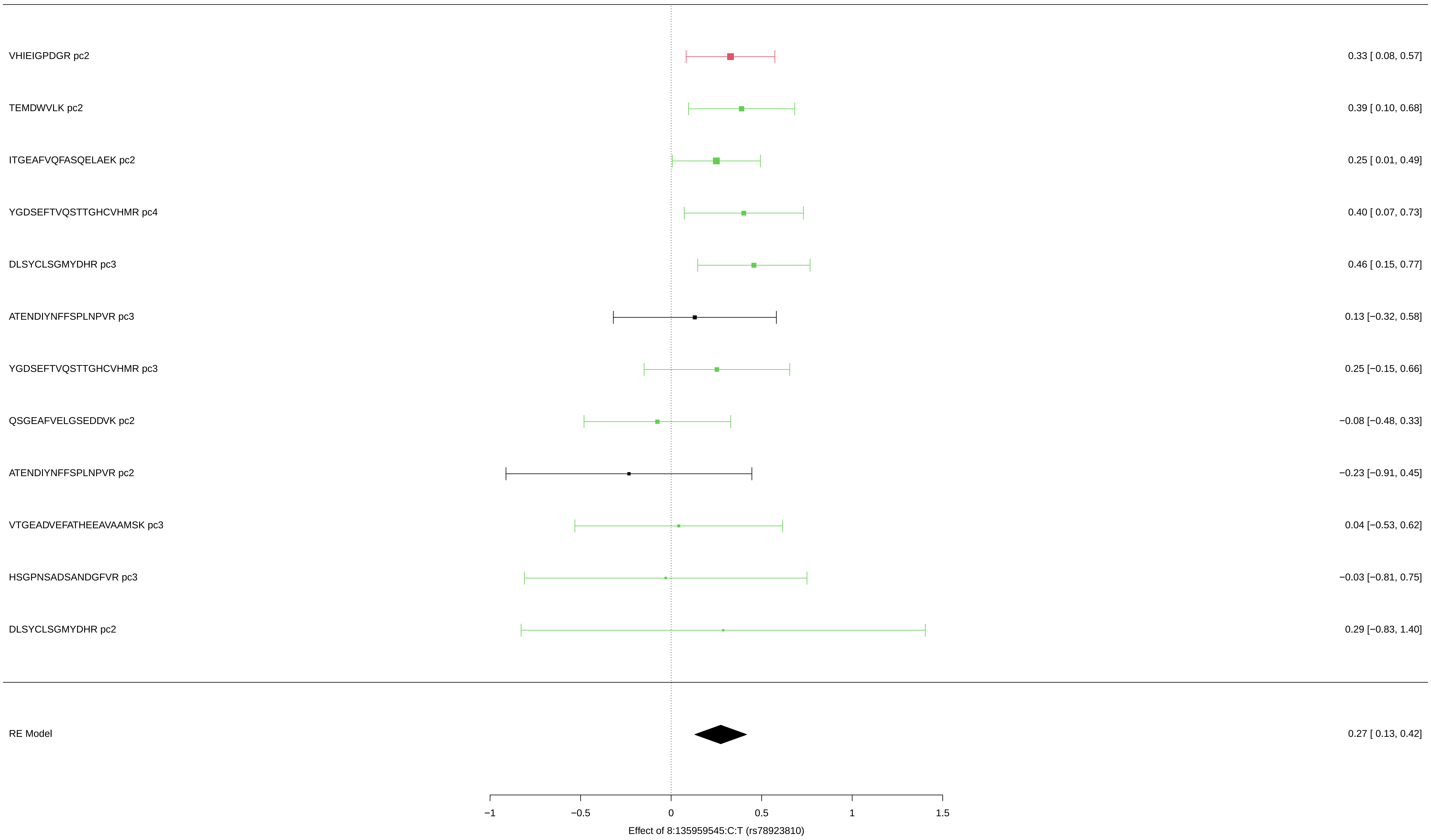

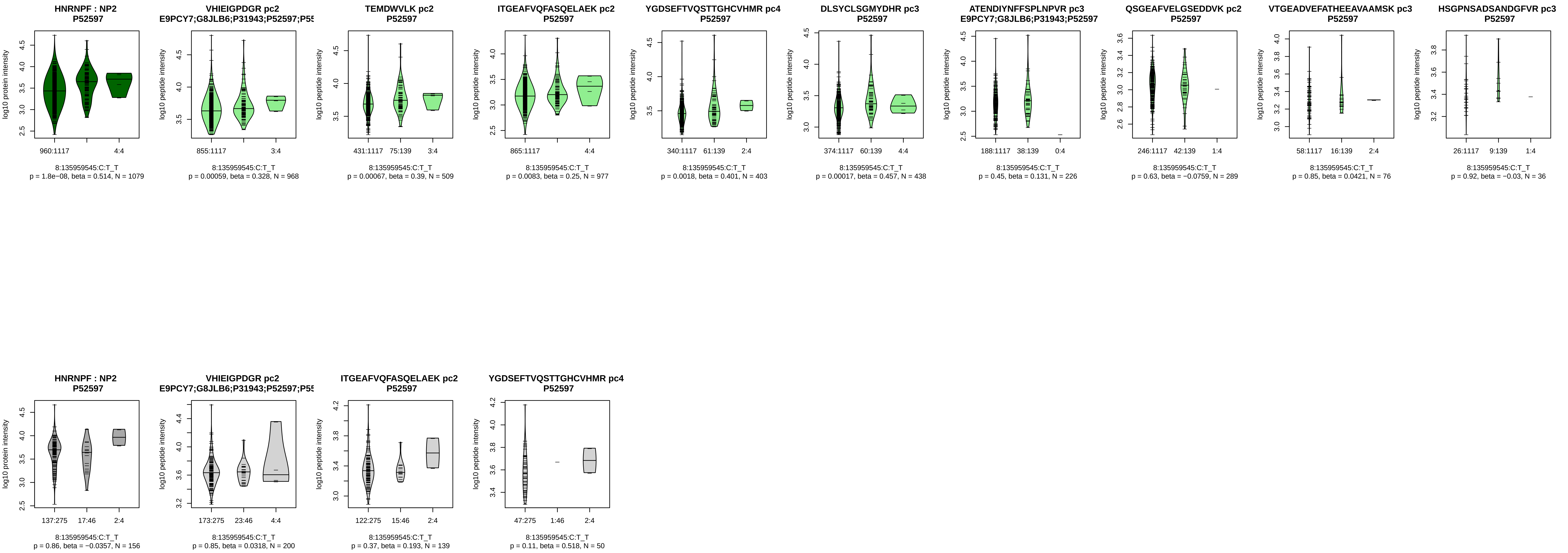

290Assay Target: HNRNPF
UniProt: P52597
rsID: rs78923810
Target SNP: 8:135959545:C:T
Proxy SNP: 8:135959545:C:T
Tarkin log10(p): 7.7
Tarkin BETA: 0.514
Tarkin MSPA: 0.758
968:509:977:403:438:226:303:2

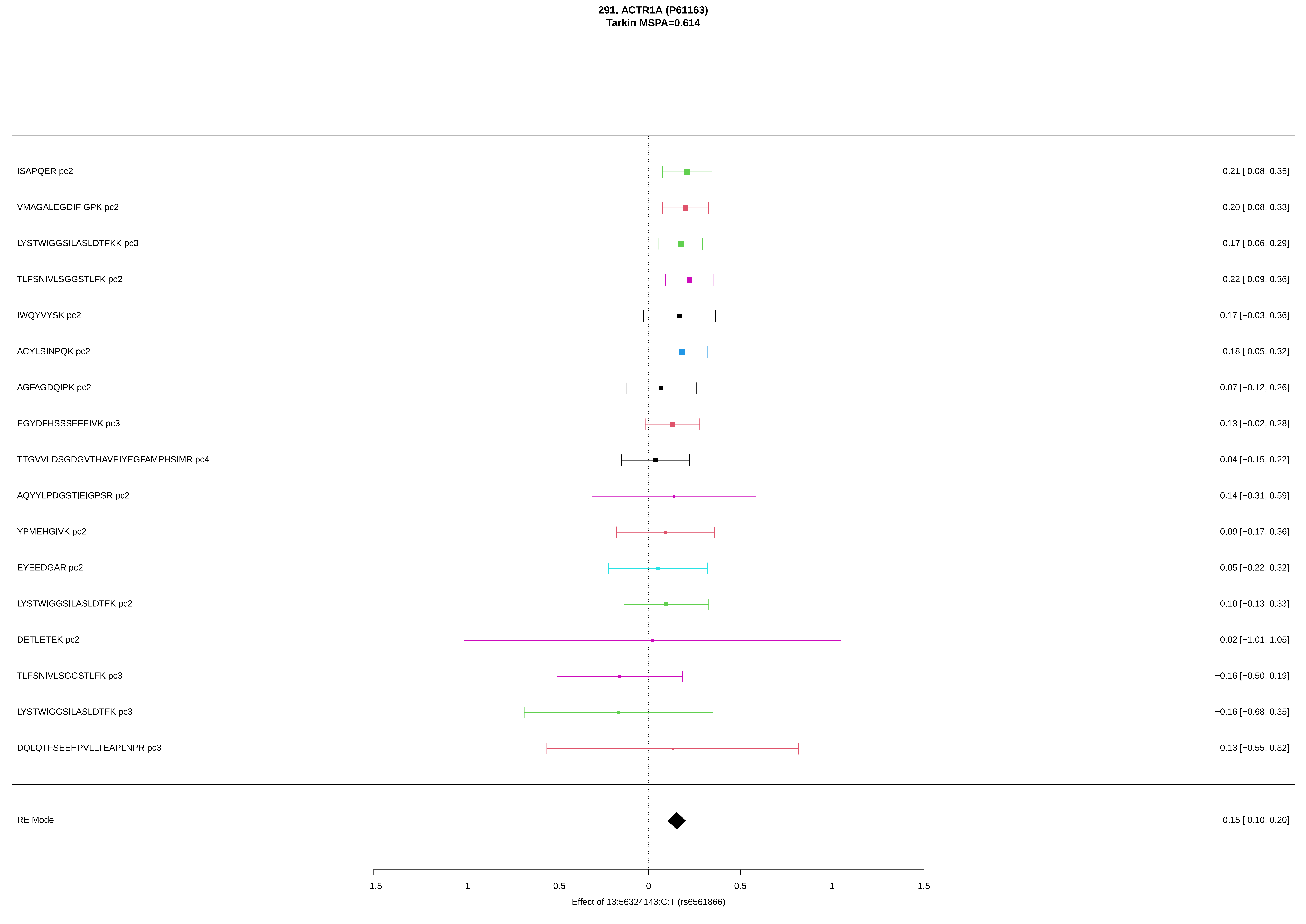
291. ACTR1A (P61163)
Tarkin MSPA=0.614
ISAPQER pc2
0.21 [0.08, 0.35]
VMAGALEGDIFIGPK pc2
0.20 [0.08, 0.33]
LYSTWIGGSILASLDTFKK pc3
0.17 [0.06, 0.29]
TLFSNIVLSGGSTLFK pc2
0.22 [0.09, 0.36]
IWQYVYSK pc2
0.17 [−0.03, 0.36]
ACYLSINPQK pc2
0.18 [0.05, 0.32]
AGFAGDQIPK pc2
0.07 [−0.12, 0.26]
EGYDFHSSSEFEIVK pc3
0.13 [−0.02, 0.28]
TTGVVLDSGDGVTHAVPIYEGFAMPHSIMR pc4
0.04 [−0.15, 0.22]
AQYYLPDGSTIEIGPSR pc2
0.14 [−0.31, 0.59]
YPMEHGIVK pc2
0.09 [−0.17, 0.36]
EYEEDGAR pc2
0.05 [−0.22, 0.32]
LYSTWIGGSILASLDTFK pc2
0.10 [−0.13, 0.33]
DETLETEK pc2
0.02 [−1.01, 1.05]
TLFSNIVLSGGSTLFK pc3
−0.16 [−0.50, 0.19]
LYSTWIGGSILASLDTFK pc3
−0.16 [−0.68, 0.35]
DQLQTFSEEHPVLLTEAPLNPR pc3
0.13 [−0.55, 0.82]
RE Model
0.15 [0.10, 0.20]
−1.5
−1
−0.5
0
0.5
1
1.5
Effect of 13:56324143:C:T (rs6561866)

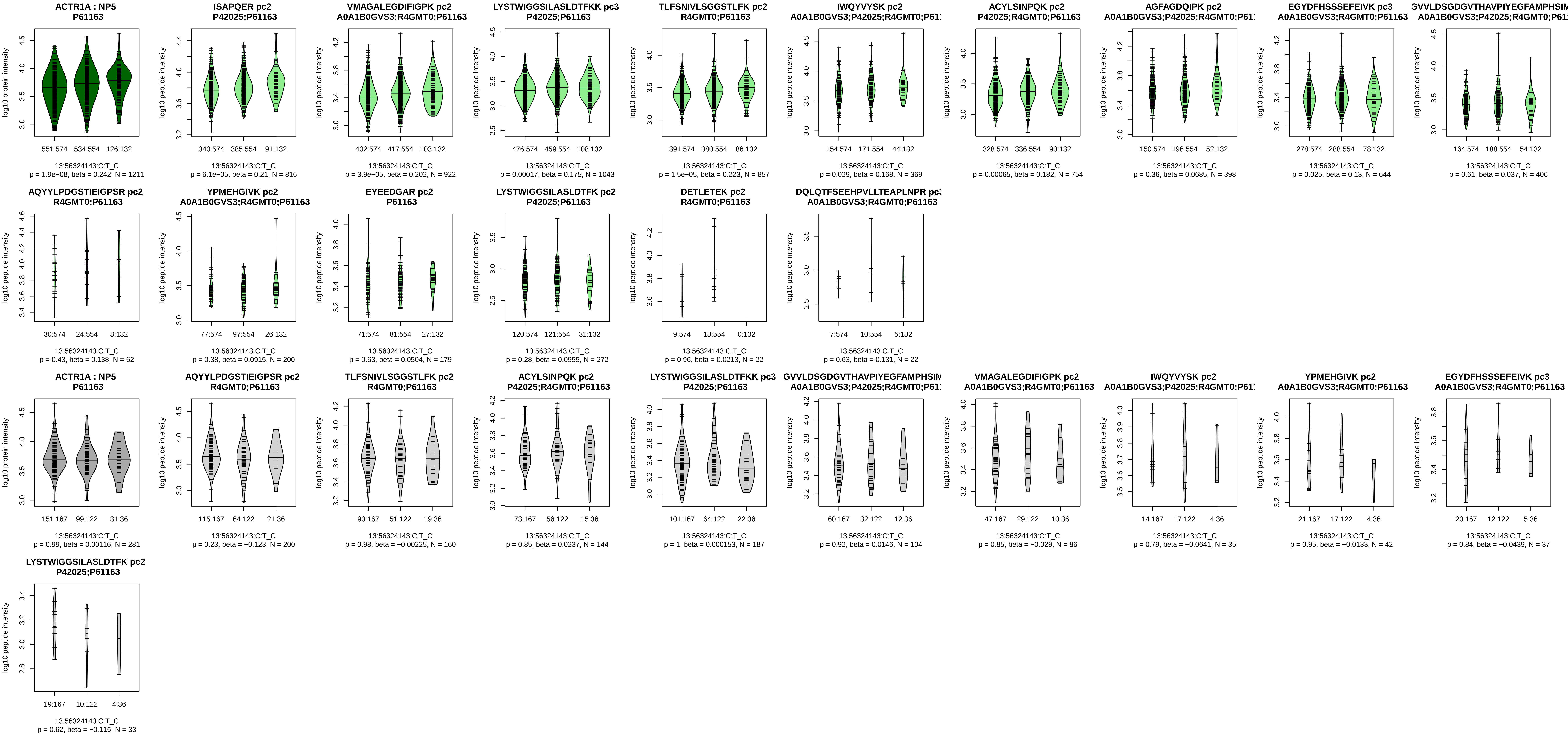

291Assay Target: ACTR1A
UniProt: P61163
rsID: rs6561866
Target SNP: 13:56324143:C:T
Proxy SNP: 13:56324143:C:T
Tarkin log10(p): 7.7
Tarkin BETA: 0.242
Tarkin MSPA: 0.614
816:922:1043:857:369:754:398:[illegible]

292. CHST11 (Q9NPF2)
Tarkin MSPA=0.938

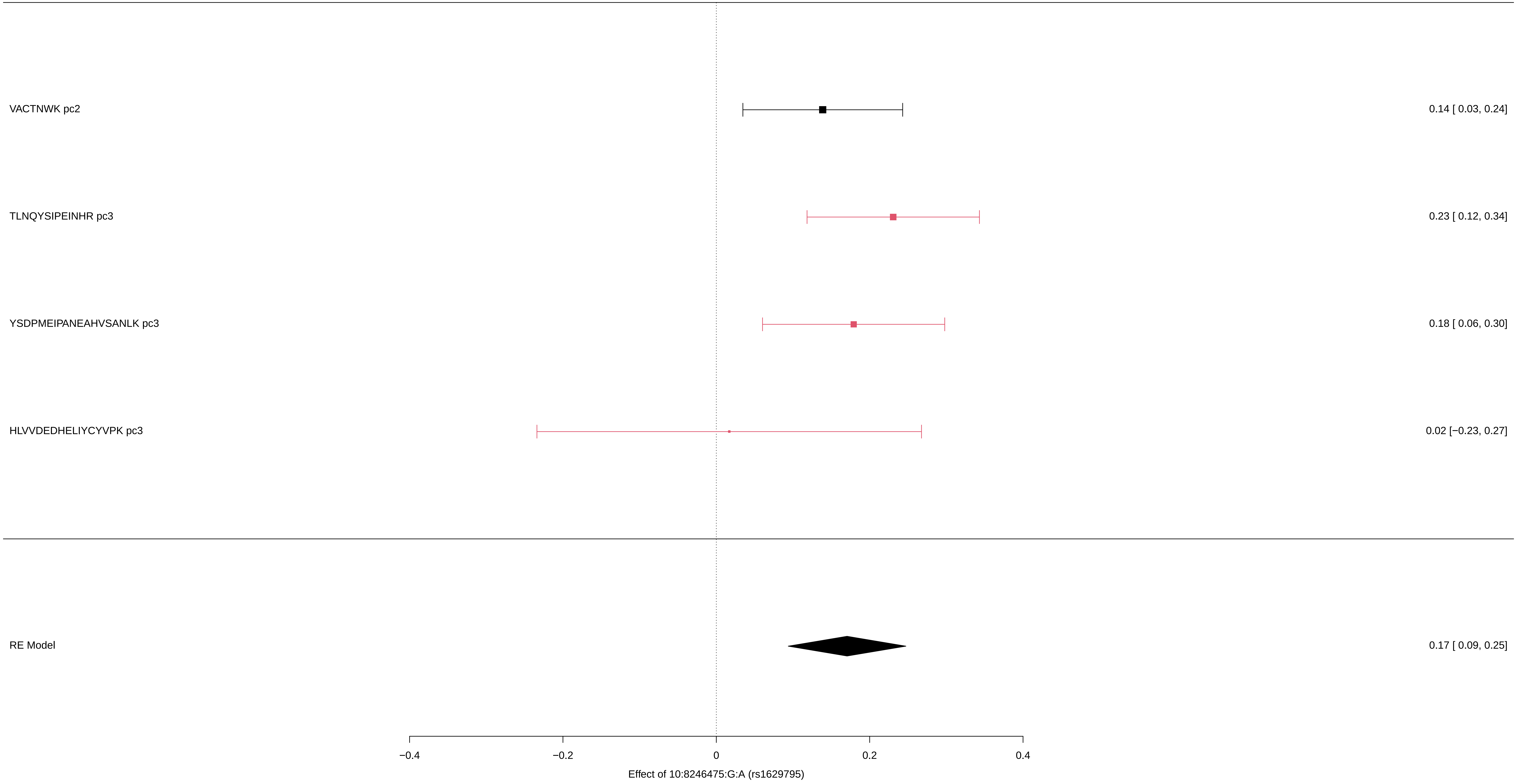

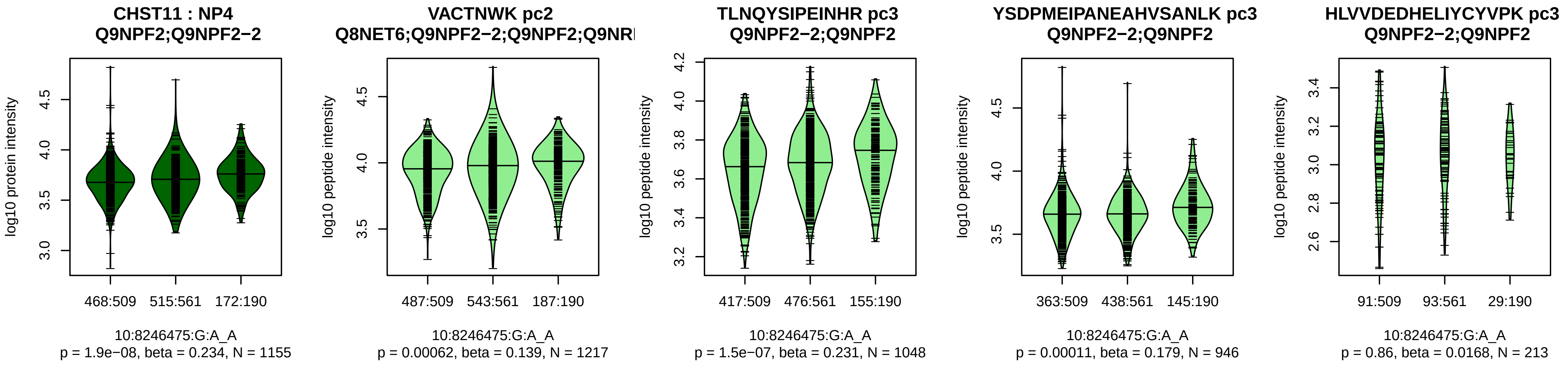

292Assay Target: CHST11
UniProt: Q9NPF2
rsID: rs1629795
Target SNP: 10:8246475:G:A
Proxy SNP: 10:8246475:G:A
Tarkin log10(p): 7.7
Tarkin BETA: 0.234
Tarkin MSPA: 0.938
::*:–
1217:1048:946:213

293. GNLY (P22749)
Tarkin MSPA=1

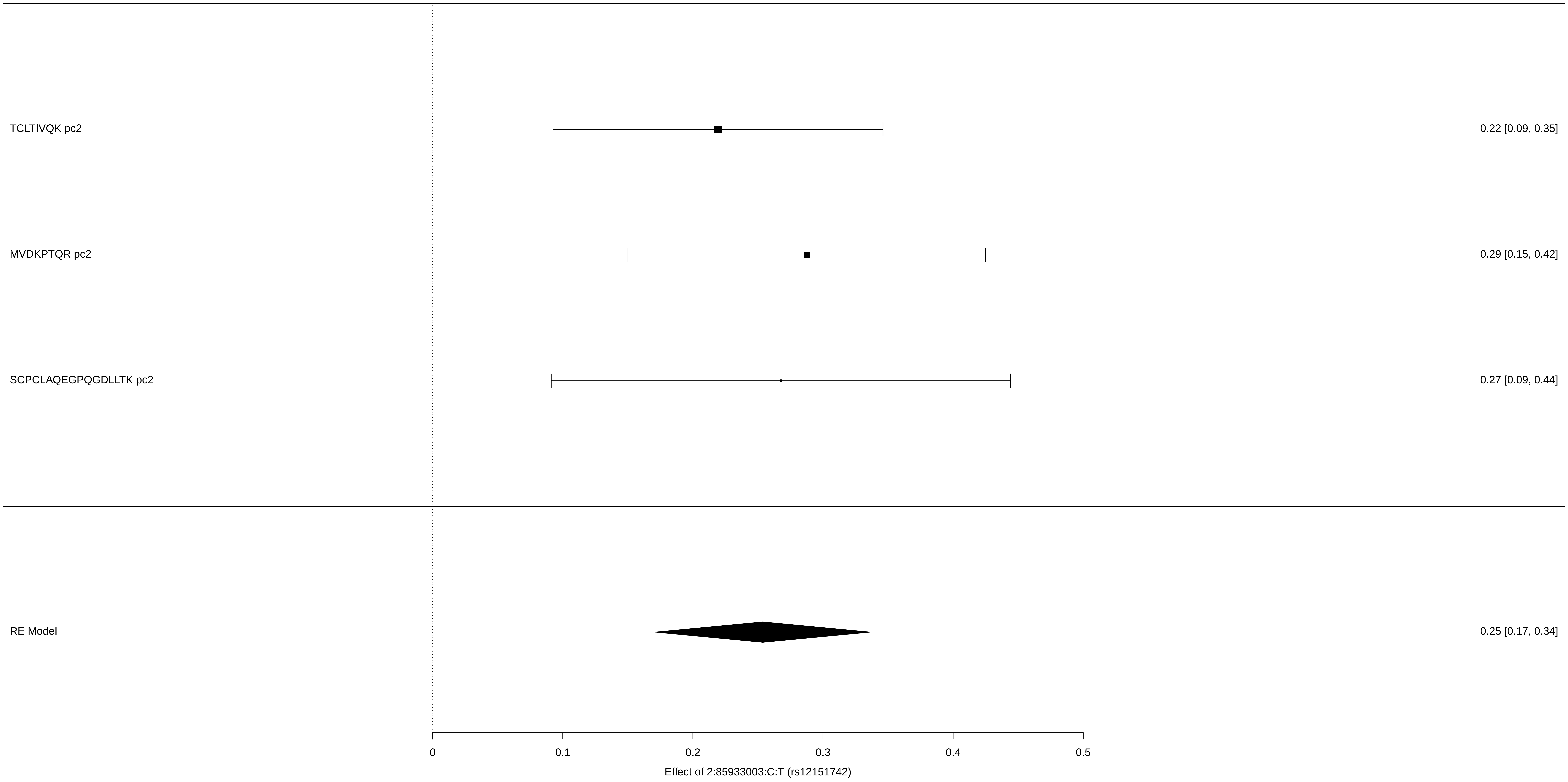

GNLY : NP4
B4E3H9;P22749;P22749−2

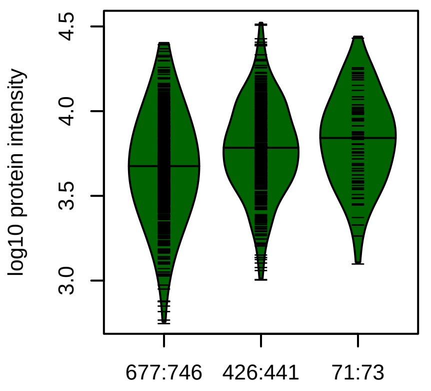

2:85933003:C:T_T
p = 1.9e−08, beta = 0.267, N = 1174

TCLTIVQK pc2
B4E3H9;P22749;P22749−2

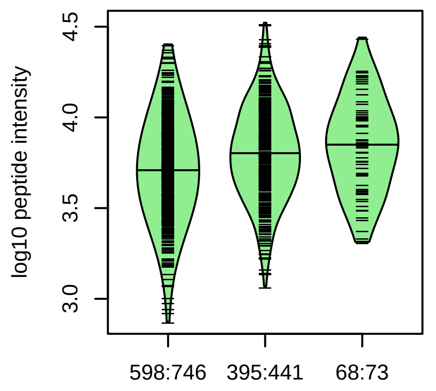

2:85933003:C:T_T
p = 9.3e−06, beta = 0.219, N = 1061

MVDKPTQR pc2
B4E3H9;P22749;P22749−2

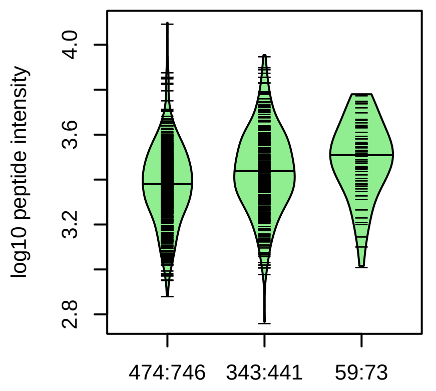

2:85933003:C:T_T
p = 9.1e−08, beta = 0.287, N = 876

SCPCLAQEGPQGDLLTK pc2
B4E3H9;P22749;P22749−2

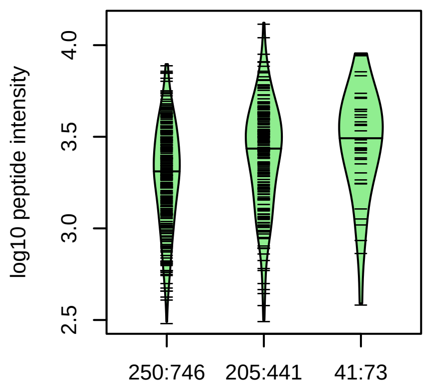

2:85933003:C:T_T
p = 0.00011, beta = 0.268, N = 496

GNLY : NP4
B4E3H9;P22749;P22749−2

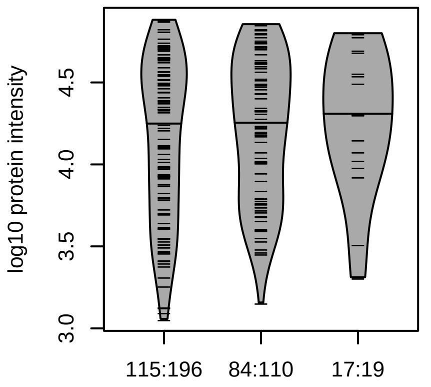

2:85933003:C:T_T
p = 0.23, beta = 0.126, N = 216

TCLTIVQK pc2
B4E3H9;P22749;P22749−2

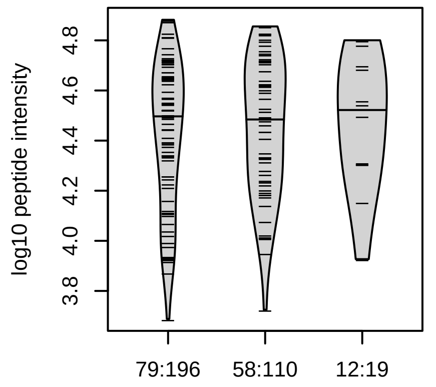

2:85933003:C:T_T
p = 0.42, beta = 0.102, N = 149

SCPCLAQEGPQGDLLTK pc2
B4E3H9;P22749;P22749−2

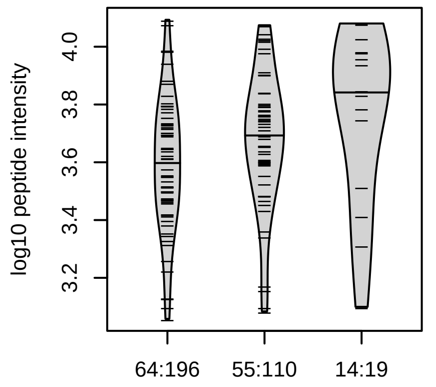

2:85933003:C:T_T
p = 0.041, beta = 0.258, N = 133

293Assay Target: GNLY
UniProt: P22749
rsID: rs12151742
Target SNP: 2:85933003:C:T
Proxy SNP: 2:85933003:C:T
Tarkin log10(p): 7.7
Tarkin BETA: 0.267
Tarkin MSPA: 1
::*
1061:876:496

VIQGLVAGETAQQICEDLR pc3
rs11127 ALT

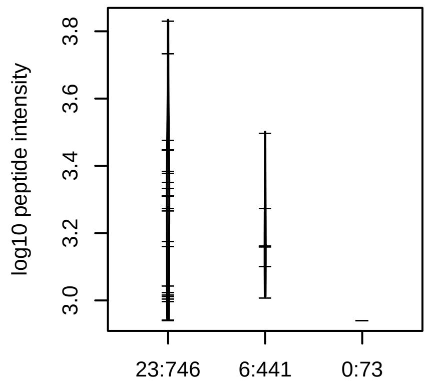

2:85933003:C:T_T
p = 0.034, model = REC, N = 29

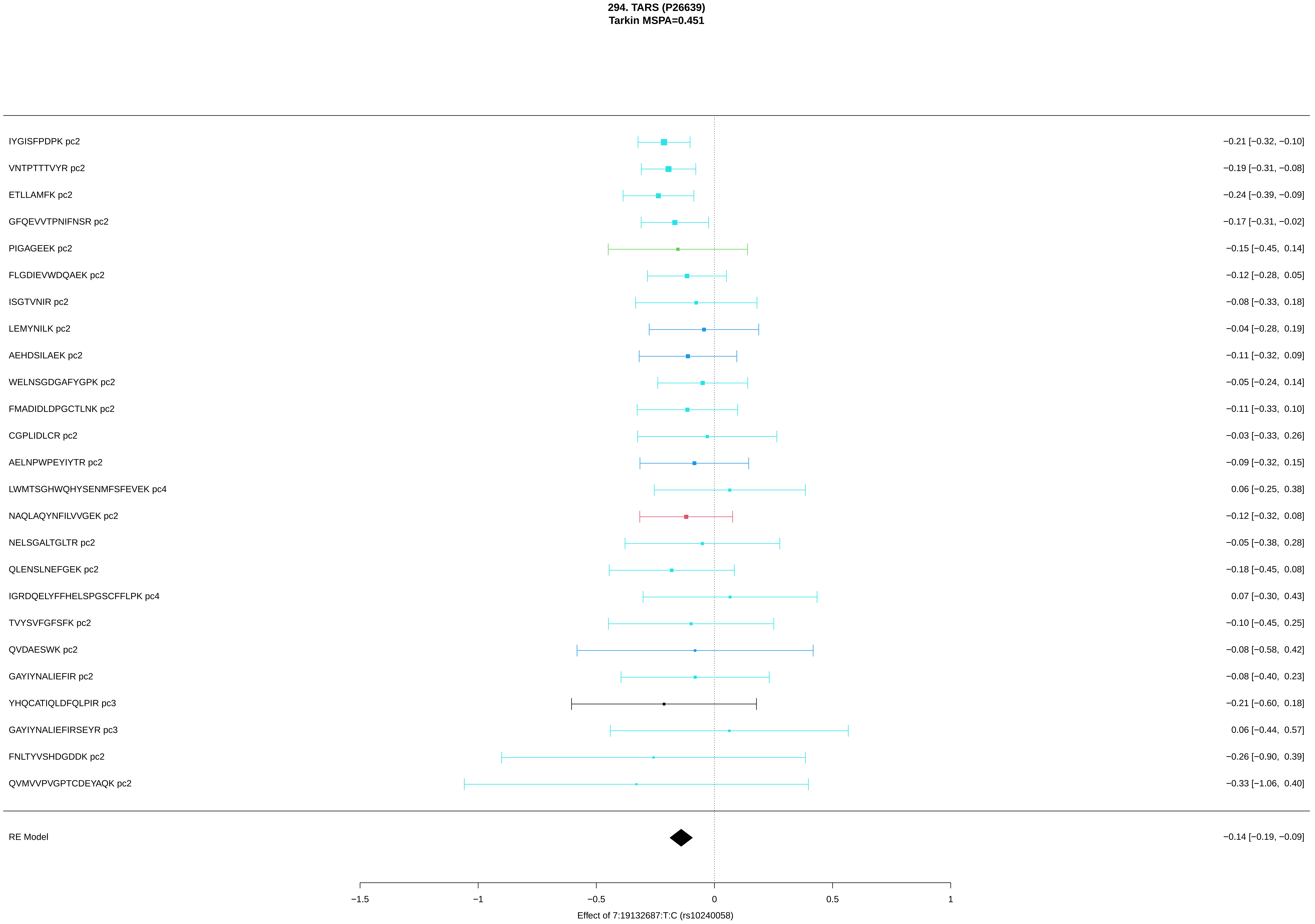

294. TARS (P26639)
Tarkin MSPA=0.451
IYGISFPDPK pc2
VNTPTTTVYR pc2
ETLLAMFK pc2
GFQEVVTPNIFNSR pc2
PIGAGEEK pc2
FLGDIEVWDQAEK pc2
ISGTVNIR pc2
LEMYNILK pc2
AEHDSILAEK pc2
WELNSGDGAFYGPK pc2
FMADIDLDPGCTLNK pc2
CGPLIDLCR pc2
AELNPWPEYIYTR pc2
LWMTSGHWQHYSENMFSFEVEK pc4
NAQLAQYNFILVVGEK pc2
NELSGALTGLTR pc2
QLENSLNEFGEK pc2
IGRDQELYFFHELSPGSCFFLPK pc4
TVYSVFGFSFK pc2
QVDAESWK pc2
GAYIYNALIEFIR pc2
YHQCATIQLDFQLPIR pc3
GAYIYNALIEFIRSEYR pc3
FNLTYVSHDGDDK pc2
QVMVVPVGPTCDEYAQK pc2
RE Model
−0.21 [−0.32, −0.10]
−0.19 [−0.31, −0.08]
−0.24 [−0.39, −0.09]
−0.17 [−0.31, −0.02]
−0.15 [−0.45, 0.14]
−0.12 [−0.28, 0.05]
−0.08 [−0.33, 0.18]
−0.04 [−0.28, 0.19]
−0.11 [−0.32, 0.09]
−0.05 [−0.24, 0.14]
−0.11 [−0.33, 0.10]
−0.03 [−0.33, 0.26]
−0.09 [−0.32, 0.15]
0.06 [−0.25, 0.38]
−0.12 [−0.32, 0.08]
−0.05 [−0.38, 0.28]
−0.18 [−0.45, 0.08]
0.07 [−0.30, 0.43]
−0.10 [−0.45, 0.25]
−0.08 [−0.58, 0.42]
−0.08 [−0.40, 0.23]
−0.21 [−0.60, 0.18]
0.06 [−0.44, 0.57]
−0.26 [−0.90, 0.39]
−0.33 [−1.06, 0.40]
−0.14 [−0.19, −0.09]
−1.5
−1
−0.5
0
0.5
1
Effect of 7:19132687:T:C (rs10240058)

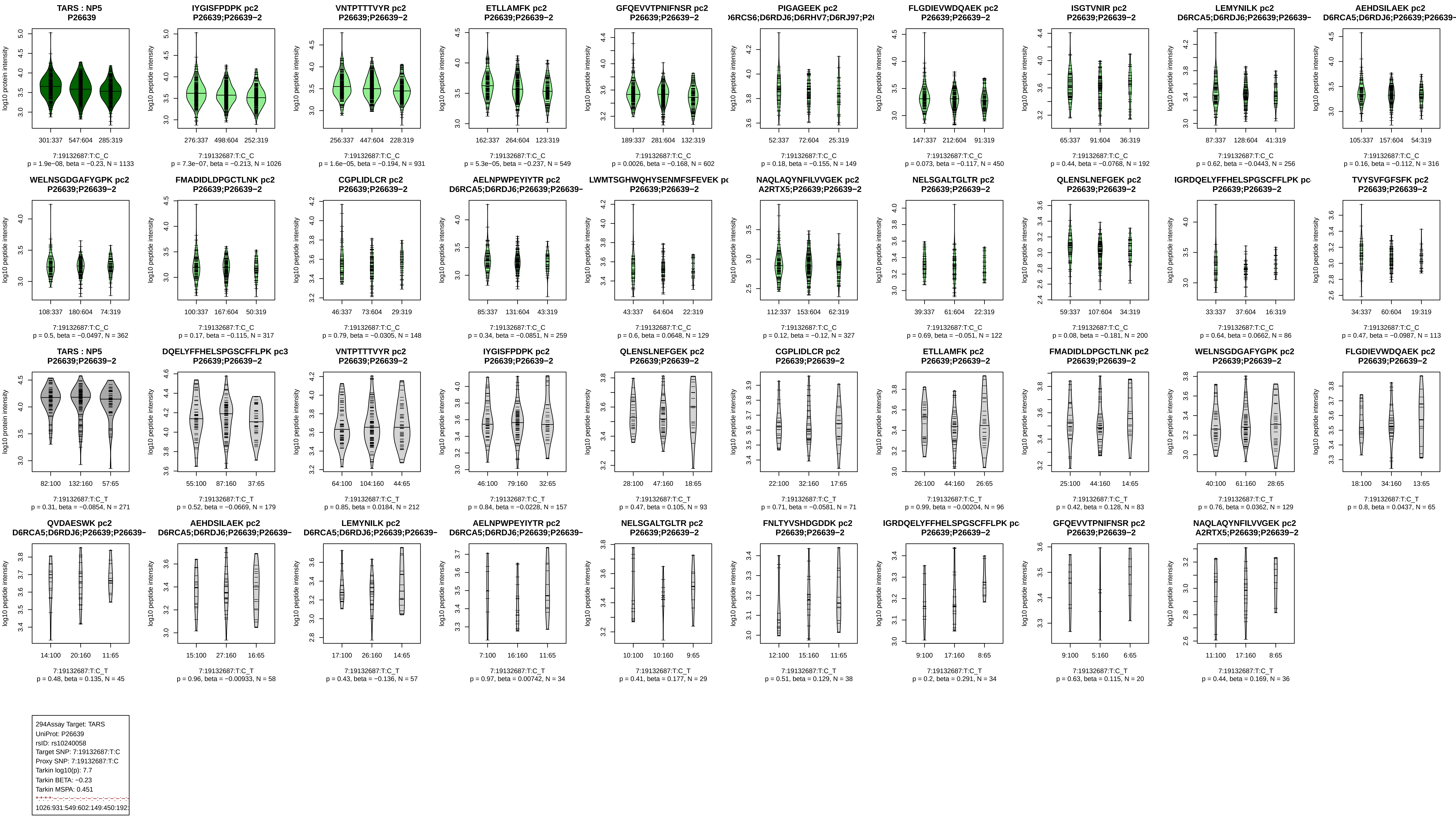
TARS : NP5
P26639
301:337 547:604 285:319
7:19132687:T:C_C
p = 1.9e−08, beta = −0.23, N = 1133
IYGISFPDPK pc2
P26639;P26639−2
7:19132687:T:C_C
p = 7.3e−07, beta = −0.213, N = 1026
VNTPTTTVYR pc2
P26639;P26639−2
7:19132687:T:C_C
p = 1.6e−05, beta = −0.194, N = 931
ETLLAMFK pc2
P26639;P26639−2
7:19132687:T:C_C
p = 5.3e−05, beta = −0.237, N = 549
GFQEVVTPNIFNSR pc2
P26639;P26639−2
7:19132687:T:C_C
p = 0.0026, beta = −0.168, N = 602
PIGAGEEK pc2
7:19132687:T:C_C
p = 0.18, beta = −0.155, N = 149
FLGDIEVWDQAEK pc2
P26639;P26639−2
7:19132687:T:C_C
p = 0.073, beta = −0.117, N = 450
ISGTVNIR pc2
P26639;P26639−2
7:19132687:T:C_C
p = 0.44, beta = −0.0768, N = 192
LEMYNILK pc2
7:19132687:T:C_C
p = 0.62, beta = −0.0443, N = 256
AEHDSILAEK pc2
7:19132687:T:C_C
p = 0.16, beta = −0.112, N = 316
WELNSGDGAFYGPK pc2
P26639;P26639−2
7:19132687:T:C_C
p = 0.5, beta = −0.0497, N = 362
FMADIDLDPGCTLNK pc2
P26639;P26639−2
7:19132687:T:C_C
p = 0.17, beta = −0.115, N = 317
CGPLIDLCR pc2
P26639;P26639−2
7:19132687:T:C_C
p = 0.79, beta = −0.0305, N = 148
AELNPWPEYIYTR pc2
7:19132687:T:C_C
p = 0.34, beta = −0.0851, N = 259
P26639;P26639−2
7:19132687:T:C_C
p = 0.6, beta = 0.0648, N = 129
NAQLAQYNFILVVGEK pc2
A2RTX5;P26639;P26639−2
7:19132687:T:C_C
p = 0.12, beta = −0.12, N = 327
NELSGALTGLTR pc2
P26639;P26639−2
7:19132687:T:C_C
p = 0.69, beta = −0.051, N = 122
QLENSLNEFGEK pc2
P26639;P26639−2
7:19132687:T:C_C
p = 0.08, beta = −0.181, N = 200
P26639;P26639−2
7:19132687:T:C_C
p = 0.64, beta = 0.0662, N = 86
TVYSVFGFSFK pc2
P26639;P26639−2
7:19132687:T:C_C
p = 0.47, beta = −0.0987, N = 113
TARS : NP5
P26639;P26639−2
7:19132687:T:C_T
p = 0.31, beta = −0.0854, N = 271
DQELYFFHELSPGSCFFLPK pc3
P26639;P26639−2
7:19132687:T:C_T
p = 0.52, beta = −0.0669, N = 179
VNTPTTTVYR pc2
P26639;P26639−2
7:19132687:T:C_T
p = 0.85, beta = 0.0184, N = 212
IYGISFPDPK pc2
P26639;P26639−2
7:19132687:T:C_T
p = 0.84, beta = −0.0228, N = 157
QLENSLNEFGEK pc2
P26639;P26639−2
7:19132687:T:C_T
p = 0.47, beta = 0.105, N = 93
CGPLIDLCR pc2
P26639;P26639−2
7:19132687:T:C_T
p = 0.71, beta = −0.0581, N = 71
ETLLAMFK pc2
P26639;P26639−2
7:19132687:T:C_T
p = 0.99, beta = −0.00204, N = 96
FMADIDLDPGCTLNK pc2
P26639;P26639−2
7:19132687:T:C_T
p = 0.42, beta = 0.128, N = 83
WELNSGDGAFYGPK pc2
P26639;P26639−2
7:19132687:T:C_T
p = 0.76, beta = 0.0362, N = 129
FLGDIEVWDQAEK pc2
P26639;P26639−2
7:19132687:T:C_T
p = 0.8, beta = 0.0437, N = 65
QVDAESWK pc2
7:19132687:T:C_T
p = 0.48, beta = 0.135, N = 45
AEHDSILAEK pc2
7:19132687:T:C_T
p = 0.96, beta = −0.00933, N = 58
LEMYNILK pc2
7:19132687:T:C_T
p = 0.43, beta = −0.136, N = 57
AELNPWPEYIYTR pc2
7:19132687:T:C_T
p = 0.97, beta = 0.00742, N = 34
NELSGALTGLTR pc2
P26639;P26639−2
7:19132687:T:C_T
p = 0.41, beta = 0.177, N = 29
FNLTYVSHDGDDK pc2
P26639;P26639−2
7:19132687:T:C_T
p = 0.51, beta = 0.129, N = 38
P26639;P26639−2
7:19132687:T:C_T
p = 0.2, beta = 0.291, N = 34
GFQEVVTPNIFNSR pc2
P26639;P26639−2
7:19132687:T:C_T
p = 0.63, beta = 0.115, N = 20
NAQLAQYNFILVVGEK pc2
A2RTX5;P26639;P26639−2
7:19132687:T:C_T
p = 0.44, beta = 0.169, N = 36
294Assay Target: TARS
UniProt: P26639
rsID: rs10240058
Target SNP: 7:19132687:T:C
Proxy SNP: 7:19132687:T:C
Tarkin log10(p): 7.7
Tarkin BETA: −0.23
Tarkin MSPA: 0.451

295. CYBB (P04839)
Tarkin MSPA=0.628

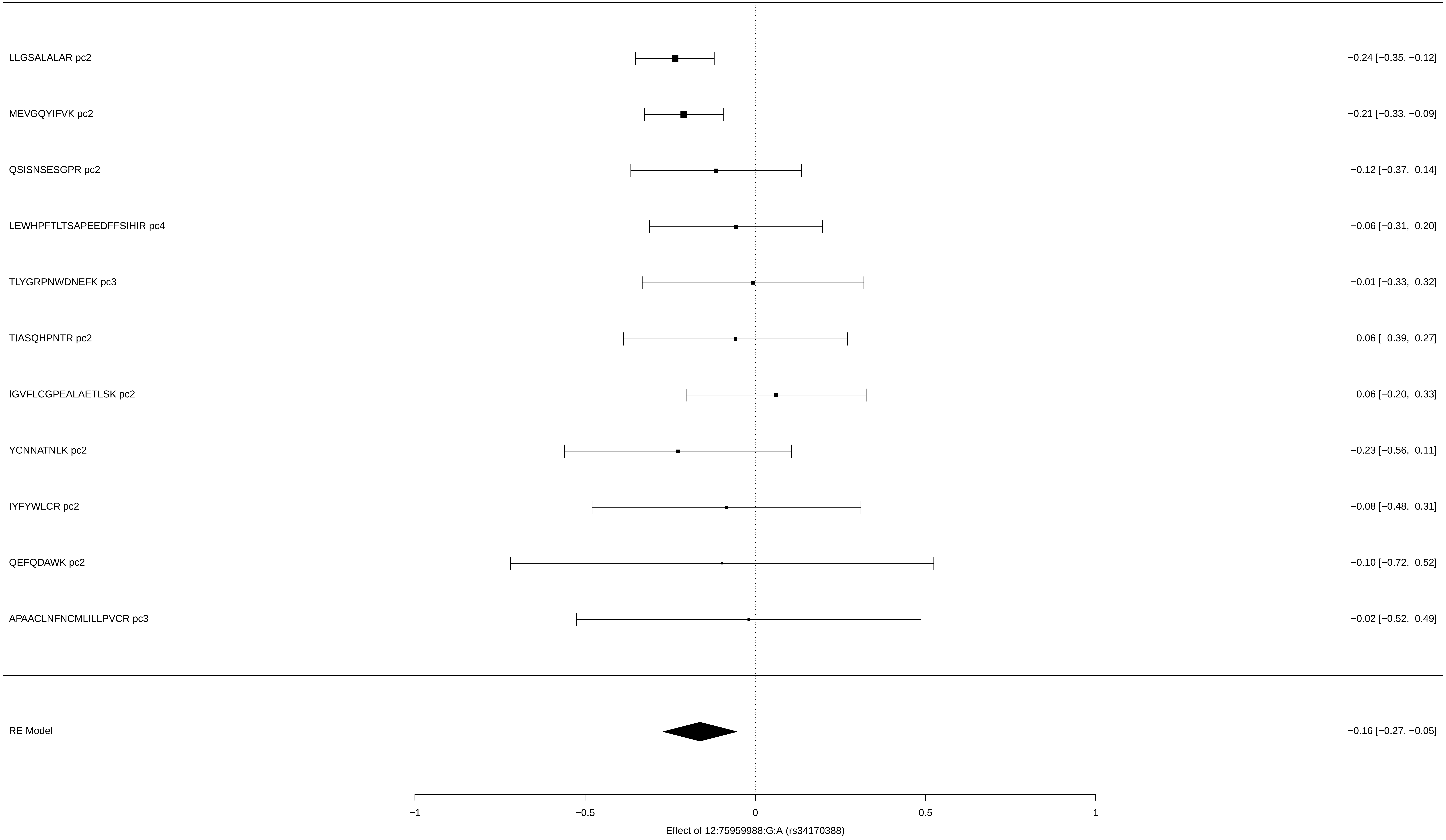

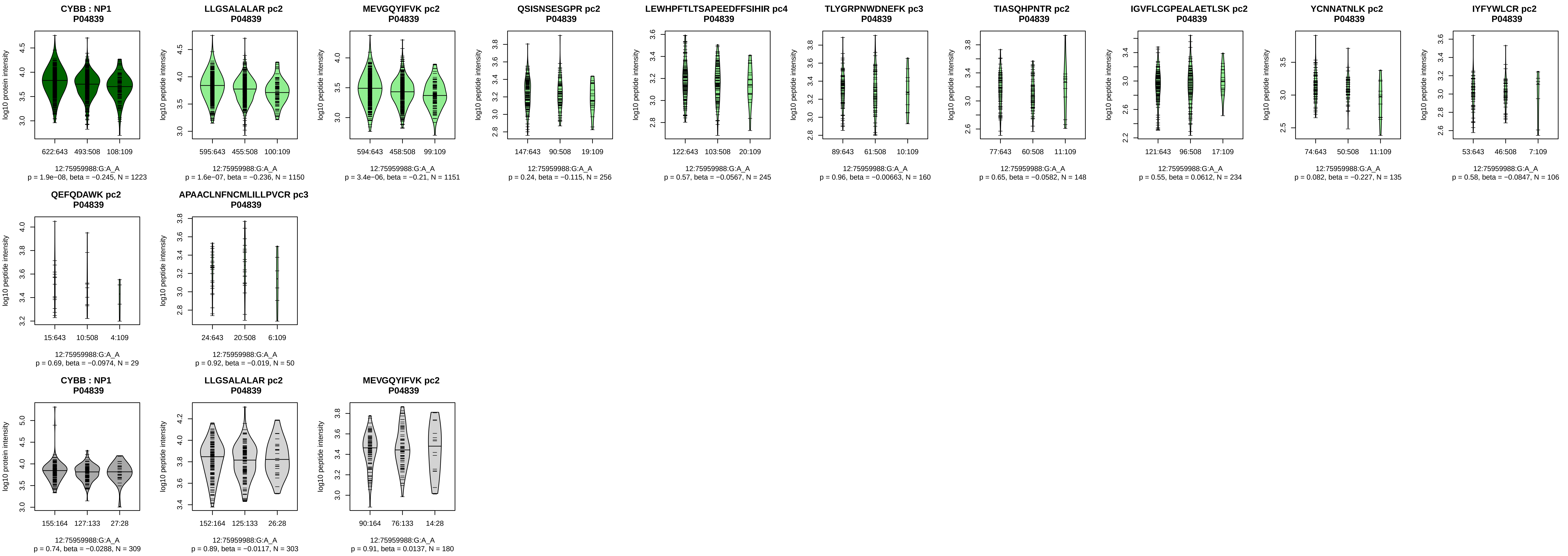

295Assay Target: CYBB
UniProt: P04839
rsID: rs34170388
Target SNP: 12:75959988:G:A
Proxy SNP: 12:75959988:G:A
Tarkin log10(p): 7.7
Tarkin BETA: −0.245
Tarkin MSPA: 0.628
::–:–:–:–:–:–:–:–:NA
1150:1151:256:245:160:148:234

296. F2 (P00734)
Tarkin MSPA=0.376

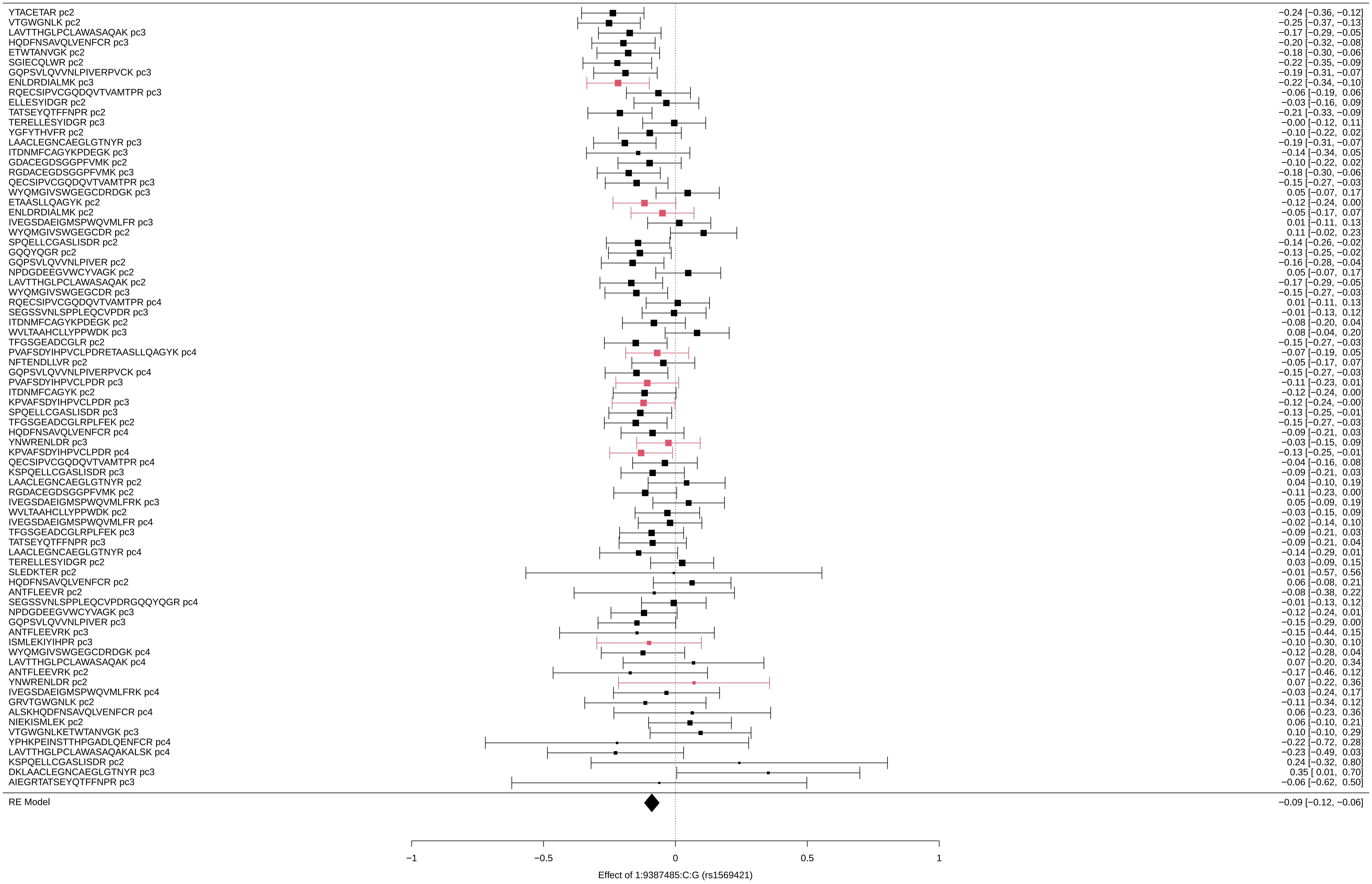

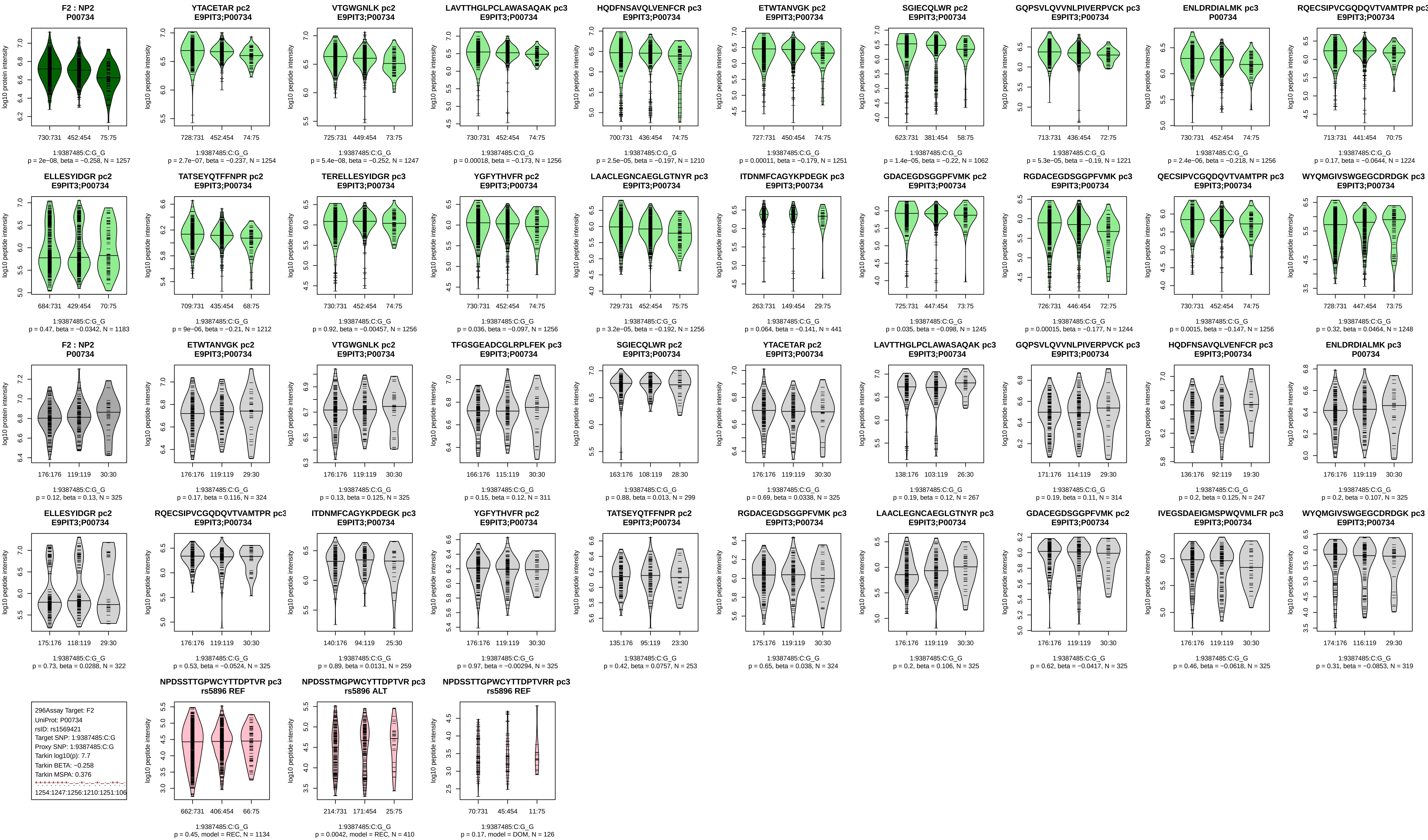
F2 : NP2
P00734
730:731 452:454 75:75
1:9387485:C:G_G
p = 2e−08, beta = −0.258, N = 1257
YTACETAR pc2
E9PIT3;P00734
728:731 452:454 74:75
1:9387485:C:G_G
p = 2.7e−07, beta = −0.237, N = 1254
VTGWGNLK pc2
E9PIT3;P00734
725:731 449:454 73:75
1:9387485:C:G_G
p = 5.4e−08, beta = −0.252, N = 1247
LAVTTHGLPCLAWASAQAK pc3
E9PIT3;P00734
730:731 452:454 74:75
1:9387485:C:G_G
p = 0.00018, beta = −0.173, N = 1256
HQDFNSAVQLVENFCR pc3
E9PIT3;P00734
700:731 436:454 74:75
1:9387485:C:G_G
p = 2.5e−05, beta = −0.197, N = 1210
ETWTANVGK pc2
E9PIT3;P00734
727:731 450:454 74:75
1:9387485:C:G_G
p = 0.00011, beta = −0.179, N = 1251
SGIECQLWR pc2
E9PIT3;P00734
623:731 381:454 58:75
1:9387485:C:G_G
p = 1.4e−05, beta = −0.22, N = 1062
GQPSVLQVVNLPIVERPVCK pc3
E9PIT3;P00734
713:731 436:454 72:75
1:9387485:C:G_G
p = 5.3e−05, beta = −0.19, N = 1221
ENLDRDIALMK pc3
P00734
730:731 452:454 74:75
1:9387485:C:G_G
p = 2.4e−06, beta = −0.218, N = 1256
RQECSIPVCGQDQVTVAMTPR pc3
E9PIT3;P00734
713:731 441:454 70:75
1:9387485:C:G_G
p = 0.17, beta = −0.0644, N = 1224
ELLESYIDGR pc2
E9PIT3;P00734
684:731 429:454 70:75
1:9387485:C:G_G
p = 0.47, beta = −0.0342, N = 1183
TATSEYQTFFNPR pc2
E9PIT3;P00734
709:731 435:454 68:75
1:9387485:C:G_G
p = 9e−06, beta = −0.21, N = 1212
TERELLESYIDGR pc3
E9PIT3;P00734
730:731 452:454 74:75
1:9387485:C:G_G
p = 0.92, beta = −0.00457, N = 1256
YGFYTHVFR pc2
E9PIT3;P00734
730:731 452:454 74:75
1:9387485:C:G_G
p = 0.036, beta = −0.097, N = 1256
LAACLEGNCAEGLGTNYR pc3
E9PIT3;P00734
729:731 452:454 75:75
1:9387485:C:G_G
p = 3.2e−05, beta = −0.192, N = 1256
ITDNMFCAGYKPDEGK pc3
E9PIT3;P00734
263:731 149:454 29:75
1:9387485:C:G_G
p = 0.064, beta = −0.141, N = 441
GDACEGDSGGPFVMK pc2
E9PIT3;P00734
725:731 447:454 73:75
1:9387485:C:G_G
p = 0.035, beta = −0.098, N = 1245
RGDACEGDSGGPFVMK pc3
E9PIT3;P00734
726:731 446:454 72:75
1:9387485:C:G_G
p = 0.00015, beta = −0.177, N = 1244
QECSIPVCGQDQVTVAMTPR pc3
E9PIT3;P00734
730:731 452:454 74:75
1:9387485:C:G_G
p = 0.0015, beta = −0.147, N = 1256
WYQMGIVSWGEGCDRDGK pc3
E9PIT3;P00734
728:731 447:454 73:75
1:9387485:C:G_G
p = 0.32, beta = 0.0464, N = 1248
F2 : NP2
P00734
176:176 119:119 30:30
1:9387485:C:G_G
p = 0.12, beta = 0.13, N = 325
ETWTANVGK pc2
E9PIT3;P00734
176:176 119:119 29:30
1:9387485:C:G_G
p = 0.17, beta = 0.116, N = 324
VTGWGNLK pc2
E9PIT3;P00734
176:176 119:119 30:30
1:9387485:C:G_G
p = 0.13, beta = 0.125, N = 325
TFGSGEADCGLRPLFEK pc3
E9PIT3;P00734
166:176 115:119 30:30
1:9387485:C:G_G
p = 0.15, beta = 0.12, N = 311
SGIECQLWR pc2
E9PIT3;P00734
163:176 108:119 28:30
1:9387485:C:G_G
p = 0.88, beta = 0.013, N = 299
YTACETAR pc2
E9PIT3;P00734
176:176 119:119 30:30
1:9387485:C:G_G
p = 0.69, beta = 0.0338, N = 325
LAVTTHGLPCLAWASAQAK pc3
E9PIT3;P00734
138:176 103:119 26:30
1:9387485:C:G_G
p = 0.19, beta = 0.12, N = 267
GQPSVLQVVNLPIVERPVCK pc3
E9PIT3;P00734
171:176 114:119 29:30
1:9387485:C:G_G
p = 0.19, beta = 0.11, N = 314
HQDFNSAVQLVENFCR pc3
E9PIT3;P00734
136:176 92:119 19:30
1:9387485:C:G_G
p = 0.2, beta = 0.125, N = 247
ENLDRDIALMK pc3
P00734
176:176 119:119 30:30
1:9387485:C:G_G
p = 0.2, beta = 0.107, N = 325
ELLESYIDGR pc2
E9PIT3;P00734
175:176 118:119 29:30
1:9387485:C:G_G
p = 0.73, beta = 0.0288, N = 322
RQECSIPVCGQDQVTVAMTPR pc3
E9PIT3;P00734
176:176 119:119 30:30
1:9387485:C:G_G
p = 0.53, beta = −0.0524, N = 325
ITDNMFCAGYKPDEGK pc3
E9PIT3;P00734
140:176 94:119 25:30
1:9387485:C:G_G
p = 0.89, beta = 0.0131, N = 259
YGFYTHVFR pc2
E9PIT3;P00734
176:176 119:119 30:30
1:9387485:C:G_G
p = 0.97, beta = −0.00294, N = 325
TATSEYQTFFNPR pc2
E9PIT3;P00734
135:176 95:119 23:30
1:9387485:C:G_G
p = 0.42, beta = 0.0757, N = 253
RGDACEGDSGGPFVMK pc3
E9PIT3;P00734
175:176 119:119 30:30
1:9387485:C:G_G
p = 0.65, beta = 0.038, N = 324
LAACLEGNCAEGLGTNYR pc3
E9PIT3;P00734
176:176 119:119 30:30
1:9387485:C:G_G
p = 0.2, beta = 0.106, N = 325
GDACEGDSGGPFVMK pc2
E9PIT3;P00734
176:176 119:119 30:30
1:9387485:C:G_G
p = 0.62, beta = −0.0417, N = 325
IVEGSDAEIGMSPWQVMLFR pc3
E9PIT3;P00734
176:176 119:119 30:30
1:9387485:C:G_G
p = 0.46, beta = −0.0618, N = 325
WYQMGIVSWGEGCDRDGK pc3
E9PIT3;P00734
174:176 116:119 29:30
1:9387485:C:G_G
p = 0.31, beta = −0.0853, N = 319
296Assay Target: F2
UniProt: P00734
rsID: rs1569421
Target SNP: 1:9387485:C:G
Proxy SNP: 1:9387485:C:G
Tarkin log10(p): 7.7
Tarkin BETA: −0.258
Tarkin MSPA: 0.376
1254:1247:1256:1210:1251:106
NPDSSTTGPWCYTTDPTVR pc3
rs5896 REF
662:731 406:454 66:75
1:9387485:C:G_G
p = 0.45, model = REC, N = 1134
NPDSSTMGPWCYTTDPTVR pc3
rs5896 ALT
214:731 171:454 25:75
1:9387485:C:G_G
p = 0.0042, model = REC, N = 410
NPDSSTTGPWCYTTDPTVRR pc3
rs5896 REF
70:731 45:454 11:75
1:9387485:C:G_G
p = 0.17, model = DOM, N = 126
log10 protein intensity
log10 peptide intensity

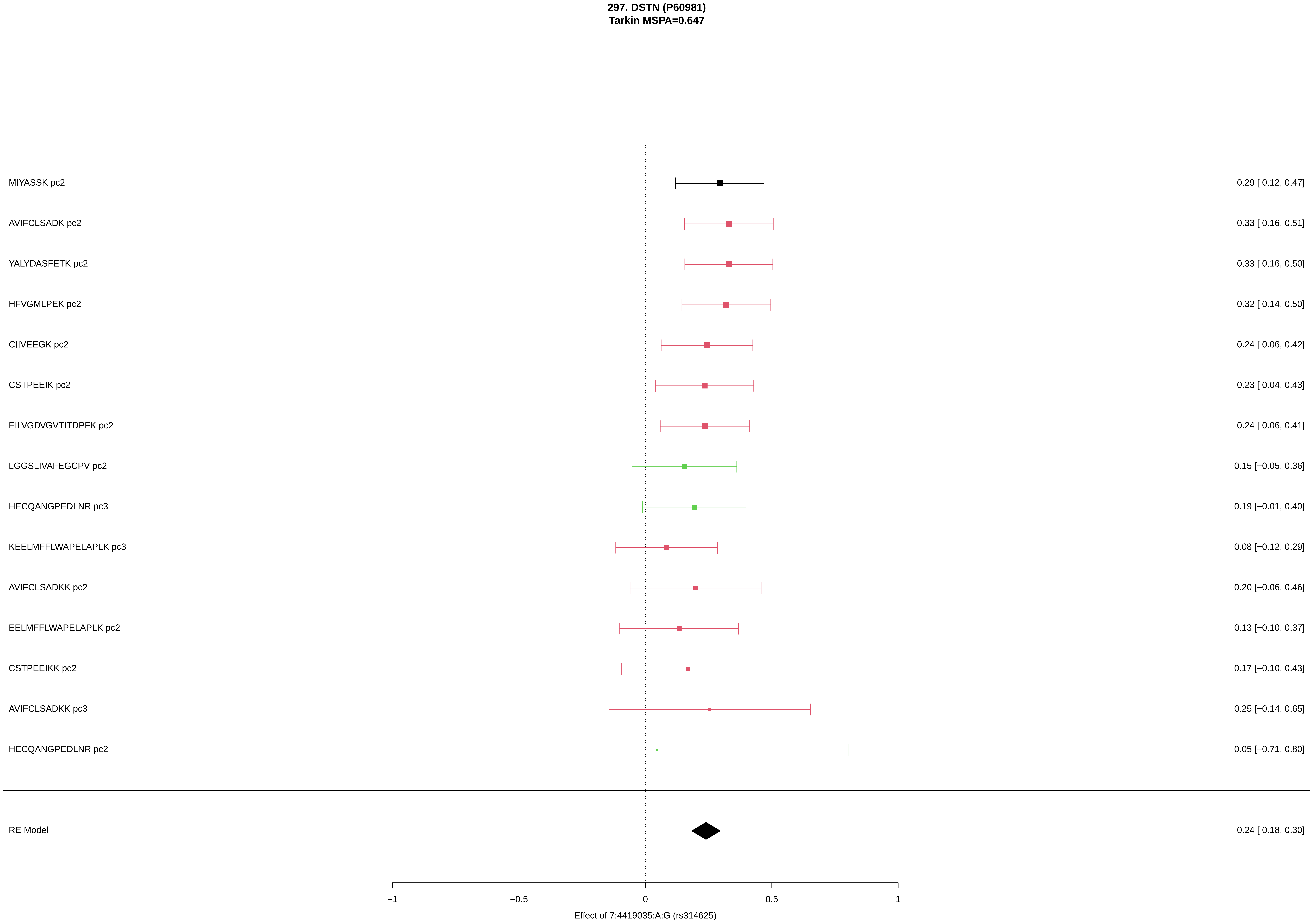
297. DSTN (P60981)
Tarkin MSPA=0.647
MIYASSK pc2
0.29 [0.12, 0.47]
AVIFCLSADK pc2
0.33 [0.16, 0.51]
YALYDASFETK pc2
0.33 [0.16, 0.50]
HFVGMLPEK pc2
0.32 [0.14, 0.50]
CIIVEEGK pc2
0.24 [0.06, 0.42]
CSTPEEIK pc2
0.23 [0.04, 0.43]
EILVGDVGVTITDPFK pc2
0.24 [0.06, 0.41]
LGGSLIVAFEGCPV pc2
0.15 [−0.05, 0.36]
HECQANGPEDLNR pc3
0.19 [−0.01, 0.40]
KEELMFFLWAPELAPLK pc3
0.08 [−0.12, 0.29]
AVIFCLSADKK pc2
0.20 [−0.06, 0.46]
EELMFFLWAPELAPLK pc2
0.13 [−0.10, 0.37]
CSTPEEIKK pc2
0.17 [−0.10, 0.43]
AVIFCLSADKK pc3
0.25 [−0.14, 0.65]
HECQANGPEDLNR pc2
0.05 [−0.71, 0.80]
RE Model
0.24 [0.18, 0.30]
−1
−0.5
0
0.5
1
Effect of 7:4419035:A:G (rs314625)

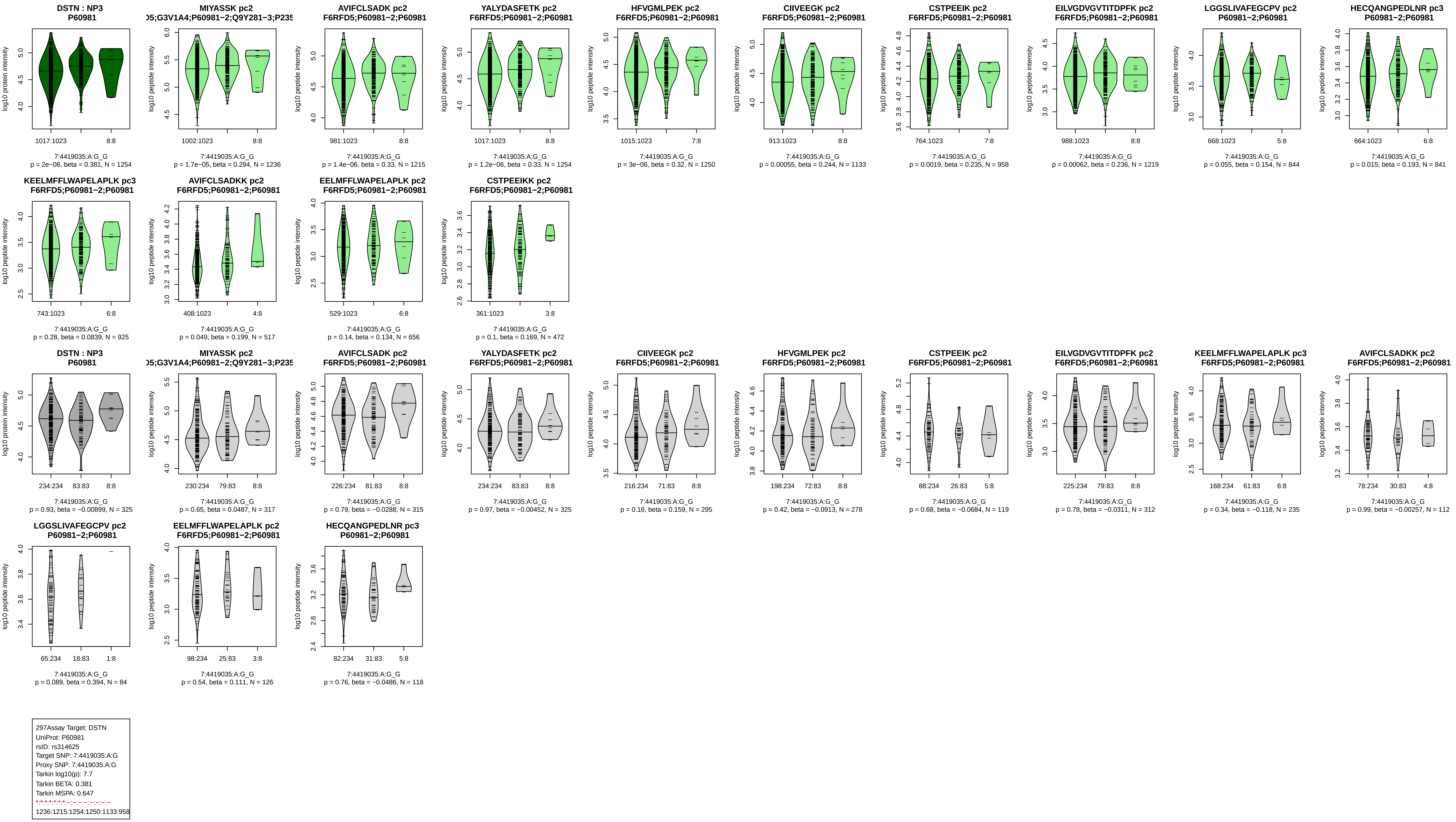
DSTN : NP3
P60981
p = 2e−08, beta = 0.381, N = 1254
MIYASSK pc2
p = 1.7e−05, beta = 0.294, N = 1236
AVIFCLSADK pc2
F6RFD5;P60981−2;P60981
p = 1.4e−06, beta = 0.33, N = 1215
YALYDASFETK pc2
F6RFD5;P60981−2;P60981
p = 1.2e−06, beta = 0.33, N = 1254
HFVGMLPEK pc2
F6RFD5;P60981−2;P60981
p = 3e−06, beta = 0.32, N = 1250
CIIVEEGK pc2
F6RFD5;P60981−2;P60981
p = 0.00055, beta = 0.244, N = 1133
CSTPEEIK pc2
F6RFD5;P60981−2;P60981
p = 0.0019, beta = 0.235, N = 958
EILVGDVGVTITDPFK pc2
F6RFD5;P60981−2;P60981
p = 0.00062, beta = 0.236, N = 1219
LGGSLIVAFEGCPV pc2
P60981−2;P60981
p = 0.055, beta = 0.154, N = 844
HECQANGPEDLNR pc3
P60981−2;P60981
p = 0.015, beta = 0.193, N = 841
KEELMFFLWAPELAPLK pc3
F6RFD5;P60981−2;P60981
p = 0.28, beta = 0.0839, N = 925
AVIFCLSADKK pc2
F6RFD5;P60981−2;P60981
p = 0.049, beta = 0.199, N = 517
EELMFFLWAPELAPLK pc2
F6RFD5;P60981−2;P60981
p = 0.14, beta = 0.134, N = 656
CSTPEEIKK pc2
F6RFD5;P60981−2;P60981
p = 0.1, beta = 0.169, N = 472
DSTN : NP3
P60981
p = 0.93, beta = −0.00899, N = 325
MIYASSK pc2
p = 0.65, beta = 0.0487, N = 317
AVIFCLSADK pc2
F6RFD5;P60981−2;P60981
p = 0.79, beta = −0.0288, N = 315
YALYDASFETK pc2
F6RFD5;P60981−2;P60981
p = 0.97, beta = −0.00452, N = 325
CIIVEEGK pc2
F6RFD5;P60981−2;P60981
p = 0.16, beta = 0.159, N = 295
HFVGMLPEK pc2
F6RFD5;P60981−2;P60981
p = 0.42, beta = −0.0913, N = 278
CSTPEEIK pc2
F6RFD5;P60981−2;P60981
p = 0.68, beta = −0.0684, N = 119
EILVGDVGVTITDPFK pc2
F6RFD5;P60981−2;P60981
p = 0.78, beta = −0.0311, N = 312
KEELMFFLWAPELAPLK pc3
F6RFD5;P60981−2;P60981
p = 0.34, beta = −0.118, N = 235
AVIFCLSADKK pc2
F6RFD5;P60981−2;P60981
p = 0.99, beta = −0.00257, N = 112
LGGSLIVAFEGCPV pc2
P60981−2;P60981
p = 0.089, beta = 0.394, N = 84
EELMFFLWAPELAPLK pc2
F6RFD5;P60981−2;P60981
p = 0.54, beta = 0.111, N = 126
HECQANGPEDLNR pc3
P60981−2;P60981
p = 0.76, beta = −0.0486, N = 118
7:4419035:A:G_G
297Assay Target: DSTN
UniProt: P60981
rsID: rs314625
Target SNP: 7:4419035:A:G
Proxy SNP: 7:4419035:A:G
Tarkin log10(p): 7.7
Tarkin BETA: 0.381
Tarkin MSPA: 0.647
1236:1215:1254:1250:1133:958

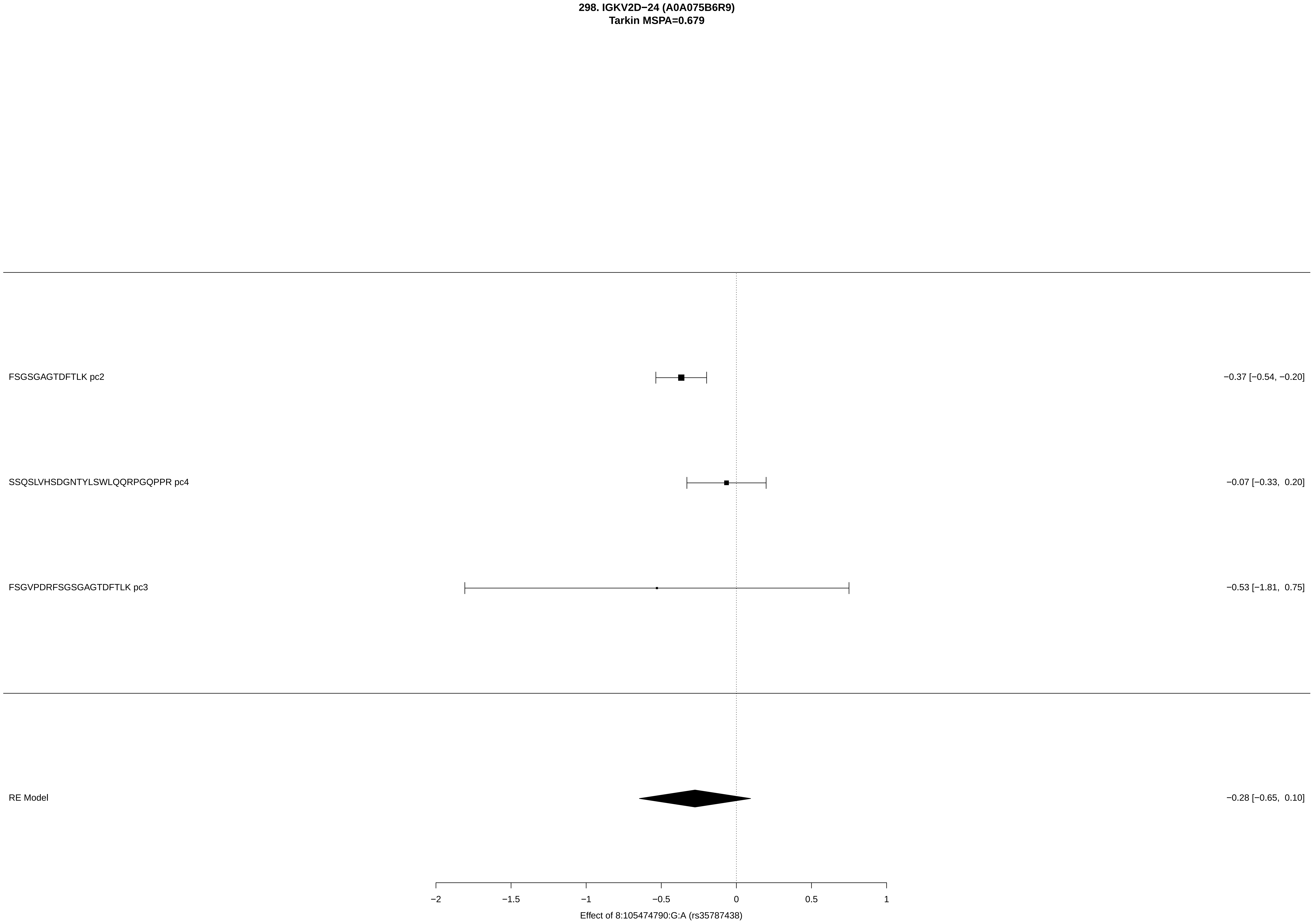

298. IGKV2D−24 (A0A075B6R9)
Tarkin MSPA=0.679
FSGSGAGTDFTLK pc2
−0.37 [−0.54, −0.20]
SSQSLVHSDGNTYLSWLQQRPGQPPR pc4
−0.07 [−0.33, 0.20]
FSGVPDRFSGSGAGTDFTLK pc3
−0.53 [−1.81, 0.75]
RE Model
−0.28 [−0.65, 0.10]
−2
−1.5
−1
−0.5
0
0.5
1
Effect of 8:105474790:G:A (rs35787438)

IGKV2D−24 : NP3
A0A075B6R9

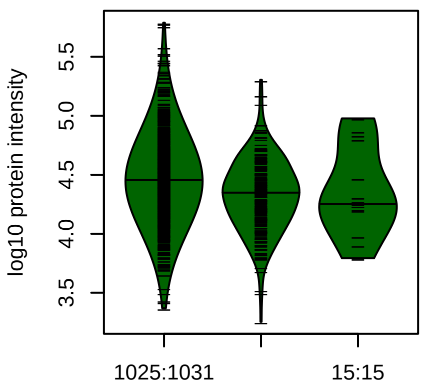

8:105474790:G:A_A
p = 2.1e−08, beta = −0.369, N = 1252

FSGSGAGTDFTLK pc2
A0A075B6R9;A0A0C4DH68

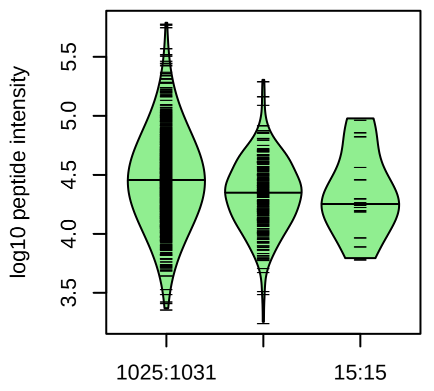

8:105474790:G:A_A
p = 2.7e−08, beta = −0.367, N = 1251

SQSLVHSDGNTYLSWLQQRPGQPPR
A0A075B6R9;A0A0C4DH68

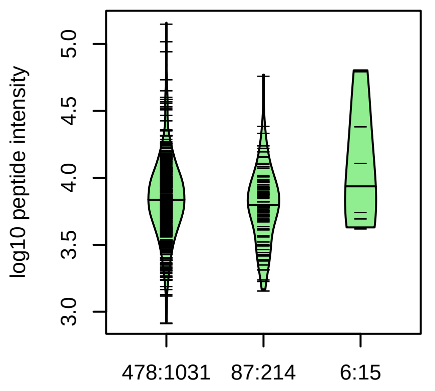

8:105474790:G:A_A
p = 0.52, beta = −0.0658, N = 571

FSGVPDRFSGSGAGTDFTLK pc3
A0A075B6R9;A0A0C4DH68

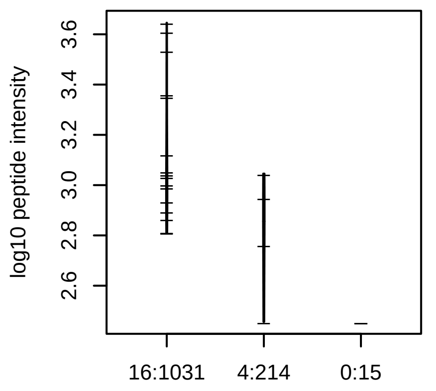

8:105474790:G:A_A
p = 0.3, beta = −0.529, N = 20

IGKV2D−24 : NP3
A0A075B6R9

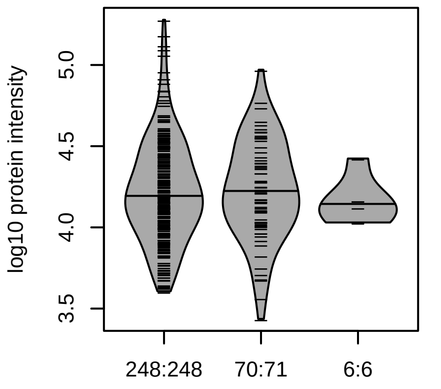

8:105474790:G:A_A
p = 0.79, beta = −0.0305, N = 324

FSGSGAGTDFTLK pc2
A0A075B6R9;A0A0C4DH68

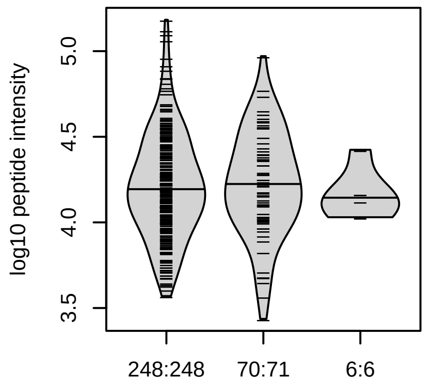

8:105474790:G:A_A
p = 0.85, beta = −0.0214, N = 324

SQSLVHSDGNTYLSWLQQRPGQPPR
A0A075B6R9;A0A0C4DH68

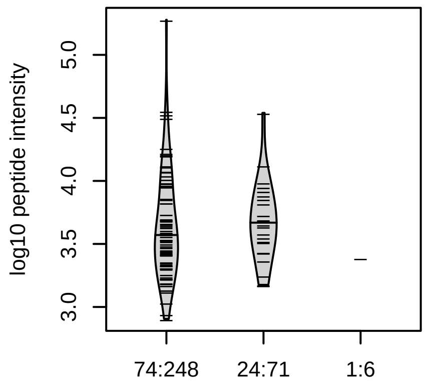

8:105474790:G:A_A
p = 0.95, beta = 0.0145, N = 99

298Assay Target: IGKV2D−24
UniProt: A0A075B6R9
rsID: rs35787438
Target SNP: 8:105474790:G:A
Proxy SNP: 8:105474790:G:A
Tarkin log10(p): 7.7
Tarkin BETA: −0.369
Tarkin MSPA: 0.679
*:−:−
1251:571:20

299. MMP9 (P14780)
Tarkin MSPA=0.233

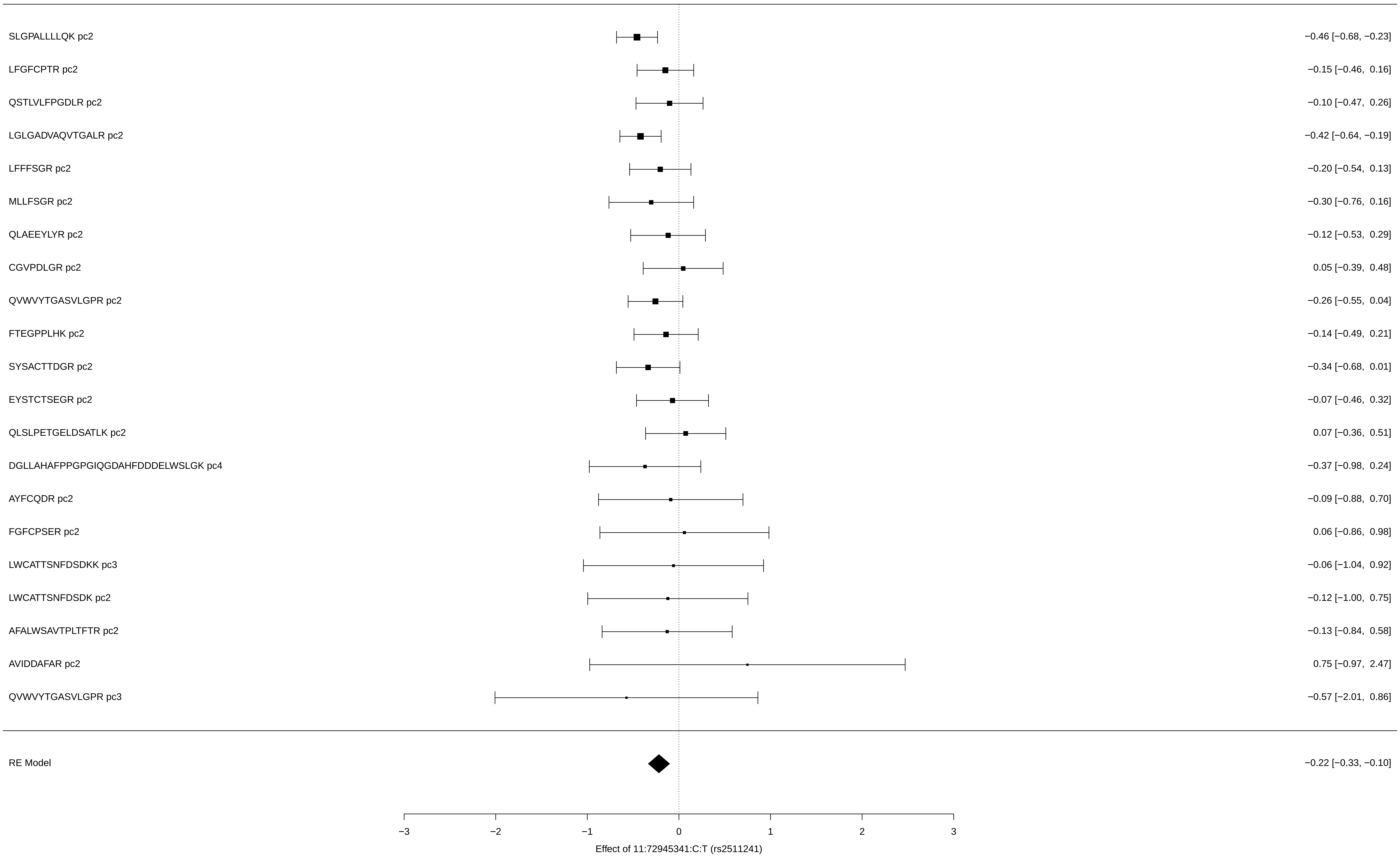

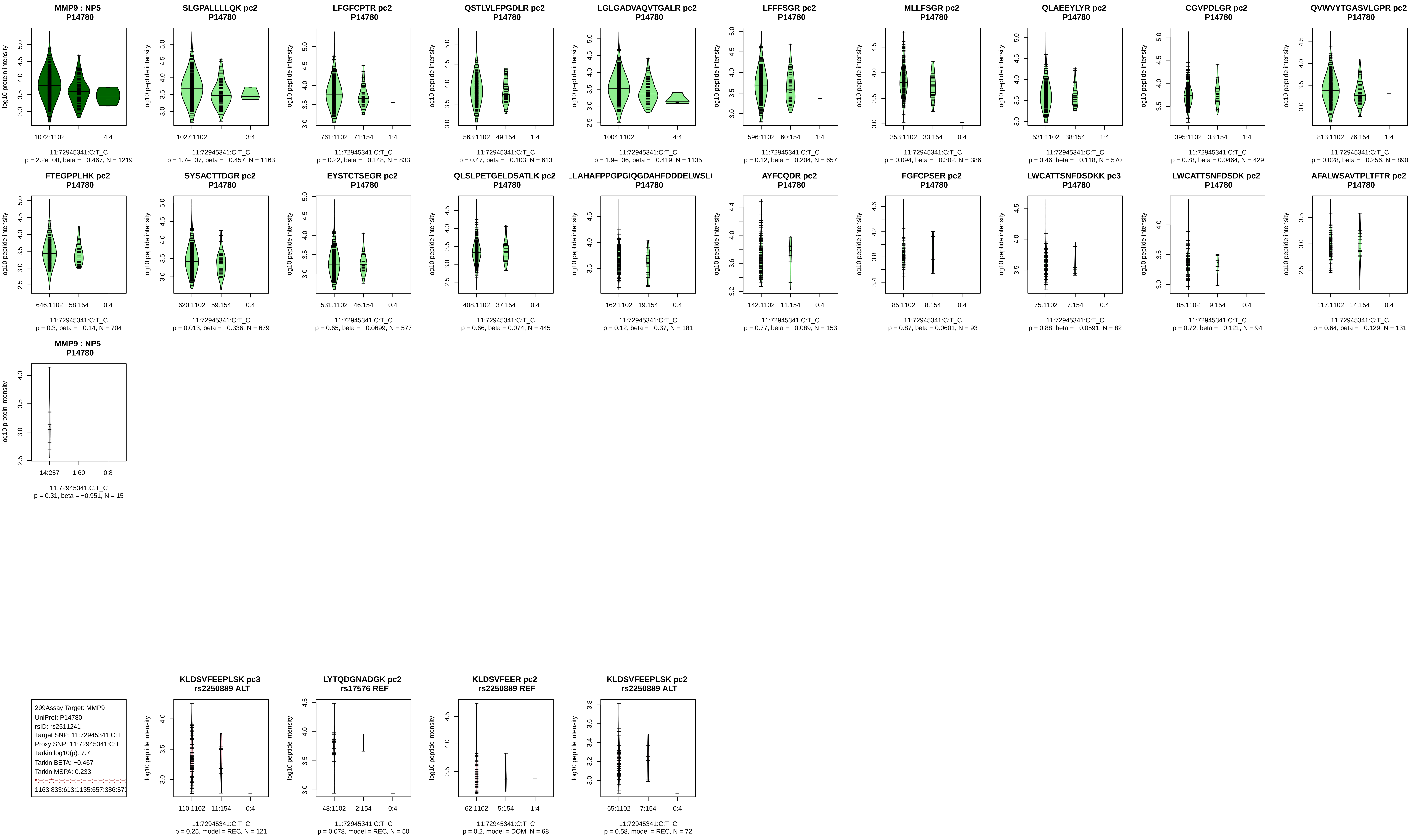
MMP9 : NP5 P14780
1072:1102 4:4
11:72945341:C:T_C
p = 2.2e−08, beta = −0.467, N = 1219
SLGPALLLLQK pc2 P14780
1027:1102 3:4
11:72945341:C:T_C
p = 1.7e−07, beta = −0.457, N = 1163
LFGFCPTR pc2 P14780
761:1102 71:154 1:4
11:72945341:C:T_C
p = 0.22, beta = −0.148, N = 833
QSTLVLFPGDLR pc2 P14780
563:1102 49:154 1:4
11:72945341:C:T_C
p = 0.47, beta = −0.103, N = 613
LGLGADVAQVTGALR pc2 P14780
1004:1102 4:4
11:72945341:C:T_C
p = 1.9e−06, beta = −0.419, N = 1135
LFFFSGR pc2 P14780
596:1102 60:154 1:4
11:72945341:C:T_C
p = 0.12, beta = −0.204, N = 657
MLLFSGR pc2 P14780
353:1102 33:154 0:4
11:72945341:C:T_C
p = 0.094, beta = −0.302, N = 386
QLAEEYLYR pc2 P14780
531:1102 38:154 1:4
11:72945341:C:T_C
p = 0.46, beta = −0.118, N = 570
CGVPDLGR pc2 P14780
395:1102 33:154 1:4
11:72945341:C:T_C
p = 0.78, beta = 0.0464, N = 429
QVWVYTGASVLGPR pc2 P14780
813:1102 76:154 1:4
11:72945341:C:T_C
p = 0.028, beta = −0.256, N = 890
FTEGPPLHK pc2 P14780
646:1102 58:154 0:4
11:72945341:C:T_C
p = 0.3, beta = −0.14, N = 704
SYSACTTDGR pc2 P14780
620:1102 59:154 0:4
11:72945341:C:T_C
p = 0.013, beta = −0.336, N = 679
EYSTCTSEGR pc2 P14780
531:1102 46:154 0:4
11:72945341:C:T_C
p = 0.65, beta = −0.0699, N = 577
QLSLPETGELDSATLK pc2 P14780
408:1102 37:154 0:4
11:72945341:C:T_C
p = 0.66, beta = 0.074, N = 445
LLAHAFPPGPGIQGDAHFDDDELWSL P14780
162:1102 19:154 0:4
11:72945341:C:T_C
p = 0.12, beta = −0.37, N = 181
AYFCQDR pc2 P14780
142:1102 11:154 0:4
11:72945341:C:T_C
p = 0.77, beta = −0.089, N = 153
FGFCPSER pc2 P14780
85:1102 8:154 0:4
11:72945341:C:T_C
p = 0.87, beta = 0.0601, N = 93
LWCATTSNFDSDKK pc3 P14780
75:1102 7:154 0:4
11:72945341:C:T_C
p = 0.88, beta = −0.0591, N = 82
LWCATTSNFDSDK pc2 P14780
85:1102 9:154 0:4
11:72945341:C:T_C
p = 0.72, beta = −0.121, N = 94
AFALWSAVTPLTFTR pc2 P14780
117:1102 14:154 0:4
11:72945341:C:T_C
p = 0.64, beta = −0.129, N = 131
MMP9 : NP5 P14780
14:257 1:60 0:8
11:72945341:C:T_C
p = 0.31, beta = −0.951, N = 15
299Assay Target: MMP9
UniProt: P14780
rsID: rs2511241
Target SNP: 11:72945341:C:T
Proxy SNP: 11:72945341:C:T
Tarkin log10(p): 7.7
Tarkin BETA: −0.467
Tarkin MSPA: 0.233
1163:833:613:1135:657:386:570
KLDSVFEEPLSK pc3 rs2250889 ALT
110:1102 11:154 0:4
11:72945341:C:T_C
p = 0.25, model = REC, N = 121
LYTQDGNADGK pc2 rs17576 REF
48:1102 2:154 0:4
11:72945341:C:T_C
p = 0.078, model = REC, N = 50
KLDSVFEER pc2 rs2250889 REF
62:1102 5:154 1:4
11:72945341:C:T_C
p = 0.2, model = DOM, N = 68
KLDSVFEEPLSK pc2 rs2250889 ALT
65:1102 7:154 0:4
11:72945341:C:T_C
p = 0.58, model = REC, N = 72

300. CPS1 (P31327)
Tarkin MSPA=0.656

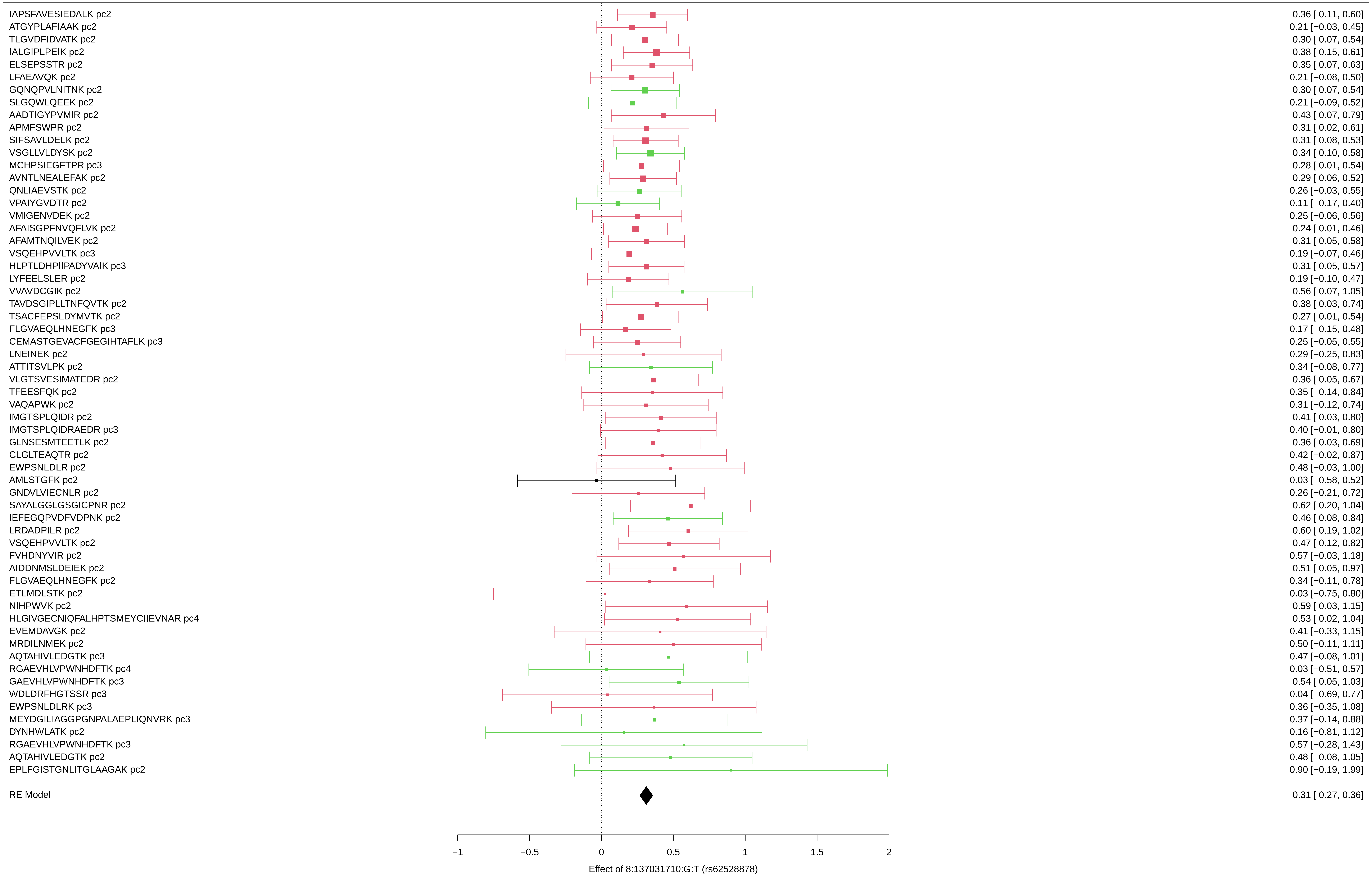

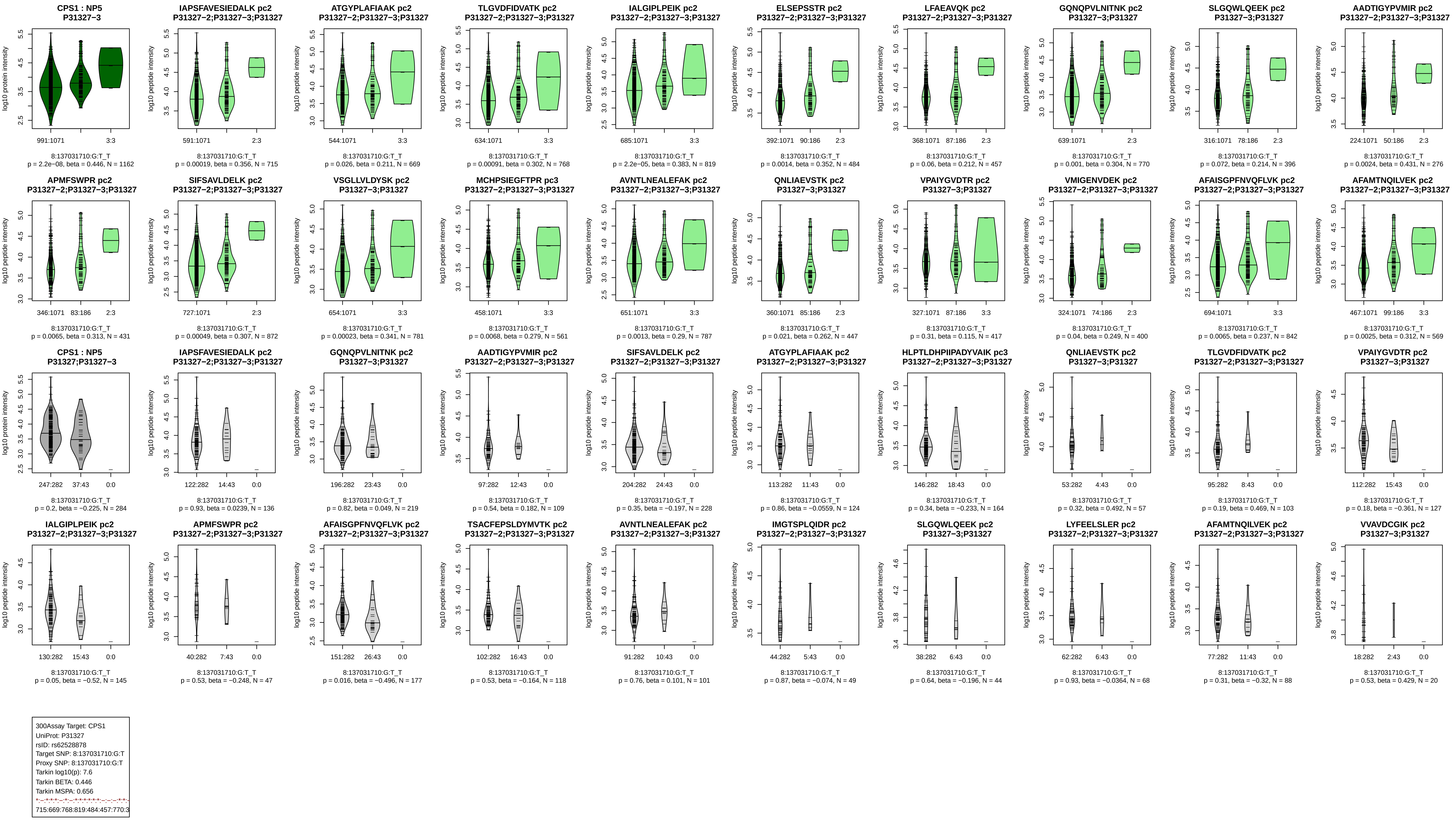
CPS1 : NP5
P31327−3
log10 protein intensity
991:1071
3:3
8:137031710:G:T_T
p = 2.2e−08, beta = 0.446, N = 1162
IAPSFAVESIEDALK pc2
P31327−2;P31327−3;P31327
log10 peptide intensity
591:1071
2:3
8:137031710:G:T_T
p = 0.00019, beta = 0.356, N = 715
ATGYPLAFIAAK pc2
P31327−2;P31327−3;P31327
544:1071
3:3
8:137031710:G:T_T
p = 0.026, beta = 0.211, N = 669
TLGVDFIDVATK pc2
P31327−2;P31327−3;P31327
634:1071
3:3
8:137031710:G:T_T
p = 0.00091, beta = 0.302, N = 768
IALGIPLPEIK pc2
P31327−2;P31327−3;P31327
685:1071
3:3
8:137031710:G:T_T
p = 2.2e−05, beta = 0.383, N = 819
ELSEPSSTR pc2
P31327−2;P31327−3;P31327
392:1071
90:186
2:3
8:137031710:G:T_T
p = 0.0014, beta = 0.352, N = 484
LFAEAVQK pc2
P31327−2;P31327−3;P31327
368:1071
87:186
2:3
8:137031710:G:T_T
p = 0.06, beta = 0.212, N = 457
GQNQPVLNITNK pc2
P31327−3;P31327
639:1071
2:3
8:137031710:G:T_T
p = 0.001, beta = 0.304, N = 770
SLGQWLQEEK pc2
P31327−3;P31327
316:1071
78:186
2:3
8:137031710:G:T_T
p = 0.072, beta = 0.214, N = 396
AADTIGYPVMIR pc2
P31327−2;P31327−3;P31327
224:1071
50:186
2:3
8:137031710:G:T_T
p = 0.0024, beta = 0.431, N = 276
APMFSWPR pc2
P31327−2;P31327−3;P31327
346:1071
83:186
2:3
8:137031710:G:T_T
p = 0.0065, beta = 0.313, N = 431
SIFSAVLDELK pc2
P31327−2;P31327−3;P31327
727:1071
2:3
8:137031710:G:T_T
p = 0.00049, beta = 0.307, N = 872
VSGLLVLDYSK pc2
P31327−3;P31327
654:1071
3:3
8:137031710:G:T_T
p = 0.00023, beta = 0.341, N = 781
MCHPSIEGFTPR pc3
P31327−2;P31327−3;P31327
458:1071
3:3
8:137031710:G:T_T
p = 0.0068, beta = 0.279, N = 561
AVNTLNEALEFAK pc2
P31327−2;P31327−3;P31327
651:1071
3:3
8:137031710:G:T_T
p = 0.0013, beta = 0.29, N = 787
QNLIAEVSTK pc2
P31327−3;P31327
360:1071
85:186
2:3
8:137031710:G:T_T
p = 0.021, beta = 0.262, N = 447
VPAIYGVDTR pc2
P31327−3;P31327
327:1071
87:186
3:3
8:137031710:G:T_T
p = 0.31, beta = 0.115, N = 417
VMIGENVDEK pc2
P31327−2;P31327−3;P31327
324:1071
74:186
2:3
8:137031710:G:T_T
p = 0.04, beta = 0.249, N = 400
AFAISGPFNVQFLVK pc2
P31327−2;P31327−3;P31327
694:1071
3:3
8:137031710:G:T_T
p = 0.0065, beta = 0.237, N = 842
AFAMTNQILVEK pc2
P31327−2;P31327−3;P31327
467:1071
99:186
3:3
8:137031710:G:T_T
p = 0.0025, beta = 0.312, N = 569
CPS1 : NP5
P31327;P31327−3
247:282
37:43
0:0
8:137031710:G:T_T
p = 0.2, beta = −0.225, N = 284
IAPSFAVESIEDALK pc2
P31327−2;P31327−3;P31327
122:282
14:43
0:0
8:137031710:G:T_T
p = 0.93, beta = 0.0239, N = 136
GQNQPVLNITNK pc2
P31327−3;P31327
196:282
23:43
0:0
8:137031710:G:T_T
p = 0.82, beta = 0.049, N = 219
AADTIGYPVMIR pc2
P31327−2;P31327−3;P31327
97:282
12:43
0:0
8:137031710:G:T_T
p = 0.54, beta = 0.182, N = 109
SIFSAVLDELK pc2
P31327−2;P31327−3;P31327
204:282
24:43
0:0
8:137031710:G:T_T
p = 0.35, beta = −0.197, N = 228
ATGYPLAFIAAK pc2
P31327−2;P31327−3;P31327
113:282
11:43
0:0
8:137031710:G:T_T
p = 0.86, beta = −0.0559, N = 124
HLPTLDHPIIPADYVAIK pc3
P31327−2;P31327−3;P31327
146:282
18:43
0:0
8:137031710:G:T_T
p = 0.34, beta = −0.233, N = 164
QNLIAEVSTK pc2
P31327−3;P31327
53:282
4:43
0:0
8:137031710:G:T_T
p = 0.32, beta = 0.492, N = 57
TLGVDFIDVATK pc2
P31327−2;P31327−3;P31327
95:282
8:43
0:0
8:137031710:G:T_T
p = 0.19, beta = 0.469, N = 103
VPAIYGVDTR pc2
P31327−3;P31327
112:282
15:43
0:0
8:137031710:G:T_T
p = 0.18, beta = −0.361, N = 127
IALGIPLPEIK pc2
P31327−2;P31327−3;P31327
130:282
15:43
0:0
8:137031710:G:T_T
p = 0.05, beta = −0.52, N = 145
APMFSWPR pc2
P31327−2;P31327−3;P31327
40:282
7:43
0:0
8:137031710:G:T_T
p = 0.53, beta = −0.248, N = 47
AFAISGPFNVQFLVK pc2
P31327−2;P31327−3;P31327
151:282
26:43
0:0
8:137031710:G:T_T
p = 0.016, beta = −0.496, N = 177
TSACFEPSLDYMVTK pc2
P31327−2;P31327−3;P31327
102:282
16:43
0:0
8:137031710:G:T_T
p = 0.53, beta = −0.164, N = 118
AVNTLNEALEFAK pc2
P31327−2;P31327−3;P31327
91:282
10:43
0:0
8:137031710:G:T_T
p = 0.76, beta = 0.101, N = 101
IMGTSPLQIDR pc2
P31327−2;P31327−3;P31327
44:282
5:43
0:0
8:137031710:G:T_T
p = 0.87, beta = −0.074, N = 49
SLGQWLQEEK pc2
P31327−3;P31327
38:282
6:43
0:0
8:137031710:G:T_T
p = 0.64, beta = −0.196, N = 44
LYFEELSLER pc2
P31327−2;P31327−3;P31327
62:282
6:43
0:0
8:137031710:G:T_T
p = 0.93, beta = −0.0364, N = 68
AFAMTNQILVEK pc2
P31327−2;P31327−3;P31327
77:282
11:43
0:0
8:137031710:G:T_T
p = 0.31, beta = −0.32, N = 88
VVAVDCGIK pc2
P31327−3;P31327
18:282
2:43
0:0
8:137031710:G:T_T
p = 0.53, beta = 0.429, N = 20
300Assay Target: CPS1
UniProt: P31327
rsID: rs62528878
Target SNP: 8:137031710:G:T
Proxy SNP: 8:137031710:G:T
Tarkin log10(p): 7.6
Tarkin BETA: 0.446
Tarkin MSPA: 0.656
715:669:768:819:484:457:770:3

301. STAB2 (Q8WWQ8)
Tarkin MSPA=0.37

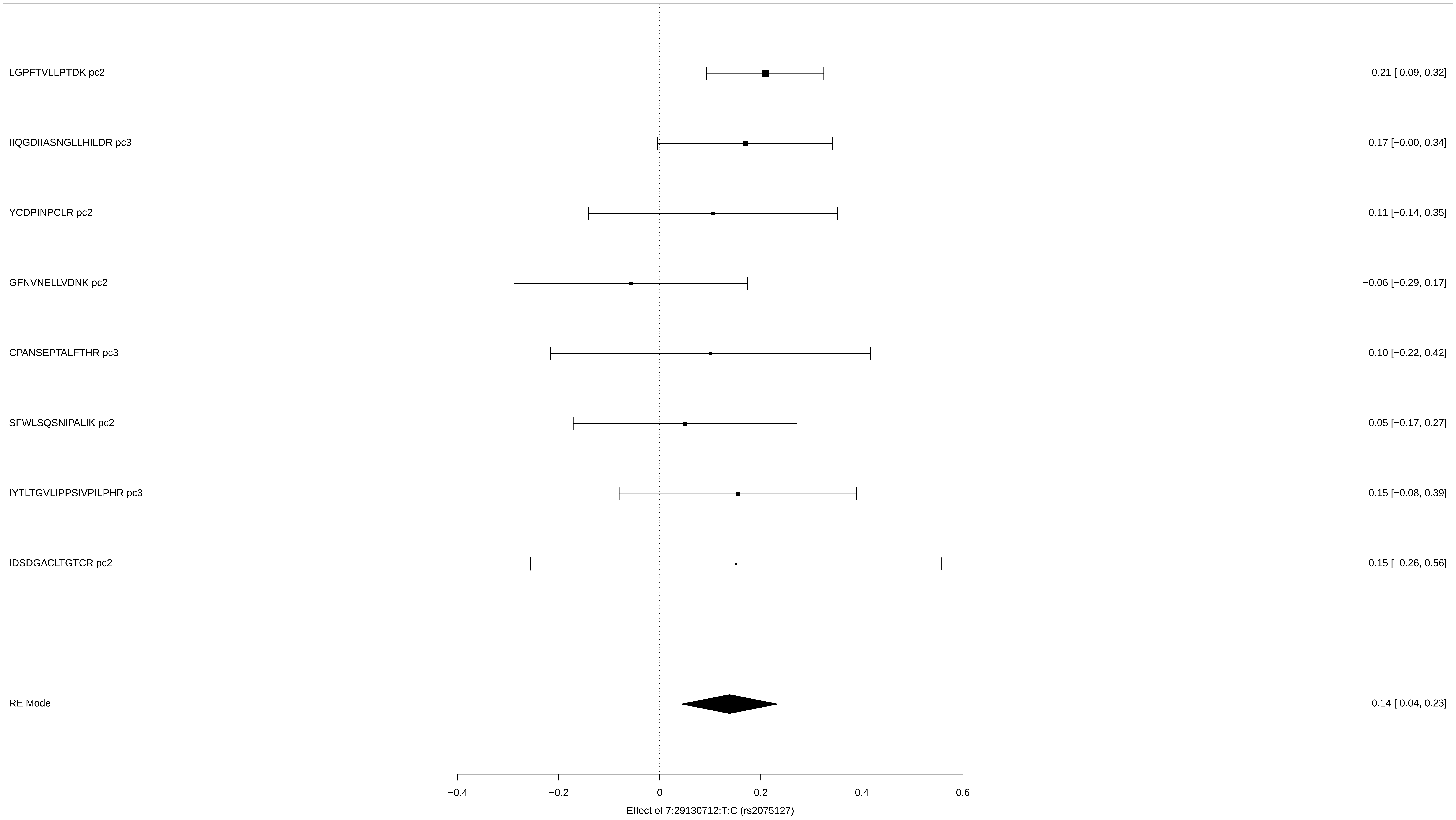

STAB2 : NP4
Q8WWQ8

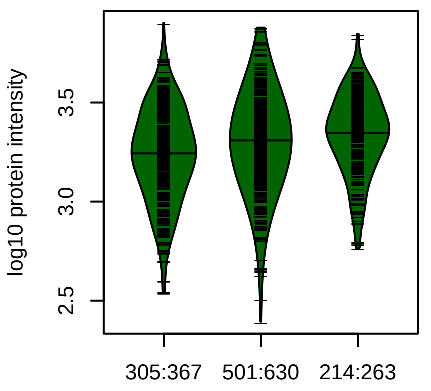

7:29130712:T:C_T
p = 2.3e−08, beta = 0.244, N = 1020

LGPFTVLLPTDK pc2
Q8WWQ8

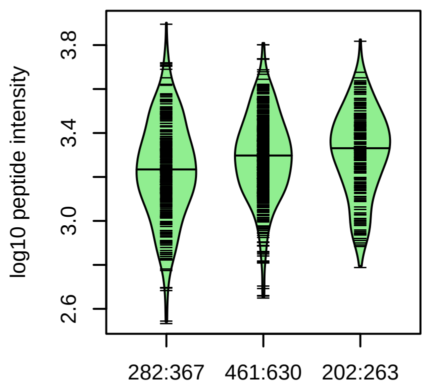

7:29130712:T:C_T
p = 4.1e−06, beta = 0.209, N = 945

IIQGDIIASNGLLHILDR pc3
Q8WWQ8

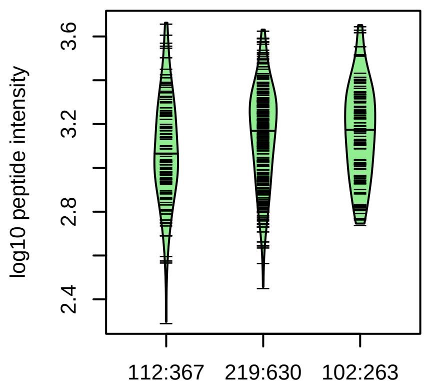

7:29130712:T:C_T
p = 0.012, beta = 0.169, N = 433

YCDPINPCLR pc2
Q8WWQ8

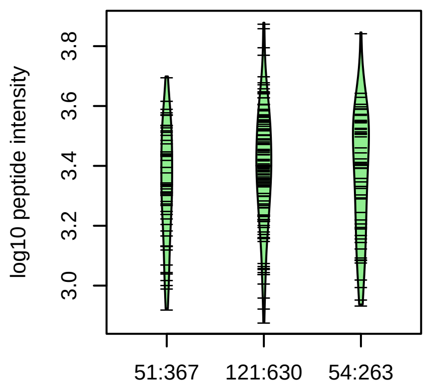

7:29130712:T:C_T
p = 0.27, beta = 0.105, N = 226

GFNVNELLVDNK pc2
Q8WWQ8

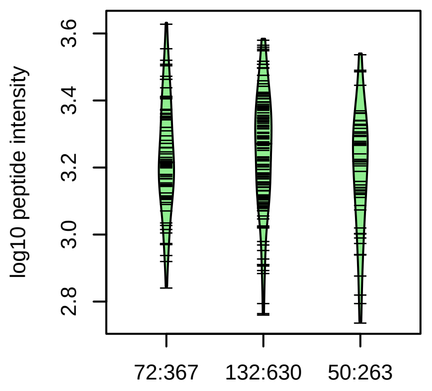

7:29130712:T:C_T
p = 0.52, beta = −0.0572, N = 254

CPANSEPTALFTHR pc3
Q8WWQ8

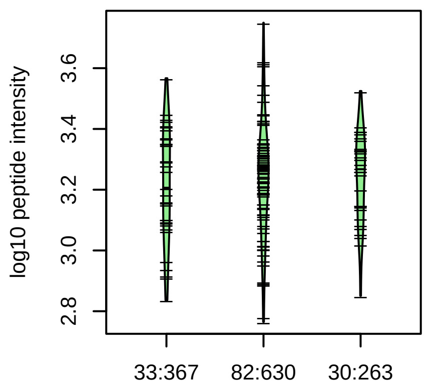

7:29130712:T:C_T
p = 0.42, beta = 0.1, N = 145

SFWLSQSNIPALIK pc2
Q8WWQ8

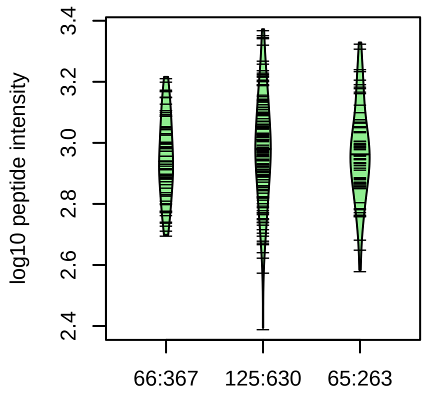

7:29130712:T:C_T
p = 0.56, beta = 0.0501, N = 256

IYTLTGVLIPPSIVPILPHR pc3
Q8WWQ8

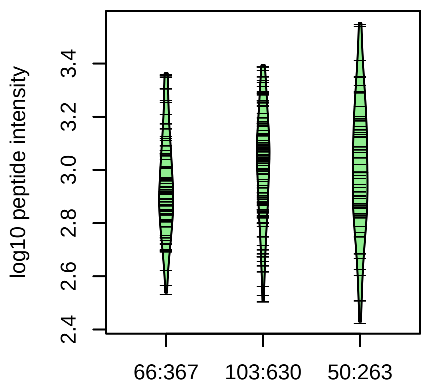

7:29130712:T:C_T
p = 0.092, beta = 0.154, N = 219

IDSDGACLTGTCR pc2
Q8WWQ8

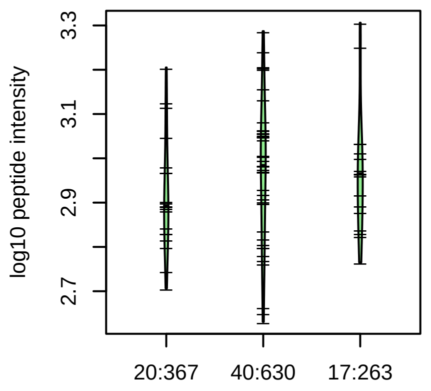

7:29130712:T:C_T
p = 0.34, beta = 0.151, N = 77

301Assay Target: STAB2
UniProt: Q8WWQ8
rsID: rs2075127
Target SNP: 7:29130712:T:C
Proxy SNP: 7:29130712:T:C
Tarkin log10(p): 7.6
Tarkin BETA: 0.244
Tarkin MSPA: 0.37
*:−:−:−:−:−:−:−
945:433:226:254:145:256:219:7

302. RALA (P11233)
Tarkin MSPA=0.706

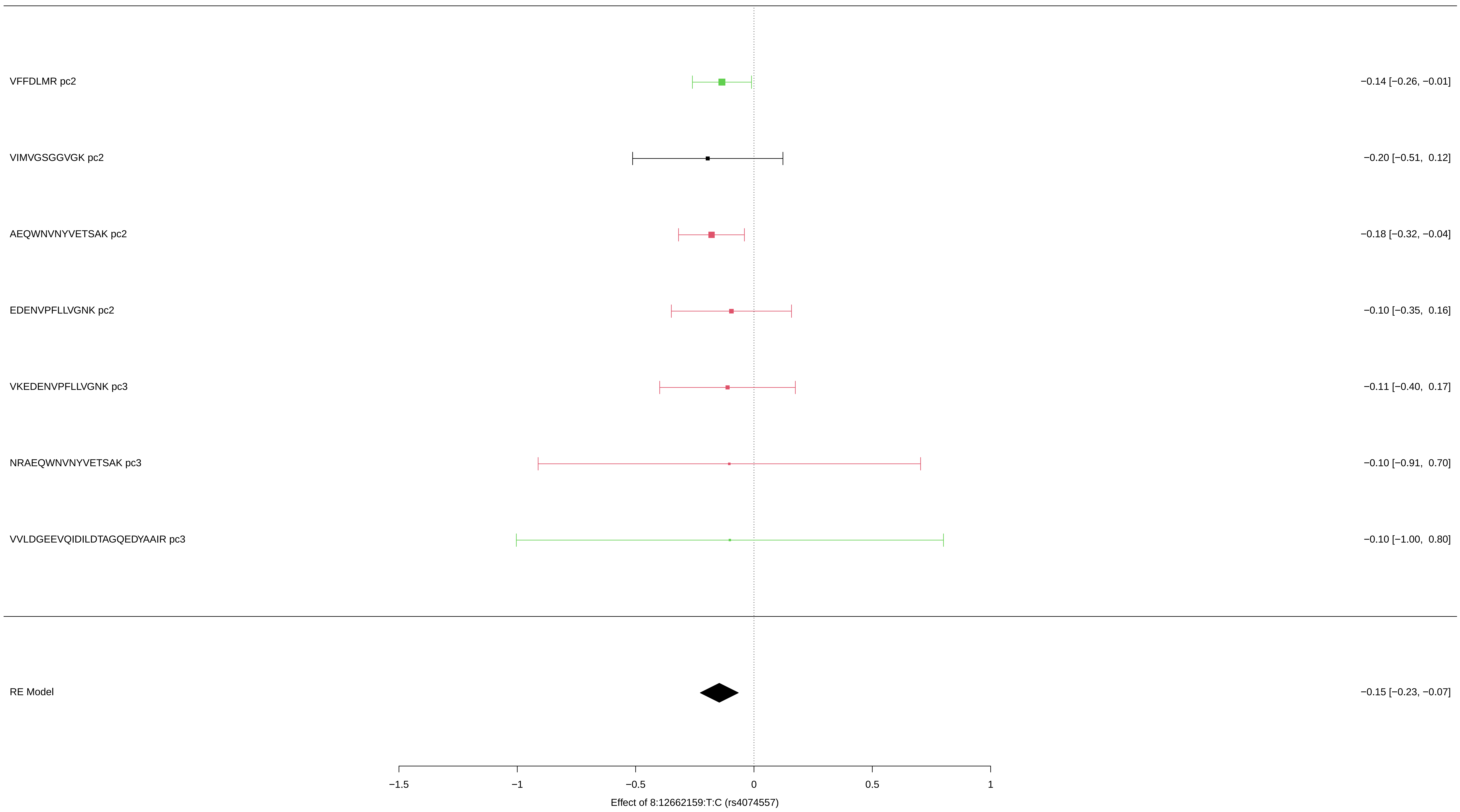

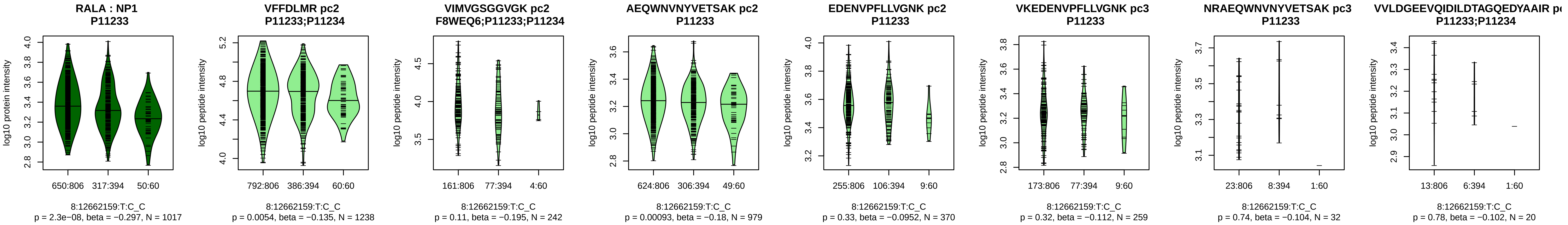

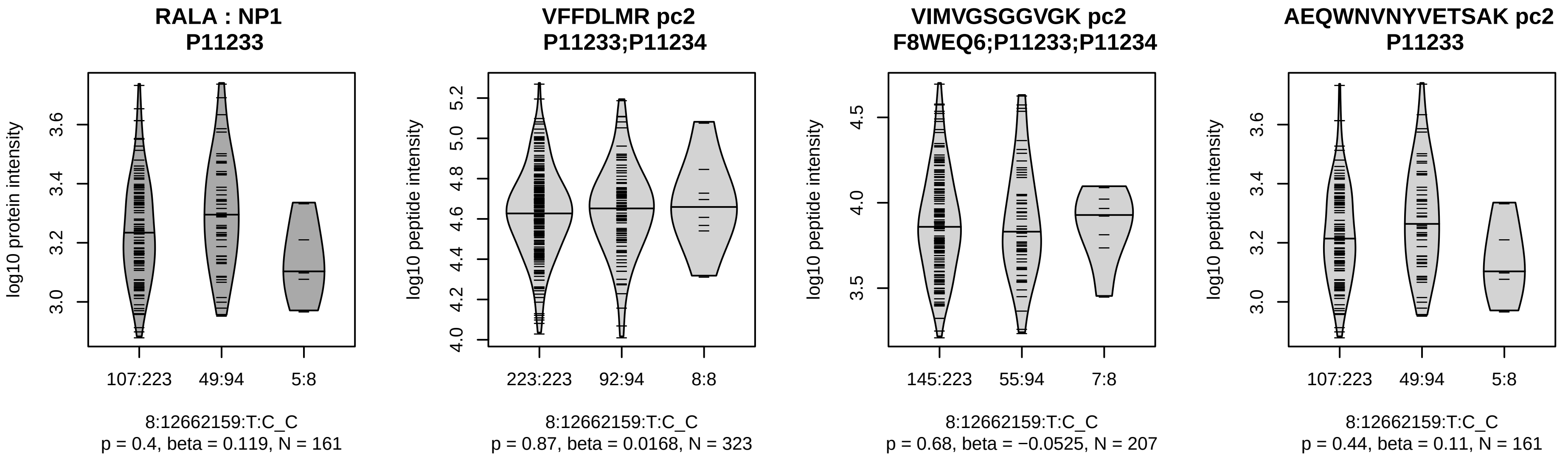

302Assay Target: RALA
UniProt: P11233
rsID: rs4074557
Target SNP: 8:12662159:T:C
Proxy SNP: 8:12662159:T:C
Tarkin log10(p): 7.6
Tarkin BETA: −0.297
Tarkin MSPA: 0.706
:−::−:−:−:−
1238:242:979:370:259:32:20

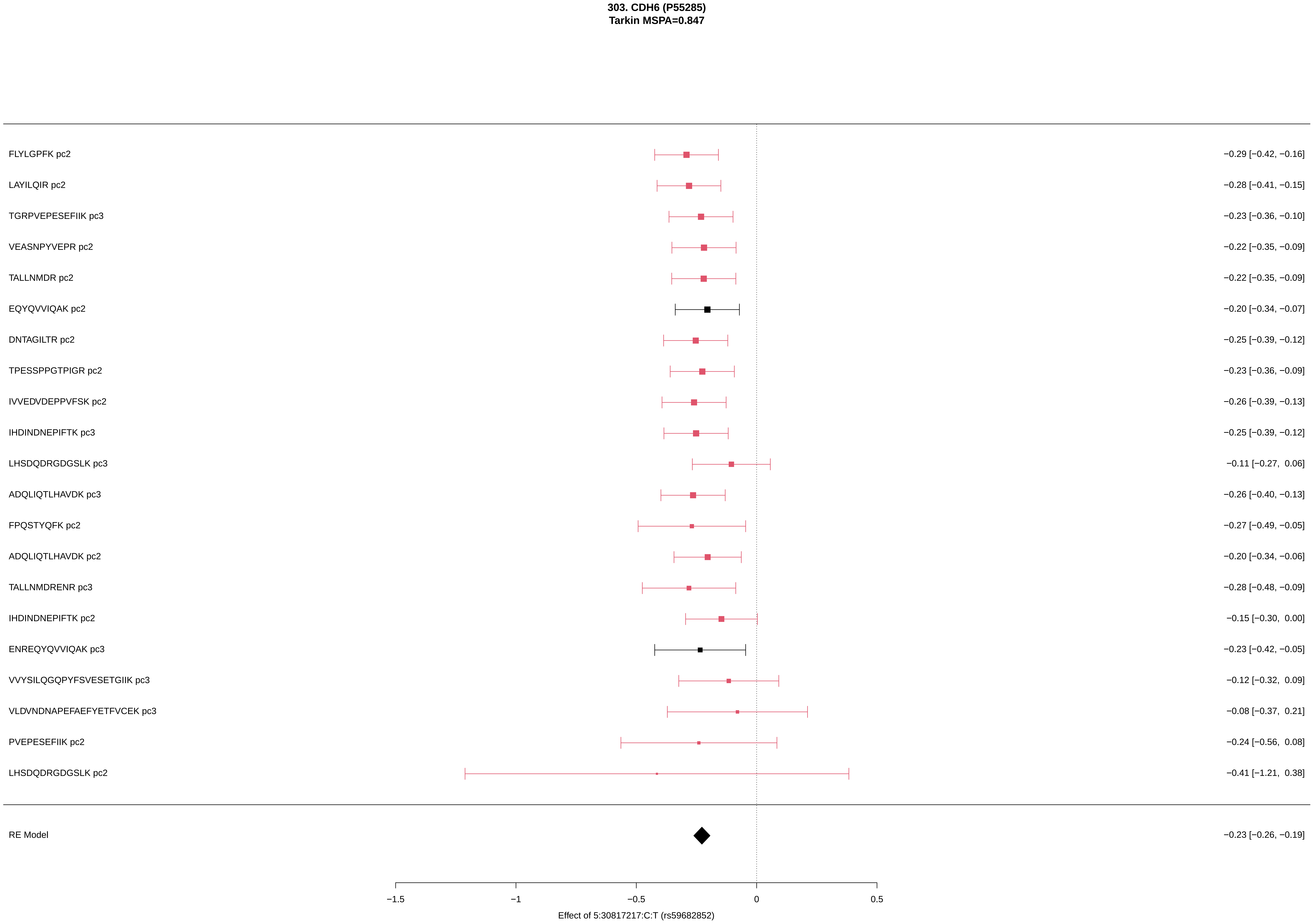

303. CDH6 (P55285)
Tarkin MSPA=0.847
FLYLGPFK pc2 −0.29 [−0.42, −0.16]
LAYILQIR pc2 −0.28 [−0.41, −0.15]
TGRPVEPESEFIIK pc3 −0.23 [−0.36, −0.10]
VEASNPYVEPR pc2 −0.22 [−0.35, −0.09]
TALLNMDR pc2 −0.22 [−0.35, −0.09]
EQYQVVIQAK pc2 −0.20 [−0.34, −0.07]
DNTAGILTR pc2 −0.25 [−0.39, −0.12]
TPESSPPGTPIGR pc2 −0.23 [−0.36, −0.09]
IVVEDVDEPPVFSK pc2 −0.26 [−0.39, −0.13]
IHDINDNEPIFTK pc3 −0.25 [−0.39, −0.12]
LHSDQDRGDGSLK pc3 −0.11 [−0.27, 0.06]
ADQLIQTLHAVDK pc3 −0.26 [−0.40, −0.13]
FPQSTYQFK pc2 −0.27 [−0.49, −0.05]
ADQLIQTLHAVDK pc2 −0.20 [−0.34, −0.06]
TALLNMDRENR pc3 −0.28 [−0.48, −0.09]
IHDINDNEPIFTK pc2 −0.15 [−0.30, 0.00]
ENREQYQVVIQAK pc3 −0.23 [−0.42, −0.05]
VVYSILQGQPYFSVESETGIIK pc3 −0.12 [−0.32, 0.09]
VLDVNDNAPEFAEFYETFVCEK pc3 −0.08 [−0.37, 0.21]
PVEPESEFIIK pc2 −0.24 [−0.56, 0.08]
LHSDQDRGDGSLK pc2 −0.41 [−1.21, 0.38]
RE Model −0.23 [−0.26, −0.19]
−1.5
−1
−0.5
0
0.5
Effect of 5:30817217:C:T (rs59682852)

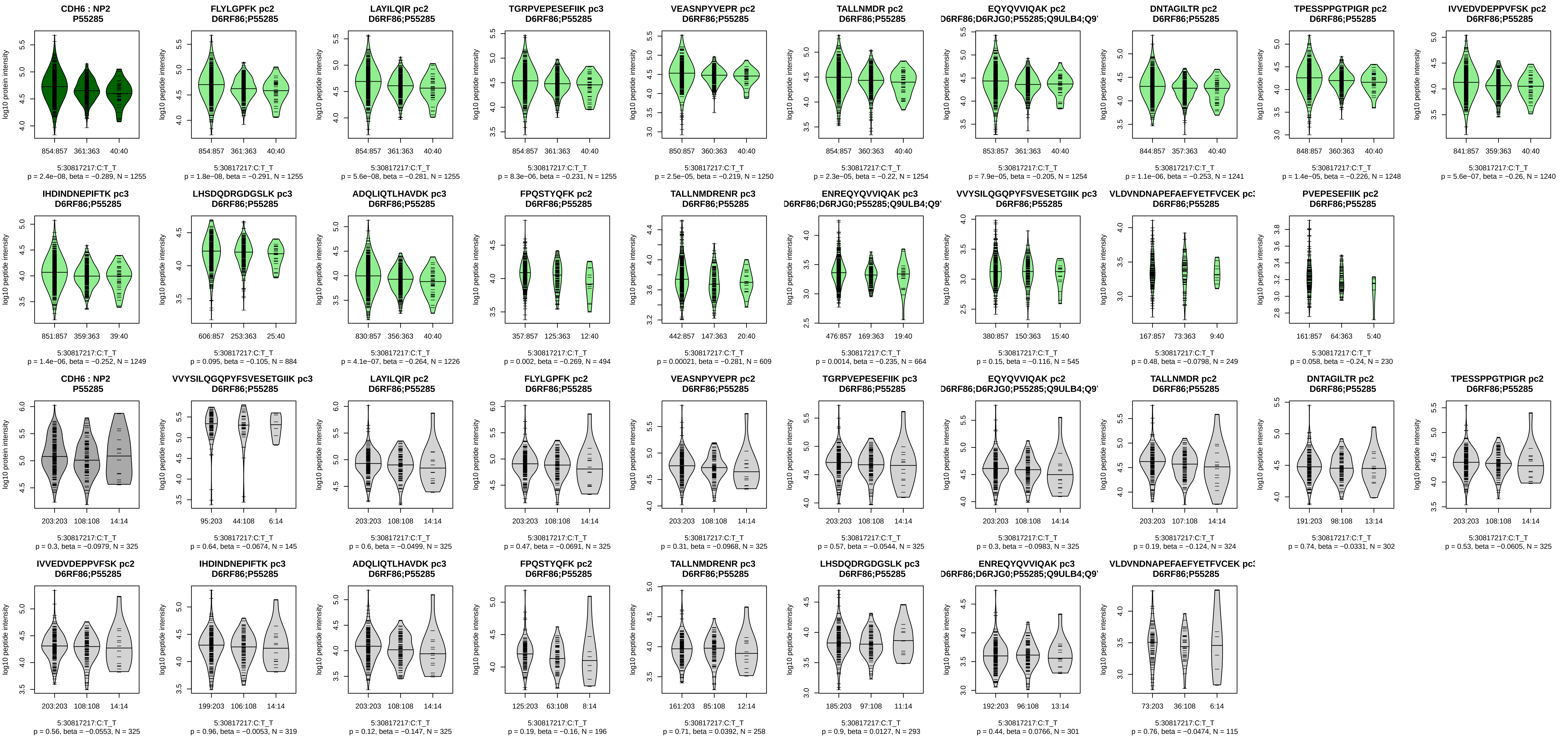

303Assay Target: CDH6
UniProt: P55285
rsID: rs59682852
Target SNP: 5:30817217:C:T
Proxy SNP: 5:30817217:C:T
Tarkin log10(p): 7.6
Tarkin BETA: −0.289
Tarkin MSPA: 0.847
[illegible]
1255:1255:1255:1250:1254:125

304. ACP1 (P24666)
Tarkin MSPA=0.85

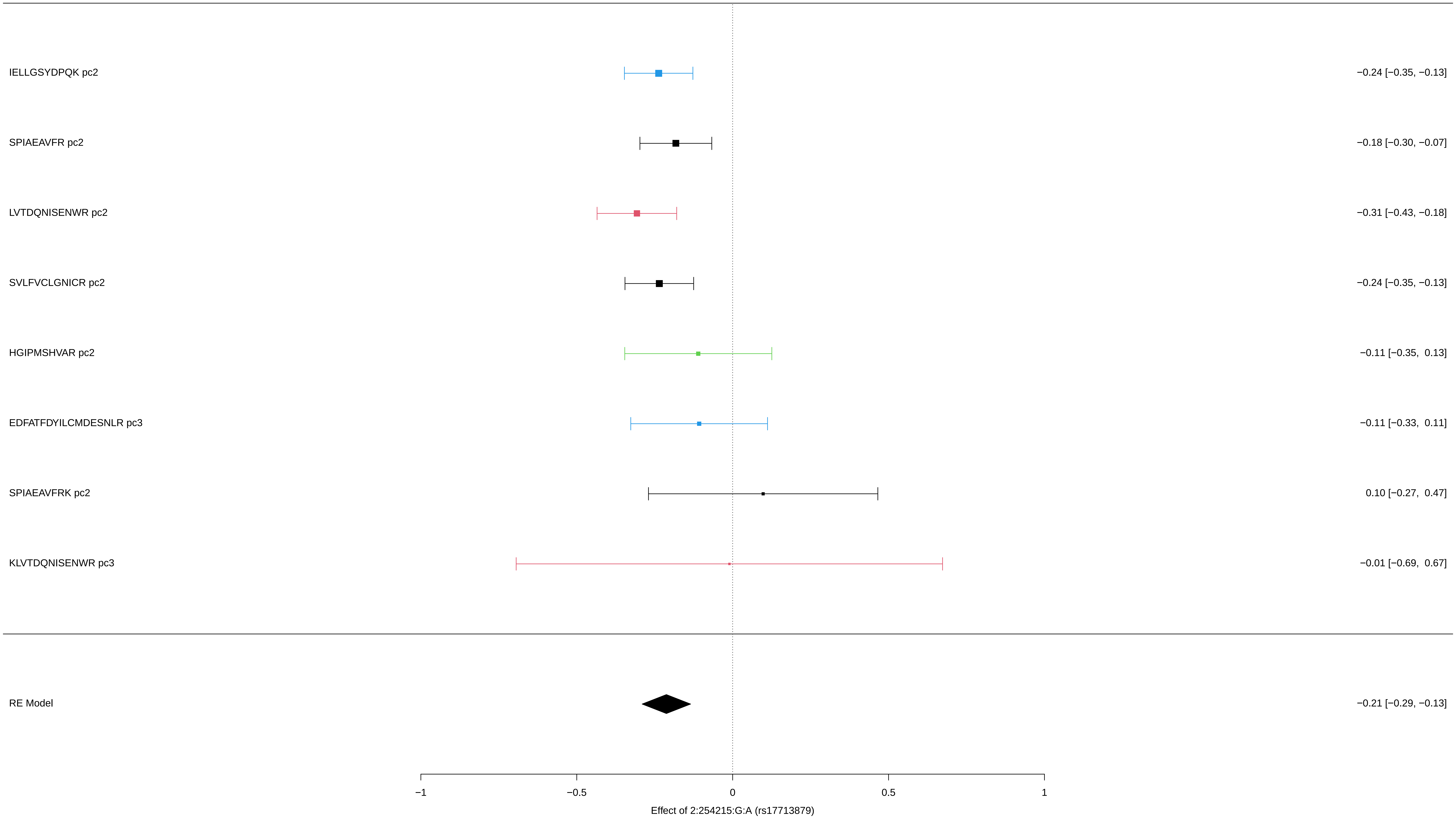

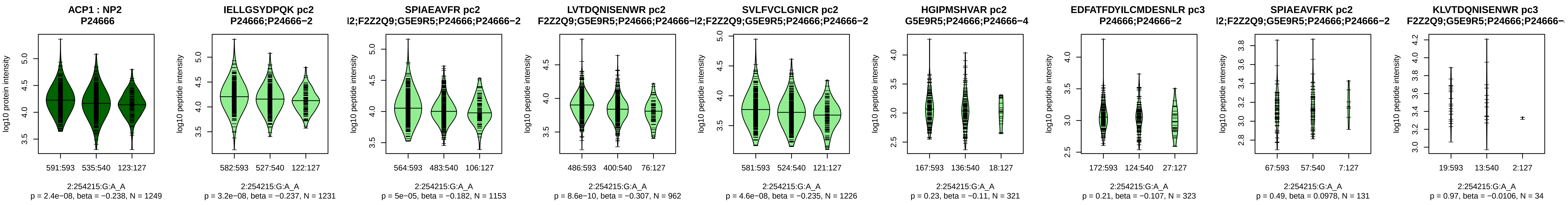

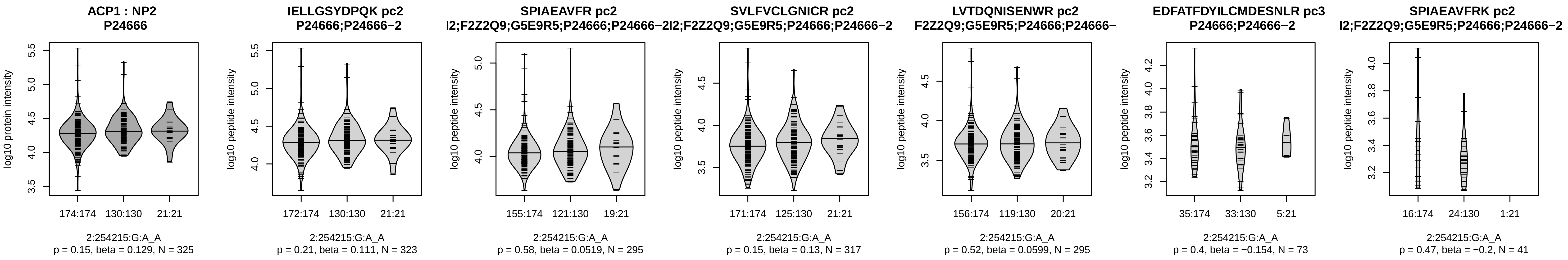

304Assay Target: ACP1
UniProt: P24666
rsID: rs17713879
Target SNP: 2:254215:G:A
Proxy SNP: 2:254215:G:A
Tarkin log10(p): 7.6
Tarkin BETA: −0.238
Tarkin MSPA: 0.85
::*:*:−:−:−:−:NA:NA:NA:NA
1231:1153:962:1226:321:323:13

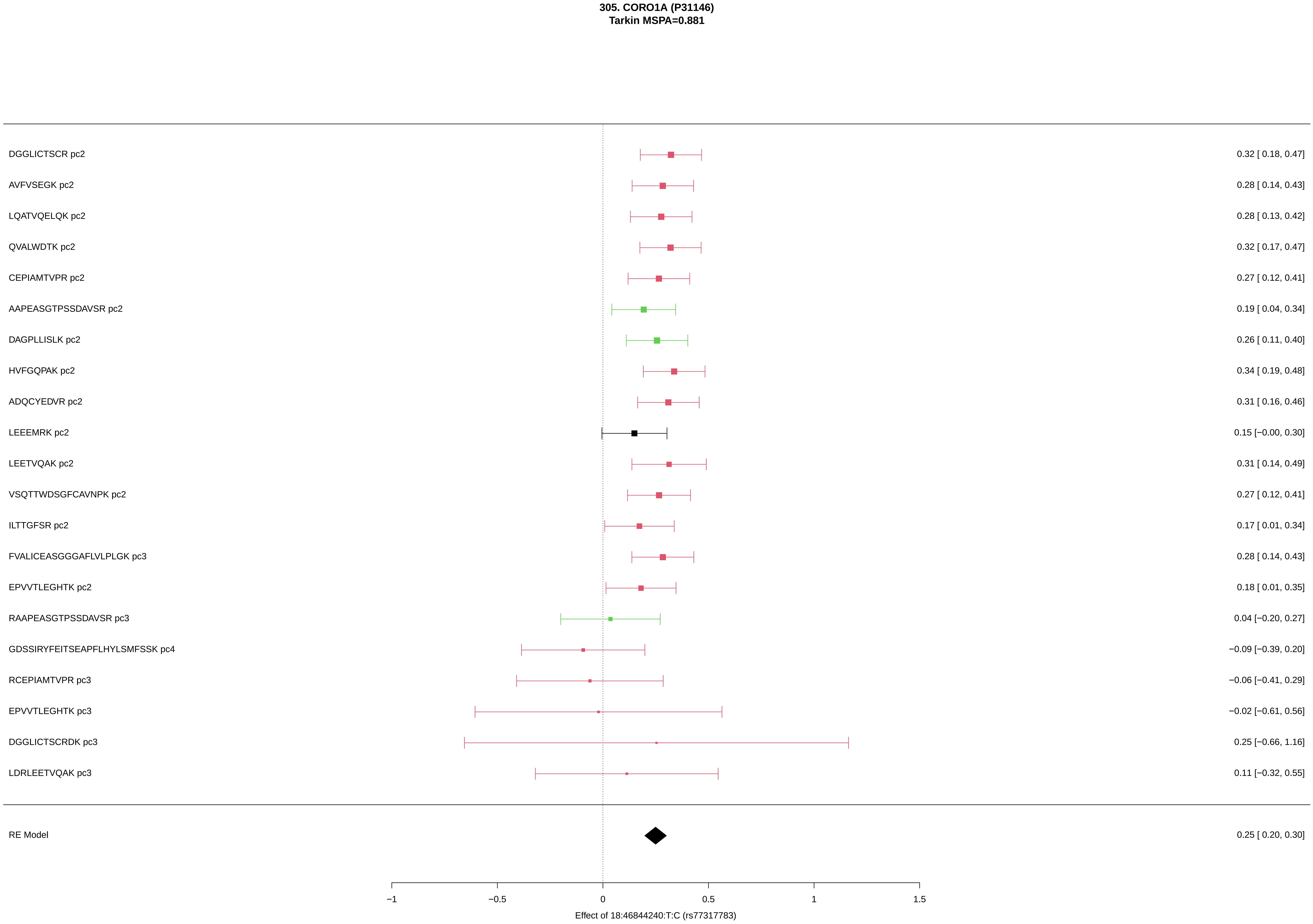

305. CORO1A (P31146)
Tarkin MSPA=0.881
DGGLICTSCR pc2
0.32 [0.18, 0.47]
AVFVSEGK pc2
0.28 [0.14, 0.43]
LQATVQELQK pc2
0.28 [0.13, 0.42]
QVALWDTK pc2
0.32 [0.17, 0.47]
CEPIAMTVPR pc2
0.27 [0.12, 0.41]
AAPEASGTPSSDAVSR pc2
0.19 [0.04, 0.34]
DAGPLLISLK pc2
0.26 [0.11, 0.40]
HVFGQPAK pc2
0.34 [0.19, 0.48]
ADQCYEDVR pc2
0.31 [0.16, 0.46]
LEEEMRK pc2
0.15 [−0.00, 0.30]
LEETVQAK pc2
0.31 [0.14, 0.49]
VSQTTWDSGFCAVNPK pc2
0.27 [0.12, 0.41]
ILTTGFSR pc2
0.17 [0.01, 0.34]
FVALICEASGGGAFLVLPLGK pc3
0.28 [0.14, 0.43]
EPVVTLEGHTK pc2
0.18 [0.01, 0.35]
RAAPEASGTPSSDAVSR pc3
0.04 [−0.20, 0.27]
GDSSIRYFEITSEAPFLHYLSMFSSK pc4
−0.09 [−0.39, 0.20]
RCEPIAMTVPR pc3
−0.06 [−0.41, 0.29]
EPVVTLEGHTK pc3
−0.02 [−0.61, 0.56]
DGGLICTSCRDK pc3
0.25 [−0.66, 1.16]
LDRLEETVQAK pc3
0.11 [−0.32, 0.55]
RE Model
0.25 [0.20, 0.30]
−1
−0.5
0
0.5
1
1.5
Effect of 18:46844240:T:C (rs77317783)

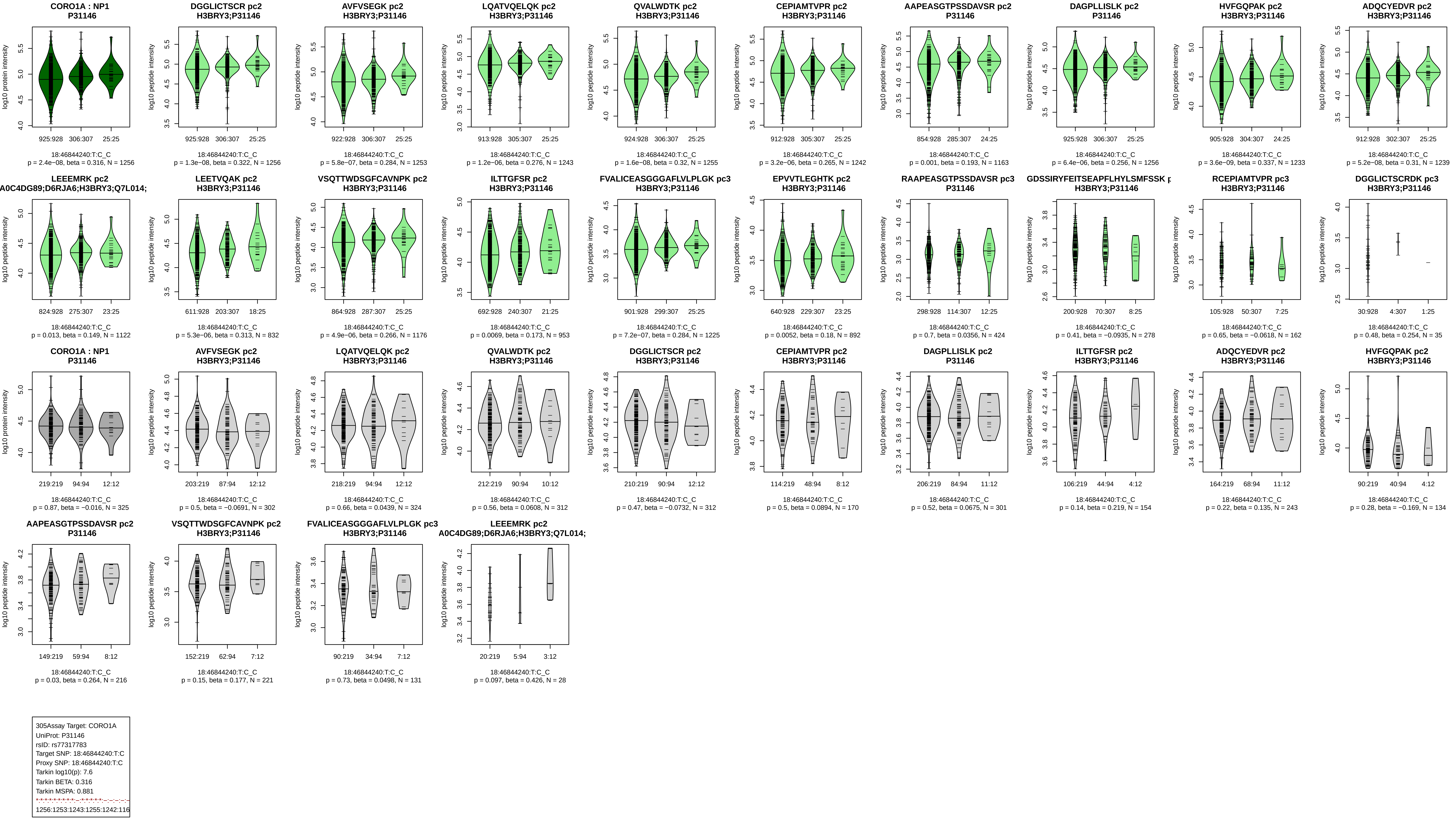
CORO1A : NP1
P31146
log10 protein intensity
925:928 306:307 25:25
18:46844240:T:C_C
p = 2.4e−08, beta = 0.316, N = 1256
DGGLICTSCR pc2
H3BRY3;P31146
log10 peptide intensity
925:928 306:307 25:25
18:46844240:T:C_C
p = 1.3e−08, beta = 0.322, N = 1256
AVFVSEGK pc2
H3BRY3;P31146
922:928 306:307 25:25
18:46844240:T:C_C
p = 5.8e−07, beta = 0.284, N = 1253
LQATVQELQK pc2
H3BRY3;P31146
913:928 305:307 25:25
18:46844240:T:C_C
p = 1.2e−06, beta = 0.276, N = 1243
QVALWDTK pc2
H3BRY3;P31146
924:928 306:307 25:25
18:46844240:T:C_C
p = 1.6e−08, beta = 0.32, N = 1255
CEPIAMTVPR pc2
H3BRY3;P31146
912:928 305:307 25:25
18:46844240:T:C_C
p = 3.2e−06, beta = 0.265, N = 1242
AAPEASGTPSSDAVSR pc2
P31146
854:928 285:307 24:25
18:46844240:T:C_C
p = 0.001, beta = 0.193, N = 1163
DAGPLLISLK pc2
P31146
925:928 306:307 25:25
18:46844240:T:C_C
p = 6.4e−06, beta = 0.256, N = 1256
HVFGQPAK pc2
H3BRY3;P31146
905:928 304:307 24:25
18:46844240:T:C_C
p = 3.6e−09, beta = 0.337, N = 1233
ADQCYEDVR pc2
H3BRY3;P31146
912:928 302:307 25:25
18:46844240:T:C_C
p = 5.2e−08, beta = 0.31, N = 1239
LEEEMRK pc2
A0C4DG89;D6RJA6;H3BRY3;Q7L014;
824:928 275:307 23:25
18:46844240:T:C_C
p = 0.013, beta = 0.149, N = 1122
LEETVQAK pc2
H3BRY3;P31146
611:928 203:307 18:25
18:46844240:T:C_C
p = 5.3e−06, beta = 0.313, N = 832
VSQTTWDSGFCAVNPK pc2
H3BRY3;P31146
864:928 287:307 25:25
18:46844240:T:C_C
p = 4.9e−06, beta = 0.266, N = 1176
ILTTGFSR pc2
H3BRY3;P31146
692:928 240:307 21:25
18:46844240:T:C_C
p = 0.0069, beta = 0.173, N = 953
FVALICEASGGGAFLVLPLGK pc3
H3BRY3;P31146
901:928 299:307 25:25
18:46844240:T:C_C
p = 7.2e−07, beta = 0.284, N = 1225
EPVVTLEGHTK pc2
H3BRY3;P31146
640:928 229:307 23:25
18:46844240:T:C_C
p = 0.0052, beta = 0.18, N = 892
RAAPEASGTPSSDAVSR pc3
P31146
298:928 114:307 12:25
18:46844240:T:C_C
p = 0.7, beta = 0.0356, N = 424
GDSSIRYFEITSEAPFLHYLSMFSSK p
H3BRY3;P31146
200:928 70:307 8:25
18:46844240:T:C_C
p = 0.41, beta = −0.0935, N = 278
RCEPIAMTVPR pc3
H3BRY3;P31146
105:928 50:307 7:25
18:46844240:T:C_C
p = 0.65, beta = −0.0618, N = 162
DGGLICTSCRDK pc3
H3BRY3;P31146
30:928 4:307 1:25
18:46844240:T:C_C
p = 0.48, beta = 0.254, N = 35
CORO1A : NP1
P31146
219:219 94:94 12:12
18:46844240:T:C_C
p = 0.87, beta = −0.016, N = 325
AVFVSEGK pc2
H3BRY3;P31146
203:219 87:94 12:12
18:46844240:T:C_C
p = 0.5, beta = −0.0691, N = 302
LQATVQELQK pc2
H3BRY3;P31146
218:219 94:94 12:12
18:46844240:T:C_C
p = 0.66, beta = 0.0439, N = 324
QVALWDTK pc2
H3BRY3;P31146
212:219 90:94 10:12
18:46844240:T:C_C
p = 0.56, beta = 0.0608, N = 312
DGGLICTSCR pc2
H3BRY3;P31146
210:219 90:94 12:12
18:46844240:T:C_C
p = 0.47, beta = −0.0732, N = 312
CEPIAMTVPR pc2
H3BRY3;P31146
114:219 48:94 8:12
18:46844240:T:C_C
p = 0.5, beta = 0.0894, N = 170
DAGPLLISLK pc2
P31146
206:219 84:94 11:12
18:46844240:T:C_C
p = 0.52, beta = 0.0675, N = 301
ILTTGFSR pc2
H3BRY3;P31146
106:219 44:94 4:12
18:46844240:T:C_C
p = 0.14, beta = 0.219, N = 154
ADQCYEDVR pc2
H3BRY3;P31146
164:219 68:94 11:12
18:46844240:T:C_C
p = 0.22, beta = 0.135, N = 243
HVFGQPAK pc2
H3BRY3;P31146
90:219 40:94 4:12
18:46844240:T:C_C
p = 0.28, beta = −0.169, N = 134
AAPEASGTPSSDAVSR pc2
P31146
149:219 59:94 8:12
18:46844240:T:C_C
p = 0.03, beta = 0.264, N = 216
VSQTTWDSGFCAVNPK pc2
H3BRY3;P31146
152:219 62:94 7:12
18:46844240:T:C_C
p = 0.15, beta = 0.177, N = 221
FVALICEASGGGAFLVLPLGK pc3
H3BRY3;P31146
90:219 34:94 7:12
18:46844240:T:C_C
p = 0.73, beta = 0.0498, N = 131
LEEEMRK pc2
A0C4DG89;D6RJA6;H3BRY3;Q7L014;
20:219 5:94 3:12
18:46844240:T:C_C
p = 0.097, beta = 0.426, N = 28
305Assay Target: CORO1A
UniProt: P31146
rsID: rs77317783
Target SNP: 18:46844240:T:C
Proxy SNP: 18:46844240:T:C
Tarkin log10(p): 7.6
Tarkin BETA: 0.316
Tarkin MSPA: 0.881
1256:1253:1243:1255:1242:116

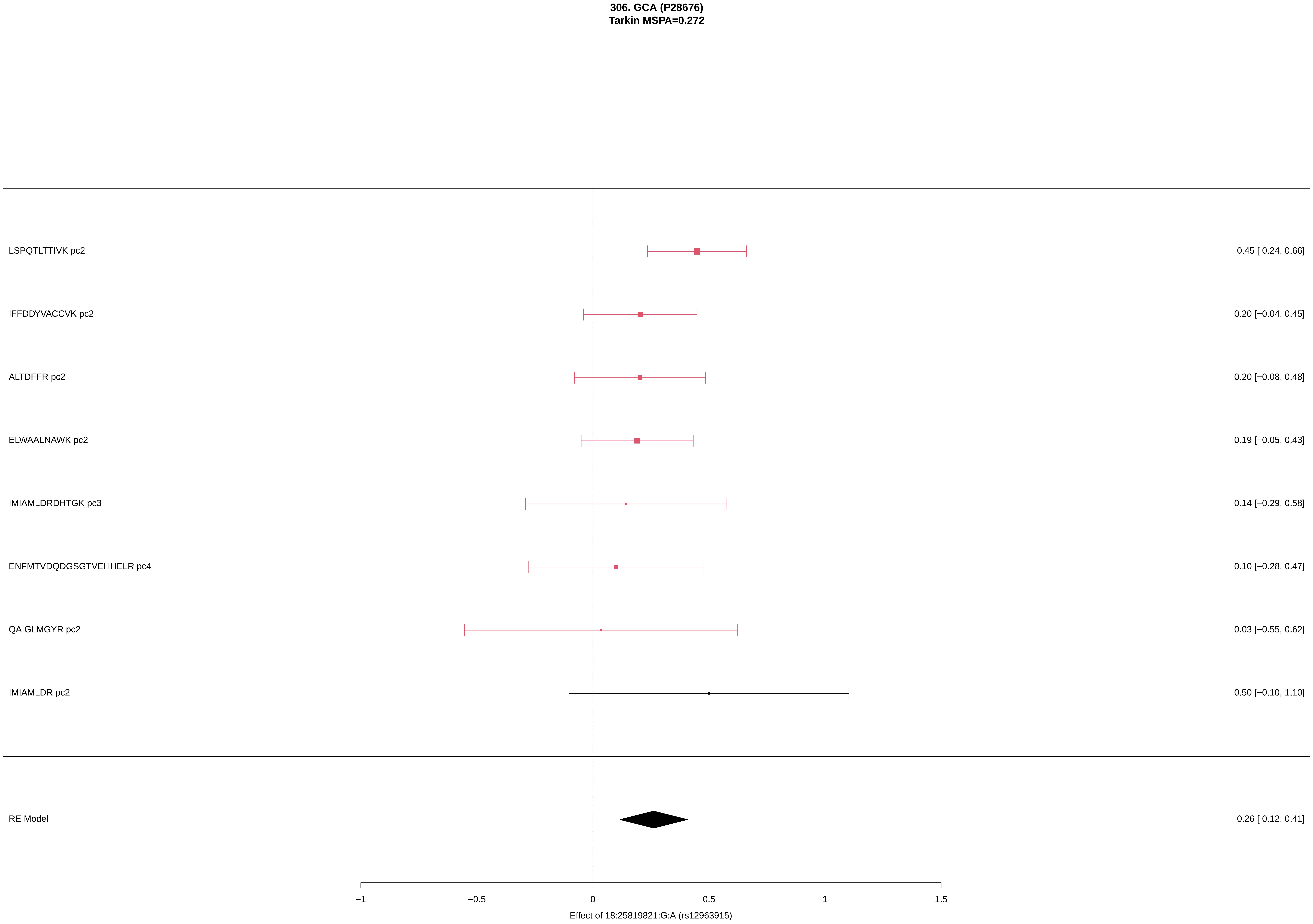
306. GCA (P28676)
Tarkin MSPA=0.272
LSPQTLTTIVK pc2
0.45 [0.24, 0.66]
IFFDDYVACCVK pc2
0.20 [−0.04, 0.45]
ALTDFFR pc2
0.20 [−0.08, 0.48]
ELWAALNAWK pc2
0.19 [−0.05, 0.43]
IMIAMLDRDHTGK pc3
0.14 [−0.29, 0.58]
ENFMTVDQDGSGTVEHHELR pc4
0.10 [−0.28, 0.47]
QAIGLMGYR pc2
0.03 [−0.55, 0.62]
IMIAMLDR pc2
0.50 [−0.10, 1.10]
RE Model
0.26 [0.12, 0.41]
−1
−0.5
0
0.5
1
1.5
Effect of 18:25819821:G:A (rs12963915)

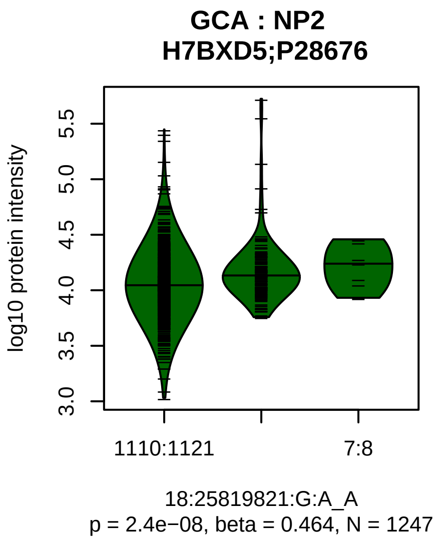

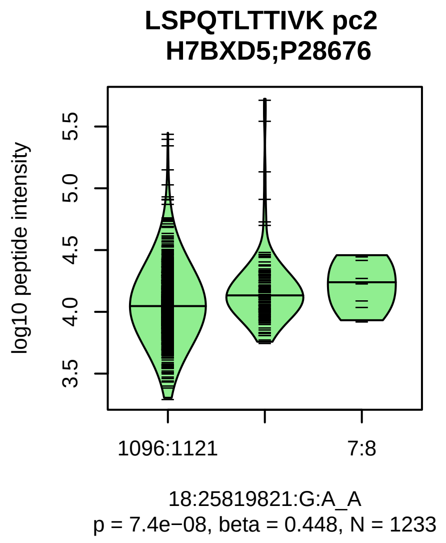

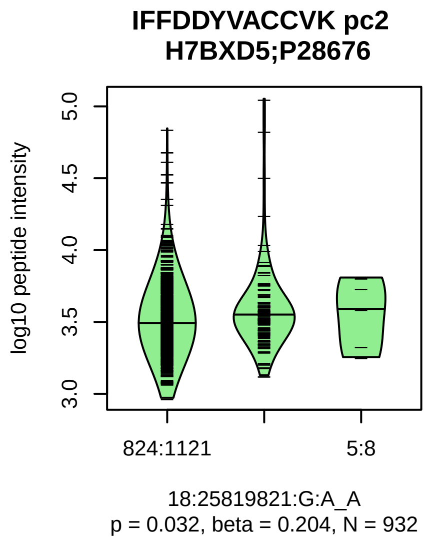

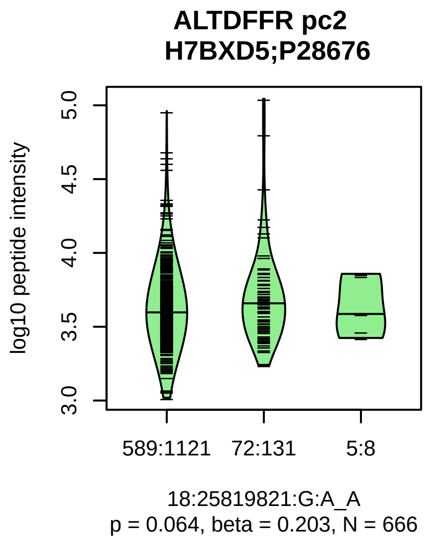

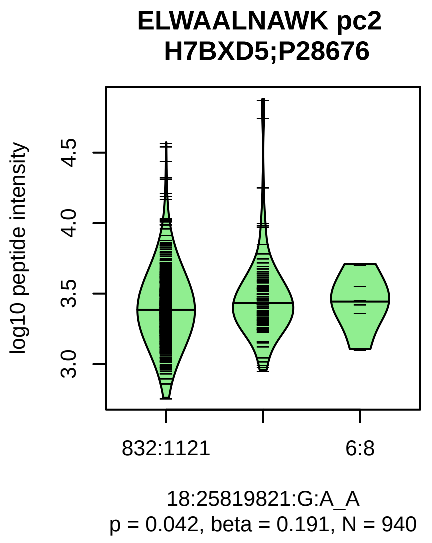

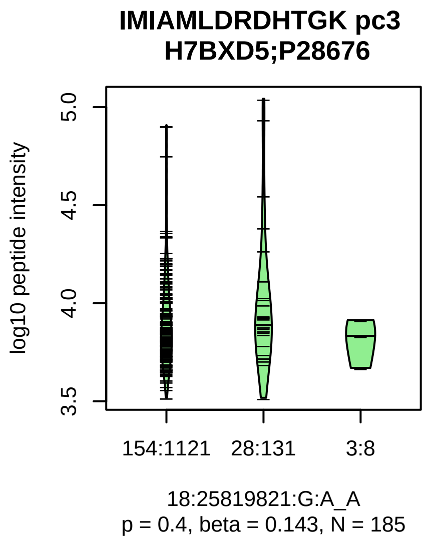

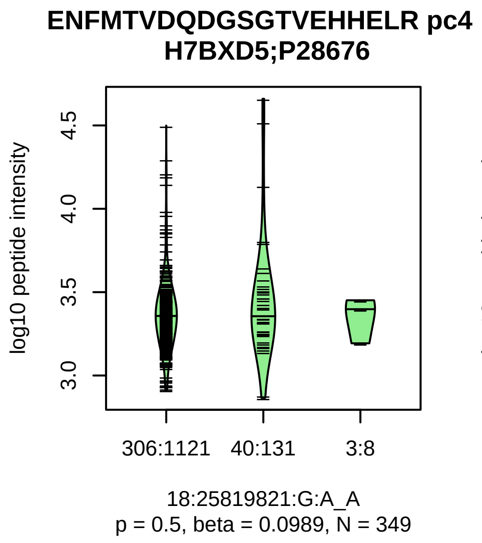

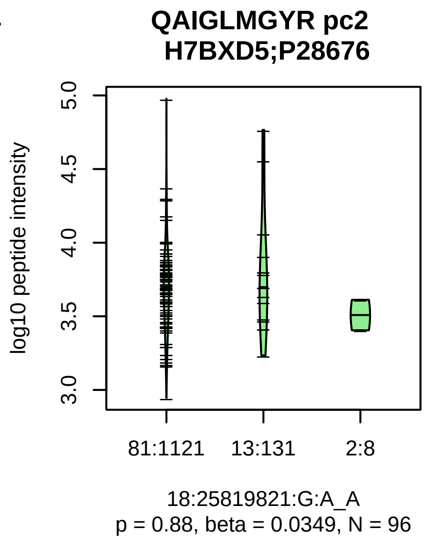

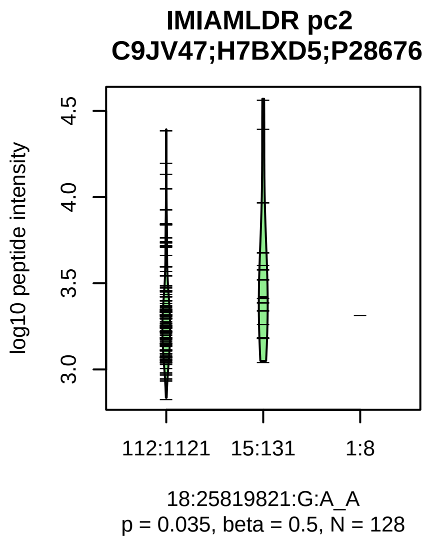

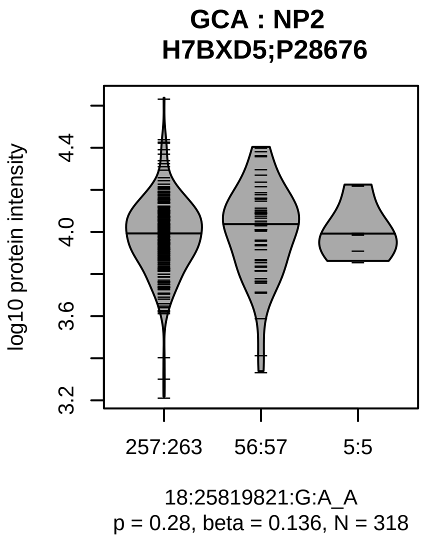

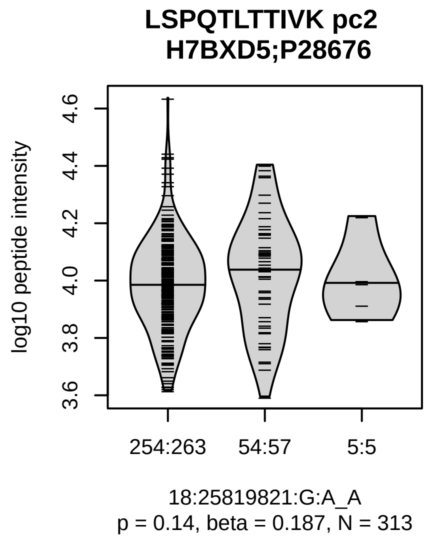

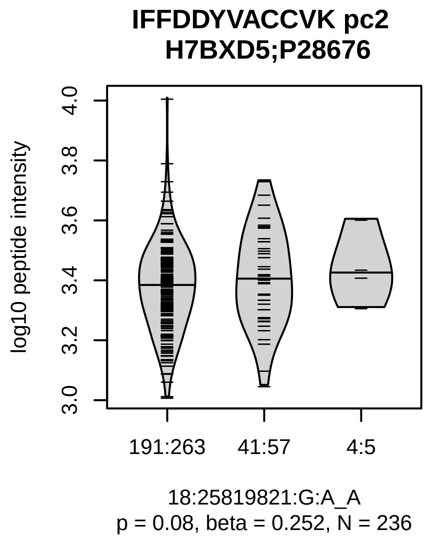

306Assay Target: GCA
UniProt: P28676
rsID: rs12963915
Target SNP: 18:25819821:G:A
Proxy SNP: 18:25819821:G:A
Tarkin log10(p): 7.6
Tarkin BETA: 0.464
Tarkin MSPA: 0.272
*!−:−:−:−:−:−:−
1233:932:666:940:185:349:96:1

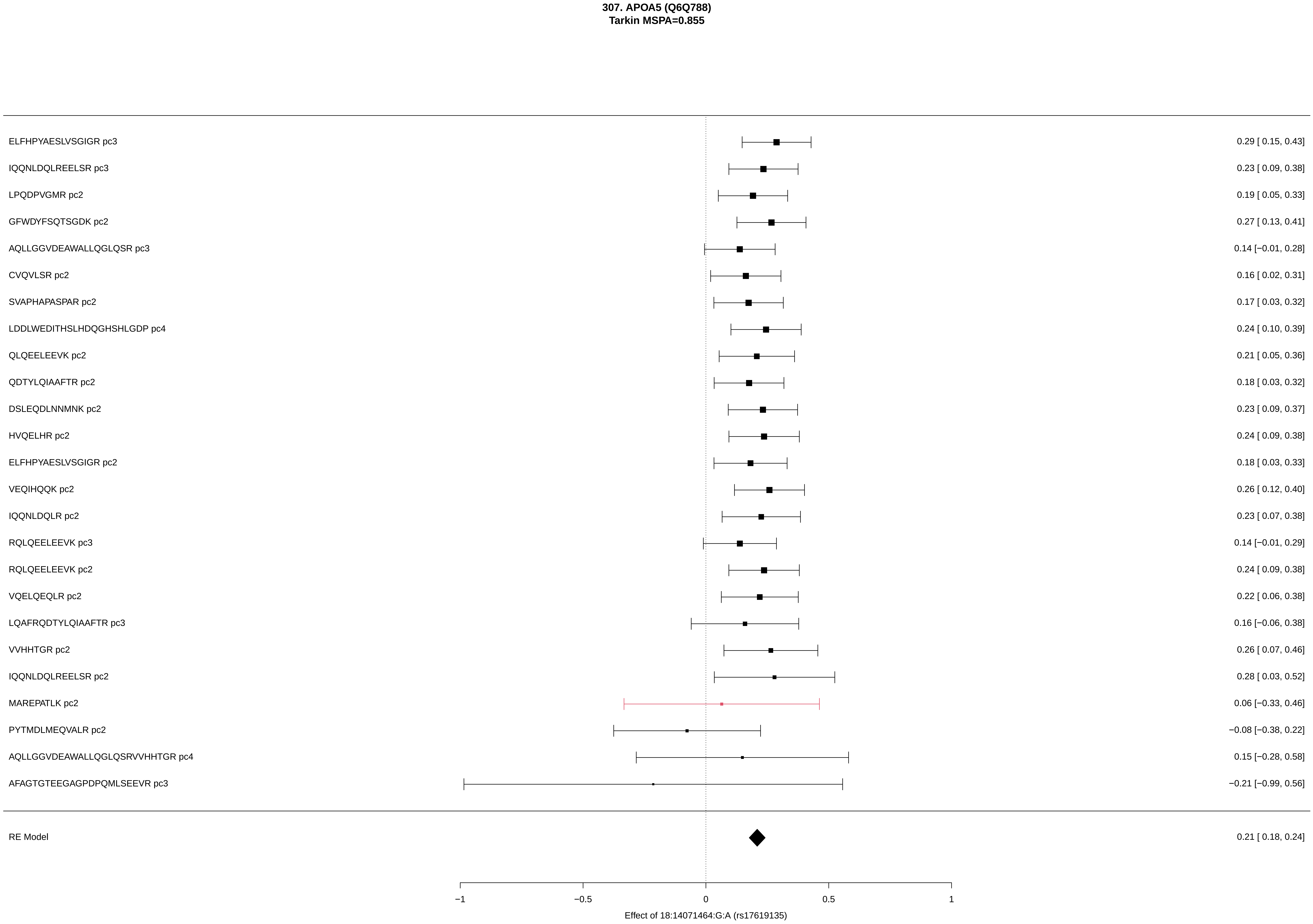

307. APOA5 (Q6Q788)
Tarkin MSPA=0.855
ELFHPYAESLVSGIGR pc3
IQQNLDQLREELSR pc3
LPQDPVGMR pc2
GFWDYFSQTSGDK pc2
AQLLGGVDEAWALLQGLQSR pc3
CVQVLSR pc2
SVAPHAPASPAR pc2
LDDLWEDITHSLHDQGHSHLGDP pc4
QLQEELEEVK pc2
QDTYLQIAAFTR pc2
DSLEQDLNNMNK pc2
HVQELHR pc2
ELFHPYAESLVSGIGR pc2
VEQIHQQK pc2
IQQNLDQLR pc2
RQLQEELEEVK pc3
RQLQEELEEVK pc2
VQELQEQLR pc2
LQAFRQDTYLQIAAFTR pc3
VVHHTGR pc2
IQQNLDQLREELSR pc2
MAREPATLK pc2
PYTMDLMEQVALR pc2
AQLLGGVDEAWALLQGLQSRVVHHTGR pc4
AFAGTGTEEGAGPDPQMLSEEVR pc3
RE Model
0.29 [0.15, 0.43]
0.23 [0.09, 0.38]
0.19 [0.05, 0.33]
0.27 [0.13, 0.41]
0.14 [−0.01, 0.28]
0.16 [0.02, 0.31]
0.17 [0.03, 0.32]
0.24 [0.10, 0.39]
0.21 [0.05, 0.36]
0.18 [0.03, 0.32]
0.23 [0.09, 0.37]
0.24 [0.09, 0.38]
0.18 [0.03, 0.33]
0.26 [0.12, 0.40]
0.23 [0.07, 0.38]
0.14 [−0.01, 0.29]
0.24 [0.09, 0.38]
0.22 [0.06, 0.38]
0.16 [−0.06, 0.38]
0.26 [0.07, 0.46]
0.28 [0.03, 0.52]
0.06 [−0.33, 0.46]
−0.08 [−0.38, 0.22]
0.15 [−0.28, 0.58]
−0.21 [−0.99, 0.56]
0.21 [0.18, 0.24]
−1
−0.5
0
0.5
1
Effect of 18:14071464:G:A (rs17619135)

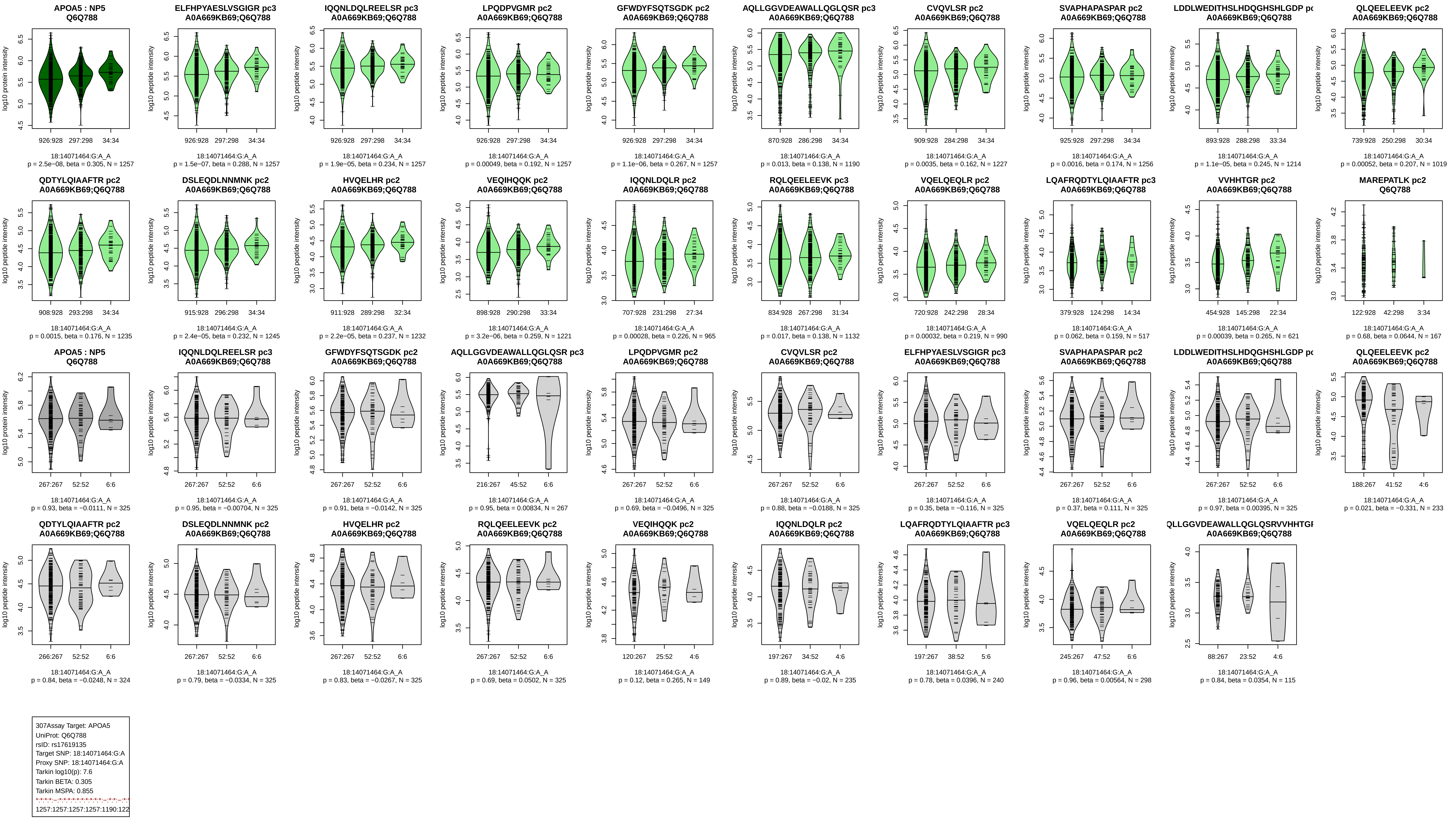
APOA5 : NP5
Q6Q788
926:928 297:298 34:34
18:14071464:G:A_A
p = 2.5e−08, beta = 0.305, N = 1257
ELFHPYAESLVSGIGR pc3
A0A669KB69;Q6Q788
926:928 297:298 34:34
18:14071464:G:A_A
p = 1.5e−07, beta = 0.288, N = 1257
IQQNLDQLREELSR pc3
A0A669KB69;Q6Q788
926:928 297:298 34:34
18:14071464:G:A_A
p = 1.9e−05, beta = 0.234, N = 1257
LPQDPVGMR pc2
A0A669KB69;Q6Q788
926:928 297:298 34:34
18:14071464:G:A_A
p = 0.00049, beta = 0.192, N = 1257
GFWDYFSQTSGDK pc2
A0A669KB69;Q6Q788
926:928 297:298 34:34
18:14071464:G:A_A
p = 1.1e−06, beta = 0.267, N = 1257
AQLLGGVDEAWALLQGLQSR pc3
A0A669KB69;Q6Q788
870:928 286:298 34:34
18:14071464:G:A_A
p = 0.013, beta = 0.138, N = 1190
CVQVLSR pc2
A0A669KB69;Q6Q788
909:928 284:298 34:34
18:14071464:G:A_A
p = 0.0035, beta = 0.162, N = 1227
SVAPHAPASPAR pc2
A0A669KB69;Q6Q788
925:928 297:298 34:34
18:14071464:G:A_A
p = 0.0016, beta = 0.174, N = 1256
LDDLWEDITHSLHDQGHSHLGDP pc
A0A669KB69;Q6Q788
893:928 288:298 33:34
18:14071464:G:A_A
p = 1.1e−05, beta = 0.245, N = 1214
QLQEELEEVK pc2
A0A669KB69;Q6Q788
739:928 250:298 30:34
18:14071464:G:A_A
p = 0.00052, beta = 0.207, N = 1019
QDTYLQIAAFTR pc2
A0A669KB69;Q6Q788
908:928 293:298 34:34
18:14071464:G:A_A
p = 0.0015, beta = 0.176, N = 1235
DSLEQDLNNMNK pc2
A0A669KB69;Q6Q788
915:928 296:298 34:34
18:14071464:G:A_A
p = 2.4e−05, beta = 0.232, N = 1245
HVQELHR pc2
A0A669KB69;Q6Q788
911:928 289:298 32:34
18:14071464:G:A_A
p = 2.2e−05, beta = 0.237, N = 1232
VEQIHQQK pc2
A0A669KB69;Q6Q788
898:928 290:298 33:34
18:14071464:G:A_A
p = 3.2e−06, beta = 0.259, N = 1221
IQQNLDQLR pc2
A0A669KB69;Q6Q788
707:928 231:298 27:34
18:14071464:G:A_A
p = 0.00028, beta = 0.226, N = 965
RQLQEELEEVK pc3
A0A669KB69;Q6Q788
834:928 267:298 31:34
18:14071464:G:A_A
p = 0.017, beta = 0.138, N = 1132
VQELQEQLR pc2
A0A669KB69;Q6Q788
720:928 242:298 28:34
18:14071464:G:A_A
p = 0.00032, beta = 0.219, N = 990
LQAFRQDTYLQIAAFTR pc3
A0A669KB69;Q6Q788
379:928 124:298 14:34
18:14071464:G:A_A
p = 0.062, beta = 0.159, N = 517
VVHHTGR pc2
A0A669KB69;Q6Q788
454:928 145:298 22:34
18:14071464:G:A_A
p = 0.00039, beta = 0.265, N = 621
MAREPATLK pc2
Q6Q788
122:928 42:298 3:34
18:14071464:G:A_A
p = 0.68, beta = 0.0644, N = 167
APOA5 : NP5
Q6Q788
267:267 52:52 6:6
18:14071464:G:A_A
p = 0.93, beta = −0.0111, N = 325
IQQNLDQLREELSR pc3
A0A669KB69;Q6Q788
267:267 52:52 6:6
18:14071464:G:A_A
p = 0.95, beta = −0.00704, N = 325
GFWDYFSQTSGDK pc2
A0A669KB69;Q6Q788
267:267 52:52 6:6
18:14071464:G:A_A
p = 0.91, beta = −0.0142, N = 325
AQLLGGVDEAWALLQGLQSR pc3
A0A669KB69;Q6Q788
216:267 45:52 6:6
18:14071464:G:A_A
p = 0.95, beta = 0.00834, N = 267
LPQDPVGMR pc2
A0A669KB69;Q6Q788
267:267 52:52 6:6
18:14071464:G:A_A
p = 0.69, beta = −0.0496, N = 325
CVQVLSR pc2
A0A669KB69;Q6Q788
267:267 52:52 6:6
18:14071464:G:A_A
p = 0.88, beta = −0.0188, N = 325
ELFHPYAESLVSGIGR pc3
A0A669KB69;Q6Q788
267:267 52:52 6:6
18:14071464:G:A_A
p = 0.35, beta = −0.116, N = 325
SVAPHAPASPAR pc2
A0A669KB69;Q6Q788
267:267 52:52 6:6
18:14071464:G:A_A
p = 0.37, beta = 0.111, N = 325
LDDLWEDITHSLHDQGHSHLGDP pc
A0A669KB69;Q6Q788
267:267 52:52 6:6
18:14071464:G:A_A
p = 0.97, beta = 0.00395, N = 325
QLQEELEEVK pc2
A0A669KB69;Q6Q788
188:267 41:52 4:6
18:14071464:G:A_A
p = 0.021, beta = −0.331, N = 233
QDTYLQIAAFTR pc2
A0A669KB69;Q6Q788
266:267 52:52 6:6
18:14071464:G:A_A
p = 0.84, beta = −0.0248, N = 324
DSLEQDLNNMNK pc2
A0A669KB69;Q6Q788
267:267 52:52 6:6
18:14071464:G:A_A
p = 0.79, beta = −0.0334, N = 325
HVQELHR pc2
A0A669KB69;Q6Q788
267:267 52:52 6:6
18:14071464:G:A_A
p = 0.83, beta = −0.0267, N = 325
RQLQEELEEVK pc2
A0A669KB69;Q6Q788
267:267 52:52 6:6
18:14071464:G:A_A
p = 0.69, beta = 0.0502, N = 325
VEQIHQQK pc2
A0A669KB69;Q6Q788
120:267 25:52 4:6
18:14071464:G:A_A
p = 0.12, beta = 0.265, N = 149
IQQNLDQLR pc2
A0A669KB69;Q6Q788
197:267 34:52 4:6
18:14071464:G:A_A
p = 0.89, beta = −0.02, N = 235
LQAFRQDTYLQIAAFTR pc3
A0A669KB69;Q6Q788
197:267 38:52 5:6
18:14071464:G:A_A
p = 0.78, beta = 0.0396, N = 240
VQELQEQLR pc2
A0A669KB69;Q6Q788
245:267 47:52 6:6
18:14071464:G:A_A
p = 0.96, beta = 0.00564, N = 298
QLLGGVDEAWALLQGLQSRVVHHTGF
A0A669KB69;Q6Q788
88:267 23:52 4:6
18:14071464:G:A_A
p = 0.84, beta = 0.0354, N = 115
307Assay Target: APOA5
UniProt: Q6Q788
rsID: rs17619135
Target SNP: 18:14071464:G:A
Proxy SNP: 18:14071464:G:A
Tarkin log10(p): 7.6
Tarkin BETA: 0.305
Tarkin MSPA: 0.855
1257:1257:1257:1257:1190:122

308. CSNK2B (Q5SRQ6)
Tarkin MSPA=0.815

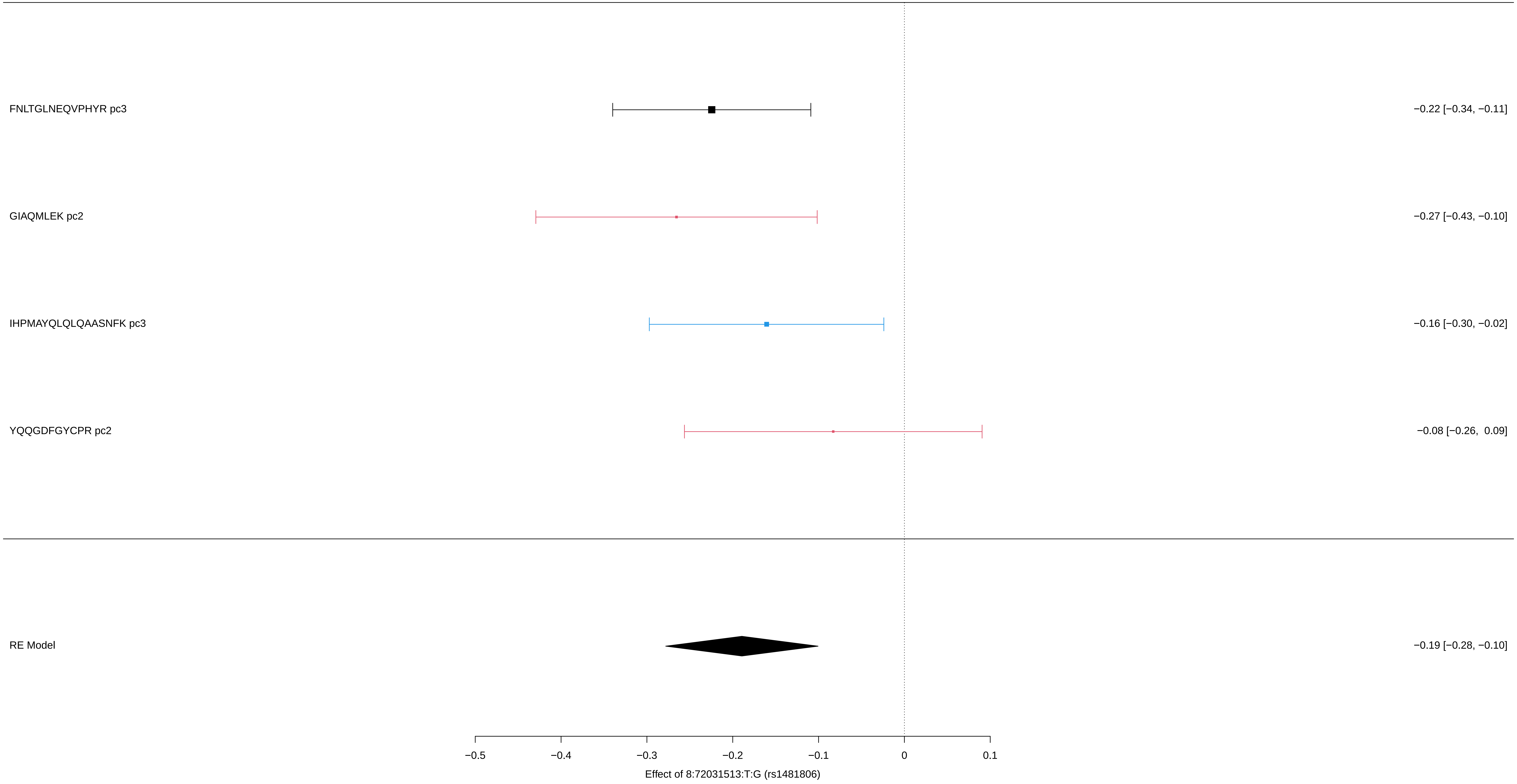

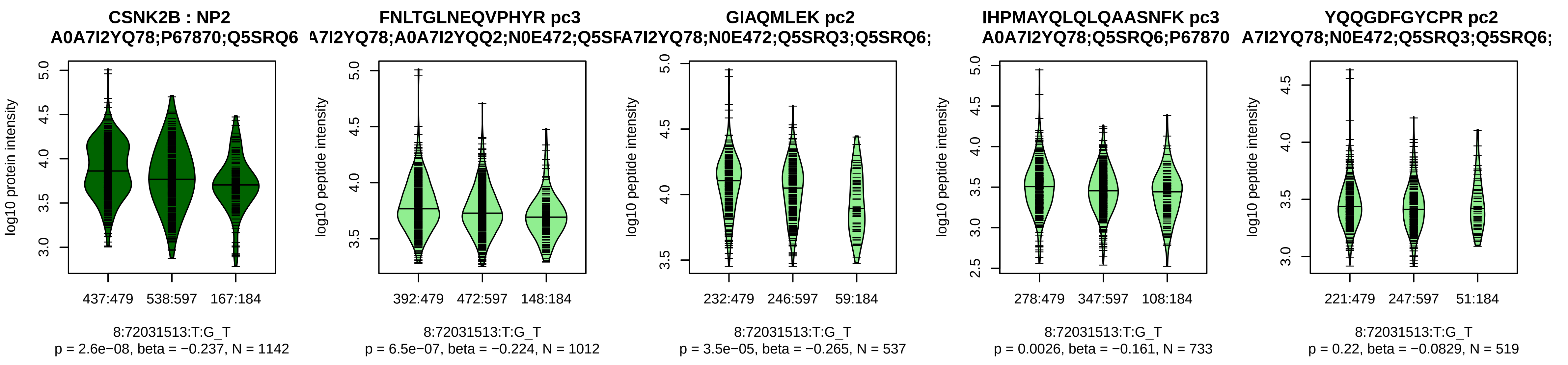

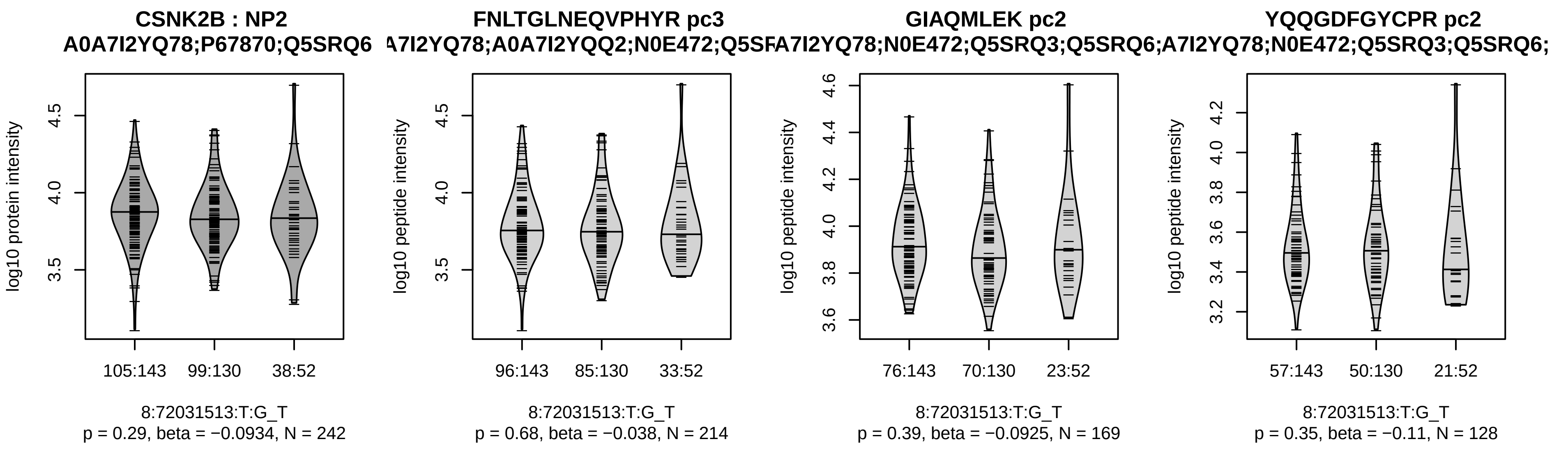

308Assay Target: CSNK2B
UniProt: Q5SRQ6
rsID: rs1481806
Target SNP: 8:72031513:T:G
Proxy SNP: 8:72031513:T:G
Tarkin log10(p): 7.6
Tarkin BETA: −0.237
Tarkin MSPA: 0.815
::*:−:NA
1012:537:733:519:9

309. C2 (P06681)
Tarkin MSPA=0.79

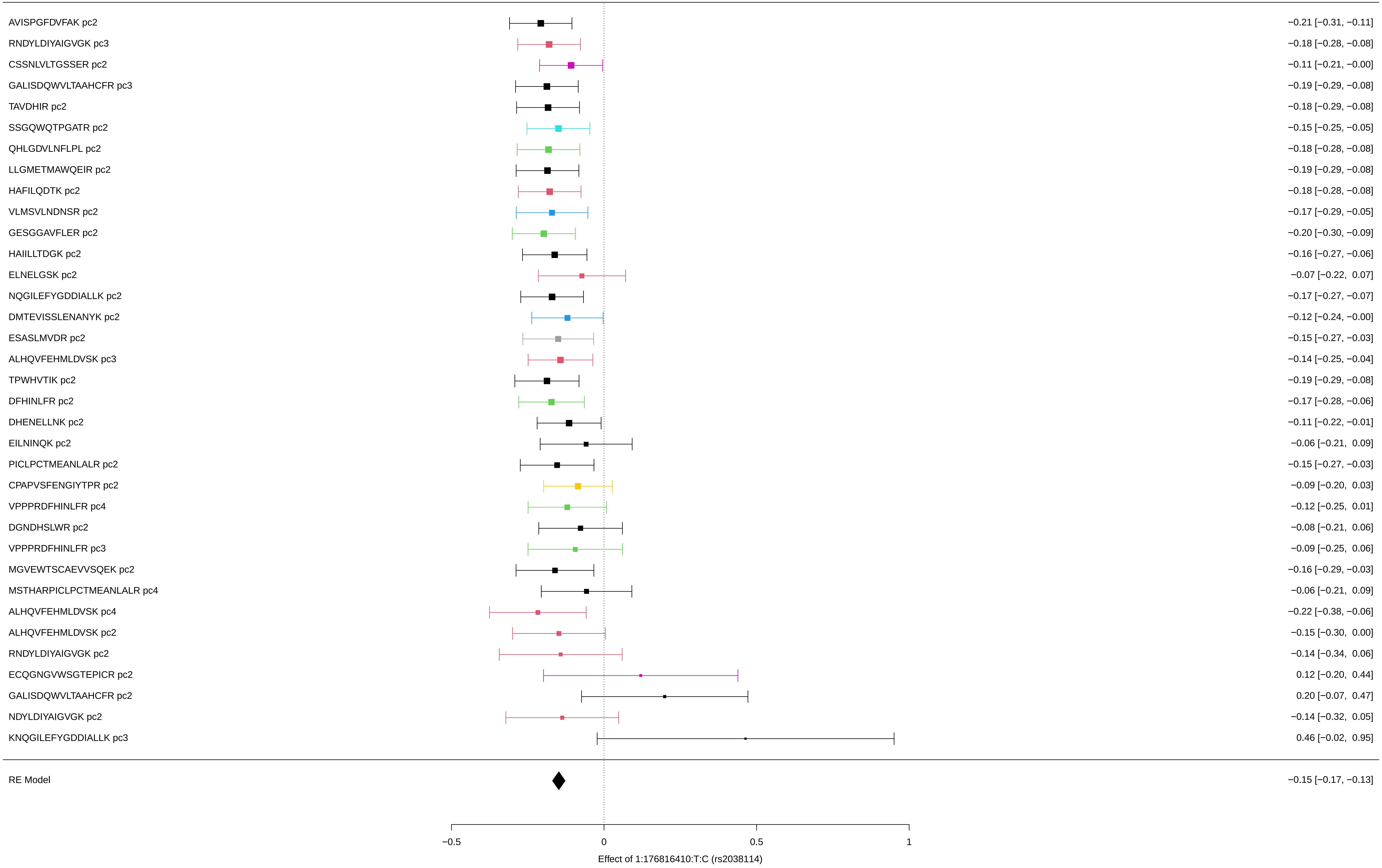

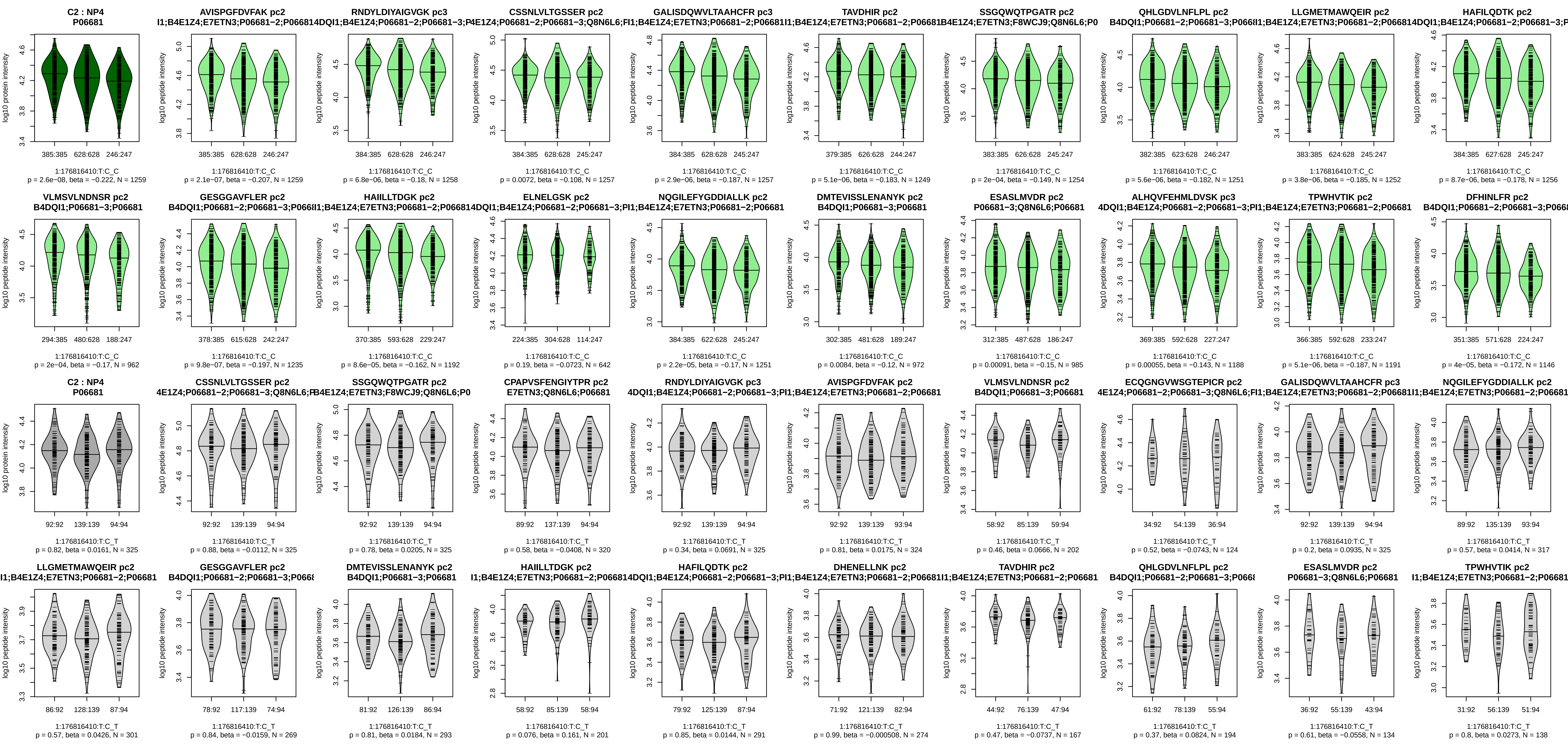

309Assay Target: C2
UniProt: P06681
rsID: rs2038114
Target SNP: 1:176816410:T:C
Proxy SNP: 1:176816410:T:C
Tarkin log10(p): 7.6
Tarkin BETA: −0.222
Tarkin MSPA: 0.79
1259:1258:1257:1257:1249:125

310. APOE (P02649)
Tarkin MSPA=0.53

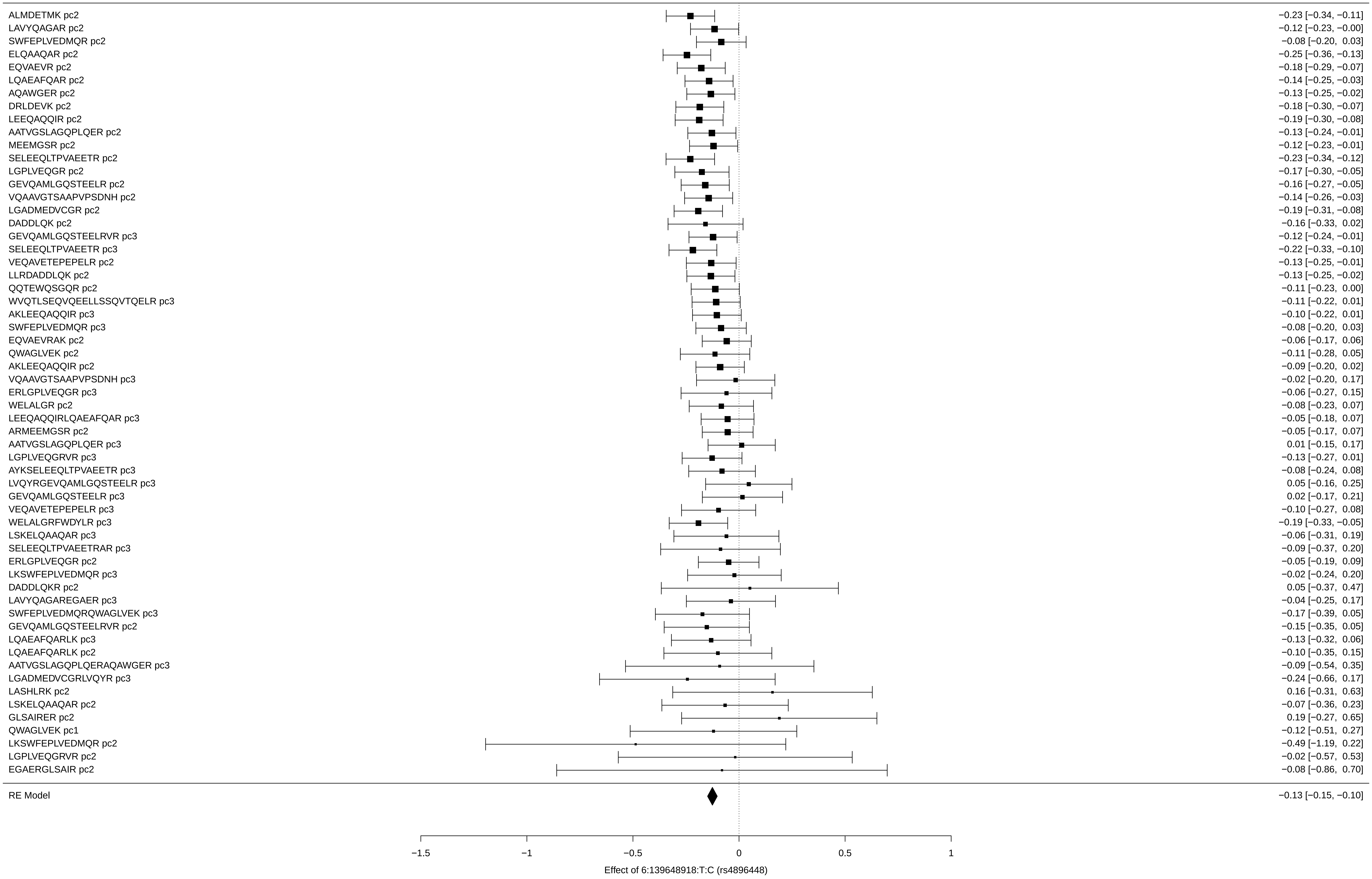

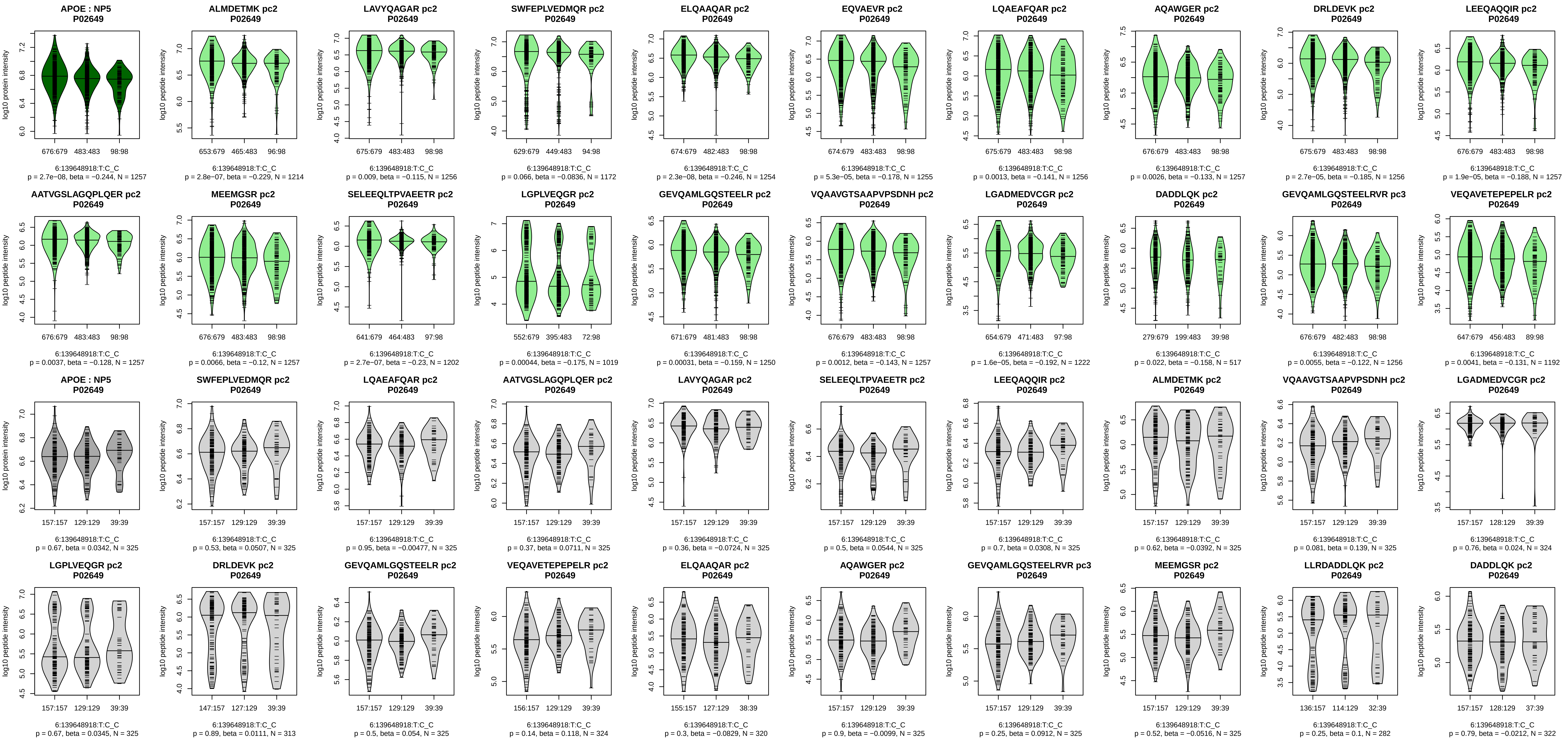

310Assay Target: APOE
UniProt: P02649
rsID: rs4896448
Target SNP: 6:139648918:T:C
Proxy SNP: 6:139648918:T:C
Tarkin log10(p): 7.6
Tarkin BETA: −0.244
Tarkin MSPA: 0.53
[illegible]
1214:1256:1172:1254:1255:125

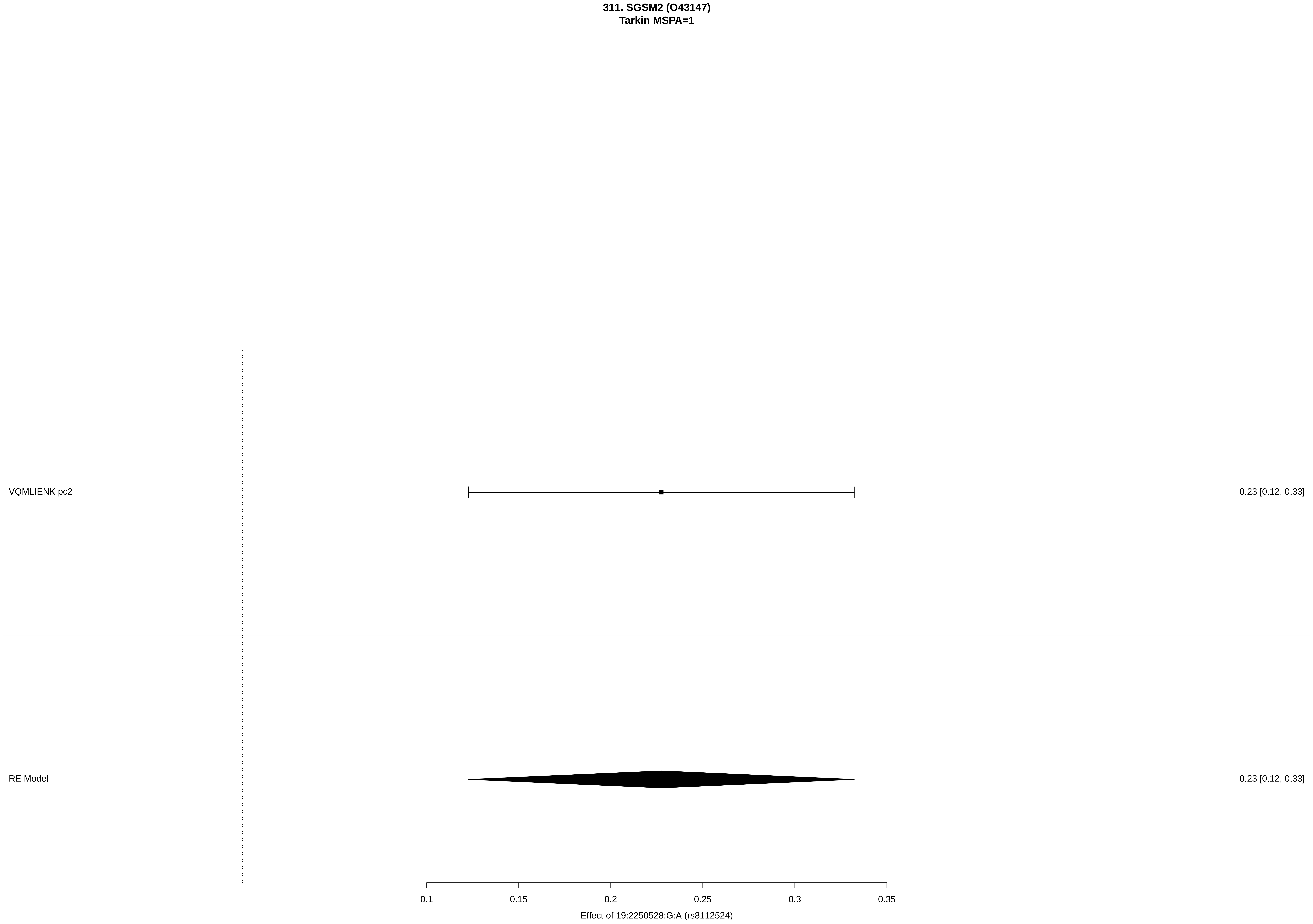
311. SGSM2 (O43147)
Tarkin MSPA=1
VQMLIENK pc2
0.23 [0.12, 0.33]
RE Model
0.23 [0.12, 0.33]
0.1
0.15
0.2
0.25
0.3
0.35
Effect of 19:2250528:G:A (rs8112524)

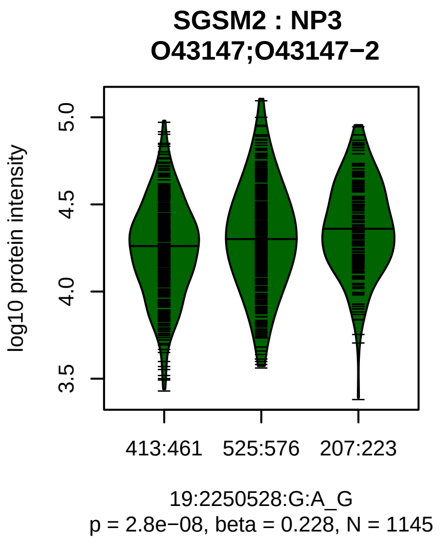

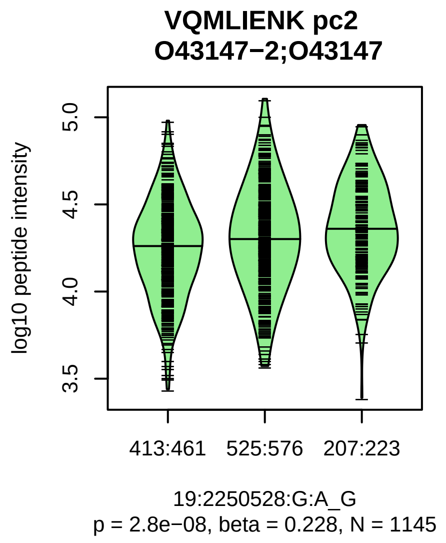

311Assay Target: SGSM2
UniProt: O43147
rsID: rs8112524
Target SNP: 19:2250528:G:A
Proxy SNP: 19:2250528:G:A
Tarkin log10(p): 7.6
Tarkin BETA: 0.228
Tarkin MSPA: 1
*
1145

312. DNAJB11 (Q9UBS4)
Tarkin MSPA=0.072

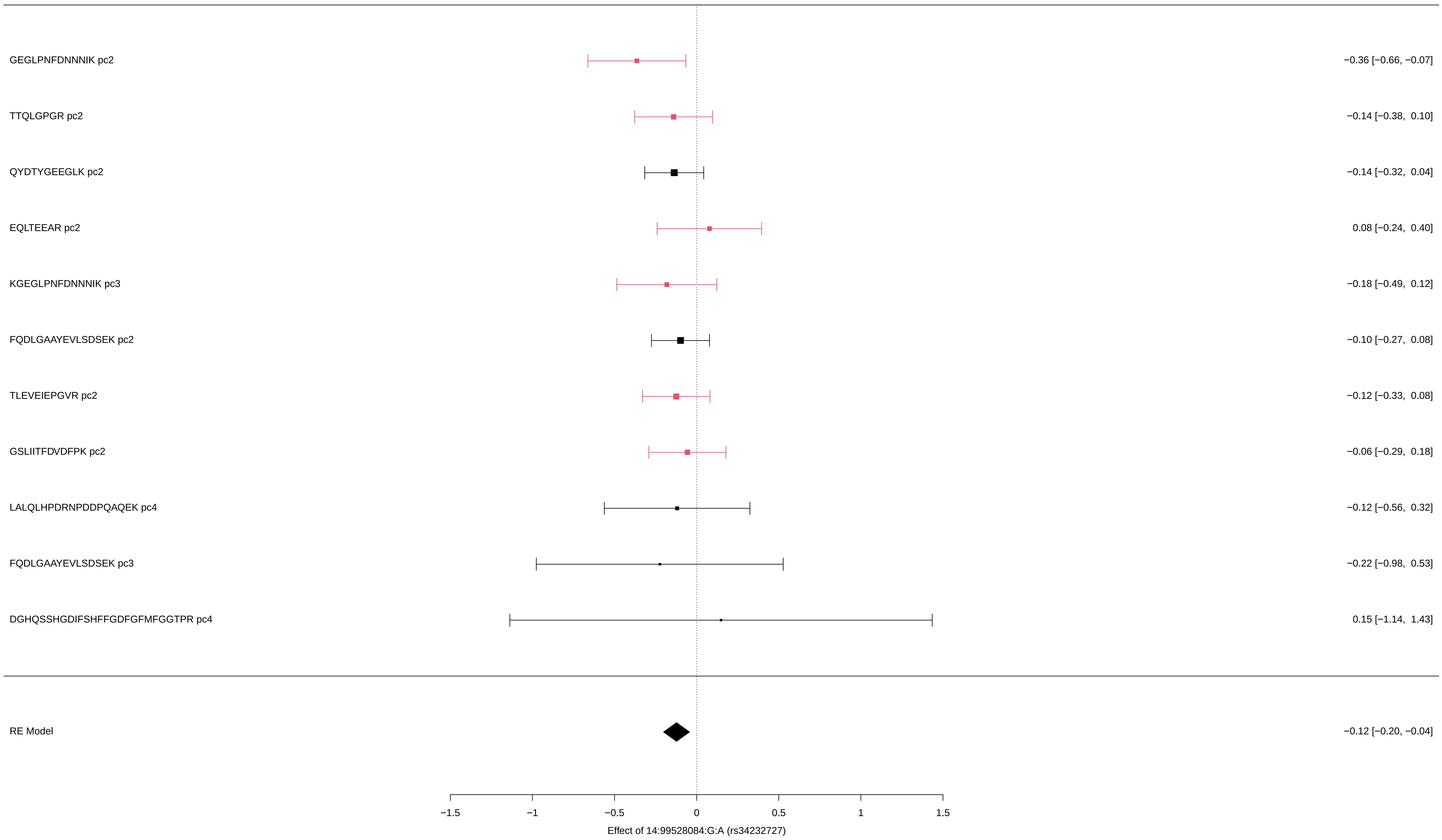

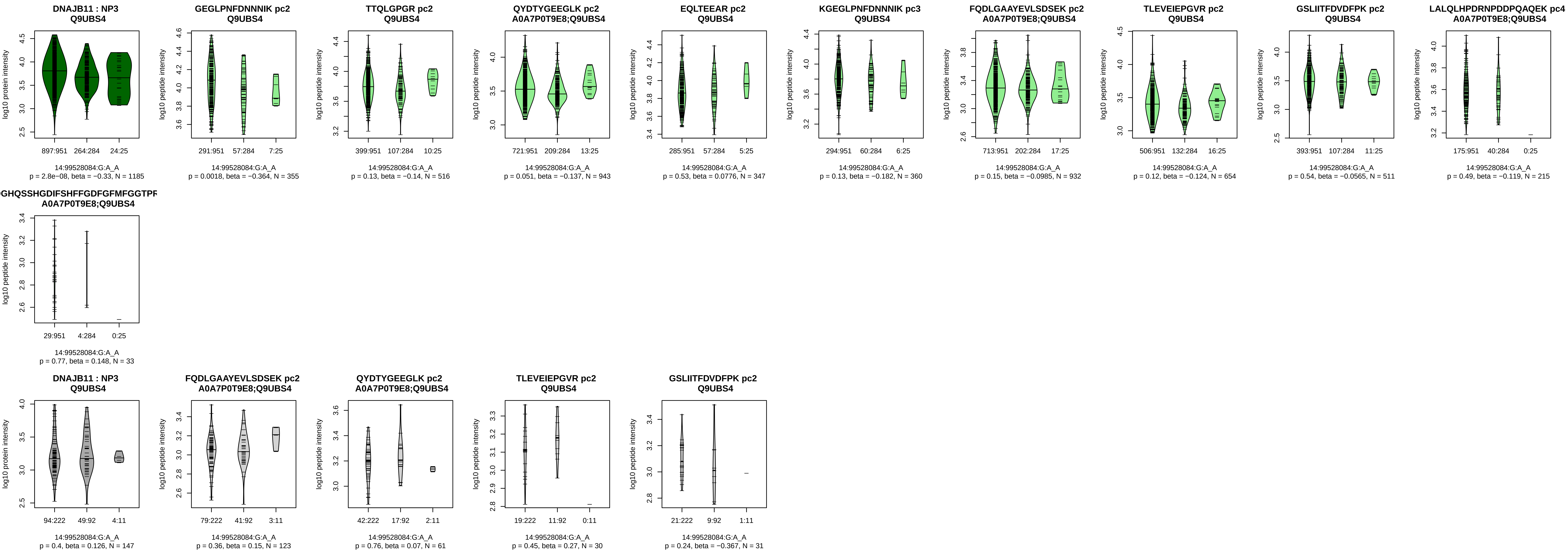

312Assay Target: DNAJB11
UniProt: Q9UBS4
rsID: rs34232727
Target SNP: 14:99528084:G:A
Proxy SNP: 14:99528084:G:A
Tarkin log10(p): 7.6
Tarkin BETA: −0.33
Tarkin MSPA: 0.072
*:−:-:-:-:-:-:-:-:-:-:NA:NA
355:516:943:347:360:932:654:5

313. NID1 (P14543)
Tarkin MSPA=0.673

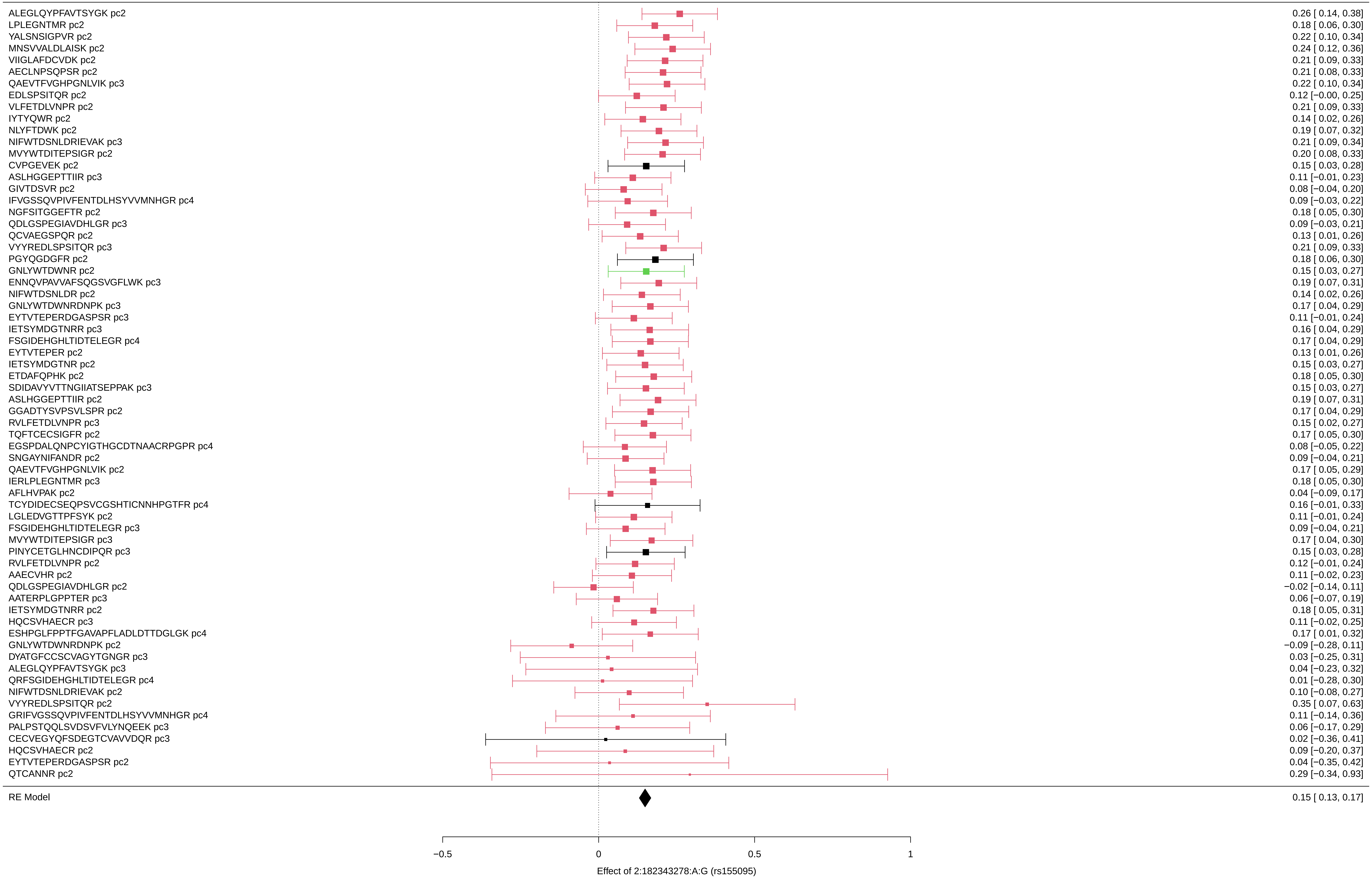

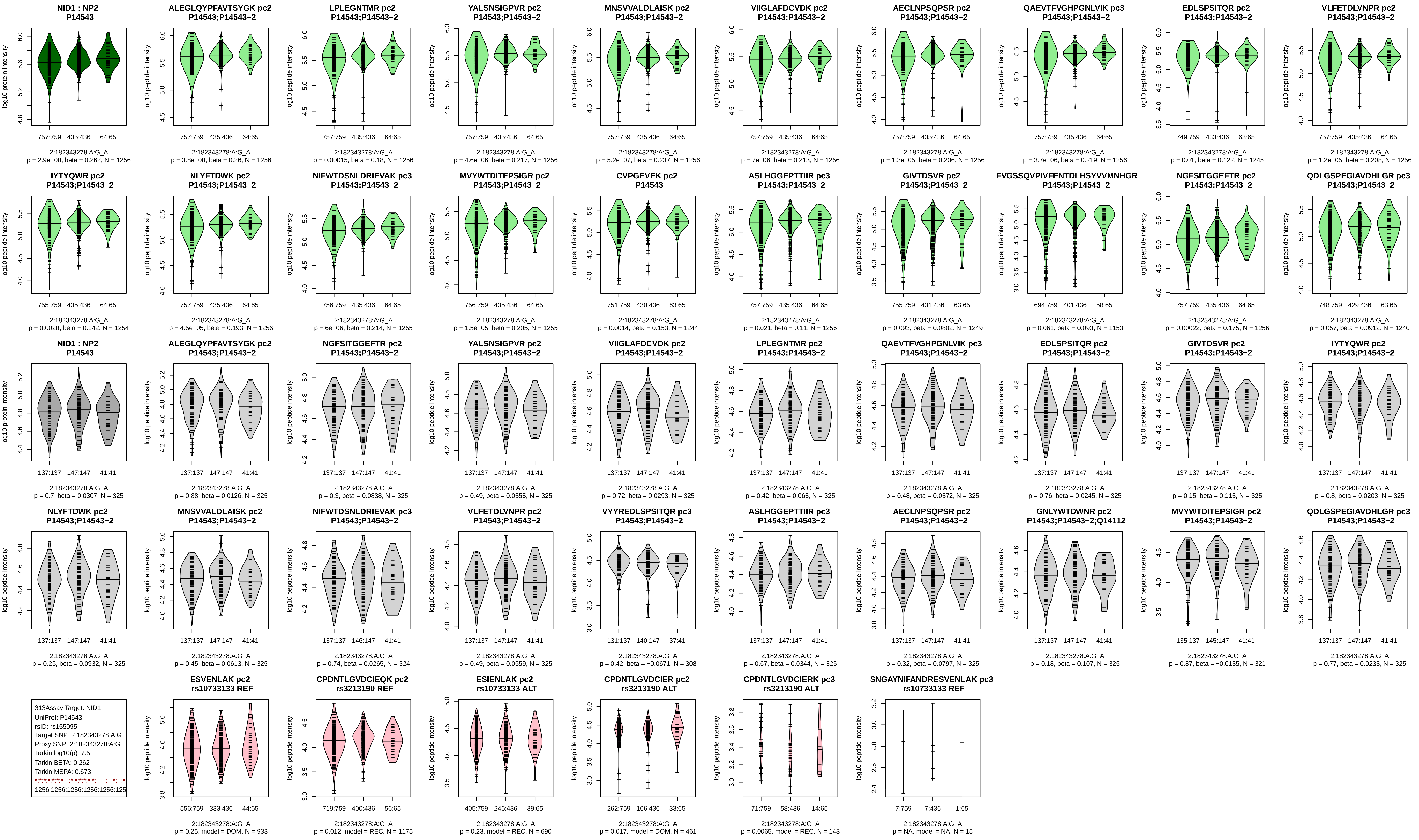
NID1 : NP2 P14543
757:759 435:436 64:65
2:182343278:A:G_A
p = 2.9e−08, beta = 0.262, N = 1256
ALEGLQYPFAVTSYGK pc2 P14543;P14543−2
p = 3.8e−08, beta = 0.26, N = 1256
LPLEGNTMR pc2 P14543;P14543−2
p = 0.00015, beta = 0.18, N = 1256
YALSNSIGPVR pc2 P14543;P14543−2
p = 4.6e−06, beta = 0.217, N = 1256
MNSVVALDLAISK pc2 P14543;P14543−2
p = 5.2e−07, beta = 0.237, N = 1256
VIIGLAFDCVDK pc2 P14543;P14543−2
p = 7e−06, beta = 0.213, N = 1256
AECLNPSQPSR pc2 P14543;P14543−2
p = 1.3e−05, beta = 0.206, N = 1256
QAEVTFVGHPGNLVIK pc3 P14543;P14543−2
p = 3.7e−06, beta = 0.219, N = 1256
EDLSPSITQR pc2 P14543;P14543−2
p = 0.01, beta = 0.122, N = 1245
VLFETDLVNPR pc2 P14543;P14543−2
p = 1.2e−05, beta = 0.208, N = 1256
IYTYQWR pc2 P14543;P14543−2
p = 0.0028, beta = 0.142, N = 1254
NLYFTDWK pc2 P14543;P14543−2
p = 4.5e−05, beta = 0.193, N = 1256
NIFWTDSNLDRIEVAK pc3 P14543;P14543−2
p = 6e−06, beta = 0.214, N = 1255
MVYWTDITEPSIGR pc2 P14543;P14543−2
p = 1.5e−05, beta = 0.205, N = 1255
CVPGEVEK pc2 P14543
p = 0.0014, beta = 0.153, N = 1244
ASLHGGEPTTIIR pc3 P14543;P14543−2
p = 0.021, beta = 0.11, N = 1256
GIVTDSVR pc2 P14543;P14543−2
p = 0.093, beta = 0.0802, N = 1249
FVGSSQVPIVFENTDLHSYVVMNHGR P14543;P14543−2
p = 0.061, beta = 0.093, N = 1153
NGFSITGGEFTR pc2 P14543;P14543−2
p = 0.00022, beta = 0.175, N = 1256
QDLGSPEGIAVDHLGR pc3 P14543;P14543−2
p = 0.057, beta = 0.0912, N = 1240
NID1 : NP2 P14543
p = 0.7, beta = 0.0307, N = 325
ALEGLQYPFAVTSYGK pc2 P14543;P14543−2
p = 0.88, beta = 0.0126, N = 325
NGFSITGGEFTR pc2 P14543;P14543−2
p = 0.3, beta = 0.0838, N = 325
YALSNSIGPVR pc2 P14543;P14543−2
p = 0.49, beta = 0.0555, N = 325
VIIGLAFDCVDK pc2 P14543;P14543−2
p = 0.72, beta = 0.0293, N = 325
LPLEGNTMR pc2 P14543;P14543−2
p = 0.42, beta = 0.065, N = 325
QAEVTFVGHPGNLVIK pc3 P14543;P14543−2
p = 0.48, beta = 0.0572, N = 325
EDLSPSITQR pc2 P14543;P14543−2
p = 0.76, beta = 0.0245, N = 325
GIVTDSVR pc2 P14543;P14543−2
p = 0.15, beta = 0.115, N = 325
IYTYQWR pc2 P14543;P14543−2
p = 0.8, beta = 0.0203, N = 325
NLYFTDWK pc2 P14543;P14543−2
p = 0.25, beta = 0.0932, N = 325
MNSVVALDLAISK pc2 P14543;P14543−2
p = 0.45, beta = 0.0613, N = 325
NIFWTDSNLDRIEVAK pc3 P14543;P14543−2
p = 0.74, beta = 0.0265, N = 324
VLFETDLVNPR pc2 P14543;P14543−2
p = 0.49, beta = 0.0559, N = 325
VYYREDLSPSITQR pc3 P14543;P14543−2
p = 0.42, beta = −0.0671, N = 308
ASLHGGEPTTIIR pc3 P14543;P14543−2
p = 0.67, beta = 0.0344, N = 325
AECLNPSQPSR pc2 P14543;P14543−2
p = 0.32, beta = 0.0797, N = 325
GNLYWTDWNR pc2 P14543;P14543−2;Q14112
p = 0.18, beta = 0.107, N = 325
MVYWTDITEPSIGR pc2 P14543;P14543−2
p = 0.87, beta = −0.0135, N = 321
QDLGSPEGIAVDHLGR pc3 P14543;P14543−2
p = 0.77, beta = 0.0233, N = 325
313Assay Target: NID1
UniProt: P14543
rsID: rs155095
Target SNP: 2:182343278:A:G
Proxy SNP: 2:182343278:A:G
Tarkin log10(p): 7.5
Tarkin BETA: 0.262
Tarkin MSPA: 0.673
1256:1256:1256:1256:1256:125
ESVENLAK pc2 rs10733133 REF
p = 0.25, model = DOM, N = 933
CPDNTLGVDCIEQK pc2 rs3213190 REF
p = 0.012, model = REC, N = 1175
ESIENLAK pc2 rs10733133 ALT
p = 0.23, model = REC, N = 690
CPDNTLGVDCIER pc2 rs3213190 ALT
p = 0.017, model = DOM, N = 461
CPDNTLGVDCIERK pc3 rs3213190 ALT
p = 0.0065, model = REC, N = 143
SNGAYNIFANDRESVENLAK pc3 rs10733133 REF
p = NA, model = NA, N = 15

314. YWHAE (P62258)
Tarkin MSPA=0.695

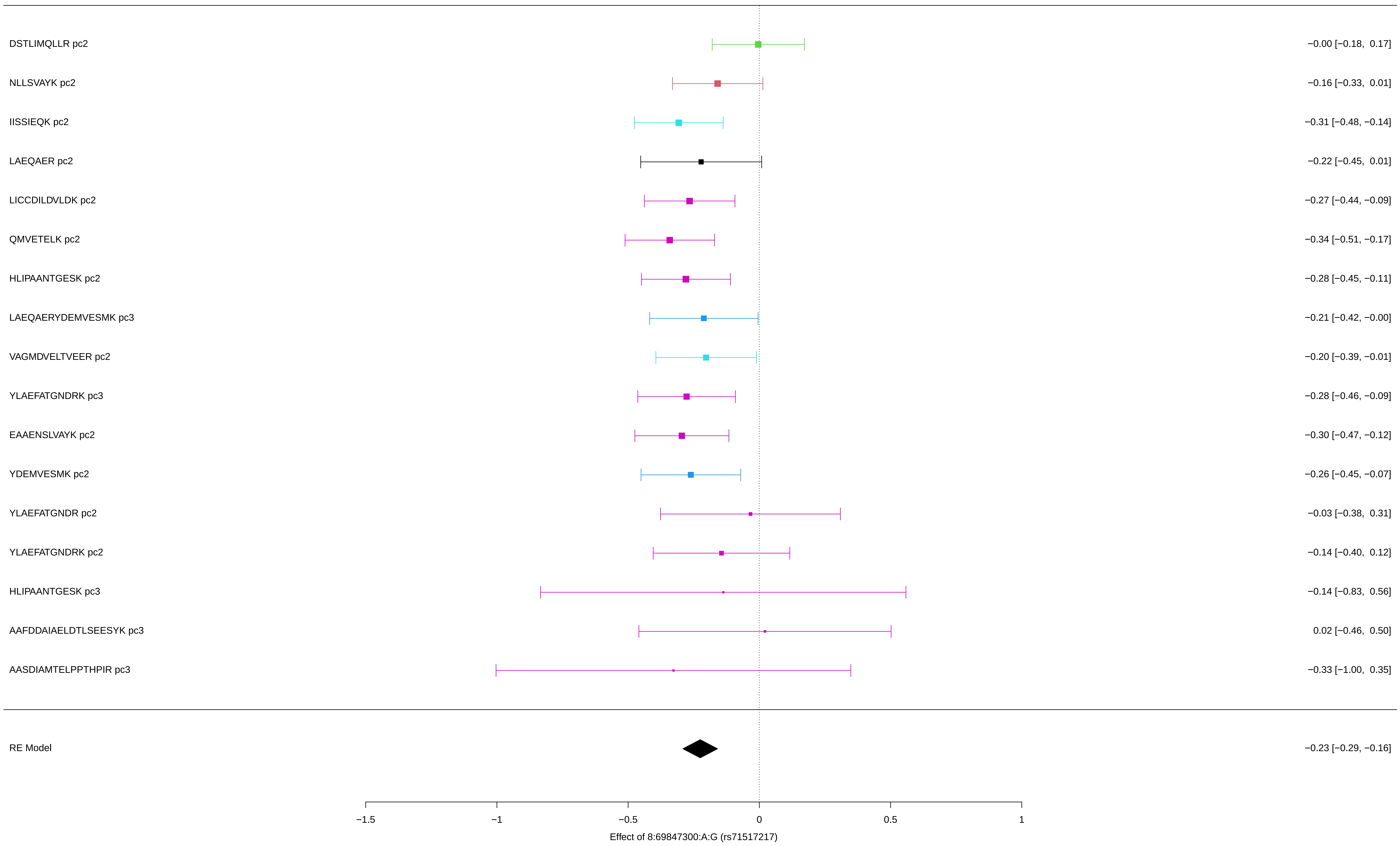

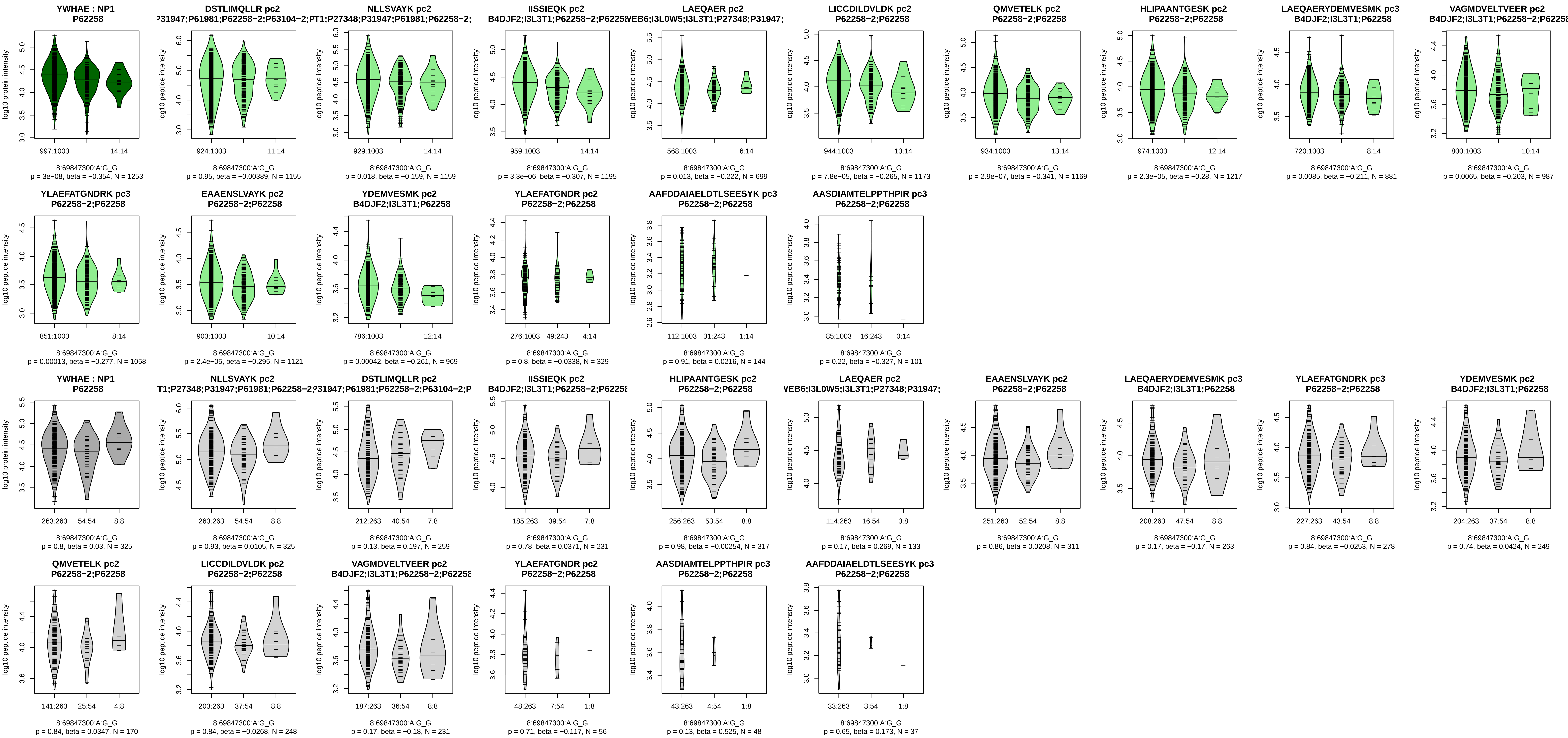

314Assay Target: YWHAE
UniProt: P62258
rsID: rs71517217
Target SNP: 8:69847300:A:G
Proxy SNP: 8:69847300:A:G
Tarkin log10(p): 7.5
Tarkin BETA: −0.354
Tarkin MSPA: 0.695
−:−:*:−:*:*:*:*:*:*:*:*:−:−:−:−:−:NA
1155:1159:1195:699:1173:1169

315. APOB (A8MUN2)
Tarkin MSPA=0.87

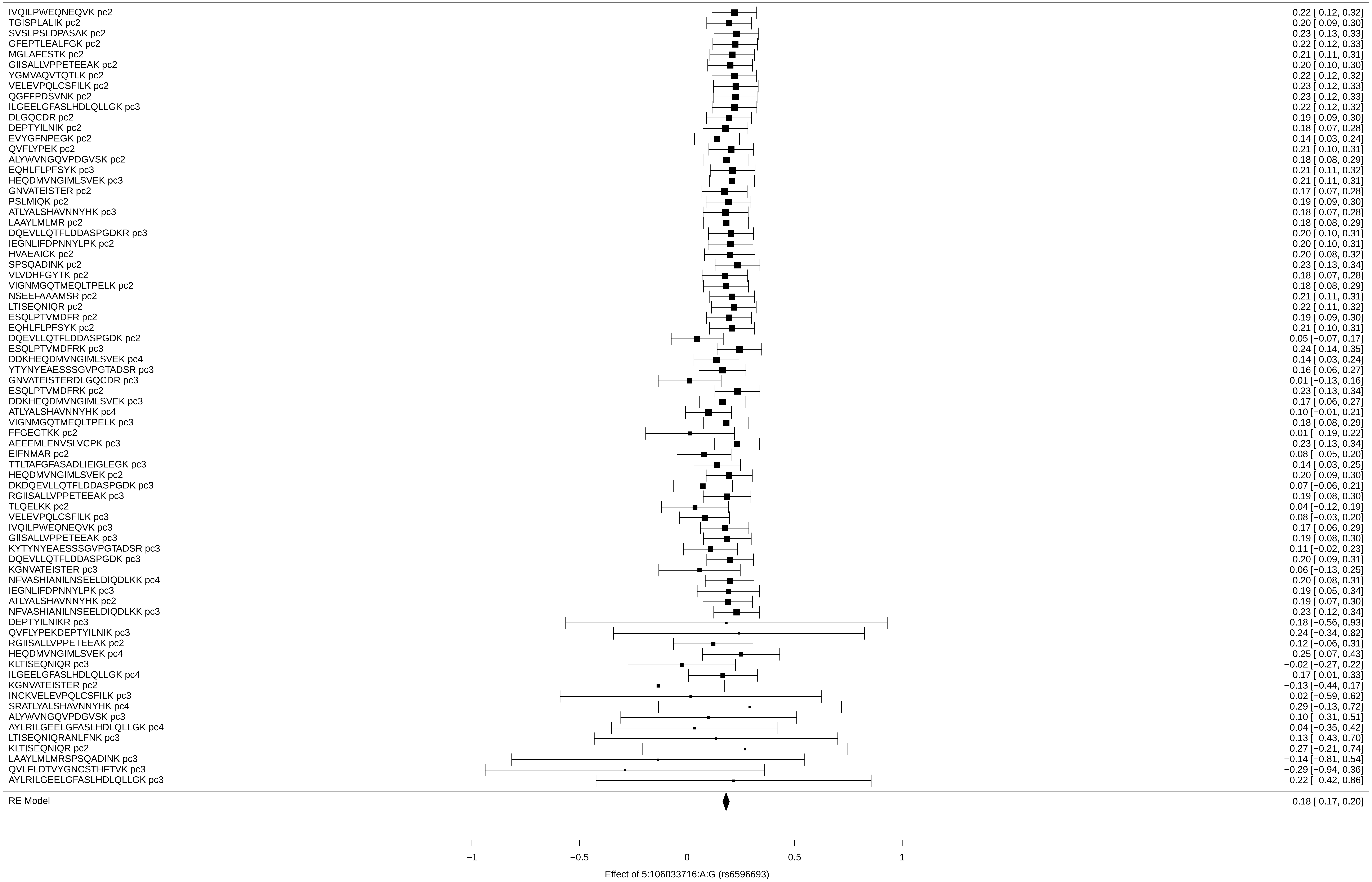

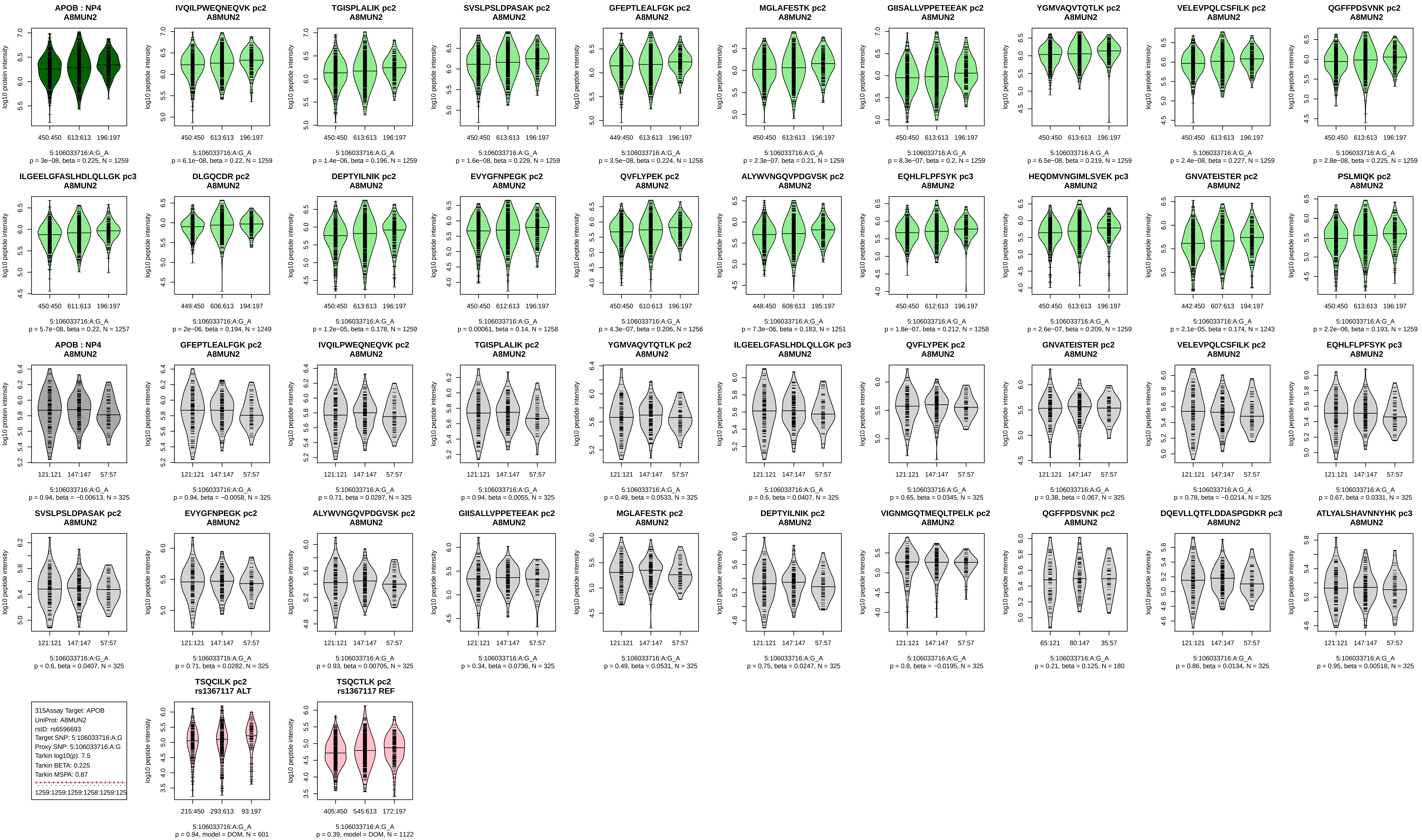

APOB : NP4
A8MUN2
log10 protein intensity
450:450 613:613 196:197
5:106033716:A:G_A
p = 3e−08, beta = 0.225, N = 1259
IVQILPWEQNEQVK pc2
A8MUN2
log10 peptide intensity
450:450 613:613 196:197
5:106033716:A:G_A
p = 6.1e−08, beta = 0.22, N = 1259
TGISPLALIK pc2
A8MUN2
450:450 613:613 196:197
5:106033716:A:G_A
p = 1.4e−06, beta = 0.196, N = 1259
SVSLPSLDPASAK pc2
A8MUN2
450:450 613:613 196:197
5:106033716:A:G_A
p = 1.6e−08, beta = 0.229, N = 1259
GFEPTLEALFGK pc2
A8MUN2
449:450 613:613 196:197
5:106033716:A:G_A
p = 3.5e−08, beta = 0.224, N = 1258
MGLAFESTK pc2
A8MUN2
450:450 613:613 196:197
5:106033716:A:G_A
p = 2.3e−07, beta = 0.21, N = 1259
GIISALLVPPETEEAK pc2
A8MUN2
450:450 613:613 196:197
5:106033716:A:G_A
p = 8.3e−07, beta = 0.2, N = 1259
YGMVAQVTQTLK pc2
A8MUN2
450:450 613:613 196:197
5:106033716:A:G_A
p = 6.5e−08, beta = 0.219, N = 1259
VELEVPQLCSFILK pc2
A8MUN2
450:450 613:613 196:197
5:106033716:A:G_A
p = 2.4e−08, beta = 0.227, N = 1259
QGFFPDSVNK pc2
A8MUN2
450:450 613:613 196:197
5:106033716:A:G_A
p = 2.8e−08, beta = 0.225, N = 1259
ILGEELGFASLHDLQLLGK pc3
A8MUN2
450:450 611:613 196:197
5:106033716:A:G_A
p = 5.7e−08, beta = 0.22, N = 1257
DLGQCDR pc2
A8MUN2
449:450 606:613 194:197
5:106033716:A:G_A
p = 2e−06, beta = 0.194, N = 1249
DEPTYILNIK pc2
A8MUN2
450:450 613:613 196:197
5:106033716:A:G_A
p = 1.2e−05, beta = 0.178, N = 1259
EVYGFNPEGK pc2
A8MUN2
450:450 612:613 196:197
5:106033716:A:G_A
p = 0.00061, beta = 0.14, N = 1258
QVFLYPEK pc2
A8MUN2
450:450 610:613 196:197
5:106033716:A:G_A
p = 4.3e−07, beta = 0.206, N = 1256
ALYWVNGQVPDGVSK pc2
A8MUN2
448:450 608:613 195:197
5:106033716:A:G_A
p = 7.3e−06, beta = 0.183, N = 1251
EQHLFLPFSYK pc3
A8MUN2
450:450 612:613 196:197
5:106033716:A:G_A
p = 1.8e−07, beta = 0.212, N = 1258
HEQDMVNGIMLSVEK pc3
A8MUN2
450:450 613:613 196:197
5:106033716:A:G_A
p = 2.6e−07, beta = 0.209, N = 1259
GNVATEISTER pc2
A8MUN2
442:450 607:613 194:197
5:106033716:A:G_A
p = 2.1e−05, beta = 0.174, N = 1243
PSLMIQK pc2
A8MUN2
450:450 613:613 196:197
5:106033716:A:G_A
p = 2.2e−06, beta = 0.193, N = 1259
APOB : NP4
A8MUN2
121:121 147:147 57:57
5:106033716:A:G_A
p = 0.94, beta = −0.00613, N = 325
GFEPTLEALFGK pc2
A8MUN2
121:121 147:147 57:57
5:106033716:A:G_A
p = 0.94, beta = −0.0058, N = 325
IVQILPWEQNEQVK pc2
A8MUN2
121:121 147:147 57:57
5:106033716:A:G_A
p = 0.71, beta = 0.0287, N = 325
TGISPLALIK pc2
A8MUN2
121:121 147:147 57:57
5:106033716:A:G_A
p = 0.94, beta = 0.0055, N = 325
YGMVAQVTQTLK pc2
A8MUN2
121:121 147:147 57:57
5:106033716:A:G_A
p = 0.49, beta = 0.0533, N = 325
ILGEELGFASLHDLQLLGK pc3
A8MUN2
121:121 147:147 57:57
5:106033716:A:G_A
p = 0.6, beta = 0.0407, N = 325
QVFLYPEK pc2
A8MUN2
121:121 147:147 57:57
5:106033716:A:G_A
p = 0.65, beta = 0.0345, N = 325
GNVATEISTER pc2
A8MUN2
121:121 147:147 57:57
5:106033716:A:G_A
p = 0.38, beta = 0.067, N = 325
VELEVPQLCSFILK pc2
A8MUN2
121:121 147:147 57:57
5:106033716:A:G_A
p = 0.78, beta = −0.0214, N = 325
EQHLFLPFSYK pc3
A8MUN2
121:121 147:147 57:57
5:106033716:A:G_A
p = 0.67, beta = 0.0331, N = 325
SVSLPSLDPASAK pc2
A8MUN2
121:121 147:147 57:57
5:106033716:A:G_A
p = 0.6, beta = 0.0407, N = 325
EVYGFNPEGK pc2
A8MUN2
121:121 147:147 57:57
5:106033716:A:G_A
p = 0.71, beta = 0.0282, N = 325
ALYWVNGQVPDGVSK pc2
A8MUN2
121:121 147:147 57:57
5:106033716:A:G_A
p = 0.93, beta = 0.00705, N = 325
GIISALLVPPETEEAK pc2
A8MUN2
121:121 147:147 57:57
5:106033716:A:G_A
p = 0.34, beta = 0.0736, N = 325
MGLAFESTK pc2
A8MUN2
121:121 147:147 57:57
5:106033716:A:G_A
p = 0.49, beta = 0.0531, N = 325
DEPTYILNIK pc2
A8MUN2
121:121 147:147 57:57
5:106033716:A:G_A
p = 0.75, beta = 0.0247, N = 325
VIGNMGQTMEQLTPELK pc2
A8MUN2
121:121 147:147 57:57
5:106033716:A:G_A
p = 0.8, beta = −0.0195, N = 325
QGFFPDSVNK pc2
A8MUN2
65:121 80:147 35:57
5:106033716:A:G_A
p = 0.21, beta = 0.125, N = 180
DQEVLLQTFLDDASPGDKR pc3
A8MUN2
121:121 147:147 57:57
5:106033716:A:G_A
p = 0.86, beta = 0.0134, N = 325
ATLYALSHAVNNYHK pc3
A8MUN2
121:121 147:147 57:57
5:106033716:A:G_A
p = 0.95, beta = 0.00518, N = 325
315Assay Target: APOB
UniProt: A8MUN2
rsID: rs6596693
Target SNP: 5:106033716:A:G
Proxy SNP: 5:106033716:A:G
Tarkin log10(p): 7.5
Tarkin BETA: 0.225
Tarkin MSPA: 0.87
1259:1259:1259:1258:1259:125
TSQCILK pc2
rs1367117 ALT
215:450 293:613 93:197
5:106033716:A:G_A
p = 0.94, model = DOM, N = 601
TSQCTLK pc2
rs1367117 REF
405:450 545:613 172:197
5:106033716:A:G_A
p = 0.39, model = DOM, N = 1122

316. OLFML2A (Q68BL7)
Tarkin MSPA=0.573

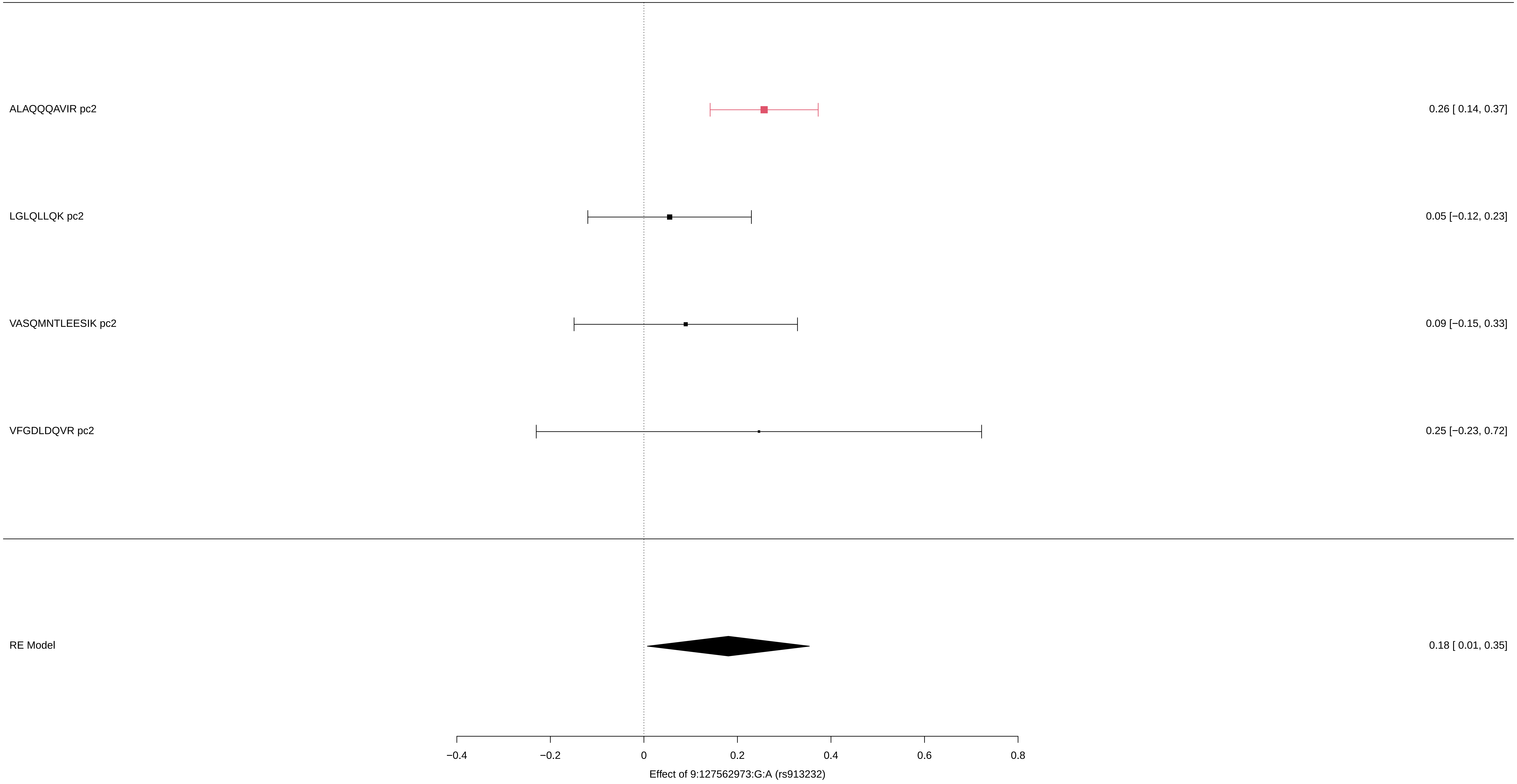

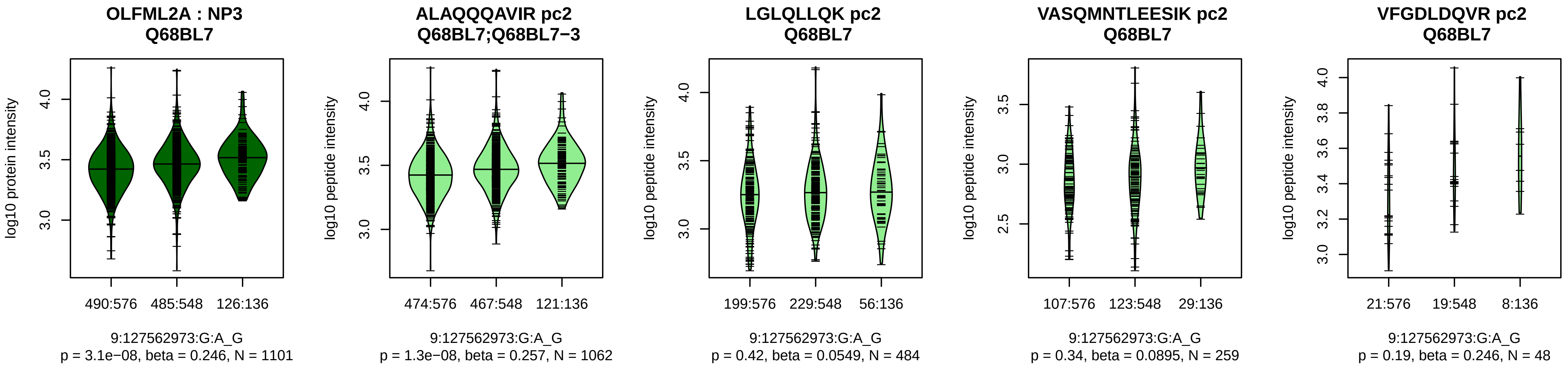

316Assay Target: OLFML2A
UniProt: Q68BL7
rsID: rs913232
Target SNP: 9:127562973:G:A
Proxy SNP: 9:127562973:G:A
Tarkin log10(p): 7.5
Tarkin BETA: 0.246
Tarkin MSPA: 0.573
*:−:−:−:NA:NA
1062:484:259:48:9:17

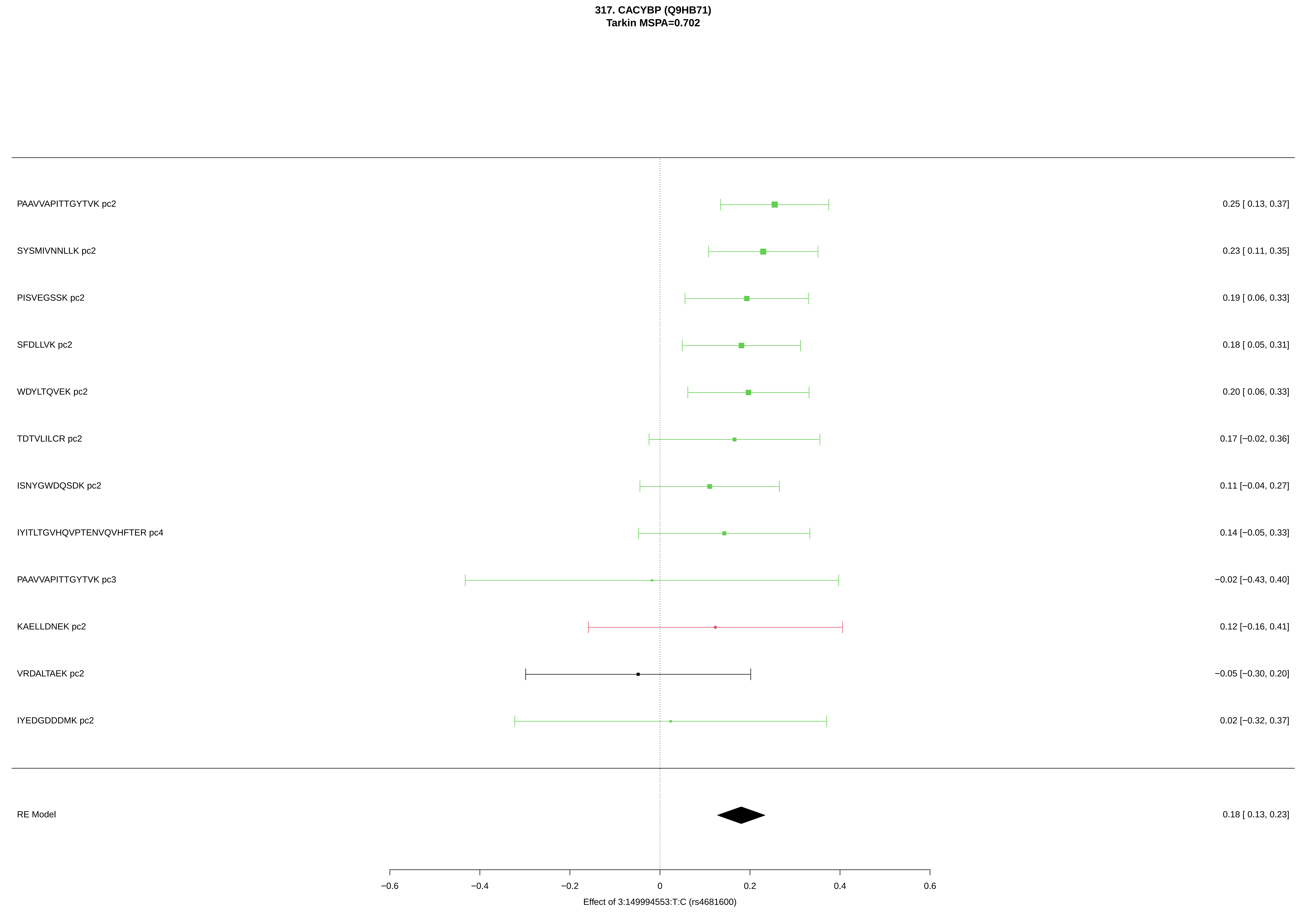
317. CACYBP (Q9HB71)
Tarkin MSPA=0.702
PAAVVAPITTGYTVK pc2
0.25 [0.13, 0.37]
SYSMIVNNLLK pc2
0.23 [0.11, 0.35]
PISVEGSSK pc2
0.19 [0.06, 0.33]
SFDLLVK pc2
0.18 [0.05, 0.31]
WDYLTQVEK pc2
0.20 [0.06, 0.33]
TDTVLILCR pc2
0.17 [−0.02, 0.36]
ISNYGWDQSDK pc2
0.11 [−0.04, 0.27]
IYITLTGVHQVPTENVQVHFTER pc4
0.14 [−0.05, 0.33]
PAAVVAPITTGYTVK pc3
−0.02 [−0.43, 0.40]
KAELLDNEK pc2
0.12 [−0.16, 0.41]
VRDALTAEK pc2
−0.05 [−0.30, 0.20]
IYEDGDDDMK pc2
0.02 [−0.32, 0.37]
RE Model
0.18 [0.13, 0.23]
−0.6
−0.4
−0.2
0
0.2
0.4
0.6
Effect of 3:149994553:T:C (rs4681600)

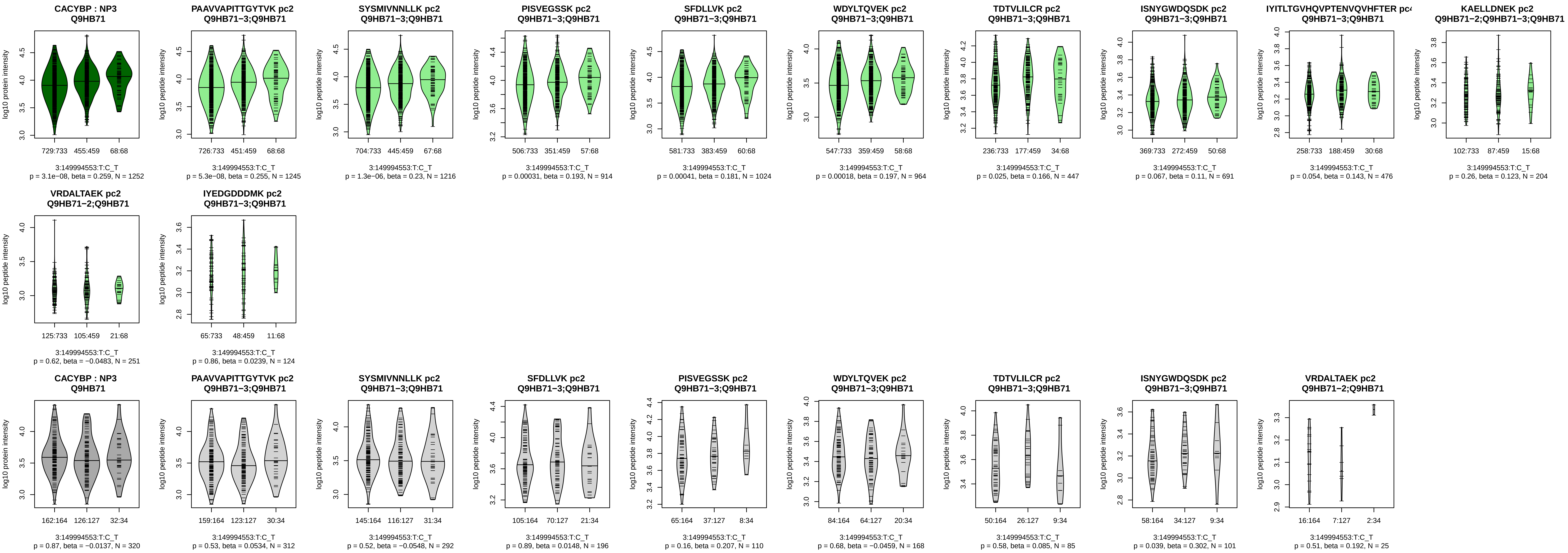

317Assay Target: CACYBP
UniProt: Q9HB71
rsID: rs4681600
Target SNP: 3:149994553:T:C
Proxy SNP: 3:149994553:T:C
Tarkin log10(p): 7.5
Tarkin BETA: 0.259
Tarkin MSPA: 0.702
::*:*:*:−:−:−:−:−:−:−:NA
1245:1216:914:1024:964:447:6[illegible]

318. KPNB1 (Q14974)
Tarkin MSPA=0.767

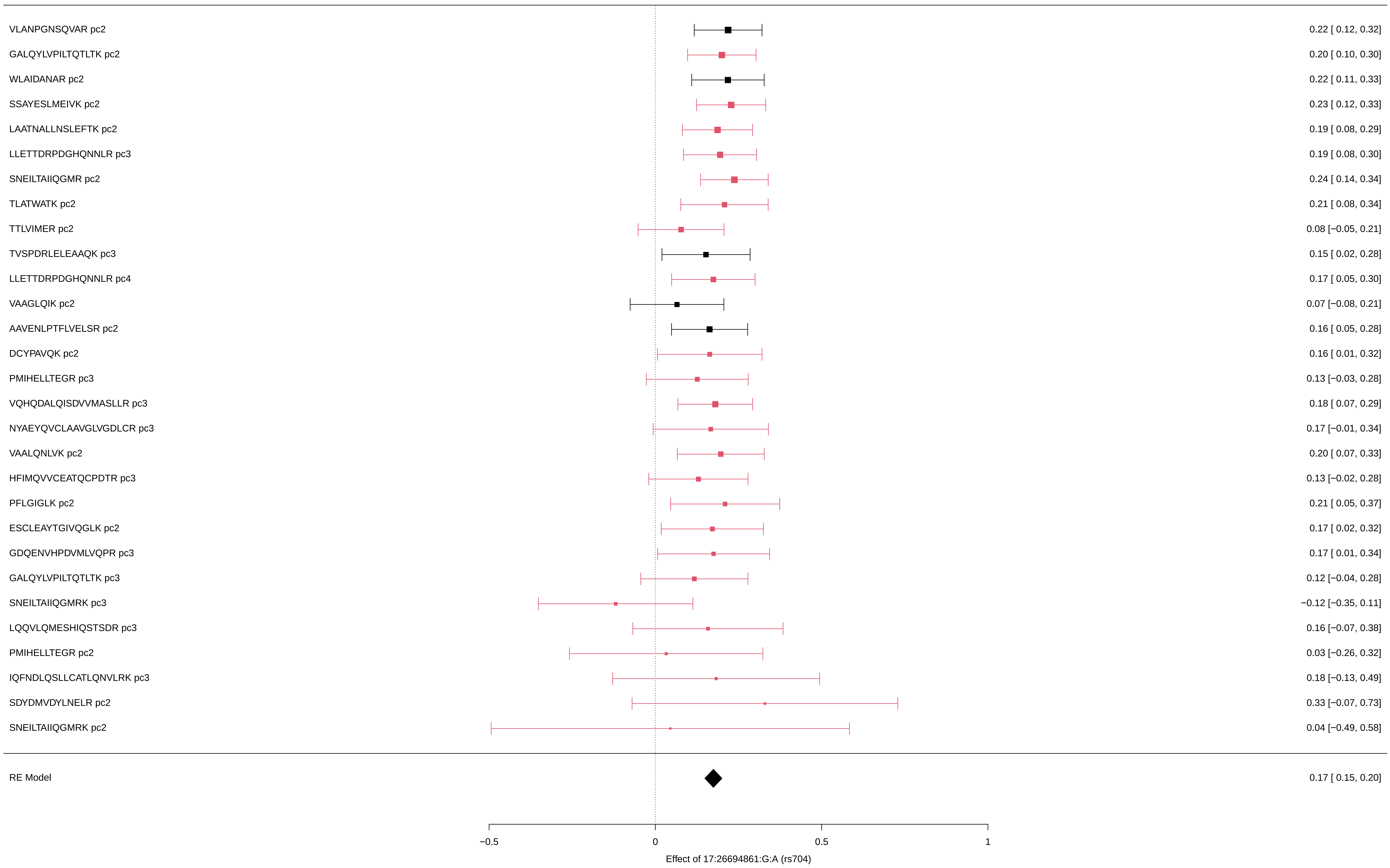

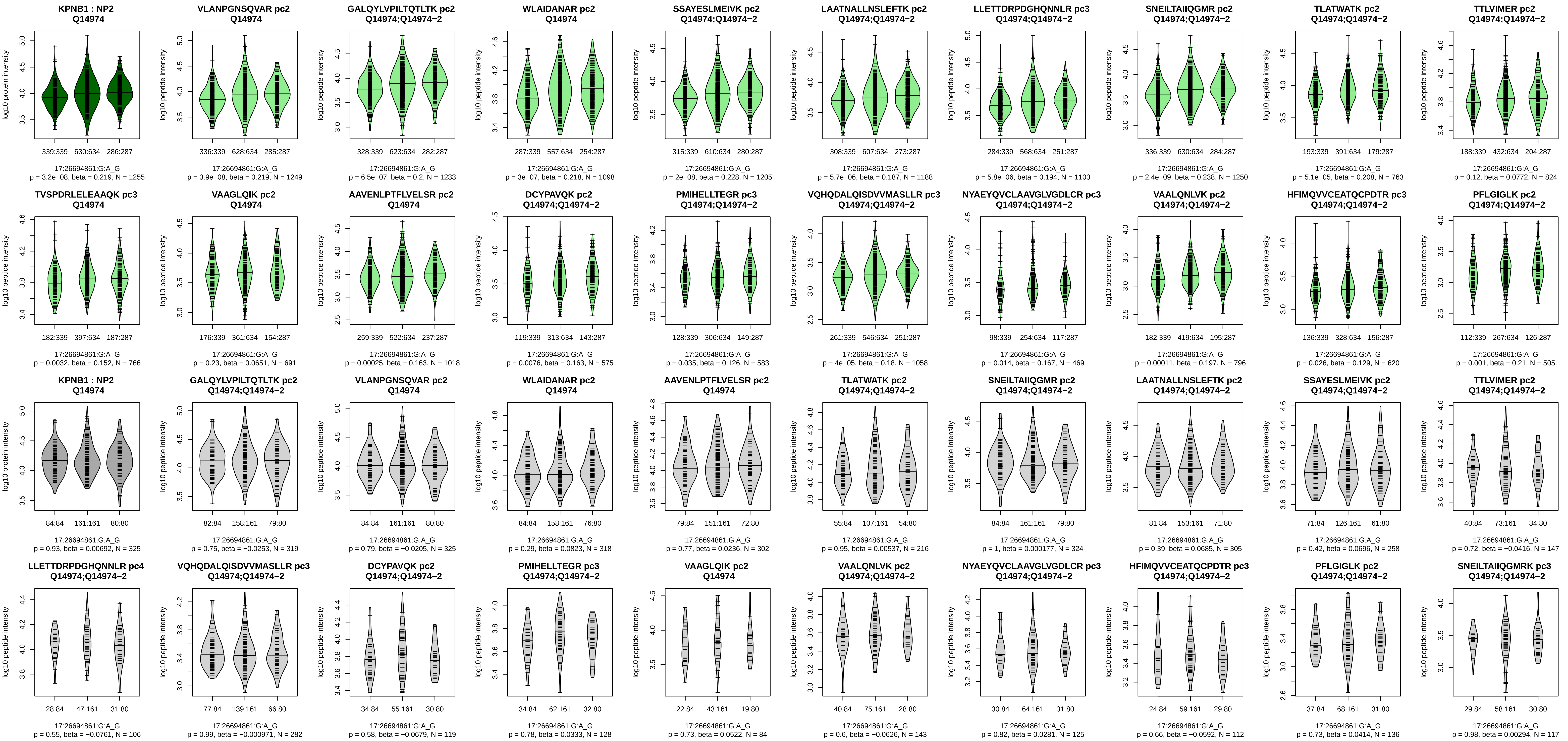

318Assay Target: KPNB1
UniProt: Q14974
rsID: rs704
Target SNP: 17:26694861:G:A
Proxy SNP: 17:26694861:G:A
Tarkin log10(p): 7.5
Tarkin BETA: 0.219
Tarkin MSPA: 0.767
1249:1233:1098:1205:1188:110

319. FABP4 (P15090)
Tarkin MSPA=0.182

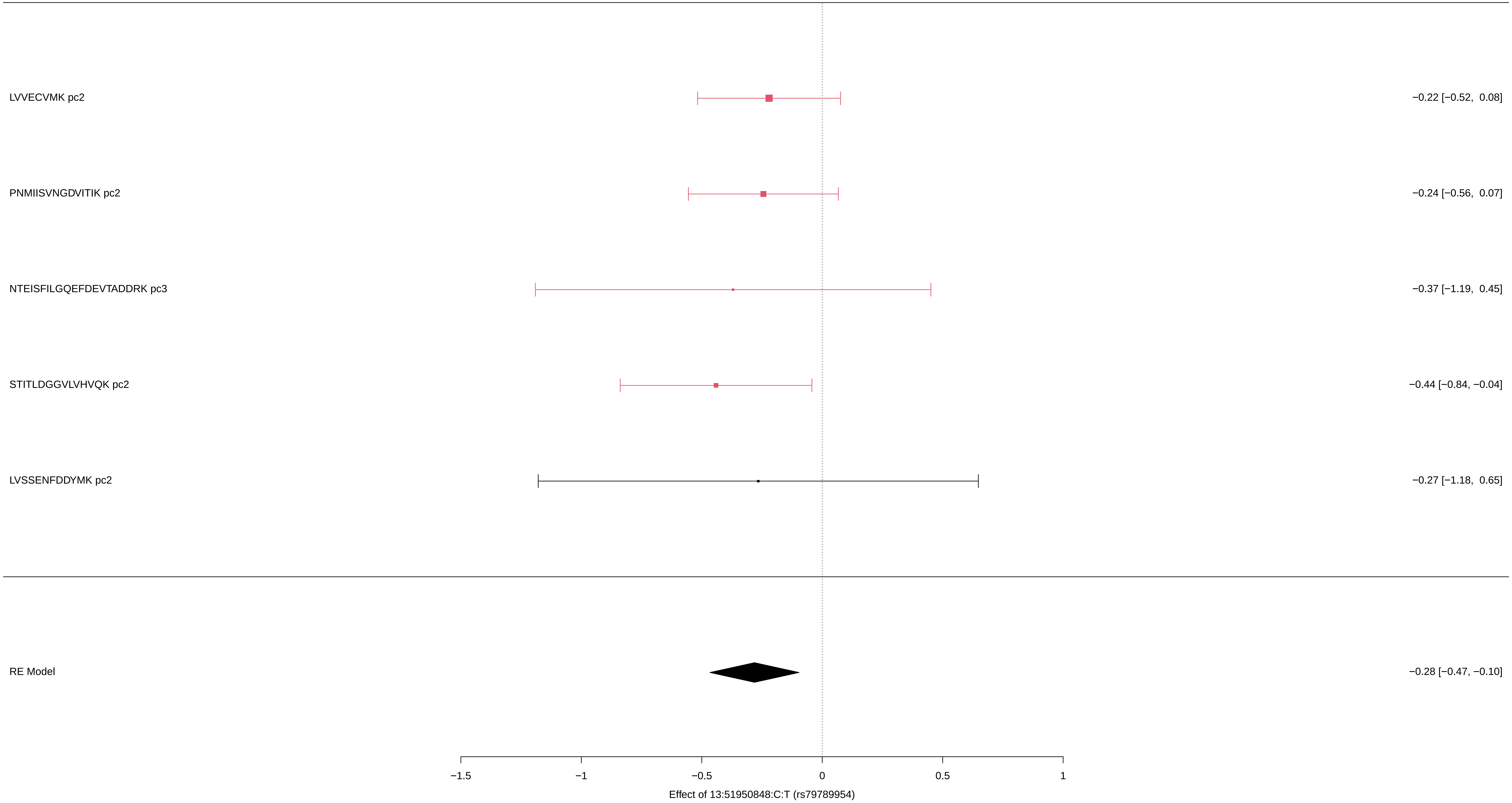

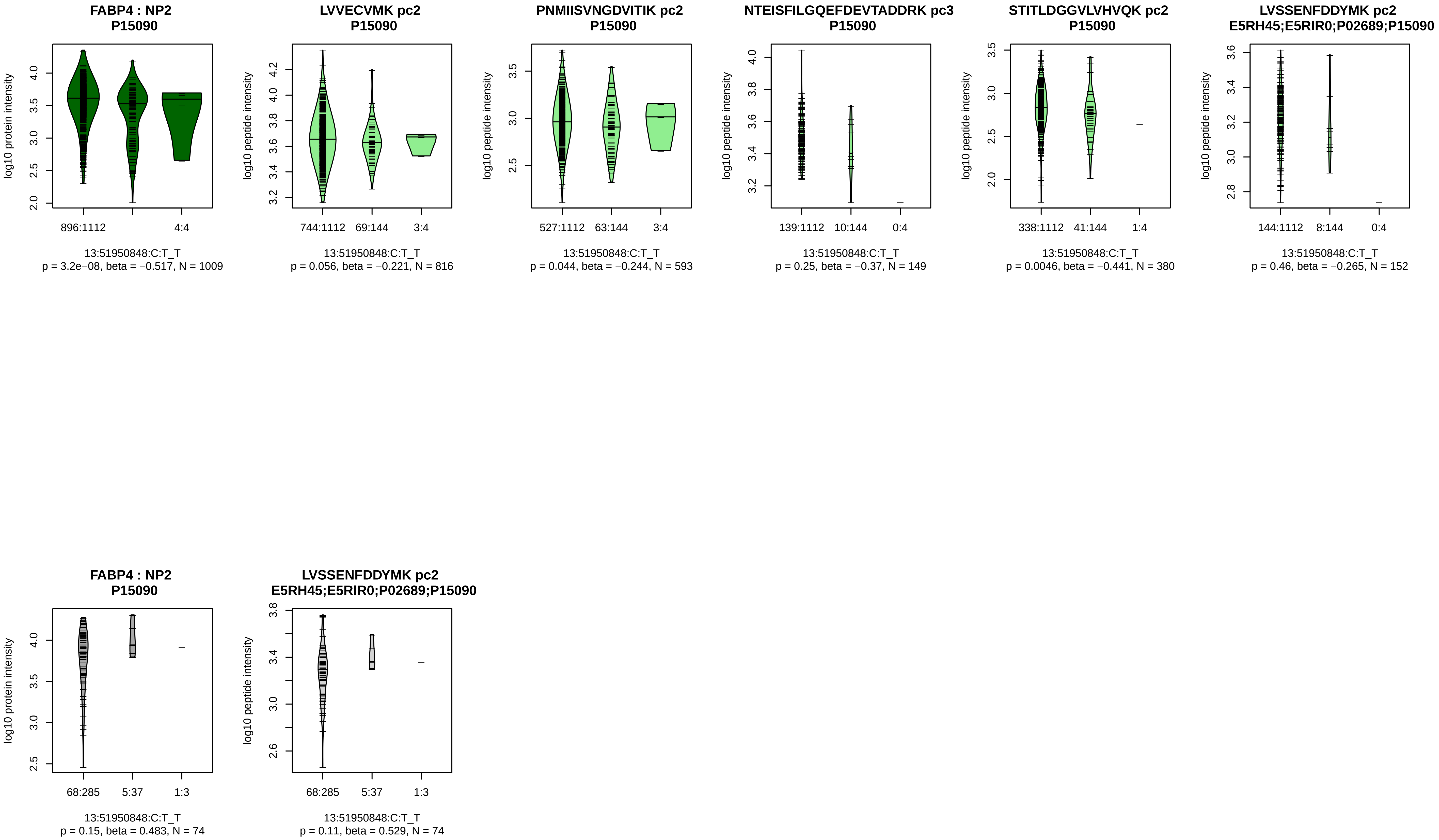

319Assay Target: FABP4
UniProt: P15090
rsID: rs79789954
Target SNP: 13:51950848:C:T
Proxy SNP: 13:51950848:C:T
Tarkin log10(p): 7.5
Tarkin BETA: −0.517
Tarkin MSPA: 0.182
–:–:–:*:–
816:593:149:380:152

320. EMC1 (Q8N766)
Tarkin MSPA=0.434

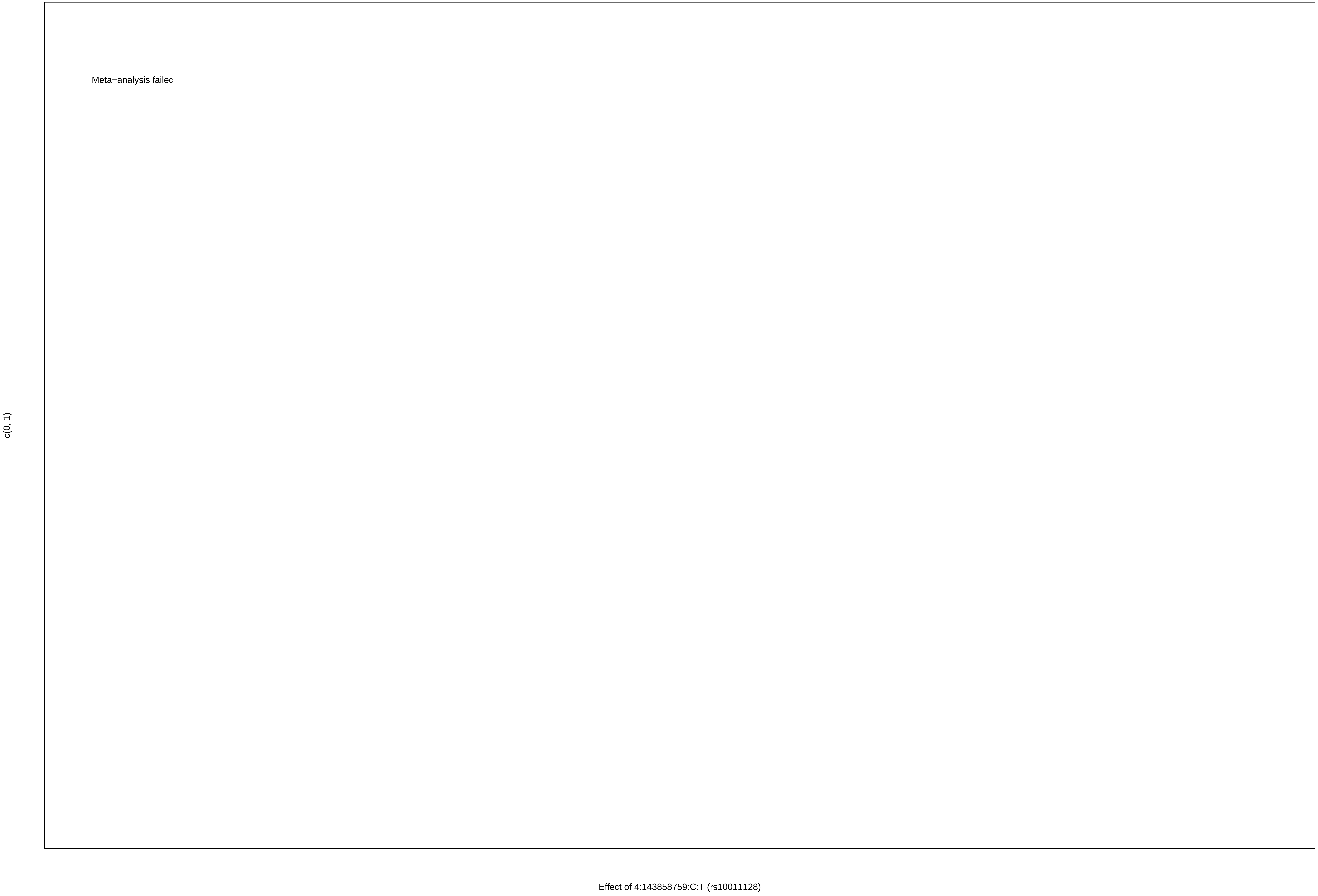

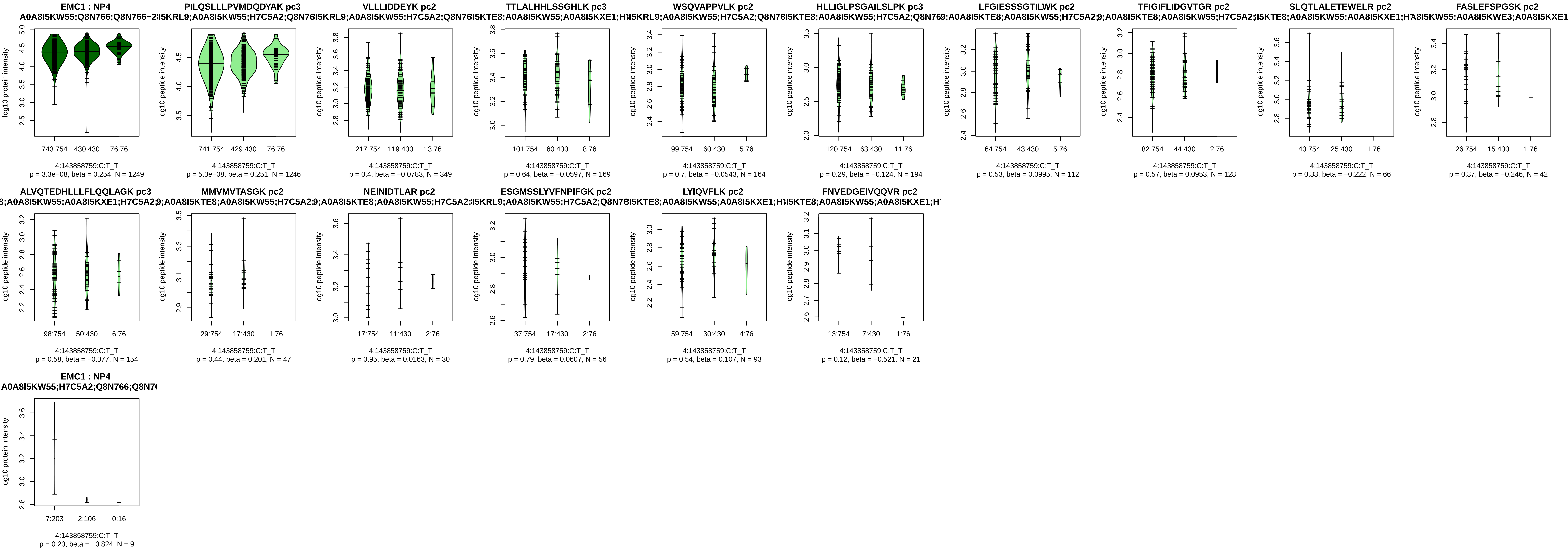

320Assay Target: EMC1
UniProt: Q8N766
rsID: rs10011128
Target SNP: 4:143858759:C:T
Proxy SNP: 4:143858759:C:T
Tarkin log10(p): 7.5
Tarkin BETA: 0.254
Tarkin MSPA: 0.434
*:−:−:−:−:−:−:−:−:−:−:−:−:−:−:NA
1246:349:169:164:194:112:128:

321. ANGPTL2 (Q9UKU9)
Tarkin MSPA=0.455

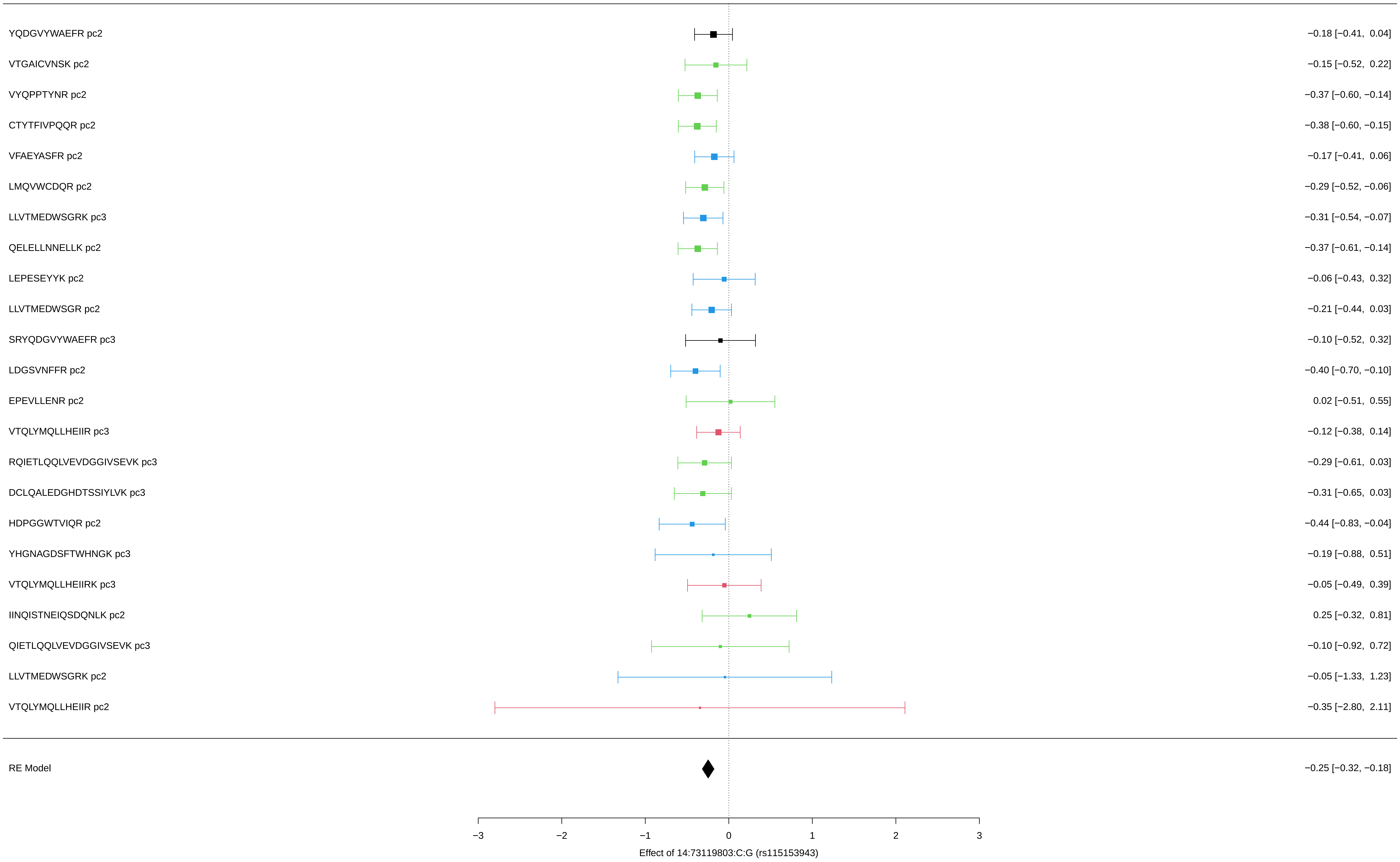

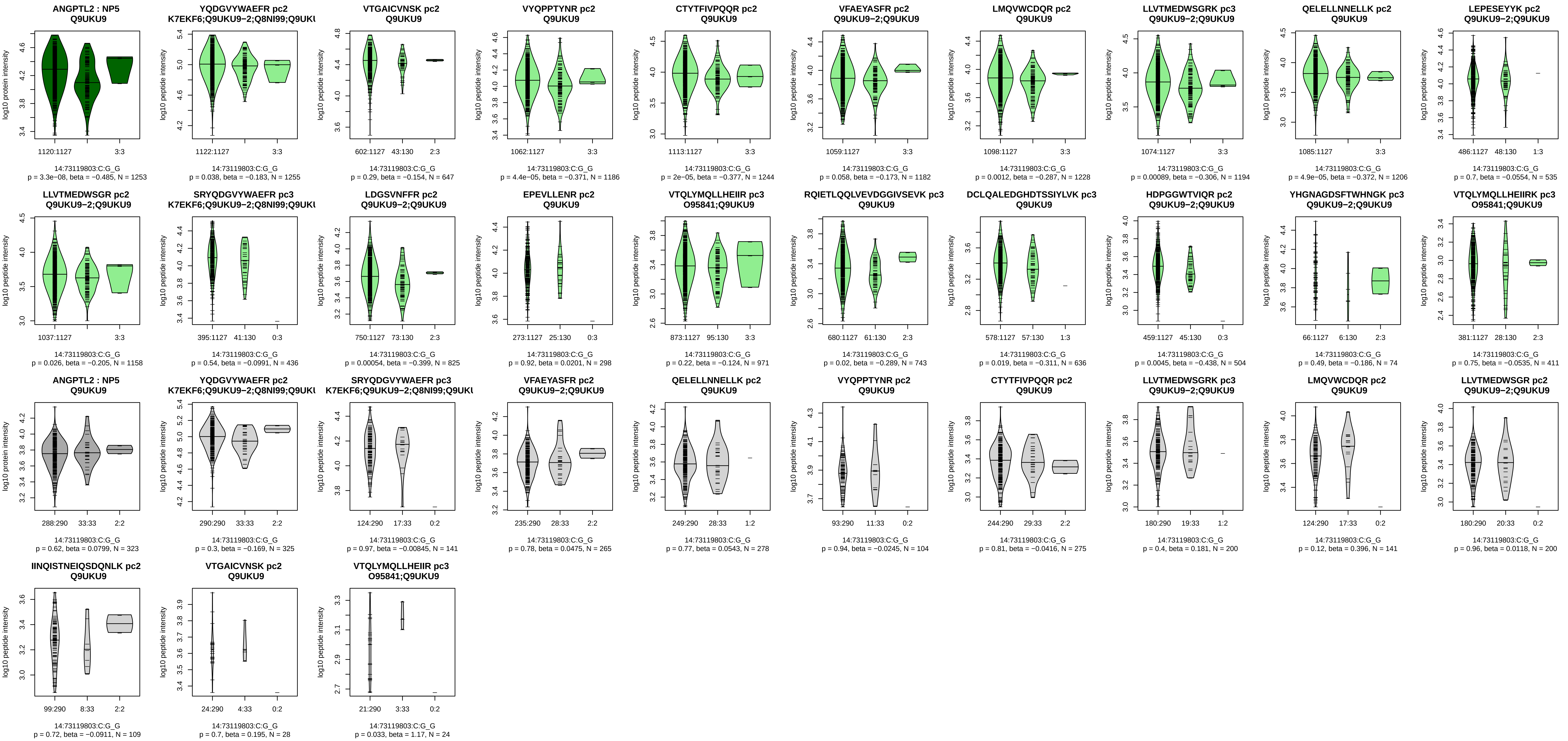

321Assay Target: ANGPTL2
UniProt: Q9UKU9
rsID: rs115153943
Target SNP: 14:73119803:C:G
Proxy SNP: 14:73119803:C:G
Tarkin log10(p): 7.5
Tarkin BETA: −0.485
Tarkin MSPA: 0.455
1255:647:1186:1244:1182:1228

322. CHMP3 (Q9Y3E7)
Tarkin MSPA=0.798

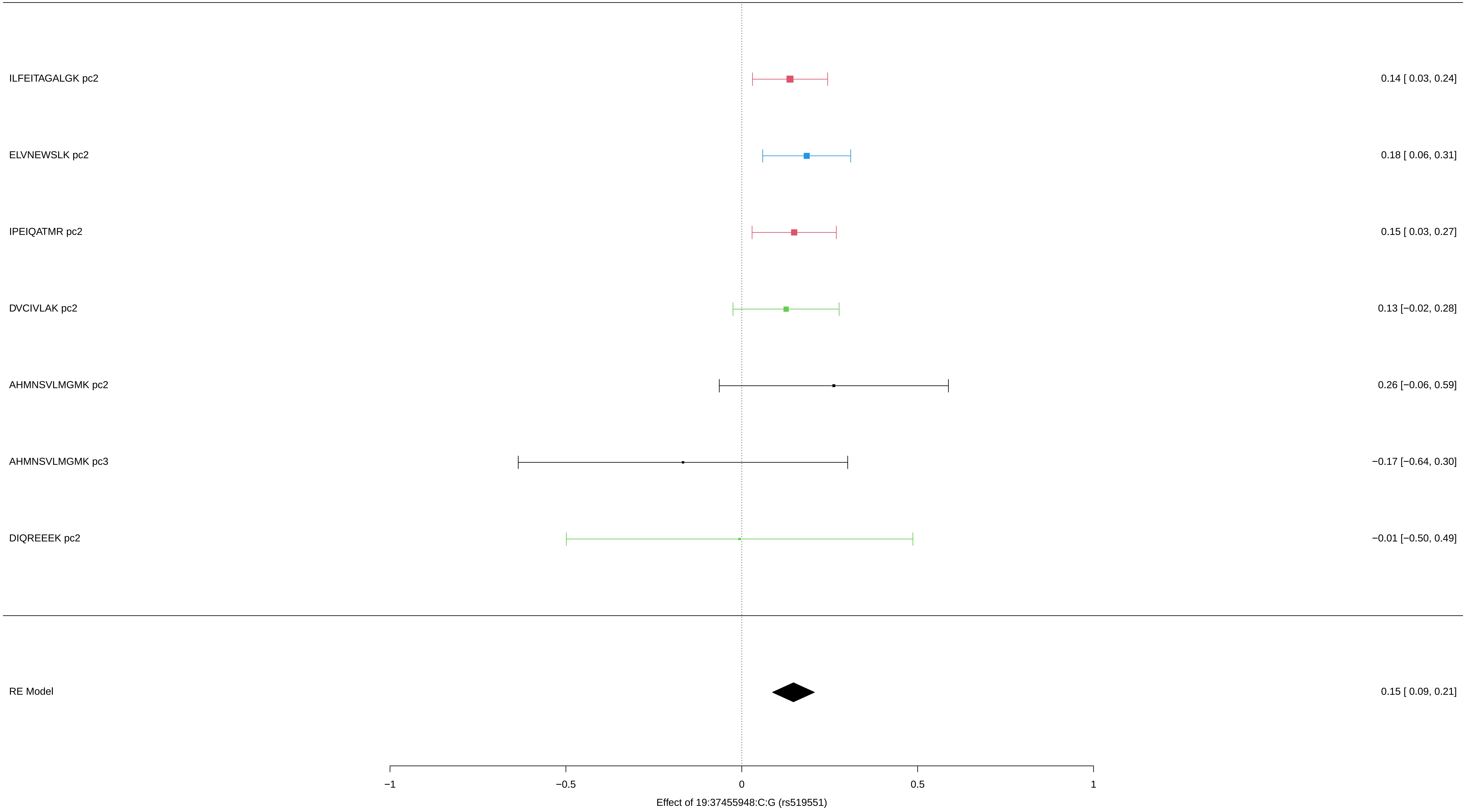

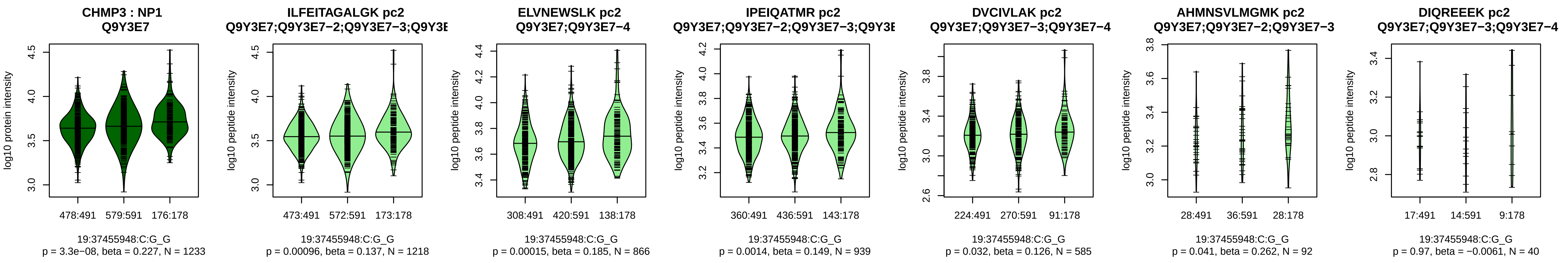

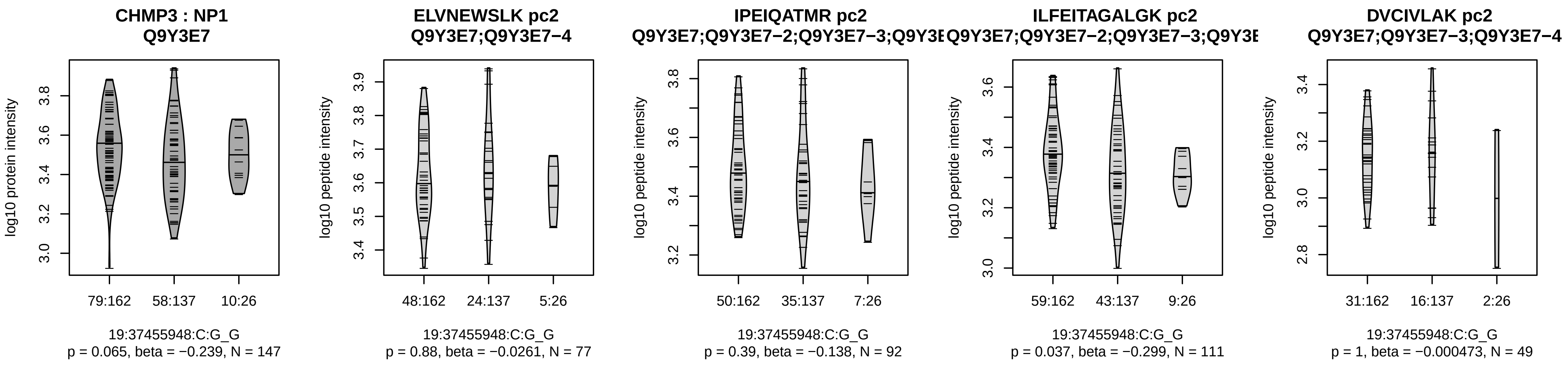

322Assay Target: CHMP3
UniProt: Q9Y3E7
rsID: rs519551
Target SNP: 19:37455948:C:G
Proxy SNP: 19:37455948:C:G
Tarkin log10(p): 7.5
Tarkin BETA: 0.227
Tarkin MSPA: 0.798
::*:–:–:–:–
1218:866:939:585:92:47:40

323. GBA2 (Q9HCG7)
Tarkin MSPA=0.984

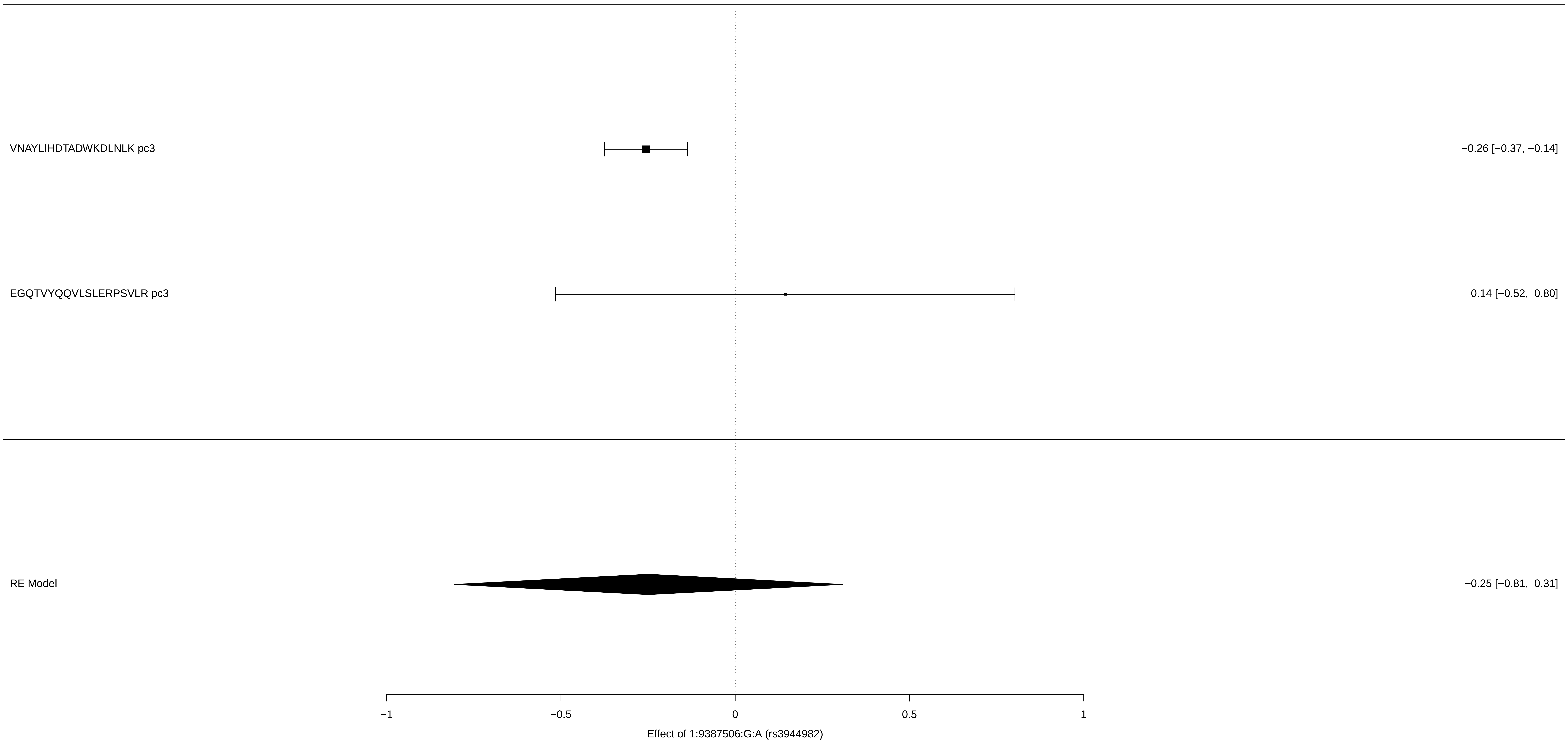

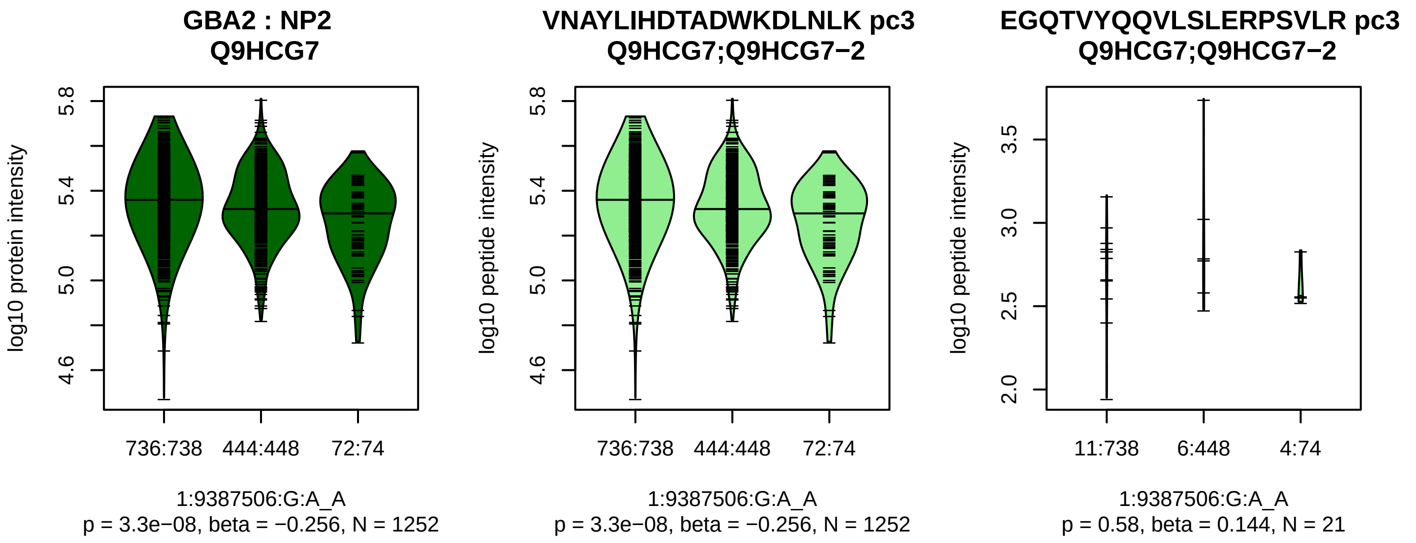

323Assay Target: GBA2
UniProt: Q9HCG7
rsID: rs3944982
Target SNP: 1:9387506:G:A
Proxy SNP: 1:9387506:G:A
Tarkin log10(p): 7.5
Tarkin BETA: −0.256
Tarkin MSPA: 0.984
*:−:NA:NA
1252:21:3:1

324. PRPSAP1 (Q14558)
Tarkin MSPA=0

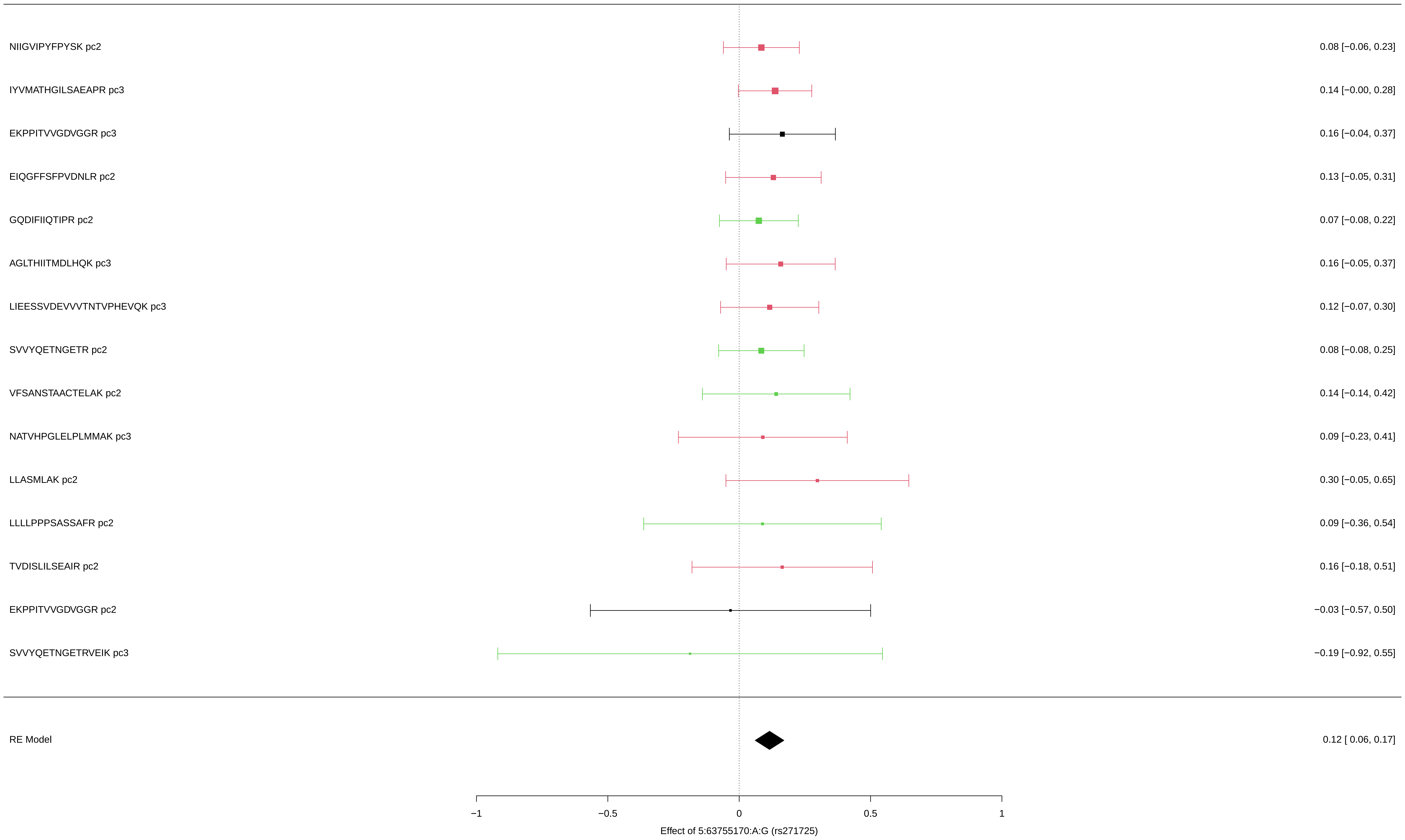

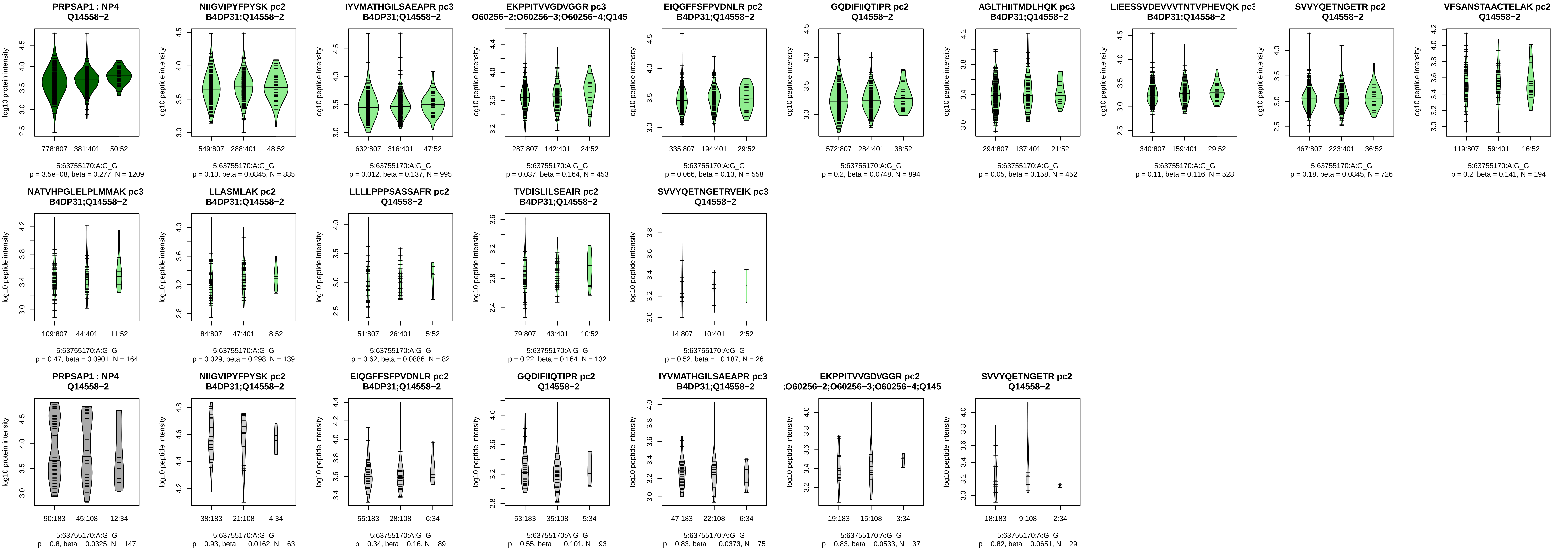

324Assay Target: PRPSAP1
UniProt: Q14558
rsID: rs271725
Target SNP: 5:63755170:A:G
Proxy SNP: 5:63755170:A:G
Tarkin log10(p): 7.5
Tarkin BETA: 0.277
Tarkin MSPA: 0
−:−:−:−:−:−:−:−:−:−:−:−:−:−:NA
885:995:453:558:894:452:528:7

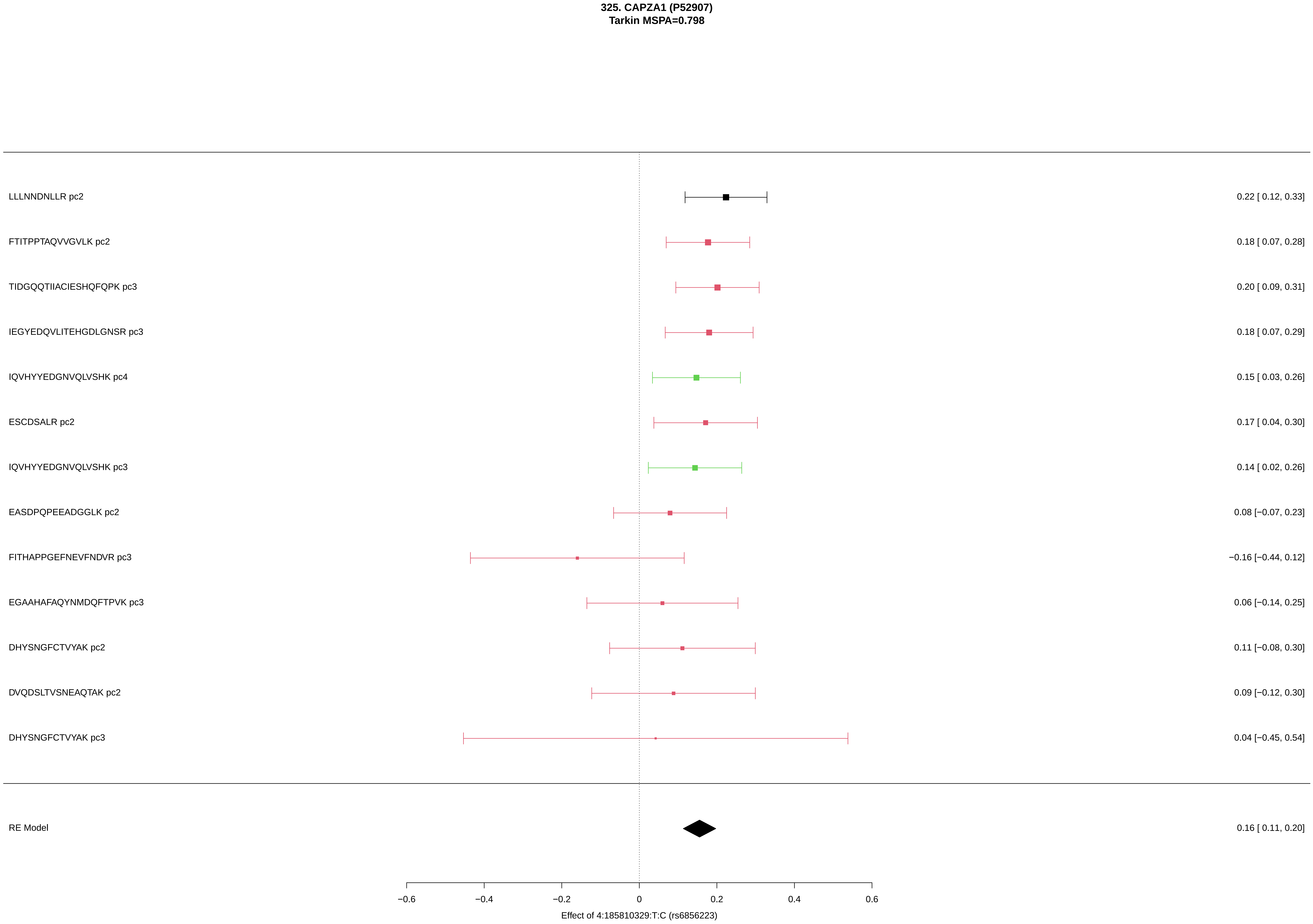
325. CAPZA1 (P52907)
Tarkin MSPA=0.798
LLLNNDNLLR pc2
0.22 [0.12, 0.33]
FTITPPTAQVVGVLK pc2
0.18 [0.07, 0.28]
TIDGQQTIIACIESHQFQPK pc3
0.20 [0.09, 0.31]
IEGYEDQVLITEHGDLGNSR pc3
0.18 [0.07, 0.29]
IQVHYYEDGNVQLVSHK pc4
0.15 [0.03, 0.26]
ESCDSALR pc2
0.17 [0.04, 0.30]
IQVHYYEDGNVQLVSHK pc3
0.14 [0.02, 0.26]
EASDPQPEEADGGLK pc2
0.08 [−0.07, 0.23]
FITHAPPGEFNEVFNDVR pc3
−0.16 [−0.44, 0.12]
EGAAHAFAQYNMDQFTPVK pc3
0.06 [−0.14, 0.25]
DHYSNGFCTVYAK pc2
0.11 [−0.08, 0.30]
DVQDSLTVSNEAQTAK pc2
0.09 [−0.12, 0.30]
DHYSNGFCTVYAK pc3
0.04 [−0.45, 0.54]
RE Model
0.16 [0.11, 0.20]
−0.6
−0.4
−0.2
0
0.2
0.4
0.6
Effect of 4:185810329:T:C (rs6856223)

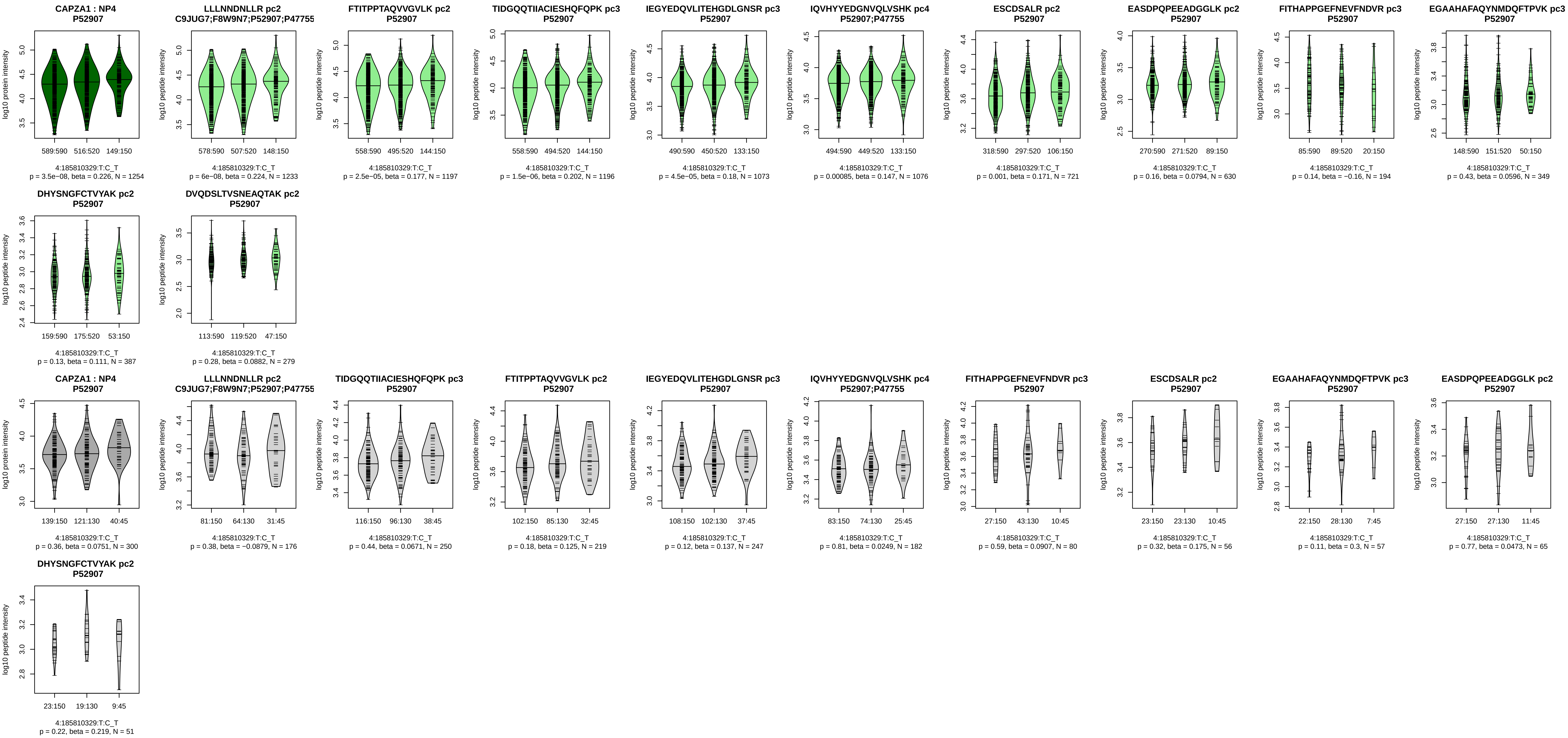

325Assay Target: CAPZA1
UniProt: P52907
rsID: rs6856223
Target SNP: 4:185810329:T:C
Proxy SNP: 4:185810329:T:C
Tarkin log10(p): 7.5
Tarkin BETA: 0.226
Tarkin MSPA: 0.798
::*:*:*:*:*:–:–:–:–:–:–
1233:1197:1196:1073:1076:721

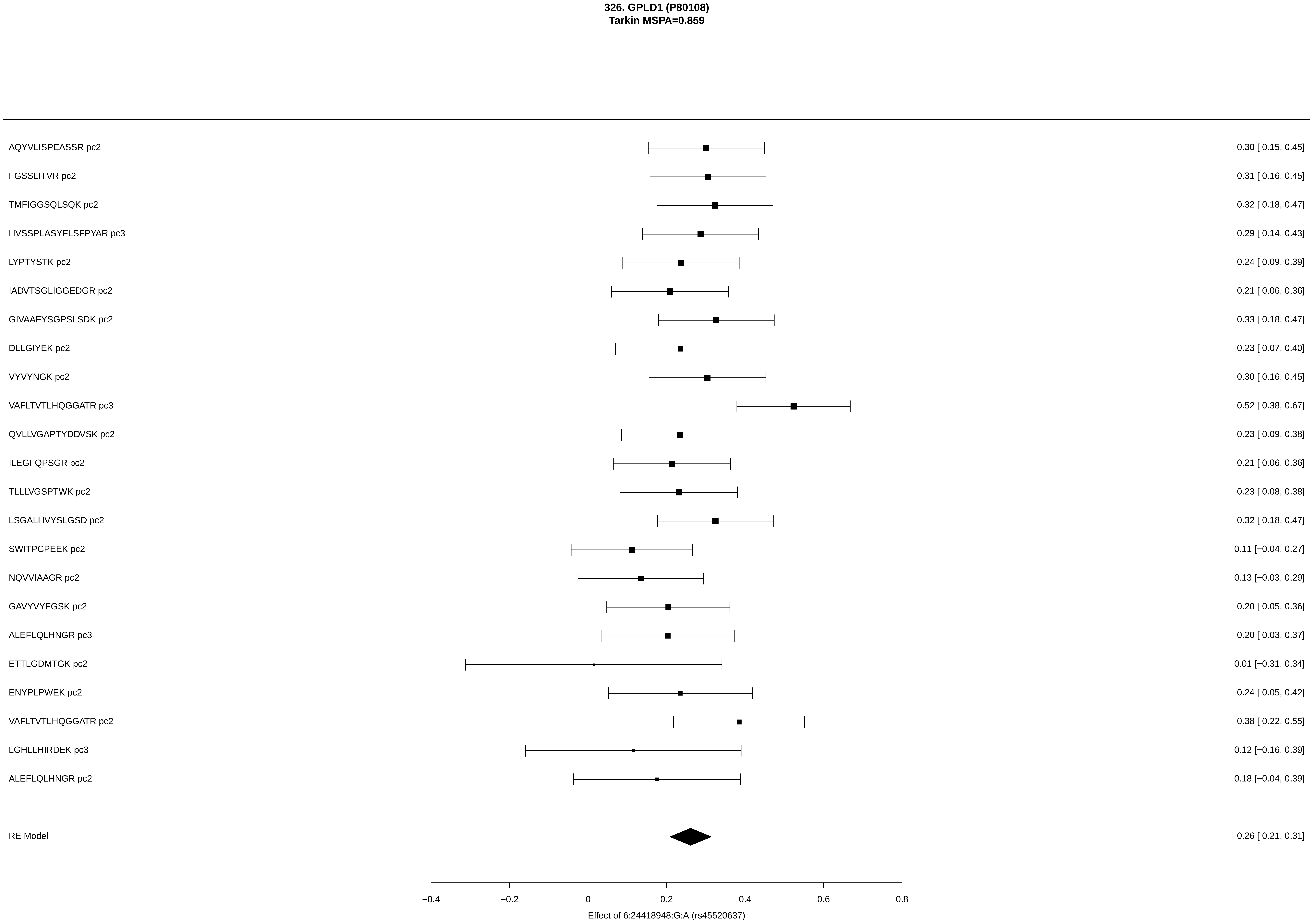

326. GPLD1 (P80108)
Tarkin MSPA=0.859
AQYVLISPEASSR pc2
0.30 [0.15, 0.45]
FGSSLITVR pc2
0.31 [0.16, 0.45]
TMFIGGSQLSQK pc2
0.32 [0.18, 0.47]
HVSSPLASYFLSFPYAR pc3
0.29 [0.14, 0.43]
LYPTYSTK pc2
0.24 [0.09, 0.39]
IADVTSGLIGGEDGR pc2
0.21 [0.06, 0.36]
GIVAAFYSGPSLSDK pc2
0.33 [0.18, 0.47]
DLLGIYEK pc2
0.23 [0.07, 0.40]
VYVYNGK pc2
0.30 [0.16, 0.45]
VAFLTVTLHQGGATR pc3
0.52 [0.38, 0.67]
QVLLVGAPTYDDVSK pc2
0.23 [0.09, 0.38]
ILEGFQPSGR pc2
0.21 [0.06, 0.36]
TLLLVGSPTWK pc2
0.23 [0.08, 0.38]
LSGALHVYSLGSD pc2
0.32 [0.18, 0.47]
SWITPCPEEK pc2
0.11 [−0.04, 0.27]
NQVVIAAGR pc2
0.13 [−0.03, 0.29]
GAVYVYFGSK pc2
0.20 [0.05, 0.36]
ALEFLQLHNGR pc3
0.20 [0.03, 0.37]
ETTLGDMTGK pc2
0.01 [−0.31, 0.34]
ENYPLPWEK pc2
0.24 [0.05, 0.42]
VAFLTVTLHQGGATR pc2
0.38 [0.22, 0.55]
LGHLLHIRDEK pc3
0.12 [−0.16, 0.39]
ALEFLQLHNGR pc2
0.18 [−0.04, 0.39]
RE Model
0.26 [0.21, 0.31]
−0.4
−0.2
0
0.2
0.4
0.6
0.8
Effect of 6:24418948:G:A (rs45520637)

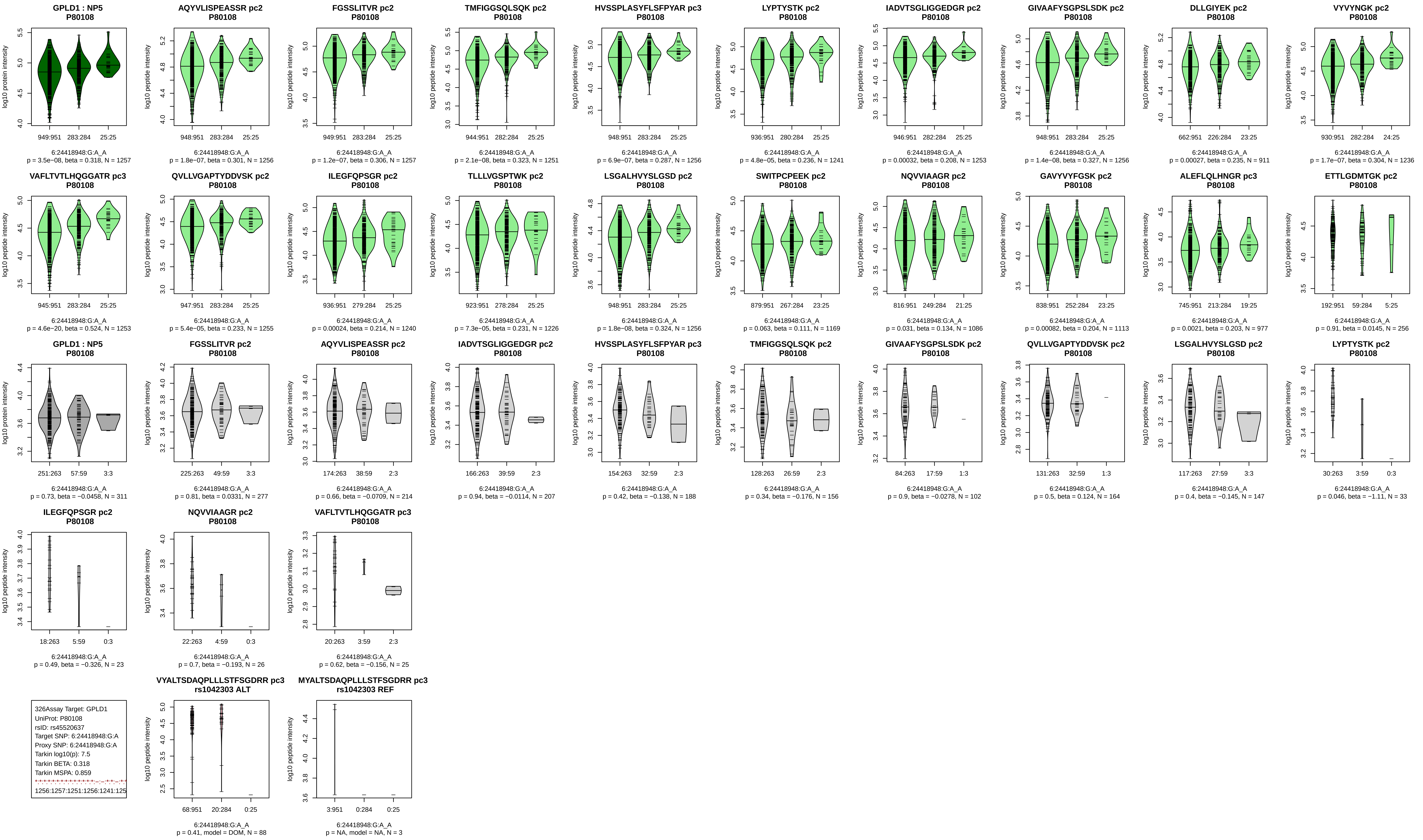
GPLD1 : NP5
P80108
log10 protein intensity
949:951 283:284 25:25
6:24418948:G:A_A
p = 3.5e−08, beta = 0.318, N = 1257
AQYVLISPEASSR pc2
P80108
log10 peptide intensity
948:951 283:284 25:25
6:24418948:G:A_A
p = 1.8e−07, beta = 0.301, N = 1256
FGSSLITVR pc2
P80108
949:951 283:284 25:25
6:24418948:G:A_A
p = 1.2e−07, beta = 0.306, N = 1257
TMFIGGSQLSQK pc2
P80108
944:951 282:284 25:25
6:24418948:G:A_A
p = 2.1e−08, beta = 0.323, N = 1251
HVSSPLASYFLSFPYAR pc3
P80108
948:951 283:284 25:25
6:24418948:G:A_A
p = 6.9e−07, beta = 0.287, N = 1256
LYPTYSTK pc2
P80108
936:951 280:284 25:25
6:24418948:G:A_A
p = 4.8e−05, beta = 0.236, N = 1241
IADVTSGLIGGEDGR pc2
P80108
946:951 282:284 25:25
6:24418948:G:A_A
p = 0.00032, beta = 0.208, N = 1253
GIVAAFYSGPSLSDK pc2
P80108
948:951 283:284 25:25
6:24418948:G:A_A
p = 1.4e−08, beta = 0.327, N = 1256
DLLGIYEK pc2
P80108
662:951 226:284 23:25
6:24418948:G:A_A
p = 0.00027, beta = 0.235, N = 911
VYVYNGK pc2
P80108
930:951 282:284 24:25
6:24418948:G:A_A
p = 1.7e−07, beta = 0.304, N = 1236
VAFLTVTLHQGGATR pc3
P80108
945:951 283:284 25:25
6:24418948:G:A_A
p = 4.6e−20, beta = 0.524, N = 1253
QVLLVGAPTYDDVSK pc2
P80108
947:951 283:284 25:25
6:24418948:G:A_A
p = 5.4e−05, beta = 0.233, N = 1255
ILEGFQPSGR pc2
P80108
936:951 279:284 25:25
6:24418948:G:A_A
p = 0.00024, beta = 0.214, N = 1240
TLLLVGSPTWK pc2
P80108
923:951 278:284 25:25
6:24418948:G:A_A
p = 7.3e−05, beta = 0.231, N = 1226
LSGALHVYSLGSD pc2
P80108
948:951 283:284 25:25
6:24418948:G:A_A
p = 1.8e−08, beta = 0.324, N = 1256
SWITPCPEEK pc2
P80108
879:951 267:284 23:25
6:24418948:G:A_A
p = 0.063, beta = 0.111, N = 1169
NQVVIAAGR pc2
P80108
816:951 249:284 21:25
6:24418948:G:A_A
p = 0.031, beta = 0.134, N = 1086
GAVYVYFGSK pc2
P80108
838:951 252:284 23:25
6:24418948:G:A_A
p = 0.00082, beta = 0.204, N = 1113
ALEFLQLHNGR pc3
P80108
745:951 213:284 19:25
6:24418948:G:A_A
p = 0.0021, beta = 0.203, N = 977
ETTLGDMTGK pc2
P80108
192:951 59:284 5:25
6:24418948:G:A_A
p = 0.91, beta = 0.0145, N = 256
GPLD1 : NP5
P80108
log10 protein intensity
251:263 57:59 3:3
6:24418948:G:A_A
p = 0.73, beta = −0.0458, N = 311
FGSSLITVR pc2
P80108
225:263 49:59 3:3
6:24418948:G:A_A
p = 0.81, beta = 0.0331, N = 277
AQYVLISPEASSR pc2
P80108
174:263 38:59 2:3
6:24418948:G:A_A
p = 0.66, beta = −0.0709, N = 214
IADVTSGLIGGEDGR pc2
P80108
166:263 39:59 2:3
6:24418948:G:A_A
p = 0.94, beta = −0.0114, N = 207
HVSSPLASYFLSFPYAR pc3
P80108
154:263 32:59 2:3
6:24418948:G:A_A
p = 0.42, beta = −0.138, N = 188
TMFIGGSQLSQK pc2
P80108
128:263 26:59 2:3
6:24418948:G:A_A
p = 0.34, beta = −0.176, N = 156
GIVAAFYSGPSLSDK pc2
P80108
84:263 17:59 1:3
6:24418948:G:A_A
p = 0.9, beta = −0.0278, N = 102
QVLLVGAPTYDDVSK pc2
P80108
131:263 32:59 1:3
6:24418948:G:A_A
p = 0.5, beta = 0.124, N = 164
LSGALHVYSLGSD pc2
P80108
117:263 27:59 3:3
6:24418948:G:A_A
p = 0.4, beta = −0.145, N = 147
LYPTYSTK pc2
P80108
30:263 3:59 0:3
6:24418948:G:A_A
p = 0.046, beta = −1.11, N = 33
ILEGFQPSGR pc2
P80108
18:263 5:59 0:3
6:24418948:G:A_A
p = 0.49, beta = −0.326, N = 23
NQVVIAAGR pc2
P80108
22:263 4:59 0:3
6:24418948:G:A_A
p = 0.7, beta = −0.193, N = 26
VAFLTVTLHQGGATR pc3
P80108
20:263 3:59 2:3
6:24418948:G:A_A
p = 0.62, beta = −0.156, N = 25
326Assay Target: GPLD1
UniProt: P80108
rsID: rs45520637
Target SNP: 6:24418948:G:A
Proxy SNP: 6:24418948:G:A
Tarkin log10(p): 7.5
Tarkin BETA: 0.318
Tarkin MSPA: 0.859
1256:1257:1251:1256:1241:125
VYALTSDAQPLLLSTFSGDRR pc3
rs1042303 ALT
68:951 20:284 0:25
6:24418948:G:A_A
p = 0.41, model = DOM, N = 88
MYALTSDAQPLLLSTFSGDRR pc3
rs1042303 REF
3:951 0:284 0:25
6:24418948:G:A_A
p = NA, model = NA, N = 3

327. COL1A2 (P08123)
Tarkin MSPA=0.652

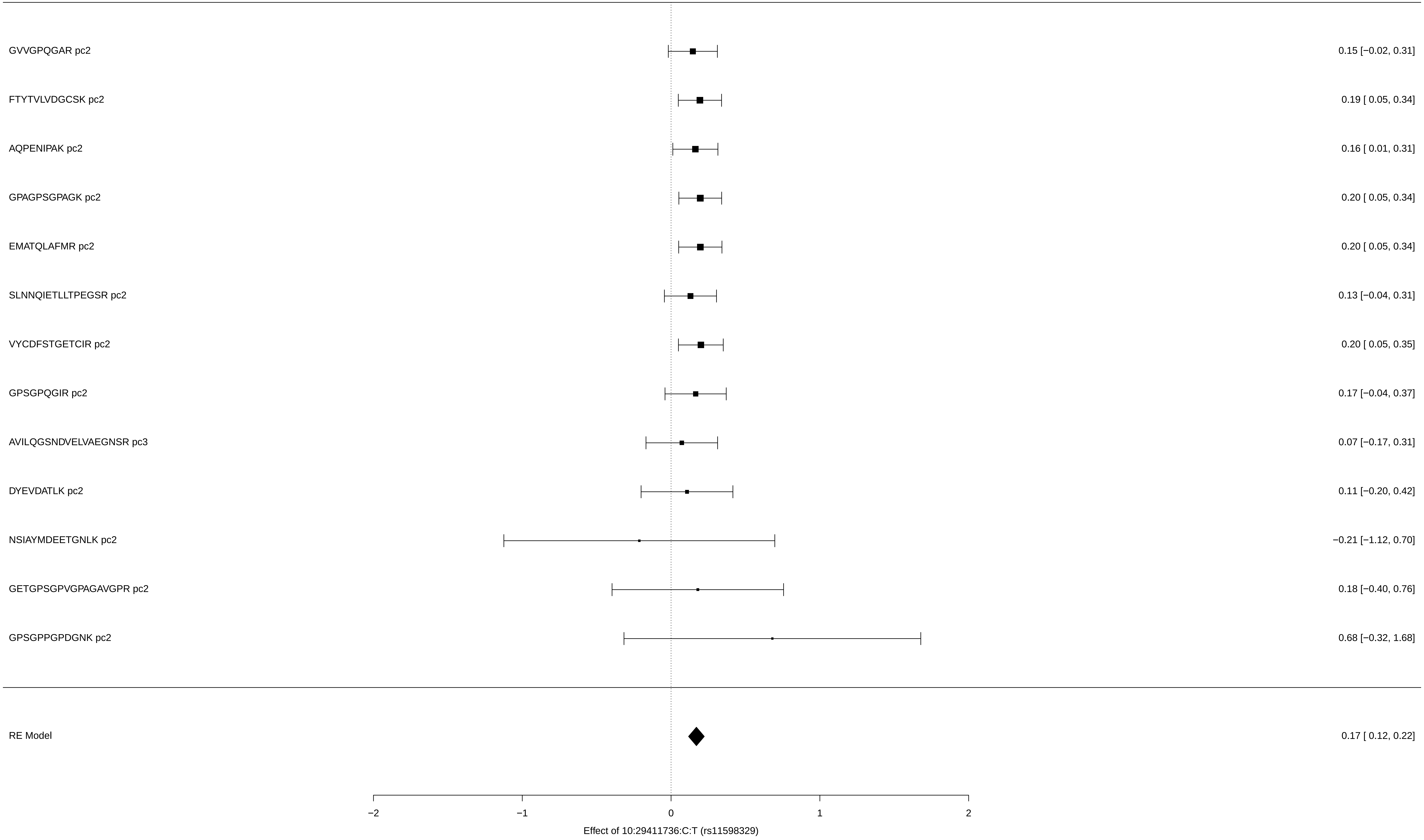

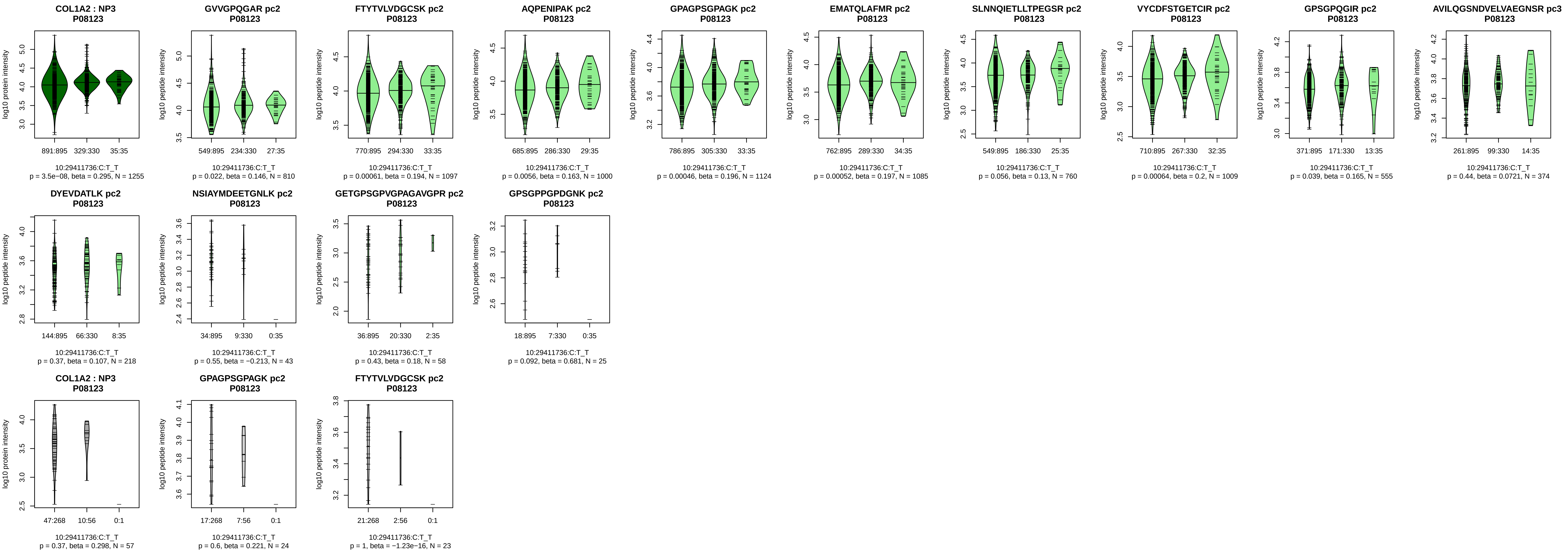

327Assay Target: COL1A2
UniProt: P08123
rsID: rs11598329
Target SNP: 10:29411736:C:T
Proxy SNP: 10:29411736:C:T
Tarkin log10(p): 7.5
Tarkin BETA: 0.295
Tarkin MSPA: 0.652
−:*:*:*:*:−:*:−:−:−:NA:−:NA:−:NA
810:1097:1000:1124:1085:760:1

328. CD109 (Q6YHK3)
Tarkin MSPA=0.786

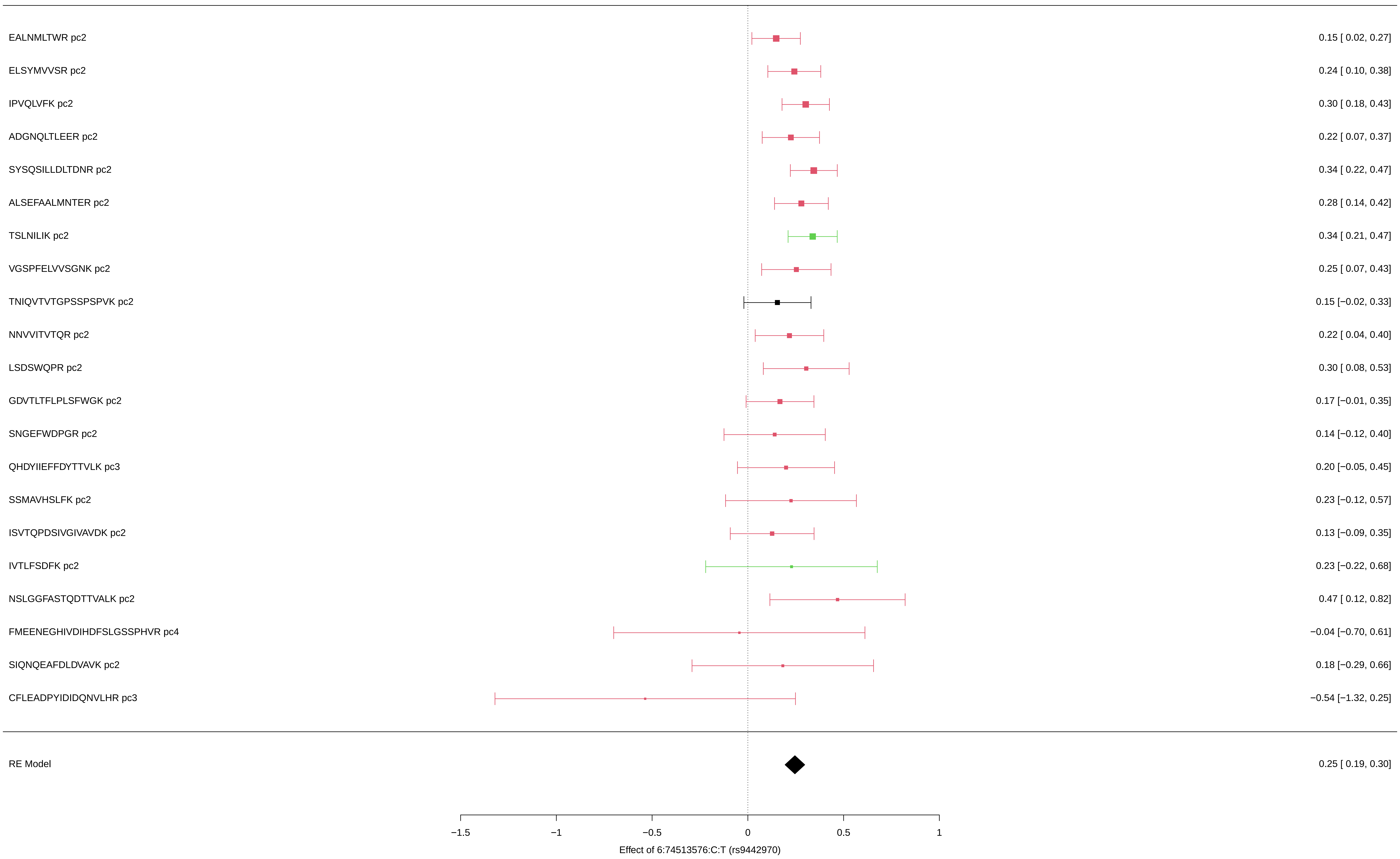

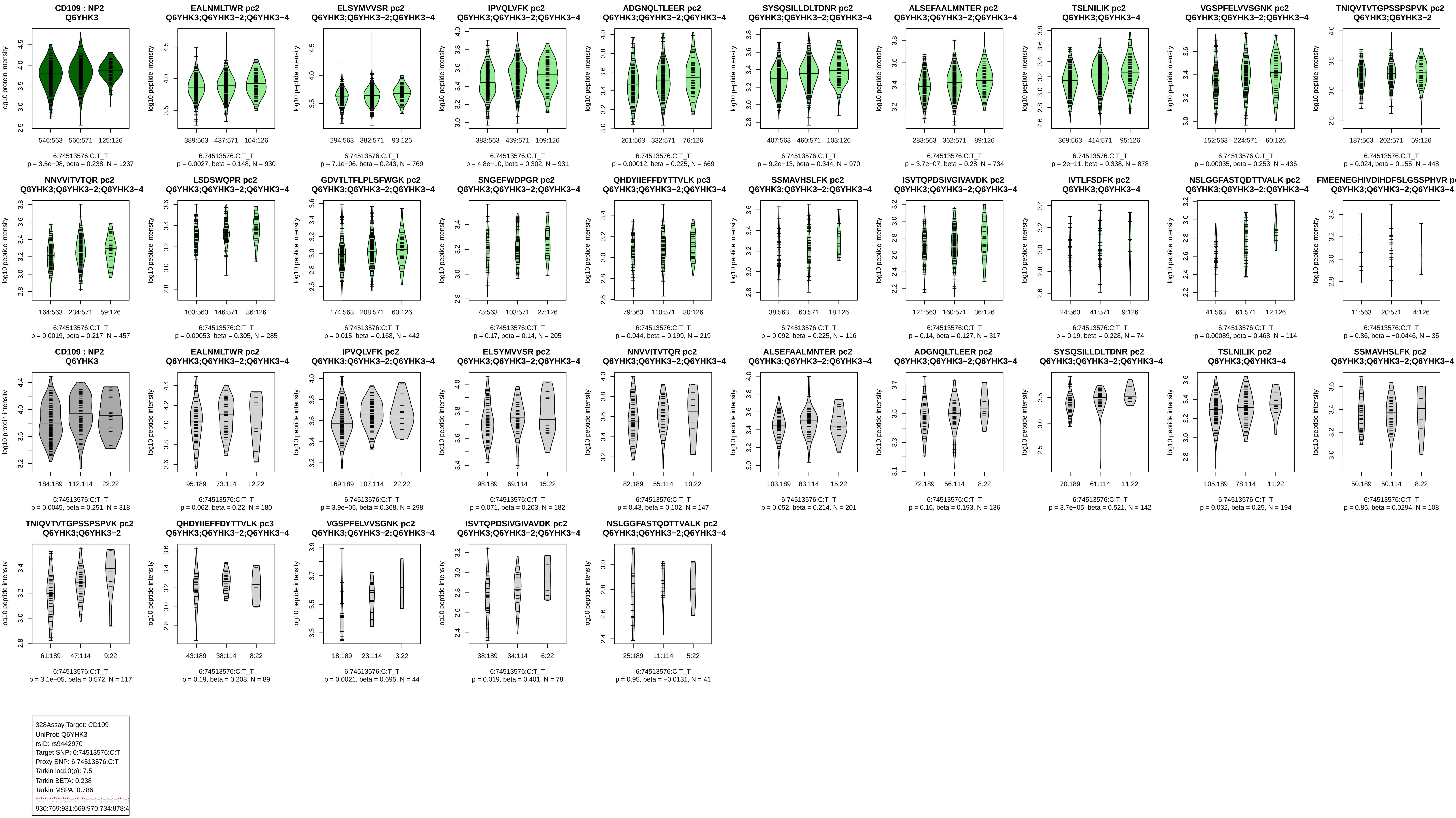
CD109 : NP2 Q6YHK3
6:74513576:C:T_T p = 3.5e−08, beta = 0.238, N = 1237
EALNMLTWR pc2 Q6YHK3;Q6YHK3−2;Q6YHK3−4
6:74513576:C:T_T p = 0.0027, beta = 0.148, N = 930
ELSYMVVSR pc2 Q6YHK3;Q6YHK3−2;Q6YHK3−4
6:74513576:C:T_T p = 7.1e−06, beta = 0.243, N = 769
IPVQLVFK pc2 Q6YHK3;Q6YHK3−2;Q6YHK3−4
6:74513576:C:T_T p = 4.8e−10, beta = 0.302, N = 931
ADGNQLTLEER pc2 Q6YHK3;Q6YHK3−2;Q6YHK3−4
6:74513576:C:T_T p = 0.00012, beta = 0.225, N = 669
SYSQSILLDLTDNR pc2 Q6YHK3;Q6YHK3−2;Q6YHK3−4
6:74513576:C:T_T p = 9.2e−13, beta = 0.344, N = 970
ALSEFAALMNTER pc2 Q6YHK3;Q6YHK3−2;Q6YHK3−4
6:74513576:C:T_T p = 3.7e−07, beta = 0.28, N = 734
TSLNILIK pc2 Q6YHK3;Q6YHK3−4
6:74513576:C:T_T p = 2e−11, beta = 0.338, N = 878
VGSPFELVVSGNK pc2 Q6YHK3;Q6YHK3−2;Q6YHK3−4
6:74513576:C:T_T p = 0.00035, beta = 0.253, N = 436
TNIQVTVTGPSSPSPVK pc2 Q6YHK3;Q6YHK3−2
6:74513576:C:T_T p = 0.024, beta = 0.155, N = 448
NNVVITVTQR pc2 Q6YHK3;Q6YHK3−2;Q6YHK3−4
6:74513576:C:T_T p = 0.0019, beta = 0.217, N = 457
LSDSWQPR pc2 Q6YHK3;Q6YHK3−2;Q6YHK3−4
6:74513576:C:T_T p = 0.00053, beta = 0.305, N = 285
GDVTLTFLPLSFWGK pc2 Q6YHK3;Q6YHK3−2;Q6YHK3−4
6:74513576:C:T_T p = 0.015, beta = 0.168, N = 442
SNGEFWDPGR pc2 Q6YHK3;Q6YHK3−2;Q6YHK3−4
6:74513576:C:T_T p = 0.17, beta = 0.14, N = 205
QHDYIIEFFDYTTVLK pc3 Q6YHK3;Q6YHK3−2;Q6YHK3−4
6:74513576:C:T_T p = 0.044, beta = 0.199, N = 219
SSMAVHSLFK pc2 Q6YHK3;Q6YHK3−2;Q6YHK3−4
6:74513576:C:T_T p = 0.092, beta = 0.225, N = 116
ISVTQPDSIVGIVAVDK pc2 Q6YHK3;Q6YHK3−2;Q6YHK3−4
6:74513576:C:T_T p = 0.14, beta = 0.127, N = 317
IVTLFSDFK pc2 Q6YHK3;Q6YHK3−4
6:74513576:C:T_T p = 0.19, beta = 0.228, N = 74
NSLGGFASTQDTTVALK pc2 Q6YHK3;Q6YHK3−2;Q6YHK3−4
6:74513576:C:T_T p = 0.00089, beta = 0.468, N = 114
FMEENEGHIVDIHDFSLGSSPHVR pc
Q6YHK3;Q6YHK3−2;Q6YHK3−4
6:74513576:C:T_T p = 0.86, beta = −0.0446, N = 35
CD109 : NP2 Q6YHK3
6:74513576:C:T_T p = 0.0045, beta = 0.251, N = 318
EALNMLTWR pc2 Q6YHK3;Q6YHK3−2;Q6YHK3−4
6:74513576:C:T_T p = 0.062, beta = 0.22, N = 180
IPVQLVFK pc2 Q6YHK3;Q6YHK3−2;Q6YHK3−4
6:74513576:C:T_T p = 3.9e−05, beta = 0.368, N = 298
ELSYMVVSR pc2 Q6YHK3;Q6YHK3−2;Q6YHK3−4
6:74513576:C:T_T p = 0.071, beta = 0.203, N = 182
NNVVITVTQR pc2 Q6YHK3;Q6YHK3−2;Q6YHK3−4
6:74513576:C:T_T p = 0.43, beta = 0.102, N = 147
ALSEFAALMNTER pc2 Q6YHK3;Q6YHK3−2;Q6YHK3−4
6:74513576:C:T_T p = 0.052, beta = 0.214, N = 201
ADGNQLTLEER pc2 Q6YHK3;Q6YHK3−2;Q6YHK3−4
6:74513576:C:T_T p = 0.16, beta = 0.193, N = 136
SYSQSILLDLTDNR pc2 Q6YHK3;Q6YHK3−2;Q6YHK3−4
6:74513576:C:T_T p = 3.7e−05, beta = 0.521, N = 142
TSLNILIK pc2 Q6YHK3;Q6YHK3−4
6:74513576:C:T_T p = 0.032, beta = 0.25, N = 194
SSMAVHSLFK pc2 Q6YHK3;Q6YHK3−2;Q6YHK3−4
6:74513576:C:T_T p = 0.85, beta = 0.0294, N = 108
TNIQVTVTGPSSPSPVK pc2 Q6YHK3;Q6YHK3−2
6:74513576:C:T_T p = 3.1e−05, beta = 0.572, N = 117
QHDYIIEFFDYTTVLK pc3 Q6YHK3;Q6YHK3−2;Q6YHK3−4
6:74513576:C:T_T p = 0.19, beta = 0.208, N = 89
VGSPFELVVSGNK pc2 Q6YHK3;Q6YHK3−2;Q6YHK3−4
6:74513576:C:T_T p = 0.0021, beta = 0.695, N = 44
ISVTQPDSIVGIVAVDK pc2 Q6YHK3;Q6YHK3−2;Q6YHK3−4
6:74513576:C:T_T p = 0.019, beta = 0.401, N = 78
NSLGGFASTQDTTVALK pc2 Q6YHK3;Q6YHK3−2;Q6YHK3−4
6:74513576:C:T_T p = 0.95, beta = −0.0131, N = 41
328Assay Target: CD109
UniProt: Q6YHK3
rsID: rs9442970
Target SNP: 6:74513576:C:T
Proxy SNP: 6:74513576:C:T
Tarkin log10(p): 7.5
Tarkin BETA: 0.238
Tarkin MSPA: 0.786
930:769:931:669:970:734:878:4

329. APLP1 (B7Z4G8)
Tarkin MSPA=0.078

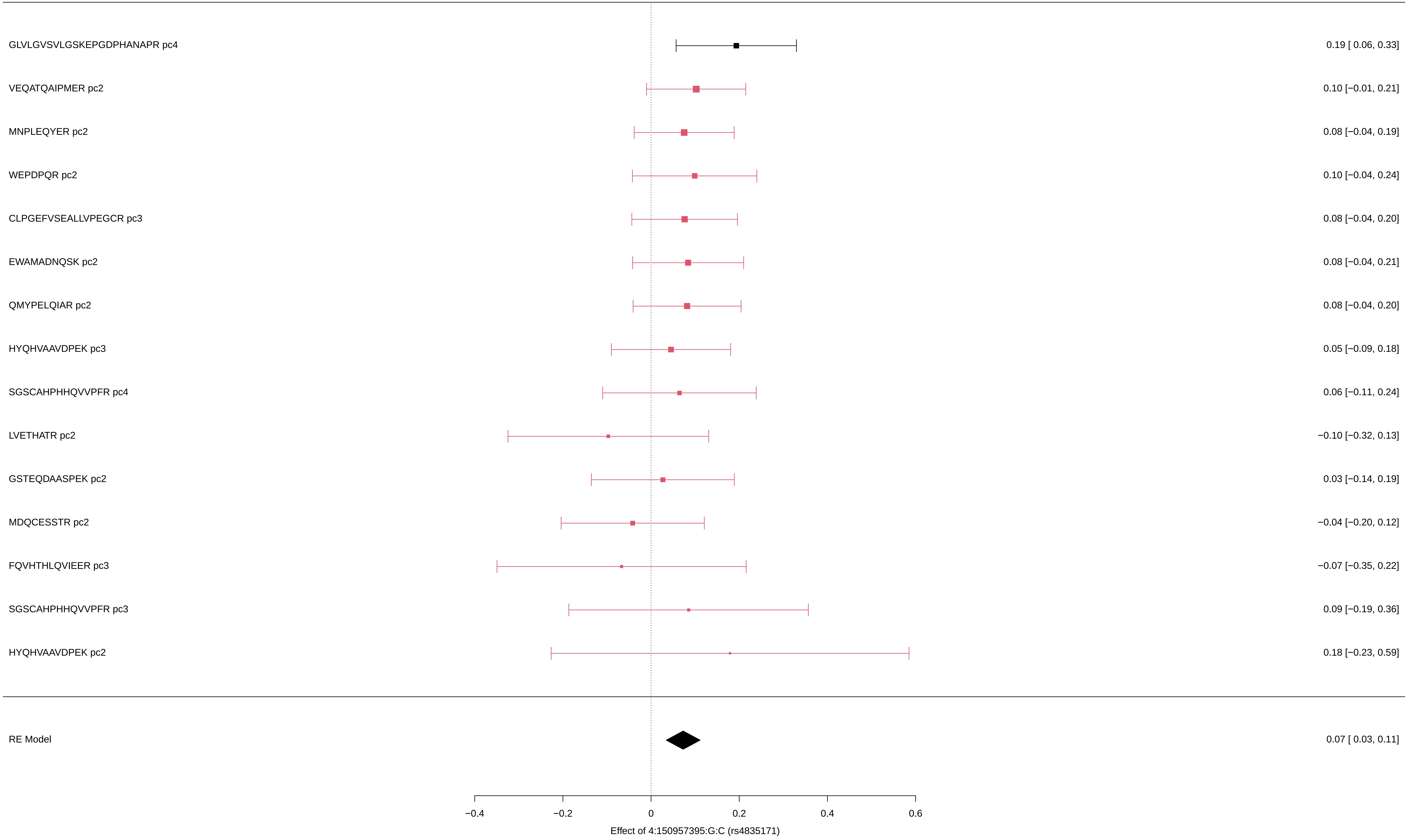

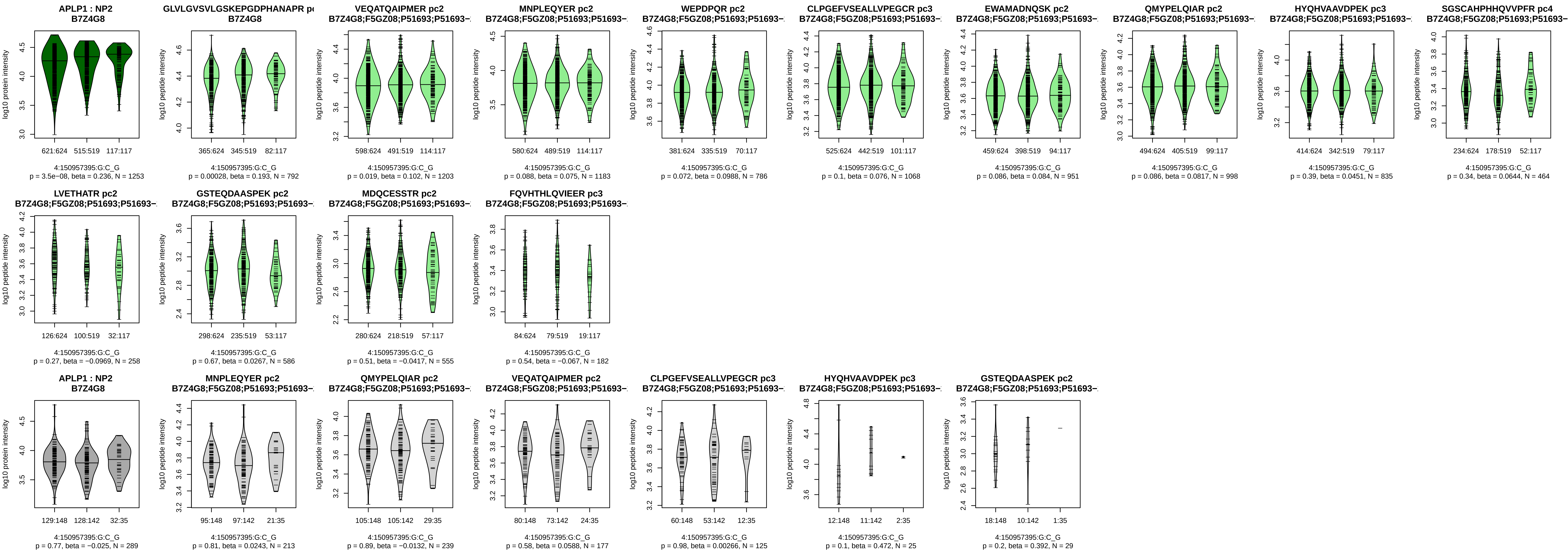

329Assay Target: APLP1
UniProt: B7Z4G8
rsID: rs4835171
Target SNP: 4:150957395:G:C
Proxy SNP: 4:150957395:G:C
Tarkin log10(p): 7.4
Tarkin BETA: 0.236
Tarkin MSPA: 0.078
*-:-:-:-:-:-:-:-:-:-:-:-
792:1203:1183:786:1068:951:9

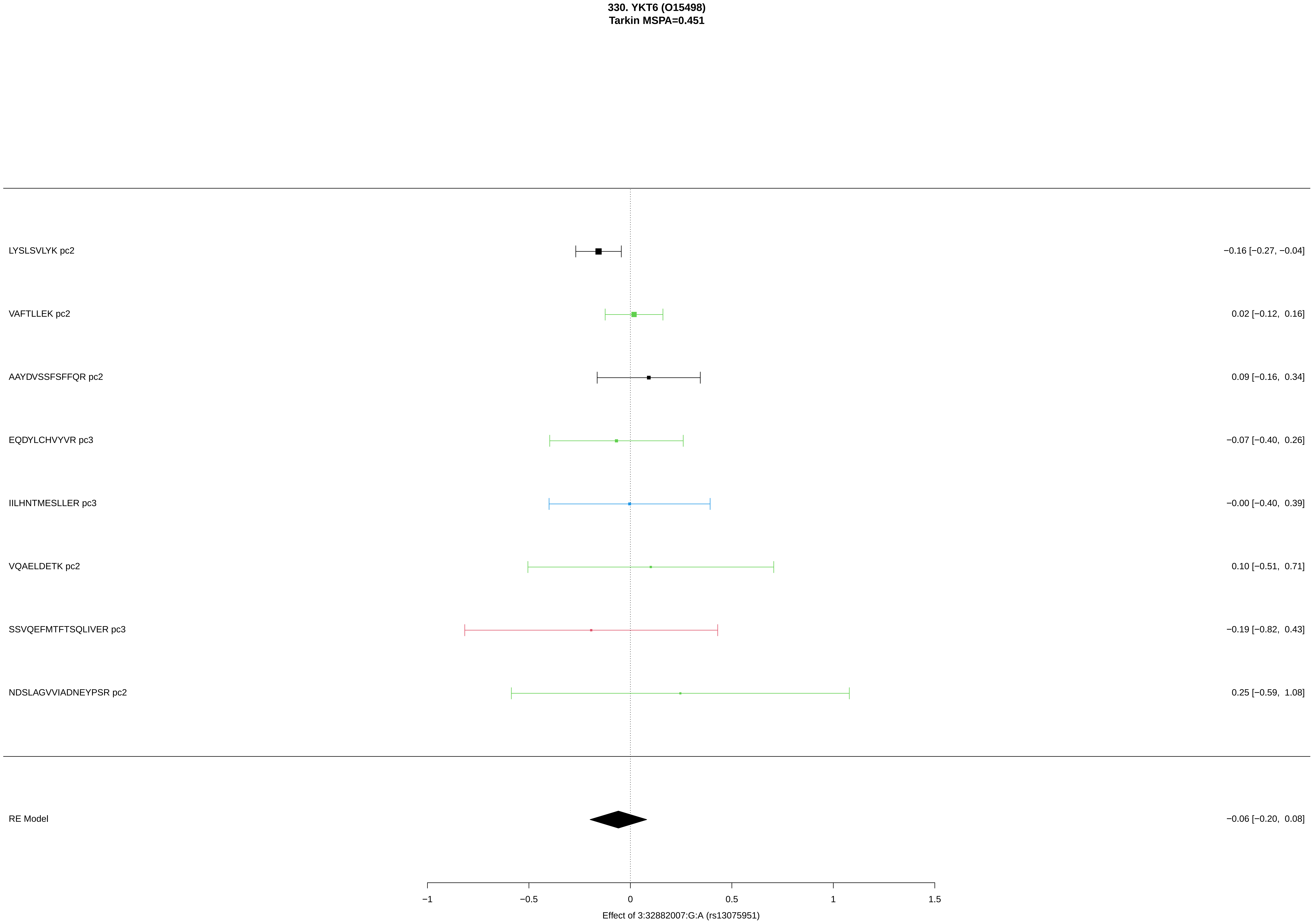
330. YKT6 (O15498)
Tarkin MSPA=0.451
LYSLSVLYK pc2
VAFTLLEK pc2
AAYDVSSFSFFQR pc2
EQDYLCHVYVR pc3
IILHNTMESLLER pc3
VQAELDETK pc2
SSVQEFMTFTSQLIVER pc3
NDSLAGVVIADNEYPSR pc2
RE Model
−0.16 [−0.27, −0.04]
0.02 [−0.12, 0.16]
0.09 [−0.16, 0.34]
−0.07 [−0.40, 0.26]
−0.00 [−0.40, 0.39]
0.10 [−0.51, 0.71]
−0.19 [−0.82, 0.43]
0.25 [−0.59, 1.08]
−0.06 [−0.20, 0.08]
−1
−0.5
0
0.5
1
1.5
Effect of 3:32882007:G:A (rs13075951)

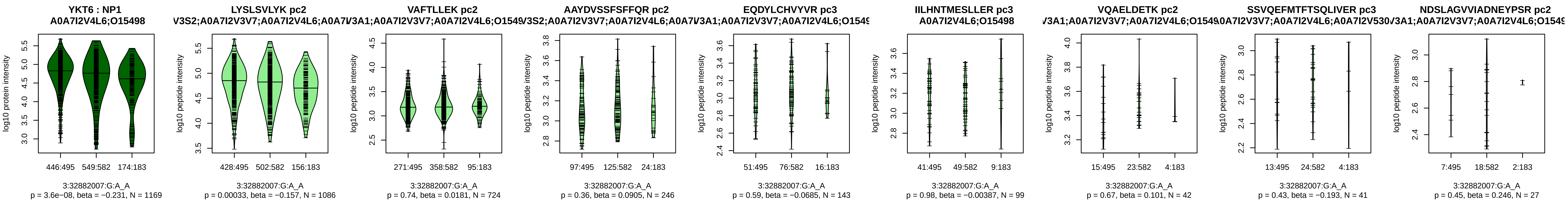

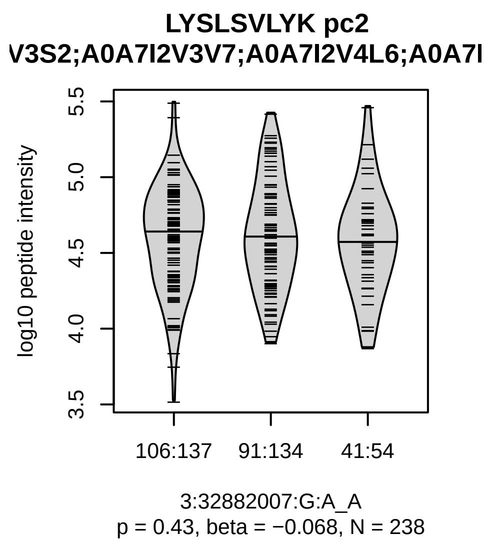

330Assay Target: YKT6
UniProt: O15498
rsID: rs13075951
Target SNP: 3:32882007:G:A
Proxy SNP: 3:32882007:G:A
Tarkin log10(p): 7.4
Tarkin BETA: −0.231
Tarkin MSPA: 0.451
*:–:–:–:–:–:–:–
1086:724:246:143:99:42:41:27

331. S100A4 (P26447)
Tarkin MSPA=0.255

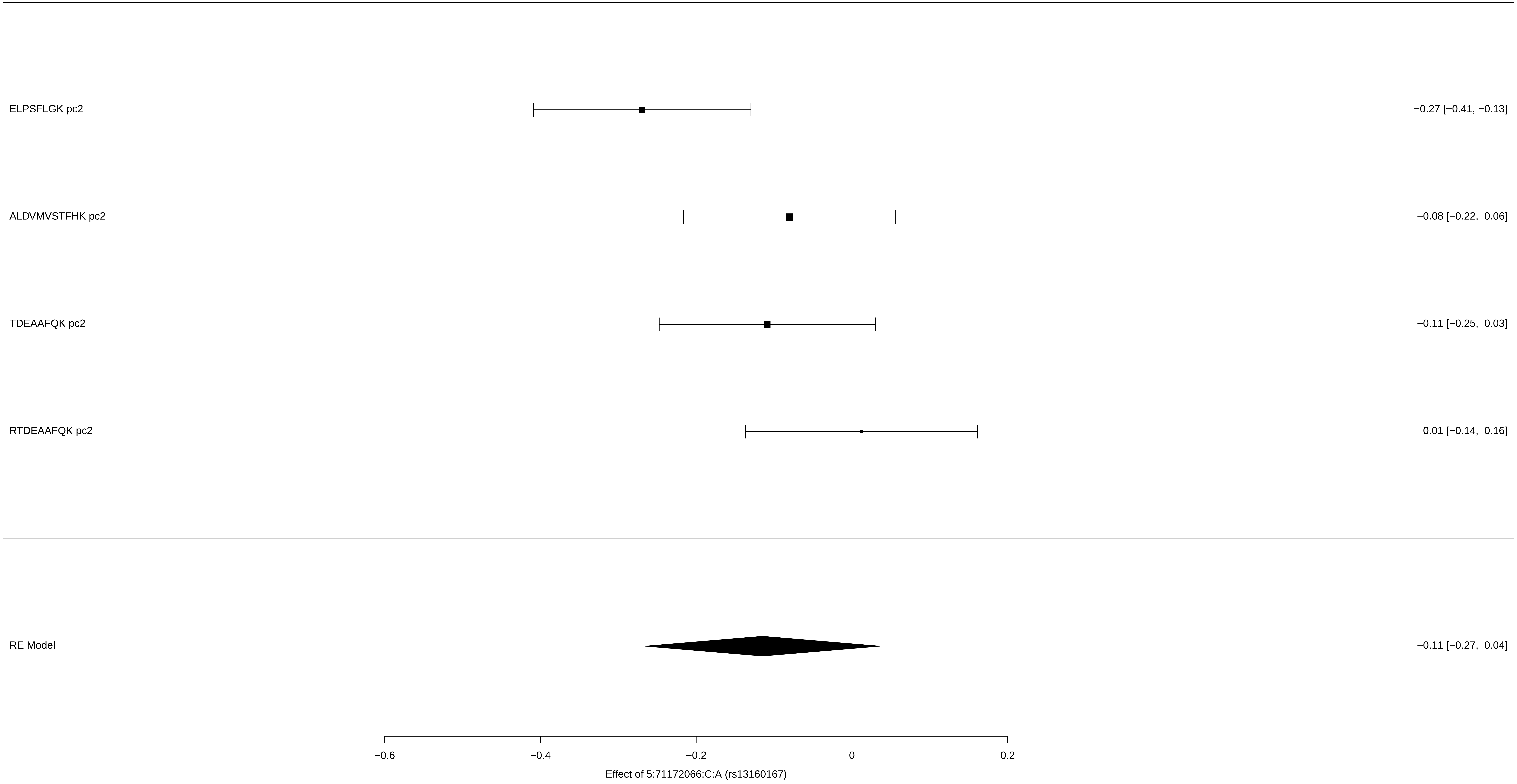

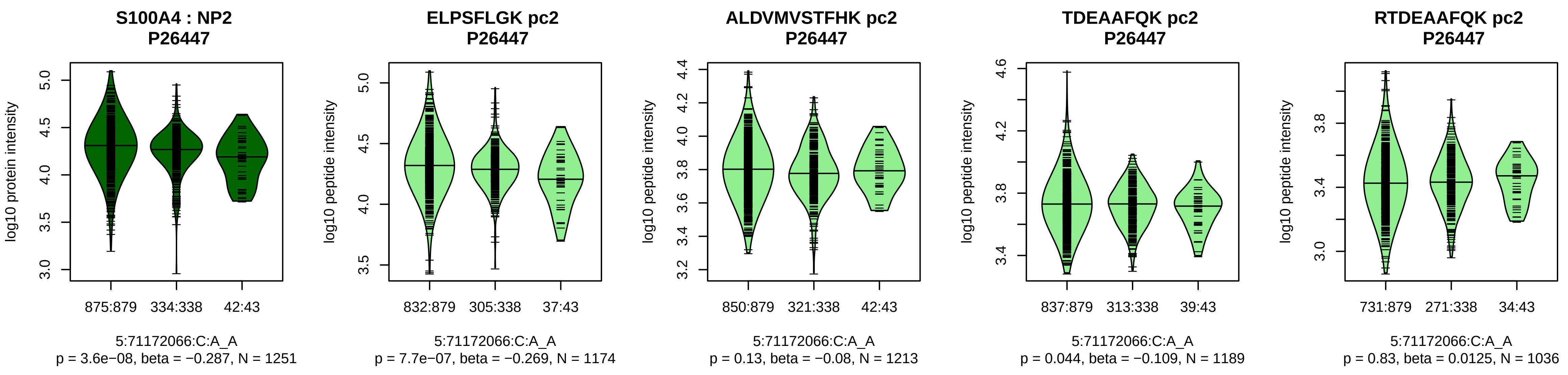

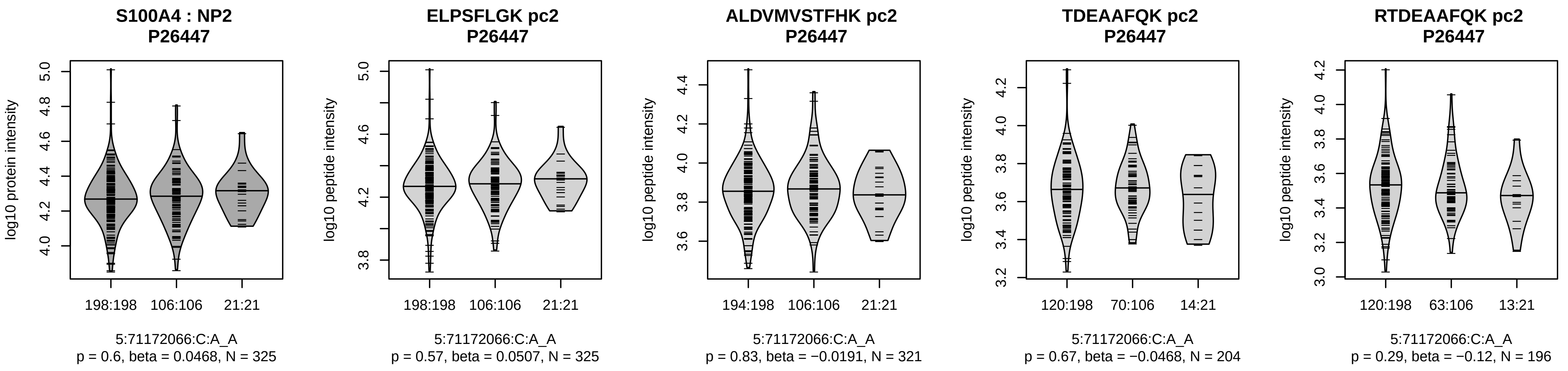

331Assay Target: S100A4
UniProt: P26447
rsID: rs13160167
Target SNP: 5:71172066:C:A
Proxy SNP: 5:71172066:C:A
Tarkin log10(p): 7.4
Tarkin BETA: −0.287
Tarkin MSPA: 0.255
*:−:−:−:NA
1174:1213:1189:1036:17

332. MAPKAPK2 (P49137)
Tarkin MSPA=0.72

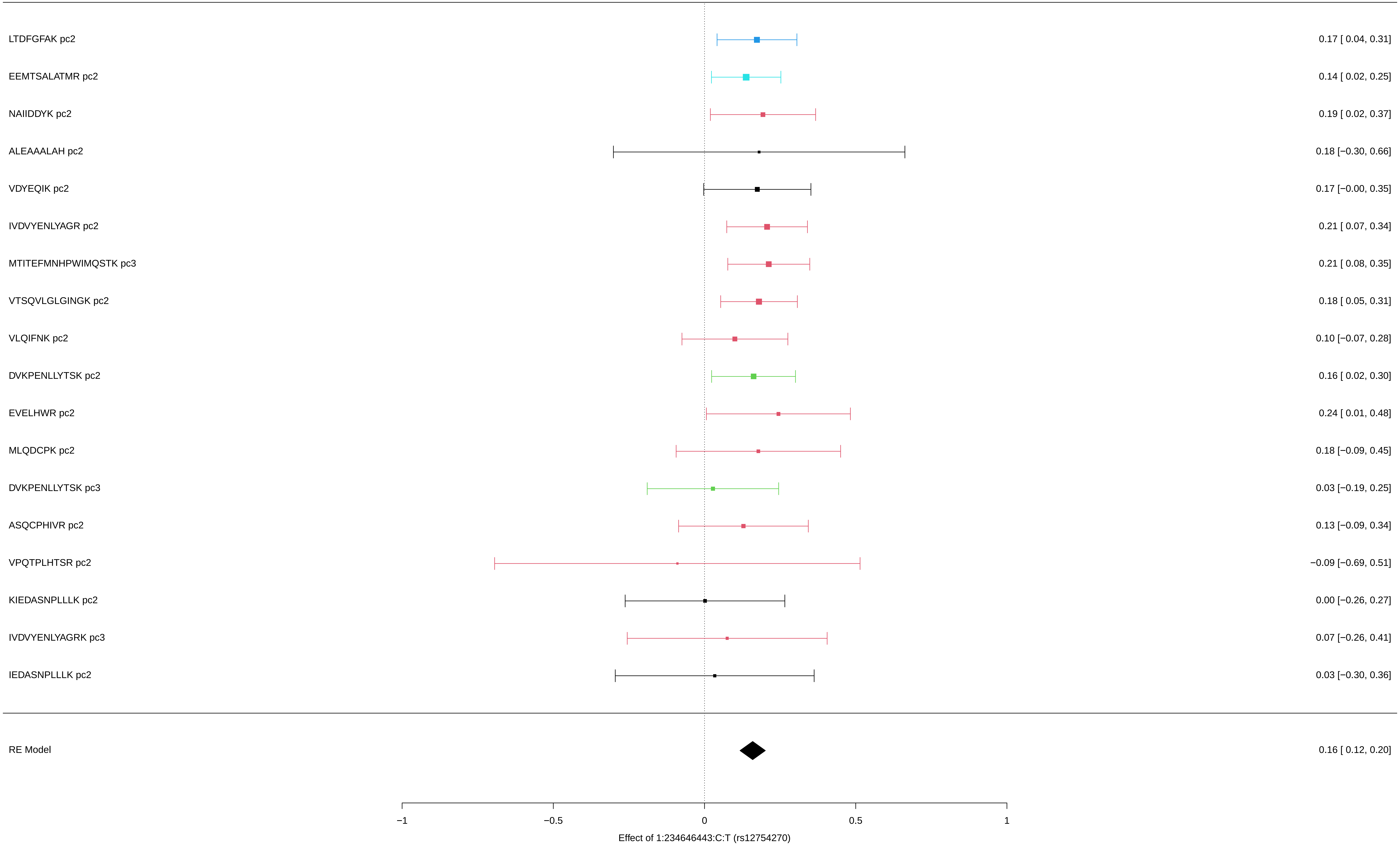

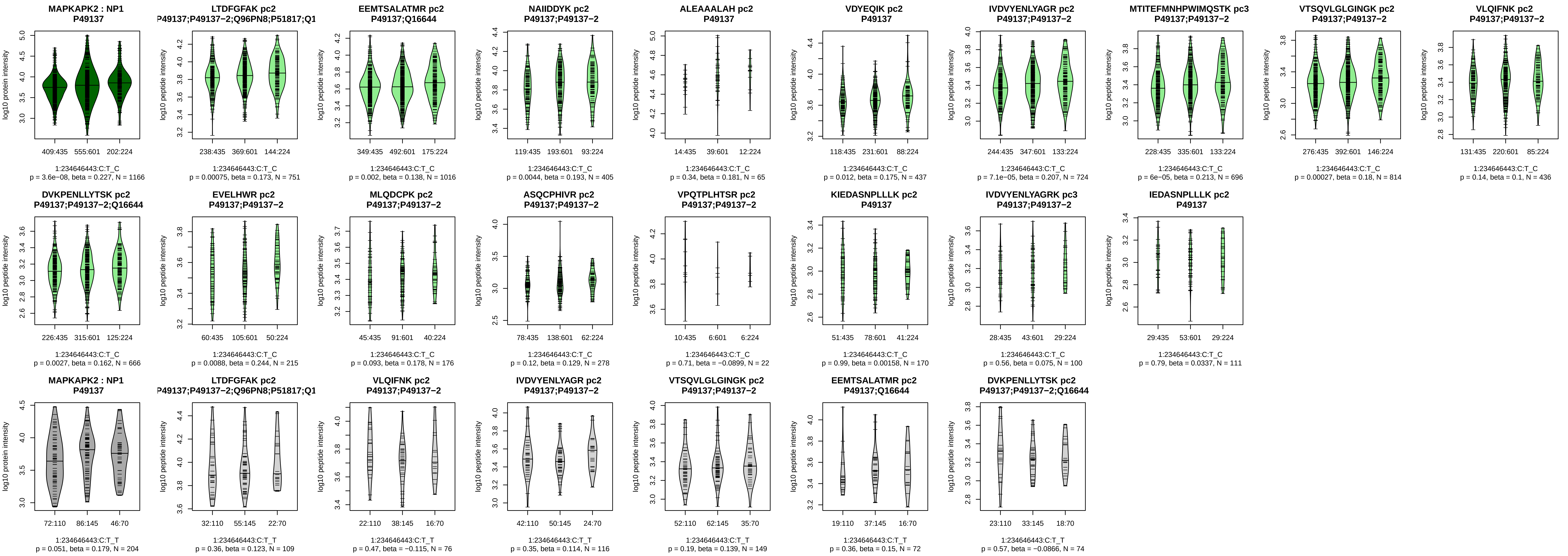

332Assay Target: MAPKAPK2
UniProt: P49137
rsID: rs12754270
Target SNP: 1:234646443:C:T
Proxy SNP: 1:234646443:C:T
Tarkin log10(p): 7.4
Tarkin BETA: 0.227
Tarkin MSPA: 0.72
::*:–:–:*:*:*:–:*:*:–:–:–:–:–:–
751:1016:405:65:437:724:696:8

333. NACA (E9PAV3)
Tarkin MSPA=0.578

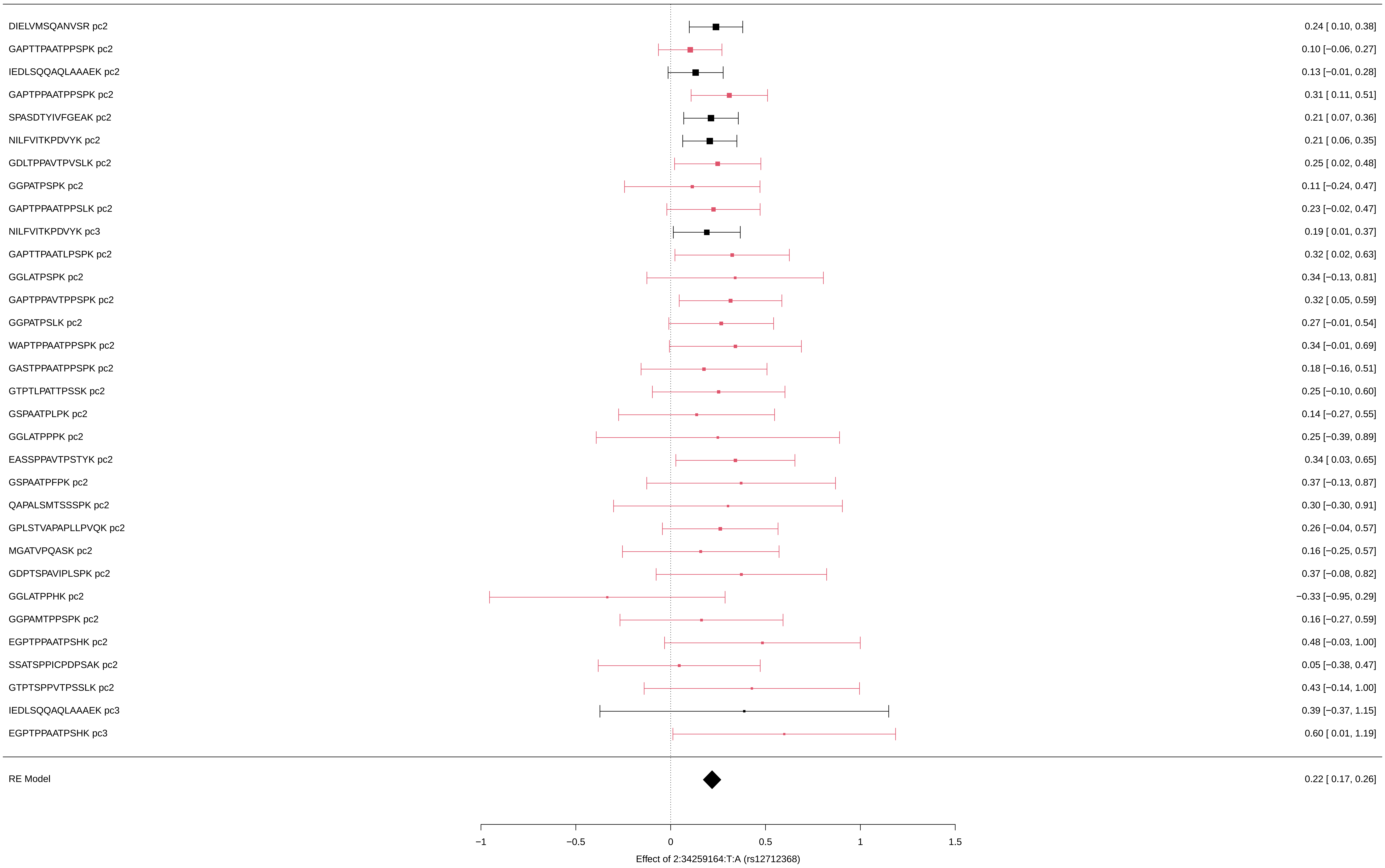

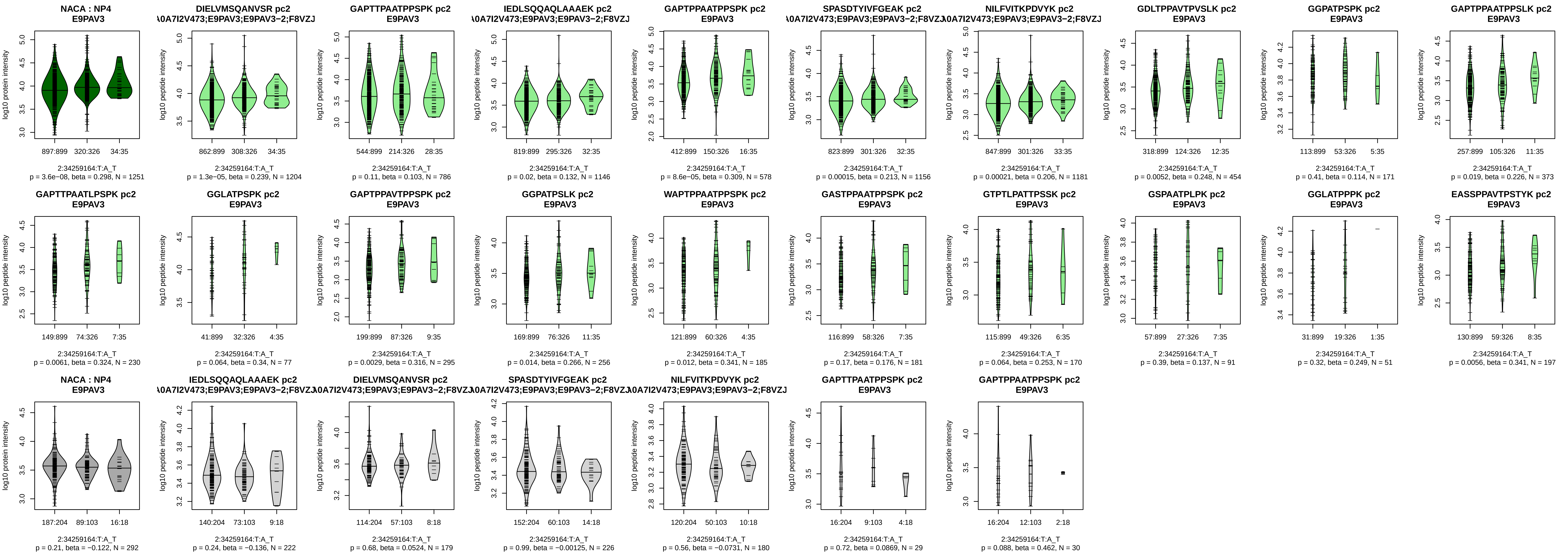

333Assay Target: NACA
UniProt: E9PAV3
rsID: rs12712368
Target SNP: 2:34259164:T:A
Proxy SNP: 2:34259164:T:A
Tarkin log10(p): 7.4
Tarkin BETA: 0.298
Tarkin MSPA: 0.578
[illegible]
1204:786:1146:578:1156:1181:4

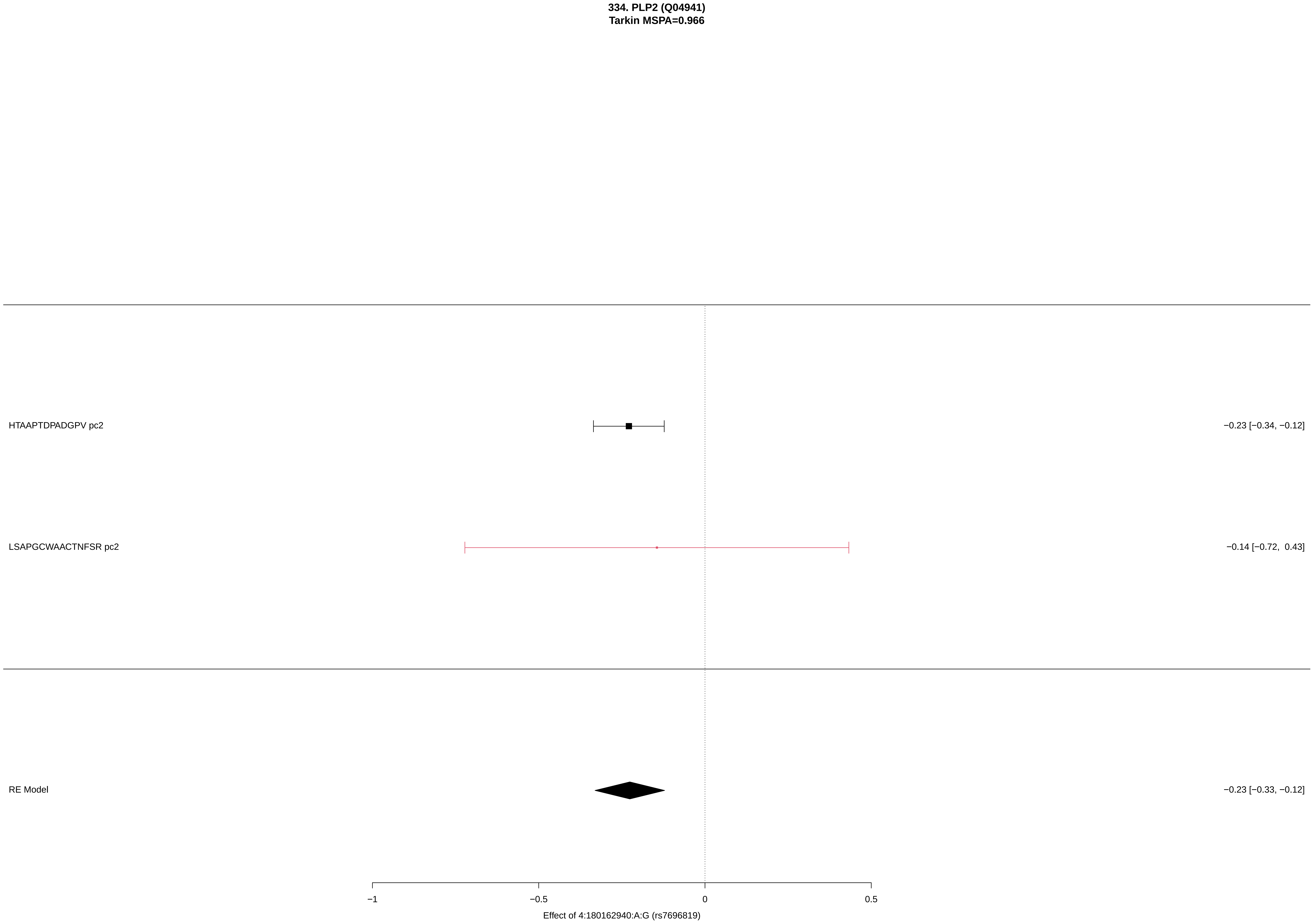

334. PLP2 (Q04941)
Tarkin MSPA=0.966
HTAAPTDPADGPV pc2
−0.23 [−0.34, −0.12]
LSAPGCWAACTNFSR pc2
−0.14 [−0.72, 0.43]
RE Model
−0.23 [−0.33, −0.12]
−1
−0.5
0
0.5
Effect of 4:180162940:A:G (rs7696819)

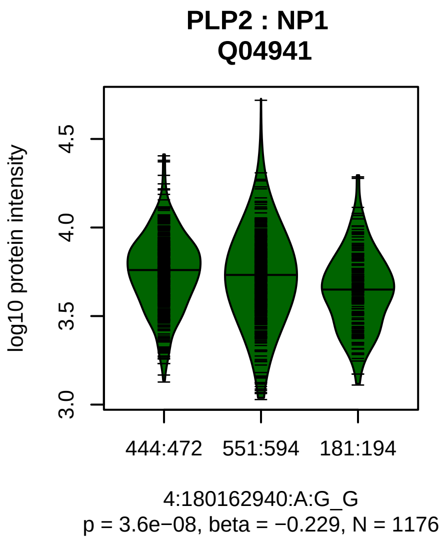

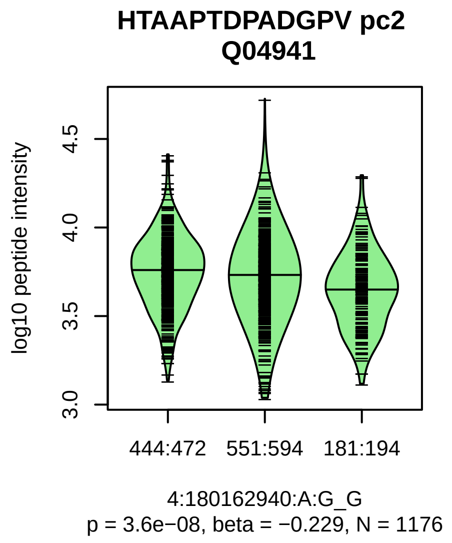

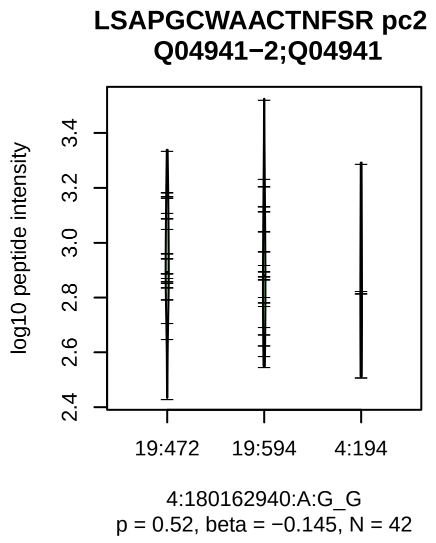

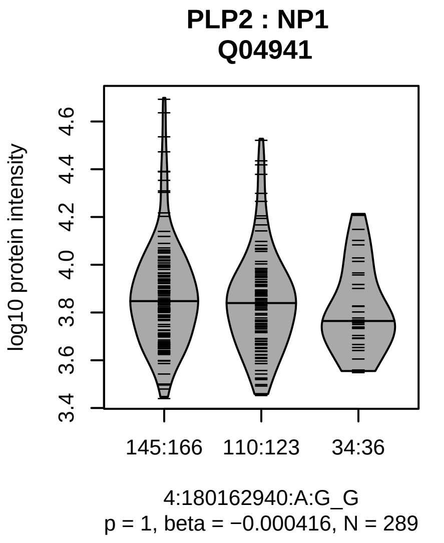

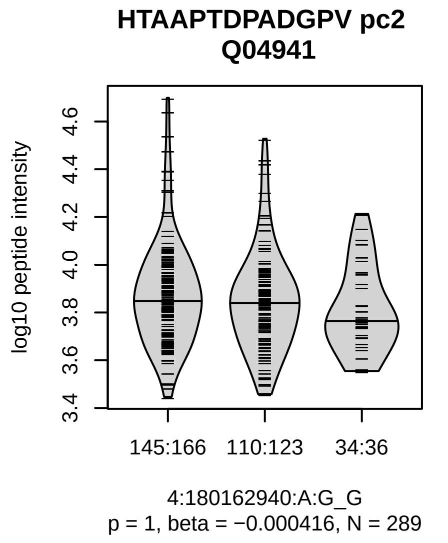

334Assay Target: PLP2
UniProt: Q04941
rsID: rs7696819
Target SNP: 4:180162940:A:G
Proxy SNP: 4:180162940:A:G
Tarkin log10(p): 7.4
Tarkin BETA: −0.229
Tarkin MSPA: 0.966
*:–
1176:42

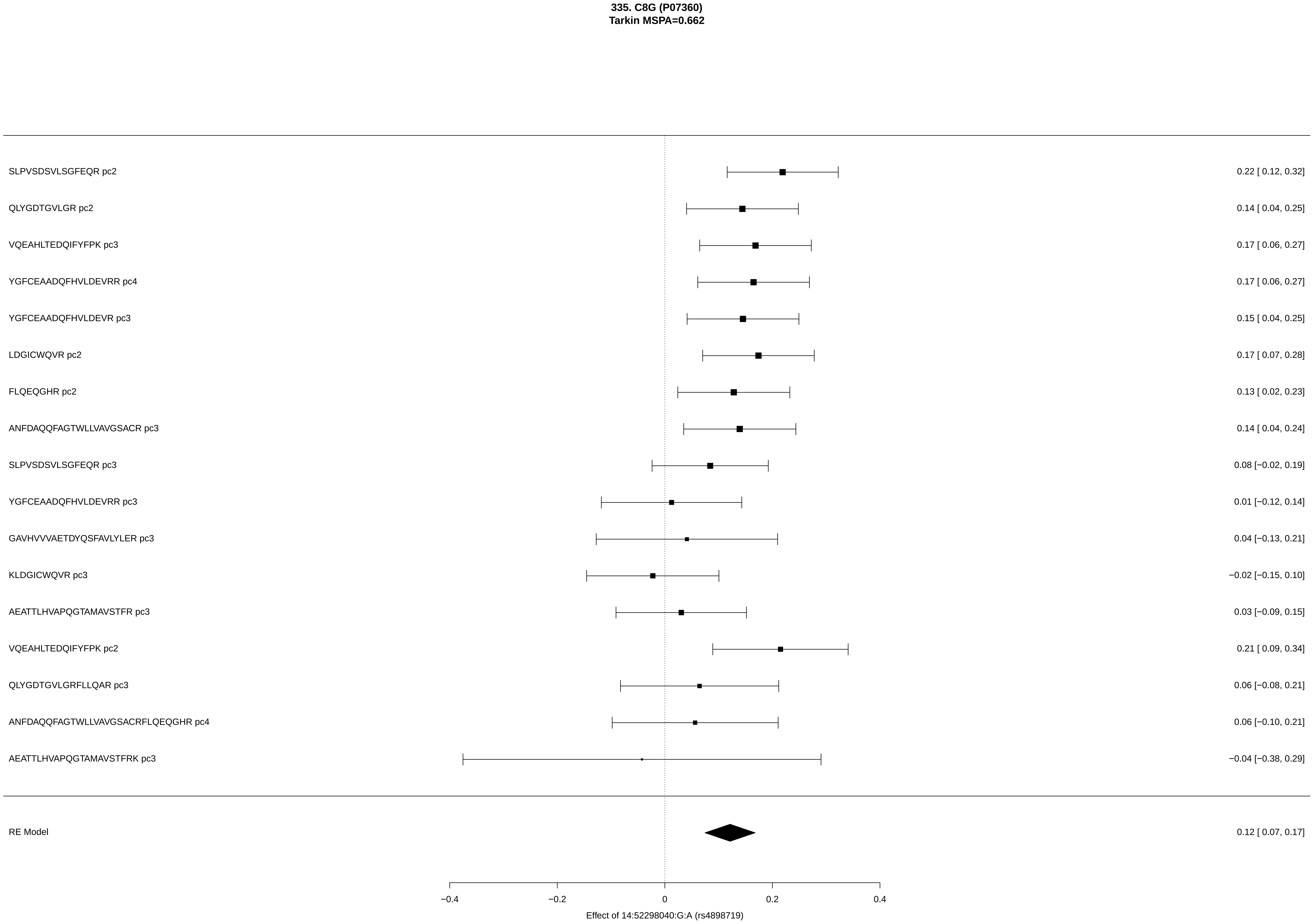
335. C8G (P07360)
Tarkin MSPA=0.662
SLPVSDSVLSGFEQR pc2
0.22 [0.12, 0.32]
QLYGDTGVLGR pc2
0.14 [0.04, 0.25]
VQEAHLTEDQIFYFPK pc3
0.17 [0.06, 0.27]
YGFCEAADQFHVLDEVRR pc4
0.17 [0.06, 0.27]
YGFCEAADQFHVLDEVR pc3
0.15 [0.04, 0.25]
LDGICWQVR pc2
0.17 [0.07, 0.28]
FLQEQGHR pc2
0.13 [0.02, 0.23]
ANFDAQQFAGTWLLVAVGSACR pc3
0.14 [0.04, 0.24]
SLPVSDSVLSGFEQR pc3
0.08 [−0.02, 0.19]
YGFCEAADQFHVLDEVRR pc3
0.01 [−0.12, 0.14]
GAVHVVVAETDYQSFAVLYLER pc3
0.04 [−0.13, 0.21]
KLDGICWQVR pc3
−0.02 [−0.15, 0.10]
AEATTLHVAPQGTAMAVSTFR pc3
0.03 [−0.09, 0.15]
VQEAHLTEDQIFYFPK pc2
0.21 [0.09, 0.34]
QLYGDTGVLGRFLLQAR pc3
0.06 [−0.08, 0.21]
ANFDAQQFAGTWLLVAVGSACRFLQEQGHR pc4
0.06 [−0.10, 0.21]
AEATTLHVAPQGTAMAVSTFRK pc3
−0.04 [−0.38, 0.29]
RE Model
0.12 [0.07, 0.17]
−0.4
−0.2
0
0.2
0.4
Effect of 14:52298040:G:A (rs4898719)

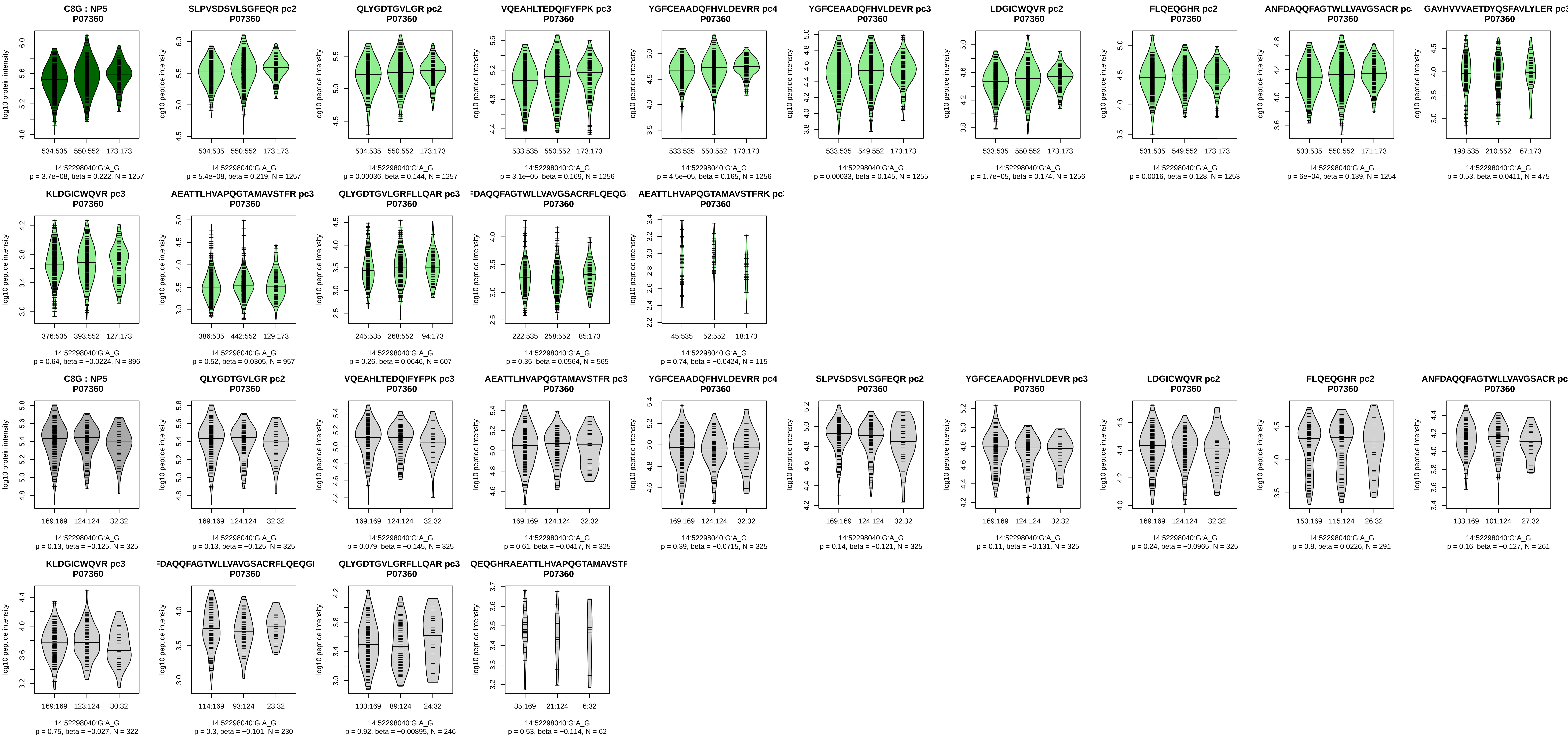

335Assay Target: C8G
UniProt: P07360
rsID: rs4898719
Target SNP: 14:52298040:G:A
Proxy SNP: 14:52298040:G:A
Tarkin log10(p): 7.4
Tarkin BETA: 0.222
Tarkin MSPA: 0.662
::*:*:*:*:*:*:–:–:–:–:–:*:–:–:–
1257:1257:1256:1256:1255:125

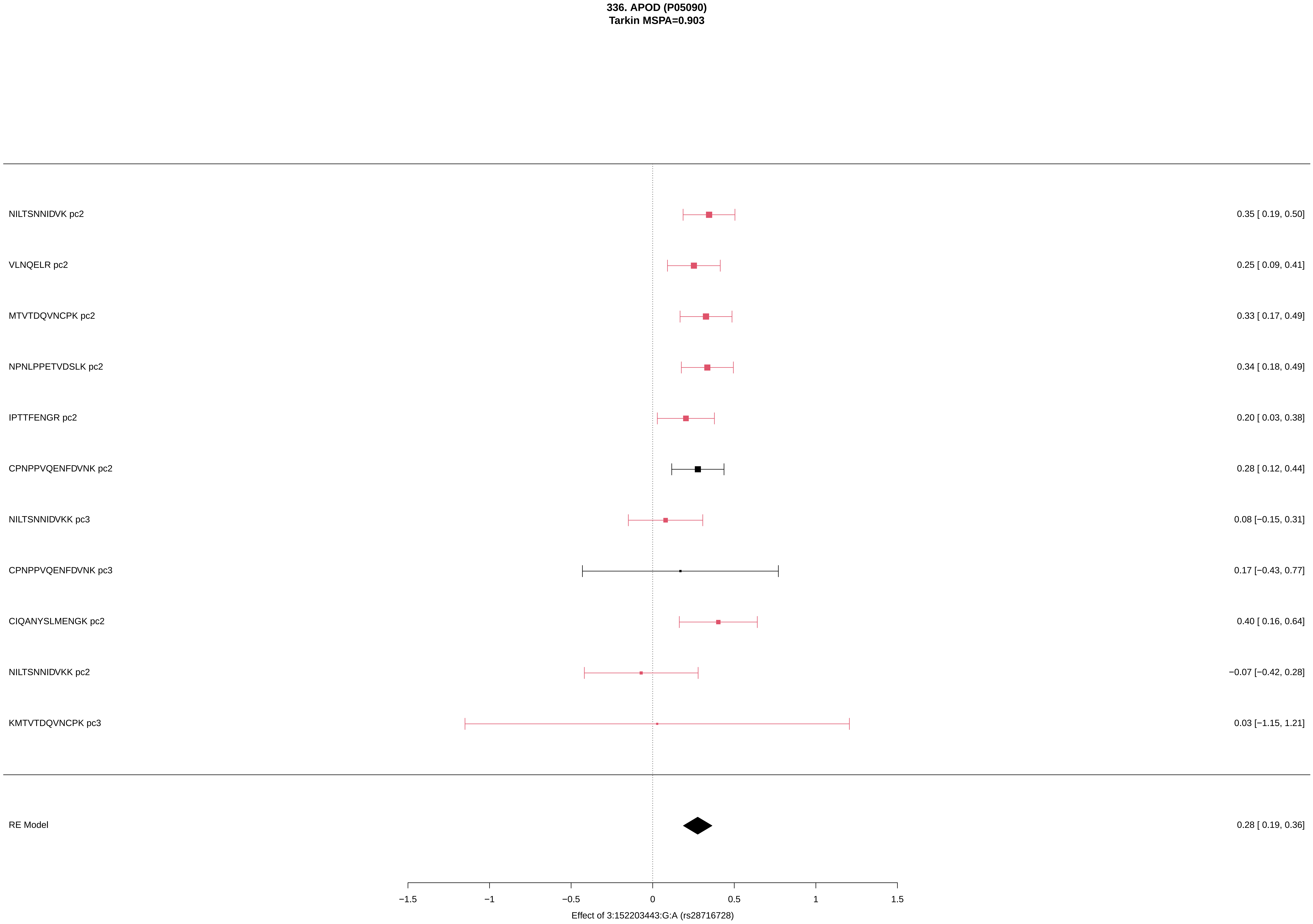
336. APOD (P05090)
Tarkin MSPA=0.903
NILTSNNIDVK pc2
VLNQELR pc2
MTVTDQVNCPK pc2
NPNLPPETVDSLK pc2
IPTTFENGR pc2
CPNPPVQENFDVNK pc2
NILTSNNIDVKK pc3
CPNPPVQENFDVNK pc3
CIQANYSLMENGK pc2
NILTSNNIDVKK pc2
KMTVTDQVNCPK pc3
RE Model
0.35 [0.19, 0.50]
0.25 [0.09, 0.41]
0.33 [0.17, 0.49]
0.34 [0.18, 0.49]
0.20 [0.03, 0.38]
0.28 [0.12, 0.44]
0.08 [−0.15, 0.31]
0.17 [−0.43, 0.77]
0.40 [0.16, 0.64]
−0.07 [−0.42, 0.28]
0.03 [−1.15, 1.21]
0.28 [0.19, 0.36]
−1.5
−1
−0.5
0
0.5
1
1.5
Effect of 3:152203443:G:A (rs28716728)

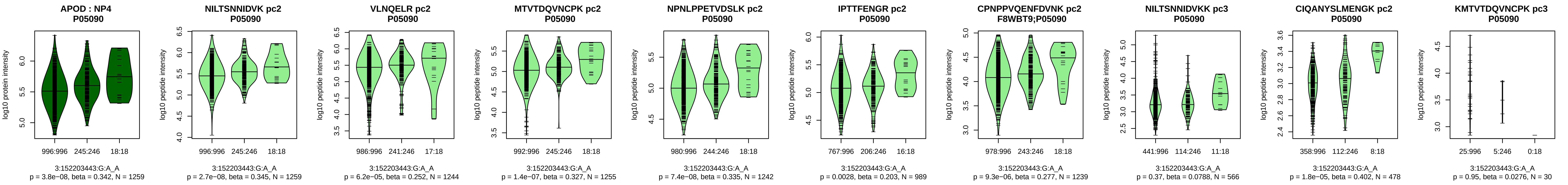

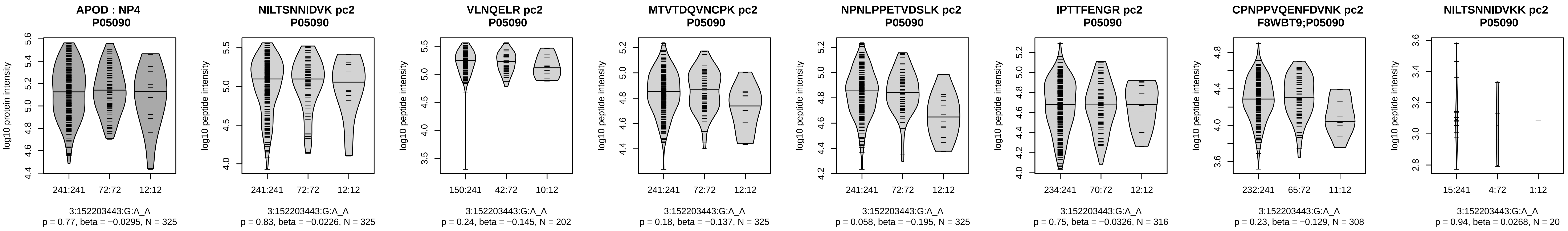

336Assay Target: APOD
UniProt: P05090
rsID: rs28716728
Target SNP: 3:152203443:G:A
Proxy SNP: 3:152203443:G:A
Tarkin log10(p): 7.4
Tarkin BETA: 0.342
Tarkin MSPA: 0.903
::*:*:*:*:–:–:*:–:–:NA
1259:1244:1255:1242:989:1239

337. CCT2 (P78371)
Tarkin MSPA=0.045

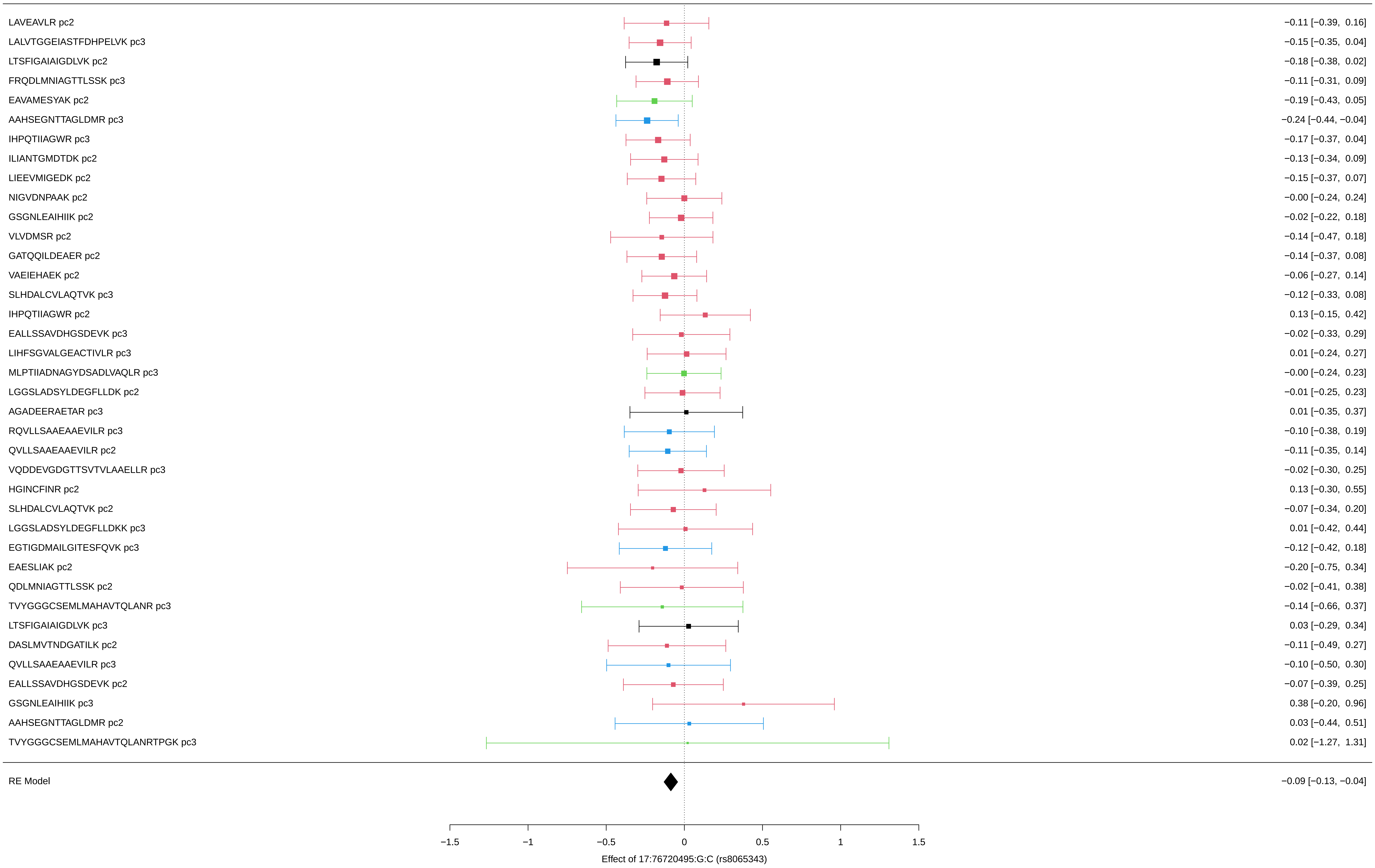

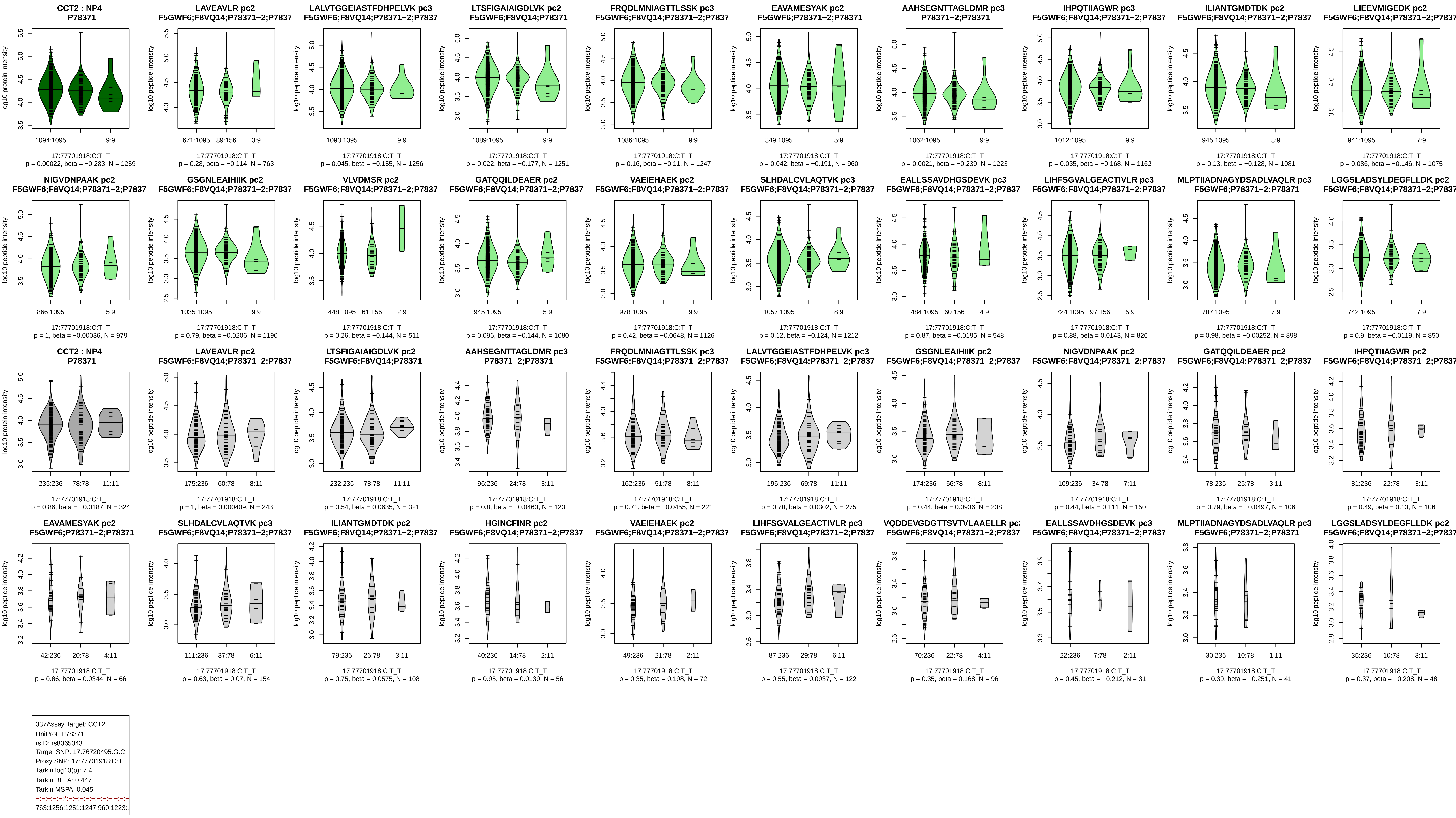
CCT2 : NP4
P78371
1094:1095 9:9
17:77701918:C:T_T
p = 0.00022, beta = −0.283, N = 1259
LAVEAVLR pc2
F5GWF6;F8VQ14;P78371−2;P78371
671:1095 89:156 3:9
17:77701918:C:T_T
p = 0.28, beta = −0.114, N = 763
LALVTGGEIASTFDHPELVK pc3
F5GWF6;F8VQ14;P78371−2;P7837
1093:1095 9:9
17:77701918:C:T_T
p = 0.045, beta = −0.155, N = 1256
LTSFIGAIAIGDLVK pc2
F5GWF6;F8VQ14;P78371
1089:1095 9:9
17:77701918:C:T_T
p = 0.022, beta = −0.177, N = 1251
FRQDLMNIAGTTLSSK pc3
F5GWF6;F8VQ14;P78371−2;P78371
1086:1095 9:9
17:77701918:C:T_T
p = 0.16, beta = −0.11, N = 1247
EAVAMESYAK pc2
F5GWF6;P78371−2;P78371
849:1095 5:9
17:77701918:C:T_T
p = 0.042, beta = −0.191, N = 960
AAHSEGNTTAGLDMR pc3
P78371−2;P78371
1062:1095 9:9
17:77701918:C:T_T
p = 0.0021, beta = −0.239, N = 1223
IHPQTIIAGWR pc3
F5GWF6;F8VQ14;P78371−2;P78371
1012:1095 9:9
17:77701918:C:T_T
p = 0.035, beta = −0.168, N = 1162
ILIANTGMDTDK pc2
F5GWF6;F8VQ14;P78371−2;P78371
945:1095 8:9
17:77701918:C:T_T
p = 0.13, beta = −0.128, N = 1081
LIEEVMIGEDK pc2
F5GWF6;F8VQ14;P78371−2;P7837
941:1095 7:9
17:77701918:C:T_T
p = 0.086, beta = −0.146, N = 1075
NIGVDNPAAK pc2
F5GWF6;F8VQ14;P78371−2;P7837
866:1095 5:9
17:77701918:C:T_T
p = 1, beta = −0.00036, N = 979
GSGNLEAIHIIK pc2
F5GWF6;F8VQ14;P78371−2;P78371
1035:1095 9:9
17:77701918:C:T_T
p = 0.79, beta = −0.0206, N = 1190
VLVDMSR pc2
F5GWF6;F8VQ14;P78371−2;P78371
448:1095 61:156 2:9
17:77701918:C:T_T
p = 0.26, beta = −0.144, N = 511
GATQQILDEAER pc2
F5GWF6;F8VQ14;P78371−2;P78371
945:1095 5:9
17:77701918:C:T_T
p = 0.096, beta = −0.144, N = 1080
VAEIEHAEK pc2
F5GWF6;F8VQ14;P78371−2;P78371
978:1095 9:9
17:77701918:C:T_T
p = 0.42, beta = −0.0648, N = 1126
SLHDALCVLAQTVK pc3
F5GWF6;F8VQ14;P78371−2;P78371
1057:1095 8:9
17:77701918:C:T_T
p = 0.12, beta = −0.124, N = 1212
EALLSSAVDHGSDEVK pc3
F5GWF6;F8VQ14;P78371−2;P78371
484:1095 60:156 4:9
17:77701918:C:T_T
p = 0.87, beta = −0.0195, N = 548
LIHFSGVALGEACTIVLR pc3
F5GWF6;F8VQ14;P78371−2;P78371
724:1095 97:156 5:9
17:77701918:C:T_T
p = 0.88, beta = 0.0143, N = 826
MLPTIIADNAGYDSADLVAQLR pc3
F5GWF6;P78371−2;P78371
787:1095 7:9
17:77701918:C:T_T
p = 0.98, beta = −0.00252, N = 898
LGGSLADSYLDEGFLLDK pc2
F5GWF6;F8VQ14;P78371−2;P7837
742:1095 7:9
17:77701918:C:T_T
p = 0.9, beta = −0.0119, N = 850
CCT2 : NP4
P78371
235:236 78:78 11:11
17:77701918:C:T_T
p = 0.86, beta = −0.0187, N = 324
LAVEAVLR pc2
F5GWF6;F8VQ14;P78371−2;P78371
175:236 60:78 8:11
17:77701918:C:T_T
p = 1, beta = 0.000409, N = 243
LTSFIGAIAIGDLVK pc2
F5GWF6;F8VQ14;P78371
232:236 78:78 11:11
17:77701918:C:T_T
p = 0.54, beta = 0.0635, N = 321
AAHSEGNTTAGLDMR pc3
P78371−2;P78371
96:236 24:78 3:11
17:77701918:C:T_T
p = 0.8, beta = −0.0463, N = 123
FRQDLMNIAGTTLSSK pc3
F5GWF6;F8VQ14;P78371−2;P78371
162:236 51:78 8:11
17:77701918:C:T_T
p = 0.71, beta = −0.0455, N = 221
LALVTGGEIASTFDHPELVK pc3
F5GWF6;F8VQ14;P78371−2;P78371
195:236 69:78 11:11
17:77701918:C:T_T
p = 0.78, beta = 0.0302, N = 275
GSGNLEAIHIIK pc2
F5GWF6;F8VQ14;P78371−2;P78371
174:236 56:78 8:11
17:77701918:C:T_T
p = 0.44, beta = 0.0936, N = 238
NIGVDNPAAK pc2
F5GWF6;F8VQ14;P78371−2;P78371
109:236 34:78 7:11
17:77701918:C:T_T
p = 0.44, beta = 0.111, N = 150
GATQQILDEAER pc2
F5GWF6;F8VQ14;P78371−2;P78371
78:236 25:78 3:11
17:77701918:C:T_T
p = 0.79, beta = −0.0497, N = 106
IHPQTIIAGWR pc2
F5GWF6;F8VQ14;P78371−2;P7837
81:236 22:78 3:11
17:77701918:C:T_T
p = 0.49, beta = 0.13, N = 106
EAVAMESYAK pc2
F5GWF6;P78371−2;P78371
42:236 20:78 4:11
17:77701918:C:T_T
p = 0.86, beta = 0.0344, N = 66
SLHDALCVLAQTVK pc3
F5GWF6;F8VQ14;P78371−2;P78371
111:236 37:78 6:11
17:77701918:C:T_T
p = 0.63, beta = 0.07, N = 154
ILIANTGMDTDK pc2
F5GWF6;F8VQ14;P78371−2;P78371
79:236 26:78 3:11
17:77701918:C:T_T
p = 0.75, beta = 0.0575, N = 108
HGINCFINR pc2
F5GWF6;F8VQ14;P78371−2;P78371
40:236 14:78 2:11
17:77701918:C:T_T
p = 0.95, beta = 0.0139, N = 56
VAEIEHAEK pc2
F5GWF6;F8VQ14;P78371−2;P78371
49:236 21:78 2:11
17:77701918:C:T_T
p = 0.35, beta = 0.198, N = 72
LIHFSGVALGEACTIVLR pc3
F5GWF6;F8VQ14;P78371−2;P78371
87:236 29:78 6:11
17:77701918:C:T_T
p = 0.55, beta = 0.0937, N = 122
VQDDEVGDGTTSVTVLAAELLR pc
F5GWF6;F8VQ14;P78371−2;P78371
70:236 22:78 4:11
17:77701918:C:T_T
p = 0.35, beta = 0.168, N = 96
EALLSSAVDHGSDEVK pc3
F5GWF6;F8VQ14;P78371−2;P78371
22:236 7:78 2:11
17:77701918:C:T_T
p = 0.45, beta = −0.212, N = 31
MLPTIIADNAGYDSADLVAQLR pc3
F5GWF6;P78371−2;P78371
30:236 10:78 1:11
17:77701918:C:T_T
p = 0.39, beta = −0.251, N = 41
LGGSLADSYLDEGFLLDK pc2
F5GWF6;F8VQ14;P78371−2;P7837
35:236 10:78 3:11
17:77701918:C:T_T
p = 0.37, beta = −0.208, N = 48
337Assay Target: CCT2
UniProt: P78371
rsID: rs8065343
Target SNP: 17:76720495:G:C
Proxy SNP: 17:77701918:C:T
Tarkin log10(p): 7.4
Tarkin BETA: 0.447
Tarkin MSPA: 0.045
763:1256:1251:1247:960:1223:

338. PPP1R14A (Q96A00)
Tarkin MSPA=0.959

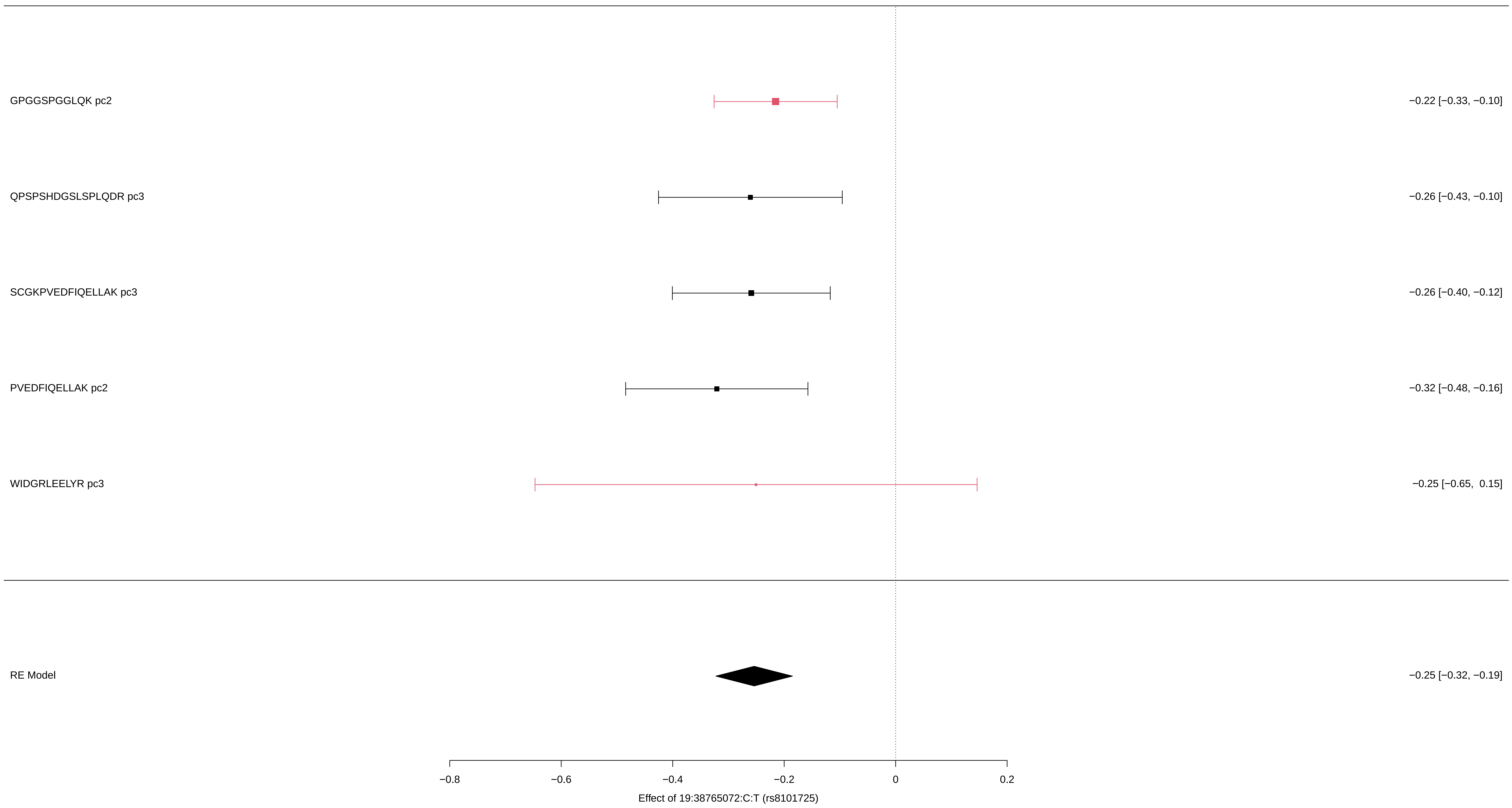

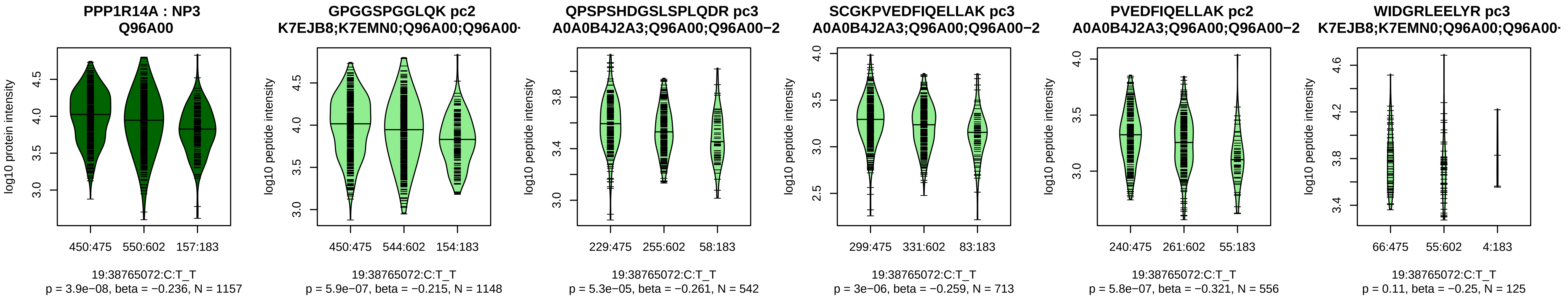

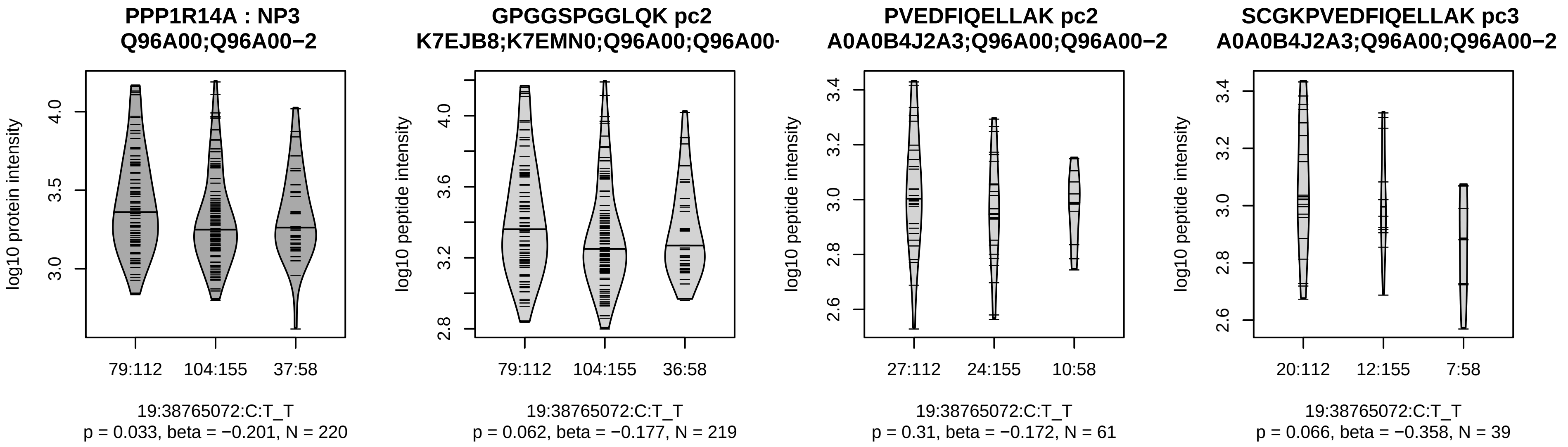

338Assay Target: PPP1R14A
UniProt: Q96A00
rsID: rs8101725
Target SNP: 19:38765072:C:T
Proxy SNP: 19:38765072:C:T
Tarkin log10(p): 7.4
Tarkin BETA: −0.236
Tarkin MSPA: 0.959
::*:*:–
1148:542:713:556:125

339. EIF3D (O15371)
Tarkin MSPA=0.231

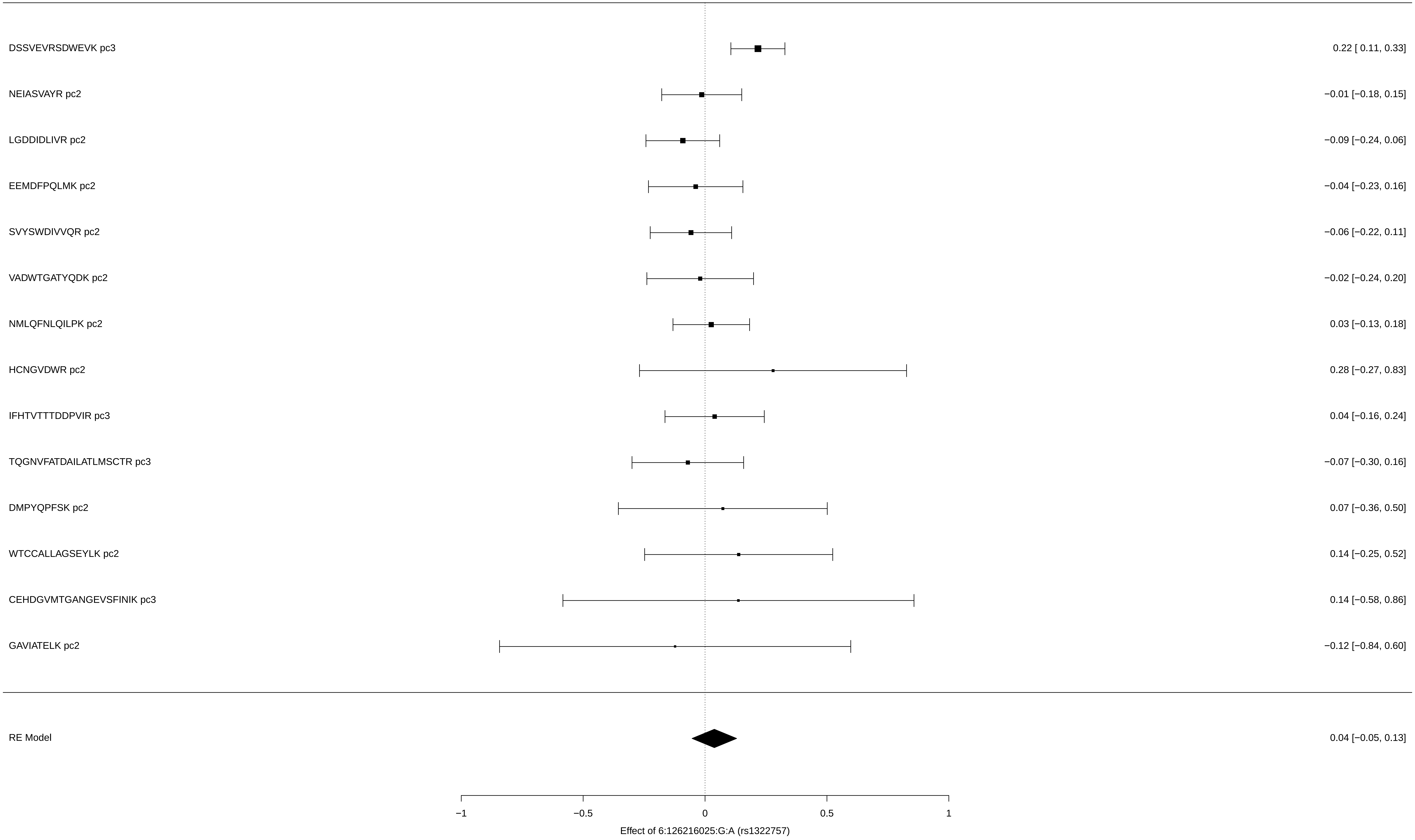

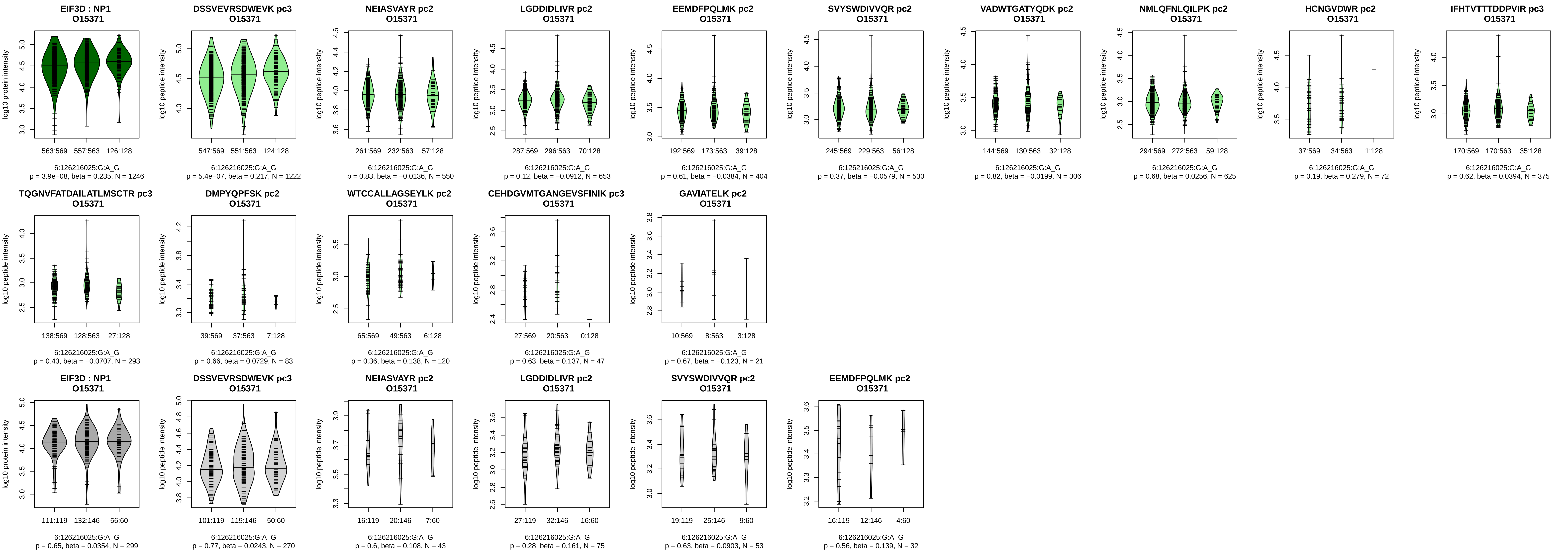

339Assay Target: EIF3D
UniProt: O15371
rsID: rs1322757
Target SNP: 6:126216025:G:A
Proxy SNP: 6:126216025:G:A
Tarkin log10(p): 7.4
Tarkin BETA: 0.235
Tarkin MSPA: 0.231
*:−:−:−:−:−:−:−:−:−:−:−:−:−:NA:N
1222:550:653:404:530:306:625:

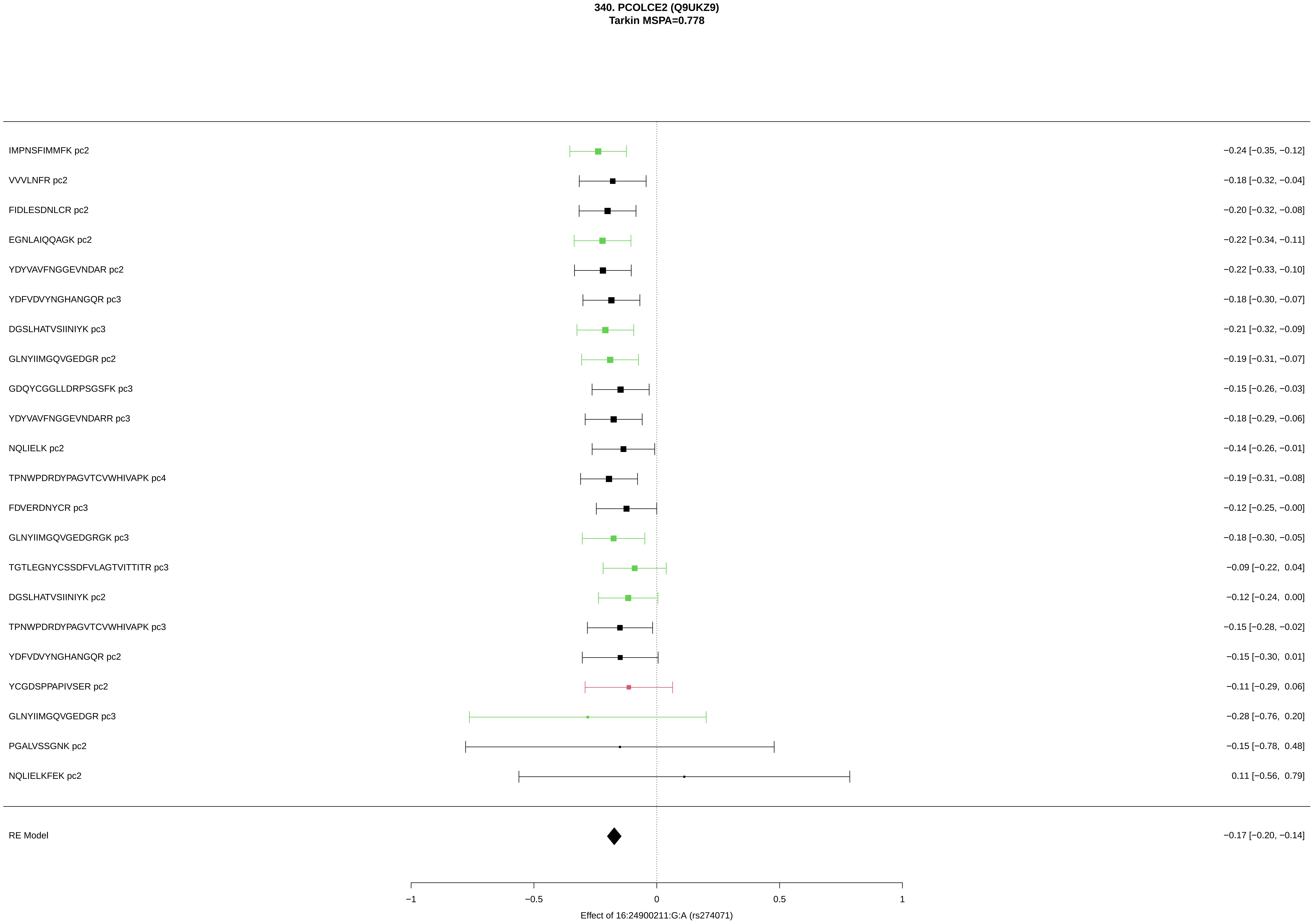
340. PCOLCE2 (Q9UKZ9)
Tarkin MSPA=0.778
IMPNSFIMMFK pc2
−0.24 [−0.35, −0.12]
VVVLNFR pc2
−0.18 [−0.32, −0.04]
FIDLESDNLCR pc2
−0.20 [−0.32, −0.08]
EGNLAIQQAGK pc2
−0.22 [−0.34, −0.11]
YDYVAVFNGGEVNDAR pc2
−0.22 [−0.33, −0.10]
YDFVDVYNGHANGQR pc3
−0.18 [−0.30, −0.07]
DGSLHATVSIINIYK pc3
−0.21 [−0.32, −0.09]
GLNYIIMGQVGEDGR pc2
−0.19 [−0.31, −0.07]
GDQYCGGLLDRPSGSFK pc3
−0.15 [−0.26, −0.03]
YDYVAVFNGGEVNDARR pc3
−0.18 [−0.29, −0.06]
NQLIELK pc2
−0.14 [−0.26, −0.01]
TPNWPDRDYPAGVTCVWHIVAPK pc4
−0.19 [−0.31, −0.08]
FDVERDNYCR pc3
−0.12 [−0.25, −0.00]
GLNYIIMGQVGEDGRGK pc3
−0.18 [−0.30, −0.05]
TGTLEGNYCSSDFVLAGTVITTITR pc3
−0.09 [−0.22, 0.04]
DGSLHATVSIINIYK pc2
−0.12 [−0.24, 0.00]
TPNWPDRDYPAGVTCVWHIVAPK pc3
−0.15 [−0.28, −0.02]
YDFVDVYNGHANGQR pc2
−0.15 [−0.30, 0.01]
YCGDSPPAPIVSER pc2
−0.11 [−0.29, 0.06]
GLNYIIMGQVGEDGR pc3
−0.28 [−0.76, 0.20]
PGALVSSGNK pc2
−0.15 [−0.78, 0.48]
NQLIELKFEK pc2
0.11 [−0.56, 0.79]
RE Model
−0.17 [−0.20, −0.14]
−1
−0.5
0
0.5
1
Effect of 16:24900211:G:A (rs274071)

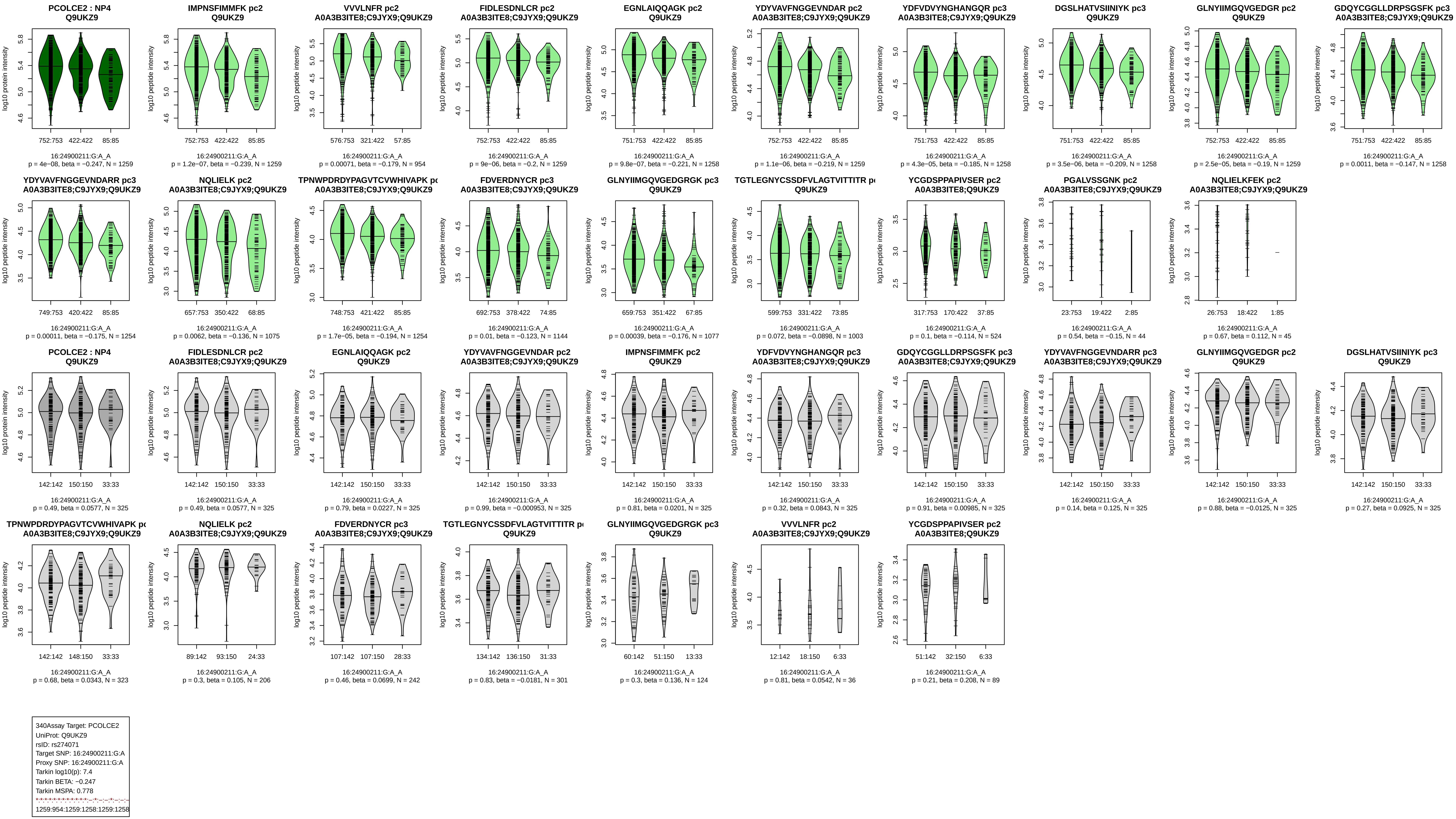

PCOLCE2 : NP4
Q9UKZ9
p = 4e−08, beta = −0.247, N = 1259
IMPNSFIMMFK pc2
Q9UKZ9
p = 1.2e−07, beta = −0.239, N = 1259
VVVLNFR pc2
A0A3B3ITE8;C9JYX9;Q9UKZ9
p = 0.00071, beta = −0.179, N = 954
FIDLESDNLCR pc2
A0A3B3ITE8;C9JYX9;Q9UKZ9
p = 9e−06, beta = −0.2, N = 1259
EGNLAIQQAGK pc2
Q9UKZ9
p = 9.8e−07, beta = −0.221, N = 1258
YDYVAVFNGGEVNDAR pc2
A0A3B3ITE8;C9JYX9;Q9UKZ9
p = 1.1e−06, beta = −0.219, N = 1259
YDFVDVYNGHANGQR pc3
A0A3B3ITE8;C9JYX9;Q9UKZ9
p = 4.3e−05, beta = −0.185, N = 1258
DGSLHATVSIINIYK pc3
Q9UKZ9
p = 3.5e−06, beta = −0.209, N = 1258
GLNYIIMGQVGEDGR pc2
Q9UKZ9
p = 2.5e−05, beta = −0.19, N = 1259
GDQYCGGLLDRPSGSFK pc3
A0A3B3ITE8;C9JYX9;Q9UKZ9
p = 0.0011, beta = −0.147, N = 1258
YDYVAVFNGGEVNDARR pc3
A0A3B3ITE8;C9JYX9;Q9UKZ9
p = 0.00011, beta = −0.175, N = 1254
NQLIELK pc2
A0A3B3ITE8;C9JYX9;Q9UKZ9
p = 0.0062, beta = −0.136, N = 1075
TPNWPDRDYPAGVTCVWHIVAPK pc
A0A3B3ITE8;C9JYX9;Q9UKZ9
p = 1.7e−05, beta = −0.194, N = 1254
FDVERDNYCR pc3
A0A3B3ITE8;C9JYX9;Q9UKZ9
p = 0.01, beta = −0.123, N = 1144
GLNYIIMGQVGEDGRGK pc3
Q9UKZ9
p = 0.00039, beta = −0.176, N = 1077
TGTLEGNYCSSDFVLAGTVITTITR pc
Q9UKZ9
p = 0.072, beta = −0.0898, N = 1003
YCGDSPPAPIVSER pc2
A0A3B3ITE8;Q9UKZ9
p = 0.1, beta = −0.114, N = 524
PGALVSSGNK pc2
A0A3B3ITE8;C9JYX9;Q9UKZ9
p = 0.54, beta = −0.15, N = 44
NQLIELKFEK pc2
A0A3B3ITE8;C9JYX9;Q9UKZ9
p = 0.67, beta = 0.112, N = 45
PCOLCE2 : NP4
Q9UKZ9
p = 0.49, beta = 0.0577, N = 325
FIDLESDNLCR pc2
A0A3B3ITE8;C9JYX9;Q9UKZ9
p = 0.49, beta = 0.0577, N = 325
EGNLAIQQAGK pc2
Q9UKZ9
p = 0.79, beta = 0.0227, N = 325
YDYVAVFNGGEVNDAR pc2
A0A3B3ITE8;C9JYX9;Q9UKZ9
p = 0.99, beta = −0.000953, N = 325
IMPNSFIMMFK pc2
Q9UKZ9
p = 0.81, beta = 0.0201, N = 325
YDFVDVYNGHANGQR pc3
A0A3B3ITE8;C9JYX9;Q9UKZ9
p = 0.32, beta = 0.0843, N = 325
GDQYCGGLLDRPSGSFK pc3
A0A3B3ITE8;C9JYX9;Q9UKZ9
p = 0.91, beta = 0.00985, N = 325
YDYVAVFNGGEVNDARR pc3
A0A3B3ITE8;C9JYX9;Q9UKZ9
p = 0.14, beta = 0.125, N = 325
GLNYIIMGQVGEDGR pc2
Q9UKZ9
p = 0.88, beta = −0.0125, N = 325
DGSLHATVSIINIYK pc3
Q9UKZ9
p = 0.27, beta = 0.0925, N = 325
TPNWPDRDYPAGVTCVWHIVAPK pc
A0A3B3ITE8;C9JYX9;Q9UKZ9
p = 0.68, beta = 0.0343, N = 323
NQLIELK pc2
A0A3B3ITE8;C9JYX9;Q9UKZ9
p = 0.3, beta = 0.105, N = 206
FDVERDNYCR pc3
A0A3B3ITE8;C9JYX9;Q9UKZ9
p = 0.46, beta = 0.0699, N = 242
TGTLEGNYCSSDFVLAGTVITTITR pc
Q9UKZ9
p = 0.83, beta = −0.0181, N = 301
GLNYIIMGQVGEDGRGK pc3
Q9UKZ9
p = 0.3, beta = 0.136, N = 124
VVVLNFR pc2
A0A3B3ITE8;C9JYX9;Q9UKZ9
p = 0.81, beta = 0.0542, N = 36
YCGDSPPAPIVSER pc2
A0A3B3ITE8;Q9UKZ9
p = 0.21, beta = 0.208, N = 89
16:24900211:G:A_A
340Assay Target: PCOLCE2
UniProt: Q9UKZ9
rsID: rs274071
Target SNP: 16:24900211:G:A
Proxy SNP: 16:24900211:G:A
Tarkin log10(p): 7.4
Tarkin BETA: −0.247
Tarkin MSPA: 0.778
1259:954:1259:1258:1259:1258

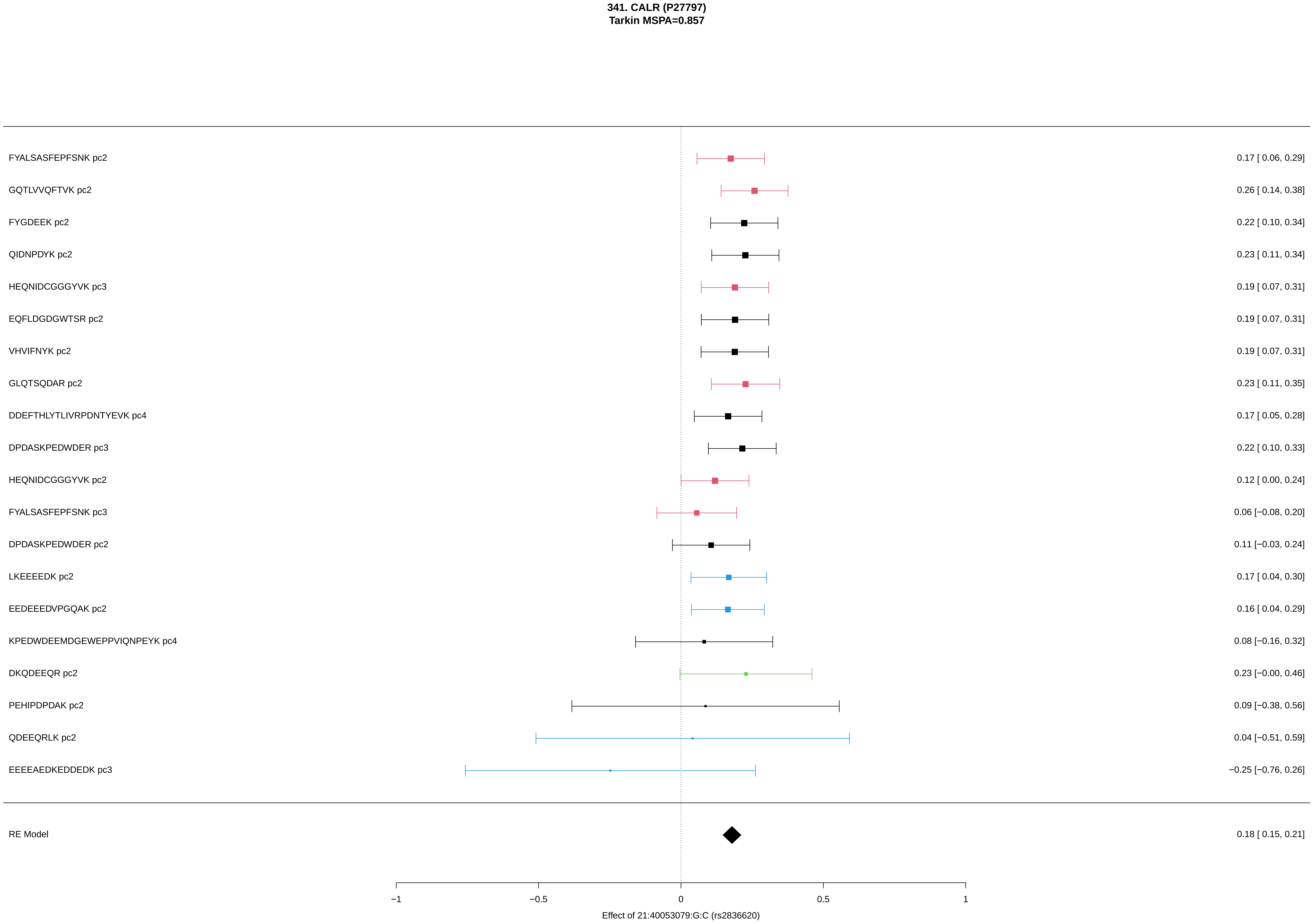

341. CALR (P27797)
Tarkin MSPA=0.857
FYALSASFEPFSNK pc2 0.17 [0.06, 0.29]
GQTLVVQFTVK pc2 0.26 [0.14, 0.38]
FYGDEEK pc2 0.22 [0.10, 0.34]
QIDNPDYK pc2 0.23 [0.11, 0.34]
HEQNIDCGGGYVK pc3 0.19 [0.07, 0.31]
EQFLDGDGWTSR pc2 0.19 [0.07, 0.31]
VHVIFNYK pc2 0.19 [0.07, 0.31]
GLQTSQDAR pc2 0.23 [0.11, 0.35]
DDEFTHLYTLIVRPDNTYEVK pc4 0.17 [0.05, 0.28]
DPDASKPEDWDER pc3 0.22 [0.10, 0.33]
HEQNIDCGGGYVK pc2 0.12 [0.00, 0.24]
FYALSASFEPFSNK pc3 0.06 [−0.08, 0.20]
DPDASKPEDWDER pc2 0.11 [−0.03, 0.24]
LKEEEEDK pc2 0.17 [0.04, 0.30]
EEDEEEDVPGQAK pc2 0.16 [0.04, 0.29]
KPEDWDEEMDGEWEPPVIQNPEYK pc4 0.08 [−0.16, 0.32]
DKQDEEQR pc2 0.23 [−0.00, 0.46]
PEHIPDPDAK pc2 0.09 [−0.38, 0.56]
QDEEQRLK pc2 0.04 [−0.51, 0.59]
EEEEAEDKEDDEDK pc3 −0.25 [−0.76, 0.26]
RE Model 0.18 [0.15, 0.21]
−1
−0.5
0
0.5
1
Effect of 21:40053079:G:C (rs2836620)

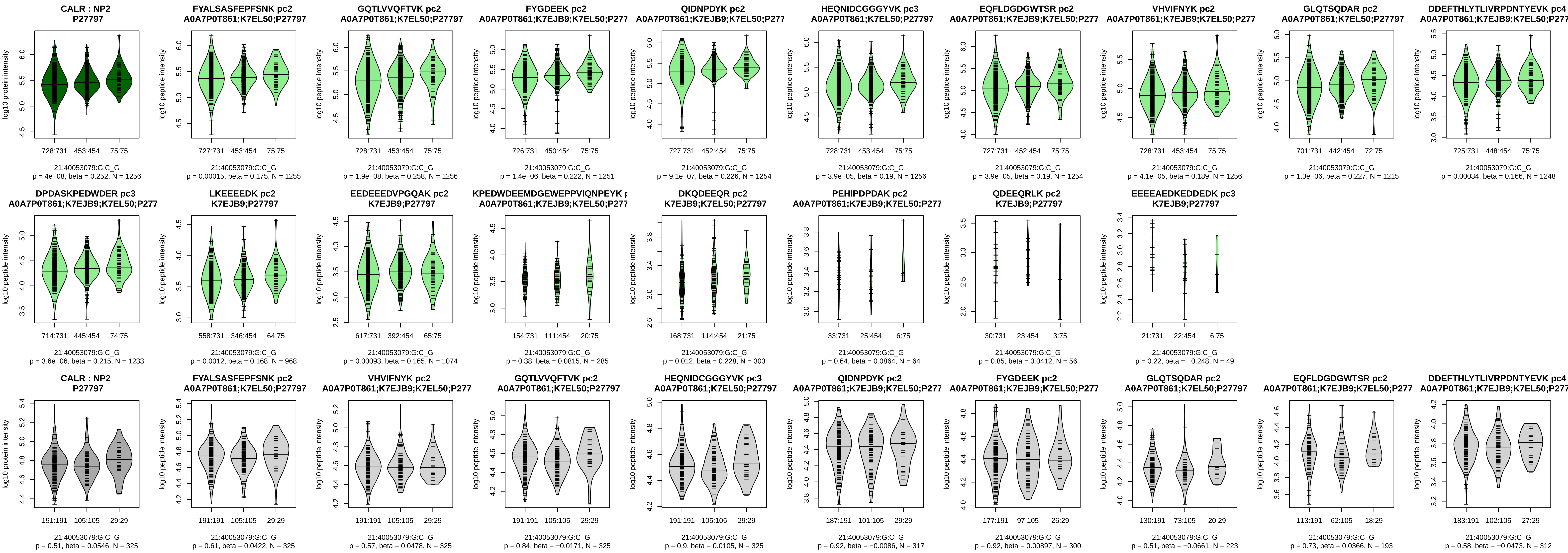

341Assay Target: CALR
UniProt: P27797
rsID: rs2836620
Target SNP: 21:40053079:G:C
Proxy SNP: 21:40053079:G:C
Tarkin log10(p): 7.4
Tarkin BETA: 0.252
Tarkin MSPA: 0.857
::*:*:*:*:*:*:*:*:*:*:–:–:*:*:–:–:–:–:NA
1255:1256:1251:1254:1256:125

342. EPRS (P07814)
Tarkin MSPA=0.082

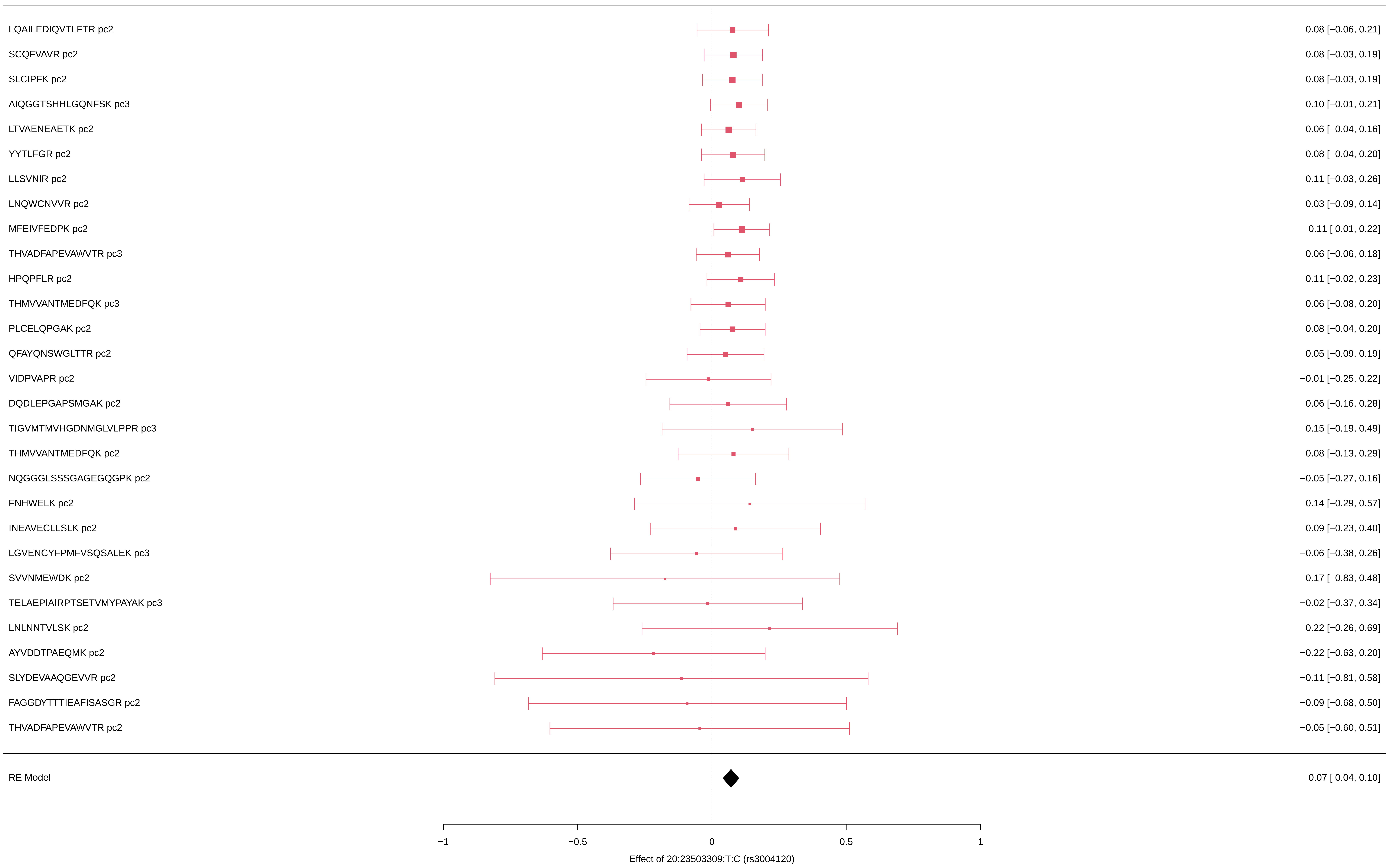

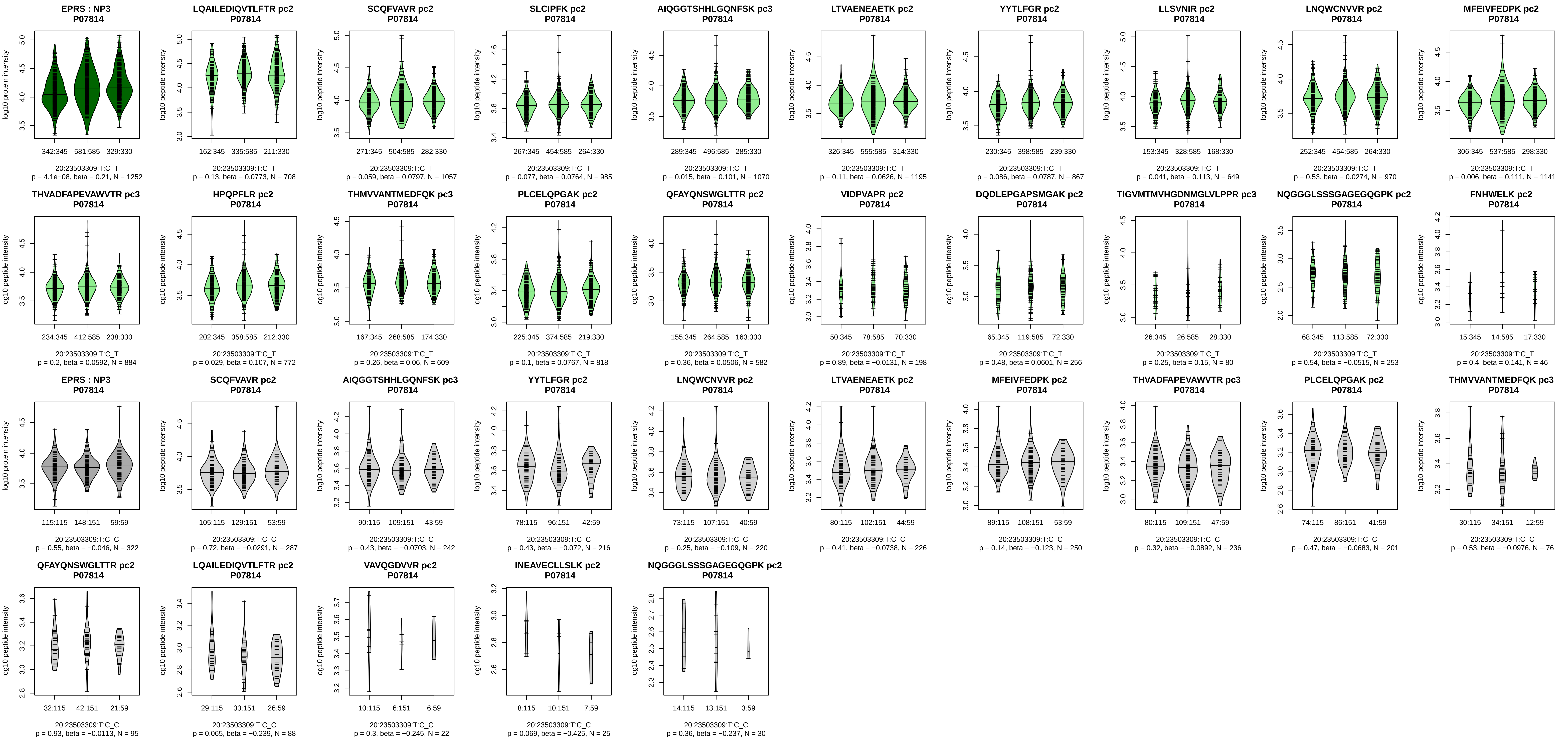

342Assay Target: EPRS
UniProt: P07814
rsID: rs3004120
Target SNP: 20:23503309:T:C
Proxy SNP: 20:23503309:T:C
Tarkin log10(p): 7.4
Tarkin BETA: 0.21
Tarkin MSPA: 0.082
-.-.-.-.-.-.-.-.-.*-.-.-.-.-.-.-.-.-
708:1057:985:1070:1195:867:64

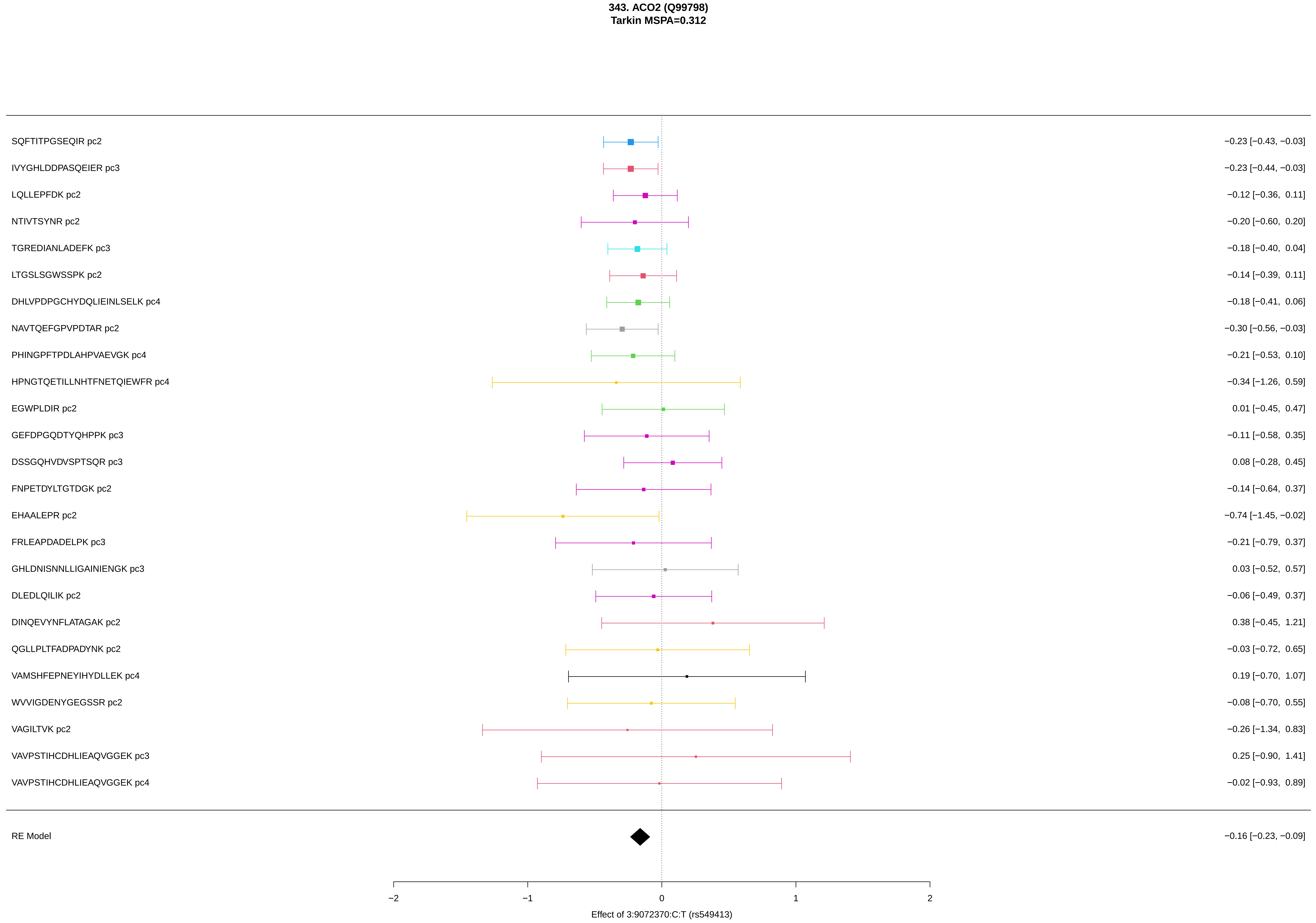
343. ACO2 (Q99798)
Tarkin MSPA=0.312
SQFTITPGSEQIR pc2
IVYGHLDDPASQEIER pc3
LQLLEPFDK pc2
NTIVTSYNR pc2
TGREDIANLADEFK pc3
LTGSLSGWSSPK pc2
DHLVPDPGCHYDQLIEINLSELK pc4
NAVTQEFGPVPDTAR pc2
PHINGPFTPDLAHPVAEVGK pc4
HPNGTQETILLNHTFNETQIEWFR pc4
EGWPLDIR pc2
GEFDPGQDTYQHPPK pc3
DSSGQHVDVSPTSQR pc3
FNPETDYLTGTDGK pc2
EHAALEPR pc2
FRLEAPDADELPK pc3
GHLDNISNNLLIGAINIENGK pc3
DLEDLQILIK pc2
DINQEVYNFLATAGAK pc2
QGLLPLTFADPADYNK pc2
VAMSHFEPNEYIHYDLLEK pc4
WVVIGDENYGEGSSR pc2
VAGILTVK pc2
VAVPSTIHCDHLIEAQVGGEK pc3
VAVPSTIHCDHLIEAQVGGEK pc4
RE Model
−0.23 [−0.43, −0.03]
−0.23 [−0.44, −0.03]
−0.12 [−0.36, 0.11]
−0.20 [−0.60, 0.20]
−0.18 [−0.40, 0.04]
−0.14 [−0.39, 0.11]
−0.18 [−0.41, 0.06]
−0.30 [−0.56, −0.03]
−0.21 [−0.53, 0.10]
−0.34 [−1.26, 0.59]
0.01 [−0.45, 0.47]
−0.11 [−0.58, 0.35]
0.08 [−0.28, 0.45]
−0.14 [−0.64, 0.37]
−0.74 [−1.45, −0.02]
−0.21 [−0.79, 0.37]
0.03 [−0.52, 0.57]
−0.06 [−0.49, 0.37]
0.38 [−0.45, 1.21]
−0.03 [−0.72, 0.65]
0.19 [−0.70, 1.07]
−0.08 [−0.70, 0.55]
−0.26 [−1.34, 0.83]
0.25 [−0.90, 1.41]
−0.02 [−0.93, 0.89]
−0.16 [−0.23, −0.09]
−2
−1
0
1
2
Effect of 3:9072370:C:T (rs549413)

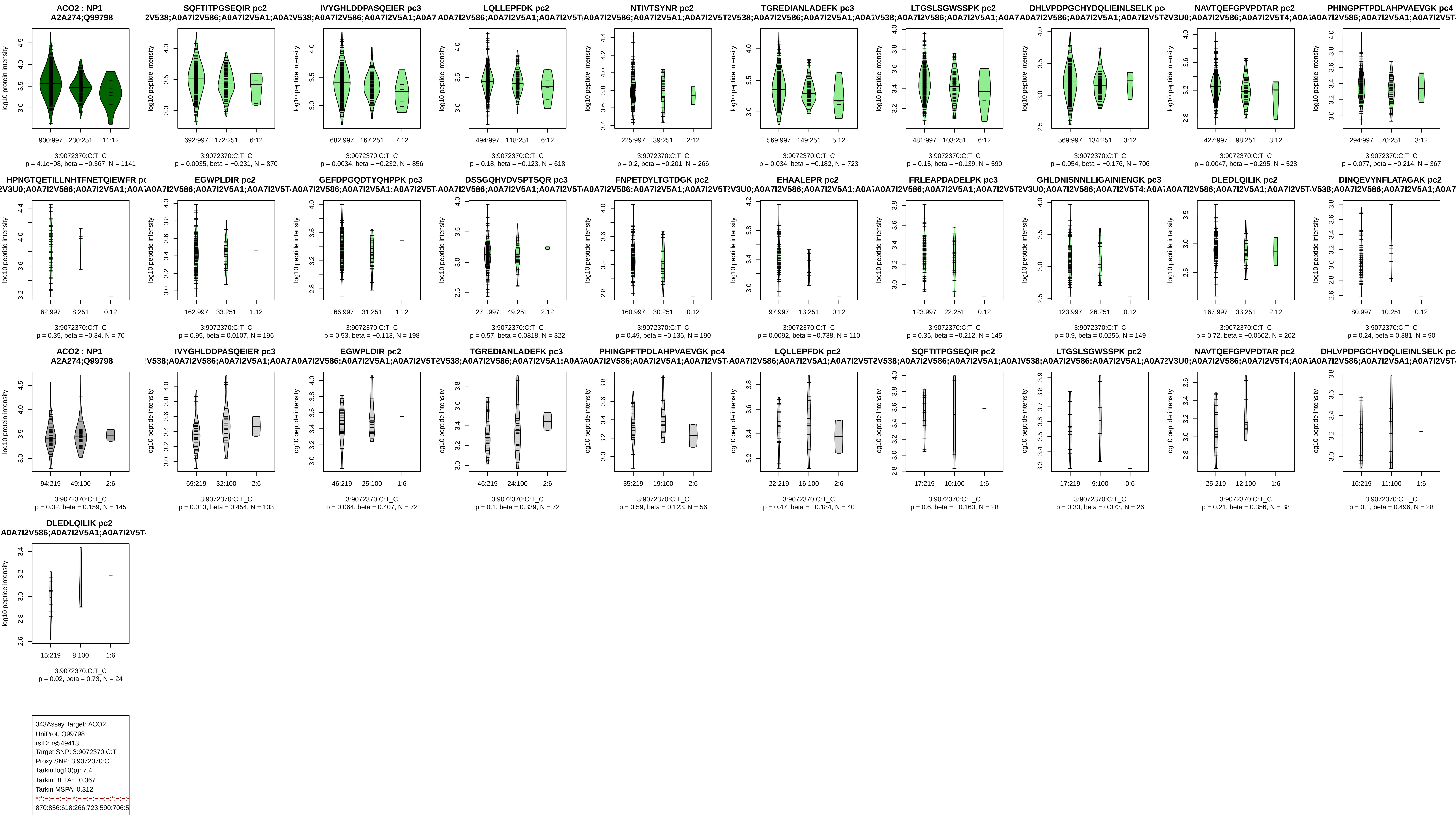
343Assay Target: ACO2
UniProt: Q99798
rsID: rs549413
Target SNP: 3:9072370:C:T
Proxy SNP: 3:9072370:C:T
Tarkin log10(p): 7.4
Tarkin BETA: −0.367
Tarkin MSPA: 0.312

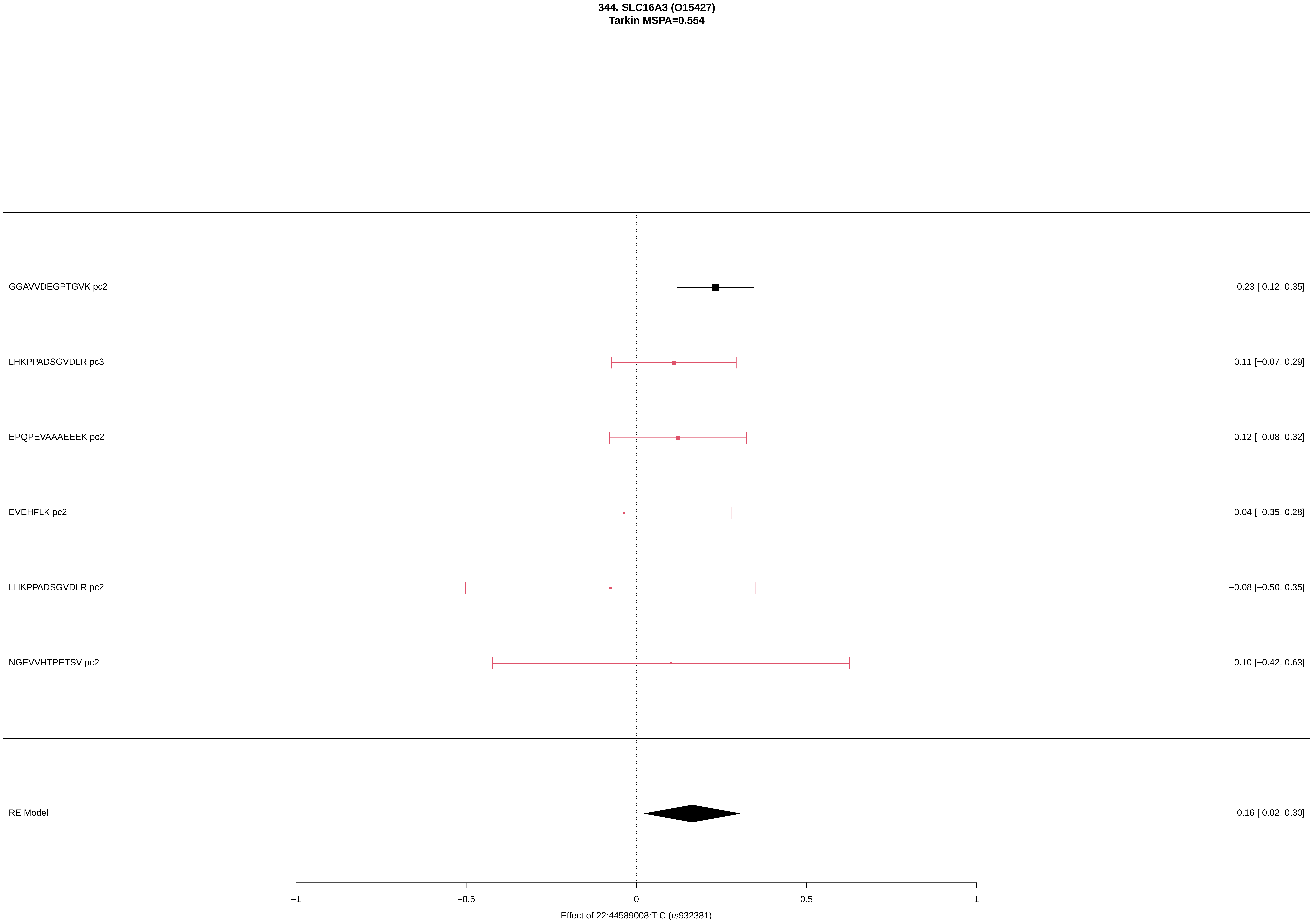
344. SLC16A3 (O15427)
Tarkin MSPA=0.554
GGAVVDEGPTGVK pc2
0.23 [0.12, 0.35]
LHKPPADSGVDLR pc3
0.11 [−0.07, 0.29]
EPQPEVAAAEEEK pc2
0.12 [−0.08, 0.32]
EVEHFLK pc2
−0.04 [−0.35, 0.28]
LHKPPADSGVDLR pc2
−0.08 [−0.50, 0.35]
NGEVVHTPETSV pc2
0.10 [−0.42, 0.63]
RE Model
0.16 [0.02, 0.30]
−1
−0.5
0
0.5
1
Effect of 22:44589008:T:C (rs932381)

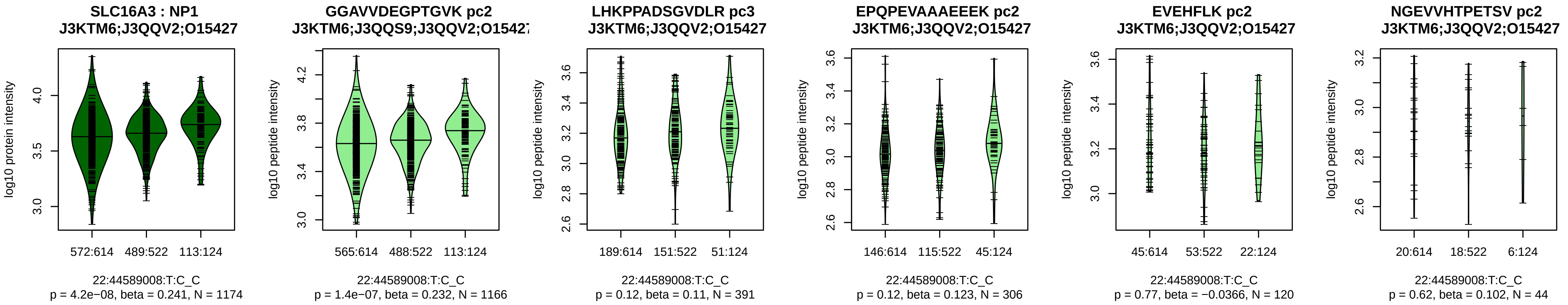

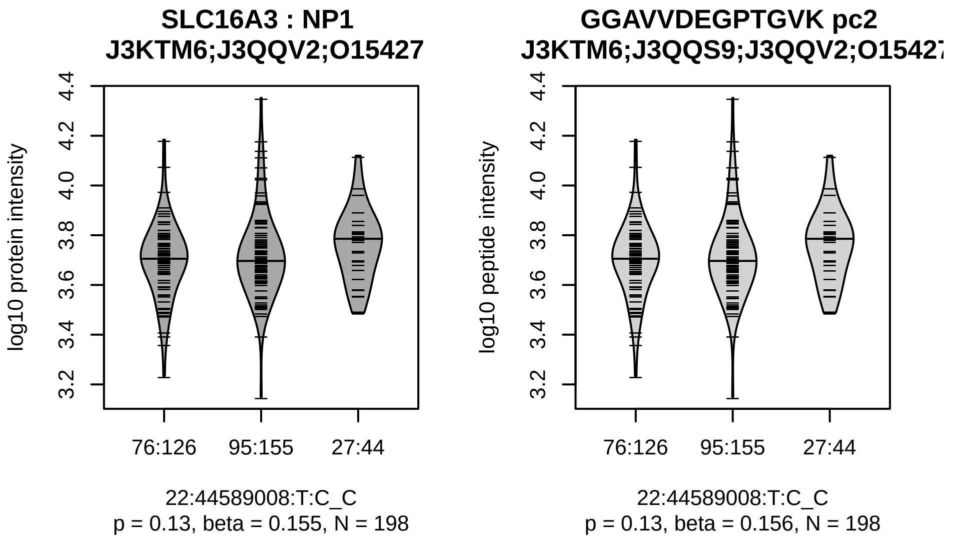

344Assay Target: SLC16A3
UniProt: O15427
rsID: rs932381
Target SNP: 22:44589008:T:C
Proxy SNP: 22:44589008:T:C
Tarkin log10(p): 7.4
Tarkin BETA: 0.241
Tarkin MSPA: 0.554
*:–:–:–:–:–
1166:391:306:120:79:44

345. PDGFA (P04085)
Tarkin MSPA=0.963

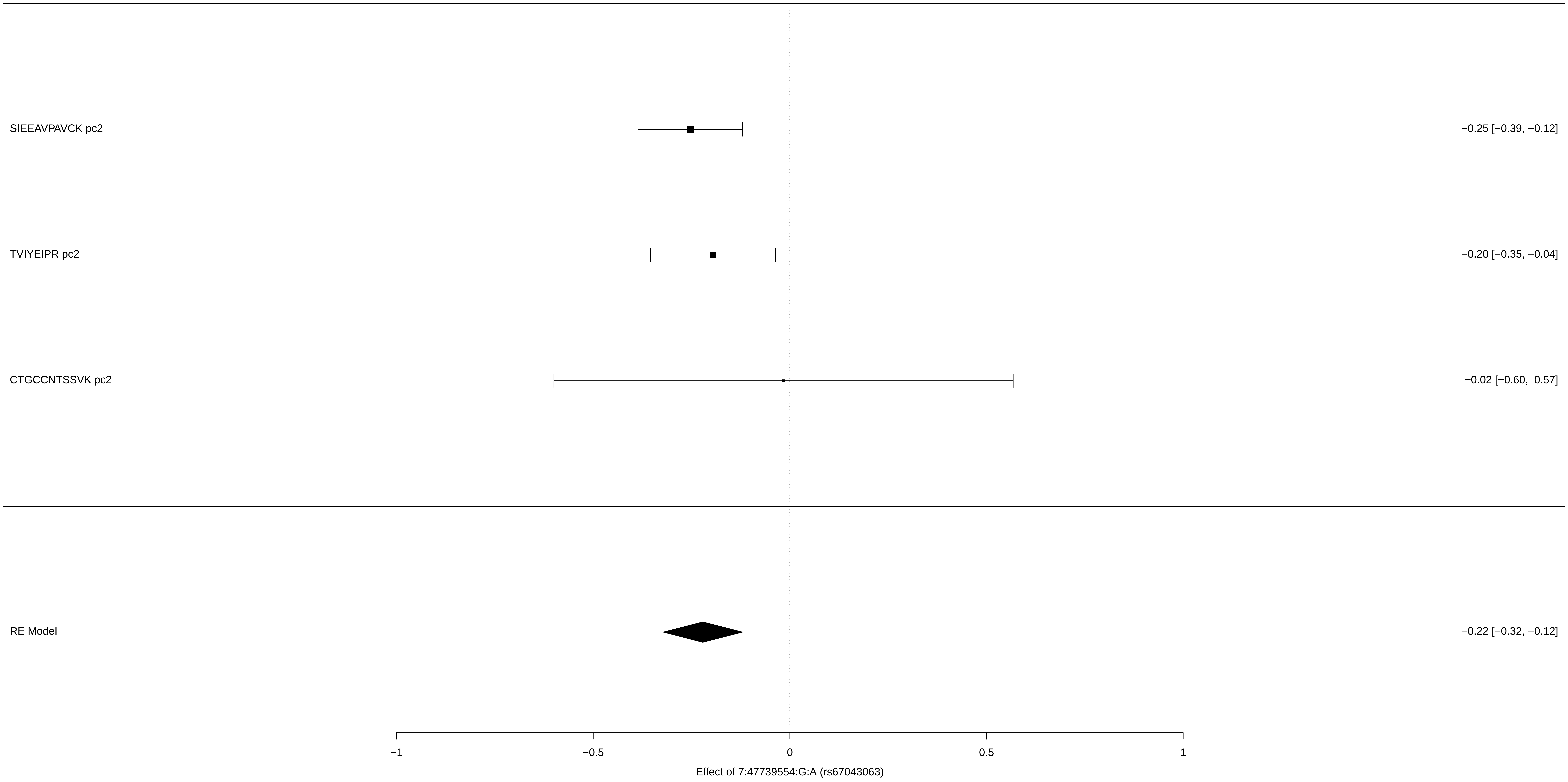

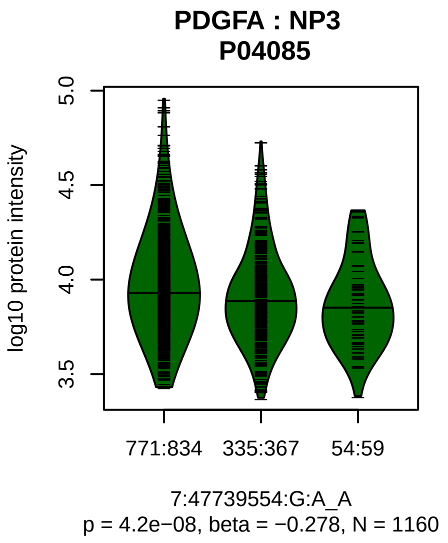

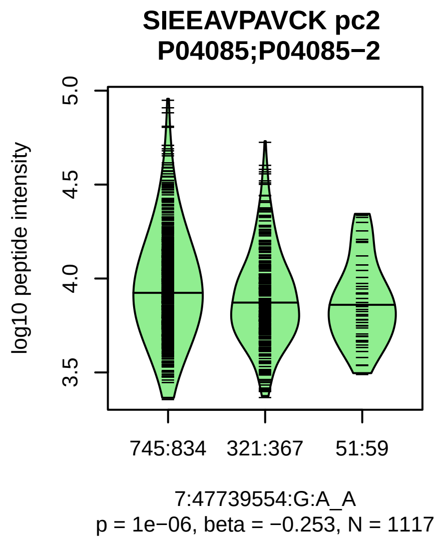

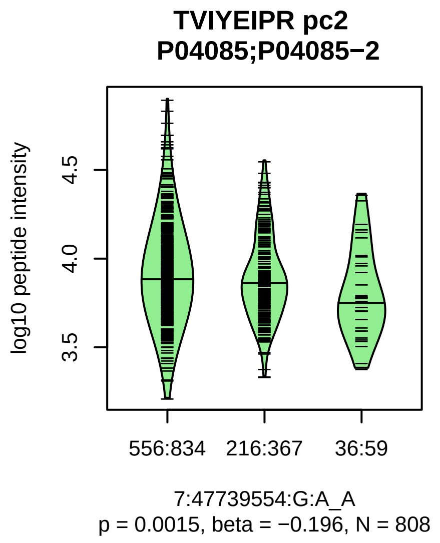

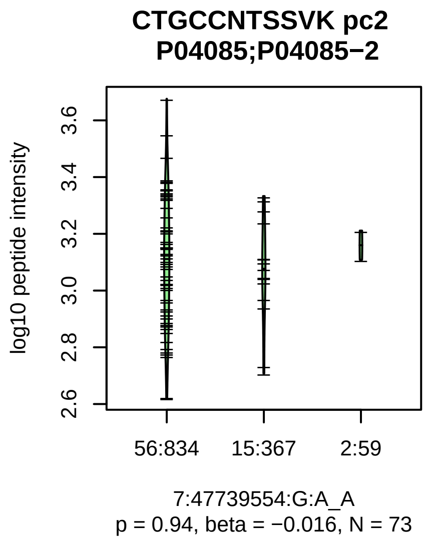

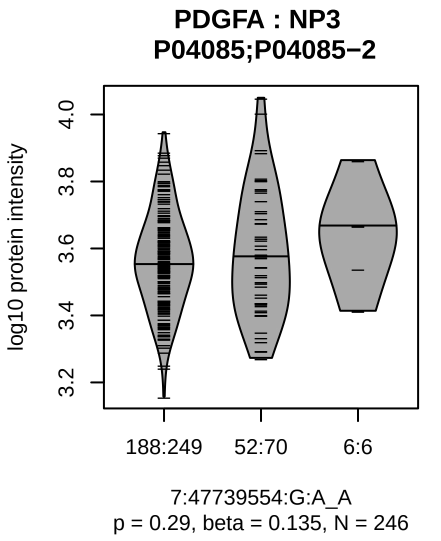

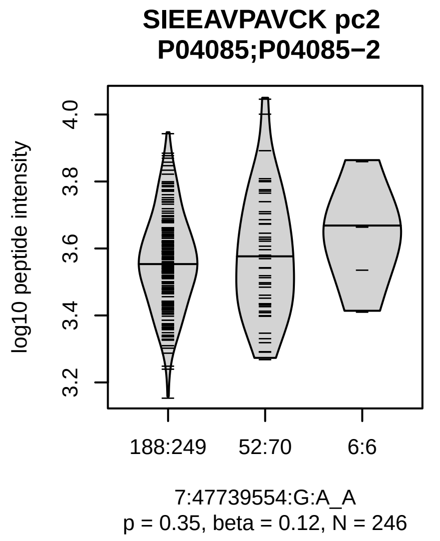

345Assay Target: PDGFA
UniProt: P04085
rsID: rs67043063
Target SNP: 7:47739554:G:A
Proxy SNP: 7:47739554:G:A
Tarkin log10(p): 7.4
Tarkin BETA: −0.278
Tarkin MSPA: 0.963
::–
1117:808:73

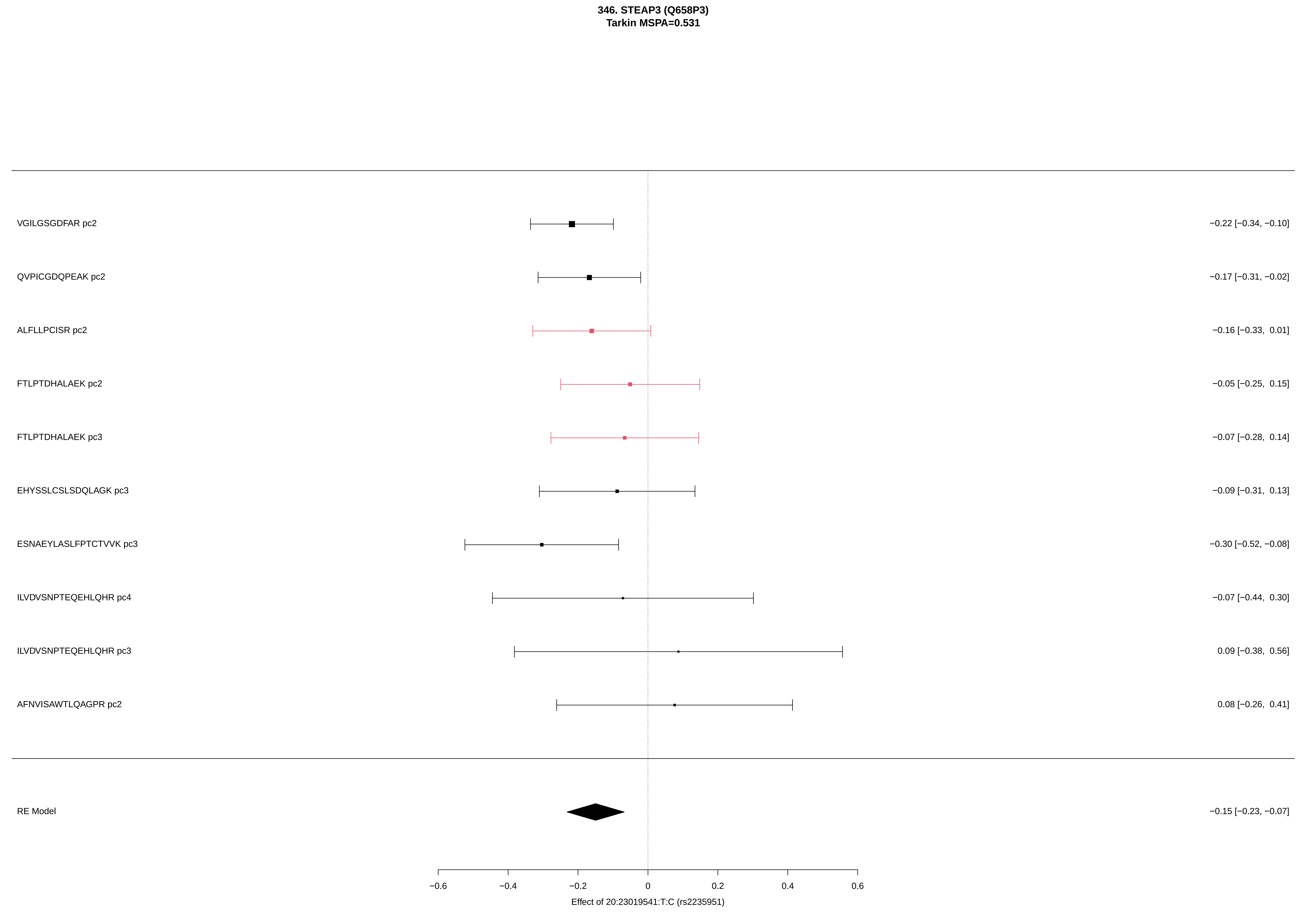

346. STEAP3 (Q658P3)
Tarkin MSPA=0.531
VGILGSGDFAR pc2
QVPICGDQPEAK pc2
ALFLLPCISR pc2
FTLPTDHALAEK pc2
FTLPTDHALAEK pc3
EHYSSLCSLSDQLAGK pc3
ESNAEYLASLFPTCTVVK pc3
ILVDVSNPTEQEHLQHR pc4
ILVDVSNPTEQEHLQHR pc3
AFNVISAWTLQAGPR pc2
RE Model
−0.22 [−0.34, −0.10]
−0.17 [−0.31, −0.02]
−0.16 [−0.33, 0.01]
−0.05 [−0.25, 0.15]
−0.07 [−0.28, 0.14]
−0.09 [−0.31, 0.13]
−0.30 [−0.52, −0.08]
−0.07 [−0.44, 0.30]
0.09 [−0.38, 0.56]
0.08 [−0.26, 0.41]
−0.15 [−0.23, −0.07]
−0.6
−0.4
−0.2
0
0.2
0.4
0.6
Effect of 20:23019541:T:C (rs2235951)

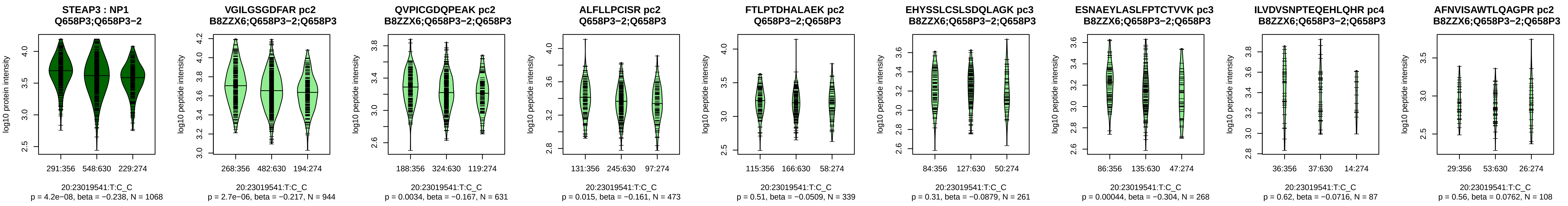

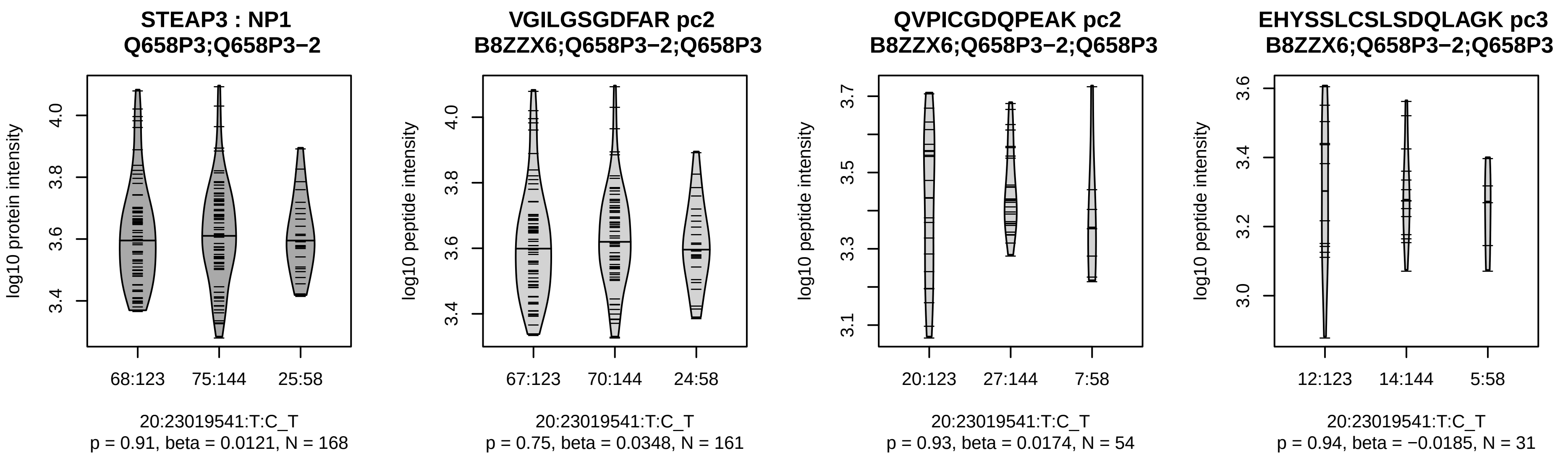

346Assay Target: STEAP3
UniProt: Q658P3
rsID: rs2235951
Target SNP: 20:23019541:T:C
Proxy SNP: 20:23019541:T:C
Tarkin log10(p): 7.4
Tarkin BETA: −0.238
Tarkin MSPA: 0.531
::−:−:−:−:*:−:−:−:NA
944:631:473:339:290:261:268:8

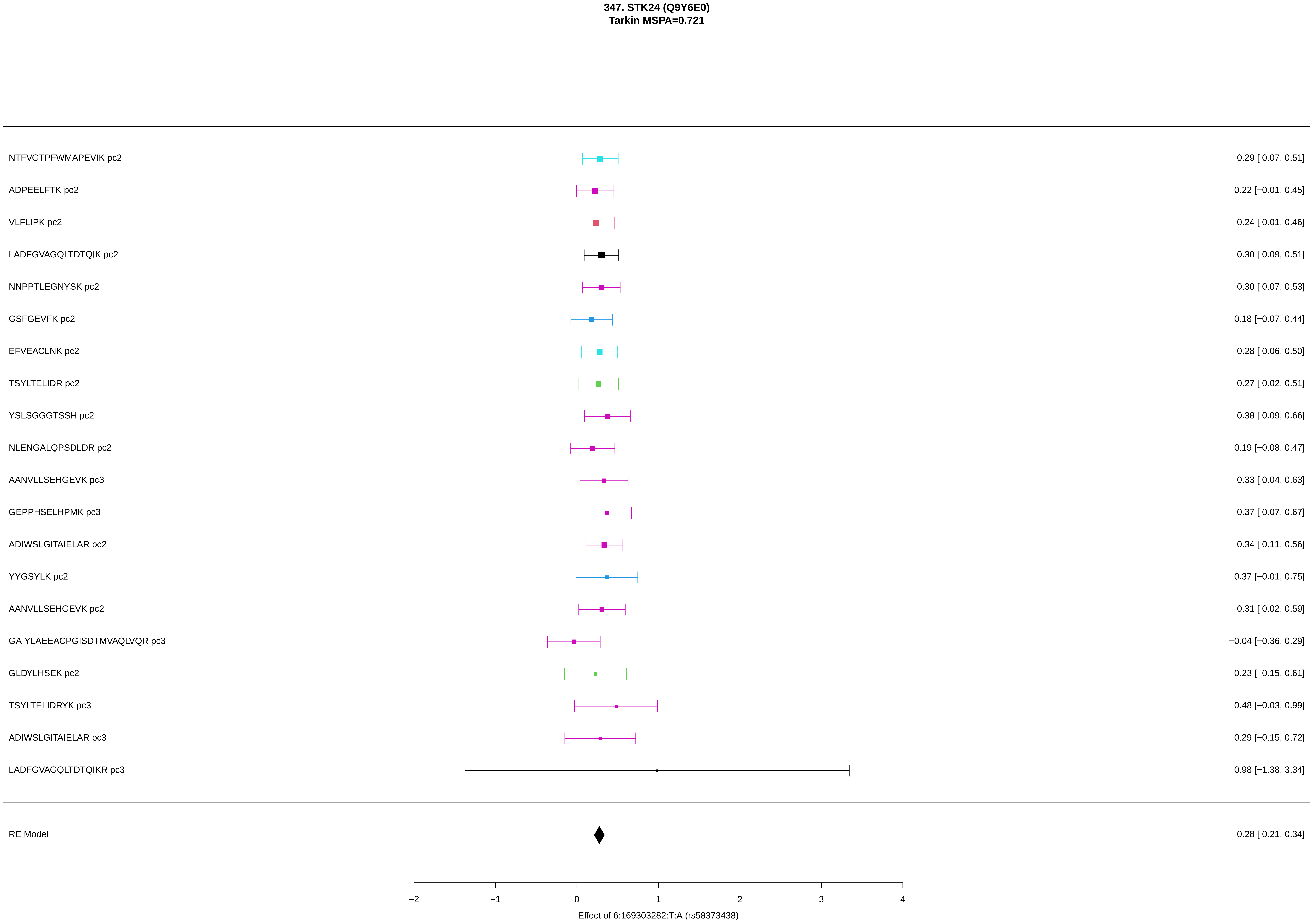
347. STK24 (Q9Y6E0)
Tarkin MSPA=0.721
NTFVGTPFWMAPEVIK pc2
0.29 [0.07, 0.51]
ADPEELFTK pc2
0.22 [−0.01, 0.45]
VLFLIPK pc2
0.24 [0.01, 0.46]
LADFGVAGQLTDTQIK pc2
0.30 [0.09, 0.51]
NNPPTLEGNYSK pc2
0.30 [0.07, 0.53]
GSFGEVFK pc2
0.18 [−0.07, 0.44]
EFVEACLNK pc2
0.28 [0.06, 0.50]
TSYLTELIDR pc2
0.27 [0.02, 0.51]
YSLSGGGTSSH pc2
0.38 [0.09, 0.66]
NLENGALQPSDLDR pc2
0.19 [−0.08, 0.47]
AANVLLSEHGEVK pc3
0.33 [0.04, 0.63]
GEPPHSELHPMK pc3
0.37 [0.07, 0.67]
ADIWSLGITAIELAR pc2
0.34 [0.11, 0.56]
YYGSYLK pc2
0.37 [−0.01, 0.75]
AANVLLSEHGEVK pc2
0.31 [0.02, 0.59]
GAIYLAEEACPGISDTMVAQLVQR pc3
−0.04 [−0.36, 0.29]
GLDYLHSEK pc2
0.23 [−0.15, 0.61]
TSYLTELIDRYK pc3
0.48 [−0.03, 0.99]
ADIWSLGITAIELAR pc3
0.29 [−0.15, 0.72]
LADFGVAGQLTDTQIKR pc3
0.98 [−1.38, 3.34]
RE Model
0.28 [0.21, 0.34]
−2
−1
0
1
2
3
4
Effect of 6:169303282:T:A (rs58373438)

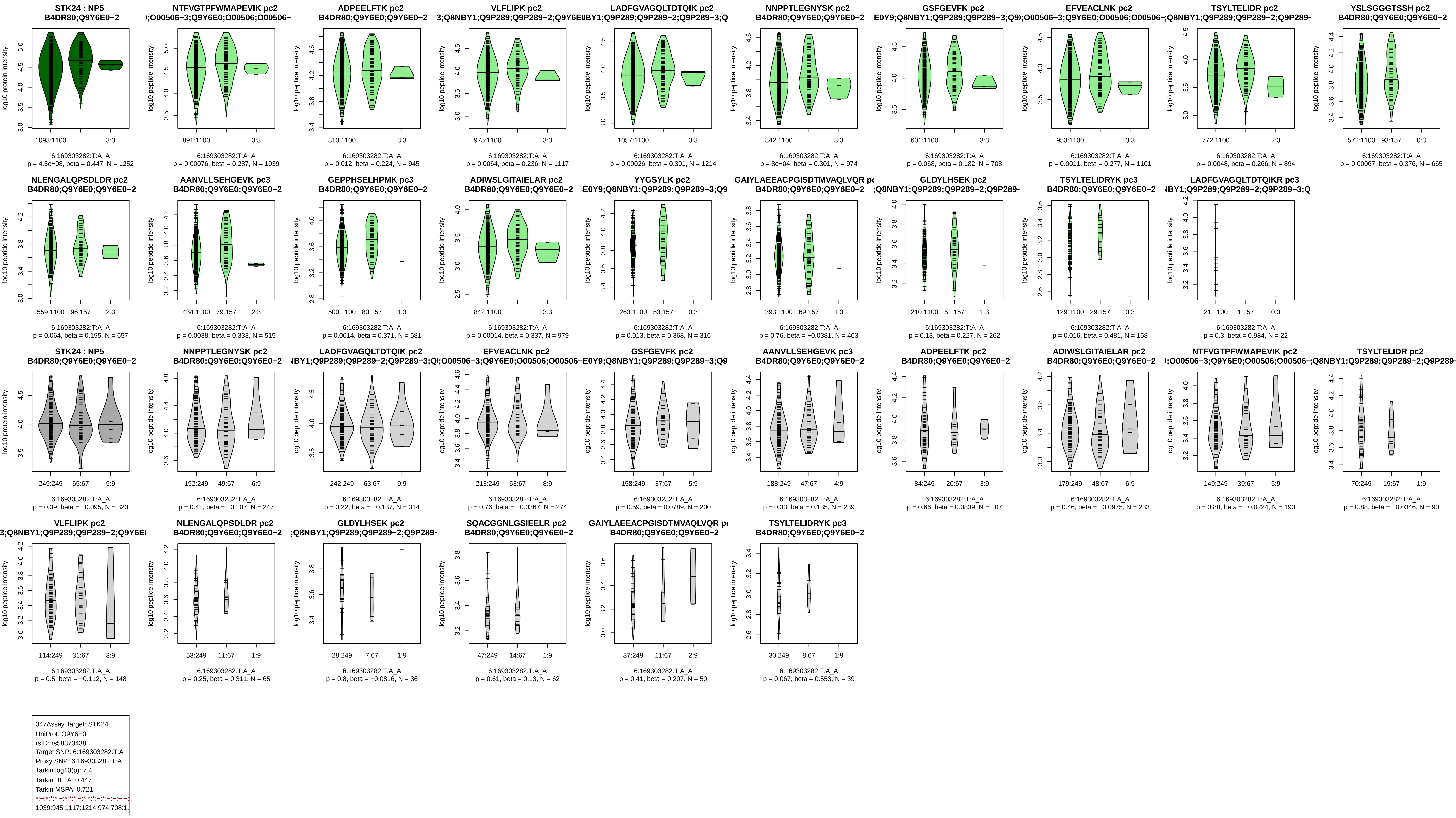
STK24 : NP5
B4DR80;Q9Y6E0;Q9Y6E0−2
log10 protein intensity
1093:1100
3:3
6:169303282:T:A_A
p = 4.3e−08, beta = 0.447, N = 1252
NTFVGTPFWMAPEVIK pc2
891:1100
p = 0.00076, beta = 0.287, N = 1039
ADPEELFTK pc2
810:1100
p = 0.012, beta = 0.224, N = 945
VLFLIPK pc2
975:1100
p = 0.0064, beta = 0.236, N = 1117
LADFGVAGQLTDTQIK pc2
1057:1100
p = 0.00026, beta = 0.301, N = 1214
NNPPTLEGNYSK pc2
842:1100
p = 8e−04, beta = 0.301, N = 974
GSFGEVFK pc2
601:1100
p = 0.068, beta = 0.182, N = 708
EFVEACLNK pc2
953:1100
p = 0.0011, beta = 0.277, N = 1101
TSYLTELIDR pc2
772:1100
2:3
p = 0.0048, beta = 0.266, N = 894
YSLSGGGTSSH pc2
572:1100
93:157
0:3
p = 0.00067, beta = 0.376, N = 665
NLENGALQPSDLDR pc2
559:1100
96:157
p = 0.064, beta = 0.195, N = 657
AANVLLSEHGEVK pc3
434:1100
79:157
p = 0.0038, beta = 0.333, N = 515
GEPPHSELHPMK pc3
500:1100
80:157
1:3
p = 0.0014, beta = 0.371, N = 581
ADIWSLGITAIELAR pc2
842:1100
p = 0.00014, beta = 0.337, N = 979
YYGSYLK pc2
263:1100
53:157
p = 0.013, beta = 0.368, N = 316
393:1100
69:157
p = 0.76, beta = −0.0311, N = 463
GLDYLHSEK pc2
210:1100
51:157
p = 0.13, beta = 0.227, N = 262
TSYLTELIDRYK pc3
129:1100
29:157
p = 0.016, beta = 0.481, N = 158
LADFGVAGQLTDTQIKR pc3
21:1100
1:157
p = 0.3, beta = 0.984, N = 22
log10 peptide intensity
249:249
65:67
9:9
p = 0.39, beta = −0.095, N = 323
192:249
49:67
6:9
p = 0.41, beta = −0.107, N = 247
242:249
63:67
p = 0.22, beta = −0.137, N = 314
213:249
53:67
8:9
p = 0.76, beta = −0.0367, N = 274
158:249
37:67
5:9
p = 0.59, beta = 0.0789, N = 200
188:249
47:67
4:9
p = 0.33, beta = 0.135, N = 239
84:249
20:67
p = 0.66, beta = 0.0839, N = 107
179:249
48:67
p = 0.46, beta = −0.0975, N = 233
149:249
39:67
p = 0.88, beta = −0.0224, N = 193
70:249
19:67
1:9
p = 0.88, beta = −0.0346, N = 90
114:249
31:67
p = 0.5, beta = −0.112, N = 148
53:249
11:67
p = 0.25, beta = 0.311, N = 65
28:249
7:67
p = 0.8, beta = −0.0816, N = 36
SQACGGNLGSIEELR pc2
47:249
14:67
p = 0.61, beta = 0.13, N = 62
37:249
2:9
p = 0.41, beta = 0.207, N = 50
30:249
8:67
p = 0.067, beta = 0.553, N = 39
347Assay Target: STK24
UniProt: Q9Y6E0
rsID: rs58373438
Target SNP: 6:169303282:T:A
Proxy SNP: 6:169303282:T:A
Tarkin log10(p): 7.4
Tarkin BETA: 0.447
Tarkin MSPA: 0.721

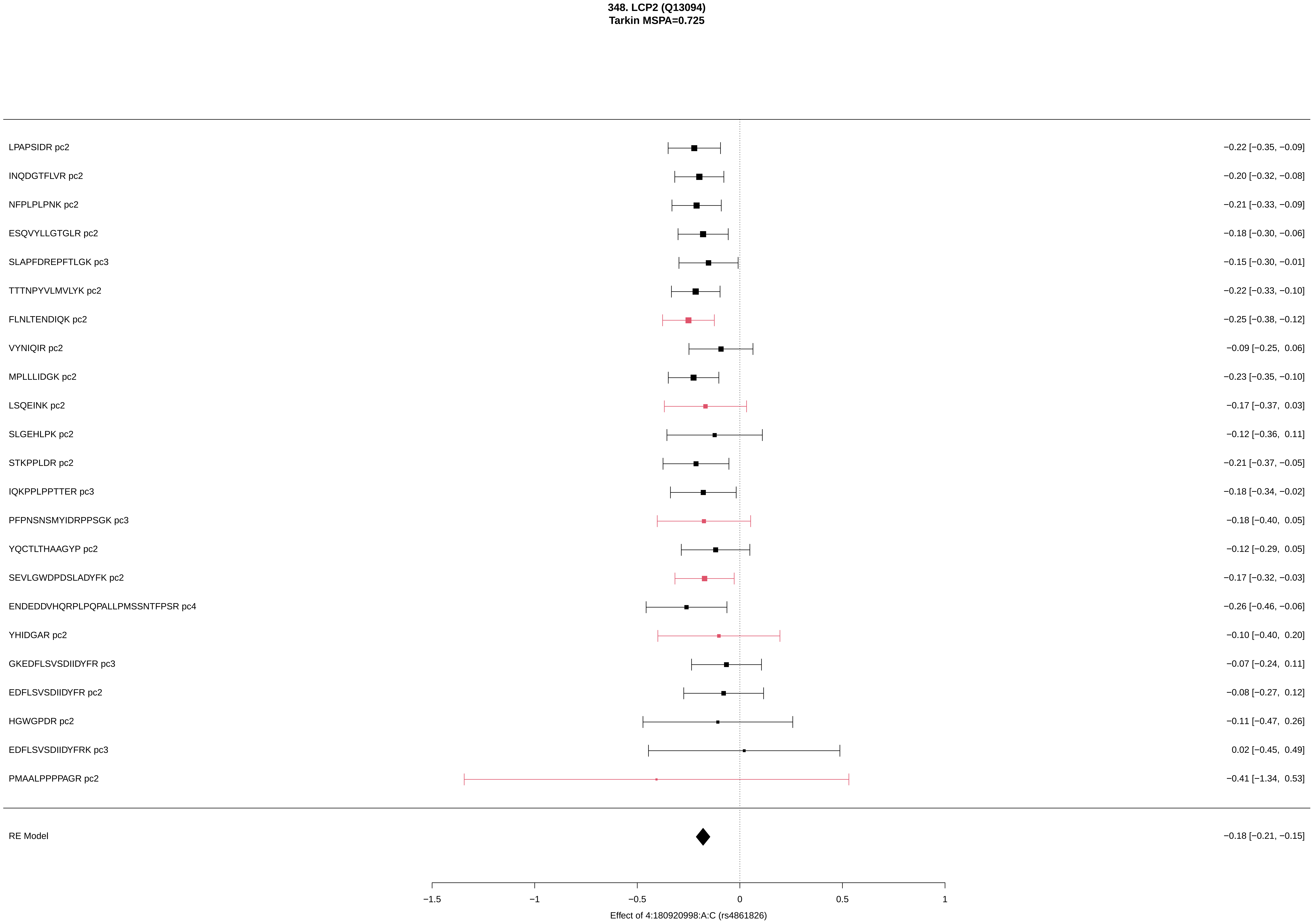
348. LCP2 (Q13094)
Tarkin MSPA=0.725
LPAPSIDR pc2 −0.22 [−0.35, −0.09]
INQDGTFLVR pc2 −0.20 [−0.32, −0.08]
NFPLPLPNK pc2 −0.21 [−0.33, −0.09]
ESQVYLLGTGLR pc2 −0.18 [−0.30, −0.06]
SLAPFDREPFTLGK pc3 −0.15 [−0.30, −0.01]
TTTNPYVLMVLYK pc2 −0.22 [−0.33, −0.10]
FLNLTENDIQK pc2 −0.25 [−0.38, −0.12]
VYNIQIR pc2 −0.09 [−0.25, 0.06]
MPLLLIDGK pc2 −0.23 [−0.35, −0.10]
LSQEINK pc2 −0.17 [−0.37, 0.03]
SLGEHLPK pc2 −0.12 [−0.36, 0.11]
STKPPLDR pc2 −0.21 [−0.37, −0.05]
IQKPPLPPTTER pc3 −0.18 [−0.34, −0.02]
PFPNSNSMYIDRPPSGK pc3 −0.18 [−0.40, 0.05]
YQCTLTHAAGYP pc2 −0.12 [−0.29, 0.05]
SEVLGWDPDSLADYFK pc2 −0.17 [−0.32, −0.03]
ENDEDDVHQRPLPQPALLPMSSNTFPSR pc4 −0.26 [−0.46, −0.06]
YHIDGAR pc2 −0.10 [−0.40, 0.20]
GKEDFLSVSDIIDYFR pc3 −0.07 [−0.24, 0.11]
EDFLSVSDIIDYFR pc2 −0.08 [−0.27, 0.12]
HGWGPDR pc2 −0.11 [−0.47, 0.26]
EDFLSVSDIIDYFRK pc3 0.02 [−0.45, 0.49]
PMAALPPPPAGR pc2 −0.41 [−1.34, 0.53]
RE Model −0.18 [−0.21, −0.15]
−1.5
−1
−0.5
0
0.5
1
Effect of 4:180920998:A:C (rs4861826)

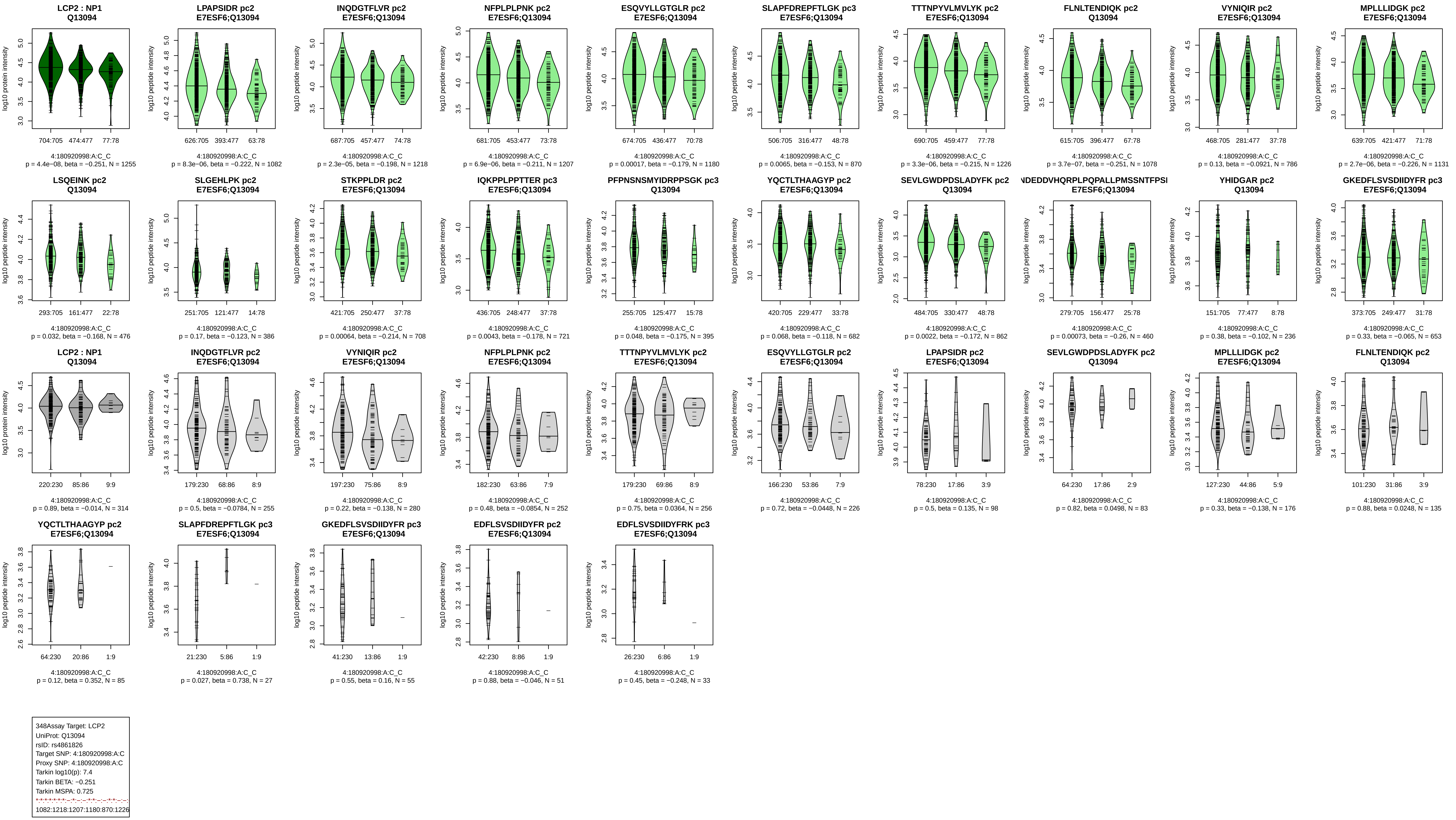
LCP2 : NP1
Q13094
log10 protein intensity
704:705 474:477 77:78
4:180920998:A:C_C
p = 4.4e−08, beta = −0.251, N = 1255
LPAPSIDR pc2
E7ESF6;Q13094
log10 peptide intensity
626:705 393:477 63:78
4:180920998:A:C_C
p = 8.3e−06, beta = −0.222, N = 1082
INQDGTFLVR pc2
E7ESF6;Q13094
log10 peptide intensity
687:705 457:477 74:78
4:180920998:A:C_C
p = 2.3e−05, beta = −0.198, N = 1218
NFPLPLPNK pc2
E7ESF6;Q13094
log10 peptide intensity
681:705 453:477 73:78
4:180920998:A:C_C
p = 6.9e−06, beta = −0.211, N = 1207
ESQVYLLGTGLR pc2
E7ESF6;Q13094
log10 peptide intensity
674:705 436:477 70:78
4:180920998:A:C_C
p = 0.00017, beta = −0.179, N = 1180
SLAPFDREPFTLGK pc3
E7ESF6;Q13094
log10 peptide intensity
506:705 316:477 48:78
4:180920998:A:C_C
p = 0.0065, beta = −0.153, N = 870
TTTNPYVLMVLYK pc2
E7ESF6;Q13094
log10 peptide intensity
690:705 459:477 77:78
4:180920998:A:C_C
p = 3.3e−06, beta = −0.215, N = 1226
FLNLTENDIQK pc2
Q13094
log10 peptide intensity
615:705 396:477 67:78
4:180920998:A:C_C
p = 3.7e−07, beta = −0.251, N = 1078
VYNIQIR pc2
E7ESF6;Q13094
log10 peptide intensity
468:705 281:477 37:78
4:180920998:A:C_C
p = 0.13, beta = −0.0921, N = 786
MPLLLIDGK pc2
E7ESF6;Q13094
log10 peptide intensity
639:705 421:477 71:78
4:180920998:A:C_C
p = 2.7e−06, beta = −0.226, N = 1131
LSQEINK pc2
Q13094
log10 peptide intensity
293:705 161:477 22:78
4:180920998:A:C_C
p = 0.032, beta = −0.168, N = 476
SLGEHLPK pc2
E7ESF6;Q13094
log10 peptide intensity
251:705 121:477 14:78
4:180920998:A:C_C
p = 0.17, beta = −0.123, N = 386
STKPPLDR pc2
E7ESF6;Q13094
log10 peptide intensity
421:705 250:477 37:78
4:180920998:A:C_C
p = 0.00064, beta = −0.214, N = 708
IQKPPLPPTTER pc3
E7ESF6;Q13094
log10 peptide intensity
436:705 248:477 37:78
4:180920998:A:C_C
p = 0.0043, beta = −0.178, N = 721
PFPNSNSMYIDRPPSGK pc3
Q13094
log10 peptide intensity
255:705 125:477 15:78
4:180920998:A:C_C
p = 0.048, beta = −0.175, N = 395
YQCTLTHAAGYP pc2
E7ESF6;Q13094
log10 peptide intensity
420:705 229:477 33:78
4:180920998:A:C_C
p = 0.068, beta = −0.118, N = 682
SEVLGWDPDSLADYFK pc2
Q13094
log10 peptide intensity
484:705 330:477 48:78
4:180920998:A:C_C
p = 0.0022, beta = −0.172, N = 862
NDEDDVHQRPLPQPALLPMSSNTFPSI
E7ESF6;Q13094
log10 peptide intensity
279:705 156:477 25:78
4:180920998:A:C_C
p = 0.00073, beta = −0.26, N = 460
YHIDGAR pc2
Q13094
log10 peptide intensity
151:705 77:477 8:78
4:180920998:A:C_C
p = 0.38, beta = −0.102, N = 236
GKEDFLSVSDIIDYFR pc3
E7ESF6;Q13094
log10 peptide intensity
373:705 249:477 31:78
4:180920998:A:C_C
p = 0.33, beta = −0.065, N = 653
LCP2 : NP1
Q13094
log10 protein intensity
220:230 85:86 9:9
4:180920998:A:C_C
p = 0.89, beta = −0.014, N = 314
INQDGTFLVR pc2
E7ESF6;Q13094
log10 peptide intensity
179:230 68:86 8:9
4:180920998:A:C_C
p = 0.5, beta = −0.0784, N = 255
VYNIQIR pc2
E7ESF6;Q13094
log10 peptide intensity
197:230 75:86 8:9
4:180920998:A:C_C
p = 0.22, beta = −0.138, N = 280
NFPLPLPNK pc2
E7ESF6;Q13094
log10 peptide intensity
182:230 63:86 7:9
4:180920998:A:C_C
p = 0.48, beta = −0.0854, N = 252
TTTNPYVLMVLYK pc2
E7ESF6;Q13094
log10 peptide intensity
179:230 69:86 8:9
4:180920998:A:C_C
p = 0.75, beta = 0.0364, N = 256
ESQVYLLGTGLR pc2
E7ESF6;Q13094
log10 peptide intensity
166:230 53:86 7:9
4:180920998:A:C_C
p = 0.72, beta = −0.0448, N = 226
LPAPSIDR pc2
E7ESF6;Q13094
log10 peptide intensity
78:230 17:86 3:9
4:180920998:A:C_C
p = 0.5, beta = 0.135, N = 98
SEVLGWDPDSLADYFK pc2
Q13094
log10 peptide intensity
64:230 17:86 2:9
4:180920998:A:C_C
p = 0.82, beta = 0.0498, N = 83
MPLLLIDGK pc2
E7ESF6;Q13094
log10 peptide intensity
127:230 44:86 5:9
4:180920998:A:C_C
p = 0.33, beta = −0.138, N = 176
FLNLTENDIQK pc2
Q13094
log10 peptide intensity
101:230 31:86 3:9
4:180920998:A:C_C
p = 0.88, beta = 0.0248, N = 135
YQCTLTHAAGYP pc2
E7ESF6;Q13094
log10 peptide intensity
64:230 20:86 1:9
4:180920998:A:C_C
p = 0.12, beta = 0.352, N = 85
SLAPFDREPFTLGK pc3
E7ESF6;Q13094
log10 peptide intensity
21:230 5:86 1:9
4:180920998:A:C_C
p = 0.027, beta = 0.738, N = 27
GKEDFLSVSDIIDYFR pc3
E7ESF6;Q13094
log10 peptide intensity
41:230 13:86 1:9
4:180920998:A:C_C
p = 0.55, beta = 0.16, N = 55
EDFLSVSDIIDYFR pc2
E7ESF6;Q13094
log10 peptide intensity
42:230 8:86 1:9
4:180920998:A:C_C
p = 0.88, beta = −0.046, N = 51
EDFLSVSDIIDYFRK pc3
E7ESF6;Q13094
log10 peptide intensity
26:230 6:86 1:9
4:180920998:A:C_C
p = 0.45, beta = −0.248, N = 33
348Assay Target: LCP2
UniProt: Q13094
rsID: rs4861826
Target SNP: 4:180920998:A:C
Proxy SNP: 4:180920998:A:C
Tarkin log10(p): 7.4
Tarkin BETA: −0.251
Tarkin MSPA: 0.725
1082:1218:1207:1180:870:1226

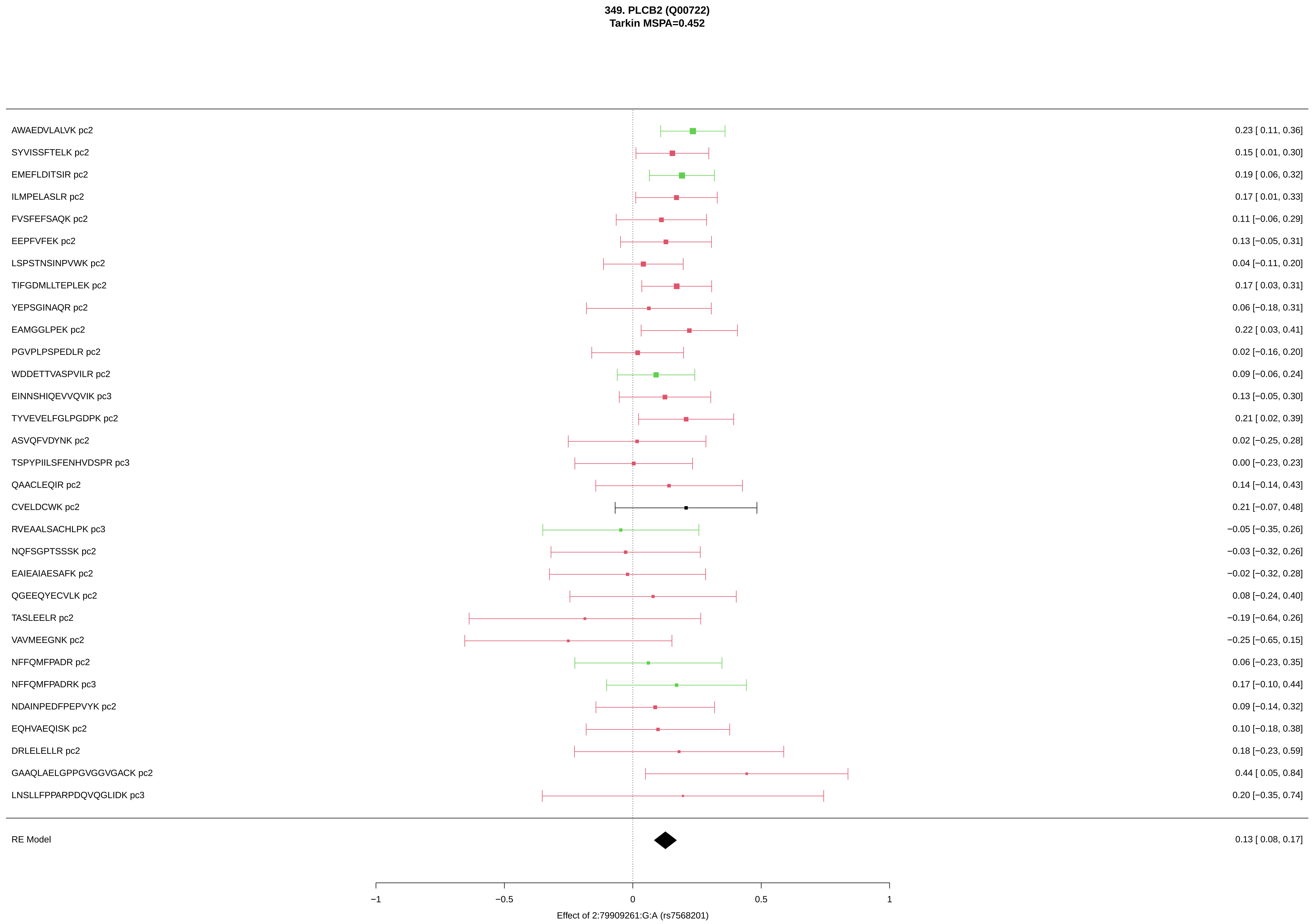

349. PLCB2 (Q00722)
Tarkin MSPA=0.452
AWAEDVLALVK pc2
SYVISSFTELK pc2
EMEFLDITSIR pc2
ILMPELASLR pc2
FVSFEFSAQK pc2
EEPFVFEK pc2
LSPSTNSINPVWK pc2
TIFGDMLLTEPLEK pc2
YEPSGINAQR pc2
EAMGGLPEK pc2
PGVPLPSPEDLR pc2
WDDETTVASPVILR pc2
EINNSHIQEVVQVIK pc3
TYVEVELFGLPGDPK pc2
ASVQFVDYNK pc2
TSPYPIILSFENHVDSPR pc3
QAACLEQIR pc2
CVELDCWK pc2
RVEAALSACHLPK pc3
NQFSGPTSSSK pc2
EAIEAIAESAFK pc2
QGEEQYECVLK pc2
TASLEELR pc2
VAVMEEGNK pc2
NFFQMFPADR pc2
NFFQMFPADRK pc3
NDAINPEDFPEPVYK pc2
EQHVAEQISK pc2
DRLELELLR pc2
GAAQLAELGPPGVGGVGACK pc2
LNSLLFPPARPDQVQGLIDK pc3
RE Model
0.23 [0.11, 0.36]
0.15 [0.01, 0.30]
0.19 [0.06, 0.32]
0.17 [0.01, 0.33]
0.11 [−0.06, 0.29]
0.13 [−0.05, 0.31]
0.04 [−0.11, 0.20]
0.17 [0.03, 0.31]
0.06 [−0.18, 0.31]
0.22 [0.03, 0.41]
0.02 [−0.16, 0.20]
0.09 [−0.06, 0.24]
0.13 [−0.05, 0.30]
0.21 [0.02, 0.39]
0.02 [−0.25, 0.28]
0.00 [−0.23, 0.23]
0.14 [−0.14, 0.43]
0.21 [−0.07, 0.48]
−0.05 [−0.35, 0.26]
−0.03 [−0.32, 0.26]
−0.02 [−0.32, 0.28]
0.08 [−0.24, 0.40]
−0.19 [−0.64, 0.26]
−0.25 [−0.65, 0.15]
0.06 [−0.23, 0.35]
0.17 [−0.10, 0.44]
0.09 [−0.14, 0.32]
0.10 [−0.18, 0.38]
0.18 [−0.23, 0.59]
0.44 [0.05, 0.84]
0.20 [−0.35, 0.74]
0.13 [0.08, 0.17]
−1
−0.5
0
0.5
1
Effect of 2:79909261:G:A (rs7568201)

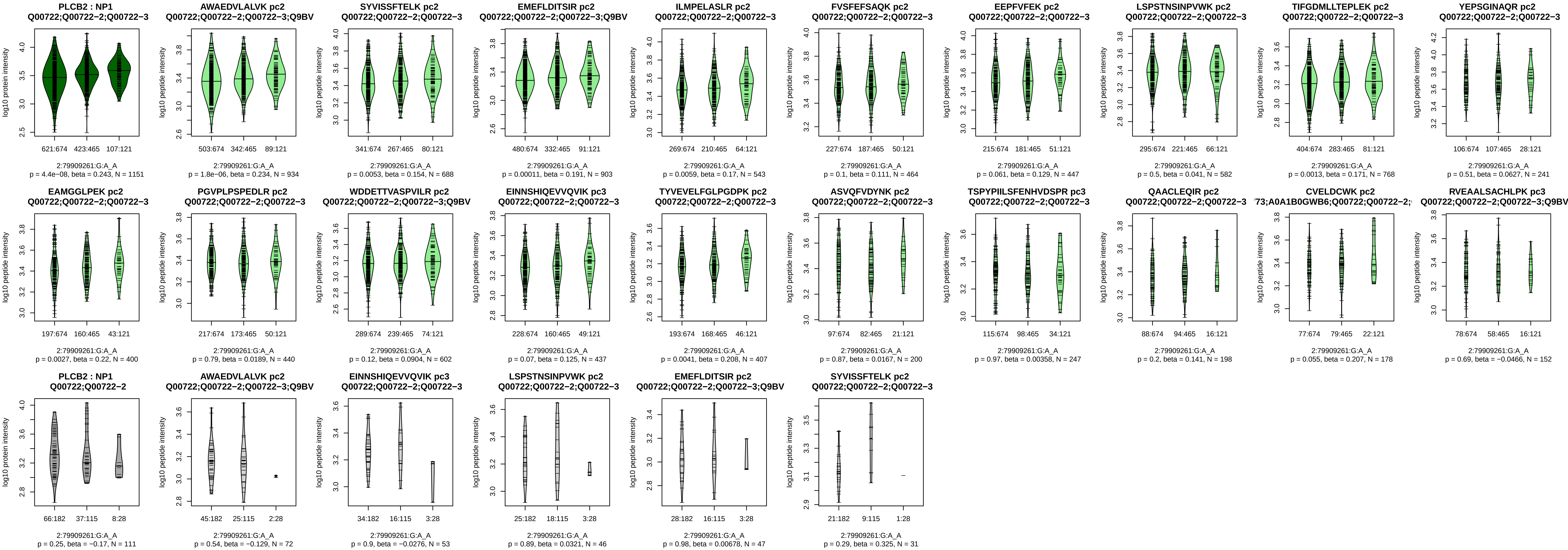

349Assay Target: PLCB2
UniProt: Q00722
rsID: rs7568201
Target SNP: 2:79909261:G:A
Proxy SNP: 2:79909261:G:A
Tarkin log10(p): 7.4
Tarkin BETA: 0.243
Tarkin MSPA: 0.452
934:688:903:543:464:447:582:7

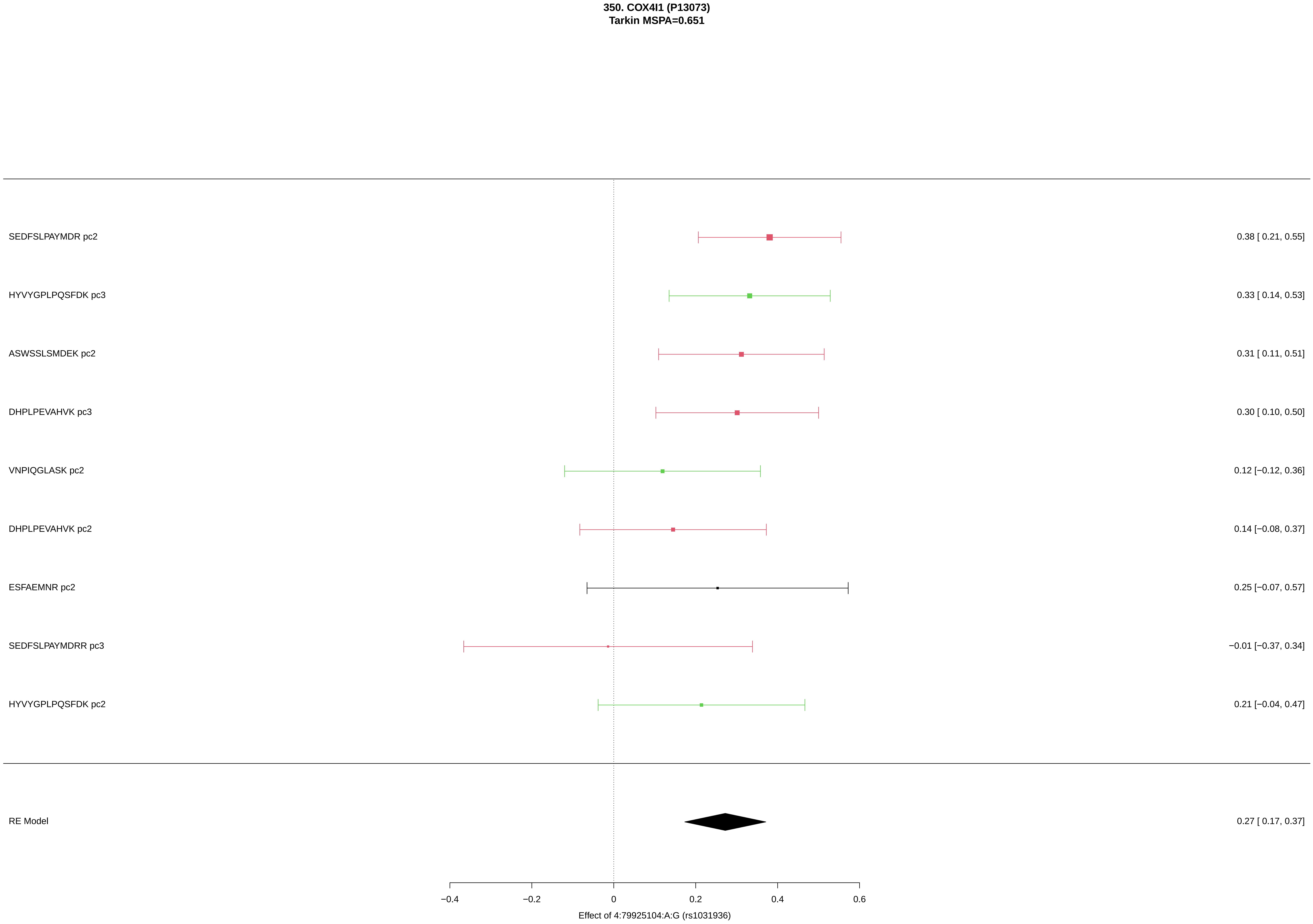
350. COX4I1 (P13073)
Tarkin MSPA=0.651
SEDFSLPAYMDR pc2
0.38 [0.21, 0.55]
HYVYGPLPQSFDK pc3
0.33 [0.14, 0.53]
ASWSSLSMDEK pc2
0.31 [0.11, 0.51]
DHPLPEVAHVK pc3
0.30 [0.10, 0.50]
VNPIQGLASK pc2
0.12 [−0.12, 0.36]
DHPLPEVAHVK pc2
0.14 [−0.08, 0.37]
ESFAEMNR pc2
0.25 [−0.07, 0.57]
SEDFSLPAYMDRR pc3
−0.01 [−0.37, 0.34]
HYVYGPLPQSFDK pc2
0.21 [−0.04, 0.47]
RE Model
0.27 [0.17, 0.37]
−0.4
−0.2
0
0.2
0.4
0.6
Effect of 4:79925104:A:G (rs1031936)

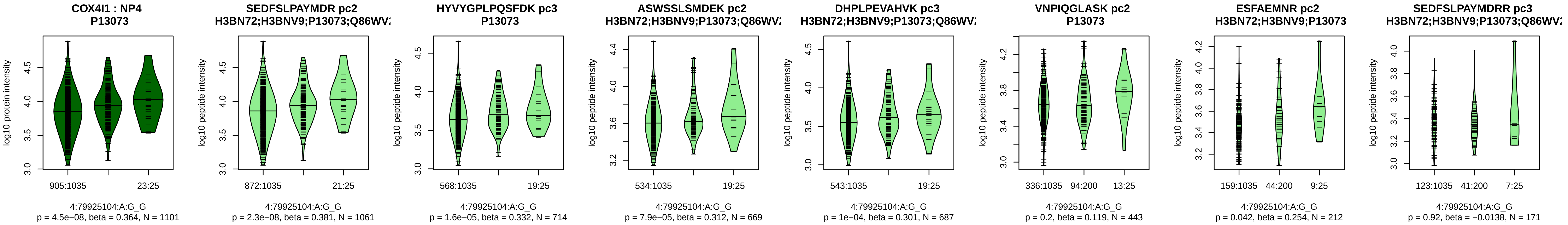

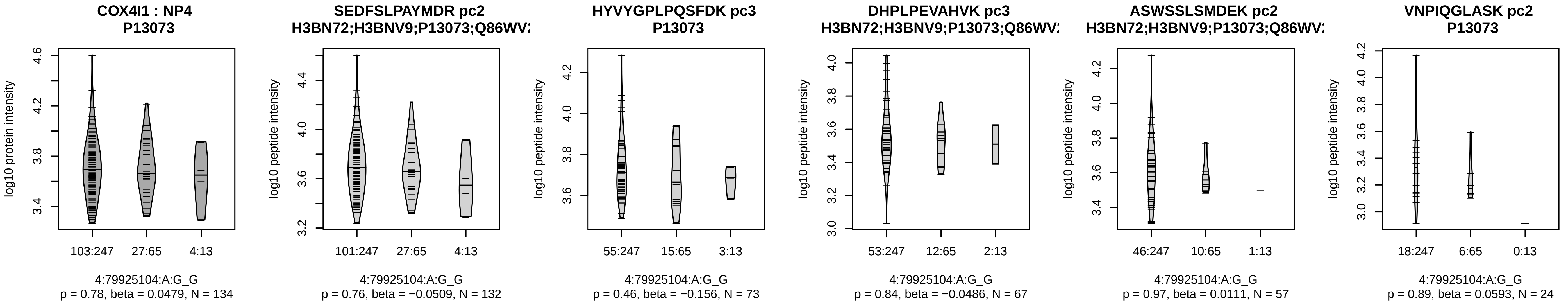

350Assay Target: COX4I1
UniProt: P13073
rsID: rs1031936
Target SNP: 4:79925104:A:G
Proxy SNP: 4:79925104:A:G
Tarkin log10(p): 7.4
Tarkin BETA: 0.364
Tarkin MSPA: 0.651
::*:*:−:−:−:−:−:NA
1061:714:669:687:443:482:212:

351. WFDC5 (Q8TCV5)
Tarkin MSPA=1

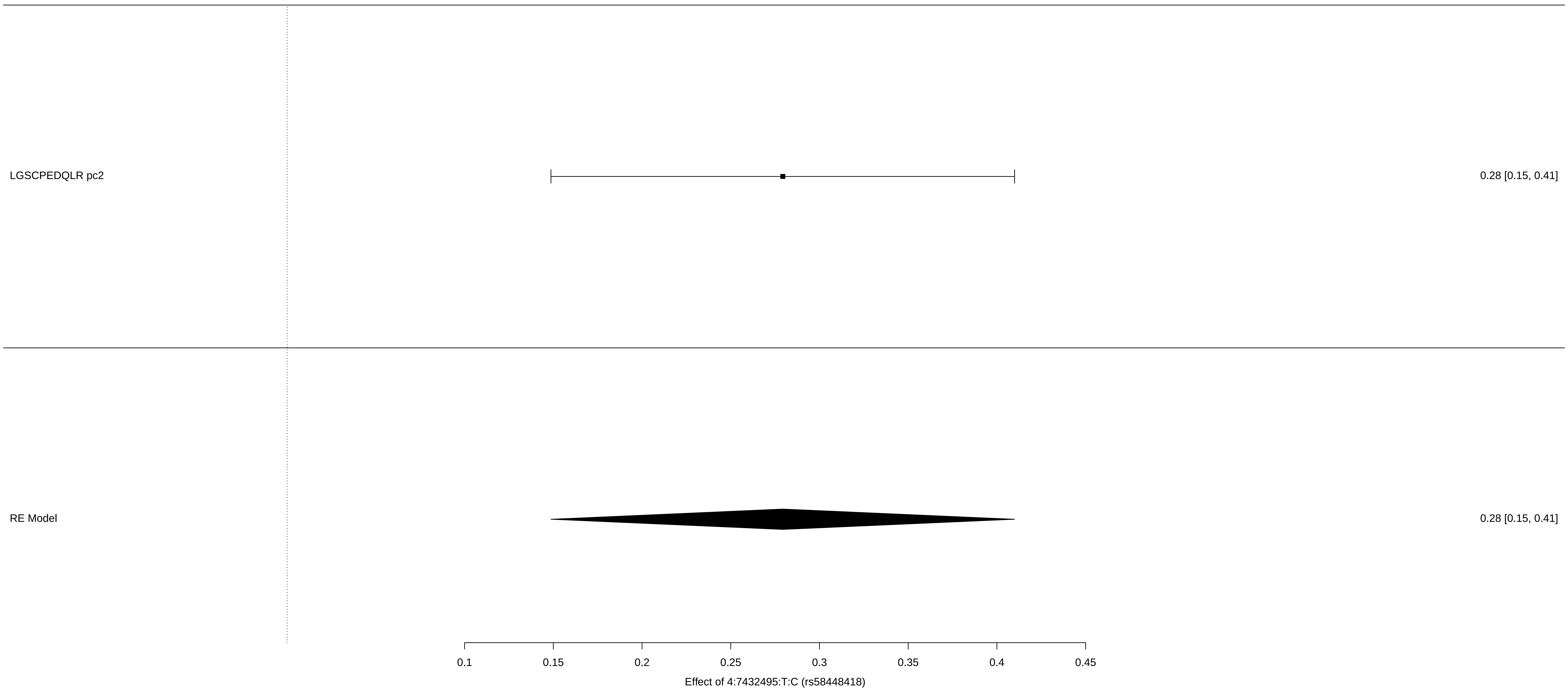

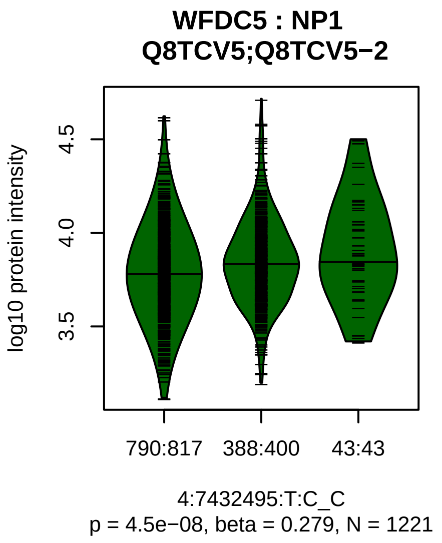

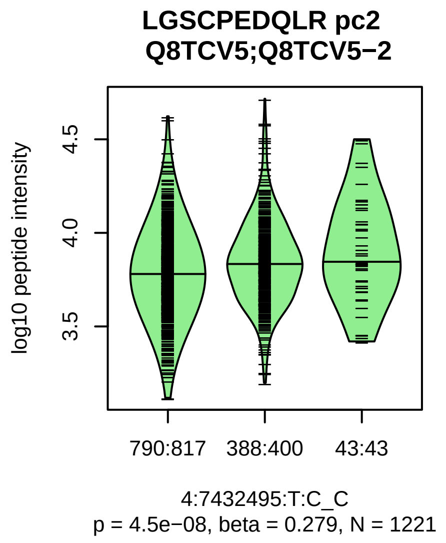

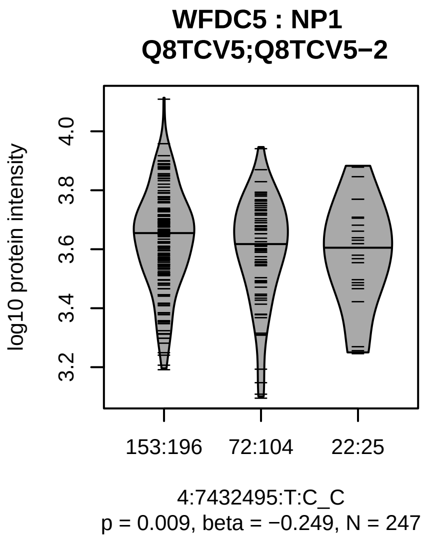

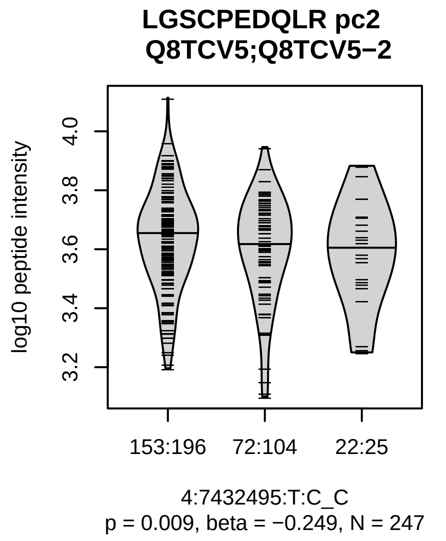

351Assay Target: WFDC5
UniProt: Q8TCV5
rsID: rs58448418
Target SNP: 4:7432495:T:C
Proxy SNP: 4:7432495:T:C
Tarkin log10(p): 7.4
Tarkin BETA: 0.279
Tarkin MSPA: 1
*
1221

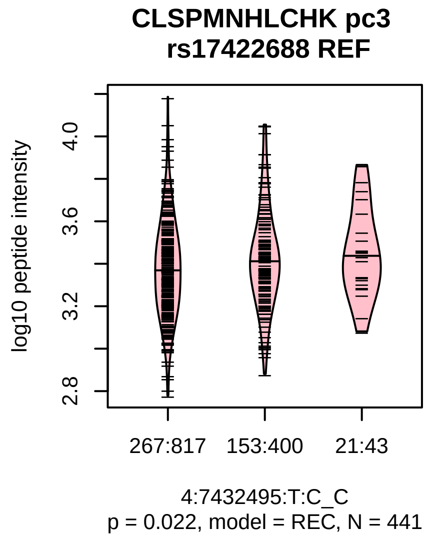

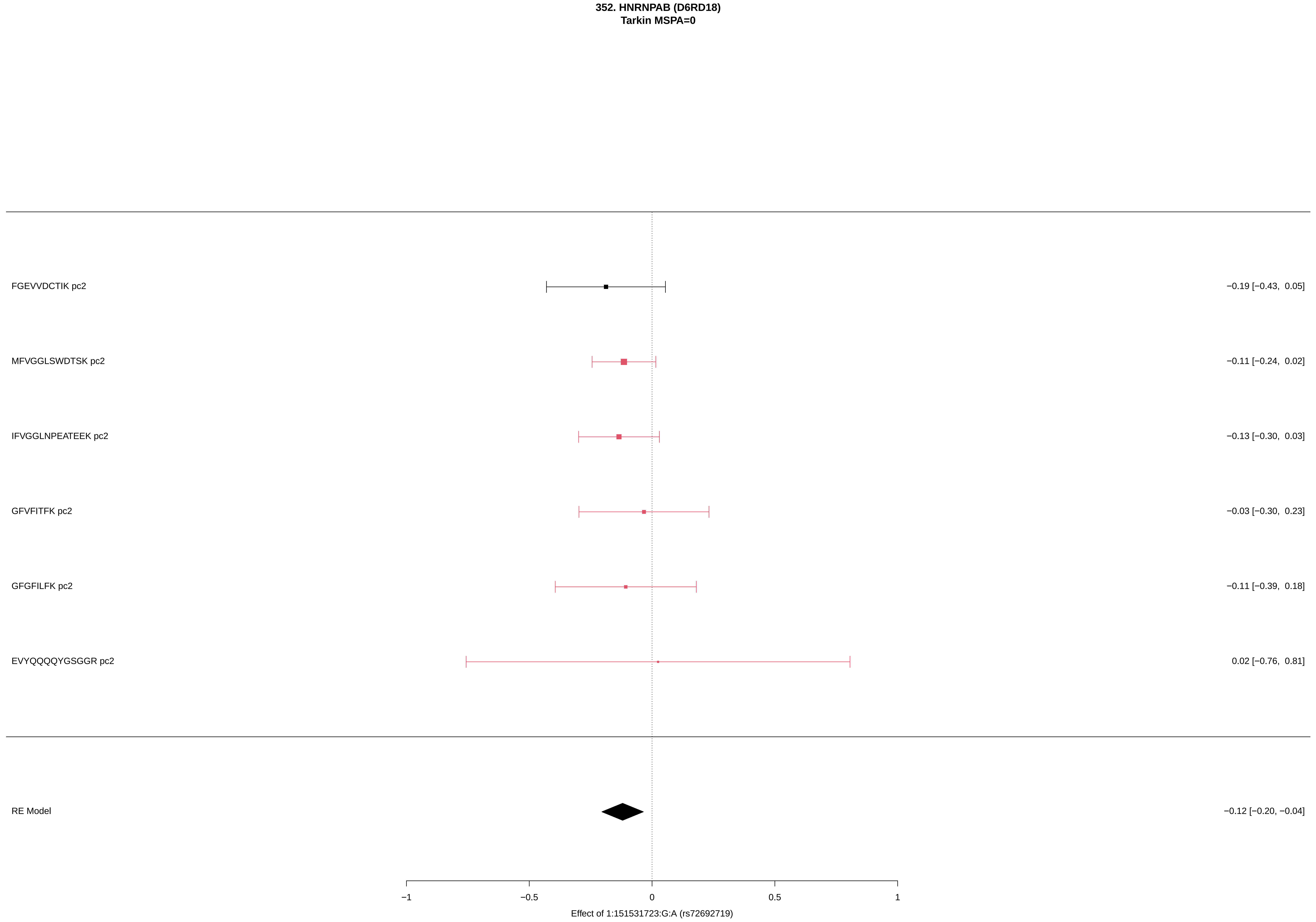

352. HNRNPAB (D6RD18)
Tarkin MSPA=0
FGEVVDCTIK pc2
−0.19 [−0.43, 0.05]
MFVGGLSWDTSK pc2
−0.11 [−0.24, 0.02]
IFVGGLNPEATEEK pc2
−0.13 [−0.30, 0.03]
GFVFITFK pc2
−0.03 [−0.30, 0.23]
GFGFILFK pc2
−0.11 [−0.39, 0.18]
EVYQQQQYGSGGR pc2
0.02 [−0.76, 0.81]
RE Model
−0.12 [−0.20, −0.04]
−1
−0.5
0
0.5
1
Effect of 1:151531723:G:A (rs72692719)

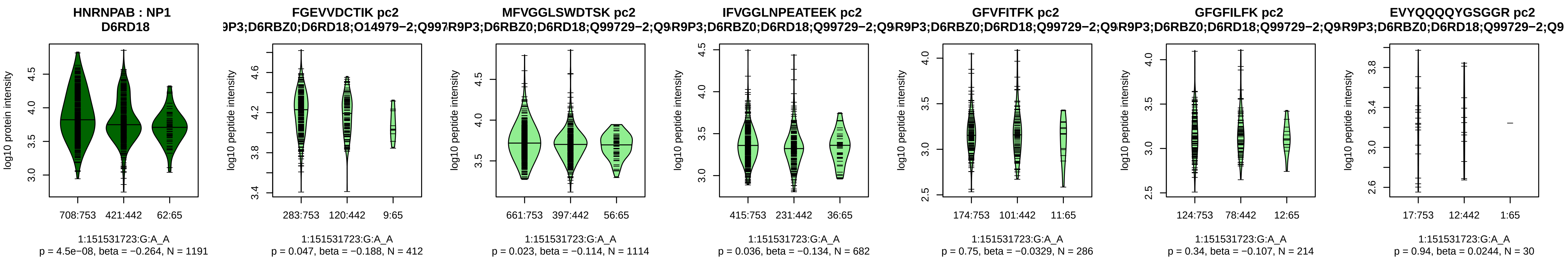

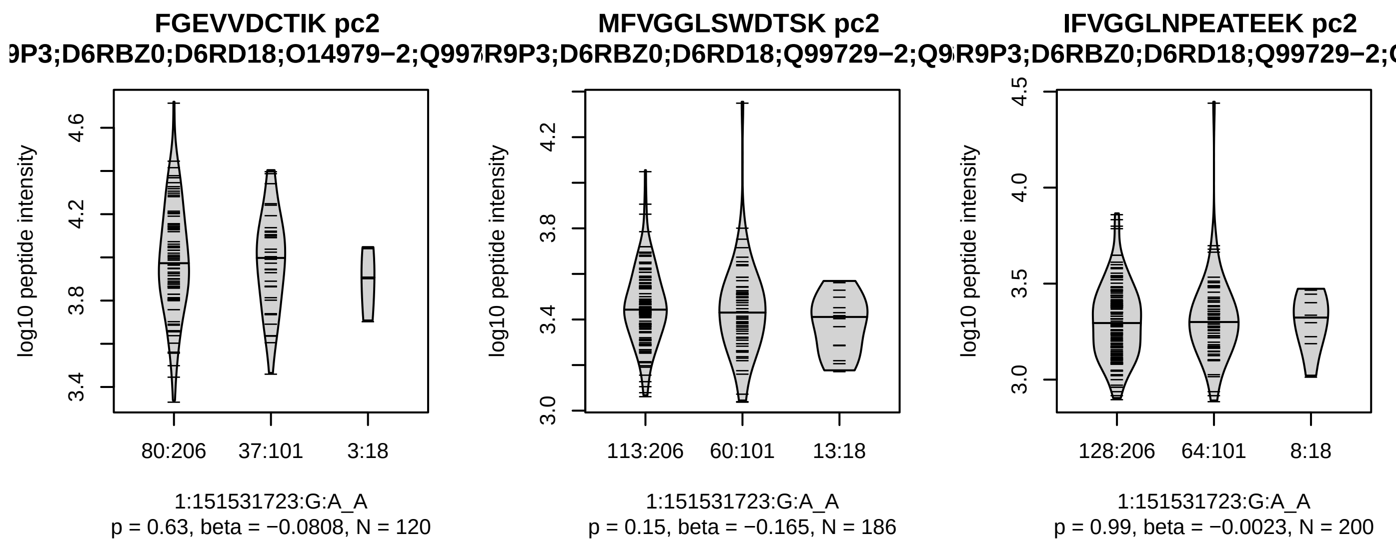

352Assay Target: HNRNPAB
UniProt: D6RD18
rsID: rs72692719
Target SNP: 1:151531723:G:A
Proxy SNP: 1:151531723:G:A
Tarkin log10(p): 7.3
Tarkin BETA: −0.264
Tarkin MSPA: 0
−:−:−:−:−:−:NA
412:1114:682:286:214:30:1

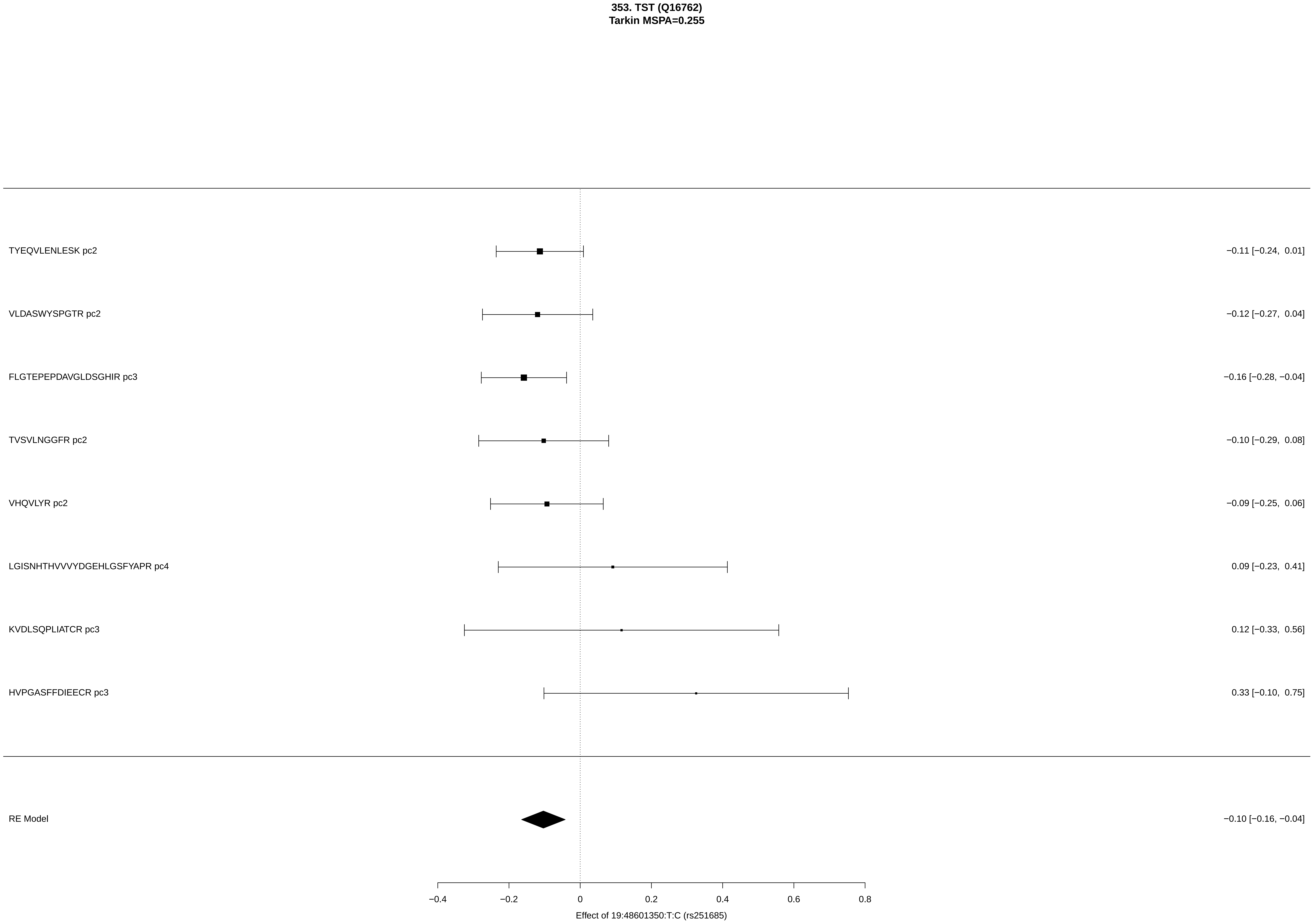
353. TST (Q16762)
Tarkin MSPA=0.255
TYEQVLENLESK pc2
−0.11 [−0.24, 0.01]
VLDASWYSPGTR pc2
−0.12 [−0.27, 0.04]
FLGTEPEPDAVGLDSGHIR pc3
−0.16 [−0.28, −0.04]
TVSVLNGGFR pc2
−0.10 [−0.29, 0.08]
VHQVLYR pc2
−0.09 [−0.25, 0.06]
LGISNHTHVVVYDGEHLGSFYAPR pc4
0.09 [−0.23, 0.41]
KVDLSQPLIATCR pc3
0.12 [−0.33, 0.56]
HVPGASFFDIEECR pc3
0.33 [−0.10, 0.75]
RE Model
−0.10 [−0.16, −0.04]
−0.4
−0.2
0
0.2
0.4
0.6
0.8
Effect of 19:48601350:T:C (rs251685)

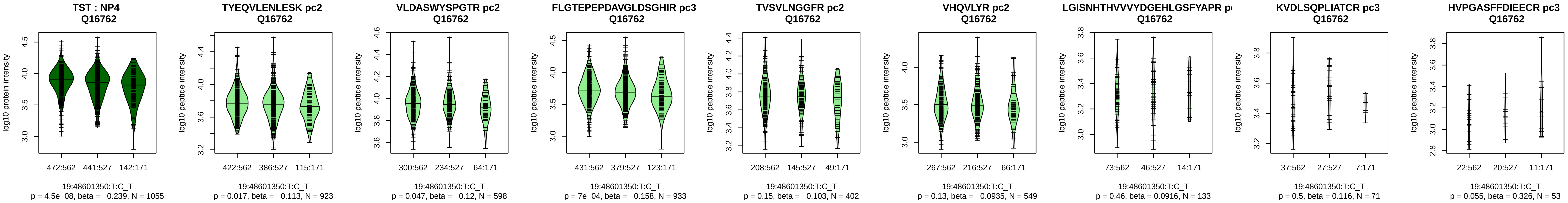

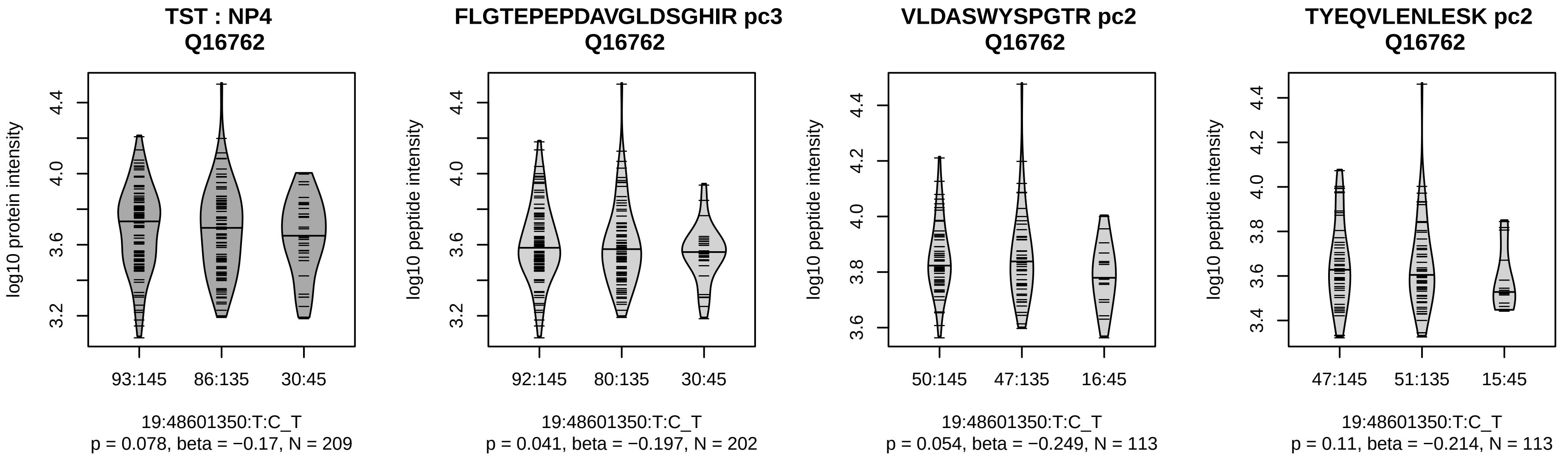

353Assay Target: TST
UniProt: Q16762
rsID: rs251685
Target SNP: 19:48601350:T:C
Proxy SNP: 19:48601350:T:C
Tarkin log10(p): 7.3
Tarkin BETA: −0.239
Tarkin MSPA: 0.255
–:–:*:–:–:–:–:–
923:598:933:402:549:133:71:53

354. ESYT1 (Q9BSJ8)
Tarkin MSPA=0.702

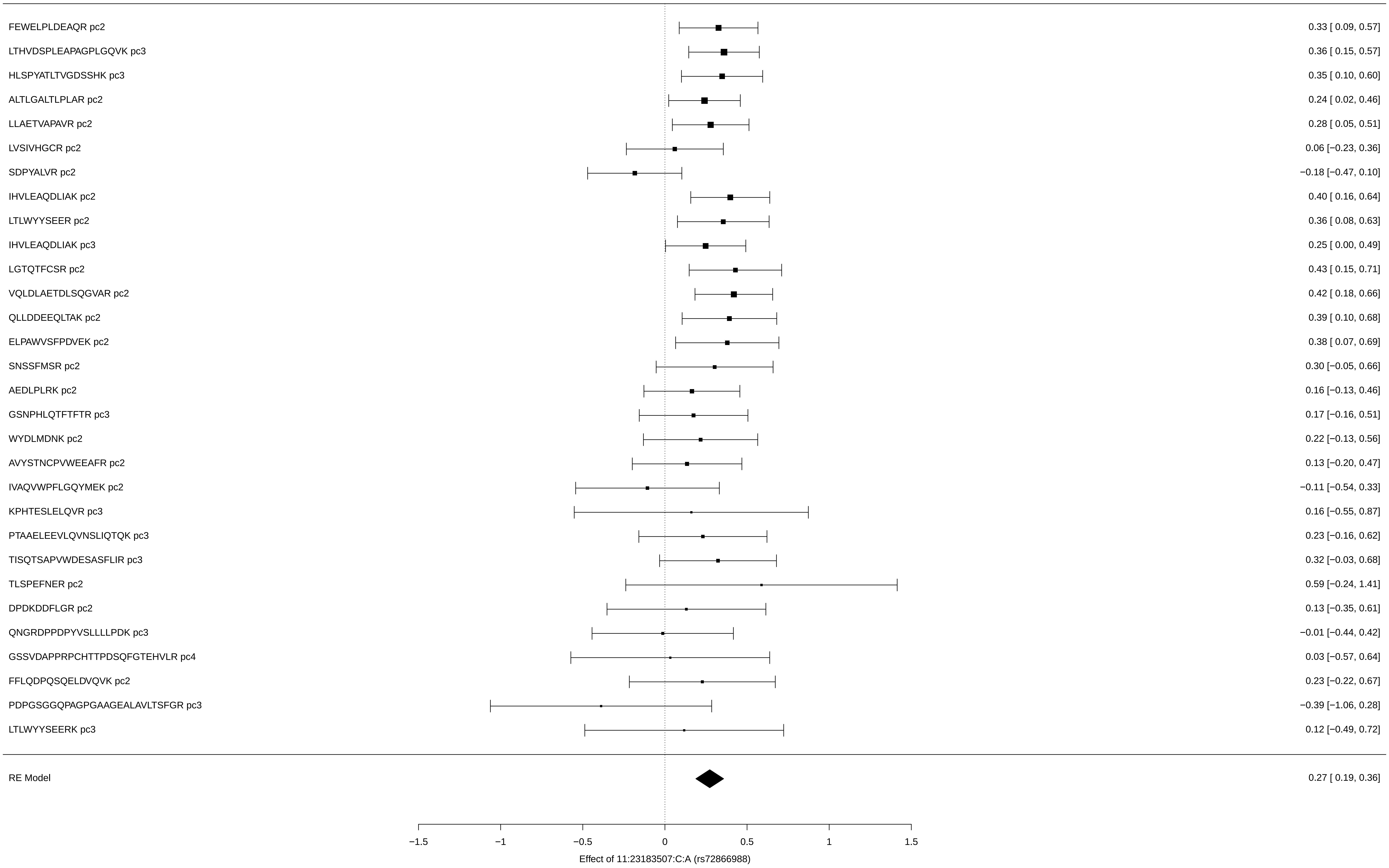

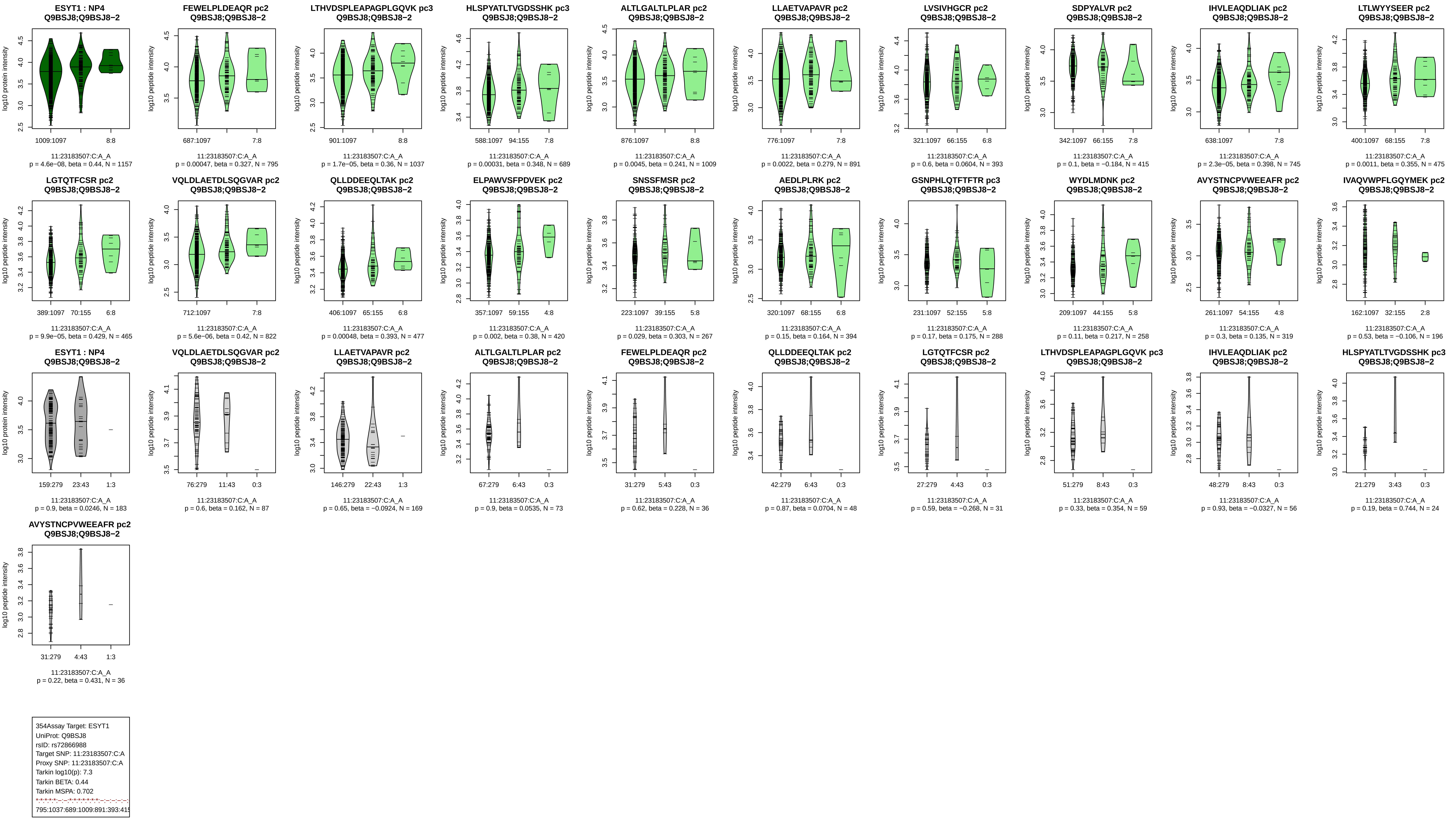
ESYT1 : NP4 Q9BSJ8;Q9BSJ8−2
1009:1097 8:8
11:23183507:C:A_A
p = 4.6e−08, beta = 0.44, N = 1157
FEWELPLDEAQR pc2 Q9BSJ8;Q9BSJ8−2
687:1097 7:8
11:23183507:C:A_A
p = 0.00047, beta = 0.327, N = 795
LTHVDSPLEAPAGPLGQVK pc3 Q9BSJ8;Q9BSJ8−2
901:1097 8:8
11:23183507:C:A_A
p = 1.7e−05, beta = 0.36, N = 1037
HLSPYATLTVGDSSHK pc3 Q9BSJ8;Q9BSJ8−2
588:1097 94:155 7:8
11:23183507:C:A_A
p = 0.00031, beta = 0.348, N = 689
ALTLGALTLPLAR pc2 Q9BSJ8;Q9BSJ8−2
876:1097 8:8
11:23183507:C:A_A
p = 0.0045, beta = 0.241, N = 1009
LLAETVAPAVR pc2 Q9BSJ8;Q9BSJ8−2
776:1097 7:8
11:23183507:C:A_A
p = 0.0022, beta = 0.279, N = 891
LVSIVHGCR pc2 Q9BSJ8;Q9BSJ8−2
321:1097 66:155 6:8
11:23183507:C:A_A
p = 0.6, beta = 0.0604, N = 393
SDPYALVR pc2 Q9BSJ8;Q9BSJ8−2
342:1097 66:155 7:8
11:23183507:C:A_A
p = 0.1, beta = −0.184, N = 415
IHVLEAQDLIAK pc2 Q9BSJ8;Q9BSJ8−2
638:1097 7:8
11:23183507:C:A_A
p = 2.3e−05, beta = 0.398, N = 745
LTLWYYSEER pc2 Q9BSJ8;Q9BSJ8−2
400:1097 68:155 7:8
11:23183507:C:A_A
p = 0.0011, beta = 0.355, N = 475
LGTQTFCSR pc2 Q9BSJ8;Q9BSJ8−2
389:1097 70:155 6:8
11:23183507:C:A_A
p = 9.9e−05, beta = 0.429, N = 465
VQLDLAETDLSQGVAR pc2 Q9BSJ8;Q9BSJ8−2
712:1097 7:8
11:23183507:C:A_A
p = 5.6e−06, beta = 0.42, N = 822
QLLDDEEQLTAK pc2 Q9BSJ8;Q9BSJ8−2
406:1097 65:155 6:8
11:23183507:C:A_A
p = 0.00048, beta = 0.393, N = 477
ELPAWVSFPDVEK pc2 Q9BSJ8;Q9BSJ8−2
357:1097 59:155 4:8
11:23183507:C:A_A
p = 0.002, beta = 0.38, N = 420
SNSSFMSR pc2 Q9BSJ8;Q9BSJ8−2
223:1097 39:155 5:8
11:23183507:C:A_A
p = 0.029, beta = 0.303, N = 267
AEDLPLRK pc2 Q9BSJ8;Q9BSJ8−2
320:1097 68:155 6:8
11:23183507:C:A_A
p = 0.15, beta = 0.164, N = 394
GSNPHLQTFTFTR pc3 Q9BSJ8;Q9BSJ8−2
231:1097 52:155 5:8
11:23183507:C:A_A
p = 0.17, beta = 0.175, N = 288
WYDLMDNK pc2 Q9BSJ8;Q9BSJ8−2
209:1097 44:155 5:8
11:23183507:C:A_A
p = 0.11, beta = 0.217, N = 258
AVYSTNCPVWEEAFR pc2 Q9BSJ8;Q9BSJ8−2
261:1097 54:155 4:8
11:23183507:C:A_A
p = 0.3, beta = 0.135, N = 319
IVAQVWPFLGQYMEK pc2 Q9BSJ8;Q9BSJ8−2
162:1097 32:155 2:8
11:23183507:C:A_A
p = 0.53, beta = −0.106, N = 196
ESYT1 : NP4 Q9BSJ8;Q9BSJ8−2
159:279 23:43 1:3
11:23183507:C:A_A
p = 0.9, beta = 0.0246, N = 183
VQLDLAETDLSQGVAR pc2 Q9BSJ8;Q9BSJ8−2
76:279 11:43 0:3
11:23183507:C:A_A
p = 0.6, beta = 0.162, N = 87
LLAETVAPAVR pc2 Q9BSJ8;Q9BSJ8−2
146:279 22:43 1:3
11:23183507:C:A_A
p = 0.65, beta = −0.0924, N = 169
ALTLGALTLPLAR pc2 Q9BSJ8;Q9BSJ8−2
67:279 6:43 0:3
11:23183507:C:A_A
p = 0.9, beta = 0.0535, N = 73
FEWELPLDEAQR pc2 Q9BSJ8;Q9BSJ8−2
31:279 5:43 0:3
11:23183507:C:A_A
p = 0.62, beta = 0.228, N = 36
QLLDDEEQLTAK pc2 Q9BSJ8;Q9BSJ8−2
42:279 6:43 0:3
11:23183507:C:A_A
p = 0.87, beta = 0.0704, N = 48
LGTQTFCSR pc2 Q9BSJ8;Q9BSJ8−2
27:279 4:43 0:3
11:23183507:C:A_A
p = 0.59, beta = −0.268, N = 31
LTHVDSPLEAPAGPLGQVK pc3 Q9BSJ8;Q9BSJ8−2
51:279 8:43 0:3
11:23183507:C:A_A
p = 0.33, beta = 0.354, N = 59
IHVLEAQDLIAK pc2 Q9BSJ8;Q9BSJ8−2
48:279 8:43 0:3
11:23183507:C:A_A
p = 0.93, beta = −0.0327, N = 56
HLSPYATLTVGDSSHK pc3 Q9BSJ8;Q9BSJ8−2
21:279 3:43 0:3
11:23183507:C:A_A
p = 0.19, beta = 0.744, N = 24
AVYSTNCPVWEEAFR pc2 Q9BSJ8;Q9BSJ8−2
31:279 4:43 1:3
11:23183507:C:A_A
p = 0.22, beta = 0.431, N = 36
log10 protein intensity
log10 peptide intensity
354Assay Target: ESYT1
UniProt: Q9BSJ8
rsID: rs72866988
Target SNP: 11:23183507:C:A
Proxy SNP: 11:23183507:C:A
Tarkin log10(p): 7.3
Tarkin BETA: 0.44
Tarkin MSPA: 0.702
795:1037:689:1009:891:393:415

355. AP3D1 (O14617)
Tarkin MSPA=0

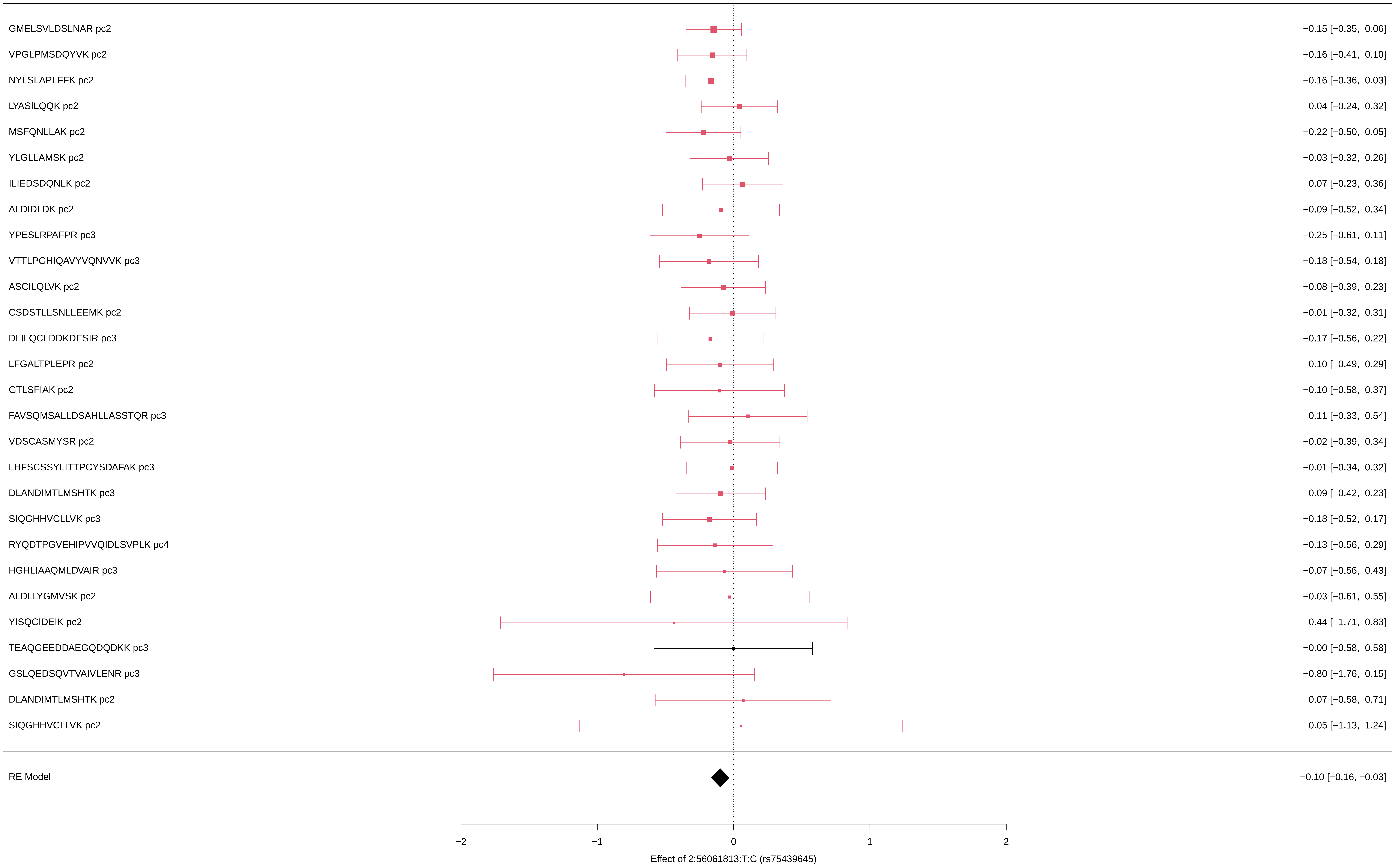

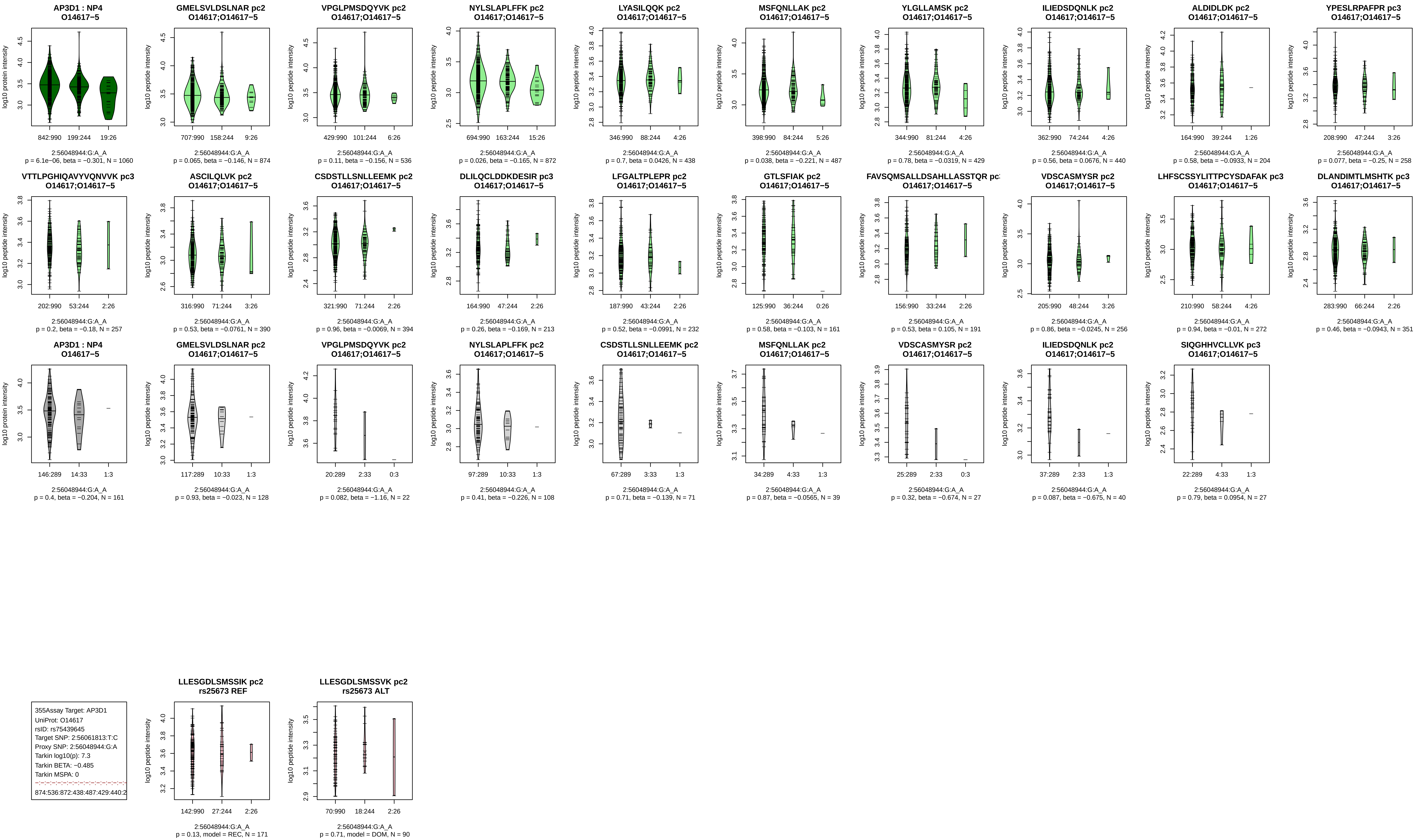
AP3D1 : NP4
O14617−5
842:990 199:244 19:26
2:56048944:G:A_A
p = 6.1e−06, beta = −0.301, N = 1060
GMELSVLDSLNAR pc2
O14617;O14617−5
707:990 158:244 9:26
2:56048944:G:A_A
p = 0.065, beta = −0.146, N = 874
VPGLPMSDQYVK pc2
O14617;O14617−5
429:990 101:244 6:26
2:56048944:G:A_A
p = 0.11, beta = −0.156, N = 536
NYLSLAPLFFK pc2
O14617;O14617−5
694:990 163:244 15:26
2:56048944:G:A_A
p = 0.026, beta = −0.165, N = 872
LYASILQQK pc2
O14617;O14617−5
346:990 88:244 4:26
2:56048944:G:A_A
p = 0.7, beta = 0.0426, N = 438
MSFQNLLAK pc2
O14617;O14617−5
398:990 84:244 5:26
2:56048944:G:A_A
p = 0.038, beta = −0.221, N = 487
YLGLLAMSK pc2
O14617;O14617−5
344:990 81:244 4:26
2:56048944:G:A_A
p = 0.78, beta = −0.0319, N = 429
ILIEDSDQNLK pc2
O14617;O14617−5
362:990 74:244 4:26
2:56048944:G:A_A
p = 0.56, beta = 0.0676, N = 440
ALDIDLDK pc2
O14617;O14617−5
164:990 39:244 1:26
2:56048944:G:A_A
p = 0.58, beta = −0.0933, N = 204
YPESLRPAFPR pc3
O14617;O14617−5
208:990 47:244 3:26
2:56048944:G:A_A
p = 0.077, beta = −0.25, N = 258
VTTLPGHIQAVYVQNVVK pc3
O14617;O14617−5
202:990 53:244 2:26
2:56048944:G:A_A
p = 0.2, beta = −0.18, N = 257
ASCILQLVK pc2
O14617;O14617−5
316:990 71:244 3:26
2:56048944:G:A_A
p = 0.53, beta = −0.0761, N = 390
CSDSTLLSNLLEEMK pc2
O14617;O14617−5
321:990 71:244 2:26
2:56048944:G:A_A
p = 0.96, beta = −0.0069, N = 394
DLILQCLDDKDESIR pc3
O14617;O14617−5
164:990 47:244 2:26
2:56048944:G:A_A
p = 0.26, beta = −0.169, N = 213
LFGALTPLEPR pc2
O14617;O14617−5
187:990 43:244 2:26
2:56048944:G:A_A
p = 0.52, beta = −0.0991, N = 232
GTLSFIAK pc2
O14617;O14617−5
125:990 36:244 0:26
2:56048944:G:A_A
p = 0.58, beta = −0.103, N = 161
FAVSQMSALLDSAHLLASSTQR pc
O14617;O14617−5
156:990 33:244 2:26
2:56048944:G:A_A
p = 0.53, beta = 0.105, N = 191
VDSCASMYSR pc2
O14617;O14617−5
205:990 48:244 3:26
2:56048944:G:A_A
p = 0.86, beta = −0.0245, N = 256
LHFSCSSYLITTPCYSDAFAK pc3
O14617;O14617−5
210:990 58:244 4:26
2:56048944:G:A_A
p = 0.94, beta = −0.01, N = 272
DLANDIMTLMSHTK pc3
O14617;O14617−5
283:990 66:244 2:26
2:56048944:G:A_A
p = 0.46, beta = −0.0943, N = 351
AP3D1 : NP4
O14617−5
146:289 14:33 1:3
2:56048944:G:A_A
p = 0.4, beta = −0.204, N = 161
GMELSVLDSLNAR pc2
O14617;O14617−5
117:289 10:33 1:3
2:56048944:G:A_A
p = 0.93, beta = −0.023, N = 128
VPGLPMSDQYVK pc2
O14617;O14617−5
20:289 2:33 0:3
2:56048944:G:A_A
p = 0.082, beta = −1.16, N = 22
NYLSLAPLFFK pc2
O14617;O14617−5
97:289 10:33 1:3
2:56048944:G:A_A
p = 0.41, beta = −0.226, N = 108
CSDSTLLSNLLEEMK pc2
O14617;O14617−5
67:289 3:33 1:3
2:56048944:G:A_A
p = 0.71, beta = −0.139, N = 71
MSFQNLLAK pc2
O14617;O14617−5
34:289 4:33 1:3
2:56048944:G:A_A
p = 0.87, beta = −0.0565, N = 39
VDSCASMYSR pc2
O14617;O14617−5
25:289 2:33 0:3
2:56048944:G:A_A
p = 0.32, beta = −0.674, N = 27
ILIEDSDQNLK pc2
O14617;O14617−5
37:289 2:33 1:3
2:56048944:G:A_A
p = 0.087, beta = −0.675, N = 40
SIQGHHVCLLVK pc3
O14617;O14617−5
22:289 4:33 1:3
2:56048944:G:A_A
p = 0.79, beta = 0.0954, N = 27
355Assay Target: AP3D1
UniProt: O14617
rsID: rs75439645
Target SNP: 2:56061813:T:C
Proxy SNP: 2:56048944:G:A
Tarkin log10(p): 7.3
Tarkin BETA: −0.485
Tarkin MSPA: 0
874:536:872:438:487:429:440:2
LLESGDLSMSSIK pc2
rs25673 REF
142:990 27:244 2:26
2:56048944:G:A_A
p = 0.13, model = REC, N = 171
LLESGDLSMSSVK pc2
rs25673 ALT
70:990 18:244 2:26
2:56048944:G:A_A
p = 0.71, model = DOM, N = 90
log10 protein intensity
log10 peptide intensity

356. PROC (E7END6)
Tarkin MSPA=0.894

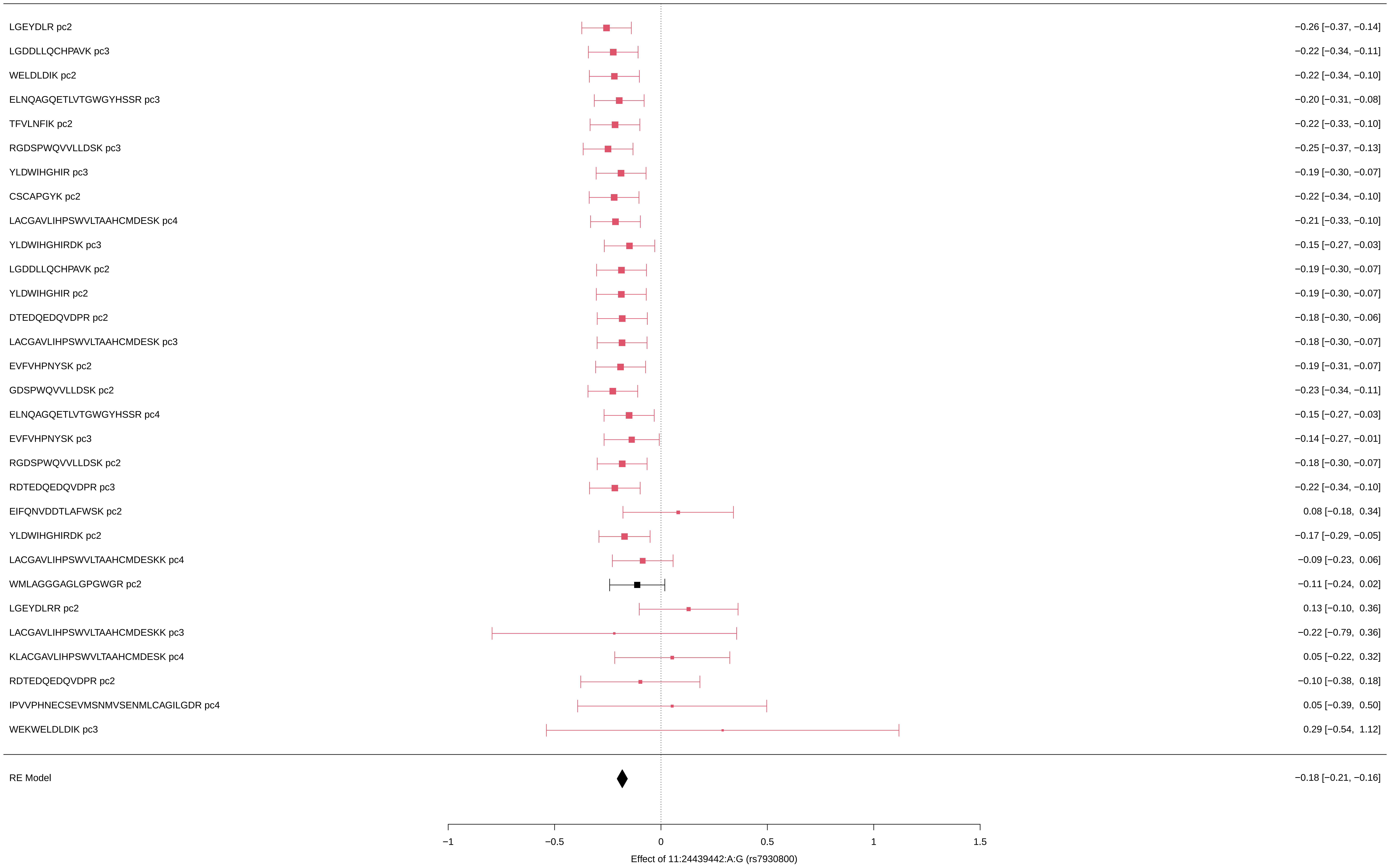

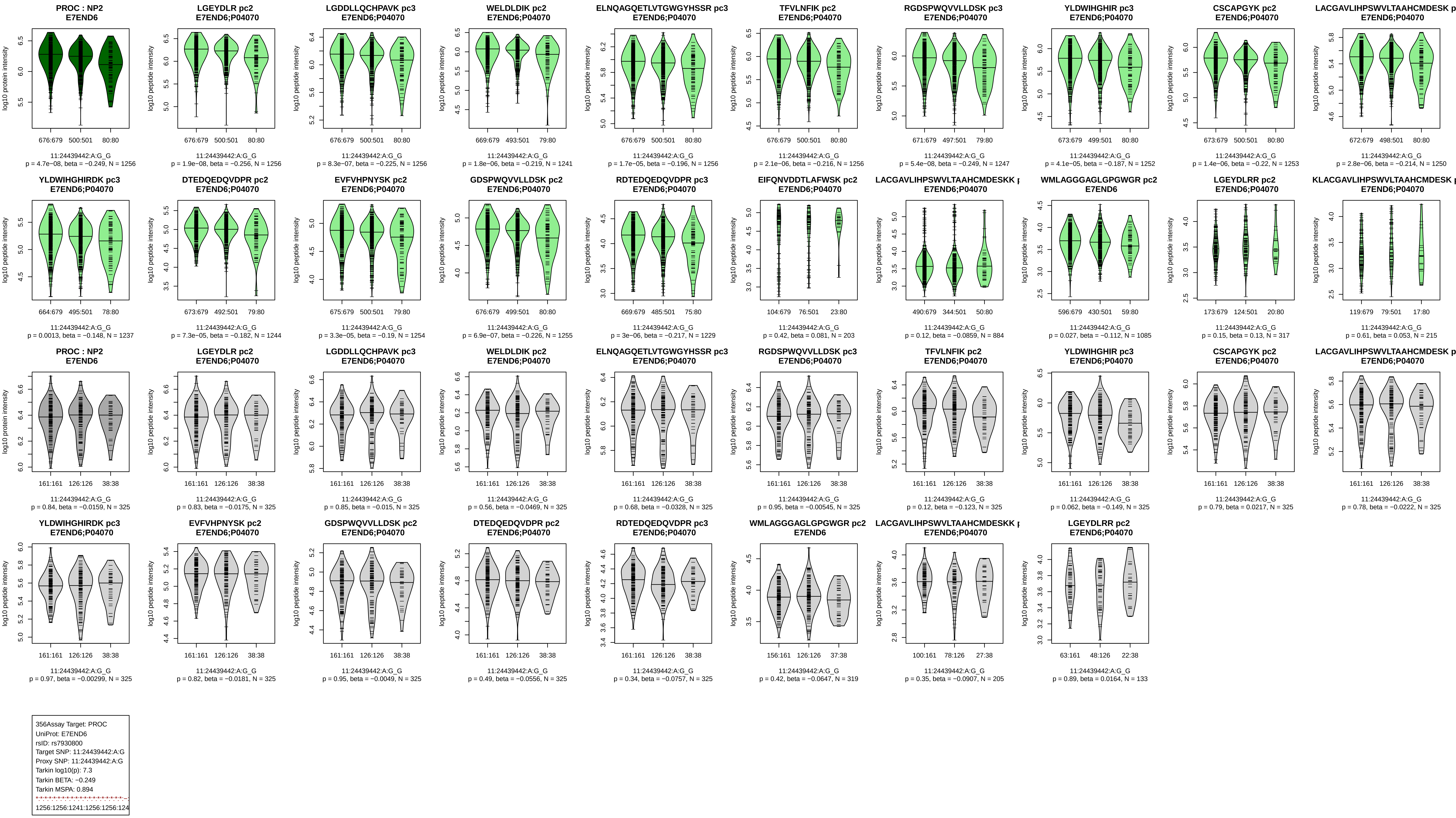

PROC : NP2 E7END6
11:24439442:A:G_G p = 4.7e−08, beta = −0.249, N = 1256
LGEYDLR pc2 E7END6;P04070
11:24439442:A:G_G p = 1.9e−08, beta = −0.256, N = 1256
LGDDLLQCHPAVK pc3 E7END6;P04070
11:24439442:A:G_G p = 8.3e−07, beta = −0.225, N = 1256
WELDLDIK pc2 E7END6;P04070
11:24439442:A:G_G p = 1.8e−06, beta = −0.219, N = 1241
ELNQAGQETLVTGWGYHSSR pc3 E7END6;P04070
11:24439442:A:G_G p = 1.7e−05, beta = −0.196, N = 1256
TFVLNFIK pc2 E7END6;P04070
11:24439442:A:G_G p = 2.1e−06, beta = −0.216, N = 1256
RGDSPWQVVLLDSK pc3 E7END6;P04070
11:24439442:A:G_G p = 5.4e−08, beta = −0.249, N = 1247
YLDWIHGHIR pc3 E7END6;P04070
11:24439442:A:G_G p = 4.1e−05, beta = −0.187, N = 1252
CSCAPGYK pc2 E7END6;P04070
11:24439442:A:G_G p = 1.4e−06, beta = −0.22, N = 1253
LACGAVLIHPSWVLTAAHCMDESK p E7END6;P04070
11:24439442:A:G_G p = 2.8e−06, beta = −0.214, N = 1250
YLDWIHGHIRDK pc3 E7END6;P04070
11:24439442:A:G_G p = 0.0013, beta = −0.148, N = 1237
DTEDQEDQVDPR pc2 E7END6;P04070
11:24439442:A:G_G p = 7.3e−05, beta = −0.182, N = 1244
EVFVHPNYSK pc2 E7END6;P04070
11:24439442:A:G_G p = 3.3e−05, beta = −0.19, N = 1254
GDSPWQVVLLDSK pc2 E7END6;P04070
11:24439442:A:G_G p = 6.9e−07, beta = −0.226, N = 1255
RDTEDQEDQVDPR pc3 E7END6;P04070
11:24439442:A:G_G p = 3e−06, beta = −0.217, N = 1229
EIFQNVDDTLAFWSK pc2 E7END6;P04070
11:24439442:A:G_G p = 0.42, beta = 0.081, N = 203
LACGAVLIHPSWVLTAAHCMDESKK p E7END6;P04070
11:24439442:A:G_G p = 0.12, beta = −0.0859, N = 884
WMLAGGGAGLGPGWGR pc2 E7END6
11:24439442:A:G_G p = 0.027, beta = −0.112, N = 1085
LGEYDLRR pc2 E7END6;P04070
11:24439442:A:G_G p = 0.15, beta = 0.13, N = 317
KLACGAVLIHPSWVLTAAHCMDESK p E7END6;P04070
11:24439442:A:G_G p = 0.61, beta = 0.053, N = 215
PROC : NP2 E7END6
11:24439442:A:G_G p = 0.84, beta = −0.0159, N = 325
LGEYDLR pc2 E7END6;P04070
11:24439442:A:G_G p = 0.83, beta = −0.0175, N = 325
LGDDLLQCHPAVK pc3 E7END6;P04070
11:24439442:A:G_G p = 0.85, beta = −0.015, N = 325
WELDLDIK pc2 E7END6;P04070
11:24439442:A:G_G p = 0.56, beta = −0.0469, N = 325
ELNQAGQETLVTGWGYHSSR pc3 E7END6;P04070
11:24439442:A:G_G p = 0.68, beta = −0.0328, N = 325
RGDSPWQVVLLDSK pc3 E7END6;P04070
11:24439442:A:G_G p = 0.95, beta = −0.00545, N = 325
TFVLNFIK pc2 E7END6;P04070
11:24439442:A:G_G p = 0.12, beta = −0.123, N = 325
YLDWIHGHIR pc3 E7END6;P04070
11:24439442:A:G_G p = 0.062, beta = −0.149, N = 325
CSCAPGYK pc2 E7END6;P04070
11:24439442:A:G_G p = 0.79, beta = 0.0217, N = 325
LACGAVLIHPSWVLTAAHCMDESK p E7END6;P04070
11:24439442:A:G_G p = 0.78, beta = −0.0222, N = 325
YLDWIHGHIRDK pc3 E7END6;P04070
11:24439442:A:G_G p = 0.97, beta = −0.00299, N = 325
EVFVHPNYSK pc2 E7END6;P04070
11:24439442:A:G_G p = 0.82, beta = −0.0181, N = 325
GDSPWQVVLLDSK pc2 E7END6;P04070
11:24439442:A:G_G p = 0.95, beta = −0.0049, N = 325
DTEDQEDQVDPR pc2 E7END6;P04070
11:24439442:A:G_G p = 0.49, beta = −0.0556, N = 325
RDTEDQEDQVDPR pc3 E7END6;P04070
11:24439442:A:G_G p = 0.34, beta = −0.0757, N = 325
WMLAGGGAGLGPGWGR pc2 E7END6
11:24439442:A:G_G p = 0.42, beta = −0.0647, N = 319
LACGAVLIHPSWVLTAAHCMDESKK p E7END6;P04070
11:24439442:A:G_G p = 0.35, beta = −0.0907, N = 205
LGEYDLRR pc2 E7END6;P04070
11:24439442:A:G_G p = 0.89, beta = 0.0164, N = 133
356Assay Target: PROC
UniProt: E7END6
rsID: rs7930800
Target SNP: 11:24439442:A:G
Proxy SNP: 11:24439442:A:G
Tarkin log10(p): 7.3
Tarkin BETA: −0.249
Tarkin MSPA: 0.894
1256:1256:1241:1256:1256:124

357. CTSF (A0A7I2V313)
Tarkin MSPA=0.631

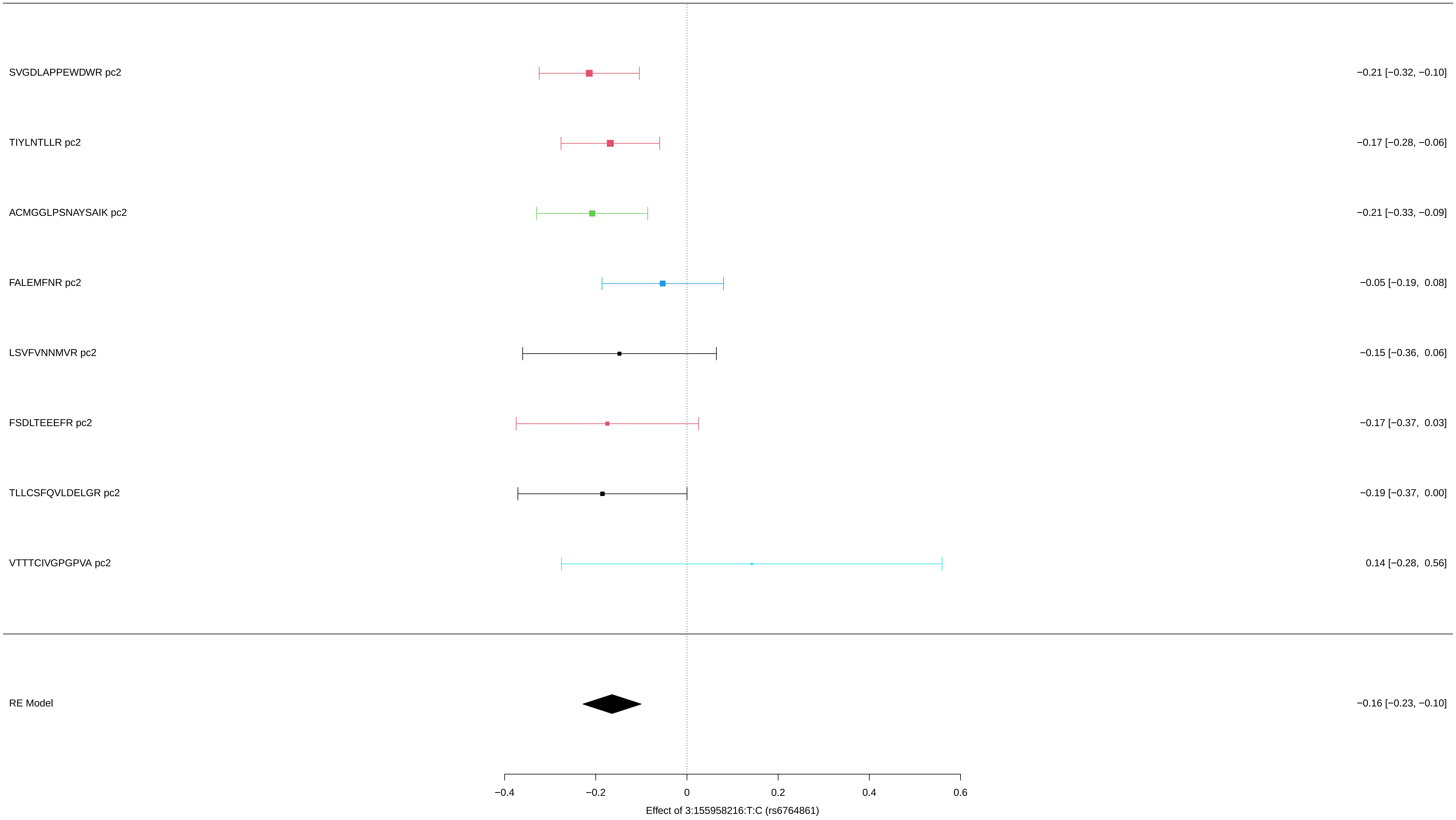

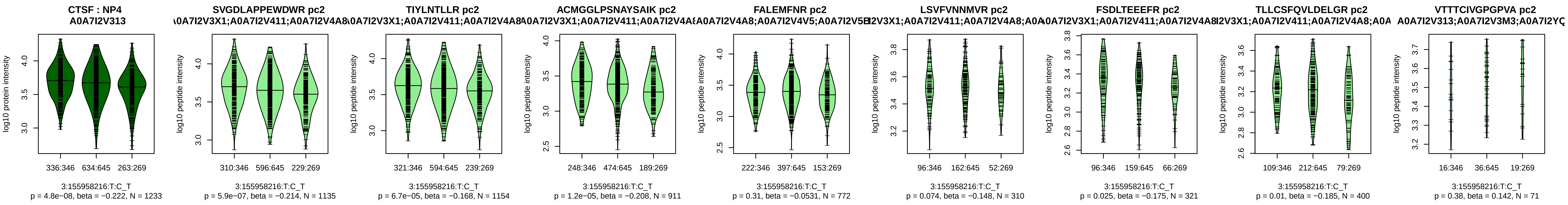

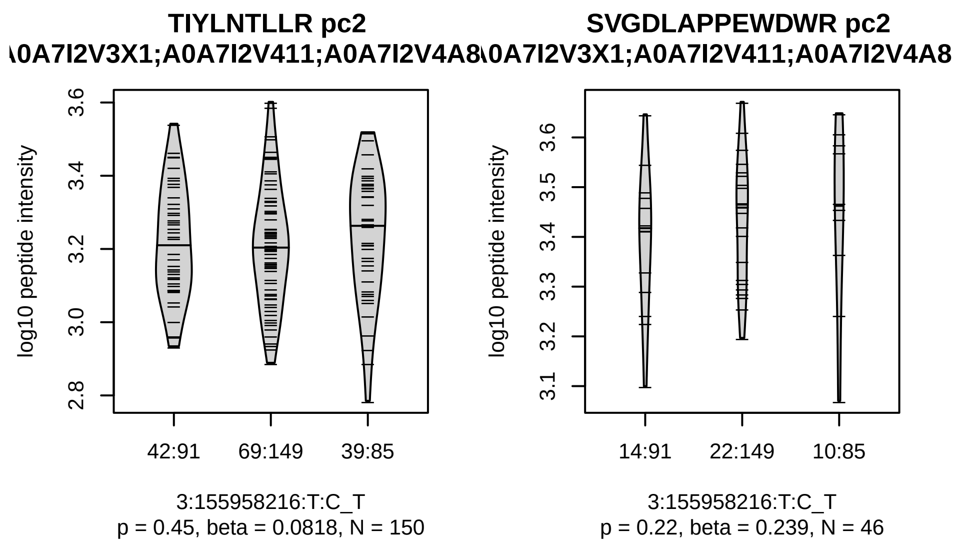

357Assay Target: CTSF
UniProt: A0A7I2V313
rsID: rs6764861
Target SNP: 3:155958216:T:C
Proxy SNP: 3:155958216:T:C
Tarkin log10(p): 7.3
Tarkin BETA: −0.222
Tarkin MSPA: 0.631
::*:–:–:–:–:–
1135:1154:911:772:310:321:400

358. GNG2 (G3V3J9)
Tarkin MSPA=1

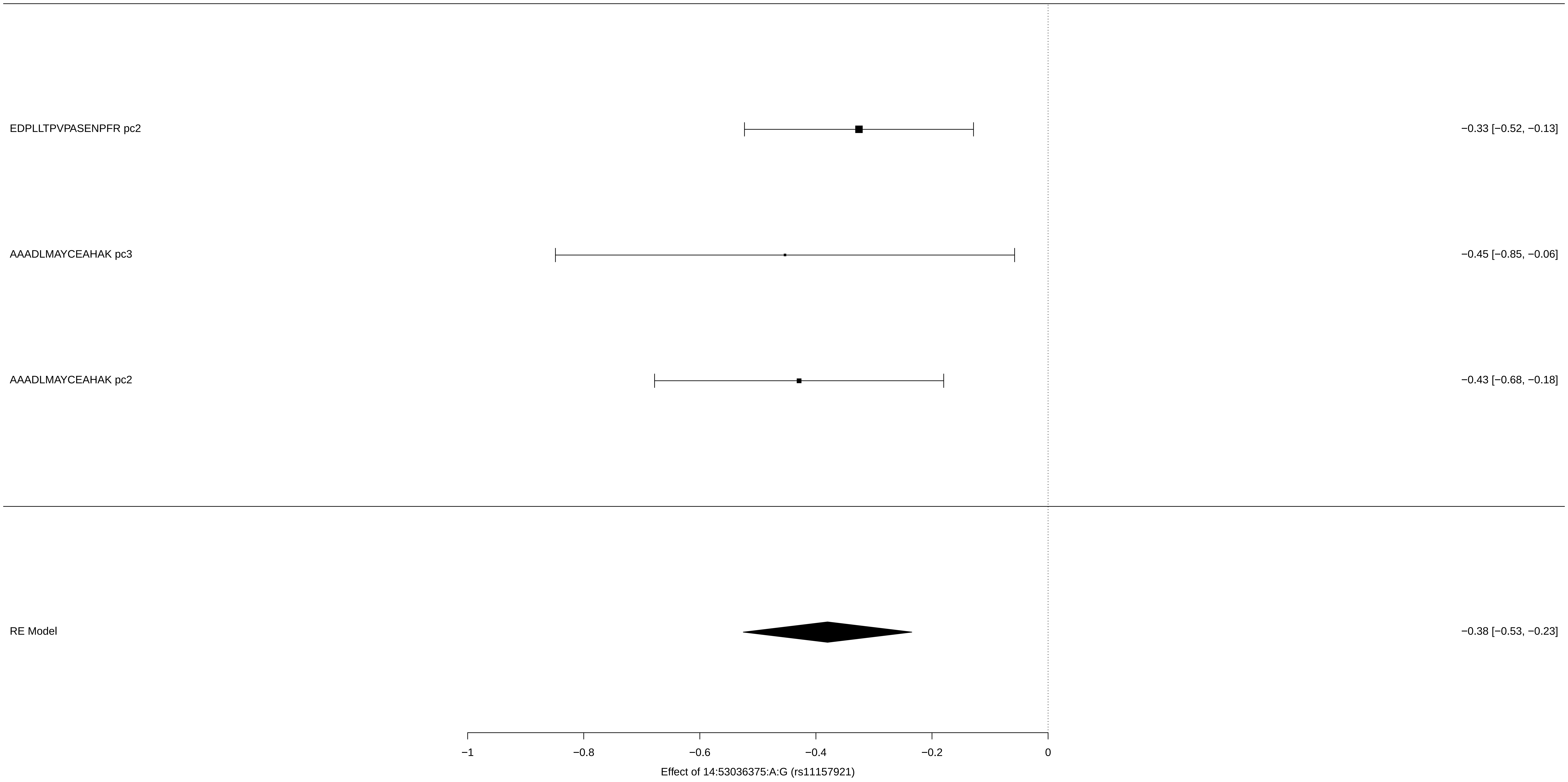

GNG2 : NP1
G3V3J9

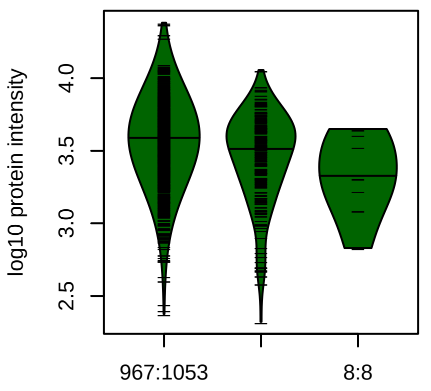

14:53036375:A:G_G
p = 4.8e−08, beta = −0.406, N = 1152

EDPLLTPVPASENPFR pc2
G3V2N0;G3V3J9;P59768

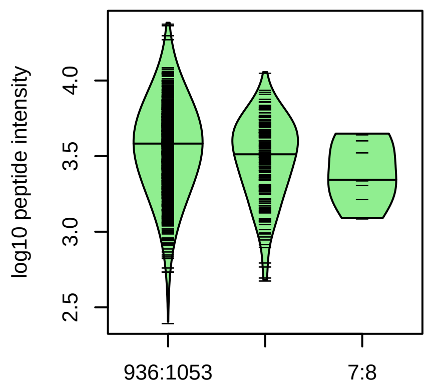

14:53036375:A:G_G
p = 2.3e−05, beta = −0.326, N = 1111

AAADLMAYCEAHAK pc3
G3V2N0;G3V3J9;P59768

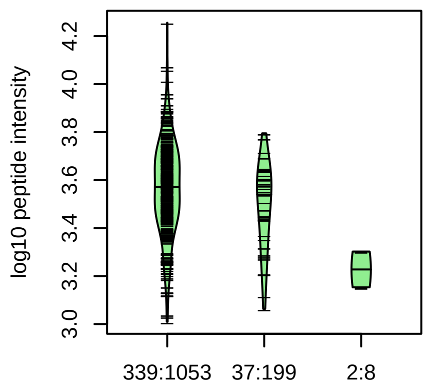

14:53036375:A:G_G
p = 0.0034, beta = −0.453, N = 378

GNG2 : NP1
G3V2N0;G3V3J9;P59768

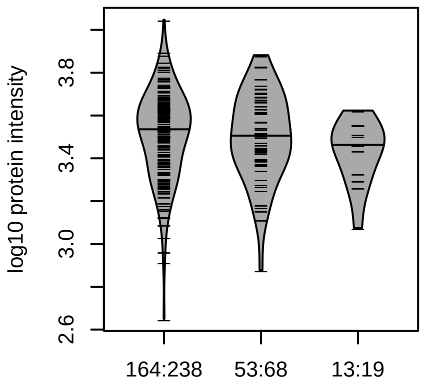

14:53036375:A:G_G
p = 0.78, beta = −0.0313, N = 230

EDPLLTPVPASENPFR pc2
G3V2N0;G3V3J9;P59768

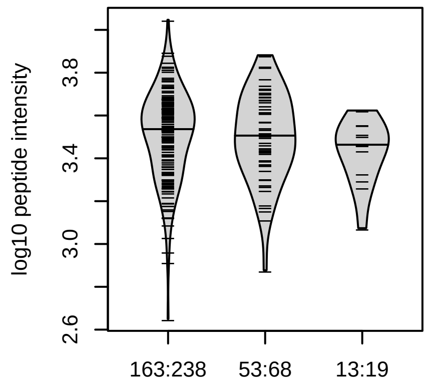

14:53036375:A:G_G
p = 0.75, beta = −0.0357, N = 229

AAADLMAYCEAHAK pc2
G3V2N0;G3V3J9;P59768

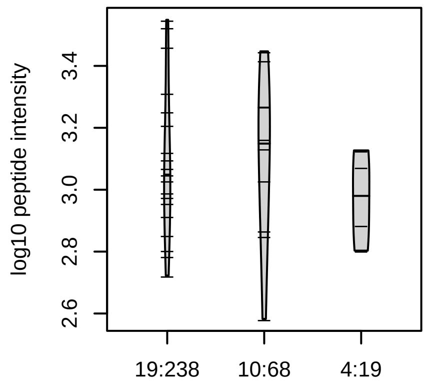

14:53036375:A:G_G
p = 0.62, beta = −0.117, N = 33

358Assay Target: GNG2
UniProt: G3V3J9
rsID: rs11157921
Target SNP: 14:53036375:A:G
Proxy SNP: 14:53036375:A:G
Tarkin log10(p): 7.3
Tarkin BETA: −0.406
Tarkin MSPA: 1
::*
1111:378:664

359. LMNB1 (P20700)
Tarkin MSPA=0.68

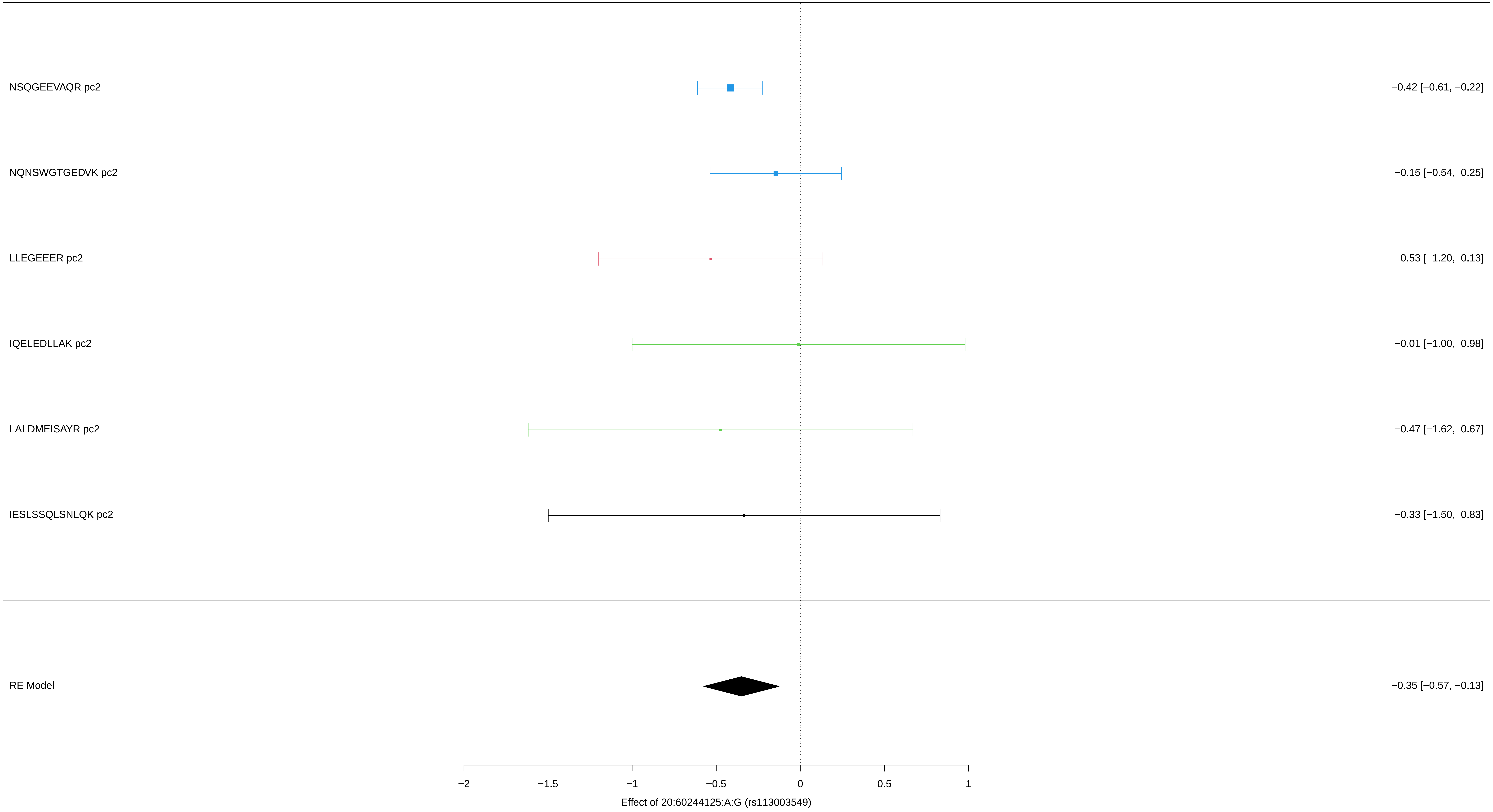

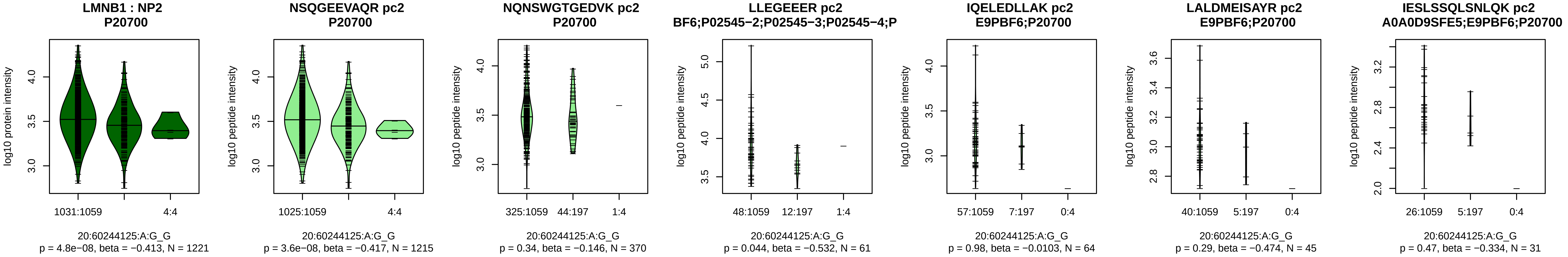

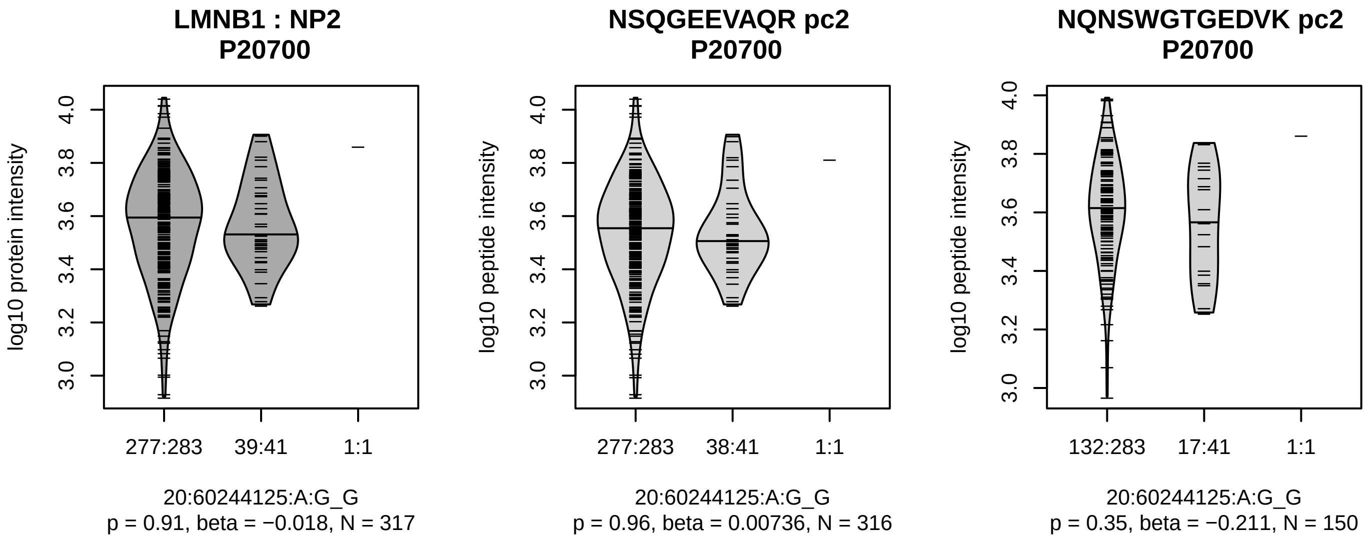

359Assay Target: LMNB1
UniProt: P20700
rsID: rs113003549
Target SNP: 20:60244125:A:G
Proxy SNP: 20:60244125:A:G
Tarkin log10(p): 7.3
Tarkin BETA: −0.413
Tarkin MSPA: 0.68
*:−:−:−:−:NA:NA:−:NA:NA:NA:N[illegible]
1215:370:61:64:45:16:8:31:8:5:[illegible]

360. PLA1A (Q53H76)
Tarkin MSPA=0.74

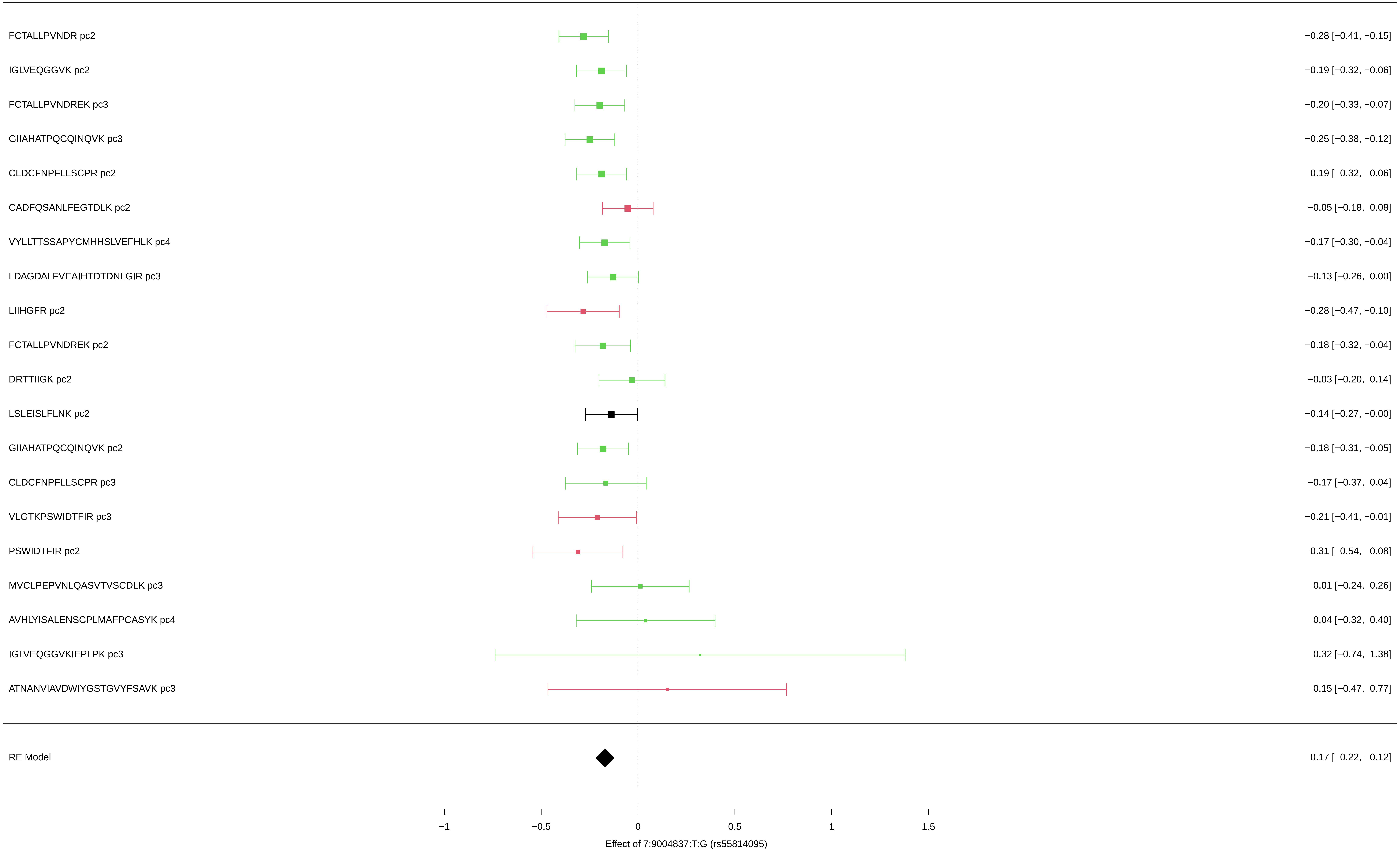

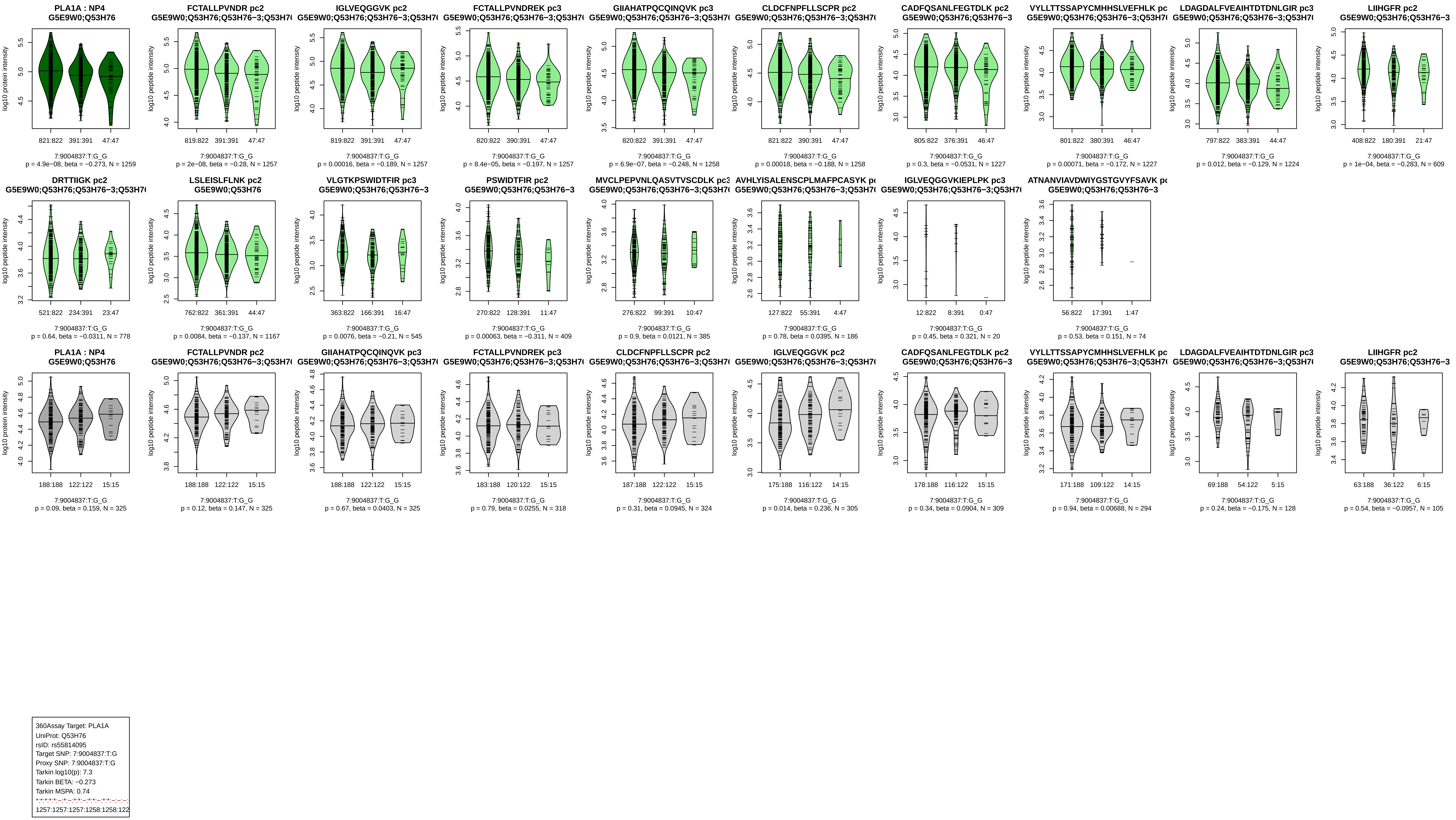

PLA1A : NP4
G5E9W0;Q53H76
7:9004837:T:G_G
p = 4.9e−08, beta = −0.273, N = 1259
FCTALLPVNDR pc2
7:9004837:T:G_G
p = 2e−08, beta = −0.28, N = 1257
IGLVEQGGVK pc2
7:9004837:T:G_G
p = 0.00016, beta = −0.189, N = 1257
FCTALLPVNDREK pc3
7:9004837:T:G_G
p = 8.4e−05, beta = −0.197, N = 1257
GIIAHATPQCQINQVK pc3
7:9004837:T:G_G
p = 6.9e−07, beta = −0.248, N = 1258
CLDCFNPFLLSCPR pc2
7:9004837:T:G_G
p = 0.00018, beta = −0.188, N = 1258
CADFQSANLFEGTDLK pc2
G5E9W0;Q53H76;Q53H76−3
7:9004837:T:G_G
p = 0.3, beta = −0.0531, N = 1227
7:9004837:T:G_G
p = 0.00071, beta = −0.172, N = 1227
7:9004837:T:G_G
p = 0.012, beta = −0.129, N = 1224
LIIHGFR pc2
7:9004837:T:G_G
p = 1e−04, beta = −0.283, N = 609
DRTTIIGK pc2
7:9004837:T:G_G
p = 0.64, beta = −0.0311, N = 778
LSLEISLFLNK pc2
G5E9W0;Q53H76
7:9004837:T:G_G
p = 0.0084, beta = −0.137, N = 1167
VLGTKPSWIDTFIR pc3
G5E9W0;Q53H76;Q53H76−3
7:9004837:T:G_G
p = 0.0076, beta = −0.21, N = 545
PSWIDTFIR pc2
G5E9W0;Q53H76;Q53H76−3
7:9004837:T:G_G
p = 0.00063, beta = −0.311, N = 409
MVCLPEPVNLQASVTVSCDLK pc3
7:9004837:T:G_G
p = 0.9, beta = 0.0121, N = 385
7:9004837:T:G_G
p = 0.78, beta = 0.0395, N = 186
IGLVEQGGVKIEPLPK pc3
7:9004837:T:G_G
p = 0.45, beta = 0.321, N = 20
G5E9W0;Q53H76;Q53H76−3
7:9004837:T:G_G
p = 0.53, beta = 0.151, N = 74
PLA1A : NP4
G5E9W0;Q53H76
7:9004837:T:G_G
p = 0.09, beta = 0.159, N = 325
FCTALLPVNDR pc2
7:9004837:T:G_G
p = 0.12, beta = 0.147, N = 325
GIIAHATPQCQINQVK pc3
7:9004837:T:G_G
p = 0.67, beta = 0.0403, N = 325
FCTALLPVNDREK pc3
7:9004837:T:G_G
p = 0.79, beta = 0.0255, N = 318
CLDCFNPFLLSCPR pc2
7:9004837:T:G_G
p = 0.31, beta = 0.0945, N = 324
IGLVEQGGVK pc2
7:9004837:T:G_G
p = 0.014, beta = 0.236, N = 305
CADFQSANLFEGTDLK pc2
G5E9W0;Q53H76;Q53H76−3
7:9004837:T:G_G
p = 0.34, beta = 0.0904, N = 309
7:9004837:T:G_G
p = 0.94, beta = 0.00688, N = 294
7:9004837:T:G_G
p = 0.24, beta = −0.175, N = 128
LIIHGFR pc2
7:9004837:T:G_G
p = 0.54, beta = −0.0957, N = 105
360Assay Target: PLA1A
UniProt: Q53H76
rsID: rs55814095
Target SNP: 7:9004837:T:G
Proxy SNP: 7:9004837:T:G
Tarkin log10(p): 7.3
Tarkin BETA: −0.273
Tarkin MSPA: 0.74

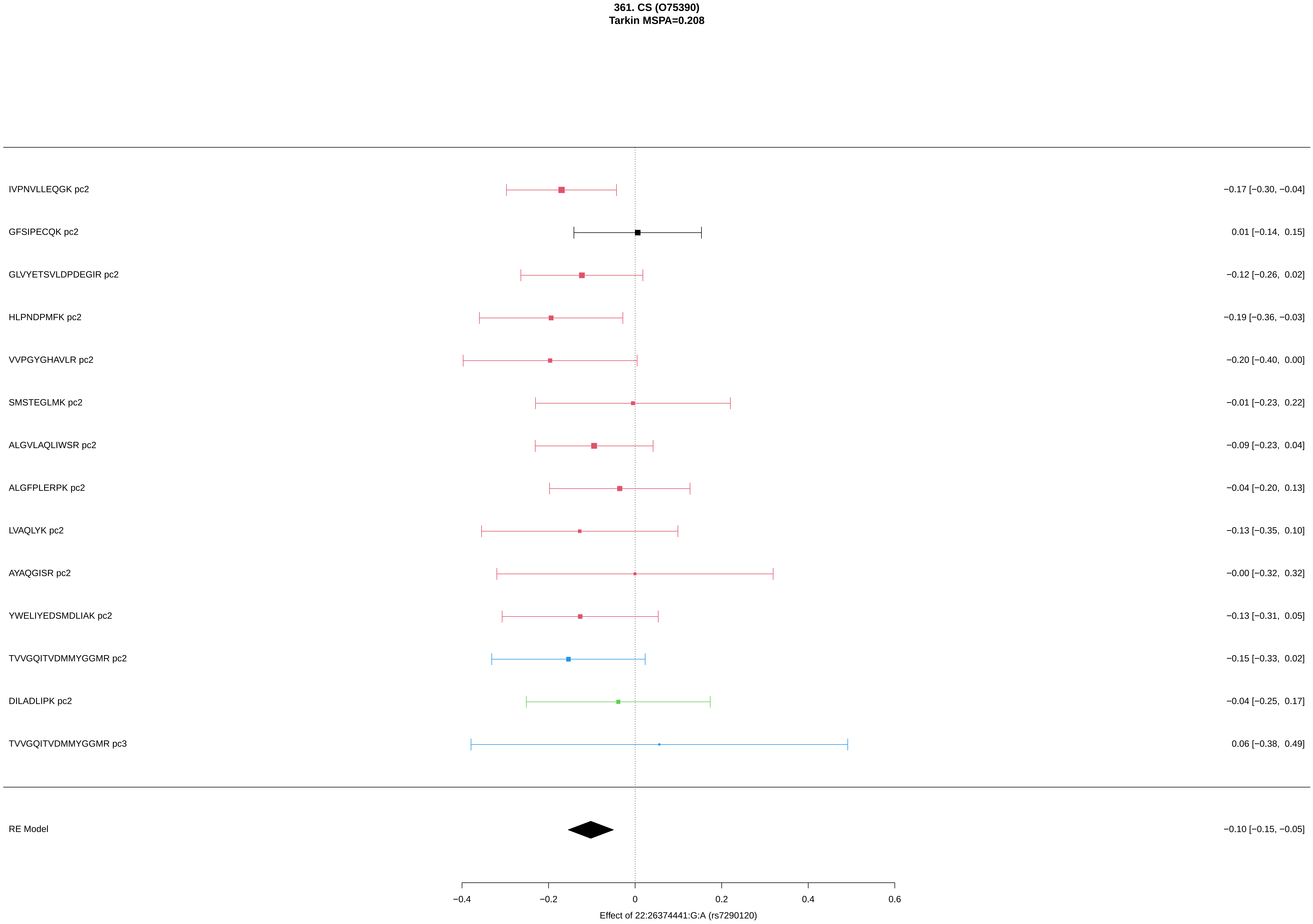
361. CS (O75390)
Tarkin MSPA=0.208
IVPNVLLEQGK pc2
GFSIPECQK pc2
GLVYETSVLDPDEGIR pc2
HLPNDPMFK pc2
VVPGYGHAVLR pc2
SMSTEGLMK pc2
ALGVLAQLIWSR pc2
ALGFPLERPK pc2
LVAQLYK pc2
AYAQGISR pc2
YWELIYEDSMDLIAK pc2
TVVGQITVDMMYGGMR pc2
DILADLIPK pc2
TVVGQITVDMMYGGMR pc3
RE Model
−0.17 [−0.30, −0.04]
0.01 [−0.14, 0.15]
−0.12 [−0.26, 0.02]
−0.19 [−0.36, −0.03]
−0.20 [−0.40, 0.00]
−0.01 [−0.23, 0.22]
−0.09 [−0.23, 0.04]
−0.04 [−0.20, 0.13]
−0.13 [−0.35, 0.10]
−0.00 [−0.32, 0.32]
−0.13 [−0.31, 0.05]
−0.15 [−0.33, 0.02]
−0.04 [−0.25, 0.17]
0.06 [−0.38, 0.49]
−0.10 [−0.15, −0.05]
−0.4
−0.2
0
0.2
0.4
0.6
Effect of 22:26374441:G:A (rs7290120)

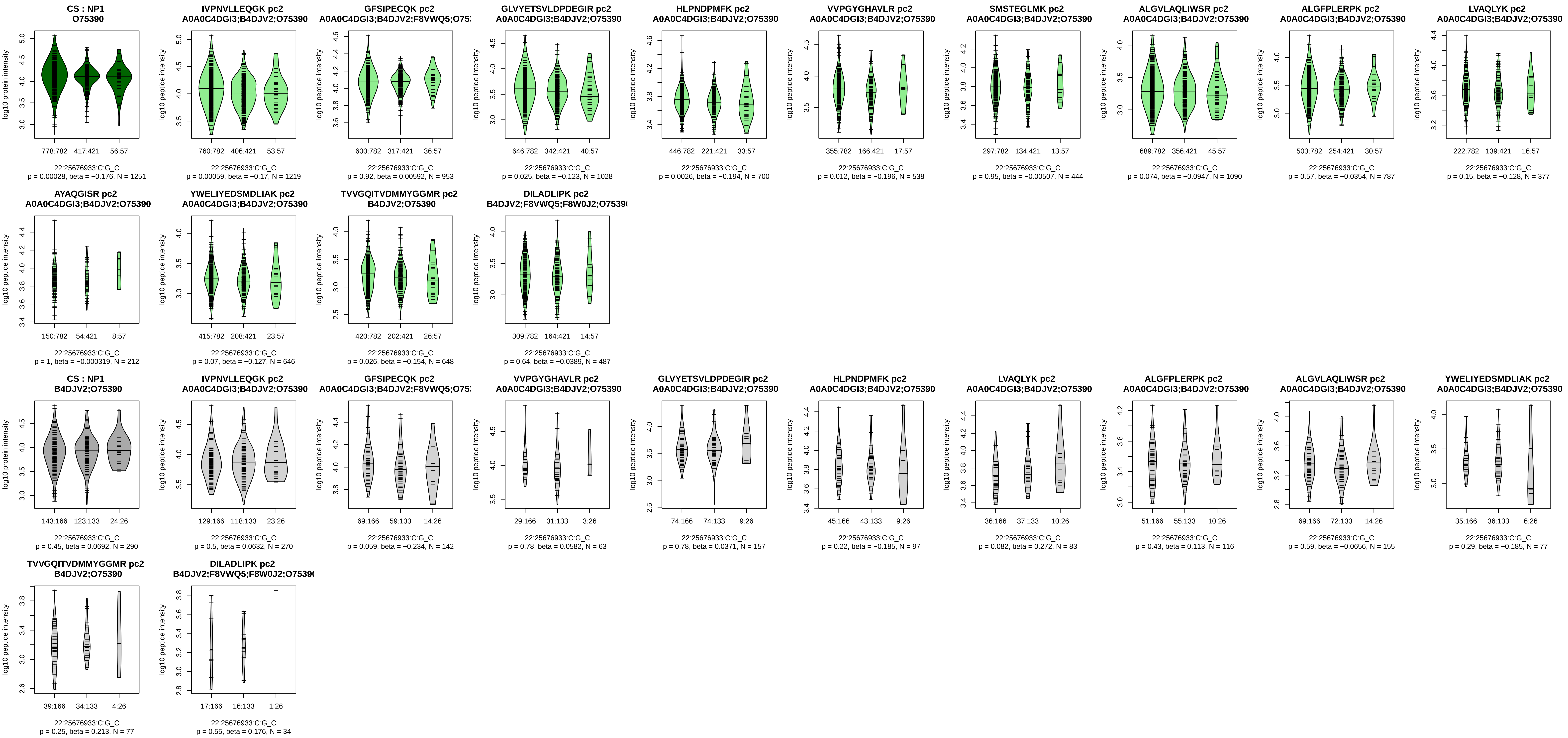

361Assay Target: CS
UniProt: O75390
rsID: rs7290120
Target SNP: 22:26374441:G:A
Proxy SNP: 22:25676933:C:G
Tarkin log10(p): 7.3
Tarkin BETA: −0.476
Tarkin MSPA: 0.208
–:–::–:–:–:–:–:–:–:–:–
1219:953:1028:700:538:444:10

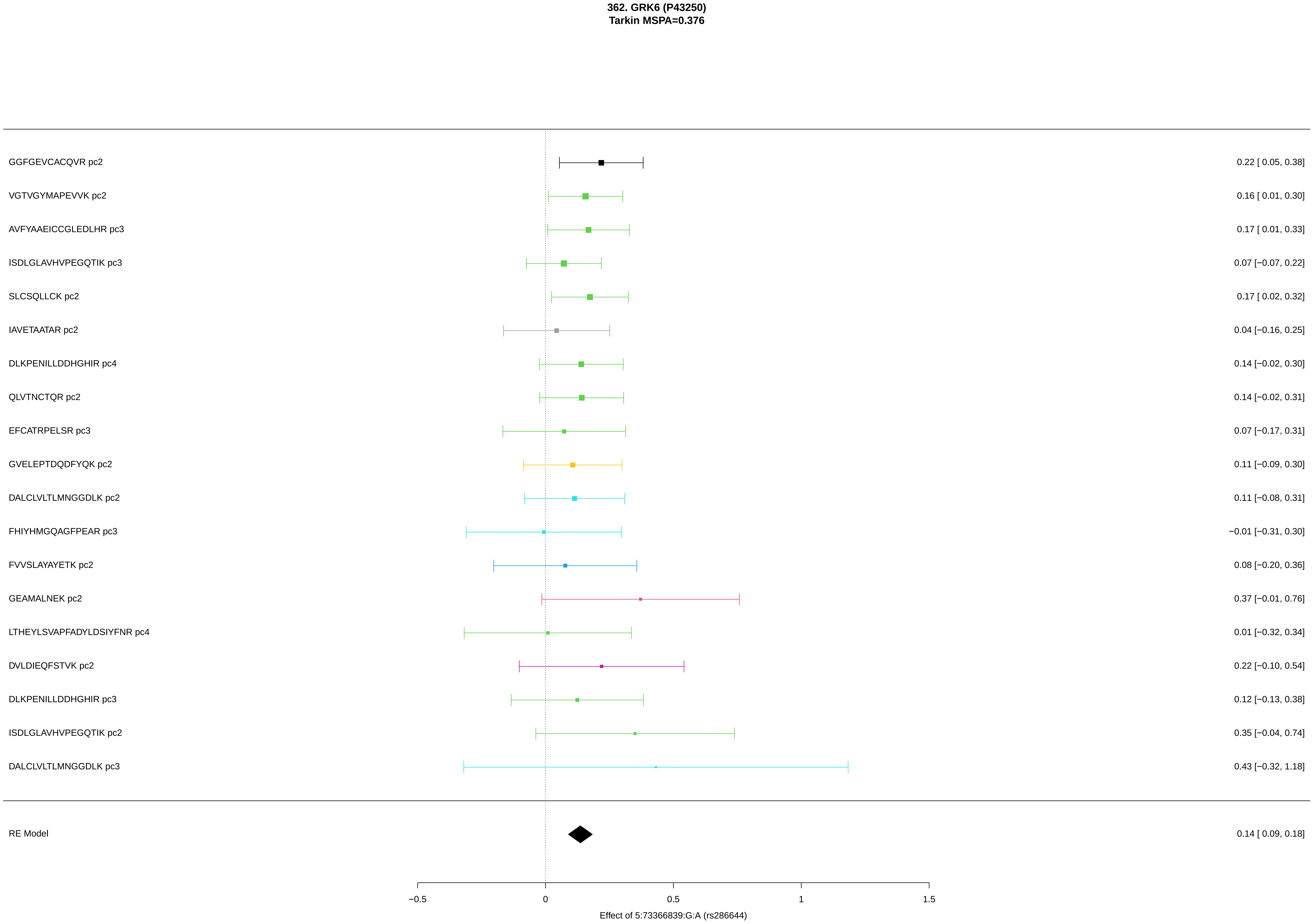
362. GRK6 (P43250)
Tarkin MSPA=0.376
GGFGEVCACQVR pc2
0.22 [0.05, 0.38]
VGTVGYMAPEVVK pc2
0.16 [0.01, 0.30]
AVFYAAEICCGLEDLHR pc3
0.17 [0.01, 0.33]
ISDLGLAVHVPEGQTIK pc3
0.07 [−0.07, 0.22]
SLCSQLLCK pc2
0.17 [0.02, 0.32]
IAVETAATAR pc2
0.04 [−0.16, 0.25]
DLKPENILLDDHGHIR pc4
0.14 [−0.02, 0.30]
QLVTNCTQR pc2
0.14 [−0.02, 0.31]
EFCATRPELSR pc3
0.07 [−0.17, 0.31]
GVELEPTDQDFYQK pc2
0.11 [−0.09, 0.30]
DALCLVLTLMNGGDLK pc2
0.11 [−0.08, 0.31]
FHIYHMGQAGFPEAR pc3
−0.01 [−0.31, 0.30]
FVVSLAYAYETK pc2
0.08 [−0.20, 0.36]
GEAMALNEK pc2
0.37 [−0.01, 0.76]
LTHEYLSVAPFADYLDSIYFNR pc4
0.01 [−0.32, 0.34]
DVLDIEQFSTVK pc2
0.22 [−0.10, 0.54]
DLKPENILLDDHGHIR pc3
0.12 [−0.13, 0.38]
ISDLGLAVHVPEGQTIK pc2
0.35 [−0.04, 0.74]
DALCLVLTLMNGGDLK pc3
0.43 [−0.32, 1.18]
RE Model
0.14 [0.09, 0.18]
−0.5
0
0.5
1
1.5
Effect of 5:73366839:G:A (rs286644)

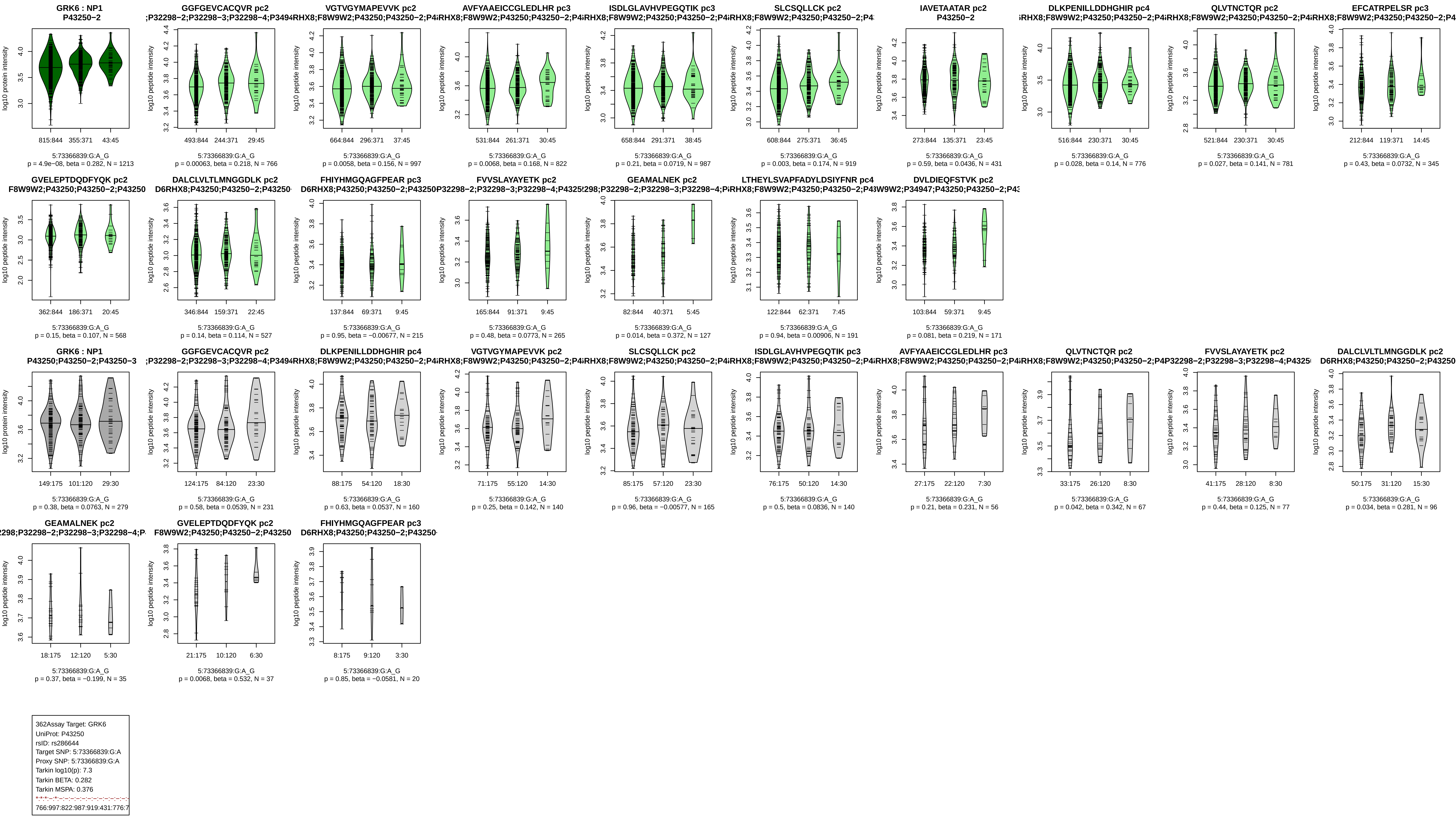
GRK6 : NP1
P43250−2
5:73366839:G:A_G
p = 4.9e−08, beta = 0.282, N = 1213
GGFGEVCACQVR pc2
p = 0.00063, beta = 0.218, N = 766
VGTVGYMAPEVVK pc2
p = 0.0058, beta = 0.156, N = 997
AVFYAAEICCGLEDLHR pc3
p = 0.0068, beta = 0.168, N = 822
ISDLGLAVHVPEGQTIK pc3
p = 0.21, beta = 0.0719, N = 987
SLCSQLLCK pc2
p = 0.003, beta = 0.174, N = 919
IAVETAATAR pc2
P43250−2
p = 0.59, beta = 0.0436, N = 431
DLKPENILLDDHGHIR pc4
p = 0.028, beta = 0.14, N = 776
QLVTNCTQR pc2
p = 0.027, beta = 0.141, N = 781
EFCATRPELSR pc3
p = 0.43, beta = 0.0732, N = 345
GVELEPTDQDFYQK pc2
p = 0.15, beta = 0.107, N = 568
DALCLVLTLMNGGDLK pc2
p = 0.14, beta = 0.114, N = 527
FHIYHMGQAGFPEAR pc3
p = 0.95, beta = −0.00677, N = 215
FVVSLAYAYETK pc2
p = 0.48, beta = 0.0773, N = 265
GEAMALNEK pc2
p = 0.014, beta = 0.372, N = 127
LTHEYLSVAPFADYLDSIYFNR pc4
p = 0.94, beta = 0.00906, N = 191
DVLDIEQFSTVK pc2
p = 0.081, beta = 0.219, N = 171
GRK6 : NP1
P43250;P43250−2;P43250−3
p = 0.38, beta = 0.0763, N = 279
GGFGEVCACQVR pc2
p = 0.58, beta = 0.0539, N = 231
DLKPENILLDDHGHIR pc4
p = 0.63, beta = 0.0537, N = 160
VGTVGYMAPEVVK pc2
p = 0.25, beta = 0.142, N = 140
SLCSQLLCK pc2
p = 0.96, beta = −0.00577, N = 165
ISDLGLAVHVPEGQTIK pc3
p = 0.5, beta = 0.0836, N = 140
AVFYAAEICCGLEDLHR pc3
p = 0.21, beta = 0.231, N = 56
QLVTNCTQR pc2
p = 0.042, beta = 0.342, N = 67
FVVSLAYAYETK pc2
p = 0.44, beta = 0.125, N = 77
DALCLVLTLMNGGDLK pc2
p = 0.034, beta = 0.281, N = 96
GEAMALNEK pc2
p = 0.37, beta = −0.199, N = 35
GVELEPTDQDFYQK pc2
p = 0.0068, beta = 0.532, N = 37
FHIYHMGQAGFPEAR pc3
p = 0.85, beta = −0.0581, N = 20
362Assay Target: GRK6
UniProt: P43250
rsID: rs286644
Target SNP: 5:73366839:G:A
Proxy SNP: 5:73366839:G:A
Tarkin log10(p): 7.3
Tarkin BETA: 0.282
Tarkin MSPA: 0.376
