# Supplementary material for: A genome-wide association study of mass spectrometry proteomics using a nanoparticle enrichment platform
Source: Nat Genet. 2025 Nov 27;57(12):2987–96. doi: 10.1038/s41588-025-02413-w (PMC12695657; doi:10.1038/s41588-025-02413-w)
Supplement: Supplementary file 7 — Meta-analysis Forest plots and violin plots of protein and up to 19 peptide levels by genotype for 319 cis-pQTLs identified by the deCODE SOMAscan study. [file 41588_2025_2413_MOESM7_ESM.pdf]

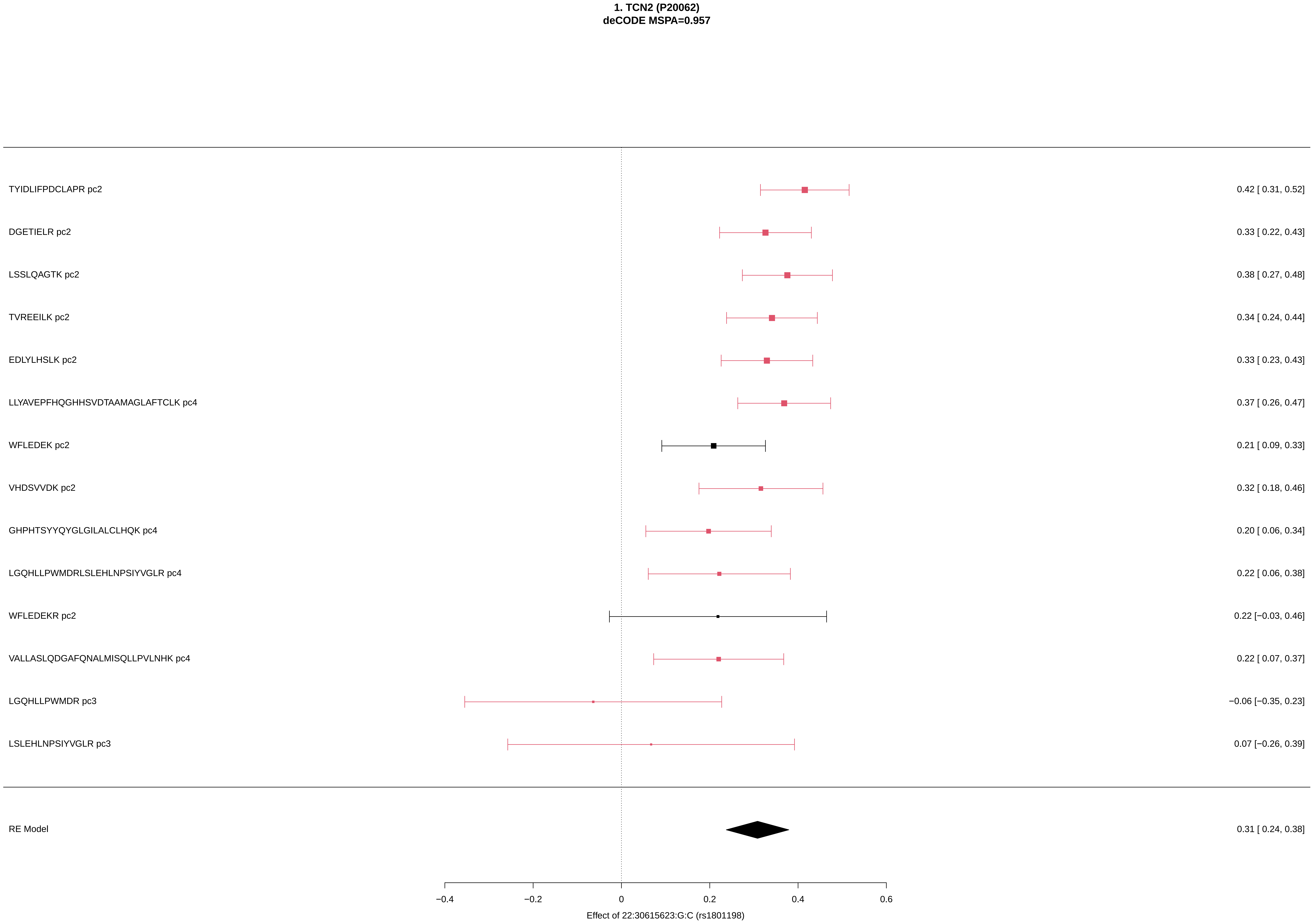
1. TCN2 (P20062)
deCODE MSPA=0.957
TYIDLIFPDCLAPR pc2
0.42 [0.31, 0.52]
DGETIELR pc2
0.33 [0.22, 0.43]
LSSLQAGTK pc2
0.38 [0.27, 0.48]
TVREEILK pc2
0.34 [0.24, 0.44]
EDLYLHSLK pc2
0.33 [0.23, 0.43]
LLYAVEPFHQGHHSVDTAAMAGLAFTCLK pc4
0.37 [0.26, 0.47]
WFLEDEK pc2
0.21 [0.09, 0.33]
VHDSVVDK pc2
0.32 [0.18, 0.46]
GHPHTSYYQYGLGILALCLHQK pc4
0.20 [0.06, 0.34]
LGQHLLPWMDRLSLEHLNPSIYVGLR pc4
0.22 [0.06, 0.38]
WFLEDEKR pc2
0.22 [−0.03, 0.46]
VALLASLQDGAFQNALMISQLLPVLNHK pc4
0.22 [0.07, 0.37]
LGQHLLPWMDR pc3
−0.06 [−0.35, 0.23]
LSLEHLNPSIYVGLR pc3
0.07 [−0.26, 0.39]
RE Model
0.31 [0.24, 0.38]
−0.4
−0.2
0
0.2
0.4
0.6
Effect of 22:30615623:G:C (rs1801198)

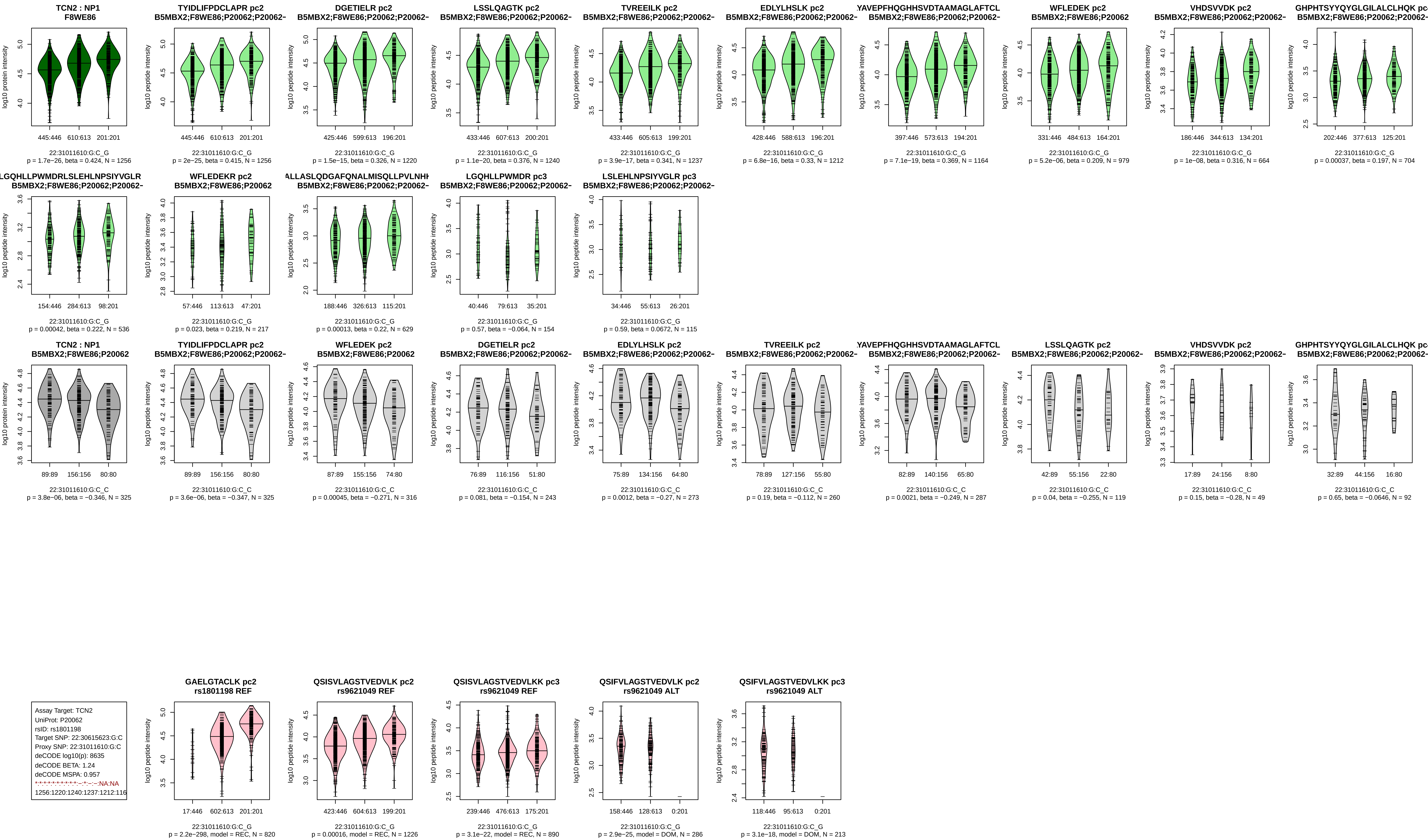
TCN2 : NP1
F8WE86
445:446 610:613 201:201
22:31011610:G:C_G
p = 1.7e−26, beta = 0.424, N = 1256
TYIDLIFPDCLAPR pc2
B5MBX2;F8WE86;P20062;P20062−
445:446 610:613 201:201
22:31011610:G:C_G
p = 2e−25, beta = 0.415, N = 1256
DGETIELR pc2
B5MBX2;F8WE86;P20062;P20062−
425:446 599:613 196:201
22:31011610:G:C_G
p = 1.5e−15, beta = 0.326, N = 1220
LSSLQAGTK pc2
B5MBX2;F8WE86;P20062;P20062−
433:446 607:613 200:201
22:31011610:G:C_G
p = 1.1e−20, beta = 0.376, N = 1240
TVREEILK pc2
B5MBX2;F8WE86;P20062;P20062−
433:446 605:613 199:201
22:31011610:G:C_G
p = 3.9e−17, beta = 0.341, N = 1237
EDLYLHSLK pc2
B5MBX2;F8WE86;P20062;P20062−
428:446 588:613 196:201
22:31011610:G:C_G
p = 6.8e−16, beta = 0.33, N = 1212
YAVEPFHQGHHSVDTAAMAGLAFTCL
B5MBX2;F8WE86;P20062;P20062−
397:446 573:613 194:201
22:31011610:G:C_G
p = 7.1e−19, beta = 0.369, N = 1164
WFLEDEK pc2
B5MBX2;F8WE86;P20062
331:446 484:613 164:201
22:31011610:G:C_G
p = 5.2e−06, beta = 0.209, N = 979
VHDSVVDK pc2
B5MBX2;F8WE86;P20062;P20062−
186:446 344:613 134:201
22:31011610:G:C_G
p = 1e−08, beta = 0.316, N = 664
GHPHTSYYQYGLGILALCLHQK pc
B5MBX2;F8WE86;P20062;P20062−
202:446 377:613 125:201
22:31011610:G:C_G
p = 0.00037, beta = 0.197, N = 704
LGQHLLPWMDRLSLEHLNPSIYVGLR
B5MBX2;F8WE86;P20062;P20062−
154:446 284:613 98:201
22:31011610:G:C_G
p = 0.00042, beta = 0.222, N = 536
WFLEDEKR pc2
B5MBX2;F8WE86;P20062
57:446 113:613 47:201
22:31011610:G:C_G
p = 0.023, beta = 0.219, N = 217
ALLASLQDGAFQNALMISQLLPVLNH
B5MBX2;F8WE86;P20062;P20062−
188:446 326:613 115:201
22:31011610:G:C_G
p = 0.00013, beta = 0.22, N = 629
LGQHLLPWMDR pc3
B5MBX2;F8WE86;P20062;P20062−
40:446 79:613 35:201
22:31011610:G:C_G
p = 0.57, beta = −0.064, N = 154
LSLEHLNPSIYVGLR pc3
B5MBX2;F8WE86;P20062;P20062−
34:446 55:613 26:201
22:31011610:G:C_G
p = 0.59, beta = 0.0672, N = 115
TCN2 : NP1
B5MBX2;F8WE86;P20062
89:89 156:156 80:80
22:31011610:G:C_C
p = 3.8e−06, beta = −0.346, N = 325
TYIDLIFPDCLAPR pc2
B5MBX2;F8WE86;P20062;P20062−
89:89 156:156 80:80
22:31011610:G:C_C
p = 3.6e−06, beta = −0.347, N = 325
WFLEDEK pc2
B5MBX2;F8WE86;P20062
87:89 155:156 74:80
22:31011610:G:C_C
p = 0.00045, beta = −0.271, N = 316
DGETIELR pc2
B5MBX2;F8WE86;P20062;P20062−
76:89 116:156 51:80
22:31011610:G:C_C
p = 0.081, beta = −0.154, N = 243
EDLYLHSLK pc2
B5MBX2;F8WE86;P20062;P20062−
75:89 134:156 64:80
22:31011610:G:C_C
p = 0.0012, beta = −0.27, N = 273
TVREEILK pc2
B5MBX2;F8WE86;P20062;P20062−
78:89 127:156 55:80
22:31011610:G:C_C
p = 0.19, beta = −0.112, N = 260
YAVEPFHQGHHSVDTAAMAGLAFTCL
B5MBX2;F8WE86;P20062;P20062−
82:89 140:156 65:80
22:31011610:G:C_C
p = 0.0021, beta = −0.249, N = 287
LSSLQAGTK pc2
B5MBX2;F8WE86;P20062;P20062−
42:89 55:156 22:80
22:31011610:G:C_C
p = 0.04, beta = −0.255, N = 119
VHDSVVDK pc2
B5MBX2;F8WE86;P20062;P20062−
17:89 24:156 8:80
22:31011610:G:C_C
p = 0.15, beta = −0.28, N = 49
GHPHTSYYQYGLGILALCLHQK pc
B5MBX2;F8WE86;P20062;P20062−
32:89 44:156 16:80
22:31011610:G:C_C
p = 0.65, beta = −0.0646, N = 92
Assay Target: TCN2
UniProt: P20062
rsID: rs1801198
Target SNP: 22:30615623:G:C
Proxy SNP: 22:31011610:G:C
deCODE log10(p): 8635
deCODE BETA: 1.24
deCODE MSPA: 0.957
::*:*:*:*:*:*:*:*:−:*:−:−:NA:NA
1256:1220:1240:1237:1212:116
GAELGTACLK pc2
rs1801198 REF
17:446 602:613 201:201
22:31011610:G:C_G
p = 2.2e−298, model = REC, N = 820
QSISVLAGSTVEDVLK pc2
rs9621049 REF
423:446 604:613 199:201
22:31011610:G:C_G
p = 0.00016, model = REC, N = 1226
QSISVLAGSTVEDVLKK pc3
rs9621049 REF
239:446 476:613 175:201
22:31011610:G:C_G
p = 3.1e−22, model = REC, N = 890
QSIFVLAGSTVEDVLK pc2
rs9621049 ALT
158:446 128:613 0:201
22:31011610:G:C_G
p = 2.9e−25, model = DOM, N = 286
QSIFVLAGSTVEDVLKK pc3
rs9621049 ALT
118:446 95:613 0:201
22:31011610:G:C_G
p = 3.1e−18, model = DOM, N = 213
log10 protein intensity
log10 peptide intensity

2. VTN (P04004)
deCODE MSPA=0.639

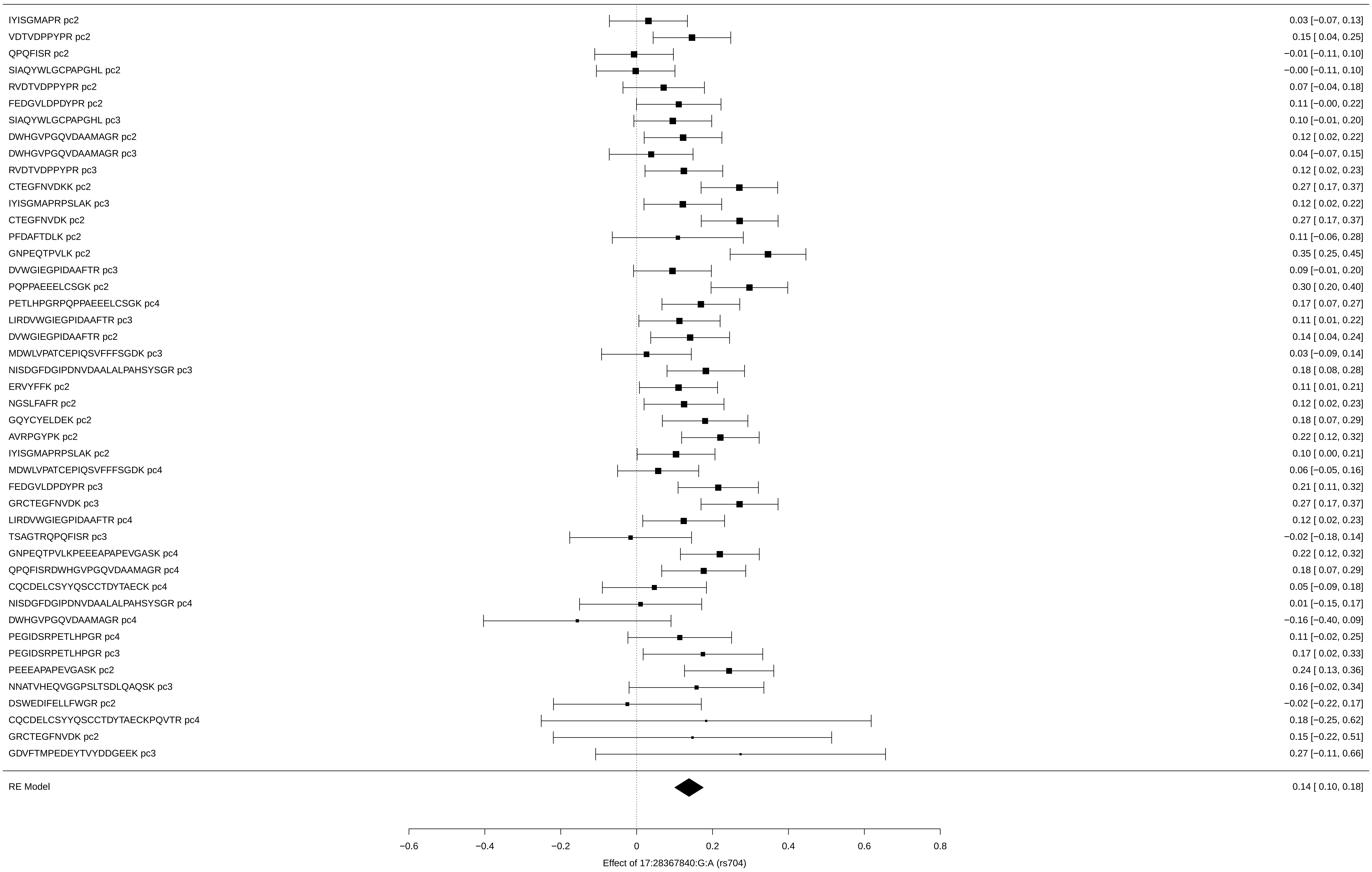

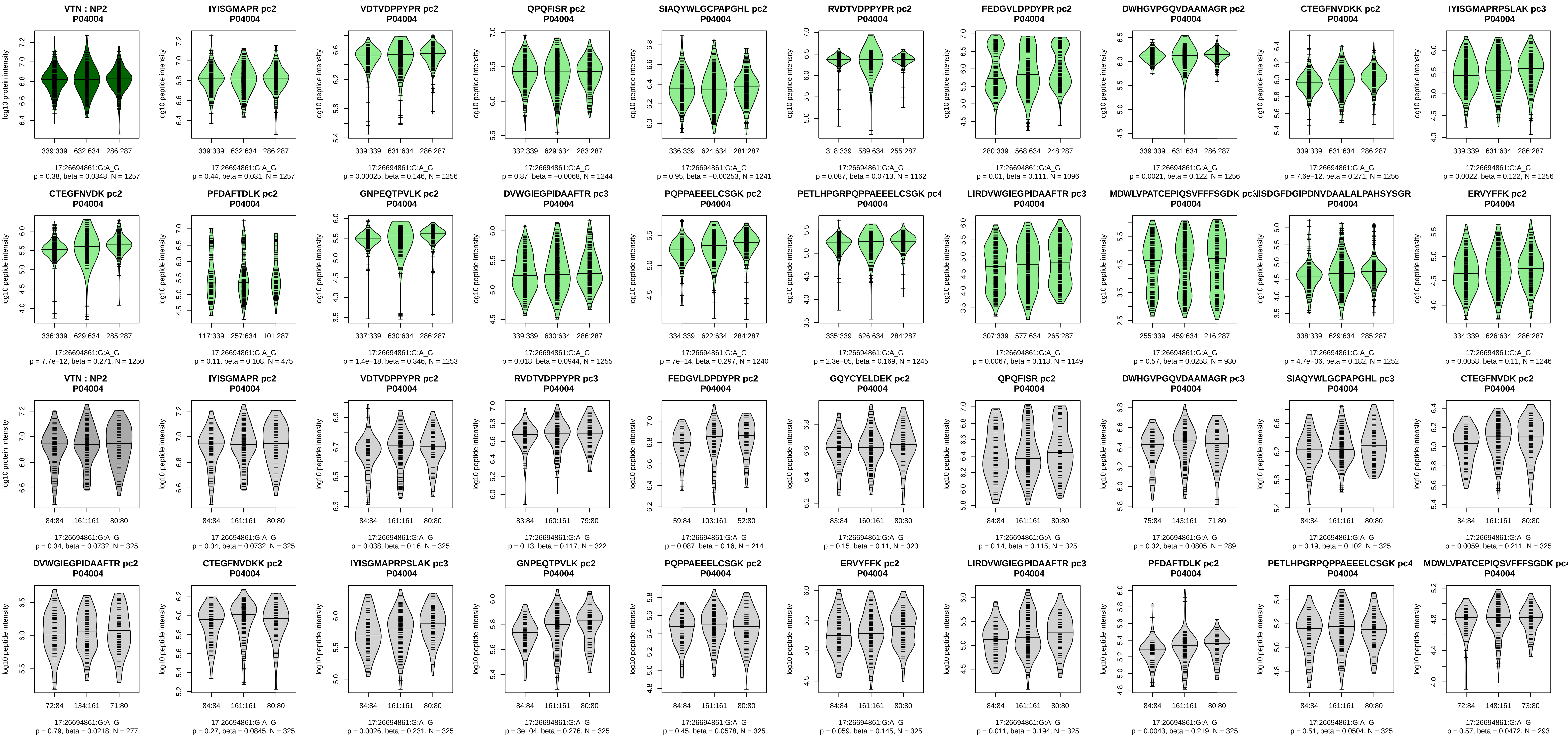

Assay Target: VTN
UniProt: P04004
rsID: rs704
Target SNP: 17:28367840:G:A
Proxy SNP: 17:26694861:G:A
deCODE log10(p): 8468.4
deCODE BETA: −1.28
deCODE MSPA: 0.639
1257:1256:1244:1241:1162:109

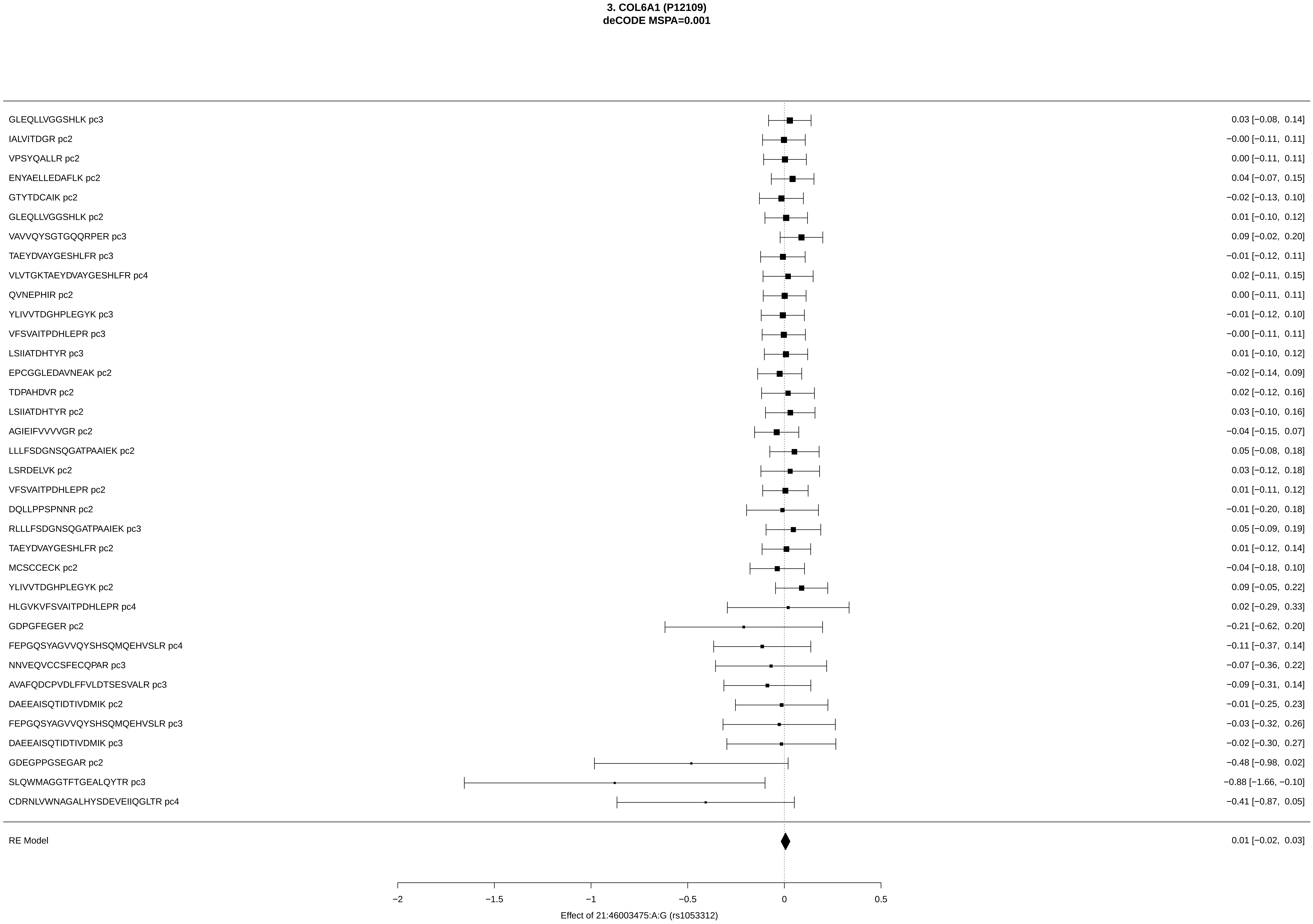

3. COL6A1 (P12109)
deCODE MSPA=0.001
GLEQLLVGGSHLK pc3 0.03 [−0.08, 0.14]
IALVITDGR pc2 −0.00 [−0.11, 0.11]
VPSYQALLR pc2 0.00 [−0.11, 0.11]
ENYAELLEDAFLK pc2 0.04 [−0.07, 0.15]
GTYTDCAIK pc2 −0.02 [−0.13, 0.10]
GLEQLLVGGSHLK pc2 0.01 [−0.10, 0.12]
VAVVQYSGTGQQRPER pc3 0.09 [−0.02, 0.20]
TAEYDVAYGESHLFR pc3 −0.01 [−0.12, 0.11]
VLVTGKTAEYDVAYGESHLFR pc4 0.02 [−0.11, 0.15]
QVNEPHIR pc2 0.00 [−0.11, 0.11]
YLIVVTDGHPLEGYK pc3 −0.01 [−0.12, 0.10]
VFSVAITPDHLEPR pc3 −0.00 [−0.11, 0.11]
LSIIATDHTYR pc3 0.01 [−0.10, 0.12]
EPCGGLEDAVNEAK pc2 −0.02 [−0.14, 0.09]
TDPAHDVR pc2 0.02 [−0.12, 0.16]
LSIIATDHTYR pc2 0.03 [−0.10, 0.16]
AGIEIFVVVVGR pc2 −0.04 [−0.15, 0.07]
LLLFSDGNSQGATPAAIEK pc2 0.05 [−0.08, 0.18]
LSRDELVK pc2 0.03 [−0.12, 0.18]
VFSVAITPDHLEPR pc2 0.01 [−0.11, 0.12]
DQLLPPSPNNR pc2 −0.01 [−0.20, 0.18]
RLLLFSDGNSQGATPAAIEK pc3 0.05 [−0.09, 0.19]
TAEYDVAYGESHLFR pc2 0.01 [−0.12, 0.14]
MCSCCECK pc2 −0.04 [−0.18, 0.10]
YLIVVTDGHPLEGYK pc2 0.09 [−0.05, 0.22]
HLGVKVFSVAITPDHLEPR pc4 0.02 [−0.29, 0.33]
GDPGFEGER pc2 −0.21 [−0.62, 0.20]
FEPGQSYAGVVQYSHSQMQEHVSLR pc4 −0.11 [−0.37, 0.14]
NNVEQVCCSFECQPAR pc3 −0.07 [−0.36, 0.22]
AVAFQDCPVDLFFVLDTSESVALR pc3 −0.09 [−0.31, 0.14]
DAEEAISQTIDTIVDMIK pc2 −0.01 [−0.25, 0.23]
FEPGQSYAGVVQYSHSQMQEHVSLR pc3 −0.03 [−0.32, 0.26]
DAEEAISQTIDTIVDMIK pc3 −0.02 [−0.30, 0.27]
GDEGPPGSEGAR pc2 −0.48 [−0.98, 0.02]
SLQWMAGGTFTGEALQYTR pc3 −0.88 [−1.66, −0.10]
CDRNLVWNAGALHYSDEVEIIQGLTR pc4 −0.41 [−0.87, 0.05]
RE Model 0.01 [−0.02, 0.03]
−2
−1.5
−1
−0.5
0
0.5
Effect of 21:46003475:A:G (rs1053312)

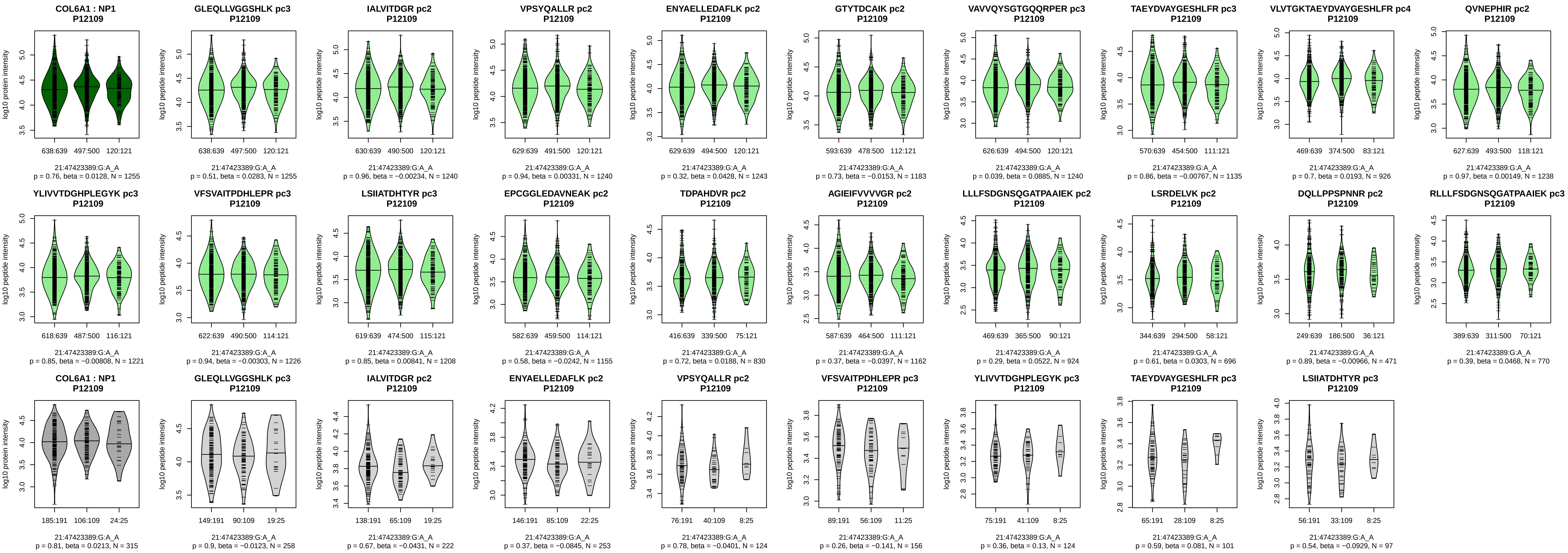

Assay Target: COL6A1
UniProt: P12109
rsID: rs1053312
Target SNP: 21:46003475:A:G
Proxy SNP: 21:47423389:G:A
deCODE log10(p): 7739.5
deCODE BETA: −1.23
deCODE MSPA: 0.001
1255:1240:1240:1243:1183:125

4. INHBC (P55103)
deCODE MSPA=0.744

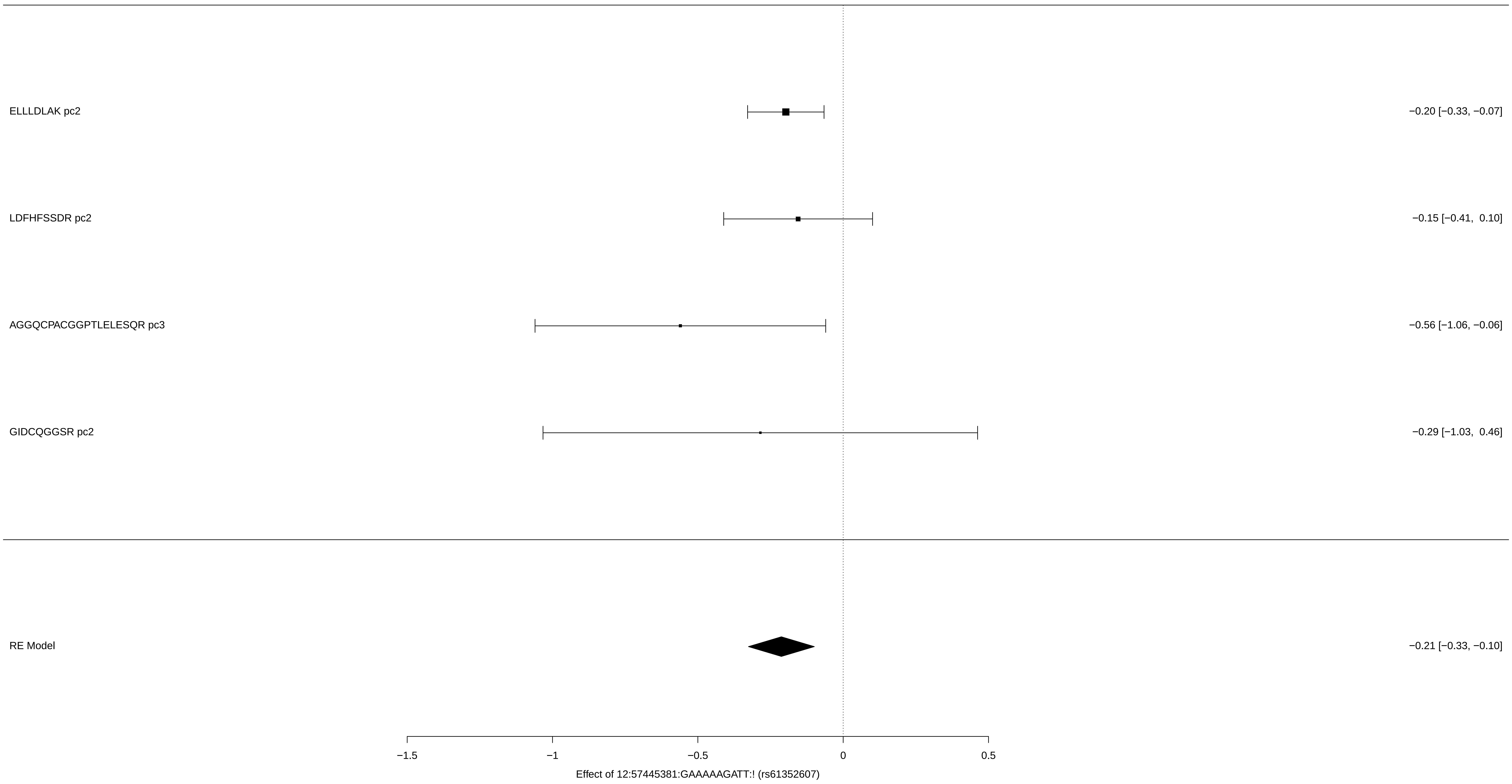

INHBC : NP3
P55103

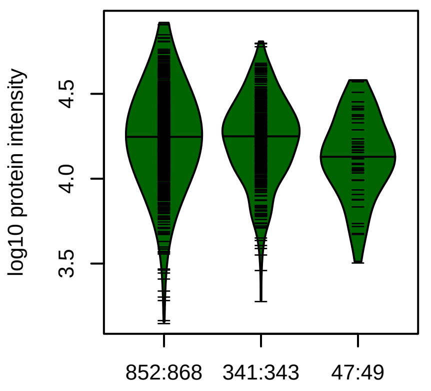

12:57839173:G:T_T
p = 2e−04, beta = −0.19, N = 1240

ELLLDLAK pc2
P55103

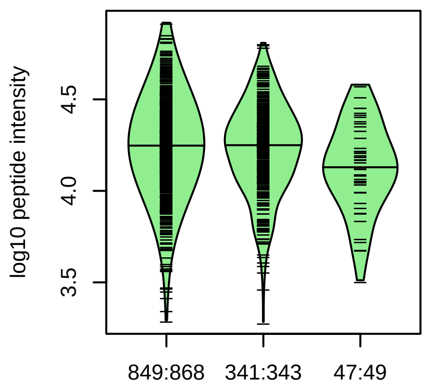

12:57839173:G:T_T
p = 0.00012, beta = −0.197, N = 1237

LDFHFSSDR pc2
P55103

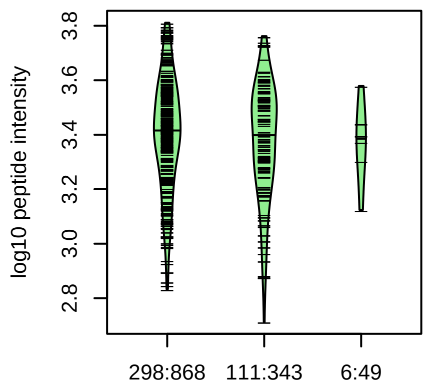

12:57839173:G:T_T
p = 0.12, beta = −0.155, N = 415

AGGQCPACGGPTLELESQR pc3
P55103

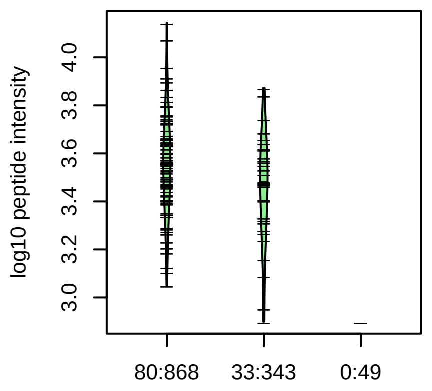

12:57839173:G:T_T
p = 0.0047, beta = −0.56, N = 113

GIDCQGGSR pc2
P55103

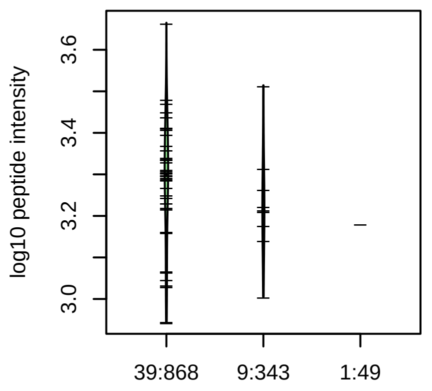

12:57839173:G:T_T
p = 0.33, beta = −0.285, N = 49

INHBC : NP3
P55103

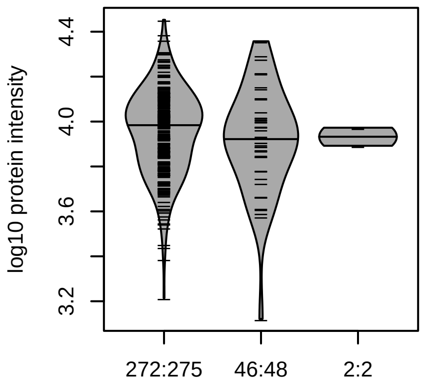

12:57839173:G:T_T
p = 0.54, beta = −0.0902, N = 320

ELLLDLAK pc2
P55103

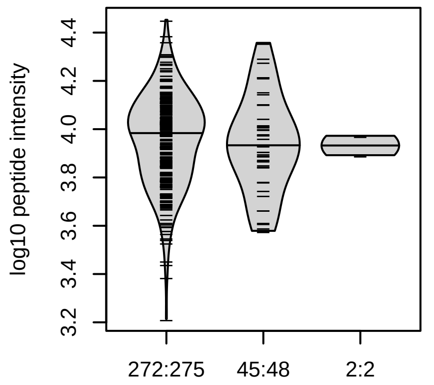

12:57839173:G:T_T
p = 0.74, beta = −0.0479, N = 319

LDFHFSSDR pc2
P55103

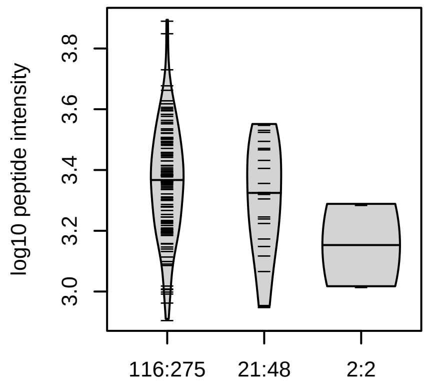

12:57839173:G:T_T
p = 0.18, beta = −0.266, N = 139

Assay Target: INHBC
UniProt: P55103
rsID: rs61352607
Target SNP: 12:57445381:GAA
Proxy SNP: 12:57839173:G:T
deCODE log10(p): 7136.9
deCODE BETA: −1.25
deCODE MSPA: 0.744
:−::−:NA
1237:415:113:49:1

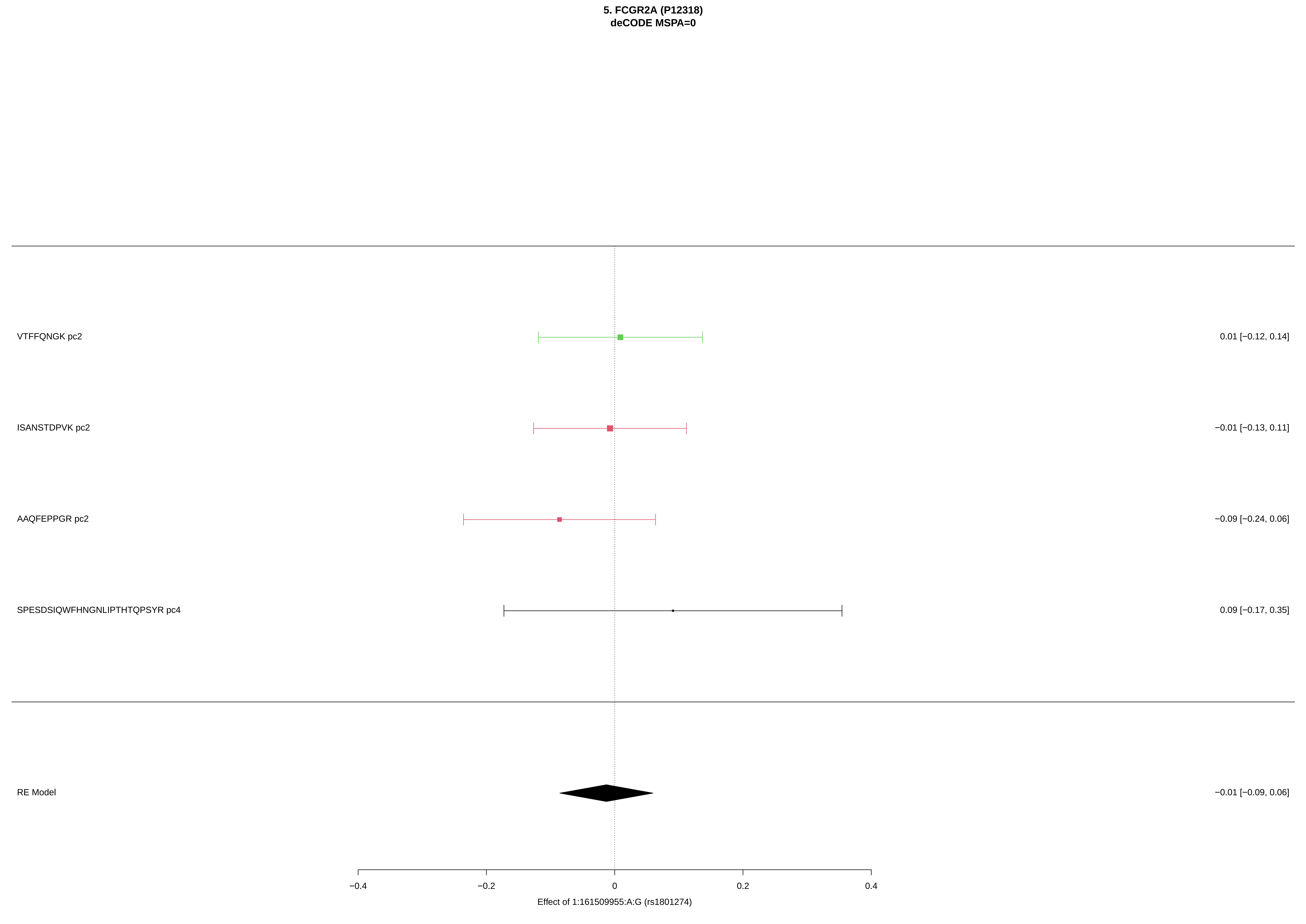
5. FCGR2A (P12318)
deCODE MSPA=0
VTFFQNGK pc2
0.01 [−0.12, 0.14]
ISANSTDPVK pc2
−0.01 [−0.13, 0.11]
AAQFEPPGR pc2
−0.09 [−0.24, 0.06]
SPESDSIQWFHNGNLIPTHTQPSYR pc4
0.09 [−0.17, 0.35]
RE Model
−0.01 [−0.09, 0.06]
−0.4
−0.2
0
0.2
0.4
Effect of 1:161509955:A:G (rs1801274)

FCGR2A : NP1
P12318−2

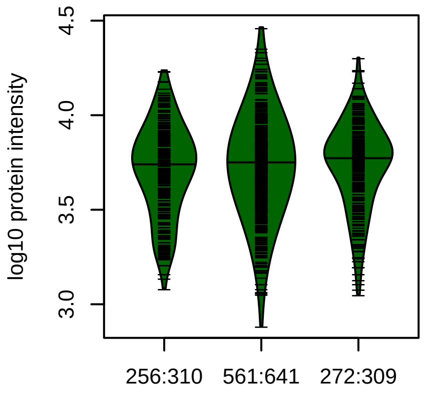

1:161479745:A:G_A
p = 0.5, beta = 0.0293, N = 1089

VTFFQNGK pc2
.2318;P12318−2;P31994;P31994−2;P3

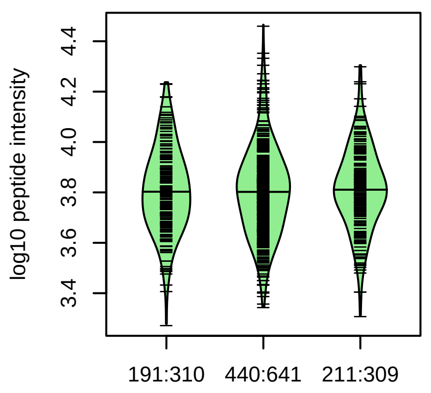

1:161479745:A:G_A
p = 0.86, beta = 0.00886, N = 842

ISANSTDPVK pc2
P12318;P12318−2

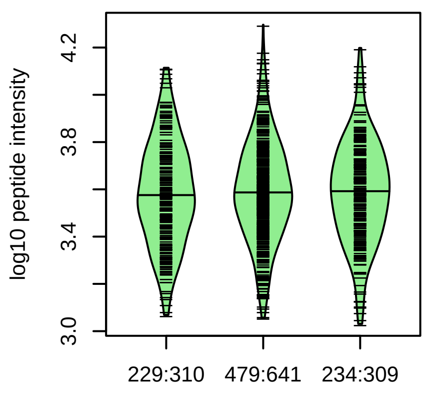

1:161479745:A:G_A
p = 0.87, beta = −0.00734, N = 942

AAQFEPPGR pc2
P12318;P12318−2

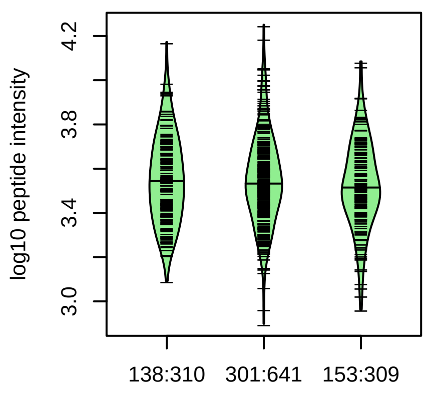

1:161479745:A:G_A
p = 0.14, beta = −0.086, N = 592

SPESDSIQWFHNGNLIPTHTQPSYR p
F5GX41;F5GXY9;P12318;P12318−

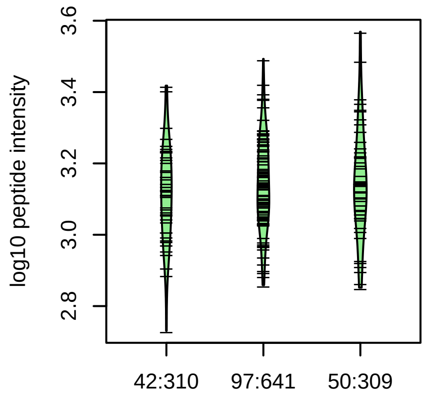

1:161479745:A:G_A
p = 0.38, beta = 0.0908, N = 189

FCGR2A : NP1
P12318−2

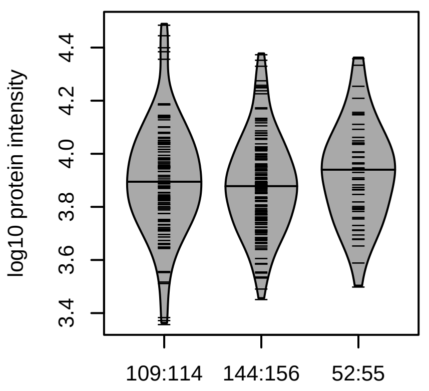

1:161479745:A:G_G
p = 0.41, beta = 0.0661, N = 305

VTFFQNGK pc2
.2318;P12318−2;P31994;P31994−2;P3

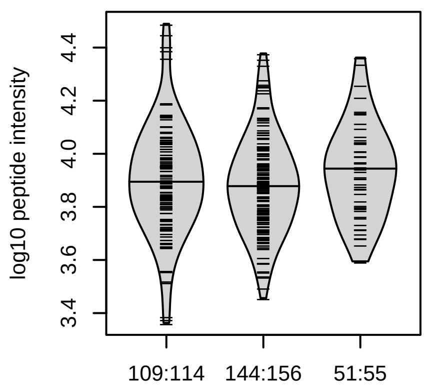

1:161479745:A:G_G
p = 0.31, beta = 0.0822, N = 304

ISANSTDPVK pc2
P12318;P12318−2

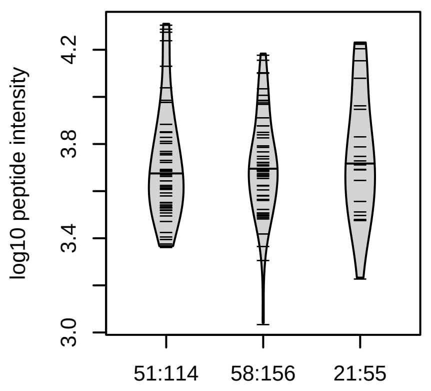

1:161479745:A:G_G
p = 0.69, beta = 0.0475, N = 130

Assay Target: FCGR2A
UniProt: P12318
rsID: rs1801274
Target SNP: 1:161509955:A:G
Proxy SNP: 1:161479745:A:G
deCODE log10(p): 6757.6
deCODE BETA: −1.24
deCODE MSPA: 0
−:−:−:−:NA
842:942:592:189:12

LEPPWINVLQEDSVTLTCQGAR pc
rs9427398 REF

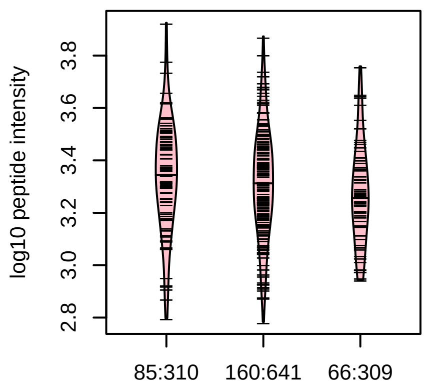

1:161479745:A:G_A
p = 0.13, model = DOM, N = 311

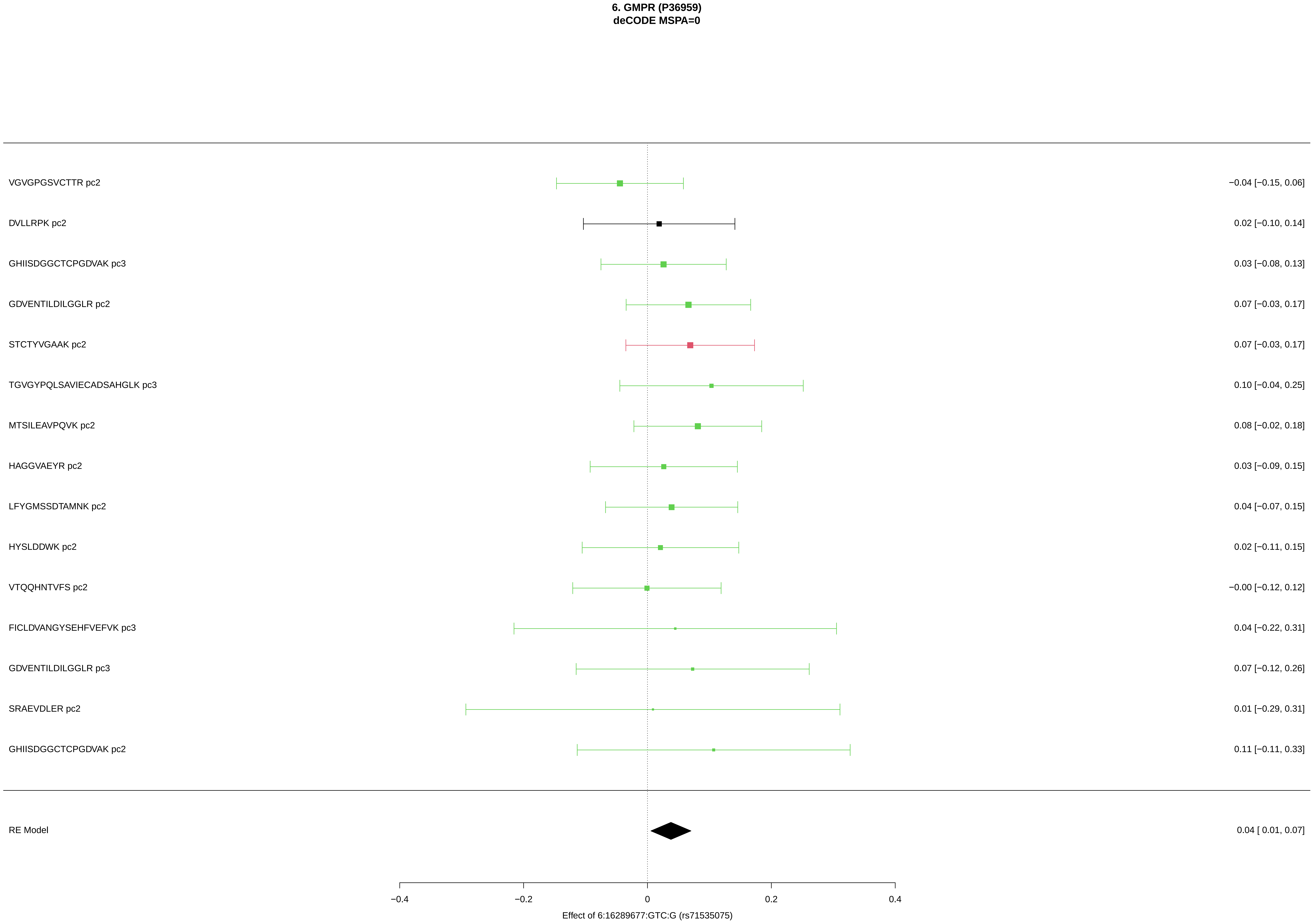
6. GMPR (P36959)
deCODE MSPA=0
VGVGPGSVCTTR pc2
−0.04 [−0.15, 0.06]
DVLLRPK pc2
0.02 [−0.10, 0.14]
GHIISDGGCTCPGDVAK pc3
0.03 [−0.08, 0.13]
GDVENTILDILGGLR pc2
0.07 [−0.03, 0.17]
STCTYVGAAK pc2
0.07 [−0.03, 0.17]
TGVGYPQLSAVIECADSAHGLK pc3
0.10 [−0.04, 0.25]
MTSILEAVPQVK pc2
0.08 [−0.02, 0.18]
HAGGVAEYR pc2
0.03 [−0.09, 0.15]
LFYGMSSDTAMNK pc2
0.04 [−0.07, 0.15]
HYSLDDWK pc2
0.02 [−0.11, 0.15]
VTQQHNTVFS pc2
−0.00 [−0.12, 0.12]
FICLDVANGYSEHFVEFVK pc3
0.04 [−0.22, 0.31]
GDVENTILDILGGLR pc3
0.07 [−0.12, 0.26]
SRAEVDLER pc2
0.01 [−0.29, 0.31]
GHIISDGGCTCPGDVAK pc2
0.11 [−0.11, 0.33]
RE Model
0.04 [0.01, 0.07]
−0.4
−0.2
0
0.2
0.4
Effect of 6:16289677:GTC:G (rs71535075)

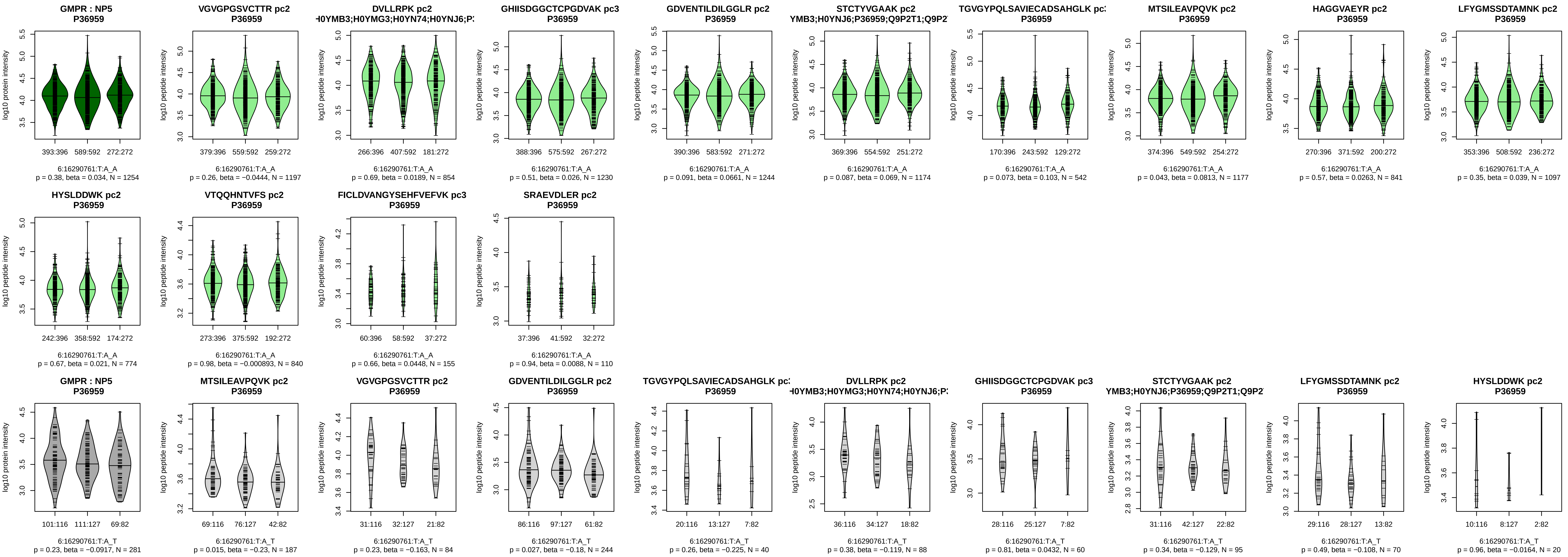

Assay Target: GMPR
UniProt: P36959
rsID: rs71535075
Target SNP: 6:16289677:GTC:G
Proxy SNP: 6:16290761:T:A
deCODE log10(p): 6478
deCODE BETA: 1.16
deCODE MSPA: 0
−:−:−:−:−:−:−:−:−:−:−:−:−:NA
1197:854:1230:1244:1174:542:1

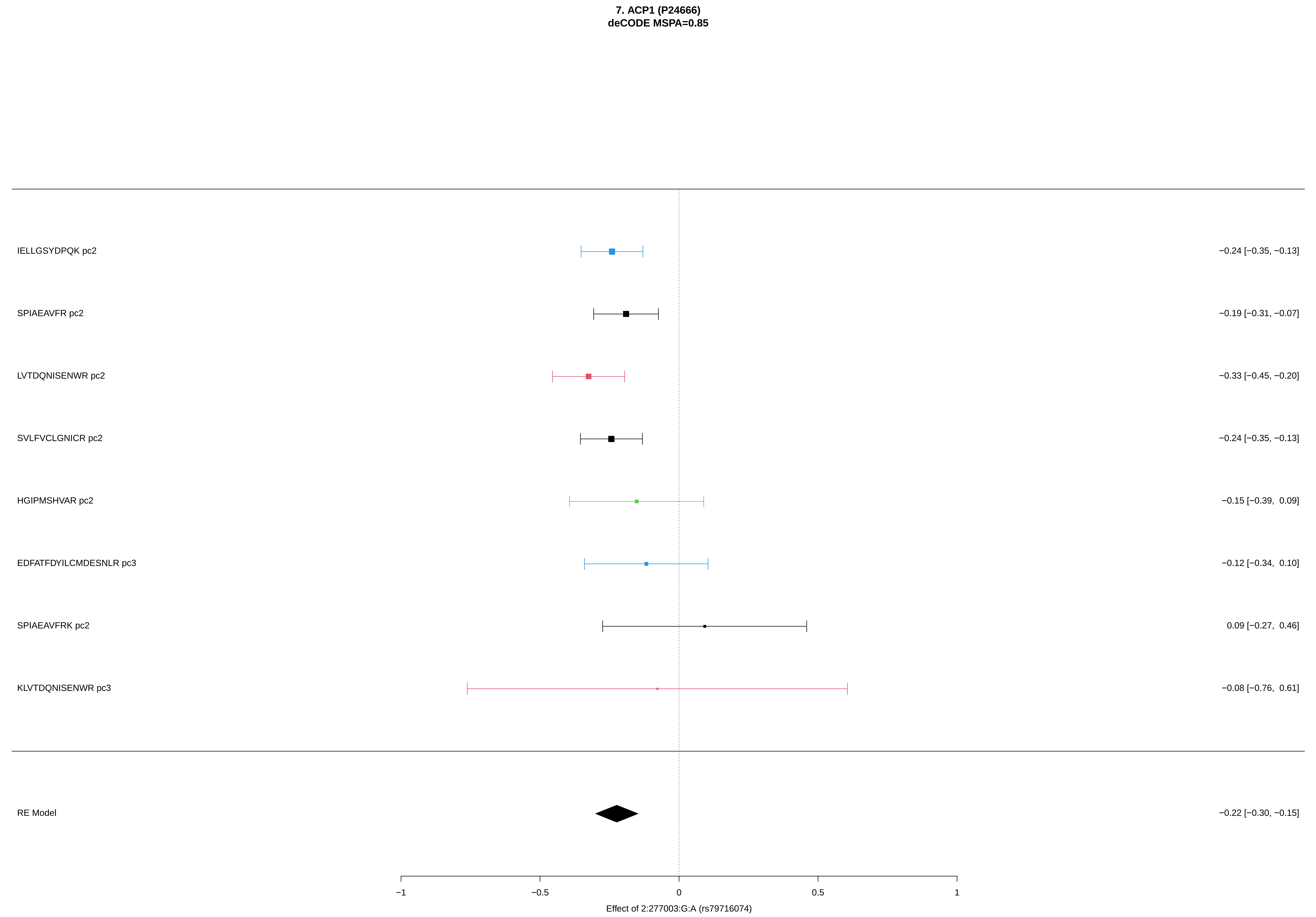
7. ACP1 (P24666)
deCODE MSPA=0.85
IELLGSYDPQK pc2
−0.24 [−0.35, −0.13]
SPIAEAVFR pc2
−0.19 [−0.31, −0.07]
LVTDQNISENWR pc2
−0.33 [−0.45, −0.20]
SVLFVCLGNICR pc2
−0.24 [−0.35, −0.13]
HGIPMSHVAR pc2
−0.15 [−0.39, 0.09]
EDFATFDYILCMDESNLR pc3
−0.12 [−0.34, 0.10]
SPIAEAVFRK pc2
0.09 [−0.27, 0.46]
KLVTDQNISENWR pc3
−0.08 [−0.76, 0.61]
RE Model
−0.22 [−0.30, −0.15]
−1
−0.5
0
0.5
1
Effect of 2:277003:G:A (rs79716074)

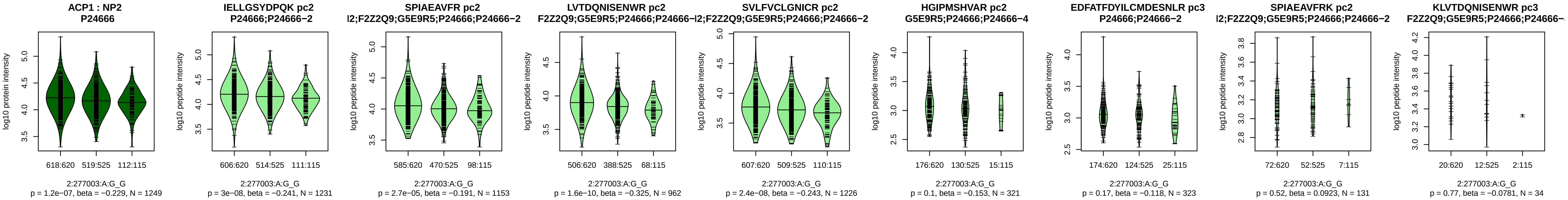

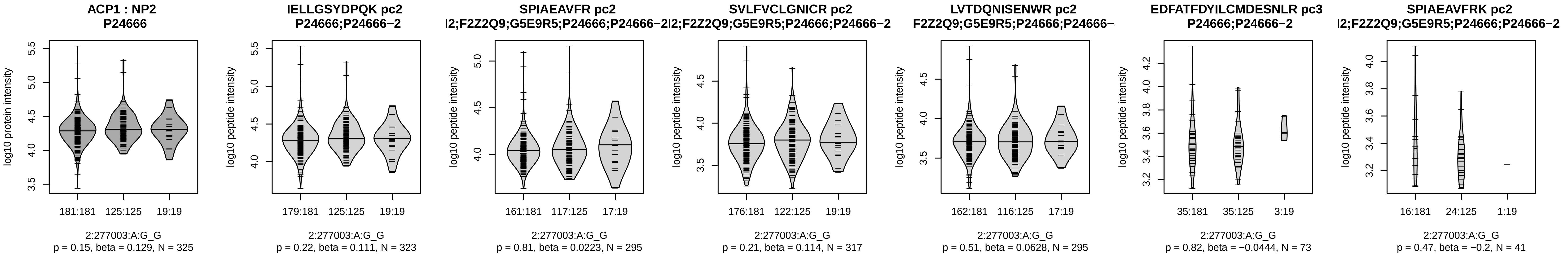

Assay Target: ACP1
UniProt: P24666
rsID: rs79716074
Target SNP: 2:277003:G:A
Proxy SNP: 2:277003:A:G
deCODE log10(p): 5886.7
deCODE BETA: 1.09
deCODE MSPA: 0.85
::*:*:−:−:−:−:NA:NA:NA:NA
1231:1153:962:1226:321:323:13

8. ICAM1 (P05362)
deCODE MSPA=0.363

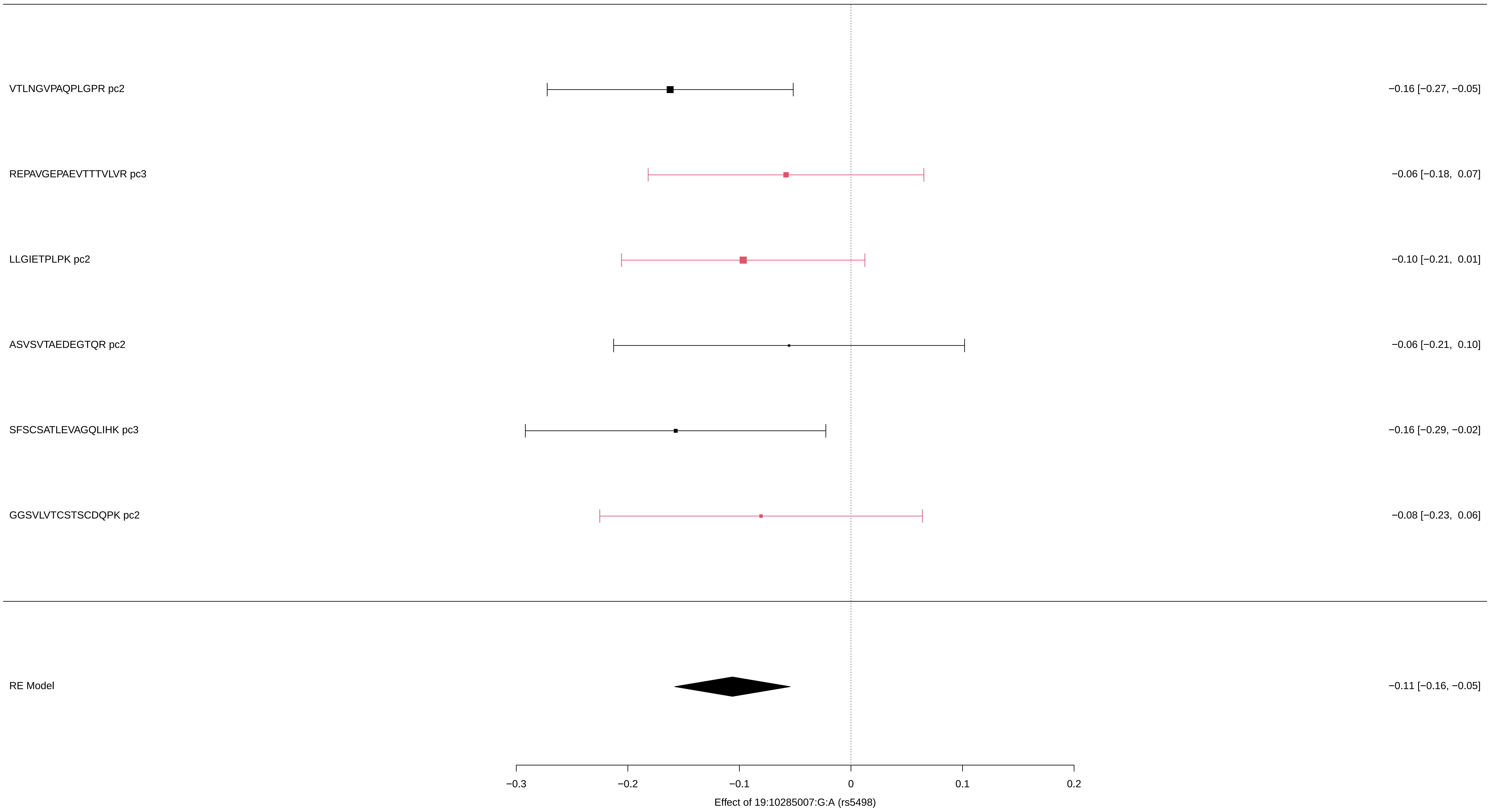

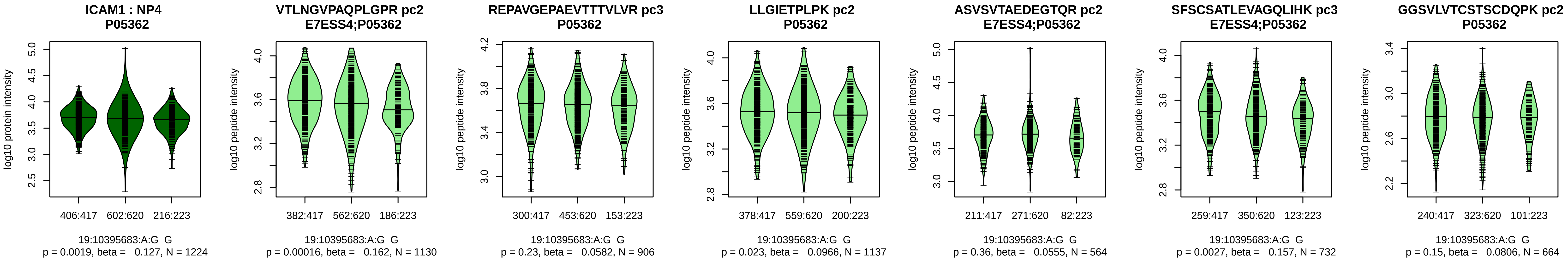

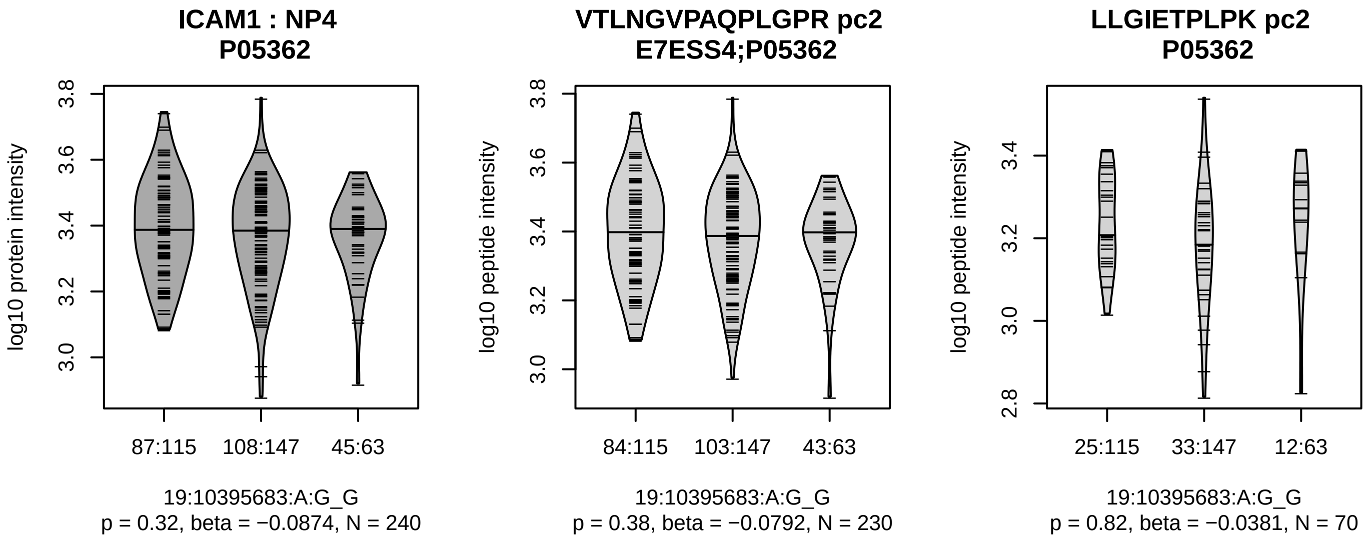

Assay Target: ICAM1
UniProt: P05362
rsID: rs5498
Target SNP: 19:10285007:G:A
Proxy SNP: 19:10395683:A:G
deCODE log10(p): 5538.2
deCODE BETA: −1.14
deCODE MSPA: 0.363
:−:−:−::−
1130:906:1137:564:732:664

9. EBI3 (Q14213)
deCODE MSPA=1

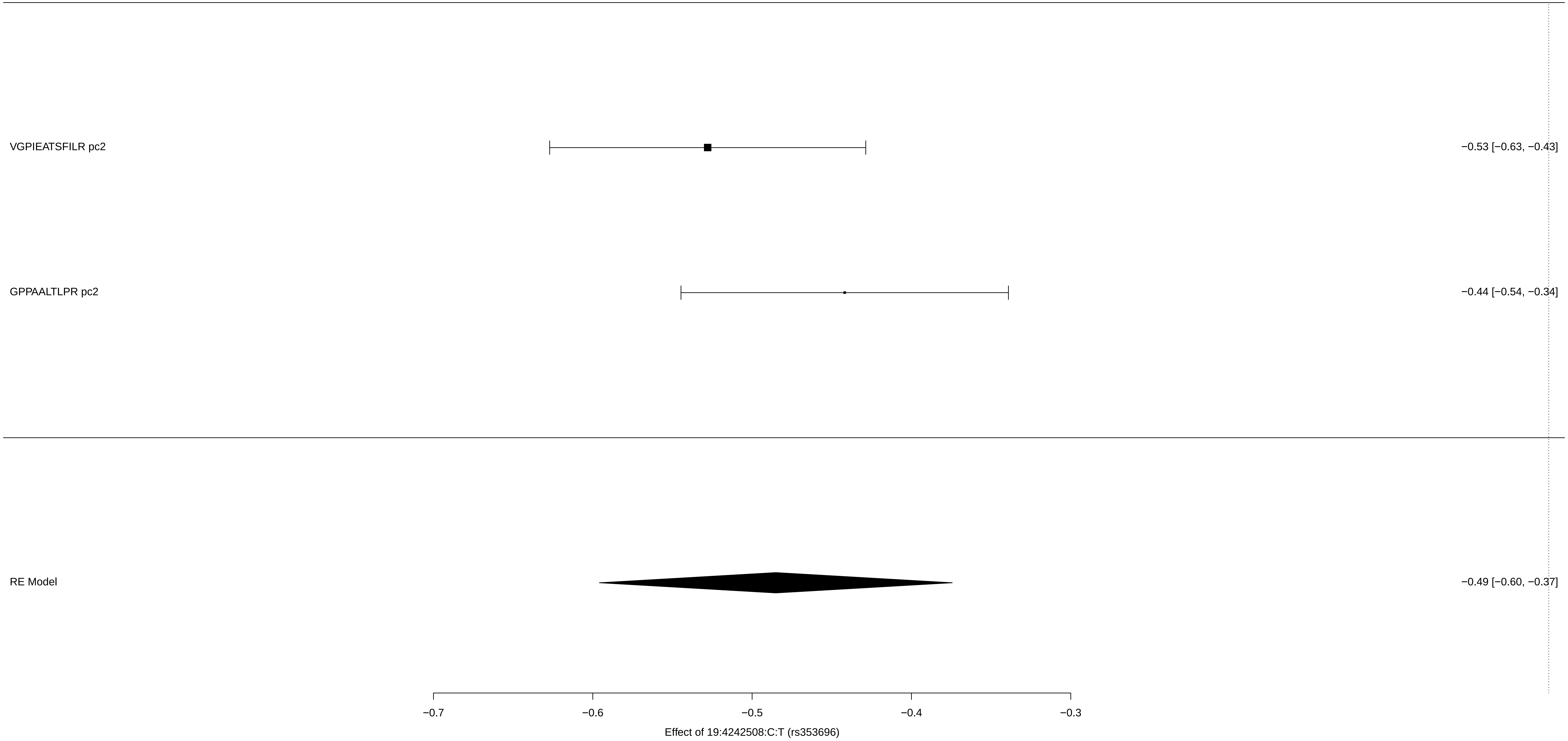

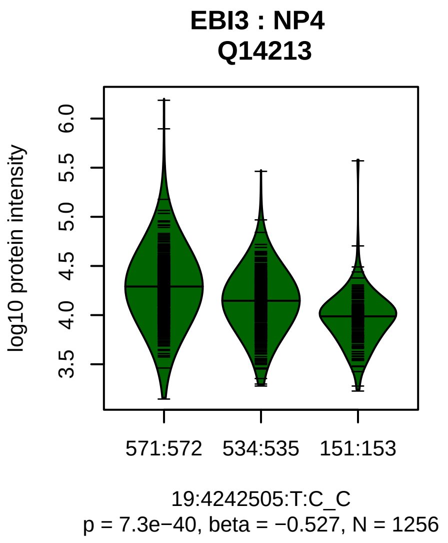

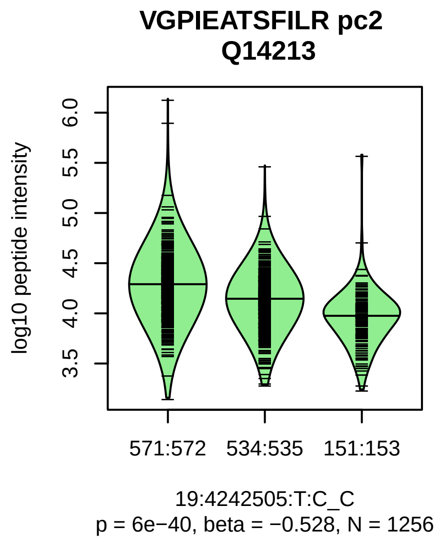

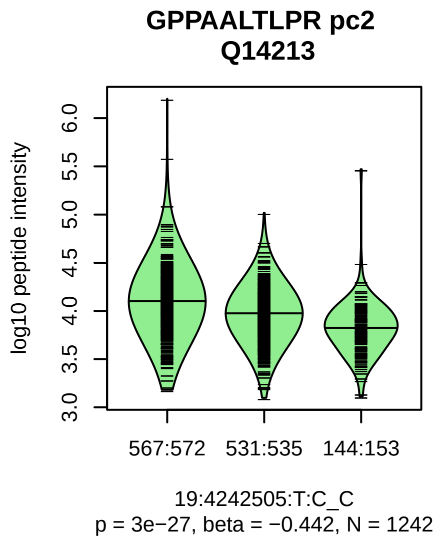

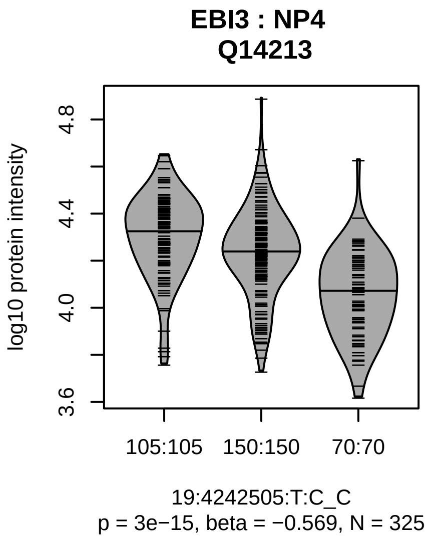

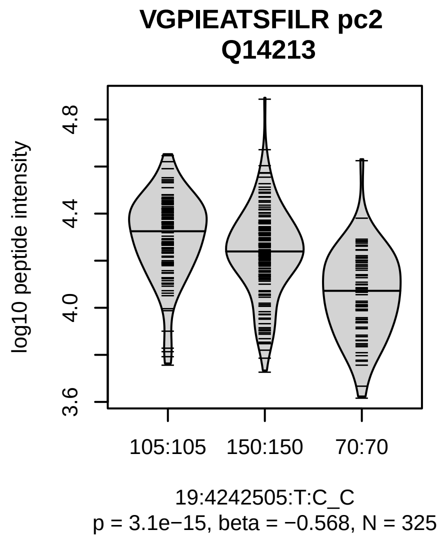

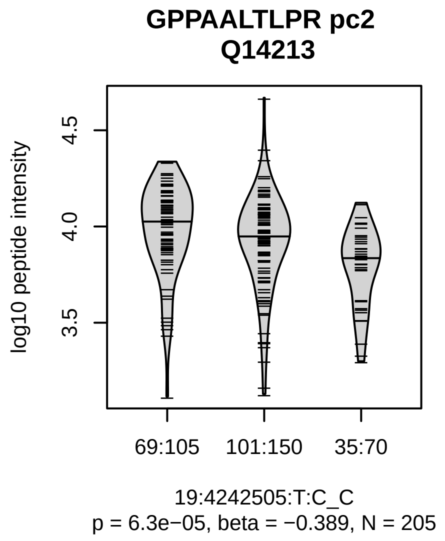

Assay Target: EBI3
UniProt: Q14213
rsID: rs353696
Target SNP: 19:4242508:C:T
Proxy SNP: 19:4242505:T:C
deCODE log10(p): 4454.4
deCODE BETA: −1.12
deCODE MSPA: 1
:
1256:1242

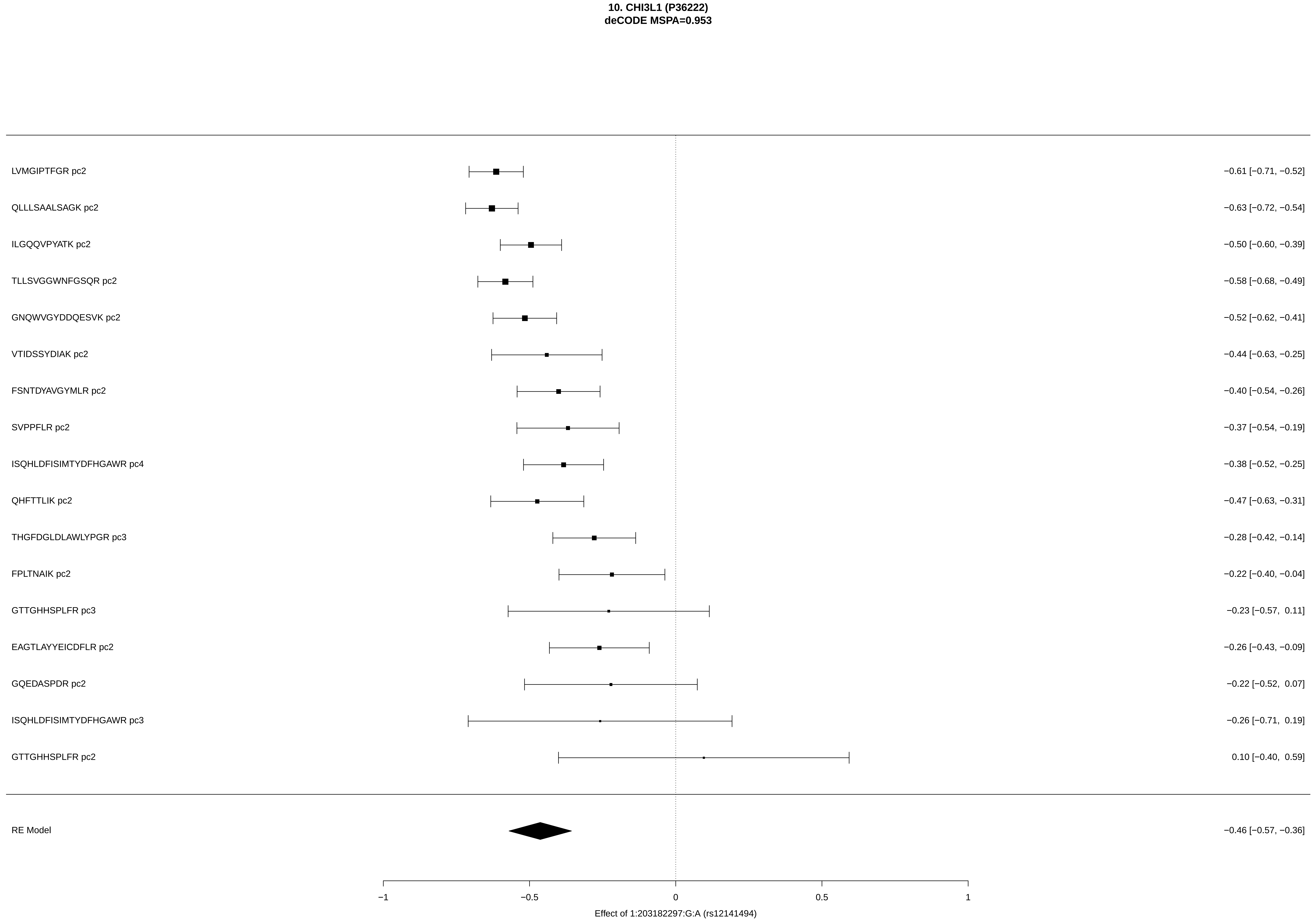
10. CHI3L1 (P36222)
deCODE MSPA=0.953
LVMGIPTFGR pc2
QLLLSAALSAGK pc2
ILGQQVPYATK pc2
TLLSVGGWNFGSQR pc2
GNQWVGYDDQESVK pc2
VTIDSSYDIAK pc2
FSNTDYAVGYMLR pc2
SVPPFLR pc2
ISQHLDFISIMTYDFHGAWR pc4
QHFTTLIK pc2
THGFDGLDLAWLYPGR pc3
FPLTNAIK pc2
GTTGHHSPLFR pc3
EAGTLAYYEICDFLR pc2
GQEDASPDR pc2
ISQHLDFISIMTYDFHGAWR pc3
GTTGHHSPLFR pc2
RE Model
−0.61 [−0.71, −0.52]
−0.63 [−0.72, −0.54]
−0.50 [−0.60, −0.39]
−0.58 [−0.68, −0.49]
−0.52 [−0.62, −0.41]
−0.44 [−0.63, −0.25]
−0.40 [−0.54, −0.26]
−0.37 [−0.54, −0.19]
−0.38 [−0.52, −0.25]
−0.47 [−0.63, −0.31]
−0.28 [−0.42, −0.14]
−0.22 [−0.40, −0.04]
−0.23 [−0.57, 0.11]
−0.26 [−0.43, −0.09]
−0.22 [−0.52, 0.07]
−0.26 [−0.71, 0.19]
0.10 [−0.40, 0.59]
−0.46 [−0.57, −0.36]
−1
−0.5
0
0.5
1
Effect of 1:203182297:G:A (rs12141494)

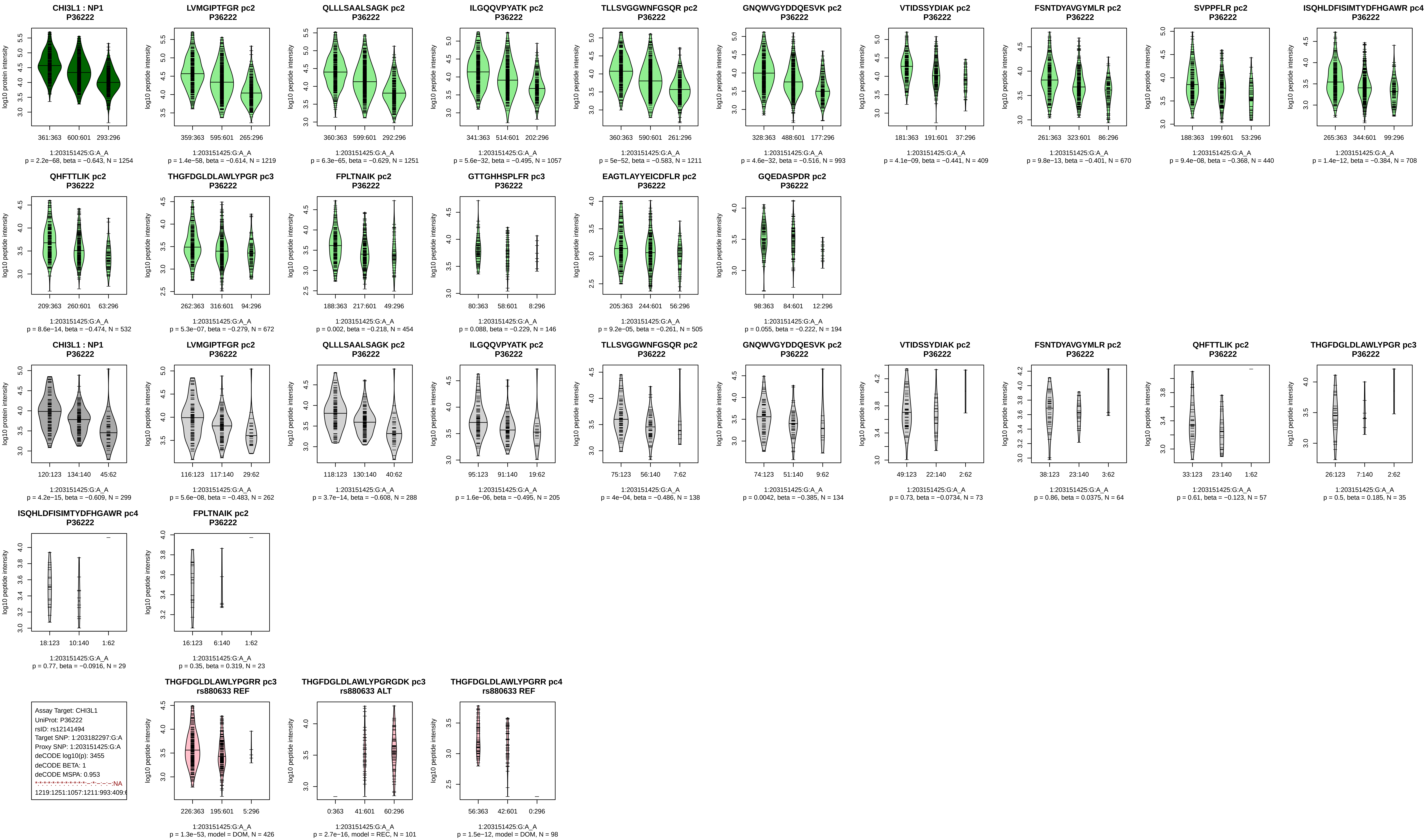

CHI3L1 : NP1 P36222
log10 protein intensity
361:363 600:601 293:296
1:203151425:G:A_A
p = 2.2e−68, beta = −0.643, N = 1254
LVMGIPTFGR pc2 P36222
log10 peptide intensity
359:363 595:601 265:296
1:203151425:G:A_A
p = 1.4e−58, beta = −0.614, N = 1219
QLLLSAALSAGK pc2 P36222
360:363 599:601 292:296
1:203151425:G:A_A
p = 6.3e−65, beta = −0.629, N = 1251
ILGQQVPYATK pc2 P36222
341:363 514:601 202:296
1:203151425:G:A_A
p = 5.6e−32, beta = −0.495, N = 1057
TLLSVGGWNFGSQR pc2 P36222
360:363 590:601 261:296
1:203151425:G:A_A
p = 5e−52, beta = −0.583, N = 1211
GNQWVGYDDQESVK pc2 P36222
328:363 488:601 177:296
1:203151425:G:A_A
p = 4.6e−32, beta = −0.516, N = 993
VTIDSSYDIAK pc2 P36222
181:363 191:601 37:296
1:203151425:G:A_A
p = 4.1e−09, beta = −0.441, N = 409
FSNTDYAVGYMLR pc2 P36222
261:363 323:601 86:296
1:203151425:G:A_A
p = 9.8e−13, beta = −0.401, N = 670
SVPPFLR pc2 P36222
188:363 199:601 53:296
1:203151425:G:A_A
p = 9.4e−08, beta = −0.368, N = 440
ISQHLDFISIMTYDFHGAWR pc4 P36222
265:363 344:601 99:296
1:203151425:G:A_A
p = 1.4e−12, beta = −0.384, N = 708
QHFTTLIK pc2 P36222
209:363 260:601 63:296
1:203151425:G:A_A
p = 8.6e−14, beta = −0.474, N = 532
THGFDGLDLAWLYPGR pc3 P36222
262:363 316:601 94:296
1:203151425:G:A_A
p = 5.3e−07, beta = −0.279, N = 672
FPLTNAIK pc2 P36222
188:363 217:601 49:296
1:203151425:G:A_A
p = 0.002, beta = −0.218, N = 454
GTTGHHSPLFR pc3 P36222
80:363 58:601 8:296
1:203151425:G:A_A
p = 0.088, beta = −0.229, N = 146
EAGTLAYYEICDFLR pc2 P36222
205:363 244:601 56:296
1:203151425:G:A_A
p = 9.2e−05, beta = −0.261, N = 505
GQEDASPDR pc2 P36222
98:363 84:601 12:296
1:203151425:G:A_A
p = 0.055, beta = −0.222, N = 194
CHI3L1 : NP1 P36222
120:123 134:140 45:62
1:203151425:G:A_A
p = 4.2e−15, beta = −0.609, N = 299
LVMGIPTFGR pc2 P36222
116:123 117:140 29:62
1:203151425:G:A_A
p = 5.6e−08, beta = −0.483, N = 262
QLLLSAALSAGK pc2 P36222
118:123 130:140 40:62
1:203151425:G:A_A
p = 3.7e−14, beta = −0.608, N = 288
ILGQQVPYATK pc2 P36222
95:123 91:140 19:62
1:203151425:G:A_A
p = 1.6e−06, beta = −0.495, N = 205
TLLSVGGWNFGSQR pc2 P36222
75:123 56:140 7:62
1:203151425:G:A_A
p = 4e−04, beta = −0.486, N = 138
GNQWVGYDDQESVK pc2 P36222
74:123 51:140 9:62
1:203151425:G:A_A
p = 0.0042, beta = −0.385, N = 134
VTIDSSYDIAK pc2 P36222
49:123 22:140 2:62
1:203151425:G:A_A
p = 0.73, beta = −0.0734, N = 73
FSNTDYAVGYMLR pc2 P36222
38:123 23:140 3:62
1:203151425:G:A_A
p = 0.86, beta = 0.0375, N = 64
QHFTTLIK pc2 P36222
33:123 23:140 1:62
1:203151425:G:A_A
p = 0.61, beta = −0.123, N = 57
THGFDGLDLAWLYPGR pc3 P36222
26:123 7:140 2:62
1:203151425:G:A_A
p = 0.5, beta = 0.185, N = 35
ISQHLDFISIMTYDFHGAWR pc4 P36222
18:123 10:140 1:62
1:203151425:G:A_A
p = 0.77, beta = −0.0916, N = 29
FPLTNAIK pc2 P36222
16:123 6:140 1:62
1:203151425:G:A_A
p = 0.35, beta = 0.319, N = 23
Assay Target: CHI3L1
UniProt: P36222
rsID: rs12141494
Target SNP: 1:203182297:G:A
Proxy SNP: 1:203151425:G:A
deCODE log10(p): 3455
deCODE BETA: 1
deCODE MSPA: 0.953
1219:1251:1057:1211:993:409:6
THGFDGLDLAWLYPGRR pc3 rs880633 REF
226:363 195:601 5:296
1:203151425:G:A_A
p = 1.3e−53, model = DOM, N = 426
THGFDGLDLAWLYPGRGDK pc3 rs880633 ALT
0:363 41:601 60:296
1:203151425:G:A_A
p = 2.7e−16, model = REC, N = 101
THGFDGLDLAWLYPGRR pc4 rs880633 REF
56:363 42:601 0:296
1:203151425:G:A_A
p = 1.5e−12, model = DOM, N = 98

11. TNC (P24821)
deCODE MSPA=0.002

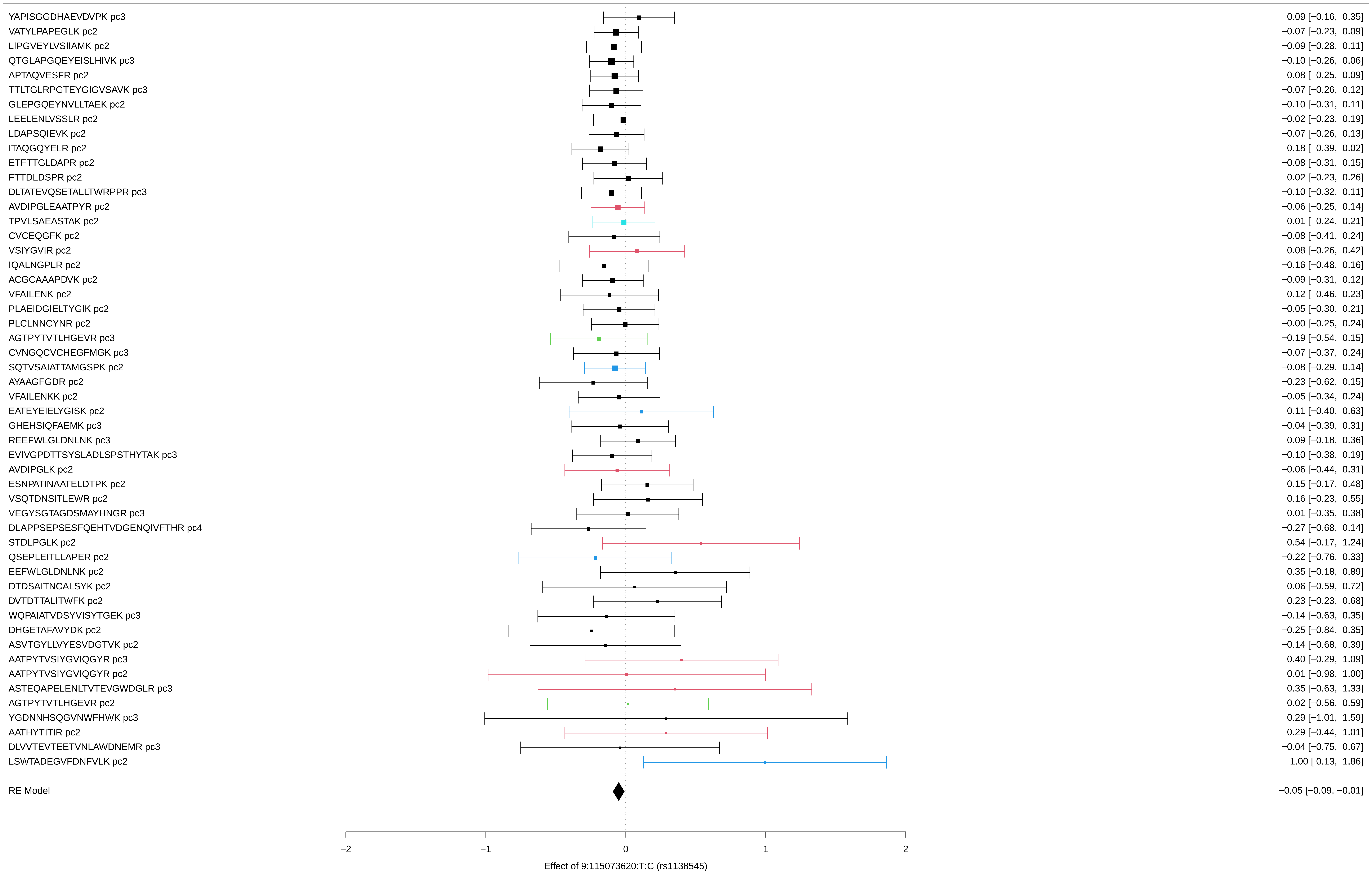

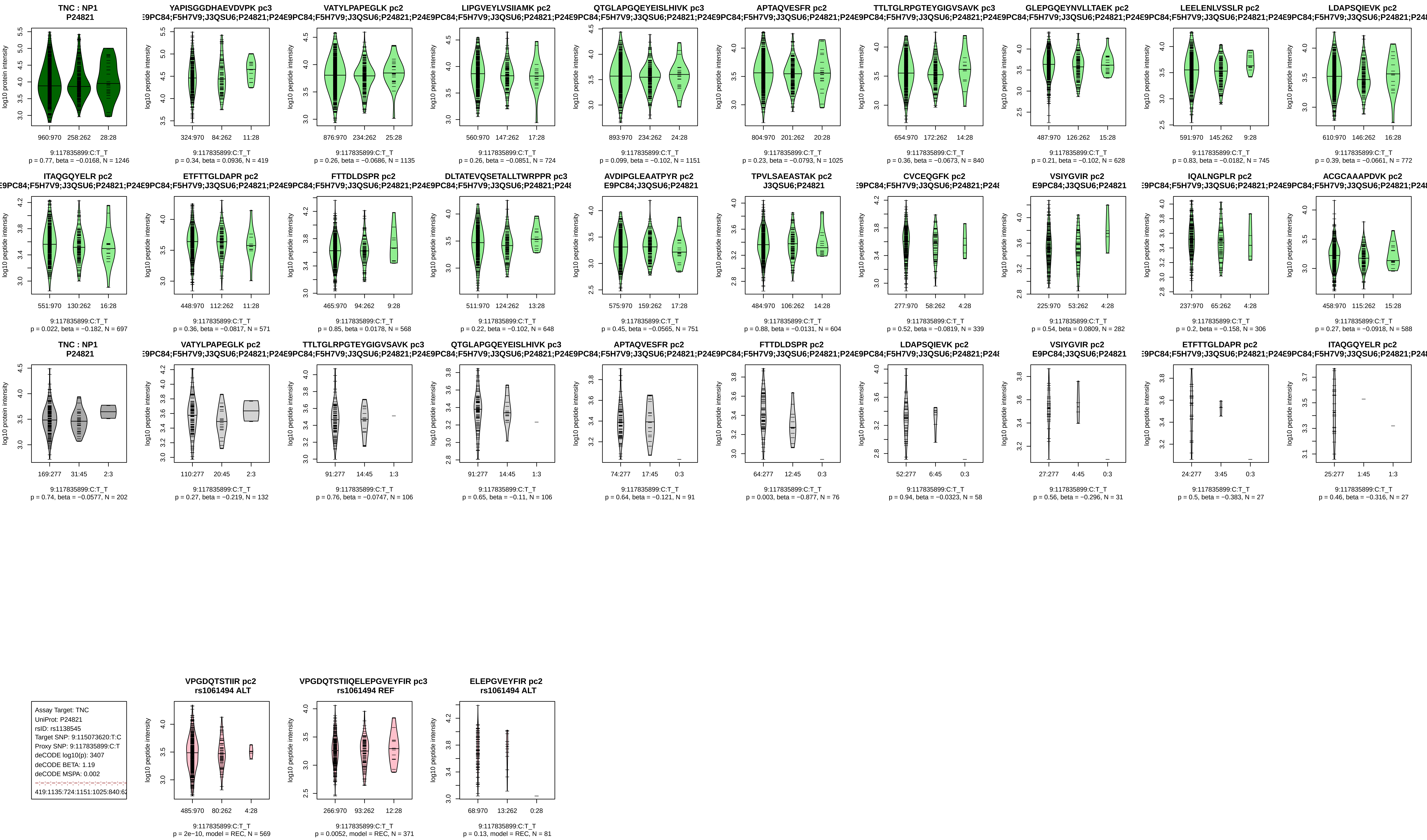

TNC : NP1
P24821
960:970 258:262 28:28
9:117835899:C:T_T
p = 0.77, beta = −0.0168, N = 1246
YAPISGGDHAEVDVPK pc3
324:970 84:262 11:28
9:117835899:C:T_T
p = 0.34, beta = 0.0936, N = 419
VATYLPAPEGLK pc2
876:970 234:262 25:28
9:117835899:C:T_T
p = 0.26, beta = −0.0686, N = 1135
LIPGVEYLVSIIAMK pc2
560:970 147:262 17:28
9:117835899:C:T_T
p = 0.26, beta = −0.0851, N = 724
QTGLAPGQEYEISLHIVK pc3
893:970 234:262 24:28
9:117835899:C:T_T
p = 0.099, beta = −0.102, N = 1151
APTAQVESFR pc2
804:970 201:262 20:28
9:117835899:C:T_T
p = 0.23, beta = −0.0793, N = 1025
TTLTGLRPGTEYGIGVSAVK pc3
654:970 172:262 14:28
9:117835899:C:T_T
p = 0.36, beta = −0.0673, N = 840
GLEPGQEYNVLLTAEK pc2
487:970 126:262 15:28
9:117835899:C:T_T
p = 0.21, beta = −0.102, N = 628
LEELENLVSSLR pc2
591:970 145:262 9:28
9:117835899:C:T_T
p = 0.83, beta = −0.0182, N = 745
LDAPSQIEVK pc2
610:970 146:262 16:28
9:117835899:C:T_T
p = 0.39, beta = −0.0661, N = 772
ITAQGQYELR pc2
551:970 130:262 16:28
9:117835899:C:T_T
p = 0.022, beta = −0.182, N = 697
ETFTTGLDAPR pc2
448:970 112:262 11:28
9:117835899:C:T_T
p = 0.36, beta = −0.0817, N = 571
FTTDLDSPR pc2
465:970 94:262 9:28
9:117835899:C:T_T
p = 0.85, beta = 0.0178, N = 568
DLTATEVQSETALLTWRPPR pc3
511:970 124:262 13:28
9:117835899:C:T_T
p = 0.22, beta = −0.102, N = 648
AVDIPGLEAATPYR pc2
E9PC84;J3QSU6;P24821
575:970 159:262 17:28
9:117835899:C:T_T
p = 0.45, beta = −0.0565, N = 751
TPVLSAEASTAK pc2
J3QSU6;P24821
484:970 106:262 14:28
9:117835899:C:T_T
p = 0.88, beta = −0.0131, N = 604
CVCEQGFK pc2
277:970 58:262 4:28
9:117835899:C:T_T
p = 0.52, beta = −0.0819, N = 339
VSIYGVIR pc2
E9PC84;J3QSU6;P24821
225:970 53:262 4:28
9:117835899:C:T_T
p = 0.54, beta = 0.0809, N = 282
IQALNGPLR pc2
237:970 65:262 4:28
9:117835899:C:T_T
p = 0.2, beta = −0.158, N = 306
ACGCAAAPDVK pc2
458:970 115:262 15:28
9:117835899:C:T_T
p = 0.27, beta = −0.0918, N = 588
TNC : NP1
P24821
169:277 31:45 2:3
9:117835899:C:T_T
p = 0.74, beta = −0.0577, N = 202
VATYLPAPEGLK pc2
110:277 20:45 2:3
9:117835899:C:T_T
p = 0.27, beta = −0.219, N = 132
TTLTGLRPGTEYGIGVSAVK pc3
91:277 14:45 1:3
9:117835899:C:T_T
p = 0.76, beta = −0.0747, N = 106
QTGLAPGQEYEISLHIVK pc3
91:277 14:45 1:3
9:117835899:C:T_T
p = 0.65, beta = −0.11, N = 106
APTAQVESFR pc2
74:277 17:45 0:3
9:117835899:C:T_T
p = 0.64, beta = −0.121, N = 91
FTTDLDSPR pc2
64:277 12:45 0:3
9:117835899:C:T_T
p = 0.003, beta = −0.877, N = 76
LDAPSQIEVK pc2
52:277 6:45 0:3
9:117835899:C:T_T
p = 0.94, beta = −0.0323, N = 58
VSIYGVIR pc2
E9PC84;J3QSU6;P24821
27:277 4:45 0:3
9:117835899:C:T_T
p = 0.56, beta = −0.296, N = 31
ETFTTGLDAPR pc2
24:277 3:45 0:3
9:117835899:C:T_T
p = 0.5, beta = −0.383, N = 27
ITAQGQYELR pc2
25:277 1:45 1:3
9:117835899:C:T_T
p = 0.46, beta = −0.316, N = 27
Assay Target: TNC
UniProt: P24821
rsID: rs1138545
Target SNP: 9:115073620:T:C
Proxy SNP: 9:117835899:C:T
deCODE log10(p): 3407
deCODE BETA: 1.19
deCODE MSPA: 0.002
419:1135:724:1151:1025:840:62
VPGDQTSTIIR pc2
rs1061494 ALT
485:970 80:262 4:28
9:117835899:C:T_T
p = 2e−10, model = REC, N = 569
VPGDQTSTIIQELEPGVEYFIR pc3
rs1061494 REF
266:970 93:262 12:28
9:117835899:C:T_T
p = 0.0052, model = REC, N = 371
ELEPGVEYFIR pc2
rs1061494 ALT
68:970 13:262 0:28
9:117835899:C:T_T
p = 0.13, model = REC, N = 81
log10 protein intensity
log10 peptide intensity

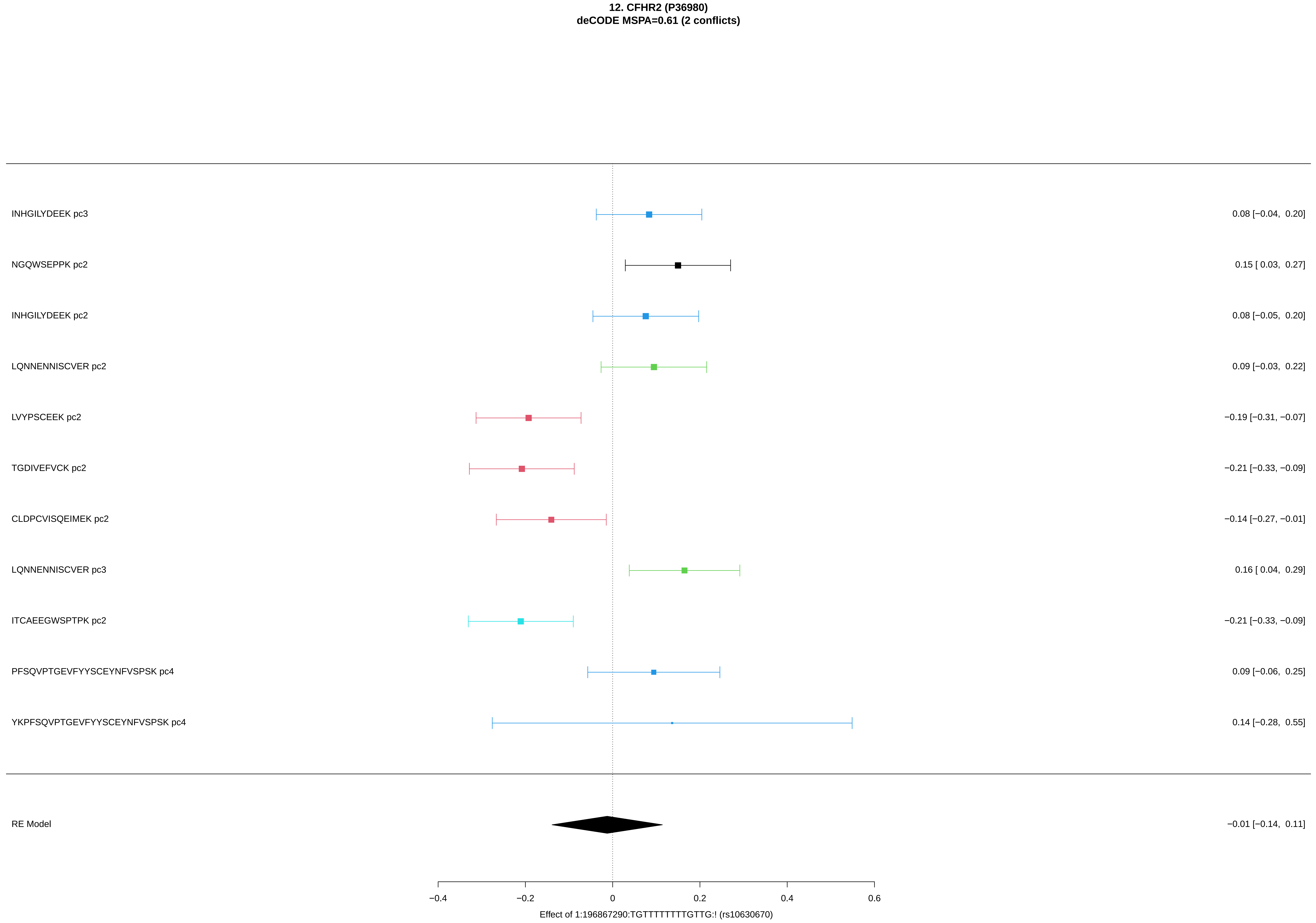
12. CFHR2 (P36980)
deCODE MSPA=0.61 (2 conflicts)
INHGILYDEEK pc3
0.08 [−0.04, 0.20]
NGQWSEPPK pc2
0.15 [0.03, 0.27]
INHGILYDEEK pc2
0.08 [−0.05, 0.20]
LQNNENNISCVER pc2
0.09 [−0.03, 0.22]
LVYPSCEEK pc2
−0.19 [−0.31, −0.07]
TGDIVEFVCK pc2
−0.21 [−0.33, −0.09]
CLDPCVISQEIMEK pc2
−0.14 [−0.27, −0.01]
LQNNENNISCVER pc3
0.16 [0.04, 0.29]
ITCAEEGWSPTPK pc2
−0.21 [−0.33, −0.09]
PFSQVPTGEVFYYSCEYNFVSPSK pc4
0.09 [−0.06, 0.25]
YKPFSQVPTGEVFYYSCEYNFVSPSK pc4
0.14 [−0.28, 0.55]
RE Model
−0.01 [−0.14, 0.11]
−0.4
−0.2
0
0.2
0.4
0.6
Effect of 1:196867290:TGTTTTTTTTGTTG:! (rs10630670)

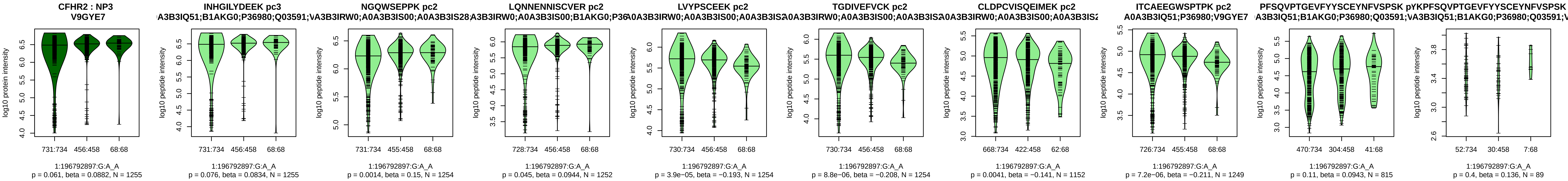

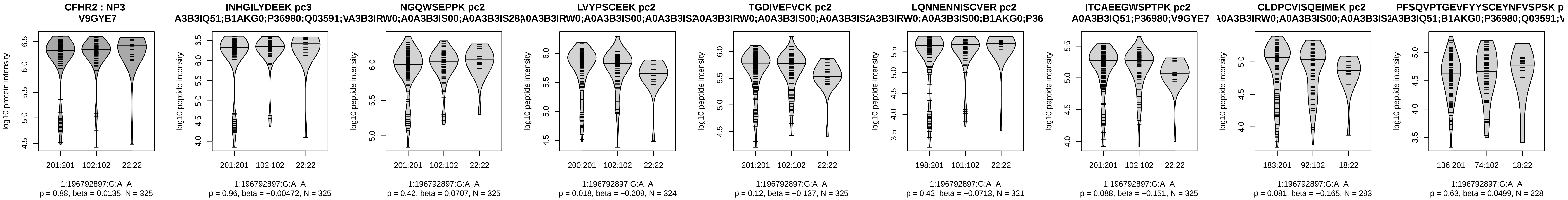

Assay Target: CFHR2
UniProt: P36980
rsID: rs10630670
Target SNP: 1:196867290:TGTT
Proxy SNP: 1:196792897:G:A
deCODE log10(p): 3225
deCODE BETA: −0.95
deCODE MSPA: 0.61 (2)
–:*:–:–:*:*:*:*:*:–:–:NA
1255:1254:1248:1252:1254:125

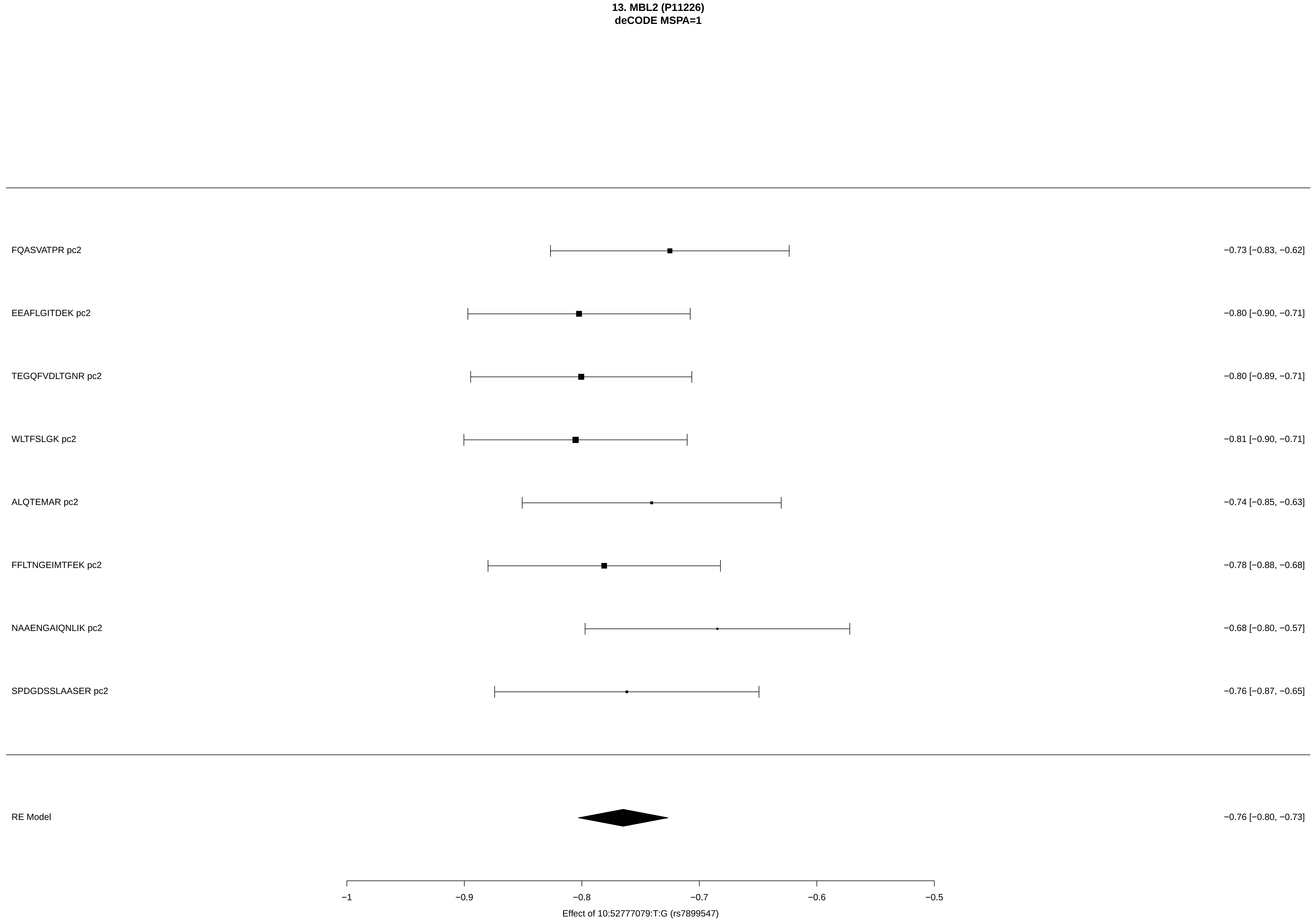

13. MBL2 (P11226)
deCODE MSPA=1
FQASVATPR pc2
−0.73 [−0.83, −0.62]
EEAFLGITDEK pc2
−0.80 [−0.90, −0.71]
TEGQFVDLTGNR pc2
−0.80 [−0.89, −0.71]
WLTFSLGK pc2
−0.81 [−0.90, −0.71]
ALQTEMAR pc2
−0.74 [−0.85, −0.63]
FFLTNGEIMTFEK pc2
−0.78 [−0.88, −0.68]
NAAENGAIQNLIK pc2
−0.68 [−0.80, −0.57]
SPDGDSSLAASER pc2
−0.76 [−0.87, −0.65]
RE Model
−0.76 [−0.80, −0.73]
−1
−0.9
−0.8
−0.7
−0.6
−0.5
Effect of 10:52777079:T:G (rs7899547)

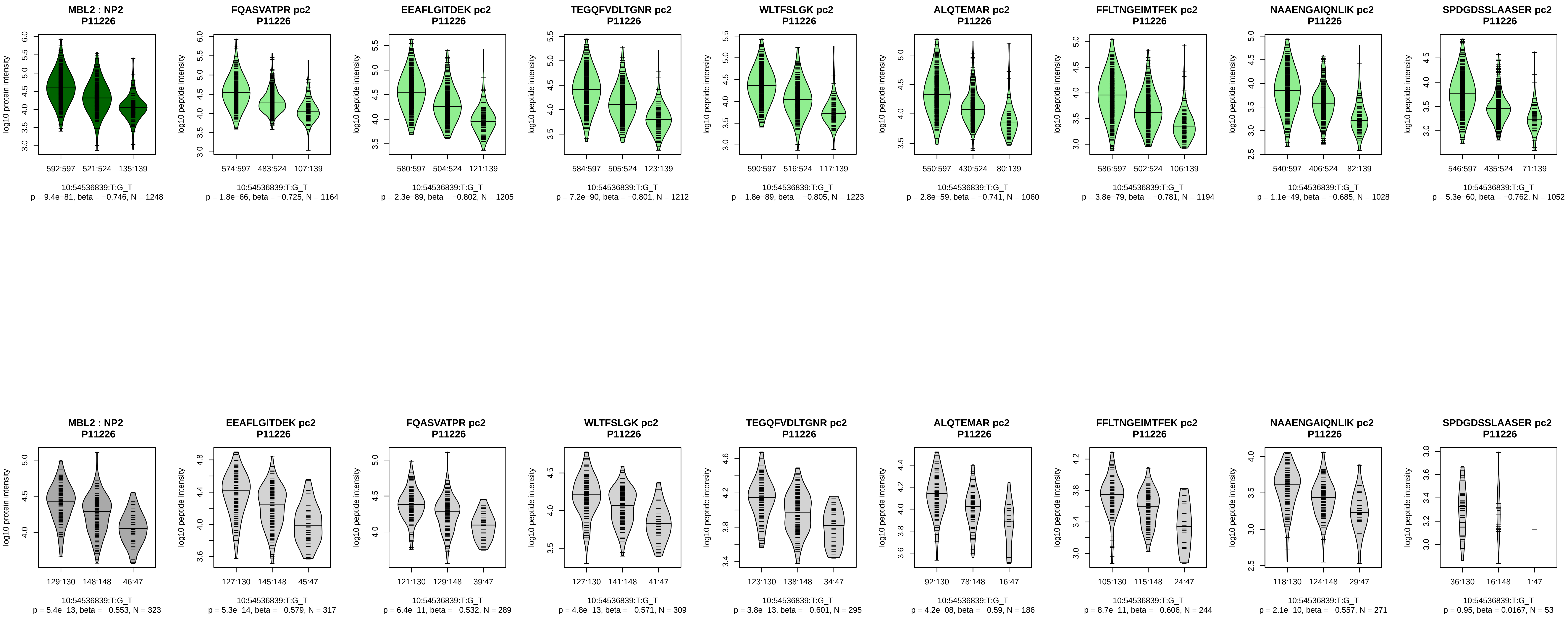

Assay Target: MBL2
UniProt: P11226
rsID: rs7899547
Target SNP: 10:52777079:T:G
Proxy SNP: 10:54536839:T:G
deCODE log10(p): 3017.5
deCODE BETA: −0.9
deCODE MSPA: 1
::*:*:*:*:*:*:NA
1164:1205:1212:1223:1060:119

14. HGFAC (Q04756)
deCODE MSPA=0.253

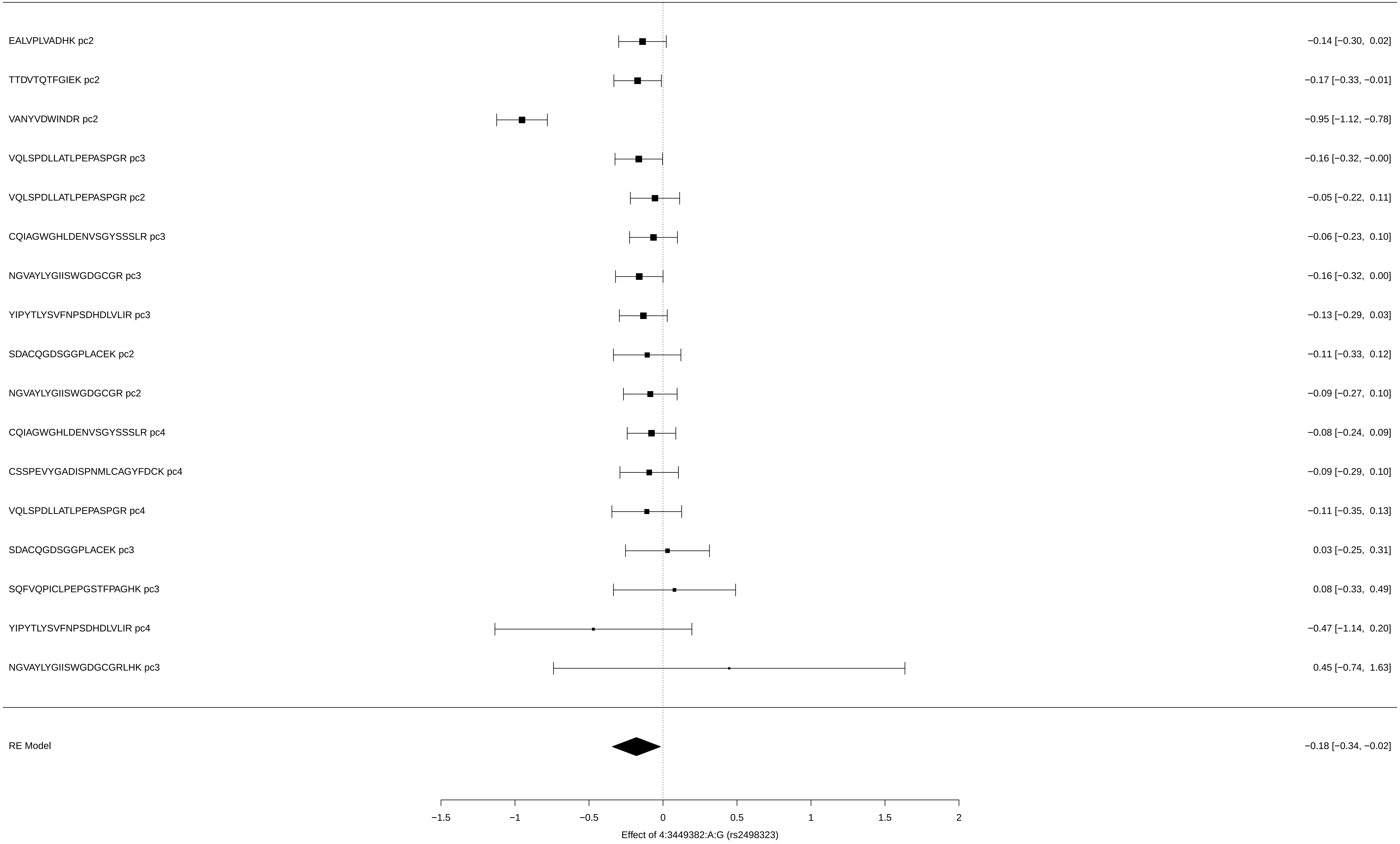

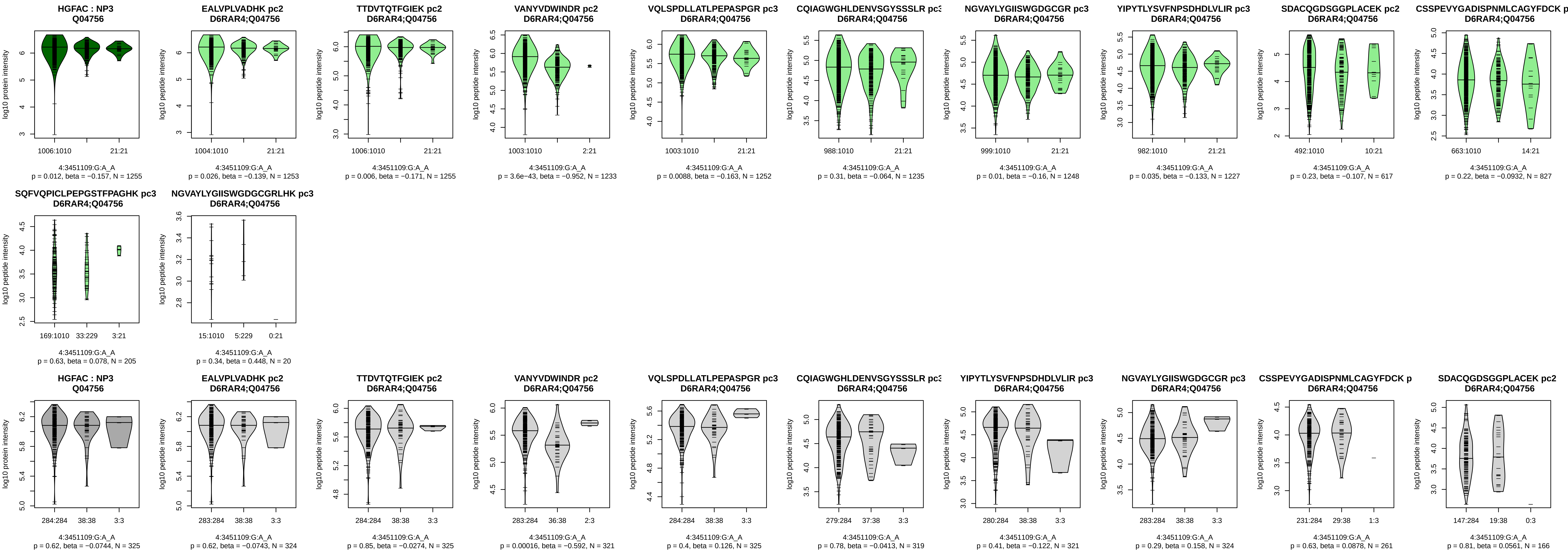

Assay Target: HGFAC
UniProt: Q04756
rsID: rs2498323
Target SNP: 4:3449382:A:G
Proxy SNP: 4:3451109:G:A
deCODE log10(p): 2946
deCODE BETA: −1.52
deCODE MSPA: 0.253
1253:1255:1233:1252:1166:123

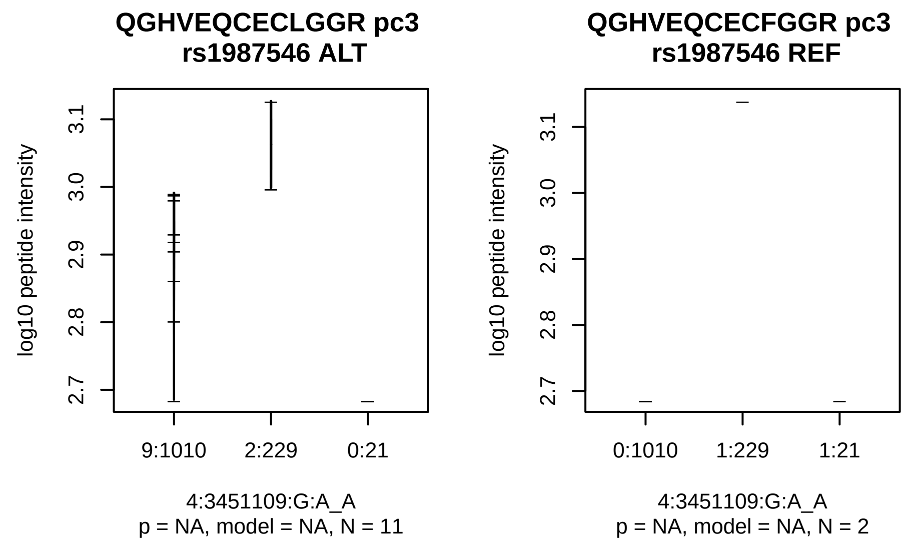

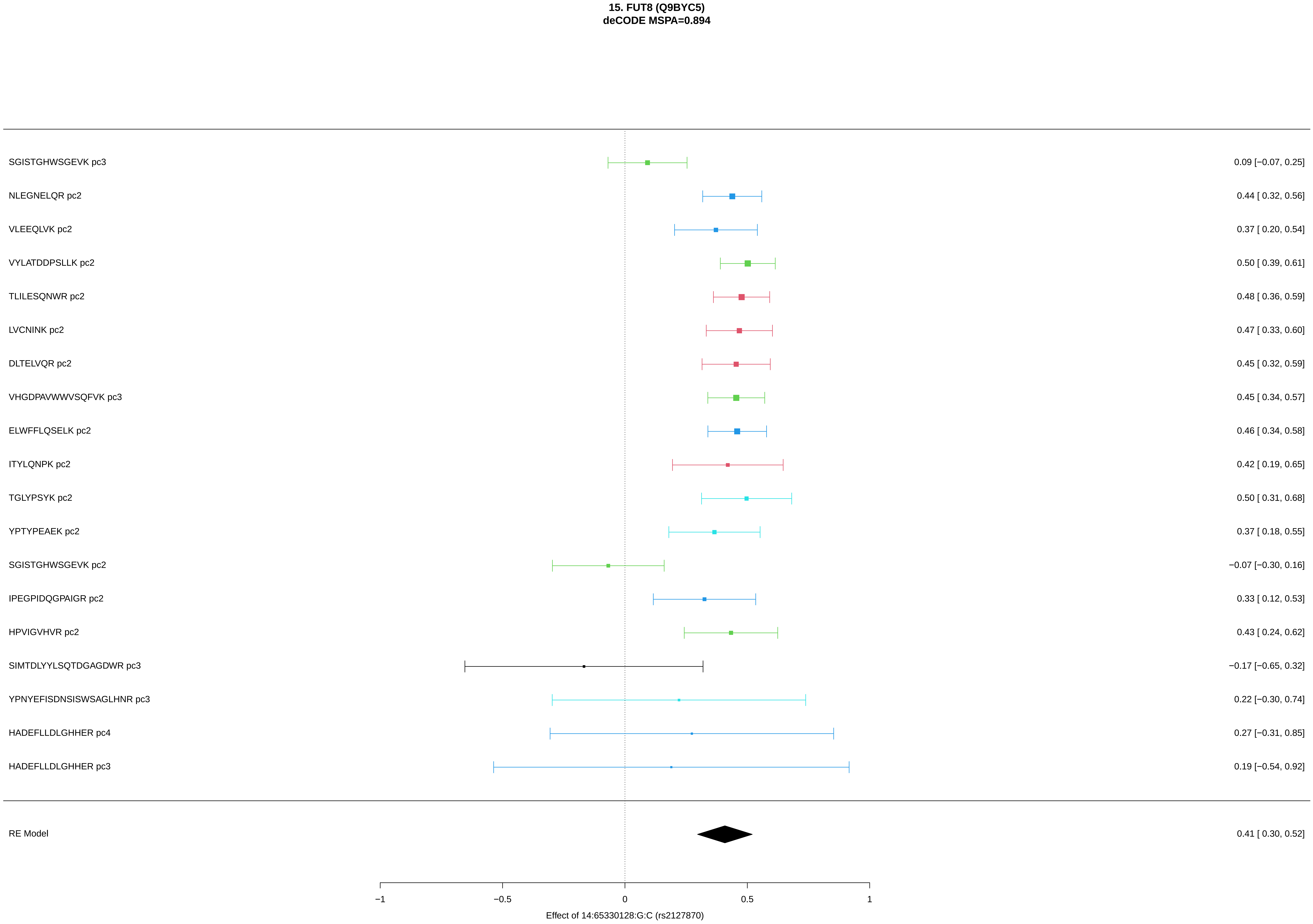
15. FUT8 (Q9BYC5)
deCODE MSPA=0.894
SGISTGHWSGEVK pc3
0.09 [−0.07, 0.25]
NLEGNELQR pc2
0.44 [0.32, 0.56]
VLEEQLVK pc2
0.37 [0.20, 0.54]
VYLATDDPSLLK pc2
0.50 [0.39, 0.61]
TLILESQNWR pc2
0.48 [0.36, 0.59]
LVCNINK pc2
0.47 [0.33, 0.60]
DLTELVQR pc2
0.45 [0.32, 0.59]
VHGDPAVWWVSQFVK pc3
0.45 [0.34, 0.57]
ELWFFLQSELK pc2
0.46 [0.34, 0.58]
ITYLQNPK pc2
0.42 [0.19, 0.65]
TGLYPSYK pc2
0.50 [0.31, 0.68]
YPTYPEAEK pc2
0.37 [0.18, 0.55]
SGISTGHWSGEVK pc2
−0.07 [−0.30, 0.16]
IPEGPIDQGPAIGR pc2
0.33 [0.12, 0.53]
HPVIGVHVR pc2
0.43 [0.24, 0.62]
SIMTDLYYLSQTDGAGDWR pc3
−0.17 [−0.65, 0.32]
YPNYEFISDNSISWSAGLHNR pc3
0.22 [−0.30, 0.74]
HADEFLLDLGHHER pc4
0.27 [−0.31, 0.85]
HADEFLLDLGHHER pc3
0.19 [−0.54, 0.92]
RE Model
0.41 [0.30, 0.52]
−1
−0.5
0
0.5
1
Effect of 14:65330128:G:C (rs2127870)

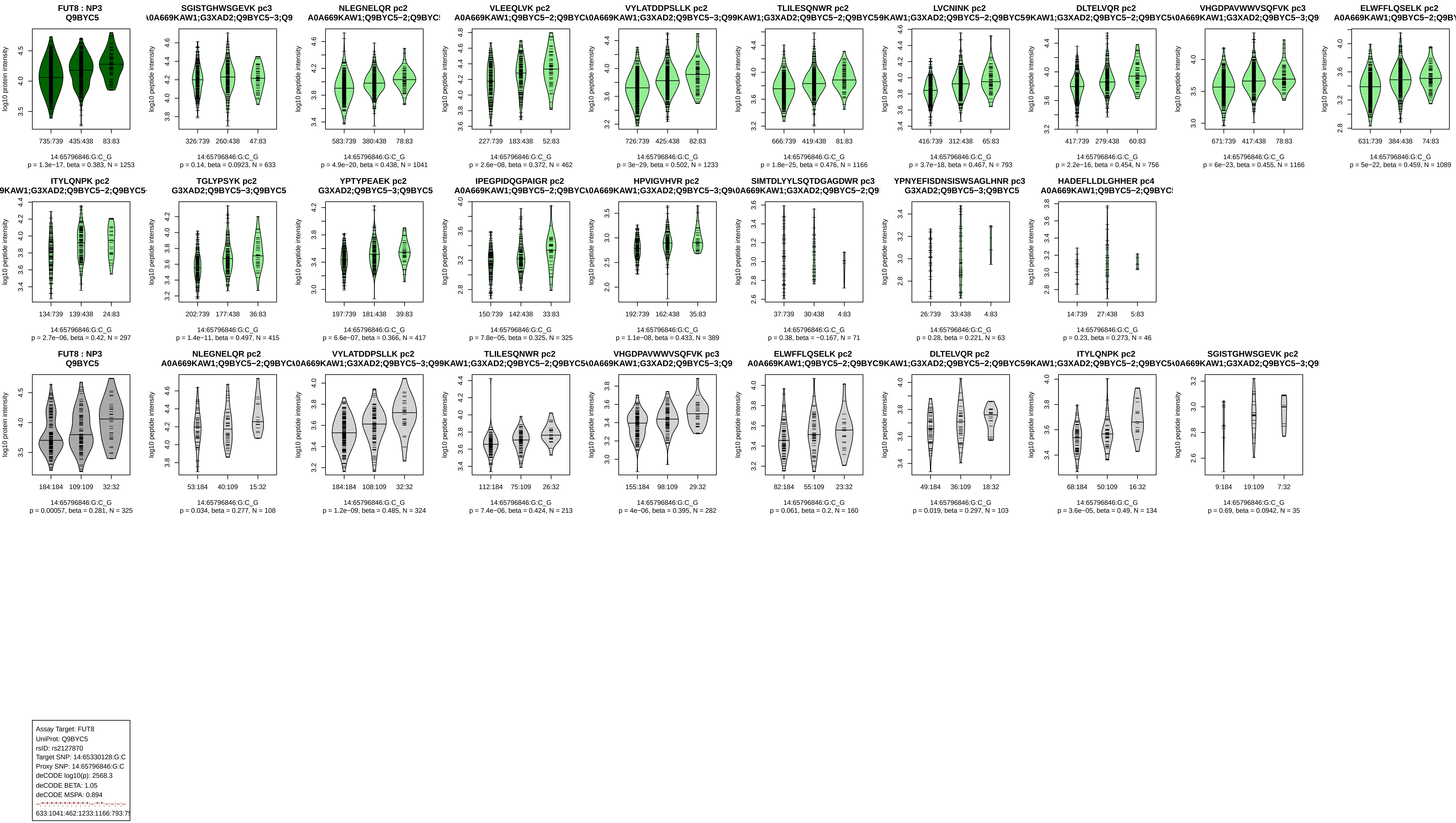
FUT8 : NP3
Q9BYC5
log10 protein intensity
log10 peptide intensity
SGISTGHWSGEVK pc3
NLEGNELQR pc2
VLEEQLVK pc2
VYLATDDPSLLK pc2
TLILESQNWR pc2
LVCNINK pc2
DLTELVQR pc2
VHGDPAVWWVSQFVK pc3
ELWFFLQSELK pc2
14:65796846:G:C_G
p = 1.3e−17, beta = 0.383, N = 1253
p = 0.14, beta = 0.0923, N = 633
p = 4.9e−20, beta = 0.438, N = 1041
p = 2.6e−08, beta = 0.372, N = 462
p = 3e−29, beta = 0.502, N = 1233
p = 1.8e−25, beta = 0.476, N = 1166
p = 3.7e−18, beta = 0.467, N = 793
p = 2.2e−16, beta = 0.454, N = 756
p = 6e−23, beta = 0.455, N = 1166
p = 5e−22, beta = 0.459, N = 1089
ITYLQNPK pc2
TGLYPSYK pc2
YPTYPEAEK pc2
IPEGPIDQGPAIGR pc2
HPVIGVHVR pc2
SIMTDLYYLSQTDGAGDWR pc3
YPNYEFISDNSISWSAGLHNR pc3
HADEFLLDLGHHER pc4
p = 2.7e−06, beta = 0.42, N = 297
p = 1.4e−11, beta = 0.497, N = 415
p = 6.6e−07, beta = 0.366, N = 417
p = 7.8e−05, beta = 0.325, N = 325
p = 1.1e−08, beta = 0.433, N = 389
p = 0.38, beta = −0.167, N = 71
p = 0.28, beta = 0.221, N = 63
p = 0.23, beta = 0.273, N = 46
FUT8 : NP3
Q9BYC5
NLEGNELQR pc2
VYLATDDPSLLK pc2
TLILESQNWR pc2
VHGDPAVWWVSQFVK pc3
ELWFFLQSELK pc2
DLTELVQR pc2
ITYLQNPK pc2
SGISTGHWSGEVK pc2
p = 0.00057, beta = 0.281, N = 325
p = 0.034, beta = 0.277, N = 108
p = 1.2e−09, beta = 0.485, N = 324
p = 7.4e−06, beta = 0.424, N = 213
p = 4e−06, beta = 0.395, N = 282
p = 0.061, beta = 0.2, N = 160
p = 0.019, beta = 0.297, N = 103
p = 3.6e−05, beta = 0.49, N = 134
p = 0.69, beta = 0.0942, N = 35
Assay Target: FUT8
UniProt: Q9BYC5
rsID: rs2127870
Target SNP: 14:65330128:G:C
Proxy SNP: 14:65796846:G:C
deCODE log10(p): 2568.3
deCODE BETA: 1.05
deCODE MSPA: 0.894

16. GSTO1 (P78417)
deCODE MSPA=0

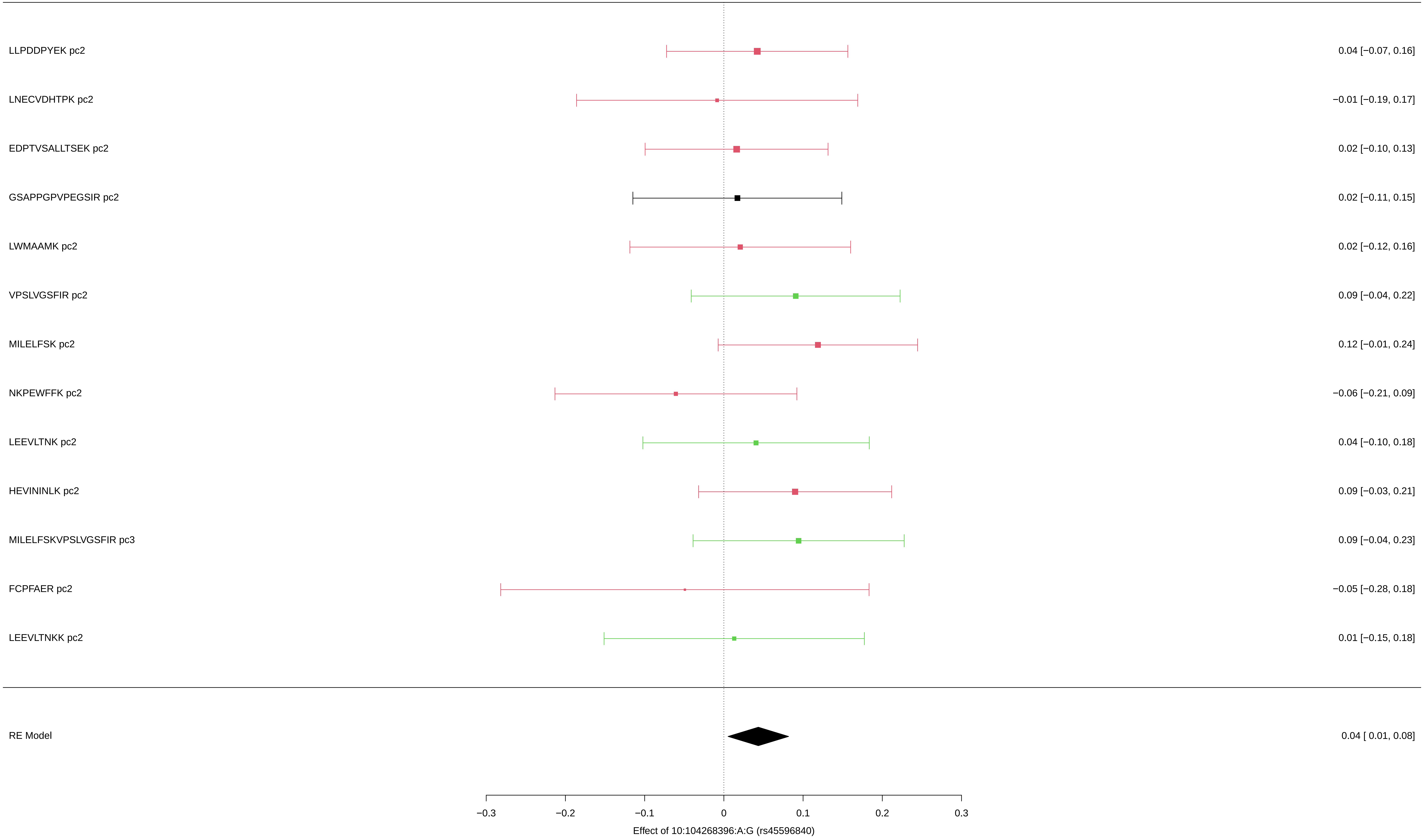

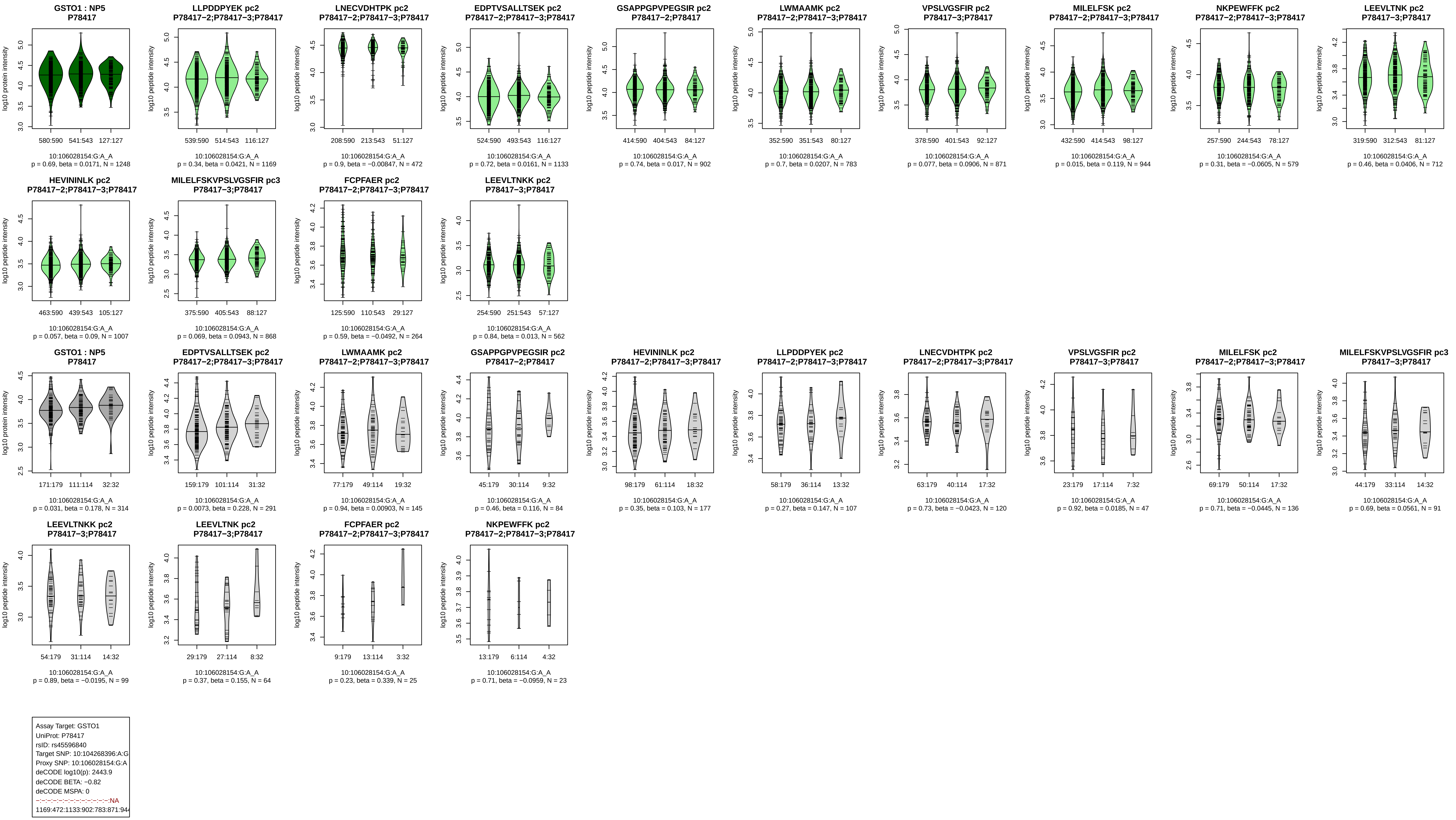
GSTO1 : NP5
P78417
580:590 541:543 127:127
10:106028154:G:A_A
p = 0.69, beta = 0.0171, N = 1248
LLPDDPYEK pc2
P78417−2;P78417−3;P78417
539:590 514:543 116:127
10:106028154:G:A_A
p = 0.34, beta = 0.0421, N = 1169
LNECVDHTPK pc2
P78417−2;P78417−3;P78417
208:590 213:543 51:127
10:106028154:G:A_A
p = 0.9, beta = −0.00847, N = 472
EDPTVSALLTSEK pc2
P78417−2;P78417−3;P78417
524:590 493:543 116:127
10:106028154:G:A_A
p = 0.72, beta = 0.0161, N = 1133
GSAPPGPVPEGSIR pc2
P78417−2;P78417
414:590 404:543 84:127
10:106028154:G:A_A
p = 0.74, beta = 0.017, N = 902
LWMAAMK pc2
P78417−2;P78417−3;P78417
352:590 351:543 80:127
10:106028154:G:A_A
p = 0.7, beta = 0.0207, N = 783
VPSLVGSFIR pc2
P78417−3;P78417
378:590 401:543 92:127
10:106028154:G:A_A
p = 0.077, beta = 0.0906, N = 871
MILELFSK pc2
P78417−2;P78417−3;P78417
432:590 414:543 98:127
10:106028154:G:A_A
p = 0.015, beta = 0.119, N = 944
NKPEWFFK pc2
P78417−2;P78417−3;P78417
257:590 244:543 78:127
10:106028154:G:A_A
p = 0.31, beta = −0.0605, N = 579
LEEVLTNK pc2
P78417−3;P78417
319:590 312:543 81:127
10:106028154:G:A_A
p = 0.46, beta = 0.0406, N = 712
HEVININLK pc2
P78417−2;P78417−3;P78417
463:590 439:543 105:127
10:106028154:G:A_A
p = 0.057, beta = 0.09, N = 1007
MILELFSKVPSLVGSFIR pc3
P78417−3;P78417
375:590 405:543 88:127
10:106028154:G:A_A
p = 0.069, beta = 0.0943, N = 868
FCPFAER pc2
P78417−2;P78417−3;P78417
125:590 110:543 29:127
10:106028154:G:A_A
p = 0.59, beta = −0.0492, N = 264
LEEVLTNKK pc2
P78417−3;P78417
254:590 251:543 57:127
10:106028154:G:A_A
p = 0.84, beta = 0.013, N = 562
GSTO1 : NP5
P78417
171:179 111:114 32:32
10:106028154:G:A_A
p = 0.031, beta = 0.178, N = 314
EDPTVSALLTSEK pc2
P78417−2;P78417−3;P78417
159:179 101:114 31:32
10:106028154:G:A_A
p = 0.0073, beta = 0.228, N = 291
LWMAAMK pc2
P78417−2;P78417−3;P78417
77:179 49:114 19:32
10:106028154:G:A_A
p = 0.94, beta = 0.00903, N = 145
GSAPPGPVPEGSIR pc2
P78417−2;P78417
45:179 30:114 9:32
10:106028154:G:A_A
p = 0.46, beta = 0.116, N = 84
HEVININLK pc2
P78417−2;P78417−3;P78417
98:179 61:114 18:32
10:106028154:G:A_A
p = 0.35, beta = 0.103, N = 177
LLPDDPYEK pc2
P78417−2;P78417−3;P78417
58:179 36:114 13:32
10:106028154:G:A_A
p = 0.27, beta = 0.147, N = 107
LNECVDHTPK pc2
P78417−2;P78417−3;P78417
63:179 40:114 17:32
10:106028154:G:A_A
p = 0.73, beta = −0.0423, N = 120
VPSLVGSFIR pc2
P78417−3;P78417
23:179 17:114 7:32
10:106028154:G:A_A
p = 0.92, beta = 0.0185, N = 47
MILELFSK pc2
P78417−2;P78417−3;P78417
69:179 50:114 17:32
10:106028154:G:A_A
p = 0.71, beta = −0.0445, N = 136
MILELFSKVPSLVGSFIR pc3
P78417−3;P78417
44:179 33:114 14:32
10:106028154:G:A_A
p = 0.69, beta = 0.0561, N = 91
LEEVLTNKK pc2
P78417−3;P78417
54:179 31:114 14:32
10:106028154:G:A_A
p = 0.89, beta = −0.0195, N = 99
LEEVLTNK pc2
P78417−3;P78417
29:179 27:114 8:32
10:106028154:G:A_A
p = 0.37, beta = 0.155, N = 64
FCPFAER pc2
P78417−2;P78417−3;P78417
9:179 13:114 3:32
10:106028154:G:A_A
p = 0.23, beta = 0.339, N = 25
NKPEWFFK pc2
P78417−2;P78417−3;P78417
13:179 6:114 4:32
10:106028154:G:A_A
p = 0.71, beta = −0.0959, N = 23
log10 protein intensity
log10 peptide intensity
Assay Target: GSTO1
UniProt: P78417
rsID: rs45596840
Target SNP: 10:104268396:A:G
Proxy SNP: 10:106028154:G:A
deCODE log10(p): 2443.9
deCODE BETA: −0.82
deCODE MSPA: 0
−:−:−:−:−:−:−:−:−:−:−:−:−:−:NA
1169:472:1133:902:783:871:94

17. HRG (P04196)
deCODE MSPA=0.064

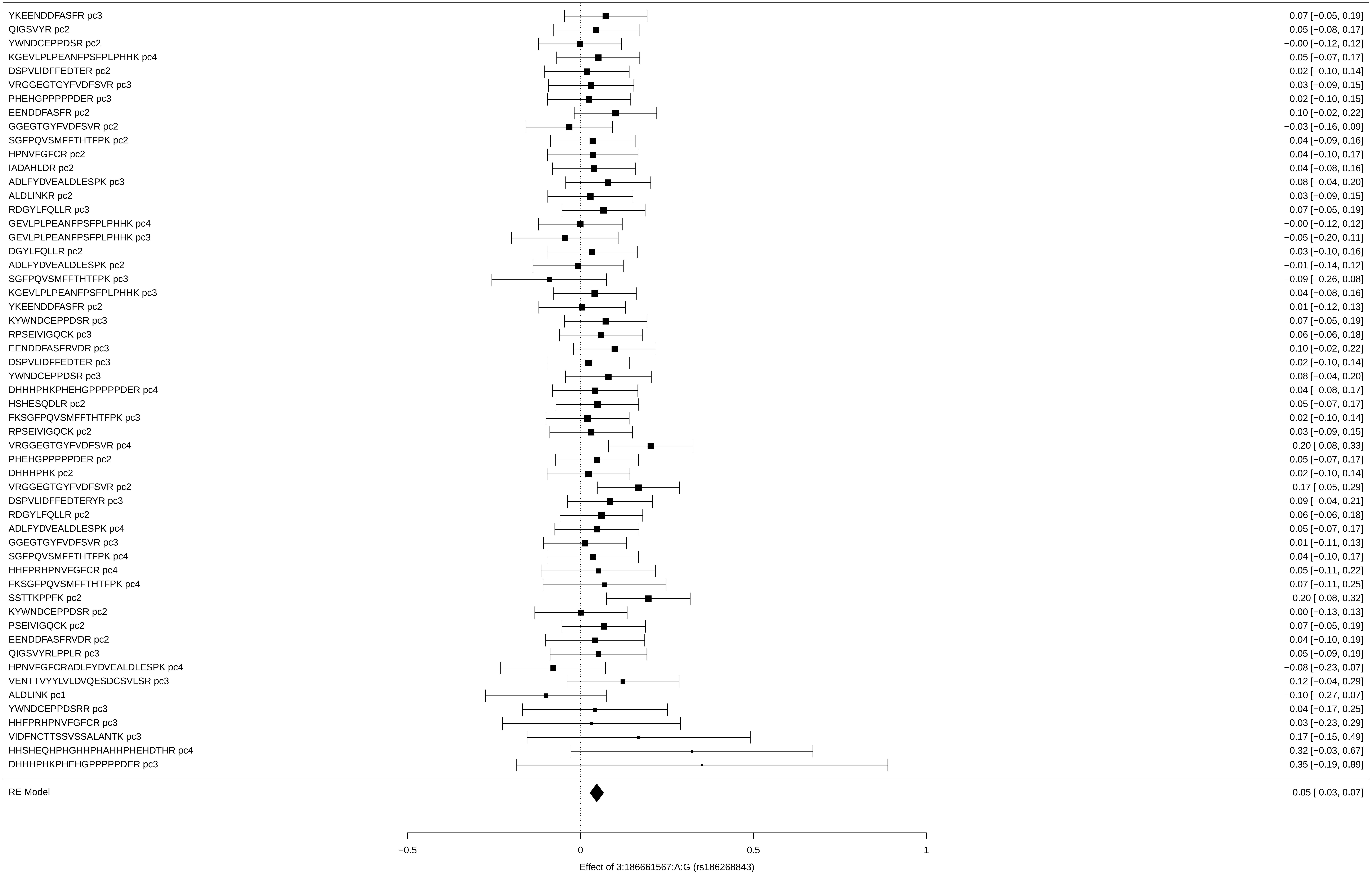

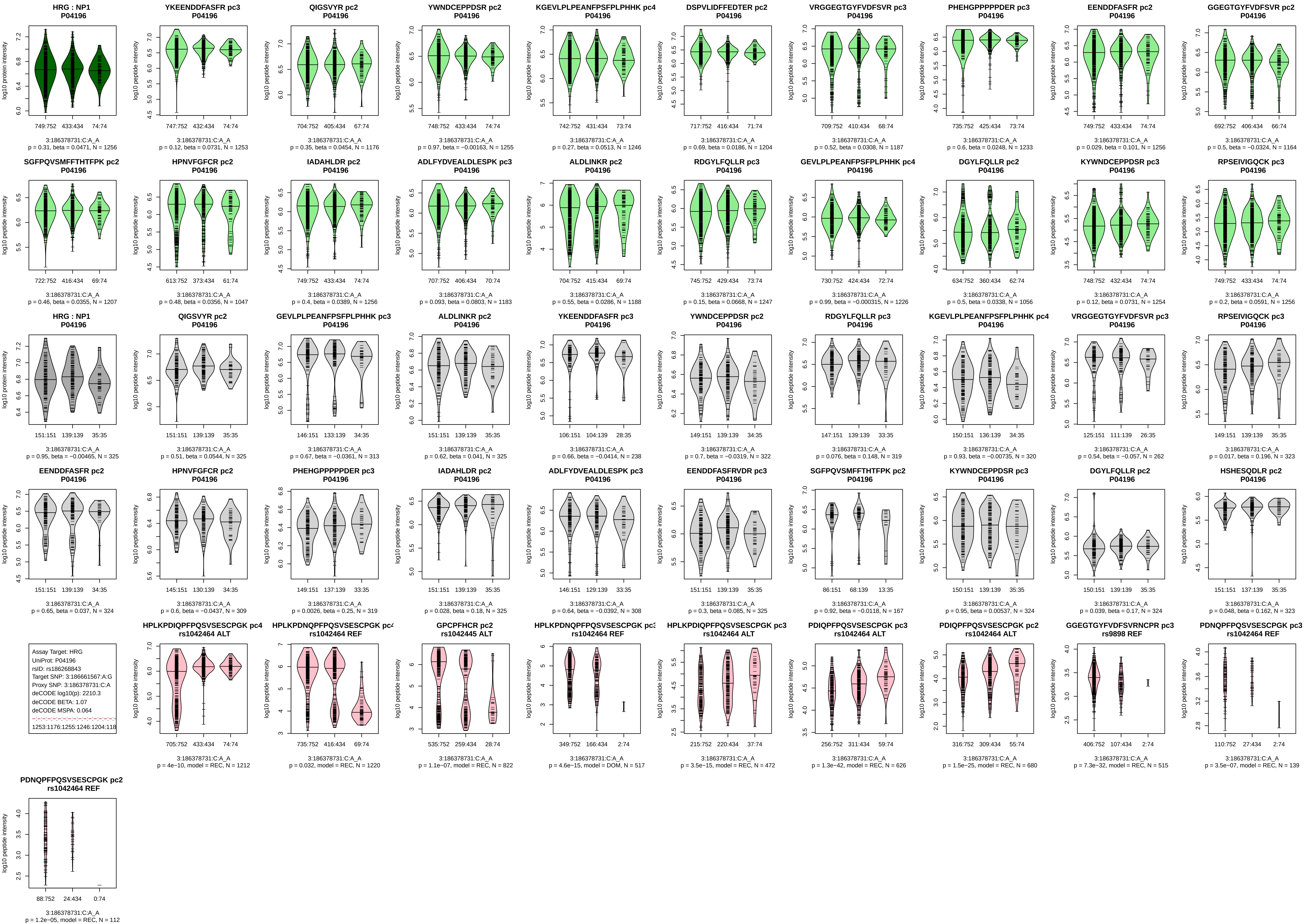
HRG : NP1
P04196
log10 protein intensity
749:752 433:434 74:74
3:186378731:C:A_A
p = 0.31, beta = 0.0471, N = 1256
YKEENDDFASFR pc3
P04196
log10 peptide intensity
747:752 432:434 74:74
3:186378731:C:A_A
p = 0.12, beta = 0.0731, N = 1253
QIGSVYR pc2
P04196
704:752 405:434 67:74
3:186378731:C:A_A
p = 0.35, beta = 0.0454, N = 1176
YWNDCEPPDSR pc2
P04196
748:752 433:434 74:74
3:186378731:C:A_A
p = 0.97, beta = −0.00163, N = 1255
KGEVLPLPEANFPSFPLPHHK pc4
P04196
742:752 431:434 73:74
3:186378731:C:A_A
p = 0.27, beta = 0.0513, N = 1246
DSPVLIDFFEDTER pc2
P04196
717:752 416:434 71:74
3:186378731:C:A_A
p = 0.69, beta = 0.0186, N = 1204
VRGGEGTGYFVDFSVR pc3
P04196
709:752 410:434 68:74
3:186378731:C:A_A
p = 0.52, beta = 0.0308, N = 1187
PHEHGPPPPPDER pc3
P04196
735:752 425:434 73:74
3:186378731:C:A_A
p = 0.6, beta = 0.0248, N = 1233
EENDDFASFR pc2
P04196
749:752 433:434 74:74
3:186378731:C:A_A
p = 0.029, beta = 0.101, N = 1256
GGEGTGYFVDFSVR pc2
P04196
692:752 406:434 66:74
3:186378731:C:A_A
p = 0.5, beta = −0.0324, N = 1164
SGFPQVSMFFTHTFPK pc2
P04196
722:752 416:434 69:74
3:186378731:C:A_A
p = 0.46, beta = 0.0355, N = 1207
HPNVFGFCR pc2
P04196
613:752 373:434 61:74
3:186378731:C:A_A
p = 0.48, beta = 0.0356, N = 1047
IADAHLDR pc2
P04196
749:752 433:434 74:74
3:186378731:C:A_A
p = 0.4, beta = 0.0389, N = 1256
ADLFYDVEALDLESPK pc3
P04196
707:752 406:434 70:74
3:186378731:C:A_A
p = 0.093, beta = 0.0803, N = 1183
ALDLINKR pc2
P04196
704:752 415:434 69:74
3:186378731:C:A_A
p = 0.55, beta = 0.0286, N = 1188
RDGYLFQLLR pc3
P04196
745:752 429:434 73:74
3:186378731:C:A_A
p = 0.15, beta = 0.0668, N = 1247
GEVLPLPEANFPSFPLPHHK pc4
P04196
730:752 424:434 72:74
3:186378731:C:A_A
p = 0.99, beta = −0.000315, N = 1226
DGYLFQLLR pc2
P04196
634:752 360:434 62:74
3:186378731:C:A_A
p = 0.5, beta = 0.0338, N = 1056
KYWNDCEPPDSR pc3
P04196
748:752 432:434 74:74
3:186378731:C:A_A
p = 0.12, beta = 0.0731, N = 1254
RPSEIVIGQCK pc3
P04196
749:752 433:434 74:74
3:186378731:C:A_A
p = 0.2, beta = 0.0591, N = 1256
HRG : NP1
P04196
151:151 139:139 35:35
3:186378731:C:A_A
p = 0.95, beta = −0.00465, N = 325
QIGSVYR pc2
P04196
151:151 139:139 35:35
3:186378731:C:A_A
p = 0.51, beta = 0.0544, N = 325
GEVLPLPEANFPSFPLPHHK pc3
P04196
146:151 133:139 34:35
3:186378731:C:A_A
p = 0.67, beta = −0.0361, N = 313
ALDLINKR pc2
P04196
151:151 139:139 35:35
3:186378731:C:A_A
p = 0.62, beta = 0.041, N = 325
YKEENDDFASFR pc3
P04196
106:151 104:139 28:35
3:186378731:C:A_A
p = 0.66, beta = −0.0414, N = 238
YWNDCEPPDSR pc2
P04196
149:151 139:139 34:35
3:186378731:C:A_A
p = 0.7, beta = −0.0319, N = 322
RDGYLFQLLR pc3
P04196
147:151 139:139 33:35
3:186378731:C:A_A
p = 0.076, beta = 0.148, N = 319
KGEVLPLPEANFPSFPLPHHK pc4
P04196
150:151 136:139 34:35
3:186378731:C:A_A
p = 0.93, beta = −0.00735, N = 320
VRGGEGTGYFVDFSVR pc3
P04196
125:151 111:139 26:35
3:186378731:C:A_A
p = 0.54, beta = −0.057, N = 262
RPSEIVIGQCK pc3
P04196
149:151 139:139 35:35
3:186378731:C:A_A
p = 0.017, beta = 0.196, N = 323
EENDDFASFR pc2
P04196
151:151 139:139 34:35
3:186378731:C:A_A
p = 0.65, beta = 0.037, N = 324
HPNVFGFCR pc2
P04196
145:151 130:139 34:35
3:186378731:C:A_A
p = 0.6, beta = −0.0437, N = 309
PHEHGPPPPPDER pc3
P04196
149:151 137:139 33:35
3:186378731:C:A_A
p = 0.0026, beta = 0.25, N = 319
IADAHLDR pc2
P04196
151:151 139:139 35:35
3:186378731:C:A_A
p = 0.028, beta = 0.18, N = 325
ADLFYDVEALDLESPK pc3
P04196
146:151 129:139 33:35
3:186378731:C:A_A
p = 0.64, beta = −0.0392, N = 308
EENDDFASFRVDR pc3
P04196
151:151 139:139 35:35
3:186378731:C:A_A
p = 0.3, beta = 0.085, N = 325
SGFPQVSMFFTHTFPK pc2
P04196
86:151 68:139 13:35
3:186378731:C:A_A
p = 0.92, beta = −0.0118, N = 167
KYWNDCEPPDSR pc3
P04196
150:151 139:139 35:35
3:186378731:C:A_A
p = 0.95, beta = 0.00537, N = 324
DGYLFQLLR pc2
P04196
150:151 139:139 35:35
3:186378731:C:A_A
p = 0.039, beta = 0.17, N = 324
HSHESQDLR pc2
P04196
151:151 137:139 35:35
3:186378731:C:A_A
p = 0.048, beta = 0.162, N = 323
Assay Target: HRG
UniProt: P04196
rsID: rs186268843
Target SNP: 3:186661567:A:G
Proxy SNP: 3:186378731:C:A
deCODE log10(p): 2210.3
deCODE BETA: 1.07
deCODE MSPA: 0.064
1253:1176:1255:1246:1204:118
HPLKPDIQPFPQSVSESCPGK pc4
rs1042464 ALT
705:752 433:434 74:74
3:186378731:C:A_A
p = 4e−10, model = REC, N = 1212
HPLKPDNQPFPQSVSESCPGK pc4
rs1042464 REF
735:752 416:434 69:74
3:186378731:C:A_A
p = 0.032, model = REC, N = 1220
GPCPFHCR pc2
rs1042445 ALT
535:752 259:434 28:74
3:186378731:C:A_A
p = 1.1e−07, model = REC, N = 822
HPLKPDNQPFPQSVSESCPGK pc3
rs1042464 REF
349:752 166:434 2:74
3:186378731:C:A_A
p = 4.6e−15, model = DOM, N = 517
HPLKPDIQPFPQSVSESCPGK pc3
rs1042464 ALT
215:752 220:434 37:74
3:186378731:C:A_A
p = 3.5e−15, model = REC, N = 472
PDIQPFPQSVSESCPGK pc3
rs1042464 ALT
256:752 311:434 59:74
3:186378731:C:A_A
p = 1.3e−42, model = REC, N = 626
PDIQPFPQSVSESCPGK pc2
rs1042464 ALT
316:752 309:434 55:74
3:186378731:C:A_A
p = 1.5e−25, model = REC, N = 680
GGEGTGYFVDFSVRNCPR pc3
rs9898 REF
406:752 107:434 2:74
3:186378731:C:A_A
p = 7.3e−32, model = REC, N = 515
PDNQPFPQSVSESCPGK pc3
rs1042464 REF
110:752 27:434 2:74
3:186378731:C:A_A
p = 3.5e−07, model = REC, N = 139
PDNQPFPQSVSESCPGK pc2
rs1042464 REF
88:752 24:434 0:74
3:186378731:C:A_A
p = 1.2e−05, model = REC, N = 112

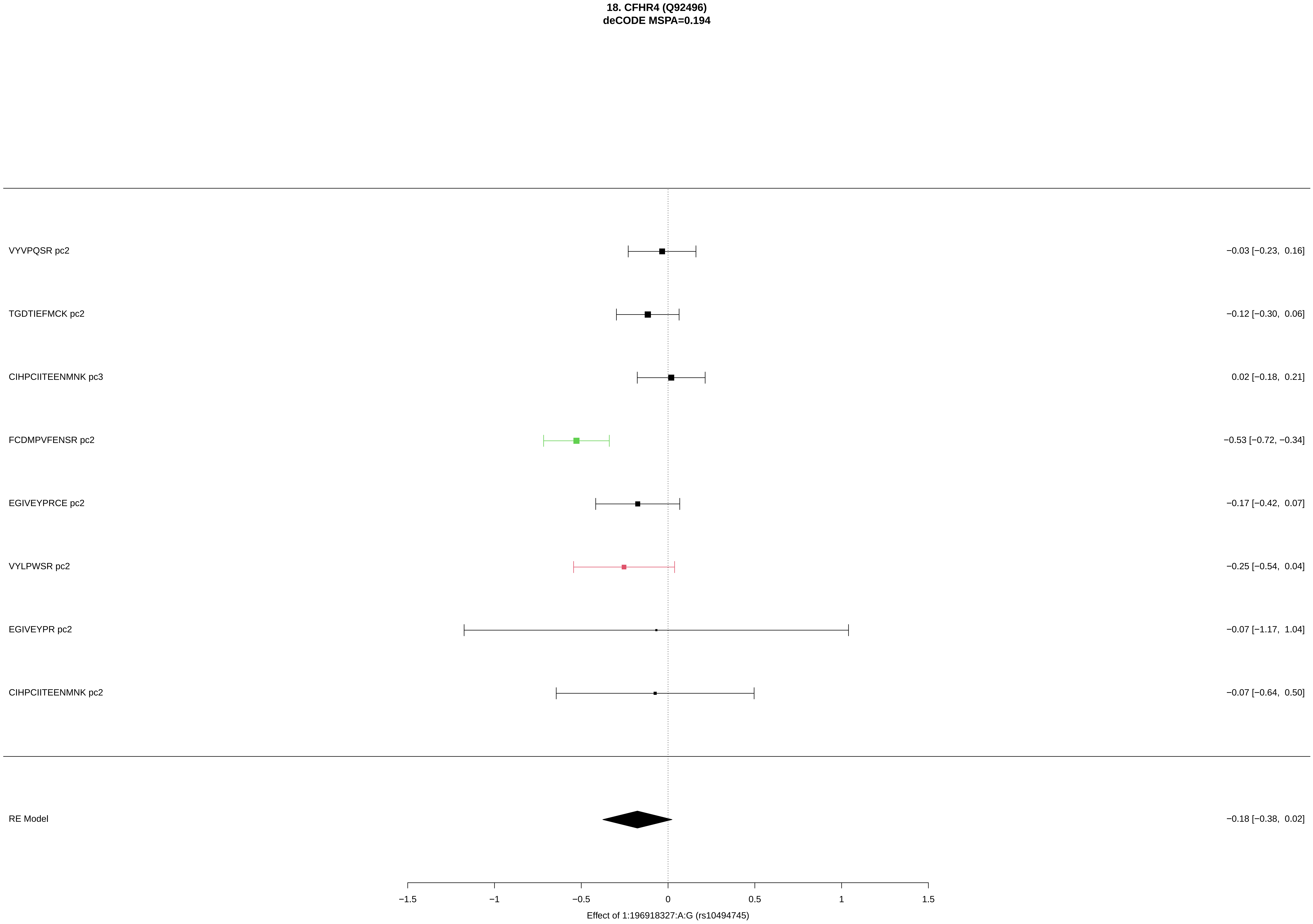
18. CFHR4 (Q92496)
deCODE MSPA=0.194
VYVPQSR pc2
−0.03 [−0.23, 0.16]
TGDTIEFMCK pc2
−0.12 [−0.30, 0.06]
CIHPCIITEENMNK pc3
0.02 [−0.18, 0.21]
FCDMPVFENSR pc2
−0.53 [−0.72, −0.34]
EGIVEYPRCE pc2
−0.17 [−0.42, 0.07]
VYLPWSR pc2
−0.25 [−0.54, 0.04]
EGIVEYPR pc2
−0.07 [−1.17, 1.04]
CIHPCIITEENMNK pc2
−0.07 [−0.64, 0.50]
RE Model
−0.18 [−0.38, 0.02]
−1.5
−1
−0.5
0
0.5
1
1.5
Effect of 1:196918327:A:G (rs10494745)

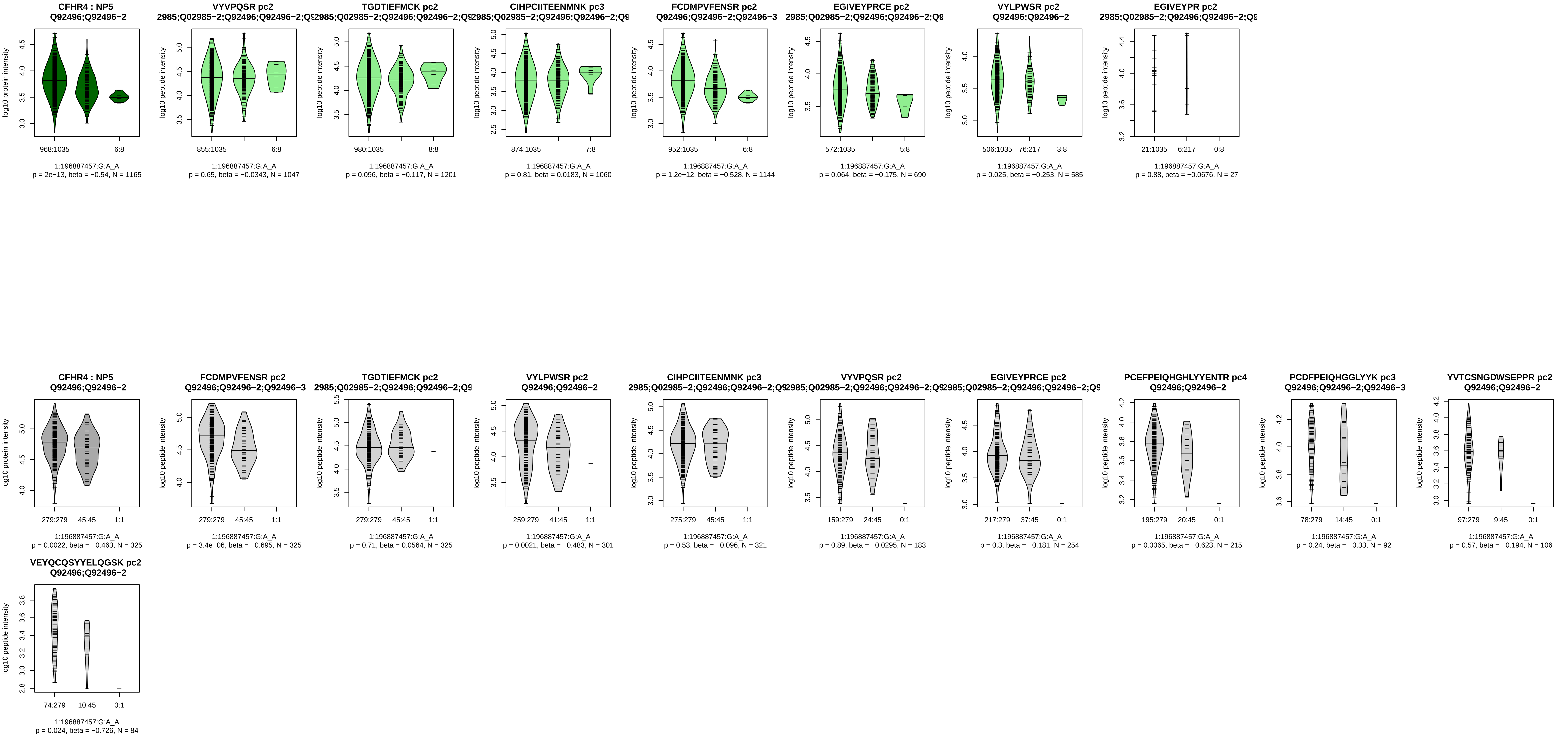

Assay Target: CFHR4
UniProt: Q92496
rsID: rs10494745
Target SNP: 1:196918327:A:G
Proxy SNP: 1:196887457:G:A
deCODE log10(p): 2098.5
deCODE BETA: −0.97
deCODE MSPA: 0.194
−:−:−:*:−:−:−:−
1047:1201:1060:1144:690:585:2

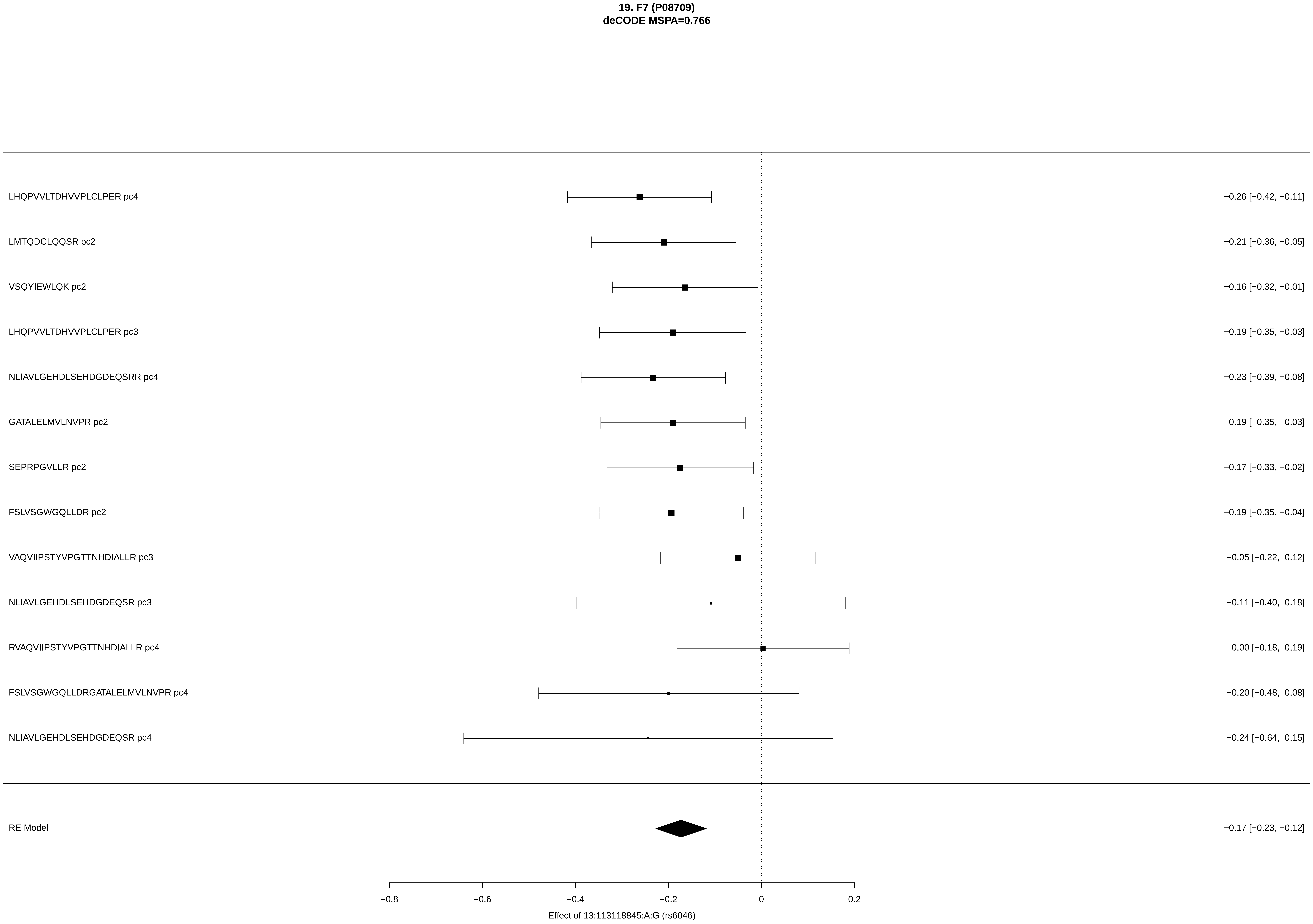
19. F7 (P08709)
deCODE MSPA=0.766
LHQPVVLTDHVVPLCLPER pc4
−0.26 [−0.42, −0.11]
LMTQDCLQQSR pc2
−0.21 [−0.36, −0.05]
VSQYIEWLQK pc2
−0.16 [−0.32, −0.01]
LHQPVVLTDHVVPLCLPER pc3
−0.19 [−0.35, −0.03]
NLIAVLGEHDLSEHDGDEQSRR pc4
−0.23 [−0.39, −0.08]
GATALELMVLNVPR pc2
−0.19 [−0.35, −0.03]
SEPRPGVLLR pc2
−0.17 [−0.33, −0.02]
FSLVSGWGQLLDR pc2
−0.19 [−0.35, −0.04]
VAQVIIPSTYVPGTTNHDIALLR pc3
−0.05 [−0.22, 0.12]
NLIAVLGEHDLSEHDGDEQSR pc3
−0.11 [−0.40, 0.18]
RVAQVIIPSTYVPGTTNHDIALLR pc4
0.00 [−0.18, 0.19]
FSLVSGWGQLLDRGATALELMVLNVPR pc4
−0.20 [−0.48, 0.08]
NLIAVLGEHDLSEHDGDEQSR pc4
−0.24 [−0.64, 0.15]
RE Model
−0.17 [−0.23, −0.12]
−0.8
−0.6
−0.4
−0.2
0
0.2
Effect of 13:113118845:A:G (rs6046)

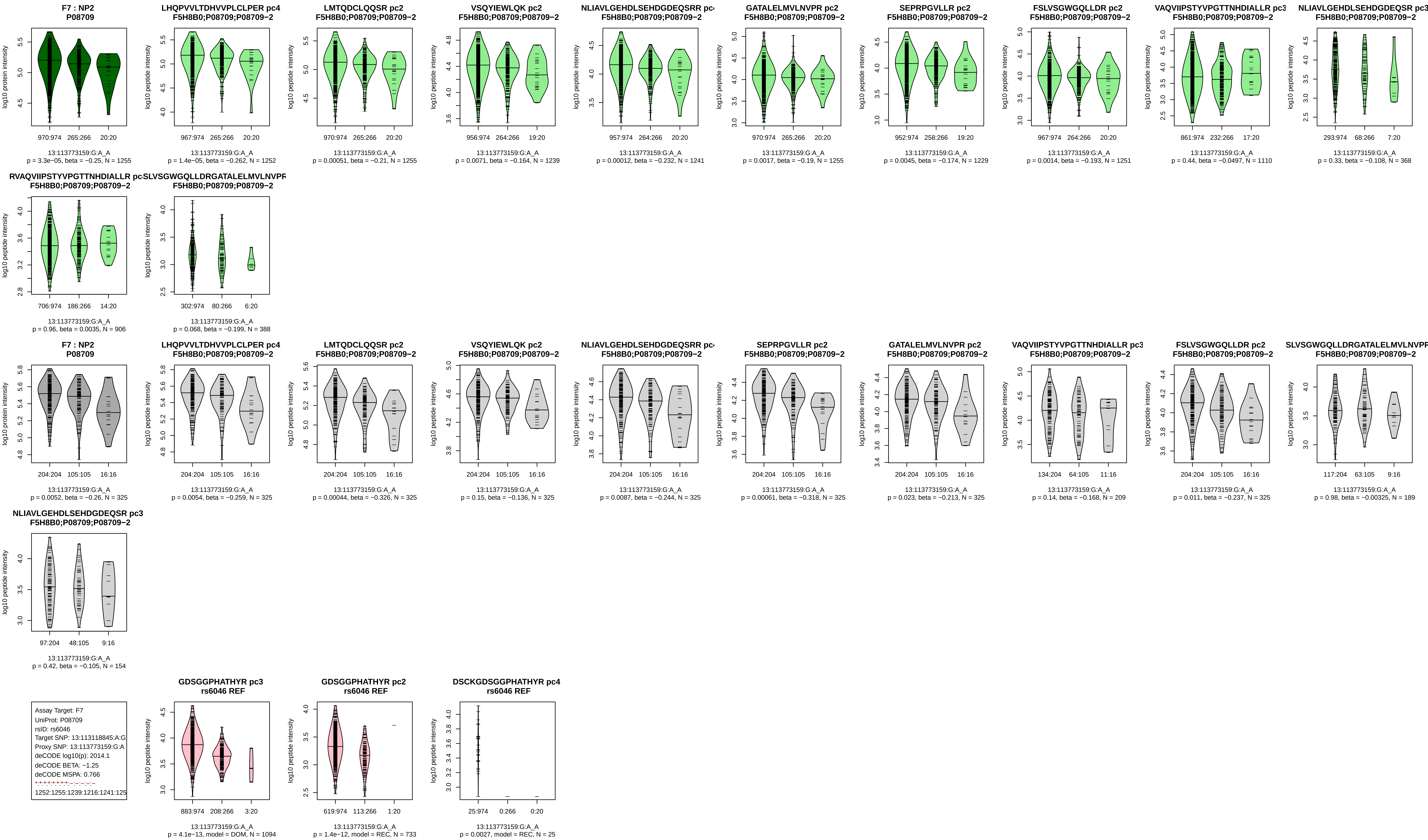
F7 : NP2
P08709
log10 protein intensity
970:974 265:266 20:20
13:113773159:G:A_A
p = 3.3e−05, beta = −0.25, N = 1255
LHQPVVLTDHVVPLCLPER pc4
F5H8B0;P08709;P08709−2
log10 peptide intensity
967:974 265:266 20:20
13:113773159:G:A_A
p = 1.4e−05, beta = −0.262, N = 1252
LMTQDCLQQSR pc2
F5H8B0;P08709;P08709−2
970:974 265:266 20:20
13:113773159:G:A_A
p = 0.00051, beta = −0.21, N = 1255
VSQYIEWLQK pc2
F5H8B0;P08709;P08709−2
956:974 264:266 19:20
13:113773159:G:A_A
p = 0.0071, beta = −0.164, N = 1239
NLIAVLGEHDLSEHDGDEQSRR pc
F5H8B0;P08709;P08709−2
957:974 264:266 20:20
13:113773159:G:A_A
p = 0.00012, beta = −0.232, N = 1241
GATALELMVLNVPR pc2
F5H8B0;P08709;P08709−2
970:974 265:266 20:20
13:113773159:G:A_A
p = 0.0017, beta = −0.19, N = 1255
SEPRPGVLLR pc2
F5H8B0;P08709;P08709−2
952:974 258:266 19:20
13:113773159:G:A_A
p = 0.0045, beta = −0.174, N = 1229
FSLVSGWGQLLDR pc2
F5H8B0;P08709;P08709−2
967:974 264:266 20:20
13:113773159:G:A_A
p = 0.0014, beta = −0.193, N = 1251
VAQVIIPSTYVPGTTNHDIALLR pc3
F5H8B0;P08709;P08709−2
861:974 232:266 17:20
13:113773159:G:A_A
p = 0.44, beta = −0.0497, N = 1110
NLIAVLGEHDLSEHDGDEQSR pc3
F5H8B0;P08709;P08709−2
293:974 68:266 7:20
13:113773159:G:A_A
p = 0.33, beta = −0.108, N = 368
RVAQVIIPSTYVPGTTNHDIALLR pc
F5H8B0;P08709;P08709−2
706:974 186:266 14:20
13:113773159:G:A_A
p = 0.96, beta = 0.0035, N = 906
SLVSGWGQLLDRGATALELMVLNVPR
F5H8B0;P08709;P08709−2
302:974 80:266 6:20
13:113773159:G:A_A
p = 0.068, beta = −0.199, N = 388
F7 : NP2
P08709
204:204 105:105 16:16
13:113773159:G:A_A
p = 0.0052, beta = −0.26, N = 325
LHQPVVLTDHVVPLCLPER pc4
F5H8B0;P08709;P08709−2
204:204 105:105 16:16
13:113773159:G:A_A
p = 0.0054, beta = −0.259, N = 325
LMTQDCLQQSR pc2
F5H8B0;P08709;P08709−2
204:204 105:105 16:16
13:113773159:G:A_A
p = 0.00044, beta = −0.326, N = 325
VSQYIEWLQK pc2
F5H8B0;P08709;P08709−2
204:204 105:105 16:16
13:113773159:G:A_A
p = 0.15, beta = −0.136, N = 325
NLIAVLGEHDLSEHDGDEQSRR pc
F5H8B0;P08709;P08709−2
204:204 105:105 16:16
13:113773159:G:A_A
p = 0.0087, beta = −0.244, N = 325
SEPRPGVLLR pc2
F5H8B0;P08709;P08709−2
204:204 105:105 16:16
13:113773159:G:A_A
p = 0.00061, beta = −0.318, N = 325
GATALELMVLNVPR pc2
F5H8B0;P08709;P08709−2
204:204 105:105 16:16
13:113773159:G:A_A
p = 0.023, beta = −0.213, N = 325
VAQVIIPSTYVPGTTNHDIALLR pc3
F5H8B0;P08709;P08709−2
134:204 64:105 11:16
13:113773159:G:A_A
p = 0.14, beta = −0.168, N = 209
FSLVSGWGQLLDR pc2
F5H8B0;P08709;P08709−2
204:204 105:105 16:16
13:113773159:G:A_A
p = 0.011, beta = −0.237, N = 325
SLVSGWGQLLDRGATALELMVLNVPR
F5H8B0;P08709;P08709−2
117:204 63:105 9:16
13:113773159:G:A_A
p = 0.98, beta = −0.00325, N = 189
NLIAVLGEHDLSEHDGDEQSR pc3
F5H8B0;P08709;P08709−2
97:204 48:105 9:16
13:113773159:G:A_A
p = 0.42, beta = −0.105, N = 154
Assay Target: F7
UniProt: P08709
rsID: rs6046
Target SNP: 13:113118845:A:G
Proxy SNP: 13:113773159:G:A
deCODE log10(p): 2014.1
deCODE BETA: −1.25
deCODE MSPA: 0.766
1252:1255:1239:1216:1241:125
GDSGGPHATHYR pc3
rs6046 REF
883:974 208:266 3:20
13:113773159:G:A_A
p = 4.1e−13, model = DOM, N = 1094
GDSGGPHATHYR pc2
rs6046 REF
619:974 113:266 1:20
13:113773159:G:A_A
p = 1.4e−12, model = REC, N = 733
DSCKGDSGGPHATHYR pc4
rs6046 REF
25:974 0:266 0:20
13:113773159:G:A_A
p = 0.0027, model = REC, N = 25

20. CTRB2 (Q6GPI1)
deCODE MSPA=0.445 (1 conflicts)

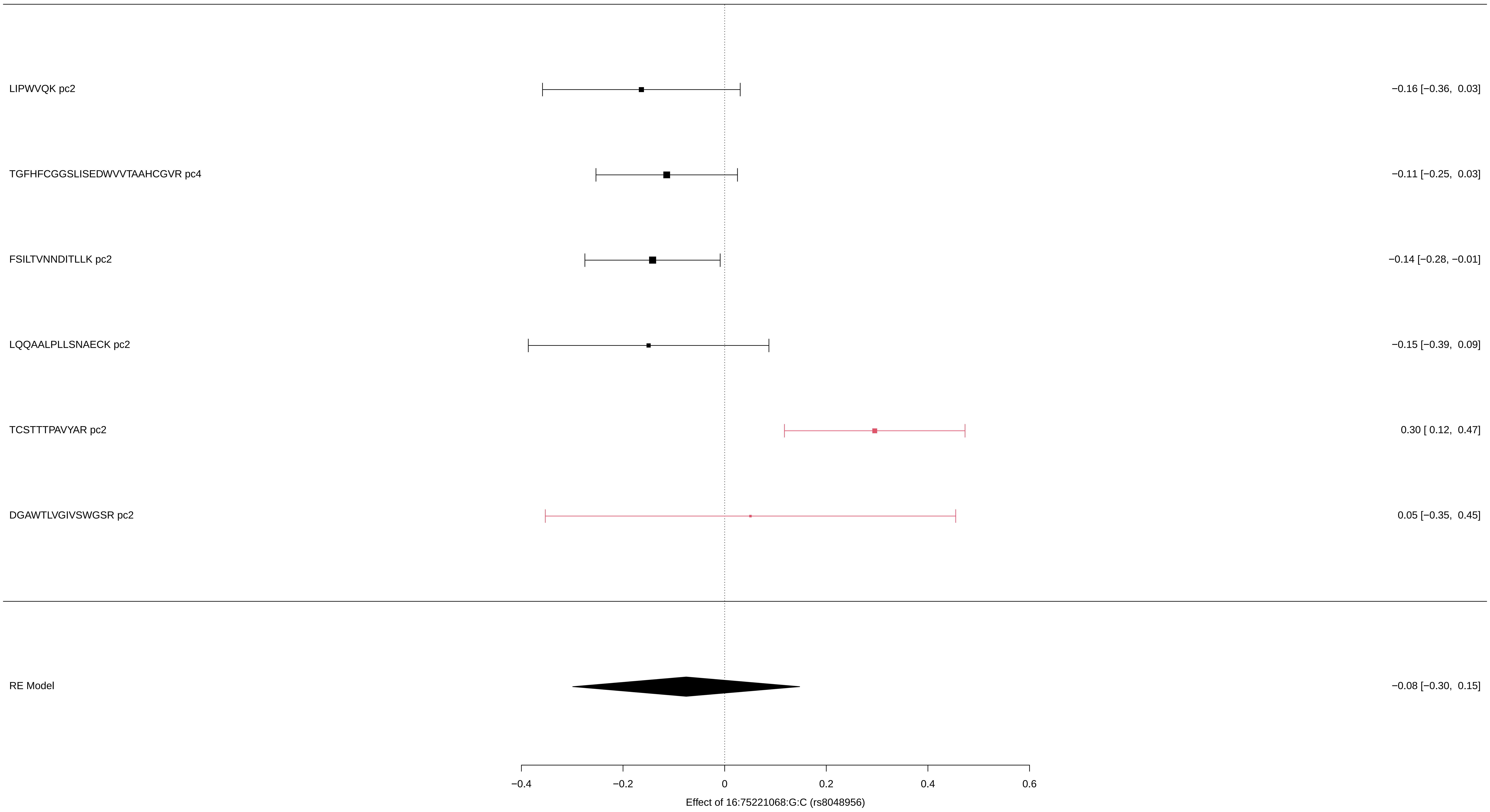

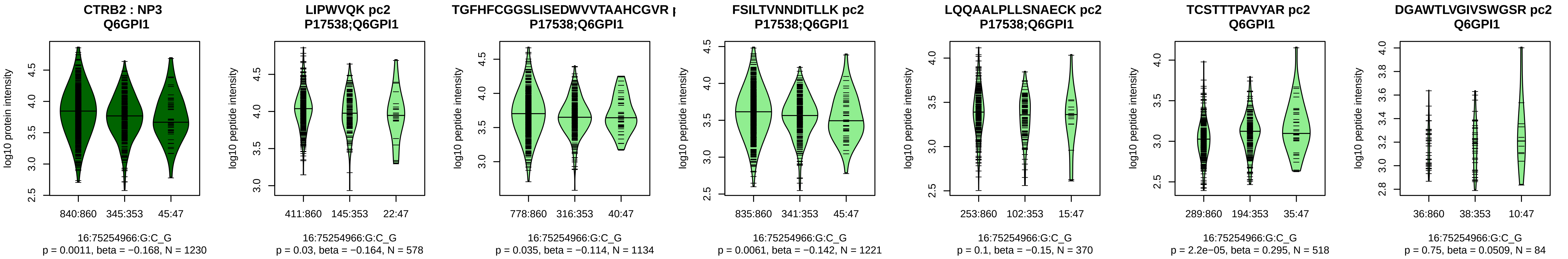

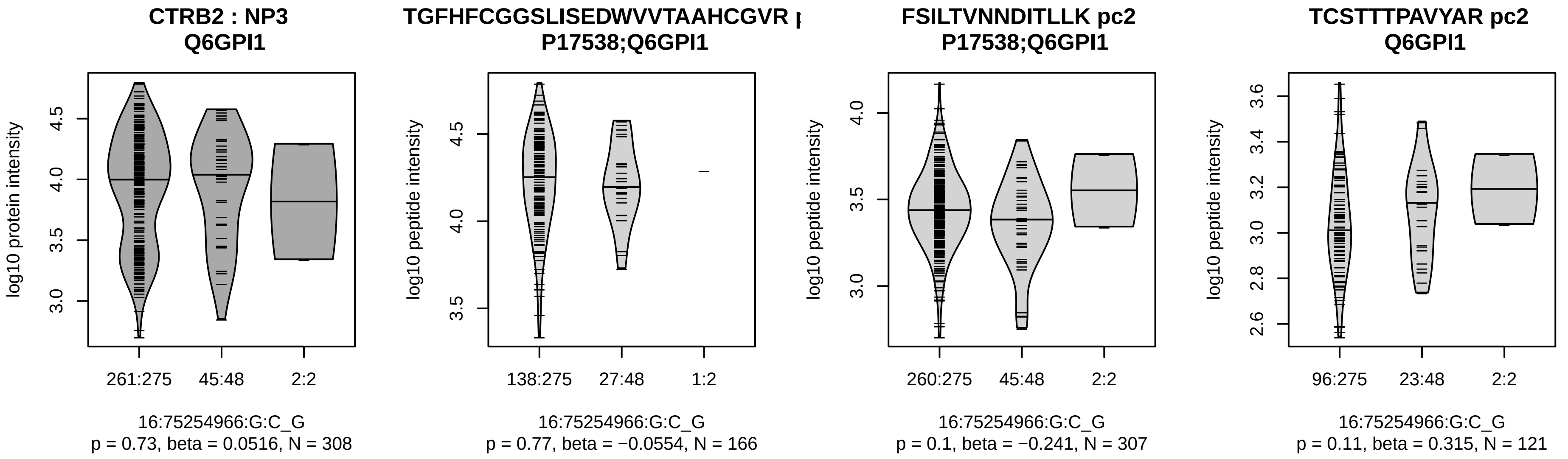

Assay Target: CTRB2
UniProt: Q6GPI1
rsID: rs8048956
Target SNP: 16:75221068:G:C
Proxy SNP: 16:75254966:G:C
deCODE log10(p): 1800.8
deCODE BETA: 0.86
deCODE MSPA: 0.445 (1)
–:–:*:–:*:–
578:1134:1221:370:518:84

21. HSP90B1 (P14625)
deCODE MSPA=0.973

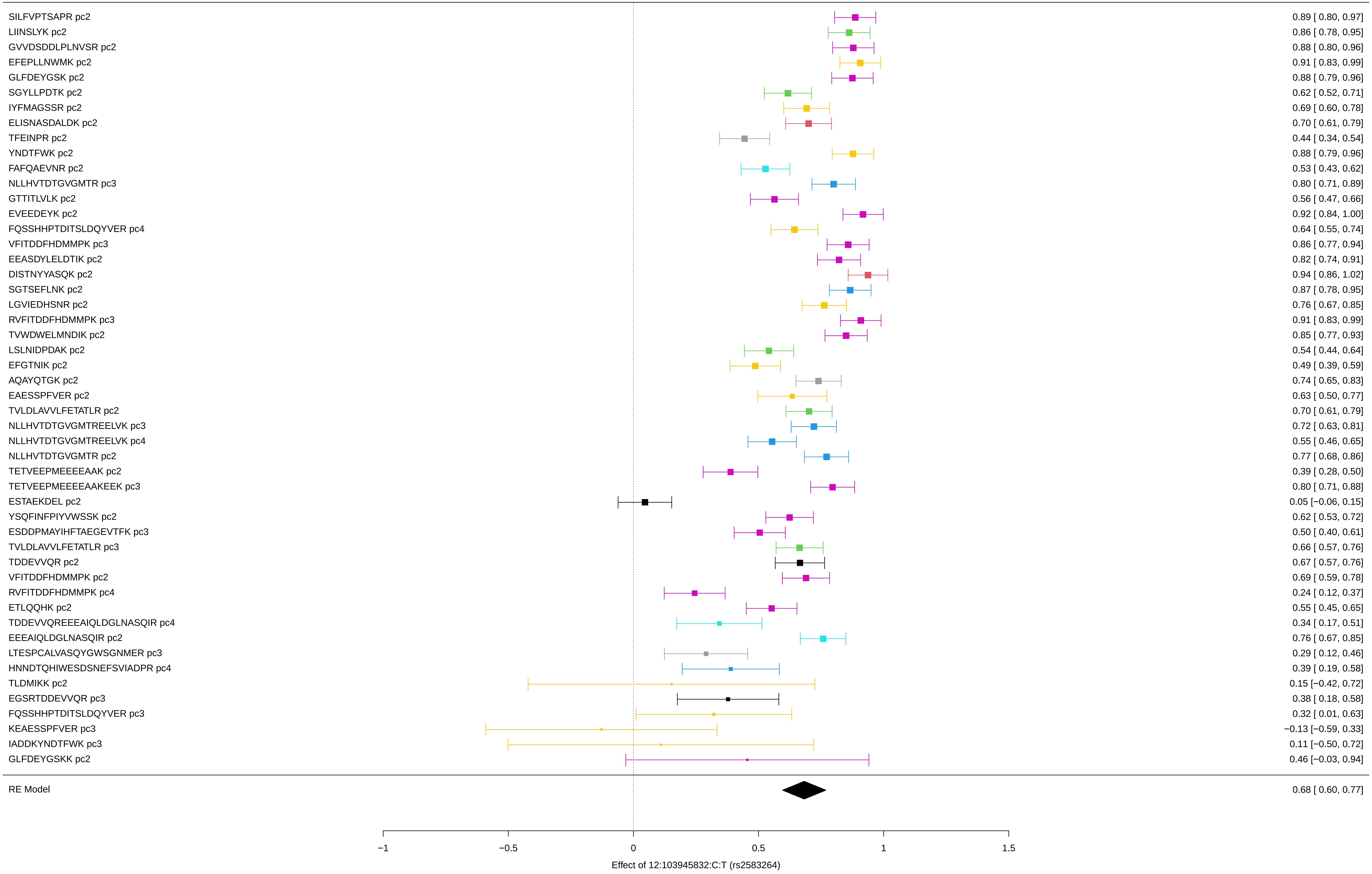

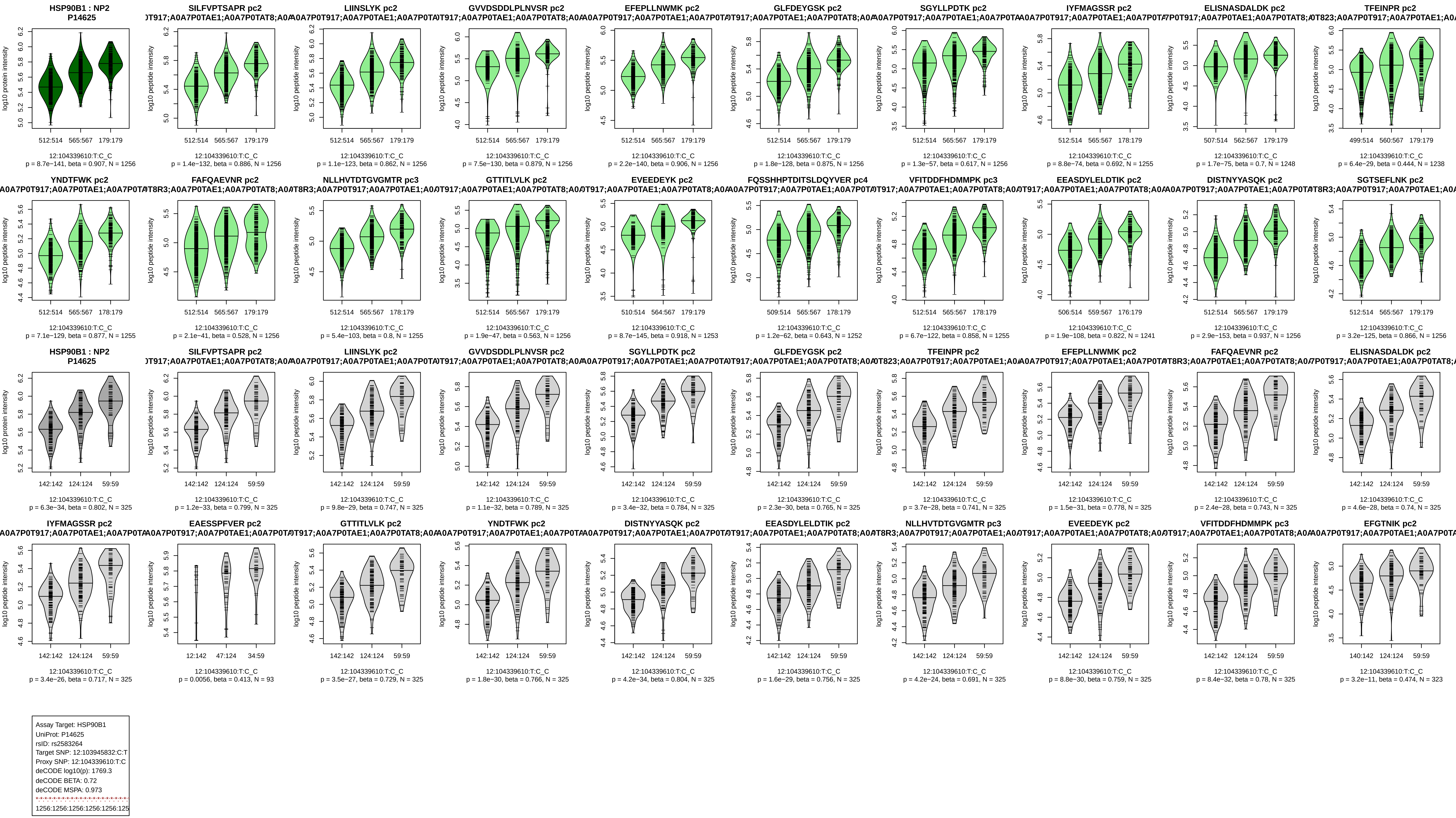
HSP90B1 : NP2
P14625
12:104339610:T:C_C
p = 8.7e−141, beta = 0.907, N = 1256
SILFVPTSAPR pc2
p = 1.4e−132, beta = 0.886, N = 1256
LIINSLYK pc2
p = 1.1e−123, beta = 0.862, N = 1256
GVVDSDDLPLNVSR pc2
p = 7.5e−130, beta = 0.879, N = 1256
EFEPLLNWMK pc2
p = 2.2e−140, beta = 0.906, N = 1256
GLFDEYGSK pc2
p = 1.8e−128, beta = 0.875, N = 1256
SGYLLPDTK pc2
p = 1.3e−57, beta = 0.617, N = 1256
IYFMAGSSR pc2
p = 8.8e−74, beta = 0.692, N = 1255
ELISNASDALDK pc2
p = 1.7e−75, beta = 0.7, N = 1248
TFEINPR pc2
p = 6.4e−29, beta = 0.444, N = 1238
YNDTFWK pc2
p = 7.1e−129, beta = 0.877, N = 1255
FAFQAEVNR pc2
p = 2.1e−41, beta = 0.528, N = 1256
NLLHVTDTGVGMTR pc3
p = 5.4e−103, beta = 0.8, N = 1255
GTTITLVLK pc2
p = 1.9e−47, beta = 0.563, N = 1256
EVEEDEYK pc2
p = 8.7e−145, beta = 0.918, N = 1253
FQSSHHPTDITSLDQYVER pc4
p = 1.2e−62, beta = 0.643, N = 1252
VFITDDFHDMMPK pc3
p = 6.7e−122, beta = 0.858, N = 1255
EEASDYLELDTIK pc2
p = 1.9e−108, beta = 0.822, N = 1241
DISTNYYASQK pc2
p = 2.9e−153, beta = 0.937, N = 1256
SGTSEFLNK pc2
p = 3.2e−125, beta = 0.866, N = 1256
HSP90B1 : NP2
P14625
p = 6.3e−34, beta = 0.802, N = 325
SILFVPTSAPR pc2
p = 1.2e−33, beta = 0.799, N = 325
LIINSLYK pc2
p = 9.8e−29, beta = 0.747, N = 325
GVVDSDDLPLNVSR pc2
p = 1.1e−32, beta = 0.789, N = 325
SGYLLPDTK pc2
p = 3.4e−32, beta = 0.784, N = 325
GLFDEYGSK pc2
p = 2.3e−30, beta = 0.765, N = 325
TFEINPR pc2
p = 3.7e−28, beta = 0.741, N = 325
EFEPLLNWMK pc2
p = 1.5e−31, beta = 0.778, N = 325
FAFQAEVNR pc2
p = 2.4e−28, beta = 0.743, N = 325
ELISNASDALDK pc2
p = 4.6e−28, beta = 0.74, N = 325
IYFMAGSSR pc2
p = 3.4e−26, beta = 0.717, N = 325
EAESSPFVER pc2
p = 0.0056, beta = 0.413, N = 93
GTTITLVLK pc2
p = 3.5e−27, beta = 0.729, N = 325
YNDTFWK pc2
p = 1.8e−30, beta = 0.766, N = 325
DISTNYYASQK pc2
p = 4.2e−34, beta = 0.804, N = 325
EEASDYLELDTIK pc2
p = 1.6e−29, beta = 0.756, N = 325
NLLHVTDTGVGMTR pc3
p = 4.2e−24, beta = 0.691, N = 325
EVEEDEYK pc2
p = 8.8e−30, beta = 0.759, N = 325
VFITDDFHDMMPK pc3
p = 8.4e−32, beta = 0.78, N = 325
EFGTNIK pc2
p = 3.2e−11, beta = 0.474, N = 323
log10 protein intensity
log10 peptide intensity
Assay Target: HSP90B1
UniProt: P14625
rsID: rs2583264
Target SNP: 12:103945832:C:T
Proxy SNP: 12:104339610:T:C
deCODE log10(p): 1769.3
deCODE BETA: 0.72
deCODE MSPA: 0.973
1256:1256:1256:1256:1256:125

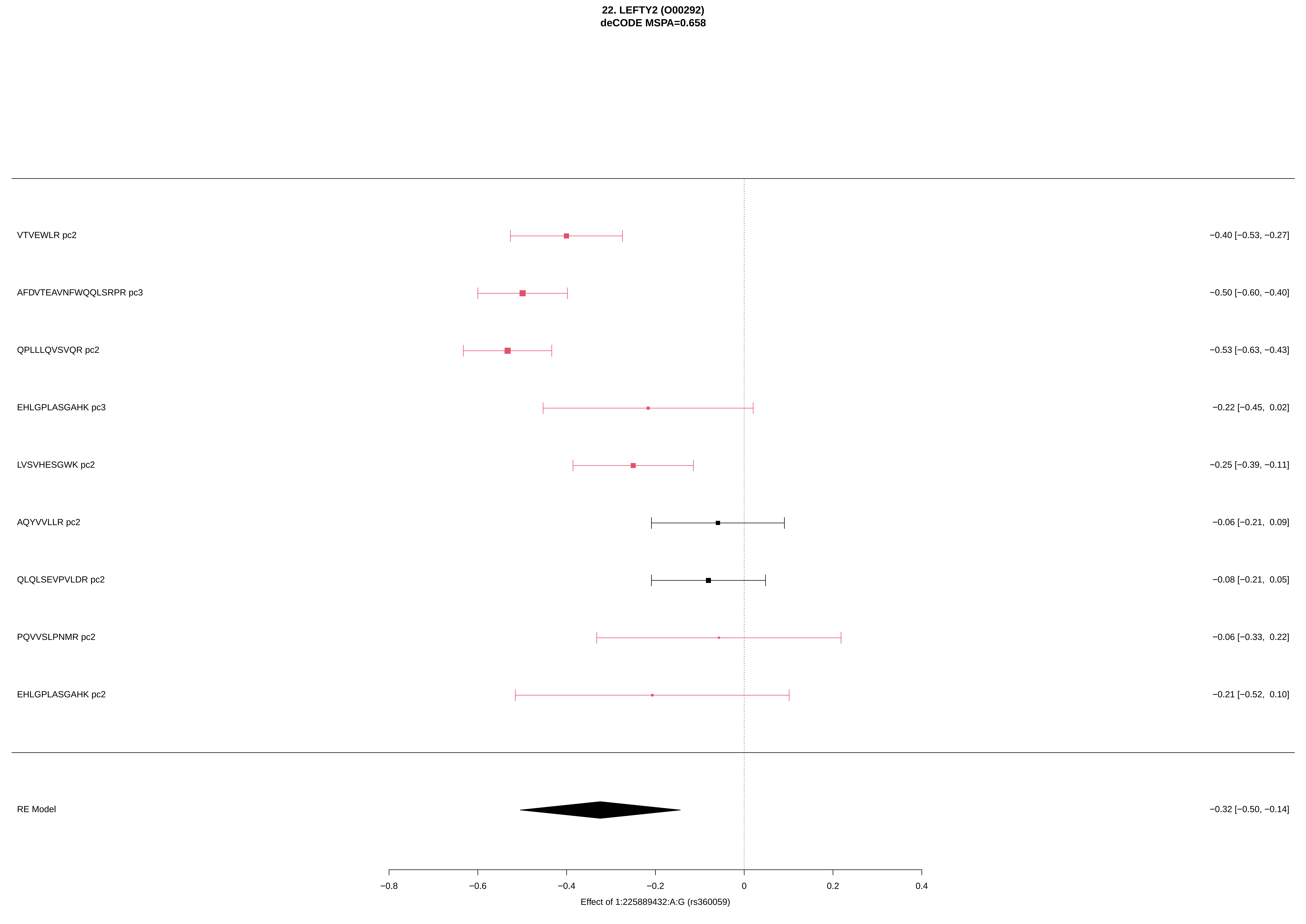
22. LEFTY2 (O00292)
deCODE MSPA=0.658
VTVEWLR pc2
−0.40 [−0.53, −0.27]
AFDVTEAVNFWQQLSRPR pc3
−0.50 [−0.60, −0.40]
QPLLLQVSVQR pc2
−0.53 [−0.63, −0.43]
EHLGPLASGAHK pc3
−0.22 [−0.45, 0.02]
LVSVHESGWK pc2
−0.25 [−0.39, −0.11]
AQYVVLLR pc2
−0.06 [−0.21, 0.09]
QLQLSEVPVLDR pc2
−0.08 [−0.21, 0.05]
PQVVSLPNMR pc2
−0.06 [−0.33, 0.22]
EHLGPLASGAHK pc2
−0.21 [−0.52, 0.10]
RE Model
−0.32 [−0.50, −0.14]
−0.8
−0.6
−0.4
−0.2
0
0.2
0.4
Effect of 1:225889432:A:G (rs360059)

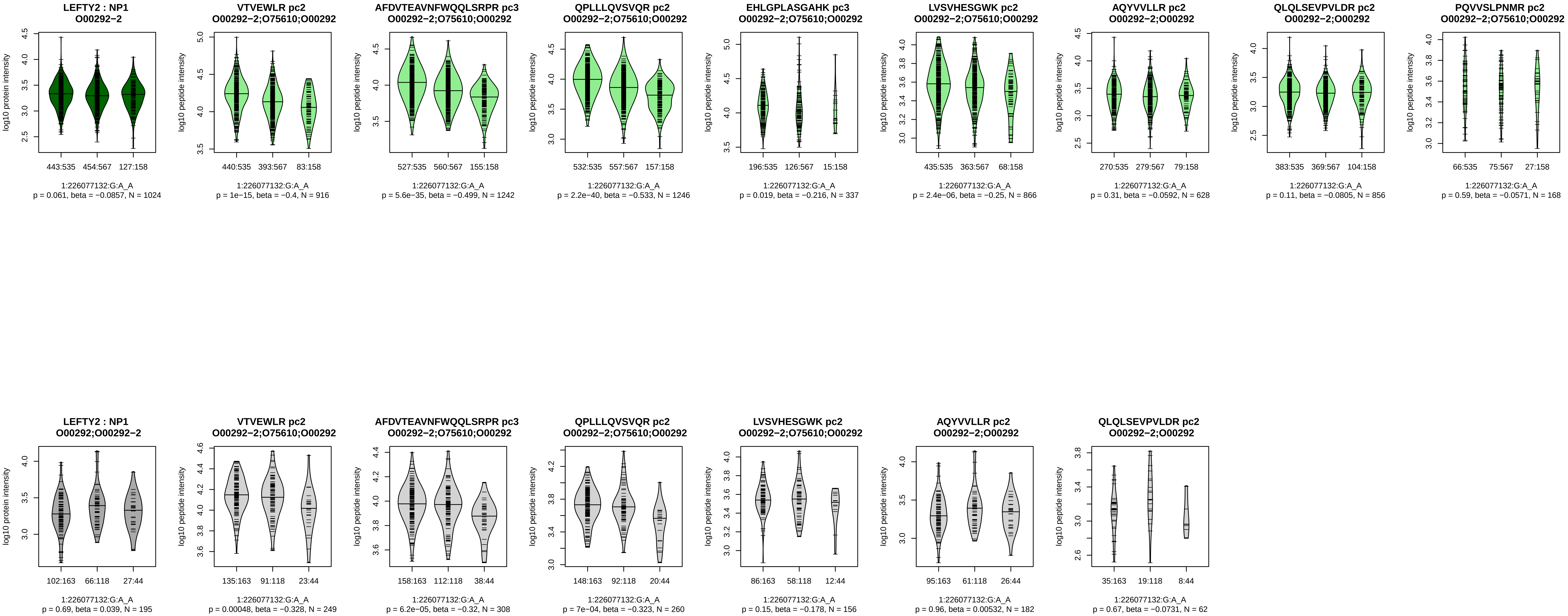

Assay Target: LEFTY2
UniProt: O00292
rsID: rs360059
Target SNP: 1:225889432:A:G
Proxy SNP: 1:226077132:G:A
deCODE log10(p): 1700
deCODE BETA: −0.71
deCODE MSPA: 0.658
::*:–:*:–:–:–:–
916:1242:1246:337:866:628:856

23. ECM1 (Q16610)
deCODE MSPA=0.77

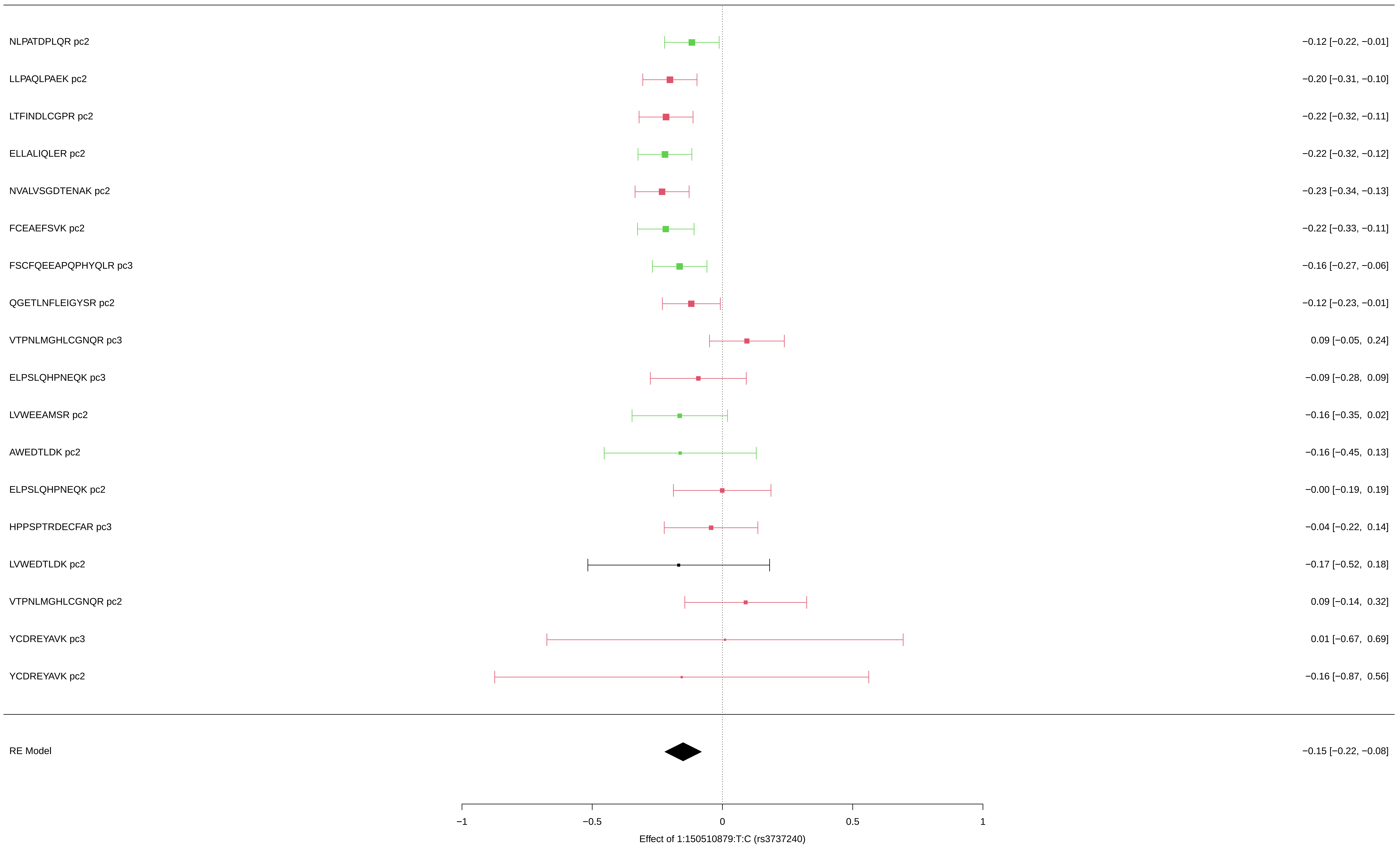

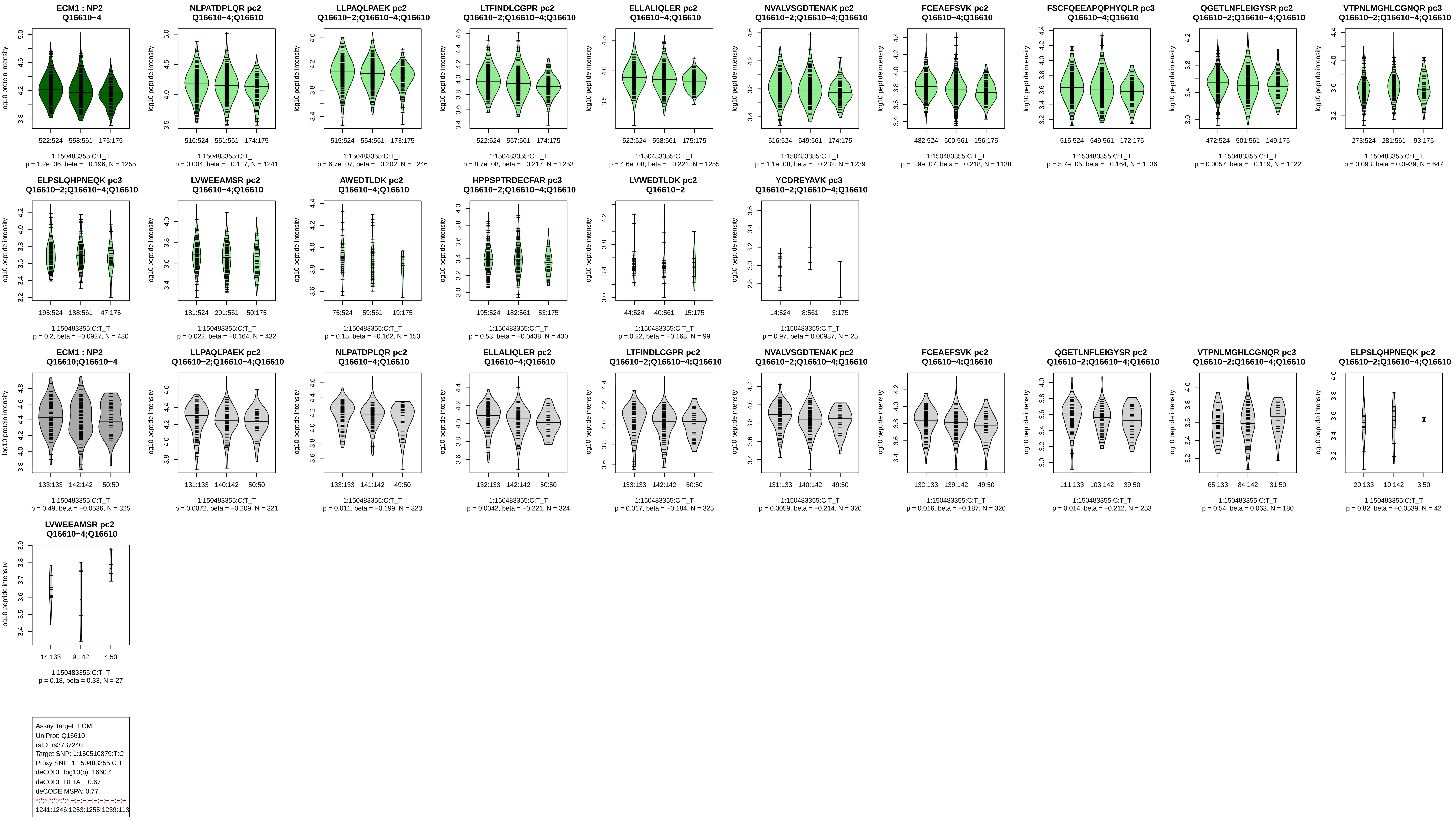
ECM1 : NP2
Q16610−4
log10 protein intensity
522:524 558:561 175:175
1:150483355:C:T_T
p = 1.2e−06, beta = −0.196, N = 1255
NLPATDPLQR pc2
Q16610−4;Q16610
log10 peptide intensity
516:524 551:561 174:175
1:150483355:C:T_T
p = 0.004, beta = −0.117, N = 1241
LLPAQLPAEK pc2
Q16610−2;Q16610−4;Q16610
519:524 554:561 173:175
1:150483355:C:T_T
p = 6.7e−07, beta = −0.202, N = 1246
LTFINDLCGPR pc2
Q16610−2;Q16610−4;Q16610
522:524 557:561 174:175
1:150483355:C:T_T
p = 8.7e−08, beta = −0.217, N = 1253
ELLALIQLER pc2
Q16610−4;Q16610
522:524 558:561 175:175
1:150483355:C:T_T
p = 4.6e−08, beta = −0.221, N = 1255
NVALVSGDTENAK pc2
Q16610−2;Q16610−4;Q16610
516:524 549:561 174:175
1:150483355:C:T_T
p = 1.1e−08, beta = −0.232, N = 1239
FCEAEFSVK pc2
Q16610−4;Q16610
482:524 500:561 156:175
1:150483355:C:T_T
p = 2.9e−07, beta = −0.218, N = 1138
FSCFQEEAPQPHYQLR pc3
Q16610−4;Q16610
515:524 549:561 172:175
1:150483355:C:T_T
p = 5.7e−05, beta = −0.164, N = 1236
QGETLNFLEIGYSR pc2
Q16610−2;Q16610−4;Q16610
472:524 501:561 149:175
1:150483355:C:T_T
p = 0.0057, beta = −0.119, N = 1122
VTPNLMGHLCGNQR pc3
Q16610−2;Q16610−4;Q16610
273:524 281:561 93:175
1:150483355:C:T_T
p = 0.093, beta = 0.0939, N = 647
ELPSLQHPNEQK pc3
Q16610−2;Q16610−4;Q16610
195:524 188:561 47:175
1:150483355:C:T_T
p = 0.2, beta = −0.0927, N = 430
LVWEEAMSR pc2
Q16610−4;Q16610
181:524 201:561 50:175
1:150483355:C:T_T
p = 0.022, beta = −0.164, N = 432
AWEDTLDK pc2
Q16610−4;Q16610
75:524 59:561 19:175
1:150483355:C:T_T
p = 0.15, beta = −0.162, N = 153
HPPSPTRDECFAR pc3
Q16610−2;Q16610−4;Q16610
195:524 182:561 53:175
1:150483355:C:T_T
p = 0.53, beta = −0.0438, N = 430
LVWEDTLDK pc2
Q16610−2
44:524 40:561 15:175
1:150483355:C:T_T
p = 0.22, beta = −0.168, N = 99
YCDREYAVK pc3
Q16610−2;Q16610−4;Q16610
14:524 8:561 3:175
1:150483355:C:T_T
p = 0.97, beta = 0.00987, N = 25
ECM1 : NP2
Q16610;Q16610−4
133:133 142:142 50:50
1:150483355:C:T_T
p = 0.49, beta = −0.0536, N = 325
LLPAQLPAEK pc2
Q16610−2;Q16610−4;Q16610
131:133 140:142 50:50
1:150483355:C:T_T
p = 0.0072, beta = −0.209, N = 321
NLPATDPLQR pc2
Q16610−4;Q16610
133:133 141:142 49:50
1:150483355:C:T_T
p = 0.011, beta = −0.199, N = 323
ELLALIQLER pc2
Q16610−4;Q16610
132:133 142:142 50:50
1:150483355:C:T_T
p = 0.0042, beta = −0.221, N = 324
LTFINDLCGPR pc2
Q16610−2;Q16610−4;Q16610
133:133 142:142 50:50
1:150483355:C:T_T
p = 0.017, beta = −0.184, N = 325
NVALVSGDTENAK pc2
Q16610−2;Q16610−4;Q16610
131:133 140:142 49:50
1:150483355:C:T_T
p = 0.0059, beta = −0.214, N = 320
FCEAEFSVK pc2
Q16610−4;Q16610
132:133 139:142 49:50
1:150483355:C:T_T
p = 0.016, beta = −0.187, N = 320
QGETLNFLEIGYSR pc2
Q16610−2;Q16610−4;Q16610
111:133 103:142 39:50
1:150483355:C:T_T
p = 0.014, beta = −0.212, N = 253
VTPNLMGHLCGNQR pc3
Q16610−2;Q16610−4;Q16610
65:133 84:142 31:50
1:150483355:C:T_T
p = 0.54, beta = 0.063, N = 180
ELPSLQHPNEQK pc2
Q16610−2;Q16610−4;Q16610
20:133 19:142 3:50
1:150483355:C:T_T
p = 0.82, beta = −0.0539, N = 42
LVWEEAMSR pc2
Q16610−4;Q16610
14:133 9:142 4:50
1:150483355:C:T_T
p = 0.18, beta = 0.33, N = 27
Assay Target: ECM1
UniProt: Q16610
rsID: rs3737240
Target SNP: 1:150510879:T:C
Proxy SNP: 1:150483355:C:T
deCODE log10(p): 1660.4
deCODE BETA: −0.67
deCODE MSPA: 0.77
1241:1246:1253:1255:1239:113

24. CFHR1 (Q03591)
deCODE MSPA=0.718

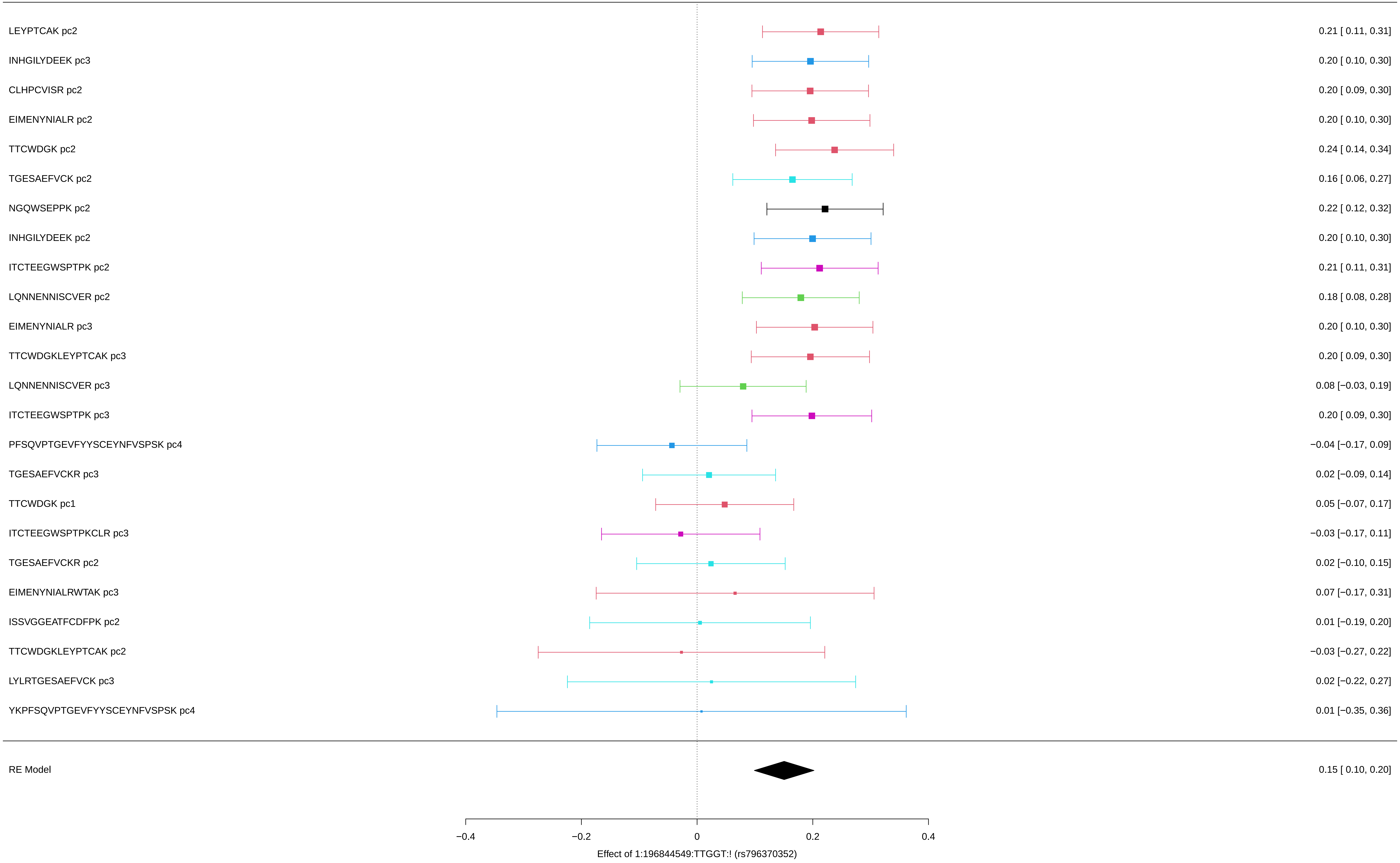

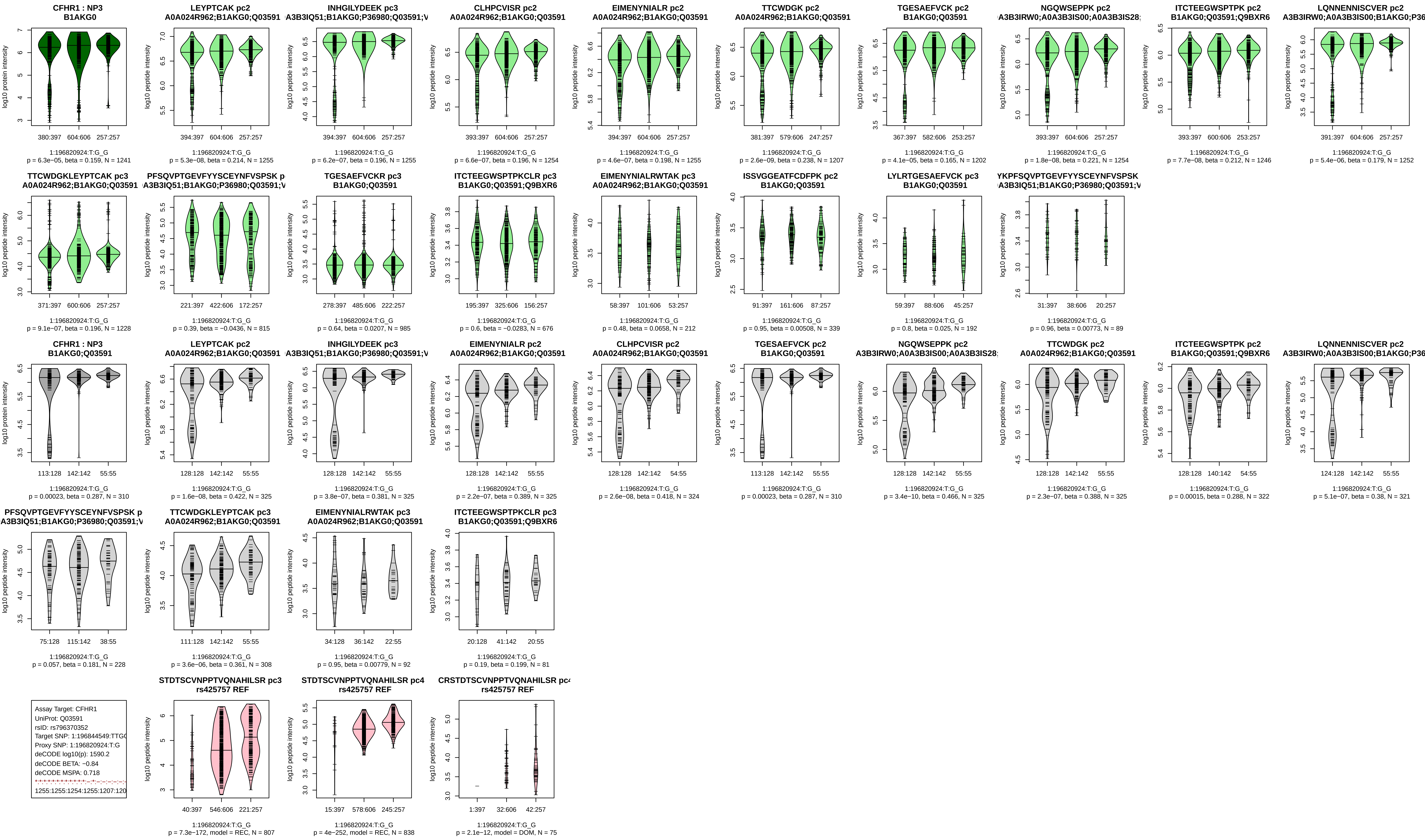
CFHR1 : NP3
B1AKG0
380:397 604:606 257:257
1:196820924:T:G_G
p = 6.3e−05, beta = 0.159, N = 1241
LEYPTCAK pc2
A0A024R962;B1AKG0;Q03591
394:397 604:606 257:257
1:196820924:T:G_G
p = 5.3e−08, beta = 0.214, N = 1255
INHGILYDEEK pc3
A3B3IQ51;B1AKG0;P36980;Q03591;\
394:397 604:606 257:257
1:196820924:T:G_G
p = 6.2e−07, beta = 0.196, N = 1255
CLHPCVISR pc2
A0A024R962;B1AKG0;Q03591
393:397 604:606 257:257
1:196820924:T:G_G
p = 6.6e−07, beta = 0.196, N = 1254
EIMENYNIALR pc2
A0A024R962;B1AKG0;Q03591
394:397 604:606 257:257
1:196820924:T:G_G
p = 4.6e−07, beta = 0.198, N = 1255
TTCWDGK pc2
A0A024R962;B1AKG0;Q03591
381:397 579:606 247:257
1:196820924:T:G_G
p = 2.6e−09, beta = 0.238, N = 1207
TGESAEFVCK pc2
B1AKG0;Q03591
367:397 582:606 253:257
1:196820924:T:G_G
p = 4.1e−05, beta = 0.165, N = 1202
NGQWSEPPK pc2
A3B3IRW0;A0A3B3IS00;A0A3B3IS28;
393:397 604:606 257:257
1:196820924:T:G_G
p = 1.8e−08, beta = 0.221, N = 1254
ITCTEEGWSPTPK pc2
B1AKG0;Q03591;Q9BXR6
393:397 600:606 253:257
1:196820924:T:G_G
p = 7.7e−08, beta = 0.212, N = 1246
LQNNENNISCVER pc2
A3B3IRW0;A0A3B3IS00;B1AKG0;P3
391:397 604:606 257:257
1:196820924:T:G_G
p = 5.4e−06, beta = 0.179, N = 1252
TTCWDGKLEYPTCAK pc3
A0A024R962;B1AKG0;Q03591
371:397 600:606 257:257
1:196820924:T:G_G
p = 9.1e−07, beta = 0.196, N = 1228
PFSQVPTGEVFYYSCEYNFVSPSK p
A3B3IQ51;B1AKG0;P36980;Q03591;\
221:397 422:606 172:257
1:196820924:T:G_G
p = 0.39, beta = −0.0436, N = 815
TGESAEFVCKR pc3
B1AKG0;Q03591
278:397 485:606 222:257
1:196820924:T:G_G
p = 0.64, beta = 0.0207, N = 985
ITCTEEGWSPTPKCLR pc3
B1AKG0;Q03591;Q9BXR6
195:397 325:606 156:257
1:196820924:T:G_G
p = 0.6, beta = −0.0283, N = 676
EIMENYNIALRWTAK pc3
A0A024R962;B1AKG0;Q03591
58:397 101:606 53:257
1:196820924:T:G_G
p = 0.48, beta = 0.0658, N = 212
ISSVGGEATFCDFPK pc2
B1AKG0;Q03591
91:397 161:606 87:257
1:196820924:T:G_G
p = 0.95, beta = 0.00508, N = 339
LYLRTGESAEFVCK pc3
B1AKG0;Q03591
59:397 88:606 45:257
1:196820924:T:G_G
p = 0.8, beta = 0.025, N = 192
YKPFSQVPTGEVFYYSCEYNFVSPSK
A3B3IQ51;B1AKG0;P36980;Q03591;\
31:397 38:606 20:257
1:196820924:T:G_G
p = 0.96, beta = 0.00773, N = 89
CFHR1 : NP3
B1AKG0;Q03591
113:128 142:142 55:55
1:196820924:T:G_G
p = 0.00023, beta = 0.287, N = 310
LEYPTCAK pc2
A0A024R962;B1AKG0;Q03591
128:128 142:142 55:55
1:196820924:T:G_G
p = 1.6e−08, beta = 0.422, N = 325
INHGILYDEEK pc3
A3B3IQ51;B1AKG0;P36980;Q03591;\
128:128 142:142 55:55
1:196820924:T:G_G
p = 3.8e−07, beta = 0.381, N = 325
EIMENYNIALR pc2
A0A024R962;B1AKG0;Q03591
128:128 142:142 55:55
1:196820924:T:G_G
p = 2.2e−07, beta = 0.389, N = 325
CLHPCVISR pc2
A0A024R962;B1AKG0;Q03591
128:128 142:142 54:55
1:196820924:T:G_G
p = 2.6e−08, beta = 0.418, N = 324
TGESAEFVCK pc2
B1AKG0;Q03591
113:128 142:142 55:55
1:196820924:T:G_G
p = 0.00023, beta = 0.287, N = 310
NGQWSEPPK pc2
A3B3IRW0;A0A3B3IS00;A0A3B3IS28;
128:128 142:142 55:55
1:196820924:T:G_G
p = 3.4e−10, beta = 0.466, N = 325
TTCWDGK pc2
A0A024R962;B1AKG0;Q03591
128:128 142:142 55:55
1:196820924:T:G_G
p = 2.3e−07, beta = 0.388, N = 325
ITCTEEGWSPTPK pc2
B1AKG0;Q03591;Q9BXR6
128:128 140:142 54:55
1:196820924:T:G_G
p = 0.00015, beta = 0.288, N = 322
LQNNENNISCVER pc2
A3B3IRW0;A0A3B3IS00;B1AKG0;P36
124:128 142:142 55:55
1:196820924:T:G_G
p = 5.1e−07, beta = 0.38, N = 321
PFSQVPTGEVFYYSCEYNFVSPSK p
A3B3IQ51;B1AKG0;P36980;Q03591;\
75:128 115:142 38:55
1:196820924:T:G_G
p = 0.057, beta = 0.181, N = 228
TTCWDGKLEYPTCAK pc3
A0A024R962;B1AKG0;Q03591
111:128 142:142 55:55
1:196820924:T:G_G
p = 3.6e−06, beta = 0.361, N = 308
EIMENYNIALRWTAK pc3
A0A024R962;B1AKG0;Q03591
34:128 36:142 22:55
1:196820924:T:G_G
p = 0.95, beta = 0.00779, N = 92
ITCTEEGWSPTPKCLR pc3
B1AKG0;Q03591;Q9BXR6
20:128 41:142 20:55
1:196820924:T:G_G
p = 0.19, beta = 0.199, N = 81
Assay Target: CFHR1
UniProt: Q03591
rsID: rs796370352
Target SNP: 1:196844549:TTGC
Proxy SNP: 1:196820924:T:G
deCODE log10(p): 1590.2
deCODE BETA: −0.84
deCODE MSPA: 0.718
1255:1255:1254:1255:1207:120
STDTSCVNPPTVQNAHILSR pc3
rs425757 REF
40:397 546:606 221:257
1:196820924:T:G_G
p = 7.3e−172, model = REC, N = 807
STDTSCVNPPTVQNAHILSR pc4
rs425757 REF
15:397 578:606 245:257
1:196820924:T:G_G
p = 4e−252, model = REC, N = 838
CRSTDTSCVNPPTVQNAHILSR pc4
rs425757 REF
1:397 32:606 42:257
1:196820924:T:G_G
p = 2.1e−12, model = DOM, N = 75
log10 protein intensity
log10 peptide intensity

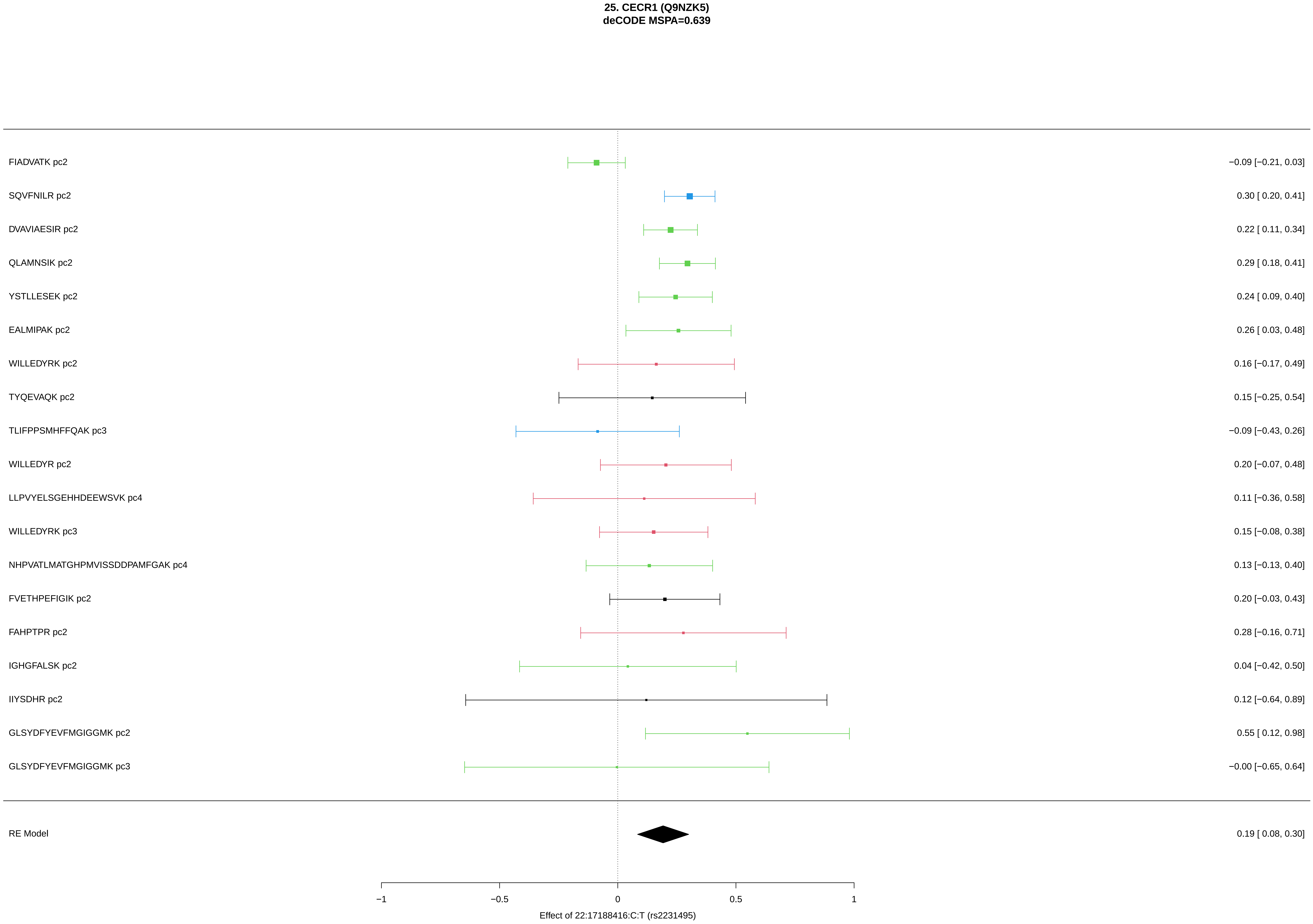

25. CECR1 (Q9NZK5)
deCODE MSPA=0.639
FIADVATK pc2
−0.09 [−0.21, 0.03]
SQVFNILR pc2
0.30 [0.20, 0.41]
DVAVIAESIR pc2
0.22 [0.11, 0.34]
QLAMNSIK pc2
0.29 [0.18, 0.41]
YSTLLESEK pc2
0.24 [0.09, 0.40]
EALMIPAK pc2
0.26 [0.03, 0.48]
WILLEDYRK pc2
0.16 [−0.17, 0.49]
TYQEVAQK pc2
0.15 [−0.25, 0.54]
TLIFPPSMHFFQAK pc3
−0.09 [−0.43, 0.26]
WILLEDYR pc2
0.20 [−0.07, 0.48]
LLPVYELSGEHHDEEWSVK pc4
0.11 [−0.36, 0.58]
WILLEDYRK pc3
0.15 [−0.08, 0.38]
NHPVATLMATGHPMVISSDDPAMFGAK pc4
0.13 [−0.13, 0.40]
FVETHPEFIGIK pc2
0.20 [−0.03, 0.43]
FAHPTPR pc2
0.28 [−0.16, 0.71]
IGHGFALSK pc2
0.04 [−0.42, 0.50]
IIYSDHR pc2
0.12 [−0.64, 0.89]
GLSYDFYEVFMGIGGMK pc2
0.55 [0.12, 0.98]
GLSYDFYEVFMGIGGMK pc3
−0.00 [−0.65, 0.64]
RE Model
0.19 [0.08, 0.30]
−1
−0.5
0
0.5
1
Effect of 22:17188416:C:T (rs2231495)

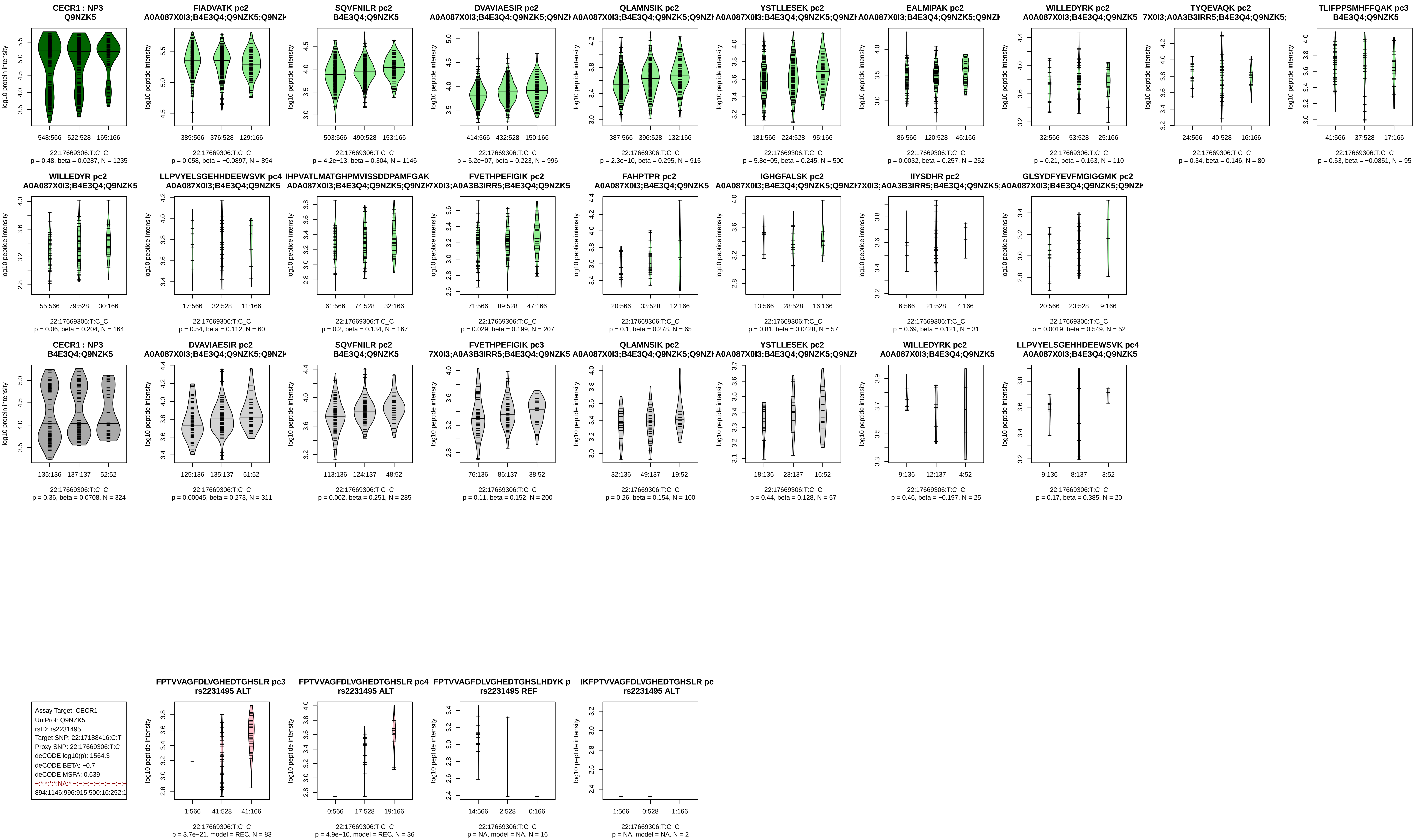
CECR1 : NP3
Q9NZK5
log10 protein intensity
548:566 522:528 165:166
22:17669306:T:C_C
p = 0.48, beta = 0.0287, N = 1235
FIADVATK pc2
A0A087X0I3;B4E3Q4;Q9NZK5;Q9NZK
log10 peptide intensity
389:566 376:528 129:166
22:17669306:T:C_C
p = 0.058, beta = −0.0897, N = 894
SQVFNILR pc2
B4E3Q4;Q9NZK5
503:566 490:528 153:166
22:17669306:T:C_C
p = 4.2e−13, beta = 0.304, N = 1146
DVAVIAESIR pc2
A0A087X0I3;B4E3Q4;Q9NZK5;Q9NZK
414:566 432:528 150:166
22:17669306:T:C_C
p = 5.2e−07, beta = 0.223, N = 996
QLAMNSIK pc2
A0A087X0I3;B4E3Q4;Q9NZK5;Q9NZK
387:566 396:528 132:166
22:17669306:T:C_C
p = 2.3e−10, beta = 0.295, N = 915
YSTLLESEK pc2
A0A087X0I3;B4E3Q4;Q9NZK5;Q9NZK
181:566 224:528 95:166
22:17669306:T:C_C
p = 5.8e−05, beta = 0.245, N = 500
EALMIPAK pc2
A0A087X0I3;B4E3Q4;Q9NZK5;Q9NZK
86:566 120:528 46:166
22:17669306:T:C_C
p = 0.0032, beta = 0.257, N = 252
WILLEDYRK pc2
A0A087X0I3;B4E3Q4;Q9NZK5
32:566 53:528 25:166
22:17669306:T:C_C
p = 0.21, beta = 0.163, N = 110
TYQEVAQK pc2
7X0I3;A0A3B3IRR5;B4E3Q4;Q9NZK5;
24:566 40:528 16:166
22:17669306:T:C_C
p = 0.34, beta = 0.146, N = 80
TLIFPPSMHFFQAK pc3
B4E3Q4;Q9NZK5
41:566 37:528 17:166
22:17669306:T:C_C
p = 0.53, beta = −0.0851, N = 95
WILLEDYR pc2
A0A087X0I3;B4E3Q4;Q9NZK5
55:566 79:528 30:166
22:17669306:T:C_C
p = 0.06, beta = 0.204, N = 164
LLPVYELSGEHHDEEWSVK pc4
A0A087X0I3;B4E3Q4;Q9NZK5
17:566 32:528 11:166
22:17669306:T:C_C
p = 0.54, beta = 0.112, N = 60
IHPVATLMATGHPMVISSDDPAMFGAK
A0A087X0I3;B4E3Q4;Q9NZK5;Q9NZK
61:566 74:528 32:166
22:17669306:T:C_C
p = 0.2, beta = 0.134, N = 167
FVETHPEFIGIK pc2
7X0I3;A0A3B3IRR5;B4E3Q4;Q9NZK5;
71:566 89:528 47:166
22:17669306:T:C_C
p = 0.029, beta = 0.199, N = 207
FAHPTPR pc2
A0A087X0I3;B4E3Q4;Q9NZK5
20:566 33:528 12:166
22:17669306:T:C_C
p = 0.1, beta = 0.278, N = 65
IGHGFALSK pc2
A0A087X0I3;B4E3Q4;Q9NZK5;Q9NZK
13:566 28:528 16:166
22:17669306:T:C_C
p = 0.81, beta = 0.0428, N = 57
IIYSDHR pc2
7X0I3;A0A3B3IRR5;B4E3Q4;Q9NZK5;
6:566 21:528 4:166
22:17669306:T:C_C
p = 0.69, beta = 0.121, N = 31
GLSYDFYEVFMGIGGMK pc2
A0A087X0I3;B4E3Q4;Q9NZK5;Q9NZK
20:566 23:528 9:166
22:17669306:T:C_C
p = 0.0019, beta = 0.549, N = 52
CECR1 : NP3
B4E3Q4;Q9NZK5
135:136 137:137 52:52
22:17669306:T:C_C
p = 0.36, beta = 0.0708, N = 324
DVAVIAESIR pc2
A0A087X0I3;B4E3Q4;Q9NZK5;Q9NZK
125:136 135:137 51:52
22:17669306:T:C_C
p = 0.00045, beta = 0.273, N = 311
SQVFNILR pc2
B4E3Q4;Q9NZK5
113:136 124:137 48:52
22:17669306:T:C_C
p = 0.002, beta = 0.251, N = 285
FVETHPEFIGIK pc3
7X0I3;A0A3B3IRR5;B4E3Q4;Q9NZK5;
76:136 86:137 38:52
22:17669306:T:C_C
p = 0.11, beta = 0.152, N = 200
QLAMNSIK pc2
A0A087X0I3;B4E3Q4;Q9NZK5;Q9NZK
32:136 49:137 19:52
22:17669306:T:C_C
p = 0.26, beta = 0.154, N = 100
YSTLLESEK pc2
A0A087X0I3;B4E3Q4;Q9NZK5;Q9NZK
18:136 23:137 16:52
22:17669306:T:C_C
p = 0.44, beta = 0.128, N = 57
WILLEDYRK pc2
A0A087X0I3;B4E3Q4;Q9NZK5
9:136 12:137 4:52
22:17669306:T:C_C
p = 0.46, beta = −0.197, N = 25
LLPVYELSGEHHDEEWSVK pc4
A0A087X0I3;B4E3Q4;Q9NZK5
9:136 8:137 3:52
22:17669306:T:C_C
p = 0.17, beta = 0.385, N = 20
Assay Target: CECR1
UniProt: Q9NZK5
rsID: rs2231495
Target SNP: 22:17188416:C:T
Proxy SNP: 22:17669306:T:C
deCODE log10(p): 1564.3
deCODE BETA: −0.7
deCODE MSPA: 0.639
−:*:*:*:NA:*:−:−:−:−:−:−:−:−:−
894:1146:996:915:500:16:252:1
FPTVVAGFDLVGHEDTGHSLR pc3
rs2231495 ALT
1:566 41:528 41:166
22:17669306:T:C_C
p = 3.7e−21, model = REC, N = 83
FPTVVAGFDLVGHEDTGHSLR pc4
rs2231495 ALT
0:566 17:528 19:166
22:17669306:T:C_C
p = 4.9e−10, model = REC, N = 36
FPTVVAGFDLVGHEDTGHSLHDYK p
rs2231495 REF
14:566 2:528 0:166
22:17669306:T:C_C
p = NA, model = NA, N = 16
IKFPTVVAGFDLVGHEDTGHSLR pc
rs2231495 ALT
1:566 0:528 1:166
22:17669306:T:C_C
p = NA, model = NA, N = 2

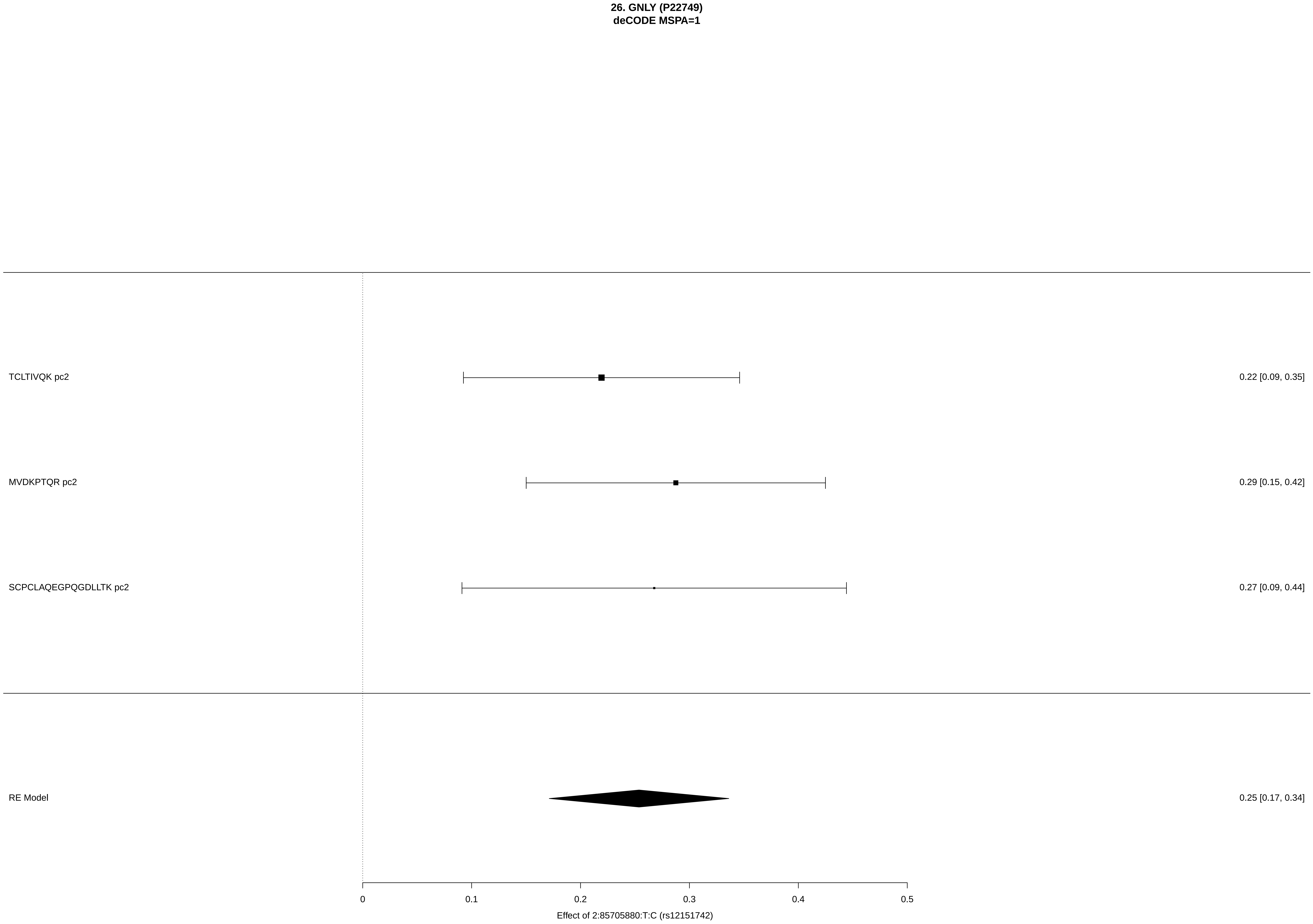
26. GNLY (P22749)
deCODE MSPA=1
TCLTIVQK pc2
0.22 [0.09, 0.35]
MVDKPTQR pc2
0.29 [0.15, 0.42]
SCPCLAQEGPQGDLLTK pc2
0.27 [0.09, 0.44]
RE Model
0.25 [0.17, 0.34]
0
0.1
0.2
0.3
0.4
0.5
Effect of 2:85705880:T:C (rs12151742)

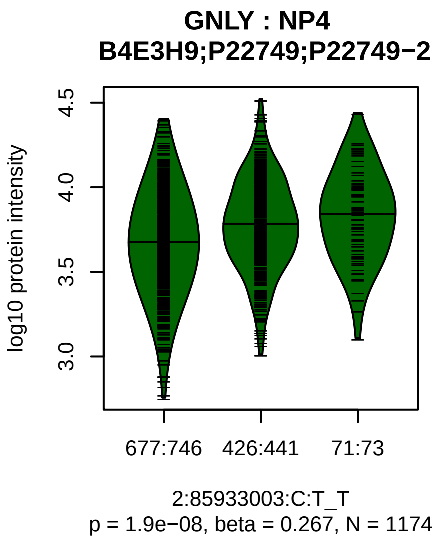

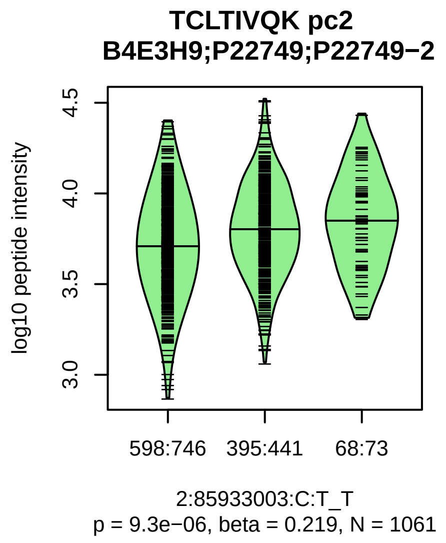

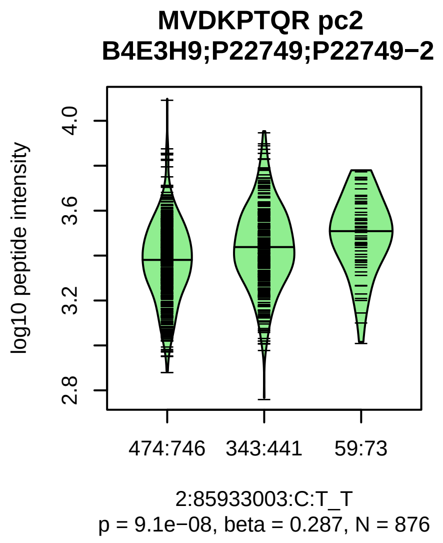

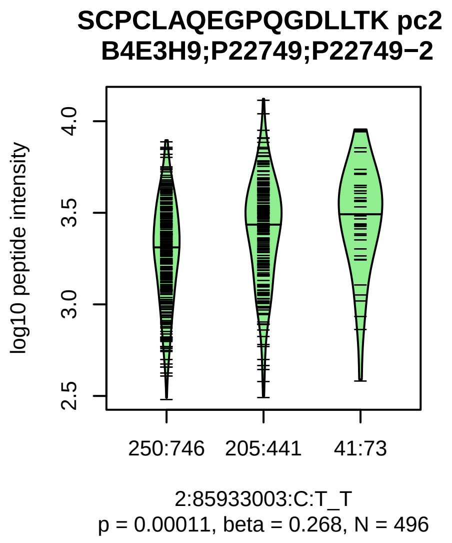

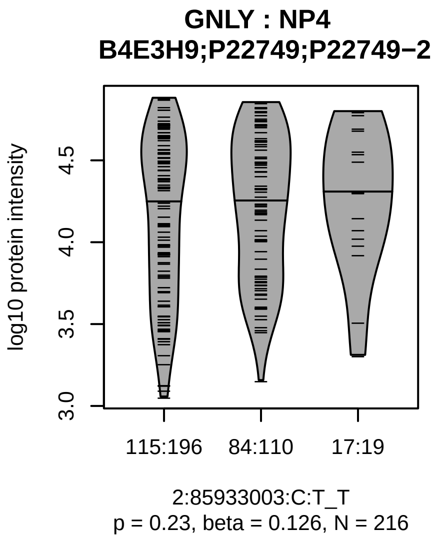

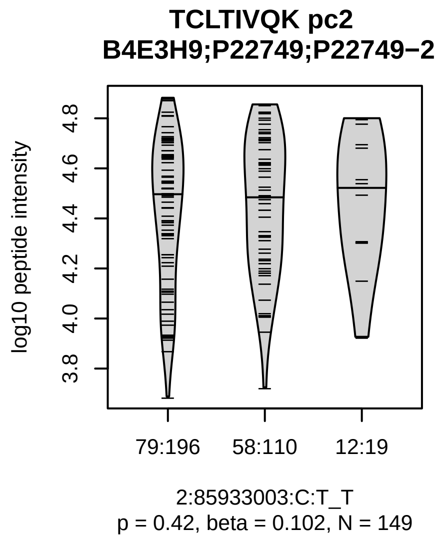

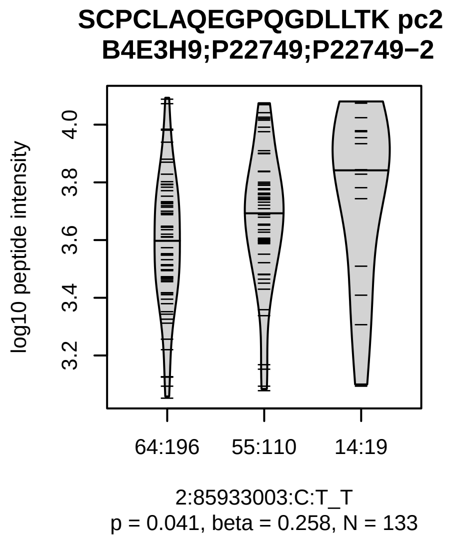

Assay Target: GNLY
UniProt: P22749
rsID: rs12151742
Target SNP: 2:85705880:T:C
Proxy SNP: 2:85933003:C:T
deCODE log10(p): 1527.6
deCODE BETA: 0.81
deCODE MSPA: 1
::*
1061:876:496

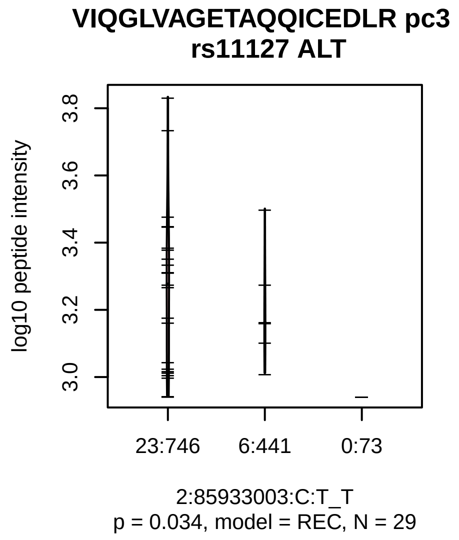

27. PLA2G2A (P14555)
deCODE MSPA=1

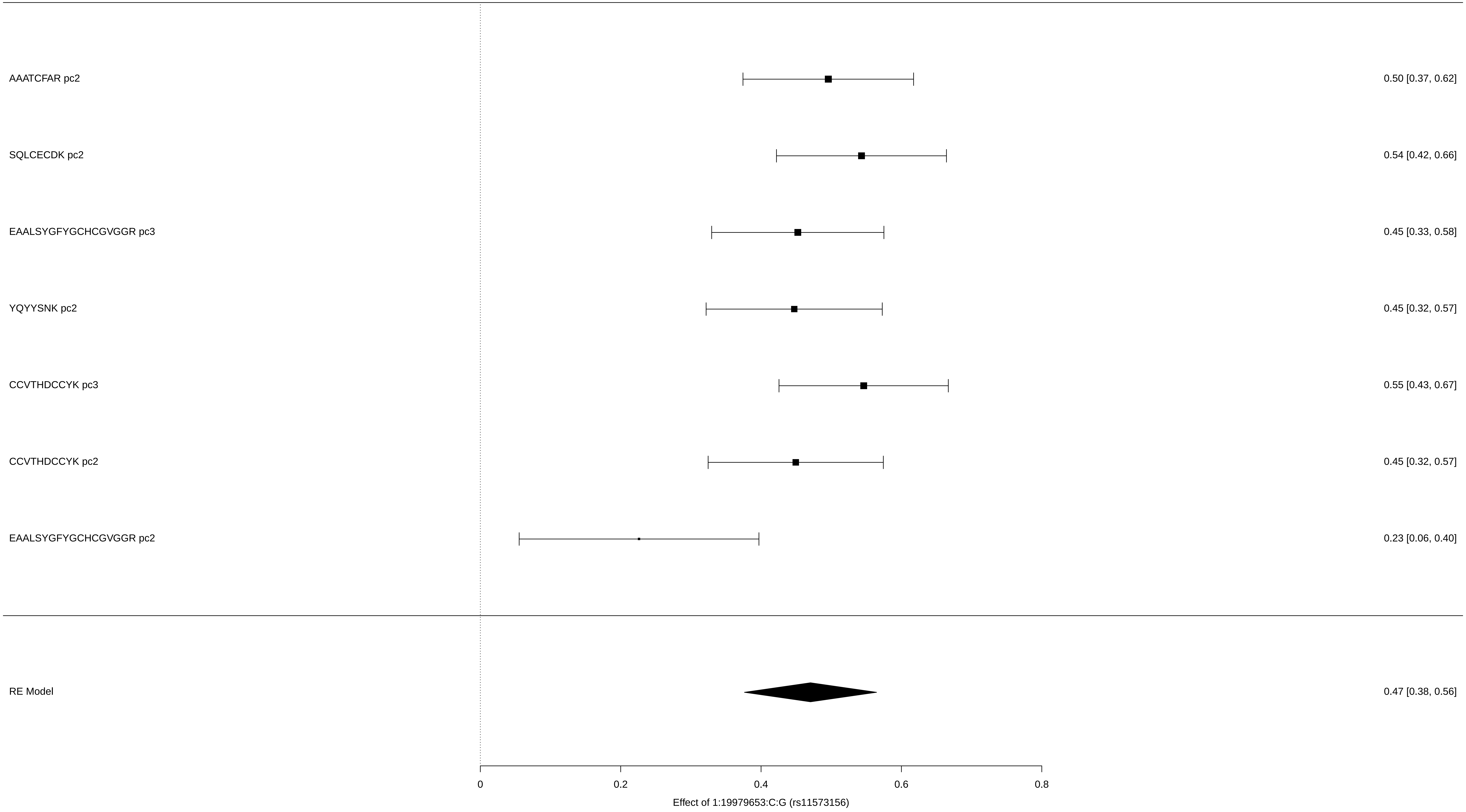

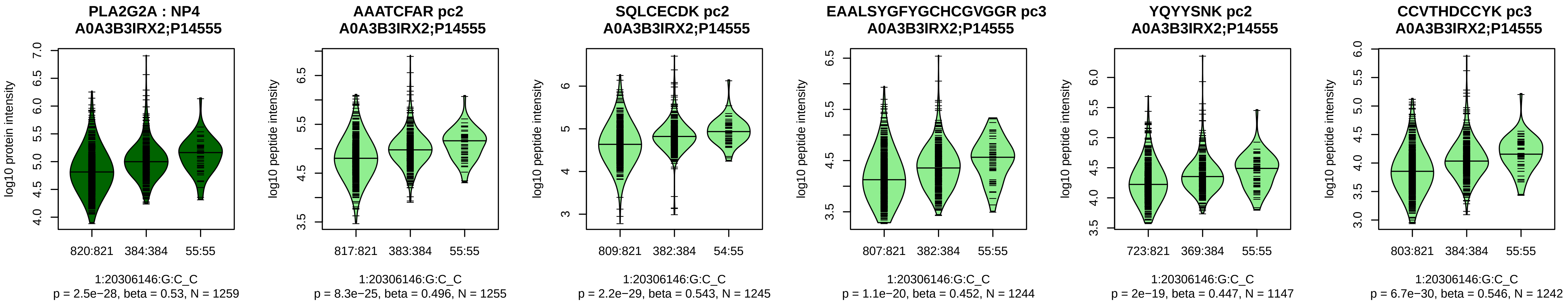

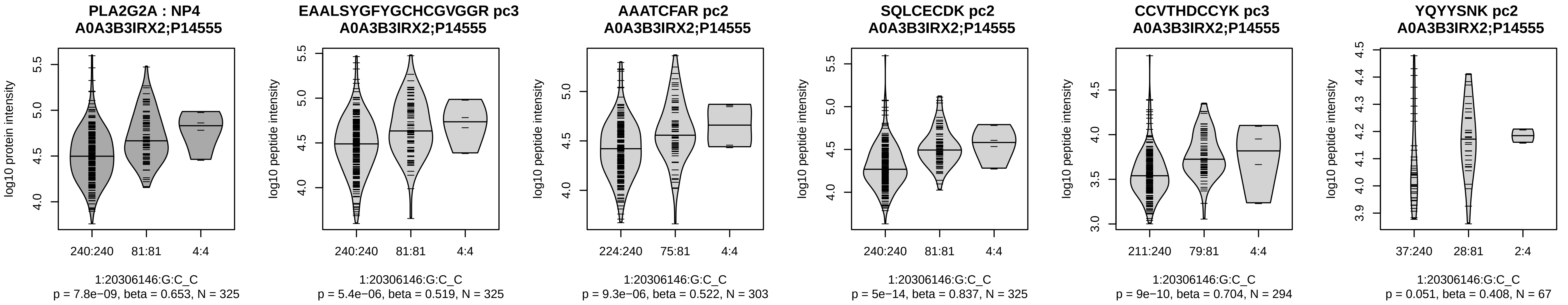

Assay Target: PLA2G2A
UniProt: P14555
rsID: rs11573156
Target SNP: 1:19979653:C:G
Proxy SNP: 1:20306146:G:C
deCODE log10(p): 1497.1
deCODE BETA: 0.77
deCODE MSPA: 1
1255:1245:1244:1147:1242:118

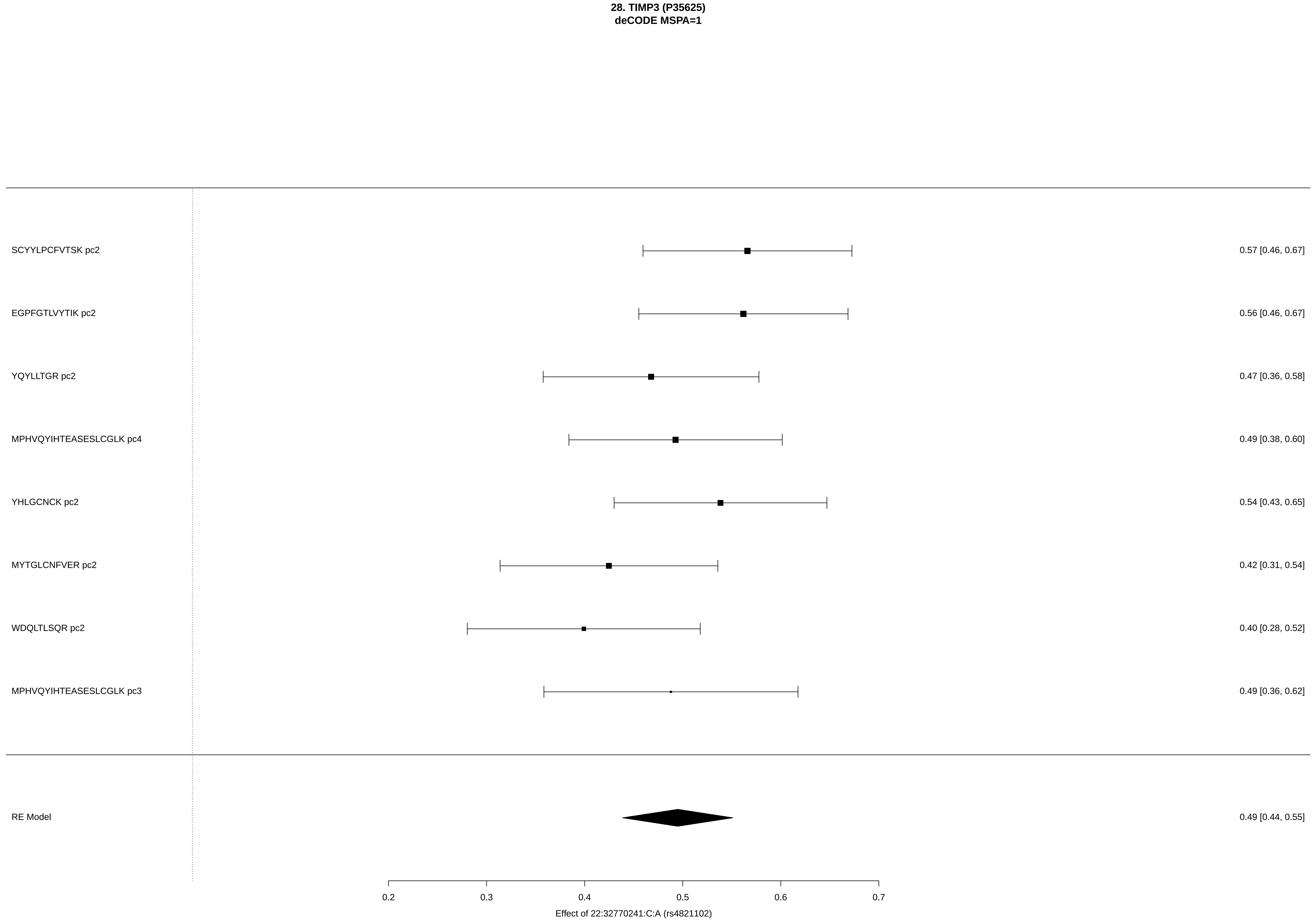
28. TIMP3 (P35625)
deCODE MSPA=1
SCYYLPCFVTSK pc2
0.57 [0.46, 0.67]
EGPFGTLVYTIK pc2
0.56 [0.46, 0.67]
YQYLLTGR pc2
0.47 [0.36, 0.58]
MPHVQYIHTEASESLCGLK pc4
0.49 [0.38, 0.60]
YHLGCNCK pc2
0.54 [0.43, 0.65]
MYTGLCNFVER pc2
0.42 [0.31, 0.54]
WDQLTLSQR pc2
0.40 [0.28, 0.52]
MPHVQYIHTEASESLCGLK pc3
0.49 [0.36, 0.62]
RE Model
0.49 [0.44, 0.55]
0.2
0.3
0.4
0.5
0.6
0.7
Effect of 22:32770241:C:A (rs4821102)

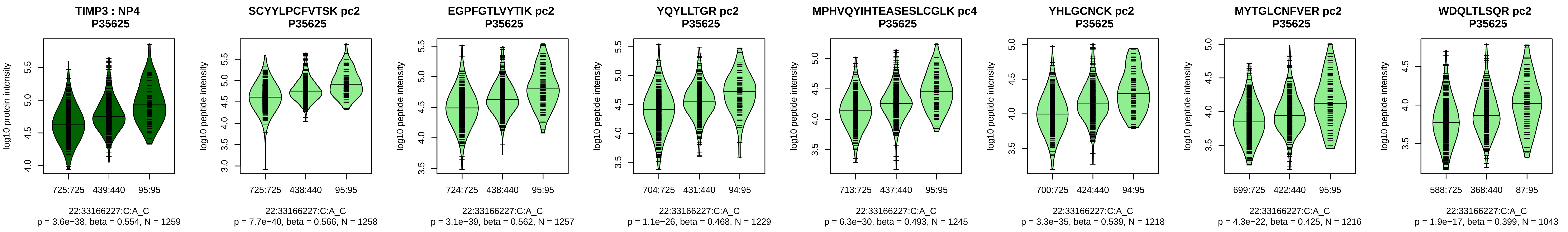

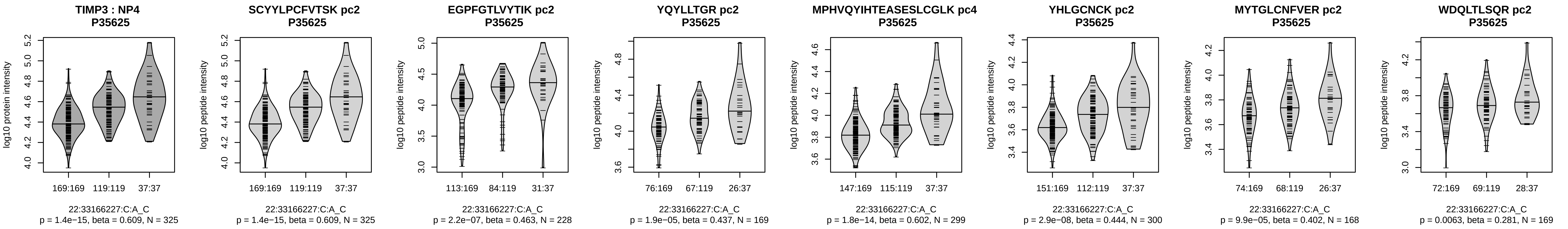

Assay Target: TIMP3
UniProt: P35625
rsID: rs4821102
Target SNP: 22:32770241:C:A
Proxy SNP: 22:33166227:C:A
deCODE log10(p): 1471
deCODE BETA: 0.78
deCODE MSPA: 1
1258:1257:1229:1245:1218:121

29. SERPINE2 (P07093)
deCODE MSPA=0.944

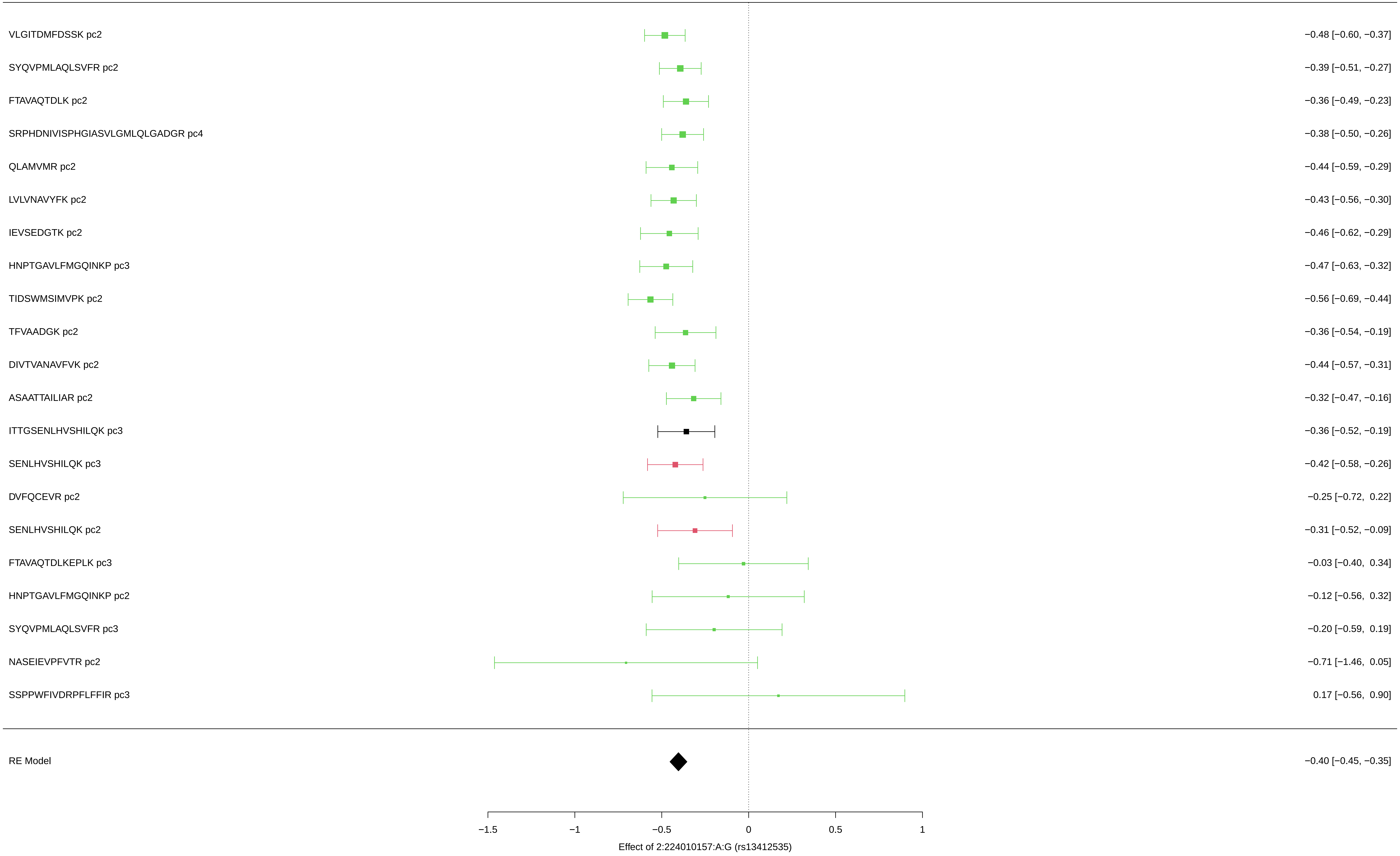

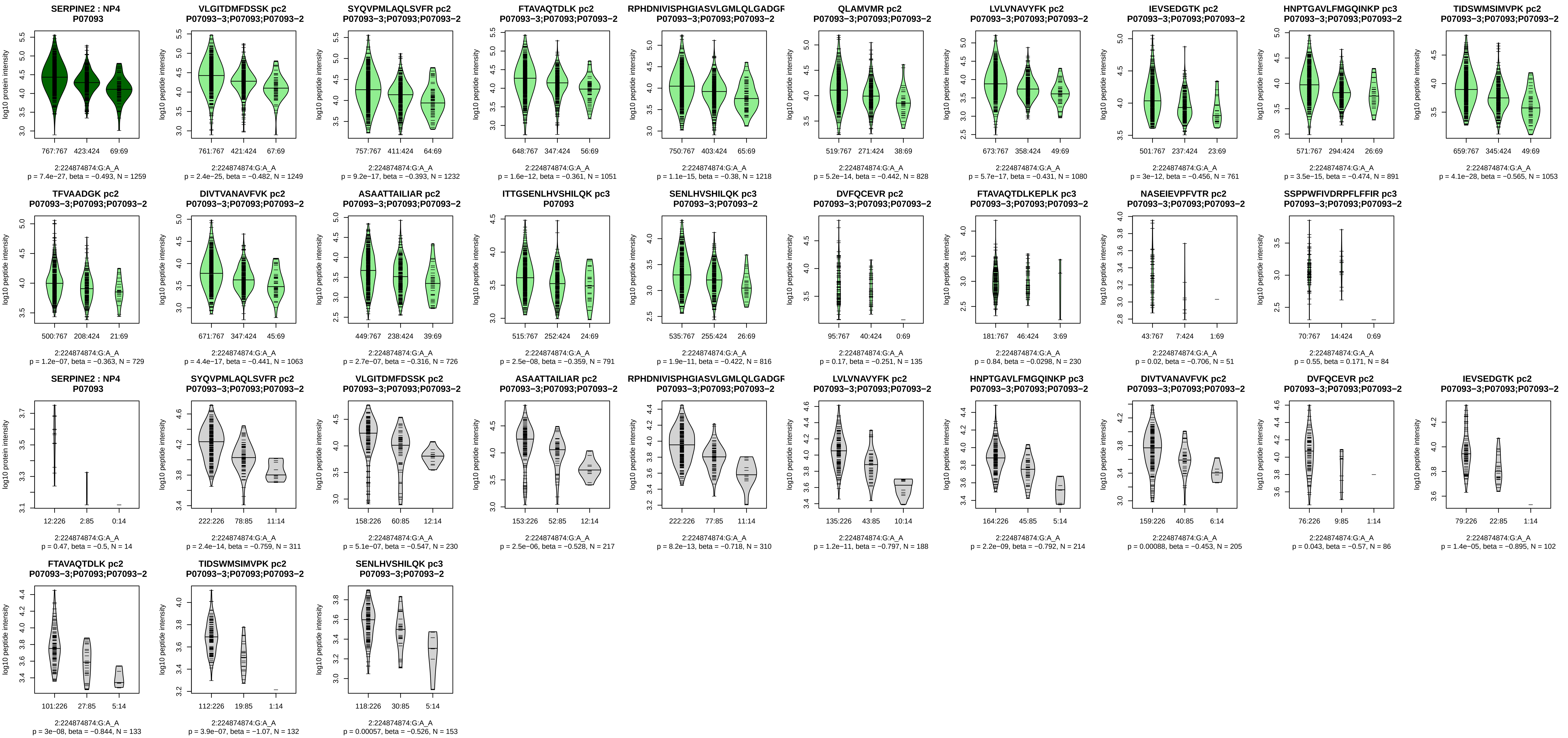

Assay Target: SERPINE2
UniProt: P07093
rsID: rs13412535
Target SNP: 2:224010157:A:G
Proxy SNP: 2:224874874:G:A
deCODE log10(p): 1403.2
deCODE BETA: −0.8
deCODE MSPA: 0.944
1249:1232:1051:1218:828:1080

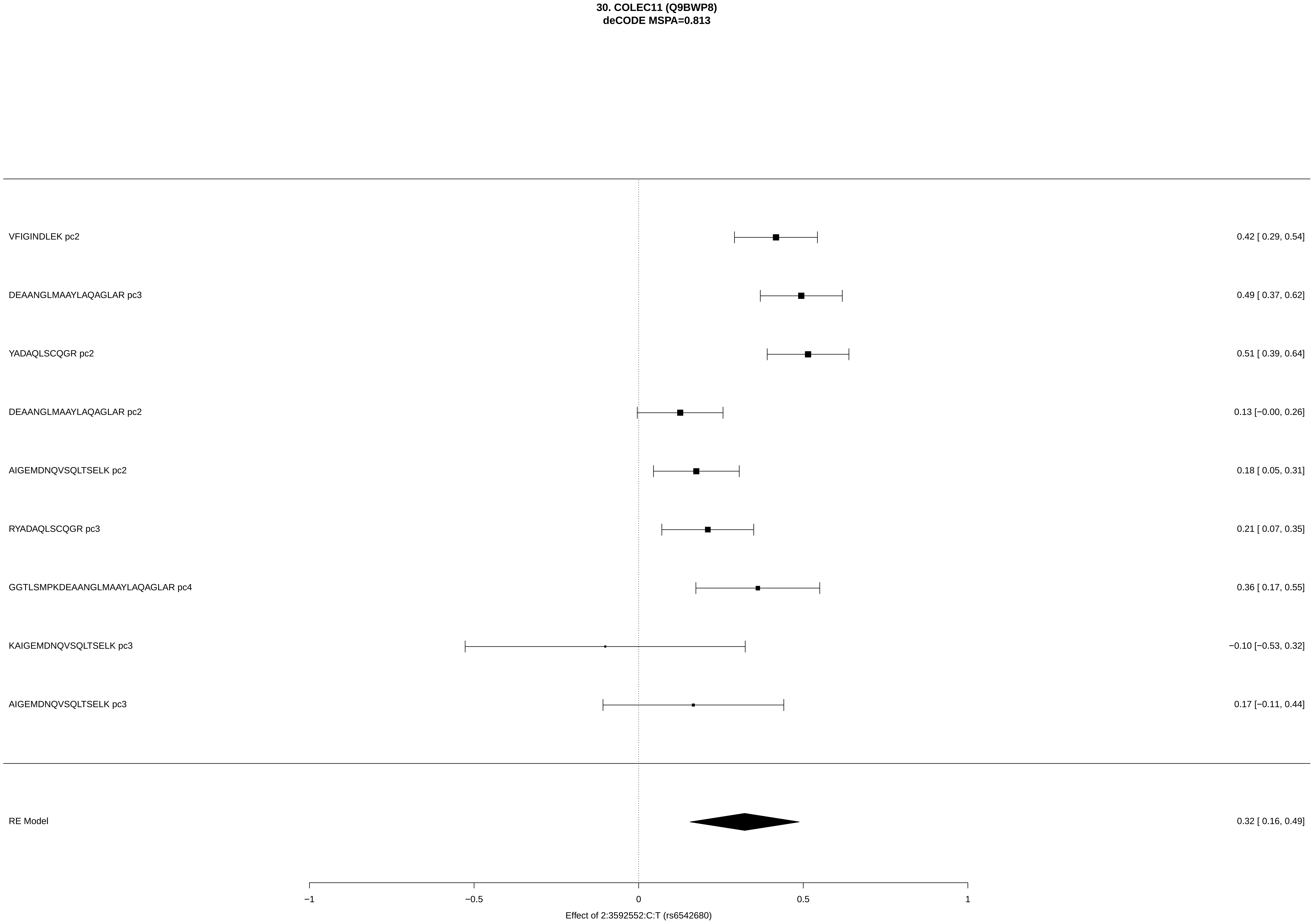

30. COLEC11 (Q9BWP8)
deCODE MSPA=0.813
VFIGINDLEK pc2
0.42 [0.29, 0.54]
DEAANGLMAAYLAQAGLAR pc3
0.49 [0.37, 0.62]
YADAQLSCQGR pc2
0.51 [0.39, 0.64]
DEAANGLMAAYLAQAGLAR pc2
0.13 [−0.00, 0.26]
AIGEMDNQVSQLTSELK pc2
0.18 [0.05, 0.31]
RYADAQLSCQGR pc3
0.21 [0.07, 0.35]
GGTLSMPKDEAANGLMAAYLAQAGLAR pc4
0.36 [0.17, 0.55]
KAIGEMDNQVSQLTSELK pc3
−0.10 [−0.53, 0.32]
AIGEMDNQVSQLTSELK pc3
0.17 [−0.11, 0.44]
RE Model
0.32 [0.16, 0.49]
−1
−0.5
0
0.5
1
Effect of 2:3592552:C:T (rs6542680)

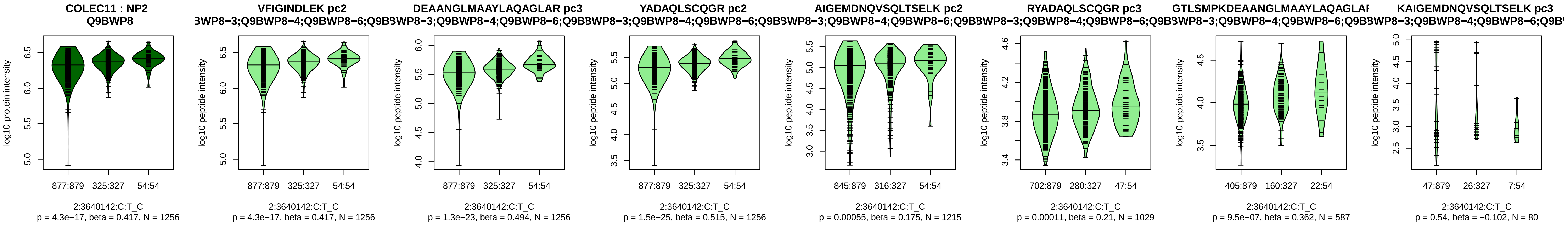

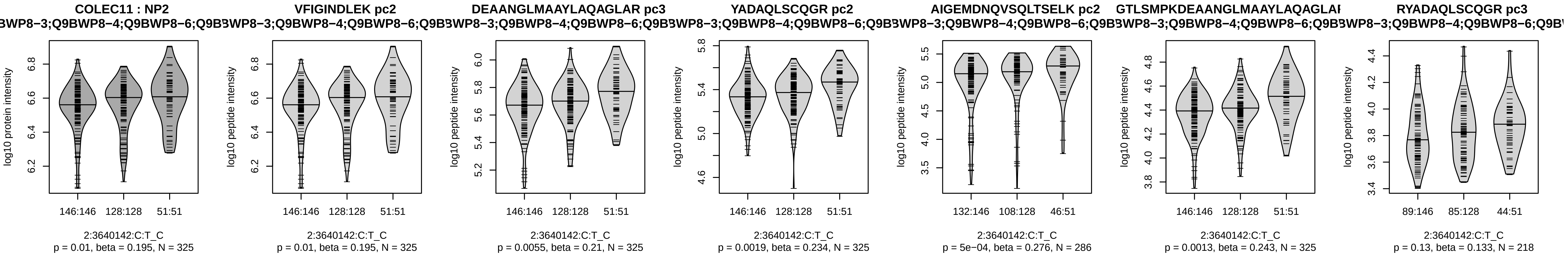

Assay Target: COLEC11
UniProt: Q9BWP8
rsID: rs6542680
Target SNP: 2:3592552:C:T
Proxy SNP: 2:3640142:C:T
deCODE log10(p): 1380.3
deCODE BETA: 0.86
deCODE MSPA: 0.813
::*:-:*:*:*:-:-:NA
1256:1256:1256:1224:1215:102

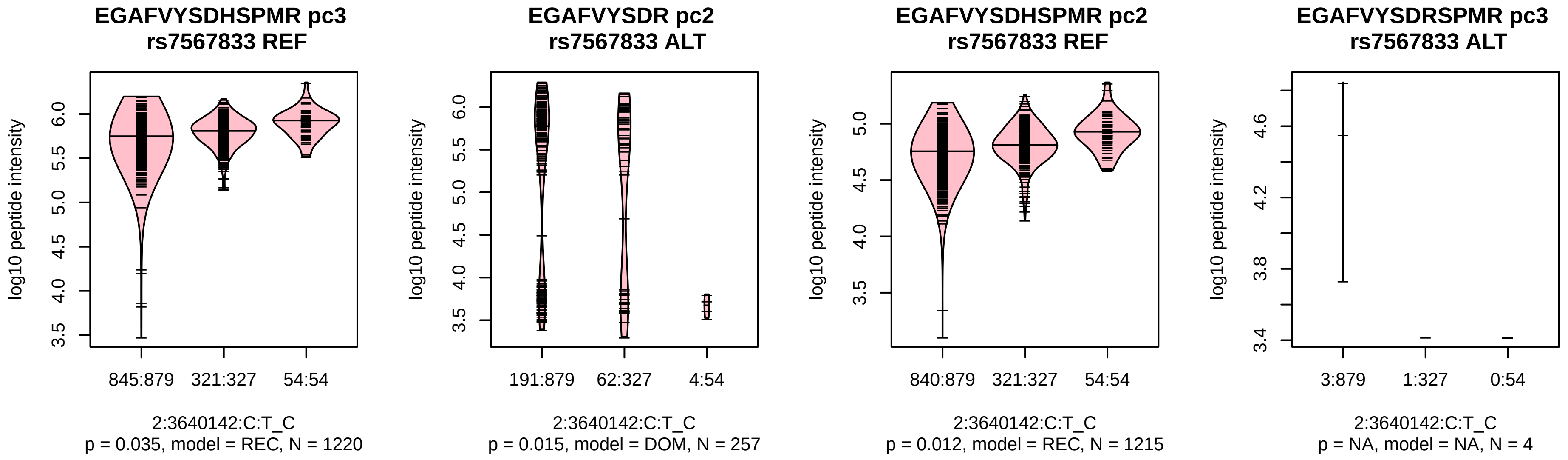

31. LBP (P18428)
deCODE MSPA=0.88

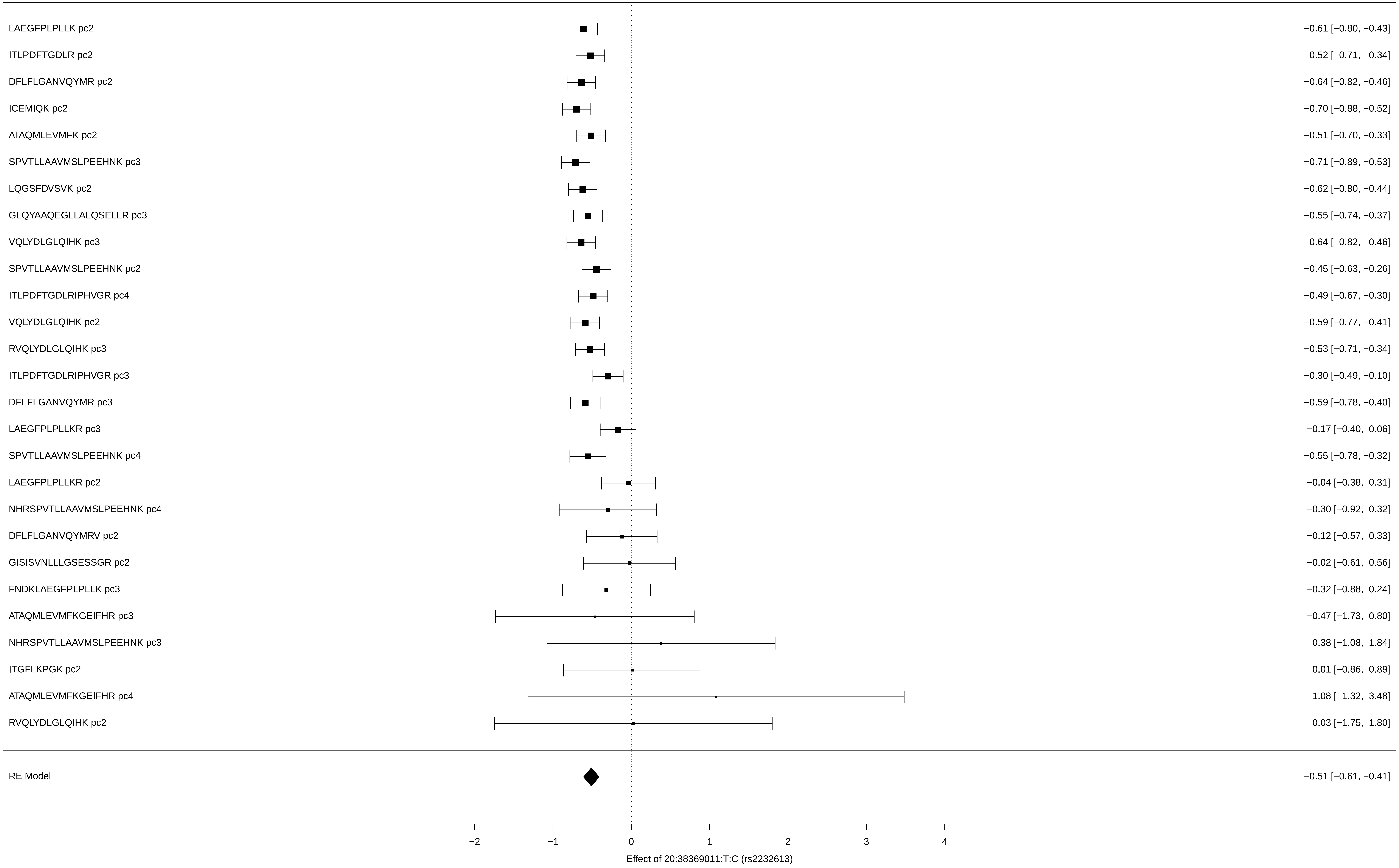

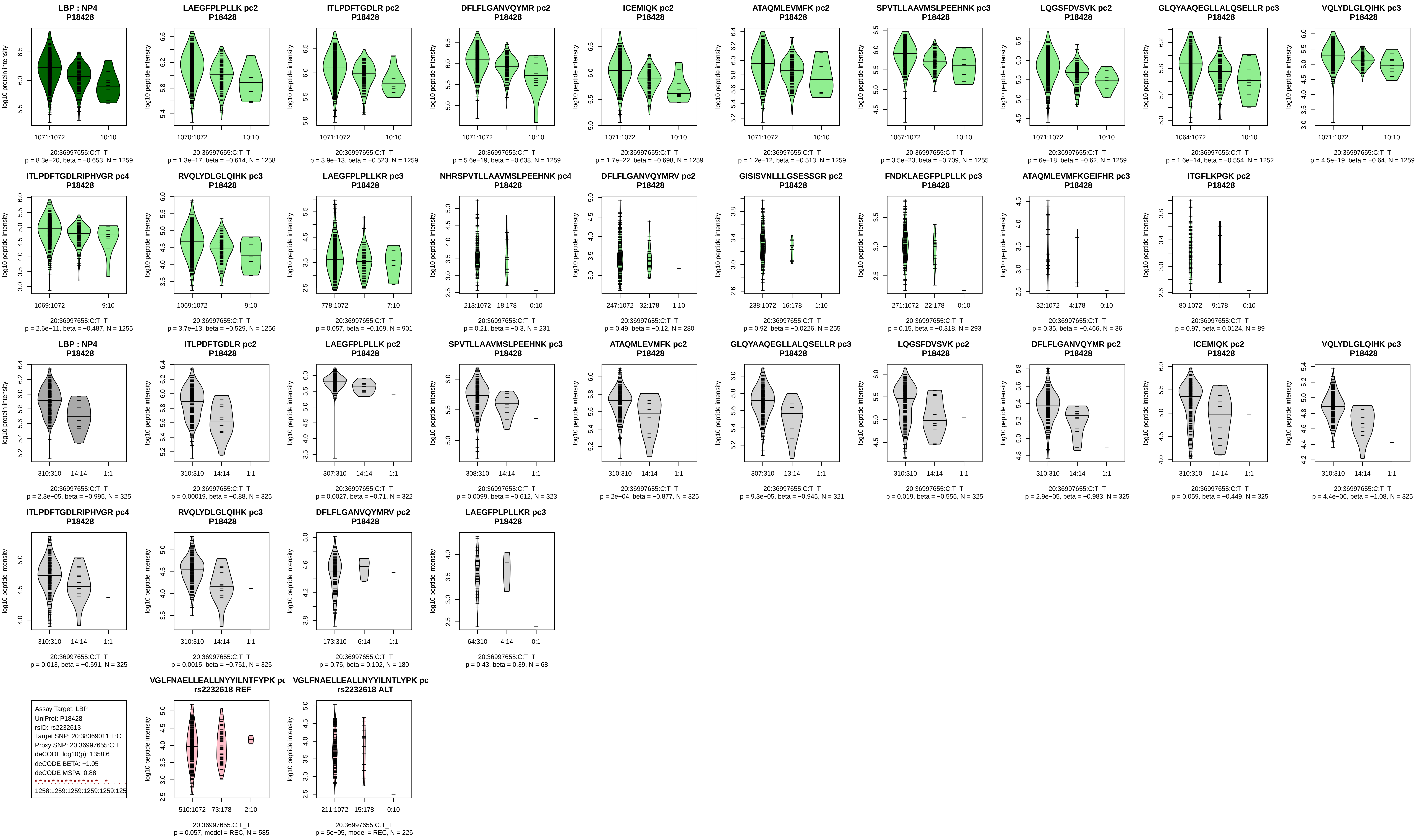

LBP : NP4
P18428
log10 protein intensity
1071:1072
10:10
20:36997655:C:T_T
p = 8.3e−20, beta = −0.653, N = 1259
LAEGFPLPLLK pc2
P18428
log10 peptide intensity
1070:1072
10:10
20:36997655:C:T_T
p = 1.3e−17, beta = −0.614, N = 1258
ITLPDFTGDLR pc2
P18428
1071:1072
10:10
20:36997655:C:T_T
p = 3.9e−13, beta = −0.523, N = 1259
DFLFLGANVQYMR pc2
P18428
1071:1072
10:10
20:36997655:C:T_T
p = 5.6e−19, beta = −0.638, N = 1259
ICEMIQK pc2
P18428
1071:1072
10:10
20:36997655:C:T_T
p = 1.7e−22, beta = −0.698, N = 1259
ATAQMLEVMFK pc2
P18428
1071:1072
10:10
20:36997655:C:T_T
p = 1.2e−12, beta = −0.513, N = 1259
SPVTLLAAVMSLPEEHNK pc3
P18428
1067:1072
10:10
20:36997655:C:T_T
p = 3.5e−23, beta = −0.709, N = 1255
LQGSFDVSVK pc2
P18428
1071:1072
10:10
20:36997655:C:T_T
p = 6e−18, beta = −0.62, N = 1259
GLQYAAQEGLLALQSELLR pc3
P18428
1064:1072
10:10
20:36997655:C:T_T
p = 1.6e−14, beta = −0.554, N = 1252
VQLYDLGLQIHK pc3
P18428
1071:1072
10:10
20:36997655:C:T_T
p = 4.5e−19, beta = −0.64, N = 1259
ITLPDFTGDLRIPHVGR pc4
P18428
1069:1072
9:10
20:36997655:C:T_T
p = 2.6e−11, beta = −0.487, N = 1255
RVQLYDLGLQIHK pc3
P18428
1069:1072
9:10
20:36997655:C:T_T
p = 3.7e−13, beta = −0.529, N = 1256
LAEGFPLPLLKR pc3
P18428
778:1072
7:10
20:36997655:C:T_T
p = 0.057, beta = −0.169, N = 901
NHRSPVTLLAAVMSLPEEHNK pc4
P18428
213:1072
18:178
0:10
20:36997655:C:T_T
p = 0.21, beta = −0.3, N = 231
DFLFLGANVQYMRV pc2
P18428
247:1072
32:178
1:10
20:36997655:C:T_T
p = 0.49, beta = −0.12, N = 280
GISISVNLLLGSESSGR pc2
P18428
238:1072
16:178
1:10
20:36997655:C:T_T
p = 0.92, beta = −0.0226, N = 255
FNDKLAEGFPLPLLK pc3
P18428
271:1072
22:178
0:10
20:36997655:C:T_T
p = 0.15, beta = −0.318, N = 293
ATAQMLEVMFKGEIFHR pc3
P18428
32:1072
4:178
0:10
20:36997655:C:T_T
p = 0.35, beta = −0.466, N = 36
ITGFLKPGK pc2
P18428
80:1072
9:178
0:10
20:36997655:C:T_T
p = 0.97, beta = 0.0124, N = 89
LBP : NP4
P18428
310:310
14:14
1:1
20:36997655:C:T_T
p = 2.3e−05, beta = −0.995, N = 325
ITLPDFTGDLR pc2
P18428
310:310
14:14
1:1
20:36997655:C:T_T
p = 0.00019, beta = −0.88, N = 325
LAEGFPLPLLK pc2
P18428
307:310
14:14
1:1
20:36997655:C:T_T
p = 0.0027, beta = −0.71, N = 322
SPVTLLAAVMSLPEEHNK pc3
P18428
308:310
14:14
1:1
20:36997655:C:T_T
p = 0.0099, beta = −0.612, N = 323
ATAQMLEVMFK pc2
P18428
310:310
14:14
1:1
20:36997655:C:T_T
p = 2e−04, beta = −0.877, N = 325
GLQYAAQEGLLALQSELLR pc3
P18428
307:310
13:14
1:1
20:36997655:C:T_T
p = 9.3e−05, beta = −0.945, N = 321
LQGSFDVSVK pc2
P18428
310:310
14:14
1:1
20:36997655:C:T_T
p = 0.019, beta = −0.555, N = 325
DFLFLGANVQYMR pc2
P18428
310:310
14:14
1:1
20:36997655:C:T_T
p = 2.9e−05, beta = −0.983, N = 325
ICEMIQK pc2
P18428
310:310
14:14
1:1
20:36997655:C:T_T
p = 0.059, beta = −0.449, N = 325
VQLYDLGLQIHK pc3
P18428
310:310
14:14
1:1
20:36997655:C:T_T
p = 4.4e−06, beta = −1.08, N = 325
ITLPDFTGDLRIPHVGR pc4
P18428
310:310
14:14
1:1
20:36997655:C:T_T
p = 0.013, beta = −0.591, N = 325
RVQLYDLGLQIHK pc3
P18428
310:310
14:14
1:1
20:36997655:C:T_T
p = 0.0015, beta = −0.751, N = 325
DFLFLGANVQYMRV pc2
P18428
173:310
6:14
1:1
20:36997655:C:T_T
p = 0.75, beta = 0.102, N = 180
LAEGFPLPLLKR pc3
P18428
64:310
4:14
0:1
20:36997655:C:T_T
p = 0.43, beta = 0.39, N = 68
Assay Target: LBP
UniProt: P18428
rsID: rs2232613
Target SNP: 20:38369011:T:C
Proxy SNP: 20:36997655:C:T
deCODE log10(p): 1358.6
deCODE BETA: −1.05
deCODE MSPA: 0.88
1258:1259:1259:1259:1259:125
VGLFNAELLEALLNYYILNTFYPK pc
rs2232618 REF
510:1072
73:178
2:10
20:36997655:C:T_T
p = 0.057, model = REC, N = 585
VGLFNAELLEALLNYYILNTLYPK pc
rs2232618 ALT
211:1072
15:178
0:10
20:36997655:C:T_T
p = 5e−05, model = REC, N = 226

32. PDIA5 (Q14554)
deCODE MSPA=0.989

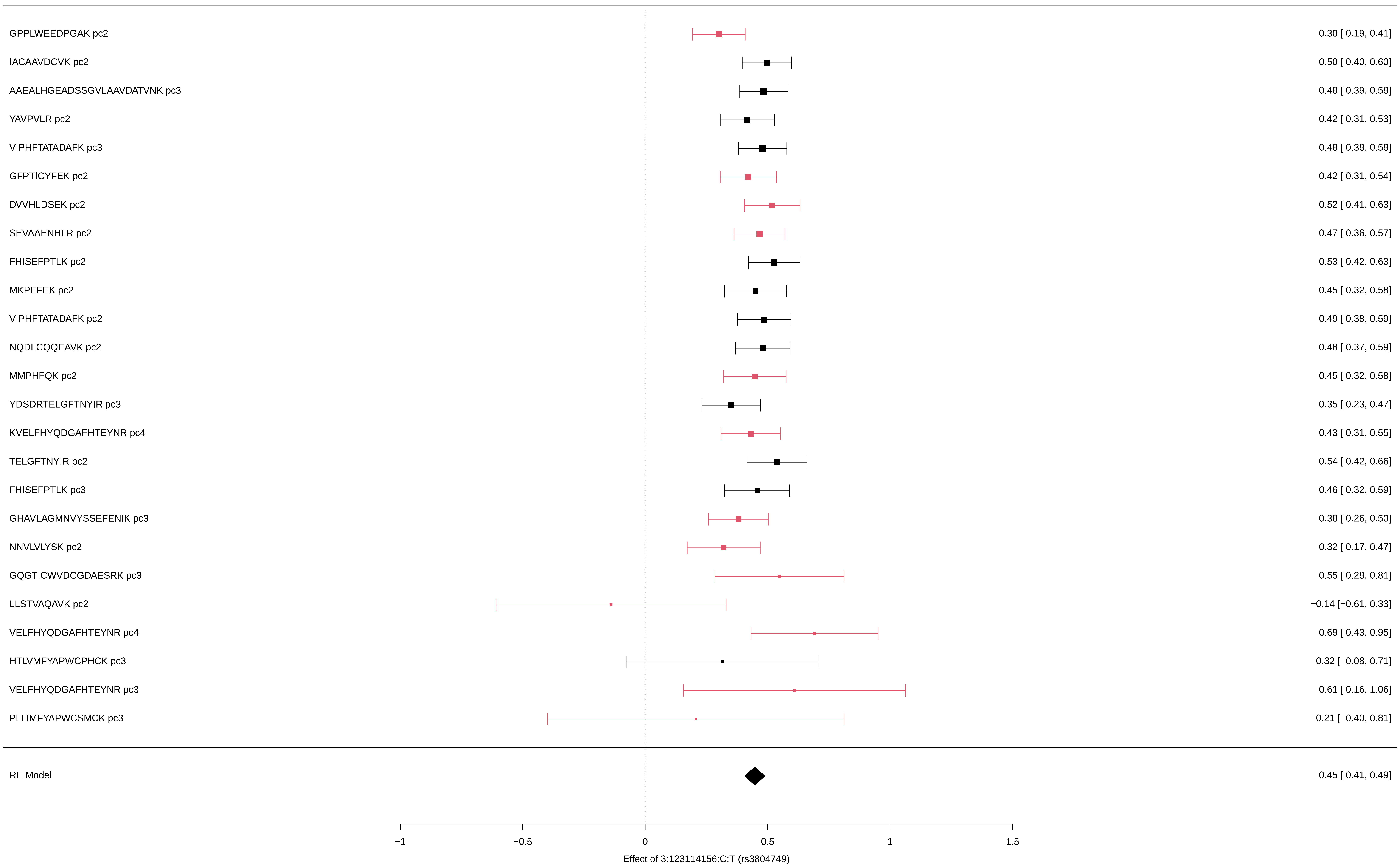

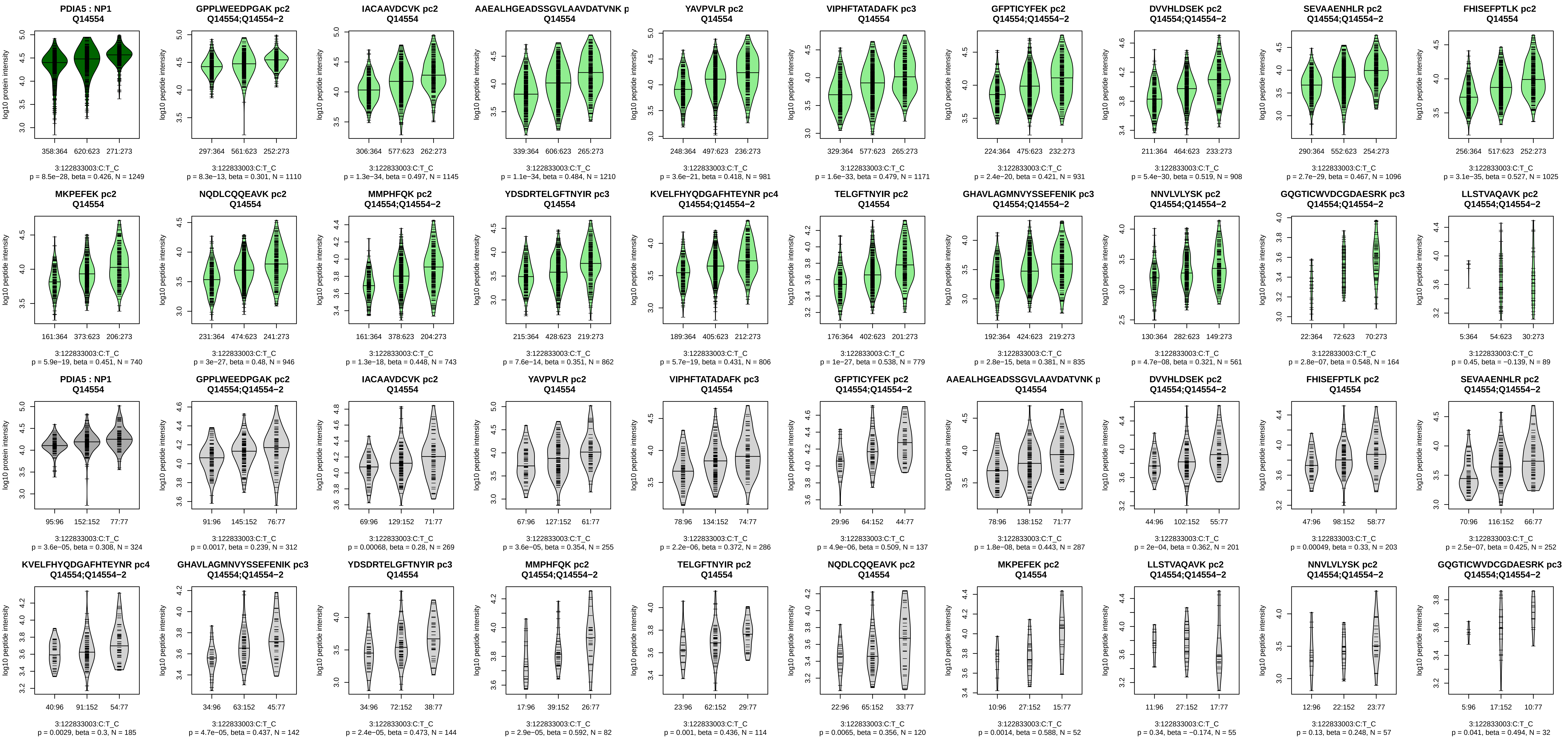

Assay Target: PDIA5
UniProt: Q14554
rsID: rs3804749
Target SNP: 3:123114156:C:T
Proxy SNP: 3:122833003:C:T
deCODE log10(p): 1276
deCODE BETA: 0.63
deCODE MSPA: 0.989
1110:1145:1210:981:1171:931:9

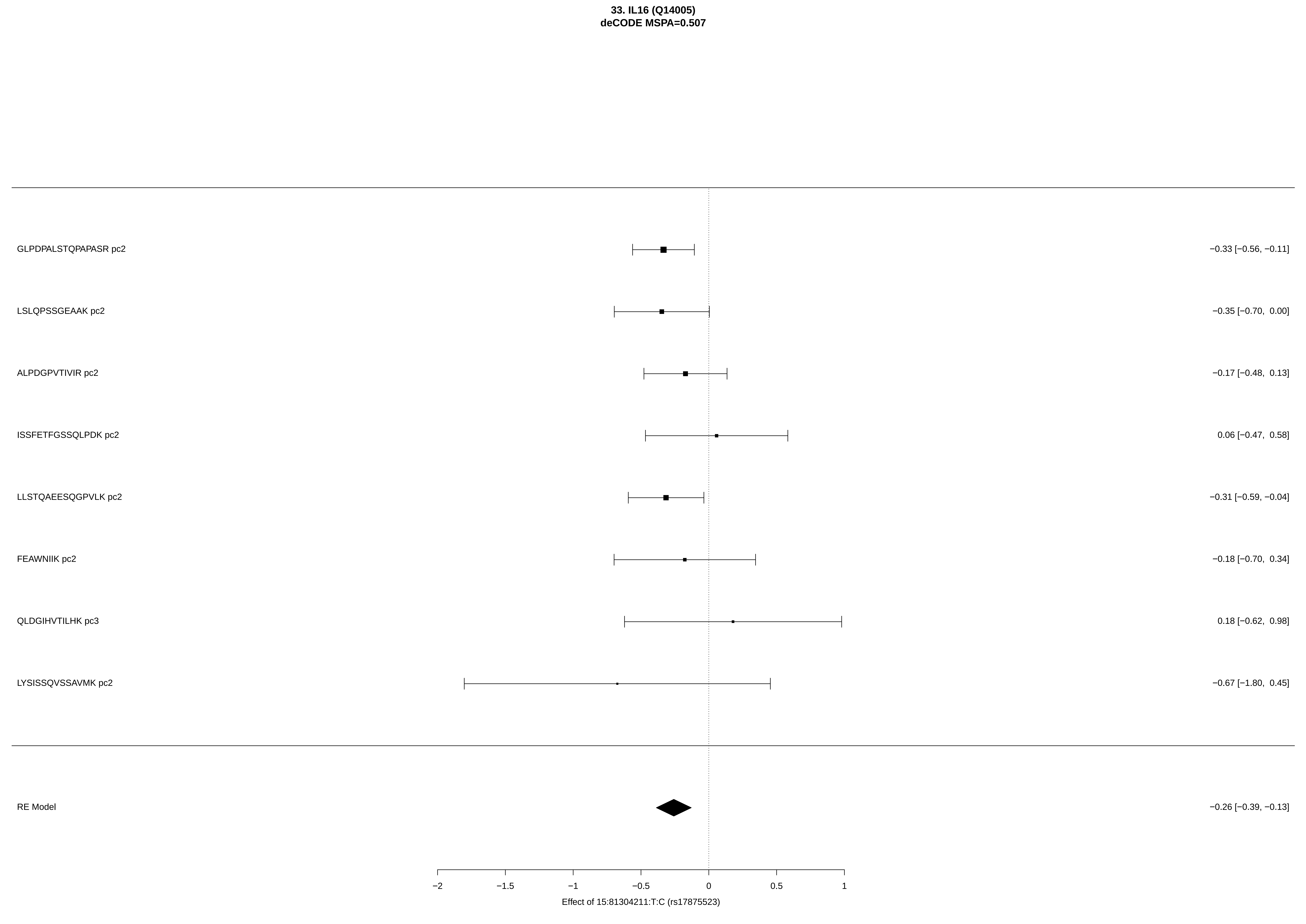
33. IL16 (Q14005)
deCODE MSPA=0.507
GLPDPALSTQPAPASR pc2
−0.33 [−0.56, −0.11]
LSLQPSSGEAAK pc2
−0.35 [−0.70, 0.00]
ALPDGPVTIVIR pc2
−0.17 [−0.48, 0.13]
ISSFETFGSSQLPDK pc2
0.06 [−0.47, 0.58]
LLSTQAEESQGPVLK pc2
−0.31 [−0.59, −0.04]
FEAWNIIK pc2
−0.18 [−0.70, 0.34]
QLDGIHVTILHK pc3
0.18 [−0.62, 0.98]
LYSISSQVSSAVMK pc2
−0.67 [−1.80, 0.45]
RE Model
−0.26 [−0.39, −0.13]
−2
−1.5
−1
−0.5
0
0.5
1
Effect of 15:81304211:T:C (rs17875523)

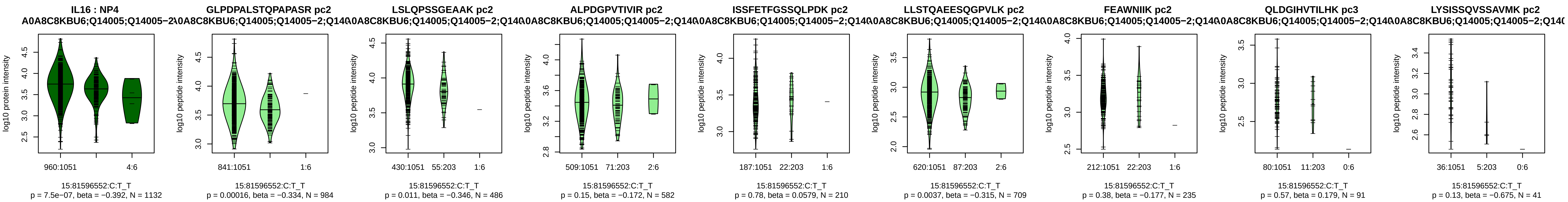

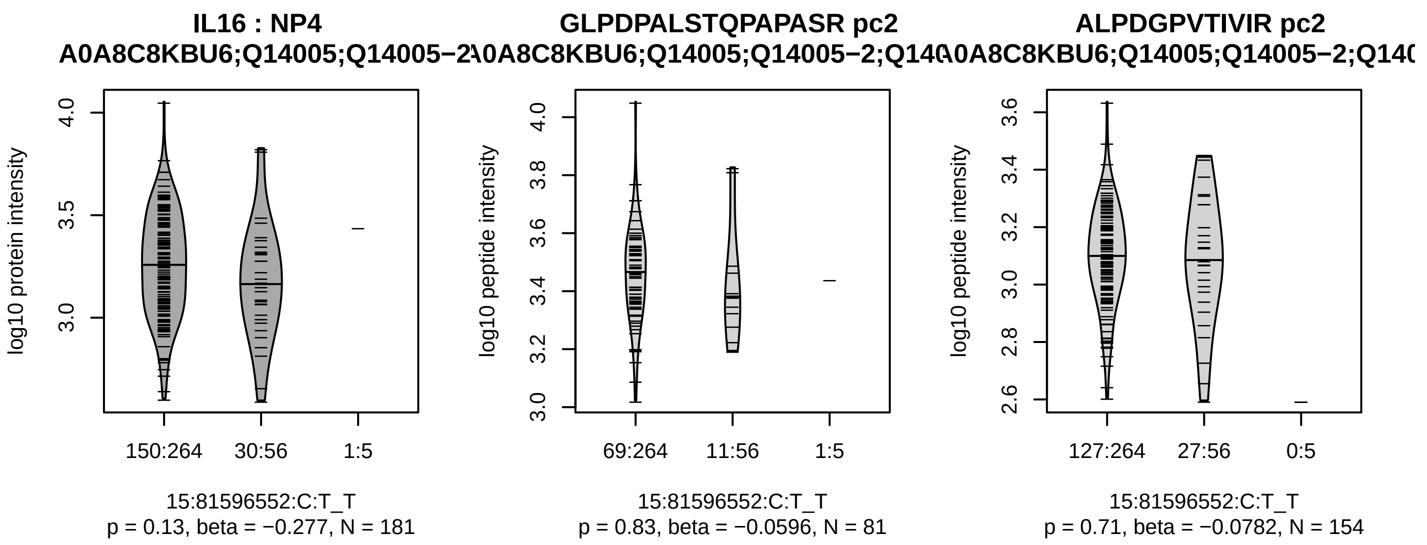

Assay Target: IL16
UniProt: Q14005
rsID: rs17875523
Target SNP: 15:81304211:T:C
Proxy SNP: 15:81596552:C:T
deCODE log10(p): 1235
deCODE BETA: −1.06
deCODE MSPA: 0.507
:−:−:−::−:−:−
984:486:582:210:709:235:91:41

34. SERPING1 (P05155)
deCODE MSPA=0

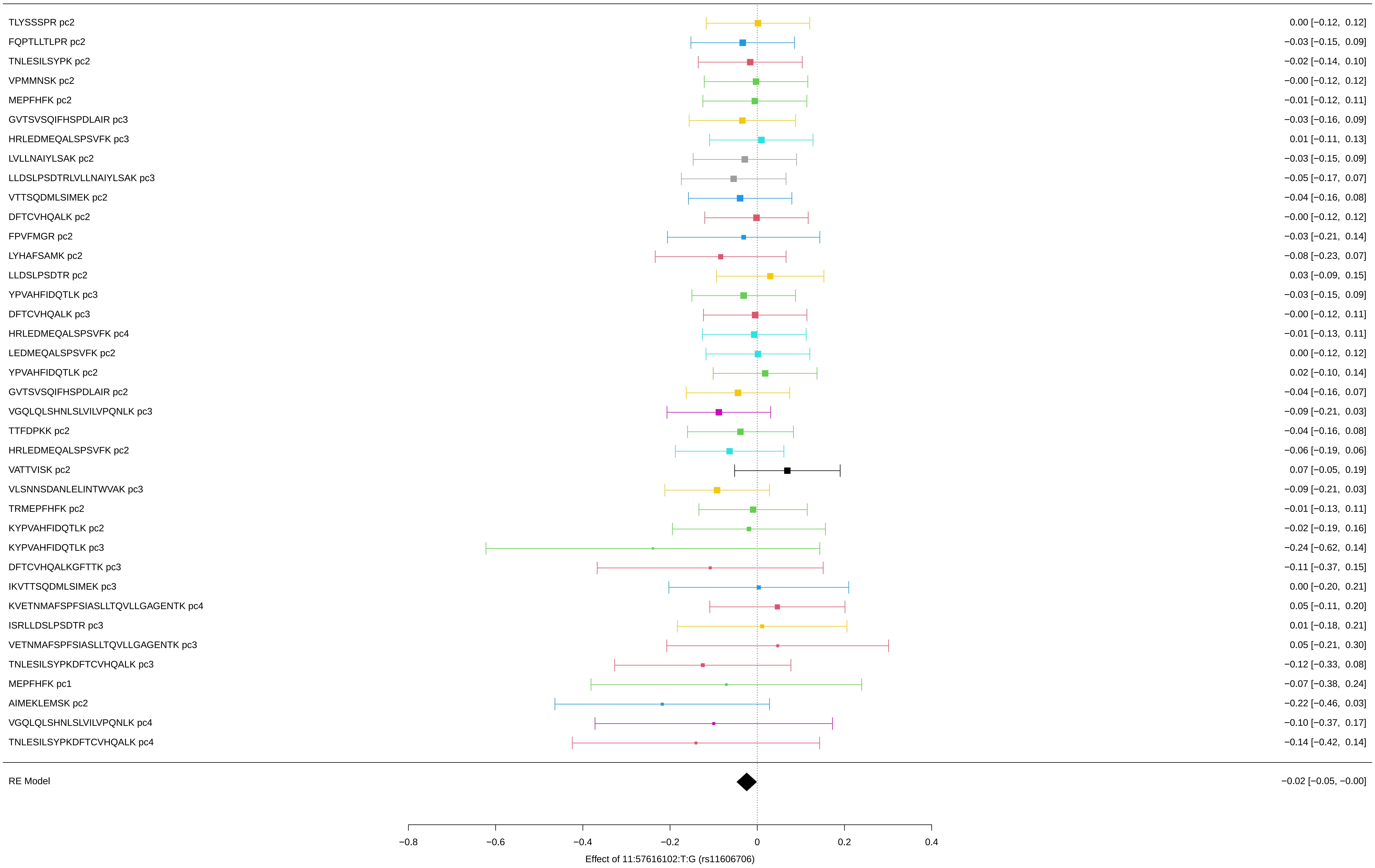

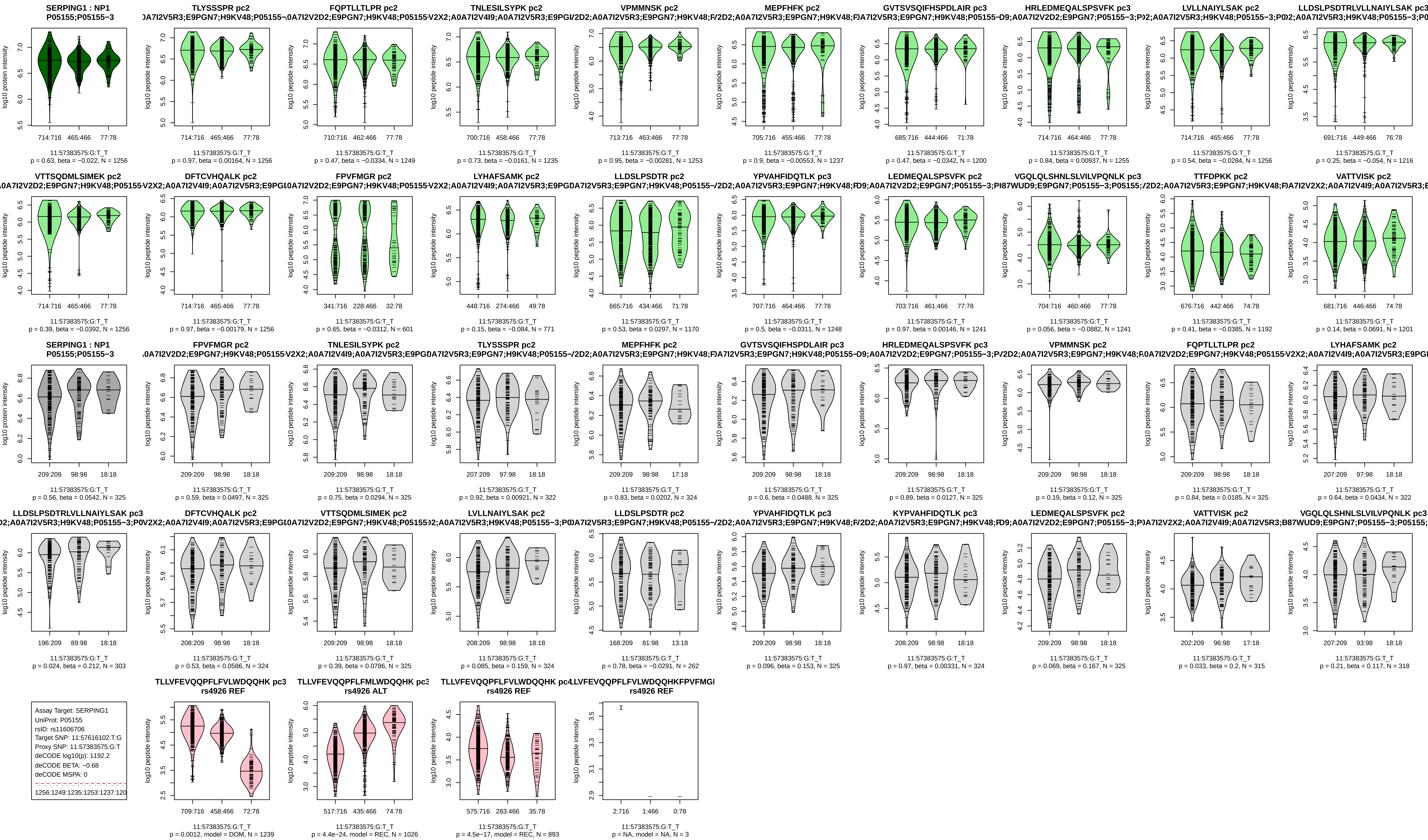
SERPING1 : NP1
P05155;P05155−3
714:716 465:466 77:78
11:57383575:G:T_T
p = 0.63, beta = −0.022, N = 1256
TLYSSSPR pc2
p = 0.97, beta = 0.00164, N = 1256
FQPTLLTLPR pc2
p = 0.47, beta = −0.0334, N = 1249
TNLESILSYPK pc2
p = 0.73, beta = −0.0161, N = 1235
VPMMNSK pc2
p = 0.95, beta = −0.00281, N = 1253
MEPFHFK pc2
p = 0.9, beta = −0.00553, N = 1237
GVTSVSQIFHSPDLAIR pc3
p = 0.47, beta = −0.0342, N = 1200
HRLEDMEQALSPSVFK pc3
p = 0.84, beta = 0.00937, N = 1255
LVLLNAIYLSAK pc2
p = 0.54, beta = −0.0284, N = 1256
LLDSLPSDTRLVLLNAIYLSAK pc3
p = 0.25, beta = −0.054, N = 1216
VTTSQDMLSIMEK pc2
p = 0.39, beta = −0.0392, N = 1256
DFTCVHQALK pc2
p = 0.97, beta = −0.00179, N = 1256
FPVFMGR pc2
p = 0.65, beta = −0.0312, N = 601
LYHAFSAMK pc2
p = 0.15, beta = −0.084, N = 771
LLDSLPSDTR pc2
p = 0.53, beta = 0.0297, N = 1170
YPVAHFIDQTLK pc3
p = 0.5, beta = −0.0311, N = 1248
LEDMEQALSPSVFK pc2
p = 0.97, beta = 0.00146, N = 1241
VGQLQLSHNLSLVILVPQNLK pc3
p = 0.056, beta = −0.0882, N = 1241
TTFDPKK pc2
p = 0.41, beta = −0.0385, N = 1192
VATTVISK pc2
p = 0.14, beta = 0.0691, N = 1201
SERPING1 : NP1
P05155;P05155−3
p = 0.56, beta = 0.0542, N = 325
FPVFMGR pc2
p = 0.59, beta = 0.0497, N = 325
TNLESILSYPK pc2
p = 0.75, beta = 0.0294, N = 325
TLYSSSPR pc2
p = 0.92, beta = 0.00921, N = 322
MEPFHFK pc2
p = 0.83, beta = 0.0202, N = 324
GVTSVSQIFHSPDLAIR pc3
p = 0.6, beta = 0.0488, N = 325
HRLEDMEQALSPSVFK pc3
p = 0.89, beta = 0.0127, N = 325
VPMMNSK pc2
p = 0.19, beta = 0.12, N = 325
FQPTLLTLPR pc2
p = 0.84, beta = 0.0185, N = 325
LYHAFSAMK pc2
p = 0.64, beta = 0.0434, N = 322
LLDSLPSDTRLVLLNAIYLSAK pc3
p = 0.024, beta = 0.212, N = 303
DFTCVHQALK pc2
p = 0.53, beta = 0.0586, N = 324
VTTSQDMLSIMEK pc2
p = 0.39, beta = 0.0786, N = 325
LVLLNAIYLSAK pc2
p = 0.085, beta = 0.159, N = 324
LLDSLPSDTR pc2
p = 0.78, beta = −0.0291, N = 262
YPVAHFIDQTLK pc3
p = 0.096, beta = 0.153, N = 325
KYPVAHFIDQTLK pc3
p = 0.97, beta = 0.00331, N = 324
LEDMEQALSPSVFK pc2
p = 0.069, beta = 0.167, N = 325
VATTVISK pc2
p = 0.033, beta = 0.2, N = 315
VGQLQLSHNLSLVILVPQNLK pc3
p = 0.21, beta = 0.117, N = 318
Assay Target: SERPING1
UniProt: P05155
rsID: rs11606706
Target SNP: 11:57616102:T:G
Proxy SNP: 11:57383575:G:T
deCODE log10(p): 1192.2
deCODE BETA: −0.68
deCODE MSPA: 0
1256:1249:1235:1253:1237:120
TLLVFEVQQPFLFVLWDQQHK pc3
rs4926 REF
p = 0.0012, model = DOM, N = 1239
TLLVFEVQQPFLFMLWDQQHK pc3
rs4926 ALT
p = 4.4e−24, model = REC, N = 1026
TLLVFEVQQPFLFVLWDQQHK pc4
rs4926 REF
p = 4.5e−17, model = REC, N = 893
LLVFEVQQPFLFVLWDQQHKFPVFMGI
rs4926 REF
p = NA, model = NA, N = 3

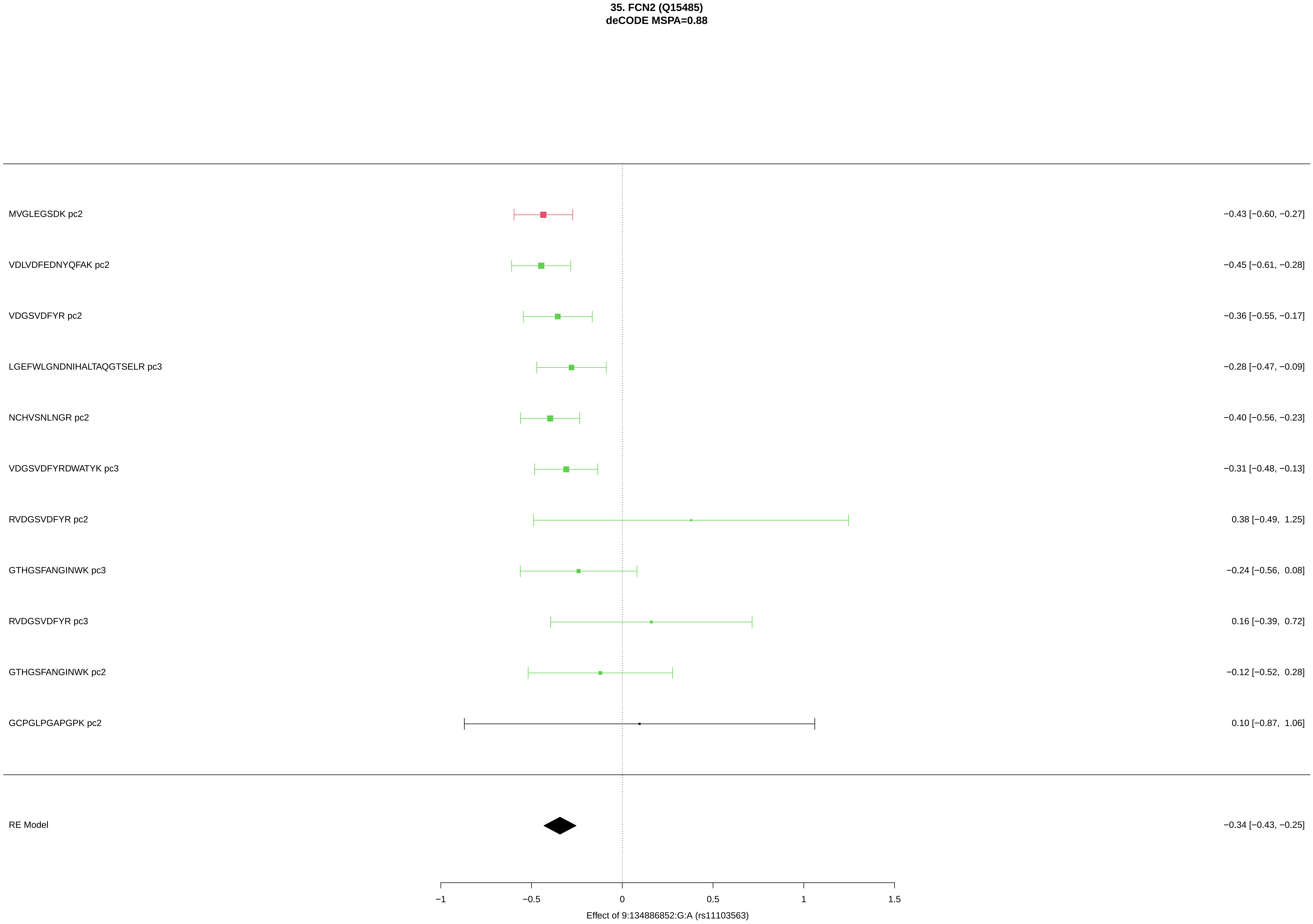
35. FCN2 (Q15485)
deCODE MSPA=0.88
MVGLEGSDK pc2
−0.43 [−0.60, −0.27]
VDLVDFEDNYQFAK pc2
−0.45 [−0.61, −0.28]
VDGSVDFYR pc2
−0.36 [−0.55, −0.17]
LGEFWLGNDNIHALTAQGTSELR pc3
−0.28 [−0.47, −0.09]
NCHVSNLNGR pc2
−0.40 [−0.56, −0.23]
VDGSVDFYRDWATYK pc3
−0.31 [−0.48, −0.13]
RVDGSVDFYR pc2
0.38 [−0.49, 1.25]
GTHGSFANGINWK pc3
−0.24 [−0.56, 0.08]
RVDGSVDFYR pc3
0.16 [−0.39, 0.72]
GTHGSFANGINWK pc2
−0.12 [−0.52, 0.28]
GCPGLPGAPGPK pc2
0.10 [−0.87, 1.06]
RE Model
−0.34 [−0.43, −0.25]
−1
−0.5
0
0.5
1
1.5
Effect of 9:134886852:G:A (rs11103563)

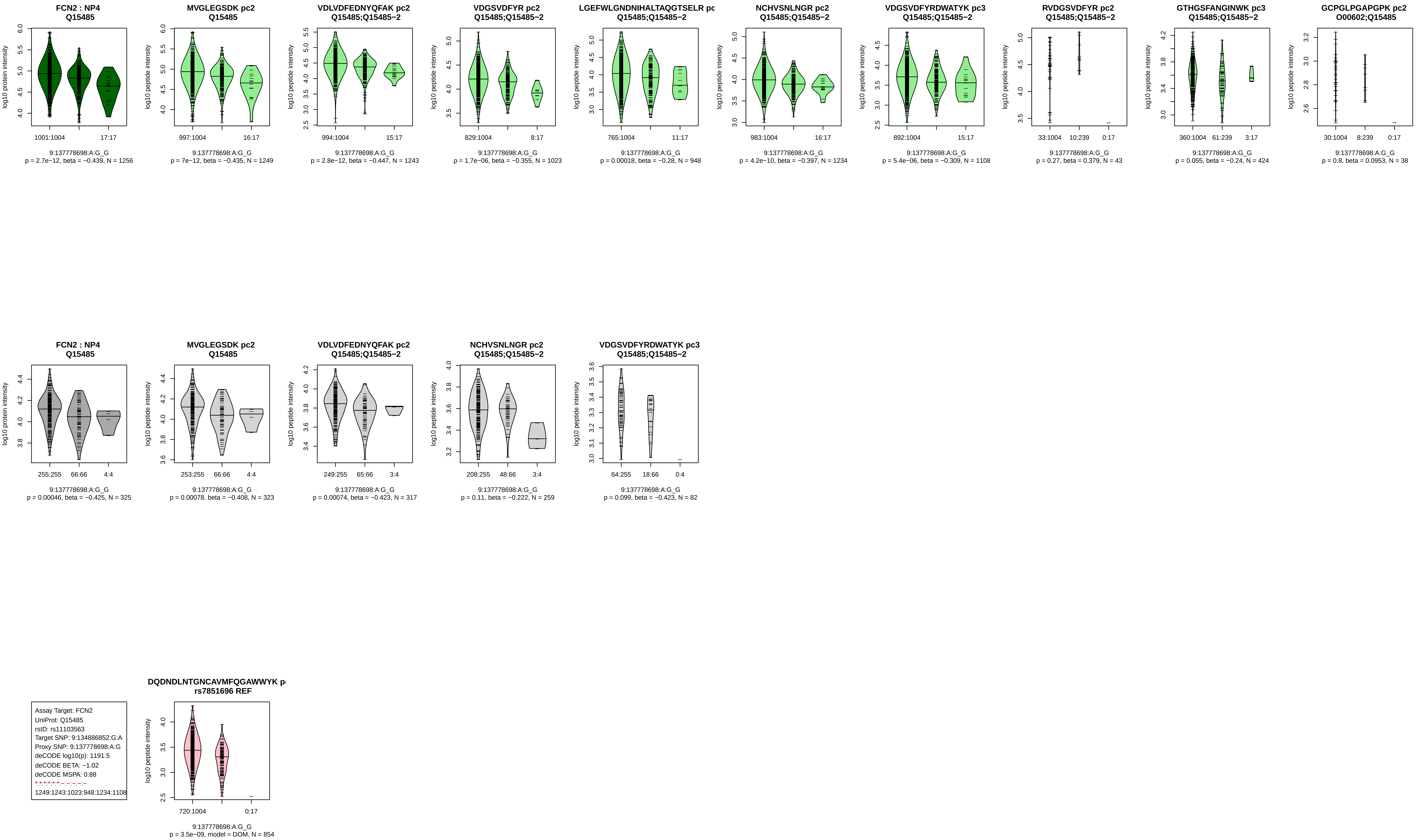
FCN2 : NP4
Q15485
log10 protein intensity
1001:1004
17:17
9:137778698:A:G_G
p = 2.7e−12, beta = −0.439, N = 1256
MVGLEGSDK pc2
Q15485
log10 peptide intensity
997:1004
16:17
9:137778698:A:G_G
p = 7e−12, beta = −0.435, N = 1249
VDLVDFEDNYQFAK pc2
Q15485;Q15485−2
994:1004
15:17
9:137778698:A:G_G
p = 2.8e−12, beta = −0.447, N = 1243
VDGSVDFYR pc2
Q15485;Q15485−2
829:1004
8:17
9:137778698:A:G_G
p = 1.7e−06, beta = −0.355, N = 1023
LGEFWLGNDNIHALTAQGTSELR pc
Q15485;Q15485−2
765:1004
11:17
9:137778698:A:G_G
p = 0.00018, beta = −0.28, N = 948
NCHVSNLNGR pc2
Q15485;Q15485−2
983:1004
16:17
9:137778698:A:G_G
p = 4.2e−10, beta = −0.397, N = 1234
VDGSVDFYRDWATYK pc3
Q15485;Q15485−2
892:1004
15:17
9:137778698:A:G_G
p = 5.4e−06, beta = −0.309, N = 1108
RVDGSVDFYR pc2
Q15485;Q15485−2
33:1004
10:239
0:17
9:137778698:A:G_G
p = 0.27, beta = 0.379, N = 43
GTHGSFANGINWK pc3
Q15485;Q15485−2
360:1004
61:239
3:17
9:137778698:A:G_G
p = 0.055, beta = −0.24, N = 424
GCPGLPGAPGPK pc2
O00602;Q15485
30:1004
8:239
0:17
9:137778698:A:G_G
p = 0.8, beta = 0.0953, N = 38
FCN2 : NP4
Q15485
log10 protein intensity
255:255
66:66
4:4
9:137778698:A:G_G
p = 0.00046, beta = −0.425, N = 325
MVGLEGSDK pc2
Q15485
253:255
66:66
4:4
9:137778698:A:G_G
p = 0.00078, beta = −0.408, N = 323
VDLVDFEDNYQFAK pc2
Q15485;Q15485−2
249:255
65:66
3:4
9:137778698:A:G_G
p = 0.00074, beta = −0.423, N = 317
NCHVSNLNGR pc2
Q15485;Q15485−2
208:255
48:66
3:4
9:137778698:A:G_G
p = 0.11, beta = −0.222, N = 259
VDGSVDFYRDWATYK pc3
Q15485;Q15485−2
64:255
18:66
0:4
9:137778698:A:G_G
p = 0.099, beta = −0.423, N = 82
Assay Target: FCN2
UniProt: Q15485
rsID: rs11103563
Target SNP: 9:134886852:G:A
Proxy SNP: 9:137778698:A:G
deCODE log10(p): 1191.5
deCODE BETA: −1.02
deCODE MSPA: 0.88
1249:1243:1023:948:1234:1108
DQDNDLNTGNCAVMFQGAWWYK p
rs7851696 REF
log10 peptide intensity
720:1004
0:17
9:137778698:A:G_G
p = 3.5e−09, model = DOM, N = 854

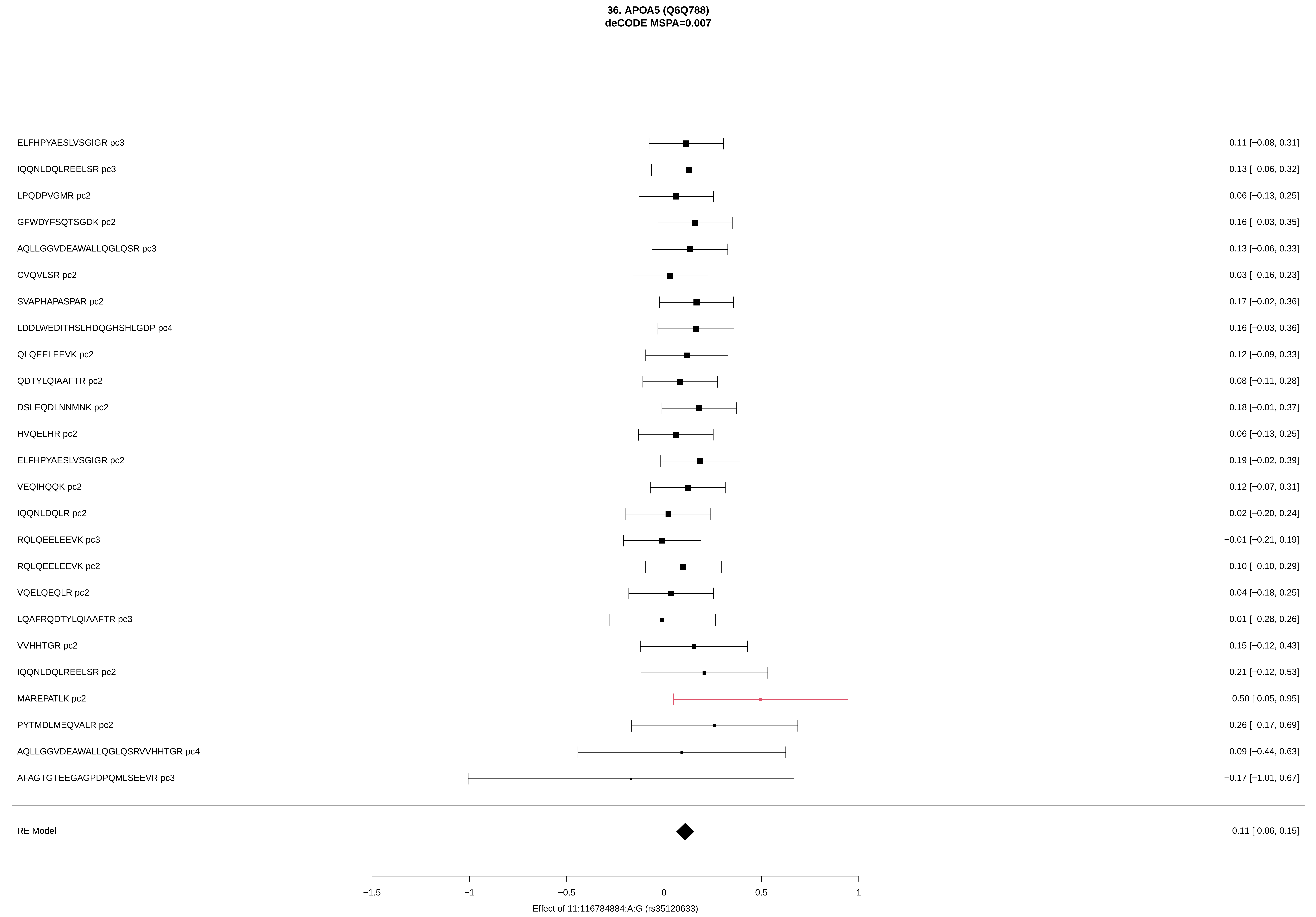
36. APOA5 (Q6Q788)
deCODE MSPA=0.007
ELFHPYAESLVSGIGR pc3
0.11 [−0.08, 0.31]
IQQNLDQLREELSR pc3
0.13 [−0.06, 0.32]
LPQDPVGMR pc2
0.06 [−0.13, 0.25]
GFWDYFSQTSGDK pc2
0.16 [−0.03, 0.35]
AQLLGGVDEAWALLQGLQSR pc3
0.13 [−0.06, 0.33]
CVQVLSR pc2
0.03 [−0.16, 0.23]
SVAPHAPASPAR pc2
0.17 [−0.02, 0.36]
LDDLWEDITHSLHDQGHSHLGDP pc4
0.16 [−0.03, 0.36]
QLQEELEEVK pc2
0.12 [−0.09, 0.33]
QDTYLQIAAFTR pc2
0.08 [−0.11, 0.28]
DSLEQDLNNMNK pc2
0.18 [−0.01, 0.37]
HVQELHR pc2
0.06 [−0.13, 0.25]
ELFHPYAESLVSGIGR pc2
0.19 [−0.02, 0.39]
VEQIHQQK pc2
0.12 [−0.07, 0.31]
IQQNLDQLR pc2
0.02 [−0.20, 0.24]
RQLQEELEEVK pc3
−0.01 [−0.21, 0.19]
RQLQEELEEVK pc2
0.10 [−0.10, 0.29]
VQELQEQLR pc2
0.04 [−0.18, 0.25]
LQAFRQDTYLQIAAFTR pc3
−0.01 [−0.28, 0.26]
VVHHTGR pc2
0.15 [−0.12, 0.43]
IQQNLDQLREELSR pc2
0.21 [−0.12, 0.53]
MAREPATLK pc2
0.50 [0.05, 0.95]
PYTMDLMEQVALR pc2
0.26 [−0.17, 0.69]
AQLLGGVDEAWALLQGLQSRVVHHTGR pc4
0.09 [−0.44, 0.63]
AFAGTGTEEGAGPDPQMLSEEVR pc3
−0.17 [−1.01, 0.67]
RE Model
0.11 [0.06, 0.15]
−1.5
−1
−0.5
0
0.5
1
Effect of 11:116784884:A:G (rs35120633)

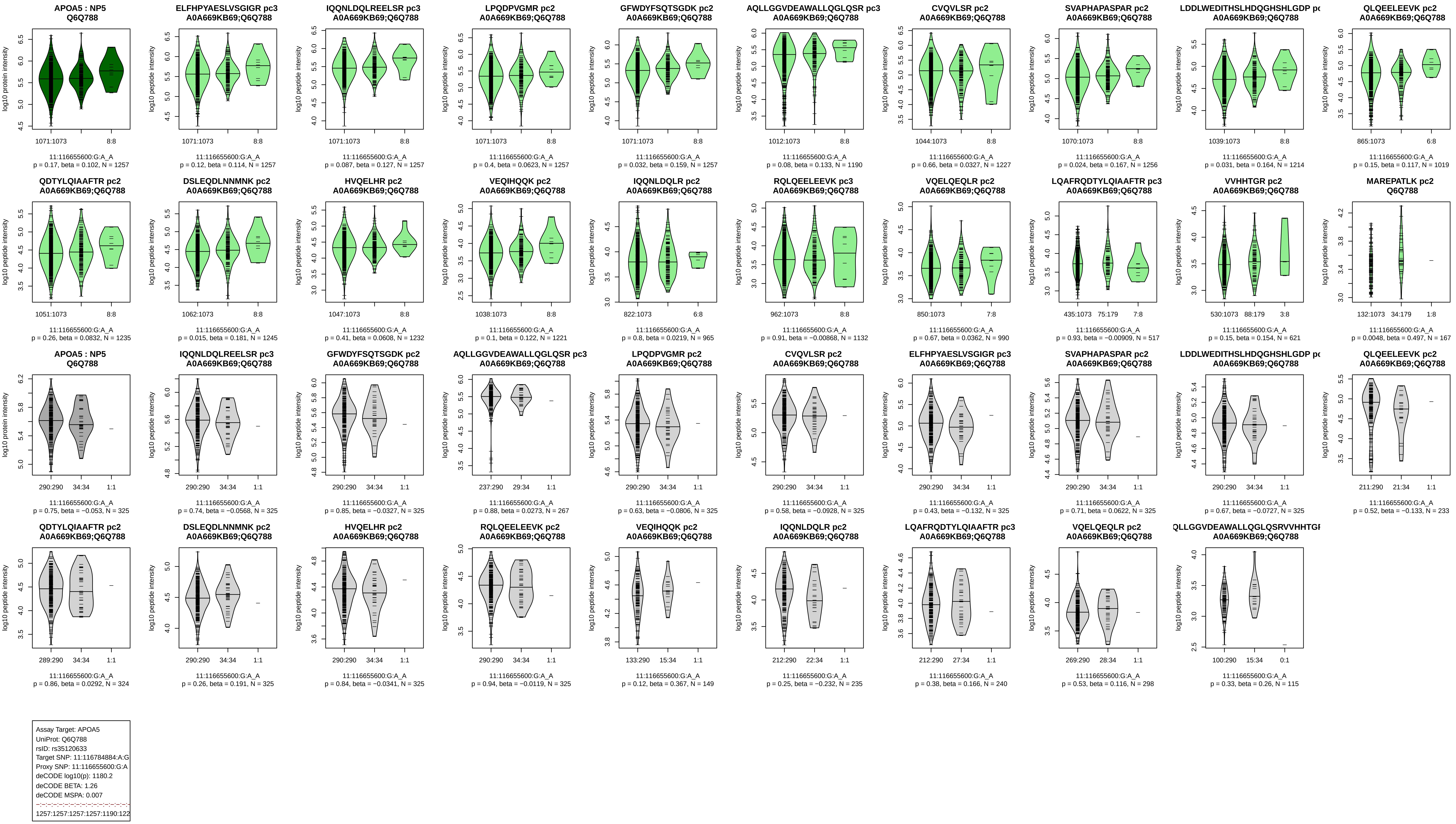
APOA5 : NP5
Q6Q788
log10 protein intensity
1071:1073
8:8
11:116655600:G:A_A
p = 0.17, beta = 0.102, N = 1257
ELFHPYAESLVSGIGR pc3
A0A669KB69;Q6Q788
log10 peptide intensity
1071:1073
8:8
11:116655600:G:A_A
p = 0.12, beta = 0.114, N = 1257
IQQNLDQLREELSR pc3
A0A669KB69;Q6Q788
1071:1073
8:8
11:116655600:G:A_A
p = 0.087, beta = 0.127, N = 1257
LPQDPVGMR pc2
A0A669KB69;Q6Q788
1071:1073
8:8
11:116655600:G:A_A
p = 0.4, beta = 0.0623, N = 1257
GFWDYFSQTSGDK pc2
A0A669KB69;Q6Q788
1071:1073
8:8
11:116655600:G:A_A
p = 0.032, beta = 0.159, N = 1257
AQLLGGVDEAWALLQGLQSR pc3
A0A669KB69;Q6Q788
1012:1073
8:8
11:116655600:G:A_A
p = 0.08, beta = 0.133, N = 1190
CVQVLSR pc2
A0A669KB69;Q6Q788
1044:1073
8:8
11:116655600:G:A_A
p = 0.66, beta = 0.0327, N = 1227
SVAPHAPASPAR pc2
A0A669KB69;Q6Q788
1070:1073
8:8
11:116655600:G:A_A
p = 0.024, beta = 0.167, N = 1256
LDDLWEDITHSLHDQGHSHLGDP pc
A0A669KB69;Q6Q788
1039:1073
8:8
11:116655600:G:A_A
p = 0.031, beta = 0.164, N = 1214
QLQEELEEVK pc2
A0A669KB69;Q6Q788
865:1073
6:8
11:116655600:G:A_A
p = 0.15, beta = 0.117, N = 1019
QDTYLQIAAFTR pc2
A0A669KB69;Q6Q788
1051:1073
8:8
11:116655600:G:A_A
p = 0.26, beta = 0.0832, N = 1235
DSLEQDLNNMNK pc2
A0A669KB69;Q6Q788
1062:1073
8:8
11:116655600:G:A_A
p = 0.015, beta = 0.181, N = 1245
HVQELHR pc2
A0A669KB69;Q6Q788
1047:1073
8:8
11:116655600:G:A_A
p = 0.41, beta = 0.0608, N = 1232
VEQIHQQK pc2
A0A669KB69;Q6Q788
1038:1073
8:8
11:116655600:G:A_A
p = 0.1, beta = 0.122, N = 1221
IQQNLDQLR pc2
A0A669KB69;Q6Q788
822:1073
6:8
11:116655600:G:A_A
p = 0.8, beta = 0.0219, N = 965
RQLQEELEEVK pc3
A0A669KB69;Q6Q788
962:1073
8:8
11:116655600:G:A_A
p = 0.91, beta = −0.00868, N = 1132
VQELQEQLR pc2
A0A669KB69;Q6Q788
850:1073
7:8
11:116655600:G:A_A
p = 0.67, beta = 0.0362, N = 990
LQAFRQDTYLQIAAFTR pc3
A0A669KB69;Q6Q788
435:1073
75:179
7:8
11:116655600:G:A_A
p = 0.93, beta = −0.00909, N = 517
VVHHTGR pc2
A0A669KB69;Q6Q788
530:1073
88:179
3:8
11:116655600:G:A_A
p = 0.15, beta = 0.154, N = 621
MAREPATLK pc2
Q6Q788
132:1073
34:179
1:8
11:116655600:G:A_A
p = 0.0048, beta = 0.497, N = 167
APOA5 : NP5
Q6Q788
290:290
34:34
1:1
11:116655600:G:A_A
p = 0.75, beta = −0.053, N = 325
IQQNLDQLREELSR pc3
A0A669KB69;Q6Q788
290:290
34:34
1:1
11:116655600:G:A_A
p = 0.74, beta = −0.0568, N = 325
GFWDYFSQTSGDK pc2
A0A669KB69;Q6Q788
290:290
34:34
1:1
11:116655600:G:A_A
p = 0.85, beta = −0.0327, N = 325
AQLLGGVDEAWALLQGLQSR pc3
A0A669KB69;Q6Q788
237:290
29:34
1:1
11:116655600:G:A_A
p = 0.88, beta = 0.0273, N = 267
LPQDPVGMR pc2
A0A669KB69;Q6Q788
290:290
34:34
1:1
11:116655600:G:A_A
p = 0.63, beta = −0.0806, N = 325
CVQVLSR pc2
A0A669KB69;Q6Q788
290:290
34:34
1:1
11:116655600:G:A_A
p = 0.58, beta = −0.0928, N = 325
ELFHPYAESLVSGIGR pc3
A0A669KB69;Q6Q788
290:290
34:34
1:1
11:116655600:G:A_A
p = 0.43, beta = −0.132, N = 325
SVAPHAPASPAR pc2
A0A669KB69;Q6Q788
290:290
34:34
1:1
11:116655600:G:A_A
p = 0.71, beta = 0.0622, N = 325
LDDLWEDITHSLHDQGHSHLGDP pc
A0A669KB69;Q6Q788
290:290
34:34
1:1
11:116655600:G:A_A
p = 0.67, beta = −0.0727, N = 325
QLQEELEEVK pc2
A0A669KB69;Q6Q788
211:290
21:34
1:1
11:116655600:G:A_A
p = 0.52, beta = −0.133, N = 233
QDTYLQIAAFTR pc2
A0A669KB69;Q6Q788
289:290
34:34
1:1
11:116655600:G:A_A
p = 0.86, beta = 0.0292, N = 324
DSLEQDLNNMNK pc2
A0A669KB69;Q6Q788
290:290
34:34
1:1
11:116655600:G:A_A
p = 0.26, beta = 0.191, N = 325
HVQELHR pc2
A0A669KB69;Q6Q788
290:290
34:34
1:1
11:116655600:G:A_A
p = 0.84, beta = −0.0341, N = 325
RQLQEELEEVK pc2
A0A669KB69;Q6Q788
290:290
34:34
1:1
11:116655600:G:A_A
p = 0.94, beta = −0.0119, N = 325
VEQIHQQK pc2
A0A669KB69;Q6Q788
133:290
15:34
1:1
11:116655600:G:A_A
p = 0.12, beta = 0.367, N = 149
IQQNLDQLR pc2
A0A669KB69;Q6Q788
212:290
22:34
1:1
11:116655600:G:A_A
p = 0.25, beta = −0.232, N = 235
LQAFRQDTYLQIAAFTR pc3
A0A669KB69;Q6Q788
212:290
27:34
1:1
11:116655600:G:A_A
p = 0.38, beta = 0.166, N = 240
VQELQEQLR pc2
A0A669KB69;Q6Q788
269:290
28:34
1:1
11:116655600:G:A_A
p = 0.53, beta = 0.116, N = 298
QLLGGVDEAWALLQGLQSRVVHHTGF
A0A669KB69;Q6Q788
100:290
15:34
0:1
11:116655600:G:A_A
p = 0.33, beta = 0.26, N = 115
Assay Target: APOA5
UniProt: Q6Q788
rsID: rs35120633
Target SNP: 11:116784884:A:G
Proxy SNP: 11:116655600:G:A
deCODE log10(p): 1180.2
deCODE BETA: 1.26
deCODE MSPA: 0.007
1257:1257:1257:1257:1190:122

37. HP (P00738)
deCODE MSPA=0.443 (2 conflicts)

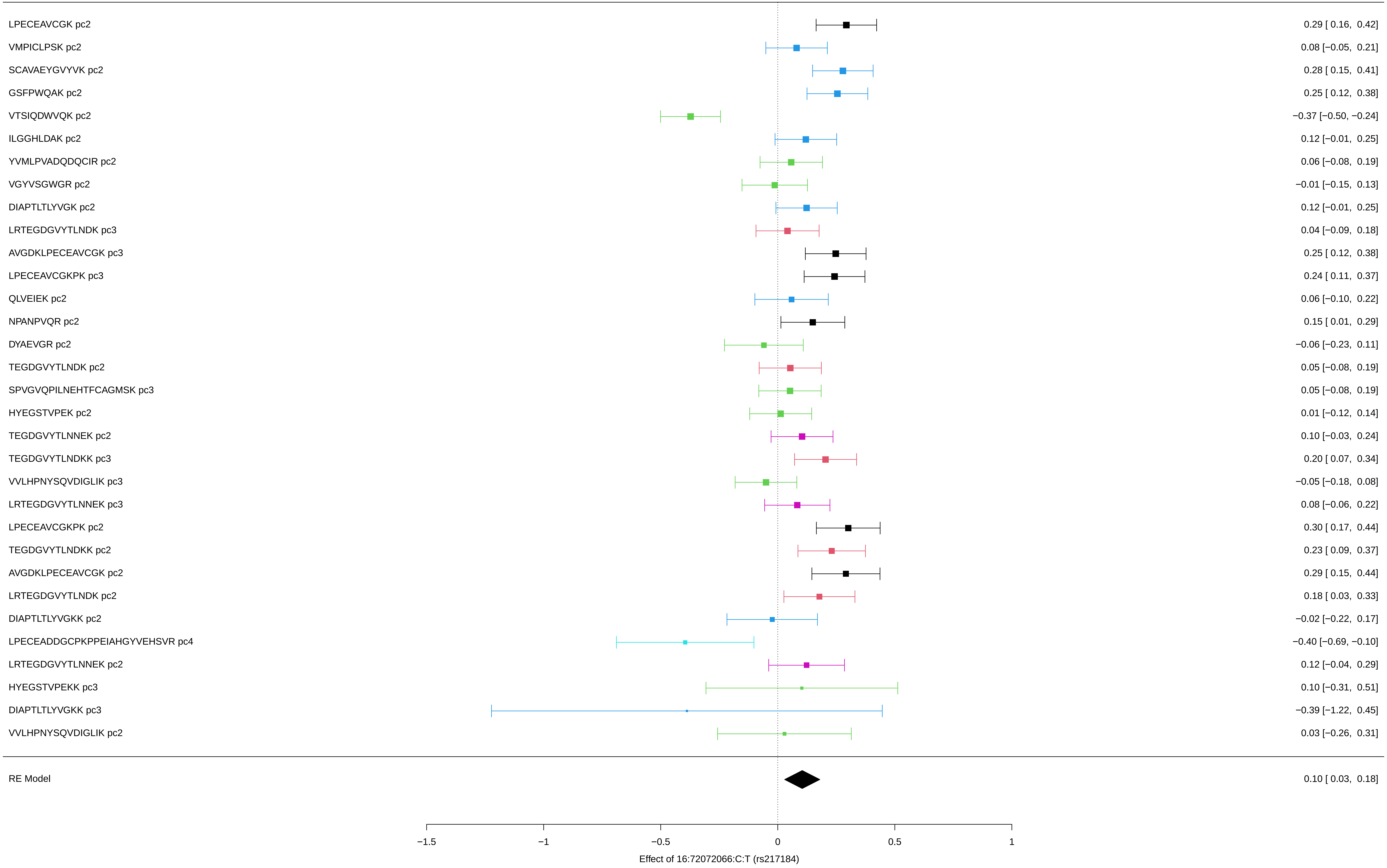

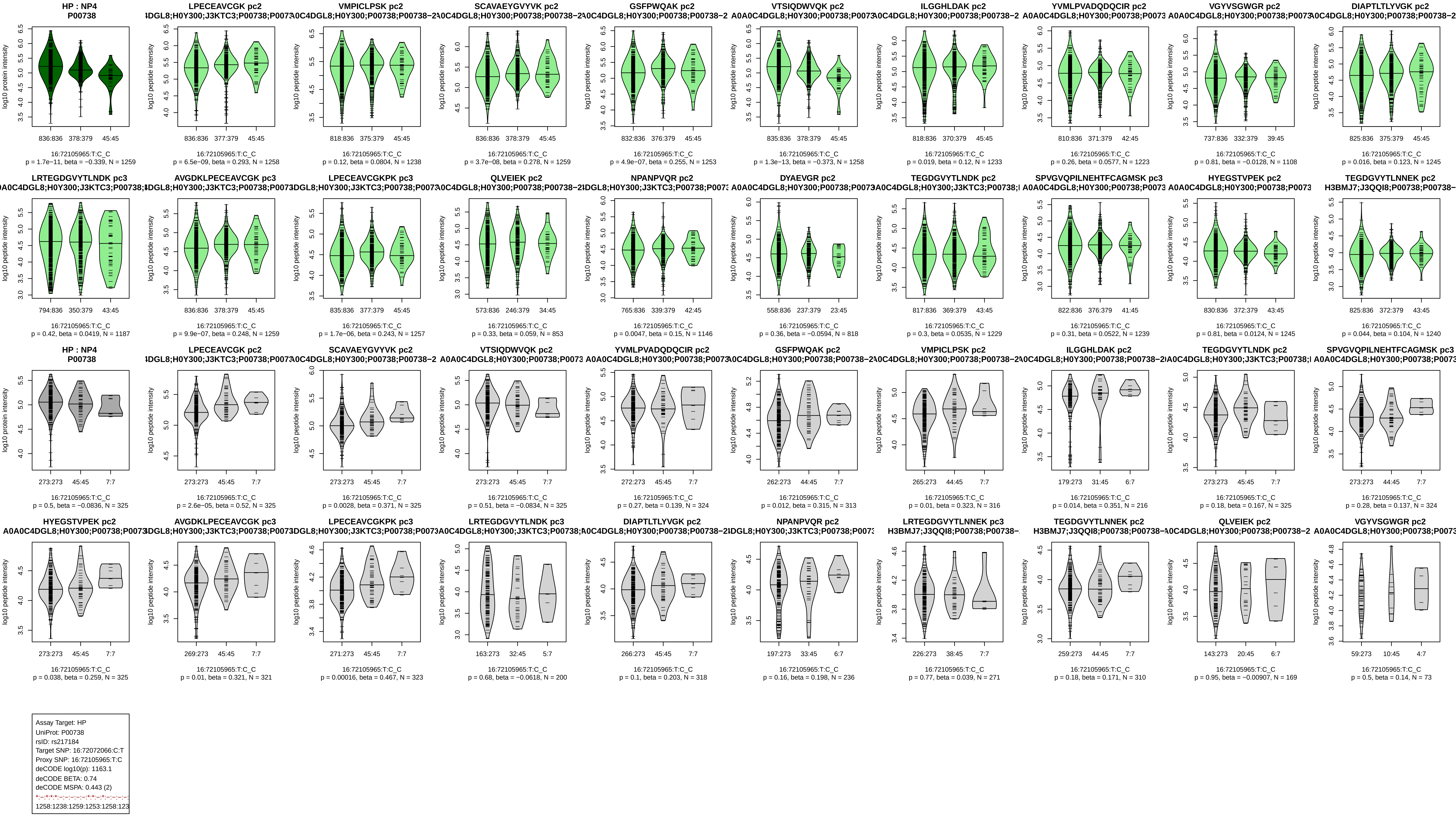
HP : NP4
P00738
16:72105965:T:C_C
p = 1.7e−11, beta = −0.339, N = 1259
LPECEAVCGK pc2
p = 6.5e−09, beta = 0.293, N = 1258
VMPICLPSK pc2
p = 0.12, beta = 0.0804, N = 1238
SCAVAEYGVYVK pc2
p = 3.7e−08, beta = 0.278, N = 1259
GSFPWQAK pc2
p = 4.9e−07, beta = 0.255, N = 1253
VTSIQDWVQK pc2
p = 1.3e−13, beta = −0.373, N = 1258
ILGGHLDAK pc2
p = 0.019, beta = 0.12, N = 1233
YVMLPVADQDQCIR pc2
p = 0.26, beta = 0.0577, N = 1223
VGYVSGWGR pc2
p = 0.81, beta = −0.0128, N = 1108
DIAPTLTLYVGK pc2
p = 0.016, beta = 0.123, N = 1245
LRTEGDGVYTLNDK pc3
p = 0.42, beta = 0.0419, N = 1187
AVGDKLPECEAVCGK pc3
p = 9.9e−07, beta = 0.248, N = 1259
LPECEAVCGKPK pc3
p = 1.7e−06, beta = 0.243, N = 1257
QLVEIEK pc2
p = 0.33, beta = 0.059, N = 853
NPANPVQR pc2
p = 0.0047, beta = 0.15, N = 1146
DYAEVGR pc2
p = 0.36, beta = −0.0594, N = 818
TEGDGVYTLNDK pc2
p = 0.3, beta = 0.0535, N = 1229
SPVGVQPILNEHTFCAGMSK pc3
p = 0.31, beta = 0.0522, N = 1239
HYEGSTVPEK pc2
p = 0.81, beta = 0.0124, N = 1245
TEGDGVYTLNNEK pc2
p = 0.044, beta = 0.104, N = 1240
HP : NP4
P00738
p = 0.5, beta = −0.0836, N = 325
LPECEAVCGK pc2
p = 2.6e−05, beta = 0.52, N = 325
SCAVAEYGVYVK pc2
p = 0.0028, beta = 0.371, N = 325
VTSIQDWVQK pc2
p = 0.51, beta = −0.0834, N = 325
YVMLPVADQDQCIR pc2
p = 0.27, beta = 0.139, N = 324
GSFPWQAK pc2
p = 0.012, beta = 0.315, N = 313
VMPICLPSK pc2
p = 0.01, beta = 0.323, N = 316
ILGGHLDAK pc2
p = 0.014, beta = 0.351, N = 216
TEGDGVYTLNDK pc2
p = 0.18, beta = 0.167, N = 325
SPVGVQPILNEHTFCAGMSK pc3
p = 0.28, beta = 0.137, N = 324
HYEGSTVPEK pc2
p = 0.038, beta = 0.259, N = 325
AVGDKLPECEAVCGK pc3
p = 0.01, beta = 0.321, N = 321
LPECEAVCGKPK pc3
p = 0.00016, beta = 0.467, N = 323
LRTEGDGVYTLNDK pc3
p = 0.68, beta = −0.0618, N = 200
DIAPTLTLYVGK pc2
p = 0.1, beta = 0.203, N = 318
NPANPVQR pc2
p = 0.16, beta = 0.198, N = 236
LRTEGDGVYTLNNEK pc3
p = 0.77, beta = 0.039, N = 271
TEGDGVYTLNNEK pc2
p = 0.18, beta = 0.171, N = 310
QLVEIEK pc2
p = 0.95, beta = −0.00907, N = 169
VGYVSGWGR pc2
p = 0.5, beta = 0.14, N = 73
Assay Target: HP
UniProt: P00738
rsID: rs217184
Target SNP: 16:72072066:C:T
Proxy SNP: 16:72105965:T:C
deCODE log10(p): 1163.1
deCODE BETA: 0.74
deCODE MSPA: 0.443 (2)
1258:1238:1259:1253:1258:123

38. PCYOX1 (Q9UHG3)
deCODE MSPA=0

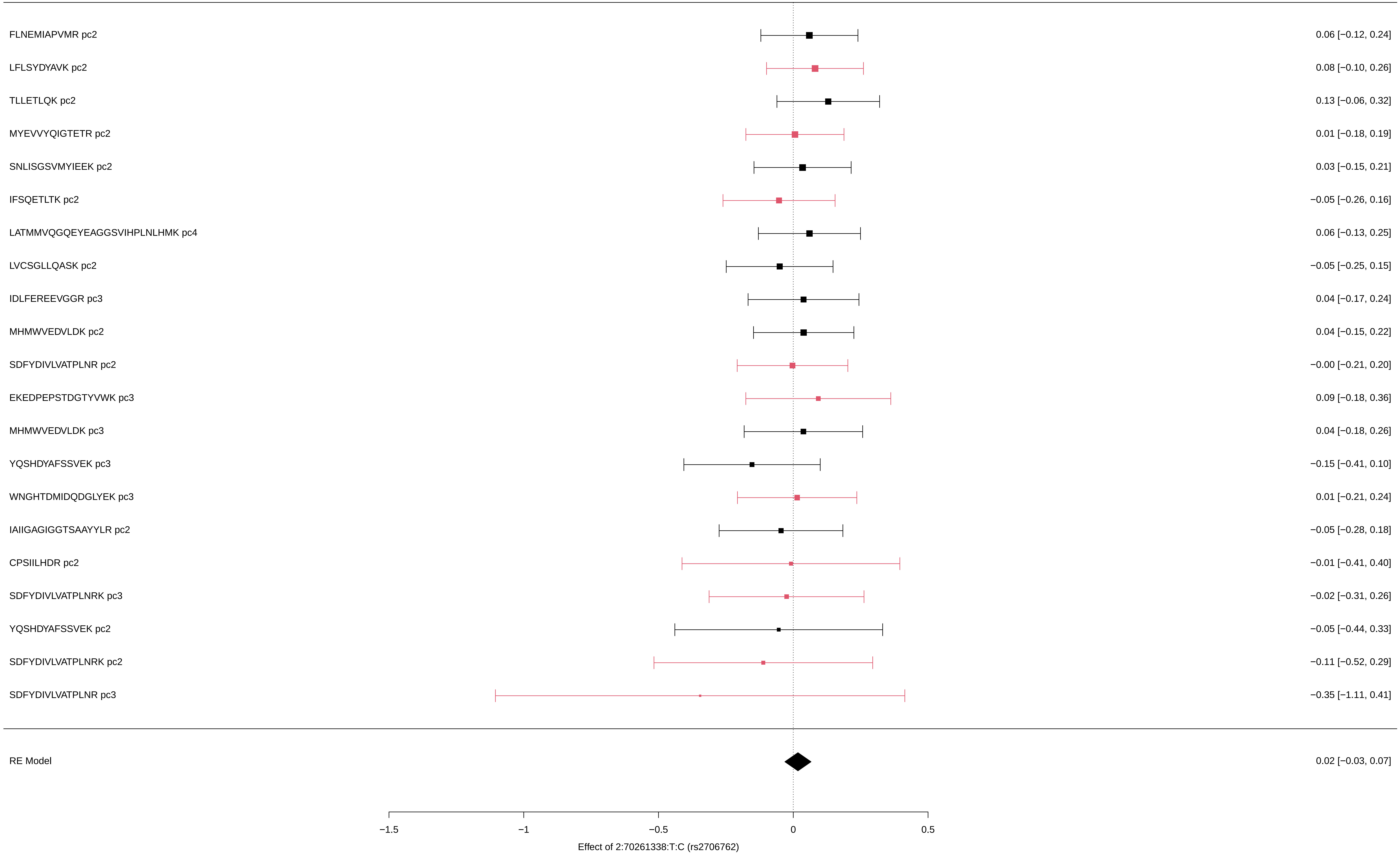

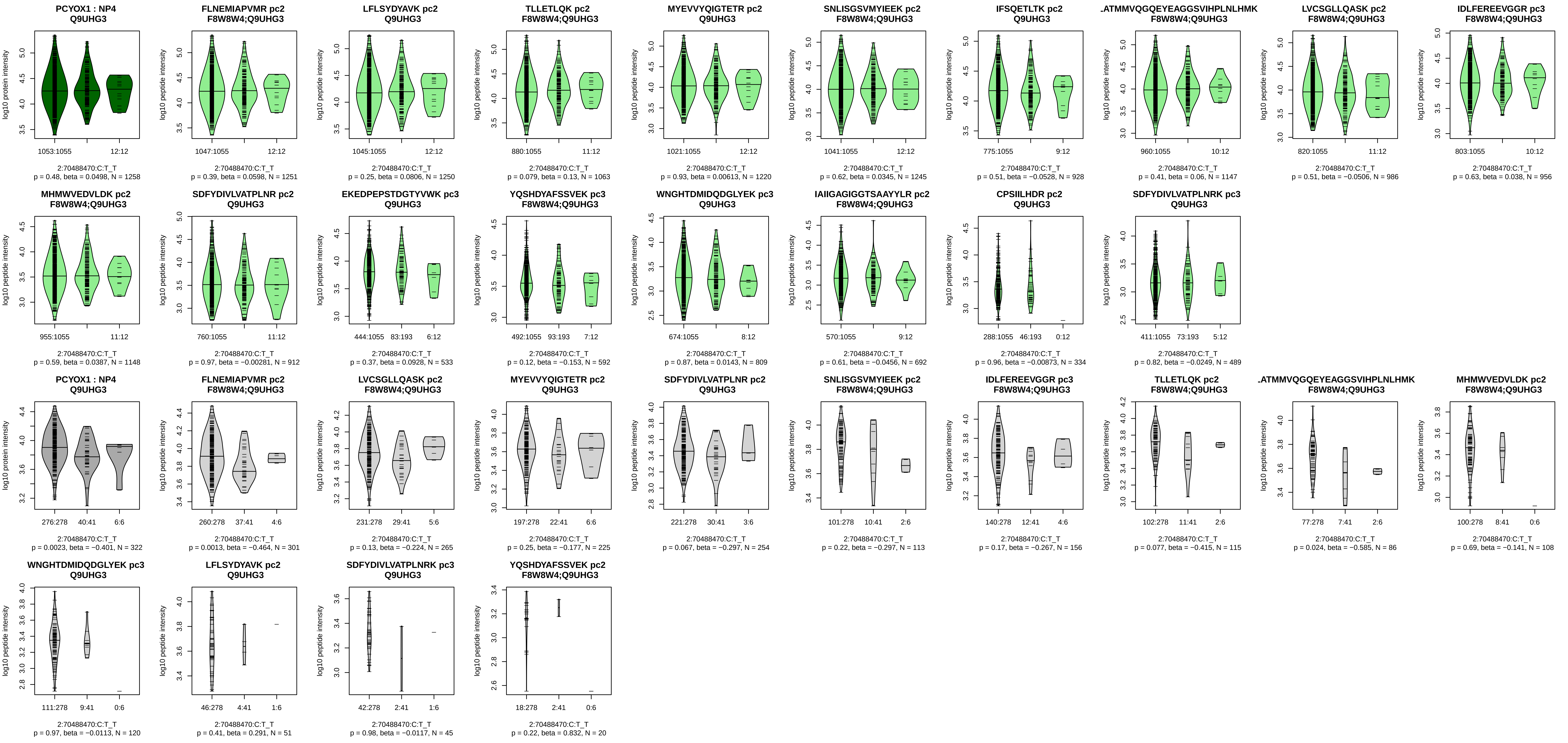

Assay Target: PCYOX1
UniProt: Q9UHG3
rsID: rs2706762
Target SNP: 2:70261338:T:C
Proxy SNP: 2:70488470:C:T
deCODE log10(p): 1115.1
deCODE BETA: −0.82
deCODE MSPA: 0

1251:1250:1063:1220:1245:928

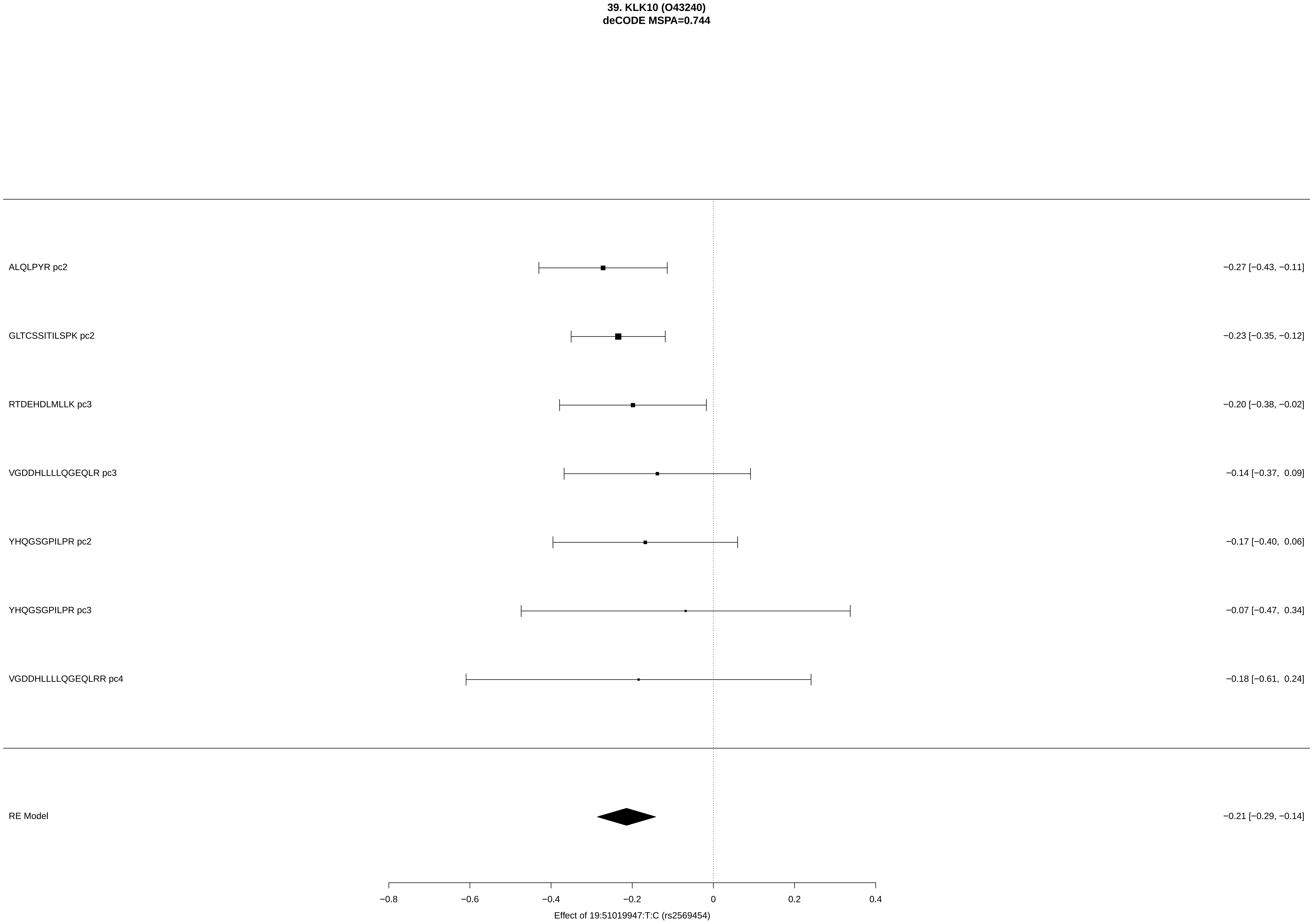

39. KLK10 (O43240)
deCODE MSPA=0.744
ALQLPYR pc2
−0.27 [−0.43, −0.11]
GLTCSSITILSPK pc2
−0.23 [−0.35, −0.12]
RTDEHDLMLLK pc3
−0.20 [−0.38, −0.02]
VGDDHLLLLQGEQLR pc3
−0.14 [−0.37, 0.09]
YHQGSGPILPR pc2
−0.17 [−0.40, 0.06]
YHQGSGPILPR pc3
−0.07 [−0.47, 0.34]
VGDDHLLLLQGEQLRR pc4
−0.18 [−0.61, 0.24]
RE Model
−0.21 [−0.29, −0.14]
−0.8
−0.6
−0.4
−0.2
0
0.2
0.4
Effect of 19:51019947:T:C (rs2569454)

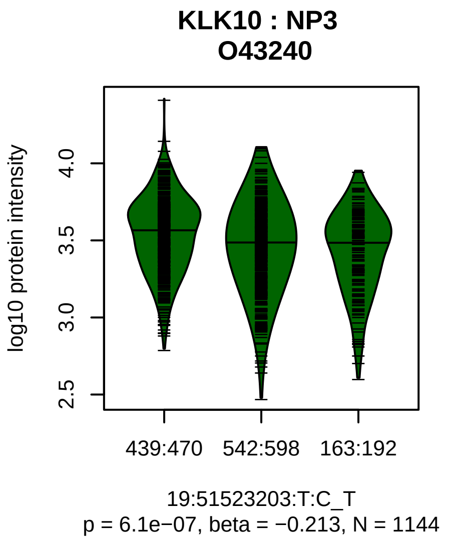

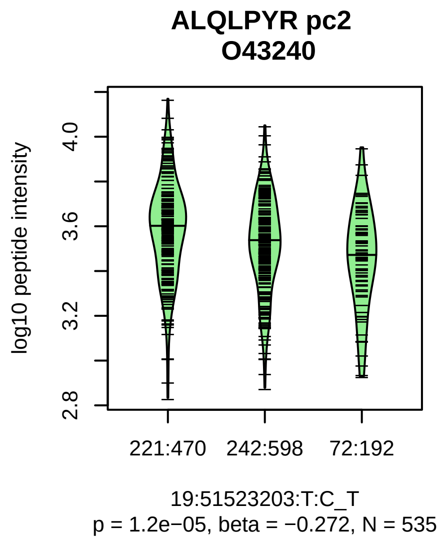

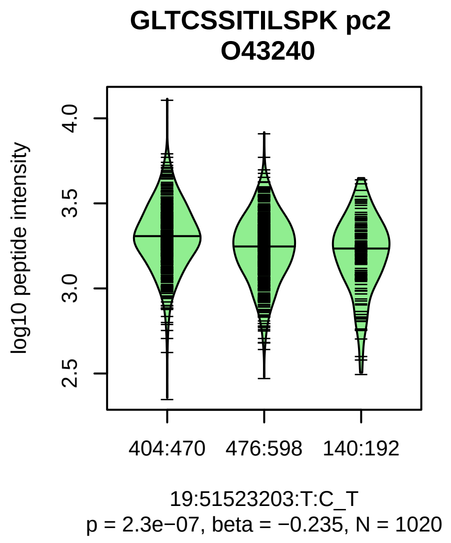

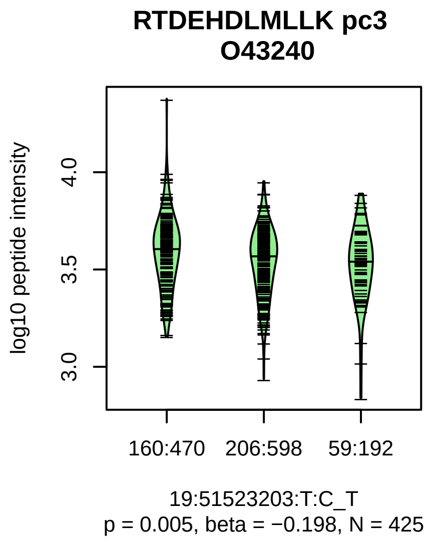

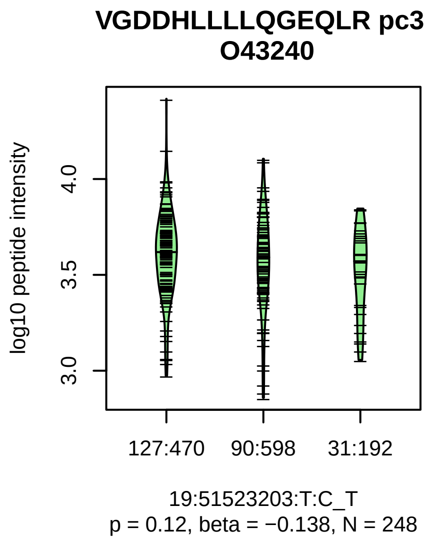

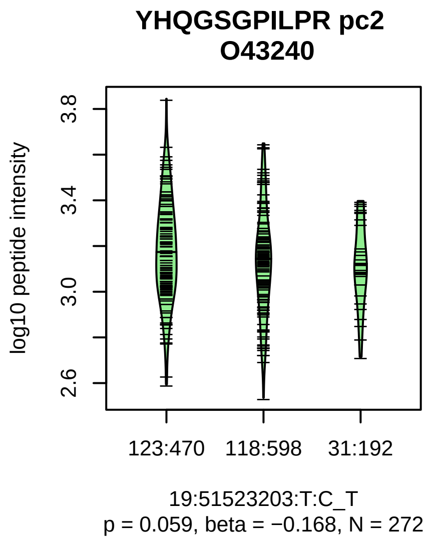

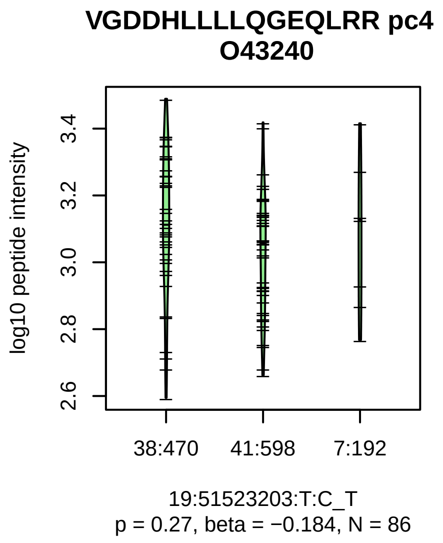

Assay Target: KLK10
UniProt: O43240
rsID: rs2569454
Target SNP: 19:51019947:T:C
Proxy SNP: 19:51523203:T:C
deCODE log10(p): 1063.6
deCODE BETA: −0.61
deCODE MSPA: 0.744
::*:−:−:−:−:NA:NA
535:1020:425:248:272:76:86:4:5

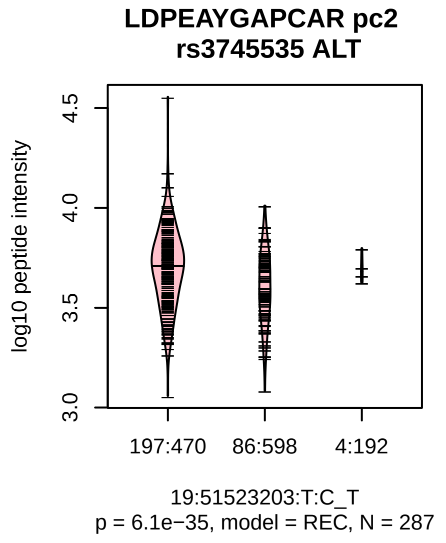

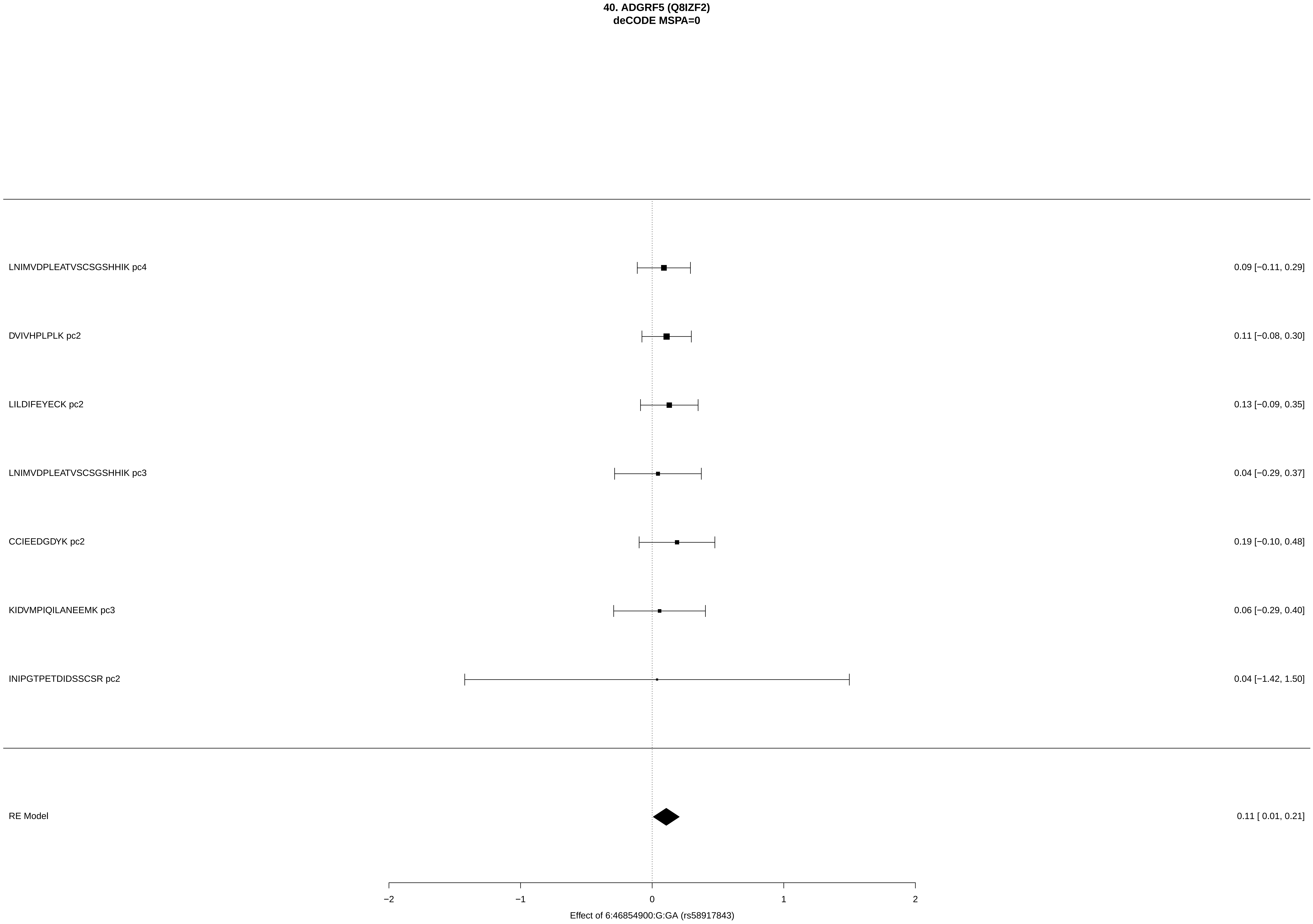
40. ADGRF5 (Q8IZF2)
deCODE MSPA=0
LNIMVDPLEATVSCSGSHHIK pc4
0.09 [−0.11, 0.29]
DVIVHPLPLK pc2
0.11 [−0.08, 0.30]
LILDIFEYECK pc2
0.13 [−0.09, 0.35]
LNIMVDPLEATVSCSGSHHIK pc3
0.04 [−0.29, 0.37]
CCIEEDGDYK pc2
0.19 [−0.10, 0.48]
KIDVMPIQILANEEMK pc3
0.06 [−0.29, 0.40]
INIPGTPETDIDSSCSR pc2
0.04 [−1.42, 1.50]
RE Model
0.11 [0.01, 0.21]
−2
−1
0
1
2
Effect of 6:46854900:G:GA (rs58917843)

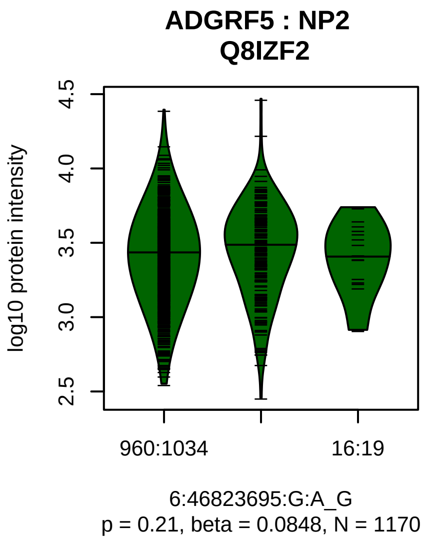

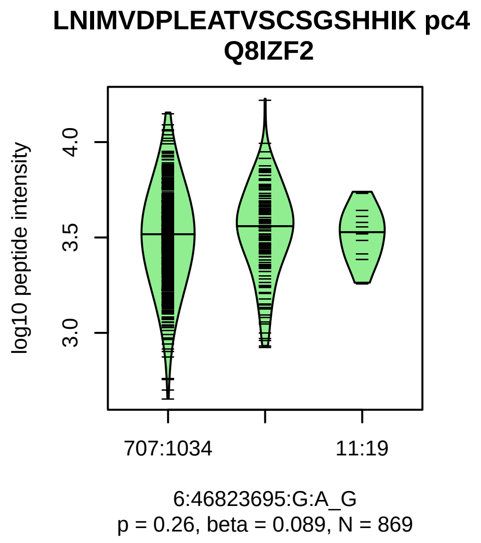

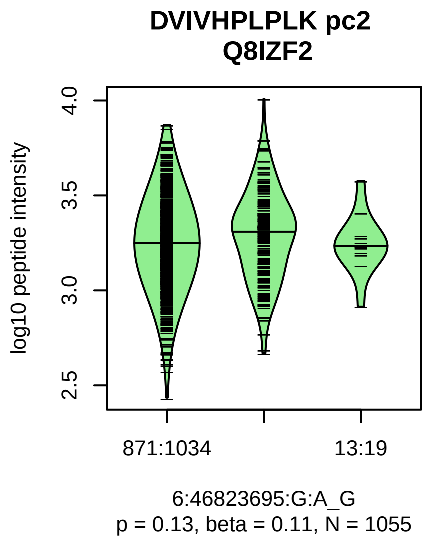

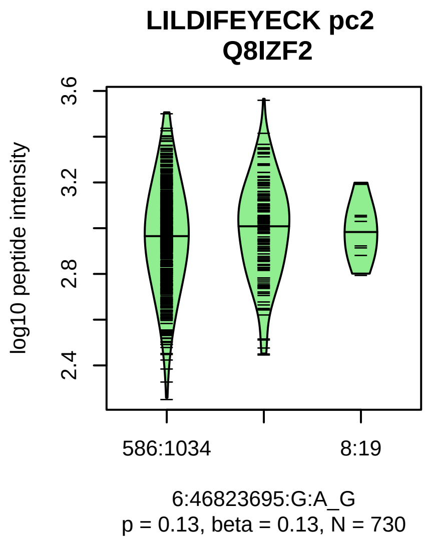

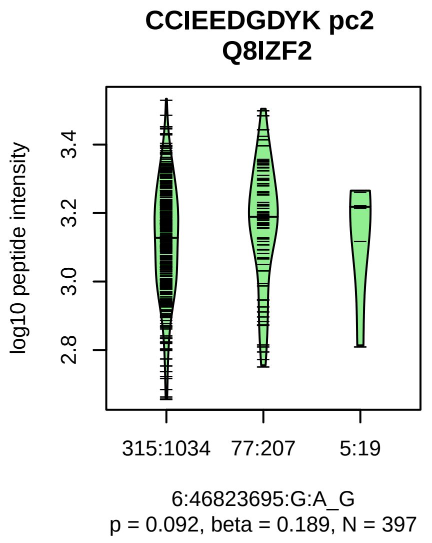

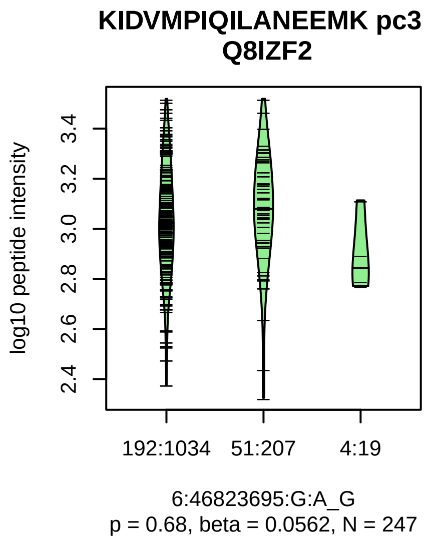

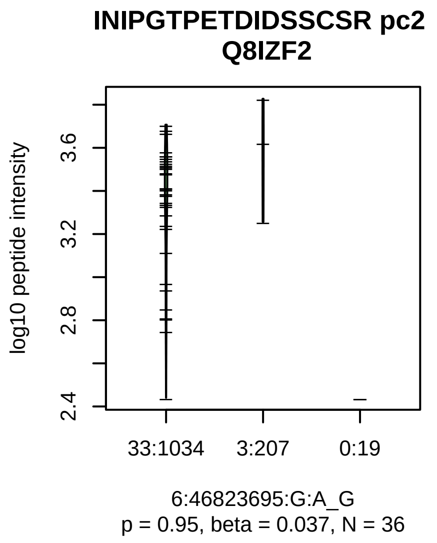

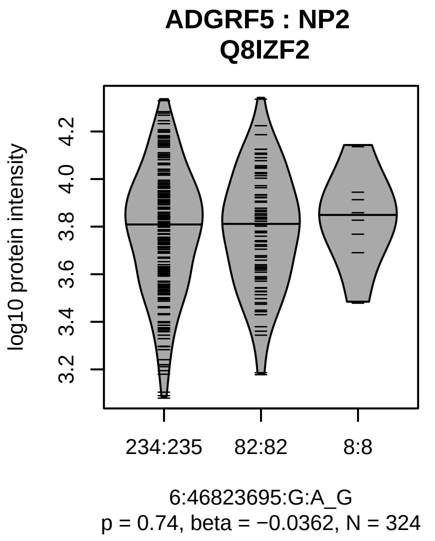

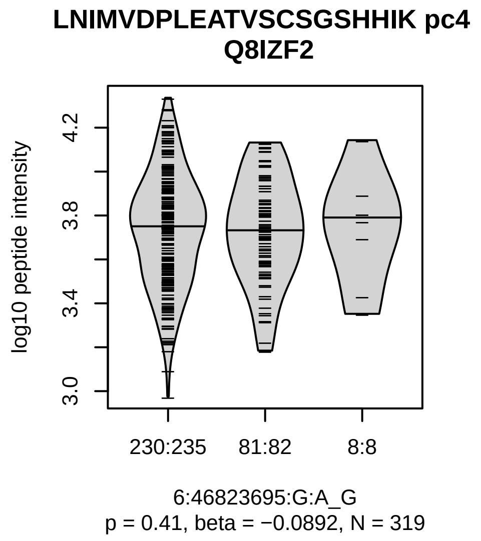

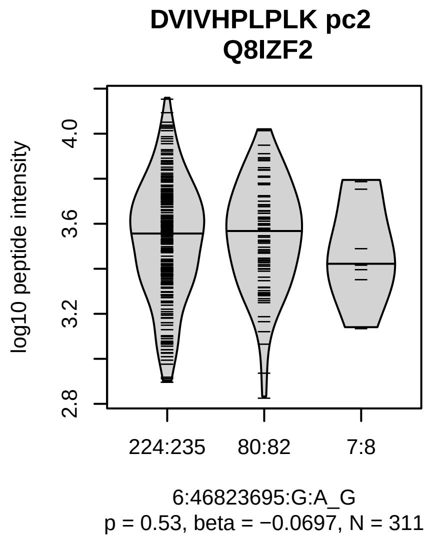

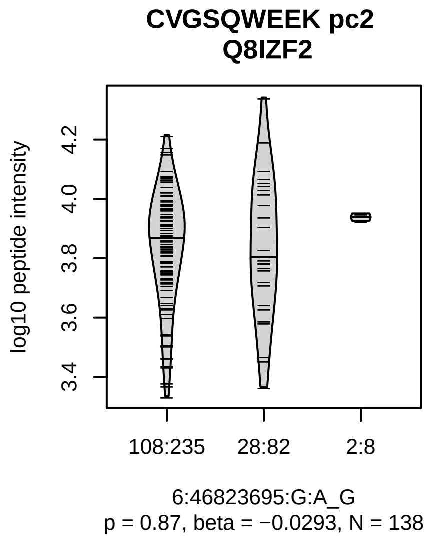

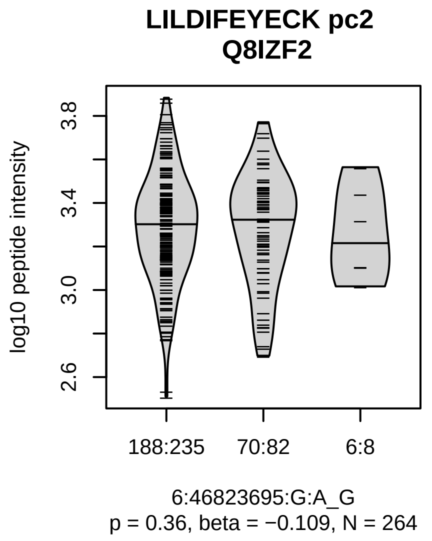

Assay Target: ADGRF5
UniProt: Q8IZF2
rsID: rs58917843
Target SNP: 6:46854900:G:GA
Proxy SNP: 6:46823695:G:A
deCODE log10(p): 1050.8
deCODE BETA: −1.49
deCODE MSPA: 0
−:−:−:−:−:−:−:NA
869:1055:730:331:397:247:36:2

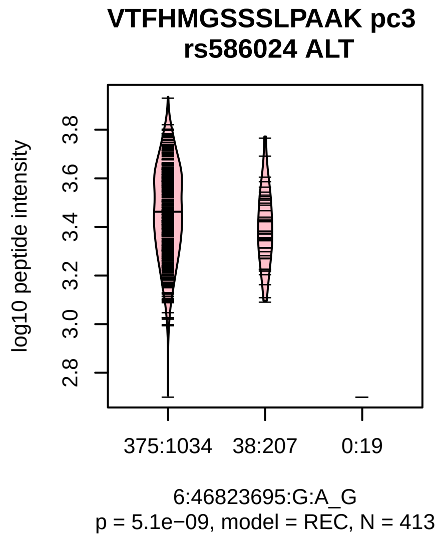

41. CTSB (P07858)
deCODE MSPA=0.881

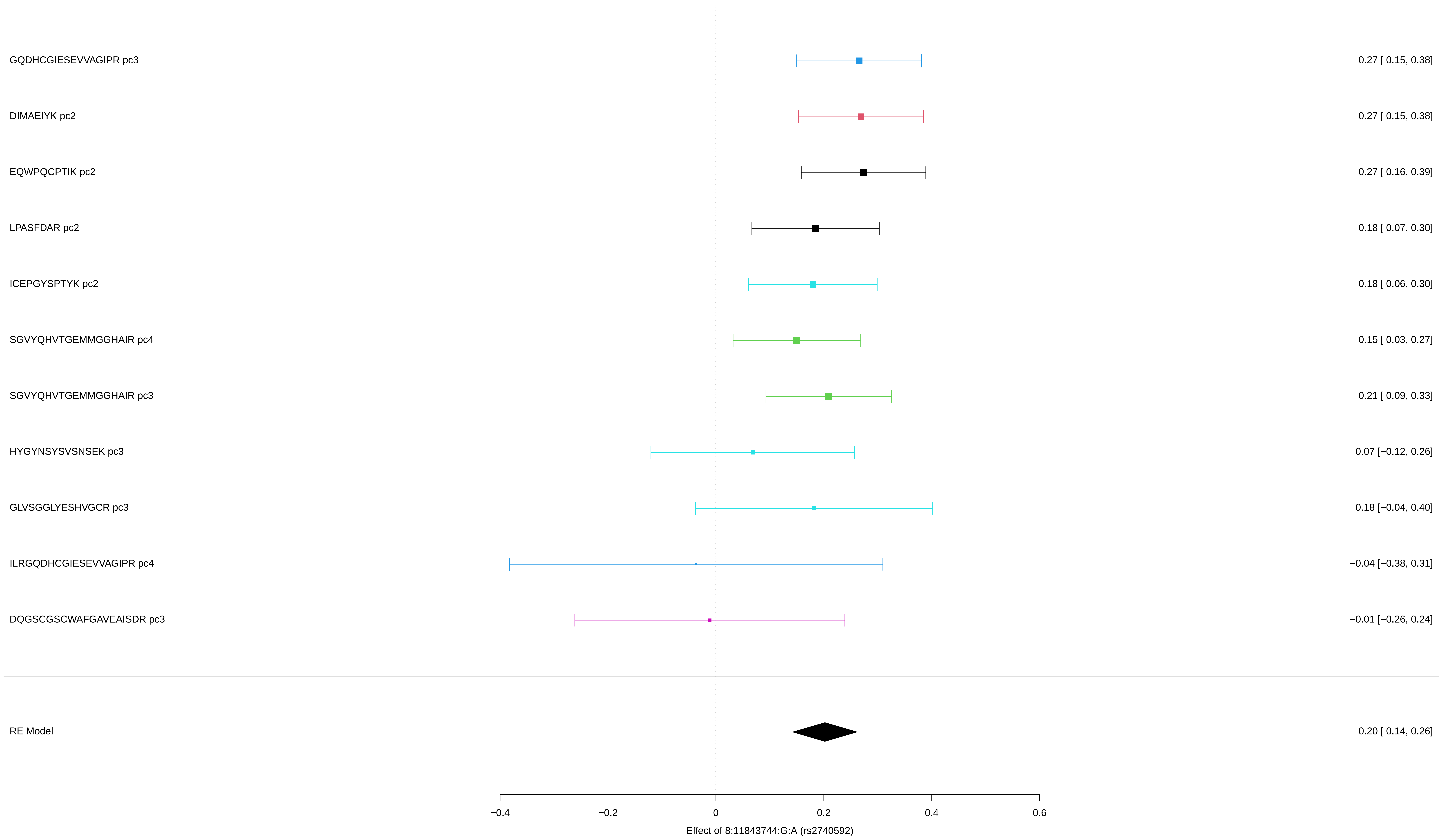

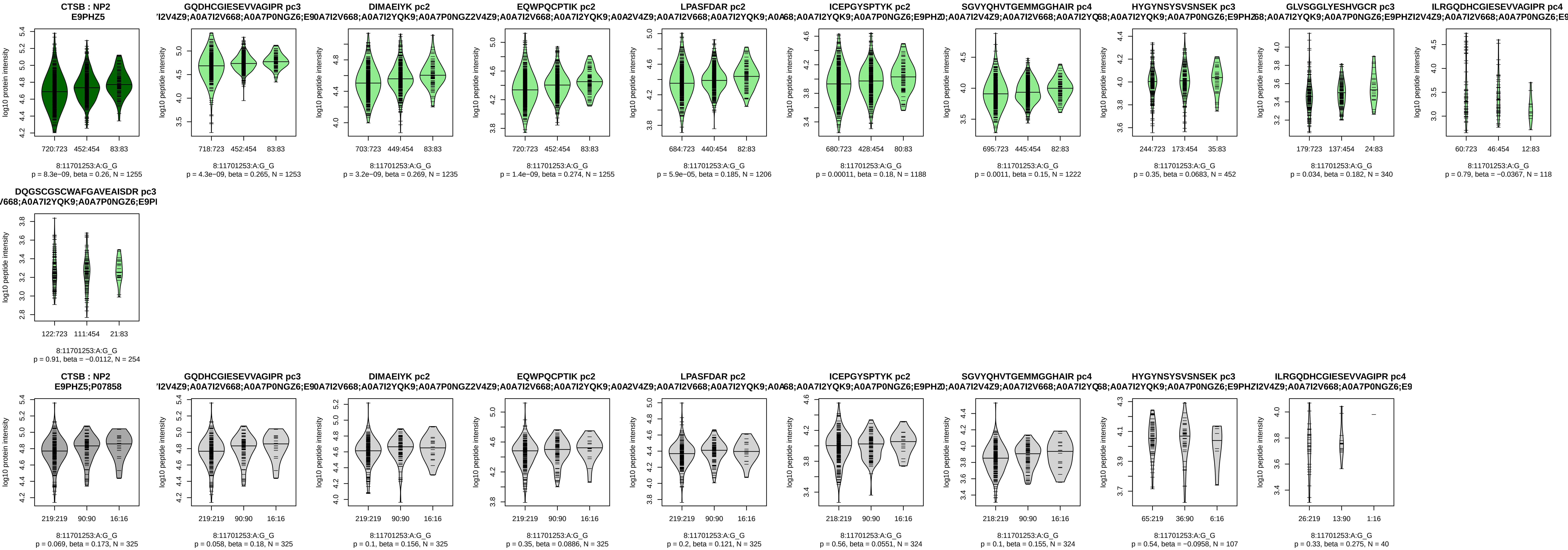

Assay Target: CTSB
UniProt: P07858
rsID: rs2740592
Target SNP: 8:11843744:G:A
Proxy SNP: 8:11701253:A:G
deCODE log10(p): 1043.8
deCODE BETA: 0.61
deCODE MSPA: 0.881
::*:*:*:*:-:-:-:-:NA
1253:1235:1255:1206:1188:122

42. SPARCL1 (Q14515)
deCODE MSPA=0.949

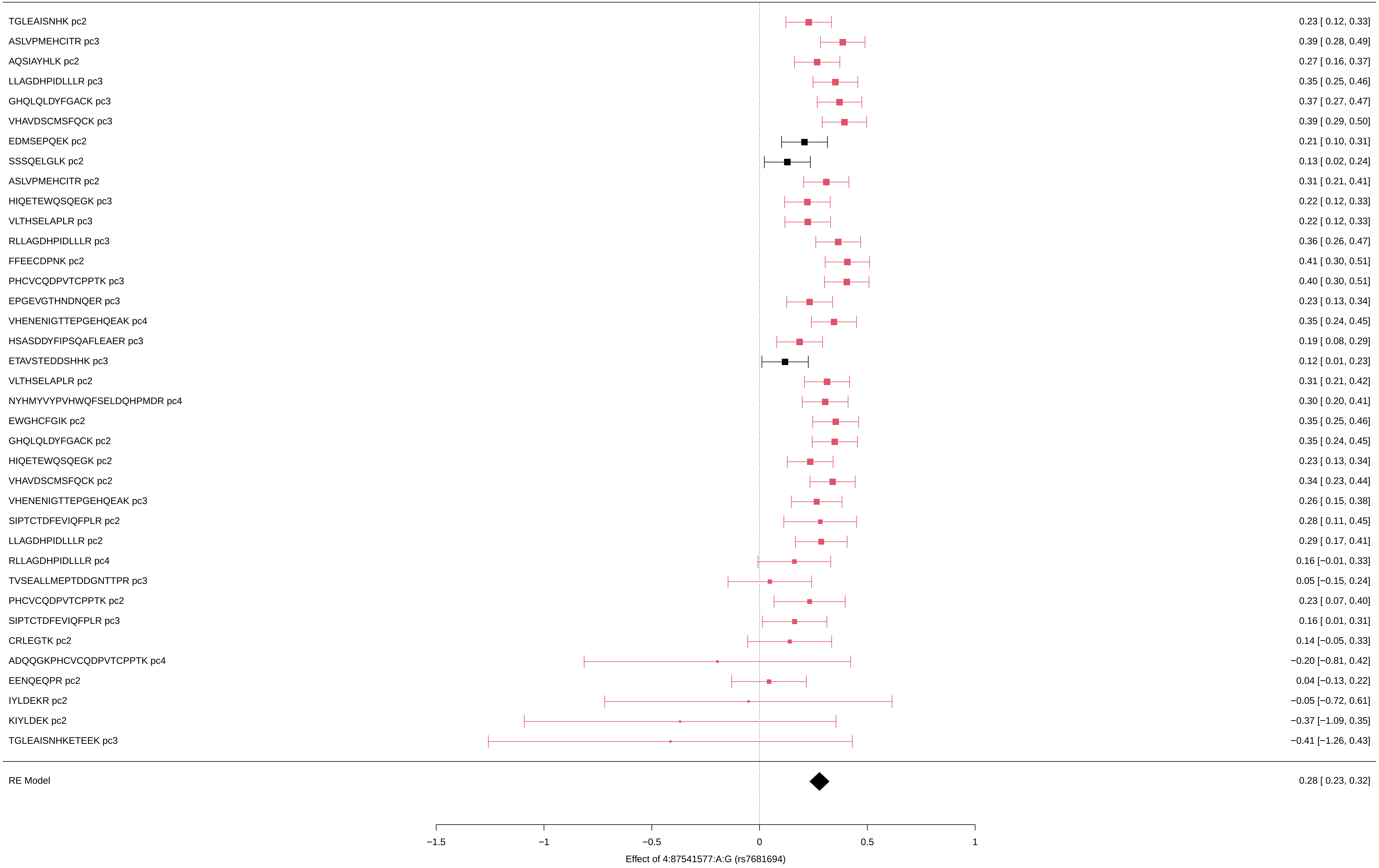

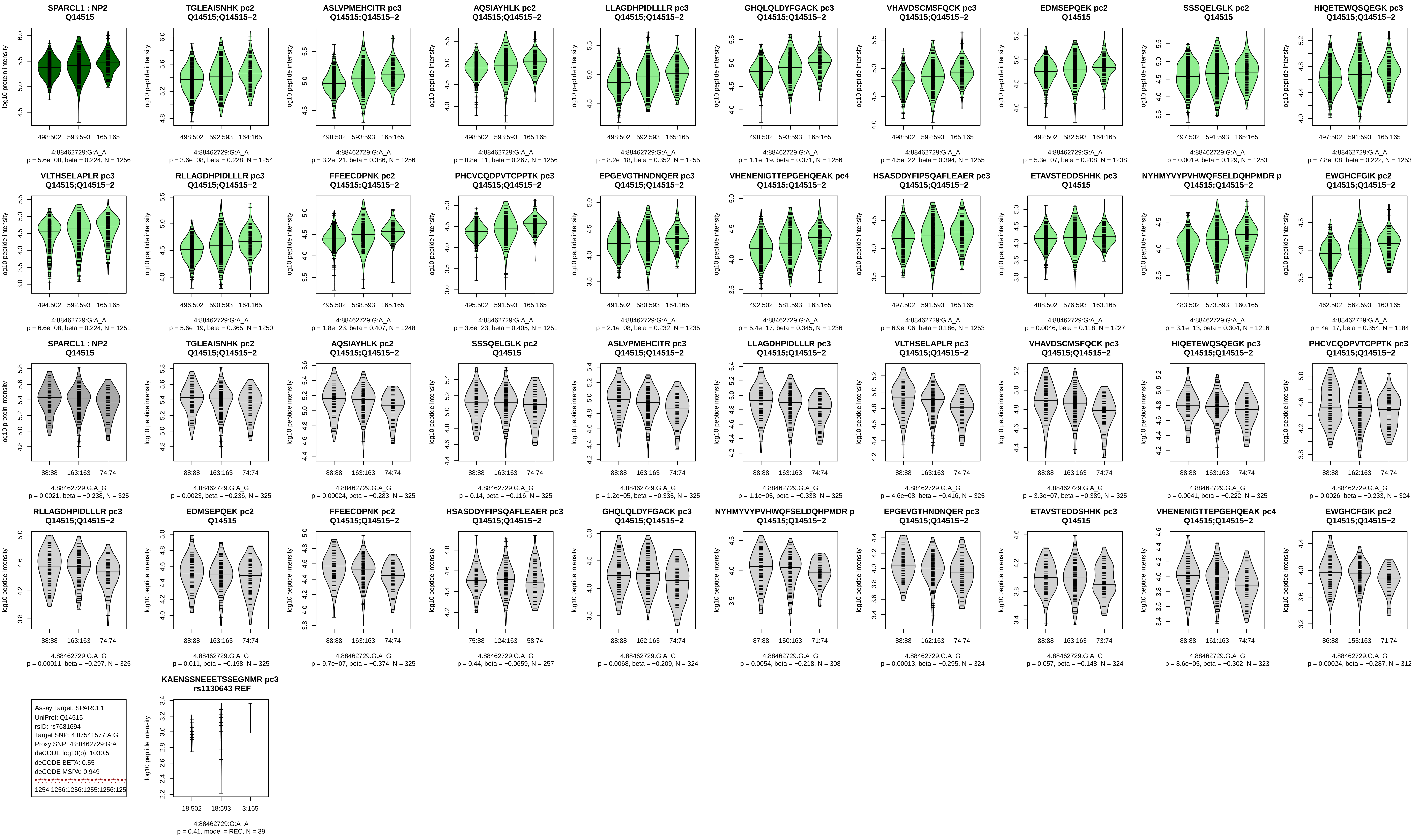
SPARCL1 : NP2
Q14515
log10 protein intensity
498:502 593:593 165:165
4:88462729:G:A_A
p = 5.6e−08, beta = 0.224, N = 1256
TGLEAISNHK pc2
Q14515;Q14515−2
log10 peptide intensity
498:502 592:593 164:165
4:88462729:G:A_A
p = 3.6e−08, beta = 0.228, N = 1254
ASLVPMEHCITR pc3
Q14515;Q14515−2
log10 peptide intensity
498:502 593:593 165:165
4:88462729:G:A_A
p = 3.2e−21, beta = 0.386, N = 1256
AQSIAYHLK pc2
Q14515;Q14515−2
log10 peptide intensity
498:502 593:593 165:165
4:88462729:G:A_A
p = 8.8e−11, beta = 0.267, N = 1256
LLAGDHPIDLLLR pc3
Q14515;Q14515−2
log10 peptide intensity
498:502 592:593 165:165
4:88462729:G:A_A
p = 8.2e−18, beta = 0.352, N = 1255
GHQLQLDYFGACK pc3
Q14515;Q14515−2
log10 peptide intensity
498:502 593:593 165:165
4:88462729:G:A_A
p = 1.1e−19, beta = 0.371, N = 1256
VHAVDSCMSFQCK pc3
Q14515;Q14515−2
log10 peptide intensity
498:502 592:593 165:165
4:88462729:G:A_A
p = 4.5e−22, beta = 0.394, N = 1255
EDMSEPQEK pc2
Q14515
log10 peptide intensity
492:502 582:593 164:165
4:88462729:G:A_A
p = 5.3e−07, beta = 0.208, N = 1238
SSSQELGLK pc2
Q14515
log10 peptide intensity
497:502 591:593 165:165
4:88462729:G:A_A
p = 0.0019, beta = 0.129, N = 1253
HIQETEWQSQEGK pc3
Q14515;Q14515−2
log10 peptide intensity
497:502 591:593 165:165
4:88462729:G:A_A
p = 7.8e−08, beta = 0.222, N = 1253
VLTHSELAPLR pc3
Q14515;Q14515−2
log10 peptide intensity
494:502 592:593 165:165
4:88462729:G:A_A
p = 6.6e−08, beta = 0.224, N = 1251
RLLAGDHPIDLLLR pc3
Q14515;Q14515−2
log10 peptide intensity
496:502 590:593 164:165
4:88462729:G:A_A
p = 5.6e−19, beta = 0.365, N = 1250
FFEECDPNK pc2
Q14515;Q14515−2
log10 peptide intensity
495:502 588:593 165:165
4:88462729:G:A_A
p = 1.8e−23, beta = 0.407, N = 1248
PHCVCQDPVTCPPTK pc3
Q14515;Q14515−2
log10 peptide intensity
495:502 591:593 165:165
4:88462729:G:A_A
p = 3.6e−23, beta = 0.405, N = 1251
EPGEVGTHNDNQER pc3
Q14515;Q14515−2
log10 peptide intensity
491:502 580:593 164:165
4:88462729:G:A_A
p = 2.1e−08, beta = 0.232, N = 1235
VHENENIGTTEPGEHQEAK pc4
Q14515;Q14515−2
log10 peptide intensity
492:502 581:593 163:165
4:88462729:G:A_A
p = 5.4e−17, beta = 0.345, N = 1236
HSASDDYFIPSQAFLEAER pc3
Q14515;Q14515−2
log10 peptide intensity
497:502 591:593 165:165
4:88462729:G:A_A
p = 6.9e−06, beta = 0.186, N = 1253
ETAVSTEDDSHHK pc3
Q14515
log10 peptide intensity
488:502 576:593 163:165
4:88462729:G:A_A
p = 0.0046, beta = 0.118, N = 1227
NYHMYVYPVHWQFSELDQHPMDR p
Q14515;Q14515−2
log10 peptide intensity
483:502 573:593 160:165
4:88462729:G:A_A
p = 3.1e−13, beta = 0.304, N = 1216
EWGHCFGIK pc2
Q14515;Q14515−2
log10 peptide intensity
462:502 562:593 160:165
4:88462729:G:A_A
p = 4e−17, beta = 0.354, N = 1184
SPARCL1 : NP2
Q14515
log10 protein intensity
88:88 163:163 74:74
4:88462729:G:A_G
p = 0.0021, beta = −0.238, N = 325
TGLEAISNHK pc2
Q14515;Q14515−2
log10 peptide intensity
88:88 163:163 74:74
4:88462729:G:A_G
p = 0.0023, beta = −0.236, N = 325
AQSIAYHLK pc2
Q14515;Q14515−2
log10 peptide intensity
88:88 163:163 74:74
4:88462729:G:A_G
p = 0.00024, beta = −0.283, N = 325
SSSQELGLK pc2
Q14515
log10 peptide intensity
88:88 163:163 74:74
4:88462729:G:A_G
p = 0.14, beta = −0.116, N = 325
ASLVPMEHCITR pc3
Q14515;Q14515−2
log10 peptide intensity
88:88 163:163 74:74
4:88462729:G:A_G
p = 1.2e−05, beta = −0.335, N = 325
LLAGDHPIDLLLR pc3
Q14515;Q14515−2
log10 peptide intensity
88:88 163:163 74:74
4:88462729:G:A_G
p = 1.1e−05, beta = −0.338, N = 325
VLTHSELAPLR pc3
Q14515;Q14515−2
log10 peptide intensity
88:88 163:163 74:74
4:88462729:G:A_G
p = 4.6e−08, beta = −0.416, N = 325
VHAVDSCMSFQCK pc3
Q14515;Q14515−2
log10 peptide intensity
88:88 163:163 74:74
4:88462729:G:A_G
p = 3.3e−07, beta = −0.389, N = 325
HIQETEWQSQEGK pc3
Q14515;Q14515−2
log10 peptide intensity
88:88 163:163 74:74
4:88462729:G:A_G
p = 0.0041, beta = −0.222, N = 325
PHCVCQDPVTCPPTK pc3
Q14515;Q14515−2
log10 peptide intensity
88:88 162:163 74:74
4:88462729:G:A_G
p = 0.0026, beta = −0.233, N = 324
RLLAGDHPIDLLLR pc3
Q14515;Q14515−2
log10 peptide intensity
88:88 163:163 74:74
4:88462729:G:A_G
p = 0.00011, beta = −0.297, N = 325
EDMSEPQEK pc2
Q14515
log10 peptide intensity
88:88 163:163 74:74
4:88462729:G:A_G
p = 0.011, beta = −0.198, N = 325
FFEECDPNK pc2
Q14515;Q14515−2
log10 peptide intensity
88:88 163:163 74:74
4:88462729:G:A_G
p = 9.7e−07, beta = −0.374, N = 325
HSASDDYFIPSQAFLEAER pc3
Q14515;Q14515−2
log10 peptide intensity
75:88 124:163 58:74
4:88462729:G:A_G
p = 0.44, beta = −0.0659, N = 257
GHQLQLDYFGACK pc3
Q14515;Q14515−2
log10 peptide intensity
88:88 162:163 74:74
4:88462729:G:A_G
p = 0.0068, beta = −0.209, N = 324
NYHMYVYPVHWQFSELDQHPMDR p
Q14515;Q14515−2
log10 peptide intensity
87:88 150:163 71:74
4:88462729:G:A_G
p = 0.0054, beta = −0.218, N = 308
EPGEVGTHNDNQER pc3
Q14515;Q14515−2
log10 peptide intensity
88:88 162:163 74:74
4:88462729:G:A_G
p = 0.00013, beta = −0.295, N = 324
ETAVSTEDDSHHK pc3
Q14515
log10 peptide intensity
88:88 163:163 73:74
4:88462729:G:A_G
p = 0.057, beta = −0.148, N = 324
VHENENIGTTEPGEHQEAK pc4
Q14515;Q14515−2
log10 peptide intensity
88:88 161:163 74:74
4:88462729:G:A_G
p = 8.6e−05, beta = −0.302, N = 323
EWGHCFGIK pc2
Q14515;Q14515−2
log10 peptide intensity
86:88 155:163 71:74
4:88462729:G:A_G
p = 0.00024, beta = −0.287, N = 312
Assay Target: SPARCL1
UniProt: Q14515
rsID: rs7681694
Target SNP: 4:87541577:A:G
Proxy SNP: 4:88462729:G:A
deCODE log10(p): 1030.5
deCODE BETA: 0.55
deCODE MSPA: 0.949
1254:1256:1256:1255:1256:125
KAENSSNEEETSSEGNMR pc3
rs1130643 REF
log10 peptide intensity
18:502 18:593 3:165
4:88462729:G:A_A
p = 0.41, model = REC, N = 39

43. ADAMTS13 (Q76LX8)
deCODE MSPA=0

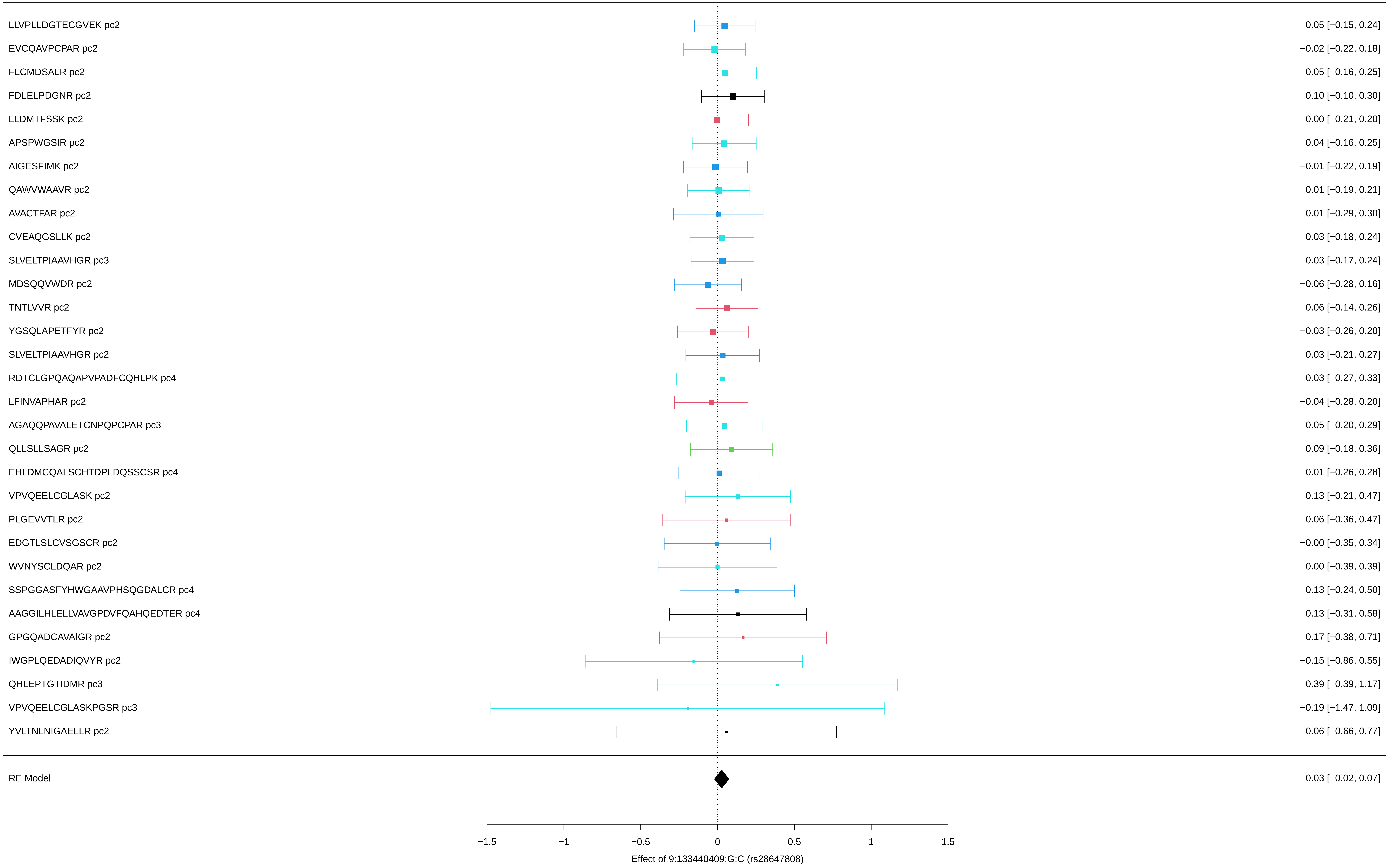

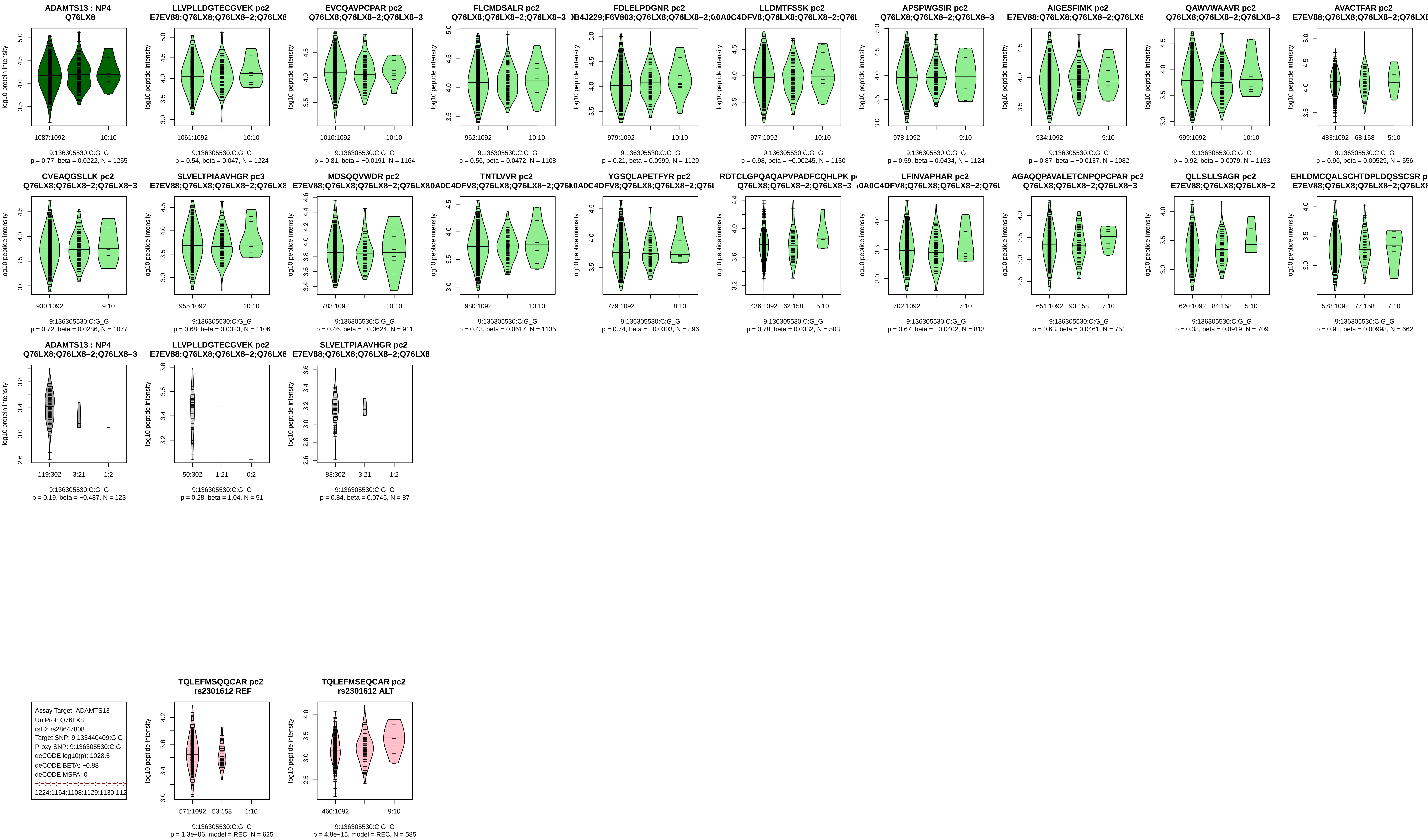
ADAMTS13 : NP4
Q76LX8
log10 protein intensity
1087:1092
10:10
9:136305530:C:G_G
p = 0.77, beta = 0.0222, N = 1255
LLVPLLDGTECGVEK pc2
E7EV88;Q76LX8;Q76LX8−2;Q76LX8
log10 peptide intensity
1061:1092
10:10
9:136305530:C:G_G
p = 0.54, beta = 0.047, N = 1224
EVCQAVPCPAR pc2
Q76LX8;Q76LX8−2;Q76LX8−3
1010:1092
10:10
9:136305530:C:G_G
p = 0.81, beta = −0.0191, N = 1164
FLCMDSALR pc2
Q76LX8;Q76LX8−2;Q76LX8−3
962:1092
10:10
9:136305530:C:G_G
p = 0.56, beta = 0.0472, N = 1108
FDLELPDGNR pc2
0B4J229;F6V803;Q76LX8;Q76LX8−2;Q
979:1092
10:10
9:136305530:C:G_G
p = 0.21, beta = 0.0999, N = 1129
LLDMTFSSK pc2
0A0C4DFV8;Q76LX8;Q76LX8−2;Q76L
977:1092
10:10
9:136305530:C:G_G
p = 0.98, beta = −0.00245, N = 1130
APSPWGSIR pc2
Q76LX8;Q76LX8−2;Q76LX8−3
978:1092
9:10
9:136305530:C:G_G
p = 0.59, beta = 0.0434, N = 1124
AIGESFIMK pc2
E7EV88;Q76LX8;Q76LX8−2;Q76LX8
934:1092
9:10
9:136305530:C:G_G
p = 0.87, beta = −0.0137, N = 1082
QAWVWAAVR pc2
Q76LX8;Q76LX8−2;Q76LX8−3
999:1092
10:10
9:136305530:C:G_G
p = 0.92, beta = 0.0079, N = 1153
AVACTFAR pc2
E7EV88;Q76LX8;Q76LX8−2;Q76LX8
483:1092
68:158
5:10
9:136305530:C:G_G
p = 0.96, beta = 0.00529, N = 556
CVEAQGSLLK pc2
Q76LX8;Q76LX8−2;Q76LX8−3
930:1092
9:10
9:136305530:C:G_G
p = 0.72, beta = 0.0286, N = 1077
SLVELTPIAAVHGR pc3
E7EV88;Q76LX8;Q76LX8−2;Q76LX8
955:1092
10:10
9:136305530:C:G_G
p = 0.68, beta = 0.0323, N = 1106
MDSQQVWDR pc2
E7EV88;Q76LX8;Q76LX8−2;Q76LX8
783:1092
10:10
9:136305530:C:G_G
p = 0.46, beta = −0.0624, N = 911
TNTLVVR pc2
0A0C4DFV8;Q76LX8;Q76LX8−2;Q76L
980:1092
10:10
9:136305530:C:G_G
p = 0.43, beta = 0.0617, N = 1135
YGSQLAPETFYR pc2
0A0C4DFV8;Q76LX8;Q76LX8−2;Q76L
779:1092
8:10
9:136305530:C:G_G
p = 0.74, beta = −0.0303, N = 896
RDTCLGPQAQAPVPADFCQHLPK pc
Q76LX8;Q76LX8−2;Q76LX8−3
436:1092
62:158
5:10
9:136305530:C:G_G
p = 0.78, beta = 0.0332, N = 503
LFINVAPHAR pc2
0A0C4DFV8;Q76LX8;Q76LX8−2;Q76L
702:1092
7:10
9:136305530:C:G_G
p = 0.67, beta = −0.0402, N = 813
AGAQQPAVALETCNPQPCPAR pc3
Q76LX8;Q76LX8−2;Q76LX8−3
651:1092
93:158
7:10
9:136305530:C:G_G
p = 0.63, beta = 0.0461, N = 751
QLLSLLSAGR pc2
E7EV88;Q76LX8;Q76LX8−2
620:1092
84:158
5:10
9:136305530:C:G_G
p = 0.38, beta = 0.0919, N = 709
EHLDMCQALSCHTDPLDQSSCSR p
E7EV88;Q76LX8;Q76LX8−2;Q76LX8
578:1092
77:158
7:10
9:136305530:C:G_G
p = 0.92, beta = 0.00998, N = 662
ADAMTS13 : NP4
Q76LX8;Q76LX8−2;Q76LX8−3
119:302
3:21
1:2
9:136305530:C:G_G
p = 0.19, beta = −0.487, N = 123
LLVPLLDGTECGVEK pc2
E7EV88;Q76LX8;Q76LX8−2;Q76LX8
50:302
1:21
0:2
9:136305530:C:G_G
p = 0.28, beta = 1.04, N = 51
SLVELTPIAAVHGR pc2
E7EV88;Q76LX8;Q76LX8−2;Q76LX8
83:302
3:21
1:2
9:136305530:C:G_G
p = 0.84, beta = 0.0745, N = 87
Assay Target: ADAMTS13
UniProt: Q76LX8
rsID: rs28647808
Target SNP: 9:133440409:G:C
Proxy SNP: 9:136305530:C:G
deCODE log10(p): 1028.5
deCODE BETA: −0.88
deCODE MSPA: 0
1224:1164:1108:1129:1130:112
TQLEFMSQQCAR pc2
rs2301612 REF
571:1092
53:158
1:10
9:136305530:C:G_G
p = 1.3e−06, model = REC, N = 625
TQLEFMSEQCAR pc2
rs2301612 ALT
460:1092
9:10
9:136305530:C:G_G
p = 4.8e−15, model = REC, N = 585

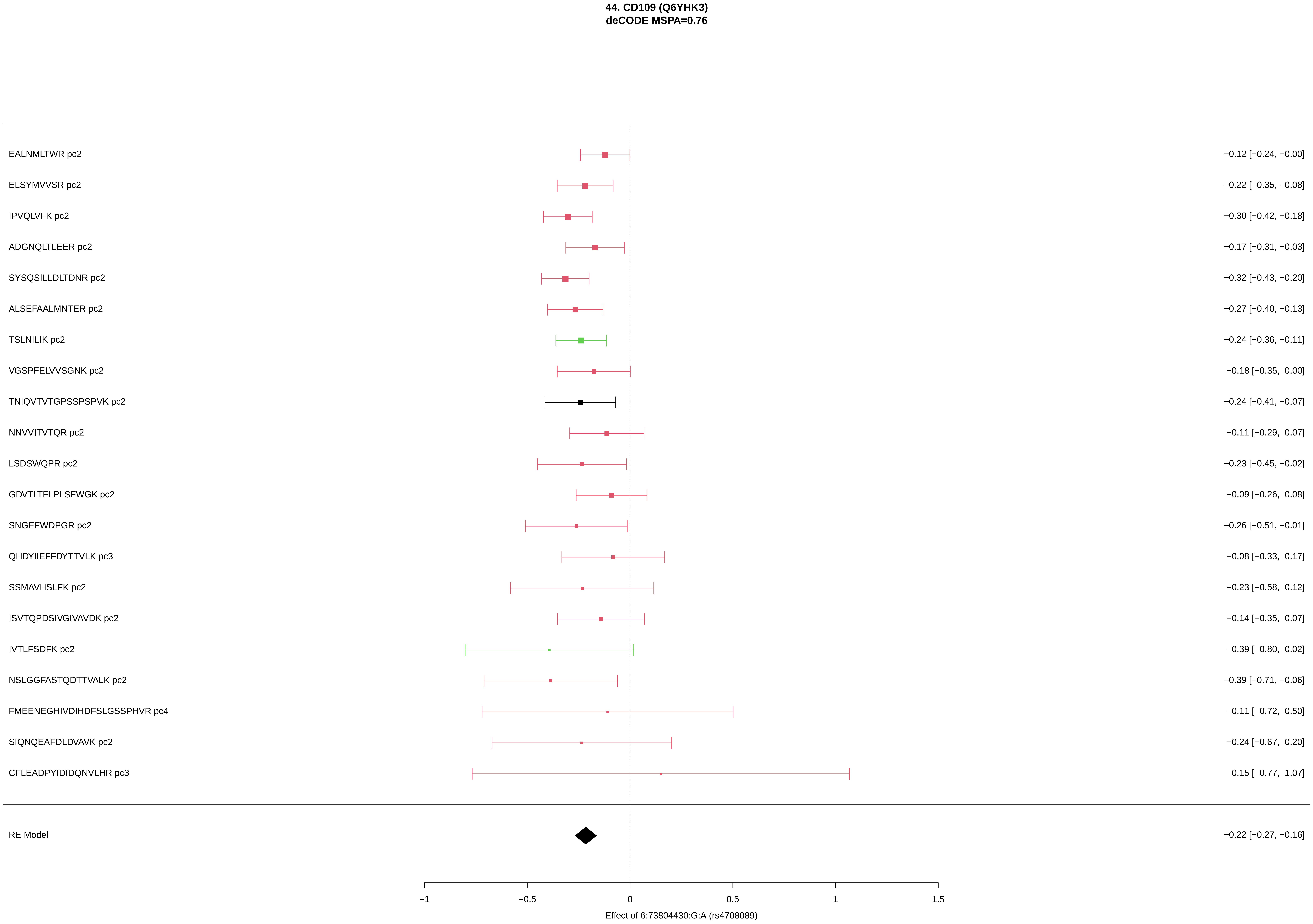
44. CD109 (Q6YHK3)
deCODE MSPA=0.76
EALNMLTWR pc2
ELSYMVVSR pc2
IPVQLVFK pc2
ADGNQLTLEER pc2
SYSQSILLDLTDNR pc2
ALSEFAALMNTER pc2
TSLNILIK pc2
VGSPFELVVSGNK pc2
TNIQVTVTGPSSPSPVK pc2
NNVVITVTQR pc2
LSDSWQPR pc2
GDVTLTFLPLSFWGK pc2
SNGEFWDPGR pc2
QHDYIIEFFDYTTVLK pc3
SSMAVHSLFK pc2
ISVTQPDSIVGIVAVDK pc2
IVTLFSDFK pc2
NSLGGFASTQDTTVALK pc2
FMEENEGHIVDIHDFSLGSSPHVR pc4
SIQNQEAFDLDVAVK pc2
CFLEADPYIDIDQNVLHR pc3
RE Model
−0.12 [−0.24, −0.00]
−0.22 [−0.35, −0.08]
−0.30 [−0.42, −0.18]
−0.17 [−0.31, −0.03]
−0.32 [−0.43, −0.20]
−0.27 [−0.40, −0.13]
−0.24 [−0.36, −0.11]
−0.18 [−0.35, 0.00]
−0.24 [−0.41, −0.07]
−0.11 [−0.29, 0.07]
−0.23 [−0.45, −0.02]
−0.09 [−0.26, 0.08]
−0.26 [−0.51, −0.01]
−0.08 [−0.33, 0.17]
−0.23 [−0.58, 0.12]
−0.14 [−0.35, 0.07]
−0.39 [−0.80, 0.02]
−0.39 [−0.71, −0.06]
−0.11 [−0.72, 0.50]
−0.24 [−0.67, 0.20]
0.15 [−0.77, 1.07]
−0.22 [−0.27, −0.16]
−1
−0.5
0
0.5
1
1.5
Effect of 6:73804430:G:A (rs4708089)

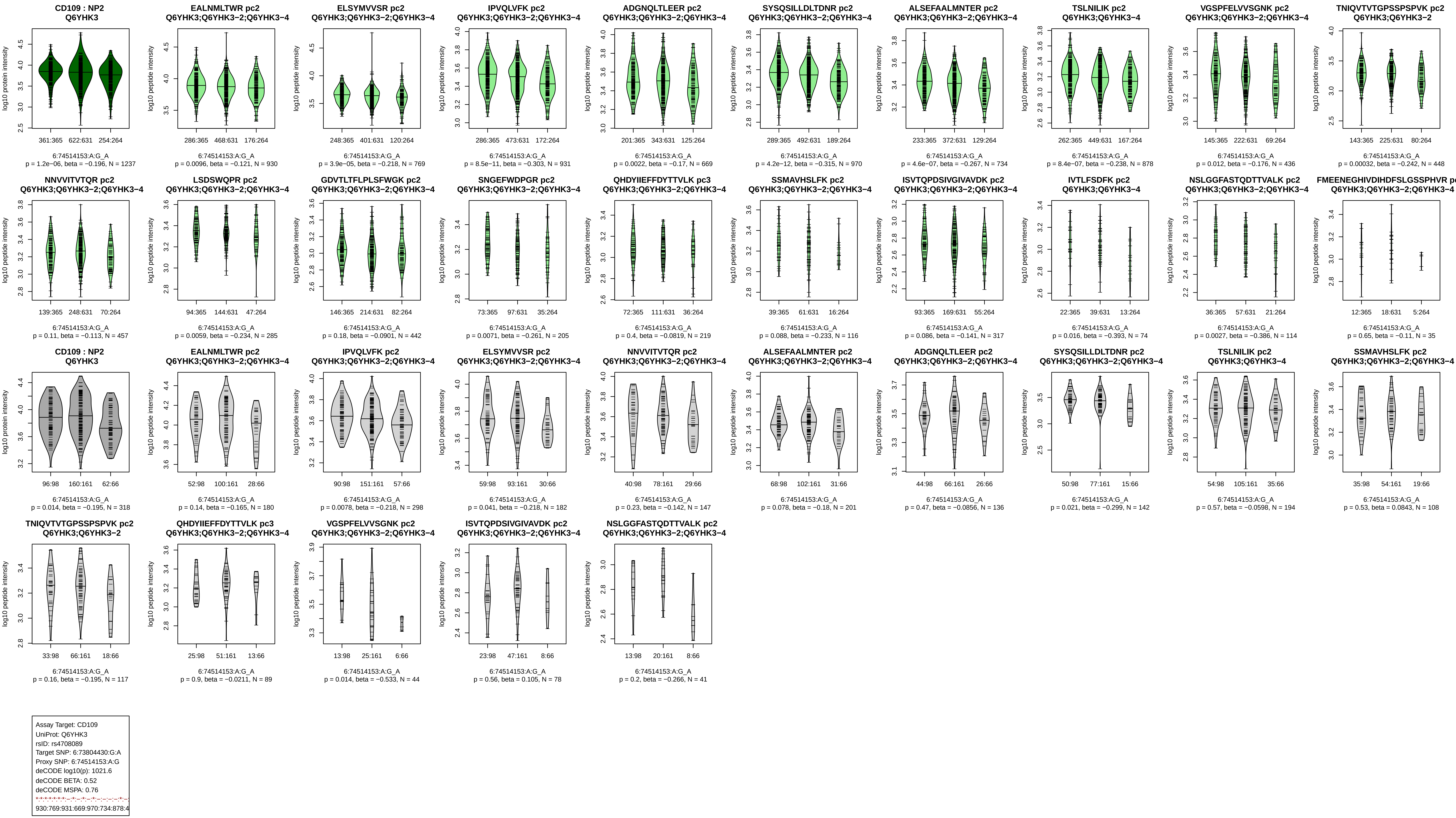
CD109 : NP2 Q6YHK3
361:365 622:631 254:264
6:74514153:A:G_A
p = 1.2e−06, beta = −0.196, N = 1237
EALNMLTWR pc2 Q6YHK3;Q6YHK3−2;Q6YHK3−4
286:365 468:631 176:264
6:74514153:A:G_A
p = 0.0096, beta = −0.121, N = 930
ELSYMVVSR pc2 Q6YHK3;Q6YHK3−2;Q6YHK3−4
248:365 401:631 120:264
6:74514153:A:G_A
p = 3.9e−05, beta = −0.218, N = 769
IPVQLVFK pc2 Q6YHK3;Q6YHK3−2;Q6YHK3−4
286:365 473:631 172:264
6:74514153:A:G_A
p = 8.5e−11, beta = −0.303, N = 931
ADGNQLTLEER pc2 Q6YHK3;Q6YHK3−2;Q6YHK3−4
201:365 343:631 125:264
6:74514153:A:G_A
p = 0.0022, beta = −0.17, N = 669
SYSQSILLDLTDNR pc2 Q6YHK3;Q6YHK3−2;Q6YHK3−4
289:365 492:631 189:264
6:74514153:A:G_A
p = 4.2e−12, beta = −0.315, N = 970
ALSEFAALMNTER pc2 Q6YHK3;Q6YHK3−2;Q6YHK3−4
233:365 372:631 129:264
6:74514153:A:G_A
p = 4.6e−07, beta = −0.267, N = 734
TSLNILIK pc2 Q6YHK3;Q6YHK3−4
262:365 449:631 167:264
6:74514153:A:G_A
p = 8.4e−07, beta = −0.238, N = 878
VGSPFELVVSGNK pc2 Q6YHK3;Q6YHK3−2;Q6YHK3−4
145:365 222:631 69:264
6:74514153:A:G_A
p = 0.012, beta = −0.176, N = 436
TNIQVTVTGPSSPSPVK pc2 Q6YHK3;Q6YHK3−2
143:365 225:631 80:264
6:74514153:A:G_A
p = 0.00032, beta = −0.242, N = 448
NNVVITVTQR pc2 Q6YHK3;Q6YHK3−2;Q6YHK3−4
139:365 248:631 70:264
6:74514153:A:G_A
p = 0.11, beta = −0.113, N = 457
LSDSWQPR pc2 Q6YHK3;Q6YHK3−2;Q6YHK3−4
94:365 144:631 47:264
6:74514153:A:G_A
p = 0.0059, beta = −0.234, N = 285
GDVTLTFLPLSFWGK pc2 Q6YHK3;Q6YHK3−2;Q6YHK3−4
146:365 214:631 82:264
6:74514153:A:G_A
p = 0.18, beta = −0.0901, N = 442
SNGEFWDPGR pc2 Q6YHK3;Q6YHK3−2;Q6YHK3−4
73:365 97:631 35:264
6:74514153:A:G_A
p = 0.0071, beta = −0.261, N = 205
QHDYIIEFFDYTTVLK pc3 Q6YHK3;Q6YHK3−2;Q6YHK3−4
72:365 111:631 36:264
6:74514153:A:G_A
p = 0.4, beta = −0.0819, N = 219
SSMAVHSLFK pc2 Q6YHK3;Q6YHK3−2;Q6YHK3−4
39:365 61:631 16:264
6:74514153:A:G_A
p = 0.088, beta = −0.233, N = 116
ISVTQPDSIVGIVAVDK pc2 Q6YHK3;Q6YHK3−2;Q6YHK3−4
93:365 169:631 55:264
6:74514153:A:G_A
p = 0.086, beta = −0.141, N = 317
IVTLFSDFK pc2 Q6YHK3;Q6YHK3−4
22:365 39:631 13:264
6:74514153:A:G_A
p = 0.016, beta = −0.393, N = 74
NSLGGFASTQDTTVALK pc2 Q6YHK3;Q6YHK3−2;Q6YHK3−4
36:365 57:631 21:264
6:74514153:A:G_A
p = 0.0027, beta = −0.386, N = 114
FMEENEGHIVDIHDFSLGSSPHVR pc Q6YHK3;Q6YHK3−2;Q6YHK3−4
12:365 18:631 5:264
6:74514153:A:G_A
p = 0.65, beta = −0.11, N = 35
CD109 : NP2 Q6YHK3
96:98 160:161 62:66
6:74514153:A:G_A
p = 0.014, beta = −0.195, N = 318
EALNMLTWR pc2 Q6YHK3;Q6YHK3−2;Q6YHK3−4
52:98 100:161 28:66
6:74514153:A:G_A
p = 0.14, beta = −0.165, N = 180
IPVQLVFK pc2 Q6YHK3;Q6YHK3−2;Q6YHK3−4
90:98 151:161 57:66
6:74514153:A:G_A
p = 0.0078, beta = −0.218, N = 298
ELSYMVVSR pc2 Q6YHK3;Q6YHK3−2;Q6YHK3−4
59:98 93:161 30:66
6:74514153:A:G_A
p = 0.041, beta = −0.218, N = 182
NNVVITVTQR pc2 Q6YHK3;Q6YHK3−2;Q6YHK3−4
40:98 78:161 29:66
6:74514153:A:G_A
p = 0.23, beta = −0.142, N = 147
ALSEFAALMNTER pc2 Q6YHK3;Q6YHK3−2;Q6YHK3−4
68:98 102:161 31:66
6:74514153:A:G_A
p = 0.078, beta = −0.18, N = 201
ADGNQLTLEER pc2 Q6YHK3;Q6YHK3−2;Q6YHK3−4
44:98 66:161 26:66
6:74514153:A:G_A
p = 0.47, beta = −0.0856, N = 136
SYSQSILLDLTDNR pc2 Q6YHK3;Q6YHK3−2;Q6YHK3−4
50:98 77:161 15:66
6:74514153:A:G_A
p = 0.021, beta = −0.299, N = 142
TSLNILIK pc2 Q6YHK3;Q6YHK3−4
54:98 105:161 35:66
6:74514153:A:G_A
p = 0.57, beta = −0.0598, N = 194
SSMAVHSLFK pc2 Q6YHK3;Q6YHK3−2;Q6YHK3−4
35:98 54:161 19:66
6:74514153:A:G_A
p = 0.53, beta = 0.0843, N = 108
TNIQVTVTGPSSPSPVK pc2 Q6YHK3;Q6YHK3−2
33:98 66:161 18:66
6:74514153:A:G_A
p = 0.16, beta = −0.195, N = 117
QHDYIIEFFDYTTVLK pc3 Q6YHK3;Q6YHK3−2;Q6YHK3−4
25:98 51:161 13:66
6:74514153:A:G_A
p = 0.9, beta = −0.0211, N = 89
VGSPFELVVSGNK pc2 Q6YHK3;Q6YHK3−2;Q6YHK3−4
13:98 25:161 6:66
6:74514153:A:G_A
p = 0.014, beta = −0.533, N = 44
ISVTQPDSIVGIVAVDK pc2 Q6YHK3;Q6YHK3−2;Q6YHK3−4
23:98 47:161 8:66
6:74514153:A:G_A
p = 0.56, beta = 0.105, N = 78
NSLGGFASTQDTTVALK pc2 Q6YHK3;Q6YHK3−2;Q6YHK3−4
13:98 20:161 8:66
6:74514153:A:G_A
p = 0.2, beta = −0.266, N = 41
log10 protein intensity
log10 peptide intensity
Assay Target: CD109
UniProt: Q6YHK3
rsID: rs4708089
Target SNP: 6:73804430:G:A
Proxy SNP: 6:74514153:A:G
deCODE log10(p): 1021.6
deCODE BETA: 0.52
deCODE MSPA: 0.76
930:769:931:669:970:734:878:4

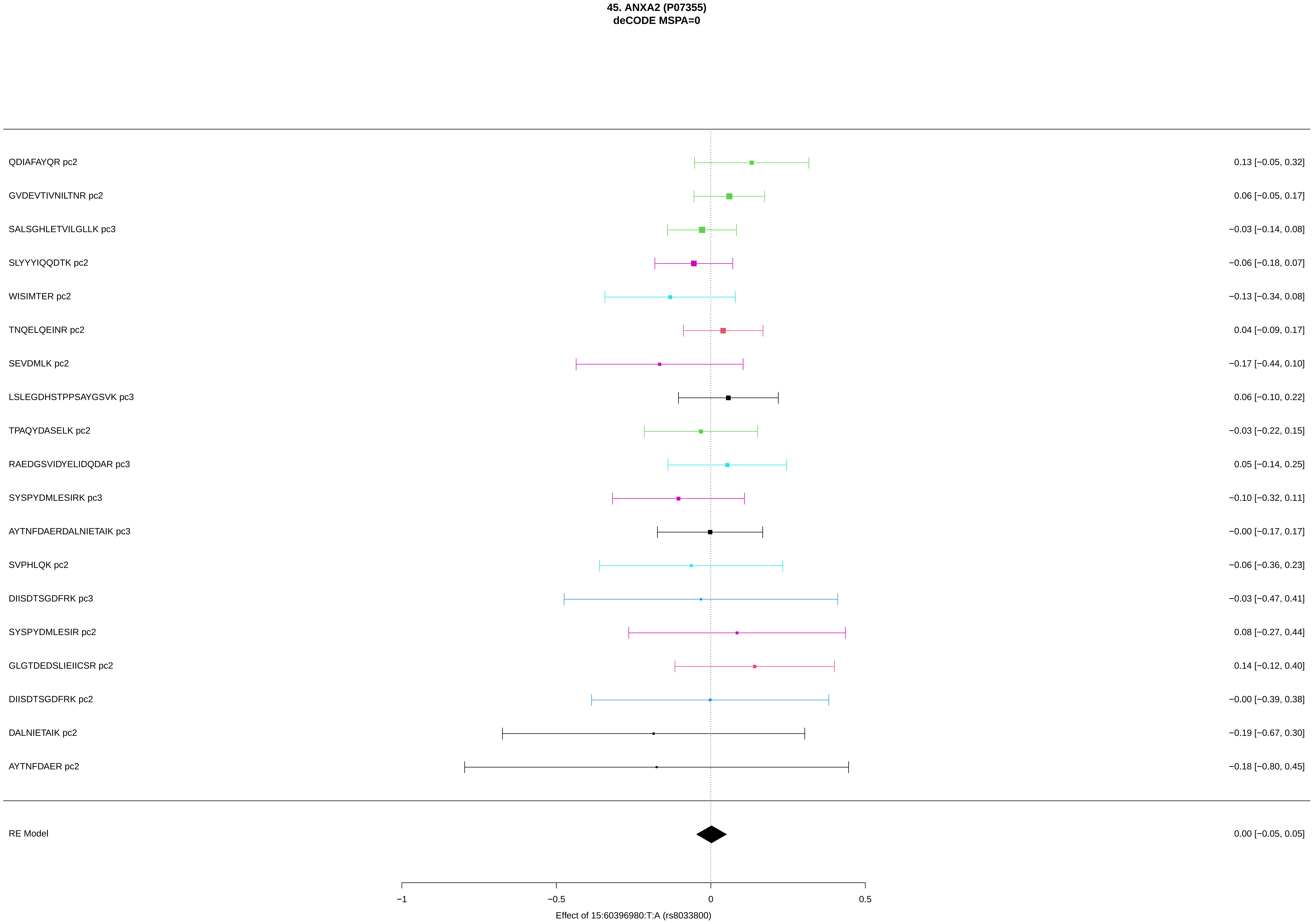
45. ANXA2 (P07355)
deCODE MSPA=0
QDIAFAYQR pc2
0.13 [−0.05, 0.32]
GVDEVTIVNILTNR pc2
0.06 [−0.05, 0.17]
SALSGHLETVILGLLK pc3
−0.03 [−0.14, 0.08]
SLYYYIQQDTK pc2
−0.06 [−0.18, 0.07]
WISIMTER pc2
−0.13 [−0.34, 0.08]
TNQELQEINR pc2
0.04 [−0.09, 0.17]
SEVDMLK pc2
−0.17 [−0.44, 0.10]
LSLEGDHSTPPSAYGSVK pc3
0.06 [−0.10, 0.22]
TPAQYDASELK pc2
−0.03 [−0.22, 0.15]
RAEDGSVIDYELIDQDAR pc3
0.05 [−0.14, 0.25]
SYSPYDMLESIRK pc3
−0.10 [−0.32, 0.11]
AYTNFDAERDALNIETAIK pc3
−0.00 [−0.17, 0.17]
SVPHLQK pc2
−0.06 [−0.36, 0.23]
DIISDTSGDFRK pc3
−0.03 [−0.47, 0.41]
SYSPYDMLESIR pc2
0.08 [−0.27, 0.44]
GLGTDEDSLIEIICSR pc2
0.14 [−0.12, 0.40]
DIISDTSGDFRK pc2
−0.00 [−0.39, 0.38]
DALNIETAIK pc2
−0.19 [−0.67, 0.30]
AYTNFDAER pc2
−0.18 [−0.80, 0.45]
RE Model
0.00 [−0.05, 0.05]
−1
−0.5
0
0.5
Effect of 15:60396980:T:A (rs8033800)

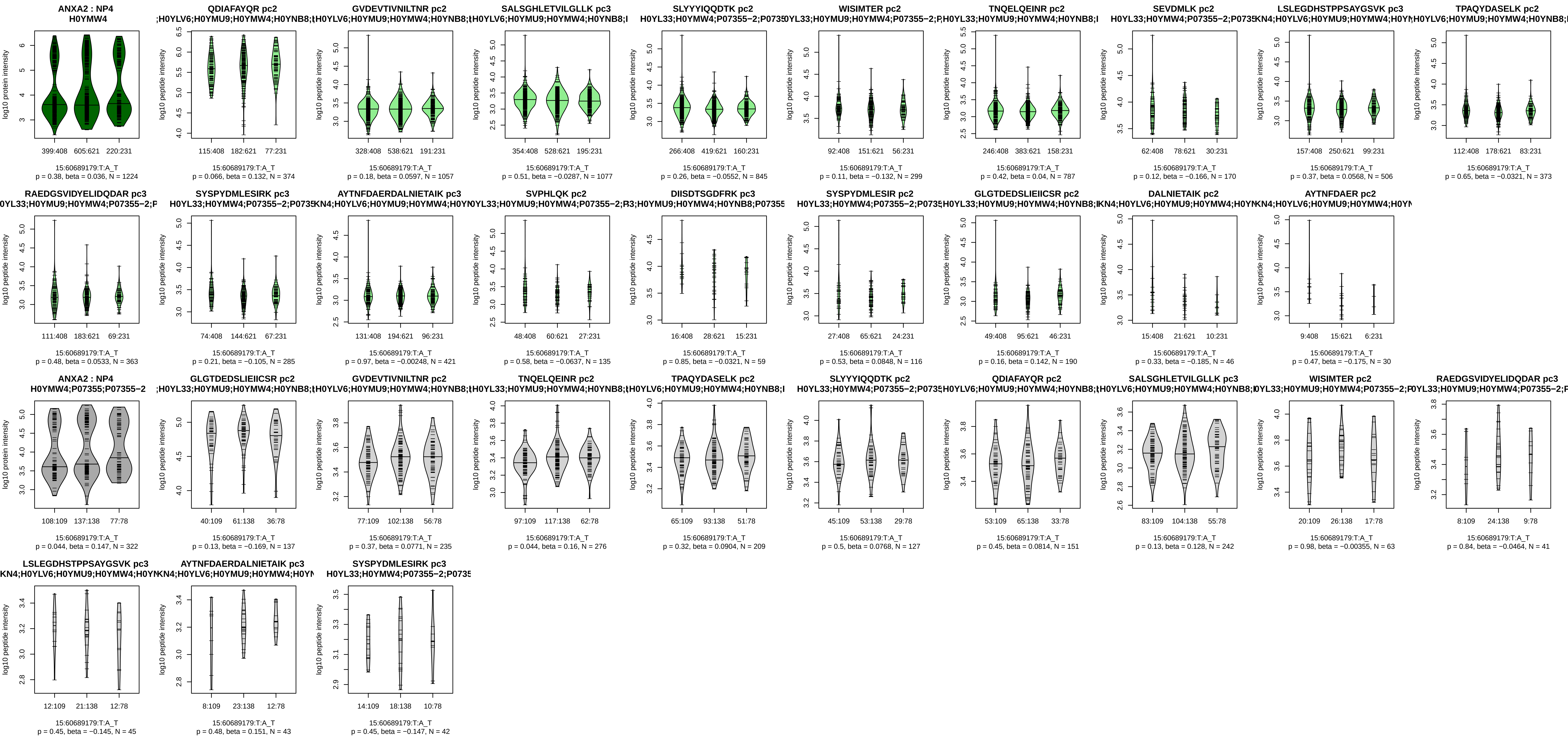

Assay Target: ANXA2
UniProt: P07355
rsID: rs8033800
Target SNP: 15:60396980:T:A
Proxy SNP: 15:60689179:T:A
deCODE log10(p): 1021.5
deCODE BETA: 0.56
deCODE MSPA: 0
374:1057:1077:845:299:787:170

46. CCL18 (P55774)
deCODE MSPA=0.937

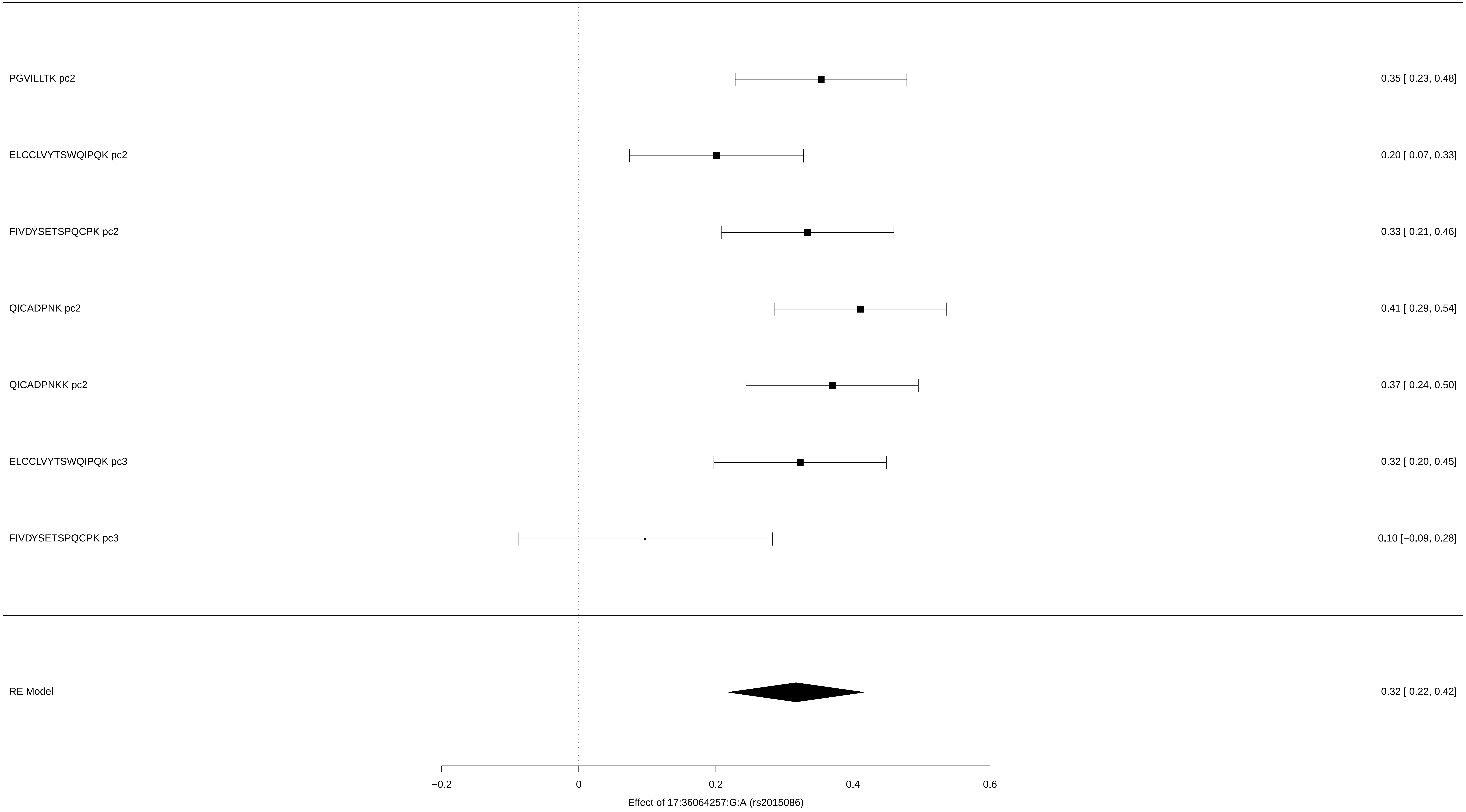

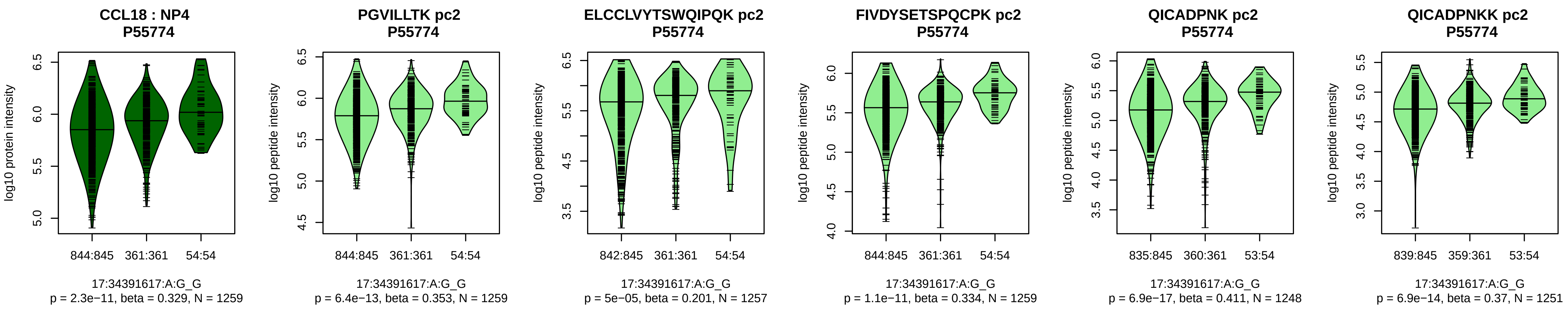

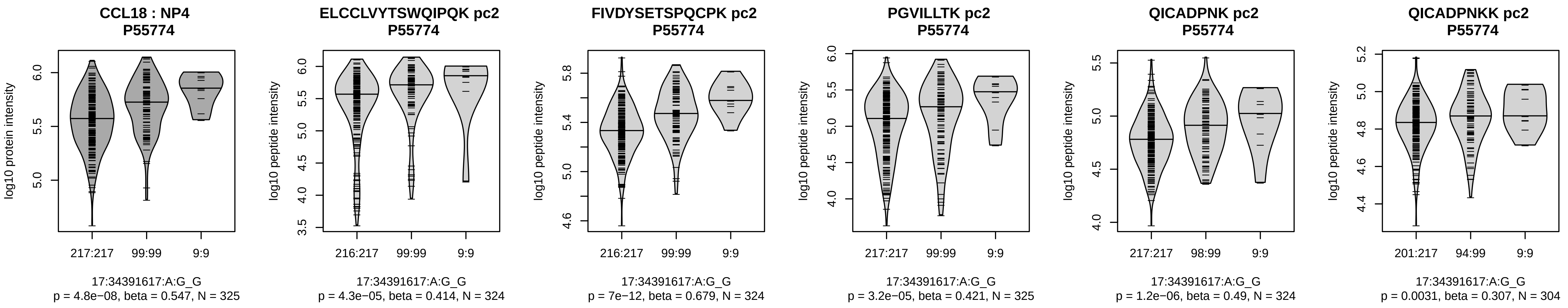

Assay Target: CCL18
UniProt: P55774
rsID: rs2015086
Target SNP: 17:36064257:G:A
Proxy SNP: 17:34391617:A:G
deCODE log10(p): 1016.6
deCODE BETA: 0.89
deCODE MSPA: 0.937
::*:*:*:*:–
1259:1257:1259:1248:1251:125

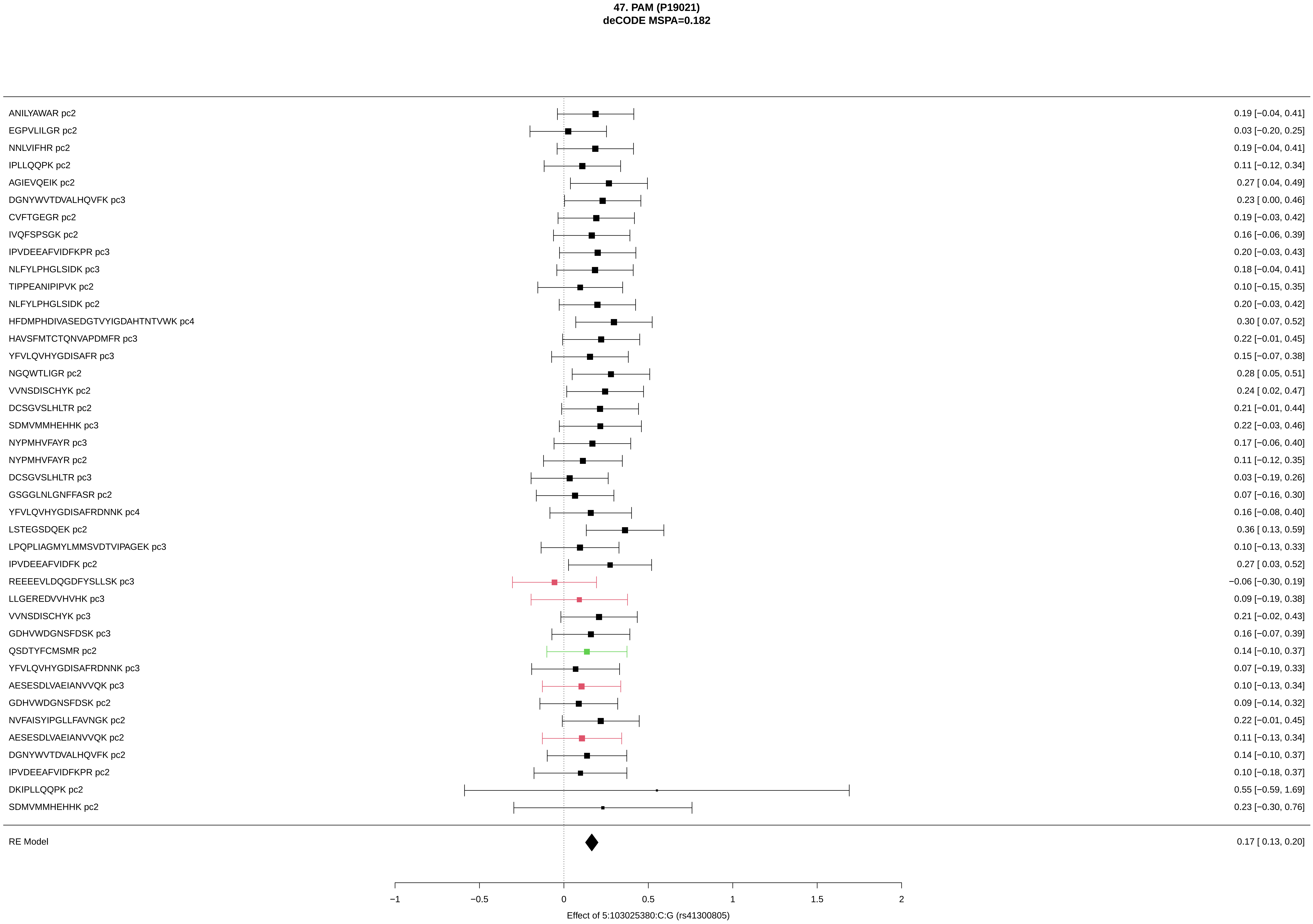
47. PAM (P19021)
deCODE MSPA=0.182
ANILYAWAR pc2 0.19 [−0.04, 0.41]
EGPVLILGR pc2 0.03 [−0.20, 0.25]
NNLVIFHR pc2 0.19 [−0.04, 0.41]
IPLLQQPK pc2 0.11 [−0.12, 0.34]
AGIEVQEIK pc2 0.27 [0.04, 0.49]
DGNYWVTDVALHQVFK pc3 0.23 [0.00, 0.46]
CVFTGEGR pc2 0.19 [−0.03, 0.42]
IVQFSPSGK pc2 0.16 [−0.06, 0.39]
IPVDEEAFVIDFKPR pc3 0.20 [−0.03, 0.43]
NLFYLPHGLSIDK pc3 0.18 [−0.04, 0.41]
TIPPEANIPIPVK pc2 0.10 [−0.15, 0.35]
NLFYLPHGLSIDK pc2 0.20 [−0.03, 0.42]
HFDMPHDIVASEDGTVYIGDAHTNTVWK pc4 0.30 [0.07, 0.52]
HAVSFMTCTQNVAPDMFR pc3 0.22 [−0.01, 0.45]
YFVLQVHYGDISAFR pc3 0.15 [−0.07, 0.38]
NGQWTLIGR pc2 0.28 [0.05, 0.51]
VVNSDISCHYK pc2 0.24 [0.02, 0.47]
DCSGVSLHLTR pc2 0.21 [−0.01, 0.44]
SDMVMMHEHHK pc3 0.22 [−0.03, 0.46]
NYPMHVFAYR pc3 0.17 [−0.06, 0.40]
NYPMHVFAYR pc2 0.11 [−0.12, 0.35]
DCSGVSLHLTR pc3 0.03 [−0.19, 0.26]
GSGGLNLGNFFASR pc2 0.07 [−0.16, 0.30]
YFVLQVHYGDISAFRDNNK pc4 0.16 [−0.08, 0.40]
LSTEGSDQEK pc2 0.36 [0.13, 0.59]
LPQPLIAGMYLMMSVDTVIPAGEK pc3 0.10 [−0.13, 0.33]
IPVDEEAFVIDFK pc2 0.27 [0.03, 0.52]
REEEEVLDQGDFYSLLSK pc3 −0.06 [−0.30, 0.19]
LLGEREDVVHVHK pc3 0.09 [−0.19, 0.38]
VVNSDISCHYK pc3 0.21 [−0.02, 0.43]
GDHVWDGNSFDSK pc3 0.16 [−0.07, 0.39]
QSDTYFCMSMR pc2 0.14 [−0.10, 0.37]
YFVLQVHYGDISAFRDNNK pc3 0.07 [−0.19, 0.33]
AESESDLVAEIANVVQK pc3 0.10 [−0.13, 0.34]
GDHVWDGNSFDSK pc2 0.09 [−0.14, 0.32]
NVFAISYIPGLLFAVNGK pc2 0.22 [−0.01, 0.45]
AESESDLVAEIANVVQK pc2 0.11 [−0.13, 0.34]
DGNYWVTDVALHQVFK pc2 0.14 [−0.10, 0.37]
IPVDEEAFVIDFKPR pc2 0.10 [−0.18, 0.37]
DKIPLLQQPK pc2 0.55 [−0.59, 1.69]
SDMVMMHEHHK pc2 0.23 [−0.30, 0.76]
RE Model 0.17 [0.13, 0.20]
−1
−0.5
0
0.5
1
1.5
2
Effect of 5:103025380:C:G (rs41300805)

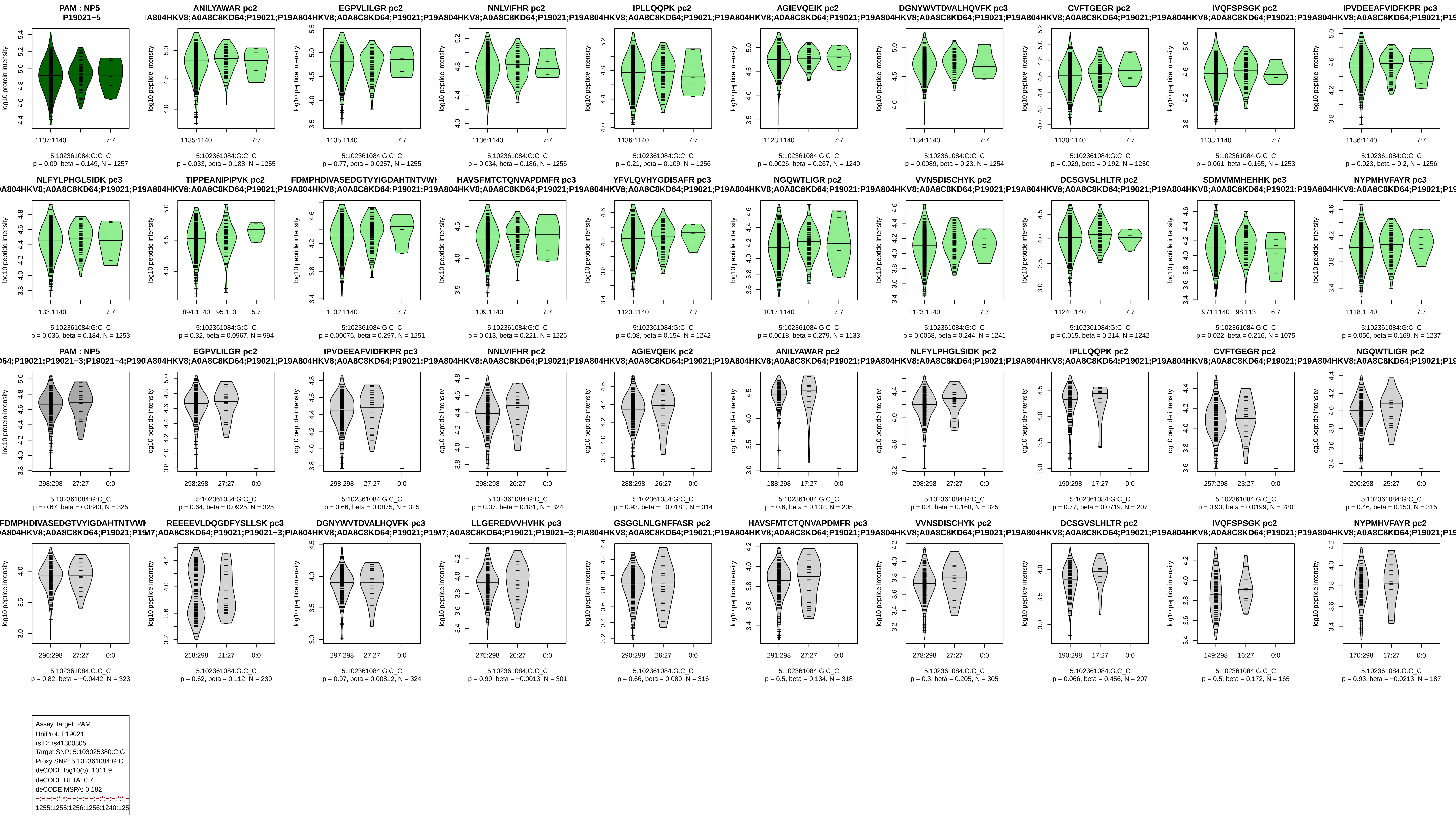
PAM : NP5
P19021−5
5:102361084:G:C_C
p = 0.09, beta = 0.149, N = 1257
ANILYAWAR pc2
p = 0.033, beta = 0.188, N = 1255
EGPVLILGR pc2
p = 0.77, beta = 0.0257, N = 1255
NNLVIFHR pc2
p = 0.034, beta = 0.186, N = 1256
IPLLQQPK pc2
p = 0.21, beta = 0.109, N = 1256
AGIEVQEIK pc2
p = 0.0026, beta = 0.267, N = 1240
DGNYWVTDVALHQVFK pc3
p = 0.0089, beta = 0.23, N = 1254
CVFTGEGR pc2
p = 0.029, beta = 0.192, N = 1250
IVQFSPSGK pc2
p = 0.061, beta = 0.165, N = 1253
IPVDEEAFVIDFKPR pc3
p = 0.023, beta = 0.2, N = 1256
NLFYLPHGLSIDK pc3
p = 0.036, beta = 0.184, N = 1253
TIPPEANIPIPVK pc2
p = 0.32, beta = 0.0967, N = 994
p = 0.00076, beta = 0.297, N = 1251
HAVSFMTCTQNVAPDMFR pc3
p = 0.013, beta = 0.221, N = 1226
YFVLQVHYGDISAFR pc3
p = 0.08, beta = 0.154, N = 1242
NGQWTLIGR pc2
p = 0.0018, beta = 0.279, N = 1133
VVNSDISCHYK pc2
p = 0.0058, beta = 0.244, N = 1241
DCSGVSLHLTR pc2
p = 0.015, beta = 0.214, N = 1242
SDMVMMHEHHK pc3
p = 0.022, beta = 0.216, N = 1075
NYPMHVFAYR pc3
p = 0.056, beta = 0.169, N = 1237
PAM : NP5
p = 0.67, beta = 0.0843, N = 325
EGPVLILGR pc2
p = 0.64, beta = 0.0925, N = 325
IPVDEEAFVIDFKPR pc3
p = 0.66, beta = 0.0875, N = 325
NNLVIFHR pc2
p = 0.37, beta = 0.181, N = 324
AGIEVQEIK pc2
p = 0.93, beta = −0.0181, N = 314
ANILYAWAR pc2
p = 0.6, beta = 0.132, N = 205
NLFYLPHGLSIDK pc2
p = 0.4, beta = 0.168, N = 325
IPLLQQPK pc2
p = 0.77, beta = 0.0719, N = 207
CVFTGEGR pc2
p = 0.93, beta = 0.0199, N = 280
NGQWTLIGR pc2
p = 0.46, beta = 0.153, N = 315
p = 0.82, beta = −0.0442, N = 323
REEEEVLDQGDFYSLLSK pc3
p = 0.62, beta = 0.112, N = 239
DGNYWVTDVALHQVFK pc3
p = 0.97, beta = 0.00812, N = 324
LLGEREDVVHVHK pc3
p = 0.99, beta = −0.0013, N = 301
GSGGLNLGNFFASR pc2
p = 0.66, beta = 0.089, N = 316
HAVSFMTCTQNVAPDMFR pc3
p = 0.5, beta = 0.134, N = 318
VVNSDISCHYK pc2
p = 0.3, beta = 0.205, N = 305
DCSGVSLHLTR pc2
p = 0.066, beta = 0.456, N = 207
IVQFSPSGK pc2
p = 0.5, beta = 0.172, N = 165
NYPMHVFAYR pc2
p = 0.93, beta = −0.0213, N = 187
Assay Target: PAM
UniProt: P19021
rsID: rs41300805
Target SNP: 5:103025380:C:G
Proxy SNP: 5:102361084:G:C
deCODE log10(p): 1011.9
deCODE BETA: 0.7
deCODE MSPA: 0.182

48. PZP (P20742)
deCODE MSPA=0.243 (1 conflicts)

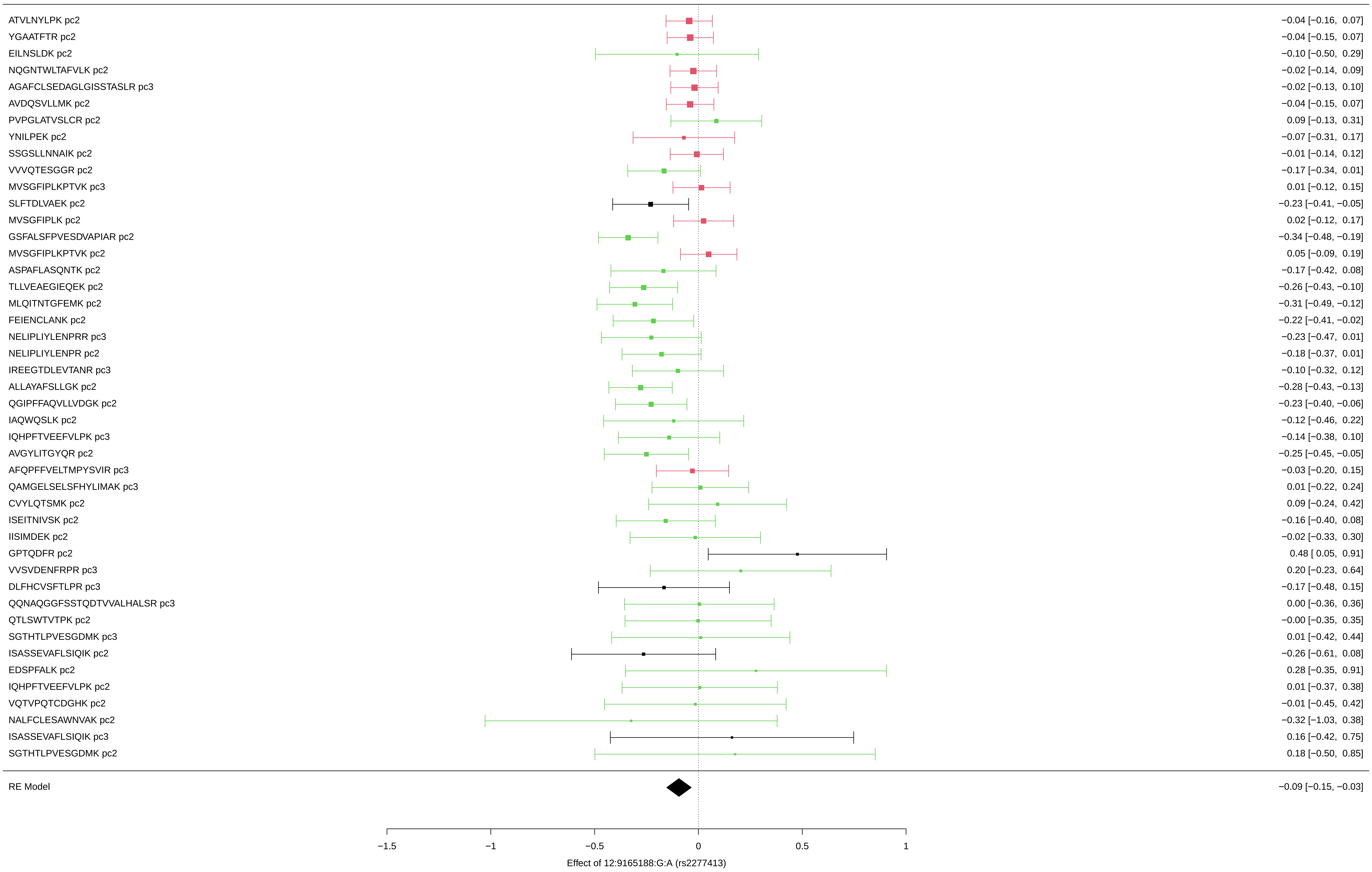

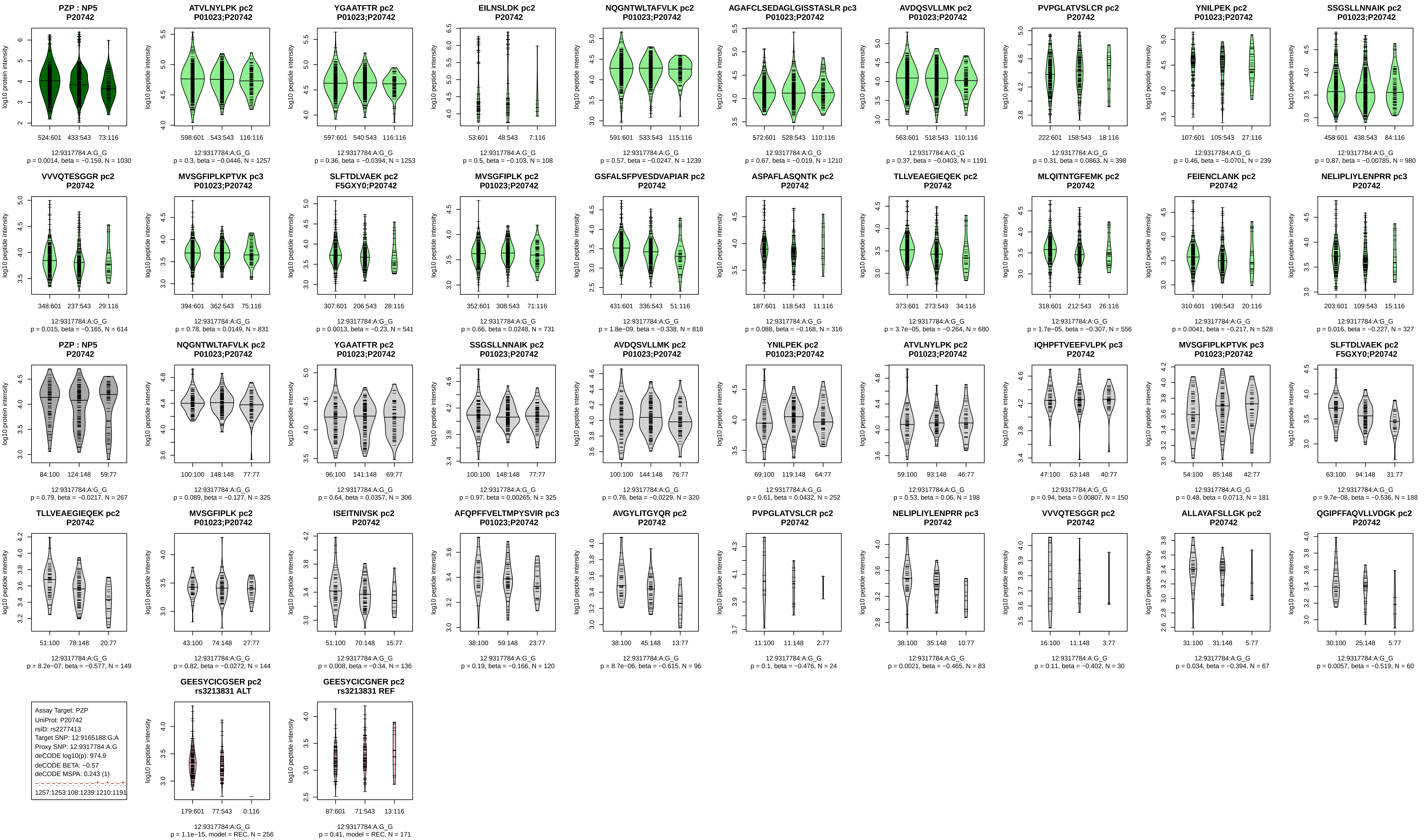
PZP : NP5 P20742
log10 protein intensity
524:601 433:543 73:116
12:9317784:A:G_G
p = 0.0014, beta = −0.159, N = 1030
ATVLNYLPK pc2 P01023;P20742
log10 peptide intensity
598:601 543:543 116:116
12:9317784:A:G_G
p = 0.3, beta = −0.0446, N = 1257
YGAATFTR pc2 P01023;P20742
597:601 540:543 116:116
12:9317784:A:G_G
p = 0.36, beta = −0.0394, N = 1253
EILNSLDK pc2 P20742
53:601 48:543 7:116
12:9317784:A:G_G
p = 0.5, beta = −0.103, N = 108
NQGNTWLTAFVLK pc2 P01023;P20742
591:601 533:543 115:116
12:9317784:A:G_G
p = 0.57, beta = −0.0247, N = 1239
AGAFCLSEDAGLGISSTASLR pc3 P01023;P20742
572:601 528:543 110:116
12:9317784:A:G_G
p = 0.67, beta = −0.019, N = 1210
AVDQSVLLMK pc2 P01023;P20742
563:601 518:543 110:116
12:9317784:A:G_G
p = 0.37, beta = −0.0403, N = 1191
PVPGLATVSLCR pc2 P20742
222:601 158:543 18:116
12:9317784:A:G_G
p = 0.31, beta = 0.0863, N = 398
YNILPEK pc2 P01023;P20742
107:601 105:543 27:116
12:9317784:A:G_G
p = 0.46, beta = −0.0701, N = 239
SSGSLLNNAIK pc2 P01023;P20742
458:601 438:543 84:116
12:9317784:A:G_G
p = 0.87, beta = −0.00785, N = 980
VVVQTESGGR pc2 P20742
348:601 237:543 29:116
12:9317784:A:G_G
p = 0.015, beta = −0.165, N = 614
MVSGFIPLKPTVK pc3 P01023;P20742
394:601 362:543 75:116
12:9317784:A:G_G
p = 0.78, beta = 0.0149, N = 831
SLFTDLVAEK pc2 F5GXY0;P20742
307:601 206:543 28:116
12:9317784:A:G_G
p = 0.0013, beta = −0.23, N = 541
MVSGFIPLK pc2 P01023;P20742
352:601 308:543 71:116
12:9317784:A:G_G
p = 0.66, beta = 0.0248, N = 731
GSFALSFPVESDVAPIAR pc2 P20742
431:601 336:543 51:116
12:9317784:A:G_G
p = 1.8e−09, beta = −0.338, N = 818
ASPAFLASQNTK pc2 P20742
187:601 118:543 11:116
12:9317784:A:G_G
p = 0.088, beta = −0.168, N = 316
TLLVEAEGIEQEK pc2 P20742
373:601 273:543 34:116
12:9317784:A:G_G
p = 3.7e−05, beta = −0.264, N = 680
MLQITNTGFEMK pc2 P20742
318:601 212:543 26:116
12:9317784:A:G_G
p = 1.7e−05, beta = −0.307, N = 556
FEIENCLANK pc2 P20742
310:601 198:543 20:116
12:9317784:A:G_G
p = 0.0041, beta = −0.217, N = 528
NELIPLIYLENPRR pc3 P20742
203:601 109:543 15:116
12:9317784:A:G_G
p = 0.016, beta = −0.227, N = 327
PZP : NP5 P20742
84:100 124:148 59:77
12:9317784:A:G_G
p = 0.79, beta = −0.0217, N = 267
NQGNTWLTAFVLK pc2 P01023;P20742
100:100 148:148 77:77
12:9317784:A:G_G
p = 0.089, beta = −0.127, N = 325
YGAATFTR pc2 P01023;P20742
96:100 141:148 69:77
12:9317784:A:G_G
p = 0.64, beta = 0.0357, N = 306
SSGSLLNNAIK pc2 P01023;P20742
100:100 148:148 77:77
12:9317784:A:G_G
p = 0.97, beta = 0.00265, N = 325
AVDQSVLLMK pc2 P01023;P20742
100:100 144:148 76:77
12:9317784:A:G_G
p = 0.76, beta = −0.0229, N = 320
YNILPEK pc2 P01023;P20742
69:100 119:148 64:77
12:9317784:A:G_G
p = 0.61, beta = 0.0432, N = 252
ATVLNYLPK pc2 P01023;P20742
59:100 93:148 46:77
12:9317784:A:G_G
p = 0.53, beta = 0.06, N = 198
IQHPFTVEEFVLPK pc3 P20742
47:100 63:148 40:77
12:9317784:A:G_G
p = 0.94, beta = 0.00807, N = 150
MVSGFIPLKPTVK pc3 P01023;P20742
54:100 85:148 42:77
12:9317784:A:G_G
p = 0.48, beta = 0.0713, N = 181
SLFTDLVAEK pc2 F5GXY0;P20742
63:100 94:148 31:77
12:9317784:A:G_G
p = 9.7e−08, beta = −0.536, N = 188
TLLVEAEGIEQEK pc2 P20742
51:100 78:148 20:77
12:9317784:A:G_G
p = 8.2e−07, beta = −0.577, N = 149
MVSGFIPLK pc2 P01023;P20742
43:100 74:148 27:77
12:9317784:A:G_G
p = 0.82, beta = −0.0272, N = 144
ISEITNIVSK pc2 P20742
51:100 70:148 15:77
12:9317784:A:G_G
p = 0.008, beta = −0.34, N = 136
AFQPFFVELTMPYSVIR pc3 P01023;P20742
38:100 59:148 23:77
12:9317784:A:G_G
p = 0.19, beta = −0.166, N = 120
AVGYLITGYQR pc2 P20742
38:100 45:148 13:77
12:9317784:A:G_G
p = 8.7e−06, beta = −0.615, N = 96
PVPGLATVSLCR pc2 P20742
11:100 11:148 2:77
12:9317784:A:G_G
p = 0.1, beta = −0.476, N = 24
NELIPLIYLENPRR pc3 P20742
38:100 35:148 10:77
12:9317784:A:G_G
p = 0.0021, beta = −0.465, N = 83
VVVQTESGGR pc2 P20742
16:100 11:148 3:77
12:9317784:A:G_G
p = 0.11, beta = −0.402, N = 30
ALLAYAFSLLGK pc2 P20742
31:100 31:148 5:77
12:9317784:A:G_G
p = 0.034, beta = −0.394, N = 67
QGIPFFAQVLLVDGK pc2 P20742
30:100 25:148 5:77
12:9317784:A:G_G
p = 0.0057, beta = −0.519, N = 60
Assay Target: PZP
UniProt: P20742
rsID: rs2277413
Target SNP: 12:9165188:G:A
Proxy SNP: 12:9317784:A:G
deCODE log10(p): 974.9
deCODE BETA: −0.57
deCODE MSPA: 0.243 (1)
1257:1253:108:1239:1210:1191
GEESYCICGSER pc2 rs3213831 ALT
179:601 77:543 0:116
12:9317784:A:G_G
p = 1.1e−15, model = REC, N = 256
GEESYCICGNER pc2 rs3213831 REF
87:601 71:543 13:116
12:9317784:A:G_G
p = 0.41, model = REC, N = 171

49. TNFAIP6 (P98066)
deCODE MSPA=0.821

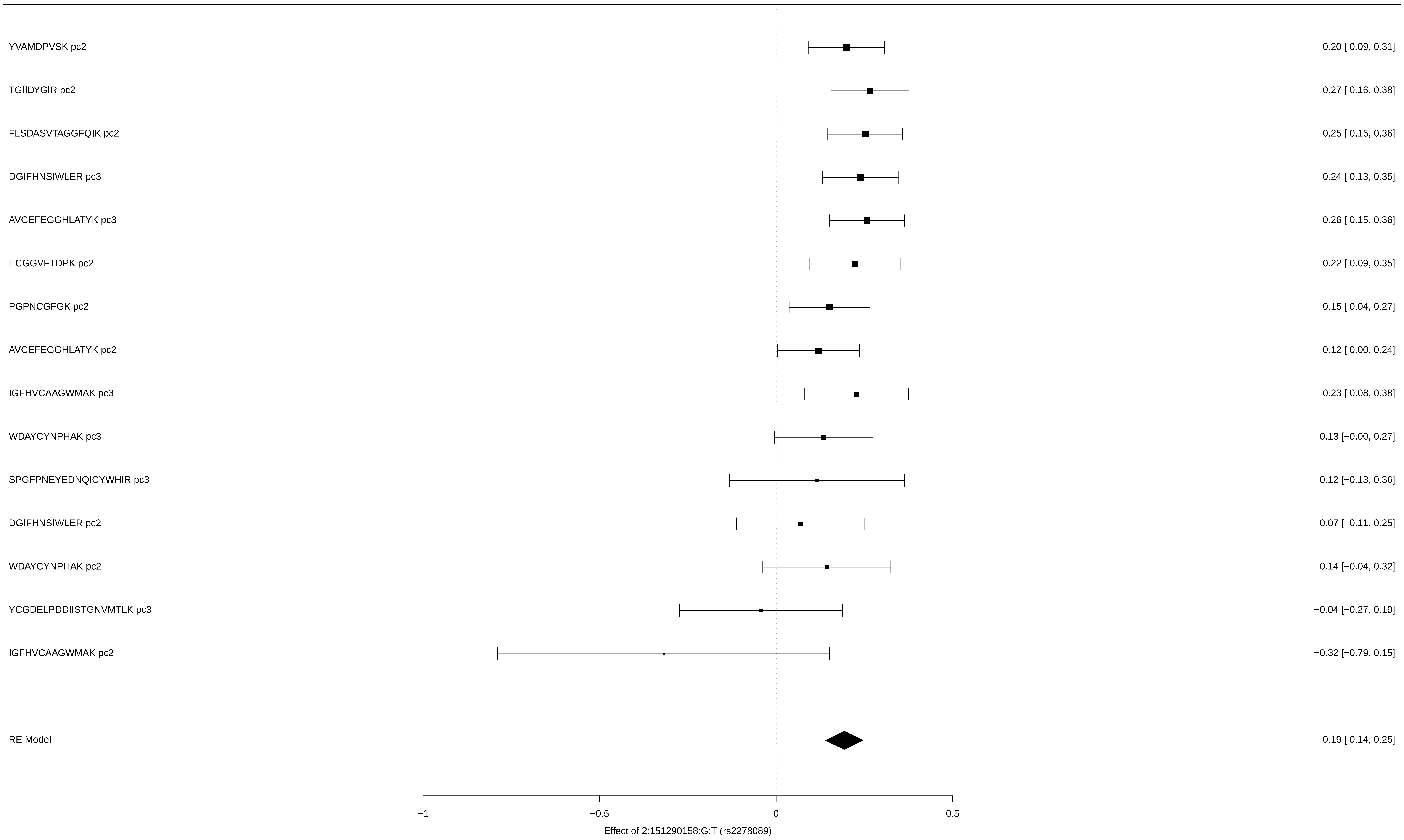

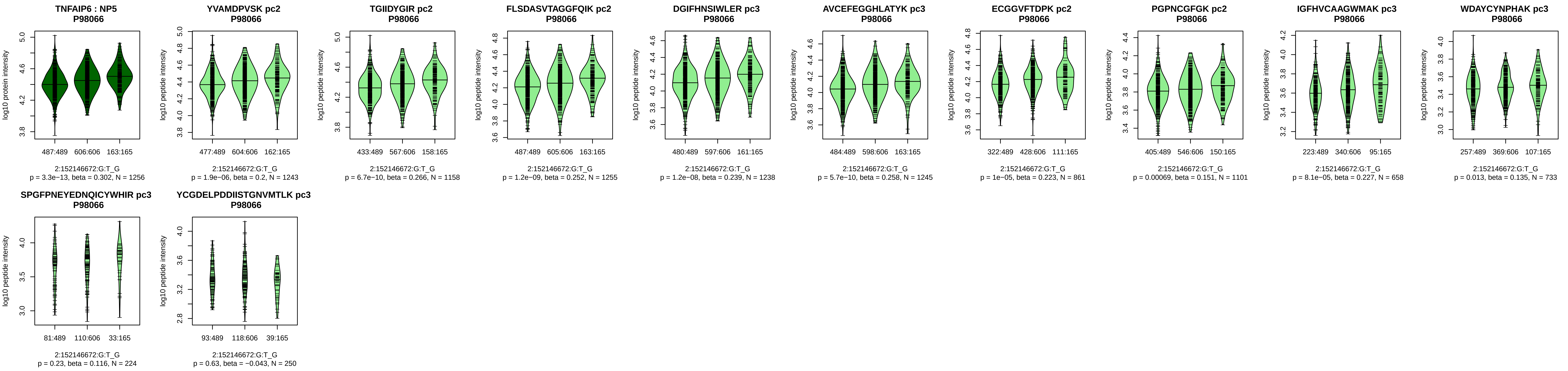

Assay Target: TNFAIP6
UniProt: P98066
rsID: rs2278089
Target SNP: 2:151290158:G:T
Proxy SNP: 2:152146672:G:T
deCODE log10(p): 959.2
deCODE BETA: 0.52
deCODE MSPA: 0.821
1243:1158:1255:1238:1245:861

50. SFTPD (P35247)
deCODE MSPA=0

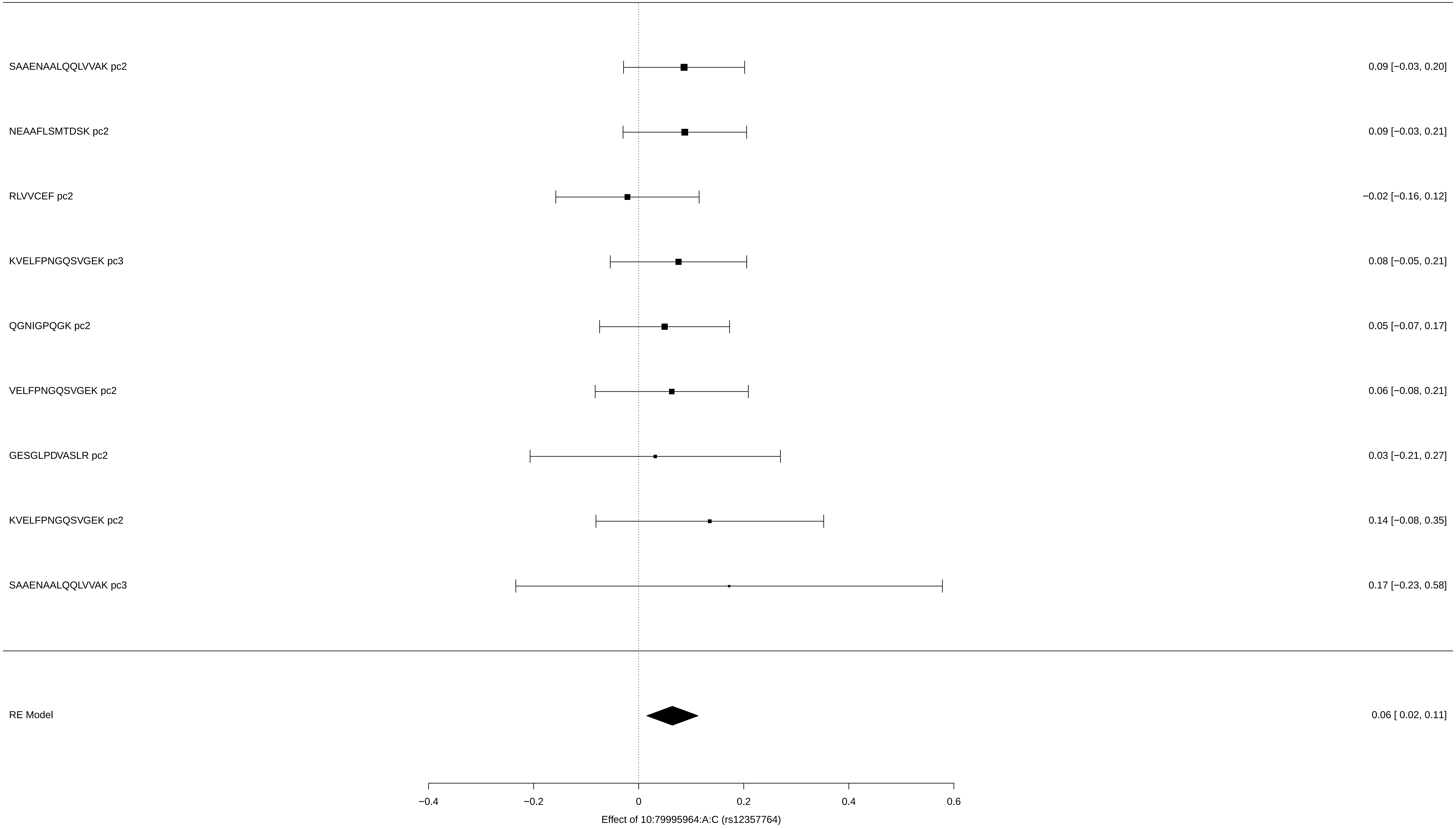

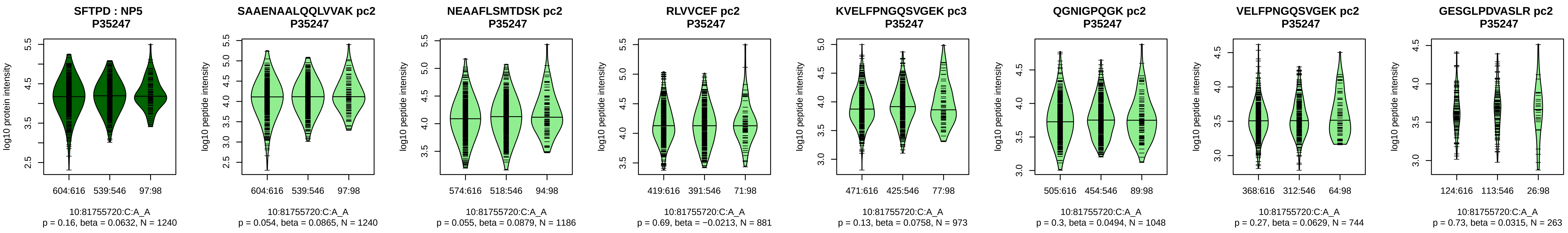

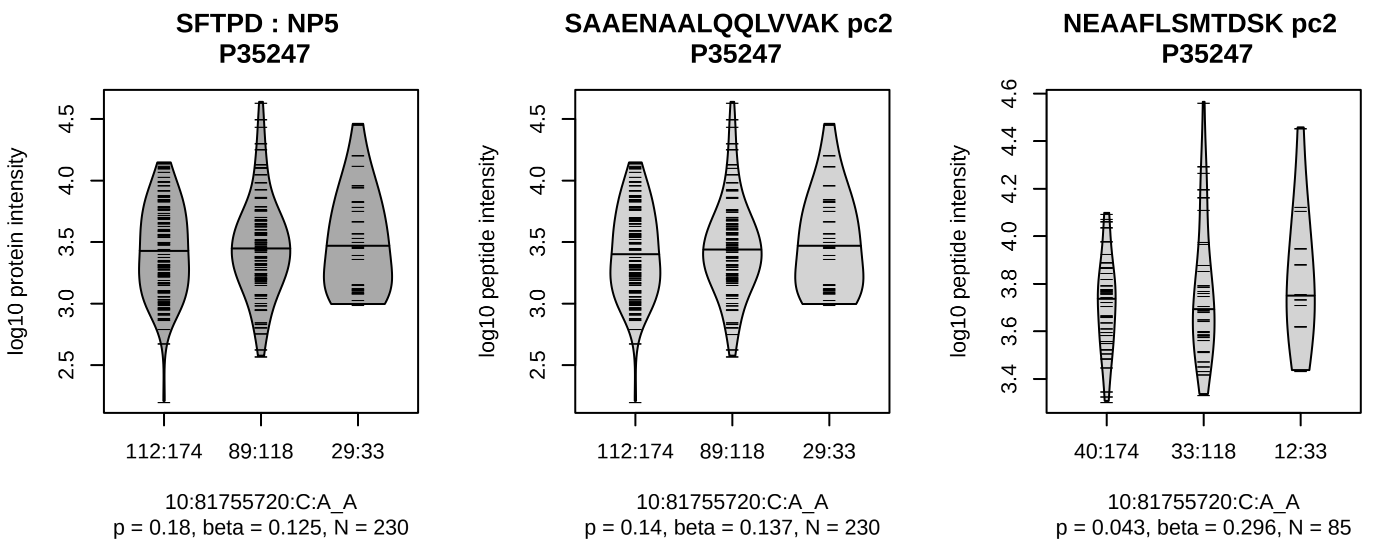

Assay Target: SFTPD
UniProt: P35247
rsID: rs12357764
Target SNP: 10:79995964:A:C
Proxy SNP: 10:81755720:C:A
deCODE log10(p): 957.5
deCODE BETA: 0.53
deCODE MSPA: 0
–:–:–:–:–:–:–:–
1240:1186:881:973:1048:744:26

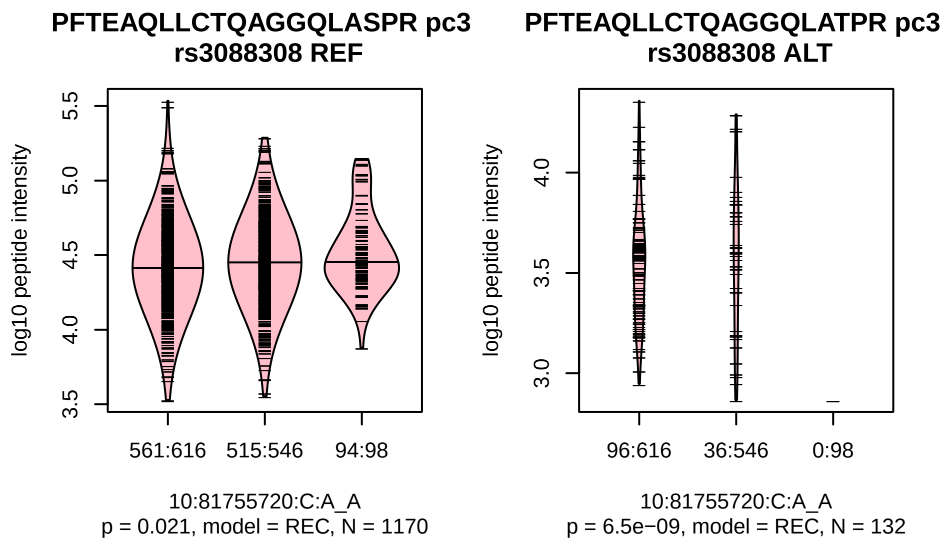

51. ITIH1 (P19827)
deCODE MSPA=0.221

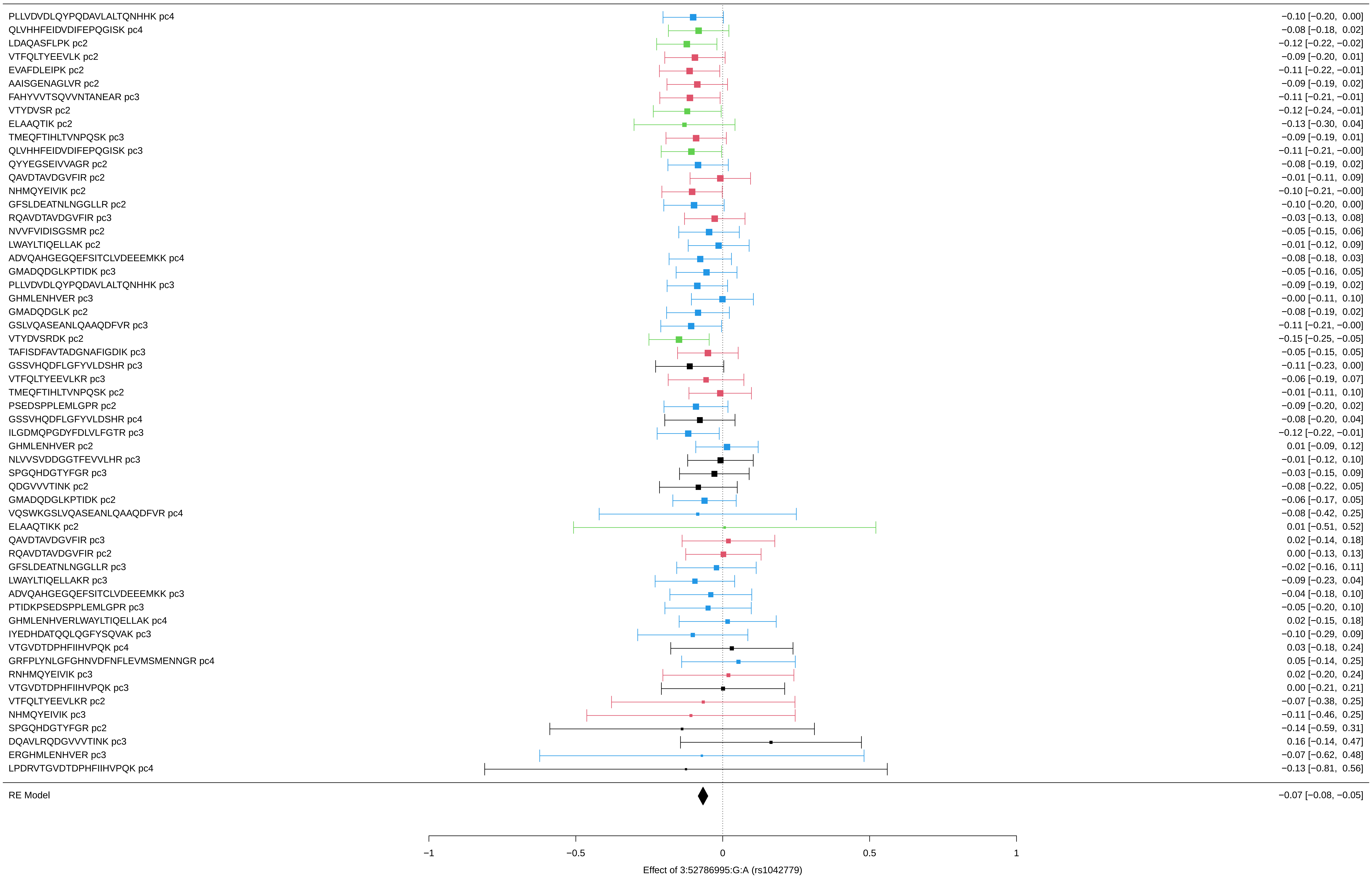

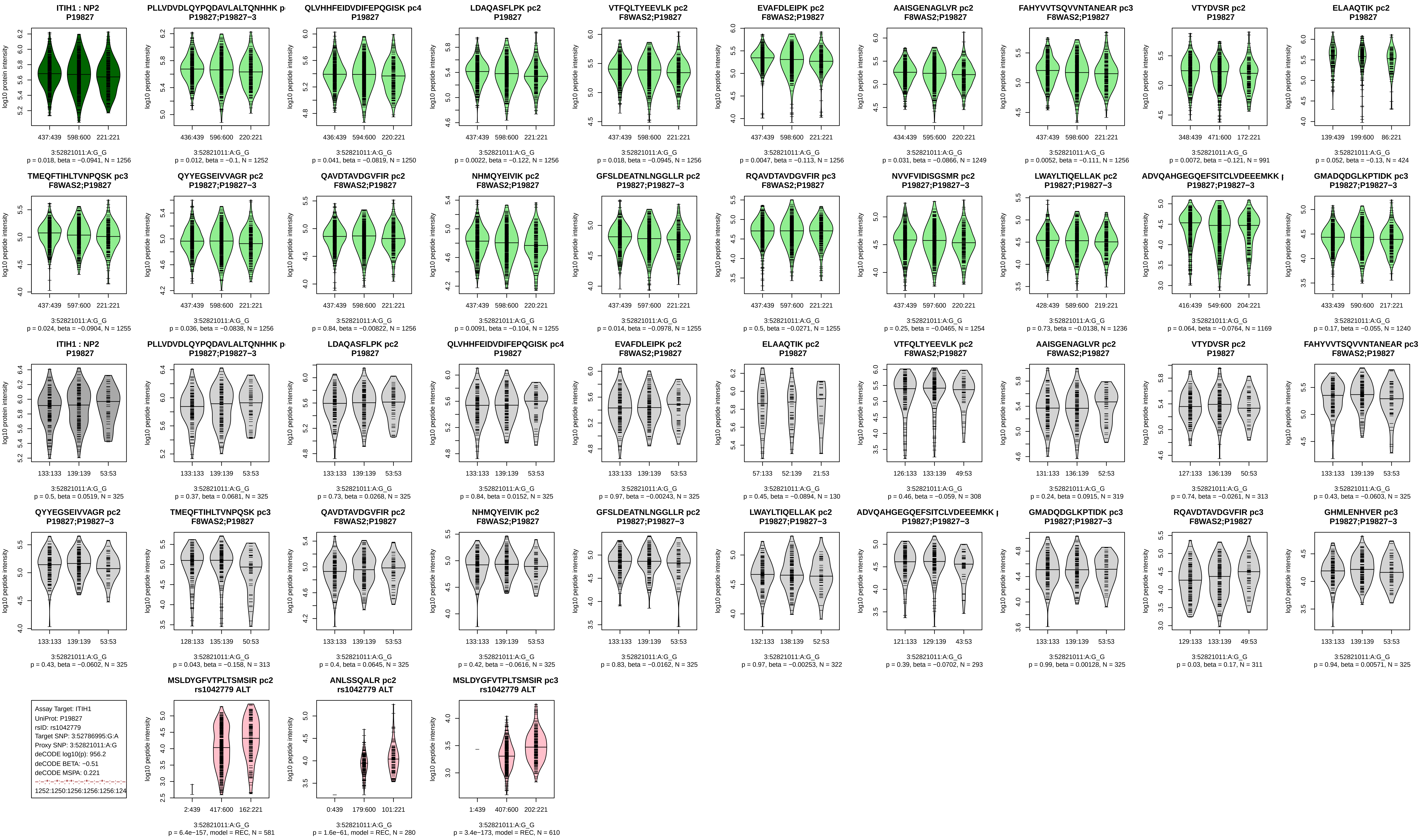
ITIH1 : NP2
P19827
log10 protein intensity
437:439 598:600 221:221
3:52821011:A:G_G
p = 0.018, beta = −0.0941, N = 1256
PLLVDVDLQYPQDAVLALTQNHHK pc
P19827;P19827−3
log10 peptide intensity
436:439 596:600 220:221
3:52821011:A:G_G
p = 0.012, beta = −0.1, N = 1252
QLVHHFEIDVDIFEPQGISK pc4
P19827
436:439 594:600 220:221
3:52821011:A:G_G
p = 0.041, beta = −0.0819, N = 1250
LDAQASFLPK pc2
P19827
437:439 598:600 221:221
3:52821011:A:G_G
p = 0.0022, beta = −0.122, N = 1256
VTFQLTYEEVLK pc2
F8WAS2;P19827
437:439 598:600 221:221
3:52821011:A:G_G
p = 0.018, beta = −0.0945, N = 1256
EVAFDLEIPK pc2
F8WAS2;P19827
437:439 598:600 221:221
3:52821011:A:G_G
p = 0.0047, beta = −0.113, N = 1256
AAISGENAGLVR pc2
F8WAS2;P19827
434:439 595:600 220:221
3:52821011:A:G_G
p = 0.031, beta = −0.0866, N = 1249
FAHYVVTSQVVNTANEAR pc3
F8WAS2;P19827
437:439 598:600 221:221
3:52821011:A:G_G
p = 0.0052, beta = −0.111, N = 1256
VTYDVSR pc2
P19827
348:439 471:600 172:221
3:52821011:A:G_G
p = 0.0072, beta = −0.121, N = 991
ELAAQTIK pc2
P19827
139:439 199:600 86:221
3:52821011:A:G_G
p = 0.052, beta = −0.13, N = 424
TMEQFTIHLTVNPQSK pc3
F8WAS2;P19827
437:439 597:600 221:221
3:52821011:A:G_G
p = 0.024, beta = −0.0904, N = 1255
QYYEGSEIVVAGR pc2
P19827;P19827−3
437:439 598:600 221:221
3:52821011:A:G_G
p = 0.036, beta = −0.0838, N = 1256
QAVDTAVDGVFIR pc2
F8WAS2;P19827
437:439 598:600 221:221
3:52821011:A:G_G
p = 0.84, beta = −0.00822, N = 1256
NHMQYEIVIK pc2
F8WAS2;P19827
437:439 598:600 220:221
3:52821011:A:G_G
p = 0.0091, beta = −0.104, N = 1255
GFSLDEATNLNGGLLR pc2
P19827;P19827−3
437:439 597:600 221:221
3:52821011:A:G_G
p = 0.014, beta = −0.0978, N = 1255
RQAVDTAVDGVFIR pc3
F8WAS2;P19827
437:439 597:600 221:221
3:52821011:A:G_G
p = 0.5, beta = −0.0271, N = 1255
NVVFVIDISGSMR pc2
P19827;P19827−3
437:439 597:600 220:221
3:52821011:A:G_G
p = 0.25, beta = −0.0465, N = 1254
LWAYLTIQELLAK pc2
P19827;P19827−3
428:439 589:600 219:221
3:52821011:A:G_G
p = 0.73, beta = −0.0138, N = 1236
ADVQAHGEGQEFSITCLVDEEEMKK p
P19827;P19827−3
416:439 549:600 204:221
3:52821011:A:G_G
p = 0.064, beta = −0.0764, N = 1169
GMADQDGLKPTIDK pc3
P19827;P19827−3
433:439 590:600 217:221
3:52821011:A:G_G
p = 0.17, beta = −0.055, N = 1240
ITIH1 : NP2
P19827
133:133 139:139 53:53
3:52821011:A:G_G
p = 0.5, beta = 0.0519, N = 325
PLLVDVDLQYPQDAVLALTQNHHK pc
P19827;P19827−3
133:133 139:139 53:53
3:52821011:A:G_G
p = 0.37, beta = 0.0681, N = 325
LDAQASFLPK pc2
P19827
133:133 139:139 53:53
3:52821011:A:G_G
p = 0.73, beta = 0.0268, N = 325
QLVHHFEIDVDIFEPQGISK pc4
P19827
133:133 139:139 53:53
3:52821011:A:G_G
p = 0.84, beta = 0.0152, N = 325
EVAFDLEIPK pc2
F8WAS2;P19827
133:133 139:139 53:53
3:52821011:A:G_G
p = 0.97, beta = −0.00243, N = 325
ELAAQTIK pc2
P19827
57:133 52:139 21:53
3:52821011:A:G_G
p = 0.45, beta = −0.0894, N = 130
VTFQLTYEEVLK pc2
F8WAS2;P19827
126:133 133:139 49:53
3:52821011:A:G_G
p = 0.46, beta = −0.059, N = 308
AAISGENAGLVR pc2
F8WAS2;P19827
131:133 136:139 52:53
3:52821011:A:G_G
p = 0.24, beta = 0.0915, N = 319
VTYDVSR pc2
P19827
127:133 136:139 50:53
3:52821011:A:G_G
p = 0.74, beta = −0.0261, N = 313
FAHYVVTSQVVNTANEAR pc3
F8WAS2;P19827
133:133 139:139 53:53
3:52821011:A:G_G
p = 0.43, beta = −0.0603, N = 325
QYYEGSEIVVAGR pc2
P19827;P19827−3
133:133 139:139 53:53
3:52821011:A:G_G
p = 0.43, beta = −0.0602, N = 325
TMEQFTIHLTVNPQSK pc3
F8WAS2;P19827
128:133 135:139 50:53
3:52821011:A:G_G
p = 0.043, beta = −0.158, N = 313
QAVDTAVDGVFIR pc2
F8WAS2;P19827
133:133 139:139 53:53
3:52821011:A:G_G
p = 0.4, beta = 0.0645, N = 325
NHMQYEIVIK pc2
F8WAS2;P19827
133:133 139:139 53:53
3:52821011:A:G_G
p = 0.42, beta = −0.0616, N = 325
GFSLDEATNLNGGLLR pc2
P19827;P19827−3
133:133 139:139 53:53
3:52821011:A:G_G
p = 0.83, beta = −0.0162, N = 325
LWAYLTIQELLAK pc2
P19827;P19827−3
132:133 138:139 52:53
3:52821011:A:G_G
p = 0.97, beta = −0.00253, N = 322
ADVQAHGEGQEFSITCLVDEEEMKK p
P19827;P19827−3
121:133 129:139 43:53
3:52821011:A:G_G
p = 0.39, beta = −0.0702, N = 293
GMADQDGLKPTIDK pc3
P19827;P19827−3
133:133 139:139 53:53
3:52821011:A:G_G
p = 0.99, beta = 0.00128, N = 325
RQAVDTAVDGVFIR pc3
F8WAS2;P19827
129:133 129:139 49:53
3:52821011:A:G_G
p = 0.03, beta = 0.17, N = 311
GHMLENHVER pc3
P19827;P19827−3
133:133 139:139 53:53
3:52821011:A:G_G
p = 0.94, beta = 0.00571, N = 325
Assay Target: ITIH1
UniProt: P19827
rsID: rs1042779
Target SNP: 3:52786995:G:A
Proxy SNP: 3:52821011:A:G
deCODE log10(p): 956.2
deCODE BETA: −0.51
deCODE MSPA: 0.221
1252:1250:1256:1256:1256:124
MSLDYGFVTPLTSMSIR pc2
rs1042779 ALT
2:439 417:600 162:221
3:52821011:A:G_G
p = 6.4e−157, model = REC, N = 581
ANLSSQALR pc2
rs1042779 ALT
0:439 179:600 101:221
3:52821011:A:G_G
p = 1.6e−61, model = REC, N = 280
MSLDYGFVTPLTSMSIR pc3
rs1042779 ALT
1:439 407:600 202:221
3:52821011:A:G_G
p = 3.4e−173, model = REC, N = 610

52. C1S (P09871)
deCODE MSPA=0.376 (2 conflicts)

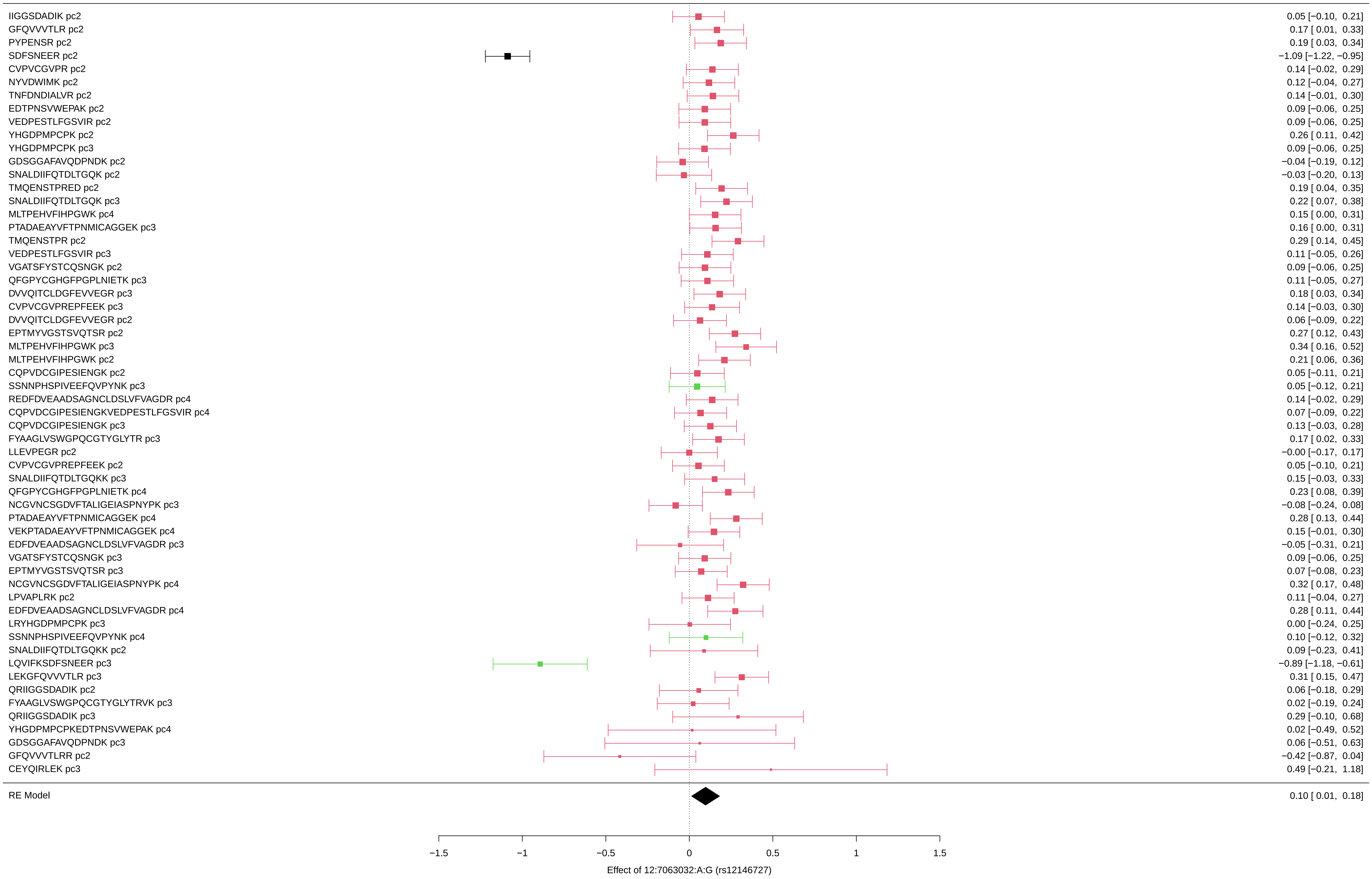

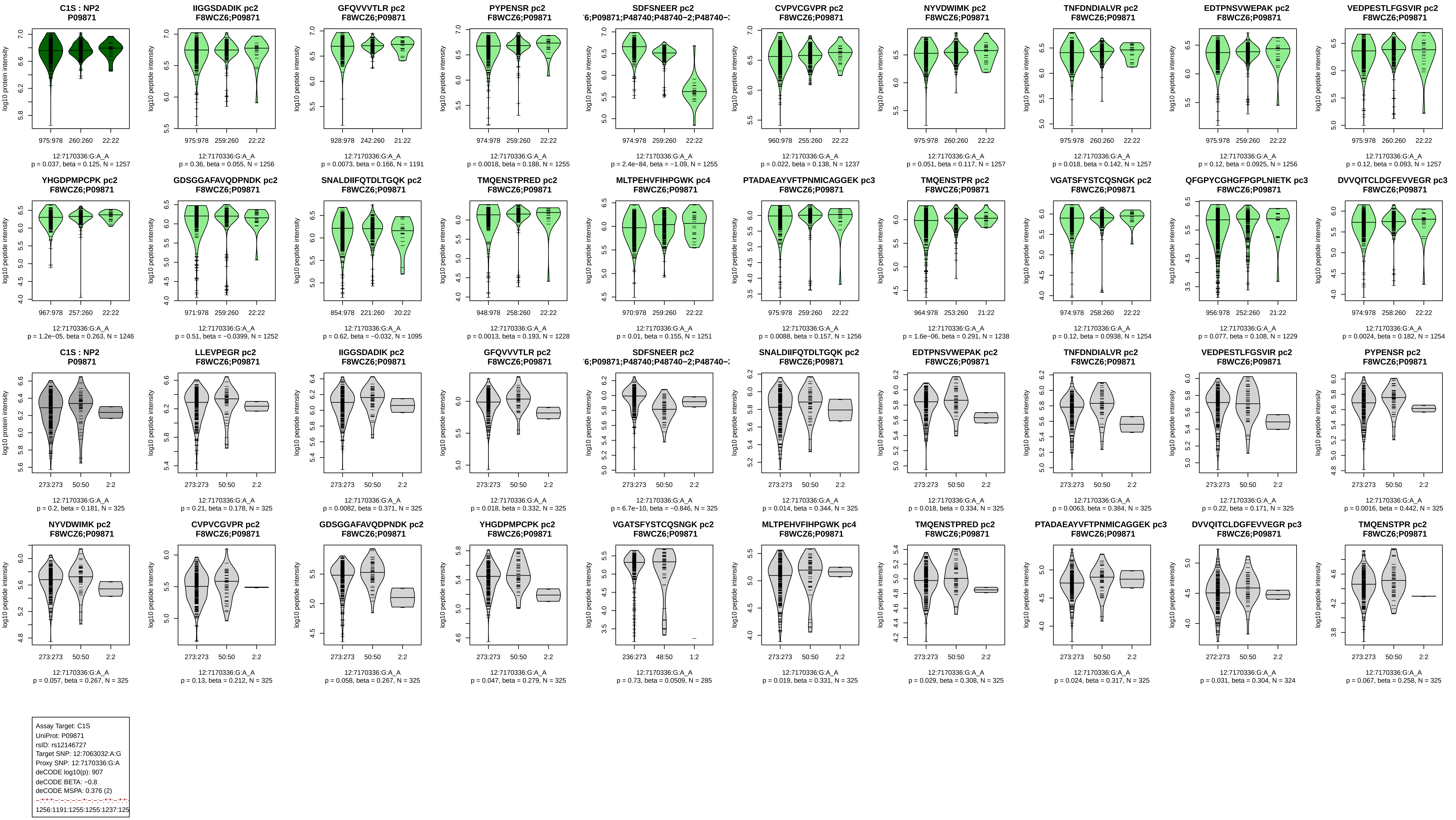

C1S : NP2
P09871
975:978 260:260 22:22
12:7170336:G:A_A
p = 0.037, beta = 0.125, N = 1257
IIGGSDADIK pc2
F8WCZ6;P09871
975:978 259:260 22:22
12:7170336:G:A_A
p = 0.36, beta = 0.055, N = 1256
GFQVVVTLR pc2
F8WCZ6;P09871
928:978 242:260 21:22
12:7170336:G:A_A
p = 0.0073, beta = 0.166, N = 1191
PYPENSR pc2
F8WCZ6;P09871
974:978 259:260 22:22
12:7170336:G:A_A
p = 0.0018, beta = 0.188, N = 1255
SDFSNEER pc2
'6;P09871;P48740;P48740−2;P48740−:
974:978 259:260 22:22
12:7170336:G:A_A
p = 2.4e−84, beta = −1.09, N = 1255
CVPVCGVPR pc2
F8WCZ6;P09871
960:978 255:260 22:22
12:7170336:G:A_A
p = 0.022, beta = 0.138, N = 1237
NYVDWIMK pc2
F8WCZ6;P09871
975:978 260:260 22:22
12:7170336:G:A_A
p = 0.051, beta = 0.117, N = 1257
TNFDNDIALVR pc2
F8WCZ6;P09871
975:978 260:260 22:22
12:7170336:G:A_A
p = 0.018, beta = 0.142, N = 1257
EDTPNSVWEPAK pc2
F8WCZ6;P09871
975:978 259:260 22:22
12:7170336:G:A_A
p = 0.12, beta = 0.0925, N = 1256
VEDPESTLFGSVIR pc2
F8WCZ6;P09871
975:978 260:260 22:22
12:7170336:G:A_A
p = 0.12, beta = 0.093, N = 1257
YHGDPMPCPK pc2
F8WCZ6;P09871
967:978 257:260 22:22
12:7170336:G:A_A
p = 1.2e−05, beta = 0.263, N = 1246
GDSGGAFAVQDPNDK pc2
F8WCZ6;P09871
971:978 259:260 22:22
12:7170336:G:A_A
p = 0.51, beta = −0.0399, N = 1252
SNALDIIFQTDLTGQK pc2
F8WCZ6;P09871
854:978 221:260 20:22
12:7170336:G:A_A
p = 0.62, beta = −0.032, N = 1095
TMQENSTPRED pc2
F8WCZ6;P09871
948:978 258:260 22:22
12:7170336:G:A_A
p = 0.0013, beta = 0.193, N = 1228
MLTPEHVFIHPGWK pc4
F8WCZ6;P09871
970:978 259:260 22:22
12:7170336:G:A_A
p = 0.01, beta = 0.155, N = 1251
PTADAEAYVFTPNMICAGGEK pc3
F8WCZ6;P09871
975:978 259:260 22:22
12:7170336:G:A_A
p = 0.0088, beta = 0.157, N = 1256
TMQENSTPR pc2
F8WCZ6;P09871
964:978 253:260 21:22
12:7170336:G:A_A
p = 1.6e−06, beta = 0.291, N = 1238
VGATSFYSTCQSNGK pc2
F8WCZ6;P09871
974:978 258:260 22:22
12:7170336:G:A_A
p = 0.12, beta = 0.0938, N = 1254
QFGPYCGHGFPGPLNIETK pc3
F8WCZ6;P09871
956:978 252:260 21:22
12:7170336:G:A_A
p = 0.077, beta = 0.108, N = 1229
DVVQITCLDGFEVVEGR pc3
F8WCZ6;P09871
974:978 258:260 22:22
12:7170336:G:A_A
p = 0.0024, beta = 0.182, N = 1254
C1S : NP2
P09871
273:273 50:50 2:2
12:7170336:G:A_A
p = 0.2, beta = 0.181, N = 325
LLEVPEGR pc2
F8WCZ6;P09871
273:273 50:50 2:2
12:7170336:G:A_A
p = 0.21, beta = 0.178, N = 325
IIGGSDADIK pc2
F8WCZ6;P09871
273:273 50:50 2:2
12:7170336:G:A_A
p = 0.0082, beta = 0.371, N = 325
GFQVVVTLR pc2
F8WCZ6;P09871
273:273 50:50 2:2
12:7170336:G:A_A
p = 0.018, beta = 0.332, N = 325
SDFSNEER pc2
'6;P09871;P48740;P48740−2;P48740−:
273:273 50:50 2:2
12:7170336:G:A_A
p = 6.7e−10, beta = −0.846, N = 325
SNALDIIFQTDLTGQK pc2
F8WCZ6;P09871
273:273 50:50 2:2
12:7170336:G:A_A
p = 0.014, beta = 0.344, N = 325
EDTPNSVWEPAK pc2
F8WCZ6;P09871
273:273 50:50 2:2
12:7170336:G:A_A
p = 0.018, beta = 0.334, N = 325
TNFDNDIALVR pc2
F8WCZ6;P09871
273:273 50:50 2:2
12:7170336:G:A_A
p = 0.0063, beta = 0.384, N = 325
VEDPESTLFGSVIR pc2
F8WCZ6;P09871
273:273 50:50 2:2
12:7170336:G:A_A
p = 0.22, beta = 0.171, N = 325
PYPENSR pc2
F8WCZ6;P09871
273:273 50:50 2:2
12:7170336:G:A_A
p = 0.0016, beta = 0.442, N = 325
NYVDWIMK pc2
F8WCZ6;P09871
273:273 50:50 2:2
12:7170336:G:A_A
p = 0.057, beta = 0.267, N = 325
CVPVCGVPR pc2
F8WCZ6;P09871
273:273 50:50 2:2
12:7170336:G:A_A
p = 0.13, beta = 0.212, N = 325
GDSGGAFAVQDPNDK pc2
F8WCZ6;P09871
273:273 50:50 2:2
12:7170336:G:A_A
p = 0.058, beta = 0.267, N = 325
YHGDPMPCPK pc2
F8WCZ6;P09871
273:273 50:50 2:2
12:7170336:G:A_A
p = 0.047, beta = 0.279, N = 325
VGATSFYSTCQSNGK pc2
F8WCZ6;P09871
236:273 48:50 1:2
12:7170336:G:A_A
p = 0.73, beta = 0.0509, N = 285
MLTPEHVFIHPGWK pc4
F8WCZ6;P09871
273:273 50:50 2:2
12:7170336:G:A_A
p = 0.019, beta = 0.331, N = 325
TMQENSTPRED pc2
F8WCZ6;P09871
273:273 50:50 2:2
12:7170336:G:A_A
p = 0.029, beta = 0.308, N = 325
PTADAEAYVFTPNMICAGGEK pc3
F8WCZ6;P09871
273:273 50:50 2:2
12:7170336:G:A_A
p = 0.024, beta = 0.317, N = 325
DVVQITCLDGFEVVEGR pc3
F8WCZ6;P09871
272:273 50:50 2:2
12:7170336:G:A_A
p = 0.031, beta = 0.304, N = 324
TMQENSTPR pc2
F8WCZ6;P09871
273:273 50:50 2:2
12:7170336:G:A_A
p = 0.067, beta = 0.258, N = 325
log10 protein intensity
log10 peptide intensity
Assay Target: C1S
UniProt: P09871
rsID: rs12146727
Target SNP: 12:7063032:A:G
Proxy SNP: 12:7170336:G:A
deCODE log10(p): 907
deCODE BETA: −0.8
deCODE MSPA: 0.376 (2)
1256:1191:1255:1255:1237:125

53. FGL1 (Q08830)
deCODE MSPA=0.982

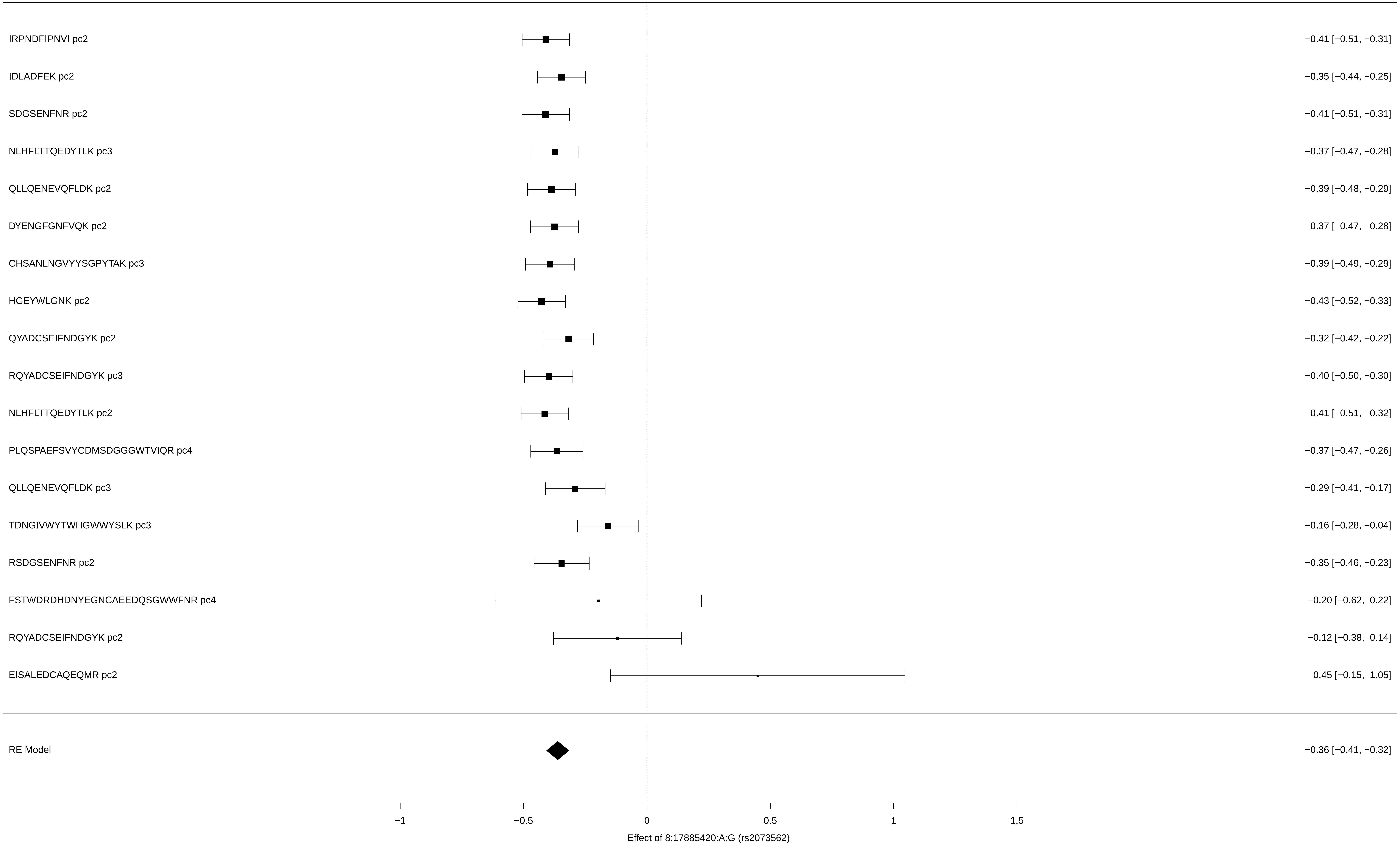

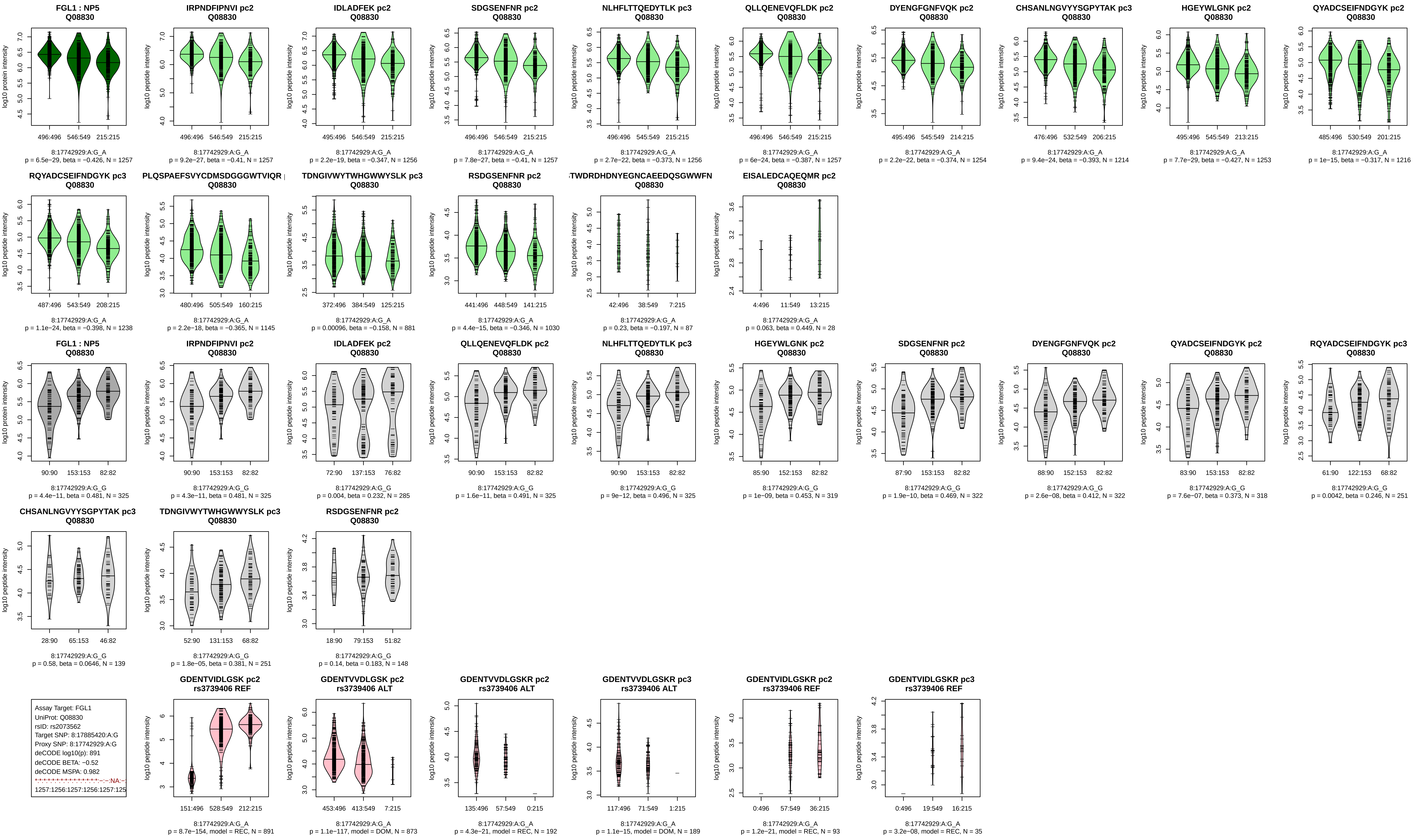
FGL1 : NP5 Q08830
496:496 546:549 215:215
8:17742929:A:G_A
p = 6.5e−29, beta = −0.426, N = 1257
IRPNDFIPNVI pc2 Q08830
496:496 546:549 215:215
8:17742929:A:G_A
p = 9.2e−27, beta = −0.41, N = 1257
IDLADFEK pc2 Q08830
495:496 546:549 215:215
8:17742929:A:G_A
p = 2.2e−19, beta = −0.347, N = 1256
SDGSENFNR pc2 Q08830
496:496 546:549 215:215
8:17742929:A:G_A
p = 7.8e−27, beta = −0.41, N = 1257
NLHFLTTQEDYTLK pc3 Q08830
496:496 545:549 215:215
8:17742929:A:G_A
p = 2.7e−22, beta = −0.373, N = 1256
QLLQENEVQFLDK pc2 Q08830
496:496 546:549 215:215
8:17742929:A:G_A
p = 6e−24, beta = −0.387, N = 1257
DYENGFGNFVQK pc2 Q08830
495:496 545:549 214:215
8:17742929:A:G_A
p = 2.2e−22, beta = −0.374, N = 1254
CHSANLNGVYYSGPYTAK pc3 Q08830
476:496 532:549 206:215
8:17742929:A:G_A
p = 9.4e−24, beta = −0.393, N = 1214
HGEYWLGNK pc2 Q08830
495:496 545:549 213:215
8:17742929:A:G_A
p = 7.7e−29, beta = −0.427, N = 1253
QYADCSEIFNDGYK pc2 Q08830
485:496 530:549 201:215
8:17742929:A:G_A
p = 1e−15, beta = −0.317, N = 1216
RQYADCSEIFNDGYK pc3 Q08830
487:496 543:549 208:215
8:17742929:A:G_A
p = 1.1e−24, beta = −0.398, N = 1238
PLQSPAEFSVYCDMSDGGGWTVIQR Q08830
480:496 505:549 160:215
8:17742929:A:G_A
p = 2.2e−18, beta = −0.365, N = 1145
TDNGIVWYTWHGWWYSLK pc3 Q08830
372:496 384:549 125:215
8:17742929:A:G_A
p = 0.00096, beta = −0.158, N = 881
RSDGSENFNR pc2 Q08830
441:496 448:549 141:215
8:17742929:A:G_A
p = 4.4e−15, beta = −0.346, N = 1030
TWDRDHDNYEGNCAEEDQSGWWFN Q08830
42:496 38:549 7:215
8:17742929:A:G_A
p = 0.23, beta = −0.197, N = 87
EISALEDCAQEQMR pc2 Q08830
4:496 11:549 13:215
8:17742929:A:G_A
p = 0.063, beta = 0.449, N = 28
FGL1 : NP5 Q08830
90:90 153:153 82:82
8:17742929:A:G_G
p = 4.4e−11, beta = 0.481, N = 325
IRPNDFIPNVI pc2 Q08830
90:90 153:153 82:82
8:17742929:A:G_G
p = 4.3e−11, beta = 0.481, N = 325
IDLADFEK pc2 Q08830
72:90 137:153 76:82
8:17742929:A:G_G
p = 0.004, beta = 0.232, N = 285
QLLQENEVQFLDK pc2 Q08830
90:90 153:153 82:82
8:17742929:A:G_G
p = 1.6e−11, beta = 0.491, N = 325
NLHFLTTQEDYTLK pc3 Q08830
90:90 153:153 82:82
8:17742929:A:G_G
p = 9e−12, beta = 0.496, N = 325
HGEYWLGNK pc2 Q08830
85:90 152:153 82:82
8:17742929:A:G_G
p = 1e−09, beta = 0.453, N = 319
SDGSENFNR pc2 Q08830
87:90 153:153 82:82
8:17742929:A:G_G
p = 1.9e−10, beta = 0.469, N = 322
DYENGFGNFVQK pc2 Q08830
88:90 152:153 82:82
8:17742929:A:G_G
p = 2.6e−08, beta = 0.412, N = 322
QYADCSEIFNDGYK pc2 Q08830
83:90 153:153 82:82
8:17742929:A:G_G
p = 7.6e−07, beta = 0.373, N = 318
RQYADCSEIFNDGYK pc3 Q08830
61:90 122:153 68:82
8:17742929:A:G_G
p = 0.0042, beta = 0.246, N = 251
CHSANLNGVYYSGPYTAK pc3 Q08830
28:90 65:153 46:82
8:17742929:A:G_G
p = 0.58, beta = 0.0646, N = 139
TDNGIVWYTWHGWWYSLK pc3 Q08830
52:90 131:153 68:82
8:17742929:A:G_G
p = 1.8e−05, beta = 0.381, N = 251
RSDGSENFNR pc2 Q08830
18:90 79:153 51:82
8:17742929:A:G_G
p = 0.14, beta = 0.183, N = 148
Assay Target: FGL1
UniProt: Q08830
rsID: rs2073562
Target SNP: 8:17885420:A:G
Proxy SNP: 8:17742929:A:G
deCODE log10(p): 891
deCODE BETA: −0.52
deCODE MSPA: 0.982
1257:1256:1257:1256:1257:125
GDENTVIDLGSK pc2 rs3739406 REF
151:496 528:549 212:215
8:17742929:A:G_A
p = 8.7e−154, model = REC, N = 891
GDENTVVDLGSK pc2 rs3739406 ALT
453:496 413:549 7:215
8:17742929:A:G_A
p = 1.1e−117, model = DOM, N = 873
GDENTVVDLGSKR pc2 rs3739406 ALT
135:496 57:549 0:215
8:17742929:A:G_A
p = 4.3e−21, model = REC, N = 192
GDENTVVDLGSKR pc3 rs3739406 ALT
117:496 71:549 1:215
8:17742929:A:G_A
p = 1.1e−15, model = DOM, N = 189
GDENTVIDLGSKR pc2 rs3739406 REF
0:496 57:549 36:215
8:17742929:A:G_A
p = 1.2e−21, model = REC, N = 93
GDENTVIDLGSKR pc3 rs3739406 REF
0:496 19:549 16:215
8:17742929:A:G_A
p = 3.2e−08, model = REC, N = 35

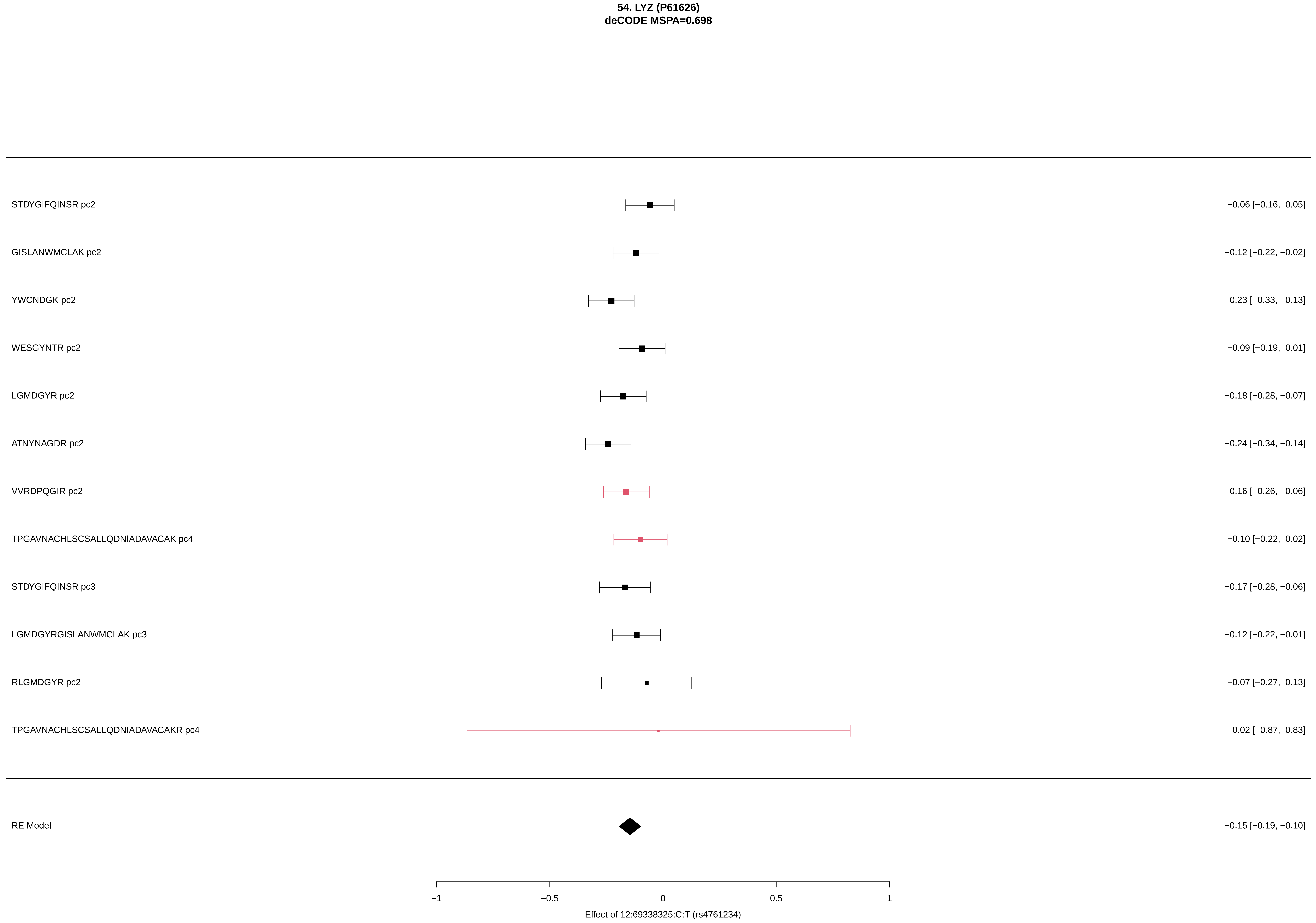

54. LYZ (P61626)
deCODE MSPA=0.698
STDYGIFQINSR pc2
−0.06 [−0.16, 0.05]
GISLANWMCLAK pc2
−0.12 [−0.22, −0.02]
YWCNDGK pc2
−0.23 [−0.33, −0.13]
WESGYNTR pc2
−0.09 [−0.19, 0.01]
LGMDGYR pc2
−0.18 [−0.28, −0.07]
ATNYNAGDR pc2
−0.24 [−0.34, −0.14]
VVRDPQGIR pc2
−0.16 [−0.26, −0.06]
TPGAVNACHLSCSALLQDNIADAVACAK pc4
−0.10 [−0.22, 0.02]
STDYGIFQINSR pc3
−0.17 [−0.28, −0.06]
LGMDGYRGISLANWMCLAK pc3
−0.12 [−0.22, −0.01]
RLGMDGYR pc2
−0.07 [−0.27, 0.13]
TPGAVNACHLSCSALLQDNIADAVACAKR pc4
−0.02 [−0.87, 0.83]
RE Model
−0.15 [−0.19, −0.10]
−1
−0.5
0
0.5
1
Effect of 12:69338325:C:T (rs4761234)

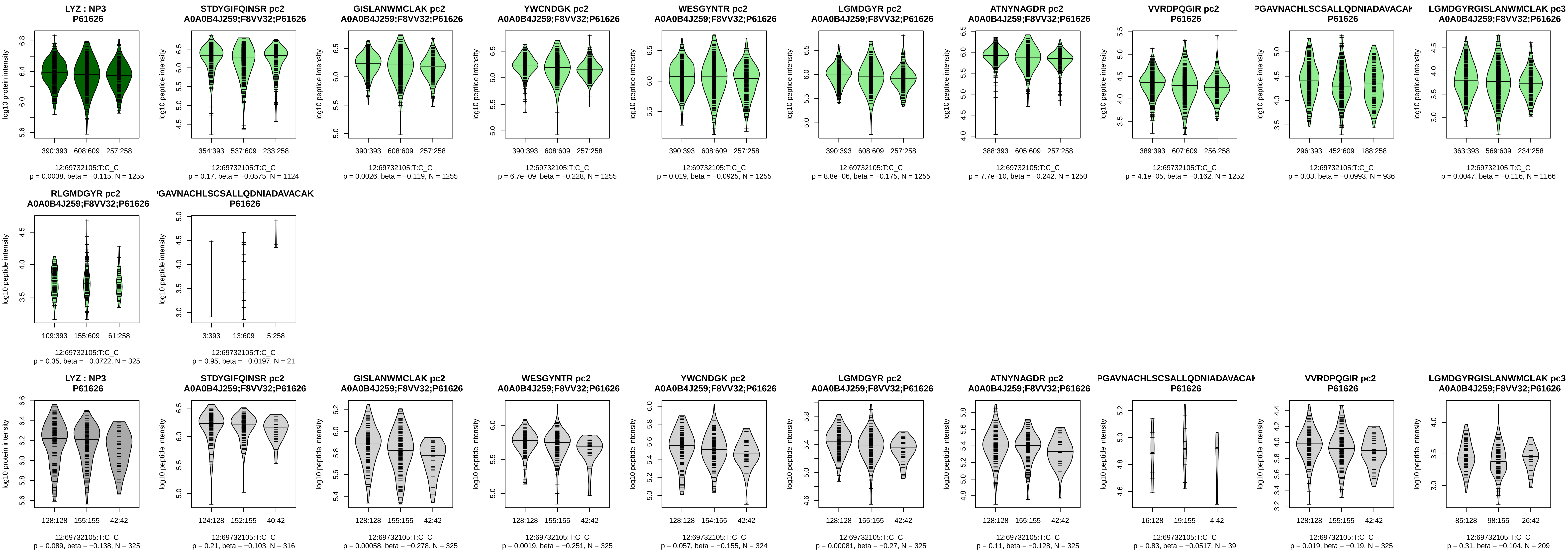

Assay Target: LYZ
UniProt: P61626
rsID: rs4761234
Target SNP: 12:69338325:C:T
Proxy SNP: 12:69732105:T:C
deCODE log10(p): 861.2
deCODE BETA: −0.49
deCODE MSPA: 0.698
–:*:*:–:*:*:*:–:*:*:–:–:NA
1124:1255:1255:1255:1255:125

55. ITIH2 (P19823)
deCODE MSPA=0

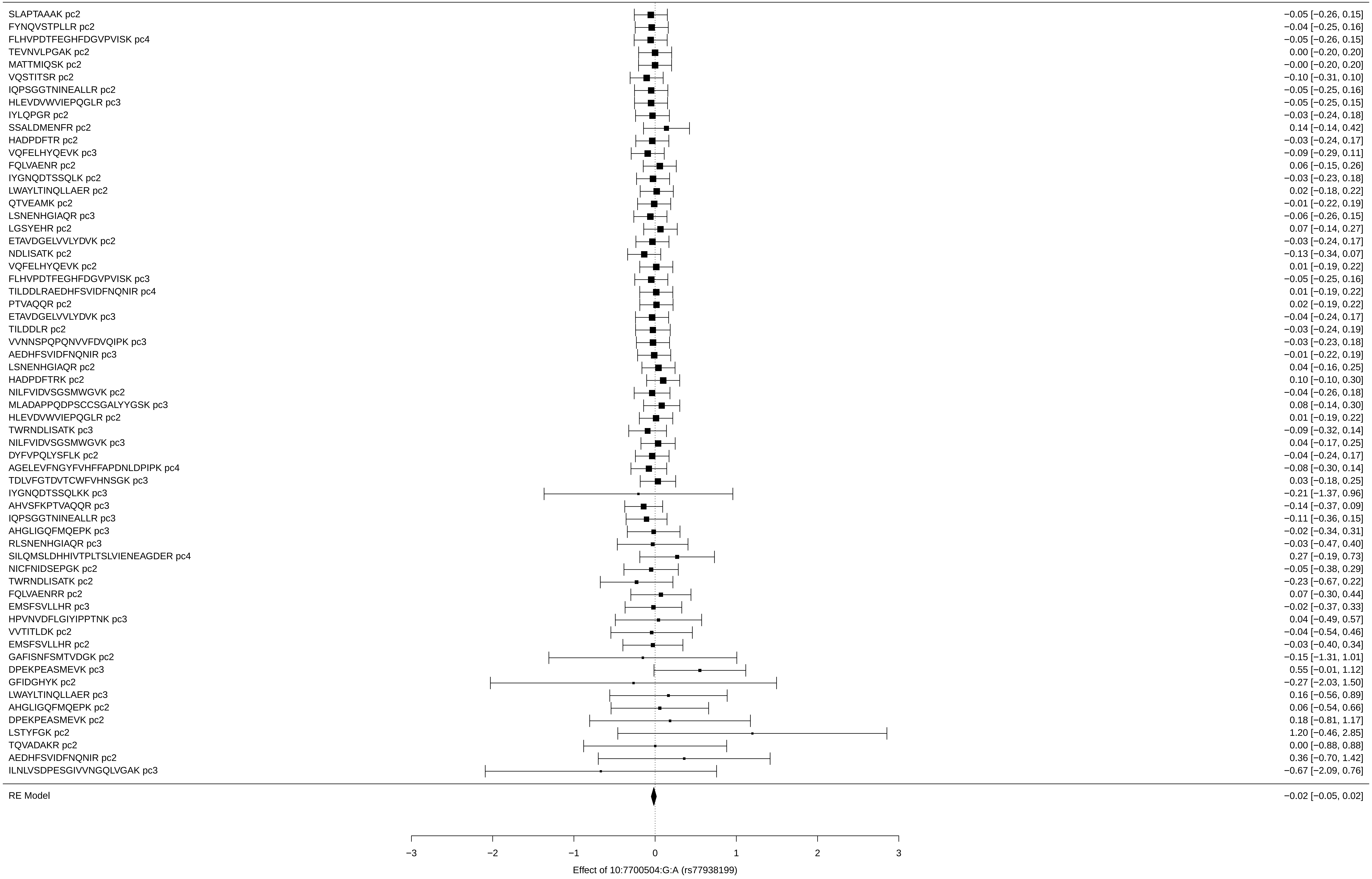

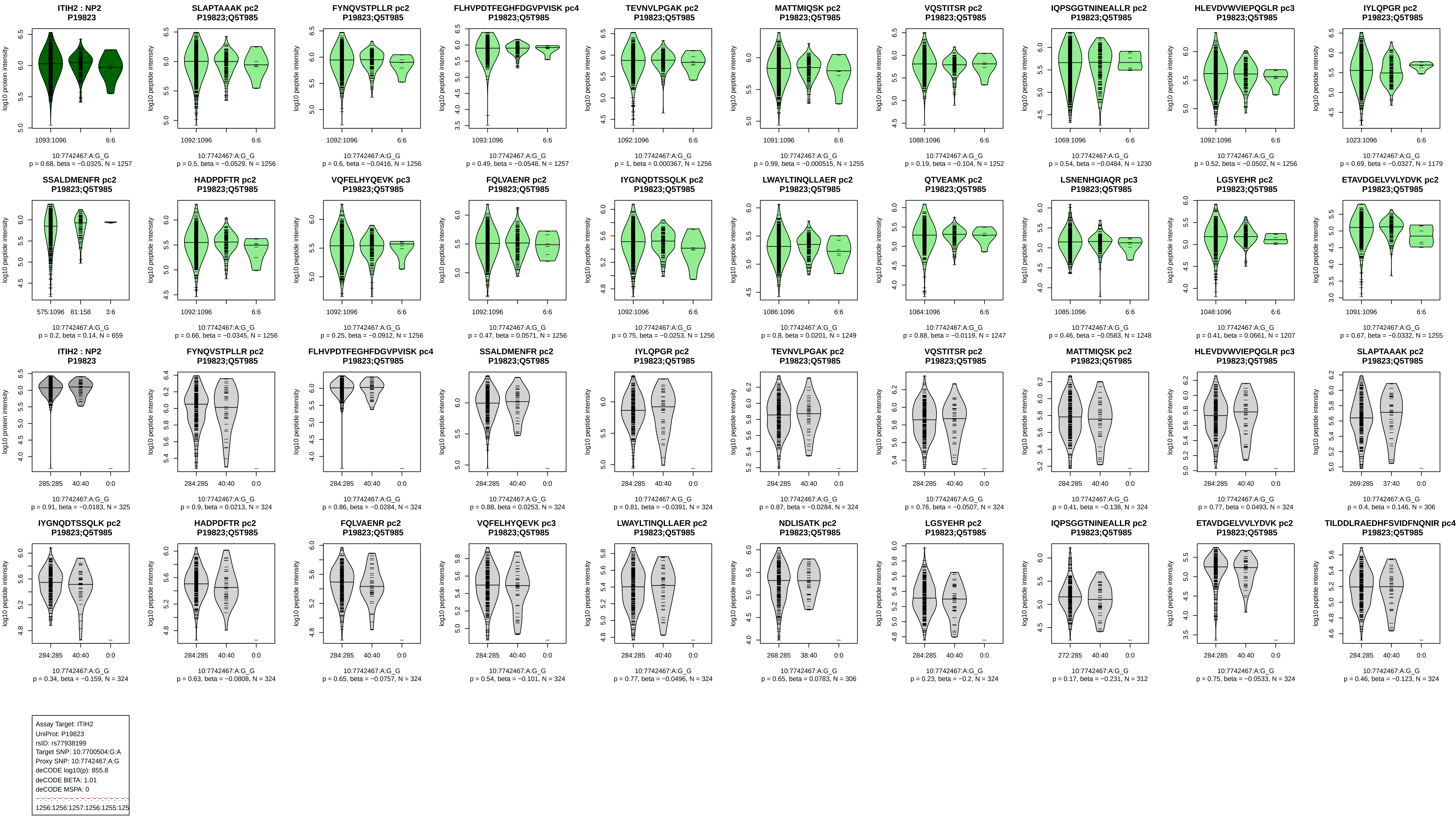
ITIH2 : NP2 P19823
10:7742467:A:G_G p = 0.68, beta = −0.0325, N = 1257
SLAPTAAAK pc2 P19823;Q5T985
10:7742467:A:G_G p = 0.5, beta = −0.0529, N = 1256
FYNQVSTPLLR pc2 P19823;Q5T985
10:7742467:A:G_G p = 0.6, beta = −0.0416, N = 1256
FLHVPDTFEGHFDGVPVISK pc4 P19823;Q5T985
10:7742467:A:G_G p = 0.49, beta = −0.0548, N = 1257
TEVNVLPGAK pc2 P19823;Q5T985
10:7742467:A:G_G p = 1, beta = 0.000367, N = 1256
MATTMIQSK pc2 P19823;Q5T985
10:7742467:A:G_G p = 0.99, beta = −0.000515, N = 1255
VQSTITSR pc2 P19823;Q5T985
10:7742467:A:G_G p = 0.19, beta = −0.104, N = 1252
IQPSGGTNINEALLR pc2 P19823;Q5T985
10:7742467:A:G_G p = 0.54, beta = −0.0484, N = 1230
HLEVDVWVIEPQGLR pc3 P19823;Q5T985
10:7742467:A:G_G p = 0.52, beta = −0.0502, N = 1256
IYLQPGR pc2 P19823;Q5T985
10:7742467:A:G_G p = 0.69, beta = −0.0327, N = 1179
SSALDMENFR pc2 P19823;Q5T985
10:7742467:A:G_G p = 0.2, beta = 0.14, N = 659
HADPDFTR pc2 P19823;Q5T985
10:7742467:A:G_G p = 0.66, beta = −0.0345, N = 1256
VQFELHYQEVK pc3 P19823;Q5T985
10:7742467:A:G_G p = 0.25, beta = −0.0912, N = 1256
FQLVAENR pc2 P19823;Q5T985
10:7742467:A:G_G p = 0.47, beta = 0.0571, N = 1256
IYGNQDTSSQLK pc2 P19823;Q5T985
10:7742467:A:G_G p = 0.75, beta = −0.0253, N = 1256
LWAYLTINQLLAER pc2 P19823;Q5T985
10:7742467:A:G_G p = 0.8, beta = 0.0201, N = 1249
QTVEAMK pc2 P19823;Q5T985
10:7742467:A:G_G p = 0.88, beta = −0.0119, N = 1247
LSNENHGIAQR pc3 P19823;Q5T985
10:7742467:A:G_G p = 0.46, beta = −0.0583, N = 1248
LGSYEHR pc2 P19823;Q5T985
10:7742467:A:G_G p = 0.41, beta = 0.0661, N = 1207
ETAVDGELVVLYDVK pc2 P19823;Q5T985
10:7742467:A:G_G p = 0.67, beta = −0.0332, N = 1255
ITIH2 : NP2 P19823
10:7742467:A:G_G p = 0.91, beta = −0.0183, N = 325
FYNQVSTPLLR pc2 P19823;Q5T985
10:7742467:A:G_G p = 0.9, beta = 0.0213, N = 324
FLHVPDTFEGHFDGVPVISK pc4 P19823;Q5T985
10:7742467:A:G_G p = 0.86, beta = −0.0284, N = 324
SSALDMENFR pc2 P19823;Q5T985
10:7742467:A:G_G p = 0.88, beta = 0.0253, N = 324
IYLQPGR pc2 P19823;Q5T985
10:7742467:A:G_G p = 0.81, beta = −0.0391, N = 324
TEVNVLPGAK pc2 P19823;Q5T985
10:7742467:A:G_G p = 0.87, beta = −0.0284, N = 324
VQSTITSR pc2 P19823;Q5T985
10:7742467:A:G_G p = 0.76, beta = −0.0507, N = 324
MATTMIQSK pc2 P19823;Q5T985
10:7742467:A:G_G p = 0.41, beta = −0.138, N = 324
HLEVDVWVIEPQGLR pc3 P19823;Q5T985
10:7742467:A:G_G p = 0.77, beta = 0.0493, N = 324
SLAPTAAAK pc2 P19823;Q5T985
10:7742467:A:G_G p = 0.4, beta = 0.146, N = 306
IYGNQDTSSQLK pc2 P19823;Q5T985
10:7742467:A:G_G p = 0.34, beta = −0.159, N = 324
HADPDFTR pc2 P19823;Q5T985
10:7742467:A:G_G p = 0.63, beta = −0.0808, N = 324
FQLVAENR pc2 P19823;Q5T985
10:7742467:A:G_G p = 0.65, beta = −0.0757, N = 324
VQFELHYQEVK pc3 P19823;Q5T985
10:7742467:A:G_G p = 0.54, beta = −0.101, N = 324
LWAYLTINQLLAER pc2 P19823;Q5T985
10:7742467:A:G_G p = 0.77, beta = −0.0496, N = 324
NDLISATK pc2 P19823;Q5T985
10:7742467:A:G_G p = 0.65, beta = 0.0783, N = 306
LGSYEHR pc2 P19823;Q5T985
10:7742467:A:G_G p = 0.23, beta = −0.2, N = 324
IQPSGGTNINEALLR pc2 P19823;Q5T985
10:7742467:A:G_G p = 0.17, beta = −0.231, N = 312
ETAVDGELVVLYDVK pc2 P19823;Q5T985
10:7742467:A:G_G p = 0.75, beta = −0.0533, N = 324
TILDDLRAEDHFSVIDFNQNIR pc4 P19823;Q5T985
10:7742467:A:G_G p = 0.46, beta = −0.123, N = 324
Assay Target: ITIH2
UniProt: P19823
rsID: rs77938199
Target SNP: 10:7700504:G:A
Proxy SNP: 10:7742467:A:G
deCODE log10(p): 855.8
deCODE BETA: 1.01
deCODE MSPA: 0
1256:1256:1257:1256:1255:125

56. ITIH3 (Q06033)
deCODE MSPA=0.107

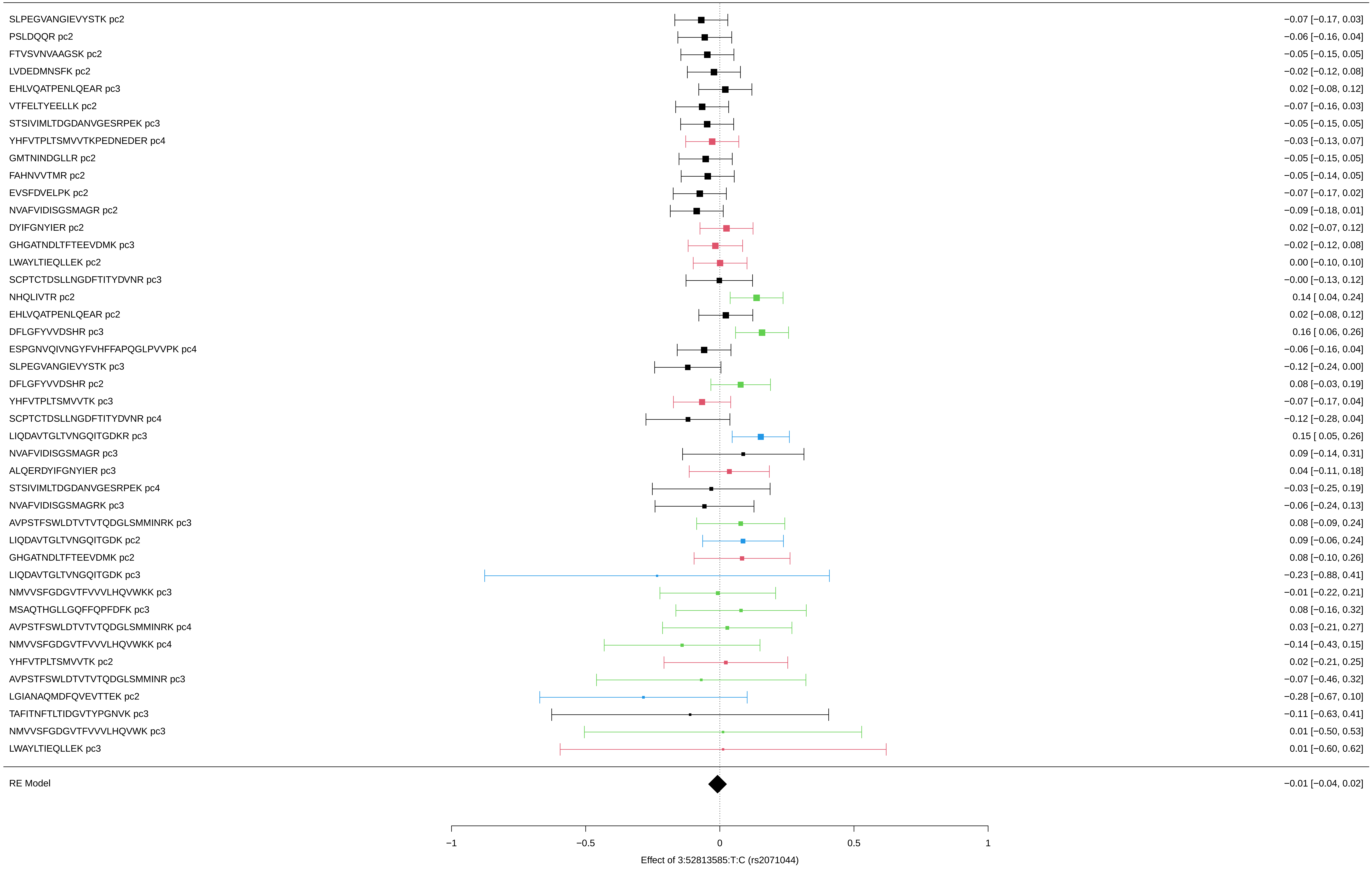

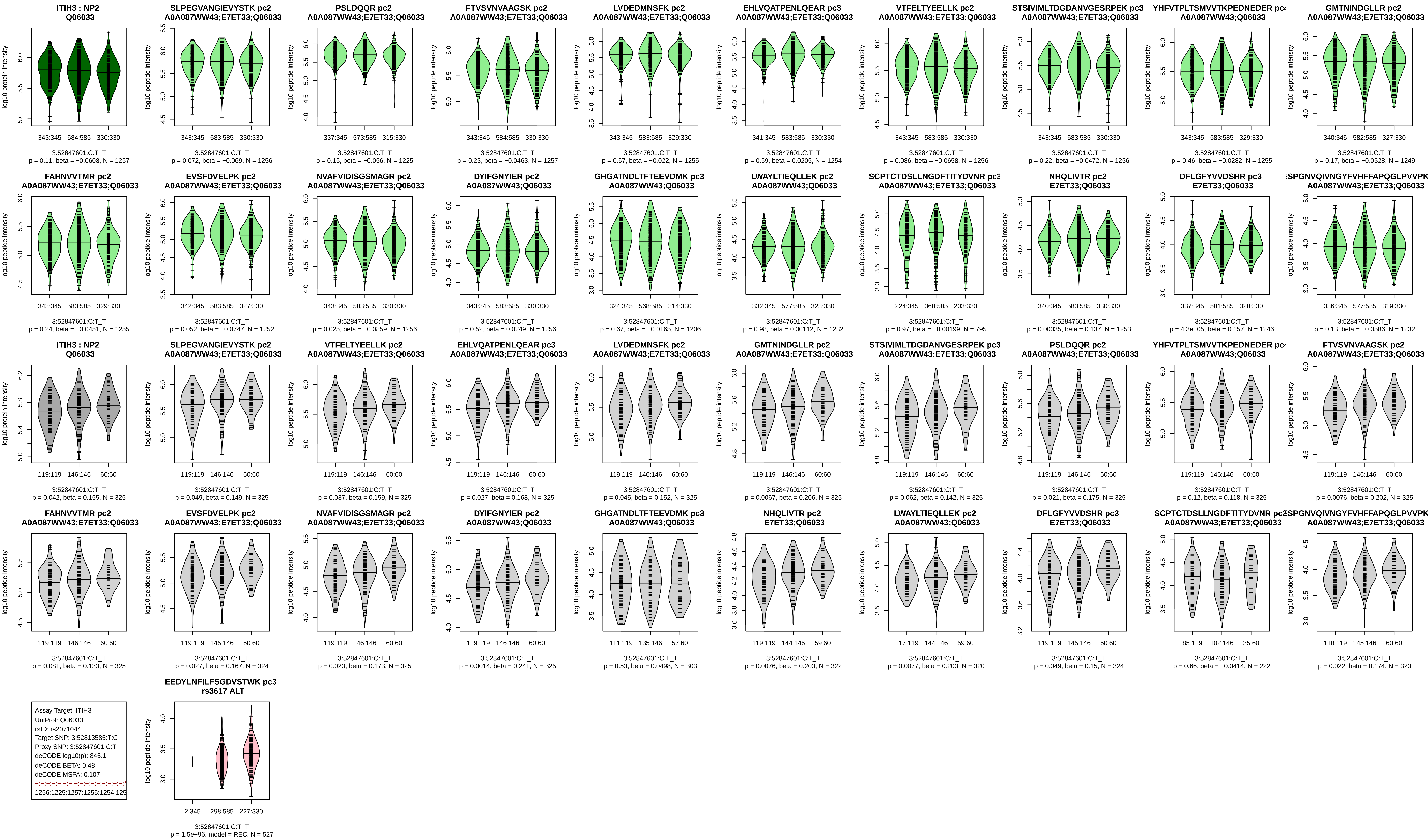
ITIH3 : NP2
Q06033
log10 protein intensity
343:345 584:585 330:330
3:52847601:C:T_T
p = 0.11, beta = −0.0608, N = 1257
SLPEGVANGIEVYSTK pc2
A0A087WW43;E7ET33;Q06033
log10 peptide intensity
343:345 583:585 330:330
3:52847601:C:T_T
p = 0.072, beta = −0.069, N = 1256
PSLDQQR pc2
A0A087WW43;E7ET33;Q06033
log10 peptide intensity
337:345 573:585 315:330
3:52847601:C:T_T
p = 0.15, beta = −0.056, N = 1225
FTVSVNVAAGSK pc2
A0A087WW43;E7ET33;Q06033
log10 peptide intensity
343:345 584:585 330:330
3:52847601:C:T_T
p = 0.23, beta = −0.0463, N = 1257
LVDEDMNSFK pc2
A0A087WW43;E7ET33;Q06033
log10 peptide intensity
343:345 583:585 329:330
3:52847601:C:T_T
p = 0.57, beta = −0.022, N = 1255
EHLVQATPENLQEAR pc3
A0A087WW43;E7ET33;Q06033
log10 peptide intensity
341:345 583:585 330:330
3:52847601:C:T_T
p = 0.59, beta = 0.0205, N = 1254
VTFELTYEELLK pc2
A0A087WW43;E7ET33;Q06033
log10 peptide intensity
343:345 583:585 330:330
3:52847601:C:T_T
p = 0.086, beta = −0.0658, N = 1256
STSIVIMLTDGDANVGESRPEK pc3
A0A087WW43;E7ET33;Q06033
log10 peptide intensity
343:345 583:585 330:330
3:52847601:C:T_T
p = 0.22, beta = −0.0472, N = 1256
YHFVTPLTSMVVTKPEDNEDER pc
A0A087WW43;Q06033
log10 peptide intensity
343:345 583:585 329:330
3:52847601:C:T_T
p = 0.46, beta = −0.0282, N = 1255
GMTNINDGLLR pc2
A0A087WW43;E7ET33;Q06033
log10 peptide intensity
340:345 582:585 327:330
3:52847601:C:T_T
p = 0.17, beta = −0.0528, N = 1249
FAHNVVTMR pc2
A0A087WW43;E7ET33;Q06033
log10 peptide intensity
343:345 583:585 329:330
3:52847601:C:T_T
p = 0.24, beta = −0.0451, N = 1255
EVSFDVELPK pc2
A0A087WW43;E7ET33;Q06033
log10 peptide intensity
342:345 583:585 327:330
3:52847601:C:T_T
p = 0.052, beta = −0.0747, N = 1252
NVAFVIDISGSMAGR pc2
A0A087WW43;E7ET33;Q06033
log10 peptide intensity
343:345 583:585 330:330
3:52847601:C:T_T
p = 0.025, beta = −0.0859, N = 1256
DYIFGNYIER pc2
A0A087WW43;Q06033
log10 peptide intensity
343:345 583:585 330:330
3:52847601:C:T_T
p = 0.52, beta = −0.0249, N = 1256
GHGATNDLTFTEEVDMK pc3
A0A087WW43;Q06033
log10 peptide intensity
324:345 568:585 314:330
3:52847601:C:T_T
p = 0.67, beta = −0.0165, N = 1206
LWAYLTIEQLLEK pc2
A0A087WW43;Q06033
log10 peptide intensity
332:345 577:585 323:330
3:52847601:C:T_T
p = 0.98, beta = 0.00112, N = 1232
SCPTCTDSLLNGDFTITYDVNR pc3
A0A087WW43;E7ET33;Q06033
log10 peptide intensity
224:345 368:585 203:330
3:52847601:C:T_T
p = 0.97, beta = −0.00199, N = 795
NHQLIVTR pc2
E7ET33;Q06033
log10 peptide intensity
340:345 583:585 330:330
3:52847601:C:T_T
p = 0.00035, beta = 0.137, N = 1253
DFLGFYVVDSHR pc3
E7ET33;Q06033
log10 peptide intensity
337:345 581:585 328:330
3:52847601:C:T_T
p = 4.3e−05, beta = 0.157, N = 1246
SPGNVQIVNGYFVHFFAPQGLPVVPK
A0A087WW43;E7ET33;Q06033
log10 peptide intensity
336:345 577:585 319:330
3:52847601:C:T_T
p = 0.13, beta = −0.0586, N = 1232
ITIH3 : NP2
Q06033
log10 protein intensity
119:119 146:146 60:60
3:52847601:C:T_T
p = 0.042, beta = 0.155, N = 325
SLPEGVANGIEVYSTK pc2
A0A087WW43;E7ET33;Q06033
log10 peptide intensity
119:119 146:146 60:60
3:52847601:C:T_T
p = 0.049, beta = 0.149, N = 325
VTFELTYEELLK pc2
A0A087WW43;E7ET33;Q06033
log10 peptide intensity
119:119 146:146 60:60
3:52847601:C:T_T
p = 0.037, beta = 0.159, N = 325
EHLVQATPENLQEAR pc3
A0A087WW43;E7ET33;Q06033
log10 peptide intensity
119:119 146:146 60:60
3:52847601:C:T_T
p = 0.027, beta = 0.168, N = 325
LVDEDMNSFK pc2
A0A087WW43;E7ET33;Q06033
log10 peptide intensity
119:119 146:146 60:60
3:52847601:C:T_T
p = 0.045, beta = 0.152, N = 325
GMTNINDGLLR pc2
A0A087WW43;E7ET33;Q06033
log10 peptide intensity
119:119 146:146 60:60
3:52847601:C:T_T
p = 0.0067, beta = 0.206, N = 325
STSIVIMLTDGDANVGESRPEK pc3
A0A087WW43;E7ET33;Q06033
log10 peptide intensity
119:119 146:146 60:60
3:52847601:C:T_T
p = 0.062, beta = 0.142, N = 325
PSLDQQR pc2
A0A087WW43;E7ET33;Q06033
log10 peptide intensity
119:119 146:146 60:60
3:52847601:C:T_T
p = 0.021, beta = 0.175, N = 325
YHFVTPLTSMVVTKPEDNEDER pc
A0A087WW43;Q06033
log10 peptide intensity
119:119 146:146 60:60
3:52847601:C:T_T
p = 0.12, beta = 0.118, N = 325
FTVSVNVAAGSK pc2
A0A087WW43;E7ET33;Q06033
log10 peptide intensity
119:119 146:146 60:60
3:52847601:C:T_T
p = 0.0076, beta = 0.202, N = 325
FAHNVVTMR pc2
A0A087WW43;E7ET33;Q06033
log10 peptide intensity
119:119 146:146 60:60
3:52847601:C:T_T
p = 0.081, beta = 0.133, N = 325
EVSFDVELPK pc2
A0A087WW43;E7ET33;Q06033
log10 peptide intensity
119:119 145:146 60:60
3:52847601:C:T_T
p = 0.027, beta = 0.167, N = 324
NVAFVIDISGSMAGR pc2
A0A087WW43;E7ET33;Q06033
log10 peptide intensity
119:119 146:146 60:60
3:52847601:C:T_T
p = 0.023, beta = 0.173, N = 325
DYIFGNYIER pc2
A0A087WW43;Q06033
log10 peptide intensity
119:119 146:146 60:60
3:52847601:C:T_T
p = 0.0014, beta = 0.241, N = 325
GHGATNDLTFTEEVDMK pc3
A0A087WW43;Q06033
log10 peptide intensity
111:119 135:146 57:60
3:52847601:C:T_T
p = 0.53, beta = 0.0498, N = 303
NHQLIVTR pc2
E7ET33;Q06033
log10 peptide intensity
119:119 144:146 59:60
3:52847601:C:T_T
p = 0.0076, beta = 0.203, N = 322
LWAYLTIEQLLEK pc2
A0A087WW43;Q06033
log10 peptide intensity
117:119 144:146 59:60
3:52847601:C:T_T
p = 0.0077, beta = 0.203, N = 320
DFLGFYVVDSHR pc3
E7ET33;Q06033
log10 peptide intensity
119:119 145:146 60:60
3:52847601:C:T_T
p = 0.049, beta = 0.15, N = 324
SCPTCTDSLLNGDFTITYDVNR pc3
A0A087WW43;E7ET33;Q06033
log10 peptide intensity
85:119 102:146 35:60
3:52847601:C:T_T
p = 0.66, beta = −0.0414, N = 222
SPGNVQIVNGYFVHFFAPQGLPVVPK
A0A087WW43;E7ET33;Q06033
log10 peptide intensity
118:119 145:146 60:60
3:52847601:C:T_T
p = 0.022, beta = 0.174, N = 323
Assay Target: ITIH3
UniProt: Q06033
rsID: rs2071044
Target SNP: 3:52813585:T:C
Proxy SNP: 3:52847601:C:T
deCODE log10(p): 845.1
deCODE BETA: 0.48
deCODE MSPA: 0.107
1256:1225:1257:1255:1254:125
EEDYLNFILFSGDVSTWK pc3
rs3617 ALT
log10 peptide intensity
2:345 298:585 227:330
3:52847601:C:T_T
p = 1.5e−96, model = REC, N = 527

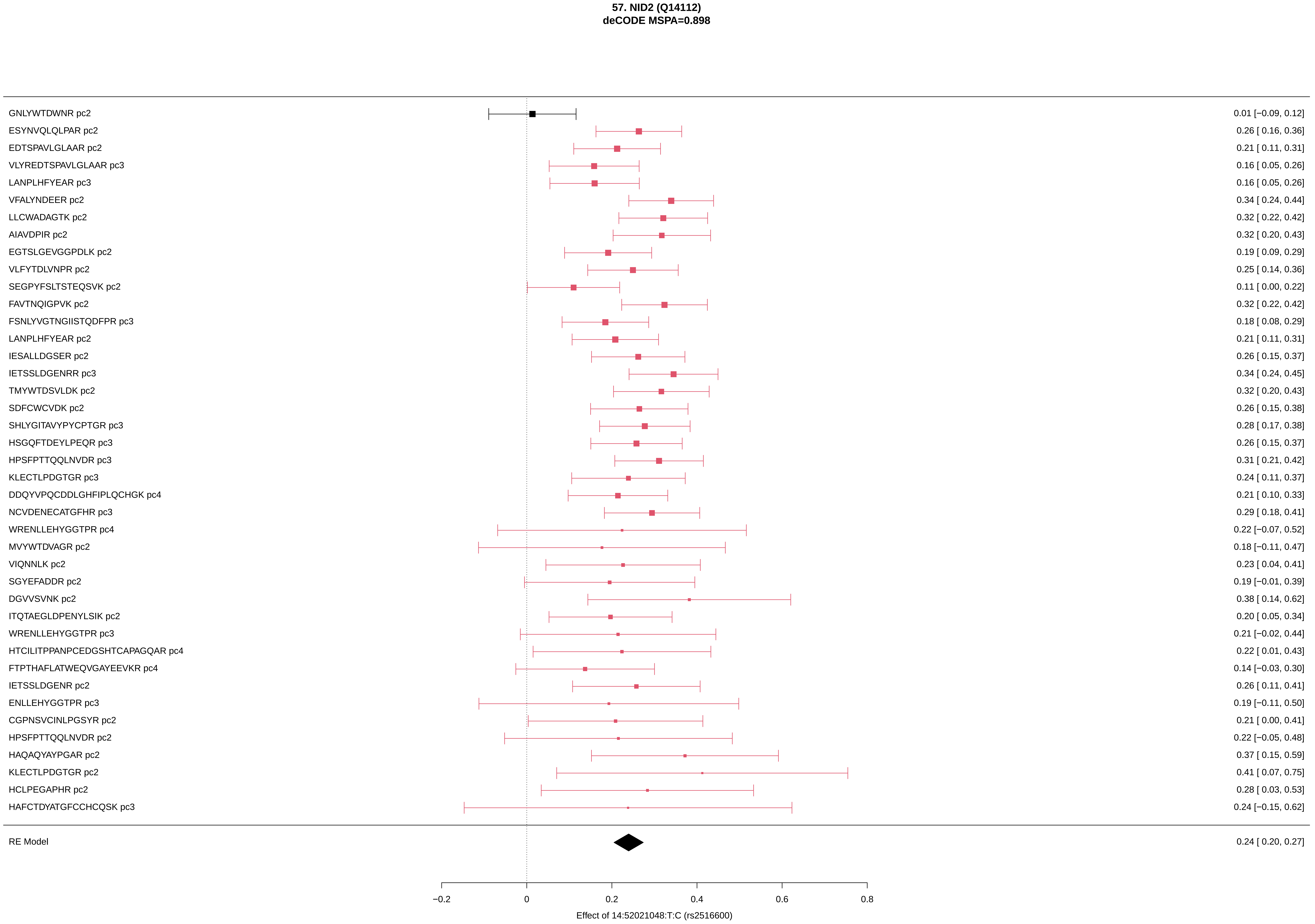
57. NID2 (Q14112)
deCODE MSPA=0.898
GNLYWTDWNR pc2
ESYNVQLQLPAR pc2
EDTSPAVLGLAAR pc2
VLYREDTSPAVLGLAAR pc3
LANPLHFYEAR pc3
VFALYNDEER pc2
LLCWADAGTK pc2
AIAVDPIR pc2
EGTSLGEVGGPDLK pc2
VLFYTDLVNPR pc2
SEGPYFSLTSTEQSVK pc2
FAVTNQIGPVK pc2
FSNLYVGTNGIISTQDFPR pc3
LANPLHFYEAR pc2
IESALLDGSER pc2
IETSSLDGENRR pc3
TMYWTDSVLDK pc2
SDFCWCVDK pc2
SHLYGITAVYPYCPTGR pc3
HSGQFTDEYLPEQR pc3
HPSFPTTQQLNVDR pc3
KLECTLPDGTGR pc3
DDQYVPQCDDLGHFIPLQCHGK pc4
NCVDENECATGFHR pc3
WRENLLEHYGGTPR pc4
MVYWTDVAGR pc2
VIQNNLK pc2
SGYEFADDR pc2
DGVVSVNK pc2
ITQTAEGLDPENYLSIK pc2
WRENLLEHYGGTPR pc3
HTCILITPPANPCEDGSHTCAPAGQAR pc4
FTPTHAFLATWEQVGAYEEVKR pc4
IETSSLDGENR pc2
ENLLEHYGGTPR pc3
CGPNSVCINLPGSYR pc2
HPSFPTTQQLNVDR pc2
HAQAQYAYPGAR pc2
KLECTLPDGTGR pc2
HCLPEGAPHR pc2
HAFCTDYATGFCCHCQSK pc3
RE Model
0.01 [−0.09, 0.12]
0.26 [0.16, 0.36]
0.21 [0.11, 0.31]
0.16 [0.05, 0.26]
0.16 [0.05, 0.26]
0.34 [0.24, 0.44]
0.32 [0.22, 0.42]
0.32 [0.20, 0.43]
0.19 [0.09, 0.29]
0.25 [0.14, 0.36]
0.11 [0.00, 0.22]
0.32 [0.22, 0.42]
0.18 [0.08, 0.29]
0.21 [0.11, 0.31]
0.26 [0.15, 0.37]
0.34 [0.24, 0.45]
0.32 [0.20, 0.43]
0.26 [0.15, 0.38]
0.28 [0.17, 0.38]
0.26 [0.15, 0.37]
0.31 [0.21, 0.42]
0.24 [0.11, 0.37]
0.21 [0.10, 0.33]
0.29 [0.18, 0.41]
0.22 [−0.07, 0.52]
0.18 [−0.11, 0.47]
0.23 [0.04, 0.41]
0.19 [−0.01, 0.39]
0.38 [0.14, 0.62]
0.20 [0.05, 0.34]
0.21 [−0.02, 0.44]
0.22 [0.01, 0.43]
0.14 [−0.03, 0.30]
0.26 [0.11, 0.41]
0.19 [−0.11, 0.50]
0.21 [0.00, 0.41]
0.22 [−0.05, 0.48]
0.37 [0.15, 0.59]
0.41 [0.07, 0.75]
0.28 [0.03, 0.53]
0.24 [−0.15, 0.62]
0.24 [0.20, 0.27]
−0.2
0
0.2
0.4
0.6
0.8
Effect of 14:52021048:T:C (rs2516600)

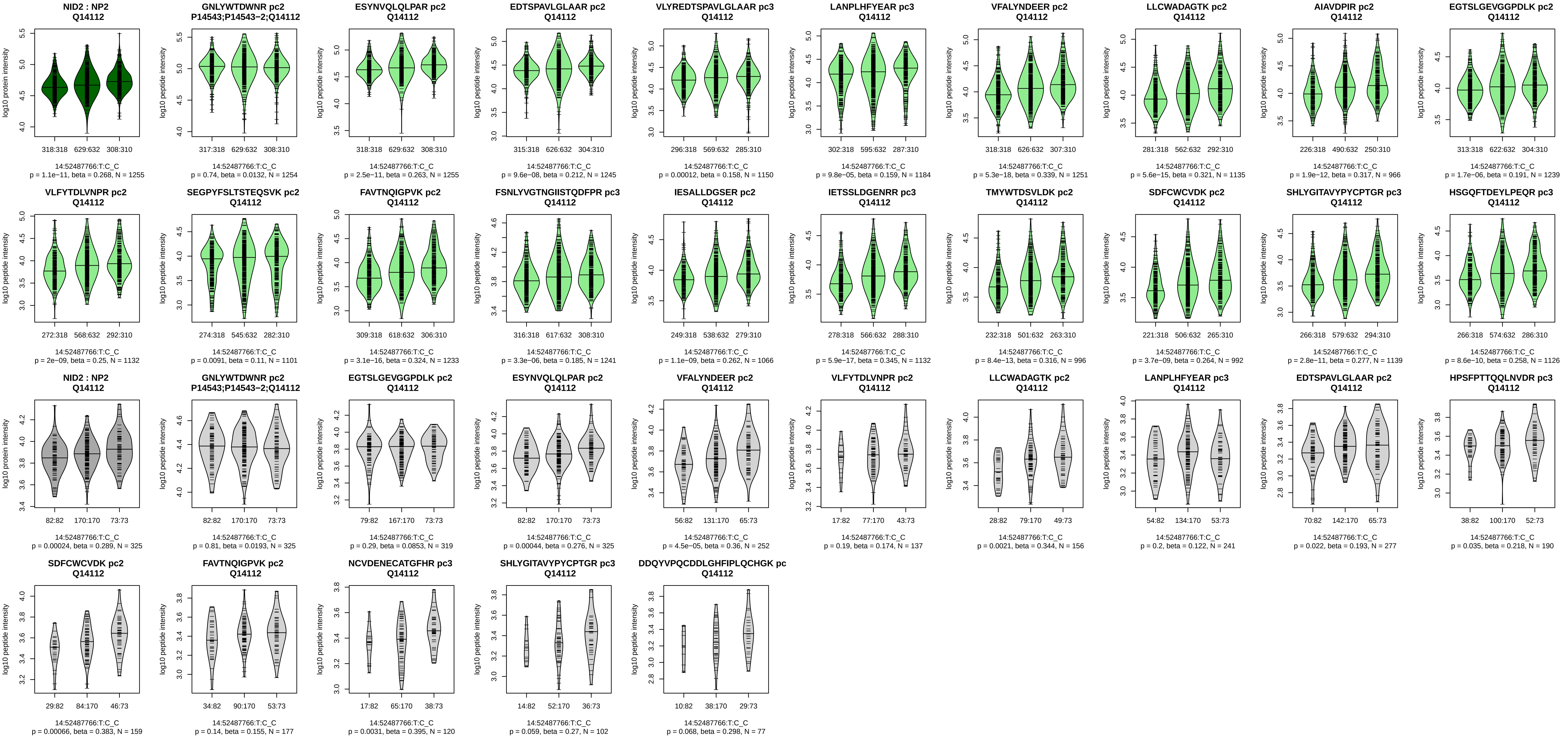

Assay Target: NID2
UniProt: Q14112
rsID: rs2516600
Target SNP: 14:52021048:T:C
Proxy SNP: 14:52487766:T:C
deCODE log10(p): 829.6
deCODE BETA: −0.49
deCODE MSPA: 0.898
1254:1255:1245:1150:1184:125

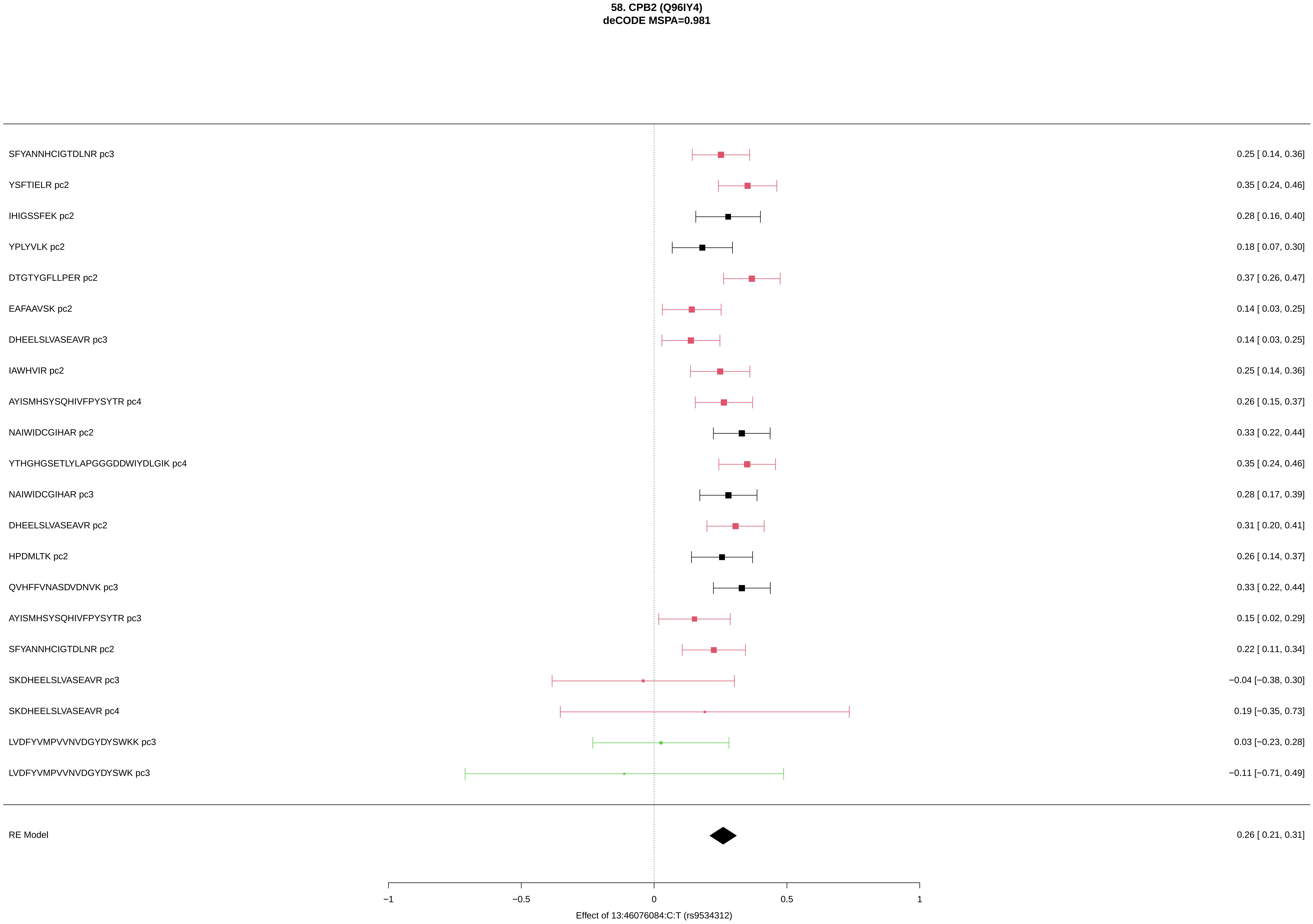

58. CPB2 (Q96IY4)
deCODE MSPA=0.981
SFYANNHCIGTDLNR pc3
0.25 [0.14, 0.36]
YSFTIELR pc2
0.35 [0.24, 0.46]
IHIGSSFEK pc2
0.28 [0.16, 0.40]
YPLYVLK pc2
0.18 [0.07, 0.30]
DTGTYGFLLPER pc2
0.37 [0.26, 0.47]
EAFAAVSK pc2
0.14 [0.03, 0.25]
DHEELSLVASEAVR pc3
0.14 [0.03, 0.25]
IAWHVIR pc2
0.25 [0.14, 0.36]
AYISMHSYSQHIVFPYSYTR pc4
0.26 [0.15, 0.37]
NAIWIDCGIHAR pc2
0.33 [0.22, 0.44]
YTHGHGSETLYLAPGGGDDWIYDLGIK pc4
0.35 [0.24, 0.46]
NAIWIDCGIHAR pc3
0.28 [0.17, 0.39]
DHEELSLVASEAVR pc2
0.31 [0.20, 0.41]
HPDMLTK pc2
0.26 [0.14, 0.37]
QVHFFVNASDVDNVK pc3
0.33 [0.22, 0.44]
AYISMHSYSQHIVFPYSYTR pc3
0.15 [0.02, 0.29]
SFYANNHCIGTDLNR pc2
0.22 [0.11, 0.34]
SKDHEELSLVASEAVR pc3
−0.04 [−0.38, 0.30]
SKDHEELSLVASEAVR pc4
0.19 [−0.35, 0.73]
LVDFYVMPVVNVDGYDYSWKK pc3
0.03 [−0.23, 0.28]
LVDFYVMPVVNVDGYDYSWK pc3
−0.11 [−0.71, 0.49]
RE Model
0.26 [0.21, 0.31]
−1
−0.5
0
0.5
1
Effect of 13:46076084:C:T (rs9534312)

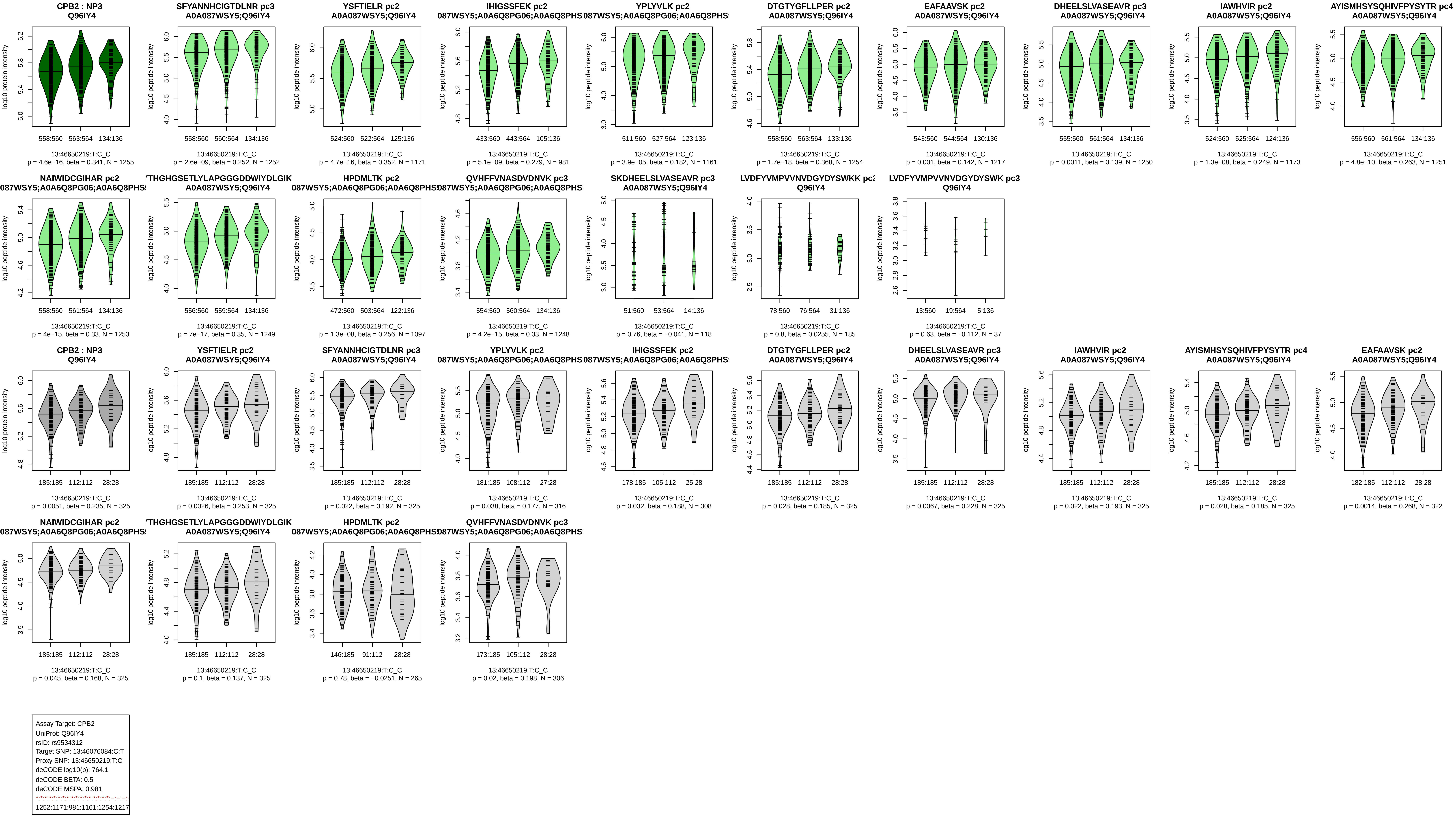

CPB2 : NP3
Q96IY4
log10 protein intensity
558:560 563:564 134:136
13:46650219:T:C_C
p = 4.6e−16, beta = 0.341, N = 1255
SFYANNHCIGTDLNR pc3
A0A087WSY5;Q96IY4
log10 peptide intensity
558:560 560:564 134:136
13:46650219:T:C_C
p = 2.6e−09, beta = 0.252, N = 1252
YSFTIELR pc2
A0A087WSY5;Q96IY4
524:560 522:564 125:136
13:46650219:T:C_C
p = 4.7e−16, beta = 0.352, N = 1171
IHIGSSFEK pc2
433:560 443:564 105:136
13:46650219:T:C_C
p = 5.1e−09, beta = 0.279, N = 981
YPLYVLK pc2
511:560 527:564 123:136
13:46650219:T:C_C
p = 3.9e−05, beta = 0.182, N = 1161
DTGTYGFLLPER pc2
A0A087WSY5;Q96IY4
558:560 563:564 133:136
13:46650219:T:C_C
p = 1.7e−18, beta = 0.368, N = 1254
EAFAAVSK pc2
A0A087WSY5;Q96IY4
543:560 544:564 130:136
13:46650219:T:C_C
p = 0.001, beta = 0.142, N = 1217
DHEELSLVASEAVR pc3
A0A087WSY5;Q96IY4
555:560 561:564 134:136
13:46650219:T:C_C
p = 0.0011, beta = 0.139, N = 1250
IAWHVIR pc2
A0A087WSY5;Q96IY4
524:560 525:564 124:136
13:46650219:T:C_C
p = 1.3e−08, beta = 0.249, N = 1173
AYISMHSYSQHIVFPYSYTR pc4
A0A087WSY5;Q96IY4
556:560 561:564 134:136
13:46650219:T:C_C
p = 4.8e−10, beta = 0.263, N = 1251
NAIWIDCGIHAR pc2
558:560 561:564 134:136
13:46650219:T:C_C
p = 4e−15, beta = 0.33, N = 1253
A0A087WSY5;Q96IY4
556:560 559:564 134:136
13:46650219:T:C_C
p = 7e−17, beta = 0.35, N = 1249
HPDMLTK pc2
472:560 503:564 122:136
13:46650219:T:C_C
p = 1.3e−08, beta = 0.256, N = 1097
QVHFFVNASDVDNVK pc3
554:560 560:564 134:136
13:46650219:T:C_C
p = 4.2e−15, beta = 0.33, N = 1248
SKDHEELSLVASEAVR pc3
A0A087WSY5;Q96IY4
51:560 53:564 14:136
13:46650219:T:C_C
p = 0.76, beta = −0.041, N = 118
Q96IY4
78:560 76:564 31:136
13:46650219:T:C_C
p = 0.8, beta = 0.0255, N = 185
LVDFYVMPVVNVDGYDYSWK pc3
Q96IY4
13:560 19:564 5:136
13:46650219:T:C_C
p = 0.63, beta = −0.112, N = 37
CPB2 : NP3
Q96IY4
185:185 112:112 28:28
13:46650219:T:C_C
p = 0.0051, beta = 0.235, N = 325
YSFTIELR pc2
A0A087WSY5;Q96IY4
185:185 112:112 28:28
13:46650219:T:C_C
p = 0.0026, beta = 0.253, N = 325
SFYANNHCIGTDLNR pc3
A0A087WSY5;Q96IY4
185:185 112:112 28:28
13:46650219:T:C_C
p = 0.022, beta = 0.192, N = 325
YPLYVLK pc2
181:185 108:112 27:28
13:46650219:T:C_C
p = 0.038, beta = 0.177, N = 316
IHIGSSFEK pc2
178:185 105:112 25:28
13:46650219:T:C_C
p = 0.032, beta = 0.188, N = 308
DTGTYGFLLPER pc2
A0A087WSY5;Q96IY4
185:185 112:112 28:28
13:46650219:T:C_C
p = 0.028, beta = 0.185, N = 325
DHEELSLVASEAVR pc3
A0A087WSY5;Q96IY4
185:185 112:112 28:28
13:46650219:T:C_C
p = 0.0067, beta = 0.228, N = 325
IAWHVIR pc2
A0A087WSY5;Q96IY4
185:185 112:112 28:28
13:46650219:T:C_C
p = 0.022, beta = 0.193, N = 325
AYISMHSYSQHIVFPYSYTR pc4
A0A087WSY5;Q96IY4
185:185 112:112 28:28
13:46650219:T:C_C
p = 0.028, beta = 0.185, N = 325
EAFAAVSK pc2
A0A087WSY5;Q96IY4
182:185 112:112 28:28
13:46650219:T:C_C
p = 0.0014, beta = 0.268, N = 322
NAIWIDCGIHAR pc2
185:185 112:112 28:28
13:46650219:T:C_C
p = 0.045, beta = 0.168, N = 325
A0A087WSY5;Q96IY4
185:185 112:112 28:28
13:46650219:T:C_C
p = 0.1, beta = 0.137, N = 325
HPDMLTK pc2
146:185 91:112 28:28
13:46650219:T:C_C
p = 0.78, beta = −0.0251, N = 265
QVHFFVNASDVDNVK pc3
173:185 105:112 28:28
13:46650219:T:C_C
p = 0.02, beta = 0.198, N = 306
Assay Target: CPB2
UniProt: Q96IY4
rsID: rs9534312
Target SNP: 13:46076084:C:T
Proxy SNP: 13:46650219:T:C
deCODE log10(p): 764.1
deCODE BETA: 0.5
deCODE MSPA: 0.981
1252:1171:981:1161:1254:1217

59. CCL14 (Q16627)
deCODE MSPA=0.86

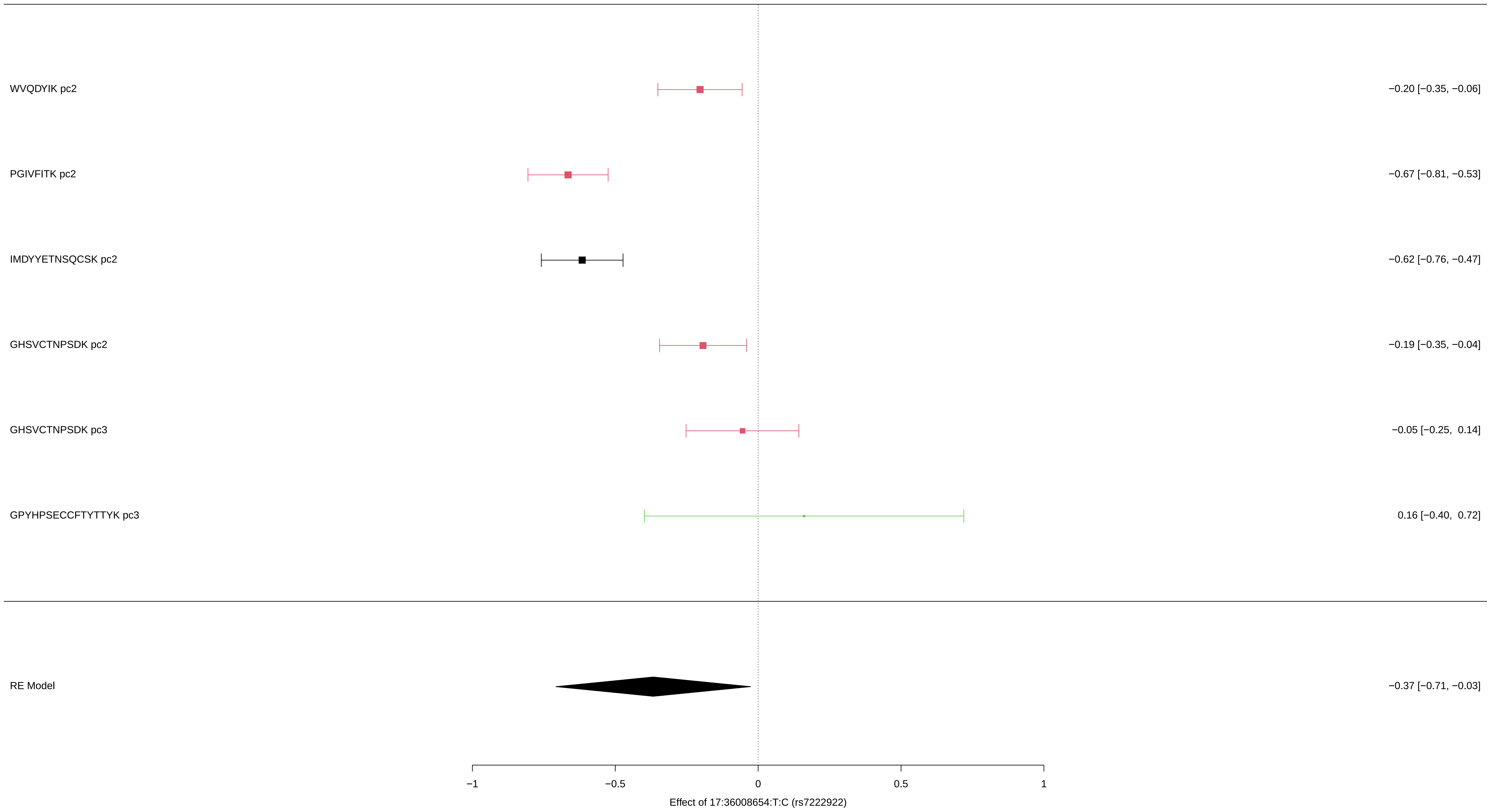

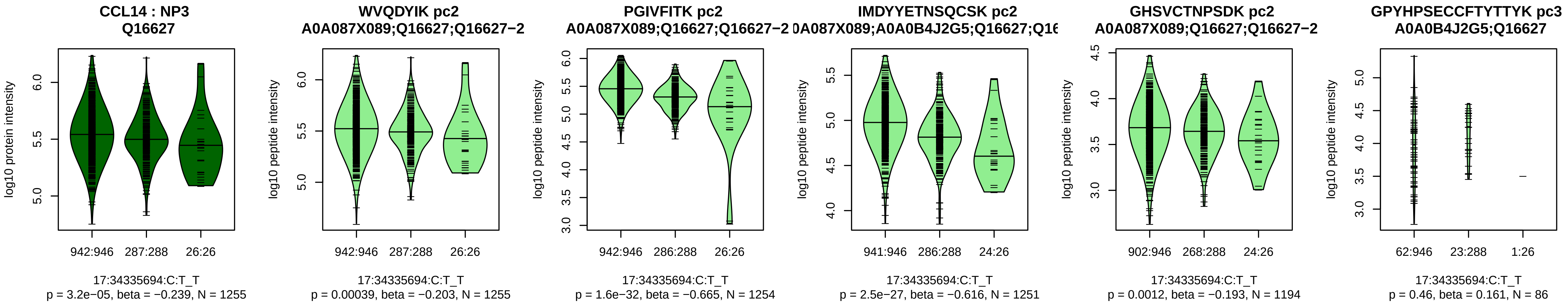

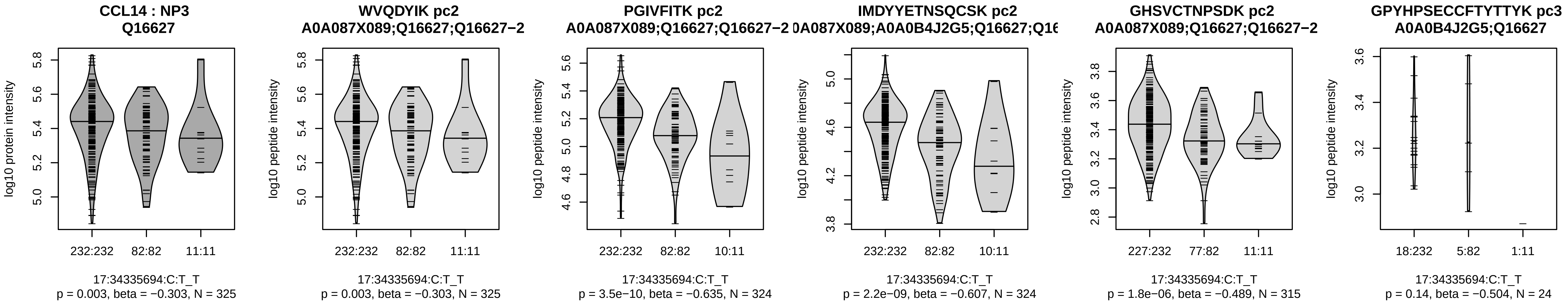

Assay Target: CCL14
UniProt: Q16627
rsID: rs7222922
Target SNP: 17:36008654:T:C
Proxy SNP: 17:34335694:C:T
deCODE log10(p): 737.6
deCODE BETA: −0.86
deCODE MSPA: 0.86
::*:*:–:–
1255:1254:1251:1194:722:86

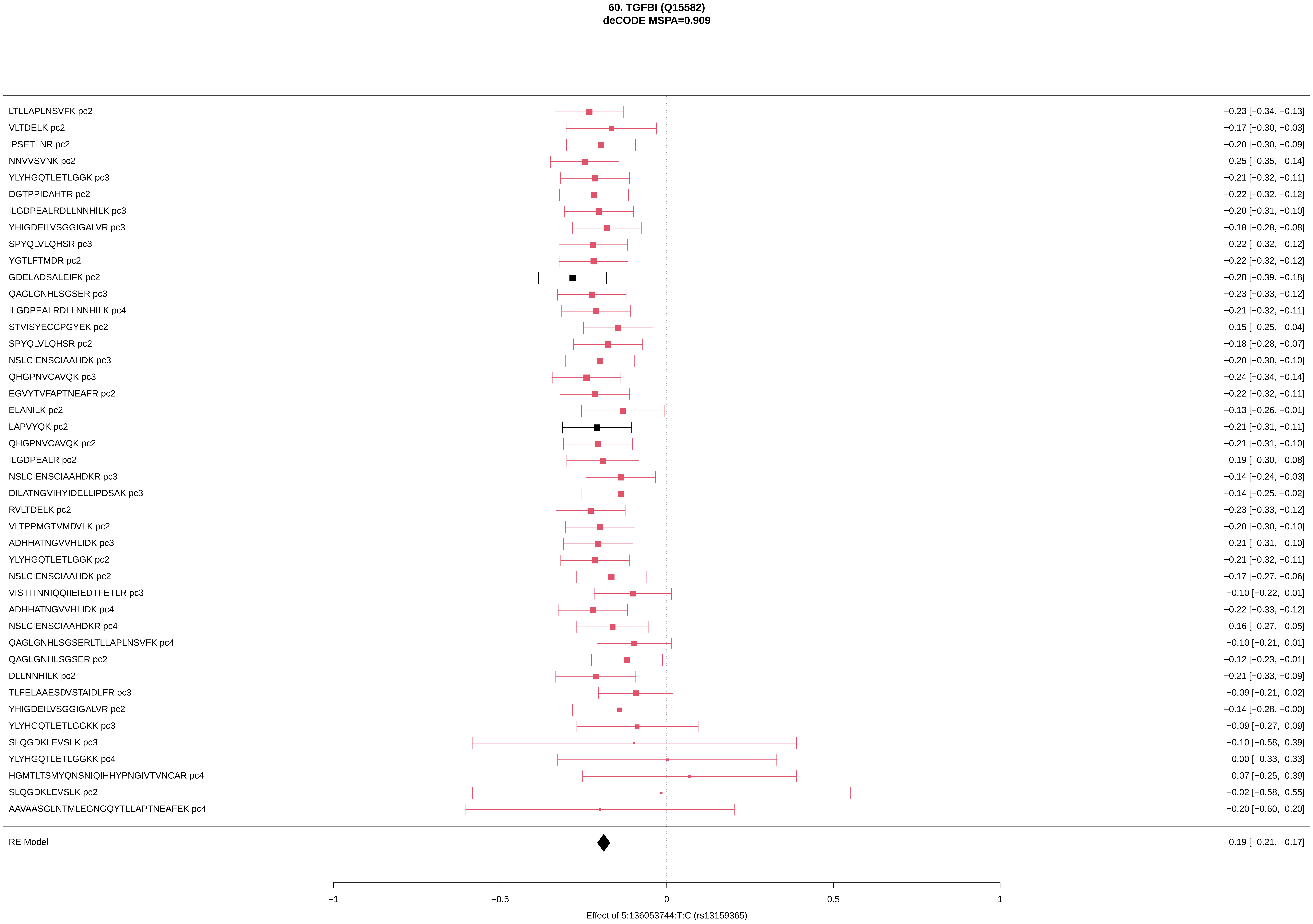
60. TGFBI (Q15582)
deCODE MSPA=0.909
LTLLAPLNSVFK pc2 −0.23 [−0.34, −0.13]
VLTDELK pc2 −0.17 [−0.30, −0.03]
IPSETLNR pc2 −0.20 [−0.30, −0.09]
NNVVSVNK pc2 −0.25 [−0.35, −0.14]
YLYHGQTLETLGGK pc3 −0.21 [−0.32, −0.11]
DGTPPIDAHTR pc2 −0.22 [−0.32, −0.12]
ILGDPEALRDLLNNHILK pc3 −0.20 [−0.31, −0.10]
YHIGDEILVSGGIGALVR pc3 −0.18 [−0.28, −0.08]
SPYQLVLQHSR pc3 −0.22 [−0.32, −0.12]
YGTLFTMDR pc2 −0.22 [−0.32, −0.12]
GDELADSALEIFK pc2 −0.28 [−0.39, −0.18]
QAGLGNHLSGSER pc3 −0.23 [−0.33, −0.12]
ILGDPEALRDLLNNHILK pc4 −0.21 [−0.32, −0.11]
STVISYECCPGYEK pc2 −0.15 [−0.25, −0.04]
SPYQLVLQHSR pc2 −0.18 [−0.28, −0.07]
NSLCIENSCIAAHDK pc3 −0.20 [−0.30, −0.10]
QHGPNVCAVQK pc3 −0.24 [−0.34, −0.14]
EGVYTVFAPTNEAFR pc2 −0.22 [−0.32, −0.11]
ELANILK pc2 −0.13 [−0.26, −0.01]
LAPVYQK pc2 −0.21 [−0.31, −0.11]
QHGPNVCAVQK pc2 −0.21 [−0.31, −0.10]
ILGDPEALR pc2 −0.19 [−0.30, −0.08]
NSLCIENSCIAAHDKR pc3 −0.14 [−0.24, −0.03]
DILATNGVIHYIDELLIPDSAK pc3 −0.14 [−0.25, −0.02]
RVLTDELK pc2 −0.23 [−0.33, −0.12]
VLTPPMGTVMDVLK pc2 −0.20 [−0.30, −0.10]
ADHHATNGVVHLIDK pc3 −0.21 [−0.31, −0.10]
YLYHGQTLETLGGK pc2 −0.21 [−0.32, −0.11]
NSLCIENSCIAAHDK pc2 −0.17 [−0.27, −0.06]
VISTITNNIQQIIEIEDTFETLR pc3 −0.10 [−0.22, 0.01]
ADHHATNGVVHLIDK pc4 −0.22 [−0.33, −0.12]
NSLCIENSCIAAHDKR pc4 −0.16 [−0.27, −0.05]
QAGLGNHLSGSERLTLLAPLNSVFK pc4 −0.10 [−0.21, 0.01]
QAGLGNHLSGSER pc2 −0.12 [−0.23, −0.01]
DLLNNHILK pc2 −0.21 [−0.33, −0.09]
TLFELAAESDVSTAIDLFR pc3 −0.09 [−0.21, 0.02]
YHIGDEILVSGGIGALVR pc2 −0.14 [−0.28, −0.00]
YLYHGQTLETLGGKK pc3 −0.09 [−0.27, 0.09]
SLQGDKLEVSLK pc3 −0.10 [−0.58, 0.39]
YLYHGQTLETLGGKK pc4 0.00 [−0.33, 0.33]
HGMTLTSMYQNSNIQIHHYPNGIVTVNCAR pc4 0.07 [−0.25, 0.39]
SLQGDKLEVSLK pc2 −0.02 [−0.58, 0.55]
AAVAASGLNTMLEGNGQYTLLAPTNEAFEK pc4 −0.20 [−0.60, 0.20]
RE Model −0.19 [−0.21, −0.17]
−1
−0.5
0
0.5
1
Effect of 5:136053744:T:C (rs13159365)

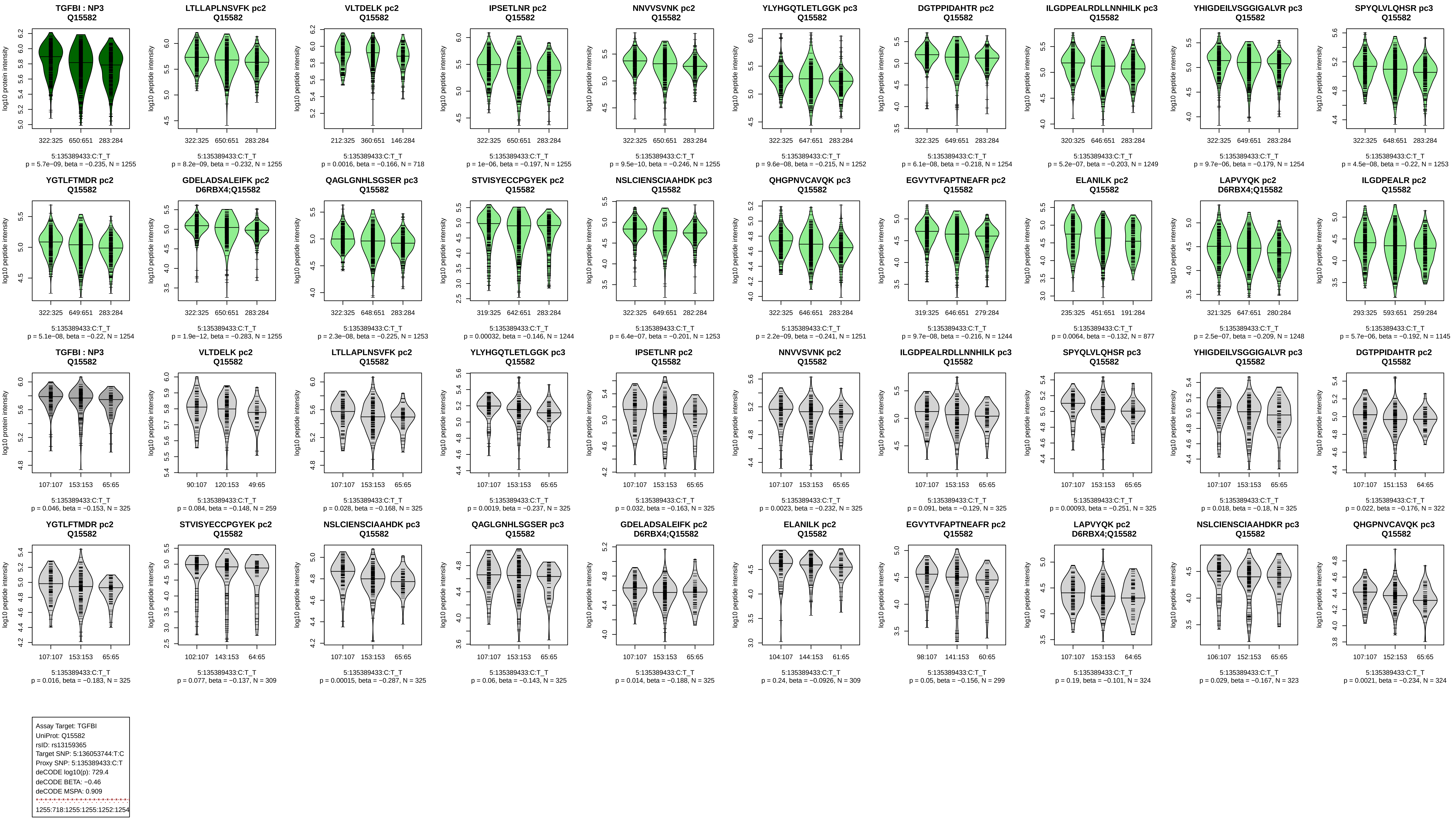
TGFBI : NP3
Q15582
322:325 650:651 283:284
5:135389433:C:T_T
p = 5.7e-09, beta = −0.235, N = 1255
LTLLAPLNSVFK pc2
Q15582
322:325 650:651 283:284
5:135389433:C:T_T
p = 8.2e-09, beta = −0.232, N = 1255
VLTDELK pc2
Q15582
212:325 360:651 146:284
5:135389433:C:T_T
p = 0.0016, beta = −0.166, N = 718
IPSETLNR pc2
Q15582
322:325 650:651 283:284
5:135389433:C:T_T
p = 1e-06, beta = −0.197, N = 1255
NNVVSVNK pc2
Q15582
322:325 650:651 283:284
5:135389433:C:T_T
p = 9.5e-10, beta = −0.246, N = 1255
YLYHGQTLETLGGK pc3
Q15582
322:325 647:651 283:284
5:135389433:C:T_T
p = 9.6e-08, beta = −0.215, N = 1252
DGTPPIDAHTR pc2
Q15582
322:325 649:651 283:284
5:135389433:C:T_T
p = 6.1e-08, beta = −0.218, N = 1254
ILGDPEALRDLLNNHILK pc3
Q15582
320:325 646:651 283:284
5:135389433:C:T_T
p = 5.2e-07, beta = −0.203, N = 1249
YHIGDEILVSGGIGALVR pc3
Q15582
322:325 649:651 283:284
5:135389433:C:T_T
p = 9.7e-06, beta = −0.179, N = 1254
SPYQLVLQHSR pc3
Q15582
322:325 648:651 283:284
5:135389433:C:T_T
p = 4.5e-08, beta = −0.22, N = 1253
YGTLFTMDR pc2
Q15582
322:325 649:651 283:284
5:135389433:C:T_T
p = 5.1e-08, beta = −0.22, N = 1254
GDELADSALEIFK pc2
D6RBX4;Q15582
322:325 650:651 283:284
5:135389433:C:T_T
p = 1.9e−12, beta = −0.283, N = 1255
QAGLGNHLSGSER pc3
Q15582
322:325 648:651 283:284
5:135389433:C:T_T
p = 2.3e−08, beta = −0.225, N = 1253
STVISYECCPGYEK pc2
Q15582
319:325 642:651 283:284
5:135389433:C:T_T
p = 0.00032, beta = −0.146, N = 1244
NSLCIENSCIAAHDK pc3
Q15582
322:325 649:651 282:284
5:135389433:C:T_T
p = 6.4e−07, beta = −0.201, N = 1253
QHGPNVCAVQK pc3
Q15582
322:325 646:651 283:284
5:135389433:C:T_T
p = 2.2e−09, beta = −0.241, N = 1251
EGVYTVFAPTNEAFR pc2
Q15582
319:325 646:651 279:284
5:135389433:C:T_T
p = 9.7e−08, beta = −0.216, N = 1244
ELANILK pc2
Q15582
235:325 451:651 191:284
5:135389433:C:T_T
p = 0.0064, beta = −0.132, N = 877
LAPVYQK pc2
D6RBX4;Q15582
321:325 647:651 280:284
5:135389433:C:T_T
p = 2.5e−07, beta = −0.209, N = 1248
ILGDPEALR pc2
Q15582
293:325 593:651 259:284
5:135389433:C:T_T
p = 5.7e−06, beta = −0.192, N = 1145
TGFBI : NP3
Q15582
107:107 153:153 65:65
5:135389433:C:T_T
p = 0.046, beta = −0.153, N = 325
VLTDELK pc2
Q15582
90:107 120:153 49:65
5:135389433:C:T_T
p = 0.084, beta = −0.148, N = 259
LTLLAPLNSVFK pc2
Q15582
107:107 153:153 65:65
5:135389433:C:T_T
p = 0.028, beta = −0.168, N = 325
YLYHGQTLETLGGK pc3
Q15582
107:107 153:153 65:65
5:135389433:C:T_T
p = 0.0019, beta = −0.237, N = 325
IPSETLNR pc2
Q15582
107:107 153:153 65:65
5:135389433:C:T_T
p = 0.032, beta = −0.163, N = 325
NNVVSVNK pc2
Q15582
107:107 153:153 65:65
5:135389433:C:T_T
p = 0.0023, beta = −0.232, N = 325
ILGDPEALRDLLNNHILK pc3
Q15582
107:107 153:153 65:65
5:135389433:C:T_T
p = 0.091, beta = −0.129, N = 325
SPYQLVLQHSR pc3
Q15582
107:107 153:153 65:65
5:135389433:C:T_T
p = 0.00093, beta = −0.251, N = 325
YHIGDEILVSGGIGALVR pc3
Q15582
107:107 153:153 65:65
5:135389433:C:T_T
p = 0.018, beta = −0.18, N = 325
DGTPPIDAHTR pc2
Q15582
107:107 151:153 64:65
5:135389433:C:T_T
p = 0.022, beta = −0.176, N = 322
YGTLFTMDR pc2
Q15582
107:107 153:153 65:65
5:135389433:C:T_T
p = 0.016, beta = −0.183, N = 325
STVISYECCPGYEK pc2
Q15582
102:107 143:153 64:65
5:135389433:C:T_T
p = 0.077, beta = −0.137, N = 309
NSLCIENSCIAAHDK pc3
Q15582
107:107 153:153 65:65
5:135389433:C:T_T
p = 0.00015, beta = −0.287, N = 325
QAGLGNHLSGSER pc3
Q15582
107:107 153:153 65:65
5:135389433:C:T_T
p = 0.06, beta = −0.143, N = 325
GDELADSALEIFK pc2
D6RBX4;Q15582
107:107 153:153 65:65
5:135389433:C:T_T
p = 0.014, beta = −0.188, N = 325
ELANILK pc2
Q15582
104:107 144:153 61:65
5:135389433:C:T_T
p = 0.24, beta = −0.0926, N = 309
EGVYTVFAPTNEAFR pc2
Q15582
98:107 141:153 60:65
5:135389433:C:T_T
p = 0.05, beta = −0.156, N = 299
LAPVYQK pc2
D6RBX4;Q15582
107:107 153:153 64:65
5:135389433:C:T_T
p = 0.19, beta = −0.101, N = 324
NSLCIENSCIAAHDKR pc3
Q15582
106:107 152:153 65:65
5:135389433:C:T_T
p = 0.029, beta = −0.167, N = 323
QHGPNVCAVQK pc3
Q15582
107:107 152:153 65:65
5:135389433:C:T_T
p = 0.0021, beta = −0.234, N = 324
Assay Target: TGFBI
UniProt: Q15582
rsID: rs13159365
Target SNP: 5:136053744:T:C
Proxy SNP: 5:135389433:C:T
deCODE log10(p): 729.4
deCODE BETA: −0.46
deCODE MSPA: 0.909
1255:718:1255:1255:1252:1254

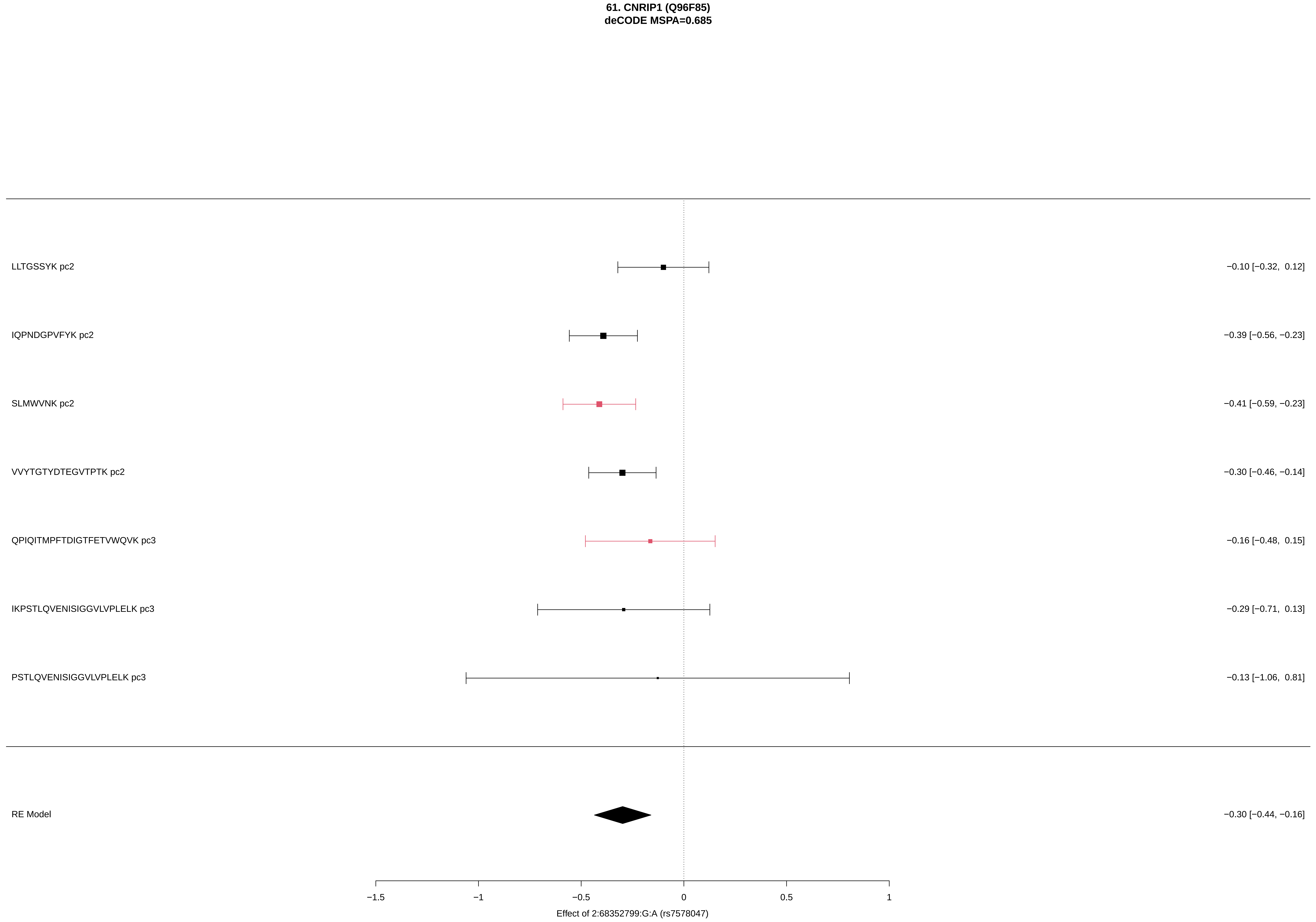
61. CNRIP1 (Q96F85)
deCODE MSPA=0.685
LLTGSSYK pc2
−0.10 [−0.32, 0.12]
IQPNDGPVFYK pc2
−0.39 [−0.56, −0.23]
SLMWVNK pc2
−0.41 [−0.59, −0.23]
VVYTGTYDTEGVTPTK pc2
−0.30 [−0.46, −0.14]
QPIQITMPFTDIGTFETVWQVK pc3
−0.16 [−0.48, 0.15]
IKPSTLQVENISIGGVLVPLELK pc3
−0.29 [−0.71, 0.13]
PSTLQVENISIGGVLVPLELK pc3
−0.13 [−1.06, 0.81]
RE Model
−0.30 [−0.44, −0.16]
−1.5
−1
−0.5
0
0.5
1
Effect of 2:68352799:G:A (rs7578047)

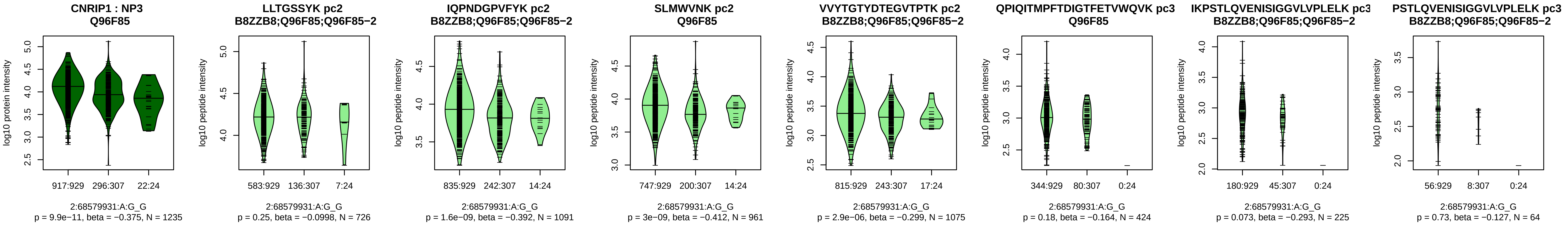

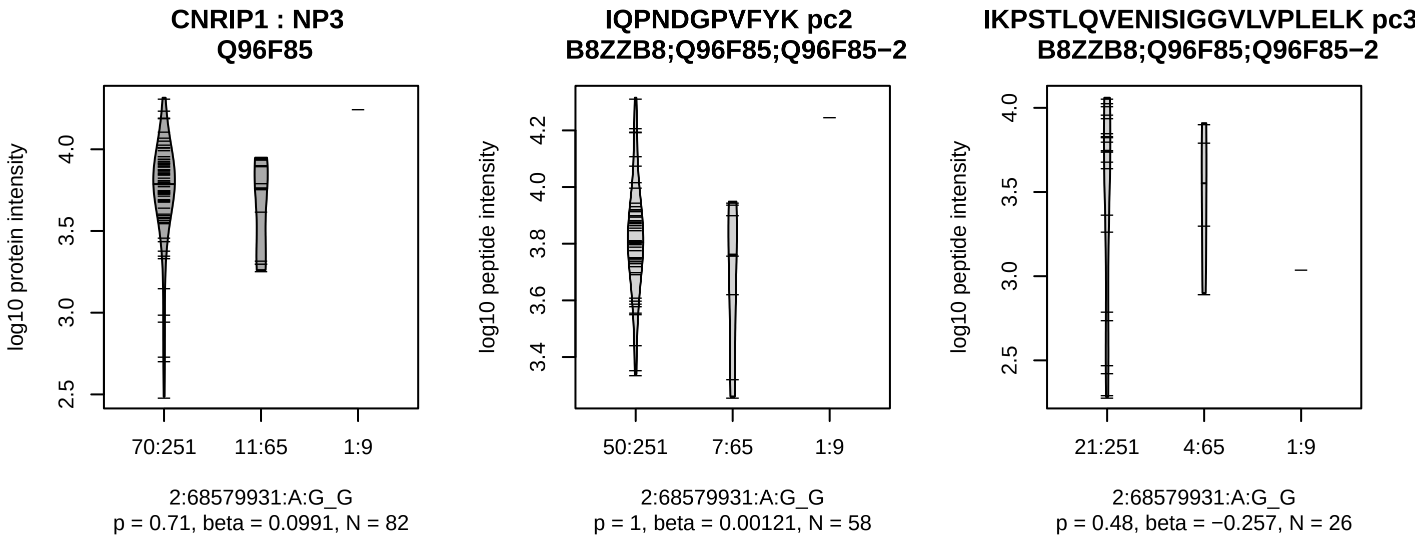

Assay Target: CNRIP1
UniProt: Q96F85
rsID: rs7578047
Target SNP: 2:68352799:G:A
Proxy SNP: 2:68579931:A:G
deCODE log10(p): 707.8
deCODE BETA: −0.66
deCODE MSPA: 0.685
−:*:*:*:−:−:−:NA
726:1091:961:1075:424:225:64:

62. ANG (P03950)
deCODE MSPA=0.993

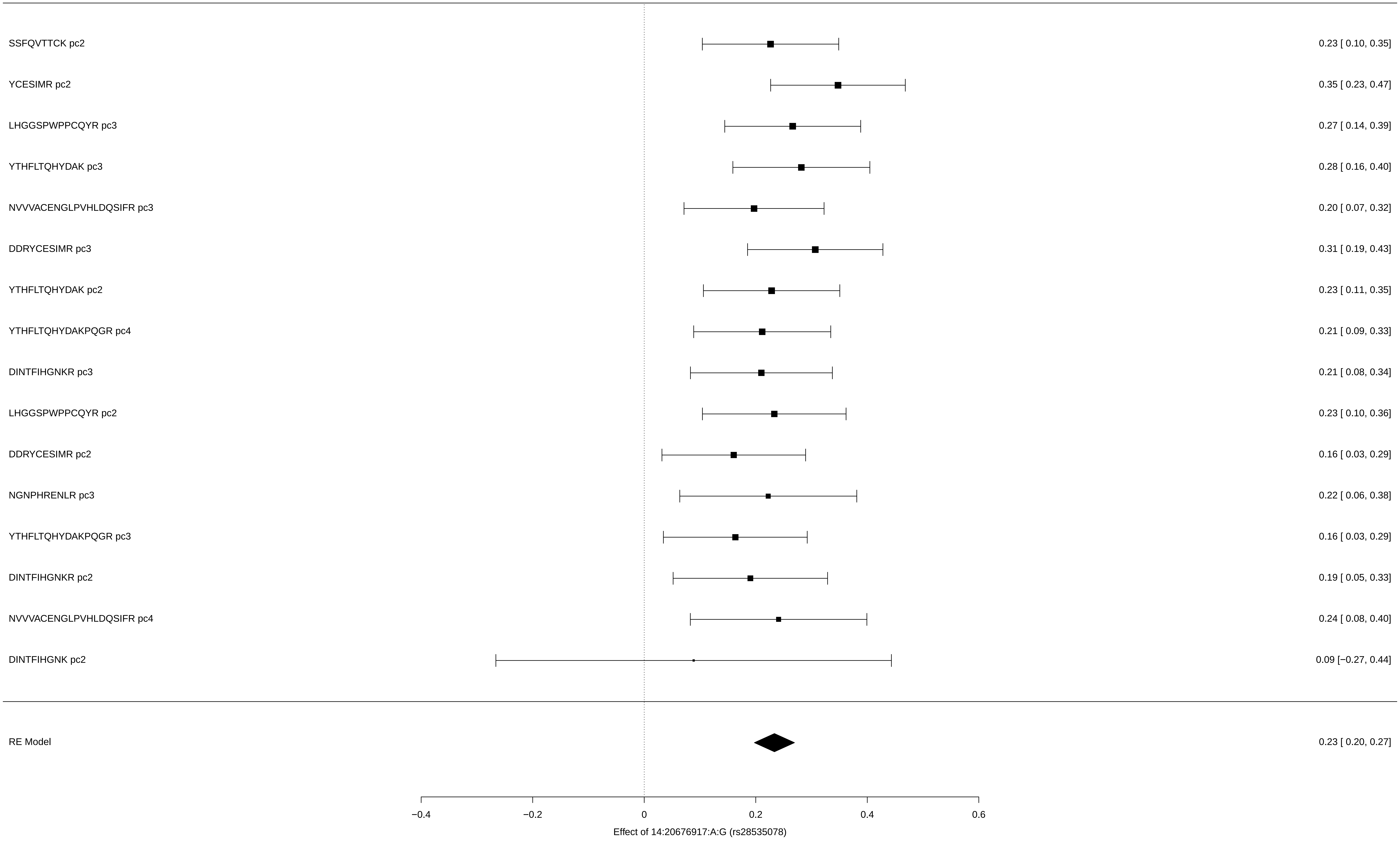

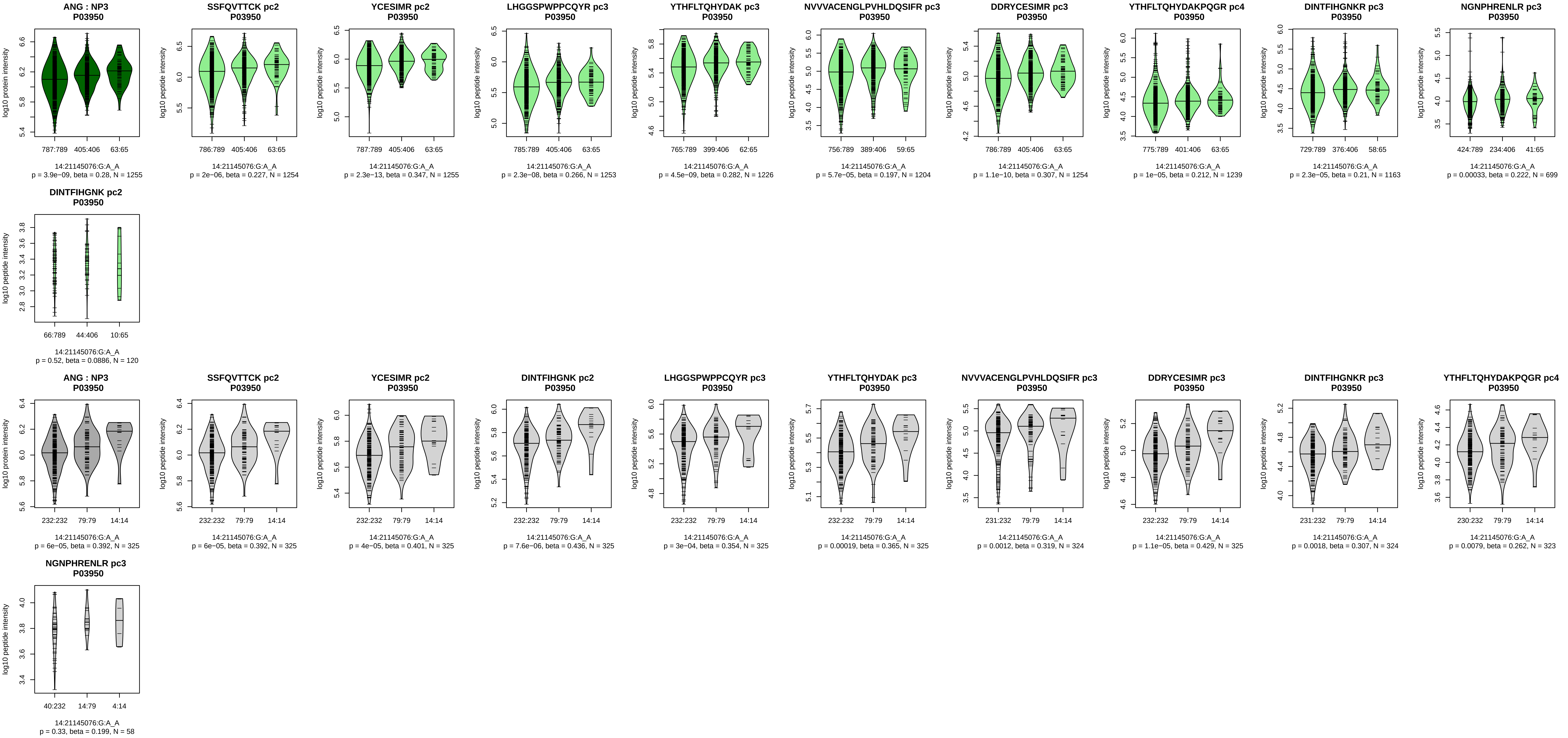

Assay Target: ANG
UniProt: P03950
rsID: rs28535078
Target SNP: 14:20676917:A:G
Proxy SNP: 14:21145076:G:A
deCODE log10(p): 686
deCODE BETA: 0.56
deCODE MSPA: 0.993
::*:*:*:*:*:*:*:*:*:*:*:*:*:–:NA
1254:1255:1253:1226:1204:125

63. PLTP (P55058)
deCODE MSPA=0.804

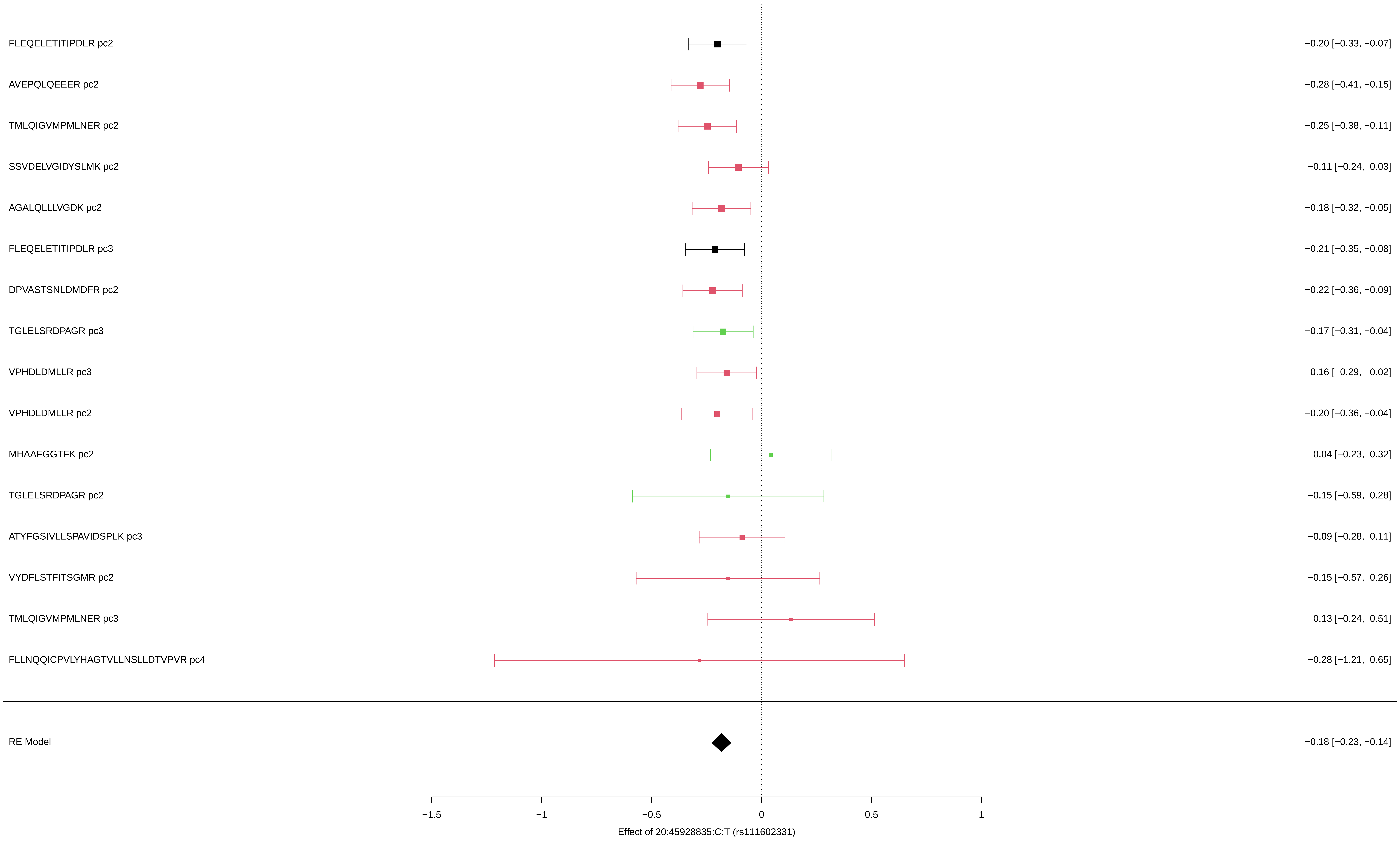

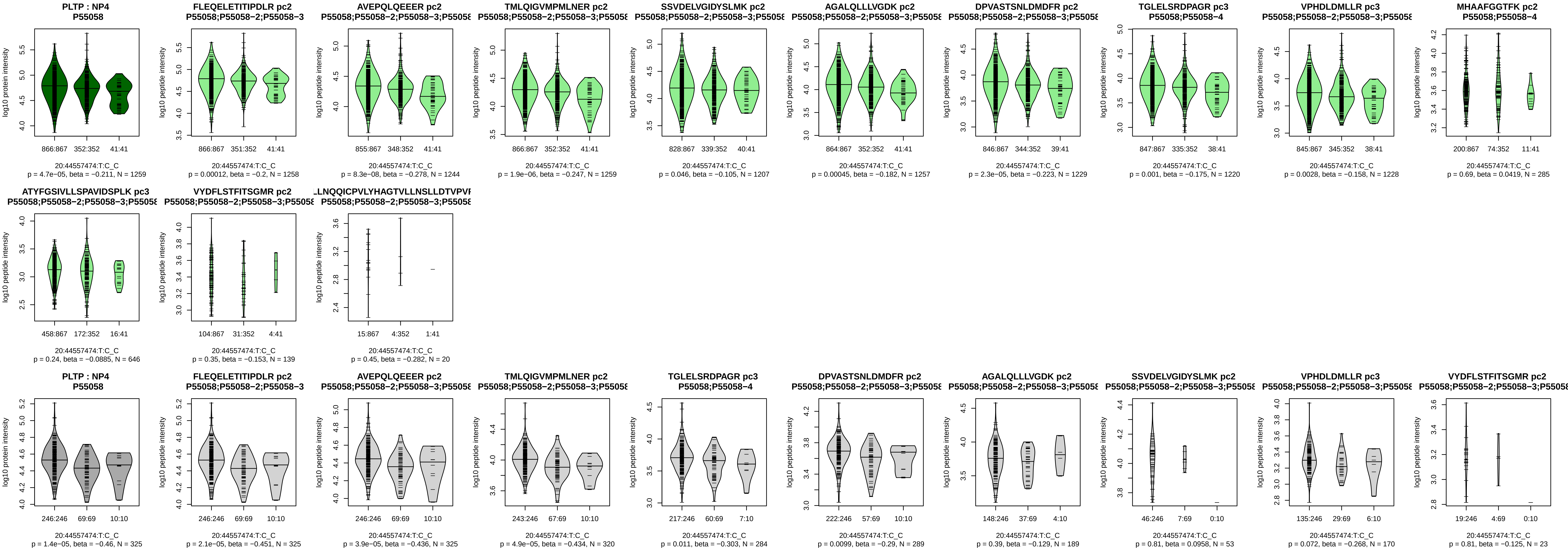

Assay Target: PLTP
UniProt: P55058
rsID: rs111602331
Target SNP: 20:45928835:C:T
Proxy SNP: 20:44557474:T:C
deCODE log10(p): 659.1
deCODE BETA: −0.54
deCODE MSPA: 0.804
::*:–:*:*:*:*:*:*:–:–:–:–:–:–
1258:1244:1259:1207:1257:123

64. MMP1 (P03956)
deCODE MSPA=0.702

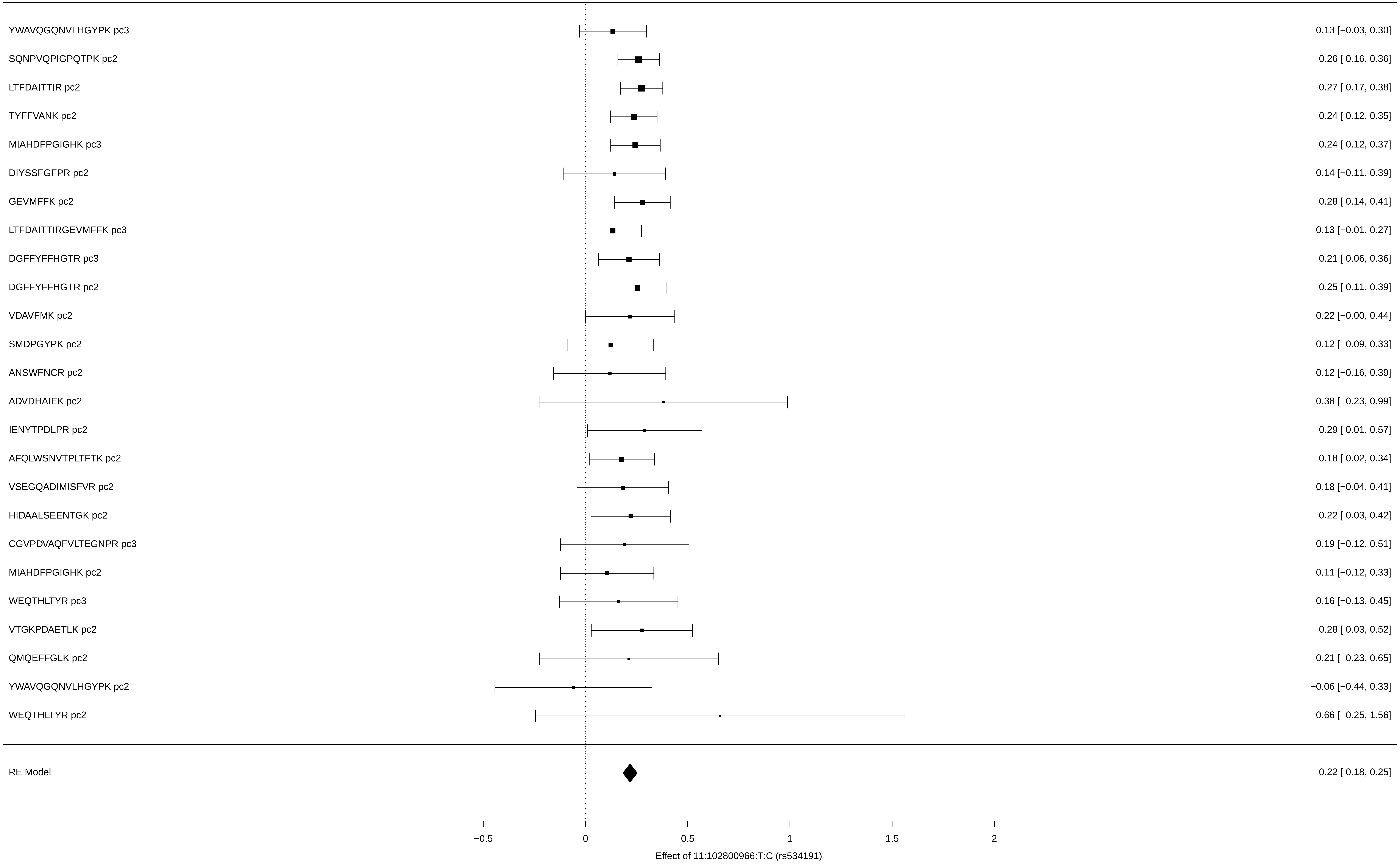

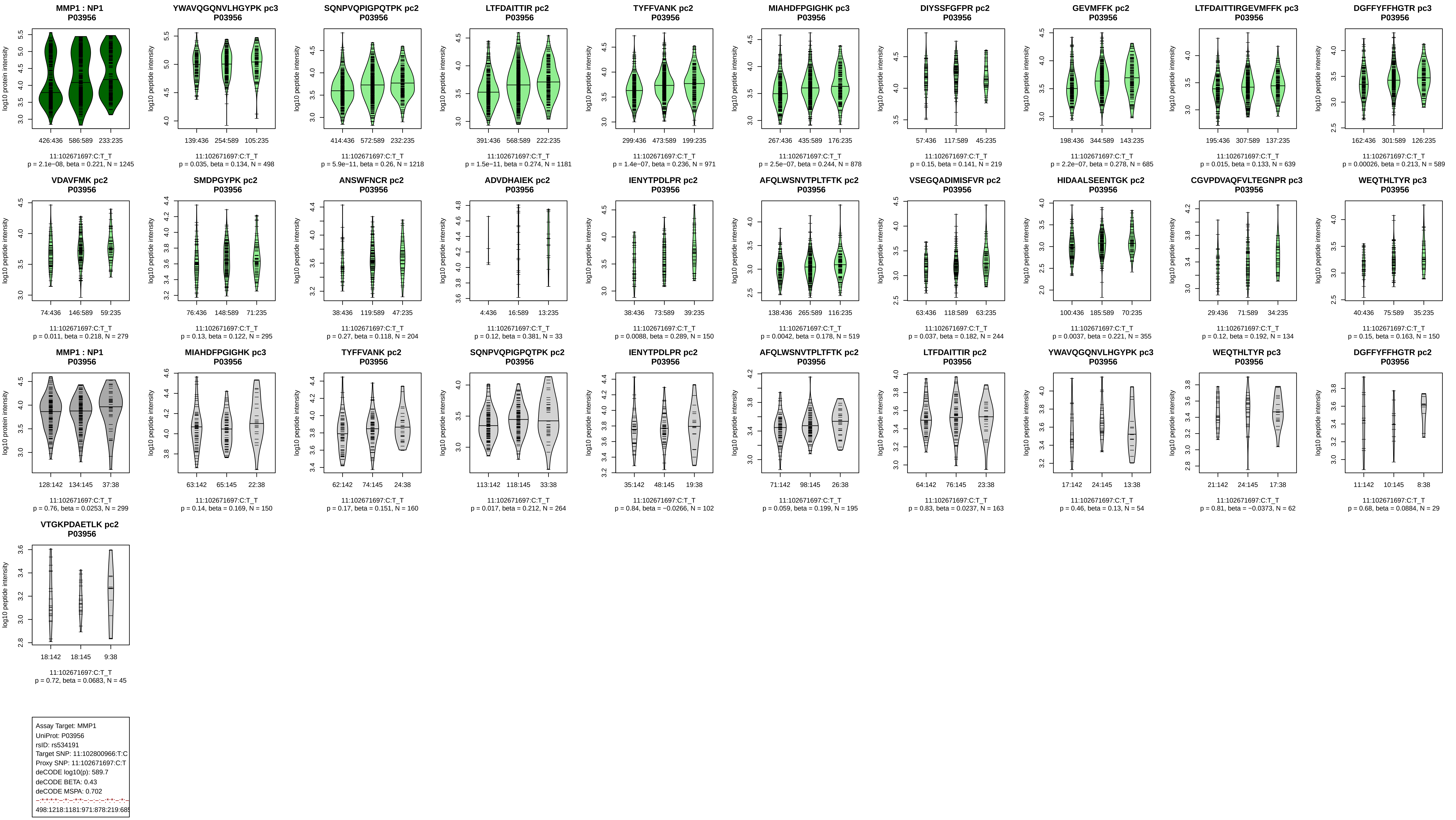
MMP1 : NP1 P03956
426:436 586:589 233:235
11:102671697:C:T_T
p = 2.1e−08, beta = 0.221, N = 1245
YWAVQGQNVLHGYPK pc3 P03956
139:436 254:589 105:235
11:102671697:C:T_T
p = 0.035, beta = 0.134, N = 498
SQNPVQPIGPQTPK pc2 P03956
414:436 572:589 232:235
11:102671697:C:T_T
p = 5.9e−11, beta = 0.26, N = 1218
LTFDAITTIR pc2 P03956
391:436 568:589 222:235
11:102671697:C:T_T
p = 1.5e−11, beta = 0.274, N = 1181
TYFFVANK pc2 P03956
299:436 473:589 199:235
11:102671697:C:T_T
p = 1.4e−07, beta = 0.236, N = 971
MIAHDFPGIGHK pc3 P03956
267:436 435:589 176:235
11:102671697:C:T_T
p = 2.5e−07, beta = 0.244, N = 878
DIYSSFGFPR pc2 P03956
57:436 117:589 45:235
11:102671697:C:T_T
p = 0.15, beta = 0.141, N = 219
GEVMFFK pc2 P03956
198:436 344:589 143:235
11:102671697:C:T_T
p = 2.2e−07, beta = 0.278, N = 685
LTFDAITTIRGEVMFFK pc3 P03956
195:436 307:589 137:235
11:102671697:C:T_T
p = 0.015, beta = 0.133, N = 639
DGFFYFFHGTR pc3 P03956
162:436 301:589 126:235
11:102671697:C:T_T
p = 0.00026, beta = 0.213, N = 589
VDAVFMK pc2 P03956
74:436 146:589 59:235
11:102671697:C:T_T
p = 0.011, beta = 0.218, N = 279
SMDPGYPK pc2 P03956
76:436 148:589 71:235
11:102671697:C:T_T
p = 0.13, beta = 0.122, N = 295
ANSWFNCR pc2 P03956
38:436 119:589 47:235
11:102671697:C:T_T
p = 0.27, beta = 0.118, N = 204
ADVDHAIEK pc2 P03956
4:436 16:589 13:235
11:102671697:C:T_T
p = 0.12, beta = 0.381, N = 33
IENYTPDLPR pc2 P03956
38:436 73:589 39:235
11:102671697:C:T_T
p = 0.0088, beta = 0.289, N = 150
AFQLWSNVTPLTFTK pc2 P03956
138:436 265:589 116:235
11:102671697:C:T_T
p = 0.0042, beta = 0.178, N = 519
VSEGQADIMISFVR pc2 P03956
63:436 118:589 63:235
11:102671697:C:T_T
p = 0.037, beta = 0.182, N = 244
HIDAALSEENTGK pc2 P03956
100:436 185:589 70:235
11:102671697:C:T_T
p = 0.0037, beta = 0.221, N = 355
CGVPDVAQFVLTEGNPR pc3 P03956
29:436 71:589 34:235
11:102671697:C:T_T
p = 0.12, beta = 0.192, N = 134
WEQTHLTYR pc3 P03956
40:436 75:589 35:235
11:102671697:C:T_T
p = 0.15, beta = 0.163, N = 150
MMP1 : NP1 P03956
128:142 134:145 37:38
11:102671697:C:T_T
p = 0.76, beta = 0.0253, N = 299
MIAHDFPGIGHK pc3 P03956
63:142 65:145 22:38
11:102671697:C:T_T
p = 0.14, beta = 0.169, N = 150
TYFFVANK pc2 P03956
62:142 74:145 24:38
11:102671697:C:T_T
p = 0.17, beta = 0.151, N = 160
SQNPVQPIGPQTPK pc2 P03956
113:142 118:145 33:38
11:102671697:C:T_T
p = 0.017, beta = 0.212, N = 264
IENYTPDLPR pc2 P03956
35:142 48:145 19:38
11:102671697:C:T_T
p = 0.84, beta = −0.0266, N = 102
AFQLWSNVTPLTFTK pc2 P03956
71:142 98:145 26:38
11:102671697:C:T_T
p = 0.059, beta = 0.199, N = 195
LTFDAITTIR pc2 P03956
64:142 76:145 23:38
11:102671697:C:T_T
p = 0.83, beta = 0.0237, N = 163
YWAVQGQNVLHGYPK pc3 P03956
17:142 24:145 13:38
11:102671697:C:T_T
p = 0.46, beta = 0.13, N = 54
WEQTHLTYR pc3 P03956
21:142 24:145 17:38
11:102671697:C:T_T
p = 0.81, beta = −0.0373, N = 62
DGFFYFFHGTR pc2 P03956
11:142 10:145 8:38
11:102671697:C:T_T
p = 0.68, beta = 0.0884, N = 29
VTGKPDAETLK pc2 P03956
18:142 18:145 9:38
11:102671697:C:T_T
p = 0.72, beta = 0.0683, N = 45
log10 protein intensity
log10 peptide intensity
Assay Target: MMP1
UniProt: P03956
rsID: rs534191
Target SNP: 11:102800966:T:C
Proxy SNP: 11:102671697:C:T
deCODE log10(p): 589.7
deCODE BETA: 0.43
deCODE MSPA: 0.702
498:1218:1181:971:878:219:685

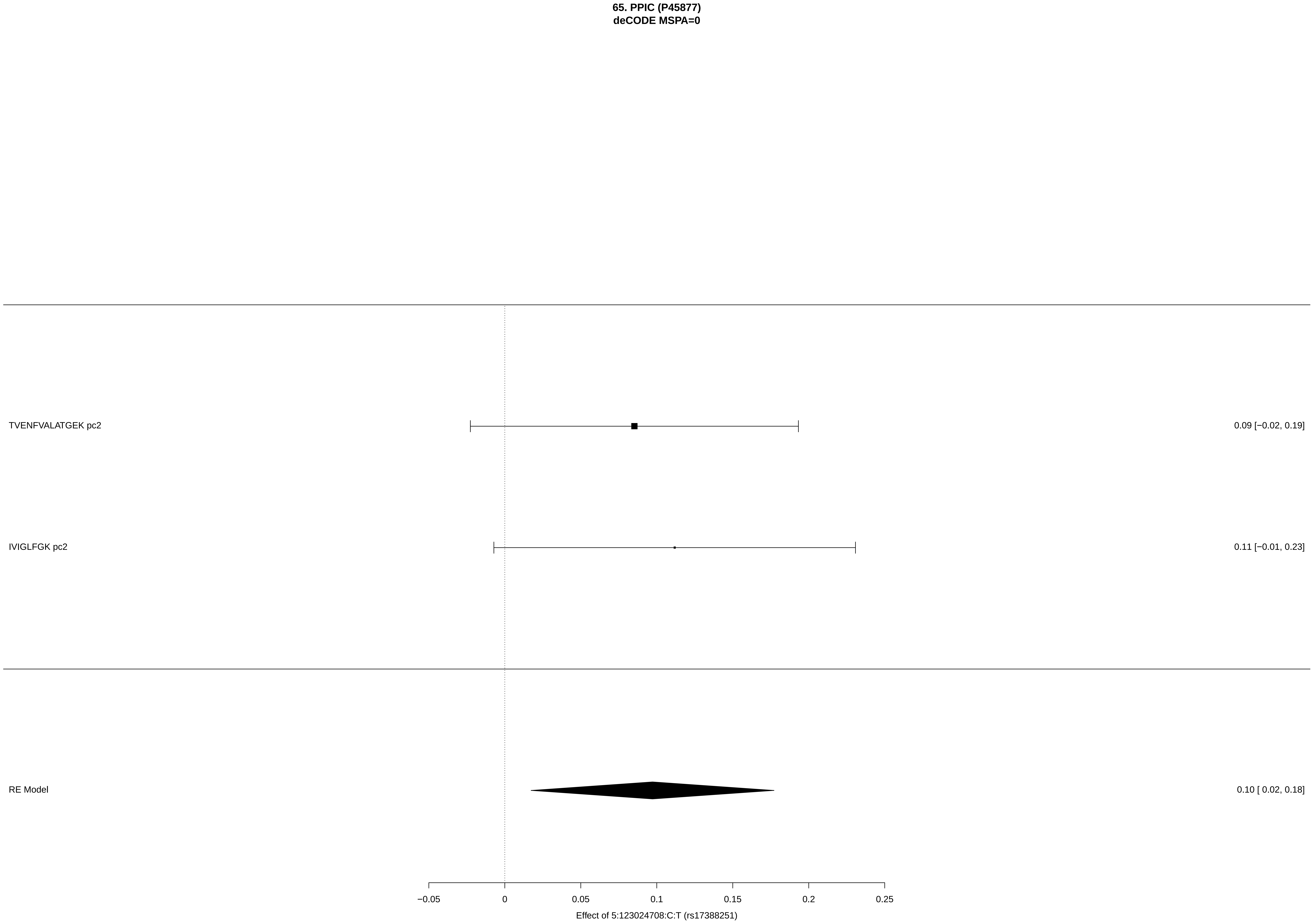
65. PPIC (P45877)
deCODE MSPA=0
TVENFVALATGEK pc2
0.09 [−0.02, 0.19]
IVIGLFGK pc2
0.11 [−0.01, 0.23]
RE Model
0.10 [0.02, 0.18]
−0.05
0
0.05
0.1
0.15
0.2
0.25
Effect of 5:123024708:C:T (rs17388251)

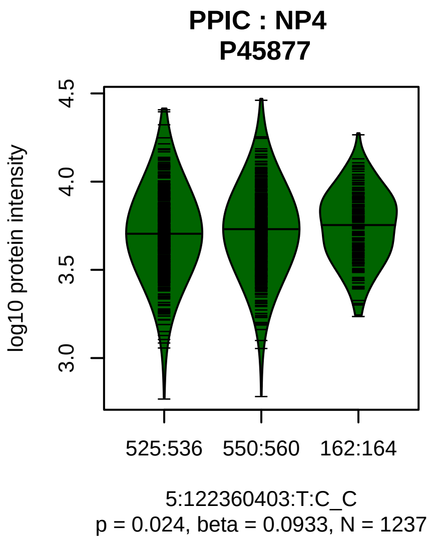

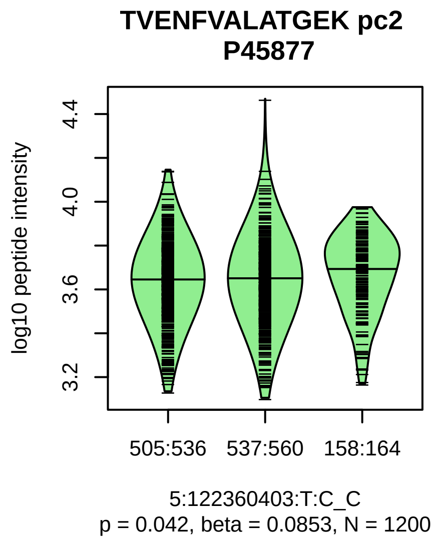

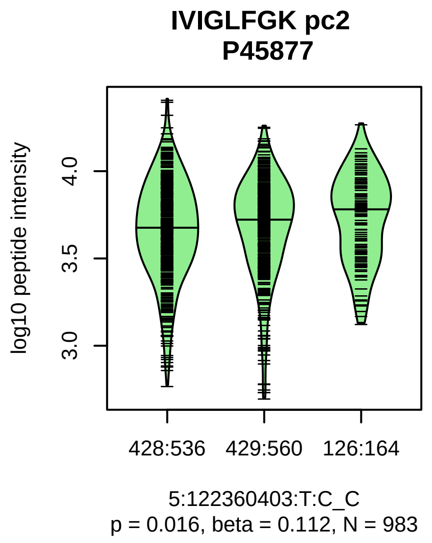

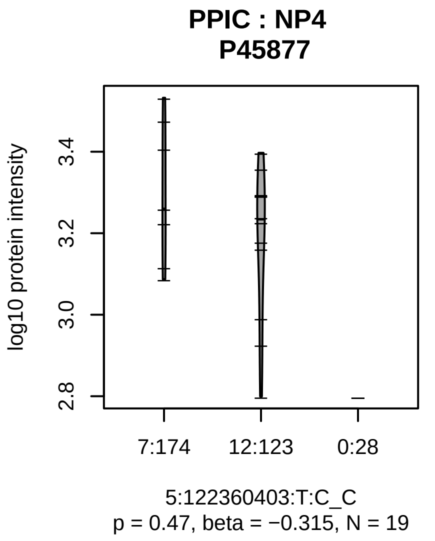

Assay Target: PPIC
UniProt: P45877
rsID: rs17388251
Target SNP: 5:123024708:C:T
Proxy SNP: 5:122360403:T:C
deCODE log10(p): 585.3
deCODE BETA: −0.42
deCODE MSPA: 0
–:–
1200:983

66. CPN2 (P22792)
deCODE MSPA=0

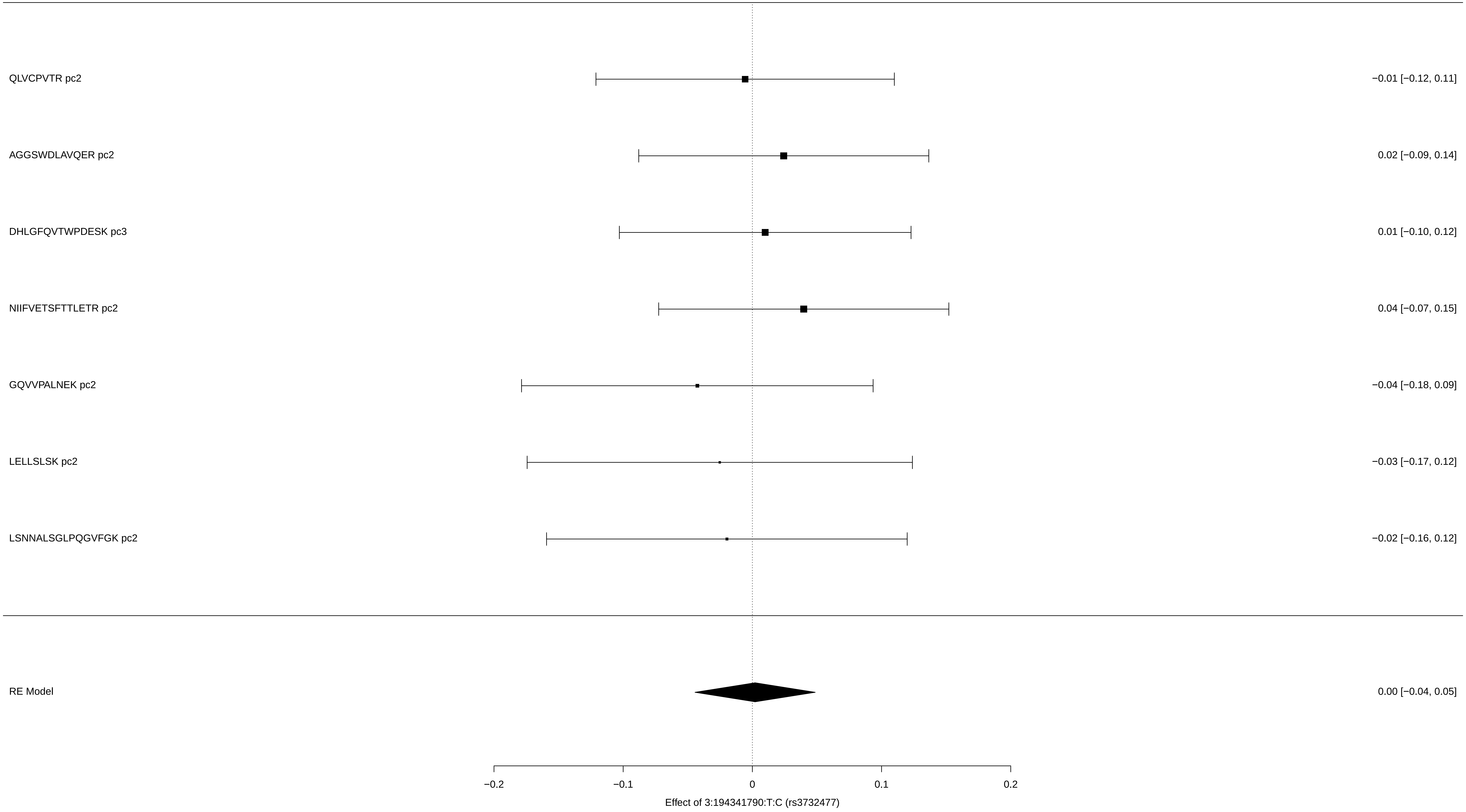

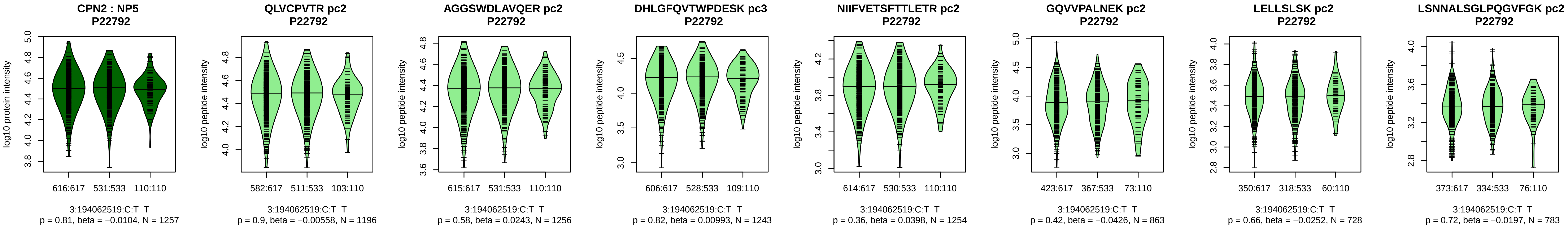

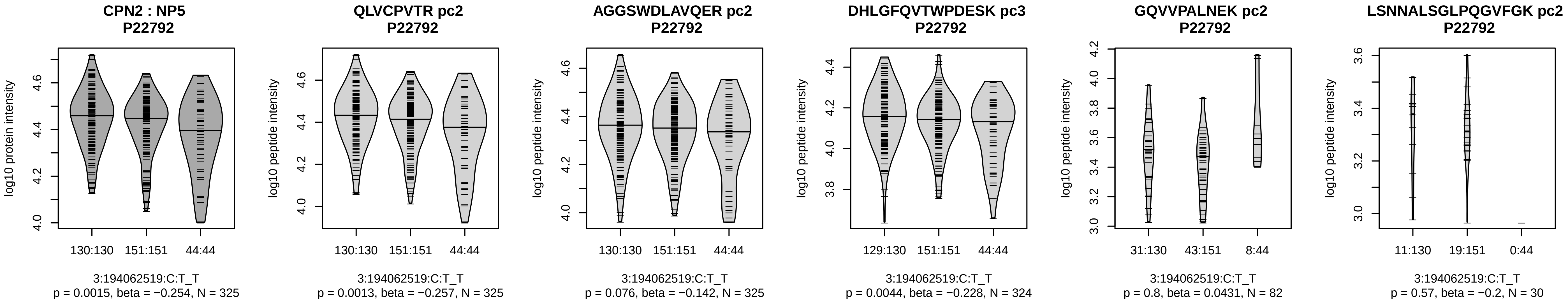

Assay Target: CPN2
UniProt: P22792
rsID: rs3732477
Target SNP: 3:194341790:T:C
Proxy SNP: 3:194062519:C:T
deCODE log10(p): 571.1
deCODE BETA: −0.5
deCODE MSPA: 0
-:-:-:-:-:-:-
1196:1256:1243:1254:863:728:7

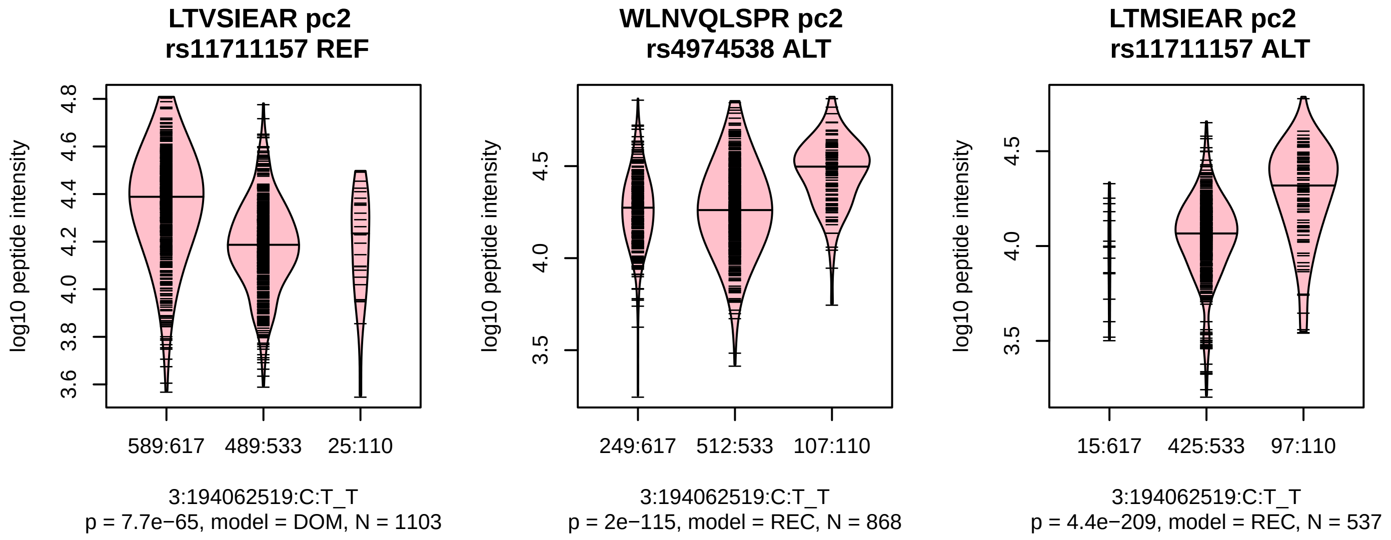

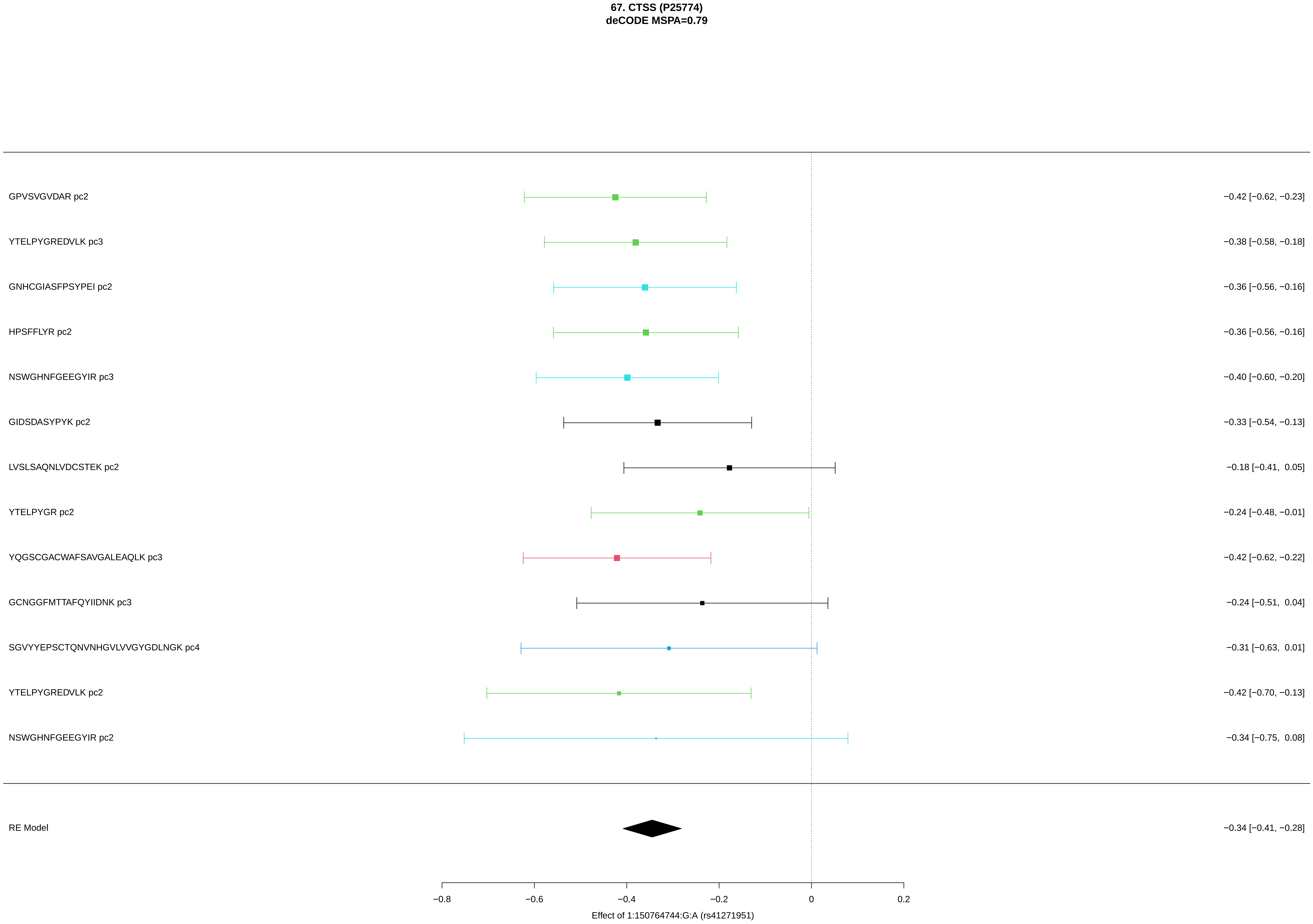
67. CTSS (P25774)
deCODE MSPA=0.79
GPVSVGVDAR pc2
−0.42 [−0.62, −0.23]
YTELPYGREDVLK pc3
−0.38 [−0.58, −0.18]
GNHCGIASFPSYPEI pc2
−0.36 [−0.56, −0.16]
HPSFFLYR pc2
−0.36 [−0.56, −0.16]
NSWGHNFGEEGYIR pc3
−0.40 [−0.60, −0.20]
GIDSDASYPYK pc2
−0.33 [−0.54, −0.13]
LVSLSAQNLVDCSTEK pc2
−0.18 [−0.41, 0.05]
YTELPYGR pc2
−0.24 [−0.48, −0.01]
YQGSCGACWAFSAVGALEAQLK pc3
−0.42 [−0.62, −0.22]
GCNGGFMTTAFQYIIDNK pc3
−0.24 [−0.51, 0.04]
SGVYYEPSCTQNVNHGVLVVGYGDLNGK pc4
−0.31 [−0.63, 0.01]
YTELPYGREDVLK pc2
−0.42 [−0.70, −0.13]
NSWGHNFGEEGYIR pc2
−0.34 [−0.75, 0.08]
RE Model
−0.34 [−0.41, −0.28]
−0.8
−0.6
−0.4
−0.2
0
0.2
Effect of 1:150764744:G:A (rs41271951)

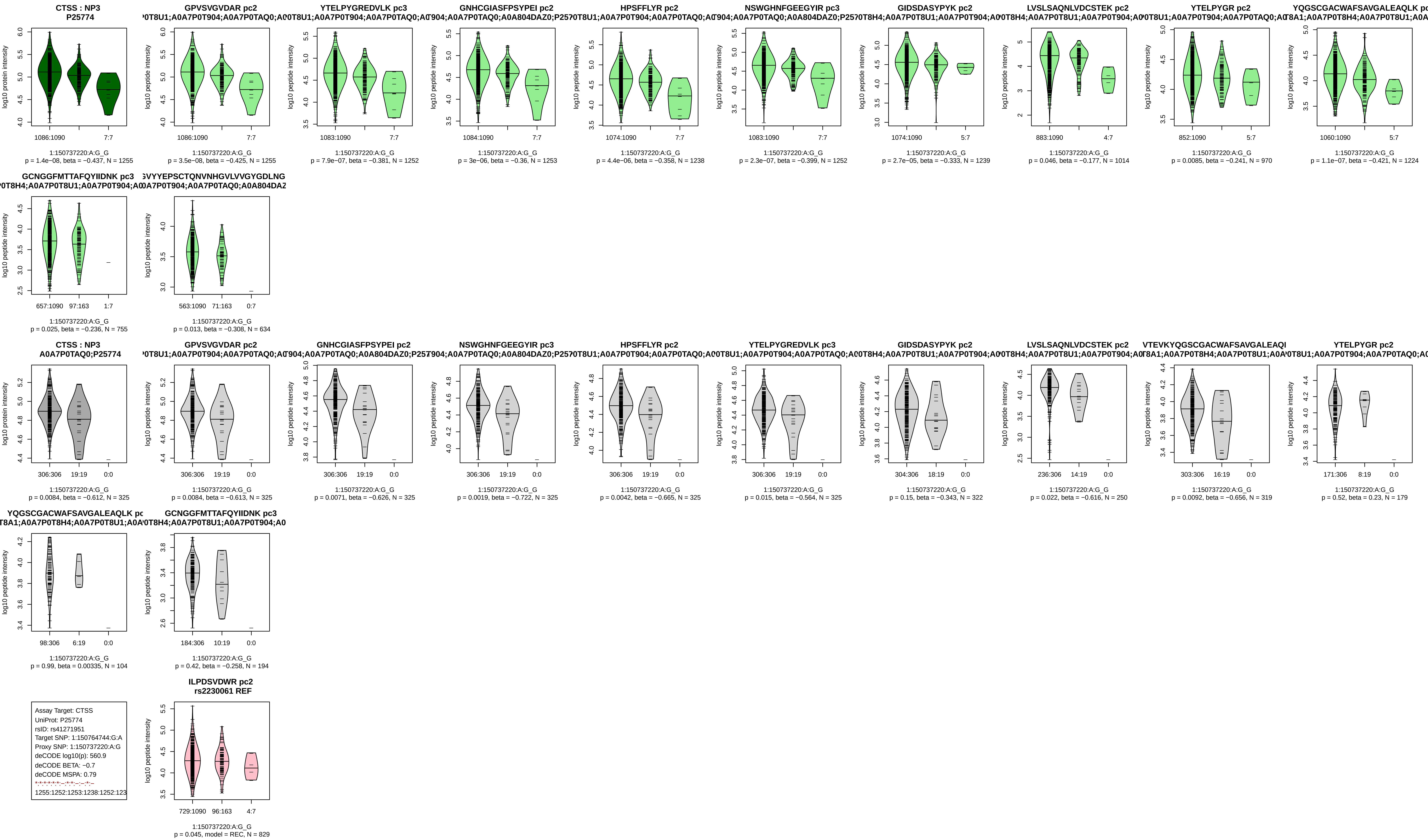
CTSS : NP3
P25774
log10 protein intensity
1086:1090
7:7
1:150737220:A:G_G
p = 1.4e−08, beta = −0.437, N = 1255
GPVSVGVDAR pc2
log10 peptide intensity
1086:1090
7:7
1:150737220:A:G_G
p = 3.5e−08, beta = −0.425, N = 1255
YTELPYGREDVLK pc3
1083:1090
7:7
1:150737220:A:G_G
p = 7.9e−07, beta = −0.381, N = 1252
GNHCGIASFPSYPEI pc2
1084:1090
7:7
1:150737220:A:G_G
p = 3e−06, beta = −0.36, N = 1253
HPSFFLYR pc2
1074:1090
7:7
1:150737220:A:G_G
p = 4.4e−06, beta = −0.358, N = 1238
NSWGHNFGEEGYIR pc3
1083:1090
7:7
1:150737220:A:G_G
p = 2.3e−07, beta = −0.399, N = 1252
GIDSDASYPYK pc2
1074:1090
5:7
1:150737220:A:G_G
p = 2.7e−05, beta = −0.333, N = 1239
LVSLSAQNLVDCSTEK pc2
883:1090
4:7
1:150737220:A:G_G
p = 0.046, beta = −0.177, N = 1014
YTELPYGR pc2
852:1090
5:7
1:150737220:A:G_G
p = 0.0085, beta = −0.241, N = 970
YQGSCGACWAFSAVGALEAQLK pc
1060:1090
5:7
1:150737220:A:G_G
p = 1.1e−07, beta = −0.421, N = 1224
GCNGGFMTTAFQYIIDNK pc3
657:1090
97:163
1:7
1:150737220:A:G_G
p = 0.025, beta = −0.236, N = 755
GVYYEPSCTQNVNHGVLVVGYGDLNG
563:1090
71:163
0:7
1:150737220:A:G_G
p = 0.013, beta = −0.308, N = 634
CTSS : NP3
A0A7P0TAQ0;P25774
306:306
19:19
0:0
1:150737220:A:G_G
p = 0.0084, beta = −0.612, N = 325
GPVSVGVDAR pc2
306:306
19:19
0:0
1:150737220:A:G_G
p = 0.0084, beta = −0.613, N = 325
GNHCGIASFPSYPEI pc2
306:306
19:19
0:0
1:150737220:A:G_G
p = 0.0071, beta = −0.626, N = 325
NSWGHNFGEEGYIR pc3
306:306
19:19
0:0
1:150737220:A:G_G
p = 0.0019, beta = −0.722, N = 325
HPSFFLYR pc2
306:306
19:19
0:0
1:150737220:A:G_G
p = 0.0042, beta = −0.665, N = 325
YTELPYGREDVLK pc3
306:306
19:19
0:0
1:150737220:A:G_G
p = 0.015, beta = −0.564, N = 325
GIDSDASYPYK pc2
304:306
18:19
0:0
1:150737220:A:G_G
p = 0.15, beta = −0.343, N = 322
LVSLSAQNLVDCSTEK pc2
236:306
14:19
0:0
1:150737220:A:G_G
p = 0.022, beta = −0.616, N = 250
VTEVKYQGSCGACWAFSAVGALEAQI
303:306
16:19
0:0
1:150737220:A:G_G
p = 0.0092, beta = −0.656, N = 319
YTELPYGR pc2
171:306
8:19
0:0
1:150737220:A:G_G
p = 0.52, beta = 0.23, N = 179
YQGSCGACWAFSAVGALEAQLK pc
98:306
6:19
0:0
1:150737220:A:G_G
p = 0.99, beta = 0.00335, N = 104
GCNGGFMTTAFQYIIDNK pc3
184:306
10:19
0:0
1:150737220:A:G_G
p = 0.42, beta = −0.258, N = 194
Assay Target: CTSS
UniProt: P25774
rsID: rs41271951
Target SNP: 1:150764744:G:A
Proxy SNP: 1:150737220:A:G
deCODE log10(p): 560.9
deCODE BETA: −0.7
deCODE MSPA: 0.79
1255:1252:1253:1238:1252:123
ILPDSVDWR pc2
rs2230061 REF
729:1090
96:163
4:7
1:150737220:A:G_G
p = 0.045, model = REC, N = 829

68. OGN (P20774)
deCODE MSPA=0.589

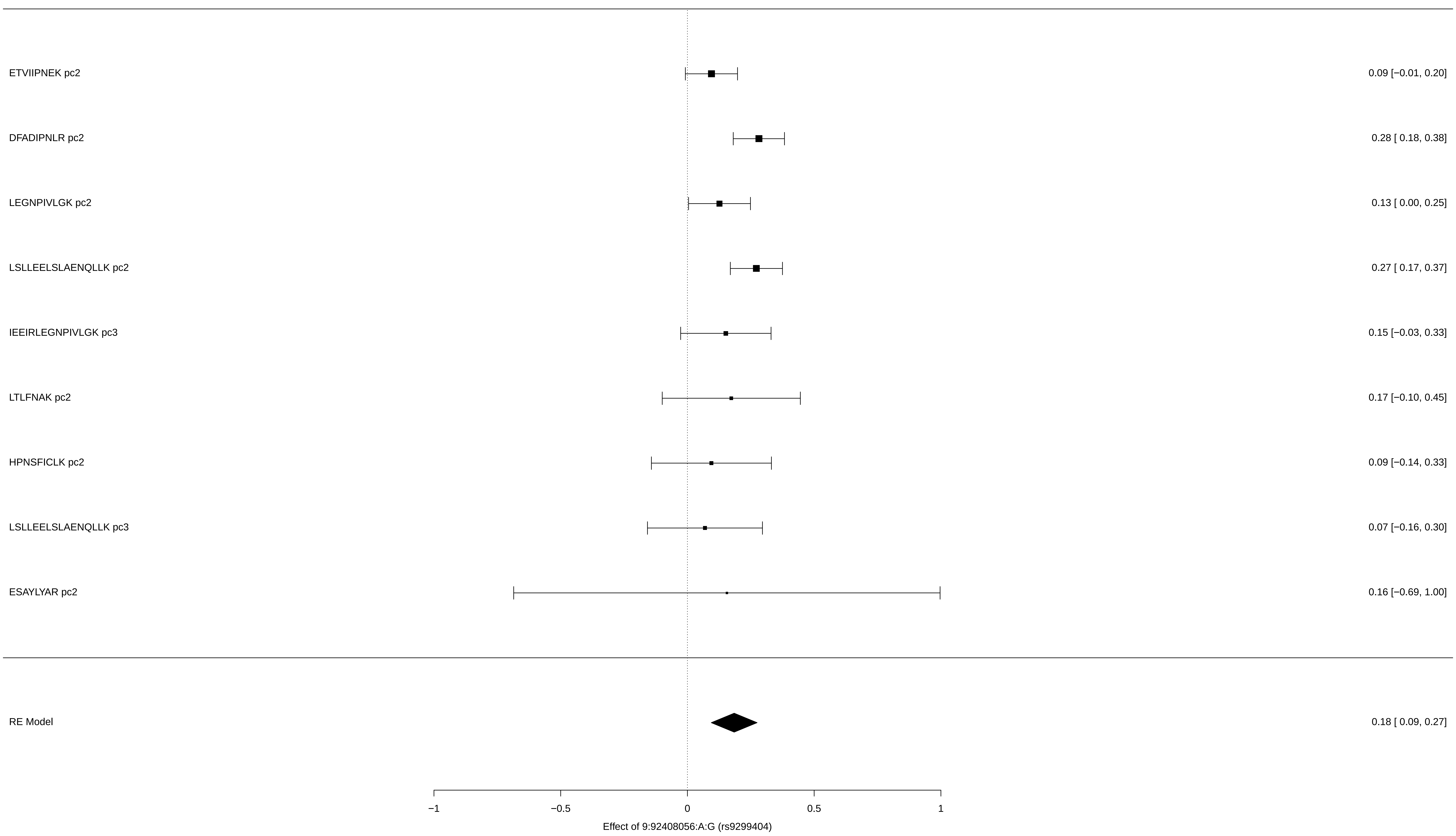

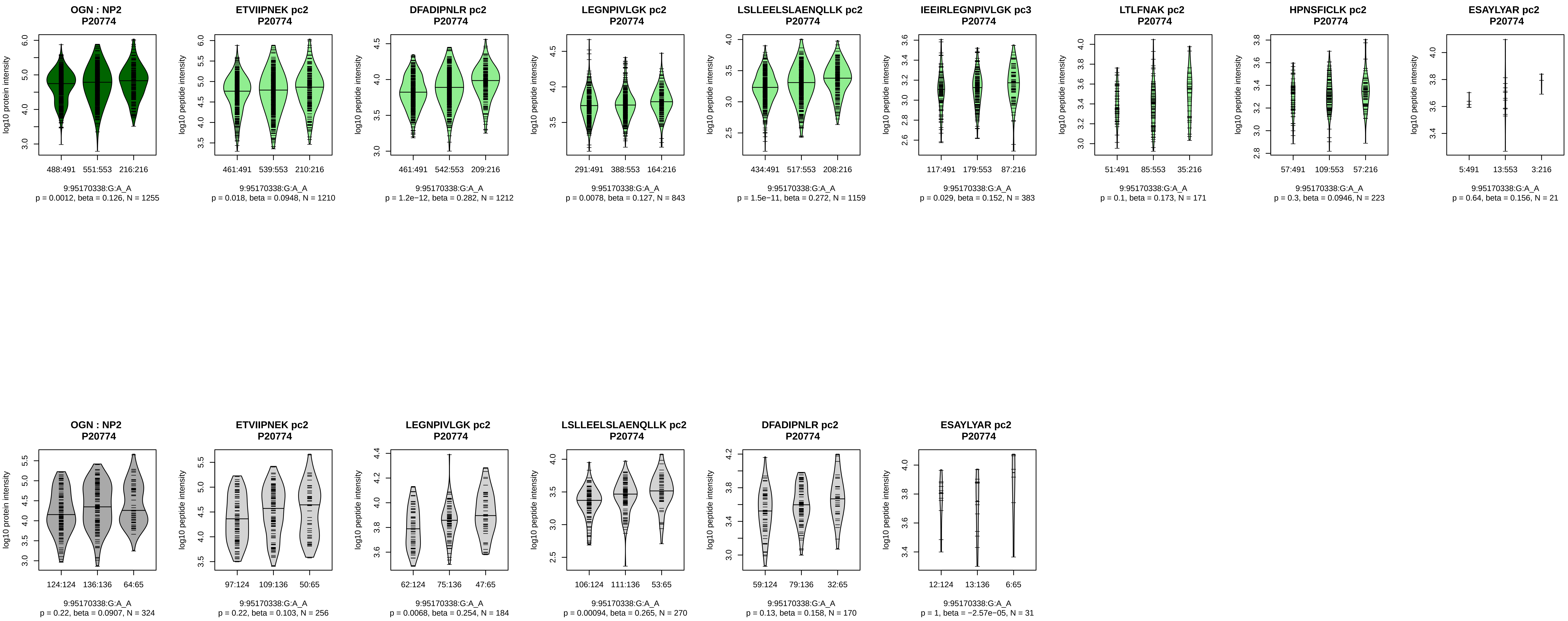

Assay Target: OGN
UniProt: P20774
rsID: rs9299404
Target SNP: 9:92408056:A:G
Proxy SNP: 9:95170338:G:A
deCODE log10(p): 550.6
deCODE BETA: 0.39
deCODE MSPA: 0.589
–:*:*:*:–:–:–:–:–
1210:1212:843:1159:383:171:22

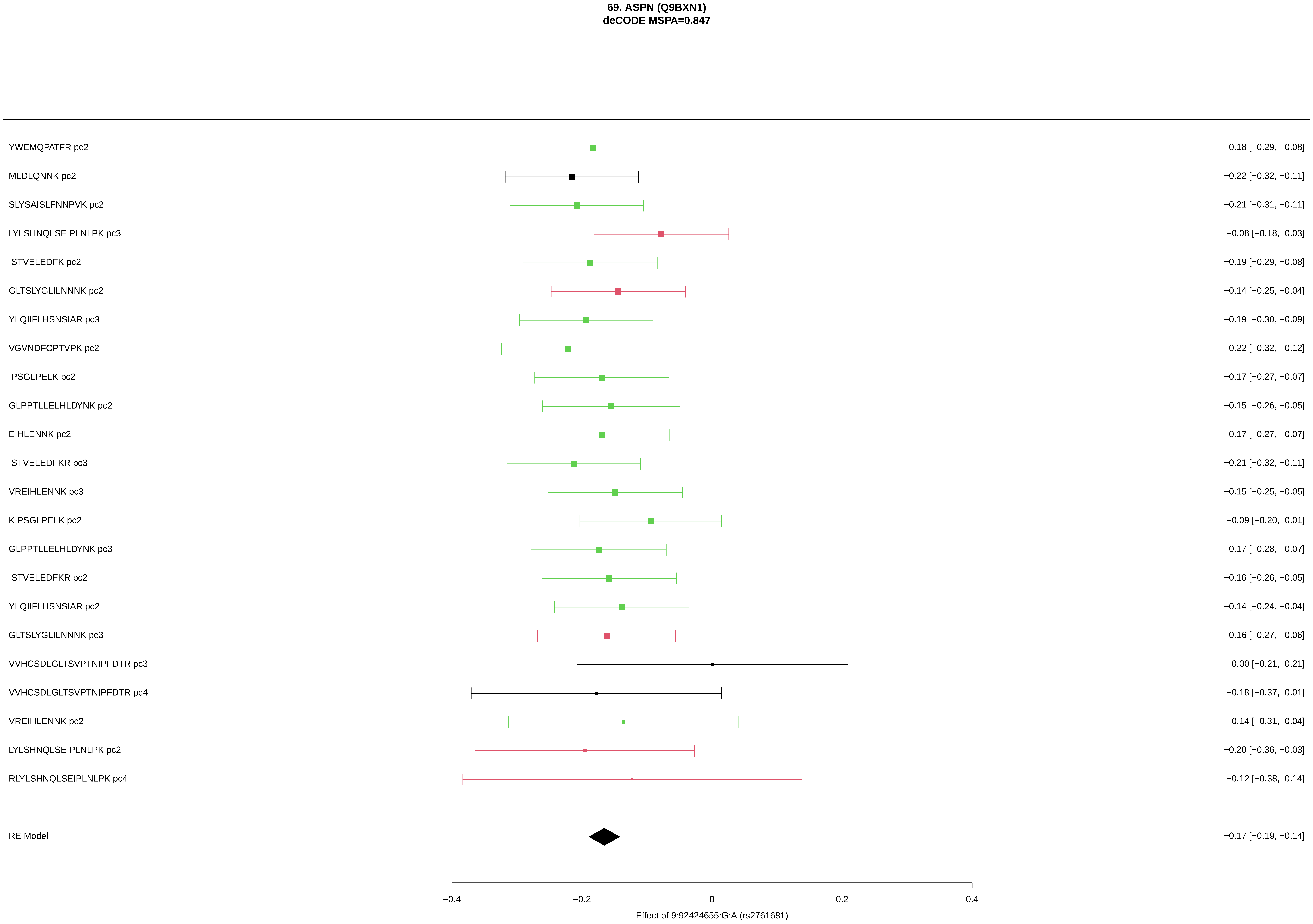
69. ASPN (Q9BXN1)
deCODE MSPA=0.847
YWEMQPATFR pc2
−0.18 [−0.29, −0.08]
MLDLQNNK pc2
−0.22 [−0.32, −0.11]
SLYSAISLFNNPVK pc2
−0.21 [−0.31, −0.11]
LYLSHNQLSEIPLNLPK pc3
−0.08 [−0.18, 0.03]
ISTVELEDFK pc2
−0.19 [−0.29, −0.08]
GLTSLYGLILNNNK pc2
−0.14 [−0.25, −0.04]
YLQIIFLHSNSIAR pc3
−0.19 [−0.30, −0.09]
VGVNDFCPTVPK pc2
−0.22 [−0.32, −0.12]
IPSGLPELK pc2
−0.17 [−0.27, −0.07]
GLPPTLLELHLDYNK pc2
−0.15 [−0.26, −0.05]
EIHLENNK pc2
−0.17 [−0.27, −0.07]
ISTVELEDFKR pc3
−0.21 [−0.32, −0.11]
VREIHLENNK pc3
−0.15 [−0.25, −0.05]
KIPSGLPELK pc2
−0.09 [−0.20, 0.01]
GLPPTLLELHLDYNK pc3
−0.17 [−0.28, −0.07]
ISTVELEDFKR pc2
−0.16 [−0.26, −0.05]
YLQIIFLHSNSIAR pc2
−0.14 [−0.24, −0.04]
GLTSLYGLILNNNK pc3
−0.16 [−0.27, −0.06]
VVHCSDLGLTSVPTNIPFDTR pc3
0.00 [−0.21, 0.21]
VVHCSDLGLTSVPTNIPFDTR pc4
−0.18 [−0.37, 0.01]
VREIHLENNK pc2
−0.14 [−0.31, 0.04]
LYLSHNQLSEIPLNLPK pc2
−0.20 [−0.36, −0.03]
RLYLSHNQLSEIPLNLPK pc4
−0.12 [−0.38, 0.14]
RE Model
−0.17 [−0.19, −0.14]
−0.4
−0.2
0
0.2
0.4
Effect of 9:92424655:G:A (rs2761681)

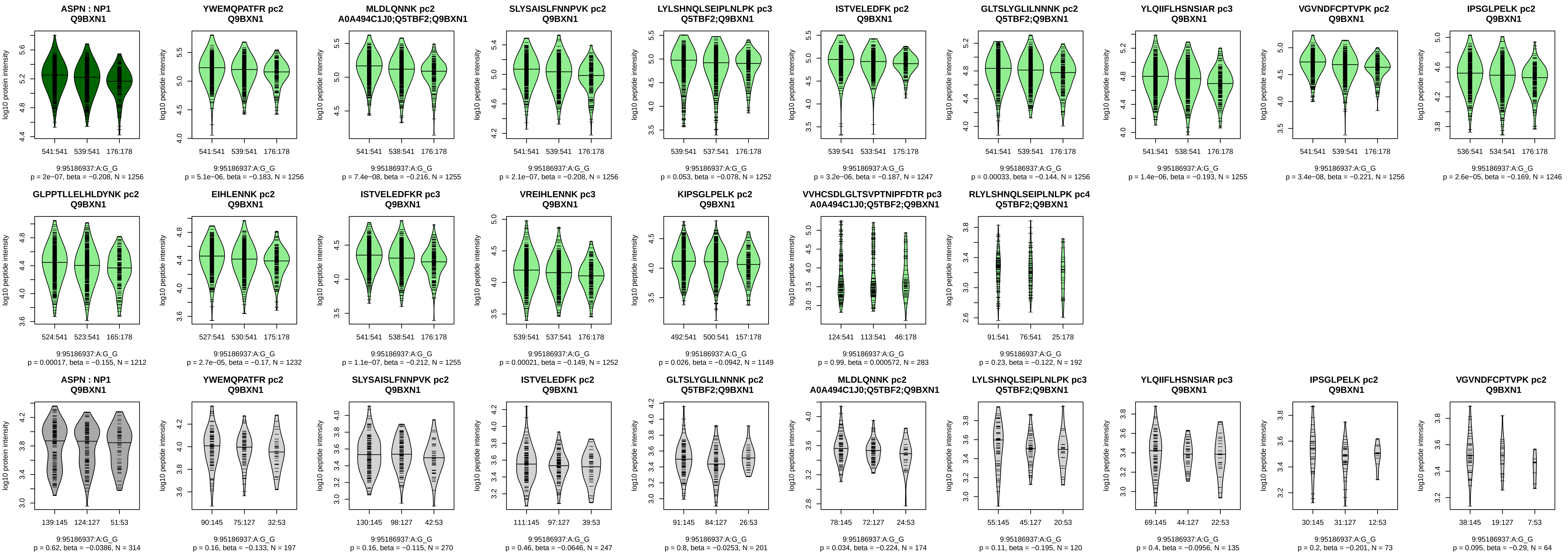

Assay Target: ASPN
UniProt: Q9BXN1
rsID: rs2761681
Target SNP: 9:92424655:G:A
Proxy SNP: 9:95186937:A:G
deCODE log10(p): 490.7
deCODE BETA: −0.39
deCODE MSPA: 0.847
[illegible]
1256:1255:1256:1252:1247:125

70. OAF (Q86UD1)
deCODE MSPA=0.977

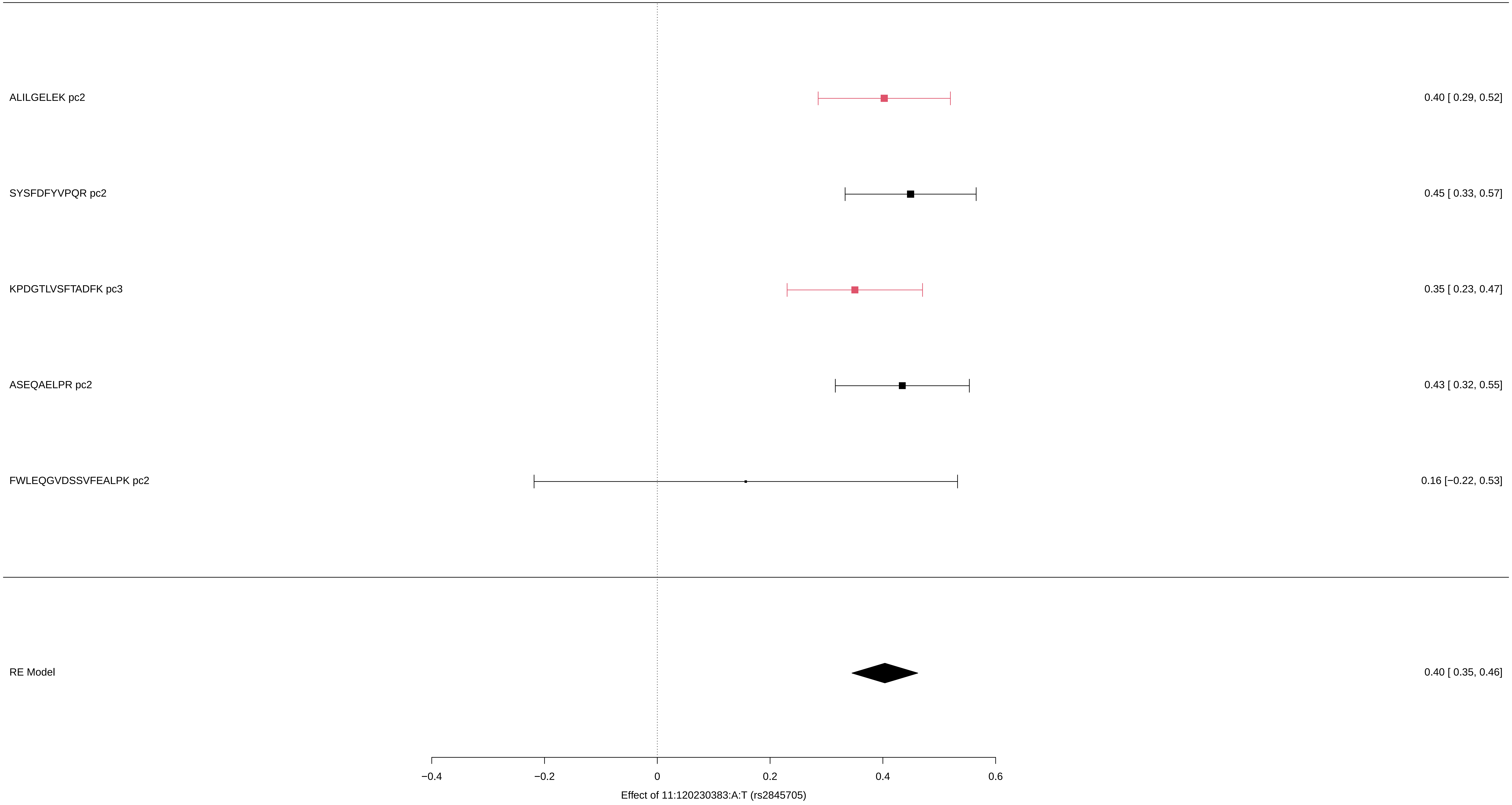

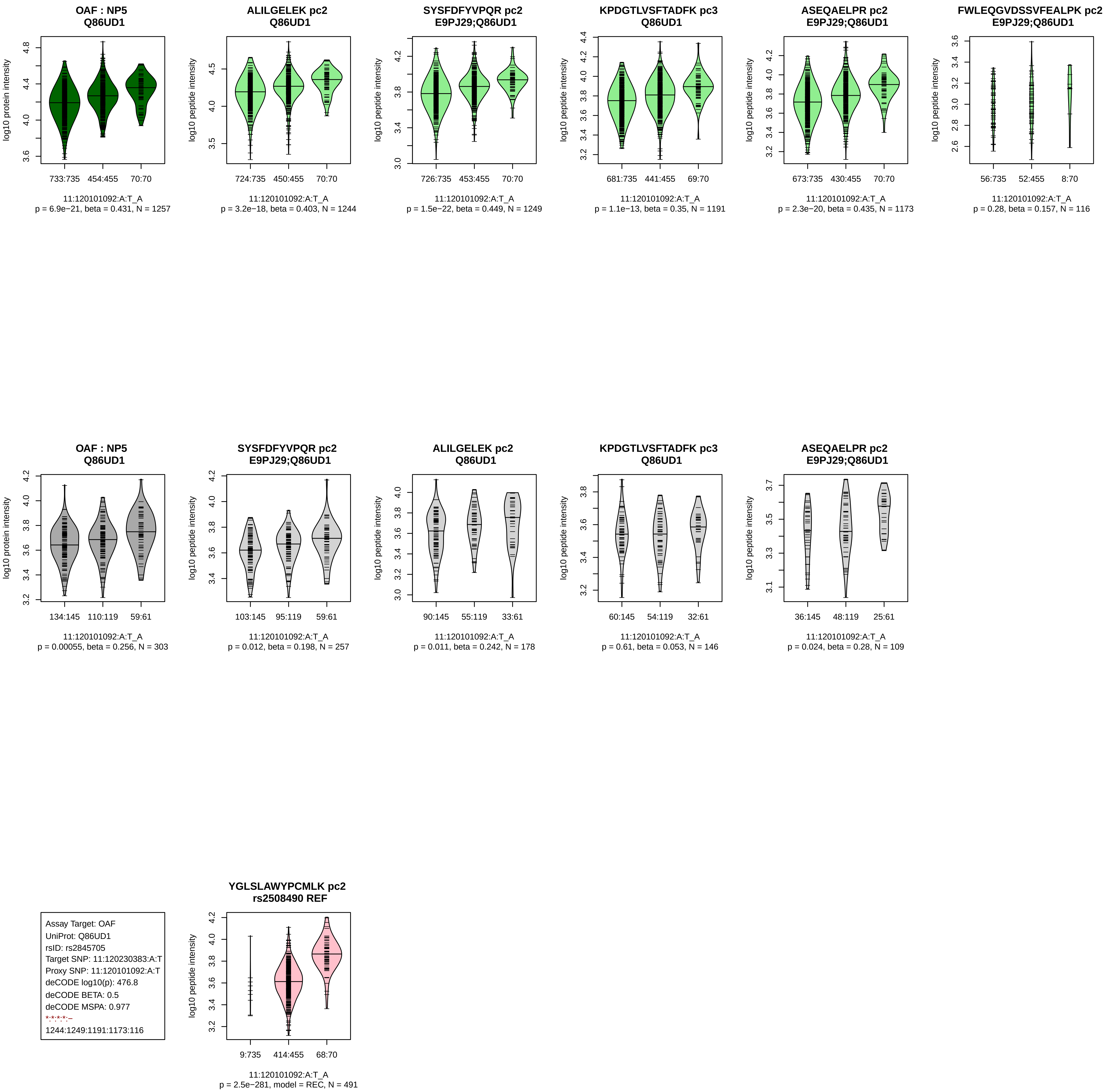
OAF : NP5
Q86UD1
log10 protein intensity
733:735 454:455 70:70
11:120101092:A:T_A
p = 6.9e−21, beta = 0.431, N = 1257
ALILGELEK pc2
Q86UD1
log10 peptide intensity
724:735 450:455 70:70
11:120101092:A:T_A
p = 3.2e−18, beta = 0.403, N = 1244
SYSFDFYVPQR pc2
E9PJ29;Q86UD1
log10 peptide intensity
726:735 453:455 70:70
11:120101092:A:T_A
p = 1.5e−22, beta = 0.449, N = 1249
KPDGTLVSFTADFK pc3
Q86UD1
log10 peptide intensity
681:735 441:455 69:70
11:120101092:A:T_A
p = 1.1e−13, beta = 0.35, N = 1191
ASEQAELPR pc2
E9PJ29;Q86UD1
log10 peptide intensity
673:735 430:455 70:70
11:120101092:A:T_A
p = 2.3e−20, beta = 0.435, N = 1173
FWLEQGVDSSVFEALPK pc2
E9PJ29;Q86UD1
log10 peptide intensity
56:735 52:455 8:70
11:120101092:A:T_A
p = 0.28, beta = 0.157, N = 116
OAF : NP5
Q86UD1
log10 protein intensity
134:145 110:119 59:61
11:120101092:A:T_A
p = 0.00055, beta = 0.256, N = 303
SYSFDFYVPQR pc2
E9PJ29;Q86UD1
log10 peptide intensity
103:145 95:119 59:61
11:120101092:A:T_A
p = 0.012, beta = 0.198, N = 257
ALILGELEK pc2
Q86UD1
log10 peptide intensity
90:145 55:119 33:61
11:120101092:A:T_A
p = 0.011, beta = 0.242, N = 178
KPDGTLVSFTADFK pc3
Q86UD1
log10 peptide intensity
60:145 54:119 32:61
11:120101092:A:T_A
p = 0.61, beta = 0.053, N = 146
ASEQAELPR pc2
E9PJ29;Q86UD1
log10 peptide intensity
36:145 48:119 25:61
11:120101092:A:T_A
p = 0.024, beta = 0.28, N = 109
Assay Target: OAF
UniProt: Q86UD1
rsID: rs2845705
Target SNP: 11:120230383:A:T
Proxy SNP: 11:120101092:A:T
deCODE log10(p): 476.8
deCODE BETA: 0.5
deCODE MSPA: 0.977
1244:1249:1191:1173:116
YGLSLAWYPCMLK pc2
rs2508490 REF
log10 peptide intensity
9:735 414:455 68:70
11:120101092:A:T_A
p = 2.5e−281, model = REC, N = 491

71. GDF15 (Q99988)
deCODE MSPA=0

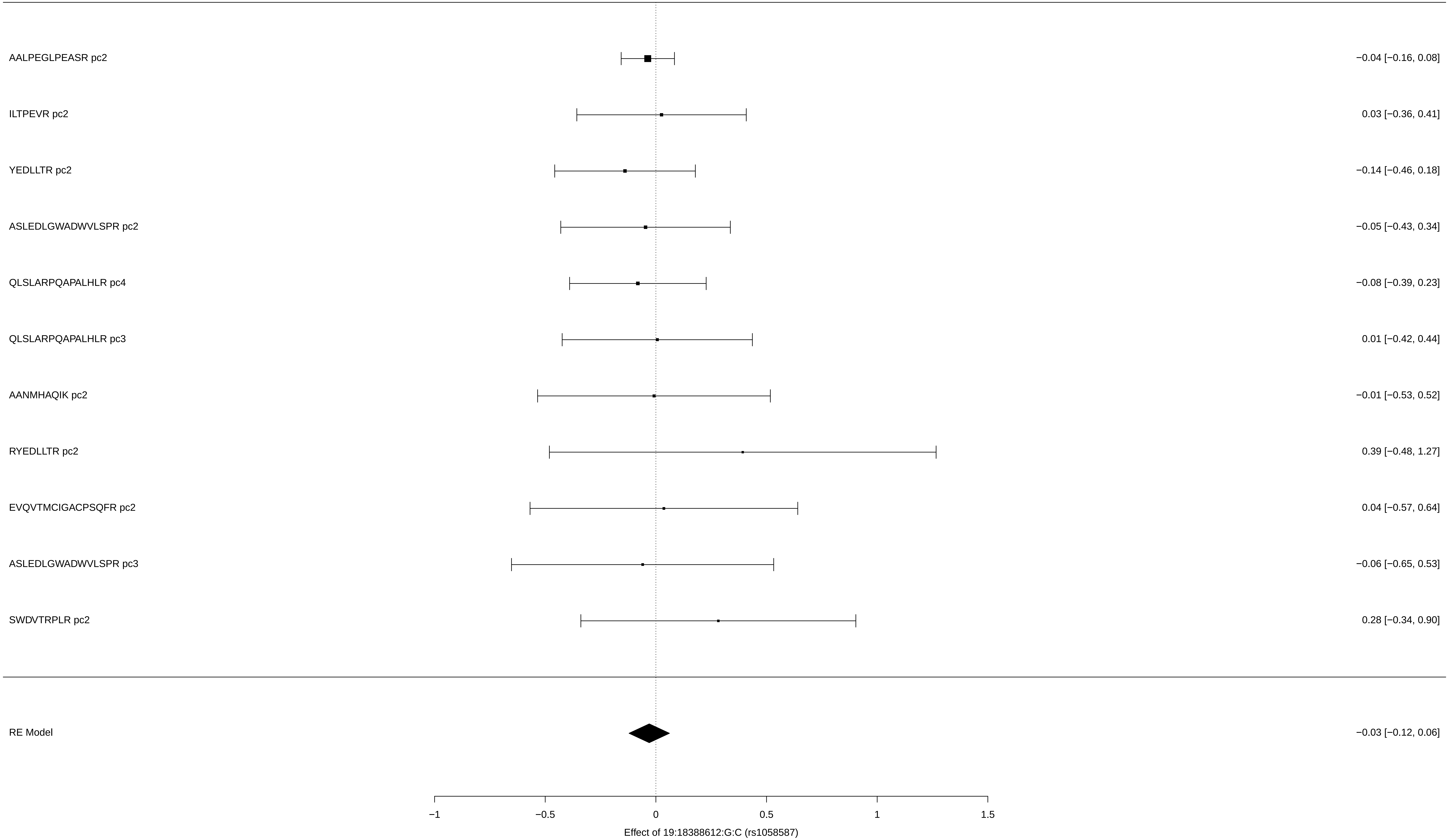

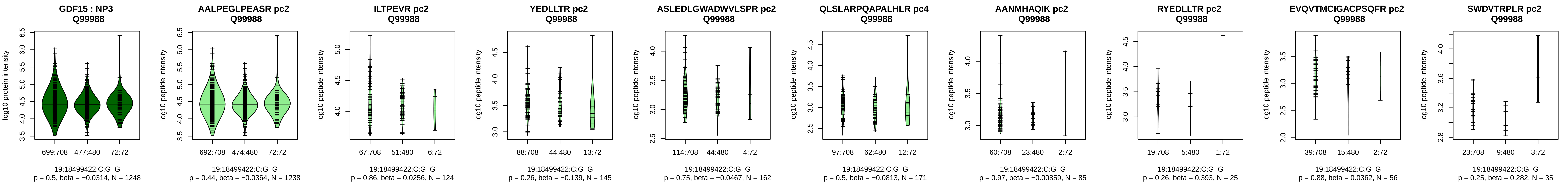

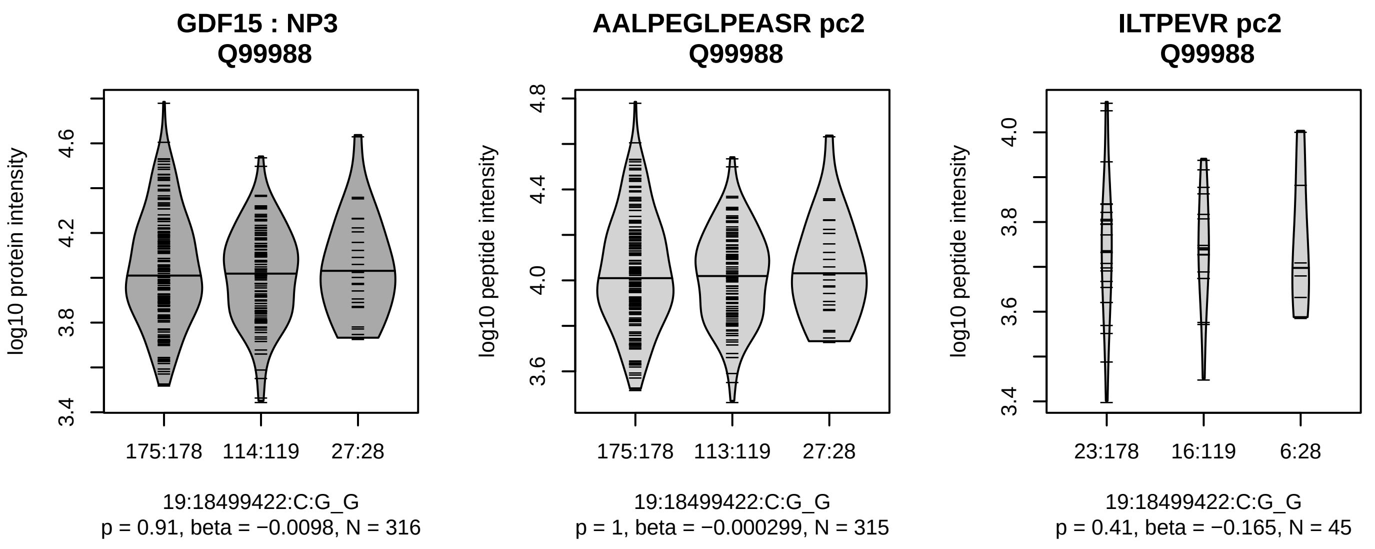

Assay Target: GDF15
UniProt: Q99988
rsID: rs1058587
Target SNP: 19:18388612:G:C
Proxy SNP: 19:18499422:C:G
deCODE log10(p): 476.4
deCODE BETA: 0.45
deCODE MSPA: 0
−:−:−:−:−:−:−:NA:−:NA:−:−:−:NA
1238:124:145:162:171:87:85:15

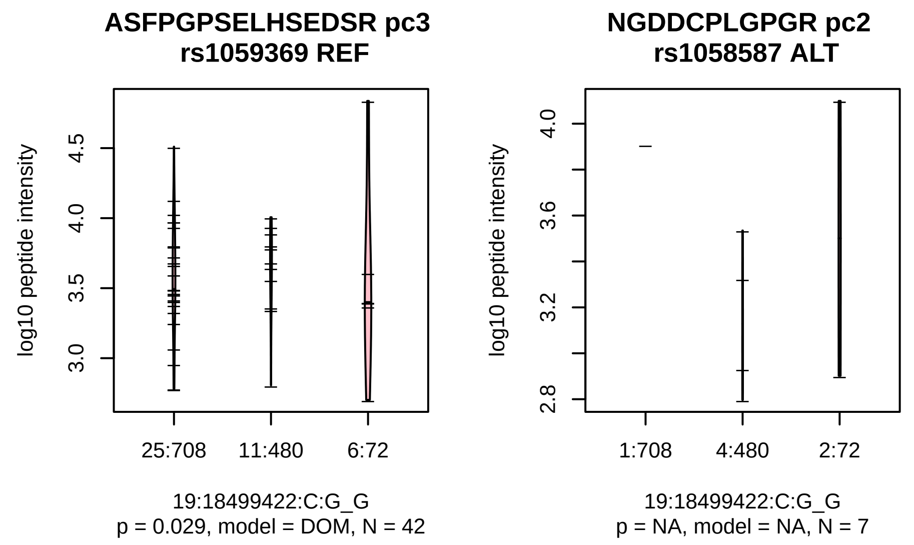

72. GPC1 (P35052)
deCODE MSPA=0

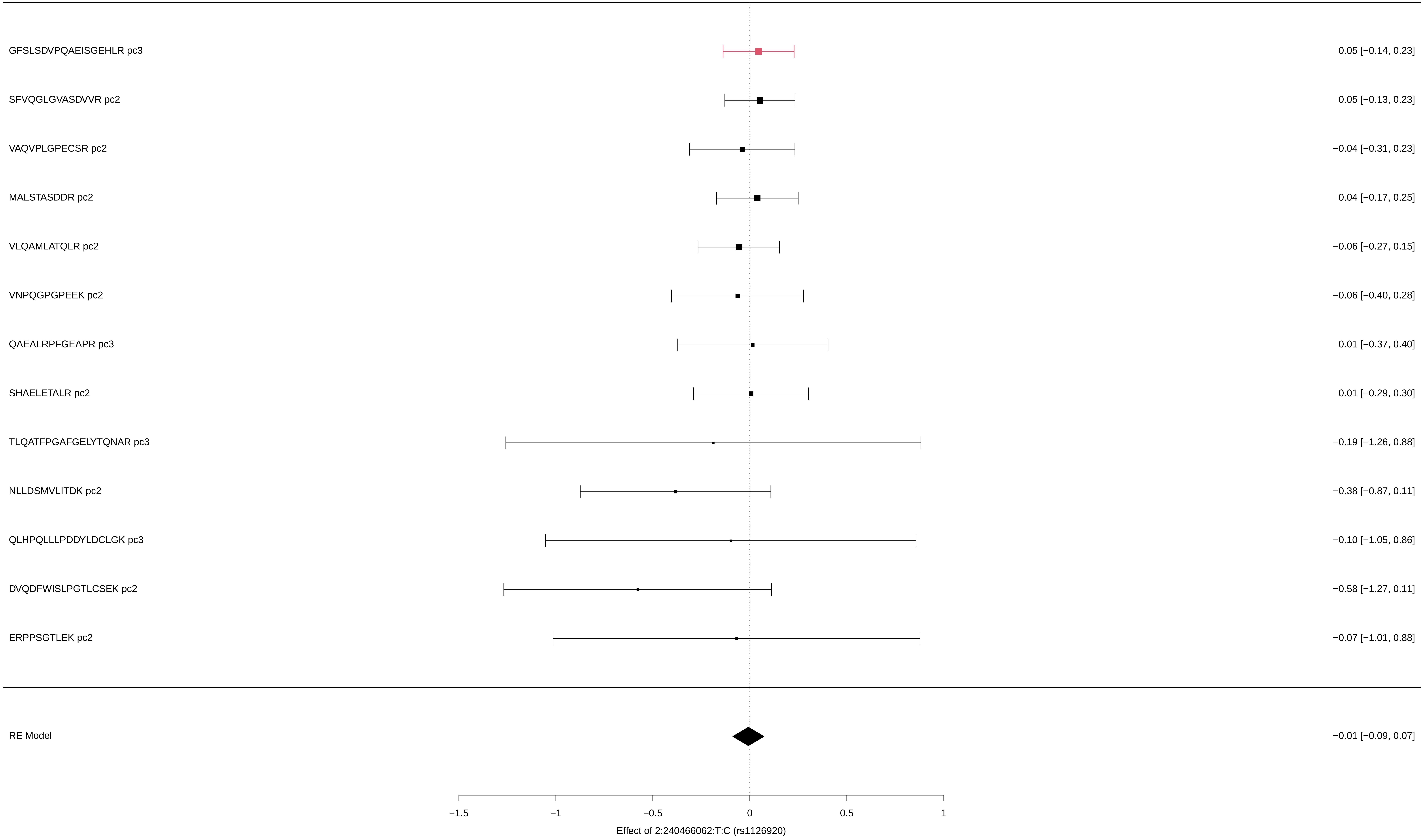

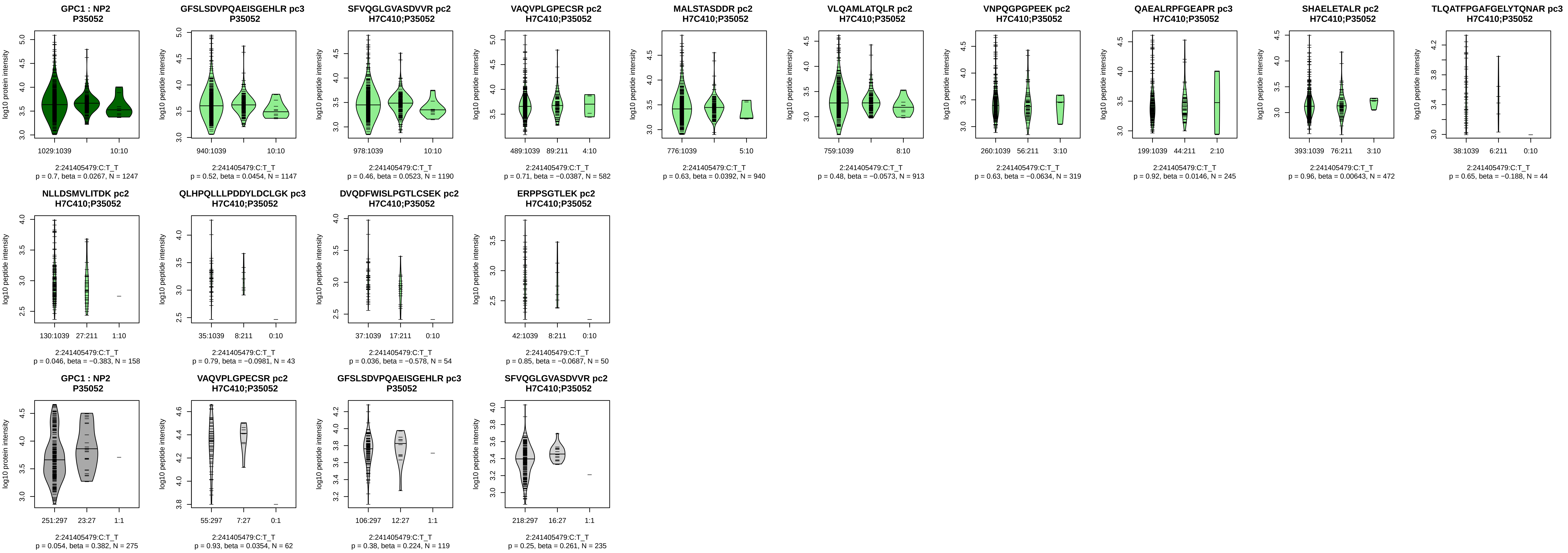

Assay Target: GPC1
UniProt: P35052
rsID: rs1126920
Target SNP: 2:240466062:T:C
Proxy SNP: 2:241405479:C:T
deCODE log10(p): 475.1
deCODE BETA: 0.63
deCODE MSPA: 0
–:–:–:–:–:–:–:–:–:–:–:–:–:NA:NA
1147:1190:582:940:913:319:245

73. ADAMTS5 (Q9UNA0)
deCODE MSPA=0

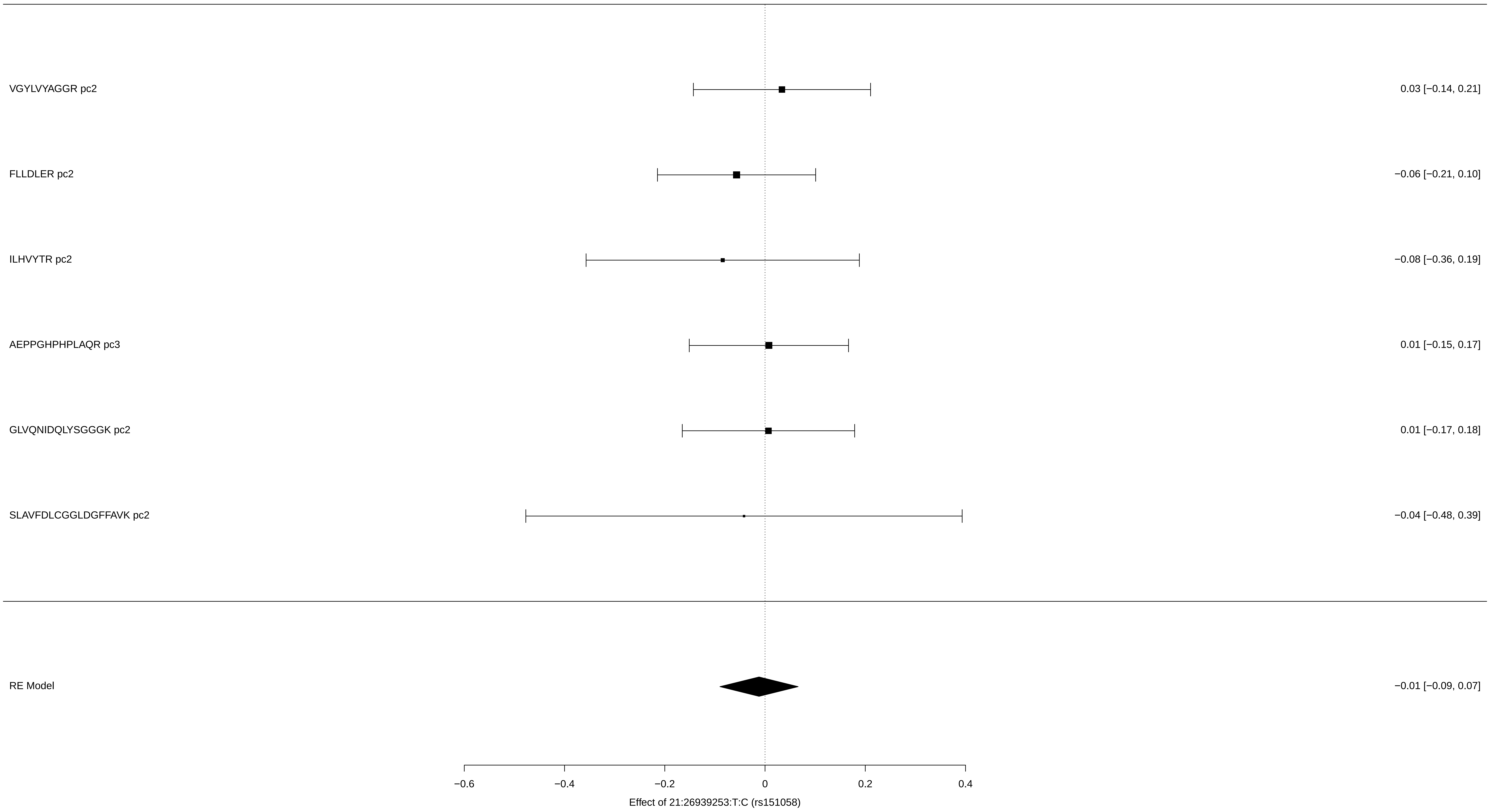

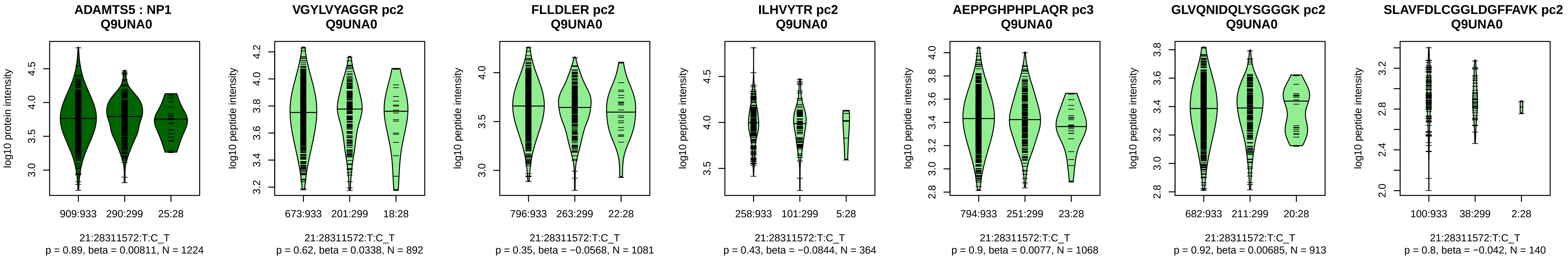

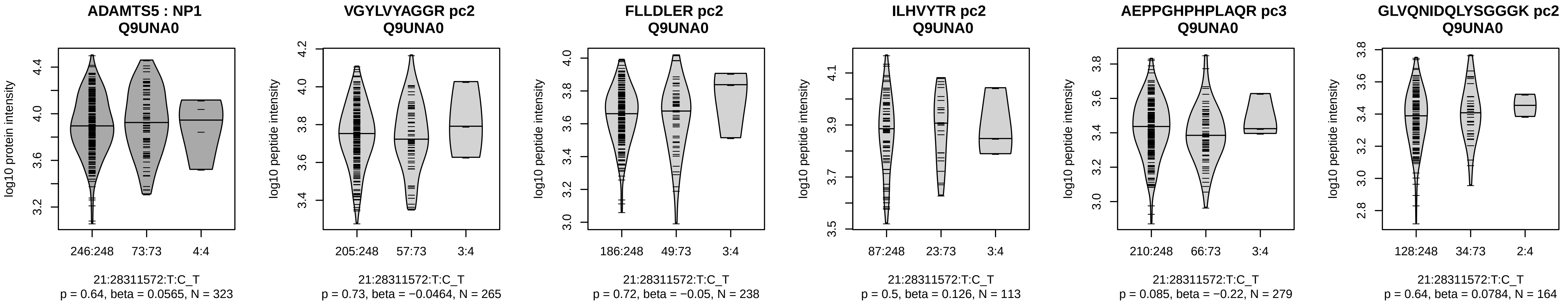

Assay Target: ADAMTS5
UniProt: Q9UNA0
rsID: rs151058
Target SNP: 21:26939253:T:C
Proxy SNP: 21:28311572:T:C
deCODE log10(p): 469
deCODE BETA: −0.56
deCODE MSPA: 0
892:1081:364:1068:913:140

74. SERPINA4 (P29622)
deCODE MSPA=0.9

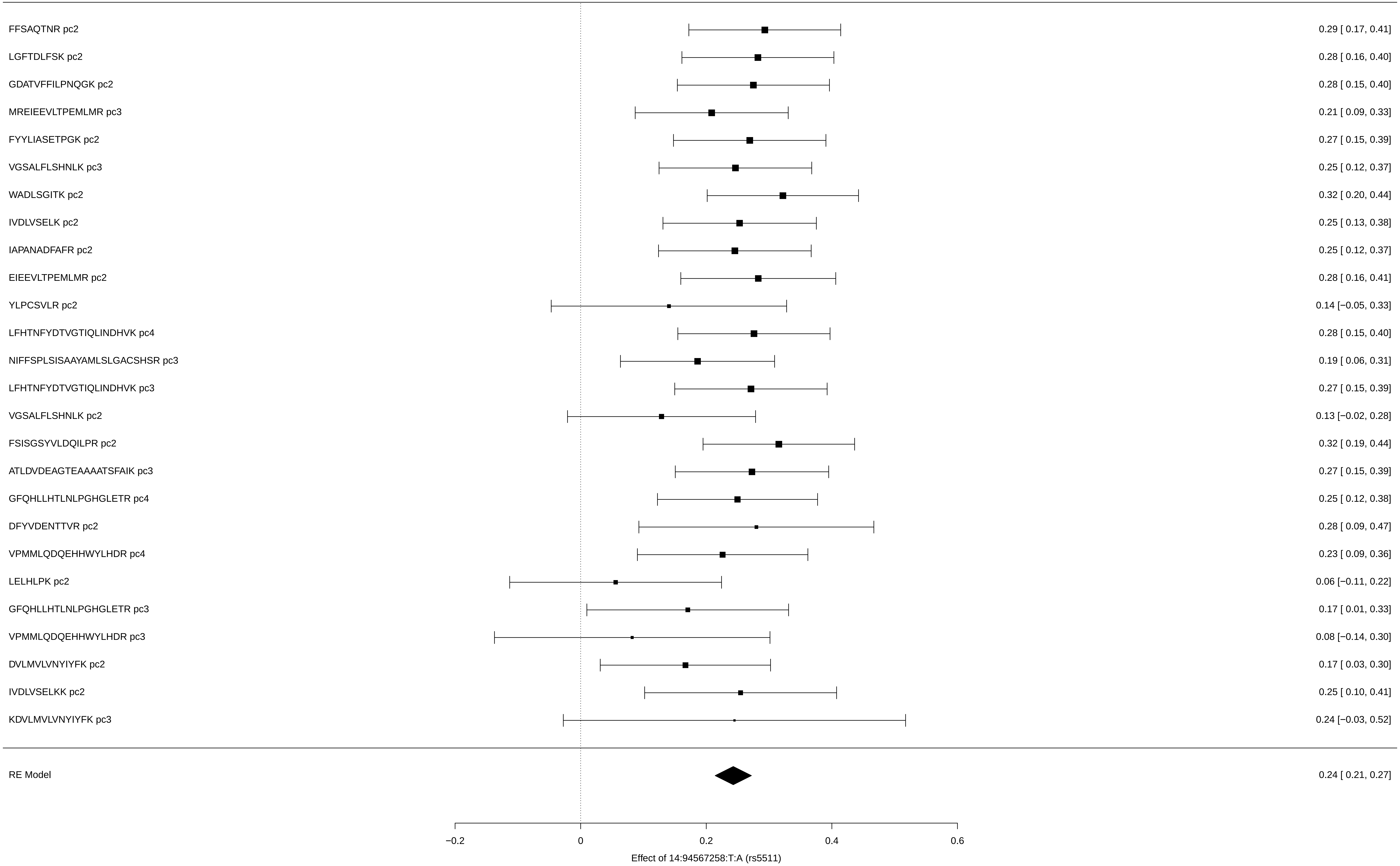

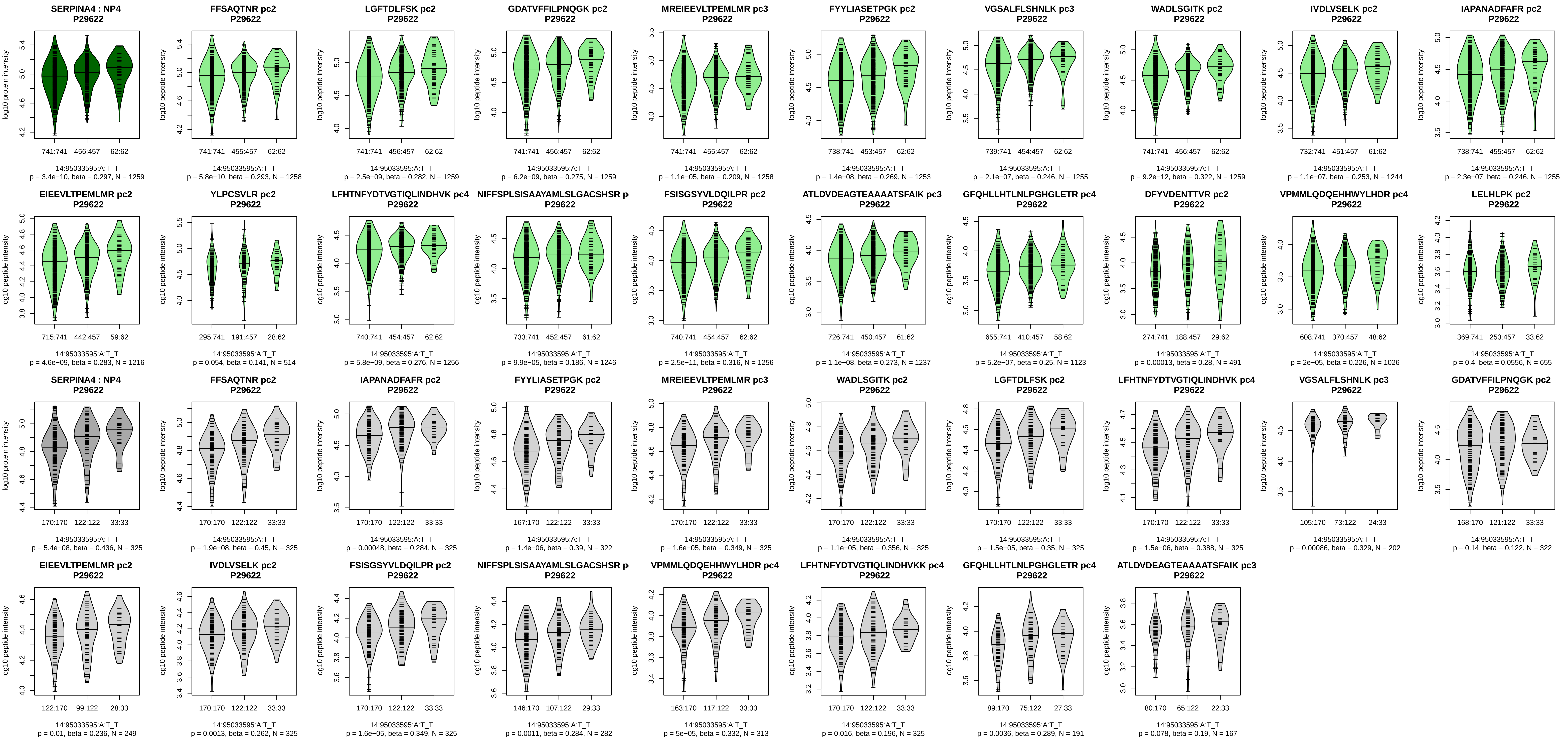

Assay Target: SERPINA4
UniProt: P29622
rsID: rs5511
Target SNP: 14:94567258:T:A
Proxy SNP: 14:95033595:A:T
deCODE log10(p): 465.8
deCODE BETA: 0.45
deCODE MSPA: 0.9
1258:1259:1259:1258:1253:125

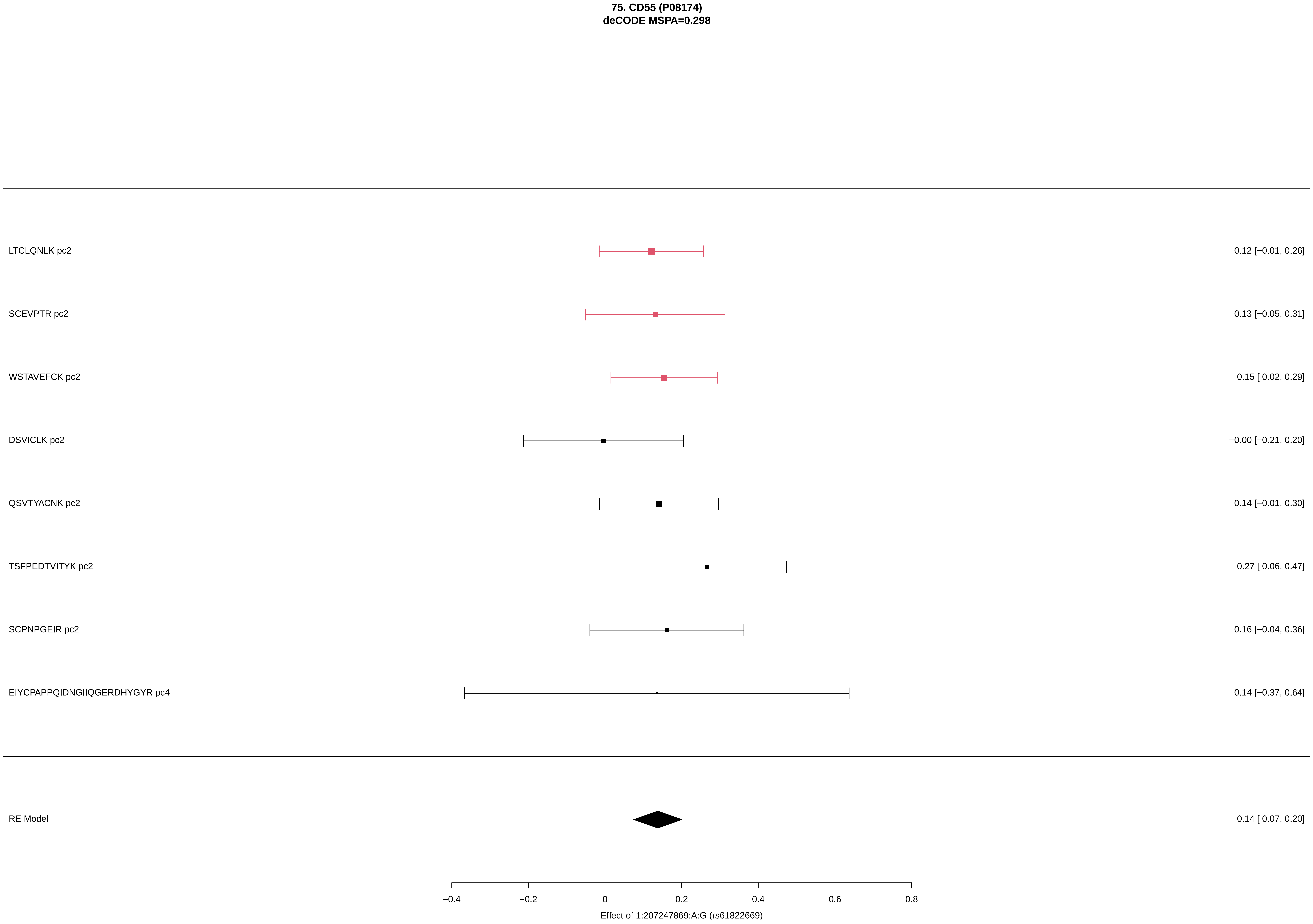

75. CD55 (P08174)
deCODE MSPA=0.298
LTCLQNLK pc2
0.12 [−0.01, 0.26]
SCEVPTR pc2
0.13 [−0.05, 0.31]
WSTAVEFCK pc2
0.15 [0.02, 0.29]
DSVICLK pc2
−0.00 [−0.21, 0.20]
QSVTYACNK pc2
0.14 [−0.01, 0.30]
TSFPEDTVITYK pc2
0.27 [0.06, 0.47]
SCPNPGEIR pc2
0.16 [−0.04, 0.36]
EIYCPAPPQIDNGIIQGERDHYGYR pc4
0.14 [−0.37, 0.64]
RE Model
0.14 [0.07, 0.20]
−0.4
−0.2
0
0.2
0.4
0.6
0.8
Effect of 1:207247869:A:G (rs61822669)

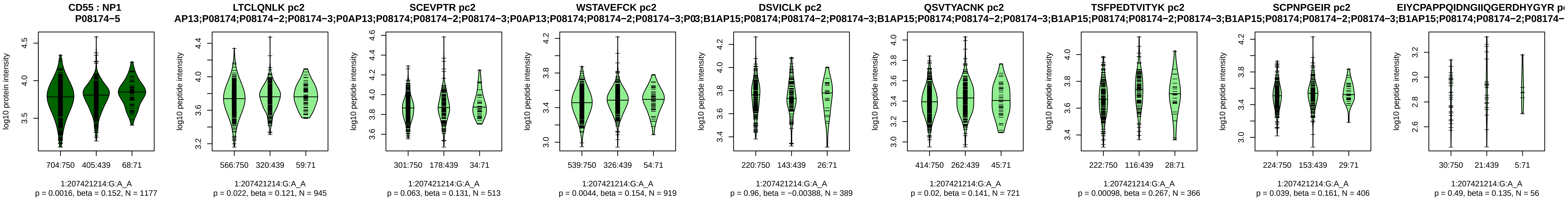

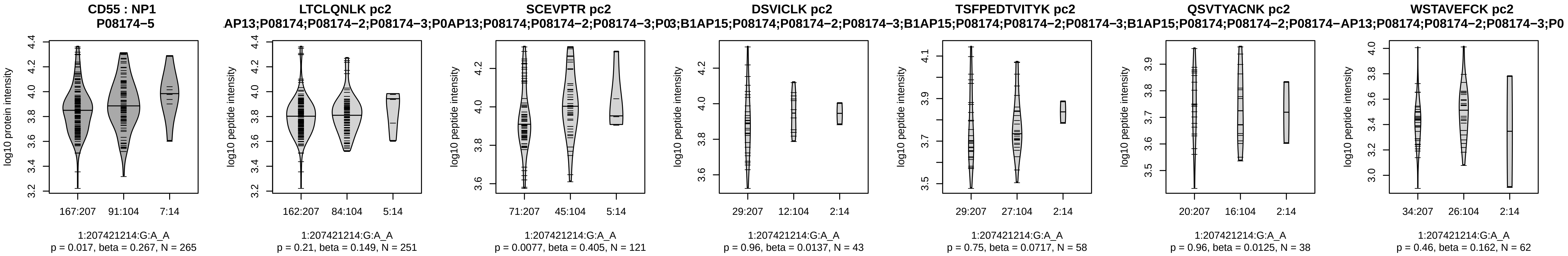

Assay Target: CD55
UniProt: P08174
rsID: rs61822669
Target SNP: 1:207247869:A:G
Proxy SNP: 1:207421214:G:A
deCODE log10(p): 433.9
deCODE BETA: −0.41
deCODE MSPA: 0.298
–:–:*:–:–:*:–:–
945:513:919:389:721:366:406:5

76. SERPINA1 (P01009)
deCODE MSPA=0

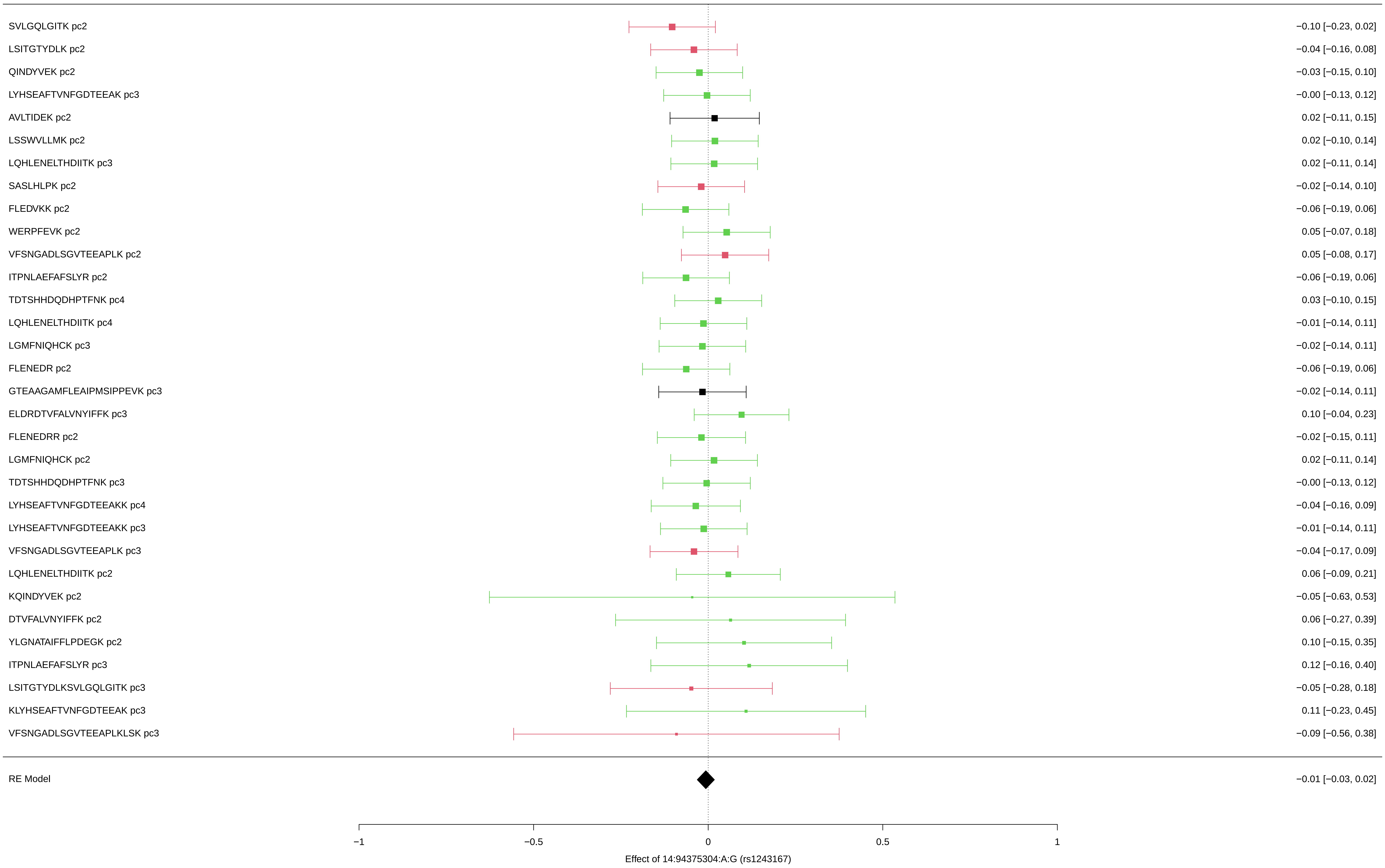

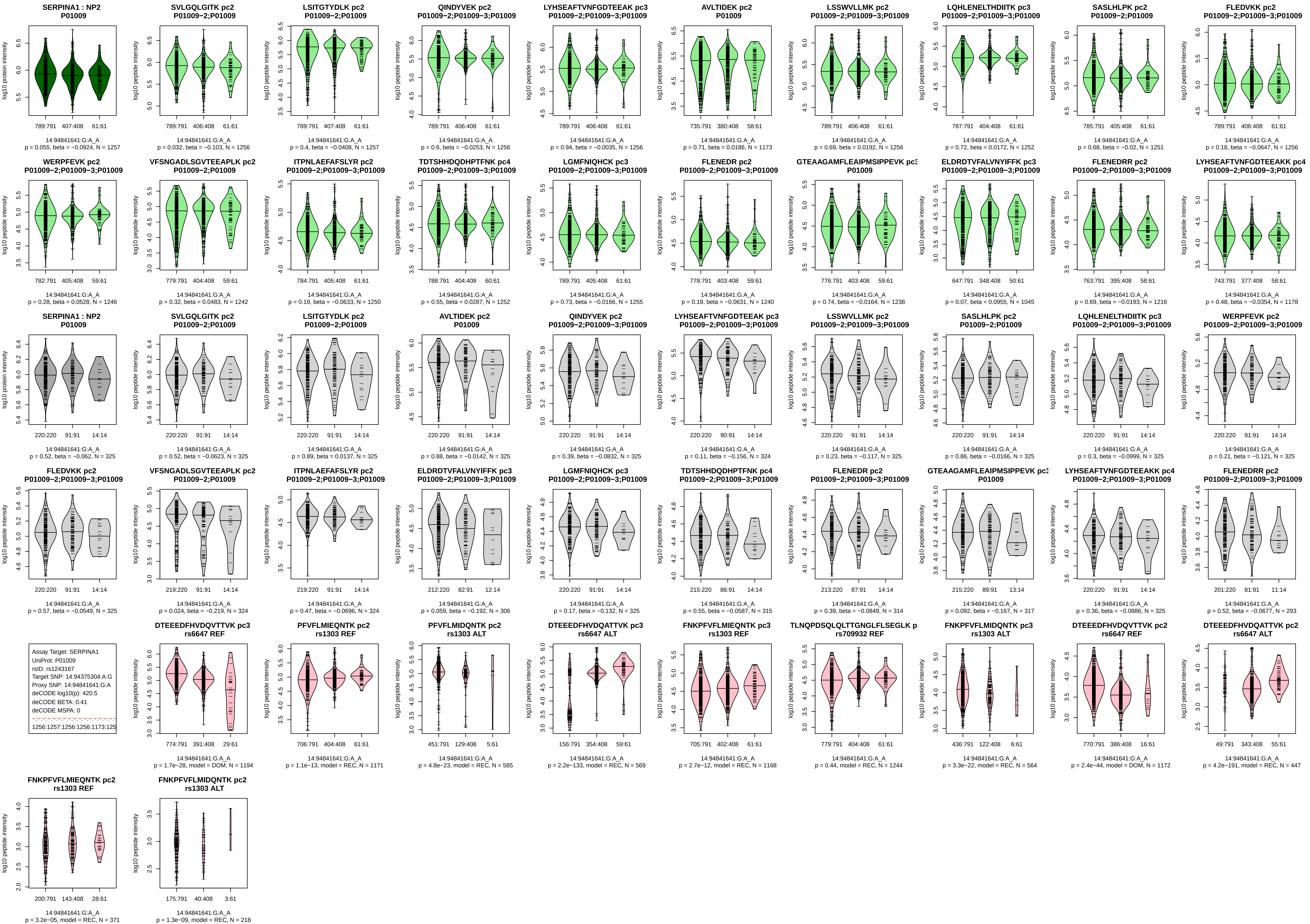
SERPINA1 : NP2
P01009
log10 protein intensity
789:791 407:408 61:61
14:94841641:G:A_A
p = 0.055, beta = −0.0924, N = 1257
SVLGQLGITK pc2
P01009−2;P01009
log10 peptide intensity
789:791 406:408 61:61
14:94841641:G:A_A
p = 0.032, beta = −0.103, N = 1256
LSITGTYDLK pc2
P01009−2;P01009
789:791 407:408 61:61
14:94841641:G:A_A
p = 0.4, beta = −0.0408, N = 1257
QINDYVEK pc2
P01009−2;P01009−3;P01009
789:791 406:408 61:61
14:94841641:G:A_A
p = 0.6, beta = −0.0253, N = 1256
LYHSEAFTVNFGDTEEAK pc3
P01009−2;P01009−3;P01009
789:791 406:408 61:61
14:94841641:G:A_A
p = 0.94, beta = −0.0035, N = 1256
AVLTIDEK pc2
P01009
735:791 380:408 58:61
14:94841641:G:A_A
p = 0.71, beta = 0.0186, N = 1173
LSSWVLLMK pc2
P01009−2;P01009−3;P01009
789:791 406:408 61:61
14:94841641:G:A_A
p = 0.69, beta = 0.0192, N = 1256
LQHLENELTHDIITK pc3
P01009−2;P01009−3;P01009
787:791 404:408 61:61
14:94841641:G:A_A
p = 0.72, beta = 0.0172, N = 1252
SASLHLPK pc2
P01009−2;P01009
785:791 405:408 61:61
14:94841641:G:A_A
p = 0.68, beta = −0.02, N = 1251
FLEDVKK pc2
P01009−2;P01009−3;P01009
789:791 406:408 61:61
14:94841641:G:A_A
p = 0.18, beta = −0.0647, N = 1256
WERPFEVK pc2
P01009−2;P01009−3;P01009
782:791 405:408 59:61
14:94841641:G:A_A
p = 0.28, beta = 0.0528, N = 1246
VFSNGADLSGVTEEAPLK pc2
P01009−2;P01009
779:791 404:408 59:61
14:94841641:G:A_A
p = 0.32, beta = 0.0483, N = 1242
ITPNLAEFAFSLYR pc2
P01009−2;P01009−3;P01009
784:791 405:408 61:61
14:94841641:G:A_A
p = 0.19, beta = −0.0633, N = 1250
TDTSHHDQDHPTFNK pc4
P01009−2;P01009−3;P01009
788:791 404:408 60:61
14:94841641:G:A_A
p = 0.55, beta = 0.0287, N = 1252
LGMFNIQHCK pc3
P01009−2;P01009−3;P01009
789:791 405:408 61:61
14:94841641:G:A_A
p = 0.73, beta = −0.0166, N = 1255
FLENEDR pc2
P01009−2;P01009−3;P01009
778:791 403:408 59:61
14:94841641:G:A_A
p = 0.19, beta = −0.0631, N = 1240
GTEAAGAMFLEAIPMSIPPEVK pc3
P01009
776:791 403:408 59:61
14:94841641:G:A_A
p = 0.74, beta = −0.0164, N = 1238
ELDRDTVFALVNYIFFK pc3
P01009−2;P01009−3;P01009
647:791 348:408 50:61
14:94841641:G:A_A
p = 0.07, beta = 0.0955, N = 1045
FLENEDRR pc2
P01009−2;P01009−3;P01009
763:791 395:408 58:61
14:94841641:G:A_A
p = 0.69, beta = −0.0193, N = 1216
LYHSEAFTVNFGDTEEAKK pc4
P01009−2;P01009−3;P01009
743:791 377:408 58:61
14:94841641:G:A_A
p = 0.48, beta = −0.0354, N = 1178
SERPINA1 : NP2
P01009
220:220 91:91 14:14
14:94841641:G:A_A
p = 0.52, beta = −0.062, N = 325
SVLGQLGITK pc2
P01009−2;P01009
220:220 91:91 14:14
14:94841641:G:A_A
p = 0.52, beta = −0.0623, N = 325
LSITGTYDLK pc2
P01009−2;P01009
220:220 91:91 14:14
14:94841641:G:A_A
p = 0.89, beta = 0.0137, N = 325
AVLTIDEK pc2
P01009
220:220 91:91 14:14
14:94841641:G:A_A
p = 0.88, beta = −0.0142, N = 325
QINDYVEK pc2
P01009−2;P01009−3;P01009
220:220 91:91 14:14
14:94841641:G:A_A
p = 0.39, beta = −0.0832, N = 325
LYHSEAFTVNFGDTEEAK pc3
P01009−2;P01009−3;P01009
220:220 90:91 14:14
14:94841641:G:A_A
p = 0.11, beta = −0.156, N = 324
LSSWVLLMK pc2
P01009−2;P01009−3;P01009
220:220 91:91 14:14
14:94841641:G:A_A
p = 0.23, beta = −0.117, N = 325
SASLHLPK pc2
P01009−2;P01009
220:220 91:91 14:14
14:94841641:G:A_A
p = 0.86, beta = −0.0166, N = 325
LQHLENELTHDIITK pc3
P01009−2;P01009−3;P01009
220:220 91:91 14:14
14:94841641:G:A_A
p = 0.3, beta = −0.0999, N = 325
WERPFEVK pc2
P01009−2;P01009−3;P01009
220:220 91:91 14:14
14:94841641:G:A_A
p = 0.21, beta = −0.121, N = 325
FLEDVKK pc2
P01009−2;P01009−3;P01009
220:220 91:91 14:14
14:94841641:G:A_A
p = 0.57, beta = −0.0549, N = 325
VFSNGADLSGVTEEAPLK pc2
P01009−2;P01009
219:220 91:91 14:14
14:94841641:G:A_A
p = 0.024, beta = −0.219, N = 324
ITPNLAEFAFSLYR pc2
P01009−2;P01009−3;P01009
219:220 91:91 14:14
14:94841641:G:A_A
p = 0.47, beta = −0.0696, N = 324
ELDRDTVFALVNYIFFK pc3
P01009−2;P01009−3;P01009
212:220 82:91 12:14
14:94841641:G:A_A
p = 0.059, beta = −0.192, N = 306
LGMFNIQHCK pc3
P01009−2;P01009−3;P01009
220:220 91:91 14:14
14:94841641:G:A_A
p = 0.17, beta = −0.132, N = 325
TDTSHHDQDHPTFNK pc4
P01009−2;P01009−3;P01009
215:220 86:91 14:14
14:94841641:G:A_A
p = 0.55, beta = −0.0587, N = 315
FLENEDR pc2
P01009−2;P01009−3;P01009
213:220 87:91 14:14
14:94841641:G:A_A
p = 0.39, beta = −0.0849, N = 314
GTEAAGAMFLEAIPMSIPPEVK pc3
P01009
215:220 89:91 13:14
14:94841641:G:A_A
p = 0.092, beta = −0.167, N = 317
LYHSEAFTVNFGDTEEAKK pc4
P01009−2;P01009−3;P01009
220:220 91:91 14:14
14:94841641:G:A_A
p = 0.36, beta = −0.0886, N = 325
FLENEDRR pc2
P01009−2;P01009−3;P01009
201:220 81:91 11:14
14:94841641:G:A_A
p = 0.52, beta = −0.0677, N = 293
Assay Target: SERPINA1
UniProt: P01009
rsID: rs1243167
Target SNP: 14:94375304:A:G
Proxy SNP: 14:94841641:G:A
deCODE log10(p): 420.5
deCODE BETA: 0.41
deCODE MSPA: 0
1256:1257:1256:1256:1173:125
DTEEEDFHVDQVTTVK pc3
rs6647 REF
774:791 391:408 29:61
14:94841641:G:A_A
p = 1.7e−28, model = DOM, N = 1194
PFVFLMIEQNTK pc2
rs1303 REF
706:791 404:408 61:61
14:94841641:G:A_A
p = 1.1e−13, model = REC, N = 1171
PFVFLMIDQNTK pc2
rs1303 ALT
451:791 129:408 5:61
14:94841641:G:A_A
p = 4.8e−23, model = REC, N = 585
DTEEEDFHVDQATTVK pc3
rs6647 ALT
156:791 354:408 59:61
14:94841641:G:A_A
p = 2.2e−133, model = REC, N = 569
FNKPFVFLMIEQNTK pc3
rs1303 REF
705:791 402:408 61:61
14:94841641:G:A_A
p = 2.7e−12, model = REC, N = 1168
TLNQPDSQLQLTTGNGLFLSEGLK p
rs709932 REF
779:791 404:408 61:61
14:94841641:G:A_A
p = 0.44, model = REC, N = 1244
FNKPFVFLMIDQNTK pc3
rs1303 ALT
436:791 122:408 6:61
14:94841641:G:A_A
p = 3.3e−22, model = REC, N = 564
DTEEEDFHVDQVTTVK pc2
rs6647 REF
770:791 386:408 16:61
14:94841641:G:A_A
p = 2.4e−44, model = DOM, N = 1172
DTEEEDFHVDQATTVK pc2
rs6647 ALT
49:791 343:408 55:61
14:94841641:G:A_A
p = 4.2e−191, model = REC, N = 447
FNKPFVFLMIEQNTK pc2
rs1303 REF
200:791 143:408 28:61
14:94841641:G:A_A
p = 3.2e−05, model = REC, N = 371
FNKPFVFLMIDQNTK pc2
rs1303 ALT
175:791 40:408 3:61
14:94841641:G:A_A
p = 1.3e−09, model = REC, N = 218

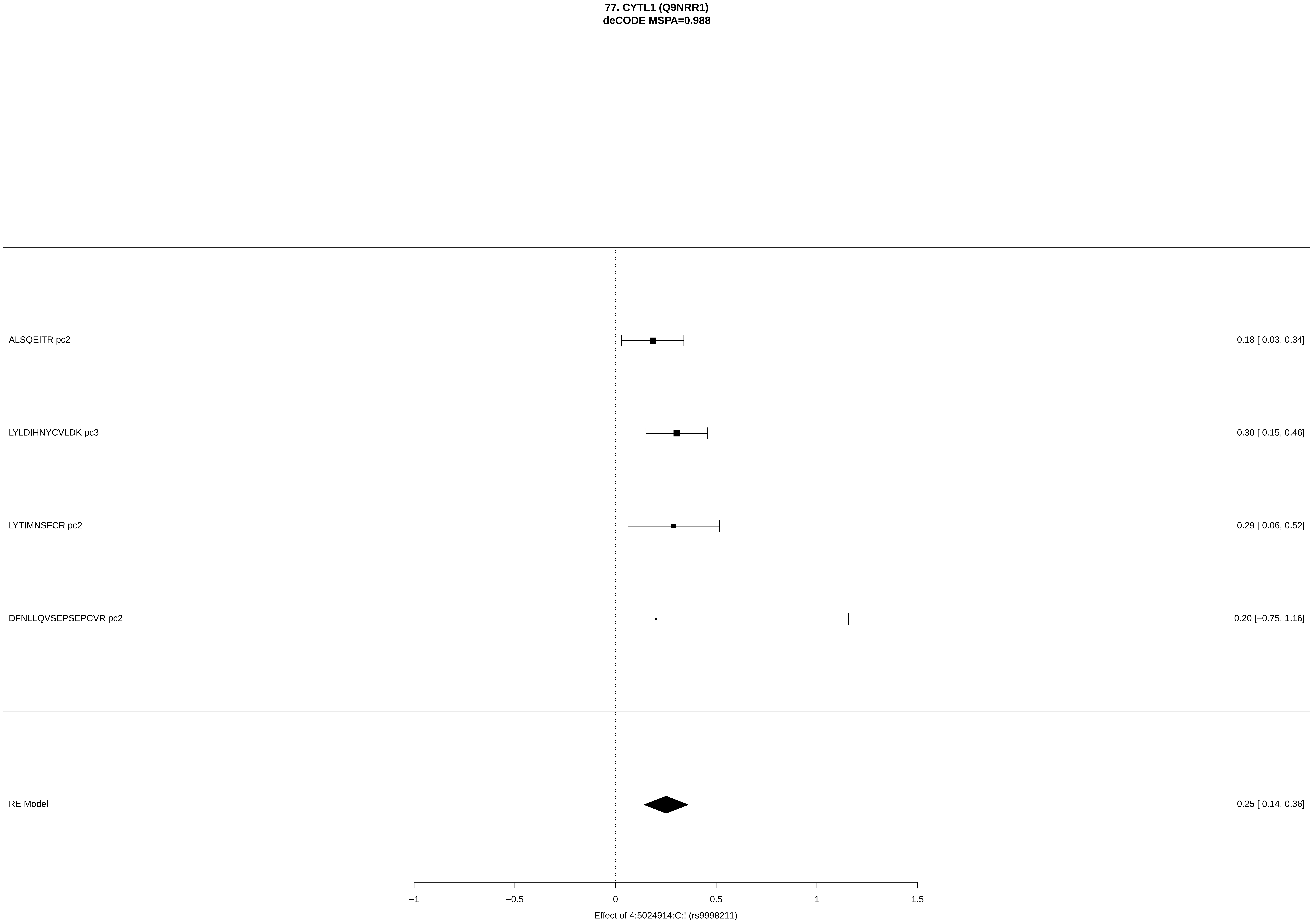

77. CYTL1 (Q9NRR1)
deCODE MSPA=0.988
ALSQEITR pc2
0.18 [0.03, 0.34]
LYLDIHNYCVLDK pc3
0.30 [0.15, 0.46]
LYTIMNSFCR pc2
0.29 [0.06, 0.52]
DFNLLQVSEPSEPCVR pc2
0.20 [−0.75, 1.16]
RE Model
0.25 [0.14, 0.36]
−1
−0.5
0
0.5
1
1.5
Effect of 4:5024914:C:! (rs9998211)

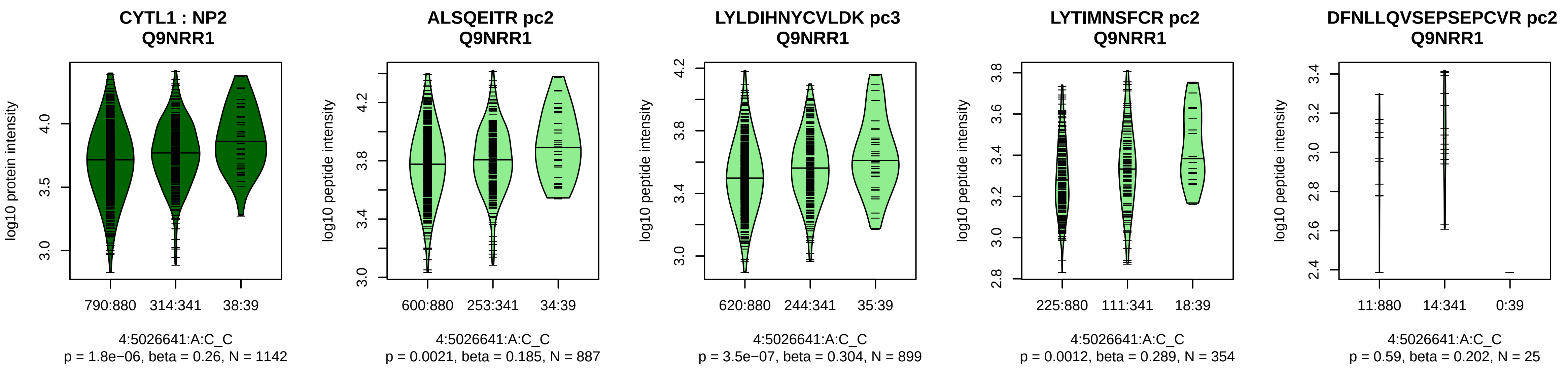

Assay Target: CYTL1
UniProt: Q9NRR1
rsID: rs9998211
Target SNP: 4:5024914:C:!
Proxy SNP: 4:5026641:A:C
deCODE log10(p): 411.7
deCODE BETA: 0.45
deCODE MSPA: 0.988
::*:–
887:899:354:25

78. KNG1 (P01042)
deCODE MSPA=0.438

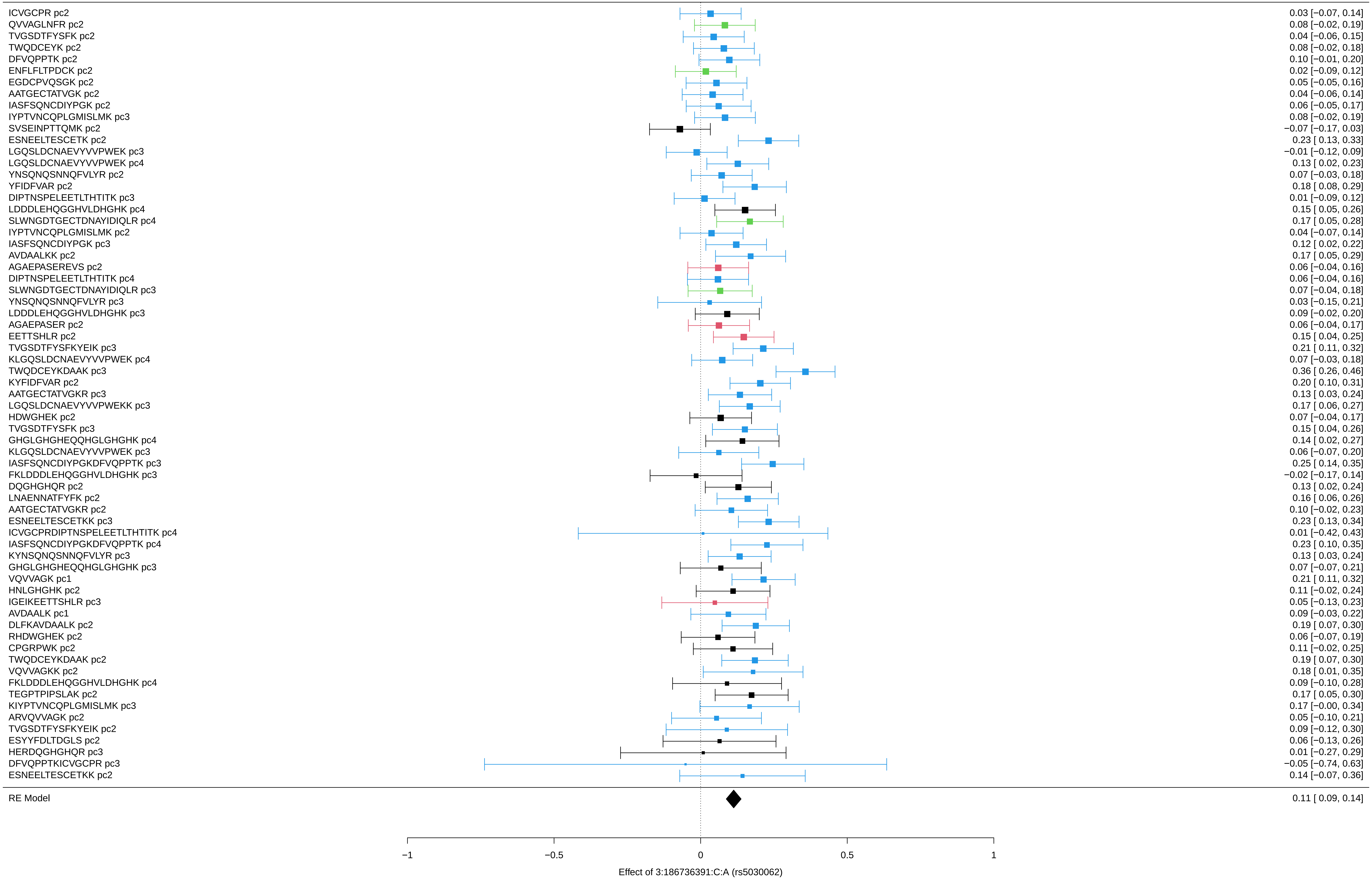

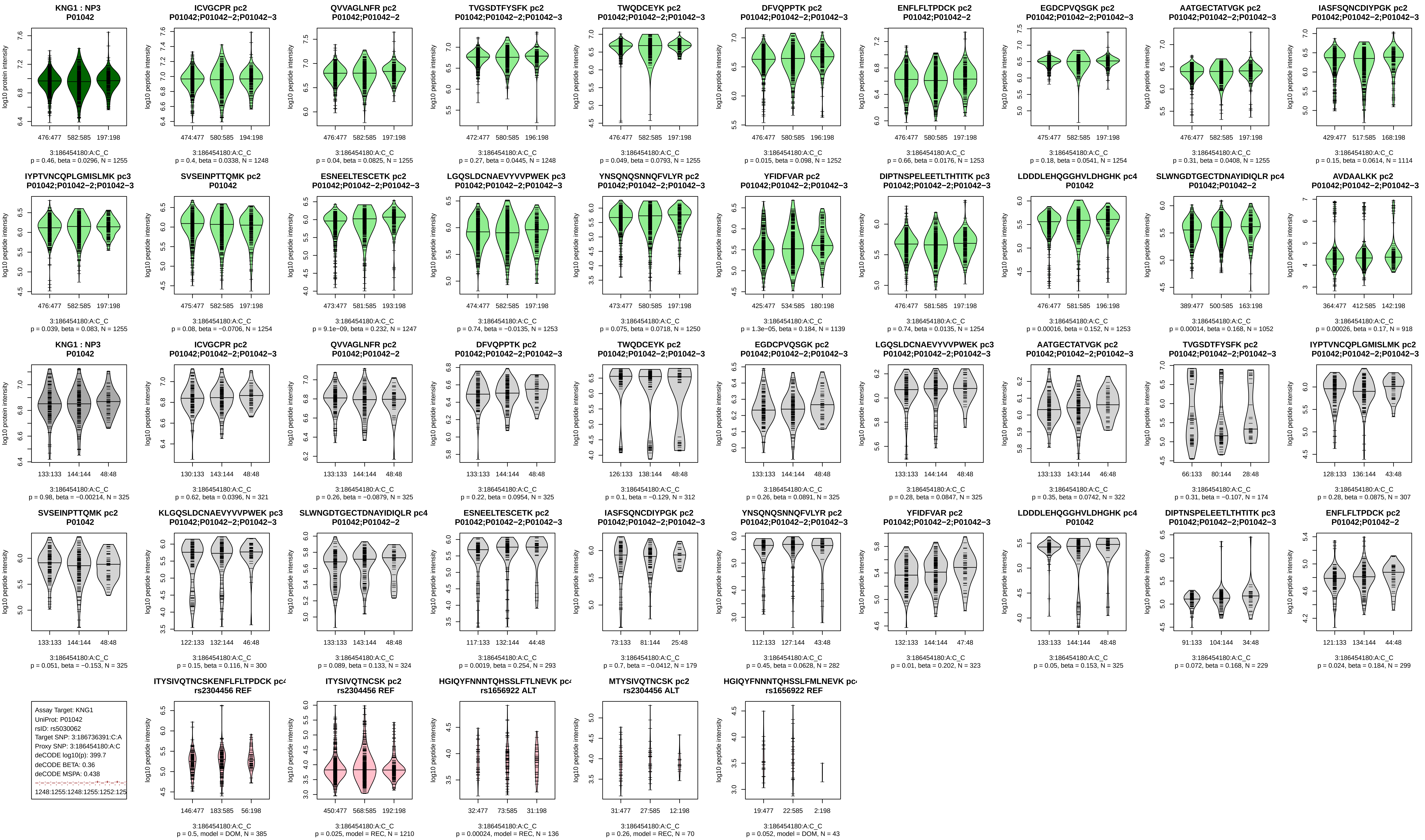
KNG1 : NP3
P01042
log10 protein intensity
476:477 582:585 197:198
3:186454180:A:C_C
p = 0.46, beta = 0.0296, N = 1255
ICVGCPR pc2
P01042;P01042−2;P01042−3
log10 peptide intensity
474:477 580:585 194:198
3:186454180:A:C_C
p = 0.4, beta = 0.0338, N = 1248
QVVAGLNFR pc2
P01042;P01042−2
476:477 582:585 197:198
3:186454180:A:C_C
p = 0.04, beta = 0.0825, N = 1255
TVGSDTFYSFK pc2
P01042;P01042−2;P01042−3
472:477 580:585 196:198
3:186454180:A:C_C
p = 0.27, beta = 0.0445, N = 1248
TWQDCEYK pc2
P01042;P01042−2;P01042−3
476:477 582:585 197:198
3:186454180:A:C_C
p = 0.049, beta = 0.0793, N = 1255
DFVQPPTK pc2
P01042;P01042−2;P01042−3
476:477 580:585 196:198
3:186454180:A:C_C
p = 0.015, beta = 0.098, N = 1252
ENFLFLTPDCK pc2
P01042;P01042−2
476:477 580:585 197:198
3:186454180:A:C_C
p = 0.66, beta = 0.0176, N = 1253
EGDCPVQSGK pc2
P01042;P01042−2;P01042−3
475:477 582:585 197:198
3:186454180:A:C_C
p = 0.18, beta = 0.0541, N = 1254
AATGECTATVGK pc2
P01042;P01042−2;P01042−3
476:477 582:585 197:198
3:186454180:A:C_C
p = 0.31, beta = 0.0408, N = 1255
IASFSQNCDIYPGK pc2
P01042;P01042−2;P01042−3
429:477 517:585 168:198
3:186454180:A:C_C
p = 0.15, beta = 0.0614, N = 1114
IYPTVNCQPLGMISLMK pc3
P01042;P01042−2;P01042−3
476:477 582:585 197:198
3:186454180:A:C_C
p = 0.039, beta = 0.083, N = 1255
SVSEINPTTQMK pc2
P01042
475:477 582:585 197:198
3:186454180:A:C_C
p = 0.08, beta = −0.0706, N = 1254
ESNEELTESCETK pc2
P01042;P01042−2;P01042−3
473:477 581:585 193:198
3:186454180:A:C_C
p = 9.1e−09, beta = 0.232, N = 1247
LGQSLDCNAEVYVVPWEK pc3
P01042;P01042−2;P01042−3
474:477 582:585 197:198
3:186454180:A:C_C
p = 0.74, beta = −0.0135, N = 1253
YNSQNQSNNQFVLYR pc2
P01042;P01042−2;P01042−3
473:477 580:585 197:198
3:186454180:A:C_C
p = 0.075, beta = 0.0718, N = 1250
YFIDFVAR pc2
P01042;P01042−2;P01042−3
425:477 534:585 180:198
3:186454180:A:C_C
p = 1.3e−05, beta = 0.184, N = 1139
DIPTNSPELEETLTHTITK pc3
P01042;P01042−2;P01042−3
476:477 581:585 197:198
3:186454180:A:C_C
p = 0.74, beta = −0.0135, N = 1254
LDDDLEHQGGHVLDHGHK pc4
P01042
476:477 581:585 196:198
3:186454180:A:C_C
p = 0.00016, beta = 0.152, N = 1253
SLWNGDTGECTDNAYIDIQLR pc4
P01042;P01042−2
389:477 500:585 163:198
3:186454180:A:C_C
p = 0.00014, beta = 0.168, N = 1052
AVDAALKK pc2
P01042;P01042−2;P01042−3
364:477 412:585 142:198
3:186454180:A:C_C
p = 0.00026, beta = 0.17, N = 918
KNG1 : NP3
P01042
133:133 144:144 48:48
3:186454180:A:C_C
p = 0.98, beta = −0.00214, N = 325
ICVGCPR pc2
P01042;P01042−2;P01042−3
130:133 143:144 48:48
3:186454180:A:C_C
p = 0.62, beta = 0.0396, N = 321
QVVAGLNFR pc2
P01042;P01042−2
133:133 144:144 48:48
3:186454180:A:C_C
p = 0.26, beta = −0.0879, N = 325
DFVQPPTK pc2
P01042;P01042−2;P01042−3
133:133 144:144 48:48
3:186454180:A:C_C
p = 0.22, beta = 0.0954, N = 325
TWQDCEYK pc2
P01042;P01042−2;P01042−3
126:133 138:144 48:48
3:186454180:A:C_C
p = 0.1, beta = −0.129, N = 312
EGDCPVQSGK pc2
P01042;P01042−2;P01042−3
133:133 144:144 48:48
3:186454180:A:C_C
p = 0.26, beta = 0.0891, N = 325
LGQSLDCNAEVYVVPWEK pc3
P01042;P01042−2;P01042−3
133:133 144:144 48:48
3:186454180:A:C_C
p = 0.28, beta = 0.0847, N = 325
AATGECTATVGK pc2
P01042;P01042−2;P01042−3
133:133 143:144 46:48
3:186454180:A:C_C
p = 0.35, beta = 0.0742, N = 322
TVGSDTFYSFK pc2
P01042;P01042−2;P01042−3
66:133 80:144 28:48
3:186454180:A:C_C
p = 0.31, beta = −0.107, N = 174
IYPTVNCQPLGMISLMK pc2
P01042;P01042−2;P01042−3
128:133 136:144 43:48
3:186454180:A:C_C
p = 0.28, beta = 0.0875, N = 307
SVSEINPTTQMK pc2
P01042
133:133 144:144 48:48
3:186454180:A:C_C
p = 0.051, beta = −0.153, N = 325
KLGQSLDCNAEVYVVPWEK pc3
P01042;P01042−2;P01042−3
122:133 132:144 46:48
3:186454180:A:C_C
p = 0.15, beta = 0.116, N = 300
SLWNGDTGECTDNAYIDIQLR pc4
P01042;P01042−2
133:133 143:144 48:48
3:186454180:A:C_C
p = 0.089, beta = 0.133, N = 324
ESNEELTESCETK pc2
P01042;P01042−2;P01042−3
117:133 132:144 44:48
3:186454180:A:C_C
p = 0.0019, beta = 0.254, N = 293
IASFSQNCDIYPGK pc2
P01042;P01042−2;P01042−3
73:133 81:144 25:48
3:186454180:A:C_C
p = 0.7, beta = −0.0412, N = 179
YNSQNQSNNQFVLYR pc2
P01042;P01042−2;P01042−3
112:133 127:144 43:48
3:186454180:A:C_C
p = 0.45, beta = 0.0628, N = 282
YFIDFVAR pc2
P01042;P01042−2;P01042−3
132:133 144:144 47:48
3:186454180:A:C_C
p = 0.01, beta = 0.202, N = 323
LDDDLEHQGGHVLDHGHK pc4
P01042
133:133 144:144 48:48
3:186454180:A:C_C
p = 0.05, beta = 0.153, N = 325
DIPTNSPELEETLTHTITK pc3
P01042;P01042−2;P01042−3
91:133 104:144 34:48
3:186454180:A:C_C
p = 0.072, beta = 0.168, N = 229
ENFLFLTPDCK pc2
P01042;P01042−2
121:133 134:144 44:48
3:186454180:A:C_C
p = 0.024, beta = 0.184, N = 299
Assay Target: KNG1
UniProt: P01042
rsID: rs5030062
Target SNP: 3:186736391:C:A
Proxy SNP: 3:186454180:A:C
deCODE log10(p): 399.7
deCODE BETA: 0.36
deCODE MSPA: 0.438
1248:1255:1248:1255:1252:125
ITYSIVQTNCSKENFLFLTPDCK pc4
rs2304456 REF
146:477 183:585 56:198
3:186454180:A:C_C
p = 0.5, model = DOM, N = 385
ITYSIVQTNCSK pc2
rs2304456 REF
450:477 568:585 192:198
3:186454180:A:C_C
p = 0.025, model = REC, N = 1210
HGIQYFNNNTQHSSLFTLNEVK pc4
rs1656922 ALT
32:477 73:585 31:198
3:186454180:A:C_C
p = 0.00024, model = REC, N = 136
MTYSIVQTNCSK pc2
rs2304456 ALT
31:477 27:585 12:198
3:186454180:A:C_C
p = 0.26, model = REC, N = 70
HGIQYFNNNTQHSSLFMLNEVK pc4
rs1656922 REF
19:477 22:585 2:198
3:186454180:A:C_C
p = 0.052, model = DOM, N = 43

79. F13B (P05160)
deCODE MSPA=0.32

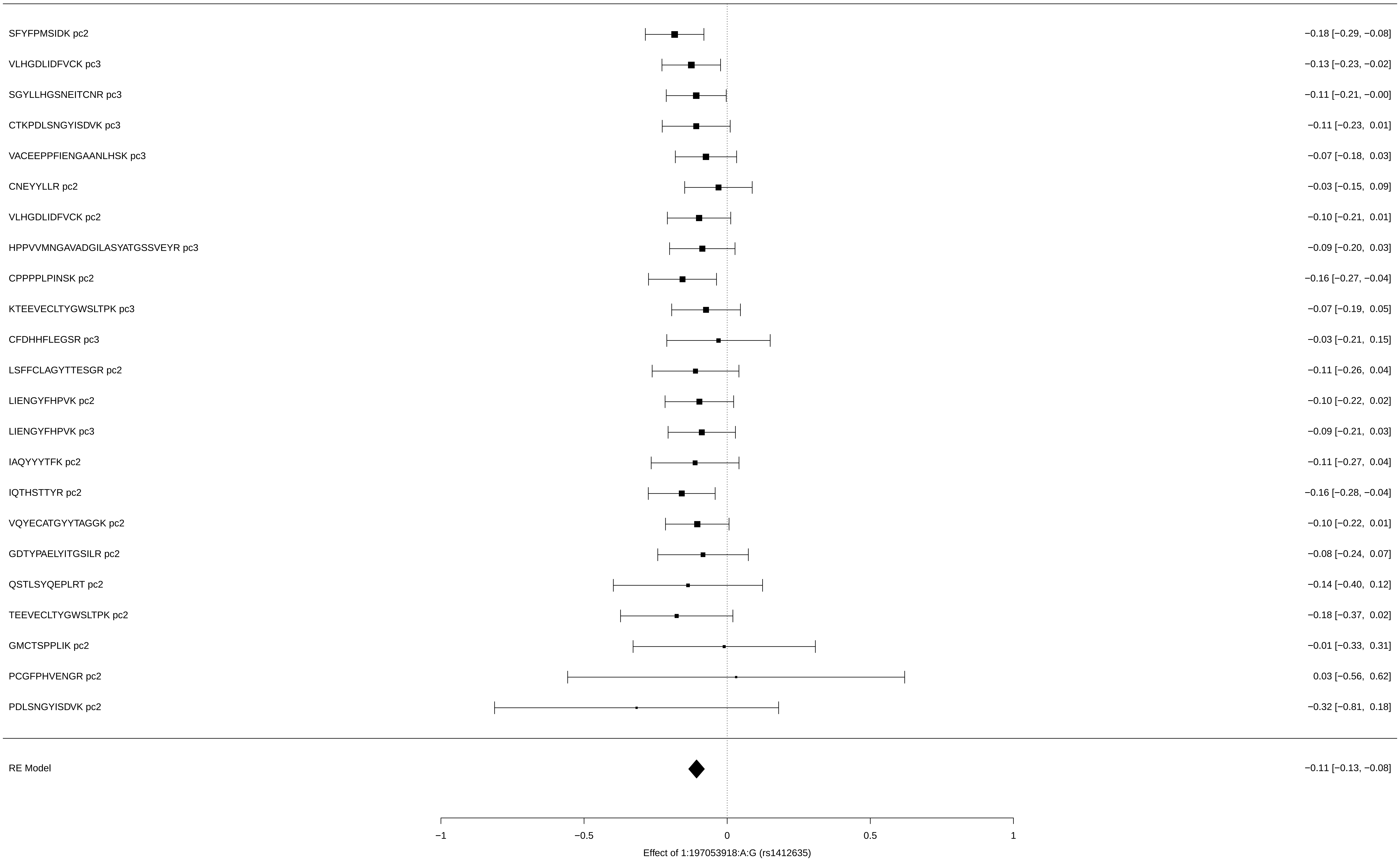

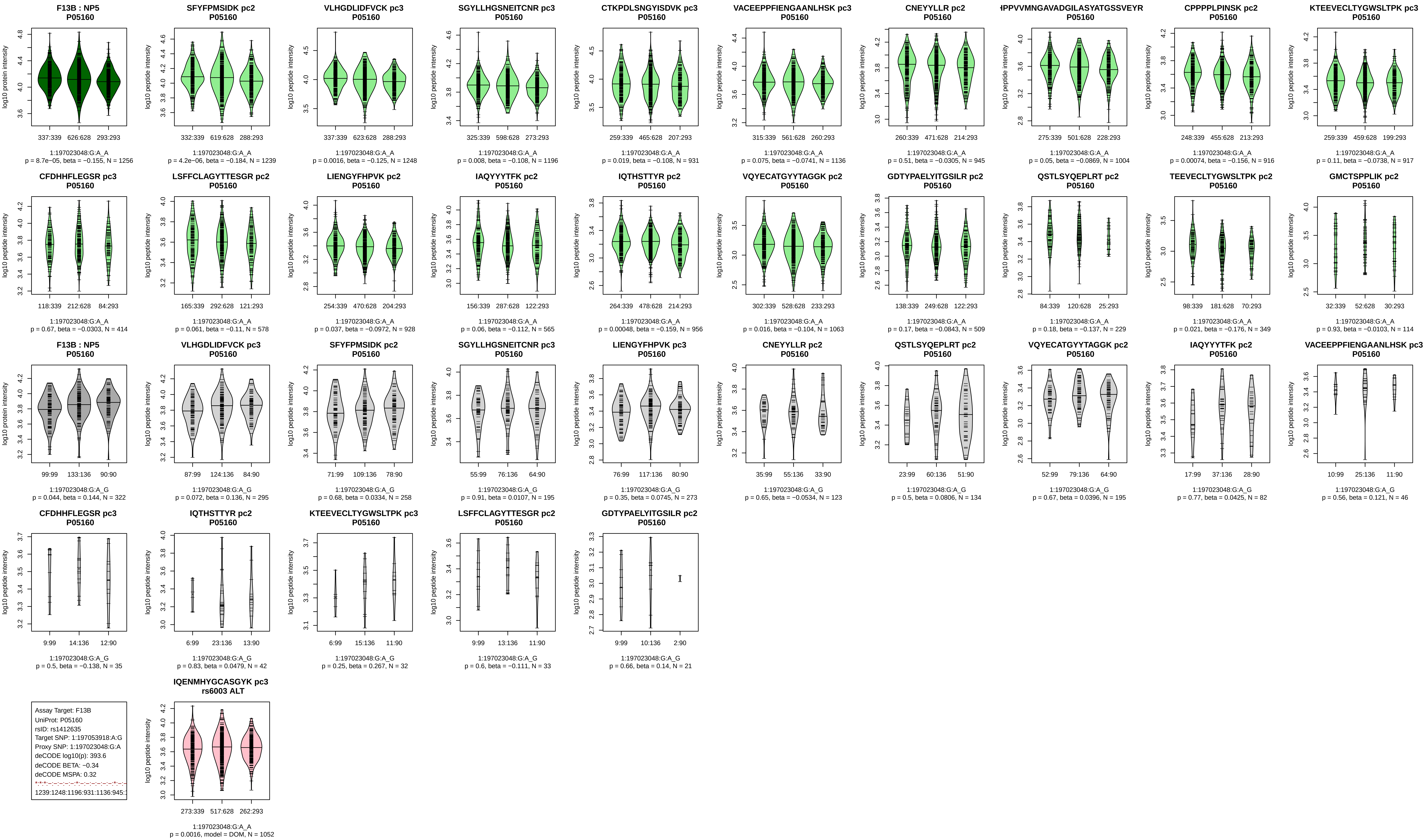

F13B : NP5
P05160
log10 protein intensity
337:339 626:628 293:293
1:197023048:G:A_A
p = 8.7e−05, beta = −0.155, N = 1256
SFYFPMSIDK pc2
P05160
log10 peptide intensity
332:339 619:628 288:293
1:197023048:G:A_A
p = 4.2e−06, beta = −0.184, N = 1239
VLHGDLIDFVCK pc3
P05160
337:339 623:628 288:293
1:197023048:G:A_A
p = 0.0016, beta = −0.125, N = 1248
SGYLLHGSNEITCNR pc3
P05160
325:339 598:628 273:293
1:197023048:G:A_A
p = 0.008, beta = −0.108, N = 1196
CTKPDLSNGYISDVK pc3
P05160
259:339 465:628 207:293
1:197023048:G:A_A
p = 0.019, beta = −0.108, N = 931
VACEEPPFIENGAANLHSK pc3
P05160
315:339 561:628 260:293
1:197023048:G:A_A
p = 0.075, beta = −0.0741, N = 1136
CNEYYLLR pc2
P05160
260:339 471:628 214:293
1:197023048:G:A_A
p = 0.51, beta = −0.0305, N = 945
IPPVVMNGAVADGILASYATGSSVEYR
P05160
275:339 501:628 228:293
1:197023048:G:A_A
p = 0.05, beta = −0.0869, N = 1004
CPPPPLPINSK pc2
P05160
248:339 455:628 213:293
1:197023048:G:A_A
p = 0.00074, beta = −0.156, N = 916
KTEEVECLTYGWSLTPK pc3
P05160
259:339 459:628 199:293
1:197023048:G:A_A
p = 0.11, beta = −0.0738, N = 917
CFDHHFLEGSR pc3
P05160
118:339 212:628 84:293
1:197023048:G:A_A
p = 0.67, beta = −0.0303, N = 414
LSFFCLAGYTTESGR pc2
P05160
165:339 292:628 121:293
1:197023048:G:A_A
p = 0.061, beta = −0.11, N = 578
LIENGYFHPVK pc2
P05160
254:339 470:628 204:293
1:197023048:G:A_A
p = 0.037, beta = −0.0972, N = 928
IAQYYYTFK pc2
P05160
156:339 287:628 122:293
1:197023048:G:A_A
p = 0.06, beta = −0.112, N = 565
IQTHSTTYR pc2
P05160
264:339 478:628 214:293
1:197023048:G:A_A
p = 0.00048, beta = −0.159, N = 956
VQYECATGYYTAGGK pc2
P05160
302:339 528:628 233:293
1:197023048:G:A_A
p = 0.016, beta = −0.104, N = 1063
GDTYPAELYITGSILR pc2
P05160
138:339 249:628 122:293
1:197023048:G:A_A
p = 0.17, beta = −0.0843, N = 509
QSTLSYQEPLRT pc2
P05160
84:339 120:628 25:293
1:197023048:G:A_A
p = 0.18, beta = −0.137, N = 229
TEEVECLTYGWSLTPK pc2
P05160
98:339 181:628 70:293
1:197023048:G:A_A
p = 0.021, beta = −0.176, N = 349
GMCTSPPLIK pc2
P05160
32:339 52:628 30:293
1:197023048:G:A_A
p = 0.93, beta = −0.0103, N = 114
F13B : NP5
P05160
99:99 133:136 90:90
1:197023048:G:A_G
p = 0.044, beta = 0.144, N = 322
VLHGDLIDFVCK pc3
P05160
87:99 124:136 84:90
1:197023048:G:A_G
p = 0.072, beta = 0.136, N = 295
SFYFPMSIDK pc2
P05160
71:99 109:136 78:90
1:197023048:G:A_G
p = 0.68, beta = 0.0334, N = 258
SGYLLHGSNEITCNR pc3
P05160
55:99 76:136 64:90
1:197023048:G:A_G
p = 0.91, beta = 0.0107, N = 195
LIENGYFHPVK pc3
P05160
76:99 117:136 80:90
1:197023048:G:A_G
p = 0.35, beta = 0.0745, N = 273
CNEYYLLR pc2
P05160
35:99 55:136 33:90
1:197023048:G:A_G
p = 0.65, beta = −0.0534, N = 123
QSTLSYQEPLRT pc2
P05160
23:99 60:136 51:90
1:197023048:G:A_G
p = 0.5, beta = 0.0806, N = 134
VQYECATGYYTAGGK pc2
P05160
52:99 79:136 64:90
1:197023048:G:A_G
p = 0.67, beta = 0.0396, N = 195
IAQYYYTFK pc2
P05160
17:99 37:136 28:90
1:197023048:G:A_G
p = 0.77, beta = 0.0425, N = 82
VACEEPPFIENGAANLHSK pc3
P05160
10:99 25:136 11:90
1:197023048:G:A_G
p = 0.56, beta = 0.121, N = 46
CFDHHFLEGSR pc3
P05160
9:99 14:136 12:90
1:197023048:G:A_G
p = 0.5, beta = −0.138, N = 35
IQTHSTTYR pc2
P05160
6:99 23:136 13:90
1:197023048:G:A_G
p = 0.83, beta = 0.0479, N = 42
KTEEVECLTYGWSLTPK pc3
P05160
6:99 15:136 11:90
1:197023048:G:A_G
p = 0.25, beta = 0.267, N = 32
LSFFCLAGYTTESGR pc2
P05160
9:99 13:136 11:90
1:197023048:G:A_G
p = 0.6, beta = −0.111, N = 33
GDTYPAELYITGSILR pc2
P05160
9:99 10:136 2:90
1:197023048:G:A_G
p = 0.66, beta = 0.14, N = 21
Assay Target: F13B
UniProt: P05160
rsID: rs1412635
Target SNP: 1:197053918:A:G
Proxy SNP: 1:197023048:G:A
deCODE log10(p): 393.6
deCODE BETA: −0.34
deCODE MSPA: 0.32
1239:1248:1196:931:1136:945:
IQENMHYGCASGYK pc3
rs6003 ALT
273:339 517:628 262:293
1:197023048:G:A_A
p = 0.0016, model = DOM, N = 1052

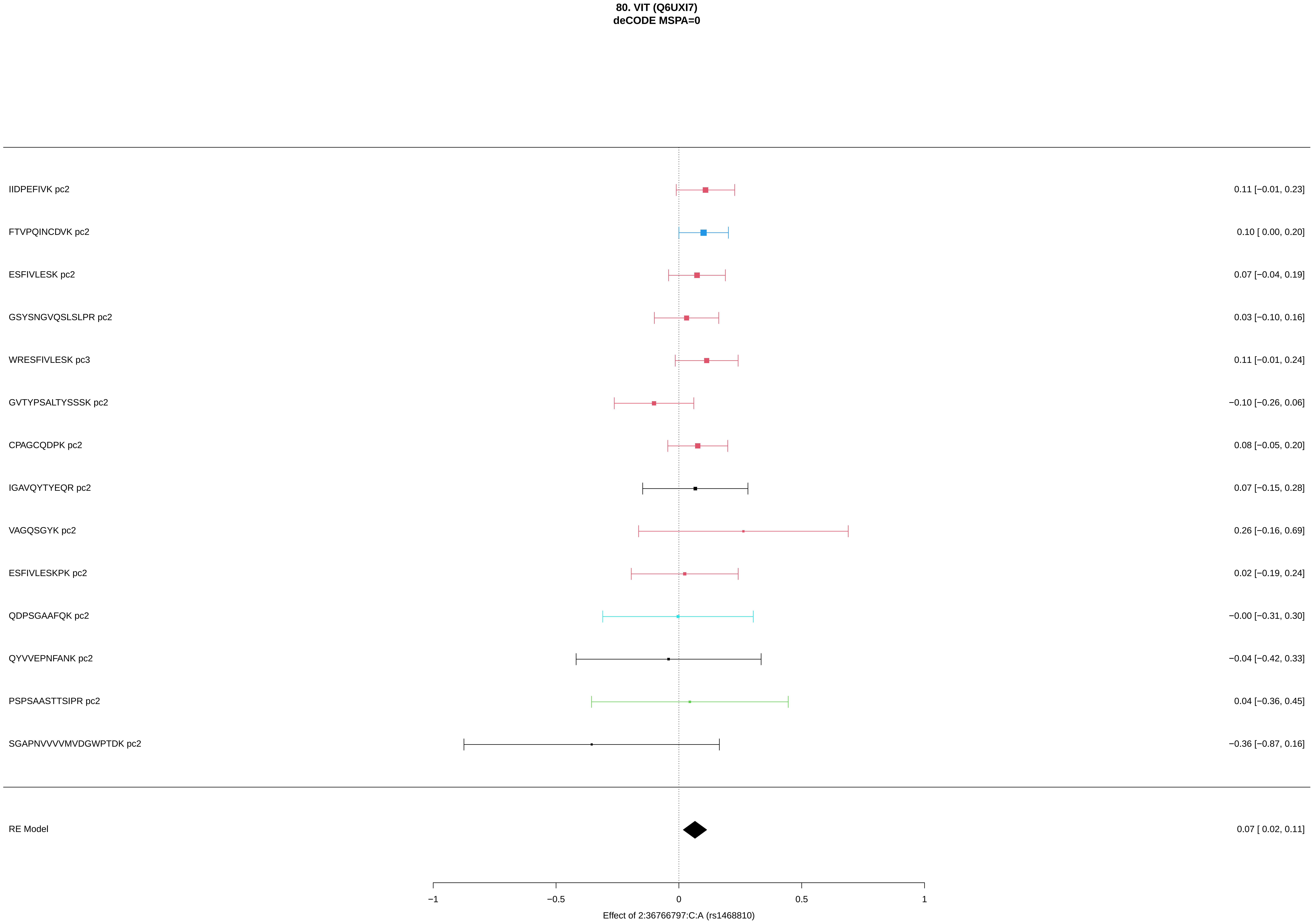
80. VIT (Q6UXI7)
deCODE MSPA=0
IIDPEFIVK pc2
0.11 [−0.01, 0.23]
FTVPQINCDVK pc2
0.10 [0.00, 0.20]
ESFIVLESK pc2
0.07 [−0.04, 0.19]
GSYSNGVQSLSLPR pc2
0.03 [−0.10, 0.16]
WRESFIVLESK pc3
0.11 [−0.01, 0.24]
GVTYPSALTYSSSK pc2
−0.10 [−0.26, 0.06]
CPAGCQDPK pc2
0.08 [−0.05, 0.20]
IGAVQYTYEQR pc2
0.07 [−0.15, 0.28]
VAGQSGYK pc2
0.26 [−0.16, 0.69]
ESFIVLESKPK pc2
0.02 [−0.19, 0.24]
QDPSGAAFQK pc2
−0.00 [−0.31, 0.30]
QYVVEPNFANK pc2
−0.04 [−0.42, 0.33]
PSPSAASTTSIPR pc2
0.04 [−0.36, 0.45]
SGAPNVVVVMVDGWPTDK pc2
−0.36 [−0.87, 0.16]
RE Model
0.07 [0.02, 0.11]
−1
−0.5
0
0.5
1
Effect of 2:36766797:C:A (rs1468810)

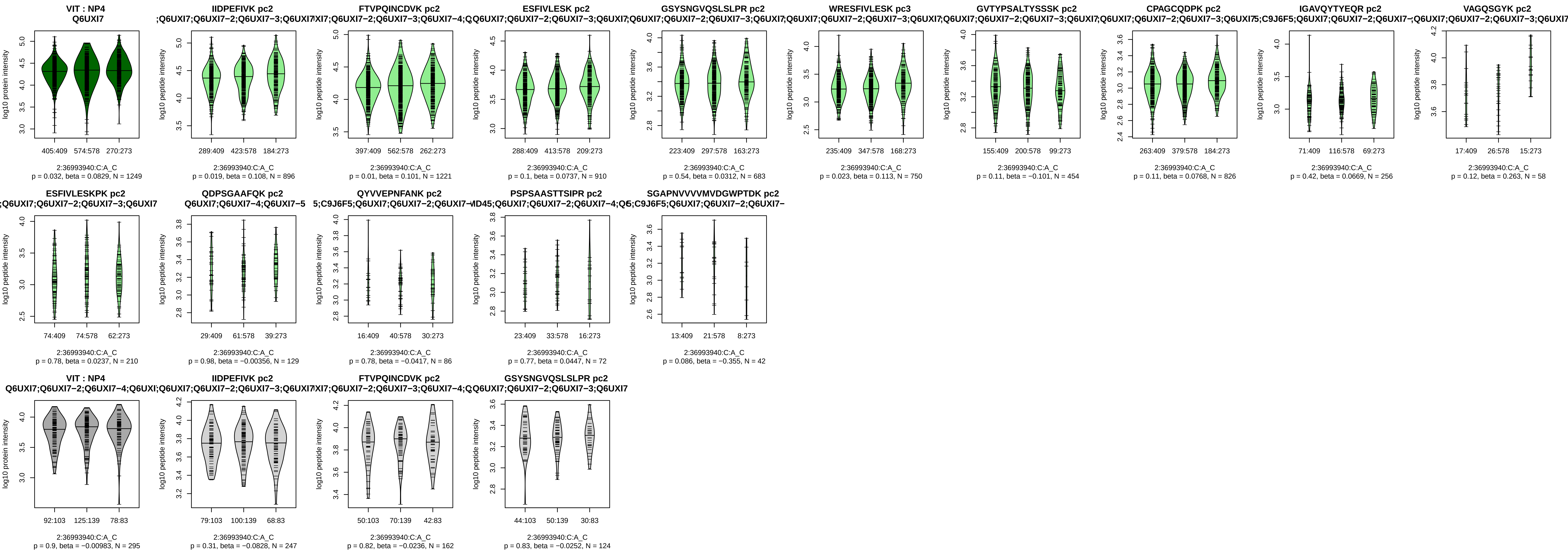

Assay Target: VIT
UniProt: Q6UXI7
rsID: rs1468810
Target SNP: 2:36766797:C:A
Proxy SNP: 2:36993940:C:A
deCODE log10(p): 389.4
deCODE BETA: 0.36
deCODE MSPA: 0
896:1221:910:683:750:454:826:

81. GPNMB (Q14956)
deCODE MSPA=0

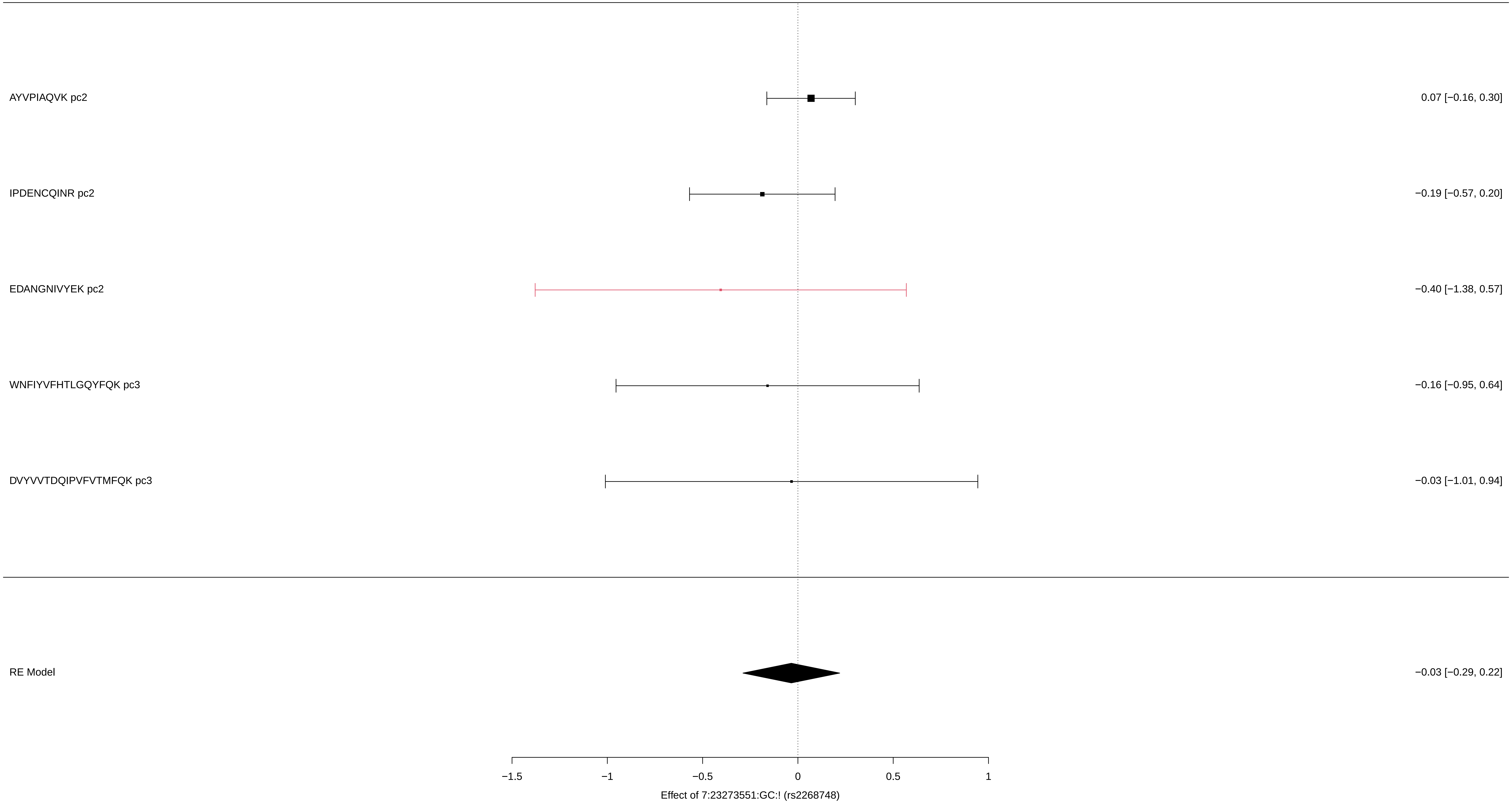

GPNMB : NP4
A0A3B3ISS6

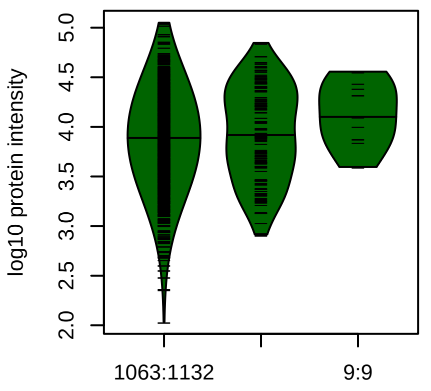

7:23313171:T:C_C
p = 0.47, beta = 0.0623, N = 1184

AYVPIAQVK pc2
A0A3B3ISS6;Q14956;Q14956−2

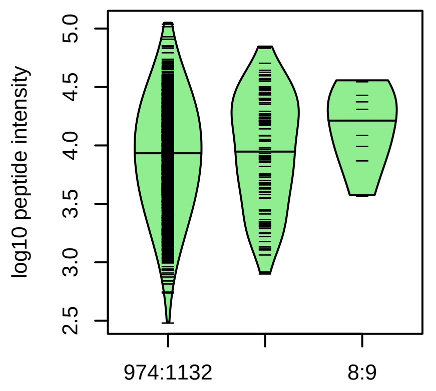

7:23313171:T:C_C
p = 0.44, beta = 0.069, N = 1085

IPDENCQINR pc2
A0A3B3ISS6;Q14956;Q14956−2

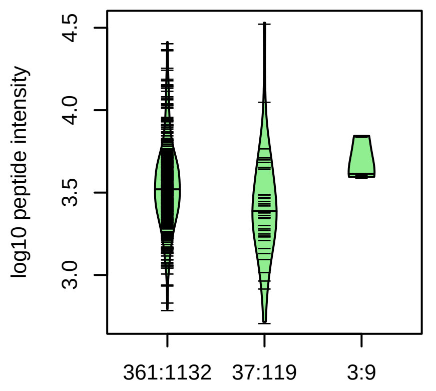

7:23313171:T:C_C
p = 0.21, beta = −0.187, N = 401

EDANGNIVYEK pc2
A0A3B3ISS6;Q14956;Q96F58;Q1495

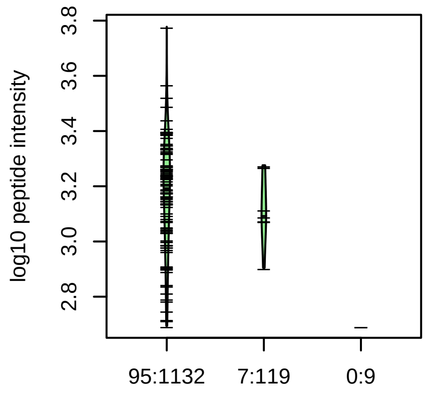

7:23313171:T:C_C
p = 0.29, beta = −0.405, N = 102

WNFIYVFHTLGQYFQK pc3
A0A3B3ISS6;Q14956;Q14956−2

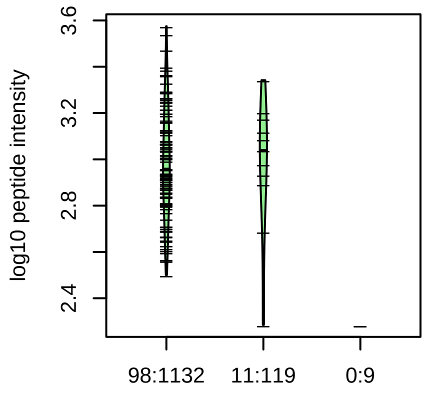

7:23313171:T:C_C
p = 0.61, beta = −0.159, N = 109

DVYVVTDQIPVFVTMFQK pc3
A0A3B3ISS6;Q14956;Q14956−2

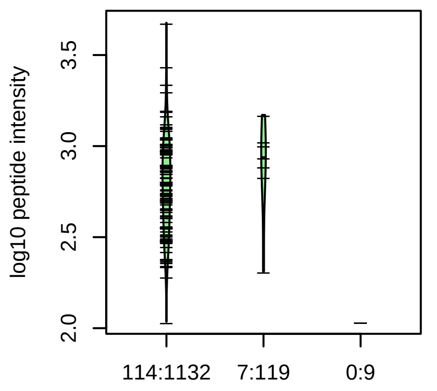

7:23313171:T:C_C
p = 0.93, beta = −0.0331, N = 121

GPNMB : NP4
A0A3B3ISS6;Q14956;Q14956−2

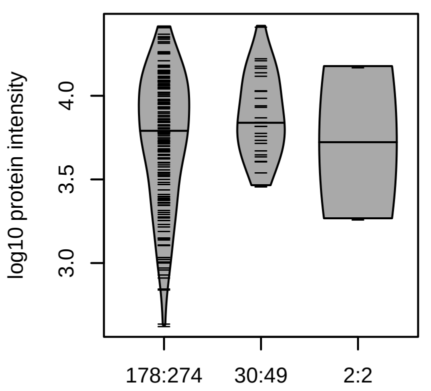

7:23313171:T:C_C
p = 0.43, beta = 0.135, N = 210

AYVPIAQVK pc2
A0A3B3ISS6;Q14956;Q14956−2

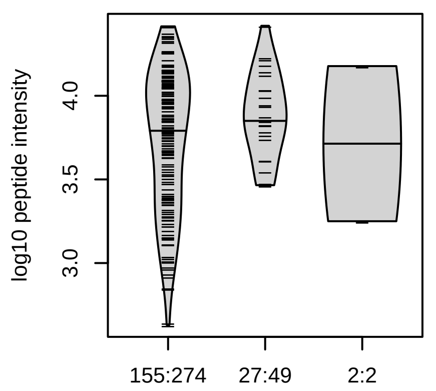

7:23313171:T:C_C
p = 0.52, beta = 0.115, N = 184

IPDENCQINR pc2
A0A3B3ISS6;Q14956;Q14956−2

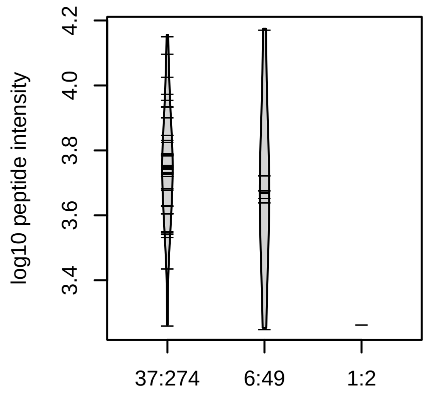

7:23313171:T:C_C
p = 0.3, beta = −0.337, N = 44

Assay Target: GPNMB
UniProt: Q14956
rsID: rs2268748
Target SNP: 7:23273551:GC:!
Proxy SNP: 7:23313171:T:C
deCODE log10(p): 388.1
deCODE BETA: 0.74
deCODE MSPA: 0
–:–:–:–:–
1085:401:102:109:121

82. IGLL1 (P15814)
deCODE MSPA=0

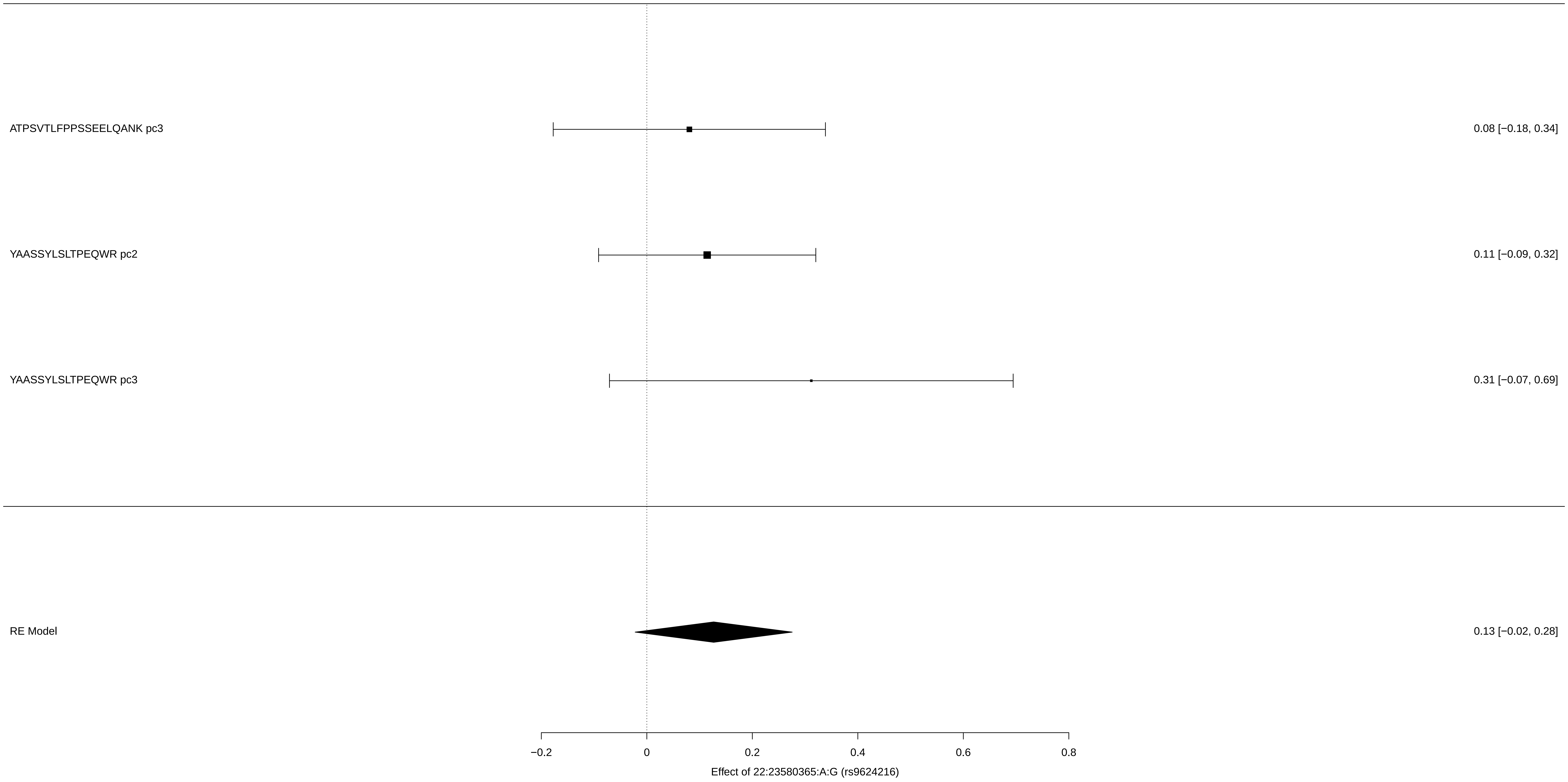

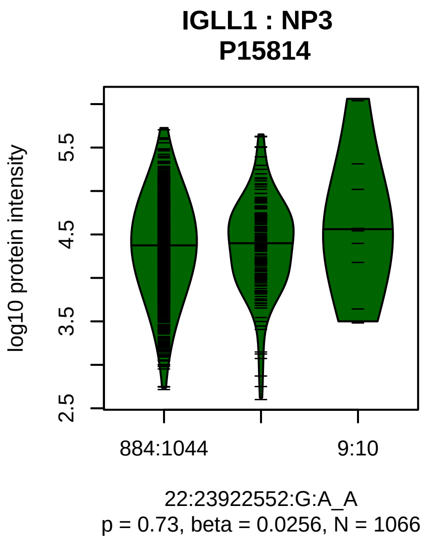

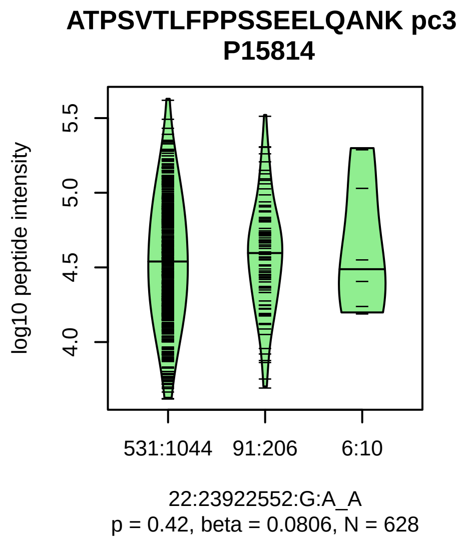

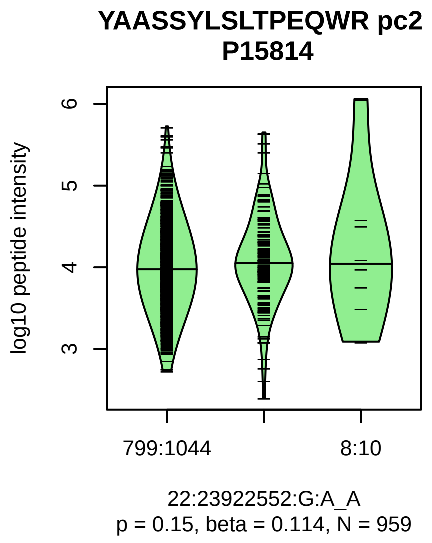

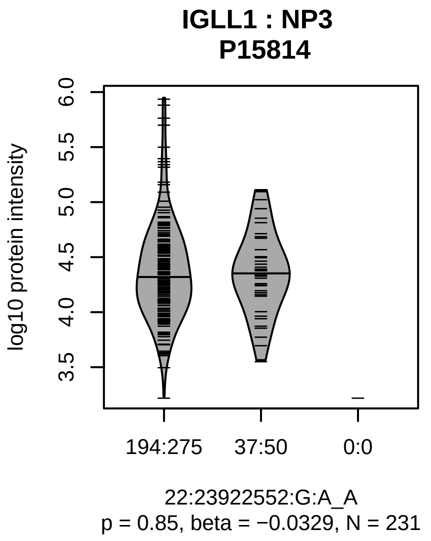

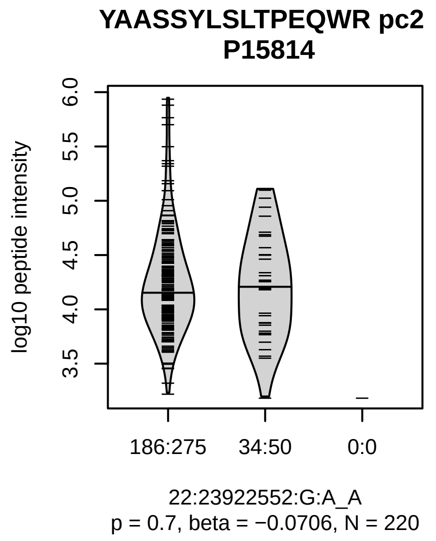

Assay Target: IGLL1
UniProt: P15814
rsID: rs9624216
Target SNP: 22:23580365:A:G
Proxy SNP: 22:23922552:G:A
deCODE log10(p): 374.6
deCODE BETA: −0.6
deCODE MSPA: 0
−:−:−:NA
628:959:220:3

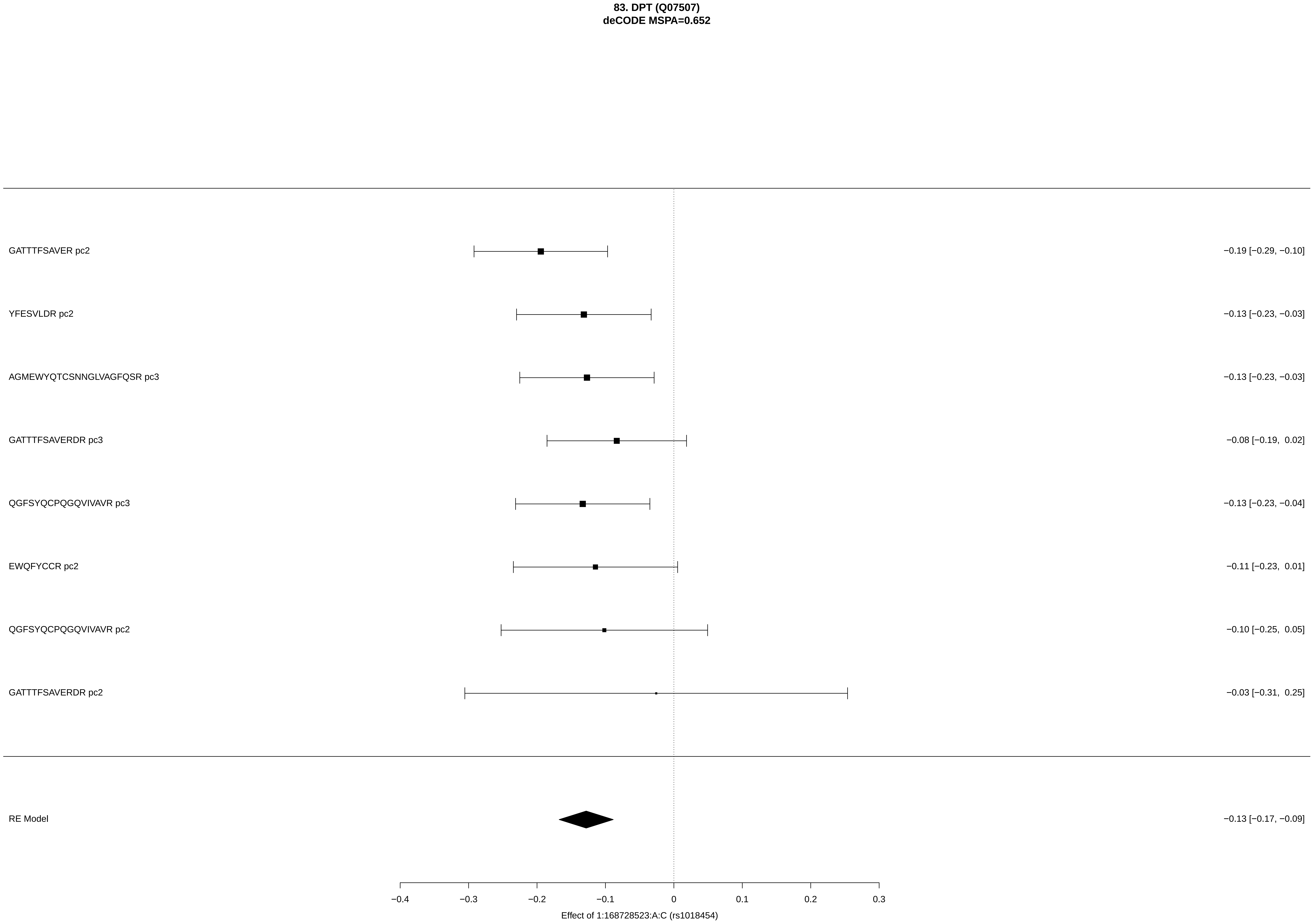

83. DPT (Q07507)
deCODE MSPA=0.652
GATTTFSAVER pc2
−0.19 [−0.29, −0.10]
YFESVLDR pc2
−0.13 [−0.23, −0.03]
AGMEWYQTCSNNGLVAGFQSR pc3
−0.13 [−0.23, −0.03]
GATTTFSAVERDR pc3
−0.08 [−0.19, 0.02]
QGFSYQCPQGQVIVAVR pc3
−0.13 [−0.23, −0.04]
EWQFYCCR pc2
−0.11 [−0.23, 0.01]
QGFSYQCPQGQVIVAVR pc2
−0.10 [−0.25, 0.05]
GATTTFSAVERDR pc2
−0.03 [−0.31, 0.25]
RE Model
−0.13 [−0.17, −0.09]
−0.4
−0.3
−0.2
−0.1
0
0.1
0.2
0.3
Effect of 1:168728523:A:C (rs1018454)

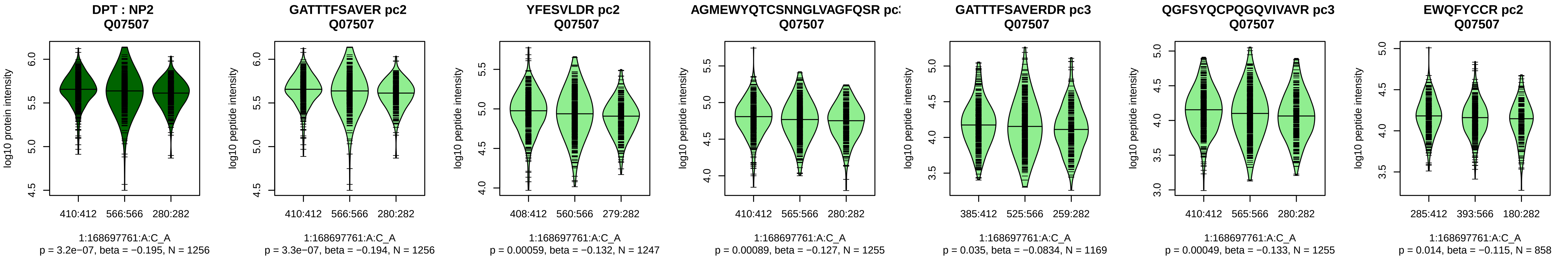

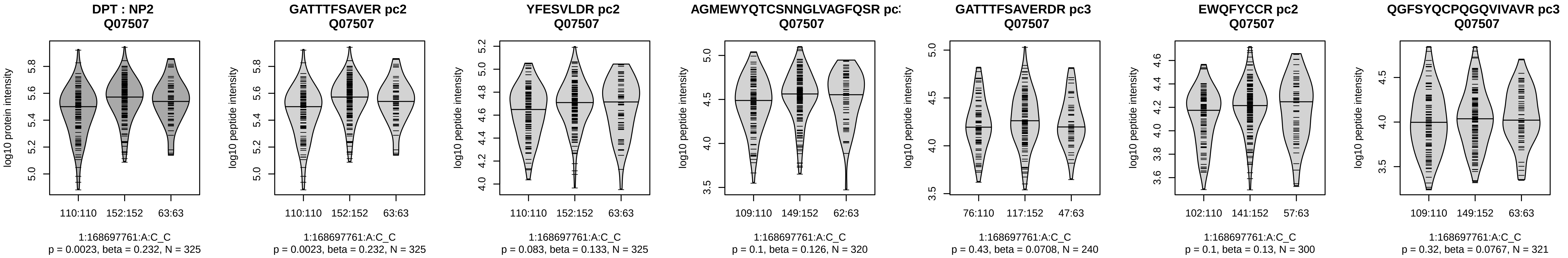

Assay Target: DPT
UniProt: Q07507
rsID: rs1018454
Target SNP: 1:168728523:A:C
Proxy SNP: 1:168697761:A:C
deCODE log10(p): 370
deCODE BETA: −0.34
deCODE MSPA: 0.652
::*:–:*:–:–:–
1256:1247:1255:1169:1255:858

84. INHBB (P09529)
deCODE MSPA=0.678

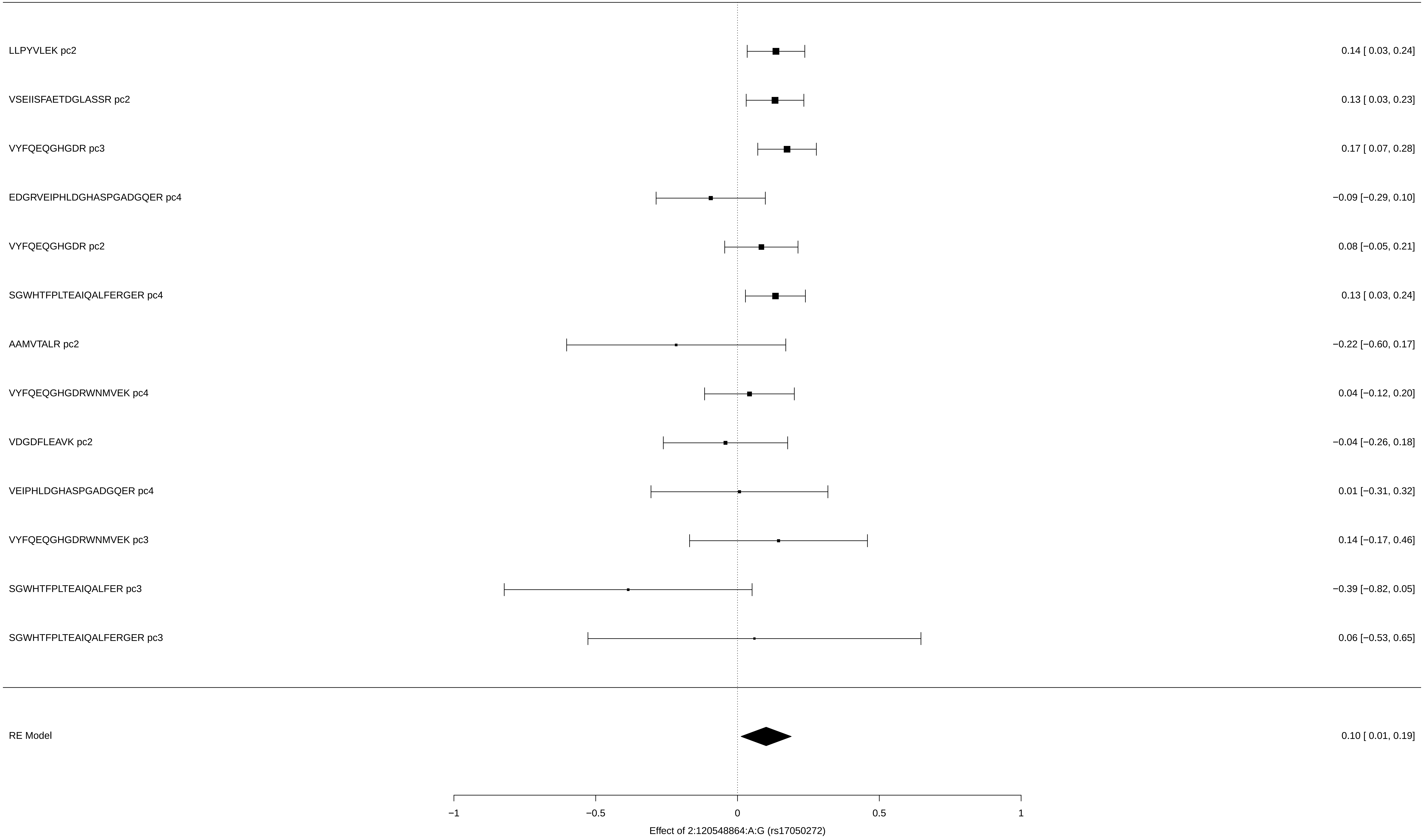

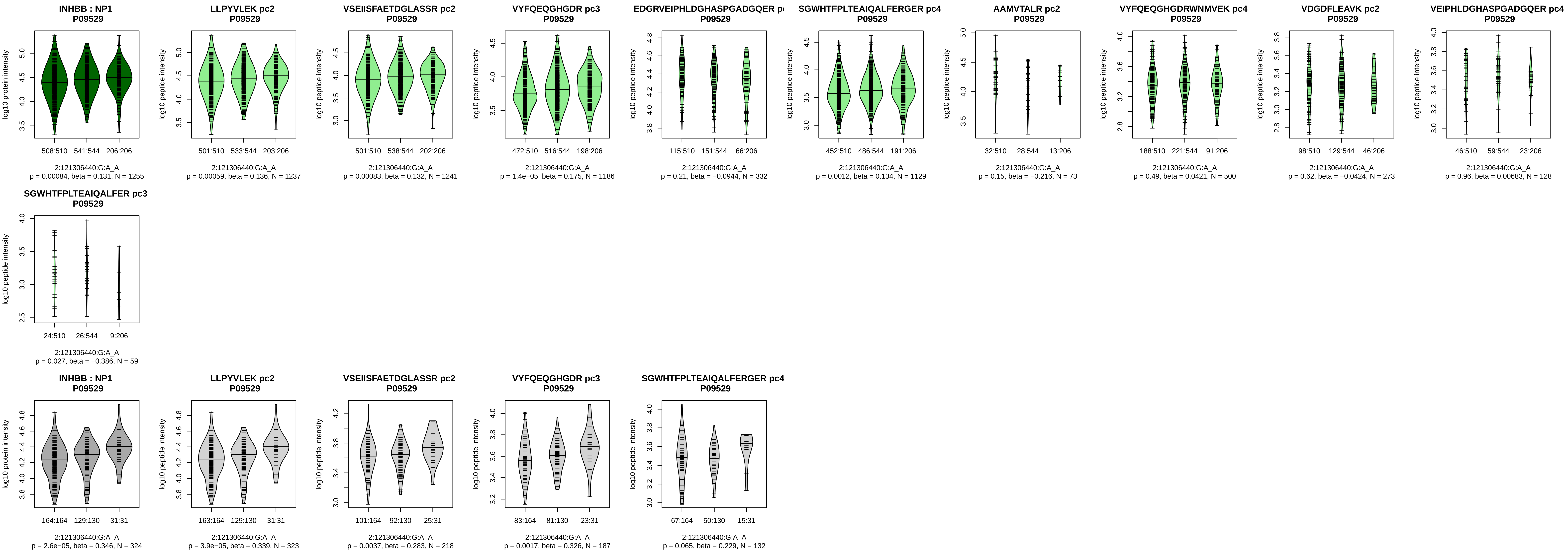

Assay Target: INHBB
UniProt: P09529
rsID: rs17050272
Target SNP: 2:120548864:A:G
Proxy SNP: 2:121306440:G:A
deCODE log10(p): 367.3
deCODE BETA: 0.34
deCODE MSPA: 0.678
::*:−:−:*:−:−:−:−:−:−:−:NA
1237:1241:1186:332:751:1129:7

85. CRTAC1 (Q9NQ79)
deCODE MSPA=0.096

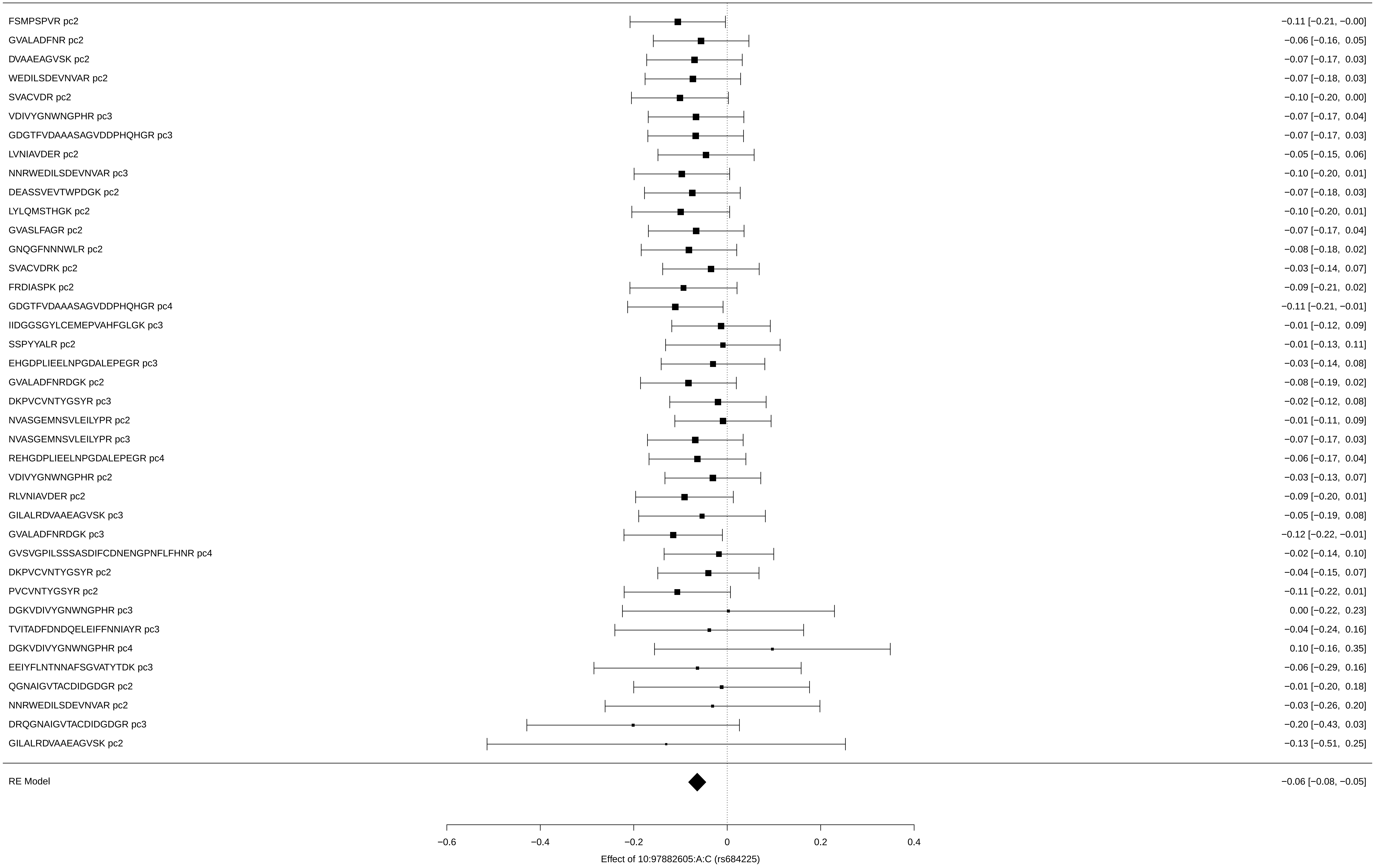

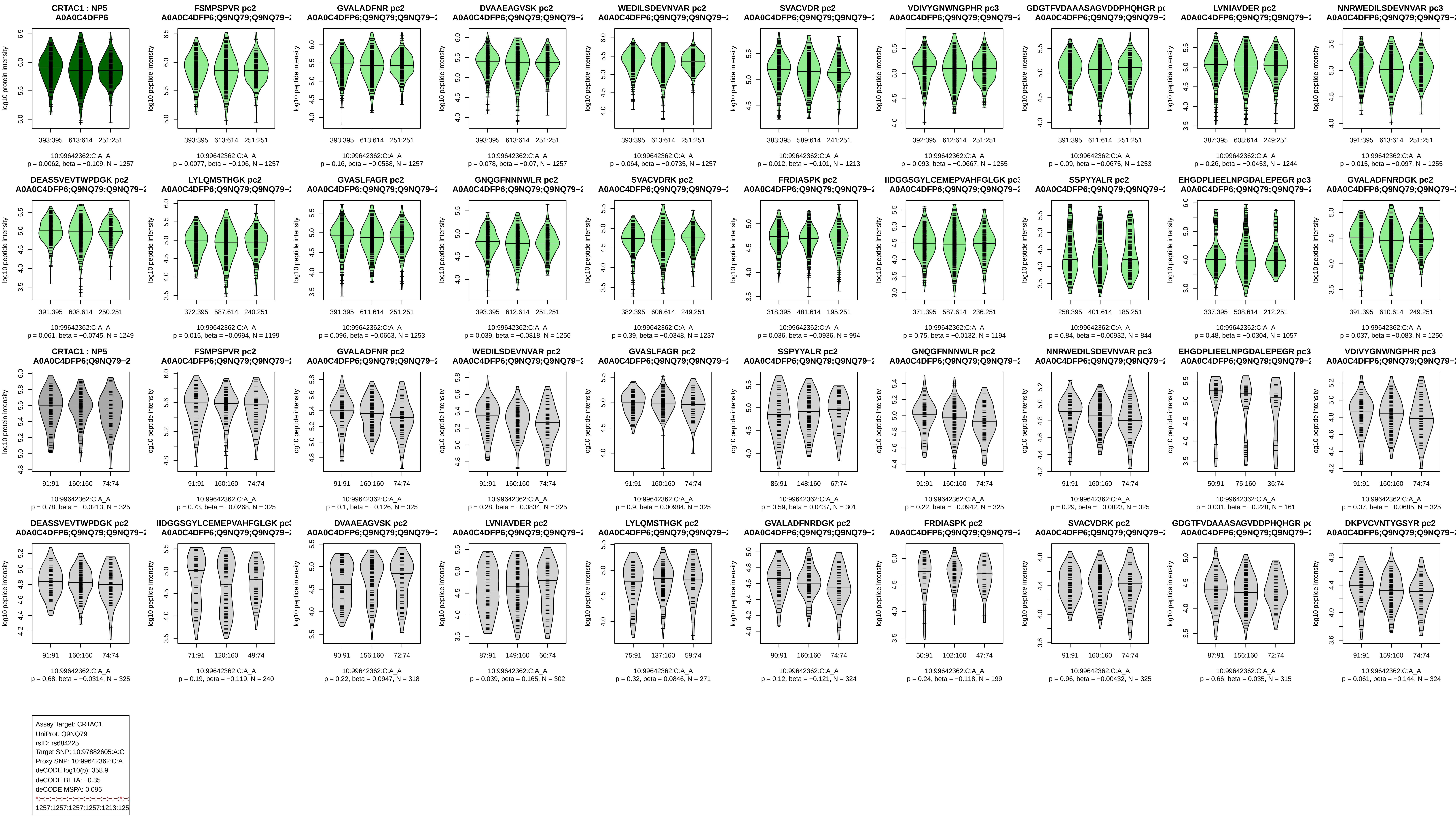
CRTAC1 : NP5
A0A0C4DFP6
log10 protein intensity
393:395 613:614 251:251
10:99642362:C:A_A
p = 0.0062, beta = −0.109, N = 1257
FSMPSPVR pc2
A0A0C4DFP6;Q9NQ79;Q9NQ79−2
log10 peptide intensity
393:395 613:614 251:251
10:99642362:C:A_A
p = 0.0077, beta = −0.106, N = 1257
GVALADFNR pc2
A0A0C4DFP6;Q9NQ79;Q9NQ79−2
393:395 613:614 251:251
10:99642362:C:A_A
p = 0.16, beta = −0.0558, N = 1257
DVAAEAGVSK pc2
A0A0C4DFP6;Q9NQ79;Q9NQ79−2
393:395 613:614 251:251
10:99642362:C:A_A
p = 0.078, beta = −0.07, N = 1257
WEDILSDEVNVAR pc2
A0A0C4DFP6;Q9NQ79;Q9NQ79−2
393:395 613:614 251:251
10:99642362:C:A_A
p = 0.064, beta = −0.0735, N = 1257
SVACVDR pc2
A0A0C4DFP6;Q9NQ79;Q9NQ79−2
383:395 589:614 241:251
10:99642362:C:A_A
p = 0.012, beta = −0.101, N = 1213
VDIVYGNWNGPHR pc3
A0A0C4DFP6;Q9NQ79;Q9NQ79−2
392:395 612:614 251:251
10:99642362:C:A_A
p = 0.093, beta = −0.0667, N = 1255
GDGTFVDAAASAGVDDPHQHGR pc
A0A0C4DFP6;Q9NQ79;Q9NQ79−2
391:395 611:614 251:251
10:99642362:C:A_A
p = 0.09, beta = −0.0675, N = 1253
LVNIAVDER pc2
A0A0C4DFP6;Q9NQ79;Q9NQ79−2
387:395 608:614 249:251
10:99642362:C:A_A
p = 0.26, beta = −0.0453, N = 1244
NNRWEDILSDEVNVAR pc3
A0A0C4DFP6;Q9NQ79;Q9NQ79−2
391:395 613:614 251:251
10:99642362:C:A_A
p = 0.015, beta = −0.097, N = 1255
DEASSVEVTWPDGK pc2
A0A0C4DFP6;Q9NQ79;Q9NQ79−2
391:395 608:614 250:251
10:99642362:C:A_A
p = 0.061, beta = −0.0745, N = 1249
LYLQMSTHGK pc2
A0A0C4DFP6;Q9NQ79;Q9NQ79−2
372:395 587:614 240:251
10:99642362:C:A_A
p = 0.015, beta = −0.0994, N = 1199
GVASLFAGR pc2
A0A0C4DFP6;Q9NQ79;Q9NQ79−2
391:395 611:614 251:251
10:99642362:C:A_A
p = 0.096, beta = −0.0663, N = 1253
GNQGFNNNWLR pc2
A0A0C4DFP6;Q9NQ79;Q9NQ79−2
393:395 612:614 251:251
10:99642362:C:A_A
p = 0.039, beta = −0.0818, N = 1256
SVACVDRK pc2
A0A0C4DFP6;Q9NQ79;Q9NQ79−2
382:395 606:614 249:251
10:99642362:C:A_A
p = 0.39, beta = −0.0348, N = 1237
FRDIASPK pc2
A0A0C4DFP6;Q9NQ79;Q9NQ79−2
318:395 481:614 195:251
10:99642362:C:A_A
p = 0.036, beta = −0.0936, N = 994
IIDGGSGYLCEMEPVAHFGLGK pc3
A0A0C4DFP6;Q9NQ79;Q9NQ79−2
371:395 587:614 236:251
10:99642362:C:A_A
p = 0.75, beta = −0.0132, N = 1194
SSPYYALR pc2
A0A0C4DFP6;Q9NQ79;Q9NQ79−2
258:395 401:614 185:251
10:99642362:C:A_A
p = 0.84, beta = −0.00932, N = 844
EHGDPLIEELNPGDALEPEGR pc3
A0A0C4DFP6;Q9NQ79;Q9NQ79−2
337:395 508:614 212:251
10:99642362:C:A_A
p = 0.48, beta = −0.0304, N = 1057
GVALADFNRDGK pc2
A0A0C4DFP6;Q9NQ79;Q9NQ79−2
391:395 610:614 249:251
10:99642362:C:A_A
p = 0.037, beta = −0.083, N = 1250
CRTAC1 : NP5
A0A0C4DFP6;Q9NQ79−2
91:91 160:160 74:74
10:99642362:C:A_A
p = 0.78, beta = −0.0213, N = 325
FSMPSPVR pc2
A0A0C4DFP6;Q9NQ79;Q9NQ79−2
91:91 160:160 74:74
10:99642362:C:A_A
p = 0.73, beta = −0.0268, N = 325
GVALADFNR pc2
A0A0C4DFP6;Q9NQ79;Q9NQ79−2
91:91 160:160 74:74
10:99642362:C:A_A
p = 0.1, beta = −0.126, N = 325
WEDILSDEVNVAR pc2
A0A0C4DFP6;Q9NQ79;Q9NQ79−2
91:91 160:160 74:74
10:99642362:C:A_A
p = 0.28, beta = −0.0834, N = 325
GVASLFAGR pc2
A0A0C4DFP6;Q9NQ79;Q9NQ79−2
91:91 160:160 74:74
10:99642362:C:A_A
p = 0.9, beta = 0.00984, N = 325
SSPYYALR pc2
A0A0C4DFP6;Q9NQ79;Q9NQ79−2
86:91 148:160 67:74
10:99642362:C:A_A
p = 0.59, beta = 0.0437, N = 301
GNQGFNNNWLR pc2
A0A0C4DFP6;Q9NQ79;Q9NQ79−2
91:91 160:160 74:74
10:99642362:C:A_A
p = 0.22, beta = −0.0942, N = 325
NNRWEDILSDEVNVAR pc3
A0A0C4DFP6;Q9NQ79;Q9NQ79−2
91:91 160:160 74:74
10:99642362:C:A_A
p = 0.29, beta = −0.0823, N = 325
EHGDPLIEELNPGDALEPEGR pc3
A0A0C4DFP6;Q9NQ79;Q9NQ79−2
50:91 75:160 36:74
10:99642362:C:A_A
p = 0.031, beta = −0.228, N = 161
VDIVYGNWNGPHR pc3
A0A0C4DFP6;Q9NQ79;Q9NQ79−2
91:91 160:160 74:74
10:99642362:C:A_A
p = 0.37, beta = −0.0685, N = 325
DEASSVEVTWPDGK pc2
A0A0C4DFP6;Q9NQ79;Q9NQ79−2
91:91 160:160 74:74
10:99642362:C:A_A
p = 0.68, beta = −0.0314, N = 325
IIDGGSGYLCEMEPVAHFGLGK pc3
A0A0C4DFP6;Q9NQ79;Q9NQ79−2
71:91 120:160 49:74
10:99642362:C:A_A
p = 0.19, beta = −0.119, N = 240
DVAAEAGVSK pc2
A0A0C4DFP6;Q9NQ79;Q9NQ79−2
90:91 156:160 72:74
10:99642362:C:A_A
p = 0.22, beta = 0.0947, N = 318
LVNIAVDER pc2
A0A0C4DFP6;Q9NQ79;Q9NQ79−2
87:91 149:160 66:74
10:99642362:C:A_A
p = 0.039, beta = 0.165, N = 302
LYLQMSTHGK pc2
A0A0C4DFP6;Q9NQ79;Q9NQ79−2
75:91 137:160 59:74
10:99642362:C:A_A
p = 0.32, beta = 0.0846, N = 271
GVALADFNRDGK pc2
A0A0C4DFP6;Q9NQ79;Q9NQ79−2
90:91 160:160 74:74
10:99642362:C:A_A
p = 0.12, beta = −0.121, N = 324
FRDIASPK pc2
A0A0C4DFP6;Q9NQ79;Q9NQ79−2
50:91 102:160 47:74
10:99642362:C:A_A
p = 0.24, beta = −0.118, N = 199
SVACVDRK pc2
A0A0C4DFP6;Q9NQ79;Q9NQ79−2
91:91 160:160 74:74
10:99642362:C:A_A
p = 0.96, beta = −0.00432, N = 325
GDGTFVDAAASAGVDDPHQHGR pc
A0A0C4DFP6;Q9NQ79;Q9NQ79−2
87:91 156:160 72:74
10:99642362:C:A_A
p = 0.66, beta = 0.035, N = 315
DKPVCVNTYGSYR pc2
A0A0C4DFP6;Q9NQ79;Q9NQ79−2
91:91 159:160 74:74
10:99642362:C:A_A
p = 0.061, beta = −0.144, N = 324
Assay Target: CRTAC1
UniProt: Q9NQ79
rsID: rs684225
Target SNP: 10:97882605:A:C
Proxy SNP: 10:99642362:C:A
deCODE log10(p): 358.9
deCODE BETA: −0.35
deCODE MSPA: 0.096
1257:1257:1257:1257:1213:125

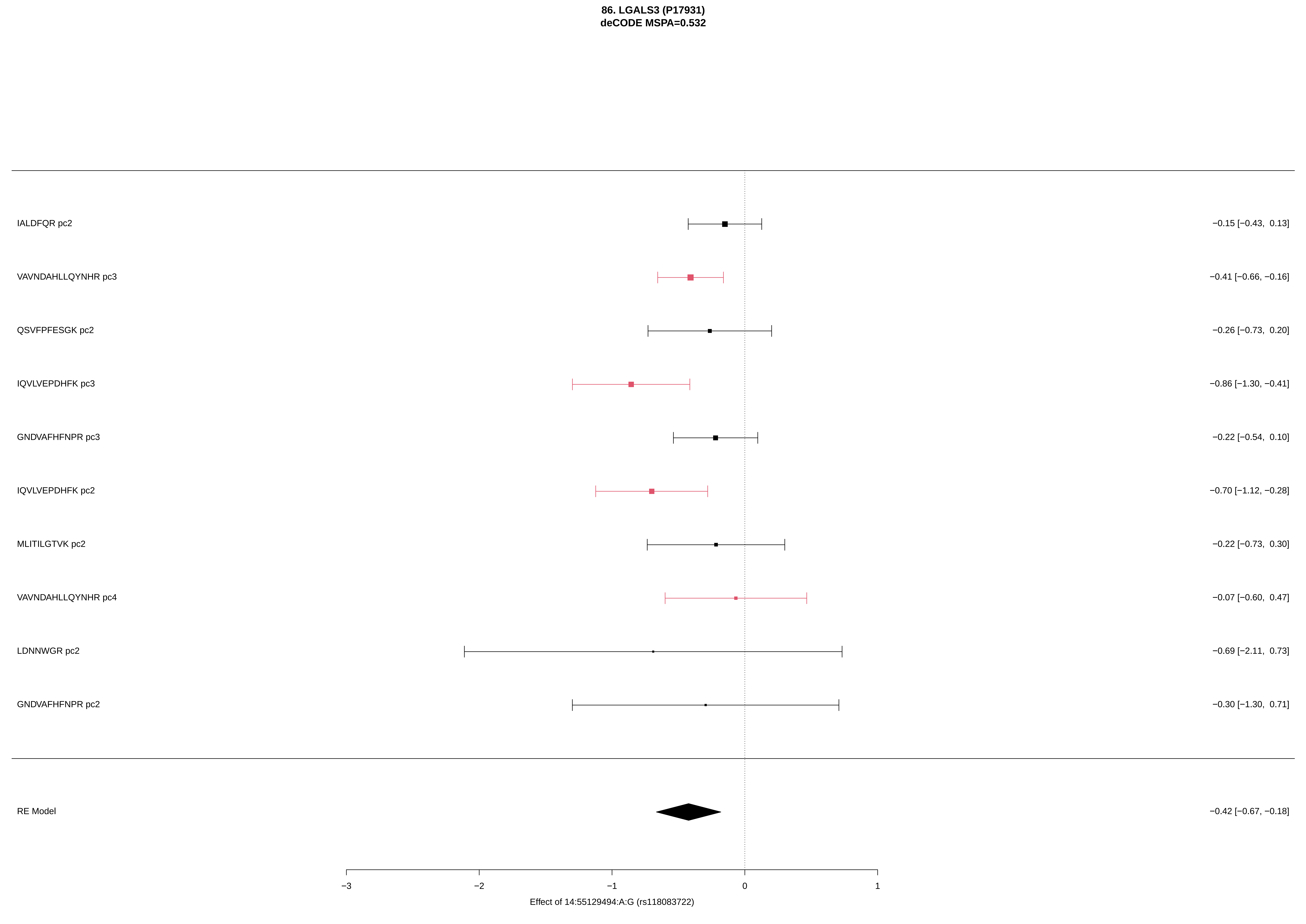
86. LGALS3 (P17931)
deCODE MSPA=0.532
IALDFQR pc2
VAVNDAHLLQYNHR pc3
QSVFPFESGK pc2
IQVLVEPDHFK pc3
GNDVAFHFNPR pc3
IQVLVEPDHFK pc2
MLITILGTVK pc2
VAVNDAHLLQYNHR pc4
LDNNWGR pc2
GNDVAFHFNPR pc2
RE Model
−0.15 [−0.43, 0.13]
−0.41 [−0.66, −0.16]
−0.26 [−0.73, 0.20]
−0.86 [−1.30, −0.41]
−0.22 [−0.54, 0.10]
−0.70 [−1.12, −0.28]
−0.22 [−0.73, 0.30]
−0.07 [−0.60, 0.47]
−0.69 [−2.11, 0.73]
−0.30 [−1.30, 0.71]
−0.42 [−0.67, −0.18]
−3
−2
−1
0
1
Effect of 14:55129494:A:G (rs118083722)

LGALS3 : NP3
P17931

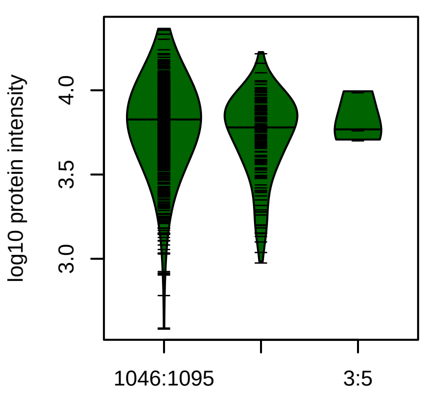

14:55596212:G:A_A
p = 0.00038, beta = −0.297, N = 1199

IALDFQR pc2
G3V3R6;P17931

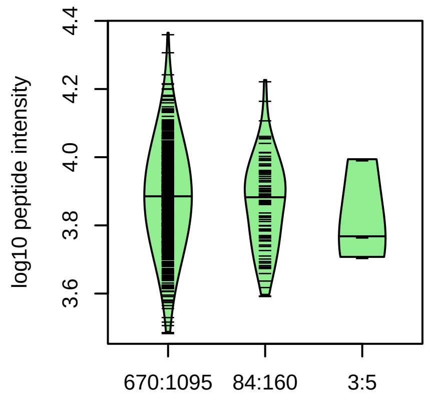

14:55596212:G:A_A
p = 0.16, beta = −0.15, N = 757

VAVNDAHLLQYNHR pc3
P17931

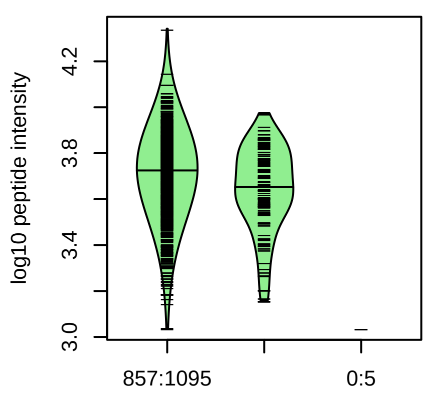

14:55596212:G:A_A
p = 2.3e−05, beta = −0.409, N = 977

QSVFPFESGK pc2
G3V3R6;P17931

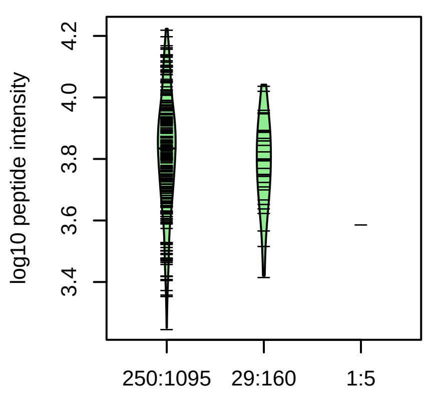

14:55596212:G:A_A
p = 0.15, beta = −0.264, N = 280

IQVLVEPDHFK pc3
P17931

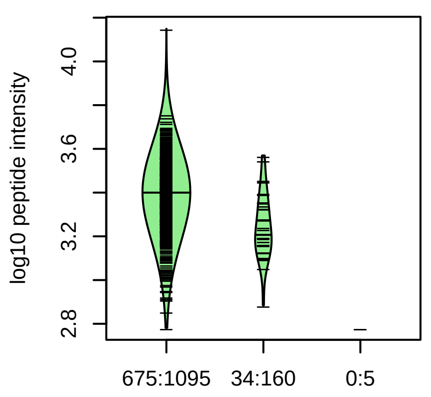

14:55596212:G:A_A
p = 7.7e−07, beta = −0.856, N = 709

GNDVAFHFNPR pc3
G3V3R6;P17931

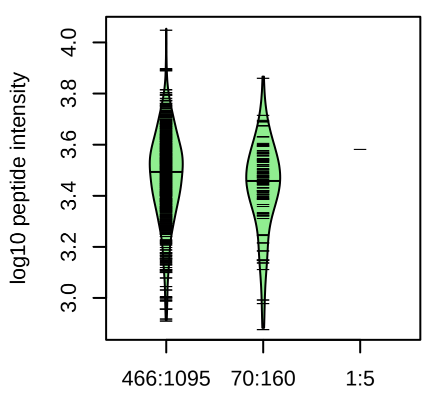

14:55596212:G:A_A
p = 0.075, beta = −0.22, N = 537

MLITILGTVK pc2
G3V3R6;P17931

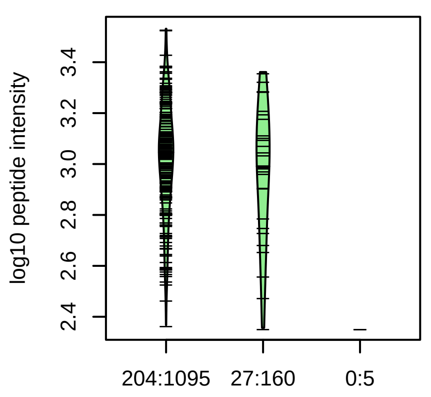

14:55596212:G:A_A
p = 0.28, beta = −0.217, N = 231

LDNNWGR pc2
G3V3R6;P17931

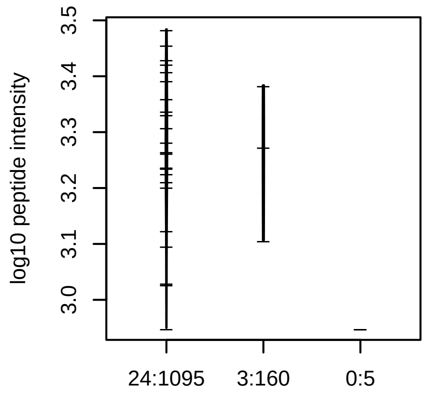

14:55596212:G:A_A
p = 0.22, beta = −0.69, N = 27

LGALS3 : NP3
P17931

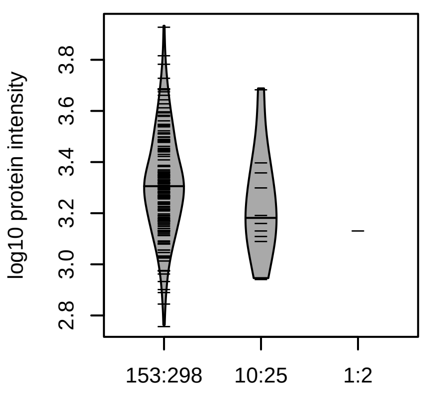

14:55596212:G:A_A
p = 0.31, beta = −0.275, N = 164

VAVNDAHLLQYNHR pc3
P17931

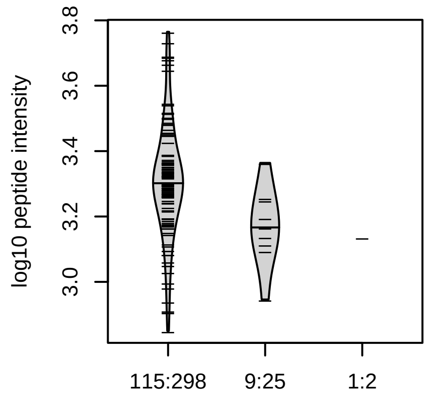

14:55596212:G:A_A
p = 0.026, beta = −0.622, N = 125

IQVLVEPDHFK pc2
P17931

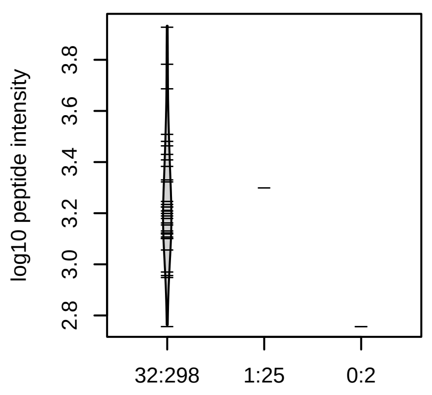

14:55596212:G:A_A
p = 0.68, beta = 0.389, N = 33

Assay Target: LGALS3
UniProt: P17931
rsID: rs118083722
Target SNP: 14:55129494:A:G
Proxy SNP: 14:55596212:G:A
deCODE log10(p): 357.8
deCODE BETA: −0.57
deCODE MSPA: 0.532
–:*:–:*:–:*:–:–:–:–
757:977:280:709:537:658:231:1

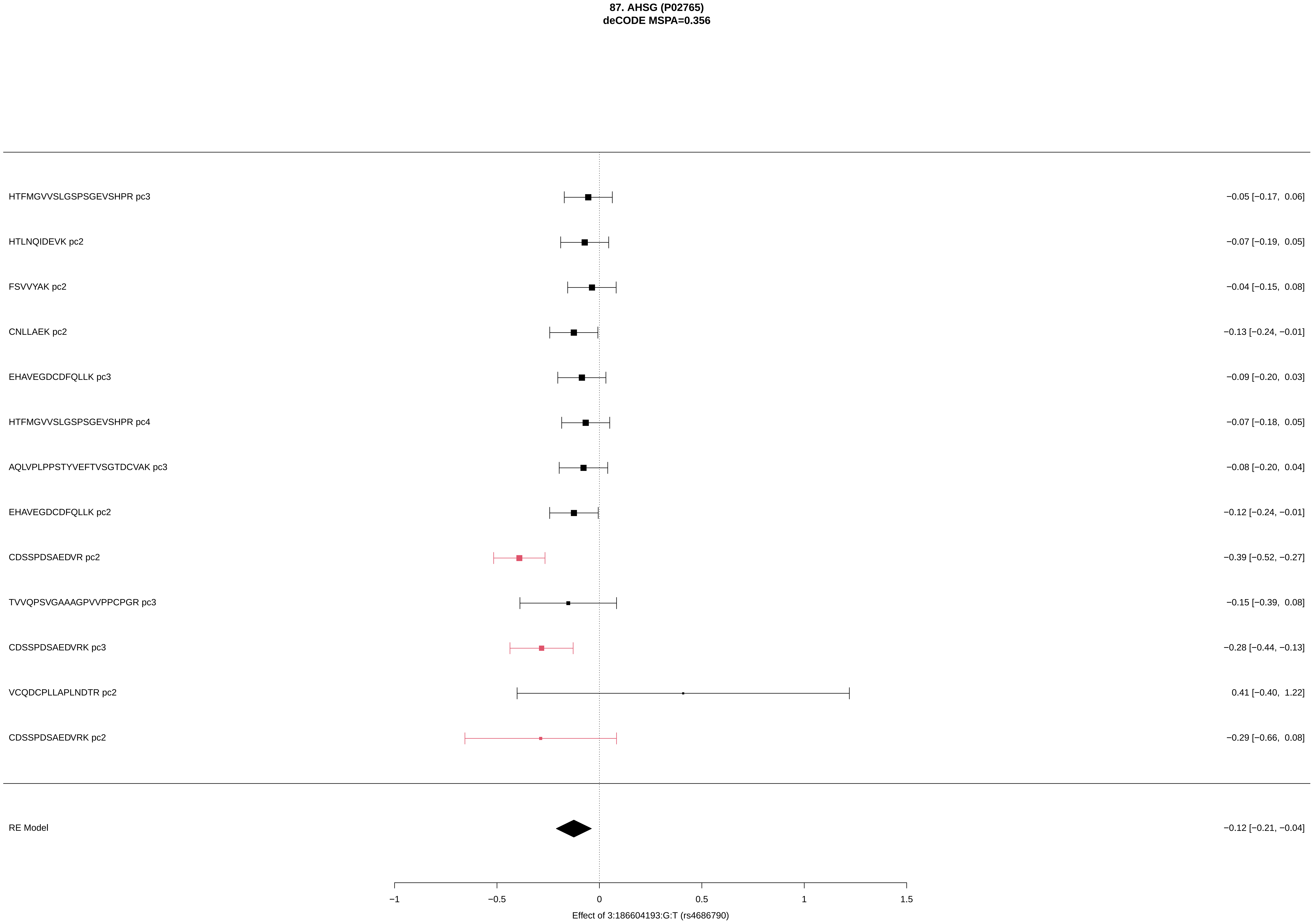
87. AHSG (P02765)
deCODE MSPA=0.356
HTFMGVVSLGSPSGEVSHPR pc3
−0.05 [−0.17, 0.06]
HTLNQIDEVK pc2
−0.07 [−0.19, 0.05]
FSVVYAK pc2
−0.04 [−0.15, 0.08]
CNLLAEK pc2
−0.13 [−0.24, −0.01]
EHAVEGDCDFQLLK pc3
−0.09 [−0.20, 0.03]
HTFMGVVSLGSPSGEVSHPR pc4
−0.07 [−0.18, 0.05]
AQLVPLPPSTYVEFTVSGTDCVAK pc3
−0.08 [−0.20, 0.04]
EHAVEGDCDFQLLK pc2
−0.12 [−0.24, −0.01]
CDSSPDSAEDVR pc2
−0.39 [−0.52, −0.27]
TVVQPSVGAAAGPVVPPCPGR pc3
−0.15 [−0.39, 0.08]
CDSSPDSAEDVRK pc3
−0.28 [−0.44, −0.13]
VCQDCPLLAPLNDTR pc2
0.41 [−0.40, 1.22]
CDSSPDSAEDVRK pc2
−0.29 [−0.66, 0.08]
RE Model
−0.12 [−0.21, −0.04]
−1
−0.5
0
0.5
1
1.5
Effect of 3:186604193:G:T (rs4686790)

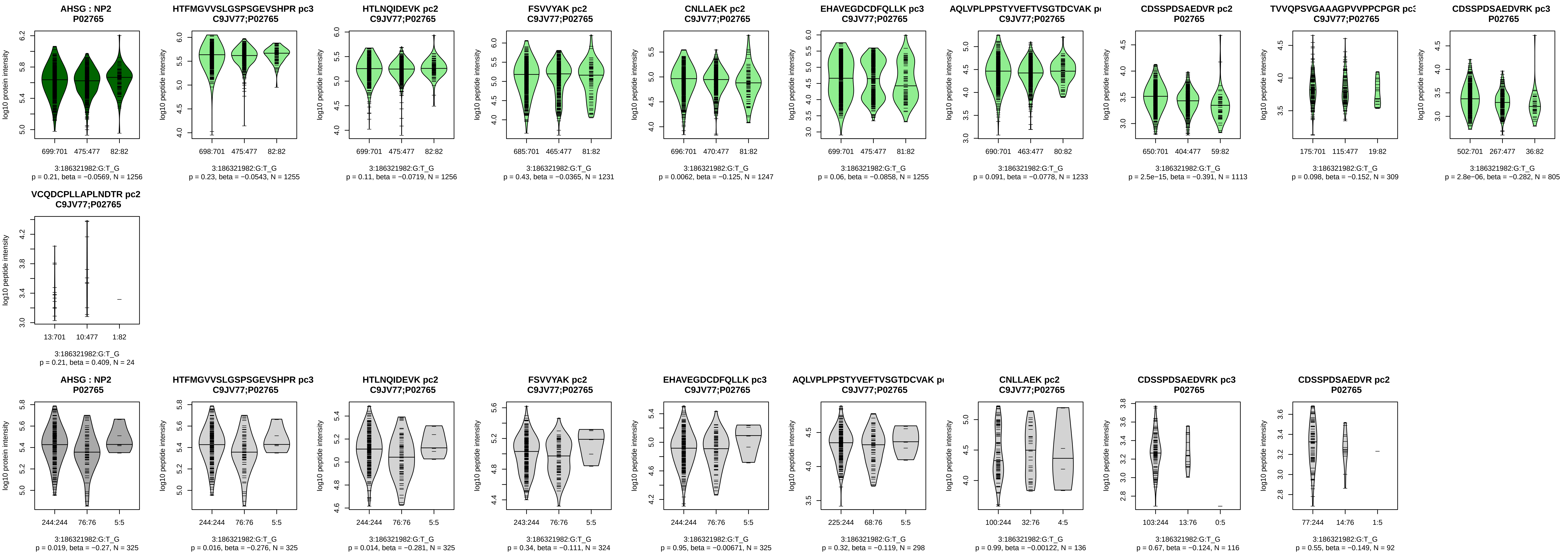

Assay Target: AHSG
UniProt: P02765
rsID: rs4686790
Target SNP: 3:186604193:G:T
Proxy SNP: 3:186321982:G:T
deCODE log10(p): 346.1
deCODE BETA: −0.36
deCODE MSPA: 0.356
−:−:−:*:−:−:−:*:*:−:*:−:−
1255:1256:1231:1247:1255:125

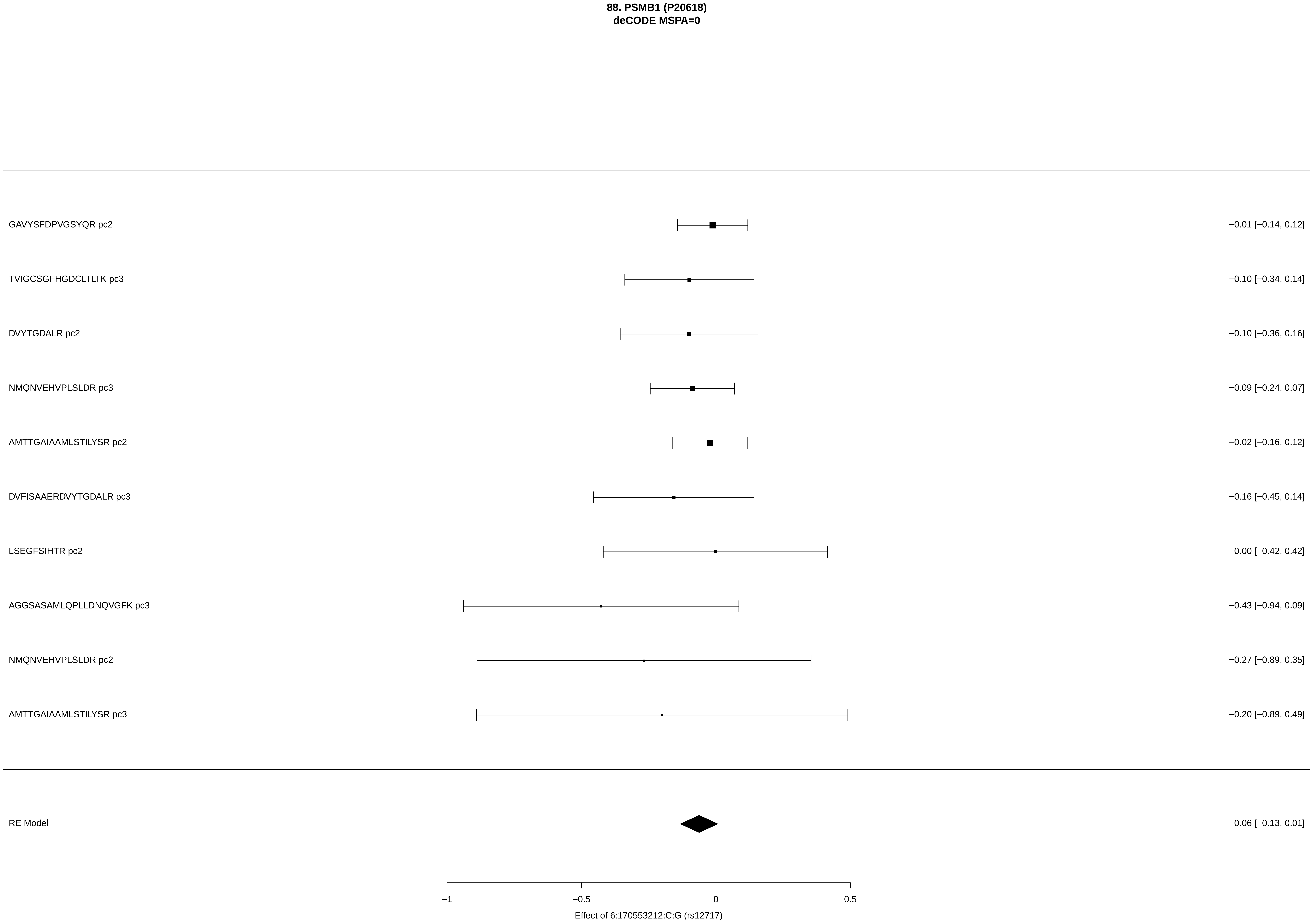
88. PSMB1 (P20618)
deCODE MSPA=0
GAVYSFDPVGSYQR pc2
−0.01 [−0.14, 0.12]
TVIGCSGFHGDCLTLTK pc3
−0.10 [−0.34, 0.14]
DVYTGDALR pc2
−0.10 [−0.36, 0.16]
NMQNVEHVPLSLDR pc3
−0.09 [−0.24, 0.07]
AMTTGAIAAMLSTILYSR pc2
−0.02 [−0.16, 0.12]
DVFISAAERDVYTGDALR pc3
−0.16 [−0.45, 0.14]
LSEGFSIHTR pc2
−0.00 [−0.42, 0.42]
AGGSASAMLQPLLDNQVGFK pc3
−0.43 [−0.94, 0.09]
NMQNVEHVPLSLDR pc2
−0.27 [−0.89, 0.35]
AMTTGAIAAMLSTILYSR pc3
−0.20 [−0.89, 0.49]
RE Model
−0.06 [−0.13, 0.01]
−1
−0.5
0
0.5
Effect of 6:170553212:C:G (rs12717)

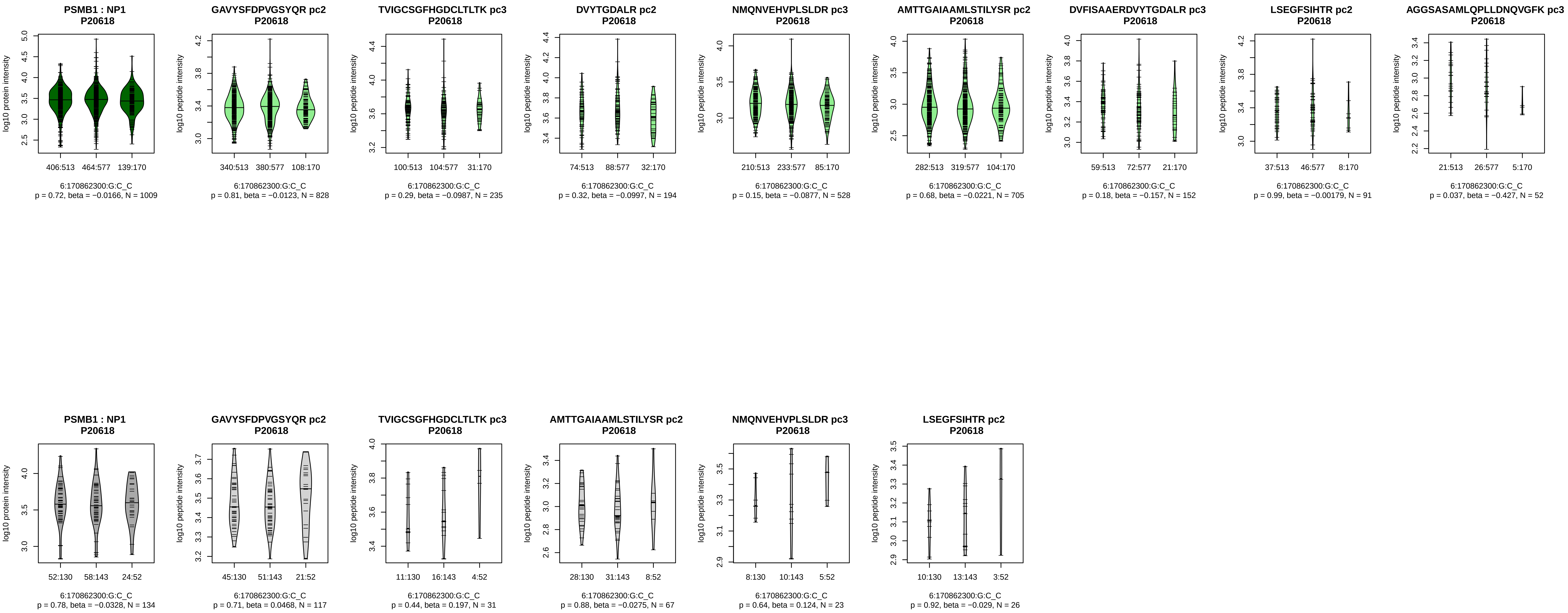

Assay Target: PSMB1
UniProt: P20618
rsID: rs12717
Target SNP: 6:170553212:C:G
Proxy SNP: 6:170862300:G:C
deCODE log10(p): 345.1
deCODE BETA: −0.31
deCODE MSPA: 0
−:−:−:−:−:NA:−:−:NA:−:−:−
828:235:194:528:705:18:152:91

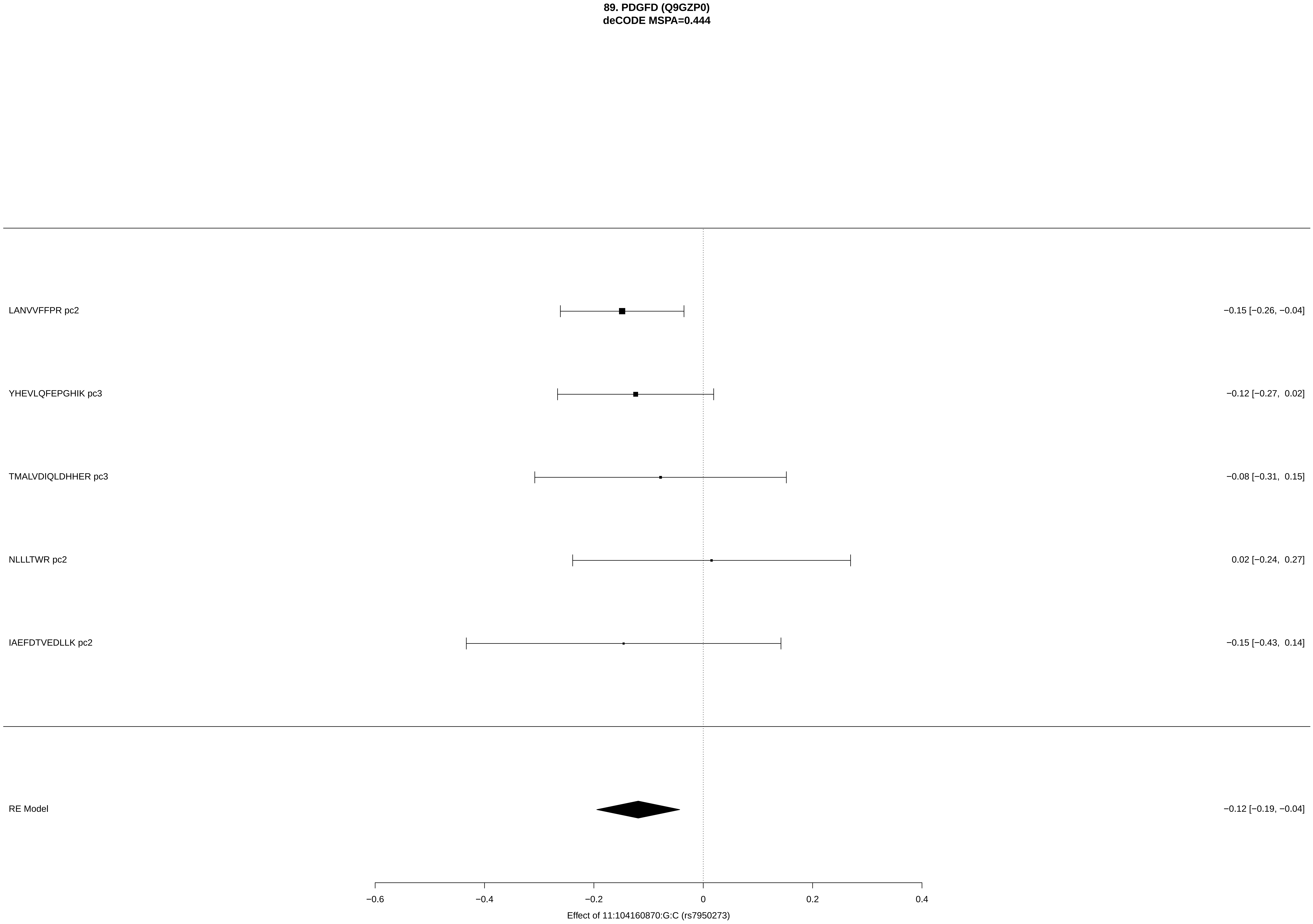
89. PDGFD (Q9GZP0)
deCODE MSPA=0.444
LANVVFFPR pc2
−0.15 [−0.26, −0.04]
YHEVLQFEPGHIK pc3
−0.12 [−0.27, 0.02]
TMALVDIQLDHHER pc3
−0.08 [−0.31, 0.15]
NLLLTWR pc2
0.02 [−0.24, 0.27]
IAEFDTVEDLLK pc2
−0.15 [−0.43, 0.14]
RE Model
−0.12 [−0.19, −0.04]
−0.6
−0.4
−0.2
0
0.2
0.4
Effect of 11:104160870:G:C (rs7950273)

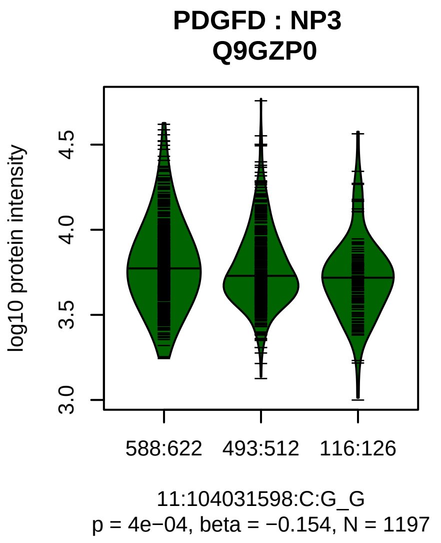

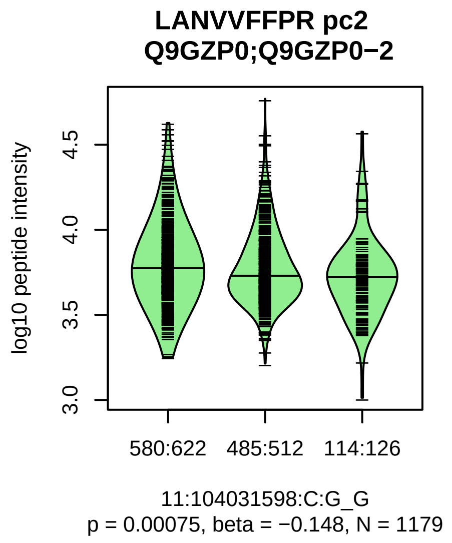

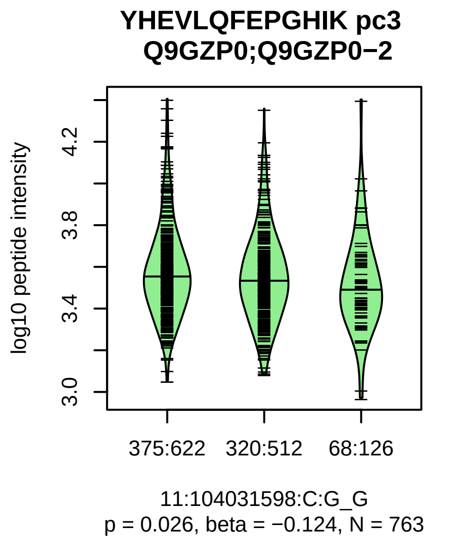

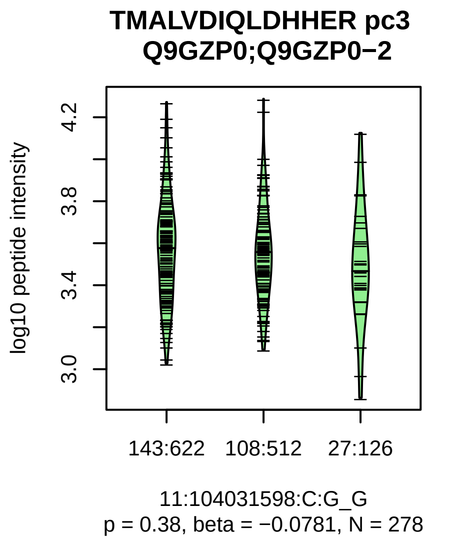

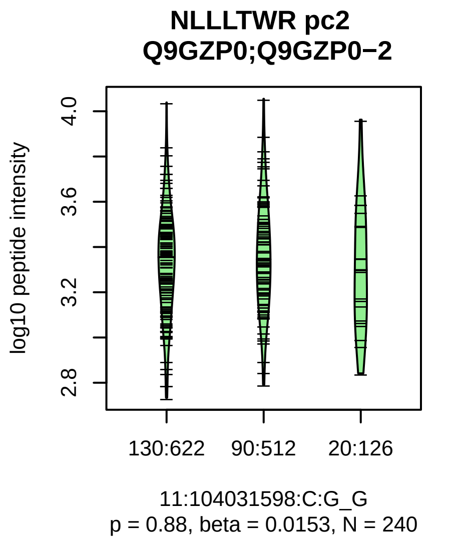

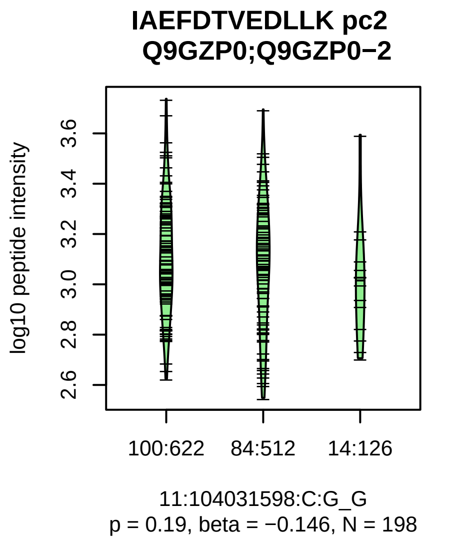

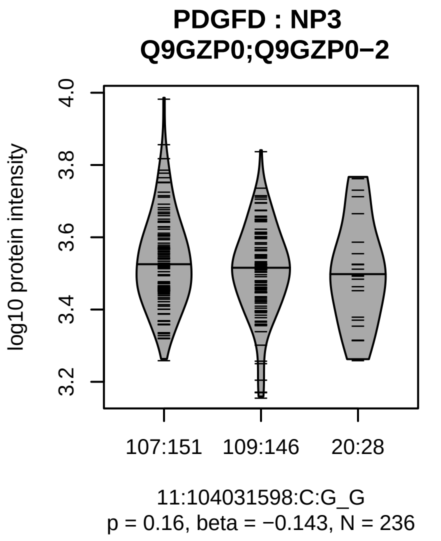

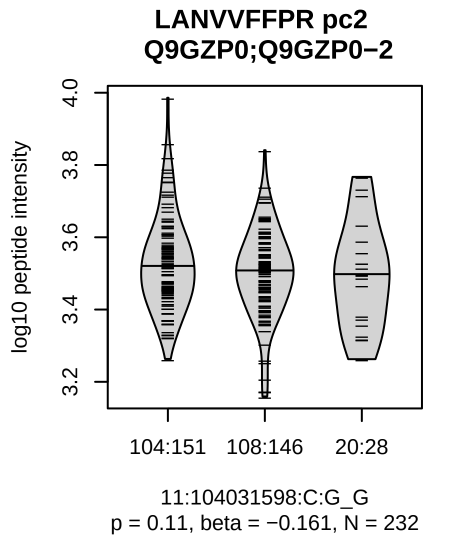

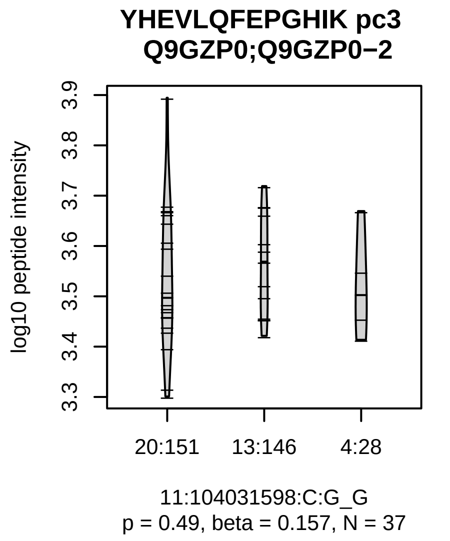

Assay Target: PDGFD
UniProt: Q9GZP0
rsID: rs7950273
Target SNP: 11:104160870:G:C
Proxy SNP: 11:104031598:C:G
deCODE log10(p): 342.9
deCODE BETA: −0.35
deCODE MSPA: 0.444
*:−:−:−:−:NA:NA:NA:NA
1179:763:278:240:198:13:8:7:3

90. F11 (P03951)
deCODE MSPA=0.856

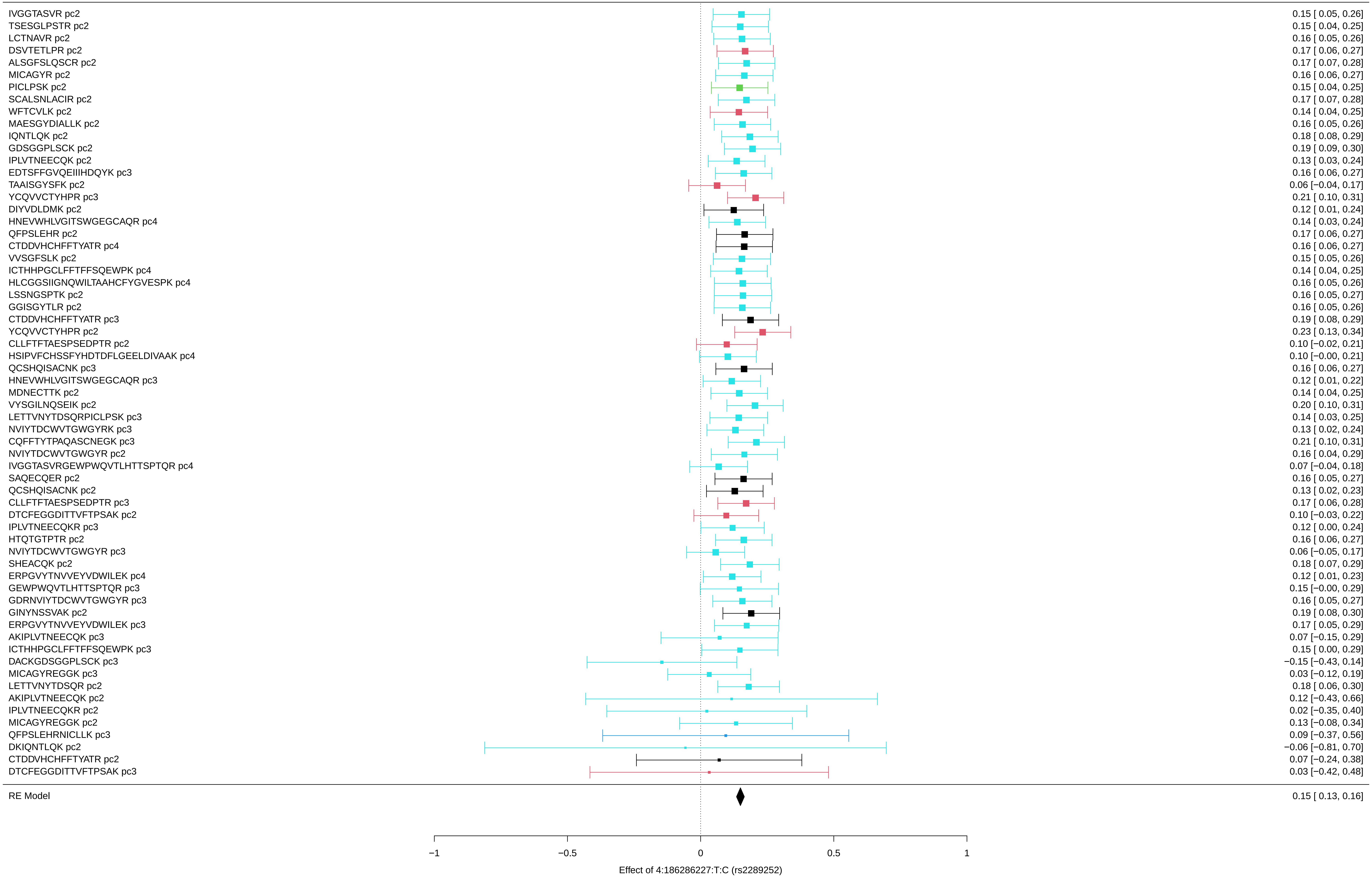

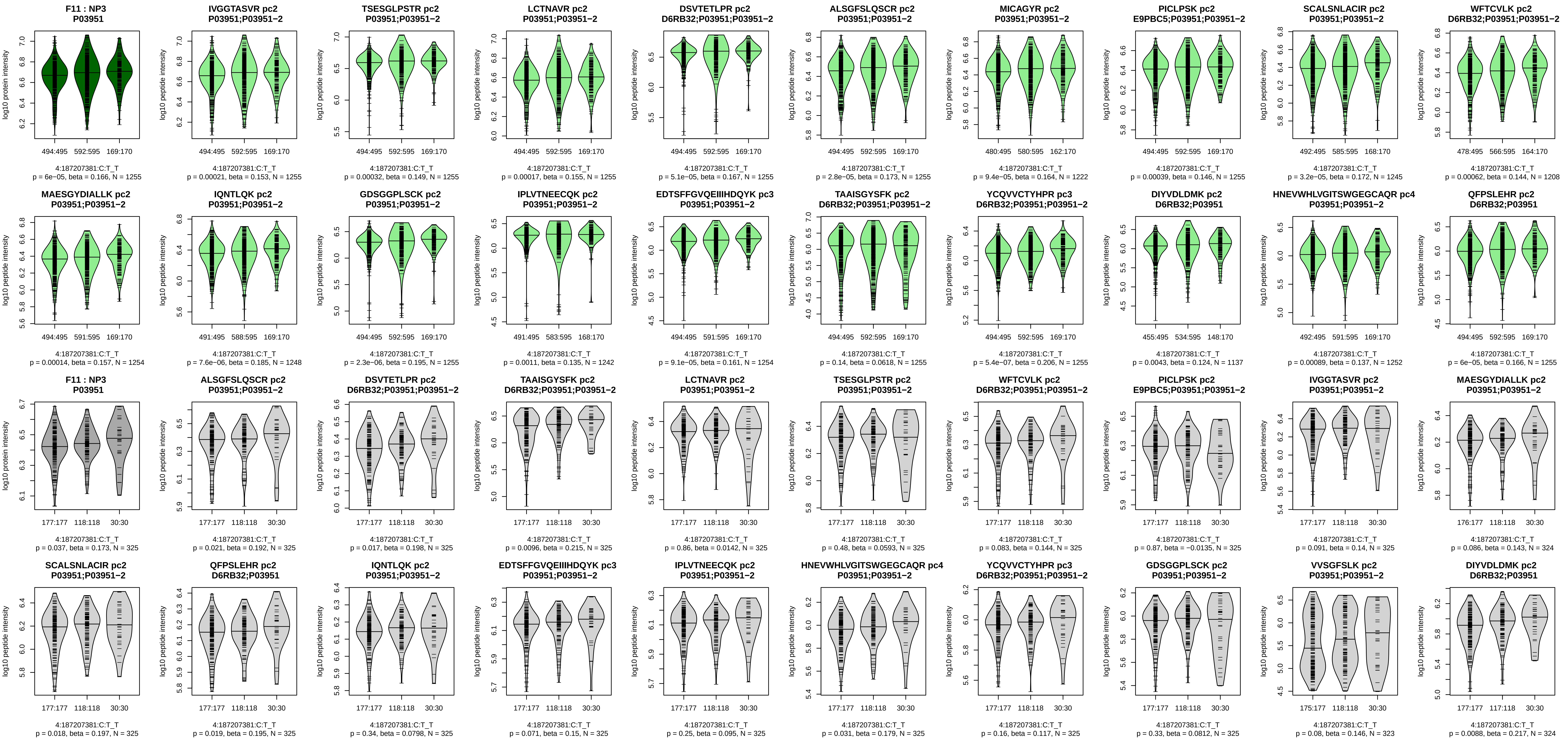

Assay Target: F11
UniProt: P03951
rsID: rs2289252
Target SNP: 4:186286227:T:C
Proxy SNP: 4:187207381:C:T
deCODE log10(p): 331.7
deCODE BETA: 0.33
deCODE MSPA: 0.856
[illegible]
1255:1255:1255:1255:1255:122

91. CD8A (P01732)
deCODE MSPA=0.818

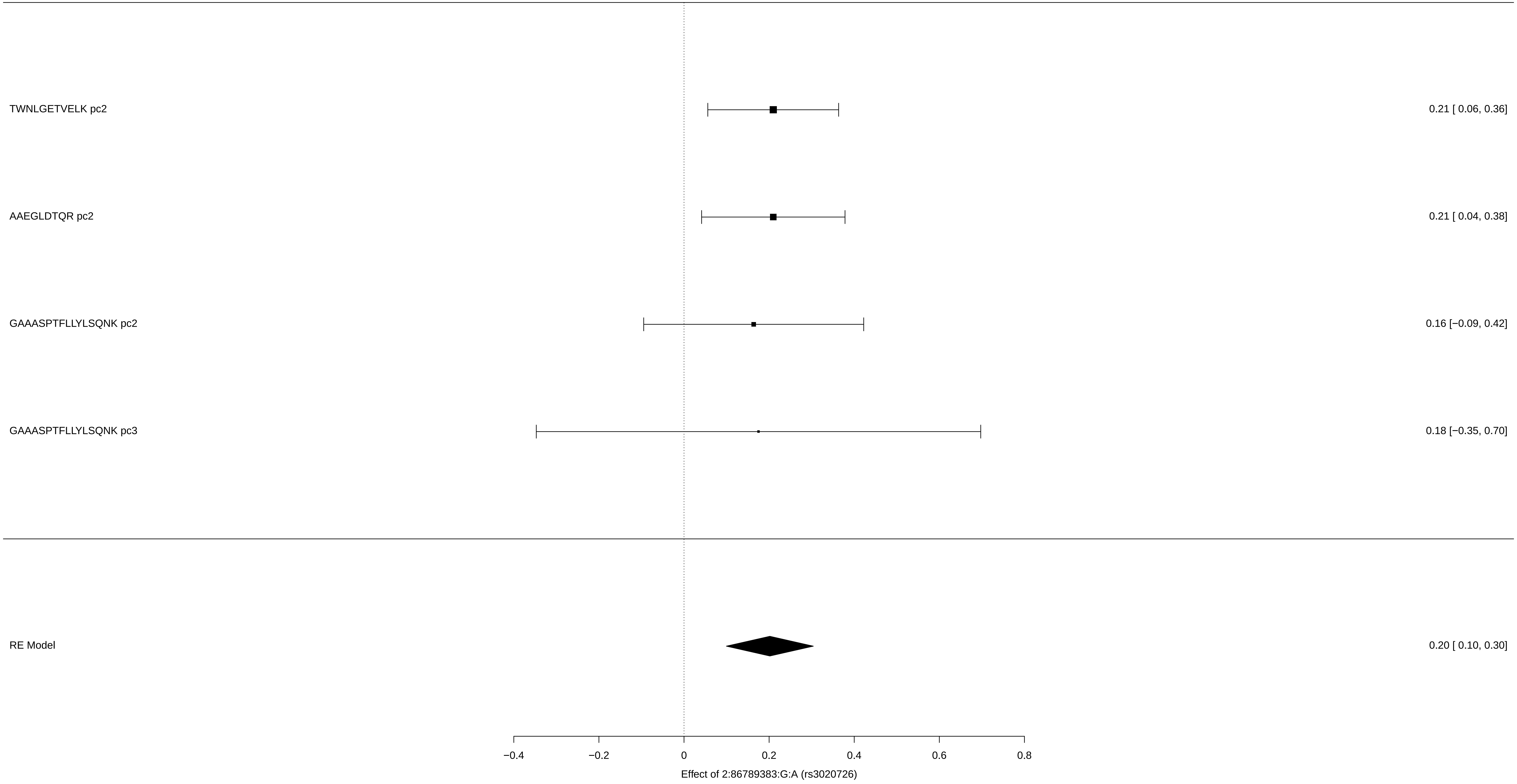

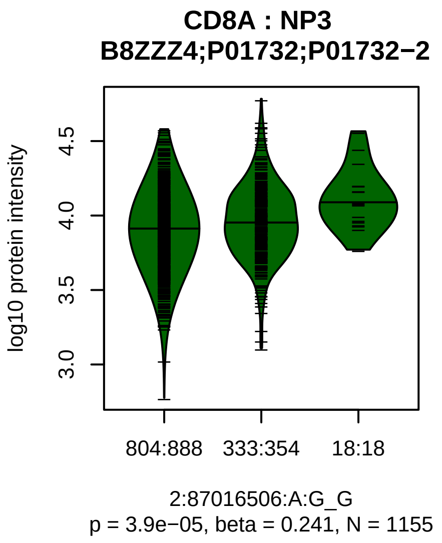

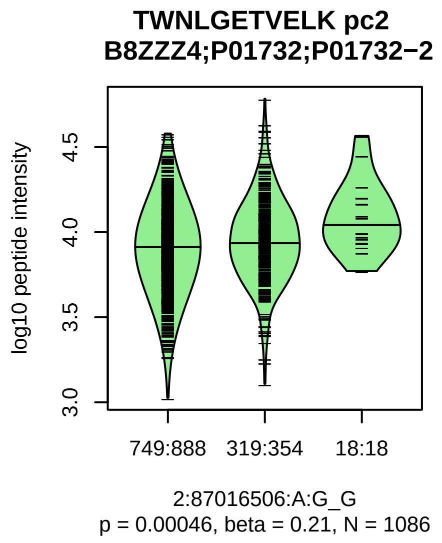

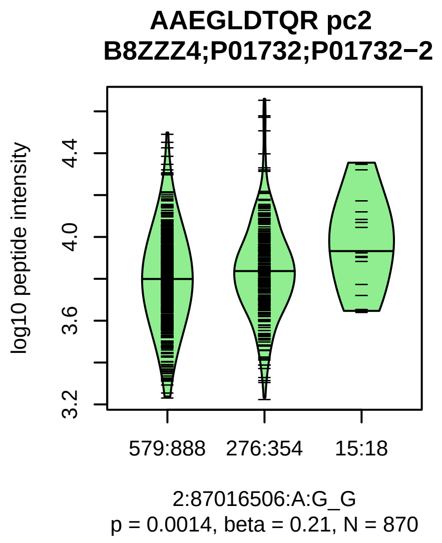

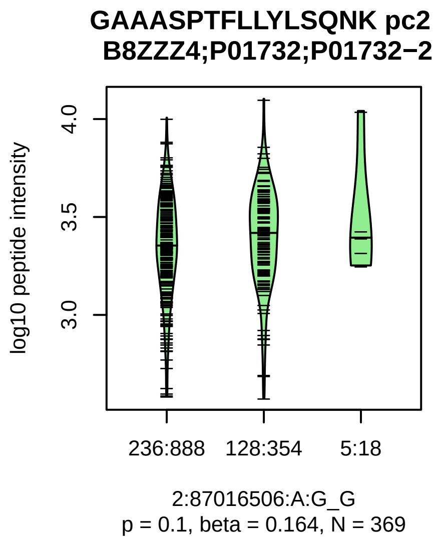

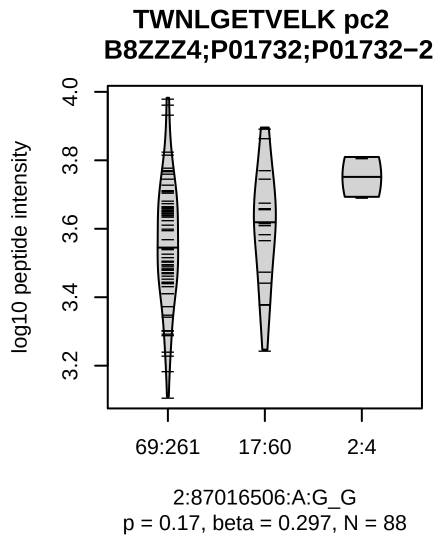

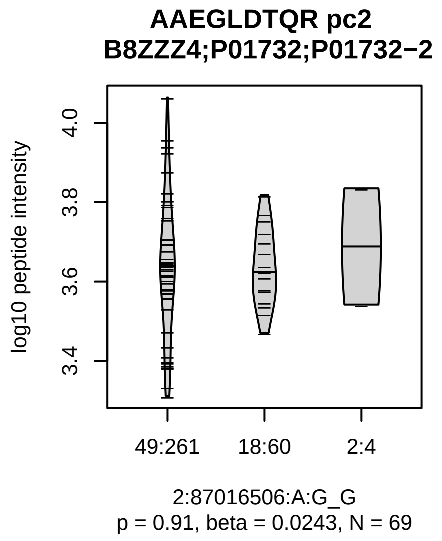

Assay Target: CD8A
UniProt: P01732
rsID: rs3020726
Target SNP: 2:86789383:G:A
Proxy SNP: 2:87016506:A:G
deCODE log10(p): 329.6
deCODE BETA: 0.4
deCODE MSPA: 0.818
::–:–
1086:870:369:65

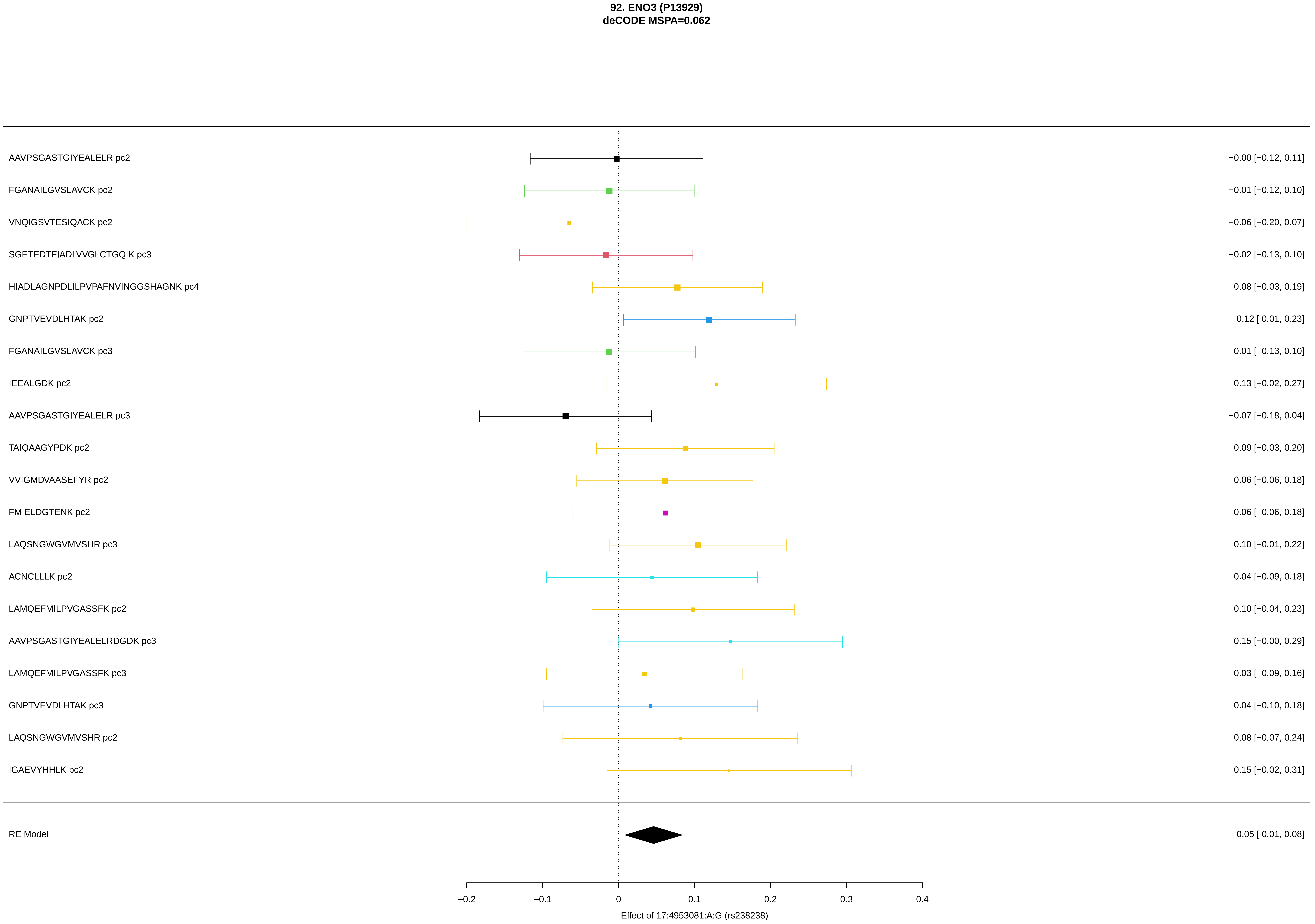

92. ENO3 (P13929)
deCODE MSPA=0.062
AAVPSGASTGIYEALELR pc2 −0.00 [−0.12, 0.11]
FGANAILGVSLAVCK pc2 −0.01 [−0.12, 0.10]
VNQIGSVTESIQACK pc2 −0.06 [−0.20, 0.07]
SGETEDTFIADLVVGLCTGQIK pc3 −0.02 [−0.13, 0.10]
HIADLAGNPDLILPVPAFNVINGGSHAGNK pc4 0.08 [−0.03, 0.19]
GNPTVEVDLHTAK pc2 0.12 [0.01, 0.23]
FGANAILGVSLAVCK pc3 −0.01 [−0.13, 0.10]
IEEALGDK pc2 0.13 [−0.02, 0.27]
AAVPSGASTGIYEALELR pc3 −0.07 [−0.18, 0.04]
TAIQAAGYPDK pc2 0.09 [−0.03, 0.20]
VVIGMDVAASEFYR pc2 0.06 [−0.06, 0.18]
FMIELDGTENK pc2 0.06 [−0.06, 0.18]
LAQSNGWGVMVSHR pc3 0.10 [−0.01, 0.22]
ACNCLLLK pc2 0.04 [−0.09, 0.18]
LAMQEFMILPVGASSFK pc2 0.10 [−0.04, 0.23]
AAVPSGASTGIYEALELRDGDK pc3 0.15 [−0.00, 0.29]
LAMQEFMILPVGASSFK pc3 0.03 [−0.09, 0.16]
GNPTVEVDLHTAK pc3 0.04 [−0.10, 0.18]
LAQSNGWGVMVSHR pc2 0.08 [−0.07, 0.24]
IGAEVYHHLK pc2 0.15 [−0.02, 0.31]
RE Model 0.05 [0.01, 0.08]
−0.2
−0.1
0
0.1
0.2
0.3
0.4
Effect of 17:4953081:A:G (rs238238)

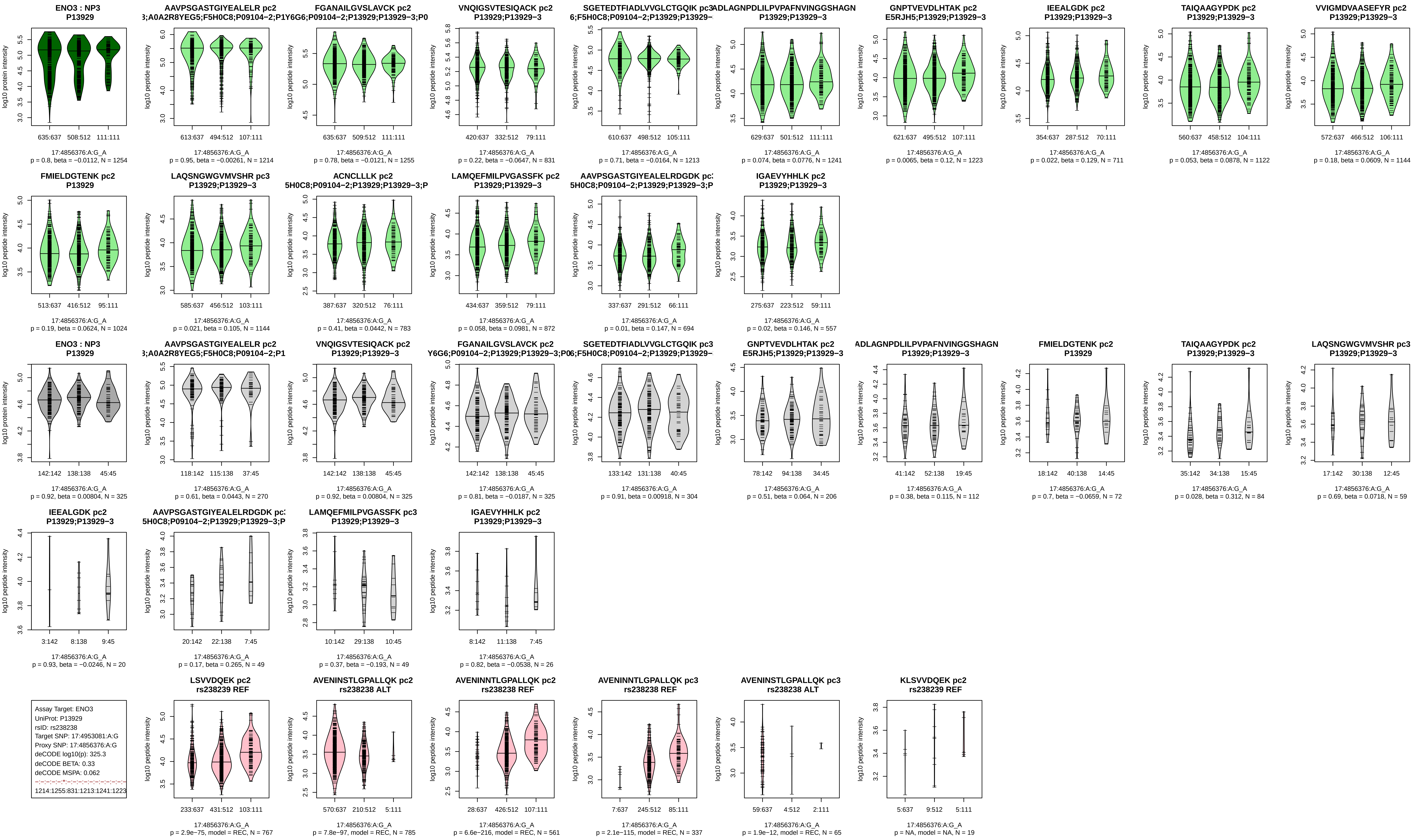

ENO3 : NP3
P13929
log10 protein intensity
635:637 508:512 111:111
17:4856376:A:G_A
p = 0.8, beta = −0.0112, N = 1254
AAVPSGASTGIYEALELR pc2
3;A0A2R8YEG5;F5H0C8;P09104−2;P1Y6G6;P09104−2;P13929;P13929−3;P0
log10 peptide intensity
613:637 494:512 107:111
17:4856376:A:G_A
p = 0.95, beta = −0.00261, N = 1214
FGANAILGVSLAVCK pc2
635:637 509:512 111:111
17:4856376:A:G_A
p = 0.78, beta = −0.0121, N = 1255
VNQIGSVTESIQACK pc2
P13929;P13929−3
420:637 332:512 79:111
17:4856376:A:G_A
p = 0.22, beta = −0.0647, N = 831
SGETEDTFIADLVVGLCTGQIK pc3
6;F5H0C8;P09104−2;P13929;P13929−
610:637 498:512 105:111
17:4856376:A:G_A
p = 0.71, beta = −0.0164, N = 1213
ADLAGNPDLILPVPAFNVINGGSHAGN
P13929;P13929−3
629:637 501:512 111:111
17:4856376:A:G_A
p = 0.074, beta = 0.0776, N = 1241
GNPTVEVDLHTAK pc2
E5RJH5;P13929;P13929−3
621:637 495:512 107:111
17:4856376:A:G_A
p = 0.0065, beta = 0.12, N = 1223
IEEALGDK pc2
P13929;P13929−3
354:637 287:512 70:111
17:4856376:A:G_A
p = 0.022, beta = 0.129, N = 711
TAIQAAGYPDK pc2
P13929;P13929−3
560:637 458:512 104:111
17:4856376:A:G_A
p = 0.053, beta = 0.0878, N = 1122
VVIGMDVAASEFYR pc2
P13929;P13929−3
572:637 466:512 106:111
17:4856376:A:G_A
p = 0.18, beta = 0.0609, N = 1144
FMIELDGTENK pc2
P13929
513:637 416:512 95:111
17:4856376:A:G_A
p = 0.19, beta = 0.0624, N = 1024
LAQSNGWGVMVSHR pc3
P13929;P13929−3
585:637 456:512 103:111
17:4856376:A:G_A
p = 0.021, beta = 0.105, N = 1144
ACNCLLLK pc2
5H0C8;P09104−2;P13929;P13929−3;P
387:637 320:512 76:111
17:4856376:A:G_A
p = 0.41, beta = 0.0442, N = 783
LAMQEFMILPVGASSFK pc2
P13929;P13929−3
434:637 359:512 79:111
17:4856376:A:G_A
p = 0.058, beta = 0.0981, N = 872
AAVPSGASTGIYEALELRDGDK pc3
5H0C8;P09104−2;P13929;P13929−3;P
337:637 291:512 66:111
17:4856376:A:G_A
p = 0.01, beta = 0.147, N = 694
IGAEVYHHLK pc2
P13929;P13929−3
275:637 223:512 59:111
17:4856376:A:G_A
p = 0.02, beta = 0.146, N = 557
ENO3 : NP3
P13929
142:142 138:138 45:45
17:4856376:A:G_A
p = 0.92, beta = 0.00804, N = 325
AAVPSGASTGIYEALELR pc2
3;A0A2R8YEG5;F5H0C8;P09104−2;P1
118:142 115:138 37:45
17:4856376:A:G_A
p = 0.61, beta = 0.0443, N = 270
VNQIGSVTESIQACK pc2
P13929;P13929−3
142:142 138:138 45:45
17:4856376:A:G_A
p = 0.92, beta = 0.00804, N = 325
FGANAILGVSLAVCK pc2
Y6G6;P09104−2;P13929;P13929−3;P0
142:142 138:138 45:45
17:4856376:A:G_A
p = 0.81, beta = −0.0187, N = 325
SGETEDTFIADLVVGLCTGQIK pc3
6;F5H0C8;P09104−2;P13929;P13929−
133:142 131:138 40:45
17:4856376:A:G_A
p = 0.91, beta = 0.00918, N = 304
GNPTVEVDLHTAK pc2
E5RJH5;P13929;P13929−3
78:142 94:138 34:45
17:4856376:A:G_A
p = 0.51, beta = 0.064, N = 206
ADLAGNPDLILPVPAFNVINGGSHAGN
P13929;P13929−3
41:142 52:138 19:45
17:4856376:A:G_A
p = 0.38, beta = 0.115, N = 112
FMIELDGTENK pc2
P13929
18:142 40:138 14:45
17:4856376:A:G_A
p = 0.7, beta = −0.0659, N = 72
TAIQAAGYPDK pc2
P13929;P13929−3
35:142 34:138 15:45
17:4856376:A:G_A
p = 0.028, beta = 0.312, N = 84
LAQSNGWGVMVSHR pc3
P13929;P13929−3
17:142 30:138 12:45
17:4856376:A:G_A
p = 0.69, beta = 0.0718, N = 59
IEEALGDK pc2
P13929;P13929−3
3:142 8:138 9:45
17:4856376:A:G_A
p = 0.93, beta = −0.0246, N = 20
AAVPSGASTGIYEALELRDGDK pc3
5H0C8;P09104−2;P13929;P13929−3;P
20:142 22:138 7:45
17:4856376:A:G_A
p = 0.17, beta = 0.265, N = 49
LAMQEFMILPVGASSFK pc3
P13929;P13929−3
10:142 29:138 10:45
17:4856376:A:G_A
p = 0.37, beta = −0.193, N = 49
IGAEVYHHLK pc2
P13929;P13929−3
8:142 11:138 7:45
17:4856376:A:G_A
p = 0.82, beta = −0.0538, N = 26
Assay Target: ENO3
UniProt: P13929
rsID: rs238238
Target SNP: 17:4953081:A:G
Proxy SNP: 17:4856376:A:G
deCODE log10(p): 325.3
deCODE BETA: 0.33
deCODE MSPA: 0.062
1214:1255:831:1213:1241:1223
LSVVDQEK pc2
rs238239 REF
233:637 431:512 103:111
17:4856376:A:G_A
p = 2.9e−75, model = REC, N = 767
AVENINSTLGPALLQK pc2
rs238238 ALT
570:637 210:512 5:111
17:4856376:A:G_A
p = 7.8e−97, model = REC, N = 785
AVENINNTLGPALLQK pc2
rs238238 REF
28:637 426:512 107:111
17:4856376:A:G_A
p = 6.6e−216, model = REC, N = 561
AVENINNTLGPALLQK pc3
rs238238 REF
7:637 245:512 85:111
17:4856376:A:G_A
p = 2.1e−115, model = REC, N = 337
AVENINSTLGPALLQK pc3
rs238238 ALT
59:637 4:512 2:111
17:4856376:A:G_A
p = 1.9e−12, model = REC, N = 65
KLSVVDQEK pc2
rs238239 REF
5:637 9:512 5:111
17:4856376:A:G_A
p = NA, model = NA, N = 19

93. APOL1 (O14791)
deCODE MSPA=0.128

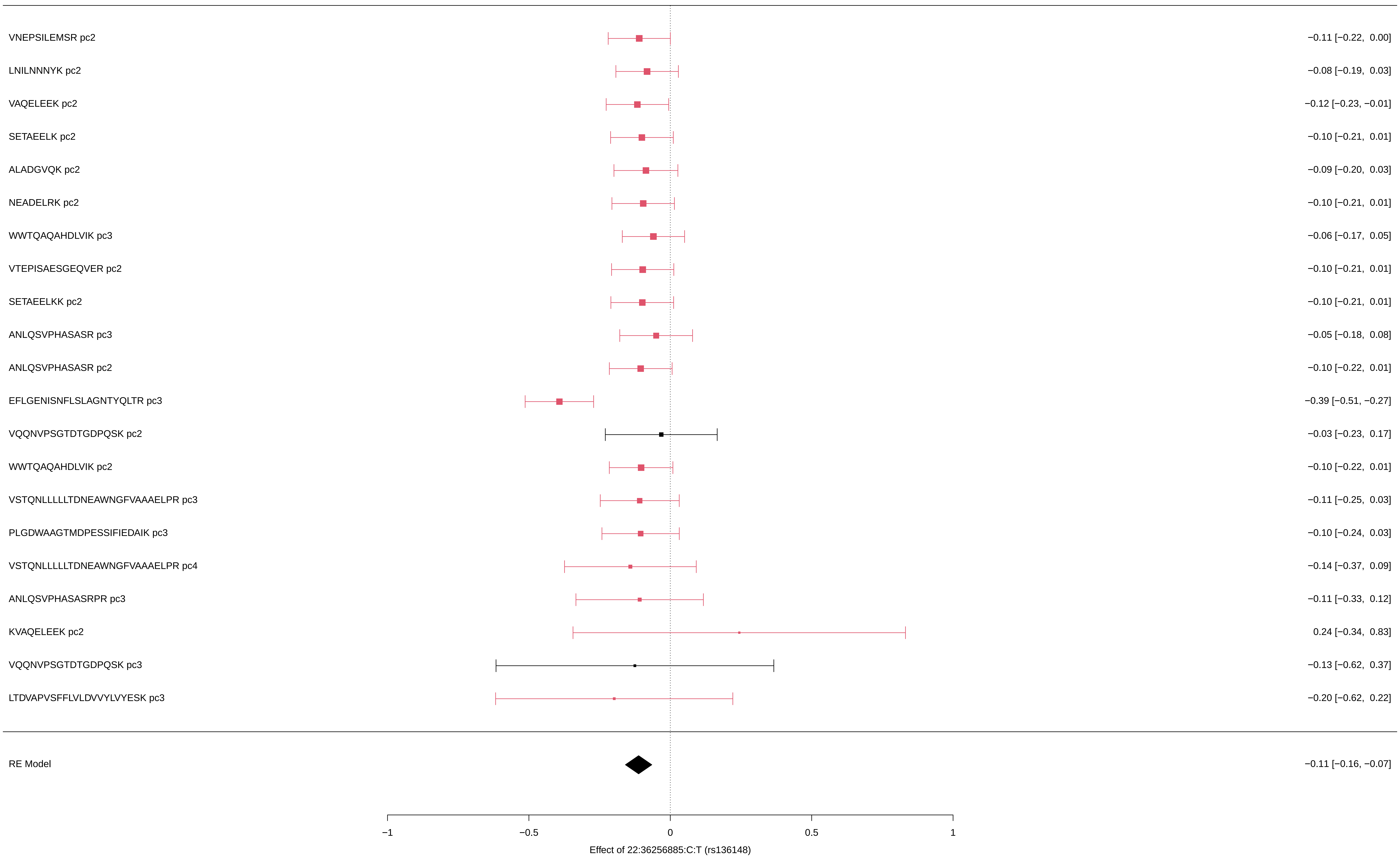

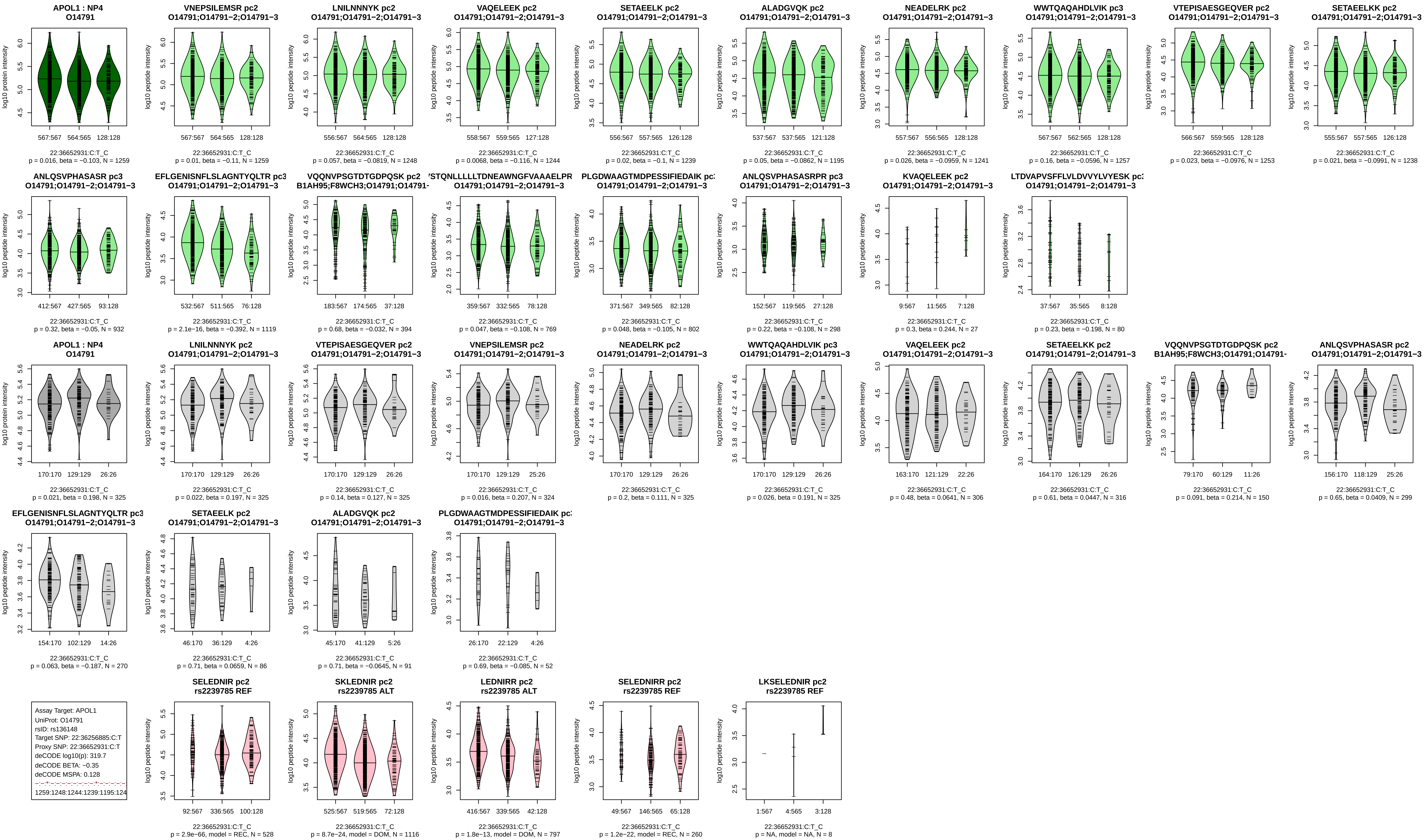
APOL1 : NP4
O14791
log10 protein intensity
567:567 564:565 128:128
22:36652931:C:T_C
p = 0.016, beta = −0.103, N = 1259
VNEPSILEMSR pc2
O14791;O14791−2;O14791−3
log10 peptide intensity
567:567 564:565 128:128
22:36652931:C:T_C
p = 0.01, beta = −0.11, N = 1259
LNILNNNYK pc2
O14791;O14791−2;O14791−3
556:567 564:565 127:128
22:36652931:C:T_C
p = 0.057, beta = −0.0819, N = 1248
VAQELEEK pc2
O14791;O14791−2;O14791−3
558:567 559:565 127:128
22:36652931:C:T_C
p = 0.0068, beta = −0.116, N = 1244
SETAEELK pc2
O14791;O14791−2;O14791−3
556:567 557:565 126:128
22:36652931:C:T_C
p = 0.02, beta = −0.1, N = 1239
ALADGVQK pc2
O14791;O14791−2;O14791−3
537:567 537:565 121:128
22:36652931:C:T_C
p = 0.05, beta = −0.0862, N = 1195
NEADELRK pc2
O14791;O14791−2;O14791−3
557:567 556:565 128:128
22:36652931:C:T_C
p = 0.026, beta = −0.0959, N = 1241
WWTQAQAHDLVIK pc3
O14791;O14791−2;O14791−3
567:567 562:565 128:128
22:36652931:C:T_C
p = 0.16, beta = −0.0596, N = 1257
VTEPISAESGEQVER pc2
O14791;O14791−2;O14791−3
566:567 559:565 128:128
22:36652931:C:T_C
p = 0.023, beta = −0.0976, N = 1253
SETAEELKK pc2
O14791;O14791−2;O14791−3
555:567 557:565 126:128
22:36652931:C:T_C
p = 0.021, beta = −0.0991, N = 1238
ANLQSVPHASASR pc3
O14791;O14791−2;O14791−3
412:567 427:565 93:128
22:36652931:C:T_C
p = 0.32, beta = −0.05, N = 932
EFLGENISNFLSLAGNTYQLTR pc3
O14791;O14791−2;O14791−3
532:567 511:565 76:128
22:36652931:C:T_C
p = 2.1e−16, beta = −0.392, N = 1119
VQQNVPSGTDTGDPQSK pc2
B1AH95;F8WCH3;O14791;O14791-
183:567 174:565 37:128
22:36652931:C:T_C
p = 0.68, beta = −0.032, N = 394
STQNLLLLTDNEAWNGFVAAAELPR
O14791;O14791−2;O14791−3
359:567 332:565 78:128
22:36652931:C:T_C
p = 0.047, beta = −0.108, N = 769
PLGDWAAGTMDPESSIFIEDAIK pc
O14791;O14791−2;O14791−3
371:567 349:565 82:128
22:36652931:C:T_C
p = 0.048, beta = −0.105, N = 802
ANLQSVPHASASRPR pc3
O14791;O14791−2;O14791−3
152:567 119:565 27:128
22:36652931:C:T_C
p = 0.22, beta = −0.108, N = 298
KVAQELEEK pc2
O14791;O14791−2;O14791−3
9:567 11:565 7:128
22:36652931:C:T_C
p = 0.3, beta = 0.244, N = 27
LTDVAPVSFFLVLDVVYLVYESK pc
O14791;O14791−2;O14791−3
37:567 35:565 8:128
22:36652931:C:T_C
p = 0.23, beta = −0.198, N = 80
APOL1 : NP4
O14791
170:170 129:129 26:26
22:36652931:C:T_C
p = 0.021, beta = 0.198, N = 325
LNILNNNYK pc2
O14791;O14791−2;O14791−3
170:170 129:129 26:26
22:36652931:C:T_C
p = 0.022, beta = 0.197, N = 325
VTEPISAESGEQVER pc2
O14791;O14791−2;O14791−3
170:170 129:129 26:26
22:36652931:C:T_C
p = 0.14, beta = 0.127, N = 325
VNEPSILEMSR pc2
O14791;O14791−2;O14791−3
170:170 129:129 25:26
22:36652931:C:T_C
p = 0.016, beta = 0.207, N = 324
NEADELRK pc2
O14791;O14791−2;O14791−3
170:170 129:129 26:26
22:36652931:C:T_C
p = 0.2, beta = 0.111, N = 325
WWTQAQAHDLVIK pc3
O14791;O14791−2;O14791−3
170:170 129:129 26:26
22:36652931:C:T_C
p = 0.026, beta = 0.191, N = 325
VAQELEEK pc2
O14791;O14791−2;O14791−3
163:170 121:129 22:26
22:36652931:C:T_C
p = 0.48, beta = 0.0641, N = 306
SETAEELKK pc2
O14791;O14791−2;O14791−3
164:170 126:129 26:26
22:36652931:C:T_C
p = 0.61, beta = 0.0447, N = 316
VQQNVPSGTDTGDPQSK pc2
B1AH95;F8WCH3;O14791;O14791-
79:170 60:129 11:26
22:36652931:C:T_C
p = 0.091, beta = 0.214, N = 150
ANLQSVPHASASR pc2
O14791;O14791−2;O14791−3
156:170 118:129 25:26
22:36652931:C:T_C
p = 0.65, beta = 0.0409, N = 299
EFLGENISNFLSLAGNTYQLTR pc3
O14791;O14791−2;O14791−3
154:170 102:129 14:26
22:36652931:C:T_C
p = 0.063, beta = −0.187, N = 270
SETAEELK pc2
O14791;O14791−2;O14791−3
46:170 36:129 4:26
22:36652931:C:T_C
p = 0.71, beta = 0.0659, N = 86
ALADGVQK pc2
O14791;O14791−2;O14791−3
45:170 41:129 5:26
22:36652931:C:T_C
p = 0.71, beta = −0.0645, N = 91
PLGDWAAGTMDPESSIFIEDAIK pc
O14791;O14791−2;O14791−3
26:170 22:129 4:26
22:36652931:C:T_C
p = 0.69, beta = −0.085, N = 52
Assay Target: APOL1
UniProt: O14791
rsID: rs136148
Target SNP: 22:36256885:C:T
Proxy SNP: 22:36652931:C:T
deCODE log10(p): 319.7
deCODE BETA: −0.35
deCODE MSPA: 0.128
1259:1248:1244:1239:1195:124
SELEDNIR pc2
rs2239785 REF
92:567 336:565 100:128
22:36652931:C:T_C
p = 2.9e−66, model = REC, N = 528
SKLEDNIR pc2
rs2239785 ALT
525:567 519:565 72:128
22:36652931:C:T_C
p = 8.7e−24, model = DOM, N = 1116
LEDNIRR pc2
rs2239785 ALT
416:567 339:565 42:128
22:36652931:C:T_C
p = 1.8e−13, model = DOM, N = 797
SELEDNIRR pc2
rs2239785 REF
49:567 146:565 65:128
22:36652931:C:T_C
p = 1.2e−22, model = REC, N = 260
LKSELEDNIR pc2
rs2239785 REF
1:567 4:565 3:128
22:36652931:C:T_C
p = NA, model = NA, N = 8

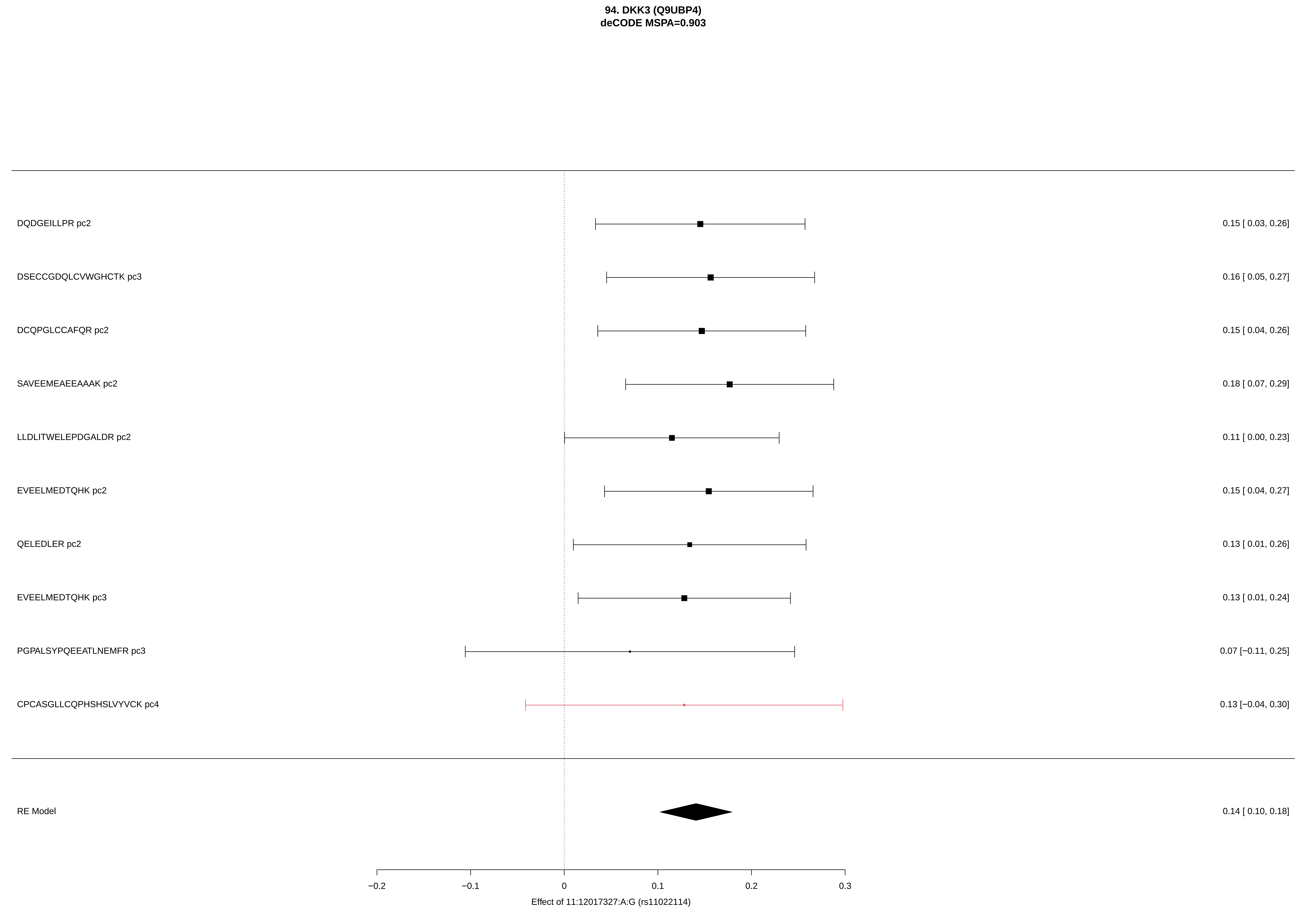
94. DKK3 (Q9UBP4)
deCODE MSPA=0.903
DQDGEILLPR pc2
0.15 [0.03, 0.26]
DSECCGDQLCVWGHCTK pc3
0.16 [0.05, 0.27]
DCQPGLCCAFQR pc2
0.15 [0.04, 0.26]
SAVEEMEAEEAAAK pc2
0.18 [0.07, 0.29]
LLDLITWELEPDGALDR pc2
0.11 [0.00, 0.23]
EVEELMEDTQHK pc2
0.15 [0.04, 0.27]
QELEDLER pc2
0.13 [0.01, 0.26]
EVEELMEDTQHK pc3
0.13 [0.01, 0.24]
PGPALSYPQEEATLNEMFR pc3
0.07 [−0.11, 0.25]
CPCASGLLCQPHSHSLVYVCK pc4
0.13 [−0.04, 0.30]
RE Model
0.14 [0.10, 0.18]
−0.2
−0.1
0
0.1
0.2
0.3
Effect of 11:12017327:A:G (rs11022114)

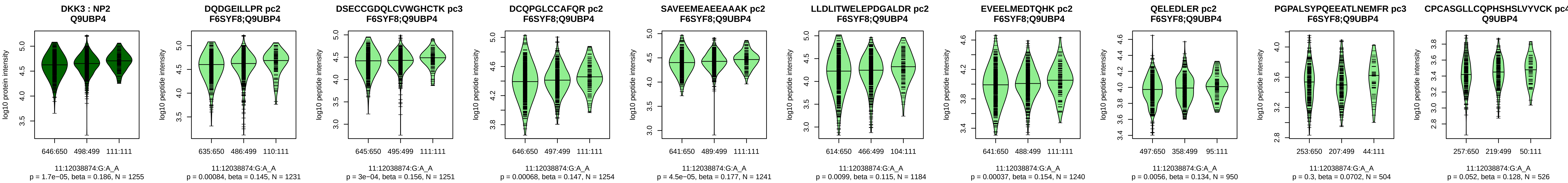

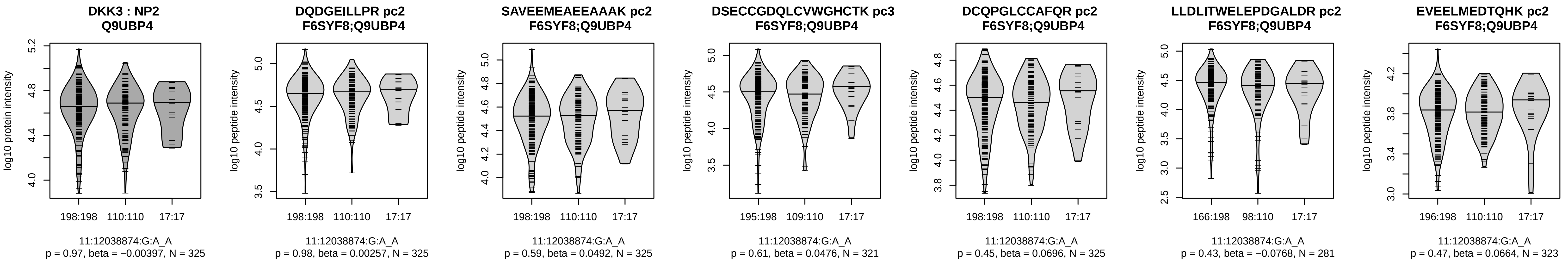

Assay Target: DKK3
UniProt: Q9UBP4
rsID: rs11022114
Target SNP: 11:12017327:A:G
Proxy SNP: 11:12038874:G:A
deCODE log10(p): 317.4
deCODE BETA: 0.33
deCODE MSPA: 0.903
::*:*:*:*:*:*:–:–
1231:1251:1254:1241:1184:124

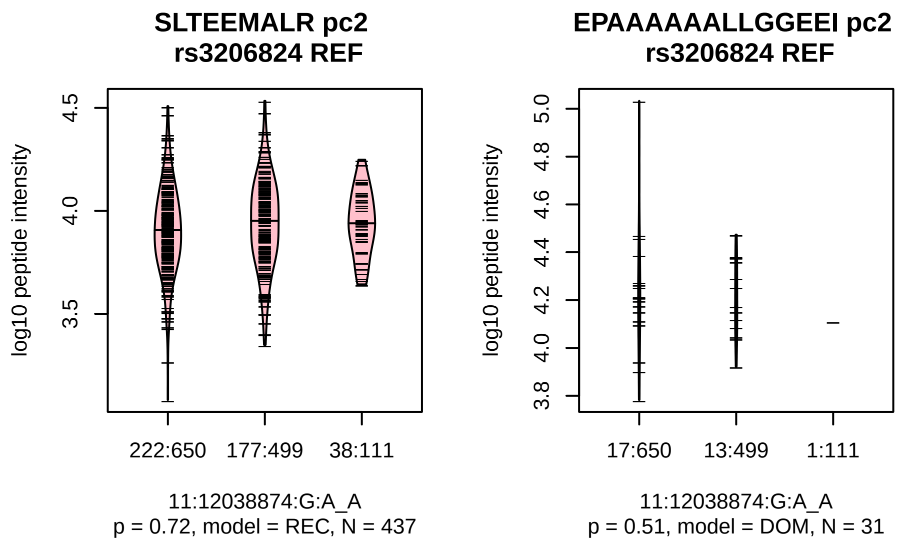

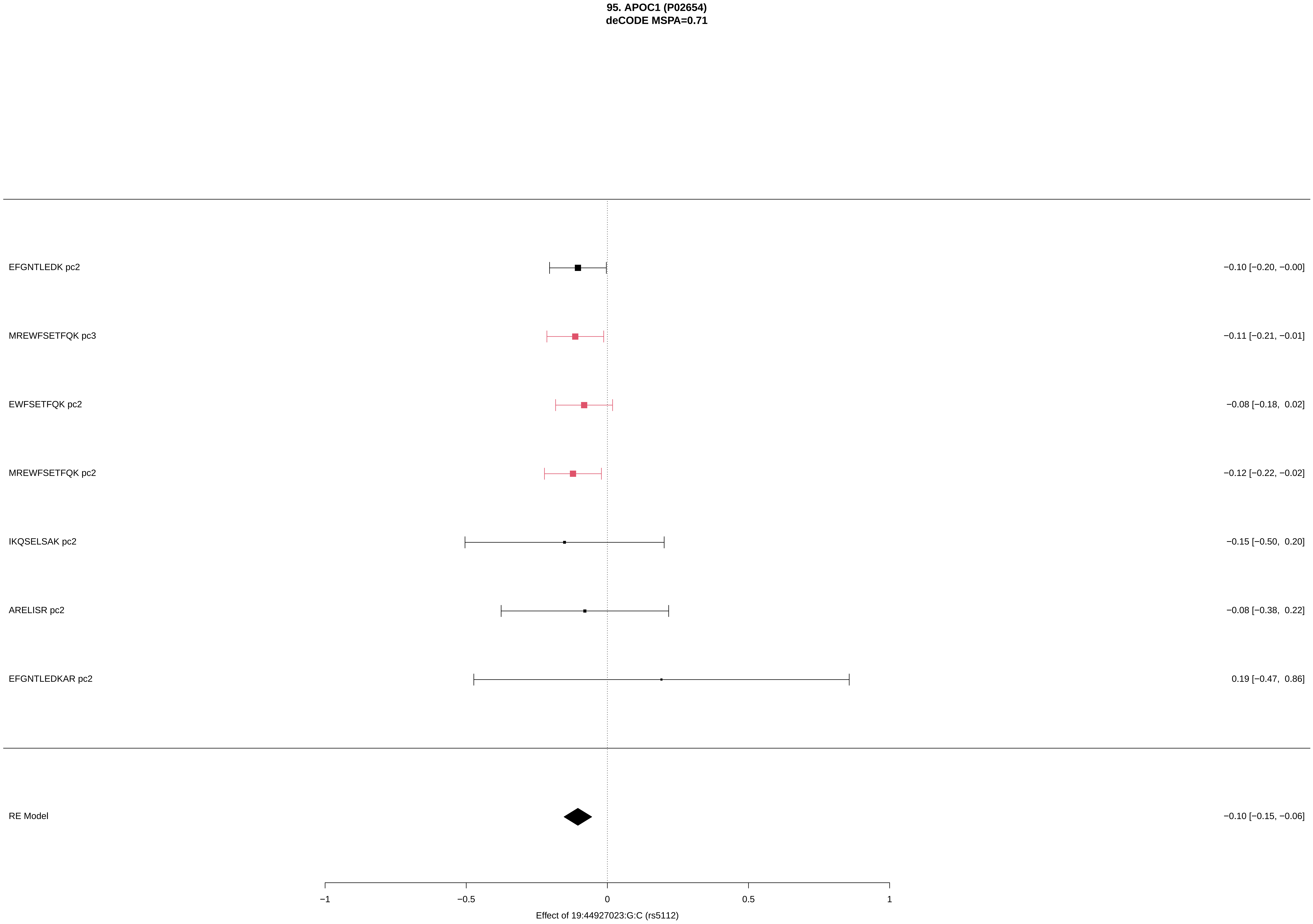
95. APOC1 (P02654)
deCODE MSPA=0.71
EFGNTLEDK pc2
−0.10 [−0.20, −0.00]
MREWFSETFQK pc3
−0.11 [−0.21, −0.01]
EWFSETFQK pc2
−0.08 [−0.18, 0.02]
MREWFSETFQK pc2
−0.12 [−0.22, −0.02]
IKQSELSAK pc2
−0.15 [−0.50, 0.20]
ARELISR pc2
−0.08 [−0.38, 0.22]
EFGNTLEDKAR pc2
0.19 [−0.47, 0.86]
RE Model
−0.10 [−0.15, −0.06]
−1
−0.5
0
0.5
1
Effect of 19:44927023:G:C (rs5112)

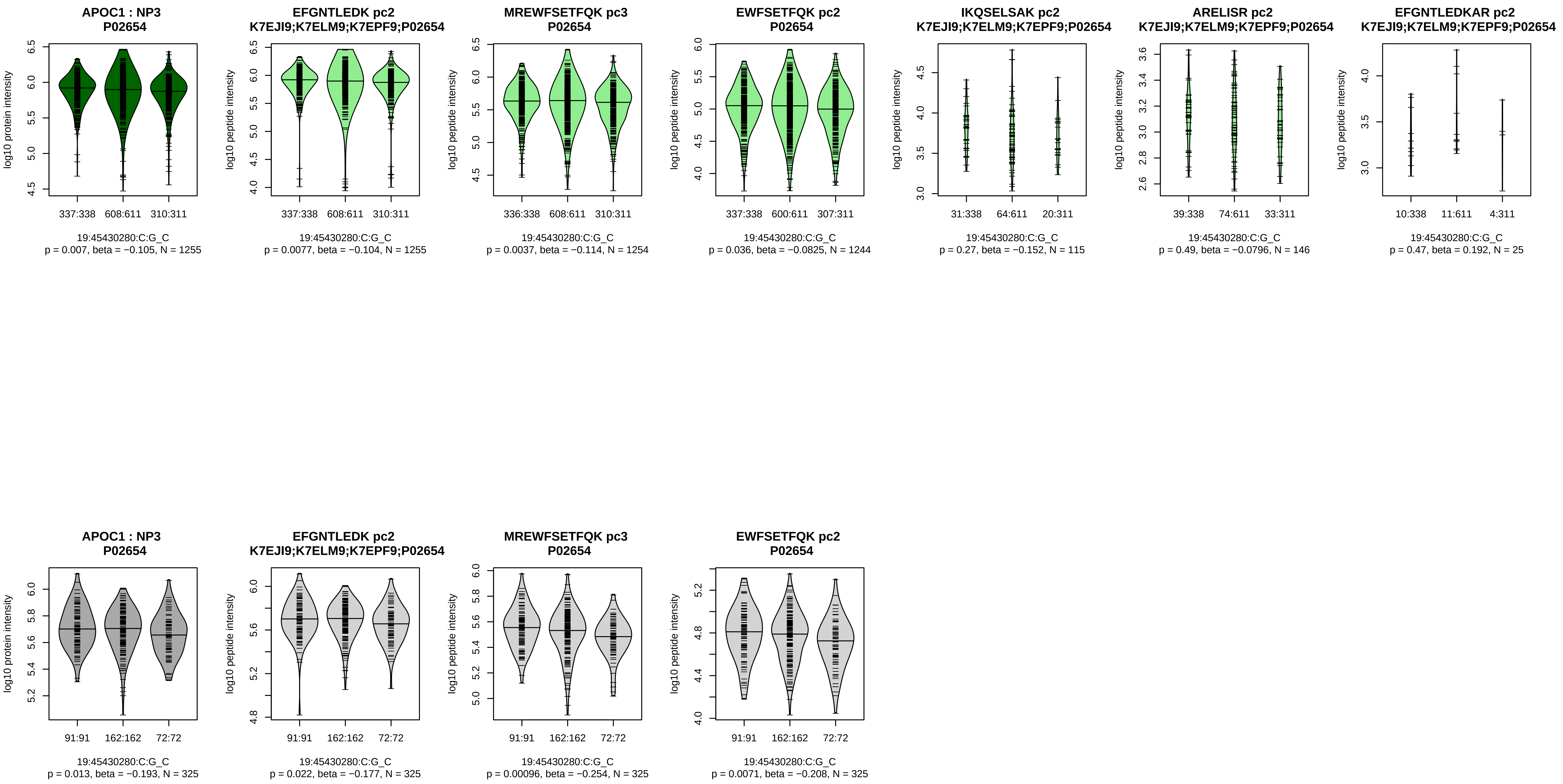

Assay Target: APOC1
UniProt: P02654
rsID: rs5112
Target SNP: 19:44927023:G:C
Proxy SNP: 19:45430280:C:G
deCODE log10(p): 298.6
deCODE BETA: 0.3
deCODE MSPA: 0.71
::–:*:–:–:NA:–
1255:1254:1244:1245:115:146:1

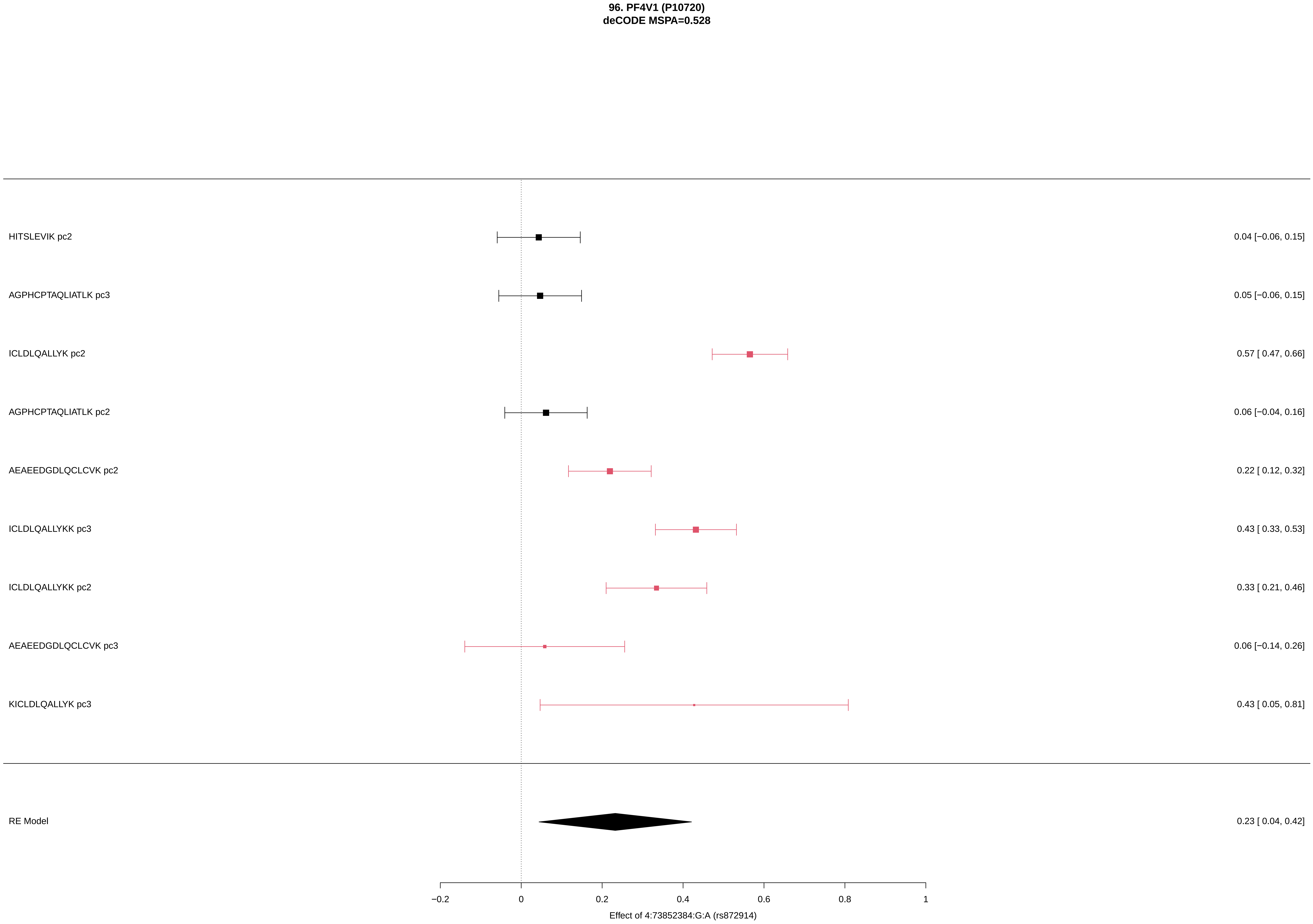
96. PF4V1 (P10720)
deCODE MSPA=0.528
HITSLEVIK pc2
AGPHCPTAQLIATLK pc3
ICLDLQALLYK pc2
AGPHCPTAQLIATLK pc2
AEAEEDGDLQCLCVK pc2
ICLDLQALLYKK pc3
ICLDLQALLYKK pc2
AEAEEDGDLQCLCVK pc3
KICLDLQALLYK pc3
RE Model
0.04 [−0.06, 0.15]
0.05 [−0.06, 0.15]
0.57 [0.47, 0.66]
0.06 [−0.04, 0.16]
0.22 [0.12, 0.32]
0.43 [0.33, 0.53]
0.33 [0.21, 0.46]
0.06 [−0.14, 0.26]
0.43 [0.05, 0.81]
0.23 [0.04, 0.42]
−0.2
0
0.2
0.4
0.6
0.8
1
Effect of 4:73852384:G:A (rs872914)

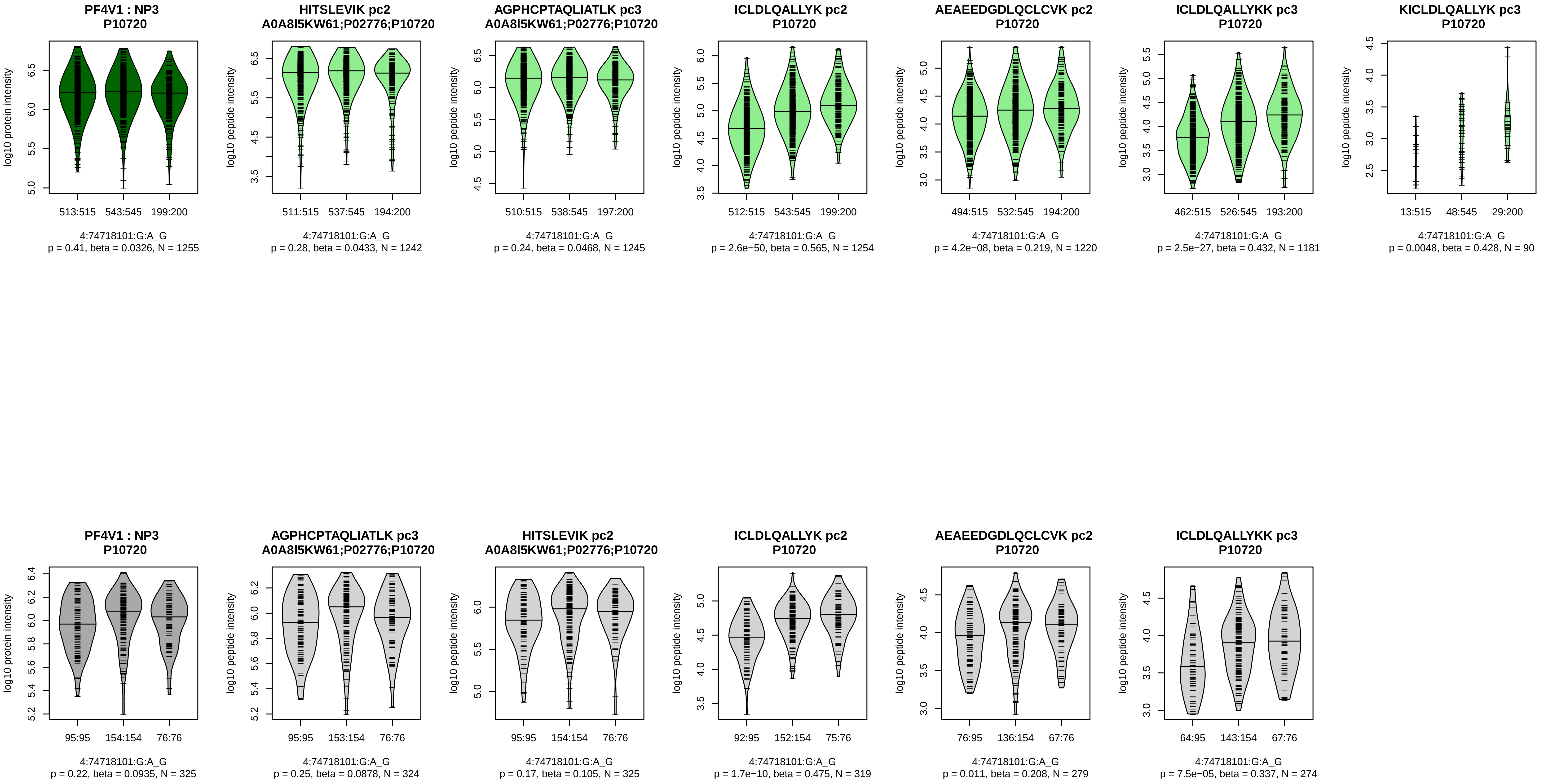

Assay Target: PF4V1
UniProt: P10720
rsID: rs872914
Target SNP: 4:73852384:G:A
Proxy SNP: 4:74718101:G:A
deCODE log10(p): 297.5
deCODE BETA: 0.32
deCODE MSPA: 0.528
–:–:*:–:*:*:*:–:*:NA
1242:1245:1254:1253:1220:118

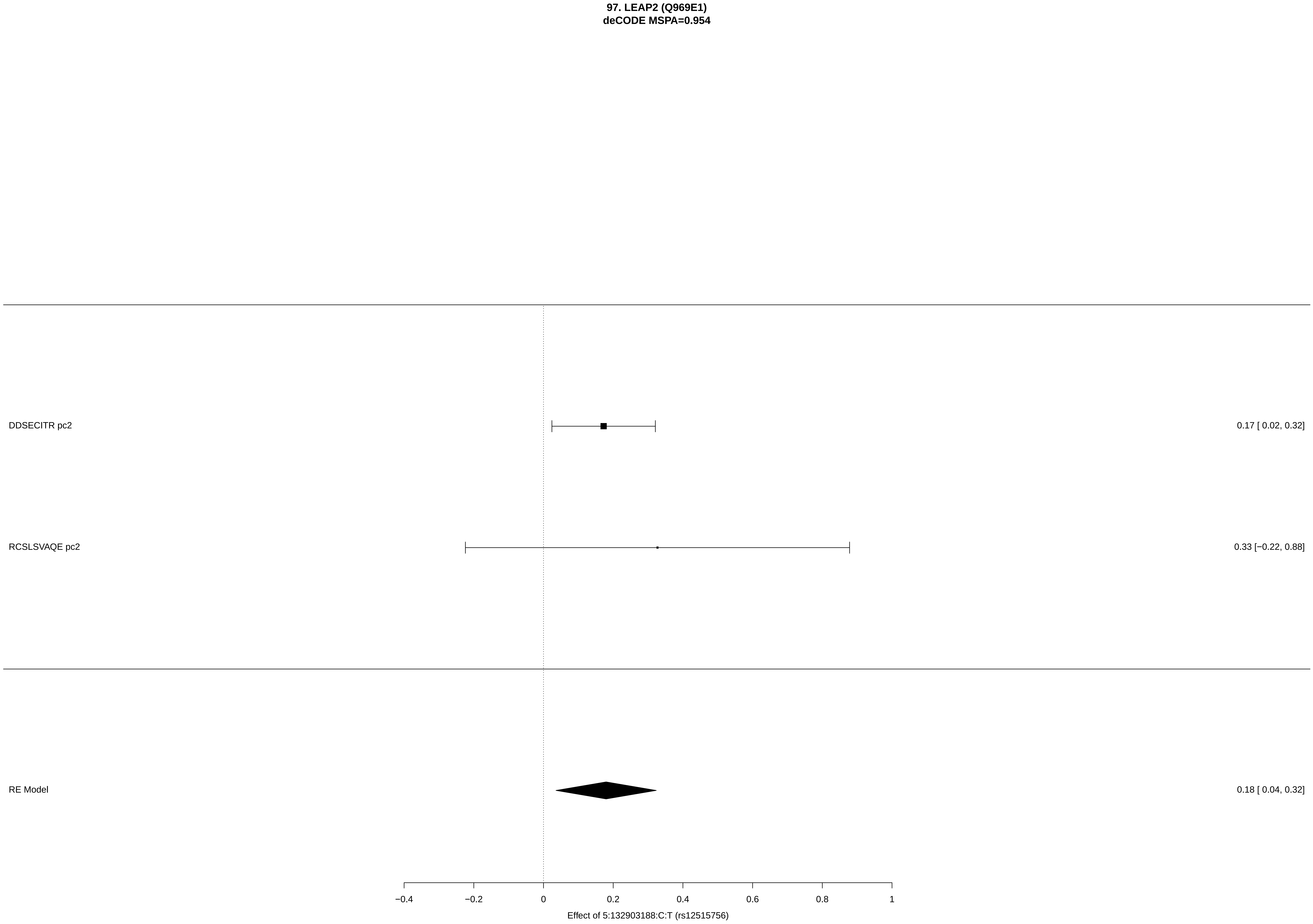

97. LEAP2 (Q969E1)
deCODE MSPA=0.954
DDSECITR pc2
0.17 [0.02, 0.32]
RCSLSVAQE pc2
0.33 [−0.22, 0.88]
RE Model
0.18 [0.04, 0.32]
−0.4
−0.2
0
0.2
0.4
0.6
0.8
1
Effect of 5:132903188:C:T (rs12515756)

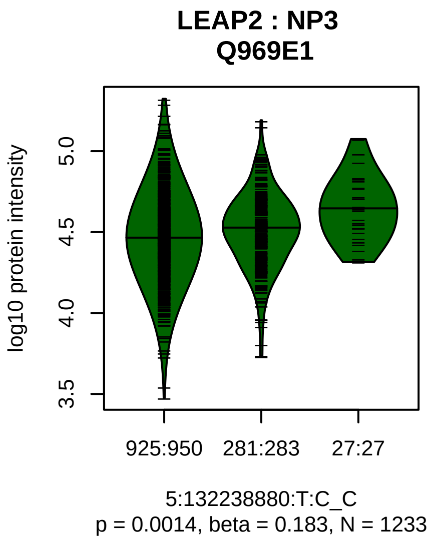

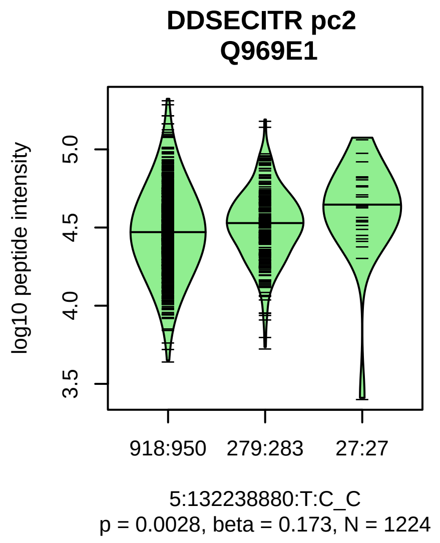

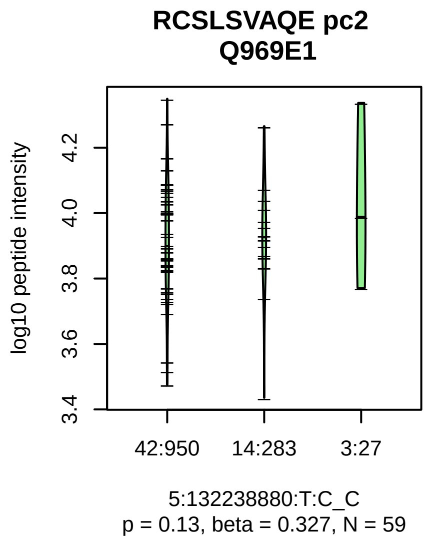

Assay Target: LEAP2
UniProt: Q969E1
rsID: rs12515756
Target SNP: 5:132903188:C:T
Proxy SNP: 5:132238880:T:C
deCODE log10(p): 296.6
deCODE BETA: 0.4
deCODE MSPA: 0.954
*:–
1224:59

98. GFRA2 (O00451)
deCODE MSPA=0

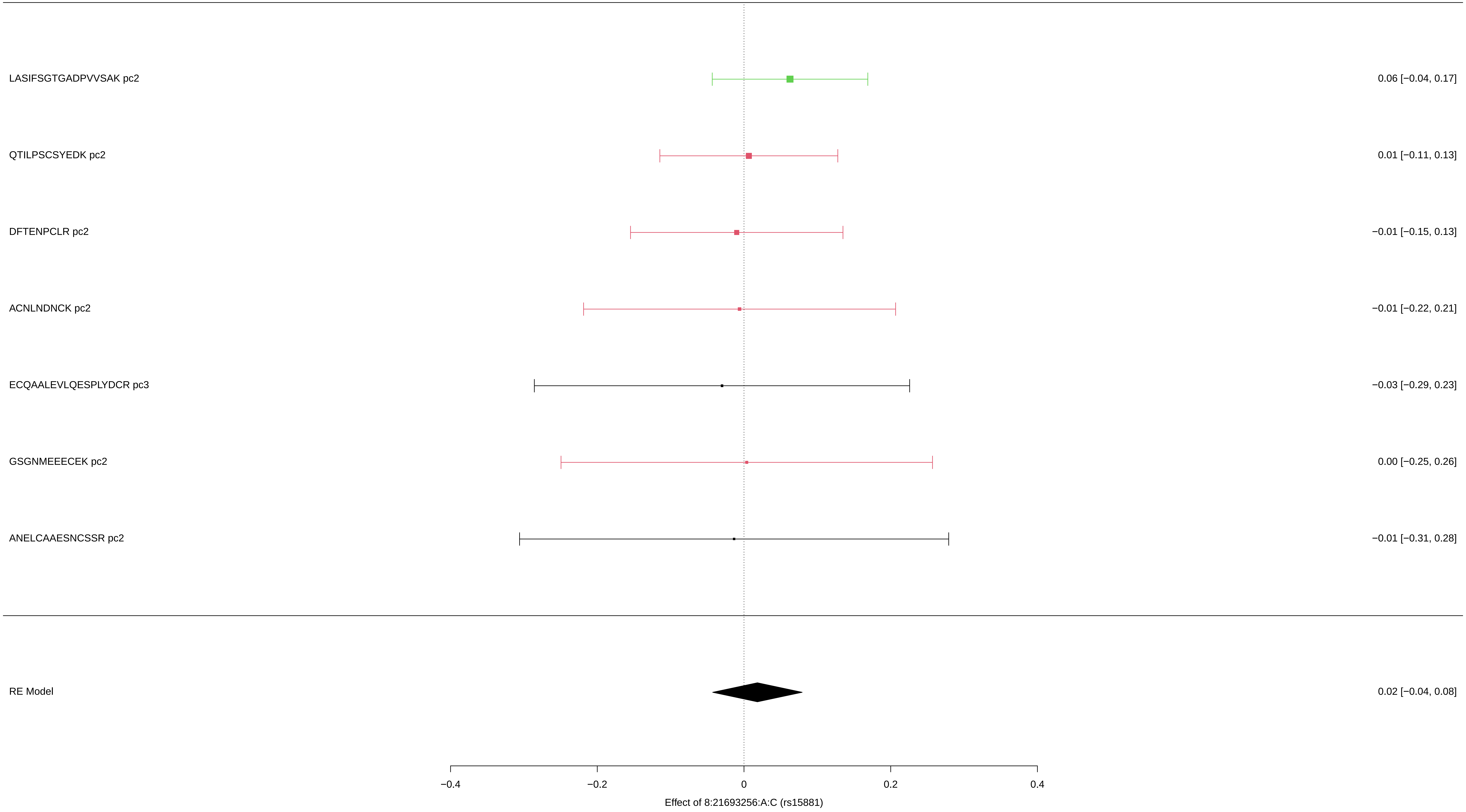

GFRA2 : NP3
O00451

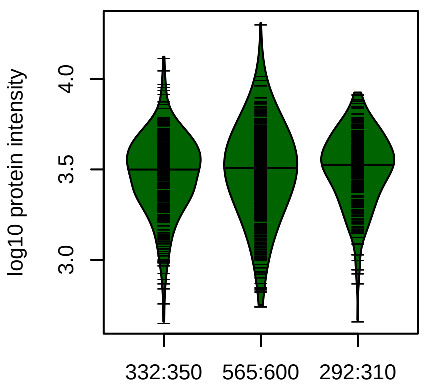

8:21550768:A:C_C
p = 0.029, beta = 0.0869, N = 1189

LASIFSGTGADPVVSAK pc2
O00451;O00451−3

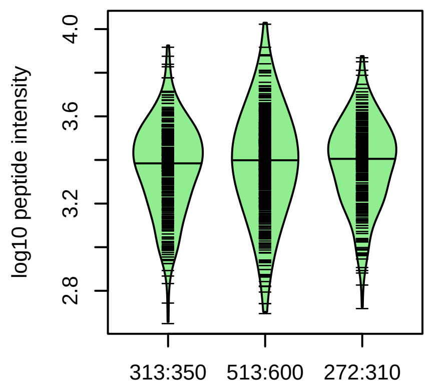

8:21550768:A:C_C
p = 0.13, beta = 0.0628, N = 1098

QTILPSCSYEDK pc2
O00451;O00451−2;O00451−3

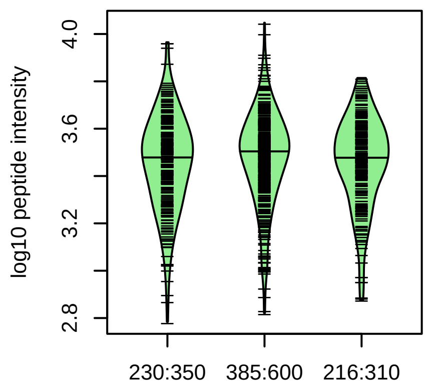

8:21550768:A:C_C
p = 0.89, beta = 0.00659, N = 831

DFTENPCLR pc2
O00451;O00451−2;O00451−3

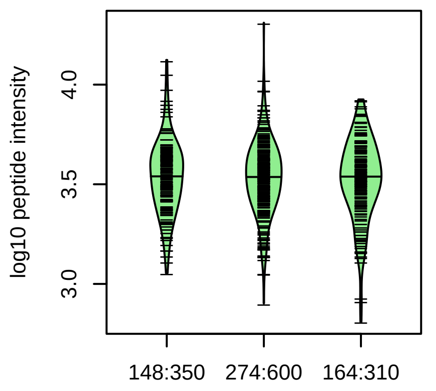

8:21550768:A:C_C
p = 0.86, beta = −0.00988, N = 586

ACNLNDNCK pc2
O00451;O00451−2;O00451−3

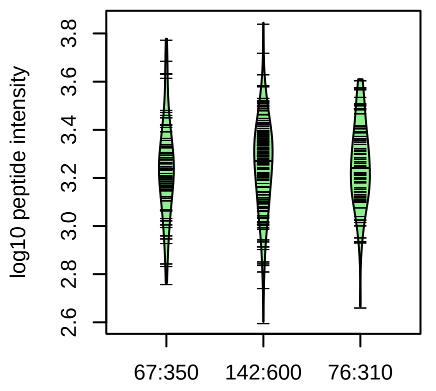

8:21550768:A:C_C
p = 0.94, beta = −0.00598, N = 285

ECQAALEVLQESPLYDCR pc3
O00451

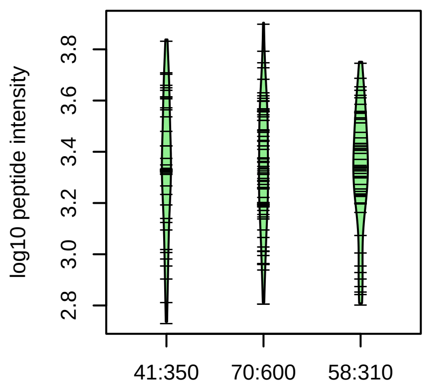

8:21550768:A:C_C
p = 0.76, beta = −0.03, N = 169

GSGNMEEECEK pc2
O00451;O00451−2;O00451−3

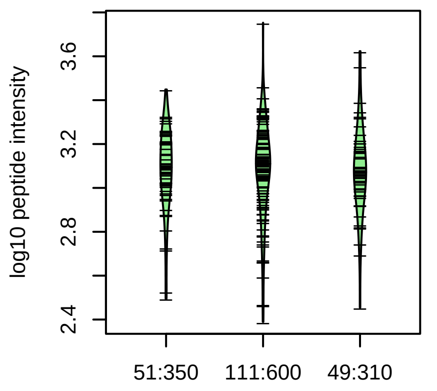

8:21550768:A:C_C
p = 0.97, beta = 0.00378, N = 211

ANELCAAESNCSSR pc2
O00451

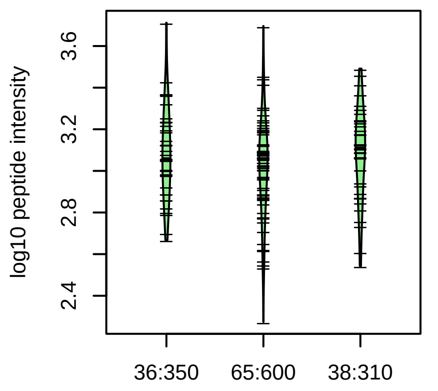

8:21550768:A:C_C
p = 0.91, beta = −0.0134, N = 139

GFRA2 : NP3
O00451

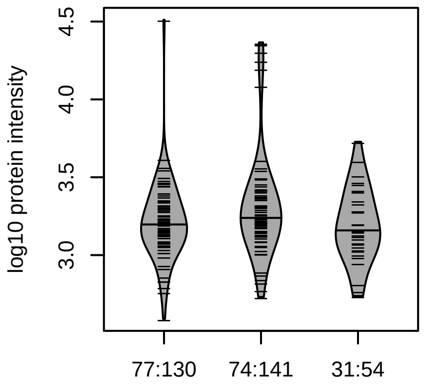

8:21550768:A:C_A
p = 0.95, beta = 0.00614, N = 182

LASIFSGTGADPVVSAK pc2
O00451;O00451−3

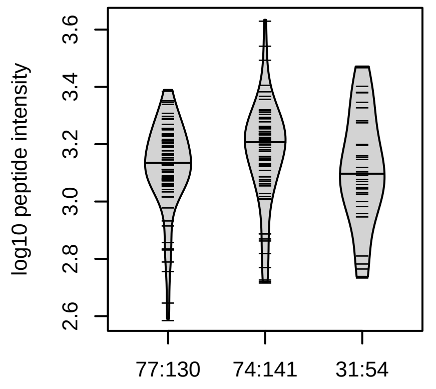

8:21550768:A:C_A
p = 0.48, beta = 0.0703, N = 182

Assay Target: GFRA2
UniProt: O00451
rsID: rs15881
Target SNP: 8:21693256:A:C
Proxy SNP: 8:21550768:A:C
deCODE log10(p): 292.7
deCODE BETA: −0.31
deCODE MSPA: 0
−:−:−:−:−:−:−
1098:831:586:285:169:211:139

99. ELANE (P08246)
deCODE MSPA=0

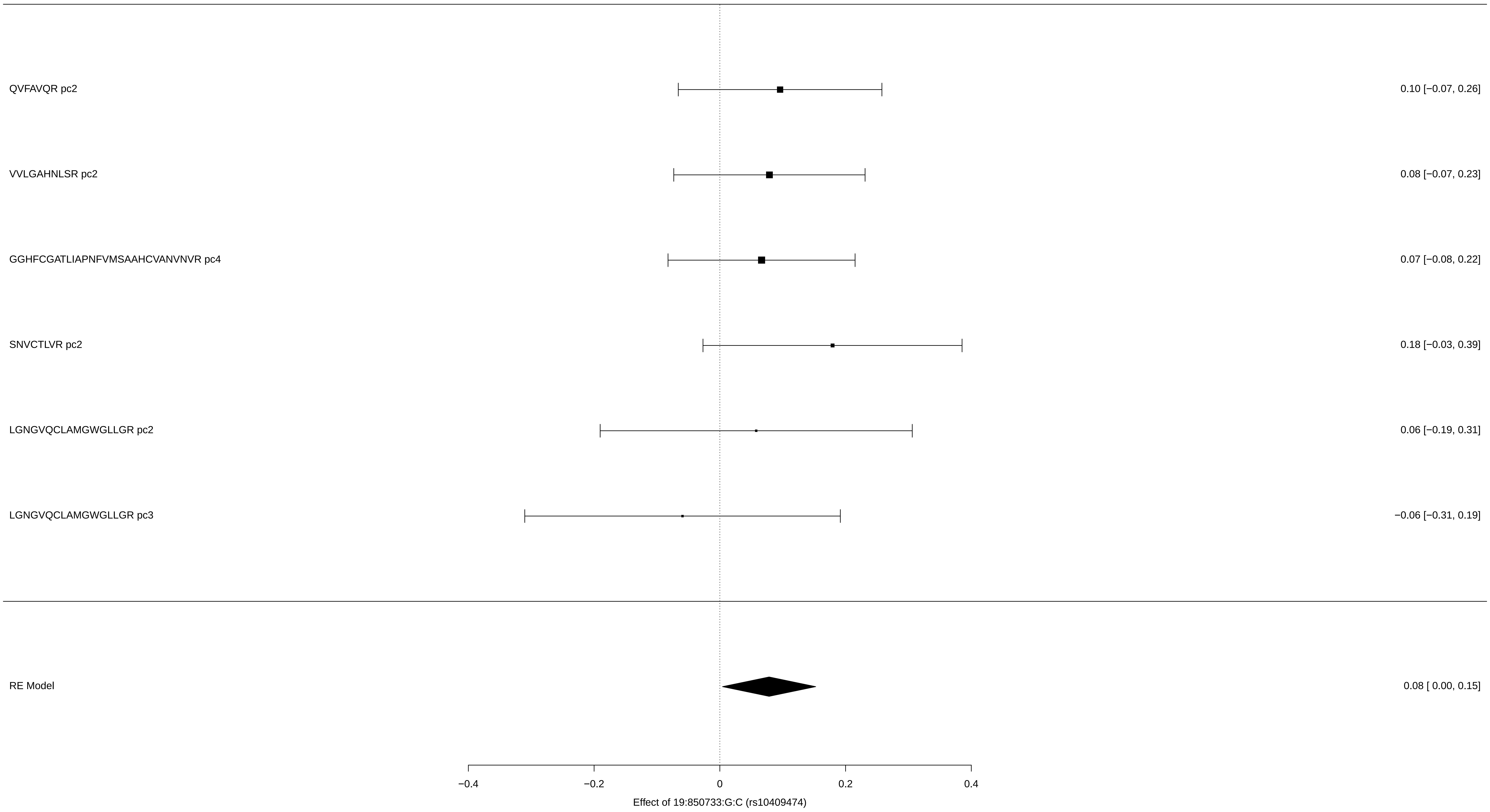

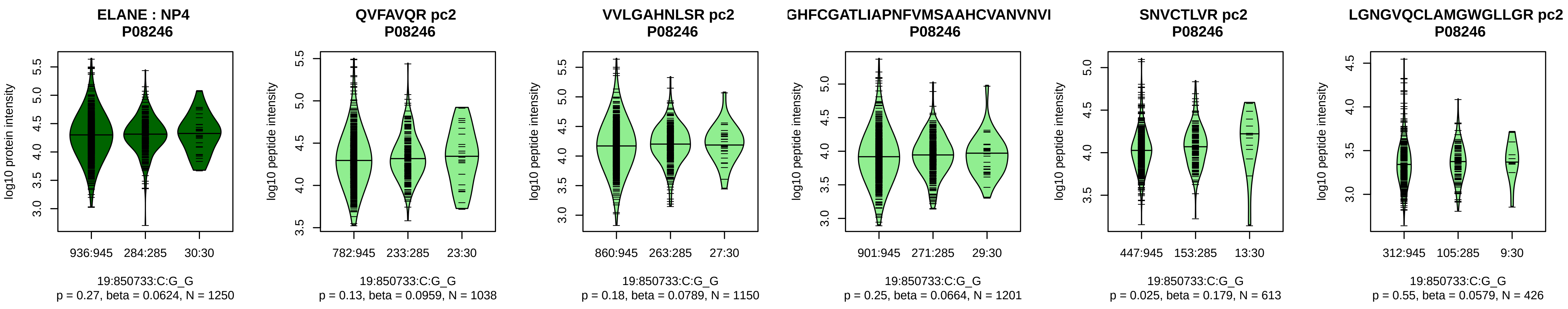

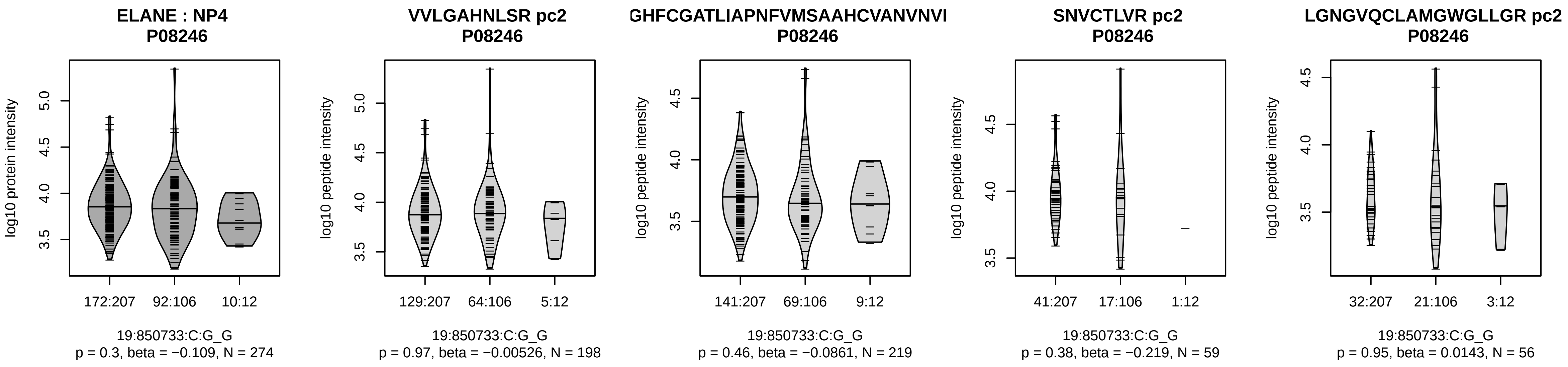

Assay Target: ELANE
UniProt: P08246
rsID: rs10409474
Target SNP: 19:850733:G:C
Proxy SNP: 19:850733:C:G
deCODE log10(p): 290.7
deCODE BETA: 0.45
deCODE MSPA: 0
–:–:–:–:–:–
1038:1150:1201:613:426:417

100. FCN1 (O00602)
deCODE MSPA=0.973

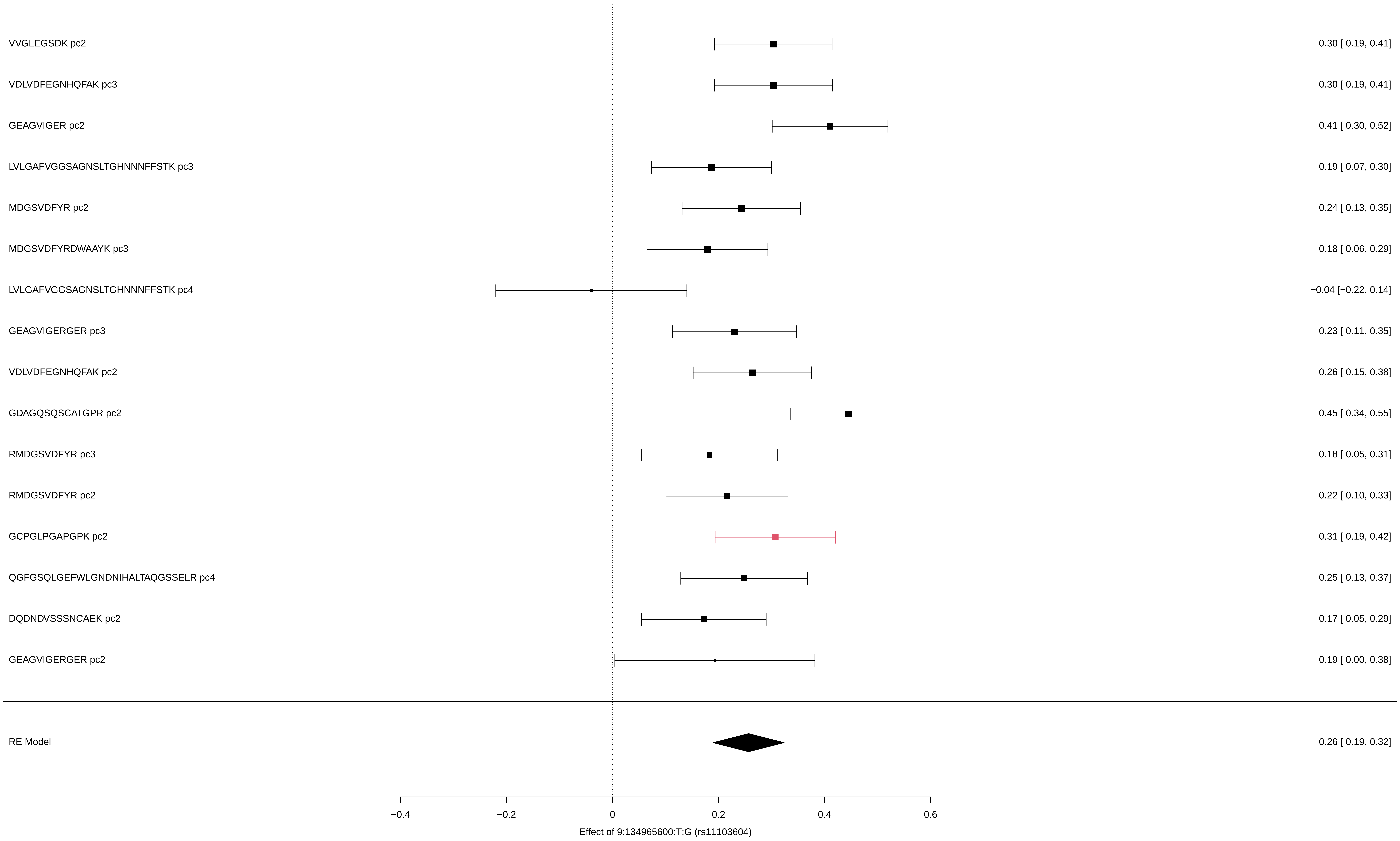

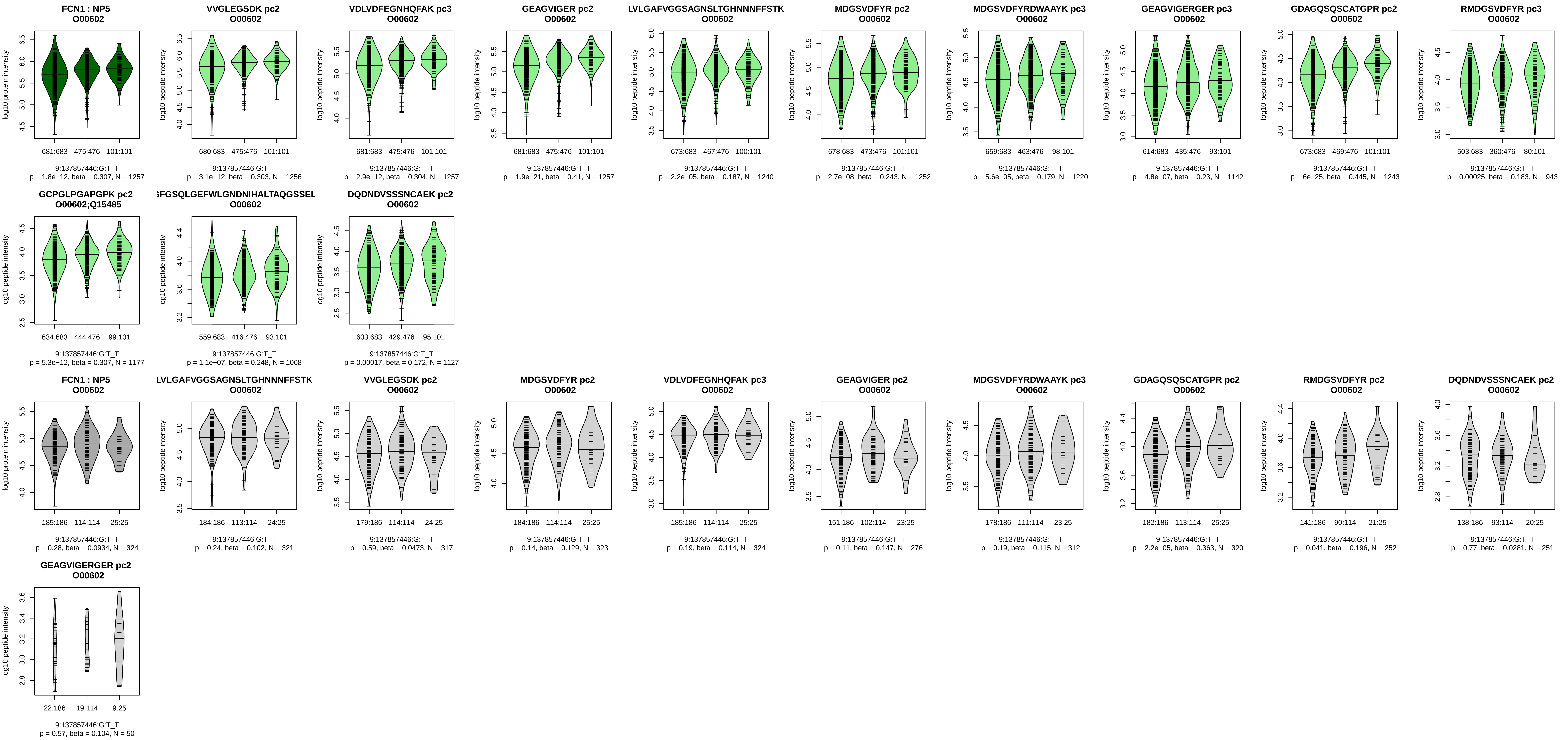

Assay Target: FCN1
UniProt: O00602
rsID: rs11103604
Target SNP: 9:134965600:T:G
Proxy SNP: 9:137857446:G:T
deCODE log10(p): 288.3
deCODE BETA: 0.34
deCODE MSPA: 0.973
::*:*:*:*:–:*:*:*:*:*:*:*:*:*:NA:NA
1256:1257:1257:1240:1252:122

101. CD5L (O43866)
deCODE MSPA=0

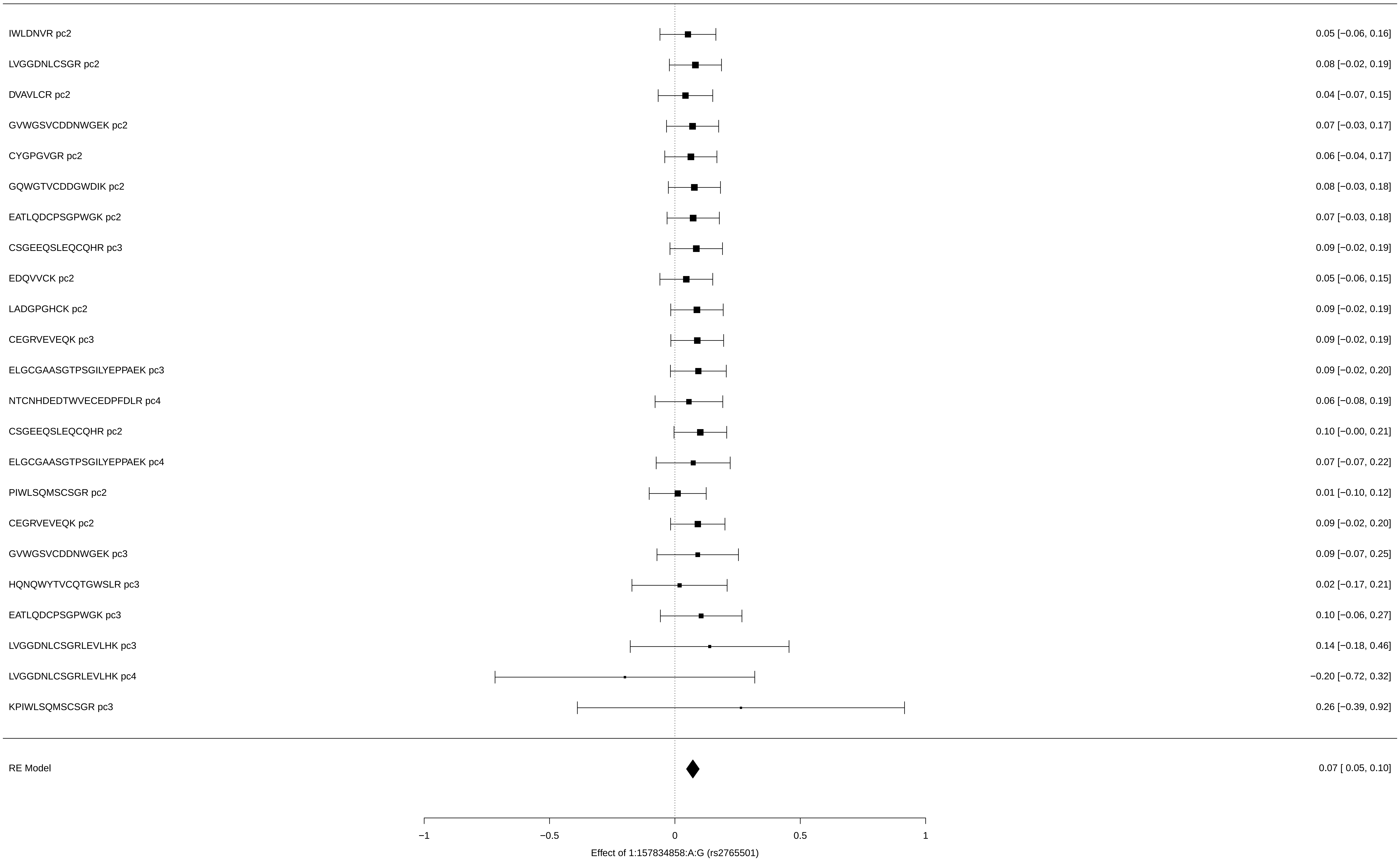

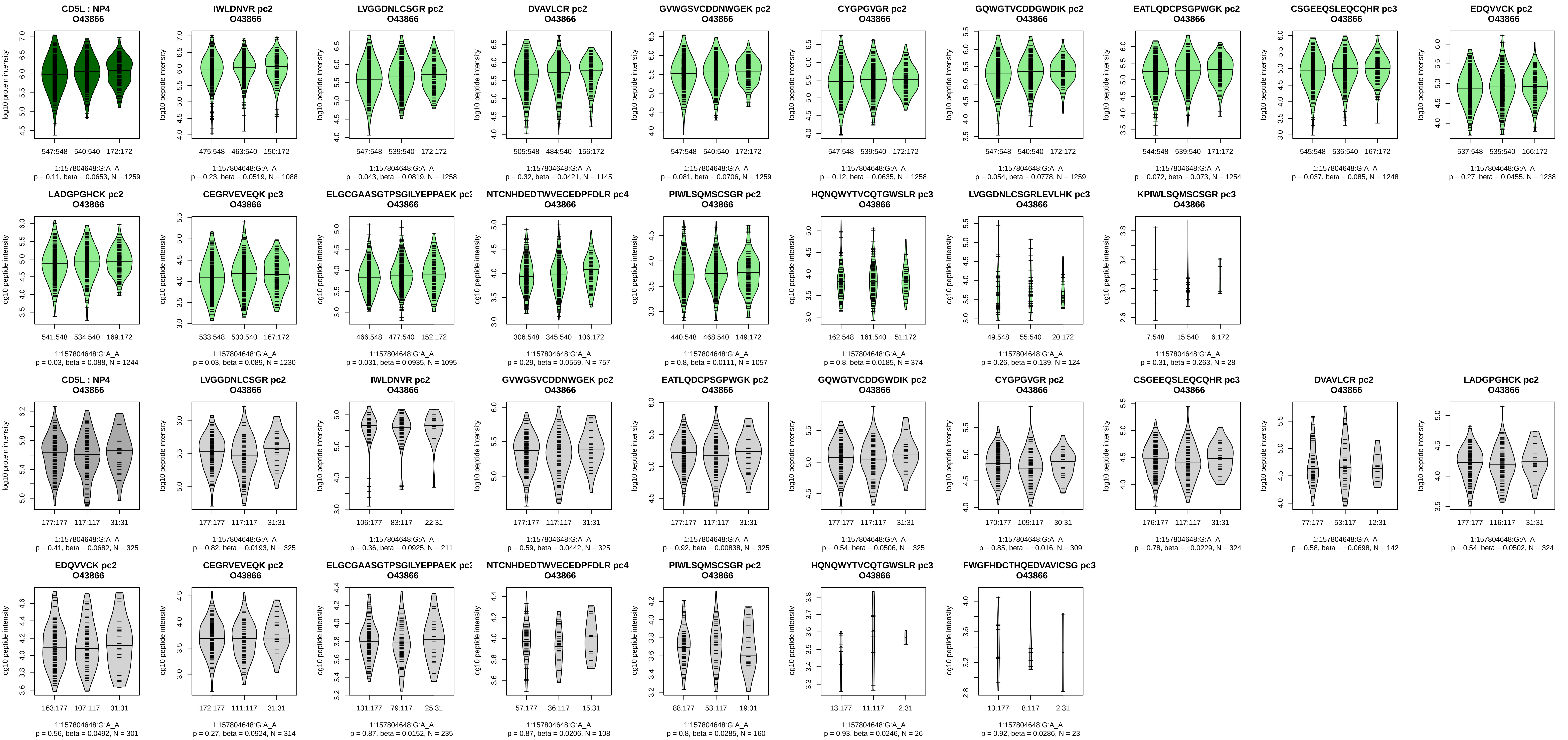

Assay Target: CD5L
UniProt: O43866
rsID: rs2765501
Target SNP: 1:157834858:A:G
Proxy SNP: 1:157804648:G:A
deCODE log10(p): 284
deCODE BETA: 0.3
deCODE MSPA: 0

1088:1258:1145:1259:1258:125

102. FRZB (Q92765)
deCODE MSPA=0.838

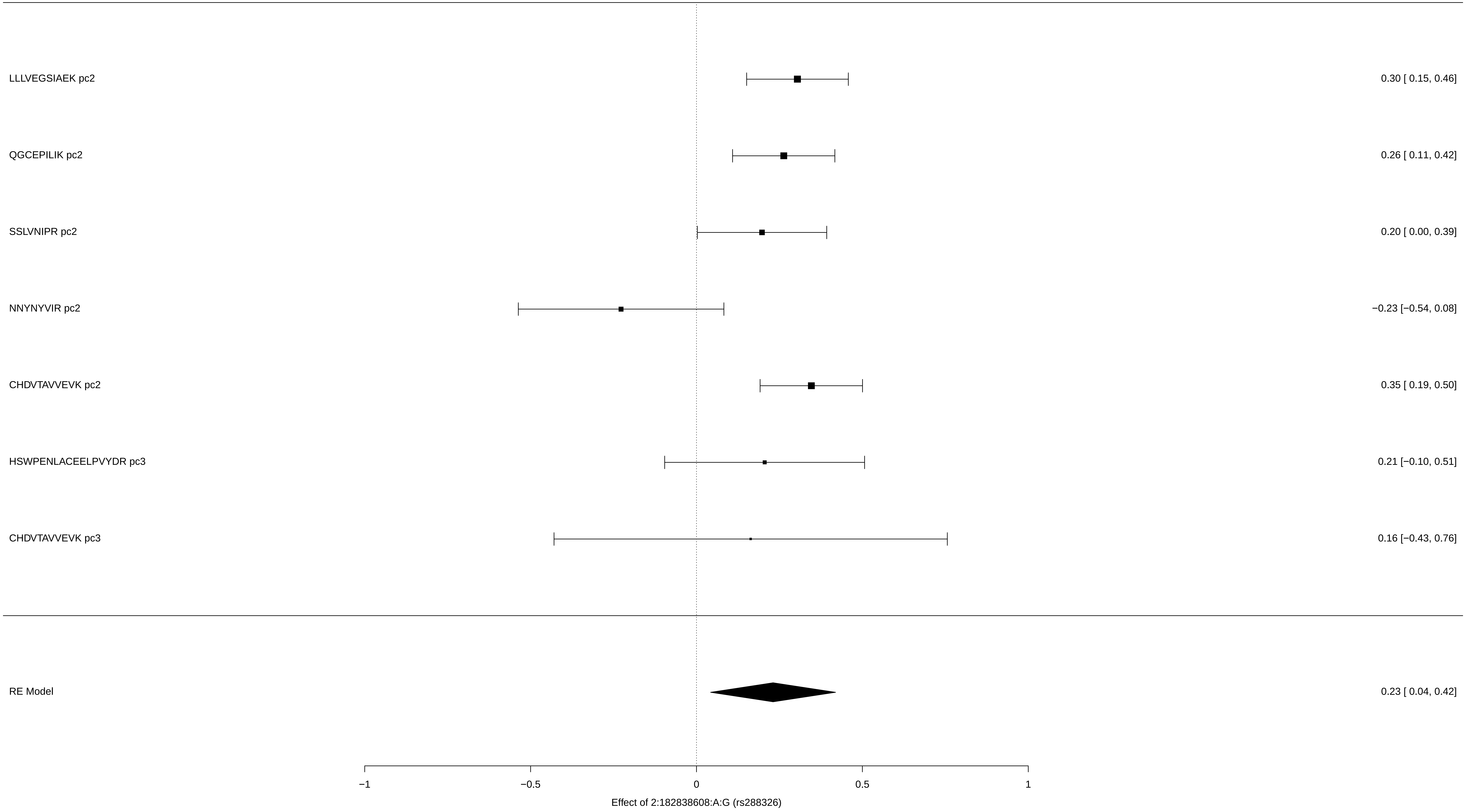

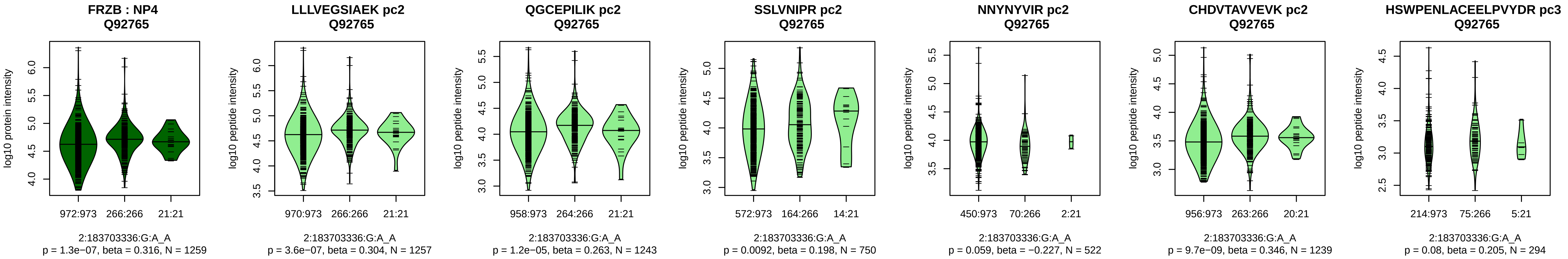

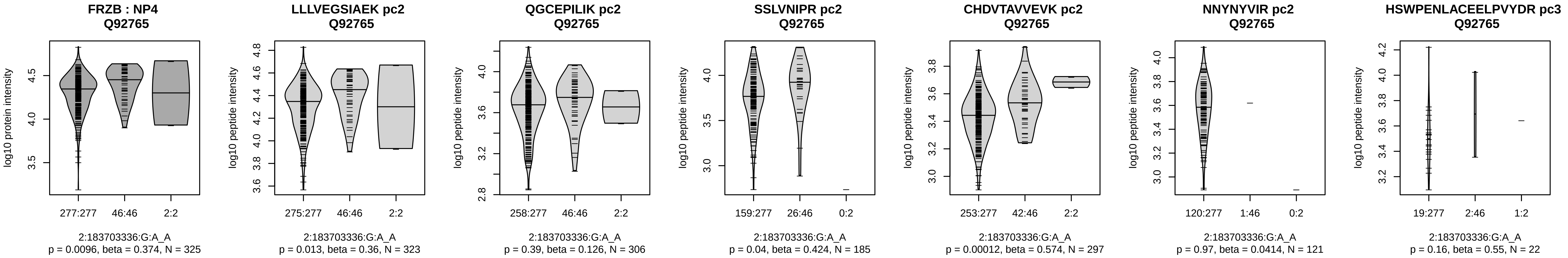

Assay Target: FRZB
UniProt: Q92765
rsID: rs288326
Target SNP: 2:182838608:A:G
Proxy SNP: 2:183703336:G:A
deCODE log10(p): 280.4
deCODE BETA: 0.54
deCODE MSPA: 0.838
::*:−:*:−:−:NA
1257:1243:750:522:1239:294:53

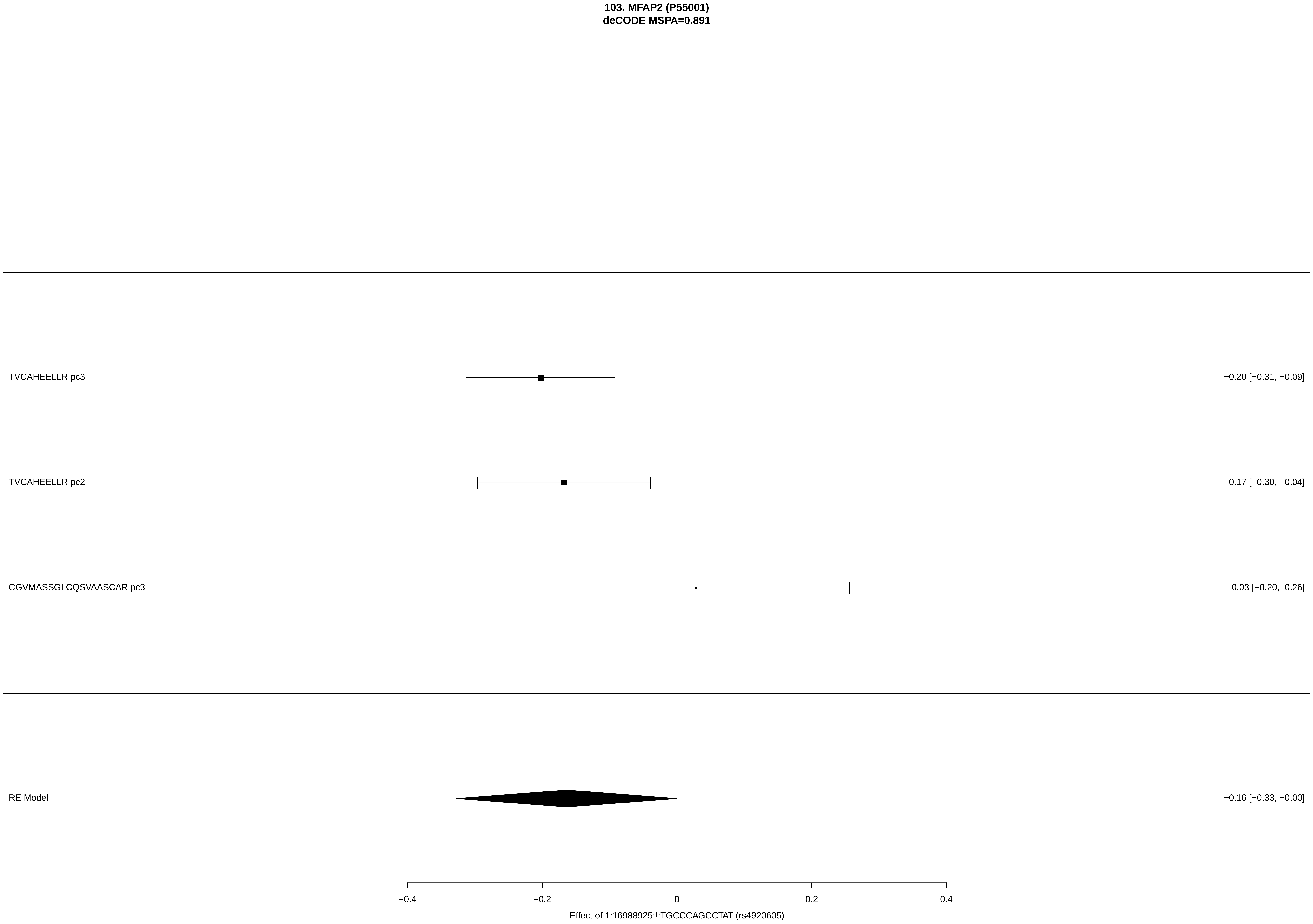
103. MFAP2 (P55001)
deCODE MSPA=0.891
TVCAHEELLR pc3
−0.20 [−0.31, −0.09]
TVCAHEELLR pc2
−0.17 [−0.30, −0.04]
CGVMASSGLCQSVAASCAR pc3
0.03 [−0.20, 0.26]
RE Model
−0.16 [−0.33, −0.00]
−0.4
−0.2
0
0.2
0.4
Effect of 1:16988925:!:TGCCCAGCCTAT (rs4920605)

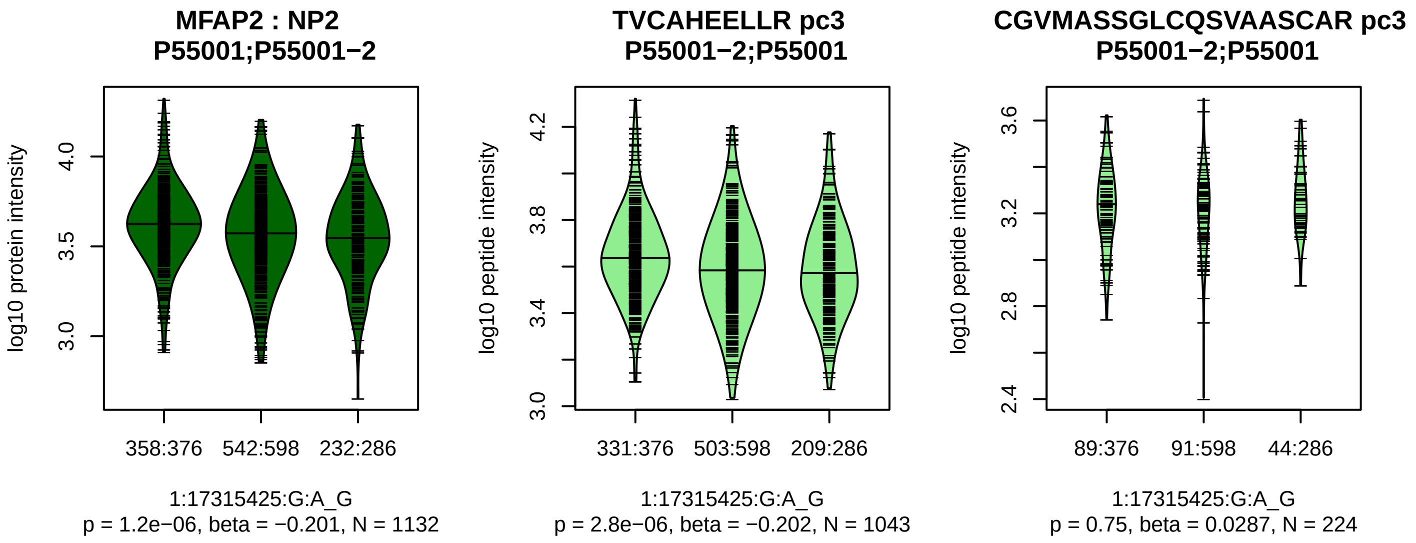

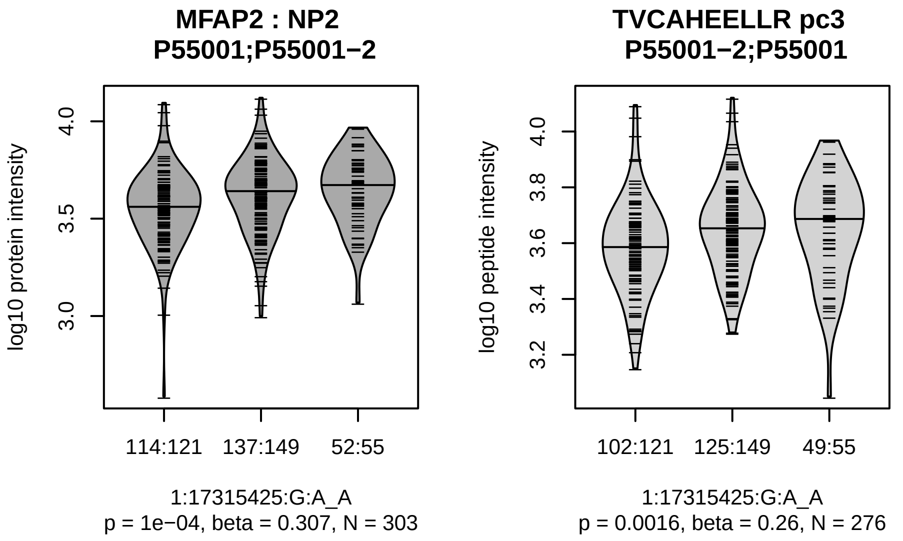

Assay Target: MFAP2
UniProt: P55001
rsID: rs4920605
Target SNP: 1:16988925:!:TGC
Proxy SNP: 1:17315425:G:A
deCODE log10(p): 275.9
deCODE BETA: −0.29
deCODE MSPA: 0.891
::–
1043:779:224

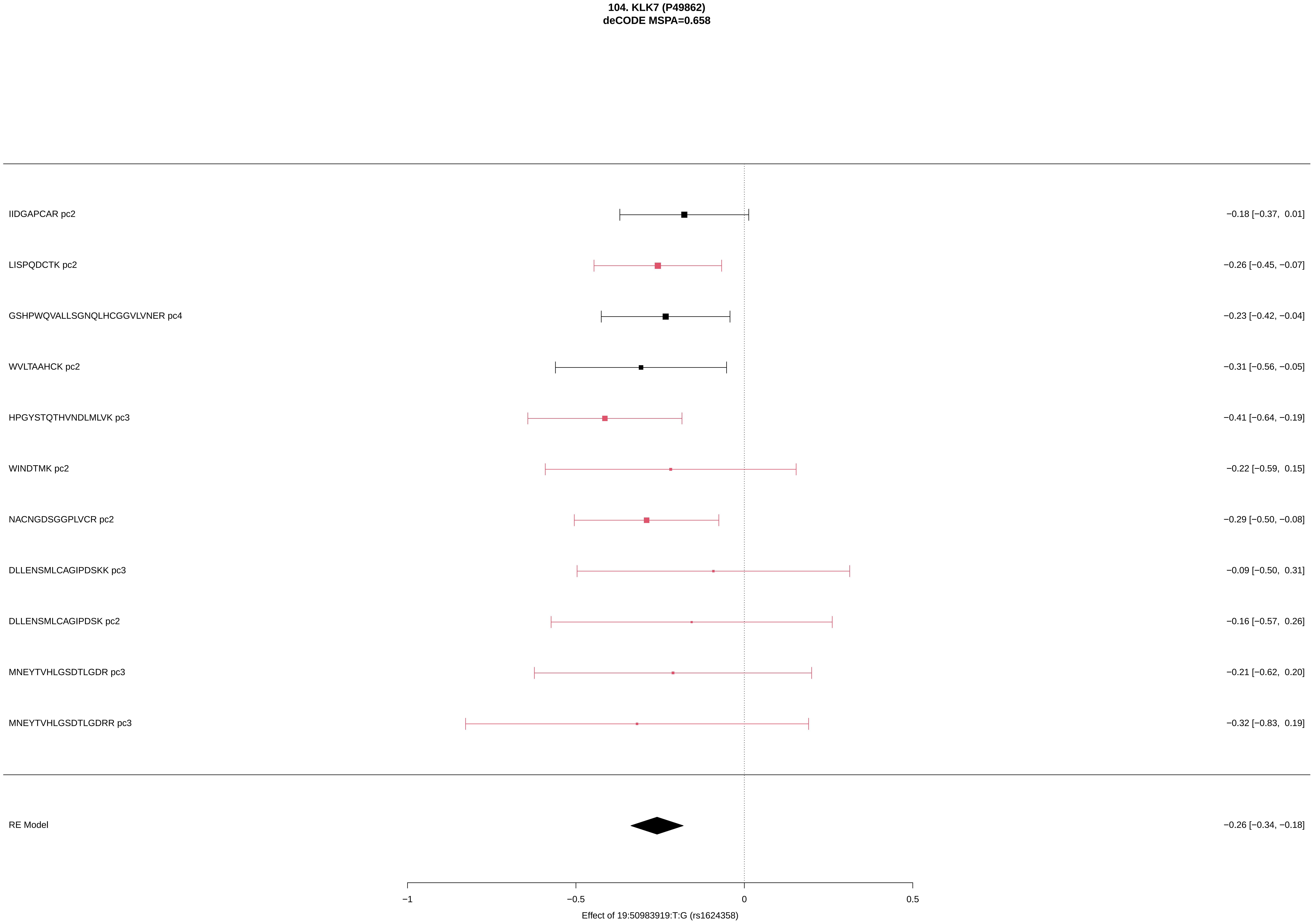
104. KLK7 (P49862)
deCODE MSPA=0.658
IIDGAPCAR pc2
−0.18 [−0.37, 0.01]
LISPQDCTK pc2
−0.26 [−0.45, −0.07]
GSHPWQVALLSGNQLHCGGVLVNER pc4
−0.23 [−0.42, −0.04]
WVLTAAHCK pc2
−0.31 [−0.56, −0.05]
HPGYSTQTHVNDLMLVK pc3
−0.41 [−0.64, −0.19]
WINDTMK pc2
−0.22 [−0.59, 0.15]
NACNGDSGGPLVCR pc2
−0.29 [−0.50, −0.08]
DLLENSMLCAGIPDSKK pc3
−0.09 [−0.50, 0.31]
DLLENSMLCAGIPDSK pc2
−0.16 [−0.57, 0.26]
MNEYTVHLGSDTLGDR pc3
−0.21 [−0.62, 0.20]
MNEYTVHLGSDTLGDRR pc3
−0.32 [−0.83, 0.19]
RE Model
−0.26 [−0.34, −0.18]
−1
−0.5
0
0.5
Effect of 19:50983919:T:G (rs1624358)

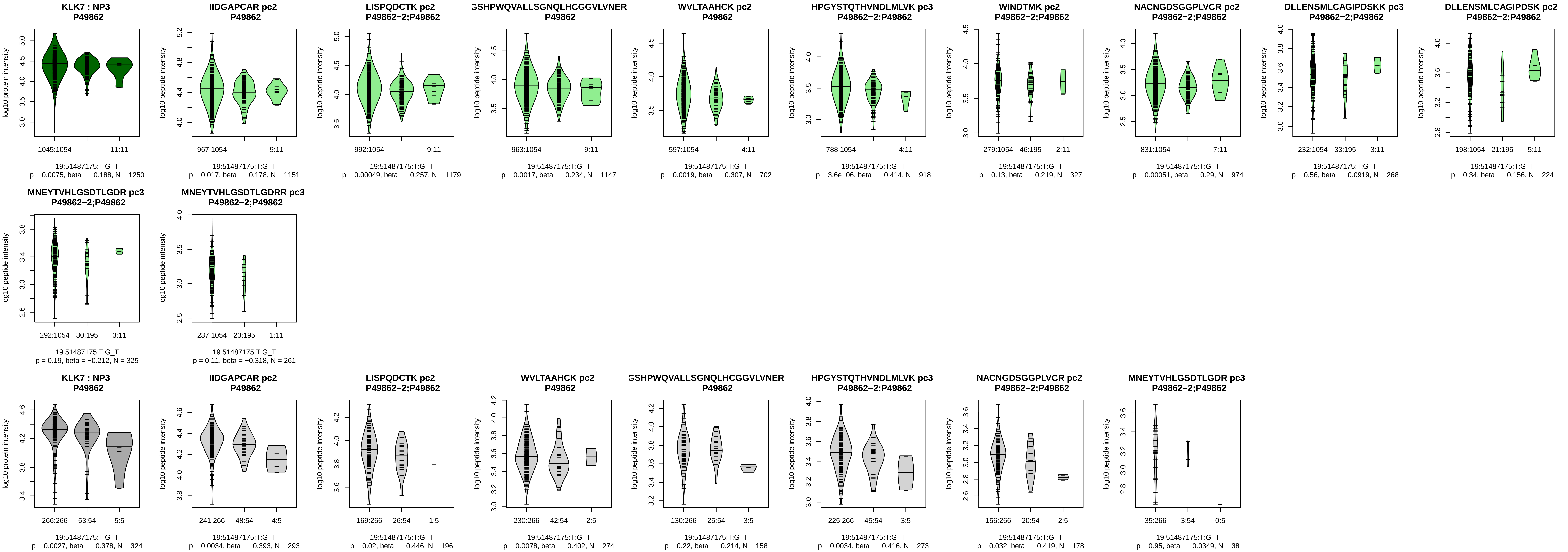

Assay Target: KLK7
UniProt: P49862
rsID: rs1624358
Target SNP: 19:50983919:T:G
Proxy SNP: 19:51487175:T:G
deCODE log10(p): 265.2
deCODE BETA: −0.53
deCODE MSPA: 0.658
–:*:*:*:*:–:*:–:–:–:–
1151:1179:1147:702:918:327:97

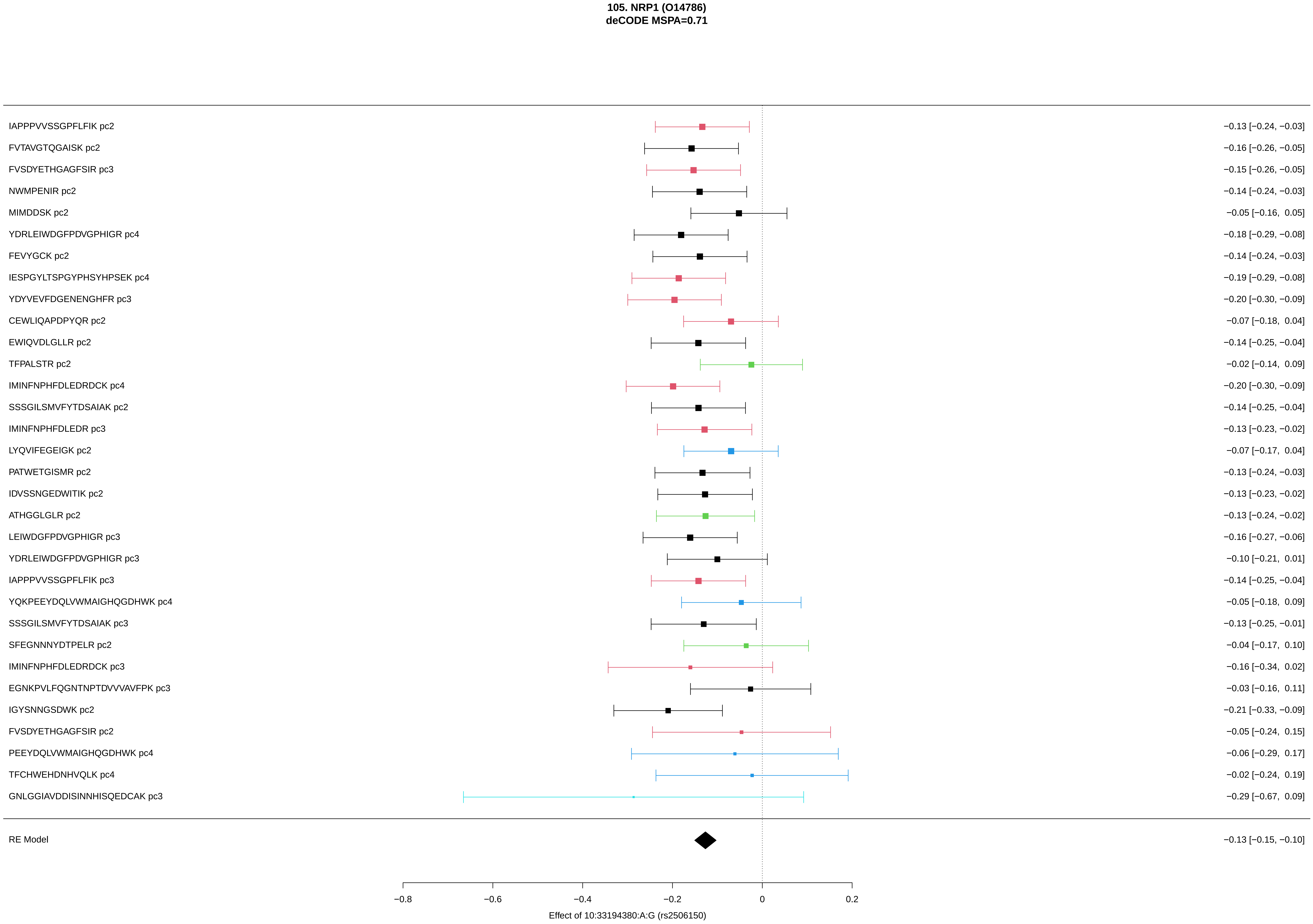

105. NRP1 (O14786)
deCODE MSPA=0.71
IAPPPVVSSGPFLFIK pc2 −0.13 [−0.24, −0.03]
FVTAVGTQGAISK pc2 −0.16 [−0.26, −0.05]
FVSDYETHGAGFSIR pc3 −0.15 [−0.26, −0.05]
NWMPENIR pc2 −0.14 [−0.24, −0.03]
MIMDDSK pc2 −0.05 [−0.16, 0.05]
YDRLEIWDGFPDVGPHIGR pc4 −0.18 [−0.29, −0.08]
FEVYGCK pc2 −0.14 [−0.24, −0.03]
IESPGYLTSPGYPHSYHPSEK pc4 −0.19 [−0.29, −0.08]
YDYVEVFDGENENGHFR pc3 −0.20 [−0.30, −0.09]
CEWLIQAPDPYQR pc2 −0.07 [−0.18, 0.04]
EWIQVDLGLLR pc2 −0.14 [−0.25, −0.04]
TFPALSTR pc2 −0.02 [−0.14, 0.09]
IMINFNPHFDLEDRDCK pc4 −0.20 [−0.30, −0.09]
SSSGILSMVFYTDSAIAK pc2 −0.14 [−0.25, −0.04]
IMINFNPHFDLEDR pc3 −0.13 [−0.23, −0.02]
LYQVIFEGEIGK pc2 −0.07 [−0.17, 0.04]
PATWETGISMR pc2 −0.13 [−0.24, −0.03]
IDVSSNGEDWITIK pc2 −0.13 [−0.23, −0.02]
ATHGGLGLR pc2 −0.13 [−0.24, −0.02]
LEIWDGFPDVGPHIGR pc3 −0.16 [−0.27, −0.06]
YDRLEIWDGFPDVGPHIGR pc3 −0.10 [−0.21, 0.01]
IAPPPVVSSGPFLFIK pc3 −0.14 [−0.25, −0.04]
YQKPEEYDQLVWMAIGHQGDHWK pc4 −0.05 [−0.18, 0.09]
SSSGILSMVFYTDSAIAK pc3 −0.13 [−0.25, −0.01]
SFEGNNNYDTPELR pc2 −0.04 [−0.17, 0.10]
IMINFNPHFDLEDRDCK pc3 −0.16 [−0.34, 0.02]
EGNKPVLFQGNTNPTDVVVAVFPK pc3 −0.03 [−0.16, 0.11]
IGYSNNGSDWK pc2 −0.21 [−0.33, −0.09]
FVSDYETHGAGFSIR pc2 −0.05 [−0.24, 0.15]
PEEYDQLVWMAIGHQGDHWK pc4 −0.06 [−0.29, 0.17]
TFCHWEHDNHVQLK pc4 −0.02 [−0.24, 0.19]
GNLGGIAVDDISINNHISQEDCAK pc3 −0.29 [−0.67, 0.09]
RE Model −0.13 [−0.15, −0.10]
−0.8
−0.6
−0.4
−0.2
0
0.2
Effect of 10:33194380:A:G (rs2506150)

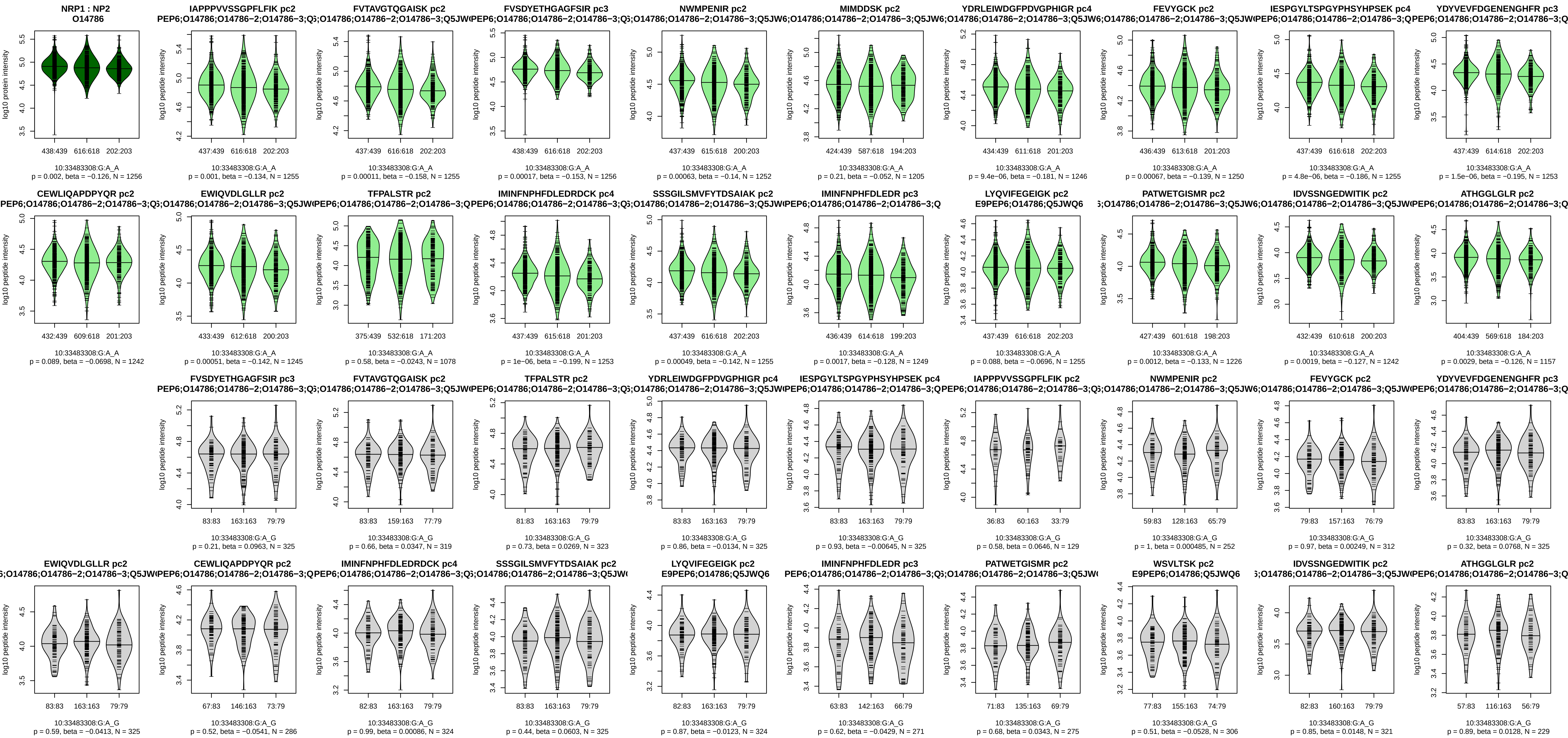

Assay Target: NRP1
UniProt: O14786
rsID: rs2506150
Target SNP: 10:33194380:A:G
Proxy SNP: 10:33483308:G:A
deCODE log10(p): 265.1
deCODE BETA: −0.3
deCODE MSPA: 0.71
1255:1255:1256:1252:1205:124

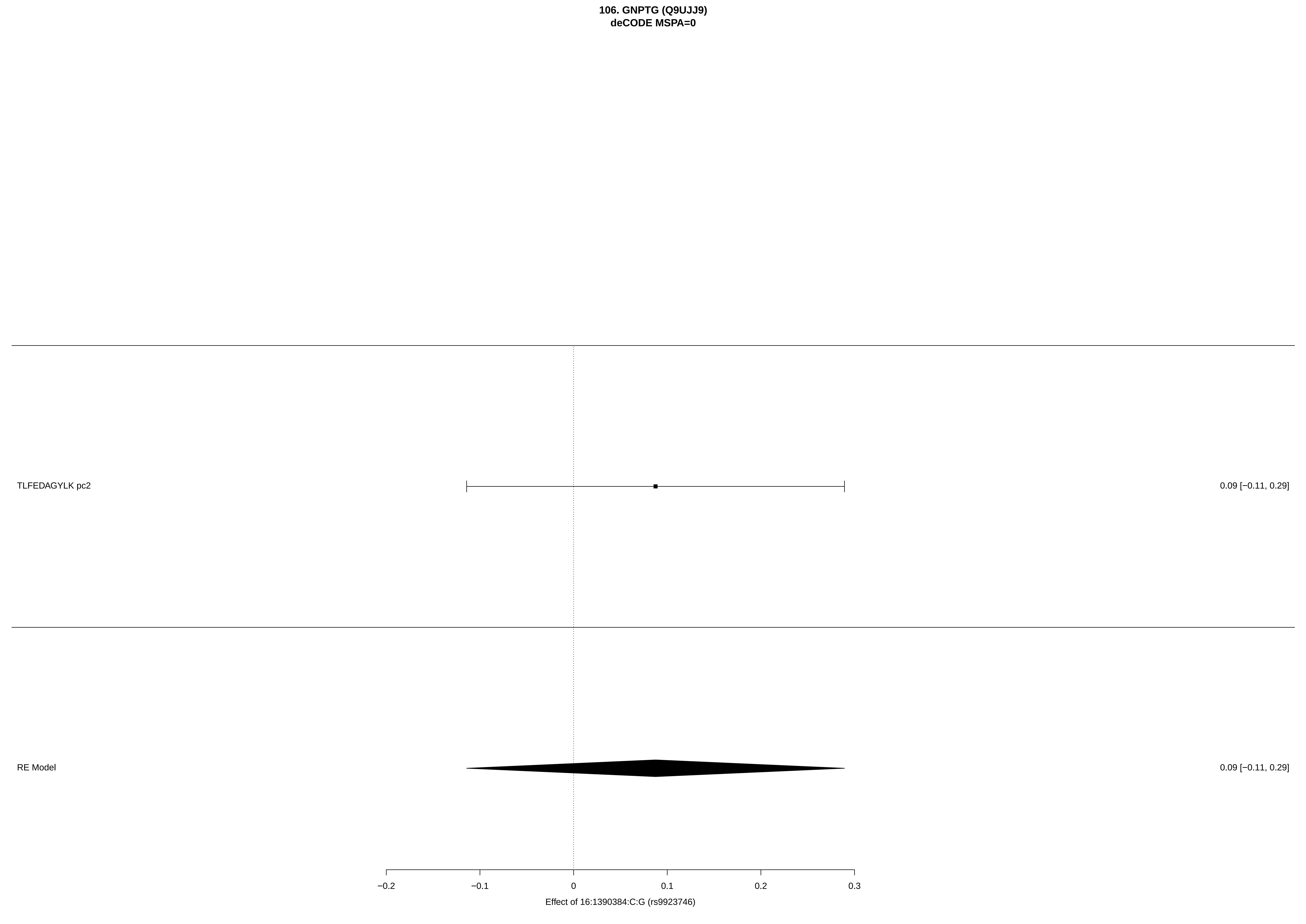
106. GNPTG (Q9UJJ9)
deCODE MSPA=0
TLFEDAGYLK pc2
0.09 [−0.11, 0.29]
RE Model
0.09 [−0.11, 0.29]
−0.2
−0.1
0
0.1
0.2
0.3
Effect of 16:1390384:C:G (rs9923746)

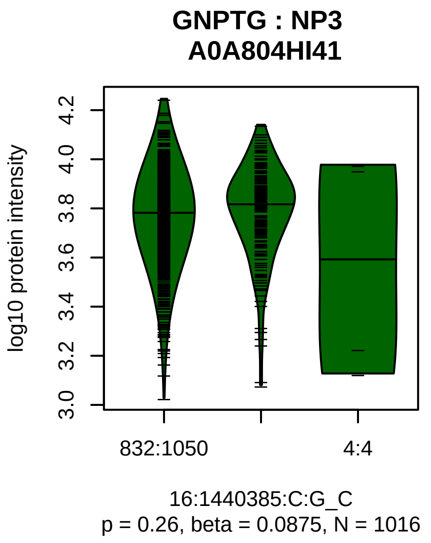

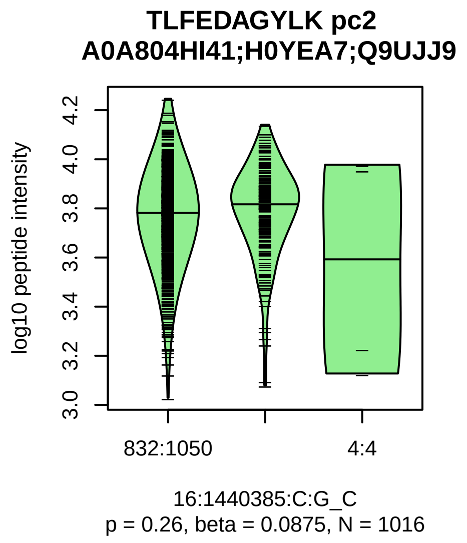

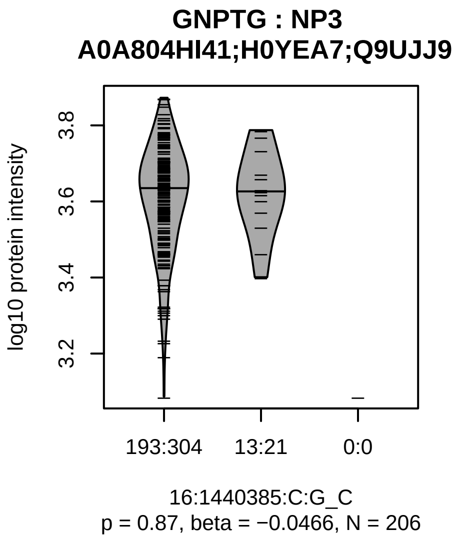

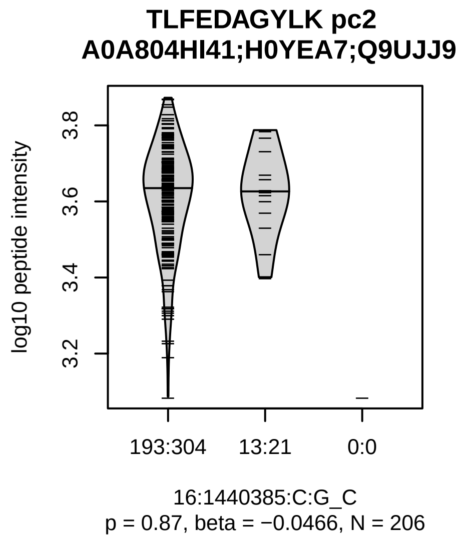

Assay Target: GNPTG
UniProt: Q9UJJ9
rsID: rs9923746
Target SNP: 16:1390384:C:G
Proxy SNP: 16:1440385:C:G
deCODE log10(p): 263
deCODE BETA: 0.52
deCODE MSPA: 0
–
1016

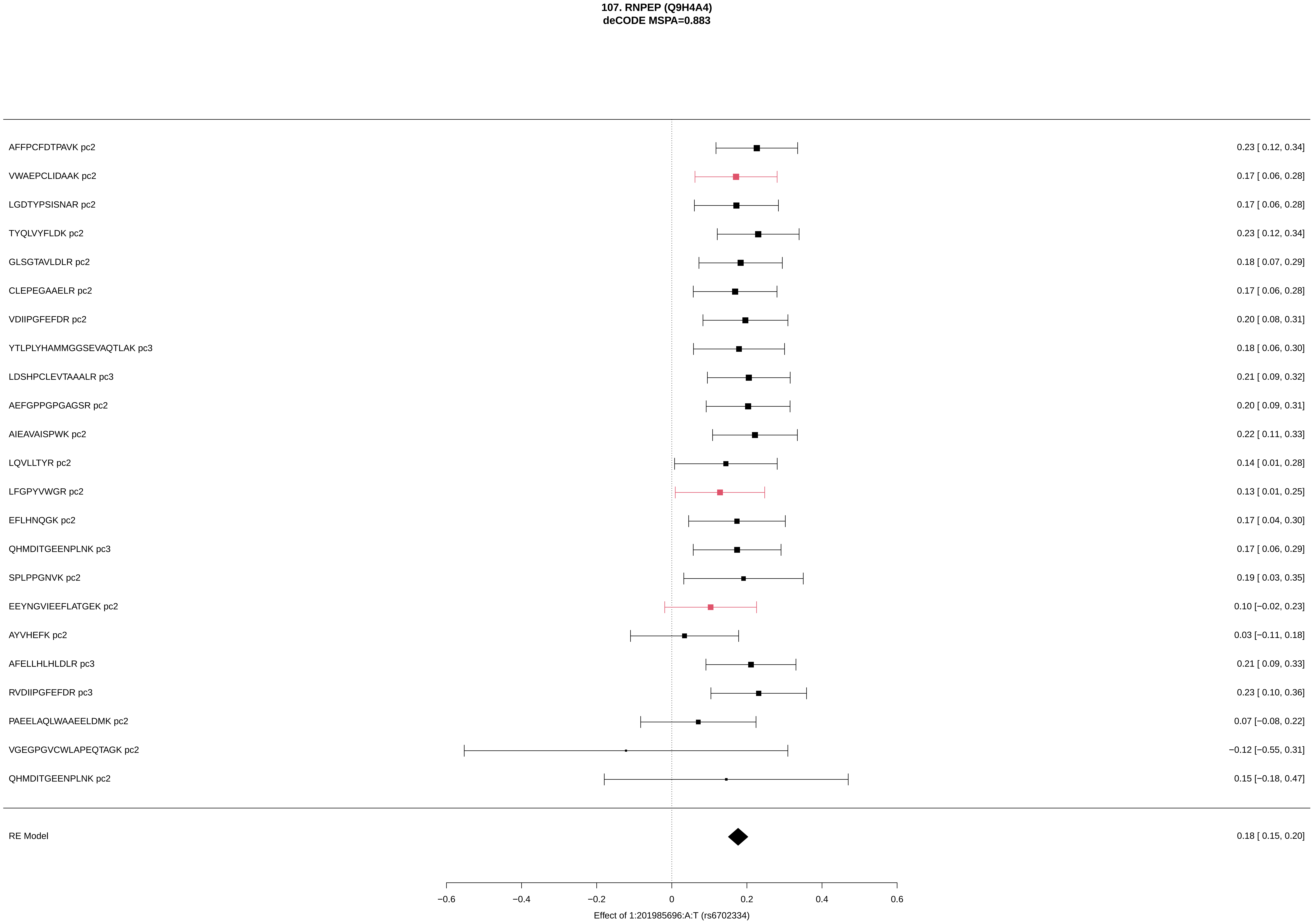

107. RNPEP (Q9H4A4)
deCODE MSPA=0.883
AFFPCFDTPAVK pc2
VWAEPCLIDAAK pc2
LGDTYPSISNAR pc2
TYQLVYFLDK pc2
GLSGTAVLDLR pc2
CLEPEGAAELR pc2
VDIIPGFEFDR pc2
YTLPLYHAMMGGSEVAQTLAK pc3
LDSHPCLEVTAAALR pc3
AEFGPPGPGAGSR pc2
AIEAVAISPWK pc2
LQVLLTYR pc2
LFGPYVWGR pc2
EFLHNQGK pc2
QHMDITGEENPLNK pc3
SPLPPGNVK pc2
EEYNGVIEEFLATGEK pc2
AYVHEFK pc2
AFELLHLHLDLR pc3
RVDIIPGFEFDR pc3
PAEELAQLWAAEELDMK pc2
VGEGPGVCWLAPEQTAGK pc2
QHMDITGEENPLNK pc2
RE Model
0.23 [0.12, 0.34]
0.17 [0.06, 0.28]
0.17 [0.06, 0.28]
0.23 [0.12, 0.34]
0.18 [0.07, 0.29]
0.17 [0.06, 0.28]
0.20 [0.08, 0.31]
0.18 [0.06, 0.30]
0.21 [0.09, 0.32]
0.20 [0.09, 0.31]
0.22 [0.11, 0.33]
0.14 [0.01, 0.28]
0.13 [0.01, 0.25]
0.17 [0.04, 0.30]
0.17 [0.06, 0.29]
0.19 [0.03, 0.35]
0.10 [−0.02, 0.23]
0.03 [−0.11, 0.18]
0.21 [0.09, 0.33]
0.23 [0.10, 0.36]
0.07 [−0.08, 0.22]
−0.12 [−0.55, 0.31]
0.15 [−0.18, 0.47]
0.18 [0.15, 0.20]
−0.6
−0.4
−0.2
0
0.2
0.4
0.6
Effect of 1:201985696:A:T (rs6702334)

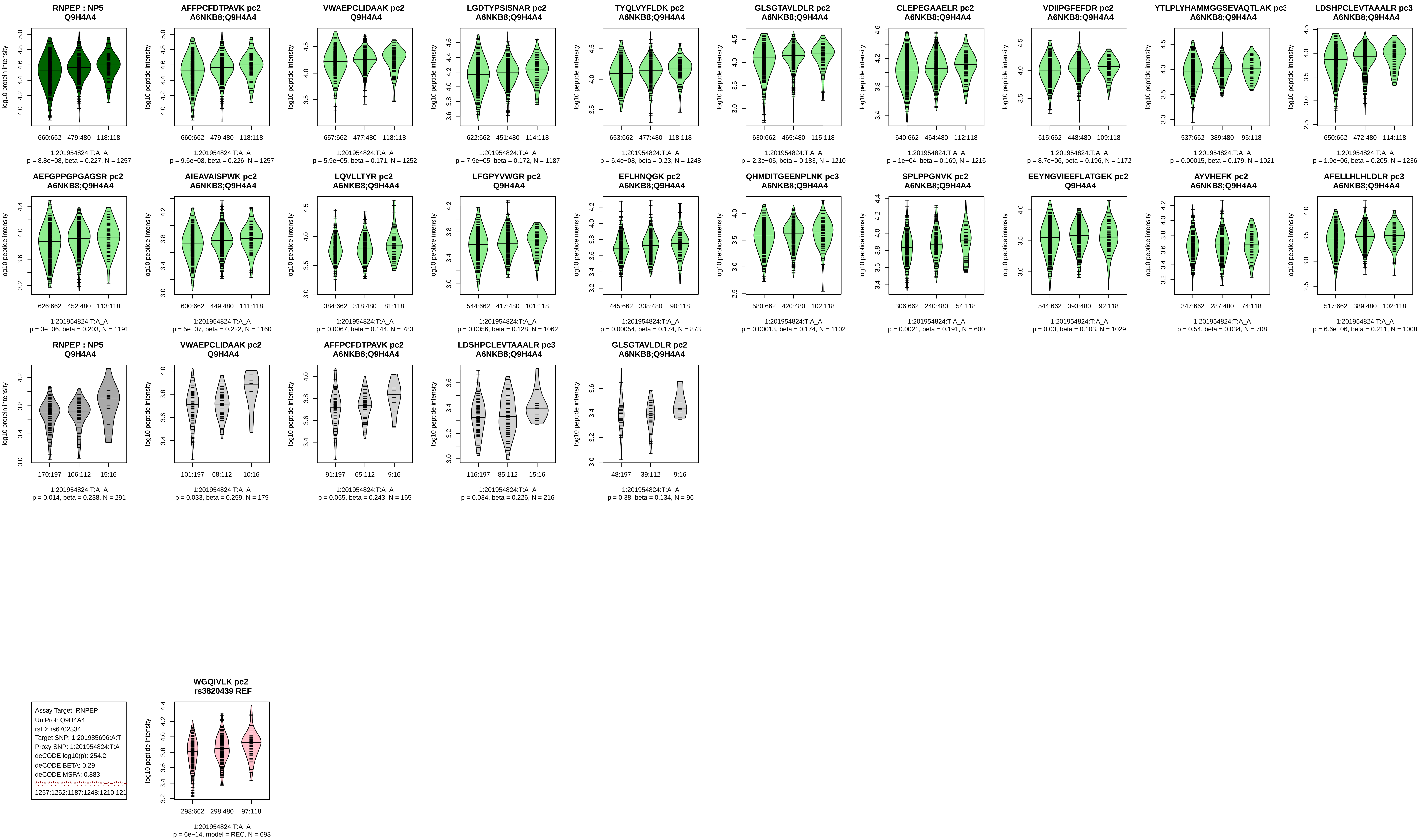
RNPEP : NP5
Q9H4A4
log10 protein intensity
660:662 479:480 118:118
1:201954824:T:A_A
p = 8.8e−08, beta = 0.227, N = 1257
AFFPCFDTPAVK pc2
A6NKB8;Q9H4A4
log10 peptide intensity
660:662 479:480 118:118
1:201954824:T:A_A
p = 9.6e−08, beta = 0.226, N = 1257
VWAEPCLIDAAK pc2
Q9H4A4
657:662 477:480 118:118
1:201954824:T:A_A
p = 5.9e−05, beta = 0.171, N = 1252
LGDTYPSISNAR pc2
A6NKB8;Q9H4A4
622:662 451:480 114:118
1:201954824:T:A_A
p = 7.9e−05, beta = 0.172, N = 1187
TYQLVYFLDK pc2
A6NKB8;Q9H4A4
653:662 477:480 118:118
1:201954824:T:A_A
p = 6.4e−08, beta = 0.23, N = 1248
GLSGTAVLDLR pc2
A6NKB8;Q9H4A4
630:662 465:480 115:118
1:201954824:T:A_A
p = 2.3e−05, beta = 0.183, N = 1210
CLEPEGAAELR pc2
A6NKB8;Q9H4A4
640:662 464:480 112:118
1:201954824:T:A_A
p = 1e−04, beta = 0.169, N = 1216
VDIIPGFEFDR pc2
A6NKB8;Q9H4A4
615:662 448:480 109:118
1:201954824:T:A_A
p = 8.7e−06, beta = 0.196, N = 1172
YTLPLYHAMMGGSEVAQTLAK pc3
A6NKB8;Q9H4A4
537:662 389:480 95:118
1:201954824:T:A_A
p = 0.00015, beta = 0.179, N = 1021
LDSHPCLEVTAAALR pc3
A6NKB8;Q9H4A4
650:662 472:480 114:118
1:201954824:T:A_A
p = 1.9e−06, beta = 0.205, N = 1236
AEFGPPGPGAGSR pc2
A6NKB8;Q9H4A4
626:662 452:480 113:118
1:201954824:T:A_A
p = 3e−06, beta = 0.203, N = 1191
AIEAVAISPWK pc2
A6NKB8;Q9H4A4
600:662 449:480 111:118
1:201954824:T:A_A
p = 5e−07, beta = 0.226, N = 1160
LQVLLTYR pc2
A6NKB8;Q9H4A4
384:662 318:480 81:118
1:201954824:T:A_A
p = 0.0067, beta = 0.144, N = 783
LFGPYVWGR pc2
Q9H4A4
544:662 417:480 101:118
1:201954824:T:A_A
p = 0.0056, beta = 0.128, N = 1062
EFLHNQGK pc2
A6NKB8;Q9H4A4
445:662 338:480 90:118
1:201954824:T:A_A
p = 0.00054, beta = 0.174, N = 873
QHMDITGEENPLNK pc3
A6NKB8;Q9H4A4
580:662 420:480 102:118
1:201954824:T:A_A
p = 0.00013, beta = 0.174, N = 1102
SPLPPGNVK pc2
A6NKB8;Q9H4A4
306:662 240:480 54:118
1:201954824:T:A_A
p = 0.0021, beta = 0.191, N = 600
EEYNGVIEEFLATGEK pc2
Q9H4A4
544:662 393:480 92:118
1:201954824:T:A_A
p = 0.03, beta = 0.103, N = 1029
AYVHEFK pc2
A6NKB8;Q9H4A4
347:662 287:480 74:118
1:201954824:T:A_A
p = 0.54, beta = 0.034, N = 708
AFELLHLHLDLR pc3
A6NKB8;Q9H4A4
517:662 389:480 102:118
1:201954824:T:A_A
p = 6.6e−06, beta = 0.211, N = 1008
RNPEP : NP5
Q9H4A4
170:197 106:112 15:16
1:201954824:T:A_A
p = 0.014, beta = 0.238, N = 291
VWAEPCLIDAAK pc2
Q9H4A4
101:197 68:112 10:16
1:201954824:T:A_A
p = 0.033, beta = 0.259, N = 179
AFFPCFDTPAVK pc2
A6NKB8;Q9H4A4
91:197 65:112 9:16
1:201954824:T:A_A
p = 0.055, beta = 0.243, N = 165
LDSHPCLEVTAAALR pc3
A6NKB8;Q9H4A4
116:197 85:112 15:16
1:201954824:T:A_A
p = 0.034, beta = 0.226, N = 216
GLSGTAVLDLR pc2
A6NKB8;Q9H4A4
48:197 39:112 9:16
1:201954824:T:A_A
p = 0.38, beta = 0.134, N = 96
Assay Target: RNPEP
UniProt: Q9H4A4
rsID: rs6702334
Target SNP: 1:201985696:A:T
Proxy SNP: 1:201954824:T:A
deCODE log10(p): 254.2
deCODE BETA: 0.29
deCODE MSPA: 0.883
1257:1252:1187:1248:1210:121
WGQIVLK pc2
rs3820439 REF
298:662 298:480 97:118
1:201954824:T:A_A
p = 6e−14, model = REC, N = 693

108. SPON1 (Q9HCB6)
deCODE MSPA=0.389

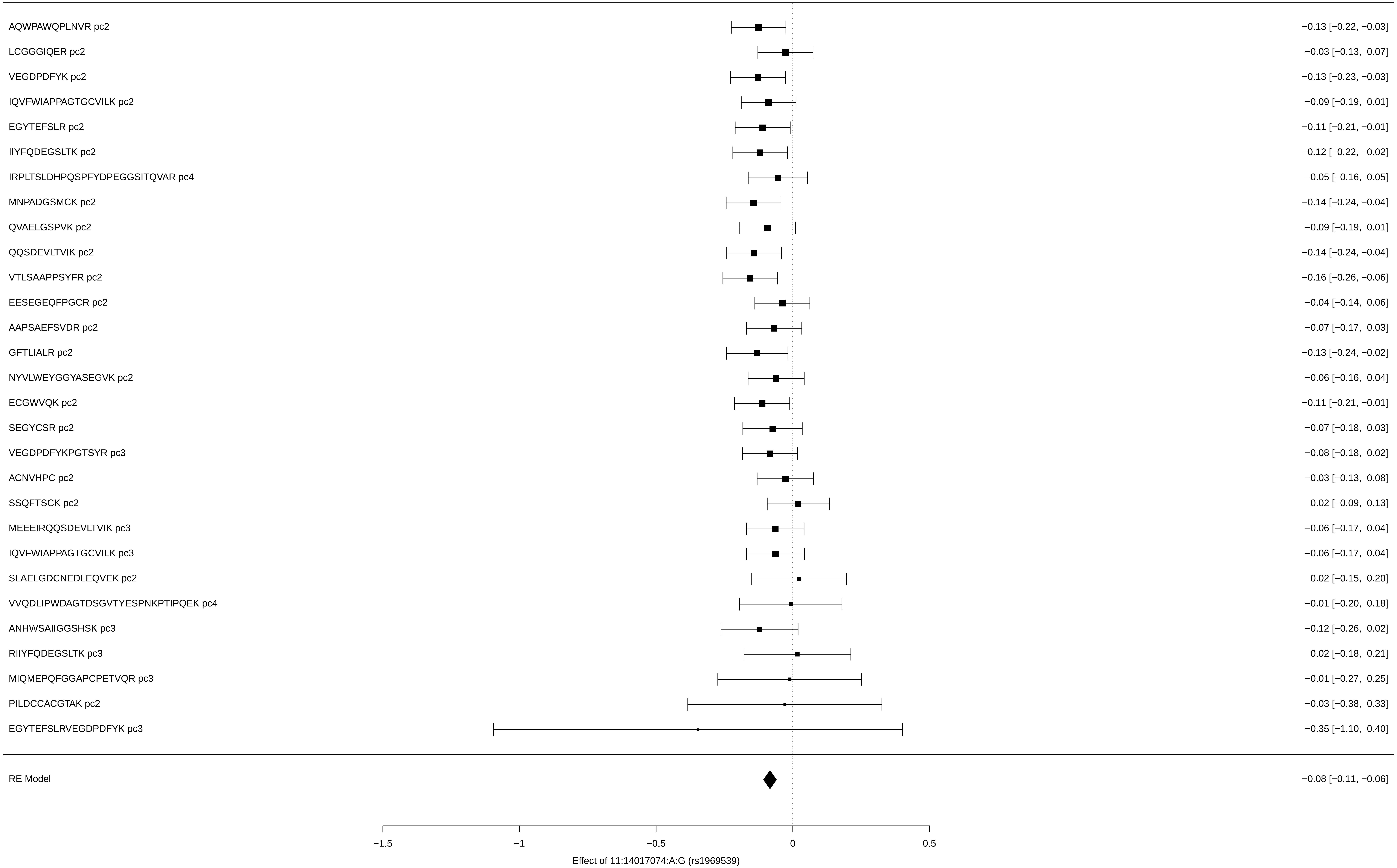

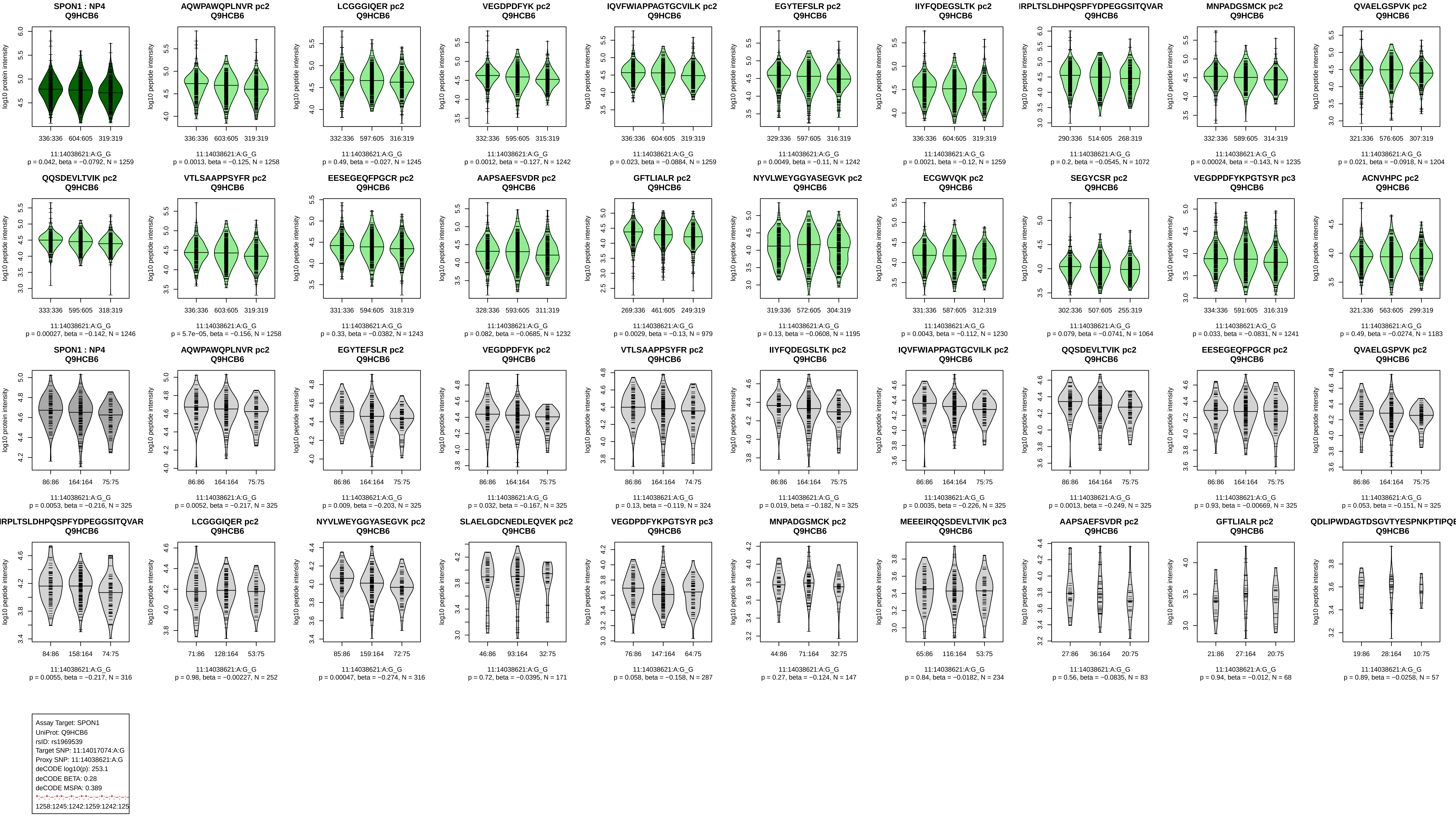
SPON1 : NP4
Q9HCB6
log10 protein intensity
336:336 604:605 319:319
11:14038621:A:G_G
p = 0.042, beta = −0.0792, N = 1259
AQWPAWQPLNVR pc2
Q9HCB6
log10 peptide intensity
336:336 603:605 319:319
11:14038621:A:G_G
p = 0.0013, beta = −0.125, N = 1258
LCGGGIQER pc2
Q9HCB6
332:336 597:605 316:319
11:14038621:A:G_G
p = 0.49, beta = −0.027, N = 1245
VEGDPDFYK pc2
Q9HCB6
332:336 595:605 315:319
11:14038621:A:G_G
p = 0.0012, beta = −0.127, N = 1242
IQVFWIAPPAGTGCVILK pc2
Q9HCB6
336:336 604:605 319:319
11:14038621:A:G_G
p = 0.023, beta = −0.0884, N = 1259
EGYTEFSLR pc2
Q9HCB6
329:336 597:605 316:319
11:14038621:A:G_G
p = 0.0049, beta = −0.11, N = 1242
IIYFQDEGSLTK pc2
Q9HCB6
336:336 604:605 319:319
11:14038621:A:G_G
p = 0.0021, beta = −0.12, N = 1259
IRPLTSLDHPQSPFYDPEGGSITQVAR
Q9HCB6
290:336 514:605 268:319
11:14038621:A:G_G
p = 0.2, beta = −0.0545, N = 1072
MNPADGSMCK pc2
Q9HCB6
332:336 589:605 314:319
11:14038621:A:G_G
p = 0.00024, beta = −0.143, N = 1235
QVAELGSPVK pc2
Q9HCB6
321:336 576:605 307:319
11:14038621:A:G_G
p = 0.021, beta = −0.0918, N = 1204
QQSDEVLTVIK pc2
Q9HCB6
333:336 595:605 318:319
11:14038621:A:G_G
p = 0.00027, beta = −0.142, N = 1246
VTLSAAPPSYFR pc2
Q9HCB6
336:336 603:605 319:319
11:14038621:A:G_G
p = 5.7e−05, beta = −0.156, N = 1258
EESEGEQFPGCR pc2
Q9HCB6
331:336 594:605 318:319
11:14038621:A:G_G
p = 0.33, beta = −0.0382, N = 1243
AAPSAEFSVDR pc2
Q9HCB6
328:336 593:605 311:319
11:14038621:A:G_G
p = 0.082, beta = −0.0685, N = 1232
GFTLIALR pc2
Q9HCB6
269:336 461:605 249:319
11:14038621:A:G_G
p = 0.0029, beta = −0.13, N = 979
NYVLWEYGGYASEGVK pc2
Q9HCB6
319:336 572:605 304:319
11:14038621:A:G_G
p = 0.13, beta = −0.0608, N = 1195
ECGWVQK pc2
Q9HCB6
331:336 587:605 312:319
11:14038621:A:G_G
p = 0.0043, beta = −0.112, N = 1230
SEGYCSR pc2
Q9HCB6
302:336 507:605 255:319
11:14038621:A:G_G
p = 0.079, beta = −0.0741, N = 1064
VEGDPDFYKPGTSYR pc3
Q9HCB6
334:336 591:605 316:319
11:14038621:A:G_G
p = 0.033, beta = −0.0831, N = 1241
ACNVHPC pc2
Q9HCB6
321:336 563:605 299:319
11:14038621:A:G_G
p = 0.49, beta = −0.0274, N = 1183
SPON1 : NP4
Q9HCB6
86:86 164:164 75:75
11:14038621:A:G_G
p = 0.0053, beta = −0.216, N = 325
AQWPAWQPLNVR pc2
Q9HCB6
86:86 164:164 75:75
11:14038621:A:G_G
p = 0.0052, beta = −0.217, N = 325
EGYTEFSLR pc2
Q9HCB6
86:86 164:164 75:75
11:14038621:A:G_G
p = 0.009, beta = −0.203, N = 325
VEGDPDFYK pc2
Q9HCB6
86:86 164:164 75:75
11:14038621:A:G_G
p = 0.032, beta = −0.167, N = 325
VTLSAAPPSYFR pc2
Q9HCB6
86:86 164:164 74:75
11:14038621:A:G_G
p = 0.13, beta = −0.119, N = 324
IIYFQDEGSLTK pc2
Q9HCB6
86:86 164:164 75:75
11:14038621:A:G_G
p = 0.019, beta = −0.182, N = 325
IQVFWIAPPAGTGCVILK pc2
Q9HCB6
86:86 164:164 75:75
11:14038621:A:G_G
p = 0.0035, beta = −0.226, N = 325
QQSDEVLTVIK pc2
Q9HCB6
86:86 164:164 75:75
11:14038621:A:G_G
p = 0.0013, beta = −0.249, N = 325
EESEGEQFPGCR pc2
Q9HCB6
86:86 164:164 75:75
11:14038621:A:G_G
p = 0.93, beta = −0.00669, N = 325
QVAELGSPVK pc2
Q9HCB6
86:86 164:164 75:75
11:14038621:A:G_G
p = 0.053, beta = −0.151, N = 325
IRPLTSLDHPQSPFYDPEGGSITQVAR
Q9HCB6
84:86 158:164 74:75
11:14038621:A:G_G
p = 0.0055, beta = −0.217, N = 316
LCGGGIQER pc2
Q9HCB6
71:86 128:164 53:75
11:14038621:A:G_G
p = 0.98, beta = −0.00227, N = 252
NYVLWEYGGYASEGVK pc2
Q9HCB6
85:86 159:164 72:75
11:14038621:A:G_G
p = 0.00047, beta = −0.274, N = 316
SLAELGDCNEDLEQVEK pc2
Q9HCB6
46:86 93:164 32:75
11:14038621:A:G_G
p = 0.72, beta = −0.0395, N = 171
VEGDPDFYKPGTSYR pc3
Q9HCB6
76:86 147:164 64:75
11:14038621:A:G_G
p = 0.058, beta = −0.158, N = 287
MNPADGSMCK pc2
Q9HCB6
44:86 71:164 32:75
11:14038621:A:G_G
p = 0.27, beta = −0.124, N = 147
MEEEIRQQSDEVLTVIK pc3
Q9HCB6
65:86 116:164 53:75
11:14038621:A:G_G
p = 0.84, beta = −0.0182, N = 234
AAPSAEFSVDR pc2
Q9HCB6
27:86 36:164 20:75
11:14038621:A:G_G
p = 0.56, beta = −0.0835, N = 83
GFTLIALR pc2
Q9HCB6
21:86 27:164 20:75
11:14038621:A:G_G
p = 0.94, beta = −0.012, N = 68
QDLIPWDAGTDSGVTYESPNKPTIPQE
Q9HCB6
19:86 28:164 10:75
11:14038621:A:G_G
p = 0.89, beta = −0.0258, N = 57
Assay Target: SPON1
UniProt: Q9HCB6
rsID: rs1969539
Target SNP: 11:14017074:A:G
Proxy SNP: 11:14038621:A:G
deCODE log10(p): 253.1
deCODE BETA: 0.28
deCODE MSPA: 0.389
1258:1245:1242:1259:1242:125

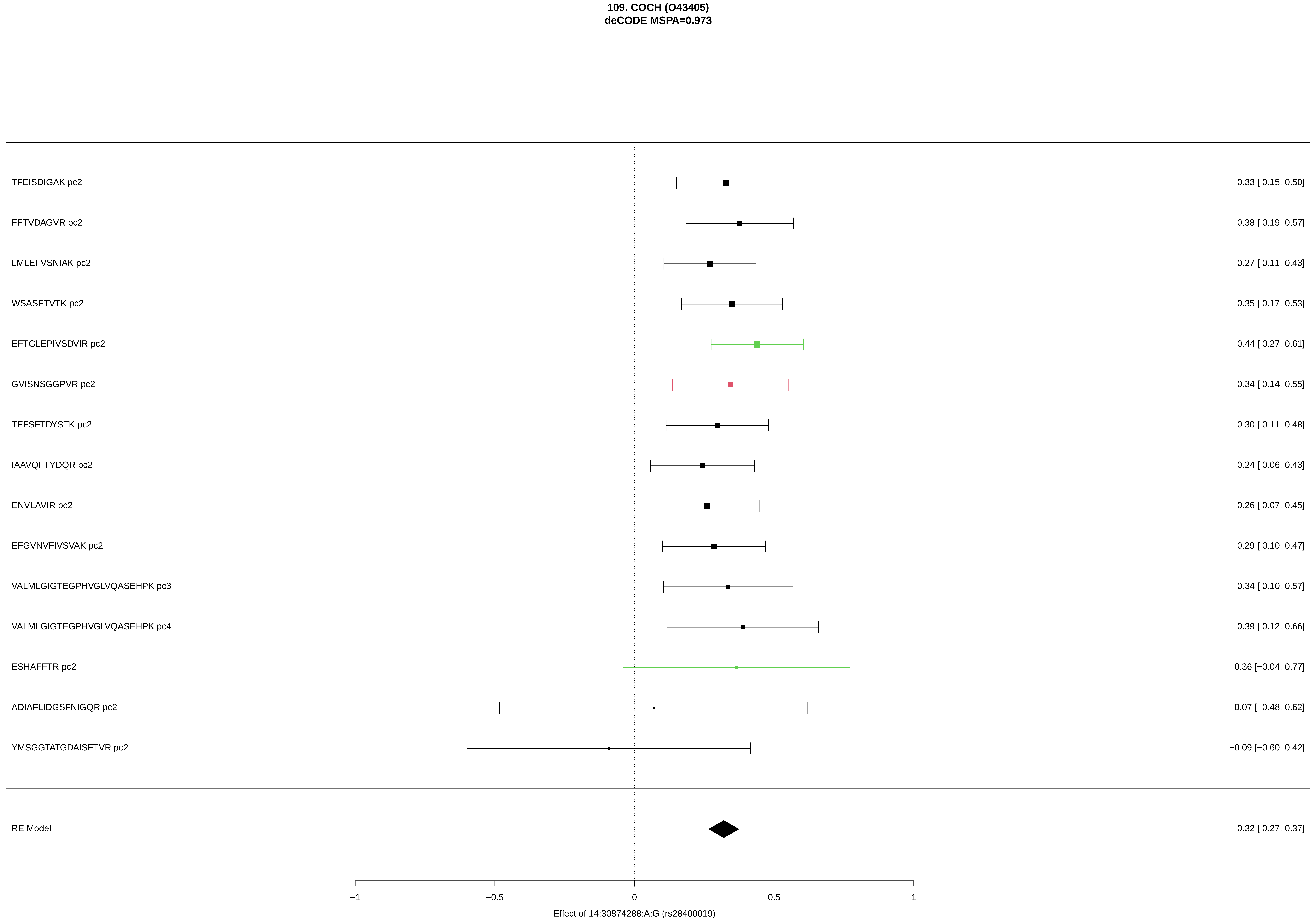
109. COCH (O43405)
deCODE MSPA=0.973
TFEISDIGAK pc2
0.33 [0.15, 0.50]
FFTVDAGVR pc2
0.38 [0.19, 0.57]
LMLEFVSNIAK pc2
0.27 [0.11, 0.43]
WSASFTVTK pc2
0.35 [0.17, 0.53]
EFTGLEPIVSDVIR pc2
0.44 [0.27, 0.61]
GVISNSGGPVR pc2
0.34 [0.14, 0.55]
TEFSFTDYSTK pc2
0.30 [0.11, 0.48]
IAAVQFTYDQR pc2
0.24 [0.06, 0.43]
ENVLAVIR pc2
0.26 [0.07, 0.45]
EFGVNVFIVSVAK pc2
0.29 [0.10, 0.47]
VALMLGIGTEGPHVGLVQASEHPK pc3
0.34 [0.10, 0.57]
VALMLGIGTEGPHVGLVQASEHPK pc4
0.39 [0.12, 0.66]
ESHAFFTR pc2
0.36 [−0.04, 0.77]
ADIAFLIDGSFNIGQR pc2
0.07 [−0.48, 0.62]
YMSGGTATGDAISFTVR pc2
−0.09 [−0.60, 0.42]
RE Model
0.32 [0.27, 0.37]
−1
−0.5
0
0.5
1
Effect of 14:30874288:A:G (rs28400019)

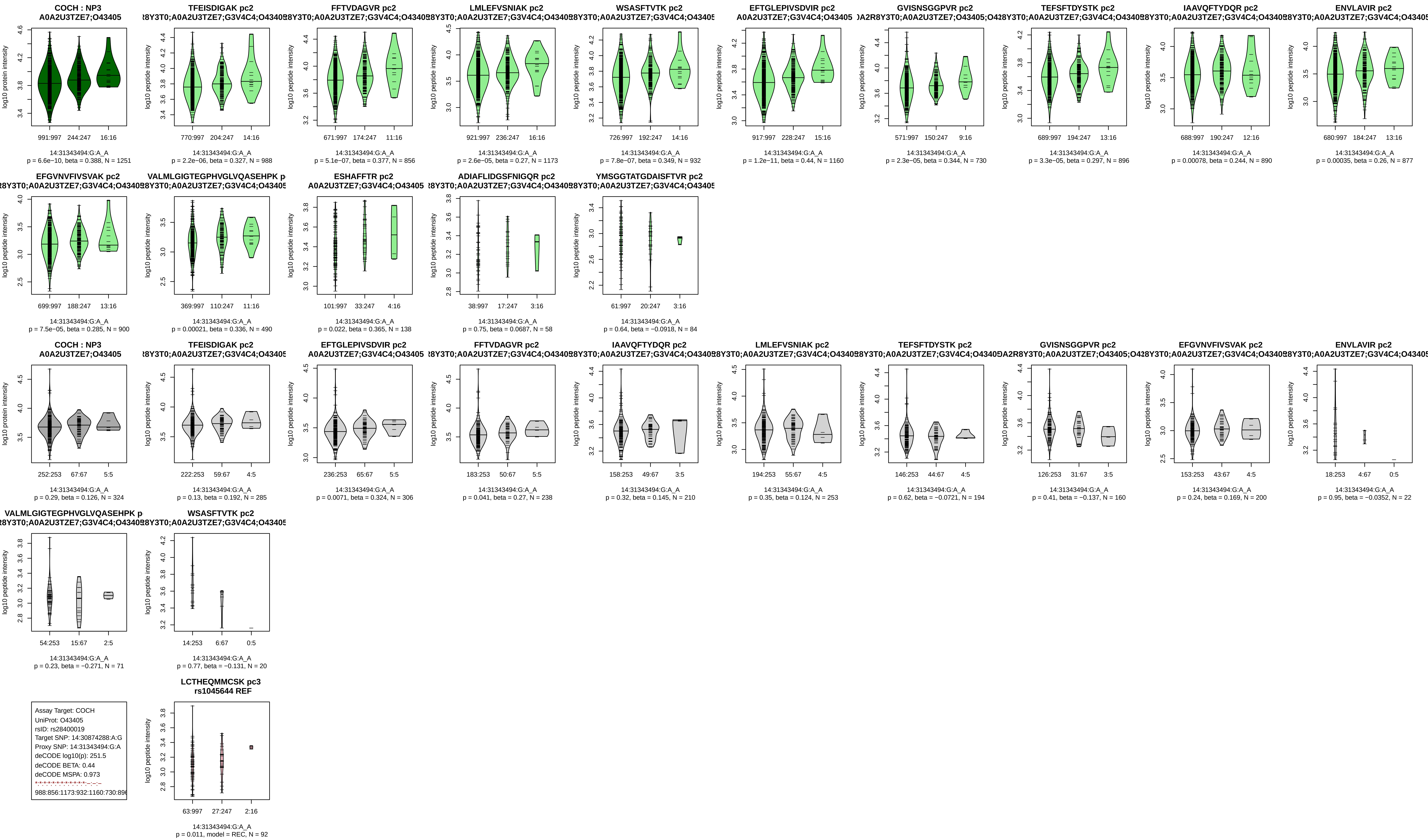

COCH : NP3
A0A2U3TZE7;O43405
log10 protein intensity
991:997 244:247 16:16
14:31343494:G:A_A
p = 6.6e−10, beta = 0.388, N = 1251
TFEISDIGAK pc2
log10 peptide intensity
770:997 204:247 14:16
14:31343494:G:A_A
p = 2.2e−06, beta = 0.327, N = 988
FFTVDAGVR pc2
671:997 174:247 11:16
14:31343494:G:A_A
p = 5.1e−07, beta = 0.377, N = 856
LMLEFVSNIAK pc2
921:997 236:247 16:16
14:31343494:G:A_A
p = 2.6e−05, beta = 0.27, N = 1173
WSASFTVTK pc2
726:997 192:247 14:16
14:31343494:G:A_A
p = 7.8e−07, beta = 0.349, N = 932
EFTGLEPIVSDVIR pc2
A0A2U3TZE7;G3V4C4;O43405
917:997 228:247 15:16
14:31343494:G:A_A
p = 1.2e−11, beta = 0.44, N = 1160
GVISNSGGPVR pc2
571:997 150:247 9:16
14:31343494:G:A_A
p = 2.3e−05, beta = 0.344, N = 730
TEFSFTDYSTK pc2
689:997 194:247 13:16
14:31343494:G:A_A
p = 3.3e−05, beta = 0.297, N = 896
IAAVQFTYDQR pc2
688:997 190:247 12:16
14:31343494:G:A_A
p = 0.00078, beta = 0.244, N = 890
ENVLAVIR pc2
680:997 184:247 13:16
14:31343494:G:A_A
p = 0.00035, beta = 0.26, N = 877
EFGVNVFIVSVAK pc2
699:997 188:247 13:16
14:31343494:G:A_A
p = 7.5e−05, beta = 0.285, N = 900
VALMLGIGTEGPHVGLVQASEHPK pc2
369:997 110:247 11:16
14:31343494:G:A_A
p = 0.00021, beta = 0.336, N = 490
ESHAFFTR pc2
A0A2U3TZE7;G3V4C4;O43405
101:997 33:247 4:16
14:31343494:G:A_A
p = 0.022, beta = 0.365, N = 138
ADIAFLIDGSFNIGQR pc2
38:997 17:247 3:16
14:31343494:G:A_A
p = 0.75, beta = 0.0687, N = 58
YMSGGTATGDAISFTVR pc2
61:997 20:247 3:16
14:31343494:G:A_A
p = 0.64, beta = −0.0918, N = 84
COCH : NP3
A0A2U3TZE7;O43405
252:253 67:67 5:5
14:31343494:G:A_A
p = 0.29, beta = 0.126, N = 324
TFEISDIGAK pc2
222:253 59:67 4:5
14:31343494:G:A_A
p = 0.13, beta = 0.192, N = 285
EFTGLEPIVSDVIR pc2
A0A2U3TZE7;G3V4C4;O43405
236:253 65:67 5:5
14:31343494:G:A_A
p = 0.0071, beta = 0.324, N = 306
FFTVDAGVR pc2
183:253 50:67 5:5
14:31343494:G:A_A
p = 0.041, beta = 0.27, N = 238
IAAVQFTYDQR pc2
158:253 49:67 3:5
14:31343494:G:A_A
p = 0.32, beta = 0.145, N = 210
LMLEFVSNIAK pc2
194:253 55:67 4:5
14:31343494:G:A_A
p = 0.35, beta = 0.124, N = 253
TEFSFTDYSTK pc2
146:253 44:67 4:5
14:31343494:G:A_A
p = 0.62, beta = −0.0721, N = 194
GVISNSGGPVR pc2
126:253 31:67 3:5
14:31343494:G:A_A
p = 0.41, beta = −0.137, N = 160
EFGVNVFIVSVAK pc2
153:253 43:67 4:5
14:31343494:G:A_A
p = 0.24, beta = 0.169, N = 200
ENVLAVIR pc2
18:253 4:67 0:5
14:31343494:G:A_A
p = 0.95, beta = −0.0352, N = 22
VALMLGIGTEGPHVGLVQASEHPK pc2
54:253 15:67 2:5
14:31343494:G:A_A
p = 0.23, beta = −0.271, N = 71
WSASFTVTK pc2
14:253 6:67 0:5
14:31343494:G:A_A
p = 0.77, beta = −0.131, N = 20
Assay Target: COCH
UniProt: O43405
rsID: rs28400019
Target SNP: 14:30874288:A:G
Proxy SNP: 14:31343494:G:A
deCODE log10(p): 251.5
deCODE BETA: 0.44
deCODE MSPA: 0.973
988:856:1173:932:1160:730:896
LCTHEQMMCSK pc3
rs1045644 REF
63:997 27:247 2:16
14:31343494:G:A_A
p = 0.011, model = REC, N = 92

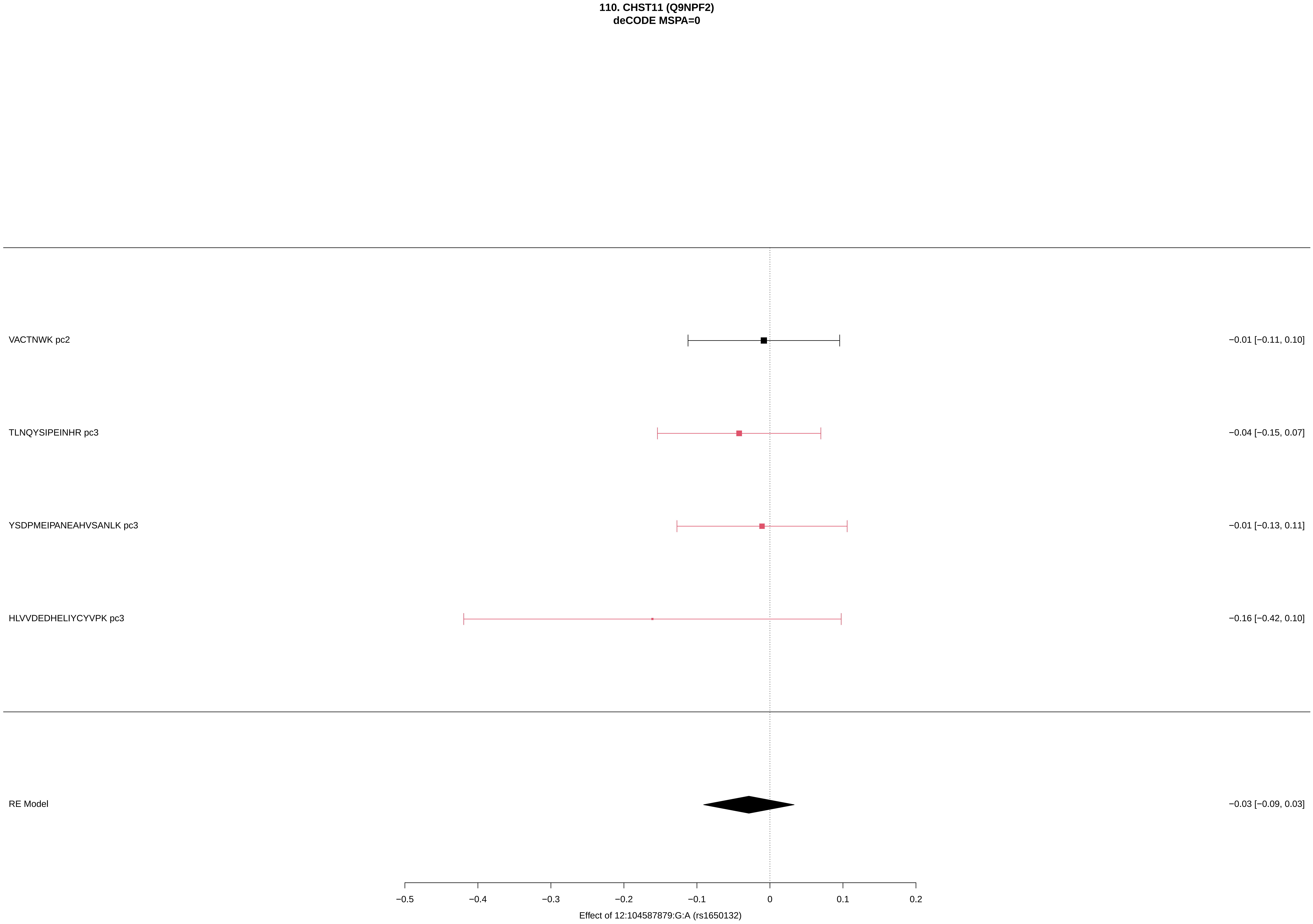
110. CHST11 (Q9NPF2)
deCODE MSPA=0
VACTNWK pc2
−0.01 [−0.11, 0.10]
TLNQYSIPEINHR pc3
−0.04 [−0.15, 0.07]
YSDPMEIPANEAHVSANLK pc3
−0.01 [−0.13, 0.11]
HLVVDEDHELIYCYVPK pc3
−0.16 [−0.42, 0.10]
RE Model
−0.03 [−0.09, 0.03]
−0.5
−0.4
−0.3
−0.2
−0.1
0
0.1
0.2
Effect of 12:104587879:G:A (rs1650132)

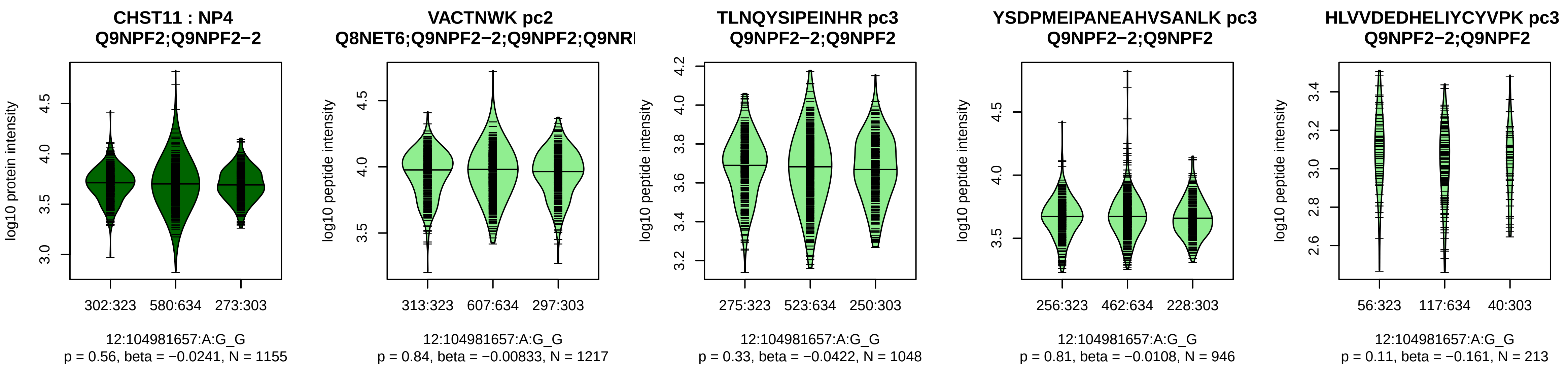

Assay Target: CHST11
UniProt: Q9NPF2
rsID: rs1650132
Target SNP: 12:104587879:G:A
Proxy SNP: 12:104981657:A:G
deCODE log10(p): 250.7
deCODE BETA: −0.28
deCODE MSPA: 0
–:–:–:–
1217:1048:946:213

111. SMOC2 (Q9H3U7)
deCODE MSPA=0.688

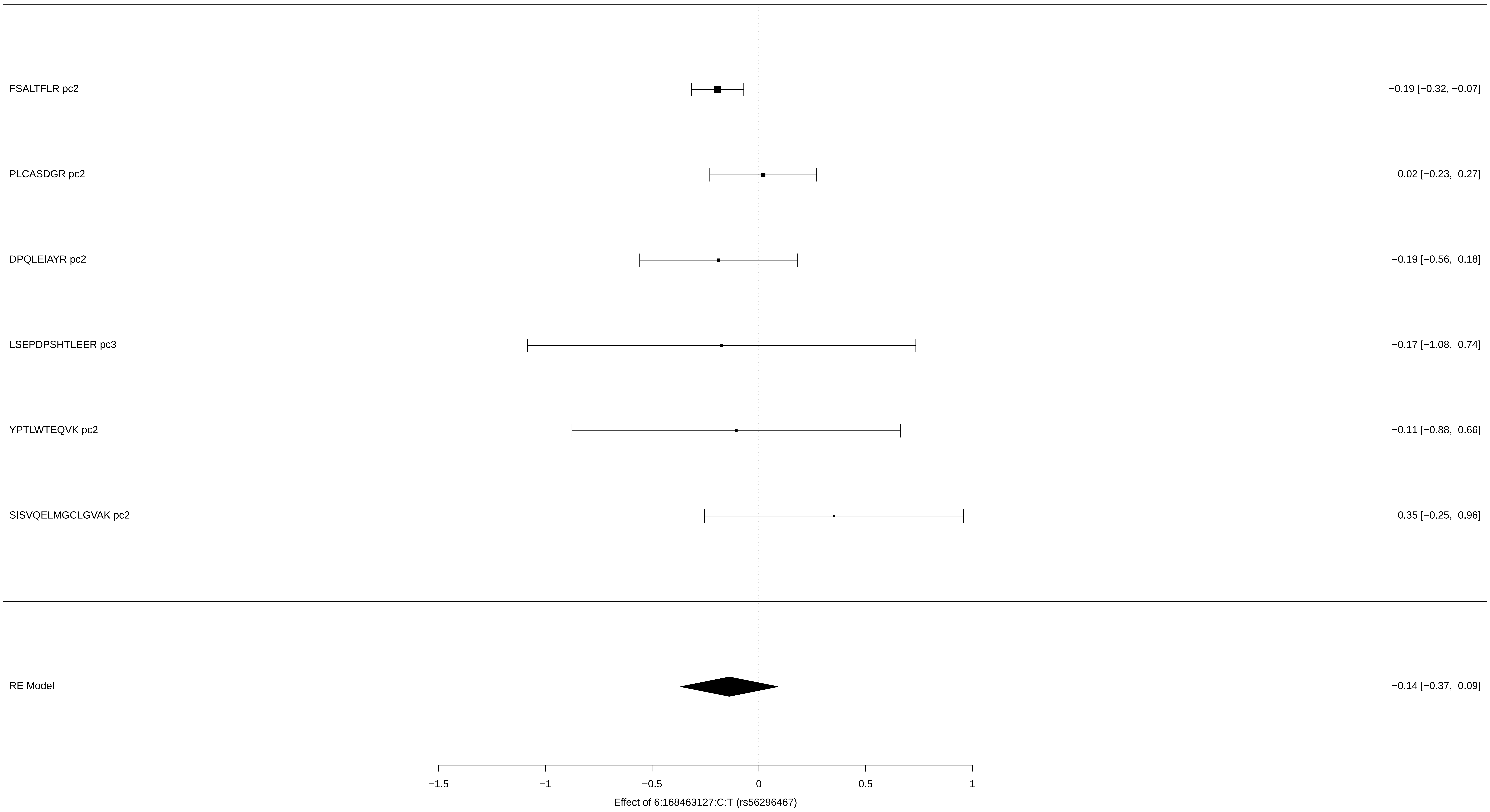

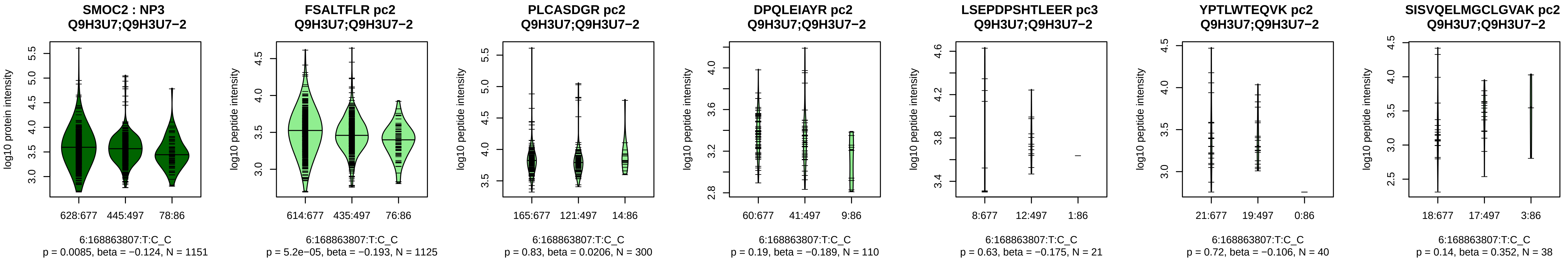

Assay Target: SMOC2
UniProt: Q9H3U7
rsID: rs56296467
Target SNP: 6:168463127:C:T
Proxy SNP: 6:168863807:T:C
deCODE log10(p): 243.7
deCODE BETA: −0.31
deCODE MSPA: 0.688
*:−:−:NA:−:−:−:NA:NA:NA:NA:N[illegible]
1125:300:110:5:21:40:38:13:15:[illegible]

112. PI3 (P19957)
deCODE MSPA=0.852

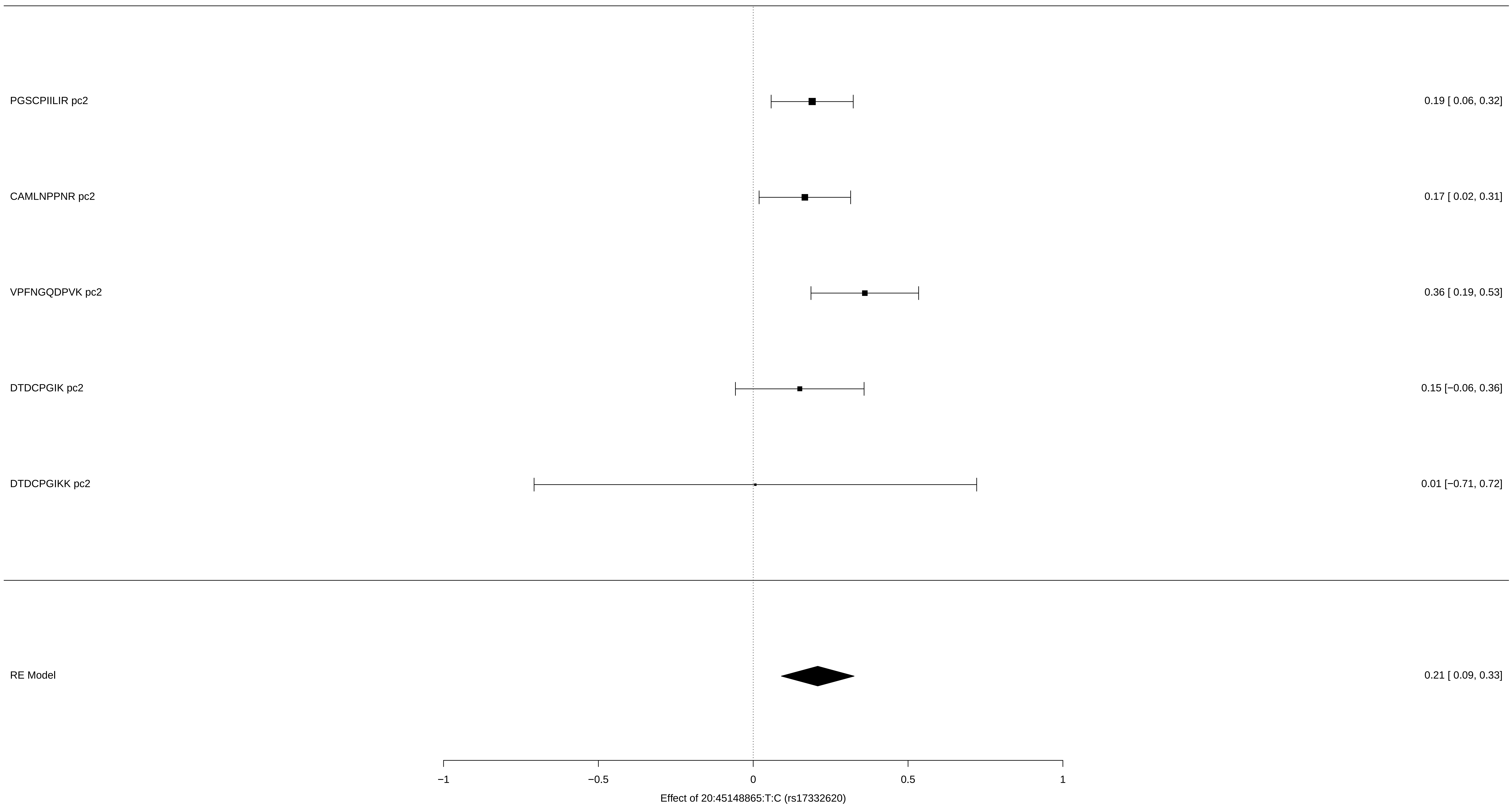

PI3 : NP3
P19957

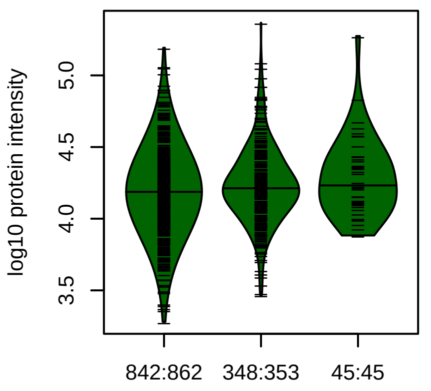

20:43777506:C:T_T
p = 7.2e−05, beta = 0.204, N = 1235

PGSCPIILIR pc2
P19957

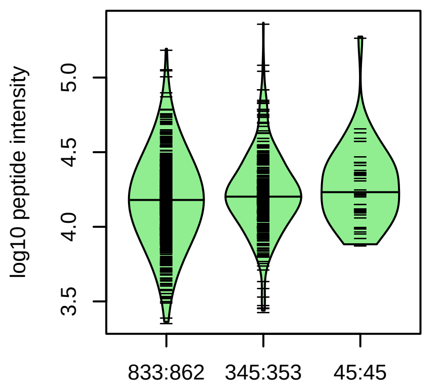

20:43777506:C:T_T
p = 0.00022, beta = 0.19, N = 1223

CAMLNPPNR pc2
P19957

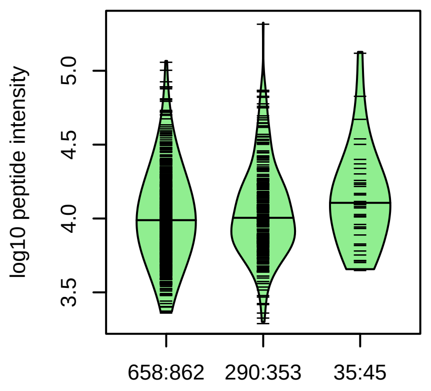

20:43777506:C:T_T
p = 0.0037, beta = 0.167, N = 983

VPFNGQDPVK pc2
P19957

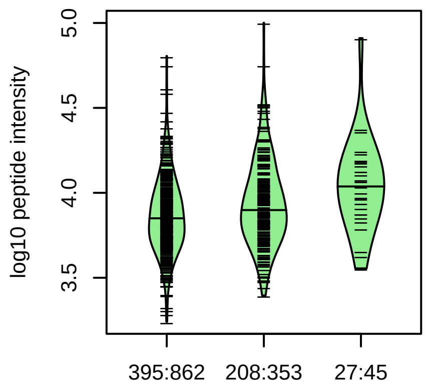

20:43777506:C:T_T
p = 1.3e−07, beta = 0.36, N = 630

DTDCPGIK pc2
P19957

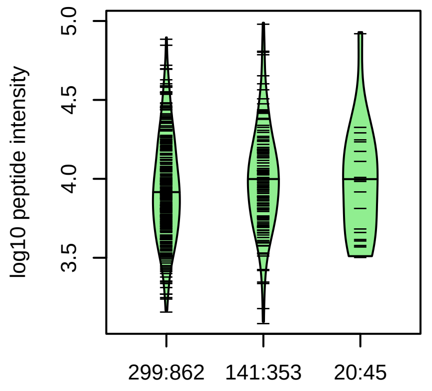

20:43777506:C:T_T
p = 0.063, beta = 0.15, N = 460

DTDCPGIKK pc2
P19957

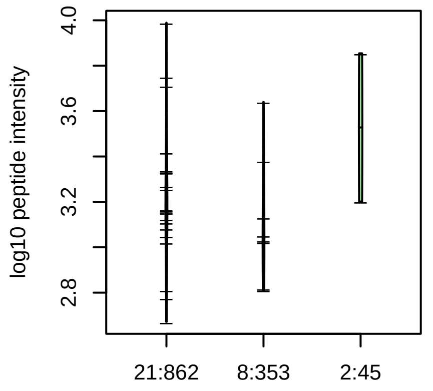

20:43777506:C:T_T
p = 0.98, beta = 0.00702, N = 31

PI3 : NP3
P19957

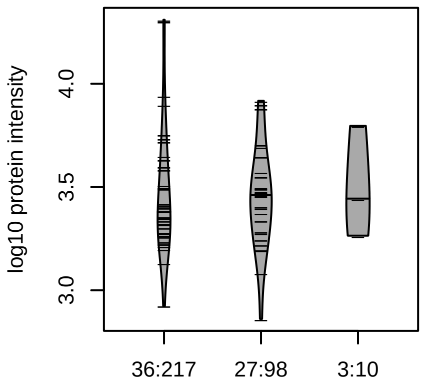

20:43777506:C:T_T
p = 0.49, beta = −0.14, N = 66

PGSCPIILIR pc2
P19957

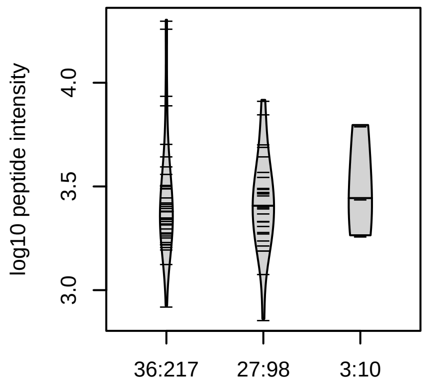

20:43777506:C:T_T
p = 0.46, beta = −0.15, N = 66

Assay Target: PI3
UniProt: P19957
rsID: rs17332620
Target SNP: 20:45148865:T:C
Proxy SNP: 20:43777506:C:T
deCODE log10(p): 241.6
deCODE BETA: 0.34
deCODE MSPA: 0.852
::*:–:–
1223:983:630:460:31

113. CNDP1 (Q96KN2)
deCODE MSPA=0.811

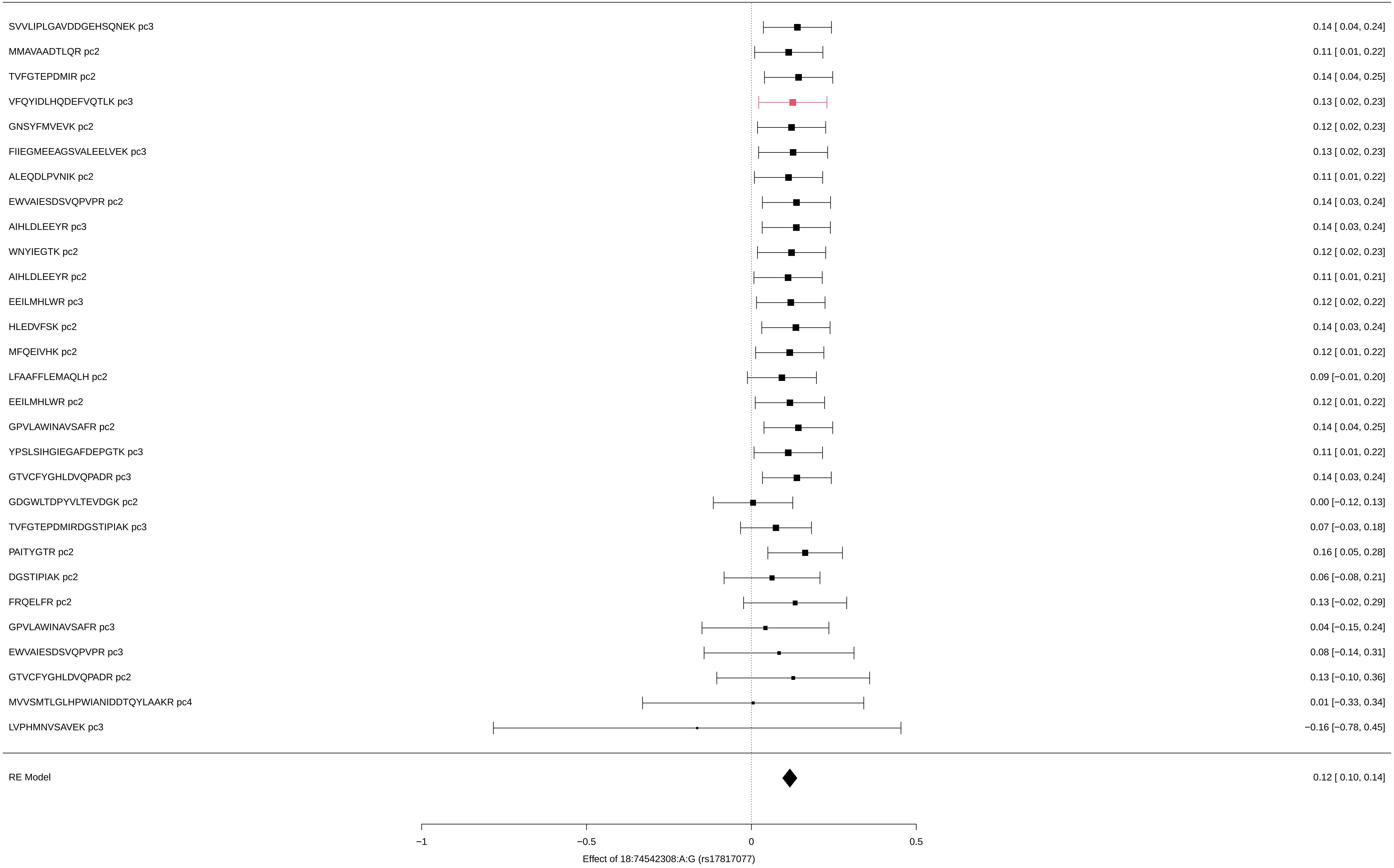

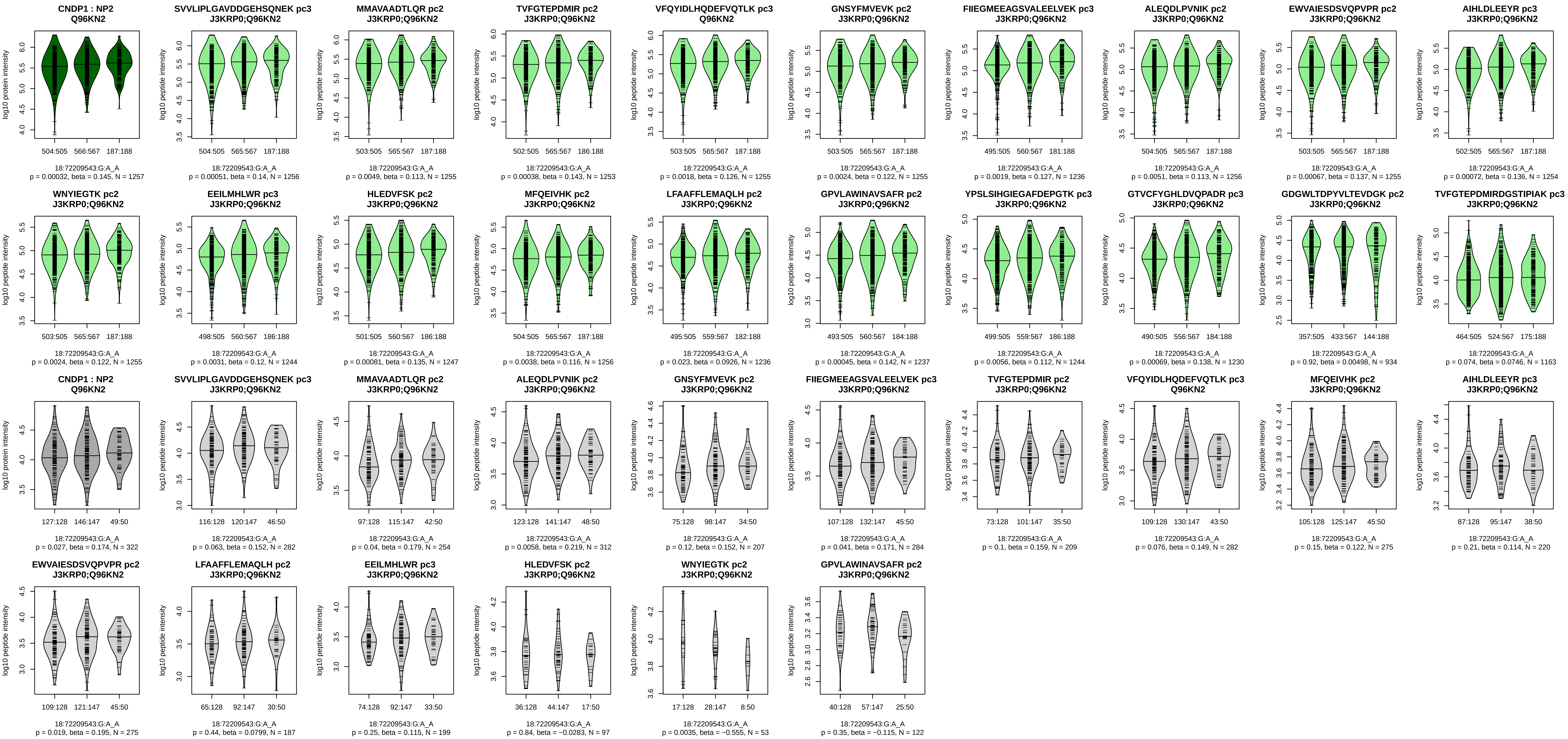

Assay Target: CNDP1
UniProt: Q96KN2
rsID: rs17817077
Target SNP: 18:74542308:A:G
Proxy SNP: 18:72209543:G:A
deCODE log10(p): 240.1
deCODE BETA: 0.28
deCODE MSPA: 0.811
1256:1255:1253:1255:1255:123

114. MYOC (Q99972)
deCODE MSPA=0.545

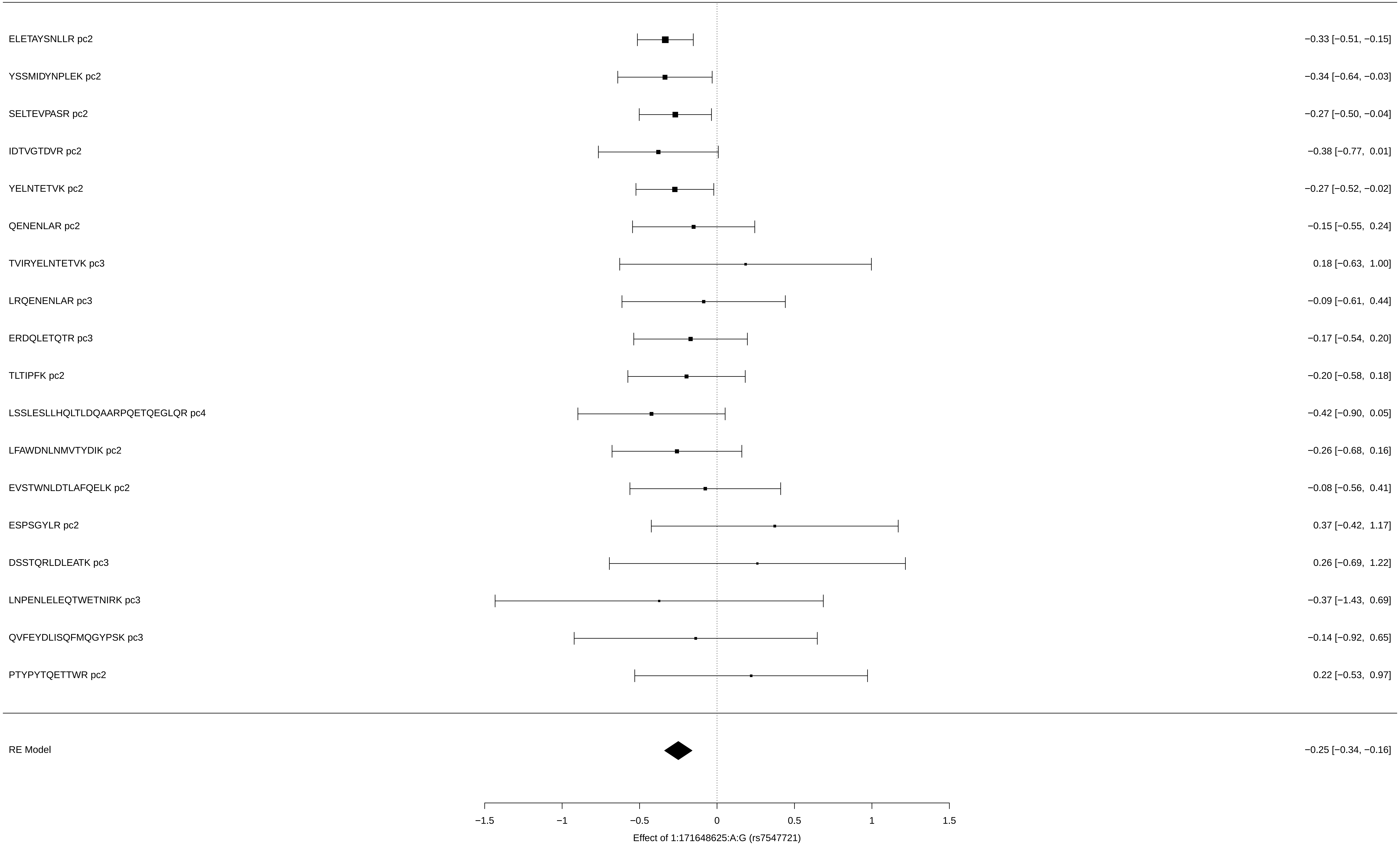

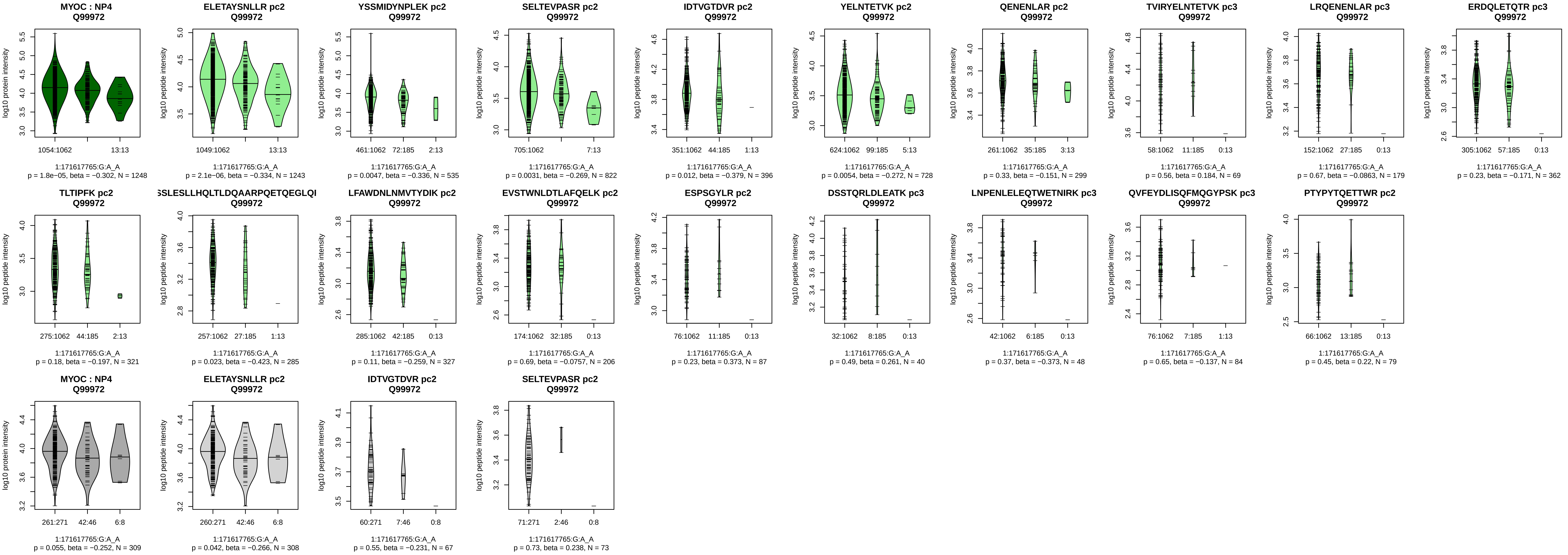

Assay Target: MYOC
UniProt: Q99972
rsID: rs7547721
Target SNP: 1:171648625:A:G
Proxy SNP: 1:171617765:G:A
deCODE log10(p): 238.1
deCODE BETA: −0.49
deCODE MSPA: 0.545
1243:535:822:396:728:299:69:1

115. IGFBP7 (Q16270)
deCODE MSPA=0.802

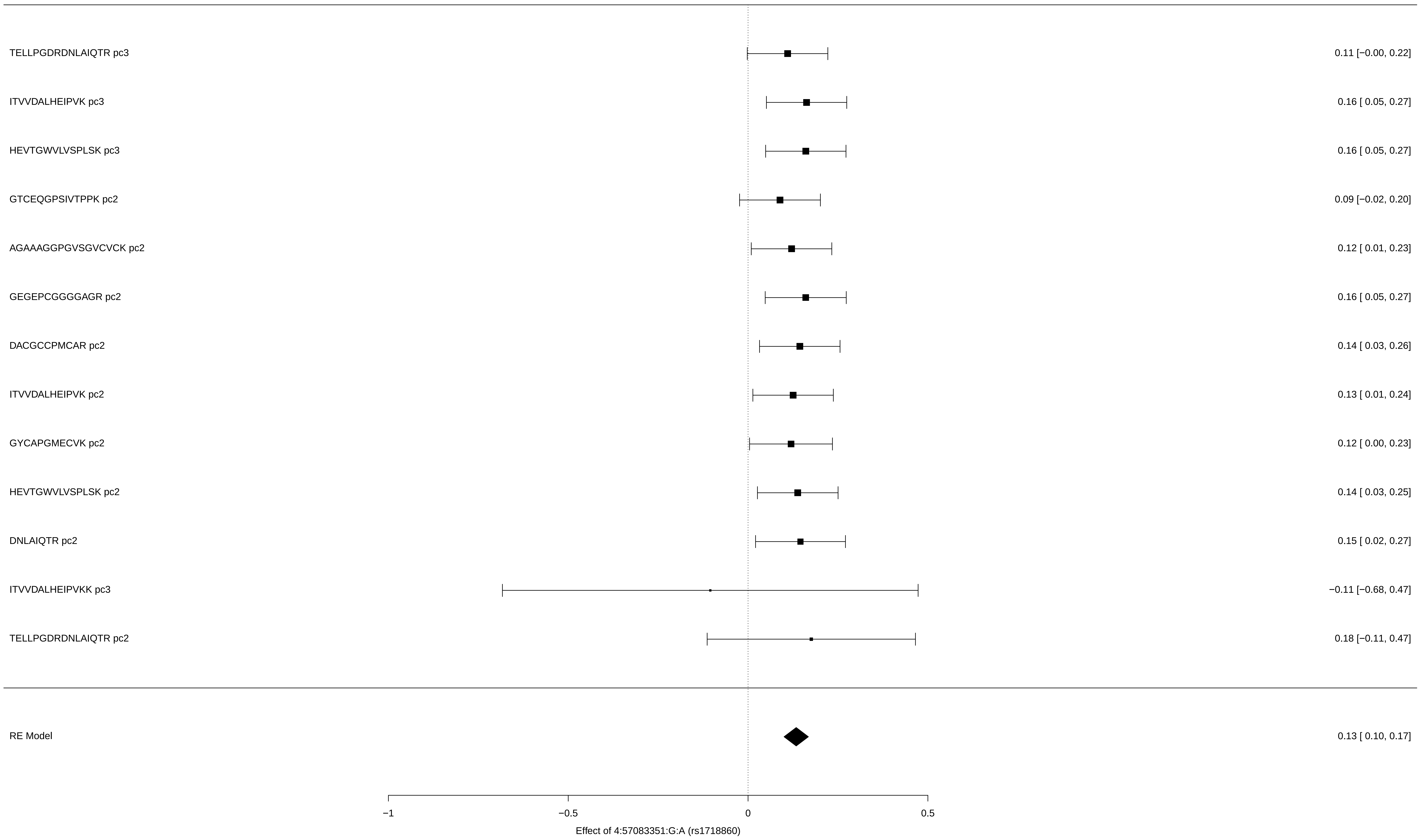

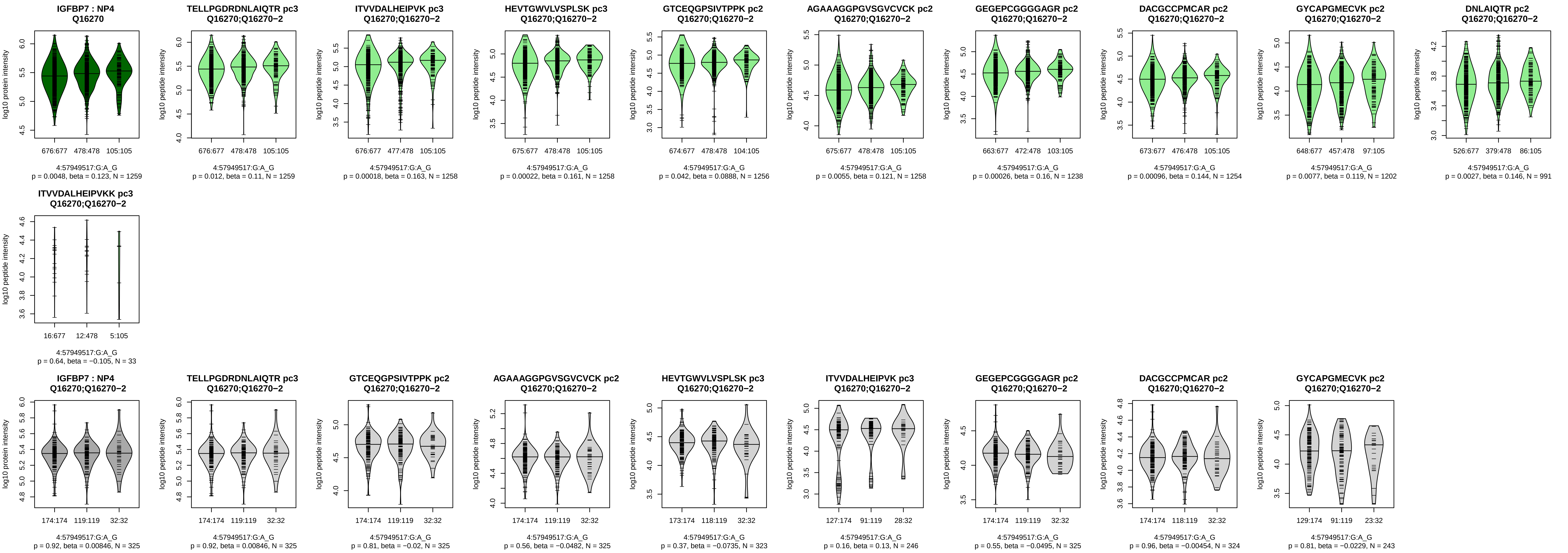

Assay Target: IGFBP7
UniProt: Q16270
rsID: rs1718860
Target SNP: 4:57083351:G:A
Proxy SNP: 4:57949517:G:A
deCODE log10(p): 235.7
deCODE BETA: 0.3
deCODE MSPA: 0.802
–:*:*:–:*:*:*:*:*:*:*:–:–:NA
1259:1258:1258:1256:1258:123

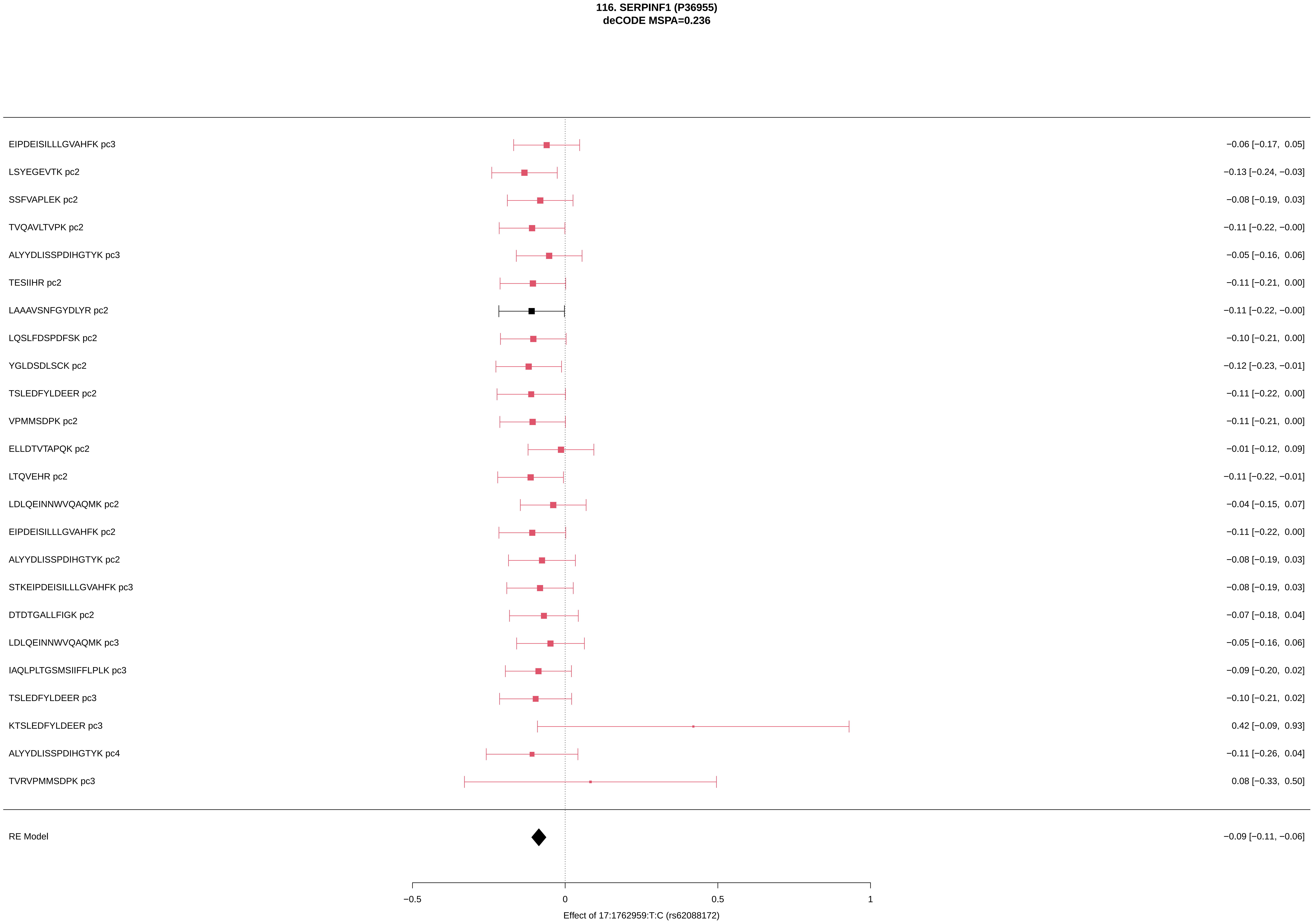
116. SERPINF1 (P36955)
deCODE MSPA=0.236
EIPDEISILLLGVAHFK pc3
−0.06 [−0.17, 0.05]
LSYEGEVTK pc2
−0.13 [−0.24, −0.03]
SSFVAPLEK pc2
−0.08 [−0.19, 0.03]
TVQAVLTVPK pc2
−0.11 [−0.22, −0.00]
ALYYDLISSPDIHGTYK pc3
−0.05 [−0.16, 0.06]
TESIIHR pc2
−0.11 [−0.21, 0.00]
LAAAVSNFGYDLYR pc2
−0.11 [−0.22, −0.00]
LQSLFDSPDFSK pc2
−0.10 [−0.21, 0.00]
YGLDSDLSCK pc2
−0.12 [−0.23, −0.01]
TSLEDFYLDEER pc2
−0.11 [−0.22, 0.00]
VPMMSDPK pc2
−0.11 [−0.21, 0.00]
ELLDTVTAPQK pc2
−0.01 [−0.12, 0.09]
LTQVEHR pc2
−0.11 [−0.22, −0.01]
LDLQEINNWVQAQMK pc2
−0.04 [−0.15, 0.07]
EIPDEISILLLGVAHFK pc2
−0.11 [−0.22, 0.00]
ALYYDLISSPDIHGTYK pc2
−0.08 [−0.19, 0.03]
STKEIPDEISILLLGVAHFK pc3
−0.08 [−0.19, 0.03]
DTDTGALLFIGK pc2
−0.07 [−0.18, 0.04]
LDLQEINNWVQAQMK pc3
−0.05 [−0.16, 0.06]
IAQLPLTGSMSIIFFLPLK pc3
−0.09 [−0.20, 0.02]
TSLEDFYLDEER pc3
−0.10 [−0.21, 0.02]
KTSLEDFYLDEER pc3
0.42 [−0.09, 0.93]
ALYYDLISSPDIHGTYK pc4
−0.11 [−0.26, 0.04]
TVRVPMMSDPK pc3
0.08 [−0.33, 0.50]
RE Model
−0.09 [−0.11, −0.06]
−0.5
0
0.5
1
Effect of 17:1762959:T:C (rs62088172)

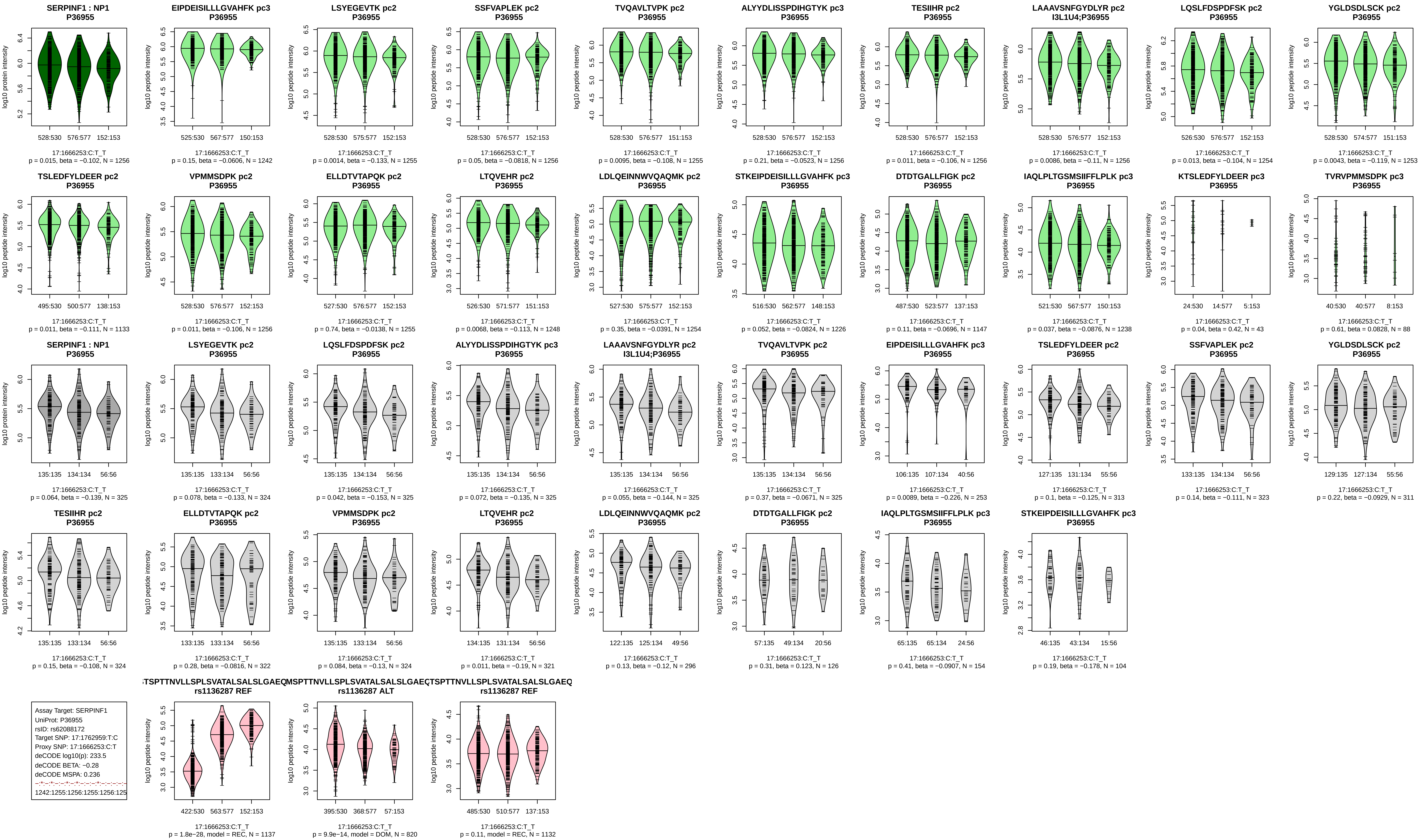

SERPINF1 : NP1
P36955
log10 protein intensity
528:530 576:577 152:153
17:1666253:C:T_T
p = 0.015, beta = −0.102, N = 1256
EIPDEISILLLGVAHFK pc3
P36955
log10 peptide intensity
525:530 567:577 150:153
17:1666253:C:T_T
p = 0.15, beta = −0.0606, N = 1242
LSYEGEVTK pc2
P36955
528:530 575:577 152:153
17:1666253:C:T_T
p = 0.0014, beta = −0.133, N = 1255
SSFVAPLEK pc2
P36955
528:530 576:577 152:153
17:1666253:C:T_T
p = 0.05, beta = −0.0818, N = 1256
TVQAVLTVPK pc2
P36955
528:530 576:577 151:153
17:1666253:C:T_T
p = 0.0095, beta = −0.108, N = 1255
ALYYDLISSPDIHGTYK pc3
P36955
528:530 576:577 152:153
17:1666253:C:T_T
p = 0.21, beta = −0.0523, N = 1256
TESIIHR pc2
P36955
528:530 576:577 152:153
17:1666253:C:T_T
p = 0.011, beta = −0.106, N = 1256
LAAAVSNFGYDLYR pc2
I3L1U4;P36955
528:530 576:577 152:153
17:1666253:C:T_T
p = 0.0086, beta = −0.11, N = 1256
LQSLFDSPDFSK pc2
P36955
526:530 576:577 152:153
17:1666253:C:T_T
p = 0.013, beta = −0.104, N = 1254
YGLDSDLSCK pc2
P36955
528:530 574:577 151:153
17:1666253:C:T_T
p = 0.0043, beta = −0.119, N = 1253
TSLEDFYLDEER pc2
P36955
495:530 500:577 138:153
17:1666253:C:T_T
p = 0.011, beta = −0.111, N = 1133
VPMMSDPK pc2
P36955
528:530 576:577 152:153
17:1666253:C:T_T
p = 0.011, beta = −0.106, N = 1256
ELLDTVTAPQK pc2
P36955
527:530 576:577 152:153
17:1666253:C:T_T
p = 0.74, beta = −0.0138, N = 1255
LTQVEHR pc2
P36955
526:530 571:577 151:153
17:1666253:C:T_T
p = 0.0068, beta = −0.113, N = 1248
LDLQEINNWVQAQMK pc2
P36955
527:530 575:577 152:153
17:1666253:C:T_T
p = 0.35, beta = −0.0391, N = 1254
STKEIPDEISILLLGVAHFK pc3
P36955
516:530 562:577 148:153
17:1666253:C:T_T
p = 0.052, beta = −0.0824, N = 1226
DTDTGALLFIGK pc2
P36955
487:530 523:577 137:153
17:1666253:C:T_T
p = 0.11, beta = −0.0696, N = 1147
IAQLPLTGSMSIIFFLPLK pc3
P36955
521:530 567:577 150:153
17:1666253:C:T_T
p = 0.037, beta = −0.0876, N = 1238
KTSLEDFYLDEER pc3
P36955
24:530 14:577 5:153
17:1666253:C:T_T
p = 0.04, beta = 0.42, N = 43
TVRVPMMSDPK pc3
P36955
40:530 40:577 8:153
17:1666253:C:T_T
p = 0.61, beta = 0.0828, N = 88
SERPINF1 : NP1
P36955
135:135 134:134 56:56
17:1666253:C:T_T
p = 0.064, beta = −0.139, N = 325
LSYEGEVTK pc2
P36955
135:135 133:134 56:56
17:1666253:C:T_T
p = 0.078, beta = −0.133, N = 324
LQSLFDSPDFSK pc2
P36955
135:135 134:134 56:56
17:1666253:C:T_T
p = 0.042, beta = −0.153, N = 325
ALYYDLISSPDIHGTYK pc3
P36955
135:135 134:134 56:56
17:1666253:C:T_T
p = 0.072, beta = −0.135, N = 325
LAAAVSNFGYDLYR pc2
I3L1U4;P36955
135:135 134:134 56:56
17:1666253:C:T_T
p = 0.055, beta = −0.144, N = 325
TVQAVLTVPK pc2
P36955
135:135 134:134 56:56
17:1666253:C:T_T
p = 0.37, beta = −0.0671, N = 325
EIPDEISILLLGVAHFK pc3
P36955
106:135 107:134 40:56
17:1666253:C:T_T
p = 0.0089, beta = −0.226, N = 253
TSLEDFYLDEER pc2
P36955
127:135 131:134 55:56
17:1666253:C:T_T
p = 0.1, beta = −0.125, N = 313
SSFVAPLEK pc2
P36955
133:135 134:134 56:56
17:1666253:C:T_T
p = 0.14, beta = −0.111, N = 323
YGLDSDLSCK pc2
P36955
129:135 127:134 55:56
17:1666253:C:T_T
p = 0.22, beta = −0.0929, N = 311
TESIIHR pc2
P36955
135:135 133:134 56:56
17:1666253:C:T_T
p = 0.15, beta = −0.108, N = 324
ELLDTVTAPQK pc2
P36955
133:135 133:134 56:56
17:1666253:C:T_T
p = 0.28, beta = −0.0816, N = 322
VPMMSDPK pc2
P36955
135:135 133:134 56:56
17:1666253:C:T_T
p = 0.084, beta = −0.13, N = 324
LTQVEHR pc2
P36955
134:135 131:134 56:56
17:1666253:C:T_T
p = 0.011, beta = −0.19, N = 321
LDLQEINNWVQAQMK pc2
P36955
122:135 125:134 49:56
17:1666253:C:T_T
p = 0.13, beta = −0.12, N = 296
DTDTGALLFIGK pc2
P36955
57:135 49:134 20:56
17:1666253:C:T_T
p = 0.31, beta = 0.123, N = 126
IAQLPLTGSMSIIFFLPLK pc3
P36955
65:135 65:134 24:56
17:1666253:C:T_T
p = 0.41, beta = −0.0907, N = 154
STKEIPDEISILLLGVAHFK pc3
P36955
46:135 43:134 15:56
17:1666253:C:T_T
p = 0.19, beta = −0.178, N = 104
Assay Target: SERPINF1
UniProt: P36955
rsID: rs62088172
Target SNP: 17:1762959:T:C
Proxy SNP: 17:1666253:C:T
deCODE log10(p): 233.5
deCODE BETA: −0.28
deCODE MSPA: 0.236
1242:1255:1256:1255:1256:125
TSPTTNVLLSPLSVATALSALSLGAEQ
rs1136287 REF
422:530 563:577 152:153
17:1666253:C:T_T
p = 1.8e−28, model = REC, N = 1137
MSPTTNVLLSPLSVATALSALSLGAEQ
rs1136287 ALT
395:530 368:577 57:153
17:1666253:C:T_T
p = 9.9e−14, model = DOM, N = 820
TSPTTNVLLSPLSVATALSALSLGAEQ
rs1136287 REF
485:530 510:577 137:153
17:1666253:C:T_T
p = 0.11, model = REC, N = 1132

117. ECH1 (Q13011)
deCODE MSPA=0.115

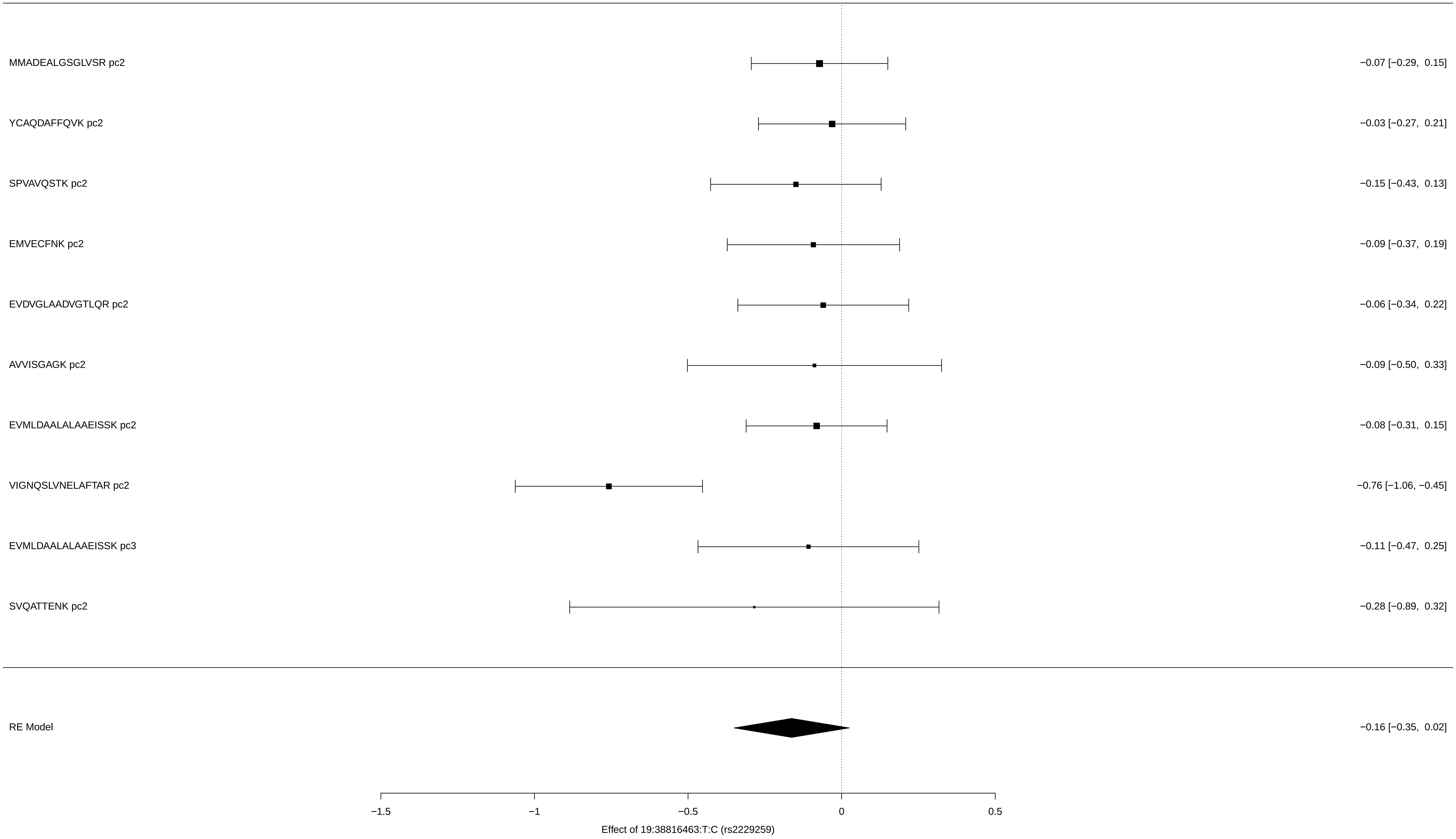

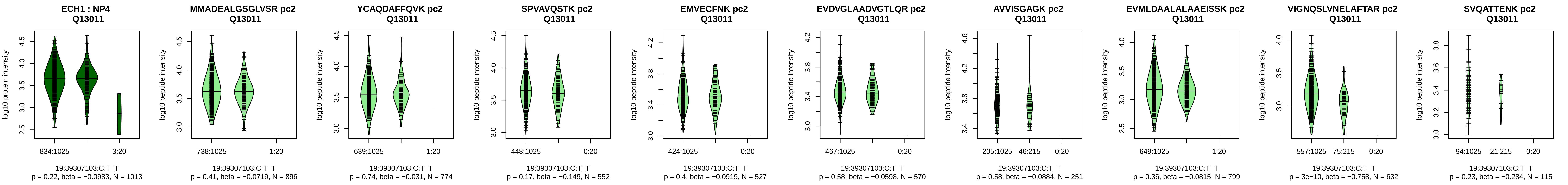

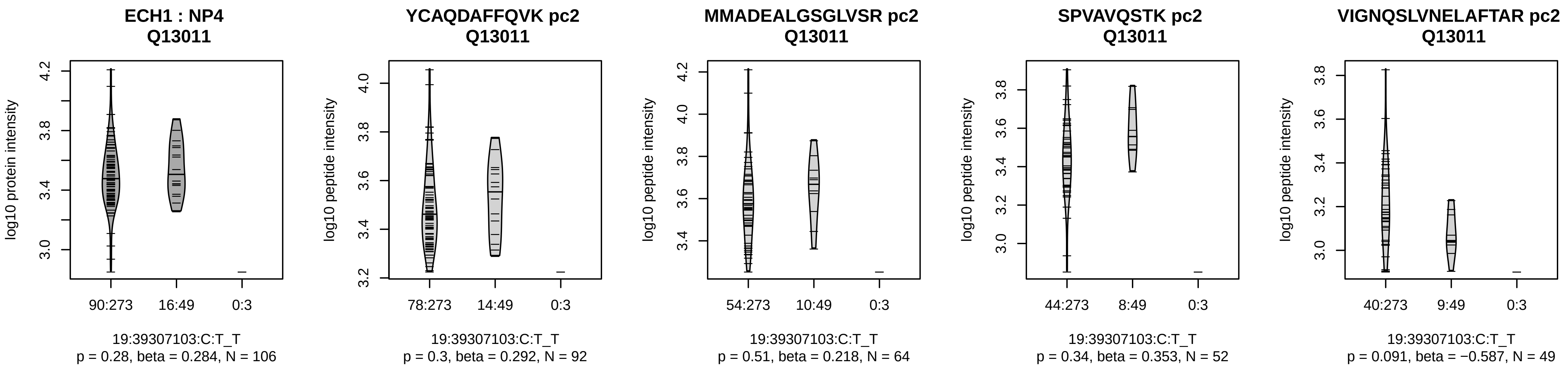

Assay Target: ECH1
UniProt: Q13011
rsID: rs2229259
Target SNP: 19:38816463:T:C
Proxy SNP: 19:39307103:C:T
deCODE log10(p): 230.5
deCODE BETA: 0.45
deCODE MSPA: 0.115
−:−:−:−:−:−:−:*:−:−:NA
896:774:552:527:570:251:799:6

118. PGLYRP2 (Q96PD5)
deCODE MSPA=0

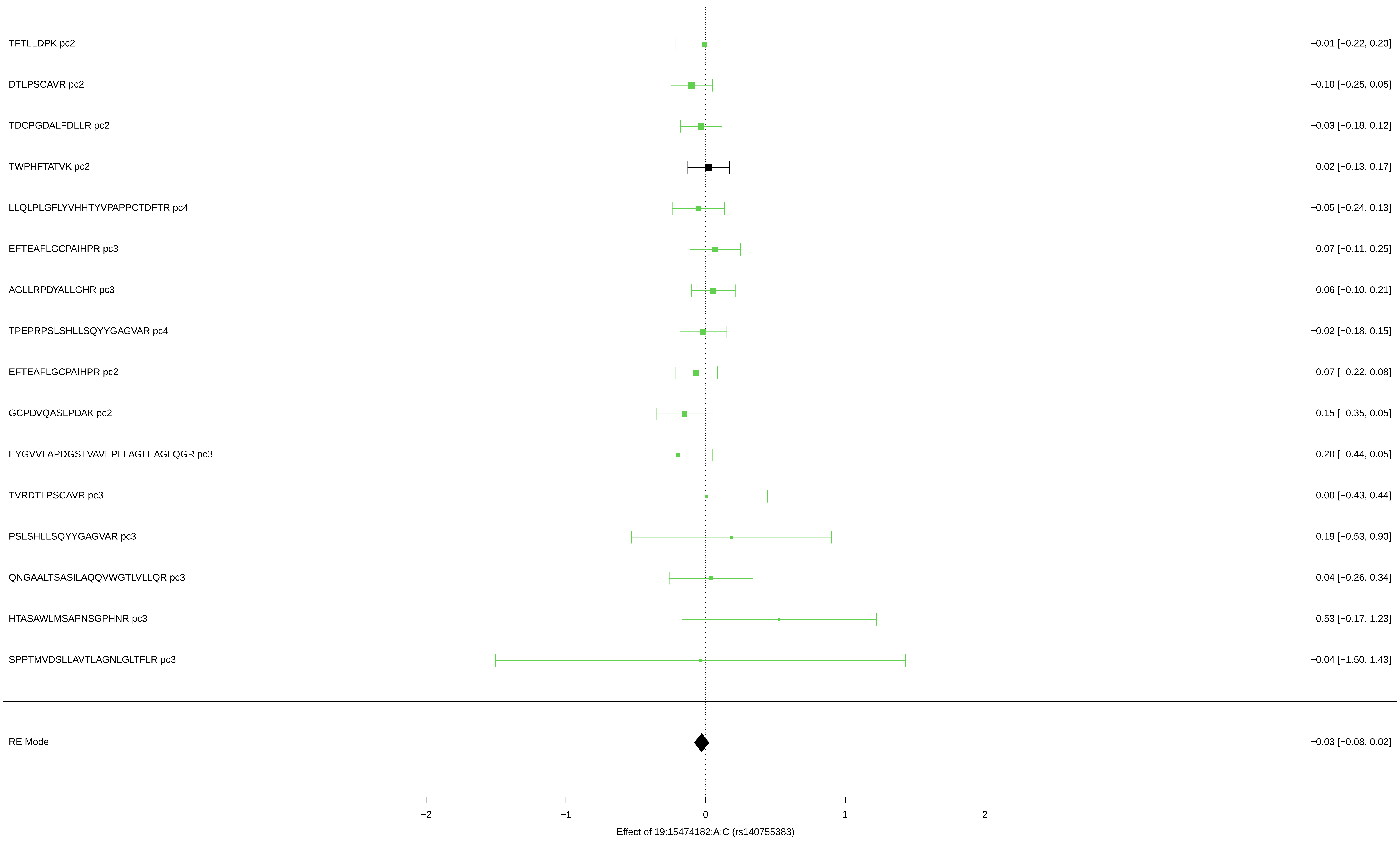

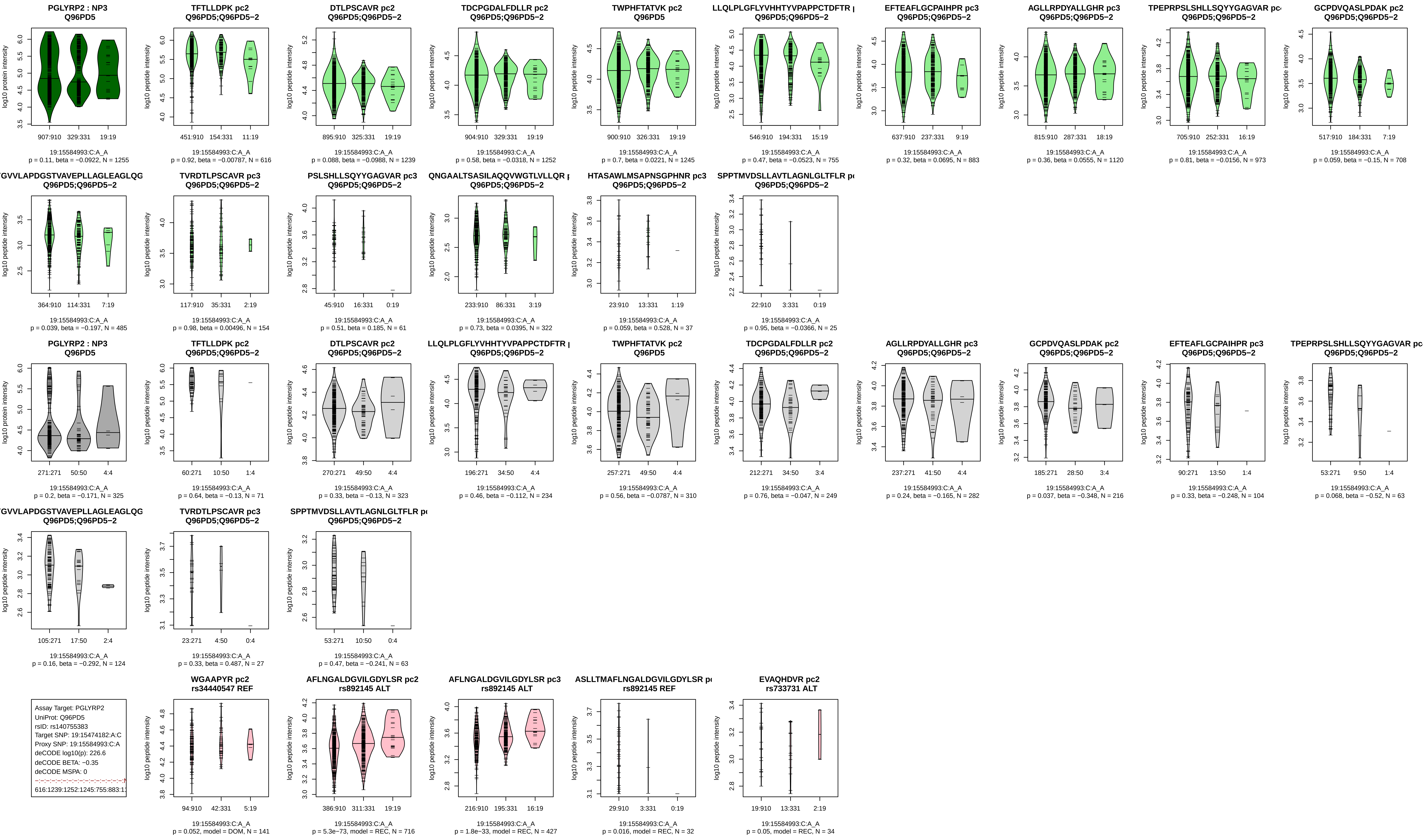
PGLYRP2 : NP3
Q96PD5
907:910 329:331 19:19
19:15584993:C:A_A
p = 0.11, beta = −0.0922, N = 1255
TFTLLDPK pc2
Q96PD5;Q96PD5−2
451:910 154:331 11:19
19:15584993:C:A_A
p = 0.92, beta = −0.00787, N = 616
DTLPSCAVR pc2
Q96PD5;Q96PD5−2
895:910 325:331 19:19
19:15584993:C:A_A
p = 0.088, beta = −0.0988, N = 1239
TDCPGDALFDLLR pc2
Q96PD5;Q96PD5−2
904:910 329:331 19:19
19:15584993:C:A_A
p = 0.58, beta = −0.0318, N = 1252
TWPHFTATVK pc2
Q96PD5
900:910 326:331 19:19
19:15584993:C:A_A
p = 0.7, beta = 0.0221, N = 1245
LLQLPLGFLYVHHTYVPAPPCTDFTR
Q96PD5;Q96PD5−2
546:910 194:331 15:19
19:15584993:C:A_A
p = 0.47, beta = −0.0523, N = 755
EFTEAFLGCPAIHPR pc3
Q96PD5;Q96PD5−2
637:910 237:331 9:19
19:15584993:C:A_A
p = 0.32, beta = 0.0695, N = 883
AGLLRPDYALLGHR pc3
Q96PD5;Q96PD5−2
815:910 287:331 18:19
19:15584993:C:A_A
p = 0.36, beta = 0.0555, N = 1120
TPEPRPSLSHLLSQYYGAGVAR pc
Q96PD5;Q96PD5−2
705:910 252:331 16:19
19:15584993:C:A_A
p = 0.81, beta = −0.0156, N = 973
GCPDVQASLPDAK pc2
Q96PD5;Q96PD5−2
517:910 184:331 7:19
19:15584993:C:A_A
p = 0.059, beta = −0.15, N = 708
GVVLAPDGSTVAVEPLLAGLEAGLQG
Q96PD5;Q96PD5−2
364:910 114:331 7:19
19:15584993:C:A_A
p = 0.039, beta = −0.197, N = 485
TVRDTLPSCAVR pc3
Q96PD5;Q96PD5−2
117:910 35:331 2:19
19:15584993:C:A_A
p = 0.98, beta = 0.00496, N = 154
PSLSHLLSQYYGAGVAR pc3
Q96PD5;Q96PD5−2
45:910 16:331 0:19
19:15584993:C:A_A
p = 0.51, beta = 0.185, N = 61
QNGAALTSASILAQQVWGTLVLLQR
Q96PD5;Q96PD5−2
233:910 86:331 3:19
19:15584993:C:A_A
p = 0.73, beta = 0.0395, N = 322
HTASAWLMSAPNSGPHNR pc3
Q96PD5;Q96PD5−2
23:910 13:331 1:19
19:15584993:C:A_A
p = 0.059, beta = 0.528, N = 37
SPPTMVDSLLAVTLAGNLGLTFLR
Q96PD5;Q96PD5−2
22:910 3:331 0:19
19:15584993:C:A_A
p = 0.95, beta = −0.0366, N = 25
PGLYRP2 : NP3
Q96PD5
271:271 50:50 4:4
19:15584993:C:A_A
p = 0.2, beta = −0.171, N = 325
TFTLLDPK pc2
Q96PD5;Q96PD5−2
60:271 10:50 1:4
19:15584993:C:A_A
p = 0.64, beta = −0.13, N = 71
DTLPSCAVR pc2
Q96PD5;Q96PD5−2
270:271 49:50 4:4
19:15584993:C:A_A
p = 0.33, beta = −0.13, N = 323
LLQLPLGFLYVHHTYVPAPPCTDFTR
Q96PD5;Q96PD5−2
196:271 34:50 4:4
19:15584993:C:A_A
p = 0.46, beta = −0.112, N = 234
TWPHFTATVK pc2
Q96PD5
257:271 49:50 4:4
19:15584993:C:A_A
p = 0.56, beta = −0.0787, N = 310
TDCPGDALFDLLR pc2
Q96PD5;Q96PD5−2
212:271 34:50 3:4
19:15584993:C:A_A
p = 0.76, beta = −0.047, N = 249
AGLLRPDYALLGHR pc3
Q96PD5;Q96PD5−2
237:271 41:50 4:4
19:15584993:C:A_A
p = 0.24, beta = −0.165, N = 282
GCPDVQASLPDAK pc2
Q96PD5;Q96PD5−2
185:271 28:50 3:4
19:15584993:C:A_A
p = 0.037, beta = −0.348, N = 216
EFTEAFLGCPAIHPR pc3
Q96PD5;Q96PD5−2
90:271 13:50 1:4
19:15584993:C:A_A
p = 0.33, beta = −0.248, N = 104
TPEPRPSLSHLLSQYYGAGVAR pc
Q96PD5;Q96PD5−2
53:271 9:50 1:4
19:15584993:C:A_A
p = 0.068, beta = −0.52, N = 63
GVVLAPDGSTVAVEPLLAGLEAGLQG
Q96PD5;Q96PD5−2
105:271 17:50 2:4
19:15584993:C:A_A
p = 0.16, beta = −0.292, N = 124
TVRDTLPSCAVR pc3
Q96PD5;Q96PD5−2
23:271 4:50 0:4
19:15584993:C:A_A
p = 0.33, beta = 0.487, N = 27
SPPTMVDSLLAVTLAGNLGLTFLR
Q96PD5;Q96PD5−2
53:271 10:50 0:4
19:15584993:C:A_A
p = 0.47, beta = −0.241, N = 63
Assay Target: PGLYRP2
UniProt: Q96PD5
rsID: rs140755383
Target SNP: 19:15474182:A:C
Proxy SNP: 19:15584993:C:A
deCODE log10(p): 226.6
deCODE BETA: −0.35
deCODE MSPA: 0
616:1239:1252:1245:755:883:1
WGAAPYR pc2
rs34440547 REF
94:910 42:331 5:19
19:15584993:C:A_A
p = 0.052, model = DOM, N = 141
AFLNGALDGVILGDYLSR pc2
rs892145 ALT
386:910 311:331 19:19
19:15584993:C:A_A
p = 5.3e−73, model = REC, N = 716
AFLNGALDGVILGDYLSR pc3
rs892145 ALT
216:910 195:331 16:19
19:15584993:C:A_A
p = 1.8e−33, model = REC, N = 427
ASLLTMAFLNGALDGVILGDYLSR
rs892145 REF
29:910 3:331 0:19
19:15584993:C:A_A
p = 0.016, model = REC, N = 32
EVAQHDVR pc2
rs733731 ALT
19:910 13:331 2:19
19:15584993:C:A_A
p = 0.05, model = REC, N = 34
log10 protein intensity
log10 peptide intensity

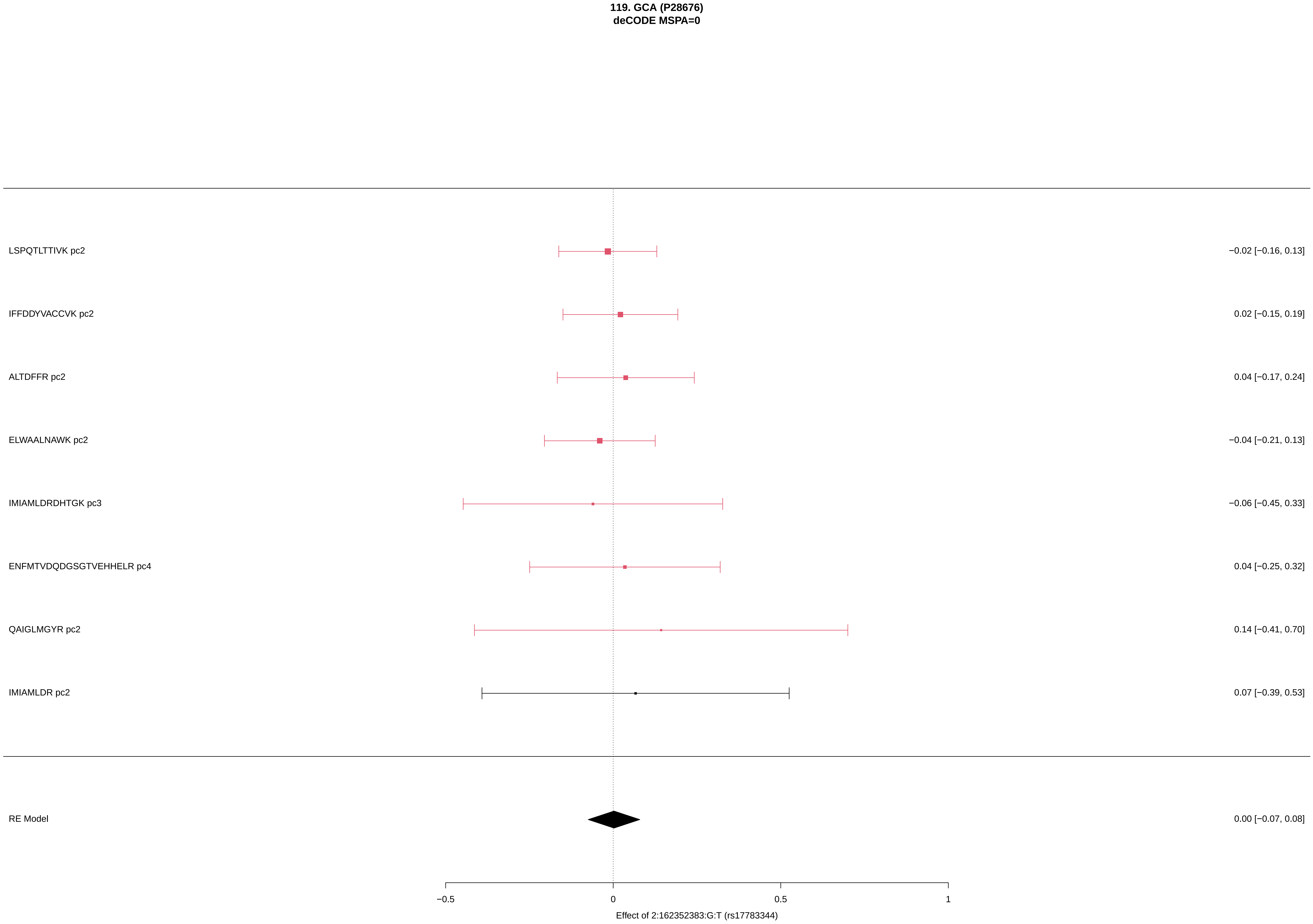
119. GCA (P28676)
deCODE MSPA=0
LSPQTLTTIVK pc2
−0.02 [−0.16, 0.13]
IFFDDYVACCVK pc2
0.02 [−0.15, 0.19]
ALTDFFR pc2
0.04 [−0.17, 0.24]
ELWAALNAWK pc2
−0.04 [−0.21, 0.13]
IMIAMLDRDHTGK pc3
−0.06 [−0.45, 0.33]
ENFMTVDQDGSGTVEHHELR pc4
0.04 [−0.25, 0.32]
QAIGLMGYR pc2
0.14 [−0.41, 0.70]
IMIAMLDR pc2
0.07 [−0.39, 0.53]
RE Model
0.00 [−0.07, 0.08]
−0.5
0
0.5
1
Effect of 2:162352383:G:T (rs17783344)

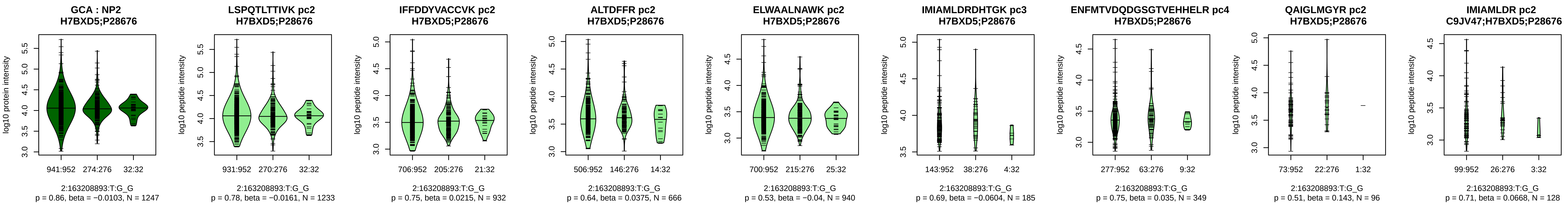

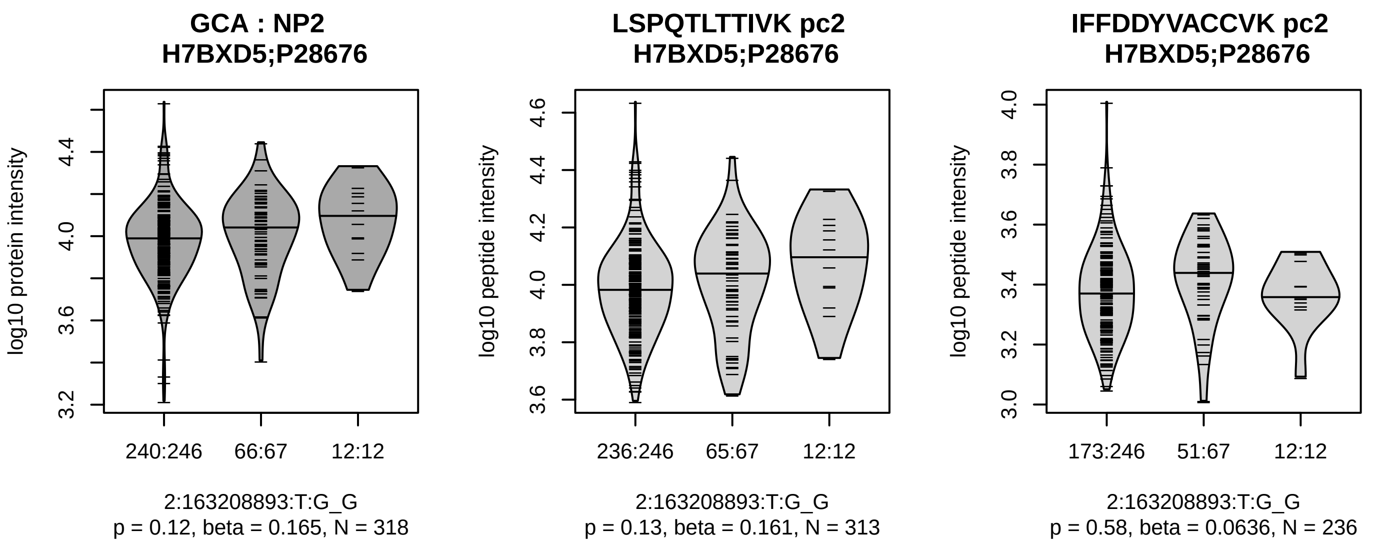

Assay Target: GCA
UniProt: P28676
rsID: rs17783344
Target SNP: 2:162352383:G:T
Proxy SNP: 2:163208893:T:G
deCODE log10(p): 222.1
deCODE BETA: −0.43
deCODE MSPA: 0
–:–:–:–:–:–:–:–
1233:932:666:940:185:349:96:1

120. FCER2 (P06734)
deCODE MSPA=0.353

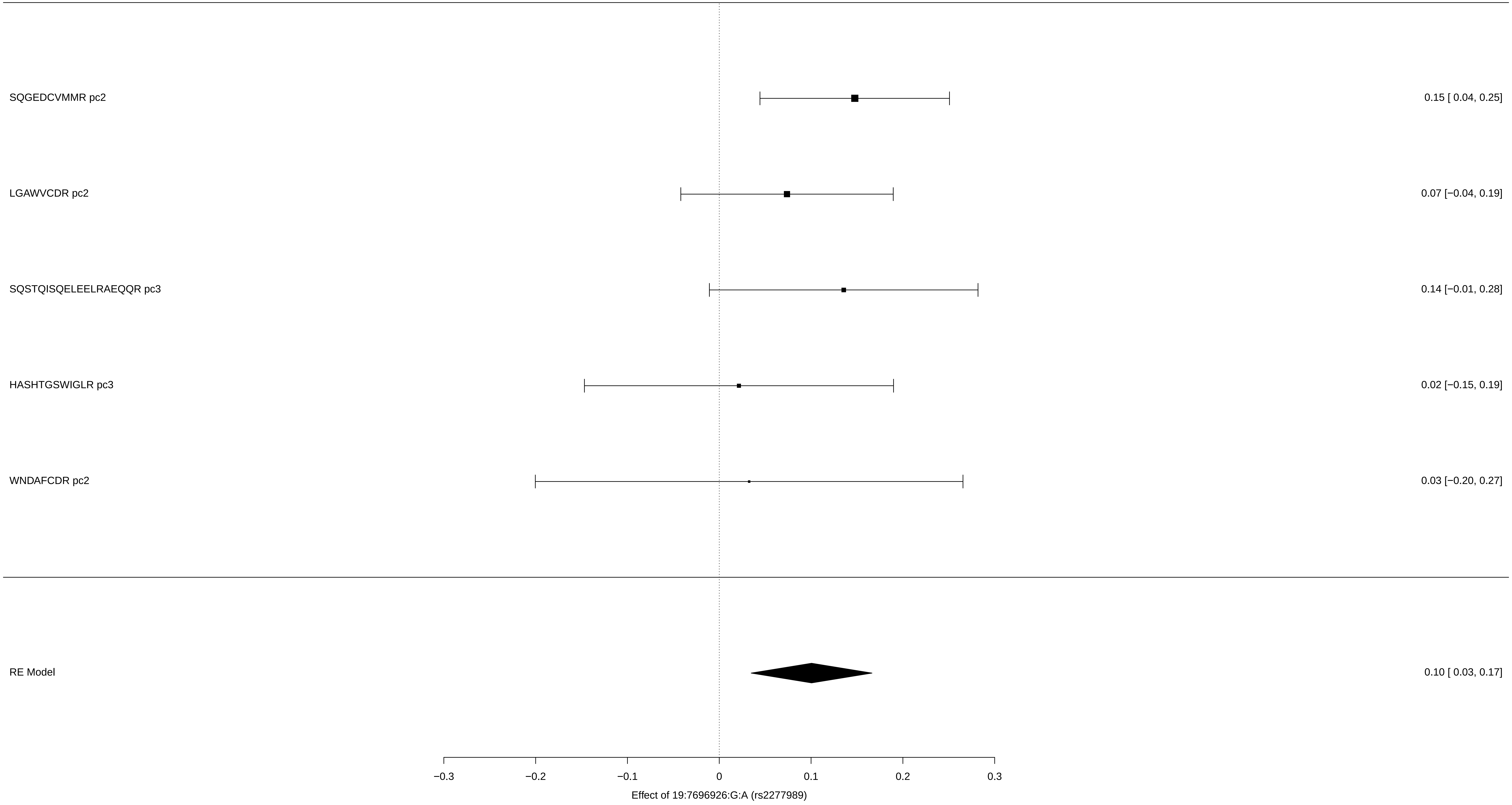

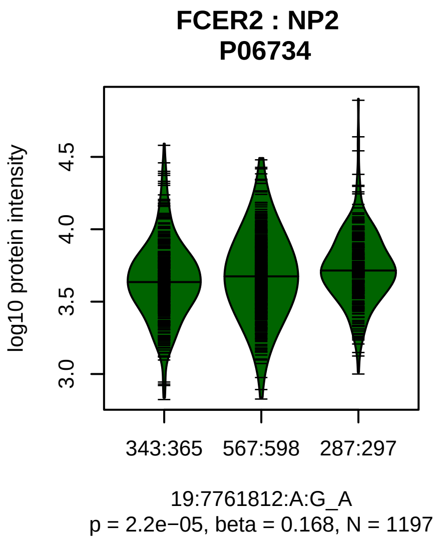

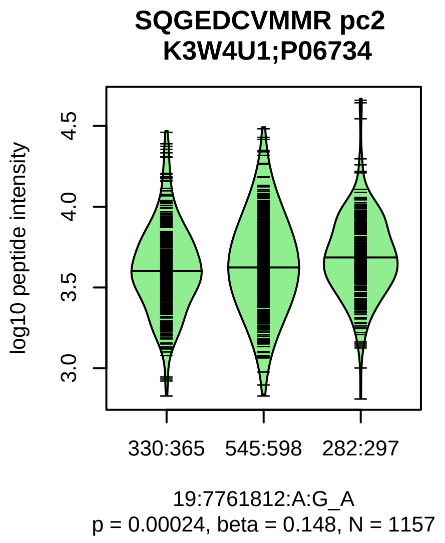

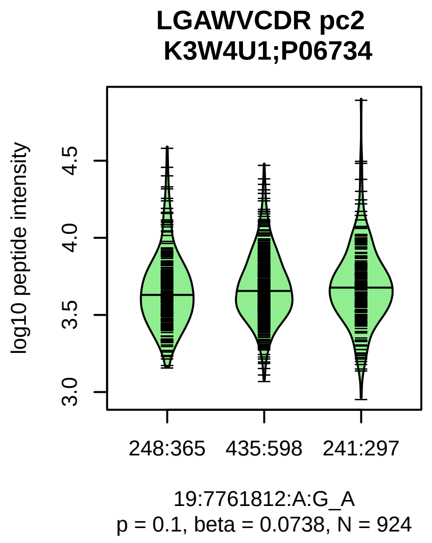

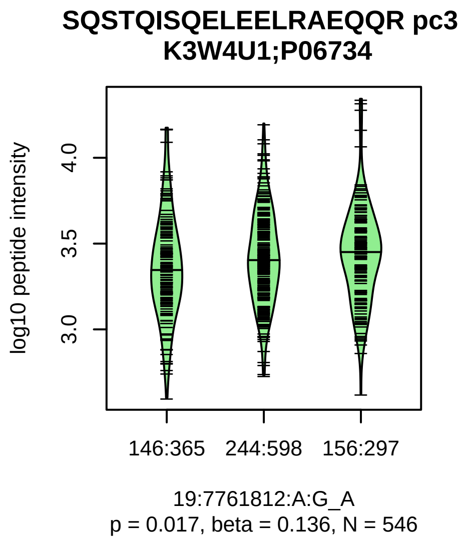

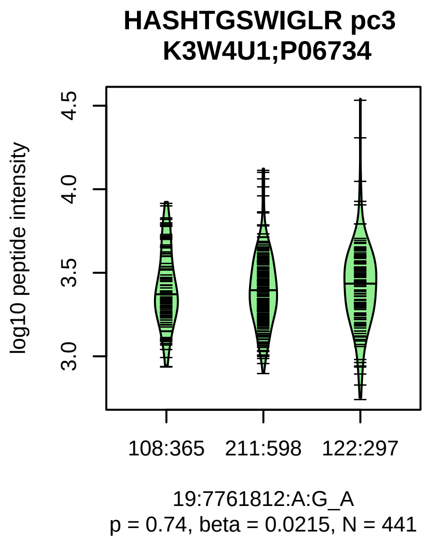

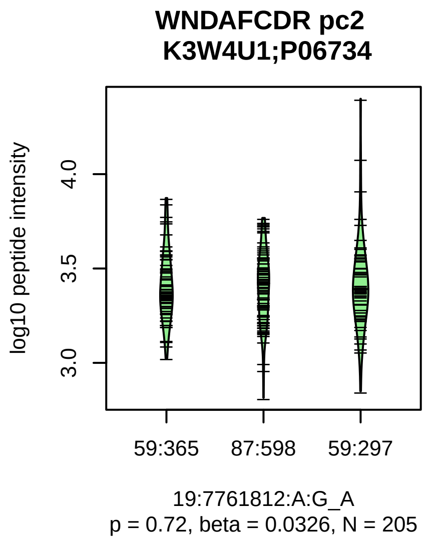

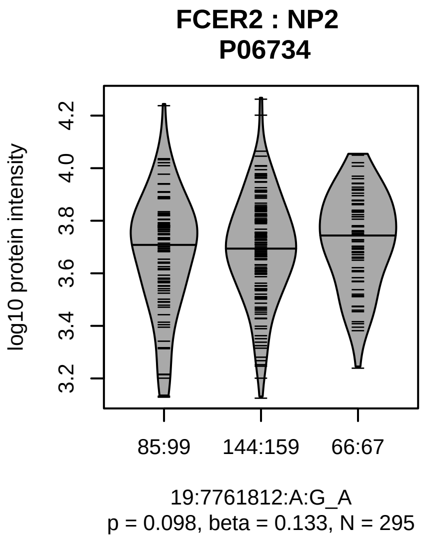

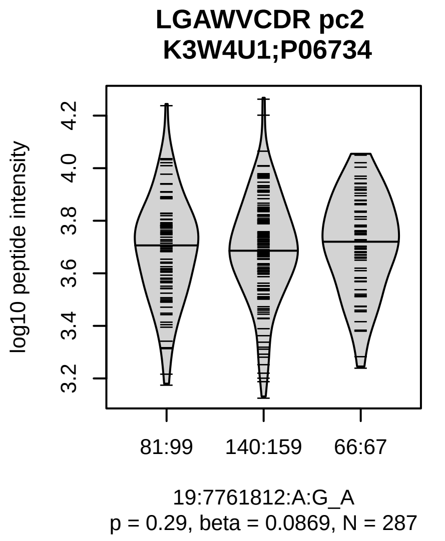

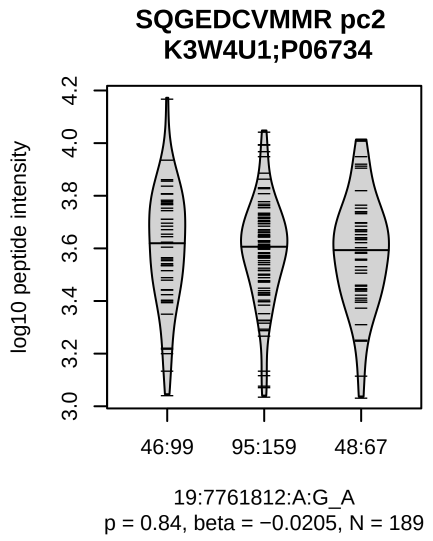

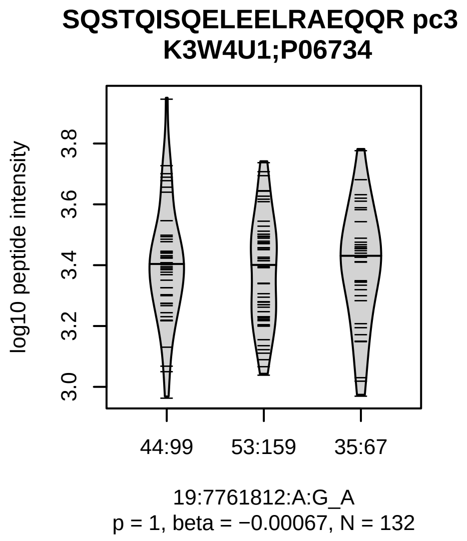

Assay Target: FCER2
UniProt: P06734
rsID: rs2277989
Target SNP: 19:7696926:G:A
Proxy SNP: 19:7761812:A:G
deCODE log10(p): 217.9
deCODE BETA: −0.27
deCODE MSPA: 0.353
*:−:−:−:−
1157:924:546:441:205

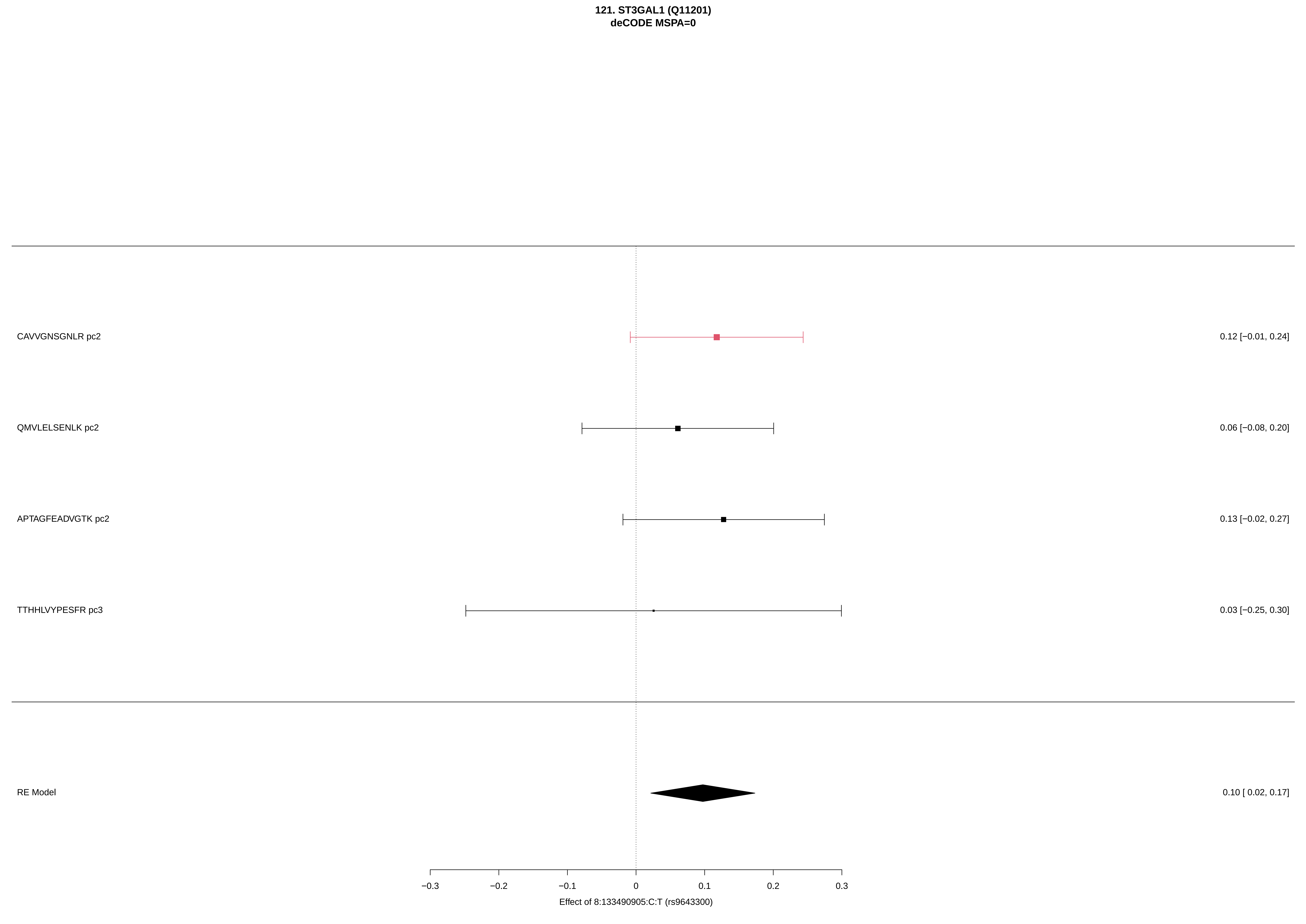
121. ST3GAL1 (Q11201)
deCODE MSPA=0
CAVVGNSGNLR pc2
0.12 [−0.01, 0.24]
QMVLELSENLK pc2
0.06 [−0.08, 0.20]
APTAGFEADVGTK pc2
0.13 [−0.02, 0.27]
TTHHLVYPESFR pc3
0.03 [−0.25, 0.30]
RE Model
0.10 [0.02, 0.17]
−0.3
−0.2
−0.1
0
0.1
0.2
0.3
Effect of 8:133490905:C:T (rs9643300)

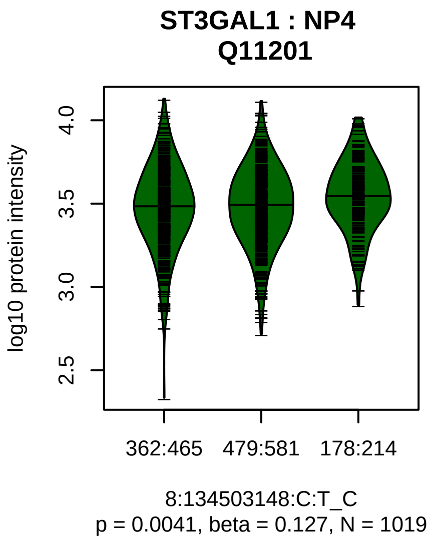

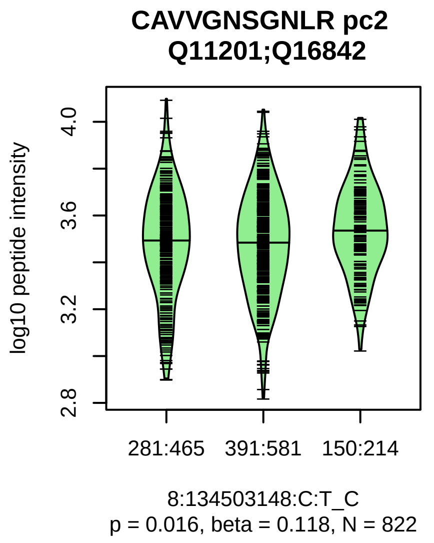

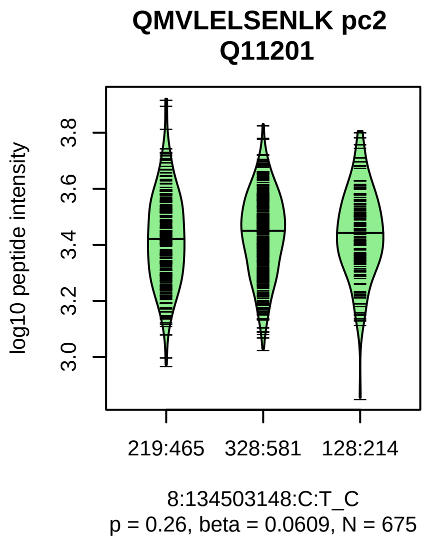

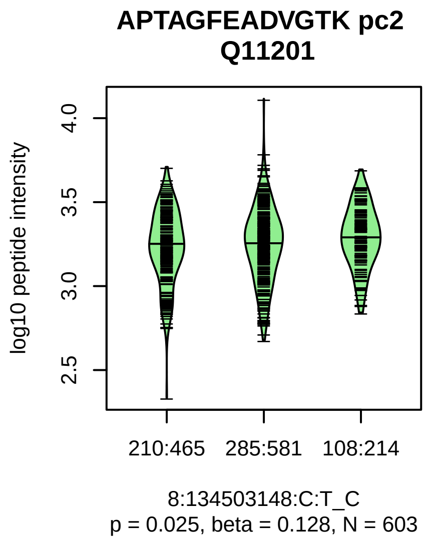

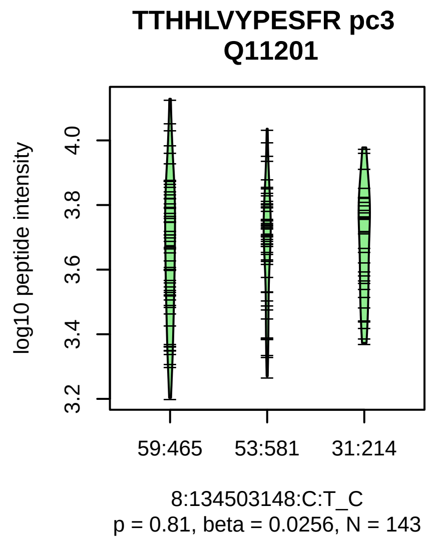

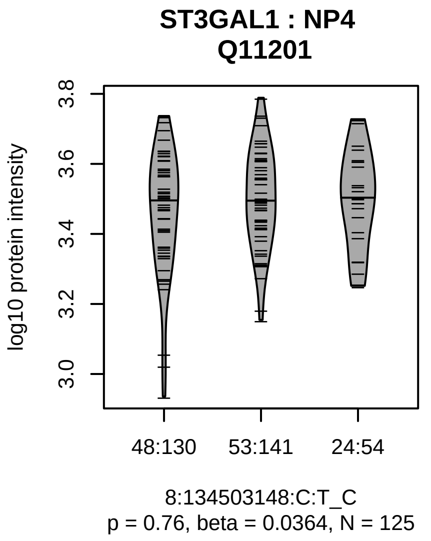

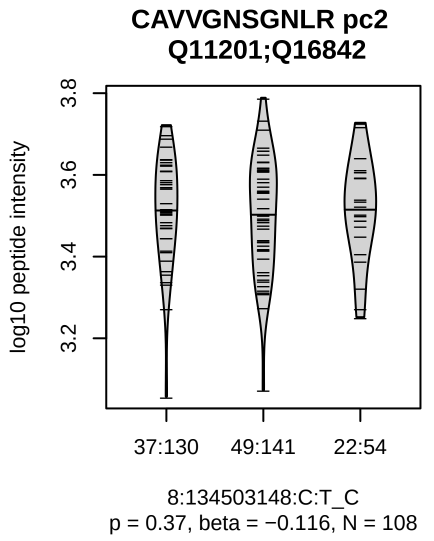

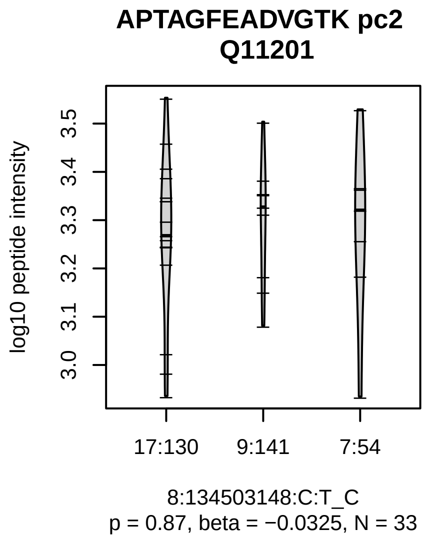

Assay Target: ST3GAL1
UniProt: Q11201
rsID: rs9643300
Target SNP: 8:133490905:C:T
Proxy SNP: 8:134503148:C:T
deCODE log10(p): 207.5
deCODE BETA: 0.25
deCODE MSPA: 0
−:−:−:−
822:675:603:143

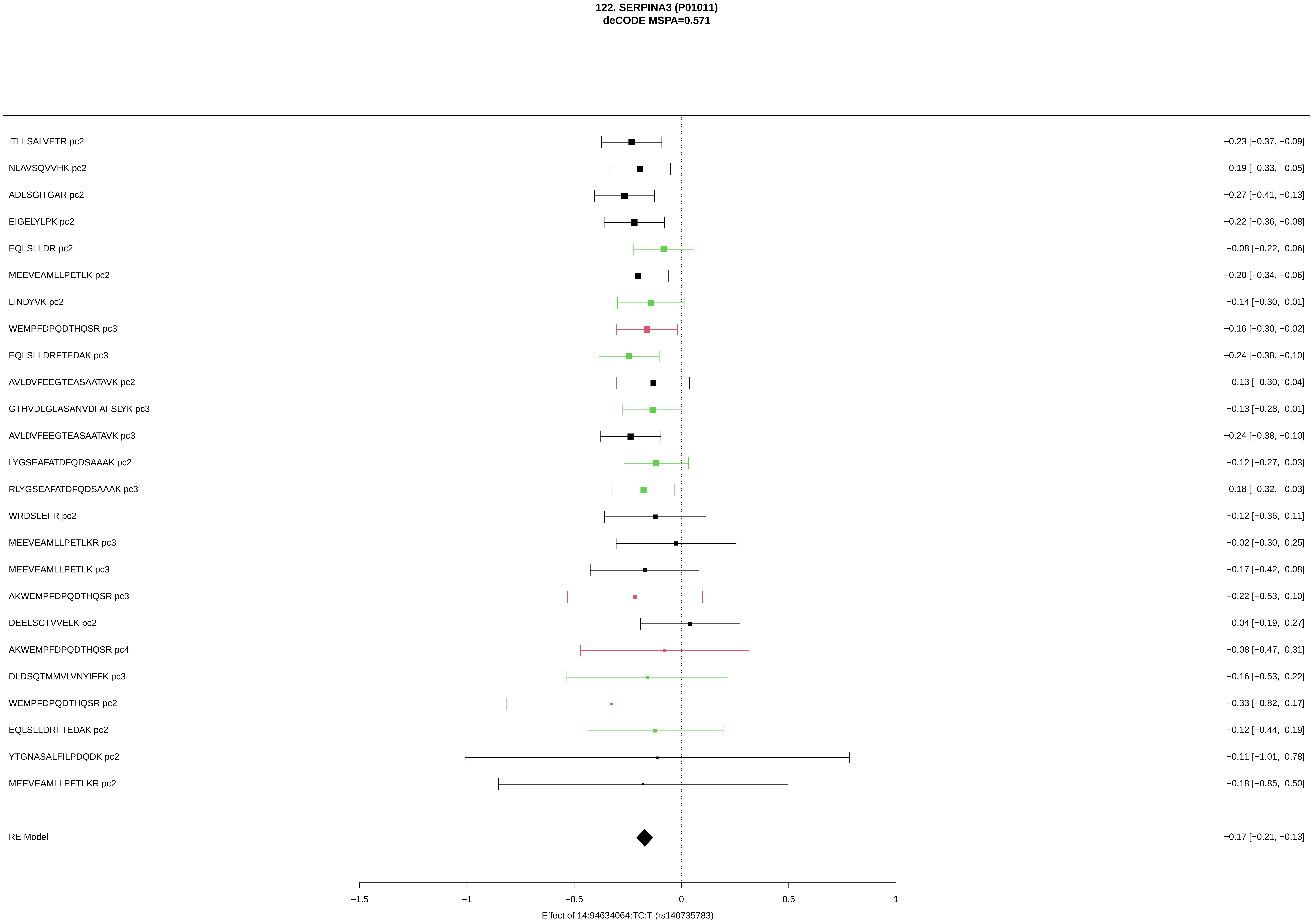
122. SERPINA3 (P01011)
deCODE MSPA=0.571
ITLLSALVETR pc2
−0.23 [−0.37, −0.09]
NLAVSQVVHK pc2
−0.19 [−0.33, −0.05]
ADLSGITGAR pc2
−0.27 [−0.41, −0.13]
EIGELYLPK pc2
−0.22 [−0.36, −0.08]
EQLSLLDR pc2
−0.08 [−0.22, 0.06]
MEEVEAMLLPETLK pc2
−0.20 [−0.34, −0.06]
LINDYVK pc2
−0.14 [−0.30, 0.01]
WEMPFDPQDTHQSR pc3
−0.16 [−0.30, −0.02]
EQLSLLDRFTEDAK pc3
−0.24 [−0.38, −0.10]
AVLDVFEEGTEASAATAVK pc2
−0.13 [−0.30, 0.04]
GTHVDLGLASANVDFAFSLYK pc3
−0.13 [−0.28, 0.01]
AVLDVFEEGTEASAATAVK pc3
−0.24 [−0.38, −0.10]
LYGSEAFATDFQDSAAAK pc2
−0.12 [−0.27, 0.03]
RLYGSEAFATDFQDSAAAK pc3
−0.18 [−0.32, −0.03]
WRDSLEFR pc2
−0.12 [−0.36, 0.11]
MEEVEAMLLPETLKR pc3
−0.02 [−0.30, 0.25]
MEEVEAMLLPETLK pc3
−0.17 [−0.42, 0.08]
AKWEMPFDPQDTHQSR pc3
−0.22 [−0.53, 0.10]
DEELSCTVVELK pc2
0.04 [−0.19, 0.27]
AKWEMPFDPQDTHQSR pc4
−0.08 [−0.47, 0.31]
DLDSQTMMVLVNYIFFK pc3
−0.16 [−0.53, 0.22]
WEMPFDPQDTHQSR pc2
−0.33 [−0.82, 0.17]
EQLSLLDRFTEDAK pc2
−0.12 [−0.44, 0.19]
YTGNASALFILPDQDK pc2
−0.11 [−1.01, 0.78]
MEEVEAMLLPETLKR pc2
−0.18 [−0.85, 0.50]
RE Model
−0.17 [−0.21, −0.13]
−1.5
−1
−0.5
0
0.5
1
Effect of 14:94634064:TC:T (rs140735783)

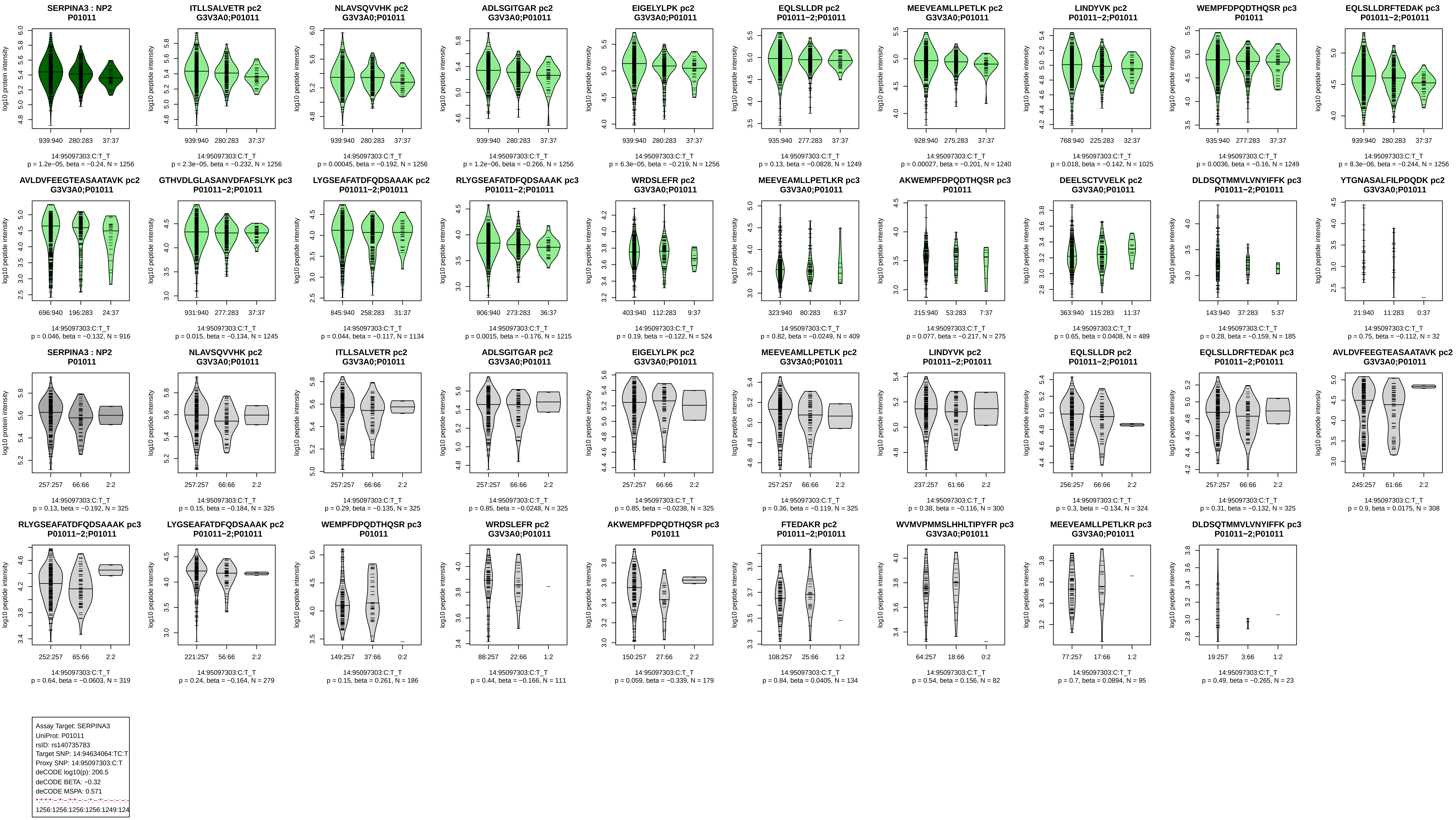

SERPINA3 : NP2 P01011
939:940 280:283 37:37
14:95097303:C:T_T
p = 1.2e−05, beta = −0.24, N = 1256
ITLLSALVETR pc2 G3V3A0;P01011
p = 2.3e−05, beta = −0.232, N = 1256
NLAVSQVVHK pc2 G3V3A0;P01011
p = 0.00045, beta = −0.192, N = 1256
ADLSGITGAR pc2 G3V3A0;P01011
p = 1.2e−06, beta = −0.266, N = 1256
EIGELYLPK pc2 G3V3A0;P01011
p = 6.3e−05, beta = −0.219, N = 1256
EQLSLLDR pc2 P01011−2;P01011
p = 0.13, beta = −0.0828, N = 1249
MEEVEAMLLPETLK pc2 G3V3A0;P01011
p = 0.00027, beta = −0.201, N = 1240
LINDYVK pc2 P01011−2;P01011
p = 0.018, beta = −0.142, N = 1025
WEMPFDPQDTHQSR pc3 P01011
p = 0.0036, beta = −0.16, N = 1249
EQLSLLDRFTEDAK pc3 P01011−2;P01011
p = 8.3e−06, beta = −0.244, N = 1256
AVLDVFEEGTEASAATAVK pc2 G3V3A0;P01011
p = 0.046, beta = −0.132, N = 916
GTHVDLGLASANVDFAFSLYK pc3 P01011−2;P01011
p = 0.015, beta = −0.134, N = 1245
LYGSEAFATDFQDSAAAK pc2 P01011−2;P01011
p = 0.044, beta = −0.117, N = 1134
RLYGSEAFATDFQDSAAAK pc3 P01011−2;P01011
p = 0.0015, beta = −0.176, N = 1215
WRDSLEFR pc2 G3V3A0;P01011
p = 0.19, beta = −0.122, N = 524
MEEVEAMLLPETLKR pc3 G3V3A0;P01011
p = 0.82, beta = −0.0249, N = 409
AKWEMPFDPQDTHQSR pc3 P01011
p = 0.077, beta = −0.217, N = 275
DEELSCTVVELK pc2 G3V3A0;P01011
p = 0.65, beta = 0.0408, N = 489
DLDSQTMMVLVNYIFFK pc3 P01011−2;P01011
p = 0.28, beta = −0.159, N = 185
YTGNASALFILPDQDK pc2 G3V3A0;P01011
p = 0.75, beta = −0.112, N = 32
SERPINA3 : NP2 P01011
p = 0.13, beta = −0.192, N = 325
NLAVSQVVHK pc2 G3V3A0;P01011
p = 0.15, beta = −0.184, N = 325
ITLLSALVETR pc2 G3V3A0;P01011
p = 0.29, beta = −0.135, N = 325
ADLSGITGAR pc2 G3V3A0;P01011
p = 0.85, beta = −0.0248, N = 325
EIGELYLPK pc2 G3V3A0;P01011
p = 0.85, beta = −0.0238, N = 325
MEEVEAMLLPETLK pc2 G3V3A0;P01011
p = 0.36, beta = −0.119, N = 325
LINDYVK pc2 P01011−2;P01011
p = 0.38, beta = −0.116, N = 300
EQLSLLDR pc2 P01011−2;P01011
p = 0.3, beta = −0.134, N = 324
EQLSLLDRFTEDAK pc3 P01011−2;P01011
p = 0.31, beta = −0.132, N = 325
AVLDVFEEGTEASAATAVK pc2 G3V3A0;P01011
p = 0.9, beta = 0.0175, N = 308
RLYGSEAFATDFQDSAAAK pc3 P01011−2;P01011
p = 0.64, beta = −0.0603, N = 319
LYGSEAFATDFQDSAAAK pc2 P01011−2;P01011
p = 0.24, beta = −0.164, N = 279
WEMPFDPQDTHQSR pc3 P01011
p = 0.15, beta = 0.261, N = 186
WRDSLEFR pc2 G3V3A0;P01011
p = 0.44, beta = −0.166, N = 111
AKWEMPFDPQDTHQSR pc3 P01011
p = 0.059, beta = −0.339, N = 179
FTEDAKR pc2 P01011−2;P01011
p = 0.84, beta = 0.0405, N = 134
WVMVPMMSLHHLTIPYFR pc3 G3V3A0;P01011
p = 0.54, beta = 0.156, N = 82
MEEVEAMLLPETLKR pc3 G3V3A0;P01011
p = 0.7, beta = 0.0894, N = 95
DLDSQTMMVLVNYIFFK pc3 P01011−2;P01011
p = 0.49, beta = −0.265, N = 23
Assay Target: SERPINA3
UniProt: P01011
rsID: rs140735783
Target SNP: 14:94634064:TC:T
Proxy SNP: 14:95097303:C:T
deCODE log10(p): 206.5
deCODE BETA: −0.32
deCODE MSPA: 0.571
1256:1256:1256:1256:1249:124

123. GLIPR2 (Q9H4G4)
deCODE MSPA=0

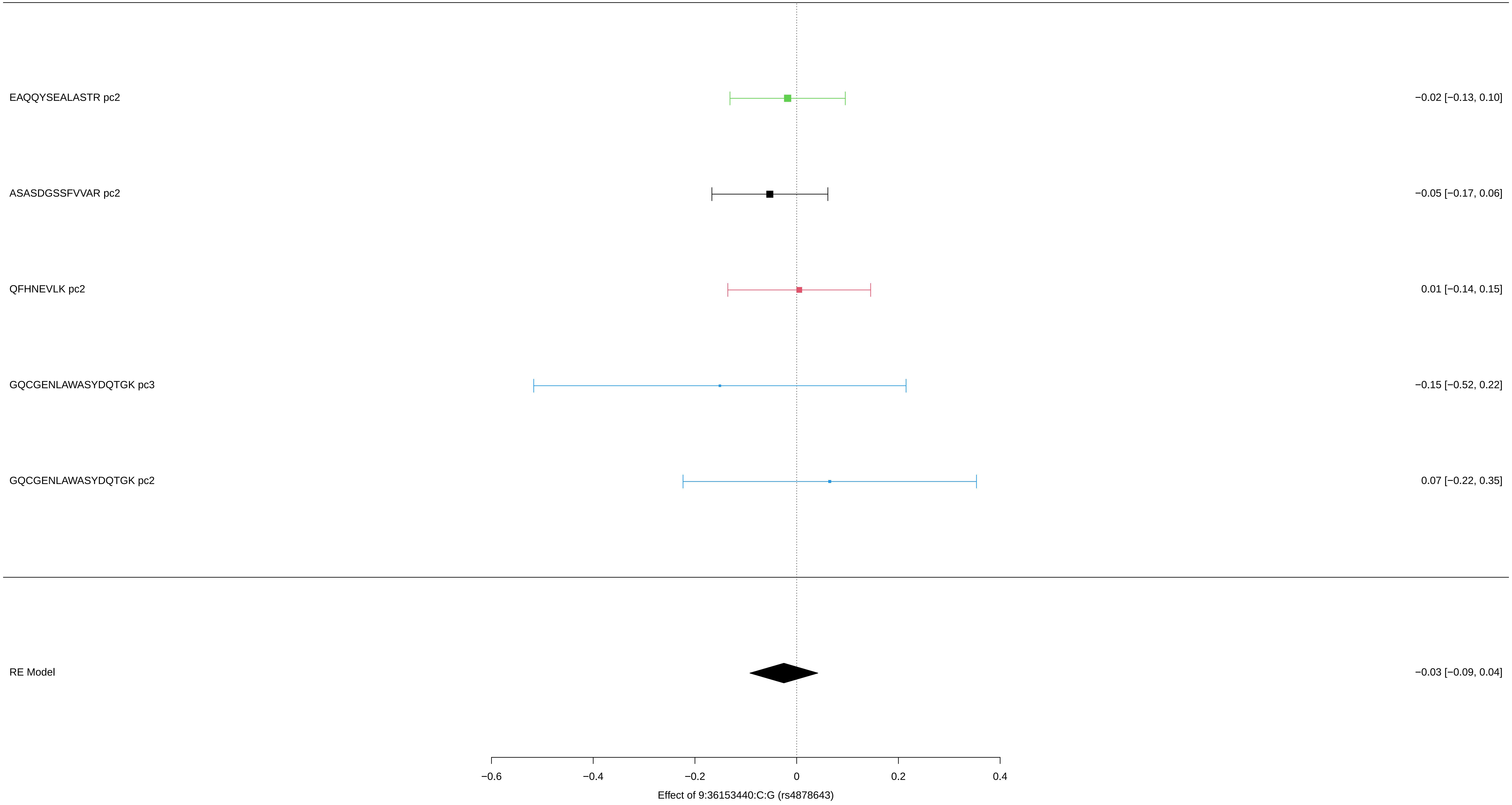

GLIPR2 : NP4
Q9H4G4

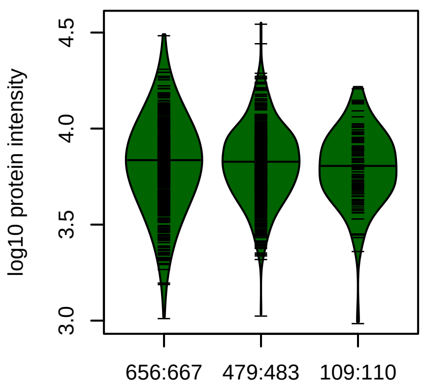

9:36153437:G:C_C
p = 0.96, beta = 0.00212, N = 1244

EAQQYSEALASTR pc2
Q5VZR0;Q9H4G4

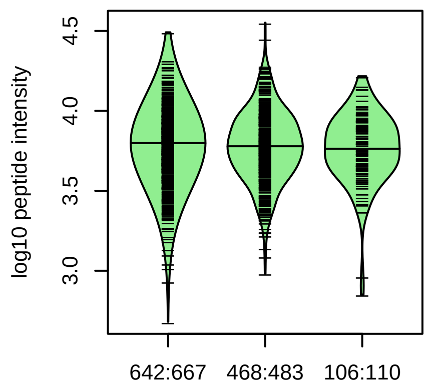

9:36153437:G:C_C
p = 0.69, beta = −0.0177, N = 1216

ASASDGSSFVVAR pc2
A0A087WUM5;Q5VZR0;Q9H4G4

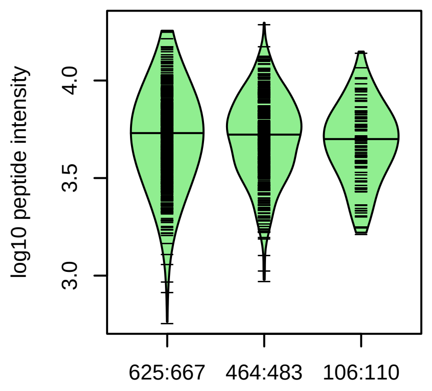

9:36153437:G:C_C
p = 0.23, beta = −0.0527, N = 1195

QFHNEVLK pc2
A0A088AWP7;Q5VZR0;Q9H4G4

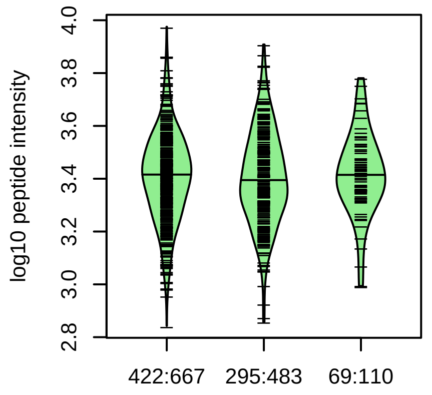

9:36153437:G:C_C
p = 0.93, beta = 0.00503, N = 786

GQCGENLAWASYDQTGK pc3
Q9H4G4

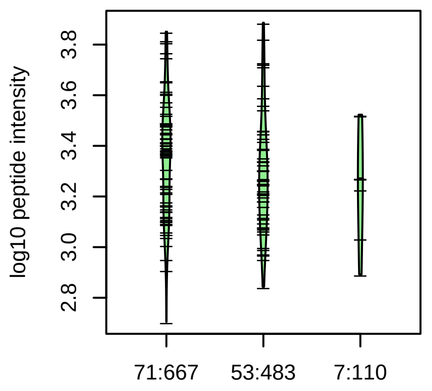

9:36153437:G:C_C
p = 0.29, beta = −0.151, N = 131

GLIPR2 : NP4
Q9H4G4

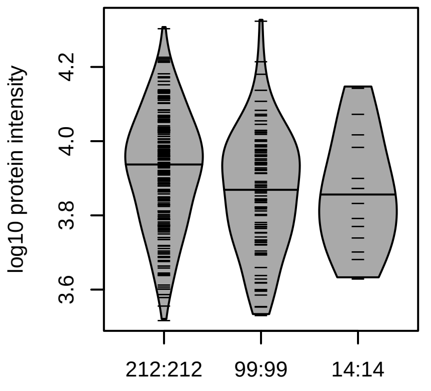

9:36153437:G:C_C
p = 0.00081, beta = −0.32, N = 325

EAQQYSEALASTR pc2
Q5VZR0;Q9H4G4

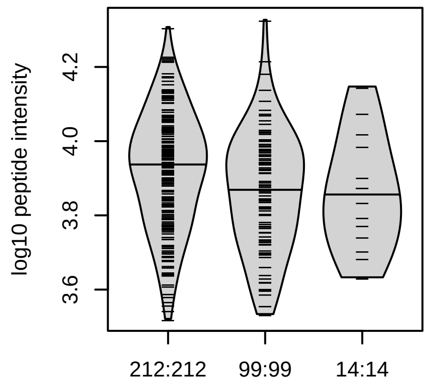

9:36153437:G:C_C
p = 0.00091, beta = −0.317, N = 325

ASASDGSSFVVAR pc2
A0A087WUM5;Q5VZR0;Q9H4G4

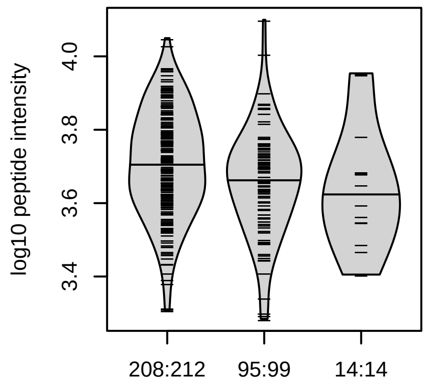

9:36153437:G:C_C
p = 0.0012, beta = −0.313, N = 317

GQCGENLAWASYDQTGK pc2
Q9H4G4

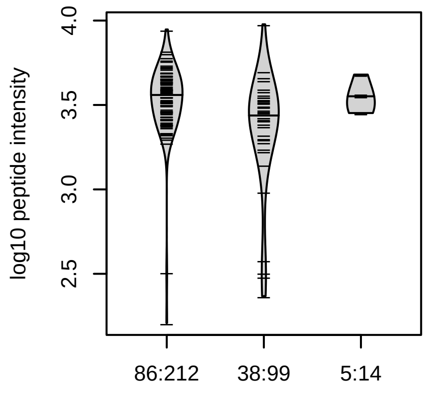

9:36153437:G:C_C
p = 0.0045, beta = −0.432, N = 129

Assay Target: GLIPR2
UniProt: Q9H4G4
rsID: rs4878643
Target SNP: 9:36153440:C:G
Proxy SNP: 9:36153437:G:C
deCODE log10(p): 204.4
deCODE BETA: −0.28
deCODE MSPA: 0
–:–:–:–:–
1216:1195:786:131:184

124. HEXB (P07686)
deCODE MSPA=0

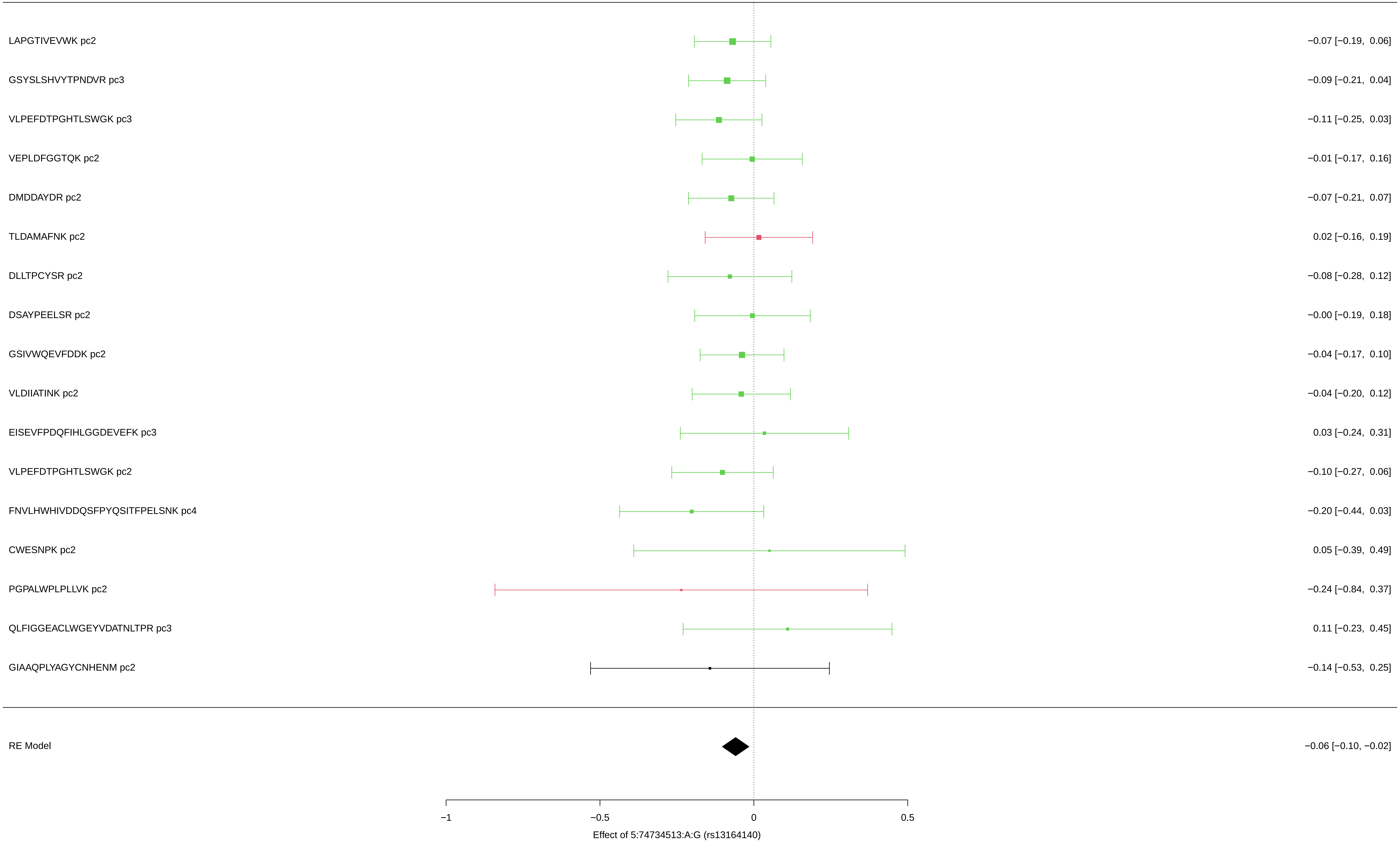

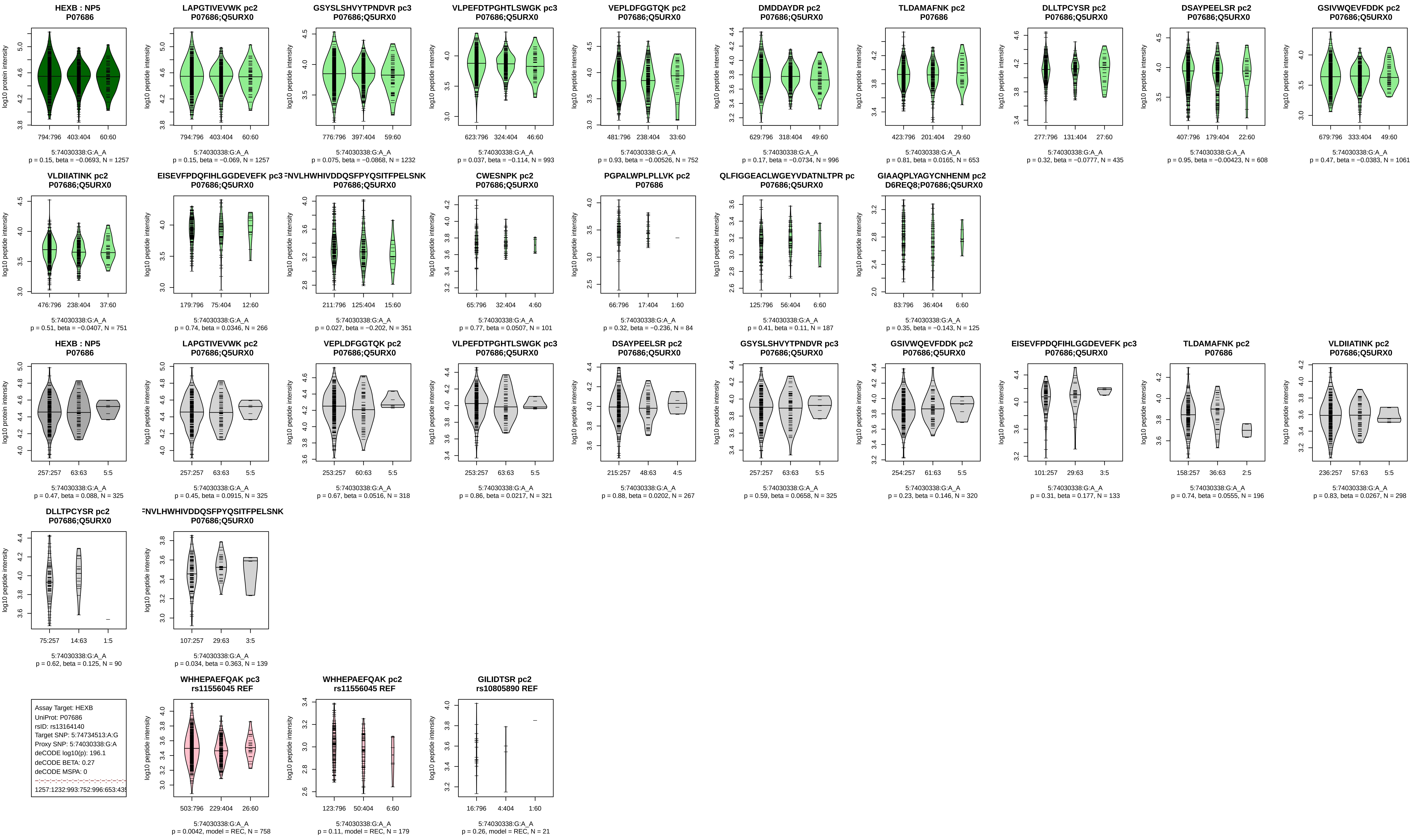
HEXB : NP5
P07686
794:796 403:404 60:60
5:74030338:G:A_A
p = 0.15, beta = −0.0693, N = 1257
LAPGTIVEVWK pc2
P07686;Q5URX0
794:796 403:404 60:60
5:74030338:G:A_A
p = 0.15, beta = −0.069, N = 1257
GSYSLSHVYTPNDVR pc3
P07686;Q5URX0
776:796 397:404 59:60
5:74030338:G:A_A
p = 0.075, beta = −0.0868, N = 1232
VLPEFDTPGHTLSWGK pc3
P07686;Q5URX0
623:796 324:404 46:60
5:74030338:G:A_A
p = 0.037, beta = −0.114, N = 993
VEPLDFGGTQK pc2
P07686;Q5URX0
481:796 238:404 33:60
5:74030338:G:A_A
p = 0.93, beta = −0.00526, N = 752
DMDDAYDR pc2
P07686;Q5URX0
629:796 318:404 49:60
5:74030338:G:A_A
p = 0.17, beta = −0.0734, N = 996
TLDAMAFNK pc2
P07686
423:796 201:404 29:60
5:74030338:G:A_A
p = 0.81, beta = 0.0165, N = 653
DLLTPCYSR pc2
P07686;Q5URX0
277:796 131:404 27:60
5:74030338:G:A_A
p = 0.32, beta = −0.0777, N = 435
DSAYPEELSR pc2
P07686;Q5URX0
407:796 179:404 22:60
5:74030338:G:A_A
p = 0.95, beta = −0.00423, N = 608
GSIVWQEVFDDK pc2
P07686;Q5URX0
679:796 333:404 49:60
5:74030338:G:A_A
p = 0.47, beta = −0.0383, N = 1061
VLDIIATINK pc2
P07686;Q5URX0
476:796 238:404 37:60
5:74030338:G:A_A
p = 0.51, beta = −0.0407, N = 751
EISEVFPDQFIHLGGDEVEFK pc3
P07686;Q5URX0
179:796 75:404 12:60
5:74030338:G:A_A
p = 0.74, beta = 0.0346, N = 266
FNVLHWHIVDDQSFPYQSITFPELSNK
P07686;Q5URX0
211:796 125:404 15:60
5:74030338:G:A_A
p = 0.027, beta = −0.202, N = 351
CWESNPK pc2
P07686;Q5URX0
65:796 32:404 4:60
5:74030338:G:A_A
p = 0.77, beta = 0.0507, N = 101
PGPALWPLPLLVK pc2
P07686
66:796 17:404 1:60
5:74030338:G:A_A
p = 0.32, beta = −0.236, N = 84
QLFIGGEACLWGEYVDATNLTPR pc
P07686;Q5URX0
125:796 56:404 6:60
5:74030338:G:A_A
p = 0.41, beta = 0.11, N = 187
GIAAQPLYAGYCNHENM pc2
D6REQ8;P07686;Q5URX0
83:796 36:404 6:60
5:74030338:G:A_A
p = 0.35, beta = −0.143, N = 125
HEXB : NP5
P07686
257:257 63:63 5:5
5:74030338:G:A_A
p = 0.47, beta = 0.088, N = 325
LAPGTIVEVWK pc2
P07686;Q5URX0
257:257 63:63 5:5
5:74030338:G:A_A
p = 0.45, beta = 0.0915, N = 325
VEPLDFGGTQK pc2
P07686;Q5URX0
253:257 60:63 5:5
5:74030338:G:A_A
p = 0.67, beta = 0.0516, N = 318
VLPEFDTPGHTLSWGK pc3
P07686;Q5URX0
253:257 63:63 5:5
5:74030338:G:A_A
p = 0.86, beta = 0.0217, N = 321
DSAYPEELSR pc2
P07686;Q5URX0
215:257 48:63 4:5
5:74030338:G:A_A
p = 0.88, beta = 0.0202, N = 267
GSYSLSHVYTPNDVR pc3
P07686;Q5URX0
257:257 63:63 5:5
5:74030338:G:A_A
p = 0.59, beta = 0.0658, N = 325
GSIVWQEVFDDK pc2
P07686;Q5URX0
254:257 61:63 5:5
5:74030338:G:A_A
p = 0.23, beta = 0.146, N = 320
EISEVFPDQFIHLGGDEVEFK pc3
P07686;Q5URX0
101:257 29:63 3:5
5:74030338:G:A_A
p = 0.31, beta = 0.177, N = 133
TLDAMAFNK pc2
P07686
158:257 36:63 2:5
5:74030338:G:A_A
p = 0.74, beta = 0.0555, N = 196
VLDIIATINK pc2
P07686;Q5URX0
236:257 57:63 5:5
5:74030338:G:A_A
p = 0.83, beta = 0.0267, N = 298
DLLTPCYSR pc2
P07686;Q5URX0
75:257 14:63 1:5
5:74030338:G:A_A
p = 0.62, beta = 0.125, N = 90
FNVLHWHIVDDQSFPYQSITFPELSNK
P07686;Q5URX0
107:257 29:63 3:5
5:74030338:G:A_A
p = 0.034, beta = 0.363, N = 139
Assay Target: HEXB
UniProt: P07686
rsID: rs13164140
Target SNP: 5:74734513:A:G
Proxy SNP: 5:74030338:G:A
deCODE log10(p): 196.1
deCODE BETA: 0.27
deCODE MSPA: 0
1257:1232:993:752:996:653:43
WHHEPAEFQAK pc3
rs11556045 REF
503:796 229:404 26:60
5:74030338:G:A_A
p = 0.0042, model = REC, N = 758
WHHEPAEFQAK pc2
rs11556045 REF
123:796 50:404 6:60
5:74030338:G:A_A
p = 0.11, model = REC, N = 179
GILIDTSR pc2
rs10805890 REF
16:796 4:404 1:60
5:74030338:G:A_A
p = 0.26, model = REC, N = 21
log10 protein intensity
log10 peptide intensity

125. GAS6 (Q14393)
deCODE MSPA=0.497

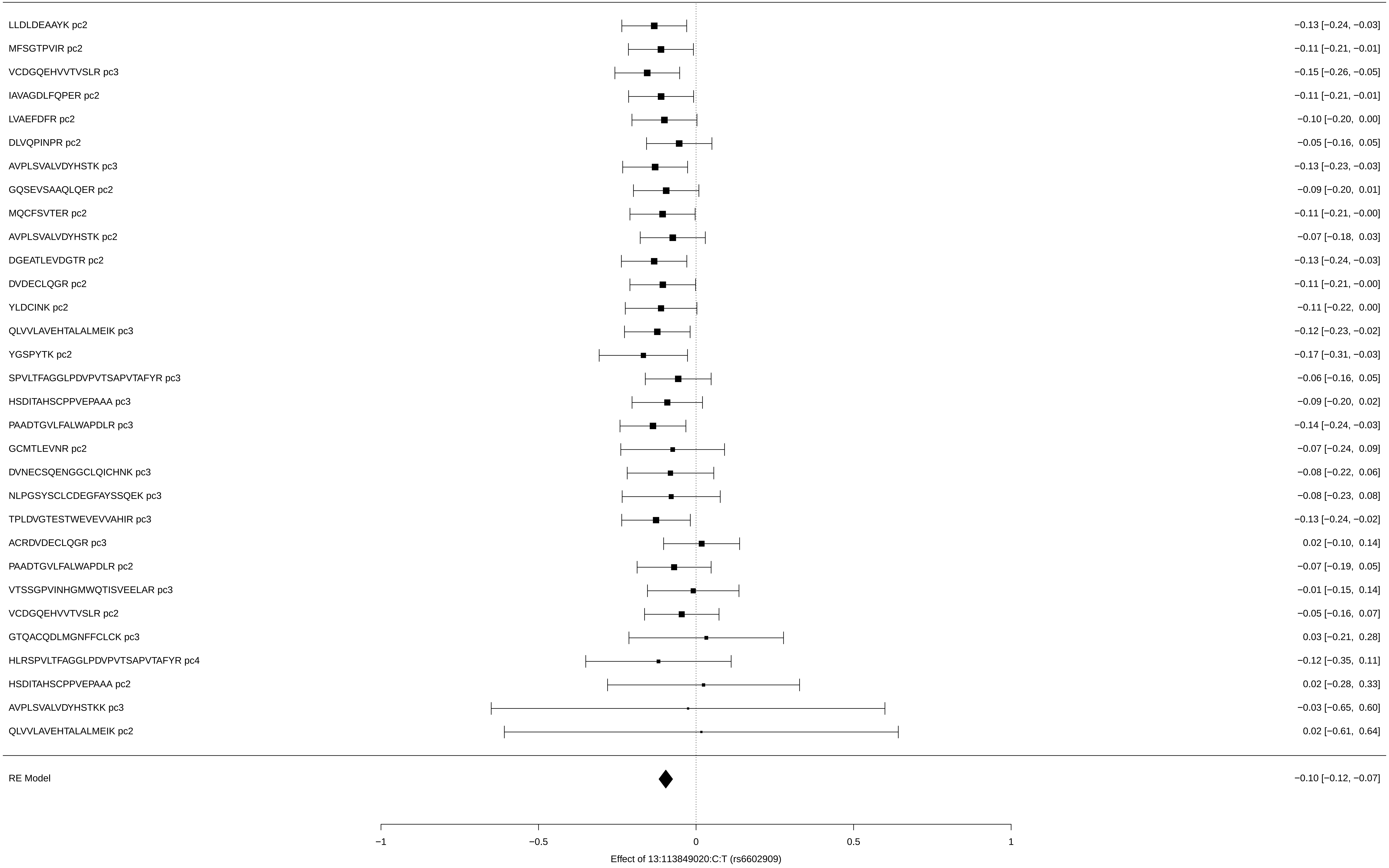

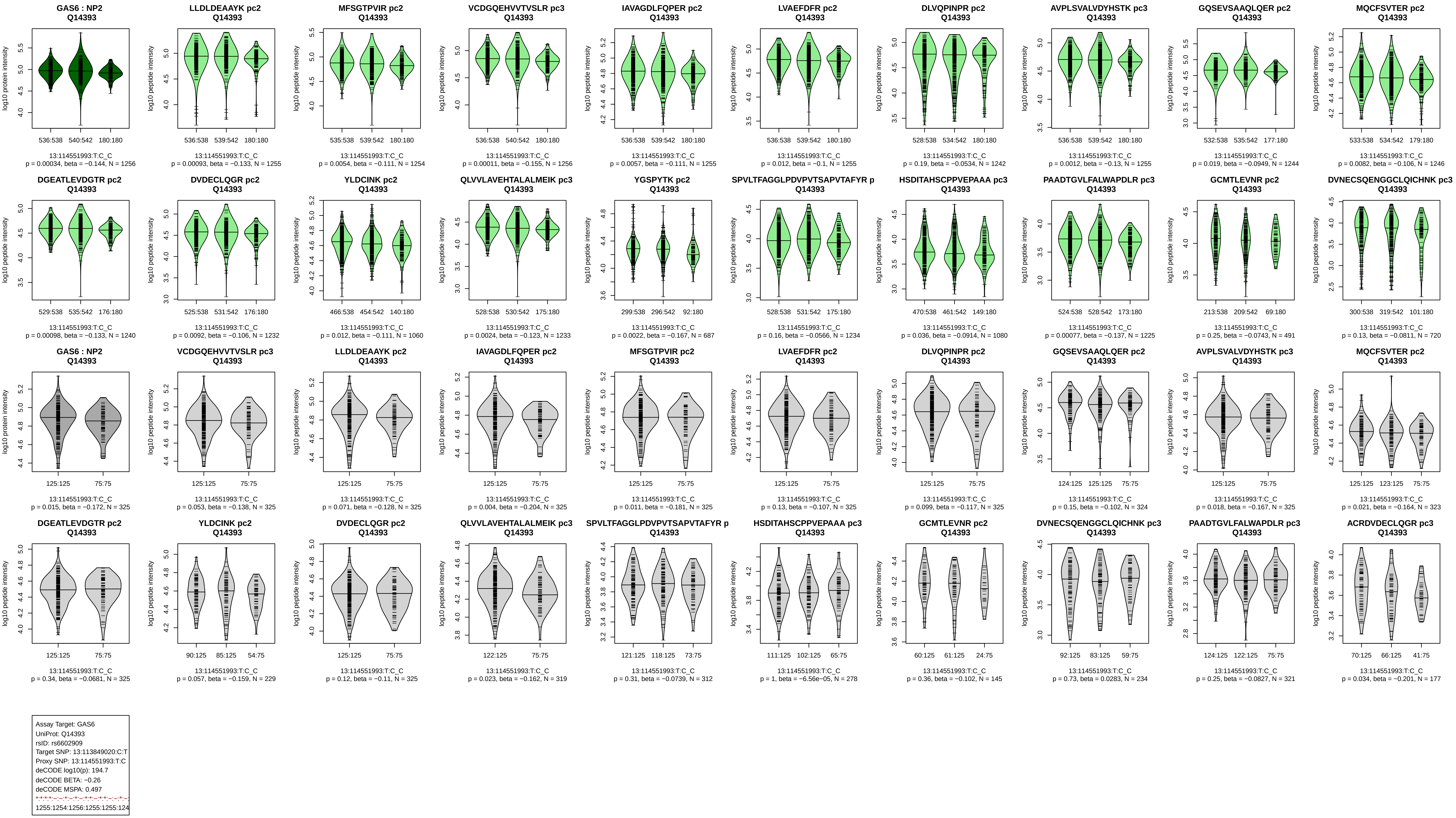
GAS6 : NP2
Q14393
536:538 540:542 180:180
13:114551993:T:C_C
p = 0.00034, beta = −0.144, N = 1256
LLDLDEAAYK pc2
Q14393
536:538 539:542 180:180
13:114551993:T:C_C
p = 0.00093, beta = −0.133, N = 1255
MFSGTPVIR pc2
Q14393
535:538 539:542 180:180
13:114551993:T:C_C
p = 0.0054, beta = −0.111, N = 1254
VCDGQEHVVTVSLR pc3
Q14393
536:538 540:542 180:180
13:114551993:T:C_C
p = 0.00011, beta = −0.155, N = 1256
IAVAGDLFQPER pc2
Q14393
536:538 539:542 180:180
13:114551993:T:C_C
p = 0.0057, beta = −0.111, N = 1255
LVAEFDFR pc2
Q14393
536:538 539:542 180:180
13:114551993:T:C_C
p = 0.012, beta = −0.1, N = 1255
DLVQPINPR pc2
Q14393
528:538 534:542 180:180
13:114551993:T:C_C
p = 0.19, beta = −0.0534, N = 1242
AVPLSVALVDYHSTK pc3
Q14393
536:538 539:542 180:180
13:114551993:T:C_C
p = 0.0012, beta = −0.13, N = 1255
GQSEVSAAQLQER pc2
Q14393
532:538 535:542 177:180
13:114551993:T:C_C
p = 0.019, beta = −0.0949, N = 1244
MQCFSVTER pc2
Q14393
533:538 534:542 179:180
13:114551993:T:C_C
p = 0.0082, beta = −0.106, N = 1246
DGEATLEVDGTR pc2
Q14393
529:538 535:542 176:180
13:114551993:T:C_C
p = 0.00098, beta = −0.133, N = 1240
DVDECLQGR pc2
Q14393
525:538 531:542 176:180
13:114551993:T:C_C
p = 0.0092, beta = −0.106, N = 1232
YLDCINK pc2
Q14393
466:538 454:542 140:180
13:114551993:T:C_C
p = 0.012, beta = −0.111, N = 1060
QLVVLAVEHTALALMEIK pc3
Q14393
528:538 530:542 175:180
13:114551993:T:C_C
p = 0.0024, beta = −0.123, N = 1233
YGSPYTK pc2
Q14393
299:538 296:542 92:180
13:114551993:T:C_C
p = 0.0022, beta = −0.167, N = 687
SPVLTFAGGLPDVPVTSAPVTAFYR p
Q14393
528:538 531:542 175:180
13:114551993:T:C_C
p = 0.16, beta = −0.0566, N = 1234
HSDITAHSCPPVEPAAA pc3
Q14393
470:538 461:542 149:180
13:114551993:T:C_C
p = 0.036, beta = −0.0914, N = 1080
PAADTGVLFALWAPDLR pc3
Q14393
524:538 528:542 173:180
13:114551993:T:C_C
p = 0.00077, beta = −0.137, N = 1225
GCMTLEVNR pc2
Q14393
213:538 209:542 69:180
13:114551993:T:C_C
p = 0.25, beta = −0.0743, N = 491
DVNECSQENGGCLQICHNK pc3
Q14393
300:538 319:542 101:180
13:114551993:T:C_C
p = 0.13, beta = −0.0811, N = 720
GAS6 : NP2
Q14393
125:125 75:75
13:114551993:T:C_C
p = 0.015, beta = −0.172, N = 325
VCDGQEHVVTVSLR pc3
Q14393
125:125 75:75
13:114551993:T:C_C
p = 0.053, beta = −0.138, N = 325
LLDLDEAAYK pc2
Q14393
125:125 75:75
13:114551993:T:C_C
p = 0.071, beta = −0.128, N = 325
IAVAGDLFQPER pc2
Q14393
125:125 75:75
13:114551993:T:C_C
p = 0.004, beta = −0.204, N = 325
MFSGTPVIR pc2
Q14393
125:125 75:75
13:114551993:T:C_C
p = 0.011, beta = −0.181, N = 325
LVAEFDFR pc2
Q14393
125:125 75:75
13:114551993:T:C_C
p = 0.13, beta = −0.107, N = 325
DLVQPINPR pc2
Q14393
125:125 75:75
13:114551993:T:C_C
p = 0.099, beta = −0.117, N = 325
GQSEVSAAQLQER pc2
Q14393
124:125 125:125 75:75
13:114551993:T:C_C
p = 0.15, beta = −0.102, N = 324
AVPLSVALVDYHSTK pc3
Q14393
125:125 75:75
13:114551993:T:C_C
p = 0.018, beta = −0.167, N = 325
MQCFSVTER pc2
Q14393
125:125 123:125 75:75
13:114551993:T:C_C
p = 0.021, beta = −0.164, N = 323
DGEATLEVDGTR pc2
Q14393
125:125 75:75
13:114551993:T:C_C
p = 0.34, beta = −0.0681, N = 325
YLDCINK pc2
Q14393
90:125 85:125 54:75
13:114551993:T:C_C
p = 0.057, beta = −0.159, N = 229
DVDECLQGR pc2
Q14393
125:125 75:75
13:114551993:T:C_C
p = 0.12, beta = −0.11, N = 325
QLVVLAVEHTALALMEIK pc3
Q14393
122:125 75:75
13:114551993:T:C_C
p = 0.023, beta = −0.162, N = 319
SPVLTFAGGLPDVPVTSAPVTAFYR p
Q14393
121:125 118:125 73:75
13:114551993:T:C_C
p = 0.31, beta = −0.0739, N = 312
HSDITAHSCPPVEPAAA pc3
Q14393
111:125 102:125 65:75
13:114551993:T:C_C
p = 1, beta = −6.56e−05, N = 278
GCMTLEVNR pc2
Q14393
60:125 61:125 24:75
13:114551993:T:C_C
p = 0.36, beta = −0.102, N = 145
DVNECSQENGGCLQICHNK pc3
Q14393
92:125 83:125 59:75
13:114551993:T:C_C
p = 0.73, beta = 0.0283, N = 234
PAADTGVLFALWAPDLR pc3
Q14393
124:125 122:125 75:75
13:114551993:T:C_C
p = 0.25, beta = −0.0827, N = 321
ACRDVDECLQGR pc3
Q14393
70:125 66:125 41:75
13:114551993:T:C_C
p = 0.034, beta = −0.201, N = 177
Assay Target: GAS6
UniProt: Q14393
rsID: rs6602909
Target SNP: 13:113849020:C:T
Proxy SNP: 13:114551993:T:C
deCODE log10(p): 194.7
deCODE BETA: −0.26
deCODE MSPA: 0.497
1255:1254:1256:1255:1255:124

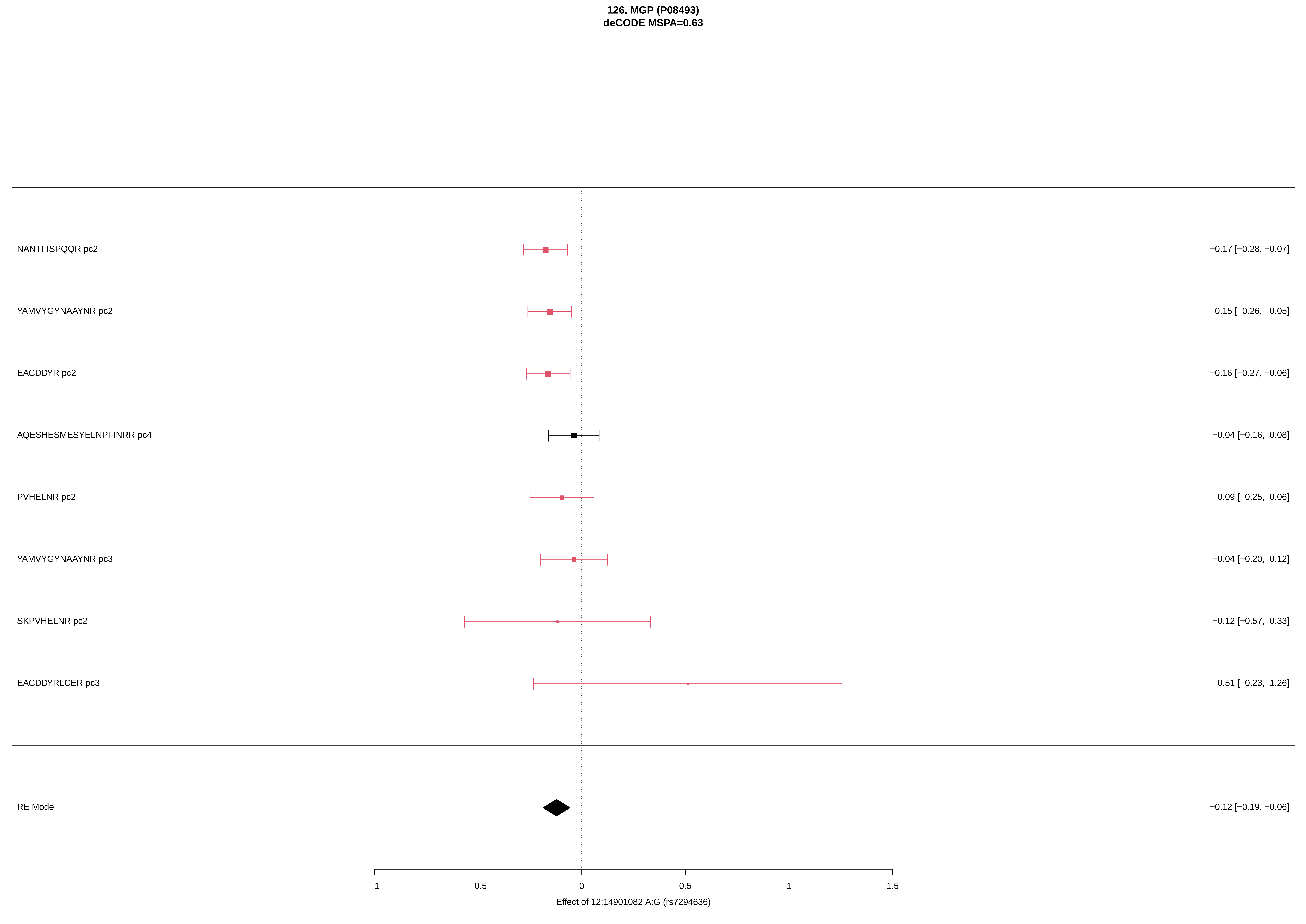
126. MGP (P08493)
deCODE MSPA=0.63
NANTFISPQQR pc2
−0.17 [−0.28, −0.07]
YAMVYGYNAAYNR pc2
−0.15 [−0.26, −0.05]
EACDDYR pc2
−0.16 [−0.27, −0.06]
AQESHESMESYELNPFINRR pc4
−0.04 [−0.16, 0.08]
PVHELNR pc2
−0.09 [−0.25, 0.06]
YAMVYGYNAAYNR pc3
−0.04 [−0.20, 0.12]
SKPVHELNR pc2
−0.12 [−0.57, 0.33]
EACDDYRLCER pc3
0.51 [−0.23, 1.26]
RE Model
−0.12 [−0.19, −0.06]
−1
−0.5
0
0.5
1
1.5
Effect of 12:14901082:A:G (rs7294636)

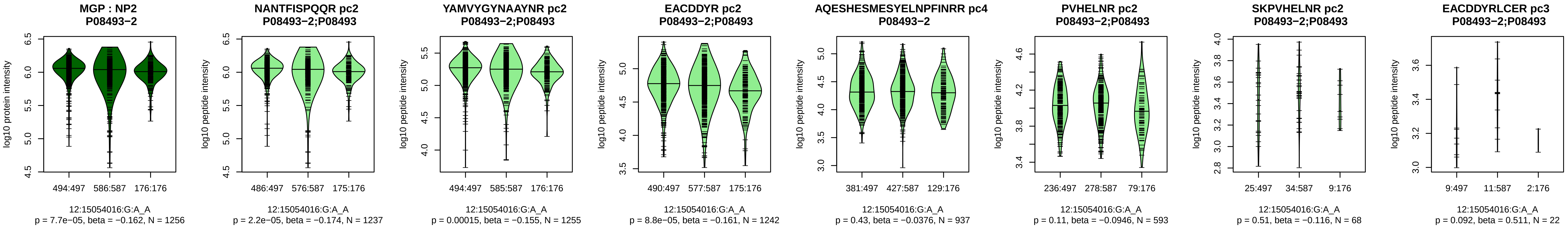

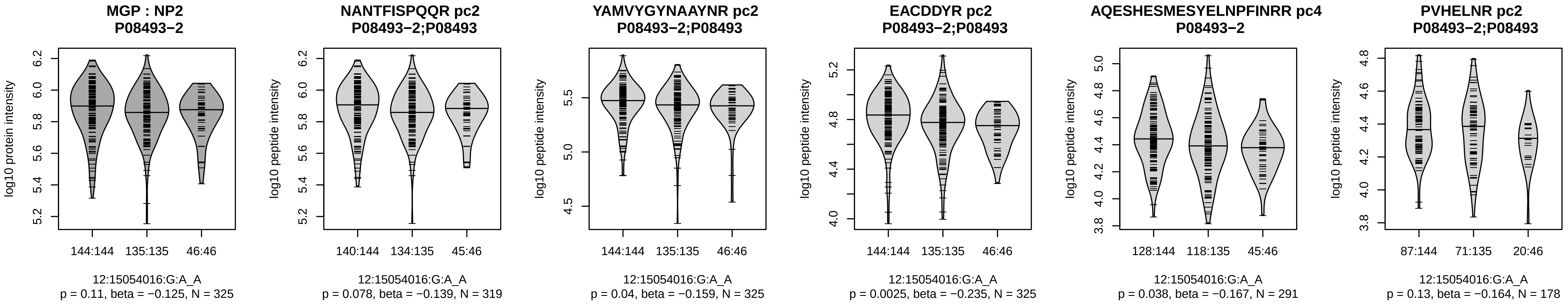

Assay Target: MGP
UniProt: P08493
rsID: rs7294636
Target SNP: 12:14901082:A:G
Proxy SNP: 12:15054016:G:A
deCODE log10(p): 192.8
deCODE BETA: −0.25
deCODE MSPA: 0.63
::*:−:−:−:−:−
1237:1255:1242:937:593:570:68

127. HSPG2 (P98160)
deCODE MSPA=0.043 (2 conflicts)

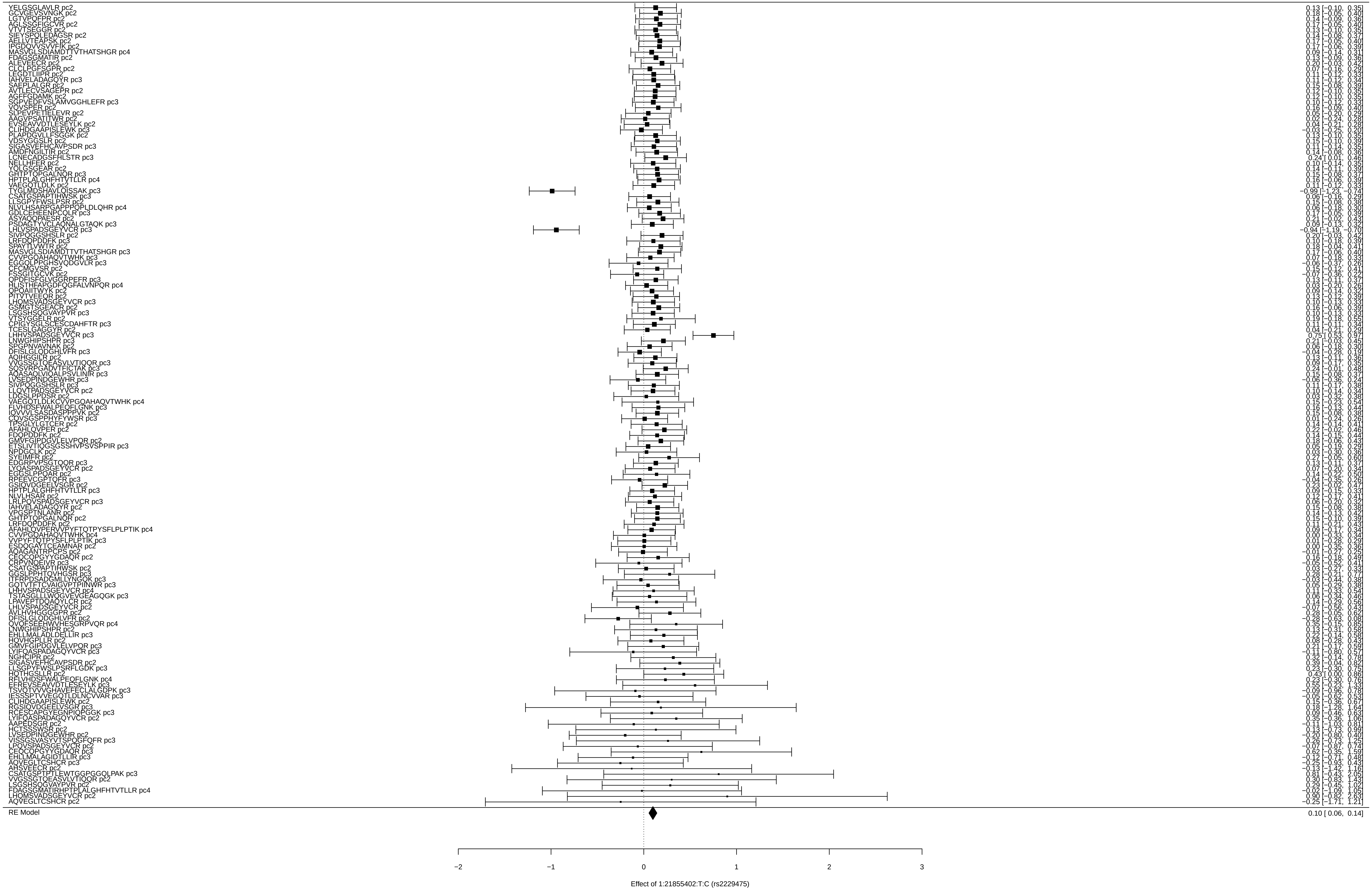

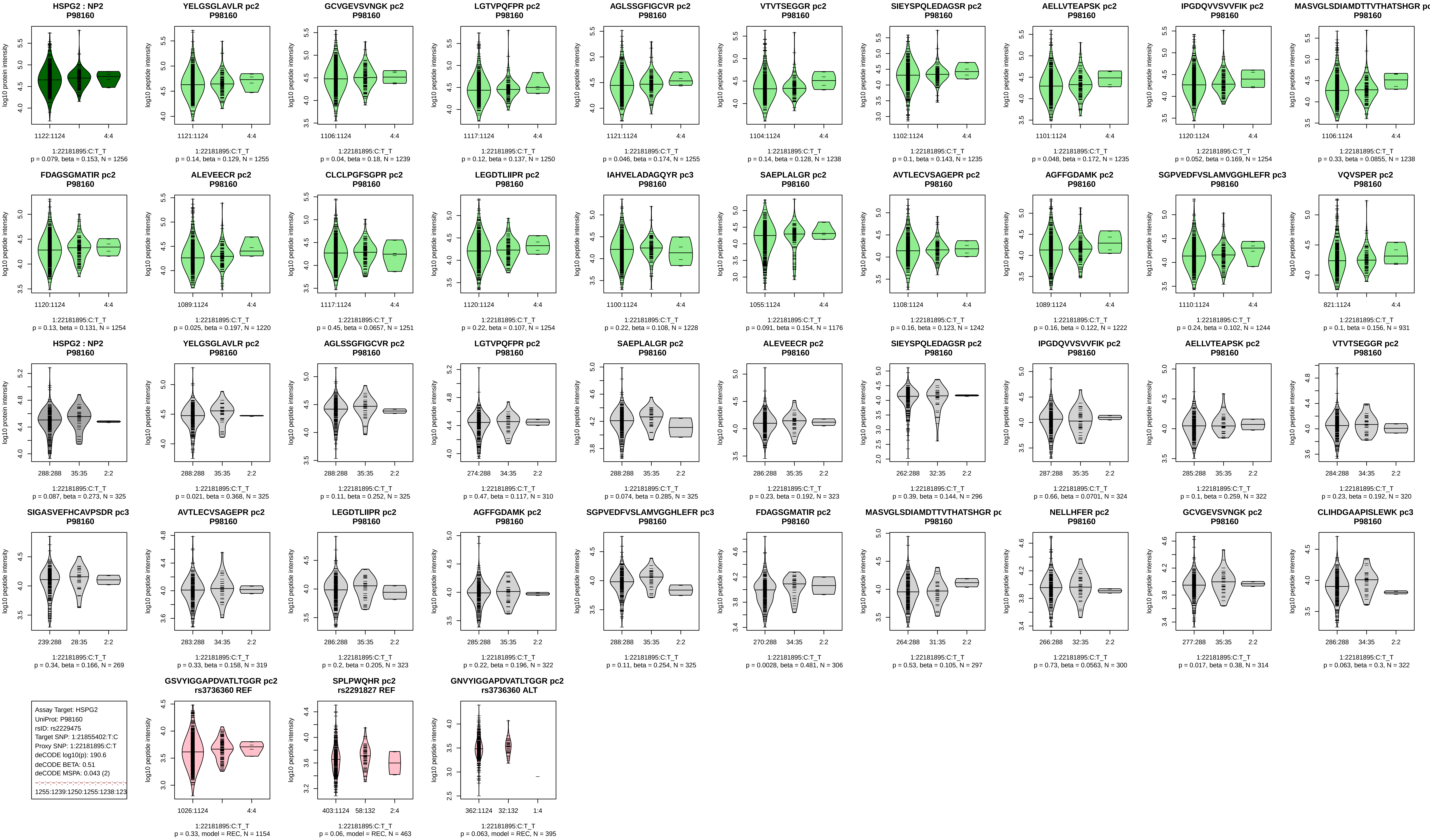
HSPG2 : NP2
P98160
log10 protein intensity
1122:1124
4:4
1:22181895:C:T_T
p = 0.079, beta = 0.153, N = 1256
YELGSGLAVLR pc2
P98160
log10 peptide intensity
1121:1124
4:4
1:22181895:C:T_T
p = 0.14, beta = 0.129, N = 1255
GCVGEVSVNGK pc2
P98160
1106:1124
4:4
1:22181895:C:T_T
p = 0.04, beta = 0.18, N = 1239
LGTVPQFPR pc2
P98160
1117:1124
4:4
1:22181895:C:T_T
p = 0.12, beta = 0.137, N = 1250
AGLSSGFIGCVR pc2
P98160
1121:1124
4:4
1:22181895:C:T_T
p = 0.046, beta = 0.174, N = 1255
VTVTSEGGR pc2
P98160
1104:1124
4:4
1:22181895:C:T_T
p = 0.14, beta = 0.128, N = 1238
SIEYSPQLEDAGSR pc2
P98160
1102:1124
4:4
1:22181895:C:T_T
p = 0.1, beta = 0.143, N = 1235
AELLVTEAPSK pc2
P98160
1101:1124
4:4
1:22181895:C:T_T
p = 0.048, beta = 0.172, N = 1235
IPGDQVVSVVFIK pc2
P98160
1120:1124
4:4
1:22181895:C:T_T
p = 0.052, beta = 0.169, N = 1254
MASVGLSDIAMDTTVTHATSHGR pc
P98160
1106:1124
4:4
1:22181895:C:T_T
p = 0.33, beta = 0.0855, N = 1238
FDAGSGMATIR pc2
P98160
1120:1124
4:4
1:22181895:C:T_T
p = 0.13, beta = 0.131, N = 1254
ALEVEECR pc2
P98160
1089:1124
4:4
1:22181895:C:T_T
p = 0.025, beta = 0.197, N = 1220
CLCLPGFSGPR pc2
P98160
1117:1124
4:4
1:22181895:C:T_T
p = 0.45, beta = 0.0657, N = 1251
LEGDTLIIPR pc2
P98160
1120:1124
4:4
1:22181895:C:T_T
p = 0.22, beta = 0.107, N = 1254
IAHVELADAGQYR pc3
P98160
1100:1124
4:4
1:22181895:C:T_T
p = 0.22, beta = 0.108, N = 1228
SAEPLALGR pc2
P98160
1055:1124
4:4
1:22181895:C:T_T
p = 0.091, beta = 0.154, N = 1176
AVTLECVSAGEPR pc2
P98160
1108:1124
4:4
1:22181895:C:T_T
p = 0.16, beta = 0.123, N = 1242
AGFFGDAMK pc2
P98160
1089:1124
4:4
1:22181895:C:T_T
p = 0.16, beta = 0.122, N = 1222
SGPVEDFVSLAMVGGHLEFR pc3
P98160
1110:1124
4:4
1:22181895:C:T_T
p = 0.24, beta = 0.102, N = 1244
VQVSPER pc2
P98160
821:1124
4:4
1:22181895:C:T_T
p = 0.1, beta = 0.156, N = 931
HSPG2 : NP2
P98160
288:288
35:35
2:2
1:22181895:C:T_T
p = 0.087, beta = 0.273, N = 325
YELGSGLAVLR pc2
P98160
288:288
35:35
2:2
1:22181895:C:T_T
p = 0.021, beta = 0.368, N = 325
AGLSSGFIGCVR pc2
P98160
288:288
35:35
2:2
1:22181895:C:T_T
p = 0.11, beta = 0.252, N = 325
LGTVPQFPR pc2
P98160
274:288
34:35
2:2
1:22181895:C:T_T
p = 0.47, beta = 0.117, N = 310
SAEPLALGR pc2
P98160
288:288
35:35
2:2
1:22181895:C:T_T
p = 0.074, beta = 0.285, N = 325
ALEVEECR pc2
P98160
286:288
35:35
2:2
1:22181895:C:T_T
p = 0.23, beta = 0.192, N = 323
SIEYSPQLEDAGSR pc2
P98160
262:288
32:35
2:2
1:22181895:C:T_T
p = 0.39, beta = 0.144, N = 296
IPGDQVVSVVFIK pc2
P98160
287:288
35:35
2:2
1:22181895:C:T_T
p = 0.66, beta = 0.0701, N = 324
AELLVTEAPSK pc2
P98160
285:288
35:35
2:2
1:22181895:C:T_T
p = 0.1, beta = 0.259, N = 322
VTVTSEGGR pc2
P98160
284:288
34:35
2:2
1:22181895:C:T_T
p = 0.23, beta = 0.192, N = 320
SIGASVEFHCAVPSDR pc3
P98160
239:288
28:35
2:2
1:22181895:C:T_T
p = 0.34, beta = 0.166, N = 269
AVTLECVSAGEPR pc2
P98160
283:288
34:35
2:2
1:22181895:C:T_T
p = 0.33, beta = 0.158, N = 319
LEGDTLIIPR pc2
P98160
286:288
35:35
2:2
1:22181895:C:T_T
p = 0.2, beta = 0.205, N = 323
AGFFGDAMK pc2
P98160
285:288
35:35
2:2
1:22181895:C:T_T
p = 0.22, beta = 0.196, N = 322
SGPVEDFVSLAMVGGHLEFR pc3
P98160
288:288
35:35
2:2
1:22181895:C:T_T
p = 0.11, beta = 0.254, N = 325
FDAGSGMATIR pc2
P98160
270:288
34:35
2:2
1:22181895:C:T_T
p = 0.0028, beta = 0.481, N = 306
MASVGLSDIAMDTTVTHATSHGR pc
P98160
264:288
31:35
2:2
1:22181895:C:T_T
p = 0.53, beta = 0.105, N = 297
NELLHFER pc2
P98160
266:288
32:35
2:2
1:22181895:C:T_T
p = 0.73, beta = 0.0563, N = 300
GCVGEVSVNGK pc2
P98160
277:288
35:35
2:2
1:22181895:C:T_T
p = 0.017, beta = 0.38, N = 314
CLIHDGAAPISLEWK pc3
P98160
286:288
34:35
2:2
1:22181895:C:T_T
p = 0.063, beta = 0.3, N = 322
Assay Target: HSPG2
UniProt: P98160
rsID: rs2229475
Target SNP: 1:21855402:T:C
Proxy SNP: 1:22181895:C:T
deCODE log10(p): 190.6
deCODE BETA: 0.51
deCODE MSPA: 0.043 (2)
1255:1239:1250:1255:1238:123
GSVYIGGAPDVATLTGGR pc2
rs3736360 REF
1026:1124
4:4
1:22181895:C:T_T
p = 0.33, model = REC, N = 1154
SPLPWQHR pc2
rs2291827 REF
403:1124
58:132
2:4
1:22181895:C:T_T
p = 0.06, model = REC, N = 463
GNVYIGGAPDVATLTGGR pc2
rs3736360 ALT
362:1124
32:132
1:4
1:22181895:C:T_T
p = 0.063, model = REC, N = 395

128. APOE (P02649)
deCODE MSPA=0.026

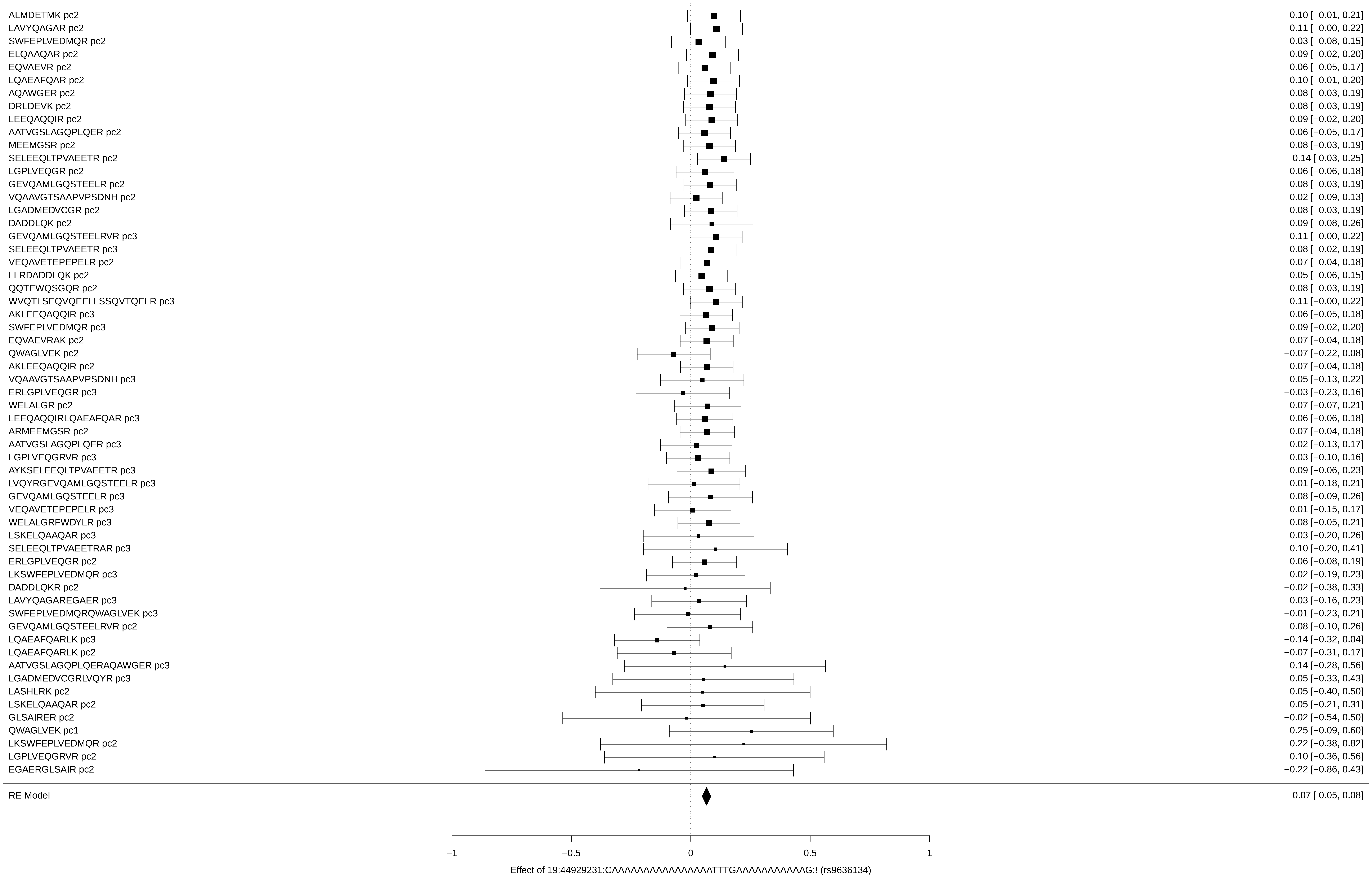

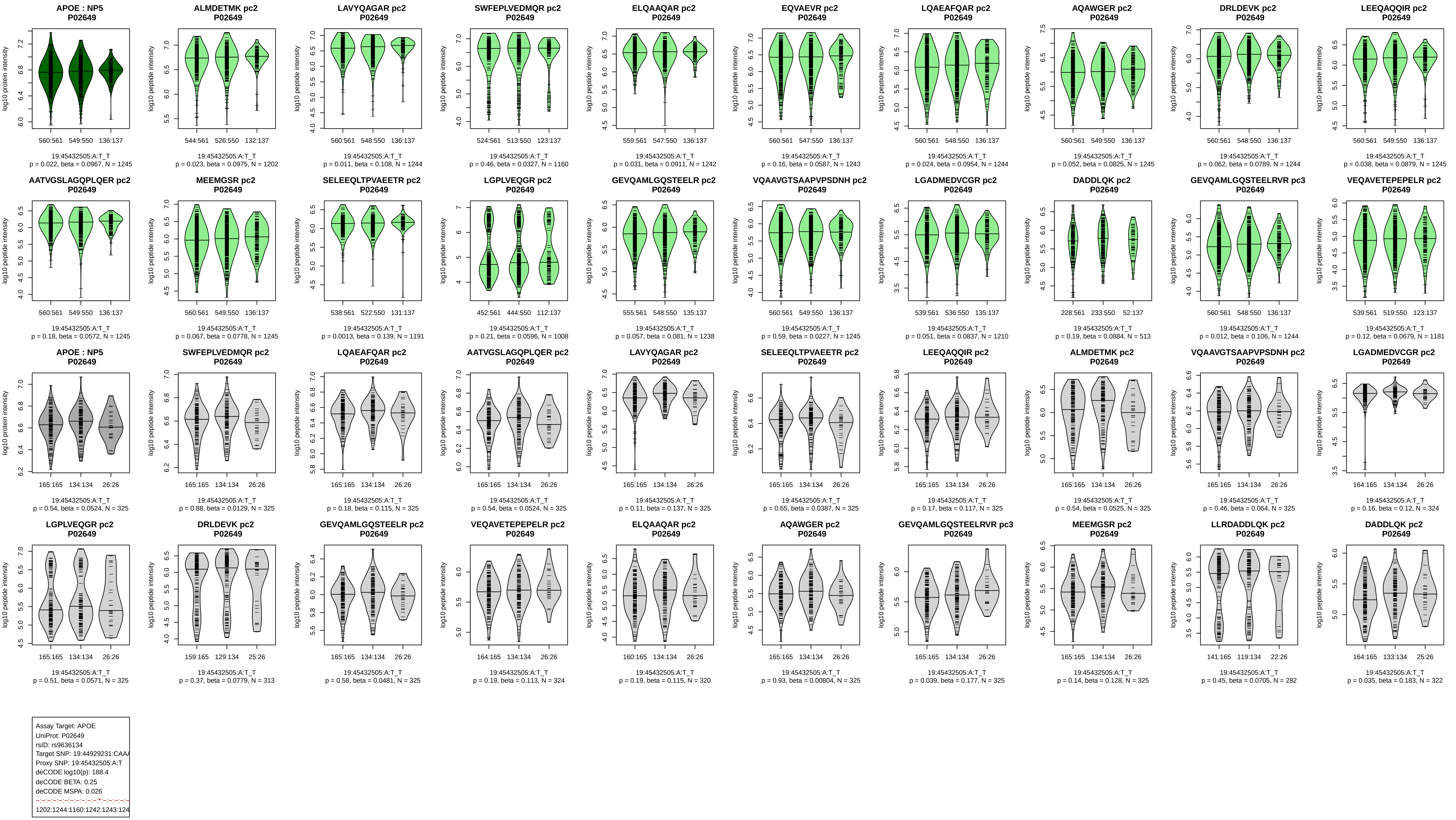
APOE : NP5
P02649
log10 protein intensity
560:561 549:550 136:137
19:45432505:A:T_T
p = 0.022, beta = 0.0967, N = 1245
ALMDETMK pc2
P02649
log10 peptide intensity
544:561 526:550 132:137
19:45432505:A:T_T
p = 0.023, beta = 0.0975, N = 1202
LAVYQAGAR pc2
P02649
log10 peptide intensity
560:561 548:550 136:137
19:45432505:A:T_T
p = 0.011, beta = 0.108, N = 1244
SWFEPLVEDMQR pc2
P02649
log10 peptide intensity
524:561 513:550 123:137
19:45432505:A:T_T
p = 0.46, beta = 0.0327, N = 1160
ELQAAQAR pc2
P02649
log10 peptide intensity
559:561 547:550 136:137
19:45432505:A:T_T
p = 0.031, beta = 0.0911, N = 1242
EQVAEVR pc2
P02649
log10 peptide intensity
560:561 547:550 136:137
19:45432505:A:T_T
p = 0.16, beta = 0.0587, N = 1243
LQAEAFQAR pc2
P02649
log10 peptide intensity
560:561 548:550 136:137
19:45432505:A:T_T
p = 0.024, beta = 0.0954, N = 1244
AQAWGER pc2
P02649
log10 peptide intensity
560:561 549:550 136:137
19:45432505:A:T_T
p = 0.052, beta = 0.0825, N = 1245
DRLDEVK pc2
P02649
log10 peptide intensity
560:561 548:550 136:137
19:45432505:A:T_T
p = 0.062, beta = 0.0789, N = 1244
LEEQAQQIR pc2
P02649
log10 peptide intensity
560:561 549:550 136:137
19:45432505:A:T_T
p = 0.038, beta = 0.0879, N = 1245
AATVGSLAGQPLQER pc2
P02649
log10 peptide intensity
560:561 549:550 136:137
19:45432505:A:T_T
p = 0.18, beta = 0.0572, N = 1245
MEEMGSR pc2
P02649
log10 peptide intensity
560:561 549:550 136:137
19:45432505:A:T_T
p = 0.067, beta = 0.0778, N = 1245
SELEEQLTPVAEETR pc2
P02649
log10 peptide intensity
538:561 522:550 131:137
19:45432505:A:T_T
p = 0.0013, beta = 0.139, N = 1191
LGPLVEQGR pc2
P02649
log10 peptide intensity
452:561 444:550 112:137
19:45432505:A:T_T
p = 0.21, beta = 0.0596, N = 1008
GEVQAMLGQSTEELR pc2
P02649
log10 peptide intensity
555:561 548:550 135:137
19:45432505:A:T_T
p = 0.057, beta = 0.081, N = 1238
VQAAVGTSAAPVPSDNH pc2
P02649
log10 peptide intensity
560:561 549:550 136:137
19:45432505:A:T_T
p = 0.59, beta = 0.0227, N = 1245
LGADMEDVCGR pc2
P02649
log10 peptide intensity
539:561 536:550 135:137
19:45432505:A:T_T
p = 0.051, beta = 0.0837, N = 1210
DADDLQK pc2
P02649
log10 peptide intensity
228:561 233:550 52:137
19:45432505:A:T_T
p = 0.19, beta = 0.0884, N = 513
GEVQAMLGQSTEELRVR pc3
P02649
log10 peptide intensity
560:561 548:550 136:137
19:45432505:A:T_T
p = 0.012, beta = 0.106, N = 1244
VEQAVETEPEPELR pc2
P02649
log10 peptide intensity
539:561 519:550 123:137
19:45432505:A:T_T
p = 0.12, beta = 0.0679, N = 1181
APOE : NP5
P02649
log10 protein intensity
165:165 134:134 26:26
19:45432505:A:T_T
p = 0.54, beta = 0.0524, N = 325
SWFEPLVEDMQR pc2
P02649
log10 peptide intensity
165:165 134:134 26:26
19:45432505:A:T_T
p = 0.88, beta = 0.0129, N = 325
LQAEAFQAR pc2
P02649
log10 peptide intensity
165:165 134:134 26:26
19:45432505:A:T_T
p = 0.18, beta = 0.115, N = 325
AATVGSLAGQPLQER pc2
P02649
log10 peptide intensity
165:165 134:134 26:26
19:45432505:A:T_T
p = 0.54, beta = 0.0524, N = 325
LAVYQAGAR pc2
P02649
log10 peptide intensity
165:165 134:134 26:26
19:45432505:A:T_T
p = 0.11, beta = 0.137, N = 325
SELEEQLTPVAEETR pc2
P02649
log10 peptide intensity
165:165 134:134 26:26
19:45432505:A:T_T
p = 0.65, beta = 0.0387, N = 325
LEEQAQQIR pc2
P02649
log10 peptide intensity
165:165 134:134 26:26
19:45432505:A:T_T
p = 0.17, beta = 0.117, N = 325
ALMDETMK pc2
P02649
log10 peptide intensity
165:165 134:134 26:26
19:45432505:A:T_T
p = 0.54, beta = 0.0525, N = 325
VQAAVGTSAAPVPSDNH pc2
P02649
log10 peptide intensity
165:165 134:134 26:26
19:45432505:A:T_T
p = 0.46, beta = 0.064, N = 325
LGADMEDVCGR pc2
P02649
log10 peptide intensity
164:165 134:134 26:26
19:45432505:A:T_T
p = 0.16, beta = 0.12, N = 324
LGPLVEQGR pc2
P02649
log10 peptide intensity
165:165 134:134 26:26
19:45432505:A:T_T
p = 0.51, beta = 0.0571, N = 325
DRLDEVK pc2
P02649
log10 peptide intensity
159:165 129:134 25:26
19:45432505:A:T_T
p = 0.37, beta = 0.0779, N = 313
GEVQAMLGQSTEELR pc2
P02649
log10 peptide intensity
165:165 134:134 26:26
19:45432505:A:T_T
p = 0.58, beta = 0.0481, N = 325
VEQAVETEPEPELR pc2
P02649
log10 peptide intensity
164:165 134:134 26:26
19:45432505:A:T_T
p = 0.19, beta = 0.113, N = 324
ELQAAQAR pc2
P02649
log10 peptide intensity
160:165 134:134 26:26
19:45432505:A:T_T
p = 0.19, beta = 0.115, N = 320
AQAWGER pc2
P02649
log10 peptide intensity
165:165 134:134 26:26
19:45432505:A:T_T
p = 0.93, beta = 0.00804, N = 325
GEVQAMLGQSTEELRVR pc3
P02649
log10 peptide intensity
165:165 134:134 26:26
19:45432505:A:T_T
p = 0.039, beta = 0.177, N = 325
MEEMGSR pc2
P02649
log10 peptide intensity
165:165 134:134 26:26
19:45432505:A:T_T
p = 0.14, beta = 0.128, N = 325
LLRDADDLQK pc2
P02649
log10 peptide intensity
141:165 119:134 22:26
19:45432505:A:T_T
p = 0.45, beta = 0.0705, N = 282
DADDLQK pc2
P02649
log10 peptide intensity
164:165 133:134 25:26
19:45432505:A:T_T
p = 0.035, beta = 0.183, N = 322
Assay Target: APOE
UniProt: P02649
rsID: rs9636134
Target SNP: 19:44929231:CAAA
Proxy SNP: 19:45432505:A:T
deCODE log10(p): 188.4
deCODE BETA: 0.25
deCODE MSPA: 0.026
1202:1244:1160:1242:1243:124

129. CHGA (P10645)
deCODE MSPA=0

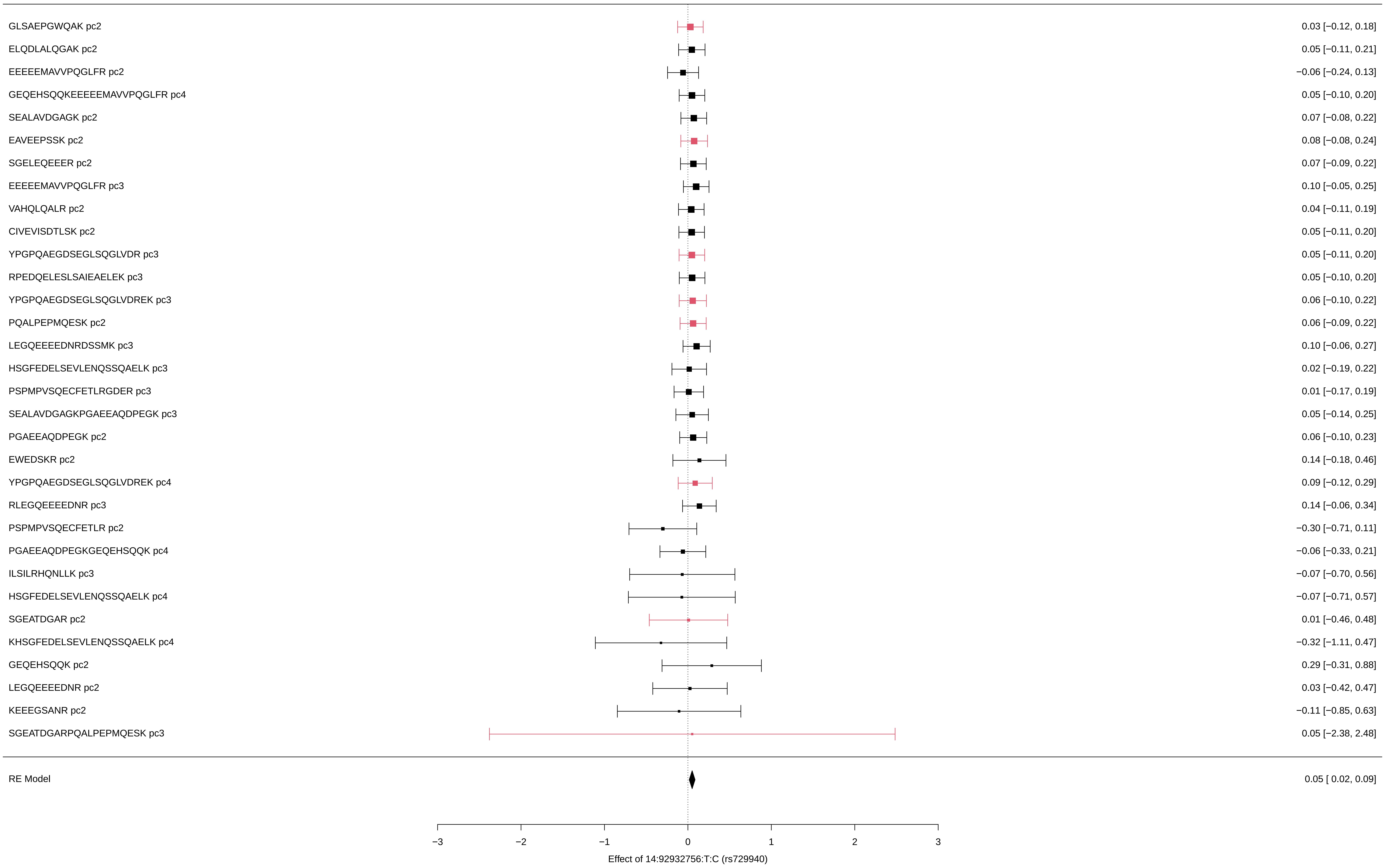

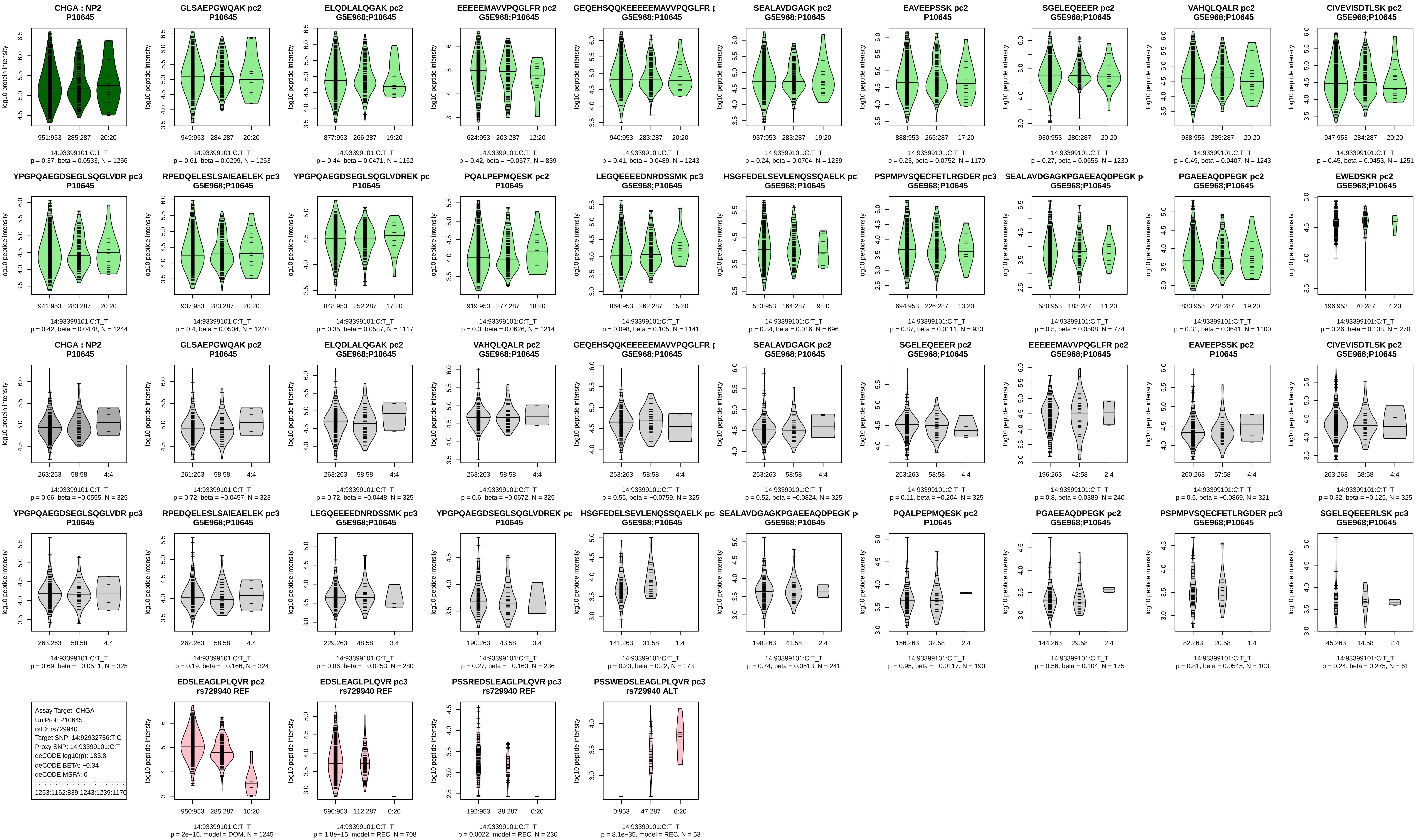
CHGA : NP2
P10645
log10 protein intensity
951:953 285:287 20:20
14:93399101:C:T_T
p = 0.37, beta = 0.0533, N = 1256
GLSAEPGWQAK pc2
P10645
log10 peptide intensity
949:953 284:287 20:20
14:93399101:C:T_T
p = 0.61, beta = 0.0299, N = 1253
ELQDLALQGAK pc2
G5E968;P10645
877:953 266:287 19:20
14:93399101:C:T_T
p = 0.44, beta = 0.0471, N = 1162
EEEEEMAVVPQGLFR pc2
G5E968;P10645
624:953 203:287 12:20
14:93399101:C:T_T
p = 0.42, beta = −0.0577, N = 839
GEQEHSQQKEEEEEMAVVPQGLFR p
G5E968;P10645
940:953 283:287 20:20
14:93399101:C:T_T
p = 0.41, beta = 0.0489, N = 1243
SEALAVDGAGK pc2
G5E968;P10645
937:953 283:287 19:20
14:93399101:C:T_T
p = 0.24, beta = 0.0704, N = 1239
EAVEEPSSK pc2
P10645
888:953 265:287 17:20
14:93399101:C:T_T
p = 0.23, beta = 0.0752, N = 1170
SGELEQEEER pc2
G5E968;P10645
930:953 280:287 20:20
14:93399101:C:T_T
p = 0.27, beta = 0.0655, N = 1230
VAHQLQALR pc2
G5E968;P10645
938:953 285:287 20:20
14:93399101:C:T_T
p = 0.49, beta = 0.0407, N = 1243
CIVEVISDTLSK pc2
G5E968;P10645
947:953 284:287 20:20
14:93399101:C:T_T
p = 0.45, beta = 0.0453, N = 1251
YPGPQAEGDSEGLSQGLVDR pc3
P10645
941:953 283:287 20:20
14:93399101:C:T_T
p = 0.42, beta = 0.0478, N = 1244
RPEDQELESLSAIEAELEK pc3
G5E968;P10645
937:953 283:287 20:20
14:93399101:C:T_T
p = 0.4, beta = 0.0504, N = 1240
YPGPQAEGDSEGLSQGLVDREK pc
P10645
848:953 252:287 17:20
14:93399101:C:T_T
p = 0.35, beta = 0.0587, N = 1117
PQALPEPMQESK pc2
P10645
919:953 277:287 18:20
14:93399101:C:T_T
p = 0.3, beta = 0.0626, N = 1214
LEGQEEEEDNRDSSMK pc3
G5E968;P10645
864:953 262:287 15:20
14:93399101:C:T_T
p = 0.098, beta = 0.105, N = 1141
HSGFEDELSEVLENQSSQAELK pc
G5E968;P10645
523:953 164:287 9:20
14:93399101:C:T_T
p = 0.84, beta = 0.016, N = 696
PSPMPVSQECFETLRGDER pc3
G5E968;P10645
694:953 226:287 13:20
14:93399101:C:T_T
p = 0.87, beta = 0.0111, N = 933
SEALAVDGAGKPGAEEAQDPEGK p
G5E968;P10645
580:953 183:287 11:20
14:93399101:C:T_T
p = 0.5, beta = 0.0508, N = 774
PGAEEAQDPEGK pc2
G5E968;P10645
833:953 248:287 19:20
14:93399101:C:T_T
p = 0.31, beta = 0.0641, N = 1100
EWEDSKR pc2
G5E968;P10645
196:953 70:287 4:20
14:93399101:C:T_T
p = 0.26, beta = 0.138, N = 270
CHGA : NP2
P10645
263:263 58:58 4:4
14:93399101:C:T_T
p = 0.66, beta = −0.0555, N = 325
GLSAEPGWQAK pc2
P10645
261:263 58:58 4:4
14:93399101:C:T_T
p = 0.72, beta = −0.0457, N = 323
ELQDLALQGAK pc2
G5E968;P10645
263:263 58:58 4:4
14:93399101:C:T_T
p = 0.72, beta = −0.0448, N = 325
VAHQLQALR pc2
G5E968;P10645
263:263 58:58 4:4
14:93399101:C:T_T
p = 0.6, beta = −0.0672, N = 325
GEQEHSQQKEEEEEMAVVPQGLFR p
G5E968;P10645
263:263 58:58 4:4
14:93399101:C:T_T
p = 0.55, beta = −0.0759, N = 325
SEALAVDGAGK pc2
G5E968;P10645
263:263 58:58 4:4
14:93399101:C:T_T
p = 0.52, beta = −0.0824, N = 325
SGELEQEEER pc2
G5E968;P10645
263:263 58:58 4:4
14:93399101:C:T_T
p = 0.11, beta = −0.204, N = 325
EEEEEMAVVPQGLFR pc2
G5E968;P10645
196:263 42:58 2:4
14:93399101:C:T_T
p = 0.8, beta = 0.0389, N = 240
EAVEEPSSK pc2
P10645
260:263 57:58 4:4
14:93399101:C:T_T
p = 0.5, beta = −0.0869, N = 321
CIVEVISDTLSK pc2
G5E968;P10645
263:263 58:58 4:4
14:93399101:C:T_T
p = 0.32, beta = −0.125, N = 325
YPGPQAEGDSEGLSQGLVDR pc3
P10645
263:263 58:58 4:4
14:93399101:C:T_T
p = 0.69, beta = −0.0511, N = 325
RPEDQELESLSAIEAELEK pc3
G5E968;P10645
262:263 58:58 4:4
14:93399101:C:T_T
p = 0.19, beta = −0.166, N = 324
LEGQEEEEDNRDSSMK pc3
G5E968;P10645
229:263 48:58 3:4
14:93399101:C:T_T
p = 0.86, beta = −0.0253, N = 280
YPGPQAEGDSEGLSQGLVDREK pc
P10645
190:263 43:58 3:4
14:93399101:C:T_T
p = 0.27, beta = −0.163, N = 236
HSGFEDELSEVLENQSSQAELK pc
G5E968;P10645
141:263 31:58 1:4
14:93399101:C:T_T
p = 0.23, beta = 0.22, N = 173
SEALAVDGAGKPGAEEAQDPEGK p
G5E968;P10645
198:263 41:58 2:4
14:93399101:C:T_T
p = 0.74, beta = 0.0513, N = 241
PQALPEPMQESK pc2
P10645
156:263 32:58 2:4
14:93399101:C:T_T
p = 0.95, beta = −0.0117, N = 190
PGAEEAQDPEGK pc2
G5E968;P10645
144:263 29:58 2:4
14:93399101:C:T_T
p = 0.56, beta = 0.104, N = 175
PSPMPVSQECFETLRGDER pc3
G5E968;P10645
82:263 20:58 1:4
14:93399101:C:T_T
p = 0.81, beta = 0.0545, N = 103
SGELEQEEERLSK pc3
G5E968;P10645
45:263 14:58 2:4
14:93399101:C:T_T
p = 0.24, beta = 0.275, N = 61
Assay Target: CHGA
UniProt: P10645
rsID: rs729940
Target SNP: 14:92932756:T:C
Proxy SNP: 14:93399101:C:T
deCODE log10(p): 183.8
deCODE BETA: −0.34
deCODE MSPA: 0
1253:1162:839:1243:1239:1170
EDSLEAGLPLQVR pc2
rs729940 REF
950:953 285:287 10:20
14:93399101:C:T_T
p = 2e−16, model = DOM, N = 1245
EDSLEAGLPLQVR pc3
rs729940 REF
596:953 112:287 0:20
14:93399101:C:T_T
p = 1.8e−15, model = REC, N = 708
PSSREDSLEAGLPLQVR pc3
rs729940 REF
192:953 38:287 0:20
14:93399101:C:T_T
p = 0.0022, model = REC, N = 230
PSSWEDSLEAGLPLQVR pc3
rs729940 ALT
0:953 47:287 6:20
14:93399101:C:T_T
p = 8.1e−35, model = REC, N = 53

130. ALAD (P13716)
deCODE MSPA=0

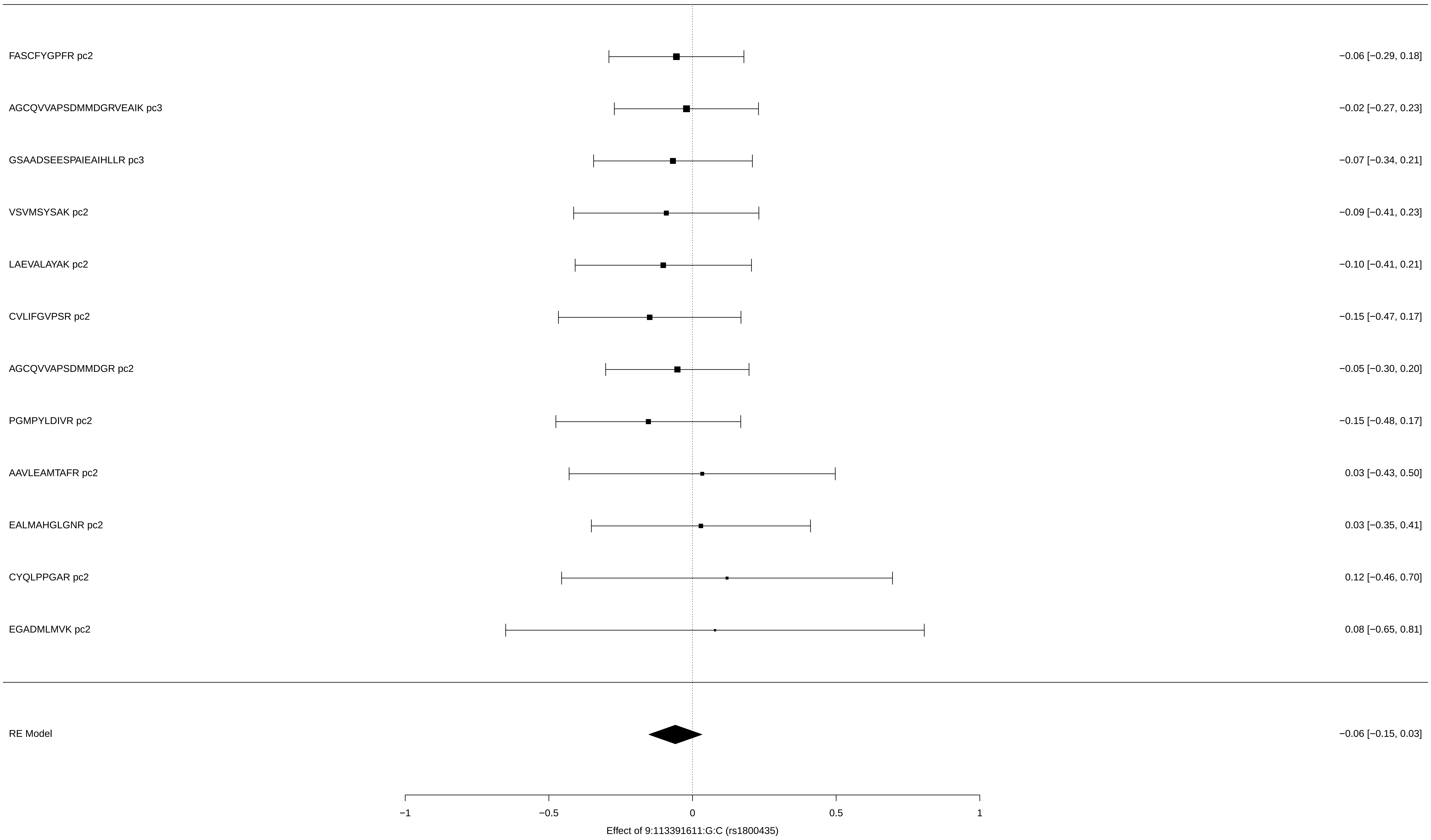

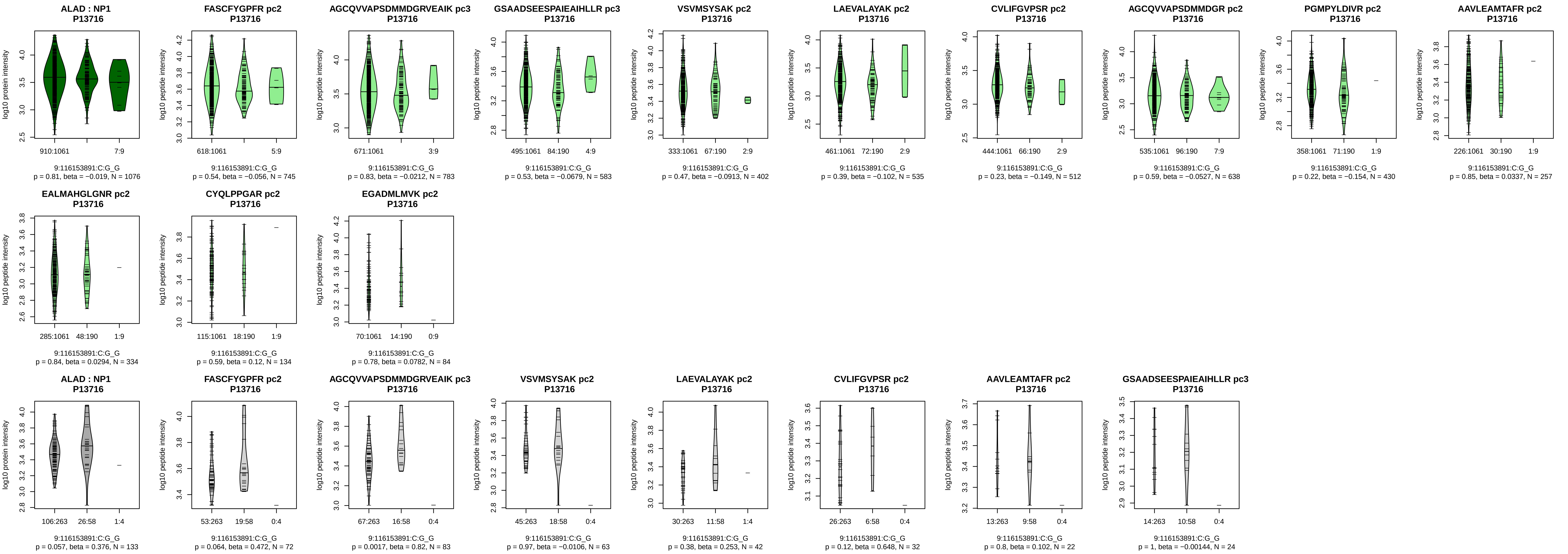

Assay Target: ALAD
UniProt: P13716
rsID: rs1800435
Target SNP: 9:113391611:G:C
Proxy SNP: 9:116153891:C:G
deCODE log10(p): 178.9
deCODE BETA: −0.42
deCODE MSPA: 0
745:783:583:402:535:512:638:4

131. MFGE8 (Q08431)
deCODE MSPA=0.195

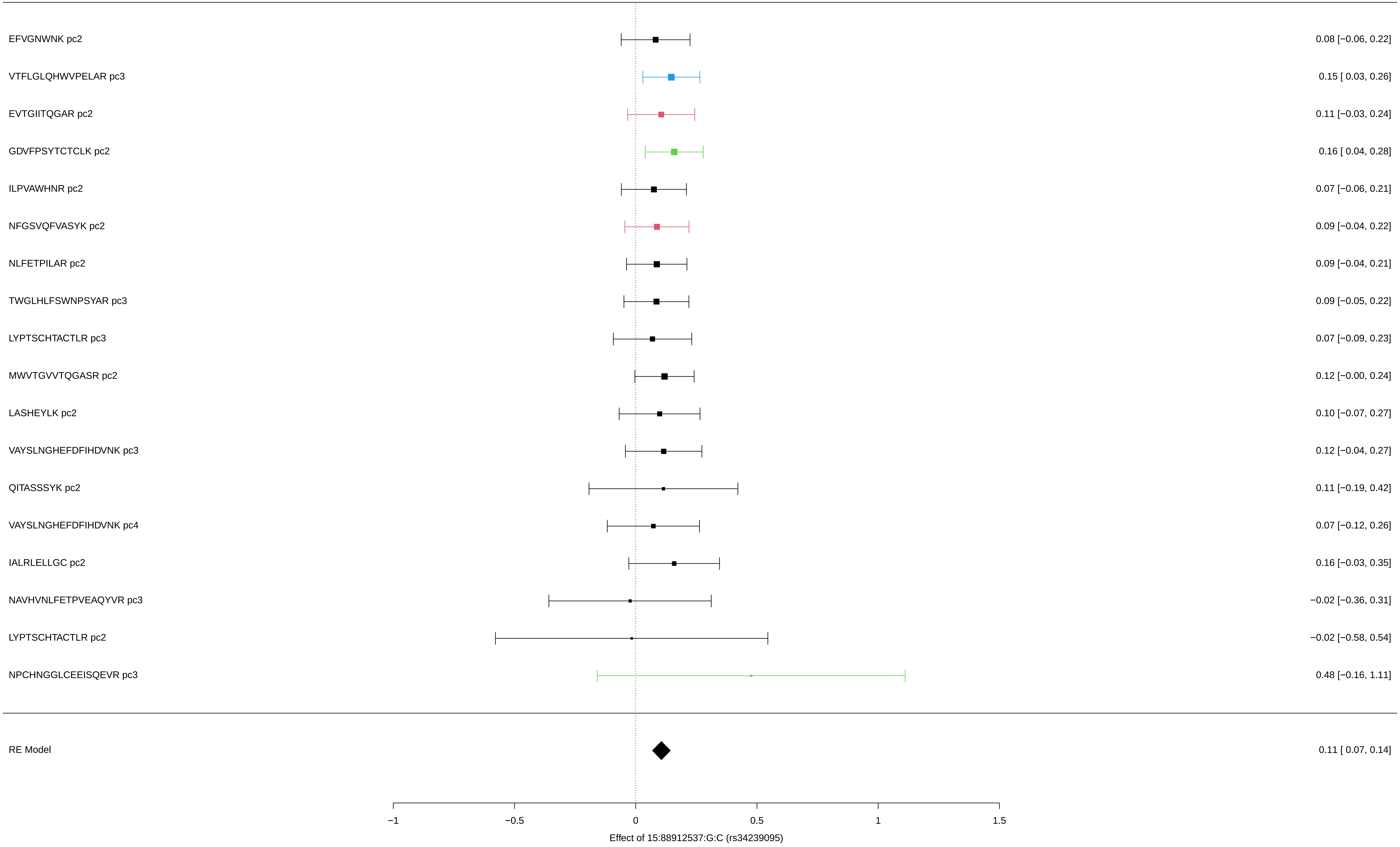

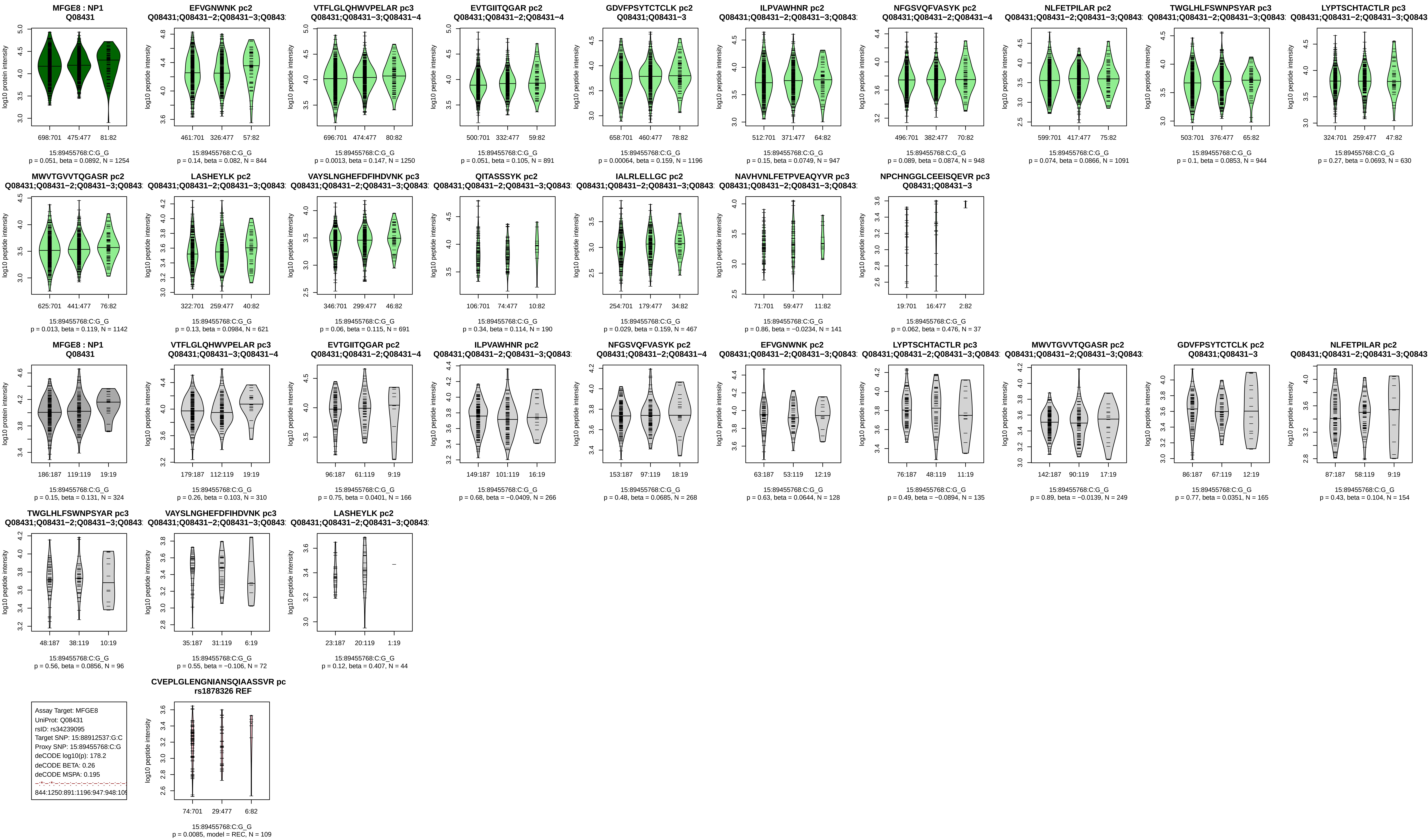

MFGE8 : NP1
Q08431
698:701 475:477 81:82
15:89455768:C:G_G
p = 0.051, beta = 0.0892, N = 1254
EFVGNWNK pc2
Q08431;Q08431−2;Q08431−3;Q0843
461:701 326:477 57:82
15:89455768:C:G_G
p = 0.14, beta = 0.082, N = 844
VTFLGLQHWVPELAR pc3
Q08431;Q08431−3;Q08431−4
696:701 474:477 80:82
15:89455768:C:G_G
p = 0.0013, beta = 0.147, N = 1250
EVTGIITQGAR pc2
Q08431;Q08431−2;Q08431−4
500:701 332:477 59:82
15:89455768:C:G_G
p = 0.051, beta = 0.105, N = 891
GDVFPSYTCTCLK pc2
Q08431;Q08431−3
658:701 460:477 78:82
15:89455768:C:G_G
p = 0.00064, beta = 0.159, N = 1196
ILPVAWHNR pc2
Q08431;Q08431−2;Q08431−3;Q0843
512:701 371:477 64:82
15:89455768:C:G_G
p = 0.15, beta = 0.0749, N = 947
NFGSVQFVASYK pc2
Q08431;Q08431−2;Q08431−4
496:701 382:477 70:82
15:89455768:C:G_G
p = 0.089, beta = 0.0874, N = 948
NLFETPILAR pc2
Q08431;Q08431−2;Q08431−3;Q0843
599:701 417:477 75:82
15:89455768:C:G_G
p = 0.074, beta = 0.0866, N = 1091
TWGLHLFSWNPSYAR pc3
Q08431;Q08431−2;Q08431−3;Q0843
503:701 376:477 65:82
15:89455768:C:G_G
p = 0.1, beta = 0.0853, N = 944
LYPTSCHTACTLR pc3
Q08431;Q08431−2;Q08431−3;Q0843
324:701 259:477 47:82
15:89455768:C:G_G
p = 0.27, beta = 0.0693, N = 630
MWVTGVVTQGASR pc2
Q08431;Q08431−2;Q08431−3;Q0843
625:701 441:477 76:82
15:89455768:C:G_G
p = 0.013, beta = 0.119, N = 1142
LASHEYLK pc2
Q08431;Q08431−2;Q08431−3;Q0843
322:701 259:477 40:82
15:89455768:C:G_G
p = 0.13, beta = 0.0984, N = 621
VAYSLNGHEFDFIHDVNK pc3
Q08431;Q08431−2;Q08431−3;Q0843
346:701 299:477 46:82
15:89455768:C:G_G
p = 0.06, beta = 0.115, N = 691
QITASSSYK pc2
Q08431;Q08431−2;Q08431−3;Q0843
106:701 74:477 10:82
15:89455768:C:G_G
p = 0.34, beta = 0.114, N = 190
IALRLELLGC pc2
Q08431;Q08431−2;Q08431−3;Q0843
254:701 179:477 34:82
15:89455768:C:G_G
p = 0.029, beta = 0.159, N = 467
NAVHVNLFETPVEAQYVR pc3
Q08431;Q08431−2;Q08431−3;Q0843
71:701 59:477 11:82
15:89455768:C:G_G
p = 0.86, beta = −0.0234, N = 141
NPCHNGGLCEEISQEVR pc3
Q08431;Q08431−3
19:701 16:477 2:82
15:89455768:C:G_G
p = 0.062, beta = 0.476, N = 37
MFGE8 : NP1
Q08431
186:187 119:119 19:19
15:89455768:C:G_G
p = 0.15, beta = 0.131, N = 324
VTFLGLQHWVPELAR pc3
Q08431;Q08431−3;Q08431−4
179:187 112:119 19:19
15:89455768:C:G_G
p = 0.26, beta = 0.103, N = 310
EVTGIITQGAR pc2
Q08431;Q08431−2;Q08431−4
96:187 61:119 9:19
15:89455768:C:G_G
p = 0.75, beta = 0.0401, N = 166
ILPVAWHNR pc2
Q08431;Q08431−2;Q08431−3;Q0843
149:187 101:119 16:19
15:89455768:C:G_G
p = 0.68, beta = −0.0409, N = 266
NFGSVQFVASYK pc2
Q08431;Q08431−2;Q08431−4
153:187 97:119 18:19
15:89455768:C:G_G
p = 0.48, beta = 0.0685, N = 268
EFVGNWNK pc2
Q08431;Q08431−2;Q08431−3;Q0843
63:187 53:119 12:19
15:89455768:C:G_G
p = 0.63, beta = 0.0644, N = 128
LYPTSCHTACTLR pc3
Q08431;Q08431−2;Q08431−3;Q0843
76:187 48:119 11:19
15:89455768:C:G_G
p = 0.49, beta = −0.0894, N = 135
MWVTGVVTQGASR pc2
Q08431;Q08431−2;Q08431−3;Q0843
142:187 90:119 17:19
15:89455768:C:G_G
p = 0.89, beta = −0.0139, N = 249
GDVFPSYTCTCLK pc2
Q08431;Q08431−3
86:187 67:119 12:19
15:89455768:C:G_G
p = 0.77, beta = 0.0351, N = 165
NLFETPILAR pc2
Q08431;Q08431−2;Q08431−3;Q0843
87:187 58:119 9:19
15:89455768:C:G_G
p = 0.43, beta = 0.104, N = 154
TWGLHLFSWNPSYAR pc3
Q08431;Q08431−2;Q08431−3;Q0843
48:187 38:119 10:19
15:89455768:C:G_G
p = 0.56, beta = 0.0856, N = 96
VAYSLNGHEFDFIHDVNK pc3
Q08431;Q08431−2;Q08431−3;Q0843
35:187 31:119 6:19
15:89455768:C:G_G
p = 0.55, beta = −0.106, N = 72
LASHEYLK pc2
Q08431;Q08431−2;Q08431−3;Q0843
23:187 20:119 1:19
15:89455768:C:G_G
p = 0.12, beta = 0.407, N = 44
Assay Target: MFGE8
UniProt: Q08431
rsID: rs34239095
Target SNP: 15:88912537:G:C
Proxy SNP: 15:89455768:C:G
deCODE log10(p): 178.2
deCODE BETA: 0.26
deCODE MSPA: 0.195
844:1250:891:1196:947:948:10
CVEPLGLENGNIANSQIAASSVR pc
rs1878326 REF
74:701 29:477 6:82
15:89455768:C:G_G
p = 0.0085, model = REC, N = 109

132. AGRN (O00468)
deCODE MSPA=0.615

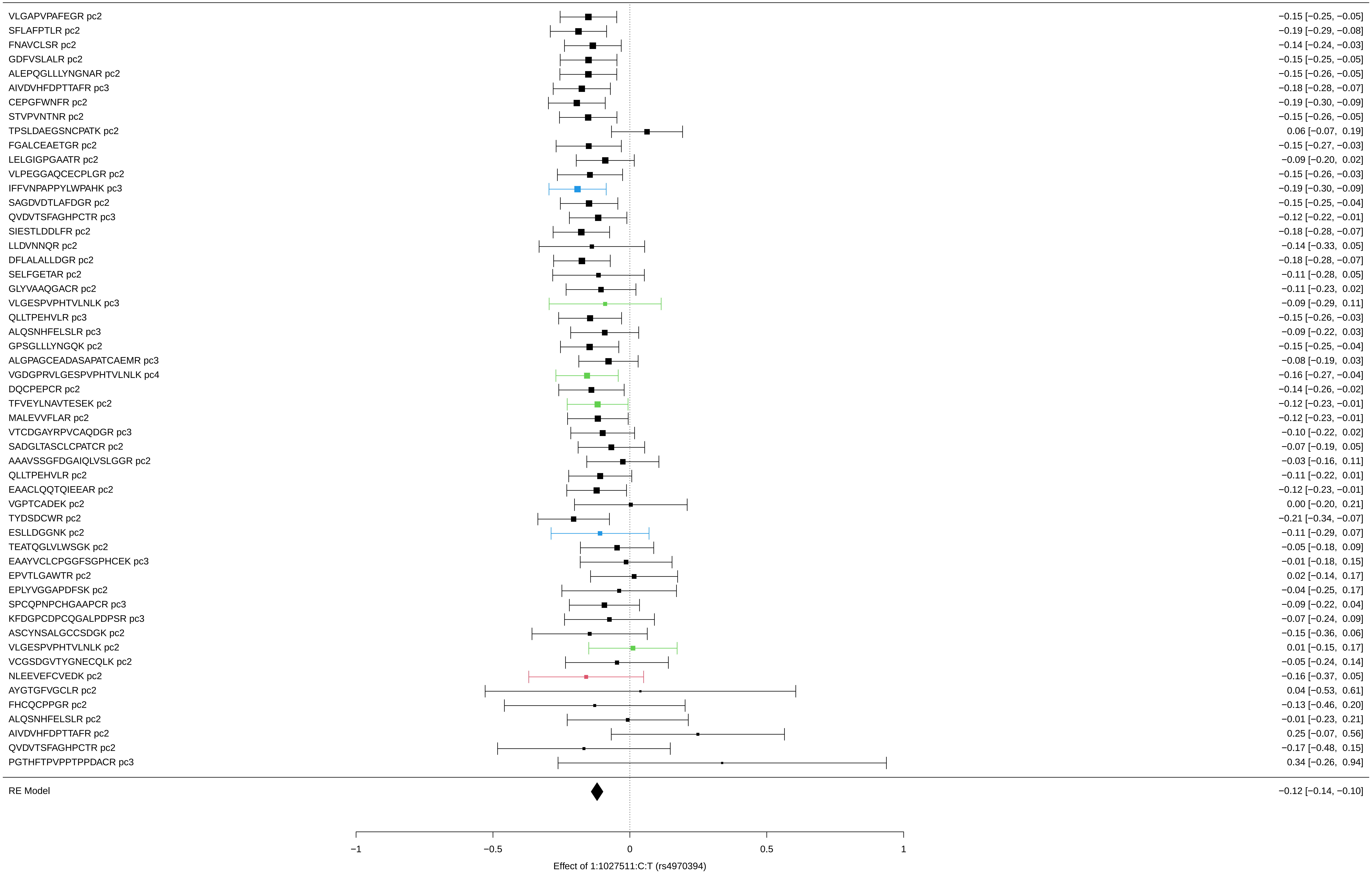

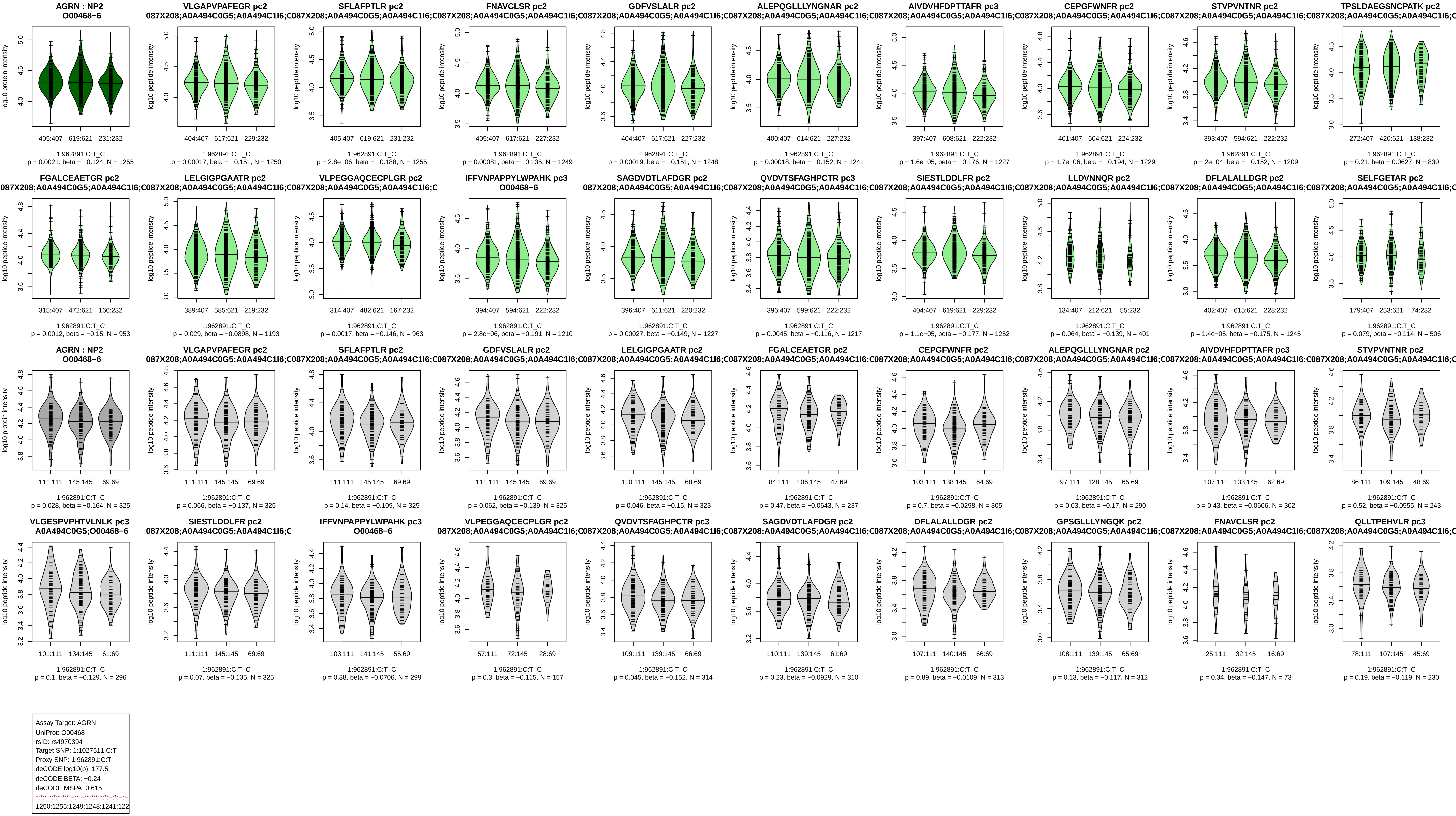
AGRN : NP2
O00468−6
log10 protein intensity
405:407 619:621 231:232
1:962891:C:T_C
p = 0.0021, beta = −0.124, N = 1255
VLGAPVPAFEGR pc2
log10 peptide intensity
404:407 617:621 229:232
1:962891:C:T_C
p = 0.00017, beta = −0.151, N = 1250
SFLAFPTLR pc2
405:407 619:621 231:232
1:962891:C:T_C
p = 2.8e−06, beta = −0.188, N = 1255
FNAVCLSR pc2
405:407 617:621 227:232
1:962891:C:T_C
p = 0.00081, beta = −0.135, N = 1249
GDFVSLALR pc2
404:407 617:621 227:232
1:962891:C:T_C
p = 0.00019, beta = −0.151, N = 1248
ALEPQGLLLYNGNAR pc2
400:407 614:621 227:232
1:962891:C:T_C
p = 0.00018, beta = −0.152, N = 1241
AIVDVHFDPTTAFR pc3
397:407 608:621 222:232
1:962891:C:T_C
p = 1.6e−05, beta = −0.176, N = 1227
CEPGFWNFR pc2
401:407 604:621 224:232
1:962891:C:T_C
p = 1.7e−06, beta = −0.194, N = 1229
STVPVNTNR pc2
393:407 594:621 222:232
1:962891:C:T_C
p = 2e−04, beta = −0.152, N = 1209
TPSLDAEGSNCPATK pc2
272:407 420:621 138:232
1:962891:C:T_C
p = 0.21, beta = 0.0627, N = 830
087X208;A0A494C0G5;A0A494C1I6;C087X208;A0A494C0G5;A0A494C1I6;C087X208;A0A494C0G5;A0A494C1I6;C087X208;A0A494C0G5;A0A494C1I6;C087X208;A0A494C0G5;A0A494C1I6;C087X208;A0A494C0G5;A0A494C1I6;C087X208;A0A494C0G5;A0A494C1I6;C087X208;A0A494C0G5;A0A494C1I6;C087X208;A0A494C0G5;A0A494C1I6;C
FGALCEAETGR pc2
315:407 472:621 166:232
1:962891:C:T_C
p = 0.0012, beta = −0.15, N = 953
LELGIGPGAATR pc2
389:407 585:621 219:232
1:962891:C:T_C
p = 0.029, beta = −0.0898, N = 1193
VLPEGGAQCECPLGR pc2
314:407 482:621 167:232
1:962891:C:T_C
p = 0.0017, beta = −0.146, N = 963
IFFVNPAPPYLWPAHK pc3
O00468−6
394:407 594:621 222:232
1:962891:C:T_C
p = 2.8e−06, beta = −0.191, N = 1210
SAGDVDTLAFDGR pc2
396:407 611:621 220:232
1:962891:C:T_C
p = 0.00027, beta = −0.149, N = 1227
QVDVTSFAGHPCTR pc3
396:407 599:621 222:232
1:962891:C:T_C
p = 0.0045, beta = −0.116, N = 1217
SIESTLDDLFR pc2
404:407 619:621 229:232
1:962891:C:T_C
p = 1.1e−05, beta = −0.175, N = 1252
LLDVNNQR pc2
134:407 212:621 55:232
1:962891:C:T_C
p = 0.064, beta = −0.139, N = 401
DFLALALLDGR pc2
402:407 615:621 228:232
1:962891:C:T_C
p = 1.4e−05, beta = −0.175, N = 1245
SELFGETAR pc2
179:407 253:621 74:232
1:962891:C:T_C
p = 0.079, beta = −0.114, N = 506
AGRN : NP2
O00468−6
111:111 145:145 69:69
1:962891:C:T_C
p = 0.028, beta = −0.164, N = 325
VLGAPVPAFEGR pc2
111:111 145:145 69:69
1:962891:C:T_C
p = 0.066, beta = −0.137, N = 325
SFLAFPTLR pc2
111:111 145:145 69:69
1:962891:C:T_C
p = 0.14, beta = −0.109, N = 325
GDFVSLALR pc2
111:111 145:145 69:69
1:962891:C:T_C
p = 0.062, beta = −0.139, N = 325
LELGIGPGAATR pc2
110:111 145:145 68:69
1:962891:C:T_C
p = 0.046, beta = −0.15, N = 323
FGALCEAETGR pc2
84:111 106:145 47:69
1:962891:C:T_C
p = 0.47, beta = −0.0643, N = 237
CEPGFWNFR pc2
103:111 138:145 64:69
1:962891:C:T_C
p = 0.7, beta = −0.0298, N = 305
ALEPQGLLLYNGNAR pc2
97:111 128:145 65:69
1:962891:C:T_C
p = 0.03, beta = −0.17, N = 290
AIVDVHFDPTTAFR pc3
107:111 133:145 62:69
1:962891:C:T_C
p = 0.43, beta = −0.0606, N = 302
STVPVNTNR pc2
86:111 109:145 48:69
1:962891:C:T_C
p = 0.52, beta = −0.0555, N = 243
VLGESPVPHTVLNLK pc3
A0A494C0G5;O00468−6
101:111 134:145 61:69
1:962891:C:T_C
p = 0.1, beta = −0.129, N = 296
SIESTLDDLFR pc2
111:111 145:145 69:69
1:962891:C:T_C
p = 0.07, beta = −0.135, N = 325
IFFVNPAPPYLWPAHK pc3
O00468−6
103:111 141:145 55:69
1:962891:C:T_C
p = 0.38, beta = −0.0706, N = 299
VLPEGGAQCECPLGR pc2
57:111 72:145 28:69
1:962891:C:T_C
p = 0.3, beta = −0.115, N = 157
QVDVTSFAGHPCTR pc3
109:111 139:145 66:69
1:962891:C:T_C
p = 0.045, beta = −0.152, N = 314
SAGDVDTLAFDGR pc2
110:111 139:145 61:69
1:962891:C:T_C
p = 0.23, beta = −0.0929, N = 310
DFLALALLDGR pc2
107:111 140:145 66:69
1:962891:C:T_C
p = 0.89, beta = −0.0109, N = 313
GPSGLLLYNGQK pc2
108:111 139:145 65:69
1:962891:C:T_C
p = 0.13, beta = −0.117, N = 312
FNAVCLSR pc2
25:111 32:145 16:69
1:962891:C:T_C
p = 0.34, beta = −0.147, N = 73
QLLTPEHVLR pc3
78:111 107:145 45:69
1:962891:C:T_C
p = 0.19, beta = −0.119, N = 230
Assay Target: AGRN
UniProt: O00468
rsID: rs4970394
Target SNP: 1:1027511:C:T
Proxy SNP: 1:962891:C:T
deCODE log10(p): 177.5
deCODE BETA: −0.24
deCODE MSPA: 0.615
1250:1255:1249:1248:1241:122

133. COL6A2 (P12110)
deCODE MSPA=0

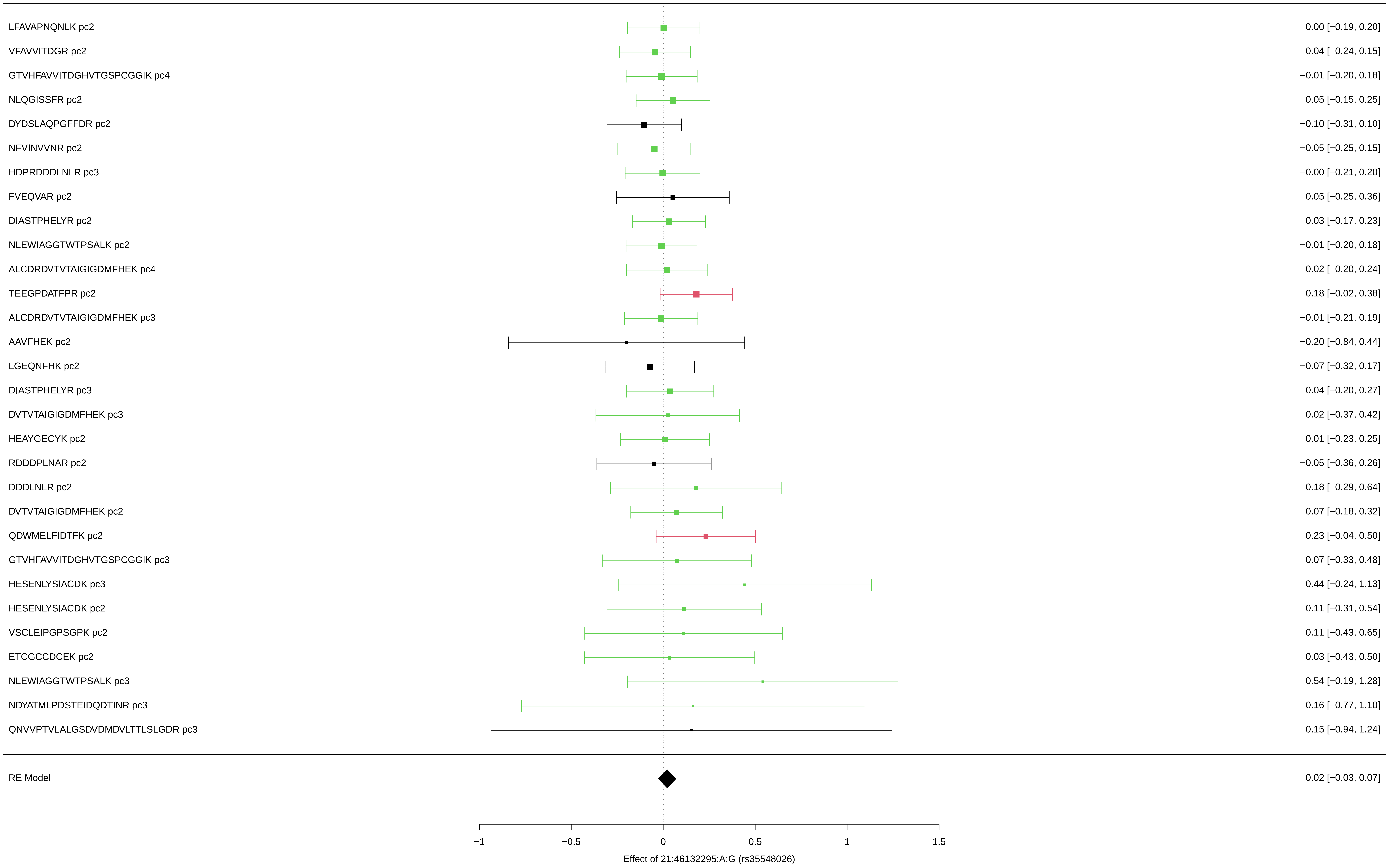

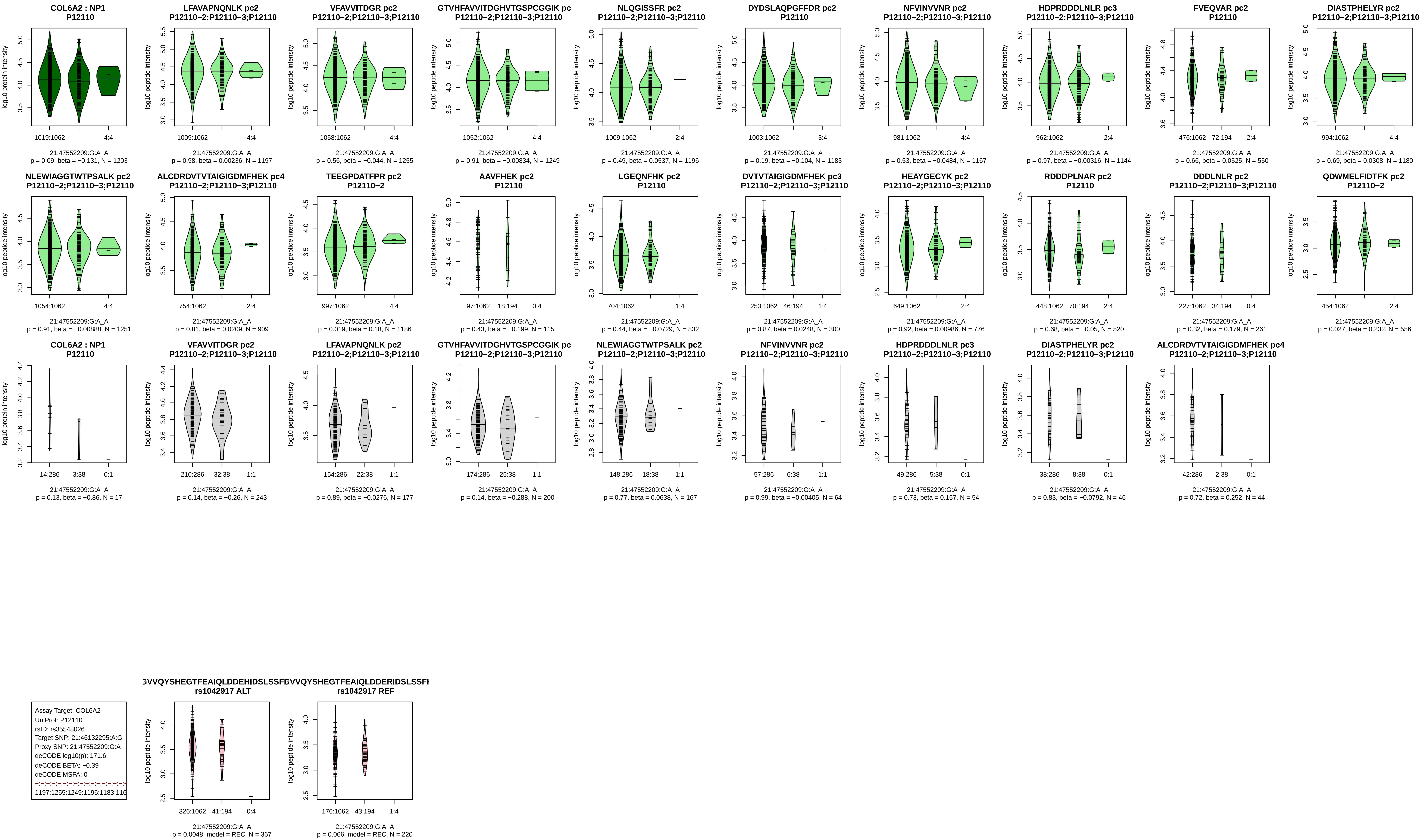
COL6A2 : NP1
P12110
log10 protein intensity
1019:1062
4:4
21:47552209:G:A_A
p = 0.09, beta = −0.131, N = 1203
LFAVAPNQNLK pc2
P12110−2;P12110−3;P12110
log10 peptide intensity
1009:1062
4:4
21:47552209:G:A_A
p = 0.98, beta = 0.00236, N = 1197
VFAVVITDGR pc2
P12110−2;P12110−3;P12110
1058:1062
4:4
21:47552209:G:A_A
p = 0.56, beta = −0.044, N = 1255
GTVHFAVVITDGHVTGSPCGGIK pc
P12110−2;P12110−3;P12110
1052:1062
4:4
21:47552209:G:A_A
p = 0.91, beta = −0.00834, N = 1249
NLQGISSFR pc2
P12110−2;P12110−3;P12110
1009:1062
2:4
21:47552209:G:A_A
p = 0.49, beta = 0.0537, N = 1196
DYDSLAQPGFFDR pc2
P12110
1003:1062
3:4
21:47552209:G:A_A
p = 0.19, beta = −0.104, N = 1183
NFVINVVNR pc2
P12110−2;P12110−3;P12110
981:1062
4:4
21:47552209:G:A_A
p = 0.53, beta = −0.0484, N = 1167
HDPRDDDLNLR pc3
P12110−2;P12110−3;P12110
962:1062
2:4
21:47552209:G:A_A
p = 0.97, beta = −0.00316, N = 1144
FVEQVAR pc2
P12110
476:1062
72:194
2:4
21:47552209:G:A_A
p = 0.66, beta = 0.0525, N = 550
DIASTPHELYR pc2
P12110−2;P12110−3;P12110
994:1062
4:4
21:47552209:G:A_A
p = 0.69, beta = 0.0308, N = 1180
NLEWIAGGTWTPSALK pc2
P12110−2;P12110−3;P12110
1054:1062
4:4
21:47552209:G:A_A
p = 0.91, beta = −0.00888, N = 1251
ALCDRDVTVTAIGIGDMFHEK pc4
P12110−2;P12110−3;P12110
754:1062
2:4
21:47552209:G:A_A
p = 0.81, beta = 0.0209, N = 909
TEEGPDATFPR pc2
P12110−2
997:1062
4:4
21:47552209:G:A_A
p = 0.019, beta = 0.18, N = 1186
AAVFHEK pc2
P12110
97:1062
18:194
0:4
21:47552209:G:A_A
p = 0.43, beta = −0.199, N = 115
LGEQNFHK pc2
P12110
704:1062
1:4
21:47552209:G:A_A
p = 0.44, beta = −0.0729, N = 832
DVTVTAIGIGDMFHEK pc3
P12110−2;P12110−3;P12110
253:1062
46:194
1:4
21:47552209:G:A_A
p = 0.87, beta = 0.0248, N = 300
HEAYGECYK pc2
P12110−2;P12110−3;P12110
649:1062
2:4
21:47552209:G:A_A
p = 0.92, beta = 0.00986, N = 776
RDDDPLNAR pc2
P12110
448:1062
70:194
2:4
21:47552209:G:A_A
p = 0.68, beta = −0.05, N = 520
DDDLNLR pc2
P12110−2;P12110−3;P12110
227:1062
34:194
0:4
21:47552209:G:A_A
p = 0.32, beta = 0.179, N = 261
QDWMELFIDTFK pc2
P12110−2
454:1062
2:4
21:47552209:G:A_A
p = 0.027, beta = 0.232, N = 556
COL6A2 : NP1
P12110
14:286
3:38
0:1
21:47552209:G:A_A
p = 0.13, beta = −0.86, N = 17
VFAVVITDGR pc2
P12110−2;P12110−3;P12110
210:286
32:38
1:1
21:47552209:G:A_A
p = 0.14, beta = −0.26, N = 243
LFAVAPNQNLK pc2
P12110−2;P12110−3;P12110
154:286
22:38
1:1
21:47552209:G:A_A
p = 0.89, beta = −0.0276, N = 177
GTVHFAVVITDGHVTGSPCGGIK pc
P12110−2;P12110−3;P12110
174:286
25:38
1:1
21:47552209:G:A_A
p = 0.14, beta = −0.288, N = 200
NLEWIAGGTWTPSALK pc2
P12110−2;P12110−3;P12110
148:286
18:38
1:1
21:47552209:G:A_A
p = 0.77, beta = 0.0638, N = 167
NFVINVVNR pc2
P12110−2;P12110−3;P12110
57:286
6:38
1:1
21:47552209:G:A_A
p = 0.99, beta = −0.00405, N = 64
HDPRDDDLNLR pc3
P12110−2;P12110−3;P12110
49:286
5:38
0:1
21:47552209:G:A_A
p = 0.73, beta = 0.157, N = 54
DIASTPHELYR pc2
P12110−2;P12110−3;P12110
38:286
8:38
0:1
21:47552209:G:A_A
p = 0.83, beta = −0.0792, N = 46
ALCDRDVTVTAIGIGDMFHEK pc4
P12110−2;P12110−3;P12110
42:286
2:38
0:1
21:47552209:G:A_A
p = 0.72, beta = 0.252, N = 44
Assay Target: COL6A2
UniProt: P12110
rsID: rs35548026
Target SNP: 21:46132295:A:G
Proxy SNP: 21:47552209:G:A
deCODE log10(p): 171.6
deCODE BETA: −0.39
deCODE MSPA: 0
1197:1255:1249:1196:1183:116
GVVQYSHEGTFEAIQLDDEHIDSLSSF
rs1042917 ALT
326:1062
41:194
0:4
21:47552209:G:A_A
p = 0.0048, model = REC, N = 367
GVVQYSHEGTFEAIQLDDERIDSLSSFI
rs1042917 REF
176:1062
43:194
1:4
21:47552209:G:A_A
p = 0.066, model = REC, N = 220

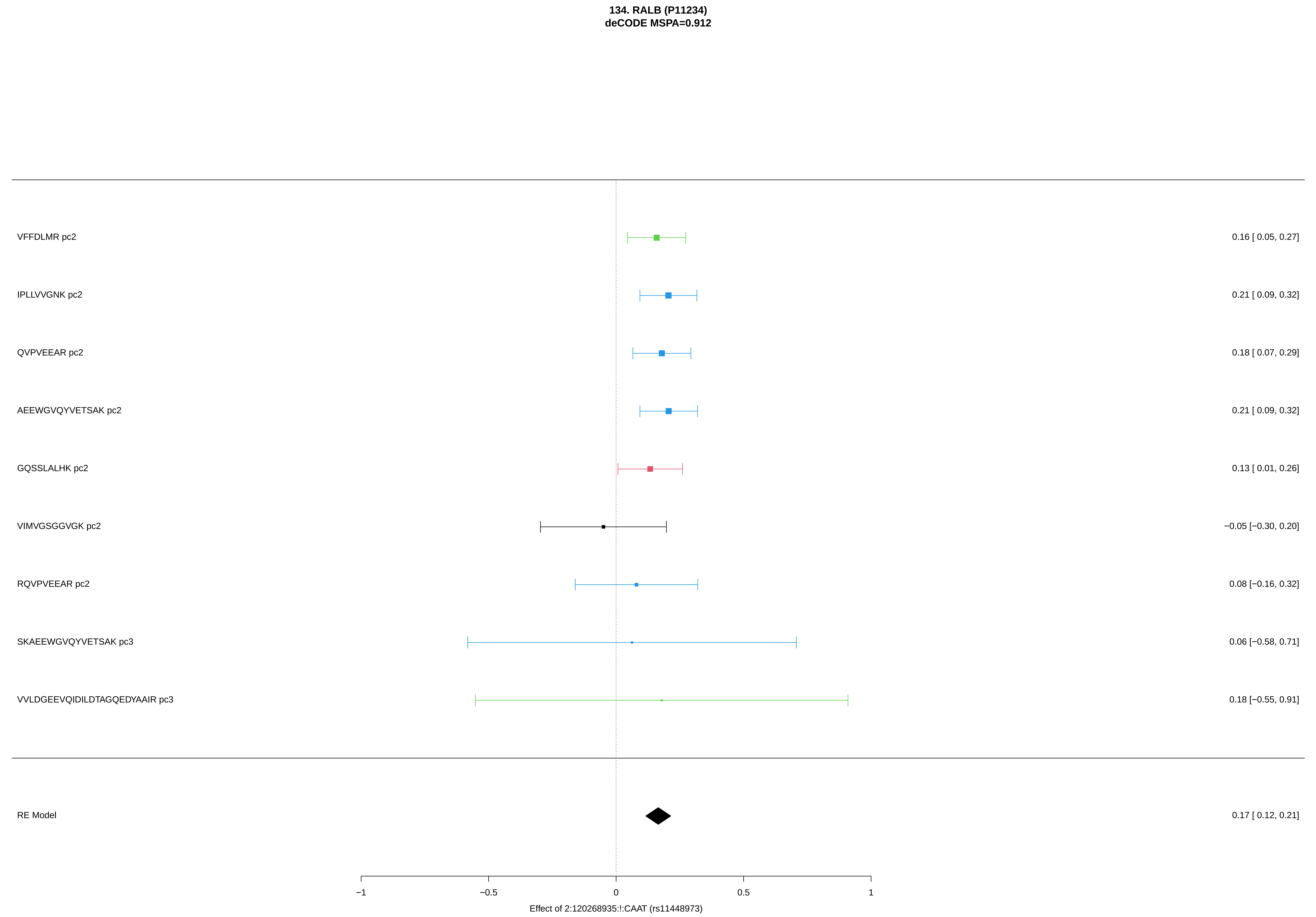

134. RALB (P11234)
deCODE MSPA=0.912
VFFDLMR pc2
IPLLVVGNK pc2
QVPVEEAR pc2
AEEWGVQYVETSAK pc2
GQSSLALHK pc2
VIMVGSGGVGK pc2
RQVPVEEAR pc2
SKAEEWGVQYVETSAK pc3
VVLDGEEVQIDILDTAGQEDYAAIR pc3
RE Model
0.16 [0.05, 0.27]
0.21 [0.09, 0.32]
0.18 [0.07, 0.29]
0.21 [0.09, 0.32]
0.13 [0.01, 0.26]
−0.05 [−0.30, 0.20]
0.08 [−0.16, 0.32]
0.06 [−0.58, 0.71]
0.18 [−0.55, 0.91]
0.17 [0.12, 0.21]
−1
−0.5
0
0.5
1
Effect of 2:120268935:!:CAAT (rs11448973)

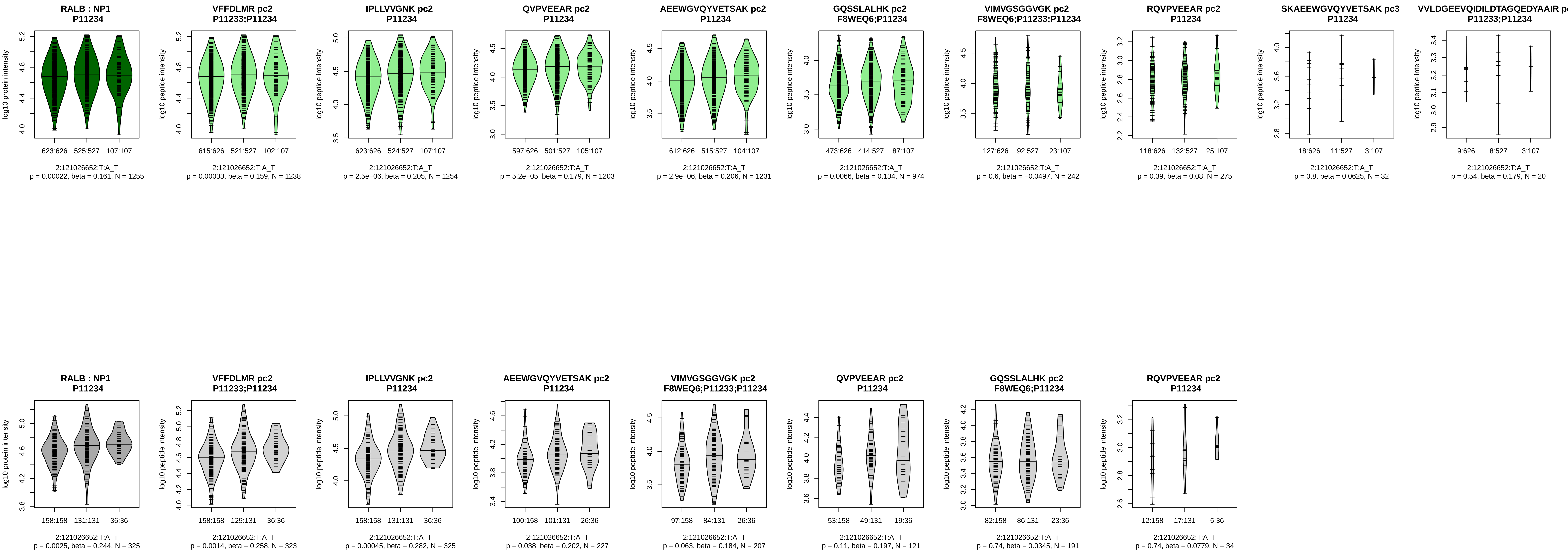

Assay Target: RALB
UniProt: P11234
rsID: rs11448973
Target SNP: 2:120268935:!:CAA
Proxy SNP: 2:121026652:T:A
deCODE log10(p): 168.9
deCODE BETA: 0.24
deCODE MSPA: 0.912
::*:*:*:–:–:–:NA:–
1238:1254:1203:1231:974:242:2

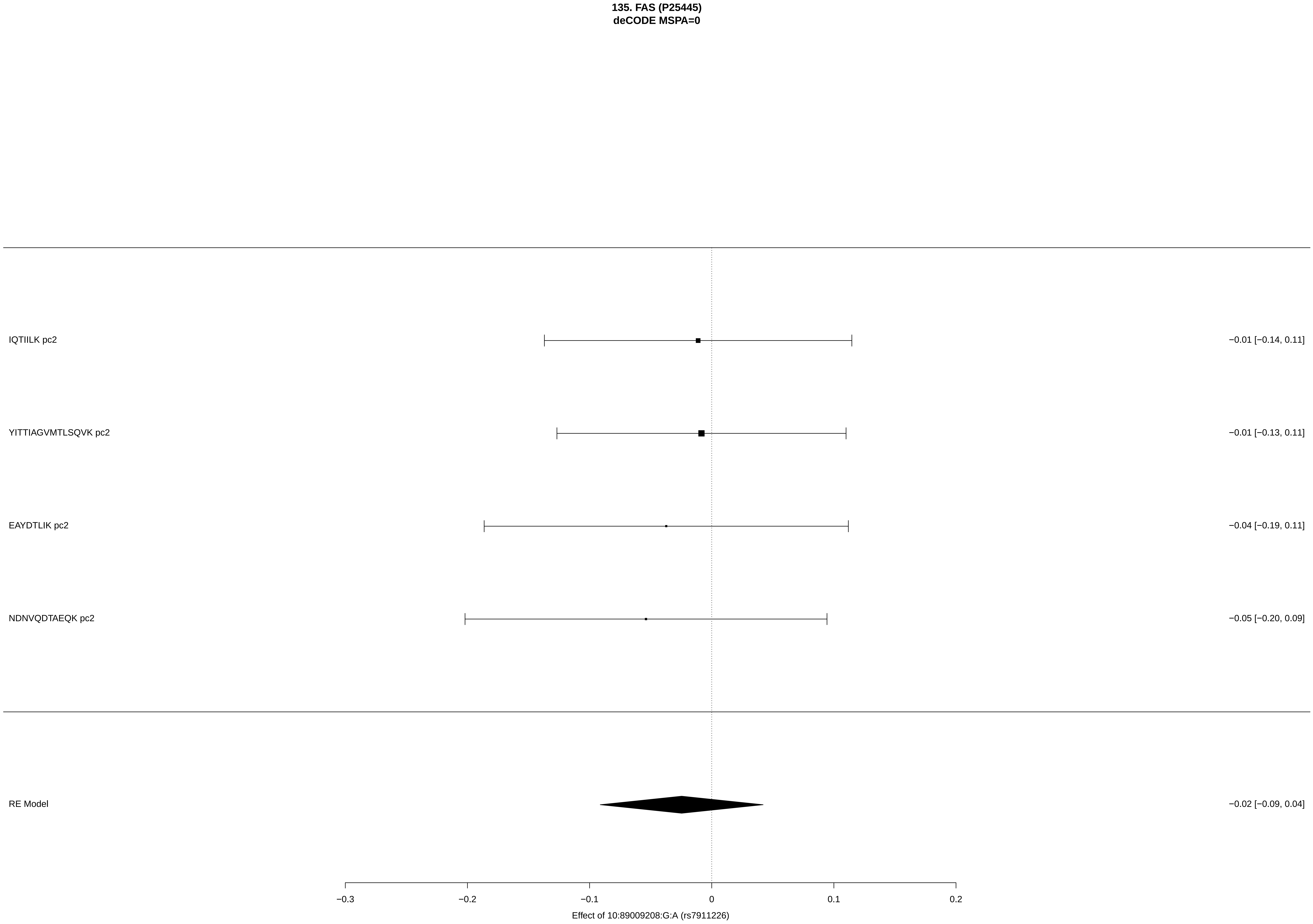
135. FAS (P25445)
deCODE MSPA=0
IQTIILK pc2
−0.01 [−0.14, 0.11]
YITTIAGVMTLSQVK pc2
−0.01 [−0.13, 0.11]
EAYDTLIK pc2
−0.04 [−0.19, 0.11]
NDNVQDTAEQK pc2
−0.05 [−0.20, 0.09]
RE Model
−0.02 [−0.09, 0.04]
−0.3
−0.2
−0.1
0
0.1
0.2
Effect of 10:89009208:G:A (rs7911226)

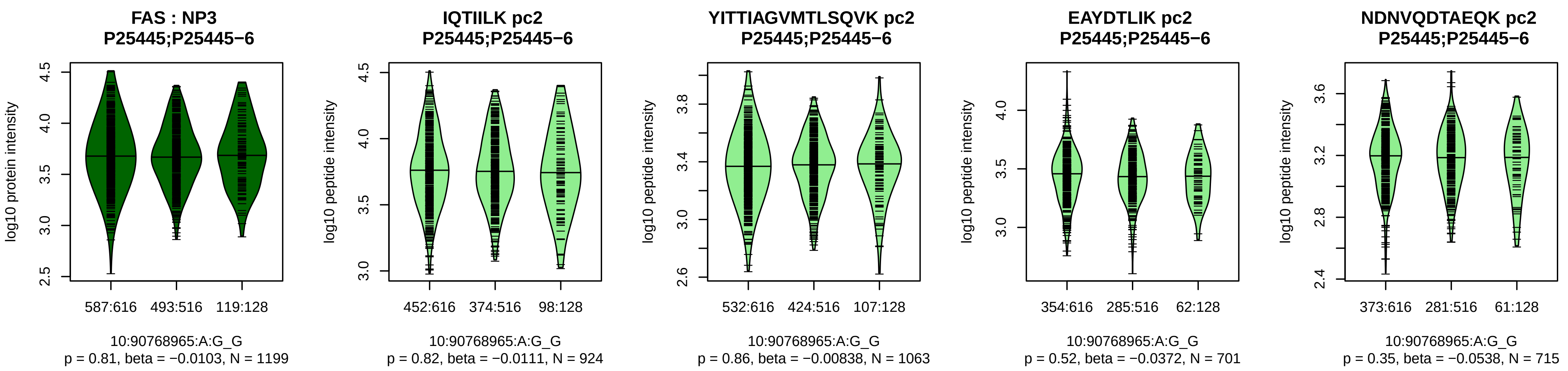

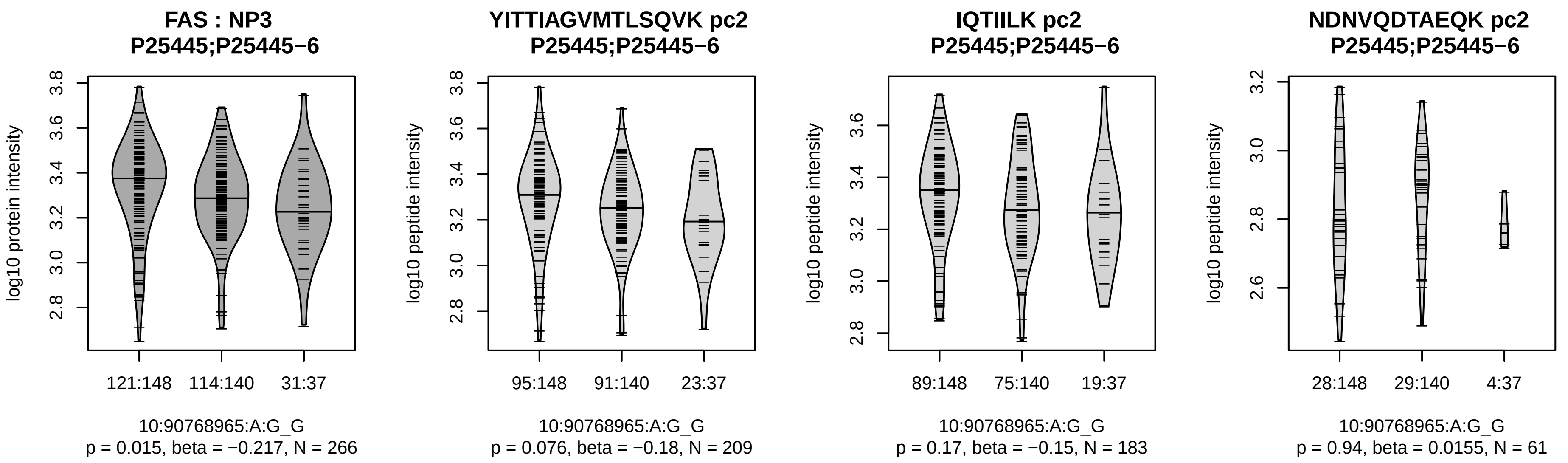

Assay Target: FAS
UniProt: P25445
rsID: rs7911226
Target SNP: 10:89009208:G:A
Proxy SNP: 10:90768965:A:G
deCODE log10(p): 166.6
deCODE BETA: −0.25
deCODE MSPA: 0
−:−:−:−
924:1063:701:715

136. SELPLG (Q14242)
deCODE MSPA=0

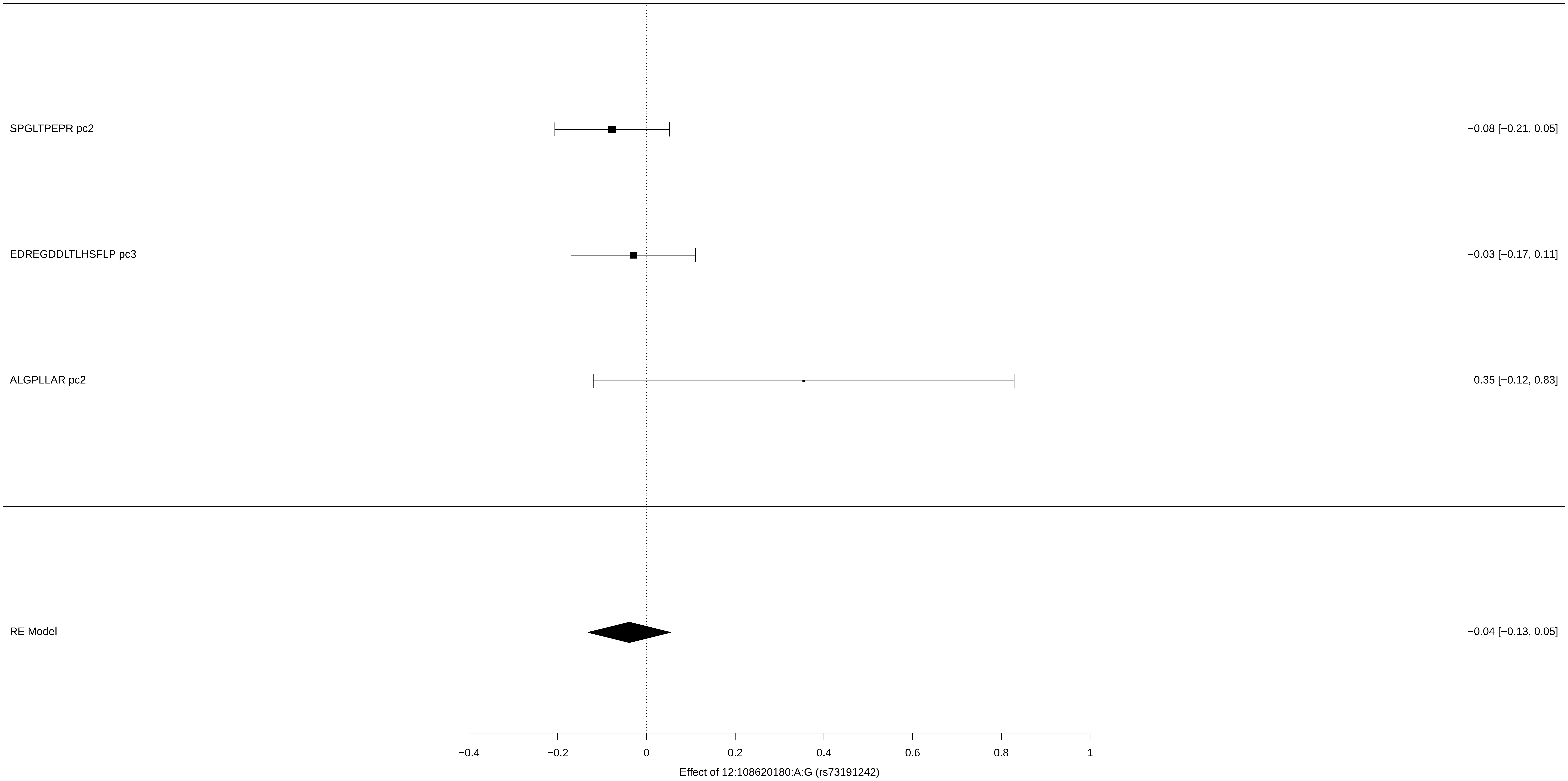

SELPLG : NP2
Q14242−2

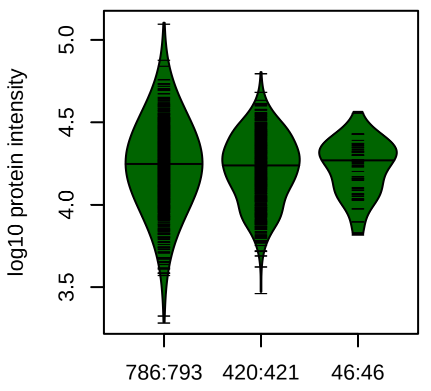

12:109013956:G:A_A
p = 0.13, beta = −0.0757, N = 1252

SPGLTPEPR pc2
A0A0C4DFY0;Q14242;Q14242−2

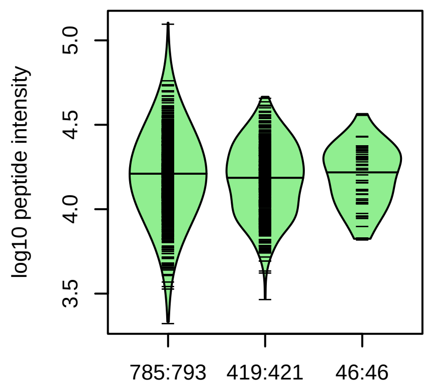

12:109013956:G:A_A
p = 0.12, beta = −0.0775, N = 1250

EDREGDDLTLHSFLP pc3
A0A0C4DFY0;Q14242;Q14242−2

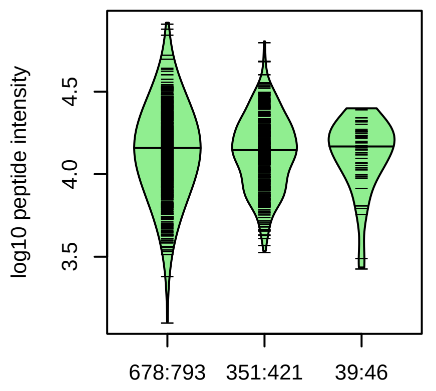

12:109013956:G:A_A
p = 0.58, beta = −0.0299, N = 1068

ALGPLLAR pc2
A0A0C4DFY0;Q14242;Q14242−2

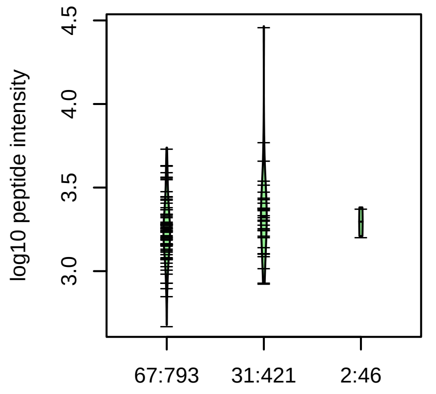

12:109013956:G:A_A
p = 0.057, beta = 0.354, N = 100

SELPLG : NP2
A0A0C4DFY0;Q14242;Q14242−2

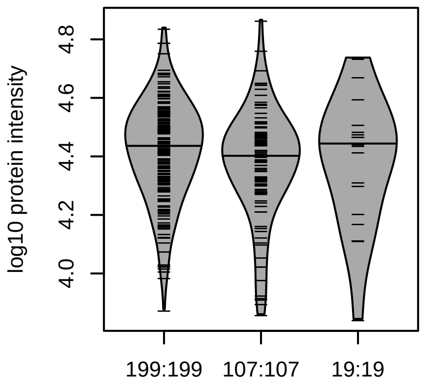

12:109013956:G:A_A
p = 0.072, beta = −0.163, N = 325

SPGLTPEPR pc2
A0A0C4DFY0;Q14242;Q14242−2

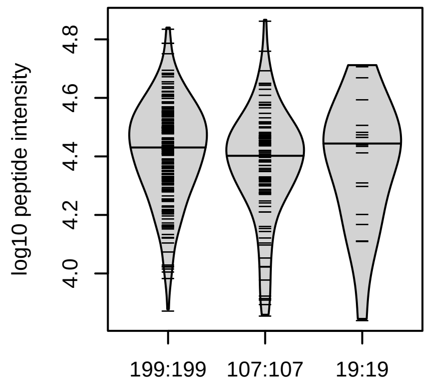

12:109013956:G:A_A
p = 0.074, beta = −0.162, N = 325

Assay Target: SELPLG
UniProt: Q14242
rsID: rs73191242
Target SNP: 12:108620180:A:G
Proxy SNP: 12:109013956:G:A
deCODE log10(p): 166.5
deCODE BETA: −0.28
deCODE MSPA: 0
−:−:−
1250:1068:100

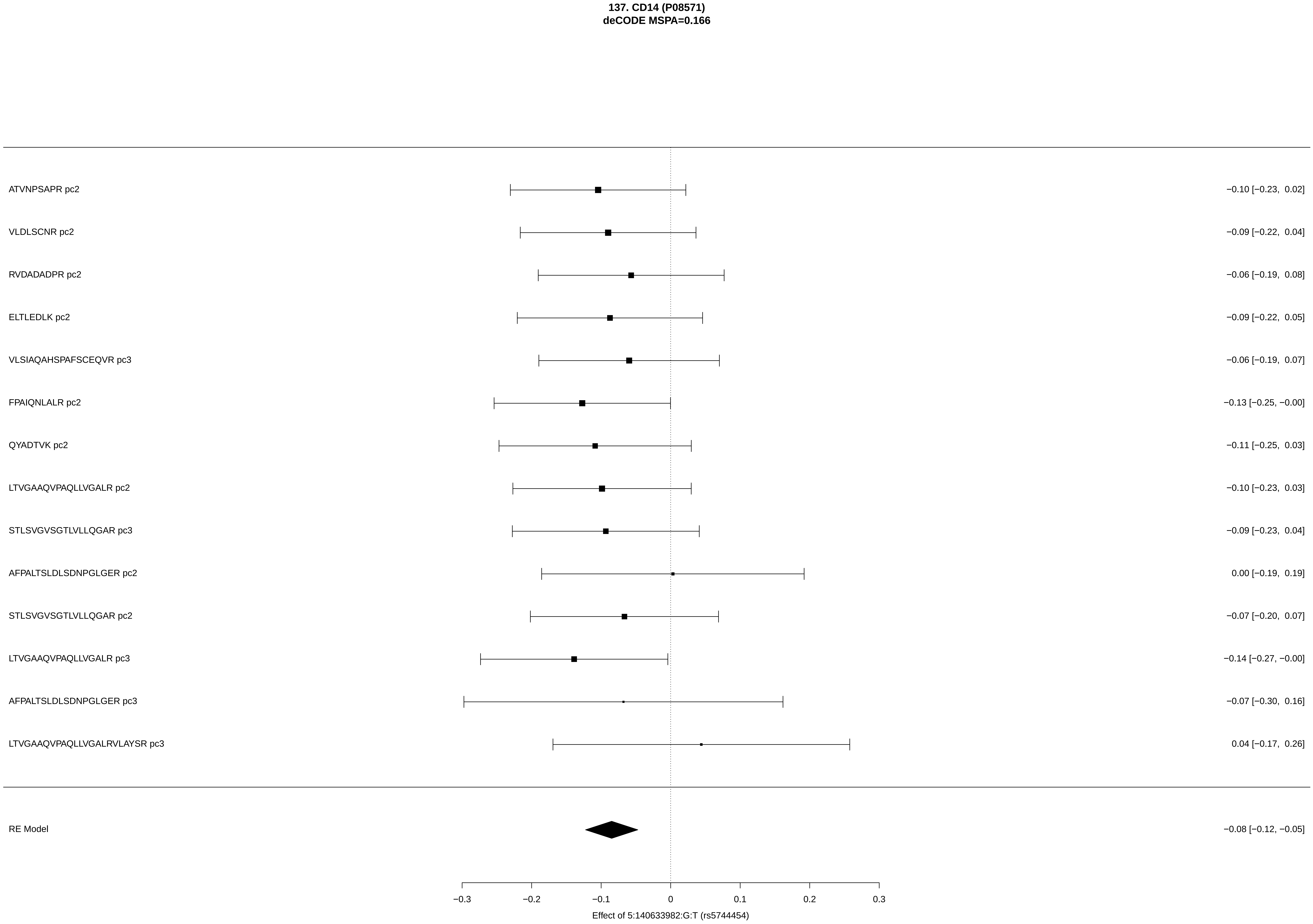
137. CD14 (P08571)
deCODE MSPA=0.166
ATVNPSAPR pc2
−0.10 [−0.23, 0.02]
VLDLSCNR pc2
−0.09 [−0.22, 0.04]
RVDADADPR pc2
−0.06 [−0.19, 0.08]
ELTLEDLK pc2
−0.09 [−0.22, 0.05]
VLSIAQAHSPAFSCEQVR pc3
−0.06 [−0.19, 0.07]
FPAIQNLALR pc2
−0.13 [−0.25, −0.00]
QYADTVK pc2
−0.11 [−0.25, 0.03]
LTVGAAQVPAQLLVGALR pc2
−0.10 [−0.23, 0.03]
STLSVGVSGTLVLLQGAR pc3
−0.09 [−0.23, 0.04]
AFPALTSLDLSDNPGLGER pc2
0.00 [−0.19, 0.19]
STLSVGVSGTLVLLQGAR pc2
−0.07 [−0.20, 0.07]
LTVGAAQVPAQLLVGALR pc3
−0.14 [−0.27, −0.00]
AFPALTSLDLSDNPGLGER pc3
−0.07 [−0.30, 0.16]
LTVGAAQVPAQLLVGALRVLAYSR pc3
0.04 [−0.17, 0.26]
RE Model
−0.08 [−0.12, −0.05]
−0.3
−0.2
−0.1
0
0.1
0.2
0.3
Effect of 5:140633982:G:T (rs5744454)

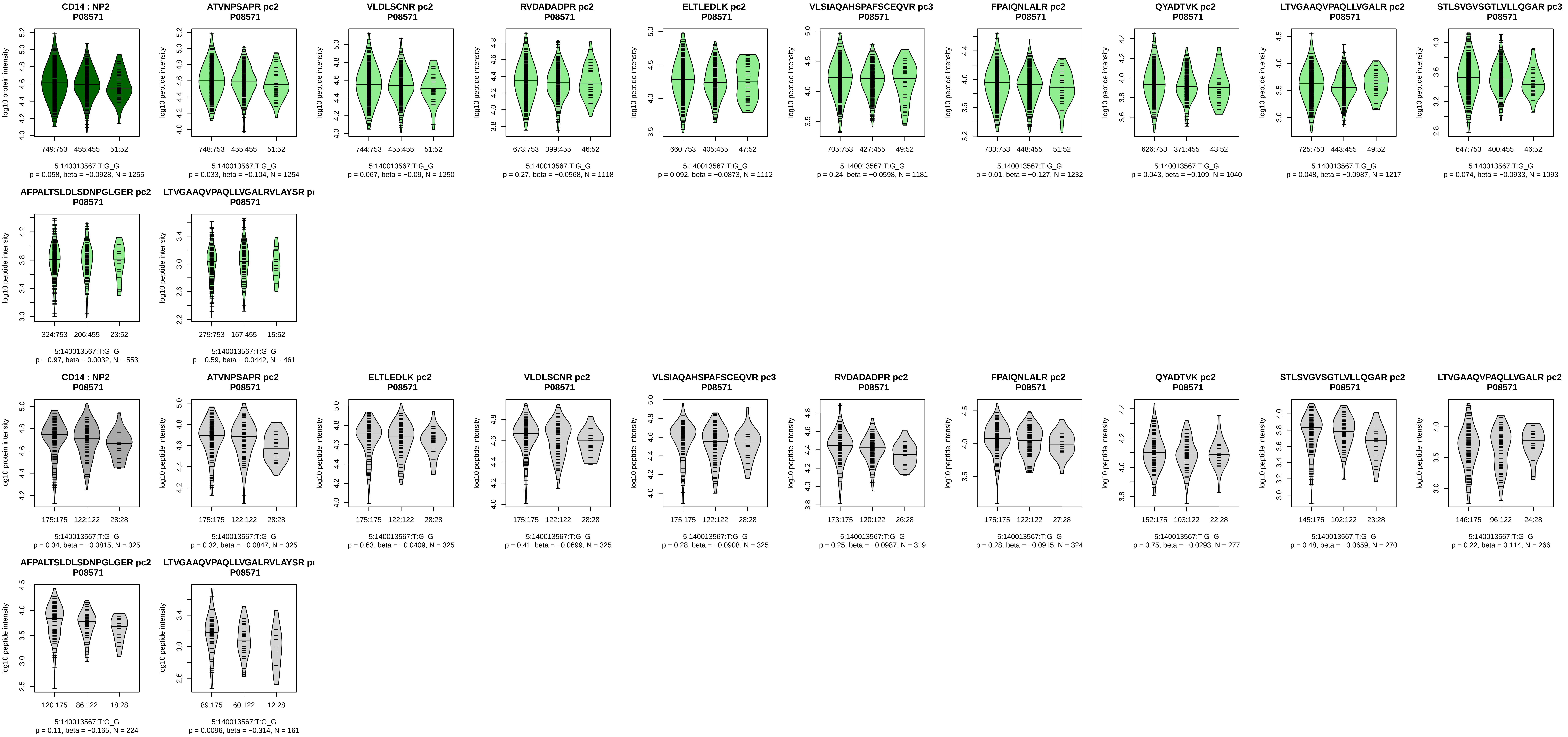

Assay Target: CD14
UniProt: P08571
rsID: rs5744454
Target SNP: 5:140633982:G:T
Proxy SNP: 5:140013567:T:G
deCODE log10(p): 160.4
deCODE BETA: −0.24
deCODE MSPA: 0.166
–:–:–:–:–:*:–:–:–:–:–:*:–:–
1254:1250:1118:1112:1181:123

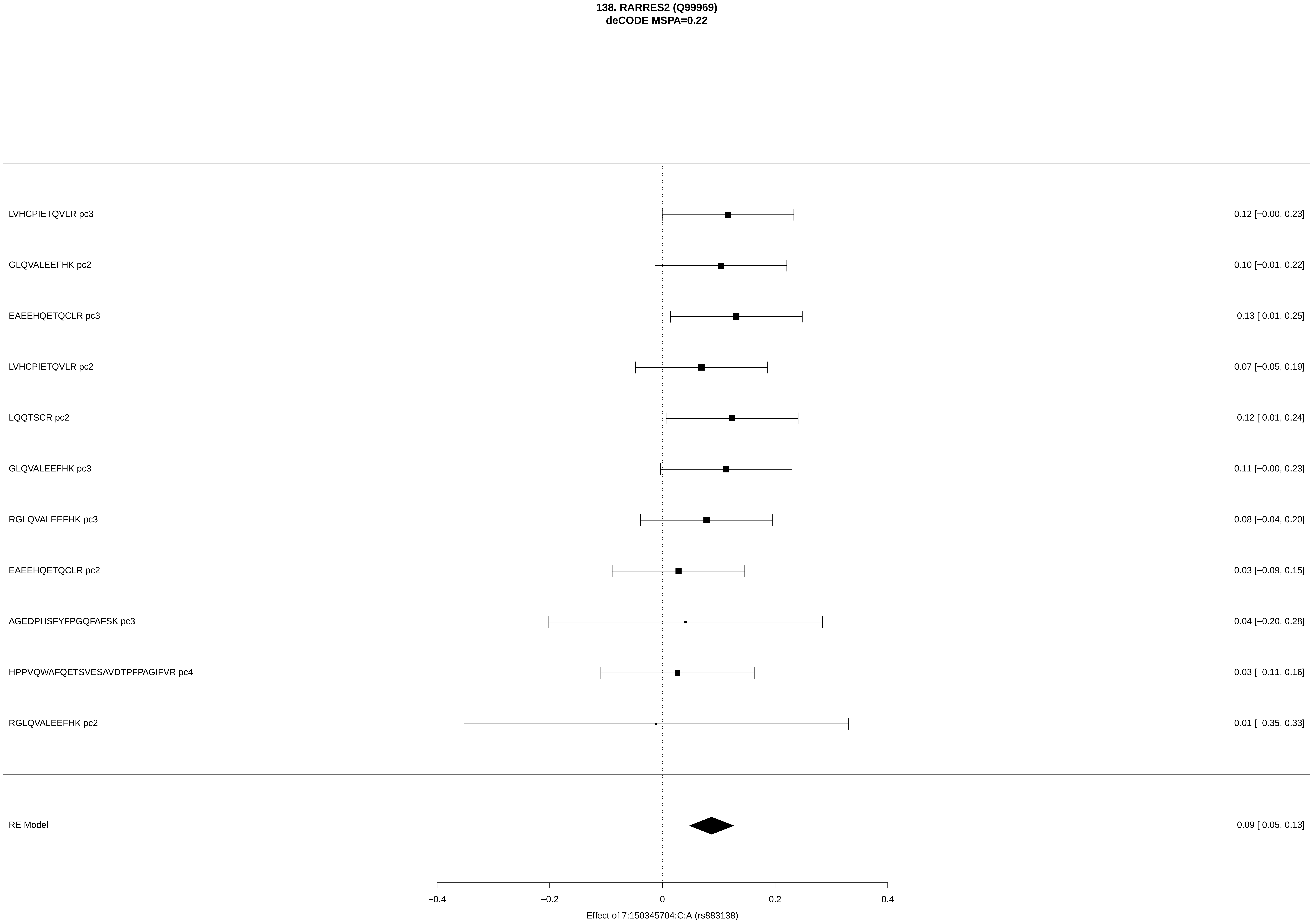
138. RARRES2 (Q99969)
deCODE MSPA=0.22
LVHCPIETQVLR pc3
0.12 [−0.00, 0.23]
GLQVALEEFHK pc2
0.10 [−0.01, 0.22]
EAEEHQETQCLR pc3
0.13 [0.01, 0.25]
LVHCPIETQVLR pc2
0.07 [−0.05, 0.19]
LQQTSCR pc2
0.12 [0.01, 0.24]
GLQVALEEFHK pc3
0.11 [−0.00, 0.23]
RGLQVALEEFHK pc3
0.08 [−0.04, 0.20]
EAEEHQETQCLR pc2
0.03 [−0.09, 0.15]
AGEDPHSFYFPGQFAFSK pc3
0.04 [−0.20, 0.28]
HPPVQWAFQETSVESAVDTPFPAGIFVR pc4
0.03 [−0.11, 0.16]
RGLQVALEEFHK pc2
−0.01 [−0.35, 0.33]
RE Model
0.09 [0.05, 0.13]
−0.4
−0.2
0
0.2
0.4
Effect of 7:150345704:C:A (rs883138)

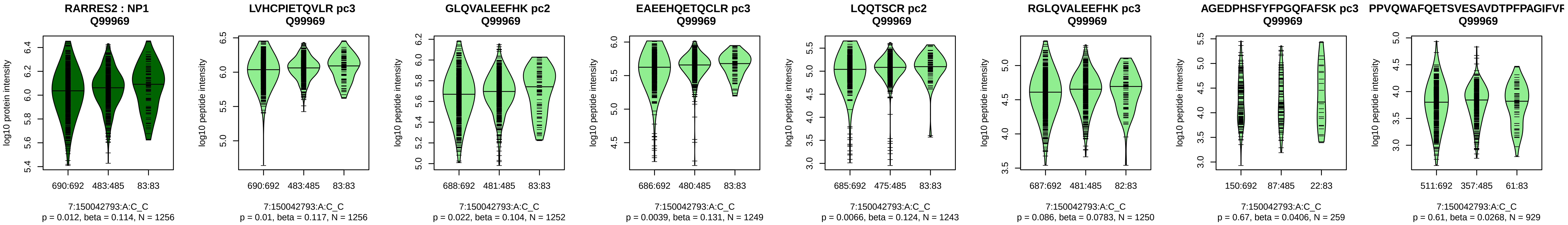

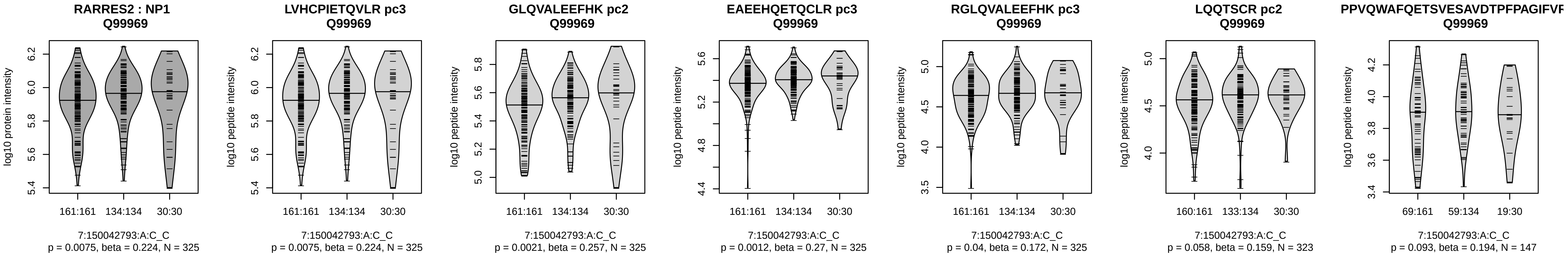

Assay Target: RARRES2
UniProt: Q99969
rsID: rs883138
Target SNP: 7:150345704:C:A
Proxy SNP: 7:150042793:A:C
deCODE log10(p): 158.7
deCODE BETA: 0.25
deCODE MSPA: 0.22
--:--:*:--:*:--:--:--:--:--:NA
1256:1252:1249:1253:1243:125

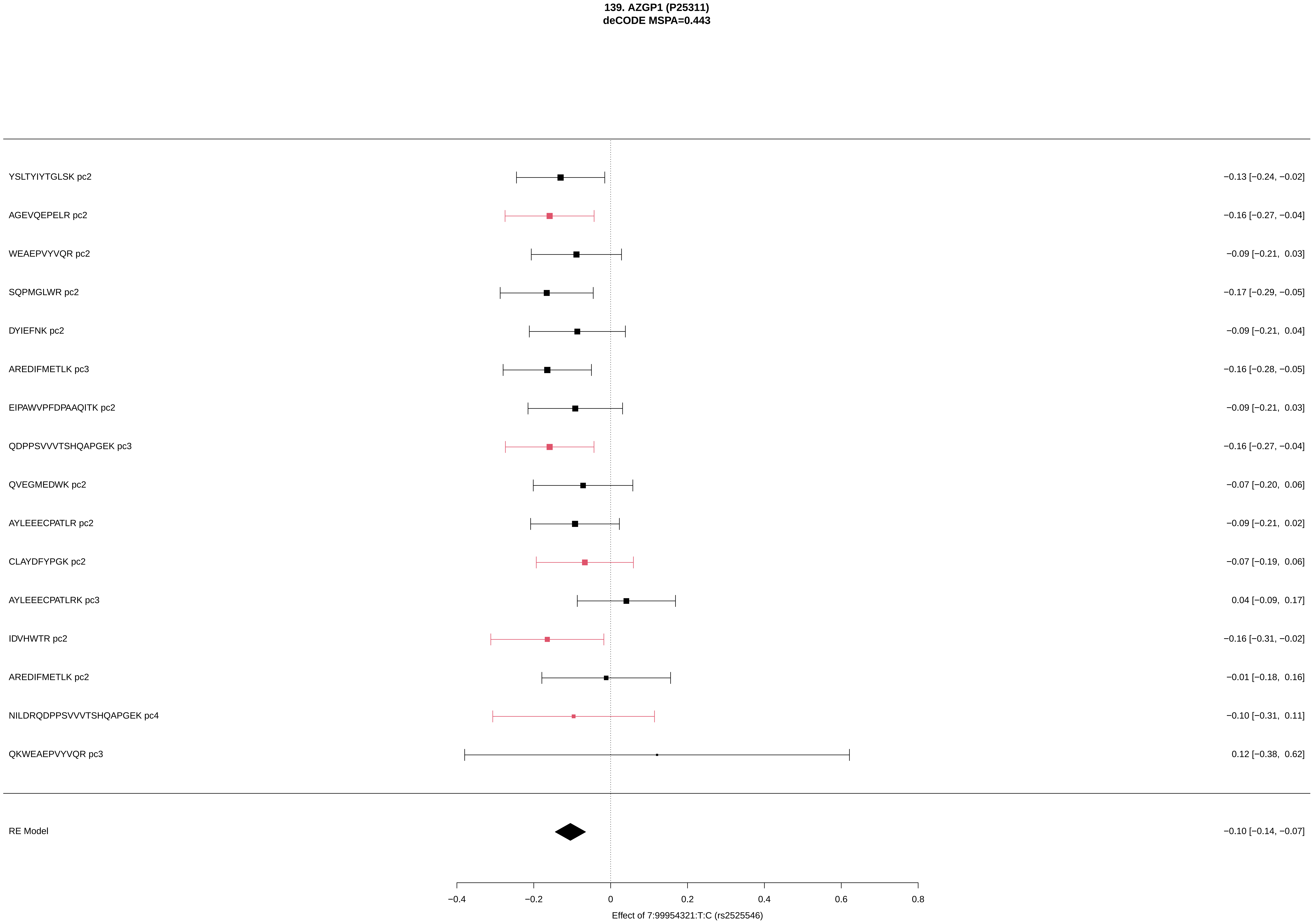

139. AZGP1 (P25311)
deCODE MSPA=0.443
YSLTYIYTGLSK pc2
AGEVQEPELR pc2
WEAEPVYVQR pc2
SQPMGLWR pc2
DYIEFNK pc2
AREDIFMETLK pc3
EIPAWVPFDPAAQITK pc2
QDPPSVVVTSHQAPGEK pc3
QVEGMEDWK pc2
AYLEEECPATLR pc2
CLAYDFYPGK pc2
AYLEEECPATLRK pc3
IDVHWTR pc2
AREDIFMETLK pc2
NILDRQDPPSVVVTSHQAPGEK pc4
QKWEAEPVYVQR pc3
RE Model
−0.13 [−0.24, −0.02]
−0.16 [−0.27, −0.04]
−0.09 [−0.21, 0.03]
−0.17 [−0.29, −0.05]
−0.09 [−0.21, 0.04]
−0.16 [−0.28, −0.05]
−0.09 [−0.21, 0.03]
−0.16 [−0.27, −0.04]
−0.07 [−0.20, 0.06]
−0.09 [−0.21, 0.02]
−0.07 [−0.19, 0.06]
0.04 [−0.09, 0.17]
−0.16 [−0.31, −0.02]
−0.01 [−0.18, 0.16]
−0.10 [−0.31, 0.11]
0.12 [−0.38, 0.62]
−0.10 [−0.14, −0.07]
−0.4
−0.2
0
0.2
0.4
0.6
0.8
Effect of 7:99954321:T:C (rs2525546)

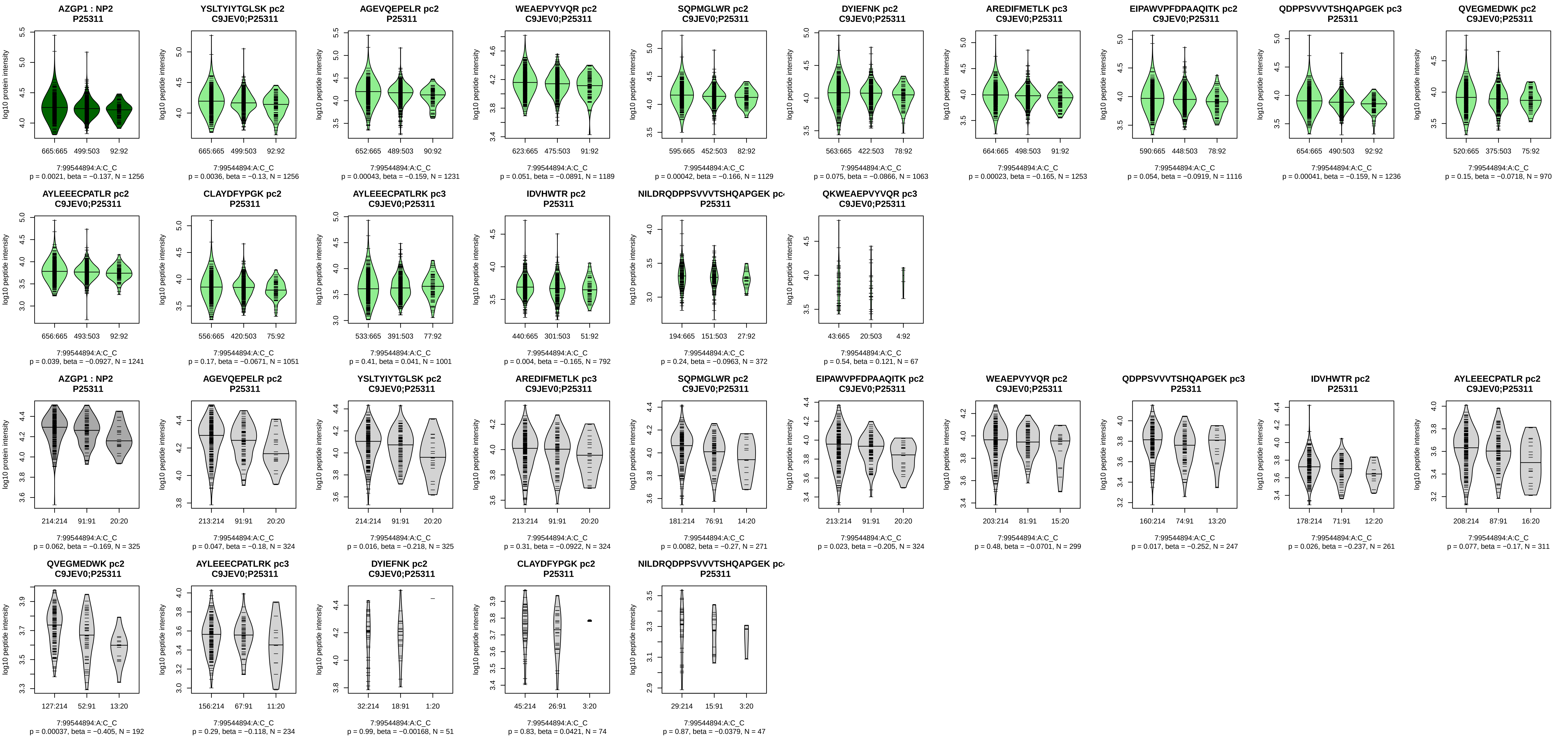

Assay Target: AZGP1
UniProt: P25311
rsID: rs2525546
Target SNP: 7:99954321:T:C
Proxy SNP: 7:99544894:A:C
deCODE log10(p): 157.9
deCODE BETA: −0.24
deCODE MSPA: 0.443
::–:*:–:*:–:*:–:–:–:*:–:–:–:NA
1256:1231:1189:1129:1063:125

140. SERPINA11 (Q86U17)
deCODE MSPA=0.721

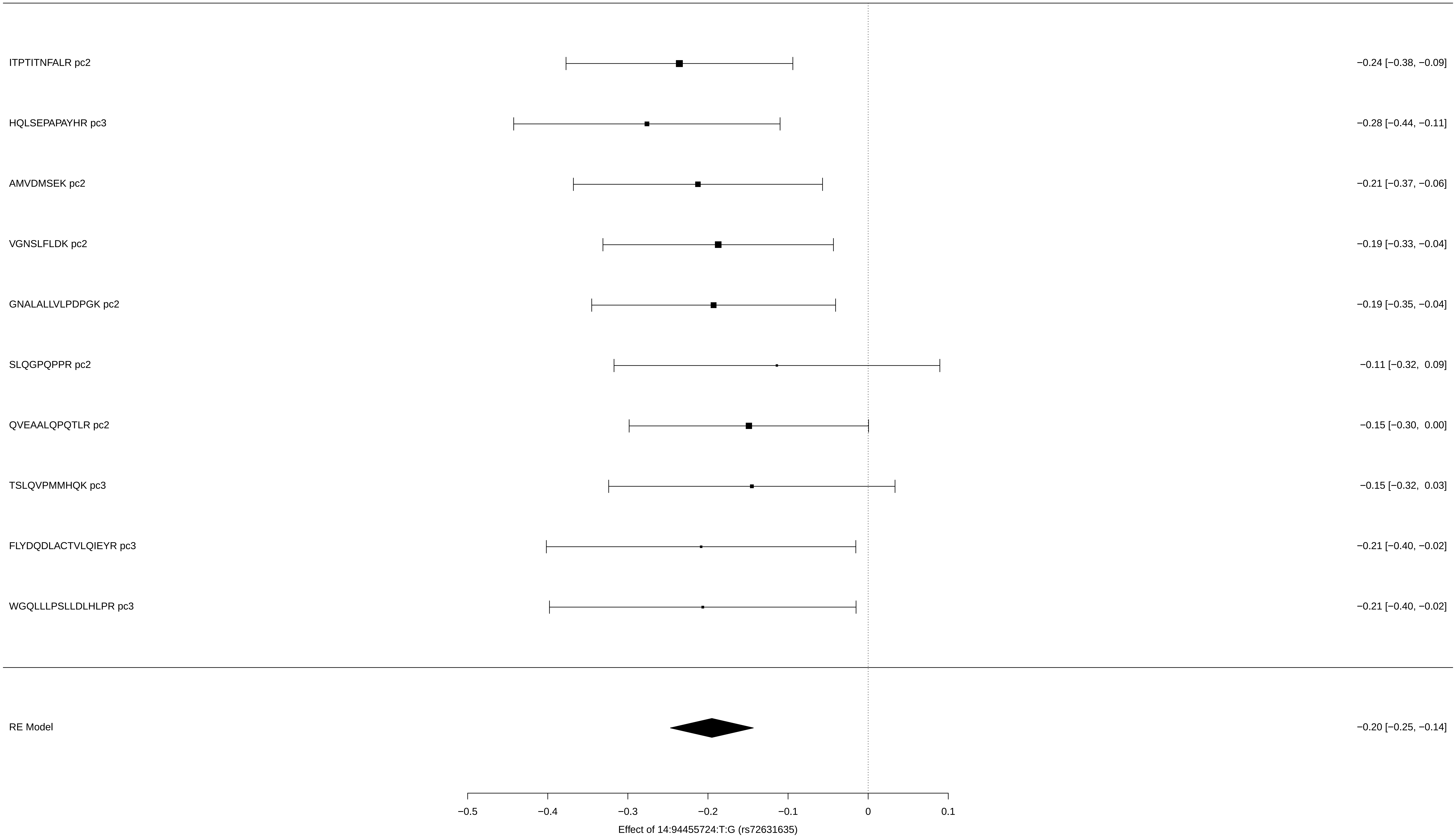

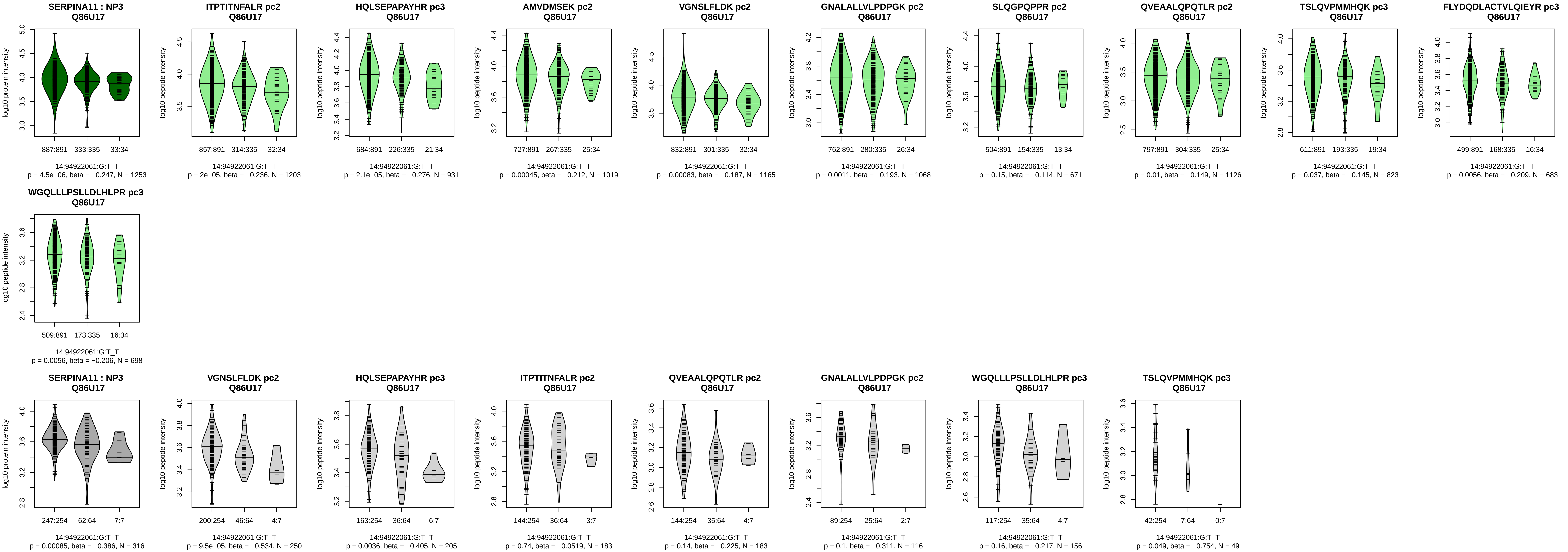

Assay Target: SERPINA11
UniProt: Q86U17
rsID: rs72631635
Target SNP: 14:94455724:T:G
Proxy SNP: 14:94922061:G:T
deCODE log10(p): 157.1
deCODE BETA: −0.3
deCODE MSPA: 0.721
::*:*:*:−:−:−:*:*:NA
1203:931:1019:1165:1068:671:1

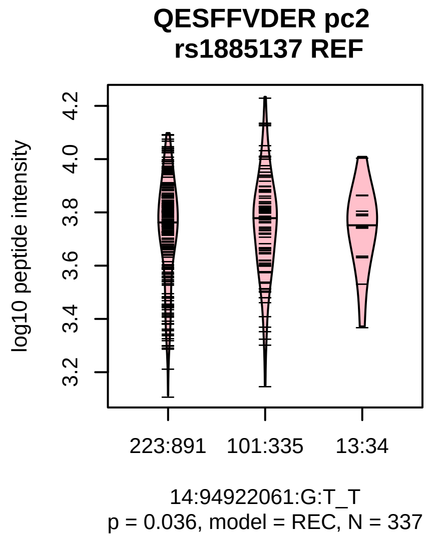

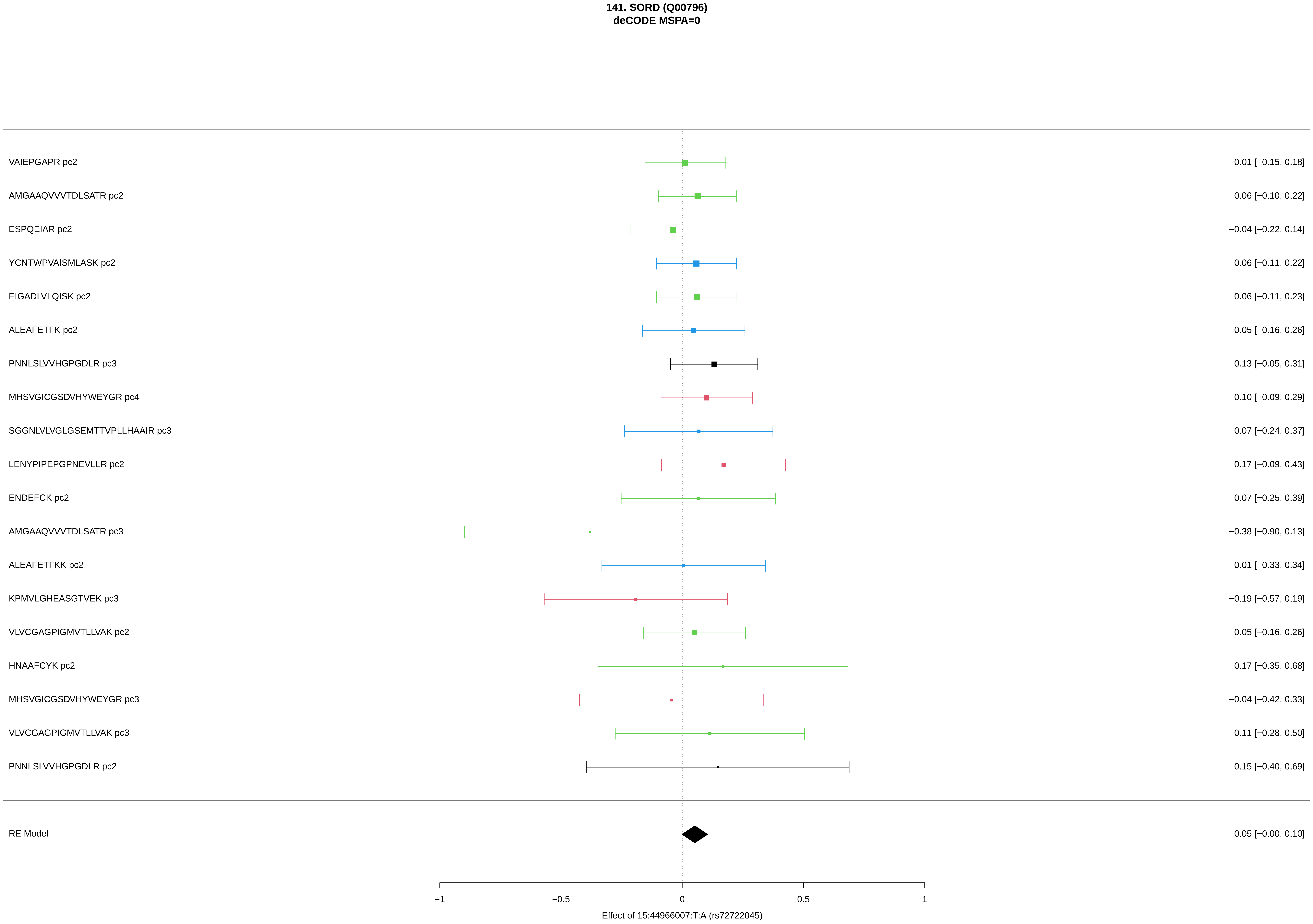

141. SORD (Q00796)
deCODE MSPA=0
VAIEPGAPR pc2
0.01 [−0.15, 0.18]
AMGAAQVVVTDLSATR pc2
0.06 [−0.10, 0.22]
ESPQEIAR pc2
−0.04 [−0.22, 0.14]
YCNTWPVAISMLASK pc2
0.06 [−0.11, 0.22]
EIGADLVLQISK pc2
0.06 [−0.11, 0.23]
ALEAFETFK pc2
0.05 [−0.16, 0.26]
PNNLSLVVHGPGDLR pc3
0.13 [−0.05, 0.31]
MHSVGICGSDVHYWEYGR pc4
0.10 [−0.09, 0.29]
SGGNLVLVGLGSEMTTVPLLHAAIR pc3
0.07 [−0.24, 0.37]
LENYPIPEPGPNEVLLR pc2
0.17 [−0.09, 0.43]
ENDEFCK pc2
0.07 [−0.25, 0.39]
AMGAAQVVVTDLSATR pc3
−0.38 [−0.90, 0.13]
ALEAFETFKK pc2
0.01 [−0.33, 0.34]
KPMVLGHEASGTVEK pc3
−0.19 [−0.57, 0.19]
VLVCGAGPIGMVTLLVAK pc2
0.05 [−0.16, 0.26]
HNAAFCYK pc2
0.17 [−0.35, 0.68]
MHSVGICGSDVHYWEYGR pc3
−0.04 [−0.42, 0.33]
VLVCGAGPIGMVTLLVAK pc3
0.11 [−0.28, 0.50]
PNNLSLVVHGPGDLR pc2
0.15 [−0.40, 0.69]
RE Model
0.05 [−0.00, 0.10]
−1
−0.5
0
0.5
1
Effect of 15:44966007:T:A (rs72722045)

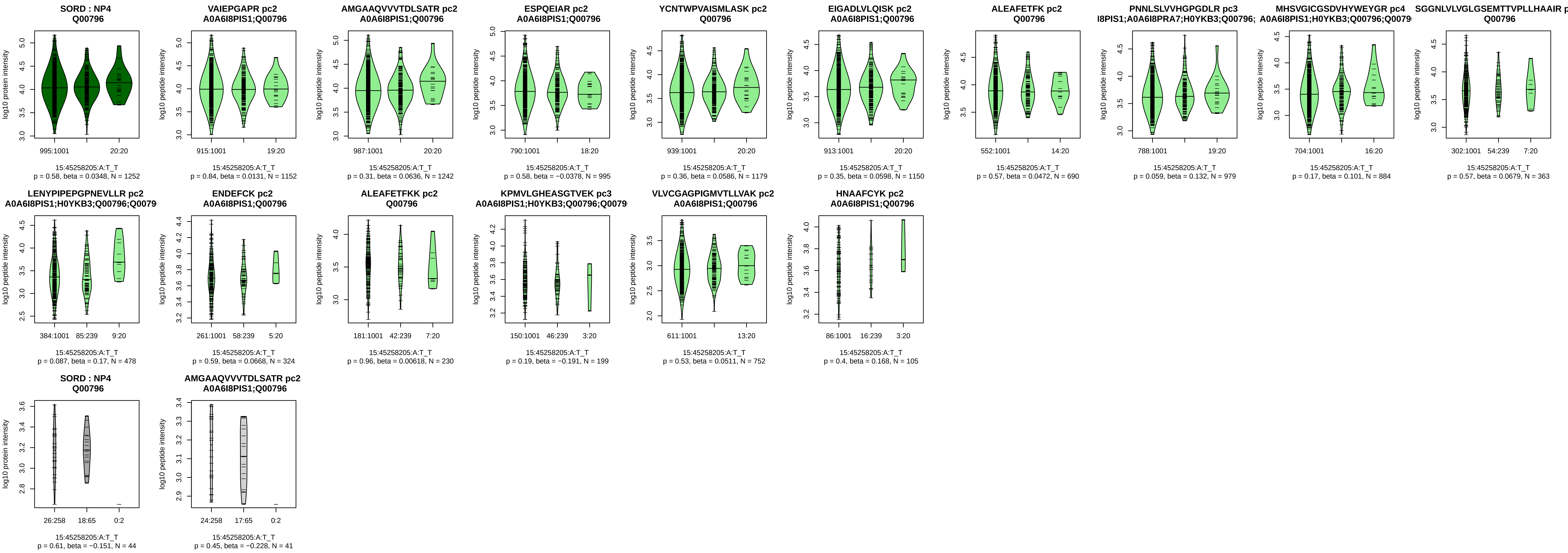

Assay Target: SORD
UniProt: Q00796
rsID: rs72722045
Target SNP: 15:44966007:T:A
Proxy SNP: 15:45258205:A:T
deCODE log10(p): 152.1
deCODE BETA: 0.3
deCODE MSPA: 0
1152:1242:995:1179:1150:690:9

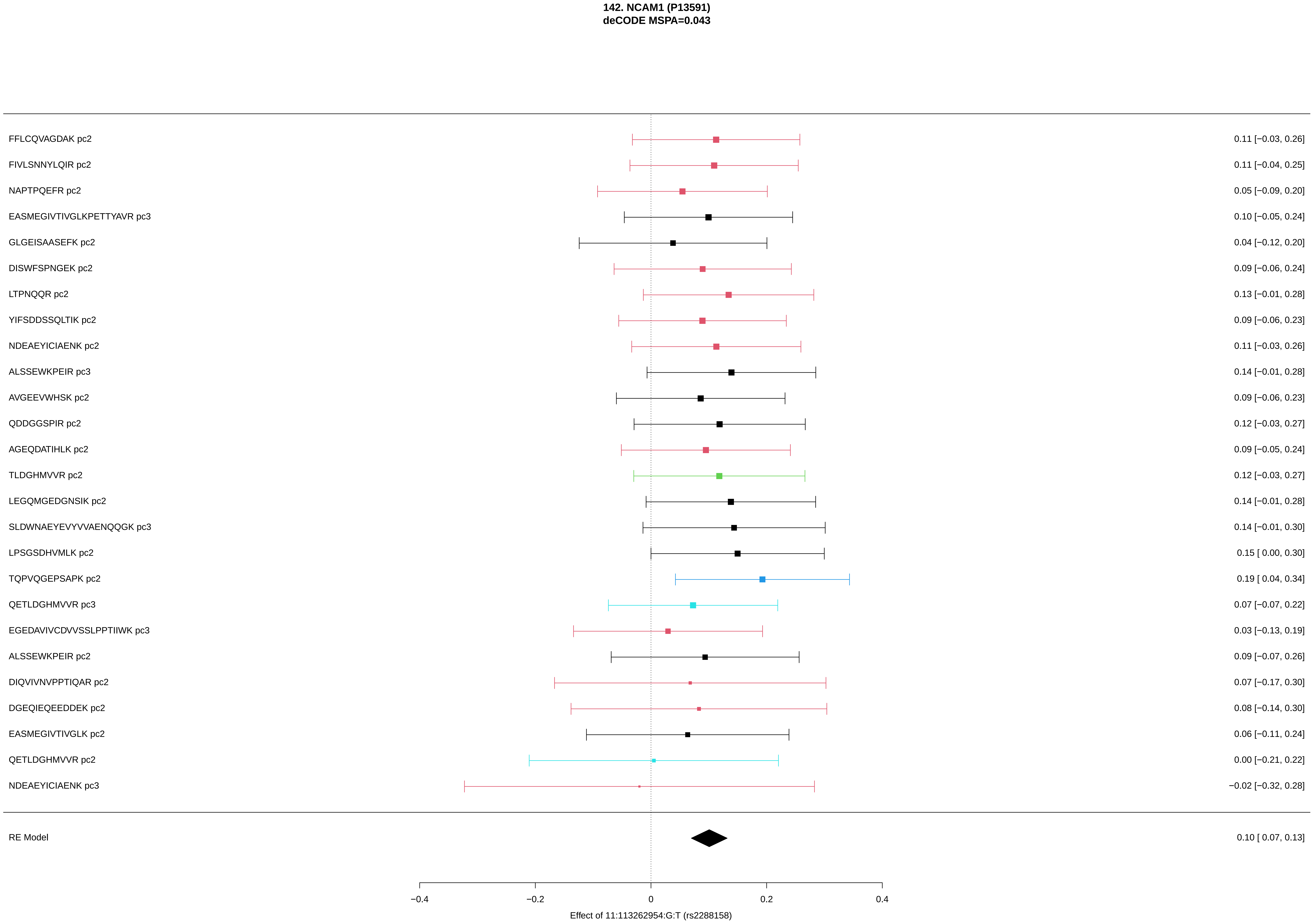
142. NCAM1 (P13591)
deCODE MSPA=0.043
FFLCQVAGDAK pc2
FIVLSNNYLQIR pc2
NAPTPQEFR pc2
EASMEGIVTIVGLKPETTYAVR pc3
GLGEISAASEFK pc2
DISWFSPNGEK pc2
LTPNQQR pc2
YIFSDDSSQLTIK pc2
NDEAEYICIAENK pc2
ALSSEWKPEIR pc3
AVGEEVWHSK pc2
QDDGGSPIR pc2
AGEQDATIHLK pc2
TLDGHMVVR pc2
LEGQMGEDGNSIK pc2
SLDWNAEYEVYVVAENQQGK pc3
LPSGSDHVMLK pc2
TQPVQGEPSAPK pc2
QETLDGHMVVR pc3
EGEDAVIVCDVVSSLPPTIIWK pc3
ALSSEWKPEIR pc2
DIQVIVNVPPTIQAR pc2
DGEQIEQEEDDEK pc2
EASMEGIVTIVGLK pc2
QETLDGHMVVR pc2
NDEAEYICIAENK pc3
RE Model
0.11 [−0.03, 0.26]
0.11 [−0.04, 0.25]
0.05 [−0.09, 0.20]
0.10 [−0.05, 0.24]
0.04 [−0.12, 0.20]
0.09 [−0.06, 0.24]
0.13 [−0.01, 0.28]
0.09 [−0.06, 0.23]
0.11 [−0.03, 0.26]
0.14 [−0.01, 0.28]
0.09 [−0.06, 0.23]
0.12 [−0.03, 0.27]
0.09 [−0.05, 0.24]
0.12 [−0.03, 0.27]
0.14 [−0.01, 0.28]
0.14 [−0.01, 0.30]
0.15 [0.00, 0.30]
0.19 [0.04, 0.34]
0.07 [−0.07, 0.22]
0.03 [−0.13, 0.19]
0.09 [−0.07, 0.26]
0.07 [−0.17, 0.30]
0.08 [−0.14, 0.30]
0.06 [−0.11, 0.24]
0.00 [−0.21, 0.22]
−0.02 [−0.32, 0.28]
0.10 [0.07, 0.13]
−0.4
−0.2
0
0.2
0.4
Effect of 11:113262954:G:T (rs2288158)

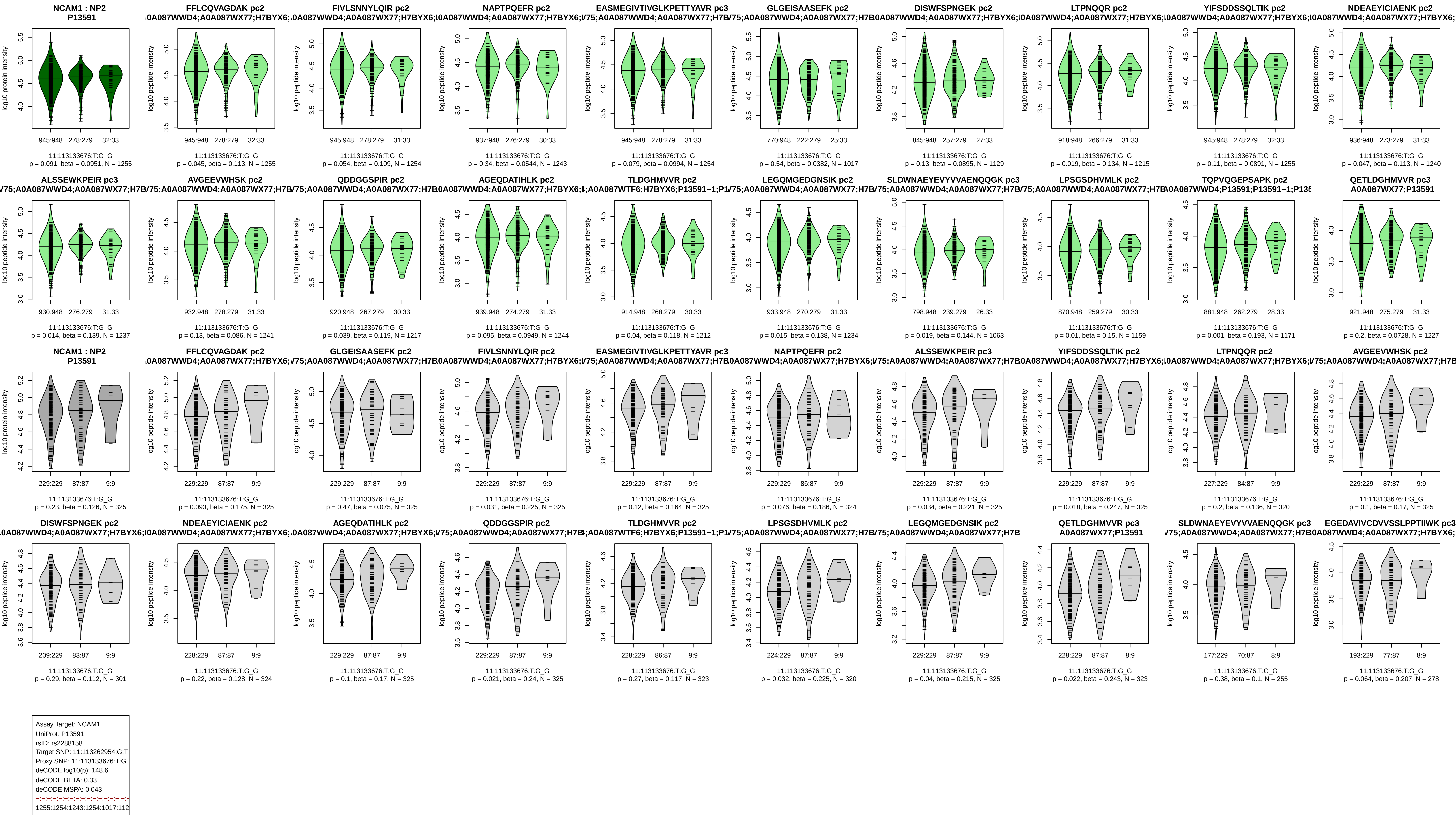
Assay Target: NCAM1
UniProt: P13591
rsID: rs2288158
Target SNP: 11:113262954:G:T
Proxy SNP: 11:113133676:T:G
deCODE log10(p): 148.6
deCODE BETA: 0.33
deCODE MSPA: 0.043
1255:1254:1243:1254:1017:112

143. SERPINA5 (P05154)
deCODE MSPA=0.198 (2 conflicts)

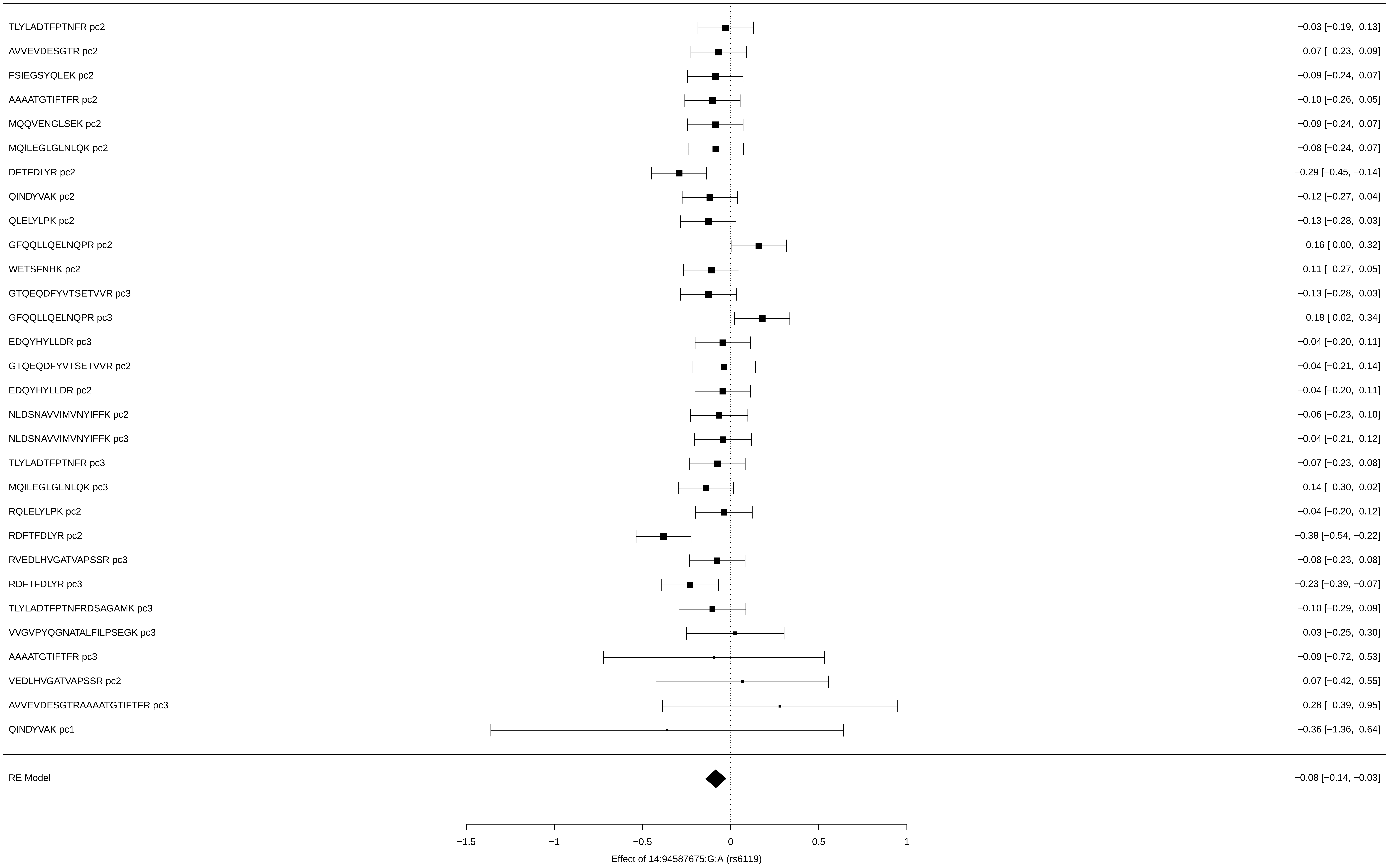

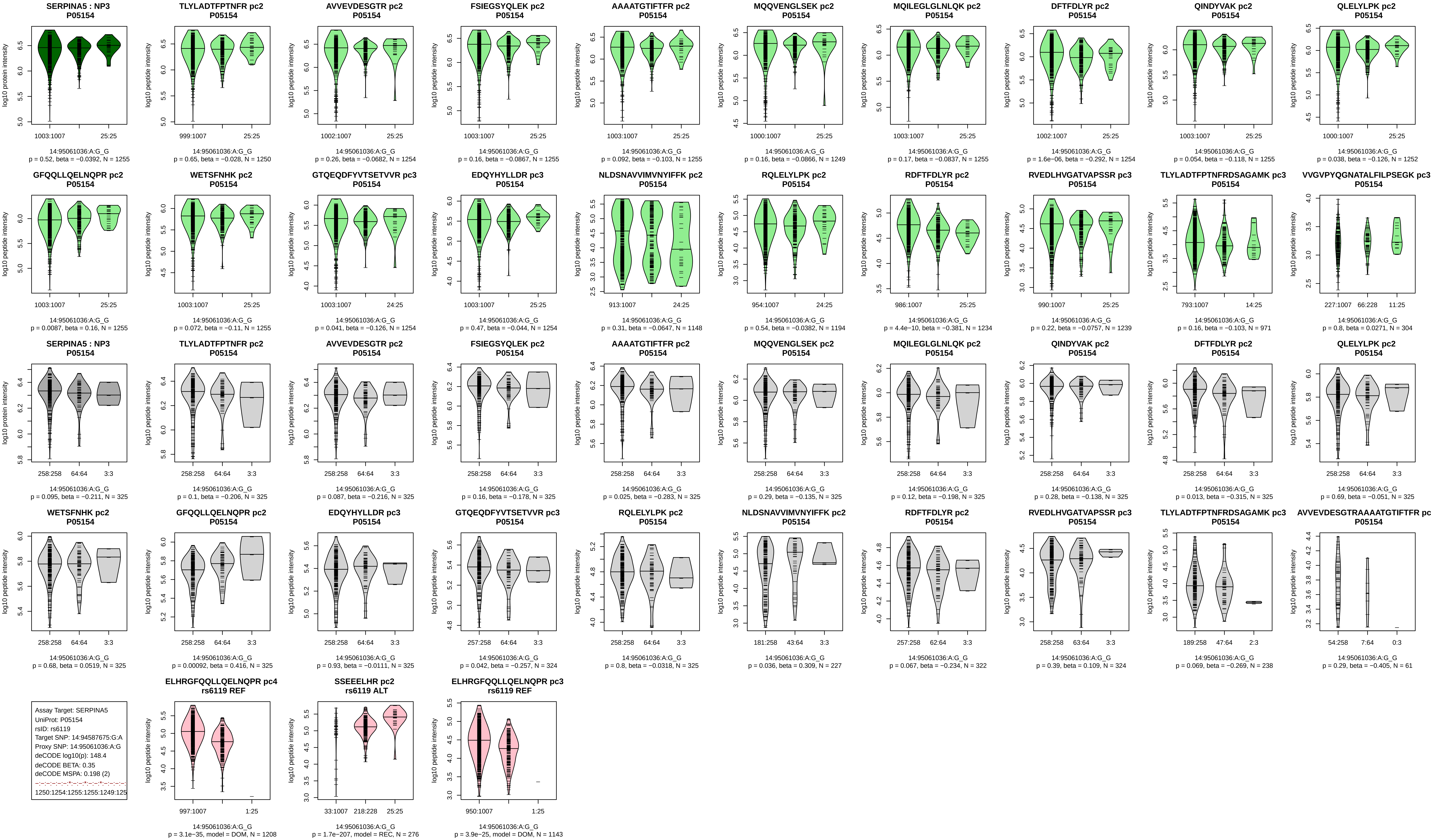
SERPINA5 : NP3
P05154
log10 protein intensity
1003:1007
25:25
14:95061036:A:G_G
p = 0.52, beta = −0.0392, N = 1255
TLYLADTFPTNFR pc2
P05154
log10 peptide intensity
999:1007
25:25
14:95061036:A:G_G
p = 0.65, beta = −0.028, N = 1250
AVVEVDESGTR pc2
P05154
1002:1007
25:25
14:95061036:A:G_G
p = 0.26, beta = −0.0682, N = 1254
FSIEGSYQLEK pc2
P05154
1003:1007
25:25
14:95061036:A:G_G
p = 0.16, beta = −0.0867, N = 1255
AAAATGTIFTFR pc2
P05154
1003:1007
25:25
14:95061036:A:G_G
p = 0.092, beta = −0.103, N = 1255
MQQVENGLSEK pc2
P05154
1000:1007
25:25
14:95061036:A:G_G
p = 0.16, beta = −0.0866, N = 1249
MQILEGLGLNLQK pc2
P05154
1003:1007
25:25
14:95061036:A:G_G
p = 0.17, beta = −0.0837, N = 1255
DFTFDLYR pc2
P05154
1002:1007
25:25
14:95061036:A:G_G
p = 1.6e−06, beta = −0.292, N = 1254
QINDYVAK pc2
P05154
1003:1007
25:25
14:95061036:A:G_G
p = 0.054, beta = −0.118, N = 1255
QLELYLPK pc2
P05154
1000:1007
25:25
14:95061036:A:G_G
p = 0.038, beta = −0.126, N = 1252
GFQQLLQELNQPR pc2
P05154
1003:1007
25:25
14:95061036:A:G_G
p = 0.0087, beta = 0.16, N = 1255
WETSFNHK pc2
P05154
1003:1007
25:25
14:95061036:A:G_G
p = 0.072, beta = −0.11, N = 1255
GTQEQDFYVTSETVVR pc3
P05154
1003:1007
24:25
14:95061036:A:G_G
p = 0.041, beta = −0.126, N = 1254
EDQYHYLLDR pc3
P05154
1003:1007
25:25
14:95061036:A:G_G
p = 0.47, beta = −0.044, N = 1254
NLDSNAVVIMVNYIFFK pc2
P05154
913:1007
24:25
14:95061036:A:G_G
p = 0.31, beta = −0.0647, N = 1148
RQLELYLPK pc2
P05154
954:1007
24:25
14:95061036:A:G_G
p = 0.54, beta = −0.0382, N = 1194
RDFTFDLYR pc2
P05154
986:1007
25:25
14:95061036:A:G_G
p = 4.4e−10, beta = −0.381, N = 1234
RVEDLHVGATVAPSSR pc3
P05154
990:1007
25:25
14:95061036:A:G_G
p = 0.22, beta = −0.0757, N = 1239
TLYLADTFPTNFRDSAGAMK pc3
P05154
793:1007
14:25
14:95061036:A:G_G
p = 0.16, beta = −0.103, N = 971
VVGVPYQGNATALFILPSEGK pc3
P05154
227:1007
66:228
11:25
14:95061036:A:G_G
p = 0.8, beta = 0.0271, N = 304
SERPINA5 : NP3
P05154
258:258
64:64
3:3
14:95061036:A:G_G
p = 0.095, beta = −0.211, N = 325
TLYLADTFPTNFR pc2
P05154
258:258
64:64
3:3
14:95061036:A:G_G
p = 0.1, beta = −0.206, N = 325
AVVEVDESGTR pc2
P05154
258:258
64:64
3:3
14:95061036:A:G_G
p = 0.087, beta = −0.216, N = 325
FSIEGSYQLEK pc2
P05154
258:258
64:64
3:3
14:95061036:A:G_G
p = 0.16, beta = −0.178, N = 325
AAAATGTIFTFR pc2
P05154
258:258
64:64
3:3
14:95061036:A:G_G
p = 0.025, beta = −0.283, N = 325
MQQVENGLSEK pc2
P05154
258:258
64:64
3:3
14:95061036:A:G_G
p = 0.29, beta = −0.135, N = 325
MQILEGLGLNLQK pc2
P05154
258:258
64:64
3:3
14:95061036:A:G_G
p = 0.12, beta = −0.198, N = 325
QINDYVAK pc2
P05154
258:258
64:64
3:3
14:95061036:A:G_G
p = 0.28, beta = −0.138, N = 325
DFTFDLYR pc2
P05154
258:258
64:64
3:3
14:95061036:A:G_G
p = 0.013, beta = −0.315, N = 325
QLELYLPK pc2
P05154
258:258
64:64
3:3
14:95061036:A:G_G
p = 0.69, beta = −0.051, N = 325
WETSFNHK pc2
P05154
258:258
64:64
3:3
14:95061036:A:G_G
p = 0.68, beta = 0.0519, N = 325
GFQQLLQELNQPR pc2
P05154
258:258
64:64
3:3
14:95061036:A:G_G
p = 0.00092, beta = 0.416, N = 325
EDQYHYLLDR pc3
P05154
258:258
64:64
3:3
14:95061036:A:G_G
p = 0.93, beta = −0.0111, N = 325
GTQEQDFYVTSETVVR pc3
P05154
257:258
64:64
3:3
14:95061036:A:G_G
p = 0.042, beta = −0.257, N = 324
RQLELYLPK pc2
P05154
258:258
64:64
3:3
14:95061036:A:G_G
p = 0.8, beta = −0.0318, N = 325
NLDSNAVVIMVNYIFFK pc2
P05154
181:258
43:64
3:3
14:95061036:A:G_G
p = 0.036, beta = 0.309, N = 227
RDFTFDLYR pc2
P05154
257:258
62:64
3:3
14:95061036:A:G_G
p = 0.067, beta = −0.234, N = 322
RVEDLHVGATVAPSSR pc3
P05154
258:258
63:64
3:3
14:95061036:A:G_G
p = 0.39, beta = 0.109, N = 324
TLYLADTFPTNFRDSAGAMK pc3
P05154
189:258
47:64
2:3
14:95061036:A:G_G
p = 0.069, beta = −0.269, N = 238
AVVEVDESGTRAAAATGTIFTFR pc
P05154
54:258
7:64
0:3
14:95061036:A:G_G
p = 0.29, beta = −0.405, N = 61
Assay Target: SERPINA5
UniProt: P05154
rsID: rs6119
Target SNP: 14:94587675:G:A
Proxy SNP: 14:95061036:A:G
deCODE log10(p): 148.4
deCODE BETA: 0.35
deCODE MSPA: 0.198 (2)
1250:1254:1255:1255:1249:125
ELHRGFQQLLQELNQPR pc4
rs6119 REF
997:1007
1:25
14:95061036:A:G_G
p = 3.1e−35, model = DOM, N = 1208
SSEEELHR pc2
rs6119 ALT
33:1007
218:228
25:25
14:95061036:A:G_G
p = 1.7e−207, model = REC, N = 276
ELHRGFQQLLQELNQPR pc3
rs6119 REF
950:1007
1:25
14:95061036:A:G_G
p = 3.9e−25, model = DOM, N = 1143

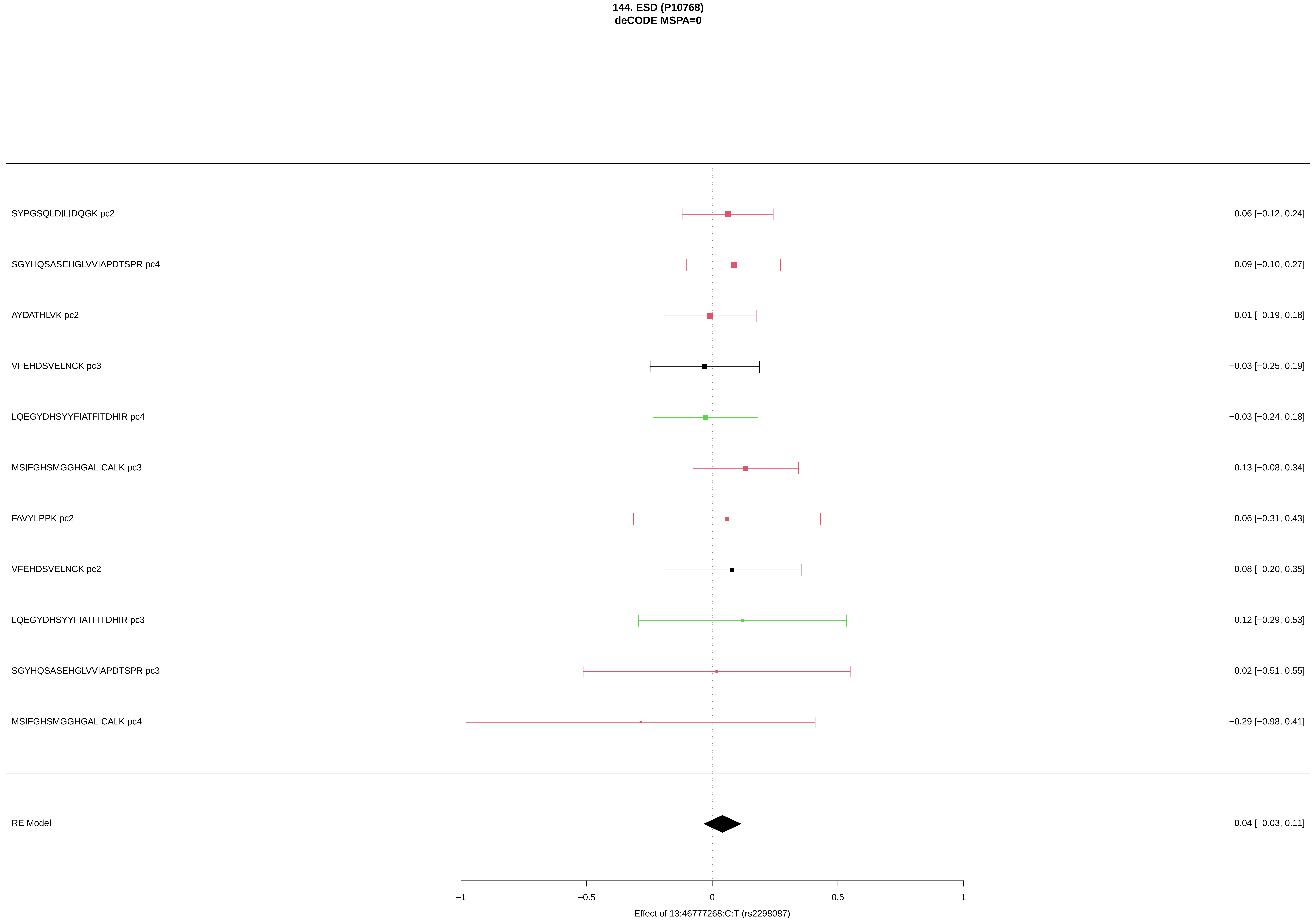
144. ESD (P10768)
deCODE MSPA=0
SYPGSQLDILIDQGK pc2
0.06 [−0.12, 0.24]
SGYHQSASEHGLVVIAPDTSPR pc4
0.09 [−0.10, 0.27]
AYDATHLVK pc2
−0.01 [−0.19, 0.18]
VFEHDSVELNCK pc3
−0.03 [−0.25, 0.19]
LQEGYDHSYYFIATFITDHIR pc4
−0.03 [−0.24, 0.18]
MSIFGHSMGGHGALICALK pc3
0.13 [−0.08, 0.34]
FAVYLPPK pc2
0.06 [−0.31, 0.43]
VFEHDSVELNCK pc2
0.08 [−0.20, 0.35]
LQEGYDHSYYFIATFITDHIR pc3
0.12 [−0.29, 0.53]
SGYHQSASEHGLVVIAPDTSPR pc3
0.02 [−0.51, 0.55]
MSIFGHSMGGHGALICALK pc4
−0.29 [−0.98, 0.41]
RE Model
0.04 [−0.03, 0.11]
−1
−0.5
0
0.5
1
Effect of 13:46777268:C:T (rs2298087)

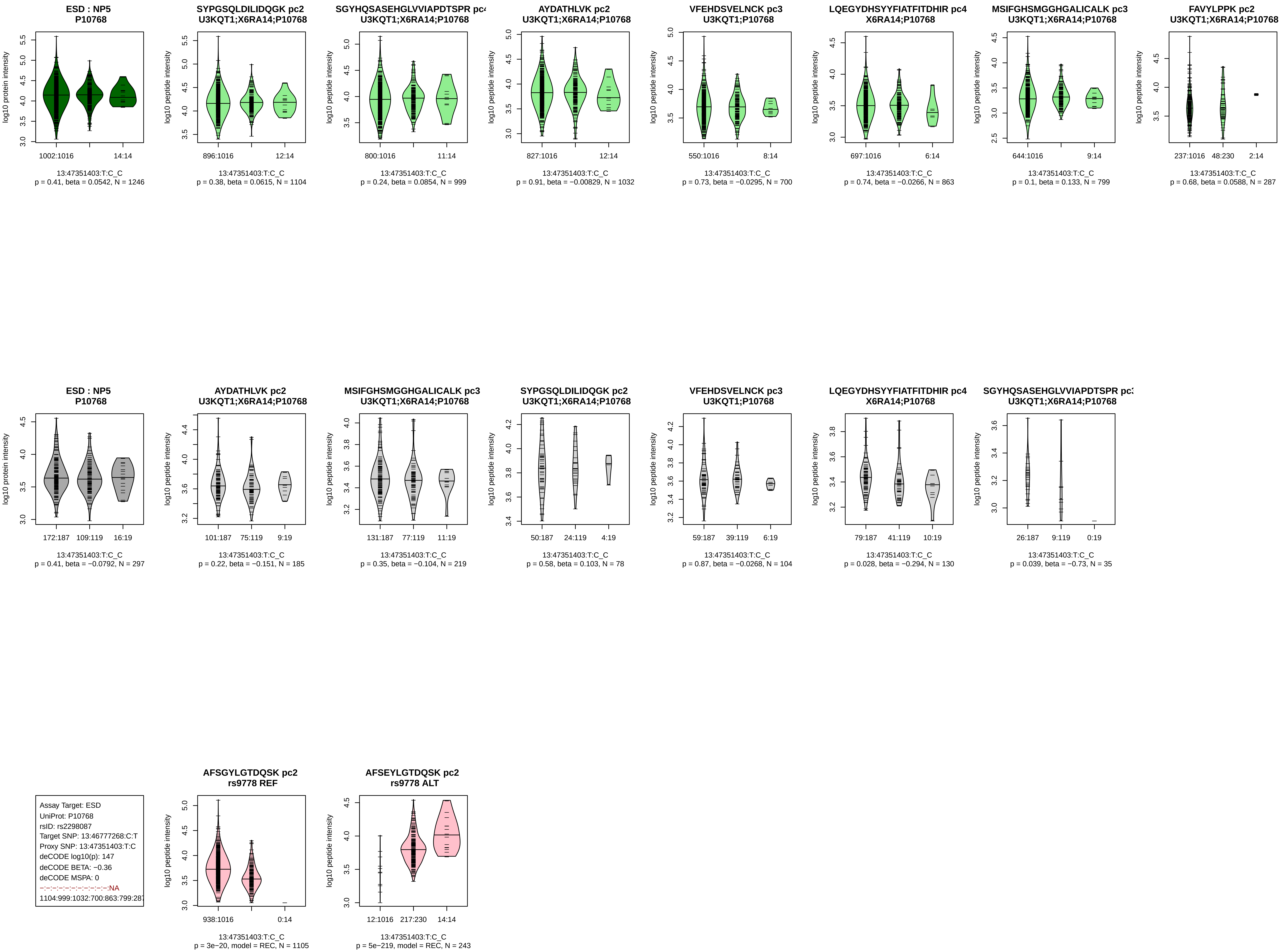
ESD : NP5
P10768
log10 protein intensity
1002:1016
14:14
13:47351403:T:C_C
p = 0.41, beta = 0.0542, N = 1246
SYPGSQLDILIDQGK pc2
U3KQT1;X6RA14;P10768
log10 peptide intensity
896:1016
12:14
13:47351403:T:C_C
p = 0.38, beta = 0.0615, N = 1104
SGYHQSASEHGLVVIAPDTSPR pc4
U3KQT1;X6RA14;P10768
800:1016
11:14
13:47351403:T:C_C
p = 0.24, beta = 0.0854, N = 999
AYDATHLVK pc2
U3KQT1;X6RA14;P10768
827:1016
12:14
13:47351403:T:C_C
p = 0.91, beta = −0.00829, N = 1032
VFEHDSVELNCK pc3
U3KQT1;P10768
550:1016
8:14
13:47351403:T:C_C
p = 0.73, beta = −0.0295, N = 700
LQEGYDHSYYFIATFITDHIR pc4
X6RA14;P10768
697:1016
6:14
13:47351403:T:C_C
p = 0.74, beta = −0.0266, N = 863
MSIFGHSMGGHGALICALK pc3
U3KQT1;X6RA14;P10768
644:1016
9:14
13:47351403:T:C_C
p = 0.1, beta = 0.133, N = 799
FAVYLPPK pc2
U3KQT1;X6RA14;P10768
237:1016
48:230
2:14
13:47351403:T:C_C
p = 0.68, beta = 0.0588, N = 287
ESD : NP5
P10768
172:187
109:119
16:19
13:47351403:T:C_C
p = 0.41, beta = −0.0792, N = 297
AYDATHLVK pc2
U3KQT1;X6RA14;P10768
101:187
75:119
9:19
13:47351403:T:C_C
p = 0.22, beta = −0.151, N = 185
MSIFGHSMGGHGALICALK pc3
U3KQT1;X6RA14;P10768
131:187
77:119
11:19
13:47351403:T:C_C
p = 0.35, beta = −0.104, N = 219
SYPGSQLDILIDQGK pc2
U3KQT1;X6RA14;P10768
50:187
24:119
4:19
13:47351403:T:C_C
p = 0.58, beta = 0.103, N = 78
VFEHDSVELNCK pc3
U3KQT1;P10768
59:187
39:119
6:19
13:47351403:T:C_C
p = 0.87, beta = −0.0268, N = 104
LQEGYDHSYYFIATFITDHIR pc4
X6RA14;P10768
79:187
41:119
10:19
13:47351403:T:C_C
p = 0.028, beta = −0.294, N = 130
SGYHQSASEHGLVVIAPDTSPR pc3
U3KQT1;X6RA14;P10768
26:187
9:119
0:19
13:47351403:T:C_C
p = 0.039, beta = −0.73, N = 35
Assay Target: ESD
UniProt: P10768
rsID: rs2298087
Target SNP: 13:46777268:C:T
Proxy SNP: 13:47351403:T:C
deCODE log10(p): 147
deCODE BETA: −0.36
deCODE MSPA: 0
−:−:−:−:−:−:−:−:−:−:NA
1104:999:1032:700:863:799:287
AFSGYLGTDQSK pc2
rs9778 REF
938:1016
0:14
13:47351403:T:C_C
p = 3e−20, model = REC, N = 1105
AFSEYLGTDQSK pc2
rs9778 ALT
12:1016
217:230
14:14
13:47351403:T:C_C
p = 5e−219, model = REC, N = 243

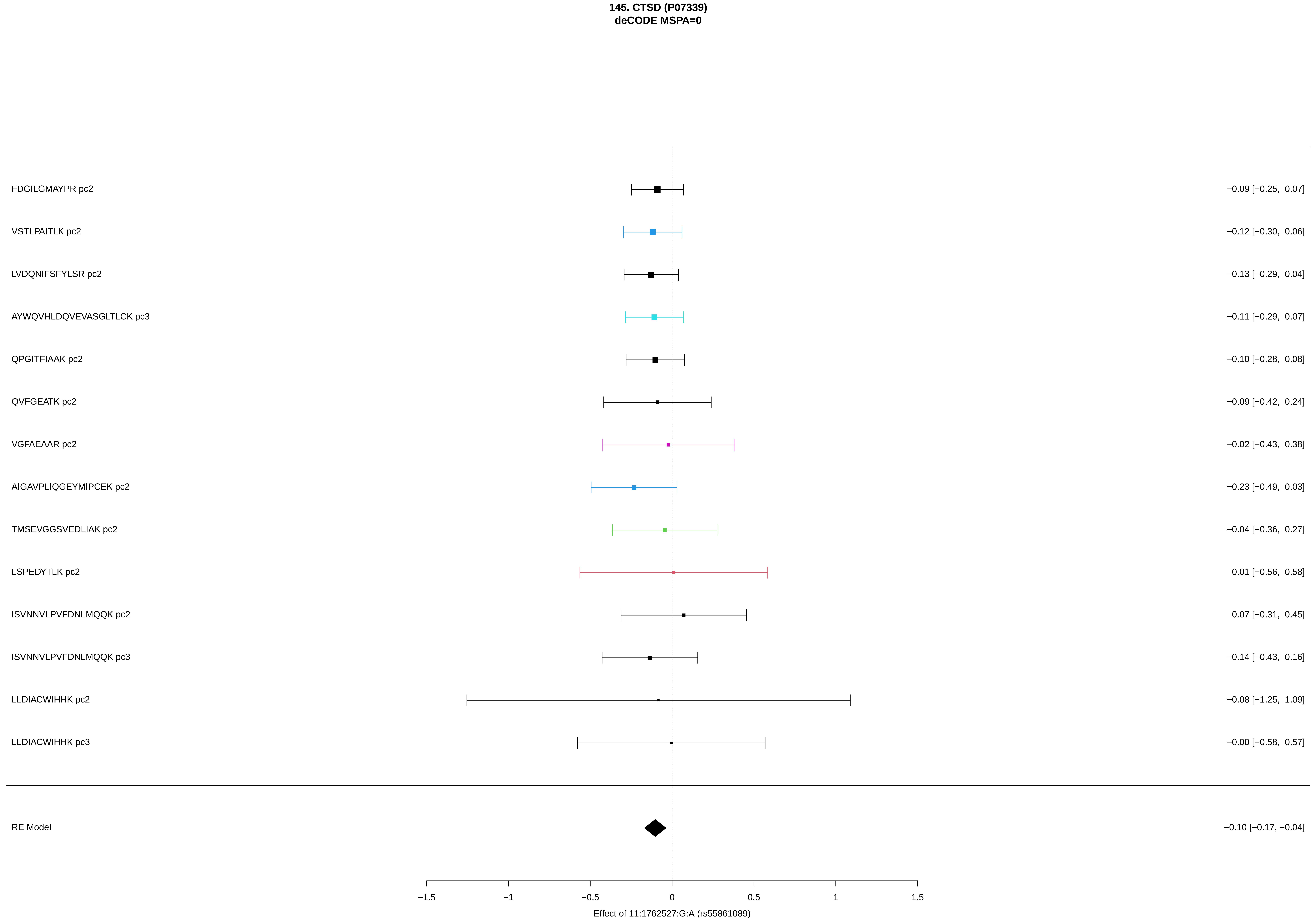

145. CTSD (P07339)
deCODE MSPA=0
FDGILGMAYPR pc2
−0.09 [−0.25, 0.07]
VSTLPAITLK pc2
−0.12 [−0.30, 0.06]
LVDQNIFSFYLSR pc2
−0.13 [−0.29, 0.04]
AYWQVHLDQVEVASGLTLCK pc3
−0.11 [−0.29, 0.07]
QPGITFIAAK pc2
−0.10 [−0.28, 0.08]
QVFGEATK pc2
−0.09 [−0.42, 0.24]
VGFAEAAR pc2
−0.02 [−0.43, 0.38]
AIGAVPLIQGEYMIPCEK pc2
−0.23 [−0.49, 0.03]
TMSEVGGSVEDLIAK pc2
−0.04 [−0.36, 0.27]
LSPEDYTLK pc2
0.01 [−0.56, 0.58]
ISVNNVLPVFDNLMQQK pc2
0.07 [−0.31, 0.45]
ISVNNVLPVFDNLMQQK pc3
−0.14 [−0.43, 0.16]
LLDIACWIHHK pc2
−0.08 [−1.25, 1.09]
LLDIACWIHHK pc3
−0.00 [−0.58, 0.57]
RE Model
−0.10 [−0.17, −0.04]
−1.5
−1
−0.5
0
0.5
1
1.5
Effect of 11:1762527:G:A (rs55861089)

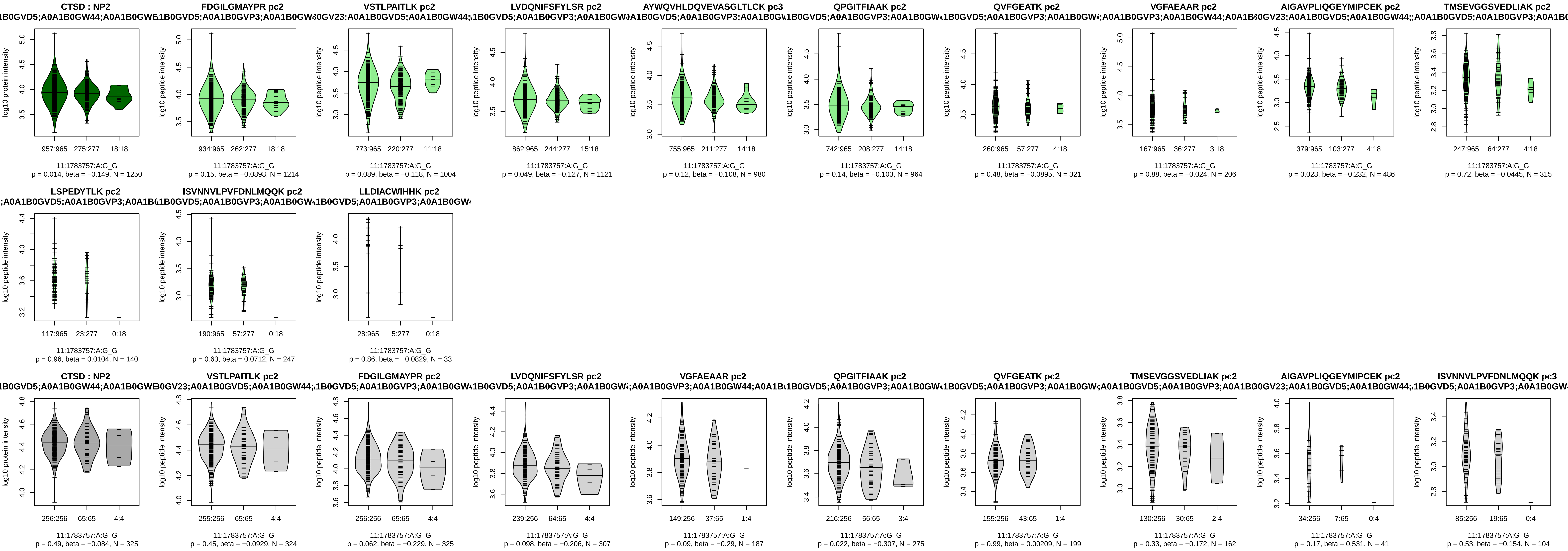

Assay Target: CTSD
UniProt: P07339
rsID: rs55861089
Target SNP: 11:1762527:G:A
Proxy SNP: 11:1783757:A:G
deCODE log10(p): 141.9
deCODE BETA: −0.33
deCODE MSPA: 0
–:–:–:–:–:–:–:–:–:–:–:–:NA
1214:1004:1121:980:964:321:2

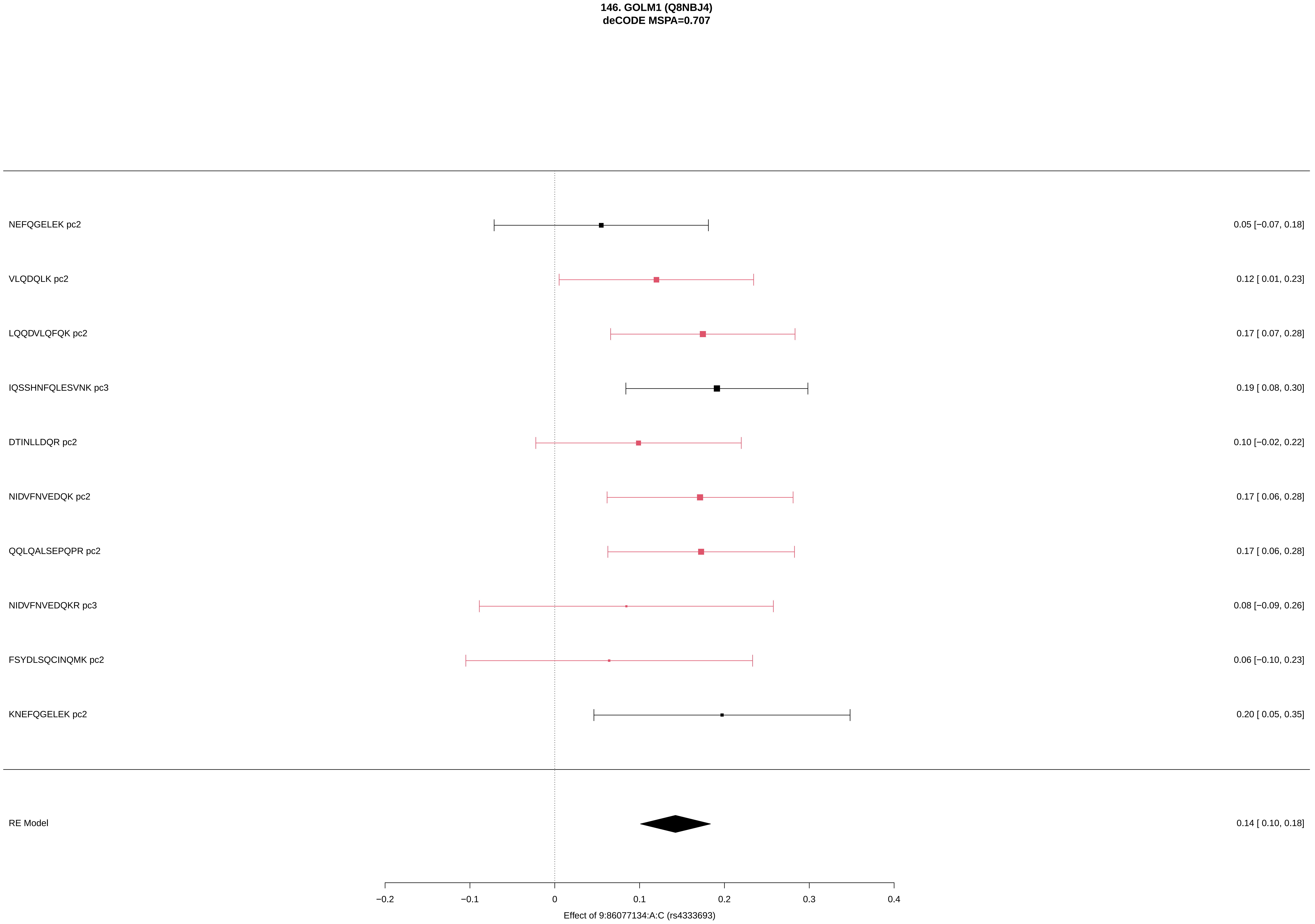

146. GOLM1 (Q8NBJ4)
deCODE MSPA=0.707
NEFQGELEK pc2
0.05 [−0.07, 0.18]
VLQDQLK pc2
0.12 [0.01, 0.23]
LQQDVLQFQK pc2
0.17 [0.07, 0.28]
IQSSHNFQLESVNK pc3
0.19 [0.08, 0.30]
DTINLLDQR pc2
0.10 [−0.02, 0.22]
NIDVFNVEDQK pc2
0.17 [0.06, 0.28]
QQLQALSEPQPR pc2
0.17 [0.06, 0.28]
NIDVFNVEDQKR pc3
0.08 [−0.09, 0.26]
FSYDLSQCINQMK pc2
0.06 [−0.10, 0.23]
KNEFQGELEK pc2
0.20 [0.05, 0.35]
RE Model
0.14 [0.10, 0.18]
−0.2
−0.1
0
0.1
0.2
0.3
0.4
Effect of 9:86077134:A:C (rs4333693)

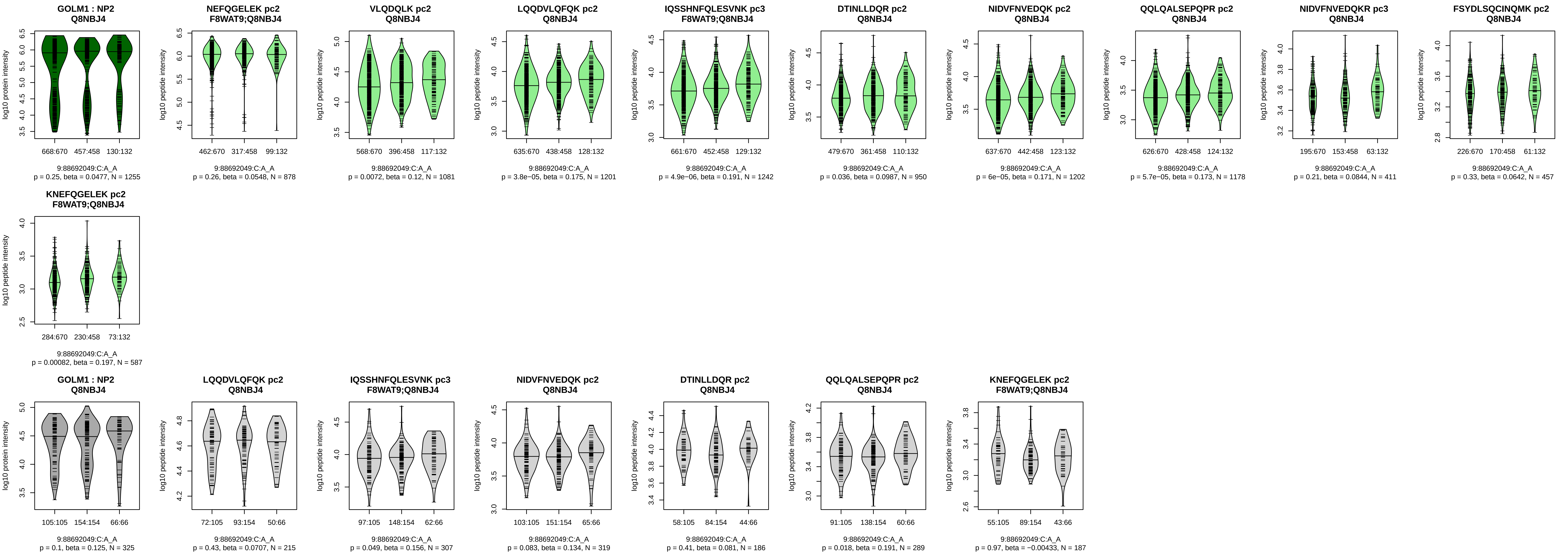

Assay Target: GOLM1
UniProt: Q8NBJ4
rsID: rs4333693
Target SNP: 9:86077134:A:C
Proxy SNP: 9:88692049:C:A
deCODE log10(p): 140.8
deCODE BETA: 0.26
deCODE MSPA: 0.707
−:*:*:*:−:*:*:−:−:*:NA
878:1081:1201:1242:950:1202:1

147. ACP5 (P13686)
deCODE MSPA=0.402

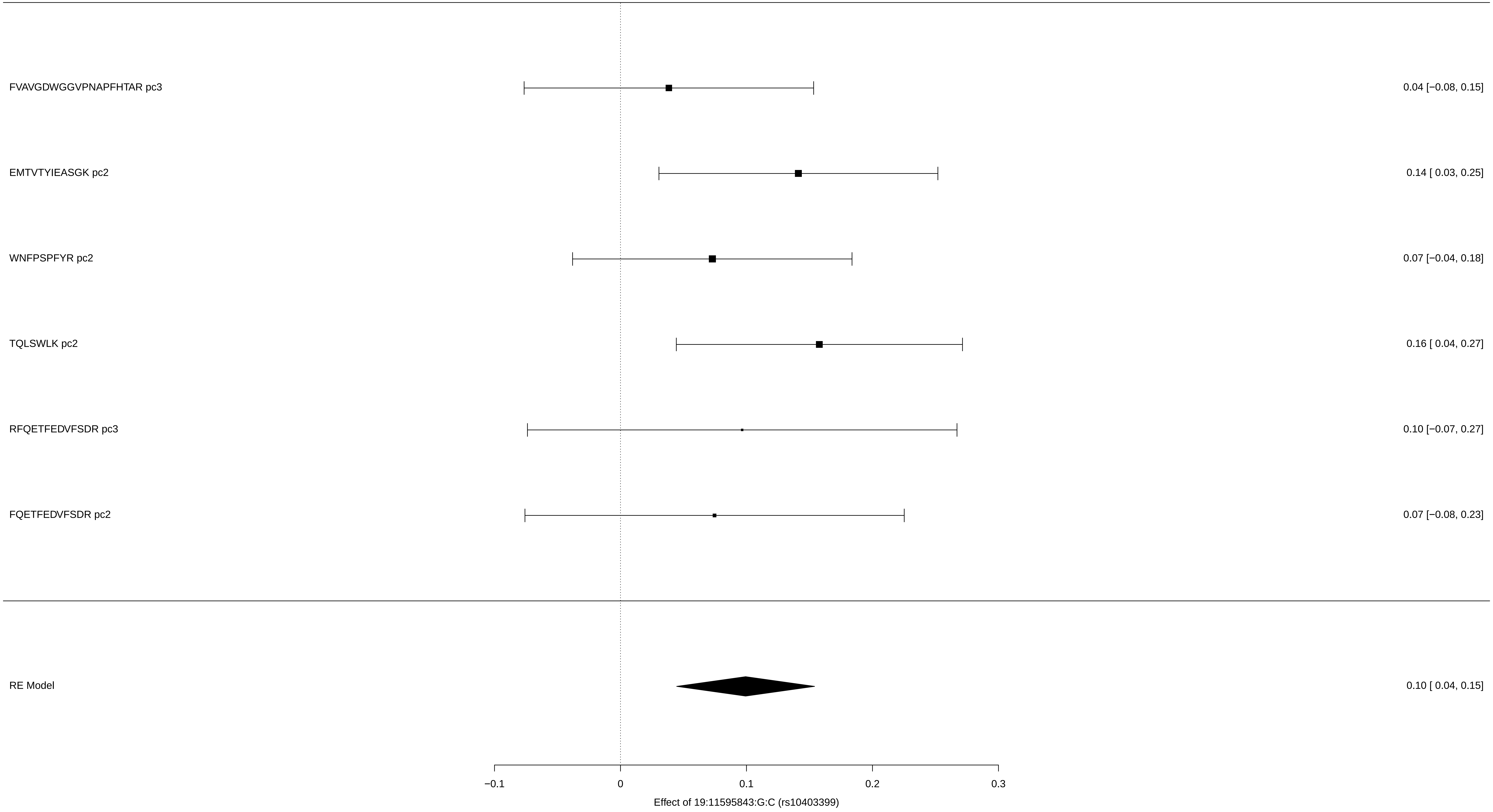

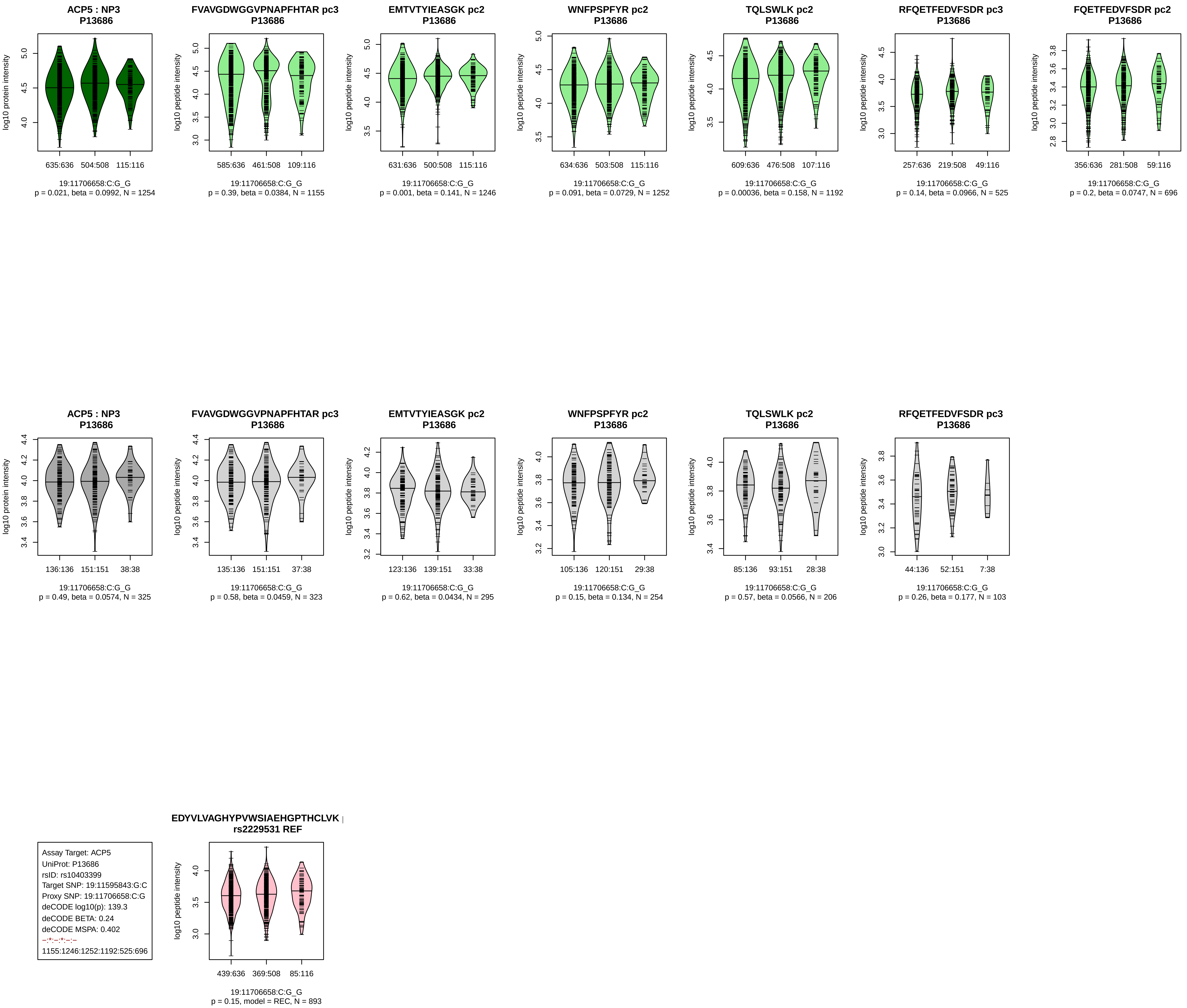
ACP5 : NP3
P13686
log10 protein intensity
635:636 504:508 115:116
19:11706658:C:G_G
p = 0.021, beta = 0.0992, N = 1254
FVAVGDWGGVPNAPFHTAR pc3
P13686
log10 peptide intensity
585:636 461:508 109:116
19:11706658:C:G_G
p = 0.39, beta = 0.0384, N = 1155
EMTVTYIEASGK pc2
P13686
631:636 500:508 115:116
19:11706658:C:G_G
p = 0.001, beta = 0.141, N = 1246
WNFPSPFYR pc2
P13686
634:636 503:508 115:116
19:11706658:C:G_G
p = 0.091, beta = 0.0729, N = 1252
TQLSWLK pc2
P13686
609:636 476:508 107:116
19:11706658:C:G_G
p = 0.00036, beta = 0.158, N = 1192
RFQETFEDVFSDR pc3
P13686
257:636 219:508 49:116
19:11706658:C:G_G
p = 0.14, beta = 0.0966, N = 525
FQETFEDVFSDR pc2
P13686
356:636 281:508 59:116
19:11706658:C:G_G
p = 0.2, beta = 0.0747, N = 696
ACP5 : NP3
P13686
136:136 151:151 38:38
19:11706658:C:G_G
p = 0.49, beta = 0.0574, N = 325
FVAVGDWGGVPNAPFHTAR pc3
P13686
135:136 151:151 37:38
19:11706658:C:G_G
p = 0.58, beta = 0.0459, N = 323
EMTVTYIEASGK pc2
P13686
123:136 139:151 33:38
19:11706658:C:G_G
p = 0.62, beta = 0.0434, N = 295
WNFPSPFYR pc2
P13686
105:136 120:151 29:38
19:11706658:C:G_G
p = 0.15, beta = 0.134, N = 254
TQLSWLK pc2
P13686
85:136 93:151 28:38
19:11706658:C:G_G
p = 0.57, beta = 0.0566, N = 206
RFQETFEDVFSDR pc3
P13686
44:136 52:151 7:38
19:11706658:C:G_G
p = 0.26, beta = 0.177, N = 103
Assay Target: ACP5
UniProt: P13686
rsID: rs10403399
Target SNP: 19:11595843:G:C
Proxy SNP: 19:11706658:C:G
deCODE log10(p): 139.3
deCODE BETA: 0.24
deCODE MSPA: 0.402
-:*:-:*:-:-
1155:1246:1252:1192:525:696
EDYVLVAGHYPVWSIAEHGPTHCLVK
rs2229531 REF
439:636 369:508 85:116
19:11706658:C:G_G
p = 0.15, model = REC, N = 893

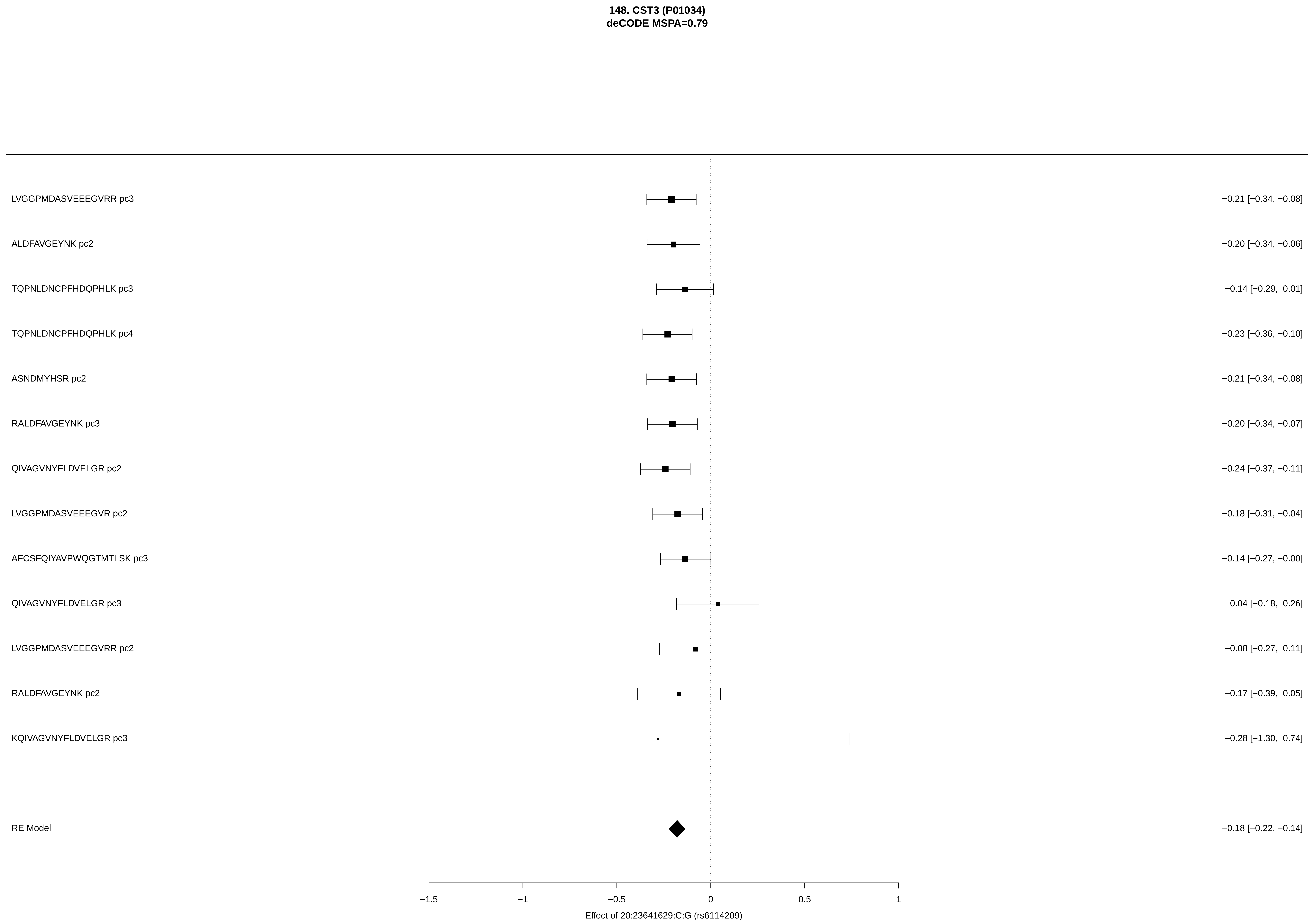
148. CST3 (P01034)
deCODE MSPA=0.79
LVGGPMDASVEEEGVRR pc3
ALDFAVGEYNK pc2
TQPNLDNCPFHDQPHLK pc3
TQPNLDNCPFHDQPHLK pc4
ASNDMYHSR pc2
RALDFAVGEYNK pc3
QIVAGVNYFLDVELGR pc2
LVGGPMDASVEEEGVR pc2
AFCSFQIYAVPWQGTMTLSK pc3
QIVAGVNYFLDVELGR pc3
LVGGPMDASVEEEGVRR pc2
RALDFAVGEYNK pc2
KQIVAGVNYFLDVELGR pc3
RE Model
−0.21 [−0.34, −0.08]
−0.20 [−0.34, −0.06]
−0.14 [−0.29, 0.01]
−0.23 [−0.36, −0.10]
−0.21 [−0.34, −0.08]
−0.20 [−0.34, −0.07]
−0.24 [−0.37, −0.11]
−0.18 [−0.31, −0.04]
−0.14 [−0.27, −0.00]
0.04 [−0.18, 0.26]
−0.08 [−0.27, 0.11]
−0.17 [−0.39, 0.05]
−0.28 [−1.30, 0.74]
−0.18 [−0.22, −0.14]
−1.5
−1
−0.5
0
0.5
1
Effect of 20:23641629:C:G (rs6114209)

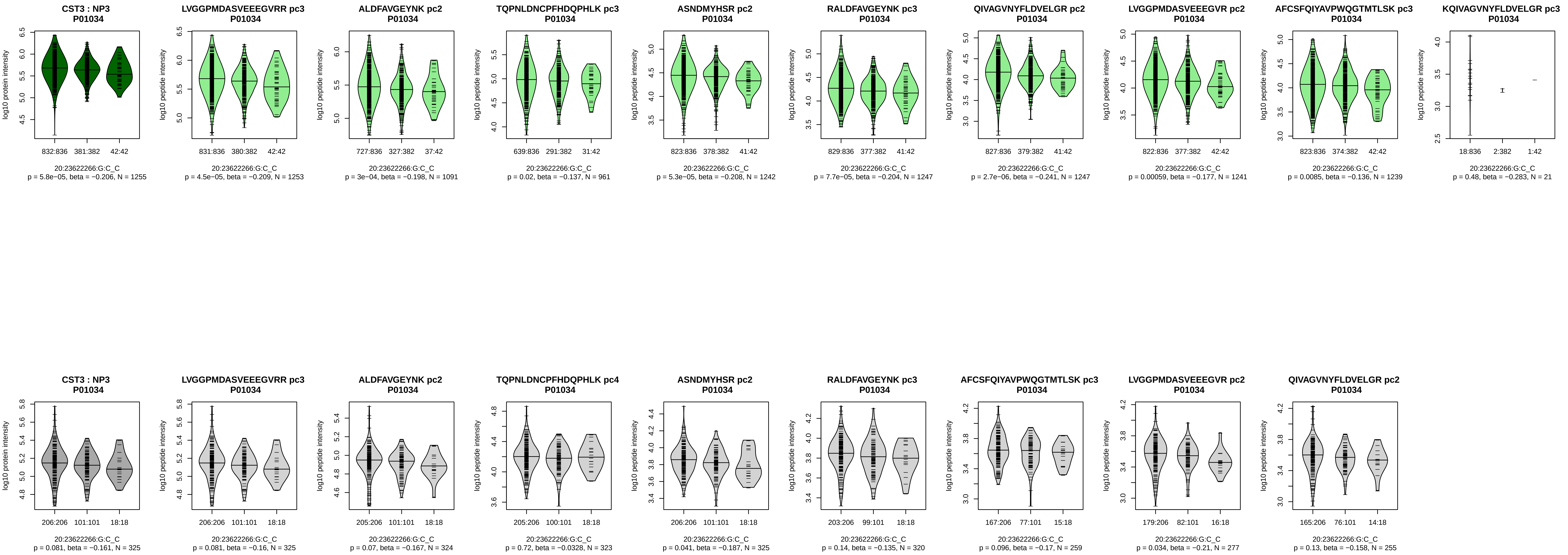

Assay Target: CST3
UniProt: P01034
rsID: rs6114209
Target SNP: 20:23641629:C:G
Proxy SNP: 20:23622266:G:C
deCODE log10(p): 137.9
deCODE BETA: −0.25
deCODE MSPA: 0.79
::−:*:*:*:*:*:*:−:−:−:NA:−
1253:1091:961:1253:1242:1247

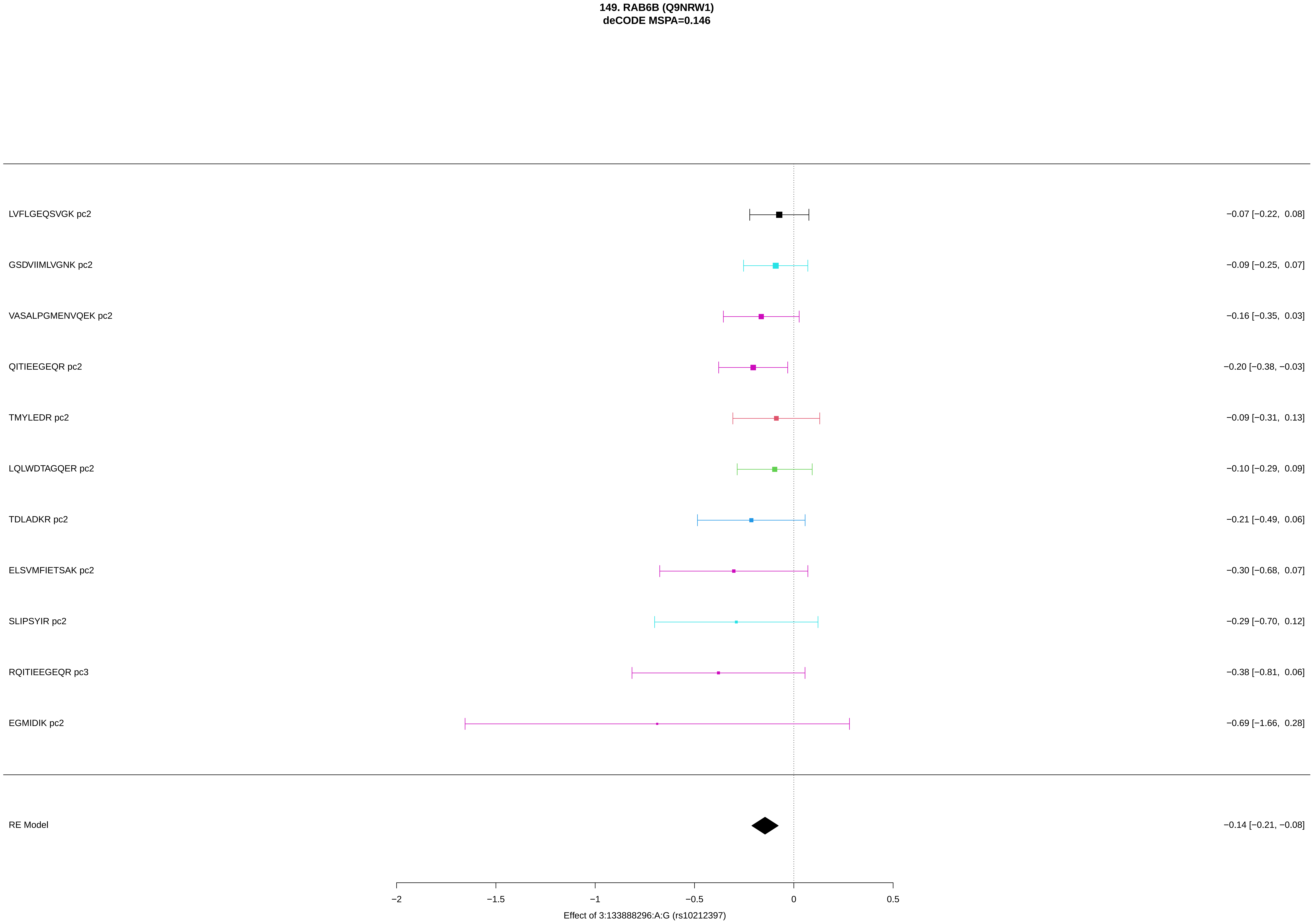
149. RAB6B (Q9NRW1)
deCODE MSPA=0.146
LVFLGEQSVGK pc2
−0.07 [−0.22, 0.08]
GSDVIIMLVGNK pc2
−0.09 [−0.25, 0.07]
VASALPGMENVQEK pc2
−0.16 [−0.35, 0.03]
QITIEEGEQR pc2
−0.20 [−0.38, −0.03]
TMYLEDR pc2
−0.09 [−0.31, 0.13]
LQLWDTAGQER pc2
−0.10 [−0.29, 0.09]
TDLADKR pc2
−0.21 [−0.49, 0.06]
ELSVMFIETSAK pc2
−0.30 [−0.68, 0.07]
SLIPSYIR pc2
−0.29 [−0.70, 0.12]
RQITIEEGEQR pc3
−0.38 [−0.81, 0.06]
EGMIDIK pc2
−0.69 [−1.66, 0.28]
RE Model
−0.14 [−0.21, −0.08]
−2
−1.5
−1
−0.5
0
0.5
Effect of 3:133888296:A:G (rs10212397)

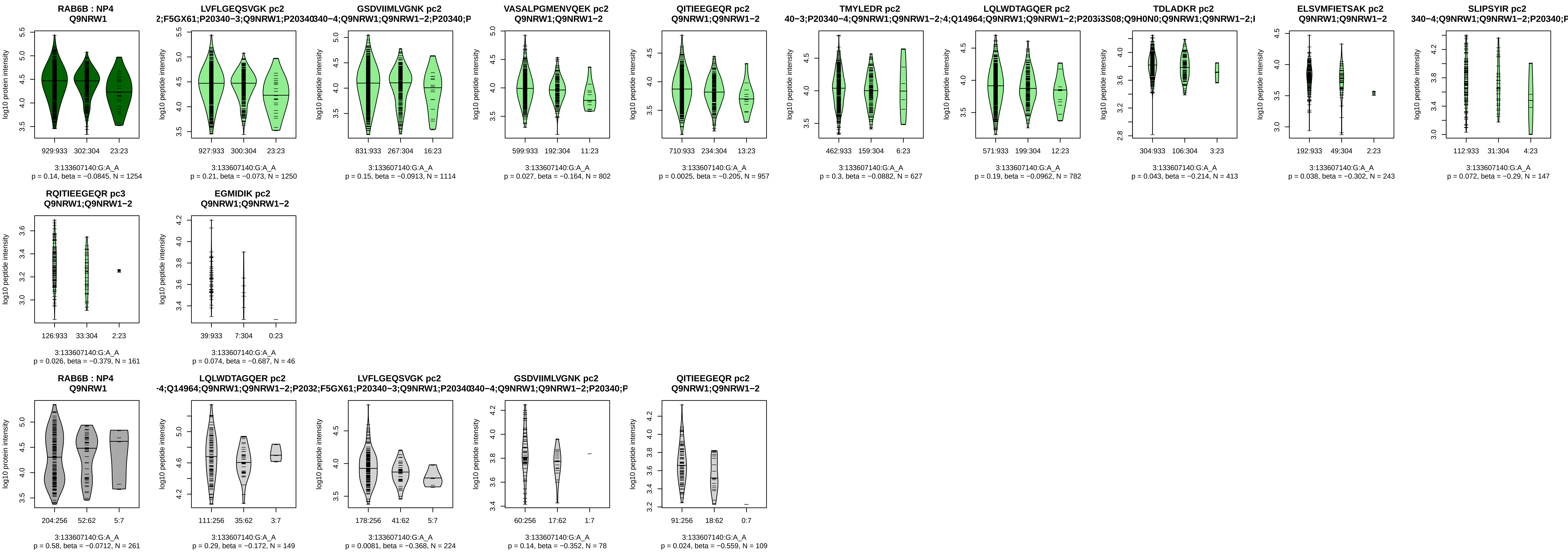

Assay Target: RAB6B
UniProt: Q9NRW1
rsID: rs10212397
Target SNP: 3:133888296:A:G
Proxy SNP: 3:133607140:G:A
deCODE log10(p): 134
deCODE BETA: −0.26
deCODE MSPA: 0.146
−:−:−:*:−:−:−:−:−:−
1250:1114:802:957:627:782:413

150. THBS4 (P35443)
deCODE MSPA=0.065 (1 conflicts)

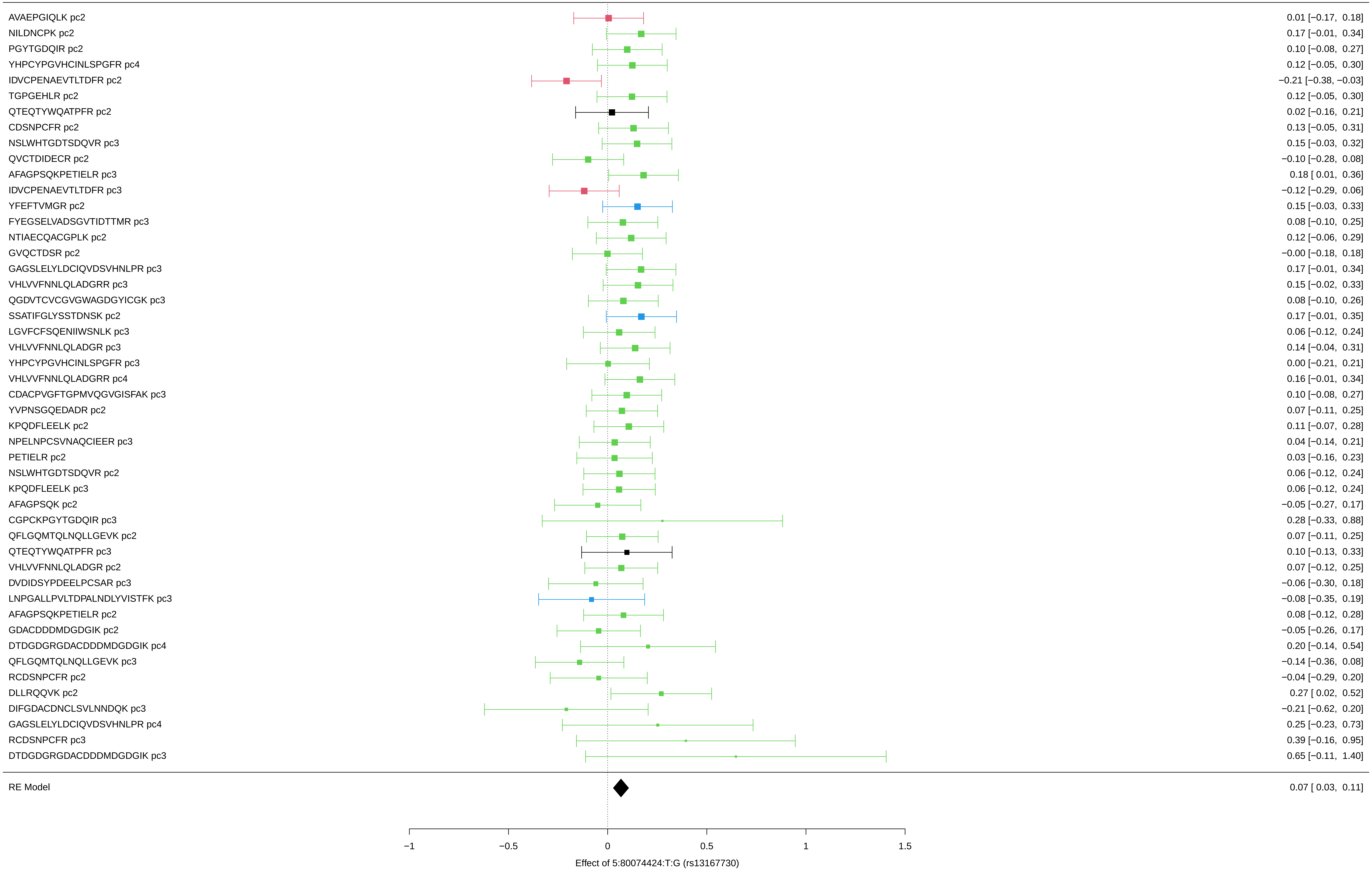

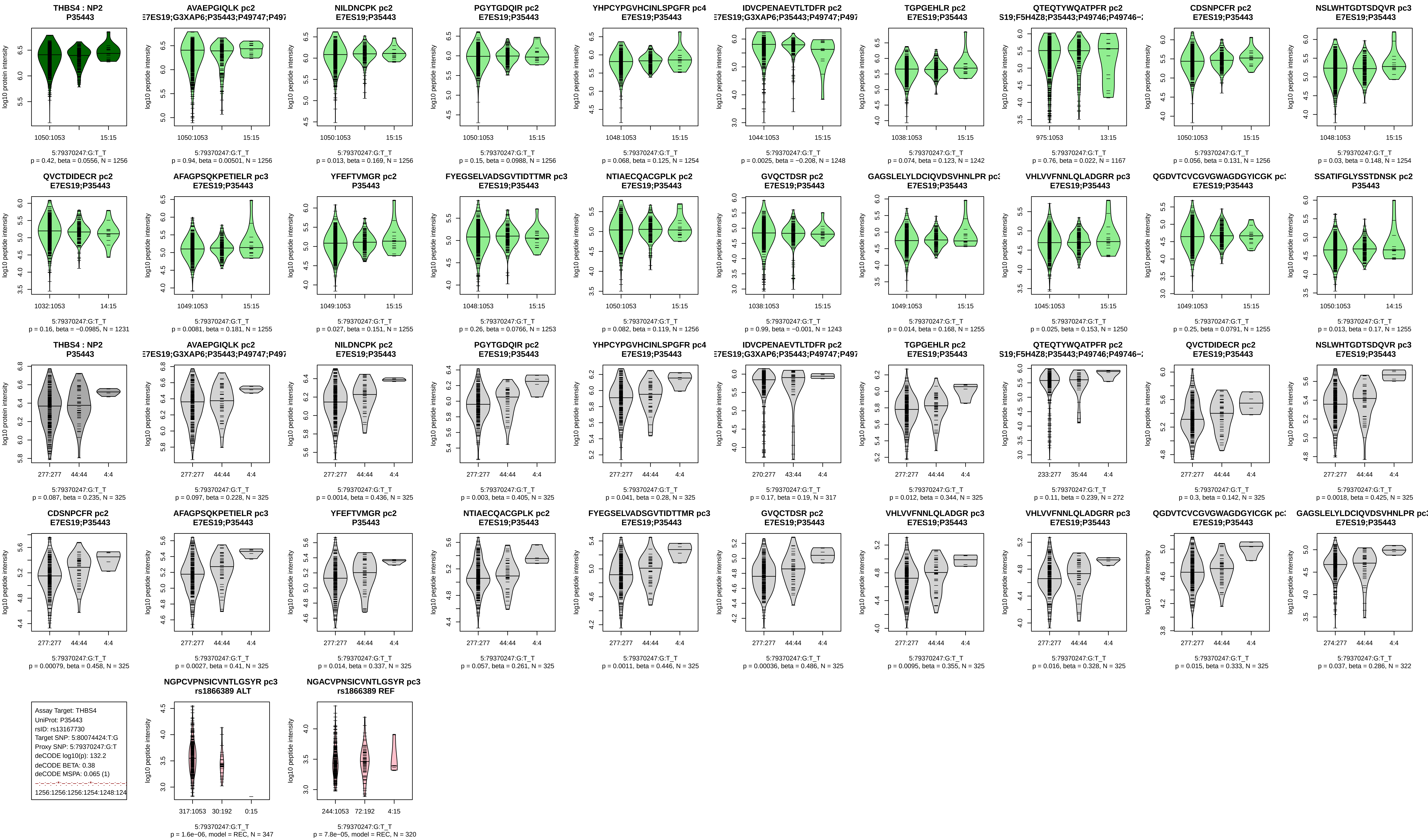
THBS4 : NP2
P35443
log10 protein intensity
1050:1053
15:15
5:79370247:G:T_T
p = 0.42, beta = 0.0556, N = 1256
AVAEPGIQLK pc2
E7ES19;G3XAP6;P35443;P49747;P497
log10 peptide intensity
1050:1053
15:15
5:79370247:G:T_T
p = 0.94, beta = 0.00501, N = 1256
NILDNCPK pc2
E7ES19;P35443
1050:1053
15:15
5:79370247:G:T_T
p = 0.013, beta = 0.169, N = 1256
PGYTGDQIR pc2
E7ES19;P35443
1050:1053
15:15
5:79370247:G:T_T
p = 0.15, beta = 0.0988, N = 1256
YHPCYPGVHCINLSPGFR pc4
E7ES19;P35443
1048:1053
15:15
5:79370247:G:T_T
p = 0.068, beta = 0.125, N = 1254
IDVCPENAEVTLTDFR pc2
E7ES19;G3XAP6;P35443;P49747;P497
1044:1053
15:15
5:79370247:G:T_T
p = 0.0025, beta = −0.208, N = 1248
TGPGEHLR pc2
E7ES19;P35443
1038:1053
15:15
5:79370247:G:T_T
p = 0.074, beta = 0.123, N = 1242
QTEQTYWQATPFR pc2
S19;F5H4Z8;P35443;P49746;P49746−
975:1053
13:15
5:79370247:G:T_T
p = 0.76, beta = 0.022, N = 1167
CDSNPCFR pc2
E7ES19;P35443
1050:1053
15:15
5:79370247:G:T_T
p = 0.056, beta = 0.131, N = 1256
NSLWHTGDTSDQVR pc3
E7ES19;P35443
1048:1053
15:15
5:79370247:G:T_T
p = 0.03, beta = 0.148, N = 1254
QVCTDIDECR pc2
E7ES19;P35443
1032:1053
14:15
5:79370247:G:T_T
p = 0.16, beta = −0.0985, N = 1231
AFAGPSQKPETIELR pc3
E7ES19;P35443
1049:1053
15:15
5:79370247:G:T_T
p = 0.0081, beta = 0.181, N = 1255
YFEFTVMGR pc2
P35443
1049:1053
15:15
5:79370247:G:T_T
p = 0.027, beta = 0.151, N = 1255
FYEGSELVADSGVTIDTTMR pc3
E7ES19;P35443
1048:1053
15:15
5:79370247:G:T_T
p = 0.26, beta = 0.0766, N = 1253
NTIAECQACGPLK pc2
E7ES19;P35443
1050:1053
15:15
5:79370247:G:T_T
p = 0.082, beta = 0.119, N = 1256
GVQCTDSR pc2
E7ES19;P35443
1038:1053
15:15
5:79370247:G:T_T
p = 0.99, beta = −0.001, N = 1243
GAGSLELYLDCIQVDSVHNLPR pc3
E7ES19;P35443
1049:1053
15:15
5:79370247:G:T_T
p = 0.014, beta = 0.168, N = 1255
VHLVVFNNLQLADGRR pc3
E7ES19;P35443
1045:1053
15:15
5:79370247:G:T_T
p = 0.025, beta = 0.153, N = 1250
QGDVTCVCGVGWAGDGYICGK pc
E7ES19;P35443
1049:1053
15:15
5:79370247:G:T_T
p = 0.25, beta = 0.0791, N = 1255
SSATIFGLYSSTDNSK pc2
P35443
1050:1053
14:15
5:79370247:G:T_T
p = 0.013, beta = 0.17, N = 1255
THBS4 : NP2
P35443
277:277
44:44
4:4
5:79370247:G:T_T
p = 0.087, beta = 0.235, N = 325
AVAEPGIQLK pc2
E7ES19;G3XAP6;P35443;P49747;P497
277:277
44:44
4:4
5:79370247:G:T_T
p = 0.097, beta = 0.228, N = 325
NILDNCPK pc2
E7ES19;P35443
277:277
44:44
4:4
5:79370247:G:T_T
p = 0.0014, beta = 0.436, N = 325
PGYTGDQIR pc2
E7ES19;P35443
277:277
44:44
4:4
5:79370247:G:T_T
p = 0.003, beta = 0.405, N = 325
YHPCYPGVHCINLSPGFR pc4
E7ES19;P35443
277:277
44:44
4:4
5:79370247:G:T_T
p = 0.041, beta = 0.28, N = 325
IDVCPENAEVTLTDFR pc2
E7ES19;G3XAP6;P35443;P49747;P497
270:277
43:44
4:4
5:79370247:G:T_T
p = 0.17, beta = 0.19, N = 317
TGPGEHLR pc2
E7ES19;P35443
277:277
44:44
4:4
5:79370247:G:T_T
p = 0.012, beta = 0.344, N = 325
QTEQTYWQATPFR pc2
S19;F5H4Z8;P35443;P49746;P49746−
233:277
35:44
4:4
5:79370247:G:T_T
p = 0.11, beta = 0.239, N = 272
QVCTDIDECR pc2
E7ES19;P35443
277:277
44:44
4:4
5:79370247:G:T_T
p = 0.3, beta = 0.142, N = 325
NSLWHTGDTSDQVR pc3
E7ES19;P35443
277:277
44:44
4:4
5:79370247:G:T_T
p = 0.0018, beta = 0.425, N = 325
CDSNPCFR pc2
E7ES19;P35443
277:277
44:44
4:4
5:79370247:G:T_T
p = 0.00079, beta = 0.458, N = 325
AFAGPSQKPETIELR pc3
E7ES19;P35443
277:277
44:44
4:4
5:79370247:G:T_T
p = 0.0027, beta = 0.41, N = 325
YFEFTVMGR pc2
P35443
277:277
44:44
4:4
5:79370247:G:T_T
p = 0.014, beta = 0.337, N = 325
NTIAECQACGPLK pc2
E7ES19;P35443
277:277
44:44
4:4
5:79370247:G:T_T
p = 0.057, beta = 0.261, N = 325
FYEGSELVADSGVTIDTTMR pc3
E7ES19;P35443
277:277
44:44
4:4
5:79370247:G:T_T
p = 0.0011, beta = 0.446, N = 325
GVQCTDSR pc2
E7ES19;P35443
277:277
44:44
4:4
5:79370247:G:T_T
p = 0.00036, beta = 0.486, N = 325
VHLVVFNNLQLADGR pc3
E7ES19;P35443
277:277
44:44
4:4
5:79370247:G:T_T
p = 0.0095, beta = 0.355, N = 325
VHLVVFNNLQLADGRR pc3
E7ES19;P35443
277:277
44:44
4:4
5:79370247:G:T_T
p = 0.016, beta = 0.328, N = 325
QGDVTCVCGVGWAGDGYICGK pc
E7ES19;P35443
277:277
44:44
4:4
5:79370247:G:T_T
p = 0.015, beta = 0.333, N = 325
GAGSLELYLDCIQVDSVHNLPR pc3
E7ES19;P35443
274:277
44:44
4:4
5:79370247:G:T_T
p = 0.037, beta = 0.286, N = 322
Assay Target: THBS4
UniProt: P35443
rsID: rs13167730
Target SNP: 5:80074424:T:G
Proxy SNP: 5:79370247:G:T
deCODE log10(p): 132.2
deCODE BETA: 0.38
deCODE MSPA: 0.065 (1)
1256:1256:1256:1254:1248:124
NGPCVPNSICVNTLGSYR pc3
rs1866389 ALT
317:1053
30:192
0:15
5:79370247:G:T_T
p = 1.6e−06, model = REC, N = 347
NGACVPNSICVNTLGSYR pc3
rs1866389 REF
244:1053
72:192
4:15
5:79370247:G:T_T
p = 7.8e−05, model = REC, N = 320

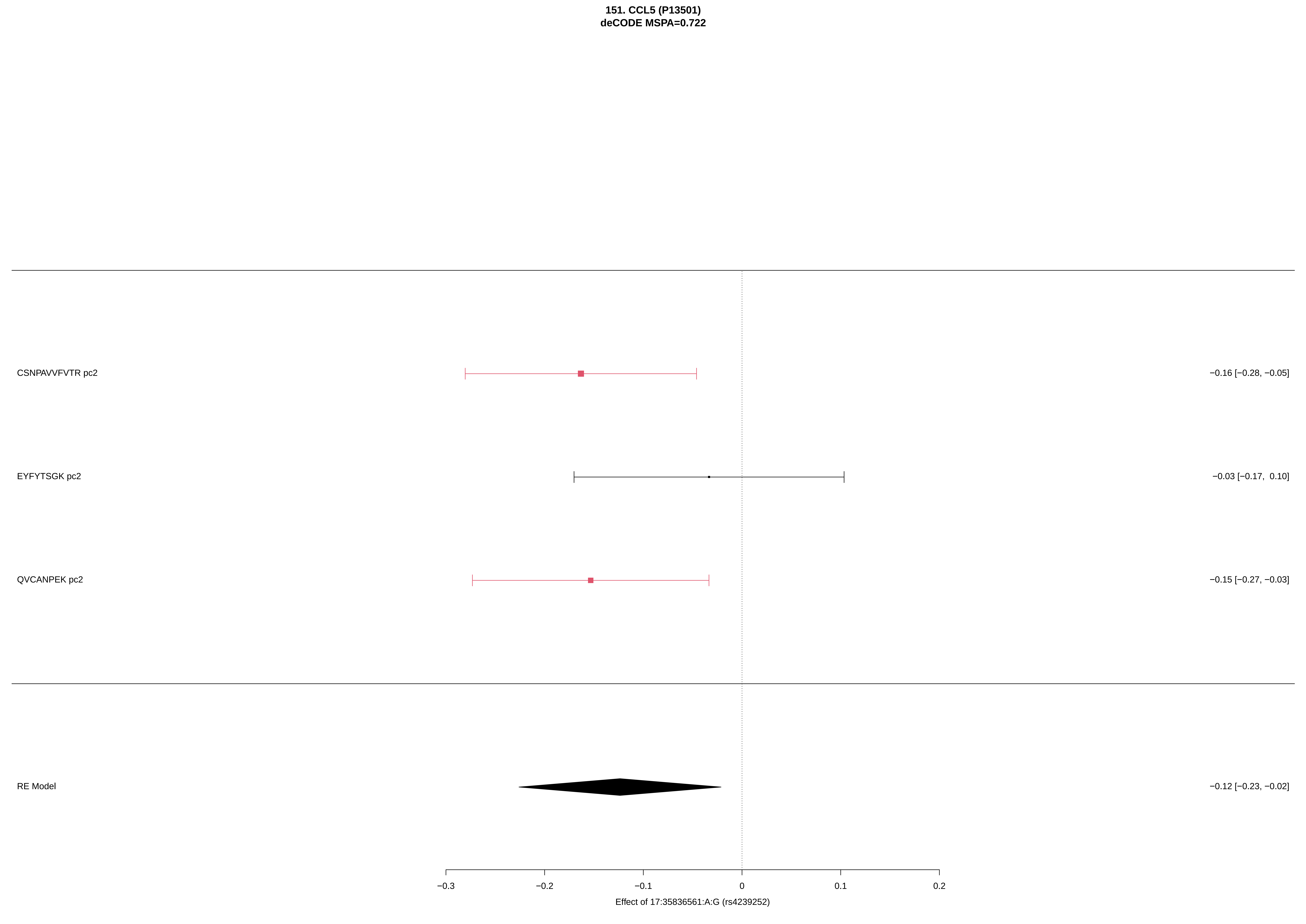
151. CCL5 (P13501)
deCODE MSPA=0.722
CSNPAVVFVTR pc2
−0.16 [−0.28, −0.05]
EYFYTSGK pc2
−0.03 [−0.17, 0.10]
QVCANPEK pc2
−0.15 [−0.27, −0.03]
RE Model
−0.12 [−0.23, −0.02]
−0.3
−0.2
−0.1
0
0.1
0.2
Effect of 17:35836561:A:G (rs4239252)

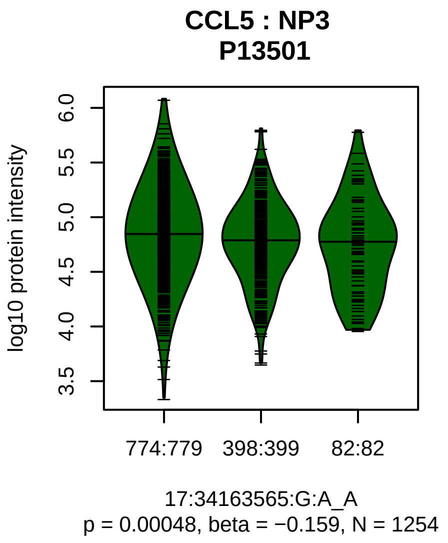

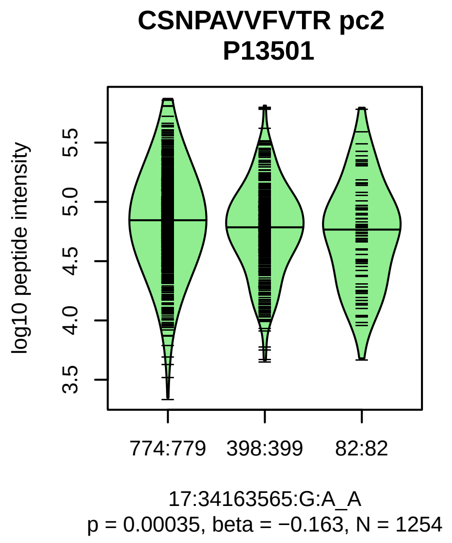

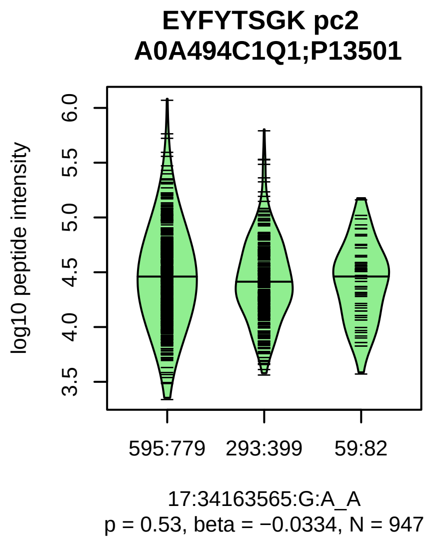

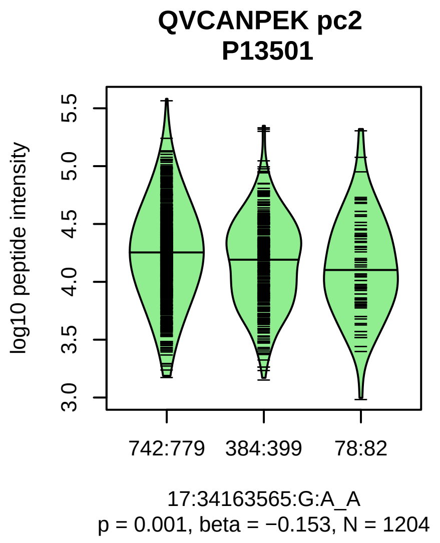

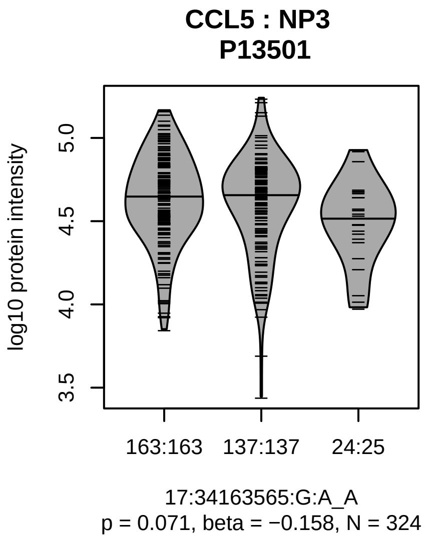

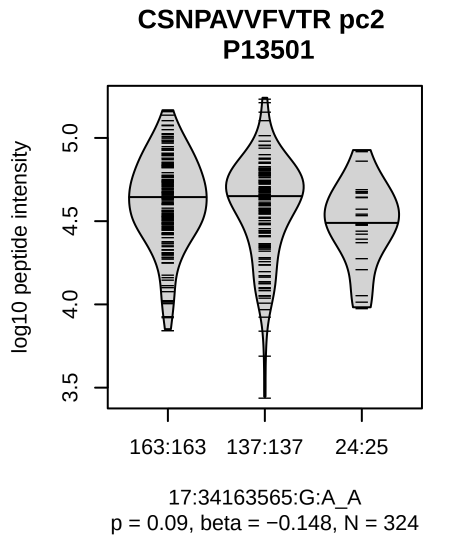

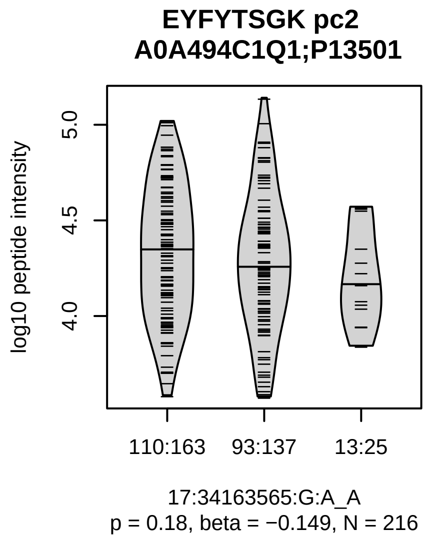

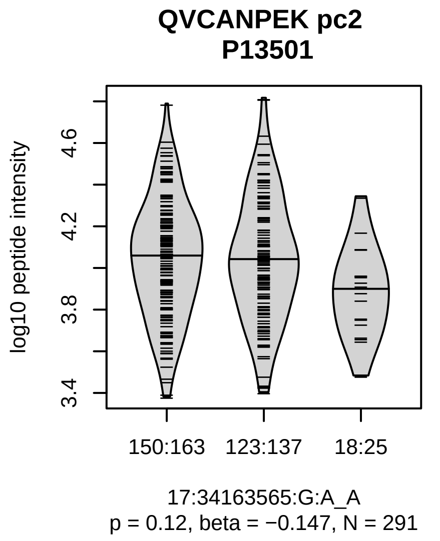

Assay Target: CCL5
UniProt: P13501
rsID: rs4239252
Target SNP: 17:35836561:A:G
Proxy SNP: 17:34163565:G:A
deCODE log10(p): 132
deCODE BETA: −0.26
deCODE MSPA: 0.722
:−:
1254:947:1204

152. RNASE1 (P07998)
deCODE MSPA=0.56

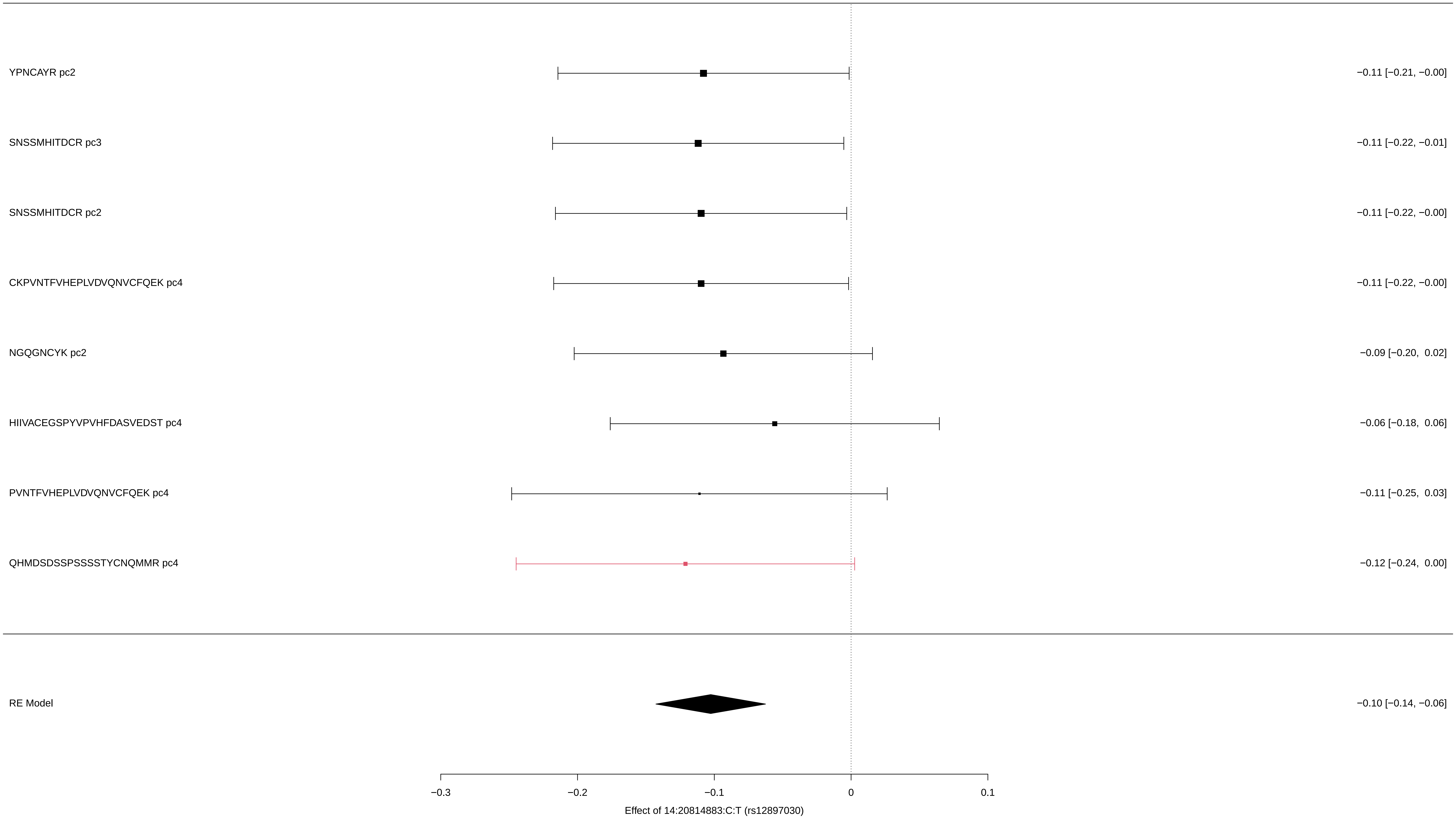

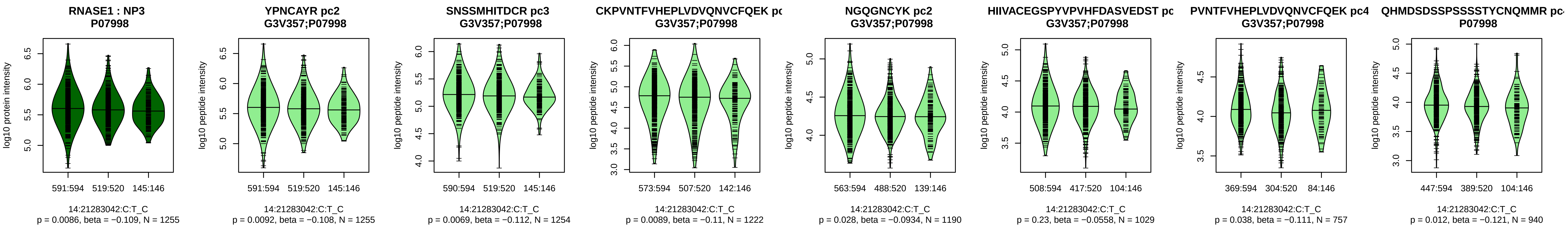

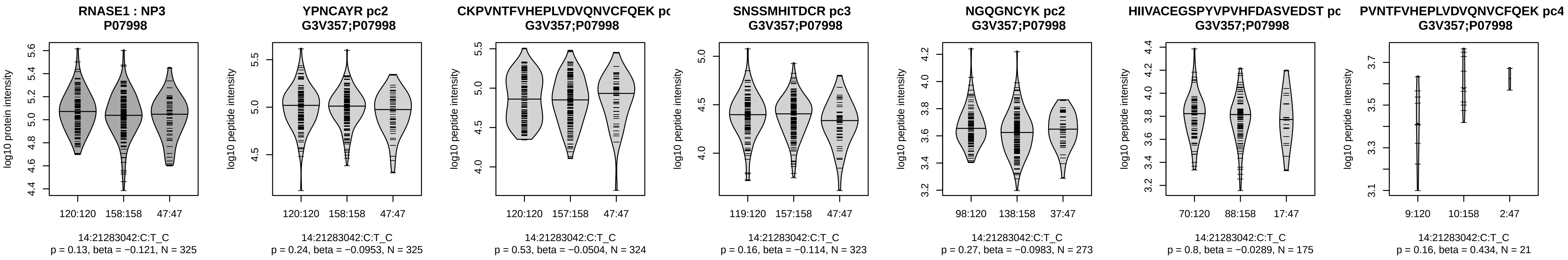

Assay Target: RNASE1
UniProt: P07998
rsID: rs12897030
Target SNP: 14:20814883:C:T
Proxy SNP: 14:21283042:C:T
deCODE log10(p): 130.1
deCODE BETA: −0.22
deCODE MSPA: 0.56
::*:*:–:–:–:–
1255:1254:1254:1222:1190:102

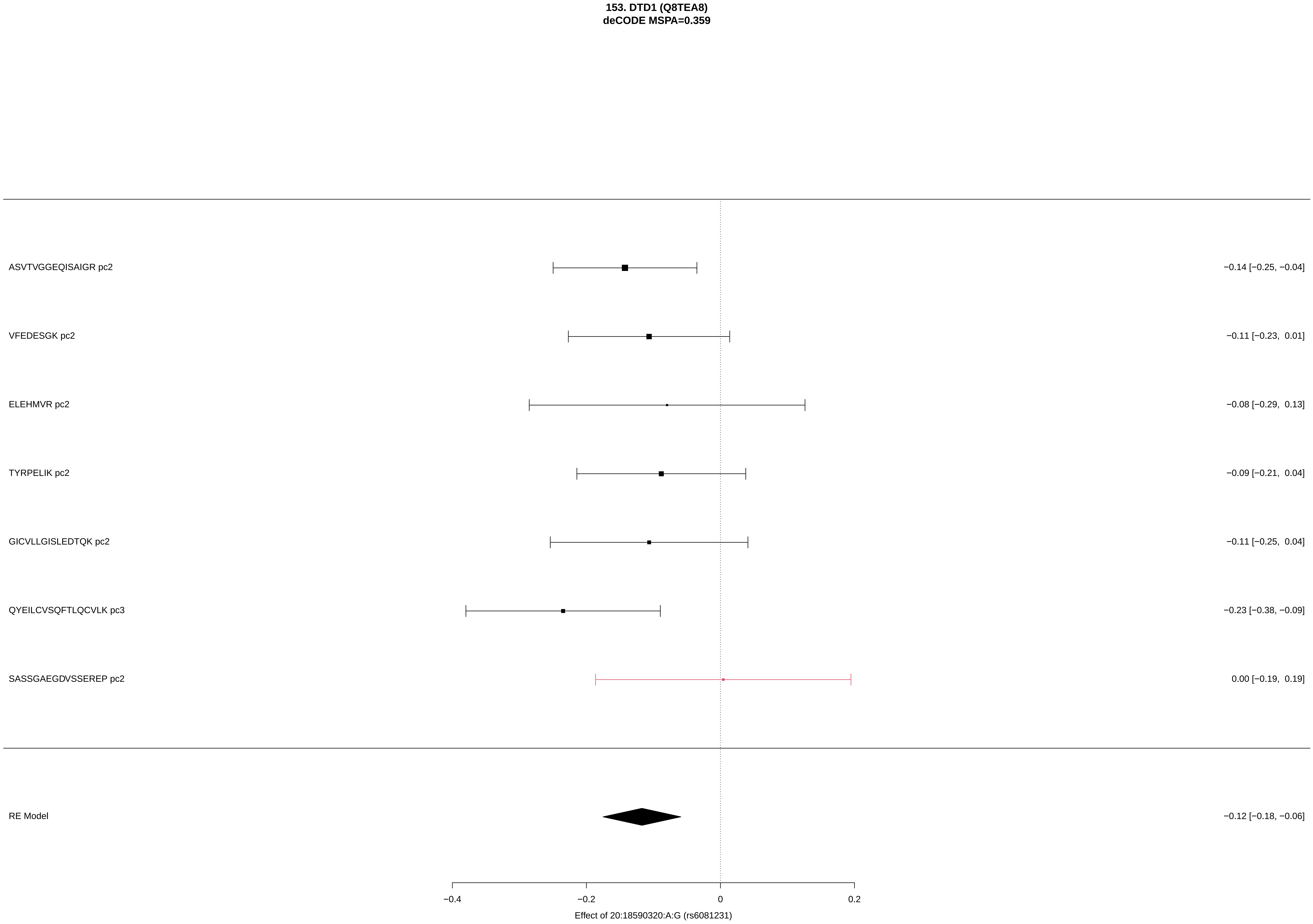

153. DTD1 (Q8TEA8)
deCODE MSPA=0.359
ASVTVGGEQISAIGR pc2
−0.14 [−0.25, −0.04]
VFEDESGK pc2
−0.11 [−0.23, 0.01]
ELEHMVR pc2
−0.08 [−0.29, 0.13]
TYRPELIK pc2
−0.09 [−0.21, 0.04]
GICVLLGISLEDTQK pc2
−0.11 [−0.25, 0.04]
QYEILCVSQFTLQCVLK pc3
−0.23 [−0.38, −0.09]
SASSGAEGDVSSEREP pc2
0.00 [−0.19, 0.19]
RE Model
−0.12 [−0.18, −0.06]
−0.4
−0.2
0
0.2
Effect of 20:18590320:A:G (rs6081231)

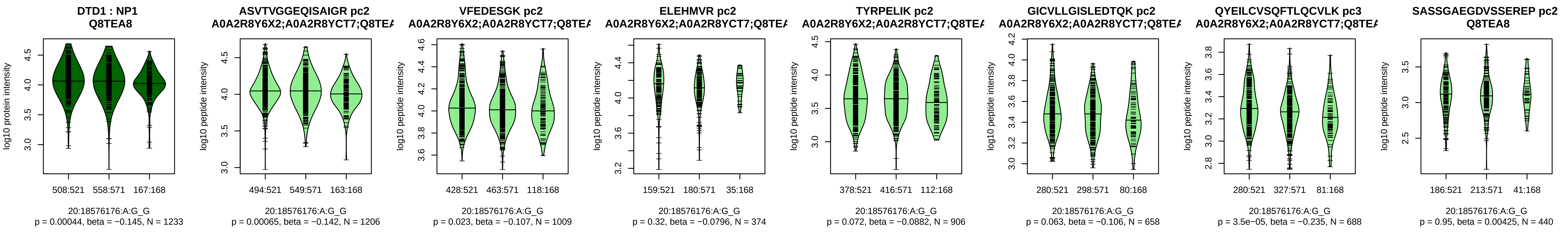

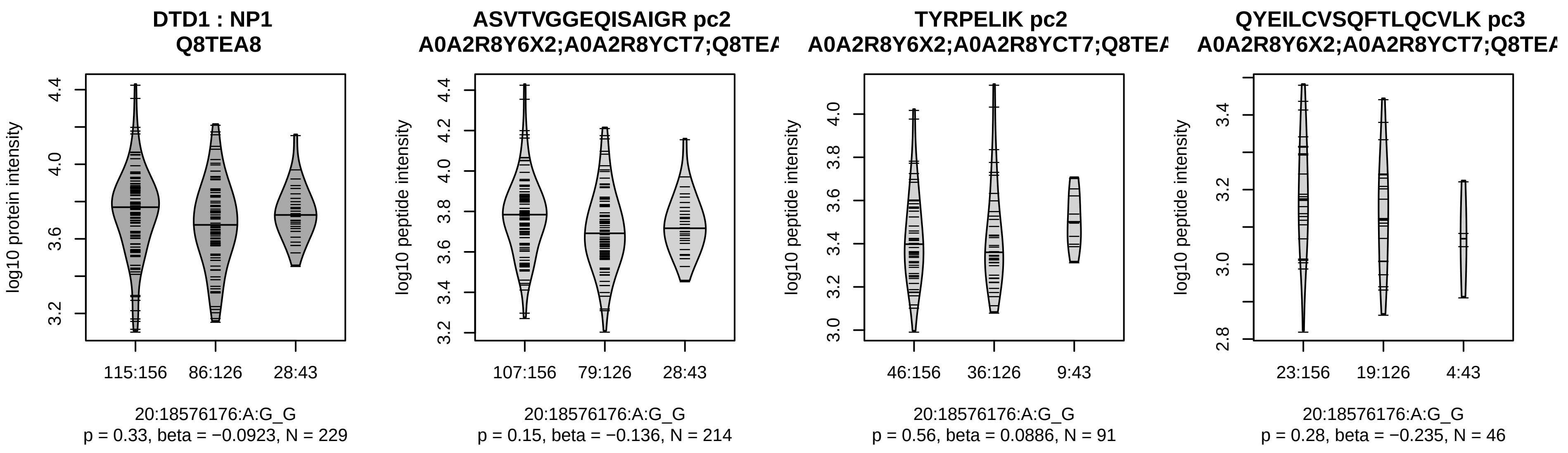

Assay Target: DTD1
UniProt: Q8TEA8
rsID: rs6081231
Target SNP: 20:18590320:A:G
Proxy SNP: 20:18576176:A:G
deCODE log10(p): 127.8
deCODE BETA: −0.2
deCODE MSPA: 0.359
:−:−:−:−::−
1206:1009:374:906:658:688:440

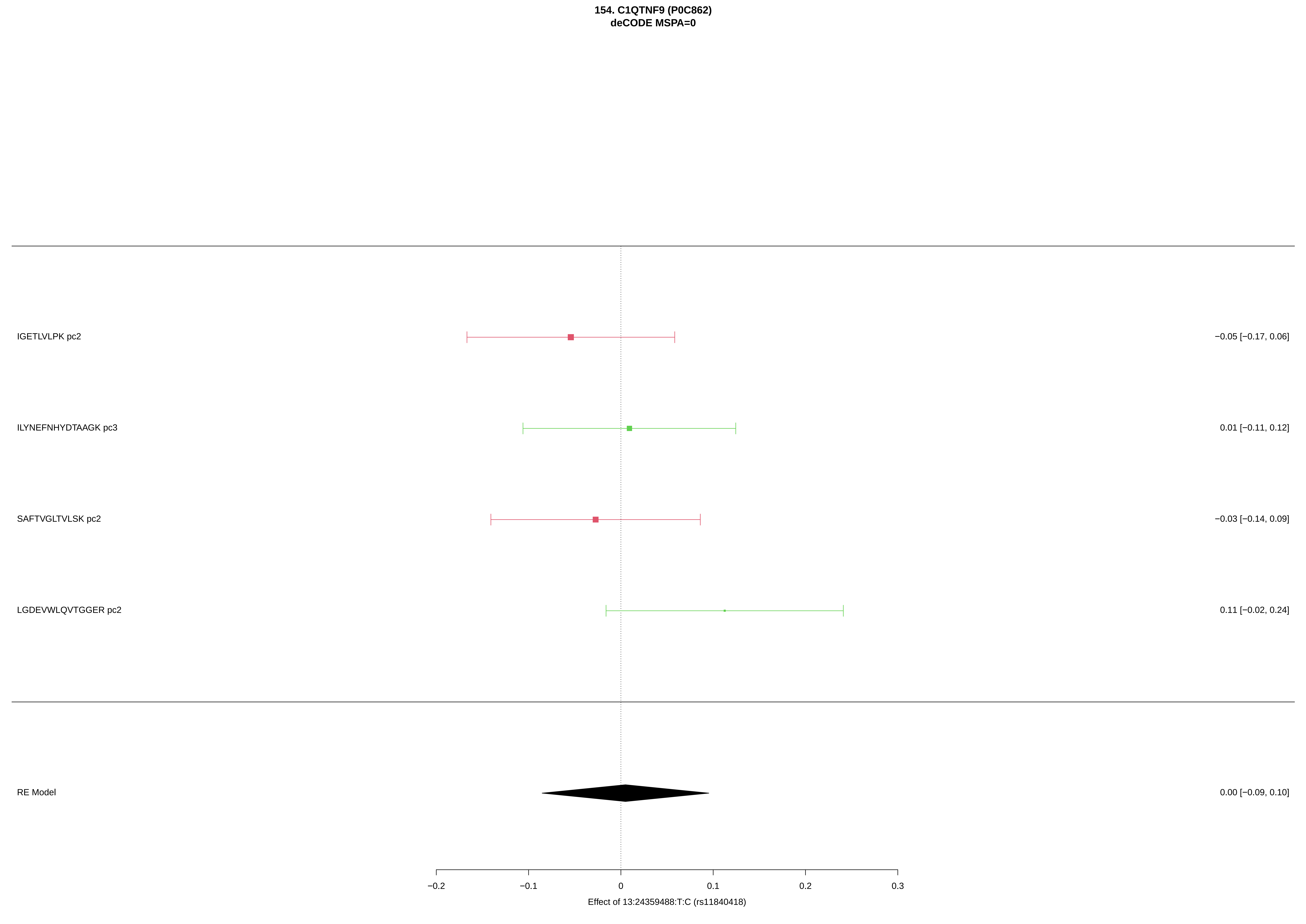
154. C1QTNF9 (P0C862)
deCODE MSPA=0
IGETLVLPK pc2
−0.05 [−0.17, 0.06]
ILYNEFNHYDTAAGK pc3
0.01 [−0.11, 0.12]
SAFTVGLTVLSK pc2
−0.03 [−0.14, 0.09]
LGDEVWLQVTGGER pc2
0.11 [−0.02, 0.24]
RE Model
0.00 [−0.09, 0.10]
−0.2
−0.1
0
0.1
0.2
0.3
Effect of 13:24359488:T:C (rs11840418)

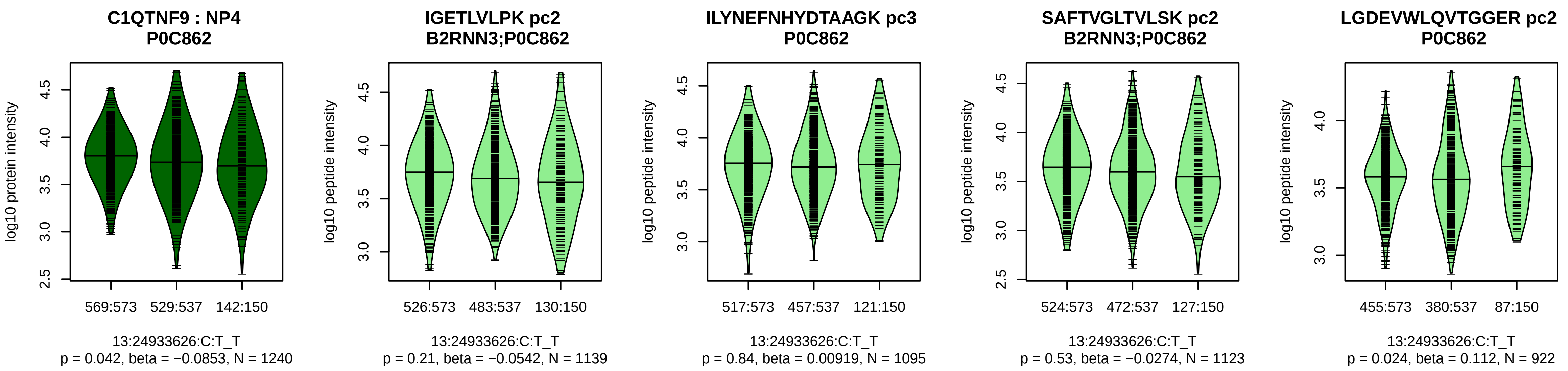

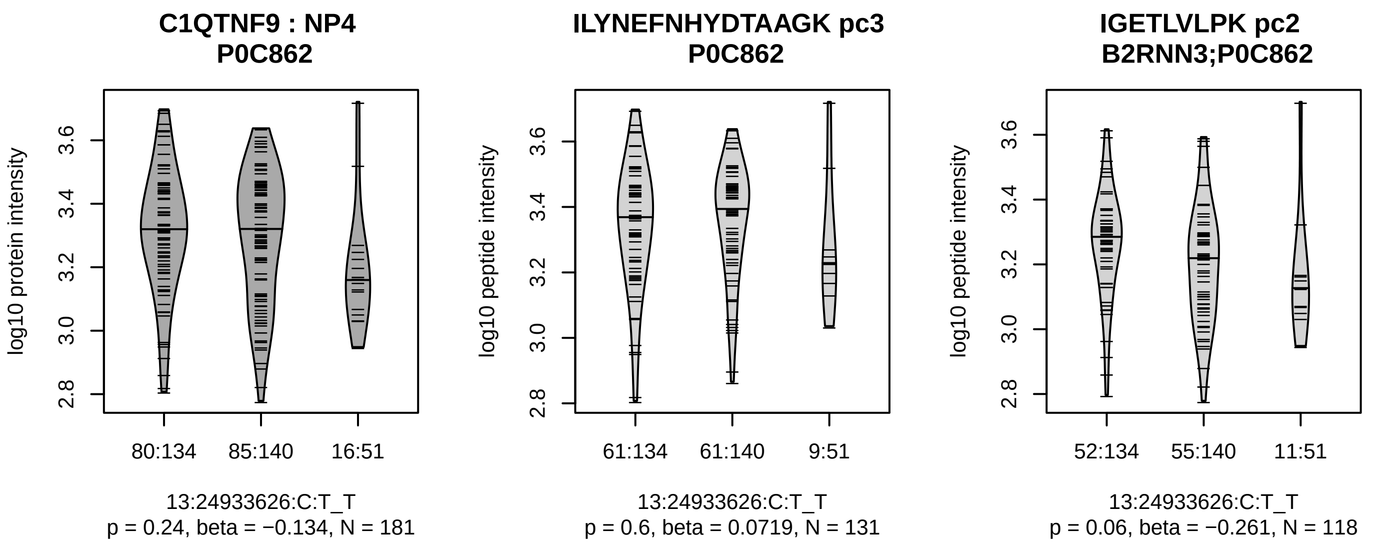

Assay Target: C1QTNF9
UniProt: P0C862
rsID: rs11840418
Target SNP: 13:24359488:T:C
Proxy SNP: 13:24933626:C:T
deCODE log10(p): 127.5
deCODE BETA: −0.21
deCODE MSPA: 0
−:−:−:−:NA
1139:1095:1123:922:3

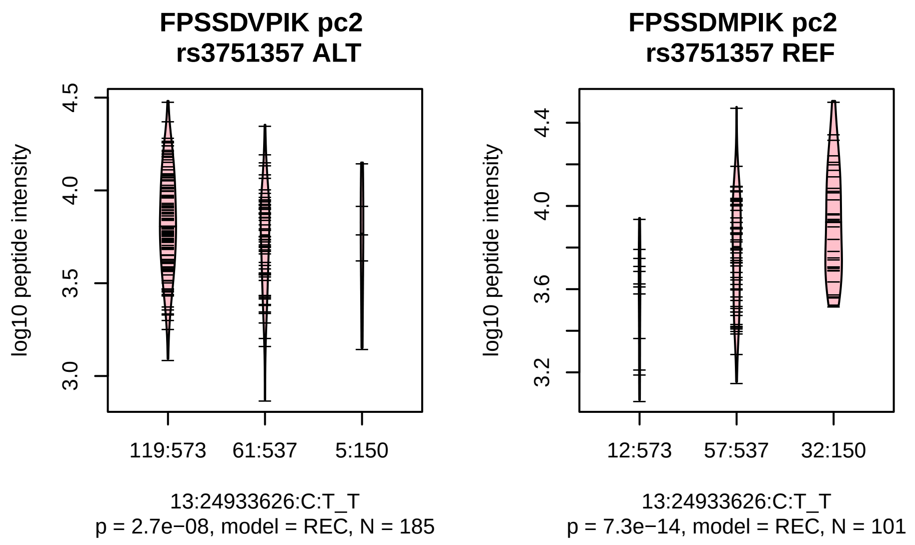

155. QPCT (Q16769)
deCODE MSPA=0

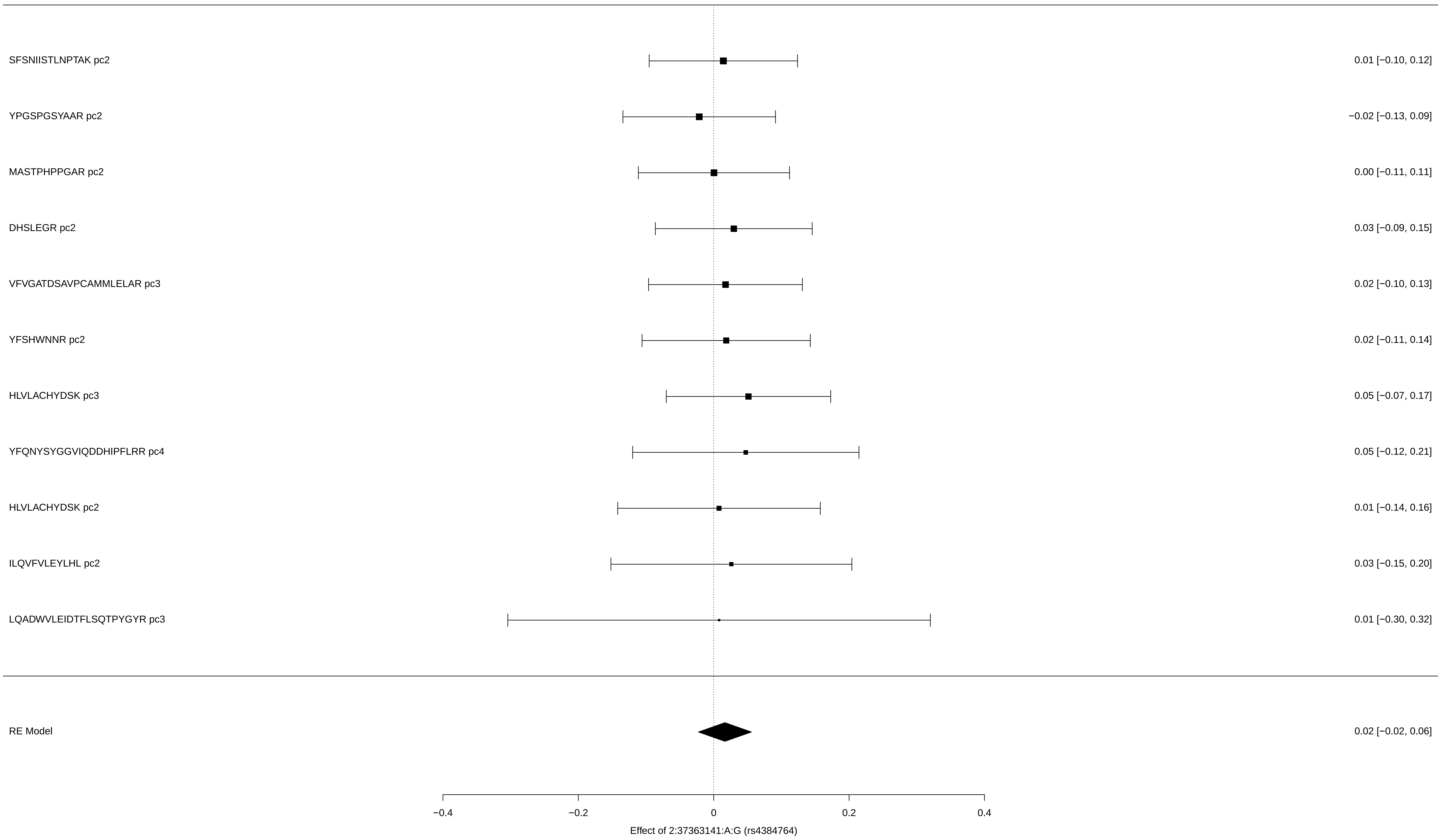

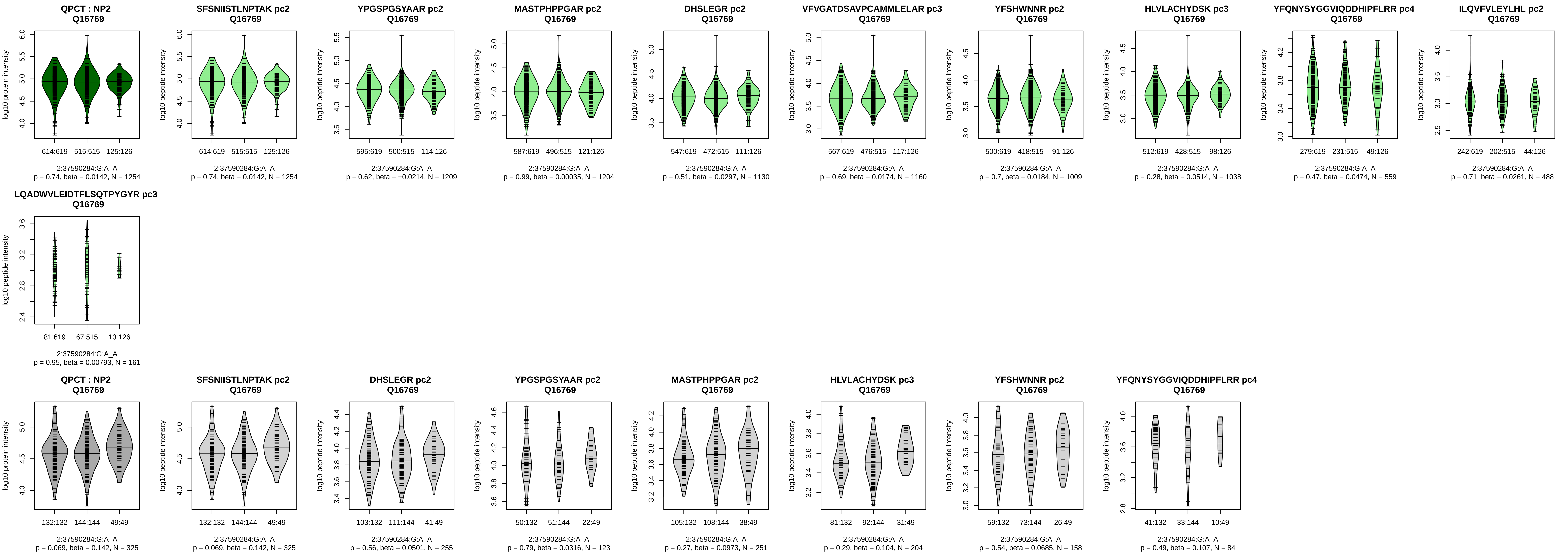

Assay Target: QPCT
UniProt: Q16769
rsID: rs4384764
Target SNP: 2:37363141:A:G
Proxy SNP: 2:37590284:G:A
deCODE log10(p): 126.5
deCODE BETA: 0.22
deCODE MSPA: 0
1254:1209:1204:1130:1160:100

156. PRSS2 (P07478)
deCODE MSPA=0

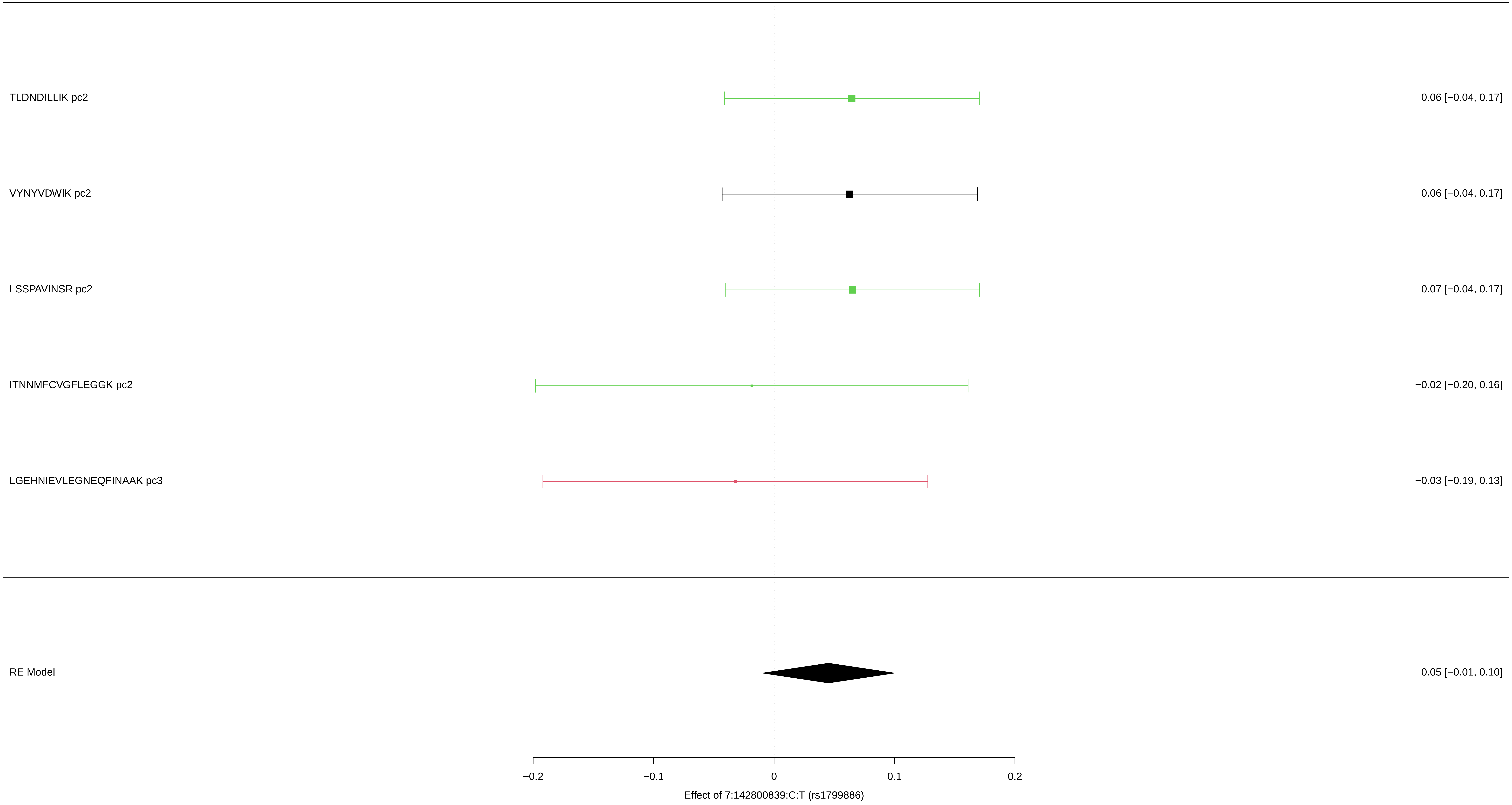

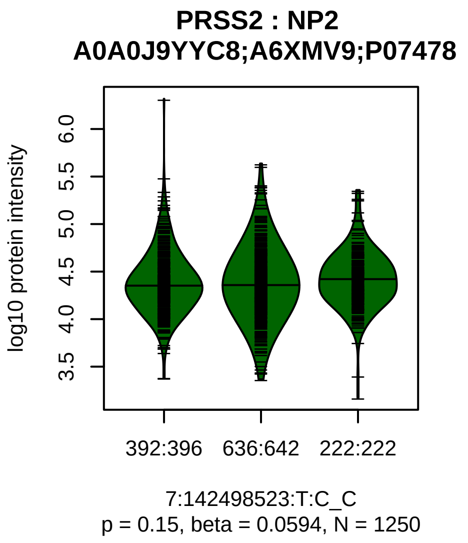

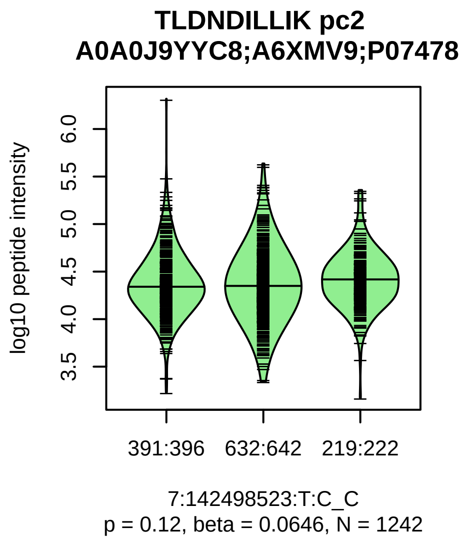

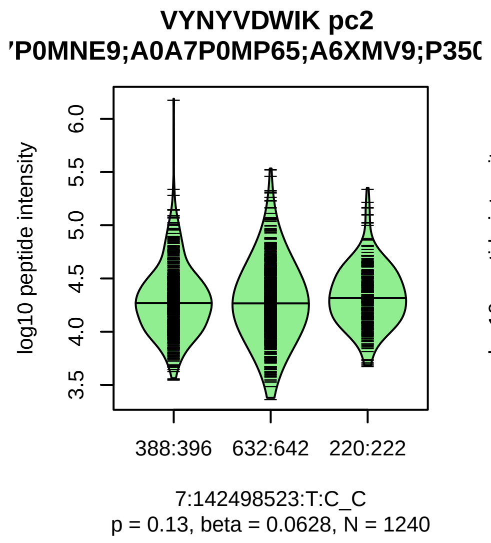

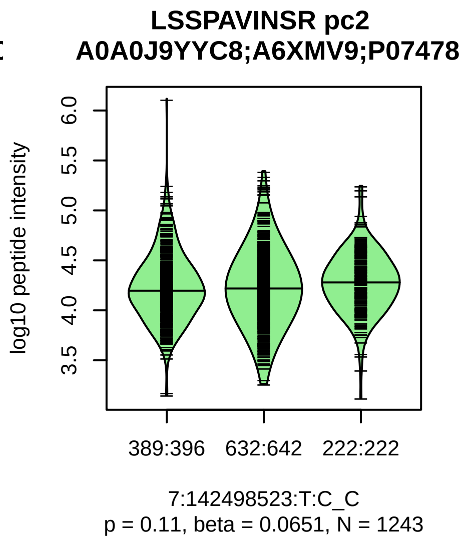

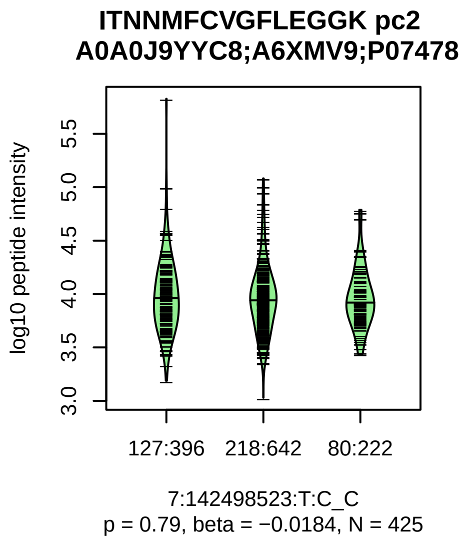

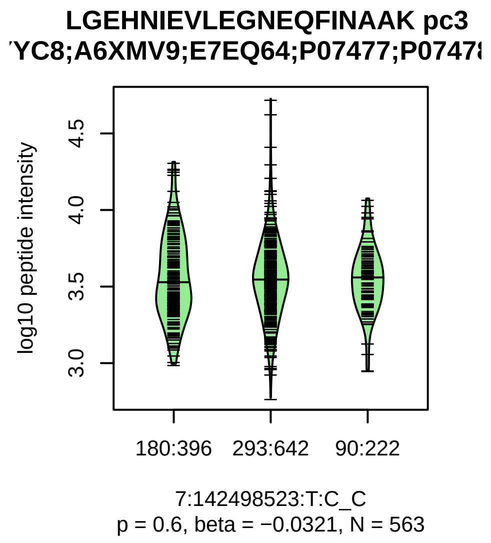

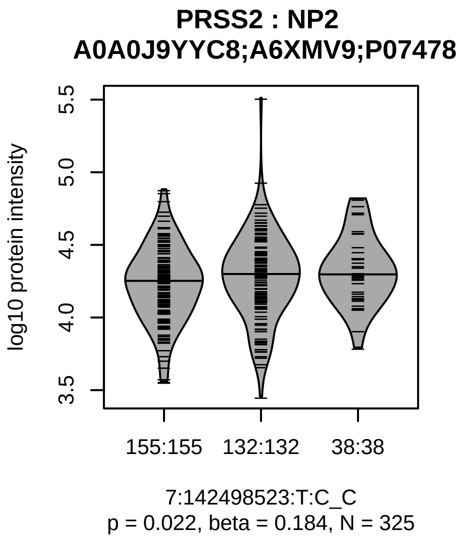

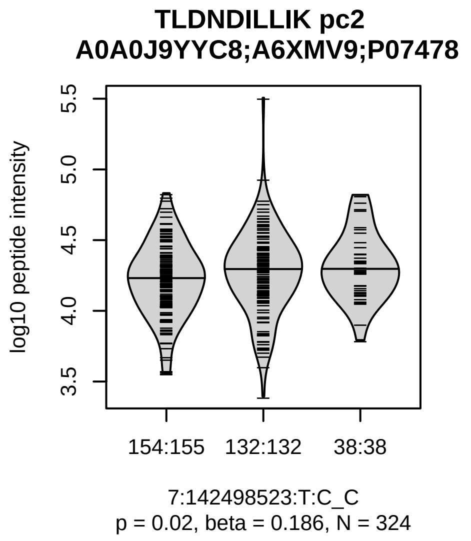

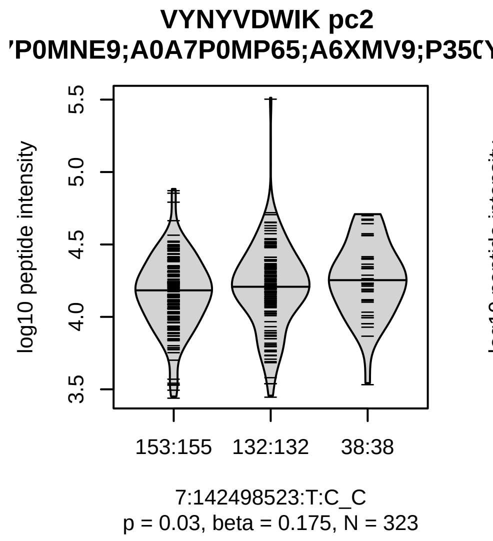

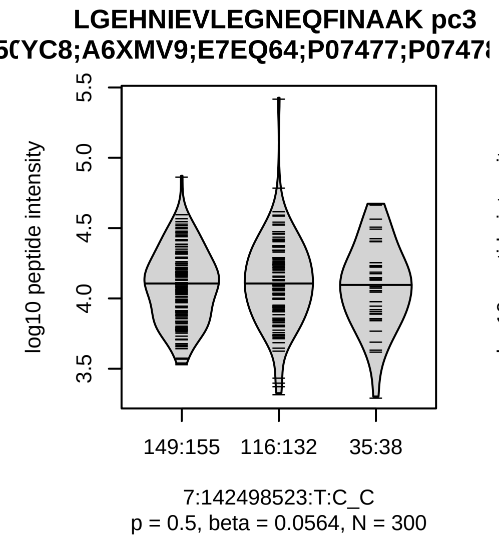

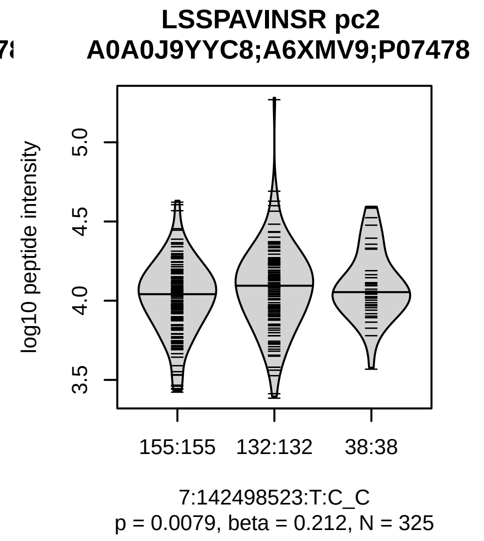

Assay Target: PRSS2
UniProt: P07478
rsID: rs1799886
Target SNP: 7:142800839:C:T
Proxy SNP: 7:142498523:T:C
deCODE log10(p): 123.9
deCODE BETA: 0.2
deCODE MSPA: 0
–:–:–:–:–
1242:1240:1243:425:563

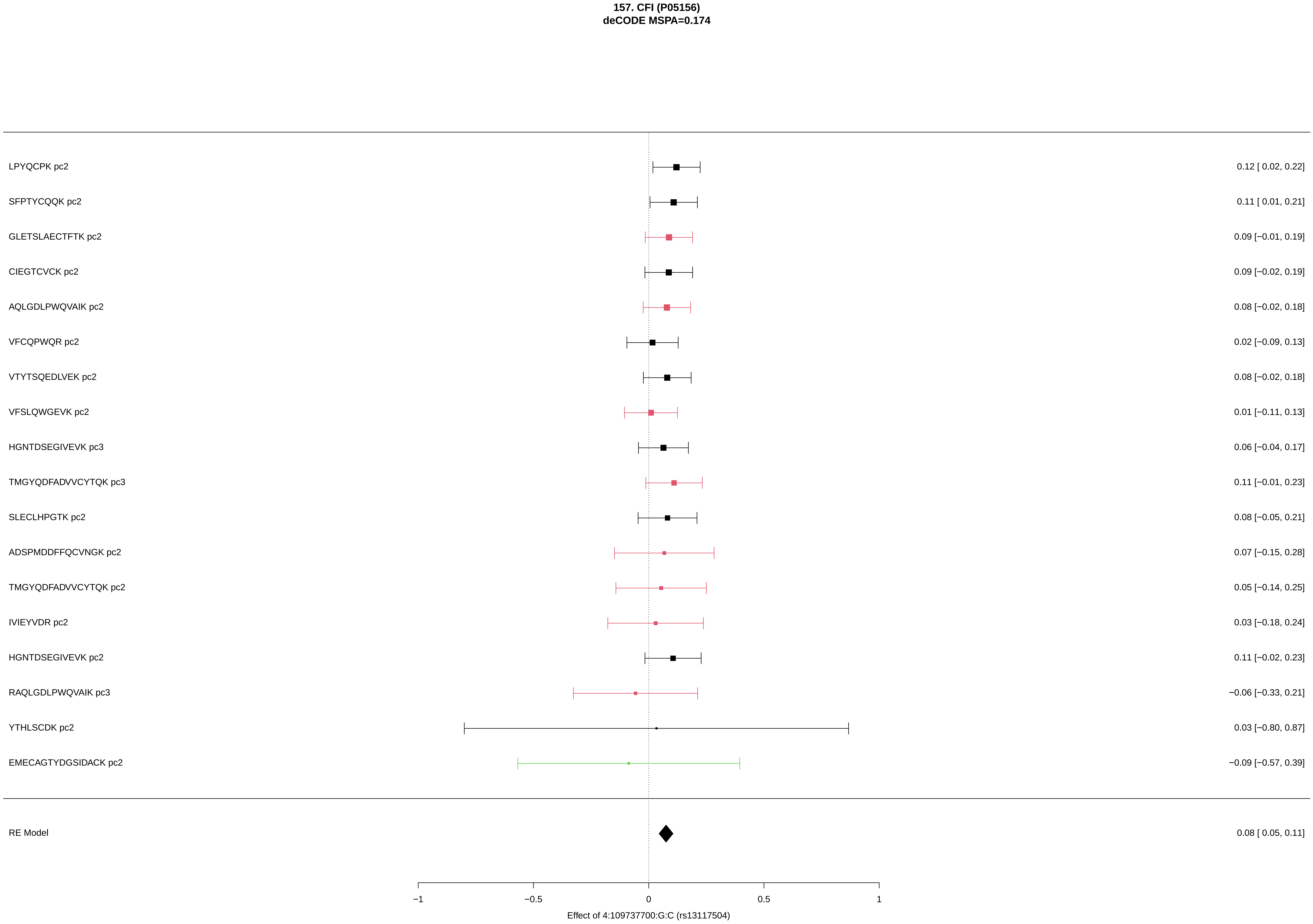
157. CFI (P05156)
deCODE MSPA=0.174
LPYQCPK pc2
0.12 [0.02, 0.22]
SFPTYCQQK pc2
0.11 [0.01, 0.21]
GLETSLAECTFTK pc2
0.09 [−0.01, 0.19]
CIEGTCVCK pc2
0.09 [−0.02, 0.19]
AQLGDLPWQVAIK pc2
0.08 [−0.02, 0.18]
VFCQPWQR pc2
0.02 [−0.09, 0.13]
VTYTSQEDLVEK pc2
0.08 [−0.02, 0.18]
VFSLQWGEVK pc2
0.01 [−0.11, 0.13]
HGNTDSEGIVEVK pc3
0.06 [−0.04, 0.17]
TMGYQDFADVVCYTQK pc3
0.11 [−0.01, 0.23]
SLECLHPGTK pc2
0.08 [−0.05, 0.21]
ADSPMDDFFQCVNGK pc2
0.07 [−0.15, 0.28]
TMGYQDFADVVCYTQK pc2
0.05 [−0.14, 0.25]
IVIEYVDR pc2
0.03 [−0.18, 0.24]
HGNTDSEGIVEVK pc2
0.11 [−0.02, 0.23]
RAQLGDLPWQVAIK pc3
−0.06 [−0.33, 0.21]
YTHLSCDK pc2
0.03 [−0.80, 0.87]
EMECAGTYDGSIDACK pc2
−0.09 [−0.57, 0.39]
RE Model
0.08 [0.05, 0.11]
−1
−0.5
0
0.5
1
Effect of 4:109737700:G:C (rs13117504)

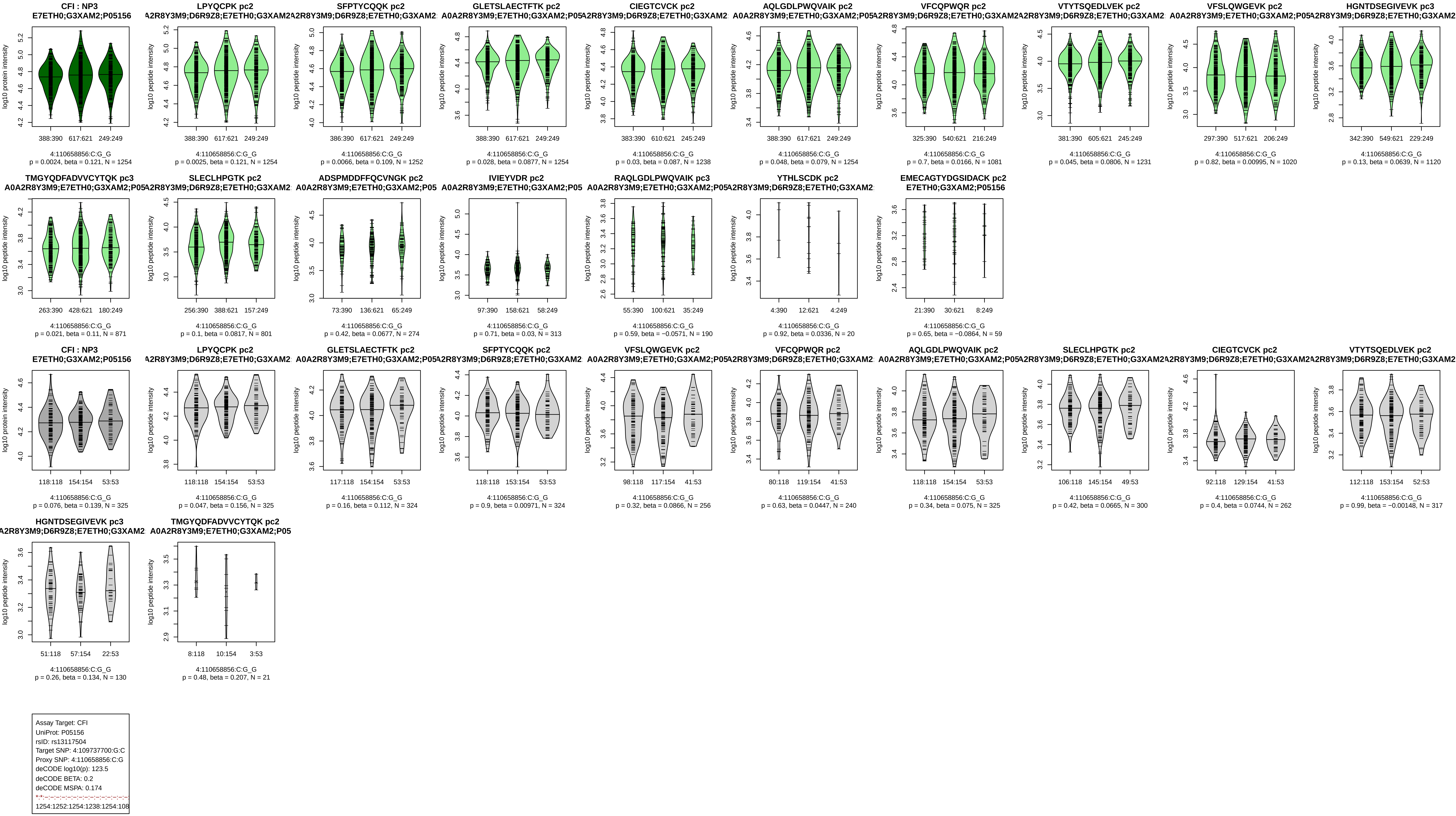
CFI : NP3
E7ETH0;G3XAM2;P05156
log10 protein intensity
388:390 617:621 249:249
4:110658856:C:G_G
p = 0.0024, beta = 0.121, N = 1254
LPYQCPK pc2
A2R8Y3M9;D6R9Z8;E7ETH0;G3XAM2
log10 peptide intensity
388:390 617:621 249:249
4:110658856:C:G_G
p = 0.0025, beta = 0.121, N = 1254
SFPTYCQQK pc2
A2R8Y3M9;D6R9Z8;E7ETH0;G3XAM2
386:390 617:621 249:249
4:110658856:C:G_G
p = 0.0066, beta = 0.109, N = 1252
GLETSLAECTFTK pc2
A0A2R8Y3M9;E7ETH0;G3XAM2;P05
388:390 617:621 249:249
4:110658856:C:G_G
p = 0.028, beta = 0.0877, N = 1254
CIEGTCVCK pc2
A2R8Y3M9;D6R9Z8;E7ETH0;G3XAM2
383:390 610:621 245:249
4:110658856:C:G_G
p = 0.03, beta = 0.087, N = 1238
AQLGDLPWQVAIK pc2
A0A2R8Y3M9;E7ETH0;G3XAM2;P05
388:390 617:621 249:249
4:110658856:C:G_G
p = 0.048, beta = 0.079, N = 1254
VFCQPWQR pc2
A2R8Y3M9;D6R9Z8;E7ETH0;G3XAM2
325:390 540:621 216:249
4:110658856:C:G_G
p = 0.7, beta = 0.0166, N = 1081
VTYTSQEDLVEK pc2
A2R8Y3M9;D6R9Z8;E7ETH0;G3XAM2
381:390 605:621 245:249
4:110658856:C:G_G
p = 0.045, beta = 0.0806, N = 1231
VFSLQWGEVK pc2
A0A2R8Y3M9;E7ETH0;G3XAM2;P05
297:390 517:621 206:249
4:110658856:C:G_G
p = 0.82, beta = 0.00995, N = 1020
HGNTDSEGIVEVK pc3
A2R8Y3M9;D6R9Z8;E7ETH0;G3XAM2
342:390 549:621 229:249
4:110658856:C:G_G
p = 0.13, beta = 0.0639, N = 1120
TMGYQDFADVVCYTQK pc3
A0A2R8Y3M9;E7ETH0;G3XAM2;P05
263:390 428:621 180:249
4:110658856:C:G_G
p = 0.021, beta = 0.11, N = 871
SLECLHPGTK pc2
A2R8Y3M9;D6R9Z8;E7ETH0;G3XAM2
256:390 388:621 157:249
4:110658856:C:G_G
p = 0.1, beta = 0.0817, N = 801
ADSPMDDFFQCVNGK pc2
A0A2R8Y3M9;E7ETH0;G3XAM2;P05
73:390 136:621 65:249
4:110658856:C:G_G
p = 0.42, beta = 0.0677, N = 274
IVIEYVDR pc2
A0A2R8Y3M9;E7ETH0;G3XAM2;P05
97:390 158:621 58:249
4:110658856:C:G_G
p = 0.71, beta = 0.03, N = 313
RAQLGDLPWQVAIK pc3
A0A2R8Y3M9;E7ETH0;G3XAM2;P05
55:390 100:621 35:249
4:110658856:C:G_G
p = 0.59, beta = −0.0571, N = 190
YTHLSCDK pc2
A2R8Y3M9;D6R9Z8;E7ETH0;G3XAM2
4:390 12:621 4:249
4:110658856:C:G_G
p = 0.92, beta = 0.0336, N = 20
EMECAGTYDGSIDACK pc2
E7ETH0;G3XAM2;P05156
21:390 30:621 8:249
4:110658856:C:G_G
p = 0.65, beta = −0.0864, N = 59
CFI : NP3
E7ETH0;G3XAM2;P05156
118:118 154:154 53:53
4:110658856:C:G_G
p = 0.076, beta = 0.139, N = 325
LPYQCPK pc2
A2R8Y3M9;D6R9Z8;E7ETH0;G3XAM2
118:118 154:154 53:53
4:110658856:C:G_G
p = 0.047, beta = 0.156, N = 325
GLETSLAECTFTK pc2
A0A2R8Y3M9;E7ETH0;G3XAM2;P05
117:118 154:154 53:53
4:110658856:C:G_G
p = 0.16, beta = 0.112, N = 324
SFPTYCQQK pc2
A2R8Y3M9;D6R9Z8;E7ETH0;G3XAM2
118:118 153:154 53:53
4:110658856:C:G_G
p = 0.9, beta = 0.00971, N = 324
VFSLQWGEVK pc2
A0A2R8Y3M9;E7ETH0;G3XAM2;P05
98:118 117:154 41:53
4:110658856:C:G_G
p = 0.32, beta = 0.0866, N = 256
VFCQPWQR pc2
A2R8Y3M9;D6R9Z8;E7ETH0;G3XAM2
80:118 119:154 41:53
4:110658856:C:G_G
p = 0.63, beta = 0.0447, N = 240
AQLGDLPWQVAIK pc2
A0A2R8Y3M9;E7ETH0;G3XAM2;P05
118:118 154:154 53:53
4:110658856:C:G_G
p = 0.34, beta = 0.075, N = 325
SLECLHPGTK pc2
A2R8Y3M9;D6R9Z8;E7ETH0;G3XAM2
106:118 145:154 49:53
4:110658856:C:G_G
p = 0.42, beta = 0.0665, N = 300
CIEGTCVCK pc2
A2R8Y3M9;D6R9Z8;E7ETH0;G3XAM2
92:118 129:154 41:53
4:110658856:C:G_G
p = 0.4, beta = 0.0744, N = 262
VTYTSQEDLVEK pc2
A2R8Y3M9;D6R9Z8;E7ETH0;G3XAM2
112:118 153:154 52:53
4:110658856:C:G_G
p = 0.99, beta = −0.00148, N = 317
HGNTDSEGIVEVK pc3
A2R8Y3M9;D6R9Z8;E7ETH0;G3XAM2
51:118 57:154 22:53
4:110658856:C:G_G
p = 0.26, beta = 0.134, N = 130
TMGYQDFADVVCYTQK pc2
A0A2R8Y3M9;E7ETH0;G3XAM2;P05
8:118 10:154 3:53
4:110658856:C:G_G
p = 0.48, beta = 0.207, N = 21
Assay Target: CFI
UniProt: P05156
rsID: rs13117504
Target SNP: 4:109737700:G:C
Proxy SNP: 4:110658856:C:G
deCODE log10(p): 123.5
deCODE BETA: 0.2
deCODE MSPA: 0.174
1254:1252:1254:1238:1254:108

158. SFRP4 (Q6FHJ7)
deCODE MSPA=0.52

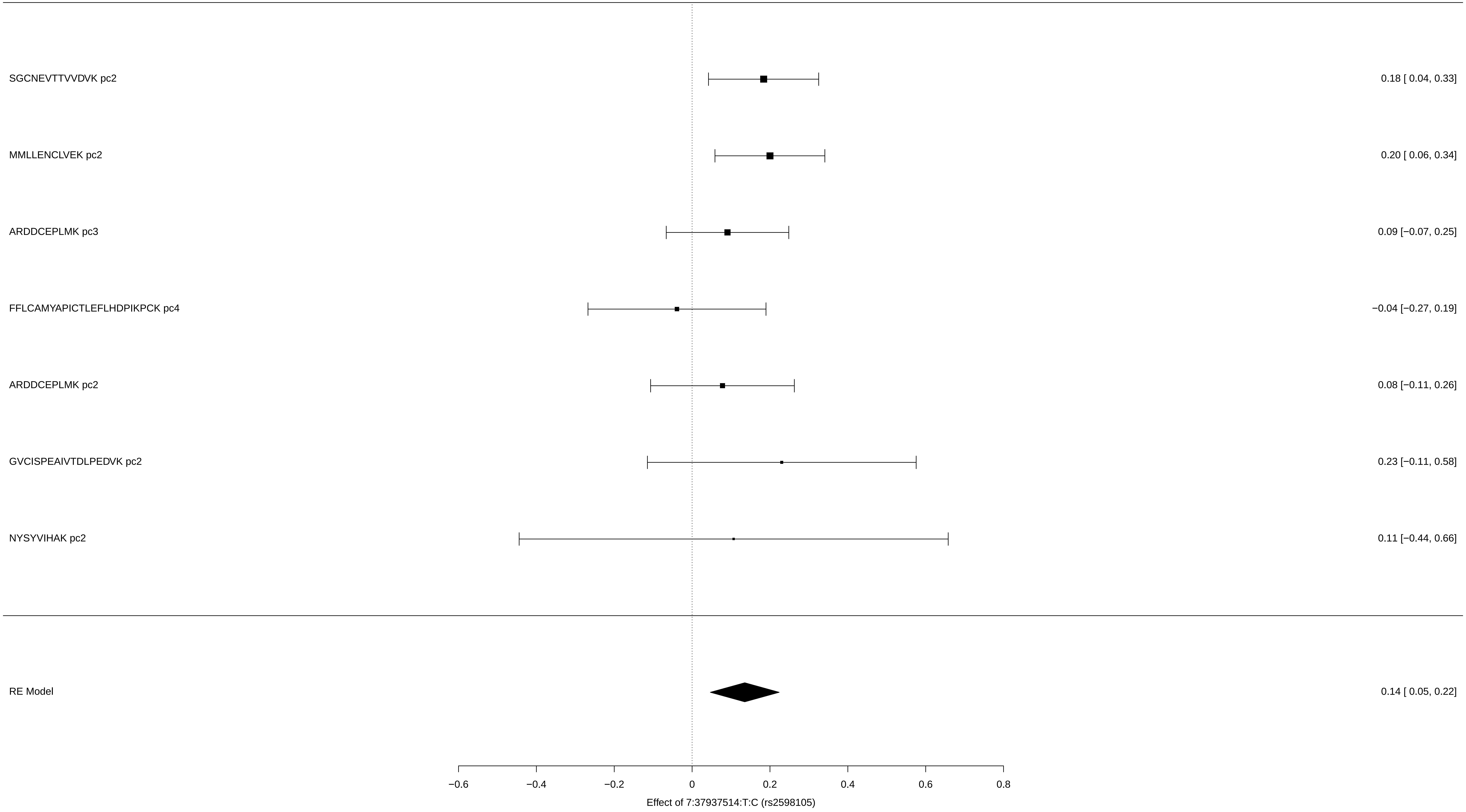

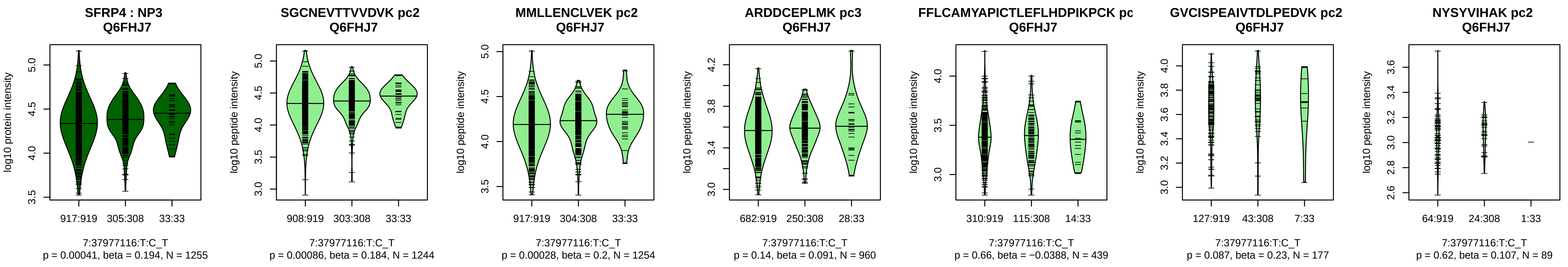

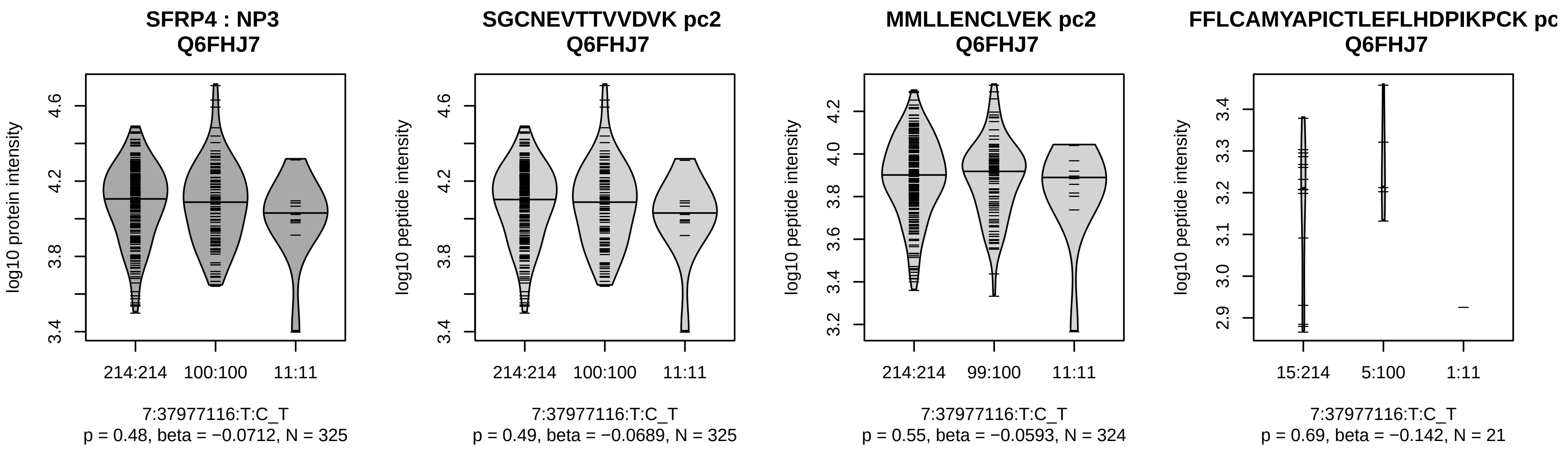

Assay Target: SFRP4
UniProt: Q6FHJ7
rsID: rs2598105
Target SNP: 7:37937514:T:C
Proxy SNP: 7:37977116:T:C
deCODE log10(p): 122.9
deCODE BETA: 0.27
deCODE MSPA: 0.52
::–:–:–:–:–
1244:1254:960:439:638:177:89

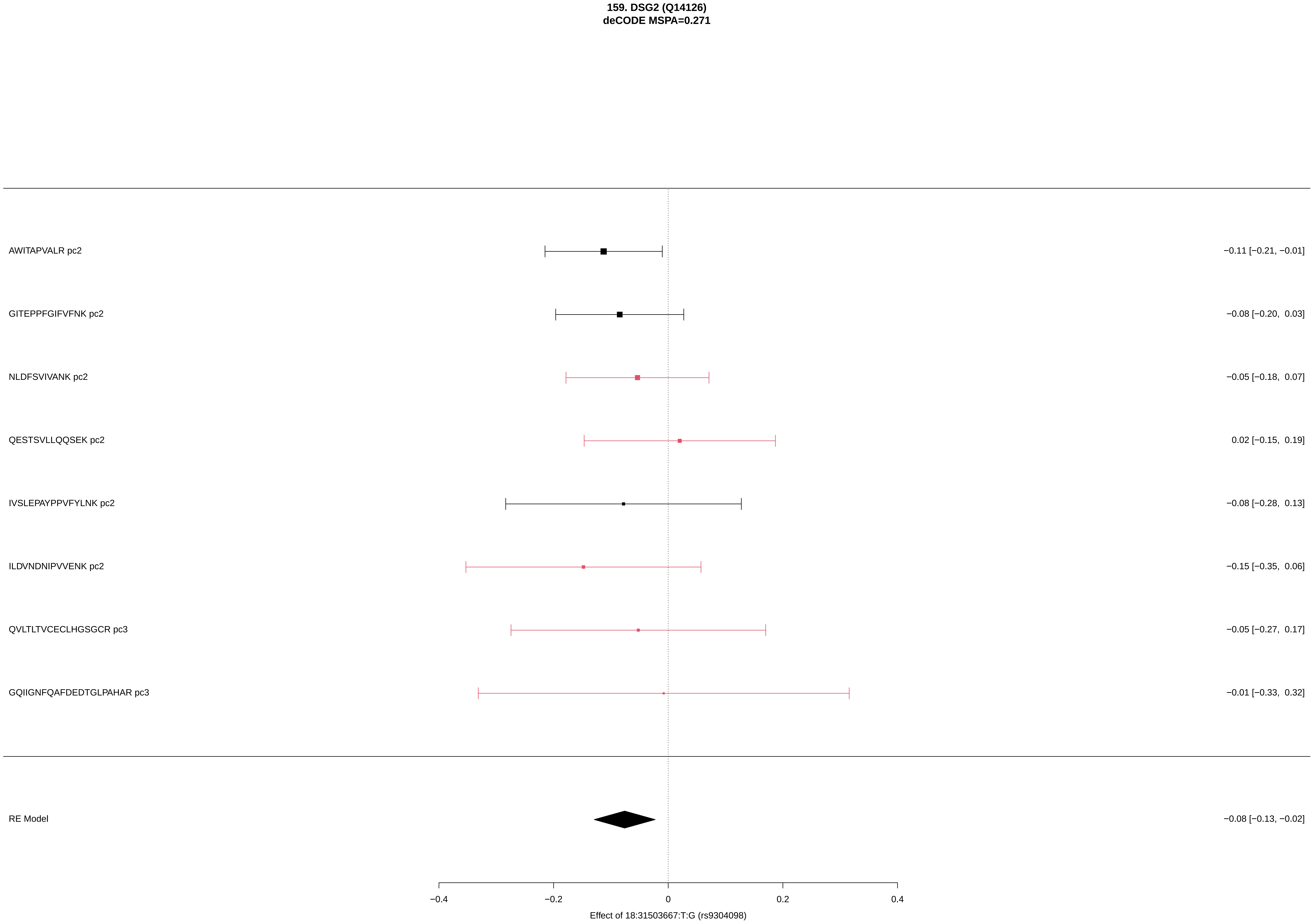

159. DSG2 (Q14126)
deCODE MSPA=0.271
AWITAPVALR pc2
−0.11 [−0.21, −0.01]
GITEPPFGIFVFNK pc2
−0.08 [−0.20, 0.03]
NLDFSVIVANK pc2
−0.05 [−0.18, 0.07]
QESTSVLLQQSEK pc2
0.02 [−0.15, 0.19]
IVSLEPAYPPVFYLNK pc2
−0.08 [−0.28, 0.13]
ILDVNDNIPVVENK pc2
−0.15 [−0.35, 0.06]
QVLTLTVCECLHGSGCR pc3
−0.05 [−0.27, 0.17]
GQIIGNFQAFDEDTGLPAHAR pc3
−0.01 [−0.33, 0.32]
RE Model
−0.08 [−0.13, −0.02]
−0.4
−0.2
0
0.2
0.4
Effect of 18:31503667:T:G (rs9304098)

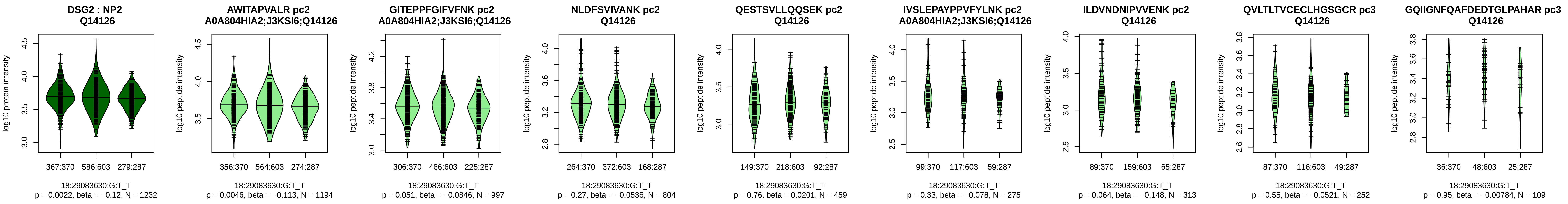

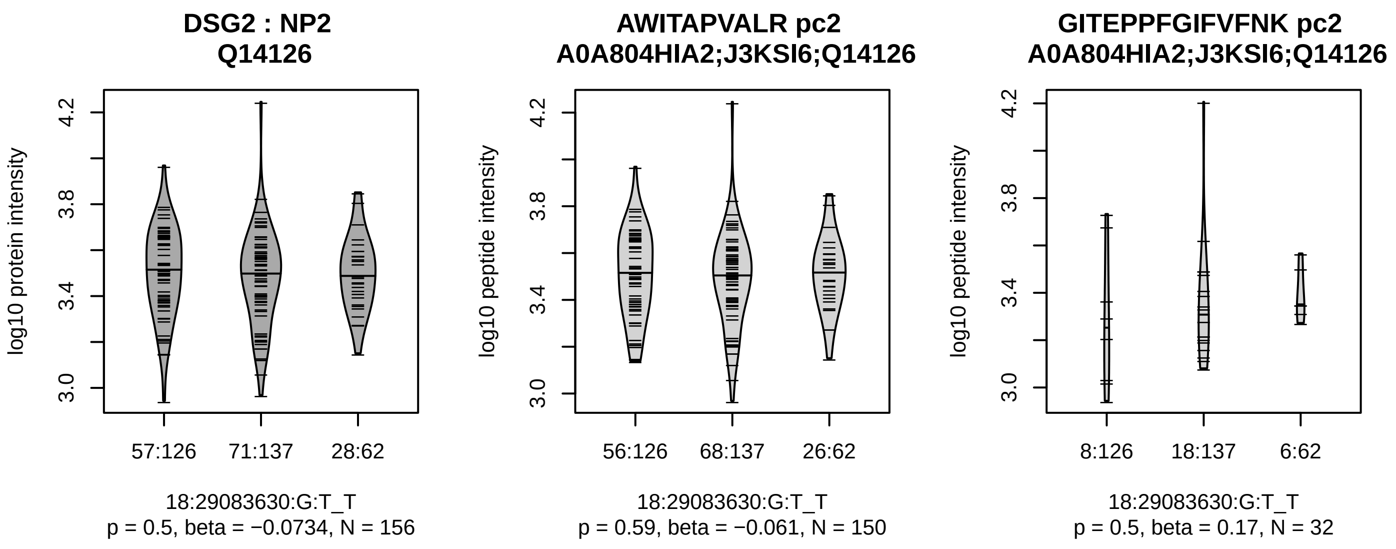

Assay Target: DSG2
UniProt: Q14126
rsID: rs9304098
Target SNP: 18:31503667:T:G
Proxy SNP: 18:29083630:G:T
deCODE log10(p): 118.8
deCODE BETA: −0.19
deCODE MSPA: 0.271
*:–:–:–:–:–:–:–
1194:997:804:459:275:313:252:

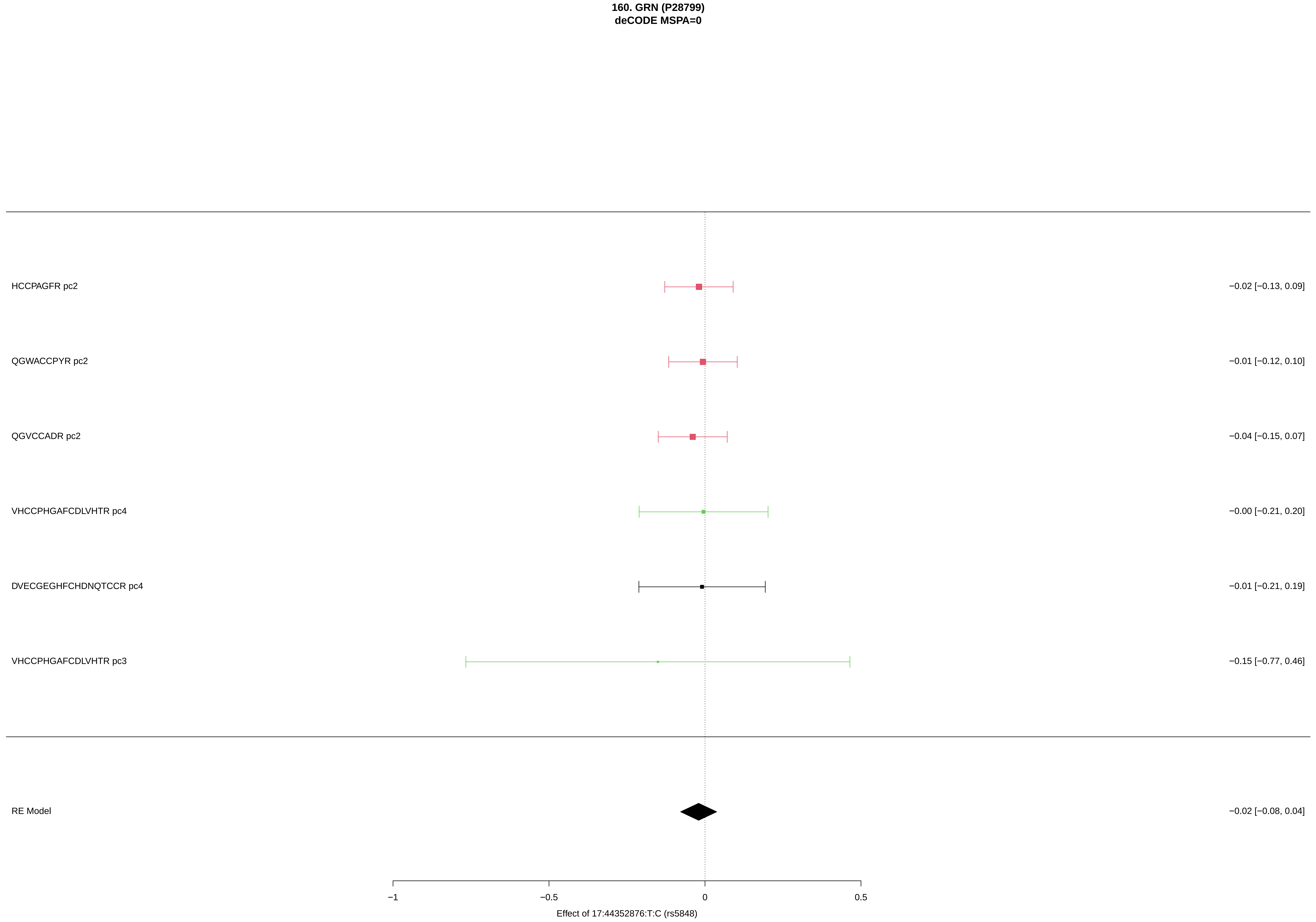
160. GRN (P28799)
deCODE MSPA=0
HCCPAGFR pc2
−0.02 [−0.13, 0.09]
QGWACCPYR pc2
−0.01 [−0.12, 0.10]
QGVCCADR pc2
−0.04 [−0.15, 0.07]
VHCCPHGAFCDLVHTR pc4
−0.00 [−0.21, 0.20]
DVECGEGHFCHDNQTCCR pc4
−0.01 [−0.21, 0.19]
VHCCPHGAFCDLVHTR pc3
−0.15 [−0.77, 0.46]
RE Model
−0.02 [−0.08, 0.04]
−1
−0.5
0
0.5
Effect of 17:44352876:T:C (rs5848)

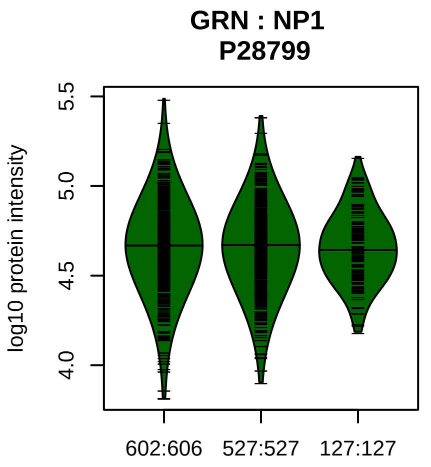

17:42430244:C:T_T
p = 0.66, beta = −0.0187, N = 1256

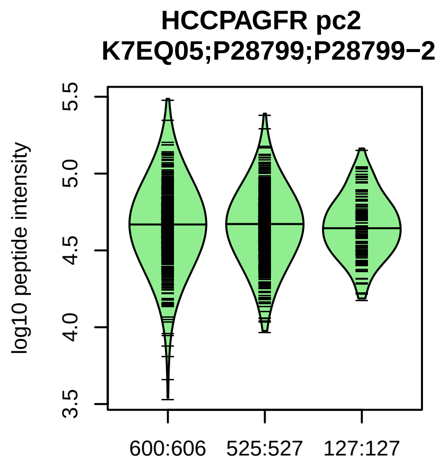

17:42430244:C:T_T
p = 0.65, beta = −0.0194, N = 1252

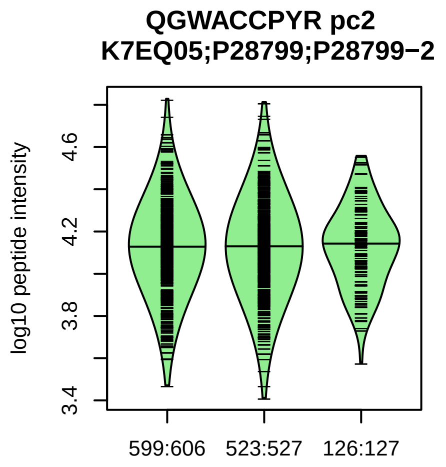

17:42430244:C:T_T
p = 0.88, beta = −0.00648, N = 1248

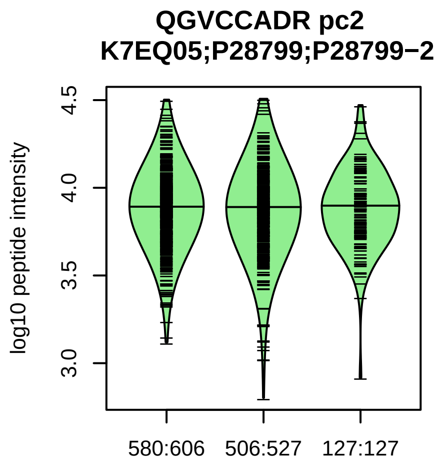

17:42430244:C:T_T
p = 0.36, beta = −0.0393, N = 1213

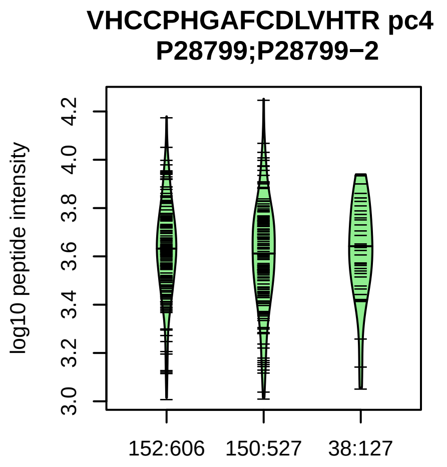

17:42430244:C:T_T
p = 0.96, beta = −0.00452, N = 340

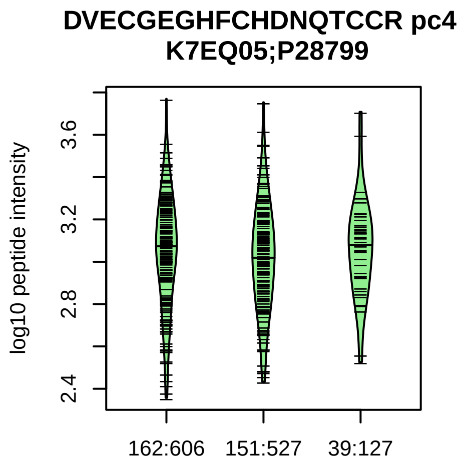

17:42430244:C:T_T
p = 0.91, beta = −0.00934, N = 352

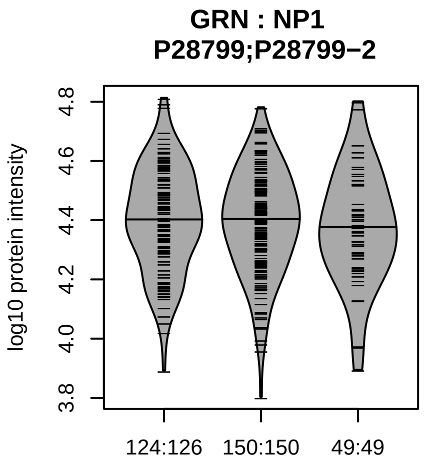

17:42430244:C:T_T
p = 0.14, beta = −0.117, N = 323

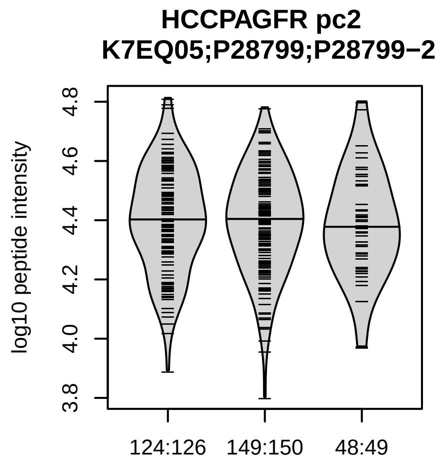

17:42430244:C:T_T
p = 0.23, beta = −0.0954, N = 321

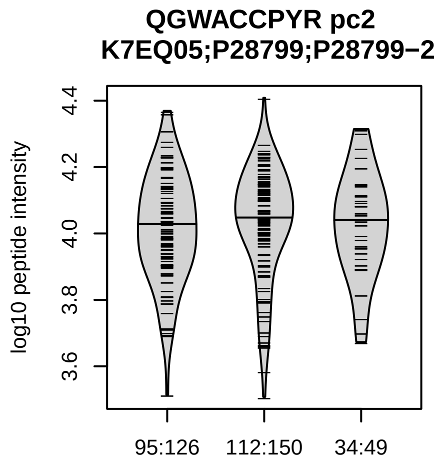

17:42430244:C:T_T
p = 0.68, beta = 0.0381, N = 241

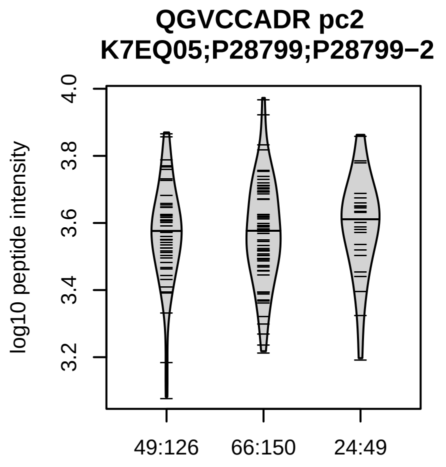

17:42430244:C:T_T
p = 0.74, beta = 0.0395, N = 139

Assay Target: GRN
UniProt: P28799
rsID: rs5848
Target SNP: 17:44352876:T:C
Proxy SNP: 17:42430244:C:T
deCODE log10(p): 118.7
deCODE BETA: −0.22
deCODE MSPA: 0
−:−:−:−:−:−:NA
1252:1248:1213:340:352:38:5

161. MAN1C1 (Q9NR34)
deCODE MSPA=0

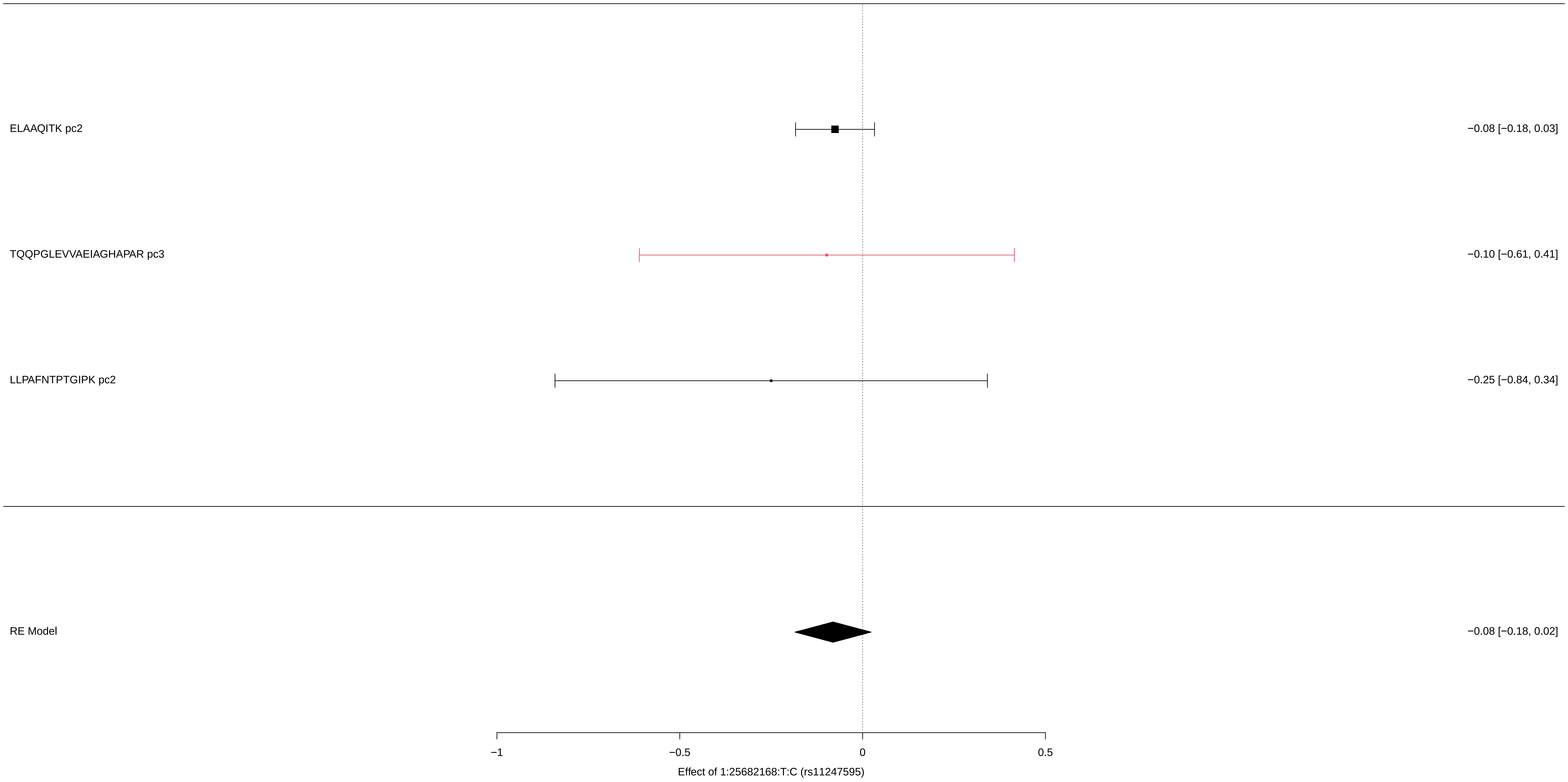

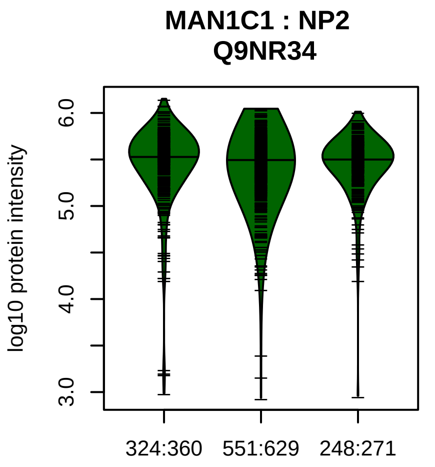

1:26008659:T:C_C
p = 0.11, beta = −0.0663, N = 1123

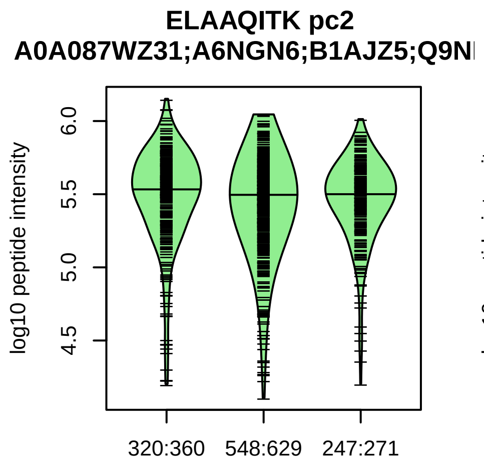

1:26008659:T:C_C
p = 0.073, beta = −0.0754, N = 1115

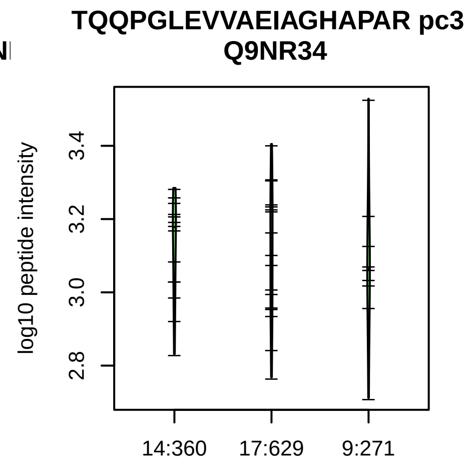

1:26008659:T:C_C
p = 0.63, beta = −0.0978, N = 40

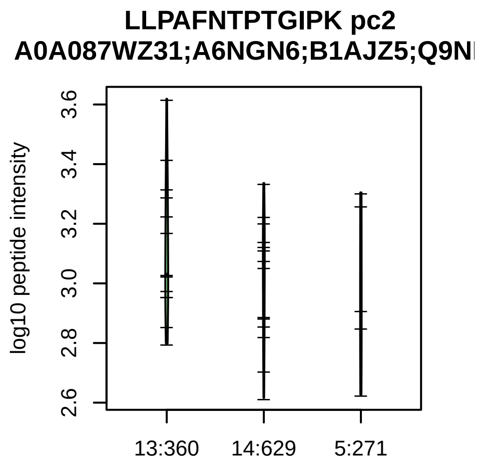

1:26008659:T:C_C
p = 0.28, beta = −0.25, N = 32

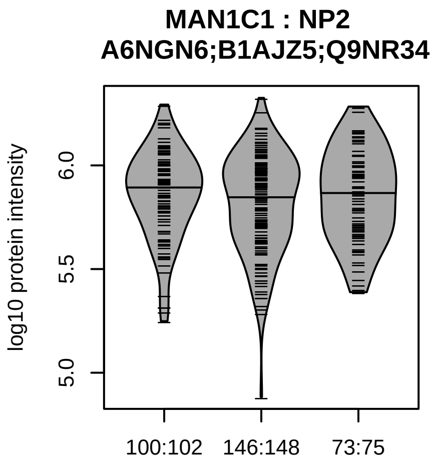

1:26008659:T:C_C
p = 0.43, beta = 0.0602, N = 319

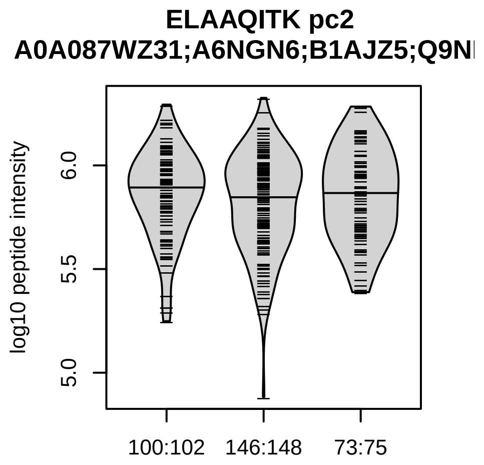

1:26008659:T:C_C
p = 0.43, beta = 0.0602, N = 319

Assay Target: MAN1C1
UniProt: Q9NR34
rsID: rs11247595
Target SNP: 1:25682168:T:C
Proxy SNP: 1:26008659:T:C
deCODE log10(p): 116.6
deCODE BETA: 0.19
deCODE MSPA: 0
–:–:–
1115:40:32

162. SPOCK2 (Q92563)
deCODE MSPA=0.528

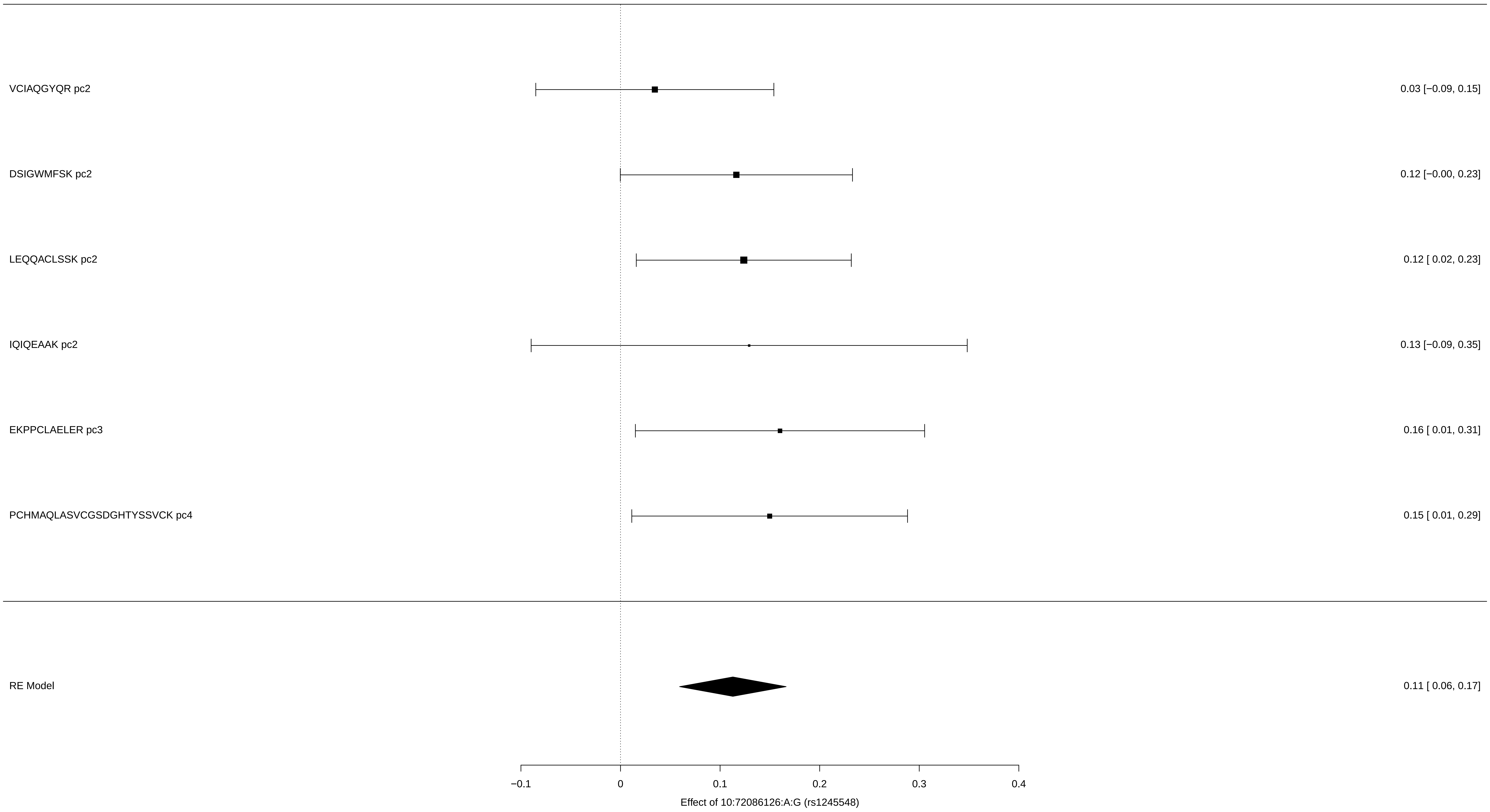

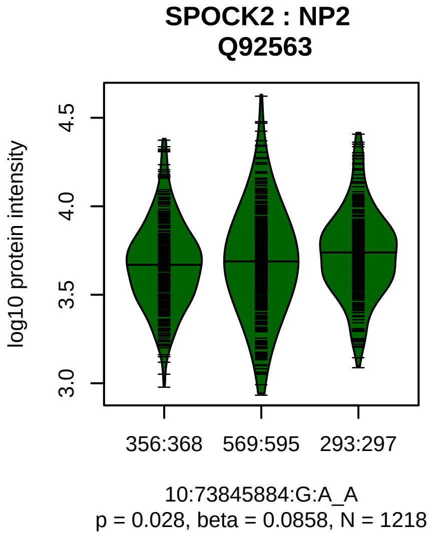

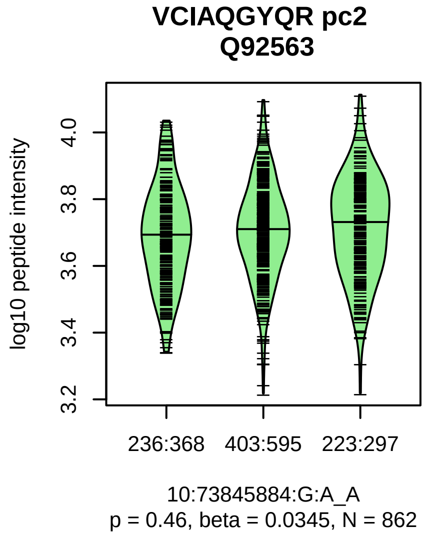

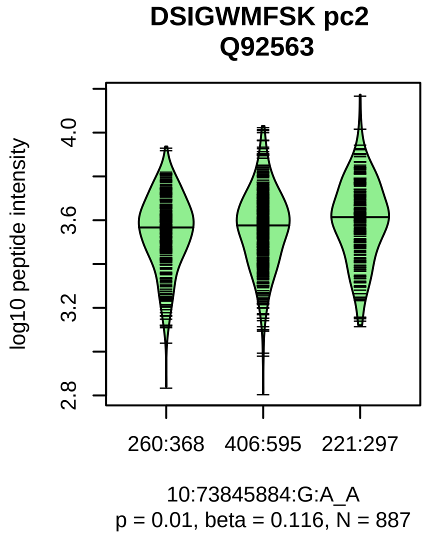

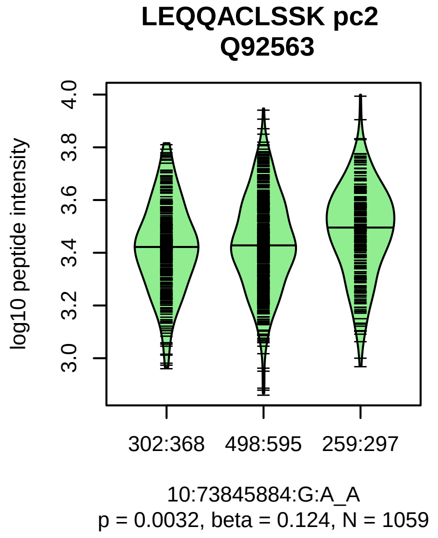

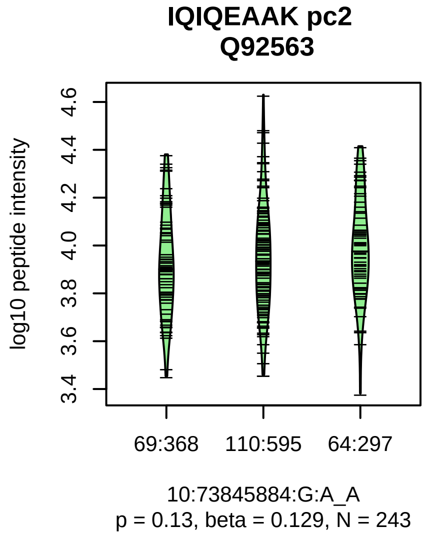

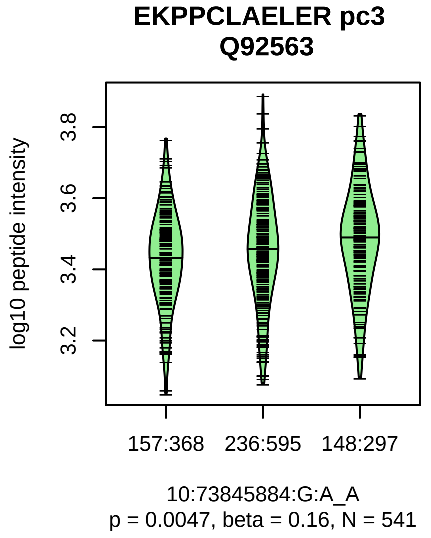

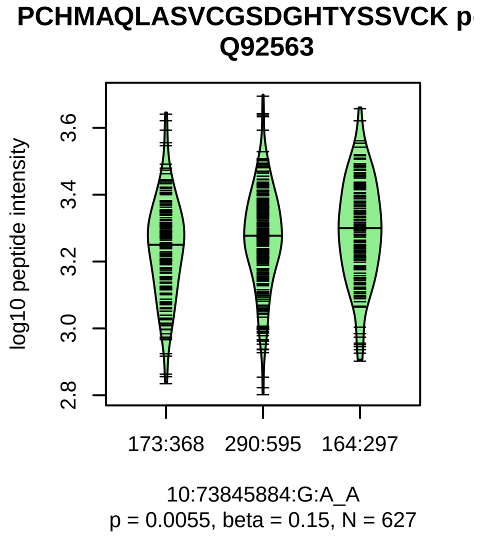

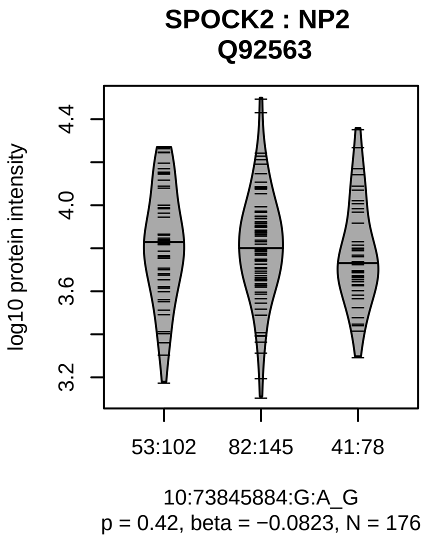

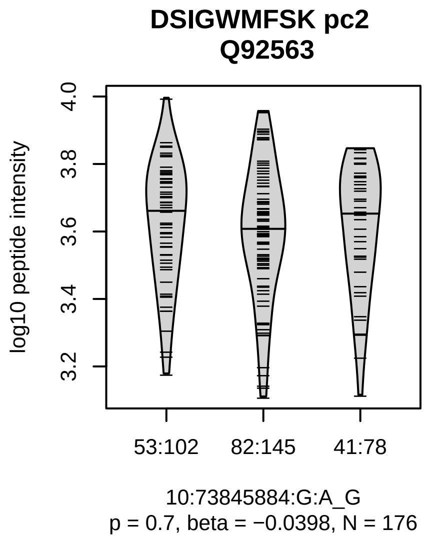

Assay Target: SPOCK2
UniProt: Q92563
rsID: rs1245548
Target SNP: 10:72086126:A:G
Proxy SNP: 10:73845884:G:A
deCODE log10(p): 116
deCODE BETA: 0.19
deCODE MSPA: 0.528
–:–:*:–:*:*
862:887:1059:243:541:627

163. S100A12 (P80511)
deCODE MSPA=0.833

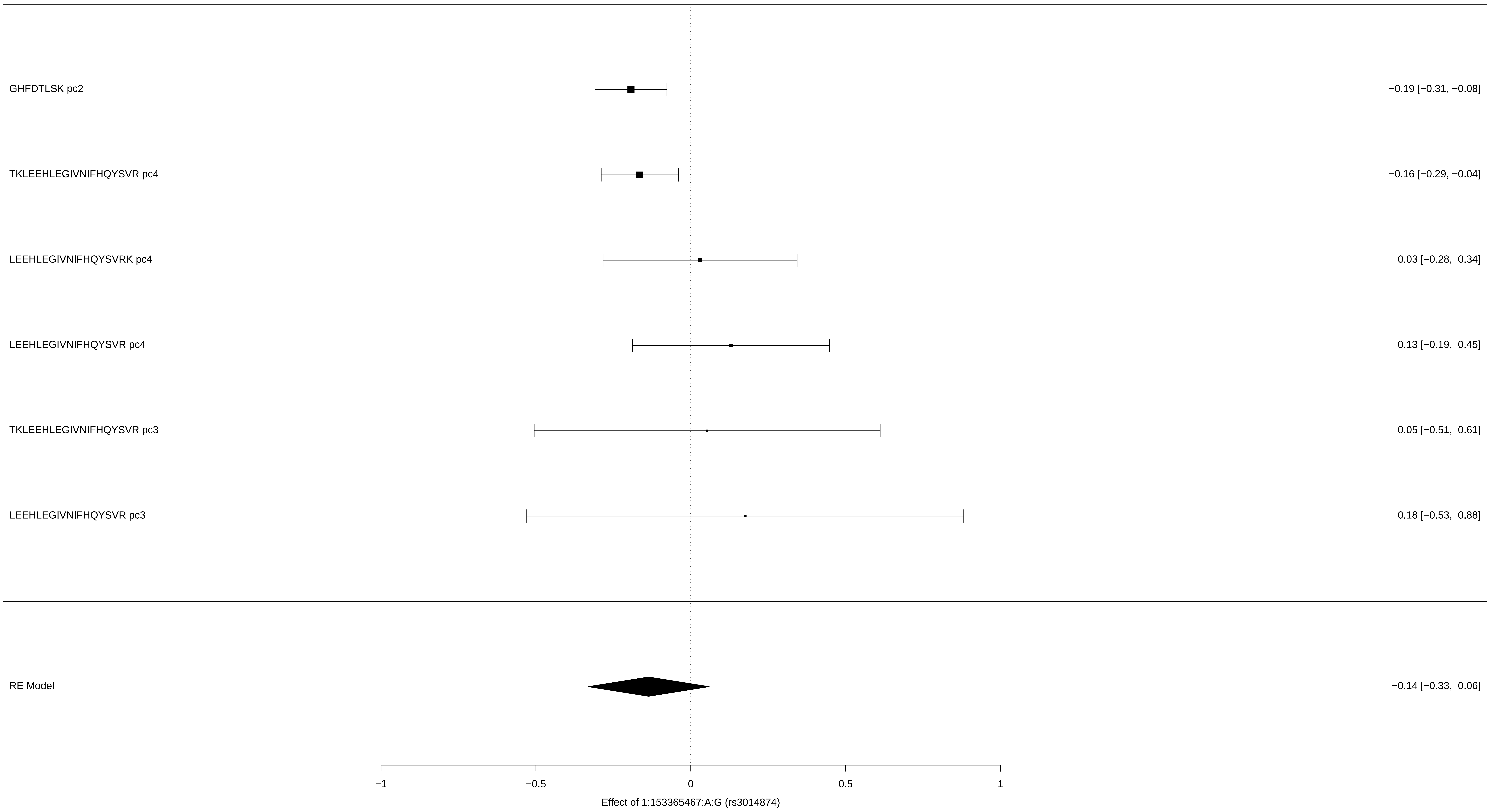

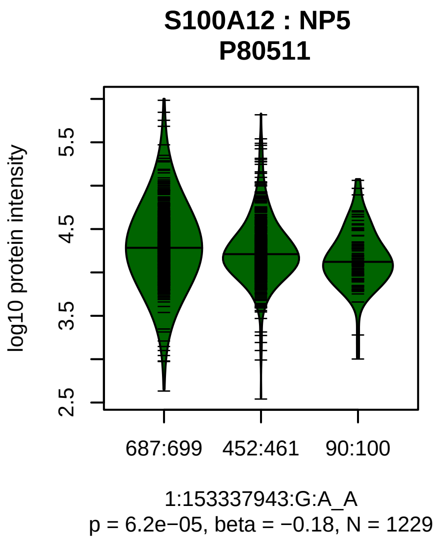

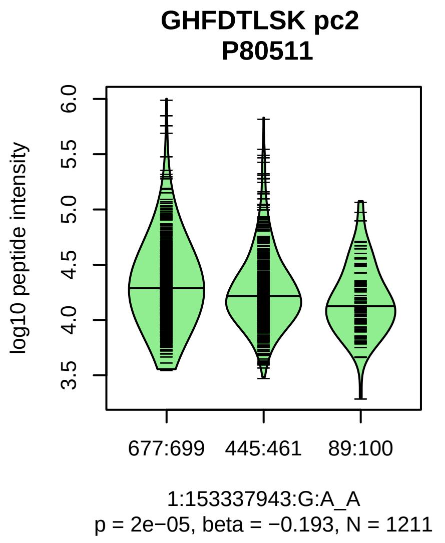

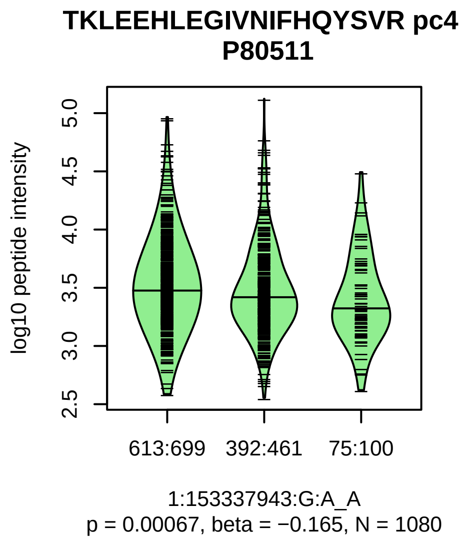

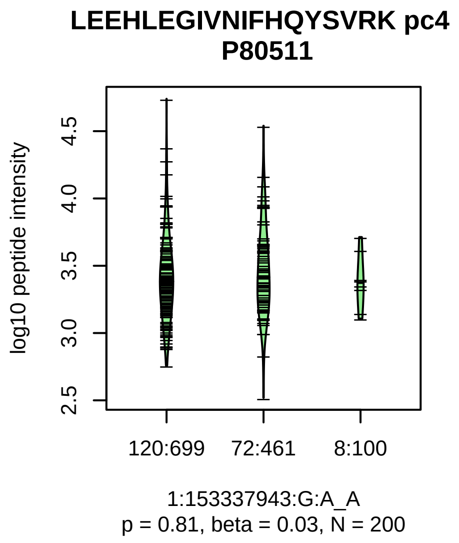

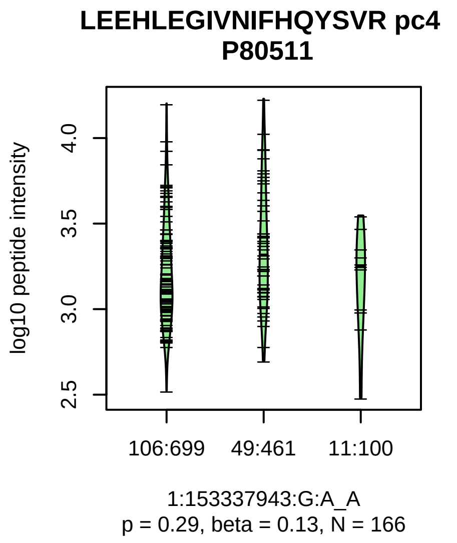

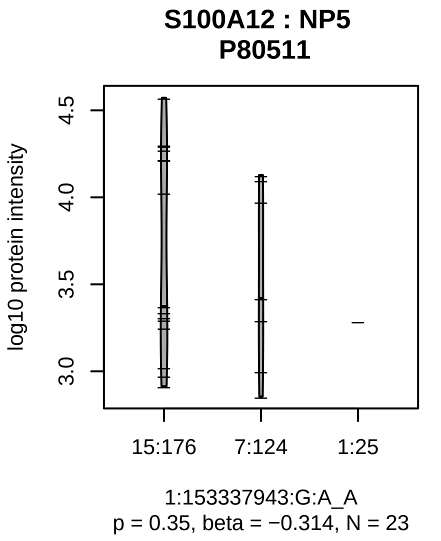

Assay Target: S100A12
UniProt: P80511
rsID: rs3014874
Target SNP: 1:153365467:A:G
Proxy SNP: 1:153337943:G:A
deCODE log10(p): 114.8
deCODE BETA: −0.22
deCODE MSPA: 0.833
::–:–:–:–
1211:1080:200:166:55:39

164. MAN1A2 (O60476)
deCODE MSPA=0.12

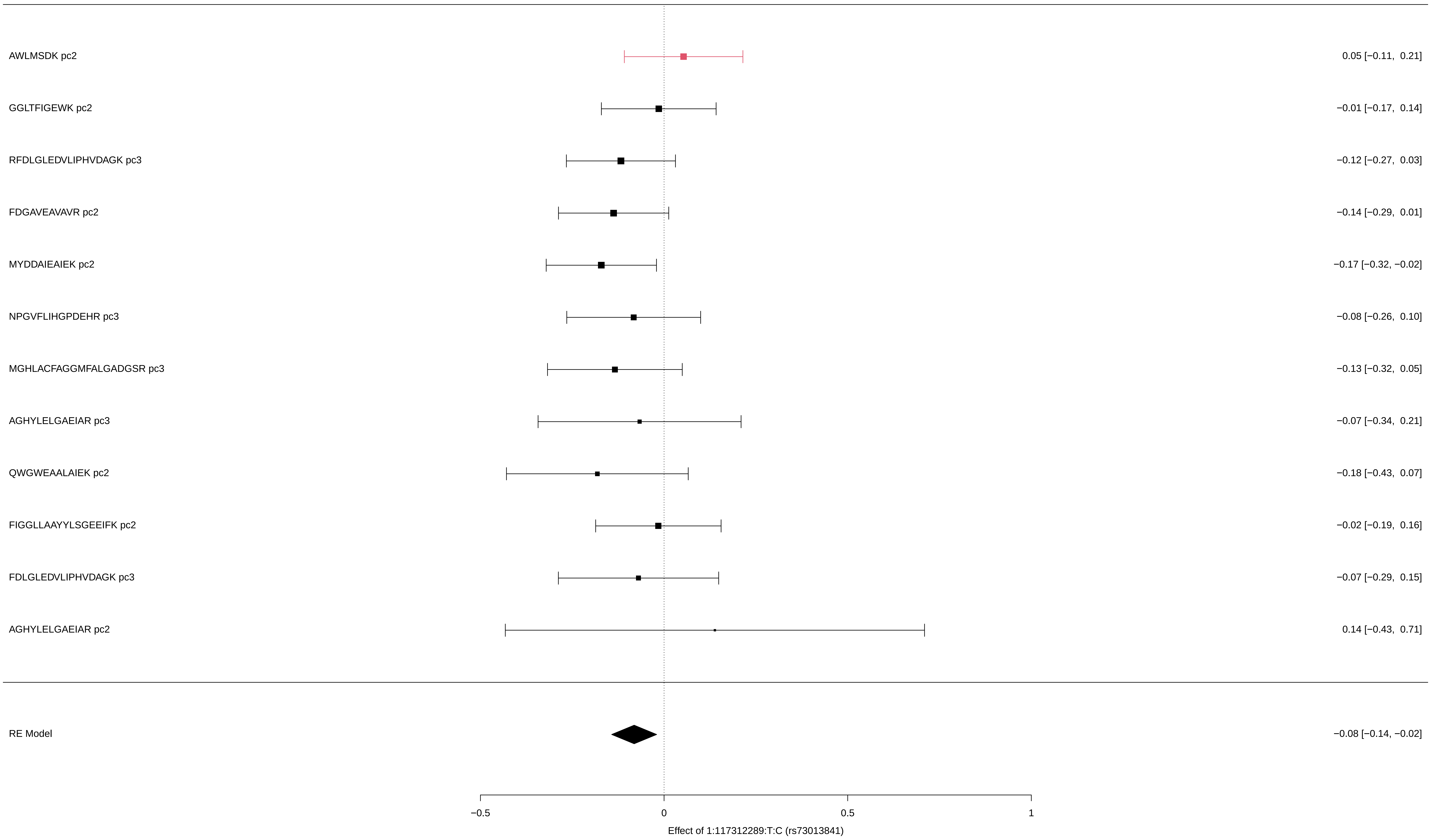

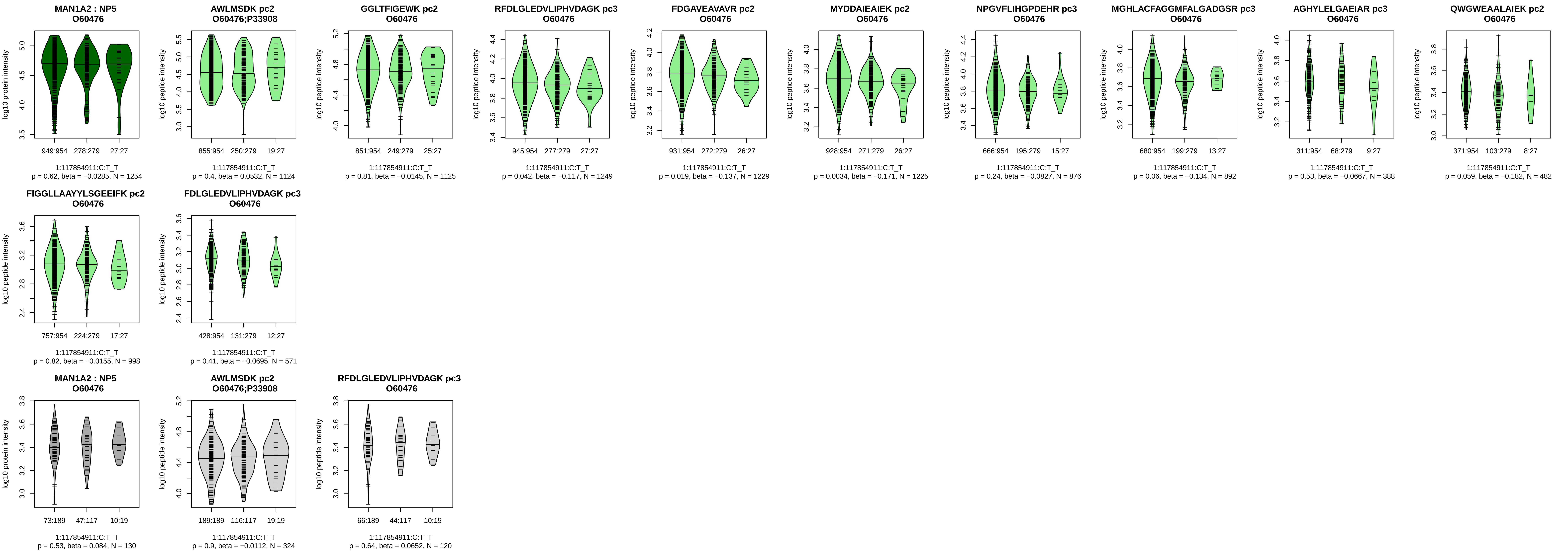

Assay Target: MAN1A2
UniProt: O60476
rsID: rs73013841
Target SNP: 1:117312289:T:C
Proxy SNP: 1:117854911:C:T
deCODE log10(p): 109.1
deCODE BETA: −0.3
deCODE MSPA: 0.12
–:–:–:–*–:–:–:–:–:–
1124:1125:1249:1229:1225:876

165. GLTPD2 (A6NH11)
deCODE MSPA=0

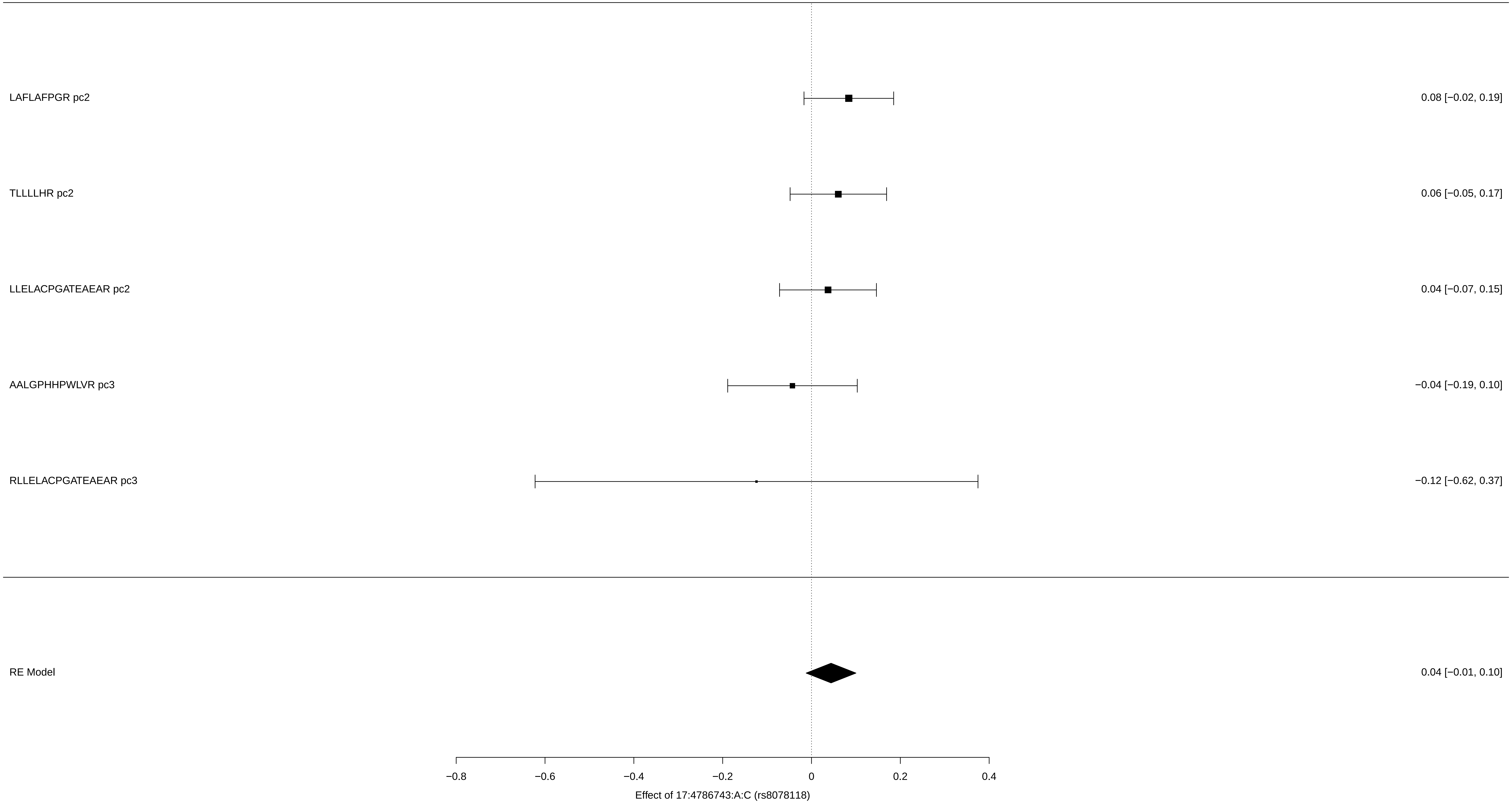

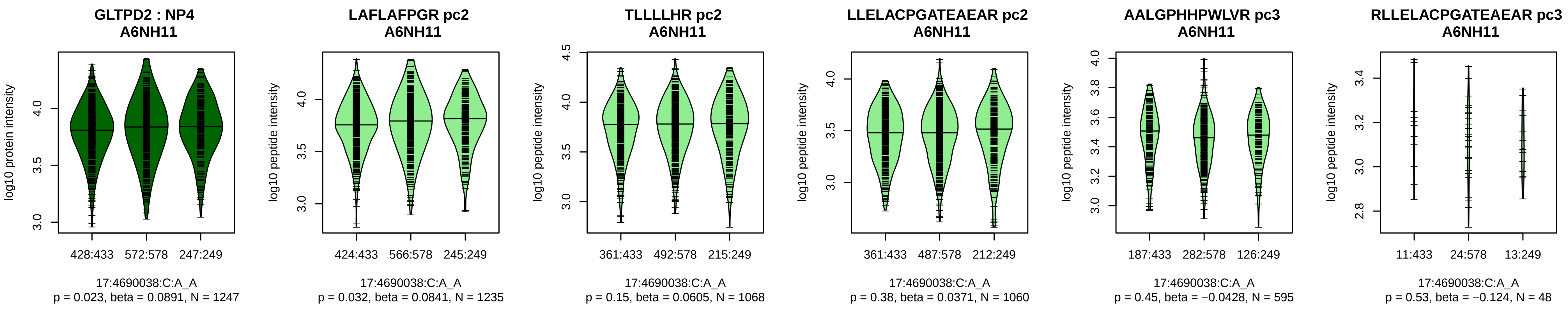

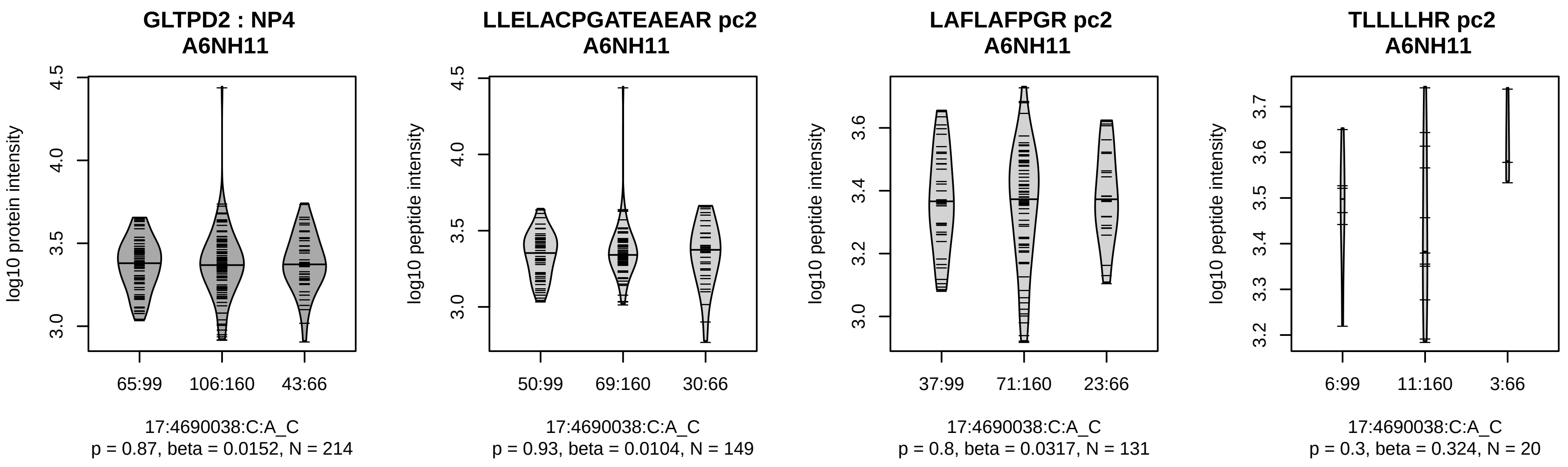

Assay Target: GLTPD2
UniProt: A6NH11
rsID: rs8078118
Target SNP: 17:4786743:A:C
Proxy SNP: 17:4690038:C:A
deCODE log10(p): 108.7
deCODE BETA: 0.19
deCODE MSPA: 0
–:–:–:–:–
1235:1068:1060:595:48

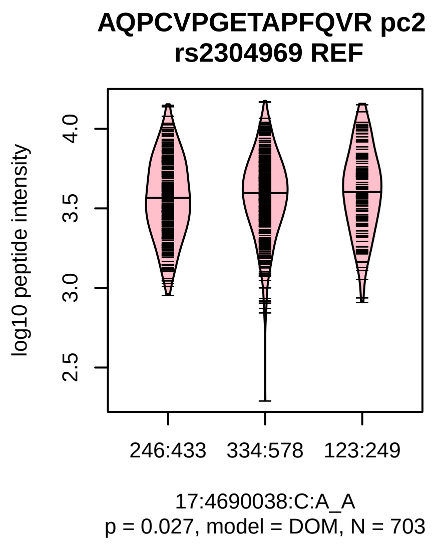

166. CAT (P04040)
deCODE MSPA=0

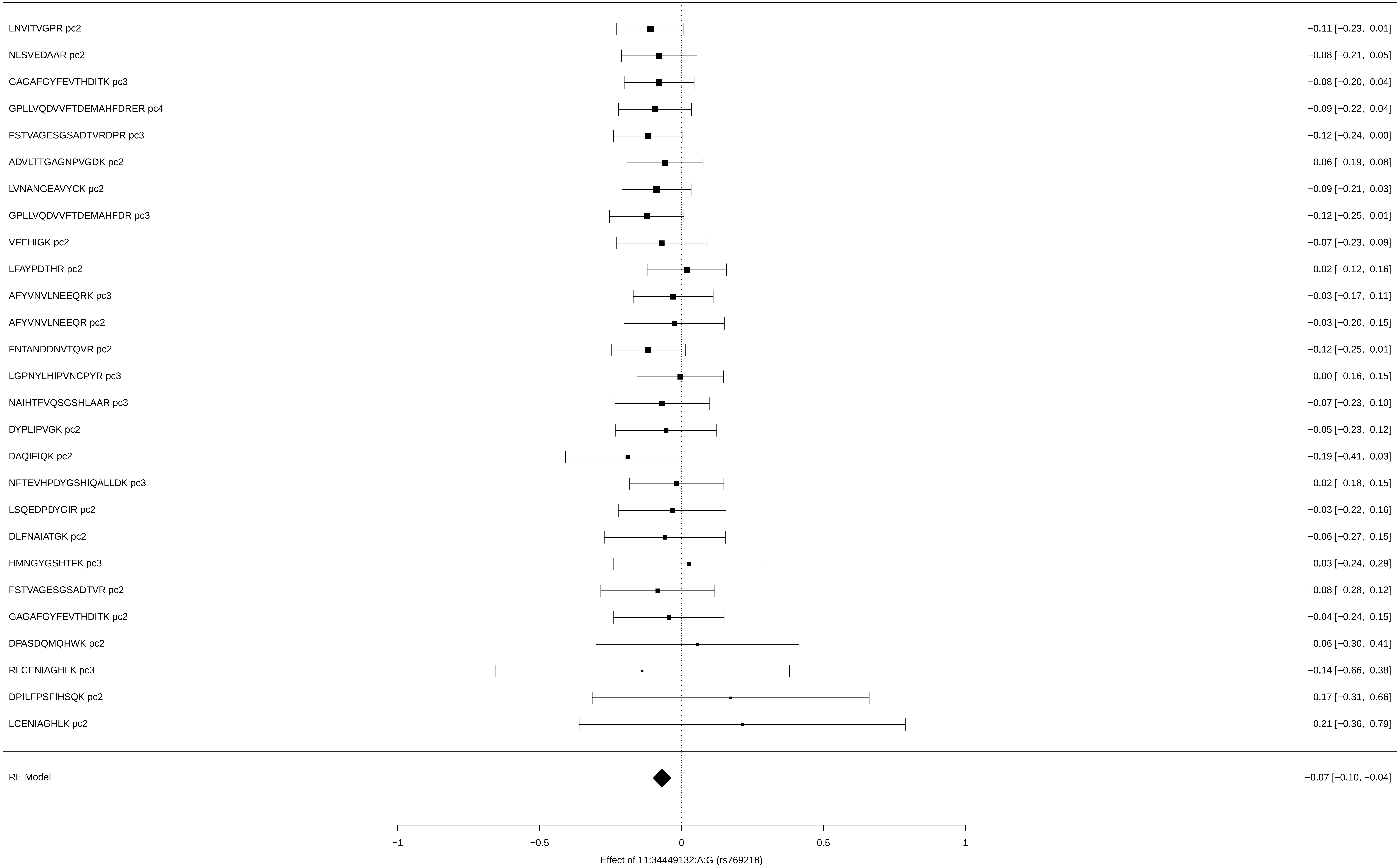

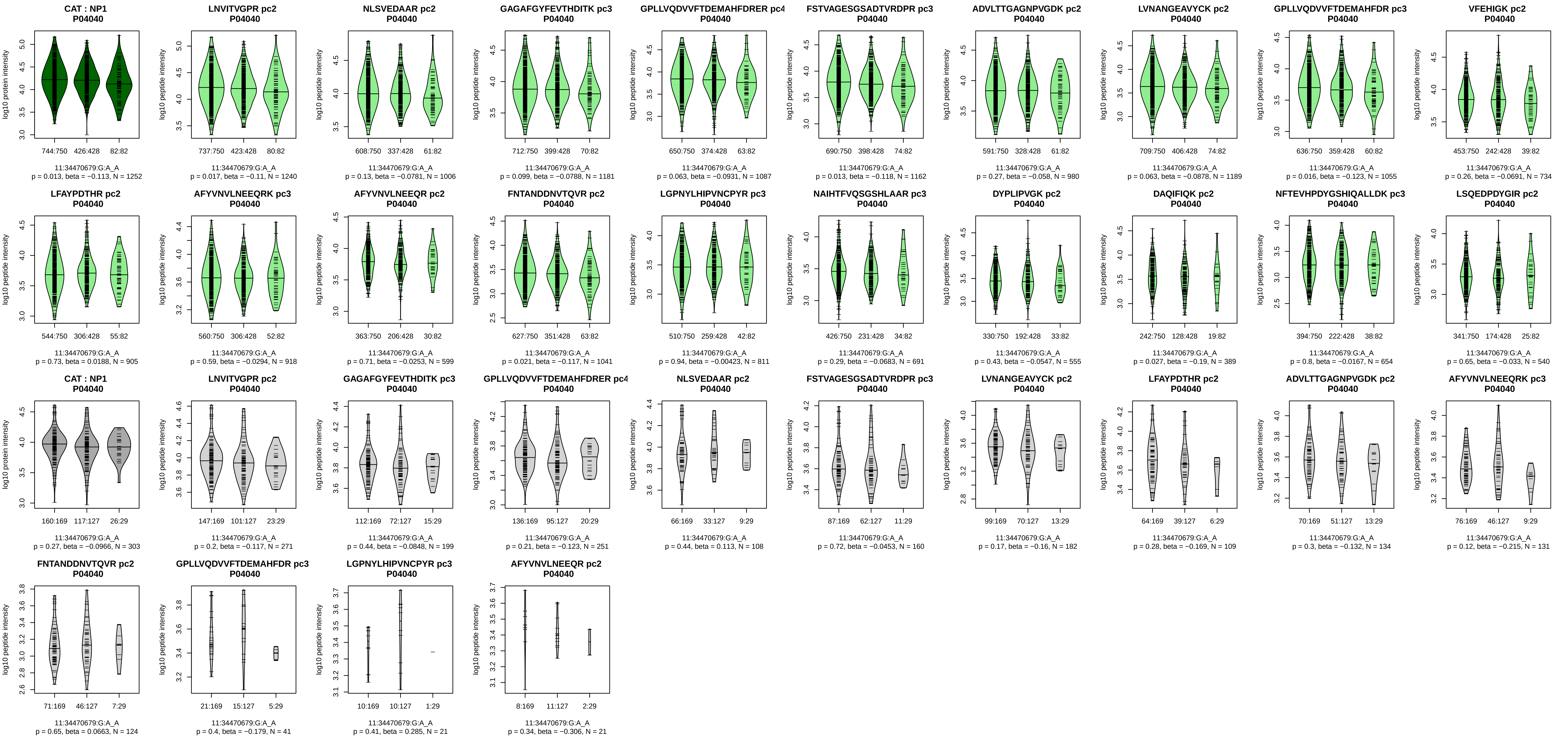

Assay Target: CAT
UniProt: P04040
rsID: rs769218
Target SNP: 11:34449132:A:G
Proxy SNP: 11:34470679:G:A
deCODE log10(p): 106.5
deCODE BETA: −0.21
deCODE MSPA: 0

1240:1006:1181:1087:1162:980

167. PPP1R14A (Q96A00)
deCODE MSPA=0.77

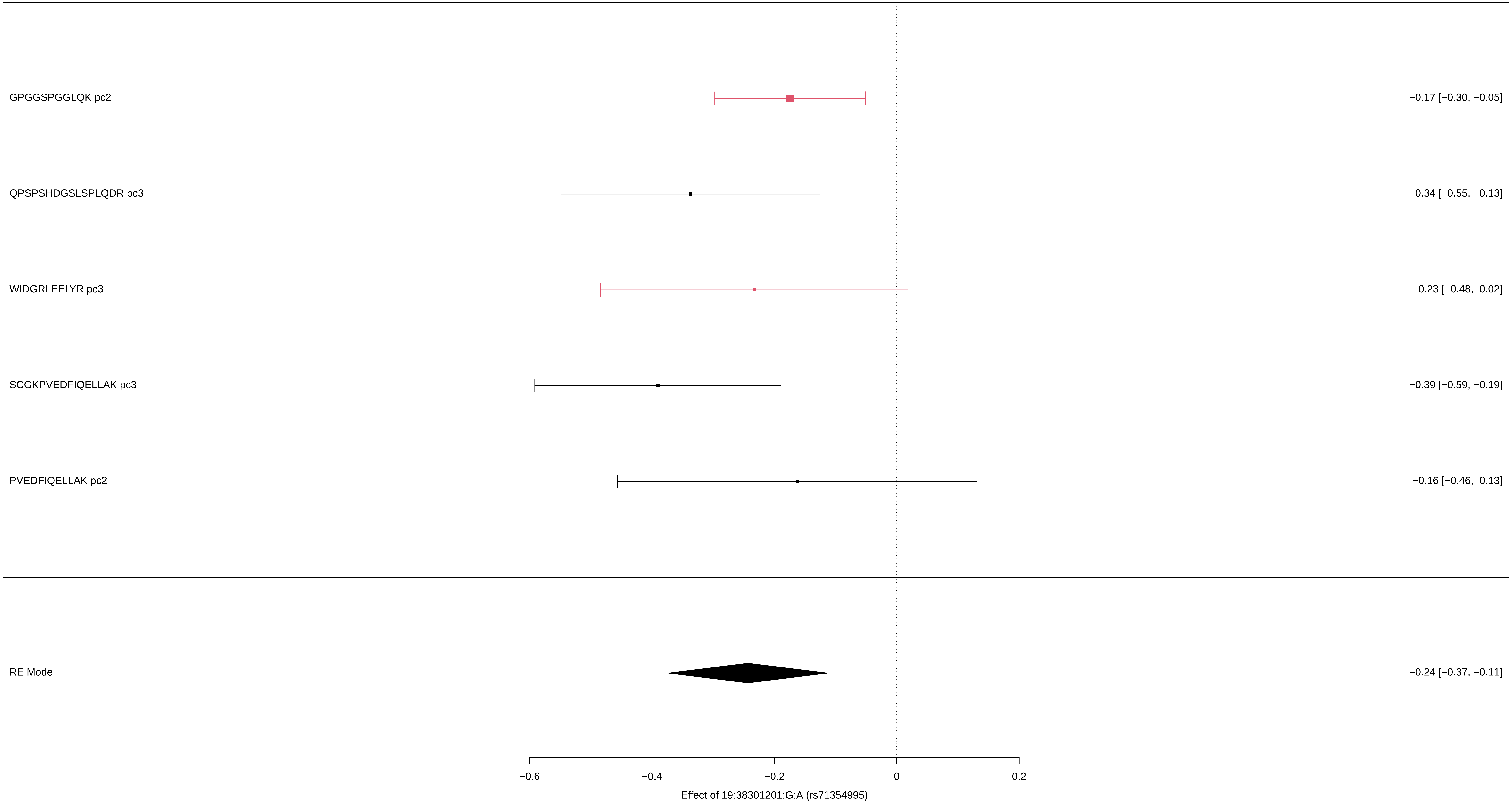

PPP1R14A : NP5
Q96A00

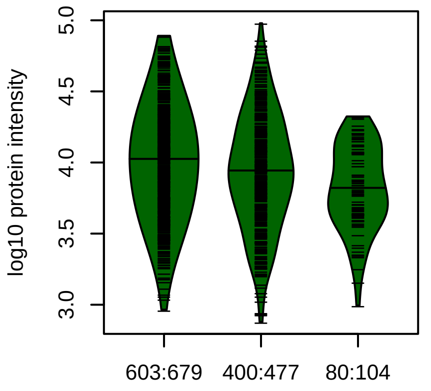

19:38791841:A:G_G
p = 5.9e−05, beta = −0.192, N = 1083

GPGGSPGGLQK pc2
K7EJB8;K7EMN0;Q96A00;Q96A00·

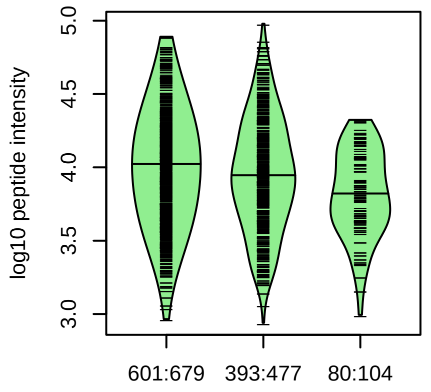

19:38791841:A:G_G
p = 0.00028, beta = −0.174, N = 1074

QPSPSHDGSLSPLQDR pc3
A0A0B4J2A3;Q96A00;Q96A00−2

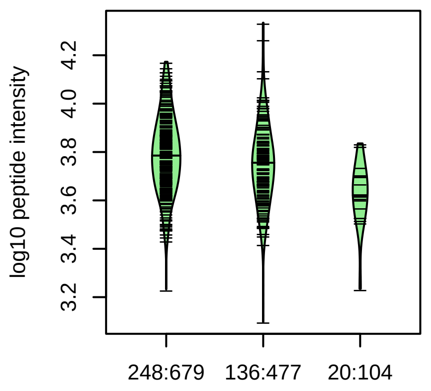

19:38791841:A:G_G
p = 4.9e−05, beta = −0.337, N = 404

WIDGRLEELYR pc3
K7EJB8;K7EMN0;Q96A00;Q96A00·

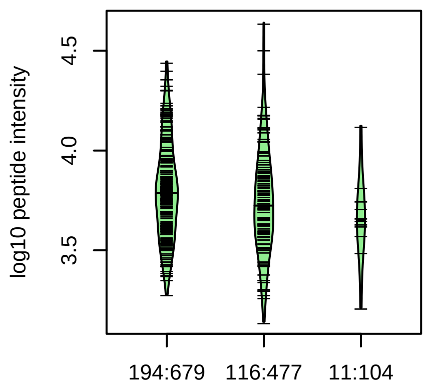

19:38791841:A:G_G
p = 0.018, beta = −0.233, N = 321

SCGKPVEDFIQELLAK pc3
A0A0B4J2A3;Q96A00;Q96A00−2

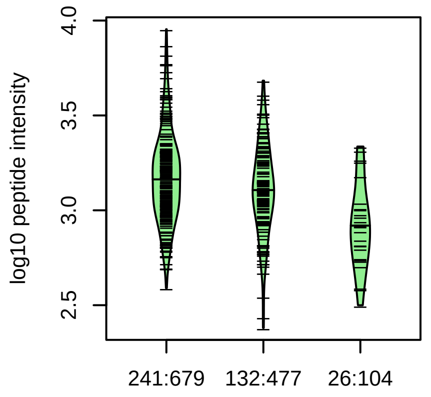

19:38791841:A:G_G
p = 8.7e−07, beta = −0.39, N = 399

PVEDFIQELLAK pc2
A0A0B4J2A3;Q96A00;Q96A00−2

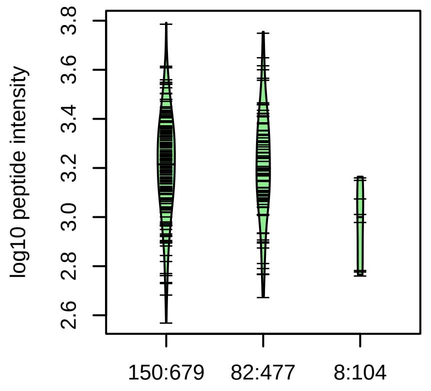

19:38791841:A:G_G
p = 0.16, beta = −0.162, N = 240

PPP1R14A : NP5
Q96A00;Q96A00−2

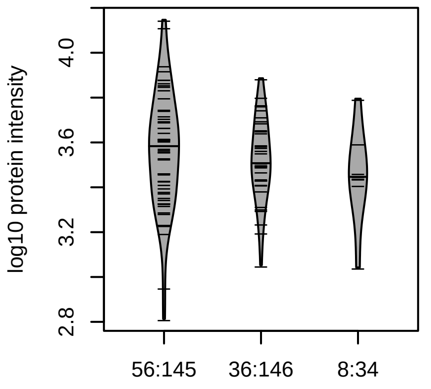

19:38791841:A:G_G
p = 0.14, beta = −0.224, N = 100

GPGGSPGGLQK pc2
K7EJB8;K7EMN0;Q96A00;Q96A00·

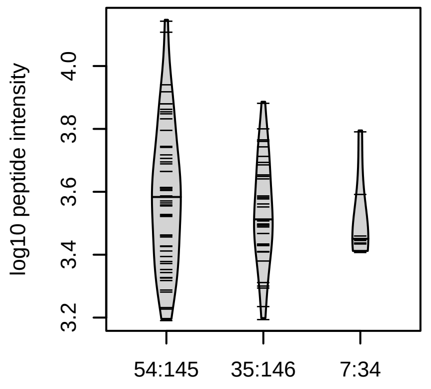

19:38791841:A:G_G
p = 0.16, beta = −0.219, N = 96

SCGKPVEDFIQELLAK pc3
A0A0B4J2A3;Q96A00;Q96A00−2

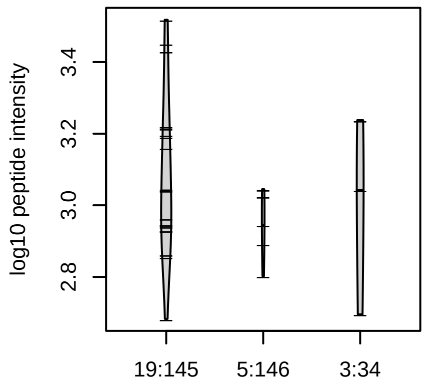

19:38791841:A:G_G
p = 0.76, beta = −0.0816, N = 27

PVEDFIQELLAK pc2
A0A0B4J2A3;Q96A00;Q96A00−2

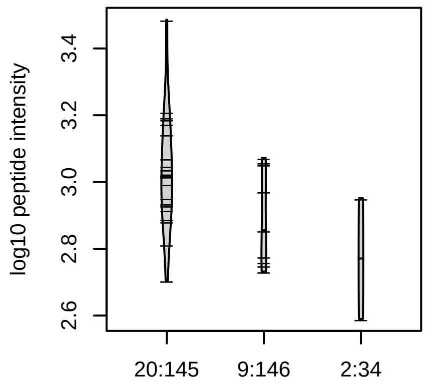

19:38791841:A:G_G
p = 0.23, beta = −0.329, N = 31

Assay Target: PPP1R14A
UniProt: Q96A00
rsID: rs71354995
Target SNP: 19:38301201:G:A
Proxy SNP: 19:38791841:A:G
deCODE log10(p): 103.5
deCODE BETA: −0.2
deCODE MSPA: 0.77
::–:*:–
1074:404:321:399:240

168. FN1 (P02751)
deCODE MSPA=0.634

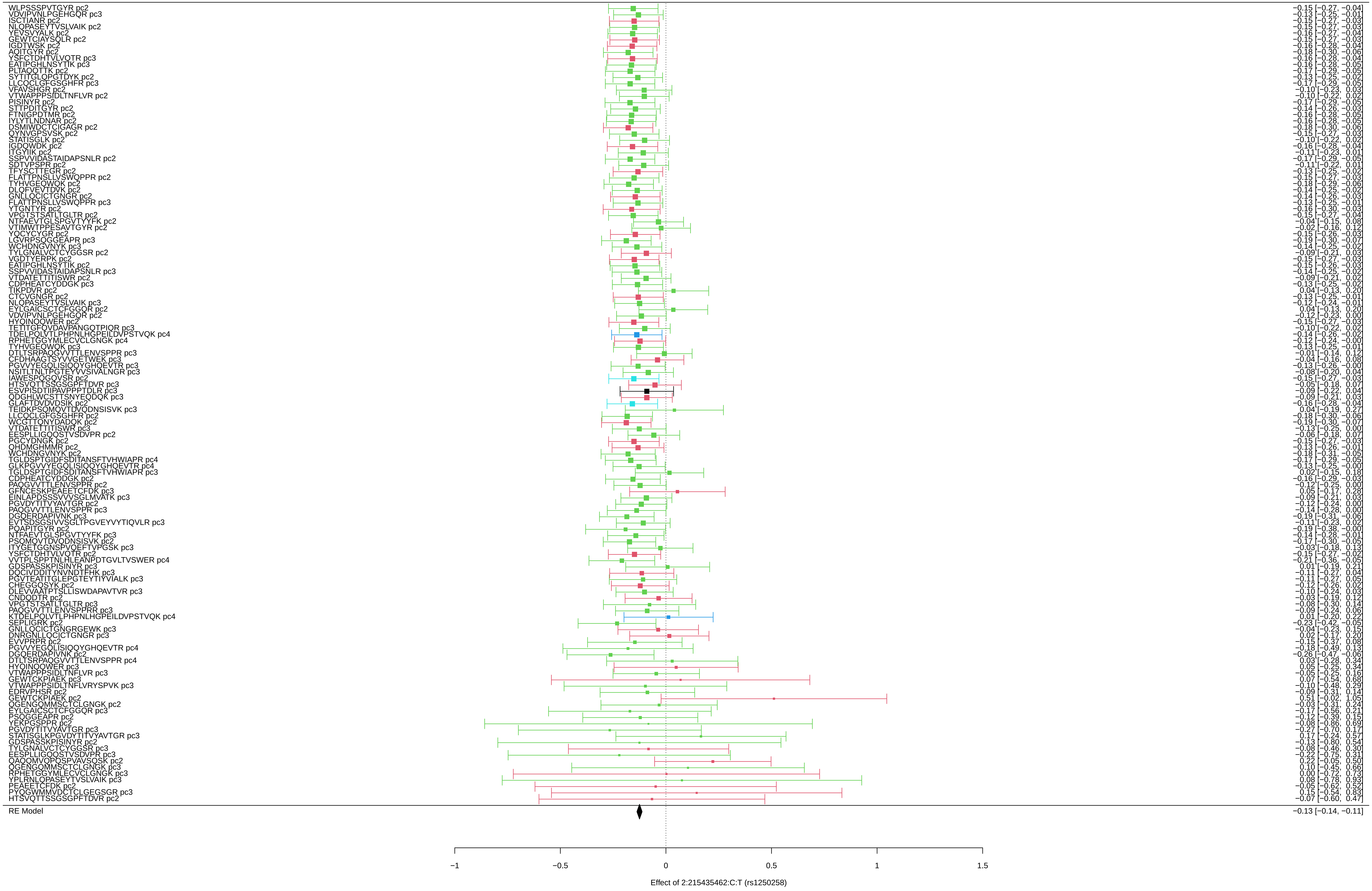

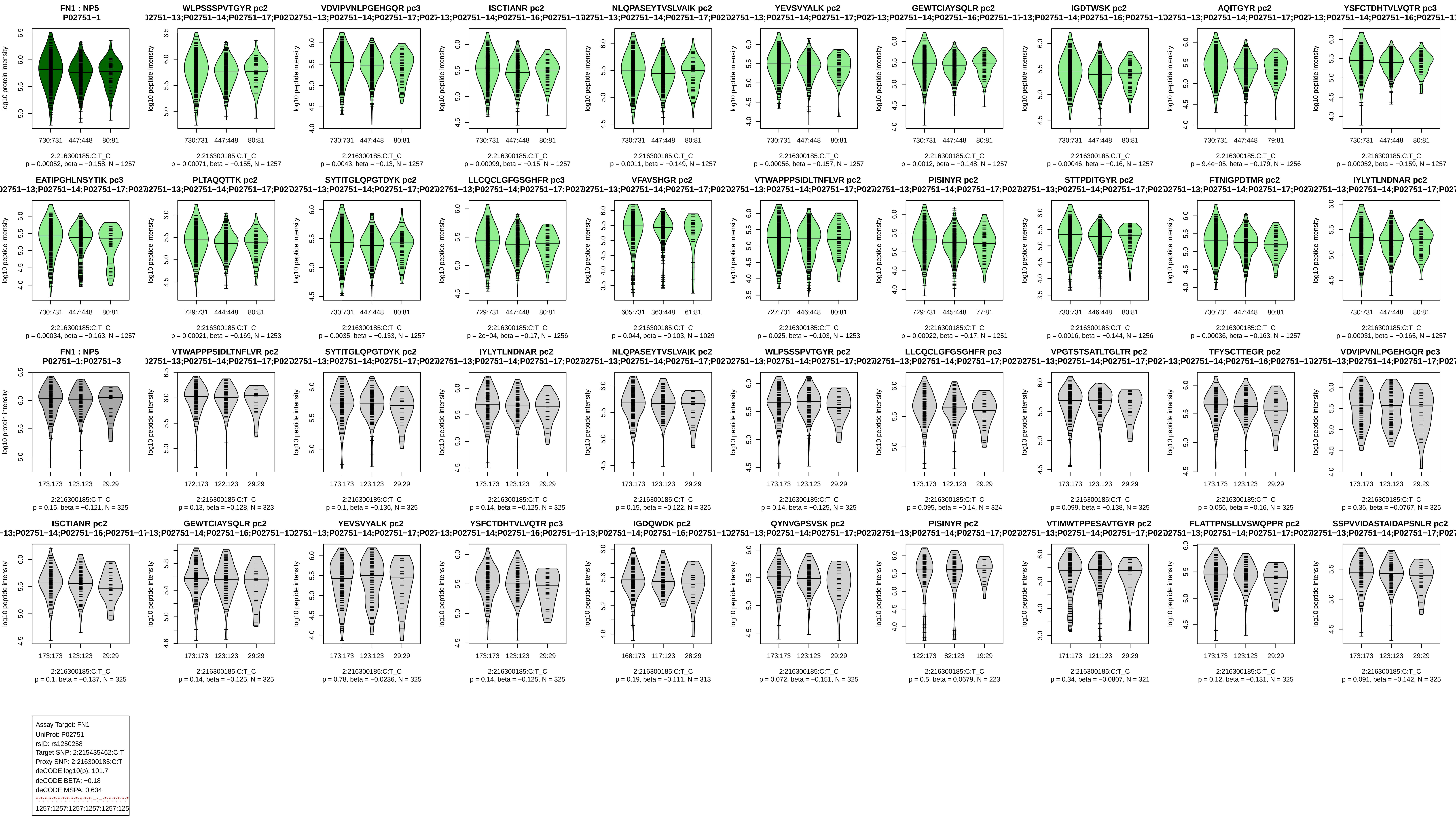
FN1 : NP5 P02751−1
p = 0.00052, beta = −0.158, N = 1257
WLPSSSPVTGYR pc2
p = 0.00071, beta = −0.155, N = 1257
VDVIPVNLPGEHGQR pc3
p = 0.0043, beta = −0.13, N = 1257
ISCTIANR pc2
p = 0.00099, beta = −0.15, N = 1257
NLQPASEYTVSLVAIK pc2
p = 0.0011, beta = −0.149, N = 1257
YEVSVYALK pc2
p = 0.00056, beta = −0.157, N = 1257
GEWTCIAYSQLR pc2
p = 0.0012, beta = −0.148, N = 1257
IGDTWSK pc2
p = 0.00046, beta = −0.16, N = 1257
AQITGYR pc2
p = 9.4e−05, beta = −0.179, N = 1256
YSFCTDHTVLVQTR pc3
p = 0.00052, beta = −0.159, N = 1257
EATIPGHLNSYTIK pc3
p = 0.00034, beta = −0.163, N = 1257
PLTAQQTTK pc2
p = 0.00021, beta = −0.169, N = 1253
SYTITGLQPGTDYK pc2
p = 0.0035, beta = −0.133, N = 1257
LLCQCLGFGSGHFR pc3
p = 2e−04, beta = −0.17, N = 1256
VFAVSHGR pc2
p = 0.044, beta = −0.103, N = 1029
VTWAPPPSIDLTNFLVR pc2
p = 0.025, beta = −0.103, N = 1253
PISINYR pc2
p = 0.00022, beta = −0.17, N = 1251
STTPDITGYR pc2
p = 0.0016, beta = −0.144, N = 1256
FTNIGPDTMR pc2
p = 0.00036, beta = −0.163, N = 1257
IYLYTLNDNAR pc2
p = 0.00031, beta = −0.165, N = 1257
FN1 : NP5 P02751−1;P02751−3
p = 0.15, beta = −0.121, N = 325
VTWAPPPSIDLTNFLVR pc2
p = 0.13, beta = −0.128, N = 323
SYTITGLQPGTDYK pc2
p = 0.1, beta = −0.136, N = 325
IYLYTLNDNAR pc2
p = 0.14, beta = −0.125, N = 325
NLQPASEYTVSLVAIK pc2
p = 0.15, beta = −0.122, N = 325
WLPSSSPVTGYR pc2
p = 0.14, beta = −0.125, N = 325
LLCQCLGFGSGHFR pc3
p = 0.095, beta = −0.14, N = 324
VPGTSTSATLTGLTR pc2
p = 0.099, beta = −0.138, N = 325
TFYSCTTEGR pc2
p = 0.056, beta = −0.16, N = 325
VDVIPVNLPGEHGQR pc3
p = 0.36, beta = −0.0767, N = 325
ISCTIANR pc2
p = 0.1, beta = −0.137, N = 325
GEWTCIAYSQLR pc2
p = 0.14, beta = −0.125, N = 325
YEVSVYALK pc2
p = 0.78, beta = −0.0236, N = 325
YSFCTDHTVLVQTR pc3
p = 0.14, beta = −0.125, N = 325
IGDQWDK pc2
p = 0.19, beta = −0.111, N = 313
QYNVGPSVSK pc2
p = 0.072, beta = −0.151, N = 325
PISINYR pc2
p = 0.5, beta = 0.0679, N = 223
VTIMWTPPESAVTGYR pc2
p = 0.34, beta = −0.0807, N = 321
FLATTPNSLLVSWQPPR pc2
p = 0.12, beta = −0.131, N = 325
SSPVVIDASTAIDAPSNLR pc2
p = 0.091, beta = −0.142, N = 325
Assay Target: FN1
UniProt: P02751
rsID: rs1250258
Target SNP: 2:215435462:C:T
Proxy SNP: 2:216300185:C:T
deCODE log10(p): 101.7
deCODE BETA: −0.18
deCODE MSPA: 0.634

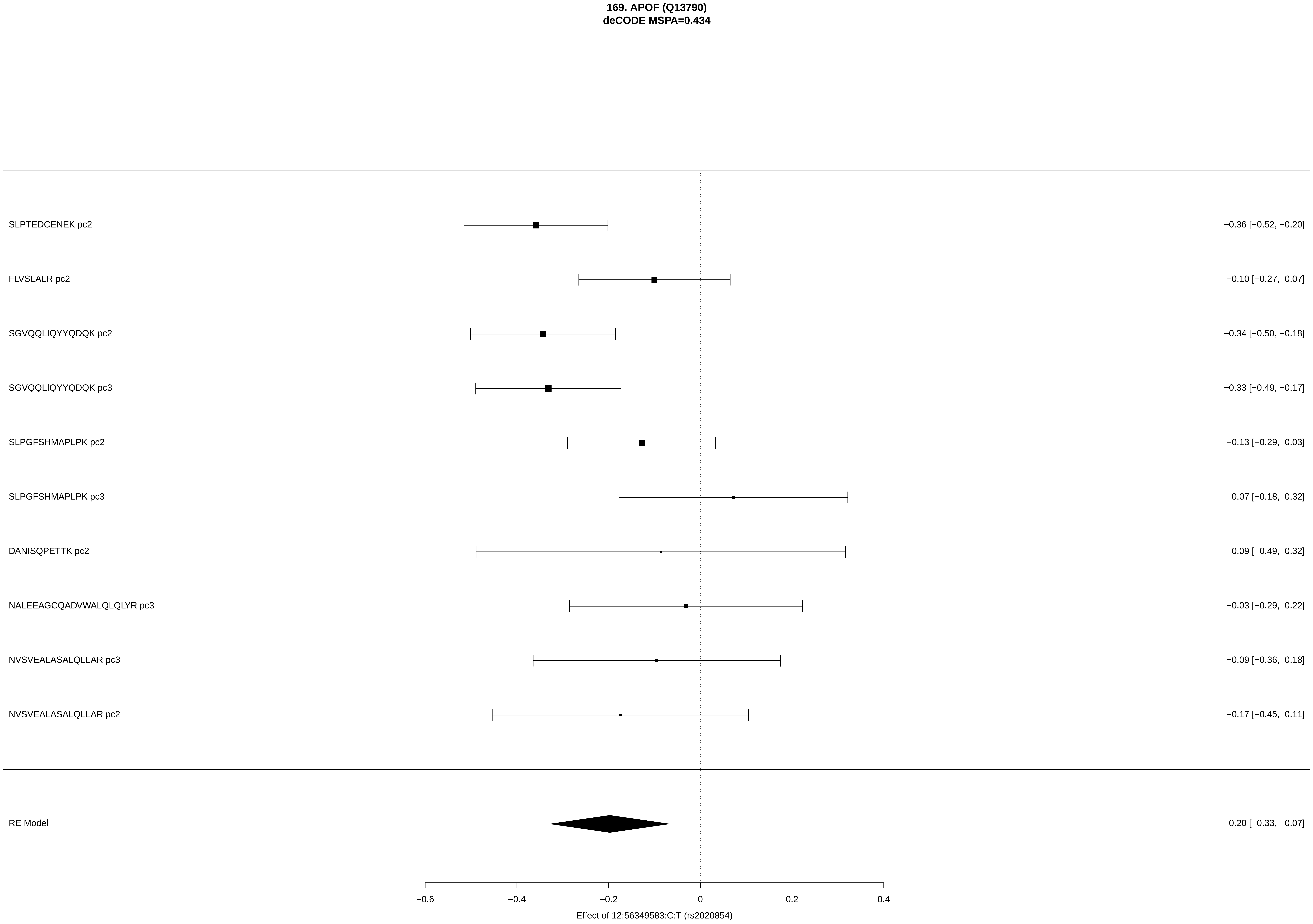

169. APOF (Q13790)
deCODE MSPA=0.434
SLPTEDCENEK pc2
−0.36 [−0.52, −0.20]
FLVSLALR pc2
−0.10 [−0.27, 0.07]
SGVQQLIQYYQDQK pc2
−0.34 [−0.50, −0.18]
SGVQQLIQYYQDQK pc3
−0.33 [−0.49, −0.17]
SLPGFSHMAPLPK pc2
−0.13 [−0.29, 0.03]
SLPGFSHMAPLPK pc3
0.07 [−0.18, 0.32]
DANISQPETTK pc2
−0.09 [−0.49, 0.32]
NALEEAGCQADVWALQLQLYR pc3
−0.03 [−0.29, 0.22]
NVSVEALASALQLLAR pc3
−0.09 [−0.36, 0.18]
NVSVEALASALQLLAR pc2
−0.17 [−0.45, 0.11]
RE Model
−0.20 [−0.33, −0.07]
−0.6
−0.4
−0.2
0
0.2
0.4
Effect of 12:56349583:C:T (rs2020854)

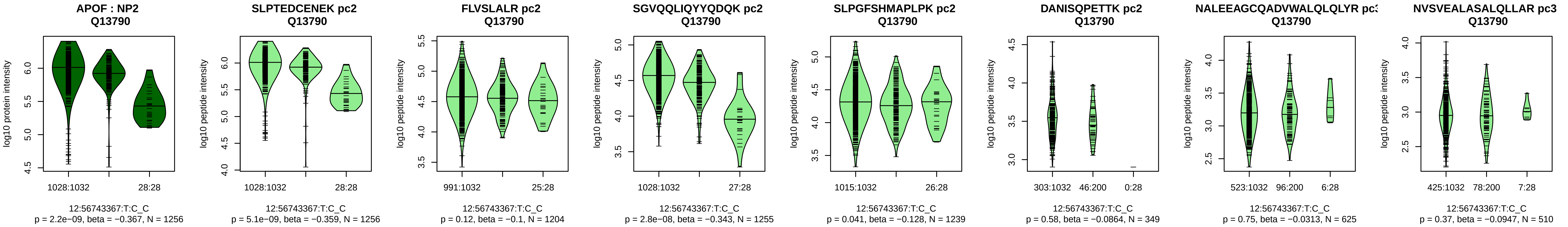

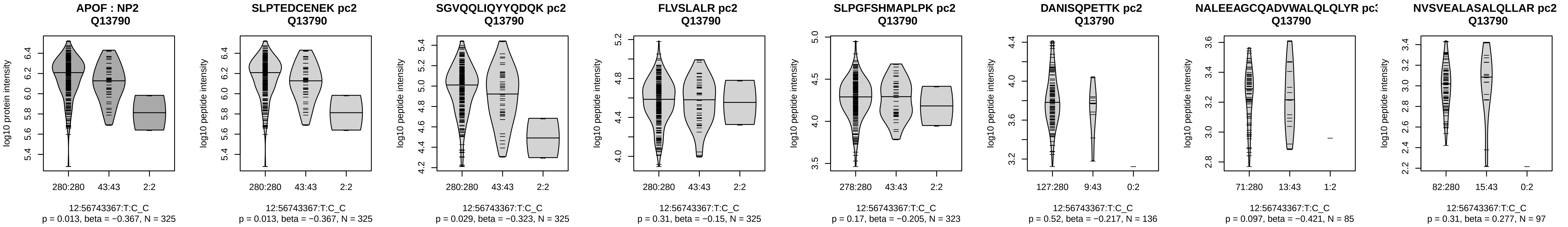

Assay Target: APOF
UniProt: Q13790
rsID: rs2020854
Target SNP: 12:56349583:C:T
Proxy SNP: 12:56743367:T:C
deCODE log10(p): 101.5
deCODE BETA: −0.31
deCODE MSPA: 0.434
:–::*:–:–:–:–:–
1256:1204:1255:1254:1239:538

170. CD59 (P13987)
deCODE MSPA=0.551

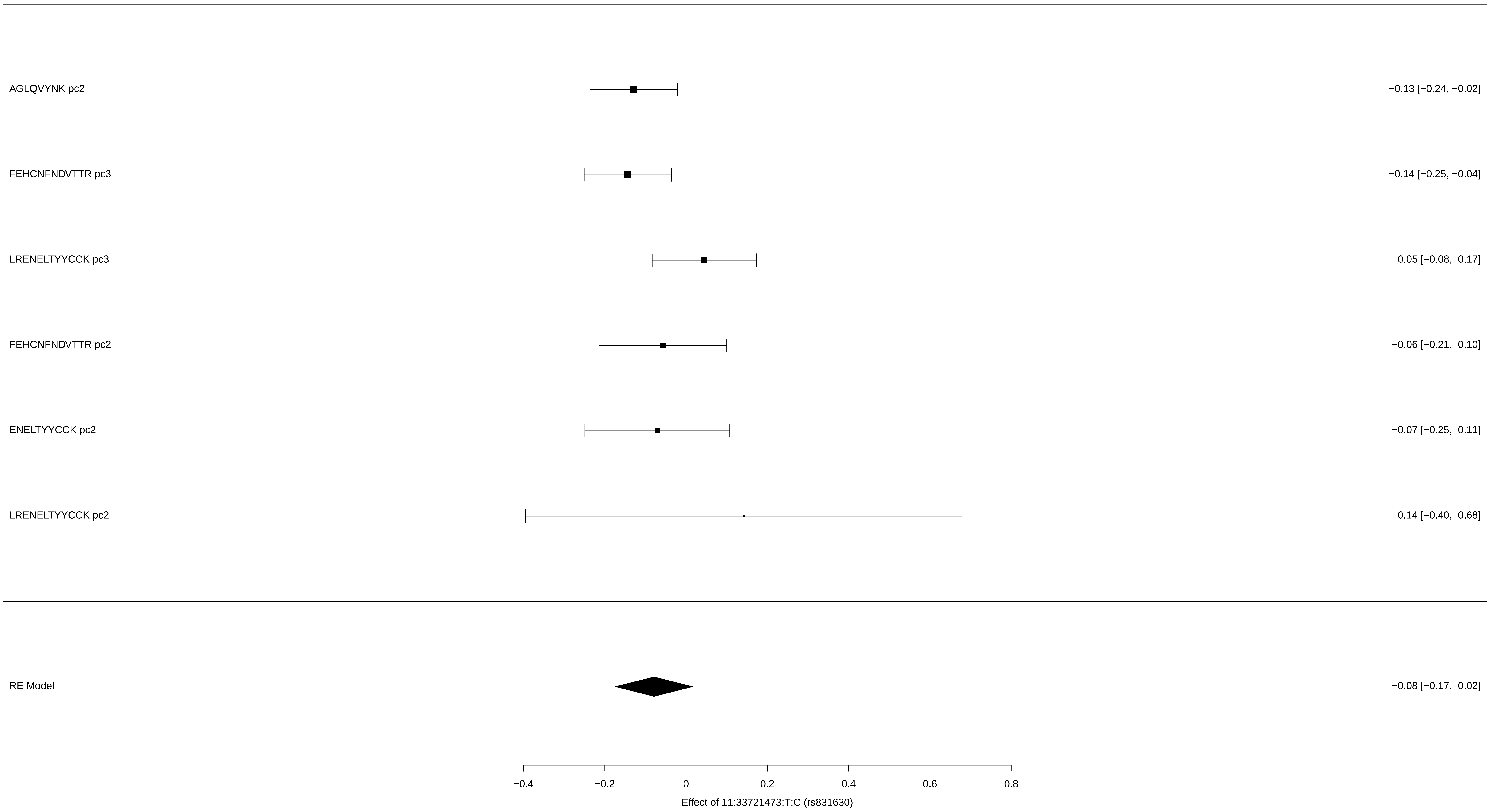

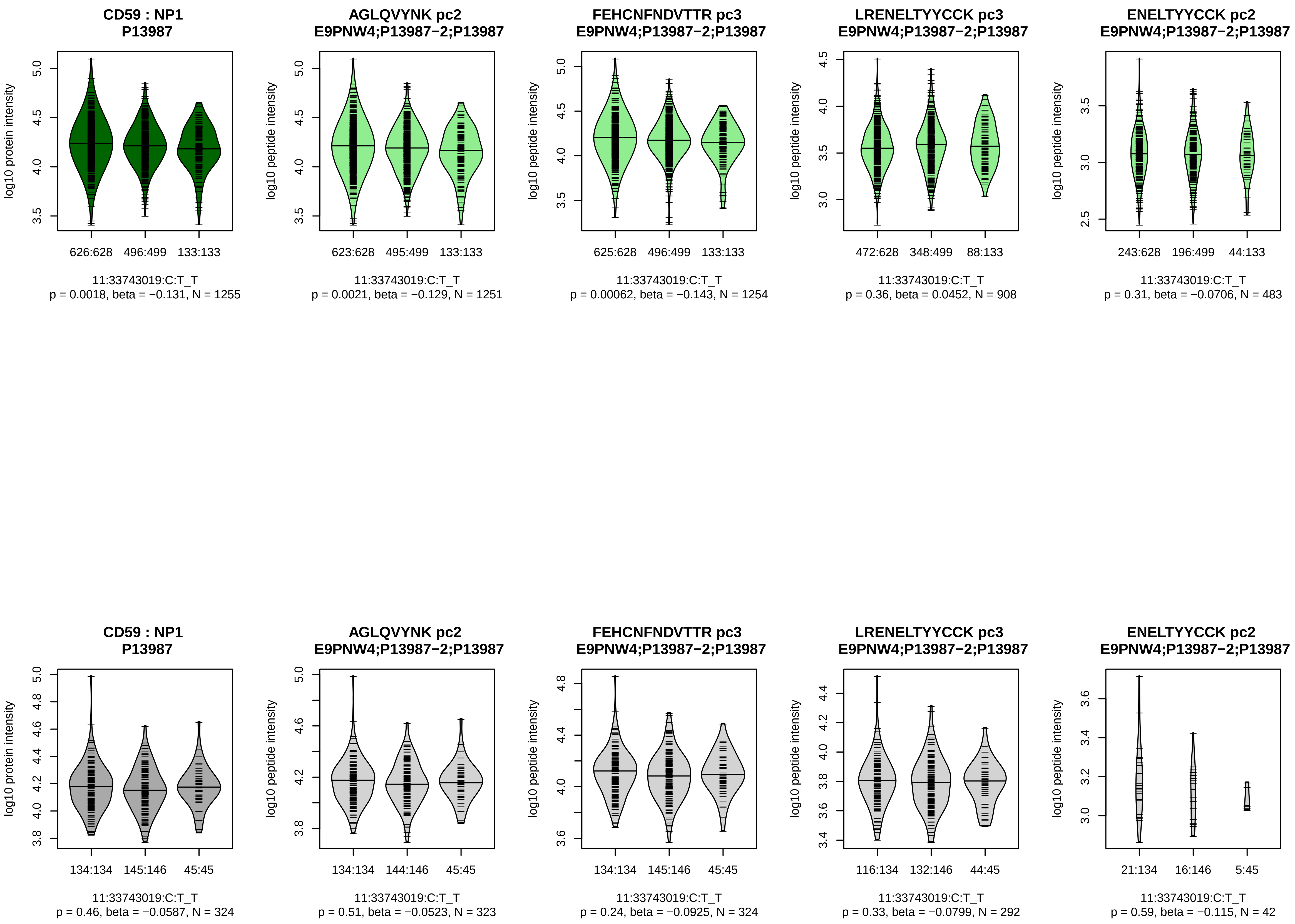

Assay Target: CD59
UniProt: P13987
rsID: rs831630
Target SNP: 11:33721473:T:C
Proxy SNP: 11:33743019:C:T
deCODE log10(p): 100.3
deCODE BETA: −0.18
deCODE MSPA: 0.551
::–:–:–:–
1251:1254:908:589:483:60

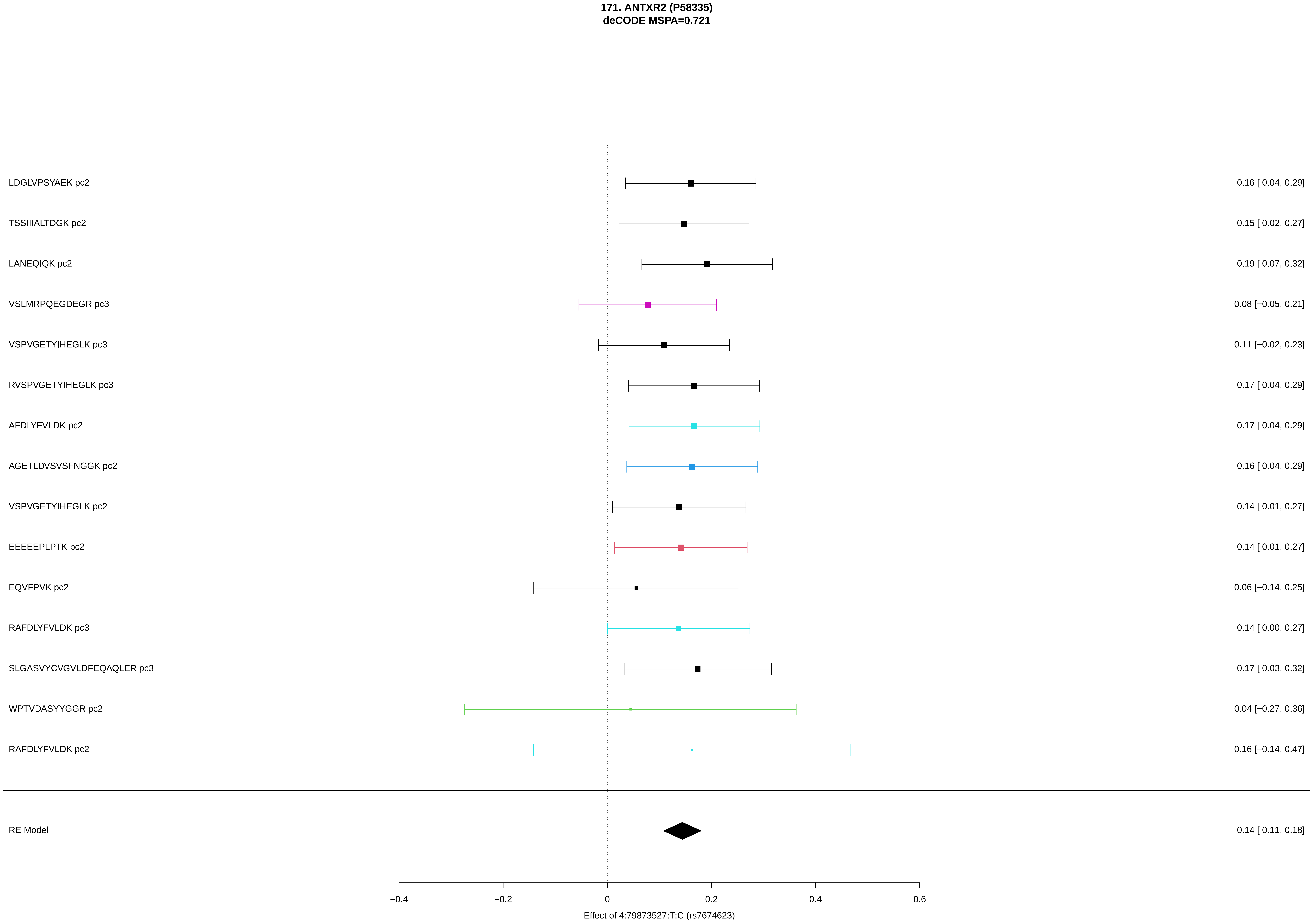
171. ANTXR2 (P58335)
deCODE MSPA=0.721
LDGLVPSYAEK pc2
0.16 [0.04, 0.29]
TSSIIIALTDGK pc2
0.15 [0.02, 0.27]
LANEQIQK pc2
0.19 [0.07, 0.32]
VSLMRPQEGDEGR pc3
0.08 [−0.05, 0.21]
VSPVGETYIHEGLK pc3
0.11 [−0.02, 0.23]
RVSPVGETYIHEGLK pc3
0.17 [0.04, 0.29]
AFDLYFVLDK pc2
0.17 [0.04, 0.29]
AGETLDVSVSFNGGK pc2
0.16 [0.04, 0.29]
VSPVGETYIHEGLK pc2
0.14 [0.01, 0.27]
EEEEEPLPTK pc2
0.14 [0.01, 0.27]
EQVFPVK pc2
0.06 [−0.14, 0.25]
RAFDLYFVLDK pc3
0.14 [0.00, 0.27]
SLGASVYCVGVLDFEQAQLER pc3
0.17 [0.03, 0.32]
WPTVDASYYGGR pc2
0.04 [−0.27, 0.36]
RAFDLYFVLDK pc2
0.16 [−0.14, 0.47]
RE Model
0.14 [0.11, 0.18]
−0.4
−0.2
0
0.2
0.4
0.6
Effect of 4:79873527:T:C (rs7674623)

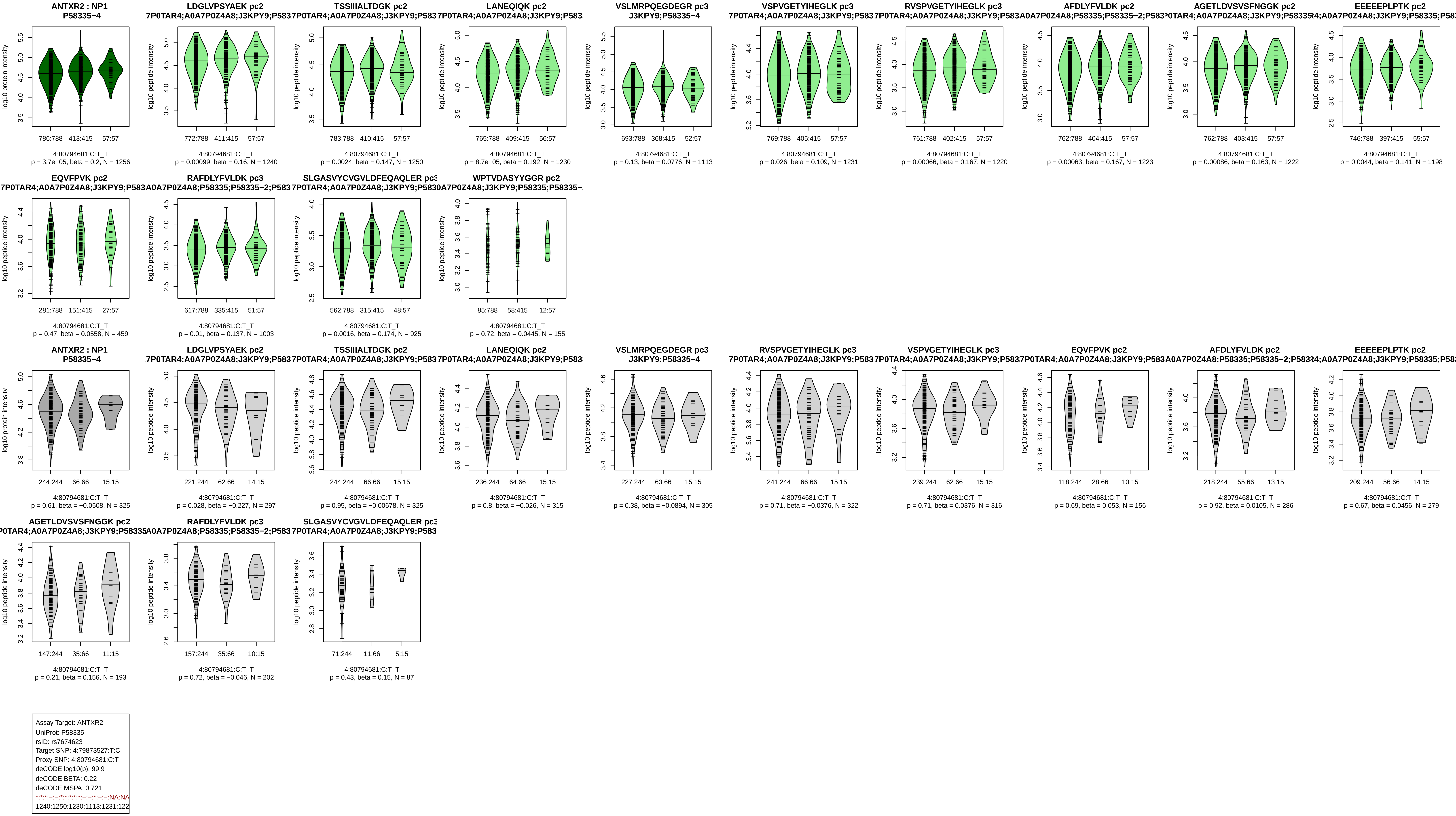
ANTXR2 : NP1
P58335−4
4:80794681:C:T_T
p = 3.7e−05, beta = 0.2, N = 1256
LDGLVPSYAEK pc2
p = 0.00099, beta = 0.16, N = 1240
TSSIIIALTDGK pc2
p = 0.0024, beta = 0.147, N = 1250
LANEQIQK pc2
p = 8.7e−05, beta = 0.192, N = 1230
VSLMRPQEGDEGR pc3
J3KPY9;P58335−4
p = 0.13, beta = 0.0776, N = 1113
VSPVGETYIHEGLK pc3
p = 0.026, beta = 0.109, N = 1231
RVSPVGETYIHEGLK pc3
p = 0.00066, beta = 0.167, N = 1220
AFDLYFVLDK pc2
p = 0.00063, beta = 0.167, N = 1223
AGETLDVSVSFNGGK pc2
p = 0.00086, beta = 0.163, N = 1222
EEEEEPLPTK pc2
p = 0.0044, beta = 0.141, N = 1198
EQVFPVK pc2
p = 0.47, beta = 0.0558, N = 459
RAFDLYFVLDK pc3
p = 0.01, beta = 0.137, N = 1003
SLGASVYCVGVLDFEQAQLER pc3
p = 0.0016, beta = 0.174, N = 925
WPTVDASYYGGR pc2
p = 0.72, beta = 0.0445, N = 155
ANTXR2 : NP1
P58335−4
p = 0.61, beta = −0.0508, N = 325
LDGLVPSYAEK pc2
p = 0.028, beta = −0.227, N = 297
TSSIIIALTDGK pc2
p = 0.95, beta = −0.00678, N = 325
LANEQIQK pc2
p = 0.8, beta = −0.026, N = 315
VSLMRPQEGDEGR pc3
p = 0.38, beta = −0.0894, N = 305
RVSPVGETYIHEGLK pc3
p = 0.71, beta = −0.0376, N = 322
VSPVGETYIHEGLK pc3
p = 0.71, beta = 0.0376, N = 316
EQVFPVK pc2
p = 0.69, beta = 0.053, N = 156
AFDLYFVLDK pc2
p = 0.92, beta = 0.0105, N = 286
EEEEEPLPTK pc2
p = 0.67, beta = 0.0456, N = 279
AGETLDVSVSFNGGK pc2
p = 0.21, beta = 0.156, N = 193
RAFDLYFVLDK pc3
p = 0.72, beta = −0.046, N = 202
SLGASVYCVGVLDFEQAQLER pc3
p = 0.43, beta = 0.15, N = 87
log10 protein intensity
log10 peptide intensity
Assay Target: ANTXR2
UniProt: P58335
rsID: rs7674623
Target SNP: 4:79873527:T:C
Proxy SNP: 4:80794681:C:T
deCODE log10(p): 99.9
deCODE BETA: 0.22
deCODE MSPA: 0.721
1240:1250:1230:1113:1231:122

172. NPNT (Q6UXI9)
deCODE MSPA=0.869

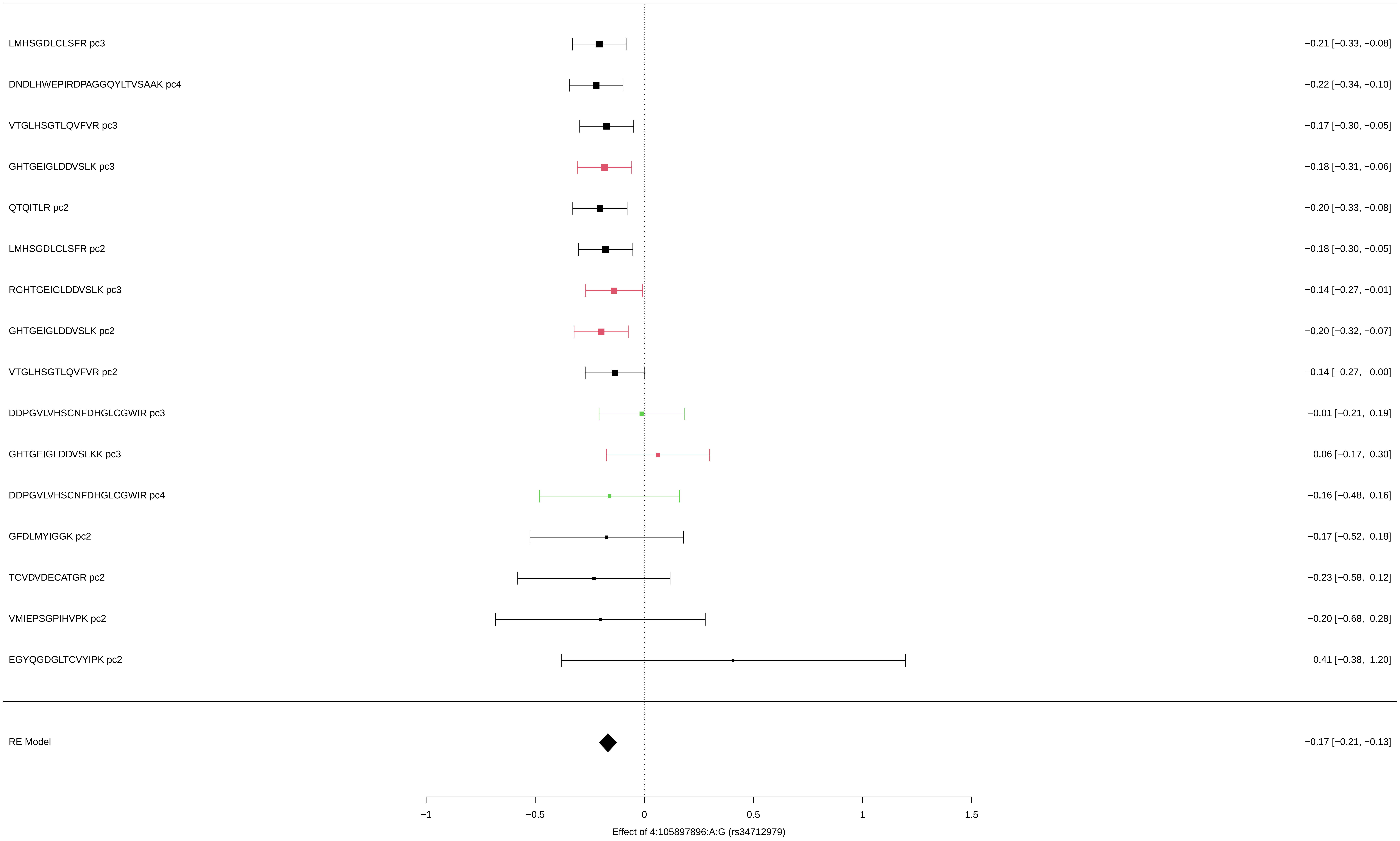

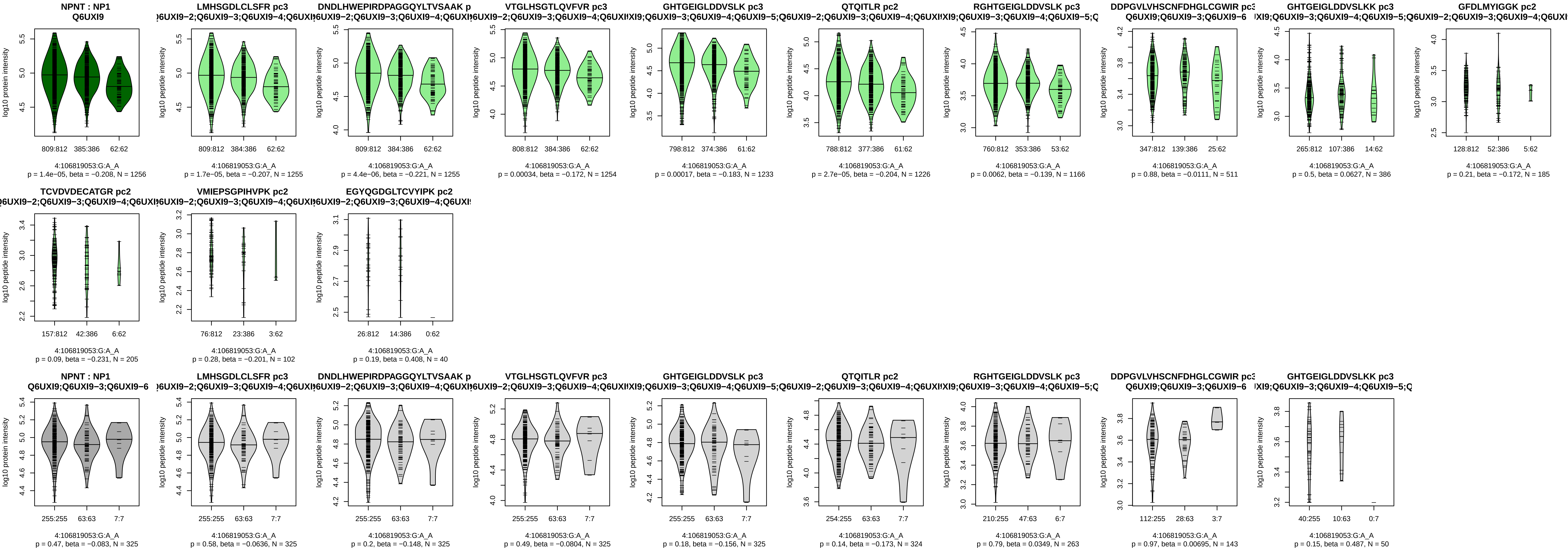

Assay Target: NPNT
UniProt: Q6UXI9
rsID: rs34712979
Target SNP: 4:105897896:A:G
Proxy SNP: 4:106819053:G:A
deCODE log10(p): 99.7
deCODE BETA: −0.2
deCODE MSPA: 0.869
::*:*:*:*:*:*:–:–:–:–:–:NA:–:–:N
1255:1255:1254:1233:1226:122

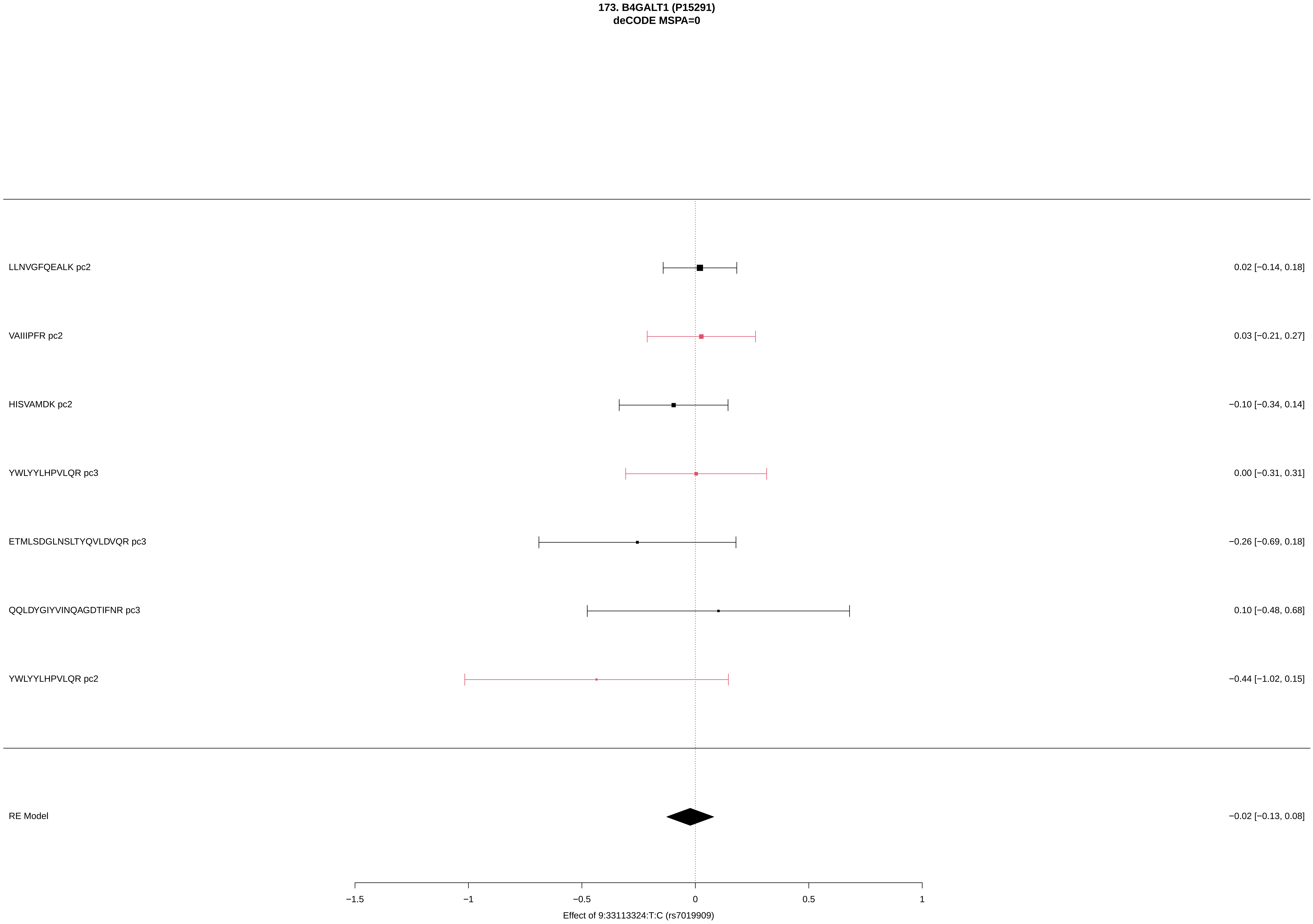
173. B4GALT1 (P15291)
deCODE MSPA=0
LLNVGFQEALK pc2
0.02 [−0.14, 0.18]
VAIIIPFR pc2
0.03 [−0.21, 0.27]
HISVAMDK pc2
−0.10 [−0.34, 0.14]
YWLYYLHPVLQR pc3
0.00 [−0.31, 0.31]
ETMLSDGLNSLTYQVLDVQR pc3
−0.26 [−0.69, 0.18]
QQLDYGIYVINQAGDTIFNR pc3
0.10 [−0.48, 0.68]
YWLYYLHPVLQR pc2
−0.44 [−1.02, 0.15]
RE Model
−0.02 [−0.13, 0.08]
−1.5
−1
−0.5
0
0.5
1
Effect of 9:33113324:T:C (rs7019909)

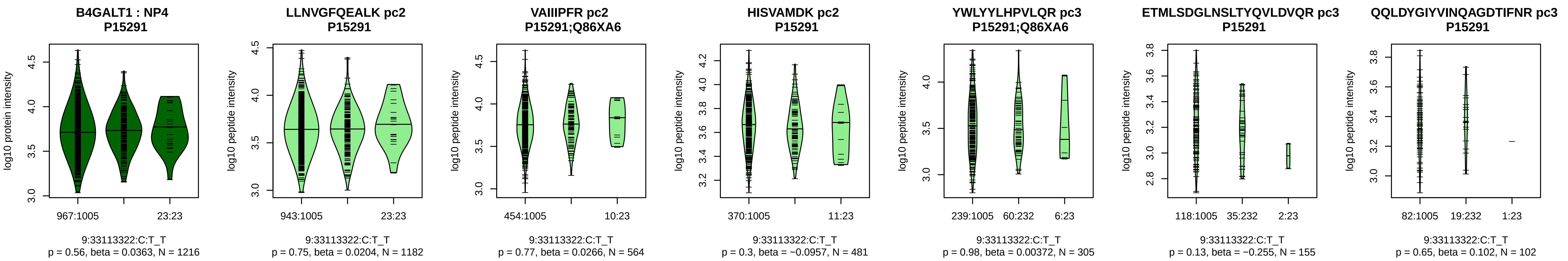

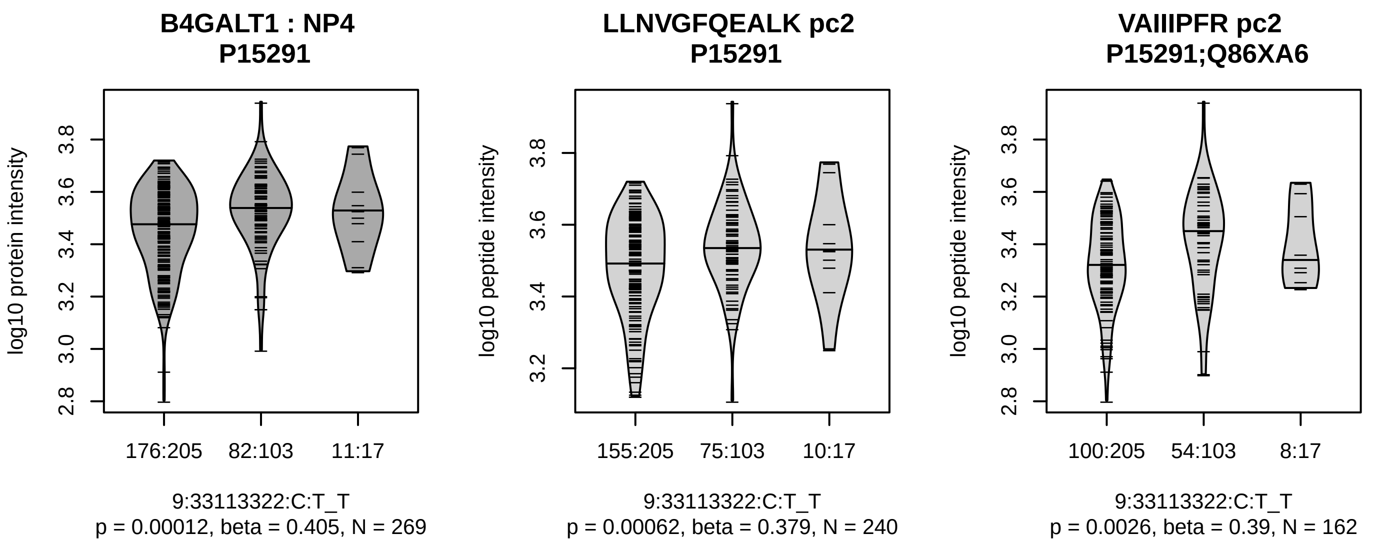

Assay Target: B4GALT1
UniProt: P15291
rsID: rs7019909
Target SNP: 9:33113324:T:C
Proxy SNP: 9:33113322:C:T
deCODE log10(p): 98.3
deCODE BETA: 0.3
deCODE MSPA: 0
–:–:–:–:–:–
1182:564:481:305:155:102:67

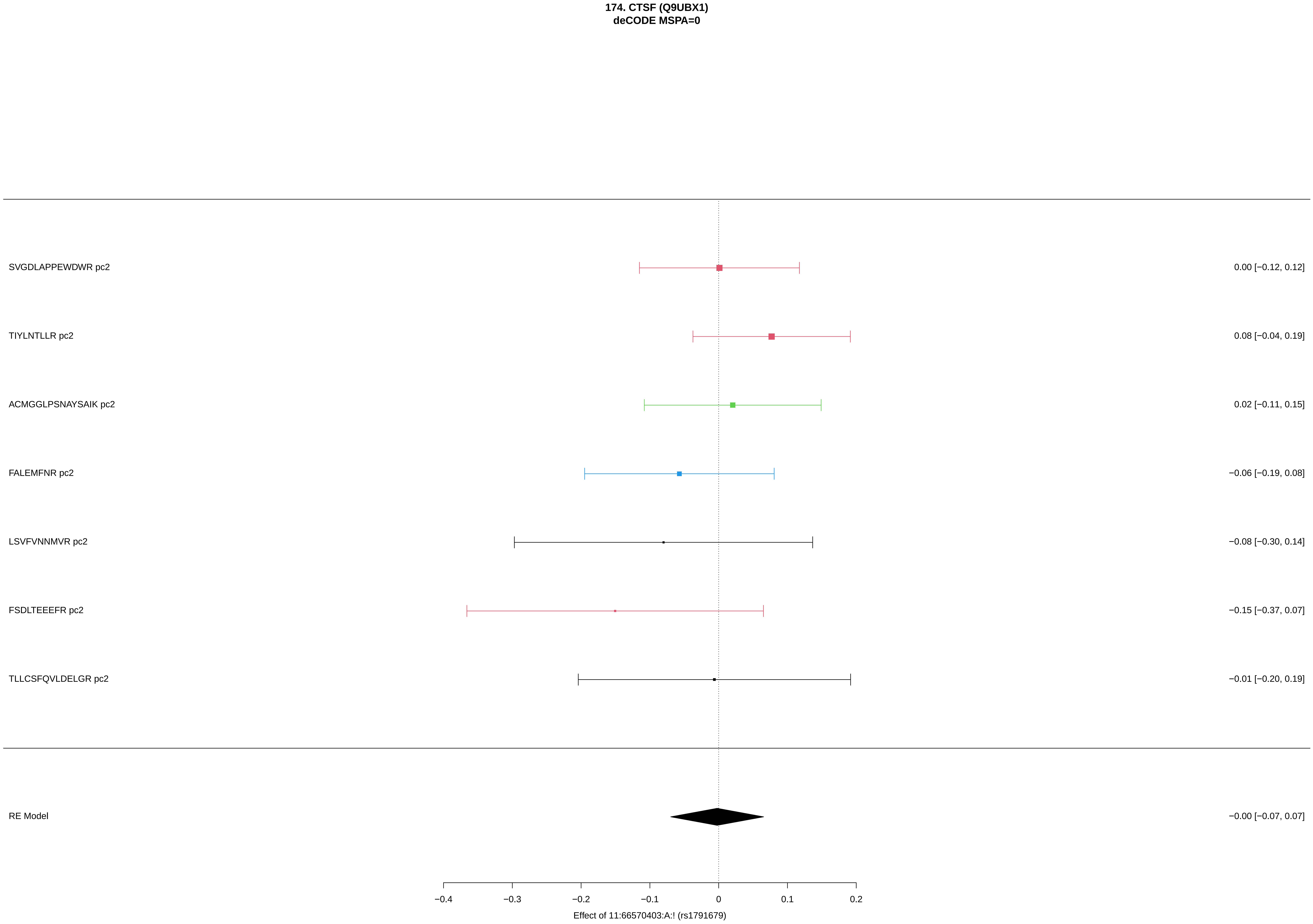

174. CTSF (Q9UBX1)
deCODE MSPA=0
SVGDLAPPEWDWR pc2
0.00 [−0.12, 0.12]
TIYLNTLLR pc2
0.08 [−0.04, 0.19]
ACMGGLPSNAYSAIK pc2
0.02 [−0.11, 0.15]
FALEMFNR pc2
−0.06 [−0.19, 0.08]
LSVFVNNMVR pc2
−0.08 [−0.30, 0.14]
FSDLTEEEFR pc2
−0.15 [−0.37, 0.07]
TLLCSFQVLDELGR pc2
−0.01 [−0.20, 0.19]
RE Model
−0.00 [−0.07, 0.07]
−0.4
−0.3
−0.2
−0.1
0
0.1
0.2
Effect of 11:66570403:A:! (rs1791679)

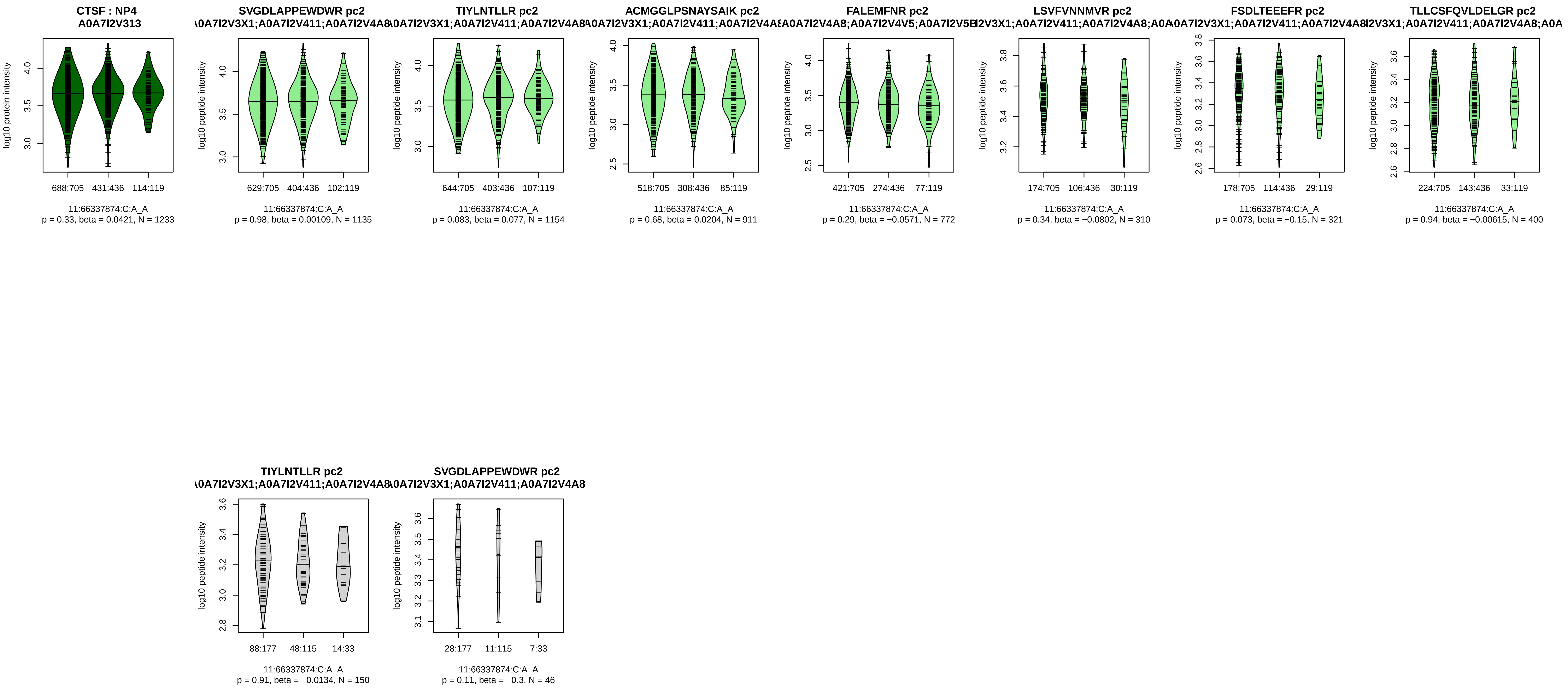

Assay Target: CTSF
UniProt: Q9UBX1
rsID: rs1791679
Target SNP: 11:66570403:A:!
Proxy SNP: 11:66337874:C:A
deCODE log10(p): 97.9
deCODE BETA: 0.18
deCODE MSPA: 0
–:–:–:–:–:–:–
1135:1154:911:772:310:321:400

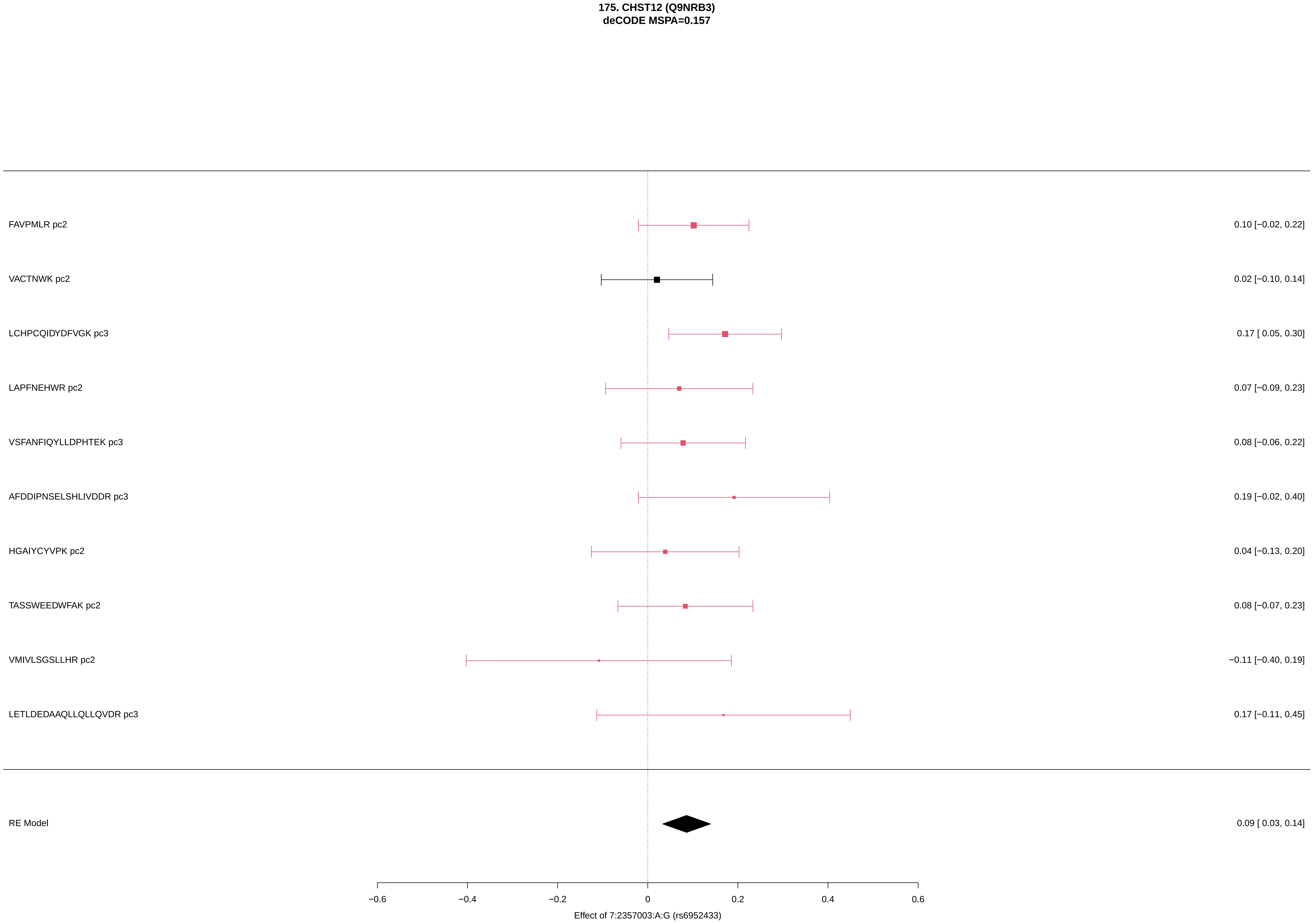
175. CHST12 (Q9NRB3)
deCODE MSPA=0.157
FAVPMLR pc2
0.10 [−0.02, 0.22]
VACTNWK pc2
0.02 [−0.10, 0.14]
LCHPCQIDYDFVGK pc3
0.17 [0.05, 0.30]
LAPFNEHWR pc2
0.07 [−0.09, 0.23]
VSFANFIQYLLDPHTEK pc3
0.08 [−0.06, 0.22]
AFDDIPNSELSHLIVDDR pc3
0.19 [−0.02, 0.40]
HGAIYCYVPK pc2
0.04 [−0.13, 0.20]
TASSWEEDWFAK pc2
0.08 [−0.07, 0.23]
VMIVLSGSLLHR pc2
−0.11 [−0.40, 0.19]
LETLDEDAAQLLQLLQVDR pc3
0.17 [−0.11, 0.45]
RE Model
0.09 [0.03, 0.14]
−0.6
−0.4
−0.2
0
0.2
0.4
0.6
Effect of 7:2357003:A:G (rs6952433)

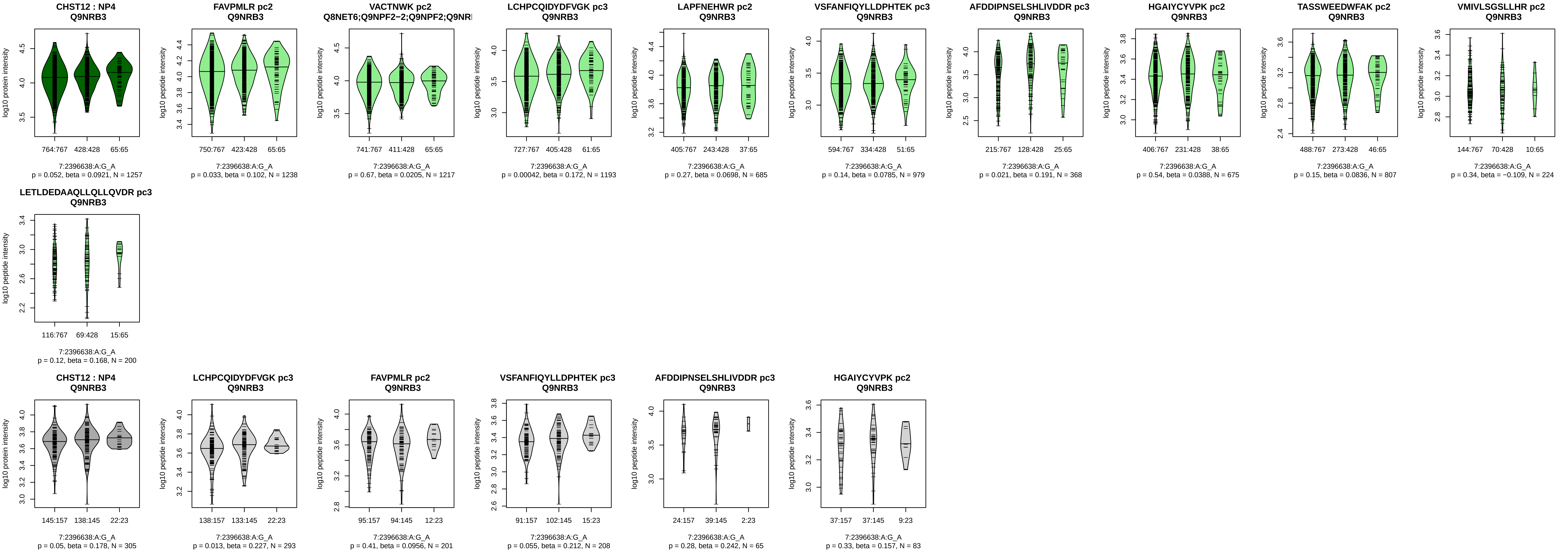

Assay Target: CHST12
UniProt: Q9NRB3
rsID: rs6952433
Target SNP: 7:2357003:A:G
Proxy SNP: 7:2396638:A:G
deCODE log10(p): 95
deCODE BETA: 0.2
deCODE MSPA: 0.157
−:−:*:−:−:−:−:−:−:−:NA
1238:1217:1193:685:979:368:67

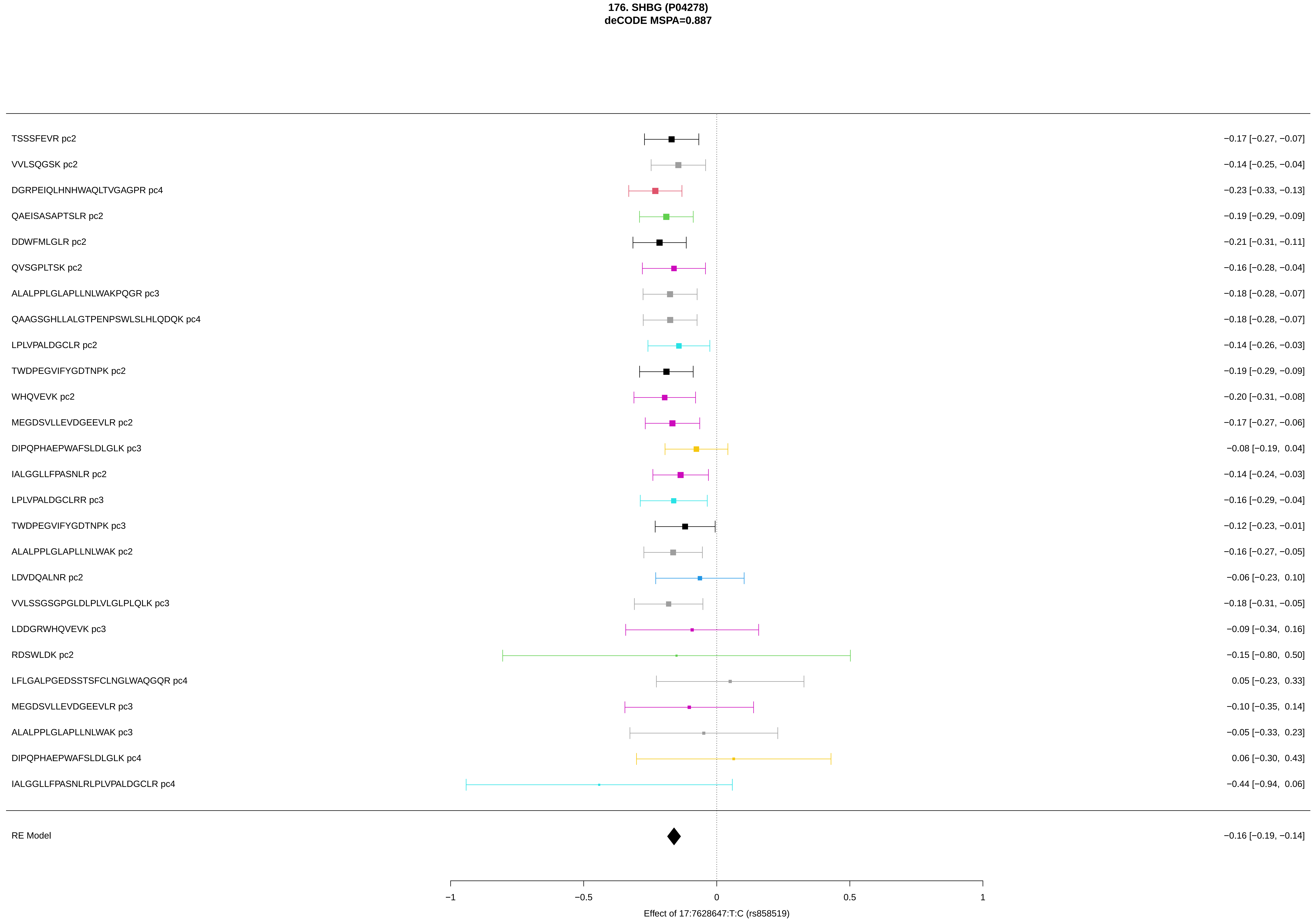

176. SHBG (P04278)
deCODE MSPA=0.887
TSSSFEVR pc2
VVLSQGSK pc2
DGRPEIQLHNHWAQLTVGAGPR pc4
QAEISASAPTSLR pc2
DDWFMLGLR pc2
QVSGPLTSK pc2
ALALPPLGLAPLLNLWAKPQGR pc3
QAAGSGHLLALGTPENPSWLSLHLQDQK pc4
LPLVPALDGCLR pc2
TWDPEGVIFYGDTNPK pc2
WHQVEVK pc2
MEGDSVLLEVDGEEVLR pc2
DIPQPHAEPWAFSLDLGLK pc3
IALGGLLFPASNLR pc2
LPLVPALDGCLRR pc3
TWDPEGVIFYGDTNPK pc3
ALALPPLGLAPLLNLWAK pc2
LDVDQALNR pc2
VVLSSGSGPGLDLPLVLGLPLQLK pc3
LDDGRWHQVEVK pc3
RDSWLDK pc2
LFLGALPGEDSSTSFCLNGLWAQGQR pc4
MEGDSVLLEVDGEEVLR pc3
ALALPPLGLAPLLNLWAK pc3
DIPQPHAEPWAFSLDLGLK pc4
IALGGLLFPASNLRLPLVPALDGCLR pc4
RE Model
−0.17 [−0.27, −0.07]
−0.14 [−0.25, −0.04]
−0.23 [−0.33, −0.13]
−0.19 [−0.29, −0.09]
−0.21 [−0.31, −0.11]
−0.16 [−0.28, −0.04]
−0.18 [−0.28, −0.07]
−0.18 [−0.28, −0.07]
−0.14 [−0.26, −0.03]
−0.19 [−0.29, −0.09]
−0.20 [−0.31, −0.08]
−0.17 [−0.27, −0.06]
−0.08 [−0.19, 0.04]
−0.14 [−0.24, −0.03]
−0.16 [−0.29, −0.04]
−0.12 [−0.23, −0.01]
−0.16 [−0.27, −0.05]
−0.06 [−0.23, 0.10]
−0.18 [−0.31, −0.05]
−0.09 [−0.34, 0.16]
−0.15 [−0.80, 0.50]
0.05 [−0.23, 0.33]
−0.10 [−0.35, 0.14]
−0.05 [−0.33, 0.23]
0.06 [−0.30, 0.43]
−0.44 [−0.94, 0.06]
−0.16 [−0.19, −0.14]
−1
−0.5
0
0.5
1
Effect of 17:7628647:T:C (rs858519)

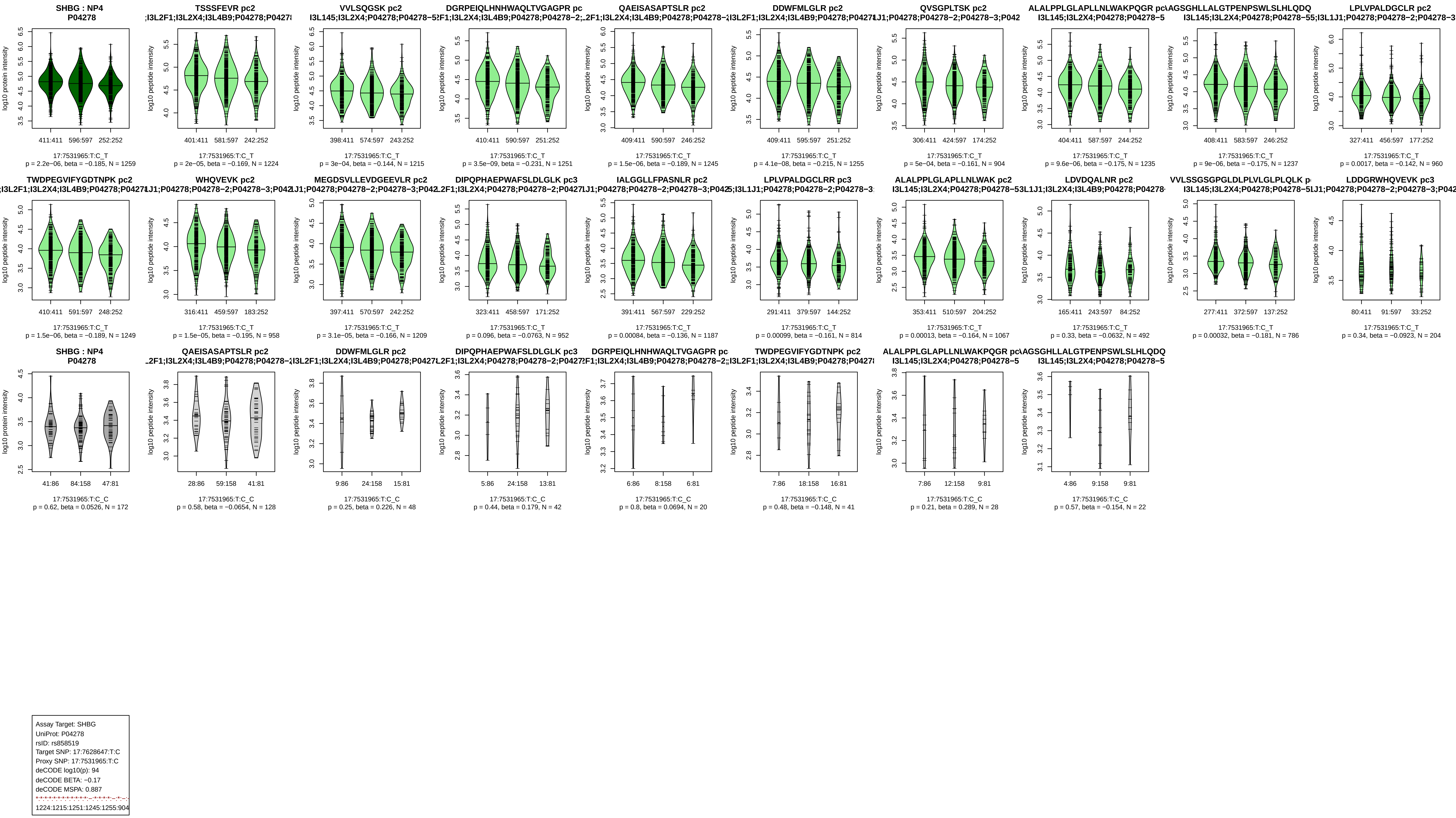
SHBG : NP4
P04278
17:7531965:T:C_T
p = 2.2e−06, beta = −0.185, N = 1259
TSSSFEVR pc2
p = 2e−05, beta = −0.169, N = 1224
VVLSQGSK pc2
p = 3e−04, beta = −0.144, N = 1215
DGRPEIQLHNHWAQLTVGAGPR pc
p = 3.5e−09, beta = −0.231, N = 1251
QAEISASAPTSLR pc2
p = 1.5e−06, beta = −0.189, N = 1245
DDWFMLGLR pc2
p = 4.1e−08, beta = −0.215, N = 1255
QVSGPLTSK pc2
p = 5e−04, beta = −0.161, N = 904
ALALPPLGLAPLLNLWAKPQGR pc
p = 9.6e−06, beta = −0.175, N = 1235
AGSGHLLALGTPENPSWLSLHLQDQ
p = 9e−06, beta = −0.175, N = 1237
LPLVPALDGCLR pc2
p = 0.0017, beta = −0.142, N = 960
TWDPEGVIFYGDTNPK pc2
p = 1.5e−06, beta = −0.189, N = 1249
WHQVEVK pc2
p = 1.5e−05, beta = −0.195, N = 958
MEGDSVLLEVDGEEVLR pc2
p = 3.1e−05, beta = −0.166, N = 1209
DIPQPHAEPWAFSLDLGLK pc3
p = 0.096, beta = −0.0763, N = 952
IALGGLLFPASNLR pc2
p = 0.00084, beta = −0.136, N = 1187
LPLVPALDGCLRR pc3
p = 0.00099, beta = −0.161, N = 814
ALALPPLGLAPLLNLWAK pc2
p = 0.00013, beta = −0.164, N = 1067
LDVDQALNR pc2
p = 0.33, beta = −0.0632, N = 492
VVLSSGSGPGLDLPLVLGLPLQLK p
p = 0.00032, beta = −0.181, N = 786
LDDGRWHQVEVK pc3
p = 0.34, beta = −0.0923, N = 204
SHBG : NP4
P04278
17:7531965:T:C_C
p = 0.62, beta = 0.0526, N = 172
QAEISASAPTSLR pc2
p = 0.58, beta = −0.0654, N = 128
DDWFMLGLR pc2
p = 0.25, beta = 0.226, N = 48
DIPQPHAEPWAFSLDLGLK pc3
p = 0.44, beta = 0.179, N = 42
DGRPEIQLHNHWAQLTVGAGPR pc
p = 0.8, beta = 0.0694, N = 20
TWDPEGVIFYGDTNPK pc2
p = 0.48, beta = −0.148, N = 41
ALALPPLGLAPLLNLWAKPQGR pc
p = 0.21, beta = 0.289, N = 28
AGSGHLLALGTPENPSWLSLHLQDQ
p = 0.57, beta = −0.154, N = 22
Assay Target: SHBG
UniProt: P04278
rsID: rs858519
Target SNP: 17:7628647:T:C
Proxy SNP: 17:7531965:T:C
deCODE log10(p): 94
deCODE BETA: −0.17
deCODE MSPA: 0.887
1224:1215:1251:1245:1255:904

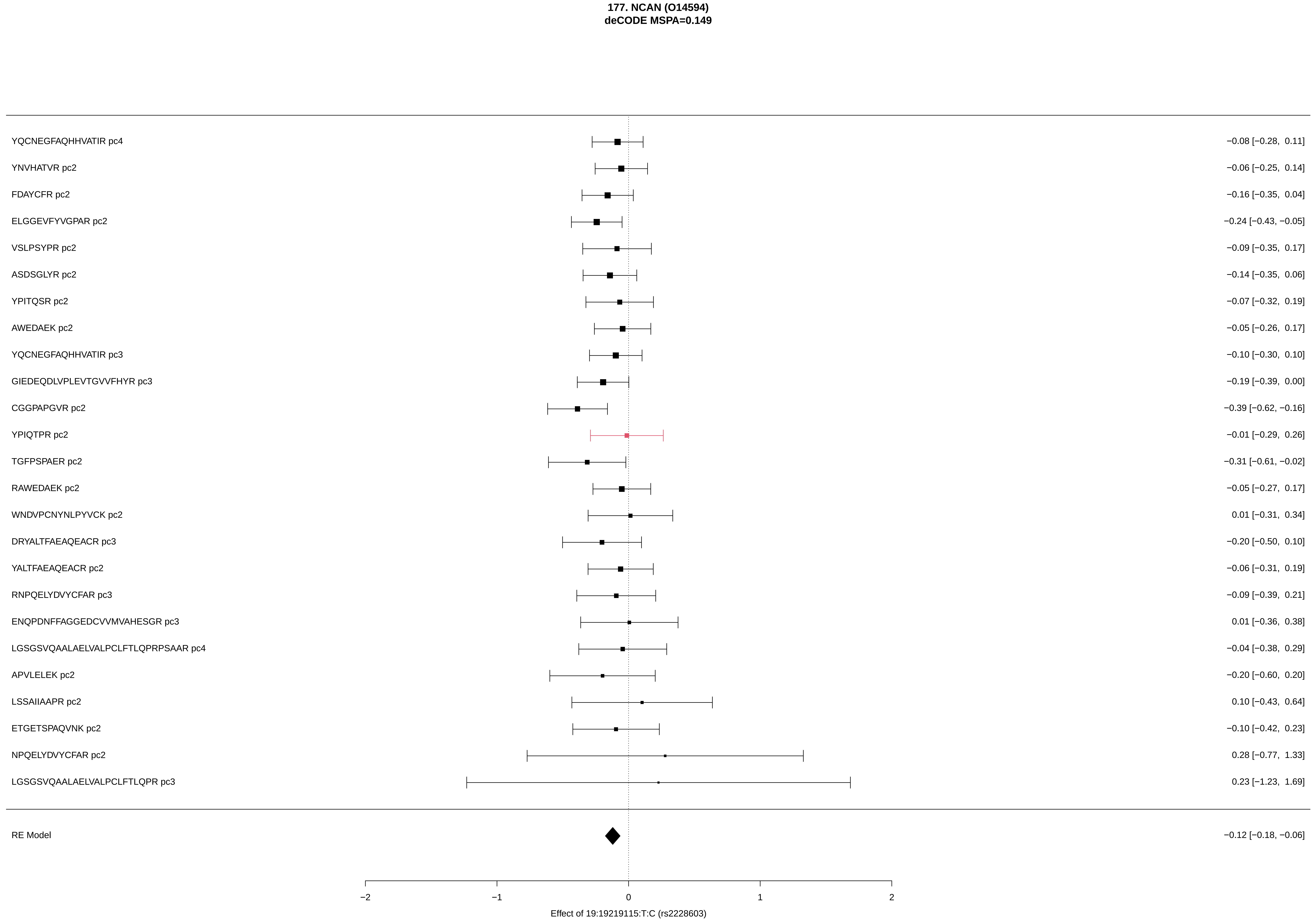

177. NCAN (O14594)
deCODE MSPA=0.149
YQCNEGFAQHHVATIR pc4
−0.08 [−0.28, 0.11]
YNVHATVR pc2
−0.06 [−0.25, 0.14]
FDAYCFR pc2
−0.16 [−0.35, 0.04]
ELGGEVFYVGPAR pc2
−0.24 [−0.43, −0.05]
VSLPSYPR pc2
−0.09 [−0.35, 0.17]
ASDSGLYR pc2
−0.14 [−0.35, 0.06]
YPITQSR pc2
−0.07 [−0.32, 0.19]
AWEDAEK pc2
−0.05 [−0.26, 0.17]
YQCNEGFAQHHVATIR pc3
−0.10 [−0.30, 0.10]
GIEDEQDLVPLEVTGVVFHYR pc3
−0.19 [−0.39, 0.00]
CGGPAPGVR pc2
−0.39 [−0.62, −0.16]
YPIQTPR pc2
−0.01 [−0.29, 0.26]
TGFPSPAER pc2
−0.31 [−0.61, −0.02]
RAWEDAEK pc2
−0.05 [−0.27, 0.17]
WNDVPCNYNLPYVCK pc2
0.01 [−0.31, 0.34]
DRYALTFAEAQEACR pc3
−0.20 [−0.50, 0.10]
YALTFAEAQEACR pc2
−0.06 [−0.31, 0.19]
RNPQELYDVYCFAR pc3
−0.09 [−0.39, 0.21]
ENQPDNFFAGGEDCVVMVAHESGR pc3
0.01 [−0.36, 0.38]
LGSGSVQAALAELVALPCLFTLQPRPSAAR pc4
−0.04 [−0.38, 0.29]
APVLELEK pc2
−0.20 [−0.60, 0.20]
LSSAIIAAPR pc2
0.10 [−0.43, 0.64]
ETGETSPAQVNK pc2
−0.10 [−0.42, 0.23]
NPQELYDVYCFAR pc2
0.28 [−0.77, 1.33]
LGSGSVQAALAELVALPCLFTLQPR pc3
0.23 [−1.23, 1.69]
RE Model
−0.12 [−0.18, −0.06]
−2
−1
0
1
2
Effect of 19:19219115:T:C (rs2228603)

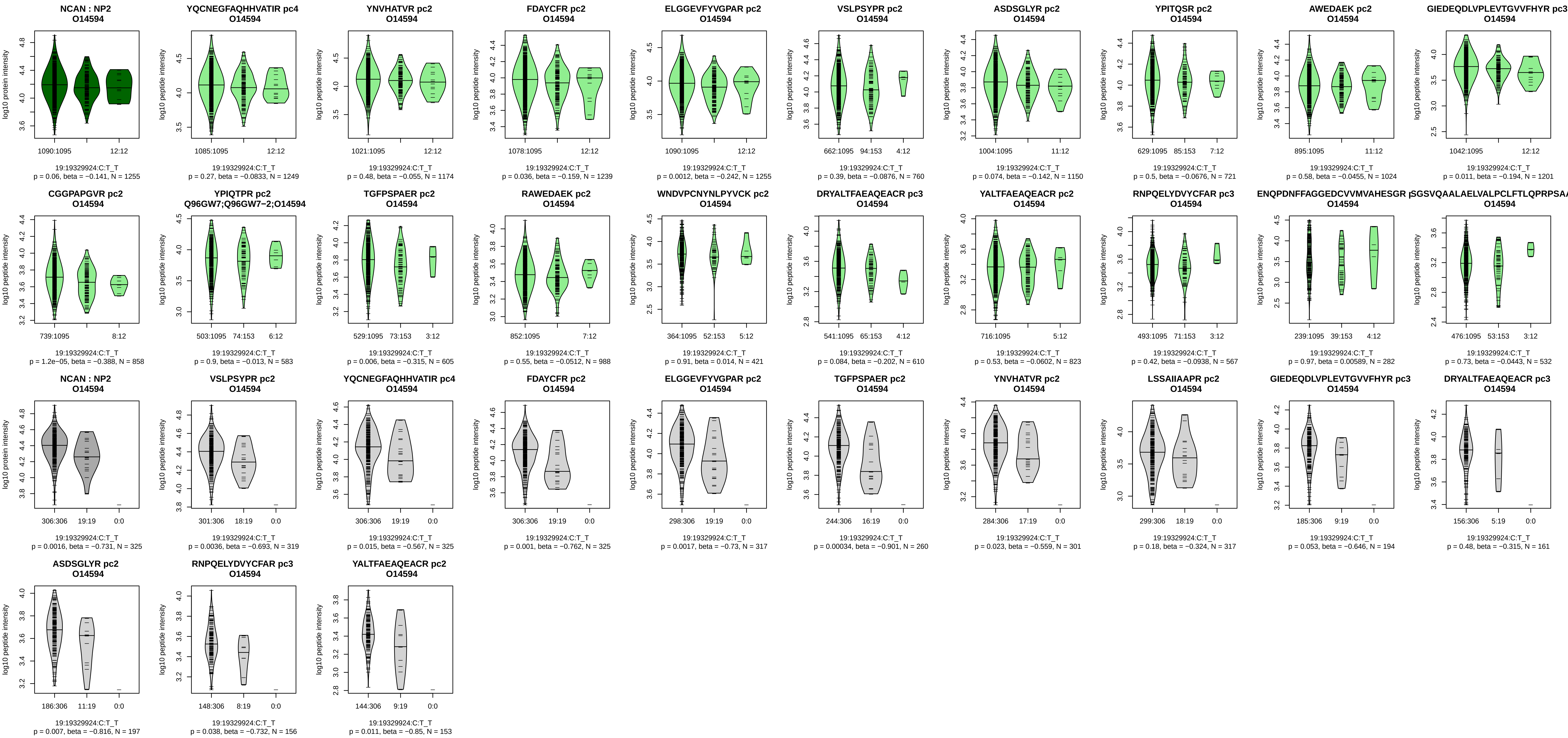

Assay Target: NCAN
UniProt: O14594
rsID: rs2228603
Target SNP: 19:19219115:T:C
Proxy SNP: 19:19329924:C:T
deCODE log10(p): 93.2
deCODE BETA: −0.33
deCODE MSPA: 0.149
1249:1174:1239:1255:760:1150

178. SMOC1 (Q9H4F8)
deCODE MSPA=0

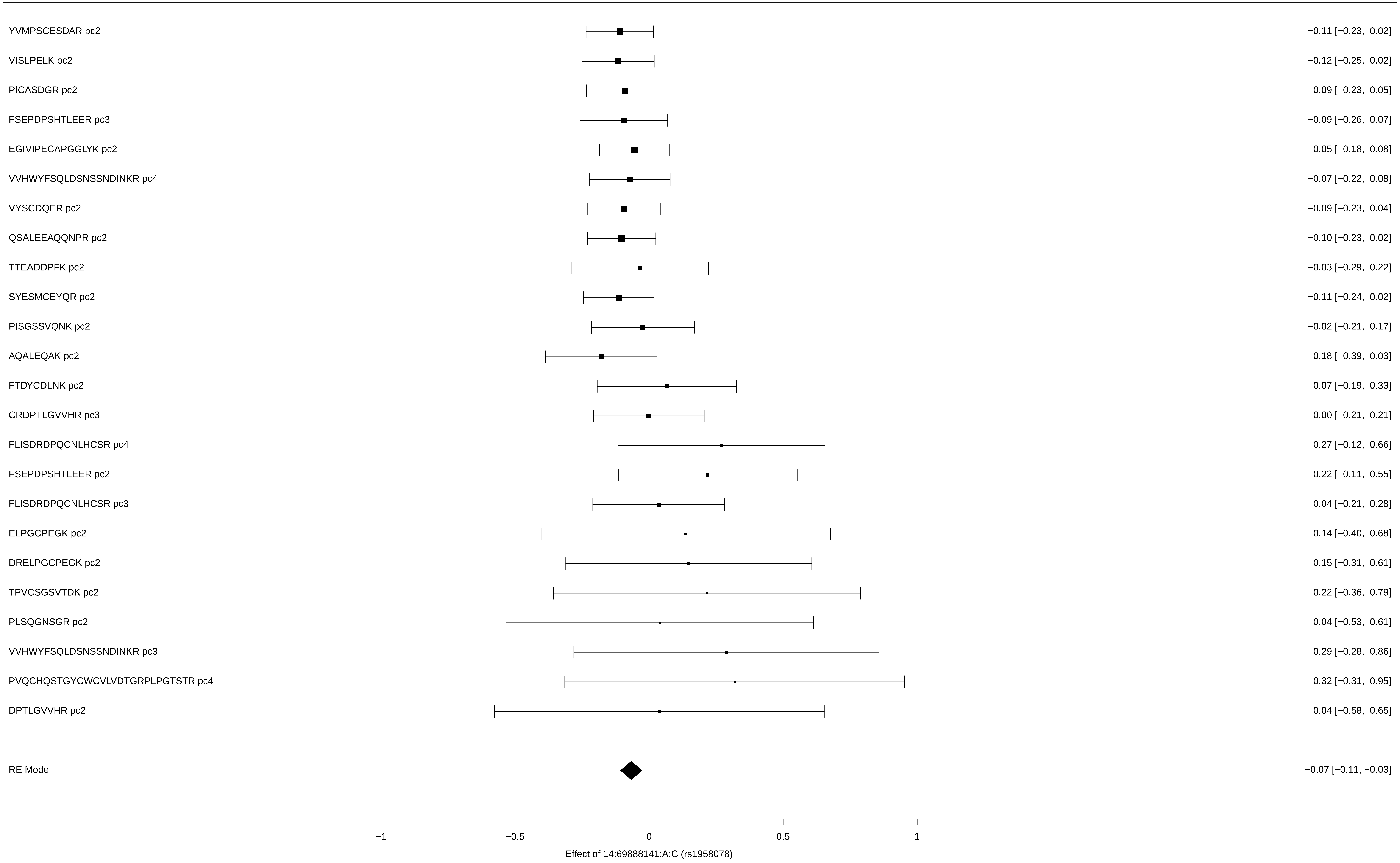

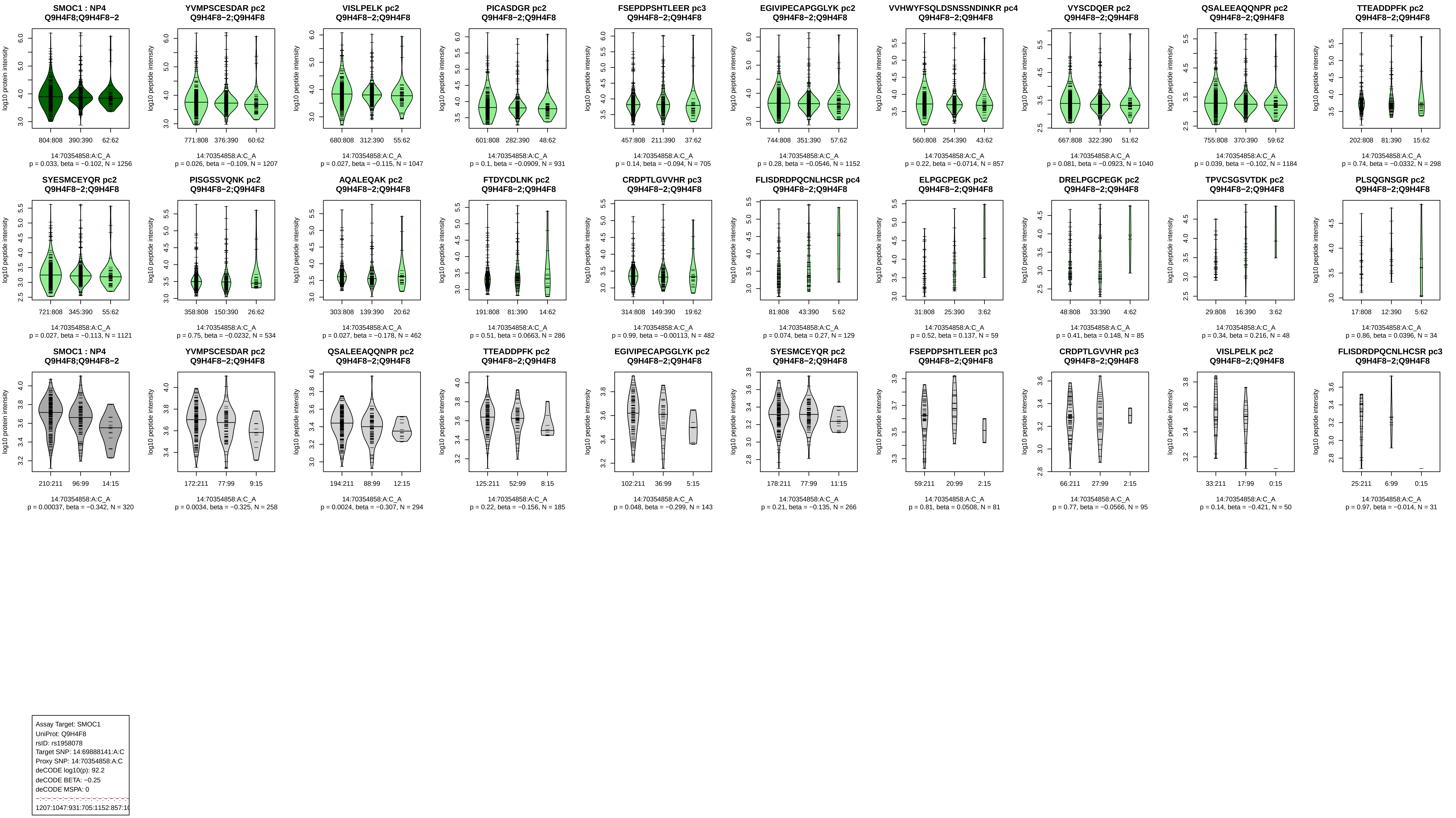
SMOC1 : NP4
Q9H4F8;Q9H4F8−2
804:808 390:390 62:62
14:70354858:A:C_A
p = 0.033, beta = −0.102, N = 1256
YVMPSCESDAR pc2
Q9H4F8−2;Q9H4F8
771:808 376:390 60:62
14:70354858:A:C_A
p = 0.026, beta = −0.109, N = 1207
VISLPELK pc2
Q9H4F8−2;Q9H4F8
680:808 312:390 55:62
14:70354858:A:C_A
p = 0.027, beta = −0.115, N = 1047
PICASDGR pc2
Q9H4F8−2;Q9H4F8
601:808 282:390 48:62
14:70354858:A:C_A
p = 0.1, beta = −0.0909, N = 931
FSEPDPSHTLEER pc3
Q9H4F8−2;Q9H4F8
457:808 211:390 37:62
14:70354858:A:C_A
p = 0.14, beta = −0.094, N = 705
EGIVIPECAPGGLYK pc2
Q9H4F8−2;Q9H4F8
744:808 351:390 57:62
14:70354858:A:C_A
p = 0.28, beta = −0.0546, N = 1152
VVHWYFSQLDSNSSNDINKR pc4
Q9H4F8−2;Q9H4F8
560:808 254:390 43:62
14:70354858:A:C_A
p = 0.22, beta = −0.0714, N = 857
VYSCDQER pc2
Q9H4F8−2;Q9H4F8
667:808 322:390 51:62
14:70354858:A:C_A
p = 0.081, beta = −0.0923, N = 1040
QSALEEAQQNPR pc2
Q9H4F8−2;Q9H4F8
755:808 370:390 59:62
14:70354858:A:C_A
p = 0.039, beta = −0.102, N = 1184
TTEADDPFK pc2
Q9H4F8−2;Q9H4F8
202:808 81:390 15:62
14:70354858:A:C_A
p = 0.74, beta = −0.0332, N = 298
SYESMCEYQR pc2
Q9H4F8−2;Q9H4F8
721:808 345:390 55:62
14:70354858:A:C_A
p = 0.027, beta = −0.113, N = 1121
PISGSSVQNK pc2
Q9H4F8−2;Q9H4F8
358:808 150:390 26:62
14:70354858:A:C_A
p = 0.75, beta = −0.0232, N = 534
AQALEQAK pc2
Q9H4F8−2;Q9H4F8
303:808 139:390 20:62
14:70354858:A:C_A
p = 0.027, beta = −0.178, N = 462
FTDYCDLNK pc2
Q9H4F8−2;Q9H4F8
191:808 81:390 14:62
14:70354858:A:C_A
p = 0.51, beta = 0.0663, N = 286
CRDPTLGVVHR pc3
Q9H4F8−2;Q9H4F8
314:808 149:390 19:62
14:70354858:A:C_A
p = 0.99, beta = −0.00113, N = 482
FLISDRDPQCNLHCSR pc4
Q9H4F8−2;Q9H4F8
81:808 43:390 5:62
14:70354858:A:C_A
p = 0.074, beta = 0.27, N = 129
ELPGCPEGK pc2
Q9H4F8−2;Q9H4F8
31:808 25:390 3:62
14:70354858:A:C_A
p = 0.52, beta = 0.137, N = 59
DRELPGCPEGK pc2
Q9H4F8−2;Q9H4F8
48:808 33:390 4:62
14:70354858:A:C_A
p = 0.41, beta = 0.148, N = 85
TPVCSGSVTDK pc2
Q9H4F8−2;Q9H4F8
29:808 16:390 3:62
14:70354858:A:C_A
p = 0.34, beta = 0.216, N = 48
PLSQGNSGR pc2
Q9H4F8−2;Q9H4F8
17:808 12:390 5:62
14:70354858:A:C_A
p = 0.86, beta = 0.0396, N = 34
SMOC1 : NP4
Q9H4F8;Q9H4F8−2
210:211 96:99 14:15
14:70354858:A:C_A
p = 0.00037, beta = −0.342, N = 320
YVMPSCESDAR pc2
Q9H4F8−2;Q9H4F8
172:211 77:99 9:15
14:70354858:A:C_A
p = 0.0034, beta = −0.325, N = 258
QSALEEAQQNPR pc2
Q9H4F8−2;Q9H4F8
194:211 88:99 12:15
14:70354858:A:C_A
p = 0.0024, beta = −0.307, N = 294
TTEADDPFK pc2
Q9H4F8−2;Q9H4F8
125:211 52:99 8:15
14:70354858:A:C_A
p = 0.22, beta = −0.156, N = 185
EGIVIPECAPGGLYK pc2
Q9H4F8−2;Q9H4F8
102:211 36:99 5:15
14:70354858:A:C_A
p = 0.048, beta = −0.299, N = 143
SYESMCEYQR pc2
Q9H4F8−2;Q9H4F8
178:211 77:99 11:15
14:70354858:A:C_A
p = 0.21, beta = −0.135, N = 266
FSEPDPSHTLEER pc3
Q9H4F8−2;Q9H4F8
59:211 20:99 2:15
14:70354858:A:C_A
p = 0.81, beta = 0.0508, N = 81
CRDPTLGVVHR pc3
Q9H4F8−2;Q9H4F8
66:211 27:99 2:15
14:70354858:A:C_A
p = 0.77, beta = −0.0566, N = 95
VISLPELK pc2
Q9H4F8−2;Q9H4F8
33:211 17:99 0:15
14:70354858:A:C_A
p = 0.14, beta = −0.421, N = 50
FLISDRDPQCNLHCSR pc3
Q9H4F8−2;Q9H4F8
25:211 6:99 0:15
14:70354858:A:C_A
p = 0.97, beta = −0.014, N = 31
log10 protein intensity
log10 peptide intensity
Assay Target: SMOC1
UniProt: Q9H4F8
rsID: rs1958078
Target SNP: 14:69888141:A:C
Proxy SNP: 14:70354858:A:C
deCODE log10(p): 92.2
deCODE BETA: −0.25
deCODE MSPA: 0
1207:1047:931:705:1152:857:1

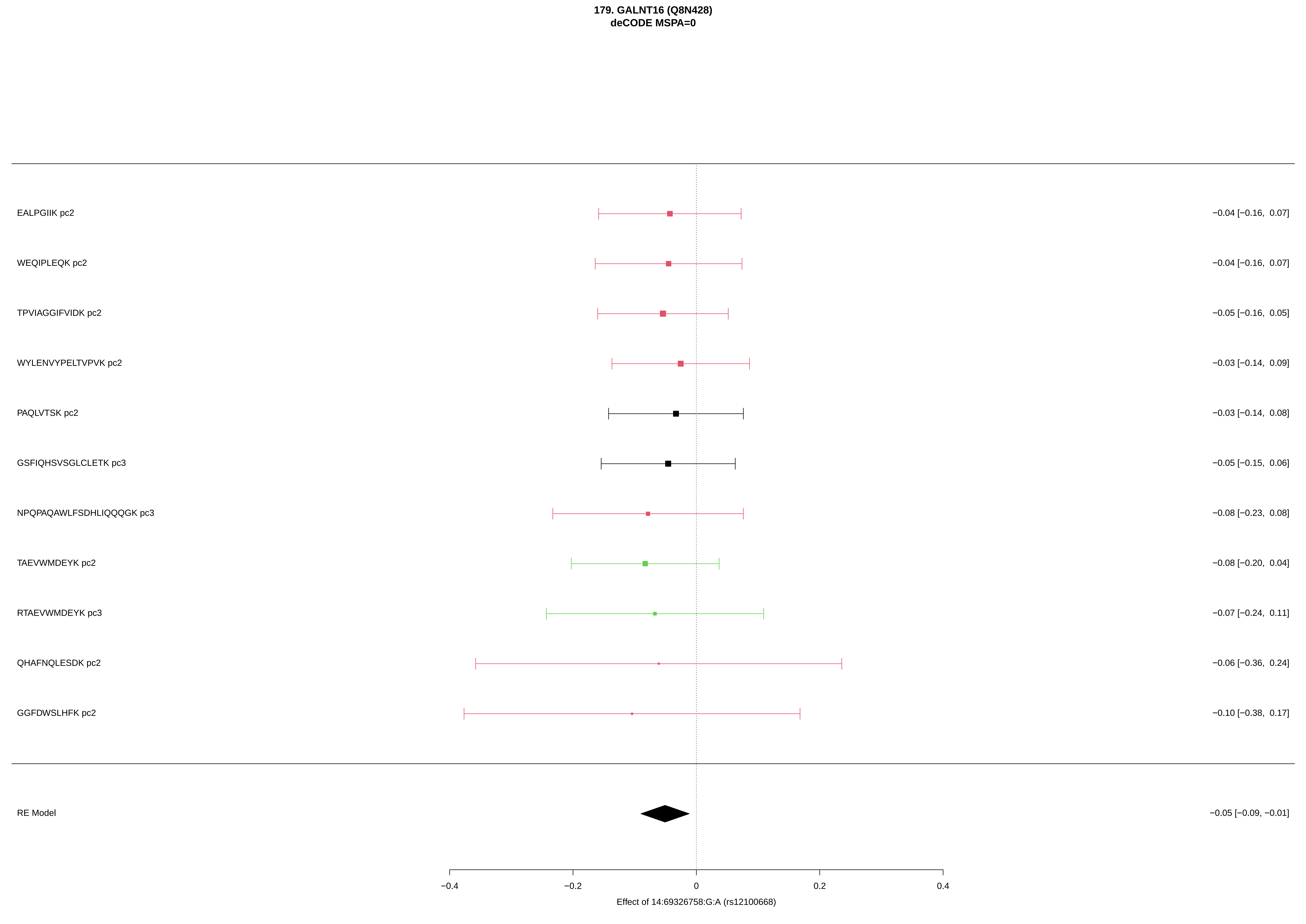

179. GALNT16 (Q8N428)
deCODE MSPA=0
EALPGIIK pc2
−0.04 [−0.16, 0.07]
WEQIPLEQK pc2
−0.04 [−0.16, 0.07]
TPVIAGGIFVIDK pc2
−0.05 [−0.16, 0.05]
WYLENVYPELTVPVK pc2
−0.03 [−0.14, 0.09]
PAQLVTSK pc2
−0.03 [−0.14, 0.08]
GSFIQHSVSGLCLETK pc3
−0.05 [−0.15, 0.06]
NPQPAQAWLFSDHLIQQQGK pc3
−0.08 [−0.23, 0.08]
TAEVWMDEYK pc2
−0.08 [−0.20, 0.04]
RTAEVWMDEYK pc3
−0.07 [−0.24, 0.11]
QHAFNQLESDK pc2
−0.06 [−0.36, 0.24]
GGFDWSLHFK pc2
−0.10 [−0.38, 0.17]
RE Model
−0.05 [−0.09, −0.01]
−0.4
−0.2
0
0.2
0.4
Effect of 14:69326758:G:A (rs12100668)

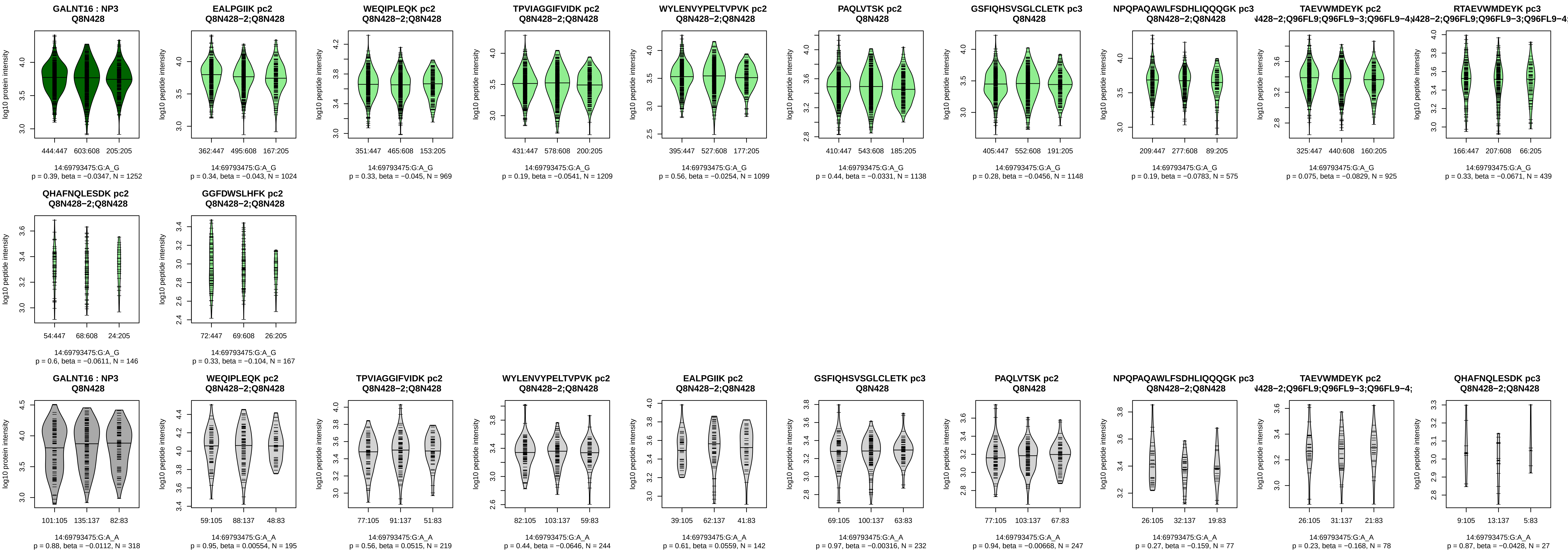

Assay Target: GALNT16
UniProt: Q8N428
rsID: rs12100668
Target SNP: 14:69326758:G:A
Proxy SNP: 14:69793475:G:A
deCODE log10(p): 90.1
deCODE BETA: −0.17
deCODE MSPA: 0
−:−:−:−:−:−:−:−:−
1024:969:1209:1099:1138:1148

180. PFKM (P08237)
deCODE MSPA=0.449

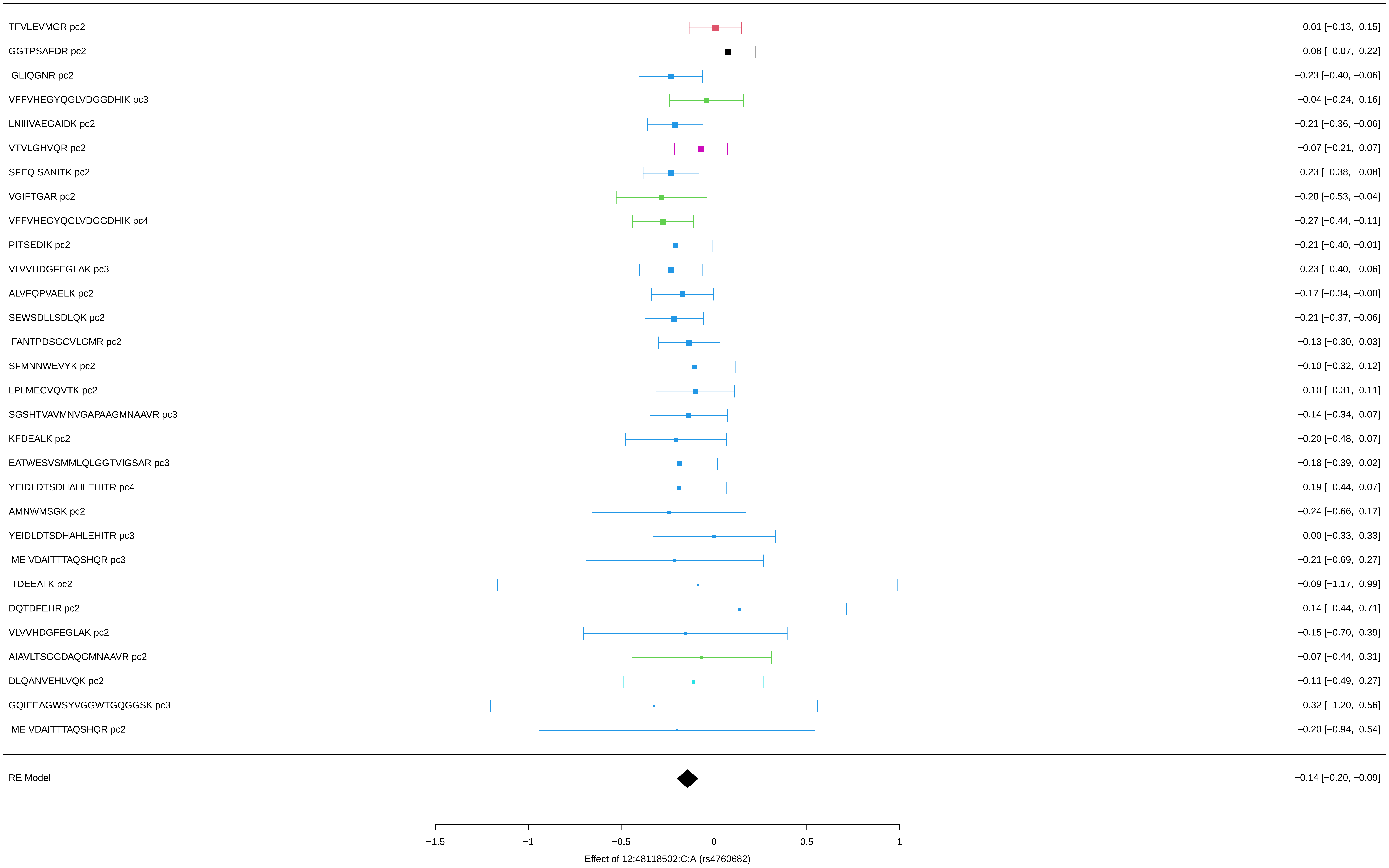

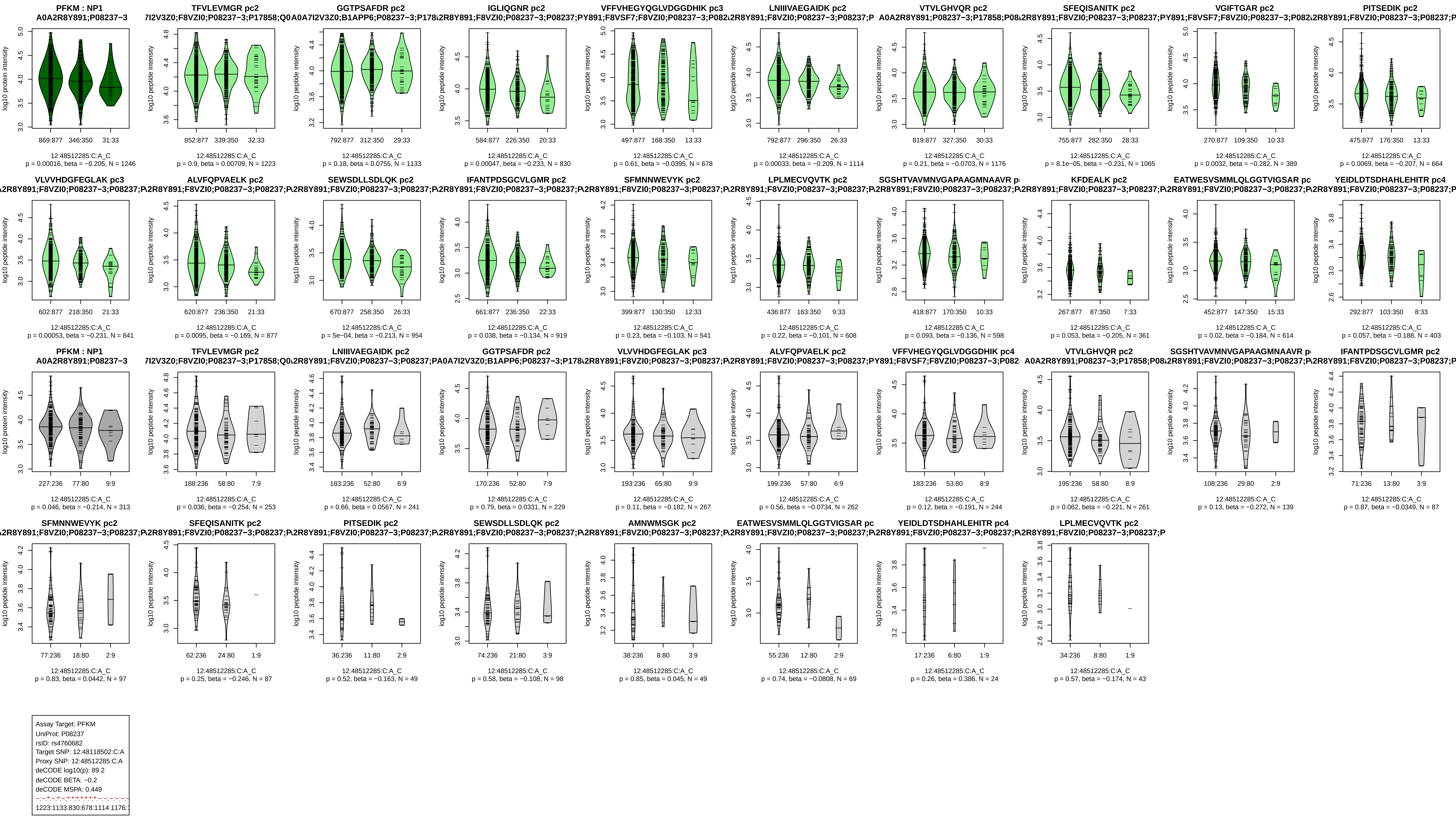
PFKM : NP1
A0A2R8Y891;P08237−3
869:877 346:350 31:33
12:48512285:C:A_C
p = 0.00016, beta = −0.205, N = 1246
TFVLEVMGR pc2
852:877 339:350 32:33
12:48512285:C:A_C
p = 0.9, beta = 0.00709, N = 1223
GGTPSAFDR pc2
792:877 312:350 29:33
12:48512285:C:A_C
p = 0.18, beta = 0.0755, N = 1133
IGLIQGNR pc2
584:877 226:350 20:33
12:48512285:C:A_C
p = 0.00047, beta = −0.233, N = 830
VFFVHEGYQGLVDGGDHIK pc3
497:877 168:350 13:33
12:48512285:C:A_C
p = 0.61, beta = −0.0395, N = 678
LNIIIVAEGAIDK pc2
792:877 296:350 26:33
12:48512285:C:A_C
p = 0.00033, beta = −0.209, N = 1114
VTVLGHVQR pc2
819:877 327:350 30:33
12:48512285:C:A_C
p = 0.21, beta = −0.0703, N = 1176
SFEQISANITK pc2
755:877 282:350 28:33
12:48512285:C:A_C
p = 8.1e−05, beta = −0.231, N = 1065
VGIFTGAR pc2
270:877 109:350 10:33
12:48512285:C:A_C
p = 0.0032, beta = −0.282, N = 389
PITSEDIK pc2
475:877 176:350 13:33
12:48512285:C:A_C
p = 0.0069, beta = −0.207, N = 664
VLVVHDGFEGLAK pc3
602:877 218:350 21:33
12:48512285:C:A_C
p = 0.00053, beta = −0.231, N = 841
ALVFQPVAELK pc2
620:877 236:350 21:33
12:48512285:C:A_C
p = 0.0095, beta = −0.169, N = 877
SEWSDLLSDLQK pc2
670:877 258:350 26:33
12:48512285:C:A_C
p = 5e−04, beta = −0.213, N = 954
IFANTPDSGCVLGMR pc2
661:877 236:350 22:33
12:48512285:C:A_C
p = 0.038, beta = −0.134, N = 919
SFMNNWEVYK pc2
399:877 130:350 12:33
12:48512285:C:A_C
p = 0.23, beta = −0.103, N = 541
LPLMECVQVTK pc2
436:877 163:350 9:33
12:48512285:C:A_C
p = 0.22, beta = −0.101, N = 608
SGSHTVAVMNVGAPAAGMNAAVR pc
418:877 170:350 10:33
12:48512285:C:A_C
p = 0.093, beta = −0.134, N = 598
KFDEALK pc2
267:877 87:350 7:33
12:48512285:C:A_C
p = 0.053, beta = −0.205, N = 361
EATWESVSMMLQLGGTVIGSAR pc
452:877 147:350 15:33
12:48512285:C:A_C
p = 0.02, beta = −0.184, N = 614
YEIDLDTSDHAHLEHITR pc4
292:877 103:350 8:33
12:48512285:C:A_C
p = 0.057, beta = −0.188, N = 403
PFKM : NP1
A0A2R8Y891;P08237−3
227:236 77:80 9:9
12:48512285:C:A_C
p = 0.046, beta = −0.214, N = 313
TFVLEVMGR pc2
188:236 58:80 7:9
12:48512285:C:A_C
p = 0.036, beta = −0.254, N = 253
LNIIIVAEGAIDK pc2
183:236 52:80 6:9
12:48512285:C:A_C
p = 0.66, beta = 0.0567, N = 241
GGTPSAFDR pc2
170:236 52:80 7:9
12:48512285:C:A_C
p = 0.79, beta = 0.0331, N = 229
VLVVHDGFEGLAK pc3
193:236 65:80 9:9
12:48512285:C:A_C
p = 0.11, beta = −0.182, N = 267
ALVFQPVAELK pc2
199:236 57:80 6:9
12:48512285:C:A_C
p = 0.56, beta = −0.0734, N = 262
VFFVHEGYQGLVDGGDHIK pc4
183:236 53:80 8:9
12:48512285:C:A_C
p = 0.12, beta = −0.191, N = 244
VTVLGHVQR pc2
195:236 58:80 8:9
12:48512285:C:A_C
p = 0.062, beta = −0.221, N = 261
SGSHTVAVMNVGAPAAGMNAAVR pc
108:236 29:80 2:9
12:48512285:C:A_C
p = 0.13, beta = −0.272, N = 139
IFANTPDSGCVLGMR pc2
71:236 13:80 3:9
12:48512285:C:A_C
p = 0.87, beta = −0.0349, N = 87
SFMNNWEVYK pc2
77:236 18:80 2:9
12:48512285:C:A_C
p = 0.83, beta = 0.0442, N = 97
SFEQISANITK pc2
62:236 24:80 1:9
12:48512285:C:A_C
p = 0.25, beta = −0.246, N = 87
PITSEDIK pc2
36:236 11:80 2:9
12:48512285:C:A_C
p = 0.52, beta = −0.163, N = 49
SEWSDLLSDLQK pc2
74:236 21:80 3:9
12:48512285:C:A_C
p = 0.58, beta = −0.108, N = 98
AMNWMSGK pc2
38:236 8:80 3:9
12:48512285:C:A_C
p = 0.85, beta = 0.045, N = 49
EATWESVSMMLQLGGTVIGSAR pc
55:236 12:80 2:9
12:48512285:C:A_C
p = 0.74, beta = −0.0808, N = 69
YEIDLDTSDHAHLEHITR pc4
17:236 6:80 1:9
12:48512285:C:A_C
p = 0.26, beta = 0.386, N = 24
LPLMECVQVTK pc2
34:236 8:80 1:9
12:48512285:C:A_C
p = 0.57, beta = −0.174, N = 43
Assay Target: PFKM
UniProt: P08237
rsID: rs4760682
Target SNP: 12:48118502:C:A
Proxy SNP: 12:48512285:C:A
deCODE log10(p): 89.2
deCODE BETA: −0.2
deCODE MSPA: 0.449

181. YWHAB (P31946)
deCODE MSPA=0.323

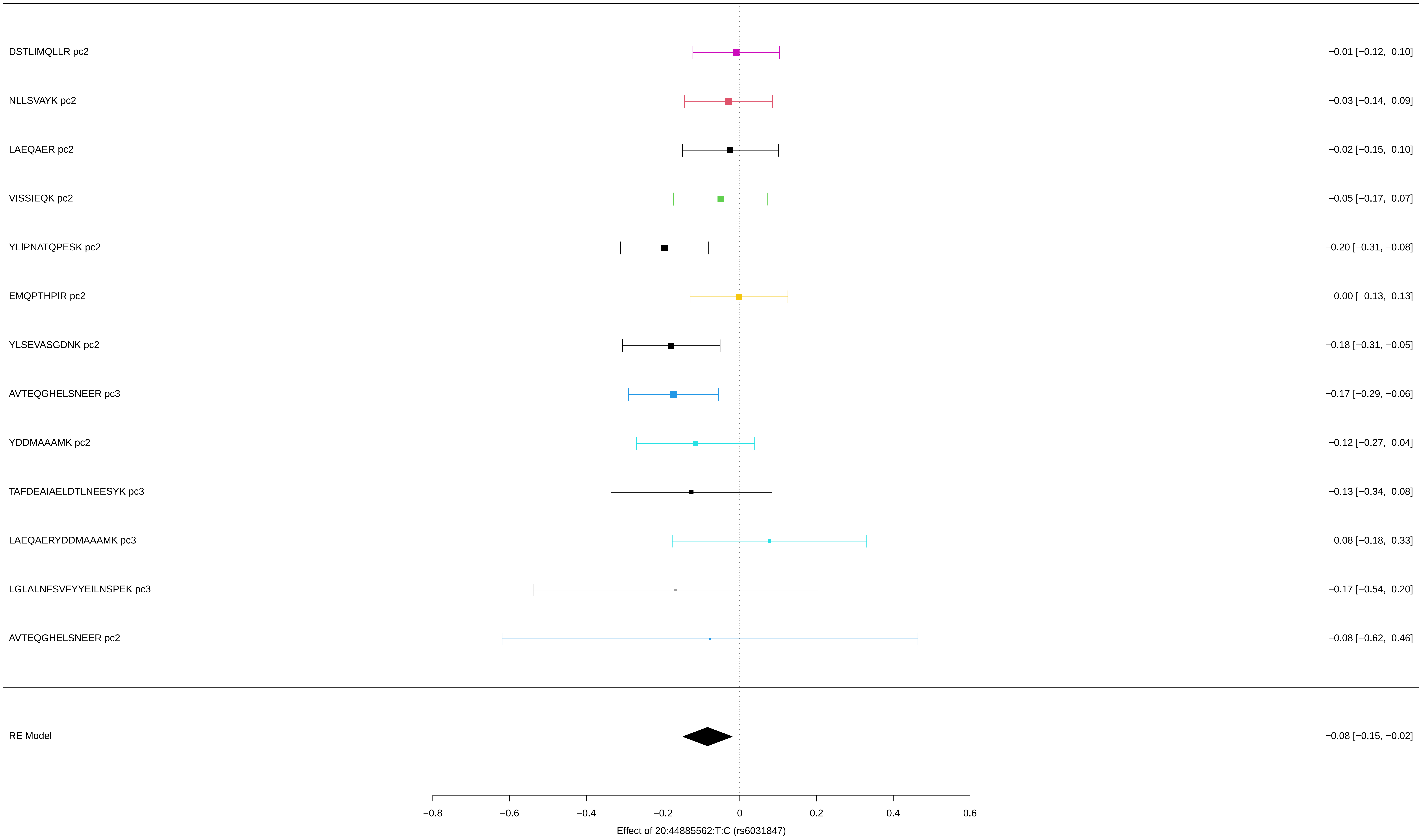

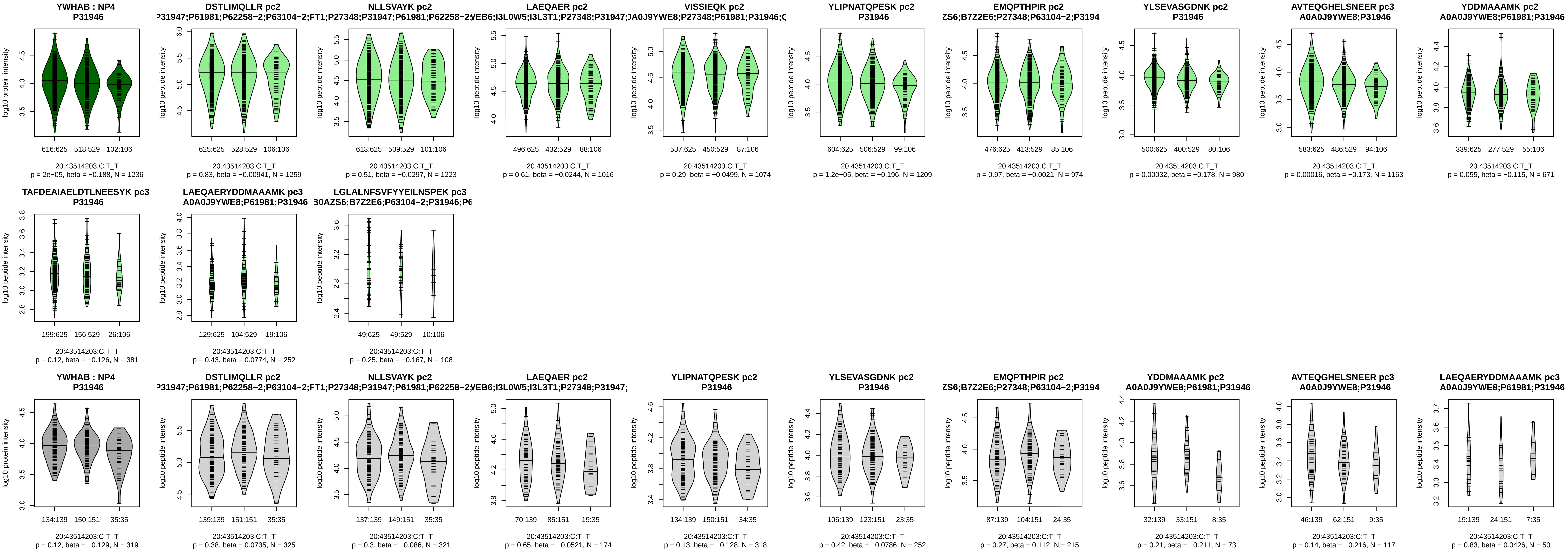

Assay Target: YWHAB
UniProt: P31946
rsID: rs6031847
Target SNP: 20:44885562:T:C
Proxy SNP: 20:43514203:C:T
deCODE log10(p): 86.5
deCODE BETA: −0.18
deCODE MSPA: 0.323
–:–:–:–:*:–:*:*:–:–:–:–:–
1259:1223:1016:1074:1209:974

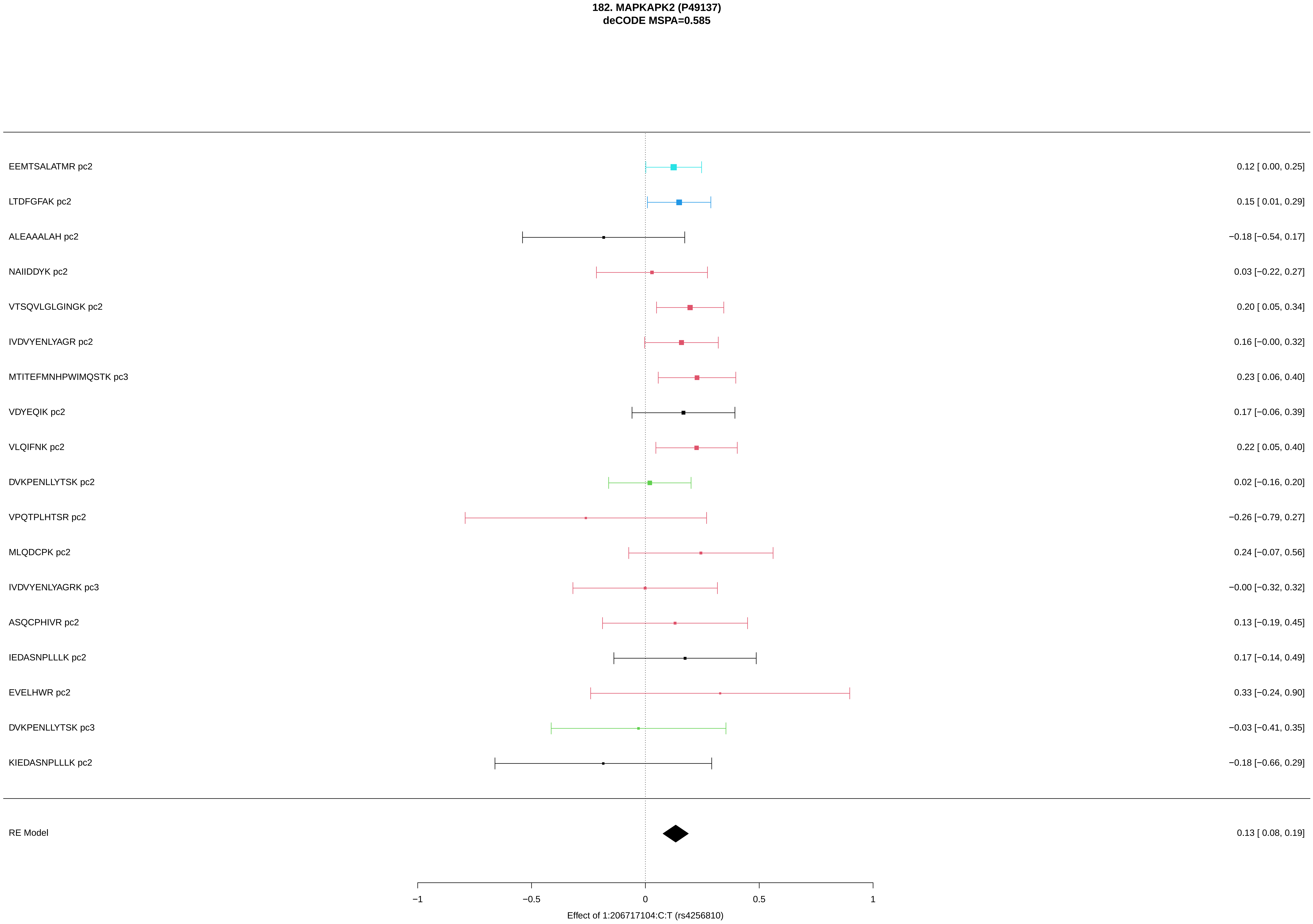

182. MAPKAPK2 (P49137)
deCODE MSPA=0.585
EEMTSALATMR pc2
0.12 [0.00, 0.25]
LTDFGFAK pc2
0.15 [0.01, 0.29]
ALEAAALAH pc2
−0.18 [−0.54, 0.17]
NAIIDDYK pc2
0.03 [−0.22, 0.27]
VTSQVLGLGINGK pc2
0.20 [0.05, 0.34]
IVDVYENLYAGR pc2
0.16 [−0.00, 0.32]
MTITEFMNHPWIMQSTK pc3
0.23 [0.06, 0.40]
VDYEQIK pc2
0.17 [−0.06, 0.39]
VLQIFNK pc2
0.22 [0.05, 0.40]
DVKPENLLYTSK pc2
0.02 [−0.16, 0.20]
VPQTPLHTSR pc2
−0.26 [−0.79, 0.27]
MLQDCPK pc2
0.24 [−0.07, 0.56]
IVDVYENLYAGRK pc3
−0.00 [−0.32, 0.32]
ASQCPHIVR pc2
0.13 [−0.19, 0.45]
IEDASNPLLLK pc2
0.17 [−0.14, 0.49]
EVELHWR pc2
0.33 [−0.24, 0.90]
DVKPENLLYTSK pc3
−0.03 [−0.41, 0.35]
KIEDASNPLLLK pc2
−0.18 [−0.66, 0.29]
RE Model
0.13 [0.08, 0.19]
−1
−0.5
0
0.5
1
Effect of 1:206717104:C:T (rs4256810)

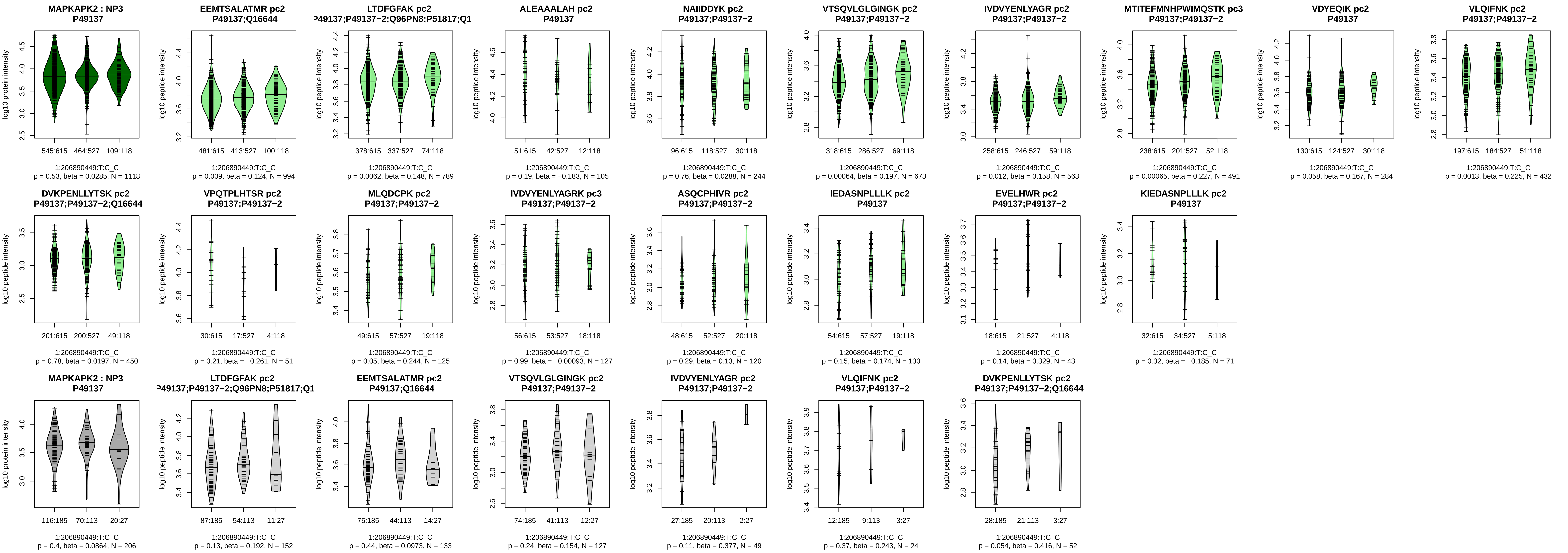

Assay Target: MAPKAPK2
UniProt: P49137
rsID: rs4256810
Target SNP: 1:206717104:C:T
Proxy SNP: 1:206890449:T:C
deCODE log10(p): 83.3
deCODE BETA: 0.18
deCODE MSPA: 0.585
994:789:105:244:673:563:491:2

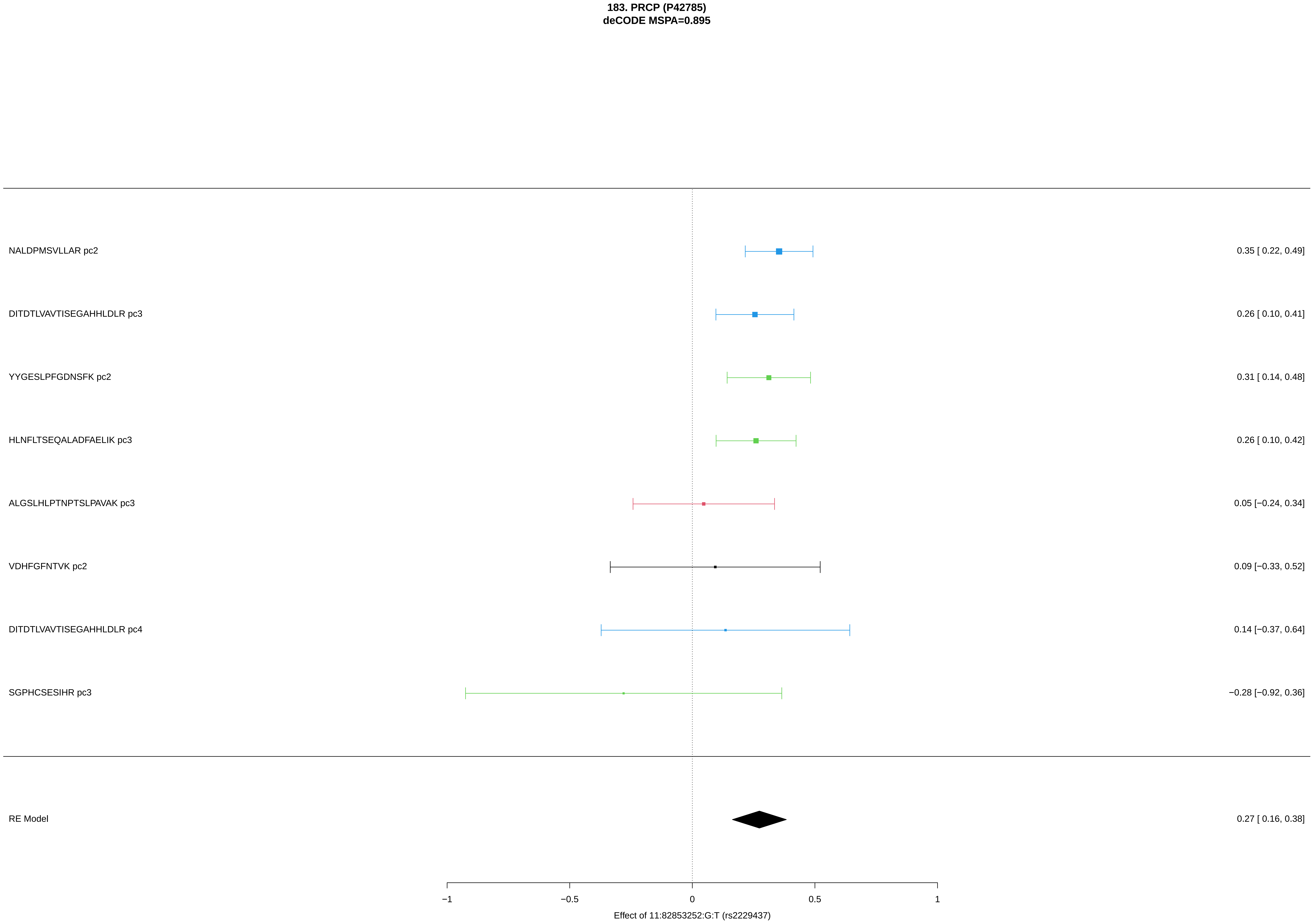

183. PRCP (P42785)
deCODE MSPA=0.895
NALDPMSVLLAR pc2
0.35 [0.22, 0.49]
DITDTLVAVTISEGAHHLDLR pc3
0.26 [0.10, 0.41]
YYGESLPFGDNSFK pc2
0.31 [0.14, 0.48]
HLNFLTSEQALADFAELIK pc3
0.26 [0.10, 0.42]
ALGSLHLPTNPTSLPAVAK pc3
0.05 [−0.24, 0.34]
VDHFGFNTVK pc2
0.09 [−0.33, 0.52]
DITDTLVAVTISEGAHHLDLR pc4
0.14 [−0.37, 0.64]
SGPHCSESIHR pc3
−0.28 [−0.92, 0.36]
RE Model
0.27 [0.16, 0.38]
−1
−0.5
0
0.5
1
Effect of 11:82853252:G:T (rs2229437)

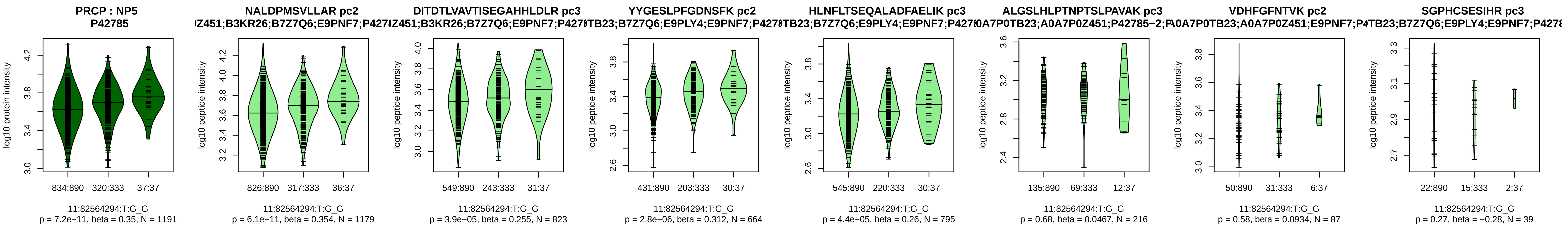

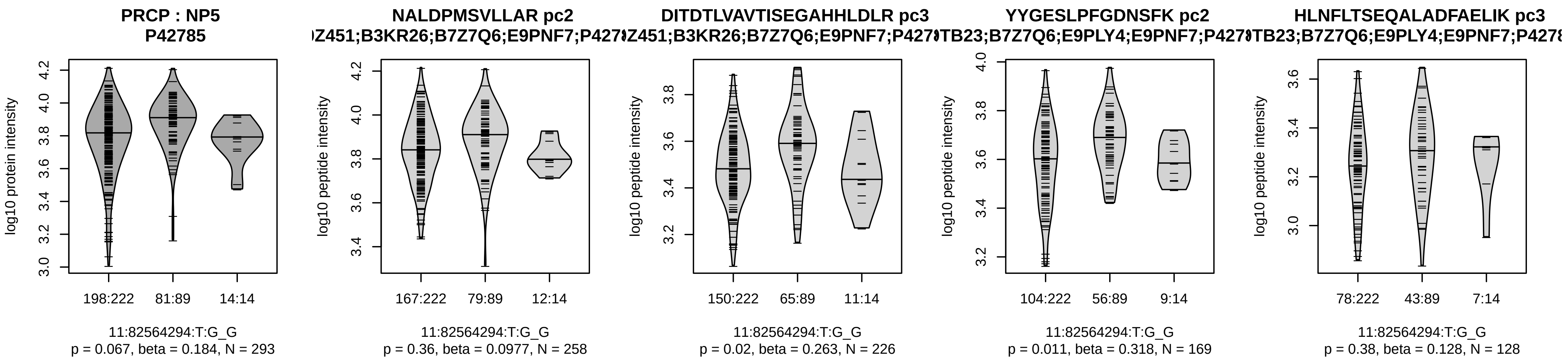

Assay Target: PRCP
UniProt: P42785
rsID: rs2229437
Target SNP: 11:82853252:G:T
Proxy SNP: 11:82564294:T:G
deCODE log10(p): 82.2
deCODE BETA: 0.22
deCODE MSPA: 0.895
::*:*:–:–:–:–
1179:823:664:795:216:87:63:39

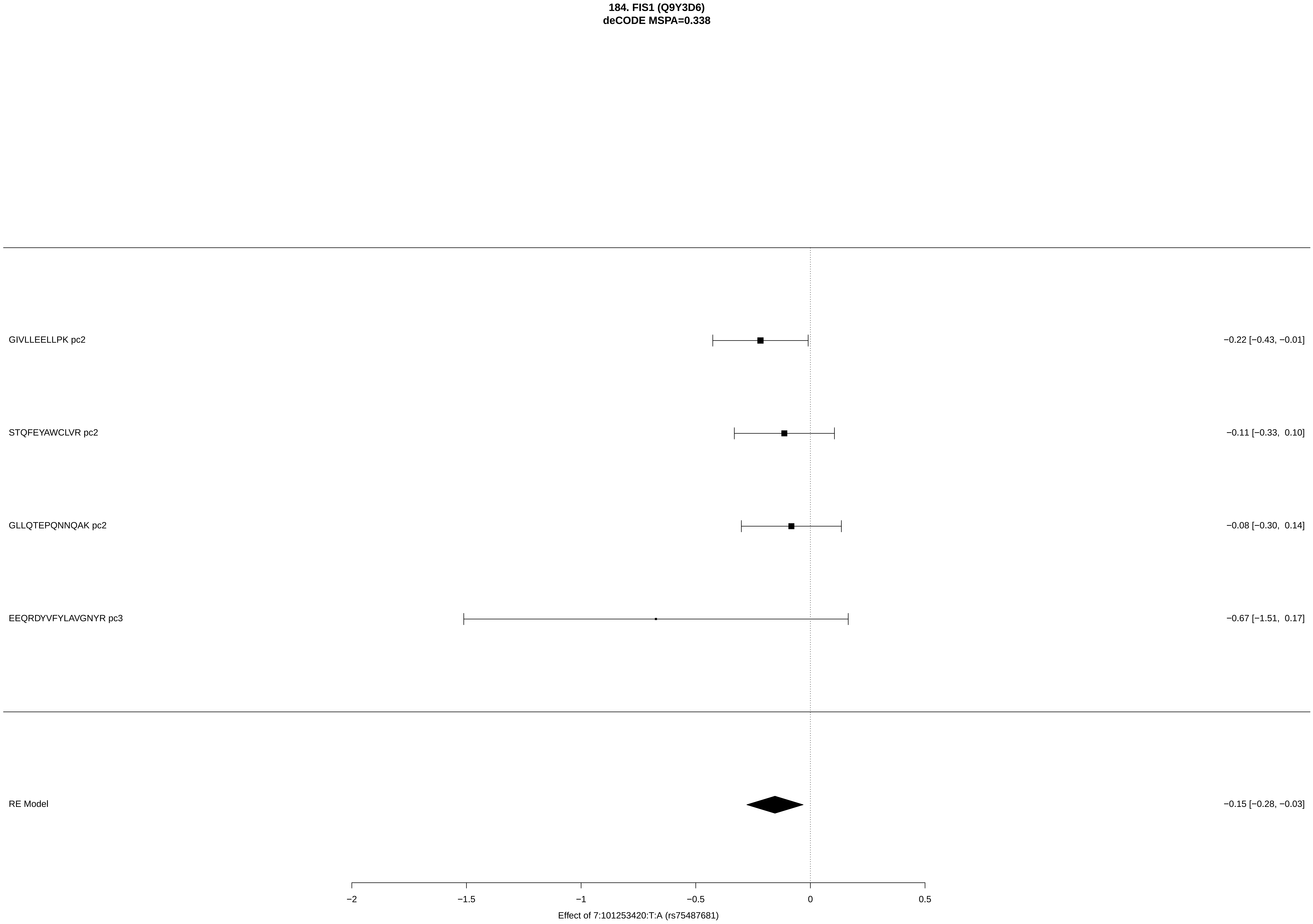
184. FIS1 (Q9Y3D6)
deCODE MSPA=0.338
GIVLLEELLPK pc2
−0.22 [−0.43, −0.01]
STQFEYAWCLVR pc2
−0.11 [−0.33, 0.10]
GLLQTEPQNNQAK pc2
−0.08 [−0.30, 0.14]
EEQRDYVFYLAVGNYR pc3
−0.67 [−1.51, 0.17]
RE Model
−0.15 [−0.28, −0.03]
−2
−1.5
−1
−0.5
0
0.5
Effect of 7:101253420:T:A (rs75487681)

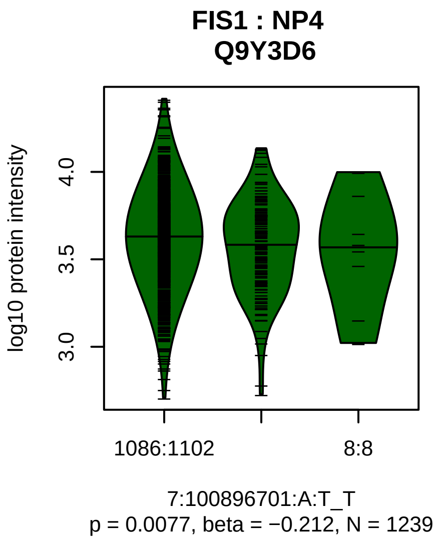

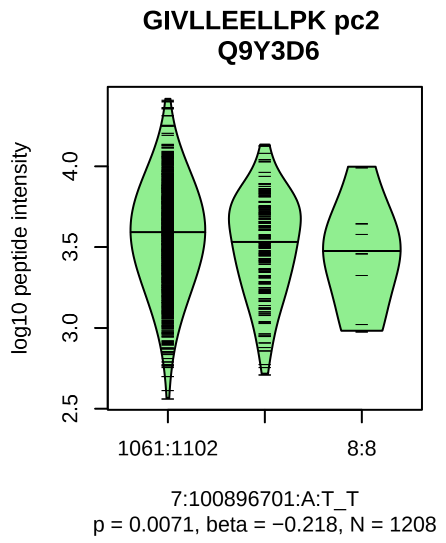

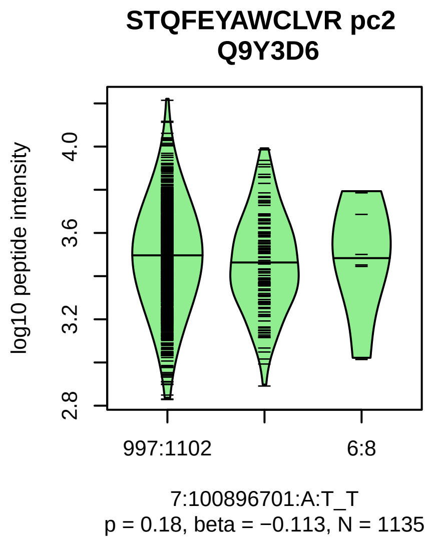

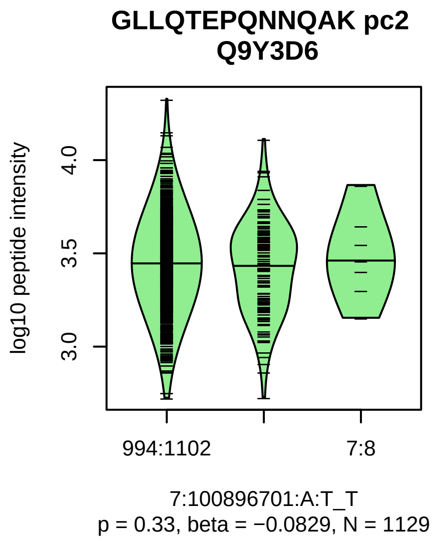

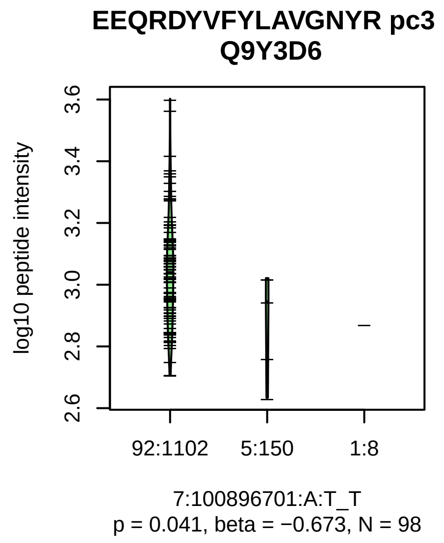

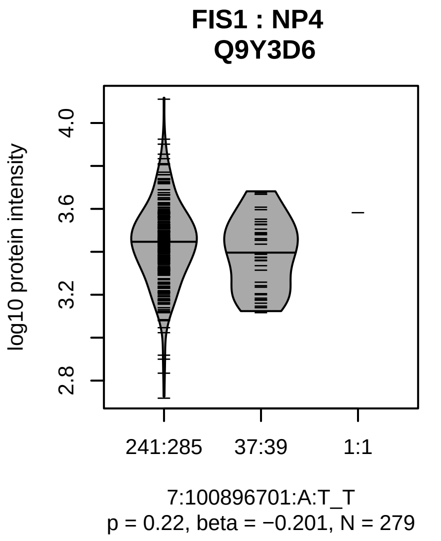

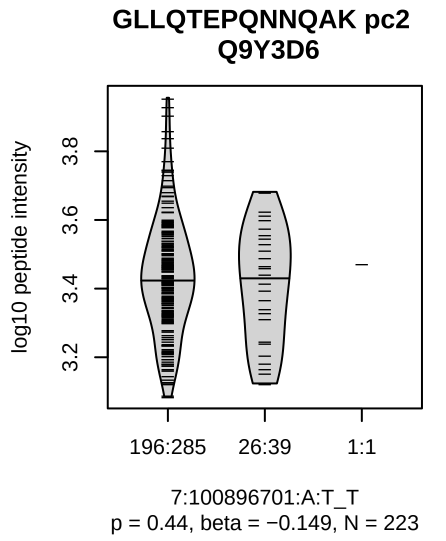

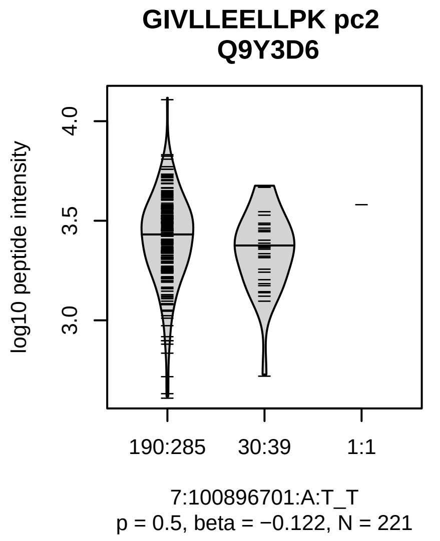

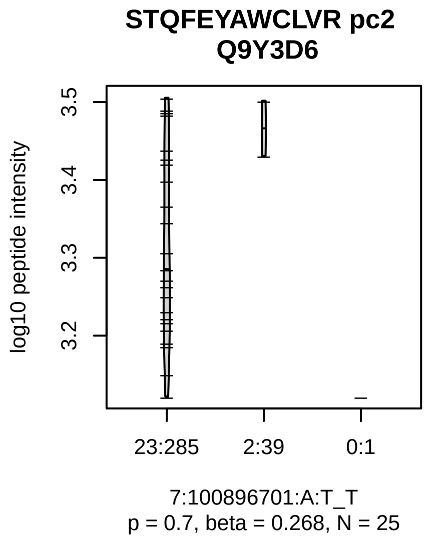

Assay Target: FIS1
UniProt: Q9Y3D6
rsID: rs75487681
Target SNP: 7:101253420:T:A
Proxy SNP: 7:100896701:A:T
deCODE log10(p): 80.3
deCODE BETA: −0.32
deCODE MSPA: 0.338
*:−:−:−
1208:1135:1129:98

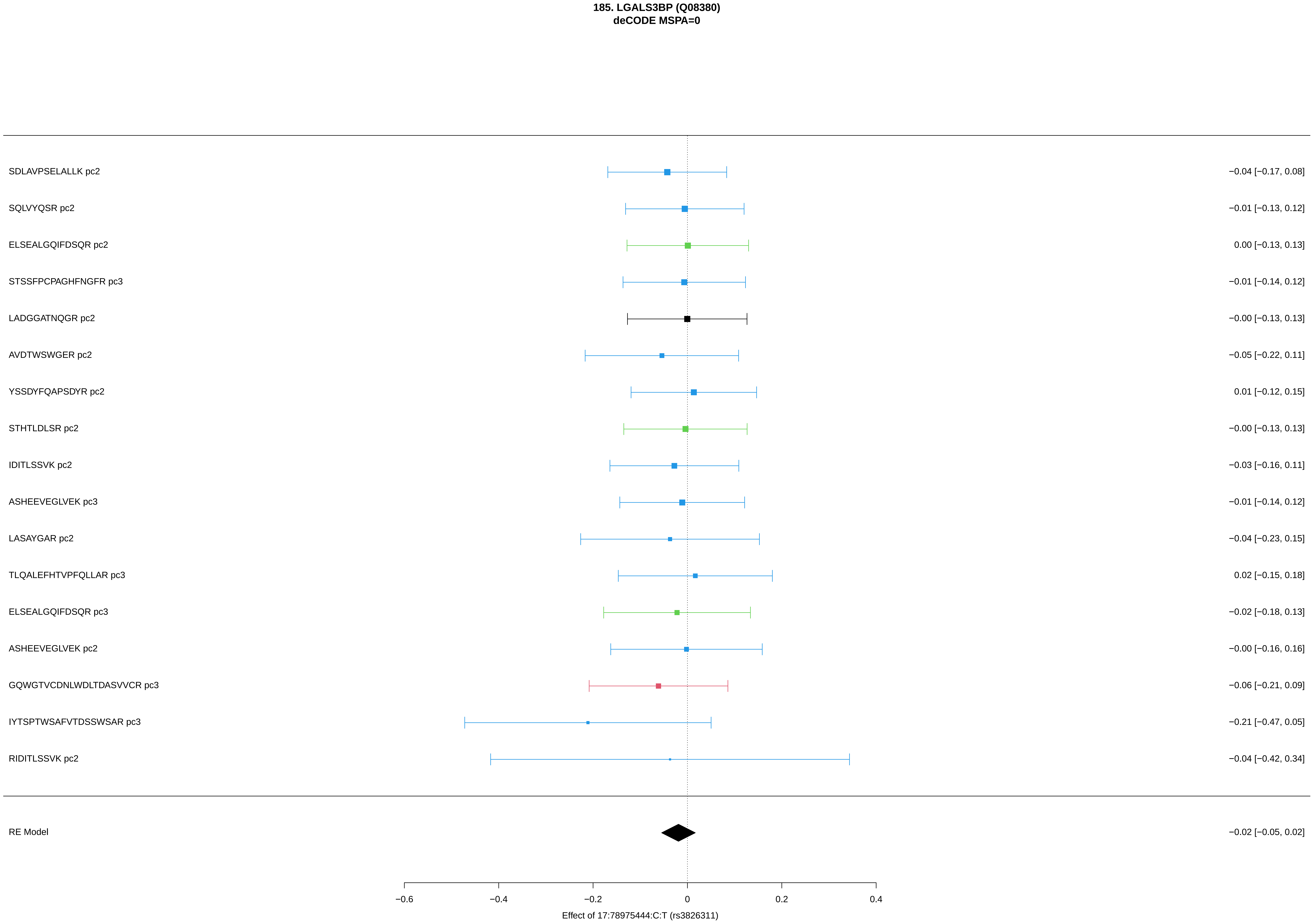

185. LGALS3BP (Q08380)
deCODE MSPA=0
SDLAVPSELALLK pc2
−0.04 [−0.17, 0.08]
SQLVYQSR pc2
−0.01 [−0.13, 0.12]
ELSEALGQIFDSQR pc2
0.00 [−0.13, 0.13]
STSSFPCPAGHFNGFR pc3
−0.01 [−0.14, 0.12]
LADGGATNQGR pc2
−0.00 [−0.13, 0.13]
AVDTWSWGER pc2
−0.05 [−0.22, 0.11]
YSSDYFQAPSDYR pc2
0.01 [−0.12, 0.15]
STHTLDLSR pc2
−0.00 [−0.13, 0.13]
IDITLSSVK pc2
−0.03 [−0.16, 0.11]
ASHEEVEGLVEK pc3
−0.01 [−0.14, 0.12]
LASAYGAR pc2
−0.04 [−0.23, 0.15]
TLQALEFHTVPFQLLAR pc3
0.02 [−0.15, 0.18]
ELSEALGQIFDSQR pc3
−0.02 [−0.18, 0.13]
ASHEEVEGLVEK pc2
−0.00 [−0.16, 0.16]
GQWGTVCDNLWDLTDASVVCR pc3
−0.06 [−0.21, 0.09]
IYTSPTWSAFVTDSSWSAR pc3
−0.21 [−0.47, 0.05]
RIDITLSSVK pc2
−0.04 [−0.42, 0.34]
RE Model
−0.02 [−0.05, 0.02]
−0.6
−0.4
−0.2
0
0.2
0.4
Effect of 17:78975444:C:T (rs3826311)

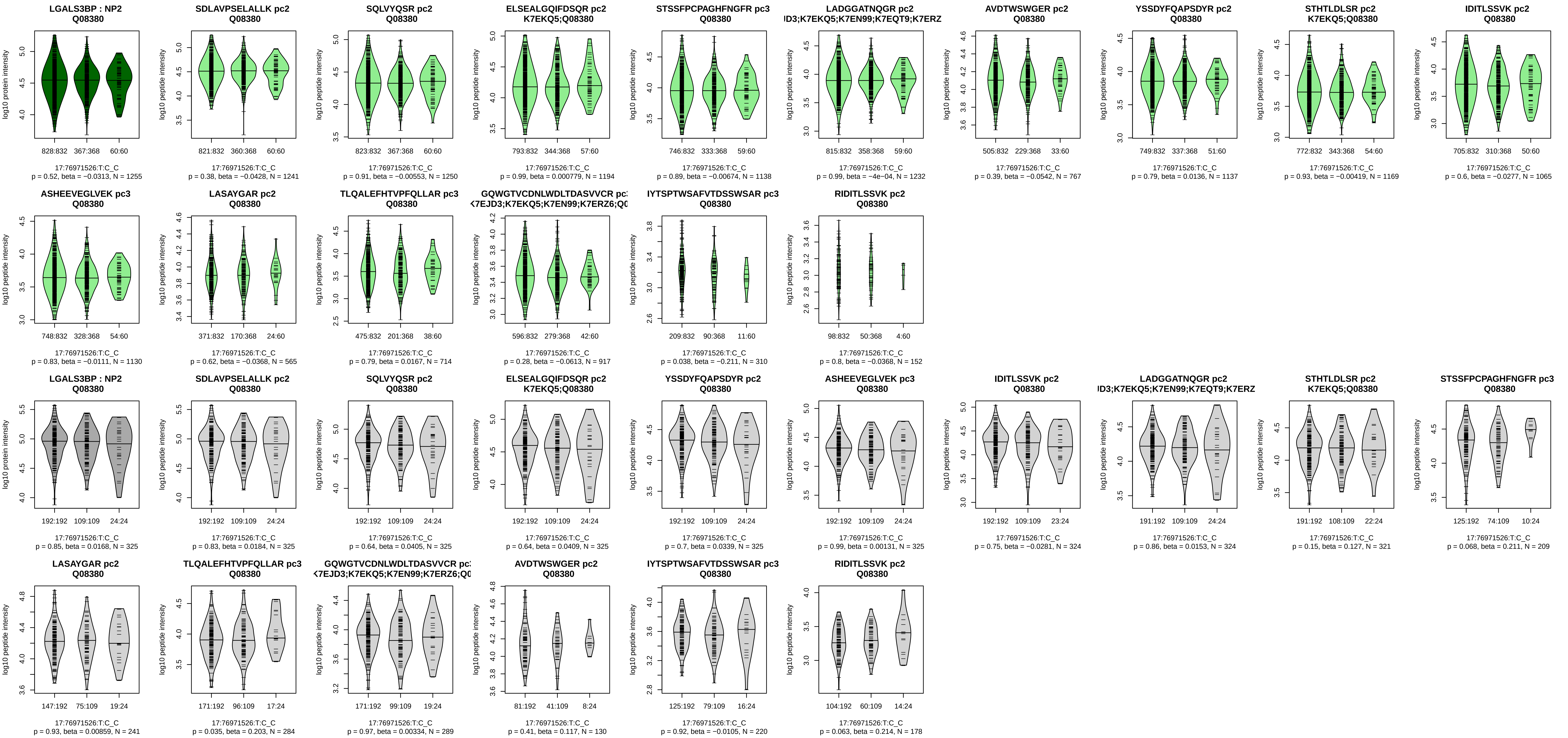

Assay Target: LGALS3BP
UniProt: Q08380
rsID: rs3826311
Target SNP: 17:78975444:C:T
Proxy SNP: 17:76971526:T:C
deCODE log10(p): 80.3
deCODE BETA: 0.2
deCODE MSPA: 0
1241:1250:1194:1138:1232:767

186. PCOLCE (Q15113)
deCODE MSPA=0.628

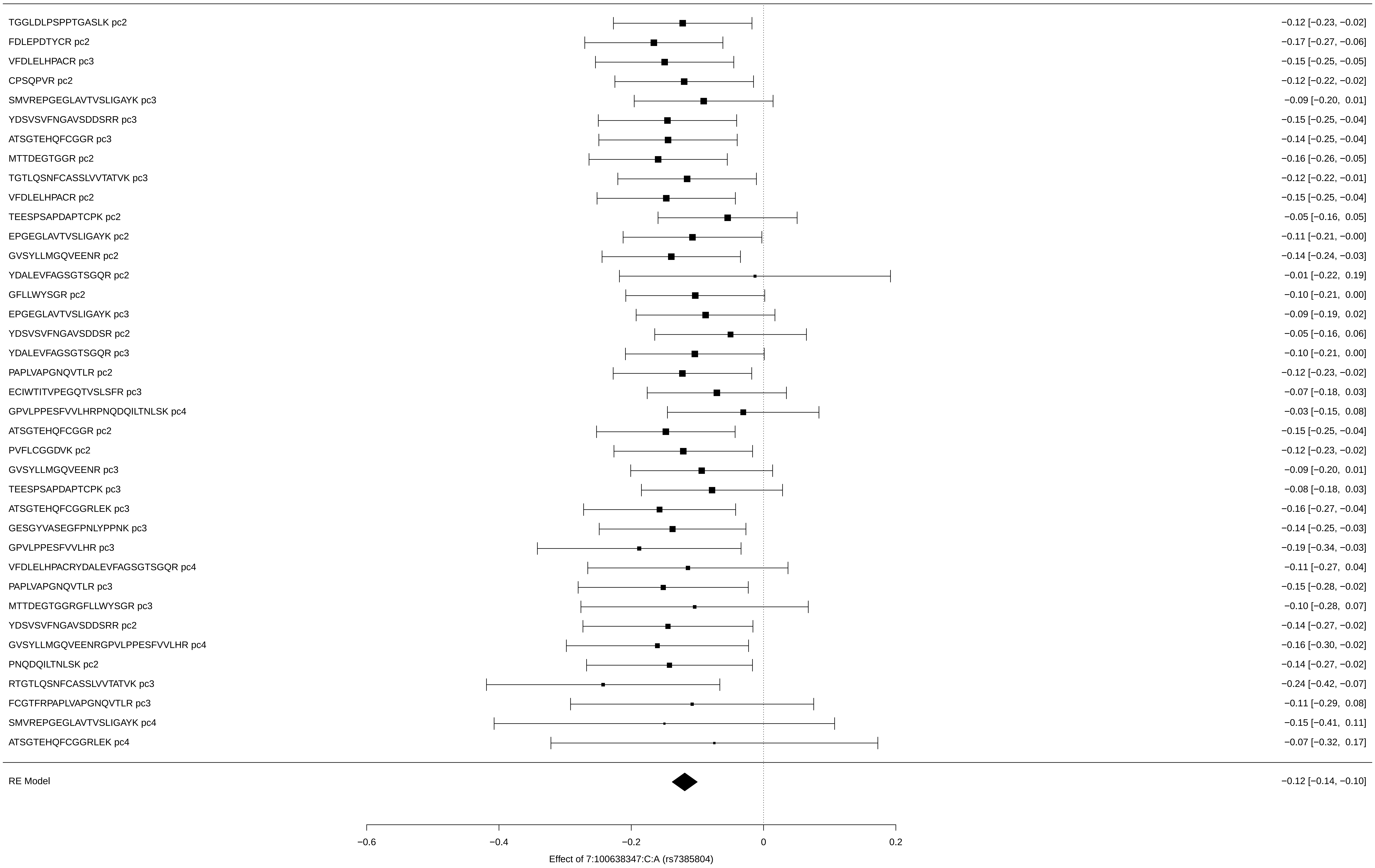

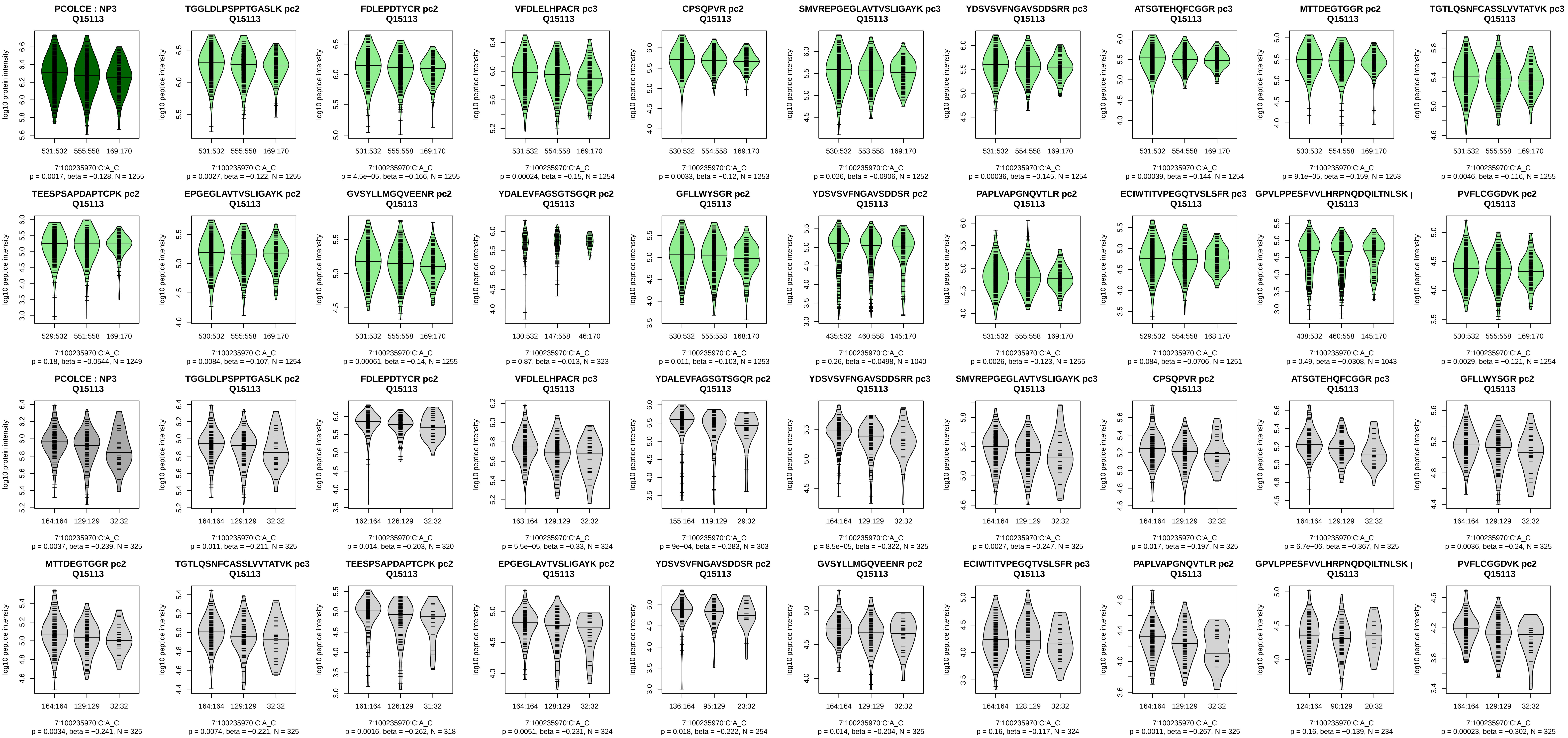

Assay Target: PCOLCE
UniProt: Q15113
rsID: rs7385804
Target SNP: 7:100638347:C:A
Proxy SNP: 7:100235970:C:A
deCODE log10(p): 77.9
deCODE BETA: −0.16
deCODE MSPA: 0.628
1255:1255:1254:1253:1252:125

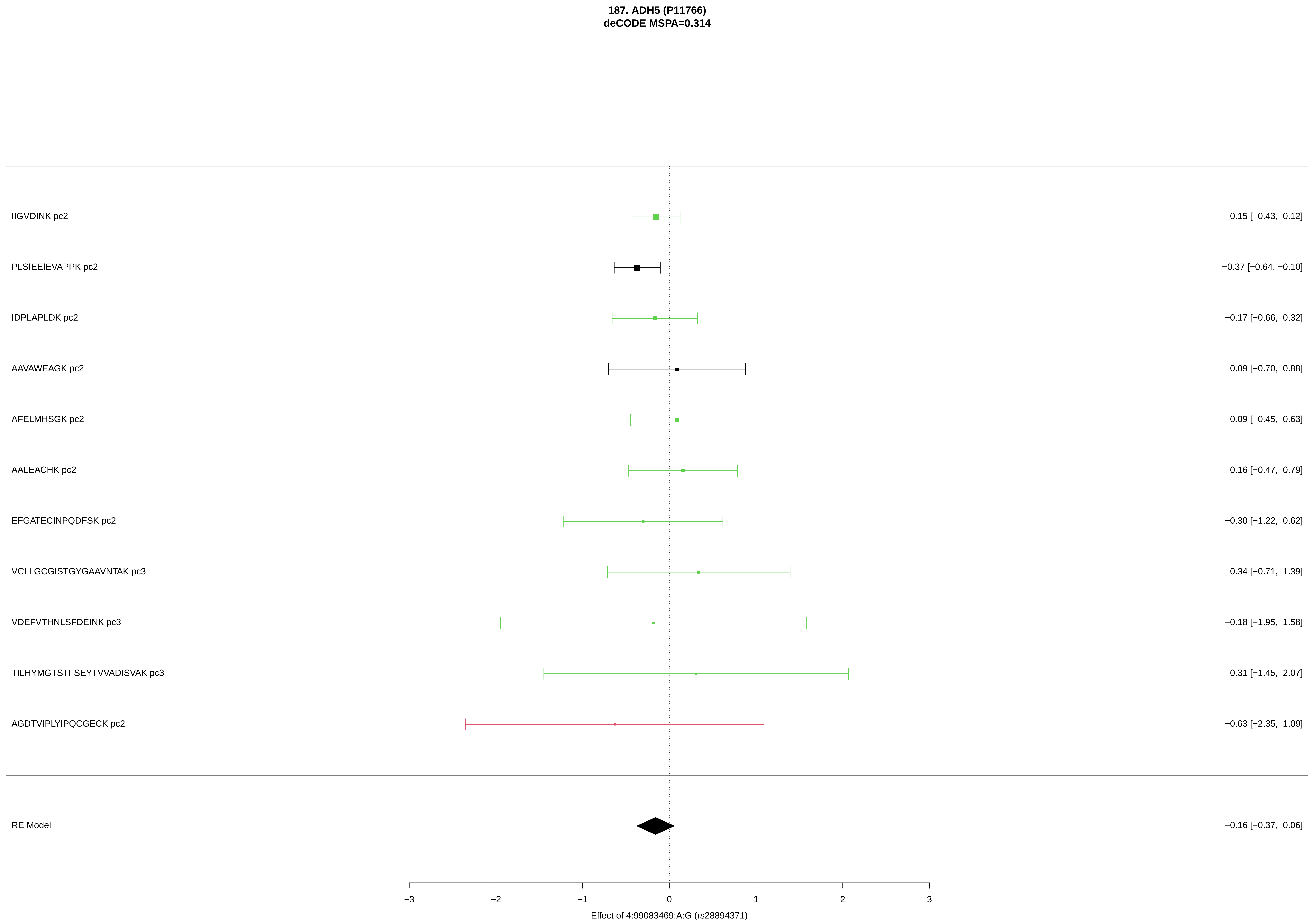

187. ADH5 (P11766)
deCODE MSPA=0.314
IIGVDINK pc2
−0.15 [−0.43, 0.12]
PLSIEEIEVAPPK pc2
−0.37 [−0.64, −0.10]
IDPLAPLDK pc2
−0.17 [−0.66, 0.32]
AAVAWEAGK pc2
0.09 [−0.70, 0.88]
AFELMHSGK pc2
0.09 [−0.45, 0.63]
AALEACHK pc2
0.16 [−0.47, 0.79]
EFGATECINPQDFSK pc2
−0.30 [−1.22, 0.62]
VCLLGCGISTGYGAAVNTAK pc3
0.34 [−0.71, 1.39]
VDEFVTHNLSFDEINK pc3
−0.18 [−1.95, 1.58]
TILHYMGTSTFSEYTVVADISVAK pc3
0.31 [−1.45, 2.07]
AGDTVIPLYIPQCGECK pc2
−0.63 [−2.35, 1.09]
RE Model
−0.16 [−0.37, 0.06]
−3
−2
−1
0
1
2
3
Effect of 4:99083469:A:G (rs28894371)

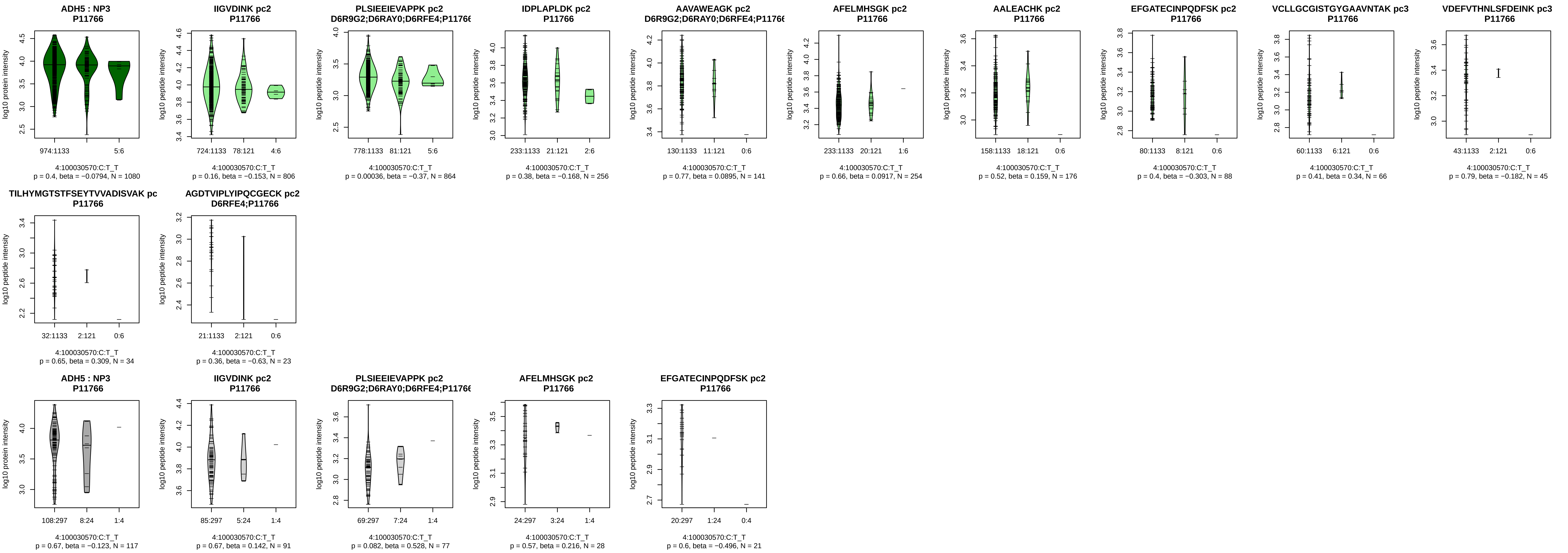

Assay Target: ADH5
UniProt: P11766
rsID: rs28894371
Target SNP: 4:99083469:A:G
Proxy SNP: 4:100030570:C:T
deCODE log10(p): 76.8
deCODE BETA: −0.36
deCODE MSPA: 0.314
−:*:−:−:−:−:−:−:−:−:NA
806:864:256:141:254:176:88:66

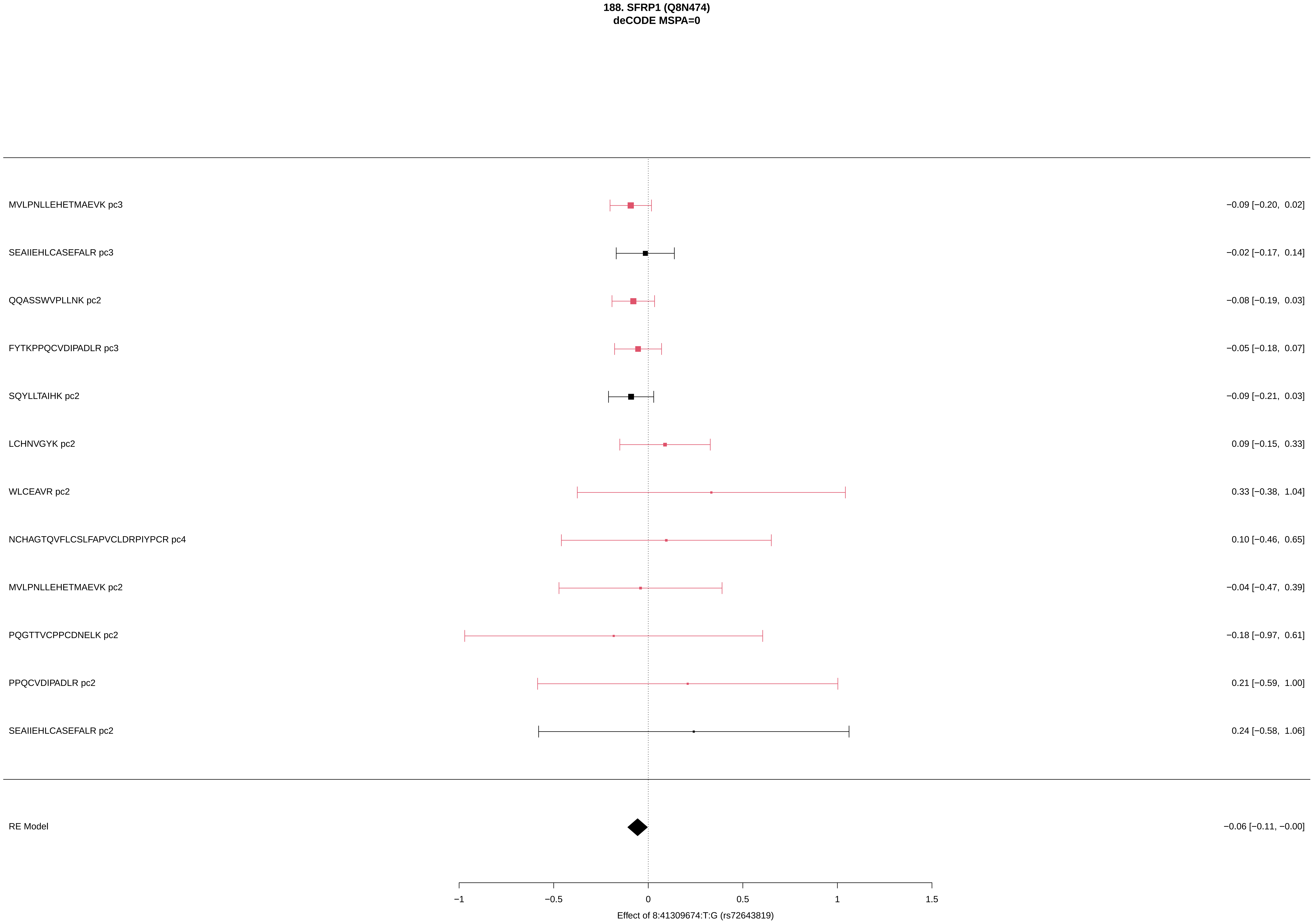
188. SFRP1 (Q8N474)
deCODE MSPA=0
MVLPNLLEHETMAEVK pc3
−0.09 [−0.20, 0.02]
SEAIIEHLCASEFALR pc3
−0.02 [−0.17, 0.14]
QQASSWVPLLNK pc2
−0.08 [−0.19, 0.03]
FYTKPPQCVDIPADLR pc3
−0.05 [−0.18, 0.07]
SQYLLTAIHK pc2
−0.09 [−0.21, 0.03]
LCHNVGYK pc2
0.09 [−0.15, 0.33]
WLCEAVR pc2
0.33 [−0.38, 1.04]
NCHAGTQVFLCSLFAPVCLDRPIYPCR pc4
0.10 [−0.46, 0.65]
MVLPNLLEHETMAEVK pc2
−0.04 [−0.47, 0.39]
PQGTTVCPPCDNELK pc2
−0.18 [−0.97, 0.61]
PPQCVDIPADLR pc2
0.21 [−0.59, 1.00]
SEAIIEHLCASEFALR pc2
0.24 [−0.58, 1.06]
RE Model
−0.06 [−0.11, −0.00]
−1
−0.5
0
0.5
1
1.5
Effect of 8:41309674:T:G (rs72643819)

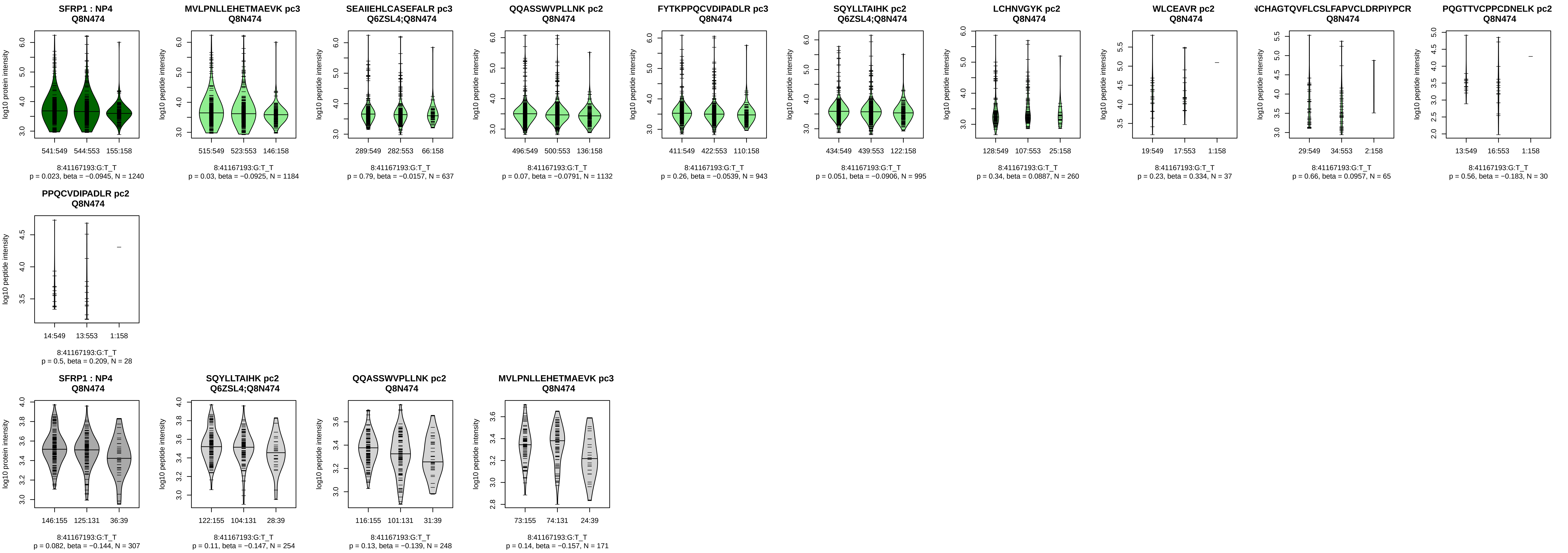

Assay Target: SFRP1
UniProt: Q8N474
rsID: rs72643819
Target SNP: 8:41309674:T:G
Proxy SNP: 8:41167193:G:T
deCODE log10(p): 76.2
deCODE BETA: −0.16
deCODE MSPA: 0
–:–:–:–:–:–:–:–:–:–:NA:NA
1184:637:1132:943:995:260:37:

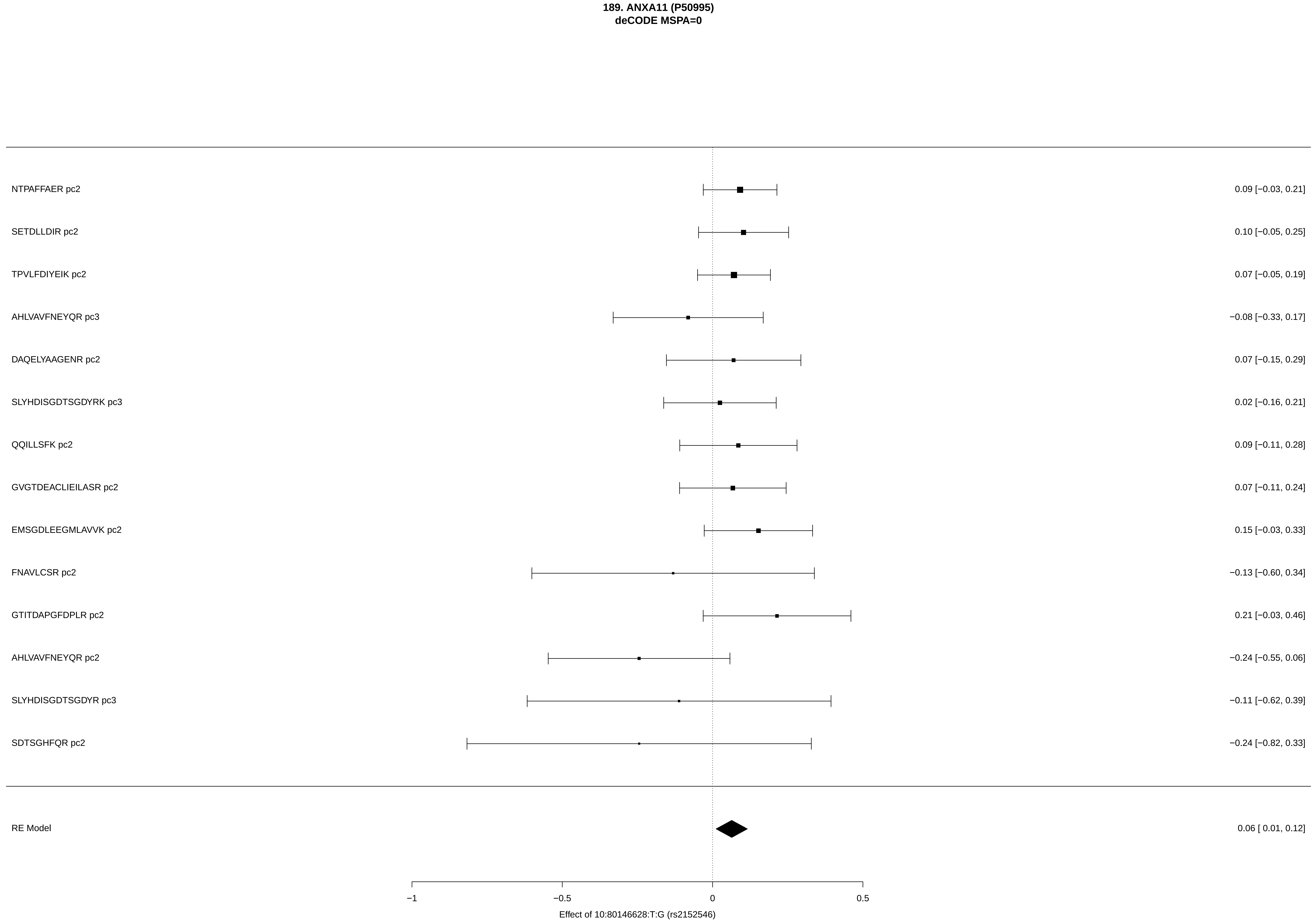
189. ANXA11 (P50995)
deCODE MSPA=0
NTPAFFAER pc2
0.09 [−0.03, 0.21]
SETDLLDIR pc2
0.10 [−0.05, 0.25]
TPVLFDIYEIK pc2
0.07 [−0.05, 0.19]
AHLVAVFNEYQR pc3
−0.08 [−0.33, 0.17]
DAQELYAAGENR pc2
0.07 [−0.15, 0.29]
SLYHDISGDTSGDYRK pc3
0.02 [−0.16, 0.21]
QQILLSFK pc2
0.09 [−0.11, 0.28]
GVGTDEACLIEILASR pc2
0.07 [−0.11, 0.24]
EMSGDLEEGMLAVVK pc2
0.15 [−0.03, 0.33]
FNAVLCSR pc2
−0.13 [−0.60, 0.34]
GTITDAPGFDPLR pc2
0.21 [−0.03, 0.46]
AHLVAVFNEYQR pc2
−0.24 [−0.55, 0.06]
SLYHDISGDTSGDYR pc3
−0.11 [−0.62, 0.39]
SDTSGHFQR pc2
−0.24 [−0.82, 0.33]
RE Model
0.06 [0.01, 0.12]
−1
−0.5
0
0.5
Effect of 10:80146628:T:G (rs2152546)

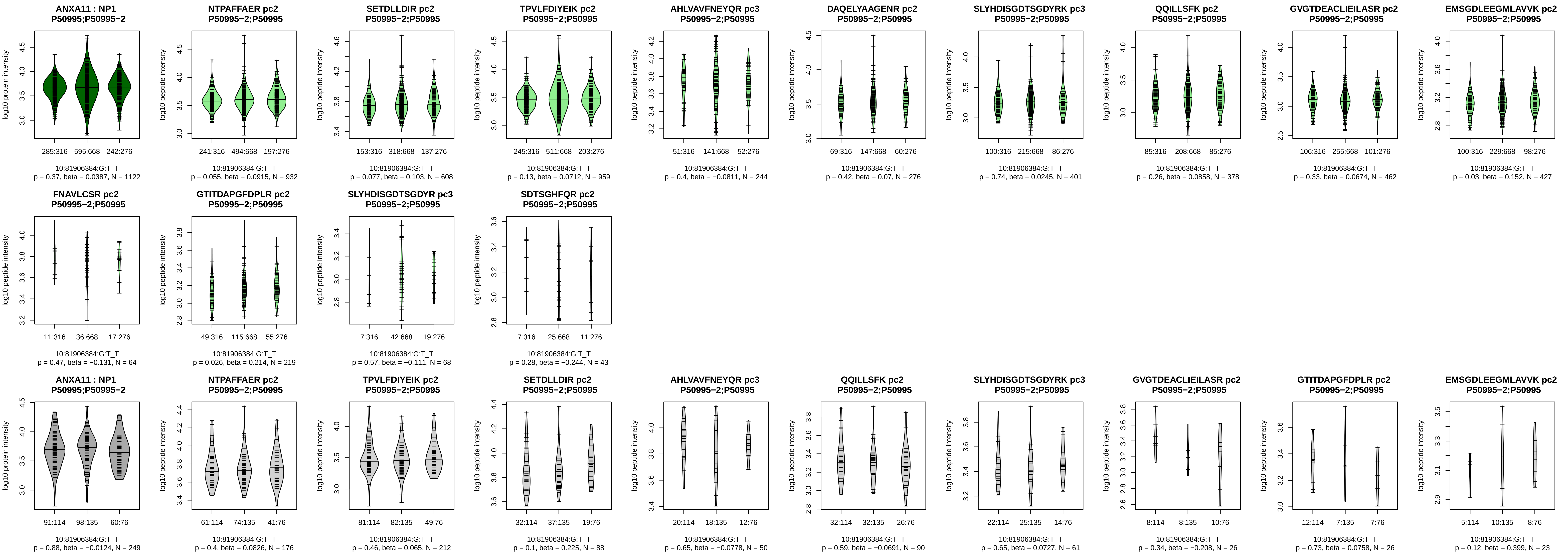

Assay Target: ANXA11
UniProt: P50995
rsID: rs2152546
Target SNP: 10:80146628:T:G
Proxy SNP: 10:81906384:G:T
deCODE log10(p): 75.8
deCODE BETA: −0.15
deCODE MSPA: 0
932:608:959:244:276:401:378:4

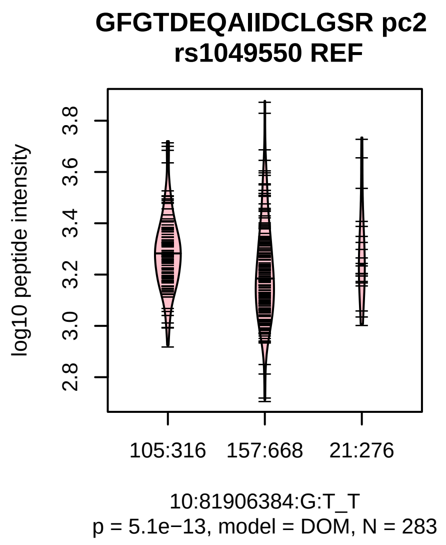

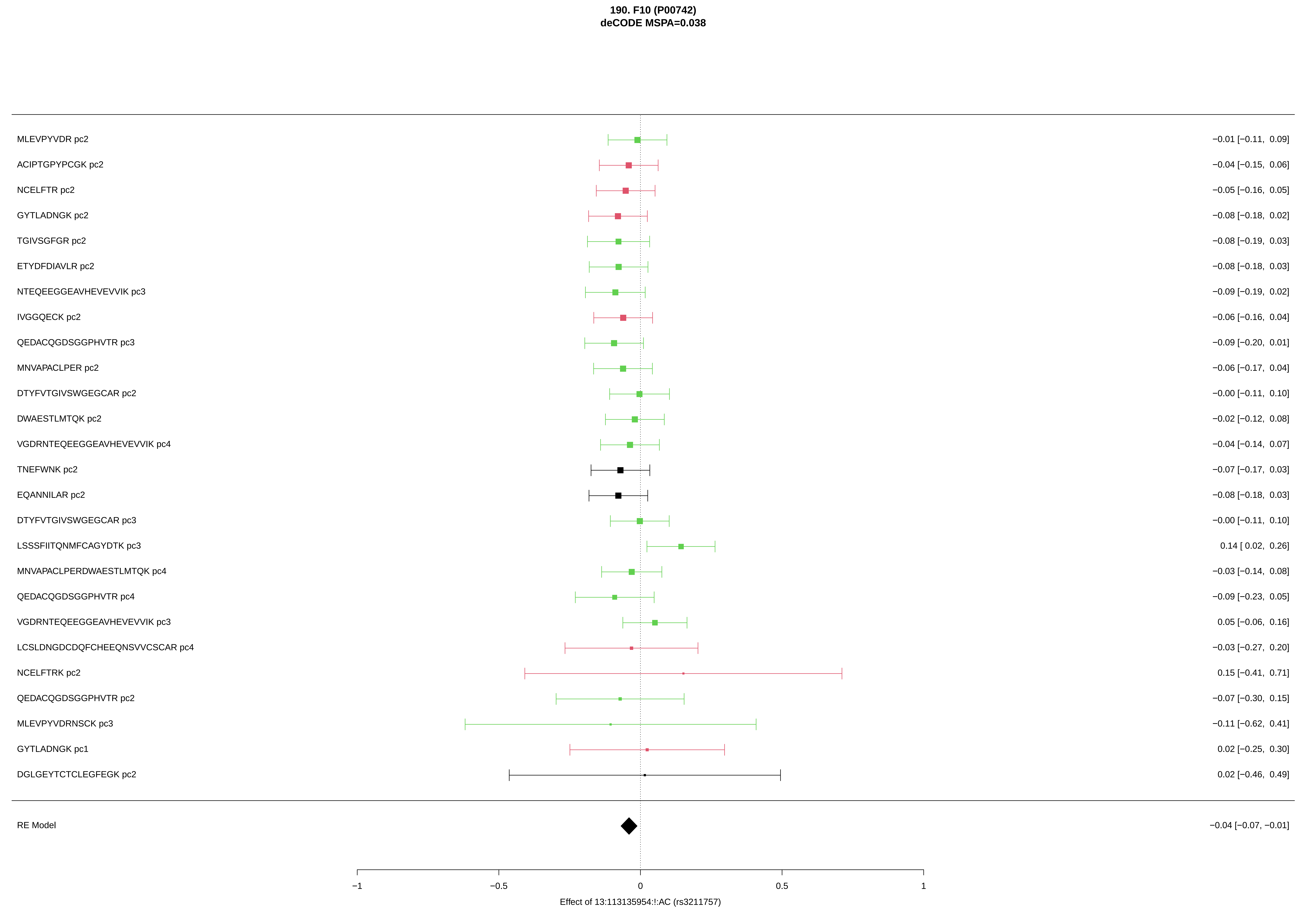
190. F10 (P00742)
deCODE MSPA=0.038
MLEVPYVDR pc2
ACIPTGPYPCGK pc2
NCELFTR pc2
GYTLADNGK pc2
TGIVSGFGR pc2
ETYDFDIAVLR pc2
NTEQEEGGEAVHEVEVVIK pc3
IVGGQECK pc2
QEDACQGDSGGPHVTR pc3
MNVAPACLPER pc2
DTYFVTGIVSWGEGCAR pc2
DWAESTLMTQK pc2
VGDRNTEQEEGGEAVHEVEVVIK pc4
TNEFWNK pc2
EQANNILAR pc2
DTYFVTGIVSWGEGCAR pc3
LSSSFIITQNMFCAGYDTK pc3
MNVAPACLPERDWAESTLMTQK pc4
QEDACQGDSGGPHVTR pc4
VGDRNTEQEEGGEAVHEVEVVIK pc3
LCSLDNGDCDQFCHEEQNSVVCSCAR pc4
NCELFTRK pc2
QEDACQGDSGGPHVTR pc2
MLEVPYVDRNSCK pc3
GYTLADNGK pc1
DGLGEYTCTCLEGFEGK pc2
RE Model
−0.01 [−0.11, 0.09]
−0.04 [−0.15, 0.06]
−0.05 [−0.16, 0.05]
−0.08 [−0.18, 0.02]
−0.08 [−0.19, 0.03]
−0.08 [−0.18, 0.03]
−0.09 [−0.19, 0.02]
−0.06 [−0.16, 0.04]
−0.09 [−0.20, 0.01]
−0.06 [−0.17, 0.04]
−0.00 [−0.11, 0.10]
−0.02 [−0.12, 0.08]
−0.04 [−0.14, 0.07]
−0.07 [−0.17, 0.03]
−0.08 [−0.18, 0.03]
−0.00 [−0.11, 0.10]
0.14 [0.02, 0.26]
−0.03 [−0.14, 0.08]
−0.09 [−0.23, 0.05]
0.05 [−0.06, 0.16]
−0.03 [−0.27, 0.20]
0.15 [−0.41, 0.71]
−0.07 [−0.30, 0.15]
−0.11 [−0.62, 0.41]
0.02 [−0.25, 0.30]
0.02 [−0.46, 0.49]
−0.04 [−0.07, −0.01]
−1
−0.5
0
0.5
1
Effect of 13:113135954:!:AC (rs3211757)

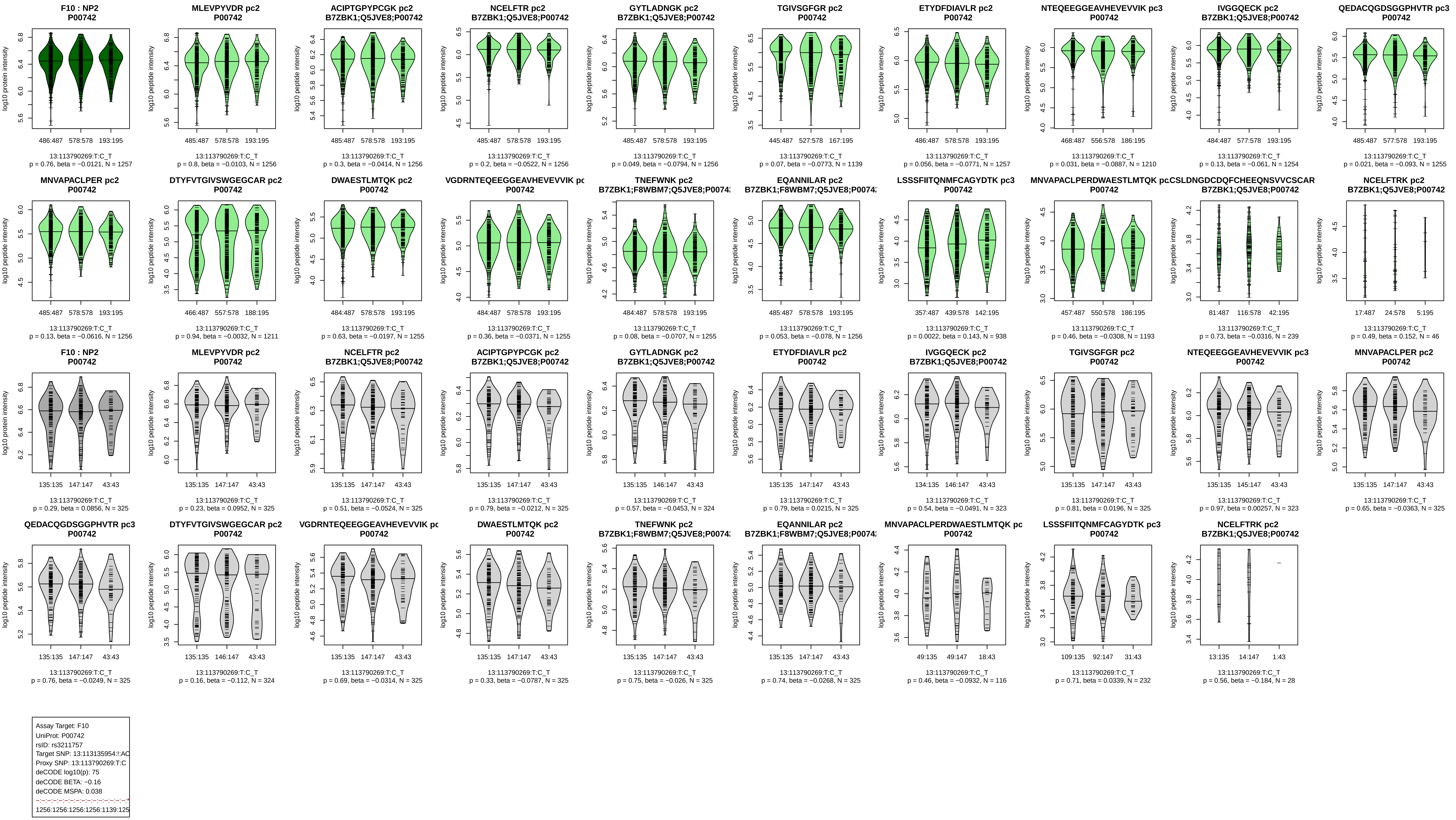
F10 : NP2
P00742
log10 protein intensity
13:113790269:T:C_T
p = 0.76, beta = −0.0121, N = 1257
MLEVPYVDR pc2
P00742
log10 peptide intensity
p = 0.8, beta = −0.0103, N = 1256
ACIPTGPYPCGK pc2
B7ZBK1;Q5JVE8;P00742
p = 0.3, beta = −0.0414, N = 1256
NCELFTR pc2
B7ZBK1;Q5JVE8;P00742
p = 0.2, beta = −0.0522, N = 1256
GYTLADNGK pc2
B7ZBK1;Q5JVE8;P00742
p = 0.049, beta = −0.0794, N = 1256
TGIVSGFGR pc2
P00742
p = 0.07, beta = −0.0773, N = 1139
ETYDFDIAVLR pc2
P00742
p = 0.056, beta = −0.0771, N = 1257
NTEQEEGGEAVHEVEVVIK pc3
P00742
p = 0.031, beta = −0.0887, N = 1210
IVGGQECK pc2
B7ZBK1;Q5JVE8;P00742
p = 0.13, beta = −0.061, N = 1254
QEDACQGDSGGPHVTR pc3
P00742
p = 0.021, beta = −0.093, N = 1255
MNVAPACLPER pc2
P00742
p = 0.13, beta = −0.0616, N = 1256
DTYFVTGIVSWGEGCAR pc2
P00742
p = 0.94, beta = −0.0032, N = 1211
DWAESTLMTQK pc2
P00742
p = 0.63, beta = −0.0197, N = 1255
VGDRNTEQEEGGEAVHEVEVVIK pc
P00742
p = 0.36, beta = −0.0371, N = 1255
TNEFWNK pc2
B7ZBK1;F8WBM7;Q5JVE8;P0074
p = 0.08, beta = −0.0707, N = 1255
EQANNILAR pc2
B7ZBK1;F8WBM7;Q5JVE8;P0074
p = 0.053, beta = −0.078, N = 1256
LSSSFIITQNMFCAGYDTK pc3
P00742
p = 0.0022, beta = 0.143, N = 938
MNVAPACLPERDWAESTLMTQK pc
P00742
p = 0.46, beta = −0.0308, N = 1193
CSLDNGDCDQFCHEEQNSVVCSCAR
B7ZBK1;Q5JVE8;P00742
p = 0.73, beta = −0.0316, N = 239
NCELFTRK pc2
B7ZBK1;Q5JVE8;P00742
p = 0.49, beta = 0.152, N = 46
F10 : NP2
P00742
p = 0.29, beta = 0.0856, N = 325
MLEVPYVDR pc2
P00742
p = 0.23, beta = 0.0952, N = 325
NCELFTR pc2
B7ZBK1;Q5JVE8;P00742
p = 0.51, beta = −0.0524, N = 325
ACIPTGPYPCGK pc2
B7ZBK1;Q5JVE8;P00742
p = 0.79, beta = −0.0212, N = 325
GYTLADNGK pc2
B7ZBK1;Q5JVE8;P00742
p = 0.57, beta = −0.0453, N = 324
ETYDFDIAVLR pc2
P00742
p = 0.79, beta = 0.0215, N = 325
IVGGQECK pc2
B7ZBK1;Q5JVE8;P00742
p = 0.54, beta = −0.0491, N = 323
TGIVSGFGR pc2
P00742
p = 0.81, beta = 0.0196, N = 325
NTEQEEGGEAVHEVEVVIK pc3
P00742
p = 0.97, beta = 0.00257, N = 323
MNVAPACLPER pc2
P00742
p = 0.65, beta = −0.0363, N = 325
QEDACQGDSGGPHVTR pc3
P00742
p = 0.76, beta = −0.0249, N = 325
DTYFVTGIVSWGEGCAR pc2
P00742
p = 0.16, beta = −0.112, N = 324
VGDRNTEQEEGGEAVHEVEVVIK pc
P00742
p = 0.69, beta = −0.0314, N = 325
DWAESTLMTQK pc2
P00742
p = 0.33, beta = −0.0787, N = 325
TNEFWNK pc2
B7ZBK1;F8WBM7;Q5JVE8;P0074
p = 0.75, beta = −0.026, N = 325
EQANNILAR pc2
B7ZBK1;F8WBM7;Q5JVE8;P0074
p = 0.74, beta = −0.0268, N = 325
MNVAPACLPERDWAESTLMTQK pc
P00742
p = 0.46, beta = −0.0932, N = 116
LSSSFIITQNMFCAGYDTK pc3
P00742
p = 0.71, beta = 0.0339, N = 232
NCELFTRK pc2
B7ZBK1;Q5JVE8;P00742
p = 0.56, beta = −0.184, N = 28
Assay Target: F10
UniProt: P00742
rsID: rs3211757
Target SNP: 13:113135954:!:AC
Proxy SNP: 13:113790269:T:C
deCODE log10(p): 75
deCODE BETA: −0.16
deCODE MSPA: 0.038
1256:1256:1256:1256:1139:125

191. FASN (P49327)
deCODE MSPA=0.319

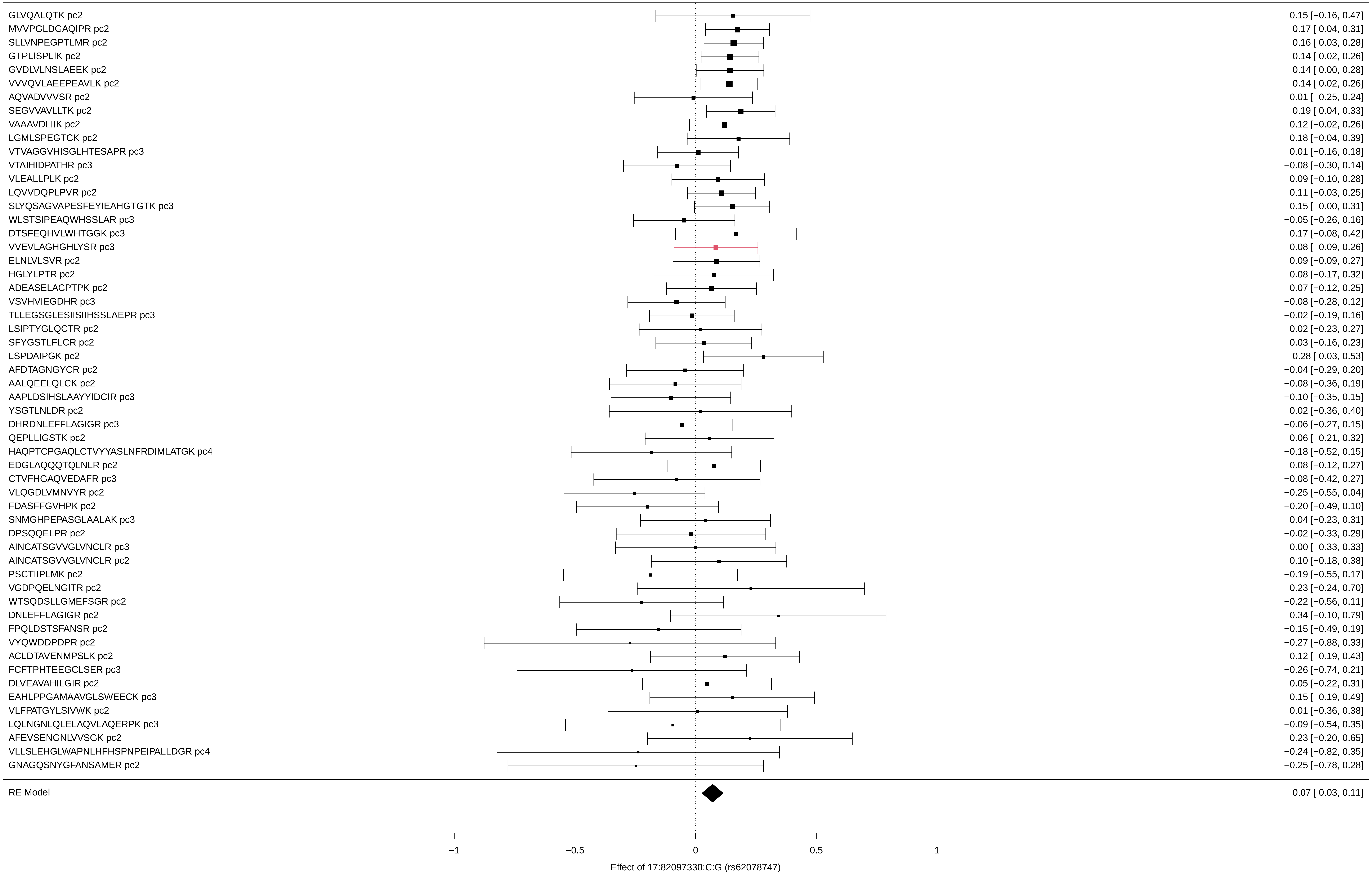

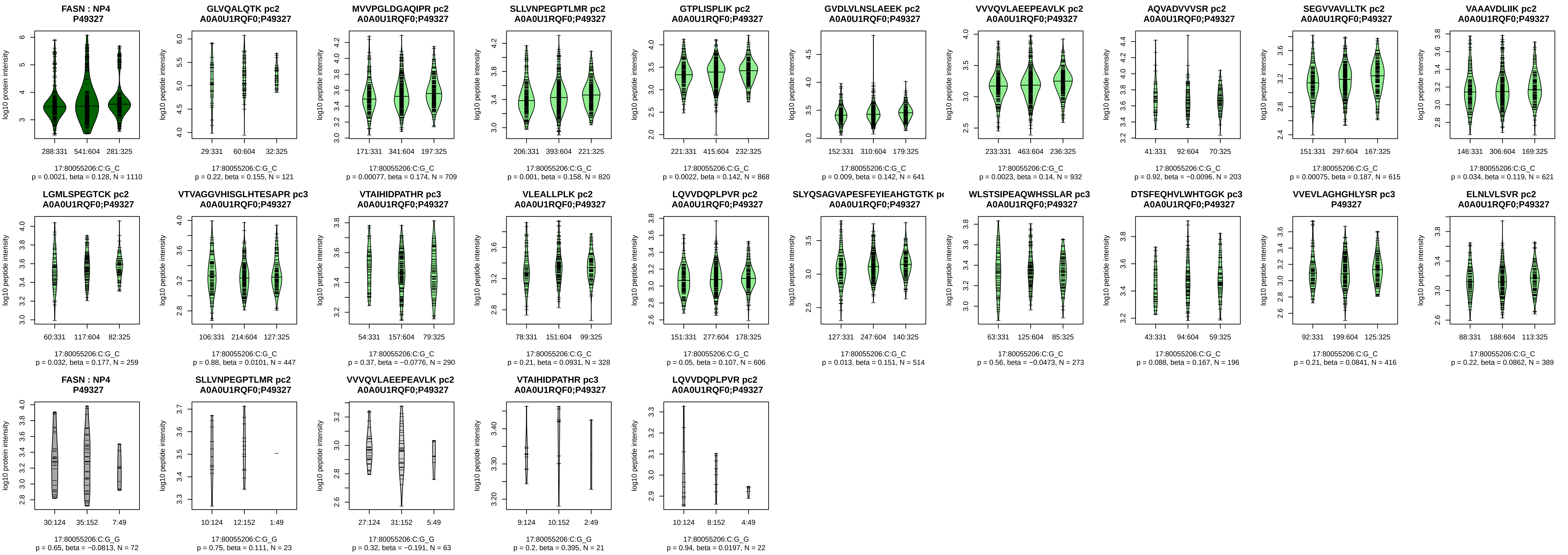

Assay Target: FASN
UniProt: P49327
rsID: rs62078747
Target SNP: 17:82097330:C:G
Proxy SNP: 17:80055206:C:G
deCODE log10(p): 74.2
deCODE BETA: 0.15
deCODE MSPA: 0.319
121:709:820:868:641:932:203:6

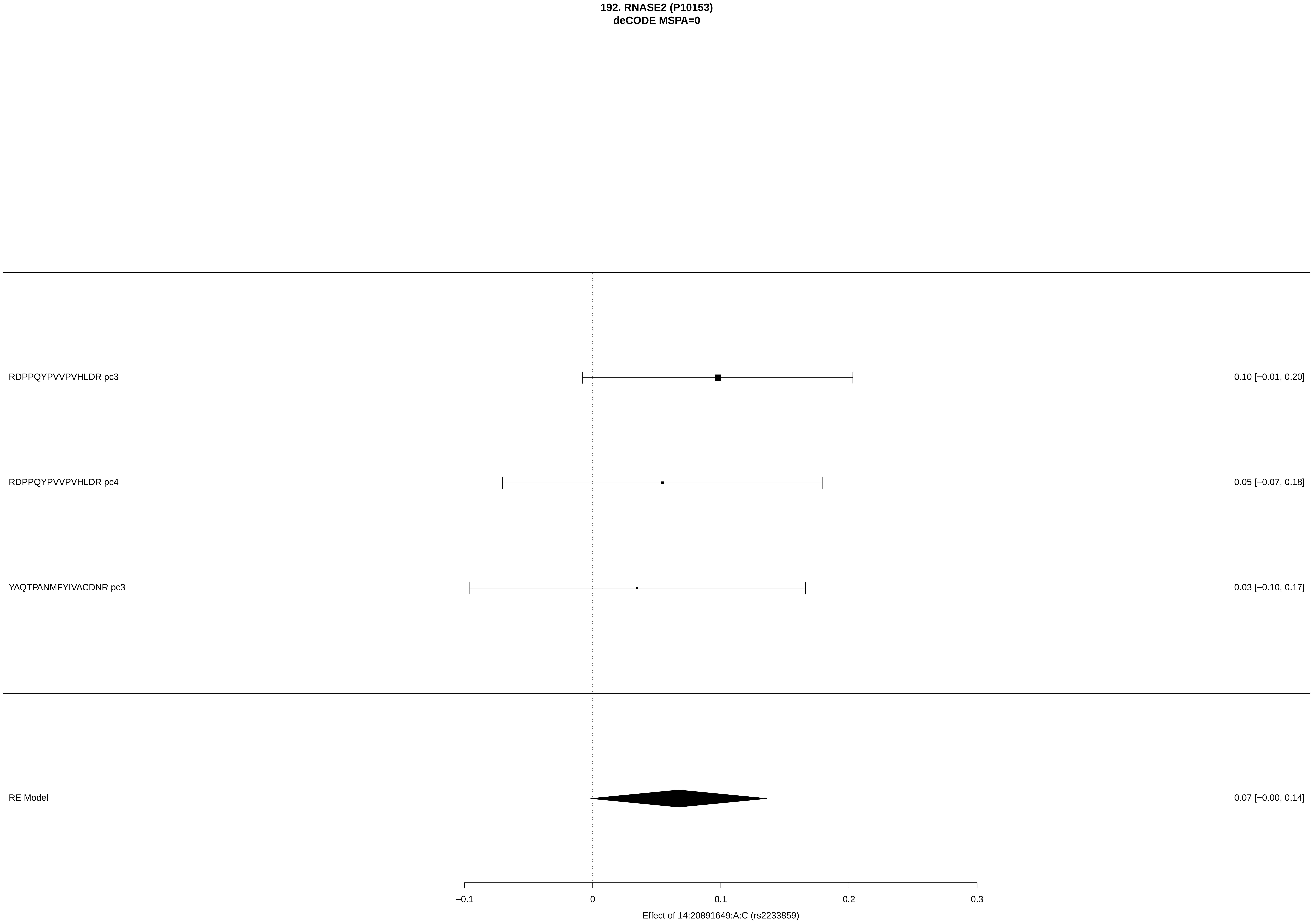

192. RNASE2 (P10153)
deCODE MSPA=0
RDPPQYPVVPVHLDR pc3
0.10 [−0.01, 0.20]
RDPPQYPVVPVHLDR pc4
0.05 [−0.07, 0.18]
YAQTPANMFYIVACDNR pc3
0.03 [−0.10, 0.17]
RE Model
0.07 [−0.00, 0.14]
−0.1
0
0.1
0.2
0.3
Effect of 14:20891649:A:C (rs2233859)

RNASE2 : NP1
P10153

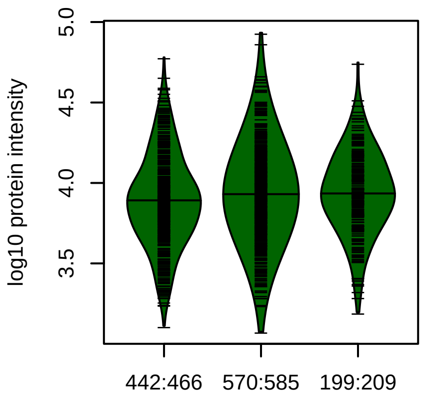

14:21359808:C:A_A
p = 0.013, beta = 0.102, N = 1211

RDPPQYPVVPVHLDR pc3
P10153

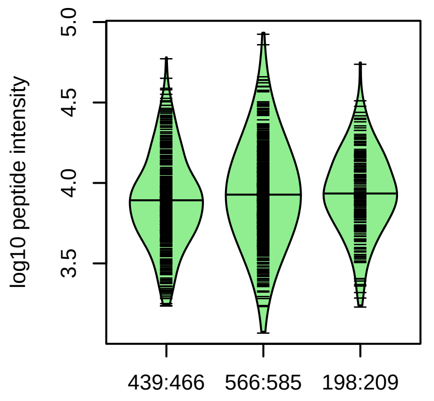

14:21359808:C:A_A
p = 0.017, beta = 0.0976, N = 1203

YAQTPANMFYIVACDNR pc3
P10153

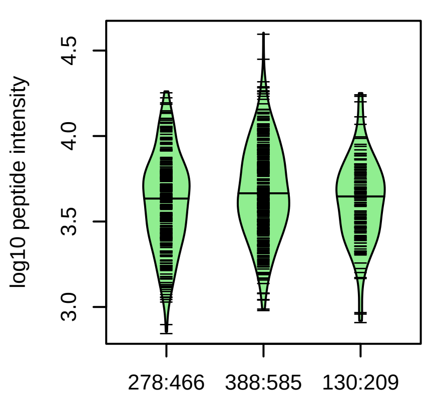

14:21359808:C:A_A
p = 0.49, beta = 0.0348, N = 796

RNASE2 : NP1
P10153

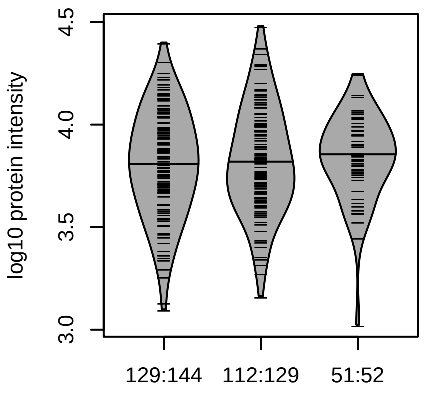

14:21359808:C:A_A
p = 0.5, beta = 0.0526, N = 292

RDPPQYPVVPVHLDR pc3
P10153

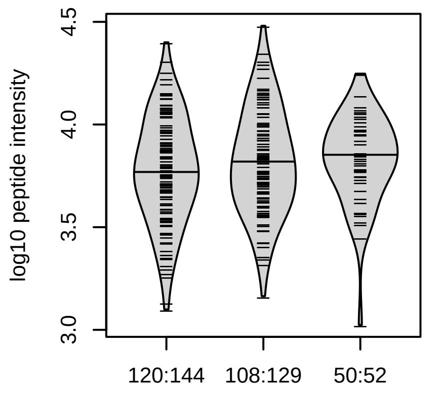

14:21359808:C:A_A
p = 0.19, beta = 0.106, N = 278

Assay Target: RNASE2
UniProt: P10153
rsID: rs2233859
Target SNP: 14:20891649:A:C
Proxy SNP: 14:21359808:C:A
deCODE log10(p): 73.8
deCODE BETA: 0.14
deCODE MSPA: 0
--:--:--
1203:862:796

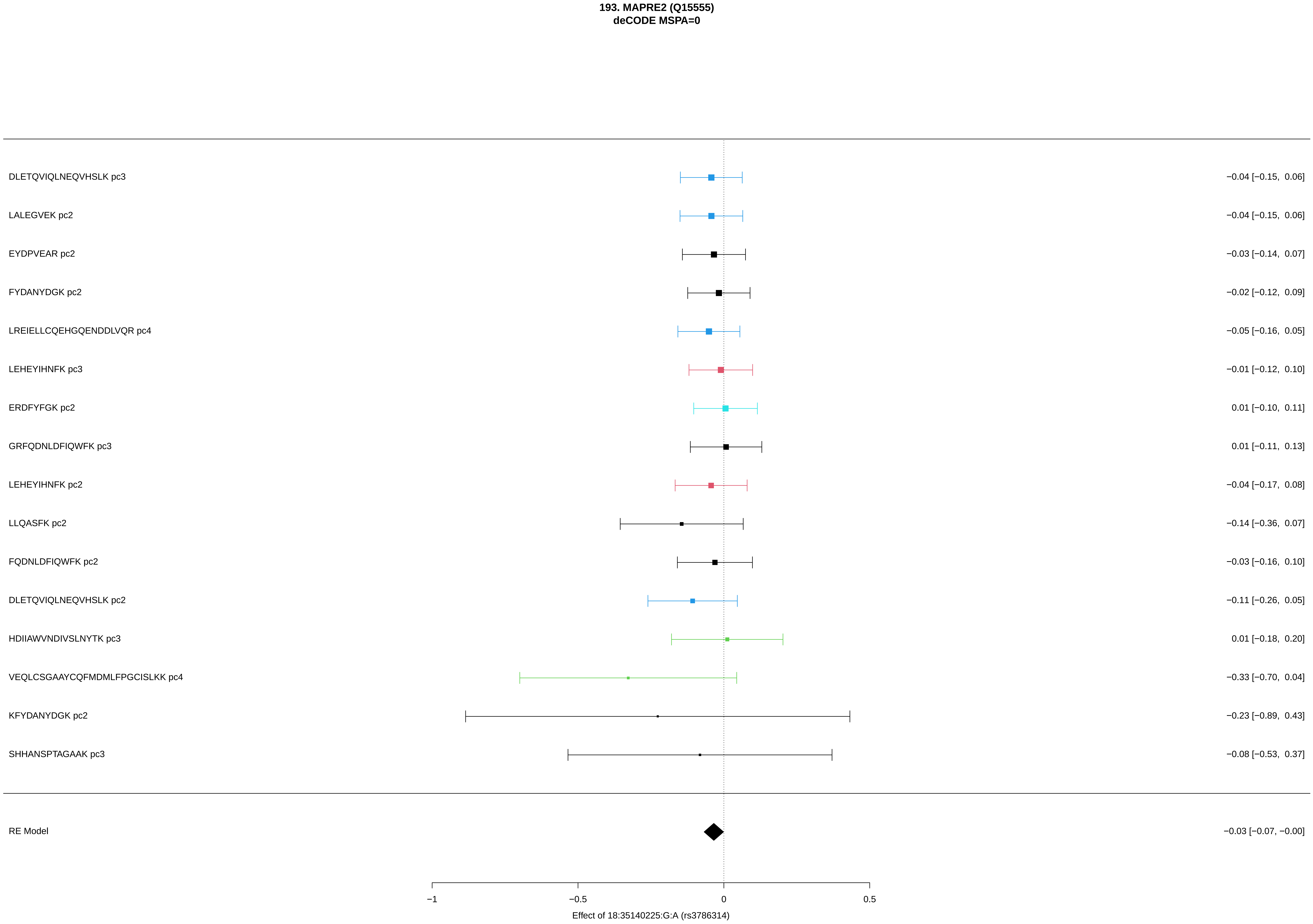
193. MAPRE2 (Q15555)
deCODE MSPA=0
DLETQVIQLNEQVHSLK pc3
LALEGVEK pc2
EYDPVEAR pc2
FYDANYDGK pc2
LREIELLCQEHGQENDDLVQR pc4
LEHEYIHNFK pc3
ERDFYFGK pc2
GRFQDNLDFIQWFK pc3
LEHEYIHNFK pc2
LLQASFK pc2
FQDNLDFIQWFK pc2
DLETQVIQLNEQVHSLK pc2
HDIIAWVNDIVSLNYTK pc3
VEQLCSGAAYCQFMDMLFPGCISLKK pc4
KFYDANYDGK pc2
SHHANSPTAGAAK pc3
RE Model
−0.04 [−0.15, 0.06]
−0.04 [−0.15, 0.06]
−0.03 [−0.14, 0.07]
−0.02 [−0.12, 0.09]
−0.05 [−0.16, 0.05]
−0.01 [−0.12, 0.10]
0.01 [−0.10, 0.11]
0.01 [−0.11, 0.13]
−0.04 [−0.17, 0.08]
−0.14 [−0.36, 0.07]
−0.03 [−0.16, 0.10]
−0.11 [−0.26, 0.05]
0.01 [−0.18, 0.20]
−0.33 [−0.70, 0.04]
−0.23 [−0.89, 0.43]
−0.08 [−0.53, 0.37]
−0.03 [−0.07, −0.00]
−1
−0.5
0
0.5
Effect of 18:35140225:G:A (rs3786314)

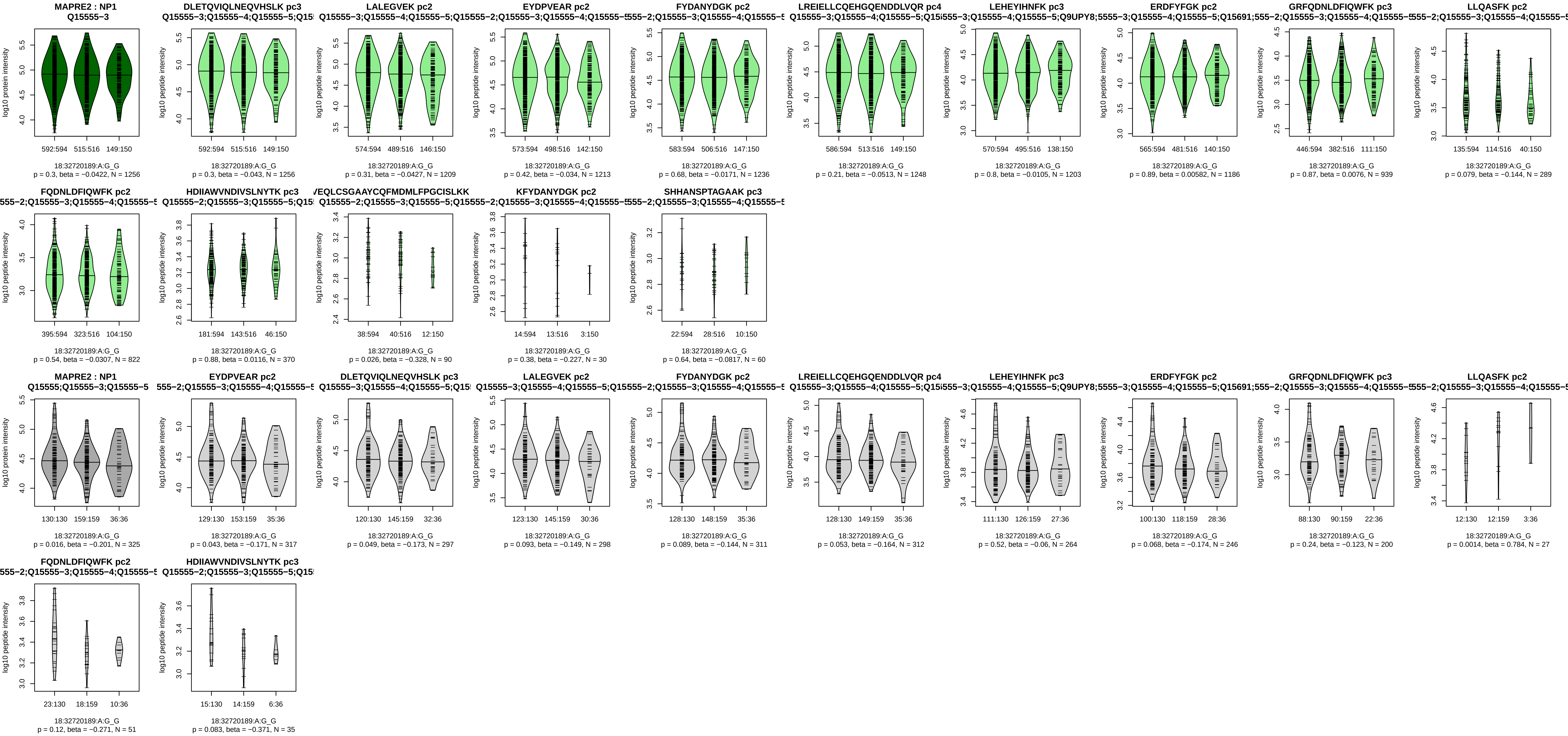

Assay Target: MAPRE2
UniProt: Q15555
rsID: rs3786314
Target SNP: 18:35140225:G:A
Proxy SNP: 18:32720189:A:G
deCODE log10(p): 72.9
deCODE BETA: −0.15
deCODE MSPA: 0
1256:1209:1213:1236:1248:120

194. SAR1A (Q9NR31)
deCODE MSPA=0.342

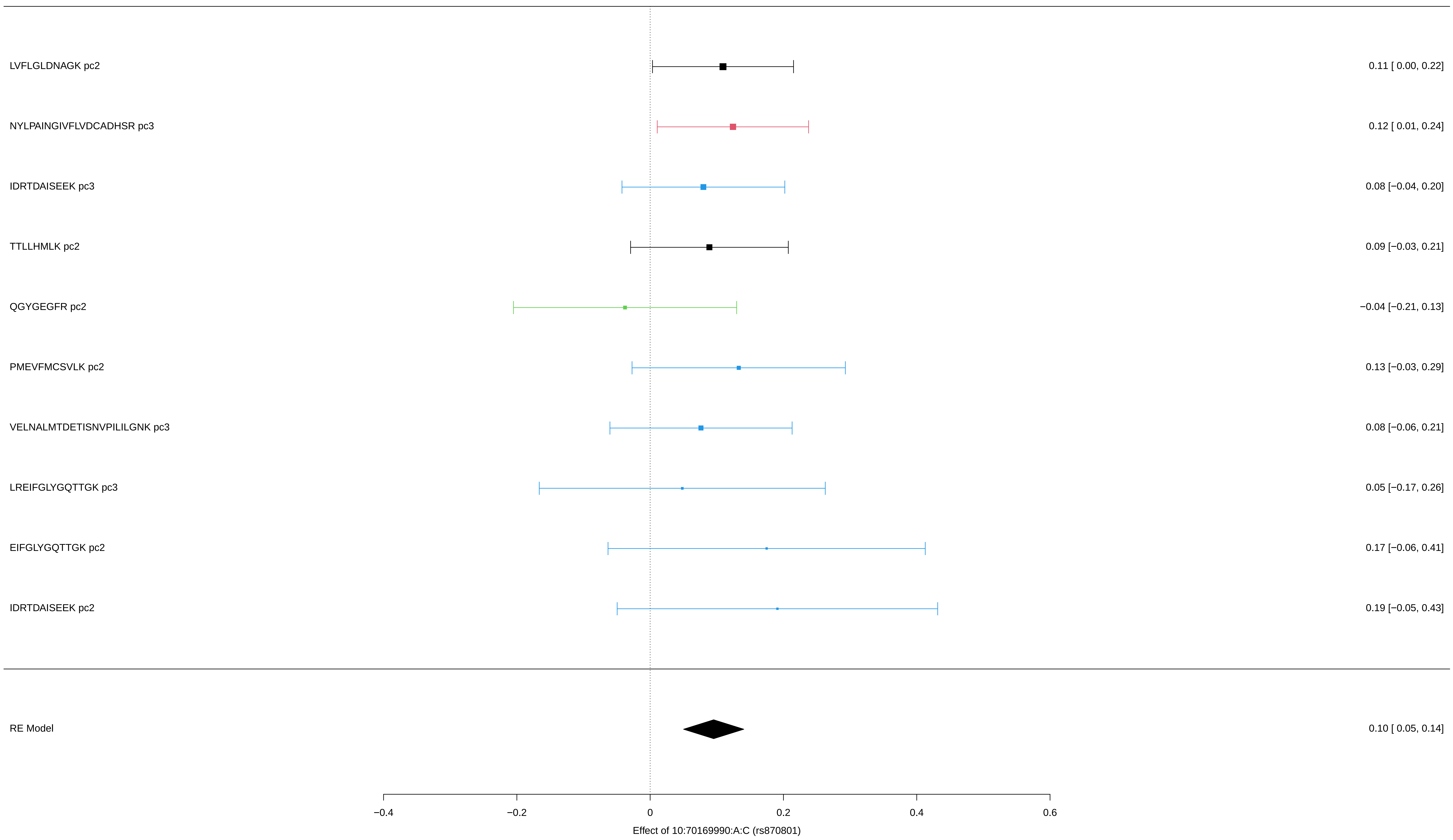

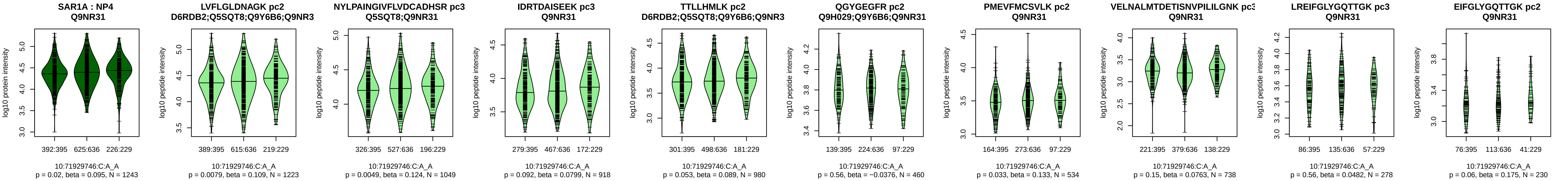

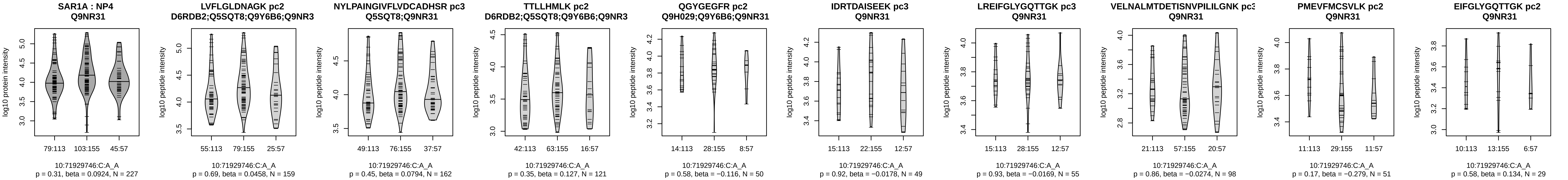

Assay Target: SAR1A
UniProt: Q9NR31
rsID: rs870801
Target SNP: 10:70169990:A:C
Proxy SNP: 10:71929746:C:A
deCODE log10(p): 72.2
deCODE BETA: 0.15
deCODE MSPA: 0.342
::–:–:–:–:–:–:–:–
1223:1049:918:980:460:534:738

195. HAGH (Q16775)
deCODE MSPA=0

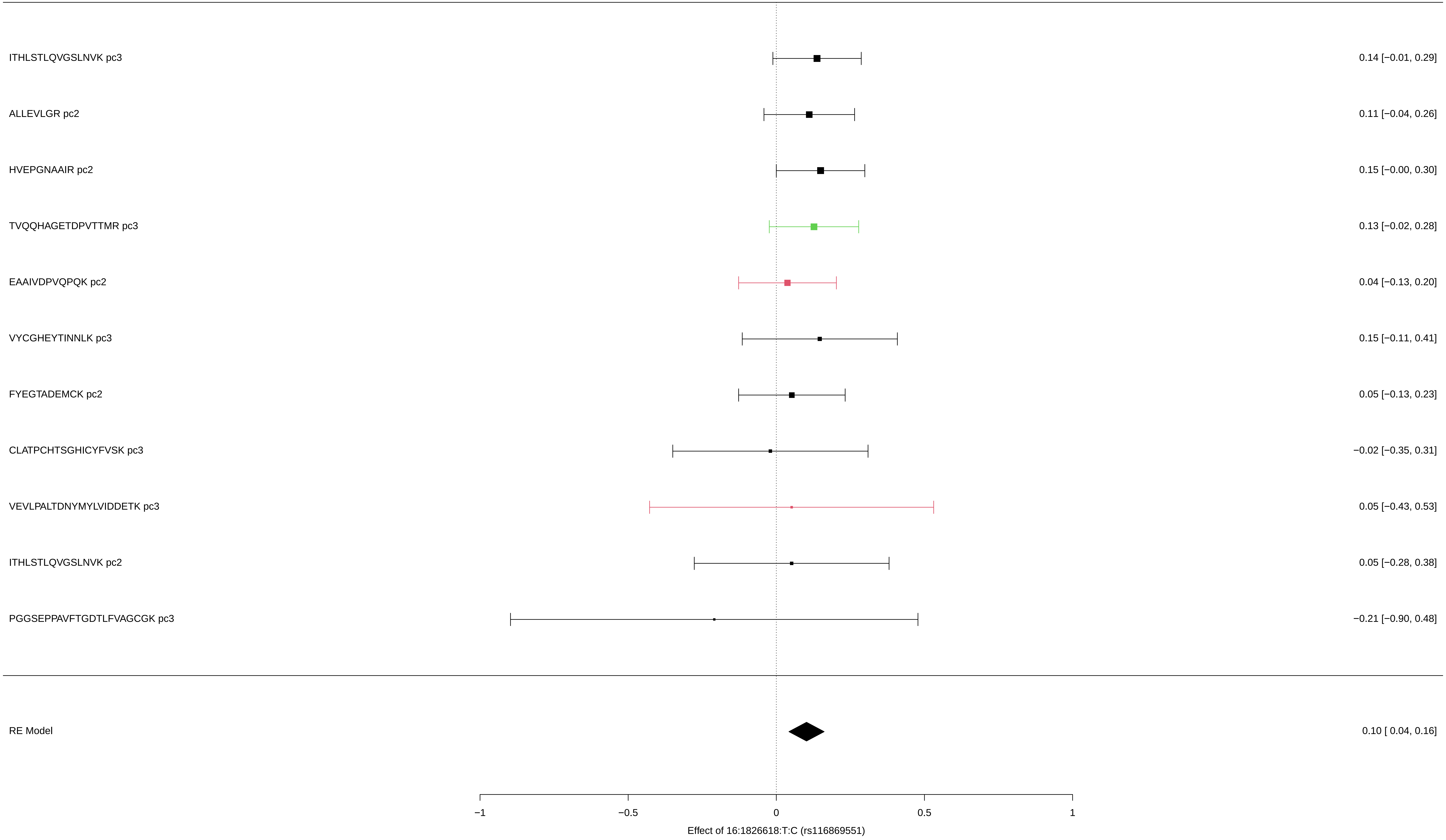

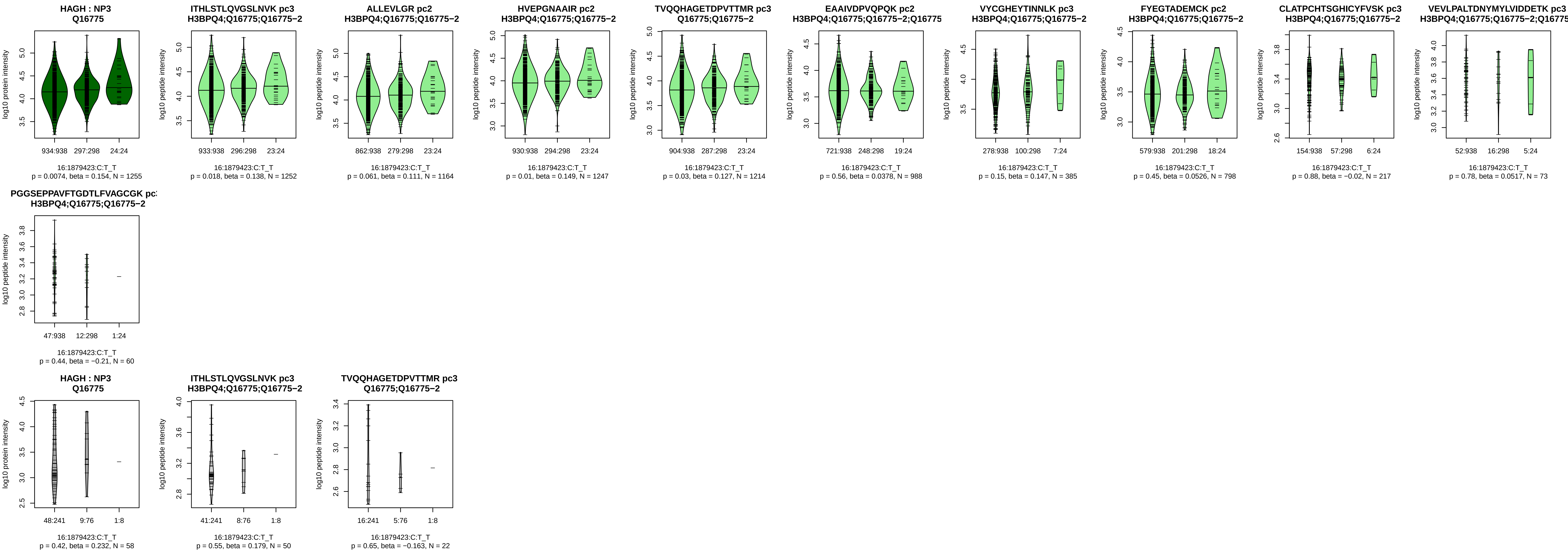

Assay Target: HAGH
UniProt: Q16775
rsID: rs116869551
Target SNP: 16:1826618:T:C
Proxy SNP: 16:1879423:C:T
deCODE log10(p): 72.2
deCODE BETA: 0.21
deCODE MSPA: 0
−:−:−:−:−:−:−:−:NA:−:NA:NA
1252:1164:1247:1214:988:385:7

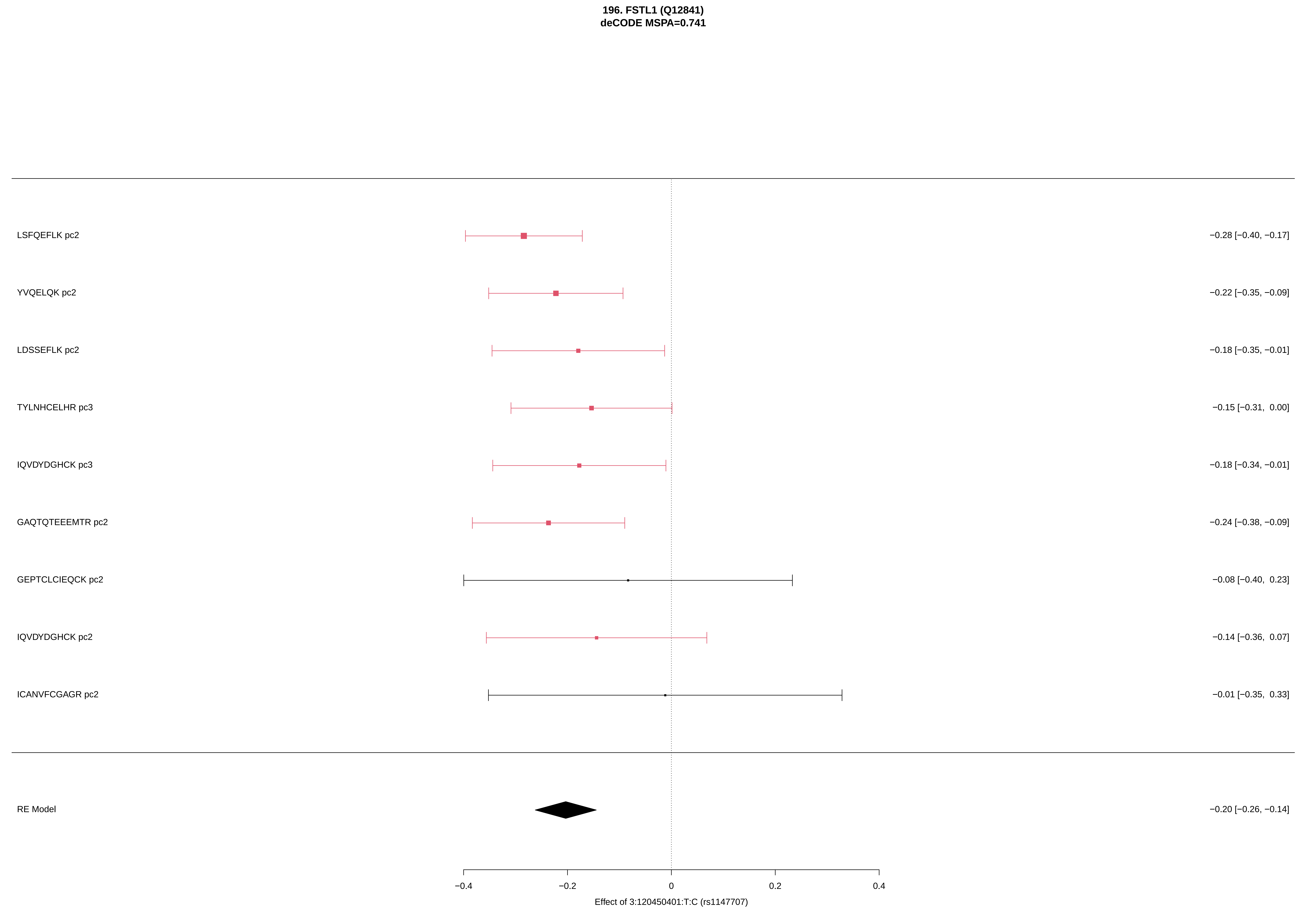
196. FSTL1 (Q12841)
deCODE MSPA=0.741
LSFQEFLK pc2
−0.28 [−0.40, −0.17]
YVQELQK pc2
−0.22 [−0.35, −0.09]
LDSSEFLK pc2
−0.18 [−0.35, −0.01]
TYLNHCELHR pc3
−0.15 [−0.31, 0.00]
IQVDYDGHCK pc3
−0.18 [−0.34, −0.01]
GAQTQTEEEMTR pc2
−0.24 [−0.38, −0.09]
GEPTCLCIEQCK pc2
−0.08 [−0.40, 0.23]
IQVDYDGHCK pc2
−0.14 [−0.36, 0.07]
ICANVFCGAGR pc2
−0.01 [−0.35, 0.33]
RE Model
−0.20 [−0.26, −0.14]
−0.4
−0.2
0
0.2
0.4
Effect of 3:120450401:T:C (rs1147707)

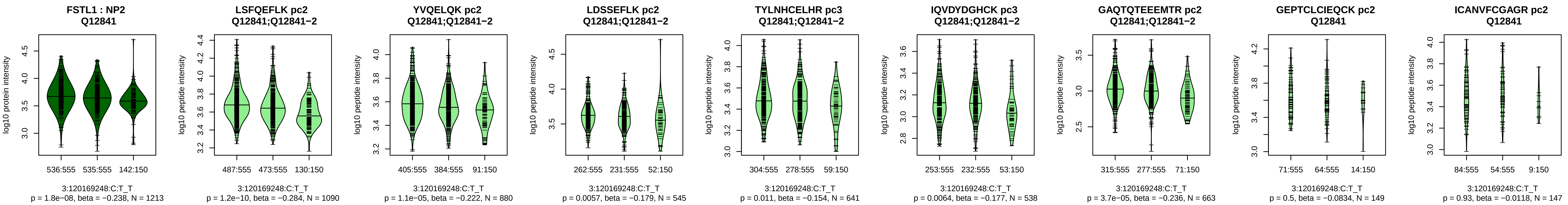

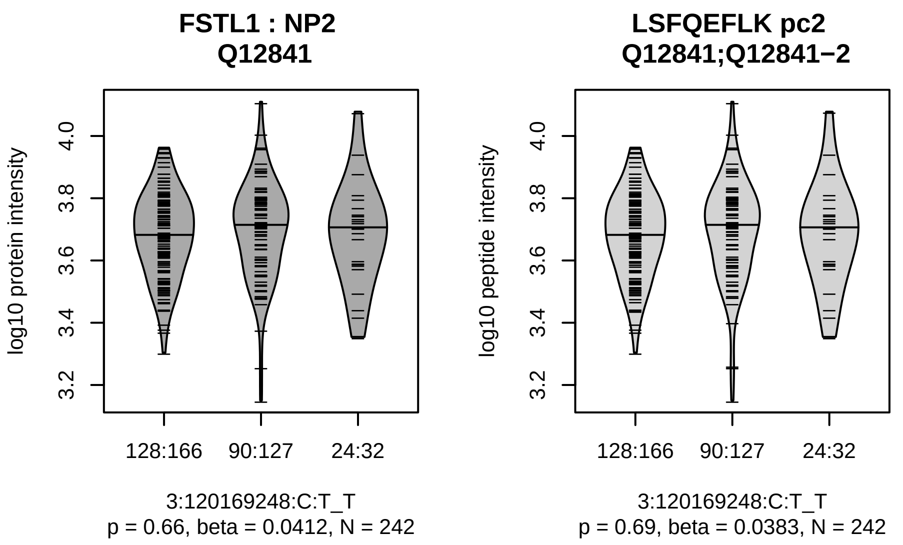

Assay Target: FSTL1
UniProt: Q12841
rsID: rs1147707
Target SNP: 3:120450401:T:C
Proxy SNP: 3:120169248:C:T
deCODE log10(p): 69.8
deCODE BETA: −0.15
deCODE MSPA: 0.741
::*:–:*:*:–:–:–
1090:880:545:641:538:663:149:

197. PROC (P04070)
deCODE MSPA=0

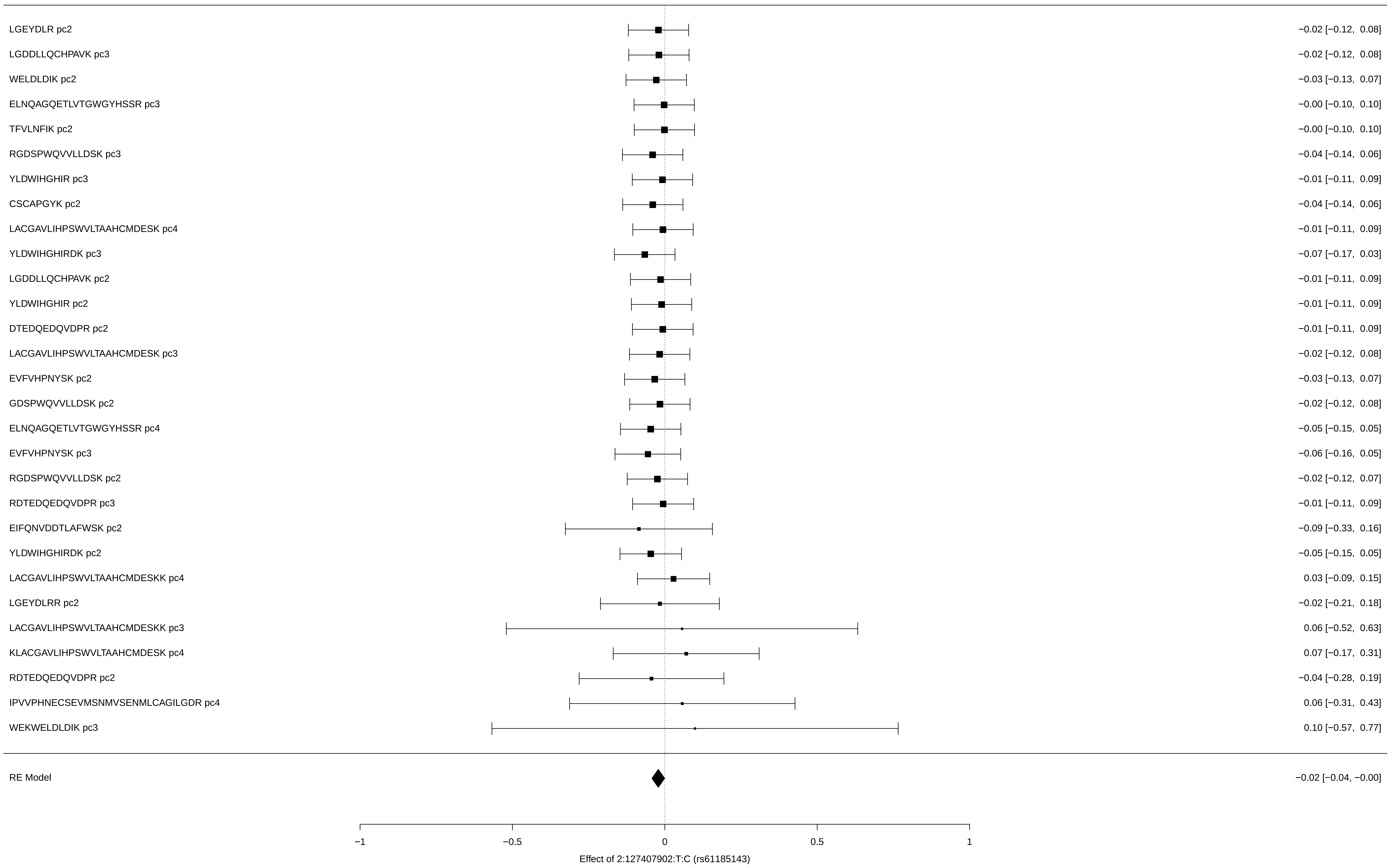

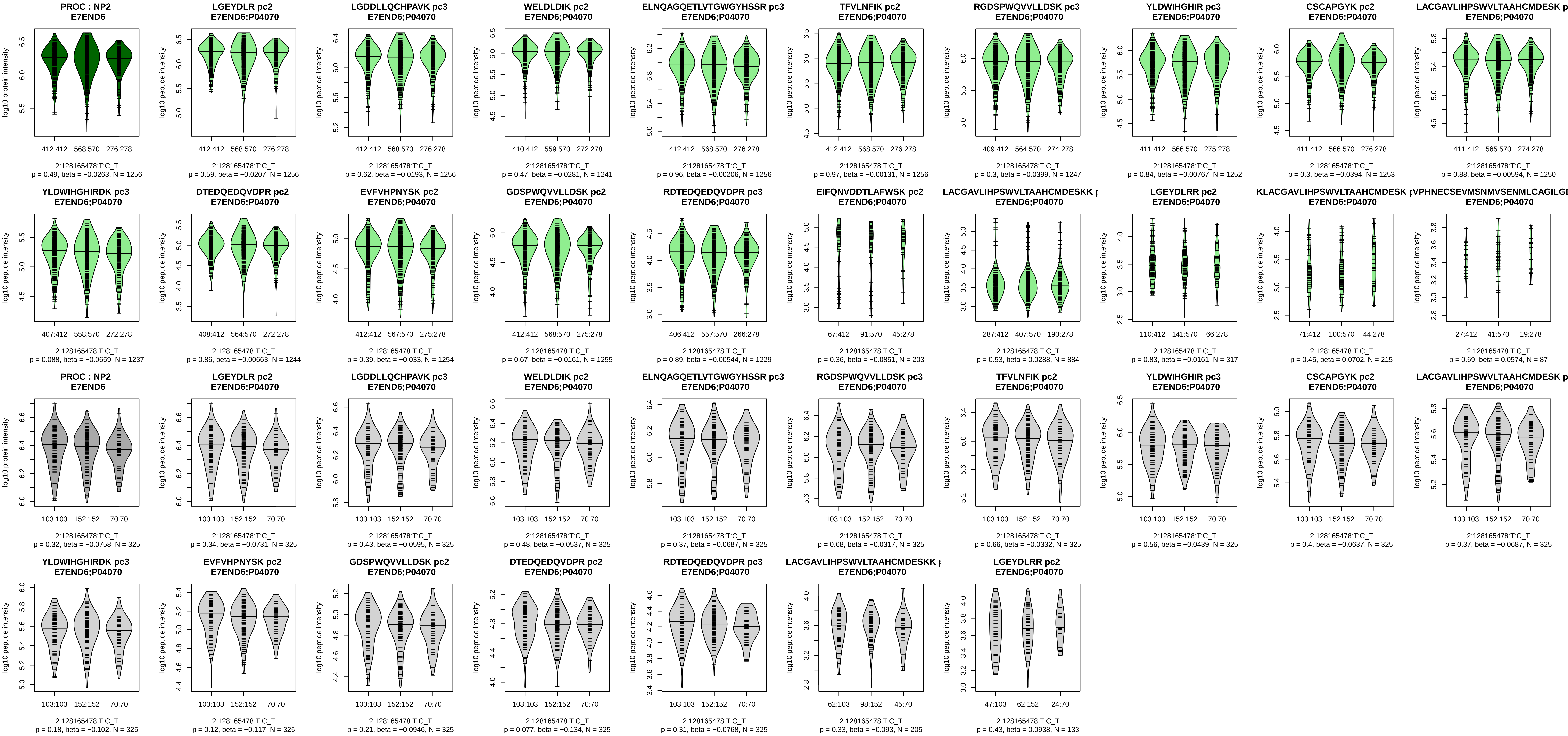

Assay Target: PROC
UniProt: P04070
rsID: rs61185143
Target SNP: 2:127407902:T:C
Proxy SNP: 2:128165478:T:C
deCODE log10(p): 69.8
deCODE BETA: −0.14
deCODE MSPA: 0
1256:1256:1241:1256:1256:124

198. HHIP (Q96QV1)
deCODE MSPA=0

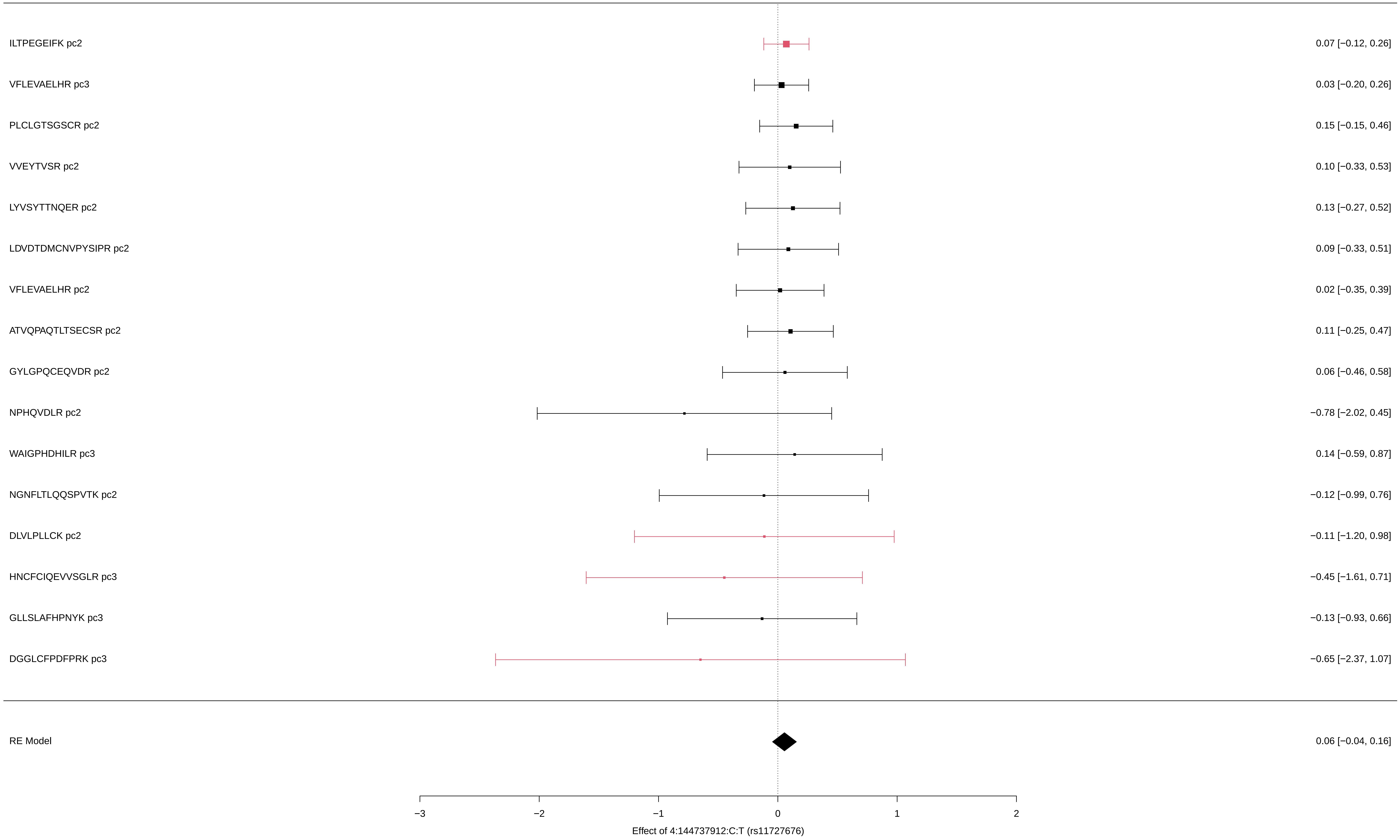

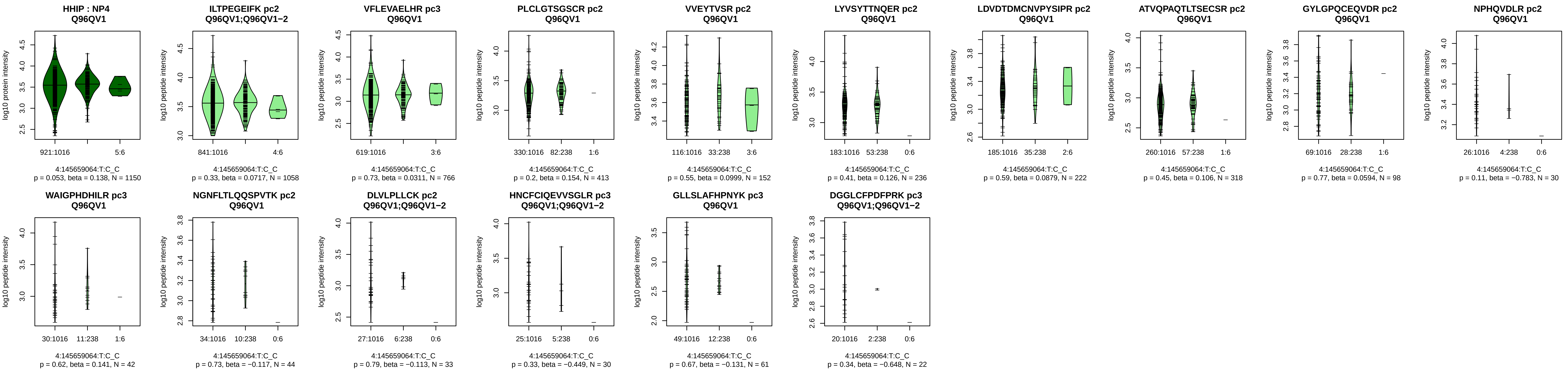

Assay Target: HHIP
UniProt: Q96QV1
rsID: rs11727676
Target SNP: 4:144737912:C:T
Proxy SNP: 4:145659064:T:C
deCODE log10(p): 69.3
deCODE BETA: 0.24
deCODE MSPA: 0
−:−:−:−:−:−:−:−:−:−:−:−:−:NA:−
1058:766:413:152:236:222:268:

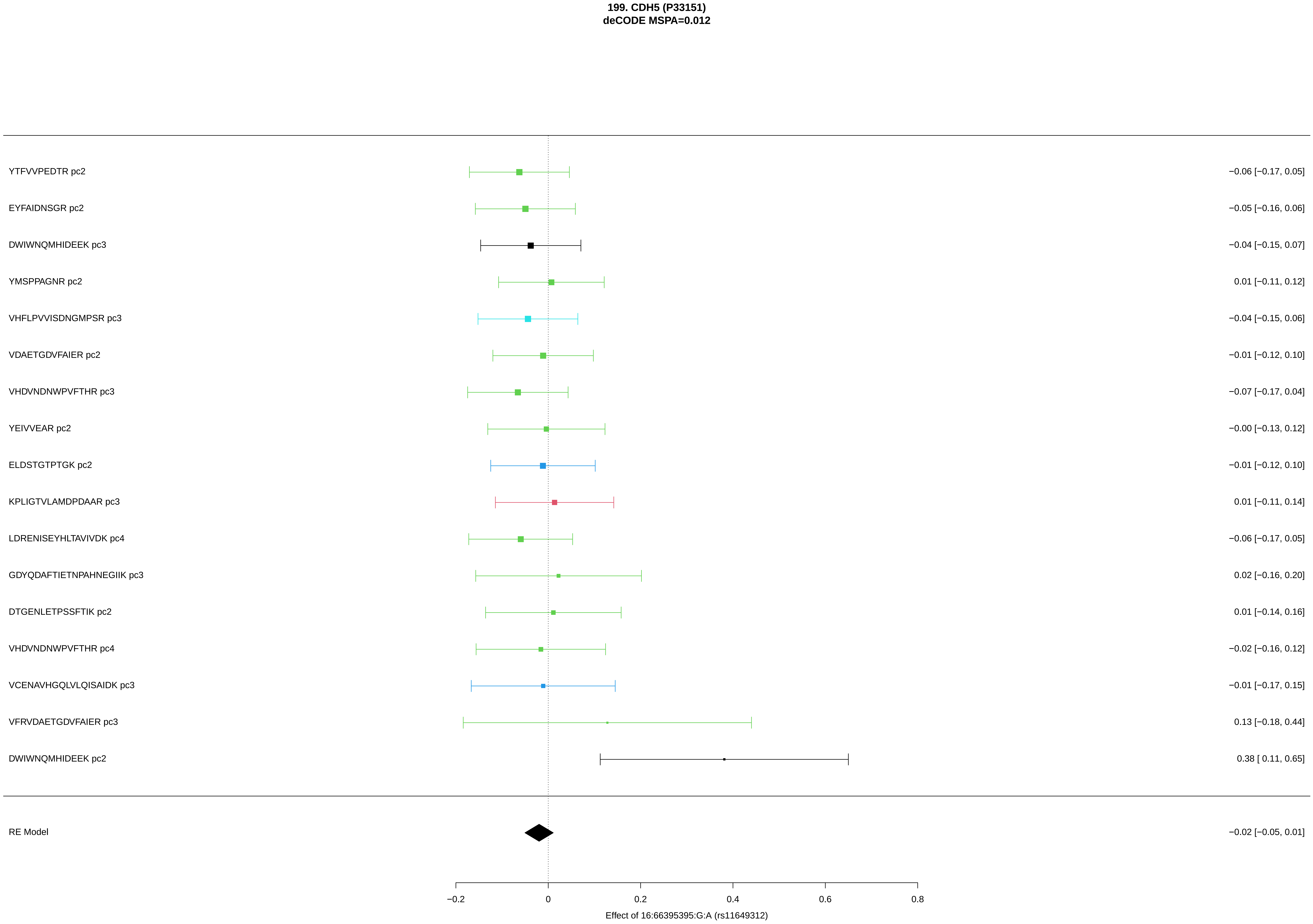

199. CDH5 (P33151)
deCODE MSPA=0.012
YTFVVPEDTR pc2
−0.06 [−0.17, 0.05]
EYFAIDNSGR pc2
−0.05 [−0.16, 0.06]
DWIWNQMHIDEEK pc3
−0.04 [−0.15, 0.07]
YMSPPAGNR pc2
0.01 [−0.11, 0.12]
VHFLPVVISDNGMPSR pc3
−0.04 [−0.15, 0.06]
VDAETGDVFAIER pc2
−0.01 [−0.12, 0.10]
VHDVNDNWPVFTHR pc3
−0.07 [−0.17, 0.04]
YEIVVEAR pc2
−0.00 [−0.13, 0.12]
ELDSTGTPTGK pc2
−0.01 [−0.12, 0.10]
KPLIGTVLAMDPDAAR pc3
0.01 [−0.11, 0.14]
LDRENISEYHLTAVIVDK pc4
−0.06 [−0.17, 0.05]
GDYQDAFTIETNPAHNEGIIK pc3
0.02 [−0.16, 0.20]
DTGENLETPSSFTIK pc2
0.01 [−0.14, 0.16]
VHDVNDNWPVFTHR pc4
−0.02 [−0.16, 0.12]
VCENAVHGQLVLQISAIDK pc3
−0.01 [−0.17, 0.15]
VFRVDAETGDVFAIER pc3
0.13 [−0.18, 0.44]
DWIWNQMHIDEEK pc2
0.38 [0.11, 0.65]
RE Model
−0.02 [−0.05, 0.01]
−0.2
0
0.2
0.4
0.6
0.8
Effect of 16:66395395:G:A (rs11649312)

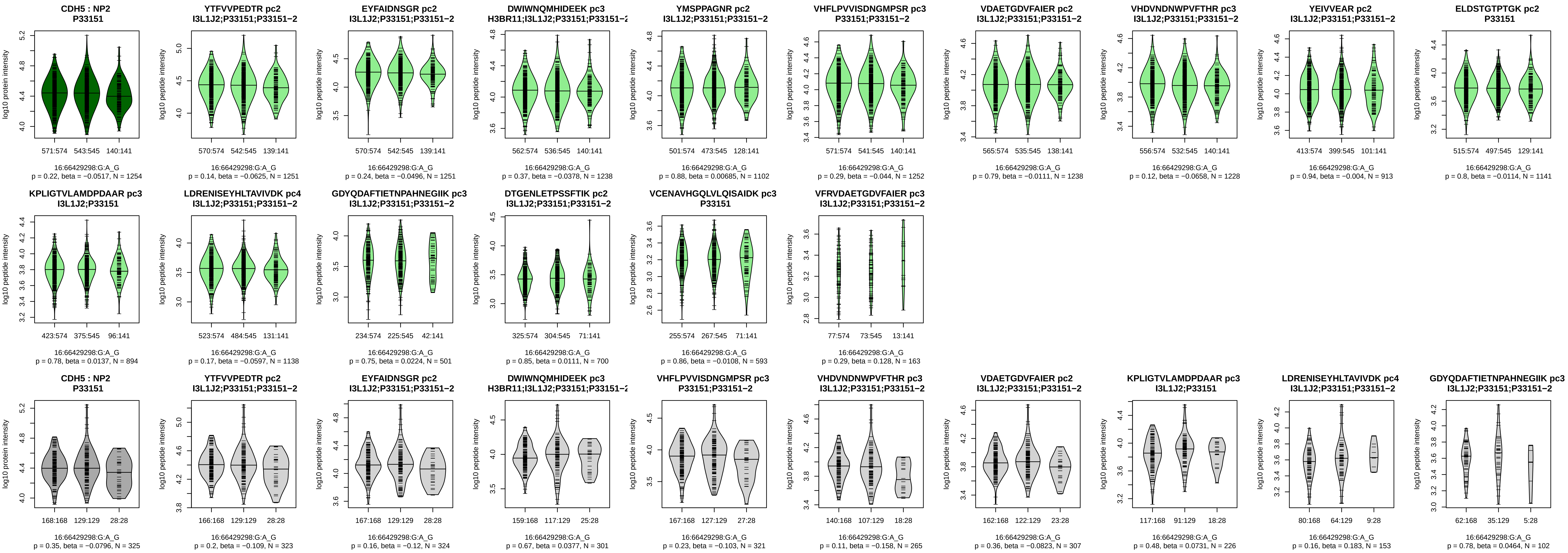

Assay Target: CDH5
UniProt: P33151
rsID: rs11649312
Target SNP: 16:66395395:G:A
Proxy SNP: 16:66429298:G:A
deCODE log10(p): 68.6
deCODE BETA: −0.15
deCODE MSPA: 0.012
1251:1251:1238:1102:1252:123

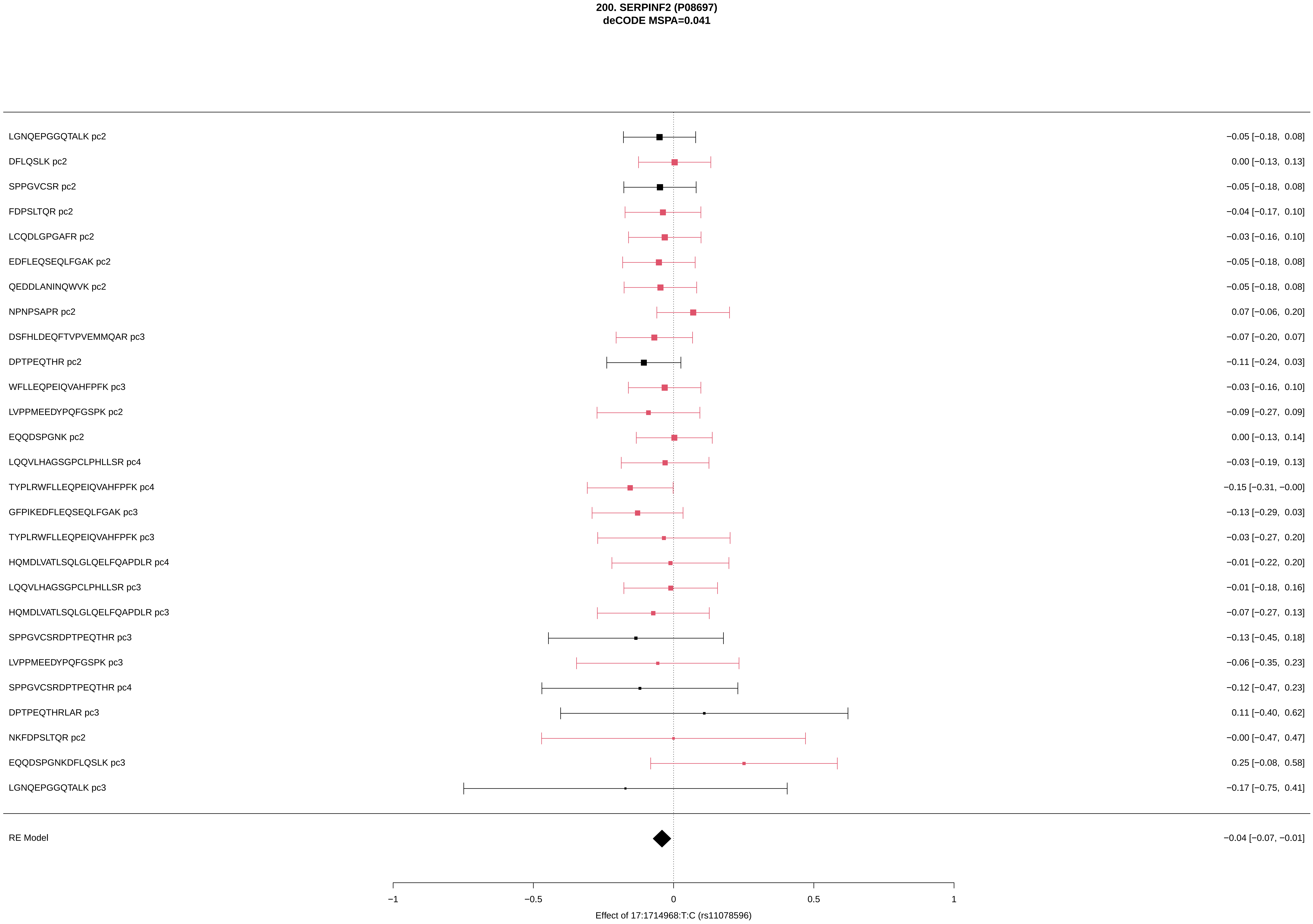
200. SERPINF2 (P08697)
deCODE MSPA=0.041
LGNQEPGGQTALK pc2 −0.05 [−0.18, 0.08]
DFLQSLK pc2 0.00 [−0.13, 0.13]
SPPGVCSR pc2 −0.05 [−0.18, 0.08]
FDPSLTQR pc2 −0.04 [−0.17, 0.10]
LCQDLGPGAFR pc2 −0.03 [−0.16, 0.10]
EDFLEQSEQLFGAK pc2 −0.05 [−0.18, 0.08]
QEDDLANINQWVK pc2 −0.05 [−0.18, 0.08]
NPNPSAPR pc2 0.07 [−0.06, 0.20]
DSFHLDEQFTVPVEMMQAR pc3 −0.07 [−0.20, 0.07]
DPTPEQTHR pc2 −0.11 [−0.24, 0.03]
WFLLEQPEIQVAHFPFK pc3 −0.03 [−0.16, 0.10]
LVPPMEEDYPQFGSPK pc2 −0.09 [−0.27, 0.09]
EQQDSPGNK pc2 0.00 [−0.13, 0.14]
LQQVLHAGSGPCLPHLLSR pc4 −0.03 [−0.19, 0.13]
TYPLRWFLLEQPEIQVAHFPFK pc4 −0.15 [−0.31, −0.00]
GFPIKEDFLEQSEQLFGAK pc3 −0.13 [−0.29, 0.03]
TYPLRWFLLEQPEIQVAHFPFK pc3 −0.03 [−0.27, 0.20]
HQMDLVATLSQLGLQELFQAPDLR pc4 −0.01 [−0.22, 0.20]
LQQVLHAGSGPCLPHLLSR pc3 −0.01 [−0.18, 0.16]
HQMDLVATLSQLGLQELFQAPDLR pc3 −0.07 [−0.27, 0.13]
SPPGVCSRDPTPEQTHR pc3 −0.13 [−0.45, 0.18]
LVPPMEEDYPQFGSPK pc3 −0.06 [−0.35, 0.23]
SPPGVCSRDPTPEQTHR pc4 −0.12 [−0.47, 0.23]
DPTPEQTHRLAR pc3 0.11 [−0.40, 0.62]
NKFDPSLTQR pc2 −0.00 [−0.47, 0.47]
EQQDSPGNKDFLQSLK pc3 0.25 [−0.08, 0.58]
LGNQEPGGQTALK pc3 −0.17 [−0.75, 0.41]
RE Model −0.04 [−0.07, −0.01]
−1
−0.5
0
0.5
1
Effect of 17:1714968:T:C (rs11078596)

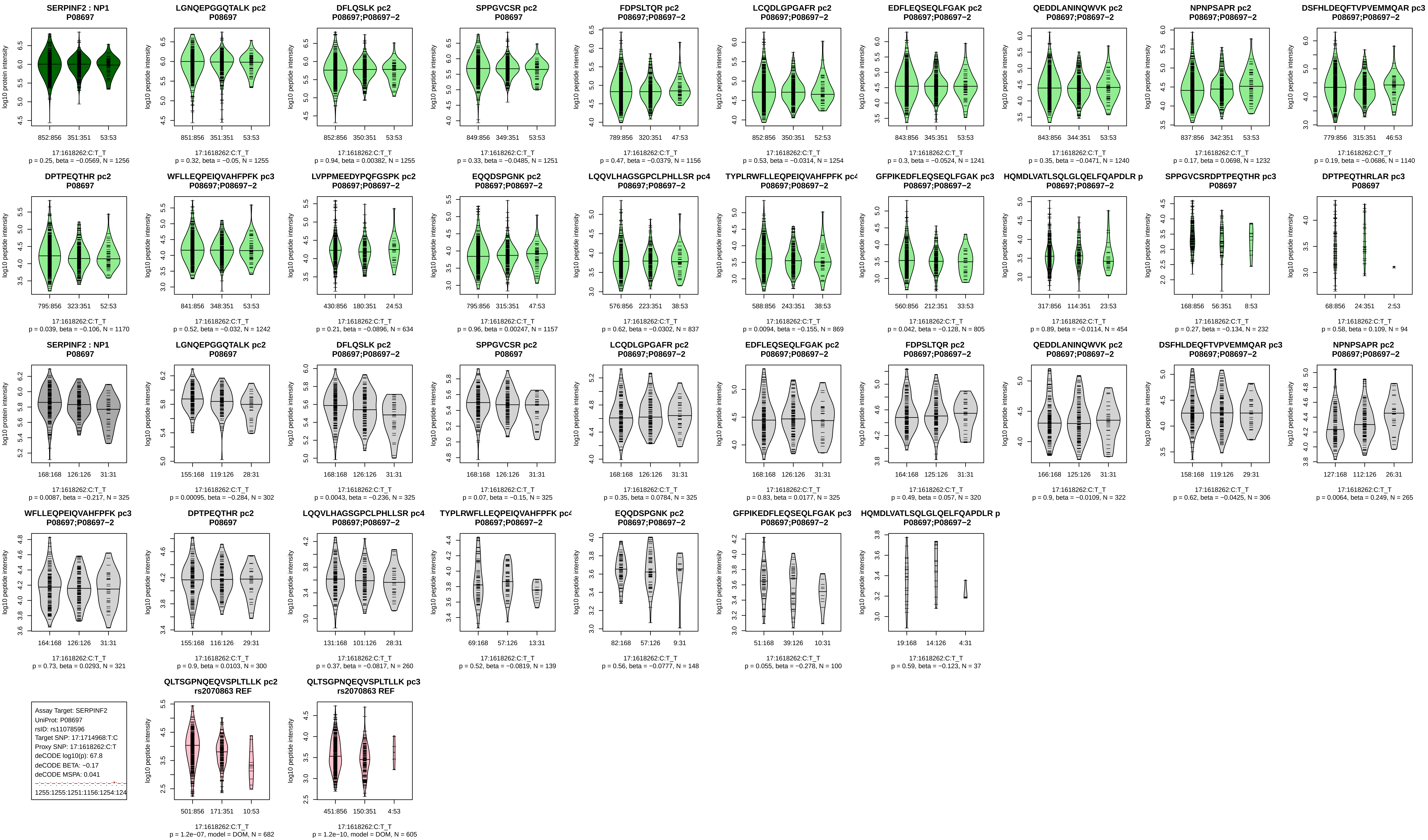

SERPINF2 : NP1
P08697
log10 protein intensity
852:856
351:351
53:53
17:1618262:C:T_T
p = 0.25, beta = −0.0569, N = 1256
LGNQEPGGQTALK pc2
P08697
log10 peptide intensity
851:856
351:351
53:53
17:1618262:C:T_T
p = 0.32, beta = −0.05, N = 1255
DFLQSLK pc2
P08697;P08697−2
852:856
350:351
53:53
17:1618262:C:T_T
p = 0.94, beta = 0.00382, N = 1255
SPPGVCSR pc2
P08697
849:856
349:351
53:53
17:1618262:C:T_T
p = 0.33, beta = −0.0485, N = 1251
FDPSLTQR pc2
P08697;P08697−2
789:856
320:351
47:53
17:1618262:C:T_T
p = 0.47, beta = −0.0379, N = 1156
LCQDLGPGAFR pc2
P08697;P08697−2
852:856
350:351
52:53
17:1618262:C:T_T
p = 0.53, beta = −0.0314, N = 1254
EDFLEQSEQLFGAK pc2
P08697;P08697−2
843:856
345:351
53:53
17:1618262:C:T_T
p = 0.3, beta = −0.0524, N = 1241
QEDDLANINQWVK pc2
P08697;P08697−2
843:856
344:351
53:53
17:1618262:C:T_T
p = 0.35, beta = −0.0471, N = 1240
NPNPSAPR pc2
P08697;P08697−2
837:856
342:351
53:53
17:1618262:C:T_T
p = 0.17, beta = 0.0698, N = 1232
DSFHLDEQFTVPVEMMQAR pc3
P08697;P08697−2
779:856
315:351
46:53
17:1618262:C:T_T
p = 0.19, beta = −0.0686, N = 1140
DPTPEQTHR pc2
P08697
795:856
323:351
52:53
17:1618262:C:T_T
p = 0.039, beta = −0.106, N = 1170
WFLLEQPEIQVAHFPFK pc3
P08697;P08697−2
841:856
348:351
53:53
17:1618262:C:T_T
p = 0.52, beta = −0.032, N = 1242
LVPPMEEDYPQFGSPK pc2
P08697;P08697−2
430:856
180:351
24:53
17:1618262:C:T_T
p = 0.21, beta = −0.0896, N = 634
EQQDSPGNK pc2
P08697;P08697−2
795:856
315:351
47:53
17:1618262:C:T_T
p = 0.96, beta = 0.00247, N = 1157
LQQVLHAGSGPCLPHLLSR pc4
P08697;P08697−2
576:856
223:351
38:53
17:1618262:C:T_T
p = 0.62, beta = −0.0302, N = 837
TYPLRWFLLEQPEIQVAHFPFK pc4
P08697;P08697−2
588:856
243:351
38:53
17:1618262:C:T_T
p = 0.0094, beta = −0.155, N = 869
GFPIKEDFLEQSEQLFGAK pc3
P08697;P08697−2
560:856
212:351
33:53
17:1618262:C:T_T
p = 0.042, beta = −0.128, N = 805
HQMDLVATLSQLGLQELFQAPDLR p
P08697;P08697−2
317:856
114:351
23:53
17:1618262:C:T_T
p = 0.89, beta = −0.0114, N = 454
SPPGVCSRDPTPEQTHR pc3
P08697
168:856
56:351
8:53
17:1618262:C:T_T
p = 0.27, beta = −0.134, N = 232
DPTPEQTHRLAR pc3
P08697
68:856
24:351
2:53
17:1618262:C:T_T
p = 0.58, beta = 0.109, N = 94
SERPINF2 : NP1
P08697
168:168
126:126
31:31
17:1618262:C:T_T
p = 0.0087, beta = −0.217, N = 325
LGNQEPGGQTALK pc2
P08697
155:168
119:126
28:31
17:1618262:C:T_T
p = 0.00095, beta = −0.284, N = 302
DFLQSLK pc2
P08697;P08697−2
168:168
126:126
31:31
17:1618262:C:T_T
p = 0.0043, beta = −0.236, N = 325
SPPGVCSR pc2
P08697
168:168
126:126
31:31
17:1618262:C:T_T
p = 0.07, beta = −0.15, N = 325
LCQDLGPGAFR pc2
P08697;P08697−2
168:168
126:126
31:31
17:1618262:C:T_T
p = 0.35, beta = 0.0784, N = 325
EDFLEQSEQLFGAK pc2
P08697;P08697−2
168:168
126:126
31:31
17:1618262:C:T_T
p = 0.83, beta = 0.0177, N = 325
FDPSLTQR pc2
P08697;P08697−2
164:168
125:126
31:31
17:1618262:C:T_T
p = 0.49, beta = 0.057, N = 320
QEDDLANINQWVK pc2
P08697;P08697−2
166:168
125:126
31:31
17:1618262:C:T_T
p = 0.9, beta = −0.0109, N = 322
DSFHLDEQFTVPVEMMQAR pc3
P08697;P08697−2
158:168
119:126
29:31
17:1618262:C:T_T
p = 0.62, beta = −0.0425, N = 306
NPNPSAPR pc2
P08697;P08697−2
127:168
112:126
26:31
17:1618262:C:T_T
p = 0.0064, beta = 0.249, N = 265
WFLLEQPEIQVAHFPFK pc3
P08697;P08697−2
164:168
126:126
31:31
17:1618262:C:T_T
p = 0.73, beta = 0.0293, N = 321
DPTPEQTHR pc2
P08697
155:168
116:126
29:31
17:1618262:C:T_T
p = 0.9, beta = 0.0103, N = 300
LQQVLHAGSGPCLPHLLSR pc4
P08697;P08697−2
131:168
101:126
28:31
17:1618262:C:T_T
p = 0.37, beta = −0.0817, N = 260
TYPLRWFLLEQPEIQVAHFPFK pc4
P08697;P08697−2
69:168
57:126
13:31
17:1618262:C:T_T
p = 0.52, beta = −0.0819, N = 139
EQQDSPGNK pc2
P08697;P08697−2
82:168
57:126
9:31
17:1618262:C:T_T
p = 0.56, beta = −0.0777, N = 148
GFPIKEDFLEQSEQLFGAK pc3
P08697;P08697−2
51:168
39:126
10:31
17:1618262:C:T_T
p = 0.055, beta = −0.278, N = 100
HQMDLVATLSQLGLQELFQAPDLR p
P08697;P08697−2
19:168
14:126
4:31
17:1618262:C:T_T
p = 0.59, beta = −0.123, N = 37
Assay Target: SERPINF2
UniProt: P08697
rsID: rs11078596
Target SNP: 17:1714968:T:C
Proxy SNP: 17:1618262:C:T
deCODE log10(p): 67.8
deCODE BETA: −0.17
deCODE MSPA: 0.041
1255:1255:1251:1156:1254:124
QLTSGPNQEQVSPLTLLK pc2
rs2070863 REF
501:856
171:351
10:53
17:1618262:C:T_T
p = 1.2e−07, model = DOM, N = 682
QLTSGPNQEQVSPLTLLK pc3
rs2070863 REF
451:856
150:351
4:53
17:1618262:C:T_T
p = 1.2e−10, model = DOM, N = 605

201. AKR7A2 (O43488)
deCODE MSPA=0.682

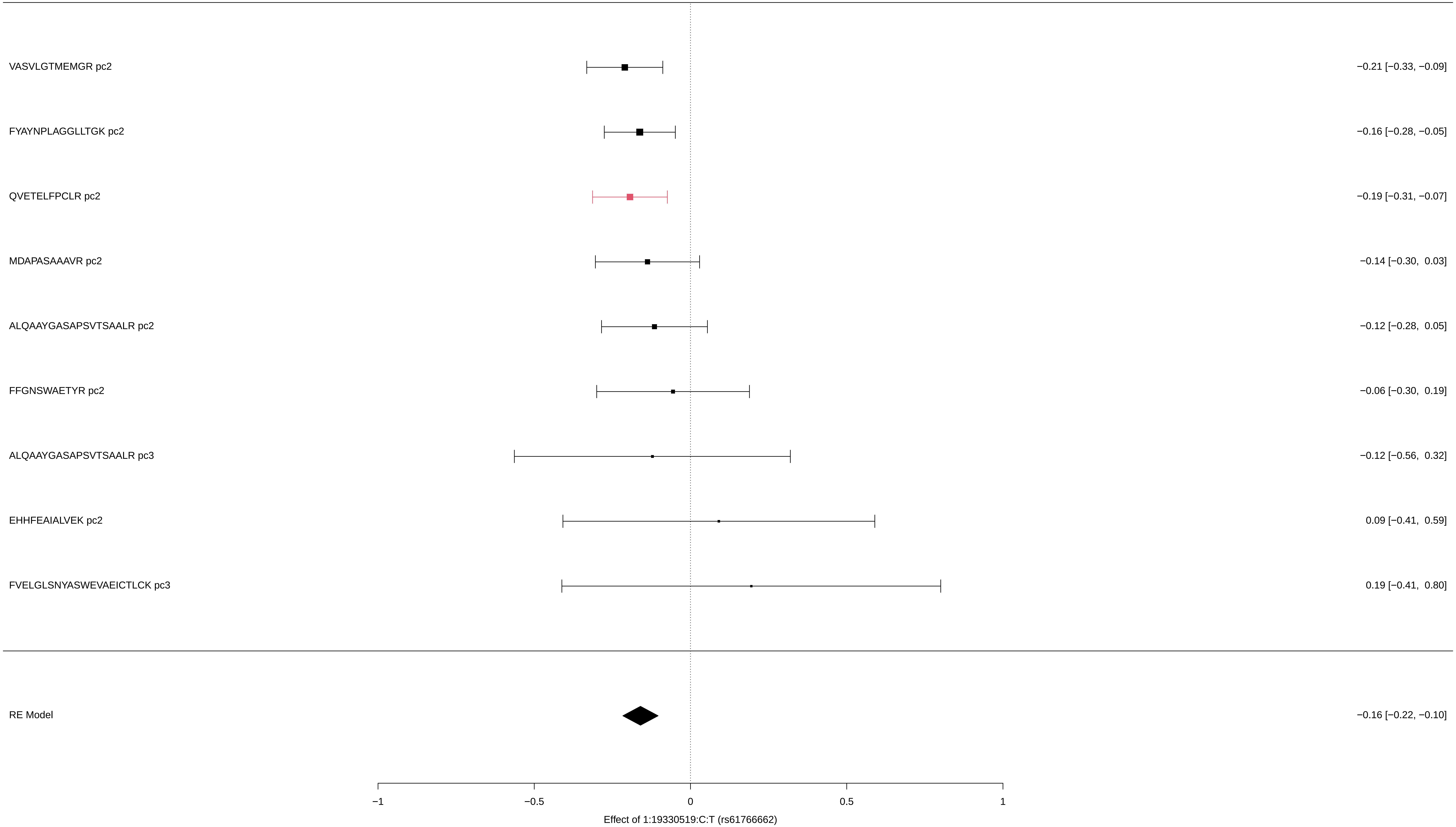

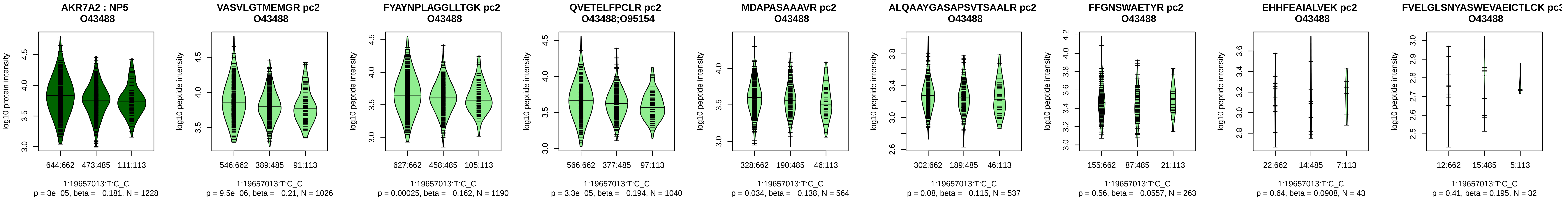

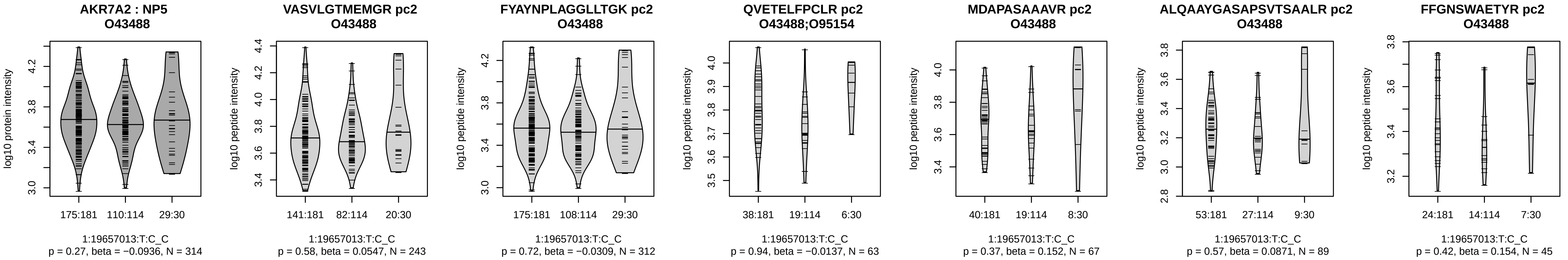

Assay Target: AKR7A2
UniProt: O43488
rsID: rs61766662
Target SNP: 1:19330519:C:T
Proxy SNP: 1:19657013:T:C
deCODE log10(p): 67.7
deCODE BETA: −0.15
deCODE MSPA: 0.682
::*:–:–:–:–:–:–
1026:1190:1040:564:537:263:78

202. TPST2 (O60704)
deCODE MSPA=0.385

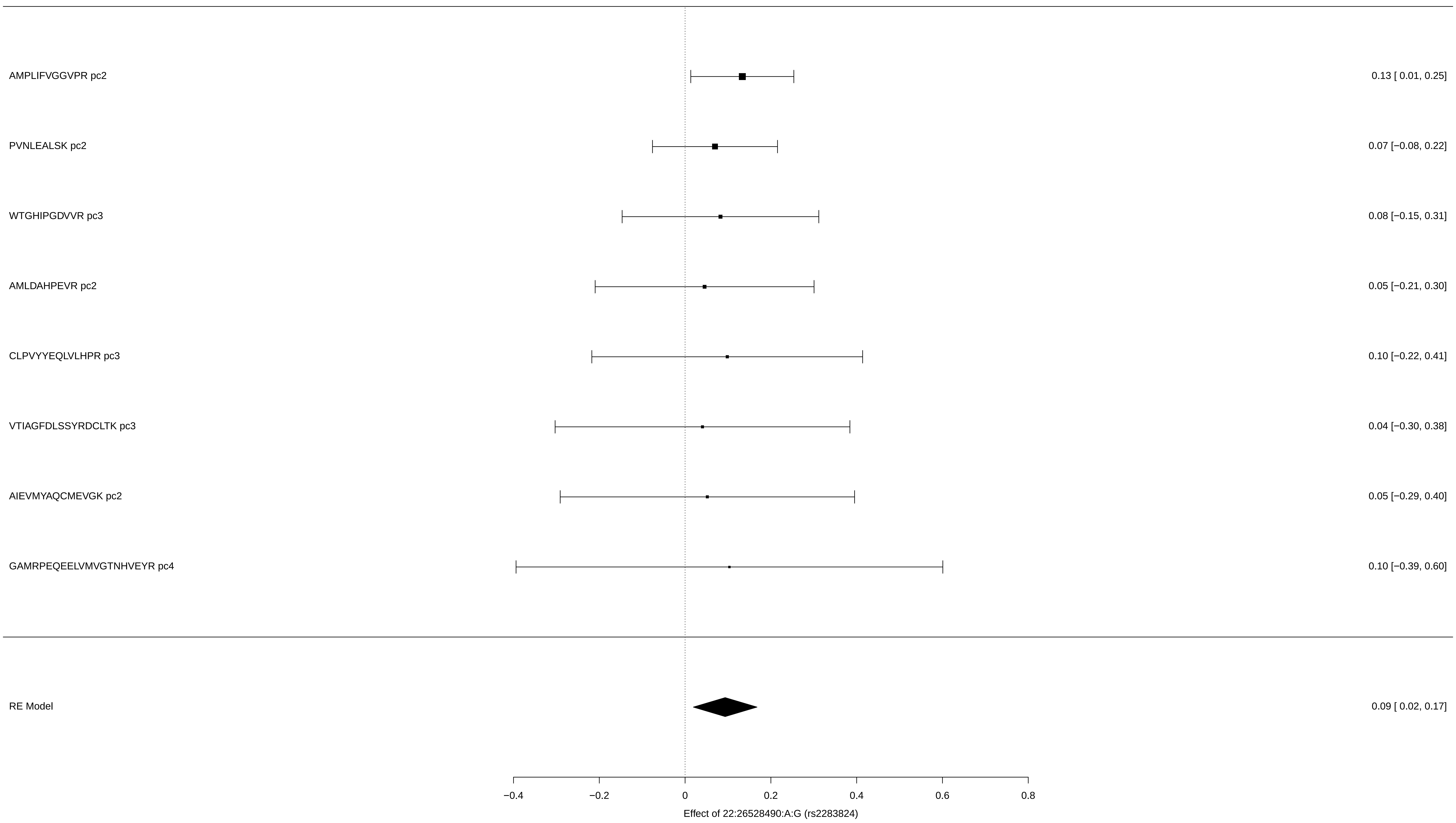

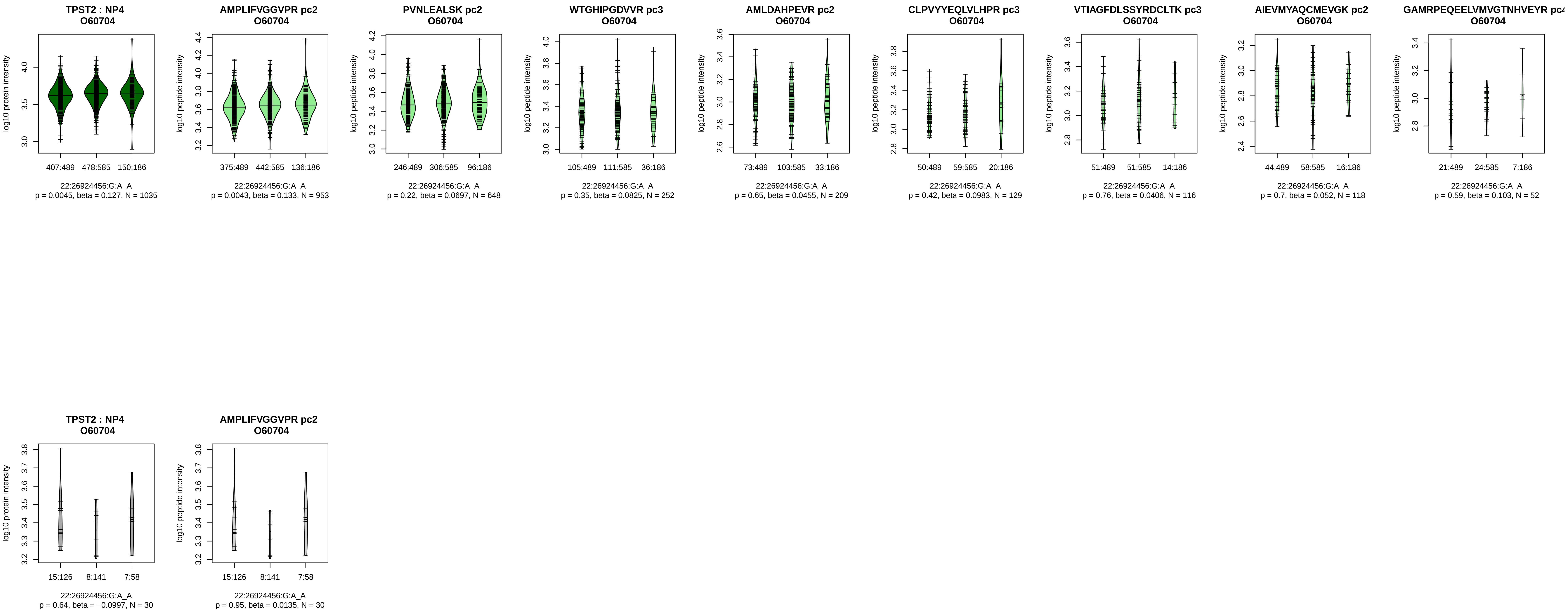

Assay Target: TPST2
UniProt: O60704
rsID: rs2283824
Target SNP: 22:26528490:A:G
Proxy SNP: 22:26924456:G:A
deCODE log10(p): 67
deCODE BETA: 0.15
deCODE MSPA: 0.385
*:–:–:–:–:–:–:–
953:648:252:209:129:116:118:5

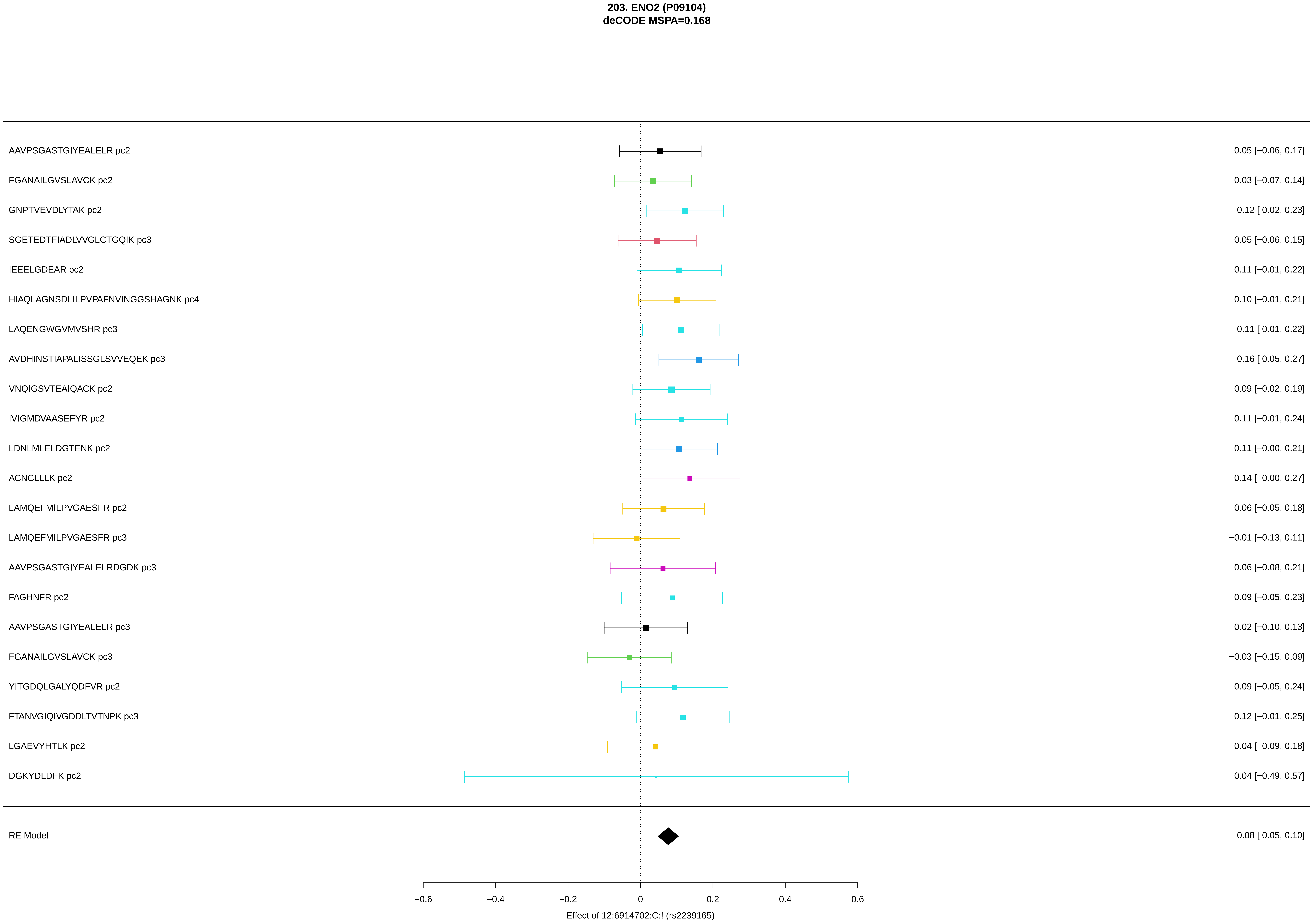

203. ENO2 (P09104)
deCODE MSPA=0.168
AAVPSGASTGIYEALELR pc2
0.05 [−0.06, 0.17]
FGANAILGVSLAVCK pc2
0.03 [−0.07, 0.14]
GNPTVEVDLYTAK pc2
0.12 [0.02, 0.23]
SGETEDTFIADLVVGLCTGQIK pc3
0.05 [−0.06, 0.15]
IEEELGDEAR pc2
0.11 [−0.01, 0.22]
HIAQLAGNSDLILPVPAFNVINGGSHAGNK pc4
0.10 [−0.01, 0.21]
LAQENGWGVMVSHR pc3
0.11 [0.01, 0.22]
AVDHINSTIAPALISSGLSVVEQEK pc3
0.16 [0.05, 0.27]
VNQIGSVTEAIQACK pc2
0.09 [−0.02, 0.19]
IVIGMDVAASEFYR pc2
0.11 [−0.01, 0.24]
LDNLMLELDGTENK pc2
0.11 [−0.00, 0.21]
ACNCLLLK pc2
0.14 [−0.00, 0.27]
LAMQEFMILPVGAESFR pc2
0.06 [−0.05, 0.18]
LAMQEFMILPVGAESFR pc3
−0.01 [−0.13, 0.11]
AAVPSGASTGIYEALELRDGDK pc3
0.06 [−0.08, 0.21]
FAGHNFR pc2
0.09 [−0.05, 0.23]
AAVPSGASTGIYEALELR pc3
0.02 [−0.10, 0.13]
FGANAILGVSLAVCK pc3
−0.03 [−0.15, 0.09]
YITGDQLGALYQDFVR pc2
0.09 [−0.05, 0.24]
FTANVGIQIVGDDLTVTNPK pc3
0.12 [−0.01, 0.25]
LGAEVYHTLK pc2
0.04 [−0.09, 0.18]
DGKYDLDFK pc2
0.04 [−0.49, 0.57]
RE Model
0.08 [0.05, 0.10]
−0.6
−0.4
−0.2
0
0.2
0.4
0.6
Effect of 12:6914702:C:! (rs2239165)

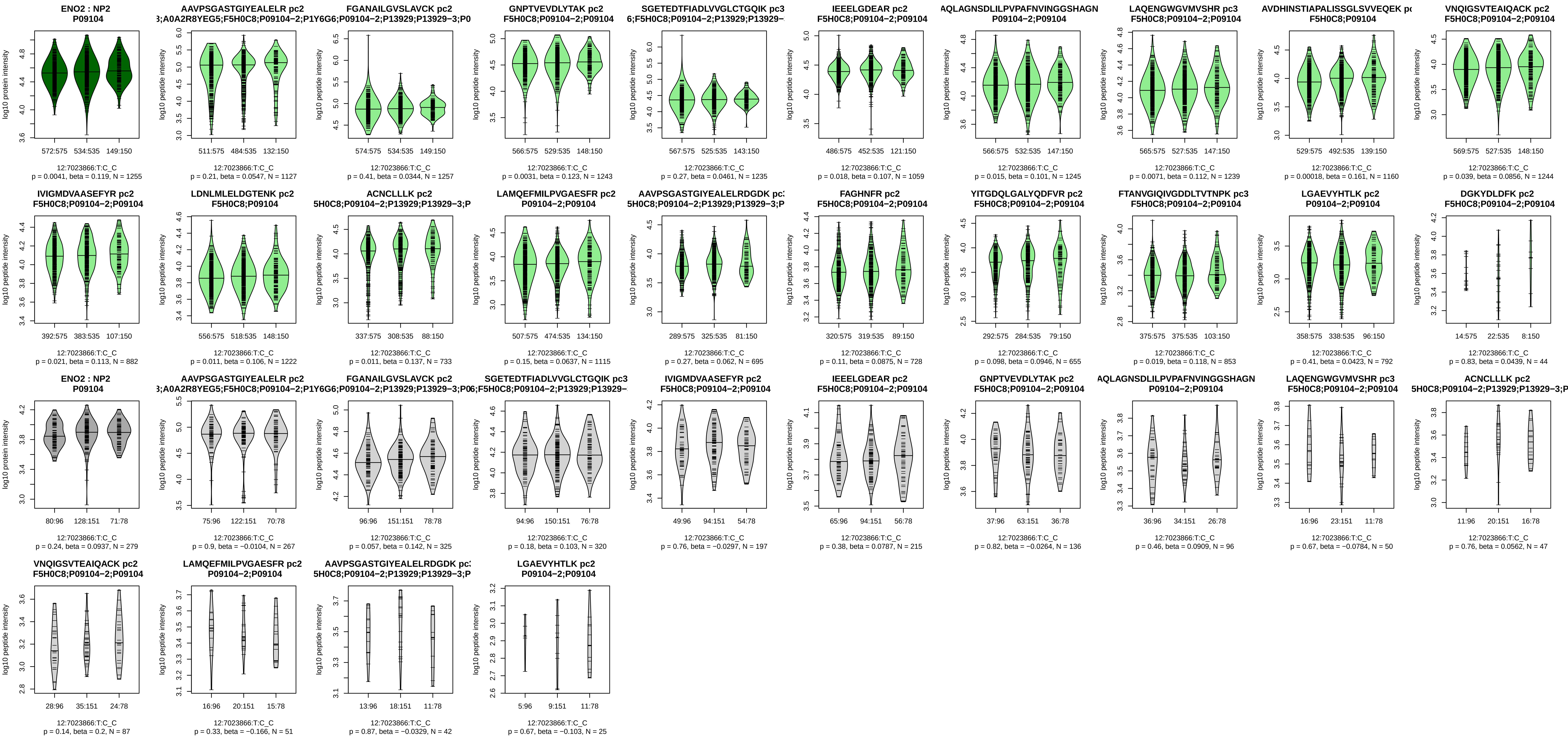

Assay Target: ENO2
UniProt: P09104
rsID: rs2239165
Target SNP: 12:6914702:C:!
Proxy SNP: 12:7023866:T:C
deCODE log10(p): 66.3
deCODE BETA: 0.16
deCODE MSPA: 0.168
−:−:*:−:−:−:*:*:−:−:−:−:−:−:−:−:
1127:1257:1243:1235:1059:124

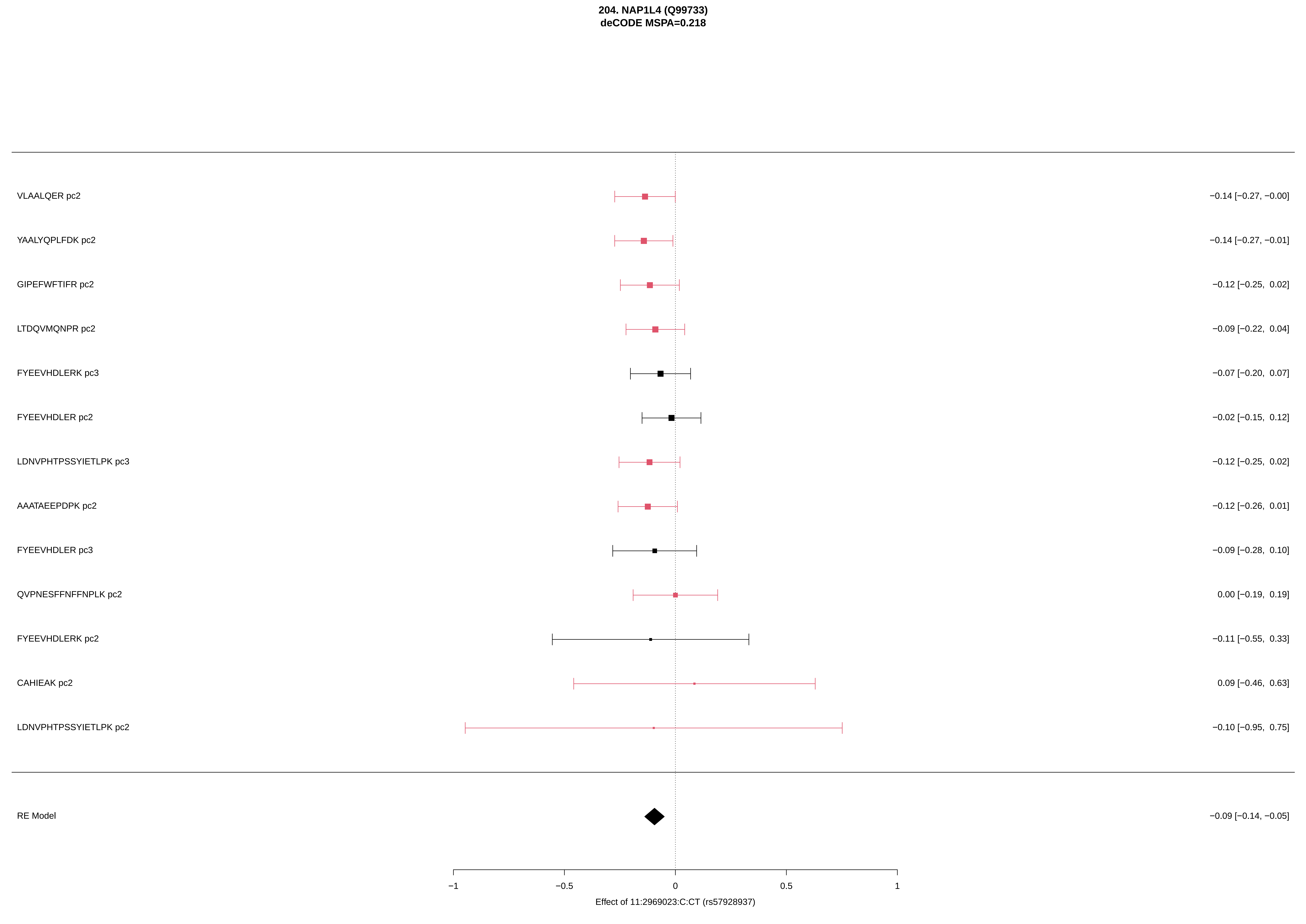
204. NAP1L4 (Q99733)
deCODE MSPA=0.218
VLAALQER pc2
−0.14 [−0.27, −0.00]
YAALYQPLFDK pc2
−0.14 [−0.27, −0.01]
GIPEFWFTIFR pc2
−0.12 [−0.25, 0.02]
LTDQVMQNPR pc2
−0.09 [−0.22, 0.04]
FYEEVHDLERK pc3
−0.07 [−0.20, 0.07]
FYEEVHDLER pc2
−0.02 [−0.15, 0.12]
LDNVPHTPSSYIETLPK pc3
−0.12 [−0.25, 0.02]
AAATAEEPDPK pc2
−0.12 [−0.26, 0.01]
FYEEVHDLER pc3
−0.09 [−0.28, 0.10]
QVPNESFFNFFNPLK pc2
0.00 [−0.19, 0.19]
FYEEVHDLERK pc2
−0.11 [−0.55, 0.33]
CAHIEAK pc2
0.09 [−0.46, 0.63]
LDNVPHTPSSYIETLPK pc2
−0.10 [−0.95, 0.75]
RE Model
−0.09 [−0.14, −0.05]
−1
−0.5
0
0.5
1
Effect of 11:2969023:C:CT (rs57928937)

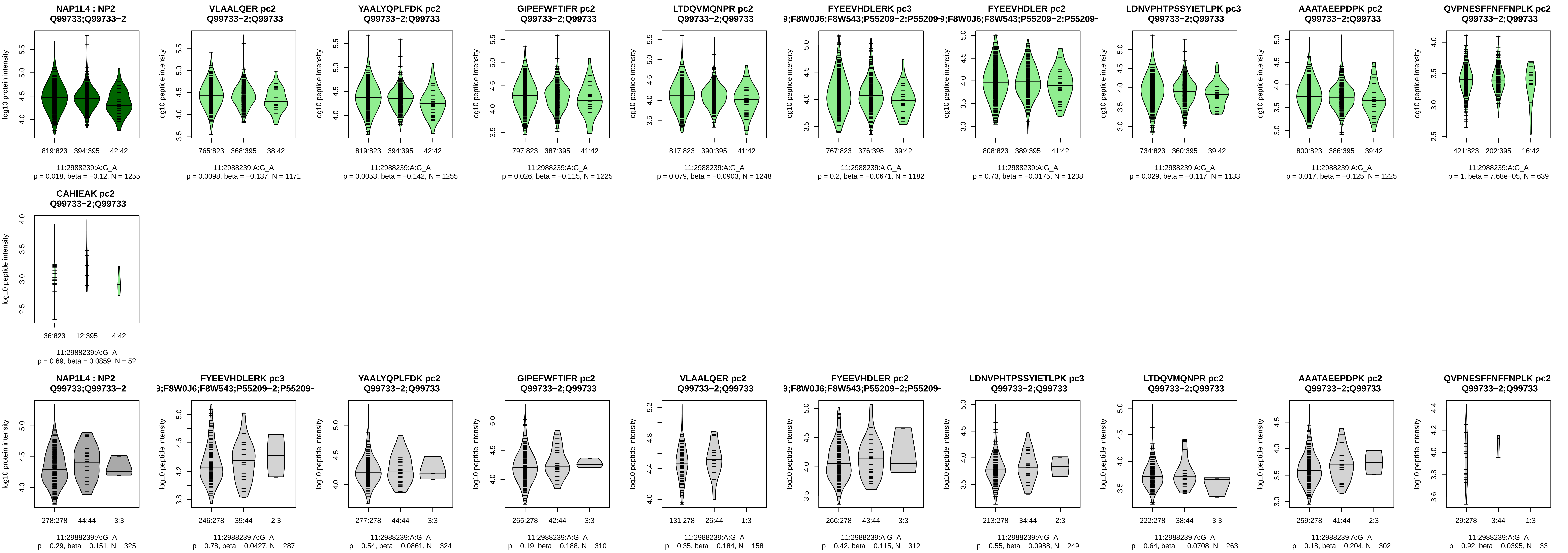

Assay Target: NAP1L4
UniProt: Q99733
rsID: rs57928937
Target SNP: 11:2969023:C:CT
Proxy SNP: 11:2988239:A:G
deCODE log10(p): 66.1
deCODE BETA: −0.18
deCODE MSPA: 0.218
::-:-:-:-:-:-:-:-:-:-:NA
1171:1255:1225:1248:1182:123

205. PIGR (P01833)
deCODE MSPA=0

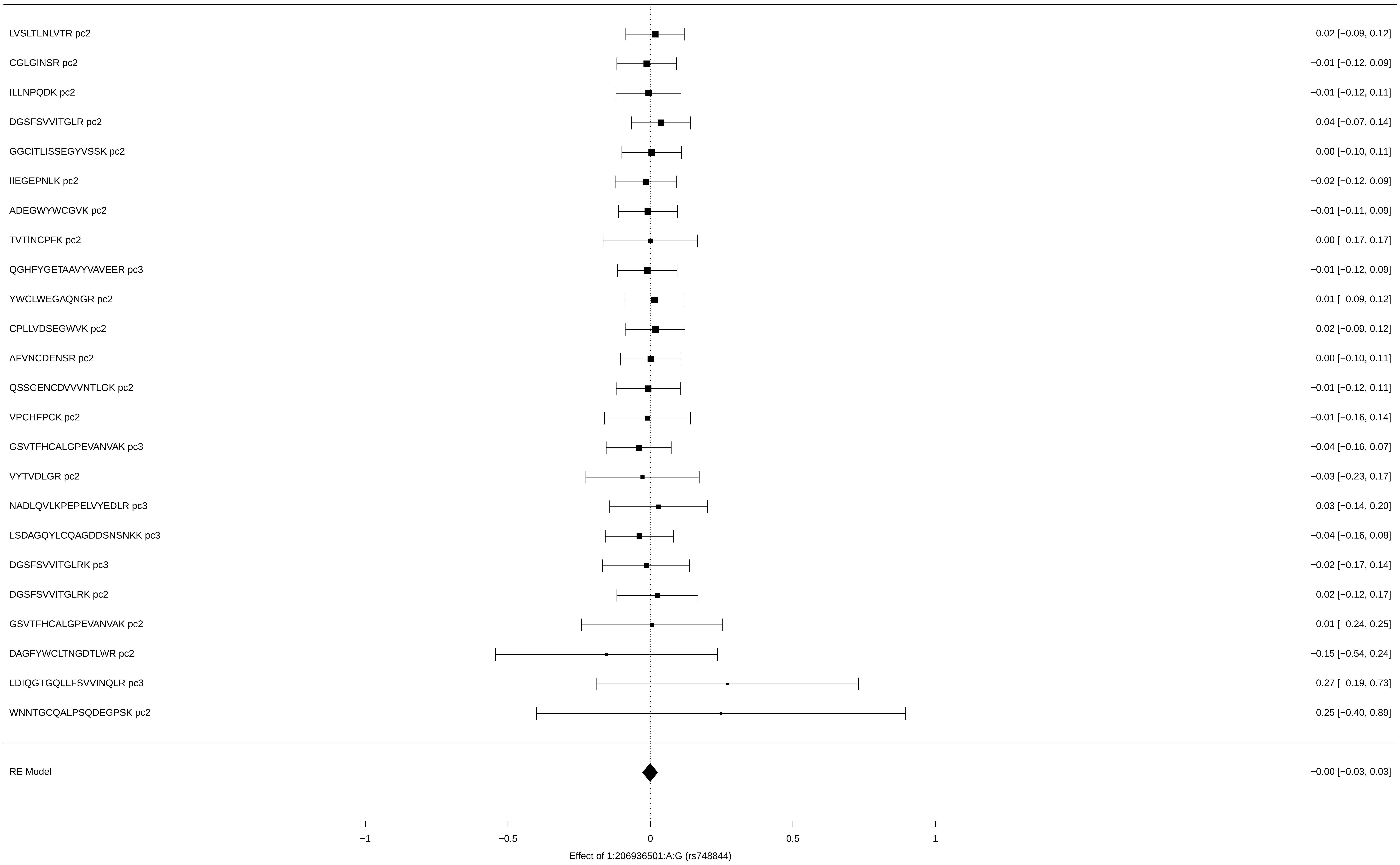

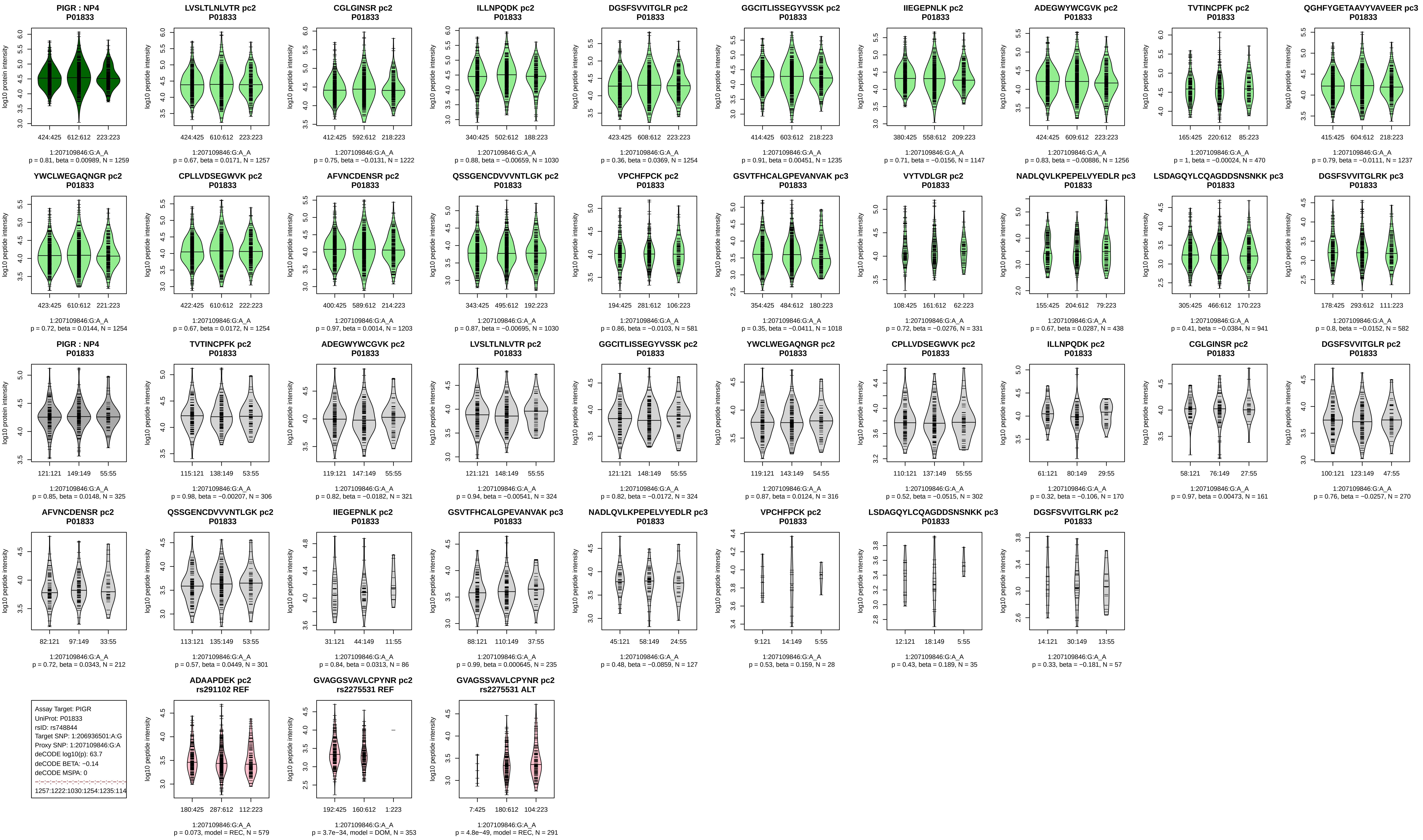
PIGR : NP4
P01833
log10 protein intensity
424:425 612:612 223:223
1:207109846:G:A_A
p = 0.81, beta = 0.00989, N = 1259
LVSLTLNLVTR pc2
P01833
log10 peptide intensity
424:425 610:612 223:223
1:207109846:G:A_A
p = 0.67, beta = 0.0171, N = 1257
CGLGINSR pc2
P01833
412:425 592:612 218:223
1:207109846:G:A_A
p = 0.75, beta = −0.0131, N = 1222
ILLNPQDK pc2
P01833
340:425 502:612 188:223
1:207109846:G:A_A
p = 0.88, beta = −0.00659, N = 1030
DGSFSVVITGLR pc2
P01833
423:425 608:612 223:223
1:207109846:G:A_A
p = 0.36, beta = 0.0369, N = 1254
GGCITLISSEGYVSSK pc2
P01833
414:425 603:612 218:223
1:207109846:G:A_A
p = 0.91, beta = 0.00451, N = 1235
IIEGEPNLK pc2
P01833
380:425 558:612 209:223
1:207109846:G:A_A
p = 0.71, beta = −0.0156, N = 1147
ADEGWYWCGVK pc2
P01833
424:425 609:612 223:223
1:207109846:G:A_A
p = 0.83, beta = −0.00886, N = 1256
TVTINCPFK pc2
P01833
165:425 220:612 85:223
1:207109846:G:A_A
p = 1, beta = −0.00024, N = 470
QGHFYGETAAVYVAVEER pc3
P01833
415:425 604:612 218:223
1:207109846:G:A_A
p = 0.79, beta = −0.0111, N = 1237
YWCLWEGAQNGR pc2
P01833
423:425 610:612 221:223
1:207109846:G:A_A
p = 0.72, beta = 0.0144, N = 1254
CPLLVDSEGWVK pc2
P01833
422:425 610:612 222:223
1:207109846:G:A_A
p = 0.67, beta = 0.0172, N = 1254
AFVNCDENSR pc2
P01833
400:425 589:612 214:223
1:207109846:G:A_A
p = 0.97, beta = 0.0014, N = 1203
QSSGENCDVVVNTLGK pc2
P01833
343:425 495:612 192:223
1:207109846:G:A_A
p = 0.87, beta = −0.00695, N = 1030
VPCHFPCK pc2
P01833
194:425 281:612 106:223
1:207109846:G:A_A
p = 0.86, beta = −0.0103, N = 581
GSVTFHCALGPEVANVAK pc3
P01833
354:425 484:612 180:223
1:207109846:G:A_A
p = 0.35, beta = −0.0411, N = 1018
VYTVDLGR pc2
P01833
108:425 161:612 62:223
1:207109846:G:A_A
p = 0.72, beta = −0.0276, N = 331
NADLQVLKPEPELVYEDLR pc3
P01833
155:425 204:612 79:223
1:207109846:G:A_A
p = 0.67, beta = 0.0287, N = 438
LSDAGQYLCQAGDDSNSNKK pc3
P01833
305:425 466:612 170:223
1:207109846:G:A_A
p = 0.41, beta = −0.0384, N = 941
DGSFSVVITGLRK pc3
P01833
178:425 293:612 111:223
1:207109846:G:A_A
p = 0.8, beta = −0.0152, N = 582
PIGR : NP4
P01833
log10 protein intensity
121:121 149:149 55:55
1:207109846:G:A_A
p = 0.85, beta = 0.0148, N = 325
TVTINCPFK pc2
P01833
115:121 138:149 53:55
1:207109846:G:A_A
p = 0.98, beta = −0.00207, N = 306
ADEGWYWCGVK pc2
P01833
119:121 147:149 55:55
1:207109846:G:A_A
p = 0.82, beta = −0.0182, N = 321
LVSLTLNLVTR pc2
P01833
121:121 148:149 55:55
1:207109846:G:A_A
p = 0.94, beta = −0.00541, N = 324
GGCITLISSEGYVSSK pc2
P01833
121:121 148:149 55:55
1:207109846:G:A_A
p = 0.82, beta = −0.0172, N = 324
YWCLWEGAQNGR pc2
P01833
119:121 143:149 54:55
1:207109846:G:A_A
p = 0.87, beta = 0.0124, N = 316
CPLLVDSEGWVK pc2
P01833
110:121 137:149 55:55
1:207109846:G:A_A
p = 0.52, beta = −0.0515, N = 302
ILLNPQDK pc2
P01833
61:121 80:149 29:55
1:207109846:G:A_A
p = 0.32, beta = −0.106, N = 170
CGLGINSR pc2
P01833
58:121 76:149 27:55
1:207109846:G:A_A
p = 0.97, beta = 0.00473, N = 161
DGSFSVVITGLR pc2
P01833
100:121 123:149 47:55
1:207109846:G:A_A
p = 0.76, beta = −0.0257, N = 270
AFVNCDENSR pc2
P01833
82:121 97:149 33:55
1:207109846:G:A_A
p = 0.72, beta = 0.0343, N = 212
QSSGENCDVVVNTLGK pc2
P01833
113:121 135:149 53:55
1:207109846:G:A_A
p = 0.57, beta = 0.0449, N = 301
IIEGEPNLK pc2
P01833
31:121 44:149 11:55
1:207109846:G:A_A
p = 0.84, beta = 0.0313, N = 86
GSVTFHCALGPEVANVAK pc3
P01833
88:121 110:149 37:55
1:207109846:G:A_A
p = 0.99, beta = 0.000645, N = 235
NADLQVLKPEPELVYEDLR pc3
P01833
45:121 58:149 24:55
1:207109846:G:A_A
p = 0.48, beta = −0.0859, N = 127
VPCHFPCK pc2
P01833
9:121 14:149 5:55
1:207109846:G:A_A
p = 0.53, beta = 0.159, N = 28
LSDAGQYLCQAGDDSNSNKK pc3
P01833
12:121 18:149 5:55
1:207109846:G:A_A
p = 0.43, beta = 0.189, N = 35
DGSFSVVITGLRK pc2
P01833
14:121 30:149 13:55
1:207109846:G:A_A
p = 0.33, beta = −0.181, N = 57
Assay Target: PIGR
UniProt: P01833
rsID: rs748844
Target SNP: 1:206936501:A:G
Proxy SNP: 1:207109846:G:A
deCODE log10(p): 63.7
deCODE BETA: −0.14
deCODE MSPA: 0
1257:1222:1030:1254:1235:114
ADAAPDEK pc2
rs291102 REF
180:425 287:612 112:223
1:207109846:G:A_A
p = 0.073, model = REC, N = 579
GVAGGSVAVLCPYNR pc2
rs2275531 REF
192:425 160:612 1:223
1:207109846:G:A_A
p = 3.7e−34, model = DOM, N = 353
GVAGSSVAVLCPYNR pc2
rs2275531 ALT
7:425 180:612 104:223
1:207109846:G:A_A
p = 4.8e−49, model = REC, N = 291

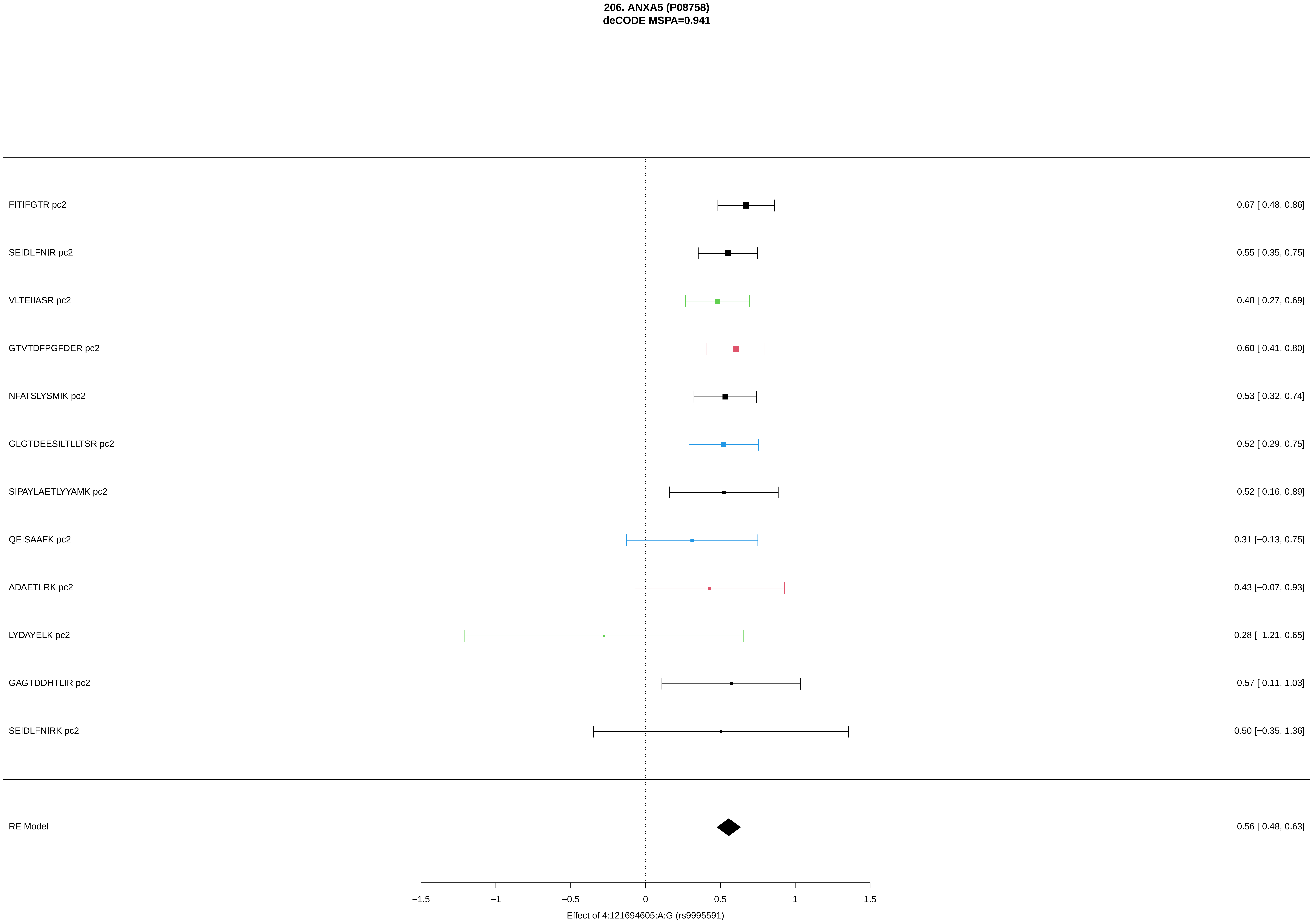
206. ANXA5 (P08758)
deCODE MSPA=0.941
FITIFGTR pc2
0.67 [0.48, 0.86]
SEIDLFNIR pc2
0.55 [0.35, 0.75]
VLTEIIASR pc2
0.48 [0.27, 0.69]
GTVTDFPGFDER pc2
0.60 [0.41, 0.80]
NFATSLYSMIK pc2
0.53 [0.32, 0.74]
GLGTDEESILTLLTSR pc2
0.52 [0.29, 0.75]
SIPAYLAETLYYAMK pc2
0.52 [0.16, 0.89]
QEISAAFK pc2
0.31 [−0.13, 0.75]
ADAETLRK pc2
0.43 [−0.07, 0.93]
LYDAYELK pc2
−0.28 [−1.21, 0.65]
GAGTDDHTLIR pc2
0.57 [0.11, 1.03]
SEIDLFNIRK pc2
0.50 [−0.35, 1.36]
RE Model
0.56 [0.48, 0.63]
−1.5
−1
−0.5
0
0.5
1
1.5
Effect of 4:121694605:A:G (rs9995591)

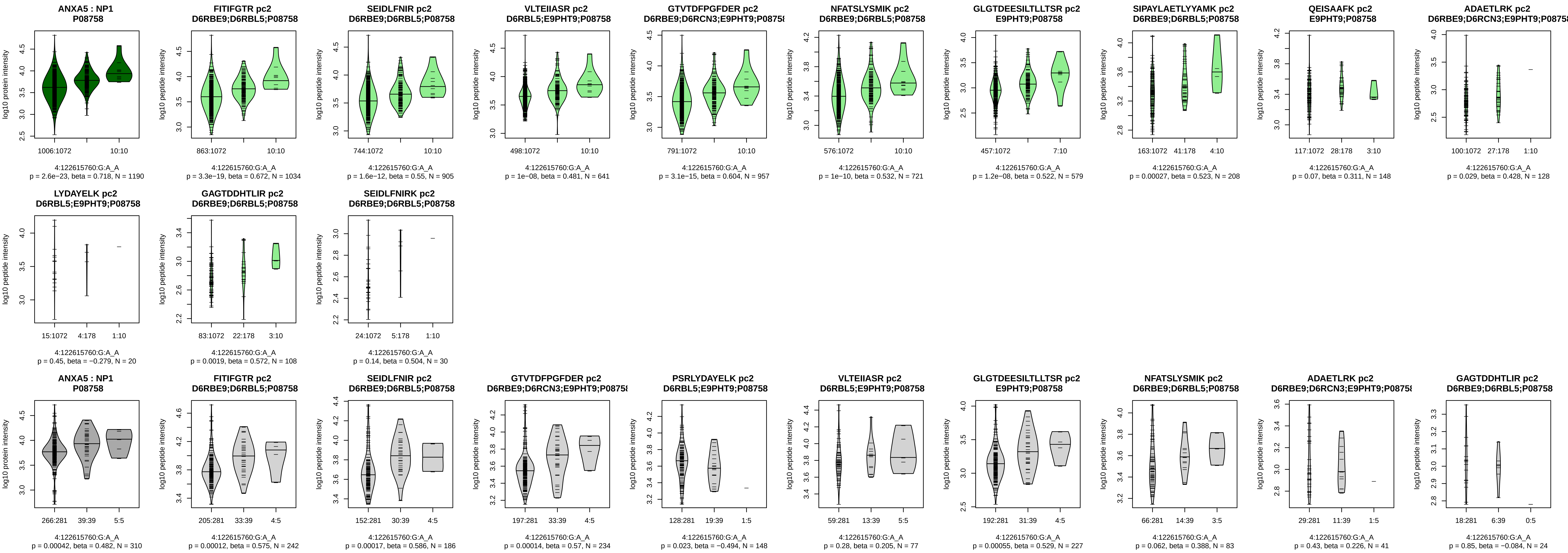

Assay Target: ANXA5
UniProt: P08758
rsID: rs9995591
Target SNP: 4:121694605:A:G
Proxy SNP: 4:122615760:G:A
deCODE log10(p): 63.4
deCODE BETA: 0.26
deCODE MSPA: 0.941
::*:*:*:*:*:−:−:−:*:−:NA
1034:905:641:957:721:579:208:

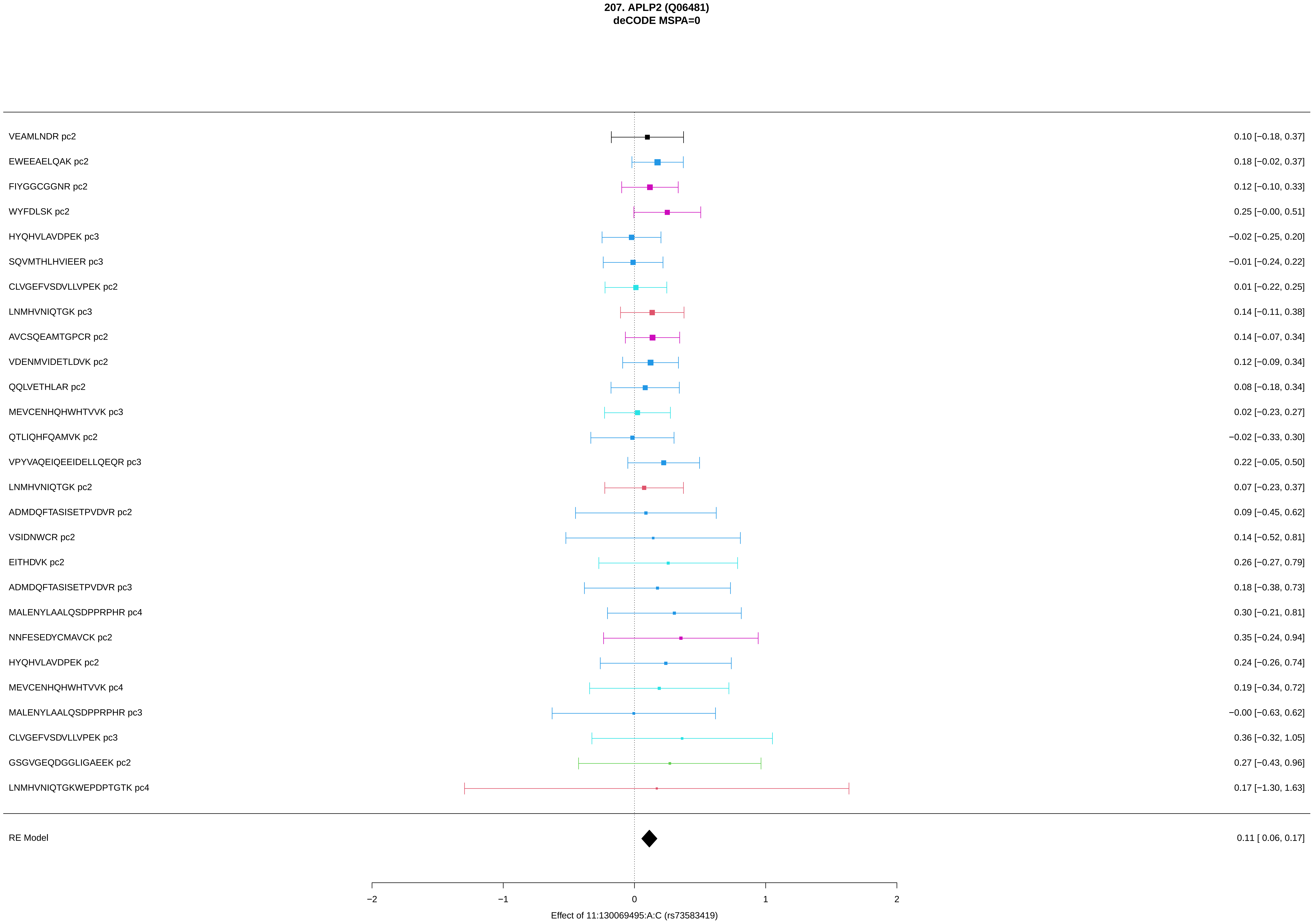
207. APLP2 (Q06481)
deCODE MSPA=0
VEAMLNDR pc2
EWEEAELQAK pc2
FIYGGCGGNR pc2
WYFDLSK pc2
HYQHVLAVDPEK pc3
SQVMTHLHVIEER pc3
CLVGEFVSDVLLVPEK pc2
LNMHVNIQTGK pc3
AVCSQEAMTGPCR pc2
VDENMVIDETLDVK pc2
QQLVETHLAR pc2
MEVCENHQHWHTVVK pc3
QTLIQHFQAMVK pc2
VPYVAQEIQEEIDELLQEQR pc3
LNMHVNIQTGK pc2
ADMDQFTASISETPVDVR pc2
VSIDNWCR pc2
EITHDVK pc2
ADMDQFTASISETPVDVR pc3
MALENYLAALQSDPPRPHR pc4
NNFESEDYCMAVCK pc2
HYQHVLAVDPEK pc2
MEVCENHQHWHTVVK pc4
MALENYLAALQSDPPRPHR pc3
CLVGEFVSDVLLVPEK pc3
GSGVGEQDGGLIGAEEK pc2
LNMHVNIQTGKWEPDPTGTK pc4
RE Model
0.10 [−0.18, 0.37]
0.18 [−0.02, 0.37]
0.12 [−0.10, 0.33]
0.25 [−0.00, 0.51]
−0.02 [−0.25, 0.20]
−0.01 [−0.24, 0.22]
0.01 [−0.22, 0.25]
0.14 [−0.11, 0.38]
0.14 [−0.07, 0.34]
0.12 [−0.09, 0.34]
0.08 [−0.18, 0.34]
0.02 [−0.23, 0.27]
−0.02 [−0.33, 0.30]
0.22 [−0.05, 0.50]
0.07 [−0.23, 0.37]
0.09 [−0.45, 0.62]
0.14 [−0.52, 0.81]
0.26 [−0.27, 0.79]
0.18 [−0.38, 0.73]
0.30 [−0.21, 0.81]
0.35 [−0.24, 0.94]
0.24 [−0.26, 0.74]
0.19 [−0.34, 0.72]
−0.00 [−0.63, 0.62]
0.36 [−0.32, 1.05]
0.27 [−0.43, 0.96]
0.17 [−1.30, 1.63]
0.11 [0.06, 0.17]
−2
−1
0
1
2
Effect of 11:130069495:A:C (rs73583419)

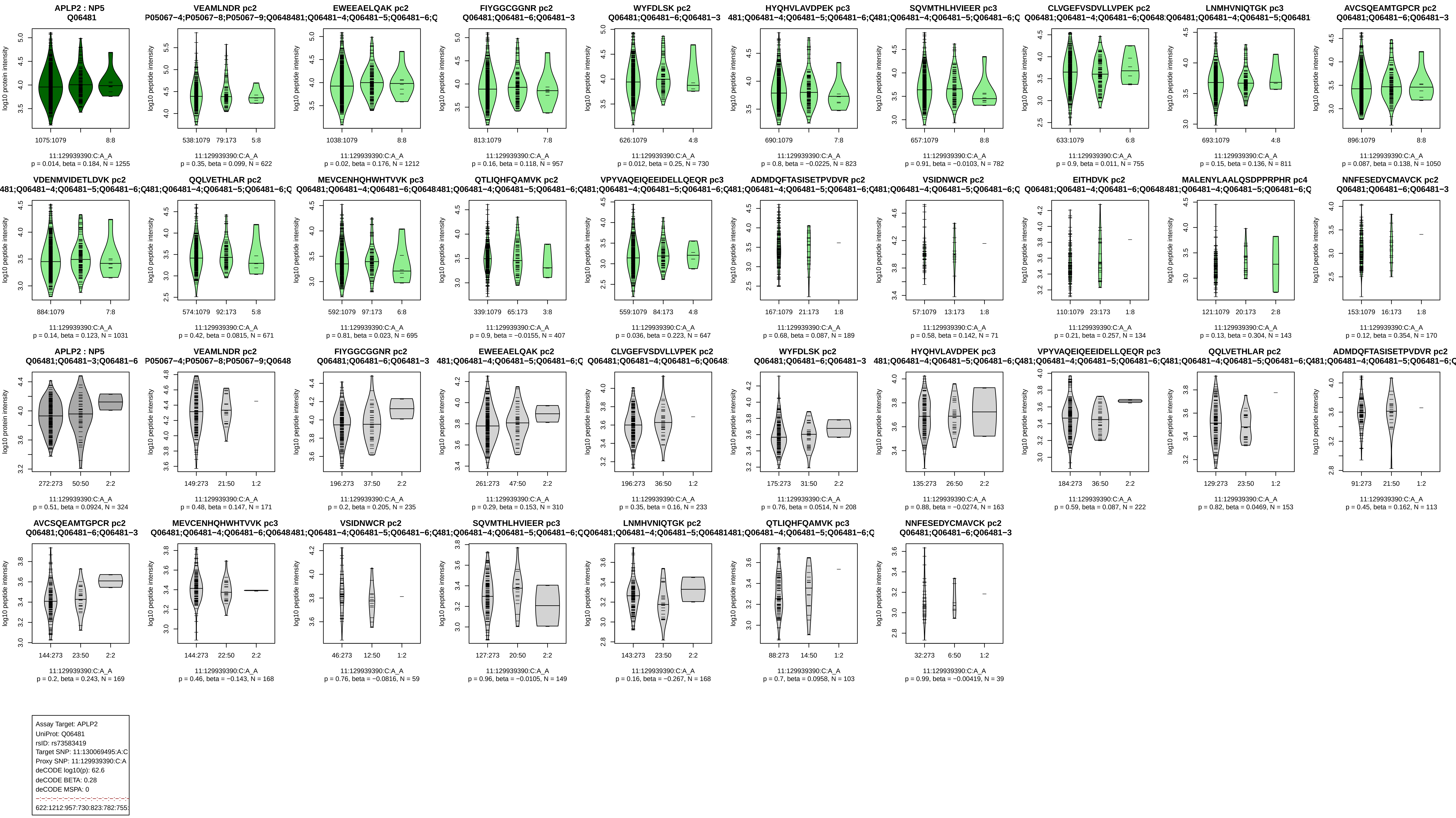
APLP2 : NP5
Q06481
1075:1079 8:8
11:129939390:C:A_A
p = 0.014, beta = 0.184, N = 1255
VEAMLNDR pc2
P05067−4;P05067−8;P05067−9;Q0648
538:1079 79:173 5:8
11:129939390:C:A_A
p = 0.35, beta = 0.099, N = 622
EWEEAELQAK pc2
481;Q06481−4;Q06481−5;Q06481−6;Q
1038:1079 8:8
11:129939390:C:A_A
p = 0.02, beta = 0.176, N = 1212
FIYGGCGGNR pc2
Q06481;Q06481−6;Q06481−3
813:1079 7:8
11:129939390:C:A_A
p = 0.16, beta = 0.118, N = 957
WYFDLSK pc2
Q06481;Q06481−6;Q06481−3
626:1079 4:8
11:129939390:C:A_A
p = 0.012, beta = 0.25, N = 730
HYQHVLAVDPEK pc3
481;Q06481−4;Q06481−5;Q06481−6;Q
690:1079 7:8
11:129939390:C:A_A
p = 0.8, beta = −0.0225, N = 823
SQVMTHLHVIEER pc3
481;Q06481−4;Q06481−5;Q06481−6;Q
657:1079 8:8
11:129939390:C:A_A
p = 0.91, beta = −0.0103, N = 782
CLVGEFVSDVLLVPEK pc2
Q06481;Q06481−4;Q06481−6;Q0648
633:1079 6:8
11:129939390:C:A_A
p = 0.9, beta = 0.011, N = 755
LNMHVNIQTGK pc3
481;Q06481−4;Q06481−5;Q06481
693:1079 4:8
11:129939390:C:A_A
p = 0.15, beta = 0.136, N = 811
AVCSQEAMTGPCR pc2
Q06481;Q06481−6;Q06481−3
896:1079 8:8
11:129939390:C:A_A
p = 0.087, beta = 0.138, N = 1050
VDENMVIDETLDVK pc2
481;Q06481−4;Q06481−5;Q06481−6;Q
884:1079 7:8
11:129939390:C:A_A
p = 0.14, beta = 0.123, N = 1031
QQLVETHLAR pc2
481;Q06481−4;Q06481−5;Q06481−6;Q
574:1079 92:173 5:8
11:129939390:C:A_A
p = 0.42, beta = 0.0815, N = 671
MEVCENHQHWHTVVK pc3
Q06481;Q06481−4;Q06481−6;Q0648
592:1079 97:173 6:8
11:129939390:C:A_A
p = 0.81, beta = 0.023, N = 695
QTLIQHFQAMVK pc2
481;Q06481−4;Q06481−5;Q06481−6;Q
339:1079 65:173 3:8
11:129939390:C:A_A
p = 0.9, beta = −0.0155, N = 407
VPYVAQEIQEEIDELLQEQR pc3
481;Q06481−4;Q06481−5;Q06481−6;Q
559:1079 84:173 4:8
11:129939390:C:A_A
p = 0.036, beta = 0.223, N = 647
ADMDQFTASISETPVDVR pc2
481;Q06481−4;Q06481−5;Q06481−6;Q
167:1079 21:173 1:8
11:129939390:C:A_A
p = 0.68, beta = 0.0837, N = 189
VSIDNWCR pc2
481;Q06481−4;Q06481−5;Q06481−6;Q
57:1079 13:173 1:8
11:129939390:C:A_A
p = 0.58, beta = 0.142, N = 71
EITHDVK pc2
Q06481;Q06481−4;Q06481−6;Q0648
110:1079 23:173 1:8
11:129939390:C:A_A
p = 0.21, beta = 0.257, N = 134
MALENYLAALQSDPPRPHR pc4
481;Q06481−4;Q06481−5;Q06481−6;Q
121:1079 20:173 2:8
11:129939390:C:A_A
p = 0.13, beta = 0.304, N = 143
NNFESEDYCMAVCK pc2
Q06481;Q06481−6;Q06481−3
153:1079 16:173 1:8
11:129939390:C:A_A
p = 0.12, beta = 0.354, N = 170
APLP2 : NP5
Q06481;Q06481−3;Q06481−6
272:273 50:50 2:2
11:129939390:C:A_A
p = 0.51, beta = 0.0924, N = 324
VEAMLNDR pc2
P05067−4;P05067−8;P05067−9;Q0648
149:273 21:50 1:2
11:129939390:C:A_A
p = 0.48, beta = 0.147, N = 171
FIYGGCGGNR pc2
Q06481;Q06481−6;Q06481−3
196:273 37:50 2:2
11:129939390:C:A_A
p = 0.2, beta = 0.205, N = 235
EWEEAELQAK pc2
481;Q06481−4;Q06481−5;Q06481−6;Q
261:273 47:50 2:2
11:129939390:C:A_A
p = 0.29, beta = 0.153, N = 310
CLVGEFVSDVLLVPEK pc2
Q06481;Q06481−4;Q06481−6;Q0648
196:273 36:50 1:2
11:129939390:C:A_A
p = 0.35, beta = 0.16, N = 233
WYFDLSK pc2
Q06481;Q06481−6;Q06481−3
175:273 31:50 2:2
11:129939390:C:A_A
p = 0.76, beta = 0.0514, N = 208
HYQHVLAVDPEK pc3
481;Q06481−4;Q06481−5;Q06481−6;Q
135:273 26:50 2:2
11:129939390:C:A_A
p = 0.88, beta = −0.0274, N = 163
VPYVAQEIQEEIDELLQEQR pc3
481;Q06481−4;Q06481−5;Q06481−6;Q
184:273 36:50 2:2
11:129939390:C:A_A
p = 0.59, beta = 0.087, N = 222
QQLVETHLAR pc2
481;Q06481−4;Q06481−5;Q06481−6;Q
129:273 23:50 1:2
11:129939390:C:A_A
p = 0.82, beta = 0.0469, N = 153
ADMDQFTASISETPVDVR pc2
481;Q06481−4;Q06481−5;Q06481−6;Q
91:273 21:50 1:2
11:129939390:C:A_A
p = 0.45, beta = 0.162, N = 113
AVCSQEAMTGPCR pc2
Q06481;Q06481−6;Q06481−3
144:273 23:50 2:2
11:129939390:C:A_A
p = 0.2, beta = 0.243, N = 169
MEVCENHQHWHTVVK pc3
Q06481;Q06481−4;Q06481−6;Q0648
144:273 22:50 2:2
11:129939390:C:A_A
p = 0.46, beta = −0.143, N = 168
VSIDNWCR pc2
481;Q06481−4;Q06481−5;Q06481−6;Q
46:273 12:50 1:2
11:129939390:C:A_A
p = 0.76, beta = −0.0816, N = 59
SQVMTHLHVIEER pc3
481;Q06481−4;Q06481−5;Q06481−6;Q
127:273 20:50 2:2
11:129939390:C:A_A
p = 0.96, beta = −0.0105, N = 149
LNMHVNIQTGK pc2
Q06481;Q06481−4;Q06481−5;Q06481
143:273 23:50 2:2
11:129939390:C:A_A
p = 0.16, beta = −0.267, N = 168
QTLIQHFQAMVK pc3
481;Q06481−4;Q06481−5;Q06481−6;Q
88:273 14:50 1:2
11:129939390:C:A_A
p = 0.7, beta = 0.0958, N = 103
NNFESEDYCMAVCK pc2
Q06481;Q06481−6;Q06481−3
32:273 6:50 1:2
11:129939390:C:A_A
p = 0.99, beta = −0.00419, N = 39
Assay Target: APLP2
UniProt: Q06481
rsID: rs73583419
Target SNP: 11:130069495:A:C
Proxy SNP: 11:129939390:C:A
deCODE log10(p): 62.6
deCODE BETA: 0.28
deCODE MSPA: 0
622:1212:957:730:823:782:755

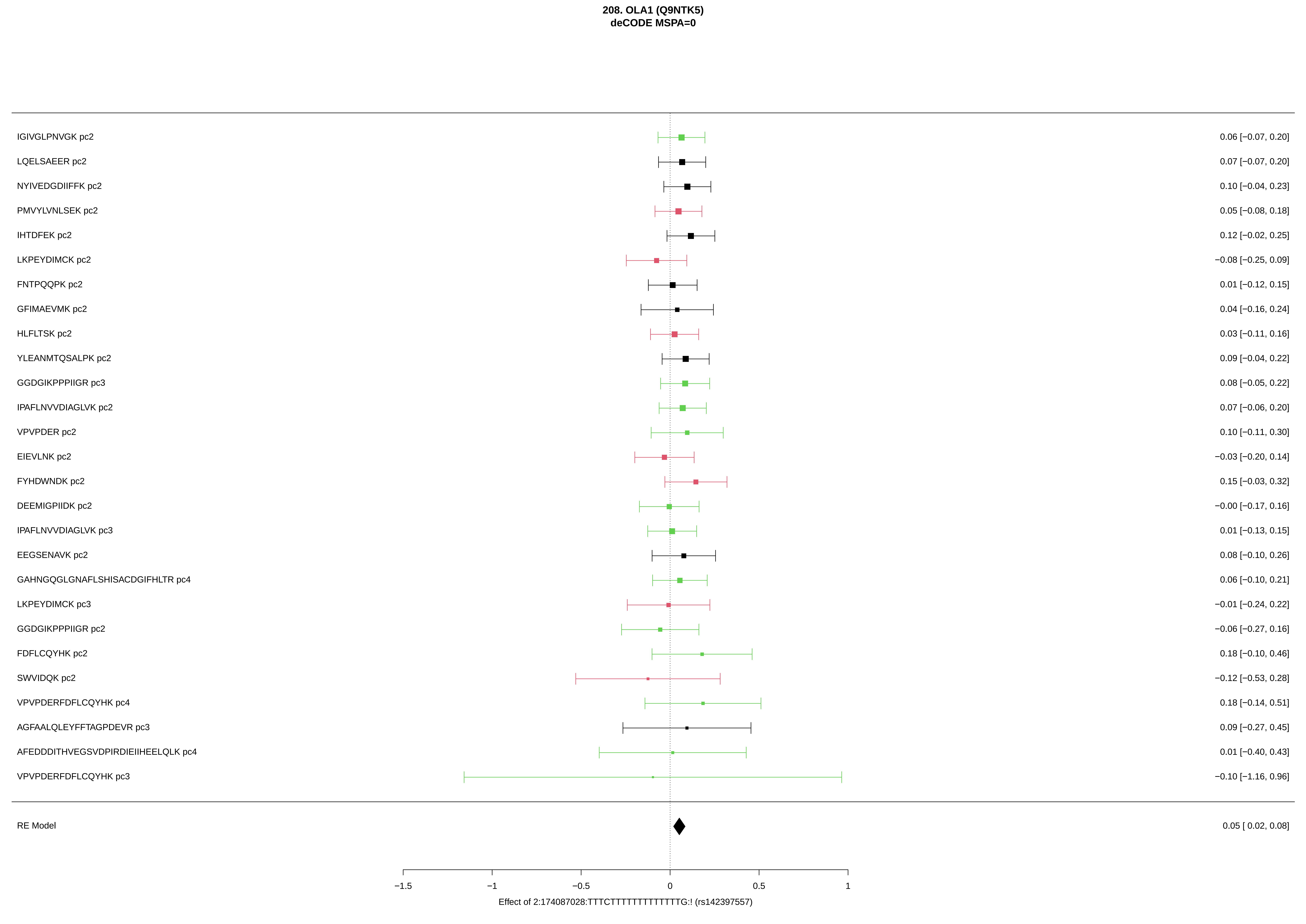
208. OLA1 (Q9NTK5)
deCODE MSPA=0
IGIVGLPNVGK pc2 0.06 [−0.07, 0.20]
LQELSAEER pc2 0.07 [−0.07, 0.20]
NYIVEDGDIIFFK pc2 0.10 [−0.04, 0.23]
PMVYLVNLSEK pc2 0.05 [−0.08, 0.18]
IHTDFEK pc2 0.12 [−0.02, 0.25]
LKPEYDIMCK pc2 −0.08 [−0.25, 0.09]
FNTPQQPK pc2 0.01 [−0.12, 0.15]
GFIMAEVMK pc2 0.04 [−0.16, 0.24]
HLFLTSK pc2 0.03 [−0.11, 0.16]
YLEANMTQSALPK pc2 0.09 [−0.04, 0.22]
GGDGIKPPPIIGR pc3 0.08 [−0.05, 0.22]
IPAFLNVVDIAGLVK pc2 0.07 [−0.06, 0.20]
VPVPDER pc2 0.10 [−0.11, 0.30]
EIEVLNK pc2 −0.03 [−0.20, 0.14]
FYHDWNDK pc2 0.15 [−0.03, 0.32]
DEEMIGPIIDK pc2 −0.00 [−0.17, 0.16]
IPAFLNVVDIAGLVK pc3 0.01 [−0.13, 0.15]
EEGSENAVK pc2 0.08 [−0.10, 0.26]
GAHNGQGLGNAFLSHISACDGIFHLTR pc4 0.06 [−0.10, 0.21]
LKPEYDIMCK pc3 −0.01 [−0.24, 0.22]
GGDGIKPPPIIGR pc2 −0.06 [−0.27, 0.16]
FDFLCQYHK pc2 0.18 [−0.10, 0.46]
SWVIDQK pc2 −0.12 [−0.53, 0.28]
VPVPDERFDFLCQYHK pc4 0.18 [−0.14, 0.51]
AGFAALQLEYFFTAGPDEVR pc3 0.09 [−0.27, 0.45]
AFEDDDITHVEGSVDPIRDIEIIHEELQLK pc4 0.01 [−0.40, 0.43]
VPVPDERFDFLCQYHK pc3 −0.10 [−1.16, 0.96]
RE Model 0.05 [0.02, 0.08]
−1.5
−1
−0.5
0
0.5
1
Effect of 2:174087028:TTTCTTTTTTTTTTTTTG:! (rs142397557)

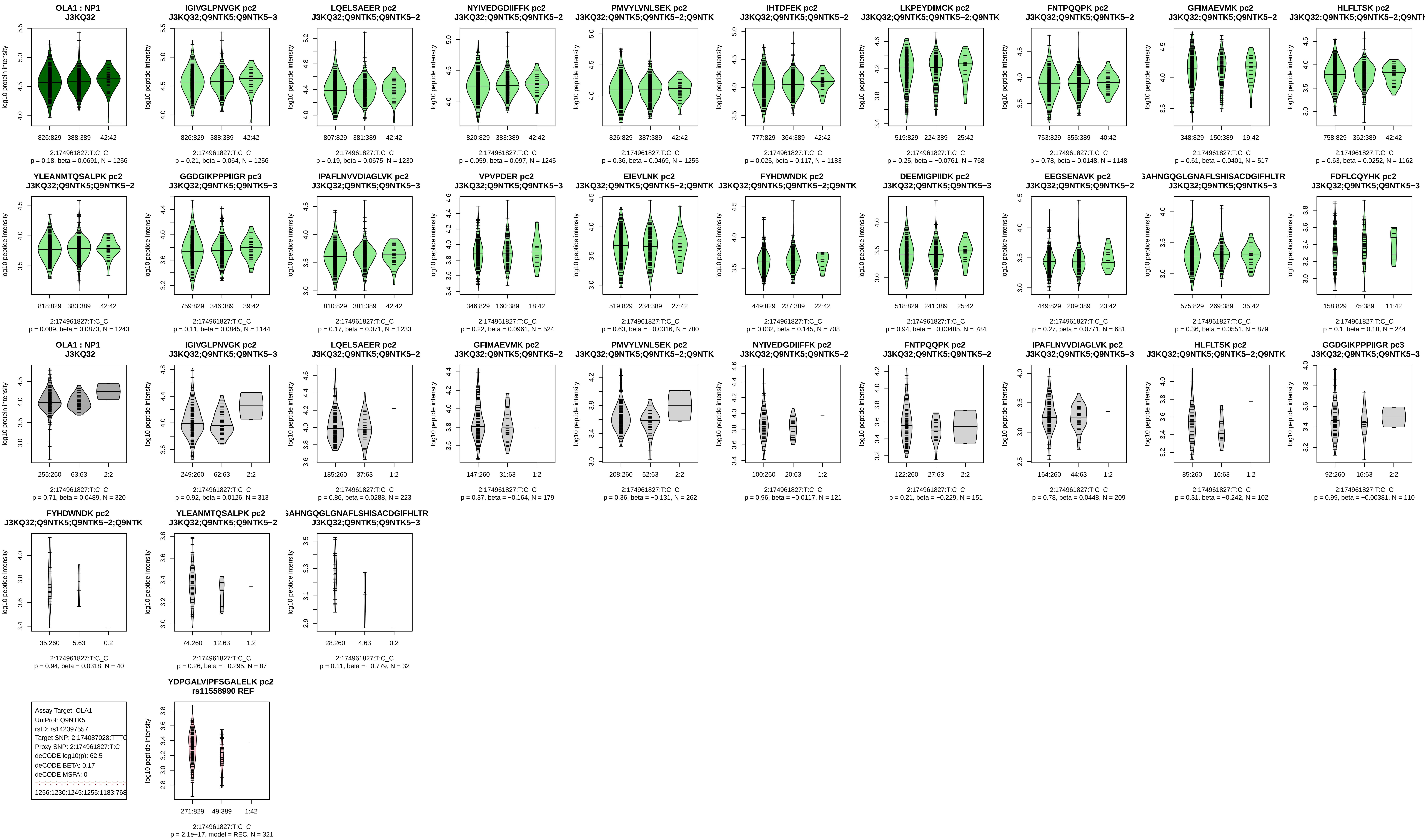

OLA1 : NP1
J3KQ32
log10 protein intensity
826:829 388:389 42:42
2:174961827:T:C_C
p = 0.18, beta = 0.0691, N = 1256
IGIVGLPNVGK pc2
J3KQ32;Q9NTK5;Q9NTK5−3
log10 peptide intensity
826:829 388:389 42:42
2:174961827:T:C_C
p = 0.21, beta = 0.064, N = 1256
LQELSAEER pc2
J3KQ32;Q9NTK5;Q9NTK5−2
807:829 381:389 42:42
2:174961827:T:C_C
p = 0.19, beta = 0.0675, N = 1230
NYIVEDGDIIFFK pc2
J3KQ32;Q9NTK5;Q9NTK5−2
820:829 383:389 42:42
2:174961827:T:C_C
p = 0.059, beta = 0.097, N = 1245
PMVYLVNLSEK pc2
J3KQ32;Q9NTK5;Q9NTK5−2;Q9NTK
826:829 387:389 42:42
2:174961827:T:C_C
p = 0.36, beta = 0.0469, N = 1255
IHTDFEK pc2
J3KQ32;Q9NTK5;Q9NTK5−2
777:829 364:389 42:42
2:174961827:T:C_C
p = 0.025, beta = 0.117, N = 1183
LKPEYDIMCK pc2
J3KQ32;Q9NTK5;Q9NTK5−2;Q9NTK
519:829 224:389 25:42
2:174961827:T:C_C
p = 0.25, beta = −0.0761, N = 768
FNTPQQPK pc2
J3KQ32;Q9NTK5;Q9NTK5−2
753:829 355:389 40:42
2:174961827:T:C_C
p = 0.78, beta = 0.0148, N = 1148
GFIMAEVMK pc2
J3KQ32;Q9NTK5;Q9NTK5−2
348:829 150:389 19:42
2:174961827:T:C_C
p = 0.61, beta = 0.0401, N = 517
HLFLTSK pc2
J3KQ32;Q9NTK5;Q9NTK5−2;Q9NTK
758:829 362:389 42:42
2:174961827:T:C_C
p = 0.63, beta = 0.0252, N = 1162
YLEANMTQSALPK pc2
J3KQ32;Q9NTK5;Q9NTK5−2
818:829 383:389 42:42
2:174961827:T:C_C
p = 0.089, beta = 0.0873, N = 1243
GGDGIKPPPIIGR pc3
J3KQ32;Q9NTK5;Q9NTK5−3
759:829 346:389 39:42
2:174961827:T:C_C
p = 0.11, beta = 0.0845, N = 1144
IPAFLNVVDIAGLVK pc2
J3KQ32;Q9NTK5;Q9NTK5−3
810:829 381:389 42:42
2:174961827:T:C_C
p = 0.17, beta = 0.071, N = 1233
VPVPDER pc2
J3KQ32;Q9NTK5;Q9NTK5−3
346:829 160:389 18:42
2:174961827:T:C_C
p = 0.22, beta = 0.0961, N = 524
EIEVLNK pc2
J3KQ32;Q9NTK5;Q9NTK5−2;Q9NTK
519:829 234:389 27:42
2:174961827:T:C_C
p = 0.63, beta = −0.0316, N = 780
FYHDWNDK pc2
J3KQ32;Q9NTK5;Q9NTK5−2;Q9NTK
449:829 237:389 22:42
2:174961827:T:C_C
p = 0.032, beta = 0.145, N = 708
DEEMIGPIIDK pc2
J3KQ32;Q9NTK5;Q9NTK5−3
518:829 241:389 25:42
2:174961827:T:C_C
p = 0.94, beta = −0.00485, N = 784
EEGSENAVK pc2
J3KQ32;Q9NTK5;Q9NTK5−2
449:829 209:389 23:42
2:174961827:T:C_C
p = 0.27, beta = 0.0771, N = 681
GAHNGQGLGNAFLSHISACDGIFHLTR
J3KQ32;Q9NTK5;Q9NTK5−3
575:829 269:389 35:42
2:174961827:T:C_C
p = 0.36, beta = 0.0551, N = 879
FDFLCQYHK pc2
J3KQ32;Q9NTK5;Q9NTK5−3
158:829 75:389 11:42
2:174961827:T:C_C
p = 0.1, beta = 0.18, N = 244
OLA1 : NP1
J3KQ32
255:260 63:63 2:2
2:174961827:T:C_C
p = 0.71, beta = 0.0489, N = 320
IGIVGLPNVGK pc2
J3KQ32;Q9NTK5;Q9NTK5−3
249:260 62:63 2:2
2:174961827:T:C_C
p = 0.92, beta = 0.0126, N = 313
LQELSAEER pc2
J3KQ32;Q9NTK5;Q9NTK5−2
185:260 37:63 1:2
2:174961827:T:C_C
p = 0.86, beta = 0.0288, N = 223
GFIMAEVMK pc2
J3KQ32;Q9NTK5;Q9NTK5−2
147:260 31:63 1:2
2:174961827:T:C_C
p = 0.37, beta = −0.164, N = 179
PMVYLVNLSEK pc2
J3KQ32;Q9NTK5;Q9NTK5−2;Q9NTK
208:260 52:63 2:2
2:174961827:T:C_C
p = 0.36, beta = −0.131, N = 262
NYIVEDGDIIFFK pc2
J3KQ32;Q9NTK5;Q9NTK5−2
100:260 20:63 1:2
2:174961827:T:C_C
p = 0.96, beta = −0.0117, N = 121
FNTPQQPK pc2
J3KQ32;Q9NTK5;Q9NTK5−2
122:260 27:63 2:2
2:174961827:T:C_C
p = 0.21, beta = −0.229, N = 151
IPAFLNVVDIAGLVK pc2
J3KQ32;Q9NTK5;Q9NTK5−3
164:260 44:63 1:2
2:174961827:T:C_C
p = 0.78, beta = 0.0448, N = 209
HLFLTSK pc2
J3KQ32;Q9NTK5;Q9NTK5−2;Q9NTK
85:260 16:63 1:2
2:174961827:T:C_C
p = 0.31, beta = −0.242, N = 102
GGDGIKPPPIIGR pc3
J3KQ32;Q9NTK5;Q9NTK5−3
92:260 16:63 2:2
2:174961827:T:C_C
p = 0.99, beta = −0.00381, N = 110
FYHDWNDK pc2
J3KQ32;Q9NTK5;Q9NTK5−2;Q9NTK
35:260 5:63 0:2
2:174961827:T:C_C
p = 0.94, beta = 0.0318, N = 40
YLEANMTQSALPK pc2
J3KQ32;Q9NTK5;Q9NTK5−2
74:260 12:63 1:2
2:174961827:T:C_C
p = 0.26, beta = −0.295, N = 87
GAHNGQGLGNAFLSHISACDGIFHLTR
J3KQ32;Q9NTK5;Q9NTK5−3
28:260 4:63 0:2
2:174961827:T:C_C
p = 0.11, beta = −0.779, N = 32
Assay Target: OLA1
UniProt: Q9NTK5
rsID: rs142397557
Target SNP: 2:174087028:TTTC
Proxy SNP: 2:174961827:T:C
deCODE log10(p): 62.5
deCODE BETA: 0.17
deCODE MSPA: 0
1256:1230:1245:1255:1183:768
YDPGALVIPFSGALELK pc2
rs11558990 REF
271:829 49:389 1:42
2:174961827:T:C_C
p = 2.1e−17, model = REC, N = 321

209. TF (P02787)
deCODE MSPA=0.142 (2 conflicts)

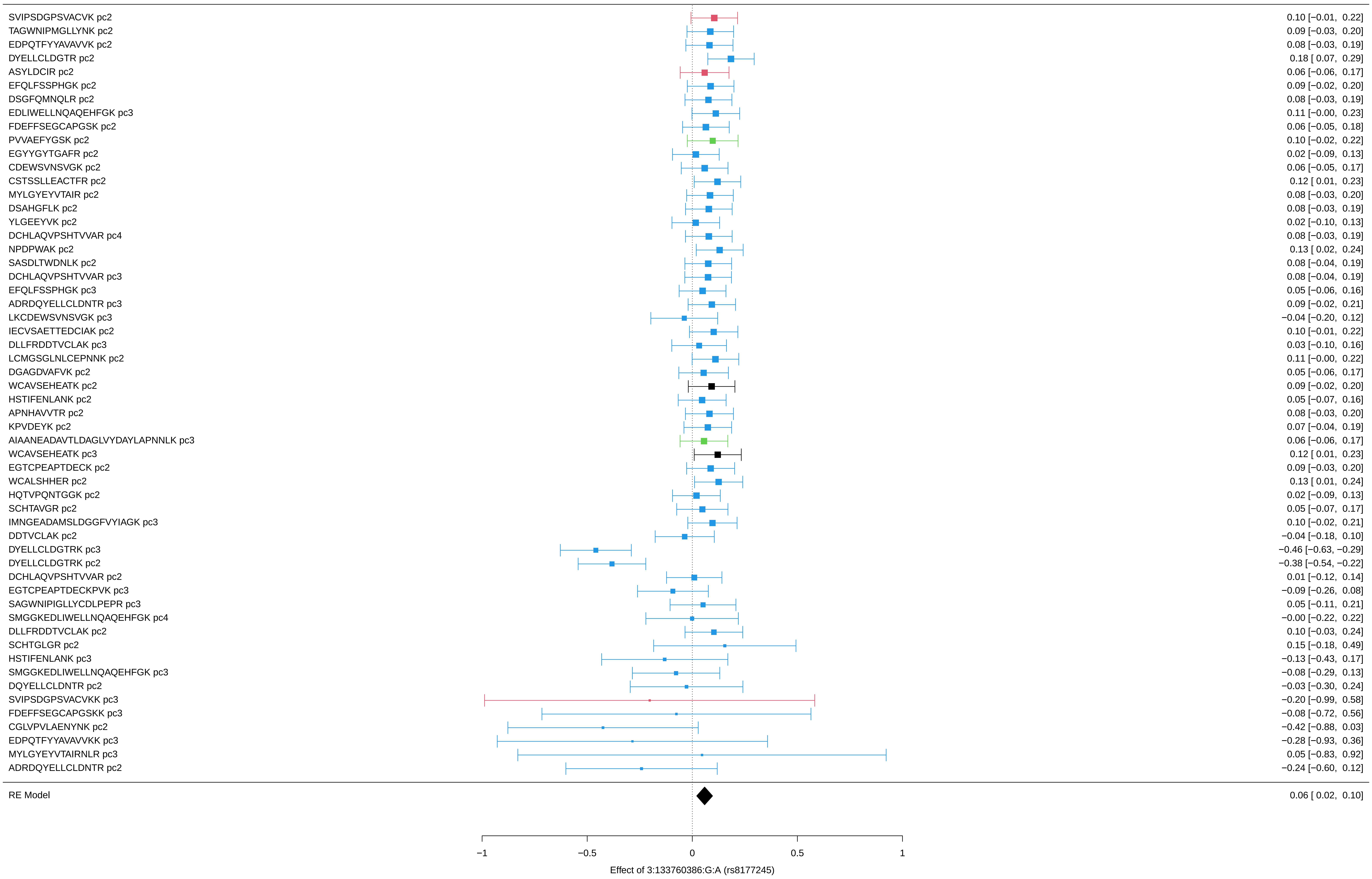

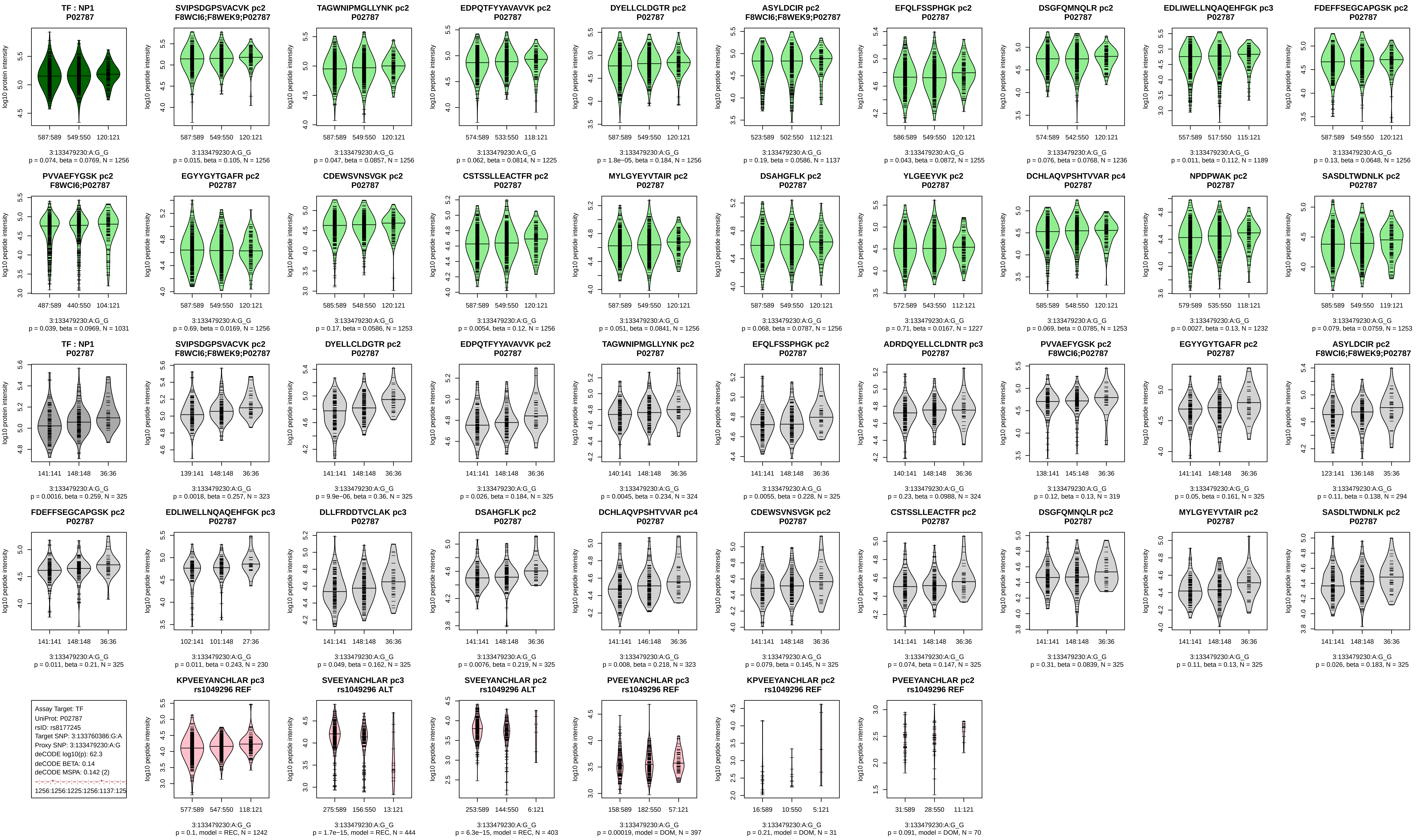
TF : NP1
P02787
log10 protein intensity
587:589 549:550 120:121
3:133479230:A:G_G
p = 0.074, beta = 0.0769, N = 1256
SVIPSDGPSVACVK pc2
F8WCI6;F8WEK9;P02787
log10 peptide intensity
587:589 549:550 120:121
3:133479230:A:G_G
p = 0.015, beta = 0.105, N = 1256
TAGWNIPMGLLYNK pc2
P02787
587:589 549:550 120:121
3:133479230:A:G_G
p = 0.047, beta = 0.0857, N = 1256
EDPQTFYYAVAVVK pc2
P02787
574:589 533:550 118:121
3:133479230:A:G_G
p = 0.062, beta = 0.0814, N = 1225
DYELLCLDGTR pc2
P02787
587:589 549:550 120:121
3:133479230:A:G_G
p = 1.8e−05, beta = 0.184, N = 1256
ASYLDCIR pc2
F8WCI6;F8WEK9;P02787
523:589 502:550 112:121
3:133479230:A:G_G
p = 0.19, beta = 0.0586, N = 1137
EFQLFSSPHGK pc2
P02787
586:589 549:550 120:121
3:133479230:A:G_G
p = 0.043, beta = 0.0872, N = 1255
DSGFQMNQLR pc2
P02787
574:589 542:550 120:121
3:133479230:A:G_G
p = 0.076, beta = 0.0768, N = 1236
EDLIWELLNQAQEHFGK pc3
P02787
557:589 517:550 115:121
3:133479230:A:G_G
p = 0.011, beta = 0.112, N = 1189
FDEFFSEGCAPGSK pc2
P02787
587:589 549:550 120:121
3:133479230:A:G_G
p = 0.13, beta = 0.0648, N = 1256
PVVAEFYGSK pc2
F8WCI6;P02787
487:589 440:550 104:121
3:133479230:A:G_G
p = 0.039, beta = 0.0969, N = 1031
EGYYGYTGAFR pc2
P02787
587:589 549:550 120:121
3:133479230:A:G_G
p = 0.69, beta = 0.0169, N = 1256
CDEWSVNSVGK pc2
P02787
585:589 548:550 120:121
3:133479230:A:G_G
p = 0.17, beta = 0.0586, N = 1253
CSTSSLLEACTFR pc2
P02787
587:589 549:550 120:121
3:133479230:A:G_G
p = 0.0054, beta = 0.12, N = 1256
MYLGYEYVTAIR pc2
P02787
587:589 549:550 120:121
3:133479230:A:G_G
p = 0.051, beta = 0.0841, N = 1256
DSAHGFLK pc2
P02787
587:589 549:550 120:121
3:133479230:A:G_G
p = 0.068, beta = 0.0787, N = 1256
YLGEEYVK pc2
P02787
572:589 543:550 112:121
3:133479230:A:G_G
p = 0.71, beta = 0.0167, N = 1227
DCHLAQVPSHTVVAR pc4
P02787
585:589 548:550 120:121
3:133479230:A:G_G
p = 0.069, beta = 0.0785, N = 1253
NPDPWAK pc2
P02787
579:589 535:550 118:121
3:133479230:A:G_G
p = 0.0027, beta = 0.13, N = 1232
SASDLTWDNLK pc2
P02787
585:589 549:550 119:121
3:133479230:A:G_G
p = 0.079, beta = 0.0759, N = 1253
TF : NP1
P02787
141:141 148:148 36:36
3:133479230:A:G_G
p = 0.0016, beta = 0.259, N = 325
SVIPSDGPSVACVK pc2
F8WCI6;F8WEK9;P02787
139:141 148:148 36:36
3:133479230:A:G_G
p = 0.0018, beta = 0.257, N = 323
DYELLCLDGTR pc2
P02787
141:141 148:148 36:36
3:133479230:A:G_G
p = 9.9e−06, beta = 0.36, N = 325
EDPQTFYYAVAVVK pc2
P02787
141:141 148:148 36:36
3:133479230:A:G_G
p = 0.026, beta = 0.184, N = 325
TAGWNIPMGLLYNK pc2
P02787
140:141 148:148 36:36
3:133479230:A:G_G
p = 0.0045, beta = 0.234, N = 324
EFQLFSSPHGK pc2
P02787
141:141 148:148 36:36
3:133479230:A:G_G
p = 0.0055, beta = 0.228, N = 325
ADRDQYELLCLDNTR pc3
P02787
140:141 148:148 36:36
3:133479230:A:G_G
p = 0.23, beta = 0.0988, N = 324
PVVAEFYGSK pc2
F8WCI6;P02787
138:141 145:148 36:36
3:133479230:A:G_G
p = 0.12, beta = 0.13, N = 319
EGYYGYTGAFR pc2
P02787
141:141 148:148 36:36
3:133479230:A:G_G
p = 0.05, beta = 0.161, N = 325
ASYLDCIR pc2
F8WCI6;F8WEK9;P02787
123:141 136:148 35:36
3:133479230:A:G_G
p = 0.11, beta = 0.138, N = 294
FDEFFSEGCAPGSK pc2
P02787
141:141 148:148 36:36
3:133479230:A:G_G
p = 0.011, beta = 0.21, N = 325
EDLIWELLNQAQEHFGK pc3
P02787
102:141 101:148 27:36
3:133479230:A:G_G
p = 0.011, beta = 0.243, N = 230
DLLFRDDTVCLAK pc3
P02787
141:141 148:148 36:36
3:133479230:A:G_G
p = 0.049, beta = 0.162, N = 325
DSAHGFLK pc2
P02787
141:141 148:148 36:36
3:133479230:A:G_G
p = 0.0076, beta = 0.219, N = 325
DCHLAQVPSHTVVAR pc4
P02787
141:141 146:148 36:36
3:133479230:A:G_G
p = 0.008, beta = 0.218, N = 323
CDEWSVNSVGK pc2
P02787
141:141 148:148 36:36
3:133479230:A:G_G
p = 0.079, beta = 0.145, N = 325
CSTSSLLEACTFR pc2
P02787
141:141 148:148 36:36
3:133479230:A:G_G
p = 0.074, beta = 0.147, N = 325
DSGFQMNQLR pc2
P02787
141:141 148:148 36:36
3:133479230:A:G_G
p = 0.31, beta = 0.0839, N = 325
MYLGYEYVTAIR pc2
P02787
141:141 148:148 36:36
3:133479230:A:G_G
p = 0.11, beta = 0.13, N = 325
SASDLTWDNLK pc2
P02787
141:141 148:148 36:36
3:133479230:A:G_G
p = 0.026, beta = 0.183, N = 325
Assay Target: TF
UniProt: P02787
rsID: rs8177245
Target SNP: 3:133760386:G:A
Proxy SNP: 3:133479230:A:G
deCODE log10(p): 62.3
deCODE BETA: 0.14
deCODE MSPA: 0.142 (2)
1256:1256:1225:1256:1137:125
KPVEEYANCHLAR pc3
rs1049296 REF
577:589 547:550 118:121
3:133479230:A:G_G
p = 0.1, model = REC, N = 1242
SVEEYANCHLAR pc3
rs1049296 ALT
275:589 156:550 13:121
3:133479230:A:G_G
p = 1.7e−15, model = REC, N = 444
SVEEYANCHLAR pc2
rs1049296 ALT
253:589 144:550 6:121
3:133479230:A:G_G
p = 6.3e−15, model = REC, N = 403
PVEEYANCHLAR pc3
rs1049296 REF
158:589 182:550 57:121
3:133479230:A:G_G
p = 0.00019, model = DOM, N = 397
KPVEEYANCHLAR pc2
rs1049296 REF
16:589 10:550 5:121
3:133479230:A:G_G
p = 0.21, model = DOM, N = 31
PVEEYANCHLAR pc2
rs1049296 REF
31:589 28:550 11:121
3:133479230:A:G_G
p = 0.091, model = DOM, N = 70

210. PXDN (Q92626)
deCODE MSPA=0

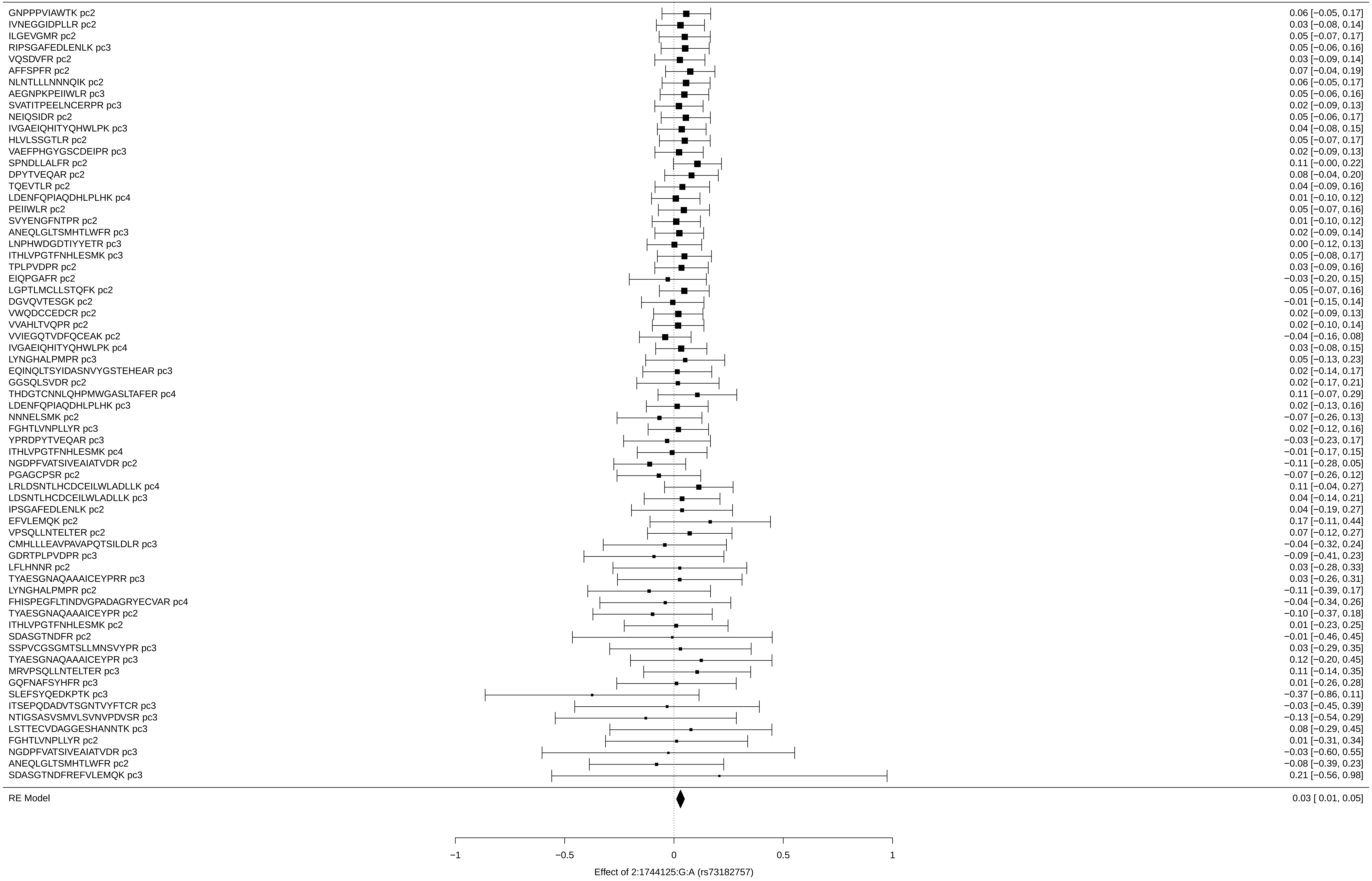

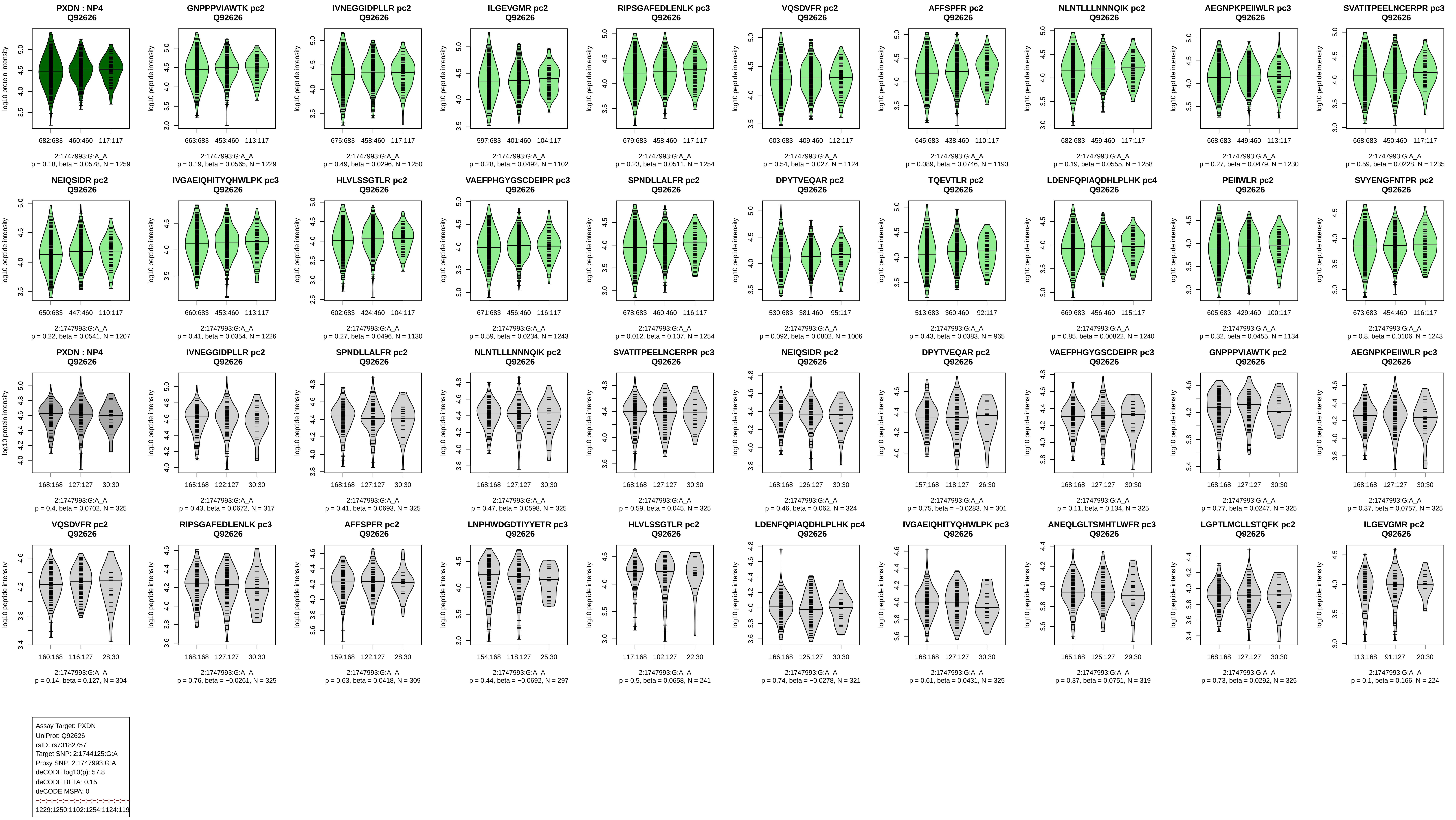
PXDN : NP4 Q92626
682:683 460:460 117:117
2:1747993:G:A_A
p = 0.18, beta = 0.0578, N = 1259
GNPPPVIAWTK pc2 Q92626
663:683 453:460 113:117
2:1747993:G:A_A
p = 0.19, beta = 0.0565, N = 1229
IVNEGGIDPLLR pc2 Q92626
675:683 458:460 117:117
2:1747993:G:A_A
p = 0.49, beta = 0.0296, N = 1250
ILGEVGMR pc2 Q92626
597:683 401:460 104:117
2:1747993:G:A_A
p = 0.28, beta = 0.0492, N = 1102
RIPSGAFEDLENLK pc3 Q92626
679:683 458:460 117:117
2:1747993:G:A_A
p = 0.23, beta = 0.0511, N = 1254
VQSDVFR pc2 Q92626
603:683 409:460 112:117
2:1747993:G:A_A
p = 0.54, beta = 0.027, N = 1124
AFFSPFR pc2 Q92626
645:683 438:460 110:117
2:1747993:G:A_A
p = 0.089, beta = 0.0746, N = 1193
NLNTLLLNNNQIK pc2 Q92626
682:683 459:460 117:117
2:1747993:G:A_A
p = 0.19, beta = 0.0555, N = 1258
AEGNPKPEIIWLR pc3 Q92626
668:683 449:460 113:117
2:1747993:G:A_A
p = 0.27, beta = 0.0479, N = 1230
SVATITPEELNCERPR pc3 Q92626
668:683 450:460 117:117
2:1747993:G:A_A
p = 0.59, beta = 0.0228, N = 1235
NEIQSIDR pc2 Q92626
650:683 447:460 110:117
2:1747993:G:A_A
p = 0.22, beta = 0.0541, N = 1207
IVGAEIQHITYQHWLPK pc3 Q92626
660:683 453:460 113:117
2:1747993:G:A_A
p = 0.41, beta = 0.0354, N = 1226
HLVLSSGTLR pc2 Q92626
602:683 424:460 104:117
2:1747993:G:A_A
p = 0.27, beta = 0.0496, N = 1130
VAEFPHGYGSCDEIPR pc3 Q92626
671:683 456:460 116:117
2:1747993:G:A_A
p = 0.59, beta = 0.0234, N = 1243
SPNDLLALFR pc2 Q92626
678:683 460:460 116:117
2:1747993:G:A_A
p = 0.012, beta = 0.107, N = 1254
DPYTVEQAR pc2 Q92626
530:683 381:460 95:117
2:1747993:G:A_A
p = 0.092, beta = 0.0802, N = 1006
TQEVTLR pc2 Q92626
513:683 360:460 92:117
2:1747993:G:A_A
p = 0.43, beta = 0.0383, N = 965
LDENFQPIAQDHLPLHK pc4 Q92626
669:683 456:460 115:117
2:1747993:G:A_A
p = 0.85, beta = 0.00822, N = 1240
PEIIWLR pc2 Q92626
605:683 429:460 100:117
2:1747993:G:A_A
p = 0.32, beta = 0.0455, N = 1134
SVYENGFNTPR pc2 Q92626
673:683 454:460 116:117
2:1747993:G:A_A
p = 0.8, beta = 0.0106, N = 1243
PXDN : NP4 Q92626
168:168 127:127 30:30
2:1747993:G:A_A
p = 0.4, beta = 0.0702, N = 325
IVNEGGIDPLLR pc2 Q92626
165:168 122:127 30:30
2:1747993:G:A_A
p = 0.43, beta = 0.0672, N = 317
SPNDLLALFR pc2 Q92626
168:168 127:127 30:30
2:1747993:G:A_A
p = 0.41, beta = 0.0693, N = 325
NLNTLLLNNNQIK pc2 Q92626
168:168 127:127 30:30
2:1747993:G:A_A
p = 0.47, beta = 0.0598, N = 325
SVATITPEELNCERPR pc3 Q92626
168:168 127:127 30:30
2:1747993:G:A_A
p = 0.59, beta = 0.045, N = 325
NEIQSIDR pc2 Q92626
168:168 126:127 30:30
2:1747993:G:A_A
p = 0.46, beta = 0.062, N = 324
DPYTVEQAR pc2 Q92626
157:168 118:127 26:30
2:1747993:G:A_A
p = 0.75, beta = −0.0283, N = 301
VAEFPHGYGSCDEIPR pc3 Q92626
168:168 127:127 30:30
2:1747993:G:A_A
p = 0.11, beta = 0.134, N = 325
GNPPPVIAWTK pc2 Q92626
168:168 127:127 30:30
2:1747993:G:A_A
p = 0.77, beta = 0.0247, N = 325
AEGNPKPEIIWLR pc3 Q92626
168:168 127:127 30:30
2:1747993:G:A_A
p = 0.37, beta = 0.0757, N = 325
VQSDVFR pc2 Q92626
160:168 116:127 28:30
2:1747993:G:A_A
p = 0.14, beta = 0.127, N = 304
RIPSGAFEDLENLK pc3 Q92626
168:168 127:127 30:30
2:1747993:G:A_A
p = 0.76, beta = −0.0261, N = 325
AFFSPFR pc2 Q92626
159:168 122:127 28:30
2:1747993:G:A_A
p = 0.63, beta = 0.0418, N = 309
LNPHWDGDTIYYETR pc3 Q92626
154:168 118:127 25:30
2:1747993:G:A_A
p = 0.44, beta = −0.0692, N = 297
HLVLSSGTLR pc2 Q92626
117:168 102:127 22:30
2:1747993:G:A_A
p = 0.5, beta = 0.0658, N = 241
LDENFQPIAQDHLPLHK pc4 Q92626
166:168 125:127 30:30
2:1747993:G:A_A
p = 0.74, beta = −0.0278, N = 321
IVGAEIQHITYQHWLPK pc3 Q92626
168:168 127:127 30:30
2:1747993:G:A_A
p = 0.61, beta = 0.0431, N = 325
ANEQLGLTSMHTLWFR pc3 Q92626
165:168 125:127 29:30
2:1747993:G:A_A
p = 0.37, beta = 0.0751, N = 319
LGPTLMCLLSTQFK pc2 Q92626
168:168 127:127 30:30
2:1747993:G:A_A
p = 0.73, beta = 0.0292, N = 325
ILGEVGMR pc2 Q92626
113:168 91:127 20:30
2:1747993:G:A_A
p = 0.1, beta = 0.166, N = 224
Assay Target: PXDN
UniProt: Q92626
rsID: rs73182757
Target SNP: 2:1744125:G:A
Proxy SNP: 2:1747993:G:A
deCODE log10(p): 57.8
deCODE BETA: 0.15
deCODE MSPA: 0
1229:1250:1102:1254:1124:119

211. NDST1 (P52848)
deCODE MSPA=0

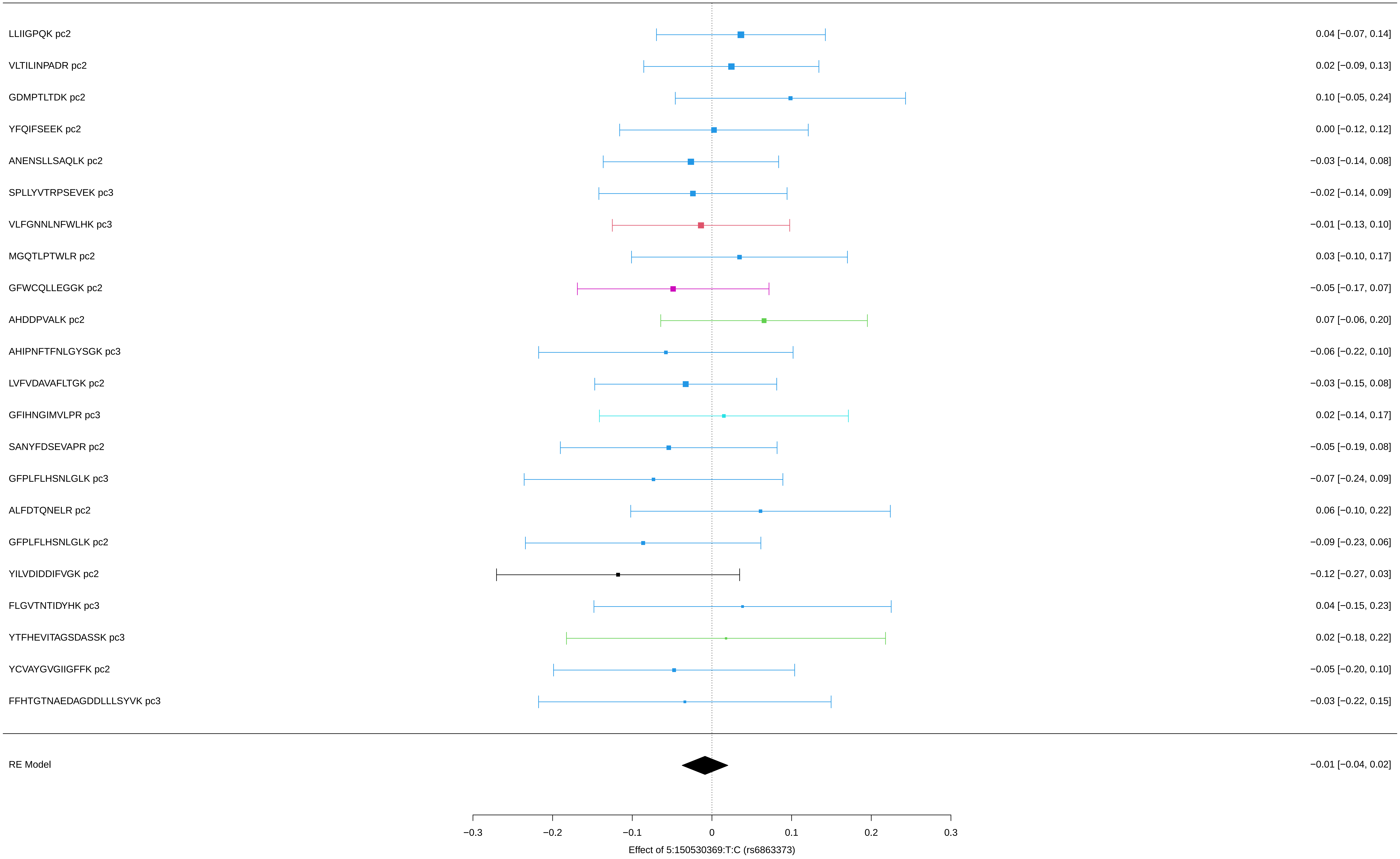

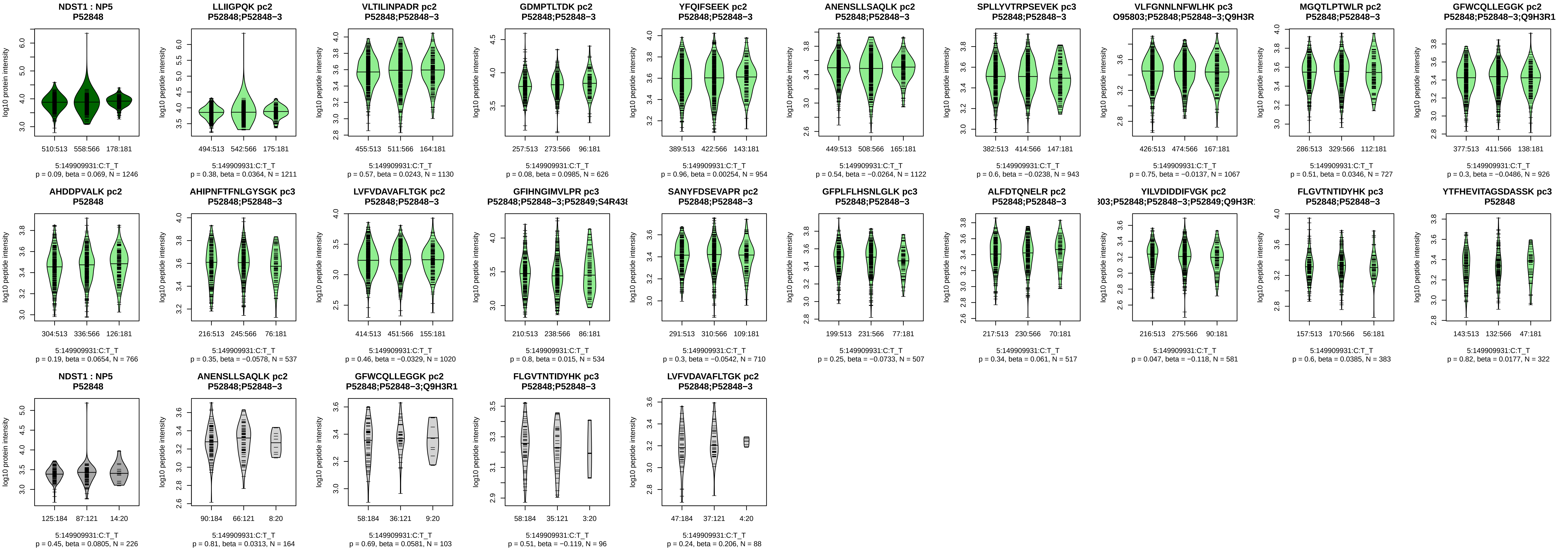

Assay Target: NDST1
UniProt: P52848
rsID: rs6863373
Target SNP: 5:150530369:T:C
Proxy SNP: 5:149909931:C:T
deCODE log10(p): 57.4
deCODE BETA: 0.13
deCODE MSPA: 0
1211:1130:626:954:1122:943:1(

212. NUCB1 (Q02818)
deCODE MSPA=0.358

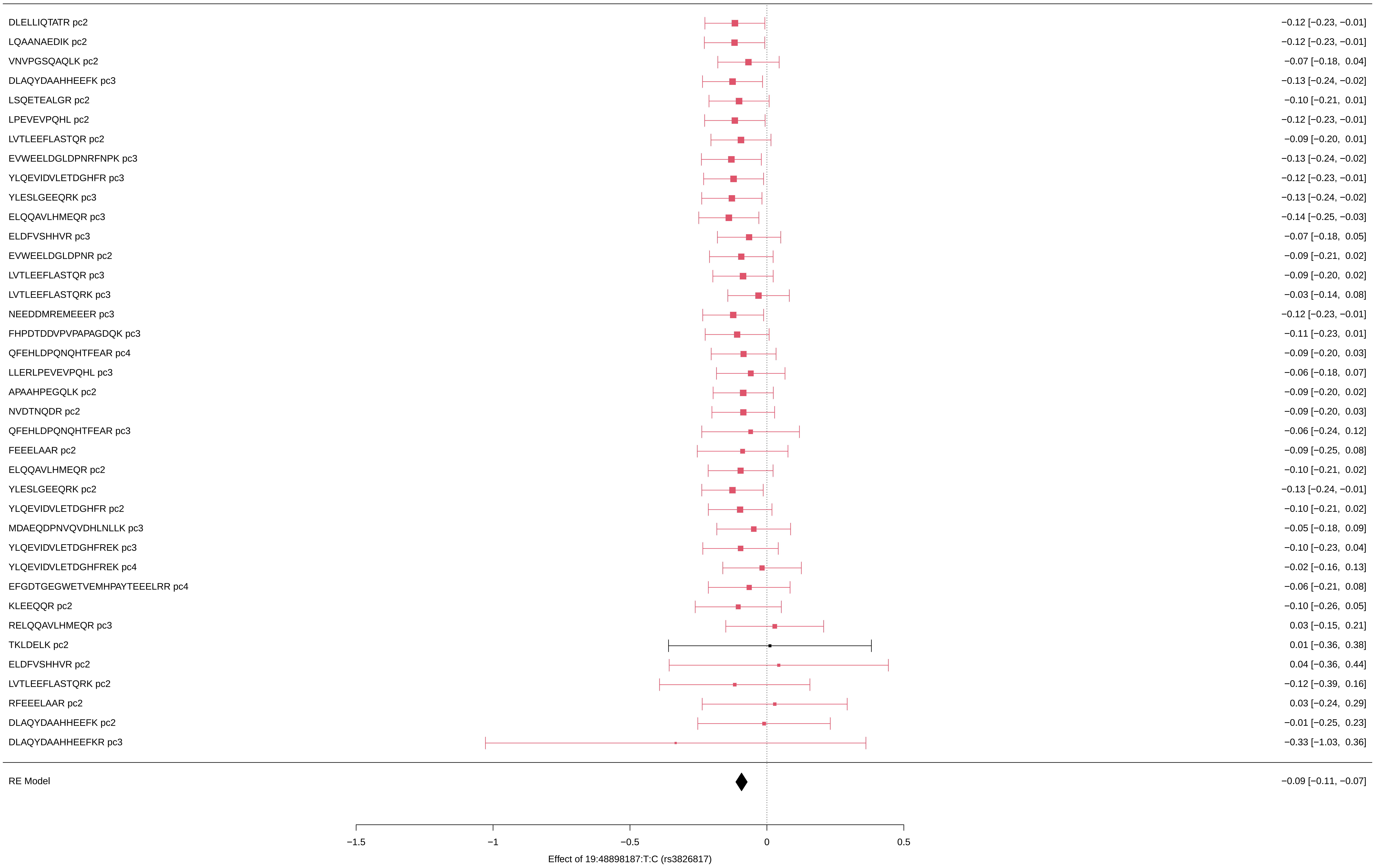

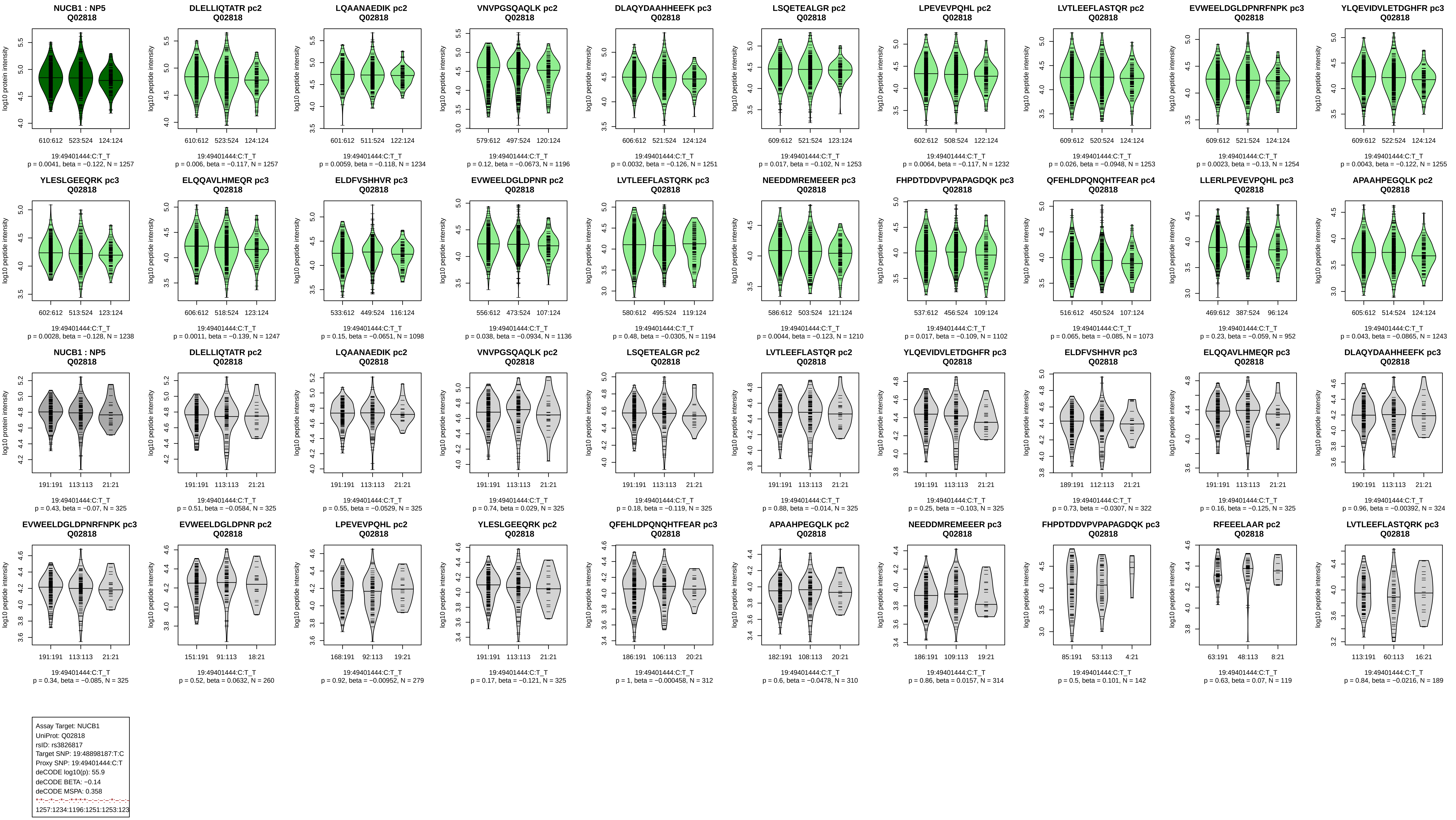
NUCB1 : NP5 Q02818
log10 protein intensity
610:612 523:524 124:124
19:49401444:C:T_T
p = 0.0041, beta = −0.122, N = 1257
DLELLIQTATR pc2 Q02818
log10 peptide intensity
610:612 523:524 124:124
19:49401444:C:T_T
p = 0.006, beta = −0.117, N = 1257
LQAANAEDIK pc2 Q02818
601:612 511:524 122:124
19:49401444:C:T_T
p = 0.0059, beta = −0.118, N = 1234
VNVPGSQAQLK pc2 Q02818
579:612 497:524 120:124
19:49401444:C:T_T
p = 0.12, beta = −0.0673, N = 1196
DLAQYDAAHHEEFK pc3 Q02818
606:612 521:524 124:124
19:49401444:C:T_T
p = 0.0032, beta = −0.126, N = 1251
LSQETEALGR pc2 Q02818
609:612 521:524 123:124
19:49401444:C:T_T
p = 0.017, beta = −0.102, N = 1253
LPEVEVPQHL pc2 Q02818
602:612 508:524 122:124
19:49401444:C:T_T
p = 0.0064, beta = −0.117, N = 1232
LVTLEEFLASTQR pc2 Q02818
609:612 520:524 124:124
19:49401444:C:T_T
p = 0.026, beta = −0.0948, N = 1253
EVWEELDGLDPNRFNPK pc3 Q02818
609:612 521:524 124:124
19:49401444:C:T_T
p = 0.0023, beta = −0.13, N = 1254
YLQEVIDVLETDGHFR pc3 Q02818
609:612 522:524 124:124
19:49401444:C:T_T
p = 0.0043, beta = −0.122, N = 1255
YLESLGEEQRK pc3 Q02818
602:612 513:524 123:124
19:49401444:C:T_T
p = 0.0028, beta = −0.128, N = 1238
ELQQAVLHMEQR pc3 Q02818
606:612 518:524 123:124
19:49401444:C:T_T
p = 0.0011, beta = −0.139, N = 1247
ELDFVSHHVR pc3 Q02818
533:612 449:524 116:124
19:49401444:C:T_T
p = 0.15, beta = −0.0651, N = 1098
EVWEELDGLDPNR pc2 Q02818
556:612 473:524 107:124
19:49401444:C:T_T
p = 0.038, beta = −0.0934, N = 1136
LVTLEEFLASTQRK pc3 Q02818
580:612 495:524 119:124
19:49401444:C:T_T
p = 0.48, beta = −0.0305, N = 1194
NEEDDMREMEEER pc3 Q02818
586:612 503:524 121:124
19:49401444:C:T_T
p = 0.0044, beta = −0.123, N = 1210
FHPDTDDVPVPAPAGDQK pc3 Q02818
537:612 456:524 109:124
19:49401444:C:T_T
p = 0.017, beta = −0.109, N = 1102
QFEHLDPQNQHTFEAR pc4 Q02818
516:612 450:524 107:124
19:49401444:C:T_T
p = 0.065, beta = −0.085, N = 1073
LLERLPEVEVPQHL pc3 Q02818
469:612 387:524 96:124
19:49401444:C:T_T
p = 0.23, beta = −0.059, N = 952
APAAHPEGQLK pc2 Q02818
605:612 514:524 124:124
19:49401444:C:T_T
p = 0.043, beta = −0.0865, N = 1243
NUCB1 : NP5 Q02818
191:191 113:113 21:21
19:49401444:C:T_T
p = 0.43, beta = −0.07, N = 325
DLELLIQTATR pc2 Q02818
191:191 113:113 21:21
19:49401444:C:T_T
p = 0.51, beta = −0.0584, N = 325
LQAANAEDIK pc2 Q02818
191:191 113:113 21:21
19:49401444:C:T_T
p = 0.55, beta = −0.0529, N = 325
VNVPGSQAQLK pc2 Q02818
191:191 113:113 21:21
19:49401444:C:T_T
p = 0.74, beta = 0.029, N = 325
LSQETEALGR pc2 Q02818
191:191 113:113 21:21
19:49401444:C:T_T
p = 0.18, beta = −0.119, N = 325
LVTLEEFLASTQR pc2 Q02818
191:191 113:113 21:21
19:49401444:C:T_T
p = 0.88, beta = −0.014, N = 325
YLQEVIDVLETDGHFR pc3 Q02818
191:191 113:113 21:21
19:49401444:C:T_T
p = 0.25, beta = −0.103, N = 325
ELDFVSHHVR pc3 Q02818
189:191 112:113 21:21
19:49401444:C:T_T
p = 0.73, beta = −0.0307, N = 322
ELQQAVLHMEQR pc3 Q02818
191:191 113:113 21:21
19:49401444:C:T_T
p = 0.16, beta = −0.125, N = 325
DLAQYDAAHHEEFK pc3 Q02818
190:191 113:113 21:21
19:49401444:C:T_T
p = 0.96, beta = −0.00392, N = 324
EVWEELDGLDPNRFNPK pc3 Q02818
191:191 113:113 21:21
19:49401444:C:T_T
p = 0.34, beta = −0.085, N = 325
EVWEELDGLDPNR pc2 Q02818
151:191 91:113 18:21
19:49401444:C:T_T
p = 0.52, beta = 0.0632, N = 260
LPEVEVPQHL pc2 Q02818
168:191 92:113 19:21
19:49401444:C:T_T
p = 0.92, beta = −0.00952, N = 279
YLESLGEEQRK pc2 Q02818
191:191 113:113 21:21
19:49401444:C:T_T
p = 0.17, beta = −0.121, N = 325
QFEHLDPQNQHTFEAR pc3 Q02818
186:191 106:113 20:21
19:49401444:C:T_T
p = 1, beta = −0.000458, N = 312
APAAHPEGQLK pc2 Q02818
182:191 108:113 20:21
19:49401444:C:T_T
p = 0.6, beta = −0.0478, N = 310
NEEDDMREMEEER pc3 Q02818
186:191 109:113 19:21
19:49401444:C:T_T
p = 0.86, beta = 0.0157, N = 314
FHPDTDDVPVPAPAGDQK pc3 Q02818
85:191 53:113 4:21
19:49401444:C:T_T
p = 0.5, beta = 0.101, N = 142
RFEEELAAR pc2 Q02818
63:191 48:113 8:21
19:49401444:C:T_T
p = 0.63, beta = 0.07, N = 119
LVTLEEFLASTQRK pc3 Q02818
113:191 60:113 16:21
19:49401444:C:T_T
p = 0.84, beta = −0.0216, N = 189
Assay Target: NUCB1
UniProt: Q02818
rsID: rs3826817
Target SNP: 19:48898187:T:C
Proxy SNP: 19:49401444:C:T
deCODE log10(p): 55.9
deCODE BETA: −0.14
deCODE MSPA: 0.358
1257:1234:1196:1251:1253:123

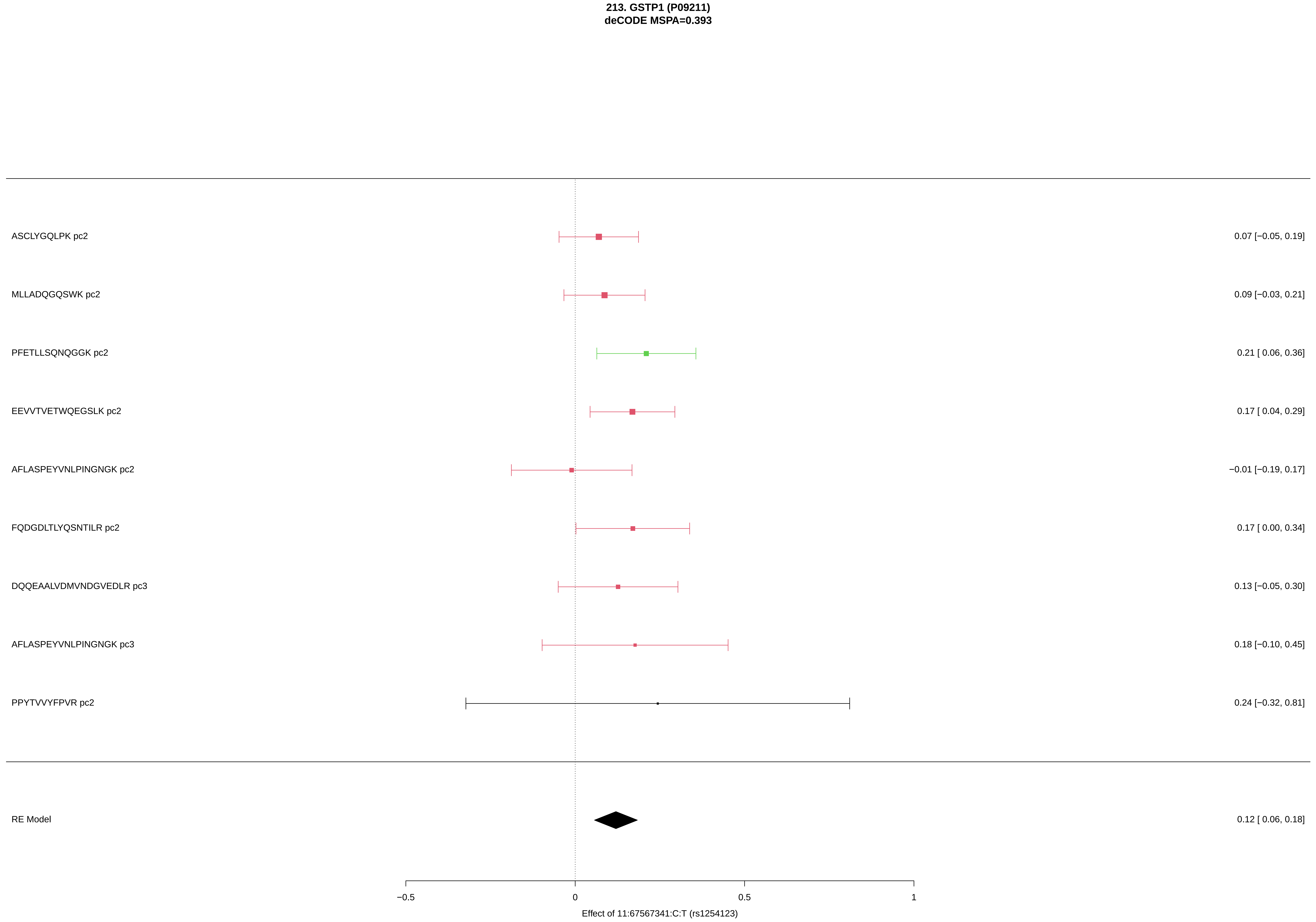
213. GSTP1 (P09211)
deCODE MSPA=0.393
ASCLYGQLPK pc2
0.07 [−0.05, 0.19]
MLLADQGQSWK pc2
0.09 [−0.03, 0.21]
PFETLLSQNQGGK pc2
0.21 [0.06, 0.36]
EEVVTVETWQEGSLK pc2
0.17 [0.04, 0.29]
AFLASPEYVNLPINGNGK pc2
−0.01 [−0.19, 0.17]
FQDGDLTLYQSNTILR pc2
0.17 [0.00, 0.34]
DQQEAALVDMVNDGVEDLR pc3
0.13 [−0.05, 0.30]
AFLASPEYVNLPINGNGK pc3
0.18 [−0.10, 0.45]
PPYTVVYFPVR pc2
0.24 [−0.32, 0.81]
RE Model
0.12 [0.06, 0.18]
−0.5
0
0.5
1
Effect of 11:67567341:C:T (rs1254123)

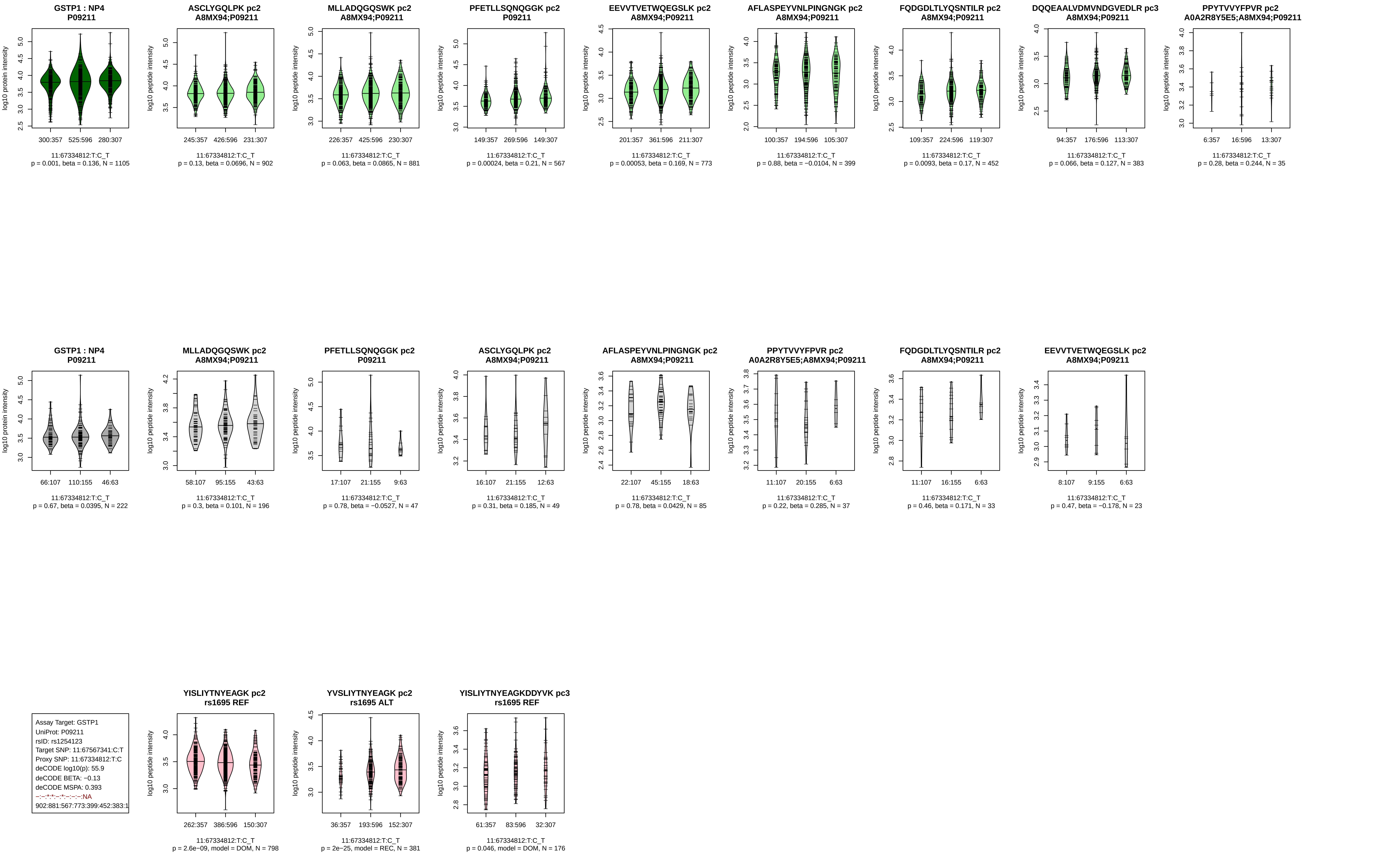

GSTP1 : NP4
P09211
300:357 525:596 280:307
11:67334812:T:C_T
p = 0.001, beta = 0.136, N = 1105
ASCLYGQLPK pc2
A8MX94;P09211
245:357 426:596 231:307
11:67334812:T:C_T
p = 0.13, beta = 0.0696, N = 902
MLLADQGQSWK pc2
A8MX94;P09211
226:357 425:596 230:307
11:67334812:T:C_T
p = 0.063, beta = 0.0865, N = 881
PFETLLSQNQGGK pc2
P09211
149:357 269:596 149:307
11:67334812:T:C_T
p = 0.00024, beta = 0.21, N = 567
EEVVTVETWQEGSLK pc2
A8MX94;P09211
201:357 361:596 211:307
11:67334812:T:C_T
p = 0.00053, beta = 0.169, N = 773
AFLASPEYVNLPINGNGK pc2
A8MX94;P09211
100:357 194:596 105:307
11:67334812:T:C_T
p = 0.88, beta = −0.0104, N = 399
FQDGDLTLYQSNTILR pc2
A8MX94;P09211
109:357 224:596 119:307
11:67334812:T:C_T
p = 0.0093, beta = 0.17, N = 452
DQQEAALVDMVNDGVEDLR pc3
A8MX94;P09211
94:357 176:596 113:307
11:67334812:T:C_T
p = 0.066, beta = 0.127, N = 383
PPYTVVYFPVR pc2
A0A2R8Y5E5;A8MX94;P09211
6:357 16:596 13:307
11:67334812:T:C_T
p = 0.28, beta = 0.244, N = 35
GSTP1 : NP4
P09211
66:107 110:155 46:63
11:67334812:T:C_T
p = 0.67, beta = 0.0395, N = 222
MLLADQGQSWK pc2
A8MX94;P09211
58:107 95:155 43:63
11:67334812:T:C_T
p = 0.3, beta = 0.101, N = 196
PFETLLSQNQGGK pc2
P09211
17:107 21:155 9:63
11:67334812:T:C_T
p = 0.78, beta = −0.0527, N = 47
ASCLYGQLPK pc2
A8MX94;P09211
16:107 21:155 12:63
11:67334812:T:C_T
p = 0.31, beta = 0.185, N = 49
AFLASPEYVNLPINGNGK pc2
A8MX94;P09211
22:107 45:155 18:63
11:67334812:T:C_T
p = 0.78, beta = 0.0429, N = 85
PPYTVVYFPVR pc2
A0A2R8Y5E5;A8MX94;P09211
11:107 20:155 6:63
11:67334812:T:C_T
p = 0.22, beta = 0.285, N = 37
FQDGDLTLYQSNTILR pc2
A8MX94;P09211
11:107 16:155 6:63
11:67334812:T:C_T
p = 0.46, beta = 0.171, N = 33
EEVVTVETWQEGSLK pc2
A8MX94;P09211
8:107 9:155 6:63
11:67334812:T:C_T
p = 0.47, beta = −0.178, N = 23
Assay Target: GSTP1
UniProt: P09211
rsID: rs1254123
Target SNP: 11:67567341:C:T
Proxy SNP: 11:67334812:T:C
deCODE log10(p): 55.9
deCODE BETA: −0.13
deCODE MSPA: 0.393
−:−:*:*:−:*:−:−:−:NA
902:881:567:773:399:452:383:1
YISLIYTNYEAGK pc2
rs1695 REF
262:357 386:596 150:307
11:67334812:T:C_T
p = 2.6e−09, model = DOM, N = 798
YVSLIYTNYEAGK pc2
rs1695 ALT
36:357 193:596 152:307
11:67334812:T:C_T
p = 2e−25, model = REC, N = 381
YISLIYTNYEAGKDDYVK pc3
rs1695 REF
61:357 83:596 32:307
11:67334812:T:C_T
p = 0.046, model = DOM, N = 176

214. PNP (P00491)
deCODE MSPA=0

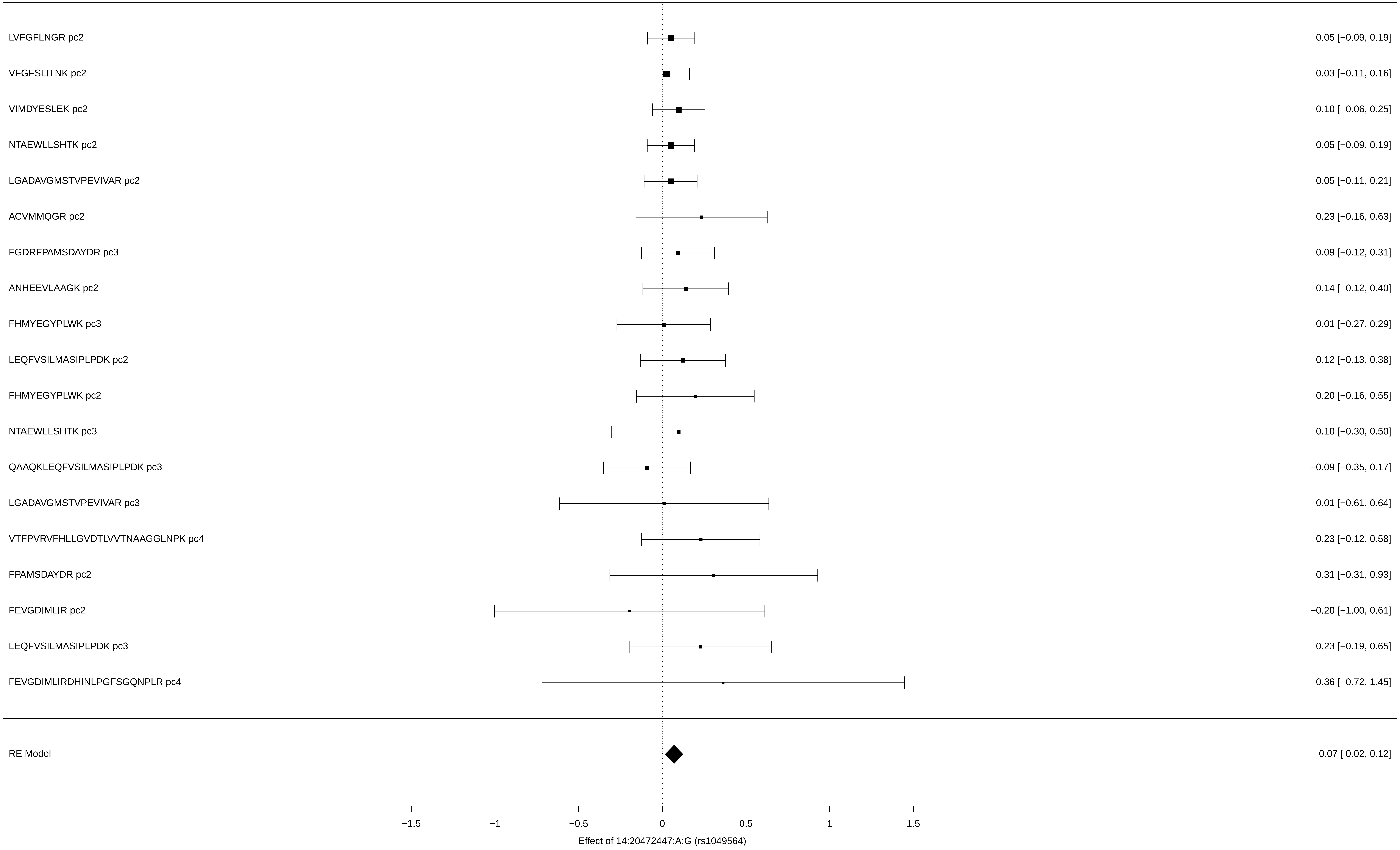

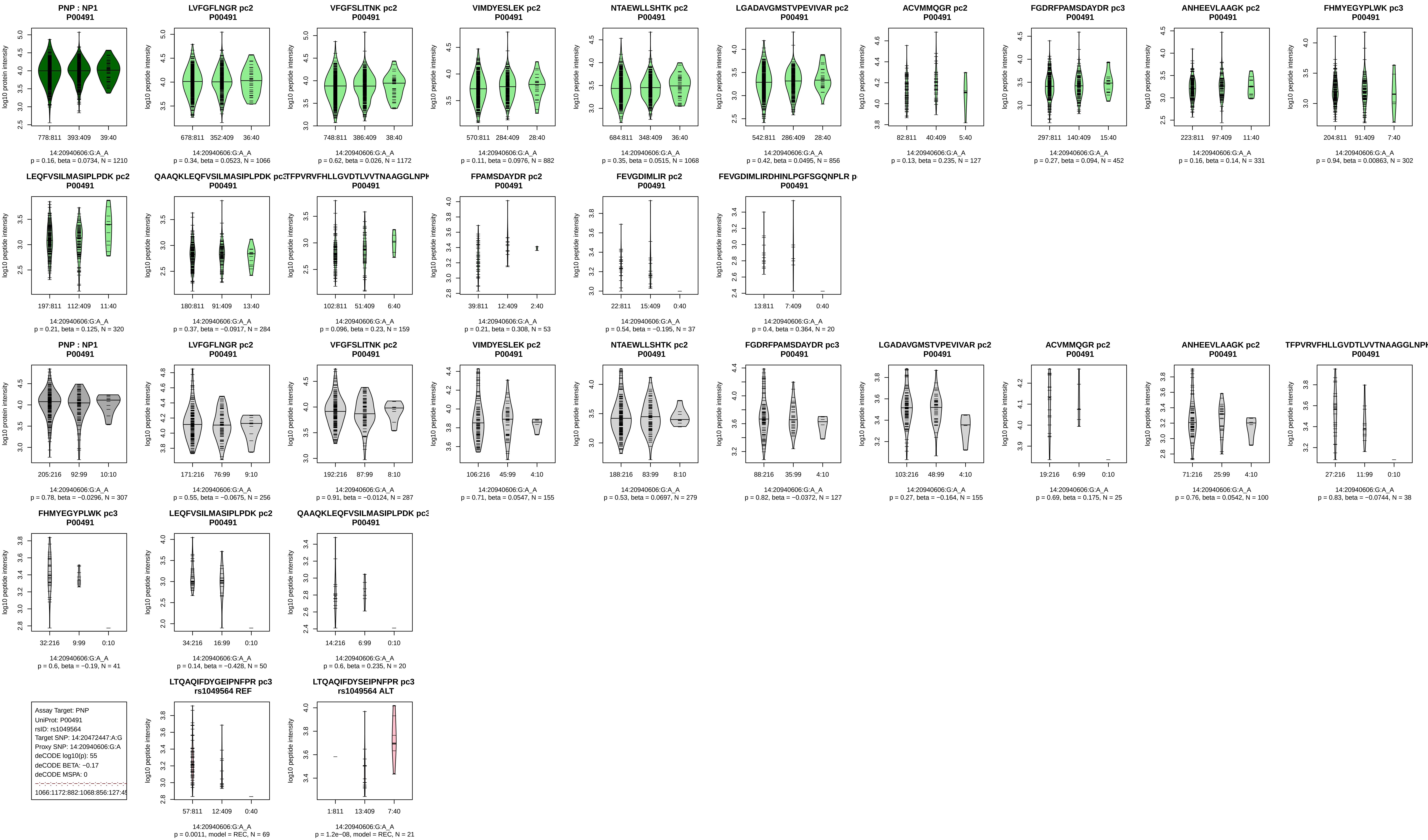
PNP : NP1
P00491
14:20940606:G:A_A
p = 0.16, beta = 0.0734, N = 1210
LVFGFLNGR pc2
P00491
p = 0.34, beta = 0.0523, N = 1066
VFGFSLITNK pc2
P00491
p = 0.62, beta = 0.026, N = 1172
VIMDYESLEK pc2
P00491
p = 0.11, beta = 0.0976, N = 882
NTAEWLLSHTK pc2
P00491
p = 0.35, beta = 0.0515, N = 1068
LGADAVGMSTVPEVIVAR pc2
P00491
p = 0.42, beta = 0.0495, N = 856
ACVMMQGR pc2
P00491
p = 0.13, beta = 0.235, N = 127
FGDRFPAMSDAYDR pc3
P00491
p = 0.27, beta = 0.094, N = 452
ANHEEVLAAGK pc2
P00491
p = 0.16, beta = 0.14, N = 331
FHMYEGYPLWK pc3
P00491
p = 0.94, beta = 0.00863, N = 302
LEQFVSILMASIPLPDK pc2
P00491
p = 0.21, beta = 0.125, N = 320
QAAQKLEQFVSILMASIPLPDK pc3
P00491
p = 0.37, beta = −0.0917, N = 284
TFPVRVFHLLGVDTLVVTNAAGGLNPK
P00491
p = 0.096, beta = 0.23, N = 159
FPAMSDAYDR pc2
P00491
p = 0.21, beta = 0.308, N = 53
FEVGDIMLIR pc2
P00491
p = 0.54, beta = −0.195, N = 37
FEVGDIMLIRDHINLPGFSGQNPLR p
P00491
p = 0.4, beta = 0.364, N = 20
PNP : NP1
P00491
p = 0.78, beta = −0.0296, N = 307
LVFGFLNGR pc2
P00491
p = 0.55, beta = −0.0675, N = 256
VFGFSLITNK pc2
P00491
p = 0.91, beta = −0.0124, N = 287
VIMDYESLEK pc2
P00491
p = 0.71, beta = 0.0547, N = 155
NTAEWLLSHTK pc2
P00491
p = 0.53, beta = 0.0697, N = 279
FGDRFPAMSDAYDR pc3
P00491
p = 0.82, beta = −0.0372, N = 127
LGADAVGMSTVPEVIVAR pc2
P00491
p = 0.27, beta = −0.164, N = 155
ACVMMQGR pc2
P00491
p = 0.69, beta = 0.175, N = 25
ANHEEVLAAGK pc2
P00491
p = 0.76, beta = 0.0542, N = 100
TFPVRVFHLLGVDTLVVTNAAGGLNPK
P00491
p = 0.83, beta = −0.0744, N = 38
FHMYEGYPLWK pc3
P00491
p = 0.6, beta = −0.19, N = 41
LEQFVSILMASIPLPDK pc2
P00491
p = 0.14, beta = −0.428, N = 50
QAAQKLEQFVSILMASIPLPDK pc3
P00491
p = 0.6, beta = 0.235, N = 20
Assay Target: PNP
UniProt: P00491
rsID: rs1049564
Target SNP: 14:20472447:A:G
Proxy SNP: 14:20940606:G:A
deCODE log10(p): 55
deCODE BETA: −0.17
deCODE MSPA: 0
1066:1172:882:1068:856:127:45
LTQAQIFDYGEIPNFPR pc3
rs1049564 REF
p = 0.0011, model = REC, N = 69
LTQAQIFDYSEIPNFPR pc3
rs1049564 ALT
p = 1.2e−08, model = REC, N = 21

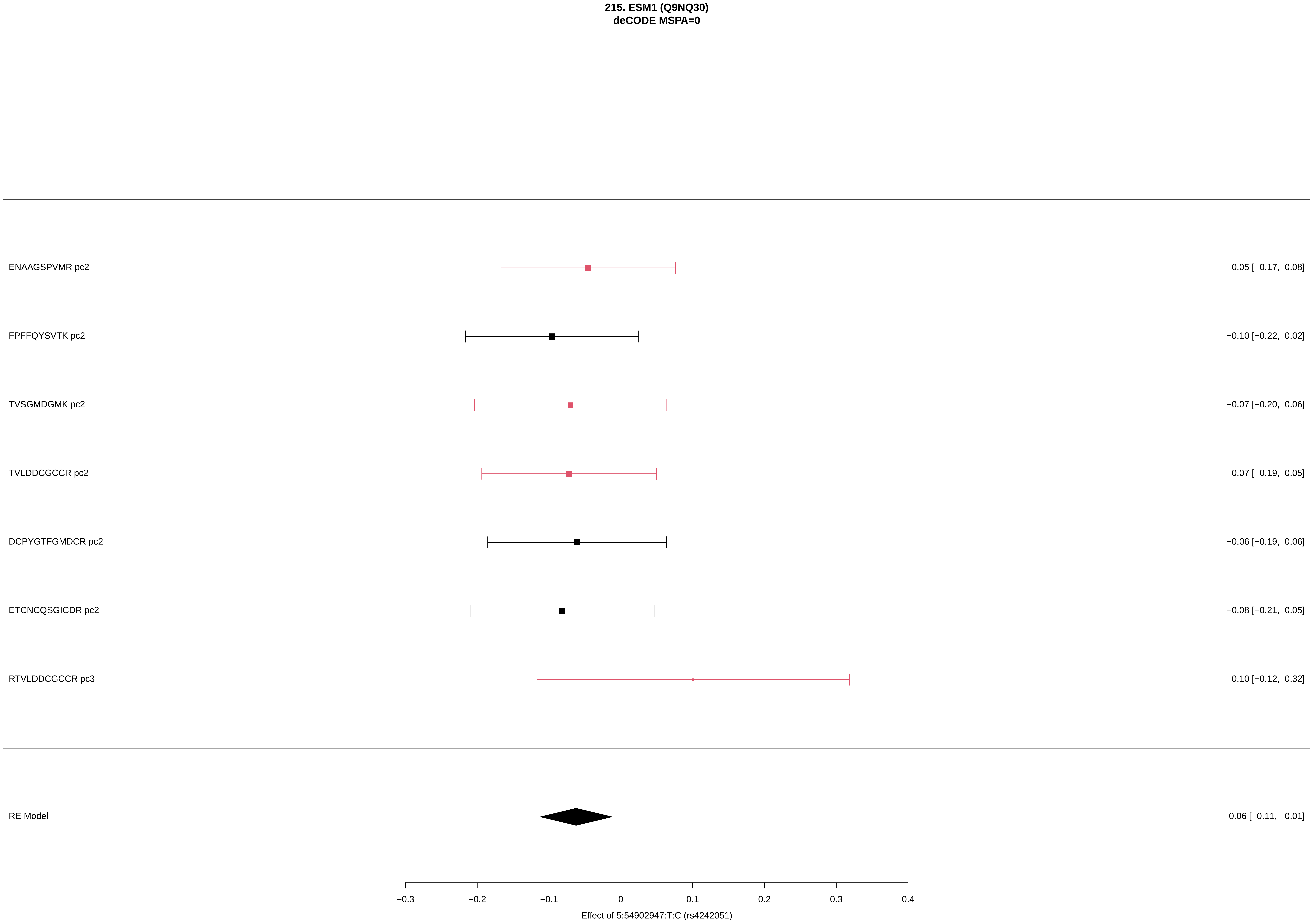

215. ESM1 (Q9NQ30)
deCODE MSPA=0
ENAAGSPVMR pc2
−0.05 [−0.17, 0.08]
FPFFQYSVTK pc2
−0.10 [−0.22, 0.02]
TVSGMDGMK pc2
−0.07 [−0.20, 0.06]
TVLDDCGCCR pc2
−0.07 [−0.19, 0.05]
DCPYGTFGMDCR pc2
−0.06 [−0.19, 0.06]
ETCNCQSGICDR pc2
−0.08 [−0.21, 0.05]
RTVLDDCGCCR pc3
0.10 [−0.12, 0.32]
RE Model
−0.06 [−0.11, −0.01]
−0.3
−0.2
−0.1
0
0.1
0.2
0.3
0.4
Effect of 5:54902947:T:C (rs4242051)

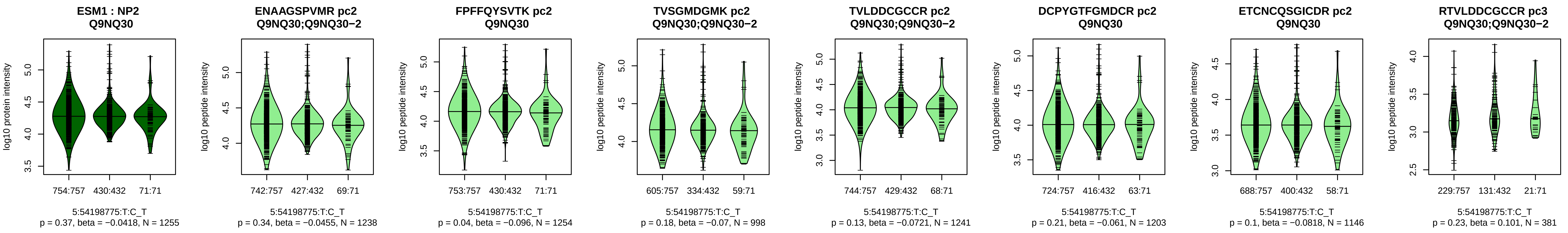

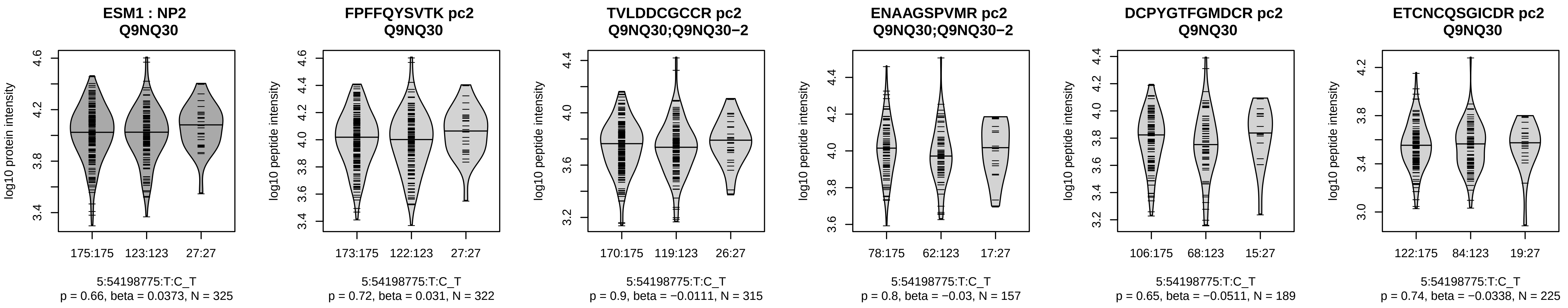

Assay Target: ESM1
UniProt: Q9NQ30
rsID: rs4242051
Target SNP: 5:54902947:T:C
Proxy SNP: 5:54198775:T:C
deCODE log10(p): 54.7
deCODE BETA: −0.15
deCODE MSPA: 0
−:−:−:−:−:−
1238:1254:998:1241:1203:1146

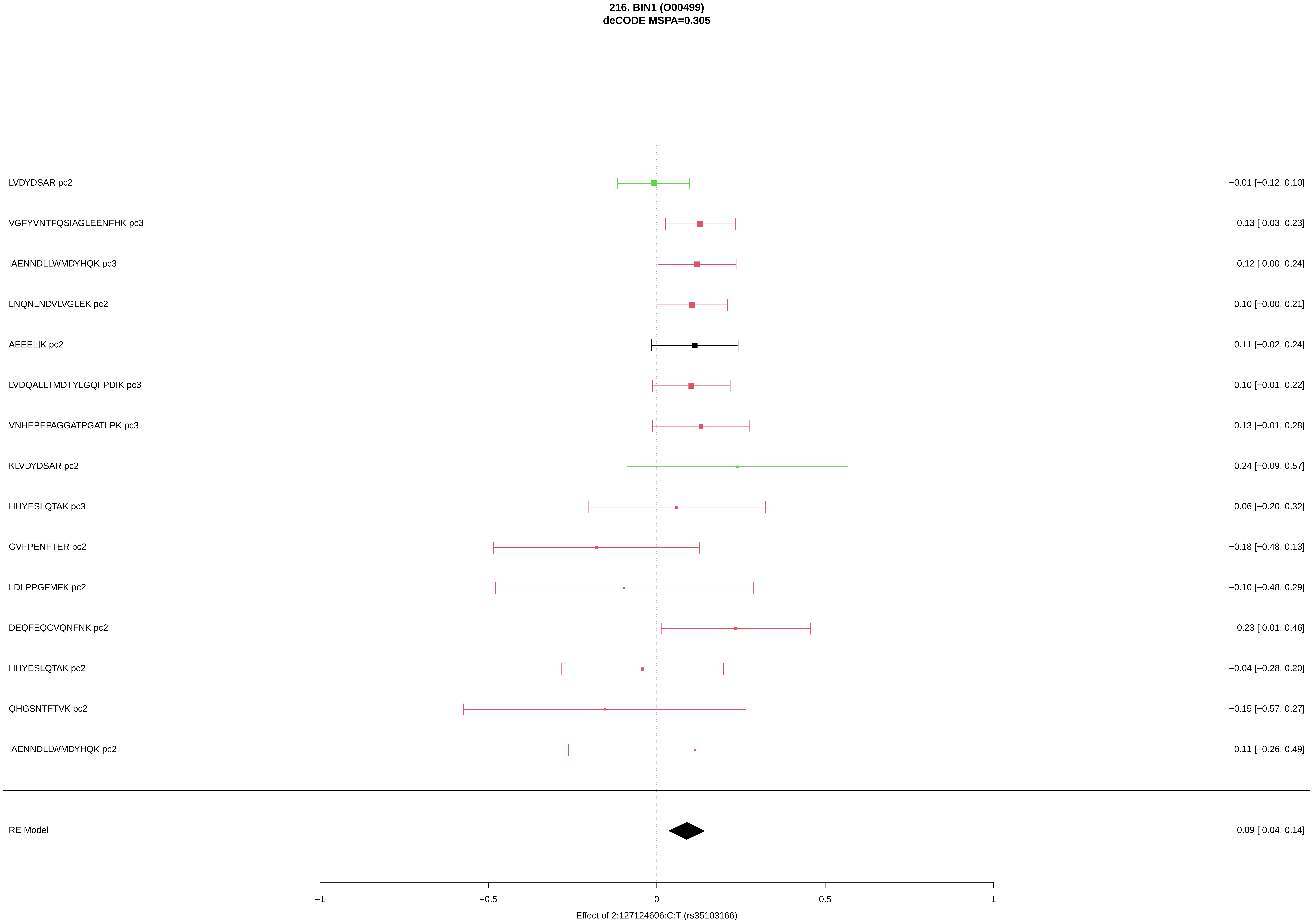

216. BIN1 (O00499)
deCODE MSPA=0.305
LVDYDSAR pc2
VGFYVNTFQSIAGLEENFHK pc3
IAENNDLLWMDYHQK pc3
LNQNLNDVLVGLEK pc2
AEEELIK pc2
LVDQALLTMDTYLGQFPDIK pc3
VNHEPEPAGGATPGATLPK pc3
KLVDYDSAR pc2
HHYESLQTAK pc3
GVFPENFTER pc2
LDLPPGFMFK pc2
DEQFEQCVQNFNK pc2
HHYESLQTAK pc2
QHGSNTFTVK pc2
IAENNDLLWMDYHQK pc2
RE Model
−0.01 [−0.12, 0.10]
0.13 [0.03, 0.23]
0.12 [0.00, 0.24]
0.10 [−0.00, 0.21]
0.11 [−0.02, 0.24]
0.10 [−0.01, 0.22]
0.13 [−0.01, 0.28]
0.24 [−0.09, 0.57]
0.06 [−0.20, 0.32]
−0.18 [−0.48, 0.13]
−0.10 [−0.48, 0.29]
0.23 [0.01, 0.46]
−0.04 [−0.28, 0.20]
−0.15 [−0.57, 0.27]
0.11 [−0.26, 0.49]
0.09 [0.04, 0.14]
−1
−0.5
0
0.5
1
Effect of 2:127124606:C:T (rs35103166)

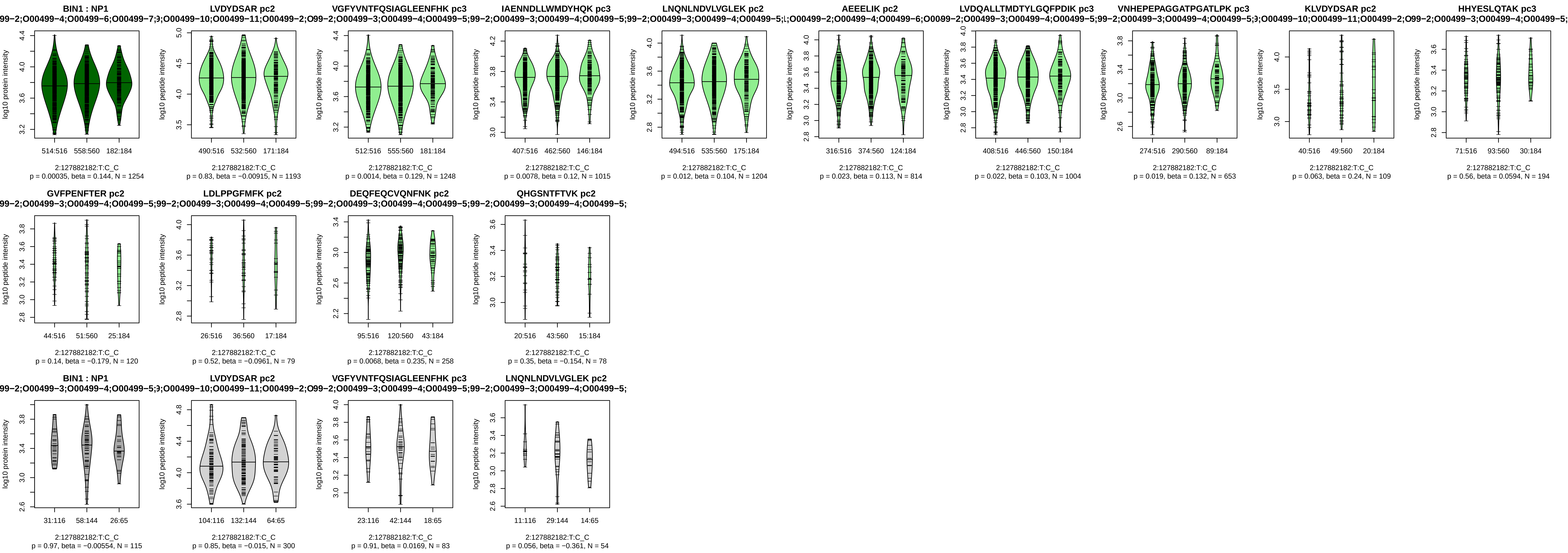

Assay Target: BIN1
UniProt: O00499
rsID: rs35103166
Target SNP: 2:127124606:C:T
Proxy SNP: 2:127882182:T:C
deCODE log10(p): 53.9
deCODE BETA: 0.13
deCODE MSPA: 0.305
−:*:*:−:−:−:−:−:−:−:−:*:−:−:−:NA
1193:1248:1015:1204:814:1004

217. FGG (P02679)
deCODE MSPA=0.023

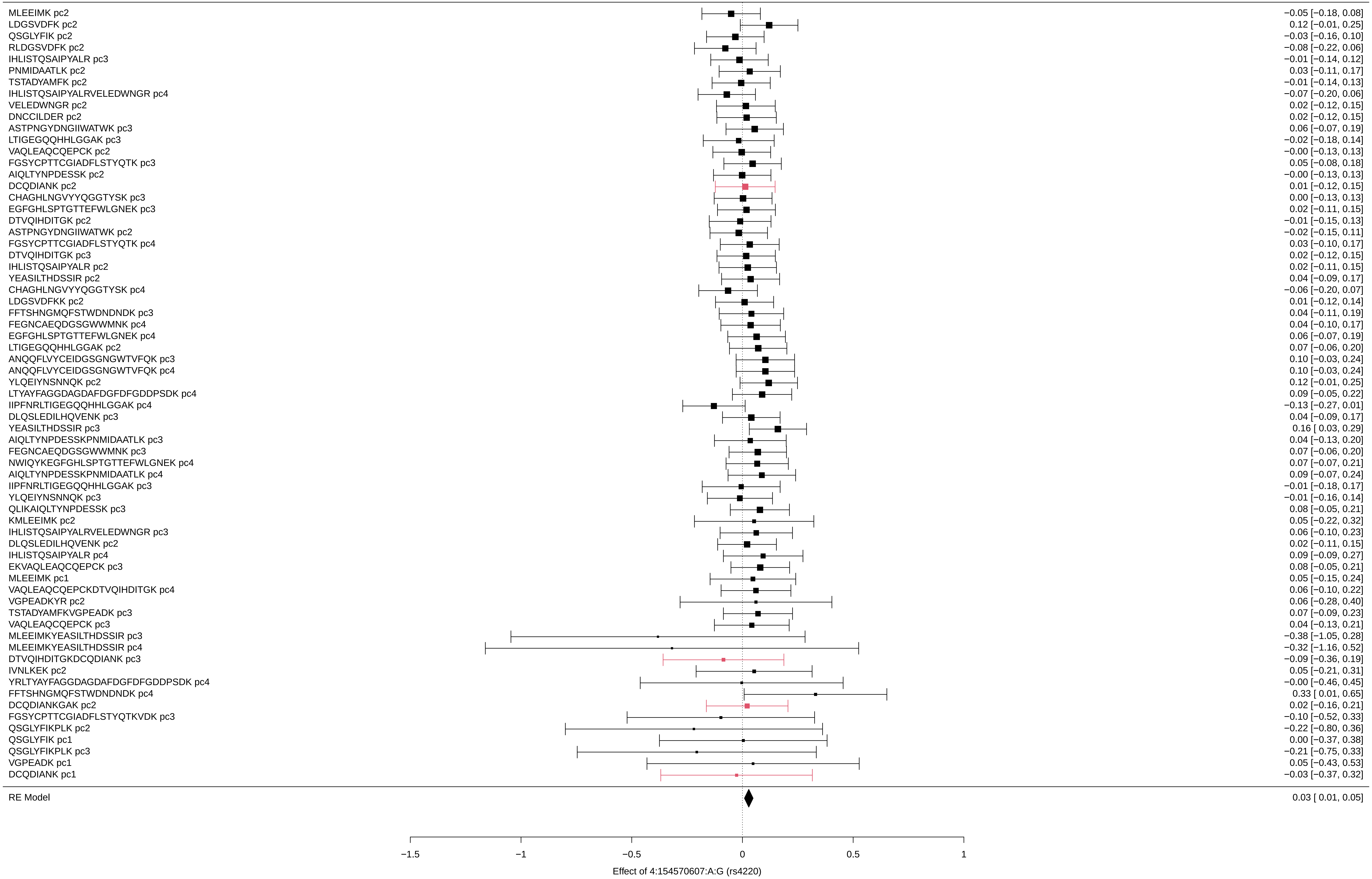

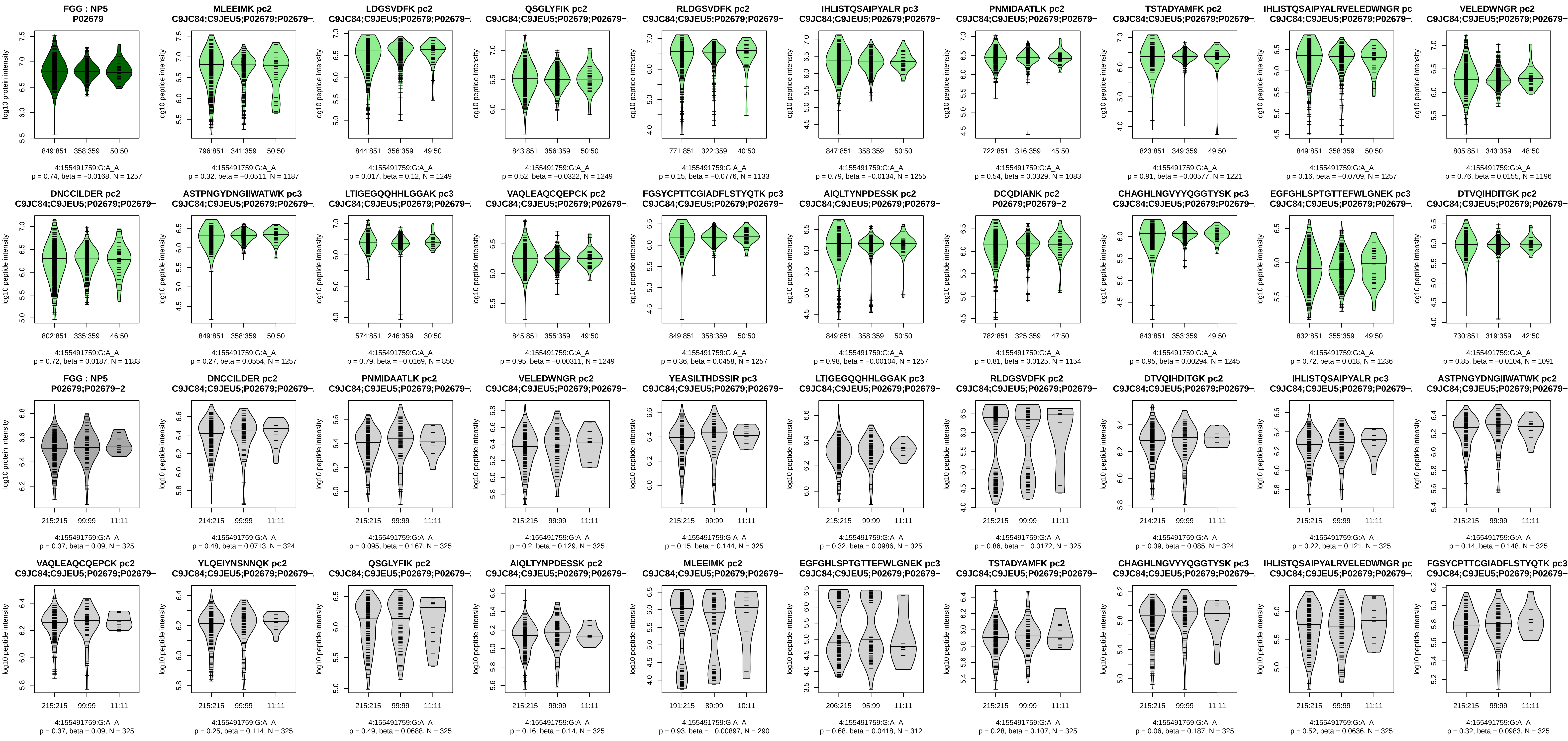

Assay Target: FGG
UniProt: P02679
rsID: rs4220
Target SNP: 4:154570607:A:G
Proxy SNP: 4:155491759:G:A
deCODE log10(p): 53.5
deCODE BETA: 0.18
deCODE MSPA: 0.023
1187:1249:1249:1133:1255:108

218. C1QTNF3 (Q9BXJ4)
deCODE MSPA=0

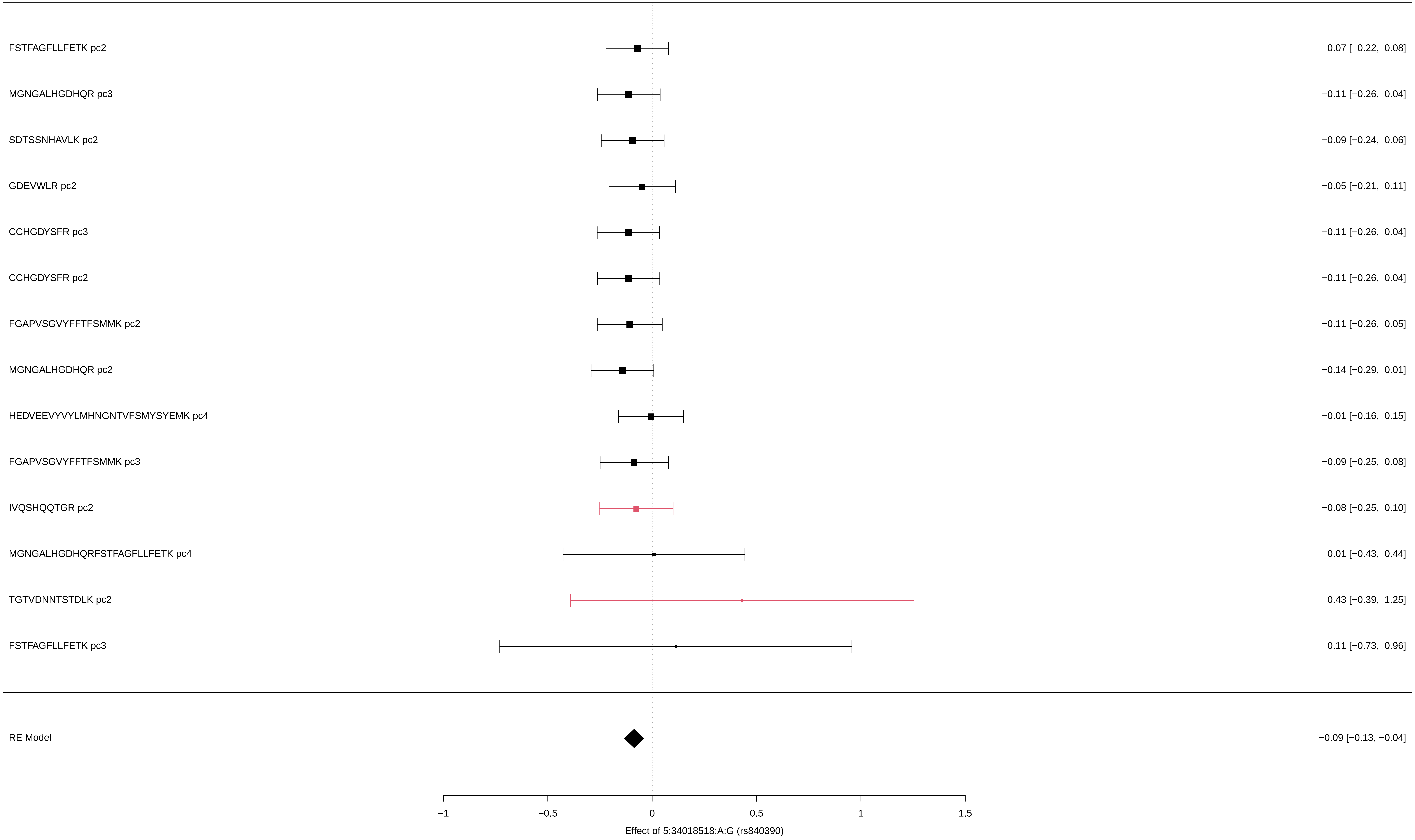

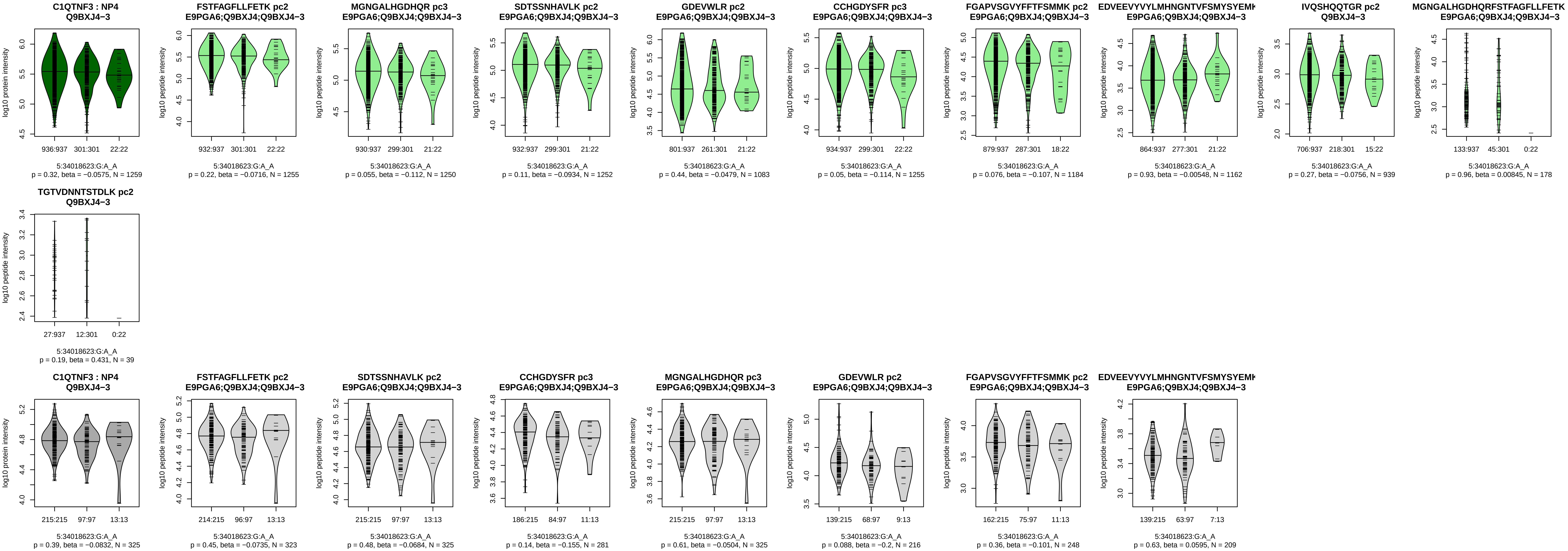

Assay Target: C1QTNF3
UniProt: Q9BXJ4
rsID: rs840390
Target SNP: 5:34018518:A:G
Proxy SNP: 5:34018623:G:A
deCODE log10(p): 53.4
deCODE BETA: −0.18
deCODE MSPA: 0
−:−:−:−:−:−:−:−:−:−:−:NA:−:−
1255:1250:1252:1083:1255:125

219. CXCL12 (P48061)
deCODE MSPA=0.307

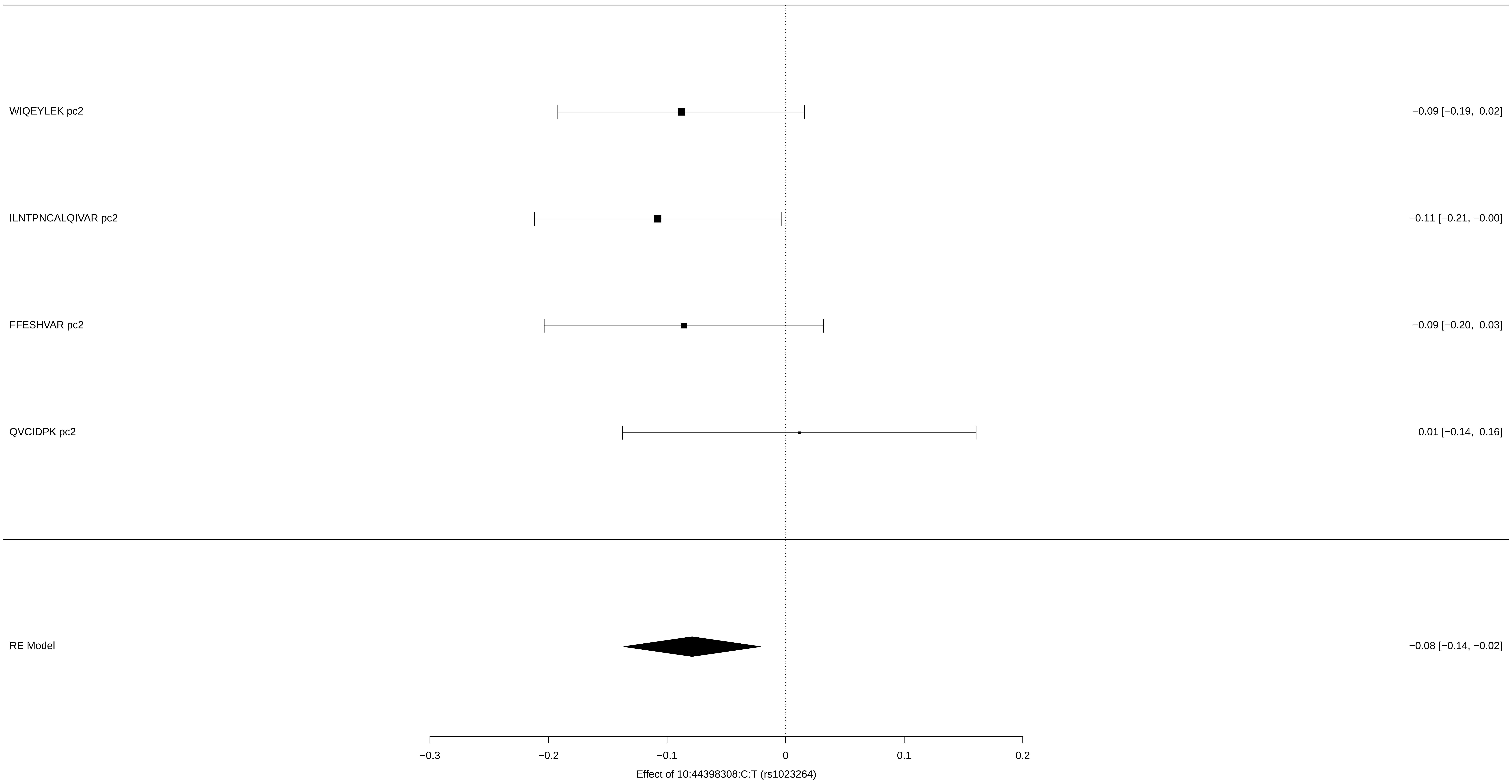

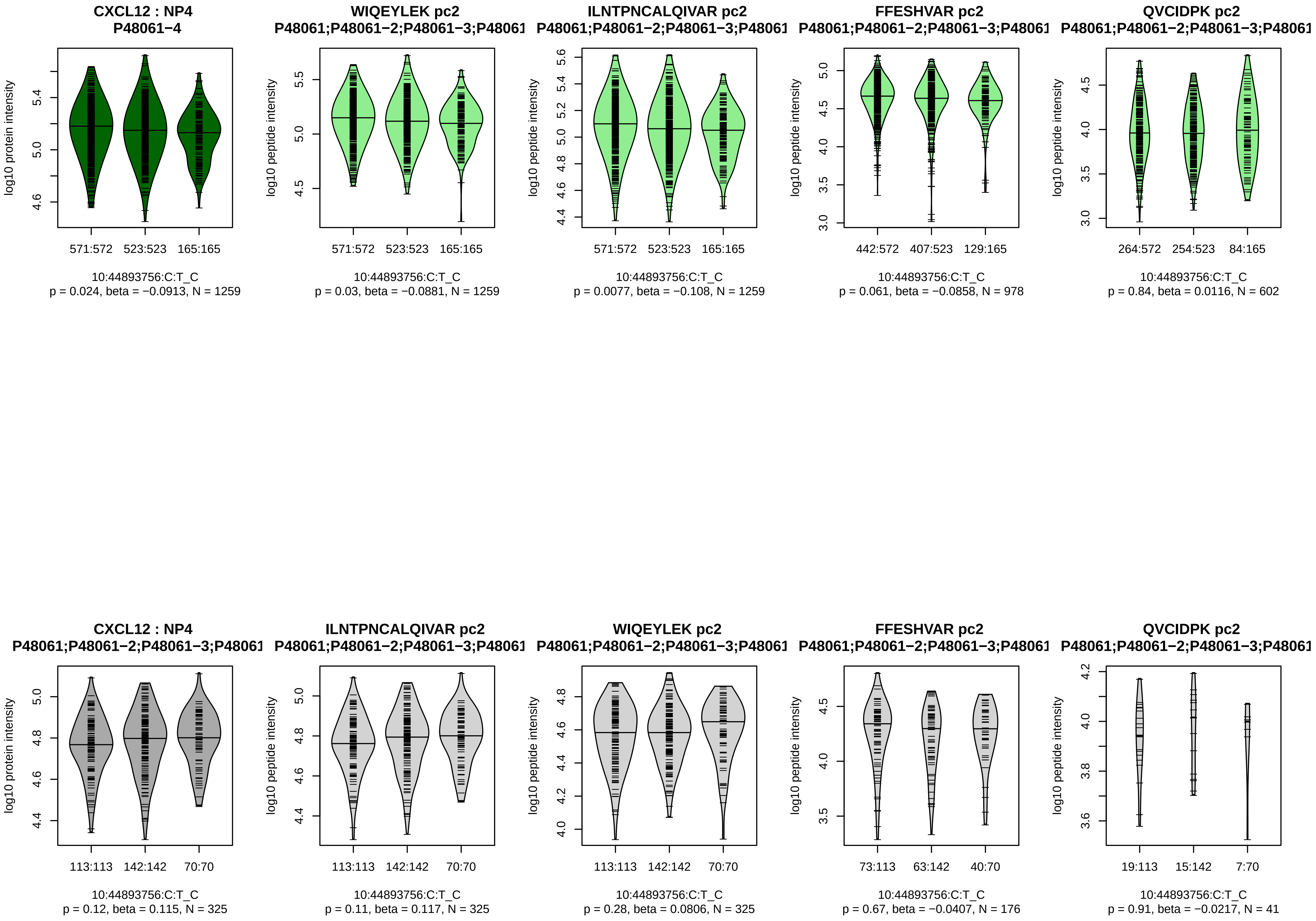

Assay Target: CXCL12
UniProt: P48061
rsID: rs1023264
Target SNP: 10:44398308:C:T
Proxy SNP: 10:44893756:C:T
deCODE log10(p): 53.1
deCODE BETA: 0.14
deCODE MSPA: 0.307
–:*:–:–
1259:1259:978:602

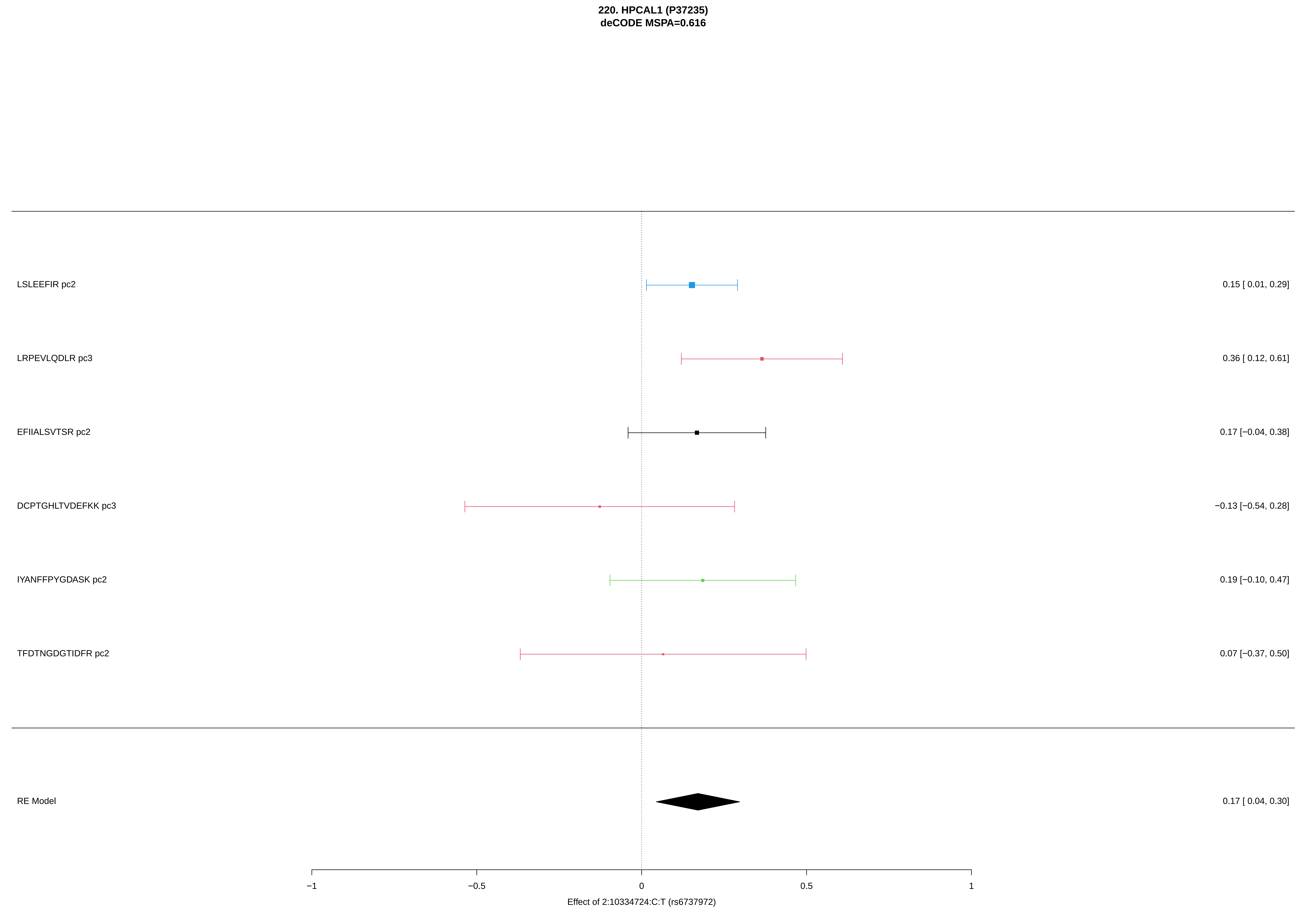
220. HPCAL1 (P37235)
deCODE MSPA=0.616
LSLEEFIR pc2
0.15 [0.01, 0.29]
LRPEVLQDLR pc3
0.36 [0.12, 0.61]
EFIIALSVTSR pc2
0.17 [−0.04, 0.38]
DCPTGHLTVDEFKK pc3
−0.13 [−0.54, 0.28]
IYANFFPYGDASK pc2
0.19 [−0.10, 0.47]
TFDTNGDGTIDFR pc2
0.07 [−0.37, 0.50]
RE Model
0.17 [0.04, 0.30]
−1
−0.5
0
0.5
1
Effect of 2:10334724:C:T (rs6737972)

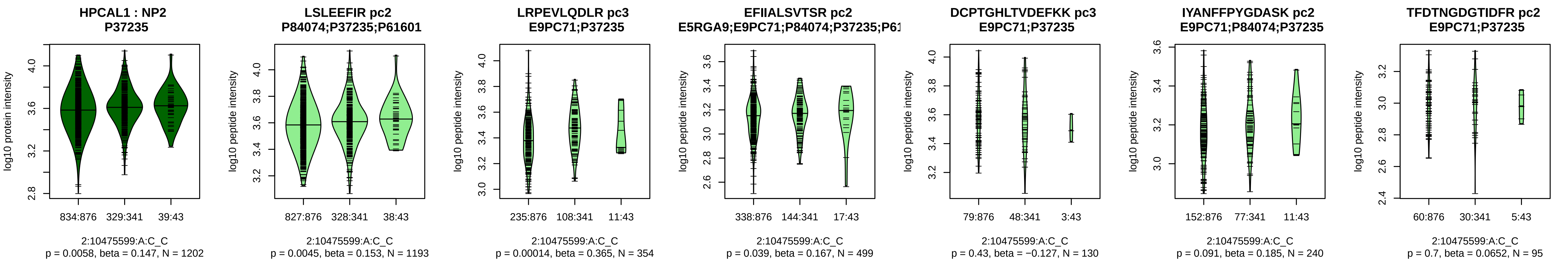

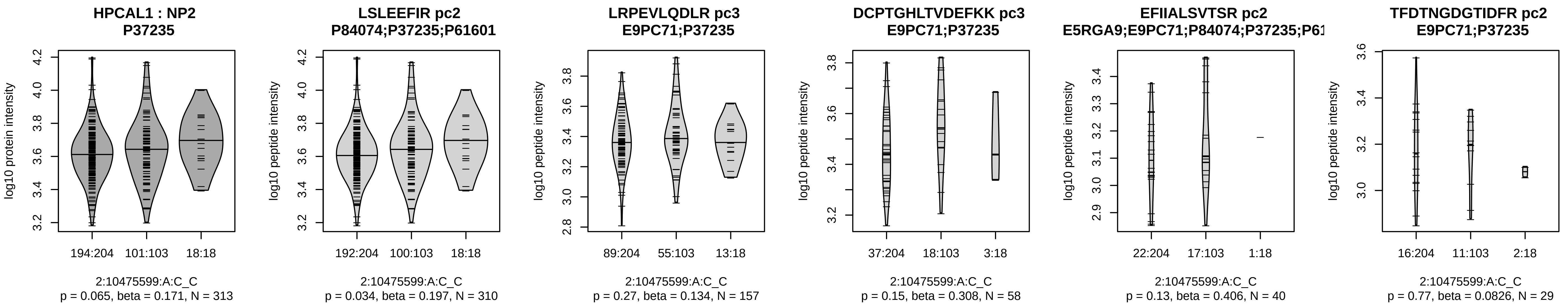

Assay Target: HPCAL1
UniProt: P37235
rsID: rs6737972
Target SNP: 2:10334724:C:T
Proxy SNP: 2:10475599:A:C
deCODE log10(p): 52.1
deCODE BETA: 0.16
deCODE MSPA: 0.616
::–:–:–:–:NA
1193:354:499:130:240:95:2

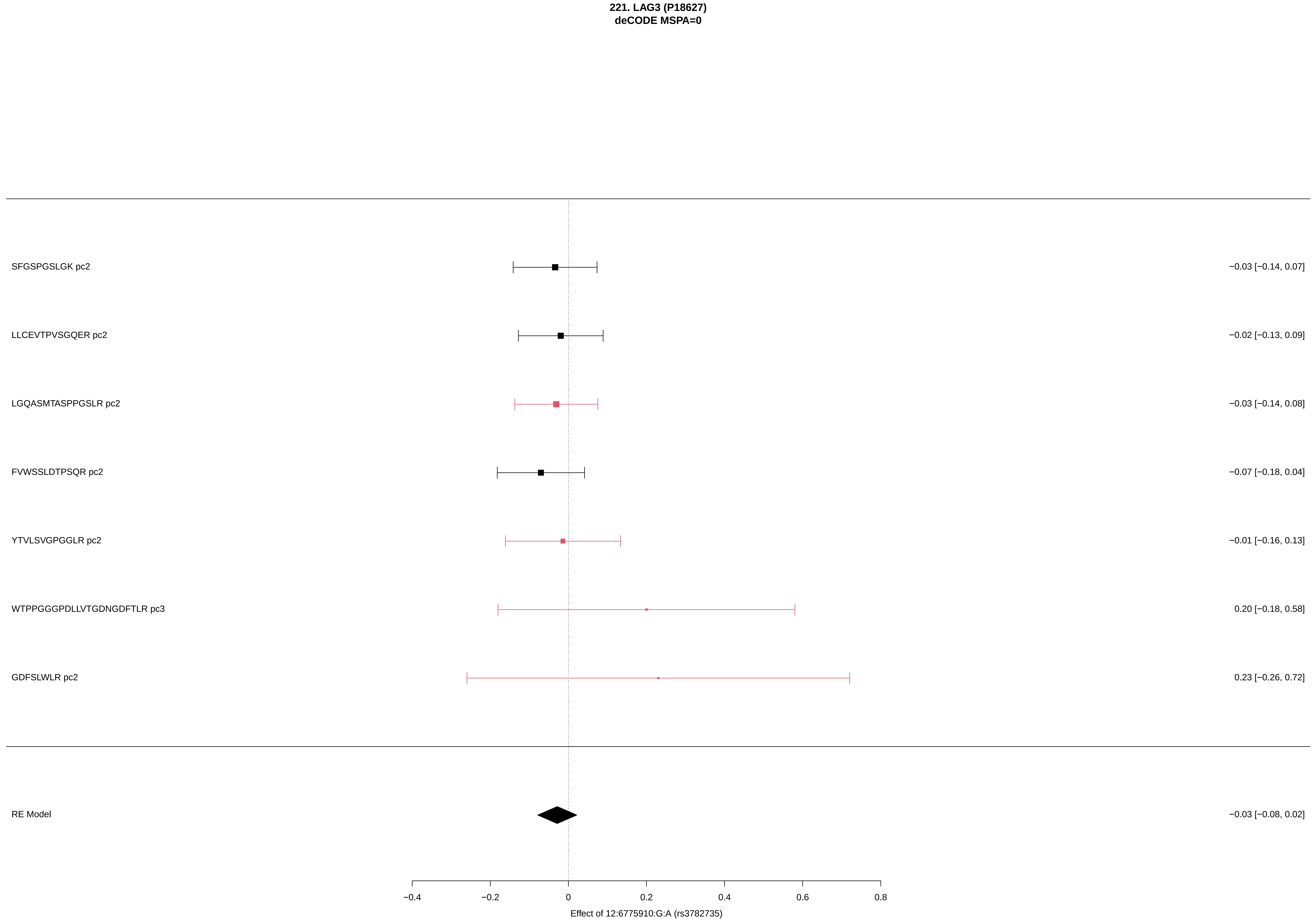
221. LAG3 (P18627)
deCODE MSPA=0
SFGSPGSLGK pc2
−0.03 [−0.14, 0.07]
LLCEVTPVSGQER pc2
−0.02 [−0.13, 0.09]
LGQASMTASPPGSLR pc2
−0.03 [−0.14, 0.08]
FVWSSLDTPSQR pc2
−0.07 [−0.18, 0.04]
YTVLSVGPGGLR pc2
−0.01 [−0.16, 0.13]
WTPPGGGPDLLVTGDNGDFTLR pc3
0.20 [−0.18, 0.58]
GDFSLWLR pc2
0.23 [−0.26, 0.72]
RE Model
−0.03 [−0.08, 0.02]
−0.4
−0.2
0
0.2
0.4
0.6
0.8
Effect of 12:6775910:G:A (rs3782735)

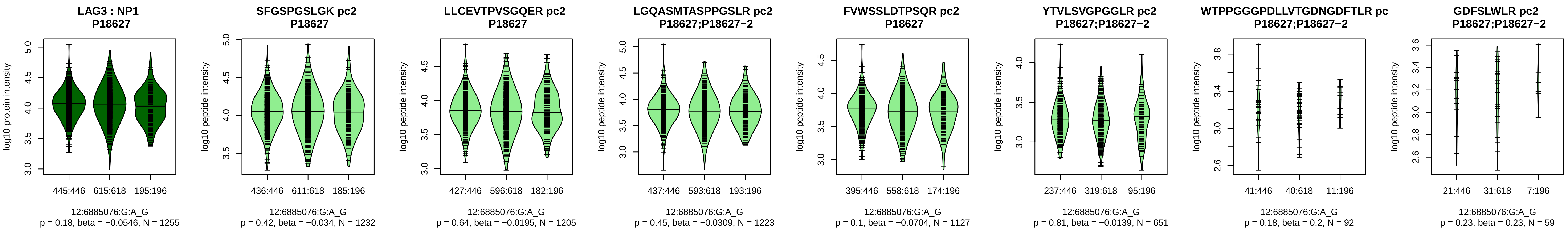

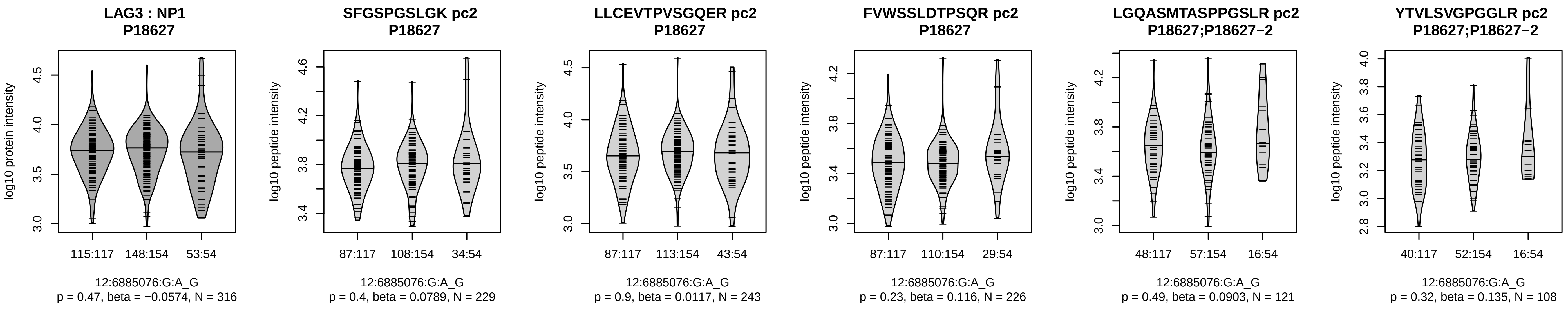

Assay Target: LAG3
UniProt: P18627
rsID: rs3782735
Target SNP: 12:6775910:G:A
Proxy SNP: 12:6885076:G:A
deCODE log10(p): 51.1
deCODE BETA: −0.13
deCODE MSPA: 0
1232:1205:1223:1127:651:92:59

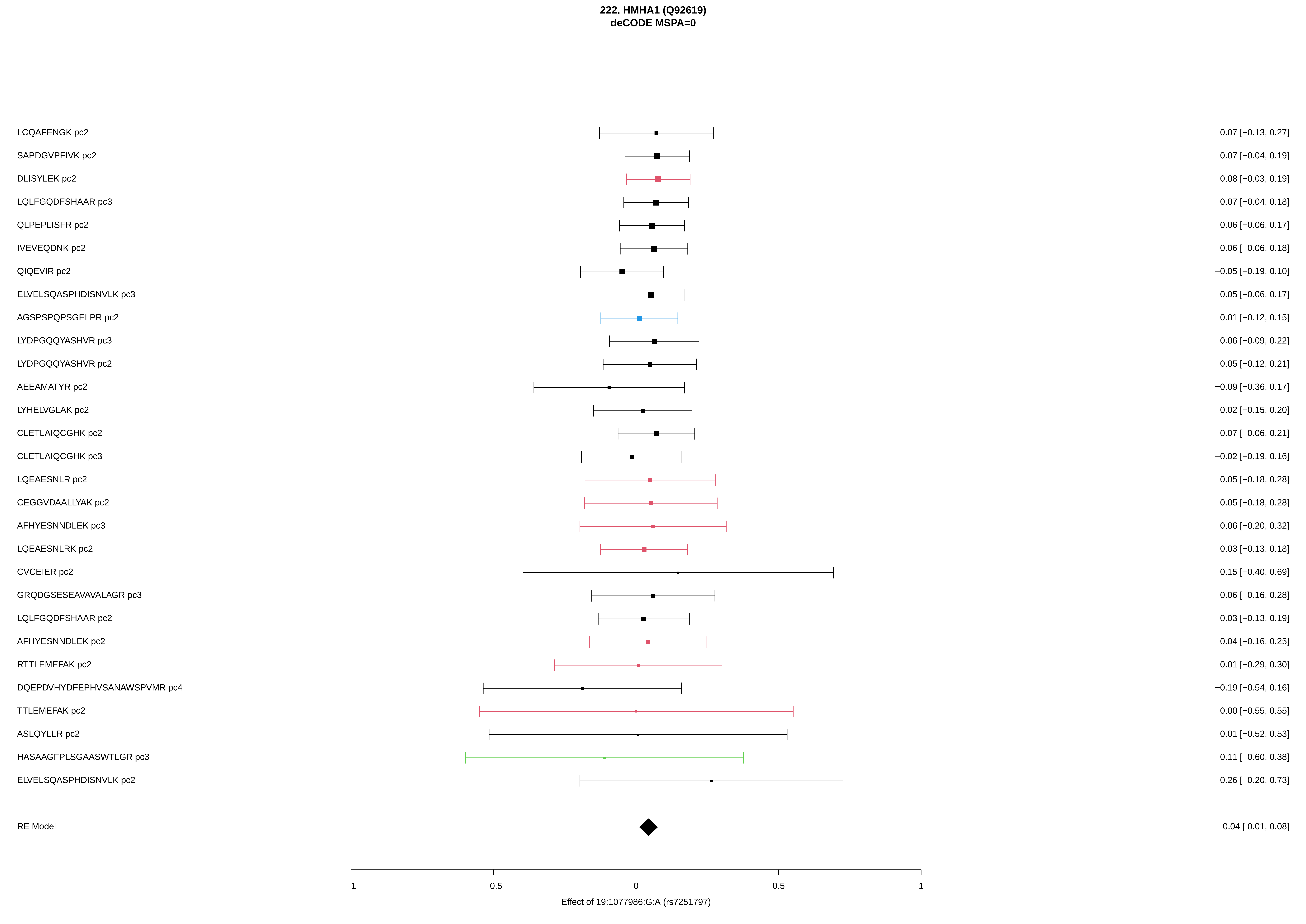
222. HMHA1 (Q92619)
deCODE MSPA=0
LCQAFENGK pc2 0.07 [−0.13, 0.27]
SAPDGVPFIVK pc2 0.07 [−0.04, 0.19]
DLISYLEK pc2 0.08 [−0.03, 0.19]
LQLFGQDFSHAAR pc3 0.07 [−0.04, 0.18]
QLPEPLISFR pc2 0.06 [−0.06, 0.17]
IVEVEQDNK pc2 0.06 [−0.06, 0.18]
QIQEVIR pc2 −0.05 [−0.19, 0.10]
ELVELSQASPHDISNVLK pc3 0.05 [−0.06, 0.17]
AGSPSPQPSGELPR pc2 0.01 [−0.12, 0.15]
LYDPGQQYASHVR pc3 0.06 [−0.09, 0.22]
LYDPGQQYASHVR pc2 0.05 [−0.12, 0.21]
AEEAMATYR pc2 −0.09 [−0.36, 0.17]
LYHELVGLAK pc2 0.02 [−0.15, 0.20]
CLETLAIQCGHK pc2 0.07 [−0.06, 0.21]
CLETLAIQCGHK pc3 −0.02 [−0.19, 0.16]
LQEAESNLR pc2 0.05 [−0.18, 0.28]
CEGGVDAALLYAK pc2 0.05 [−0.18, 0.28]
AFHYESNNDLEK pc3 0.06 [−0.20, 0.32]
LQEAESNLRK pc2 0.03 [−0.13, 0.18]
CVCEIER pc2 0.15 [−0.40, 0.69]
GRQDGSESEAVAVALAGR pc3 0.06 [−0.16, 0.28]
LQLFGQDFSHAAR pc2 0.03 [−0.13, 0.19]
AFHYESNNDLEK pc2 0.04 [−0.16, 0.25]
RTTLEMEFAK pc2 0.01 [−0.29, 0.30]
DQEPDVHYDFEPHVSANAWSPVMR pc4 −0.19 [−0.54, 0.16]
TTLEMEFAK pc2 0.00 [−0.55, 0.55]
ASLQYLLR pc2 0.01 [−0.52, 0.53]
HASAAGFPLSGAASWTLGR pc3 −0.11 [−0.60, 0.38]
ELVELSQASPHDISNVLK pc2 0.26 [−0.20, 0.73]
RE Model 0.04 [0.01, 0.08]
−1
−0.5
0
0.5
1
Effect of 19:1077986:G:A (rs7251797)

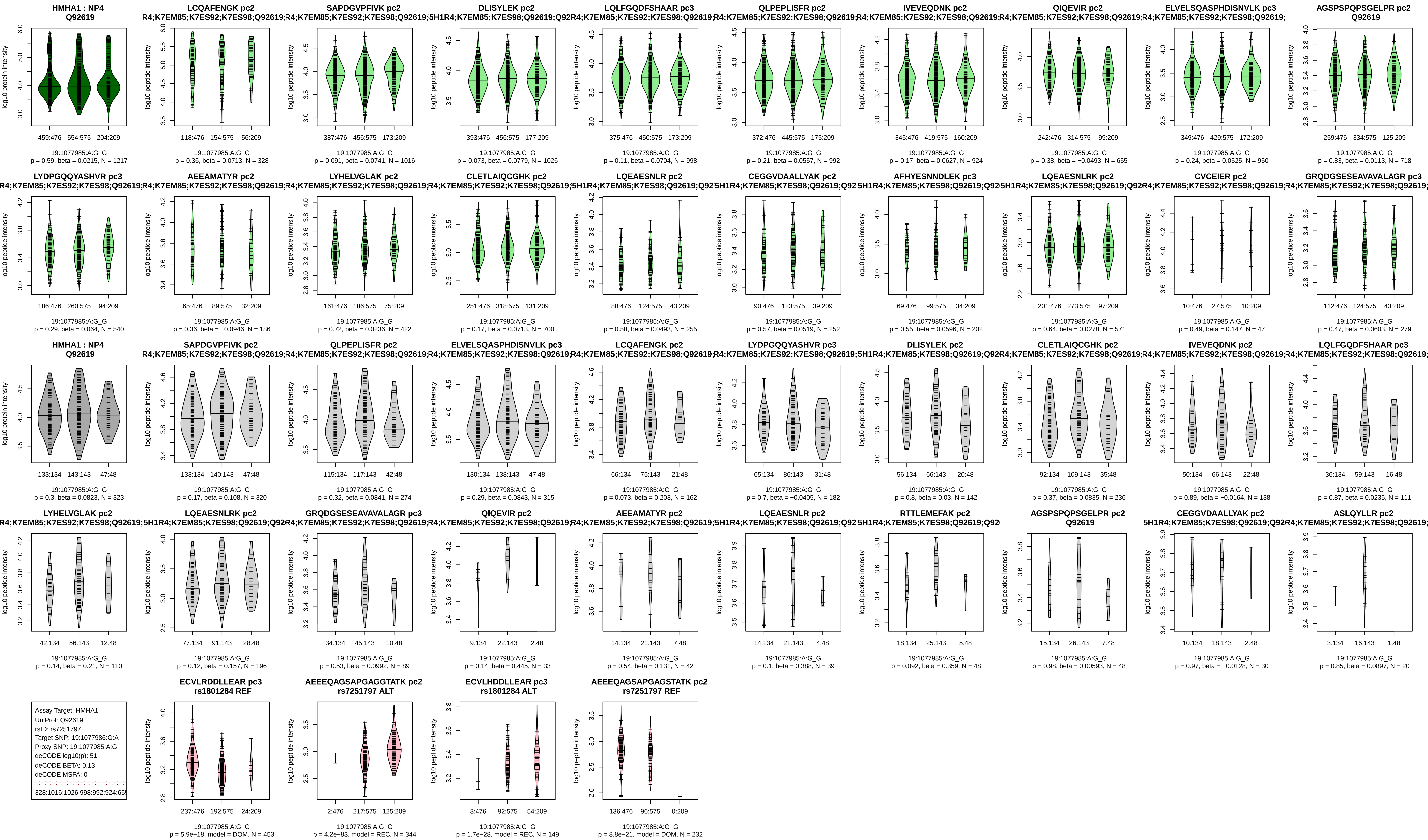
HMHA1 : NP4
Q92619
log10 protein intensity
459:476 554:575 204:209
19:1077985:A:G_G
p = 0.59, beta = 0.0215, N = 1217
LCQAFENGK pc2
log10 peptide intensity
118:476 154:575 56:209
19:1077985:A:G_G
p = 0.36, beta = 0.0713, N = 328
SAPDGVPFIVK pc2
387:476 456:575 173:209
19:1077985:A:G_G
p = 0.091, beta = 0.0741, N = 1016
DLISYLEK pc2
393:476 456:575 177:209
19:1077985:A:G_G
p = 0.073, beta = 0.0779, N = 1026
LQLFGQDFSHAAR pc3
375:476 450:575 173:209
19:1077985:A:G_G
p = 0.11, beta = 0.0704, N = 998
QLPEPLISFR pc2
372:476 445:575 175:209
19:1077985:A:G_G
p = 0.21, beta = 0.0557, N = 992
IVEVEQDNK pc2
345:476 419:575 160:209
19:1077985:A:G_G
p = 0.17, beta = 0.0627, N = 924
QIQEVIR pc2
242:476 314:575 99:209
19:1077985:A:G_G
p = 0.38, beta = −0.0493, N = 655
ELVELSQASPHDISNVLK pc3
349:476 429:575 172:209
19:1077985:A:G_G
p = 0.24, beta = 0.0525, N = 950
AGSPSPQPSGELPR pc2
Q92619
259:476 334:575 125:209
19:1077985:A:G_G
p = 0.83, beta = 0.0113, N = 718
LYDPGQQYASHVR pc3
186:476 260:575 94:209
19:1077985:A:G_G
p = 0.29, beta = 0.064, N = 540
AEEAMATYR pc2
65:476 89:575 32:209
19:1077985:A:G_G
p = 0.36, beta = −0.0946, N = 186
LYHELVGLAK pc2
161:476 186:575 75:209
19:1077985:A:G_G
p = 0.72, beta = 0.0236, N = 422
CLETLAIQCGHK pc2
251:476 318:575 131:209
19:1077985:A:G_G
p = 0.17, beta = 0.0713, N = 700
LQEAESNLR pc2
88:476 124:575 43:209
19:1077985:A:G_G
p = 0.58, beta = 0.0493, N = 255
CEGGVDAALLYAK pc2
90:476 123:575 39:209
19:1077985:A:G_G
p = 0.57, beta = 0.0519, N = 252
AFHYESNNDLEK pc3
69:476 99:575 34:209
19:1077985:A:G_G
p = 0.55, beta = 0.0596, N = 202
LQEAESNLRK pc2
201:476 273:575 97:209
19:1077985:A:G_G
p = 0.64, beta = 0.0278, N = 571
CVCEIER pc2
10:476 27:575 10:209
19:1077985:A:G_G
p = 0.49, beta = 0.147, N = 47
GRQDGSESEAVAVALAGR pc3
112:476 124:575 43:209
19:1077985:A:G_G
p = 0.47, beta = 0.0603, N = 279
HMHA1 : NP4
Q92619
133:134 143:143 47:48
19:1077985:A:G_G
p = 0.3, beta = 0.0823, N = 323
SAPDGVPFIVK pc2
133:134 140:143 47:48
19:1077985:A:G_G
p = 0.17, beta = 0.108, N = 320
QLPEPLISFR pc2
115:134 117:143 42:48
19:1077985:A:G_G
p = 0.32, beta = 0.0841, N = 274
ELVELSQASPHDISNVLK pc3
130:134 138:143 47:48
19:1077985:A:G_G
p = 0.29, beta = 0.0843, N = 315
LCQAFENGK pc2
66:134 75:143 21:48
19:1077985:A:G_G
p = 0.073, beta = 0.203, N = 162
LYDPGQQYASHVR pc3
65:134 86:143 31:48
19:1077985:A:G_G
p = 0.7, beta = −0.0405, N = 182
DLISYLEK pc2
56:134 66:143 20:48
19:1077985:A:G_G
p = 0.8, beta = 0.03, N = 142
CLETLAIQCGHK pc2
92:134 109:143 35:48
19:1077985:A:G_G
p = 0.37, beta = 0.0835, N = 236
IVEVEQDNK pc2
50:134 66:143 22:48
19:1077985:A:G_G
p = 0.89, beta = −0.0164, N = 138
LQLFGQDFSHAAR pc3
36:134 59:143 16:48
19:1077985:A:G_G
p = 0.87, beta = 0.0235, N = 111
LYHELVGLAK pc2
42:134 56:143 12:48
19:1077985:A:G_G
p = 0.14, beta = 0.21, N = 110
LQEAESNLRK pc2
77:134 91:143 28:48
19:1077985:A:G_G
p = 0.12, beta = 0.157, N = 196
GRQDGSESEAVAVALAGR pc3
34:134 45:143 10:48
19:1077985:A:G_G
p = 0.53, beta = 0.0992, N = 89
QIQEVIR pc2
9:134 22:143 2:48
19:1077985:A:G_G
p = 0.14, beta = 0.445, N = 33
AEEAMATYR pc2
14:134 21:143 7:48
19:1077985:A:G_G
p = 0.54, beta = 0.131, N = 42
LQEAESNLR pc2
14:134 21:143 4:48
19:1077985:A:G_G
p = 0.1, beta = 0.388, N = 39
RTTLEMEFAK pc2
18:134 25:143 5:48
19:1077985:A:G_G
p = 0.092, beta = 0.359, N = 48
AGSPSPQPSGELPR pc2
Q92619
15:134 26:143 7:48
19:1077985:A:G_G
p = 0.98, beta = 0.00593, N = 48
CEGGVDAALLYAK pc2
10:134 18:143 2:48
19:1077985:A:G_G
p = 0.97, beta = −0.0128, N = 30
ASLQYLLR pc2
3:134 16:143 1:48
19:1077985:A:G_G
p = 0.85, beta = 0.0897, N = 20
Assay Target: HMHA1
UniProt: Q92619
rsID: rs7251797
Target SNP: 19:1077986:G:A
Proxy SNP: 19:1077985:A:G
deCODE log10(p): 51
deCODE BETA: 0.13
deCODE MSPA: 0
ECVLRDDLLEAR pc3
rs1801284 REF
237:476 192:575 24:209
19:1077985:A:G_G
p = 5.9e−18, model = DOM, N = 453
AEEEQAGSAPGAGGTATK pc2
rs7251797 ALT
2:476 217:575 125:209
19:1077985:A:G_G
p = 4.2e−83, model = REC, N = 344
ECVLHDDLLEAR pc3
rs1801284 ALT
3:476 92:575 54:209
19:1077985:A:G_G
p = 1.7e−28, model = REC, N = 149
AEEEQAGSAPGAGSTATK pc2
rs7251797 REF
136:476 96:575 0:209
19:1077985:A:G_G
p = 8.8e−21, model = DOM, N = 232

223. RCN1 (Q15293)
deCODE MSPA=0.638

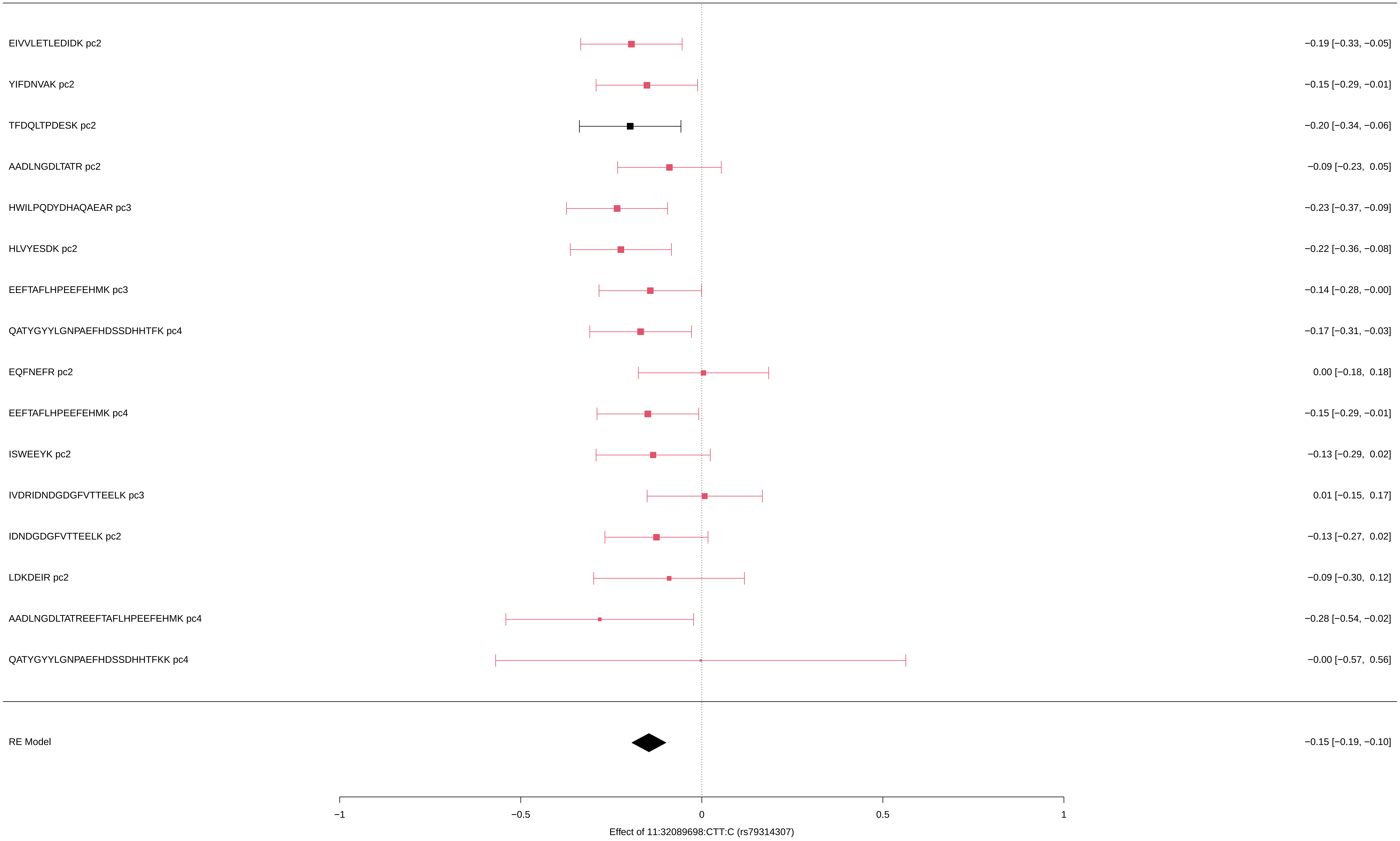

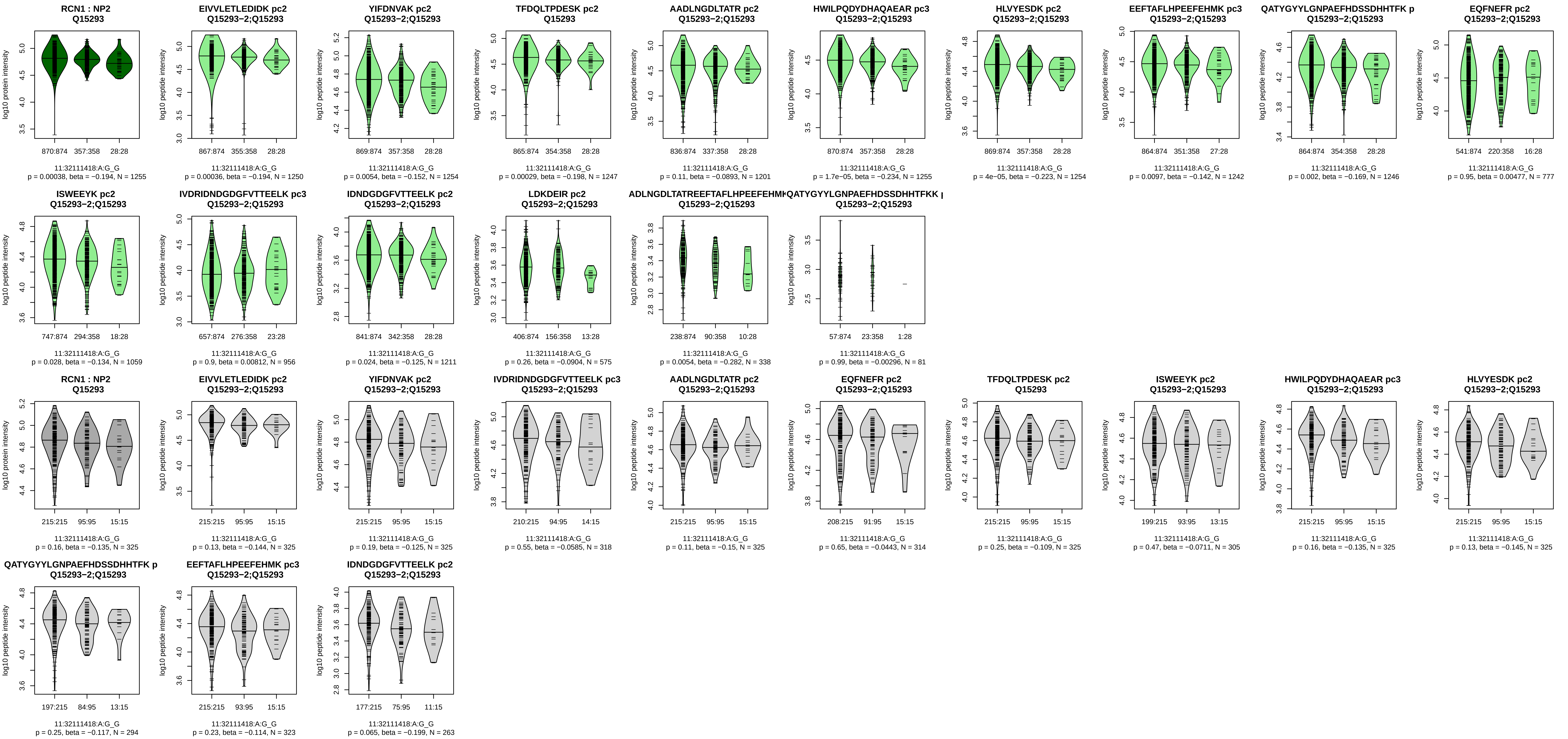

Assay Target: RCN1
UniProt: Q15293
rsID: rs79314307
Target SNP: 11:32089698:CTT:(
Proxy SNP: 11:32111418:A:G
deCODE log10(p): 50.5
deCODE BETA: −0.17
deCODE MSPA: 0.638
::*:-:*:*:*:*:-:*:-:-:-:-:-:*:-:NA
1250:1254:1247:1201:1255:125

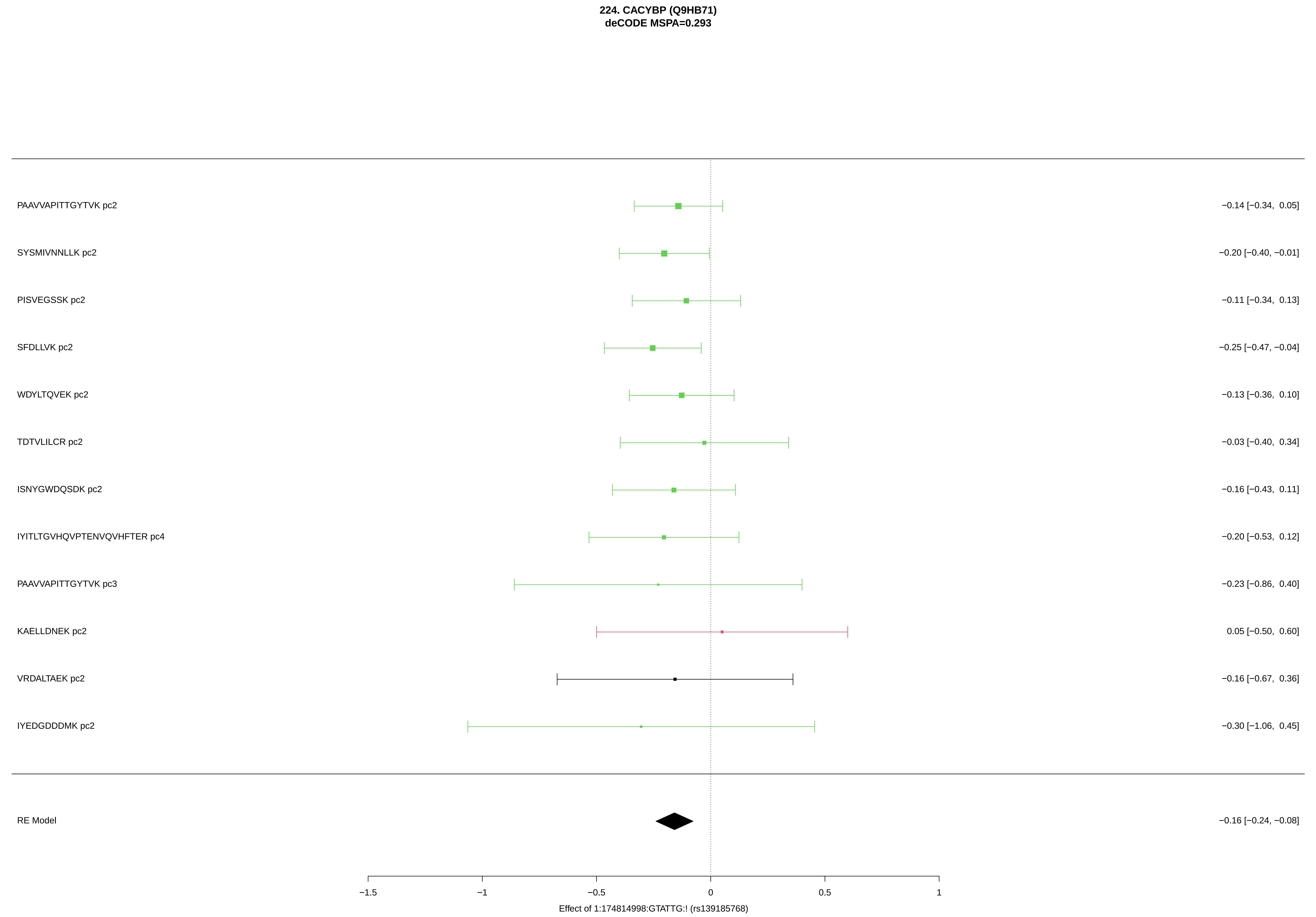
224. CACYBP (Q9HB71)
deCODE MSPA=0.293
PAAVVAPITTGYTVK pc2
SYSMIVNNLLK pc2
PISVEGSSK pc2
SFDLLVK pc2
WDYLTQVEK pc2
TDTVLILCR pc2
ISNYGWDQSDK pc2
IYITLTGVHQVPTENVQVHFTER pc4
PAAVVAPITTGYTVK pc3
KAELLDNEK pc2
VRDALTAEK pc2
IYEDGDDDMK pc2
RE Model
−0.14 [−0.34, 0.05]
−0.20 [−0.40, −0.01]
−0.11 [−0.34, 0.13]
−0.25 [−0.47, −0.04]
−0.13 [−0.36, 0.10]
−0.03 [−0.40, 0.34]
−0.16 [−0.43, 0.11]
−0.20 [−0.53, 0.12]
−0.23 [−0.86, 0.40]
0.05 [−0.50, 0.60]
−0.16 [−0.67, 0.36]
−0.30 [−1.06, 0.45]
−0.16 [−0.24, −0.08]
−1.5
−1
−0.5
0
0.5
1
Effect of 1:174814998:GTATTG:! (rs139185768)

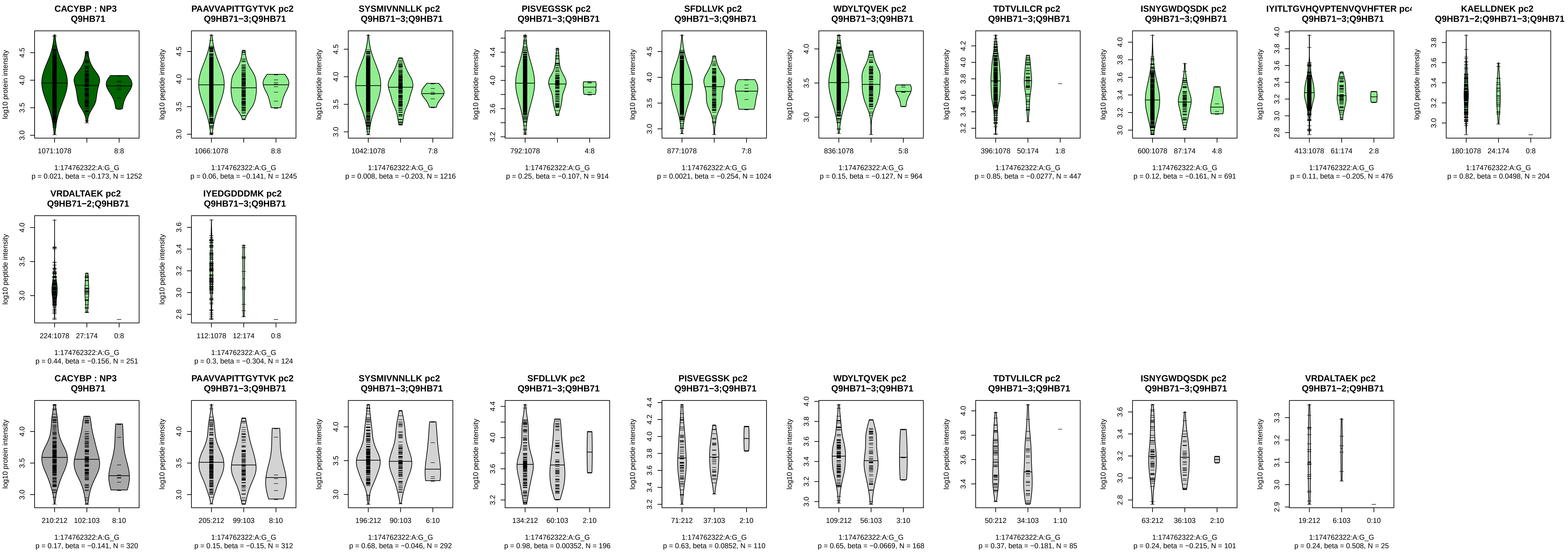

Assay Target: CACYBP
UniProt: Q9HB71
rsID: rs139185768
Target SNP: 1:174814998:GTAT
Proxy SNP: 1:174762322:A:G
deCODE log10(p): 49.5
deCODE BETA: −0.2
deCODE MSPA: 0.293
−:*:−:*:−:−:−:−:−:−:−:NA
1245:1216:914:1024:964:447:69

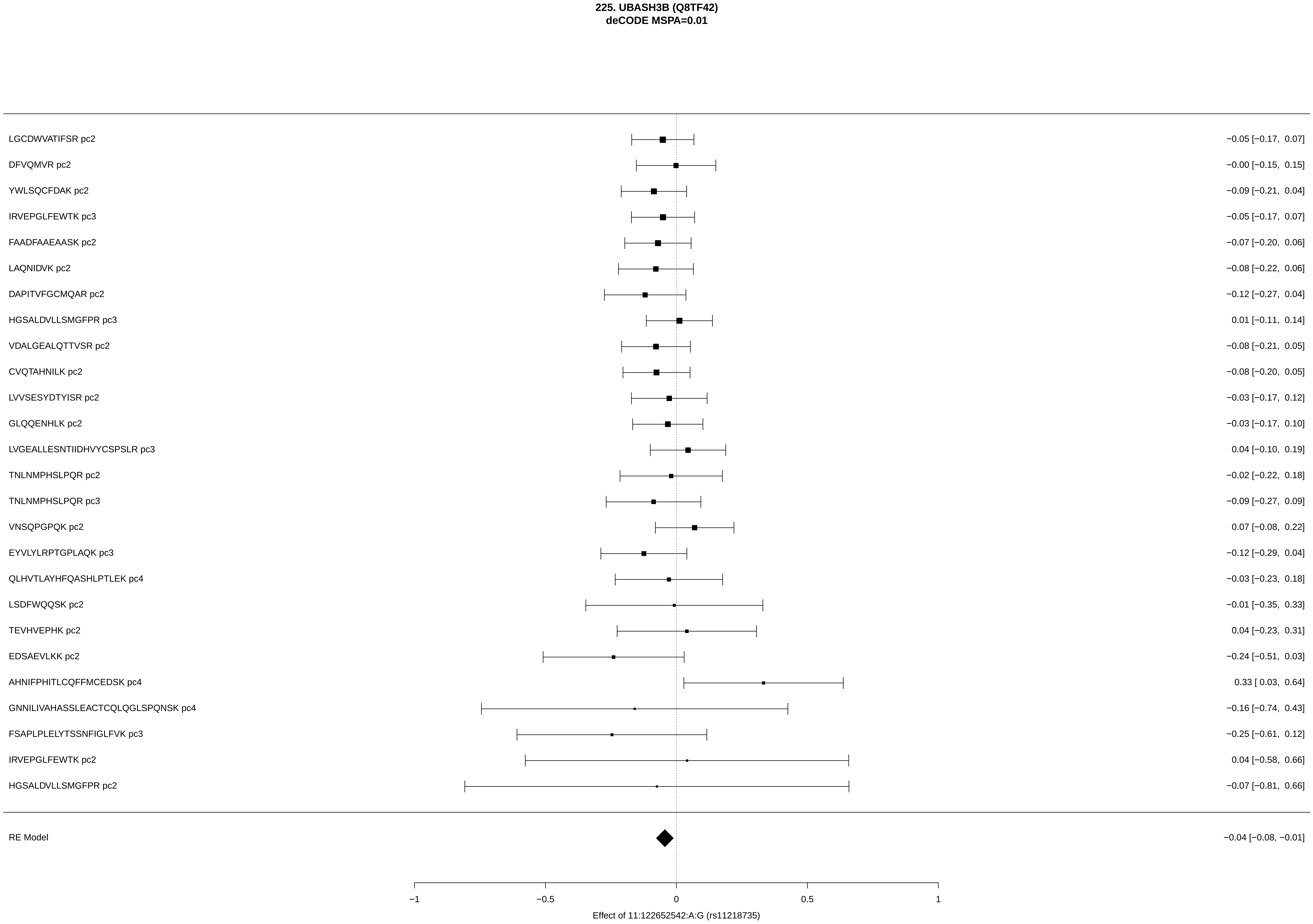

225. UBASH3B (Q8TF42)
deCODE MSPA=0.01
LGCDWVATIFSR pc2 −0.05 [−0.17, 0.07]
DFVQMVR pc2 −0.00 [−0.15, 0.15]
YWLSQCFDAK pc2 −0.09 [−0.21, 0.04]
IRVEPGLFEWTK pc3 −0.05 [−0.17, 0.07]
FAADFAAEAASK pc2 −0.07 [−0.20, 0.06]
LAQNIDVK pc2 −0.08 [−0.22, 0.06]
DAPITVFGCMQAR pc2 −0.12 [−0.27, 0.04]
HGSALDVLLSMGFPR pc3 0.01 [−0.11, 0.14]
VDALGEALQTTVSR pc2 −0.08 [−0.21, 0.05]
CVQTAHNILK pc2 −0.08 [−0.20, 0.05]
LVVSESYDTYISR pc2 −0.03 [−0.17, 0.12]
GLQQENHLK pc2 −0.03 [−0.17, 0.10]
LVGEALLESNTIIDHVYCSPSLR pc3 0.04 [−0.10, 0.19]
TNLNMPHSLPQR pc2 −0.02 [−0.22, 0.18]
TNLNMPHSLPQR pc3 −0.09 [−0.27, 0.09]
VNSQPGPQK pc2 0.07 [−0.08, 0.22]
EYVLYLRPTGPLAQK pc3 −0.12 [−0.29, 0.04]
QLHVTLAYHFQASHLPTLEK pc4 −0.03 [−0.23, 0.18]
LSDFWQQSK pc2 −0.01 [−0.35, 0.33]
TEVHVEPHK pc2 0.04 [−0.23, 0.31]
EDSAEVLKK pc2 −0.24 [−0.51, 0.03]
AHNIFPHITLCQFFMCEDSK pc4 0.33 [0.03, 0.64]
GNNILIVAHASSLEACTCQLQGLSPQNSK pc4 −0.16 [−0.74, 0.43]
FSAPLPLELYTSSNFIGLFVK pc3 −0.25 [−0.61, 0.12]
IRVEPGLFEWTK pc2 0.04 [−0.58, 0.66]
HGSALDVLLSMGFPR pc2 −0.07 [−0.81, 0.66]
RE Model −0.04 [−0.08, −0.01]
−1
−0.5
0
0.5
1
Effect of 11:122652542:A:G (rs11218735)

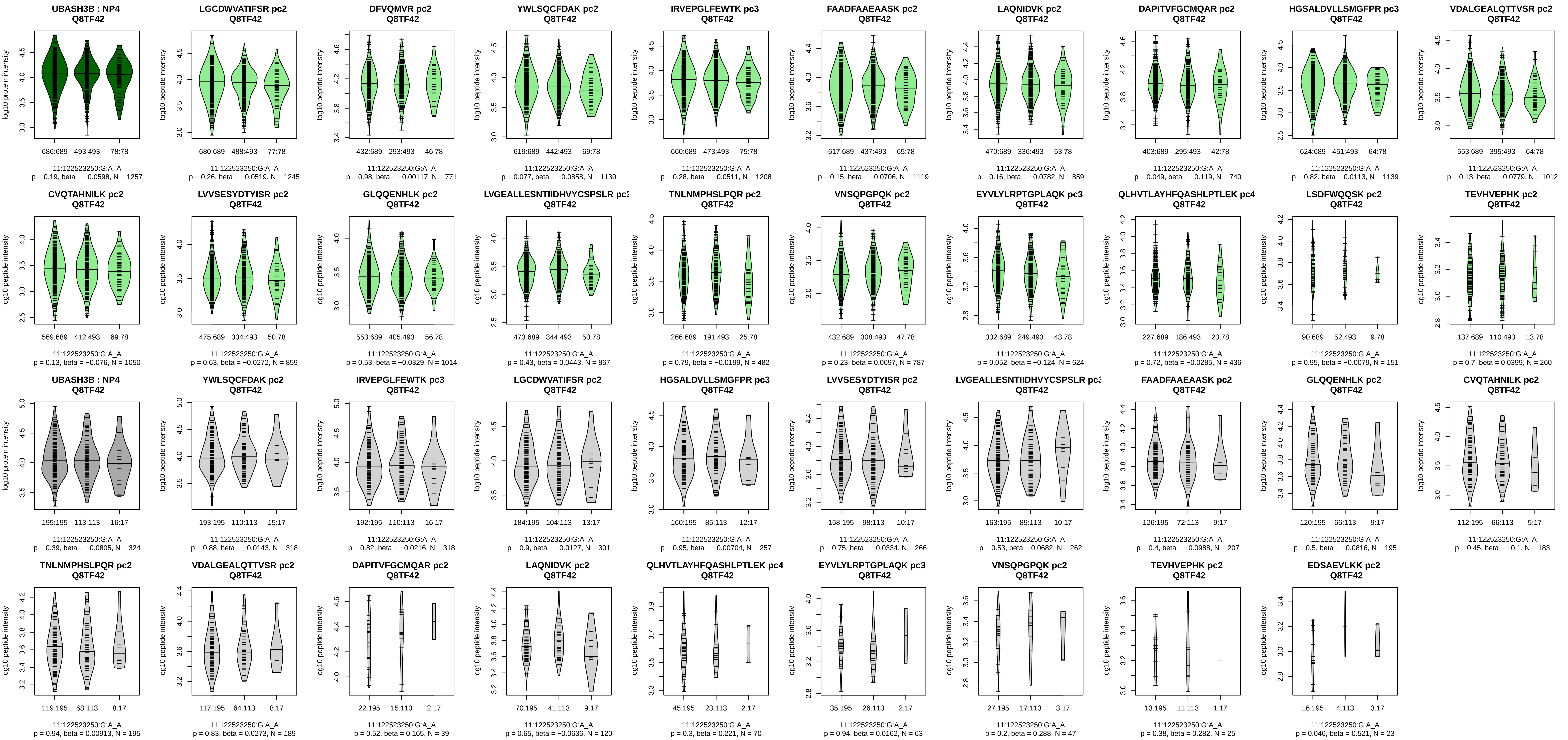

Assay Target: UBASH3B
UniProt: Q8TF42
rsID: rs11218735
Target SNP: 11:122652542:A:G
Proxy SNP: 11:122523250:G:A
deCODE log10(p): 48.5
deCODE BETA: −0.13
deCODE MSPA: 0.01
1245:771:1130:1208:1119:859:[illegible]

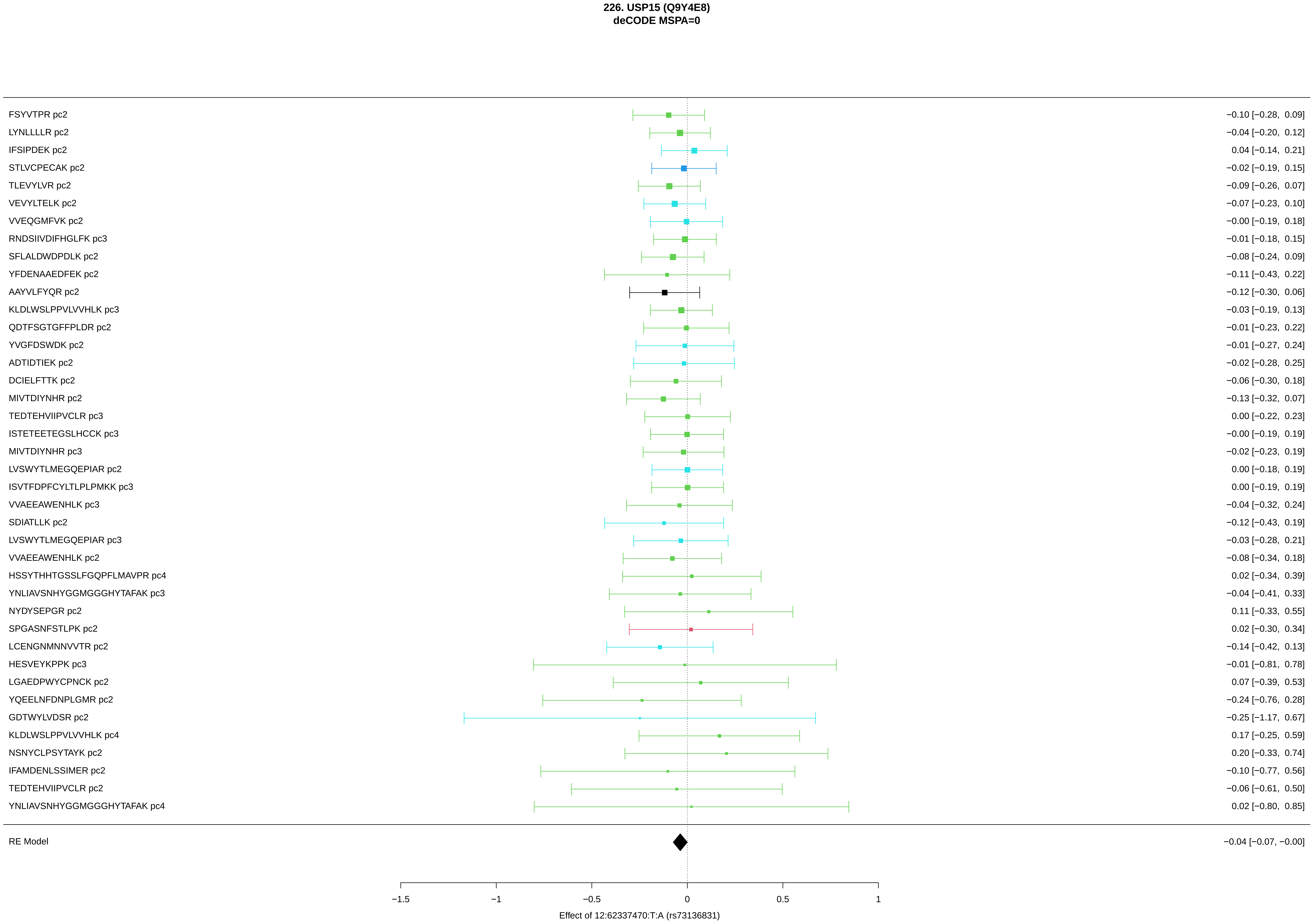
226. USP15 (Q9Y4E8)
deCODE MSPA=0
FSYVTPR pc2
LYNLLLLR pc2
IFSIPDEK pc2
STLVCPECAK pc2
TLEVYLVR pc2
VEVYLTELK pc2
VVEQGMFVK pc2
RNDSIIVDIFHGLFK pc3
SFLALDWDPDLK pc2
YFDENAAEDFEK pc2
AAYVLFYQR pc2
KLDLWSLPPVLVVHLK pc3
QDTFSGTGFFPLDR pc2
YVGFDSWDK pc2
ADTIDTIEK pc2
DCIELFTTK pc2
MIVTDIYNHR pc2
TEDTEHVIIPVCLR pc3
ISTETEETEGSLHCCK pc3
MIVTDIYNHR pc3
LVSWYTLMEGQEPIAR pc2
ISVTFDPFCYLTLPLPMKK pc3
VVAEEAWENHLK pc3
SDIATLLK pc2
LVSWYTLMEGQEPIAR pc3
VVAEEAWENHLK pc2
HSSYTHHTGSSLFGQPFLMAVPR pc4
YNLIAVSNHYGGMGGGHYTAFAK pc3
NYDYSEPGR pc2
SPGASNFSTLPK pc2
LCENGNMNNVVTR pc2
HESVEYKPPK pc3
LGAEDPWYCPNCK pc2
YQEELNFDNPLGMR pc2
GDTWYLVDSR pc2
KLDLWSLPPVLVVHLK pc4
NSNYCLPSYTAYK pc2
IFAMDENLSSIMER pc2
TEDTEHVIIPVCLR pc2
YNLIAVSNHYGGMGGGHYTAFAK pc4
RE Model
−0.10 [−0.28, 0.09]
−0.04 [−0.20, 0.12]
0.04 [−0.14, 0.21]
−0.02 [−0.19, 0.15]
−0.09 [−0.26, 0.07]
−0.07 [−0.23, 0.10]
−0.00 [−0.19, 0.18]
−0.01 [−0.18, 0.15]
−0.08 [−0.24, 0.09]
−0.11 [−0.43, 0.22]
−0.12 [−0.30, 0.06]
−0.03 [−0.19, 0.13]
−0.01 [−0.23, 0.22]
−0.01 [−0.27, 0.24]
−0.02 [−0.28, 0.25]
−0.06 [−0.30, 0.18]
−0.13 [−0.32, 0.07]
0.00 [−0.22, 0.23]
−0.00 [−0.19, 0.19]
−0.02 [−0.23, 0.19]
0.00 [−0.18, 0.19]
0.00 [−0.19, 0.19]
−0.04 [−0.32, 0.24]
−0.12 [−0.43, 0.19]
−0.03 [−0.28, 0.21]
−0.08 [−0.34, 0.18]
0.02 [−0.34, 0.39]
−0.04 [−0.41, 0.33]
0.11 [−0.33, 0.55]
0.02 [−0.30, 0.34]
−0.14 [−0.42, 0.13]
−0.01 [−0.81, 0.78]
0.07 [−0.39, 0.53]
−0.24 [−0.76, 0.28]
−0.25 [−1.17, 0.67]
0.17 [−0.25, 0.59]
0.20 [−0.33, 0.74]
−0.10 [−0.77, 0.56]
−0.06 [−0.61, 0.50]
0.02 [−0.80, 0.85]
−0.04 [−0.07, −0.00]
−1.5
−1
−0.5
0
0.5
1
Effect of 12:62337470:T:A (rs73136831)

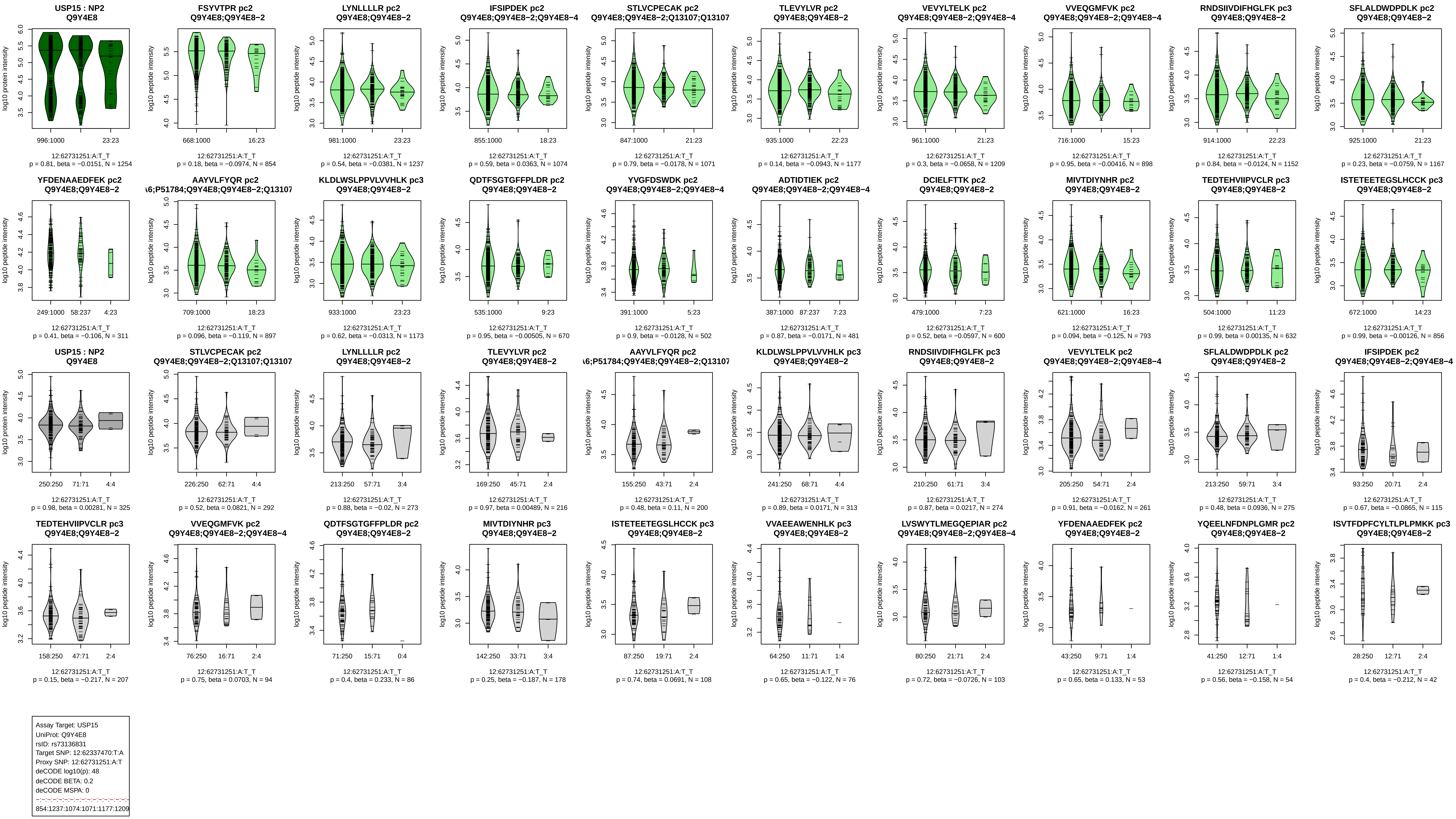

USP15 : NP2 Q9Y4E8
996:1000 23:23
12:62731251:A:T_T
p = 0.81, beta = −0.0151, N = 1254
FSYVTPR pc2 Q9Y4E8;Q9Y4E8−2
668:1000 16:23
12:62731251:A:T_T
p = 0.18, beta = −0.0974, N = 854
LYNLLLLR pc2 Q9Y4E8;Q9Y4E8−2
981:1000 23:23
12:62731251:A:T_T
p = 0.54, beta = −0.0381, N = 1237
IFSIPDEK pc2 Q9Y4E8;Q9Y4E8−2;Q9Y4E8−4
855:1000 18:23
12:62731251:A:T_T
p = 0.59, beta = 0.0363, N = 1074
STLVCPECAK pc2 Q9Y4E8;Q9Y4E8−2;Q13107;Q13107
847:1000 21:23
12:62731251:A:T_T
p = 0.79, beta = −0.0178, N = 1071
TLEVYLVR pc2 Q9Y4E8;Q9Y4E8−2
935:1000 22:23
12:62731251:A:T_T
p = 0.14, beta = −0.0943, N = 1177
VEVYLTELK pc2 Q9Y4E8;Q9Y4E8−2;Q9Y4E8−4
961:1000 21:23
12:62731251:A:T_T
p = 0.3, beta = −0.0658, N = 1209
VVEQGMFVK pc2 Q9Y4E8;Q9Y4E8−2;Q9Y4E8−4
716:1000 15:23
12:62731251:A:T_T
p = 0.95, beta = −0.00416, N = 898
RNDSIIVDIFHGLFK pc3 Q9Y4E8;Q9Y4E8−2
914:1000 22:23
12:62731251:A:T_T
p = 0.84, beta = −0.0124, N = 1152
SFLALDWDPDLK pc2 Q9Y4E8;Q9Y4E8−2
925:1000 21:23
12:62731251:A:T_T
p = 0.23, beta = −0.0759, N = 1167
YFDENAAEDFEK pc2 Q9Y4E8;Q9Y4E8−2
249:1000 58:237 4:23
12:62731251:A:T_T
p = 0.41, beta = −0.106, N = 311
AAYVLFYQR pc2 \6;P51784;Q9Y4E8;Q9Y4E8−2;Q13107
709:1000 18:23
12:62731251:A:T_T
p = 0.096, beta = −0.119, N = 897
KLDLWSLPPVLVVHLK pc3 Q9Y4E8;Q9Y4E8−2
933:1000 23:23
12:62731251:A:T_T
p = 0.62, beta = −0.0313, N = 1173
QDTFSGTGFFPLDR pc2 Q9Y4E8;Q9Y4E8−2
535:1000 9:23
12:62731251:A:T_T
p = 0.95, beta = −0.00505, N = 670
YVGFDSWDK pc2 Q9Y4E8;Q9Y4E8−2;Q9Y4E8−4
391:1000 5:23
12:62731251:A:T_T
p = 0.9, beta = −0.0128, N = 502
ADTIDTIEK pc2 Q9Y4E8;Q9Y4E8−2;Q9Y4E8−4
387:1000 87:237 7:23
12:62731251:A:T_T
p = 0.87, beta = −0.0171, N = 481
DCIELFTTK pc2 Q9Y4E8;Q9Y4E8−2
479:1000 7:23
12:62731251:A:T_T
p = 0.52, beta = −0.0597, N = 600
MIVTDIYNHR pc2 Q9Y4E8;Q9Y4E8−2
621:1000 16:23
12:62731251:A:T_T
p = 0.094, beta = −0.125, N = 793
TEDTEHVIIPVCLR pc3 Q9Y4E8;Q9Y4E8−2
504:1000 11:23
12:62731251:A:T_T
p = 0.99, beta = 0.00135, N = 632
ISTETEETEGSLHCCK pc3 Q9Y4E8;Q9Y4E8−2
672:1000 14:23
12:62731251:A:T_T
p = 0.99, beta = −0.00126, N = 856
USP15 : NP2 Q9Y4E8
250:250 71:71 4:4
12:62731251:A:T_T
p = 0.98, beta = 0.00281, N = 325
STLVCPECAK pc2 Q9Y4E8;Q9Y4E8−2;Q13107;Q13107
226:250 62:71 4:4
12:62731251:A:T_T
p = 0.52, beta = 0.0821, N = 292
LYNLLLLR pc2 Q9Y4E8;Q9Y4E8−2
213:250 57:71 3:4
12:62731251:A:T_T
p = 0.88, beta = −0.02, N = 273
TLEVYLVR pc2 Q9Y4E8;Q9Y4E8−2
169:250 45:71 2:4
12:62731251:A:T_T
p = 0.97, beta = 0.00489, N = 216
AAYVLFYQR pc2 \6;P51784;Q9Y4E8;Q9Y4E8−2;Q13107
155:250 43:71 2:4
12:62731251:A:T_T
p = 0.48, beta = 0.11, N = 200
KLDLWSLPPVLVVHLK pc3 Q9Y4E8;Q9Y4E8−2
241:250 68:71 4:4
12:62731251:A:T_T
p = 0.89, beta = 0.0171, N = 313
RNDSIIVDIFHGLFK pc3 Q9Y4E8;Q9Y4E8−2
210:250 61:71 3:4
12:62731251:A:T_T
p = 0.87, beta = 0.0217, N = 274
VEVYLTELK pc2 Q9Y4E8;Q9Y4E8−2;Q9Y4E8−4
205:250 54:71 2:4
12:62731251:A:T_T
p = 0.91, beta = −0.0162, N = 261
SFLALDWDPDLK pc2 Q9Y4E8;Q9Y4E8−2
213:250 59:71 3:4
12:62731251:A:T_T
p = 0.48, beta = 0.0936, N = 275
IFSIPDEK pc2 Q9Y4E8;Q9Y4E8−2;Q9Y4E8−4
93:250 20:71 2:4
12:62731251:A:T_T
p = 0.67, beta = −0.0865, N = 115
TEDTEHVIIPVCLR pc3 Q9Y4E8;Q9Y4E8−2
158:250 47:71 2:4
12:62731251:A:T_T
p = 0.15, beta = −0.217, N = 207
VVEQGMFVK pc2 Q9Y4E8;Q9Y4E8−2;Q9Y4E8−4
76:250 16:71 2:4
12:62731251:A:T_T
p = 0.75, beta = 0.0703, N = 94
QDTFSGTGFFPLDR pc2 Q9Y4E8;Q9Y4E8−2
71:250 15:71 0:4
12:62731251:A:T_T
p = 0.4, beta = 0.233, N = 86
MIVTDIYNHR pc3 Q9Y4E8;Q9Y4E8−2
142:250 33:71 3:4
12:62731251:A:T_T
p = 0.25, beta = −0.187, N = 178
ISTETEETEGSLHCCK pc3 Q9Y4E8;Q9Y4E8−2
87:250 19:71 2:4
12:62731251:A:T_T
p = 0.74, beta = 0.0691, N = 108
VVAEEAWENHLK pc3 Q9Y4E8;Q9Y4E8−2
64:250 11:71 1:4
12:62731251:A:T_T
p = 0.65, beta = −0.122, N = 76
LVSWYTLMEGQEPIAR pc2 Q9Y4E8;Q9Y4E8−2;Q9Y4E8−4
80:250 21:71 2:4
12:62731251:A:T_T
p = 0.72, beta = −0.0726, N = 103
YFDENAAEDFEK pc2 Q9Y4E8;Q9Y4E8−2
43:250 9:71 1:4
12:62731251:A:T_T
p = 0.65, beta = 0.133, N = 53
YQEELNFDNPLGMR pc2 Q9Y4E8;Q9Y4E8−2
41:250 12:71 1:4
12:62731251:A:T_T
p = 0.56, beta = −0.158, N = 54
ISVTFDPFCYLTLPLPMKK pc3 Q9Y4E8;Q9Y4E8−2
28:250 12:71 2:4
12:62731251:A:T_T
p = 0.4, beta = −0.212, N = 42
log10 protein intensity
log10 peptide intensity
Assay Target: USP15
UniProt: Q9Y4E8
rsID: rs73136831
Target SNP: 12:62337470:T:A
Proxy SNP: 12:62731251:A:T
deCODE log10(p): 48
deCODE BETA: 0.2
deCODE MSPA: 0
854:1237:1074:1071:1177:1209

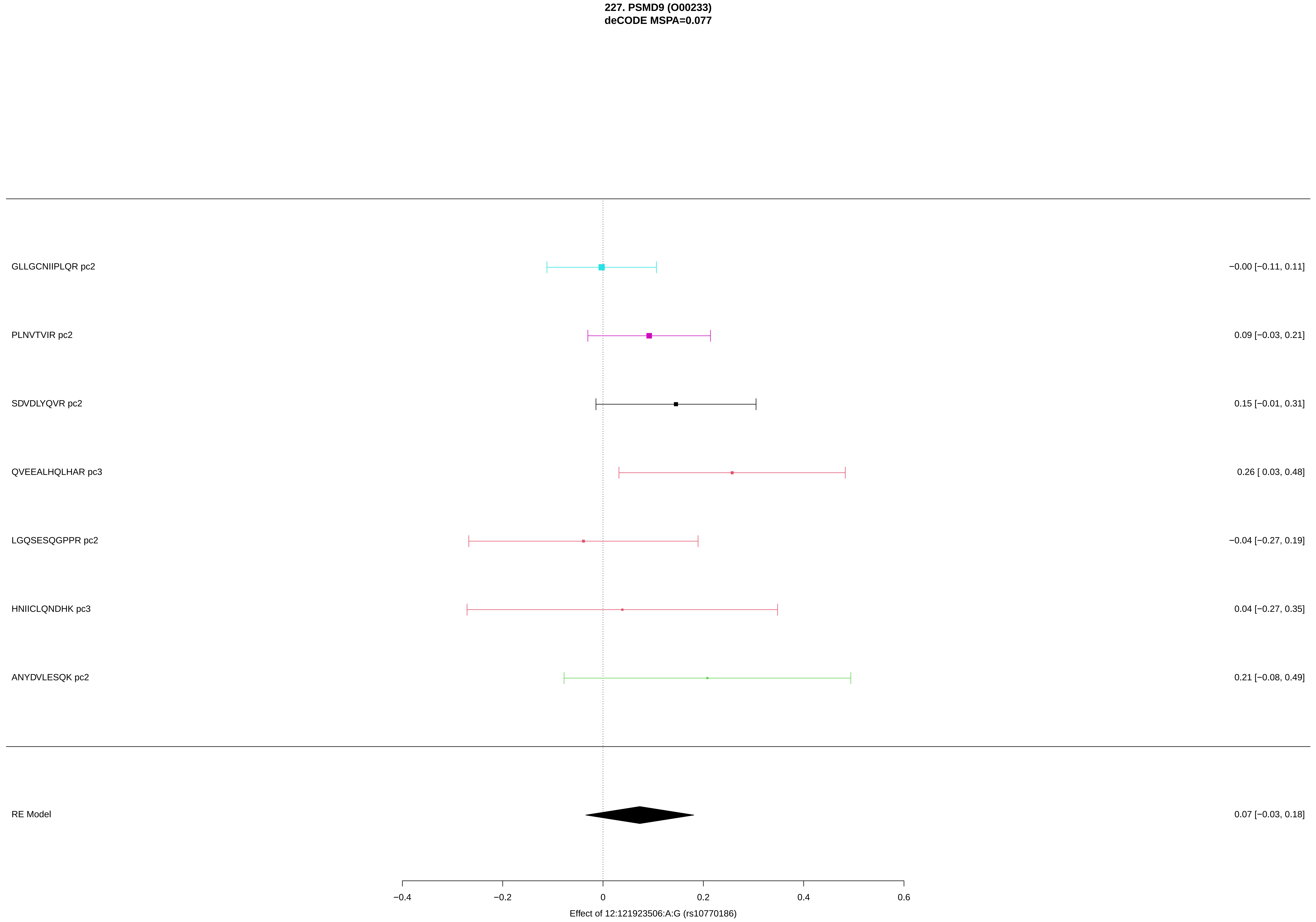
227. PSMD9 (O00233)
deCODE MSPA=0.077
GLLGCNIIPLQR pc2
PLNVTVIR pc2
SDVDLYQVR pc2
QVEEALHQLHAR pc3
LGQSESQGPPR pc2
HNIICLQNDHK pc3
ANYDVLESQK pc2
RE Model
−0.00 [−0.11, 0.11]
0.09 [−0.03, 0.21]
0.15 [−0.01, 0.31]
0.26 [0.03, 0.48]
−0.04 [−0.27, 0.19]
0.04 [−0.27, 0.35]
0.21 [−0.08, 0.49]
0.07 [−0.03, 0.18]
−0.4
−0.2
0
0.2
0.4
0.6
Effect of 12:121923506:A:G (rs10770186)

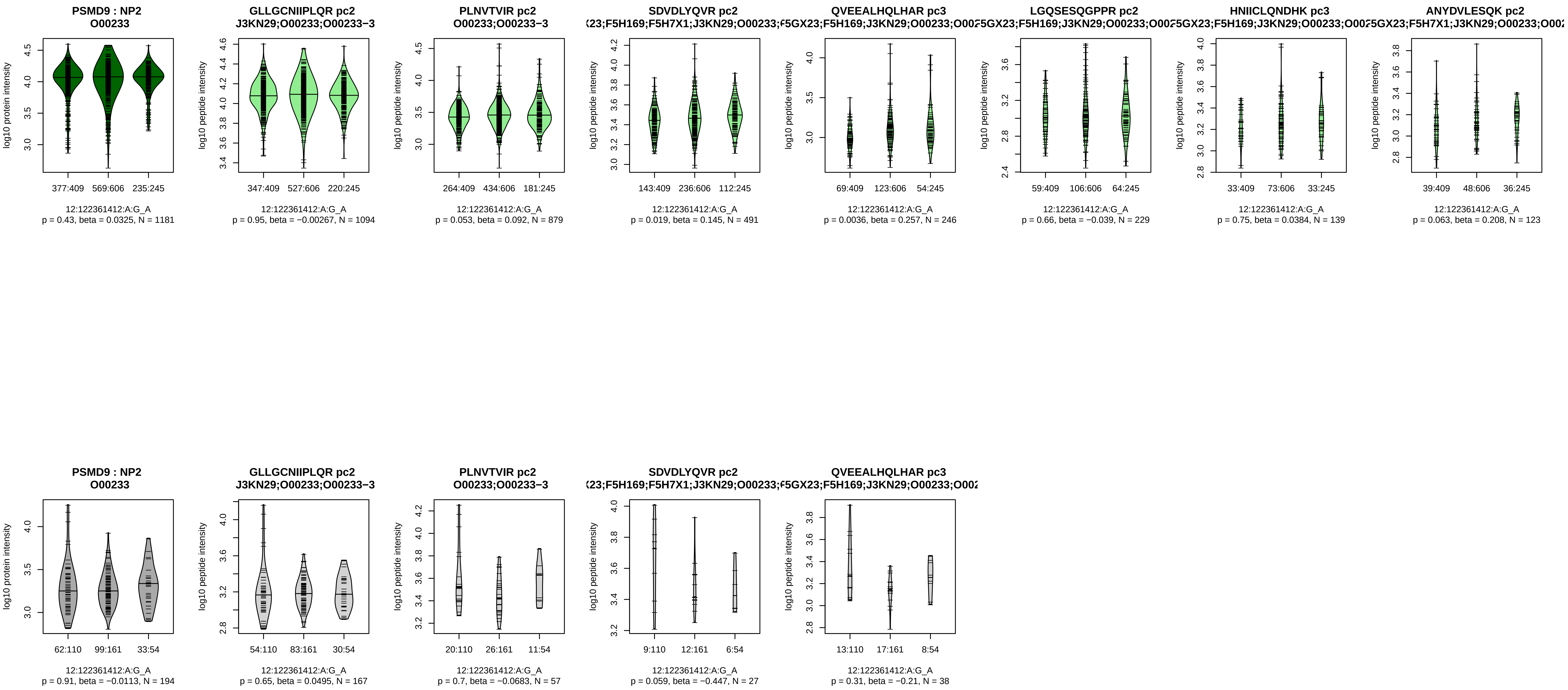

Assay Target: PSMD9
UniProt: O00233
rsID: rs10770186
Target SNP: 12:121923506:A:G
Proxy SNP: 12:122361412:A:G
deCODE log10(p): 47.8
deCODE BETA: −0.12
deCODE MSPA: 0.077
−:−:−:*:−:−:−:−:NA:NA:NA:NA
1094:879:491:246:229:139:123:

228. VARS (P26640)
deCODE MSPA=0.081

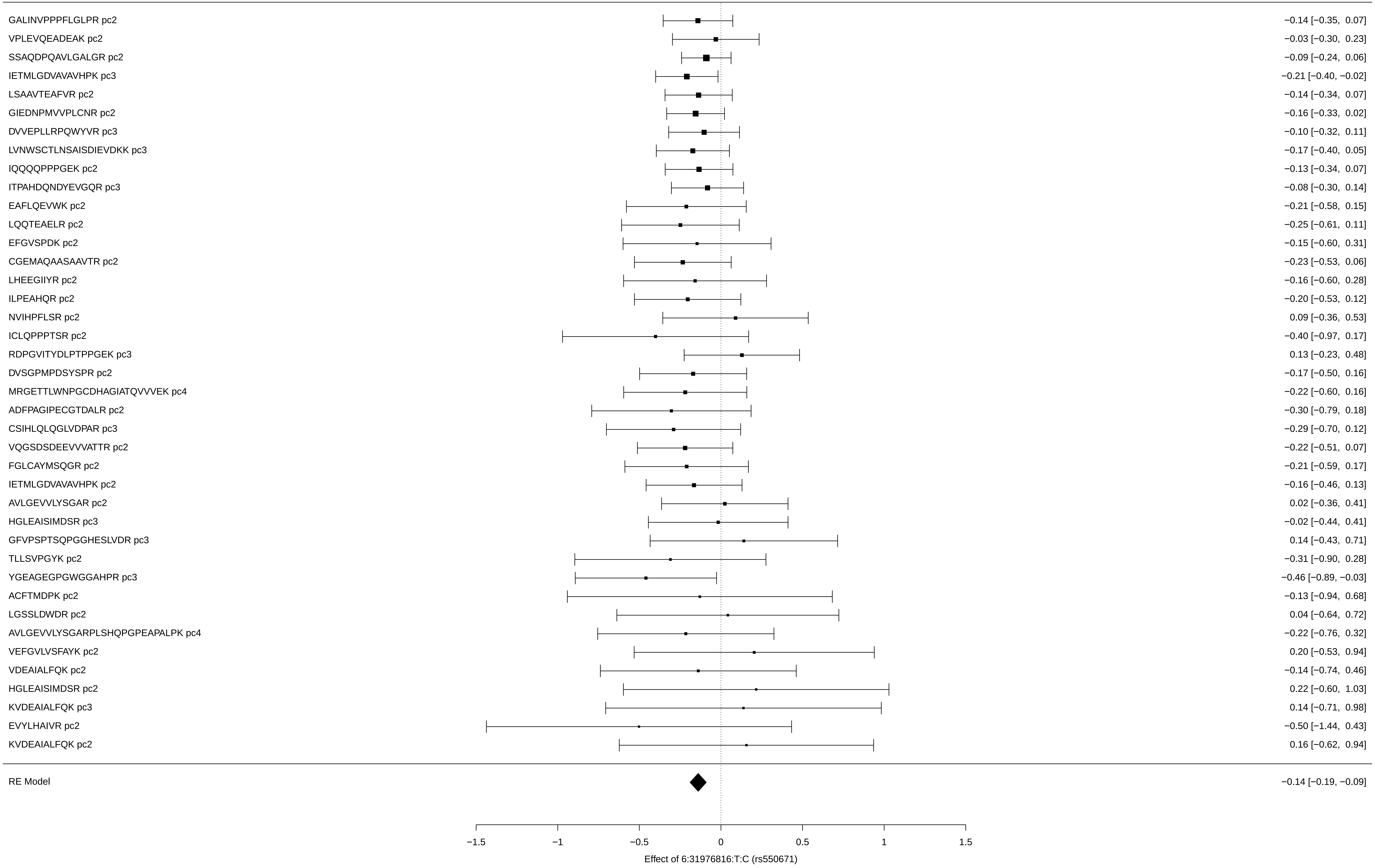

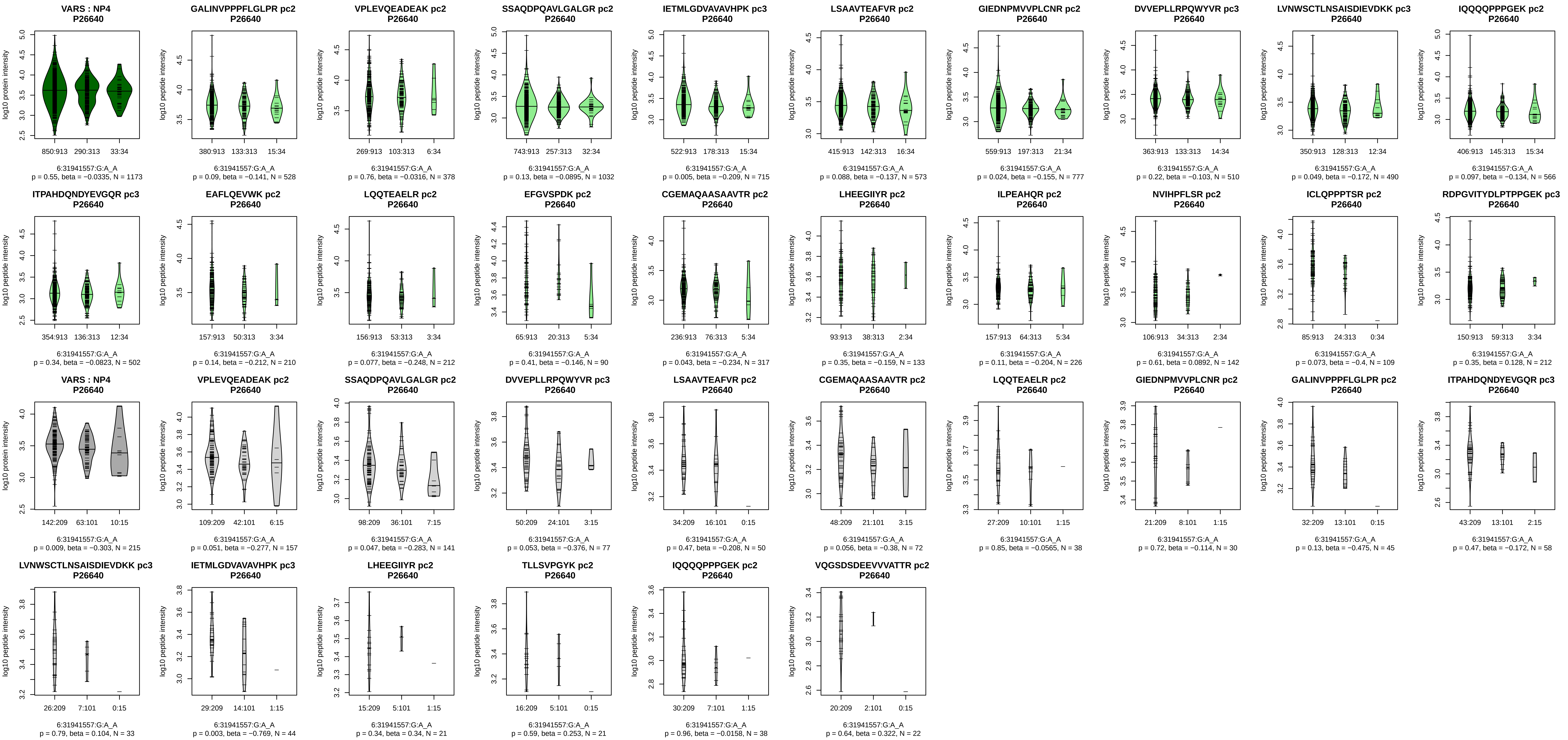

Assay Target: VARS
UniProt: P26640
rsID: rs550671
Target SNP: 6:31976816:T:C
Proxy SNP: 6:31941557:G:A
deCODE log10(p): 47.3
deCODE BETA: 0.17
deCODE MSPA: 0.081
528:378:1032:715:573:777:510:

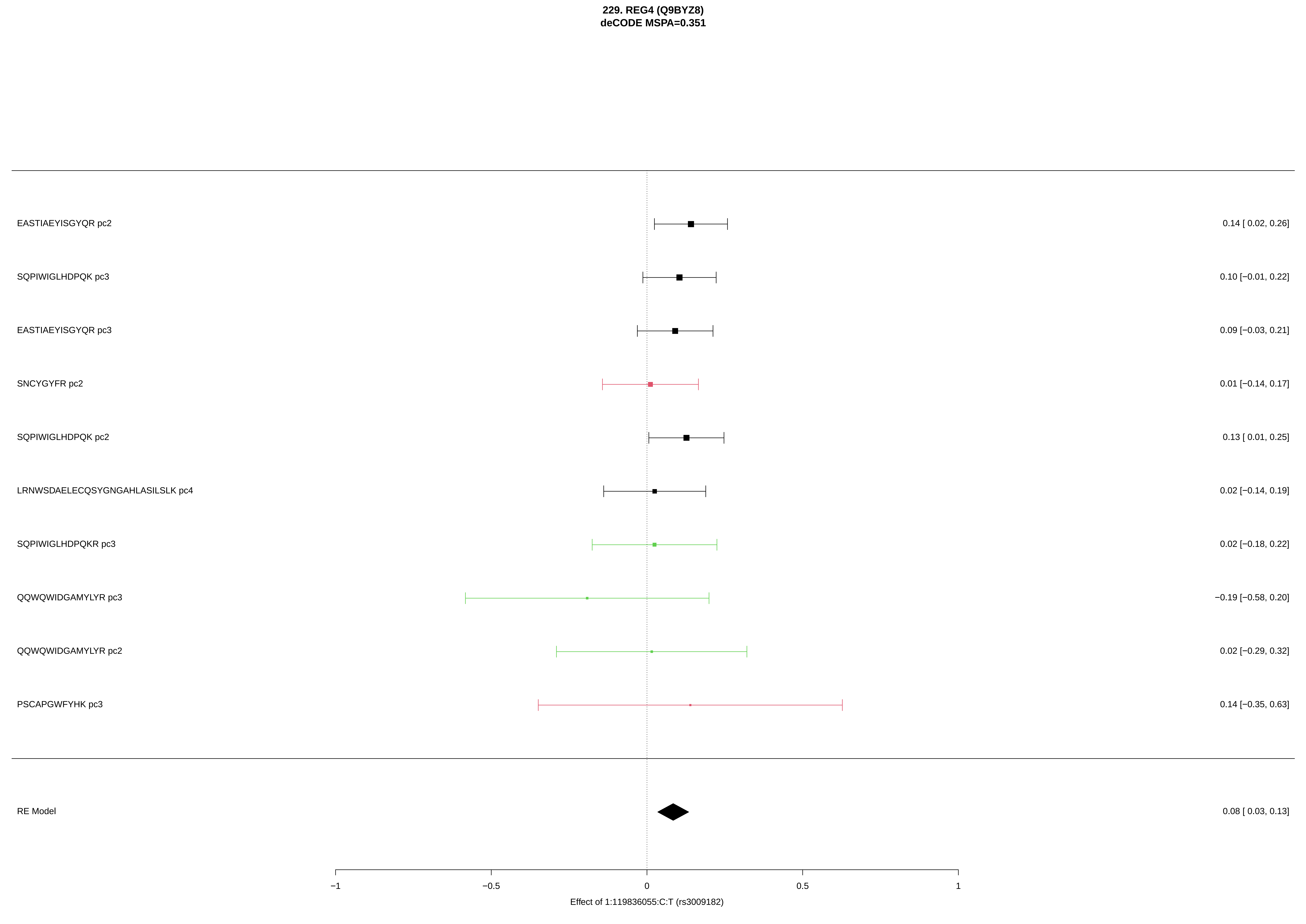

229. REG4 (Q9BYZ8)
deCODE MSPA=0.351
EASTIAEYISGYQR pc2
0.14 [0.02, 0.26]
SQPIWIGLHDPQK pc3
0.10 [−0.01, 0.22]
EASTIAEYISGYQR pc3
0.09 [−0.03, 0.21]
SNCYGYFR pc2
0.01 [−0.14, 0.17]
SQPIWIGLHDPQK pc2
0.13 [0.01, 0.25]
LRNWSDAELECQSYGNGAHLASILSLK pc4
0.02 [−0.14, 0.19]
SQPIWIGLHDPQKR pc3
0.02 [−0.18, 0.22]
QQWQWIDGAMYLYR pc3
−0.19 [−0.58, 0.20]
QQWQWIDGAMYLYR pc2
0.02 [−0.29, 0.32]
PSCAPGWFYHK pc3
0.14 [−0.35, 0.63]
RE Model
0.08 [0.03, 0.13]
−1
−0.5
0
0.5
1
Effect of 1:119836055:C:T (rs3009182)

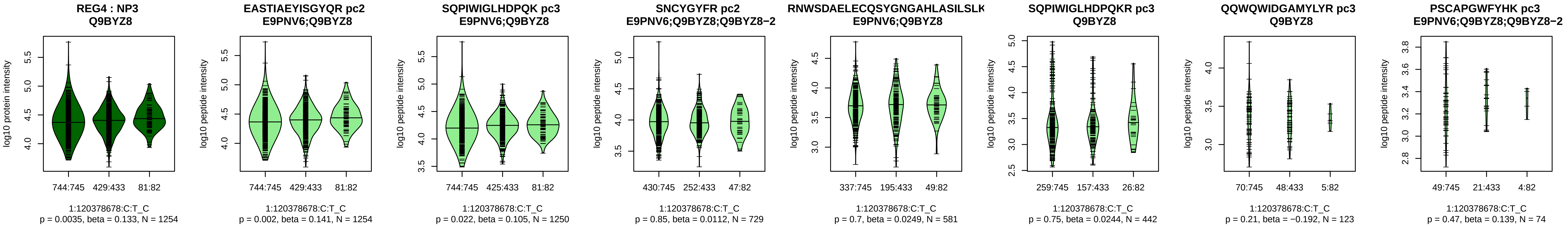

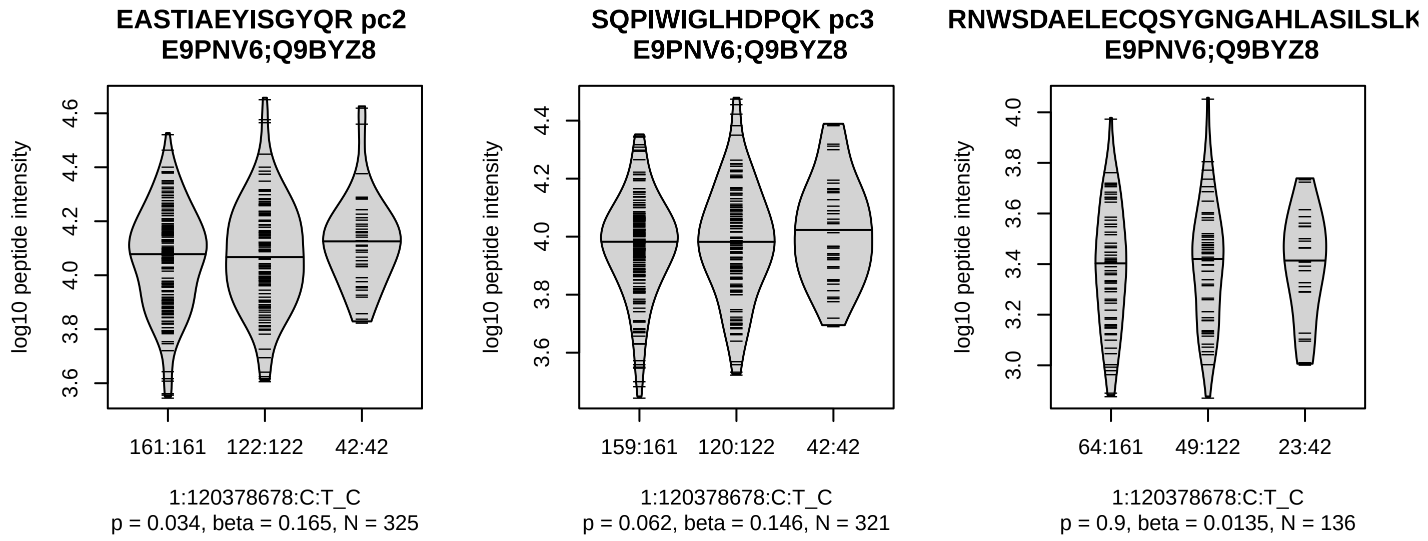

Assay Target: REG4
UniProt: Q9BYZ8
rsID: rs3009182
Target SNP: 1:119836055:C:T
Proxy SNP: 1:120378678:C:T
deCODE log10(p): 45.6
deCODE BETA: 0.14
deCODE MSPA: 0.351
:−:−:−::−:−:−:−:−:NA
1254:1250:1156:729:1175:581:4

230. SRL (Q86TD4)
deCODE MSPA=0

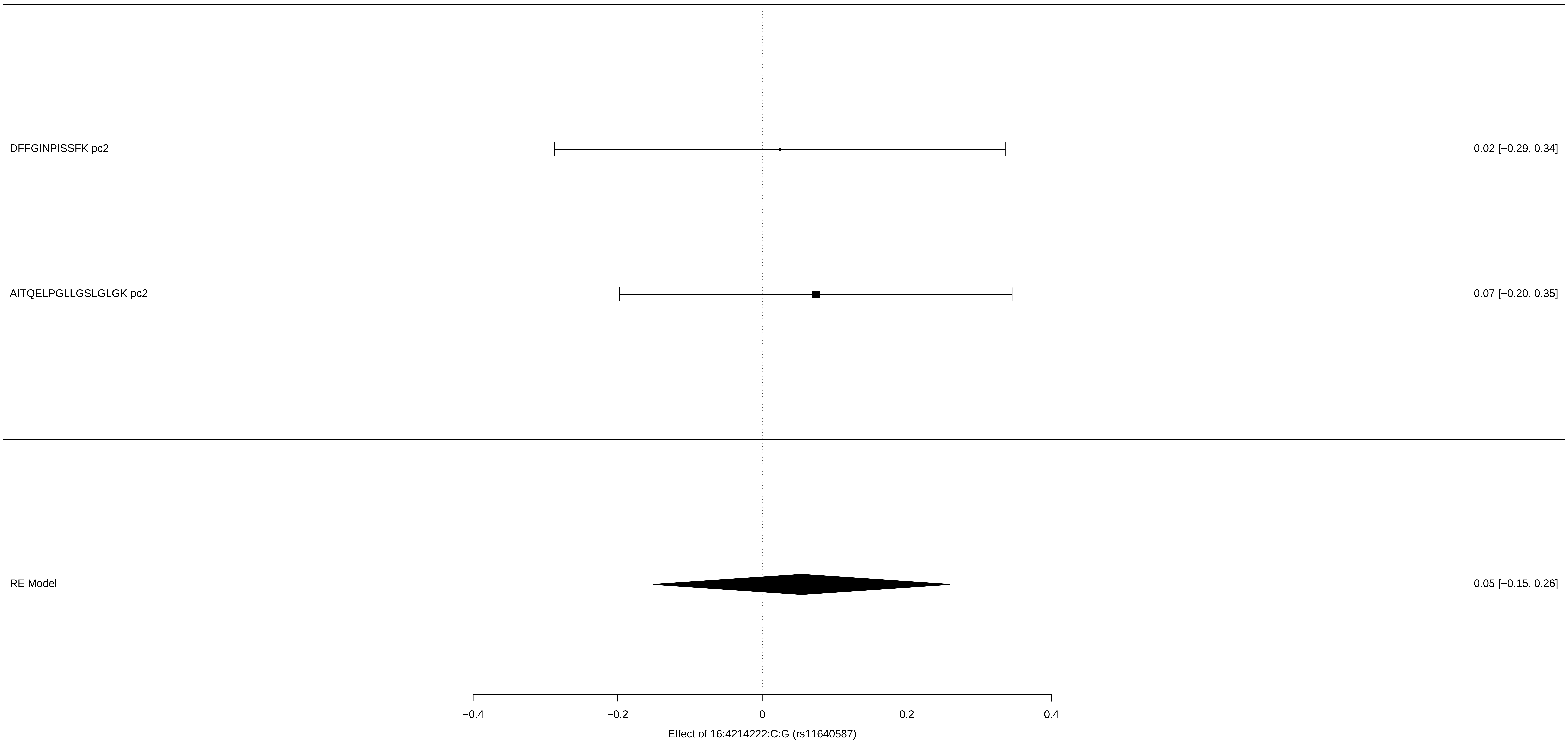

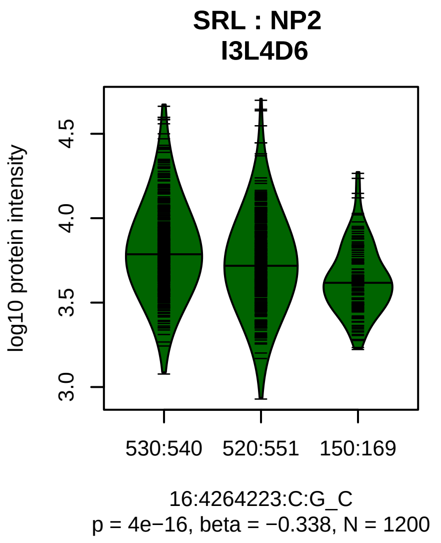

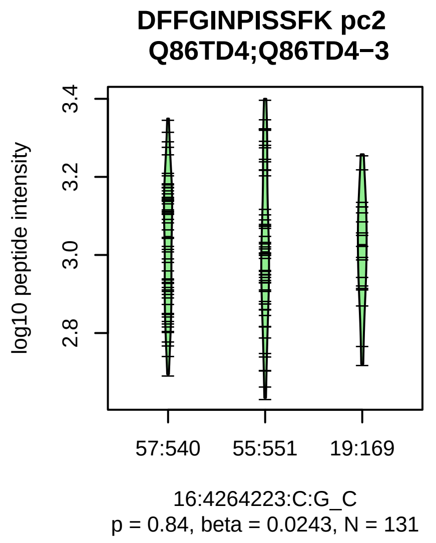

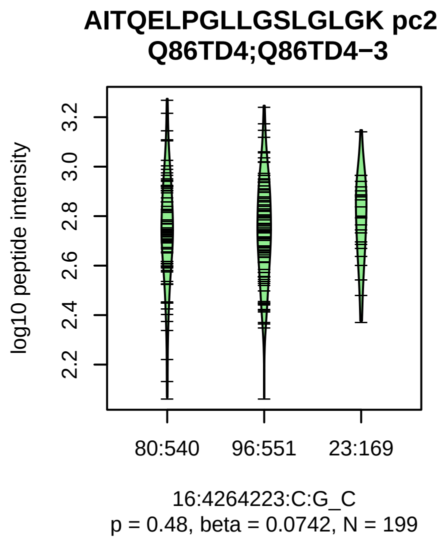

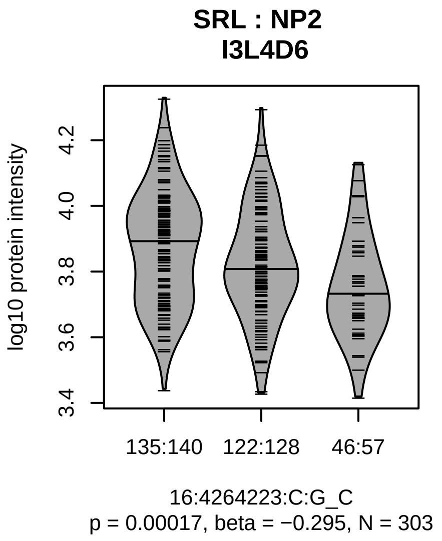

Assay Target: SRL
UniProt: Q86TD4
rsID: rs11640587
Target SNP: 16:4214222:C:G
Proxy SNP: 16:4264223:C:G
deCODE log10(p): 45.2
deCODE BETA: 0.12
deCODE MSPA: 0
−:−:NA:NA:NA
131:199:19:7:4

231. STX7 (O15400)
deCODE MSPA=0.922

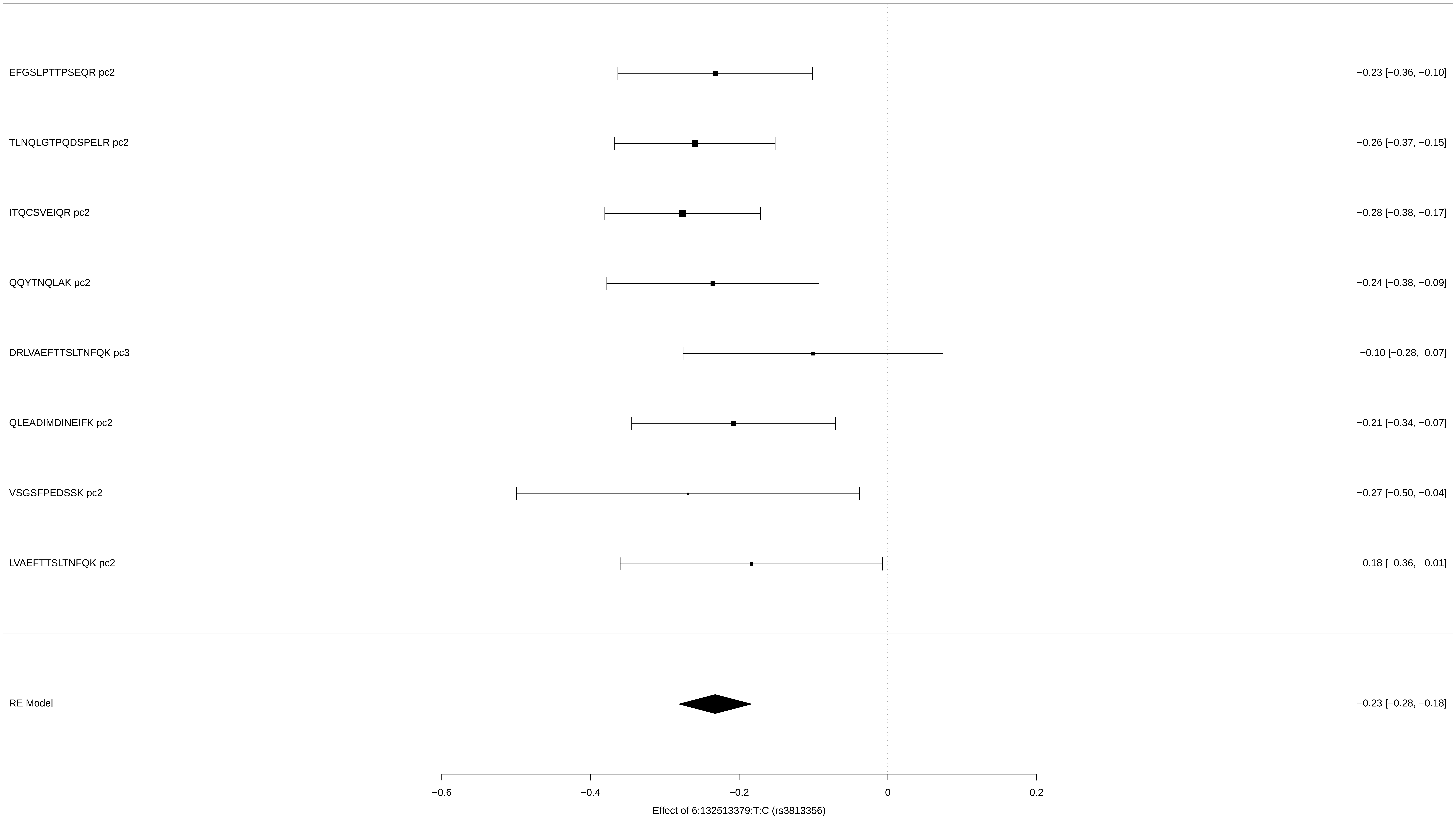

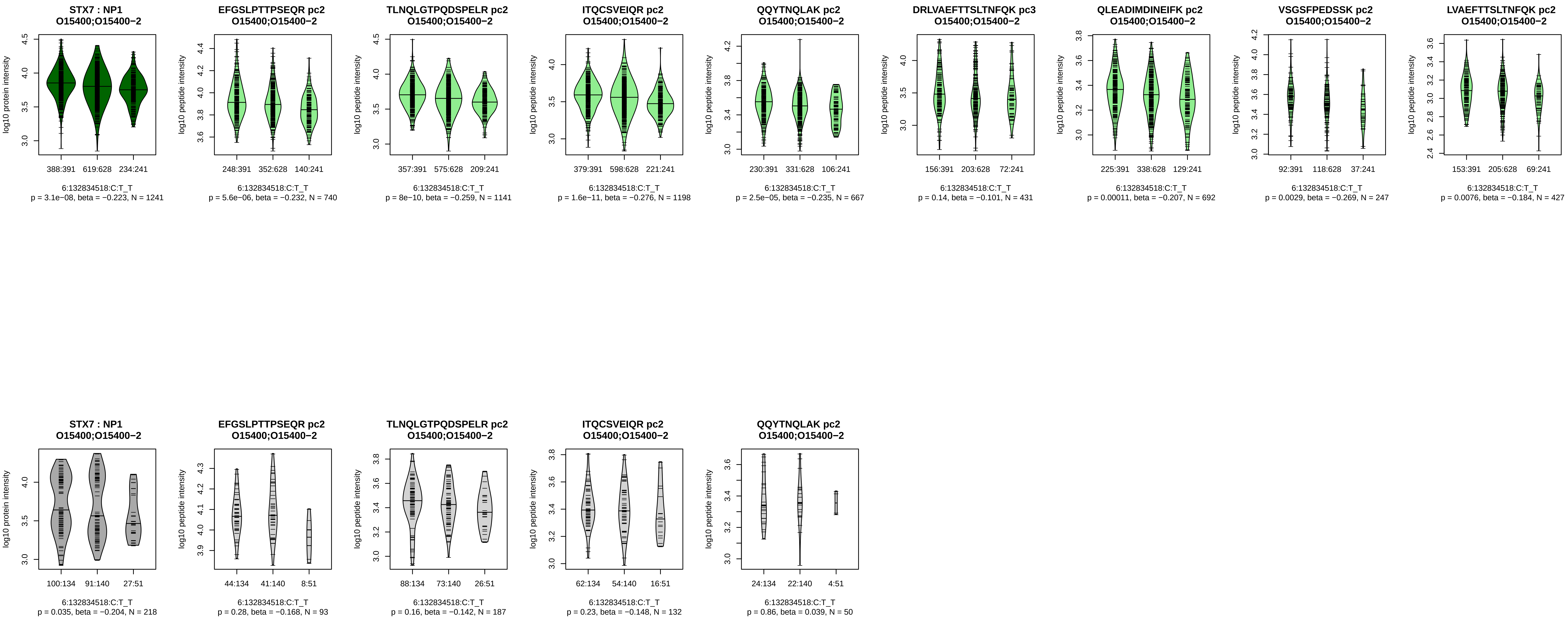

Assay Target: STX7
UniProt: O15400
rsID: rs3813356
Target SNP: 6:132513379:T:C
Proxy SNP: 6:132834518:C:T
deCODE log10(p): 45.1
deCODE BETA: −0.12
deCODE MSPA: 0.922
::*:*:–:*:*:*
740:1141:1198:667:431:692:247

232. HPSE (Q9Y251)
deCODE MSPA=0

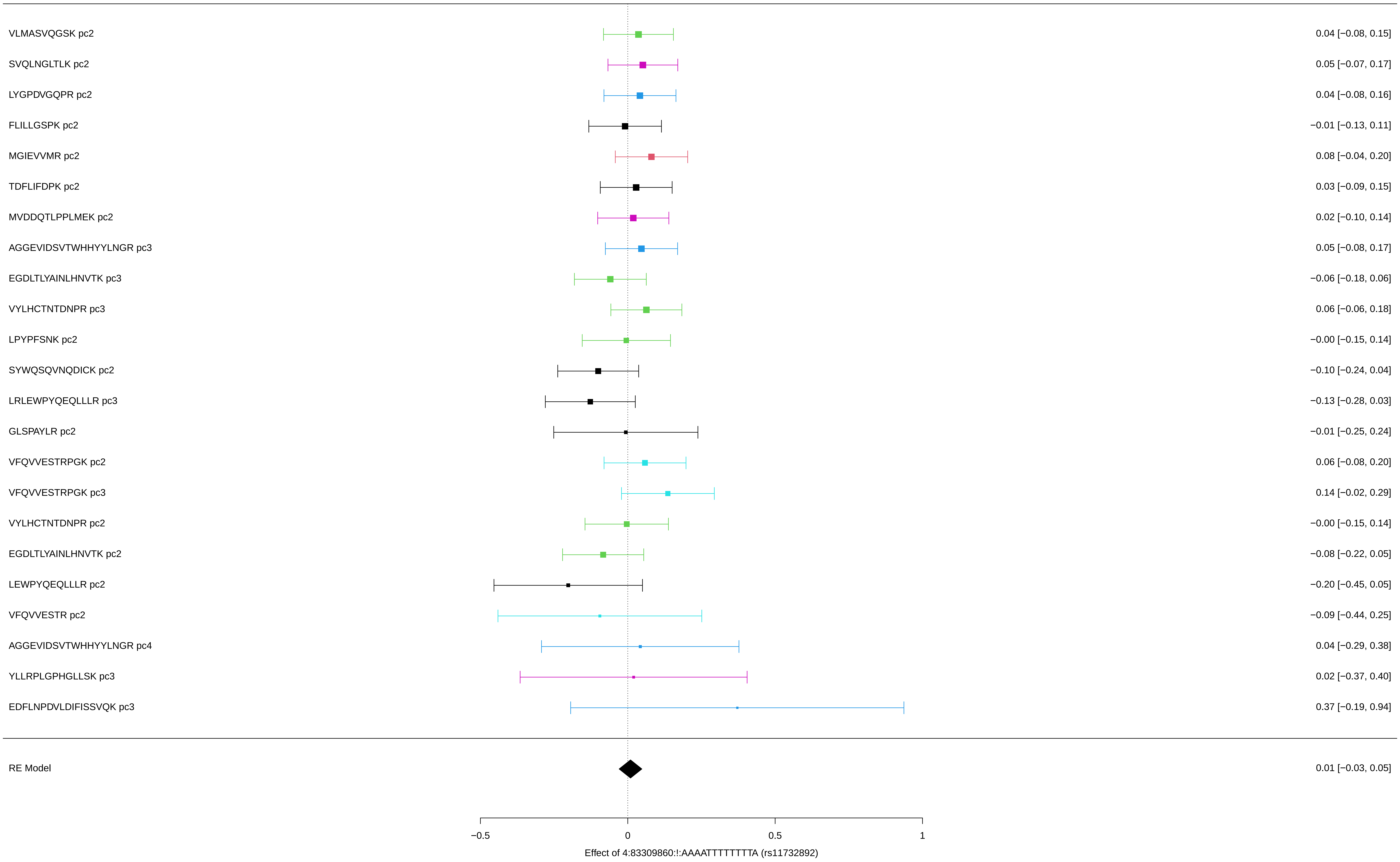

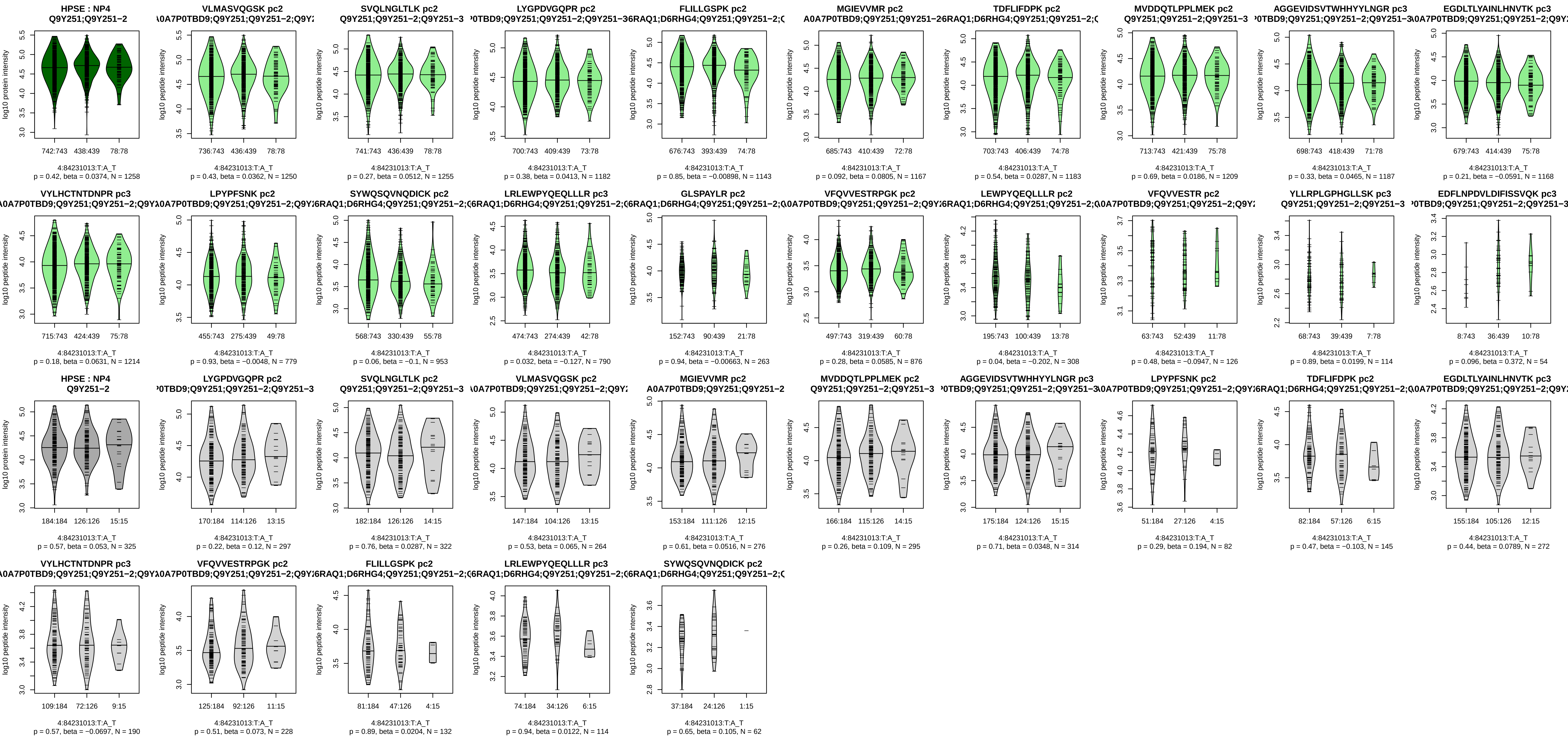

Assay Target: HPSE
UniProt: Q9Y251
rsID: rs11732892
Target SNP: 4:83309860:I:AAAA
Proxy SNP: 4:84231013:T:A
deCODE log10(p): 43
deCODE BETA: −0.13
deCODE MSPA: 0
1250:1255:1182:1143:1167:118

233. PLCG2 (P16885)
deCODE MSPA=0.242

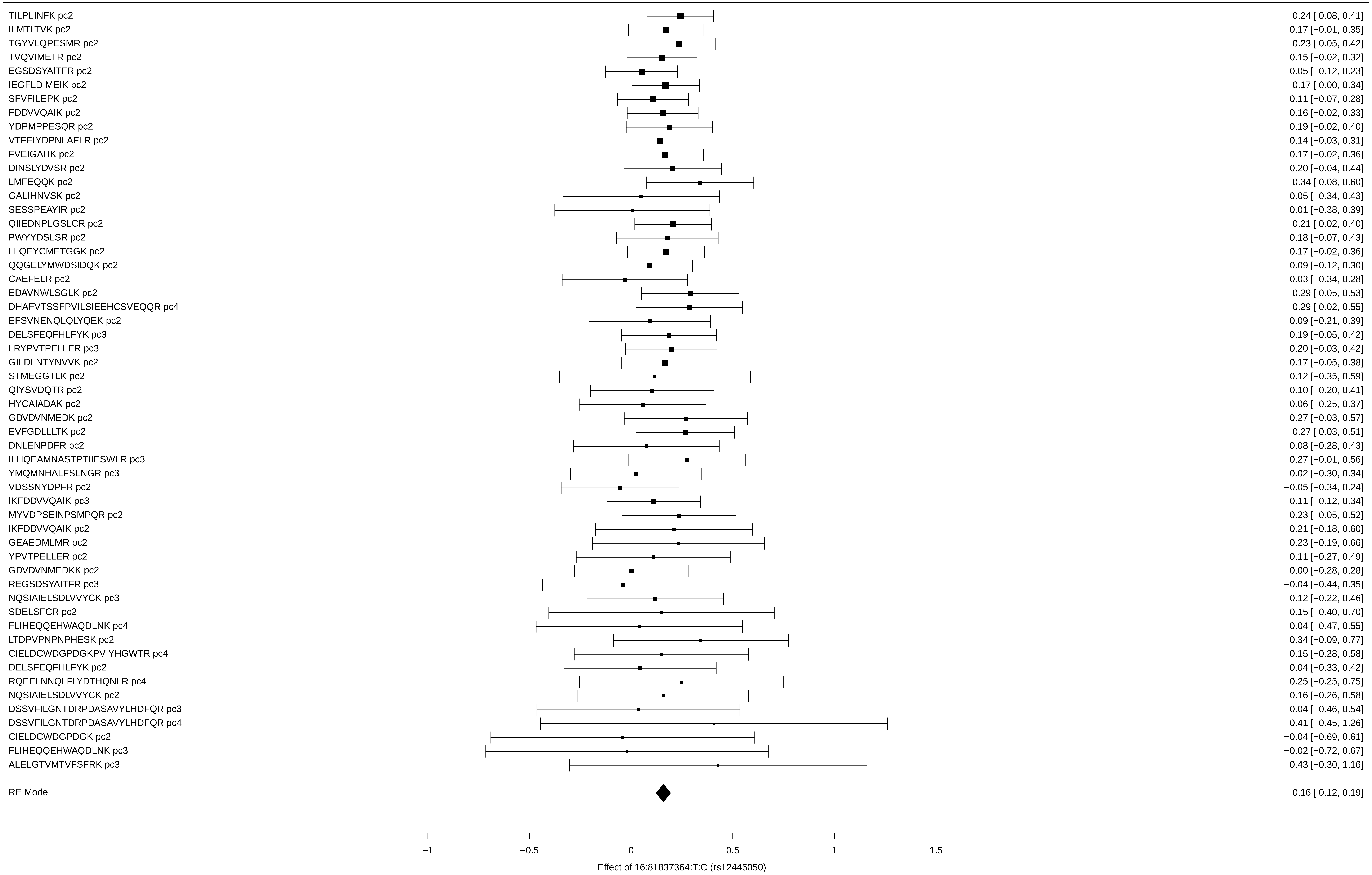

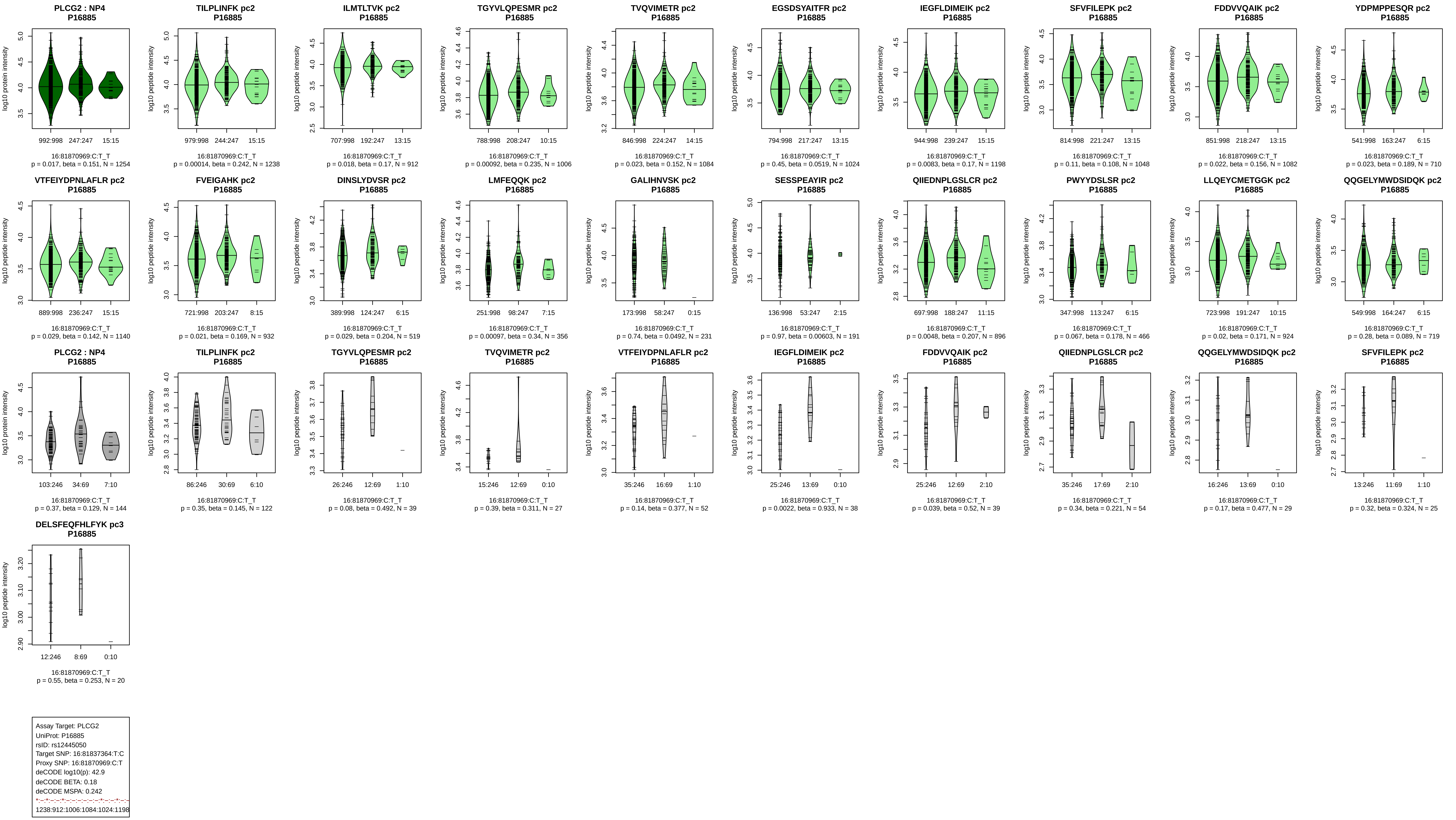
PLCG2 : NP4
P16885
log10 protein intensity
992:998 247:247 15:15
16:81870969:C:T_T
p = 0.017, beta = 0.151, N = 1254
TILPLINFK pc2
P16885
log10 peptide intensity
979:998 244:247 15:15
16:81870969:C:T_T
p = 0.00014, beta = 0.242, N = 1238
ILMTLTVK pc2
P16885
707:998 192:247 13:15
16:81870969:C:T_T
p = 0.018, beta = 0.17, N = 912
TGYVLQPESMR pc2
P16885
788:998 208:247 10:15
16:81870969:C:T_T
p = 0.00092, beta = 0.235, N = 1006
TVQVIMETR pc2
P16885
846:998 224:247 14:15
16:81870969:C:T_T
p = 0.023, beta = 0.152, N = 1084
EGSDSYAITFR pc2
P16885
794:998 217:247 13:15
16:81870969:C:T_T
p = 0.45, beta = 0.0519, N = 1024
IEGFLDIMEIK pc2
P16885
944:998 239:247 15:15
16:81870969:C:T_T
p = 0.0083, beta = 0.17, N = 1198
SFVFILEPK pc2
P16885
814:998 221:247 13:15
16:81870969:C:T_T
p = 0.11, beta = 0.108, N = 1048
FDDVVQAIK pc2
P16885
851:998 218:247 13:15
16:81870969:C:T_T
p = 0.022, beta = 0.156, N = 1082
YDPMPPESQR pc2
P16885
541:998 163:247 6:15
16:81870969:C:T_T
p = 0.023, beta = 0.189, N = 710
VTFEIYDPNLAFLR pc2
P16885
889:998 236:247 15:15
16:81870969:C:T_T
p = 0.029, beta = 0.142, N = 1140
FVEIGAHK pc2
P16885
721:998 203:247 8:15
16:81870969:C:T_T
p = 0.021, beta = 0.169, N = 932
DINSLYDVSR pc2
P16885
389:998 124:247 6:15
16:81870969:C:T_T
p = 0.029, beta = 0.204, N = 519
LMFEQQK pc2
P16885
251:998 98:247 7:15
16:81870969:C:T_T
p = 0.00097, beta = 0.34, N = 356
GALIHNVSK pc2
P16885
173:998 58:247 0:15
16:81870969:C:T_T
p = 0.74, beta = 0.0492, N = 231
SESSPEAYIR pc2
P16885
136:998 53:247 2:15
16:81870969:C:T_T
p = 0.97, beta = 0.00603, N = 191
QIIEDNPLGSLCR pc2
P16885
697:998 188:247 11:15
16:81870969:C:T_T
p = 0.0048, beta = 0.207, N = 896
PWYYDSLSR pc2
P16885
347:998 113:247 6:15
16:81870969:C:T_T
p = 0.067, beta = 0.178, N = 466
LLQEYCMETGGK pc2
P16885
723:998 191:247 10:15
16:81870969:C:T_T
p = 0.02, beta = 0.171, N = 924
QQGELYMWDSIDQK pc2
P16885
549:998 164:247 6:15
16:81870969:C:T_T
p = 0.28, beta = 0.089, N = 719
PLCG2 : NP4
P16885
103:246 34:69 7:10
16:81870969:C:T_T
p = 0.37, beta = 0.129, N = 144
TILPLINFK pc2
P16885
86:246 30:69 6:10
16:81870969:C:T_T
p = 0.35, beta = 0.145, N = 122
TGYVLQPESMR pc2
P16885
26:246 12:69 1:10
16:81870969:C:T_T
p = 0.08, beta = 0.492, N = 39
TVQVIMETR pc2
P16885
15:246 12:69 0:10
16:81870969:C:T_T
p = 0.39, beta = 0.311, N = 27
VTFEIYDPNLAFLR pc2
P16885
35:246 16:69 1:10
16:81870969:C:T_T
p = 0.14, beta = 0.377, N = 52
IEGFLDIMEIK pc2
P16885
25:246 13:69 0:10
16:81870969:C:T_T
p = 0.0022, beta = 0.933, N = 38
FDDVVQAIK pc2
P16885
25:246 12:69 2:10
16:81870969:C:T_T
p = 0.039, beta = 0.52, N = 39
QIIEDNPLGSLCR pc2
P16885
35:246 17:69 2:10
16:81870969:C:T_T
p = 0.34, beta = 0.221, N = 54
QQGELYMWDSIDQK pc2
P16885
16:246 13:69 0:10
16:81870969:C:T_T
p = 0.17, beta = 0.477, N = 29
SFVFILEPK pc2
P16885
13:246 11:69 1:10
16:81870969:C:T_T
p = 0.32, beta = 0.324, N = 25
DELSFEQFHLFYK pc3
P16885
12:246 8:69 0:10
16:81870969:C:T_T
p = 0.55, beta = 0.253, N = 20
Assay Target: PLCG2
UniProt: P16885
rsID: rs12445050
Target SNP: 16:81837364:T:C
Proxy SNP: 16:81870969:C:T
deCODE log10(p): 42.9
deCODE BETA: 0.18
deCODE MSPA: 0.242
1238:912:1006:1084:1024:1198

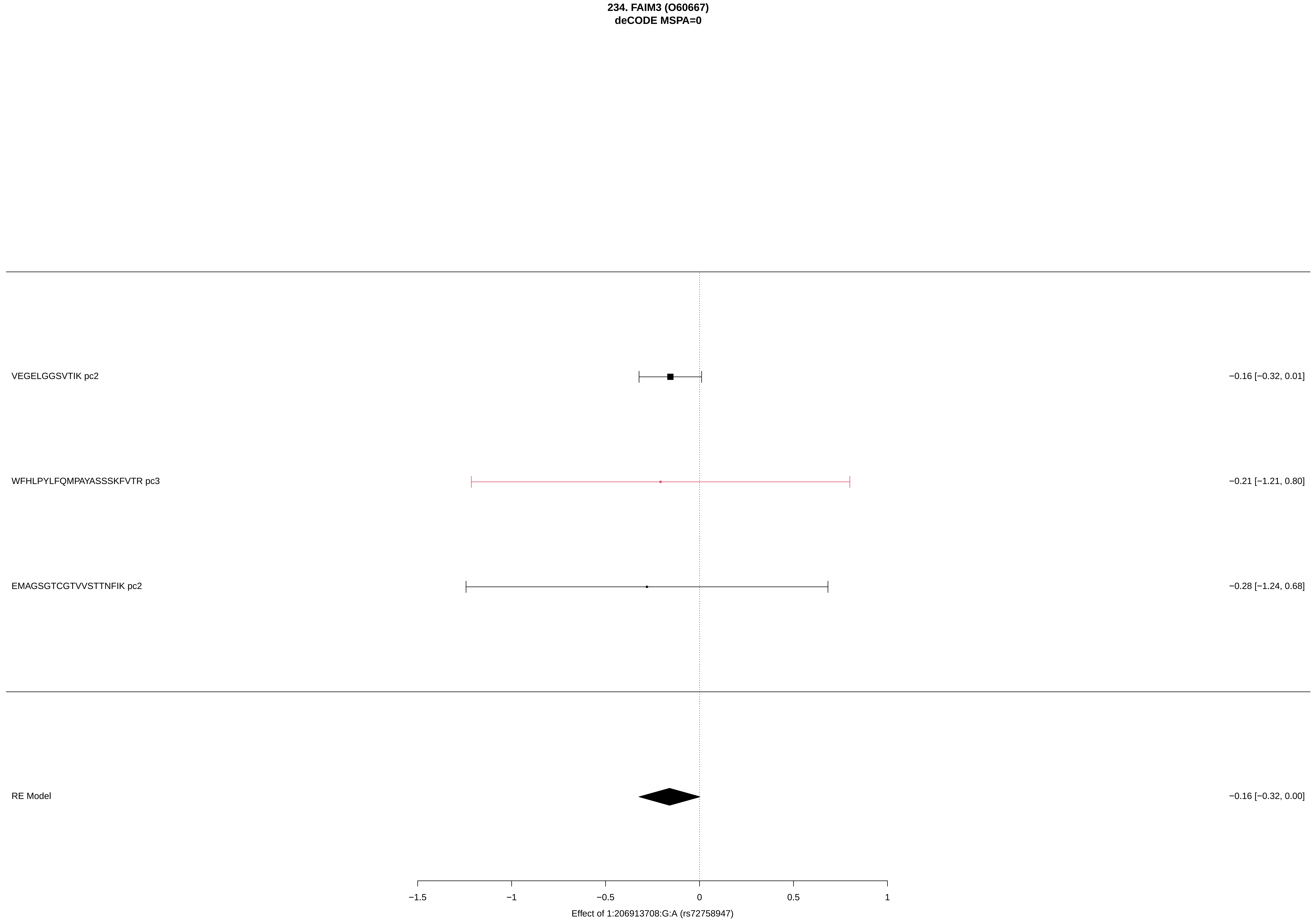
234. FAIM3 (O60667)
deCODE MSPA=0
VEGELGGSVTIK pc2
−0.16 [−0.32, 0.01]
WFHLPYLFQMPAYASSSKFVTR pc3
−0.21 [−1.21, 0.80]
EMAGSGTCGTVVSTTNFIK pc2
−0.28 [−1.24, 0.68]
RE Model
−0.16 [−0.32, 0.00]
−1.5
−1
−0.5
0
0.5
1
Effect of 1:206913708:G:A (rs72758947)

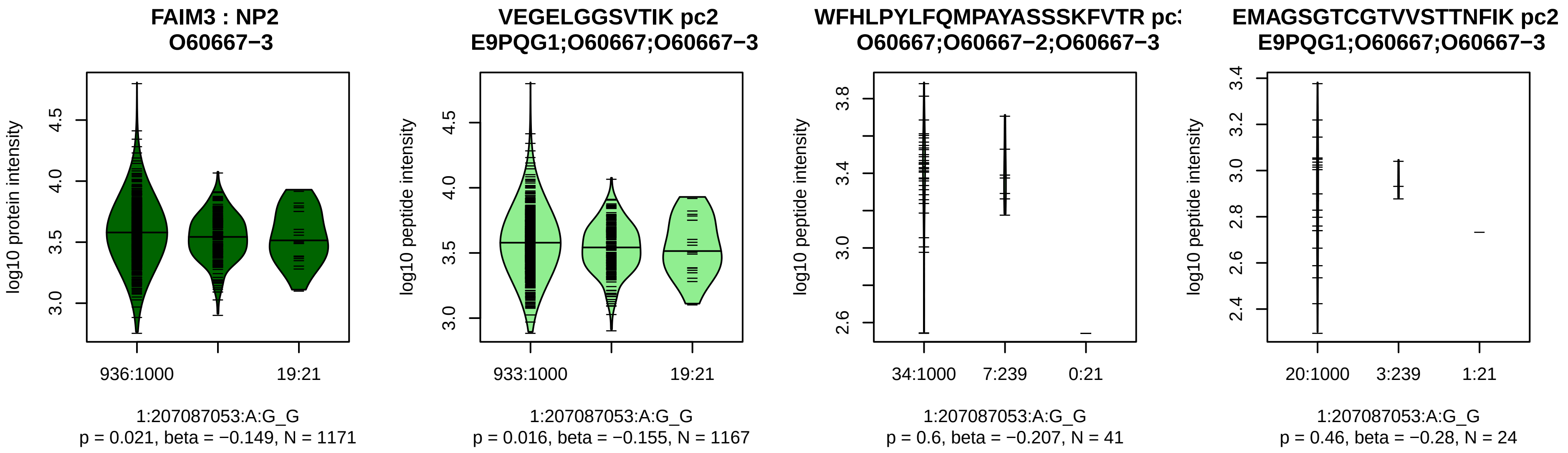

Assay Target: FAIM3
UniProt: O60667
rsID: rs72758947
Target SNP: 1:206913708:G:A
Proxy SNP: 1:207087053:A:G
deCODE log10(p): 42.2
deCODE BETA: −0.16
deCODE MSPA: 0
−:−:−
1167:41:24

235. CFHR3 (Q02985)
deCODE MSPA=0

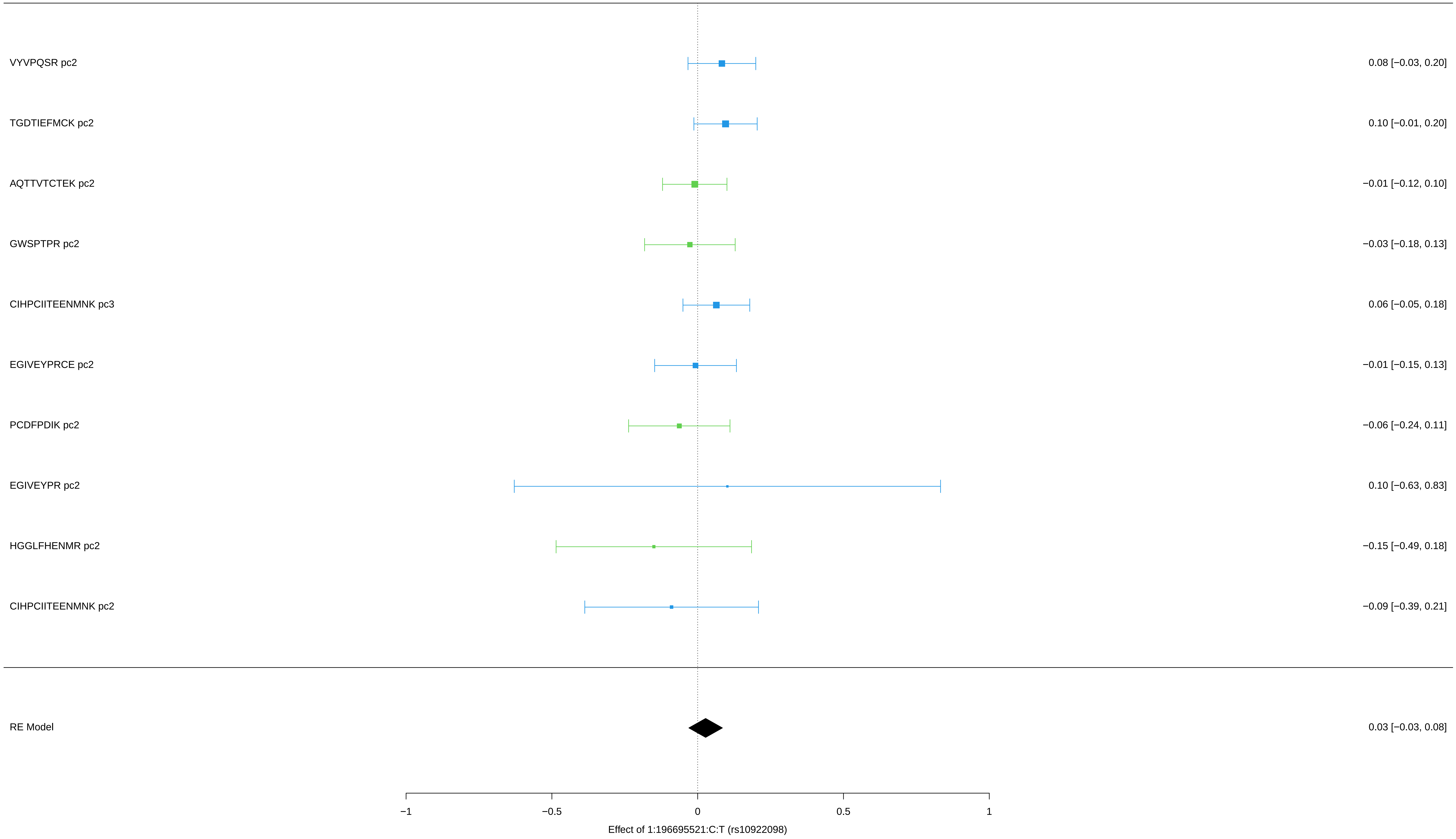

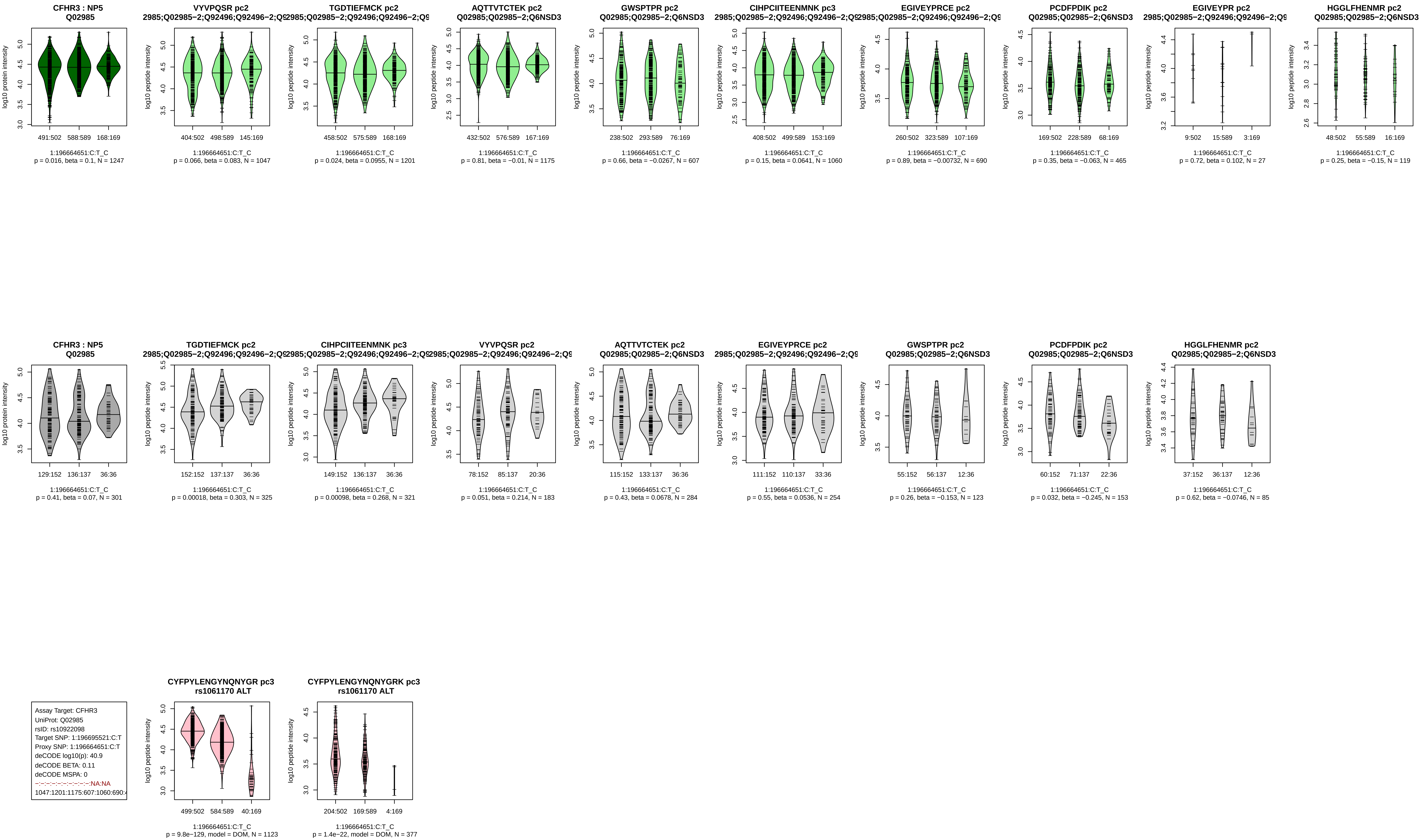

CFHR3 : NP5
Q02985
log10 protein intensity
491:502 588:589 168:169
1:196664651:C:T_C
p = 0.016, beta = 0.1, N = 1247
VYVPQSR pc2
2985;Q02985−2;Q92496;Q92496−2;Q9
log10 peptide intensity
404:502 498:589 145:169
1:196664651:C:T_C
p = 0.066, beta = 0.083, N = 1047
TGDTIEFMCK pc2
2985;Q02985−2;Q92496;Q92496−2;Q9
458:502 575:589 168:169
1:196664651:C:T_C
p = 0.024, beta = 0.0955, N = 1201
AQTTVTCTEK pc2
Q02985;Q02985−2;Q6NSD3
432:502 576:589 167:169
1:196664651:C:T_C
p = 0.81, beta = −0.01, N = 1175
GWSPTPR pc2
Q02985;Q02985−2;Q6NSD3
238:502 293:589 76:169
1:196664651:C:T_C
p = 0.66, beta = −0.0267, N = 607
CIHPCIITEENMNK pc3
2985;Q02985−2;Q92496;Q92496−2;Q9
408:502 499:589 153:169
1:196664651:C:T_C
p = 0.15, beta = 0.0641, N = 1060
EGIVEYPRCE pc2
2985;Q02985−2;Q92496;Q92496−2;Q9
260:502 323:589 107:169
1:196664651:C:T_C
p = 0.89, beta = −0.00732, N = 690
PCDFPDIK pc2
Q02985;Q02985−2;Q6NSD3
169:502 228:589 68:169
1:196664651:C:T_C
p = 0.35, beta = −0.063, N = 465
EGIVEYPR pc2
2985;Q02985−2;Q92496;Q92496−2;Q9
9:502 15:589 3:169
1:196664651:C:T_C
p = 0.72, beta = 0.102, N = 27
HGGLFHENMR pc2
Q02985;Q02985−2;Q6NSD3
48:502 55:589 16:169
1:196664651:C:T_C
p = 0.25, beta = −0.15, N = 119
CFHR3 : NP5
Q02985
129:152 136:137 36:36
1:196664651:C:T_C
p = 0.41, beta = 0.07, N = 301
TGDTIEFMCK pc2
2985;Q02985−2;Q92496;Q92496−2;Q9
152:152 137:137 36:36
1:196664651:C:T_C
p = 0.00018, beta = 0.303, N = 325
CIHPCIITEENMNK pc3
2985;Q02985−2;Q92496;Q92496−2;Q9
149:152 136:137 36:36
1:196664651:C:T_C
p = 0.00098, beta = 0.268, N = 321
VYVPQSR pc2
2985;Q02985−2;Q92496;Q92496−2;Q9
78:152 85:137 20:36
1:196664651:C:T_C
p = 0.051, beta = 0.214, N = 183
AQTTVTCTEK pc2
Q02985;Q02985−2;Q6NSD3
115:152 133:137 36:36
1:196664651:C:T_C
p = 0.43, beta = 0.0678, N = 284
EGIVEYPRCE pc2
2985;Q02985−2;Q92496;Q92496−2;Q9
111:152 110:137 33:36
1:196664651:C:T_C
p = 0.55, beta = 0.0536, N = 254
GWSPTPR pc2
Q02985;Q02985−2;Q6NSD3
55:152 56:137 12:36
1:196664651:C:T_C
p = 0.26, beta = −0.153, N = 123
PCDFPDIK pc2
Q02985;Q02985−2;Q6NSD3
60:152 71:137 22:36
1:196664651:C:T_C
p = 0.032, beta = −0.245, N = 153
HGGLFHENMR pc2
Q02985;Q02985−2;Q6NSD3
37:152 36:137 12:36
1:196664651:C:T_C
p = 0.62, beta = −0.0746, N = 85
Assay Target: CFHR3
UniProt: Q02985
rsID: rs10922098
Target SNP: 1:196695521:C:T
Proxy SNP: 1:196664651:C:T
deCODE log10(p): 40.9
deCODE BETA: 0.11
deCODE MSPA: 0
−:−:−:−:−:−:−:−:−:−:NA:NA
1047:1201:1175:607:1060:690:4
CYFPYLENGYNQNYGR pc3
rs1061170 ALT
499:502 584:589 40:169
1:196664651:C:T_C
p = 9.8e−129, model = DOM, N = 1123
CYFPYLENGYNQNYGRK pc3
rs1061170 ALT
204:502 169:589 4:169
1:196664651:C:T_C
p = 1.4e−22, model = DOM, N = 377

236. F2 (P00734)
deCODE MSPA=0

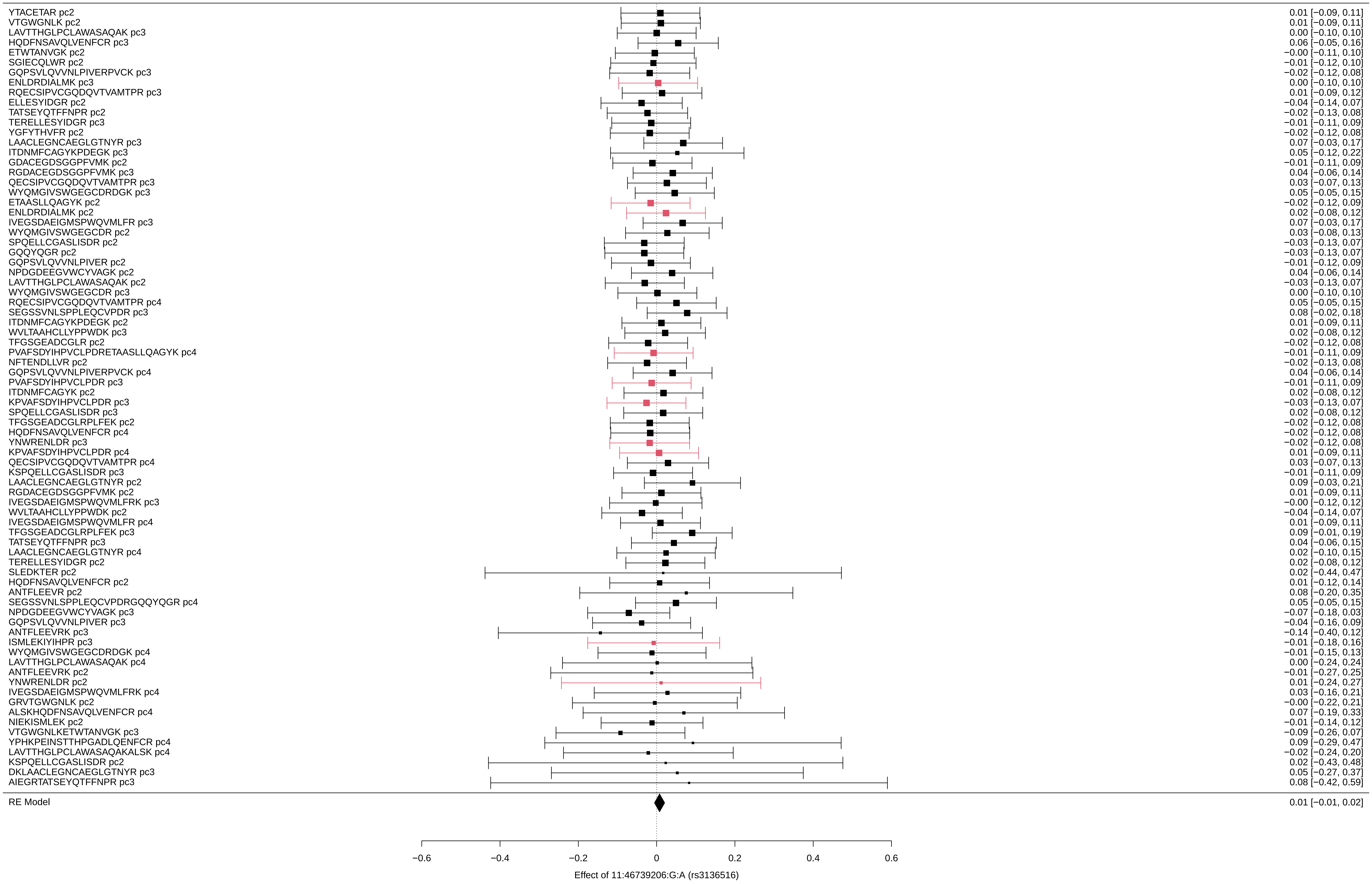

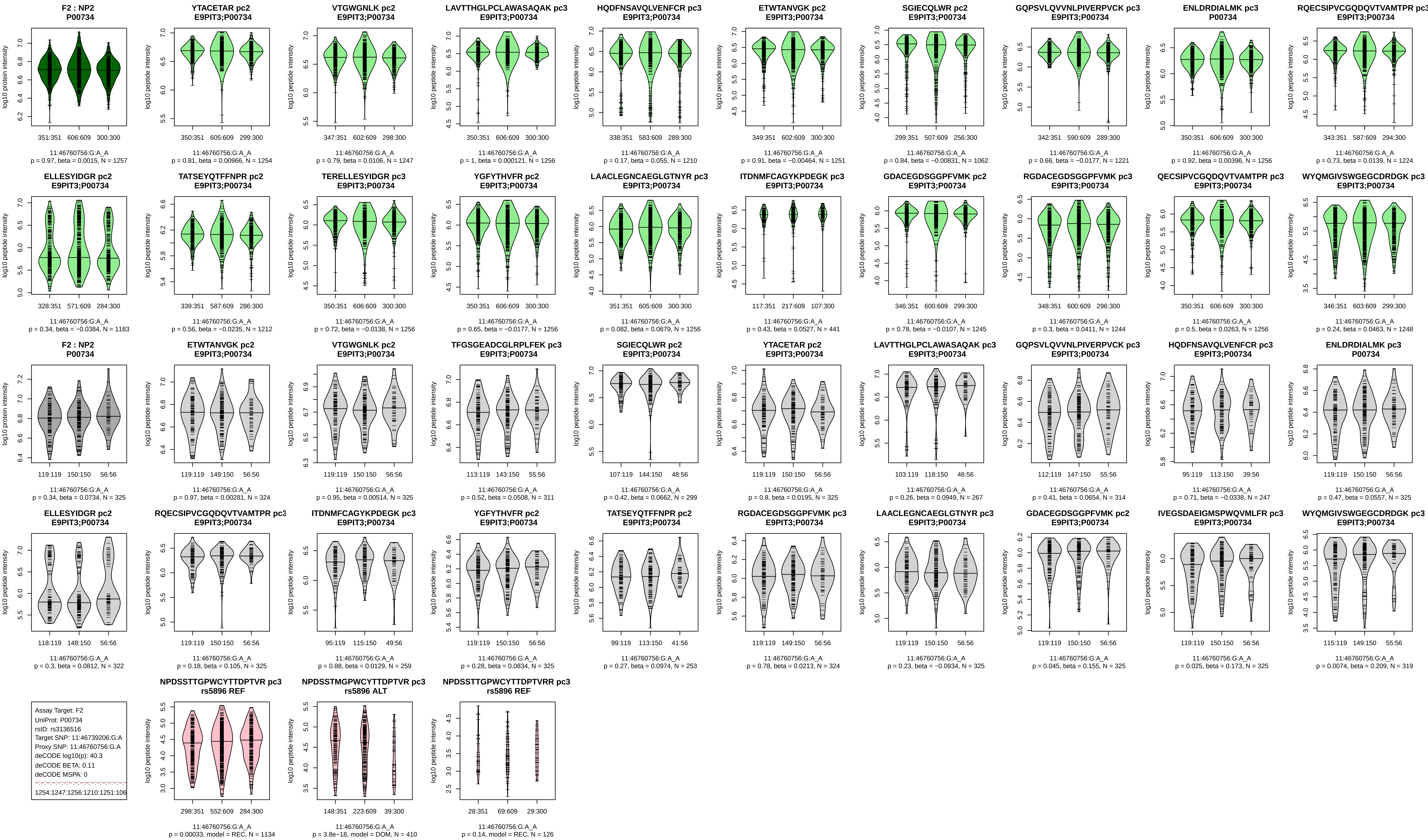
F2 : NP2
P00734
log10 protein intensity
351:351 606:609 300:300
11:46760756:G:A_A
p = 0.97, beta = 0.0015, N = 1257
YTACETAR pc2
E9PIT3;P00734
log10 peptide intensity
350:351 605:609 299:300
11:46760756:G:A_A
p = 0.81, beta = 0.00966, N = 1254
VTGWGNLK pc2
E9PIT3;P00734
347:351 602:609 298:300
11:46760756:G:A_A
p = 0.79, beta = 0.0106, N = 1247
LAVTTHGLPCLAWASAQAK pc3
E9PIT3;P00734
350:351 606:609 300:300
11:46760756:G:A_A
p = 1, beta = 0.000121, N = 1256
HQDFNSAVQLVENFCR pc3
E9PIT3;P00734
338:351 583:609 289:300
11:46760756:G:A_A
p = 0.17, beta = 0.055, N = 1210
ETWTANVGK pc2
E9PIT3;P00734
349:351 602:609 300:300
11:46760756:G:A_A
p = 0.91, beta = −0.00464, N = 1251
SGIECQLWR pc2
E9PIT3;P00734
299:351 507:609 256:300
11:46760756:G:A_A
p = 0.84, beta = −0.00831, N = 1062
GQPSVLQVVNLPIVERPVCK pc3
E9PIT3;P00734
342:351 590:609 289:300
11:46760756:G:A_A
p = 0.66, beta = −0.0177, N = 1221
ENLDRDIALMK pc3
P00734
350:351 606:609 300:300
11:46760756:G:A_A
p = 0.92, beta = 0.00396, N = 1256
RQECSIPVCGQDQVTVAMTPR pc3
E9PIT3;P00734
343:351 587:609 294:300
11:46760756:G:A_A
p = 0.73, beta = 0.0139, N = 1224
ELLESYIDGR pc2
E9PIT3;P00734
328:351 571:609 284:300
11:46760756:G:A_A
p = 0.34, beta = −0.0384, N = 1183
TATSEYQTFFNPR pc2
E9PIT3;P00734
339:351 587:609 286:300
11:46760756:G:A_A
p = 0.56, beta = −0.0235, N = 1212
TERELLESYIDGR pc3
E9PIT3;P00734
350:351 606:609 300:300
11:46760756:G:A_A
p = 0.72, beta = −0.0138, N = 1256
YGFYTHVFR pc2
E9PIT3;P00734
350:351 606:609 300:300
11:46760756:G:A_A
p = 0.65, beta = −0.0177, N = 1256
LAACLEGNCAEGLGTNYR pc3
E9PIT3;P00734
351:351 605:609 300:300
11:46760756:G:A_A
p = 0.082, beta = 0.0679, N = 1256
ITDNMFCAGYKPDEGK pc3
E9PIT3;P00734
117:351 217:609 107:300
11:46760756:G:A_A
p = 0.43, beta = 0.0527, N = 441
GDACEGDSGGPFVMK pc2
E9PIT3;P00734
346:351 600:609 299:300
11:46760756:G:A_A
p = 0.78, beta = −0.0107, N = 1245
RGDACEGDSGGPFVMK pc3
E9PIT3;P00734
348:351 600:609 296:300
11:46760756:G:A_A
p = 0.3, beta = 0.0411, N = 1244
QECSIPVCGQDQVTVAMTPR pc3
E9PIT3;P00734
350:351 606:609 300:300
11:46760756:G:A_A
p = 0.5, beta = 0.0263, N = 1256
WYQMGIVSWGEGCDRDGK pc3
E9PIT3;P00734
346:351 603:609 299:300
11:46760756:G:A_A
p = 0.24, beta = 0.0463, N = 1248
F2 : NP2
P00734
119:119 150:150 56:56
11:46760756:G:A_A
p = 0.34, beta = 0.0734, N = 325
ETWTANVGK pc2
E9PIT3;P00734
119:119 149:150 56:56
11:46760756:G:A_A
p = 0.97, beta = 0.00281, N = 324
VTGWGNLK pc2
E9PIT3;P00734
119:119 150:150 56:56
11:46760756:G:A_A
p = 0.95, beta = 0.00514, N = 325
TFGSGEADCGLRPLFEK pc3
E9PIT3;P00734
113:119 143:150 55:56
11:46760756:G:A_A
p = 0.52, beta = 0.0508, N = 311
SGIECQLWR pc2
E9PIT3;P00734
107:119 144:150 48:56
11:46760756:G:A_A
p = 0.42, beta = 0.0662, N = 299
YTACETAR pc2
E9PIT3;P00734
119:119 150:150 56:56
11:46760756:G:A_A
p = 0.8, beta = 0.0195, N = 325
LAVTTHGLPCLAWASAQAK pc3
E9PIT3;P00734
103:119 118:150 46:56
11:46760756:G:A_A
p = 0.26, beta = 0.0949, N = 267
GQPSVLQVVNLPIVERPVCK pc3
E9PIT3;P00734
112:119 147:150 55:56
11:46760756:G:A_A
p = 0.41, beta = 0.0654, N = 314
HQDFNSAVQLVENFCR pc3
E9PIT3;P00734
95:119 113:150 39:56
11:46760756:G:A_A
p = 0.71, beta = −0.0338, N = 247
ENLDRDIALMK pc3
P00734
119:119 150:150 56:56
11:46760756:G:A_A
p = 0.47, beta = 0.0557, N = 325
ELLESYIDGR pc2
E9PIT3;P00734
118:119 148:150 56:56
11:46760756:G:A_A
p = 0.3, beta = 0.0812, N = 322
RQECSIPVCGQDQVTVAMTPR pc3
E9PIT3;P00734
119:119 150:150 56:56
11:46760756:G:A_A
p = 0.18, beta = 0.105, N = 325
ITDNMFCAGYKPDEGK pc3
E9PIT3;P00734
95:119 115:150 49:56
11:46760756:G:A_A
p = 0.88, beta = 0.0129, N = 259
YGFYTHVFR pc2
E9PIT3;P00734
119:119 150:150 56:56
11:46760756:G:A_A
p = 0.28, beta = 0.0834, N = 325
TATSEYQTFFNPR pc2
E9PIT3;P00734
99:119 113:150 41:56
11:46760756:G:A_A
p = 0.27, beta = 0.0974, N = 253
RGDACEGDSGGPFVMK pc3
E9PIT3;P00734
119:119 149:150 56:56
11:46760756:G:A_A
p = 0.78, beta = 0.0213, N = 324
LAACLEGNCAEGLGTNYR pc3
E9PIT3;P00734
119:119 150:150 56:56
11:46760756:G:A_A
p = 0.23, beta = −0.0934, N = 325
GDACEGDSGGPFVMK pc2
E9PIT3;P00734
119:119 150:150 56:56
11:46760756:G:A_A
p = 0.045, beta = 0.155, N = 325
IVEGSDAEIGMSPWQVMLFR pc3
E9PIT3;P00734
119:119 150:150 56:56
11:46760756:G:A_A
p = 0.025, beta = 0.173, N = 325
WYQMGIVSWGEGCDRDGK pc3
E9PIT3;P00734
115:119 149:150 55:56
11:46760756:G:A_A
p = 0.0074, beta = 0.209, N = 319
Assay Target: F2
UniProt: P00734
rsID: rs3136516
Target SNP: 11:46739206:G:A
Proxy SNP: 11:46760756:G:A
deCODE log10(p): 40.3
deCODE BETA: 0.11
deCODE MSPA: 0
1254:1247:1256:1210:1251:106
NPDSSTTGPWCYTTDPTVR pc3
rs5896 REF
298:351 552:609 284:300
11:46760756:G:A_A
p = 0.00033, model = REC, N = 1134
NPDSSTMGPWCYTTDPTVR pc3
rs5896 ALT
148:351 223:609 39:300
11:46760756:G:A_A
p = 3.8e−18, model = DOM, N = 410
NPDSSTTGPWCYTTDPTVRR pc3
rs5896 REF
28:351 69:609 29:300
11:46760756:G:A_A
p = 0.14, model = REC, N = 126

237. ANXA1 (P04083)
deCODE MSPA=0

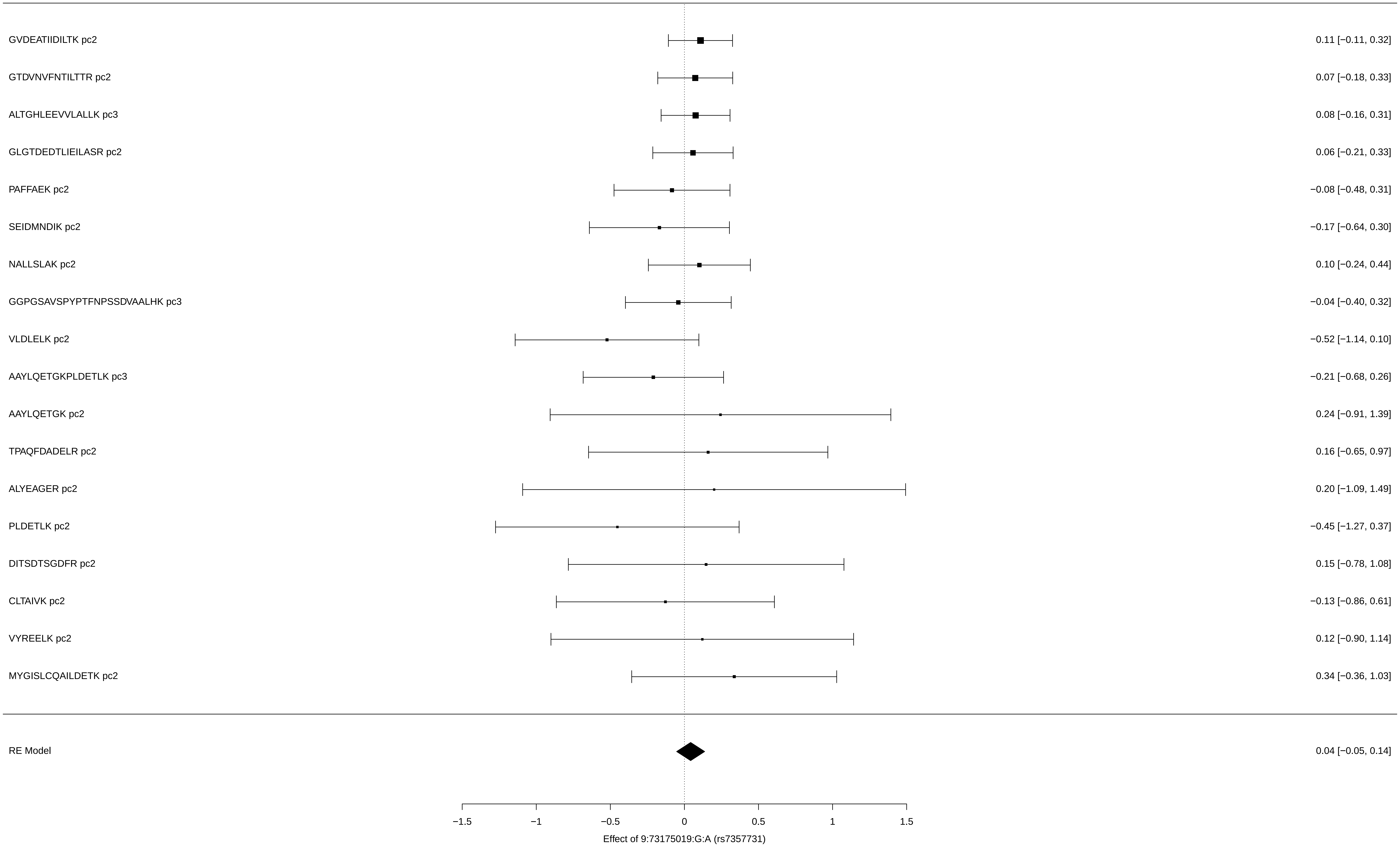

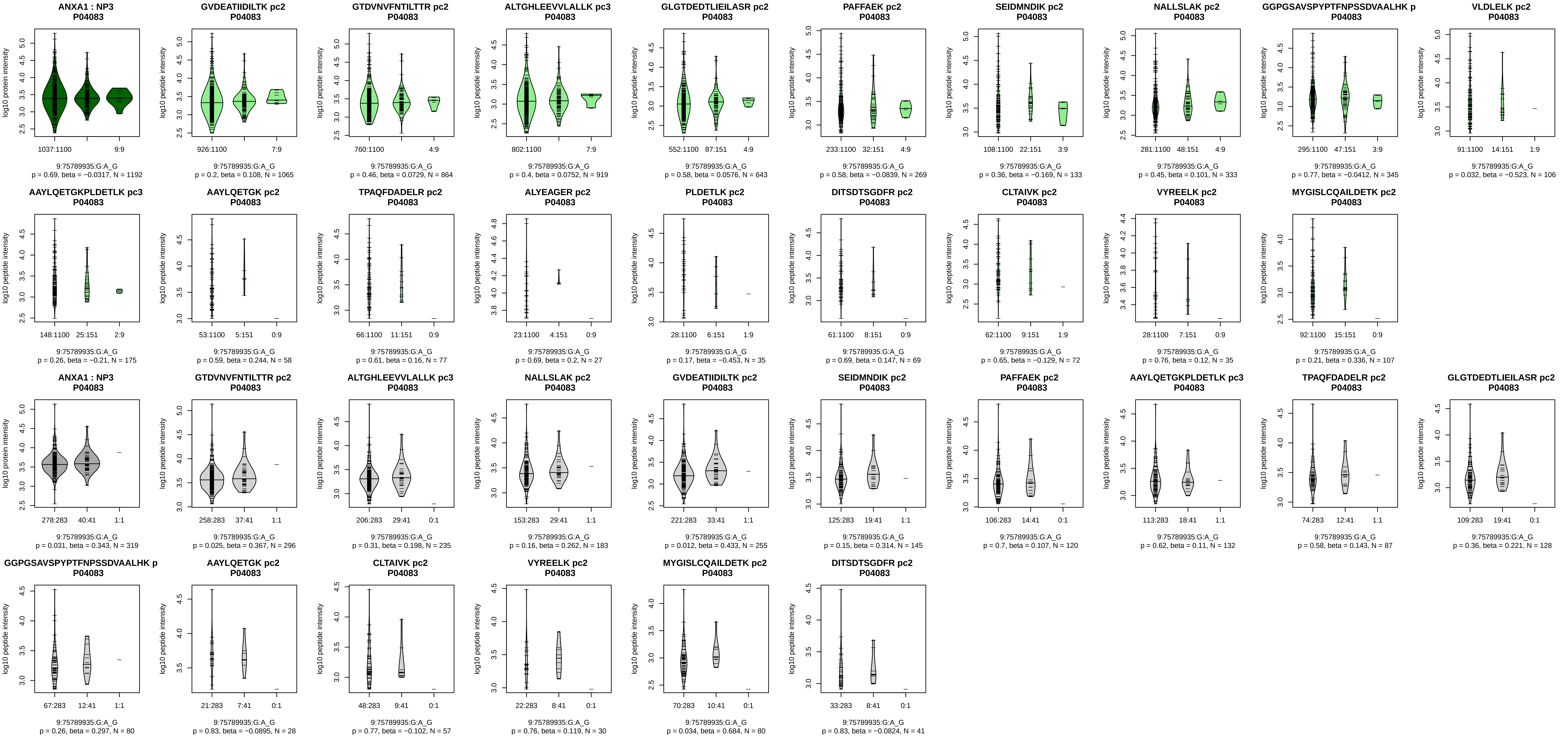

Assay Target: ANXA1
UniProt: P04083
rsID: rs7357731
Target SNP: 9:73175019:G:A
Proxy SNP: 9:75789935:G:A
deCODE log10(p): 39.6
deCODE BETA: 0.34
deCODE MSPA: 0
1065:864:919:643:269:133:333:

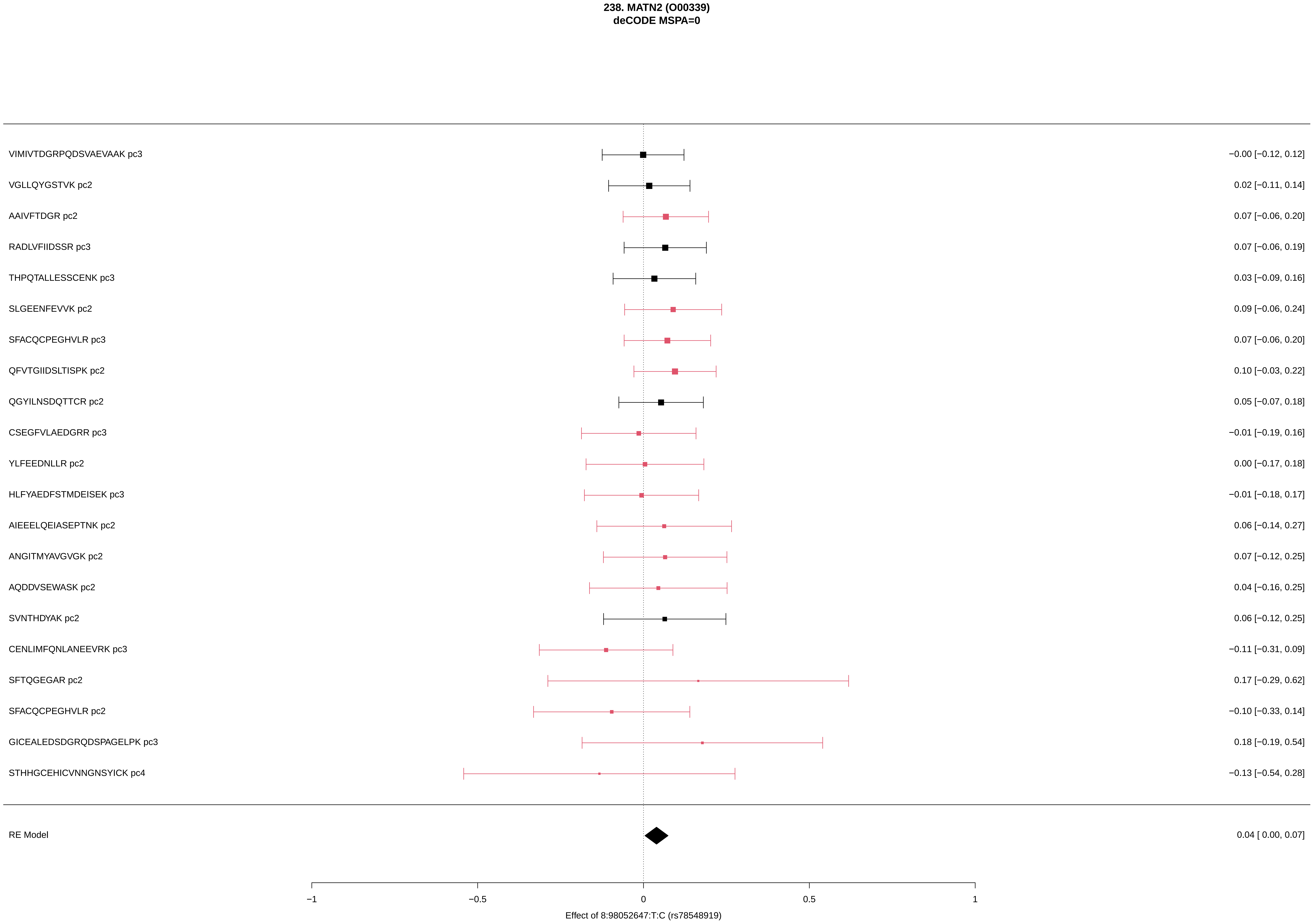
238. MATN2 (O00339)
deCODE MSPA=0
VIMIVTDGRPQDSVAEVAAK pc3
VGLLQYGSTVK pc2
AAIVFTDGR pc2
RADLVFIIDSSR pc3
THPQTALLESSCENK pc3
SLGEENFEVVK pc2
SFACQCPEGHVLR pc3
QFVTGIIDSLTISPK pc2
QGYILNSDQTTCR pc2
CSEGFVLAEDGRR pc3
YLFEEDNLLR pc2
HLFYAEDFSTMDEISEK pc3
AIEEELQEIASEPTNK pc2
ANGITMYAVGVGK pc2
AQDDVSEWASK pc2
SVNTHDYAK pc2
CENLIMFQNLANEEVRK pc3
SFTQGEGAR pc2
SFACQCPEGHVLR pc2
GICEALEDSDGRQDSPAGELPK pc3
STHHGCEHICVNNGNSYICK pc4
RE Model
−0.00 [−0.12, 0.12]
0.02 [−0.11, 0.14]
0.07 [−0.06, 0.20]
0.07 [−0.06, 0.19]
0.03 [−0.09, 0.16]
0.09 [−0.06, 0.24]
0.07 [−0.06, 0.20]
0.10 [−0.03, 0.22]
0.05 [−0.07, 0.18]
−0.01 [−0.19, 0.16]
0.00 [−0.17, 0.18]
−0.01 [−0.18, 0.17]
0.06 [−0.14, 0.27]
0.07 [−0.12, 0.25]
0.04 [−0.16, 0.25]
0.06 [−0.12, 0.25]
−0.11 [−0.31, 0.09]
0.17 [−0.29, 0.62]
−0.10 [−0.33, 0.14]
0.18 [−0.19, 0.54]
−0.13 [−0.54, 0.28]
0.04 [0.00, 0.07]
−1
−0.5
0
0.5
1
Effect of 8:98052647:T:C (rs78548919)

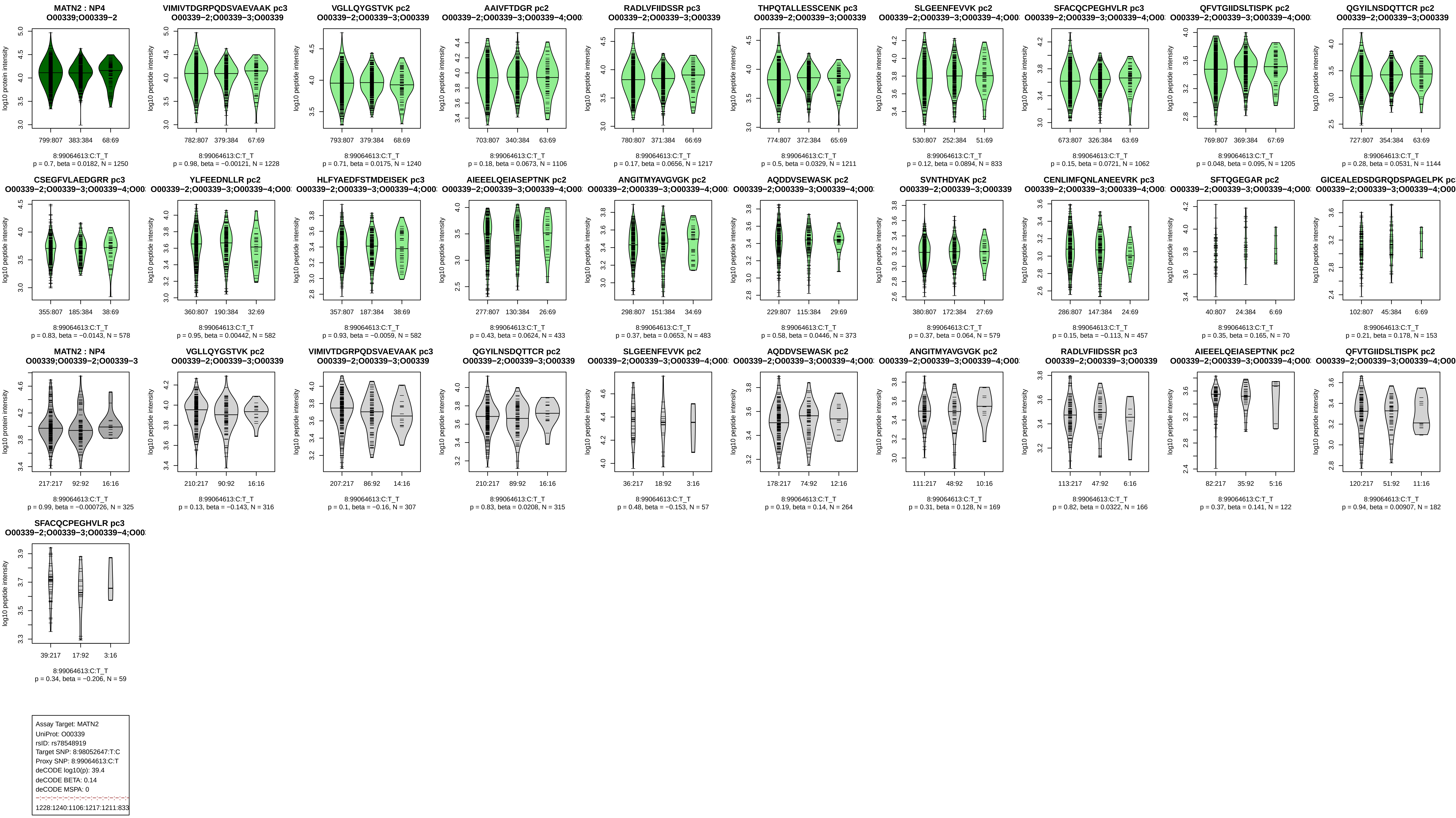
MATN2 : NP4
O00339;O00339−2
log10 protein intensity
799:807 383:384 68:69
8:99064613:C:T_T
p = 0.7, beta = 0.0182, N = 1250
VIMIVTDGRPQDSVAEVAAK pc3
O00339−2;O00339−3;O00339
log10 peptide intensity
782:807 379:384 67:69
8:99064613:C:T_T
p = 0.98, beta = −0.00121, N = 1228
VGLLQYGSTVK pc2
O00339−2;O00339−3;O00339
793:807 379:384 68:69
8:99064613:C:T_T
p = 0.71, beta = 0.0175, N = 1240
AAIVFTDGR pc2
703:807 340:384 63:69
8:99064613:C:T_T
p = 0.18, beta = 0.0673, N = 1106
RADLVFIIDSSR pc3
O00339−2;O00339−3;O00339
780:807 371:384 66:69
8:99064613:C:T_T
p = 0.17, beta = 0.0656, N = 1217
THPQTALLESSCENK pc3
O00339−2;O00339−3;O00339
774:807 372:384 65:69
8:99064613:C:T_T
p = 0.5, beta = 0.0329, N = 1211
SLGEENFEVVK pc2
530:807 252:384 51:69
8:99064613:C:T_T
p = 0.12, beta = 0.0894, N = 833
SFACQCPEGHVLR pc3
673:807 326:384 63:69
8:99064613:C:T_T
p = 0.15, beta = 0.0721, N = 1062
QFVTGIIDSLTISPK pc2
769:807 369:384 67:69
8:99064613:C:T_T
p = 0.048, beta = 0.095, N = 1205
QGYILNSDQTTCR pc2
O00339−2;O00339−3;O00339
727:807 354:384 63:69
8:99064613:C:T_T
p = 0.28, beta = 0.0531, N = 1144
CSEGFVLAEDGRR pc3
355:807 185:384 38:69
8:99064613:C:T_T
p = 0.83, beta = −0.0143, N = 578
YLFEEDNLLR pc2
360:807 190:384 32:69
8:99064613:C:T_T
p = 0.95, beta = 0.00442, N = 582
HLFYAEDFSTMDEISEK pc3
357:807 187:384 38:69
8:99064613:C:T_T
p = 0.93, beta = −0.0059, N = 582
AIEEELQEIASEPTNK pc2
277:807 130:384 26:69
8:99064613:C:T_T
p = 0.43, beta = 0.0624, N = 433
ANGITMYAVGVGK pc2
298:807 151:384 34:69
8:99064613:C:T_T
p = 0.37, beta = 0.0653, N = 483
AQDDVSEWASK pc2
229:807 115:384 29:69
8:99064613:C:T_T
p = 0.58, beta = 0.0446, N = 373
SVNTHDYAK pc2
O00339−2;O00339−3;O00339
380:807 172:384 27:69
8:99064613:C:T_T
p = 0.37, beta = 0.064, N = 579
CENLIMFQNLANEEVRK pc3
286:807 147:384 24:69
8:99064613:C:T_T
p = 0.15, beta = −0.113, N = 457
SFTQGEGAR pc2
40:807 24:384 6:69
8:99064613:C:T_T
p = 0.35, beta = 0.165, N = 70
102:807 45:384 6:69
8:99064613:C:T_T
p = 0.21, beta = 0.178, N = 153
MATN2 : NP4
O00339;O00339−2;O00339−3
217:217 92:92 16:16
8:99064613:C:T_T
p = 0.99, beta = −0.000726, N = 325
VGLLQYGSTVK pc2
O00339−2;O00339−3;O00339
210:217 90:92 16:16
8:99064613:C:T_T
p = 0.13, beta = −0.143, N = 316
VIMIVTDGRPQDSVAEVAAK pc3
O00339−2;O00339−3;O00339
207:217 86:92 14:16
8:99064613:C:T_T
p = 0.1, beta = −0.16, N = 307
QGYILNSDQTTCR pc2
O00339−2;O00339−3;O00339
210:217 89:92 16:16
8:99064613:C:T_T
p = 0.83, beta = 0.0208, N = 315
SLGEENFEVVK pc2
36:217 18:92 3:16
8:99064613:C:T_T
p = 0.48, beta = −0.153, N = 57
AQDDVSEWASK pc2
178:217 74:92 12:16
8:99064613:C:T_T
p = 0.19, beta = 0.14, N = 264
ANGITMYAVGVGK pc2
111:217 48:92 10:16
8:99064613:C:T_T
p = 0.31, beta = 0.128, N = 169
RADLVFIIDSSR pc3
O00339−2;O00339−3;O00339
113:217 47:92 6:16
8:99064613:C:T_T
p = 0.82, beta = 0.0322, N = 166
AIEEELQEIASEPTNK pc2
82:217 35:92 5:16
8:99064613:C:T_T
p = 0.37, beta = 0.141, N = 122
QFVTGIIDSLTISPK pc2
120:217 51:92 11:16
8:99064613:C:T_T
p = 0.94, beta = 0.00907, N = 182
SFACQCPEGHVLR pc3
39:217 17:92 3:16
8:99064613:C:T_T
p = 0.34, beta = −0.206, N = 59
Assay Target: MATN2
UniProt: O00339
rsID: rs78548919
Target SNP: 8:98052647:T:C
Proxy SNP: 8:99064613:C:T
deCODE log10(p): 39.4
deCODE BETA: 0.14
deCODE MSPA: 0
1228:1240:1106:1217:1211:833

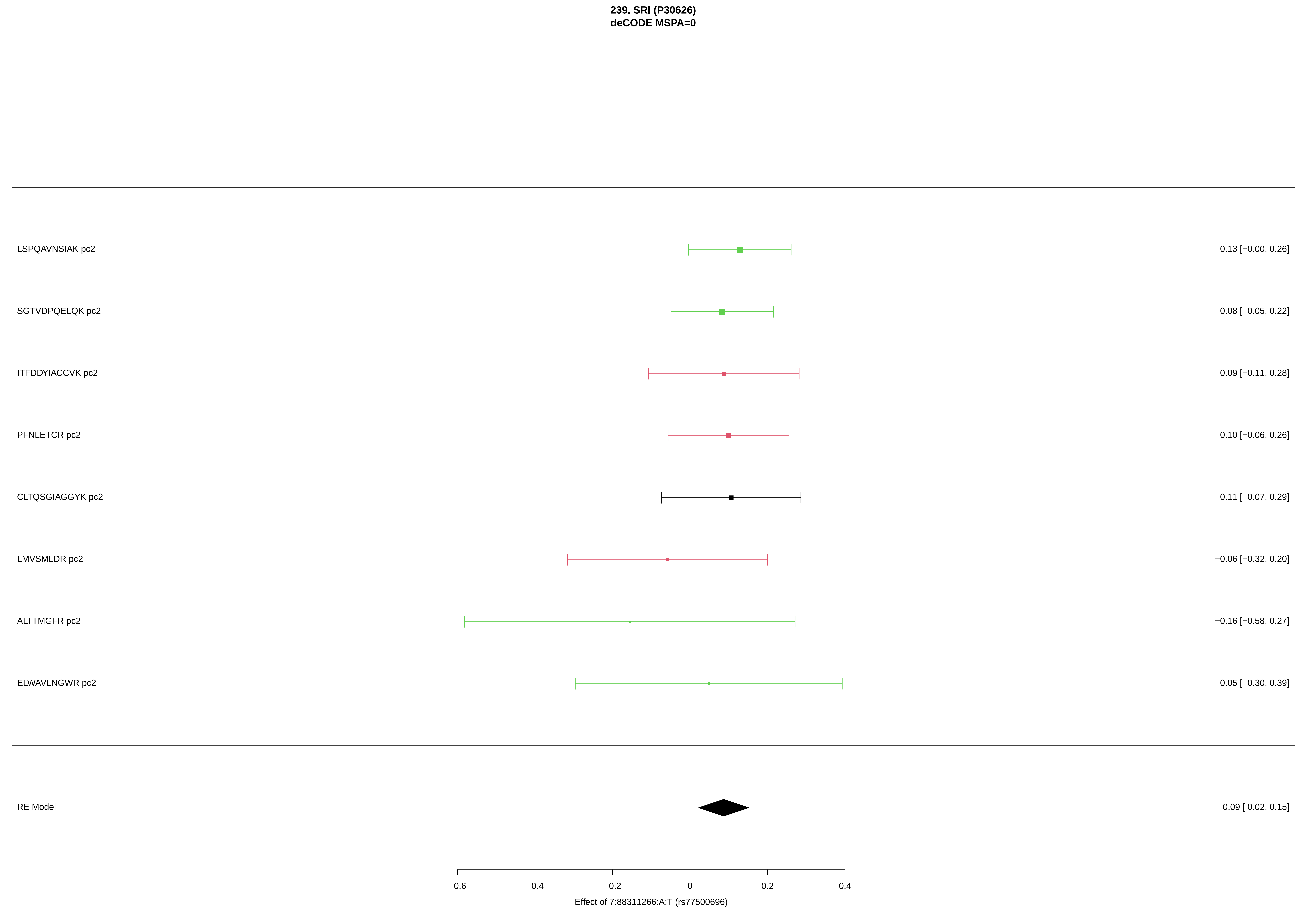
239. SRI (P30626)
deCODE MSPA=0
LSPQAVNSIAK pc2
0.13 [−0.00, 0.26]
SGTVDPQELQK pc2
0.08 [−0.05, 0.22]
ITFDDYIACCVK pc2
0.09 [−0.11, 0.28]
PFNLETCR pc2
0.10 [−0.06, 0.26]
CLTQSGIAGGYK pc2
0.11 [−0.07, 0.29]
LMVSMLDR pc2
−0.06 [−0.32, 0.20]
ALTTMGFR pc2
−0.16 [−0.58, 0.27]
ELWAVLNGWR pc2
0.05 [−0.30, 0.39]
RE Model
0.09 [0.02, 0.15]
−0.6
−0.4
−0.2
0
0.2
0.4
Effect of 7:88311266:A:T (rs77500696)

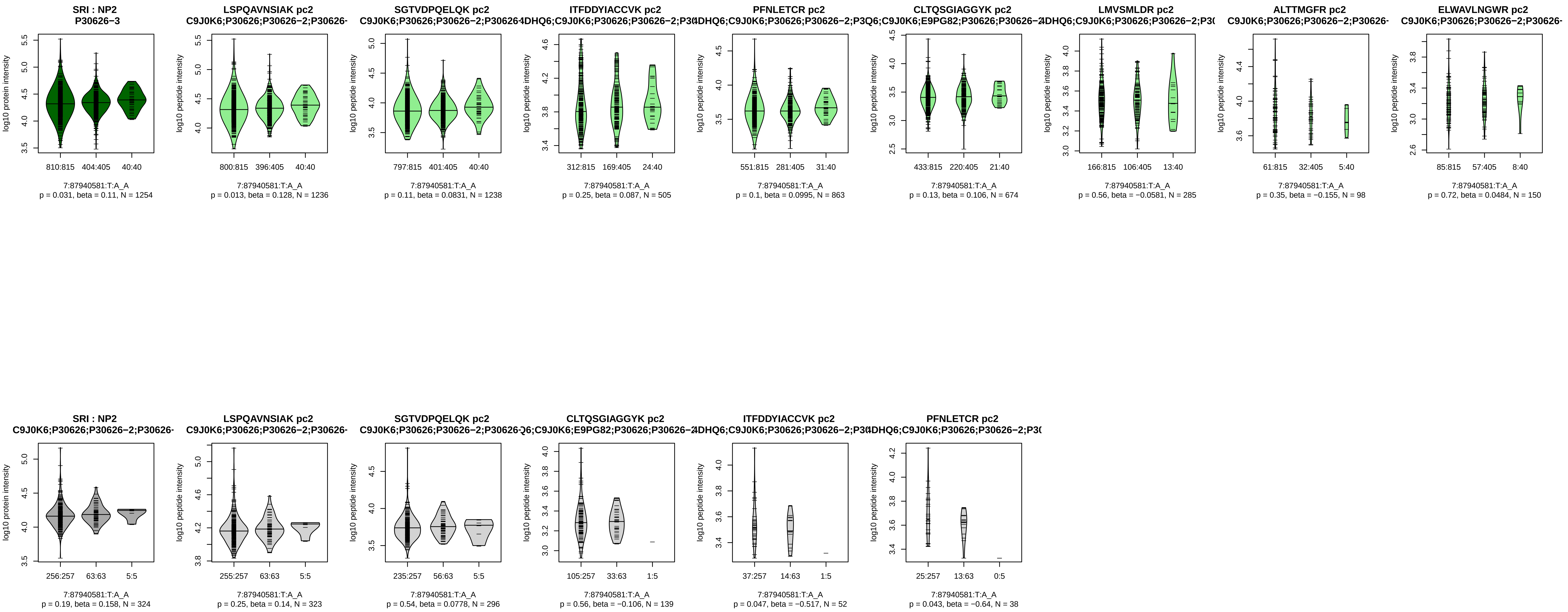

Assay Target: SRI
UniProt: P30626
rsID: rs77500696
Target SNP: 7:88311266:A:T
Proxy SNP: 7:87940581:T:A
deCODE log10(p): 39.2
deCODE BETA: 0.14
deCODE MSPA: 0
−:−:−:−:−:−:−:−:NA
1236:1238:505:863:674:285:98:

240. ADH4 (P08319)
deCODE MSPA=0.082

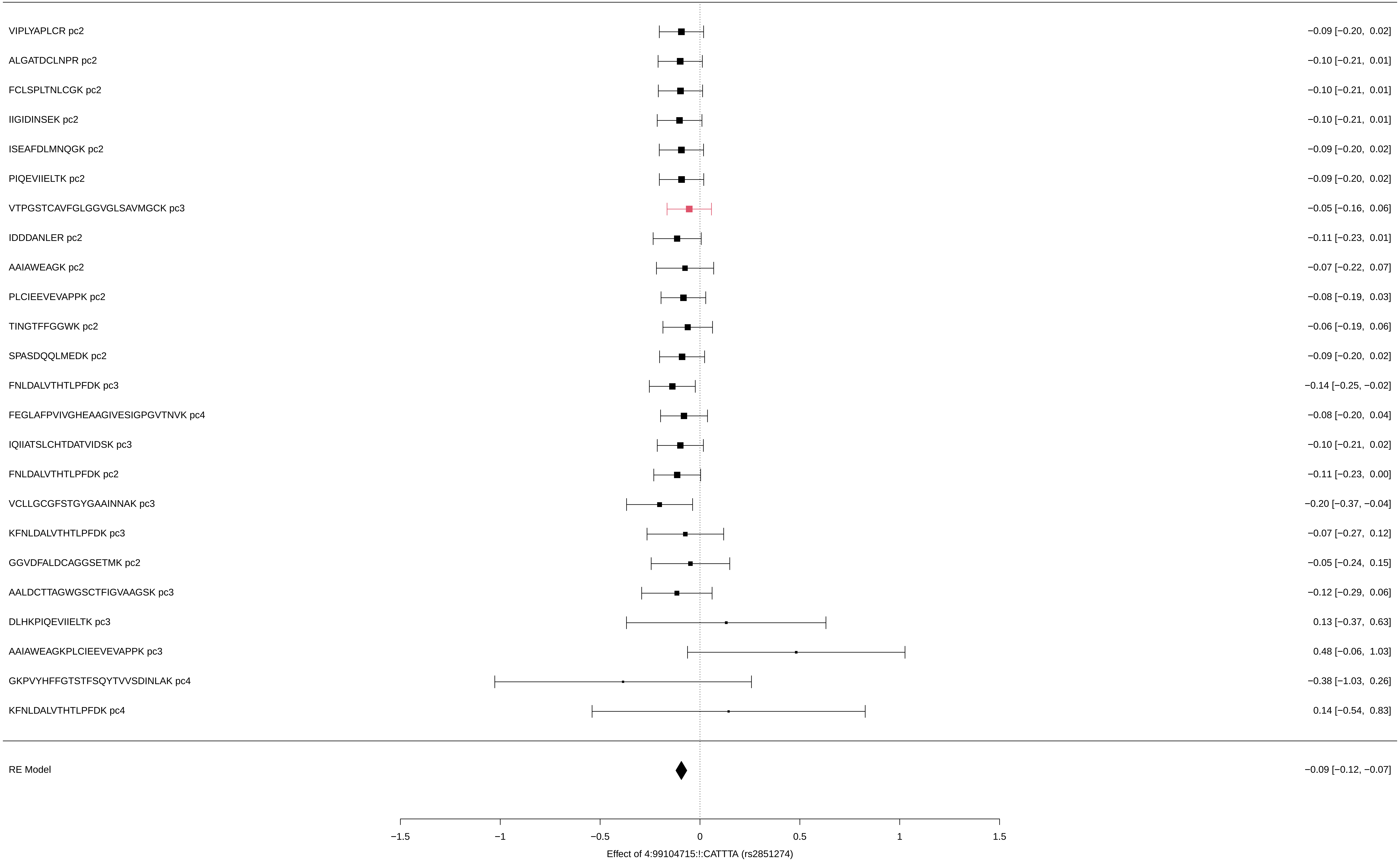

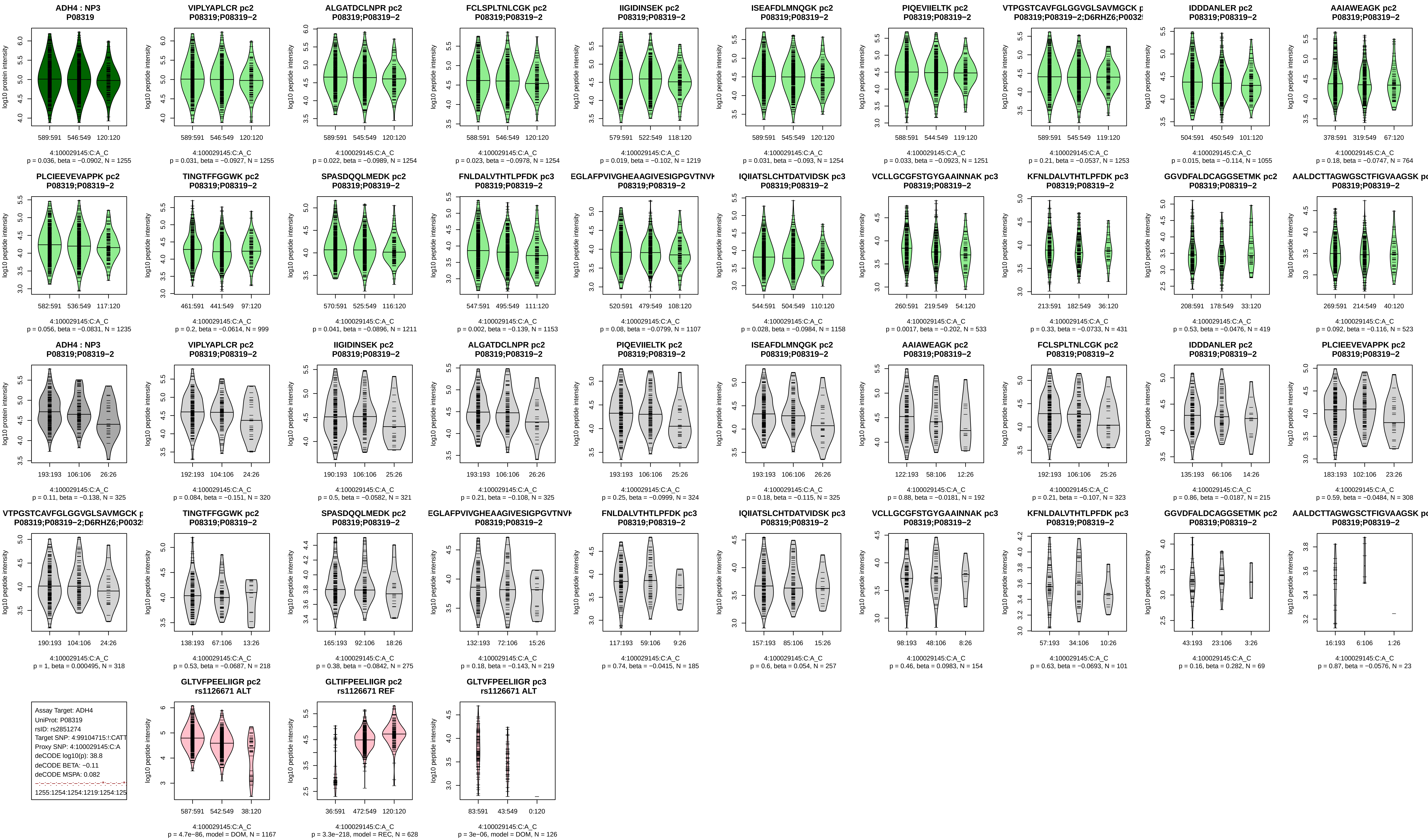

ADH4 : NP3
P08319
log10 protein intensity
589:591 546:549 120:120
4:100029145:C:A_C
p = 0.036, beta = −0.0902, N = 1255
VIPLYAPLCR pc2
P08319;P08319−2
log10 peptide intensity
589:591 546:549 120:120
4:100029145:C:A_C
p = 0.031, beta = −0.0927, N = 1255
ALGATDCLNPR pc2
P08319;P08319−2
589:591 545:549 120:120
4:100029145:C:A_C
p = 0.022, beta = −0.0989, N = 1254
FCLSPLTNLCGK pc2
P08319;P08319−2
588:591 546:549 120:120
4:100029145:C:A_C
p = 0.023, beta = −0.0978, N = 1254
IIGIDINSEK pc2
P08319;P08319−2
579:591 522:549 118:120
4:100029145:C:A_C
p = 0.019, beta = −0.102, N = 1219
ISEAFDLMNQGK pc2
P08319;P08319−2
589:591 545:549 120:120
4:100029145:C:A_C
p = 0.031, beta = −0.093, N = 1254
PIQEVIIELTK pc2
P08319;P08319−2
588:591 544:549 119:120
4:100029145:C:A_C
p = 0.033, beta = −0.0923, N = 1251
VTPGSTCAVFGLGGVGLSAVMGCK p
P08319;P08319−2;D6RHZ6;P0032
589:591 545:549 119:120
4:100029145:C:A_C
p = 0.21, beta = −0.0537, N = 1253
IDDDANLER pc2
P08319;P08319−2
504:591 450:549 101:120
4:100029145:C:A_C
p = 0.015, beta = −0.114, N = 1055
AAIAWEAGK pc2
P08319;P08319−2
378:591 319:549 67:120
4:100029145:C:A_C
p = 0.18, beta = −0.0747, N = 764
PLCIEEVEVAPPK pc2
P08319;P08319−2
582:591 536:549 117:120
4:100029145:C:A_C
p = 0.056, beta = −0.0831, N = 1235
TINGTFFGGWK pc2
P08319;P08319−2
461:591 441:549 97:120
4:100029145:C:A_C
p = 0.2, beta = −0.0614, N = 999
SPASDQQLMEDK pc2
P08319;P08319−2
570:591 525:549 116:120
4:100029145:C:A_C
p = 0.041, beta = −0.0896, N = 1211
FNLDALVTHTLPFDK pc3
P08319;P08319−2
547:591 495:549 111:120
4:100029145:C:A_C
p = 0.002, beta = −0.139, N = 1153
EGLAFPVIVGHEAAGIVESIGPGVTNVK
P08319;P08319−2
520:591 479:549 108:120
4:100029145:C:A_C
p = 0.08, beta = −0.0799, N = 1107
IQIIATSLCHTDATVIDSK pc3
P08319;P08319−2
544:591 504:549 110:120
4:100029145:C:A_C
p = 0.028, beta = −0.0984, N = 1158
VCLLGCGFSTGYGAAINNAK pc3
P08319;P08319−2
260:591 219:549 54:120
4:100029145:C:A_C
p = 0.0017, beta = −0.202, N = 533
KFNLDALVTHTLPFDK pc3
P08319;P08319−2
213:591 182:549 36:120
4:100029145:C:A_C
p = 0.33, beta = −0.0733, N = 431
GGVDFALDCAGGSETMK pc2
P08319;P08319−2
208:591 178:549 33:120
4:100029145:C:A_C
p = 0.53, beta = −0.0476, N = 419
AALDCTTAGWGSCTFIGVAAGSK pc
P08319;P08319−2
269:591 214:549 40:120
4:100029145:C:A_C
p = 0.092, beta = −0.116, N = 523
ADH4 : NP3
P08319;P08319−2
193:193 106:106 26:26
4:100029145:C:A_C
p = 0.11, beta = −0.138, N = 325
VIPLYAPLCR pc2
P08319;P08319−2
192:193 104:106 24:26
4:100029145:C:A_C
p = 0.084, beta = −0.151, N = 320
IIGIDINSEK pc2
P08319;P08319−2
190:193 106:106 25:26
4:100029145:C:A_C
p = 0.5, beta = −0.0582, N = 321
ALGATDCLNPR pc2
P08319;P08319−2
193:193 106:106 26:26
4:100029145:C:A_C
p = 0.21, beta = −0.108, N = 325
PIQEVIIELTK pc2
P08319;P08319−2
193:193 106:106 25:26
4:100029145:C:A_C
p = 0.25, beta = −0.0999, N = 324
ISEAFDLMNQGK pc2
P08319;P08319−2
193:193 106:106 26:26
4:100029145:C:A_C
p = 0.18, beta = −0.115, N = 325
AAIAWEAGK pc2
P08319;P08319−2
122:193 58:106 12:26
4:100029145:C:A_C
p = 0.88, beta = −0.0181, N = 192
FCLSPLTNLCGK pc2
P08319;P08319−2
192:193 106:106 25:26
4:100029145:C:A_C
p = 0.21, beta = −0.107, N = 323
IDDDANLER pc2
P08319;P08319−2
135:193 66:106 14:26
4:100029145:C:A_C
p = 0.86, beta = −0.0187, N = 215
PLCIEEVEVAPPK pc2
P08319;P08319−2
183:193 102:106 23:26
4:100029145:C:A_C
p = 0.59, beta = −0.0484, N = 308
VTPGSTCAVFGLGGVGLSAVMGCK p
P08319;P08319−2;D6RHZ6;P0032
190:193 104:106 24:26
4:100029145:C:A_C
p = 1, beta = 0.000495, N = 318
TINGTFFGGWK pc2
P08319;P08319−2
138:193 67:106 13:26
4:100029145:C:A_C
p = 0.53, beta = −0.0687, N = 218
SPASDQQLMEDK pc2
P08319;P08319−2
165:193 92:106 18:26
4:100029145:C:A_C
p = 0.38, beta = −0.0842, N = 275
EGLAFPVIVGHEAAGIVESIGPGVTNVK
P08319;P08319−2
132:193 72:106 15:26
4:100029145:C:A_C
p = 0.18, beta = −0.143, N = 219
FNLDALVTHTLPFDK pc3
P08319;P08319−2
117:193 59:106 9:26
4:100029145:C:A_C
p = 0.74, beta = −0.0415, N = 185
IQIIATSLCHTDATVIDSK pc3
P08319;P08319−2
157:193 85:106 15:26
4:100029145:C:A_C
p = 0.6, beta = 0.054, N = 257
VCLLGCGFSTGYGAAINNAK pc3
P08319;P08319−2
98:193 48:106 8:26
4:100029145:C:A_C
p = 0.46, beta = 0.0983, N = 154
KFNLDALVTHTLPFDK pc3
P08319;P08319−2
57:193 34:106 10:26
4:100029145:C:A_C
p = 0.63, beta = −0.0693, N = 101
GGVDFALDCAGGSETMK pc2
P08319;P08319−2
43:193 23:106 3:26
4:100029145:C:A_C
p = 0.16, beta = 0.282, N = 69
AALDCTTAGWGSCTFIGVAAGSK pc
P08319;P08319−2
16:193 6:106 1:26
4:100029145:C:A_C
p = 0.87, beta = −0.0576, N = 23
Assay Target: ADH4
UniProt: P08319
rsID: rs2851274
Target SNP: 4:99104715:!:CATT
Proxy SNP: 4:100029145:C:A
deCODE log10(p): 38.8
deCODE BETA: −0.11
deCODE MSPA: 0.082
1255:1254:1254:1219:1254:125
GLTVFPEELIIGR pc2
rs1126671 ALT
587:591 542:549 38:120
4:100029145:C:A_C
p = 4.7e−86, model = DOM, N = 1167
GLTIFPEELIIGR pc2
rs1126671 REF
36:591 472:549 120:120
4:100029145:C:A_C
p = 3.3e−218, model = REC, N = 628
GLTVFPEELIIGR pc3
rs1126671 ALT
83:591 43:549 0:120
4:100029145:C:A_C
p = 3e−06, model = DOM, N = 126

241. ACAN (P16112)
deCODE MSPA=0.184

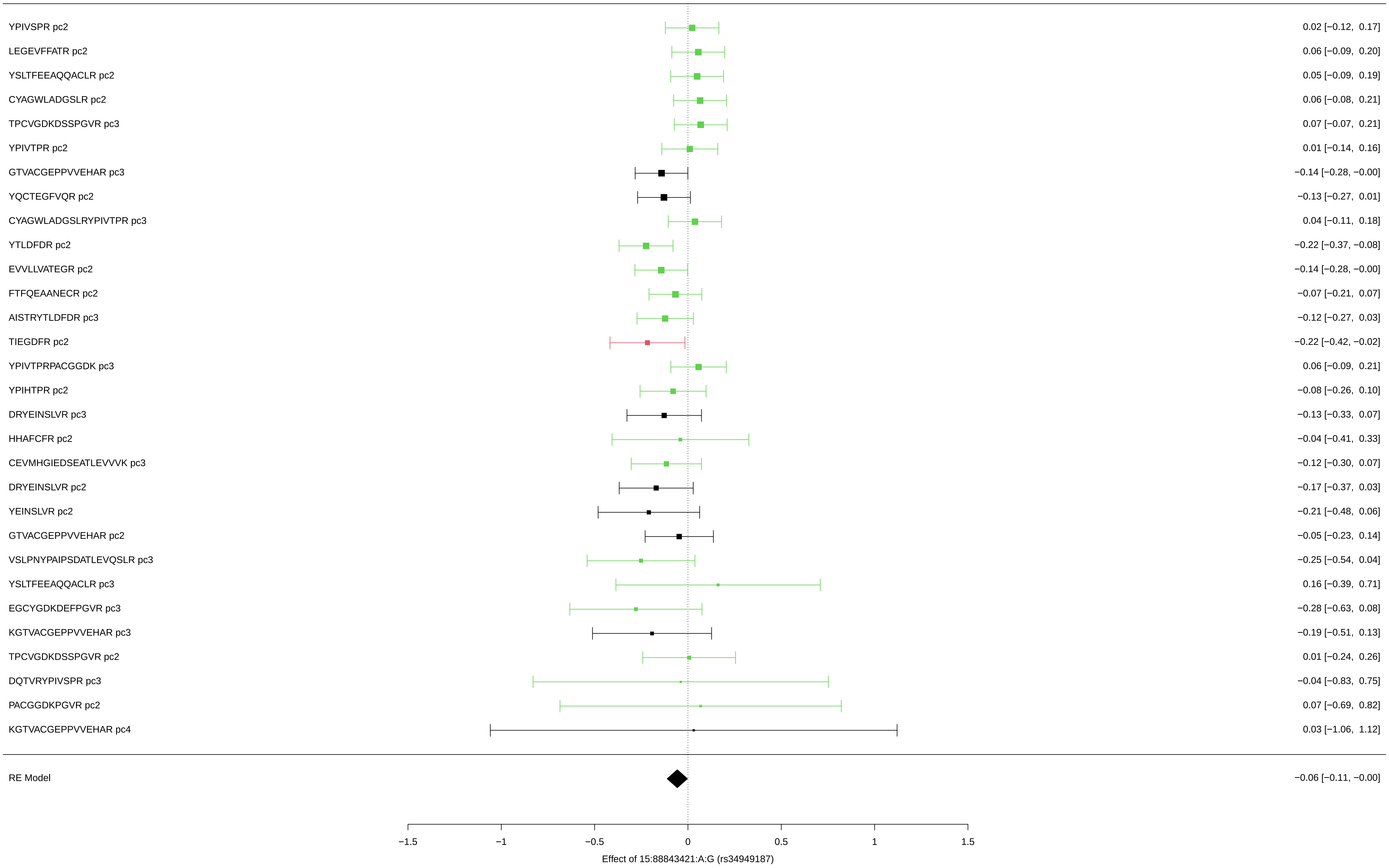

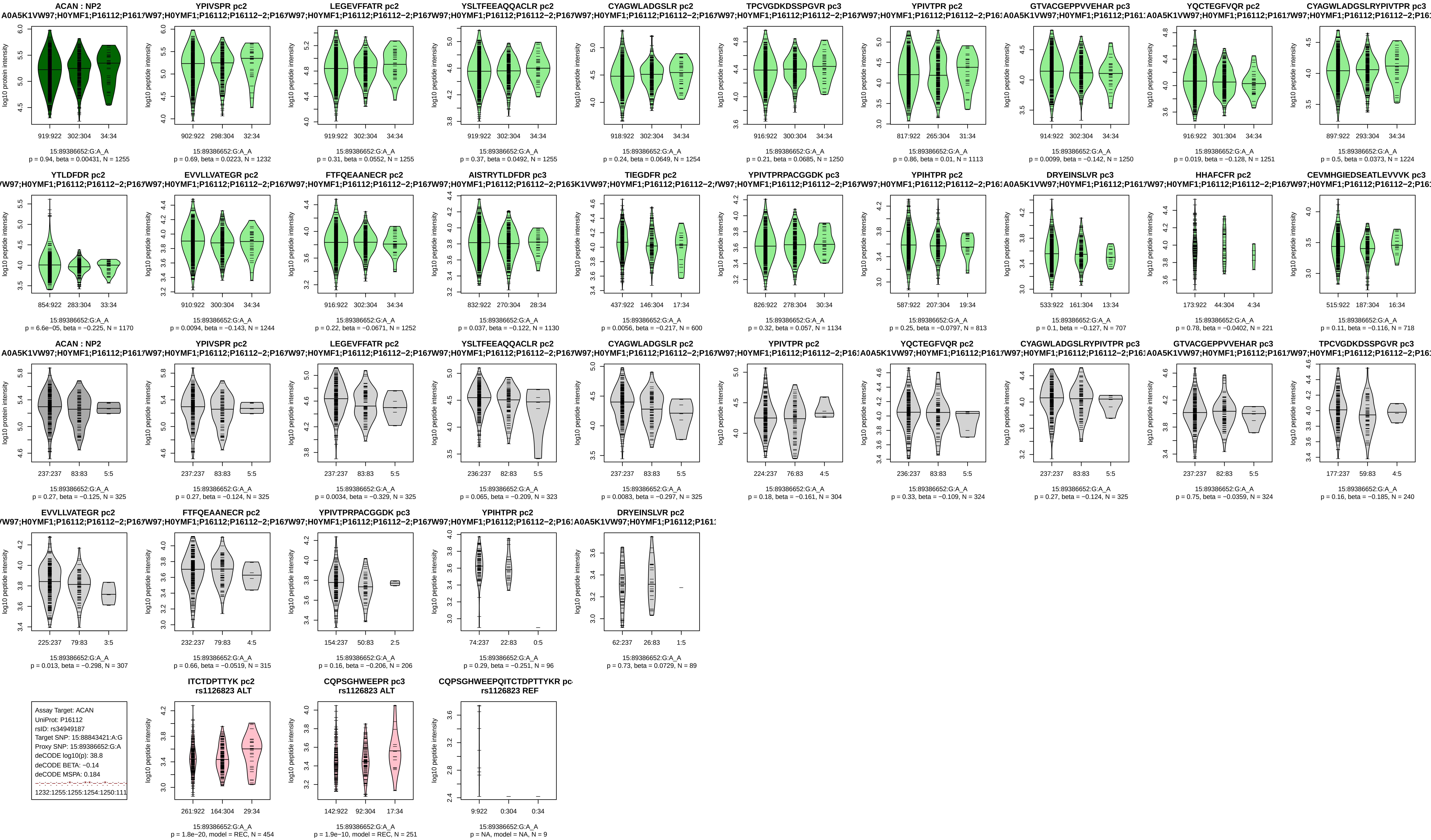

ACAN : NP2
p = 0.94, beta = 0.00431, N = 1255
YPIVSPR pc2
p = 0.69, beta = 0.0223, N = 1232
LEGEVFFATR pc2
p = 0.31, beta = 0.0552, N = 1255
YSLTFEEAQQACLR pc2
p = 0.37, beta = 0.0492, N = 1255
CYAGWLADGSLR pc2
p = 0.24, beta = 0.0649, N = 1254
TPCVGDKDSSPGVR pc3
p = 0.21, beta = 0.0685, N = 1250
YPIVTPR pc2
p = 0.86, beta = 0.01, N = 1113
GTVACGEPPVVEHAR pc3
p = 0.0099, beta = −0.142, N = 1250
YQCTEGFVQR pc2
p = 0.019, beta = −0.128, N = 1251
CYAGWLADGSLRYPIVTPR pc3
p = 0.5, beta = 0.0373, N = 1224
YTLDFDR pc2
p = 6.6e−05, beta = −0.225, N = 1170
EVVLLVATEGR pc2
p = 0.0094, beta = −0.143, N = 1244
FTFQEAANECR pc2
p = 0.22, beta = −0.0671, N = 1252
AISTRYTLDFDR pc3
p = 0.037, beta = −0.122, N = 1130
TIEGDFR pc2
p = 0.0056, beta = −0.217, N = 600
YPIVTPRPACGGDK pc3
p = 0.32, beta = 0.057, N = 1134
YPIHTPR pc2
p = 0.25, beta = −0.0797, N = 813
DRYEINSLVR pc3
p = 0.1, beta = −0.127, N = 707
HHAFCFR pc2
p = 0.78, beta = −0.0402, N = 221
CEVMHGIEDSEATLEVVVK pc3
p = 0.11, beta = −0.116, N = 718
ACAN : NP2
p = 0.27, beta = −0.125, N = 325
YPIVSPR pc2
p = 0.27, beta = −0.124, N = 325
LEGEVFFATR pc2
p = 0.0034, beta = −0.329, N = 325
YSLTFEEAQQACLR pc2
p = 0.065, beta = −0.209, N = 323
CYAGWLADGSLR pc2
p = 0.0083, beta = −0.297, N = 325
YPIVTPR pc2
p = 0.18, beta = −0.161, N = 304
YQCTEGFVQR pc2
p = 0.33, beta = −0.109, N = 324
CYAGWLADGSLRYPIVTPR pc3
p = 0.27, beta = −0.124, N = 325
GTVACGEPPVVEHAR pc3
p = 0.75, beta = −0.0359, N = 324
TPCVGDKDSSPGVR pc3
p = 0.16, beta = −0.185, N = 240
EVVLLVATEGR pc2
p = 0.013, beta = −0.298, N = 307
FTFQEAANECR pc2
p = 0.66, beta = −0.0519, N = 315
YPIVTPRPACGGDK pc3
p = 0.16, beta = −0.206, N = 206
YPIHTPR pc2
p = 0.29, beta = −0.251, N = 96
DRYEINSLVR pc2
p = 0.73, beta = 0.0729, N = 89
15:89386652:G:A_A
Assay Target: ACAN
UniProt: P16112
rsID: rs34949187
Target SNP: 15:88843421:A:G
Proxy SNP: 15:89386652:G:A
deCODE log10(p): 38.8
deCODE BETA: −0.14
deCODE MSPA: 0.184
1232:1255:1255:1254:1250:111
ITCTDPTTYK pc2
rs1126823 ALT
p = 1.8e−20, model = REC, N = 454
CQPSGHWEEPR pc3
rs1126823 ALT
p = 1.9e−10, model = REC, N = 251
CQPSGHWEEPQITCTDPTTYKR pc
rs1126823 REF
p = NA, model = NA, N = 9
log10 protein intensity
log10 peptide intensity

242. APMAP (Q9HDC9)
deCODE MSPA=0.875

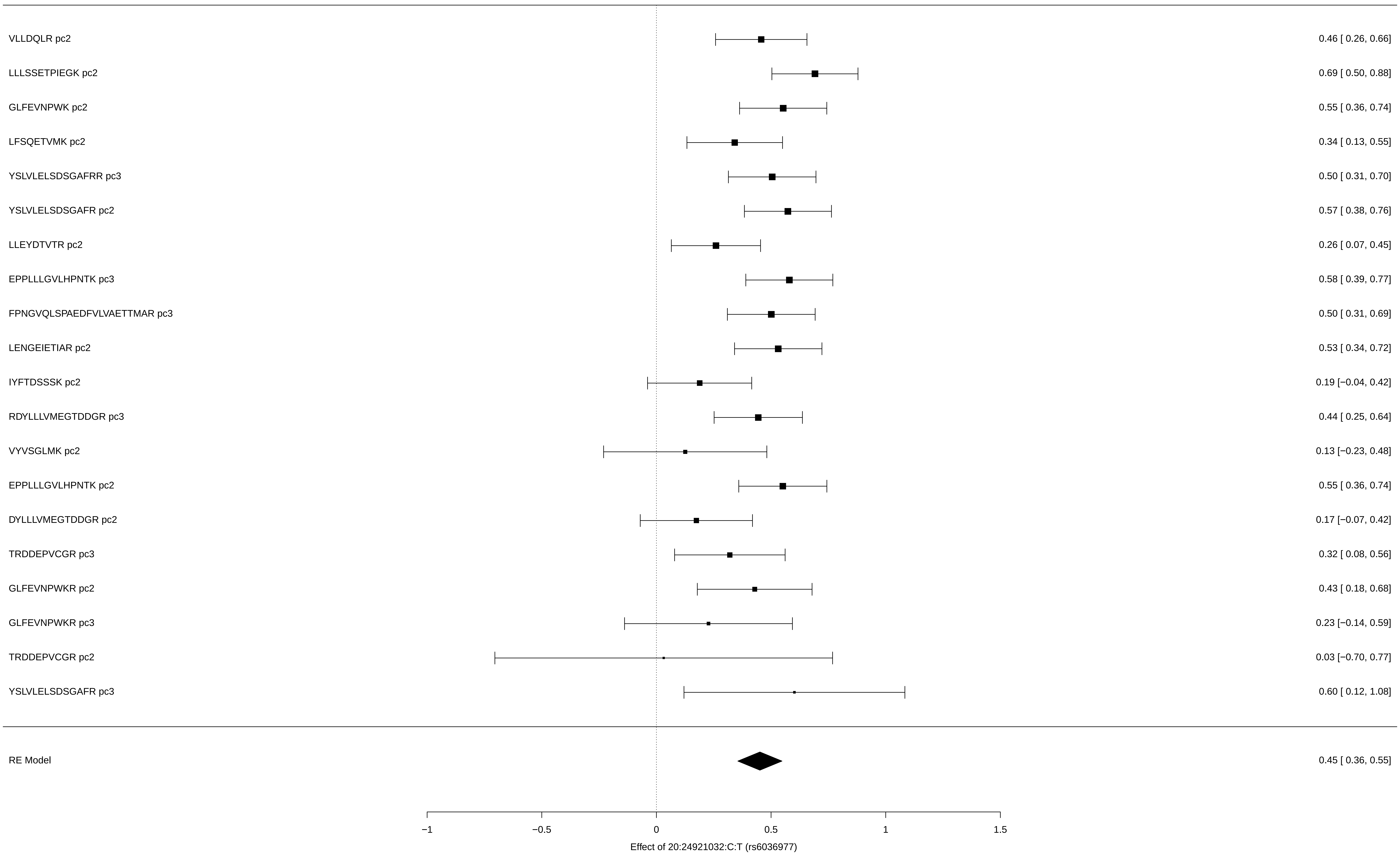

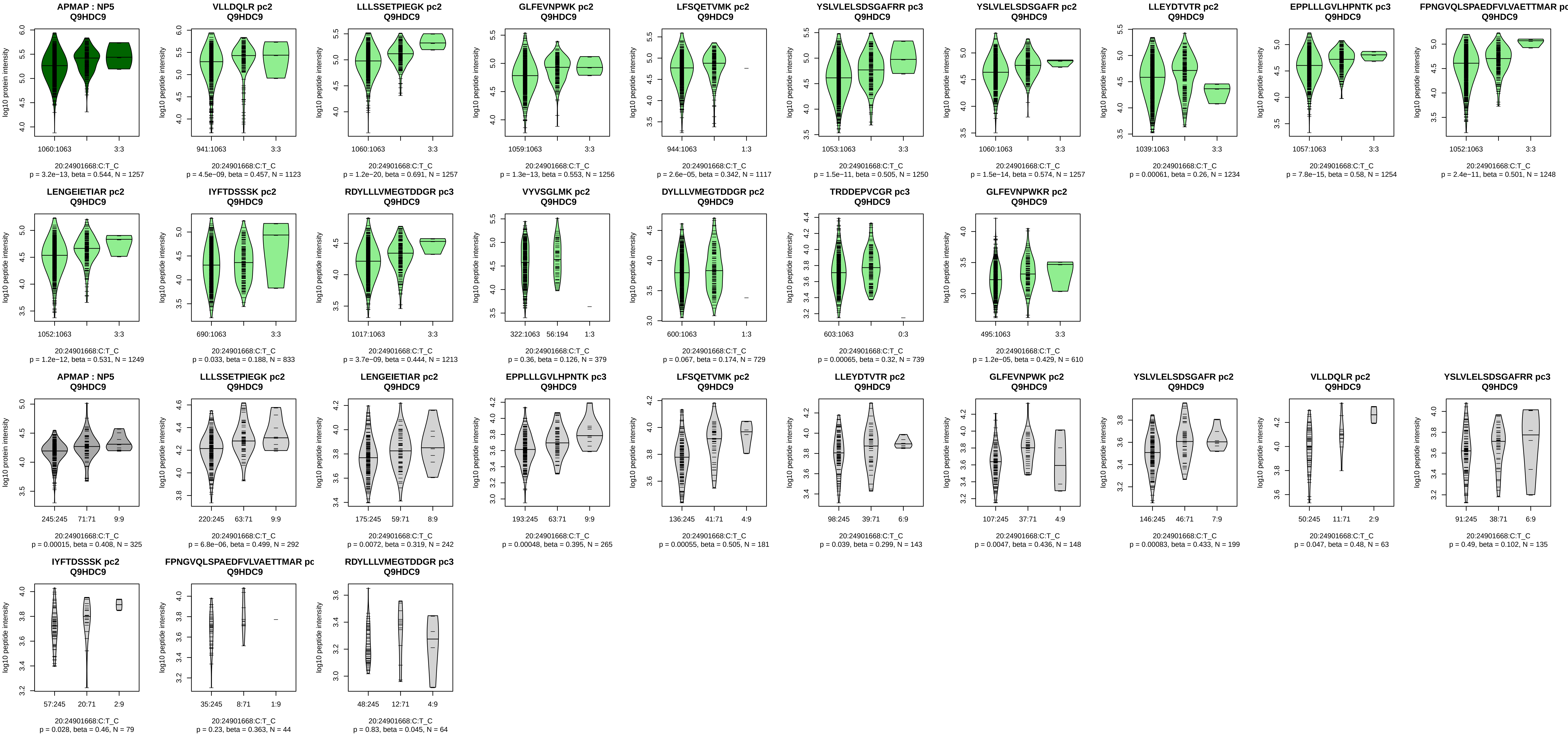

Assay Target: APMAP
UniProt: Q9HDC9
rsID: rs6036977
Target SNP: 20:24921032:C:T
Proxy SNP: 20:24901668:C:T
deCODE log10(p): 37.6
deCODE BETA: 0.16
deCODE MSPA: 0.875
1123:1257:1256:1117:1250:125

243. UXS1 (Q8NBZ7)
deCODE MSPA=0

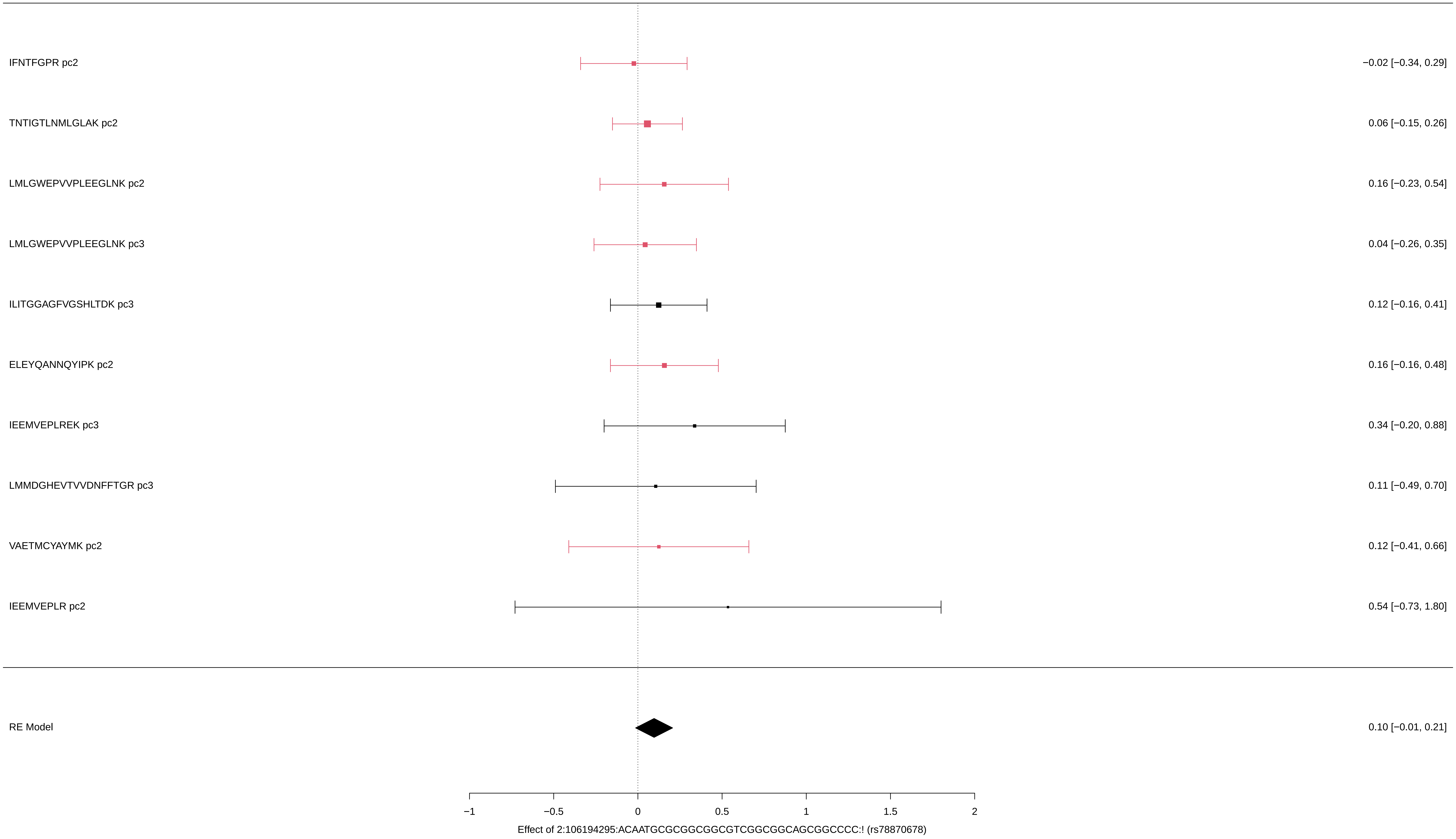

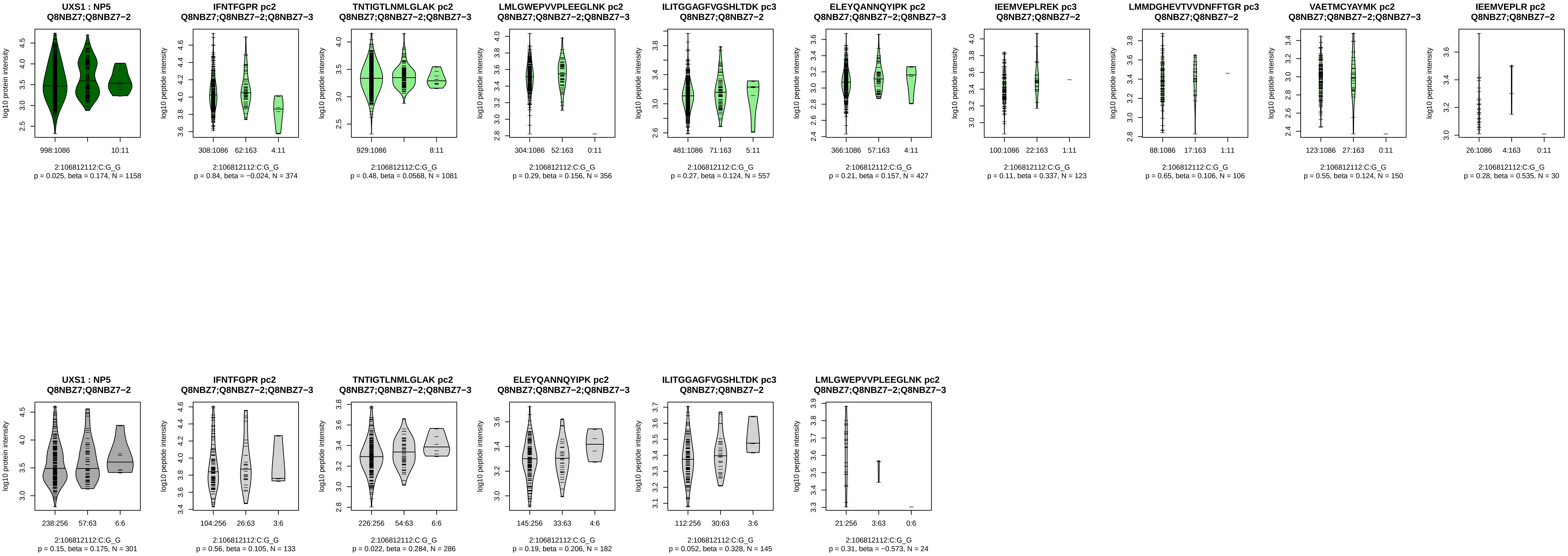

Assay Target: UXS1
UniProt: Q8NBZ7
rsID: rs78870678
Target SNP: 2:106194295:ACAA
Proxy SNP: 2:106812112:C:G
deCODE log10(p): 37
deCODE BETA: 0.17
deCODE MSPA: 0
−:−:−:−:−:−:−:−:−:NA:−
374:1081:356:444:557:427:123:

244. ADAMTSL2 (Q86TH1)
deCODE MSPA=0

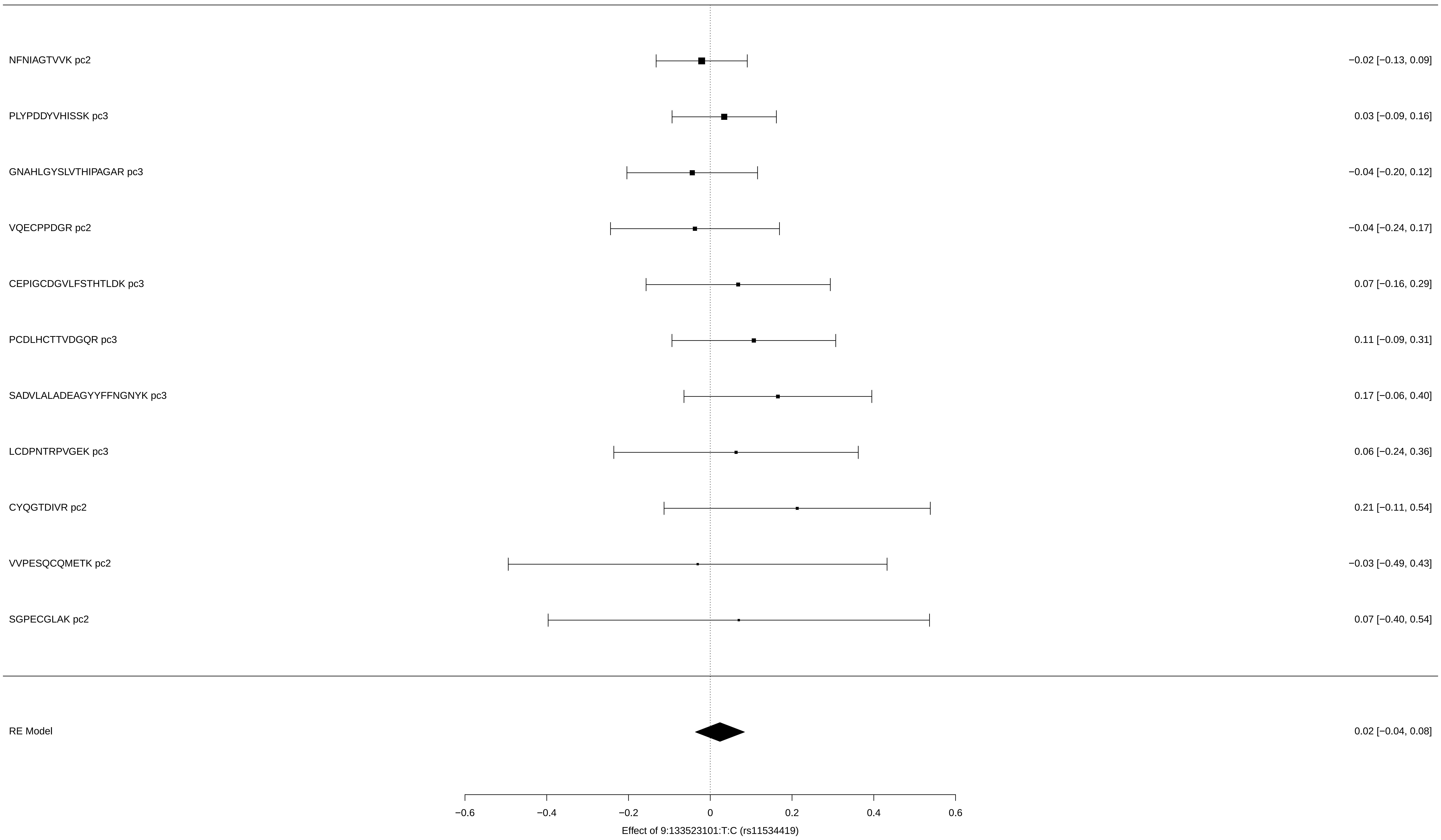

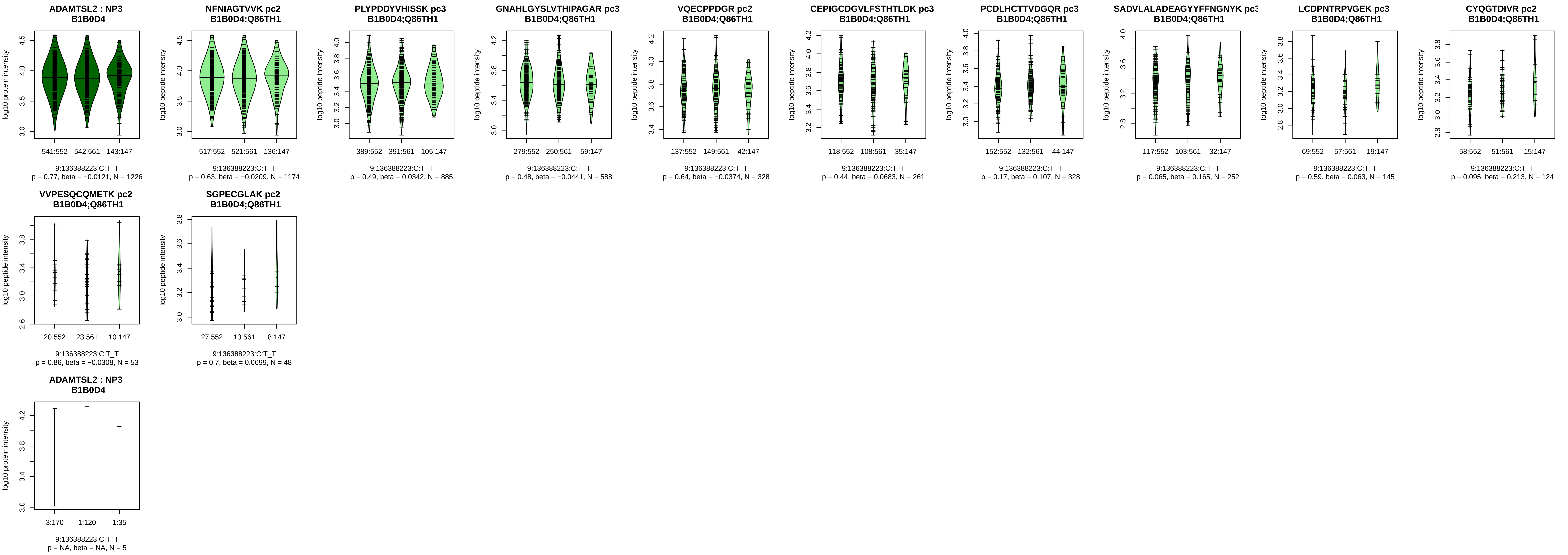

Assay Target: ADAMTSL2
UniProt: Q86TH1
rsID: rs11534419
Target SNP: 9:133523101:T:C
Proxy SNP: 9:136388223:C:T
deCODE log10(p): 36.9
deCODE BETA: 0.11
deCODE MSPA: 0
−:−:−:−:−:−:−:−:−:NA:−:NA:NA
1174:885:588:328:261:328:252:

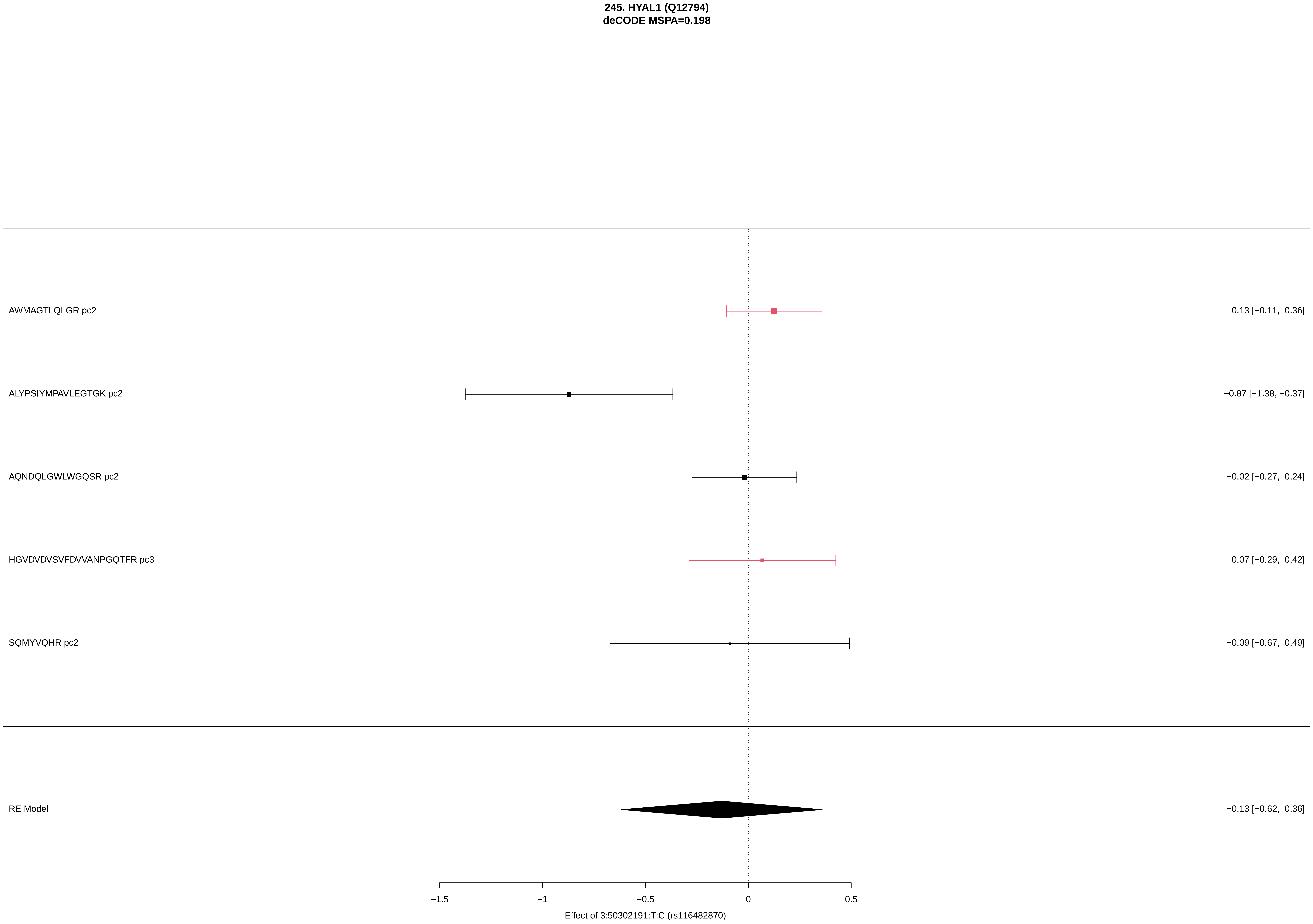
245. HYAL1 (Q12794)
deCODE MSPA=0.198
AWMAGTLQLGR pc2
0.13 [−0.11, 0.36]
ALYPSIYMPAVLEGTGK pc2
−0.87 [−1.38, −0.37]
AQNDQLGWLWGQSR pc2
−0.02 [−0.27, 0.24]
HGVDVDVSVFDVVANPGQTFR pc3
0.07 [−0.29, 0.42]
SQMYVQHR pc2
−0.09 [−0.67, 0.49]
RE Model
−0.13 [−0.62, 0.36]
−1.5
−1
−0.5
0
0.5
Effect of 3:50302191:T:C (rs116482870)

HYAL1 : NP5
Q12794

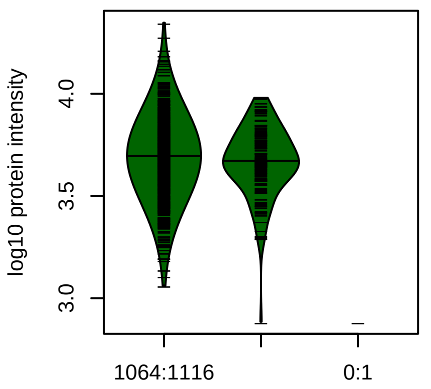

3:50339622:C:T_T
p = 0.25, beta = −0.104, N = 1204

AWMAGTLQLGR pc2
Q12794;Q12794−2

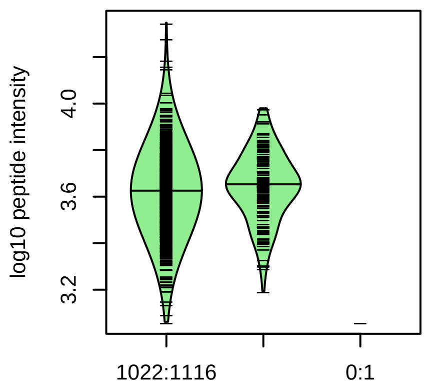

3:50339622:C:T_T
p = 0.17, beta = 0.125, N = 1160

ALYPSIYMPAVLEGTGK pc2
Q12794−3;Q12794;Q12794−2

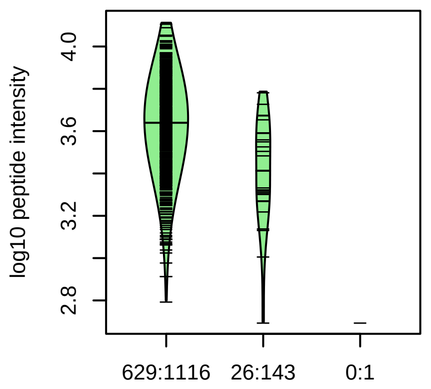

3:50339622:C:T_T
p = 1e−05, beta = −0.871, N = 655

AQNDQLGWLWGQSR pc2
Q12794−3;Q12794;Q12794−2

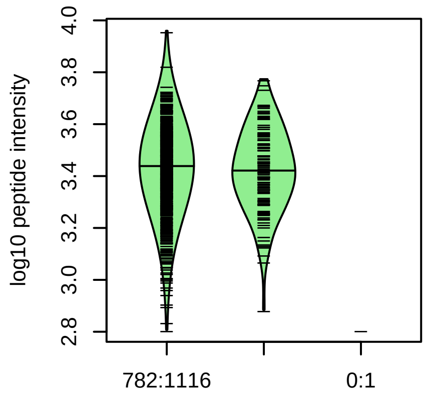

3:50339622:C:T_T
p = 0.84, beta = −0.0196, N = 898

HGVDVDVSVFDVVANPGQTFR pc3
Q12794;Q12794−2

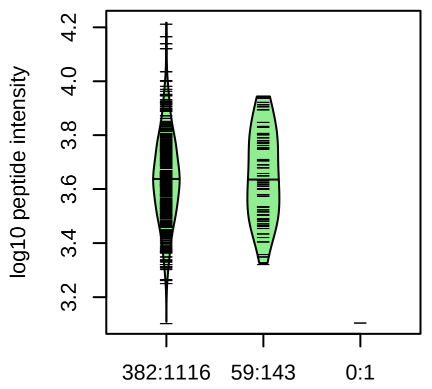

3:50339622:C:T_T
p = 0.62, beta = 0.0681, N = 441

SQMYVQHR pc2
Q12794−3;Q12794;Q12794−2

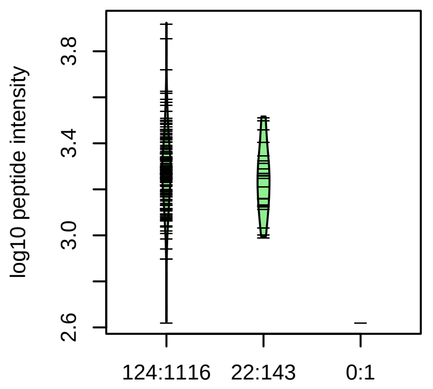

3:50339622:C:T_T
p = 0.69, beta = −0.0904, N = 146

HYAL1 : NP5
Q12794;Q12794−2

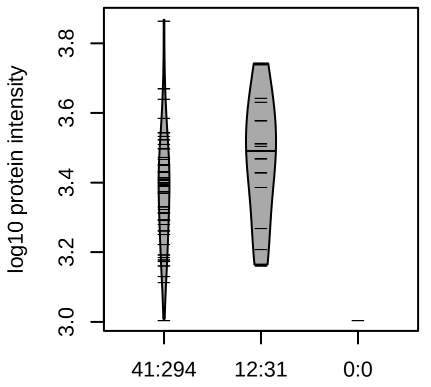

3:50339622:C:T_T
p = 0.13, beta = 0.466, N = 53

AQNDQLGWLWGQSR pc2
Q12794−3;Q12794;Q12794−2

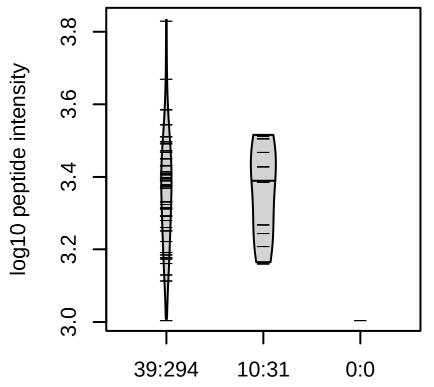

3:50339622:C:T_T
p = 0.79, beta = 0.0894, N = 49

Assay Target: HYAL1
UniProt: Q12794
rsID: rs116482870
Target SNP: 3:50302191:T:C
Proxy SNP: 3:50339622:C:T
deCODE log10(p): 35.8
deCODE BETA: −0.24
deCODE MSPA: 0.198
−:*:−:−:−
1160:655:898:441:146

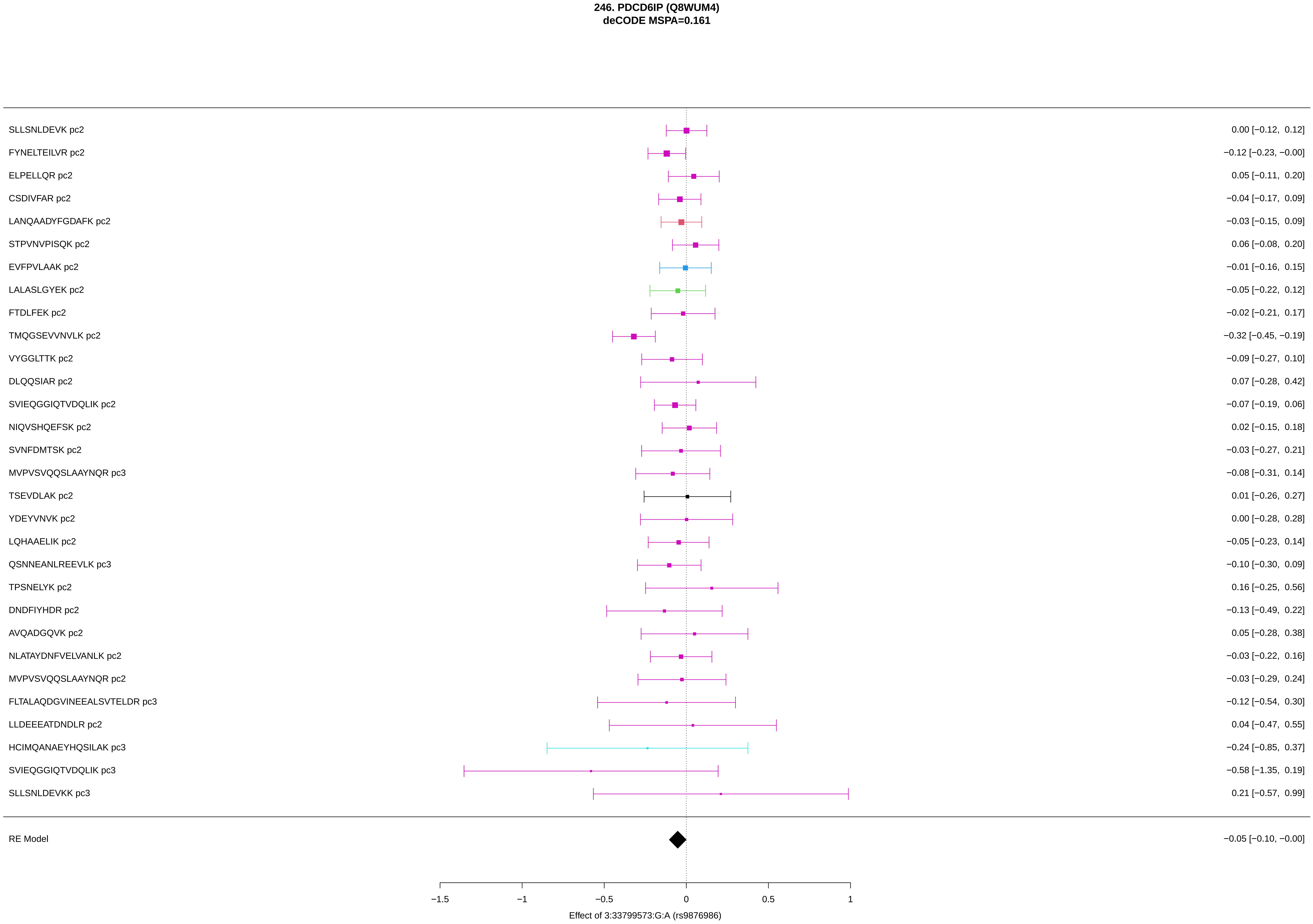
246. PDCD6IP (Q8WUM4)
deCODE MSPA=0.161
SLLSNLDEVK pc2
0.00 [−0.12, 0.12]
FYNELTEILVR pc2
−0.12 [−0.23, −0.00]
ELPELLQR pc2
0.05 [−0.11, 0.20]
CSDIVFAR pc2
−0.04 [−0.17, 0.09]
LANQAADYFGDAFK pc2
−0.03 [−0.15, 0.09]
STPVNVPISQK pc2
0.06 [−0.08, 0.20]
EVFPVLAAK pc2
−0.01 [−0.16, 0.15]
LALASLGYEK pc2
−0.05 [−0.22, 0.12]
FTDLFEK pc2
−0.02 [−0.21, 0.17]
TMQGSEVVNVLK pc2
−0.32 [−0.45, −0.19]
VYGGLTTK pc2
−0.09 [−0.27, 0.10]
DLQQSIAR pc2
0.07 [−0.28, 0.42]
SVIEQGGIQTVDQLIK pc2
−0.07 [−0.19, 0.06]
NIQVSHQEFSK pc2
0.02 [−0.15, 0.18]
SVNFDMTSK pc2
−0.03 [−0.27, 0.21]
MVPVSVQQSLAAYNQR pc3
−0.08 [−0.31, 0.14]
TSEVDLAK pc2
0.01 [−0.26, 0.27]
YDEYVNVK pc2
0.00 [−0.28, 0.28]
LQHAAELIK pc2
−0.05 [−0.23, 0.14]
QSNNEANLREEVLK pc3
−0.10 [−0.30, 0.09]
TPSNELYK pc2
0.16 [−0.25, 0.56]
DNDFIYHDR pc2
−0.13 [−0.49, 0.22]
AVQADGQVK pc2
0.05 [−0.28, 0.38]
NLATAYDNFVELVANLK pc2
−0.03 [−0.22, 0.16]
MVPVSVQQSLAAYNQR pc2
−0.03 [−0.29, 0.24]
FLTALAQDGVINEEALSVTELDR pc3
−0.12 [−0.54, 0.30]
LLDEEEATDNDLR pc2
0.04 [−0.47, 0.55]
HCIMQANAEYHQSILAK pc3
−0.24 [−0.85, 0.37]
SVIEQGGIQTVDQLIK pc3
−0.58 [−1.35, 0.19]
SLLSNLDEVKK pc3
0.21 [−0.57, 0.99]
RE Model
−0.05 [−0.10, −0.00]
−1.5
−1
−0.5
0
0.5
1
Effect of 3:33799573:G:A (rs9876986)

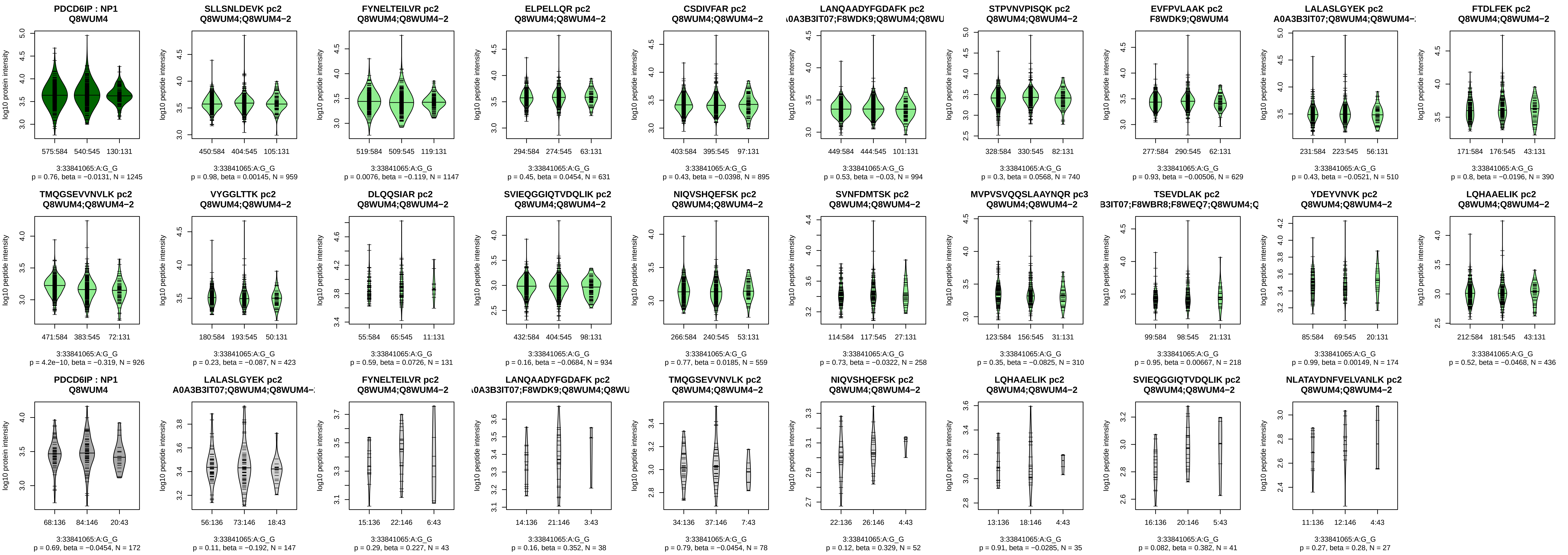

Assay Target: PDCD6IP
UniProt: Q8WUM4
rsID: rs9876986
Target SNP: 3:33799573:G:A
Proxy SNP: 3:33841065:A:G
deCODE log10(p): 34.2
deCODE BETA: −0.11
deCODE MSPA: 0.161
959:1147:631:895:994:740:629:

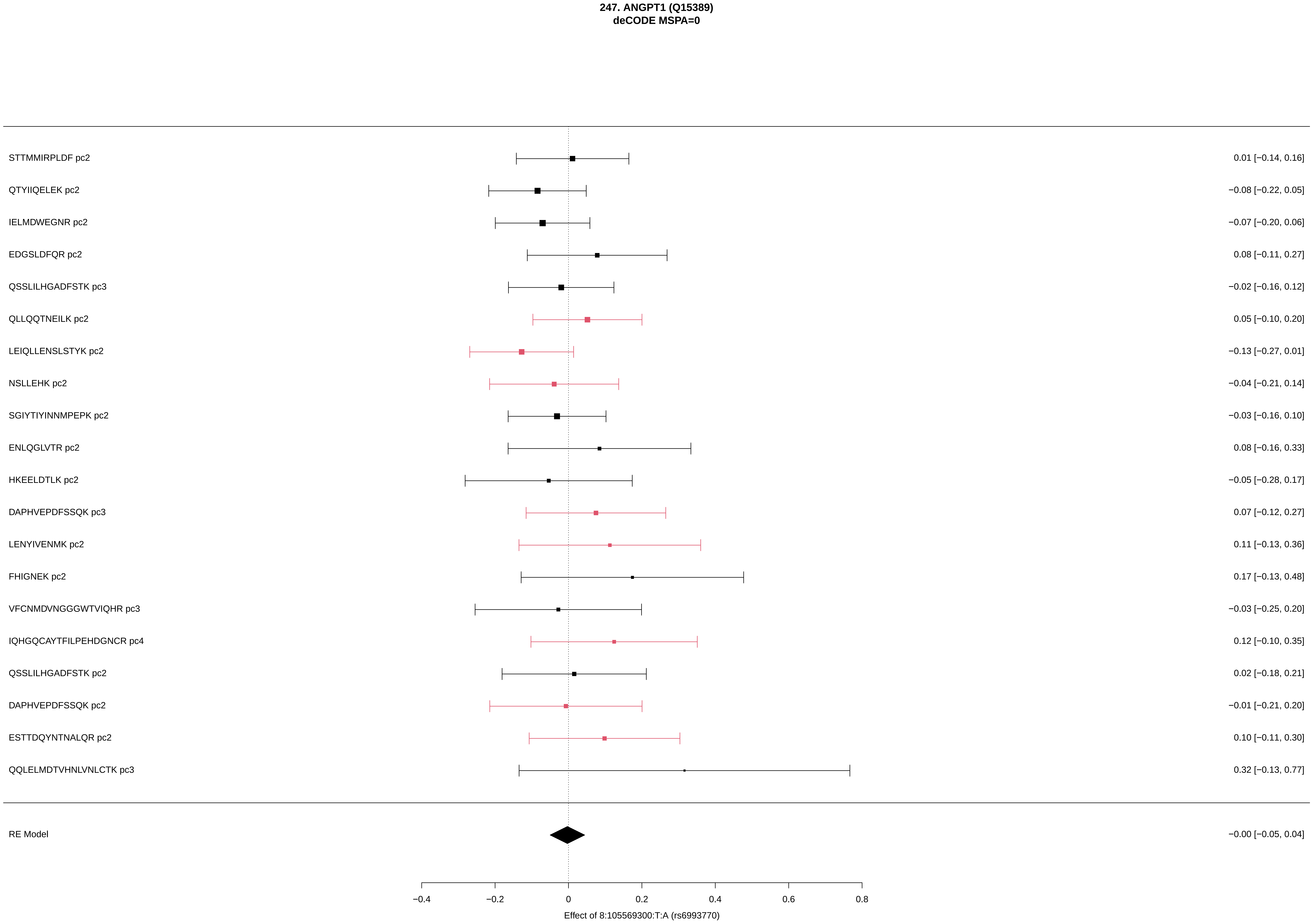
247. ANGPT1 (Q15389)
deCODE MSPA=0
STTMMIRPLDF pc2
0.01 [−0.14, 0.16]
QTYIIQELEK pc2
−0.08 [−0.22, 0.05]
IELMDWEGNR pc2
−0.07 [−0.20, 0.06]
EDGSLDFQR pc2
0.08 [−0.11, 0.27]
QSSLILHGADFSTK pc3
−0.02 [−0.16, 0.12]
QLLQQTNEILK pc2
0.05 [−0.10, 0.20]
LEIQLLENSLSTYK pc2
−0.13 [−0.27, 0.01]
NSLLEHK pc2
−0.04 [−0.21, 0.14]
SGIYTIYINNMPEPK pc2
−0.03 [−0.16, 0.10]
ENLQGLVTR pc2
0.08 [−0.16, 0.33]
HKEELDTLK pc2
−0.05 [−0.28, 0.17]
DAPHVEPDFSSQK pc3
0.07 [−0.12, 0.27]
LENYIVENMK pc2
0.11 [−0.13, 0.36]
FHIGNEK pc2
0.17 [−0.13, 0.48]
VFCNMDVNGGGWTVIQHR pc3
−0.03 [−0.25, 0.20]
IQHGQCAYTFILPEHDGNCR pc4
0.12 [−0.10, 0.35]
QSSLILHGADFSTK pc2
0.02 [−0.18, 0.21]
DAPHVEPDFSSQK pc2
−0.01 [−0.21, 0.20]
ESTTDQYNTNALQR pc2
0.10 [−0.11, 0.30]
QQLELMDTVHNLVNLCTK pc3
0.32 [−0.13, 0.77]
RE Model
−0.00 [−0.05, 0.04]
−0.4
−0.2
0
0.2
0.4
0.6
0.8
Effect of 8:105569300:T:A (rs6993770)

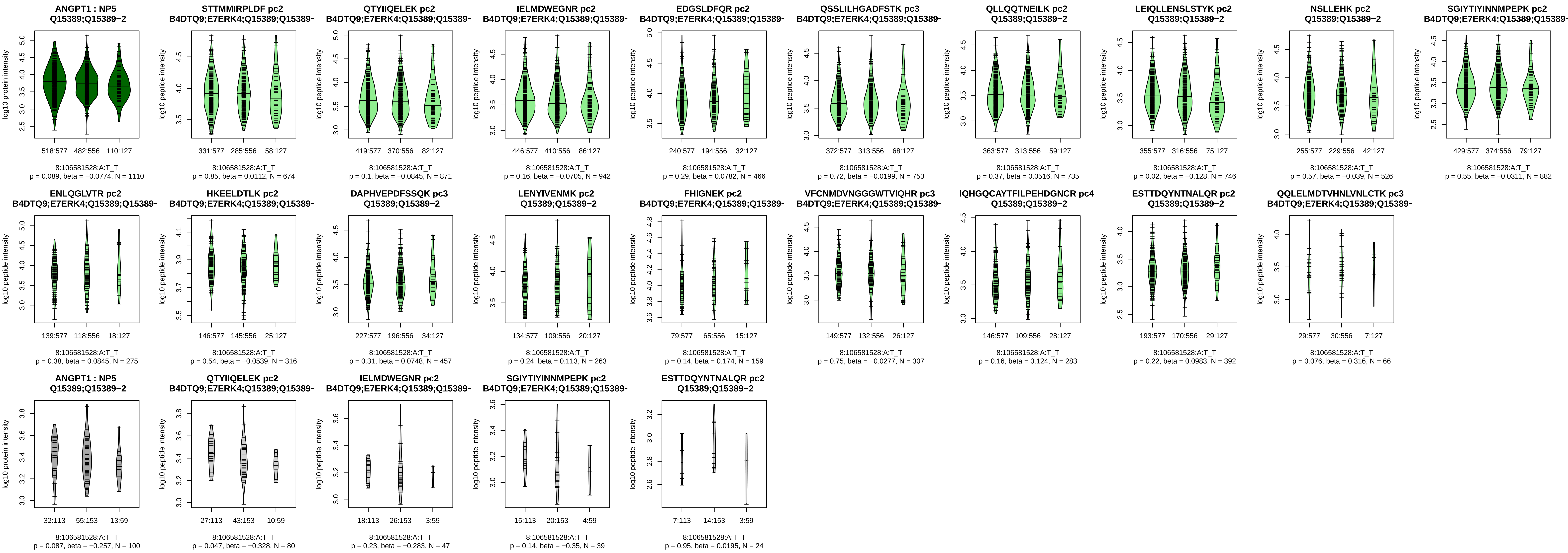

Assay Target: ANGPT1
UniProt: Q15389
rsID: rs6993770
Target SNP: 8:105569300:T:A
Proxy SNP: 8:106581528:A:T
deCODE log10(p): 31.9
deCODE BETA: −0.12
deCODE MSPA: 0

674:871:942:466:753:735:746:5

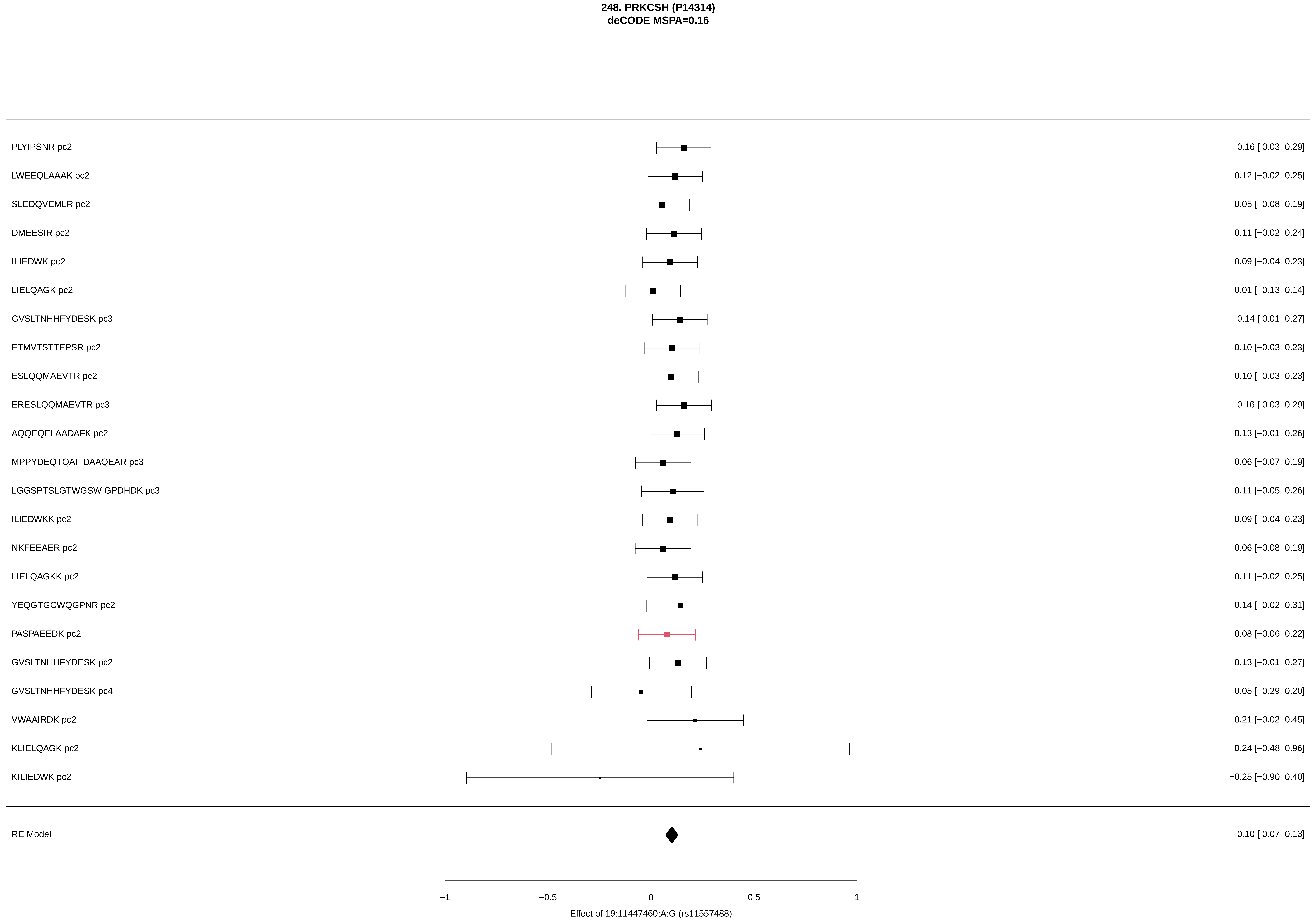

248. PRKCSH (P14314)
deCODE MSPA=0.16
PLYIPSNR pc2
0.16 [0.03, 0.29]
LWEEQLAAAK pc2
0.12 [−0.02, 0.25]
SLEDQVEMLR pc2
0.05 [−0.08, 0.19]
DMEESIR pc2
0.11 [−0.02, 0.24]
ILIEDWK pc2
0.09 [−0.04, 0.23]
LIELQAGK pc2
0.01 [−0.13, 0.14]
GVSLTNHHFYDESK pc3
0.14 [0.01, 0.27]
ETMVTSTTEPSR pc2
0.10 [−0.03, 0.23]
ESLQQMAEVTR pc2
0.10 [−0.03, 0.23]
ERESLQQMAEVTR pc3
0.16 [0.03, 0.29]
AQQEQELAADAFK pc2
0.13 [−0.01, 0.26]
MPPYDEQTQAFIDAAQEAR pc3
0.06 [−0.07, 0.19]
LGGSPTSLGTWGSWIGPDHDK pc3
0.11 [−0.05, 0.26]
ILIEDWKK pc2
0.09 [−0.04, 0.23]
NKFEEAER pc2
0.06 [−0.08, 0.19]
LIELQAGKK pc2
0.11 [−0.02, 0.25]
YEQGTGCWQGPNR pc2
0.14 [−0.02, 0.31]
PASPAEEDK pc2
0.08 [−0.06, 0.22]
GVSLTNHHFYDESK pc2
0.13 [−0.01, 0.27]
GVSLTNHHFYDESK pc4
−0.05 [−0.29, 0.20]
VWAAIRDK pc2
0.21 [−0.02, 0.45]
KLIELQAGK pc2
0.24 [−0.48, 0.96]
KILIEDWK pc2
−0.25 [−0.90, 0.40]
RE Model
0.10 [0.07, 0.13]
−1
−0.5
0
0.5
1
Effect of 19:11447460:A:G (rs11557488)

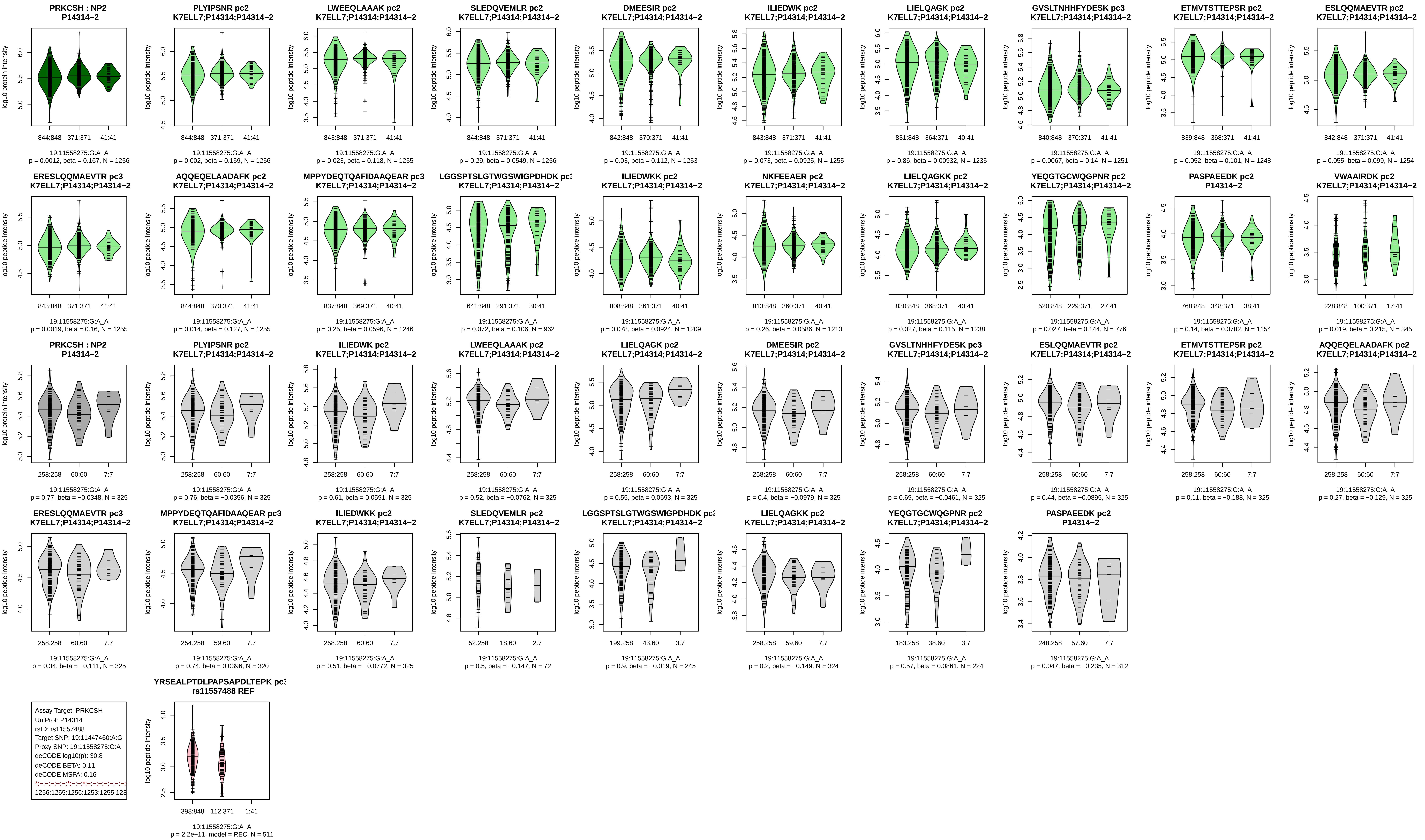
PRKCSH : NP2
P14314−2
844:848 371:371 41:41
19:11558275:G:A_A
p = 0.0012, beta = 0.167, N = 1256
PLYIPSNR pc2
K7ELL7;P14314;P14314−2
844:848 371:371 41:41
19:11558275:G:A_A
p = 0.002, beta = 0.159, N = 1256
LWEEQLAAAK pc2
K7ELL7;P14314;P14314−2
843:848 371:371 41:41
19:11558275:G:A_A
p = 0.023, beta = 0.118, N = 1255
SLEDQVEMLR pc2
K7ELL7;P14314;P14314−2
844:848 371:371 41:41
19:11558275:G:A_A
p = 0.29, beta = 0.0549, N = 1256
DMEESIR pc2
K7ELL7;P14314;P14314−2
842:848 370:371 41:41
19:11558275:G:A_A
p = 0.03, beta = 0.112, N = 1253
ILIEDWK pc2
K7ELL7;P14314;P14314−2
843:848 371:371 41:41
19:11558275:G:A_A
p = 0.073, beta = 0.0925, N = 1255
LIELQAGK pc2
K7ELL7;P14314;P14314−2
831:848 364:371 40:41
19:11558275:G:A_A
p = 0.86, beta = 0.00932, N = 1235
GVSLTNHHFYDESK pc3
K7ELL7;P14314;P14314−2
840:848 370:371 41:41
19:11558275:G:A_A
p = 0.0067, beta = 0.14, N = 1251
ETMVTSTTEPSR pc2
K7ELL7;P14314;P14314−2
839:848 368:371 41:41
19:11558275:G:A_A
p = 0.052, beta = 0.101, N = 1248
ESLQQMAEVTR pc2
K7ELL7;P14314;P14314−2
842:848 371:371 41:41
19:11558275:G:A_A
p = 0.055, beta = 0.099, N = 1254
ERESLQQMAEVTR pc3
K7ELL7;P14314;P14314−2
843:848 371:371 41:41
19:11558275:G:A_A
p = 0.0019, beta = 0.16, N = 1255
AQQEQELAADAFK pc2
K7ELL7;P14314;P14314−2
844:848 370:371 41:41
19:11558275:G:A_A
p = 0.014, beta = 0.127, N = 1255
MPPYDEQTQAFIDAAQEAR pc3
K7ELL7;P14314;P14314−2
837:848 369:371 40:41
19:11558275:G:A_A
p = 0.25, beta = 0.0596, N = 1246
LGGSPTSLGTWGSWIGPDHDK pc3
K7ELL7;P14314;P14314−2
641:848 291:371 30:41
19:11558275:G:A_A
p = 0.072, beta = 0.106, N = 962
ILIEDWKK pc2
K7ELL7;P14314;P14314−2
808:848 361:371 40:41
19:11558275:G:A_A
p = 0.078, beta = 0.0924, N = 1209
NKFEEAER pc2
K7ELL7;P14314;P14314−2
813:848 360:371 40:41
19:11558275:G:A_A
p = 0.26, beta = 0.0586, N = 1213
LIELQAGKK pc2
K7ELL7;P14314;P14314−2
830:848 368:371 40:41
19:11558275:G:A_A
p = 0.027, beta = 0.115, N = 1238
YEQGTGCWQGPNR pc2
K7ELL7;P14314;P14314−2
520:848 229:371 27:41
19:11558275:G:A_A
p = 0.027, beta = 0.144, N = 776
PASPAEEDK pc2
P14314−2
768:848 348:371 38:41
19:11558275:G:A_A
p = 0.14, beta = 0.0782, N = 1154
VWAAIRDK pc2
K7ELL7;P14314;P14314−2
228:848 100:371 17:41
19:11558275:G:A_A
p = 0.019, beta = 0.215, N = 345
PRKCSH : NP2
P14314−2
258:258 60:60 7:7
19:11558275:G:A_A
p = 0.77, beta = −0.0348, N = 325
PLYIPSNR pc2
K7ELL7;P14314;P14314−2
258:258 60:60 7:7
19:11558275:G:A_A
p = 0.76, beta = −0.0356, N = 325
ILIEDWK pc2
K7ELL7;P14314;P14314−2
258:258 60:60 7:7
19:11558275:G:A_A
p = 0.61, beta = 0.0591, N = 325
LWEEQLAAAK pc2
K7ELL7;P14314;P14314−2
258:258 60:60 7:7
19:11558275:G:A_A
p = 0.52, beta = −0.0762, N = 325
LIELQAGK pc2
K7ELL7;P14314;P14314−2
258:258 60:60 7:7
19:11558275:G:A_A
p = 0.55, beta = 0.0693, N = 325
DMEESIR pc2
K7ELL7;P14314;P14314−2
258:258 60:60 7:7
19:11558275:G:A_A
p = 0.4, beta = −0.0979, N = 325
GVSLTNHHFYDESK pc3
K7ELL7;P14314;P14314−2
258:258 60:60 7:7
19:11558275:G:A_A
p = 0.69, beta = −0.0461, N = 325
ESLQQMAEVTR pc2
K7ELL7;P14314;P14314−2
258:258 60:60 7:7
19:11558275:G:A_A
p = 0.44, beta = −0.0895, N = 325
ETMVTSTTEPSR pc2
K7ELL7;P14314;P14314−2
258:258 60:60 7:7
19:11558275:G:A_A
p = 0.11, beta = −0.188, N = 325
AQQEQELAADAFK pc2
K7ELL7;P14314;P14314−2
258:258 60:60 7:7
19:11558275:G:A_A
p = 0.27, beta = −0.129, N = 325
ERESLQQMAEVTR pc3
K7ELL7;P14314;P14314−2
258:258 60:60 7:7
19:11558275:G:A_A
p = 0.34, beta = −0.111, N = 325
MPPYDEQTQAFIDAAQEAR pc3
K7ELL7;P14314;P14314−2
254:258 59:60 7:7
19:11558275:G:A_A
p = 0.74, beta = 0.0396, N = 320
ILIEDWKK pc2
K7ELL7;P14314;P14314−2
258:258 60:60 7:7
19:11558275:G:A_A
p = 0.51, beta = −0.0772, N = 325
SLEDQVEMLR pc2
K7ELL7;P14314;P14314−2
52:258 18:60 2:7
19:11558275:G:A_A
p = 0.5, beta = −0.147, N = 72
LGGSPTSLGTWGSWIGPDHDK pc3
K7ELL7;P14314;P14314−2
199:258 43:60 3:7
19:11558275:G:A_A
p = 0.9, beta = −0.019, N = 245
LIELQAGKK pc2
K7ELL7;P14314;P14314−2
258:258 59:60 7:7
19:11558275:G:A_A
p = 0.2, beta = −0.149, N = 324
YEQGTGCWQGPNR pc2
K7ELL7;P14314;P14314−2
183:258 38:60 3:7
19:11558275:G:A_A
p = 0.57, beta = 0.0861, N = 224
PASPAEEDK pc2
P14314−2
248:258 57:60 7:7
19:11558275:G:A_A
p = 0.047, beta = −0.235, N = 312
Assay Target: PRKCSH
UniProt: P14314
rsID: rs11557488
Target SNP: 19:11447460:A:G
Proxy SNP: 19:11558275:G:A
deCODE log10(p): 30.8
deCODE BETA: 0.11
deCODE MSPA: 0.16
1256:1255:1256:1253:1255:123
YRSEALPTDLPAPSAPDLTEPK pc3
rs11557488 REF
398:848 112:371 1:41
19:11558275:G:A_A
p = 2.2e−11, model = REC, N = 511
log10 protein intensity
log10 peptide intensity

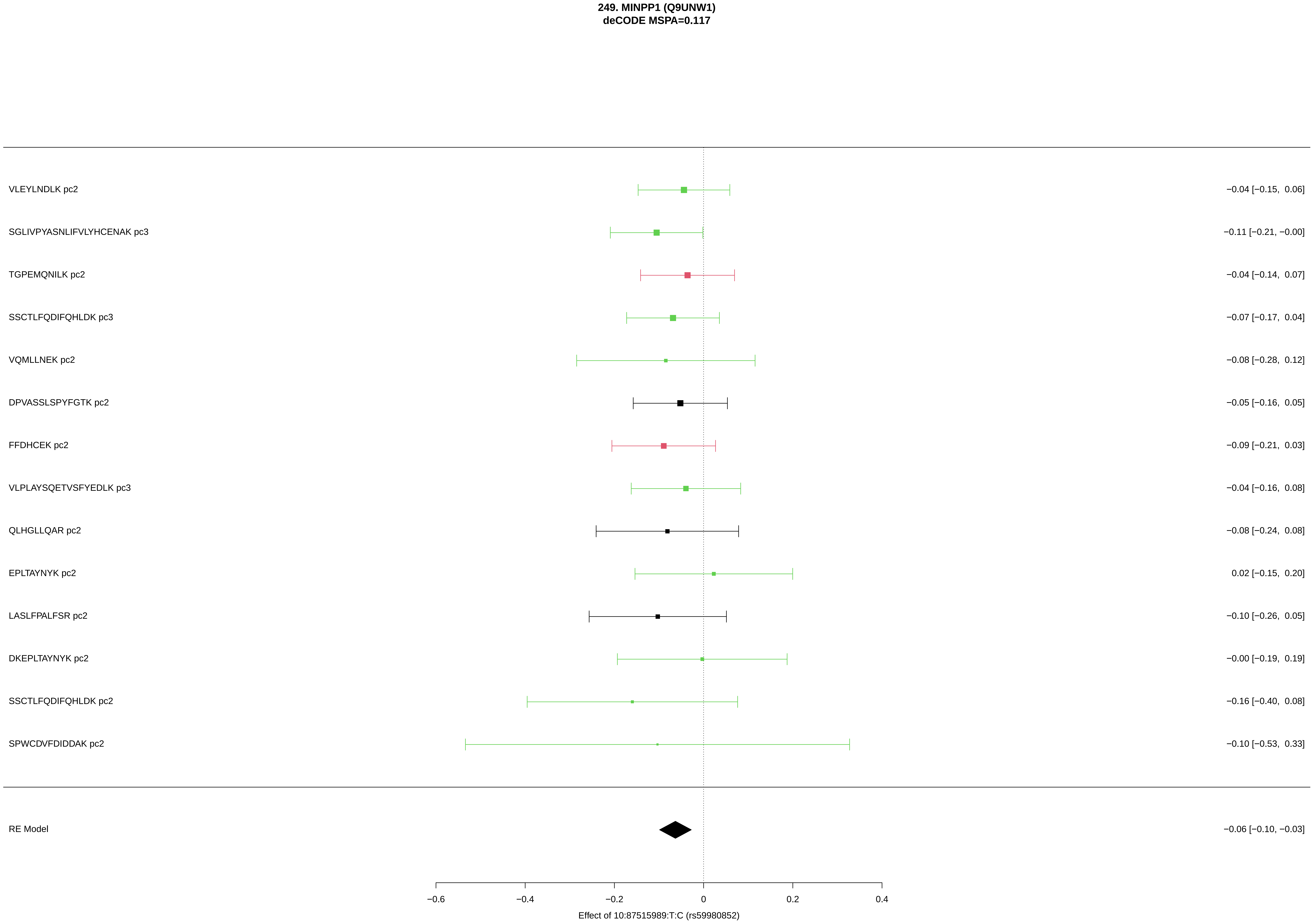

249. MINPP1 (Q9UNW1)
deCODE MSPA=0.117
VLEYLNDLK pc2
−0.04 [−0.15, 0.06]
SGLIVPYASNLIFVLYHCENAK pc3
−0.11 [−0.21, −0.00]
TGPEMQNILK pc2
−0.04 [−0.14, 0.07]
SSCTLFQDIFQHLDK pc3
−0.07 [−0.17, 0.04]
VQMLLNEK pc2
−0.08 [−0.28, 0.12]
DPVASSLSPYFGTK pc2
−0.05 [−0.16, 0.05]
FFDHCEK pc2
−0.09 [−0.21, 0.03]
VLPLAYSQETVSFYEDLK pc3
−0.04 [−0.16, 0.08]
QLHGLLQAR pc2
−0.08 [−0.24, 0.08]
EPLTAYNYK pc2
0.02 [−0.15, 0.20]
LASLFPALFSR pc2
−0.10 [−0.26, 0.05]
DKEPLTAYNYK pc2
−0.00 [−0.19, 0.19]
SSCTLFQDIFQHLDK pc2
−0.16 [−0.40, 0.08]
SPWCDVFDIDDAK pc2
−0.10 [−0.53, 0.33]
RE Model
−0.06 [−0.10, −0.03]
−0.6
−0.4
−0.2
0
0.2
0.4
Effect of 10:87515989:T:C (rs59980852)

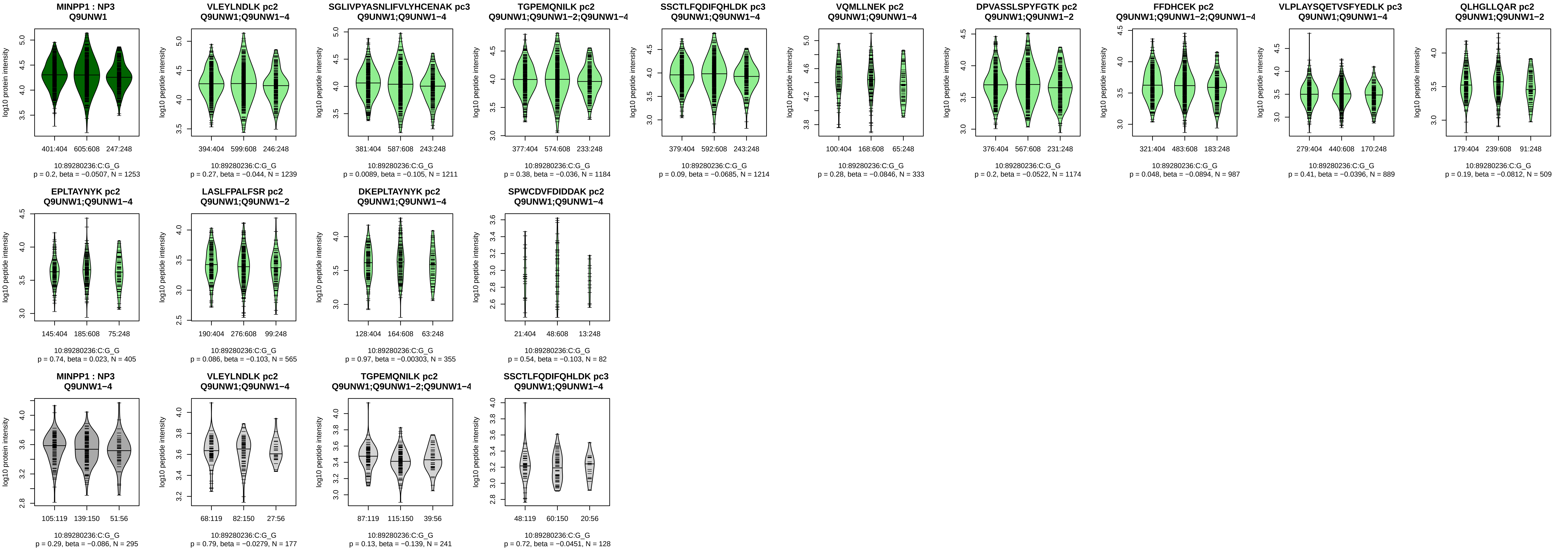

Assay Target: MINPP1
UniProt: Q9UNW1
rsID: rs59980852
Target SNP: 10:87515989:T:C
Proxy SNP: 10:89280236:C:G
deCODE log10(p): 30.2
deCODE BETA: −0.1
deCODE MSPA: 0.117
–:*:–:–:–:–:–:–:–:–:–:–
1239:1211:1184:1214:333:1174

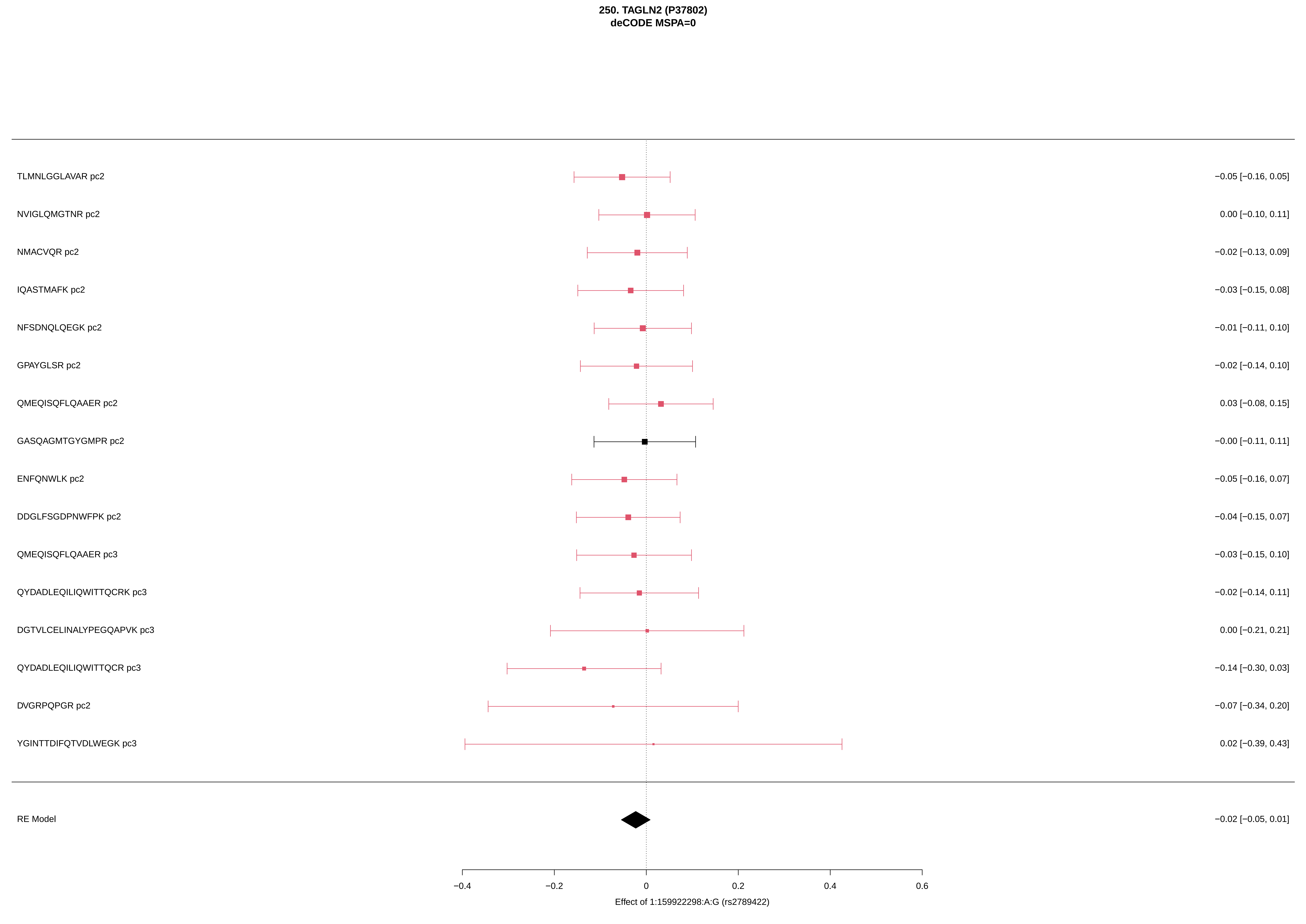

250. TAGLN2 (P37802)
deCODE MSPA=0
TLMNLGGLAVAR pc2
−0.05 [−0.16, 0.05]
NVIGLQMGTNR pc2
0.00 [−0.10, 0.11]
NMACVQR pc2
−0.02 [−0.13, 0.09]
IQASTMAFK pc2
−0.03 [−0.15, 0.08]
NFSDNQLQEGK pc2
−0.01 [−0.11, 0.10]
GPAYGLSR pc2
−0.02 [−0.14, 0.10]
QMEQISQFLQAAER pc2
0.03 [−0.08, 0.15]
GASQAGMTGYGMPR pc2
−0.00 [−0.11, 0.11]
ENFQNWLK pc2
−0.05 [−0.16, 0.07]
DDGLFSGDPNWFPK pc2
−0.04 [−0.15, 0.07]
QMEQISQFLQAAER pc3
−0.03 [−0.15, 0.10]
QYDADLEQILIQWITTQCRK pc3
−0.02 [−0.14, 0.11]
DGTVLCELINALYPEGQAPVK pc3
0.00 [−0.21, 0.21]
QYDADLEQILIQWITTQCR pc3
−0.14 [−0.30, 0.03]
DVGRPQPGR pc2
−0.07 [−0.34, 0.20]
YGINTTDIFQTVDLWEGK pc3
0.02 [−0.39, 0.43]
RE Model
−0.02 [−0.05, 0.01]
−0.4
−0.2
0
0.2
0.4
0.6
Effect of 1:159922298:A:G (rs2789422)

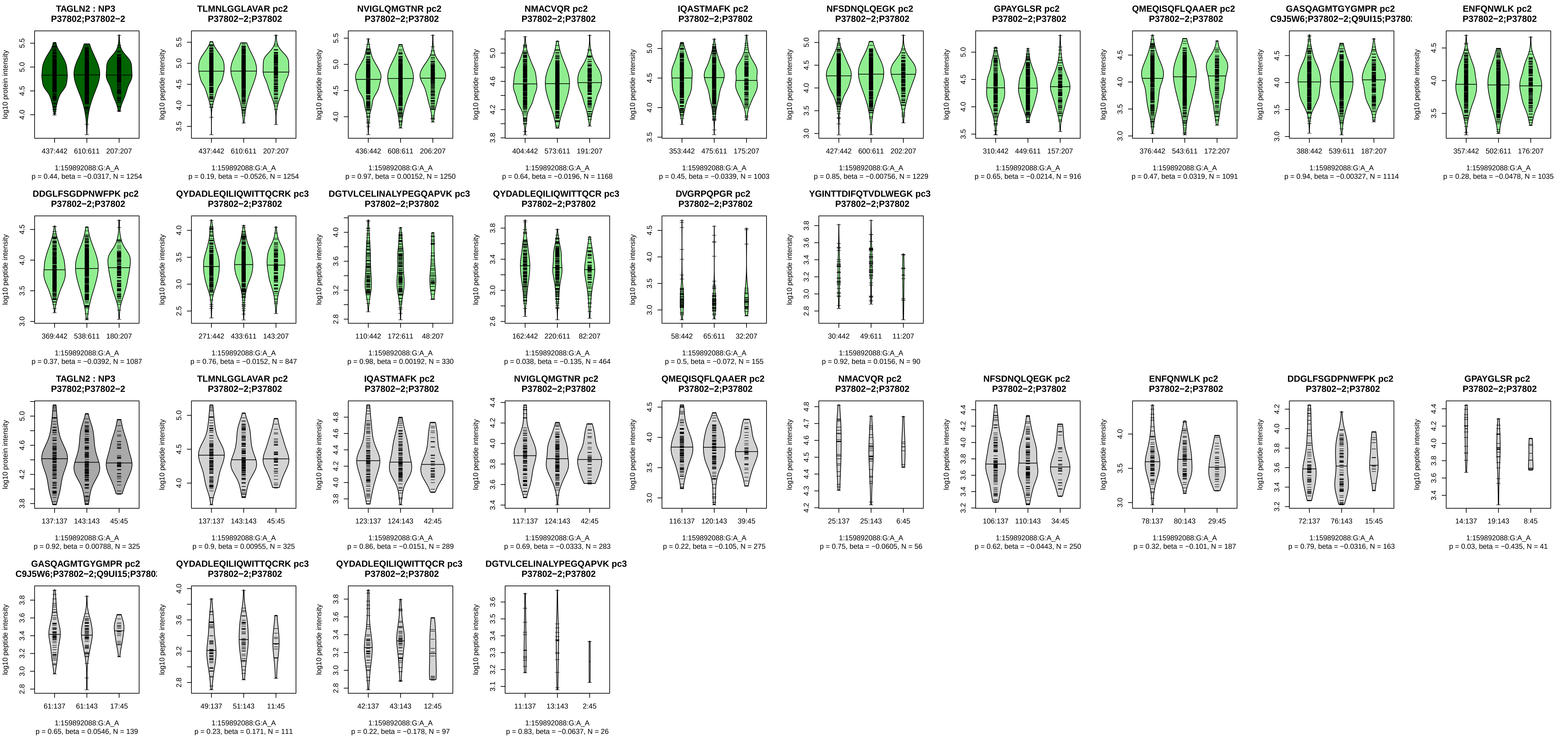

Assay Target: TAGLN2
UniProt: P37802
rsID: rs2789422
Target SNP: 1:159922298:A:G
Proxy SNP: 1:159892088:G:A
deCODE log10(p): 28.7
deCODE BETA: −0.09
deCODE MSPA: 0
1254:1250:1168:1003:1229:916

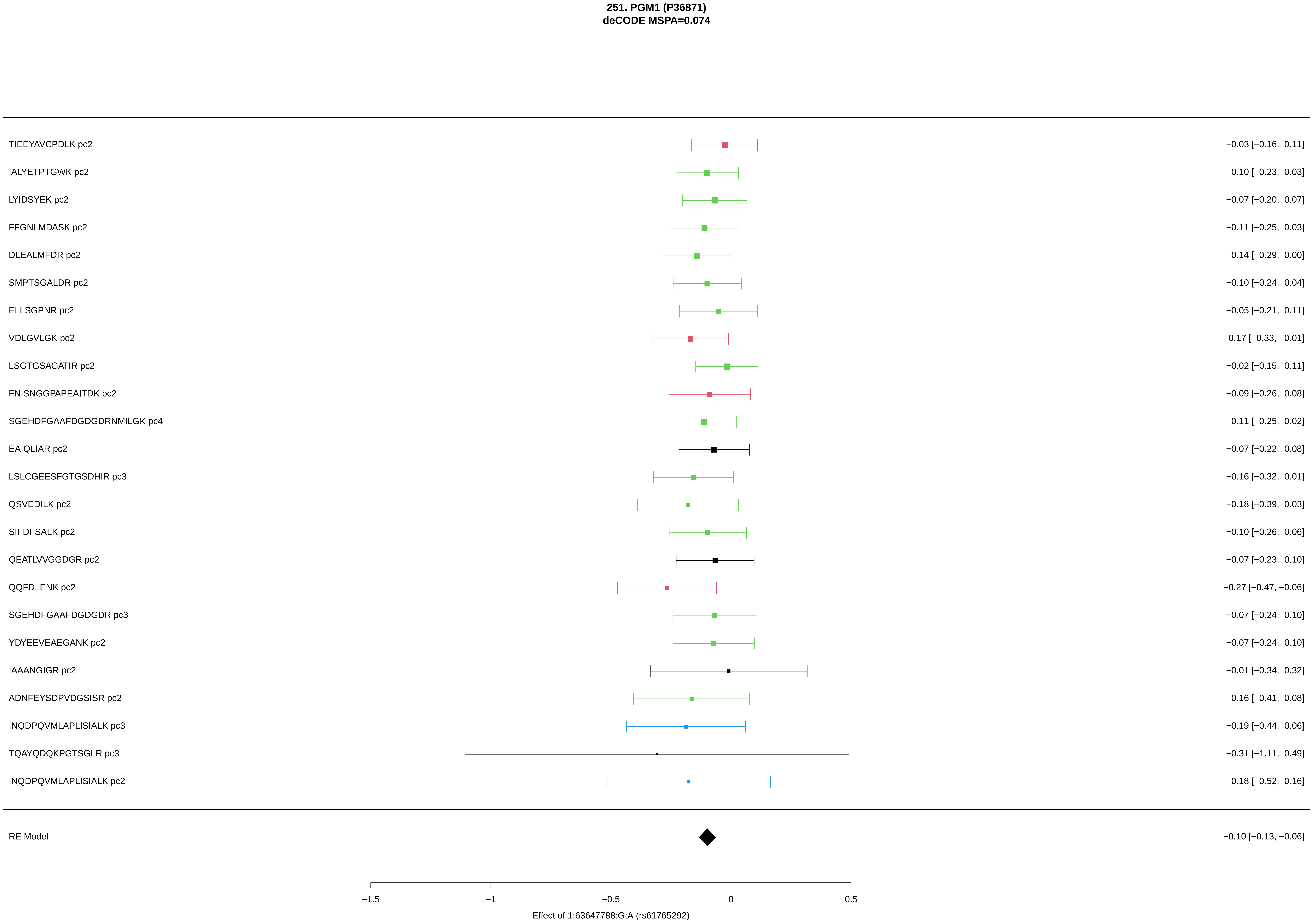

251. PGM1 (P36871)
deCODE MSPA=0.074
TIEEYAVCPDLK pc2
−0.03 [−0.16, 0.11]
IALYETPTGWK pc2
−0.10 [−0.23, 0.03]
LYIDSYEK pc2
−0.07 [−0.20, 0.07]
FFGNLMDASK pc2
−0.11 [−0.25, 0.03]
DLEALMFDR pc2
−0.14 [−0.29, 0.00]
SMPTSGALDR pc2
−0.10 [−0.24, 0.04]
ELLSGPNR pc2
−0.05 [−0.21, 0.11]
VDLGVLGK pc2
−0.17 [−0.33, −0.01]
LSGTGSAGATIR pc2
−0.02 [−0.15, 0.11]
FNISNGGPAPEAITDK pc2
−0.09 [−0.26, 0.08]
SGEHDFGAAFDGDGDRNMILGK pc4
−0.11 [−0.25, 0.02]
EAIQLIAR pc2
−0.07 [−0.22, 0.08]
LSLCGEESFGTGSDHIR pc3
−0.16 [−0.32, 0.01]
QSVEDILK pc2
−0.18 [−0.39, 0.03]
SIFDFSALK pc2
−0.10 [−0.26, 0.06]
QEATLVVGGDGR pc2
−0.07 [−0.23, 0.10]
QQFDLENK pc2
−0.27 [−0.47, −0.06]
SGEHDFGAAFDGDGDR pc3
−0.07 [−0.24, 0.10]
YDYEEVEAEGANK pc2
−0.07 [−0.24, 0.10]
IAAANGIGR pc2
−0.01 [−0.34, 0.32]
ADNFEYSDPVDGSISR pc2
−0.16 [−0.41, 0.08]
INQDPQVMLAPLISIALK pc3
−0.19 [−0.44, 0.06]
TQAYQDQKPGTSGLR pc3
−0.31 [−1.11, 0.49]
INQDPQVMLAPLISIALK pc2
−0.18 [−0.52, 0.16]
RE Model
−0.10 [−0.13, −0.06]
−1.5
−1
−0.5
0
0.5
Effect of 1:63647788:G:A (rs61765292)

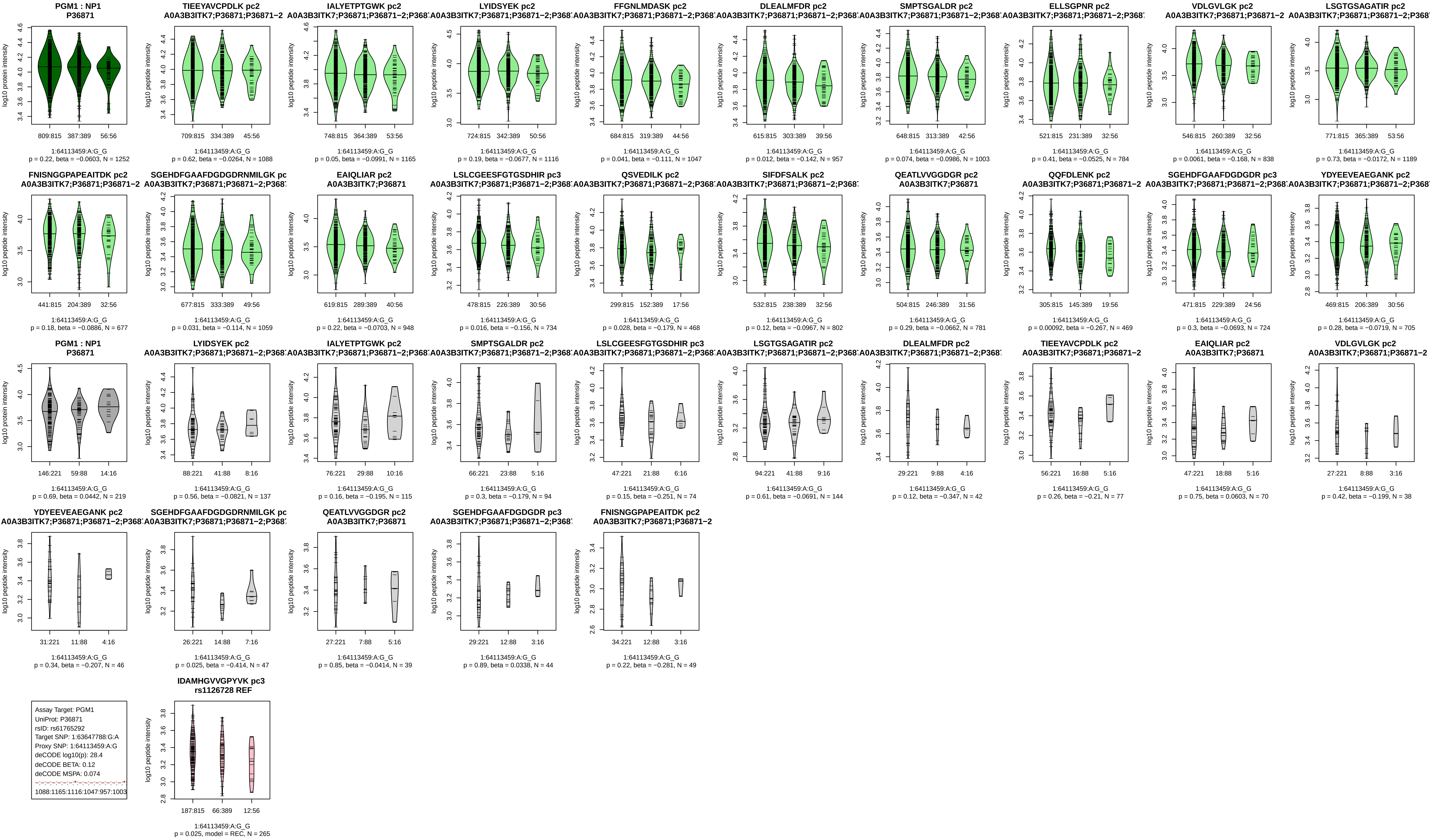
PGM1 : NP1
P36871
809:815 387:389 56:56
1:64113459:A:G_G
p = 0.22, beta = −0.0603, N = 1252
TIEEYAVCPDLK pc2
A0A3B3ITK7;P36871;P36871−2
709:815 334:389 45:56
1:64113459:A:G_G
p = 0.62, beta = −0.0264, N = 1088
IALYETPTGWK pc2
A0A3B3ITK7;P36871;P36871−2;P368
748:815 364:389 53:56
1:64113459:A:G_G
p = 0.05, beta = −0.0991, N = 1165
LYIDSYEK pc2
A0A3B3ITK7;P36871;P36871−2;P368
724:815 342:389 50:56
1:64113459:A:G_G
p = 0.19, beta = −0.0677, N = 1116
FFGNLMDASK pc2
A0A3B3ITK7;P36871;P36871−2;P368
684:815 319:389 44:56
1:64113459:A:G_G
p = 0.041, beta = −0.111, N = 1047
DLEALMFDR pc2
A0A3B3ITK7;P36871;P36871−2;P368
615:815 303:389 39:56
1:64113459:A:G_G
p = 0.012, beta = −0.142, N = 957
SMPTSGALDR pc2
A0A3B3ITK7;P36871;P36871−2;P368
648:815 313:389 42:56
1:64113459:A:G_G
p = 0.074, beta = −0.0986, N = 1003
ELLSGPNR pc2
A0A3B3ITK7;P36871;P36871−2;P368
521:815 231:389 32:56
1:64113459:A:G_G
p = 0.41, beta = −0.0525, N = 784
VDLGVLGK pc2
A0A3B3ITK7;P36871;P36871−2
546:815 260:389 32:56
1:64113459:A:G_G
p = 0.0061, beta = −0.168, N = 838
LSGTGSAGATIR pc2
A0A3B3ITK7;P36871;P36871−2;P368
771:815 365:389 53:56
1:64113459:A:G_G
p = 0.73, beta = −0.0172, N = 1189
FNISNGGPAPEAITDK pc2
A0A3B3ITK7;P36871;P36871−2
441:815 204:389 32:56
1:64113459:A:G_G
p = 0.18, beta = −0.0886, N = 677
SGEHDFGAAFDGDGDRNMILGK pc
A0A3B3ITK7;P36871;P36871−2;P368
677:815 333:389 49:56
1:64113459:A:G_G
p = 0.031, beta = −0.114, N = 1059
EAIQLIAR pc2
A0A3B3ITK7;P36871
619:815 289:389 40:56
1:64113459:A:G_G
p = 0.22, beta = −0.0703, N = 948
LSLCGEESFGTGSDHIR pc3
A0A3B3ITK7;P36871;P36871−2;P368
478:815 226:389 30:56
1:64113459:A:G_G
p = 0.016, beta = −0.156, N = 734
QSVEDILK pc2
A0A3B3ITK7;P36871;P36871−2;P368
299:815 152:389 17:56
1:64113459:A:G_G
p = 0.028, beta = −0.179, N = 468
SIFDFSALK pc2
A0A3B3ITK7;P36871;P36871−2;P368
532:815 238:389 32:56
1:64113459:A:G_G
p = 0.12, beta = −0.0967, N = 802
QEATLVVGGDGR pc2
A0A3B3ITK7;P36871
504:815 246:389 31:56
1:64113459:A:G_G
p = 0.29, beta = −0.0662, N = 781
QQFDLENK pc2
A0A3B3ITK7;P36871;P36871−2
305:815 145:389 19:56
1:64113459:A:G_G
p = 0.00092, beta = −0.267, N = 469
SGEHDFGAAFDGDGDR pc3
A0A3B3ITK7;P36871;P36871−2;P368
471:815 229:389 24:56
1:64113459:A:G_G
p = 0.3, beta = −0.0693, N = 724
YDYEEVEAEGANK pc2
A0A3B3ITK7;P36871;P36871−2;P368
469:815 206:389 30:56
1:64113459:A:G_G
p = 0.28, beta = −0.0719, N = 705
PGM1 : NP1
P36871
146:221 59:88 14:16
1:64113459:A:G_G
p = 0.69, beta = 0.0442, N = 219
LYIDSYEK pc2
A0A3B3ITK7;P36871;P36871−2;P368
88:221 41:88 8:16
1:64113459:A:G_G
p = 0.56, beta = −0.0821, N = 137
IALYETPTGWK pc2
A0A3B3ITK7;P36871;P36871−2;P368
76:221 29:88 10:16
1:64113459:A:G_G
p = 0.16, beta = −0.195, N = 115
SMPTSGALDR pc2
A0A3B3ITK7;P36871;P36871−2;P368
66:221 23:88 5:16
1:64113459:A:G_G
p = 0.3, beta = −0.179, N = 94
LSLCGEESFGTGSDHIR pc3
A0A3B3ITK7;P36871;P36871−2;P368
47:221 21:88 6:16
1:64113459:A:G_G
p = 0.15, beta = −0.251, N = 74
LSGTGSAGATIR pc2
A0A3B3ITK7;P36871;P36871−2;P368
94:221 41:88 9:16
1:64113459:A:G_G
p = 0.61, beta = −0.0691, N = 144
DLEALMFDR pc2
A0A3B3ITK7;P36871;P36871−2;P368
29:221 9:88 4:16
1:64113459:A:G_G
p = 0.12, beta = −0.347, N = 42
TIEEYAVCPDLK pc2
A0A3B3ITK7;P36871;P36871−2
56:221 16:88 5:16
1:64113459:A:G_G
p = 0.26, beta = −0.21, N = 77
EAIQLIAR pc2
A0A3B3ITK7;P36871
47:221 18:88 5:16
1:64113459:A:G_G
p = 0.75, beta = 0.0603, N = 70
VDLGVLGK pc2
A0A3B3ITK7;P36871;P36871−2
27:221 8:88 3:16
1:64113459:A:G_G
p = 0.42, beta = −0.199, N = 38
YDYEEVEAEGANK pc2
A0A3B3ITK7;P36871;P36871−2;P368
31:221 11:88 4:16
1:64113459:A:G_G
p = 0.34, beta = −0.207, N = 46
SGEHDFGAAFDGDGDRNMILGK pc
A0A3B3ITK7;P36871;P36871−2;P368
26:221 14:88 7:16
1:64113459:A:G_G
p = 0.025, beta = −0.414, N = 47
QEATLVVGGDGR pc2
A0A3B3ITK7;P36871
27:221 7:88 5:16
1:64113459:A:G_G
p = 0.85, beta = −0.0414, N = 39
SGEHDFGAAFDGDGDR pc3
A0A3B3ITK7;P36871;P36871−2;P368
29:221 12:88 3:16
1:64113459:A:G_G
p = 0.89, beta = 0.0338, N = 44
FNISNGGPAPEAITDK pc2
A0A3B3ITK7;P36871;P36871−2
34:221 12:88 3:16
1:64113459:A:G_G
p = 0.22, beta = −0.281, N = 49
Assay Target: PGM1
UniProt: P36871
rsID: rs61765292
Target SNP: 1:63647788:G:A
Proxy SNP: 1:64113459:A:G
deCODE log10(p): 28.4
deCODE BETA: 0.12
deCODE MSPA: 0.074
1088:1165:1116:1047:957:1003
IDAMHGVVGPYVK pc3
rs1126728 REF
187:815 66:389 12:56
1:64113459:A:G_G
p = 0.025, model = REC, N = 265

252. STIM1 (Q13586)
deCODE MSPA=0.029

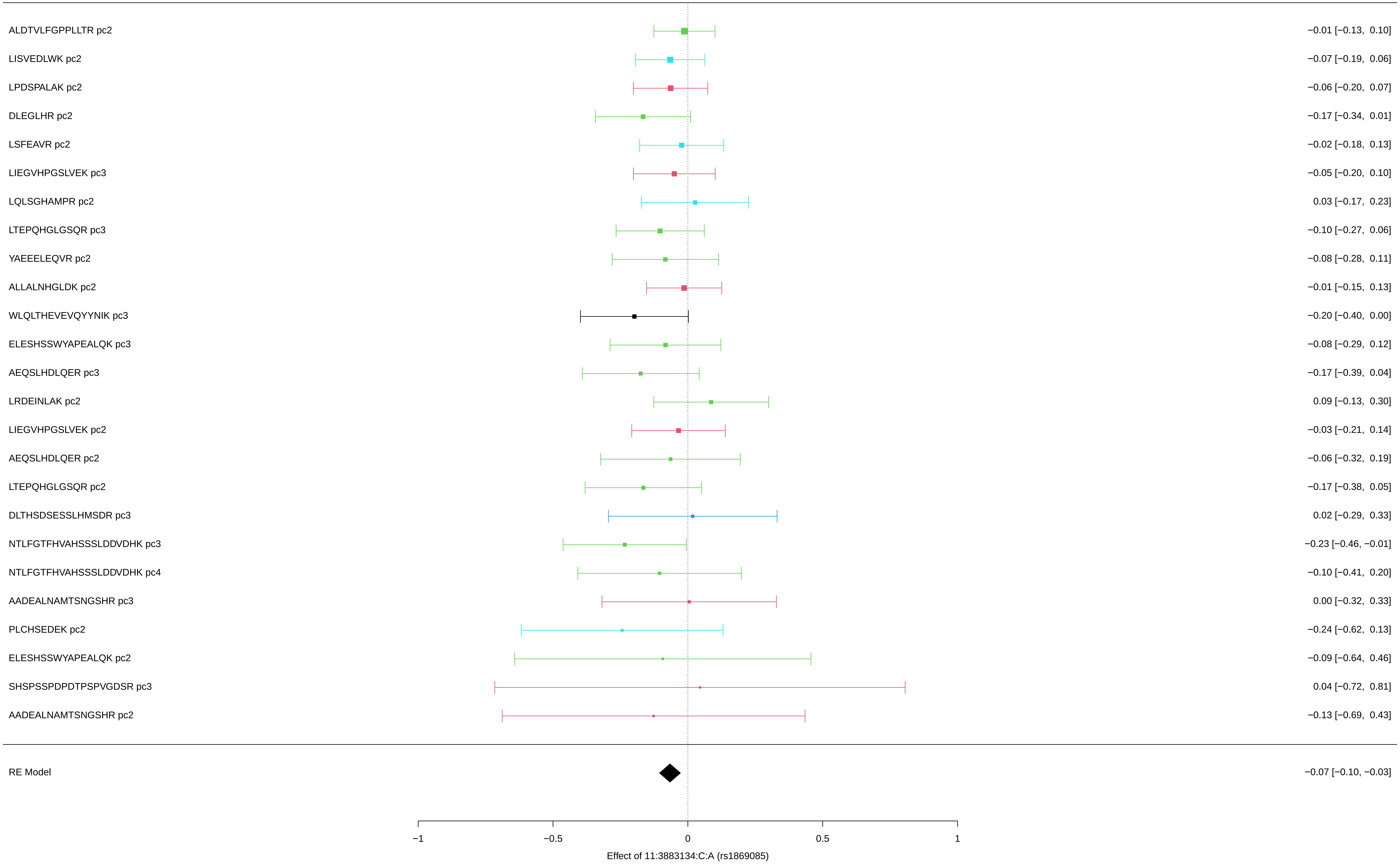

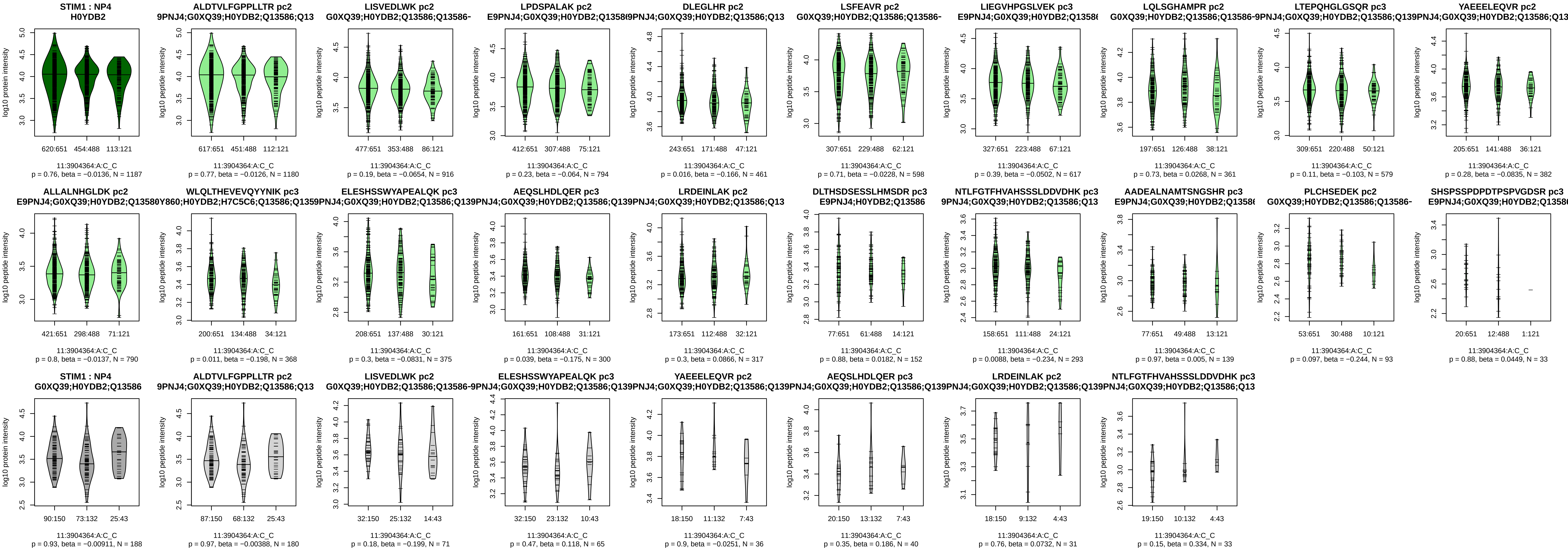

Assay Target: STIM1
UniProt: Q13586
rsID: rs1869085
Target SNP: 11:3883134:C:A
Proxy SNP: 11:3904364:A:C
deCODE log10(p): 28.3
deCODE BETA: −0.1
deCODE MSPA: 0.029
1180:916:794:461:598:617:361:

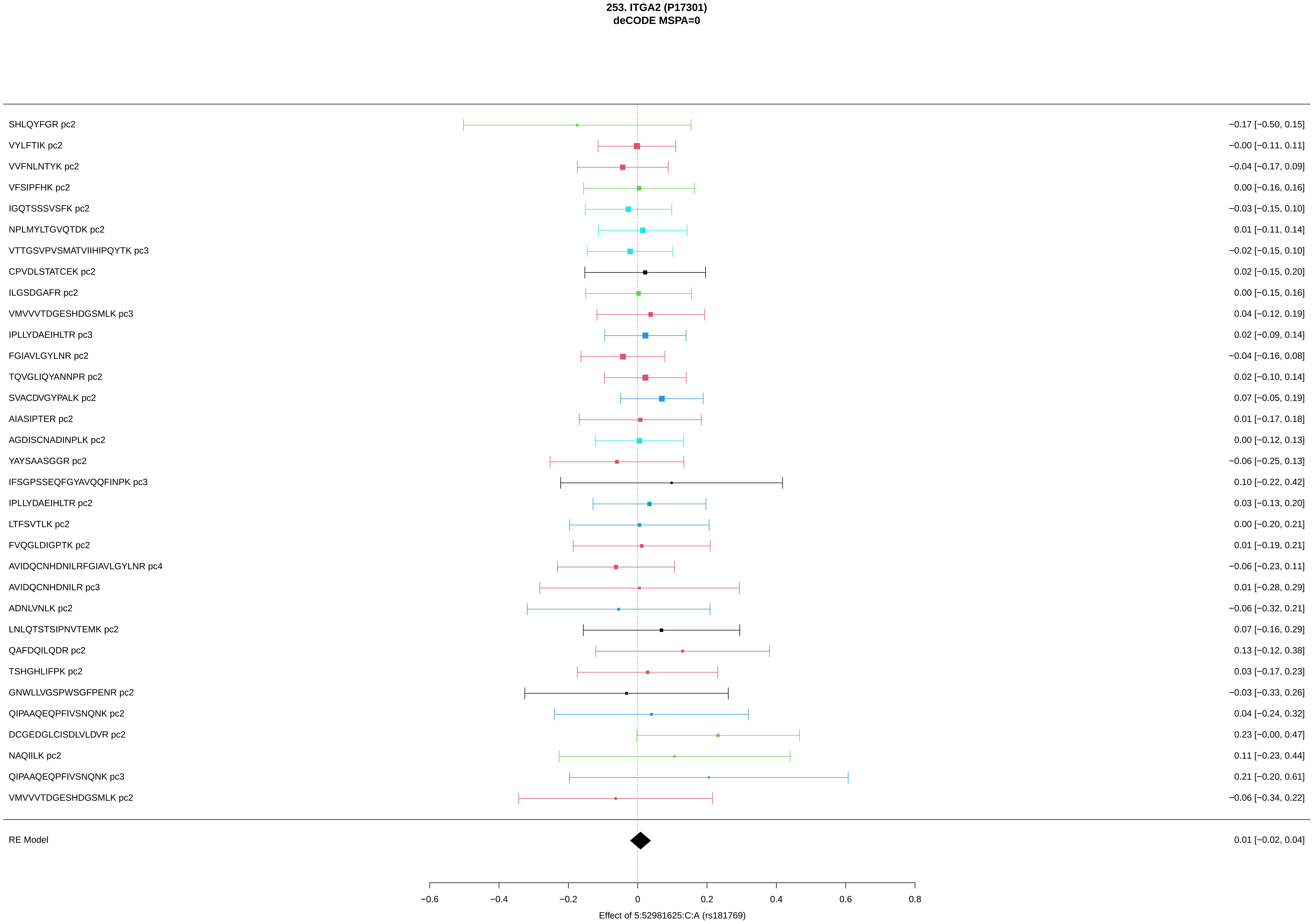

253. ITGA2 (P17301)
deCODE MSPA=0
SHLQYFGR pc2 −0.17 [−0.50, 0.15]
VYLFTIK pc2 −0.00 [−0.11, 0.11]
VVFNLNTYK pc2 −0.04 [−0.17, 0.09]
VFSIPFHK pc2 0.00 [−0.16, 0.16]
IGQTSSSVSFK pc2 −0.03 [−0.15, 0.10]
NPLMYLTGVQTDK pc2 0.01 [−0.11, 0.14]
VTTGSVPVSMATVIIHIPQYTK pc3 −0.02 [−0.15, 0.10]
CPVDLSTATCEK pc2 0.02 [−0.15, 0.20]
ILGSDGAFR pc2 0.00 [−0.15, 0.16]
VMVVVTDGESHDGSMLK pc3 0.04 [−0.12, 0.19]
IPLLYDAEIHLTR pc3 0.02 [−0.09, 0.14]
FGIAVLGYLNR pc2 −0.04 [−0.16, 0.08]
TQVGLIQYANNPR pc2 0.02 [−0.10, 0.14]
SVACDVGYPALK pc2 0.07 [−0.05, 0.19]
AIASIPTER pc2 0.01 [−0.17, 0.18]
AGDISCNADINPLK pc2 0.00 [−0.12, 0.13]
YAYSAASGGR pc2 −0.06 [−0.25, 0.13]
IFSGPSSEQFGYAVQQFINPK pc3 0.10 [−0.22, 0.42]
IPLLYDAEIHLTR pc2 0.03 [−0.13, 0.20]
LTFSVTLK pc2 0.00 [−0.20, 0.21]
FVQGLDIGPTK pc2 0.01 [−0.19, 0.21]
AVIDQCNHDNILRFGIAVLGYLNR pc4 −0.06 [−0.23, 0.11]
AVIDQCNHDNILR pc3 0.01 [−0.28, 0.29]
ADNLVNLK pc2 −0.06 [−0.32, 0.21]
LNLQTSTSIPNVTEMK pc2 0.07 [−0.16, 0.29]
QAFDQILQDR pc2 0.13 [−0.12, 0.38]
TSHGHLIFPK pc2 0.03 [−0.17, 0.23]
GNWLLVGSPWSGFPENR pc2 −0.03 [−0.33, 0.26]
QIPAAQEQPFIVSNQNK pc2 0.04 [−0.24, 0.32]
DCGEDGLCISDLVLDVR pc2 0.23 [−0.00, 0.47]
NAQIILK pc2 0.11 [−0.23, 0.44]
QIPAAQEQPFIVSNQNK pc3 0.21 [−0.20, 0.61]
VMVVVTDGESHDGSMLK pc2 −0.06 [−0.34, 0.22]
RE Model 0.01 [−0.02, 0.04]
−0.6
−0.4
−0.2
0
0.2
0.4
0.6
0.8
Effect of 5:52981625:C:A (rs181769)

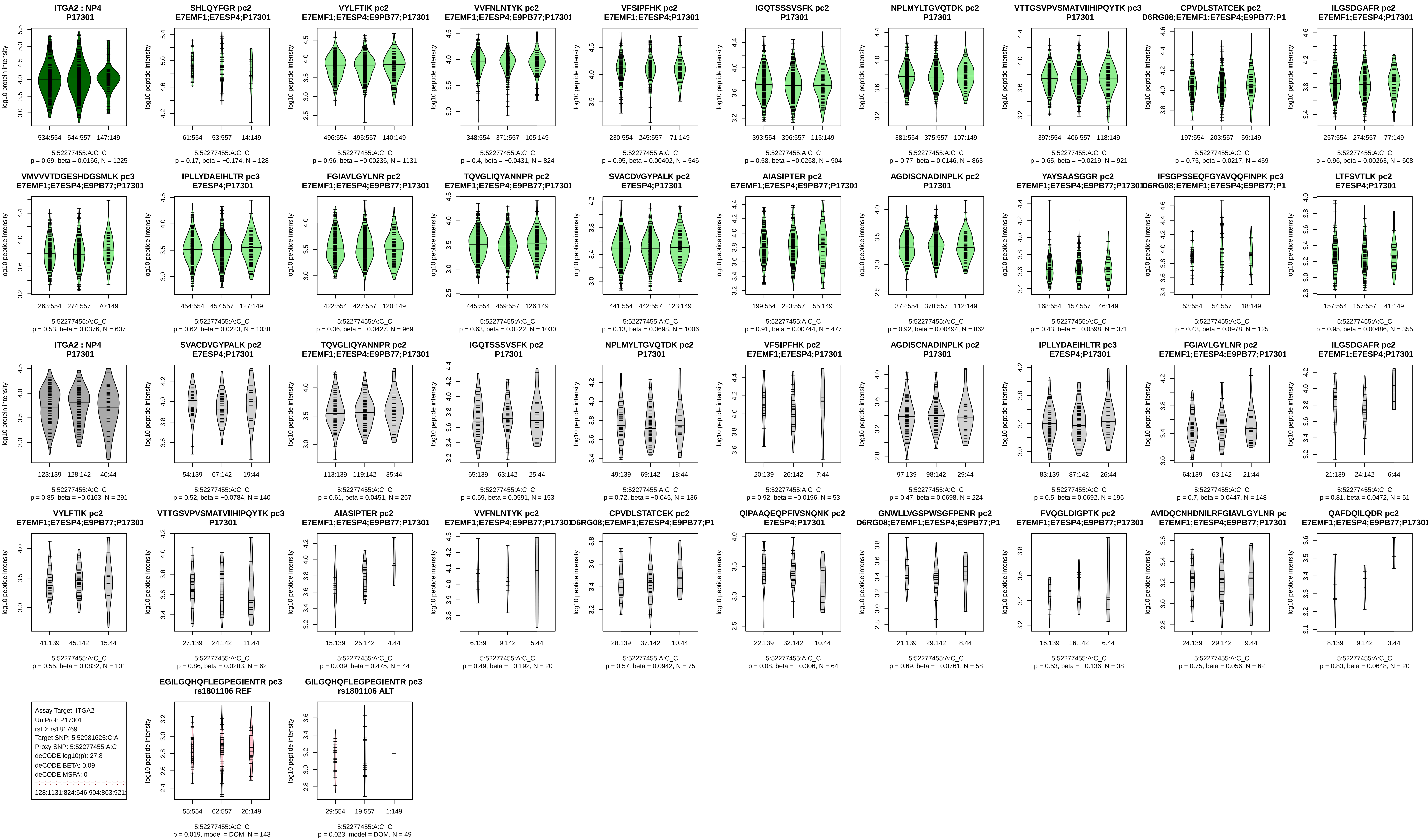
ITGA2 : NP4 P17301
p = 0.69, beta = 0.0166, N = 1225
SHLQYFGR pc2 E7EMF1;E7ESP4;P17301
p = 0.17, beta = −0.174, N = 128
VYLFTIK pc2 E7EMF1;E7ESP4;E9PB77;P17301
p = 0.96, beta = −0.00236, N = 1131
VVFNLNTYK pc2 E7EMF1;E7ESP4;E9PB77;P17301
p = 0.4, beta = −0.0431, N = 824
VFSIPFHK pc2 E7EMF1;E7ESP4;P17301
p = 0.95, beta = 0.00402, N = 546
IGQTSSSVSFK pc2 P17301
p = 0.58, beta = −0.0268, N = 904
NPLMYLTGVQTDK pc2 P17301
p = 0.77, beta = 0.0146, N = 863
VTTGSVPVSMATVIIHIPQYTK pc3 P17301
p = 0.65, beta = −0.0219, N = 921
CPVDLSTATCEK pc2 D6RG08;E7EMF1;E7ESP4;E9PB77;P1
p = 0.75, beta = 0.0217, N = 459
ILGSDGAFR pc2 E7EMF1;E7ESP4;P17301
p = 0.96, beta = 0.00263, N = 608
VMVVVTDGESHDGSMLK pc3 E7EMF1;E7ESP4;E9PB77;P17301
p = 0.53, beta = 0.0376, N = 607
IPLLYDAEIHLTR pc3 E7ESP4;P17301
p = 0.62, beta = 0.0223, N = 1038
FGIAVLGYLNR pc2 E7EMF1;E7ESP4;E9PB77;P17301
p = 0.36, beta = −0.0427, N = 969
TQVGLIQYANNPR pc2 E7EMF1;E7ESP4;E9PB77;P17301
p = 0.63, beta = 0.0222, N = 1030
SVACDVGYPALK pc2 E7ESP4;P17301
p = 0.13, beta = 0.0698, N = 1006
AIASIPTER pc2 E7EMF1;E7ESP4;E9PB77;P17301
p = 0.91, beta = 0.00744, N = 477
AGDISCNADINPLK pc2 P17301
p = 0.92, beta = 0.00494, N = 862
YAYSAASGGR pc2 E7EMF1;E7ESP4;E9PB77;P17301
p = 0.43, beta = −0.0598, N = 371
IFSGPSSEQFGYAVQQFINPK pc3 D6RG08;E7EMF1;E7ESP4;E9PB77;P1
p = 0.43, beta = 0.0978, N = 125
LTFSVTLK pc2 E7ESP4;P17301
p = 0.95, beta = 0.00486, N = 355
ITGA2 : NP4 P17301
p = 0.85, beta = −0.0163, N = 291
SVACDVGYPALK pc2 E7ESP4;P17301
p = 0.52, beta = −0.0784, N = 140
TQVGLIQYANNPR pc2 E7EMF1;E7ESP4;E9PB77;P17301
p = 0.61, beta = 0.0451, N = 267
IGQTSSSVSFK pc2 P17301
p = 0.59, beta = 0.0591, N = 153
NPLMYLTGVQTDK pc2 P17301
p = 0.72, beta = −0.045, N = 136
VFSIPFHK pc2 E7EMF1;E7ESP4;P17301
p = 0.92, beta = −0.0196, N = 53
AGDISCNADINPLK pc2 P17301
p = 0.47, beta = 0.0698, N = 224
IPLLYDAEIHLTR pc3 E7ESP4;P17301
p = 0.5, beta = 0.0692, N = 196
FGIAVLGYLNR pc2 E7EMF1;E7ESP4;E9PB77;P17301
p = 0.7, beta = 0.0447, N = 148
ILGSDGAFR pc2 E7EMF1;E7ESP4;P17301
p = 0.81, beta = 0.0472, N = 51
VYLFTIK pc2 E7EMF1;E7ESP4;E9PB77;P17301
p = 0.55, beta = 0.0832, N = 101
VTTGSVPVSMATVIIHIPQYTK pc3 P17301
p = 0.86, beta = 0.0283, N = 62
AIASIPTER pc2 E7EMF1;E7ESP4;E9PB77;P17301
p = 0.039, beta = 0.475, N = 44
VVFNLNTYK pc2 E7EMF1;E7ESP4;E9PB77;P17301
p = 0.49, beta = −0.192, N = 20
CPVDLSTATCEK pc2 D6RG08;E7EMF1;E7ESP4;E9PB77;P1
p = 0.57, beta = 0.0942, N = 75
QIPAAQEQPFIVSNQNK pc2 E7ESP4;P17301
p = 0.08, beta = −0.306, N = 64
GNWLLVGSPWSGFPENR pc2 D6RG08;E7EMF1;E7ESP4;E9PB77;P1
p = 0.69, beta = −0.0761, N = 58
FVQGLDIGPTK pc2 E7EMF1;E7ESP4;E9PB77;P17301
p = 0.53, beta = −0.136, N = 38
AVIDQCNHDNILRFGIAVLGYLNR pc E7EMF1;E7ESP4;E9PB77;P17301
p = 0.75, beta = 0.056, N = 62
QAFDQILQDR pc2 E7EMF1;E7ESP4;E9PB77;P17301
p = 0.83, beta = 0.0648, N = 20
5:52277455:A:C_C
Assay Target: ITGA2
UniProt: P17301
rsID: rs181769
Target SNP: 5:52981625:C:A
Proxy SNP: 5:52277455:A:C
deCODE log10(p): 27.8
deCODE BETA: 0.09
deCODE MSPA: 0
128:1131:824:546:904:863:921:
EGILGQHQFLEGPEGIENTR pc3 rs1801106 REF
p = 0.019, model = DOM, N = 143
GILGQHQFLEGPEGIENTR pc3 rs1801106 ALT
p = 0.023, model = DOM, N = 49
log10 protein intensity
log10 peptide intensity

254. C3 (P01024)
deCODE MSPA=0.699 (1 conflicts)

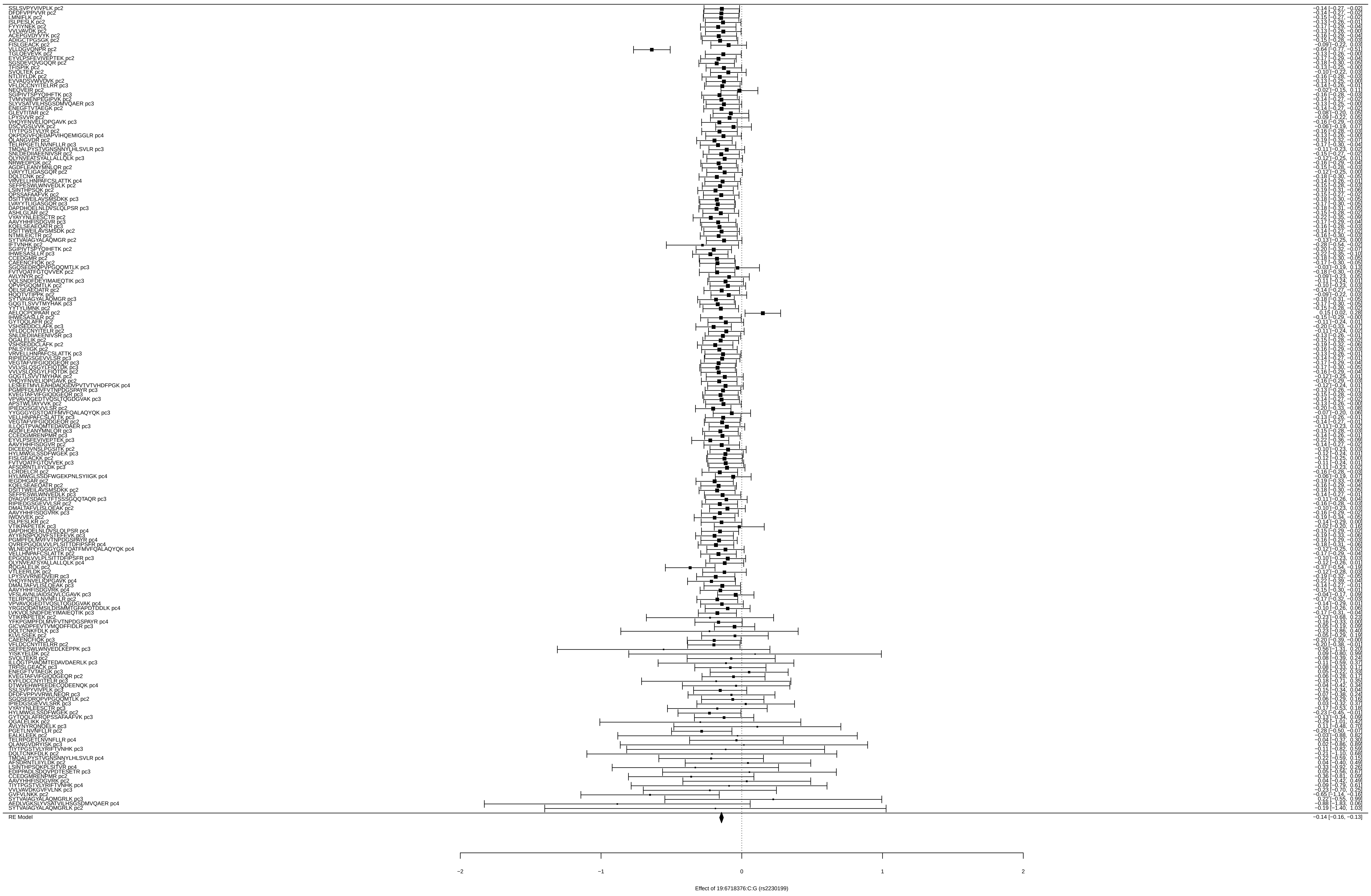

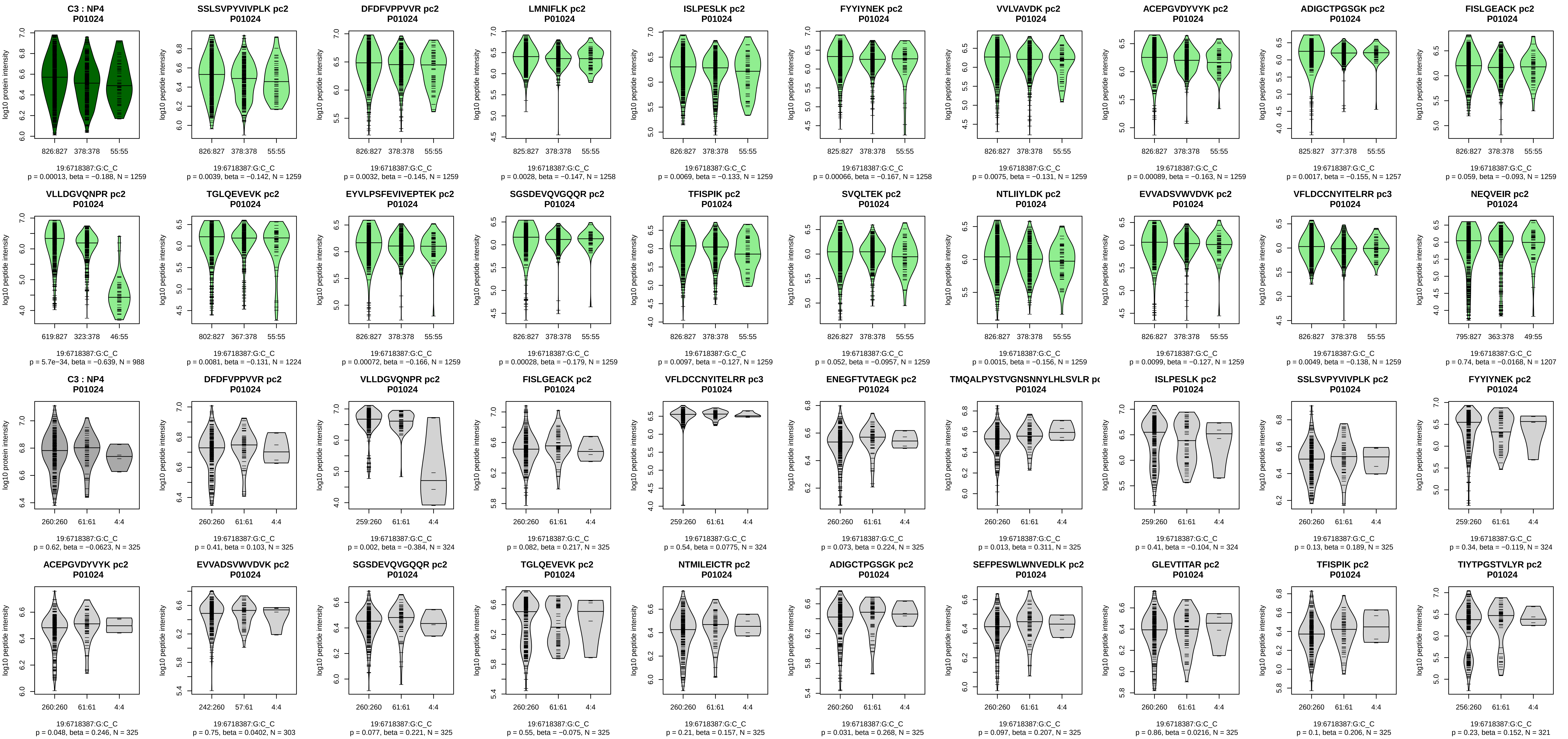

Assay Target: C3
UniProt: P01024
rsID: rs2230199
Target SNP: 19:6718376:C:G
Proxy SNP: 19:6718387:G:C
deCODE log10(p): 27.4
deCODE BETA: 0.1
deCODE MSPA: 0.699 (1)
[illegible]
1259:1259:1258:1259:1258:125

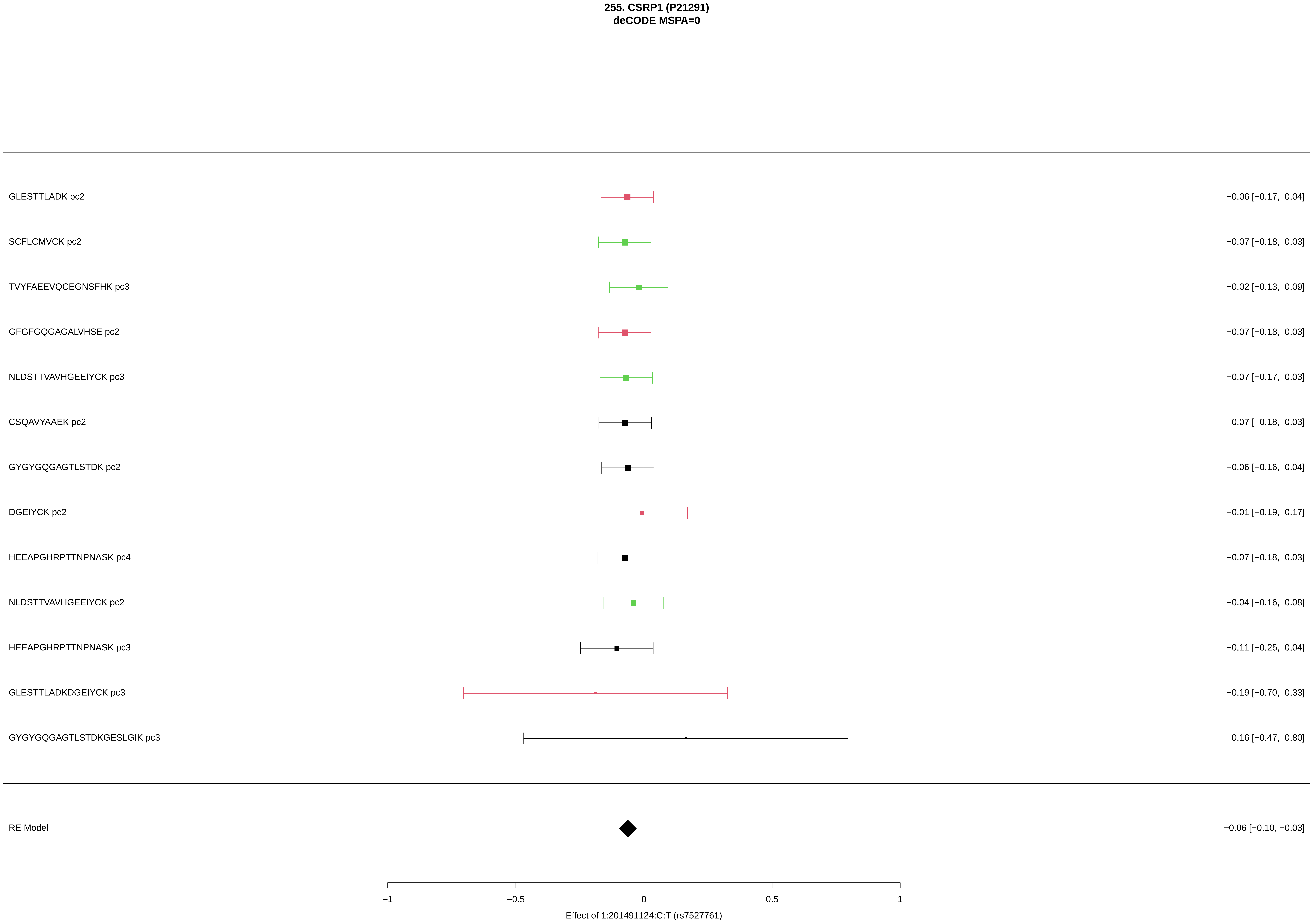
255. CSRP1 (P21291)
deCODE MSPA=0
GLESTTLADK pc2
−0.06 [−0.17, 0.04]
SCFLCMVCK pc2
−0.07 [−0.18, 0.03]
TVYFAEEVQCEGNSFHK pc3
−0.02 [−0.13, 0.09]
GFGFGQGAGALVHSE pc2
−0.07 [−0.18, 0.03]
NLDSTTVAVHGEEIYCK pc3
−0.07 [−0.17, 0.03]
CSQAVYAAEK pc2
−0.07 [−0.18, 0.03]
GYGYGQGAGTLSTDK pc2
−0.06 [−0.16, 0.04]
DGEIYCK pc2
−0.01 [−0.19, 0.17]
HEEAPGHRPTTNPNASK pc4
−0.07 [−0.18, 0.03]
NLDSTTVAVHGEEIYCK pc2
−0.04 [−0.16, 0.08]
HEEAPGHRPTTNPNASK pc3
−0.11 [−0.25, 0.04]
GLESTTLADKDGEIYCK pc3
−0.19 [−0.70, 0.33]
GYGYGQGAGTLSTDKGESLGIK pc3
0.16 [−0.47, 0.80]
RE Model
−0.06 [−0.10, −0.03]
−1
−0.5
0
0.5
1
Effect of 1:201491124:C:T (rs7527761)

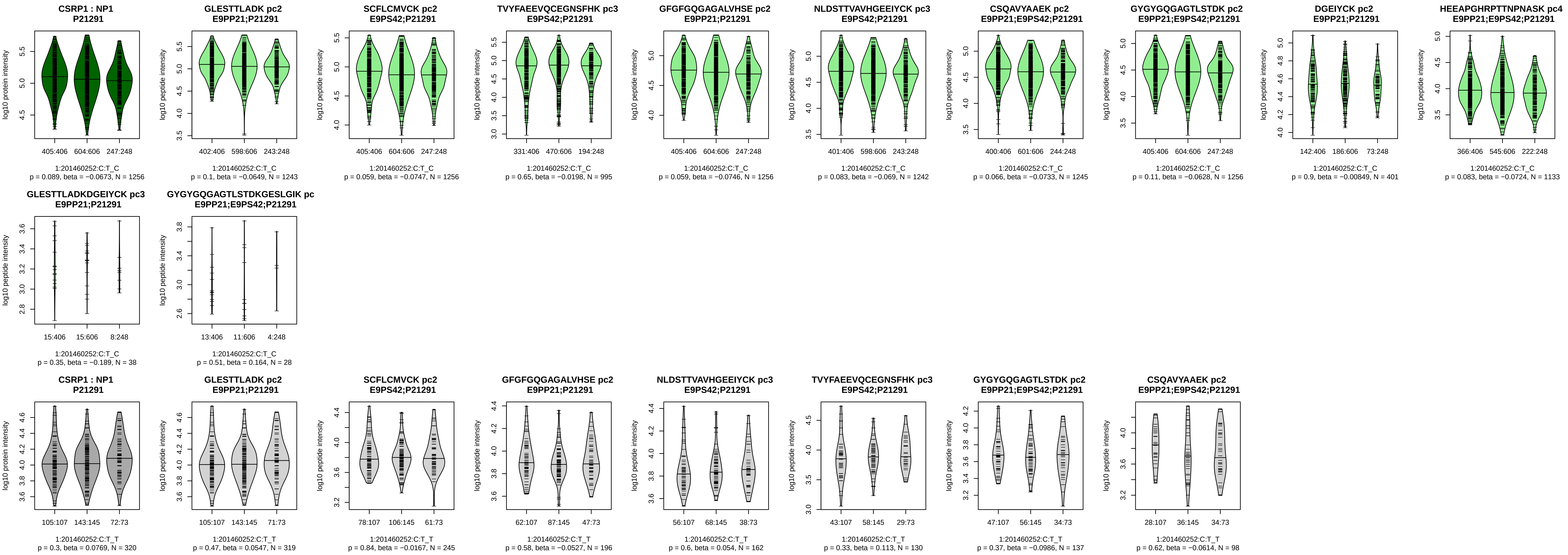

Assay Target: CSRP1
UniProt: P21291
rsID: rs7527761
Target SNP: 1:201491124:C:T
Proxy SNP: 1:201460252:C:T
deCODE log10(p): 26.9
deCODE BETA: −0.1
deCODE MSPA: 0
1243:1256:995:1256:1242:1245

256. PLA2G12B (Q9BX93)
deCODE MSPA=0

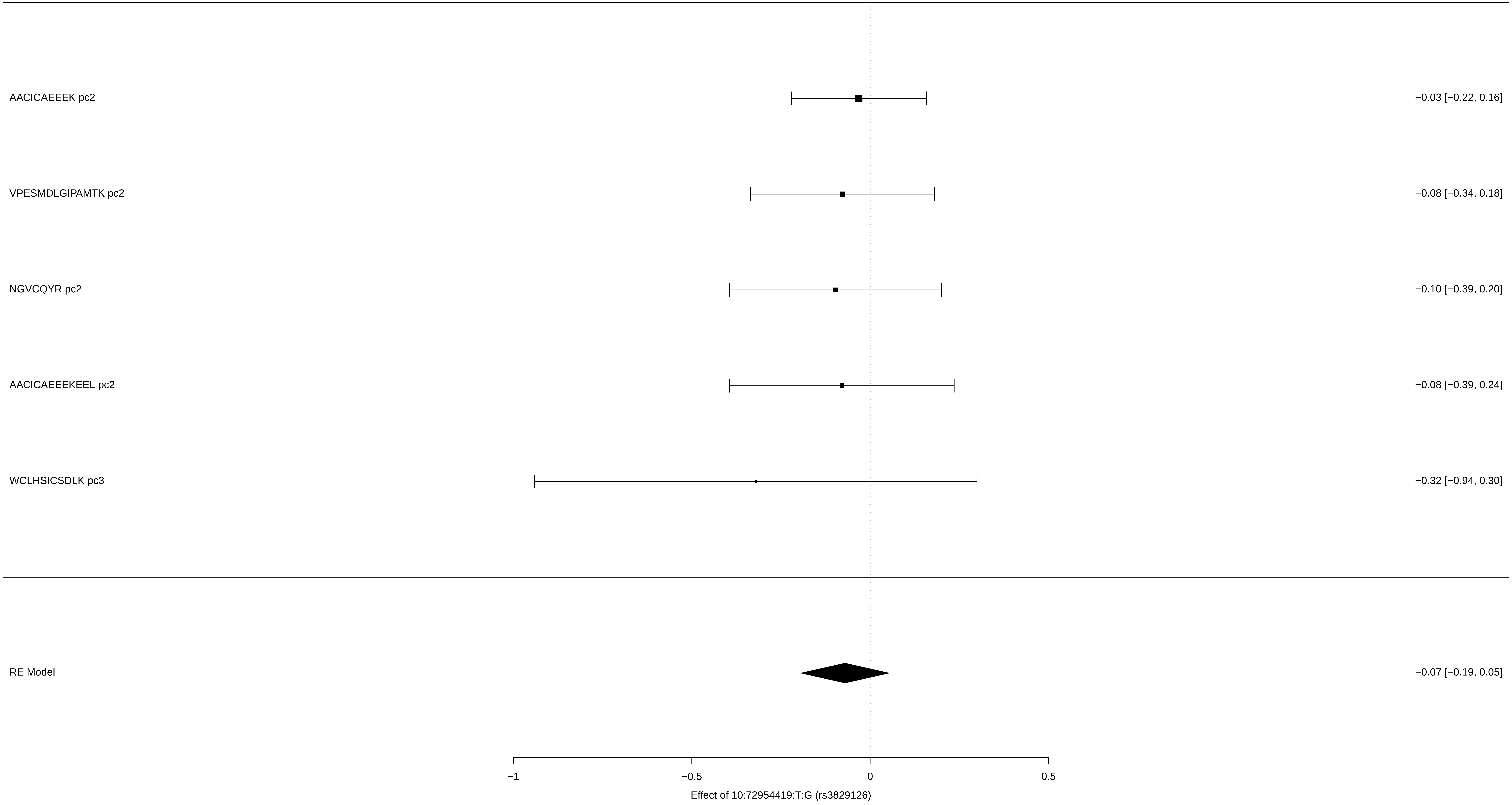

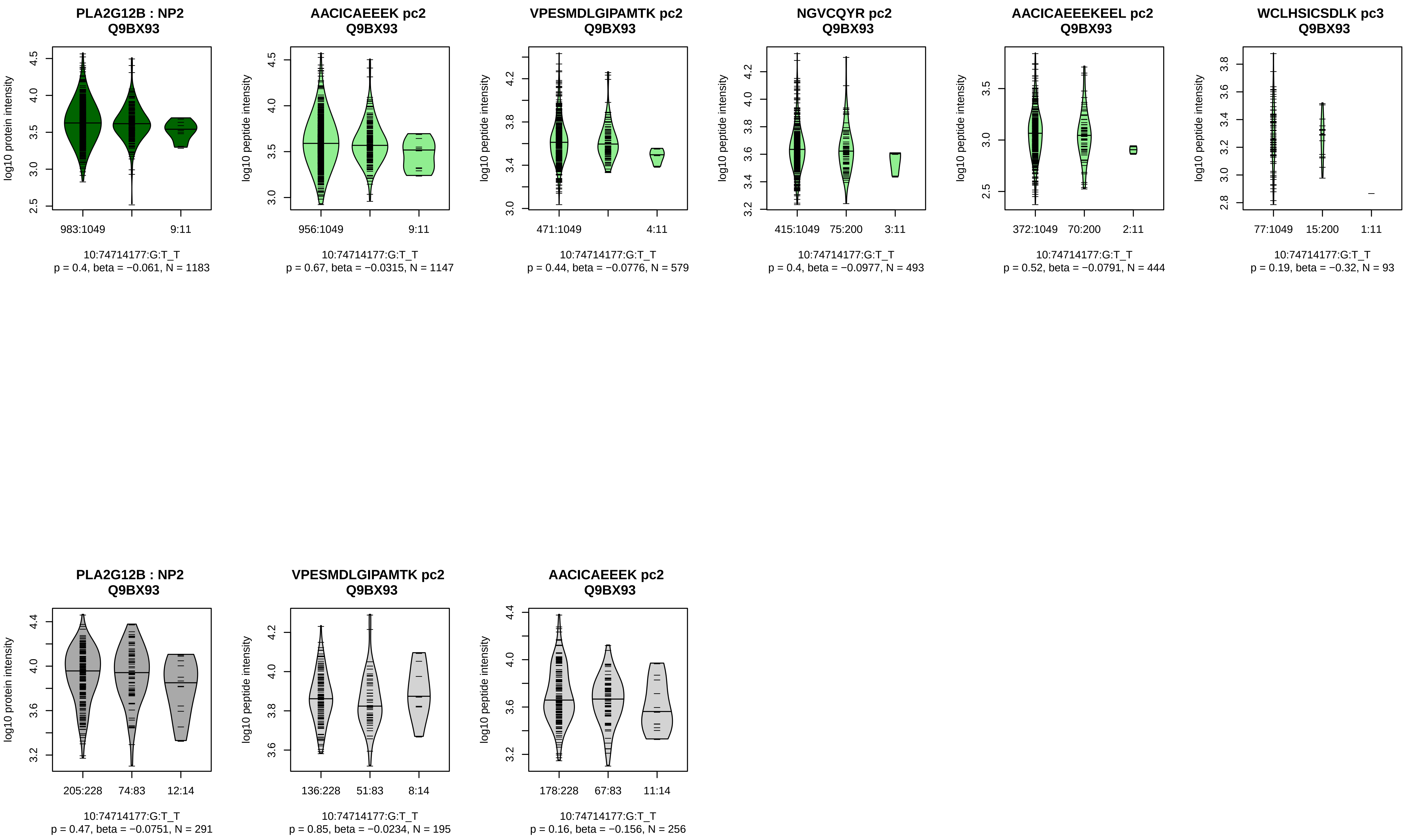

Assay Target: PLA2G12B
UniProt: Q9BX93
rsID: rs3829126
Target SNP: 10:72954419:T:G
Proxy SNP: 10:74714177:G:T
deCODE log10(p): 26.9
deCODE BETA: −0.15
deCODE MSPA: 0
–:–:–:–:–
1147:579:493:444:93

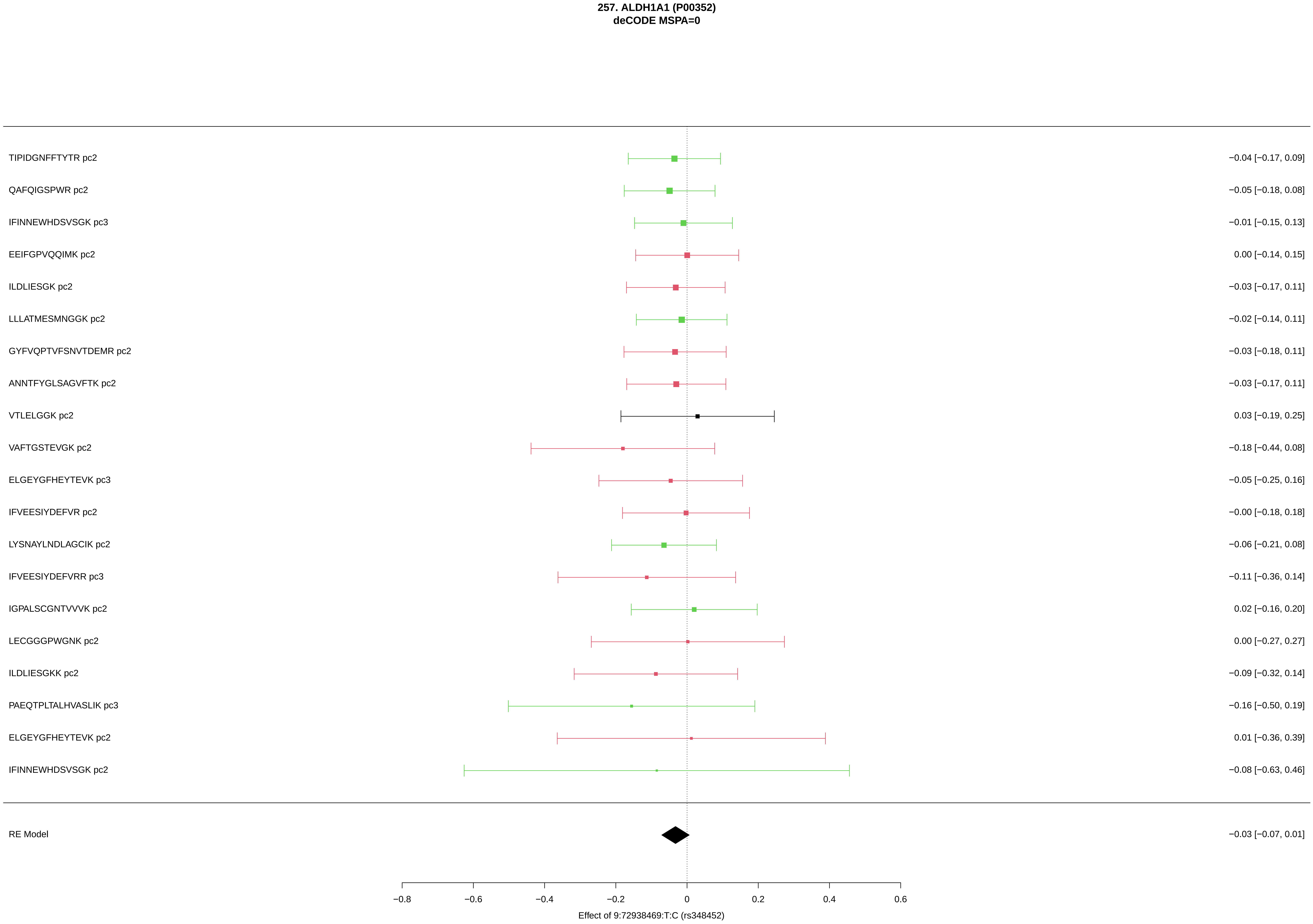

257. ALDH1A1 (P00352)
deCODE MSPA=0
TIPIDGNFFTYTR pc2
−0.04 [−0.17, 0.09]
QAFQIGSPWR pc2
−0.05 [−0.18, 0.08]
IFINNEWHDSVSGK pc3
−0.01 [−0.15, 0.13]
EEIFGPVQQIMK pc2
0.00 [−0.14, 0.15]
ILDLIESGK pc2
−0.03 [−0.17, 0.11]
LLLATMESMNGGK pc2
−0.02 [−0.14, 0.11]
GYFVQPTVFSNVTDEMR pc2
−0.03 [−0.18, 0.11]
ANNTFYGLSAGVFTK pc2
−0.03 [−0.17, 0.11]
VTLELGGK pc2
0.03 [−0.19, 0.25]
VAFTGSTEVGK pc2
−0.18 [−0.44, 0.08]
ELGEYGFHEYTEVK pc3
−0.05 [−0.25, 0.16]
IFVEESIYDEFVR pc2
−0.00 [−0.18, 0.18]
LYSNAYLNDLAGCIK pc2
−0.06 [−0.21, 0.08]
IFVEESIYDEFVRR pc3
−0.11 [−0.36, 0.14]
IGPALSCGNTVVVK pc2
0.02 [−0.16, 0.20]
LECGGGPWGNK pc2
0.00 [−0.27, 0.27]
ILDLIESGKK pc2
−0.09 [−0.32, 0.14]
PAEQTPLTALHVASLIK pc3
−0.16 [−0.50, 0.19]
ELGEYGFHEYTEVK pc2
0.01 [−0.36, 0.39]
IFINNEWHDSVSGK pc2
−0.08 [−0.63, 0.46]
RE Model
−0.03 [−0.07, 0.01]
−0.8
−0.6
−0.4
−0.2
0
0.2
0.4
0.6
Effect of 9:72938469:T:C (rs348452)

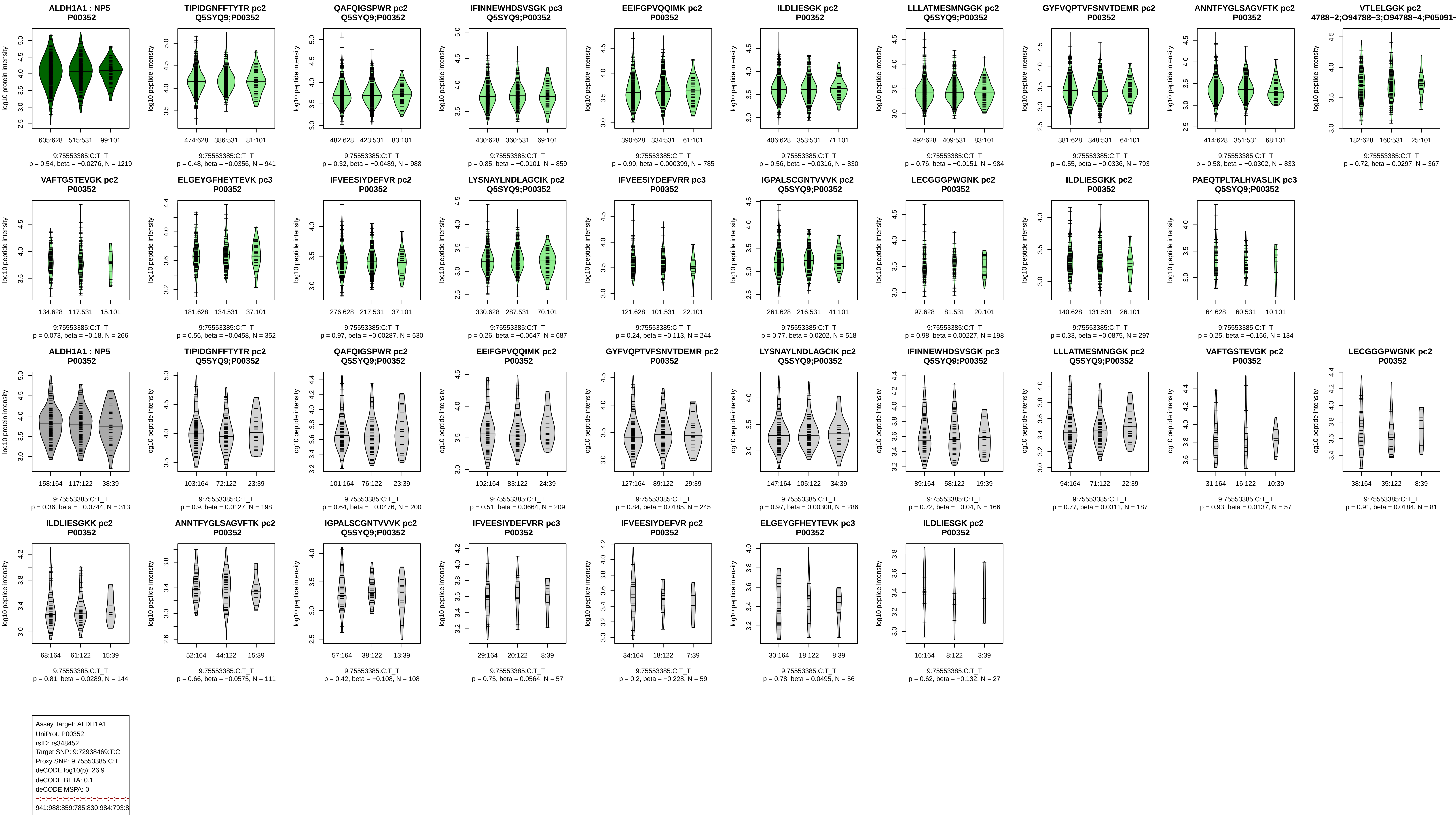
ALDH1A1 : NP5
P00352
log10 protein intensity
605:628 515:531 99:101
9:75553385:C:T_T
p = 0.54, beta = −0.0276, N = 1219
TIPIDGNFFTYTR pc2
Q5SYQ9;P00352
log10 peptide intensity
474:628 386:531 81:101
9:75553385:C:T_T
p = 0.48, beta = −0.0356, N = 941
QAFQIGSPWR pc2
Q5SYQ9;P00352
482:628 423:531 83:101
9:75553385:C:T_T
p = 0.32, beta = −0.0489, N = 988
IFINNEWHDSVSGK pc3
Q5SYQ9;P00352
430:628 360:531 69:101
9:75553385:C:T_T
p = 0.85, beta = −0.0101, N = 859
EEIFGPVQQIMK pc2
P00352
390:628 334:531 61:101
9:75553385:C:T_T
p = 0.99, beta = 0.000399, N = 785
ILDLIESGK pc2
P00352
406:628 353:531 71:101
9:75553385:C:T_T
p = 0.56, beta = −0.0316, N = 830
LLLATMESMNGGK pc2
Q5SYQ9;P00352
492:628 409:531 83:101
9:75553385:C:T_T
p = 0.76, beta = −0.0151, N = 984
GYFVQPTVFSNVTDEMR pc2
P00352
381:628 348:531 64:101
9:75553385:C:T_T
p = 0.55, beta = −0.0336, N = 793
ANNTFYGLSAGVFTK pc2
P00352
414:628 351:531 68:101
9:75553385:C:T_T
p = 0.58, beta = −0.0302, N = 833
VTLELGGK pc2
4788−2;O94788−3;O94788−4;P05091−
182:628 160:531 25:101
9:75553385:C:T_T
p = 0.72, beta = 0.0297, N = 367
VAFTGSTEVGK pc2
P00352
134:628 117:531 15:101
9:75553385:C:T_T
p = 0.073, beta = −0.18, N = 266
ELGEYGFHEYTEVK pc3
P00352
181:628 134:531 37:101
9:75553385:C:T_T
p = 0.56, beta = −0.0458, N = 352
IFVEESIYDEFVR pc2
P00352
276:628 217:531 37:101
9:75553385:C:T_T
p = 0.97, beta = −0.00287, N = 530
LYSNAYLNDLAGCIK pc2
Q5SYQ9;P00352
330:628 287:531 70:101
9:75553385:C:T_T
p = 0.26, beta = −0.0647, N = 687
IFVEESIYDEFVRR pc3
P00352
121:628 101:531 22:101
9:75553385:C:T_T
p = 0.24, beta = −0.113, N = 244
IGPALSCGNTVVVK pc2
Q5SYQ9;P00352
261:628 216:531 41:101
9:75553385:C:T_T
p = 0.77, beta = 0.0202, N = 518
LECGGGPWGNK pc2
P00352
97:628 81:531 20:101
9:75553385:C:T_T
p = 0.98, beta = 0.00227, N = 198
ILDLIESGKK pc2
P00352
140:628 131:531 26:101
9:75553385:C:T_T
p = 0.33, beta = −0.0875, N = 297
PAEQTPLTALHVASLIK pc3
Q5SYQ9;P00352
64:628 60:531 10:101
9:75553385:C:T_T
p = 0.25, beta = −0.156, N = 134
ALDH1A1 : NP5
P00352
158:164 117:122 38:39
9:75553385:C:T_T
p = 0.36, beta = −0.0744, N = 313
TIPIDGNFFTYTR pc2
Q5SYQ9;P00352
103:164 72:122 23:39
9:75553385:C:T_T
p = 0.9, beta = 0.0127, N = 198
QAFQIGSPWR pc2
Q5SYQ9;P00352
101:164 76:122 23:39
9:75553385:C:T_T
p = 0.64, beta = −0.0476, N = 200
EEIFGPVQQIMK pc2
P00352
102:164 83:122 24:39
9:75553385:C:T_T
p = 0.51, beta = 0.0664, N = 209
GYFVQPTVFSNVTDEMR pc2
P00352
127:164 89:122 29:39
9:75553385:C:T_T
p = 0.84, beta = 0.0185, N = 245
LYSNAYLNDLAGCIK pc2
Q5SYQ9;P00352
147:164 105:122 34:39
9:75553385:C:T_T
p = 0.97, beta = 0.00308, N = 286
IFINNEWHDSVSGK pc3
Q5SYQ9;P00352
89:164 58:122 19:39
9:75553385:C:T_T
p = 0.72, beta = −0.04, N = 166
LLLATMESMNGGK pc2
Q5SYQ9;P00352
94:164 71:122 22:39
9:75553385:C:T_T
p = 0.77, beta = 0.0311, N = 187
VAFTGSTEVGK pc2
P00352
31:164 16:122 10:39
9:75553385:C:T_T
p = 0.93, beta = 0.0137, N = 57
LECGGGPWGNK pc2
P00352
38:164 35:122 8:39
9:75553385:C:T_T
p = 0.91, beta = 0.0184, N = 81
ILDLIESGKK pc2
P00352
68:164 61:122 15:39
9:75553385:C:T_T
p = 0.81, beta = 0.0289, N = 144
ANNTFYGLSAGVFTK pc2
P00352
52:164 44:122 15:39
9:75553385:C:T_T
p = 0.66, beta = −0.0575, N = 111
IGPALSCGNTVVVK pc2
Q5SYQ9;P00352
57:164 38:122 13:39
9:75553385:C:T_T
p = 0.42, beta = −0.108, N = 108
IFVEESIYDEFVRR pc3
P00352
29:164 20:122 8:39
9:75553385:C:T_T
p = 0.75, beta = 0.0564, N = 57
IFVEESIYDEFVR pc2
P00352
34:164 18:122 7:39
9:75553385:C:T_T
p = 0.2, beta = −0.228, N = 59
ELGEYGFHEYTEVK pc3
P00352
30:164 18:122 8:39
9:75553385:C:T_T
p = 0.78, beta = 0.0495, N = 56
ILDLIESGK pc2
P00352
16:164 8:122 3:39
9:75553385:C:T_T
p = 0.62, beta = −0.132, N = 27
Assay Target: ALDH1A1
UniProt: P00352
rsID: rs348452
Target SNP: 9:72938469:T:C
Proxy SNP: 9:75553385:C:T
deCODE log10(p): 26.9
deCODE BETA: 0.1
deCODE MSPA: 0
941:988:859:785:830:984:793:8

258. S100A4 (P26447)
deCODE MSPA=0.745

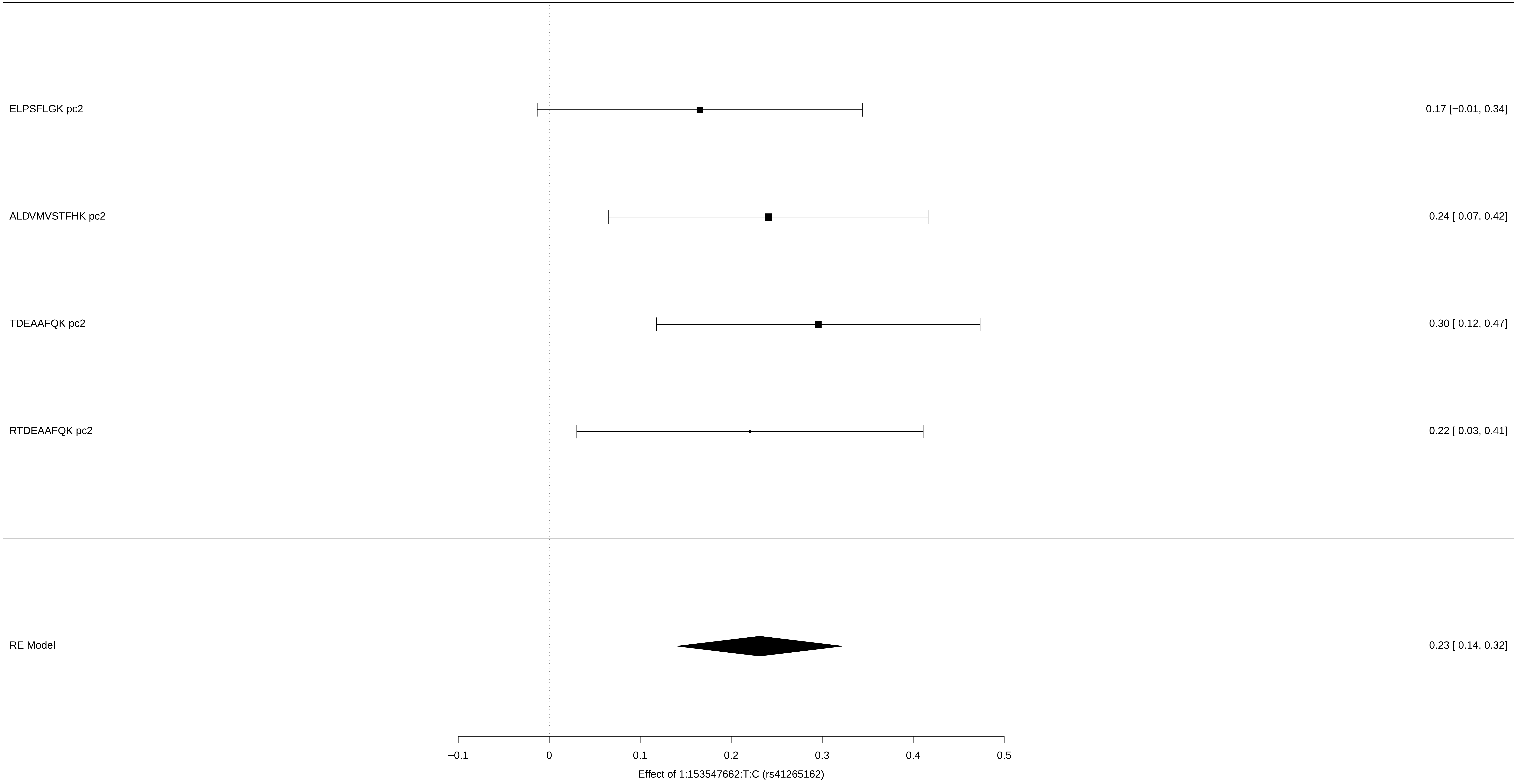

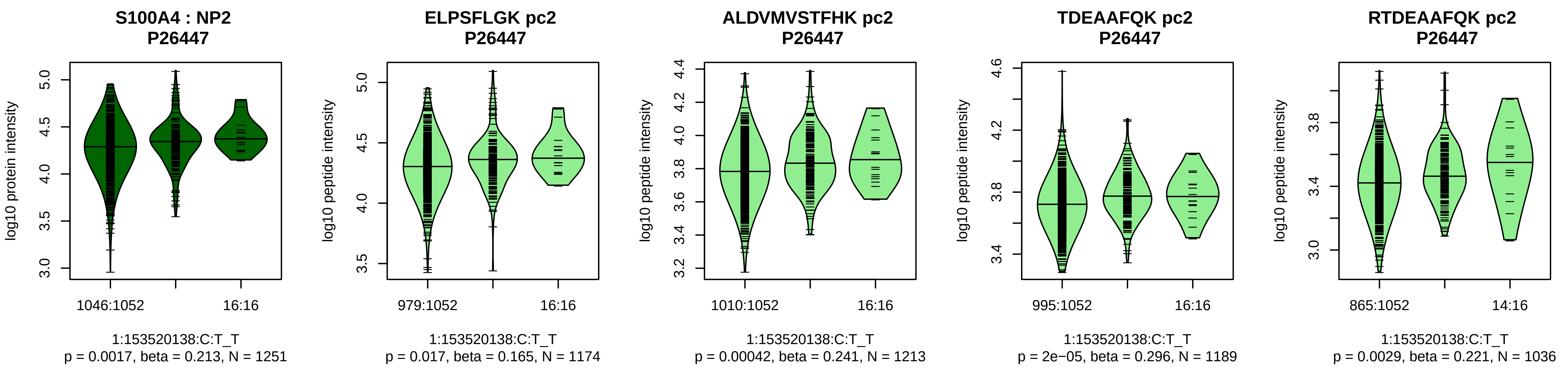

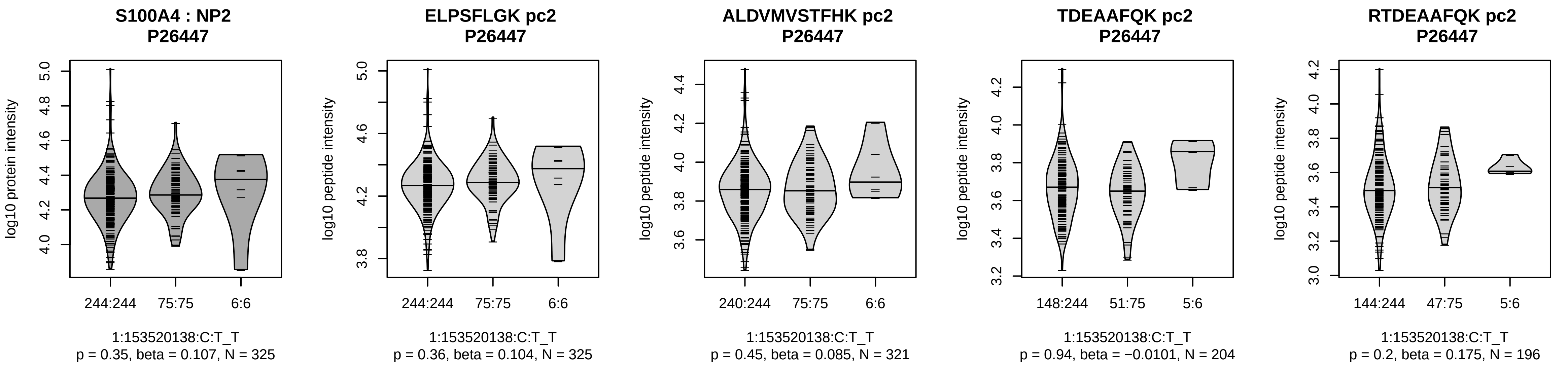

Assay Target: S100A4
UniProt: P26447
rsID: rs41265162
Target SNP: 1:153547662:T:C
Proxy SNP: 1:153520138:C:T
deCODE log10(p): 26.4
deCODE BETA: 0.21
deCODE MSPA: 0.745
−:*:*:*:NA
1174:1213:1189:1036:17

259. SYK (P43405)
deCODE MSPA=0.037

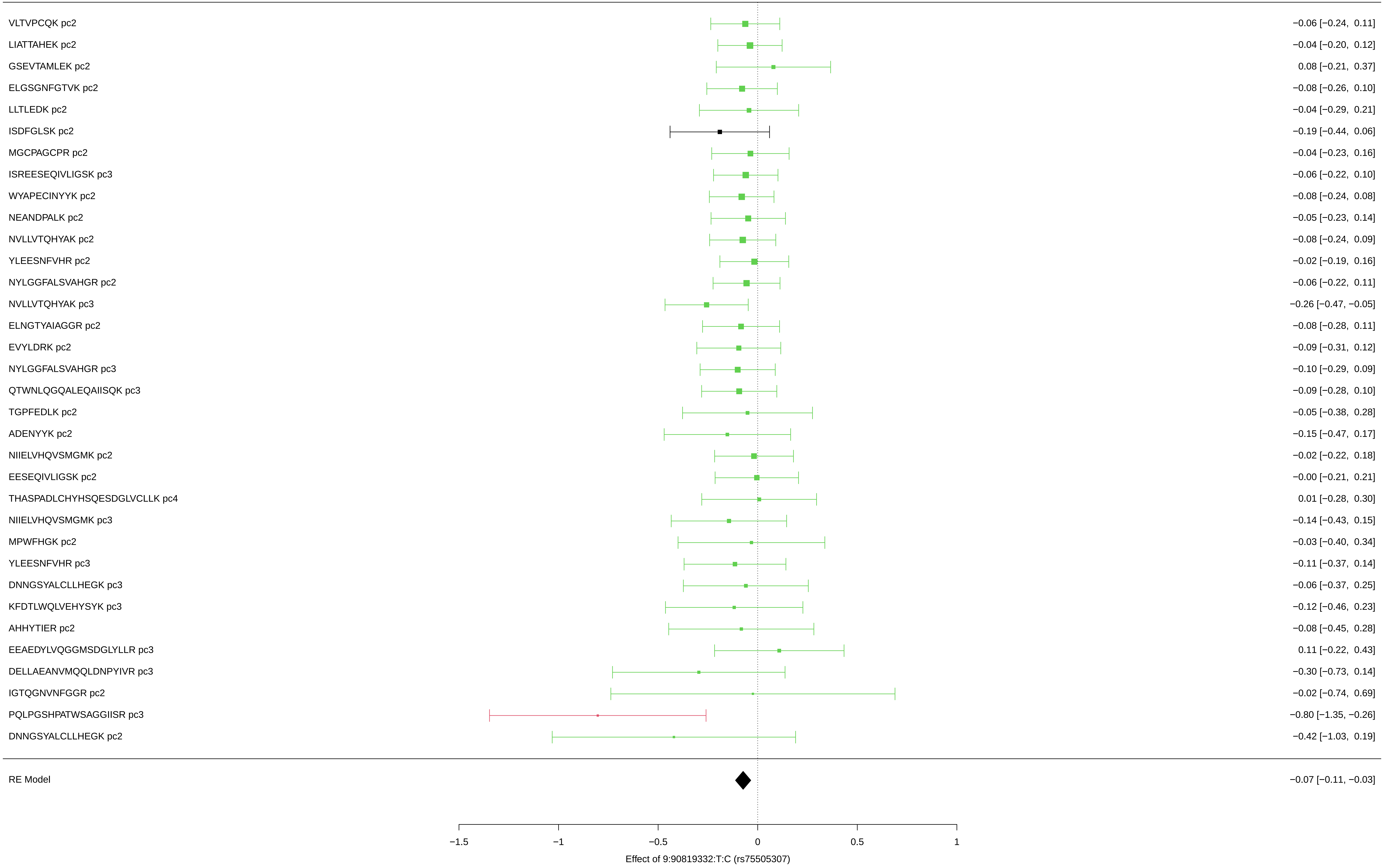

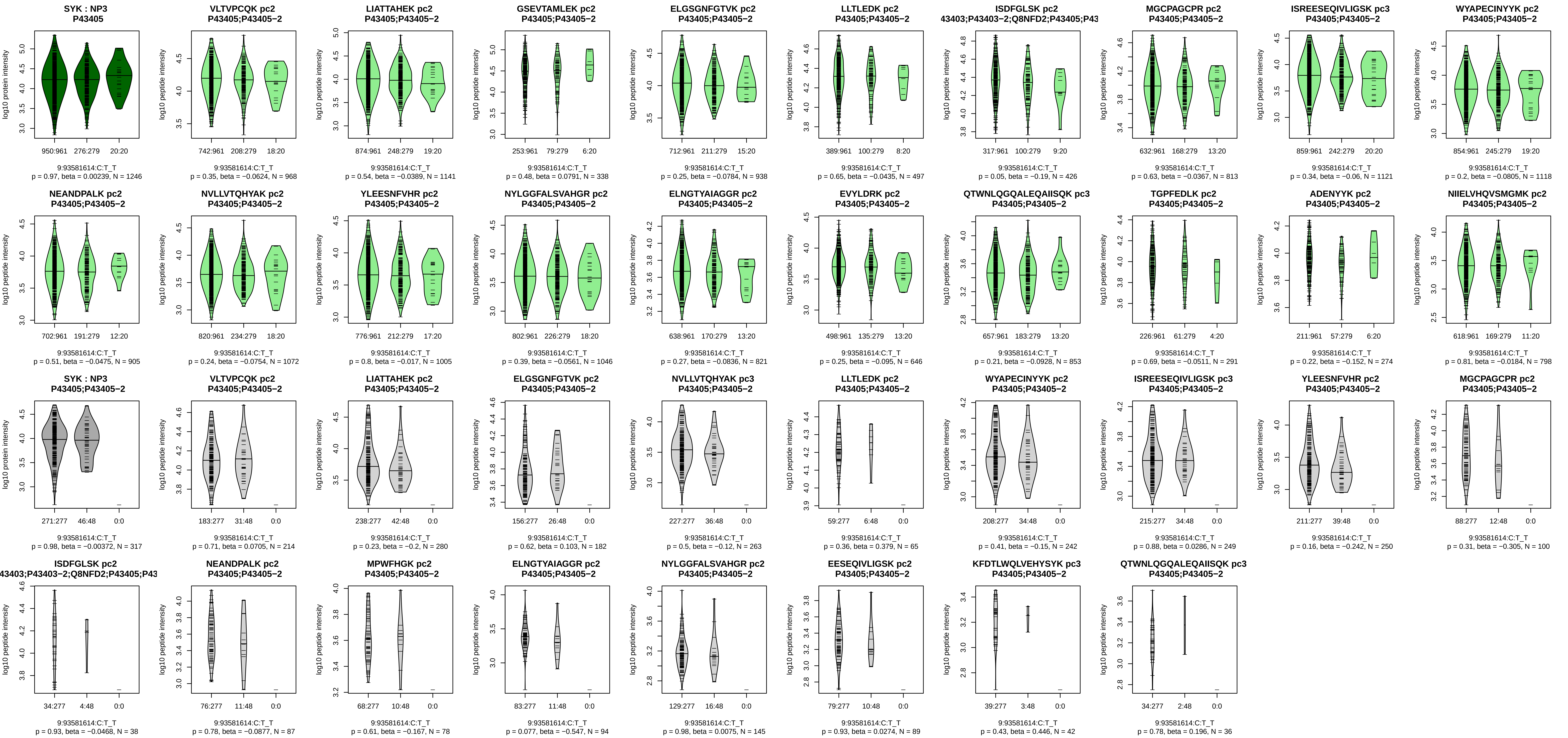

Assay Target: SYK
UniProt: P43405
rsID: rs75505307
Target SNP: 9:90819332:T:C
Proxy SNP: 9:93581614:C:T
deCODE log10(p): 26.1
deCODE BETA: −0.13
deCODE MSPA: 0.037
968:1141:338:938:497:426:813:

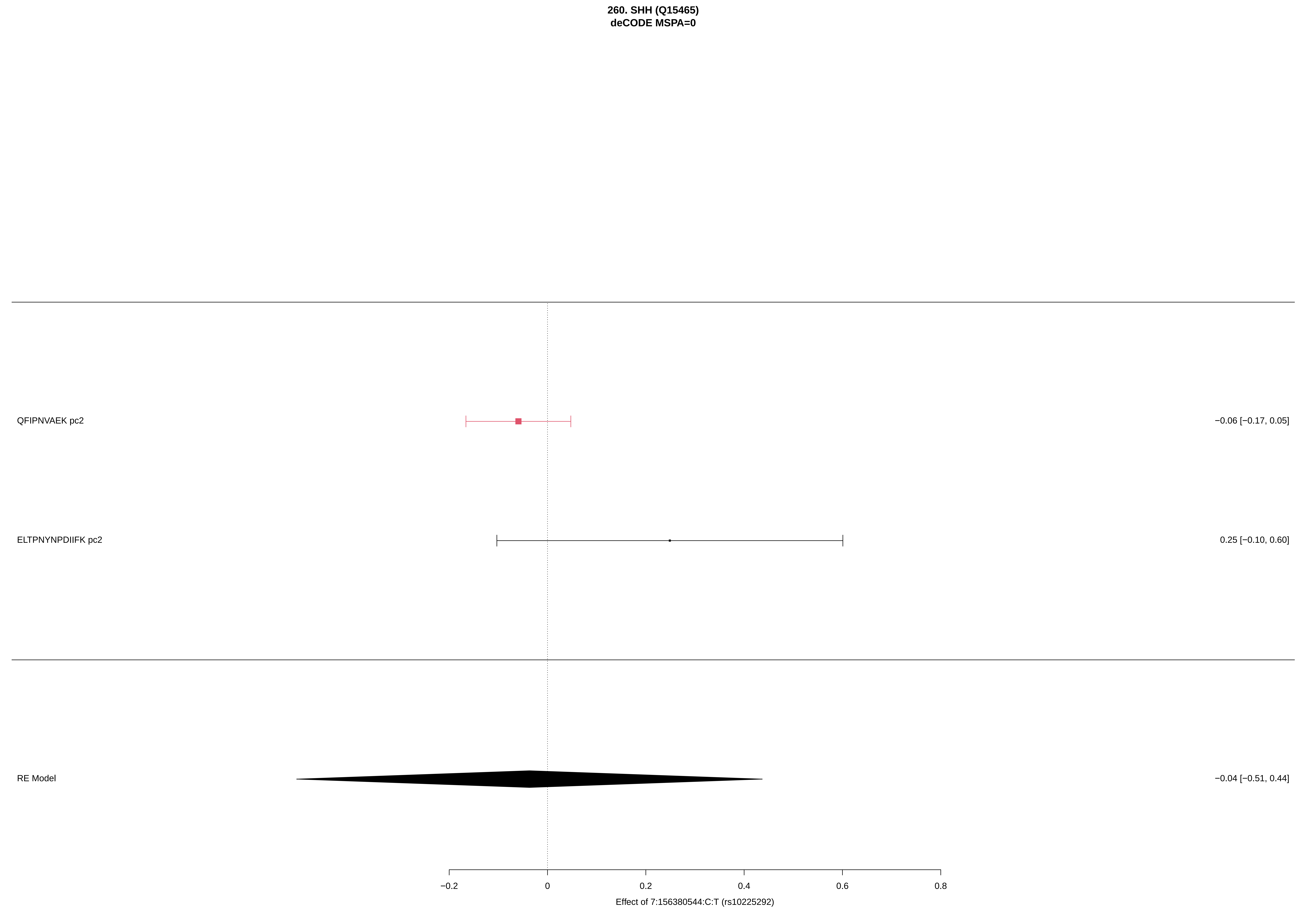
260. SHH (Q15465)
deCODE MSPA=0
QFIPNVAEK pc2
−0.06 [−0.17, 0.05]
ELTPNYNPDIIFK pc2
0.25 [−0.10, 0.60]
RE Model
−0.04 [−0.51, 0.44]
−0.2
0
0.2
0.4
0.6
0.8
Effect of 7:156380544:C:T (rs10225292)

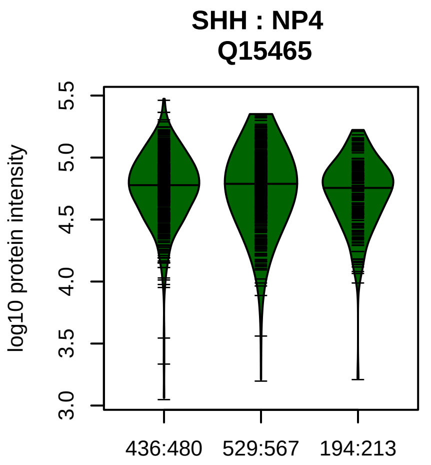

7:156173238:T:C_C
p = 0.13, beta = −0.0618, N = 1159

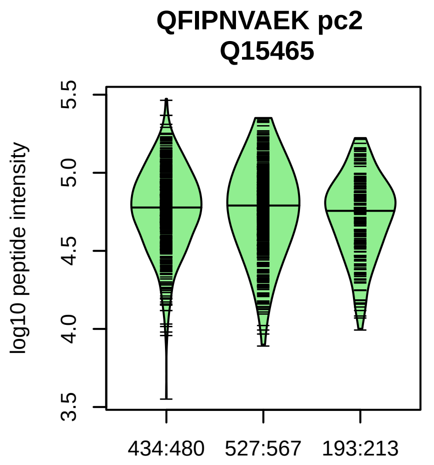

7:156173238:T:C_C
p = 0.15, beta = −0.0593, N = 1154

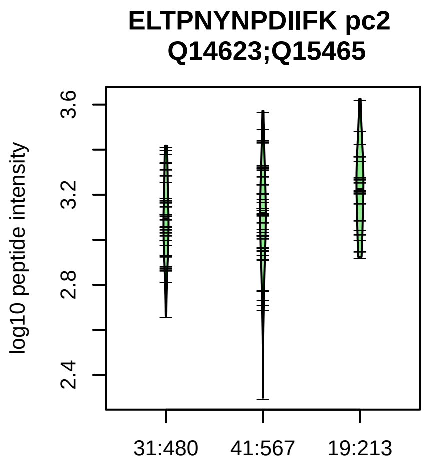

7:156173238:T:C_C
p = 0.072, beta = 0.249, N = 91

Assay Target: SHH
UniProt: Q15465
rsID: rs10225292
Target SNP: 7:156380544:C:T
Proxy SNP: 7:156173238:T:C
deCODE log10(p): 25.7
deCODE BETA: −0.09
deCODE MSPA: 0
–:–
1154:91

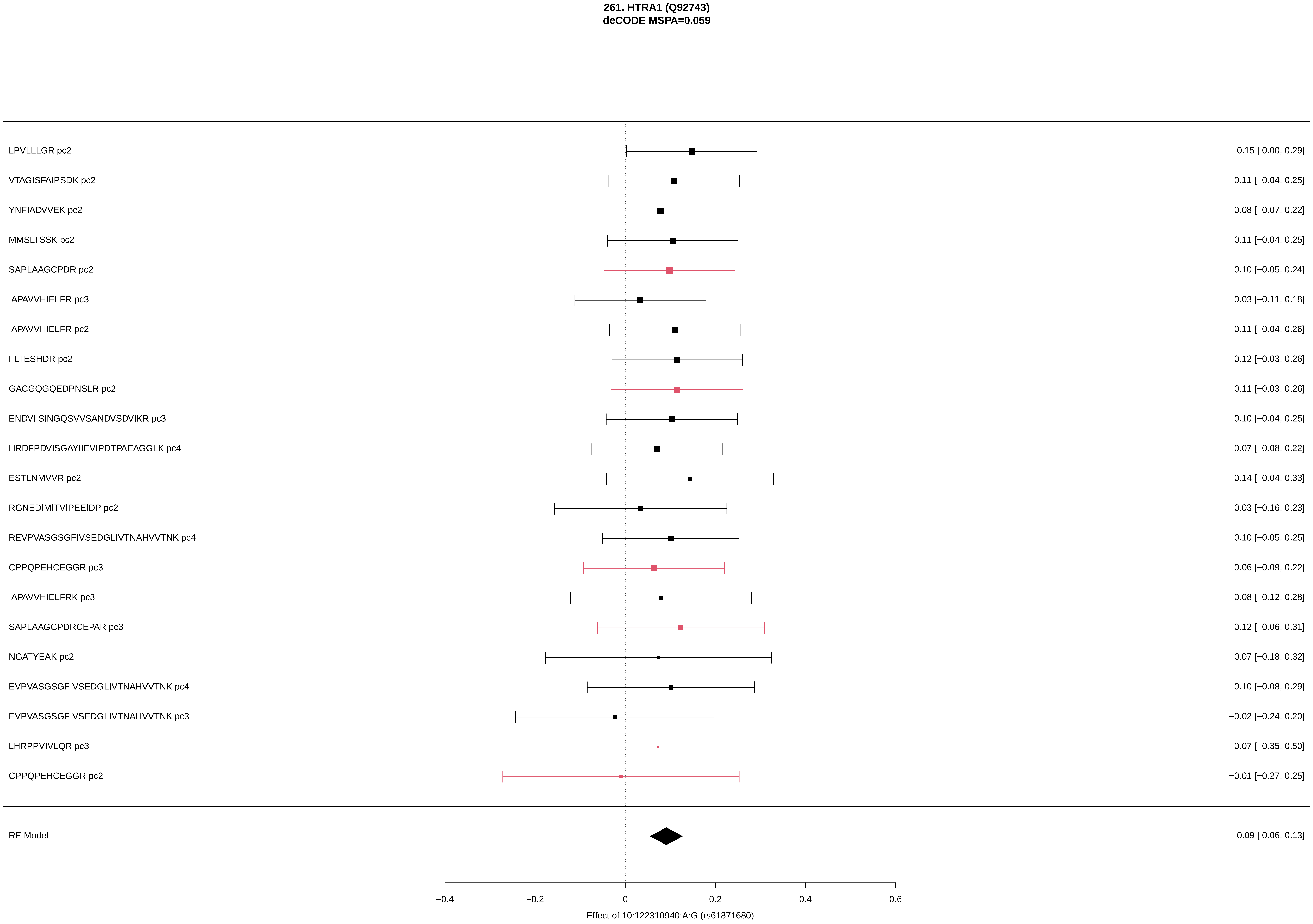
261. HTRA1 (Q92743)
deCODE MSPA=0.059
LPVLLLGR pc2 0.15 [0.00, 0.29]
VTAGISFAIPSDK pc2 0.11 [−0.04, 0.25]
YNFIADVVEK pc2 0.08 [−0.07, 0.22]
MMSLTSSK pc2 0.11 [−0.04, 0.25]
SAPLAAGCPDR pc2 0.10 [−0.05, 0.24]
IAPAVVHIELFR pc3 0.03 [−0.11, 0.18]
IAPAVVHIELFR pc2 0.11 [−0.04, 0.26]
FLTESHDR pc2 0.12 [−0.03, 0.26]
GACGQGQEDPNSLR pc2 0.11 [−0.03, 0.26]
ENDVIISINGQSVVSANDVSDVIKR pc3 0.10 [−0.04, 0.25]
HRDFPDVISGAYIIEVIPDTPAEAGGLK pc4 0.07 [−0.08, 0.22]
ESTLNMVVR pc2 0.14 [−0.04, 0.33]
RGNEDIMITVIPEEIDP pc2 0.03 [−0.16, 0.23]
REVPVASGSGFIVSEDGLIVTNAHVVTNK pc4 0.10 [−0.05, 0.25]
CPPQPEHCEGGR pc3 0.06 [−0.09, 0.22]
IAPAVVHIELFRK pc3 0.08 [−0.12, 0.28]
SAPLAAGCPDRCEPAR pc3 0.12 [−0.06, 0.31]
NGATYEAK pc2 0.07 [−0.18, 0.32]
EVPVASGSGFIVSEDGLIVTNAHVVTNK pc4 0.10 [−0.08, 0.29]
EVPVASGSGFIVSEDGLIVTNAHVVTNK pc3 −0.02 [−0.24, 0.20]
LHRPPVIVLQR pc3 0.07 [−0.35, 0.50]
CPPQPEHCEGGR pc2 −0.01 [−0.27, 0.25]
RE Model 0.09 [0.06, 0.13]
−0.4
−0.2
0
0.2
0.4
0.6
Effect of 10:122310940:A:G (rs61871680)

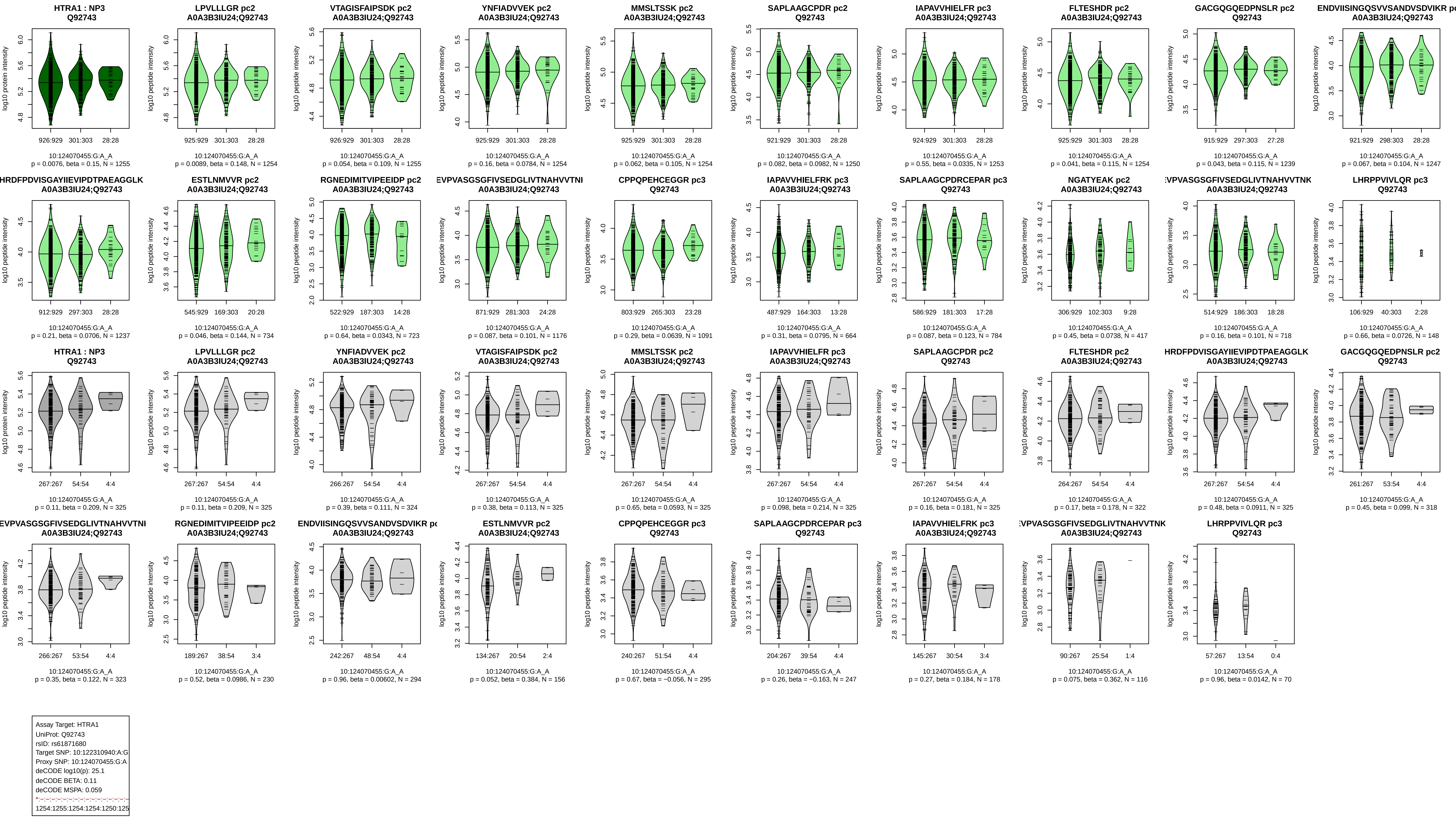
HTRA1 : NP3
Q92743
p = 0.0076, beta = 0.15, N = 1255
LPVLLLGR pc2
A0A3B3IU24;Q92743
p = 0.0089, beta = 0.148, N = 1254
VTAGISFAIPSDK pc2
A0A3B3IU24;Q92743
p = 0.054, beta = 0.109, N = 1255
YNFIADVVEK pc2
A0A3B3IU24;Q92743
p = 0.16, beta = 0.0784, N = 1254
MMSLTSSK pc2
A0A3B3IU24;Q92743
p = 0.062, beta = 0.105, N = 1254
SAPLAAGCPDR pc2
Q92743
p = 0.082, beta = 0.0982, N = 1250
IAPAVVHIELFR pc3
A0A3B3IU24;Q92743
p = 0.55, beta = 0.0335, N = 1253
FLTESHDR pc2
A0A3B3IU24;Q92743
p = 0.041, beta = 0.115, N = 1254
GACGQGQEDPNSLR pc2
Q92743
p = 0.043, beta = 0.115, N = 1239
ENDVIISINGQSVVSANDVSDVIKR pc
A0A3B3IU24;Q92743
p = 0.067, beta = 0.104, N = 1247
HRDFPDVISGAYIIEVIPDTPAEAGGLK
A0A3B3IU24;Q92743
p = 0.21, beta = 0.0706, N = 1237
ESTLNMVVR pc2
A0A3B3IU24;Q92743
p = 0.046, beta = 0.144, N = 734
RGNEDIMITVIPEEIDP pc2
A0A3B3IU24;Q92743
p = 0.64, beta = 0.0343, N = 723
EVPVASGSGFIVSEDGLIVTNAHVVTNI
A0A3B3IU24;Q92743
p = 0.087, beta = 0.101, N = 1176
CPPQPEHCEGGR pc3
Q92743
p = 0.29, beta = 0.0639, N = 1091
IAPAVVHIELFRK pc3
A0A3B3IU24;Q92743
p = 0.31, beta = 0.0795, N = 664
SAPLAAGCPDRCEPAR pc3
Q92743
p = 0.087, beta = 0.123, N = 784
NGATYEAK pc2
A0A3B3IU24;Q92743
p = 0.45, beta = 0.0738, N = 417
EVPVASGSGFIVSEDGLIVTNAHVVTNK
A0A3B3IU24;Q92743
p = 0.16, beta = 0.101, N = 718
LHRPPVIVLQR pc3
Q92743
p = 0.66, beta = 0.0726, N = 148
HTRA1 : NP3
Q92743
p = 0.11, beta = 0.209, N = 325
LPVLLLGR pc2
A0A3B3IU24;Q92743
p = 0.11, beta = 0.209, N = 325
YNFIADVVEK pc2
A0A3B3IU24;Q92743
p = 0.39, beta = 0.111, N = 324
VTAGISFAIPSDK pc2
A0A3B3IU24;Q92743
p = 0.38, beta = 0.113, N = 325
MMSLTSSK pc2
A0A3B3IU24;Q92743
p = 0.65, beta = 0.0593, N = 325
IAPAVVHIELFR pc3
A0A3B3IU24;Q92743
p = 0.098, beta = 0.214, N = 325
SAPLAAGCPDR pc2
Q92743
p = 0.16, beta = 0.181, N = 325
FLTESHDR pc2
A0A3B3IU24;Q92743
p = 0.17, beta = 0.178, N = 322
HRDFPDVISGAYIIEVIPDTPAEAGGLK
A0A3B3IU24;Q92743
p = 0.48, beta = 0.0911, N = 325
GACGQGQEDPNSLR pc2
Q92743
p = 0.45, beta = 0.099, N = 318
EVPVASGSGFIVSEDGLIVTNAHVVTNI
A0A3B3IU24;Q92743
p = 0.35, beta = 0.122, N = 323
RGNEDIMITVIPEEIDP pc2
A0A3B3IU24;Q92743
p = 0.52, beta = 0.0986, N = 230
ENDVIISINGQSVVSANDVSDVIKR pc
A0A3B3IU24;Q92743
p = 0.96, beta = 0.00602, N = 294
ESTLNMVVR pc2
A0A3B3IU24;Q92743
p = 0.052, beta = 0.384, N = 156
CPPQPEHCEGGR pc3
Q92743
p = 0.67, beta = −0.056, N = 295
SAPLAAGCPDRCEPAR pc3
Q92743
p = 0.26, beta = −0.163, N = 247
IAPAVVHIELFRK pc3
A0A3B3IU24;Q92743
p = 0.27, beta = 0.184, N = 178
EVPVASGSGFIVSEDGLIVTNAHVVTNK
A0A3B3IU24;Q92743
p = 0.075, beta = 0.362, N = 116
LHRPPVIVLQR pc3
Q92743
p = 0.96, beta = 0.0142, N = 70
10:124070455:G:A_A
log10 protein intensity
log10 peptide intensity
Assay Target: HTRA1
UniProt: Q92743
rsID: rs61871680
Target SNP: 10:122310940:A:G
Proxy SNP: 10:124070455:G:A
deCODE log10(p): 25.1
deCODE BETA: 0.11
deCODE MSPA: 0.059
1254:1255:1254:1254:1250:125

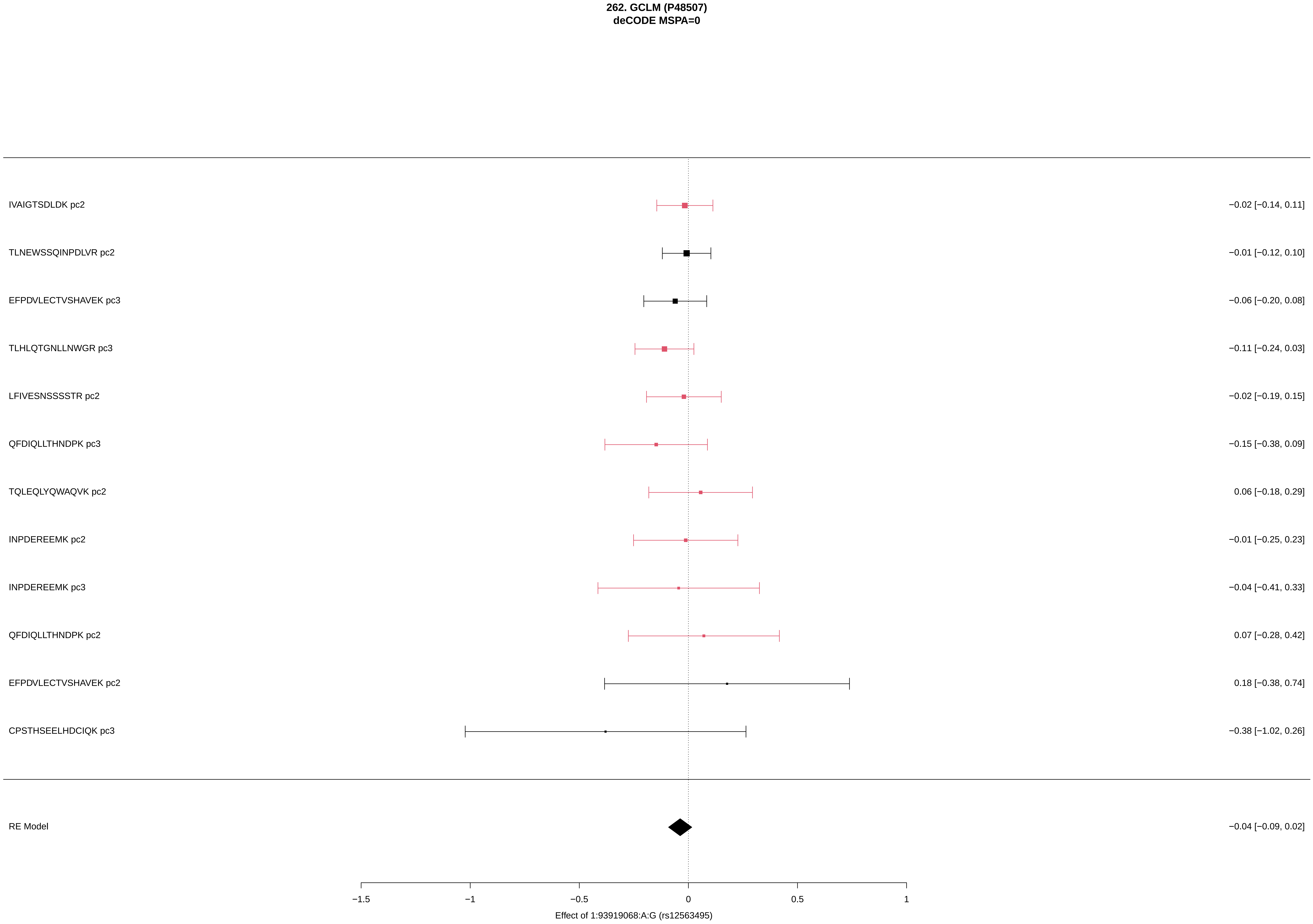
262. GCLM (P48507)
deCODE MSPA=0
IVAIGTSDLDK pc2
TLNEWSSQINPDLVR pc2
EFPDVLECTVSHAVEK pc3
TLHLQTGNLLNWGR pc3
LFIVESNSSSSTR pc2
QFDIQLLTHNDPK pc3
TQLEQLYQWAQVK pc2
INPDEREEMK pc2
INPDEREEMK pc3
QFDIQLLTHNDPK pc2
EFPDVLECTVSHAVEK pc2
CPSTHSEELHDCIQK pc3
RE Model
−0.02 [−0.14, 0.11]
−0.01 [−0.12, 0.10]
−0.06 [−0.20, 0.08]
−0.11 [−0.24, 0.03]
−0.02 [−0.19, 0.15]
−0.15 [−0.38, 0.09]
0.06 [−0.18, 0.29]
−0.01 [−0.25, 0.23]
−0.04 [−0.41, 0.33]
0.07 [−0.28, 0.42]
0.18 [−0.38, 0.74]
−0.38 [−1.02, 0.26]
−0.04 [−0.09, 0.02]
−1.5
−1
−0.5
0
0.5
1
Effect of 1:93919068:A:G (rs12563495)

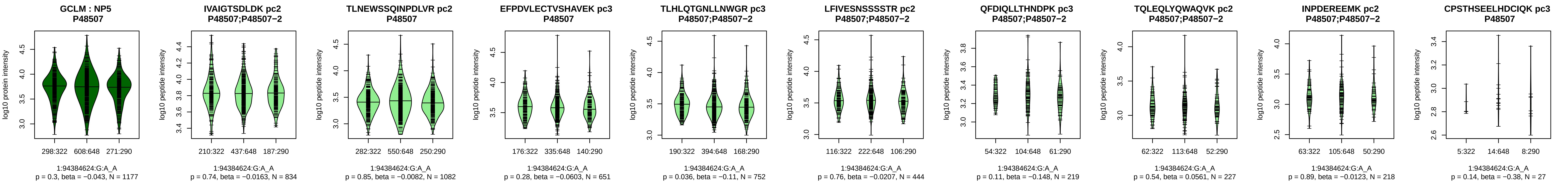

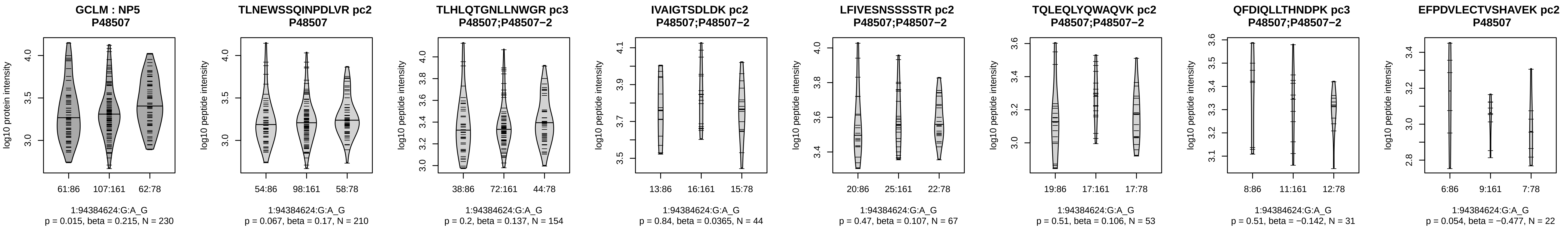

Assay Target: GCLM
UniProt: P48507
rsID: rs12563495
Target SNP: 1:93919068:A:G
Proxy SNP: 1:94384624:G:A
deCODE log10(p): 25
deCODE BETA: −0.08
deCODE MSPA: 0
−:−:−:−:−:−:−:−:−:−:NA:NA
834:1082:651:752:444:219:227:

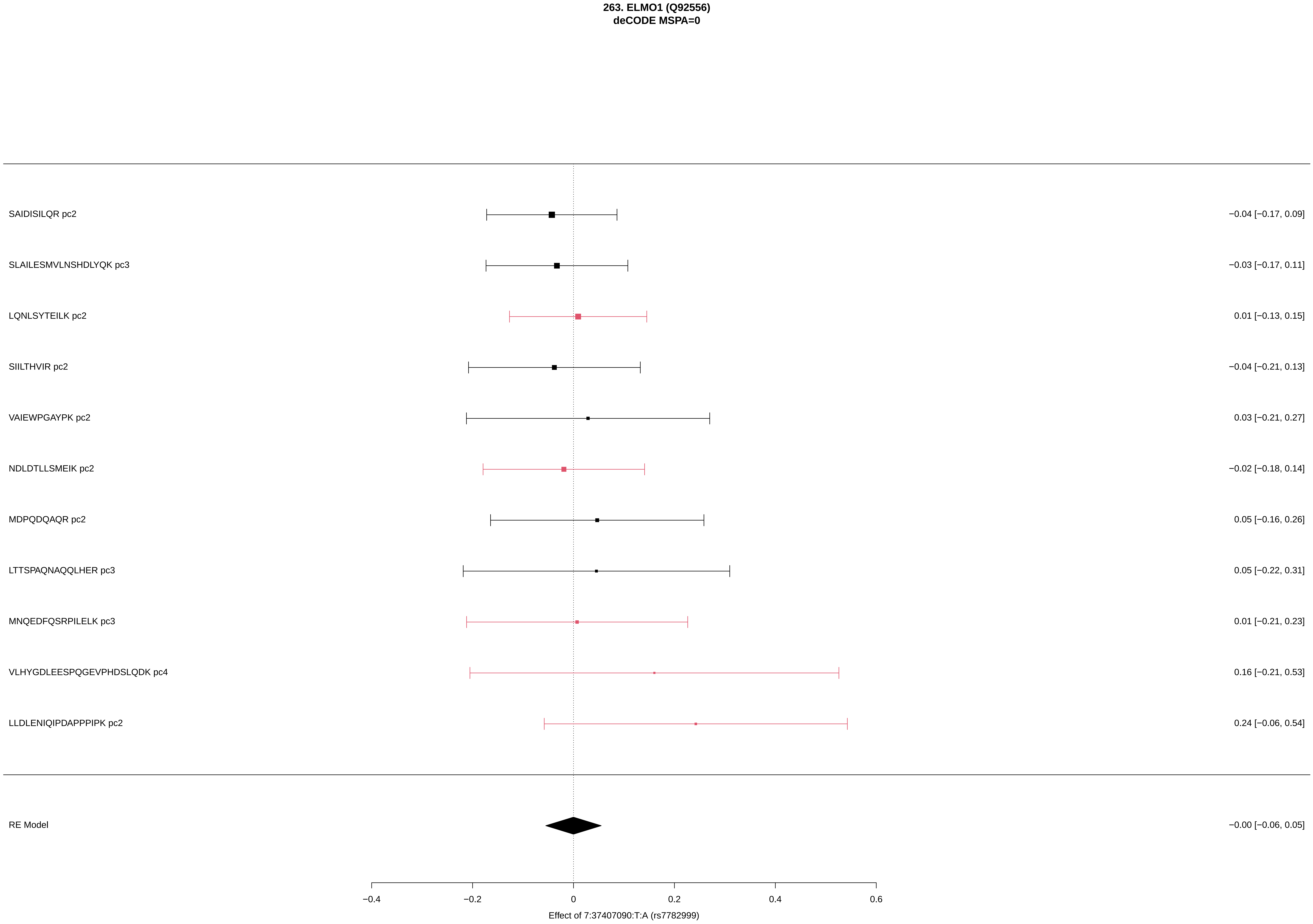

263. ELMO1 (Q92556)
deCODE MSPA=0
SAIDISILQR pc2
−0.04 [−0.17, 0.09]
SLAILESMVLNSHDLYQK pc3
−0.03 [−0.17, 0.11]
LQNLSYTEILK pc2
0.01 [−0.13, 0.15]
SIILTHVIR pc2
−0.04 [−0.21, 0.13]
VAIEWPGAYPK pc2
0.03 [−0.21, 0.27]
NDLDTLLSMEIK pc2
−0.02 [−0.18, 0.14]
MDPQDQAQR pc2
0.05 [−0.16, 0.26]
LTTSPAQNAQQLHER pc3
0.05 [−0.22, 0.31]
MNQEDFQSRPILELK pc3
0.01 [−0.21, 0.23]
VLHYGDLEESPQGEVPHDSLQDK pc4
0.16 [−0.21, 0.53]
LLDLENIQIPDAPPPIPK pc2
0.24 [−0.06, 0.54]
RE Model
−0.00 [−0.06, 0.05]
−0.4
−0.2
0
0.2
0.4
0.6
Effect of 7:37407090:T:A (rs7782999)

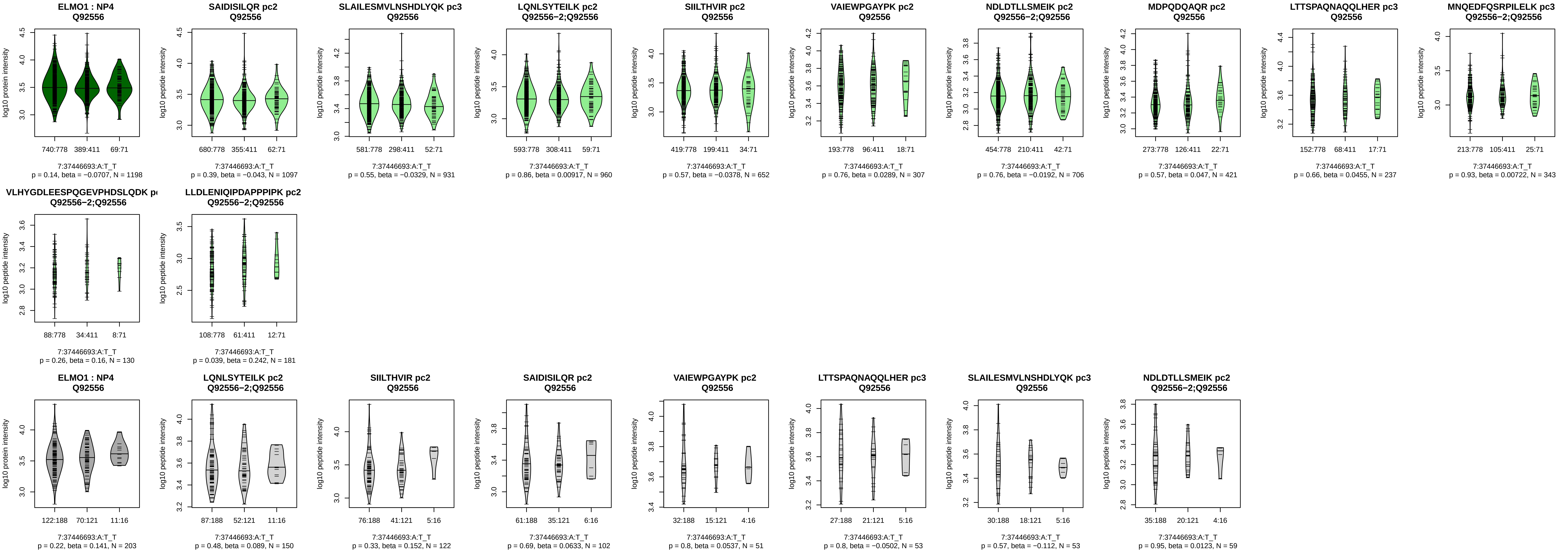

Assay Target: ELMO1
UniProt: Q92556
rsID: rs7782999
Target SNP: 7:37407090:T:A
Proxy SNP: 7:37446693:A:T
deCODE log10(p): 24.4
deCODE BETA: −0.1
deCODE MSPA: 0
−:−:−:−:−:−:−:−:−
1097:931:960:652:307:706:421:

264. PCSK2 (P16519)
deCODE MSPA=0.585

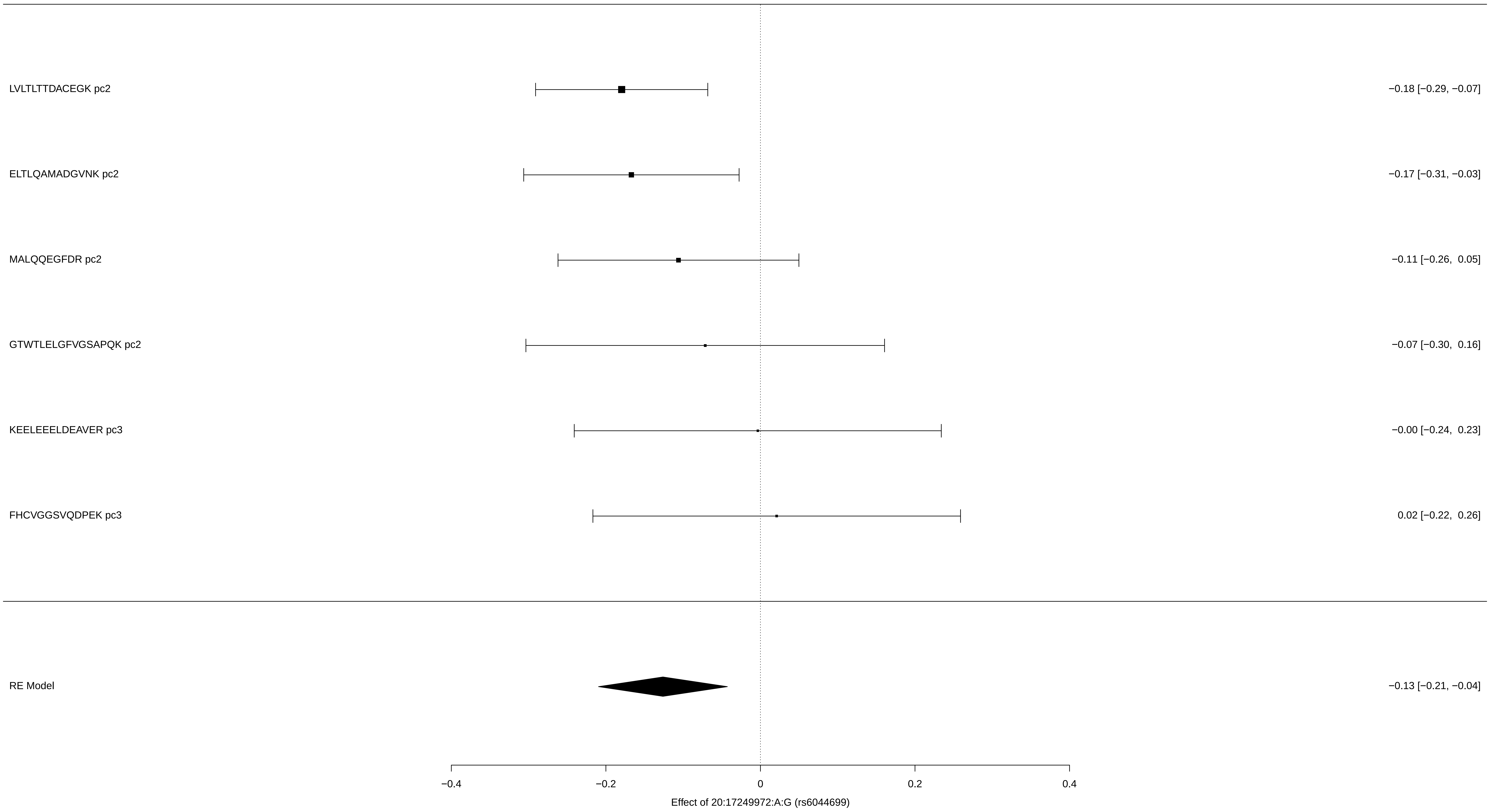

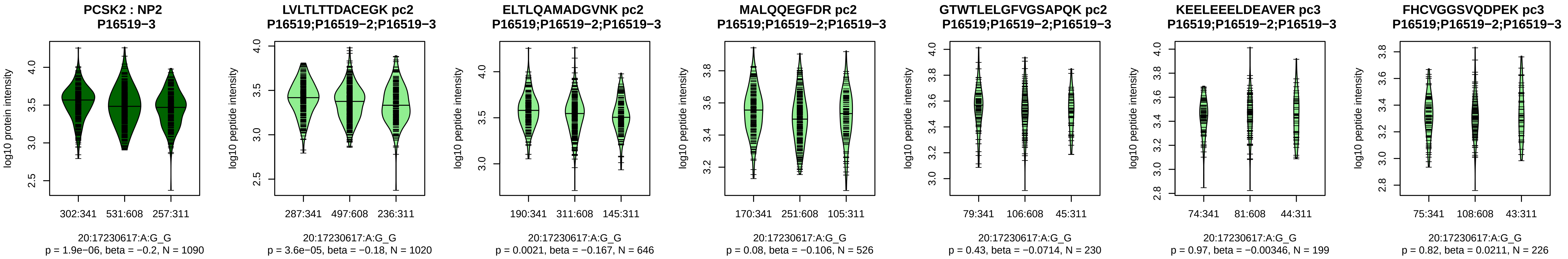

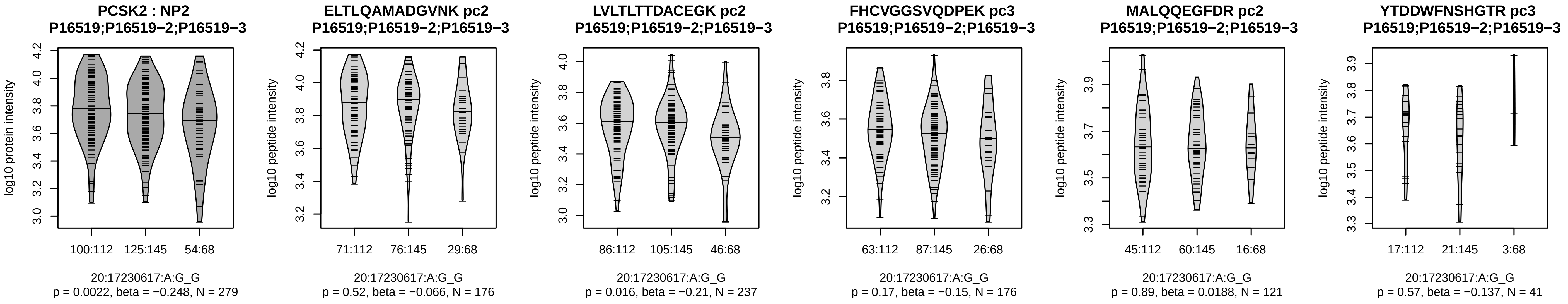

Assay Target: PCSK2
UniProt: P16519
rsID: rs6044699
Target SNP: 20:17249972:A:G
Proxy SNP: 20:17230617:A:G
deCODE log10(p): 23.9
deCODE BETA: 0.08
deCODE MSPA: 0.585
::–:–:–:–
1020:646:526:230:199:226

265. KLC1 (Q07866)
deCODE MSPA=0

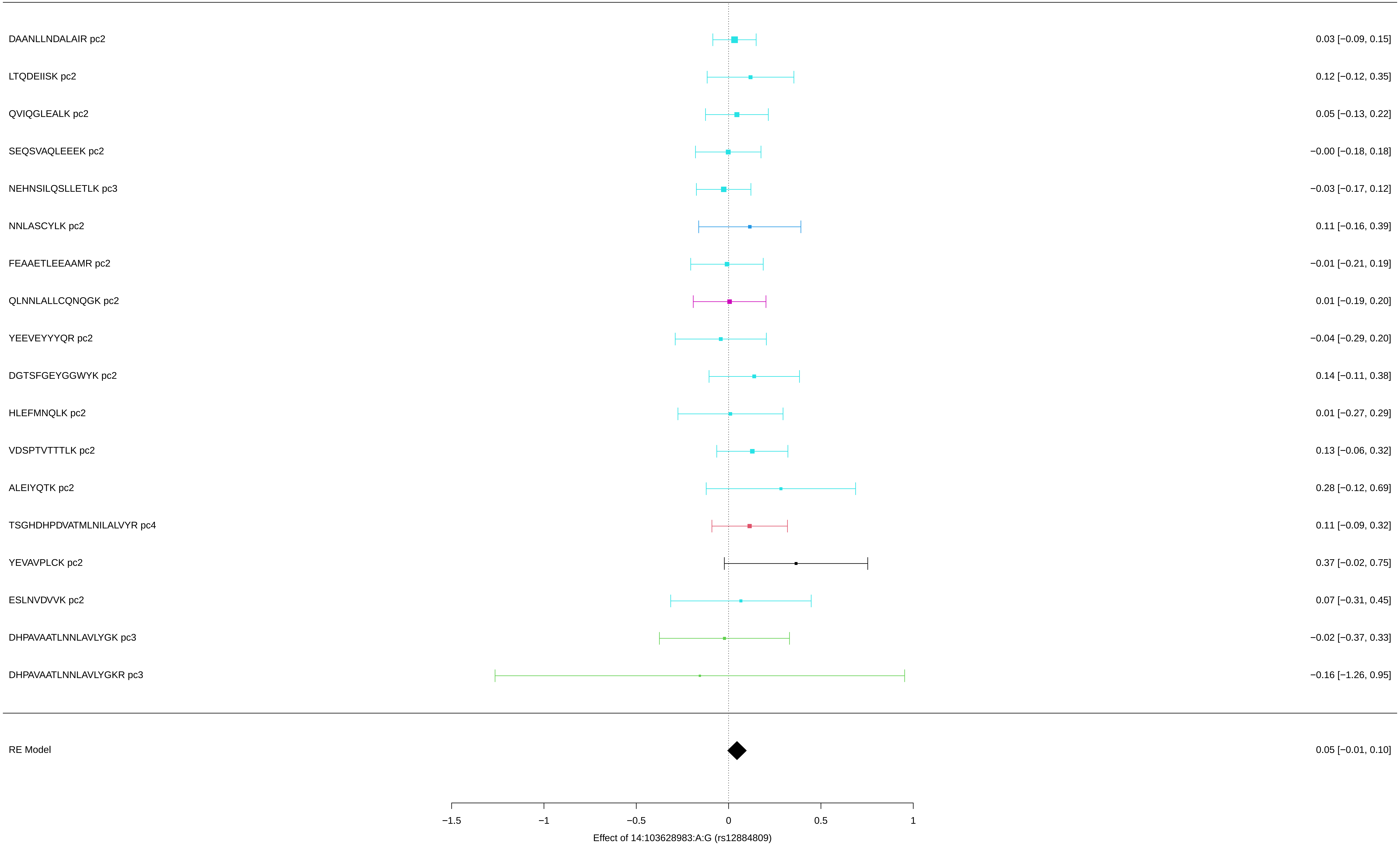

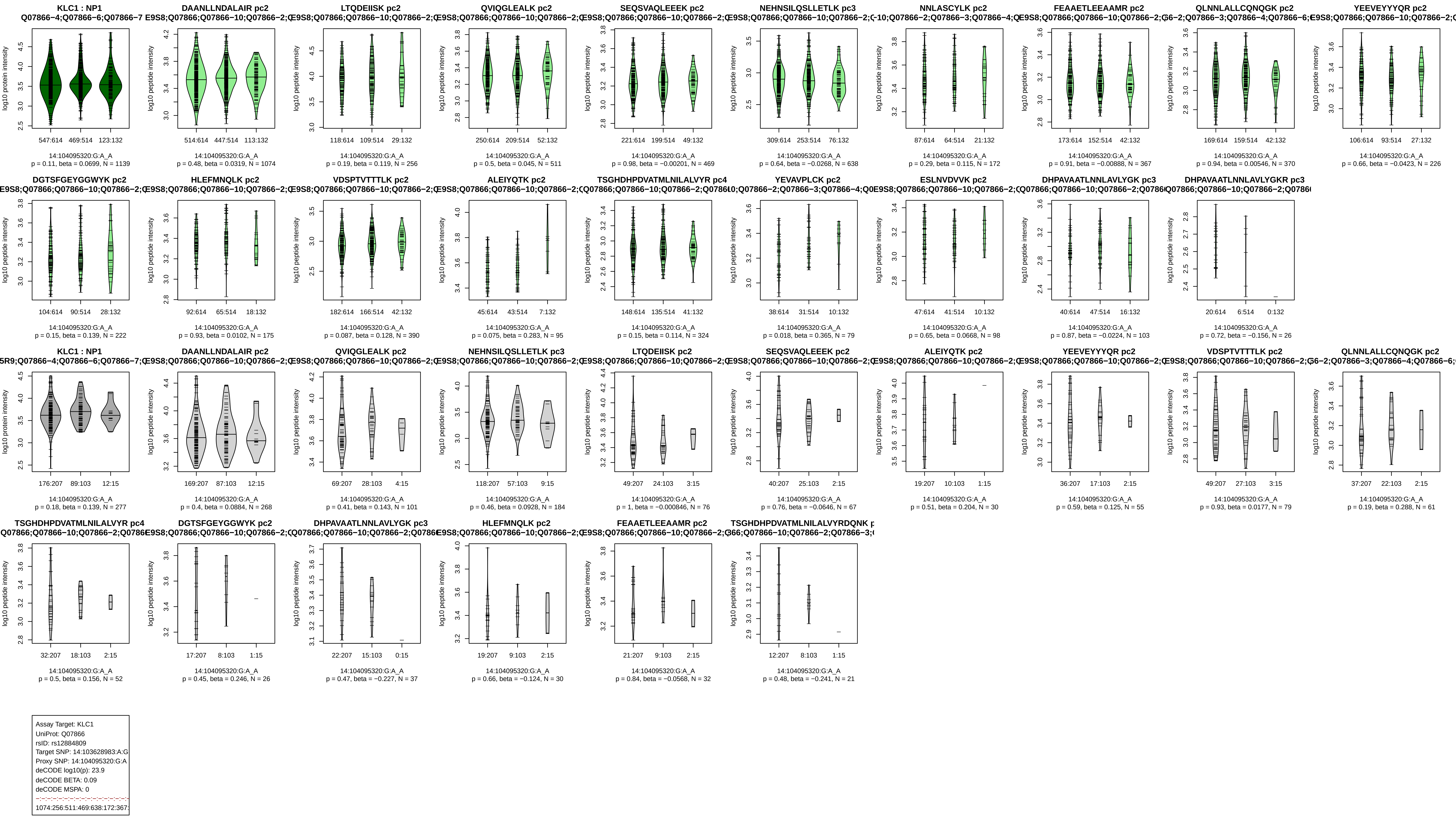

KLC1 : NP1
14:104095320:G:A_A
p = 0.11, beta = 0.0699, N = 1139
DAANLLNDALAIR pc2
p = 0.48, beta = 0.0319, N = 1074
LTQDEIISK pc2
p = 0.19, beta = 0.119, N = 256
QVIQGLEALK pc2
p = 0.5, beta = 0.045, N = 511
SEQSVAQLEEEK pc2
p = 0.98, beta = −0.00201, N = 469
NEHNSILQSLLETLK pc3
p = 0.64, beta = −0.0268, N = 638
NNLASCYLK pc2
p = 0.29, beta = 0.115, N = 172
FEAAETLEEAAMR pc2
p = 0.91, beta = −0.00888, N = 367
QLNNLALLCQNQGK pc2
p = 0.94, beta = 0.00546, N = 370
YEEVEYYYQR pc2
p = 0.66, beta = −0.0423, N = 226
DGTSFGEYGGWYK pc2
p = 0.15, beta = 0.139, N = 222
HLEFMNQLK pc2
p = 0.93, beta = 0.0102, N = 175
VDSPTVTTTLK pc2
p = 0.087, beta = 0.128, N = 390
ALEIYQTK pc2
p = 0.075, beta = 0.283, N = 95
TSGHDHPDVATMLNILALVYR pc4
p = 0.15, beta = 0.114, N = 324
YEVAVPLCK pc2
p = 0.018, beta = 0.365, N = 79
ESLNVDVVK pc2
p = 0.65, beta = 0.0668, N = 98
DHPAVAATLNNLAVLYGK pc3
p = 0.87, beta = −0.0224, N = 103
DHPAVAATLNNLAVLYGKR pc3
p = 0.72, beta = −0.156, N = 26
KLC1 : NP1
p = 0.18, beta = 0.139, N = 277
DAANLLNDALAIR pc2
p = 0.4, beta = 0.0884, N = 268
QVIQGLEALK pc2
p = 0.41, beta = 0.143, N = 101
NEHNSILQSLLETLK pc3
p = 0.46, beta = 0.0928, N = 184
LTQDEIISK pc2
p = 1, beta = −0.000846, N = 76
SEQSVAQLEEEK pc2
p = 0.76, beta = −0.0646, N = 67
ALEIYQTK pc2
p = 0.51, beta = 0.204, N = 30
YEEVEYYYQR pc2
p = 0.59, beta = 0.125, N = 55
VDSPTVTTTLK pc2
p = 0.93, beta = 0.0177, N = 79
QLNNLALLCQNQGK pc2
p = 0.19, beta = 0.288, N = 61
TSGHDHPDVATMLNILALVYR pc4
p = 0.5, beta = 0.156, N = 52
DGTSFGEYGGWYK pc2
p = 0.45, beta = 0.246, N = 26
DHPAVAATLNNLAVLYGK pc3
p = 0.47, beta = −0.227, N = 37
HLEFMNQLK pc2
p = 0.66, beta = −0.124, N = 30
FEAAETLEEAAMR pc2
p = 0.84, beta = −0.0568, N = 32
TSGHDHPDVATMLNILALVYRDQNK
p = 0.48, beta = −0.241, N = 21
Assay Target: KLC1
UniProt: Q07866
rsID: rs12884809
Target SNP: 14:103628983:A:G
Proxy SNP: 14:104095320:G:A
deCODE log10(p): 23.9
deCODE BETA: 0.09
deCODE MSPA: 0

266. OLFML3 (Q9NRN5)
deCODE MSPA=0.058

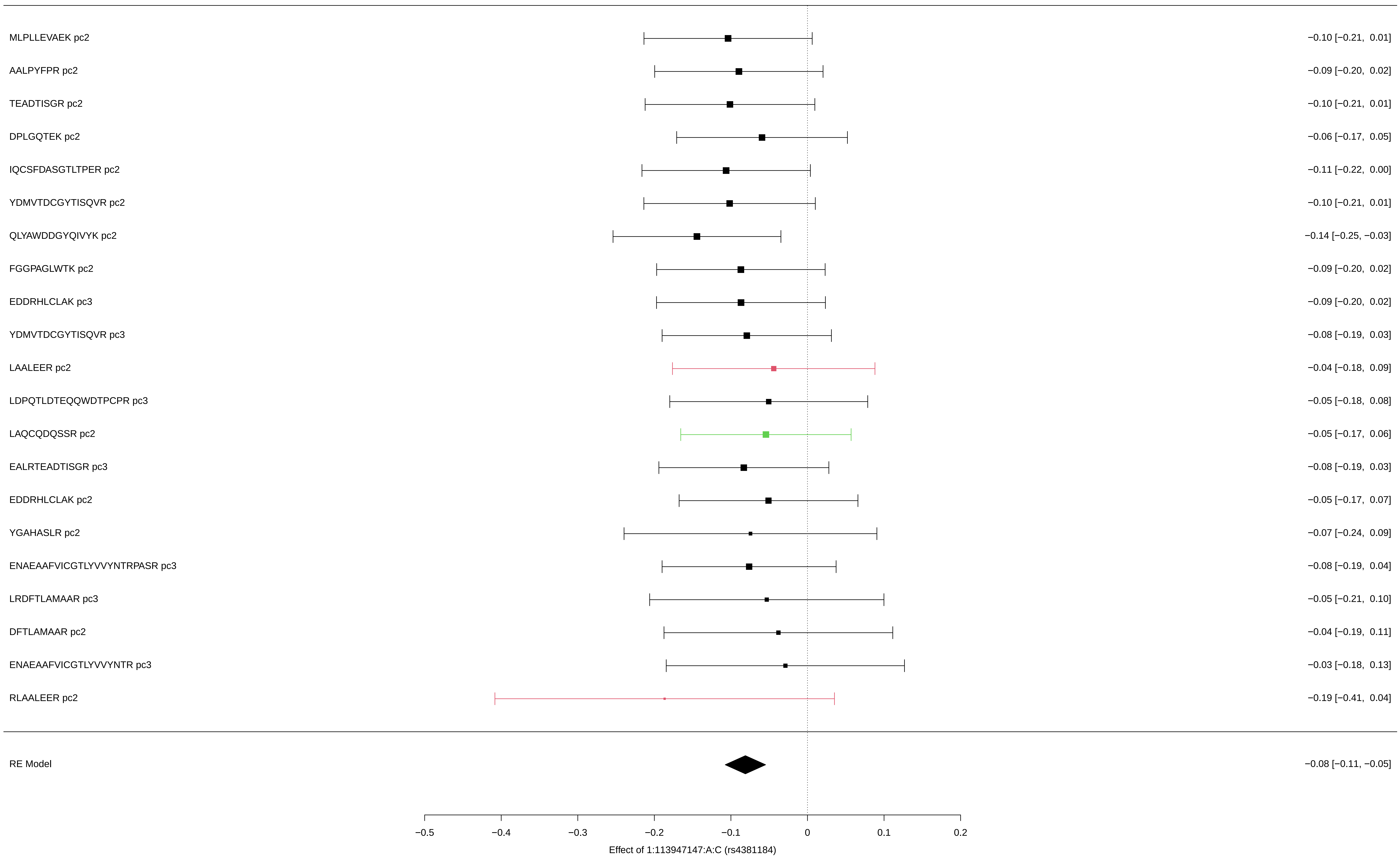

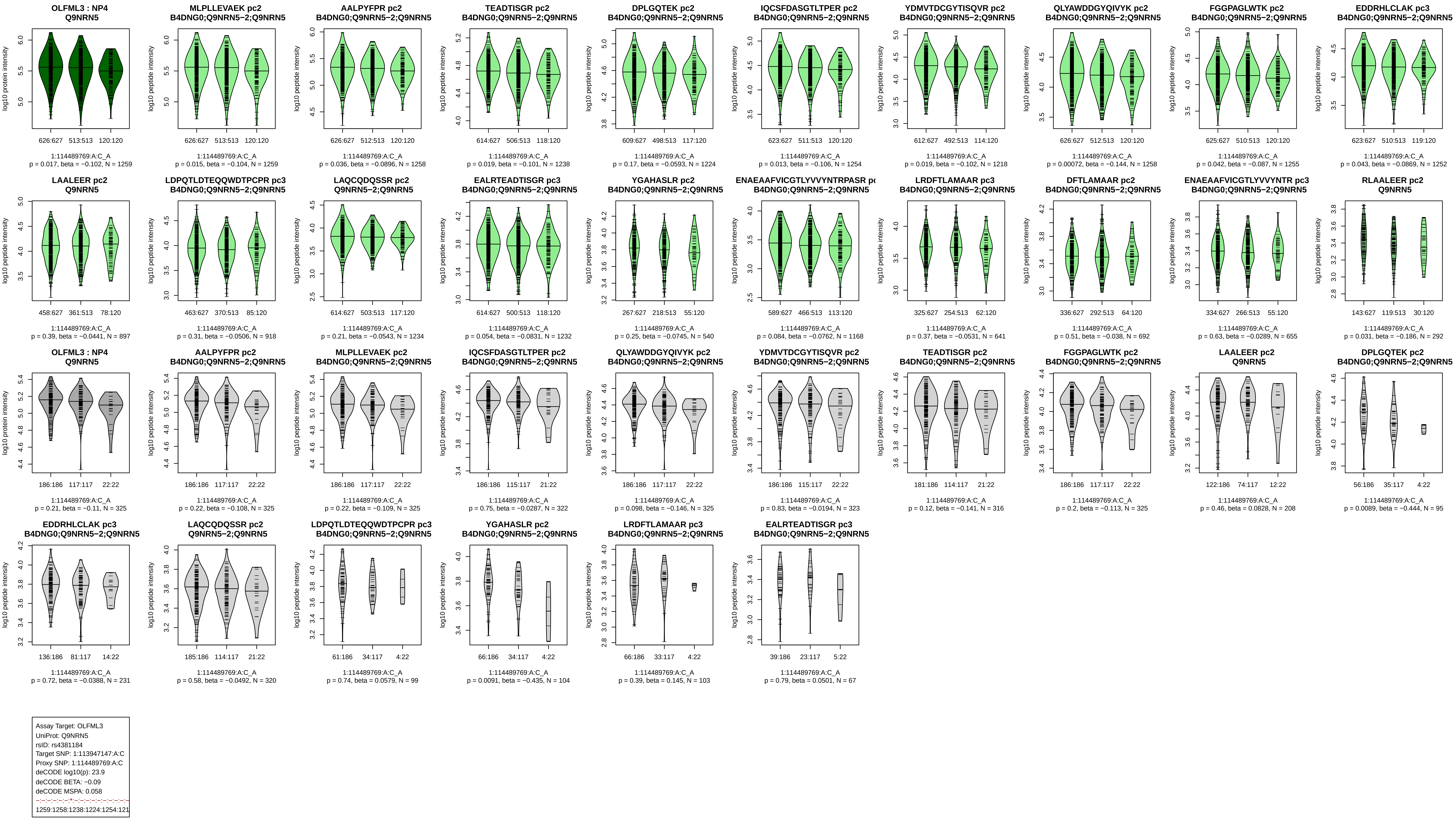
OLFML3 : NP4 Q9NRN5
1:114489769:A:C_A
p = 0.017, beta = −0.102, N = 1259
MLPLLEVAEK pc2 B4DNG0;Q9NRN5−2;Q9NRN5
p = 0.015, beta = −0.104, N = 1259
AALPYFPR pc2 B4DNG0;Q9NRN5−2;Q9NRN5
p = 0.036, beta = −0.0896, N = 1258
TEADTISGR pc2 B4DNG0;Q9NRN5−2;Q9NRN5
p = 0.019, beta = −0.101, N = 1238
DPLGQTEK pc2 B4DNG0;Q9NRN5−2;Q9NRN5
p = 0.17, beta = −0.0593, N = 1224
IQCSFDASGTLTPER pc2 B4DNG0;Q9NRN5−2;Q9NRN5
p = 0.013, beta = −0.106, N = 1254
YDMVTDCGYTISQVR pc2 B4DNG0;Q9NRN5−2;Q9NRN5
p = 0.019, beta = −0.102, N = 1218
QLYAWDDGYQIVYK pc2 B4DNG0;Q9NRN5−2;Q9NRN5
p = 0.00072, beta = −0.144, N = 1258
FGGPAGLWTK pc2 B4DNG0;Q9NRN5−2;Q9NRN5
p = 0.042, beta = −0.087, N = 1255
EDDRHLCLAK pc3 B4DNG0;Q9NRN5−2;Q9NRN5
p = 0.043, beta = −0.0869, N = 1252
LAALEER pc2 Q9NRN5
p = 0.39, beta = −0.0441, N = 897
LDPQTLDTEQQWDTPCPR pc3 B4DNG0;Q9NRN5−2;Q9NRN5
p = 0.31, beta = −0.0506, N = 918
LAQCQDQSSR pc2 Q9NRN5−2;Q9NRN5
p = 0.21, beta = −0.0543, N = 1234
EALRTEADTISGR pc3 B4DNG0;Q9NRN5−2;Q9NRN5
p = 0.054, beta = −0.0831, N = 1232
YGAHASLR pc2 B4DNG0;Q9NRN5−2;Q9NRN5
p = 0.25, beta = −0.0745, N = 540
ENAEAAFVICGTLYVVYNTRPASR pc B4DNG0;Q9NRN5−2;Q9NRN5
p = 0.084, beta = −0.0762, N = 1168
LRDFTLAMAAR pc3 B4DNG0;Q9NRN5−2;Q9NRN5
p = 0.37, beta = −0.0531, N = 641
DFTLAMAAR pc2 B4DNG0;Q9NRN5−2;Q9NRN5
p = 0.51, beta = −0.038, N = 692
ENAEAAFVICGTLYVVYNTR pc3 B4DNG0;Q9NRN5−2;Q9NRN5
p = 0.63, beta = −0.0289, N = 655
RLAALEER pc2 Q9NRN5
p = 0.031, beta = −0.186, N = 292
OLFML3 : NP4 Q9NRN5
p = 0.21, beta = −0.11, N = 325
AALPYFPR pc2 B4DNG0;Q9NRN5−2;Q9NRN5
p = 0.22, beta = −0.108, N = 325
MLPLLEVAEK pc2 B4DNG0;Q9NRN5−2;Q9NRN5
p = 0.22, beta = −0.109, N = 325
IQCSFDASGTLTPER pc2 B4DNG0;Q9NRN5−2;Q9NRN5
p = 0.75, beta = −0.0287, N = 322
QLYAWDDGYQIVYK pc2 B4DNG0;Q9NRN5−2;Q9NRN5
p = 0.098, beta = −0.146, N = 325
YDMVTDCGYTISQVR pc2 B4DNG0;Q9NRN5−2;Q9NRN5
p = 0.83, beta = −0.0194, N = 323
TEADTISGR pc2 B4DNG0;Q9NRN5−2;Q9NRN5
p = 0.12, beta = −0.141, N = 316
FGGPAGLWTK pc2 B4DNG0;Q9NRN5−2;Q9NRN5
p = 0.2, beta = −0.113, N = 325
LAALEER pc2 Q9NRN5
p = 0.46, beta = 0.0828, N = 208
DPLGQTEK pc2 B4DNG0;Q9NRN5−2;Q9NRN5
p = 0.0089, beta = −0.444, N = 95
EDDRHLCLAK pc3 B4DNG0;Q9NRN5−2;Q9NRN5
p = 0.72, beta = −0.0388, N = 231
LAQCQDQSSR pc2 Q9NRN5−2;Q9NRN5
p = 0.58, beta = −0.0492, N = 320
LDPQTLDTEQQWDTPCPR pc3 B4DNG0;Q9NRN5−2;Q9NRN5
p = 0.74, beta = 0.0579, N = 99
YGAHASLR pc2 B4DNG0;Q9NRN5−2;Q9NRN5
p = 0.0091, beta = −0.435, N = 104
LRDFTLAMAAR pc3 B4DNG0;Q9NRN5−2;Q9NRN5
p = 0.39, beta = 0.145, N = 103
EALRTEADTISGR pc3 B4DNG0;Q9NRN5−2;Q9NRN5
p = 0.79, beta = 0.0501, N = 67
Assay Target: OLFML3
UniProt: Q9NRN5
rsID: rs4381184
Target SNP: 1:113947147:A:C
Proxy SNP: 1:114489769:A:C
deCODE log10(p): 23.9
deCODE BETA: −0.09
deCODE MSPA: 0.058
1259:1258:1238:1224:1254:121

267. ARF4 (P18085)
deCODE MSPA=0

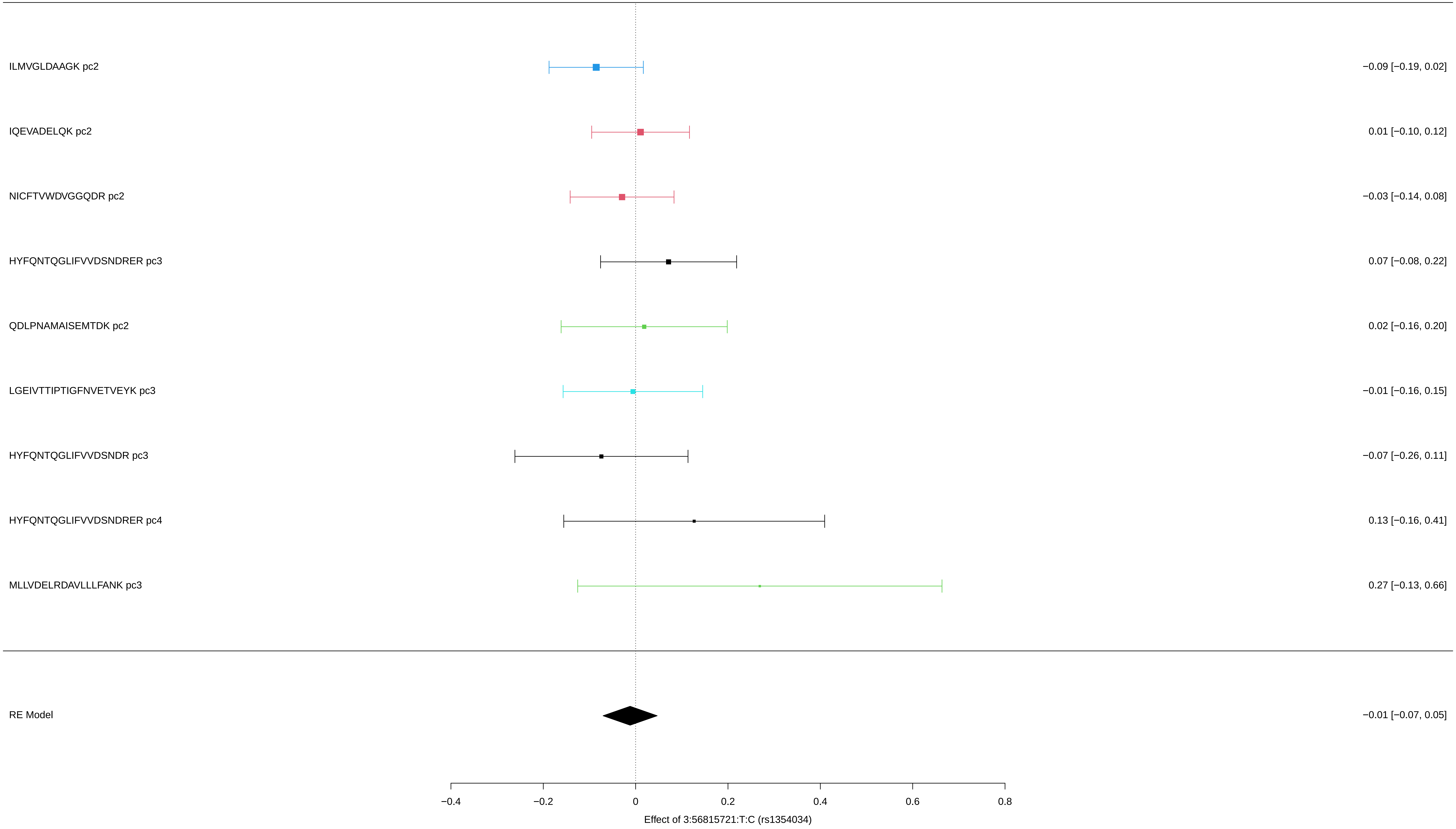

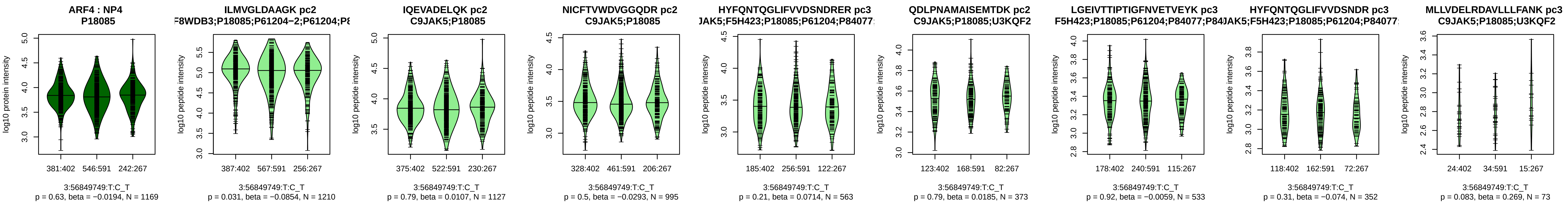

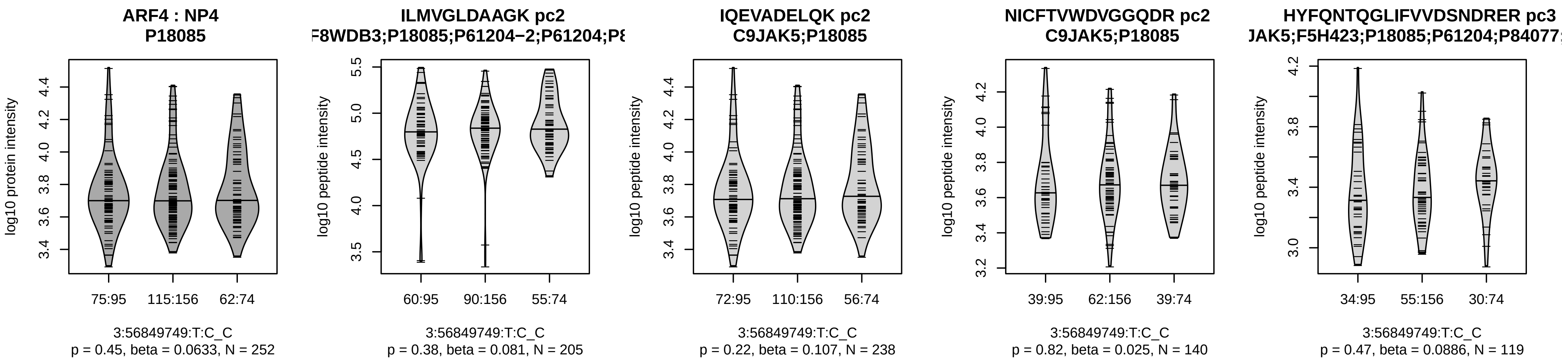

Assay Target: ARF4
UniProt: P18085
rsID: rs1354034
Target SNP: 3:56815721:T:C
Proxy SNP: 3:56849749:T:C
deCODE log10(p): 23.8
deCODE BETA: −0.08
deCODE MSPA: 0
−:−:−:−:−:−:−:−:−
1210:1127:995:563:373:533:352

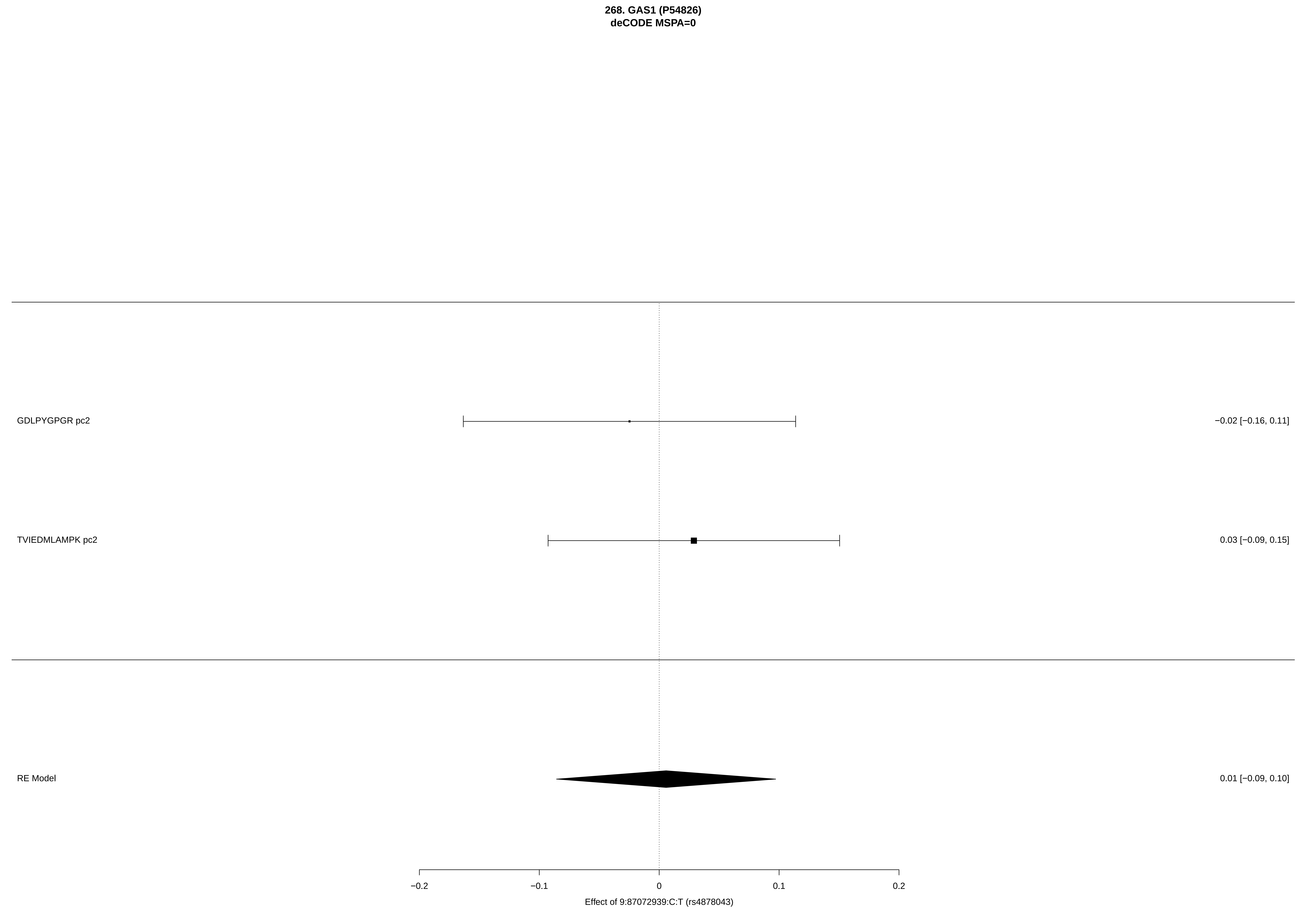
268. GAS1 (P54826)
deCODE MSPA=0
GDLPYGPGR pc2
−0.02 [−0.16, 0.11]
TVIEDMLAMPK pc2
0.03 [−0.09, 0.15]
RE Model
0.01 [−0.09, 0.10]
−0.2
−0.1
0
0.1
0.2
Effect of 9:87072939:C:T (rs4878043)

GAS1 : NP2
P54826

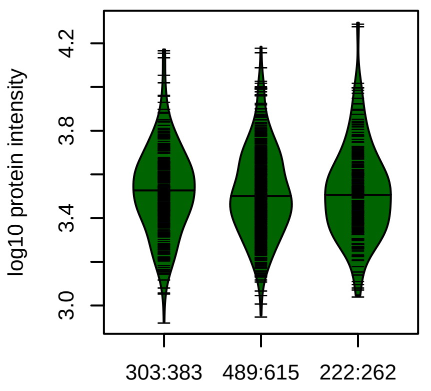

9:89687854:C:T_C
p = 0.46, beta = −0.0323, N = 1014

GDLPYGPGR pc2
P54826

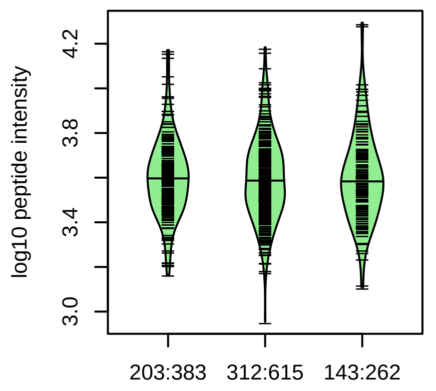

9:89687854:C:T_C
p = 0.65, beta = −0.0248, N = 658

TVIEDMLAMPK pc2
P54826

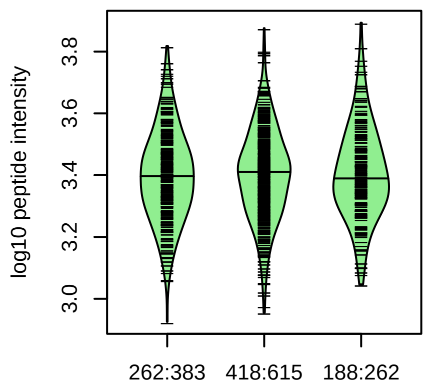

9:89687854:C:T_C
p = 0.54, beta = 0.0289, N = 868

Assay Target: GAS1
UniProt: P54826
rsID: rs4878043
Target SNP: 9:87072939:C:T
Proxy SNP: 9:89687854:C:T
deCODE log10(p): 23.1
deCODE BETA: −0.08
deCODE MSPA: 0
−:−
658:868

269. SERPINB1 (P30740)
deCODE MSPA=0

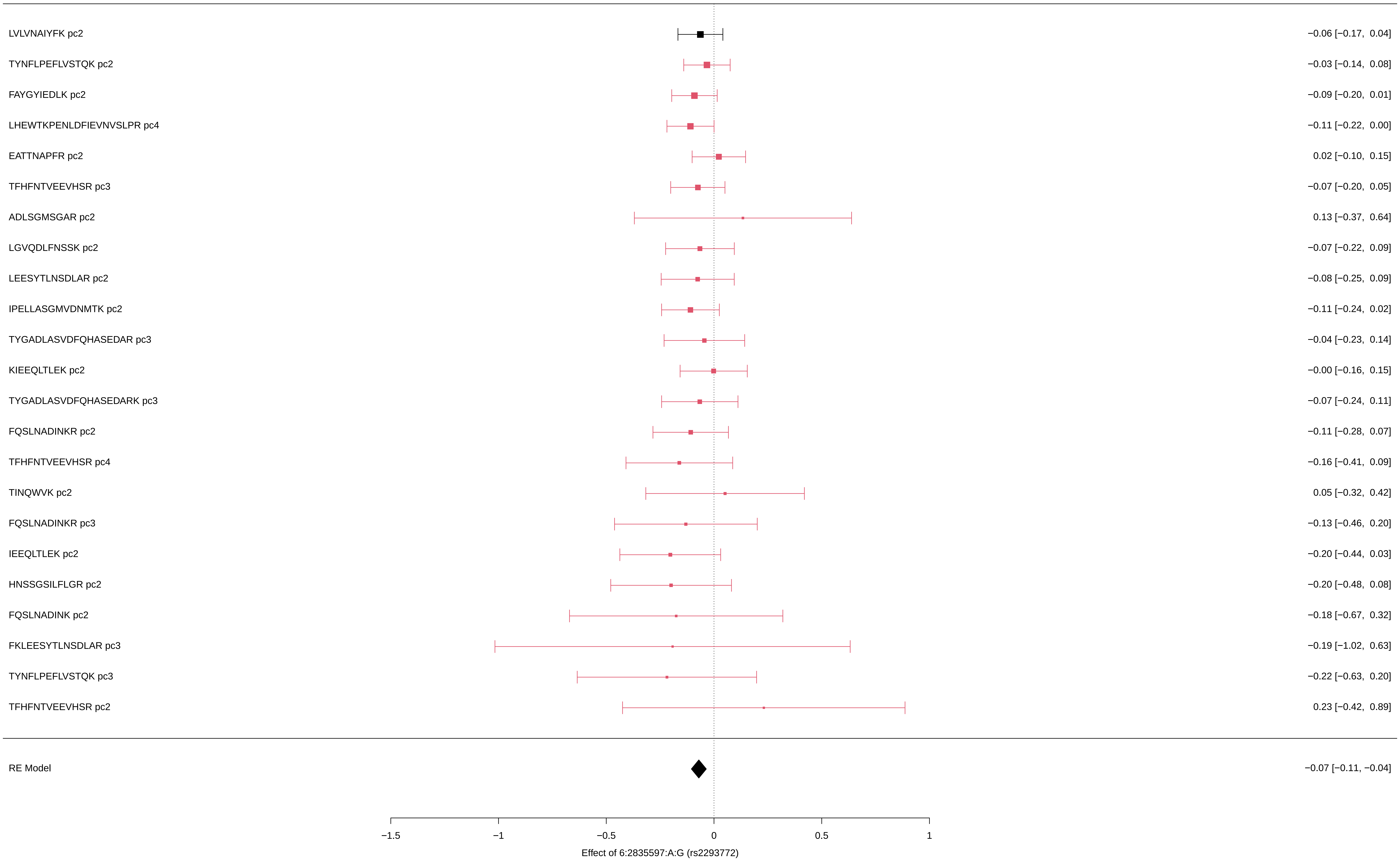

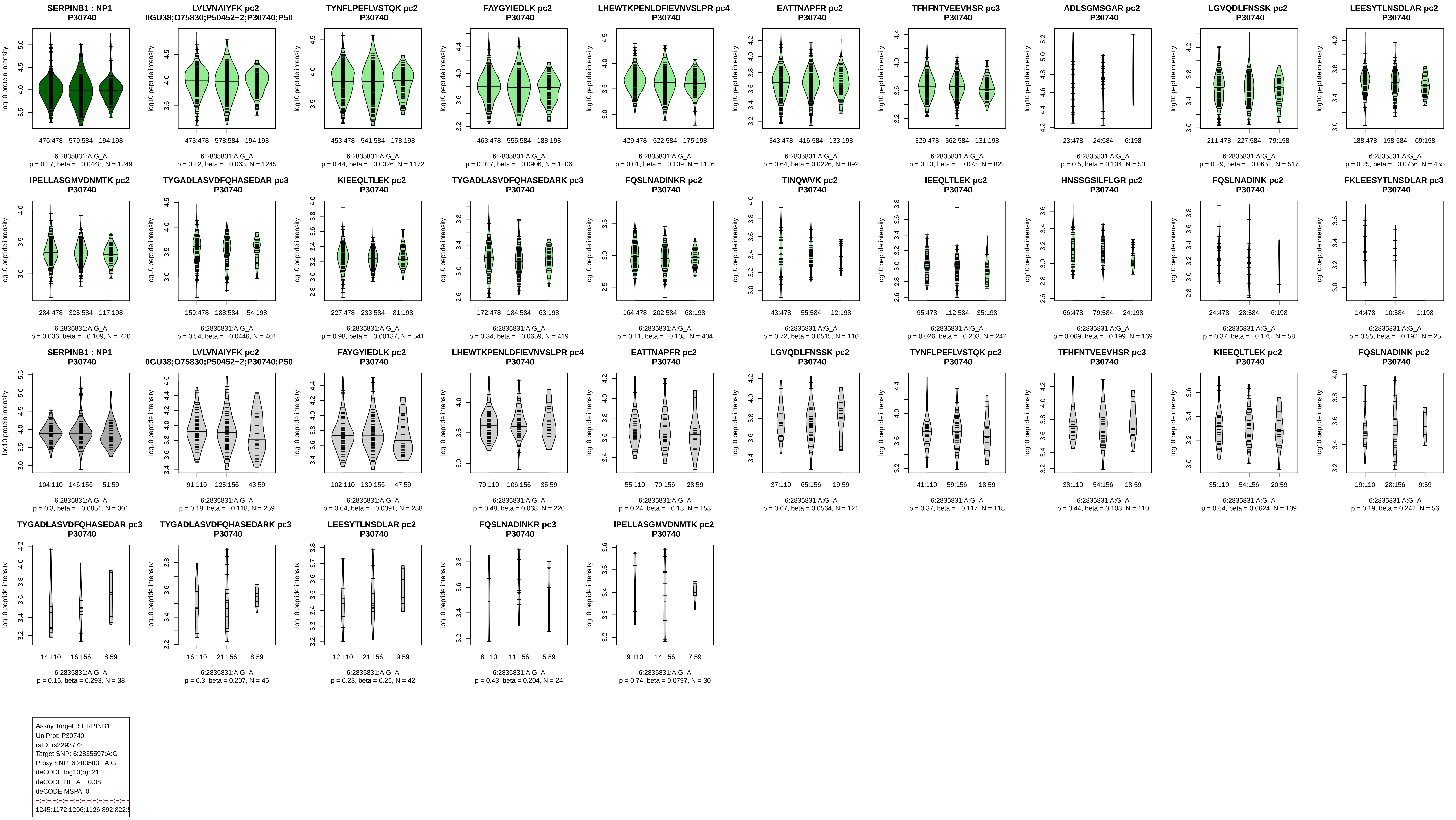

SERPINB1 : NP1
P30740
log10 protein intensity
476:478 579:584 194:198
6:2835831:A:G_A
p = 0.27, beta = −0.0448, N = 1249
LVLVNAIYFK pc2
0GU38;O75830;P50452−2;P30740;P50
log10 peptide intensity
473:478 578:584 194:198
6:2835831:A:G_A
p = 0.12, beta = −0.063, N = 1245
TYNFLPEFLVSTQK pc2
P30740
453:478 541:584 178:198
6:2835831:A:G_A
p = 0.44, beta = −0.0326, N = 1172
FAYGYIEDLK pc2
P30740
463:478 555:584 188:198
6:2835831:A:G_A
p = 0.027, beta = −0.0906, N = 1206
LHEWTKPENLDFIEVNVSLPR pc4
P30740
429:478 522:584 175:198
6:2835831:A:G_A
p = 0.01, beta = −0.109, N = 1126
EATTNAPFR pc2
P30740
343:478 416:584 133:198
6:2835831:A:G_A
p = 0.64, beta = 0.0226, N = 892
TFHFNTVEEVHSR pc3
P30740
329:478 362:584 131:198
6:2835831:A:G_A
p = 0.13, beta = −0.075, N = 822
ADLSGMSGAR pc2
P30740
23:478 24:584 6:198
6:2835831:A:G_A
p = 0.5, beta = 0.134, N = 53
LGVQDLFNSSK pc2
P30740
211:478 227:584 79:198
6:2835831:A:G_A
p = 0.29, beta = −0.0651, N = 517
LEESYTLNSDLAR pc2
P30740
188:478 198:584 69:198
6:2835831:A:G_A
p = 0.25, beta = −0.0756, N = 455
IPELLASGMVDNMTK pc2
P30740
284:478 325:584 117:198
6:2835831:A:G_A
p = 0.036, beta = −0.109, N = 726
TYGADLASVDFQHASEDAR pc3
P30740
159:478 188:584 54:198
6:2835831:A:G_A
p = 0.54, beta = −0.0446, N = 401
KIEEQLTLEK pc2
P30740
227:478 233:584 81:198
6:2835831:A:G_A
p = 0.98, beta = −0.00137, N = 541
TYGADLASVDFQHASEDARK pc3
P30740
172:478 184:584 63:198
6:2835831:A:G_A
p = 0.34, beta = −0.0659, N = 419
FQSLNADINKR pc2
P30740
164:478 202:584 68:198
6:2835831:A:G_A
p = 0.11, beta = −0.108, N = 434
TINQWVK pc2
P30740
43:478 55:584 12:198
6:2835831:A:G_A
p = 0.72, beta = 0.0515, N = 110
IEEQLTLEK pc2
P30740
95:478 112:584 35:198
6:2835831:A:G_A
p = 0.026, beta = −0.203, N = 242
HNSSGSILFLGR pc2
P30740
66:478 79:584 24:198
6:2835831:A:G_A
p = 0.069, beta = −0.199, N = 169
FQSLNADINK pc2
P30740
24:478 28:584 6:198
6:2835831:A:G_A
p = 0.37, beta = −0.175, N = 58
FKLEESYTLNSDLAR pc3
P30740
14:478 10:584 1:198
6:2835831:A:G_A
p = 0.55, beta = −0.192, N = 25
SERPINB1 : NP1
P30740
104:110 146:156 51:59
6:2835831:A:G_A
p = 0.3, beta = −0.0851, N = 301
LVLVNAIYFK pc2
0GU38;O75830;P50452−2;P30740;P50
91:110 125:156 43:59
6:2835831:A:G_A
p = 0.18, beta = −0.118, N = 259
FAYGYIEDLK pc2
P30740
102:110 139:156 47:59
6:2835831:A:G_A
p = 0.64, beta = −0.0391, N = 288
LHEWTKPENLDFIEVNVSLPR pc4
P30740
79:110 106:156 35:59
6:2835831:A:G_A
p = 0.48, beta = 0.068, N = 220
EATTNAPFR pc2
P30740
55:110 70:156 28:59
6:2835831:A:G_A
p = 0.24, beta = −0.13, N = 153
LGVQDLFNSSK pc2
P30740
37:110 65:156 19:59
6:2835831:A:G_A
p = 0.67, beta = 0.0564, N = 121
TYNFLPEFLVSTQK pc2
P30740
41:110 59:156 18:59
6:2835831:A:G_A
p = 0.37, beta = −0.117, N = 118
TFHFNTVEEVHSR pc3
P30740
38:110 54:156 18:59
6:2835831:A:G_A
p = 0.44, beta = 0.103, N = 110
KIEEQLTLEK pc2
P30740
35:110 54:156 20:59
6:2835831:A:G_A
p = 0.64, beta = 0.0624, N = 109
FQSLNADINK pc2
P30740
19:110 28:156 9:59
6:2835831:A:G_A
p = 0.19, beta = 0.242, N = 56
TYGADLASVDFQHASEDAR pc3
P30740
14:110 16:156 8:59
6:2835831:A:G_A
p = 0.15, beta = 0.293, N = 38
TYGADLASVDFQHASEDARK pc3
P30740
16:110 21:156 8:59
6:2835831:A:G_A
p = 0.3, beta = 0.207, N = 45
LEESYTLNSDLAR pc2
P30740
12:110 21:156 9:59
6:2835831:A:G_A
p = 0.23, beta = 0.25, N = 42
FQSLNADINKR pc3
P30740
8:110 11:156 5:59
6:2835831:A:G_A
p = 0.43, beta = 0.204, N = 24
IPELLASGMVDNMTK pc2
P30740
9:110 14:156 7:59
6:2835831:A:G_A
p = 0.74, beta = 0.0797, N = 30
Assay Target: SERPINB1
UniProt: P30740
rsID: rs2293772
Target SNP: 6:2835597:A:G
Proxy SNP: 6:2835831:A:G
deCODE log10(p): 21.2
deCODE BETA: −0.08
deCODE MSPA: 0
1245:1172:1206:1126:892:822:5

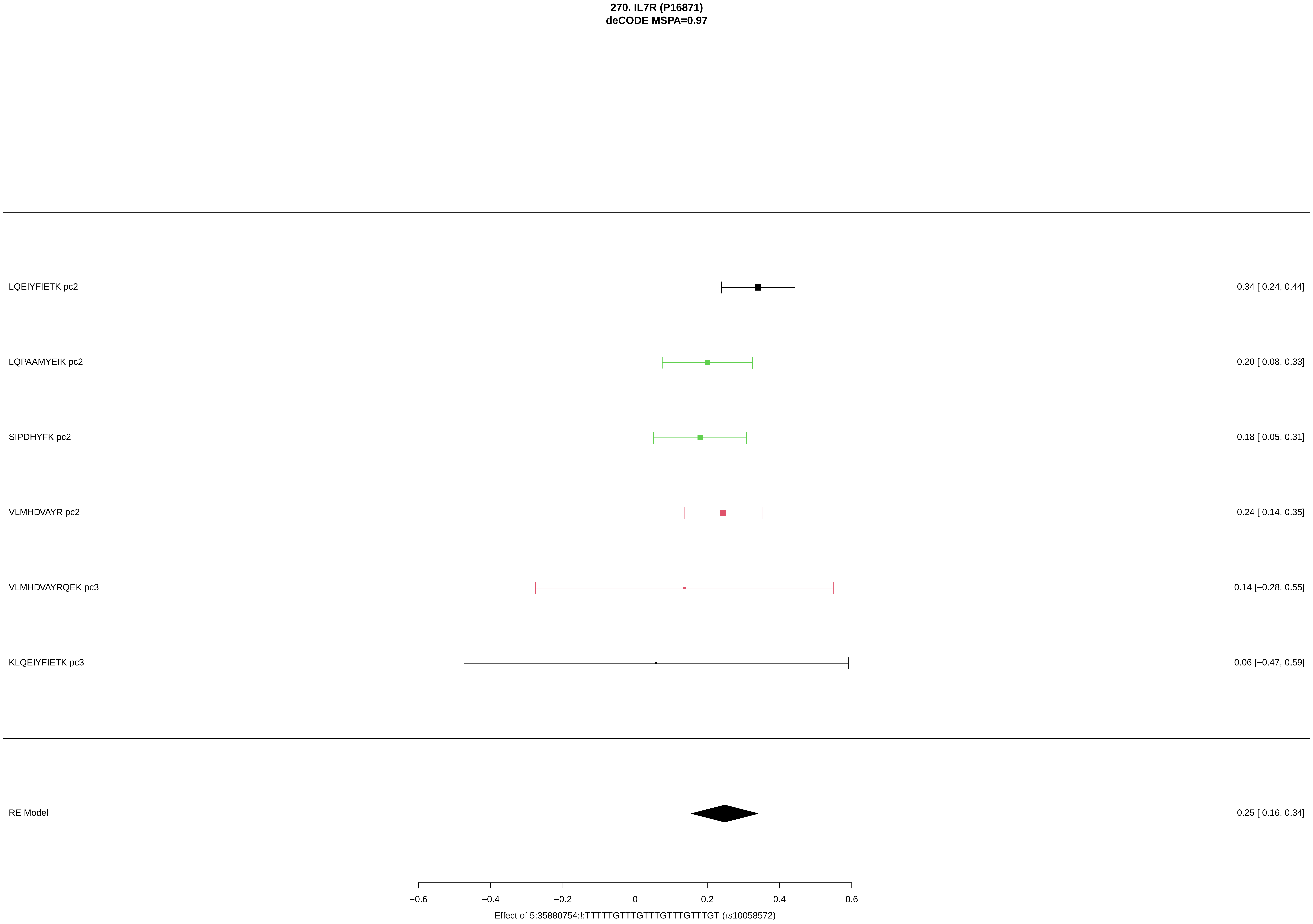
270. IL7R (P16871)
deCODE MSPA=0.97
LQEIYFIETK pc2
0.34 [0.24, 0.44]
LQPAAMYEIK pc2
0.20 [0.08, 0.33]
SIPDHYFK pc2
0.18 [0.05, 0.31]
VLMHDVAYR pc2
0.24 [0.14, 0.35]
VLMHDVAYRQEK pc3
0.14 [−0.28, 0.55]
KLQEIYFIETK pc3
0.06 [−0.47, 0.59]
RE Model
0.25 [0.16, 0.34]
−0.6
−0.4
−0.2
0
0.2
0.4
0.6
Effect of 5:35880754:!:TTTTTGTTTGTTTGTTTGTTTGT (rs10058572)

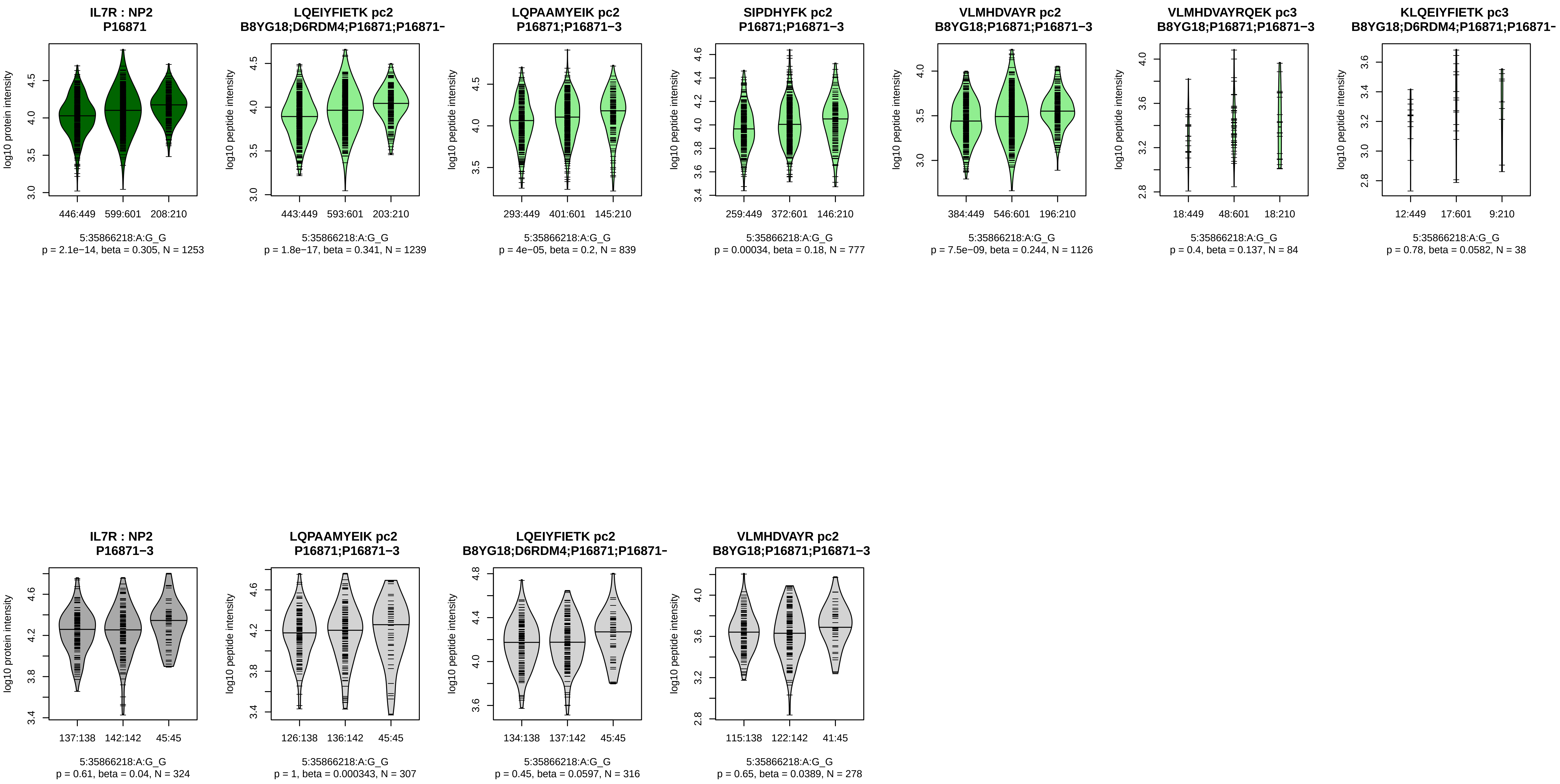

Assay Target: IL7R
UniProt: P16871
rsID: rs10058572
Target SNP: 5:35880754:!:TTTT
Proxy SNP: 5:35866218:A:G
deCODE log10(p): 20.9
deCODE BETA: 0.08
deCODE MSPA: 0.97
::*:*:–:–
1239:839:777:1126:84:38

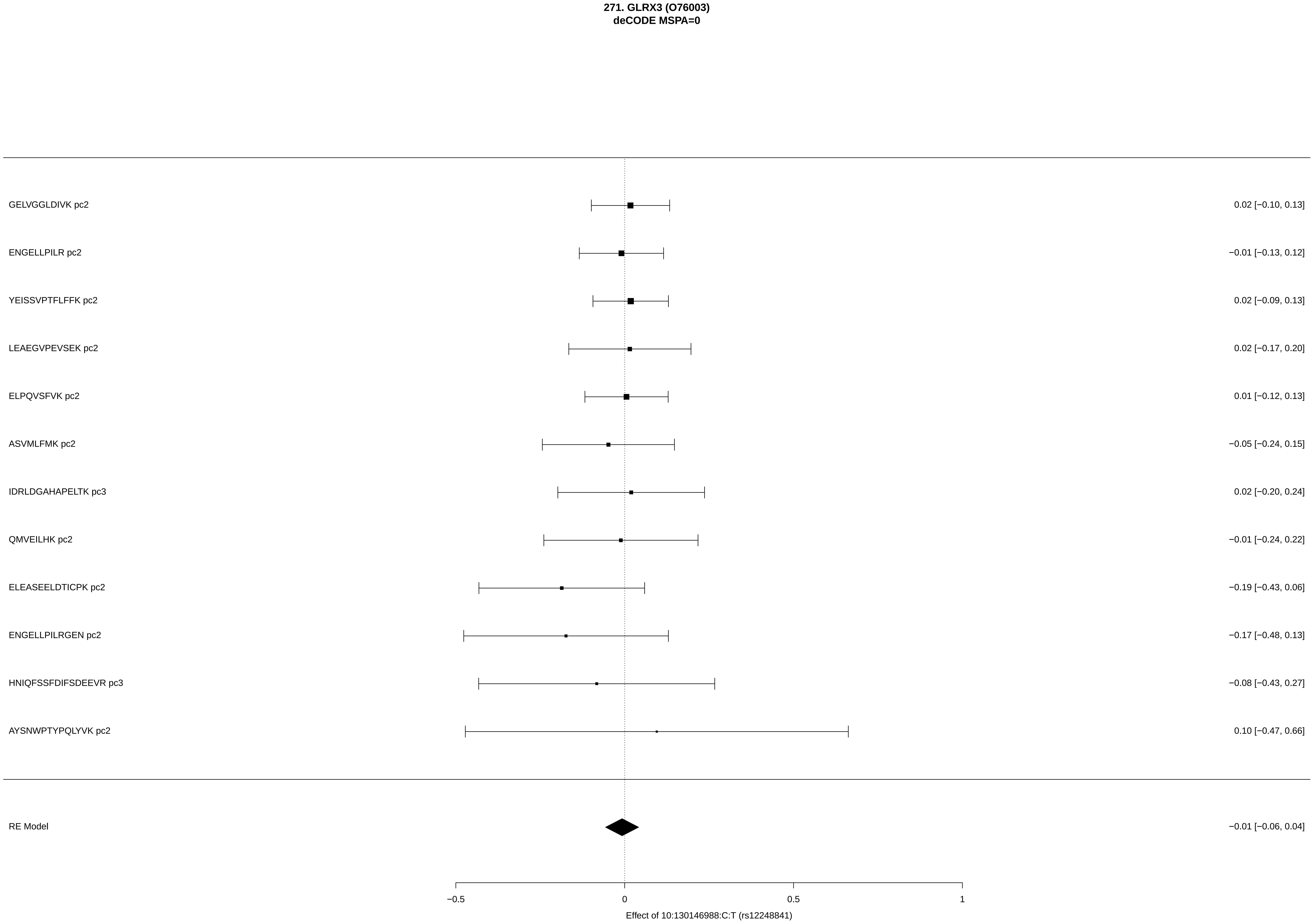

271. GLRX3 (O76003)
deCODE MSPA=0
GELVGGLDIVK pc2
0.02 [−0.10, 0.13]
ENGELLPILR pc2
−0.01 [−0.13, 0.12]
YEISSVPTFLFFK pc2
0.02 [−0.09, 0.13]
LEAEGVPEVSEK pc2
0.02 [−0.17, 0.20]
ELPQVSFVK pc2
0.01 [−0.12, 0.13]
ASVMLFMK pc2
−0.05 [−0.24, 0.15]
IDRLDGAHAPELTK pc3
0.02 [−0.20, 0.24]
QMVEILHK pc2
−0.01 [−0.24, 0.22]
ELEASEELDTICPK pc2
−0.19 [−0.43, 0.06]
ENGELLPILRGEN pc2
−0.17 [−0.48, 0.13]
HNIQFSSFDIFSDEEVR pc3
−0.08 [−0.43, 0.27]
AYSNWPTYPQLYVK pc2
0.10 [−0.47, 0.66]
RE Model
−0.01 [−0.06, 0.04]
−0.5
0
0.5
1
Effect of 10:130146988:C:T (rs12248841)

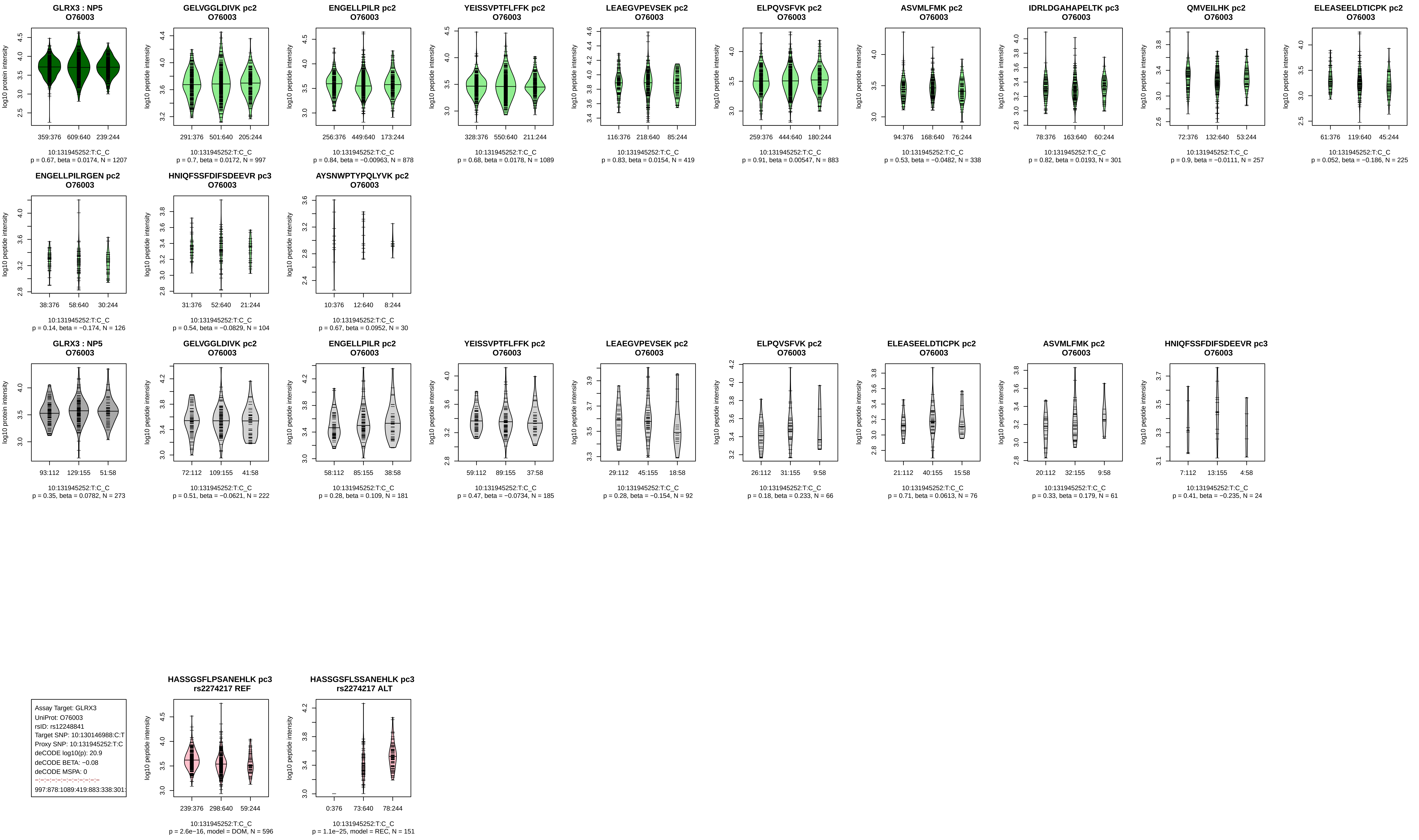
GLRX3 : NP5
O76003
359:376 609:640 239:244
10:131945252:T:C_C
p = 0.67, beta = 0.0174, N = 1207
GELVGGLDIVK pc2
O76003
291:376 501:640 205:244
10:131945252:T:C_C
p = 0.7, beta = 0.0172, N = 997
ENGELLPILR pc2
O76003
256:376 449:640 173:244
10:131945252:T:C_C
p = 0.84, beta = −0.00963, N = 878
YEISSVPTFLFFK pc2
O76003
328:376 550:640 211:244
10:131945252:T:C_C
p = 0.68, beta = 0.0178, N = 1089
LEAEGVPEVSEK pc2
O76003
116:376 218:640 85:244
10:131945252:T:C_C
p = 0.83, beta = 0.0154, N = 419
ELPQVSFVK pc2
O76003
259:376 444:640 180:244
10:131945252:T:C_C
p = 0.91, beta = 0.00547, N = 883
ASVMLFMK pc2
O76003
94:376 168:640 76:244
10:131945252:T:C_C
p = 0.53, beta = −0.0482, N = 338
IDRLDGAHAPELTK pc3
O76003
78:376 163:640 60:244
10:131945252:T:C_C
p = 0.82, beta = 0.0193, N = 301
QMVEILHK pc2
O76003
72:376 132:640 53:244
10:131945252:T:C_C
p = 0.9, beta = −0.0111, N = 257
ELEASEELDTICPK pc2
O76003
61:376 119:640 45:244
10:131945252:T:C_C
p = 0.052, beta = −0.186, N = 225
ENGELLPILRGEN pc2
O76003
38:376 58:640 30:244
10:131945252:T:C_C
p = 0.14, beta = −0.174, N = 126
HNIQFSSFDIFSDEEVR pc3
O76003
31:376 52:640 21:244
10:131945252:T:C_C
p = 0.54, beta = −0.0829, N = 104
AYSNWPTYPQLYVK pc2
O76003
10:376 12:640 8:244
10:131945252:T:C_C
p = 0.67, beta = 0.0952, N = 30
GLRX3 : NP5
O76003
93:112 129:155 51:58
10:131945252:T:C_C
p = 0.35, beta = 0.0782, N = 273
GELVGGLDIVK pc2
O76003
72:112 109:155 41:58
10:131945252:T:C_C
p = 0.51, beta = −0.0621, N = 222
ENGELLPILR pc2
O76003
58:112 85:155 38:58
10:131945252:T:C_C
p = 0.28, beta = 0.109, N = 181
YEISSVPTFLFFK pc2
O76003
59:112 89:155 37:58
10:131945252:T:C_C
p = 0.47, beta = −0.0734, N = 185
LEAEGVPEVSEK pc2
O76003
29:112 45:155 18:58
10:131945252:T:C_C
p = 0.28, beta = −0.154, N = 92
ELPQVSFVK pc2
O76003
26:112 31:155 9:58
10:131945252:T:C_C
p = 0.18, beta = 0.233, N = 66
ELEASEELDTICPK pc2
O76003
21:112 40:155 15:58
10:131945252:T:C_C
p = 0.71, beta = 0.0613, N = 76
ASVMLFMK pc2
O76003
20:112 32:155 9:58
10:131945252:T:C_C
p = 0.33, beta = 0.179, N = 61
HNIQFSSFDIFSDEEVR pc3
O76003
7:112 13:155 4:58
10:131945252:T:C_C
p = 0.41, beta = −0.235, N = 24
Assay Target: GLRX3
UniProt: O76003
rsID: rs12248841
Target SNP: 10:130146988:C:T
Proxy SNP: 10:131945252:T:C
deCODE log10(p): 20.9
deCODE BETA: −0.08
deCODE MSPA: 0
997:878:1089:419:883:338:301:
HASSGSFLPSANEHLK pc3
rs2274217 REF
239:376 298:640 59:244
10:131945252:T:C_C
p = 2.6e−16, model = DOM, N = 596
HASSGSFLSSANEHLK pc3
rs2274217 ALT
0:376 73:640 78:244
10:131945252:T:C_C
p = 1.1e−25, model = REC, N = 151

272. PRKCA (P17252)
deCODE MSPA=0

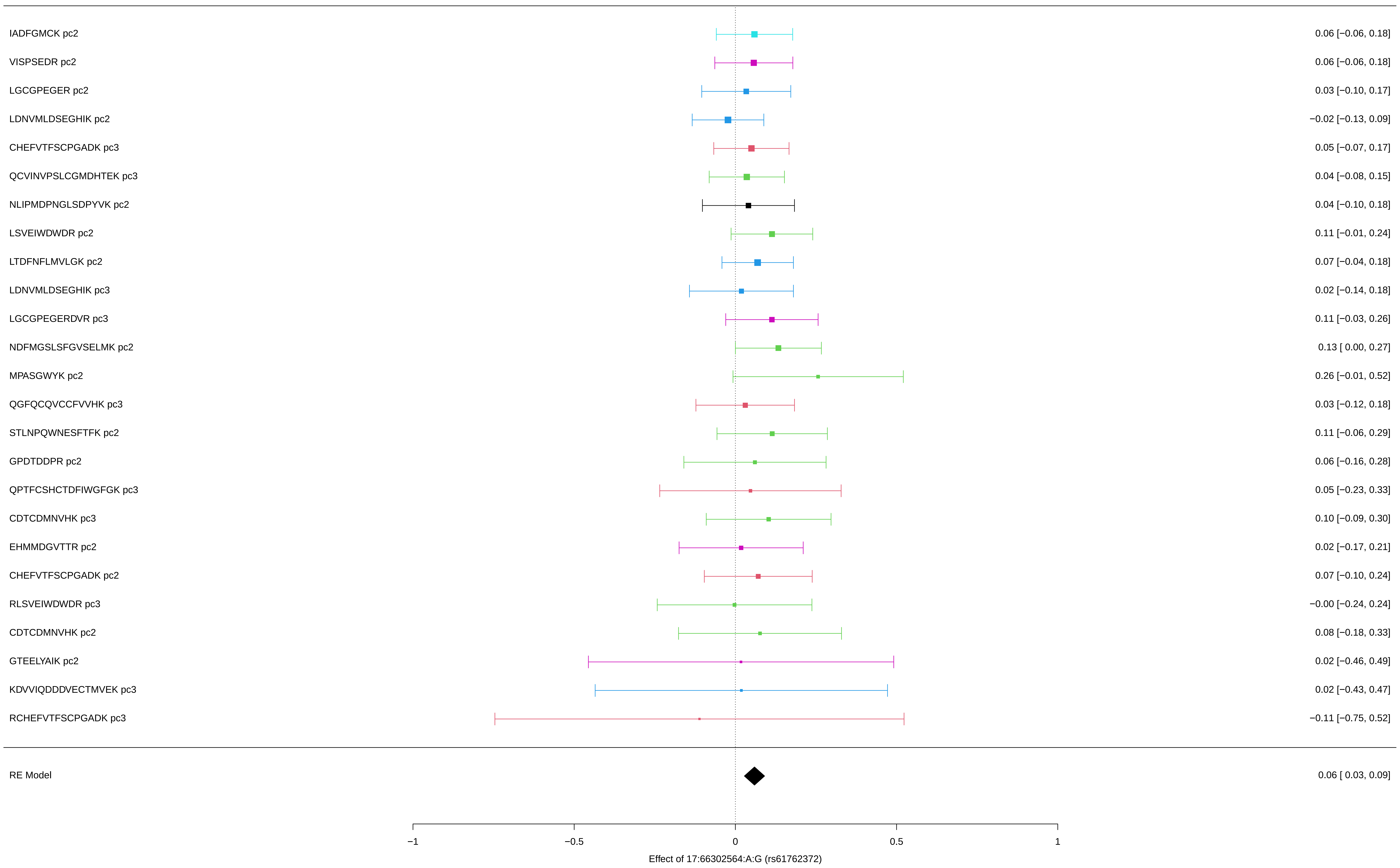

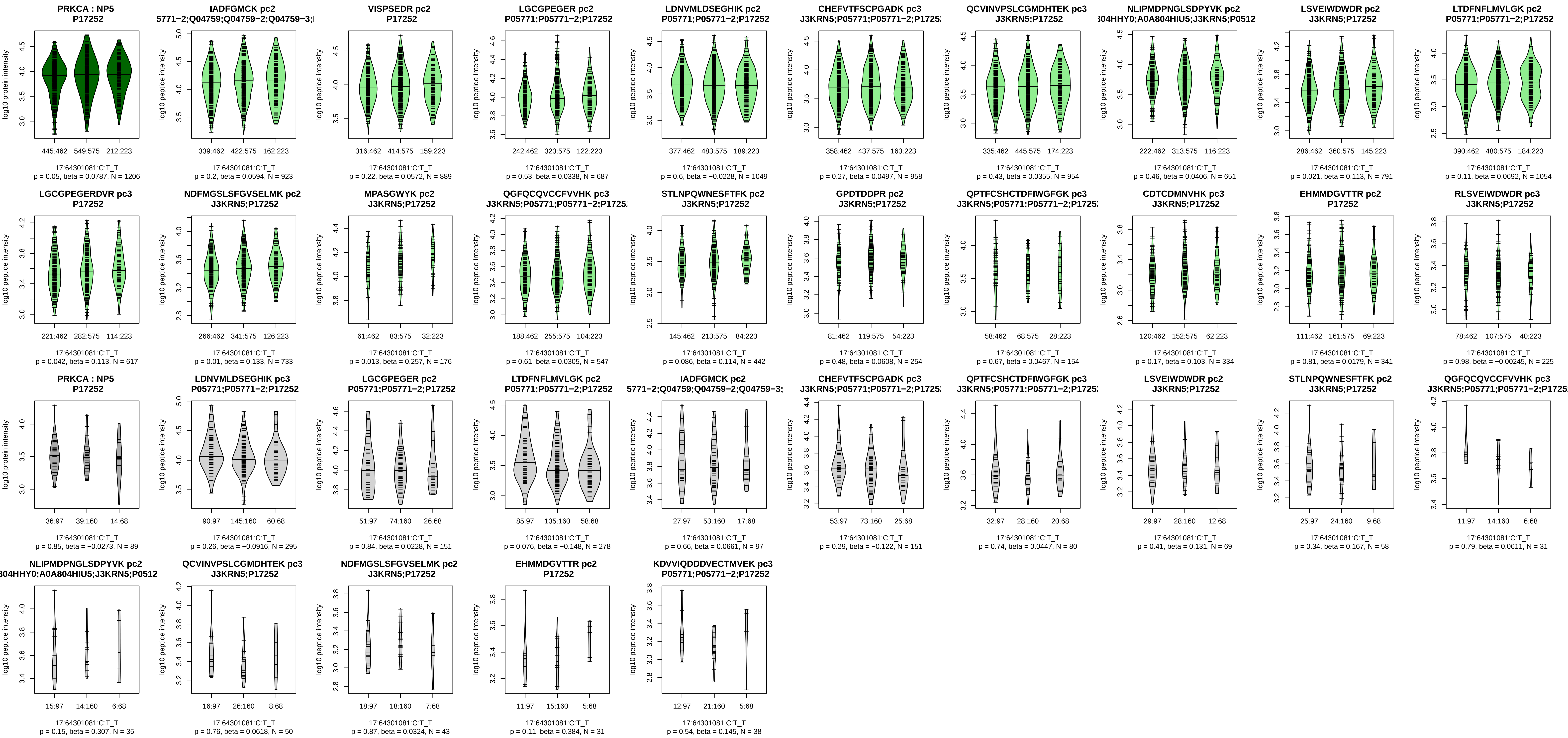

Assay Target: PRKCA
UniProt: P17252
rsID: rs61762372
Target SNP: 17:66302564:A:G
Proxy SNP: 17:64301081:C:T
deCODE log10(p): 20.8
deCODE BETA: 0.08
deCODE MSPA: 0

923:889:687:1049:958:954:651:

273. VIM (P08670)
deCODE MSPA=0

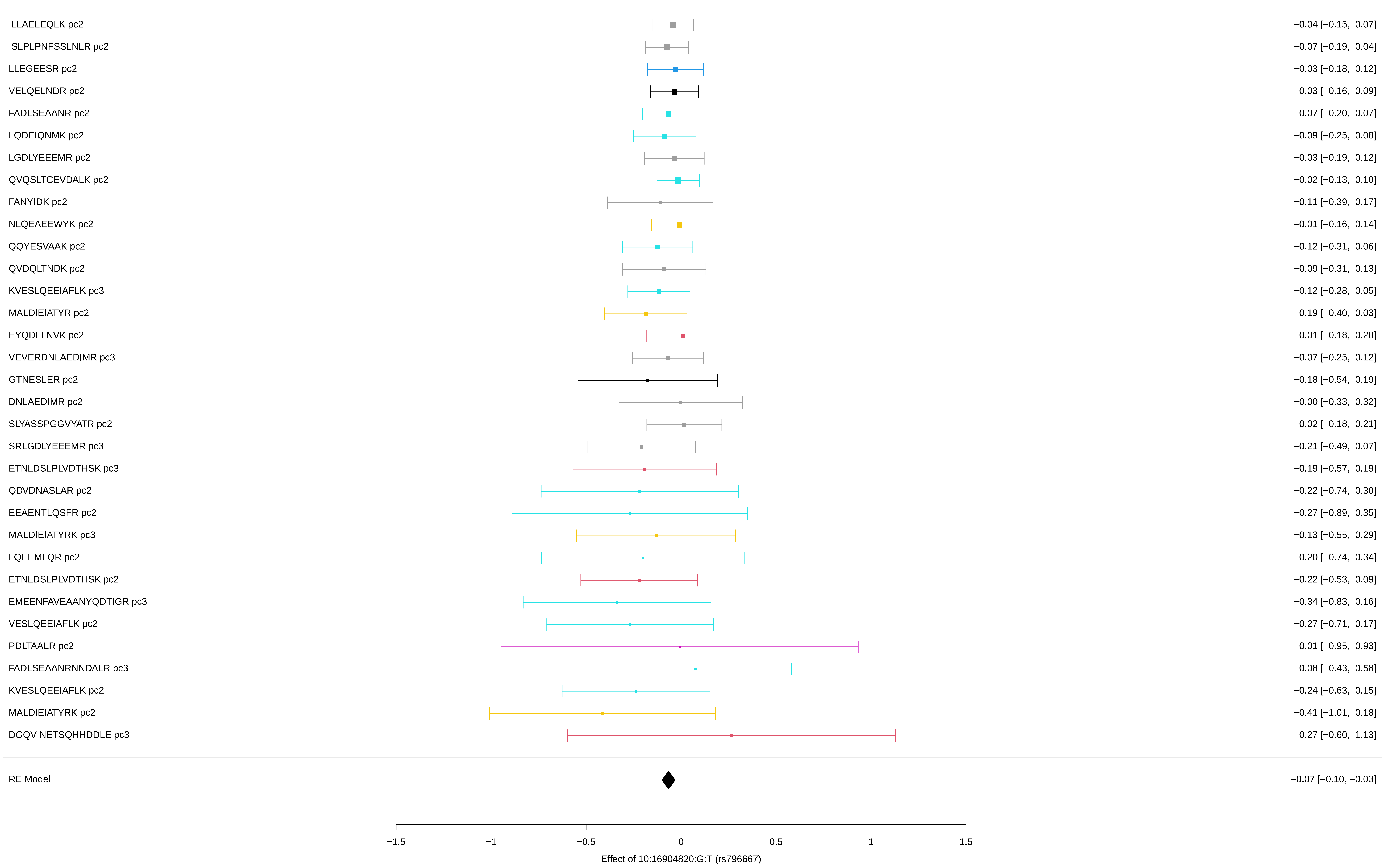

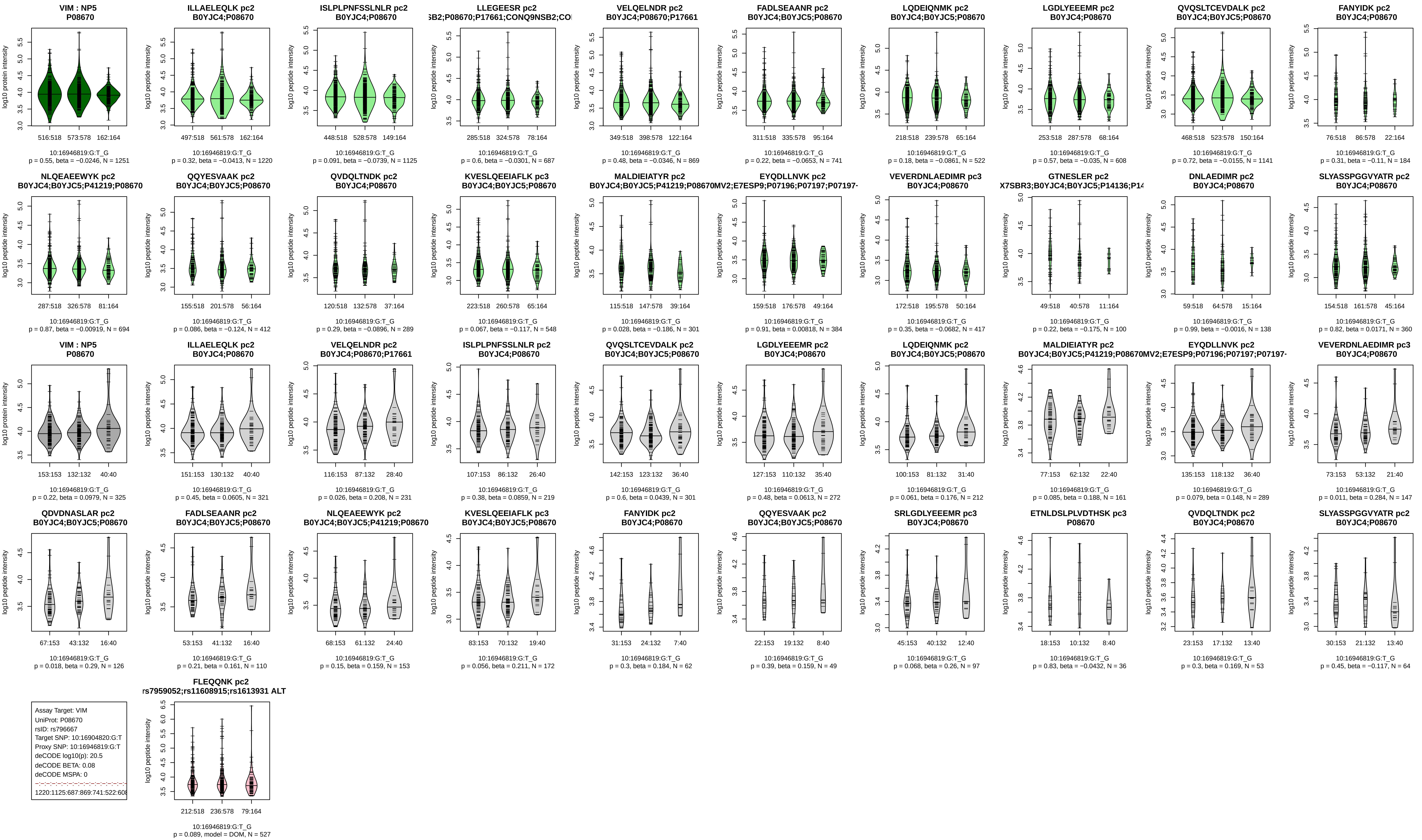
VIM : NP5
P08670
p = 0.55, beta = −0.0246, N = 1251
ILLAELEQLK pc2
B0YJC4;P08670
p = 0.32, beta = −0.0413, N = 1220
ISLPLPNFSSLNLR pc2
B0YJC4;P08670
p = 0.091, beta = −0.0739, N = 1125
LLEGEESR pc2
p = 0.6, beta = −0.0301, N = 687
VELQELNDR pc2
B0YJC4;P08670;P17661
p = 0.48, beta = −0.0346, N = 869
FADLSEAANR pc2
B0YJC4;B0YJC5;P08670
p = 0.22, beta = −0.0653, N = 741
LQDEIQNMK pc2
B0YJC4;B0YJC5;P08670
p = 0.18, beta = −0.0861, N = 522
LGDLYEEEMR pc2
B0YJC4;P08670
p = 0.57, beta = −0.035, N = 608
QVQSLTCEVDALK pc2
B0YJC4;B0YJC5;P08670
p = 0.72, beta = −0.0155, N = 1141
FANYIDK pc2
B0YJC4;P08670
p = 0.31, beta = −0.11, N = 184
NLQEAEEWYK pc2
B0YJC4;B0YJC5;P41219;P08670
p = 0.87, beta = −0.00919, N = 694
QQYESVAAK pc2
B0YJC4;B0YJC5;P08670
p = 0.086, beta = −0.124, N = 412
QVDQLTNDK pc2
B0YJC4;P08670
p = 0.29, beta = −0.0896, N = 289
KVESLQEEIAFLK pc3
B0YJC4;B0YJC5;P08670
p = 0.067, beta = −0.117, N = 548
MALDIEIATYR pc2
p = 0.028, beta = −0.186, N = 301
EYQDLLNVK pc2
p = 0.91, beta = 0.00818, N = 384
VEVERDNLAEDIMR pc3
B0YJC4;P08670
p = 0.35, beta = −0.0861, N = 417
GTNESLER pc2
p = 0.22, beta = −0.175, N = 100
DNLAEDIMR pc2
B0YJC4;P08670
p = 0.99, beta = −0.0016, N = 138
SLYASSPGGVYATR pc2
B0YJC4;P08670
p = 0.82, beta = 0.0171, N = 360
VIM : NP5
P08670
p = 0.22, beta = 0.0979, N = 325
ILLAELEQLK pc2
B0YJC4;P08670
p = 0.45, beta = 0.0605, N = 321
VELQELNDR pc2
B0YJC4;P08670;P17661
p = 0.026, beta = 0.208, N = 231
ISLPLPNFSSLNLR pc2
B0YJC4;P08670
p = 0.38, beta = 0.0859, N = 219
QVQSLTCEVDALK pc2
B0YJC4;B0YJC5;P08670
p = 0.6, beta = 0.0439, N = 301
LGDLYEEEMR pc2
B0YJC4;P08670
p = 0.48, beta = 0.0613, N = 272
LQDEIQNMK pc2
B0YJC4;B0YJC5;P08670
p = 0.061, beta = 0.176, N = 212
MALDIEIATYR pc2
p = 0.085, beta = 0.188, N = 161
EYQDLLNVK pc2
p = 0.079, beta = 0.148, N = 289
VEVERDNLAEDIMR pc3
B0YJC4;P08670
p = 0.011, beta = 0.284, N = 147
QDVDNASLAR pc2
B0YJC4;B0YJC5;P08670
p = 0.018, beta = 0.29, N = 126
FADLSEAANR pc2
B0YJC4;B0YJC5;P08670
p = 0.21, beta = 0.161, N = 110
NLQEAEEWYK pc2
B0YJC4;B0YJC5;P41219;P08670
p = 0.15, beta = 0.159, N = 153
KVESLQEEIAFLK pc3
B0YJC4;B0YJC5;P08670
p = 0.056, beta = 0.211, N = 172
FANYIDK pc2
B0YJC4;P08670
p = 0.3, beta = 0.184, N = 62
QQYESVAAK pc2
B0YJC4;B0YJC5;P08670
p = 0.39, beta = 0.159, N = 49
SRLGDLYEEEMR pc3
B0YJC4;P08670
p = 0.068, beta = 0.26, N = 97
ETNLDSLPLVDTHSK pc3
P08670
p = 0.83, beta = −0.0432, N = 36
QVDQLTNDK pc2
B0YJC4;P08670
p = 0.3, beta = 0.169, N = 53
SLYASSPGGVYATR pc2
B0YJC4;P08670
p = 0.45, beta = −0.117, N = 64
Assay Target: VIM
UniProt: P08670
rsID: rs796667
Target SNP: 10:16904820:G:T
Proxy SNP: 10:16946819:G:T
deCODE log10(p): 20.5
deCODE BETA: 0.08
deCODE MSPA: 0
FLEQQNK pc2
rs7959052;rs11608915;rs1613931 ALT
p = 0.089, model = DOM, N = 527

274. IGFBP1 (P08833)
deCODE MSPA=0

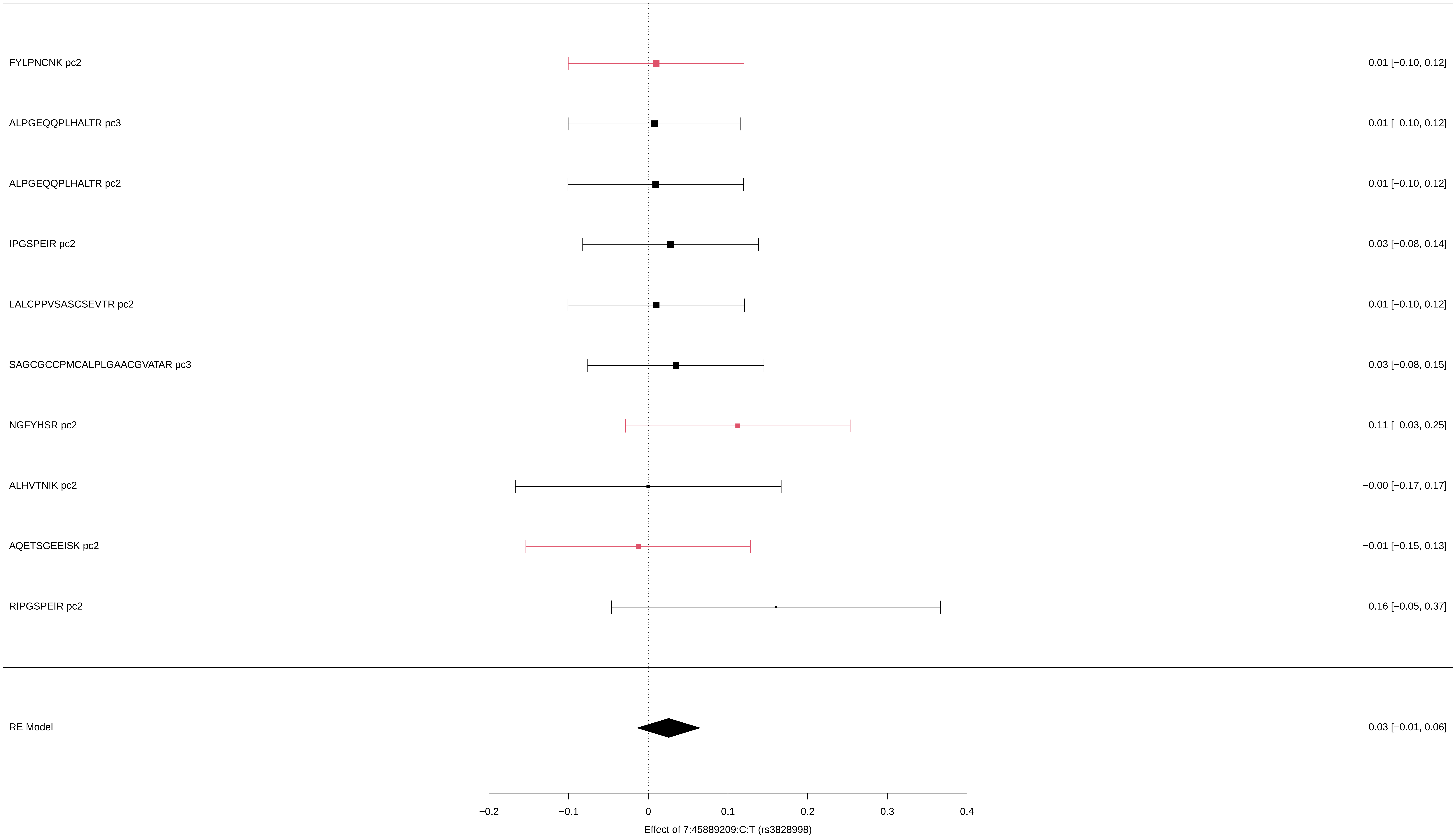

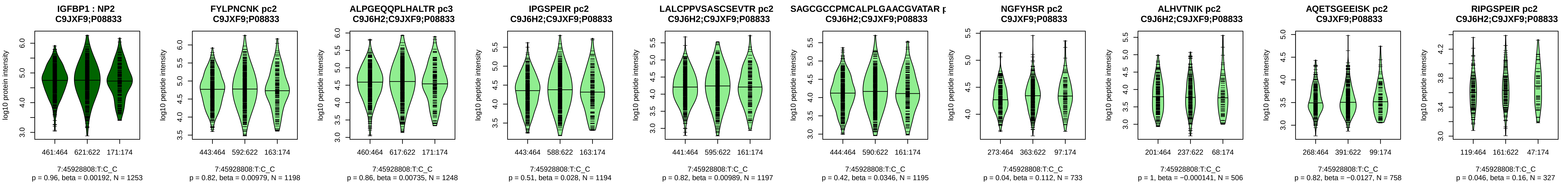

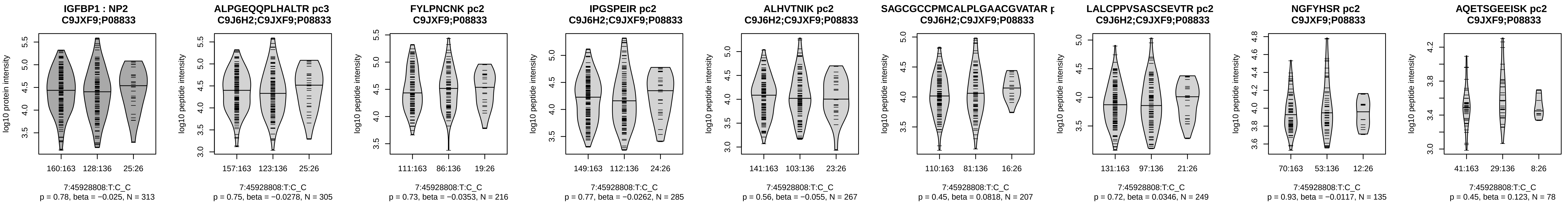

Assay Target: IGFBP1
UniProt: P08833
rsID: rs3828998
Target SNP: 7:45889209:C:T
Proxy SNP: 7:45928808:T:C
deCODE log10(p): 20.4
deCODE BETA: −0.08
deCODE MSPA: 0
–:–:–:–:–:–:–
1198:1248:1206:1194:1197:119

275. RPE (Q96AT9)
deCODE MSPA=0

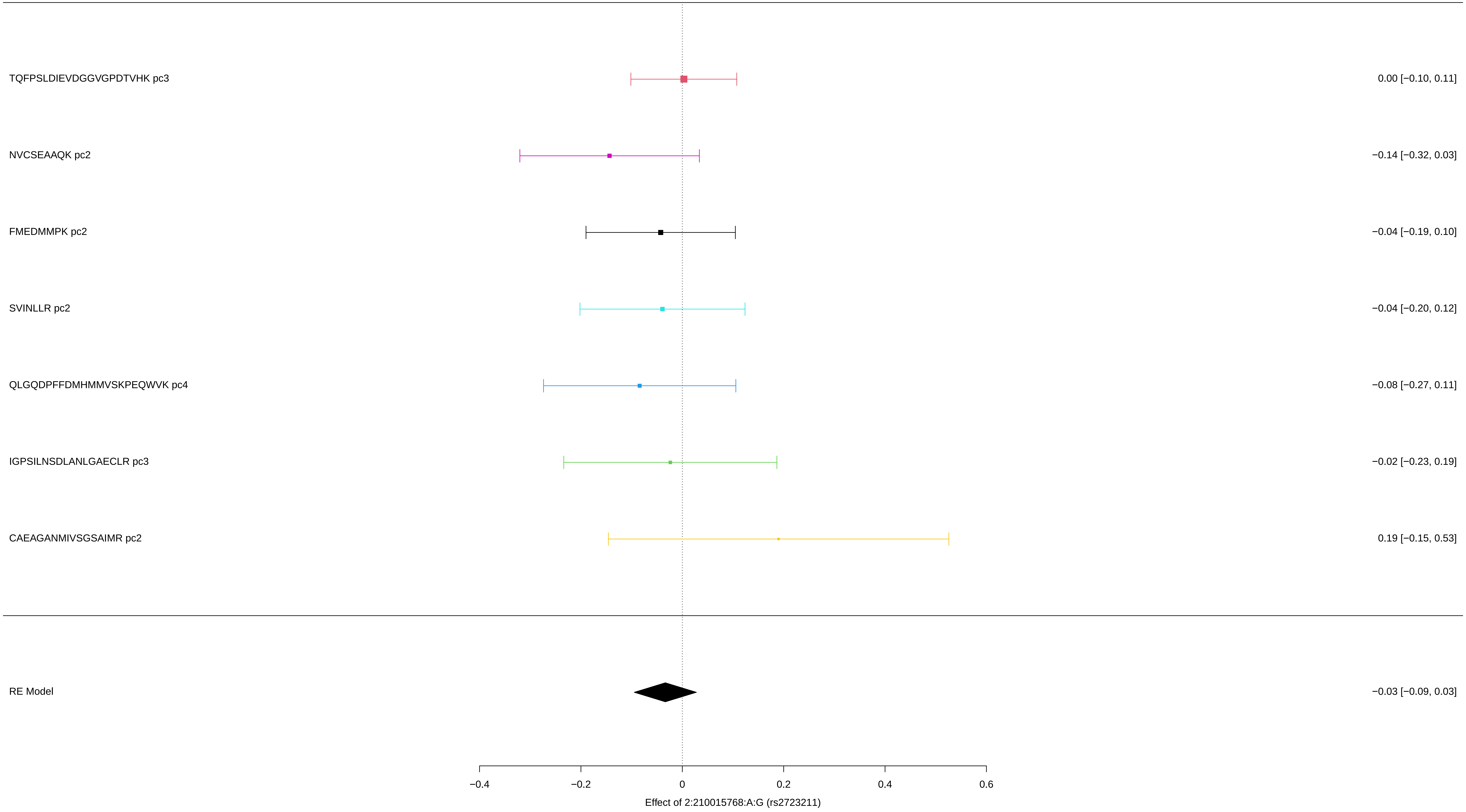

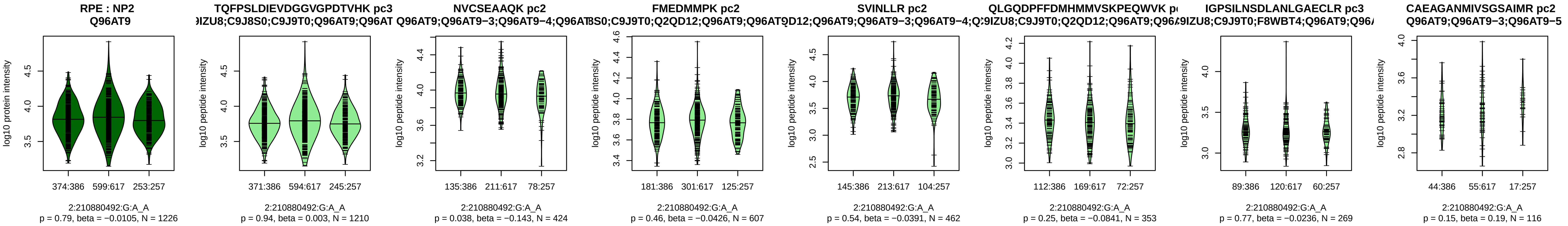

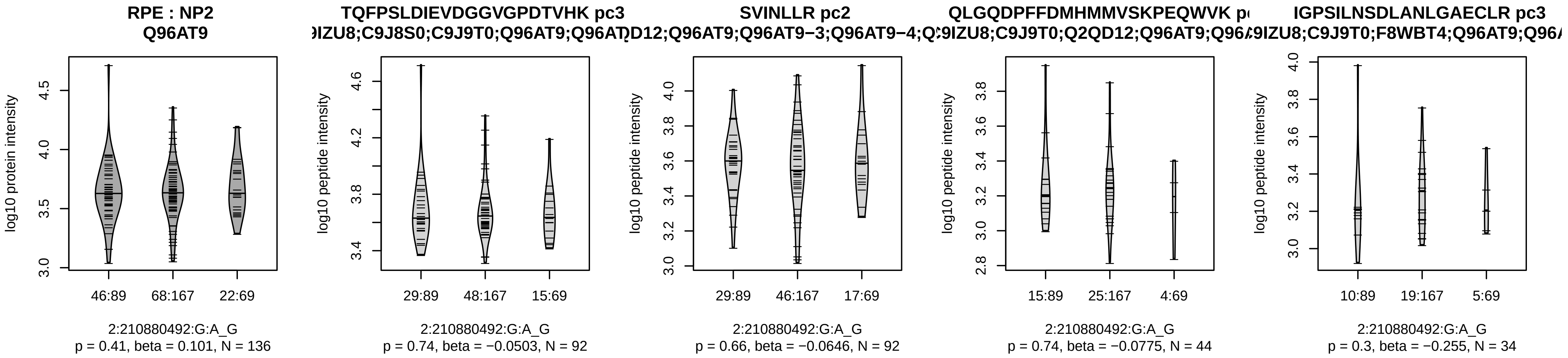

Assay Target: RPE
UniProt: Q96AT9
rsID: rs2723211
Target SNP: 2:210015768:A:G
Proxy SNP: 2:210880492:G:A
deCODE log10(p): 20.1
deCODE BETA: 0.08
deCODE MSPA: 0
−:−:−:−:−:−:−:NA
1210:424:607:462:353:269:116:

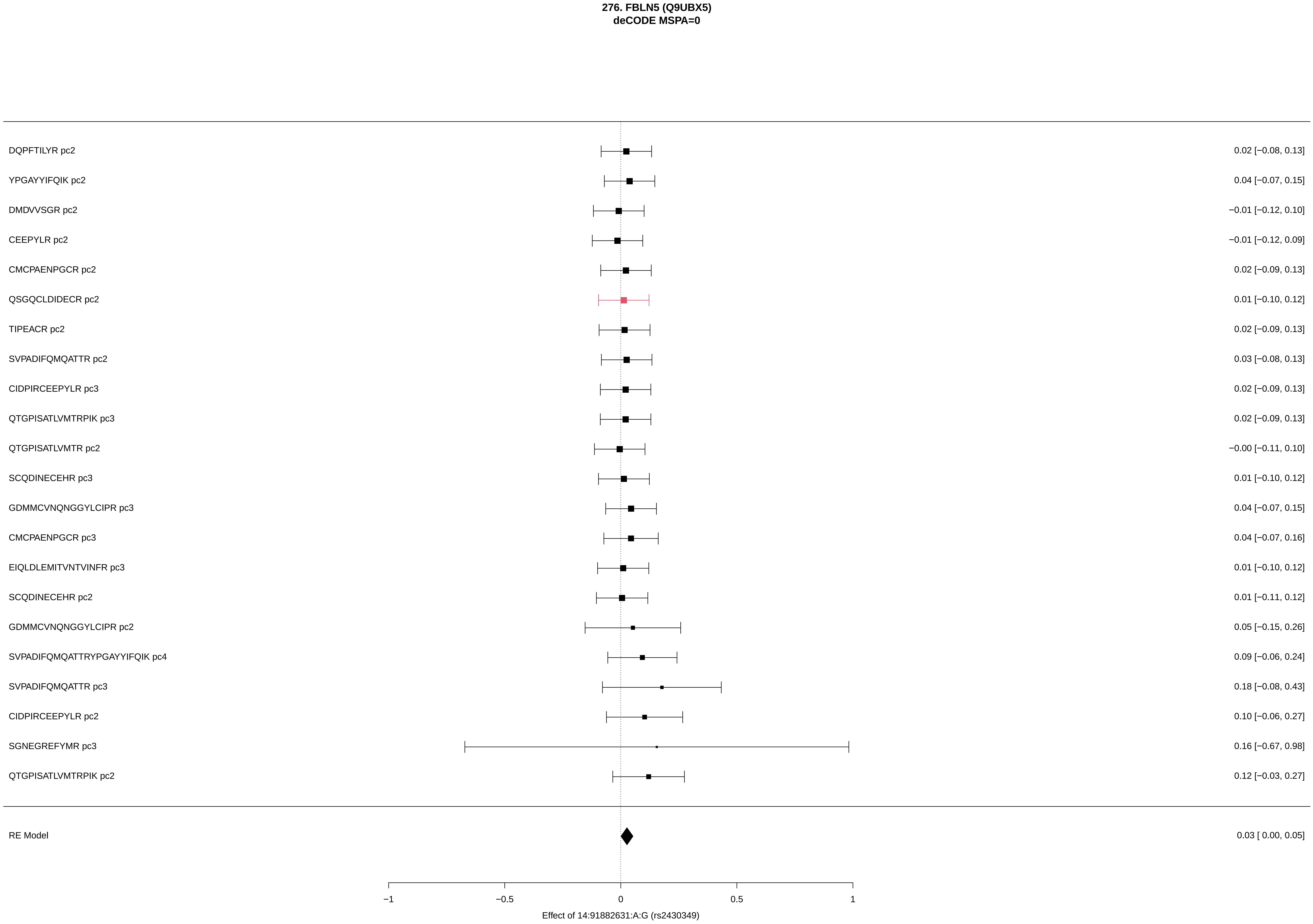

276. FBLN5 (Q9UBX5)
deCODE MSPA=0
DQPFTILYR pc2
0.02 [−0.08, 0.13]
YPGAYYIFQIK pc2
0.04 [−0.07, 0.15]
DMDVVSGR pc2
−0.01 [−0.12, 0.10]
CEEPYLR pc2
−0.01 [−0.12, 0.09]
CMCPAENPGCR pc2
0.02 [−0.09, 0.13]
QSGQCLDIDECR pc2
0.01 [−0.10, 0.12]
TIPEACR pc2
0.02 [−0.09, 0.13]
SVPADIFQMQATTR pc2
0.03 [−0.08, 0.13]
CIDPIRCEEPYLR pc3
0.02 [−0.09, 0.13]
QTGPISATLVMTRPIK pc3
0.02 [−0.09, 0.13]
QTGPISATLVMTR pc2
−0.00 [−0.11, 0.10]
SCQDINECEHR pc3
0.01 [−0.10, 0.12]
GDMMCVNQNGGYLCIPR pc3
0.04 [−0.07, 0.15]
CMCPAENPGCR pc3
0.04 [−0.07, 0.16]
EIQLDLEMITVNTVINFR pc3
0.01 [−0.10, 0.12]
SCQDINECEHR pc2
0.01 [−0.11, 0.12]
GDMMCVNQNGGYLCIPR pc2
0.05 [−0.15, 0.26]
SVPADIFQMQATTRYPGAYYIFQIK pc4
0.09 [−0.06, 0.24]
SVPADIFQMQATTR pc3
0.18 [−0.08, 0.43]
CIDPIRCEEPYLR pc2
0.10 [−0.06, 0.27]
SGNEGREFYMR pc3
0.16 [−0.67, 0.98]
QTGPISATLVMTRPIK pc2
0.12 [−0.03, 0.27]
RE Model
0.03 [0.00, 0.05]
−1
−0.5
0
0.5
1
Effect of 14:91882631:A:G (rs2430349)

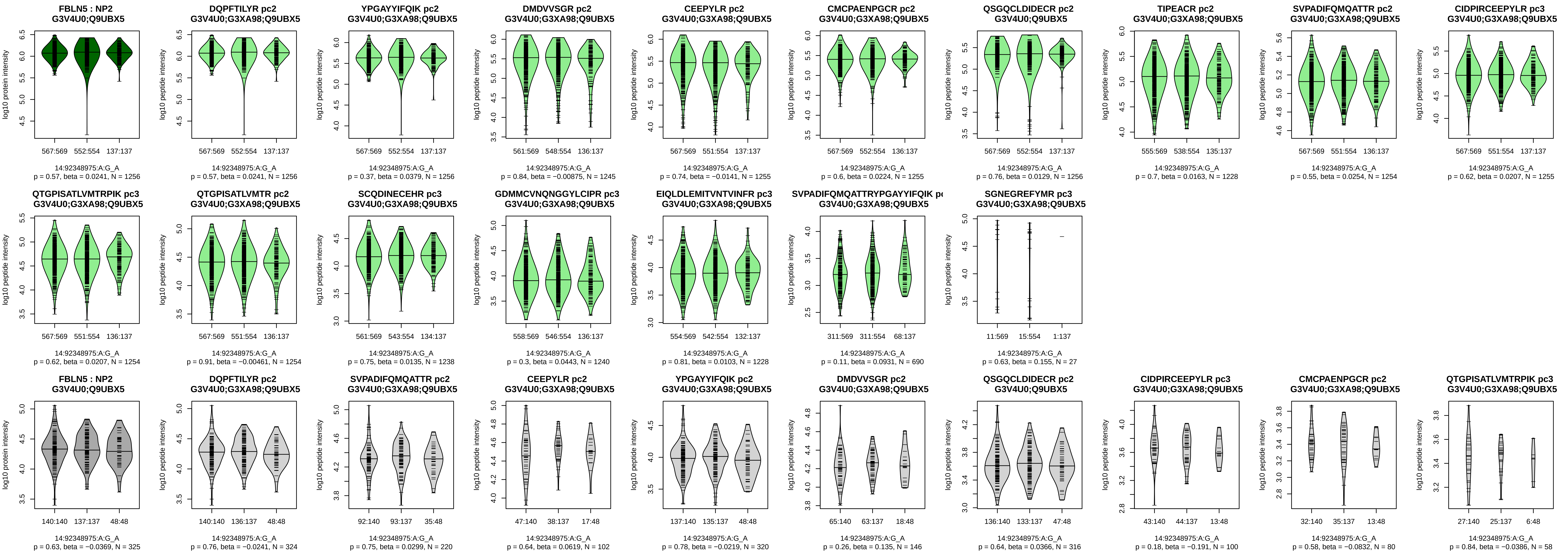

Assay Target: FBLN5
UniProt: Q9UBX5
rsID: rs2430349
Target SNP: 14:91882631:A:G
Proxy SNP: 14:92348975:A:G
deCODE log10(p): 19.1
deCODE BETA: 0.08
deCODE MSPA: 0
1256:1256:1245:1255:1255:125

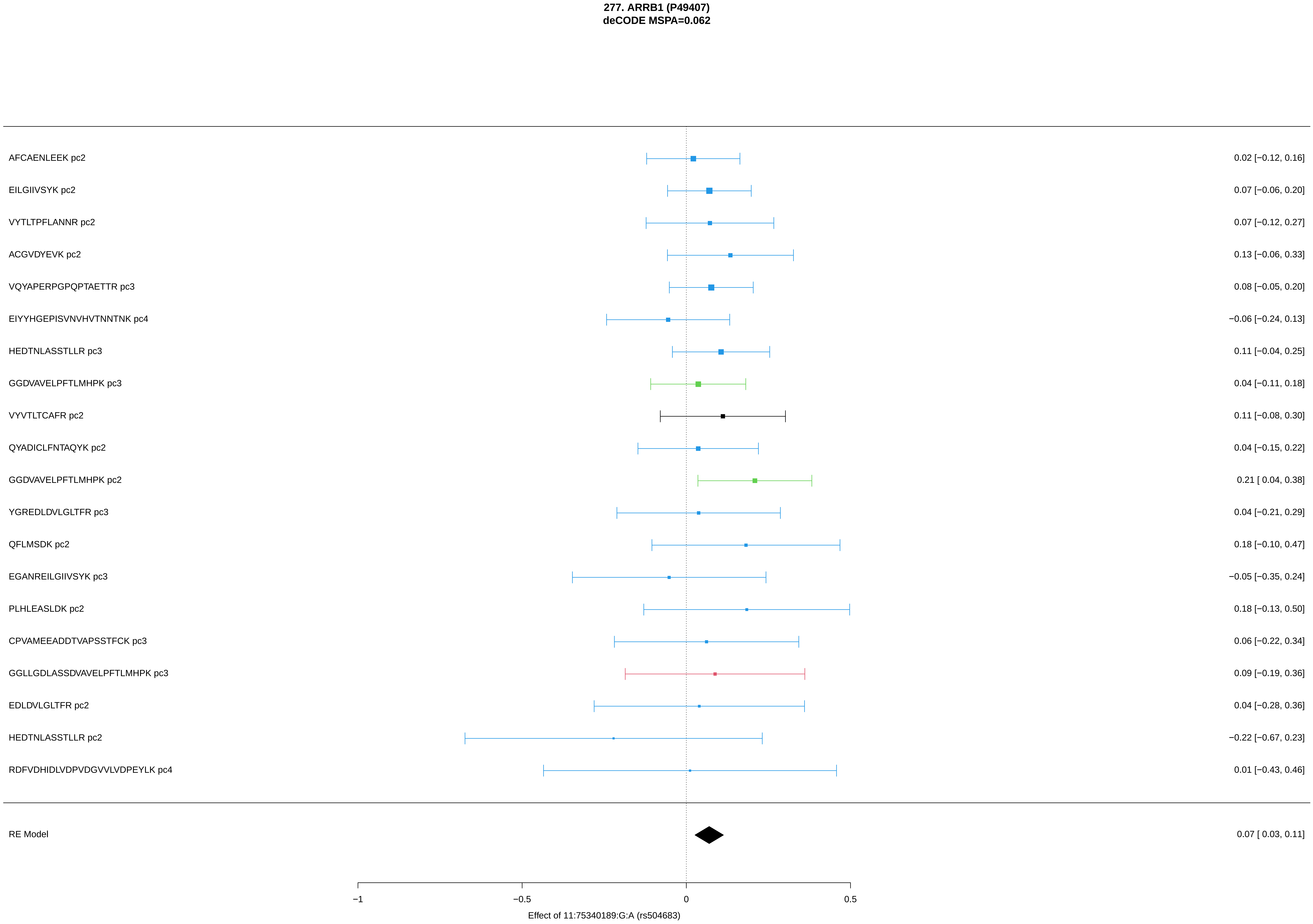
277. ARRB1 (P49407)
deCODE MSPA=0.062
AFCAENLEEK pc2
0.02 [−0.12, 0.16]
EILGIIVSYK pc2
0.07 [−0.06, 0.20]
VYTLTPFLANNR pc2
0.07 [−0.12, 0.27]
ACGVDYEVK pc2
0.13 [−0.06, 0.33]
VQYAPERPGPQPTAETTR pc3
0.08 [−0.05, 0.20]
EIYYHGEPISVNVHVTNNTNK pc4
−0.06 [−0.24, 0.13]
HEDTNLASSTLLR pc3
0.11 [−0.04, 0.25]
GGDVAVELPFTLMHPK pc3
0.04 [−0.11, 0.18]
VYVTLTCAFR pc2
0.11 [−0.08, 0.30]
QYADICLFNTAQYK pc2
0.04 [−0.15, 0.22]
GGDVAVELPFTLMHPK pc2
0.21 [0.04, 0.38]
YGREDLDVLGLTFR pc3
0.04 [−0.21, 0.29]
QFLMSDK pc2
0.18 [−0.10, 0.47]
EGANREILGIIVSYK pc3
−0.05 [−0.35, 0.24]
PLHLEASLDK pc2
0.18 [−0.13, 0.50]
CPVAMEEADDTVAPSSTFCK pc3
0.06 [−0.22, 0.34]
GGLLGDLASSDVAVELPFTLMHPK pc3
0.09 [−0.19, 0.36]
EDLDVLGLTFR pc2
0.04 [−0.28, 0.36]
HEDTNLASSTLLR pc2
−0.22 [−0.67, 0.23]
RDFVDHIDLVDPVDGVVLVDPEYLK pc4
0.01 [−0.43, 0.46]
RE Model
0.07 [0.03, 0.11]
−1
−0.5
0
0.5
Effect of 11:75340189:G:A (rs504683)

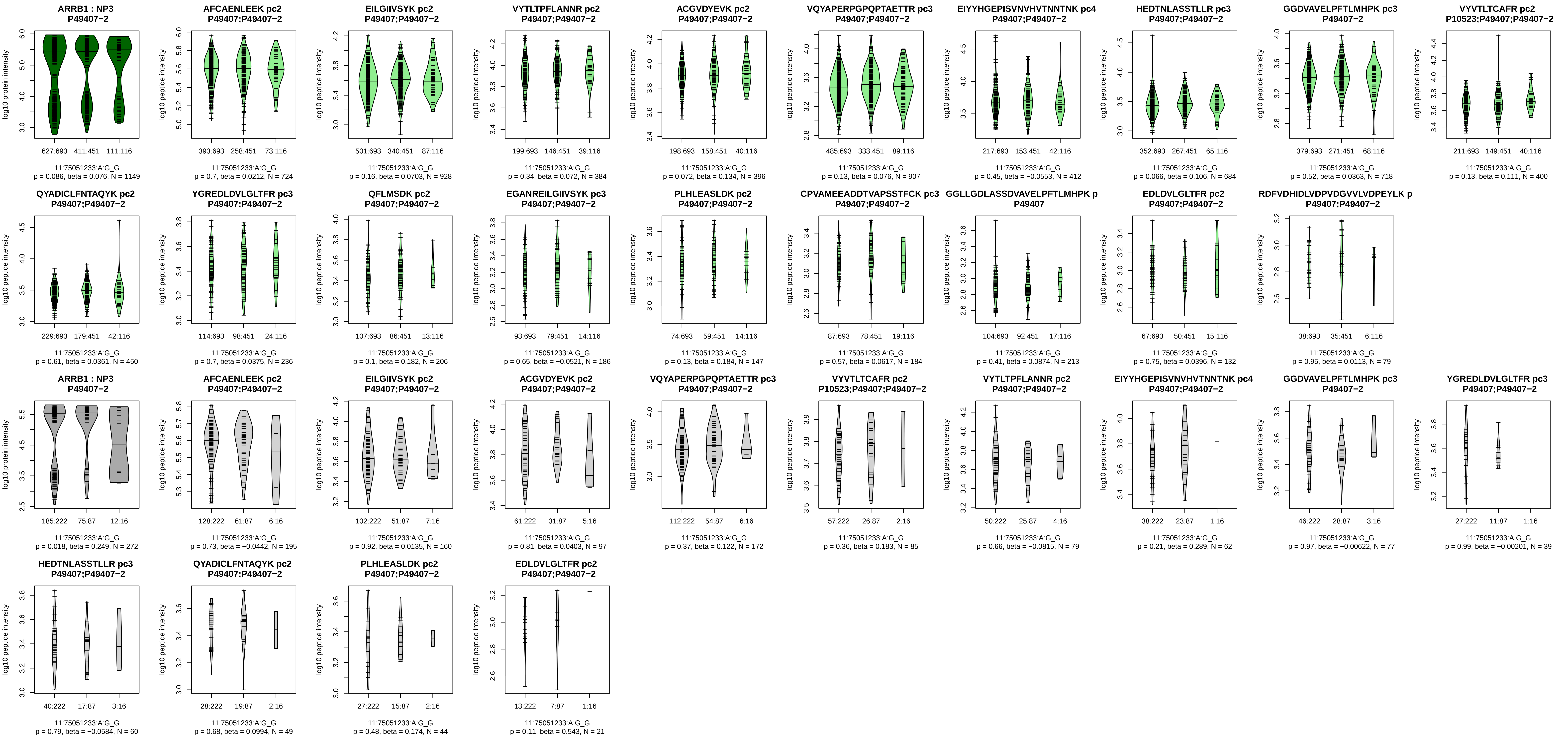

Assay Target: ARRB1
UniProt: P49407
rsID: rs504683
Target SNP: 11:75340189:G:A
Proxy SNP: 11:75051233:A:G
deCODE log10(p): 19
deCODE BETA: 0.08
deCODE MSPA: 0.062
724:928:384:396:907:412:684:7

278. CNP (P09543)
deCODE MSPA=0

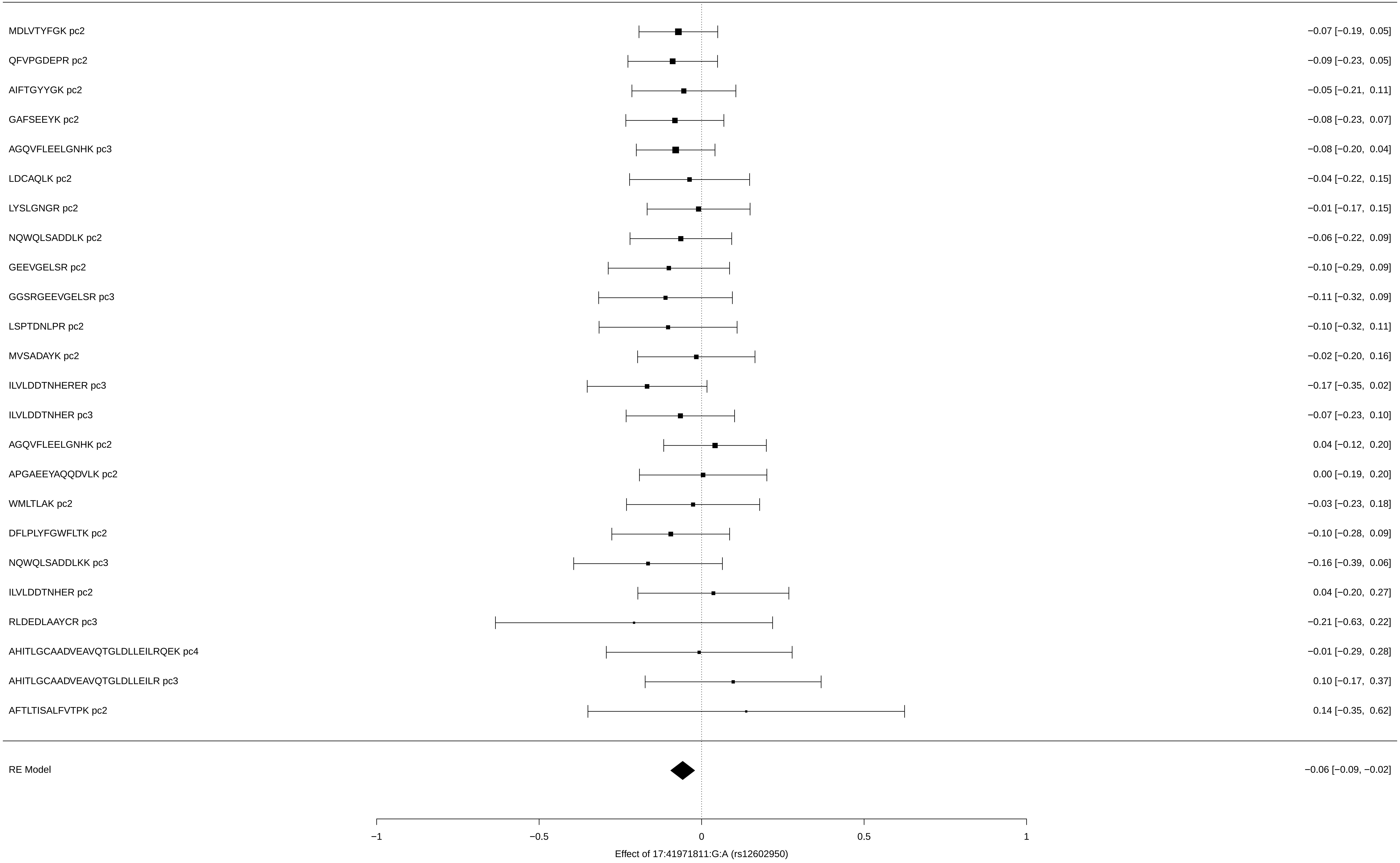

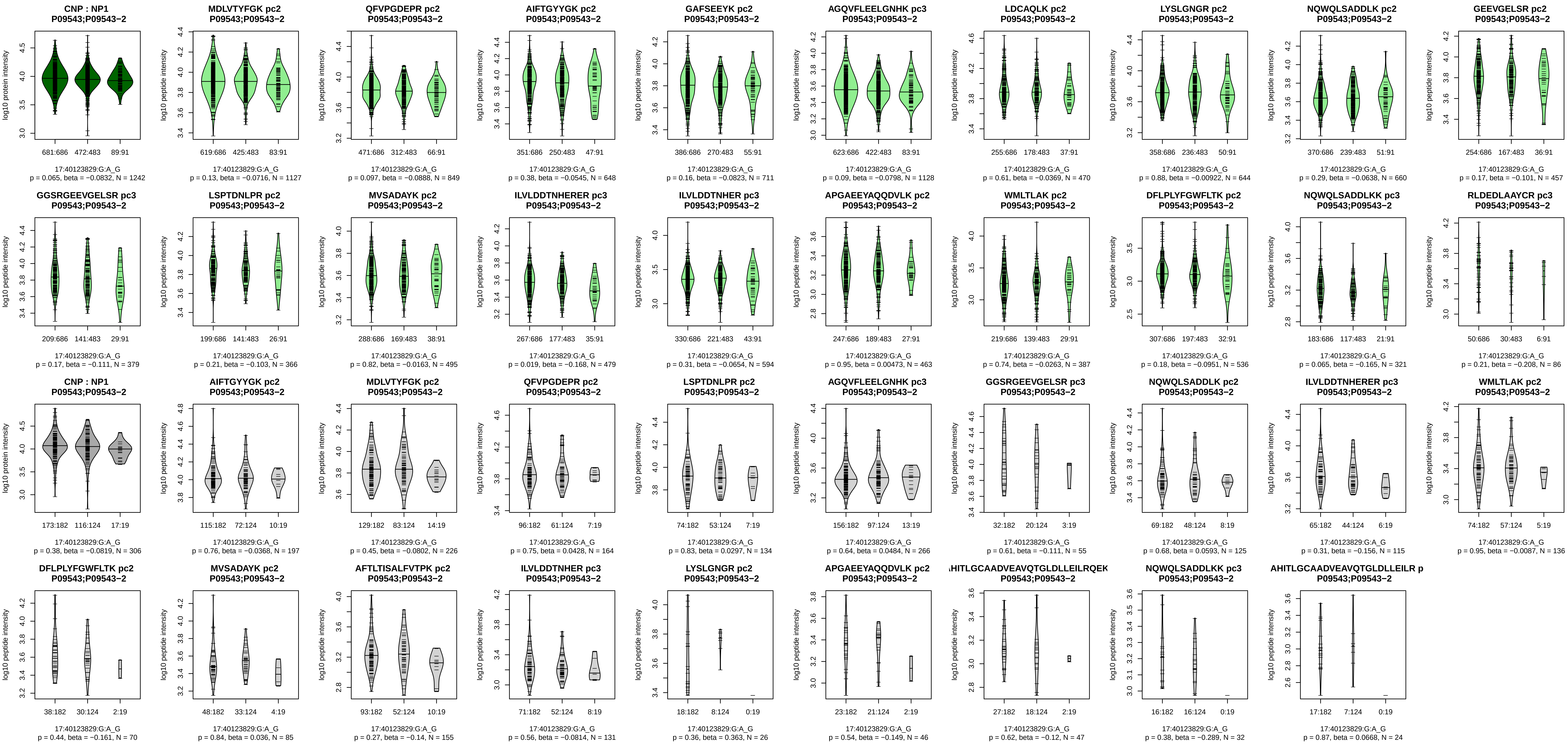

Assay Target: CNP
UniProt: P09543
rsID: rs12602950
Target SNP: 17:41971811:G:A
Proxy SNP: 17:40123829:G:A
deCODE log10(p): 19
deCODE BETA: −0.08
deCODE MSPA: 0
1127:849:648:711:1128:470:644

279. NAPG (Q99747)
deCODE MSPA=0

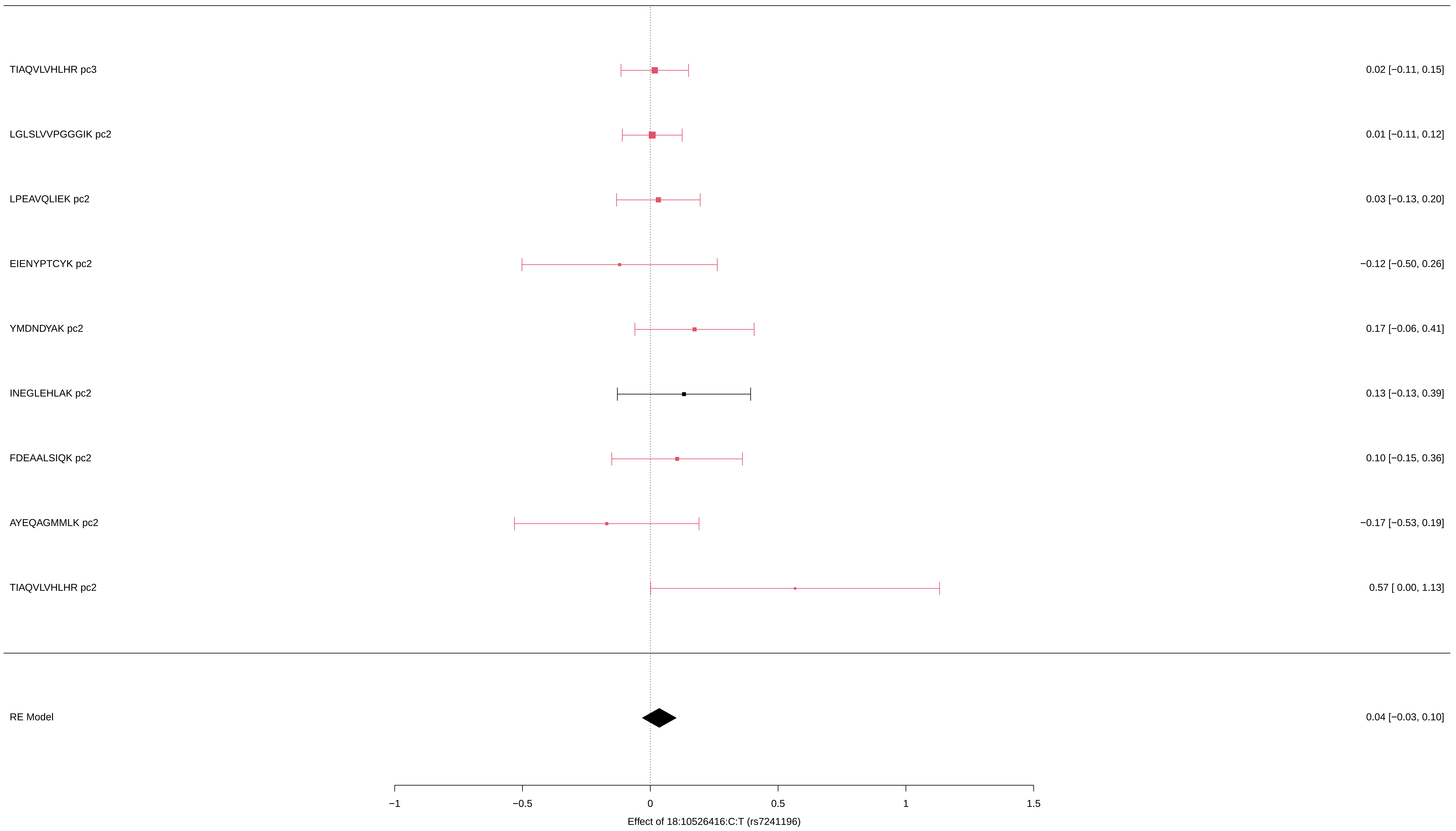

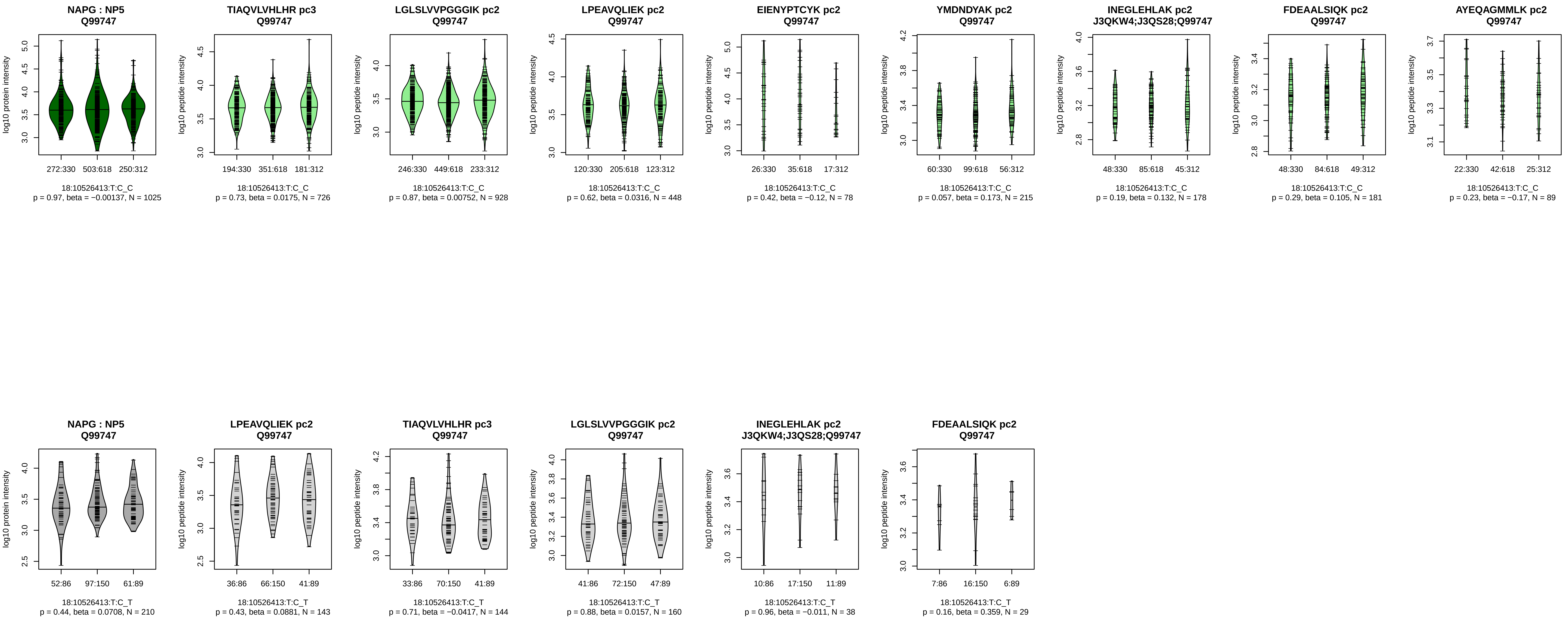

Assay Target: NAPG
UniProt: Q99747
rsID: rs7241196
Target SNP: 18:10526416:C:T
Proxy SNP: 18:10526413:T:C
deCODE log10(p): 18.2
deCODE BETA: −0.07
deCODE MSPA: 0
−:−:−:−:−:−:−:−:NA:NA:−
726:928:448:78:215:178:181:89

280. PAICS (P22234)
deCODE MSPA=0.017

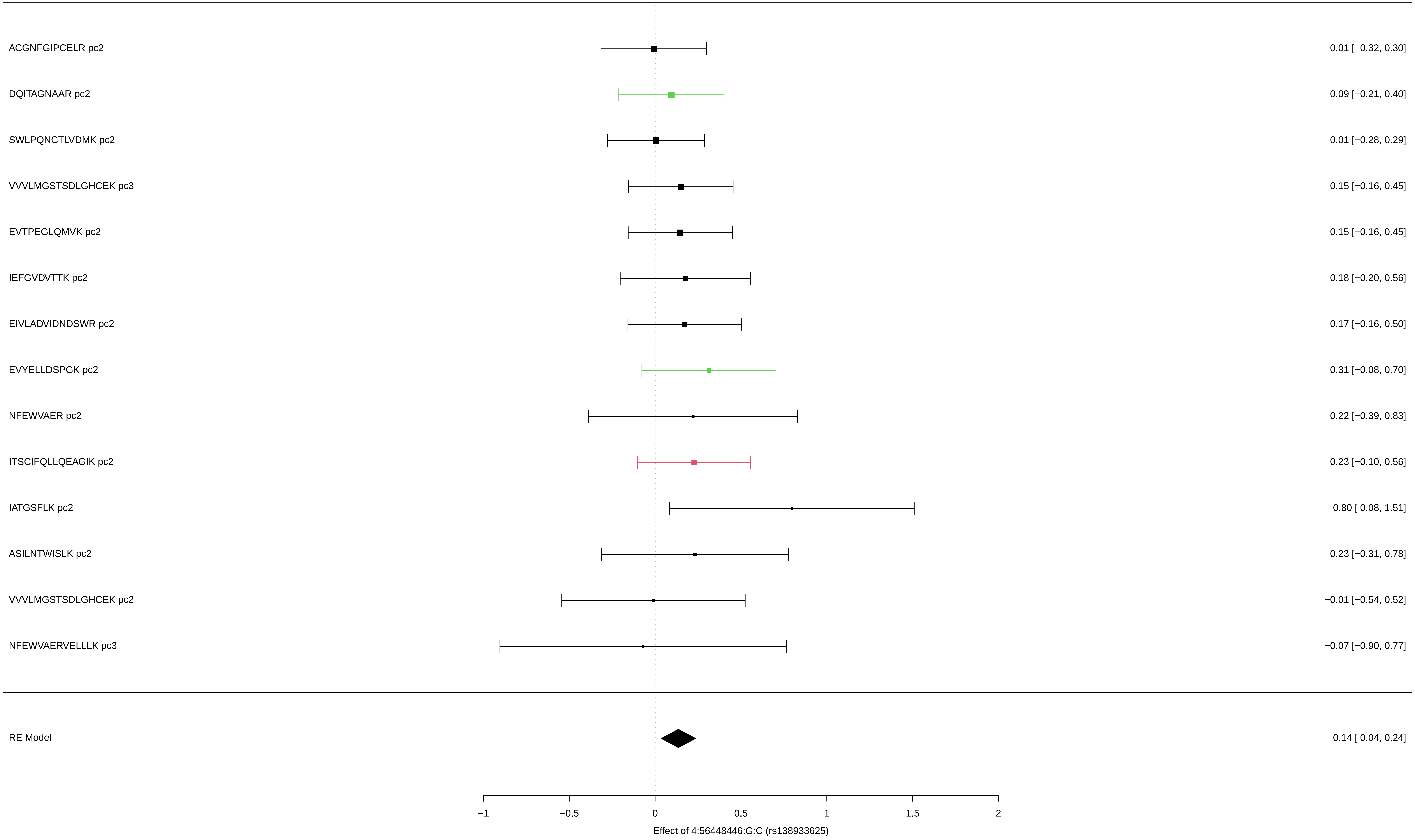

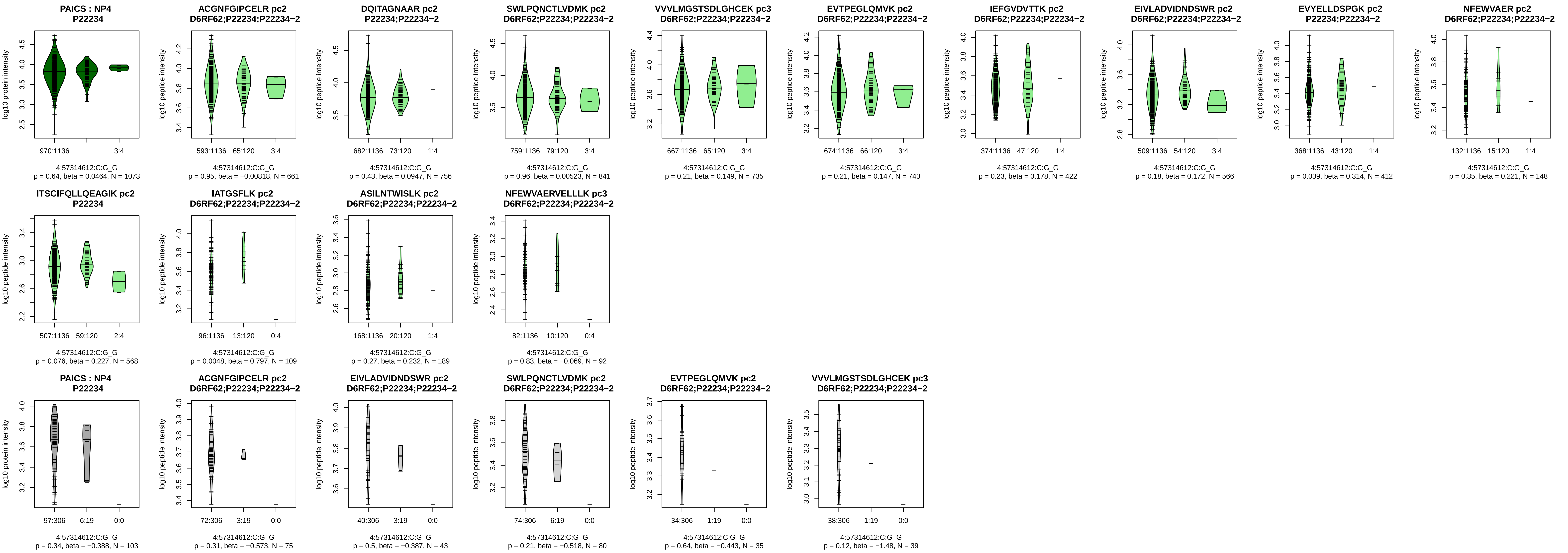

Assay Target: PAICS
UniProt: P22234
rsID: rs138933625
Target SNP: 4:56448446:G:C
Proxy SNP: 4:57314612:C:G
deCODE log10(p): 18.2
deCODE BETA: −0.19
deCODE MSPA: 0.017
661:756:841:735:743:422:566:4

281. APP (P05067)
deCODE MSPA=0

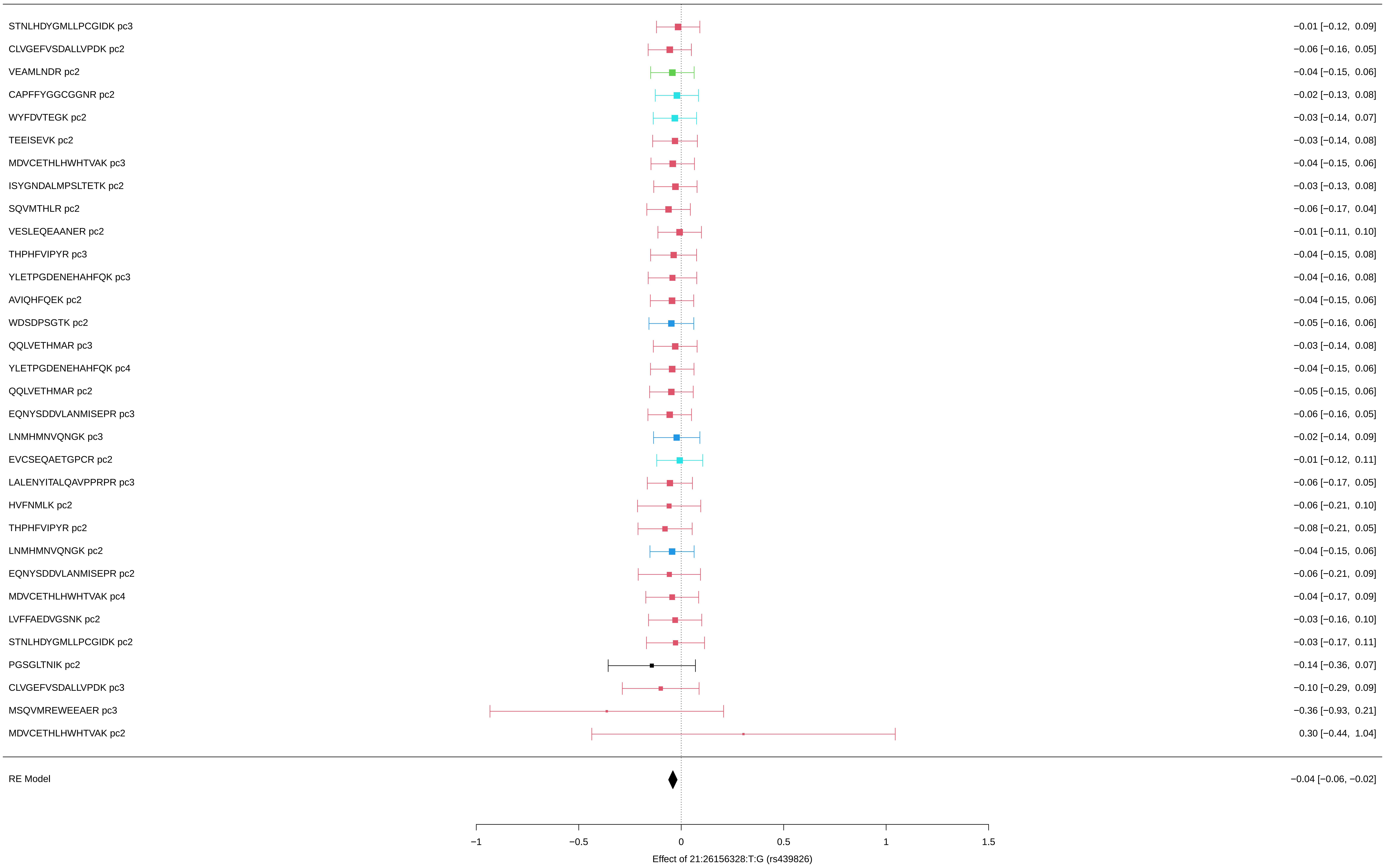

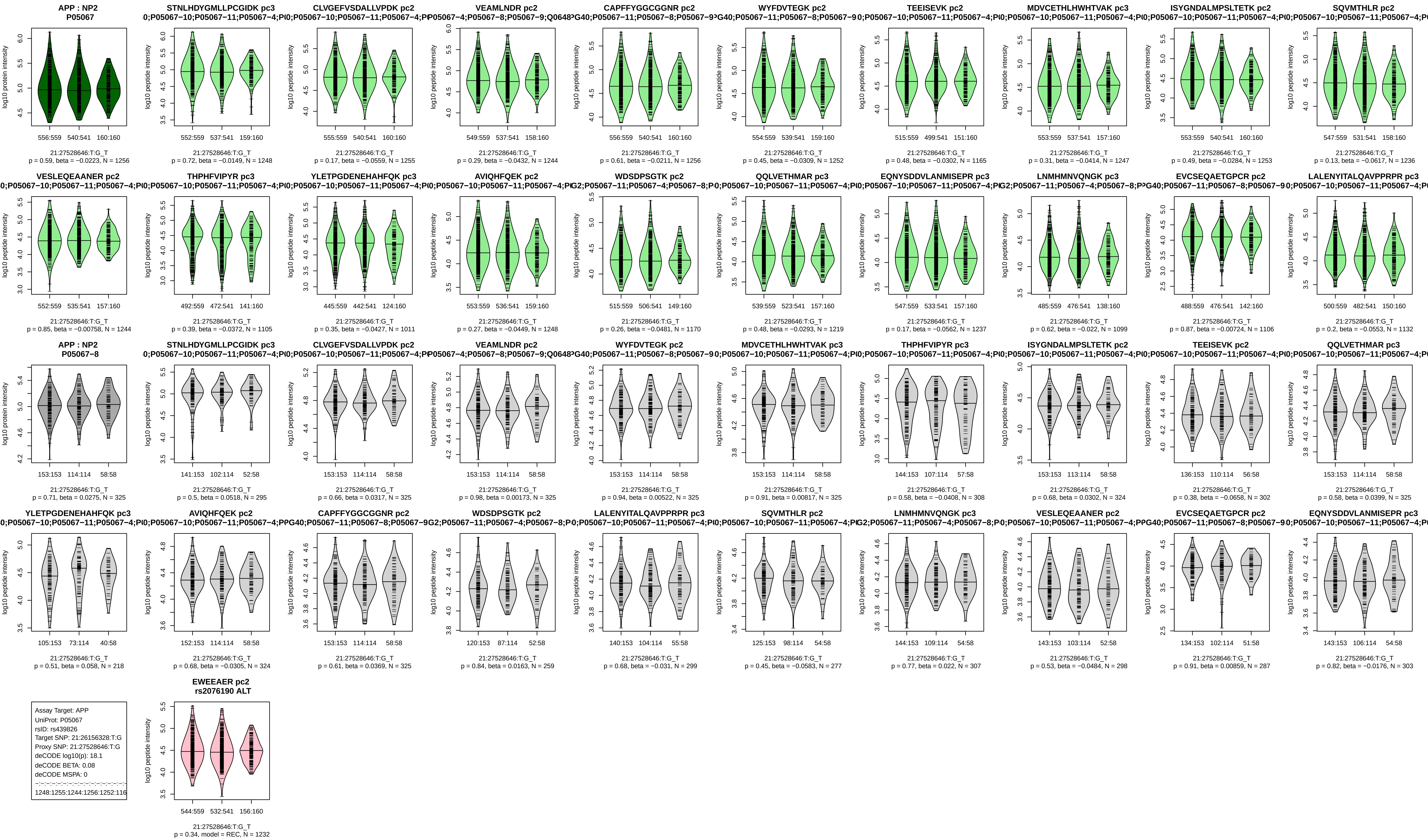

APP : NP2
P05067
21:27528646:T:G_T
p = 0.59, beta = −0.0223, N = 1256
STNLHDYGMLLPCGIDK pc3
p = 0.72, beta = −0.0149, N = 1248
CLVGEFVSDALLVPDK pc2
p = 0.17, beta = −0.0559, N = 1255
VEAMLNDR pc2
p = 0.29, beta = −0.0432, N = 1244
CAPFFYGGCGGNR pc2
p = 0.61, beta = −0.0211, N = 1256
WYFDVTEGK pc2
p = 0.45, beta = −0.0309, N = 1252
TEEISEVK pc2
p = 0.48, beta = −0.0302, N = 1165
MDVCETHLHWHTVAK pc3
p = 0.31, beta = −0.0414, N = 1247
ISYGNDALMPSLTETK pc2
p = 0.49, beta = −0.0284, N = 1253
SQVMTHLR pc2
p = 0.13, beta = −0.0617, N = 1236
VESLEQEAANER pc2
p = 0.85, beta = −0.00758, N = 1244
THPHFVIPYR pc3
p = 0.39, beta = −0.0372, N = 1105
YLETPGDENEHAHFQK pc3
p = 0.35, beta = −0.0427, N = 1011
AVIQHFQEK pc2
p = 0.27, beta = −0.0449, N = 1248
WDSDPSGTK pc2
p = 0.26, beta = −0.0481, N = 1170
QQLVETHMAR pc3
p = 0.48, beta = −0.0293, N = 1219
EQNYSDDVLANMISEPR pc3
p = 0.17, beta = −0.0562, N = 1237
LNMHMNVQNGK pc3
p = 0.62, beta = −0.022, N = 1099
EVCSEQAETGPCR pc2
p = 0.87, beta = −0.00724, N = 1106
LALENYITALQAVPPRPR pc3
p = 0.2, beta = −0.0553, N = 1132
APP : NP2
P05067−8
p = 0.71, beta = 0.0275, N = 325
STNLHDYGMLLPCGIDK pc3
p = 0.5, beta = 0.0518, N = 295
CLVGEFVSDALLVPDK pc2
p = 0.66, beta = 0.0317, N = 325
VEAMLNDR pc2
p = 0.98, beta = 0.00173, N = 325
WYFDVTEGK pc2
p = 0.94, beta = 0.00522, N = 325
MDVCETHLHWHTVAK pc3
p = 0.91, beta = 0.00817, N = 325
THPHFVIPYR pc3
p = 0.58, beta = −0.0408, N = 308
ISYGNDALMPSLTETK pc2
p = 0.68, beta = 0.0302, N = 324
TEEISEVK pc2
p = 0.38, beta = −0.0658, N = 302
QQLVETHMAR pc3
p = 0.58, beta = 0.0399, N = 325
YLETPGDENEHAHFQK pc3
p = 0.51, beta = 0.058, N = 218
AVIQHFQEK pc2
p = 0.68, beta = −0.0305, N = 324
CAPFFYGGCGGNR pc2
p = 0.61, beta = 0.0369, N = 325
WDSDPSGTK pc2
p = 0.84, beta = 0.0163, N = 259
LALENYITALQAVPPRPR pc3
p = 0.68, beta = −0.031, N = 299
SQVMTHLR pc2
p = 0.45, beta = −0.0583, N = 277
LNMHMNVQNGK pc3
p = 0.77, beta = 0.022, N = 307
VESLEQEAANER pc2
p = 0.53, beta = −0.0484, N = 298
EVCSEQAETGPCR pc2
p = 0.91, beta = 0.00859, N = 287
EQNYSDDVLANMISEPR pc3
p = 0.82, beta = −0.0176, N = 303
Assay Target: APP
UniProt: P05067
rsID: rs439826
Target SNP: 21:26156328:T:G
Proxy SNP: 21:27528646:T:G
deCODE log10(p): 18.1
deCODE BETA: 0.08
deCODE MSPA: 0
1248:1255:1244:1256:1252:116
EWEEAER pc2
rs2076190 ALT
544:559 532:541 156:160
21:27528646:T:G_T
p = 0.34, model = REC, N = 1232

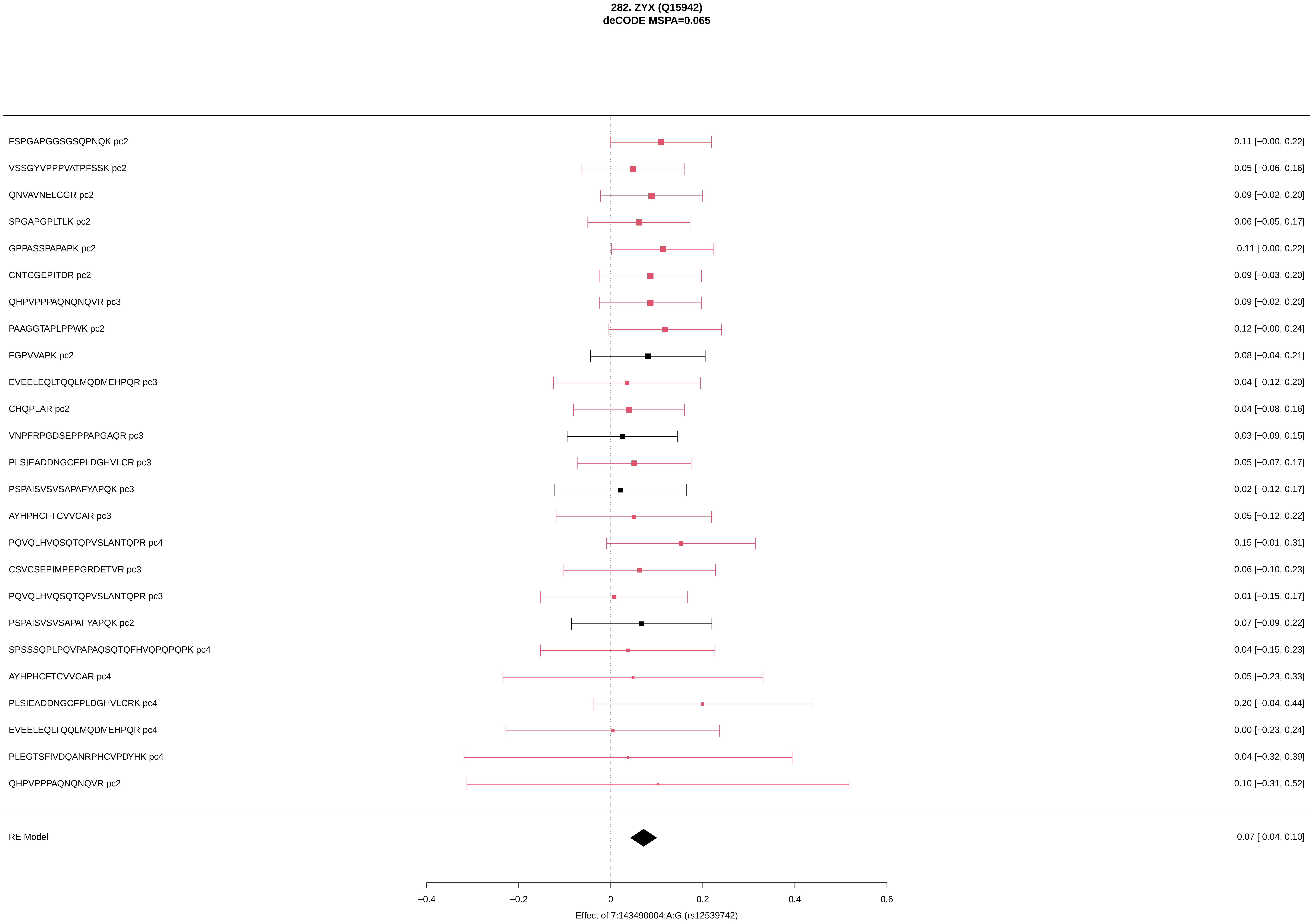

282. ZYX (Q15942)
deCODE MSPA=0.065
FSPGAPGGSGSQPNQK pc2
VSSGYVPPPVATPFSSK pc2
QNVAVNELCGR pc2
SPGAPGPLTLK pc2
GPPASSPAPAPK pc2
CNTCGEPITDR pc2
QHPVPPPAQNQNQVR pc3
PAAGGTAPLPPWK pc2
FGPVVAPK pc2
EVEELEQLTQQLMQDMEHPQR pc3
CHQPLAR pc2
VNPFRPGDSEPPPAPGAQR pc3
PLSIEADDNGCFPLDGHVLCR pc3
PSPAISVSVSAPAFYAPQK pc3
AYHPHCFTCVVCAR pc3
PQVQLHVQSQTQPVSLANTQPR pc4
CSVCSEPIMPEPGRDETVR pc3
PQVQLHVQSQTQPVSLANTQPR pc3
PSPAISVSVSAPAFYAPQK pc2
SPSSSQPLPQVPAPAQSQTQFHVQPQPQPK pc4
AYHPHCFTCVVCAR pc4
PLSIEADDNGCFPLDGHVLCRK pc4
EVEELEQLTQQLMQDMEHPQR pc4
PLEGTSFIVDQANRPHCVPDYHK pc4
QHPVPPPAQNQNQVR pc2
RE Model
0.11 [−0.00, 0.22]
0.05 [−0.06, 0.16]
0.09 [−0.02, 0.20]
0.06 [−0.05, 0.17]
0.11 [0.00, 0.22]
0.09 [−0.03, 0.20]
0.09 [−0.02, 0.20]
0.12 [−0.00, 0.24]
0.08 [−0.04, 0.21]
0.04 [−0.12, 0.20]
0.04 [−0.08, 0.16]
0.03 [−0.09, 0.15]
0.05 [−0.07, 0.17]
0.02 [−0.12, 0.17]
0.05 [−0.12, 0.22]
0.15 [−0.01, 0.31]
0.06 [−0.10, 0.23]
0.01 [−0.15, 0.17]
0.07 [−0.09, 0.22]
0.04 [−0.15, 0.23]
0.05 [−0.23, 0.33]
0.20 [−0.04, 0.44]
0.00 [−0.23, 0.24]
0.04 [−0.32, 0.39]
0.10 [−0.31, 0.52]
0.07 [0.04, 0.10]
−0.4
−0.2
0
0.2
0.4
0.6
Effect of 7:143490004:A:G (rs12539742)

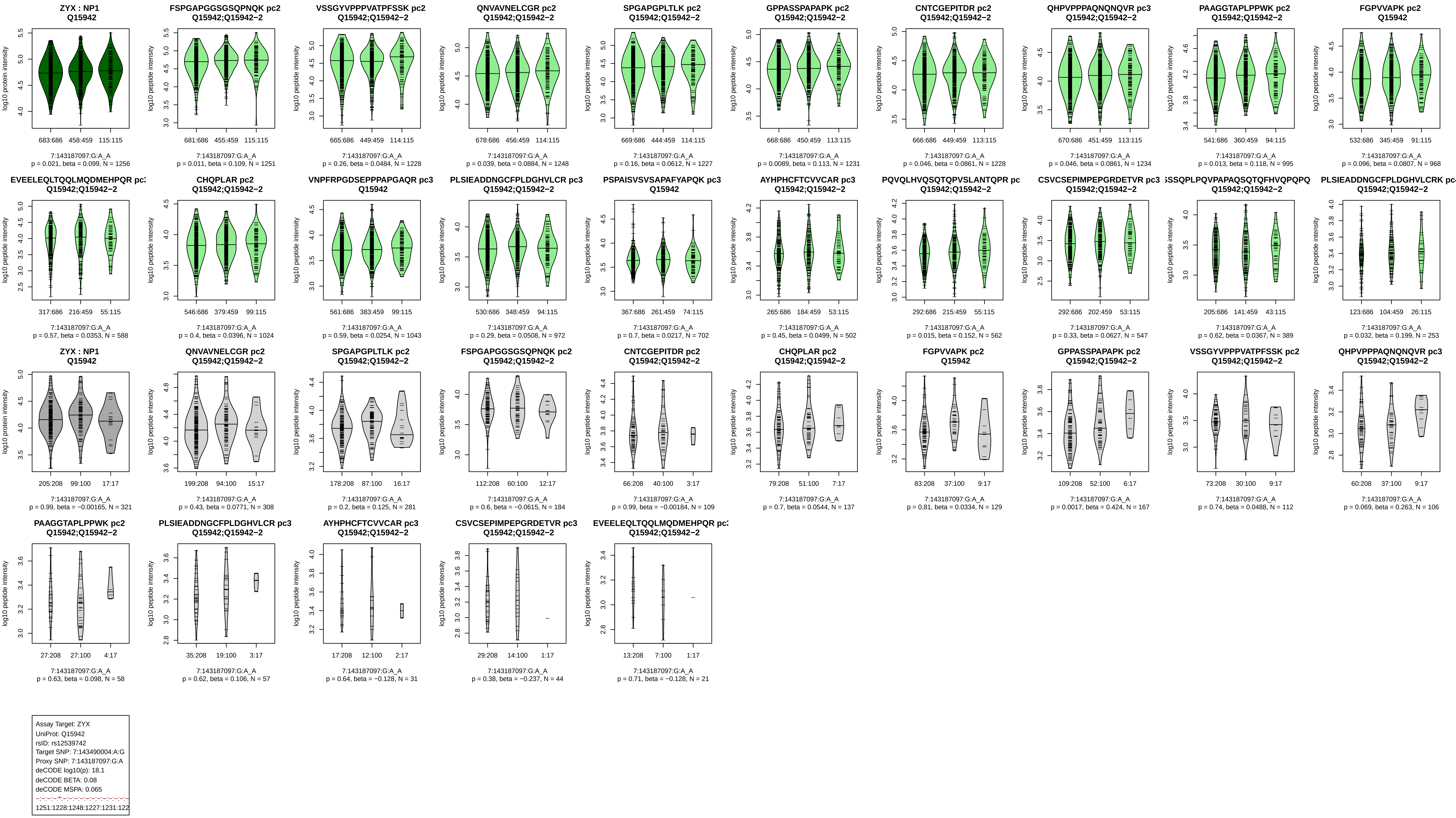
ZYX : NP1 Q15942
683:686 458:459 115:115
7:143187097:G:A_A p = 0.021, beta = 0.099, N = 1256
FSPGAPGGSGSQPNQK pc2 Q15942;Q15942−2
681:686 455:459 115:115
7:143187097:G:A_A p = 0.011, beta = 0.109, N = 1251
VSSGYVPPPVATPFSSK pc2 Q15942;Q15942−2
665:686 449:459 114:115
7:143187097:G:A_A p = 0.26, beta = 0.0484, N = 1228
QNVAVNELCGR pc2 Q15942;Q15942−2
678:686 456:459 114:115
7:143187097:G:A_A p = 0.039, beta = 0.0884, N = 1248
SPGAPGPLTLK pc2 Q15942;Q15942−2
669:686 444:459 114:115
7:143187097:G:A_A p = 0.16, beta = 0.0612, N = 1227
GPPASSPAPAPK pc2 Q15942;Q15942−2
668:686 450:459 113:115
7:143187097:G:A_A p = 0.0089, beta = 0.113, N = 1231
CNTCGEPITDR pc2 Q15942;Q15942−2
666:686 449:459 113:115
7:143187097:G:A_A p = 0.046, beta = 0.0861, N = 1228
QHPVPPPAQNQNQVR pc3 Q15942;Q15942−2
670:686 451:459 113:115
7:143187097:G:A_A p = 0.046, beta = 0.0861, N = 1234
PAAGGTAPLPPWK pc2 Q15942;Q15942−2
541:686 360:459 94:115
7:143187097:G:A_A p = 0.013, beta = 0.118, N = 995
FGPVVAPK pc2 Q15942
532:686 345:459 91:115
7:143187097:G:A_A p = 0.096, beta = 0.0807, N = 968
EVEELEQLTQQLMQDMEHPQR pc3 Q15942;Q15942−2
317:686 216:459 55:115
7:143187097:G:A_A p = 0.57, beta = 0.0353, N = 588
CHQPLAR pc2 Q15942;Q15942−2
546:686 379:459 99:115
7:143187097:G:A_A p = 0.4, beta = 0.0396, N = 1024
VNPFRPGDSEPPPAPGAQR pc3 Q15942
561:686 383:459 99:115
7:143187097:G:A_A p = 0.59, beta = 0.0254, N = 1043
PLSIEADDNGCFPLDGHVLCR pc3 Q15942;Q15942−2
530:686 348:459 94:115
7:143187097:G:A_A p = 0.29, beta = 0.0508, N = 972
PSPAISVSVSAPAFYAPQK pc3 Q15942
367:686 261:459 74:115
7:143187097:G:A_A p = 0.7, beta = 0.0217, N = 702
AYHPHCFTCVVCAR pc3 Q15942;Q15942−2
265:686 184:459 53:115
7:143187097:G:A_A p = 0.45, beta = 0.0499, N = 502
PQVQLHVQSQTQPVSLANTQPR pc Q15942;Q15942−2
292:686 215:459 55:115
7:143187097:G:A_A p = 0.015, beta = 0.152, N = 562
CSVCSEPIMPEPGRDETVR pc3 Q15942;Q15942−2
292:686 202:459 53:115
7:143187097:G:A_A p = 0.33, beta = 0.0627, N = 547
SSQPLPQVPAPAQSQTQFHVQPQPQ Q15942;Q15942−2
205:686 141:459 43:115
7:143187097:G:A_A p = 0.62, beta = 0.0367, N = 389
PLSIEADDNGCFPLDGHVLCRK pc Q15942;Q15942−2
123:686 104:459 26:115
7:143187097:G:A_A p = 0.032, beta = 0.199, N = 253
ZYX : NP1 Q15942
205:208 99:100 17:17
7:143187097:G:A_A p = 0.99, beta = −0.00165, N = 321
QNVAVNELCGR pc2 Q15942;Q15942−2
199:208 94:100 15:17
7:143187097:G:A_A p = 0.43, beta = 0.0771, N = 308
SPGAPGPLTLK pc2 Q15942;Q15942−2
178:208 87:100 16:17
7:143187097:G:A_A p = 0.2, beta = 0.125, N = 281
FSPGAPGGSGSQPNQK pc2 Q15942;Q15942−2
112:208 60:100 12:17
7:143187097:G:A_A p = 0.6, beta = −0.0615, N = 184
CNTCGEPITDR pc2 Q15942;Q15942−2
66:208 40:100 3:17
7:143187097:G:A_A p = 0.99, beta = −0.00184, N = 109
CHQPLAR pc2 Q15942;Q15942−2
79:208 51:100 7:17
7:143187097:G:A_A p = 0.7, beta = 0.0544, N = 137
FGPVVAPK pc2 Q15942
83:208 37:100 9:17
7:143187097:G:A_A p = 0.81, beta = 0.0334, N = 129
GPPASSPAPAPK pc2 Q15942;Q15942−2
109:208 52:100 6:17
7:143187097:G:A_A p = 0.0017, beta = 0.424, N = 167
VSSGYVPPPVATPFSSK pc2 Q15942;Q15942−2
73:208 30:100 9:17
7:143187097:G:A_A p = 0.74, beta = 0.0488, N = 112
QHPVPPPAQNQNQVR pc3 Q15942;Q15942−2
60:208 37:100 9:17
7:143187097:G:A_A p = 0.069, beta = 0.263, N = 106
PAAGGTAPLPPWK pc2 Q15942;Q15942−2
27:208 27:100 4:17
7:143187097:G:A_A p = 0.63, beta = 0.098, N = 58
PLSIEADDNGCFPLDGHVLCR pc3 Q15942;Q15942−2
35:208 19:100 3:17
7:143187097:G:A_A p = 0.62, beta = 0.106, N = 57
AYHPHCFTCVVCAR pc3 Q15942;Q15942−2
17:208 12:100 2:17
7:143187097:G:A_A p = 0.64, beta = −0.128, N = 31
CSVCSEPIMPEPGRDETVR pc3 Q15942;Q15942−2
29:208 14:100 1:17
7:143187097:G:A_A p = 0.38, beta = −0.237, N = 44
EVEELEQLTQQLMQDMEHPQR pc3 Q15942;Q15942−2
13:208 7:100 1:17
7:143187097:G:A_A p = 0.71, beta = −0.128, N = 21
log10 protein intensity
log10 peptide intensity
Assay Target: ZYX
UniProt: Q15942
rsID: rs12539742
Target SNP: 7:143490004:A:G
Proxy SNP: 7:143187097:G:A
deCODE log10(p): 18.1
deCODE BETA: 0.08
deCODE MSPA: 0.065
1251:1228:1248:1227:1231:122

283. FABP4 (P15090)
deCODE MSPA=0.071

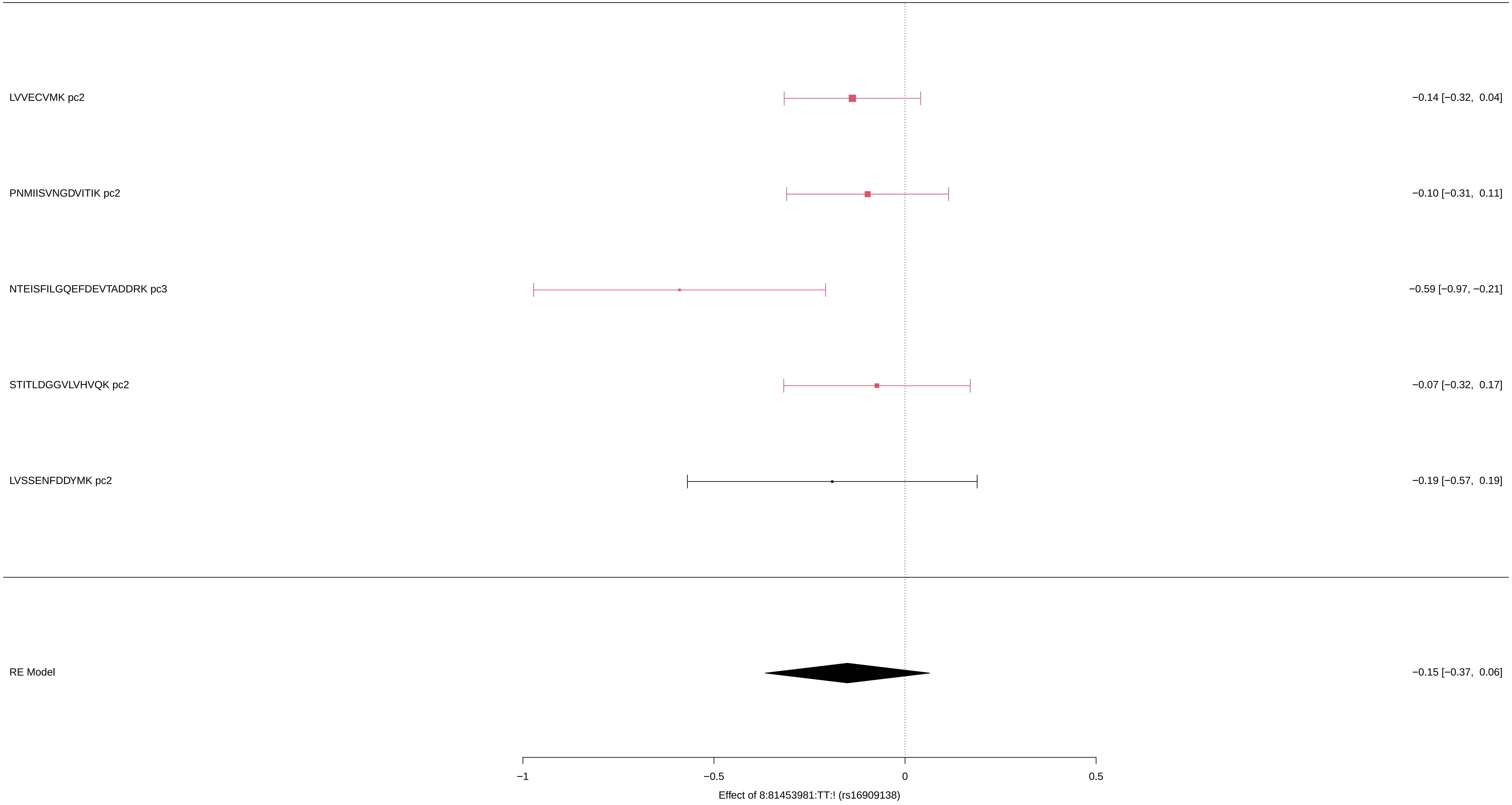

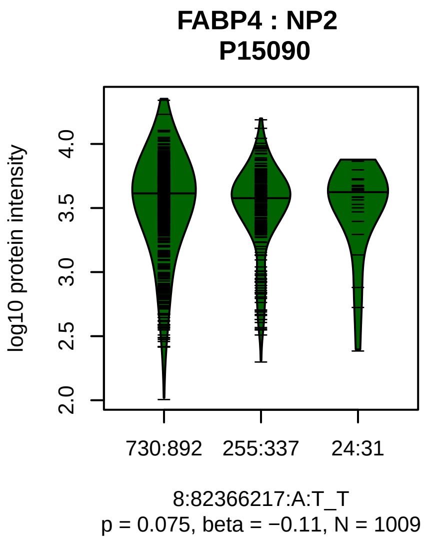

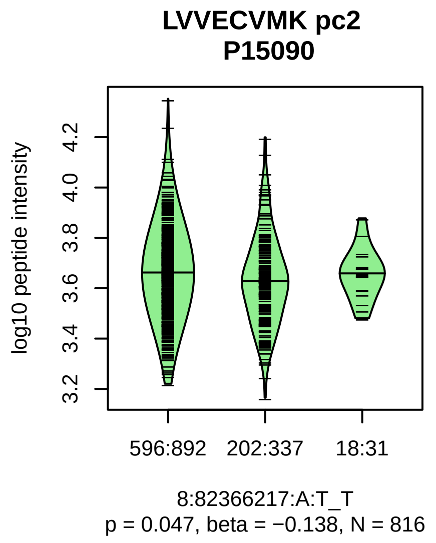

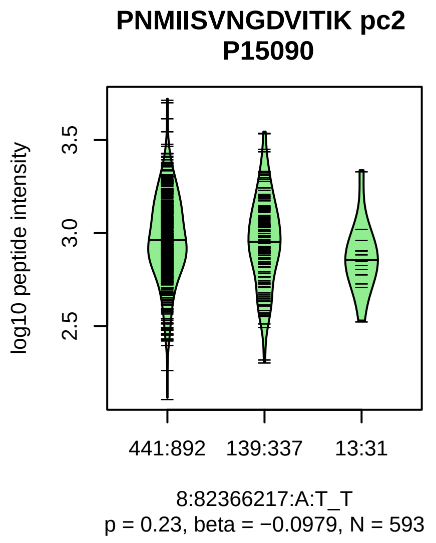

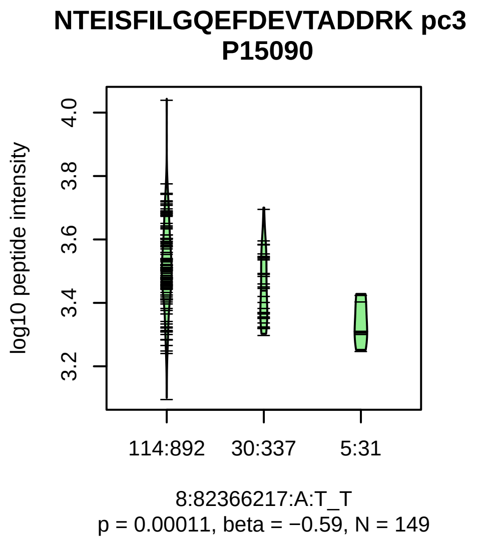

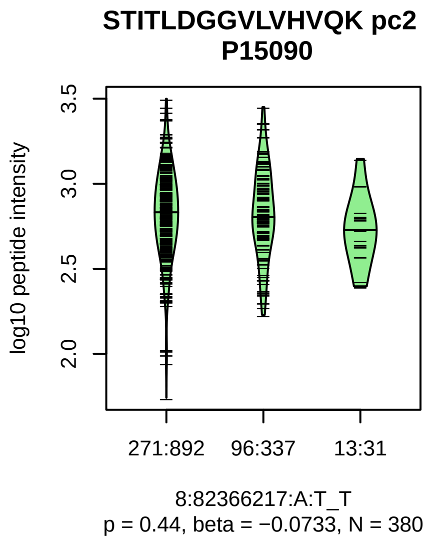

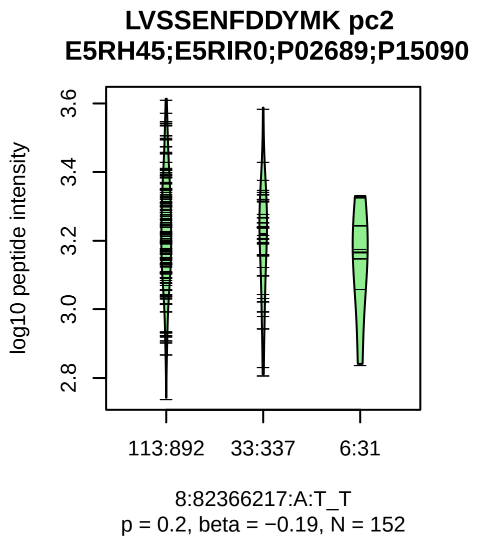

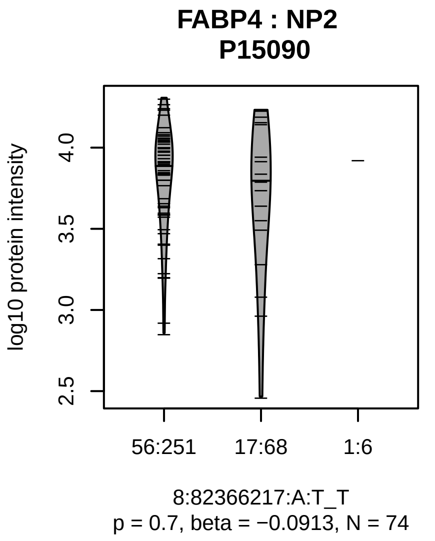

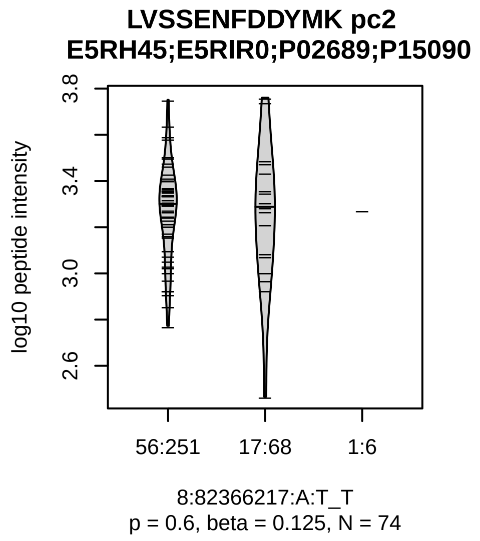

Assay Target: FABP4
UniProt: P15090
rsID: rs16909138
Target SNP: 8:81453981:TT:!
Proxy SNP: 8:82366217:A:T
deCODE log10(p): 17.4
deCODE BETA: −0.1
deCODE MSPA: 0.071
–:–:*:–:–
816:593:149:380:152

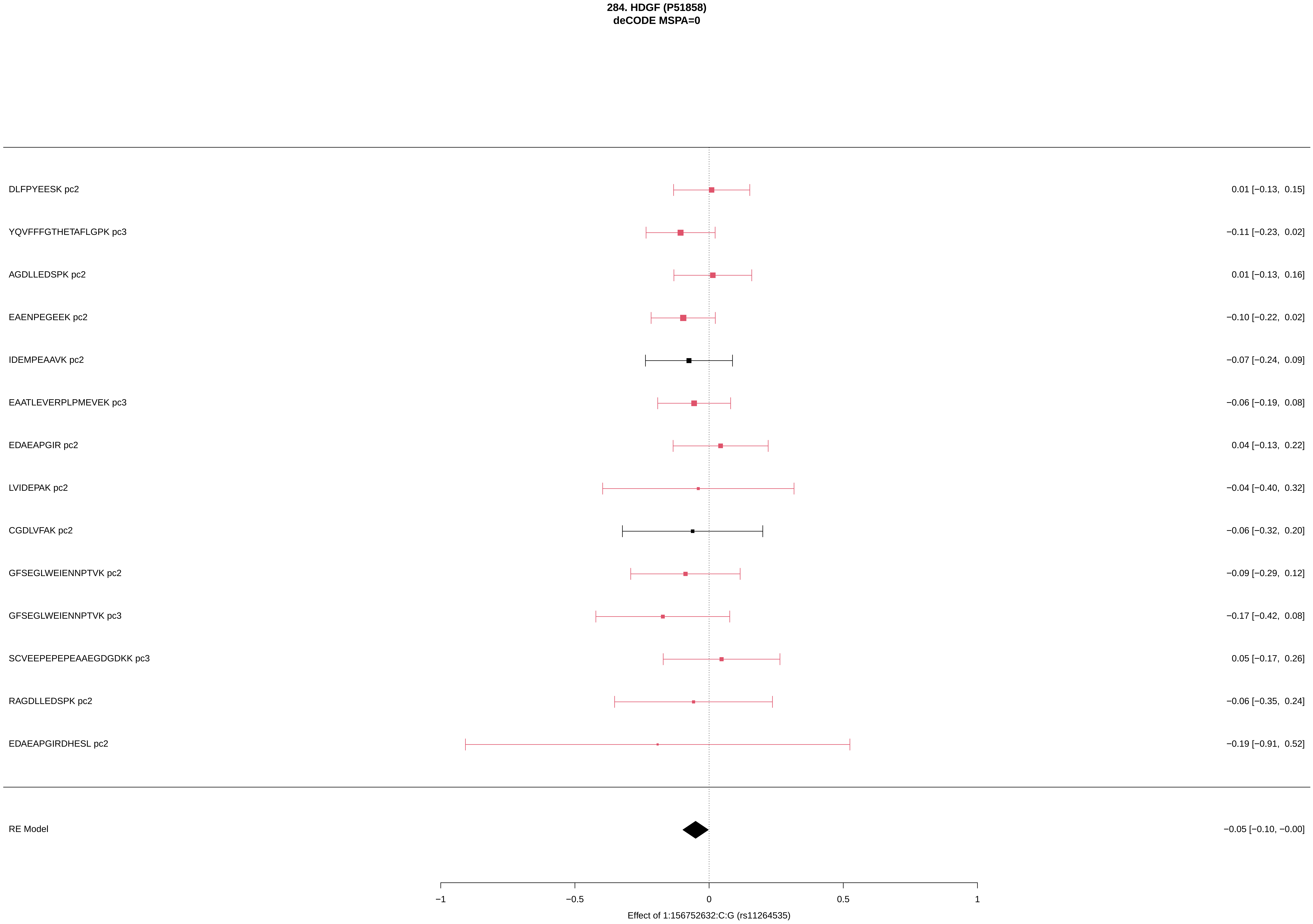

284. HDGF (P51858)
deCODE MSPA=0
DLFPYEESK pc2
0.01 [−0.13, 0.15]
YQVFFFGTHETAFLGPK pc3
−0.11 [−0.23, 0.02]
AGDLLEDSPK pc2
0.01 [−0.13, 0.16]
EAENPEGEEK pc2
−0.10 [−0.22, 0.02]
IDEMPEAAVK pc2
−0.07 [−0.24, 0.09]
EAATLEVERPLPMEVEK pc3
−0.06 [−0.19, 0.08]
EDAEAPGIR pc2
0.04 [−0.13, 0.22]
LVIDEPAK pc2
−0.04 [−0.40, 0.32]
CGDLVFAK pc2
−0.06 [−0.32, 0.20]
GFSEGLWEIENNPTVK pc2
−0.09 [−0.29, 0.12]
GFSEGLWEIENNPTVK pc3
−0.17 [−0.42, 0.08]
SCVEEPEPEPEAAEGDGDKK pc3
0.05 [−0.17, 0.26]
RAGDLLEDSPK pc2
−0.06 [−0.35, 0.24]
EDAEAPGIRDHESL pc2
−0.19 [−0.91, 0.52]
RE Model
−0.05 [−0.10, −0.00]
−1
−0.5
0
0.5
1
Effect of 1:156752632:C:G (rs11264535)

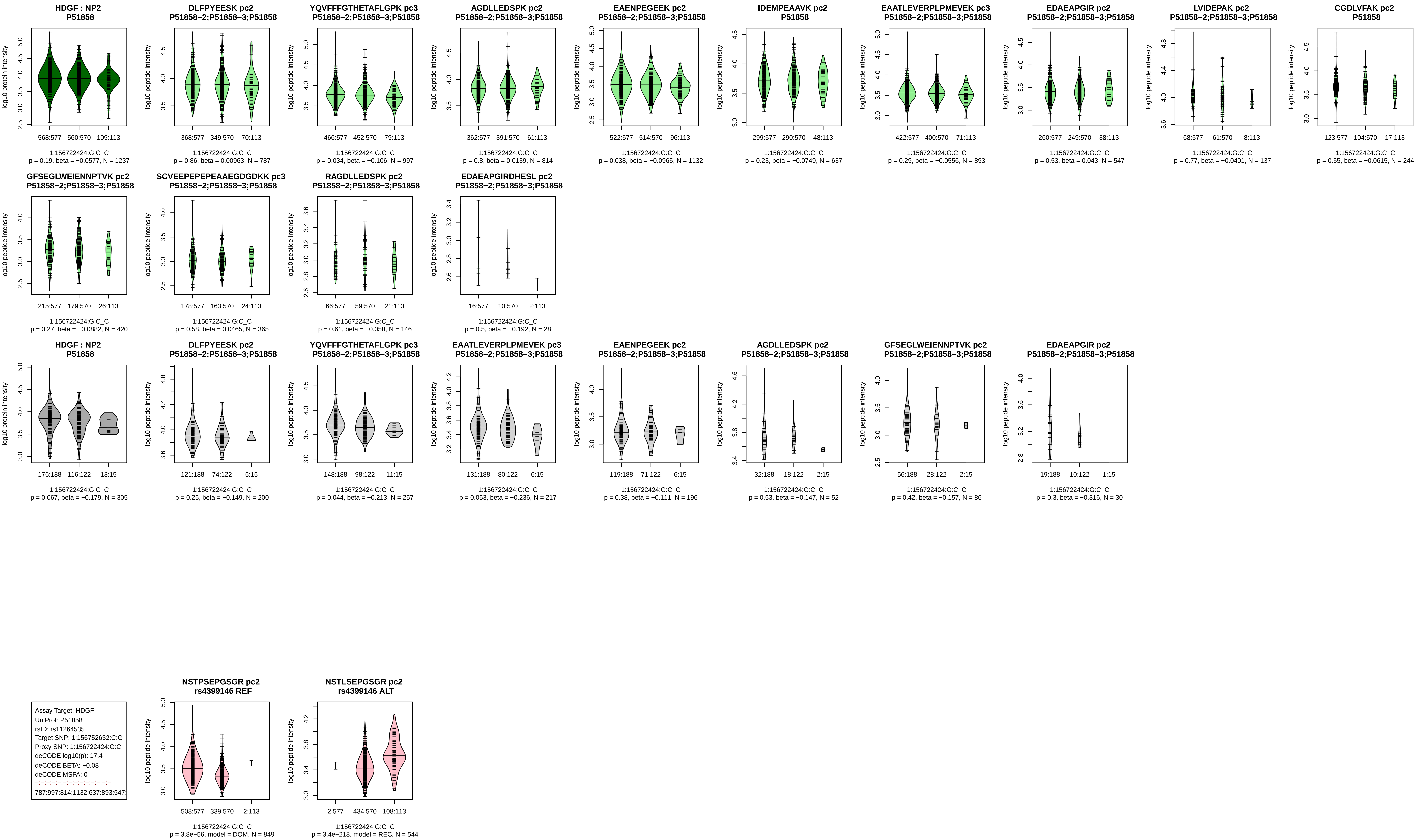
HDGF : NP2 P51858
568:577 560:570 109:113
1:156722424:G:C_C
p = 0.19, beta = −0.0577, N = 1237
DLFPYEESK pc2 P51858−2;P51858−3;P51858
368:577 349:570 70:113
1:156722424:G:C_C
p = 0.86, beta = 0.00963, N = 787
YQVFFFGTHETAFLGPK pc3 P51858−2;P51858−3;P51858
466:577 452:570 79:113
1:156722424:G:C_C
p = 0.034, beta = −0.106, N = 997
AGDLLEDSPK pc2 P51858−2;P51858−3;P51858
362:577 391:570 61:113
1:156722424:G:C_C
p = 0.8, beta = 0.0139, N = 814
EAENPEGEEK pc2 P51858−2;P51858−3;P51858
522:577 514:570 96:113
1:156722424:G:C_C
p = 0.038, beta = −0.0965, N = 1132
IDEMPEAAVK pc2 P51858
299:577 290:570 48:113
1:156722424:G:C_C
p = 0.23, beta = −0.0749, N = 637
EAATLEVERPLPMEVEK pc3 P51858−2;P51858−3;P51858
422:577 400:570 71:113
1:156722424:G:C_C
p = 0.29, beta = −0.0556, N = 893
EDAEAPGIR pc2 P51858−2;P51858−3;P51858
260:577 249:570 38:113
1:156722424:G:C_C
p = 0.53, beta = 0.043, N = 547
LVIDEPAK pc2 P51858−2;P51858−3;P51858
68:577 61:570 8:113
1:156722424:G:C_C
p = 0.77, beta = −0.0401, N = 137
CGDLVFAK pc2 P51858
123:577 104:570 17:113
1:156722424:G:C_C
p = 0.55, beta = −0.0615, N = 244
GFSEGLWEIENNPTVK pc2 P51858−2;P51858−3;P51858
215:577 179:570 26:113
1:156722424:G:C_C
p = 0.27, beta = −0.0882, N = 420
SCVEEPEPEPEAAEGDGDKK pc3 P51858−2;P51858−3;P51858
178:577 163:570 24:113
1:156722424:G:C_C
p = 0.58, beta = 0.0465, N = 365
RAGDLLEDSPK pc2 P51858−2;P51858−3;P51858
66:577 59:570 21:113
1:156722424:G:C_C
p = 0.61, beta = −0.058, N = 146
EDAEAPGIRDHESL pc2 P51858−2;P51858−3;P51858
16:577 10:570 2:113
1:156722424:G:C_C
p = 0.5, beta = −0.192, N = 28
HDGF : NP2 P51858
176:188 116:122 13:15
1:156722424:G:C_C
p = 0.067, beta = −0.179, N = 305
DLFPYEESK pc2 P51858−2;P51858−3;P51858
121:188 74:122 5:15
1:156722424:G:C_C
p = 0.25, beta = −0.149, N = 200
YQVFFFGTHETAFLGPK pc3 P51858−2;P51858−3;P51858
148:188 98:122 11:15
1:156722424:G:C_C
p = 0.044, beta = −0.213, N = 257
EAATLEVERPLPMEVEK pc3 P51858−2;P51858−3;P51858
131:188 80:122 6:15
1:156722424:G:C_C
p = 0.053, beta = −0.236, N = 217
EAENPEGEEK pc2 P51858−2;P51858−3;P51858
119:188 71:122 6:15
1:156722424:G:C_C
p = 0.38, beta = −0.111, N = 196
AGDLLEDSPK pc2 P51858−2;P51858−3;P51858
32:188 18:122 2:15
1:156722424:G:C_C
p = 0.53, beta = −0.147, N = 52
GFSEGLWEIENNPTVK pc2 P51858−2;P51858−3;P51858
56:188 28:122 2:15
1:156722424:G:C_C
p = 0.42, beta = −0.157, N = 86
EDAEAPGIR pc2 P51858−2;P51858−3;P51858
19:188 10:122 1:15
1:156722424:G:C_C
p = 0.3, beta = −0.316, N = 30
Assay Target: HDGF
UniProt: P51858
rsID: rs11264535
Target SNP: 1:156752632:C:G
Proxy SNP: 1:156722424:G:C
deCODE log10(p): 17.4
deCODE BETA: −0.08
deCODE MSPA: 0
787:997:814:1132:637:893:547:
NSTPSEPGSGR pc2 rs4399146 REF
508:577 339:570 2:113
1:156722424:G:C_C
p = 3.8e−56, model = DOM, N = 849
NSTLSEPGSGR pc2 rs4399146 ALT
2:577 434:570 108:113
1:156722424:G:C_C
p = 3.4e−218, model = REC, N = 544

285. MIF (P14174)
deCODE MSPA=0

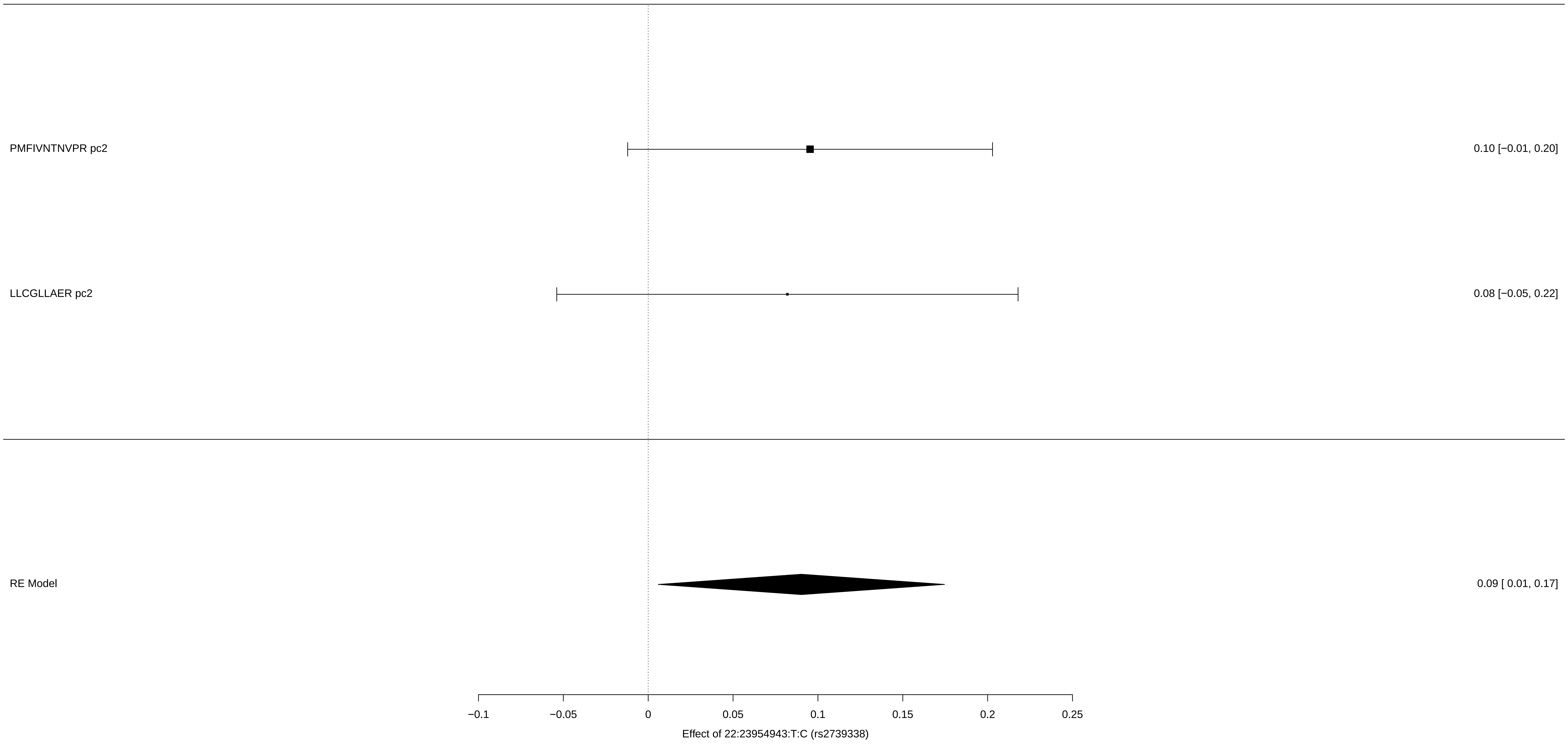

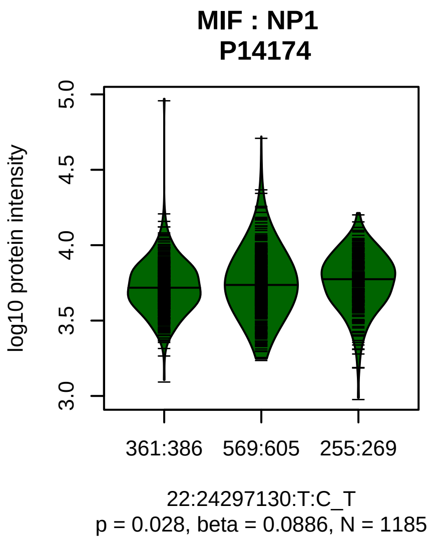

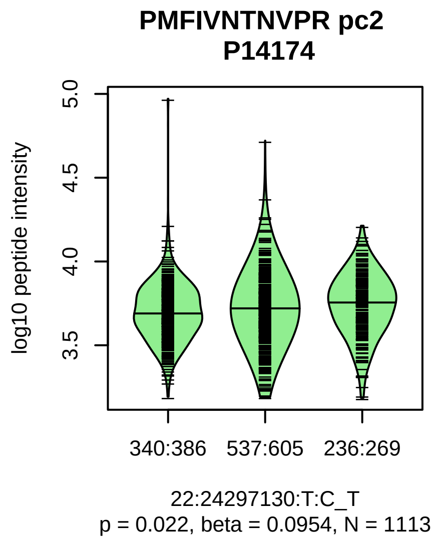

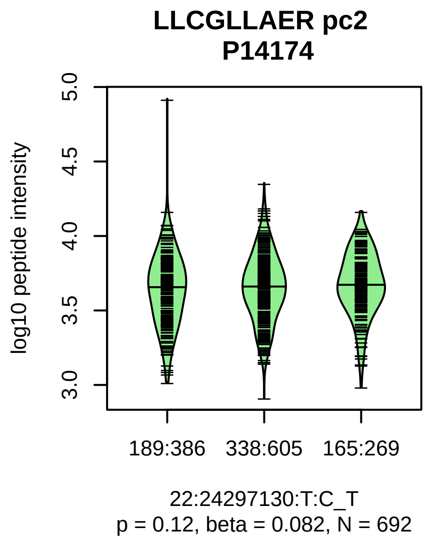

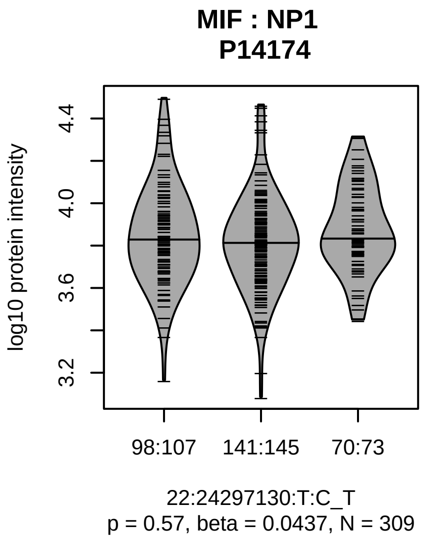

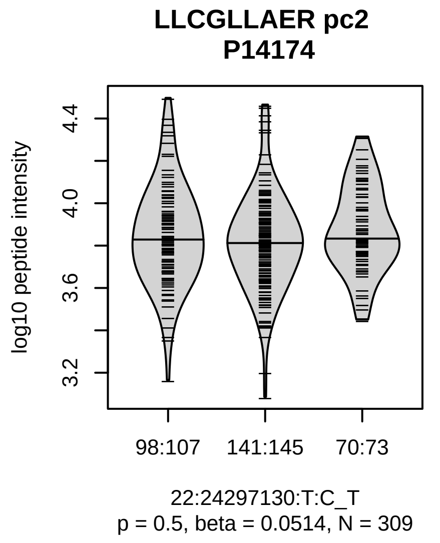

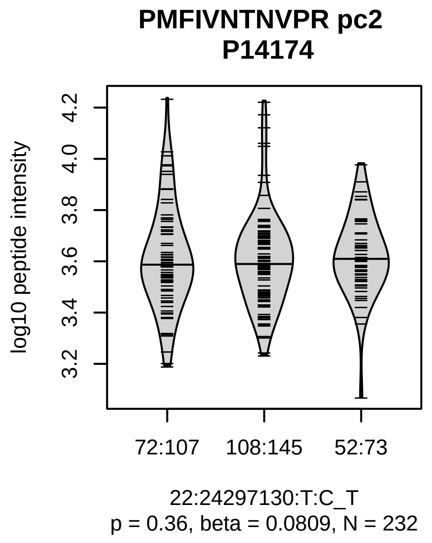

Assay Target: MIF
UniProt: P14174
rsID: rs2739338
Target SNP: 22:23954943:T:C
Proxy SNP: 22:24297130:T:C
deCODE log10(p): 17.3
deCODE BETA: 0.07
deCODE MSPA: 0
–:–
1113:692

286. DBNL (Q9UJU6)
deCODE MSPA=0

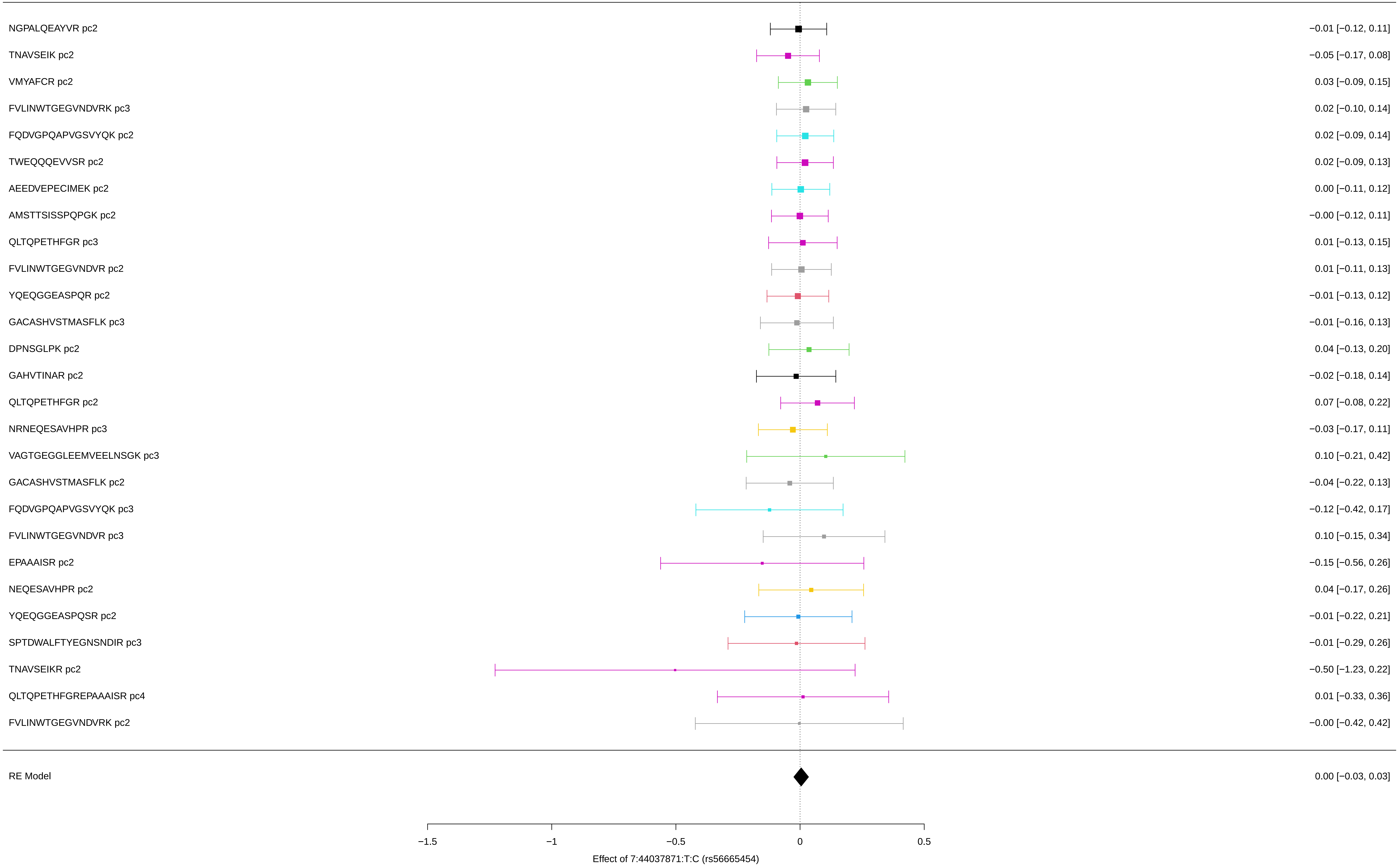

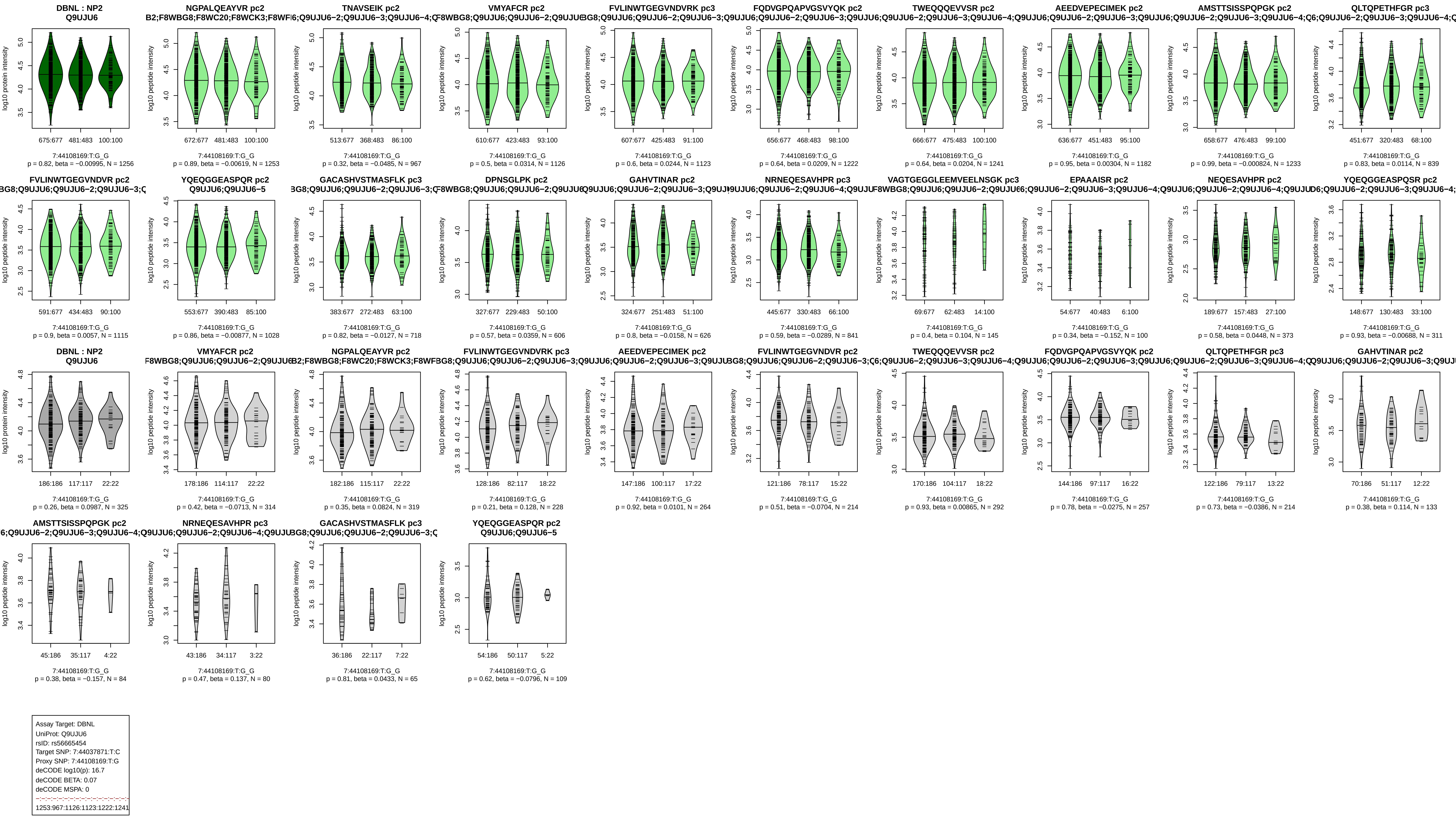
DBNL : NP2
Q9UJU6
675:677 481:483 100:100
7:44108169:T:G_G
p = 0.82, beta = −0.00995, N = 1256
NGPALQEAYVR pc2
672:677 481:483 100:100
7:44108169:T:G_G
p = 0.89, beta = −0.00619, N = 1253
TNAVSEIK pc2
513:677 368:483 86:100
7:44108169:T:G_G
p = 0.32, beta = −0.0485, N = 967
VMYAFCR pc2
610:677 423:483 93:100
7:44108169:T:G_G
p = 0.5, beta = 0.0314, N = 1126
FVLINWTGEGVNDVRK pc3
607:677 425:483 91:100
7:44108169:T:G_G
p = 0.6, beta = 0.0244, N = 1123
FQDVGPQAPVGSVYQK pc2
656:677 468:483 98:100
7:44108169:T:G_G
p = 0.64, beta = 0.0209, N = 1222
TWEQQQEVVSR pc2
666:677 475:483 100:100
7:44108169:T:G_G
p = 0.64, beta = 0.0204, N = 1241
AEEDVEPECIMEK pc2
636:677 451:483 95:100
7:44108169:T:G_G
p = 0.95, beta = 0.00304, N = 1182
AMSTTSISSPQPGK pc2
658:677 476:483 99:100
7:44108169:T:G_G
p = 0.99, beta = −0.000824, N = 1233
QLTQPETHFGR pc3
451:677 320:483 68:100
7:44108169:T:G_G
p = 0.83, beta = 0.0114, N = 839
FVLINWTGEGVNDVR pc2
591:677 434:483 90:100
7:44108169:T:G_G
p = 0.9, beta = 0.0057, N = 1115
YQEQGGEASPQR pc2
Q9UJU6;Q9UJU6−5
553:677 390:483 85:100
7:44108169:T:G_G
p = 0.86, beta = −0.0359, N = 1028
GACASHVSTMASFLK pc3
383:677 272:483 63:100
7:44108169:T:G_G
p = 0.82, beta = −0.0127, N = 718
DPNSGLPK pc2
327:677 229:483 50:100
7:44108169:T:G_G
p = 0.57, beta = 0.0359, N = 606
GAHVTINAR pc2
324:677 251:483 51:100
7:44108169:T:G_G
p = 0.8, beta = −0.0158, N = 626
NRNEQESAVHPR pc3
445:677 330:483 66:100
7:44108169:T:G_G
p = 0.59, beta = −0.0289, N = 841
VAGTGEGGLEEMVEELNSGK pc3
69:677 62:483 14:100
7:44108169:T:G_G
p = 0.4, beta = 0.104, N = 145
EPAAAISR pc2
54:677 40:483 6:100
7:44108169:T:G_G
p = 0.34, beta = −0.152, N = 100
NEQESAVHPR pc2
189:677 157:483 27:100
7:44108169:T:G_G
p = 0.58, beta = 0.0448, N = 373
YQEQGGEASPQSR pc2
148:677 130:483 33:100
7:44108169:T:G_G
p = 0.93, beta = −0.00688, N = 311
DBNL : NP2
Q9UJU6
186:186 117:117 22:22
7:44108169:T:G_G
p = 0.26, beta = 0.0987, N = 325
VMYAFCR pc2
178:186 114:117 22:22
7:44108169:T:G_G
p = 0.42, beta = −0.0713, N = 314
NGPALQEAYVR pc2
182:186 115:117 22:22
7:44108169:T:G_G
p = 0.35, beta = 0.0824, N = 319
FVLINWTGEGVNDVRK pc3
128:186 82:117 18:22
7:44108169:T:G_G
p = 0.21, beta = 0.128, N = 228
AEEDVEPECIMEK pc2
147:186 100:117 17:22
7:44108169:T:G_G
p = 0.92, beta = 0.0101, N = 264
FVLINWTGEGVNDVR pc2
121:186 78:117 15:22
7:44108169:T:G_G
p = 0.51, beta = −0.0704, N = 214
TWEQQQEVVSR pc2
170:186 104:117 18:22
7:44108169:T:G_G
p = 0.93, beta = 0.00865, N = 292
FQDVGPQAPVGSVYQK pc2
144:186 97:117 16:22
7:44108169:T:G_G
p = 0.78, beta = −0.0275, N = 257
QLTQPETHFGR pc3
122:186 79:117 13:22
7:44108169:T:G_G
p = 0.73, beta = −0.0386, N = 214
GAHVTINAR pc2
70:186 51:117 12:22
7:44108169:T:G_G
p = 0.38, beta = 0.114, N = 133
AMSTTSISSPQPGK pc2
45:186 35:117 4:22
7:44108169:T:G_G
p = 0.38, beta = −0.157, N = 84
NRNEQESAVHPR pc3
43:186 34:117 3:22
7:44108169:T:G_G
p = 0.47, beta = 0.137, N = 80
GACASHVSTMASFLK pc3
36:186 22:117 7:22
7:44108169:T:G_G
p = 0.81, beta = 0.0433, N = 65
YQEQGGEASPQR pc2
Q9UJU6;Q9UJU6−5
54:186 50:117 5:22
7:44108169:T:G_G
p = 0.62, beta = −0.0796, N = 109
Assay Target: DBNL
UniProt: Q9UJU6
rsID: rs56665454
Target SNP: 7:44037871:T:C
Proxy SNP: 7:44108169:T:G
deCODE log10(p): 16.7
deCODE BETA: 0.07
deCODE MSPA: 0
1253:967:1126:1123:1222:1241

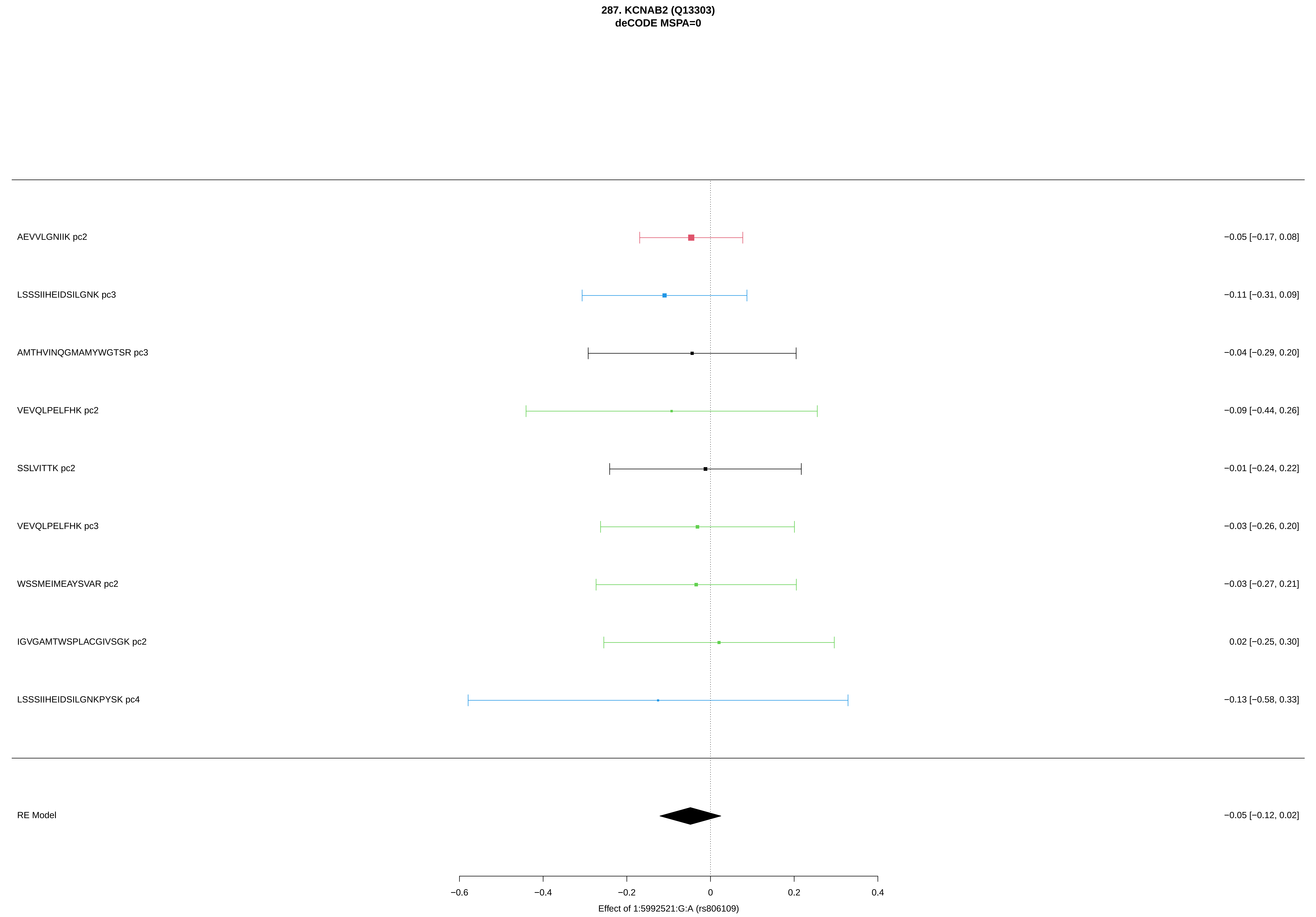

287. KCNAB2 (Q13303)
deCODE MSPA=0
AEVVLGNIIK pc2
−0.05 [−0.17, 0.08]
LSSSIIHEIDSILGNK pc3
−0.11 [−0.31, 0.09]
AMTHVINQGMAMYWGTSR pc3
−0.04 [−0.29, 0.20]
VEVQLPELFHK pc2
−0.09 [−0.44, 0.26]
SSLVITTK pc2
−0.01 [−0.24, 0.22]
VEVQLPELFHK pc3
−0.03 [−0.26, 0.20]
WSSMEIMEAYSVAR pc2
−0.03 [−0.27, 0.21]
IGVGAMTWSPLACGIVSGK pc2
0.02 [−0.25, 0.30]
LSSSIIHEIDSILGNKPYSK pc4
−0.13 [−0.58, 0.33]
RE Model
−0.05 [−0.12, 0.02]
−0.6
−0.4
−0.2
0
0.2
0.4
Effect of 1:5992521:G:A (rs806109)

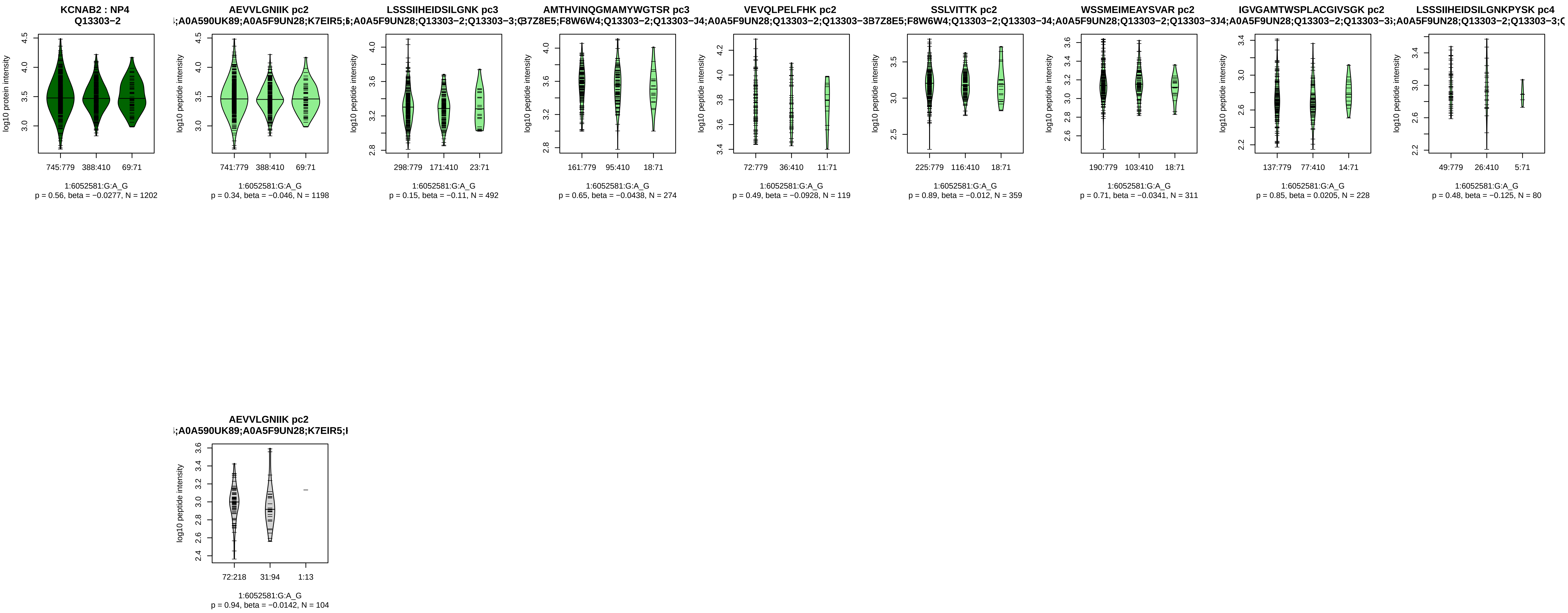

Assay Target: KCNAB2
UniProt: Q13303
rsID: rs806109
Target SNP: 1:5992521:G:A
Proxy SNP: 1:6052581:G:A
deCODE log10(p): 16.2
deCODE BETA: −0.08
deCODE MSPA: 0
–:–:–:–:–:–:–:–
1198:492:274:119:359:320:311:

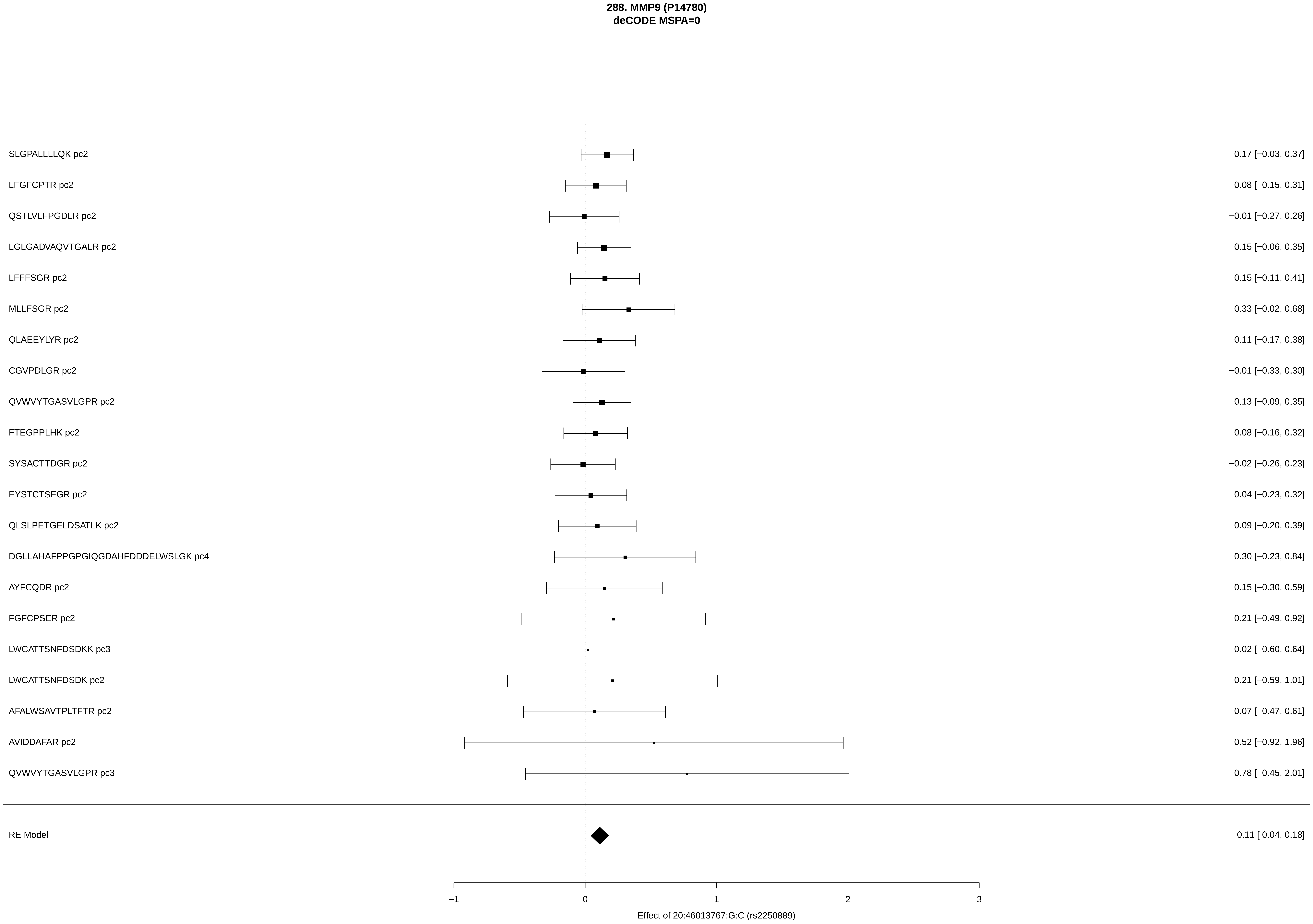
288. MMP9 (P14780)
deCODE MSPA=0
SLGPALLLLQK pc2
0.17 [−0.03, 0.37]
LFGFCPTR pc2
0.08 [−0.15, 0.31]
QSTLVLFPGDLR pc2
−0.01 [−0.27, 0.26]
LGLGADVAQVTGALR pc2
0.15 [−0.06, 0.35]
LFFFSGR pc2
0.15 [−0.11, 0.41]
MLLFSGR pc2
0.33 [−0.02, 0.68]
QLAEEYLYR pc2
0.11 [−0.17, 0.38]
CGVPDLGR pc2
−0.01 [−0.33, 0.30]
QVWVYTGASVLGPR pc2
0.13 [−0.09, 0.35]
FTEGPPLHK pc2
0.08 [−0.16, 0.32]
SYSACTTDGR pc2
−0.02 [−0.26, 0.23]
EYSTCTSEGR pc2
0.04 [−0.23, 0.32]
QLSLPETGELDSATLK pc2
0.09 [−0.20, 0.39]
DGLLAHAFPPGPGIQGDAHFDDDELWSLGK pc4
0.30 [−0.23, 0.84]
AYFCQDR pc2
0.15 [−0.30, 0.59]
FGFCPSER pc2
0.21 [−0.49, 0.92]
LWCATTSNFDSDKK pc3
0.02 [−0.60, 0.64]
LWCATTSNFDSDK pc2
0.21 [−0.59, 1.01]
AFALWSAVTPLTFTR pc2
0.07 [−0.47, 0.61]
AVIDDAFAR pc2
0.52 [−0.92, 1.96]
QVWVYTGASVLGPR pc3
0.78 [−0.45, 2.01]
RE Model
0.11 [0.04, 0.18]
−1
0
1
2
3
Effect of 20:46013767:G:C (rs2250889)

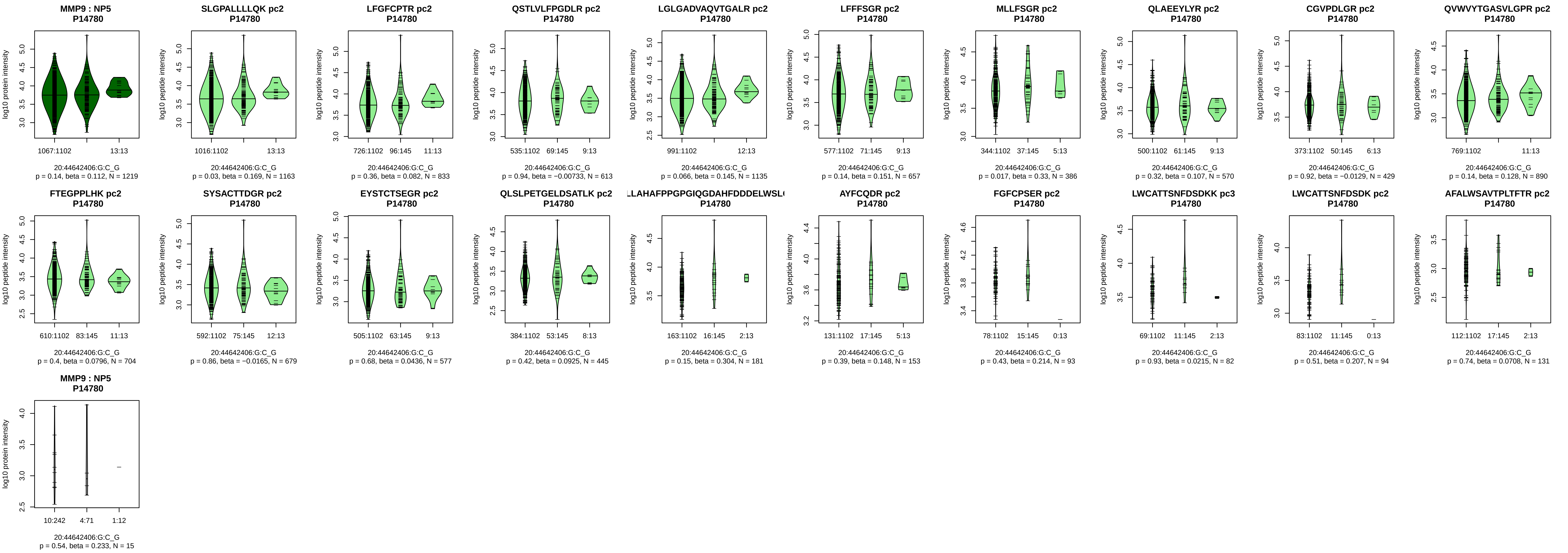
MMP9 : NP5 P14780
p = 0.14, beta = 0.112, N = 1219
SLGPALLLLQK pc2 P14780
p = 0.03, beta = 0.169, N = 1163
LFGFCPTR pc2 P14780
p = 0.36, beta = 0.082, N = 833
QSTLVLFPGDLR pc2 P14780
p = 0.94, beta = −0.00733, N = 613
LGLGADVAQVTGALR pc2 P14780
p = 0.066, beta = 0.145, N = 1135
LFFFSGR pc2 P14780
p = 0.14, beta = 0.151, N = 657
MLLFSGR pc2 P14780
p = 0.017, beta = 0.33, N = 386
QLAEEYLYR pc2 P14780
p = 0.32, beta = 0.107, N = 570
CGVPDLGR pc2 P14780
p = 0.92, beta = −0.0129, N = 429
QVWVYTGASVLGPR pc2 P14780
p = 0.14, beta = 0.128, N = 890
FTEGPPLHK pc2 P14780
p = 0.4, beta = 0.0796, N = 704
SYSACTTDGR pc2 P14780
p = 0.86, beta = −0.0165, N = 679
EYSTCTSEGR pc2 P14780
p = 0.68, beta = 0.0436, N = 577
QLSLPETGELDSATLK pc2 P14780
p = 0.42, beta = 0.0925, N = 445
LLAHAFPPGPGIQGDAHFDDDELWSL P14780
p = 0.15, beta = 0.304, N = 181
AYFCQDR pc2 P14780
p = 0.39, beta = 0.148, N = 153
FGFCPSER pc2 P14780
p = 0.43, beta = 0.214, N = 93
LWCATTSNFDSDKK pc3 P14780
p = 0.93, beta = 0.0215, N = 82
LWCATTSNFDSDK pc2 P14780
p = 0.51, beta = 0.207, N = 94
AFALWSAVTPLTFTR pc2 P14780
p = 0.74, beta = 0.0708, N = 131
MMP9 : NP5 P14780
p = 0.54, beta = 0.233, N = 15
20:44642406:G:C_G
log10 protein intensity
log10 peptide intensity

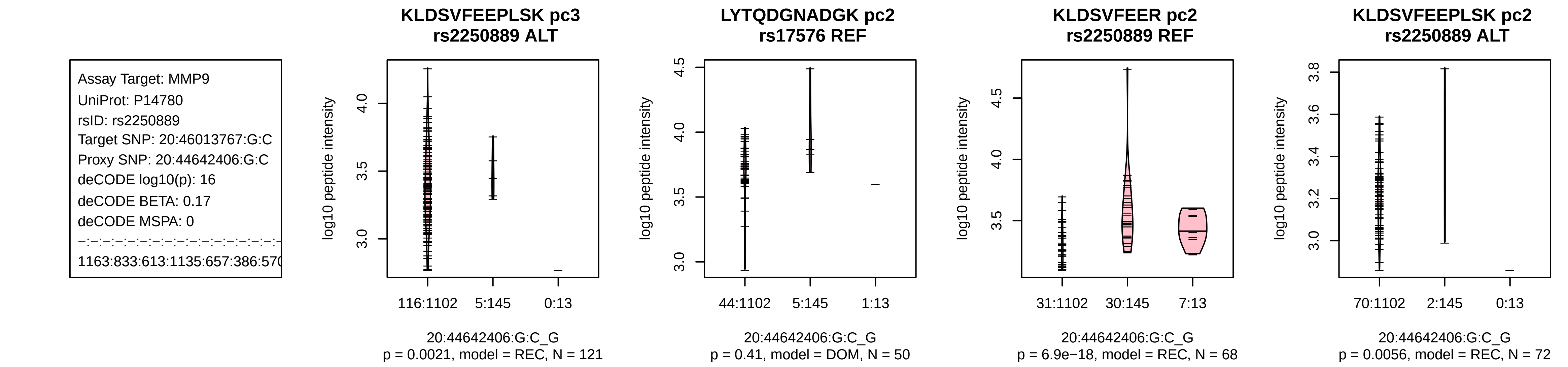
Assay Target: MMP9
UniProt: P14780
rsID: rs2250889
Target SNP: 20:46013767:G:C
Proxy SNP: 20:44642406:G:C
deCODE log10(p): 16
deCODE BETA: 0.17
deCODE MSPA: 0
1163:833:613:1135:657:386:57
KLDSVFEEPLSK pc3 rs2250889 ALT
p = 0.0021, model = REC, N = 121
LYTQDGNADGK pc2 rs17576 REF
p = 0.41, model = DOM, N = 50
KLDSVFEER pc2 rs2250889 REF
p = 6.9e−18, model = REC, N = 68
KLDSVFEEPLSK pc2 rs2250889 ALT
p = 0.0056, model = REC, N = 72
20:44642406:G:C_G
log10 peptide intensity

289. TIMP2 (P16035)
deCODE MSPA=0

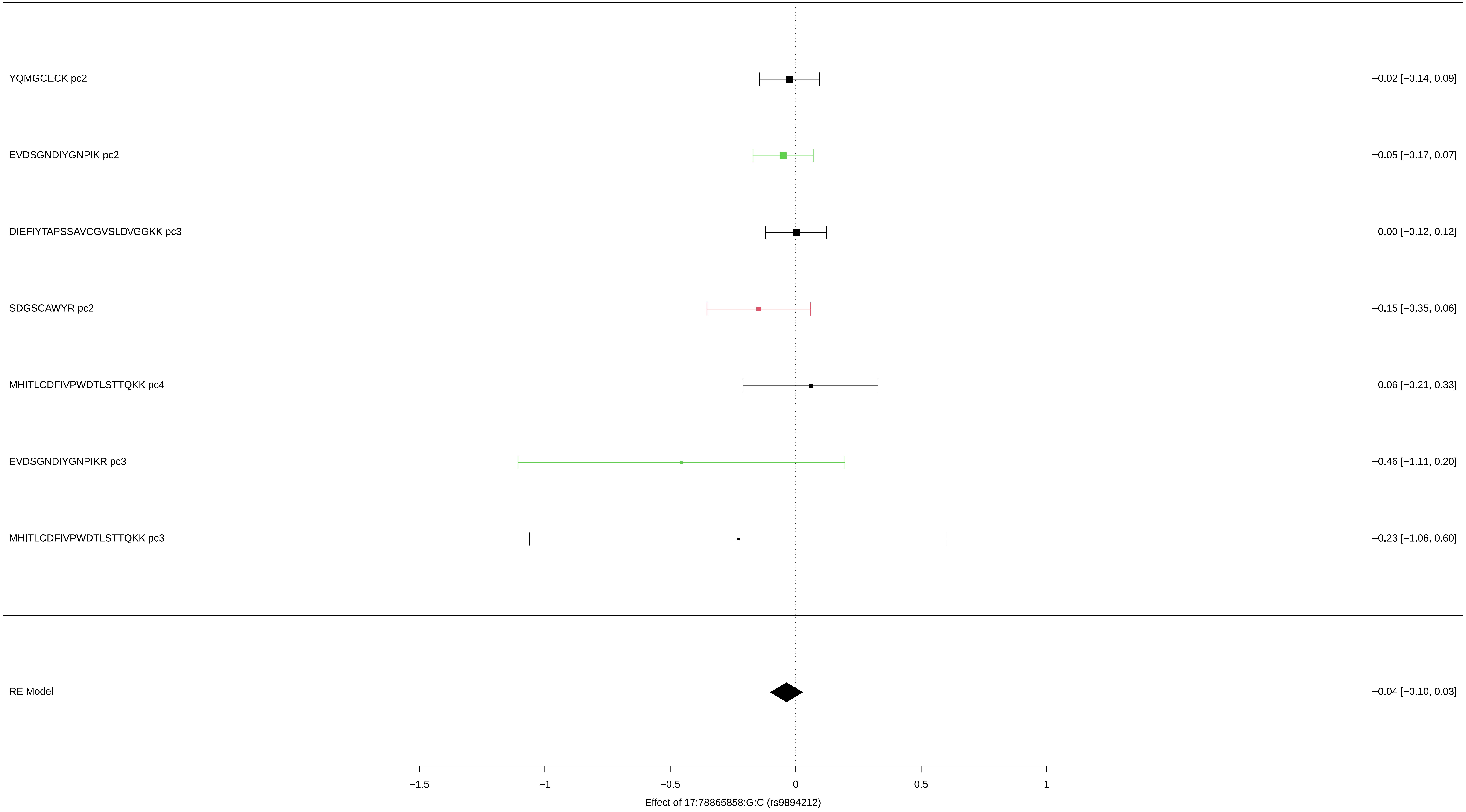

TIMP2 : NP5
P16035

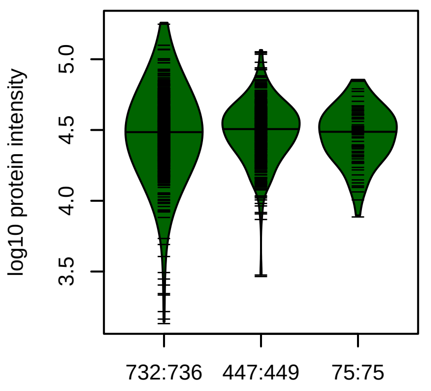

17:76861940:C:G_G
p = 0.63, beta = −0.0225, N = 1254

YQMGCECK pc2
B4DFW2;K7EIX4;P16035

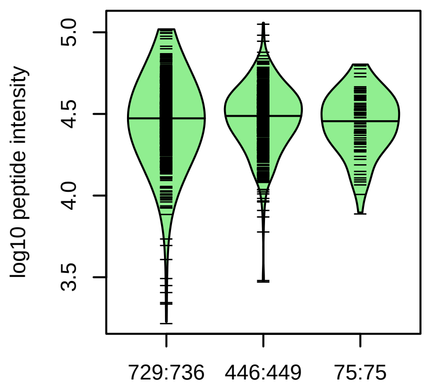

17:76861940:C:G_G
p = 0.6, beta = −0.0244, N = 1250

EVDSGNDIYGNPIK pc2
P16035

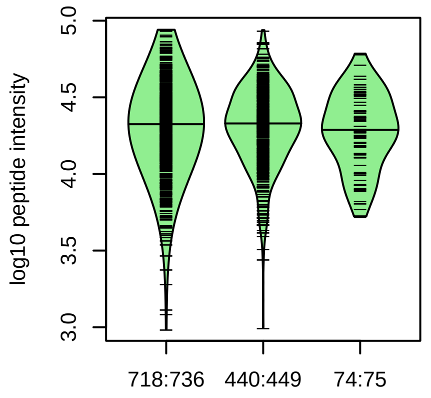

17:76861940:C:G_G
p = 0.28, beta = −0.05, N = 1232

DIEFIYTAPSSAVCGVSLDVGGKK pc
B4DFW2;K7EIX4;P16035

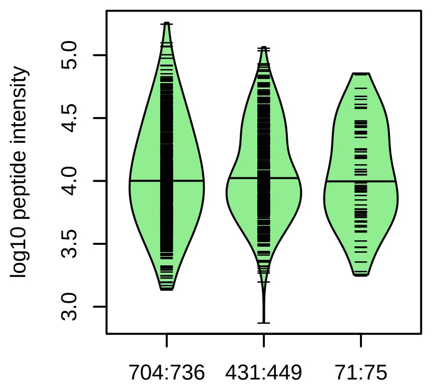

17:76861940:C:G_G
p = 0.97, beta = 0.0019, N = 1206

SDGSCAWYR pc2
B4DFW2;P16035

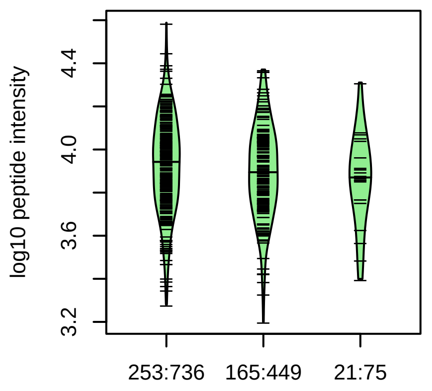

17:76861940:C:G_G
p = 0.067, beta = −0.147, N = 439

MHITLCDFIVPWDTLSTTQKK pc4
B4DFW2;K7EIX4;P16035

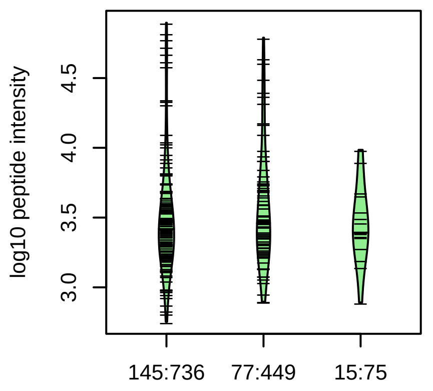

17:76861940:C:G_G
p = 0.57, beta = 0.0591, N = 237

EVDSGNDIYGNPIKR pc3
P16035

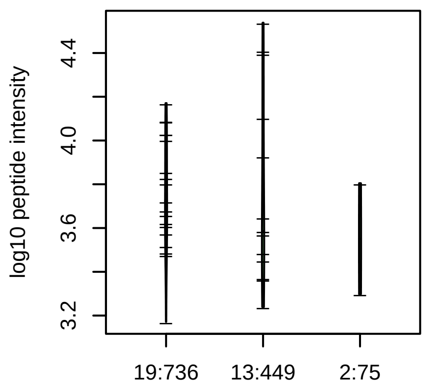

17:76861940:C:G_G
p = 0.081, beta = −0.456, N = 34

TIMP2 : NP5
P16035

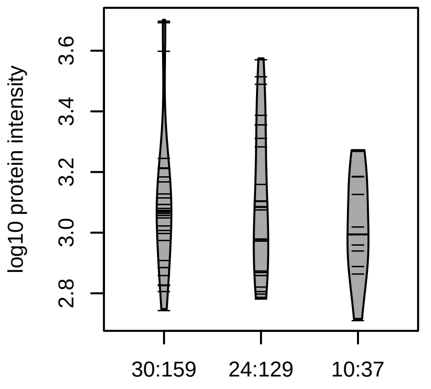

17:76861940:C:G_G
p = 0.69, beta = −0.0656, N = 64

EVDSGNDIYGNPIK pc2
P16035

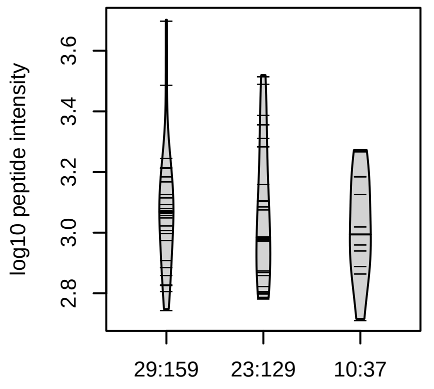

17:76861940:C:G_G
p = 0.86, beta = −0.0305, N = 62

Assay Target: TIMP2
UniProt: P16035
rsID: rs9894212
Target SNP: 17:78865858:G:C
Proxy SNP: 17:76861940:C:G
deCODE log10(p): 15.8
deCODE BETA: −0.08
deCODE MSPA: 0
−:−:−:−:−:NA:−
1250:1232:1206:439:237:34:17:

290. PROS1 (P07225)
deCODE MSPA=0

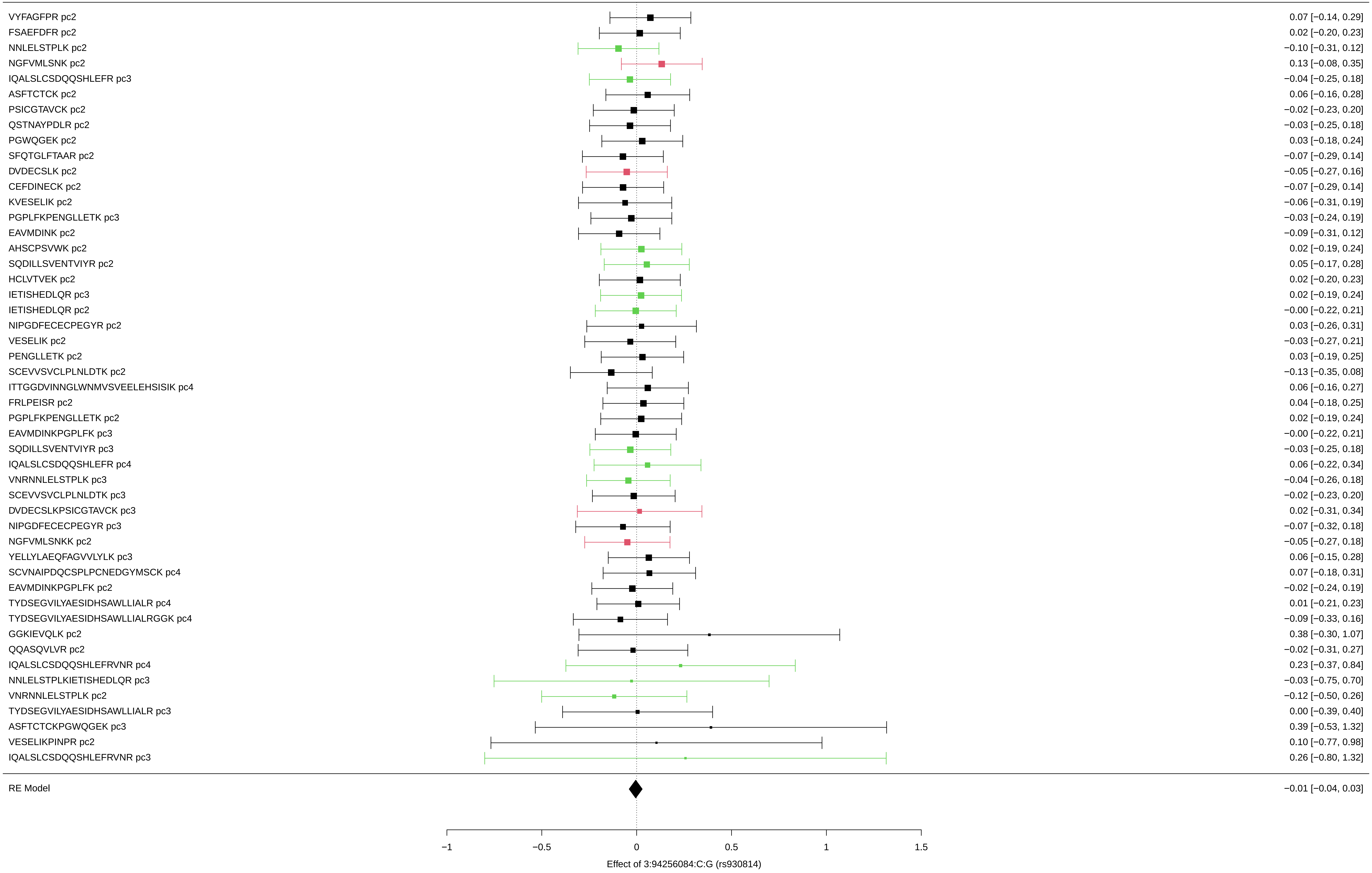

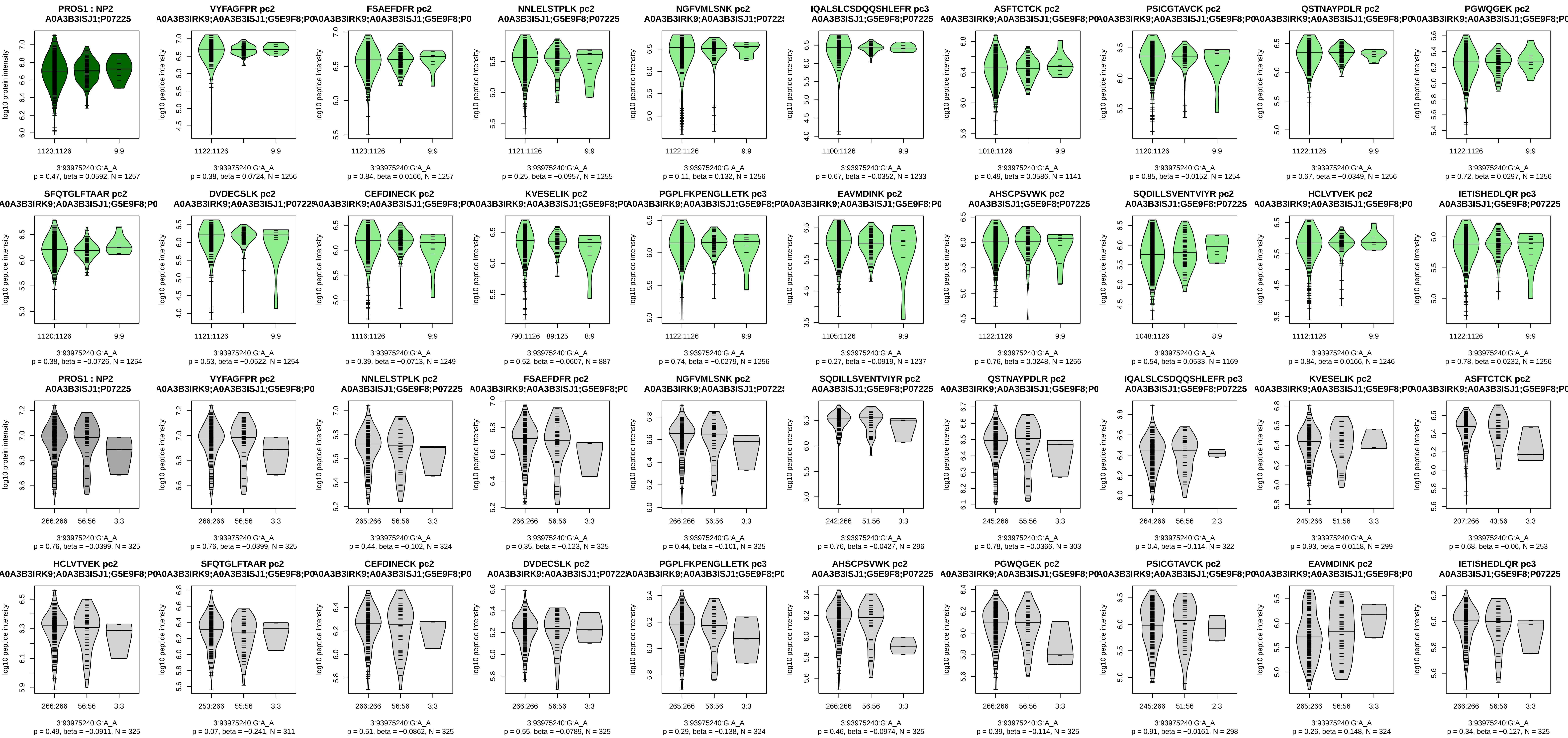

Assay Target: PROS1
UniProt: P07225
rsID: rs930814
Target SNP: 3:94256084:C:G
Proxy SNP: 3:93975240:G:A
deCODE log10(p): 14.5
deCODE BETA: −0.18
deCODE MSPA: 0
1256:1257:1255:1256:1233:114

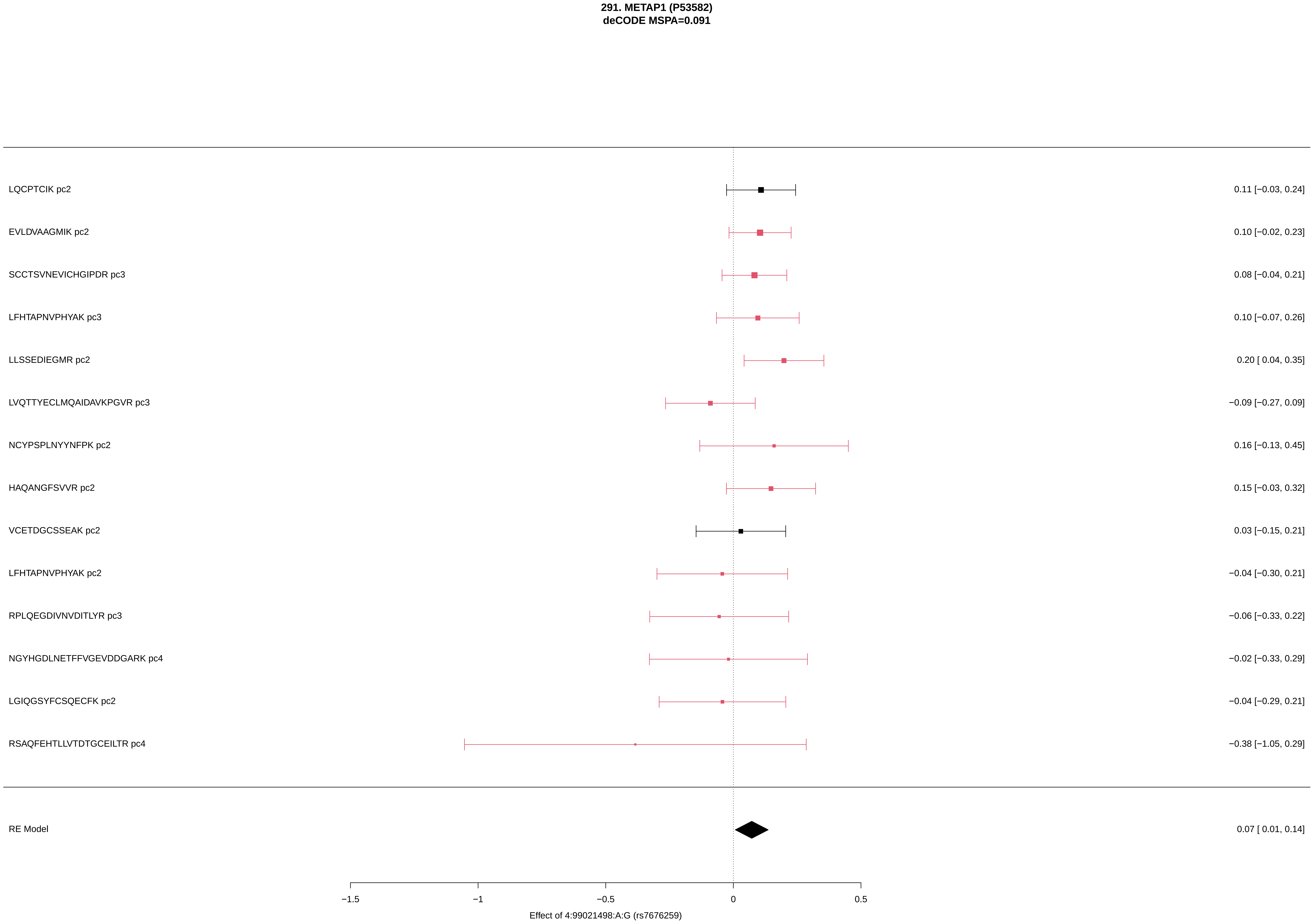
291. METAP1 (P53582)
deCODE MSPA=0.091
LQCPTCIK pc2
0.11 [−0.03, 0.24]
EVLDVAAGMIK pc2
0.10 [−0.02, 0.23]
SCCTSVNEVICHGIPDR pc3
0.08 [−0.04, 0.21]
LFHTAPNVPHYAK pc3
0.10 [−0.07, 0.26]
LLSSEDIEGMR pc2
0.20 [0.04, 0.35]
LVQTTYECLMQAIDAVKPGVR pc3
−0.09 [−0.27, 0.09]
NCYPSPLNYYNFPK pc2
0.16 [−0.13, 0.45]
HAQANGFSVVR pc2
0.15 [−0.03, 0.32]
VCETDGCSSEAK pc2
0.03 [−0.15, 0.21]
LFHTAPNVPHYAK pc2
−0.04 [−0.30, 0.21]
RPLQEGDIVNVDITLYR pc3
−0.06 [−0.33, 0.22]
NGYHGDLNETFFVGEVDDGARK pc4
−0.02 [−0.33, 0.29]
LGIQGSYFCSQECFK pc2
−0.04 [−0.29, 0.21]
RSAQFEHTLLVTDTGCEILTR pc4
−0.38 [−1.05, 0.29]
RE Model
0.07 [0.01, 0.14]
−1.5
−1
−0.5
0
0.5
Effect of 4:99021498:A:G (rs7676259)

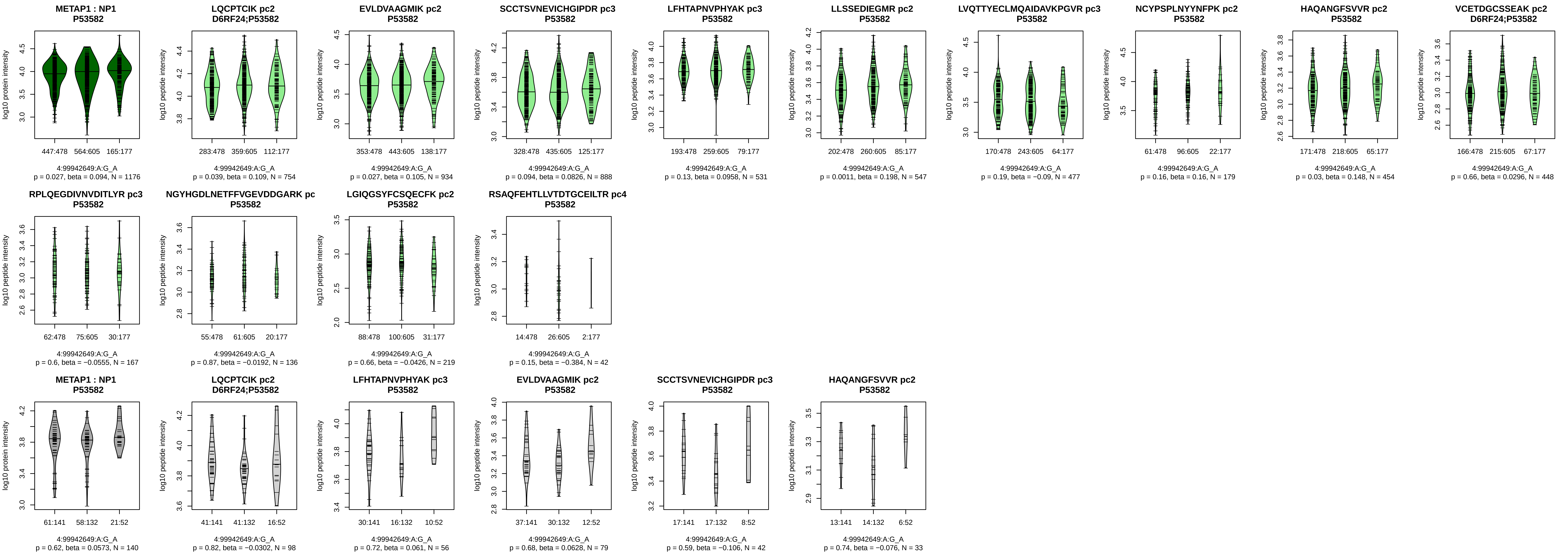

Assay Target: METAP1
UniProt: P53582
rsID: rs7676259
Target SNP: 4:99021498:A:G
Proxy SNP: 4:99942649:A:G
deCODE log10(p): 14.4
deCODE BETA: 0.06
deCODE MSPA: 0.091
–:–:–:–:*:–:–:–:–:–:–:–:–:–
754:934:888:531:547:477:179:4

292. EPB41 (P11171)
deCODE MSPA=0

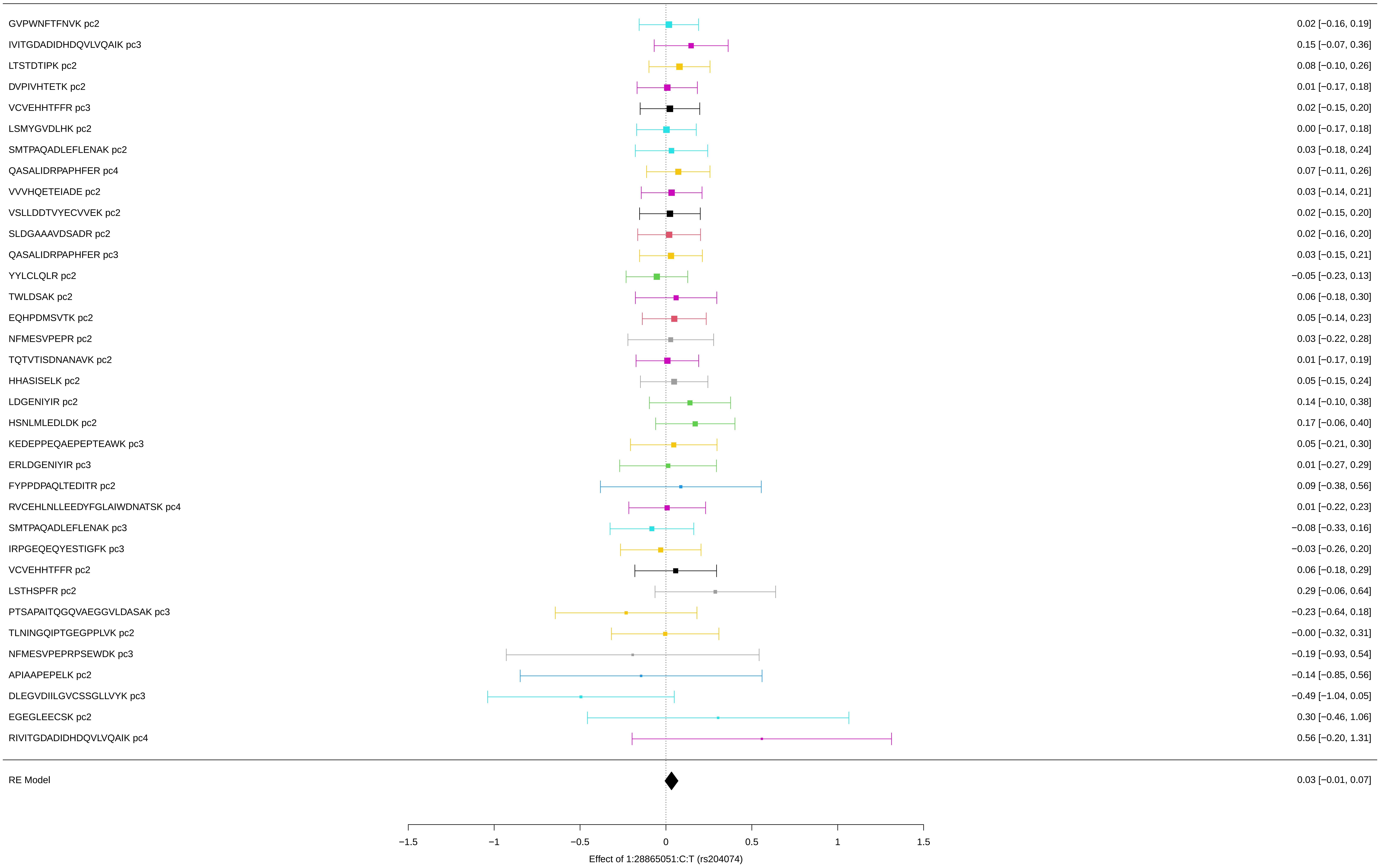

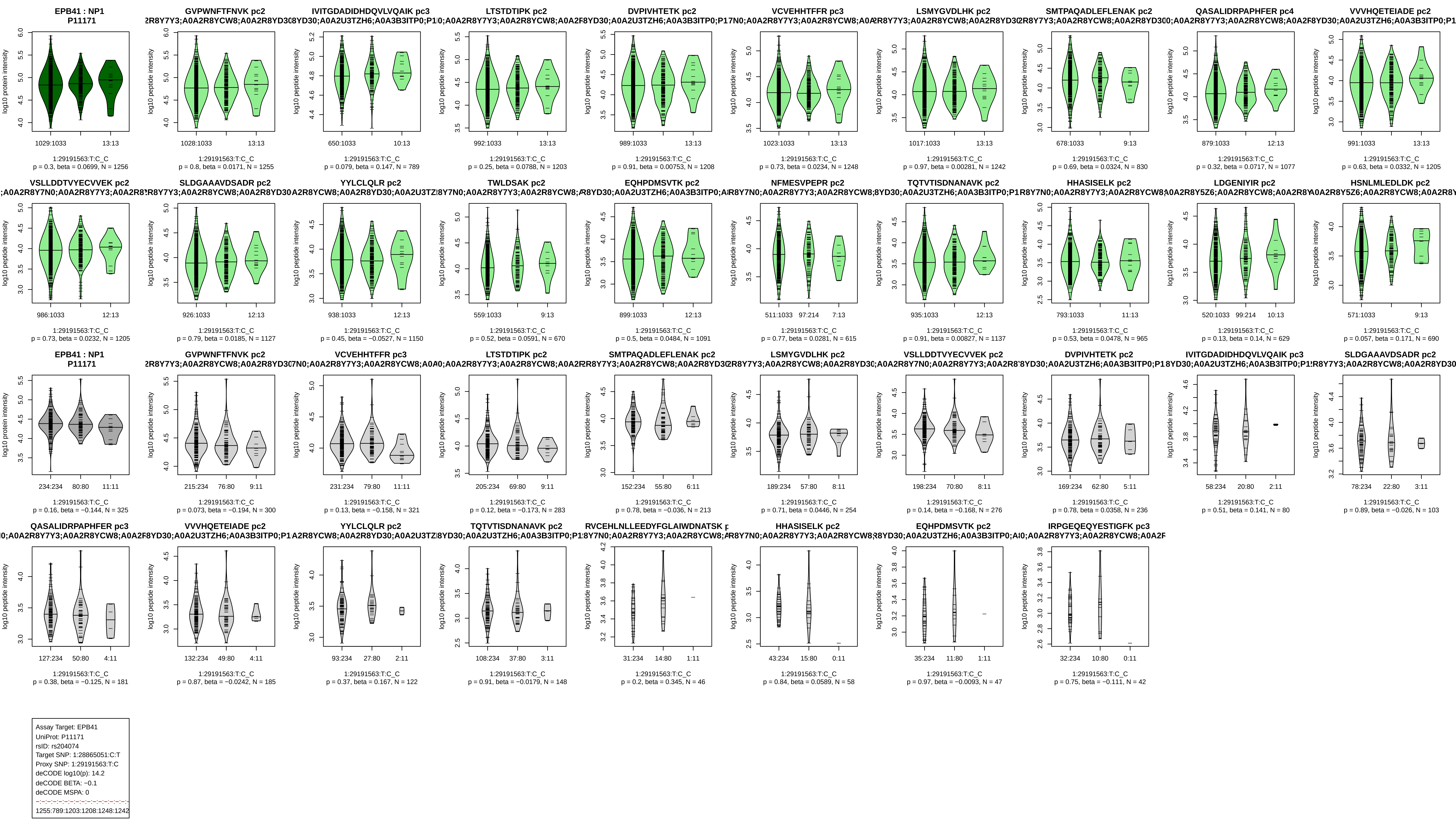
EPB41 : NP1
P11171
1:29191563:T:C_C
p = 0.3, beta = 0.0699, N = 1256
GVPWNFTFNVK pc2
p = 0.8, beta = 0.0171, N = 1255
IVITGDADIDHDQVLVQAIK pc3
p = 0.079, beta = 0.147, N = 789
LTSTDTIPK pc2
p = 0.25, beta = 0.0788, N = 1203
DVPIVHTETK pc2
p = 0.91, beta = 0.00753, N = 1208
VCVEHHTFFR pc3
p = 0.73, beta = 0.0234, N = 1248
LSMYGVDLHK pc2
p = 0.97, beta = 0.00281, N = 1242
SMTPAQADLEFLENAK pc2
p = 0.69, beta = 0.0324, N = 830
QASALIDRPAPHFER pc4
p = 0.32, beta = 0.0717, N = 1077
VVVHQETEIADE pc2
p = 0.63, beta = 0.0332, N = 1205
VSLLDDTVYECVVEK pc2
p = 0.73, beta = 0.0232, N = 1205
SLDGAAAVDSADR pc2
p = 0.79, beta = 0.0185, N = 1127
YYLCLQLR pc2
p = 0.45, beta = −0.0527, N = 1150
TWLDSAK pc2
p = 0.52, beta = 0.0591, N = 670
EQHPDMSVTK pc2
p = 0.5, beta = 0.0484, N = 1091
NFMESVPEPR pc2
p = 0.77, beta = 0.0281, N = 615
TQTVTISDNANAVK pc2
p = 0.91, beta = 0.00827, N = 1137
HHASISELK pc2
p = 0.53, beta = 0.0478, N = 965
LDGENIYIR pc2
p = 0.13, beta = 0.14, N = 629
HSNLMLEDLDK pc2
p = 0.057, beta = 0.171, N = 690
EPB41 : NP1
P11171
p = 0.16, beta = −0.144, N = 325
GVPWNFTFNVK pc2
p = 0.073, beta = −0.194, N = 300
VCVEHHTFFR pc3
p = 0.13, beta = −0.158, N = 321
LTSTDTIPK pc2
p = 0.12, beta = −0.173, N = 283
SMTPAQADLEFLENAK pc2
p = 0.78, beta = −0.036, N = 213
LSMYGVDLHK pc2
p = 0.71, beta = 0.0446, N = 254
VSLLDDTVYECVVEK pc2
p = 0.14, beta = −0.168, N = 276
DVPIVHTETK pc2
p = 0.78, beta = 0.0358, N = 236
IVITGDADIDHDQVLVQAIK pc3
p = 0.51, beta = 0.141, N = 80
SLDGAAAVDSADR pc2
p = 0.89, beta = −0.026, N = 103
QASALIDRPAPHFER pc3
p = 0.38, beta = −0.125, N = 181
VVVHQETEIADE pc2
p = 0.87, beta = −0.0242, N = 185
YYLCLQLR pc2
p = 0.37, beta = 0.167, N = 122
TQTVTISDNANAVK pc2
p = 0.91, beta = −0.0179, N = 148
RVCEHLNLLEEDYFGLAIWDNATSK
p = 0.2, beta = 0.345, N = 46
HHASISELK pc2
p = 0.84, beta = 0.0589, N = 58
EQHPDMSVTK pc2
p = 0.97, beta = −0.0093, N = 47
IRPGEQEQYESTIGFK pc3
p = 0.75, beta = −0.111, N = 42
Assay Target: EPB41
UniProt: P11171
rsID: rs204074
Target SNP: 1:28865051:C:T
Proxy SNP: 1:29191563:T:C
deCODE log10(p): 14.2
deCODE BETA: −0.1
deCODE MSPA: 0
1255:789:1203:1208:1248:1242

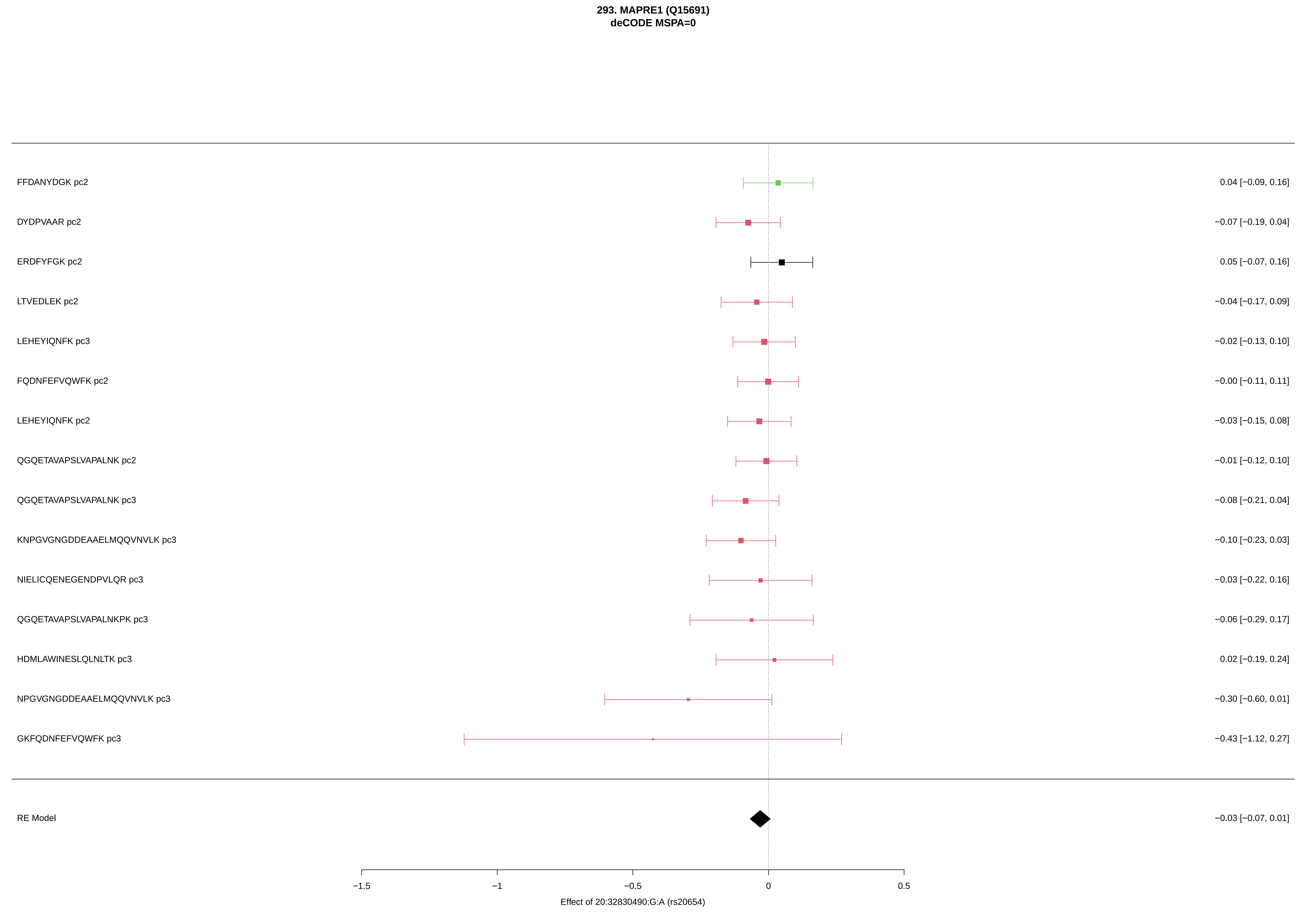
293. MAPRE1 (Q15691)
deCODE MSPA=0
FFDANYDGK pc2
0.04 [−0.09, 0.16]
DYDPVAAR pc2
−0.07 [−0.19, 0.04]
ERDFYFGK pc2
0.05 [−0.07, 0.16]
LTVEDLEK pc2
−0.04 [−0.17, 0.09]
LEHEYIQNFK pc3
−0.02 [−0.13, 0.10]
FQDNFEFVQWFK pc2
−0.00 [−0.11, 0.11]
LEHEYIQNFK pc2
−0.03 [−0.15, 0.08]
QGQETAVAPSLVAPALNK pc2
−0.01 [−0.12, 0.10]
QGQETAVAPSLVAPALNK pc3
−0.08 [−0.21, 0.04]
KNPGVGNGDDEAAELMQQVNVLK pc3
−0.10 [−0.23, 0.03]
NIELICQENEGENDPVLQR pc3
−0.03 [−0.22, 0.16]
QGQETAVAPSLVAPALNKPK pc3
−0.06 [−0.29, 0.17]
HDMLAWINESLQLNLTK pc3
0.02 [−0.19, 0.24]
NPGVGNGDDEAAELMQQVNVLK pc3
−0.30 [−0.60, 0.01]
GKFQDNFEFVQWFK pc3
−0.43 [−1.12, 0.27]
RE Model
−0.03 [−0.07, 0.01]
−1.5
−1
−0.5
0
0.5
Effect of 20:32830490:G:A (rs20654)

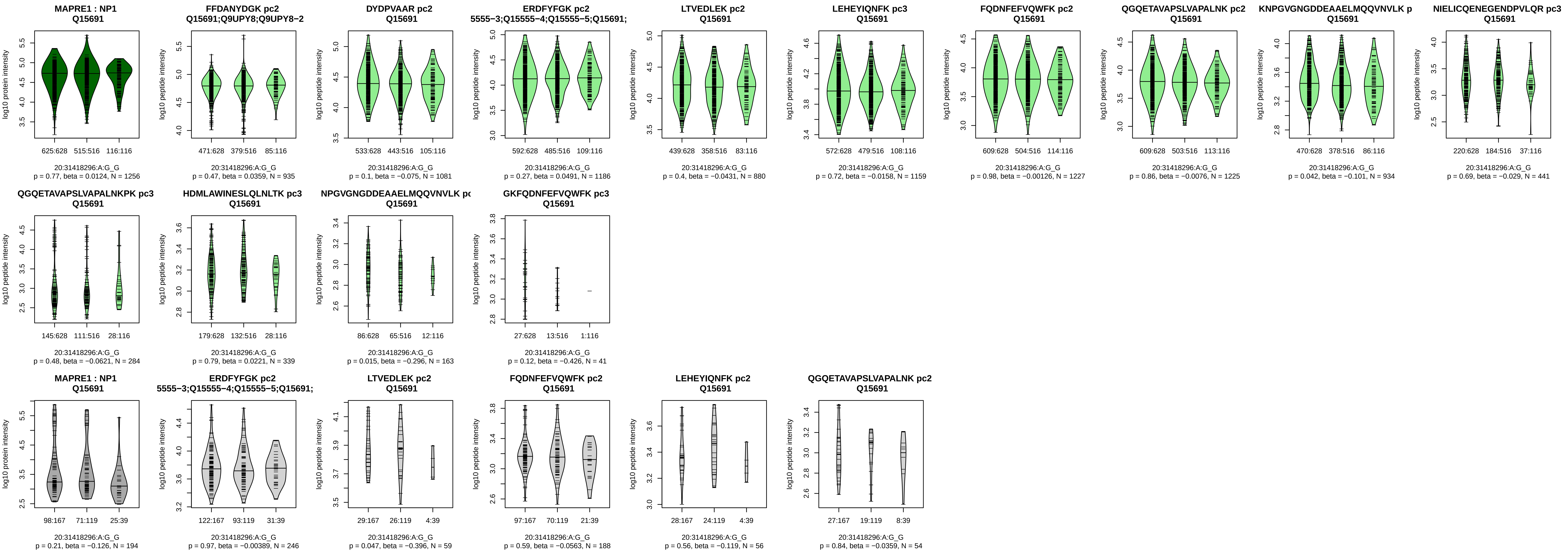

Assay Target: MAPRE1
UniProt: Q15691
rsID: rs20654
Target SNP: 20:32830490:G:A
Proxy SNP: 20:31418296:A:G
deCODE log10(p): 13.4
deCODE BETA: −0.07
deCODE MSPA: 0
−:−:−:−:−:−:−:−:−:−:−:−:−:−:NA
935:1081:1186:880:1159:1227:1

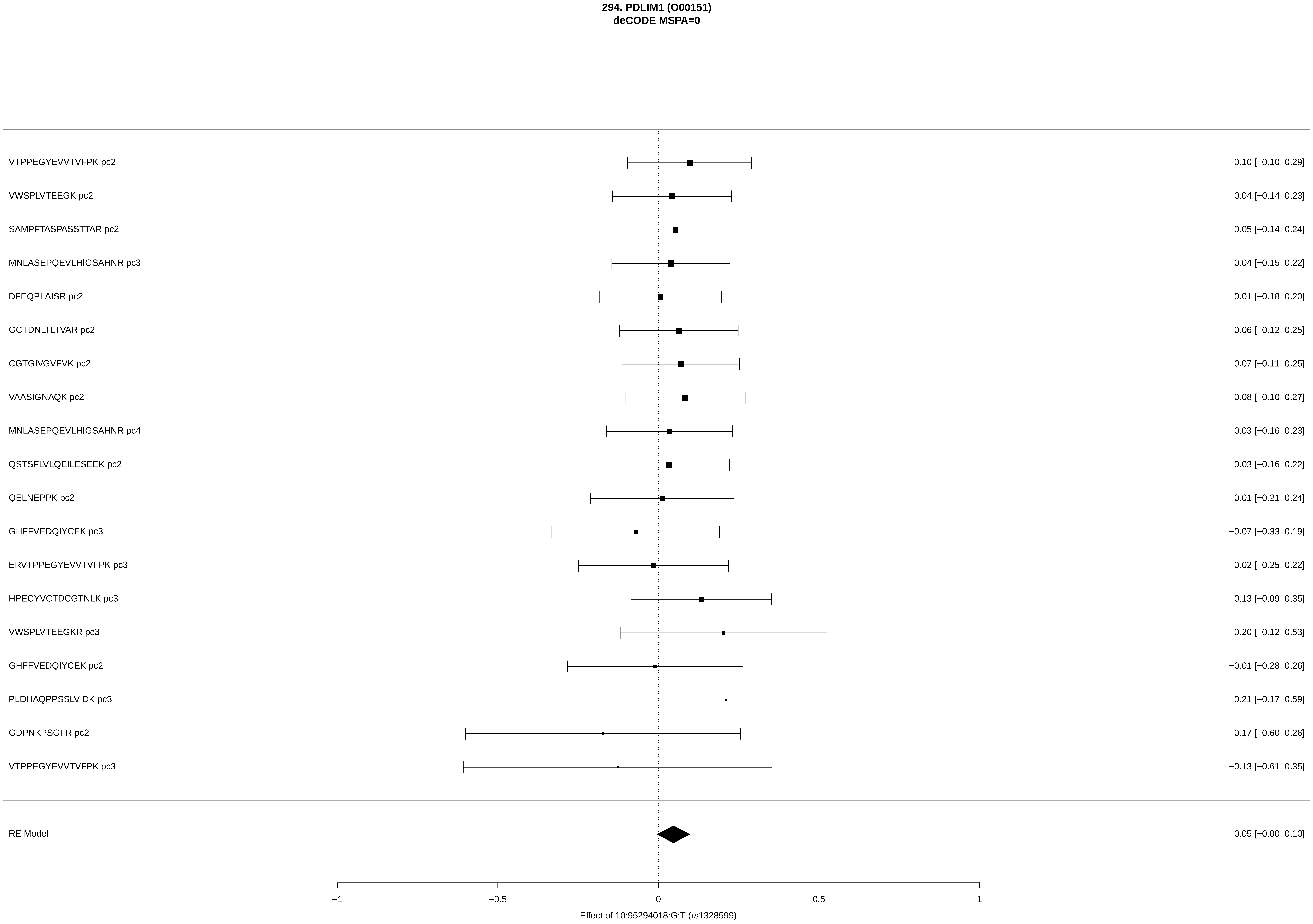
294. PDLIM1 (O00151)
deCODE MSPA=0
VTPPEGYEVVTVFPK pc2
0.10 [−0.10, 0.29]
VWSPLVTEEGK pc2
0.04 [−0.14, 0.23]
SAMPFTASPASSTTAR pc2
0.05 [−0.14, 0.24]
MNLASEPQEVLHIGSAHNR pc3
0.04 [−0.15, 0.22]
DFEQPLAISR pc2
0.01 [−0.18, 0.20]
GCTDNLTLTVAR pc2
0.06 [−0.12, 0.25]
CGTGIVGVFVK pc2
0.07 [−0.11, 0.25]
VAASIGNAQK pc2
0.08 [−0.10, 0.27]
MNLASEPQEVLHIGSAHNR pc4
0.03 [−0.16, 0.23]
QSTSFLVLQEILESEEK pc2
0.03 [−0.16, 0.22]
QELNEPPK pc2
0.01 [−0.21, 0.24]
GHFFVEDQIYCEK pc3
−0.07 [−0.33, 0.19]
ERVTPPEGYEVVTVFPK pc3
−0.02 [−0.25, 0.22]
HPECYVCTDCGTNLK pc3
0.13 [−0.09, 0.35]
VWSPLVTEEGKR pc3
0.20 [−0.12, 0.53]
GHFFVEDQIYCEK pc2
−0.01 [−0.28, 0.26]
PLDHAQPPSSLVIDK pc3
0.21 [−0.17, 0.59]
GDPNKPSGFR pc2
−0.17 [−0.60, 0.26]
VTPPEGYEVVTVFPK pc3
−0.13 [−0.61, 0.35]
RE Model
0.05 [−0.00, 0.10]
−1
−0.5
0
0.5
1
Effect of 10:95294018:G:T (rs1328599)

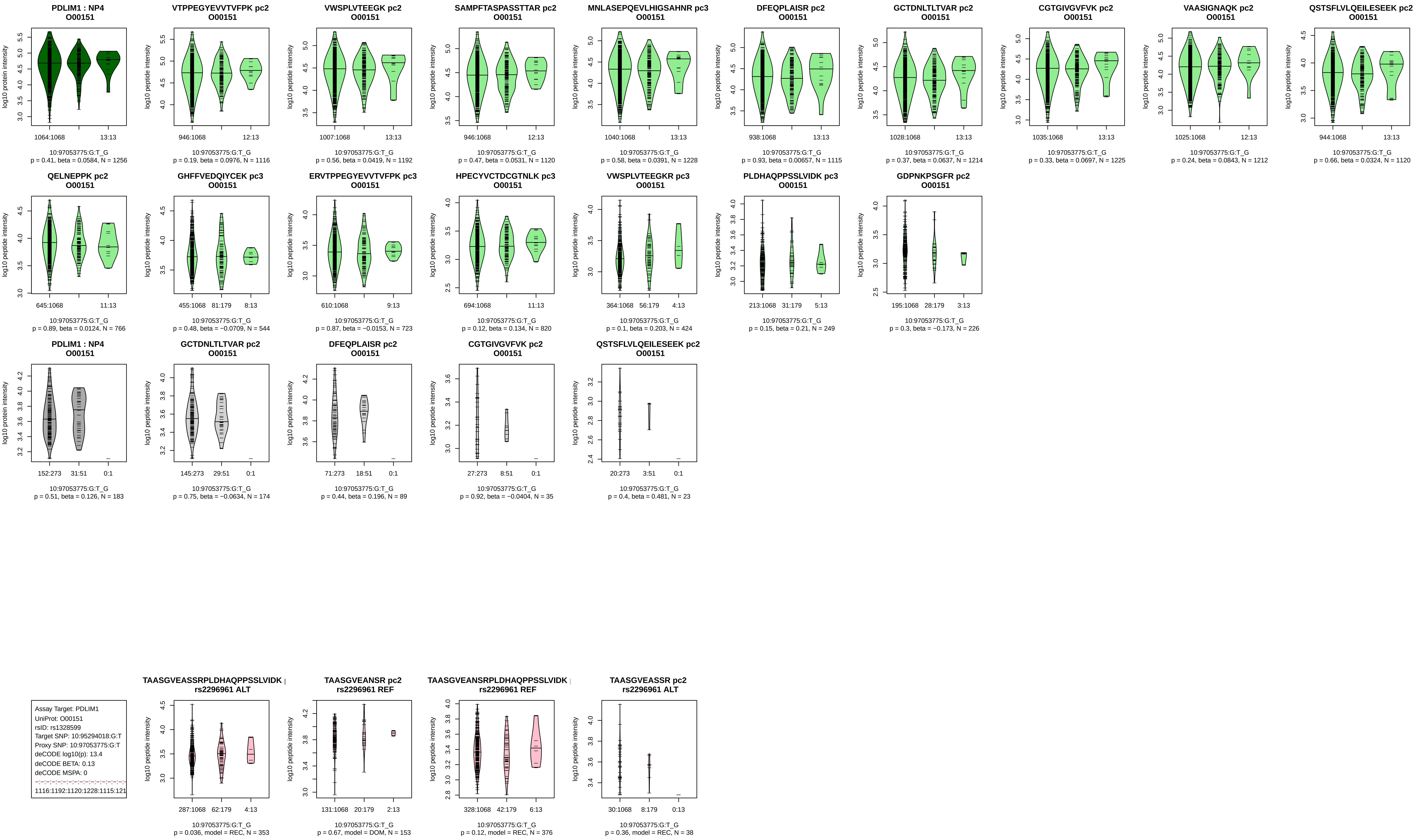

PDLIM1 : NP4 O00151
10:97053775:G:T_G
p = 0.41, beta = 0.0584, N = 1256
VTPPEGYEVVTVFPK pc2 O00151
10:97053775:G:T_G
p = 0.19, beta = 0.0976, N = 1116
VWSPLVTEEGK pc2 O00151
10:97053775:G:T_G
p = 0.56, beta = 0.0419, N = 1192
SAMPFTASPASSTTAR pc2 O00151
10:97053775:G:T_G
p = 0.47, beta = 0.0531, N = 1120
MNLASEPQEVLHIGSAHNR pc3 O00151
10:97053775:G:T_G
p = 0.58, beta = 0.0391, N = 1228
DFEQPLAISR pc2 O00151
10:97053775:G:T_G
p = 0.93, beta = 0.00657, N = 1115
GCTDNLTLTVAR pc2 O00151
10:97053775:G:T_G
p = 0.37, beta = 0.0637, N = 1214
CGTGIVGVFVK pc2 O00151
10:97053775:G:T_G
p = 0.33, beta = 0.0697, N = 1225
VAASIGNAQK pc2 O00151
10:97053775:G:T_G
p = 0.24, beta = 0.0843, N = 1212
QSTSFLVLQEILESEEK pc2 O00151
10:97053775:G:T_G
p = 0.66, beta = 0.0324, N = 1120
QELNEPPK pc2 O00151
10:97053775:G:T_G
p = 0.89, beta = 0.0124, N = 766
GHFFVEDQIYCEK pc3 O00151
10:97053775:G:T_G
p = 0.48, beta = −0.0709, N = 544
ERVTPPEGYEVVTVFPK pc3 O00151
10:97053775:G:T_G
p = 0.87, beta = −0.0153, N = 723
HPECYVCTDCGTNLK pc3 O00151
10:97053775:G:T_G
p = 0.12, beta = 0.134, N = 820
VWSPLVTEEGKR pc3 O00151
10:97053775:G:T_G
p = 0.1, beta = 0.203, N = 424
PLDHAQPPSSLVIDK pc3 O00151
10:97053775:G:T_G
p = 0.15, beta = 0.21, N = 249
GDPNKPSGFR pc2 O00151
10:97053775:G:T_G
p = 0.3, beta = −0.173, N = 226
PDLIM1 : NP4 O00151
10:97053775:G:T_G
p = 0.51, beta = 0.126, N = 183
GCTDNLTLTVAR pc2 O00151
10:97053775:G:T_G
p = 0.75, beta = −0.0634, N = 174
DFEQPLAISR pc2 O00151
10:97053775:G:T_G
p = 0.44, beta = 0.196, N = 89
CGTGIVGVFVK pc2 O00151
10:97053775:G:T_G
p = 0.92, beta = −0.0404, N = 35
QSTSFLVLQEILESEEK pc2 O00151
10:97053775:G:T_G
p = 0.4, beta = 0.481, N = 23
Assay Target: PDLIM1
UniProt: O00151
rsID: rs1328599
Target SNP: 10:95294018:G:T
Proxy SNP: 10:97053775:G:T
deCODE log10(p): 13.4
deCODE BETA: 0.13
deCODE MSPA: 0
1116:1192:1120:1228:1115:121
TAASGVEASSRPLDHAQPPSSLVIDK rs2296961 ALT
10:97053775:G:T_G
p = 0.036, model = REC, N = 353
TAASGVEANSR pc2 rs2296961 REF
10:97053775:G:T_G
p = 0.67, model = DOM, N = 153
TAASGVEANSRPLDHAQPPSSLVIDK rs2296961 REF
10:97053775:G:T_G
p = 0.12, model = REC, N = 376
TAASGVEASSR pc2 rs2296961 ALT
10:97053775:G:T_G
p = 0.36, model = REC, N = 38

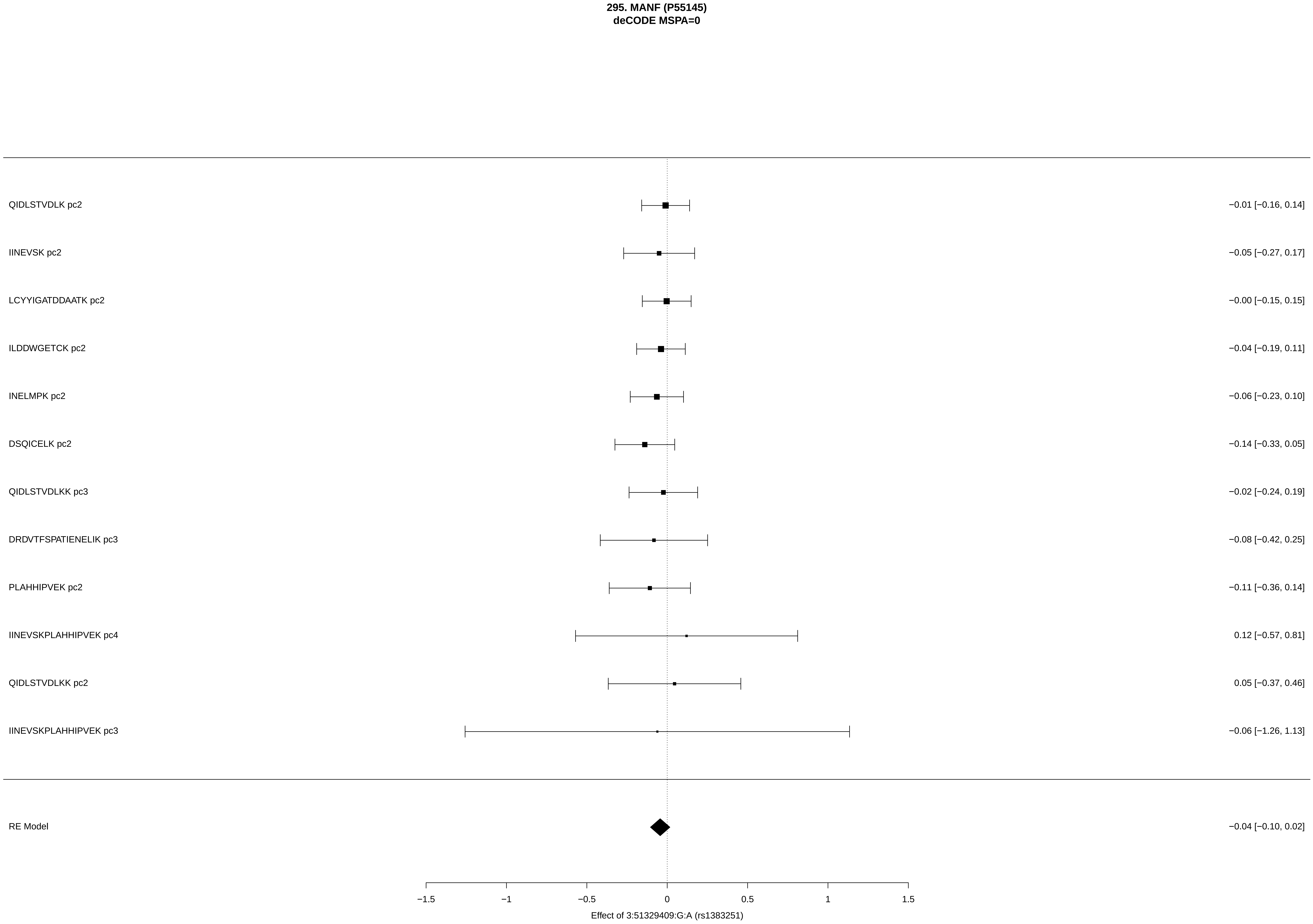

295. MANF (P55145)
deCODE MSPA=0
QIDLSTVDLK pc2
−0.01 [−0.16, 0.14]
IINEVSK pc2
−0.05 [−0.27, 0.17]
LCYYIGATDDAATK pc2
−0.00 [−0.15, 0.15]
ILDDWGETCK pc2
−0.04 [−0.19, 0.11]
INELMPK pc2
−0.06 [−0.23, 0.10]
DSQICELK pc2
−0.14 [−0.33, 0.05]
QIDLSTVDLKK pc3
−0.02 [−0.24, 0.19]
DRDVTFSPATIENELIK pc3
−0.08 [−0.42, 0.25]
PLAHHIPVEK pc2
−0.11 [−0.36, 0.14]
IINEVSKPLAHHIPVEK pc4
0.12 [−0.57, 0.81]
QIDLSTVDLKK pc2
0.05 [−0.37, 0.46]
IINEVSKPLAHHIPVEK pc3
−0.06 [−1.26, 1.13]
RE Model
−0.04 [−0.10, 0.02]
−1.5
−1
−0.5
0
0.5
1
1.5
Effect of 3:51329409:G:A (rs1383251)

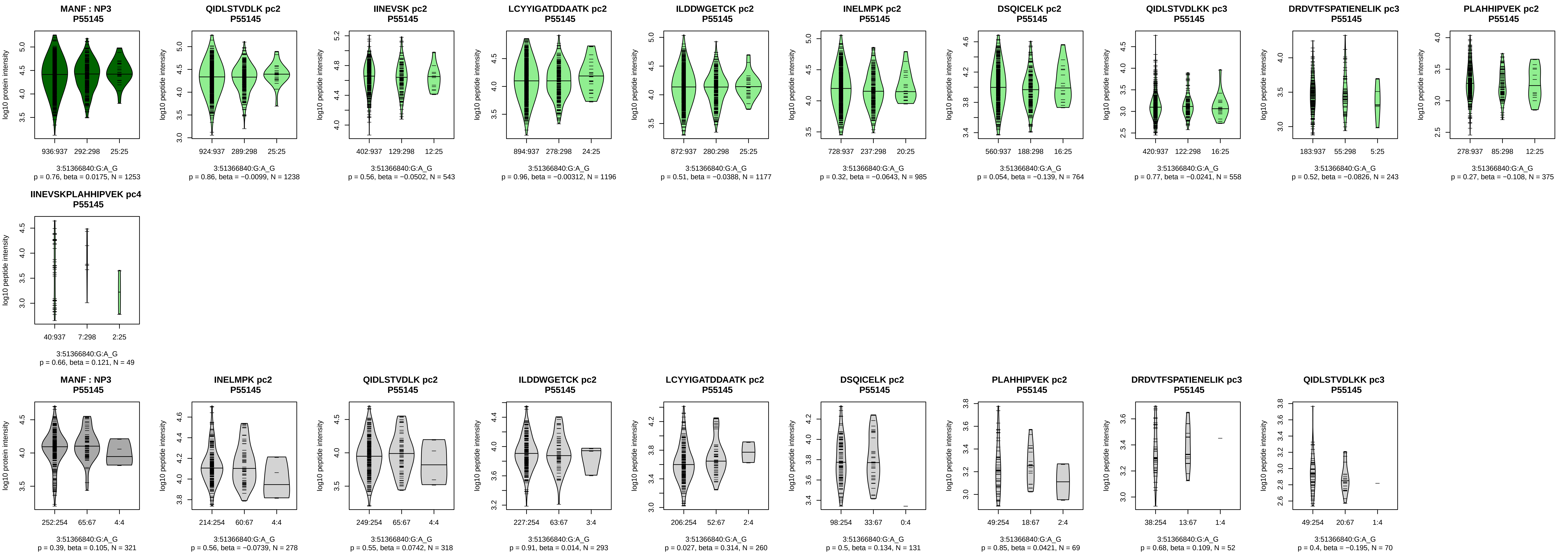

Assay Target: MANF
UniProt: P55145
rsID: rs1383251
Target SNP: 3:51329409:G:A
Proxy SNP: 3:51366840:G:A
deCODE log10(p): 12.5
deCODE BETA: −0.09
deCODE MSPA: 0
–:–:–:–:–:–:–:–:–:–:NA
1238:543:1196:1177:985:764:55

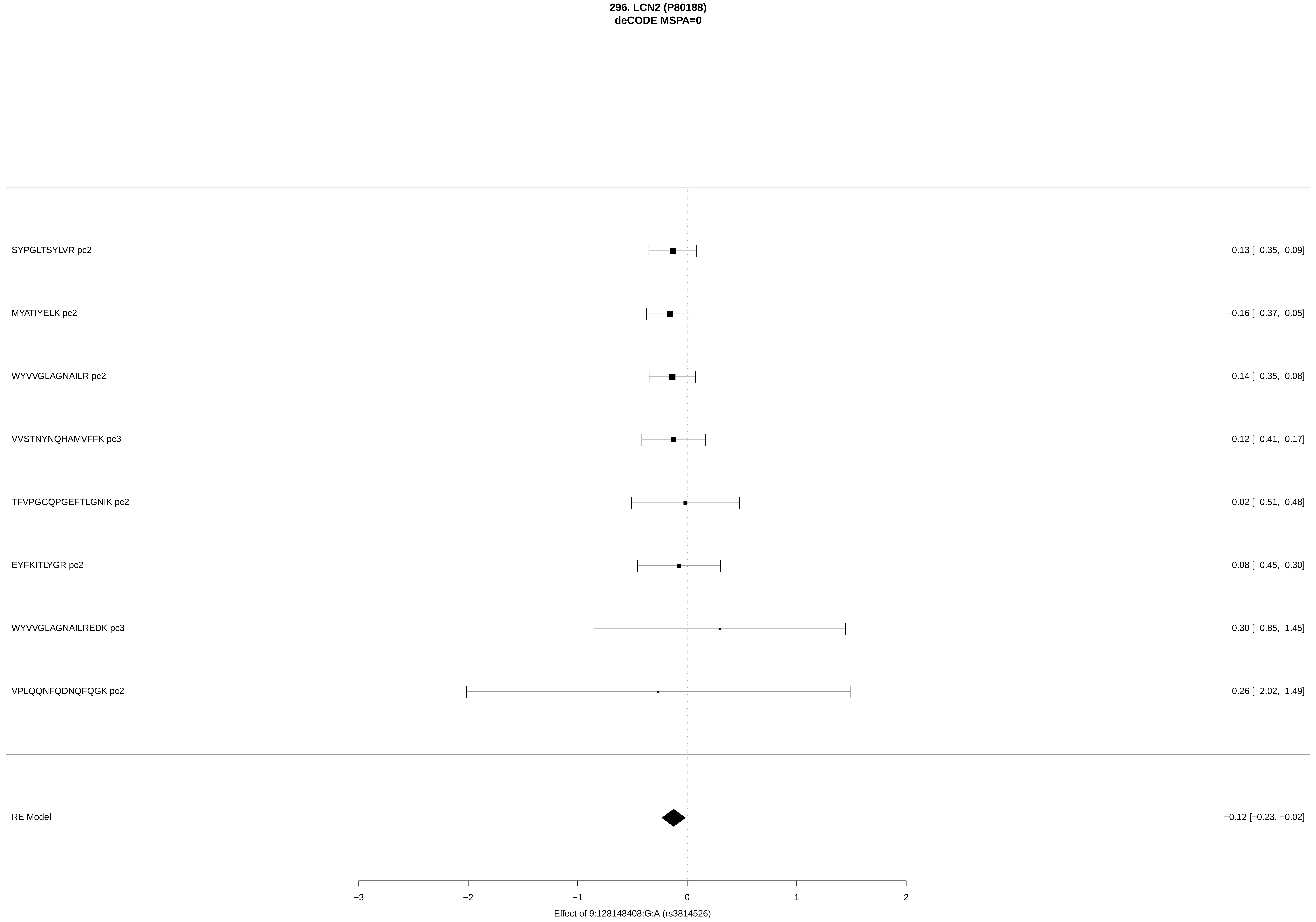
296. LCN2 (P80188)
deCODE MSPA=0
SYPGLTSYLVR pc2
−0.13 [−0.35, 0.09]
MYATIYELK pc2
−0.16 [−0.37, 0.05]
WYVVGLAGNAILR pc2
−0.14 [−0.35, 0.08]
VVSTNYNQHAMVFFK pc3
−0.12 [−0.41, 0.17]
TFVPGCQPGEFTLGNIK pc2
−0.02 [−0.51, 0.48]
EYFKITLYGR pc2
−0.08 [−0.45, 0.30]
WYVVGLAGNAILREDK pc3
0.30 [−0.85, 1.45]
VPLQQNFQDNQFQGK pc2
−0.26 [−2.02, 1.49]
RE Model
−0.12 [−0.23, −0.02]
−3
−2
−1
0
1
2
Effect of 9:128148408:G:A (rs3814526)

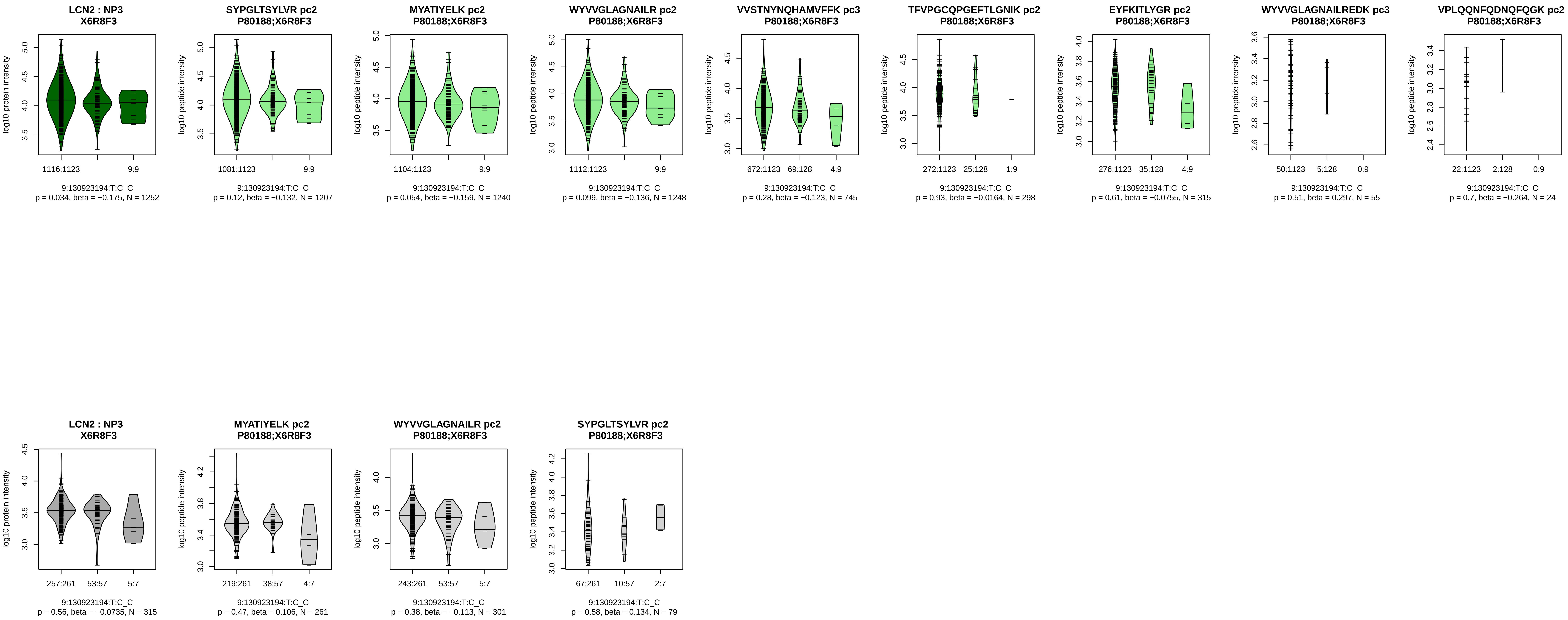

Assay Target: LCN2
UniProt: P80188
rsID: rs3814526
Target SNP: 9:128148408:G:A
Proxy SNP: 9:130923194:T:C
deCODE log10(p): 12.3
deCODE BETA: −0.15
deCODE MSPA: 0
−:−:−:−:−:−:−:NA
1207:1240:1248:745:298:315:5

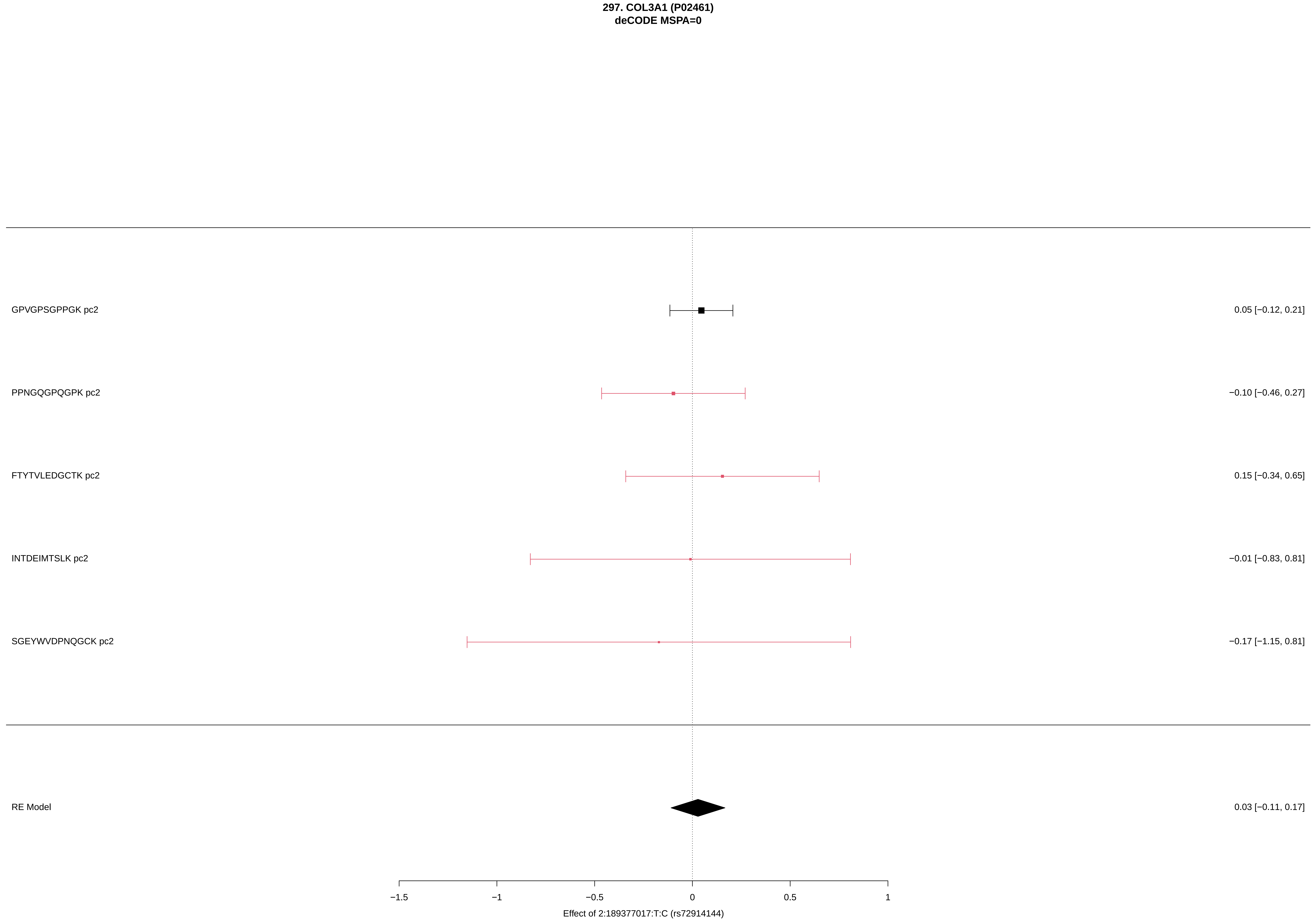
297. COL3A1 (P02461)
deCODE MSPA=0
GPVGPSGPPGK pc2
0.05 [−0.12, 0.21]
PPNGQGPQGPK pc2
−0.10 [−0.46, 0.27]
FTYTVLEDGCTK pc2
0.15 [−0.34, 0.65]
INTDEIMTSLK pc2
−0.01 [−0.83, 0.81]
SGEYWVDPNQGCK pc2
−0.17 [−1.15, 0.81]
RE Model
0.03 [−0.11, 0.17]
−1.5
−1
−0.5
0
0.5
1
Effect of 2:189377017:T:C (rs72914144)

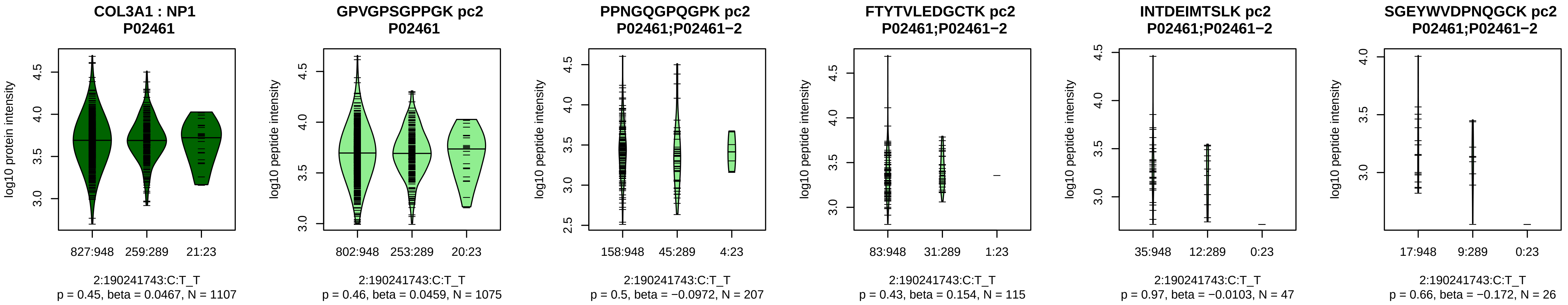

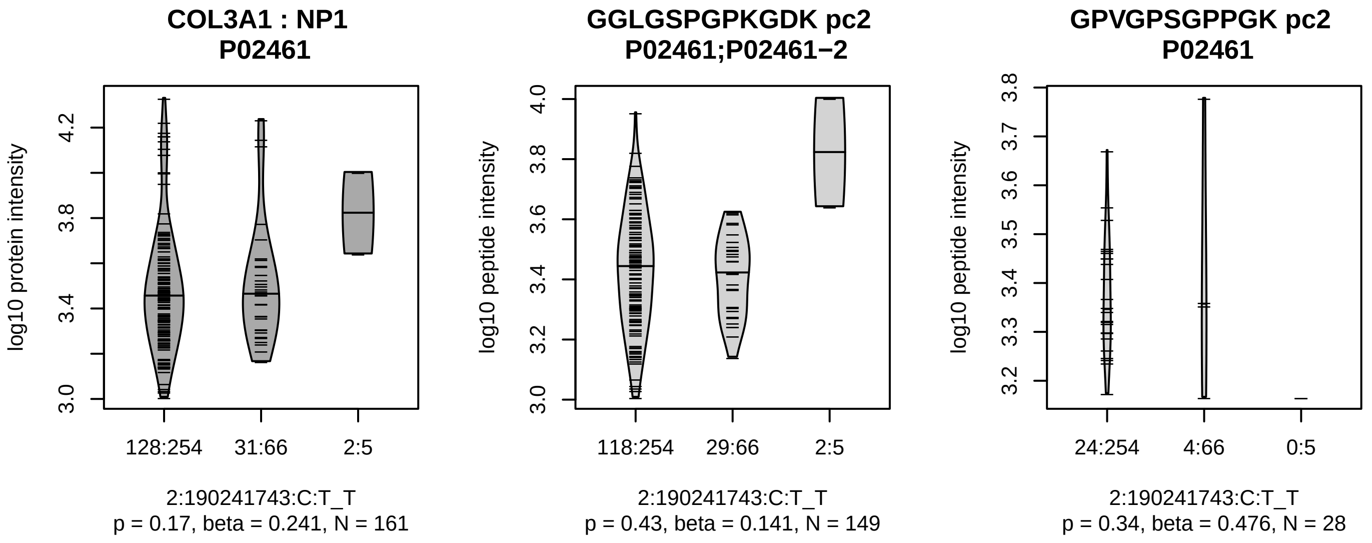

Assay Target: COL3A1
UniProt: P02461
rsID: rs72914144
Target SNP: 2:189377017:T:C
Proxy SNP: 2:190241743:C:T
deCODE log10(p): 11.9
deCODE BETA: 0.08
deCODE MSPA: 0
−:−:−:−:NA:−:NA:NA
1075:207:115:47:8:26:11:6

298. MGAT1 (P26572)
deCODE MSPA=0.543

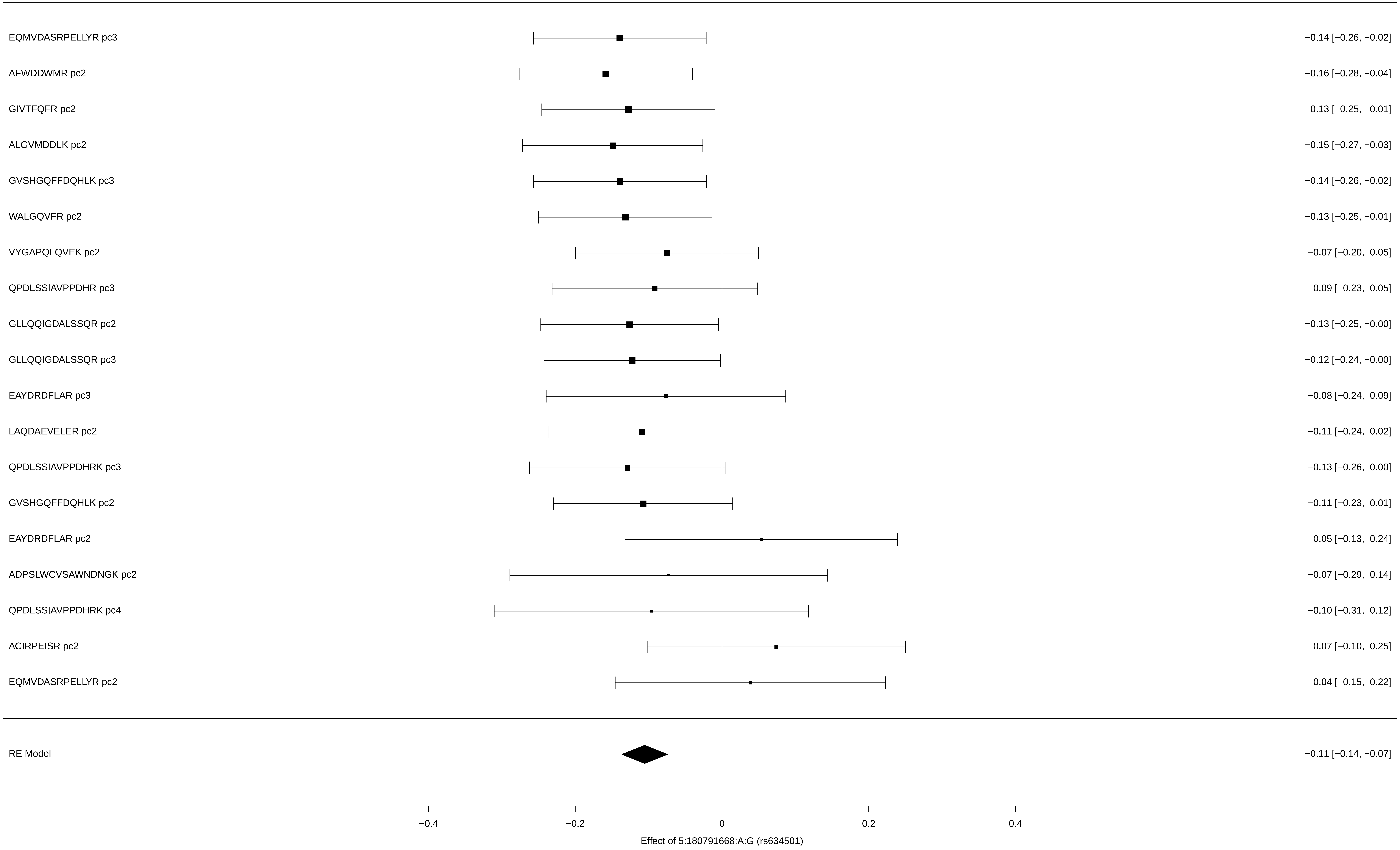

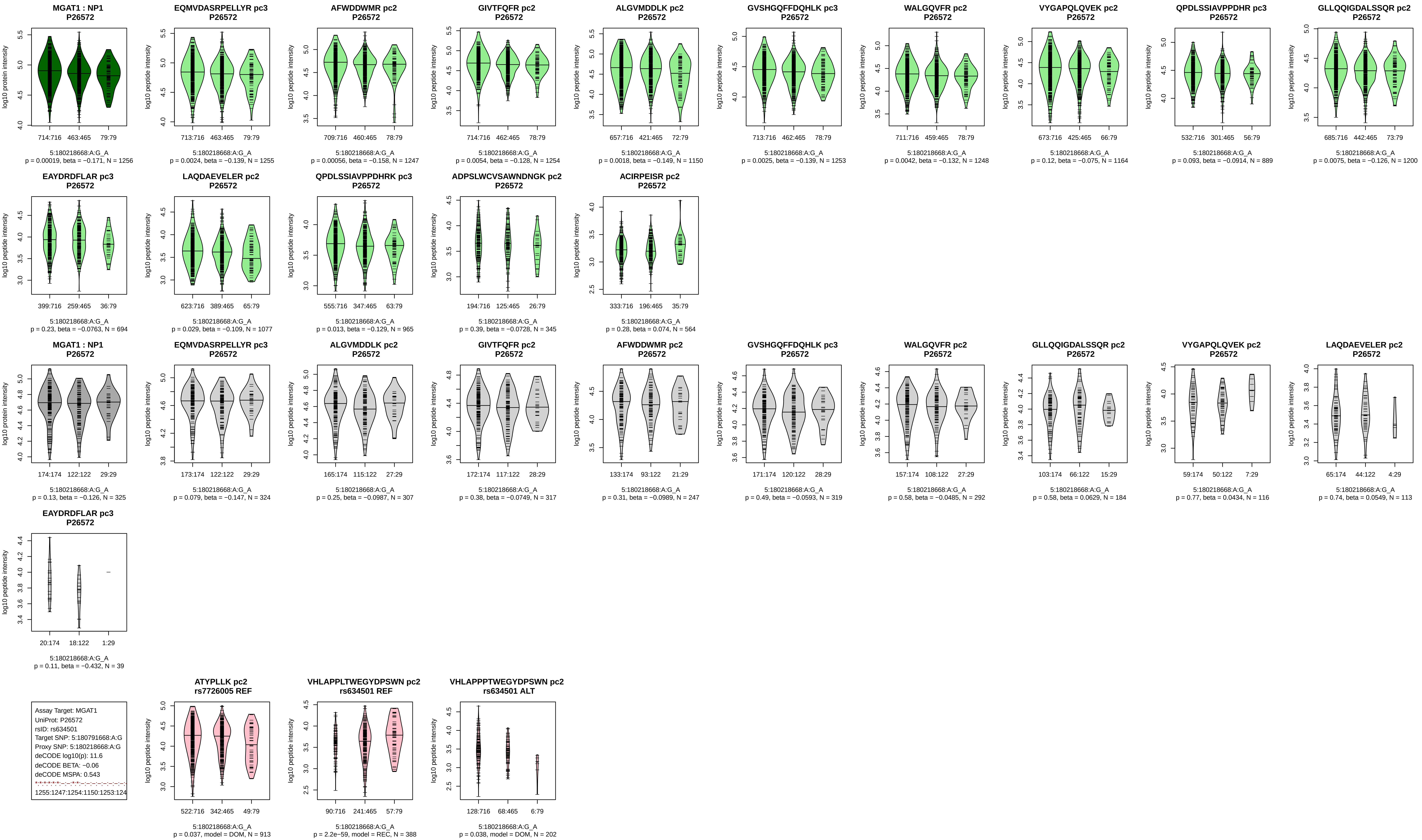
MGAT1 : NP1
P26572
log10 protein intensity
714:716 463:465 79:79
5:180218668:A:G_A
p = 0.00019, beta = −0.171, N = 1256
EQMVDASRPELLYR pc3
P26572
log10 peptide intensity
713:716 463:465 79:79
5:180218668:A:G_A
p = 0.0024, beta = −0.139, N = 1255
AFWDDWMR pc2
P26572
709:716 460:465 78:79
5:180218668:A:G_A
p = 0.00056, beta = −0.158, N = 1247
GIVTFQFR pc2
P26572
714:716 462:465 78:79
5:180218668:A:G_A
p = 0.0054, beta = −0.128, N = 1254
ALGVMDDLK pc2
P26572
657:716 421:465 72:79
5:180218668:A:G_A
p = 0.0018, beta = −0.149, N = 1150
GVSHGQFFDQHLK pc3
P26572
713:716 462:465 78:79
5:180218668:A:G_A
p = 0.0025, beta = −0.139, N = 1253
WALGQVFR pc2
P26572
711:716 459:465 78:79
5:180218668:A:G_A
p = 0.0042, beta = −0.132, N = 1248
VYGAPQLQVEK pc2
P26572
673:716 425:465 66:79
5:180218668:A:G_A
p = 0.12, beta = −0.075, N = 1164
QPDLSSIAVPPDHR pc3
P26572
532:716 301:465 56:79
5:180218668:A:G_A
p = 0.093, beta = −0.0914, N = 889
GLLQQIGDALSSQR pc2
P26572
685:716 442:465 73:79
5:180218668:A:G_A
p = 0.0075, beta = −0.126, N = 1200
EAYDRDFLAR pc3
P26572
399:716 259:465 36:79
5:180218668:A:G_A
p = 0.23, beta = −0.0763, N = 694
LAQDAEVELER pc2
P26572
623:716 389:465 65:79
5:180218668:A:G_A
p = 0.029, beta = −0.109, N = 1077
QPDLSSIAVPPDHRK pc3
P26572
555:716 347:465 63:79
5:180218668:A:G_A
p = 0.013, beta = −0.129, N = 965
ADPSLWCVSAWNDNGK pc2
P26572
194:716 125:465 26:79
5:180218668:A:G_A
p = 0.39, beta = −0.0728, N = 345
ACIRPEISR pc2
P26572
333:716 196:465 35:79
5:180218668:A:G_A
p = 0.28, beta = 0.074, N = 564
MGAT1 : NP1
P26572
174:174 122:122 29:29
5:180218668:A:G_A
p = 0.13, beta = −0.126, N = 325
EQMVDASRPELLYR pc3
P26572
173:174 122:122 29:29
5:180218668:A:G_A
p = 0.079, beta = −0.147, N = 324
ALGVMDDLK pc2
P26572
165:174 115:122 27:29
5:180218668:A:G_A
p = 0.25, beta = −0.0987, N = 307
GIVTFQFR pc2
P26572
172:174 117:122 28:29
5:180218668:A:G_A
p = 0.38, beta = −0.0749, N = 317
AFWDDWMR pc2
P26572
133:174 93:122 21:29
5:180218668:A:G_A
p = 0.31, beta = −0.0989, N = 247
GVSHGQFFDQHLK pc3
P26572
171:174 120:122 28:29
5:180218668:A:G_A
p = 0.49, beta = −0.0593, N = 319
WALGQVFR pc2
P26572
157:174 108:122 27:29
5:180218668:A:G_A
p = 0.58, beta = −0.0485, N = 292
GLLQQIGDALSSQR pc2
P26572
103:174 66:122 15:29
5:180218668:A:G_A
p = 0.58, beta = 0.0629, N = 184
VYGAPQLQVEK pc2
P26572
59:174 50:122 7:29
5:180218668:A:G_A
p = 0.77, beta = 0.0434, N = 116
LAQDAEVELER pc2
P26572
65:174 44:122 4:29
5:180218668:A:G_A
p = 0.74, beta = 0.0549, N = 113
EAYDRDFLAR pc3
P26572
20:174 18:122 1:29
5:180218668:A:G_A
p = 0.11, beta = −0.432, N = 39
Assay Target: MGAT1
UniProt: P26572
rsID: rs634501
Target SNP: 5:180791668:A:G
Proxy SNP: 5:180218668:A:G
deCODE log10(p): 11.6
deCODE BETA: −0.06
deCODE MSPA: 0.543
1255:1247:1254:1150:1253:124
ATYPLLK pc2
rs7726005 REF
522:716 342:465 49:79
5:180218668:A:G_A
p = 0.037, model = DOM, N = 913
VHLAPPLTWEGYDPSWN pc2
rs634501 REF
90:716 241:465 57:79
5:180218668:A:G_A
p = 2.2e−59, model = REC, N = 388
VHLAPPPTWEGYDPSWN pc2
rs634501 ALT
128:716 68:465 6:79
5:180218668:A:G_A
p = 0.038, model = DOM, N = 202

299. SUMO3 (P55854)
deCODE MSPA=0

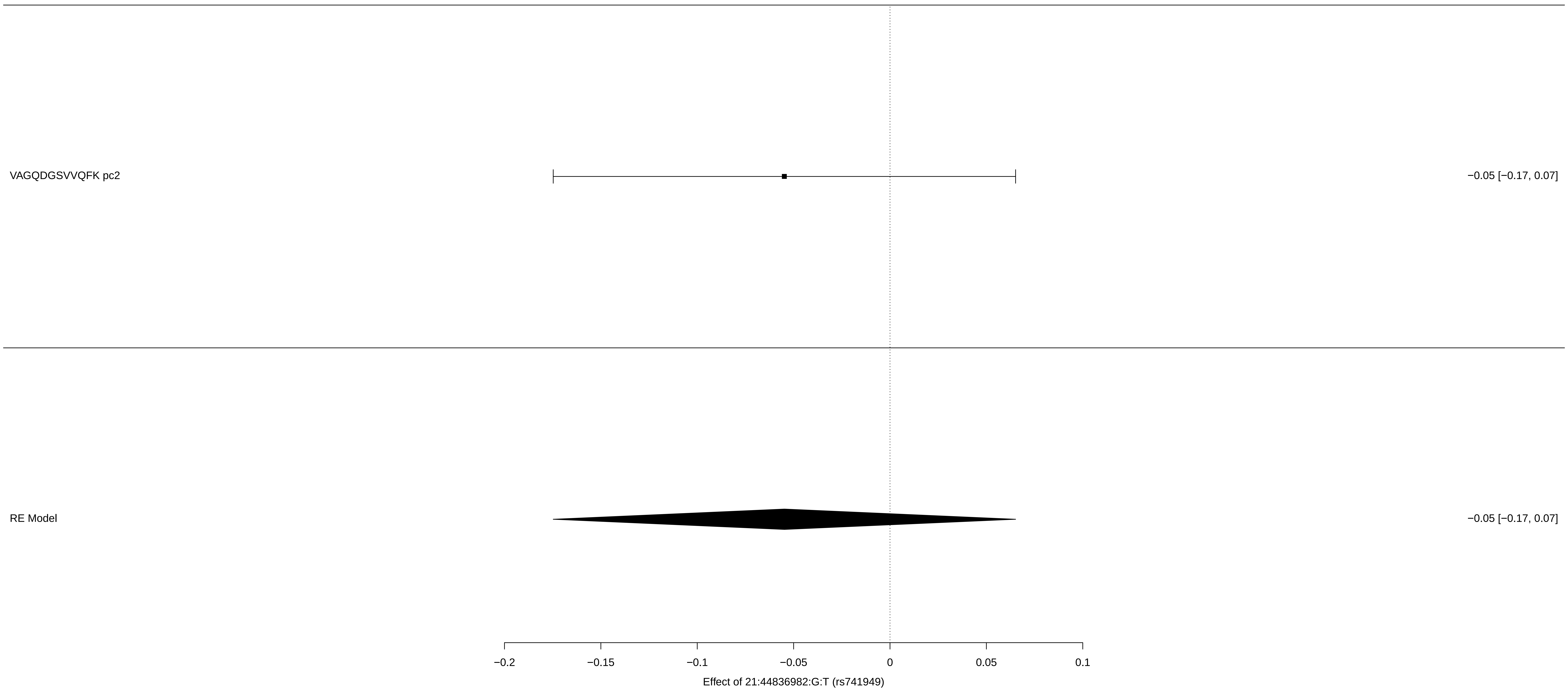

SUMO3 : NP2
A8MUA9

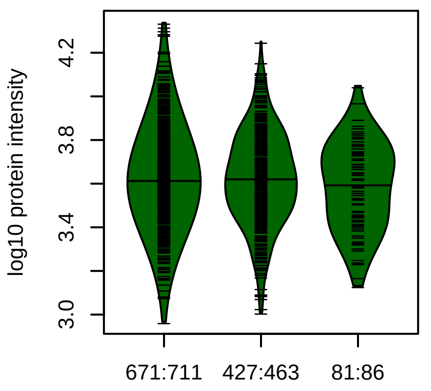

21:46256897:T:G_G
p = 0.24, beta = −0.0548, N = 1179

VAGQDGSVVQFK pc2
8MUA9;P55854;P55854−2;P61956−2;C

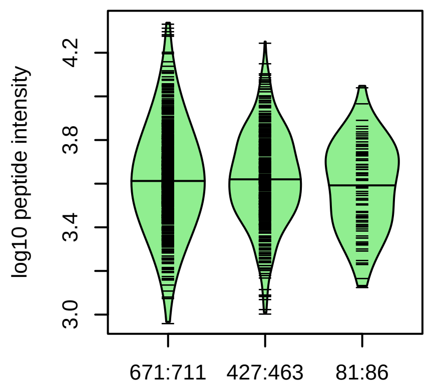

21:46256897:T:G_G
p = 0.24, beta = −0.0548, N = 1179

SUMO3 : NP2
A8MUA9

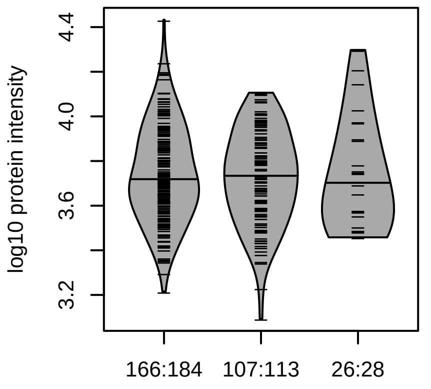

21:46256897:T:G_G
p = 0.54, beta = −0.0532, N = 299

VAGQDGSVVQFK pc2
8MUA9;P55854;P55854−2;P61956−2;C

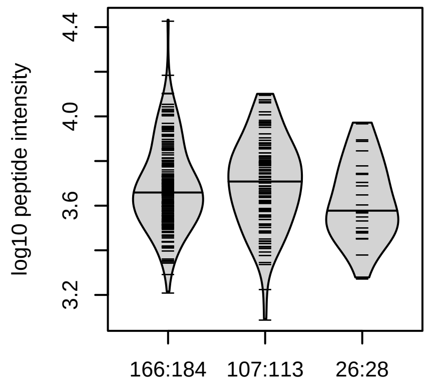

21:46256897:T:G_G
p = 0.41, beta = −0.0729, N = 299

Assay Target: SUMO3
UniProt: P55854
rsID: rs741949
Target SNP: 21:44836982:G:T
Proxy SNP: 21:46256897:T:G
deCODE log10(p): 11.5
deCODE BETA: 0.06
deCODE MSPA: 0
–
1179

300. ADH1B (P00325)
deCODE MSPA=0

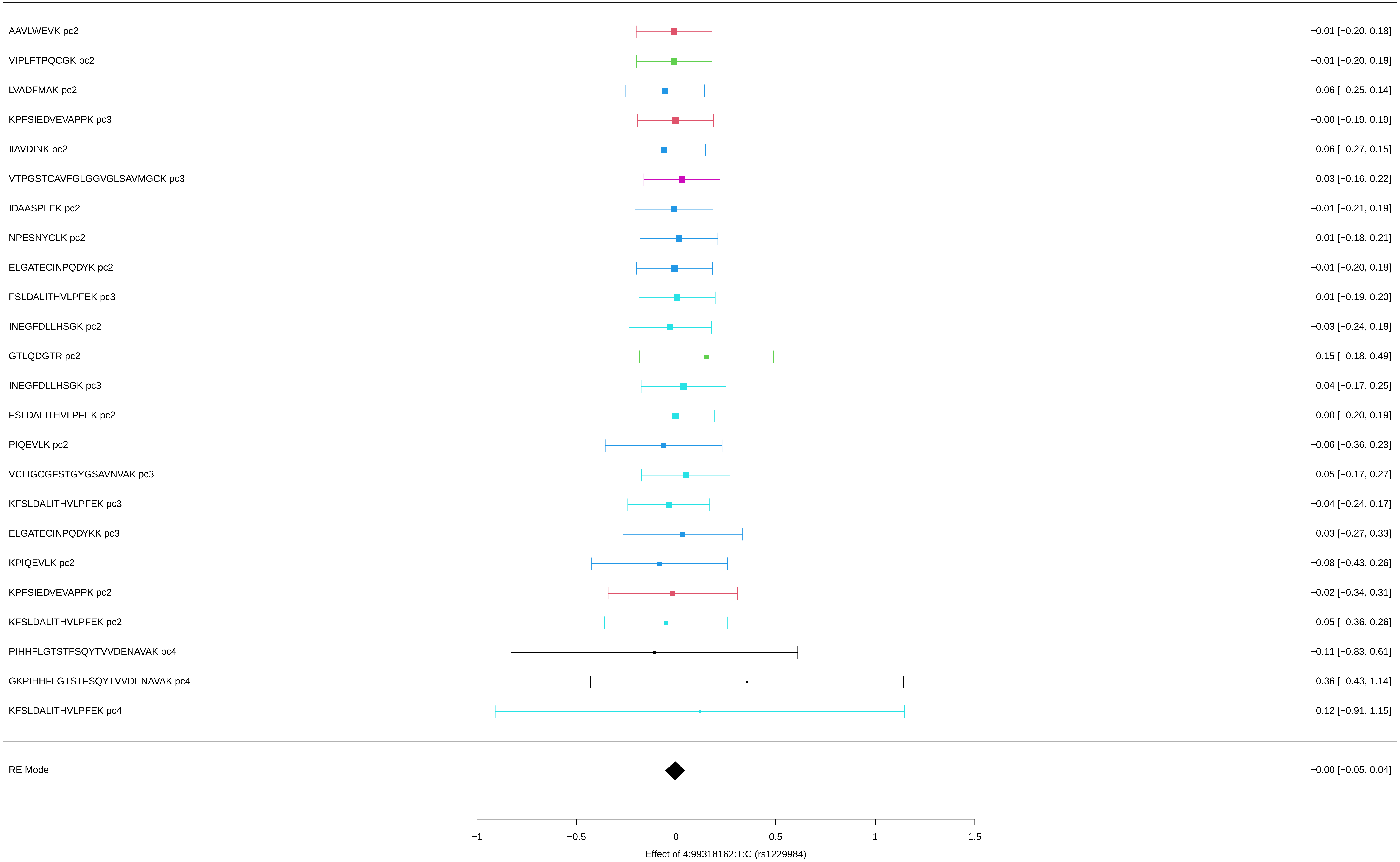

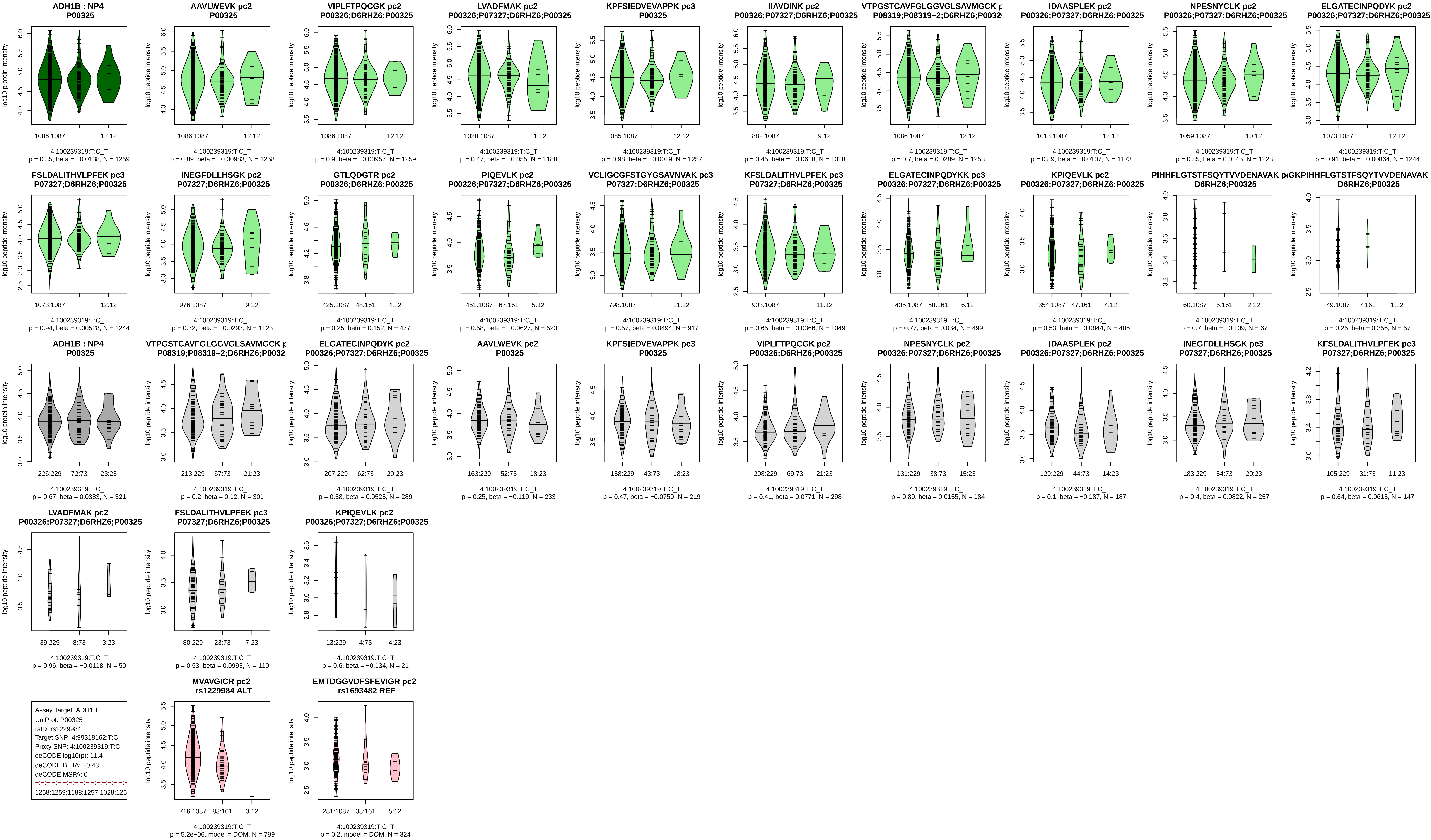
ADH1B : NP4
P00325
log10 protein intensity
1086:1087
12:12
4:100239319:T:C_T
p = 0.85, beta = −0.0138, N = 1259
AAVLWEVK pc2
P00325
log10 peptide intensity
1086:1087
12:12
4:100239319:T:C_T
p = 0.89, beta = −0.00983, N = 1258
VIPLFTPQCGK pc2
P00326;D6RHZ6;P00325
1086:1087
12:12
4:100239319:T:C_T
p = 0.9, beta = −0.00957, N = 1259
LVADFMAK pc2
P00326;P07327;D6RHZ6;P00325
1028:1087
11:12
4:100239319:T:C_T
p = 0.47, beta = −0.055, N = 1188
KPFSIEDVEVAPPK pc3
P00325
1085:1087
12:12
4:100239319:T:C_T
p = 0.98, beta = −0.0019, N = 1257
IIAVDINK pc2
P00326;P07327;D6RHZ6;P00325
882:1087
9:12
4:100239319:T:C_T
p = 0.45, beta = −0.0618, N = 1028
VTPGSTCAVFGLGGVGLSAVMGCK p
P08319;P08319−2;D6RHZ6;P0032
1086:1087
12:12
4:100239319:T:C_T
p = 0.7, beta = 0.0289, N = 1258
IDAASPLEK pc2
P00326;P07327;D6RHZ6;P00325
1013:1087
12:12
4:100239319:T:C_T
p = 0.89, beta = −0.0107, N = 1173
NPESNYCLK pc2
P00326;P07327;D6RHZ6;P00325
1059:1087
10:12
4:100239319:T:C_T
p = 0.85, beta = 0.0145, N = 1228
ELGATECINPQDYK pc2
P00326;P07327;D6RHZ6;P00325
1073:1087
12:12
4:100239319:T:C_T
p = 0.91, beta = −0.00864, N = 1244
FSLDALITHVLPFEK pc3
P07327;D6RHZ6;P00325
1073:1087
12:12
4:100239319:T:C_T
p = 0.94, beta = 0.00528, N = 1244
INEGFDLLHSGK pc2
P07327;D6RHZ6;P00325
976:1087
9:12
4:100239319:T:C_T
p = 0.72, beta = −0.0293, N = 1123
GTLQDGTR pc2
P00326;D6RHZ6;P00325
425:1087
48:161
4:12
4:100239319:T:C_T
p = 0.25, beta = 0.152, N = 477
PIQEVLK pc2
P00326;P07327;D6RHZ6;P00325
451:1087
67:161
5:12
4:100239319:T:C_T
p = 0.58, beta = −0.0627, N = 523
VCLIGCGFSTGYGSAVNVAK pc3
P07327;D6RHZ6;P00325
798:1087
11:12
4:100239319:T:C_T
p = 0.57, beta = −0.0494, N = 917
KFSLDALITHVLPFEK pc3
P07327;D6RHZ6;P00325
903:1087
11:12
4:100239319:T:C_T
p = 0.65, beta = −0.0366, N = 1049
ELGATECINPQDYKK pc3
P00326;P07327;D6RHZ6;P00325
435:1087
58:161
6:12
4:100239319:T:C_T
p = 0.77, beta = 0.034, N = 499
KPIQEVLK pc2
P00326;P07327;D6RHZ6;P00325
354:1087
47:161
4:12
4:100239319:T:C_T
p = 0.53, beta = −0.0844, N = 405
PIHHFLGTSTFSQYTVVDENAVAK pc
D6RHZ6;P00325
60:1087
5:161
2:12
4:100239319:T:C_T
p = 0.7, beta = −0.109, N = 67
GKPIHHFLGTSTFSQYTVVDENAVAK
D6RHZ6;P00325
49:1087
7:161
1:12
4:100239319:T:C_T
p = 0.25, beta = 0.356, N = 57
ADH1B : NP4
P00325
226:229
72:73
23:23
4:100239319:T:C_T
p = 0.67, beta = 0.0383, N = 321
VTPGSTCAVFGLGGVGLSAVMGCK p
P08319;P08319−2;D6RHZ6;P0032
213:229
67:73
21:23
4:100239319:T:C_T
p = 0.2, beta = 0.12, N = 301
ELGATECINPQDYK pc2
P00326;P07327;D6RHZ6;P00325
207:229
62:73
20:23
4:100239319:T:C_T
p = 0.58, beta = 0.0525, N = 289
AAVLWEVK pc2
P00325
163:229
52:73
18:23
4:100239319:T:C_T
p = 0.25, beta = −0.119, N = 233
KPFSIEDVEVAPPK pc3
P00325
158:229
43:73
18:23
4:100239319:T:C_T
p = 0.47, beta = −0.0759, N = 219
VIPLFTPQCGK pc2
P00326;D6RHZ6;P00325
208:229
69:73
21:23
4:100239319:T:C_T
p = 0.41, beta = 0.0771, N = 298
NPESNYCLK pc2
P00326;P07327;D6RHZ6;P00325
131:229
38:73
15:23
4:100239319:T:C_T
p = 0.89, beta = 0.0155, N = 184
IDAASPLEK pc2
P00326;P07327;D6RHZ6;P00325
129:229
44:73
14:23
4:100239319:T:C_T
p = 0.1, beta = −0.187, N = 187
INEGFDLLHSGK pc3
P07327;D6RHZ6;P00325
183:229
54:73
20:23
4:100239319:T:C_T
p = 0.4, beta = 0.0822, N = 257
KFSLDALITHVLPFEK pc3
P07327;D6RHZ6;P00325
105:229
31:73
11:23
4:100239319:T:C_T
p = 0.64, beta = 0.0615, N = 147
LVADFMAK pc2
P00326;P07327;D6RHZ6;P00325
39:229
8:73
3:23
4:100239319:T:C_T
p = 0.96, beta = −0.0118, N = 50
FSLDALITHVLPFEK pc3
P07327;D6RHZ6;P00325
80:229
23:73
7:23
4:100239319:T:C_T
p = 0.53, beta = 0.0993, N = 110
KPIQEVLK pc2
P00326;P07327;D6RHZ6;P00325
13:229
4:73
4:23
4:100239319:T:C_T
p = 0.6, beta = −0.134, N = 21
Assay Target: ADH1B
UniProt: P00325
rsID: rs1229984
Target SNP: 4:99318162:T:C
Proxy SNP: 4:100239319:T:C
deCODE log10(p): 11.4
deCODE BETA: −0.43
deCODE MSPA: 0
1258:1259:1188:1257:1028:125
MVAVGICR pc2
rs1229984 ALT
716:1087
83:161
0:12
4:100239319:T:C_T
p = 5.2e−06, model = DOM, N = 799
EMTDGGVDFSFEVIGR pc2
rs1693482 REF
281:1087
38:161
5:12
4:100239319:T:C_T
p = 0.2, model = DOM, N = 324

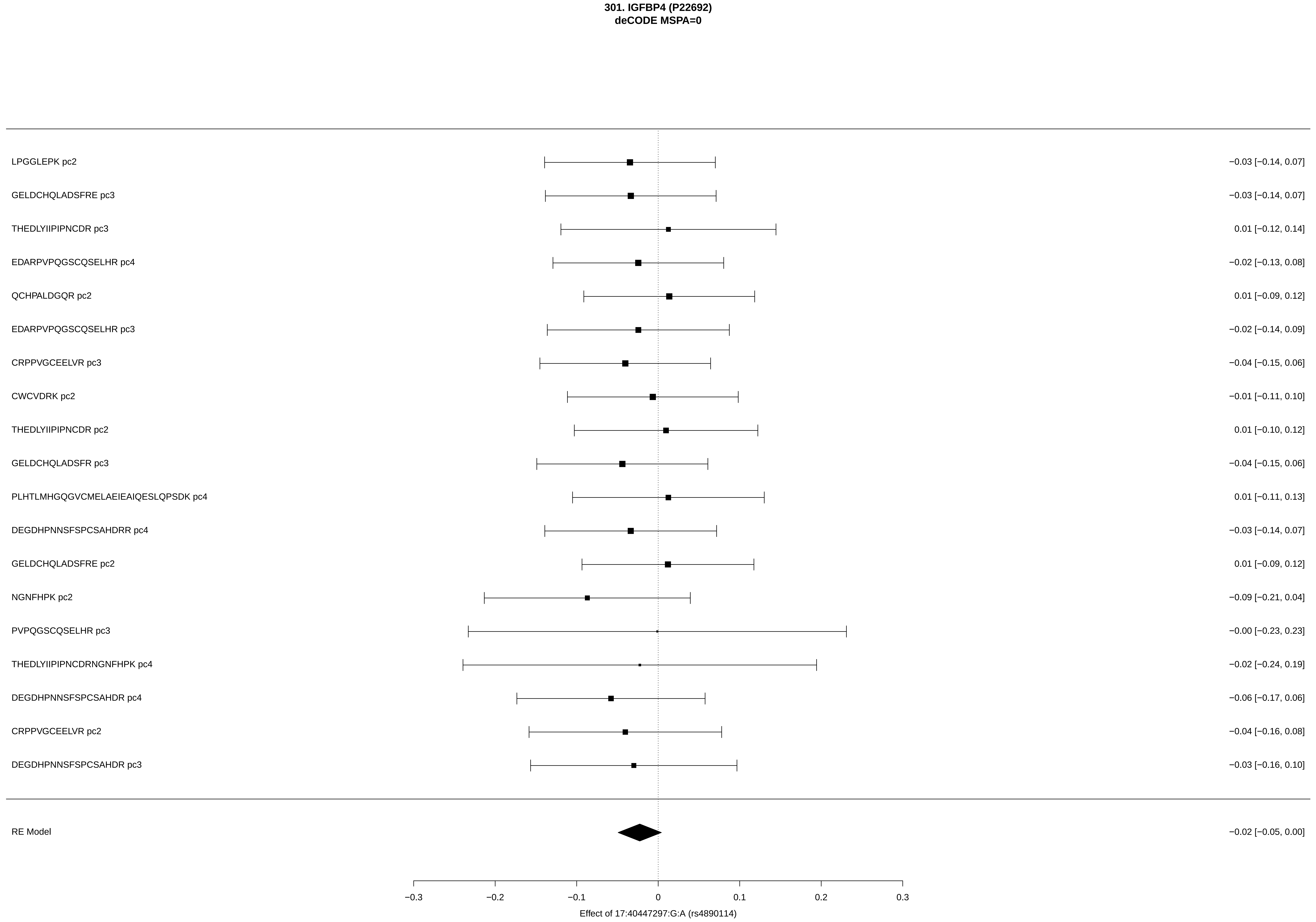

301. IGFBP4 (P22692)
deCODE MSPA=0
LPGGLEPK pc2
GELDCHQLADSFRE pc3
THEDLYIIPIPNCDR pc3
EDARPVPQGSCQSELHR pc4
QCHPALDGQR pc2
EDARPVPQGSCQSELHR pc3
CRPPVGCEELVR pc3
CWCVDRK pc2
THEDLYIIPIPNCDR pc2
GELDCHQLADSFR pc3
PLHTLMHGQGVCMELAEIEAIQESLQPSDK pc4
DEGDHPNNSFSPCSAHDRR pc4
GELDCHQLADSFRE pc2
NGNFHPK pc2
PVPQGSCQSELHR pc3
THEDLYIIPIPNCDRNGNFHPK pc4
DEGDHPNNSFSPCSAHDR pc4
CRPPVGCEELVR pc2
DEGDHPNNSFSPCSAHDR pc3
RE Model
−0.03 [−0.14, 0.07]
−0.03 [−0.14, 0.07]
0.01 [−0.12, 0.14]
−0.02 [−0.13, 0.08]
0.01 [−0.09, 0.12]
−0.02 [−0.14, 0.09]
−0.04 [−0.15, 0.06]
−0.01 [−0.11, 0.10]
0.01 [−0.10, 0.12]
−0.04 [−0.15, 0.06]
0.01 [−0.11, 0.13]
−0.03 [−0.14, 0.07]
0.01 [−0.09, 0.12]
−0.09 [−0.21, 0.04]
−0.00 [−0.23, 0.23]
−0.02 [−0.24, 0.19]
−0.06 [−0.17, 0.06]
−0.04 [−0.16, 0.08]
−0.03 [−0.16, 0.10]
−0.02 [−0.05, 0.00]
−0.3
−0.2
−0.1
0
0.1
0.2
0.3
Effect of 17:40447297:G:A (rs4890114)

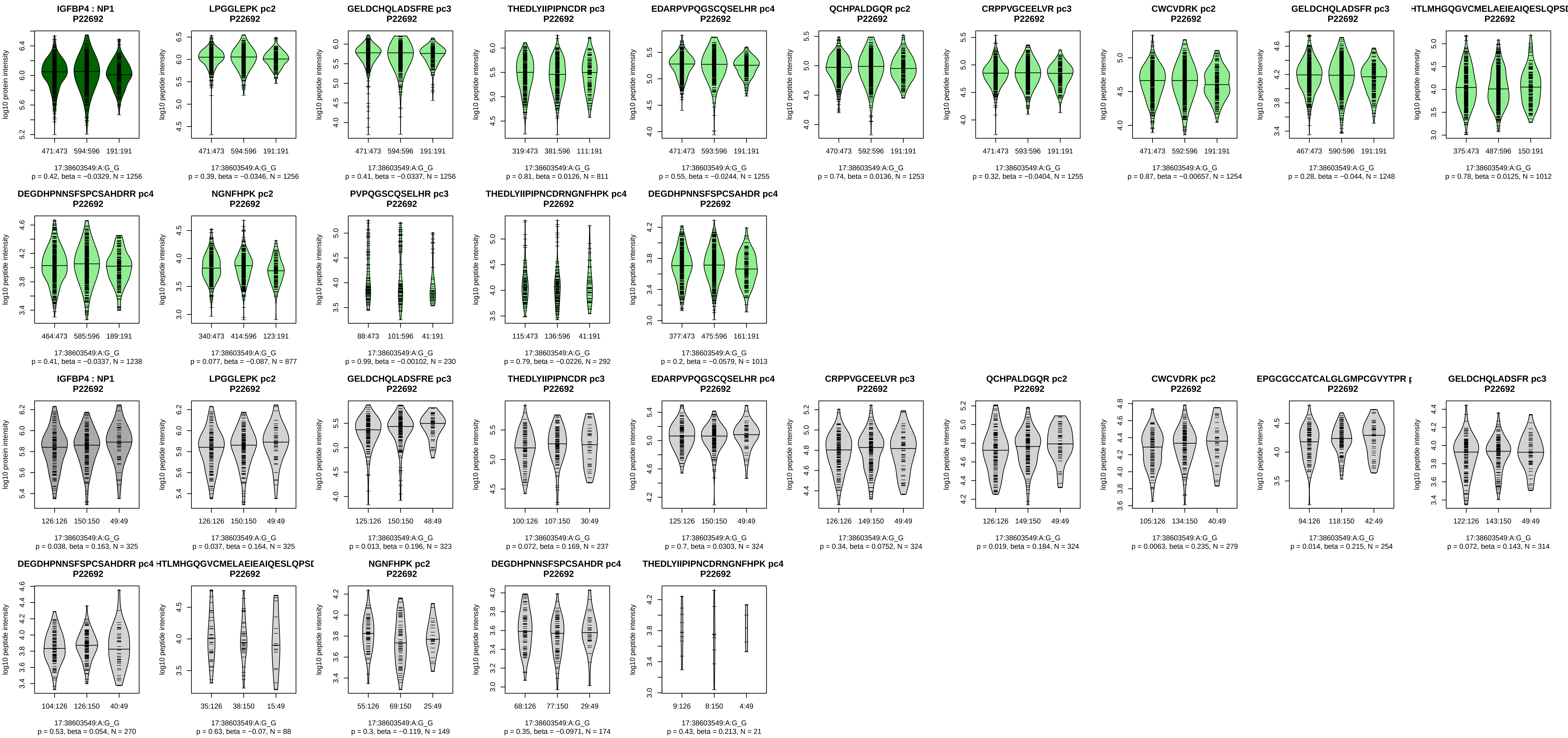

Assay Target: IGFBP4
UniProt: P22692
rsID: rs4890114
Target SNP: 17:40447297:G:A
Proxy SNP: 17:38603549:A:G
deCODE log10(p): 11.3
deCODE BETA: 0.06
deCODE MSPA: 0
1256:1256:811:1255:1253:1107

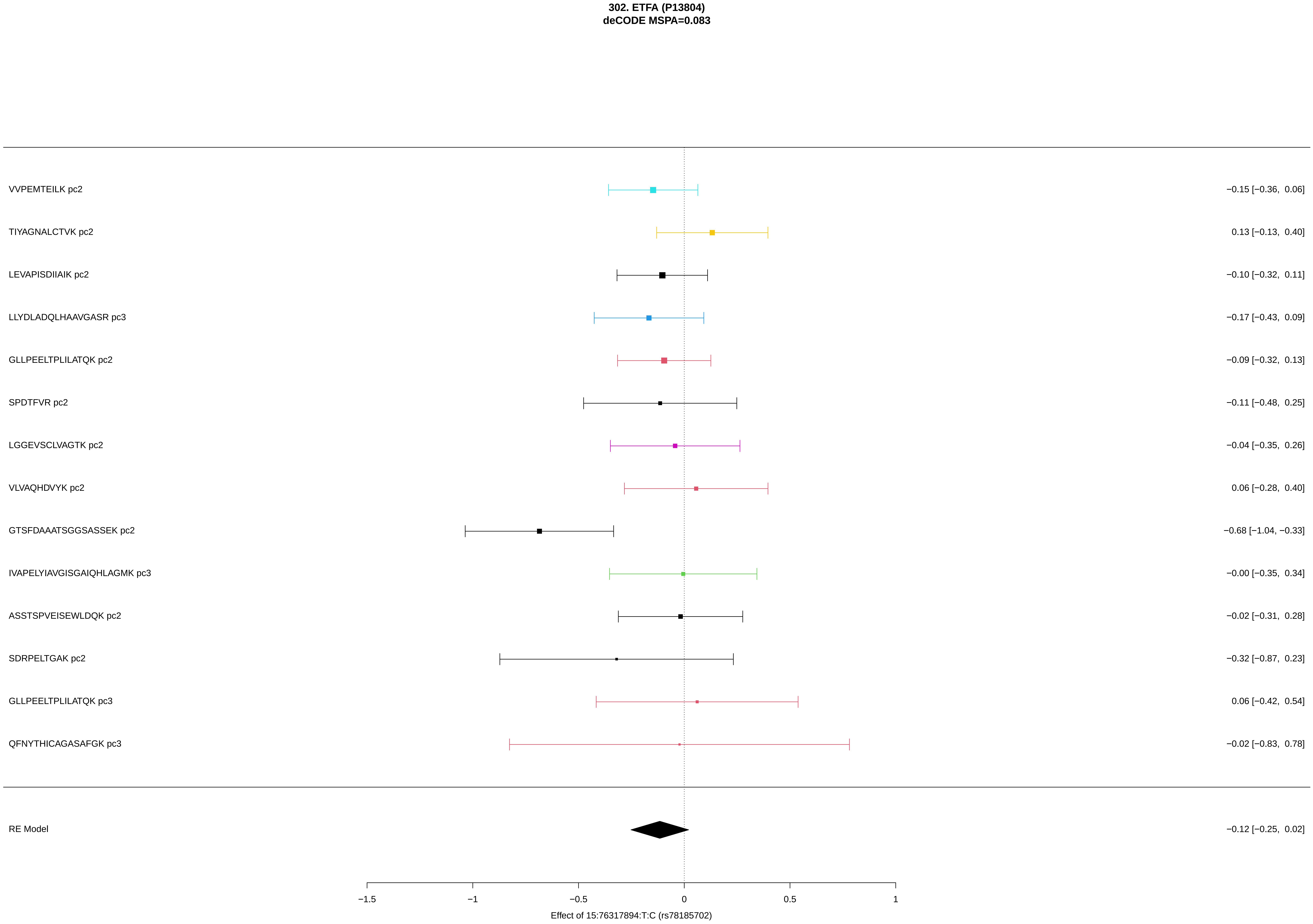
302. ETFA (P13804)
deCODE MSPA=0.083
VVPEMTEILK pc2
TIYAGNALCTVK pc2
LEVAPISDIIAIK pc2
LLYDLADQLHAAVGASR pc3
GLLPEELTPLILATQK pc2
SPDTFVR pc2
LGGEVSCLVAGTK pc2
VLVAQHDVYK pc2
GTSFDAAATSGGSASSEK pc2
IVAPELYIAVGISGAIQHLAGMK pc3
ASSTSPVEISEWLDQK pc2
SDRPELTGAK pc2
GLLPEELTPLILATQK pc3
QFNYTHICAGASAFGK pc3
RE Model
−0.15 [−0.36, 0.06]
0.13 [−0.13, 0.40]
−0.10 [−0.32, 0.11]
−0.17 [−0.43, 0.09]
−0.09 [−0.32, 0.13]
−0.11 [−0.48, 0.25]
−0.04 [−0.35, 0.26]
0.06 [−0.28, 0.40]
−0.68 [−1.04, −0.33]
−0.00 [−0.35, 0.34]
−0.02 [−0.31, 0.28]
−0.32 [−0.87, 0.23]
0.06 [−0.42, 0.54]
−0.02 [−0.83, 0.78]
−0.12 [−0.25, 0.02]
−1.5
−1
−0.5
0
0.5
1
Effect of 15:76317894:T:C (rs78185702)

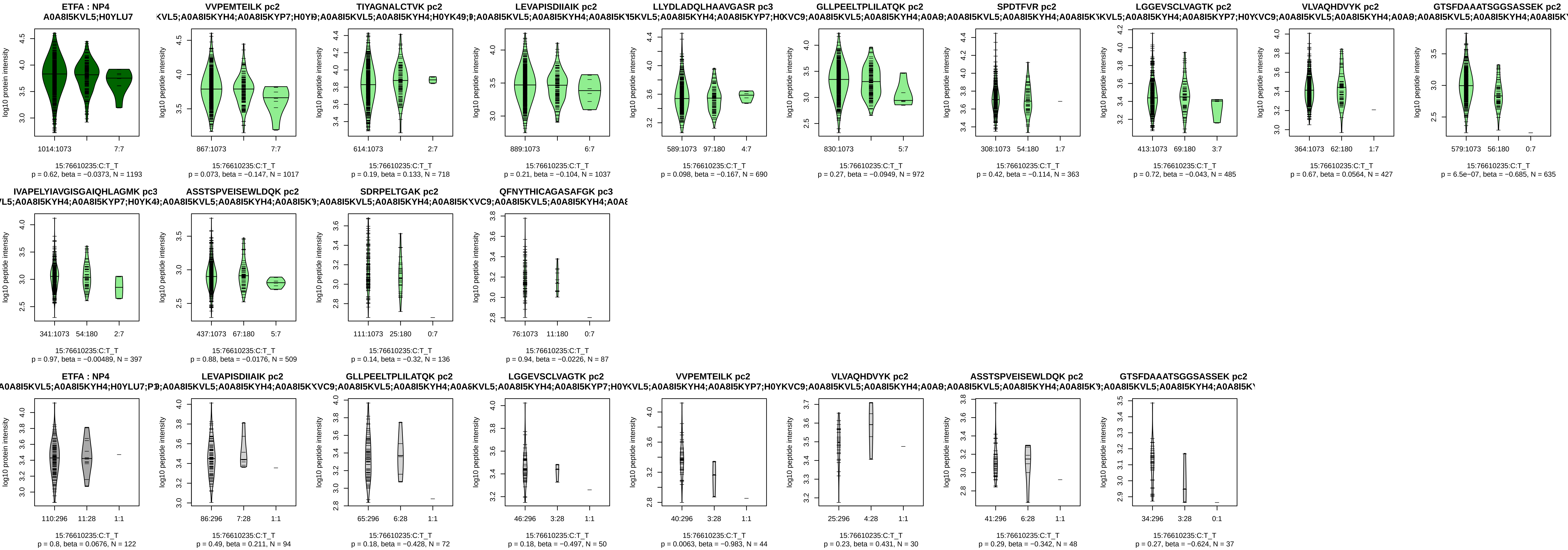

Assay Target: ETFA
UniProt: P13804
rsID: rs78185702
Target SNP: 15:76317894:T:C
Proxy SNP: 15:76610235:C:T
deCODE log10(p): 11.1
deCODE BETA: 0.09
deCODE MSPA: 0.083
–:–:–:–:–:–:–:–:*:–:–:–:–
1017:718:1037:690:972:363:485

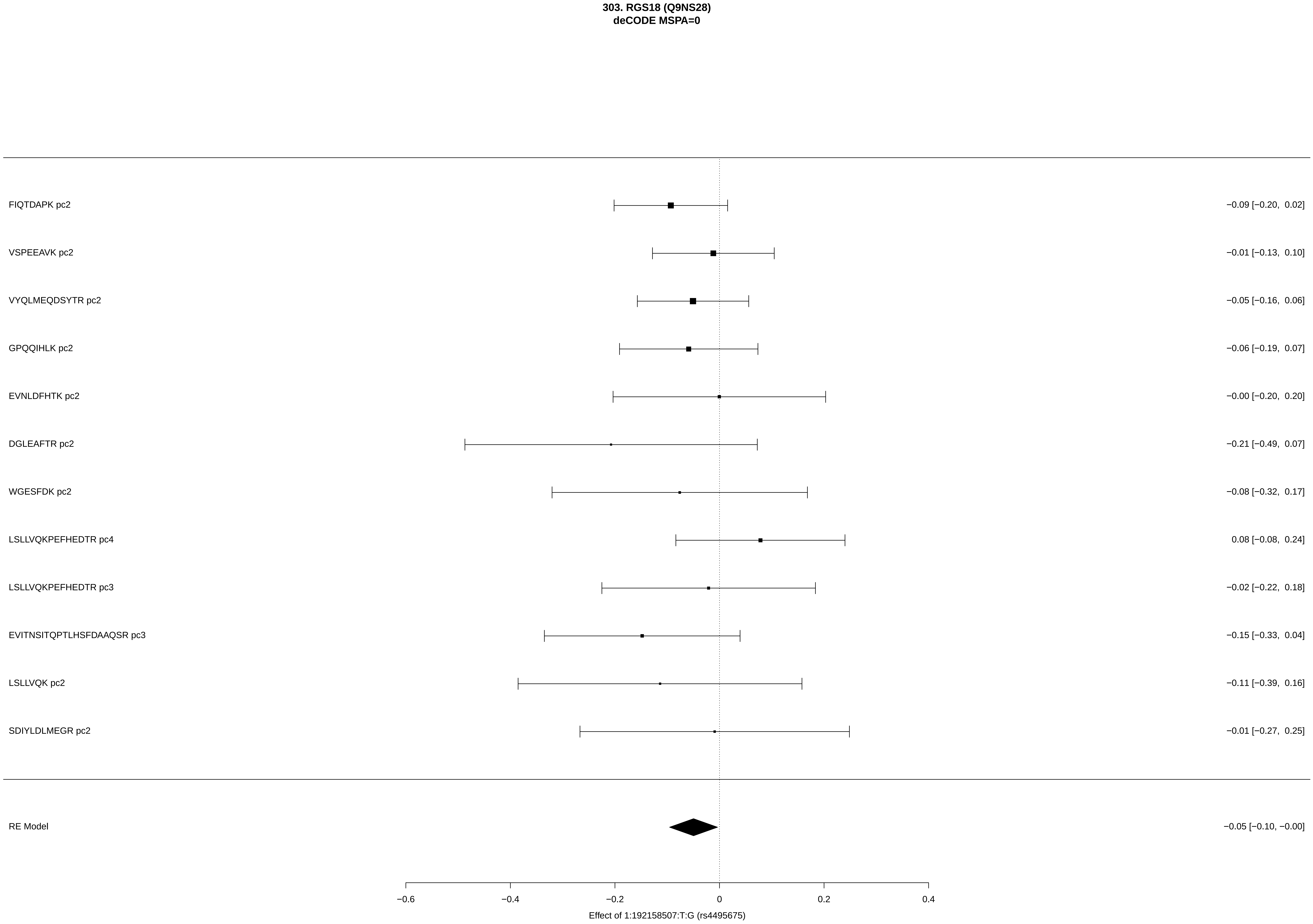

303. RGS18 (Q9NS28)
deCODE MSPA=0
FIQTDAPK pc2
−0.09 [−0.20, 0.02]
VSPEEAVK pc2
−0.01 [−0.13, 0.10]
VYQLMEQDSYTR pc2
−0.05 [−0.16, 0.06]
GPQQIHLK pc2
−0.06 [−0.19, 0.07]
EVNLDFHTK pc2
−0.00 [−0.20, 0.20]
DGLEAFTR pc2
−0.21 [−0.49, 0.07]
WGESFDK pc2
−0.08 [−0.32, 0.17]
LSLLVQKPEFHEDTR pc4
0.08 [−0.08, 0.24]
LSLLVQKPEFHEDTR pc3
−0.02 [−0.22, 0.18]
EVITNSITQPTLHSFDAAQSR pc3
−0.15 [−0.33, 0.04]
LSLLVQK pc2
−0.11 [−0.39, 0.16]
SDIYLDLMEGR pc2
−0.01 [−0.27, 0.25]
RE Model
−0.05 [−0.10, −0.00]
−0.6
−0.4
−0.2
0
0.2
0.4
Effect of 1:192158507:T:G (rs4495675)

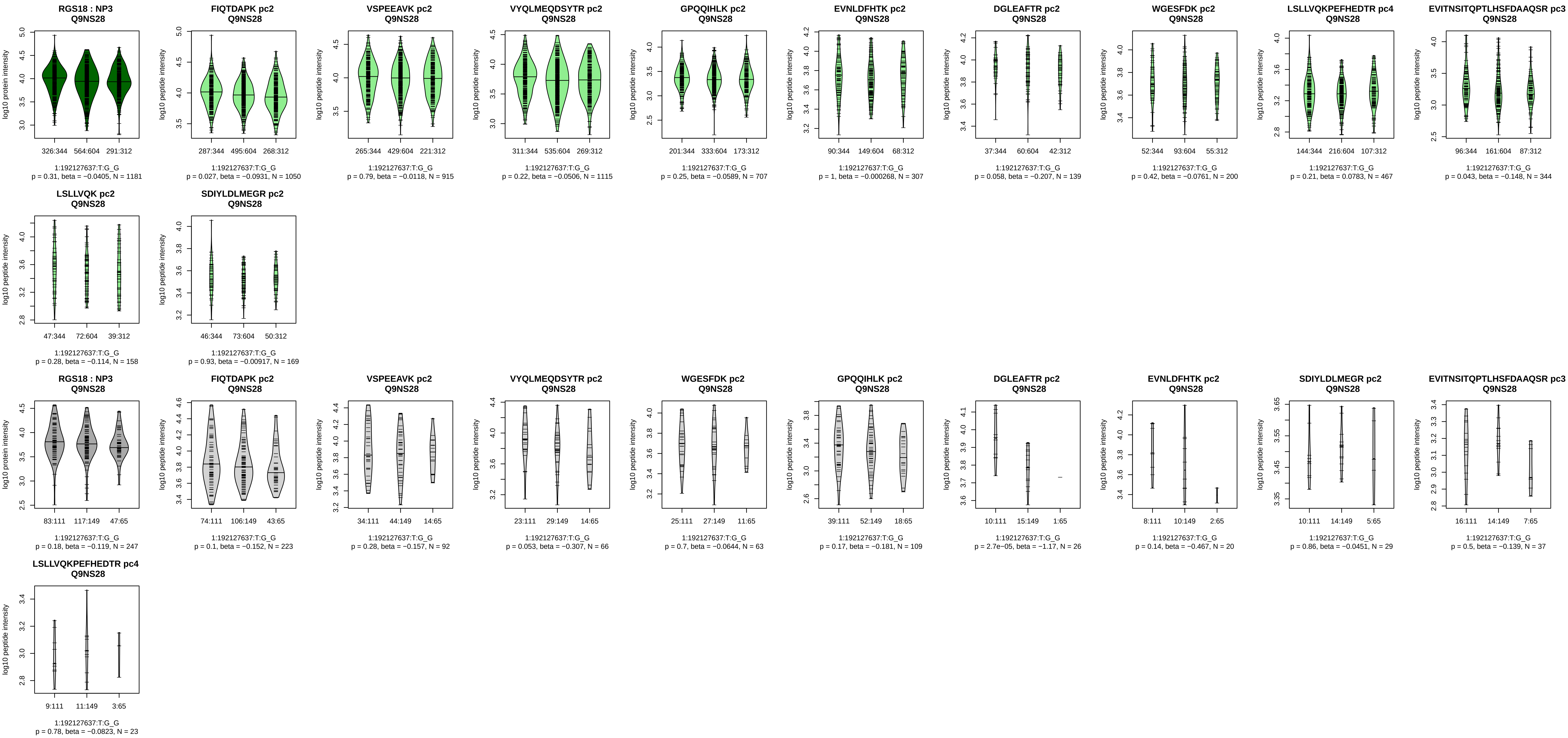

Assay Target: RGS18
UniProt: Q9NS28
rsID: rs4495675
Target SNP: 1:192158507:T:G
Proxy SNP: 1:192127637:T:G
deCODE log10(p): 11.1
deCODE BETA: 0.06
deCODE MSPA: 0
1050:915:1115:707:307:139:200

304. TLL1 (O43897)
deCODE MSPA=0

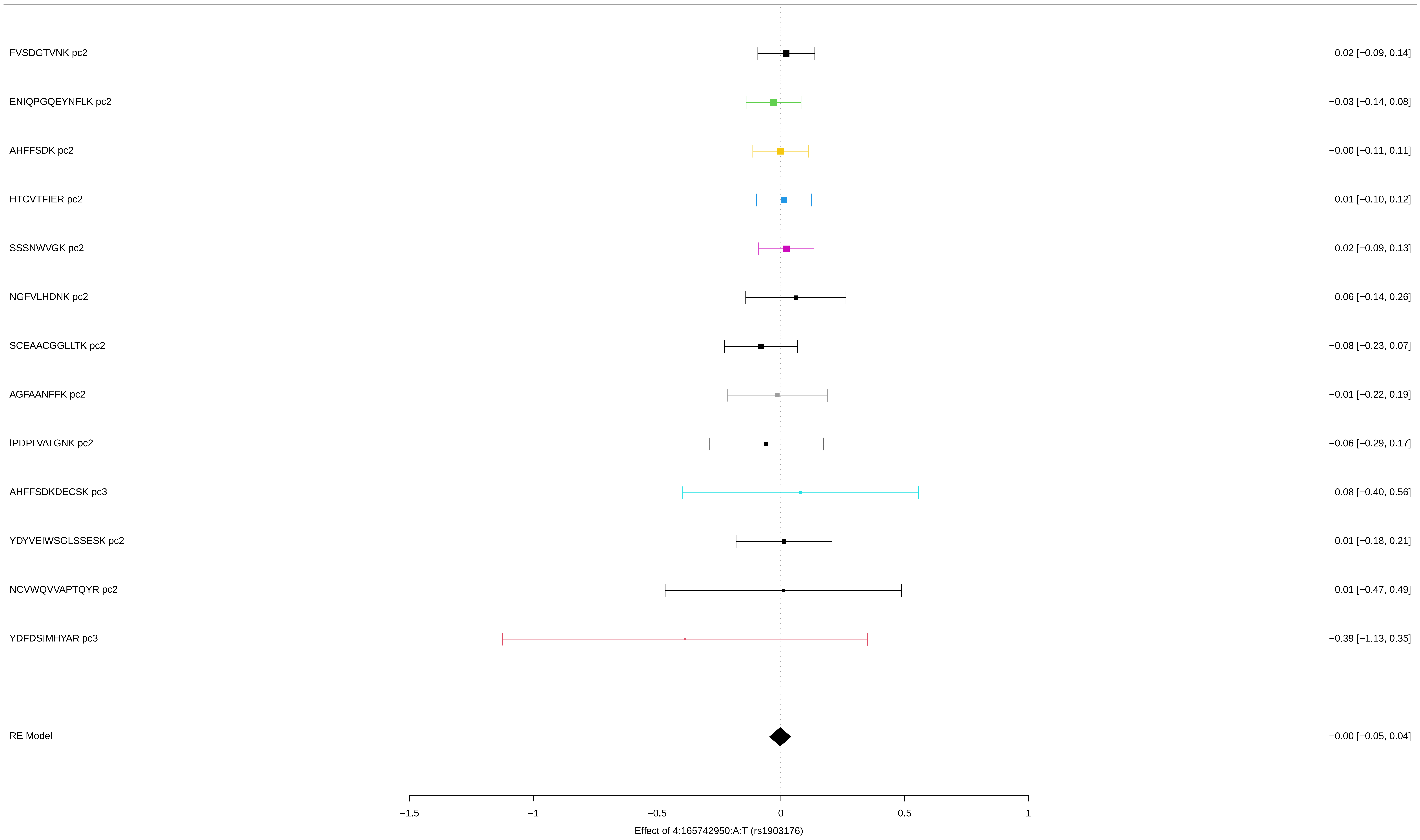

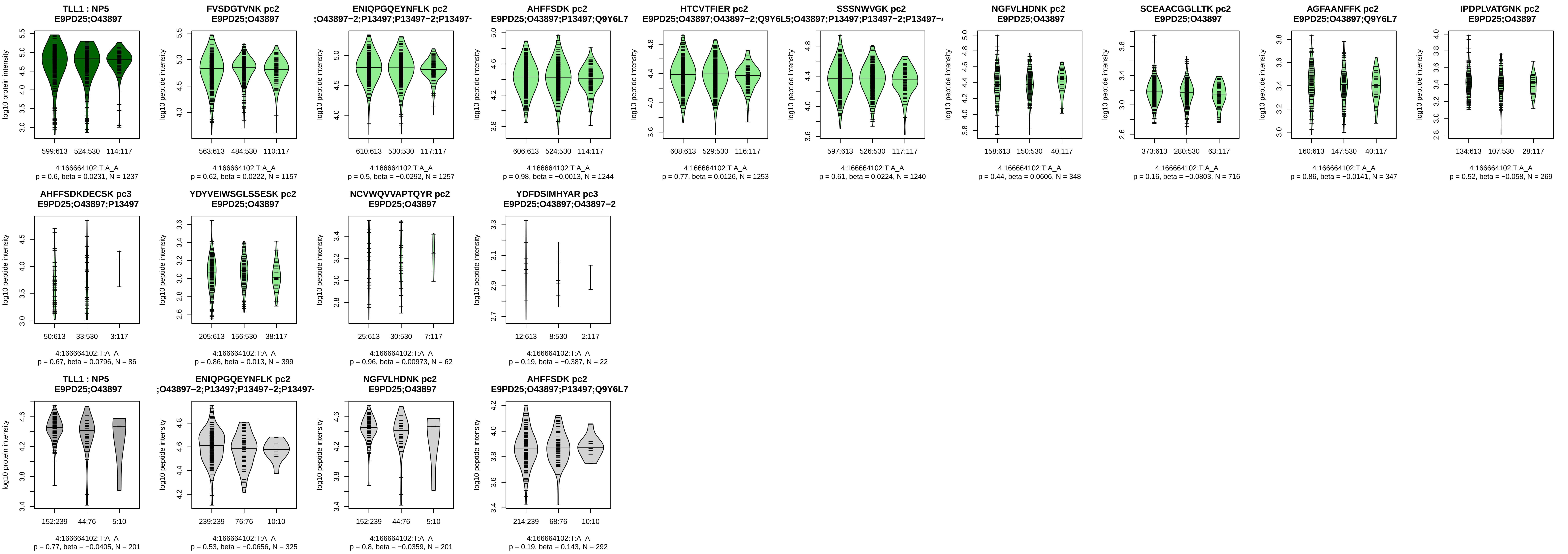

Assay Target: TLL1
UniProt: O43897
rsID: rs1903176
Target SNP: 4:165742950:A:T
Proxy SNP: 4:166664102:T:A
deCODE log10(p): 10.9
deCODE BETA: −0.06
deCODE MSPA: 0
1157:1257:1244:1253:1240:348

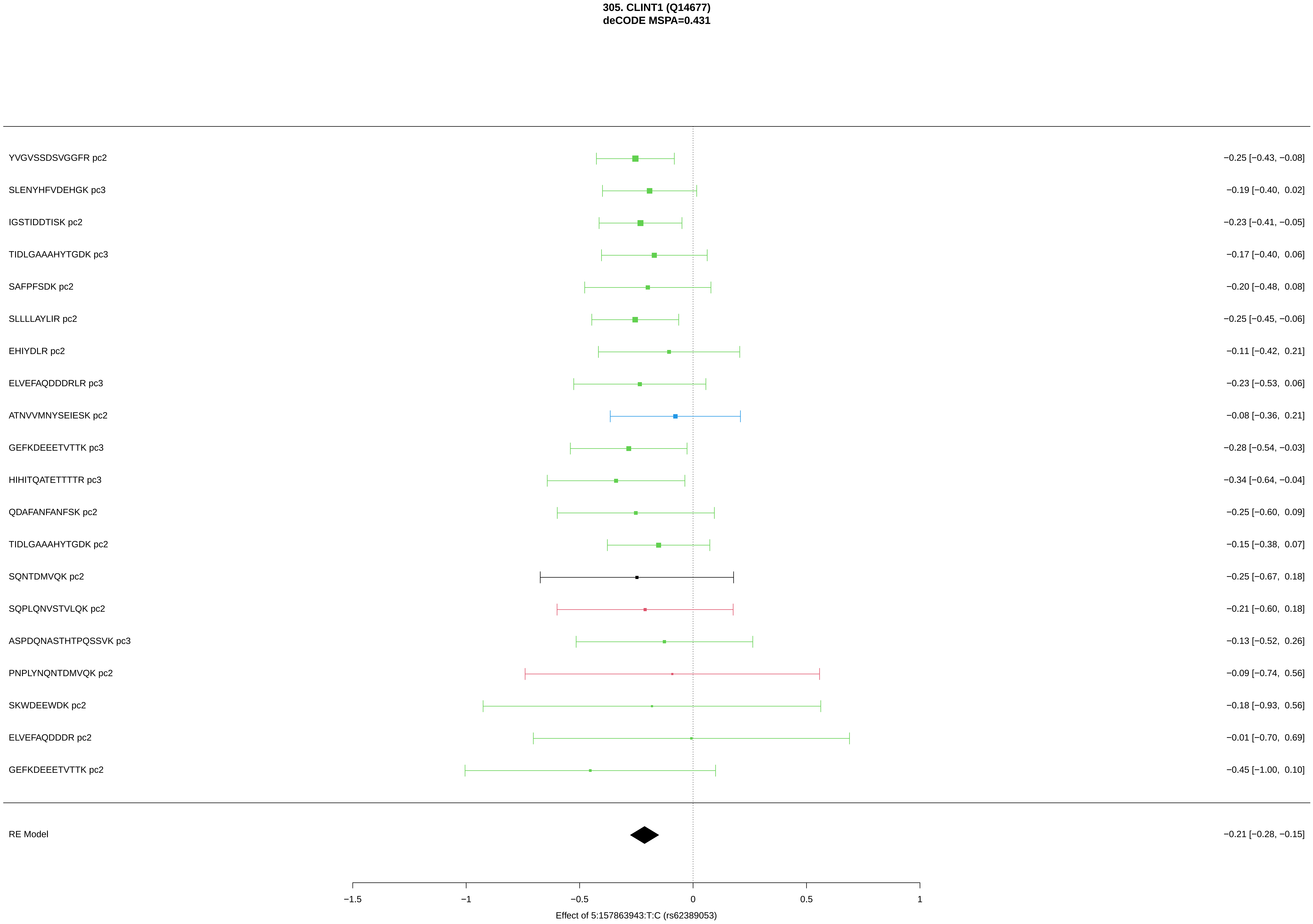
305. CLINT1 (Q14677)
deCODE MSPA=0.431
YVGVSSDSVGGFR pc2
−0.25 [−0.43, −0.08]
SLENYHFVDEHGK pc3
−0.19 [−0.40, 0.02]
IGSTIDDTISK pc2
−0.23 [−0.41, −0.05]
TIDLGAAAHYTGDK pc3
−0.17 [−0.40, 0.06]
SAFPFSDK pc2
−0.20 [−0.48, 0.08]
SLLLLAYLIR pc2
−0.25 [−0.45, −0.06]
EHIYDLR pc2
−0.11 [−0.42, 0.21]
ELVEFAQDDDRLR pc3
−0.23 [−0.53, 0.06]
ATNVVMNYSEIESK pc2
−0.08 [−0.36, 0.21]
GEFKDEEETVTTK pc3
−0.28 [−0.54, −0.03]
HIHITQATETTTTR pc3
−0.34 [−0.64, −0.04]
QDAFANFANFSK pc2
−0.25 [−0.60, 0.09]
TIDLGAAAHYTGDK pc2
−0.15 [−0.38, 0.07]
SQNTDMVQK pc2
−0.25 [−0.67, 0.18]
SQPLQNVSTVLQK pc2
−0.21 [−0.60, 0.18]
ASPDQNASTHTPQSSVK pc3
−0.13 [−0.52, 0.26]
PNPLYNQNTDMVQK pc2
−0.09 [−0.74, 0.56]
SKWDEEWDK pc2
−0.18 [−0.93, 0.56]
ELVEFAQDDDR pc2
−0.01 [−0.70, 0.69]
GEFKDEEETVTTK pc2
−0.45 [−1.00, 0.10]
RE Model
−0.21 [−0.28, −0.15]
−1.5
−1
−0.5
0
0.5
1
Effect of 5:157863943:T:C (rs62389053)

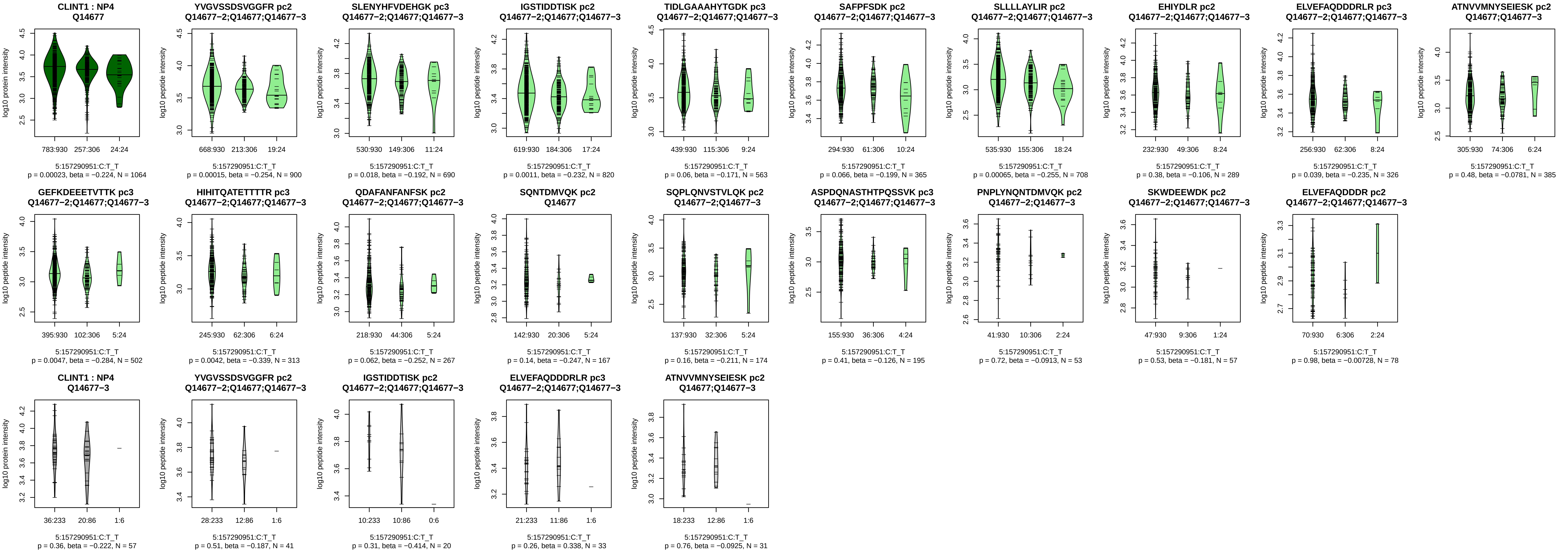

Assay Target: CLINT1
UniProt: Q14677
rsID: rs62389053
Target SNP: 5:157863943:T:C
Proxy SNP: 5:157290951:C:T
deCODE log10(p): 10.7
deCODE BETA: −0.08
deCODE MSPA: 0.431
900:690:820:563:365:708:289:3

306. LGALS4 (P56470)
deCODE MSPA=0.095

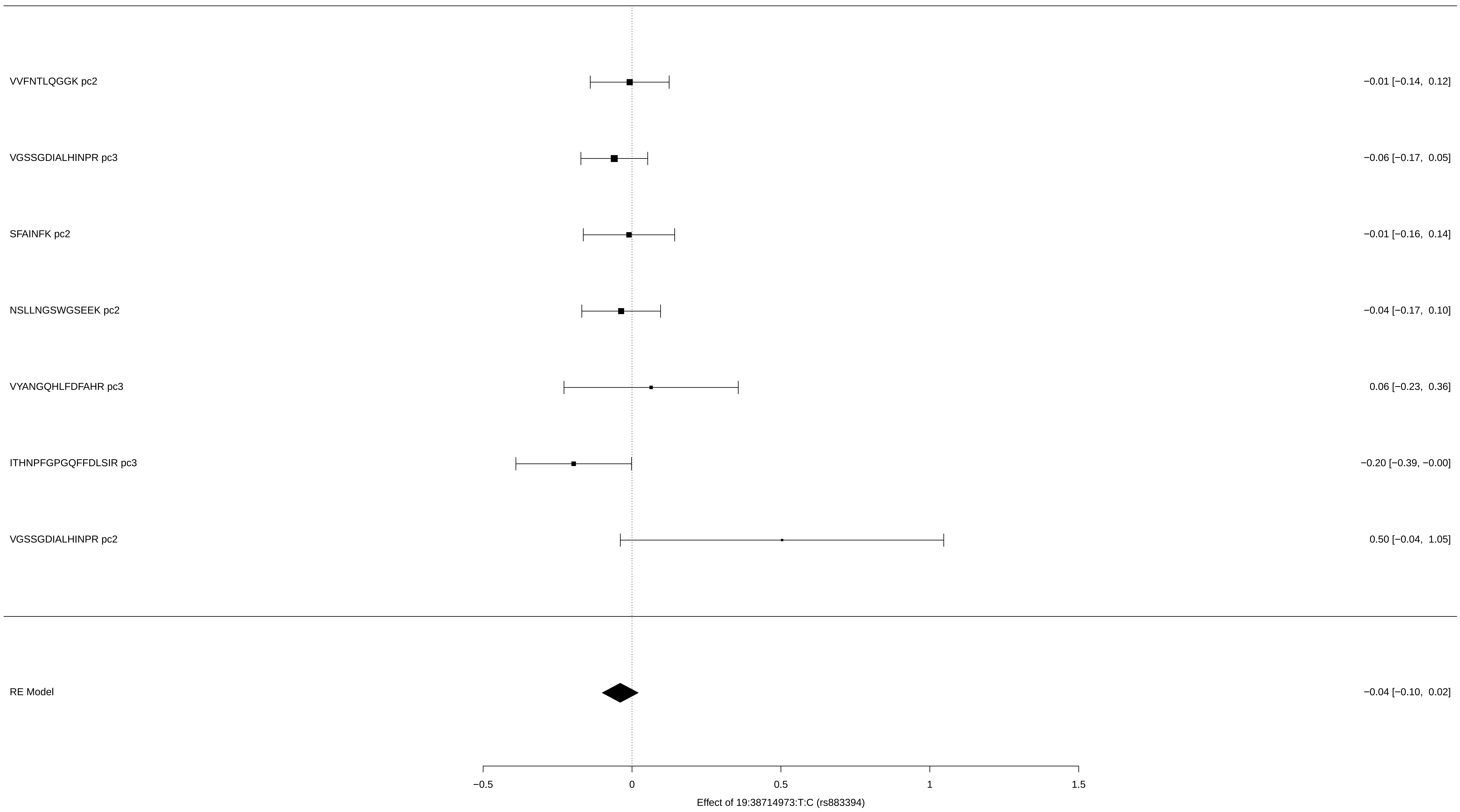

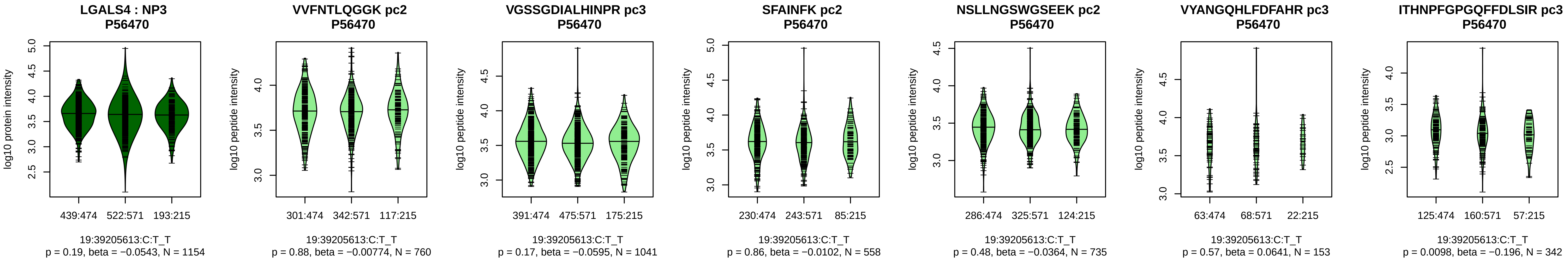

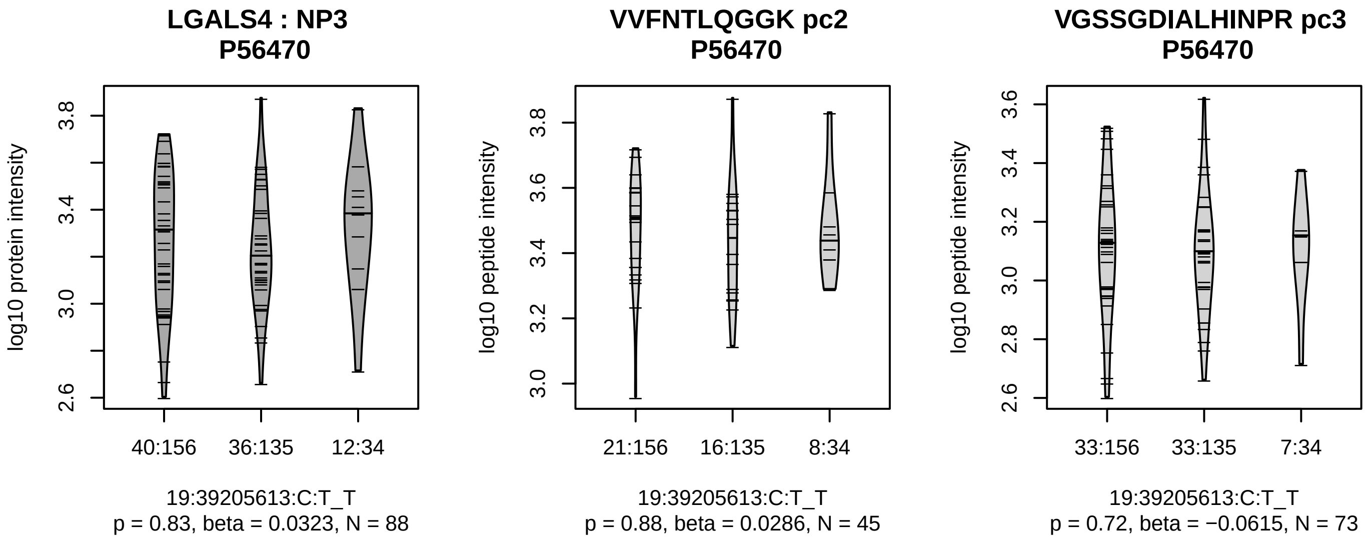

Assay Target: LGALS4
UniProt: P56470
rsID: rs883394
Target SNP: 19:38714973:T:C
Proxy SNP: 19:39205613:C:T
deCODE log10(p): 10.4
deCODE BETA: −0.05
deCODE MSPA: 0.095
–:–:–:–:–:*:–
760:1041:558:735:153:342:29

307. WFDC5 (Q8TCV5)
deCODE MSPA=1

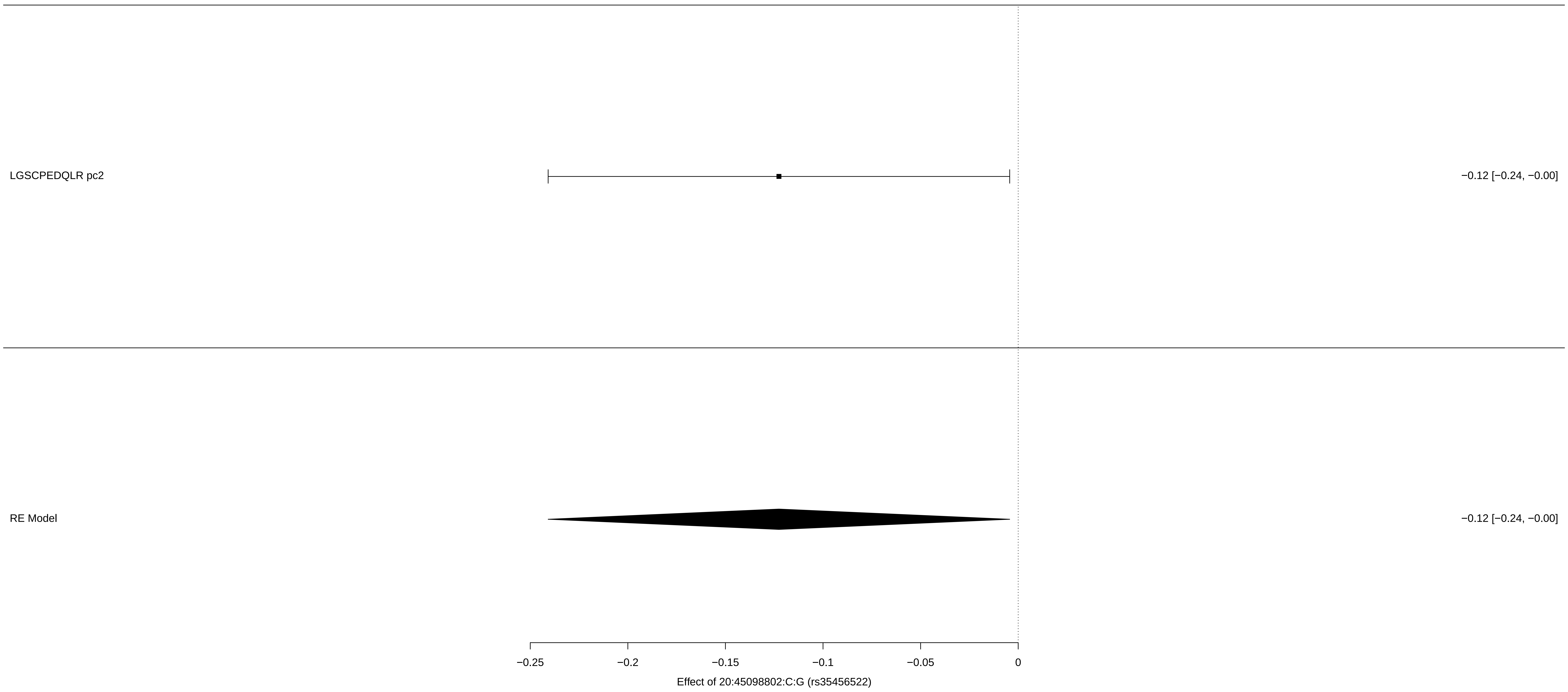

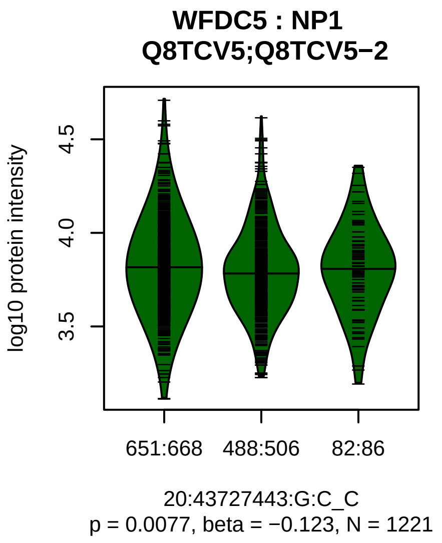

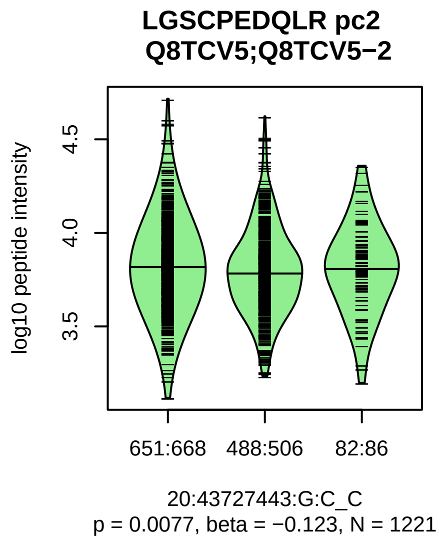

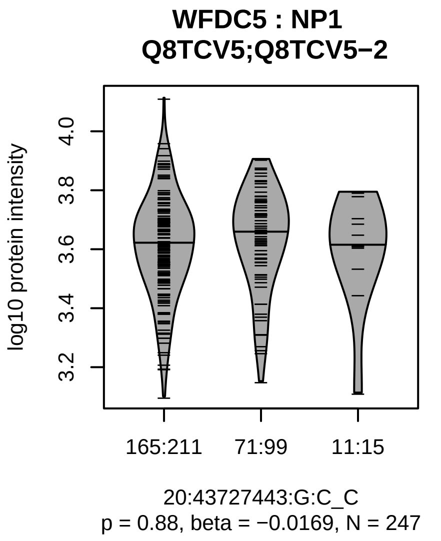

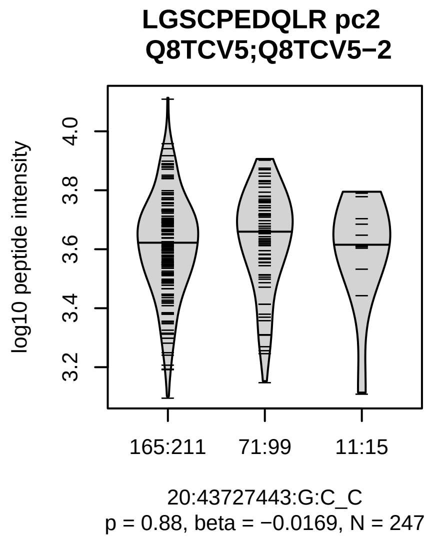

Assay Target: WFDC5
UniProt: Q8TCV5
rsID: rs35456522
Target SNP: 20:45098802:C:G
Proxy SNP: 20:43727443:G:C
deCODE log10(p): 10.2
deCODE BETA: −0.06
deCODE MSPA: 1
*
1221

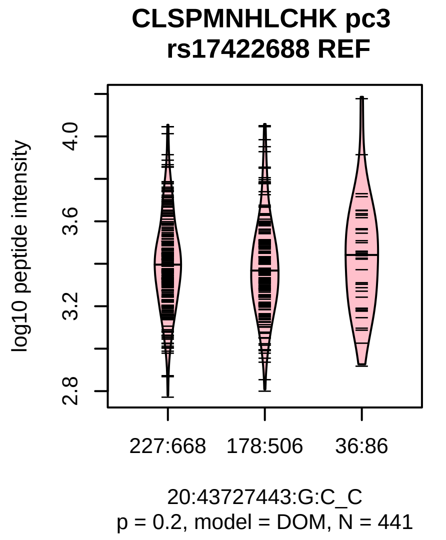

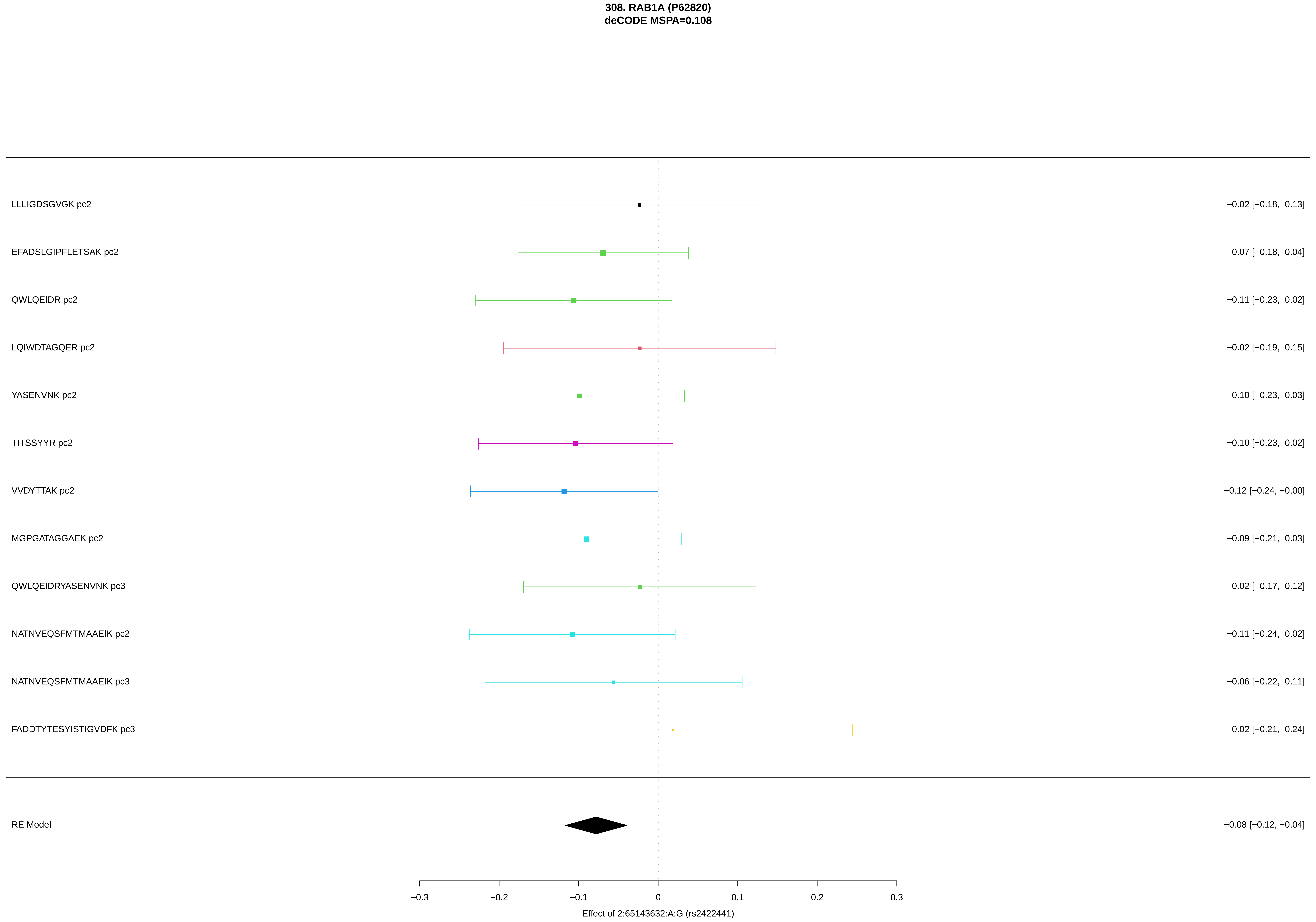
308. RAB1A (P62820)
deCODE MSPA=0.108
LLLIGDSGVGK pc2
−0.02 [−0.18, 0.13]
EFADSLGIPFLETSAK pc2
−0.07 [−0.18, 0.04]
QWLQEIDR pc2
−0.11 [−0.23, 0.02]
LQIWDTAGQER pc2
−0.02 [−0.19, 0.15]
YASENVNK pc2
−0.10 [−0.23, 0.03]
TITSSYYR pc2
−0.10 [−0.23, 0.02]
VVDYTTAK pc2
−0.12 [−0.24, −0.00]
MGPGATAGGAEK pc2
−0.09 [−0.21, 0.03]
QWLQEIDRYASENVNK pc3
−0.02 [−0.17, 0.12]
NATNVEQSFMTMAAEIK pc2
−0.11 [−0.24, 0.02]
NATNVEQSFMTMAAEIK pc3
−0.06 [−0.22, 0.11]
FADDTYTESYISTIGVDFK pc3
0.02 [−0.21, 0.24]
RE Model
−0.08 [−0.12, −0.04]
−0.3
−0.2
−0.1
0
0.1
0.2
0.3
Effect of 2:65143632:A:G (rs2422441)

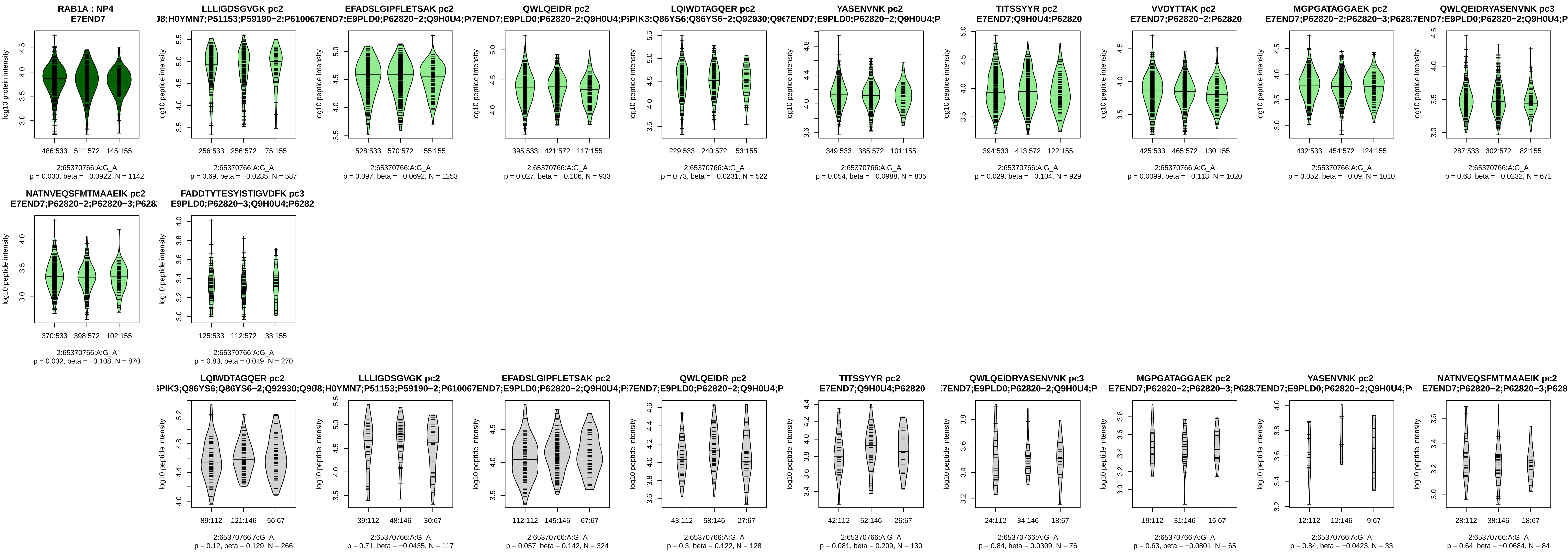

Assay Target: RAB1A
UniProt: P62820
rsID: rs2422441
Target SNP: 2:65143632:A:G
Proxy SNP: 2:65370766:A:G
deCODE log10(p): 10
deCODE BETA: −0.06
deCODE MSPA: 0.108
−:−:−:−:−:−:*:−:−:−:−:−
587:1253:933:522:835:929:1020

309. PSME1 (Q06323)
deCODE MSPA=0.262

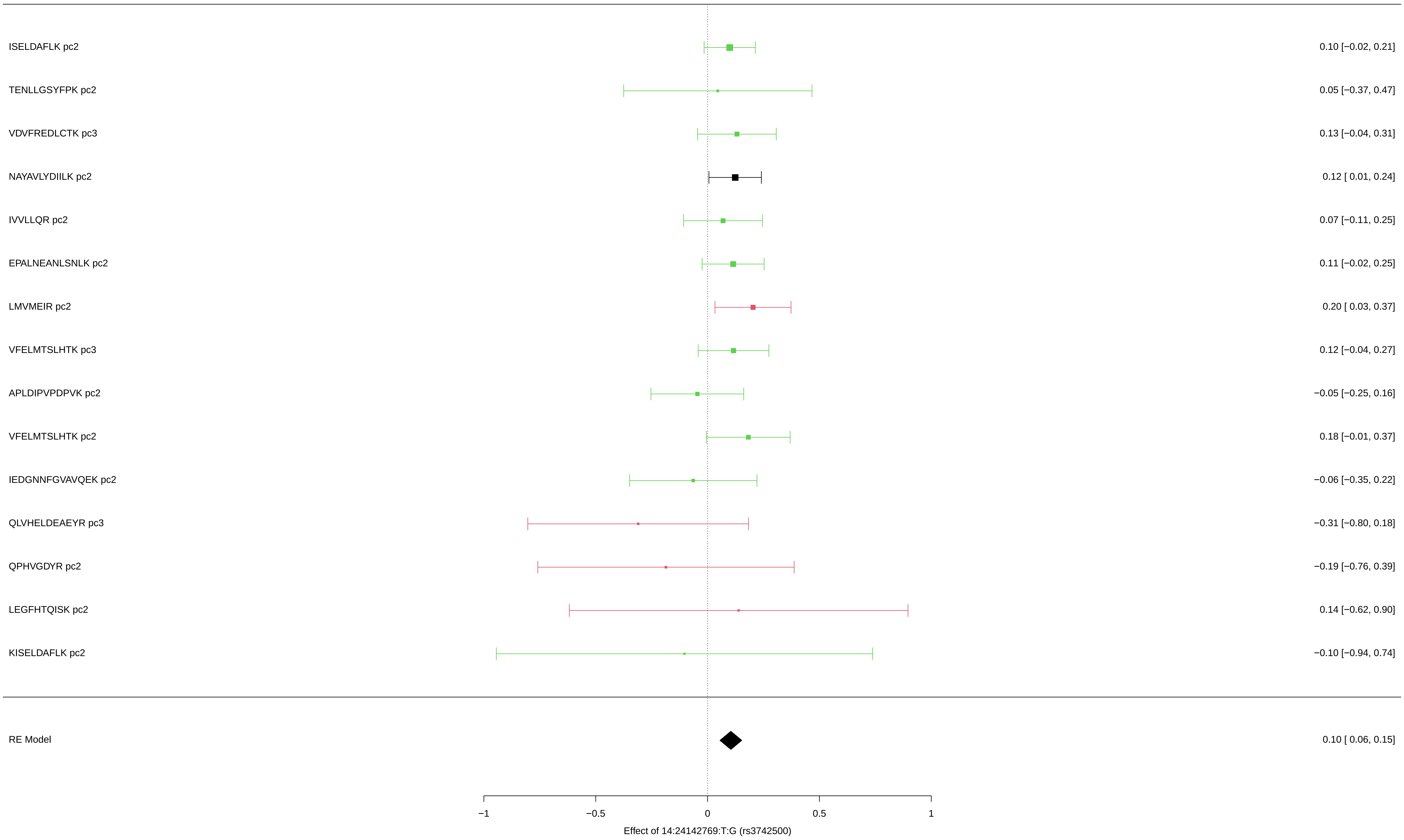

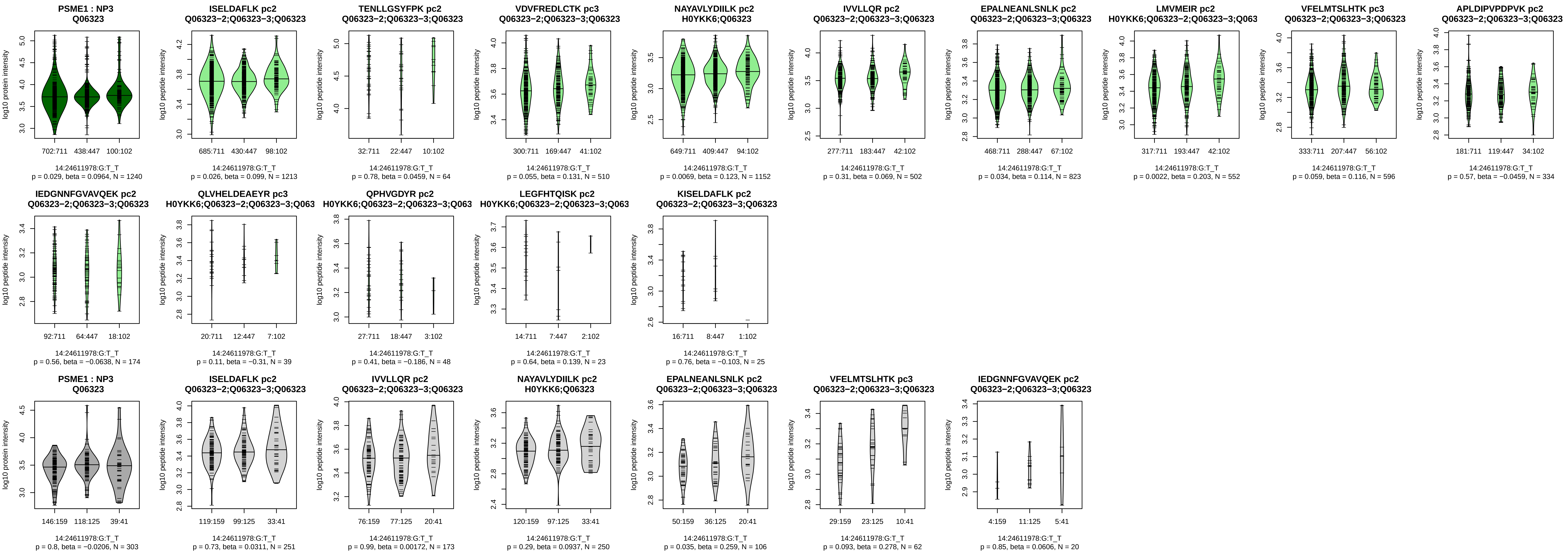

Assay Target: PSME1
UniProt: Q06323
rsID: rs3742500
Target SNP: 14:24142769:T:G
Proxy SNP: 14:24611978:G:T
deCODE log10(p): 9.9
deCODE BETA: 0.06
deCODE MSPA: 0.262
−:−:−:*:−:−:*:−:−:−:−:−:−:−:NA
1213:64:510:1152:502:823:552:

310. NBL1 (P41271)
deCODE MSPA=0

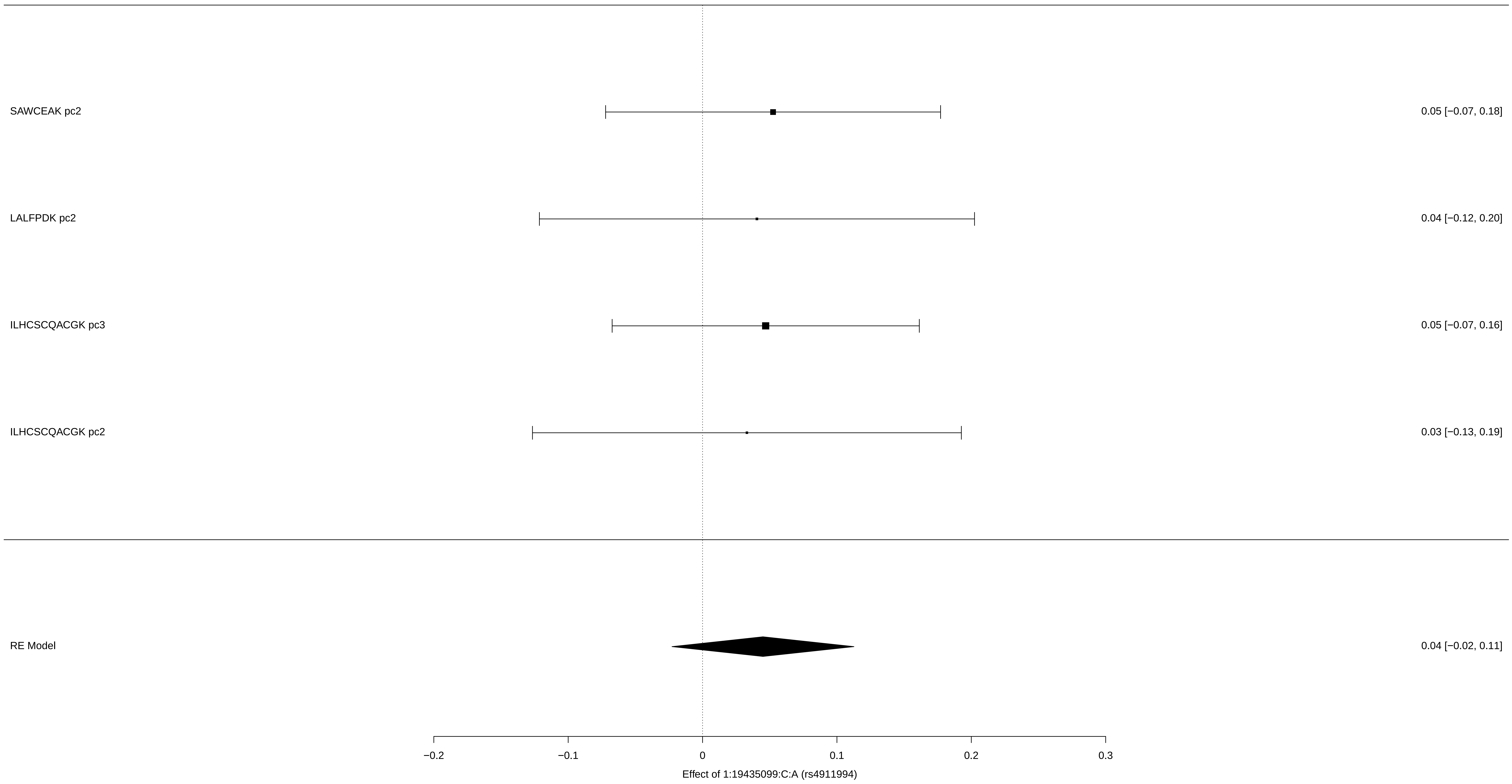

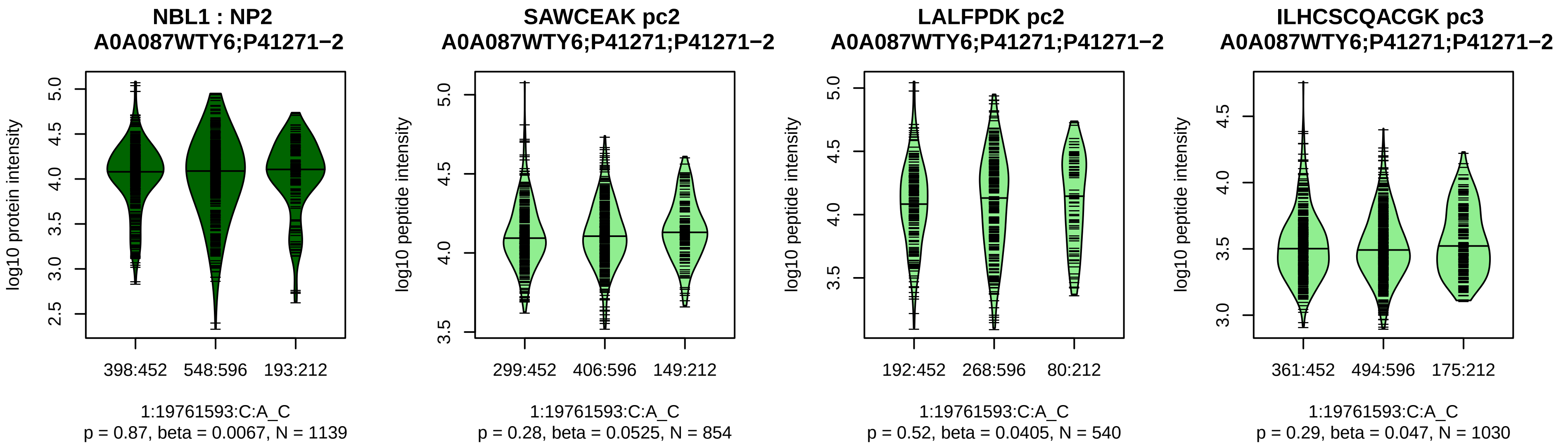

Assay Target: NBL1
UniProt: P41271
rsID: rs4911994
Target SNP: 1:19435099:C:A
Proxy SNP: 1:19761593:C:A
deCODE log10(p): 9.8
deCODE BETA: 0.05
deCODE MSPA: 0
–:–:–:–
854:540:1030:517

311. DNAJB1 (P25685)
deCODE MSPA=0

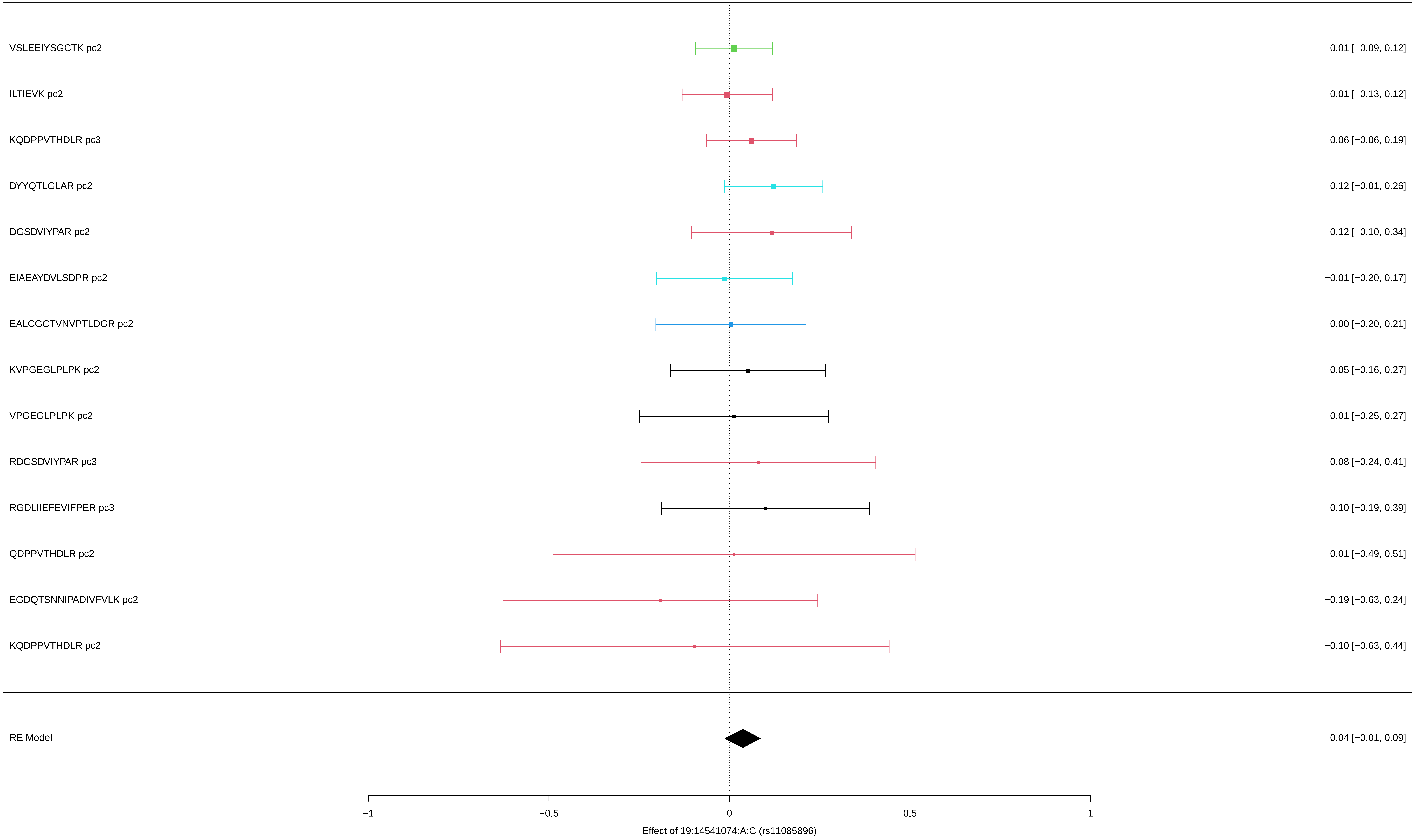

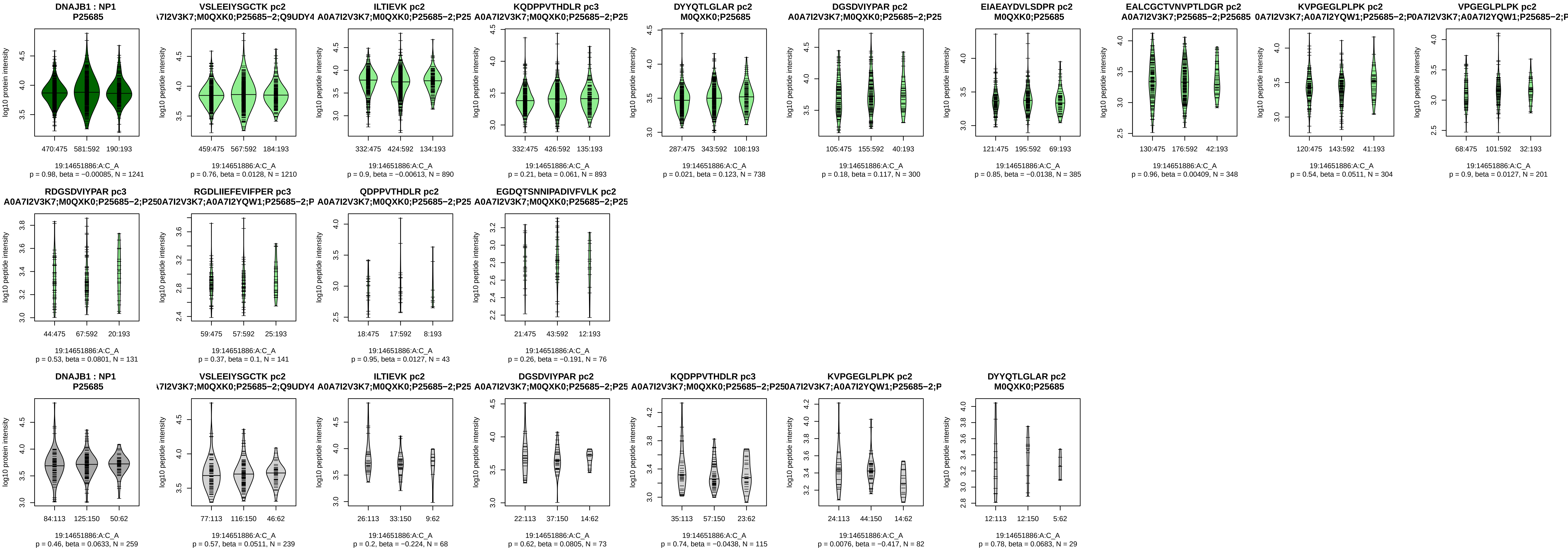

Assay Target: DNAJB1
UniProt: P25685
rsID: rs11085896
Target SNP: 19:14541074:A:C
Proxy SNP: 19:14651886:A:C
deCODE log10(p): 9.7
deCODE BETA: 0.05
deCODE MSPA: 0
−:−:−:−:−:−:−:−:−:−:−:−:−:−:NA:N
1210:890:893:738:300:385:348:

312. AK1 (P00568)
deCODE MSPA=0

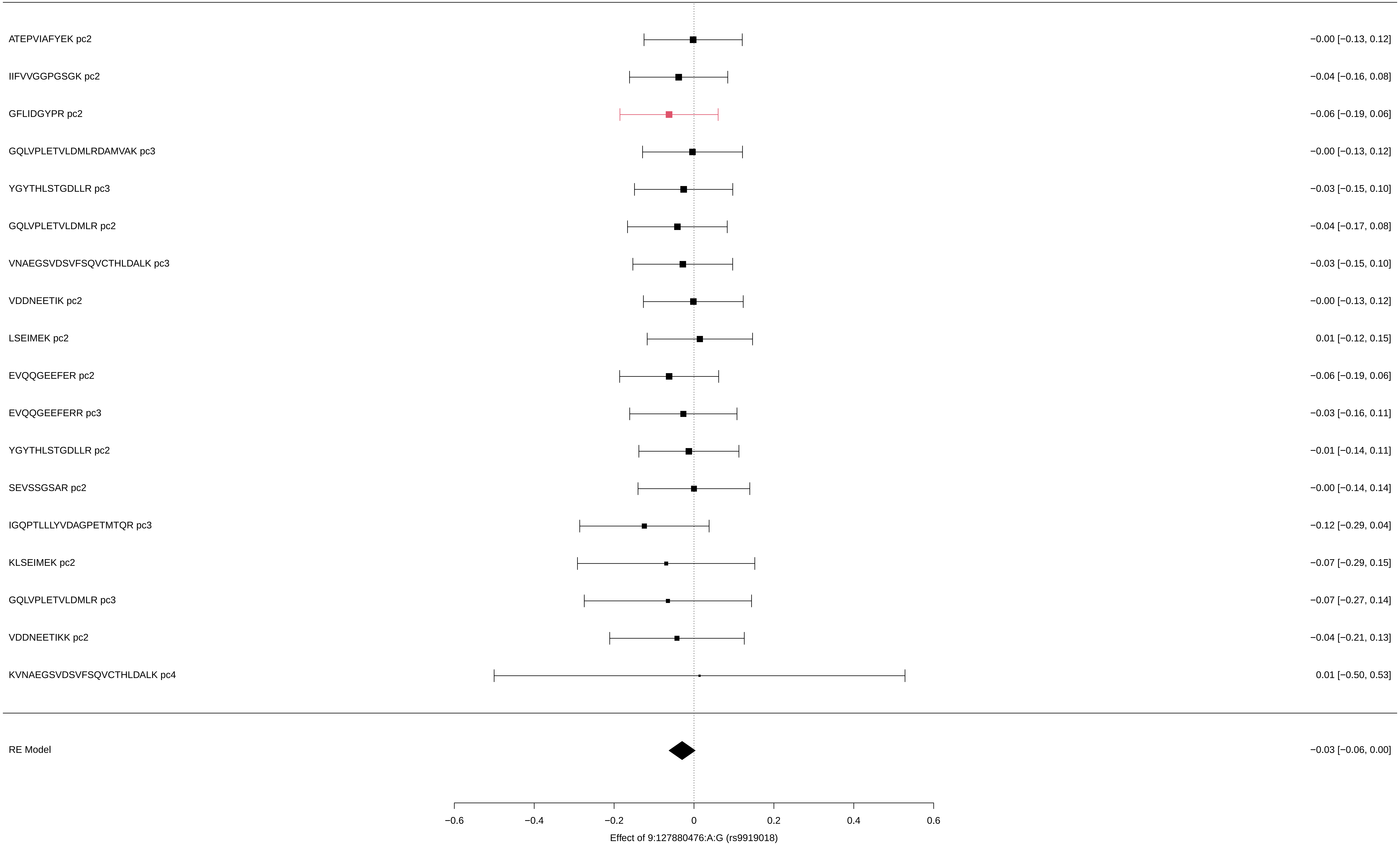

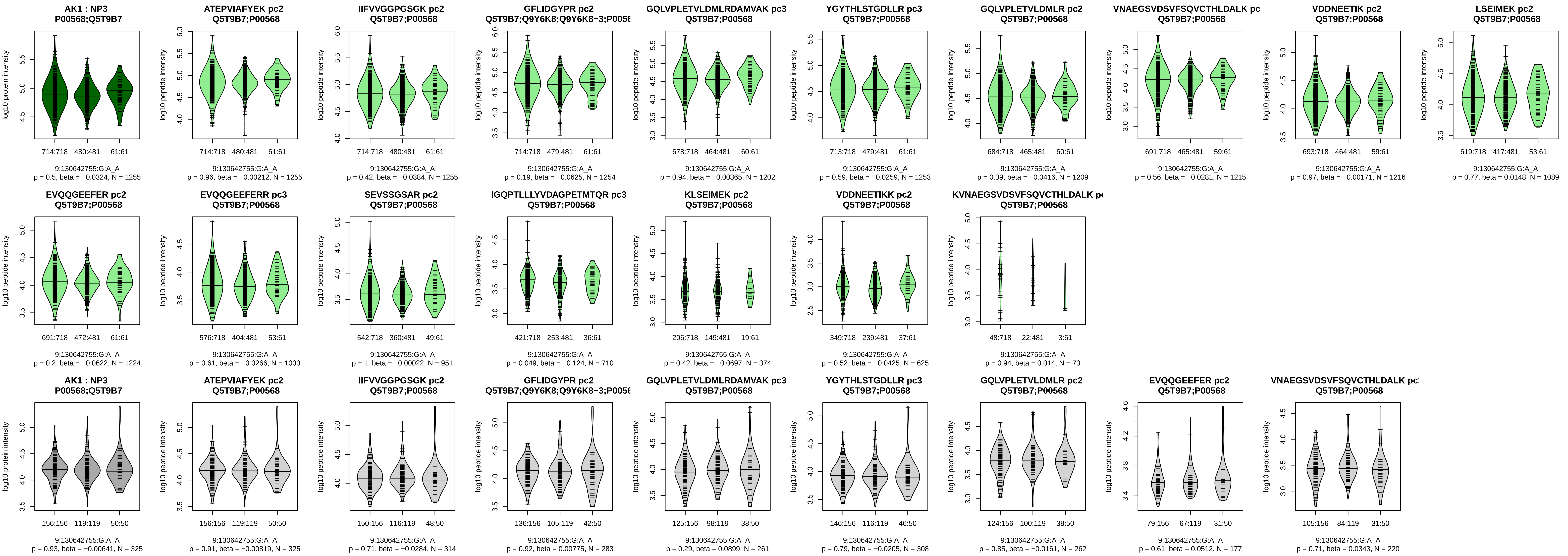

Assay Target: AK1
UniProt: P00568
rsID: rs9919018
Target SNP: 9:127880476:A:G
Proxy SNP: 9:130642755:G:A
deCODE log10(p): 9.7
deCODE BETA: −0.06
deCODE MSPA: 0
1255:1255:1254:1202:1253:120

313. TPI1 (P60174)
deCODE MSPA=0

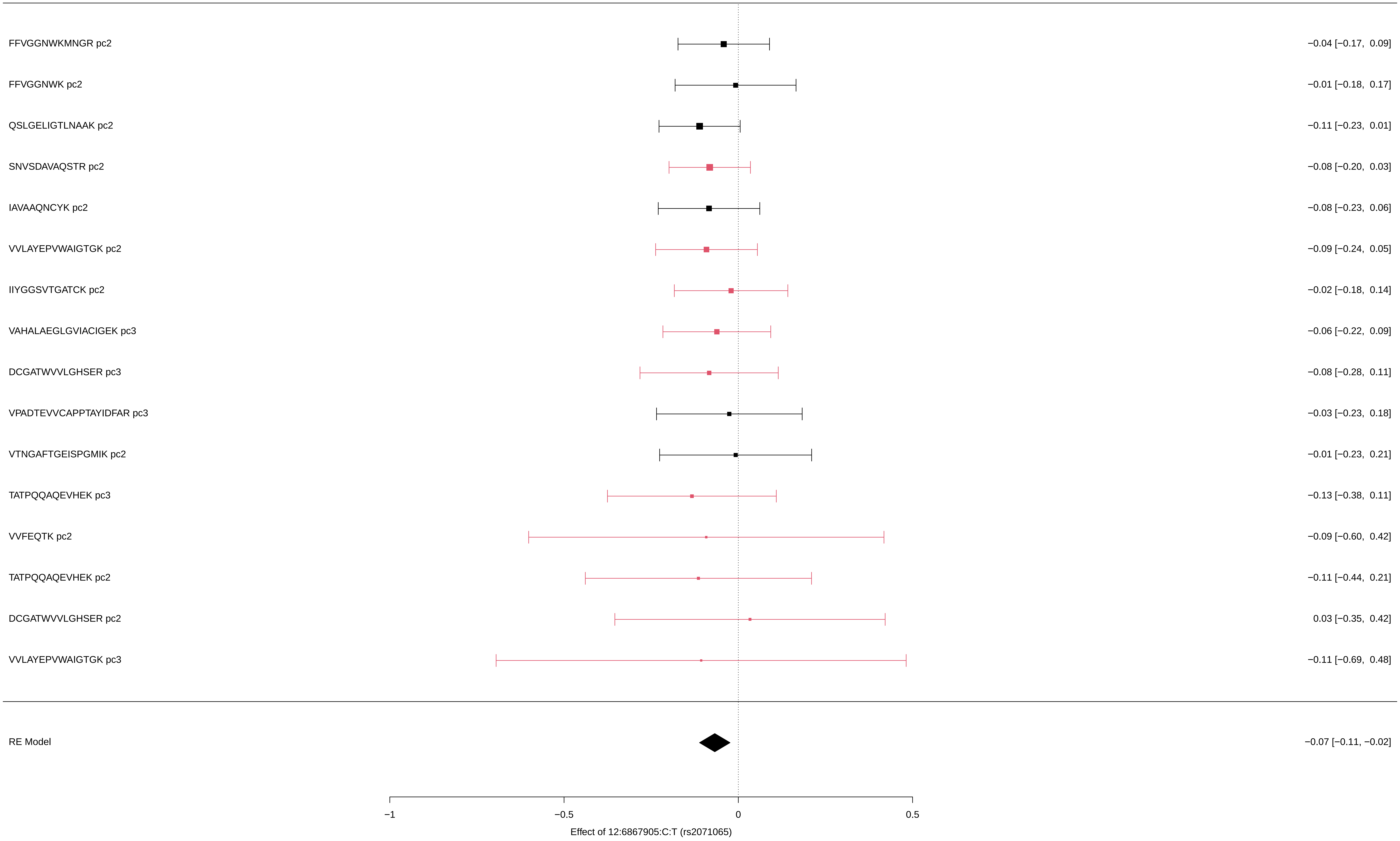

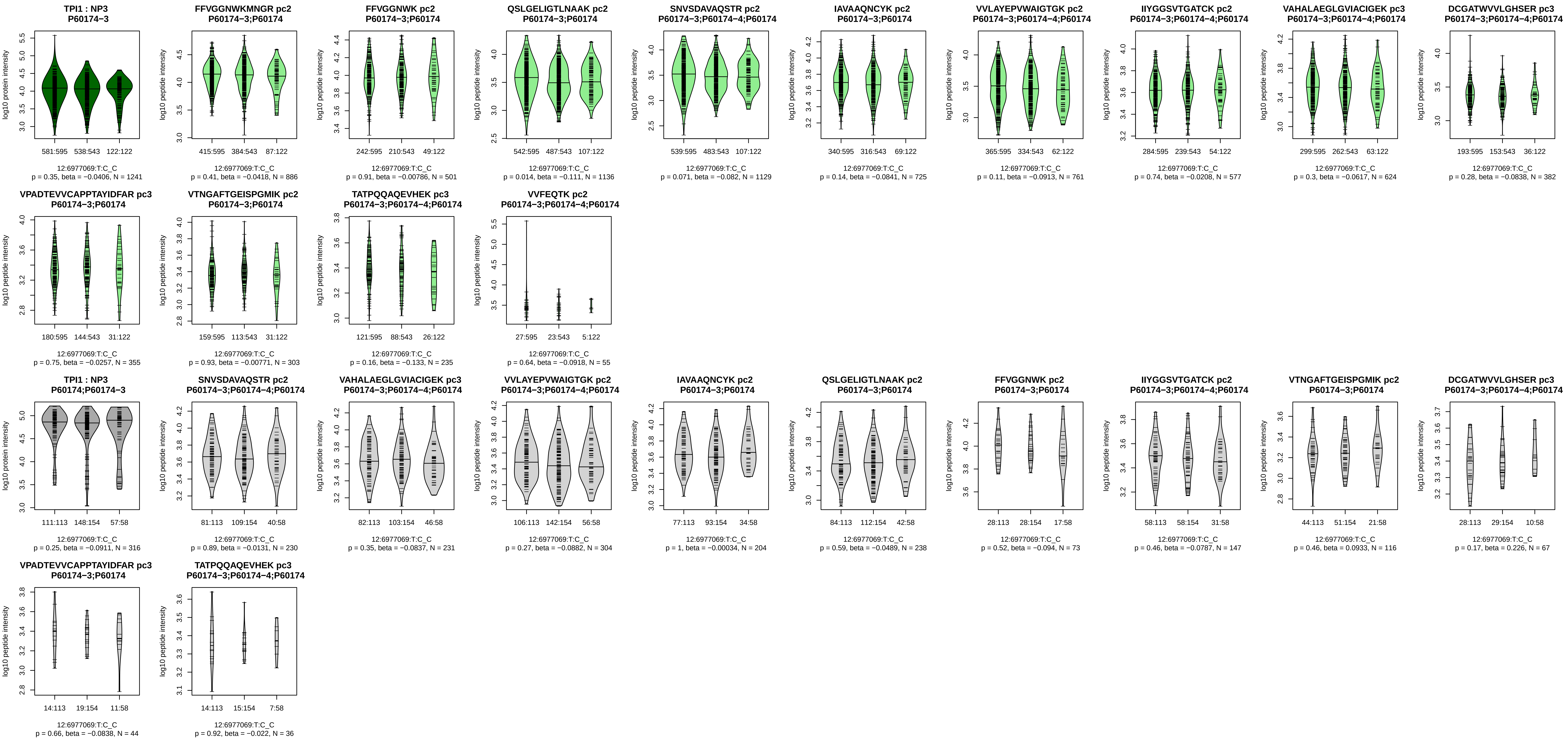

Assay Target: TPI1
UniProt: P60174
rsID: rs2071065
Target SNP: 12:6867905:C:T
Proxy SNP: 12:6977069:T:C
deCODE log10(p): 9.7
deCODE BETA: −0.06
deCODE MSPA: 0
−:−:−:−:−:−:−:−:−:−:−:−:−:N
886:501:1136:1129:725:761:577

314. IGFBP5 (P24593)
deCODE MSPA=0

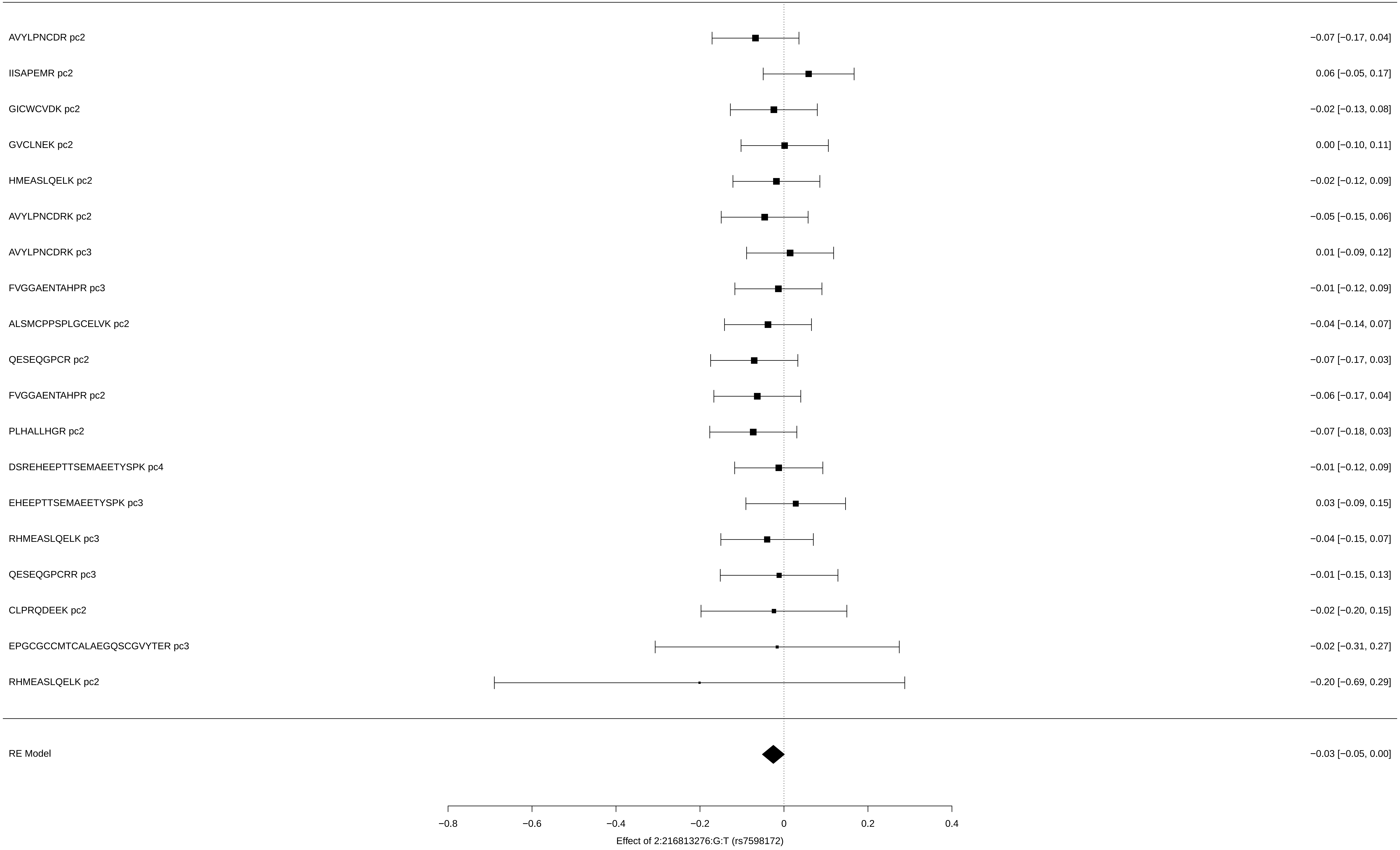

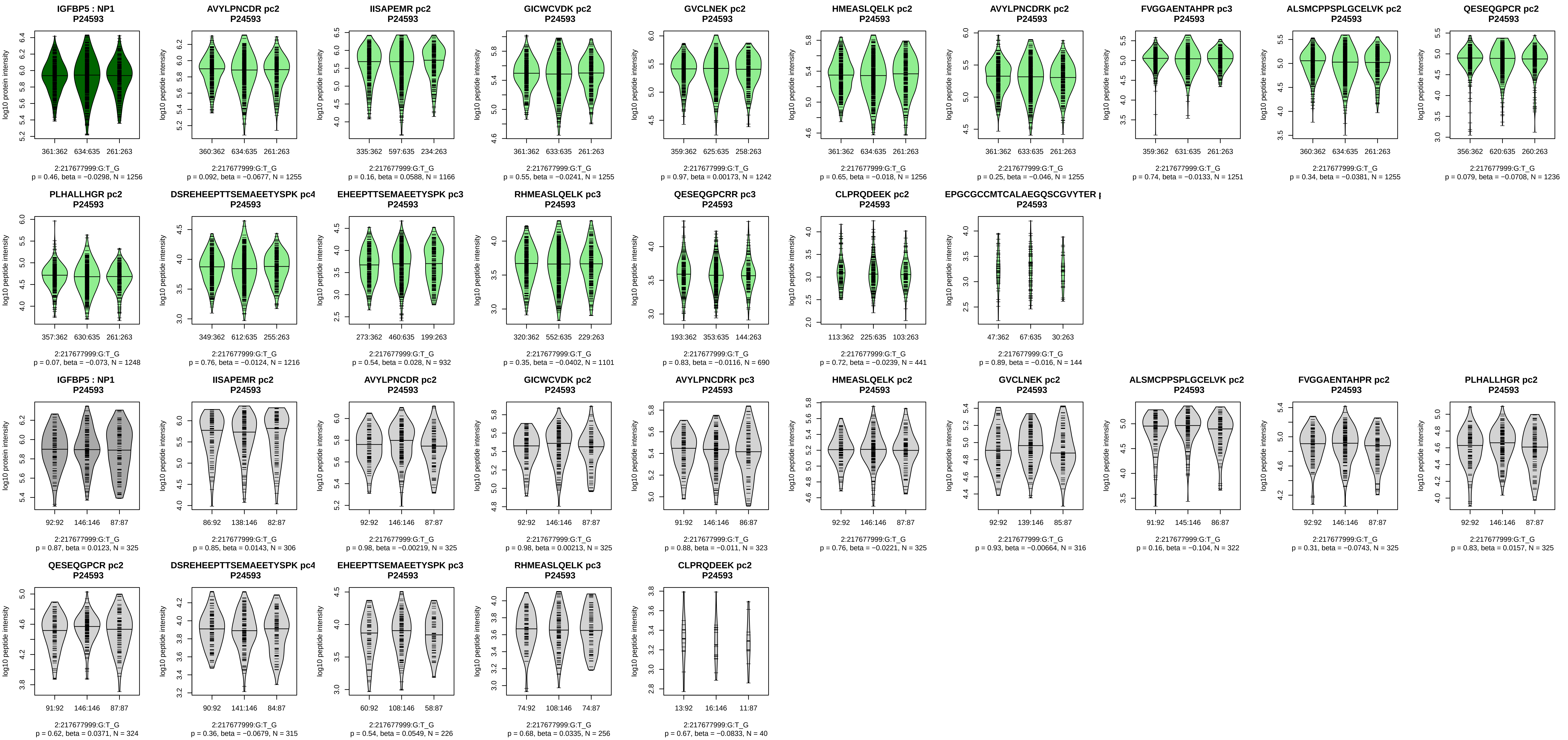

Assay Target: IGFBP5
UniProt: P24593
rsID: rs7598172
Target SNP: 2:216813276:G:T
Proxy SNP: 2:217677999:G:T
deCODE log10(p): 9.6
deCODE BETA: 0.05
deCODE MSPA: 0
1255:1166:1255:1242:1256:125

315. B3GALT6 (Q96L58)
deCODE MSPA=0

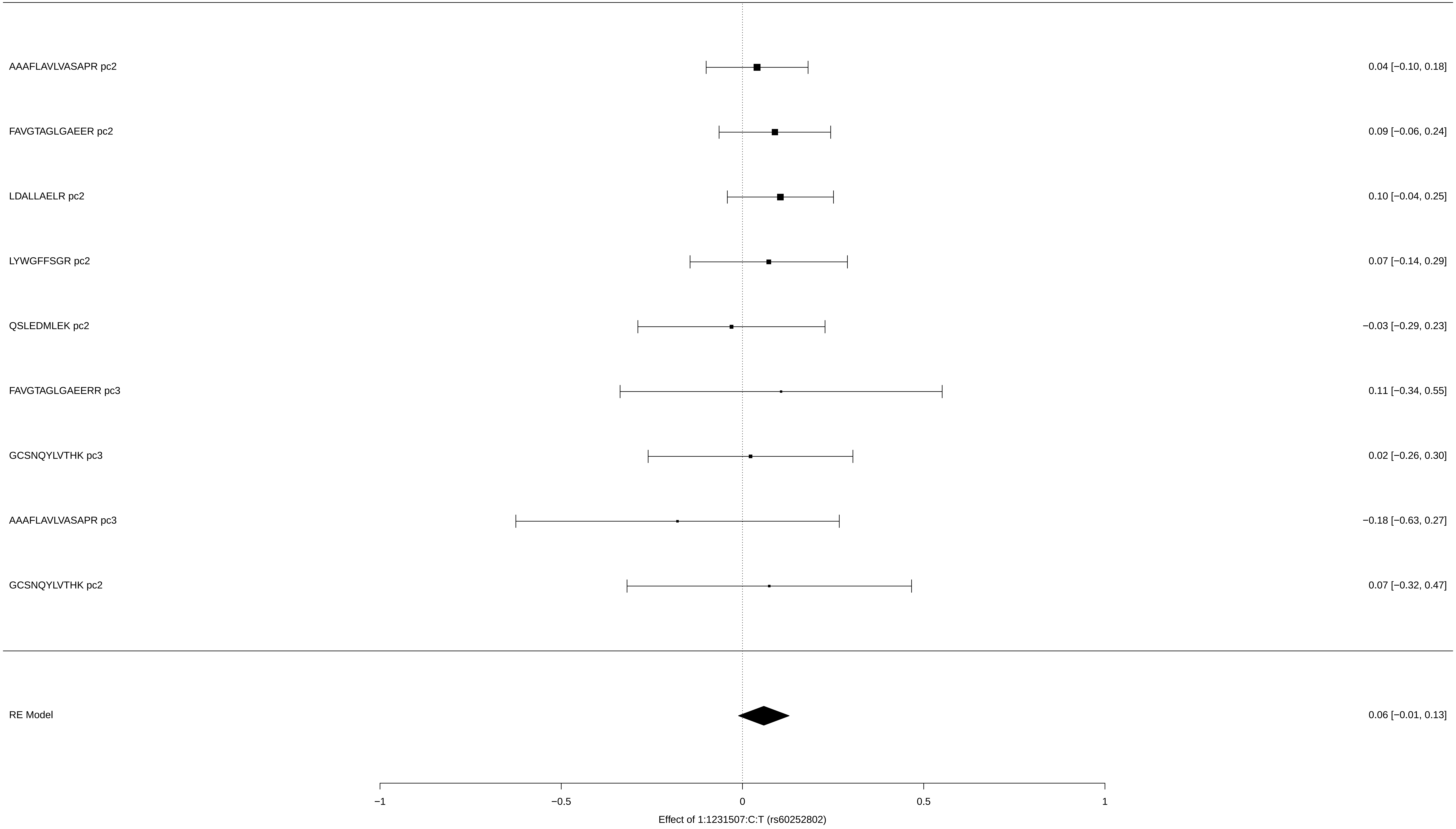

B3GALT6 : NP1
Q96L58

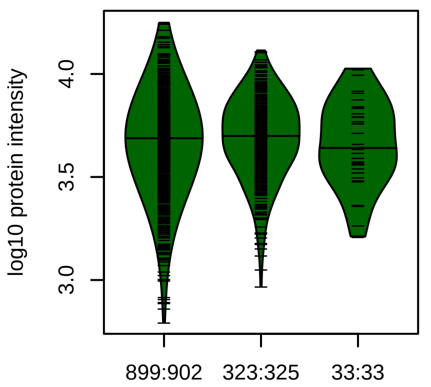

1:1166887:T:C_C
p = 0.17, beta = 0.0744, N = 1255

AAAFLAVLVASAPR pc2
Q96L58

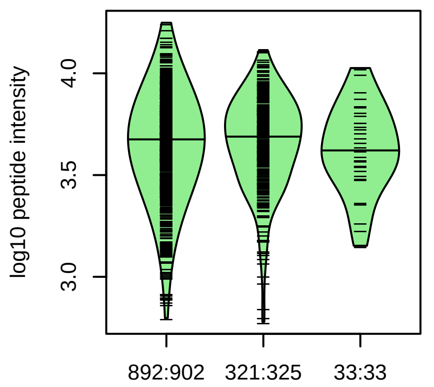

1:1166887:T:C_C
p = 0.46, beta = 0.0404, N = 1246

FAVGTAGLGAEER pc2
Q96L58

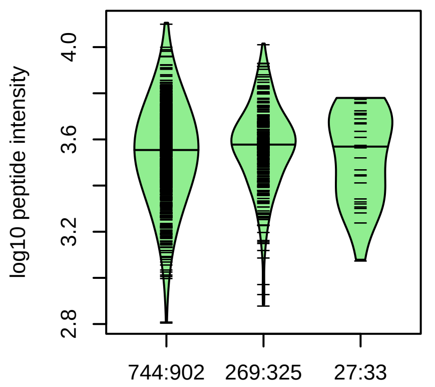

1:1166887:T:C_C
p = 0.13, beta = 0.0894, N = 1040

LDALLAELR pc2
Q96L58

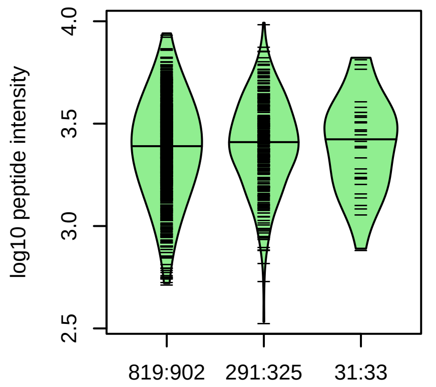

1:1166887:T:C_C
p = 0.066, beta = 0.105, N = 1141

LYWGFFSGR pc2
Q96L58

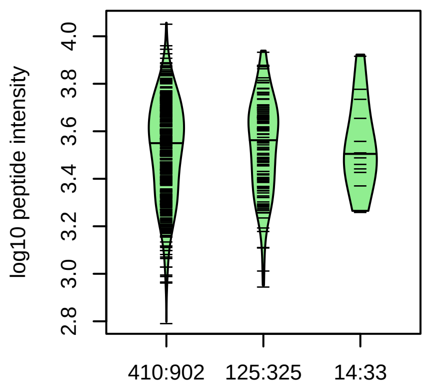

1:1166887:T:C_C
p = 0.39, beta = 0.0725, N = 549

QSLEDMLEK pc2
Q96L58

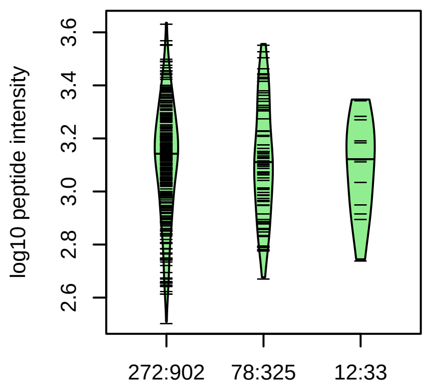

1:1166887:T:C_C
p = 0.76, beta = −0.0304, N = 362

FAVGTAGLGAEERR pc3
Q96L58

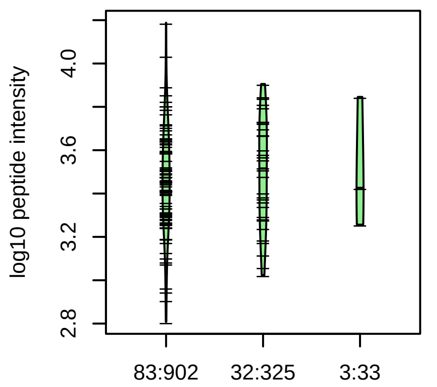

1:1166887:T:C_C
p = 0.54, beta = 0.107, N = 118

GCSNQYLVTHK pc3
Q96L58

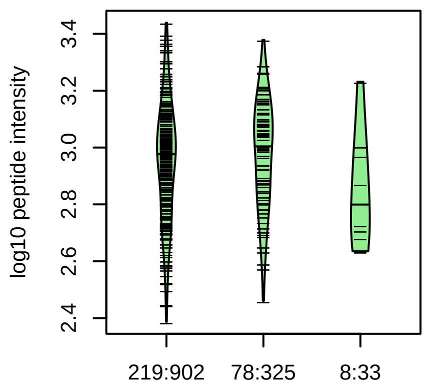

1:1166887:T:C_C
p = 0.84, beta = 0.0222, N = 305

B3GALT6 : NP1
Q96L58

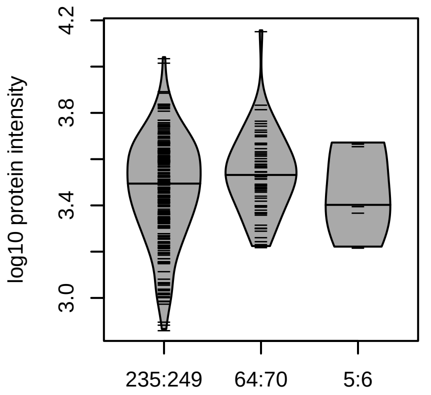

1:1166887:T:C_C
p = 0.2, beta = 0.155, N = 304

AAAFLAVLVASAPR pc2
Q96L58

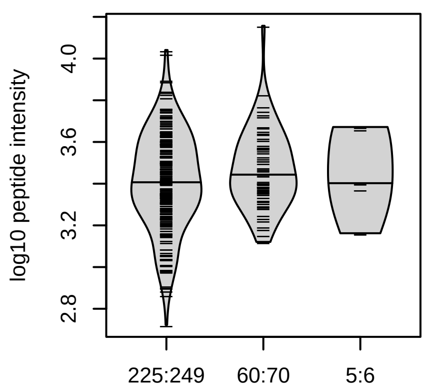

1:1166887:T:C_C
p = 0.23, beta = 0.149, N = 290

FAVGTAGLGAEER pc2
Q96L58

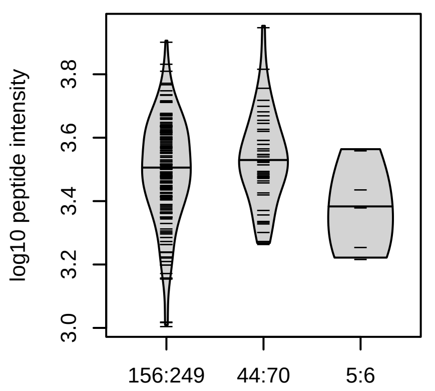

1:1166887:T:C_C
p = 0.93, beta = 0.0126, N = 205

LDALLAELR pc2
Q96L58

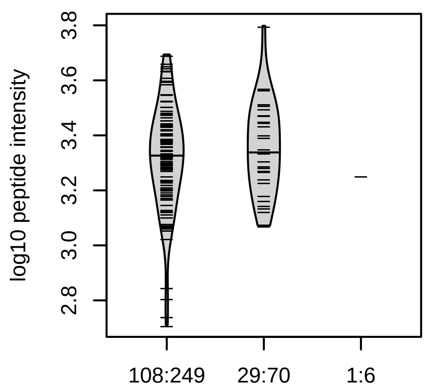

1:1166887:T:C_C
p = 0.87, beta = −0.0318, N = 138

Assay Target: B3GALT6
UniProt: Q96L58
rsID: rs60252802
Target SNP: 1:1231507:C:T
Proxy SNP: 1:1166887:T:C
deCODE log10(p): 9.4
deCODE BETA: −0.07
deCODE MSPA: 0
–:–:–:–:–:–:–:–
1246:1040:1141:549:362:118:30

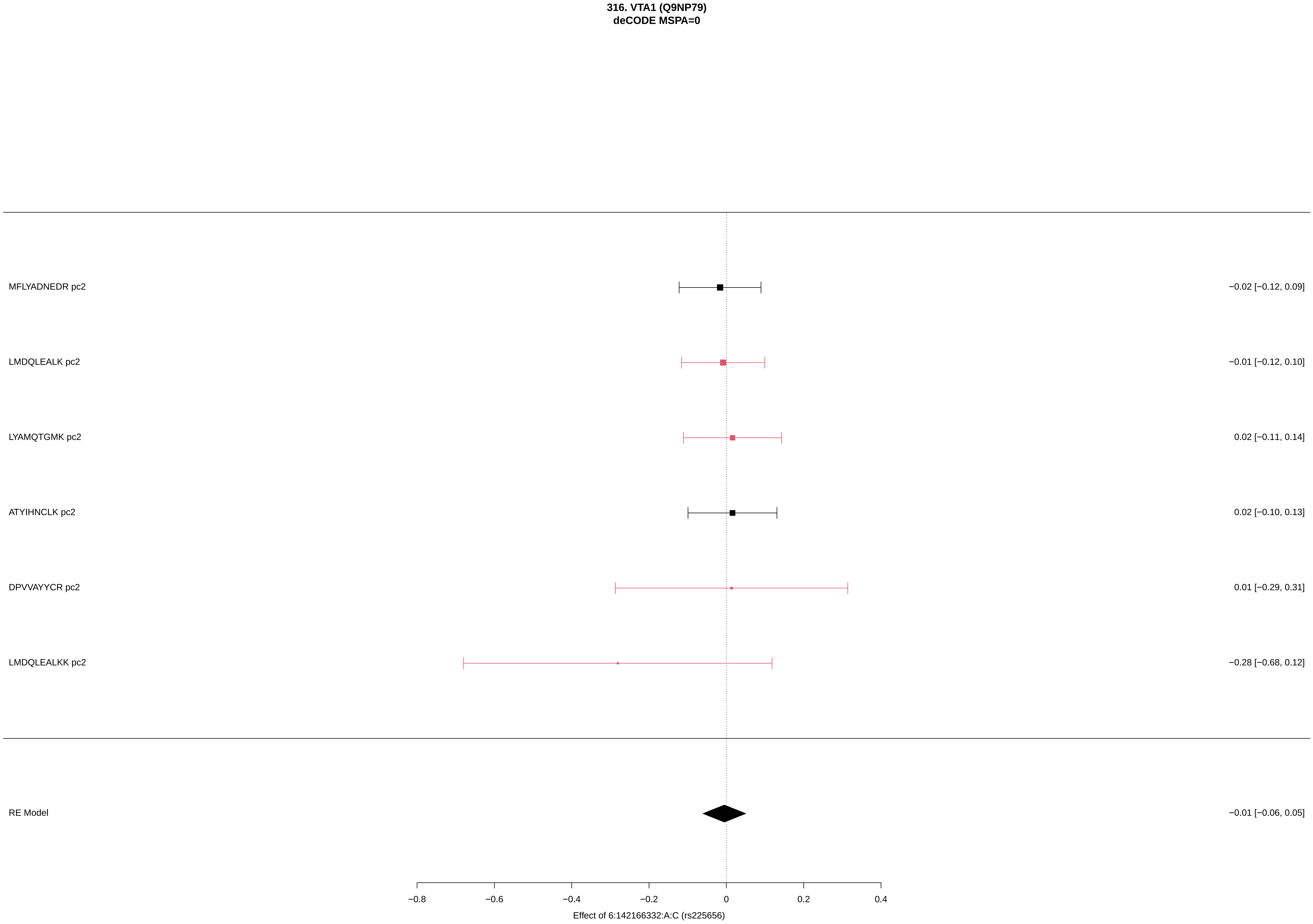

316. VTA1 (Q9NP79)
deCODE MSPA=0
MFLYADNEDR pc2
−0.02 [−0.12, 0.09]
LMDQLEALK pc2
−0.01 [−0.12, 0.10]
LYAMQTGMK pc2
0.02 [−0.11, 0.14]
ATYIHNCLK pc2
0.02 [−0.10, 0.13]
DPVVAYYCR pc2
0.01 [−0.29, 0.31]
LMDQLEALKK pc2
−0.28 [−0.68, 0.12]
RE Model
−0.01 [−0.06, 0.05]
−0.8
−0.6
−0.4
−0.2
0
0.2
0.4
Effect of 6:142166332:A:C (rs225656)

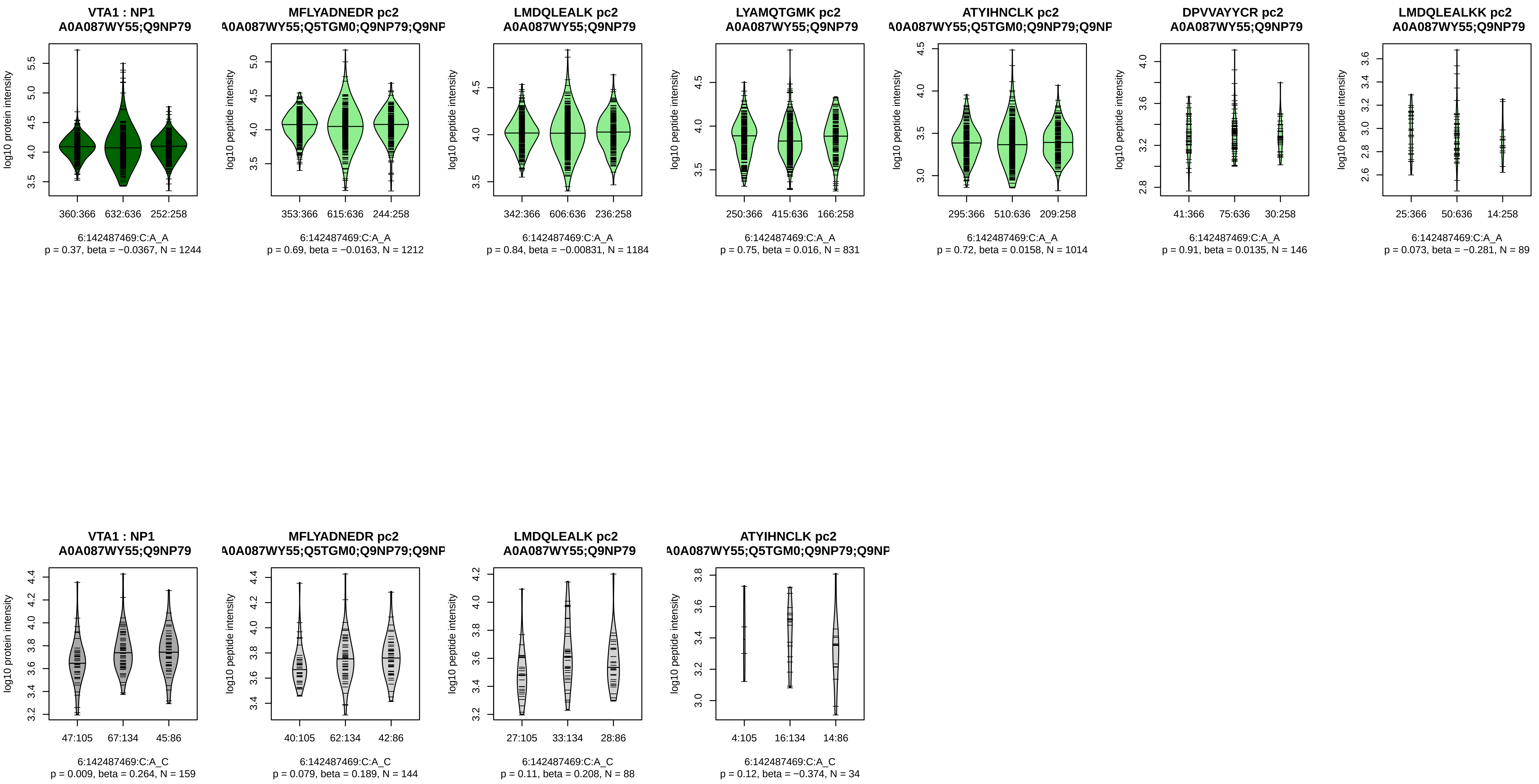

Assay Target: VTA1
UniProt: Q9NP79
rsID: rs225656
Target SNP: 6:142166332:A:C
Proxy SNP: 6:142487469:C:A
deCODE log10(p): 9.1
deCODE BETA: −0.05
deCODE MSPA: 0
−:−:−:−:NA:−:−:NA
1212:1184:831:1014:12:146:89:

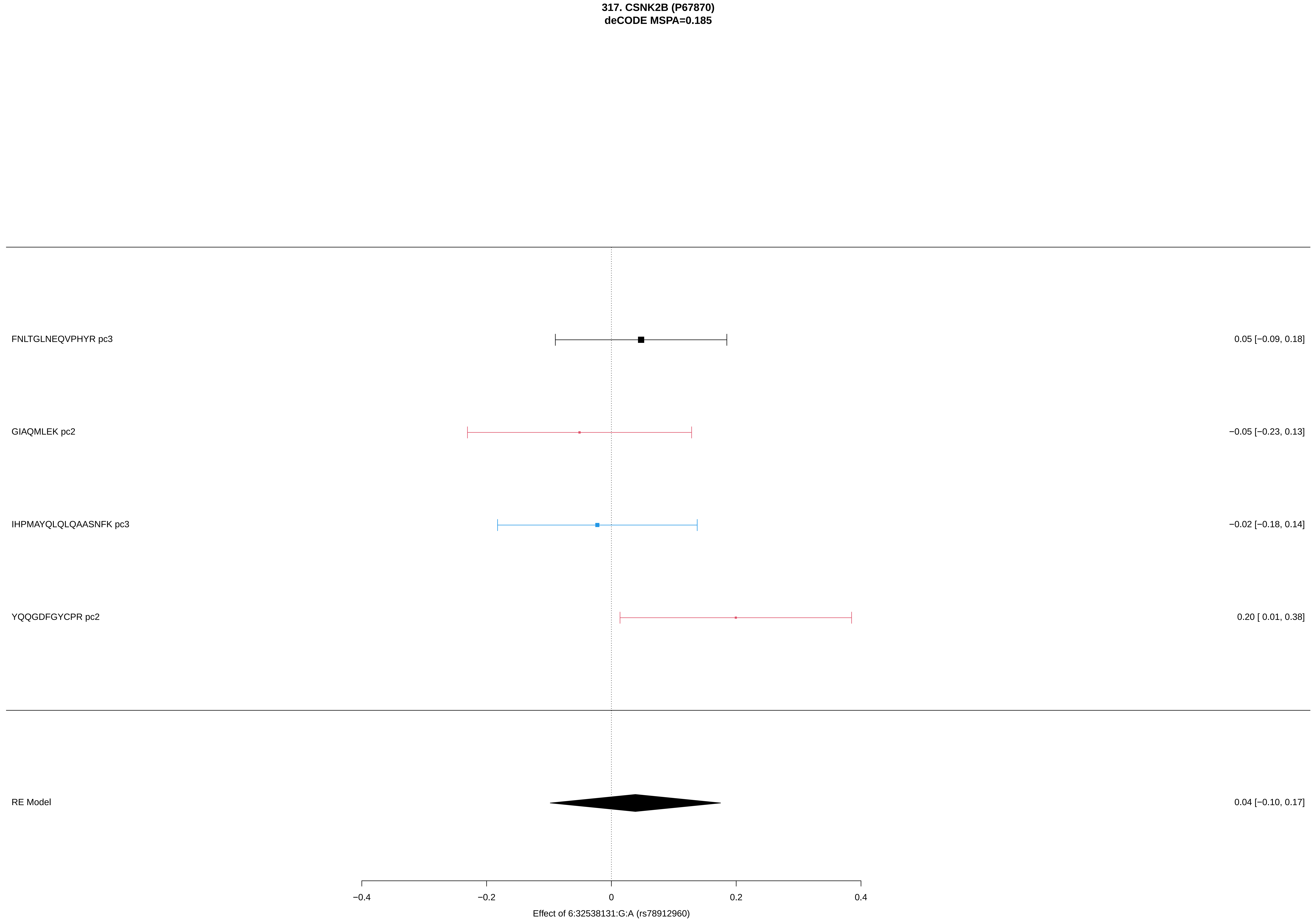
317. CSNK2B (P67870)
deCODE MSPA=0.185
FNLTGLNEQVPHYR pc3
0.05 [−0.09, 0.18]
GIAQMLEK pc2
−0.05 [−0.23, 0.13]
IHPMAYQLQLQAASNFK pc3
−0.02 [−0.18, 0.14]
YQQGDFGYCPR pc2
0.20 [0.01, 0.38]
RE Model
0.04 [−0.10, 0.17]
−0.4
−0.2
0
0.2
0.4
Effect of 6:32538131:G:A (rs78912960)

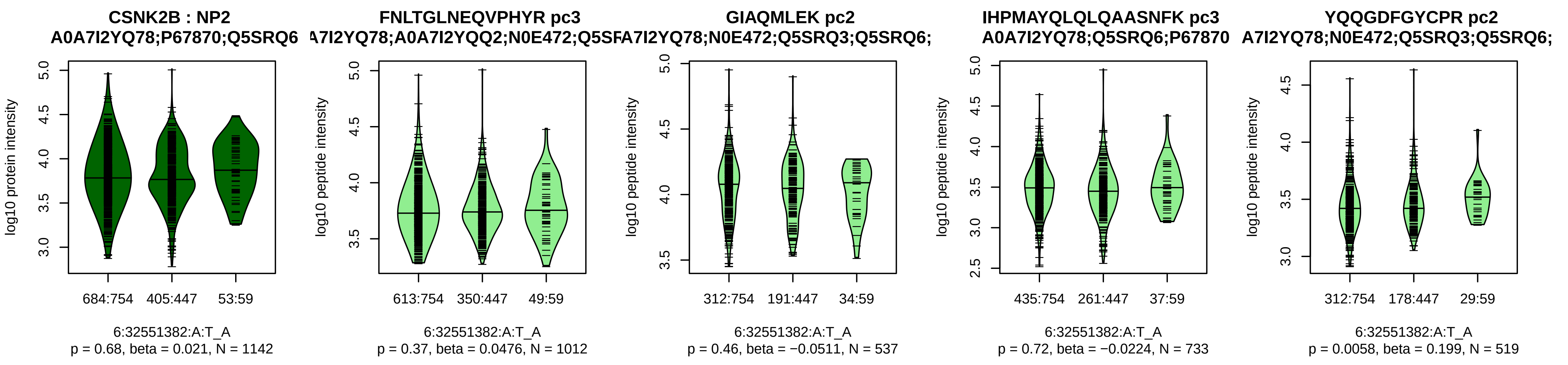

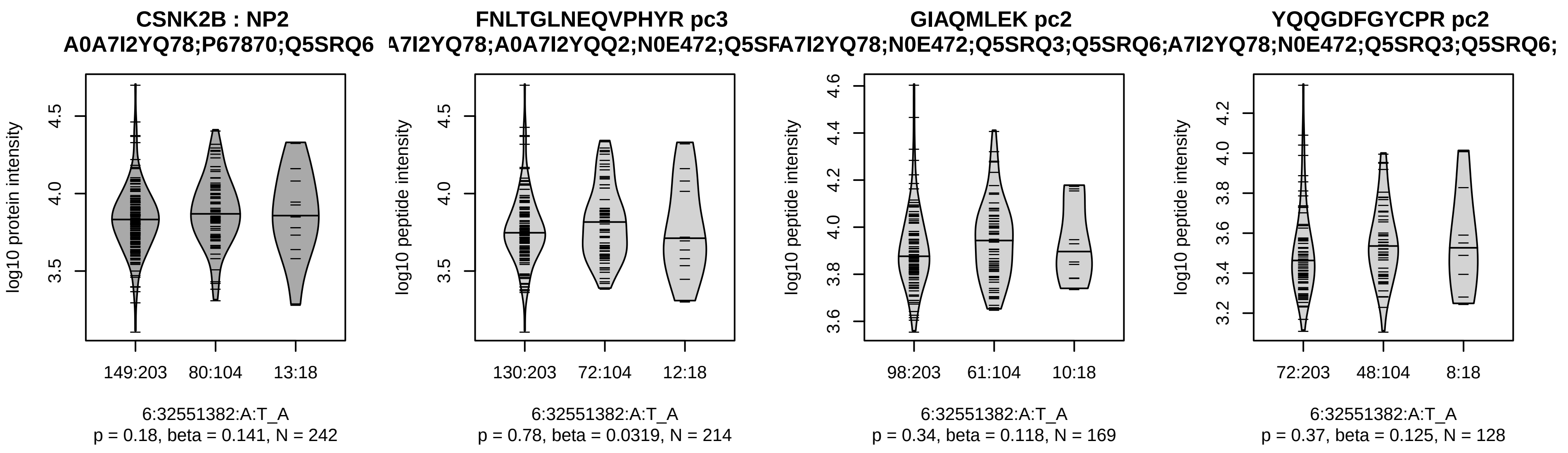

Assay Target: CSNK2B
UniProt: P67870
rsID: rs78912960
Target SNP: 6:32538131:G:A
Proxy SNP: 6:32551382:A:T
deCODE log10(p): 9
deCODE BETA: −0.05
deCODE MSPA: 0.185
−:−:−:*:NA
1012:537:733:519:9

318. FKBP2 (P26885)
deCODE MSPA=0

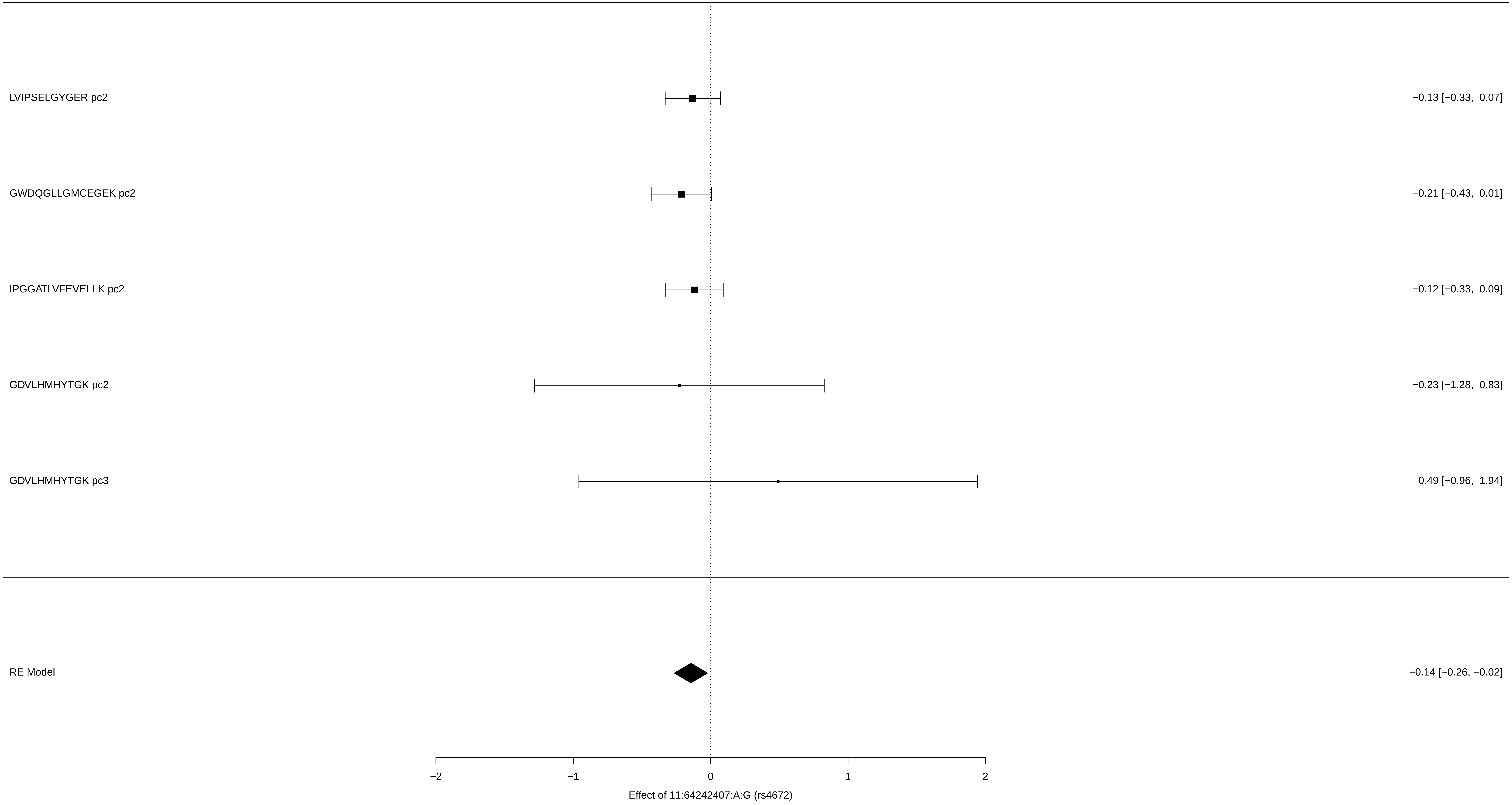

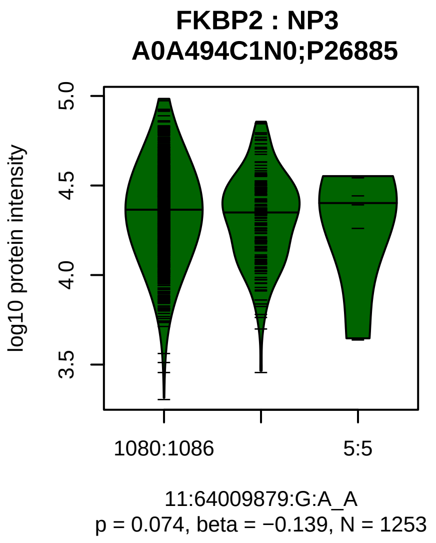

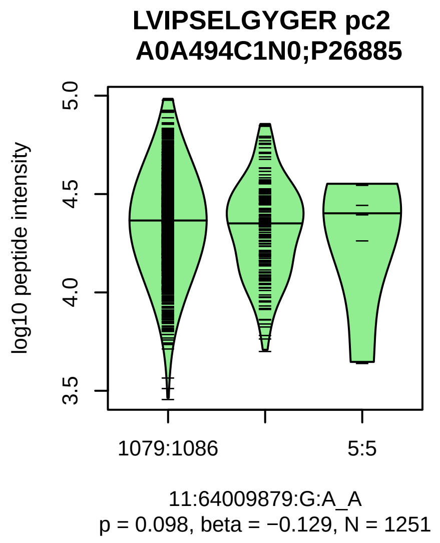

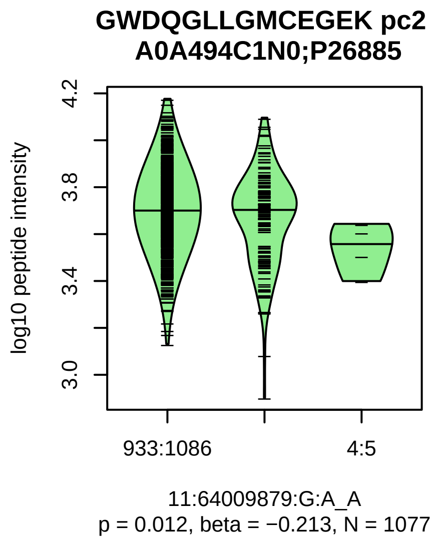

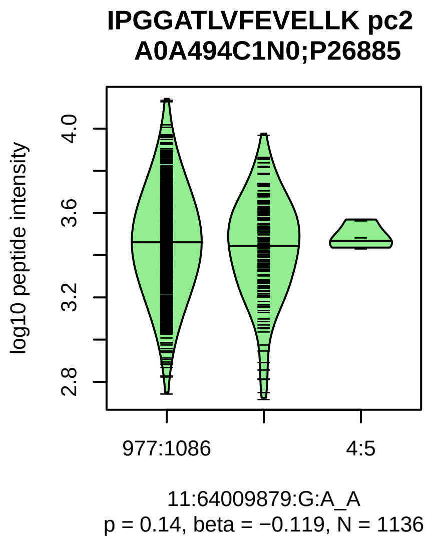

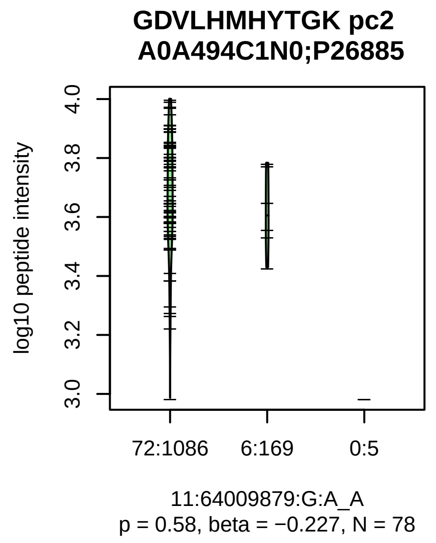

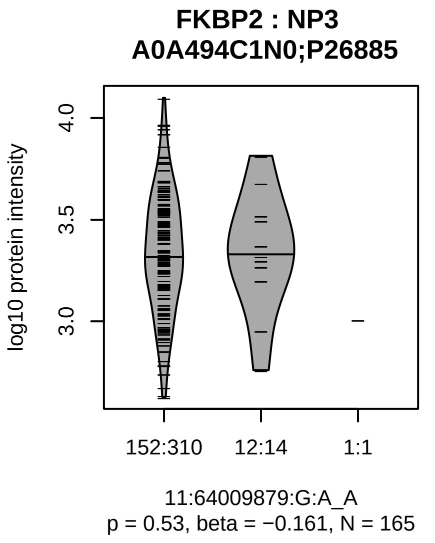

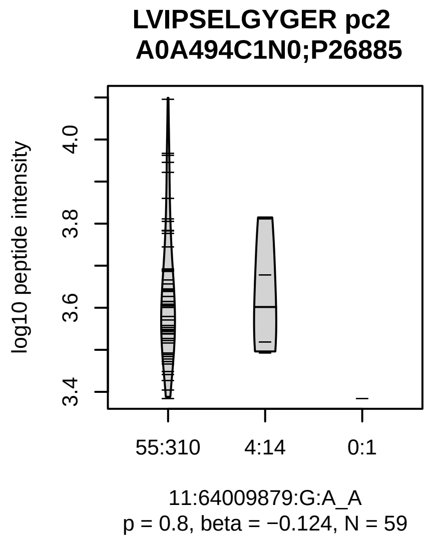

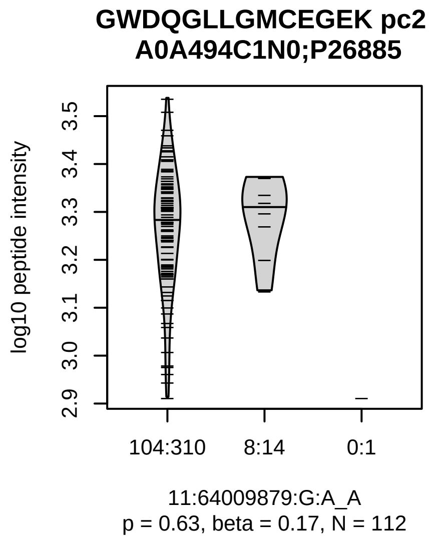

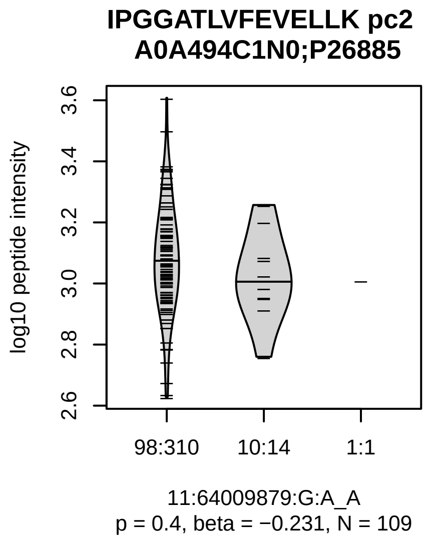

Assay Target: FKBP2
UniProt: P26885
rsID: rs4672
Target SNP: 11:64242407:A:G
Proxy SNP: 11:64009879:G:A
deCODE log10(p): 8.9
deCODE BETA: −0.08
deCODE MSPA: 0
–:–:–:–:–
1251:1077:1136:78:57

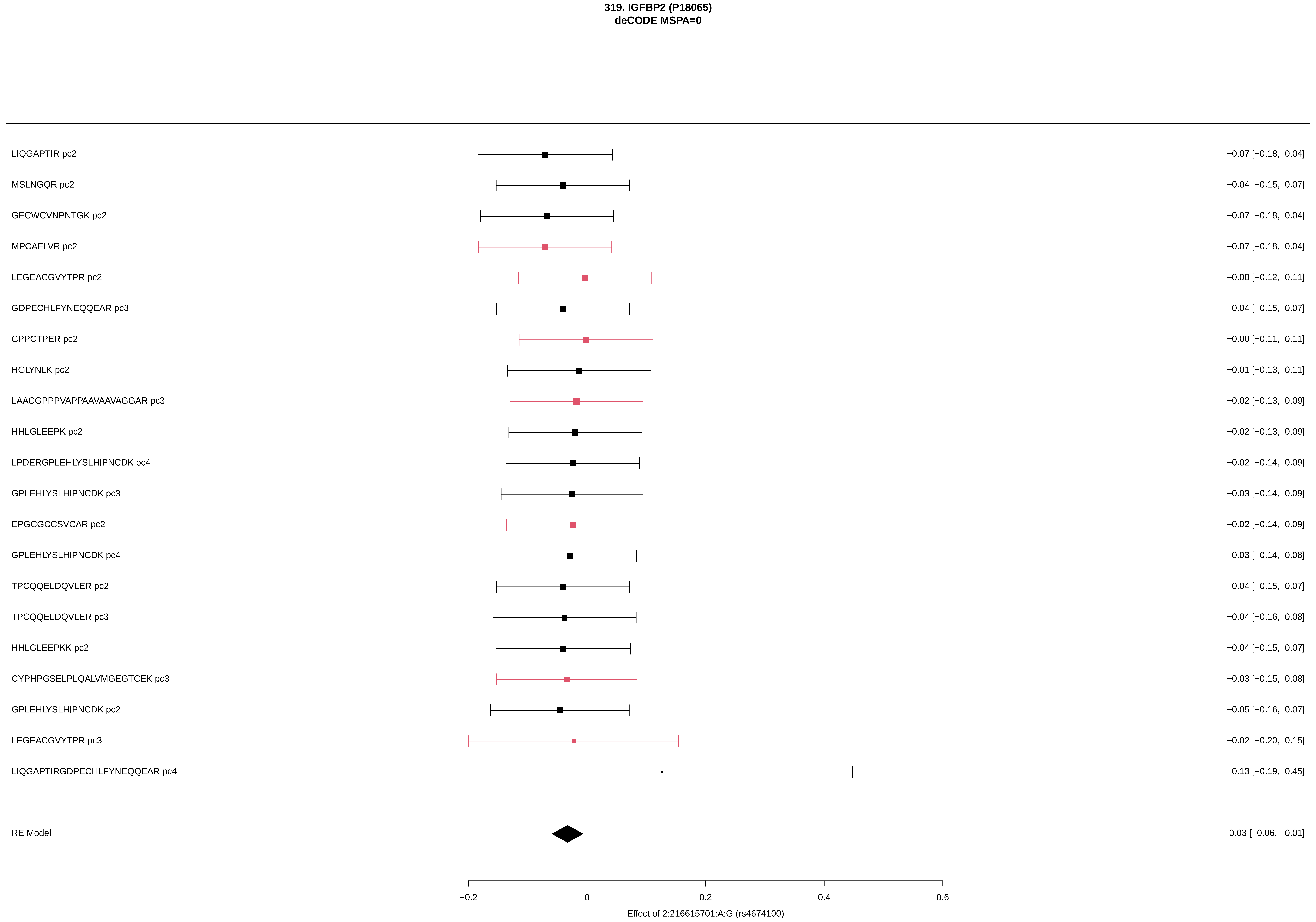

319. IGFBP2 (P18065)
deCODE MSPA=0
LIQGAPTIR pc2
−0.07 [−0.18, 0.04]
MSLNGQR pc2
−0.04 [−0.15, 0.07]
GECWCVNPNTGK pc2
−0.07 [−0.18, 0.04]
MPCAELVR pc2
−0.07 [−0.18, 0.04]
LEGEACGVYTPR pc2
−0.00 [−0.12, 0.11]
GDPECHLFYNEQQEAR pc3
−0.04 [−0.15, 0.07]
CPPCTPER pc2
−0.00 [−0.11, 0.11]
HGLYNLK pc2
−0.01 [−0.13, 0.11]
LAACGPPPVAPPAAVAAVAGGAR pc3
−0.02 [−0.13, 0.09]
HHLGLEEPK pc2
−0.02 [−0.13, 0.09]
LPDERGPLEHLYSLHIPNCDK pc4
−0.02 [−0.14, 0.09]
GPLEHLYSLHIPNCDK pc3
−0.03 [−0.14, 0.09]
EPGCGCCSVCAR pc2
−0.02 [−0.14, 0.09]
GPLEHLYSLHIPNCDK pc4
−0.03 [−0.14, 0.08]
TPCQQELDQVLER pc2
−0.04 [−0.15, 0.07]
TPCQQELDQVLER pc3
−0.04 [−0.16, 0.08]
HHLGLEEPKK pc2
−0.04 [−0.15, 0.07]
CYPHPGSELPLQALVMGEGTCEK pc3
−0.03 [−0.15, 0.08]
GPLEHLYSLHIPNCDK pc2
−0.05 [−0.16, 0.07]
LEGEACGVYTPR pc3
−0.02 [−0.20, 0.15]
LIQGAPTIRGDPECHLFYNEQQEAR pc4
0.13 [−0.19, 0.45]
RE Model
−0.03 [−0.06, −0.01]
−0.2
0
0.2
0.4
0.6
Effect of 2:216615701:A:G (rs4674100)

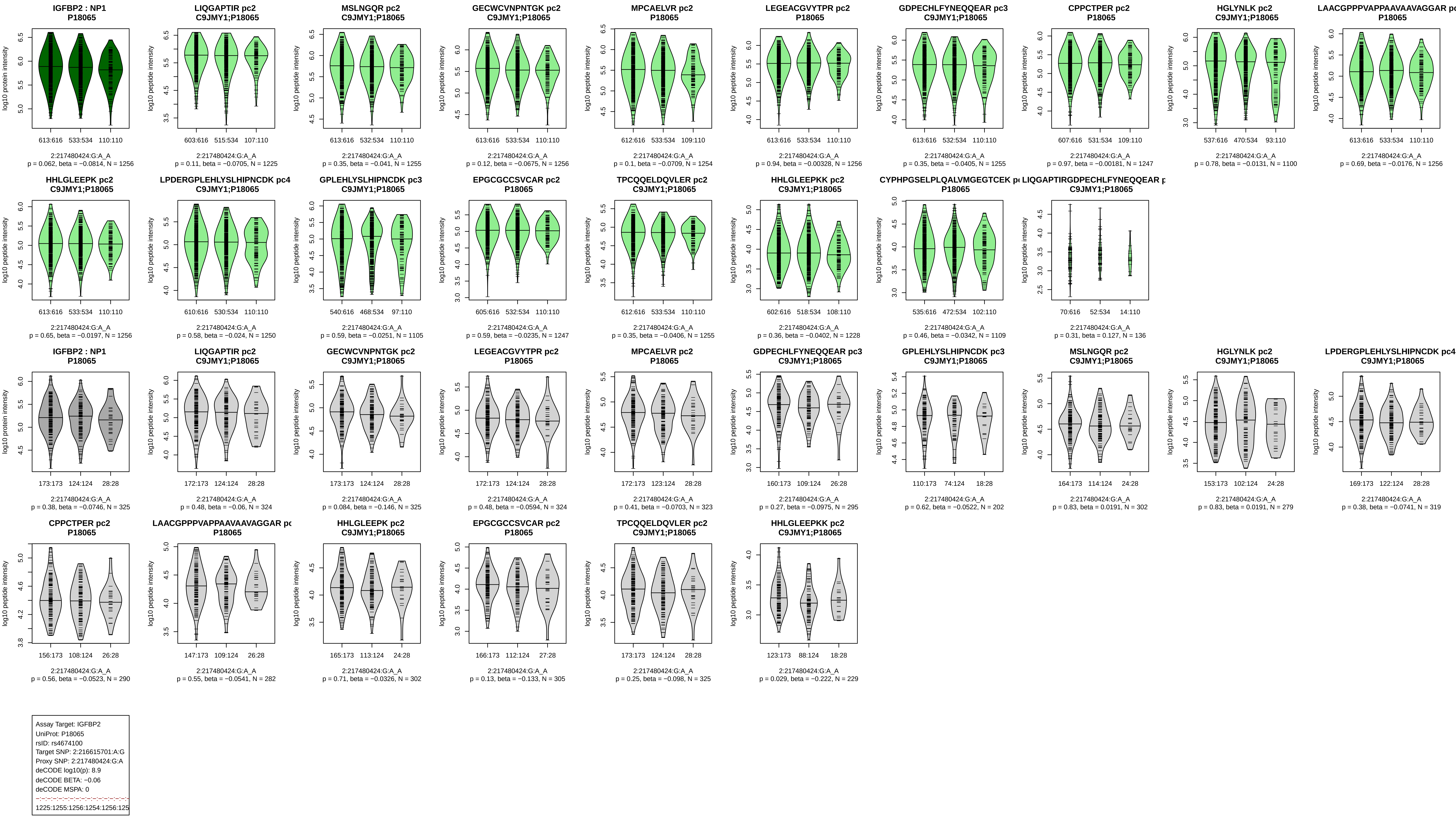
IGFBP2 : NP1
P18065
2:217480424:G:A_A
p = 0.062, beta = −0.0814, N = 1256
LIQGAPTIR pc2
C9JMY1;P18065
2:217480424:G:A_A
p = 0.11, beta = −0.0705, N = 1225
MSLNGQR pc2
C9JMY1;P18065
2:217480424:G:A_A
p = 0.35, beta = −0.041, N = 1255
GECWCVNPNTGK pc2
C9JMY1;P18065
2:217480424:G:A_A
p = 0.12, beta = −0.0675, N = 1256
MPCAELVR pc2
P18065
2:217480424:G:A_A
p = 0.1, beta = −0.0709, N = 1254
LEGEACGVYTPR pc2
P18065
2:217480424:G:A_A
p = 0.94, beta = −0.00328, N = 1256
GDPECHLFYNEQQEAR pc3
C9JMY1;P18065
2:217480424:G:A_A
p = 0.35, beta = −0.0405, N = 1255
CPPCTPER pc2
P18065
2:217480424:G:A_A
p = 0.97, beta = −0.00181, N = 1247
HGLYNLK pc2
C9JMY1;P18065
2:217480424:G:A_A
p = 0.78, beta = −0.0131, N = 1100
LAACGPPPVAPPAAVAAVAGGAR pc
P18065
2:217480424:G:A_A
p = 0.69, beta = −0.0176, N = 1256
HHLGLEEPK pc2
C9JMY1;P18065
2:217480424:G:A_A
p = 0.65, beta = −0.0197, N = 1256
LPDERGPLEHLYSLHIPNCDK pc4
C9JMY1;P18065
2:217480424:G:A_A
p = 0.58, beta = −0.024, N = 1250
GPLEHLYSLHIPNCDK pc3
C9JMY1;P18065
2:217480424:G:A_A
p = 0.59, beta = −0.0251, N = 1105
EPGCGCCSVCAR pc2
P18065
2:217480424:G:A_A
p = 0.59, beta = −0.0235, N = 1247
TPCQQELDQVLER pc2
C9JMY1;P18065
2:217480424:G:A_A
p = 0.35, beta = −0.0406, N = 1255
HHLGLEEPKK pc2
C9JMY1;P18065
2:217480424:G:A_A
p = 0.36, beta = −0.0402, N = 1228
CYPHPGSELPLQALVMGEGTCEK pc
P18065
2:217480424:G:A_A
p = 0.46, beta = −0.0342, N = 1109
LIQGAPTIRGDPECHLFYNEQQEAR p
C9JMY1;P18065
2:217480424:G:A_A
p = 0.31, beta = 0.127, N = 136
IGFBP2 : NP1
P18065
2:217480424:G:A_A
p = 0.38, beta = −0.0746, N = 325
LIQGAPTIR pc2
C9JMY1;P18065
2:217480424:G:A_A
p = 0.48, beta = −0.06, N = 324
GECWCVNPNTGK pc2
C9JMY1;P18065
2:217480424:G:A_A
p = 0.084, beta = −0.146, N = 325
LEGEACGVYTPR pc2
P18065
2:217480424:G:A_A
p = 0.48, beta = −0.0594, N = 324
MPCAELVR pc2
P18065
2:217480424:G:A_A
p = 0.41, beta = −0.0703, N = 323
GDPECHLFYNEQQEAR pc3
C9JMY1;P18065
2:217480424:G:A_A
p = 0.27, beta = −0.0975, N = 295
GPLEHLYSLHIPNCDK pc3
C9JMY1;P18065
2:217480424:G:A_A
p = 0.62, beta = −0.0522, N = 202
MSLNGQR pc2
C9JMY1;P18065
2:217480424:G:A_A
p = 0.83, beta = 0.0191, N = 302
HGLYNLK pc2
C9JMY1;P18065
2:217480424:G:A_A
p = 0.83, beta = 0.0191, N = 279
LPDERGPLEHLYSLHIPNCDK pc4
C9JMY1;P18065
2:217480424:G:A_A
p = 0.38, beta = −0.0741, N = 319
CPPCTPER pc2
P18065
2:217480424:G:A_A
p = 0.56, beta = −0.0523, N = 290
LAACGPPPVAPPAAVAAVAGGAR pc
P18065
2:217480424:G:A_A
p = 0.55, beta = −0.0541, N = 282
HHLGLEEPK pc2
C9JMY1;P18065
2:217480424:G:A_A
p = 0.71, beta = −0.0326, N = 302
EPGCGCCSVCAR pc2
P18065
2:217480424:G:A_A
p = 0.13, beta = −0.133, N = 305
TPCQQELDQVLER pc2
C9JMY1;P18065
2:217480424:G:A_A
p = 0.25, beta = −0.098, N = 325
HHLGLEEPKK pc2
C9JMY1;P18065
2:217480424:G:A_A
p = 0.029, beta = −0.222, N = 229
Assay Target: IGFBP2
UniProt: P18065
rsID: rs4674100
Target SNP: 2:216615701:A:G
Proxy SNP: 2:217480424:G:A
deCODE log10(p): 8.9
deCODE BETA: −0.06
deCODE MSPA: 0
1225:1255:1256:1254:1256:125
