# Supplementary material for: A genome-wide association study of mass spectrometry proteomics using a nanoparticle enrichment platform
Source: Nat Genet. 2025 Nov 27;57(12):2987–96. doi: 10.1038/s41588-025-02413-w (PMC12695657; doi:10.1038/s41588-025-02413-w)
Supplement: Supplementary file 8 — Meta-analysis Forest plots and violin plots of protein and up to 19 peptide levels by genotype for 392 cis-pQTLs identified by the UKB-PPP Olink study. [file 41588_2025_2413_MOESM8_ESM.pdf]

1. GC (P02774)
UKBPPP MSPA=0.715

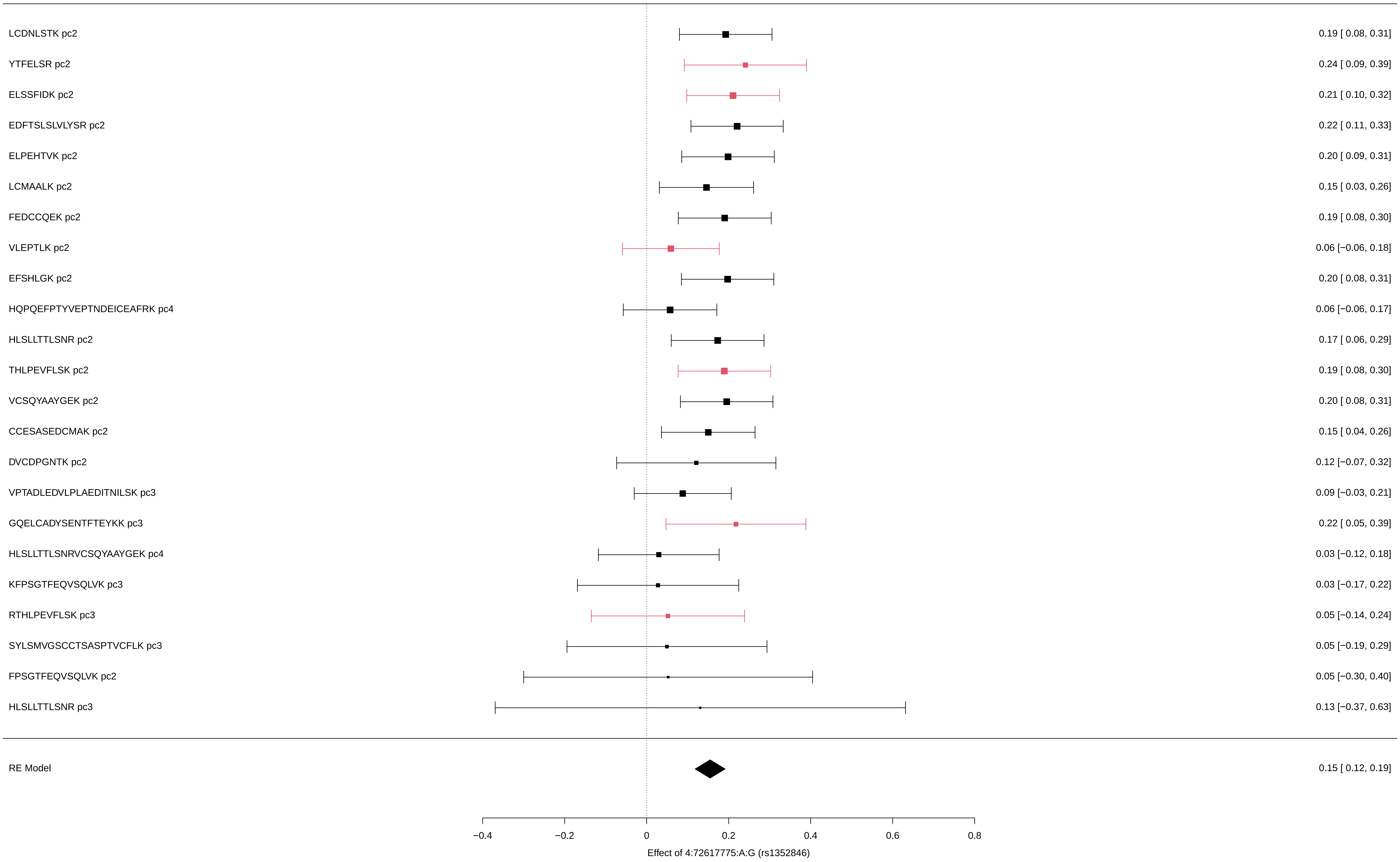

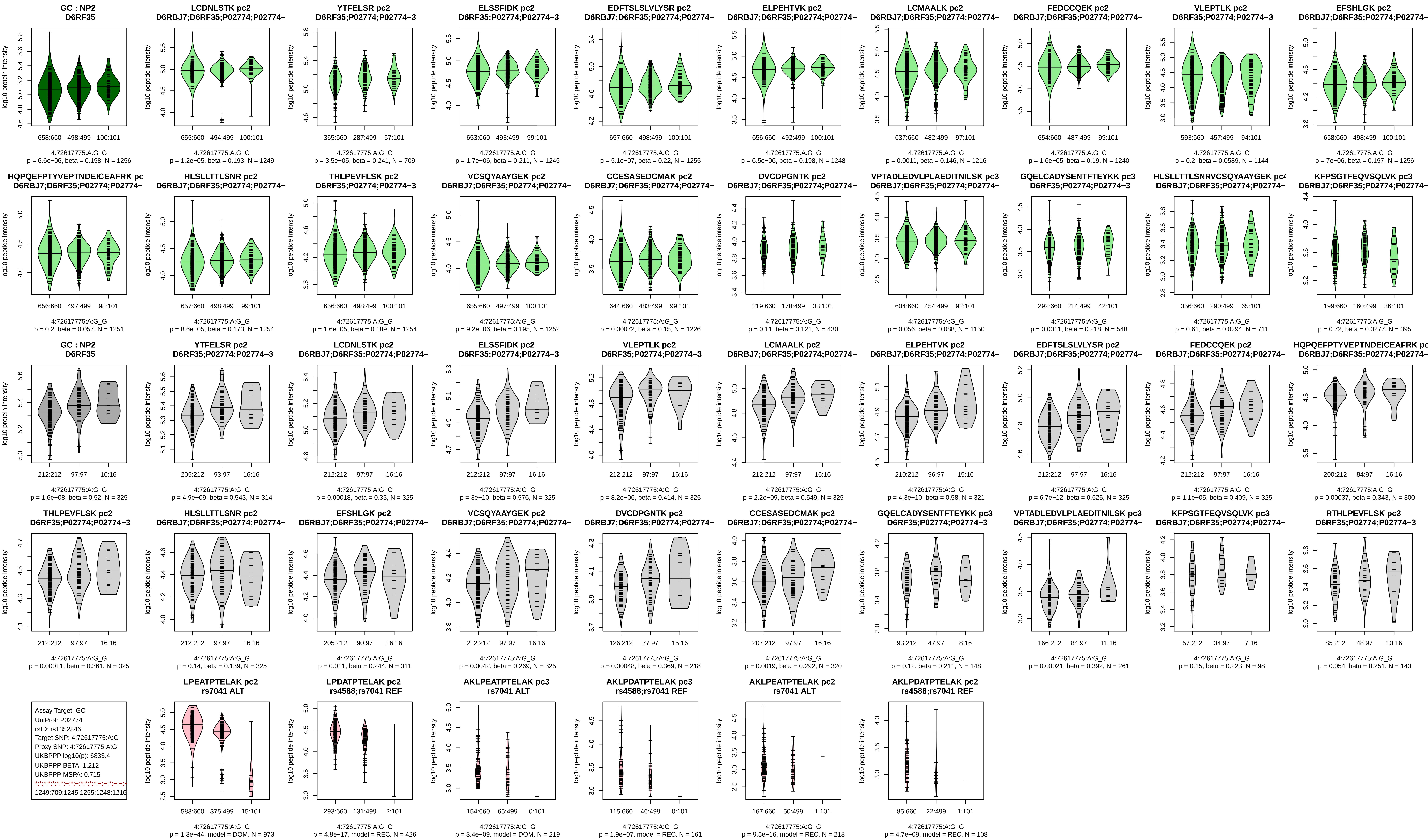
GC : NP2
D6RF35
4:72617775:A:G_G
p = 6.6e-06, beta = 0.198, N = 1256
LCDNLSTK pc2
D6RBJ7;D6RF35;P02774;P02774−
p = 1.2e-05, beta = 0.193, N = 1249
YTFELSR pc2
D6RF35;P02774;P02774−3
p = 3.5e-05, beta = 0.241, N = 709
ELSSFIDK pc2
D6RF35;P02774;P02774−3
p = 1.7e-06, beta = 0.211, N = 1245
EDFTSLSLVLYSR pc2
D6RBJ7;D6RF35;P02774;P02774−
p = 5.1e-07, beta = 0.22, N = 1255
ELPEHTVK pc2
D6RBJ7;D6RF35;P02774;P02774−
p = 6.5e-06, beta = 0.198, N = 1248
LCMAALK pc2
D6RBJ7;D6RF35;P02774;P02774−
p = 0.0011, beta = 0.146, N = 1216
FEDCCQEK pc2
D6RBJ7;D6RF35;P02774;P02774−
p = 1.6e-05, beta = 0.19, N = 1240
VLEPTLK pc2
D6RF35;P02774;P02774−3
p = 0.2, beta = 0.0589, N = 1144
EFSHLGK pc2
D6RBJ7;D6RF35;P02774;P02774−
p = 7e-06, beta = 0.197, N = 1256
HQPQEFPTYVEPTNDEICEAFRK pc
D6RBJ7;D6RF35;P02774;P02774−
p = 0.2, beta = 0.057, N = 1251
HLSLLTTLSNR pc2
D6RBJ7;D6RF35;P02774;P02774−
p = 8.6e-05, beta = 0.173, N = 1254
THLPEVFLSK pc2
D6RF35;P02774;P02774−3
p = 1.6e-05, beta = 0.189, N = 1254
VCSQYAAYGEK pc2
D6RBJ7;D6RF35;P02774;P02774−
p = 9.2e-06, beta = 0.195, N = 1252
CCESASEDCMAK pc2
D6RBJ7;D6RF35;P02774;P02774−
p = 0.00072, beta = 0.15, N = 1226
DVCDPGNTK pc2
D6RBJ7;D6RF35;P02774;P02774−
p = 0.11, beta = 0.121, N = 430
VPTADLEDVLPLAEDITNILSK pc3
D6RBJ7;D6RF35;P02774;P02774−
p = 0.056, beta = 0.088, N = 1150
GQELCADYSENTFTEYKK pc3
D6RF35;P02774;P02774−3
p = 0.0011, beta = 0.218, N = 548
HLSLLTTLSNRVCSQYAAYGEK pc4
D6RBJ7;D6RF35;P02774;P02774−
p = 0.61, beta = 0.0294, N = 711
KFPSGTFEQVSQLVK pc3
D6RBJ7;D6RF35;P02774;P02774−
p = 0.72, beta = 0.0277, N = 395
GC : NP2
D6RF35
p = 1.6e-08, beta = 0.52, N = 325
YTFELSR pc2
D6RF35;P02774;P02774−3
p = 4.9e-09, beta = 0.543, N = 314
LCDNLSTK pc2
D6RBJ7;D6RF35;P02774;P02774−
p = 0.00018, beta = 0.35, N = 325
ELSSFIDK pc2
D6RF35;P02774;P02774−3
p = 3e-10, beta = 0.576, N = 325
VLEPTLK pc2
D6RF35;P02774;P02774−3
p = 8.2e-06, beta = 0.414, N = 325
LCMAALK pc2
D6RBJ7;D6RF35;P02774;P02774−
p = 2.2e-09, beta = 0.549, N = 325
ELPEHTVK pc2
D6RBJ7;D6RF35;P02774;P02774−
p = 4.3e-10, beta = 0.58, N = 321
EDFTSLSLVLYSR pc2
D6RBJ7;D6RF35;P02774;P02774−
p = 6.7e-12, beta = 0.625, N = 325
FEDCCQEK pc2
D6RBJ7;D6RF35;P02774;P02774−
p = 1.1e-05, beta = 0.409, N = 325
HQPQEFPTYVEPTNDEICEAFRK pc
D6RBJ7;D6RF35;P02774;P02774−
p = 0.00037, beta = 0.343, N = 300
THLPEVFLSK pc2
D6RF35;P02774;P02774−3
p = 0.00011, beta = 0.361, N = 325
HLSLLTTLSNR pc2
D6RBJ7;D6RF35;P02774;P02774−
p = 0.14, beta = 0.139, N = 325
EFSHLGK pc2
D6RBJ7;D6RF35;P02774;P02774−
p = 0.011, beta = 0.244, N = 311
VCSQYAAYGEK pc2
D6RBJ7;D6RF35;P02774;P02774−
p = 0.0042, beta = 0.269, N = 325
DVCDPGNTK pc2
D6RBJ7;D6RF35;P02774;P02774−
p = 0.00048, beta = 0.369, N = 218
CCESASEDCMAK pc2
D6RBJ7;D6RF35;P02774;P02774−
p = 0.0019, beta = 0.292, N = 320
GQELCADYSENTFTEYKK pc3
D6RF35;P02774;P02774−3
p = 0.12, beta = 0.211, N = 148
VPTADLEDVLPLAEDITNILSK pc3
D6RBJ7;D6RF35;P02774;P02774−
p = 0.00021, beta = 0.392, N = 261
KFPSGTFEQVSQLVK pc3
D6RBJ7;D6RF35;P02774;P02774−
p = 0.15, beta = 0.223, N = 98
RTHLPEVFLSK pc3
D6RF35;P02774;P02774−3
p = 0.054, beta = 0.251, N = 143
Assay Target: GC
UniProt: P02774
rsID: rs1352846
Target SNP: 4:72617775:A:G
Proxy SNP: 4:72617775:A:G
UKBPPP log10(p): 6833.4
UKBPPP BETA: 1.212
UKBPPP MSPA: 0.715
1249:709:1245:1255:1248:1216
LPEATPTELAK pc2
rs7041 ALT
p = 1.3e-44, model = DOM, N = 973
LPDATPTELAK pc2
rs4588;rs7041 REF
p = 4.8e-17, model = REC, N = 426
AKLPEATPTELAK pc3
rs7041 ALT
p = 3.4e-09, model = DOM, N = 219
AKLPDATPTELAK pc3
rs4588;rs7041 REF
p = 1.9e-07, model = REC, N = 161
AKLPEATPTELAK pc2
rs7041 ALT
p = 9.5e-16, model = REC, N = 218
AKLPDATPTELAK pc2
rs4588;rs7041 REF
p = 4.7e-09, model = REC, N = 108

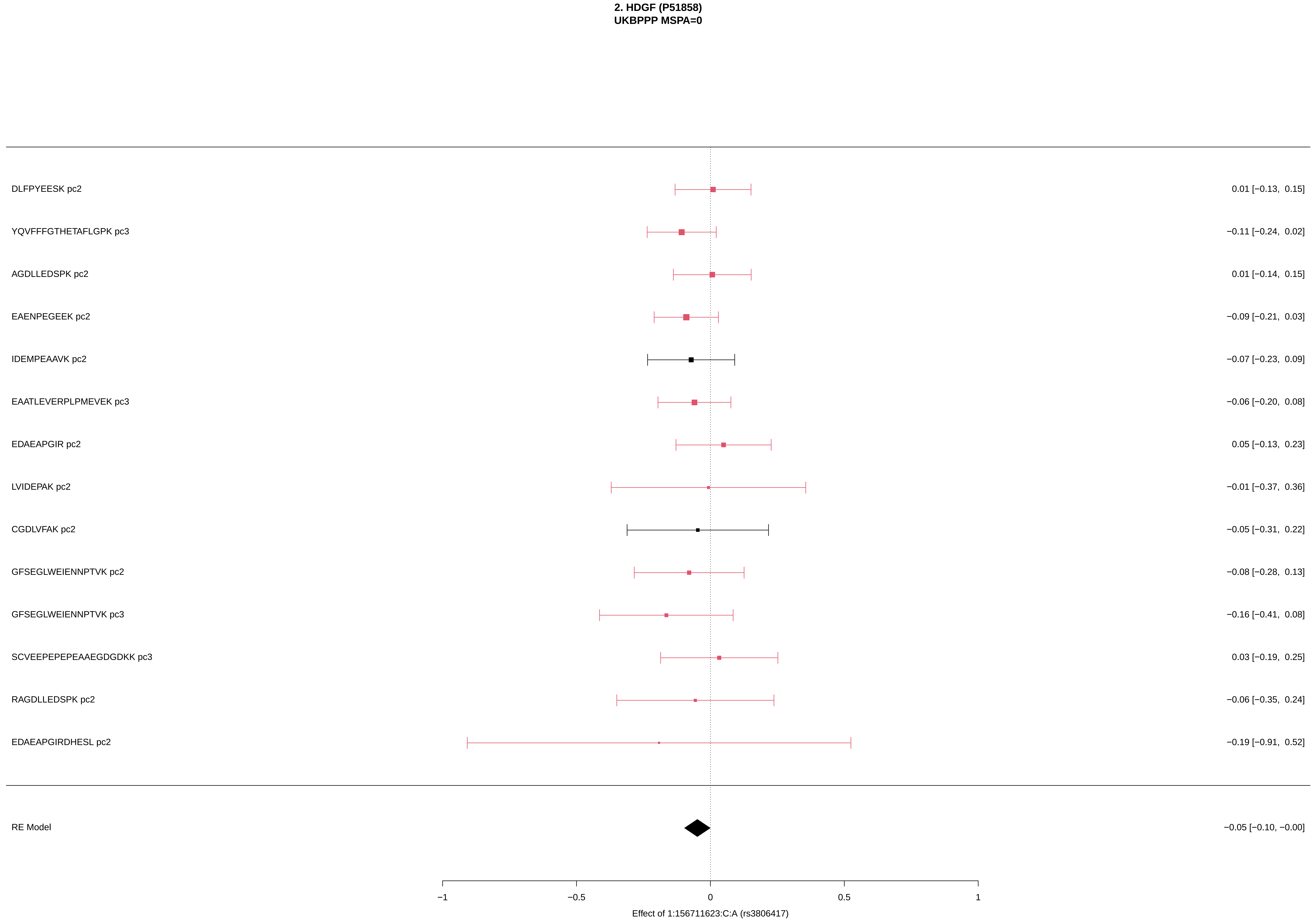
2. HDGF (P51858)
UKBPPP MSPA=0
DLFPYEESK pc2
0.01 [−0.13, 0.15]
YQVFFFGTHETAFLGPK pc3
−0.11 [−0.24, 0.02]
AGDLLEDSPK pc2
0.01 [−0.14, 0.15]
EAENPEGEEK pc2
−0.09 [−0.21, 0.03]
IDEMPEAAVK pc2
−0.07 [−0.23, 0.09]
EAATLEVERPLPMEVEK pc3
−0.06 [−0.20, 0.08]
EDAEAPGIR pc2
0.05 [−0.13, 0.23]
LVIDEPAK pc2
−0.01 [−0.37, 0.36]
CGDLVFAK pc2
−0.05 [−0.31, 0.22]
GFSEGLWEIENNPTVK pc2
−0.08 [−0.28, 0.13]
GFSEGLWEIENNPTVK pc3
−0.16 [−0.41, 0.08]
SCVEEPEPEPEAAEGDGDKK pc3
0.03 [−0.19, 0.25]
RAGDLLEDSPK pc2
−0.06 [−0.35, 0.24]
EDAEAPGIRDHESL pc2
−0.19 [−0.91, 0.52]
RE Model
−0.05 [−0.10, −0.00]
−1
−0.5
0
0.5
1
Effect of 1:156711623:C:A (rs3806417)

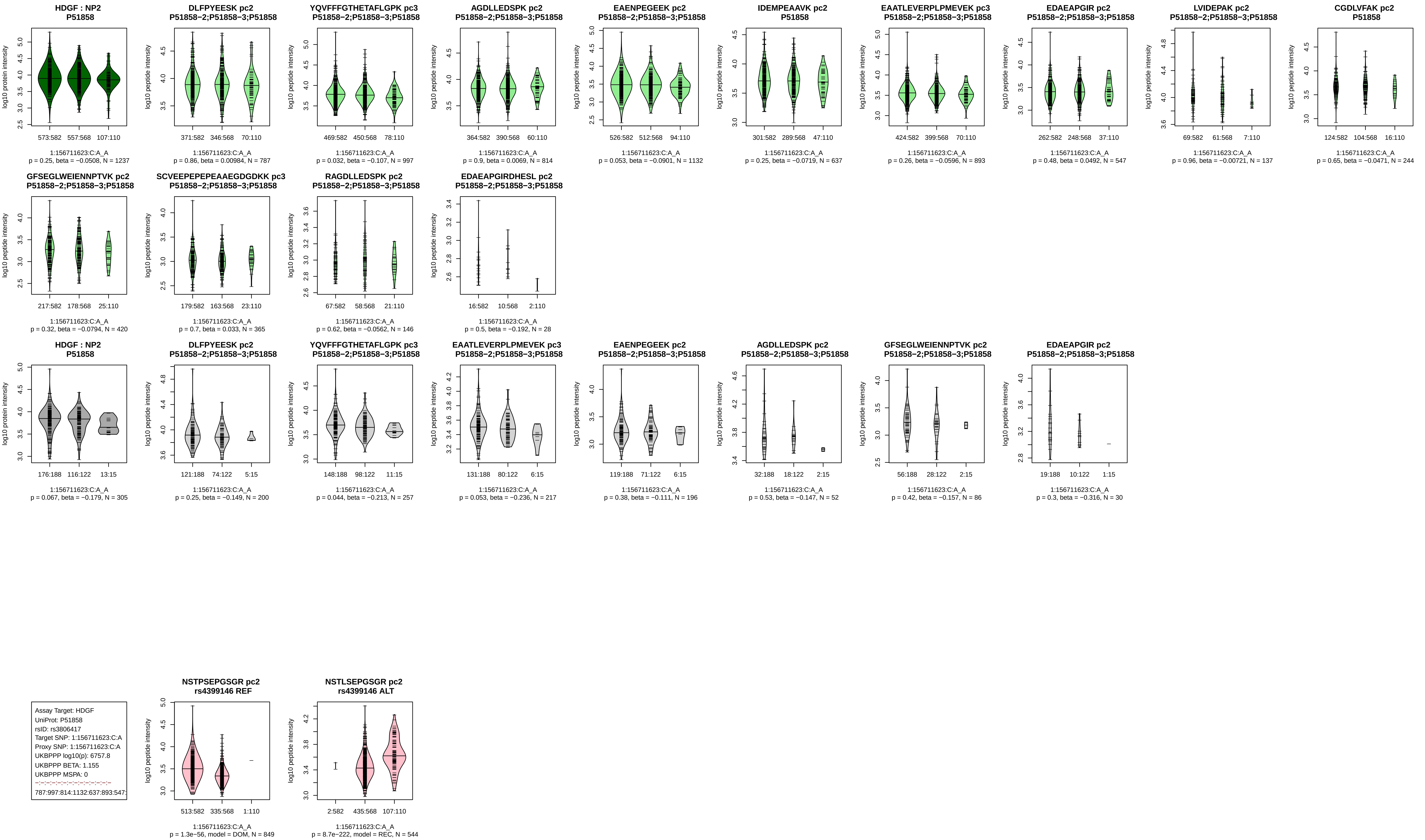
HDGF : NP2
P51858
log10 protein intensity
573:582 557:568 107:110
1:156711623:C:A_A
p = 0.25, beta = −0.0508, N = 1237
DLFPYEESK pc2
P51858−2;P51858−3;P51858
log10 peptide intensity
371:582 346:568 70:110
1:156711623:C:A_A
p = 0.86, beta = 0.00984, N = 787
YQVFFFGTHETAFLGPK pc3
P51858−2;P51858−3;P51858
469:582 450:568 78:110
1:156711623:C:A_A
p = 0.032, beta = −0.107, N = 997
AGDLLEDSPK pc2
P51858−2;P51858−3;P51858
364:582 390:568 60:110
1:156711623:C:A_A
p = 0.9, beta = 0.0069, N = 814
EAENPEGEEK pc2
P51858−2;P51858−3;P51858
526:582 512:568 94:110
1:156711623:C:A_A
p = 0.053, beta = −0.0901, N = 1132
IDEMPEAAVK pc2
P51858
301:582 289:568 47:110
1:156711623:C:A_A
p = 0.25, beta = −0.0719, N = 637
EAATLEVERPLPMEVEK pc3
P51858−2;P51858−3;P51858
424:582 399:568 70:110
1:156711623:C:A_A
p = 0.26, beta = −0.0596, N = 893
EDAEAPGIR pc2
P51858−2;P51858−3;P51858
262:582 248:568 37:110
1:156711623:C:A_A
p = 0.48, beta = 0.0492, N = 547
LVIDEPAK pc2
P51858−2;P51858−3;P51858
69:582 61:568 7:110
1:156711623:C:A_A
p = 0.96, beta = −0.00721, N = 137
CGDLVFAK pc2
P51858
124:582 104:568 16:110
1:156711623:C:A_A
p = 0.65, beta = −0.0471, N = 244
GFSEGLWEIENNPTVK pc2
P51858−2;P51858−3;P51858
217:582 178:568 25:110
1:156711623:C:A_A
p = 0.32, beta = −0.0794, N = 420
SCVEEPEPEPEAAEGDGDKK pc3
P51858−2;P51858−3;P51858
179:582 163:568 23:110
1:156711623:C:A_A
p = 0.7, beta = 0.033, N = 365
RAGDLLEDSPK pc2
P51858−2;P51858−3;P51858
67:582 58:568 21:110
1:156711623:C:A_A
p = 0.62, beta = −0.0562, N = 146
EDAEAPGIRDHESL pc2
P51858−2;P51858−3;P51858
16:582 10:568 2:110
1:156711623:C:A_A
p = 0.5, beta = −0.192, N = 28
HDGF : NP2
P51858
176:188 116:122 13:15
1:156711623:C:A_A
p = 0.067, beta = −0.179, N = 305
DLFPYEESK pc2
P51858−2;P51858−3;P51858
121:188 74:122 5:15
1:156711623:C:A_A
p = 0.25, beta = −0.149, N = 200
YQVFFFGTHETAFLGPK pc3
P51858−2;P51858−3;P51858
148:188 98:122 11:15
1:156711623:C:A_A
p = 0.044, beta = −0.213, N = 257
EAATLEVERPLPMEVEK pc3
P51858−2;P51858−3;P51858
131:188 80:122 6:15
1:156711623:C:A_A
p = 0.053, beta = −0.236, N = 217
EAENPEGEEK pc2
P51858−2;P51858−3;P51858
119:188 71:122 6:15
1:156711623:C:A_A
p = 0.38, beta = −0.111, N = 196
AGDLLEDSPK pc2
P51858−2;P51858−3;P51858
32:188 18:122 2:15
1:156711623:C:A_A
p = 0.53, beta = −0.147, N = 52
GFSEGLWEIENNPTVK pc2
P51858−2;P51858−3;P51858
56:188 28:122 2:15
1:156711623:C:A_A
p = 0.42, beta = −0.157, N = 86
EDAEAPGIR pc2
P51858−2;P51858−3;P51858
19:188 10:122 1:15
1:156711623:C:A_A
p = 0.3, beta = −0.316, N = 30
Assay Target: HDGF
UniProt: P51858
rsID: rs3806417
Target SNP: 1:156711623:C:A
Proxy SNP: 1:156711623:C:A
UKBPPP log10(p): 6757.8
UKBPPP BETA: 1.155
UKBPPP MSPA: 0
787:997:814:1132:637:893:547:
NSTPSEPGSGR pc2
rs4399146 REF
513:582 335:568 1:110
1:156711623:C:A_A
p = 1.3e−56, model = DOM, N = 849
NSTLSEPGSGR pc2
rs4399146 ALT
2:582 435:568 107:110
1:156711623:C:A_A
p = 8.7e−222, model = REC, N = 544

3. APOBR (Q0VD83)
UKBPPP MSPA=0.117

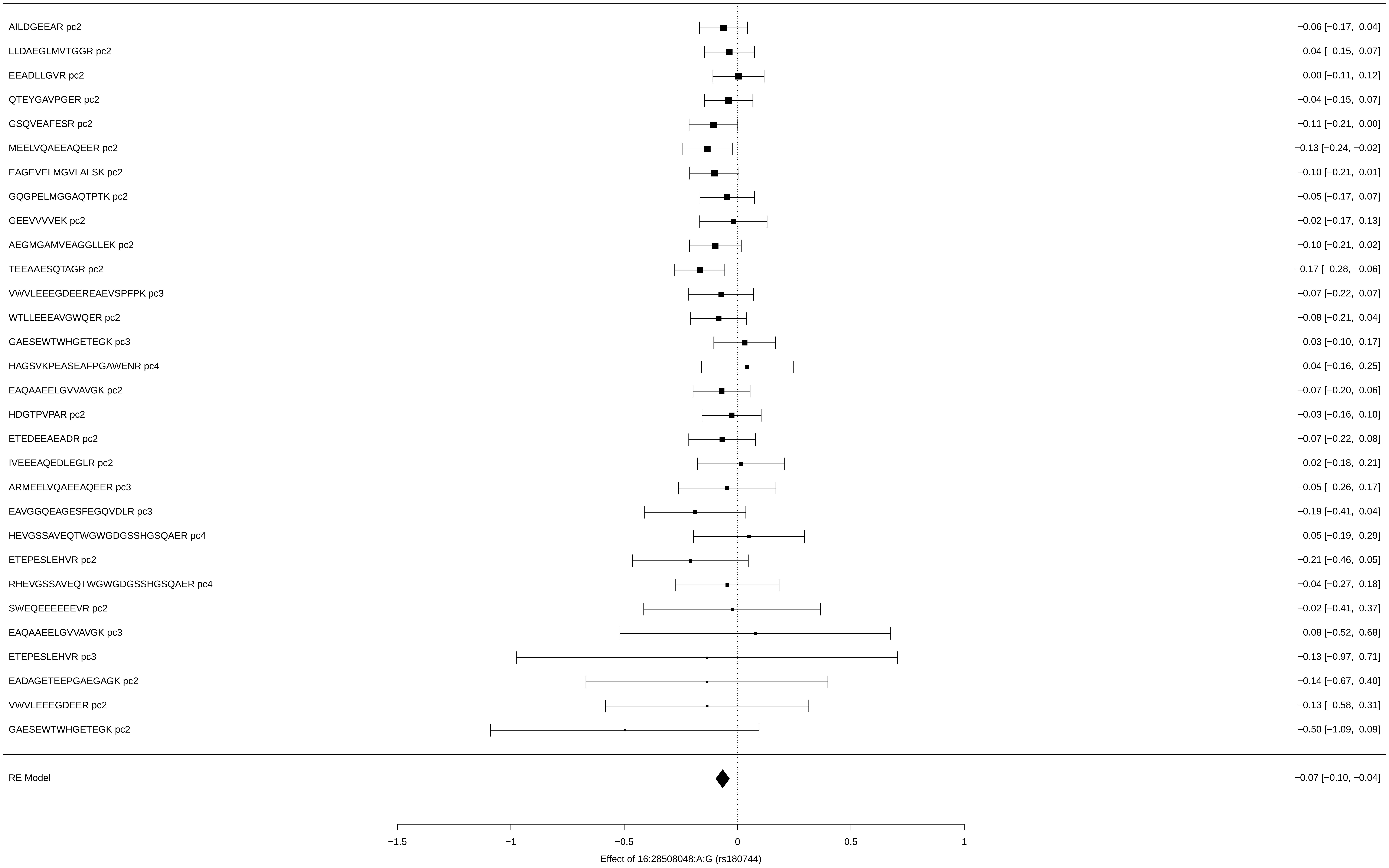

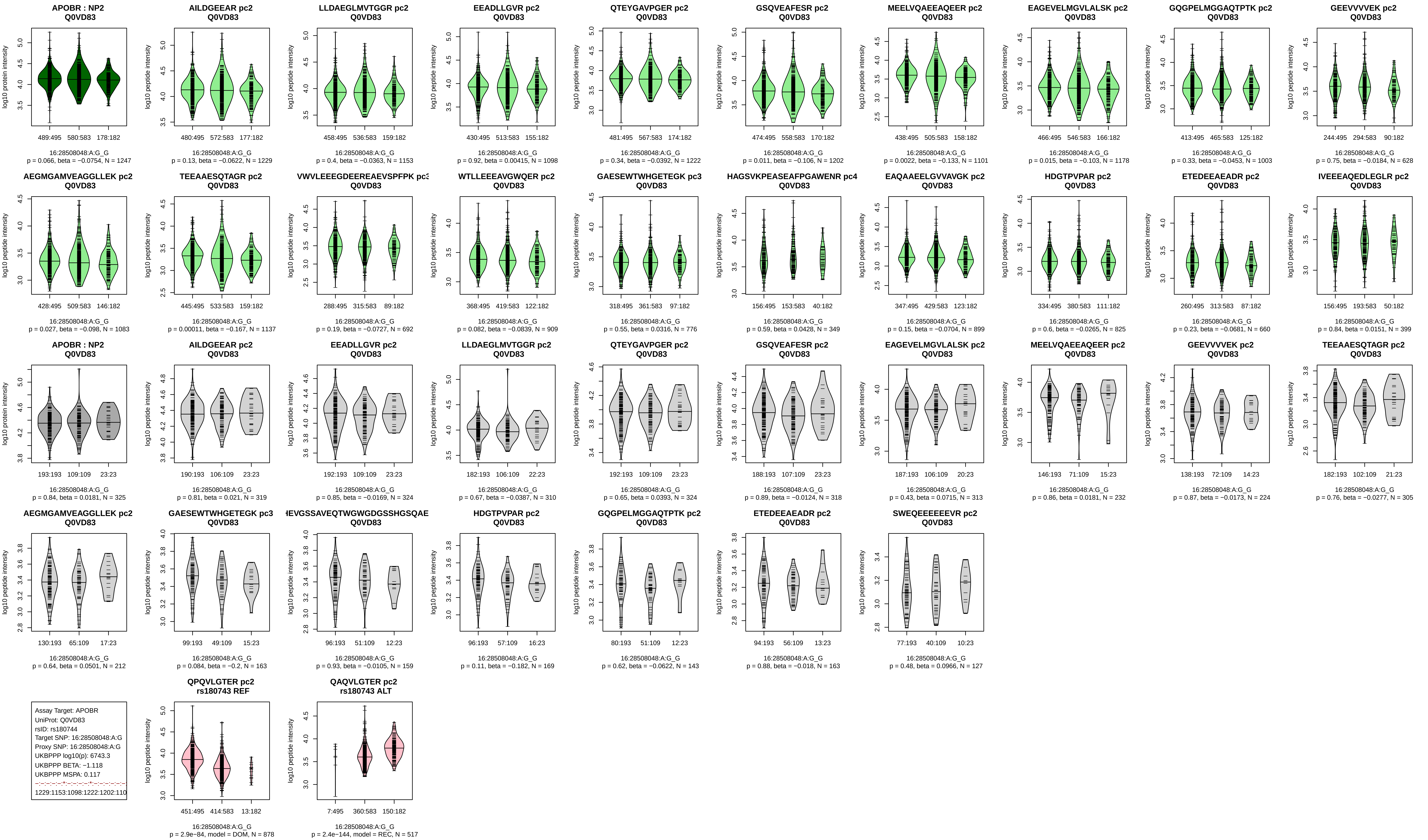
APOBR : NP2 Q0VD83
489:495 580:583 178:182
16:28508048:A:G_G
p = 0.066, beta = −0.0754, N = 1247
AILDGEEAR pc2 Q0VD83
480:495 572:583 177:182
16:28508048:A:G_G
p = 0.13, beta = −0.0622, N = 1229
LLDAEGLMVTGGR pc2 Q0VD83
458:495 536:583 159:182
16:28508048:A:G_G
p = 0.4, beta = −0.0363, N = 1153
EEADLLGVR pc2 Q0VD83
430:495 513:583 155:182
16:28508048:A:G_G
p = 0.92, beta = 0.00415, N = 1098
QTEYGAVPGER pc2 Q0VD83
481:495 567:583 174:182
16:28508048:A:G_G
p = 0.34, beta = −0.0392, N = 1222
GSQVEAFESR pc2 Q0VD83
474:495 558:583 170:182
16:28508048:A:G_G
p = 0.011, beta = −0.106, N = 1202
MEELVQAEEAQEER pc2 Q0VD83
438:495 505:583 158:182
16:28508048:A:G_G
p = 0.0022, beta = −0.133, N = 1101
EAGEVELMGVLALSK pc2 Q0VD83
466:495 546:583 166:182
16:28508048:A:G_G
p = 0.015, beta = −0.103, N = 1178
GQGPELMGGAQTPTK pc2 Q0VD83
413:495 465:583 125:182
16:28508048:A:G_G
p = 0.33, beta = −0.0453, N = 1003
GEEVVVVEK pc2 Q0VD83
244:495 294:583 90:182
16:28508048:A:G_G
p = 0.75, beta = −0.0184, N = 628
AEGMGAMVEAGGLLEK pc2 Q0VD83
428:495 509:583 146:182
16:28508048:A:G_G
p = 0.027, beta = −0.098, N = 1083
TEEAAESQTAGR pc2 Q0VD83
445:495 533:583 159:182
16:28508048:A:G_G
p = 0.00011, beta = −0.167, N = 1137
VWVLEEEGDEEREAEVSPFPK pc3 Q0VD83
288:495 315:583 89:182
16:28508048:A:G_G
p = 0.19, beta = −0.0727, N = 692
WTLLEEEAVGWQER pc2 Q0VD83
368:495 419:583 122:182
16:28508048:A:G_G
p = 0.082, beta = −0.0839, N = 909
GAESEWTWHGETEGK pc3 Q0VD83
318:495 361:583 97:182
16:28508048:A:G_G
p = 0.55, beta = 0.0316, N = 776
HAGSVKPEASEAFPGAWENR pc4 Q0VD83
156:495 153:583 40:182
16:28508048:A:G_G
p = 0.59, beta = 0.0428, N = 349
EAQAAEELGVVAVGK pc2 Q0VD83
347:495 429:583 123:182
16:28508048:A:G_G
p = 0.15, beta = −0.0704, N = 899
HDGTPVPAR pc2 Q0VD83
334:495 380:583 111:182
16:28508048:A:G_G
p = 0.6, beta = −0.0265, N = 825
ETEDEEAEADR pc2 Q0VD83
260:495 313:583 87:182
16:28508048:A:G_G
p = 0.23, beta = −0.0681, N = 660
IVEEEAQEDLEGLR pc2 Q0VD83
156:495 193:583 50:182
16:28508048:A:G_G
p = 0.84, beta = 0.0151, N = 399
APOBR : NP2 Q0VD83
193:193 109:109 23:23
16:28508048:A:G_G
p = 0.84, beta = 0.0181, N = 325
AILDGEEAR pc2 Q0VD83
190:193 106:109 23:23
16:28508048:A:G_G
p = 0.81, beta = 0.021, N = 319
EEADLLGVR pc2 Q0VD83
192:193 109:109 23:23
16:28508048:A:G_G
p = 0.85, beta = −0.0169, N = 324
LLDAEGLMVTGGR pc2 Q0VD83
182:193 106:109 22:23
16:28508048:A:G_G
p = 0.67, beta = −0.0387, N = 310
QTEYGAVPGER pc2 Q0VD83
192:193 109:109 23:23
16:28508048:A:G_G
p = 0.65, beta = 0.0393, N = 324
GSQVEAFESR pc2 Q0VD83
188:193 107:109 23:23
16:28508048:A:G_G
p = 0.89, beta = −0.0124, N = 318
EAGEVELMGVLALSK pc2 Q0VD83
187:193 106:109 20:23
16:28508048:A:G_G
p = 0.43, beta = 0.0715, N = 313
MEELVQAEEAQEER pc2 Q0VD83
146:193 71:109 15:23
16:28508048:A:G_G
p = 0.86, beta = 0.0181, N = 232
GEEVVVVEK pc2 Q0VD83
138:193 72:109 14:23
16:28508048:A:G_G
p = 0.87, beta = −0.0173, N = 224
TEEAAESQTAGR pc2 Q0VD83
182:193 102:109 21:23
16:28508048:A:G_G
p = 0.76, beta = −0.0277, N = 305
AEGMGAMVEAGGLLEK pc2 Q0VD83
130:193 65:109 17:23
16:28508048:A:G_G
p = 0.64, beta = 0.0501, N = 212
GAESEWTWHGETEGK pc3 Q0VD83
99:193 49:109 15:23
16:28508048:A:G_G
p = 0.084, beta = −0.2, N = 163
HEVGSSAVEQTWGWGDGSSHGSQAE Q0VD83
96:193 51:109 12:23
16:28508048:A:G_G
p = 0.93, beta = −0.0105, N = 159
HDGTPVPAR pc2 Q0VD83
96:193 57:109 16:23
16:28508048:A:G_G
p = 0.11, beta = −0.182, N = 169
GQGPELMGGAQTPTK pc2 Q0VD83
80:193 51:109 12:23
16:28508048:A:G_G
p = 0.62, beta = −0.0622, N = 143
ETEDEEAEADR pc2 Q0VD83
94:193 56:109 13:23
16:28508048:A:G_G
p = 0.88, beta = −0.018, N = 163
SWEQEEEEEEVR pc2 Q0VD83
77:193 40:109 10:23
16:28508048:A:G_G
p = 0.48, beta = 0.0966, N = 127
Assay Target: APOBR
UniProt: Q0VD83
rsID: rs180744
Target SNP: 16:28508048:A:G
Proxy SNP: 16:28508048:A:G
UKBPPP log10(p): 6743.3
UKBPPP BETA: −1.118
UKBPPP MSPA: 0.117
1229:1153:1098:1222:1202:110
QPQVLGTER pc2 rs180743 REF
451:495 414:583 13:182
16:28508048:A:G_G
p = 2.9e−84, model = DOM, N = 878
QAQVLGTER pc2 rs180743 ALT
7:495 360:583 150:182
16:28508048:A:G_G
p = 2.4e−144, model = REC, N = 517
log10 protein intensity
log10 peptide intensity

4. F12 (P00748)
UKBPPP MSPA=0.925

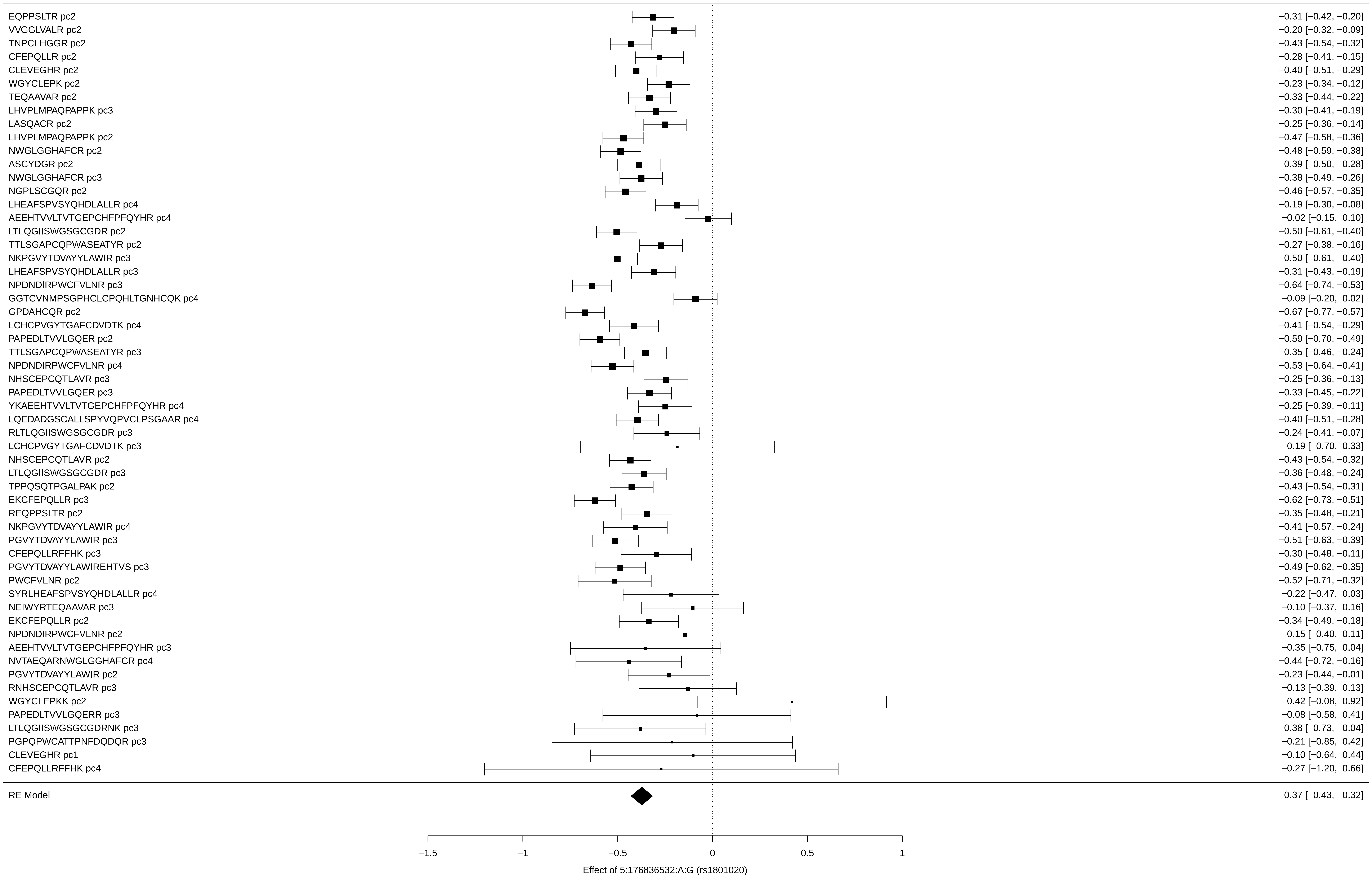

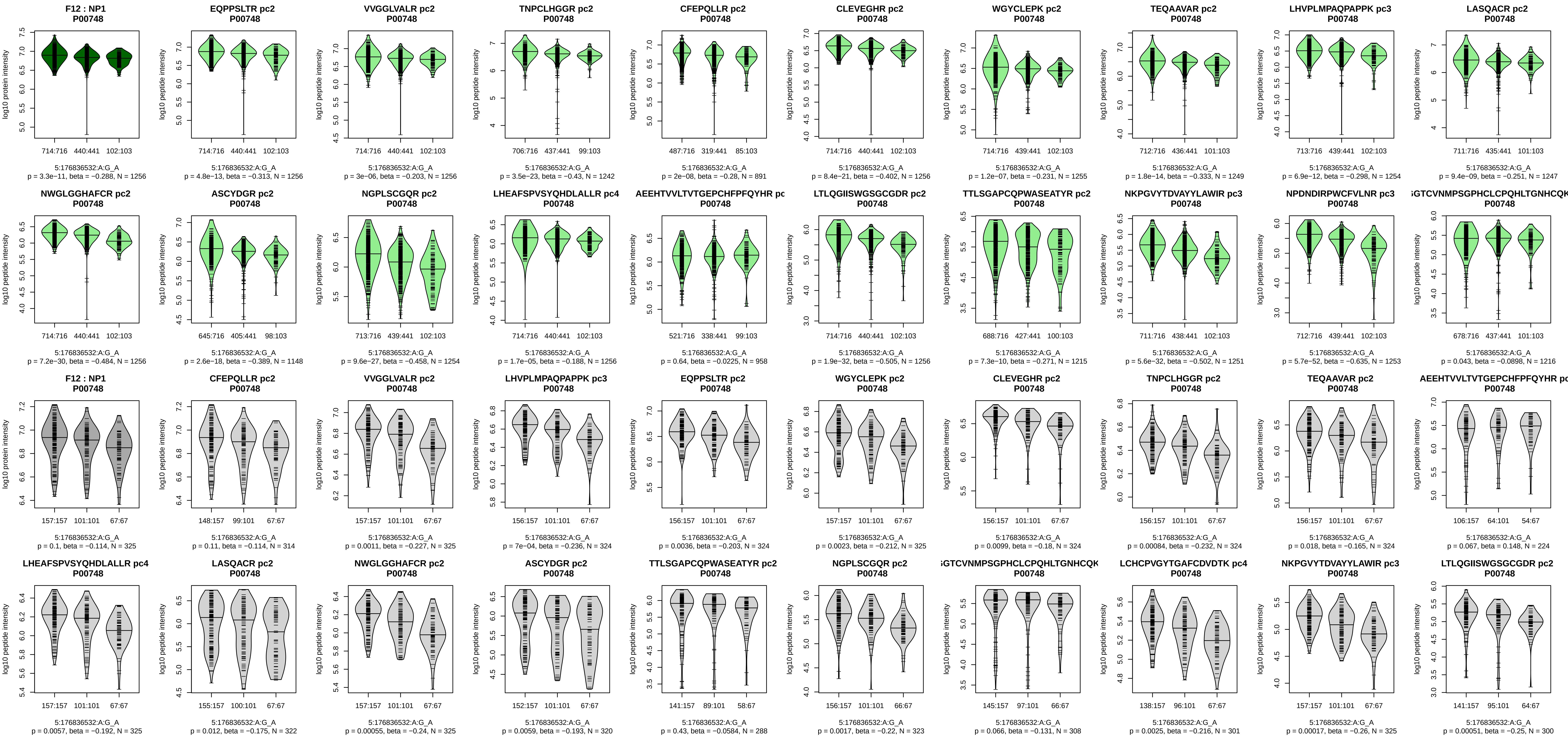

Assay Target: F12
UniProt: P00748
rsID: rs1801020
Target SNP: 5:176836532:A:G
Proxy SNP: 5:176836532:A:G
UKBPPP log10(p): 6066.9
UKBPPP BETA: 1.167
UKBPPP MSPA: 0.925
1256:1256:1242:891:1256:1255

5. PDCD6 (O75340)
UKBPPP MSPA=0

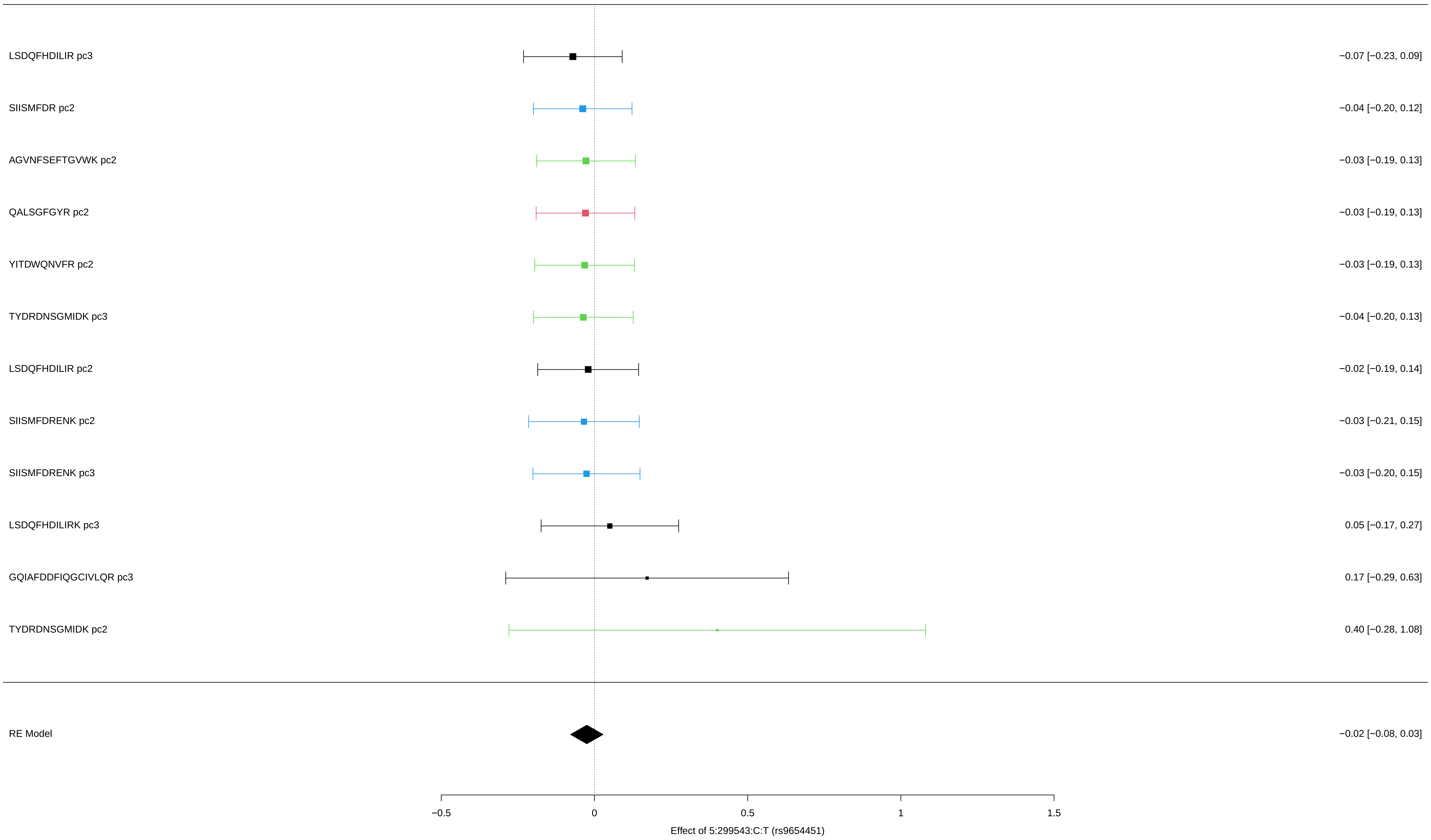

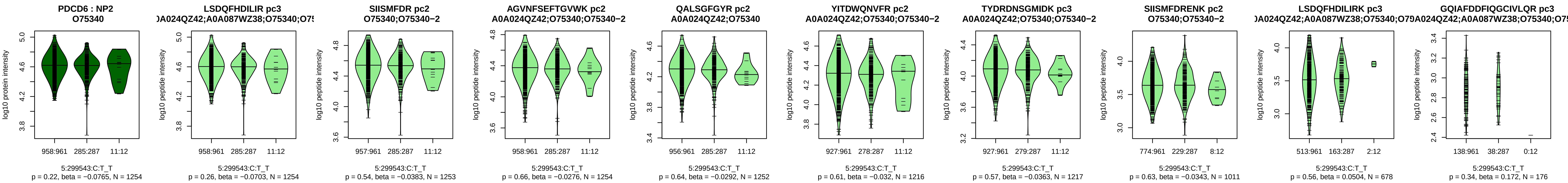

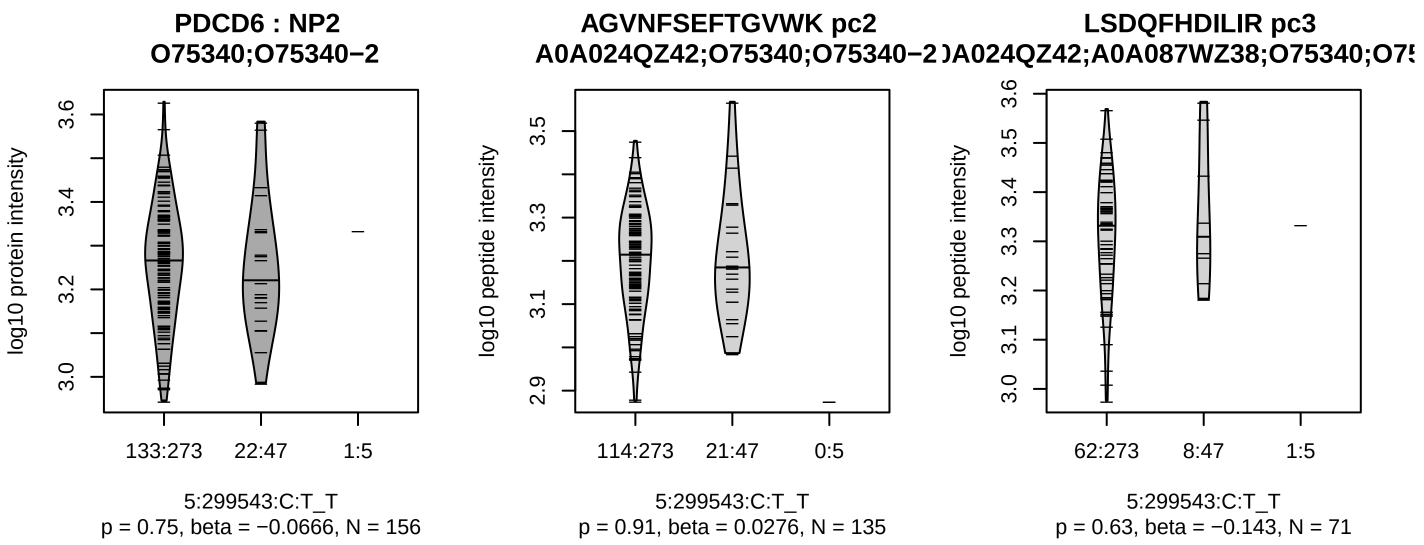

Assay Target: PDCD6
UniProt: O75340
rsID: rs9654451
Target SNP: 5:299543:C:T
Proxy SNP: 5:299543:C:T
UKBPPP log10(p): 5682.7
UKBPPP BETA: −1.506
UKBPPP MSPA: 0
−:−:−:−:−:−:−:−:−:−
1254:1253:1254:1252:1216:121

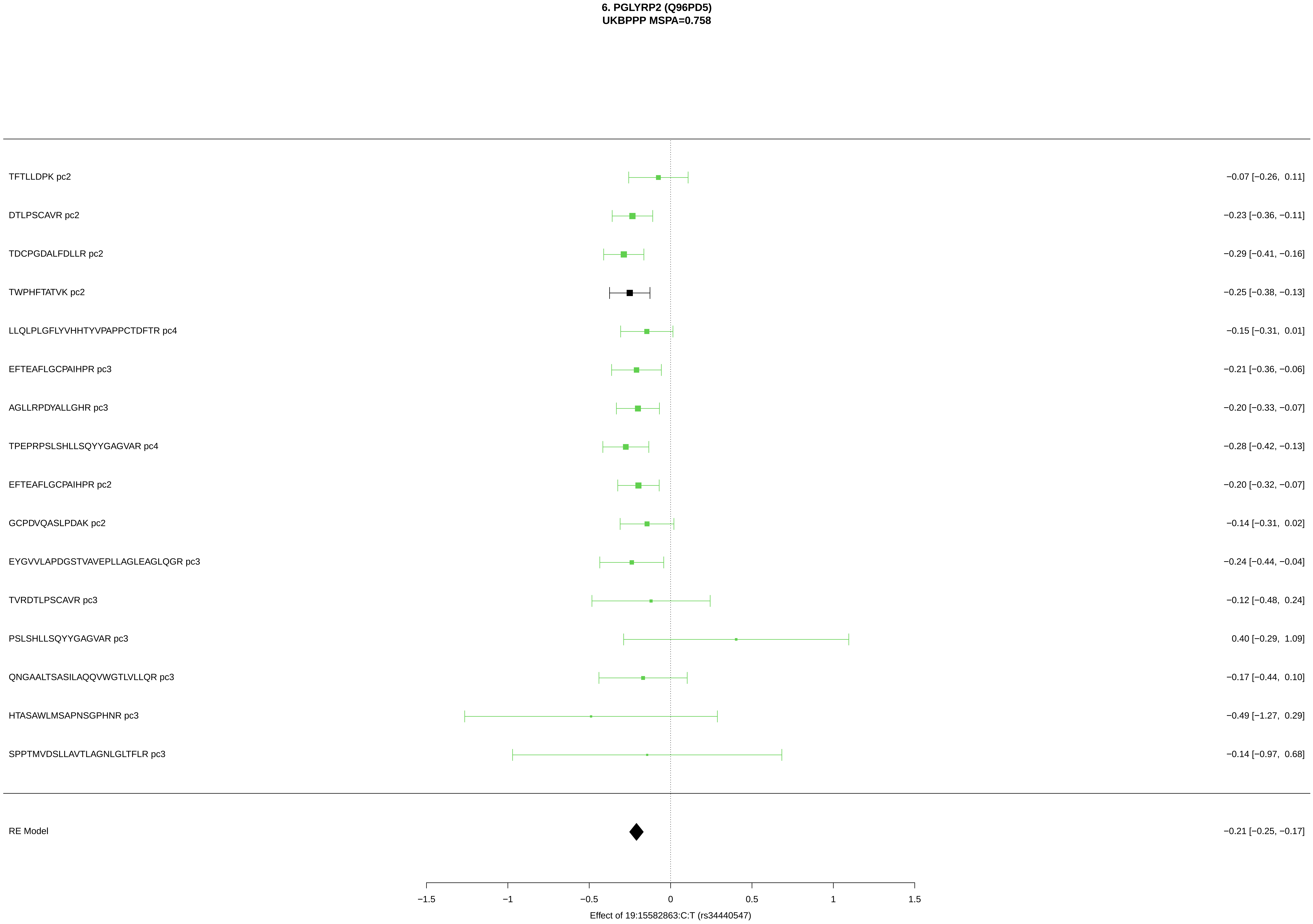

6. PGLYRP2 (Q96PD5)
UKBPPP MSPA=0.758
TFTLLDPK pc2
−0.07 [−0.26, 0.11]
DTLPSCAVR pc2
−0.23 [−0.36, −0.11]
TDCPGDALFDLLR pc2
−0.29 [−0.41, −0.16]
TWPHFTATVK pc2
−0.25 [−0.38, −0.13]
LLQLPLGFLYVHHTYVPAPPCTDFTR pc4
−0.15 [−0.31, 0.01]
EFTEAFLGCPAIHPR pc3
−0.21 [−0.36, −0.06]
AGLLRPDYALLGHR pc3
−0.20 [−0.33, −0.07]
TPEPRPSLSHLLSQYYGAGVAR pc4
−0.28 [−0.42, −0.13]
EFTEAFLGCPAIHPR pc2
−0.20 [−0.32, −0.07]
GCPDVQASLPDAK pc2
−0.14 [−0.31, 0.02]
EYGVVLAPDGSTVAVEPLLAGLEAGLQGR pc3
−0.24 [−0.44, −0.04]
TVRDTLPSCAVR pc3
−0.12 [−0.48, 0.24]
PSLSHLLSQYYGAGVAR pc3
0.40 [−0.29, 1.09]
QNGAALTSASILAQQVWGTLVLLQR pc3
−0.17 [−0.44, 0.10]
HTASAWLMSAPNSGPHNR pc3
−0.49 [−1.27, 0.29]
SPPTMVDSLLAVTLAGNLGLTFLR pc3
−0.14 [−0.97, 0.68]
RE Model
−0.21 [−0.25, −0.17]
−1.5
−1
−0.5
0
0.5
1
1.5
Effect of 19:15582863:C:T (rs34440547)

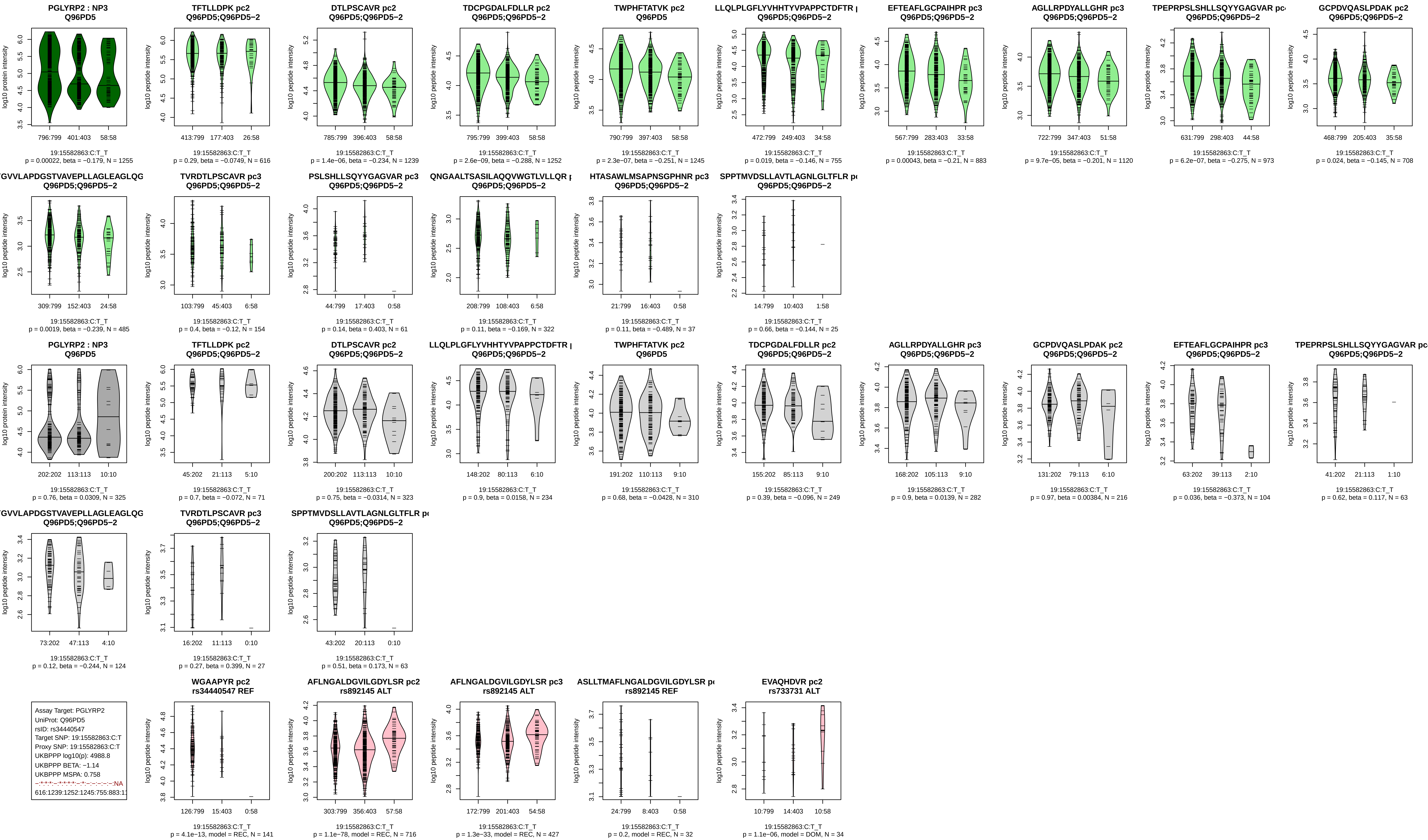
PGLYRP2 : NP3
Q96PD5
log10 protein intensity
796:799 401:403 58:58
19:15582863:C:T_T
p = 0.00022, beta = −0.179, N = 1255
TFTLLDPK pc2
Q96PD5;Q96PD5−2
log10 peptide intensity
413:799 177:403 26:58
19:15582863:C:T_T
p = 0.29, beta = −0.0749, N = 616
DTLPSCAVR pc2
Q96PD5;Q96PD5−2
785:799 396:403 58:58
19:15582863:C:T_T
p = 1.4e−06, beta = −0.234, N = 1239
TDCPGDALFDLLR pc2
Q96PD5;Q96PD5−2
795:799 399:403 58:58
19:15582863:C:T_T
p = 2.6e−09, beta = −0.288, N = 1252
TWPHFTATVK pc2
Q96PD5
790:799 397:403 58:58
19:15582863:C:T_T
p = 2.3e−07, beta = −0.251, N = 1245
Q96PD5;Q96PD5−2
472:799 249:403 34:58
19:15582863:C:T_T
p = 0.019, beta = −0.146, N = 755
EFTEAFLGCPAIHPR pc3
Q96PD5;Q96PD5−2
567:799 283:403 33:58
19:15582863:C:T_T
p = 0.00043, beta = −0.21, N = 883
AGLLRPDYALLGHR pc3
Q96PD5;Q96PD5−2
722:799 347:403 51:58
19:15582863:C:T_T
p = 9.7e−05, beta = −0.201, N = 1120
Q96PD5;Q96PD5−2
631:799 298:403 44:58
19:15582863:C:T_T
p = 6.2e−07, beta = −0.275, N = 973
GCPDVQASLPDAK pc2
Q96PD5;Q96PD5−2
468:799 205:403 35:58
19:15582863:C:T_T
p = 0.024, beta = −0.145, N = 708
Q96PD5;Q96PD5−2
309:799 152:403 24:58
19:15582863:C:T_T
p = 0.0019, beta = −0.239, N = 485
TVRDTLPSCAVR pc3
Q96PD5;Q96PD5−2
103:799 45:403 6:58
19:15582863:C:T_T
p = 0.4, beta = −0.12, N = 154
PSLSHLLSQYYGAGVAR pc3
Q96PD5;Q96PD5−2
44:799 17:403 0:58
19:15582863:C:T_T
p = 0.14, beta = 0.403, N = 61
Q96PD5;Q96PD5−2
208:799 108:403 6:58
19:15582863:C:T_T
p = 0.11, beta = −0.169, N = 322
HTASAWLMSAPNSGPHNR pc3
Q96PD5;Q96PD5−2
21:799 16:403 0:58
19:15582863:C:T_T
p = 0.11, beta = −0.489, N = 37
Q96PD5;Q96PD5−2
14:799 10:403 1:58
19:15582863:C:T_T
p = 0.66, beta = −0.144, N = 25
PGLYRP2 : NP3
Q96PD5
202:202 113:113 10:10
19:15582863:C:T_T
p = 0.76, beta = 0.0309, N = 325
TFTLLDPK pc2
Q96PD5;Q96PD5−2
45:202 21:113 5:10
19:15582863:C:T_T
p = 0.7, beta = −0.072, N = 71
DTLPSCAVR pc2
Q96PD5;Q96PD5−2
200:202 113:113 10:10
19:15582863:C:T_T
p = 0.75, beta = −0.0314, N = 323
Q96PD5;Q96PD5−2
148:202 80:113 6:10
19:15582863:C:T_T
p = 0.9, beta = 0.0158, N = 234
TWPHFTATVK pc2
Q96PD5
191:202 110:113 9:10
19:15582863:C:T_T
p = 0.68, beta = −0.0428, N = 310
TDCPGDALFDLLR pc2
Q96PD5;Q96PD5−2
155:202 85:113 9:10
19:15582863:C:T_T
p = 0.39, beta = −0.096, N = 249
AGLLRPDYALLGHR pc3
Q96PD5;Q96PD5−2
168:202 105:113 9:10
19:15582863:C:T_T
p = 0.9, beta = 0.0139, N = 282
GCPDVQASLPDAK pc2
Q96PD5;Q96PD5−2
131:202 79:113 6:10
19:15582863:C:T_T
p = 0.97, beta = 0.00384, N = 216
EFTEAFLGCPAIHPR pc3
Q96PD5;Q96PD5−2
63:202 39:113 2:10
19:15582863:C:T_T
p = 0.036, beta = −0.373, N = 104
Q96PD5;Q96PD5−2
41:202 21:113 1:10
19:15582863:C:T_T
p = 0.62, beta = 0.117, N = 63
Q96PD5;Q96PD5−2
73:202 47:113 4:10
19:15582863:C:T_T
p = 0.12, beta = −0.244, N = 124
TVRDTLPSCAVR pc3
Q96PD5;Q96PD5−2
16:202 11:113 0:10
19:15582863:C:T_T
p = 0.27, beta = 0.399, N = 27
Q96PD5;Q96PD5−2
43:202 20:113 0:10
19:15582863:C:T_T
p = 0.51, beta = 0.173, N = 63
Assay Target: PGLYRP2
UniProt: Q96PD5
rsID: rs34440547
Target SNP: 19:15582863:C:T
Proxy SNP: 19:15582863:C:T
UKBPPP log10(p): 4988.8
UKBPPP BETA: −1.14
UKBPPP MSPA: 0.758
WGAAPYR pc2
rs34440547 REF
126:799 15:403 0:58
19:15582863:C:T_T
p = 4.1e−13, model = REC, N = 141
AFLNGALDGVILGDYLSR pc2
rs892145 ALT
303:799 356:403 57:58
19:15582863:C:T_T
p = 1.1e−78, model = REC, N = 716
AFLNGALDGVILGDYLSR pc3
rs892145 ALT
172:799 201:403 54:58
19:15582863:C:T_T
p = 1.3e−33, model = REC, N = 427
rs892145 REF
24:799 8:403 0:58
19:15582863:C:T_T
p = 0.2, model = REC, N = 32
EVAQHDVR pc2
rs733731 ALT
10:799 14:403 10:58
19:15582863:C:T_T
p = 1.1e−06, model = DOM, N = 34

7. FCGR2A (P12318)
UKBPPP MSPA=0

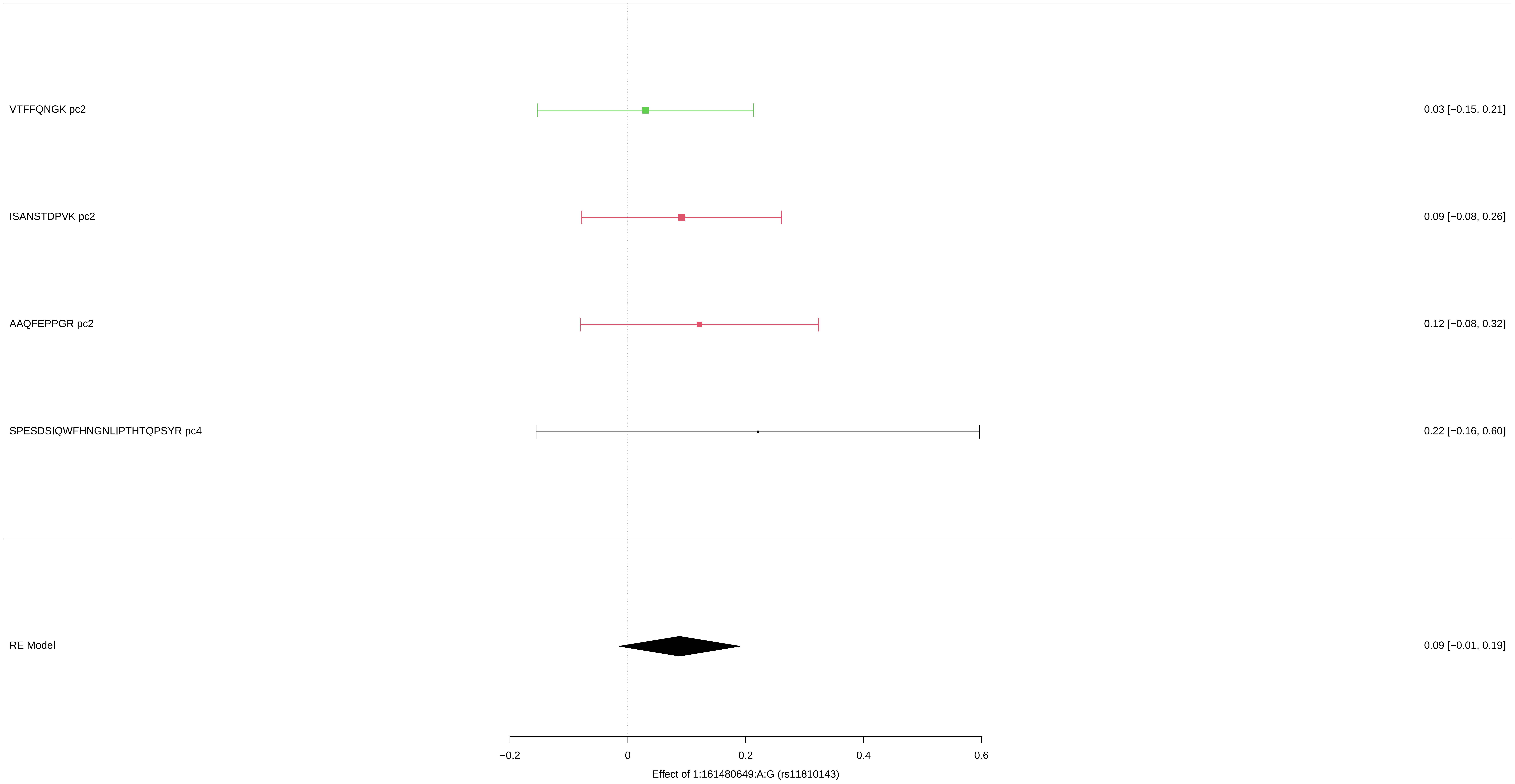

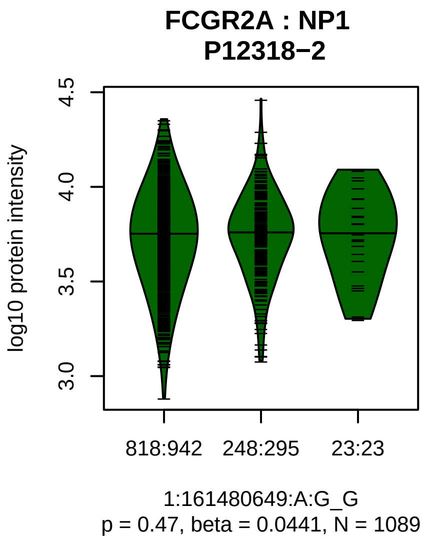

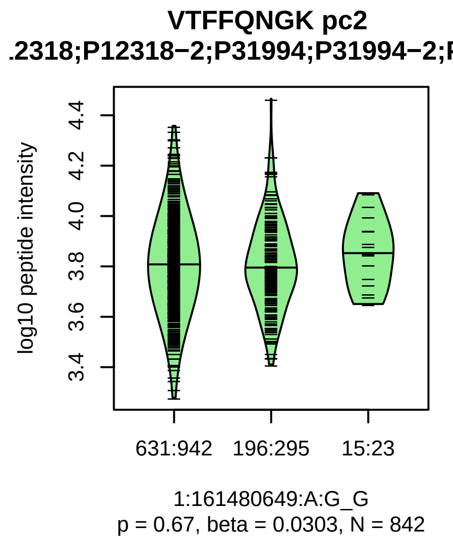

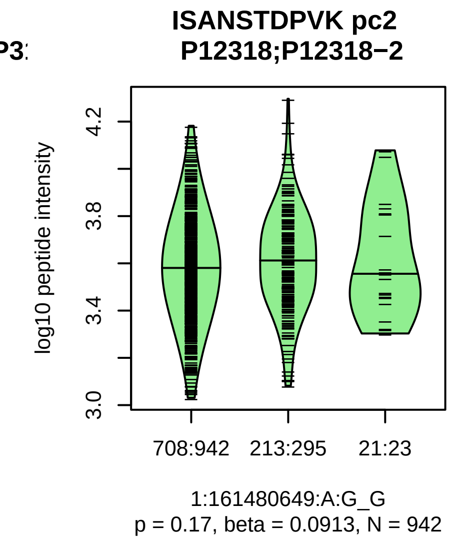

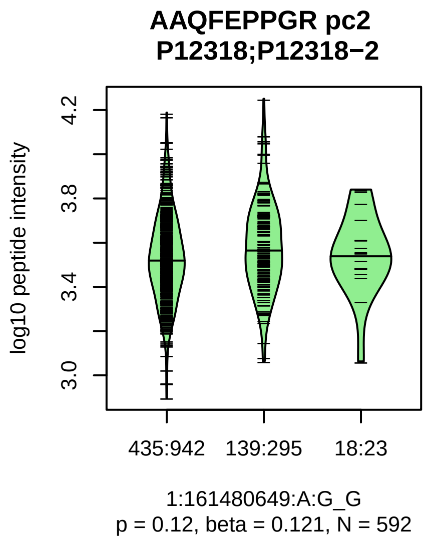

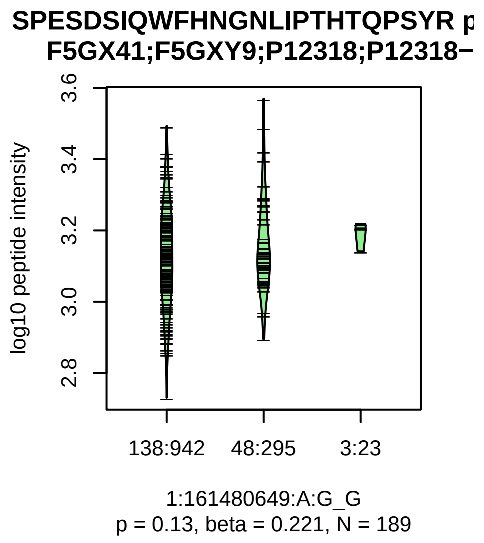

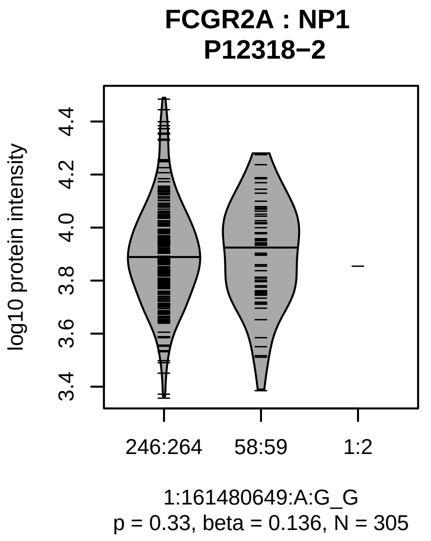

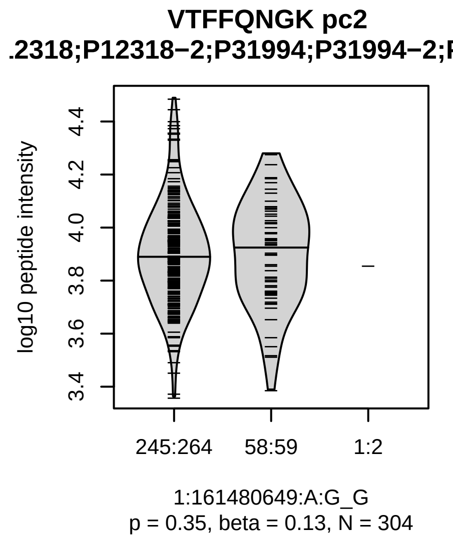

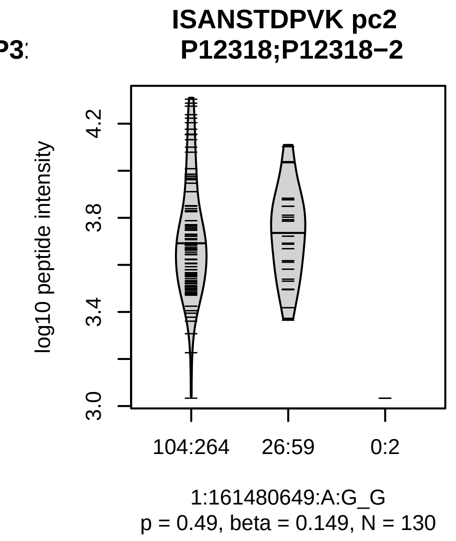

Assay Target: FCGR2A
UniProt: P12318
rsID: rs11810143
Target SNP: 1:161480649:A:G
Proxy SNP: 1:161480649:A:G
UKBPPP log10(p): 4980.3
UKBPPP BETA: −1.453
UKBPPP MSPA: 0
−:−:−:−:NA
842:942:592:189:12

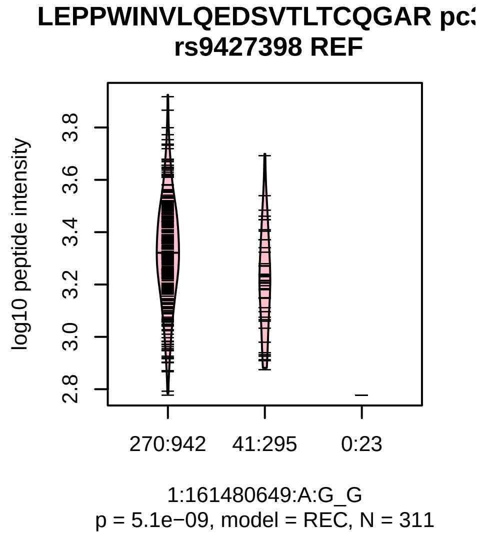

8. C1QTNF9 (P0C862)
UKBPPP MSPA=1

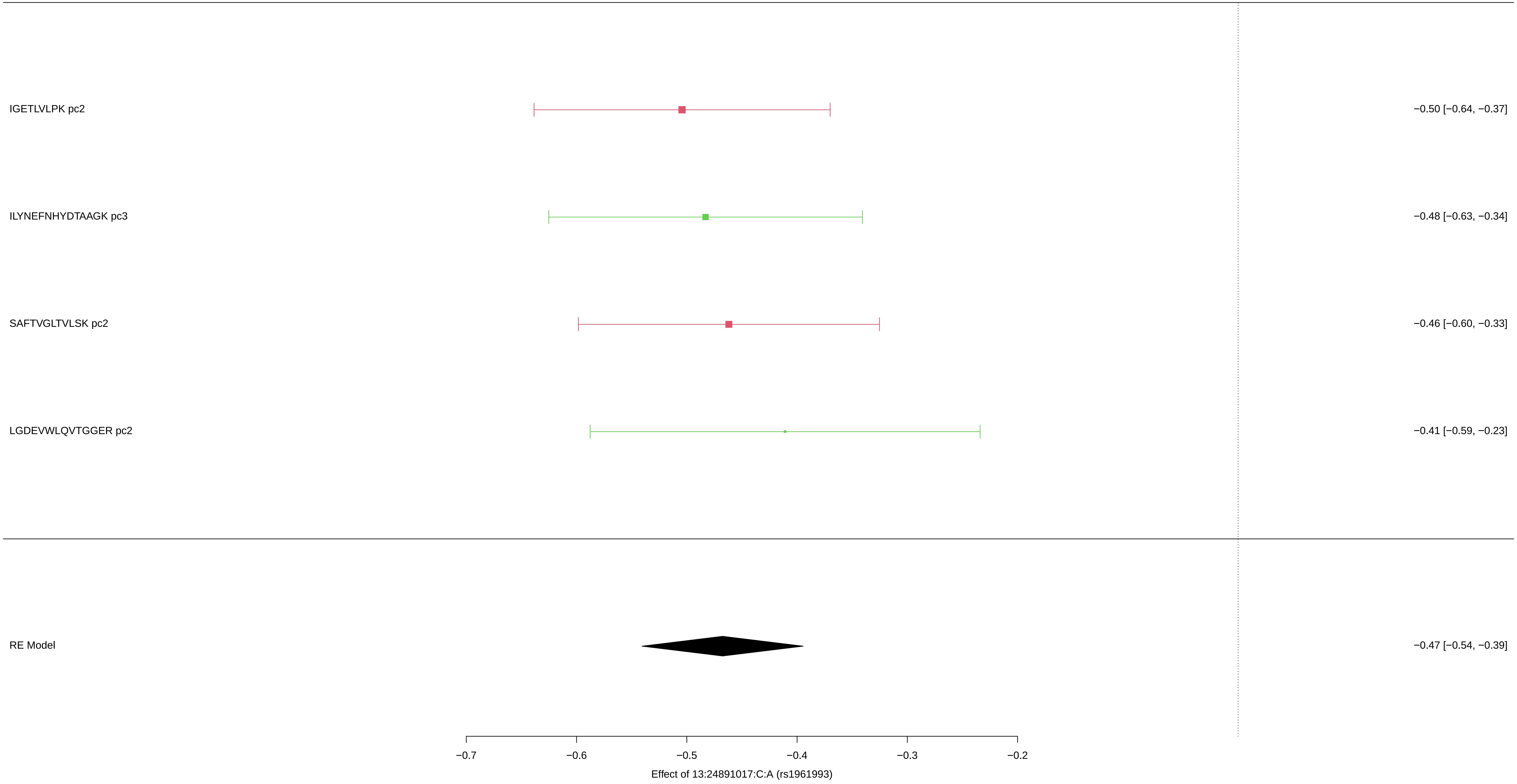

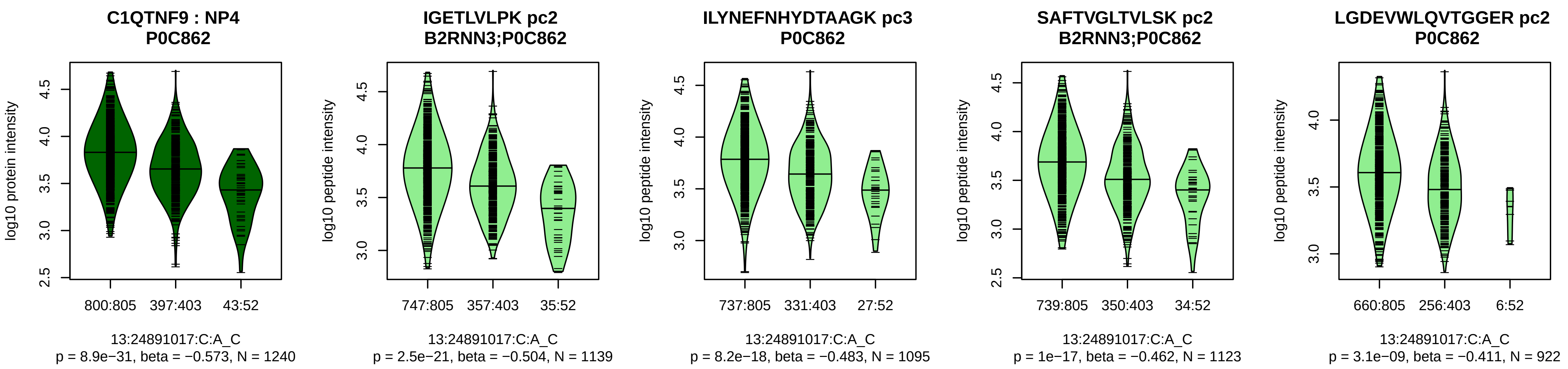

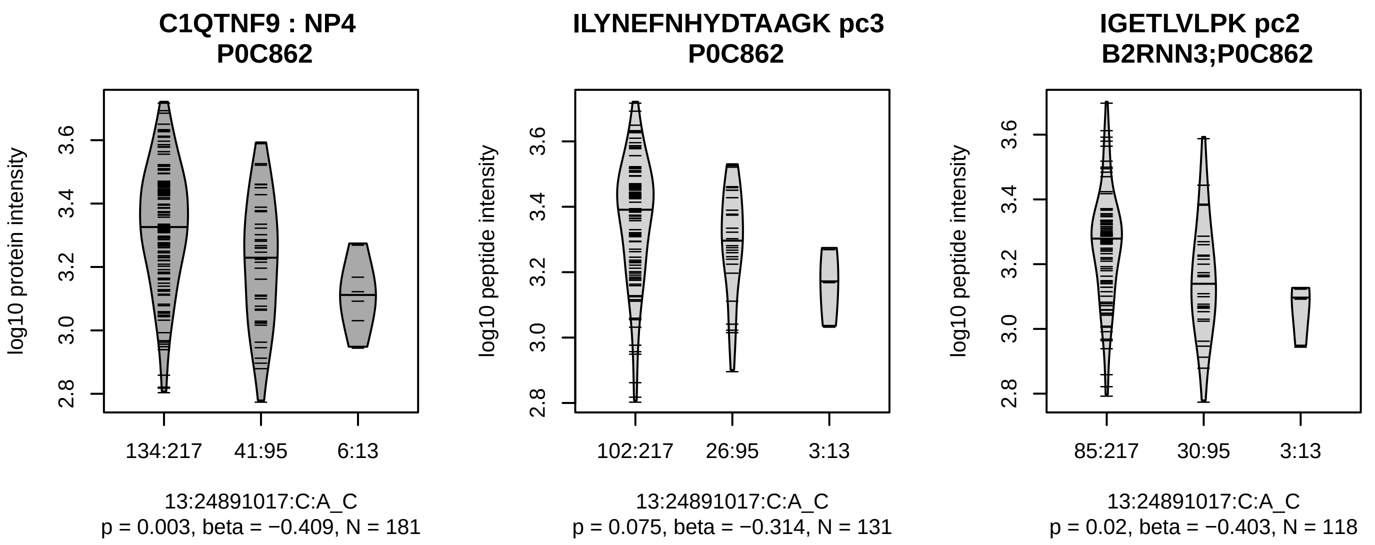

Assay Target: C1QTNF9
UniProt: P0C862
rsID: rs1961993
Target SNP: 13:24891017:C:A
Proxy SNP: 13:24891017:C:A
UKBPPP log10(p): 4552.4
UKBPPP BETA: 1.129
UKBPPP MSPA: 1
::*:*:NA
1139:1095:1123:922:3

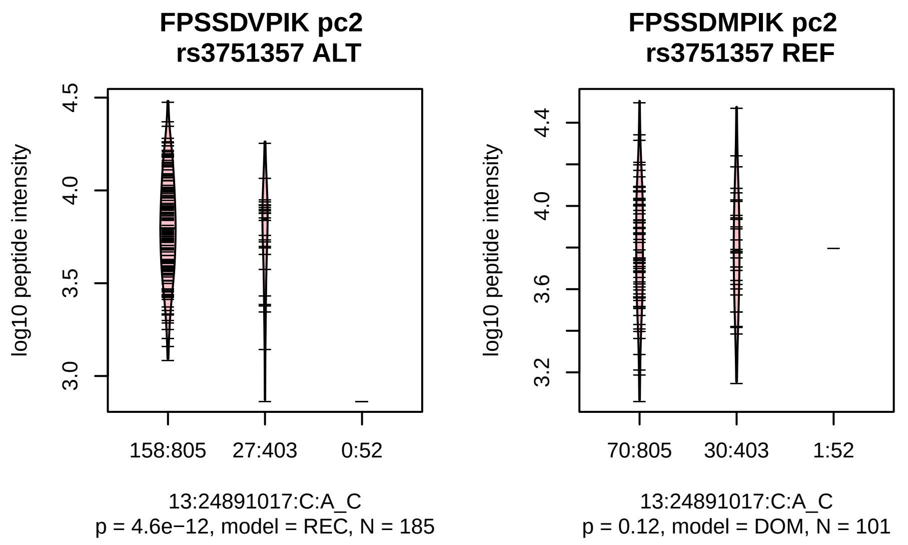

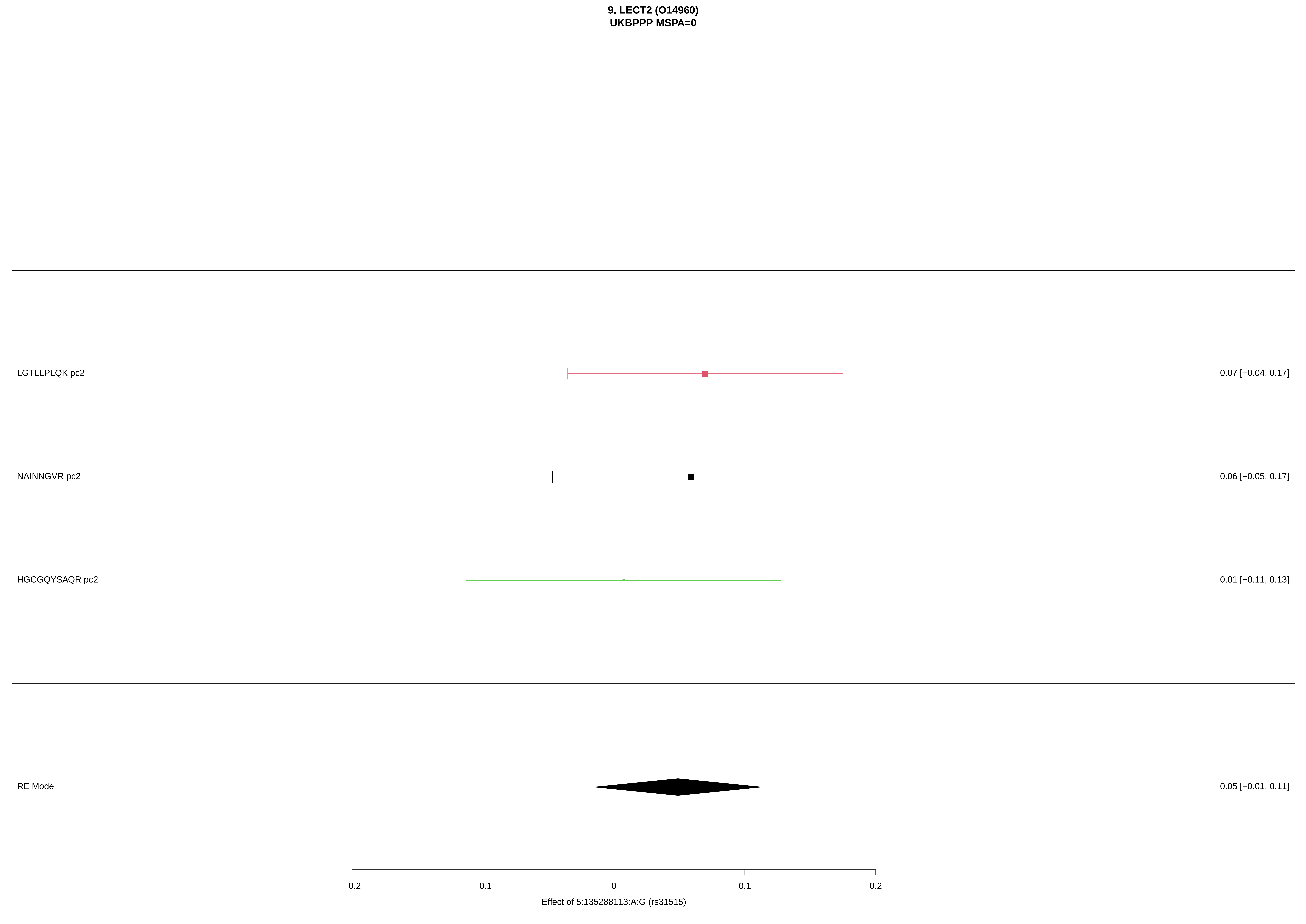
9. LECT2 (O14960)
UKBPPP MSPA=0
LGTLLPLQK pc2
0.07 [−0.04, 0.17]
NAINNGVR pc2
0.06 [−0.05, 0.17]
HGCGQYSAQR pc2
0.01 [−0.11, 0.13]
RE Model
0.05 [−0.01, 0.11]
−0.2
−0.1
0
0.1
0.2
Effect of 5:135288113:A:G (rs31515)

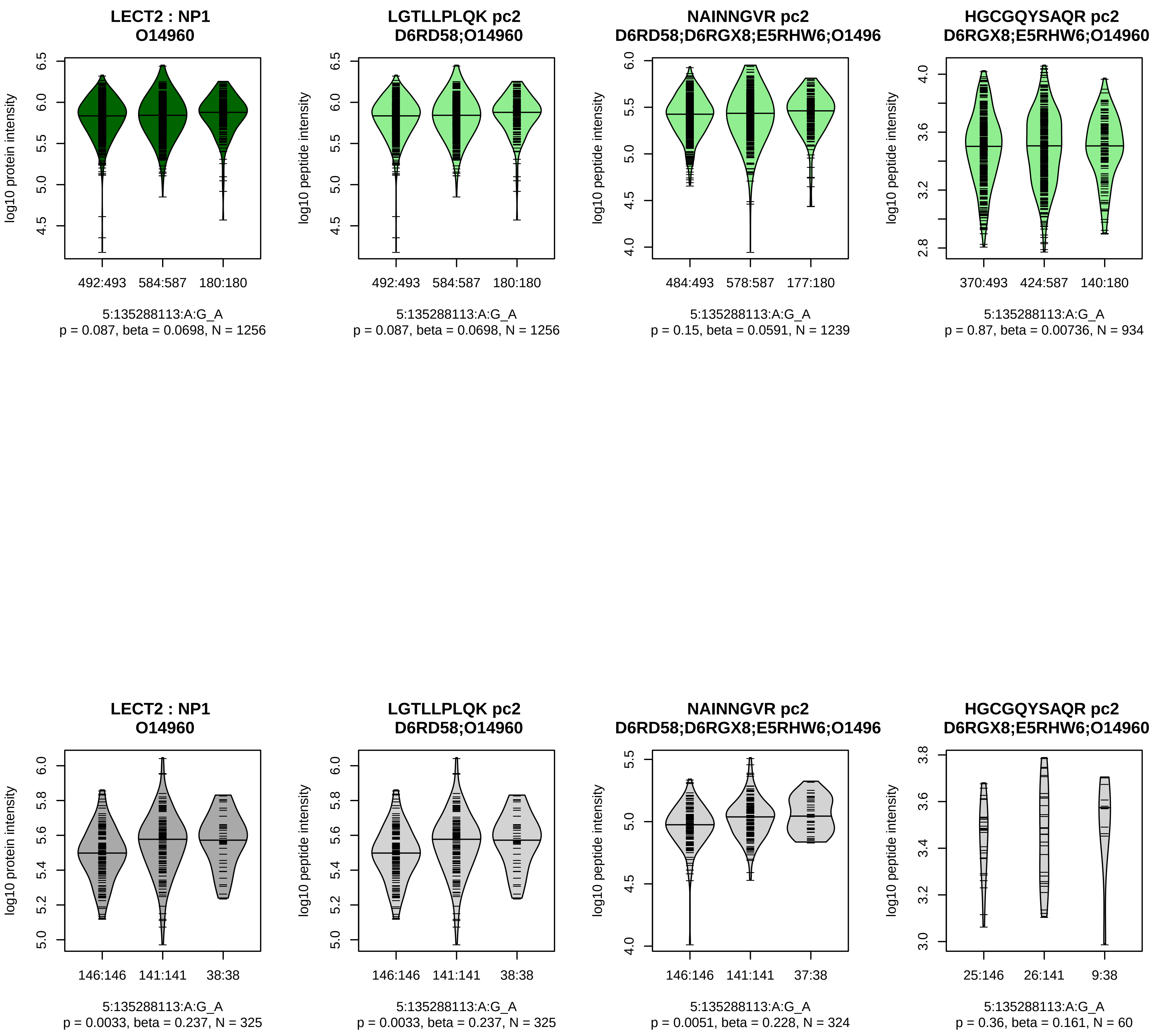

Assay Target: LECT2
UniProt: O14960
rsID: rs31515
Target SNP: 5:135288113:A:G
Proxy SNP: 5:135288113:A:G
UKBPPP log10(p): 4434.6
UKBPPP BETA: −0.789
UKBPPP MSPA: 0
–:–:–
1256:1239:934

10. CFHR2 (P36980)
UKBPPP MSPA=0.924 (4 conflicts)

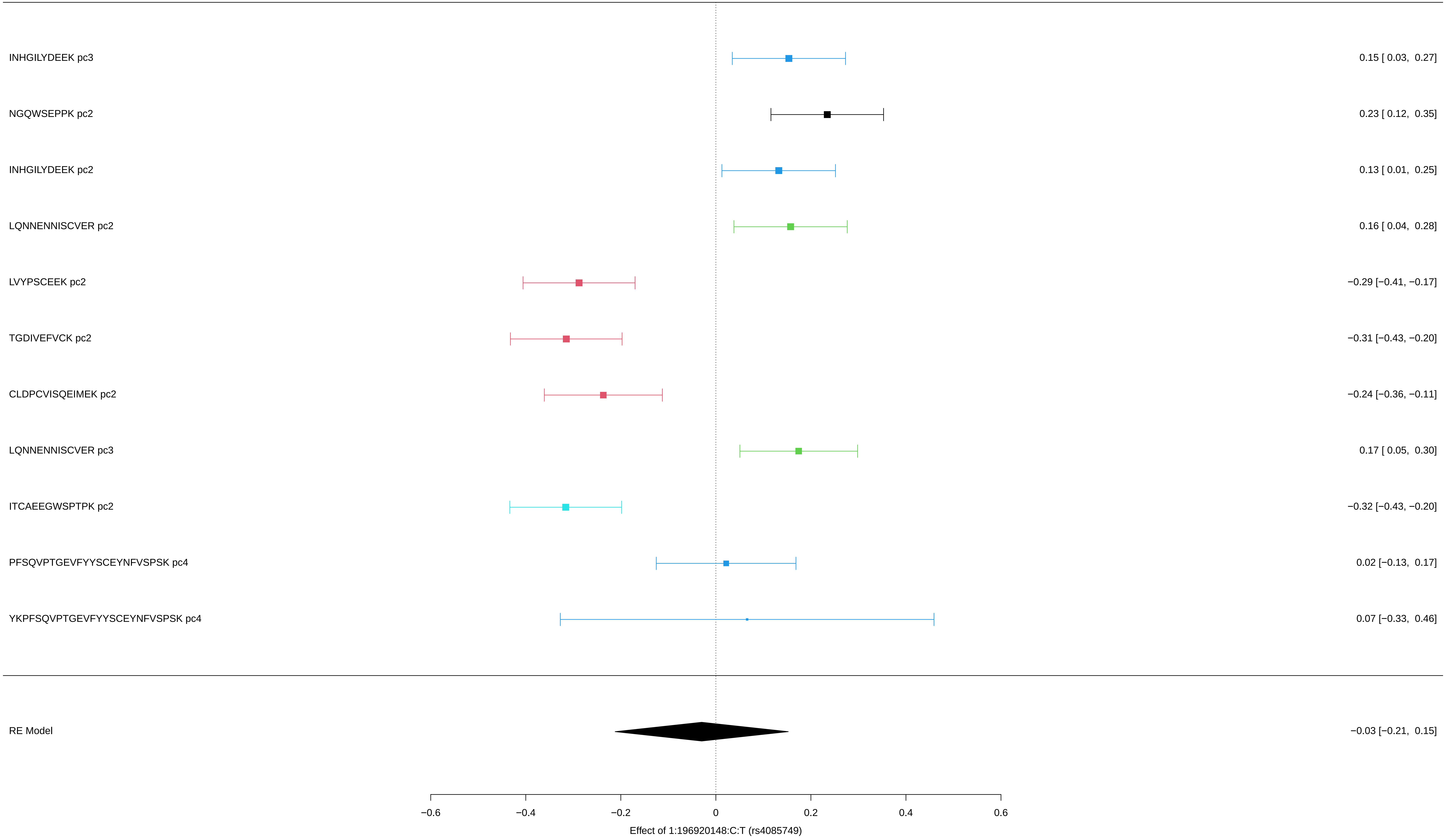

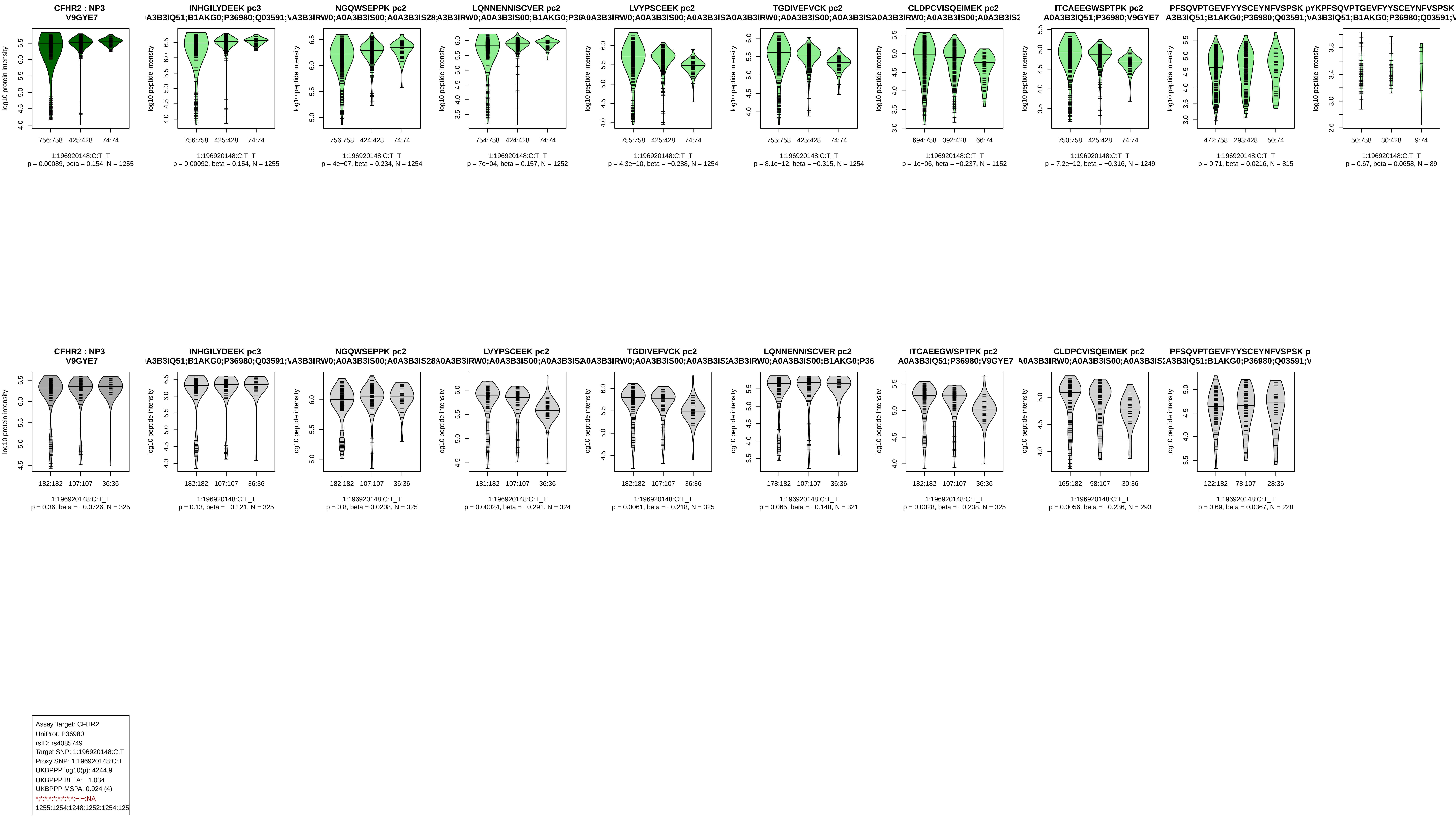

CFHR2 : NP3
V9GYE7
INHGILYDEEK pc3
NGQWSEPPK pc2
LQNNENNISCVER pc2
LVYPSCEEK pc2
TGDIVEFVCK pc2
CLDPCVISQEIMEK pc2
ITCAEEGWSPTPK pc2
A0A3B3IQ51;P36980;V9GYE7
PFSQVPTGEVFYYSCEYNFVSPSK
log10 protein intensity
log10 peptide intensity
1:196920148:C:T_T
p = 0.00089, beta = 0.154, N = 1255
p = 0.00092, beta = 0.154, N = 1255
p = 4e−07, beta = 0.234, N = 1254
p = 7e−04, beta = 0.157, N = 1252
p = 4.3e−10, beta = −0.288, N = 1254
p = 8.1e−12, beta = −0.315, N = 1254
p = 1e−06, beta = −0.237, N = 1152
p = 7.2e−12, beta = −0.316, N = 1249
p = 0.71, beta = 0.0216, N = 815
p = 0.67, beta = 0.0658, N = 89
p = 0.36, beta = −0.0726, N = 325
p = 0.13, beta = −0.121, N = 325
p = 0.8, beta = 0.0208, N = 325
p = 0.00024, beta = −0.291, N = 324
p = 0.0061, beta = −0.218, N = 325
p = 0.065, beta = −0.148, N = 321
p = 0.0028, beta = −0.238, N = 325
p = 0.0056, beta = −0.236, N = 293
p = 0.69, beta = 0.0367, N = 228
Assay Target: CFHR2
UniProt: P36980
rsID: rs4085749
Target SNP: 1:196920148:C:T
Proxy SNP: 1:196920148:C:T
UKBPPP log10(p): 4244.9
UKBPPP BETA: −1.034
UKBPPP MSPA: 0.924 (4)
1255:1254:1248:1252:1254:125

11. MBL2 (P11226)
UKBPPP MSPA=1

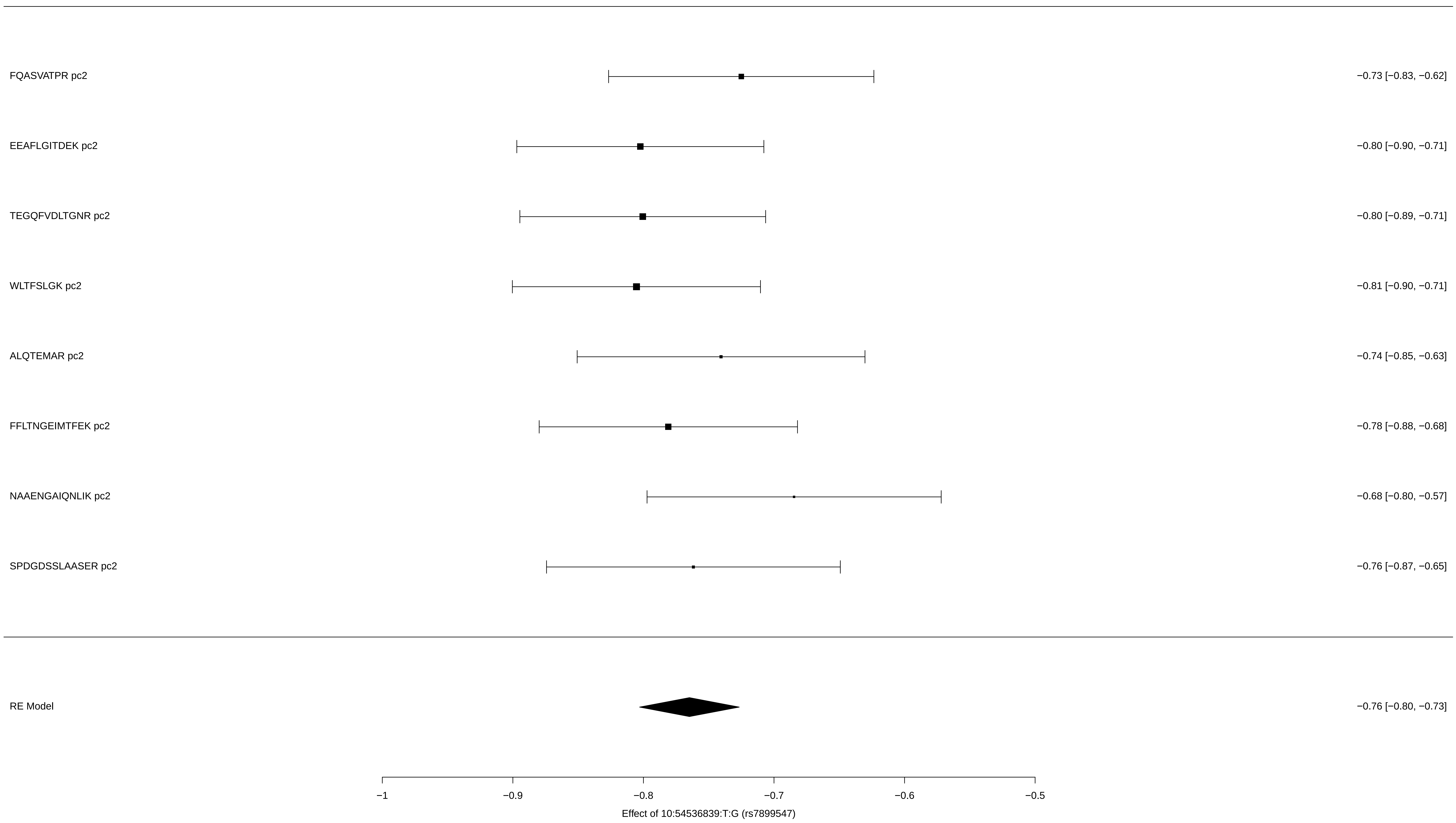

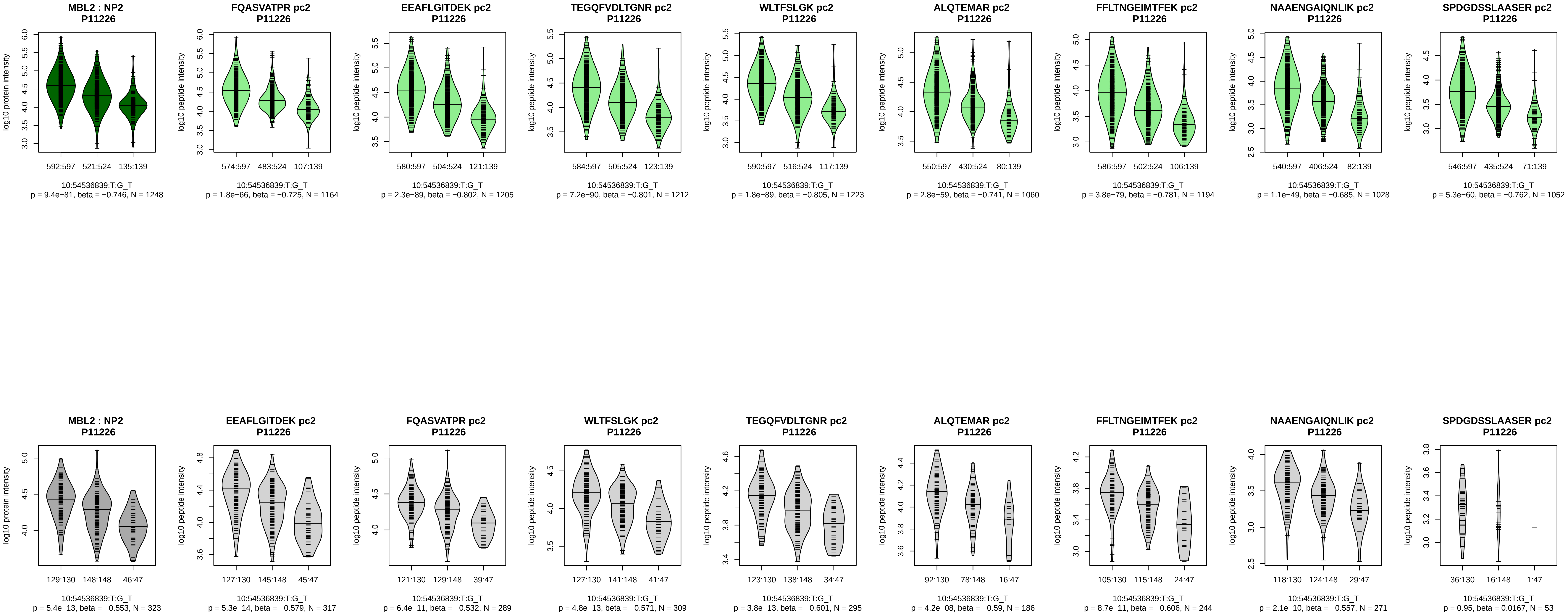

Assay Target: MBL2
UniProt: P11226
rsID: rs7899547
Target SNP: 10:54536839:T:G
Proxy SNP: 10:54536839:T:G
UKBPPP log10(p): 4214.8
UKBPPP BETA: 0.905
UKBPPP MSPA: 1
::*:*:*:*:*:*:NA
1164:1205:1212:1223:1060:119

12. LCP1 (P13796)
UKBPPP MSPA=0.196

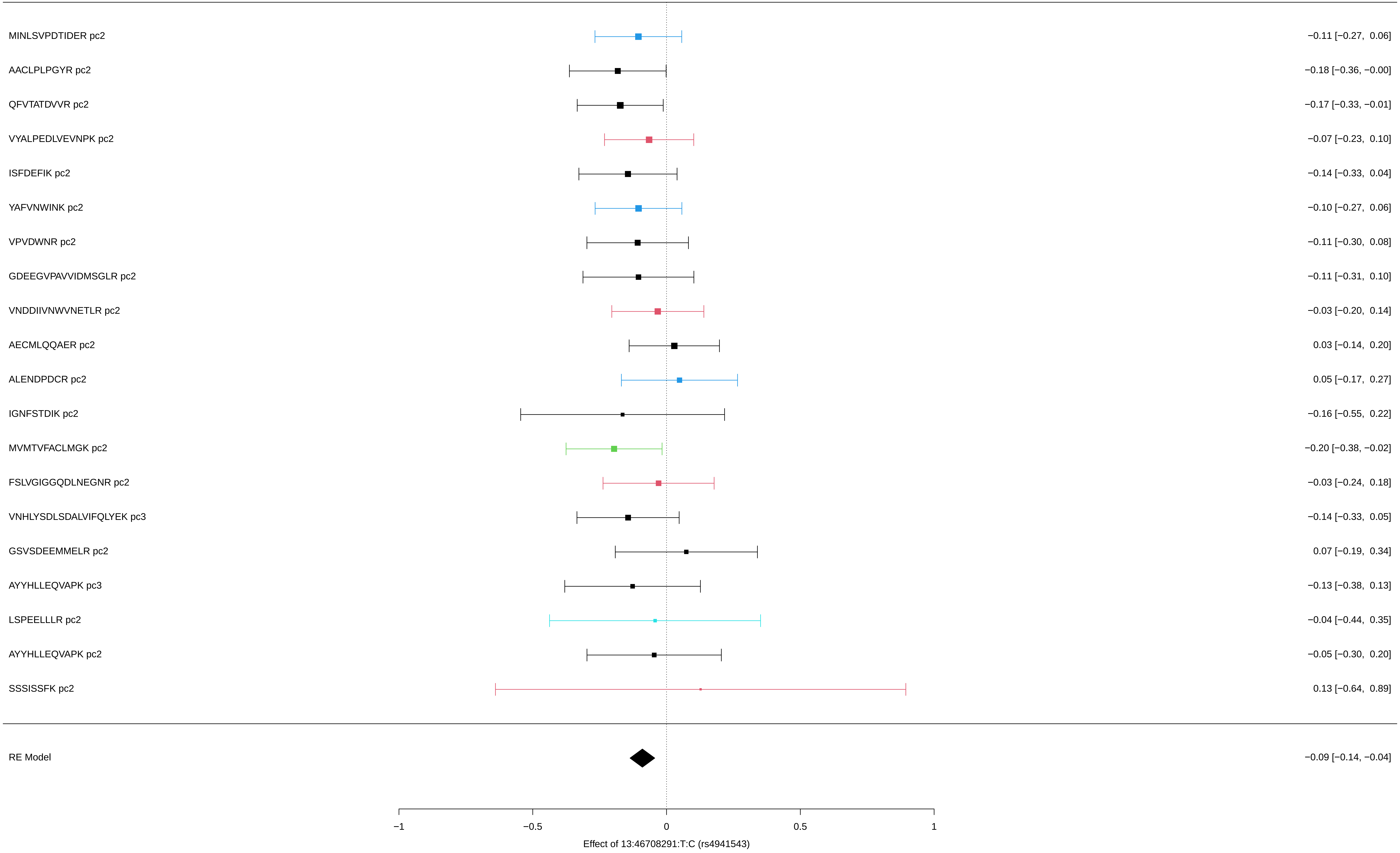

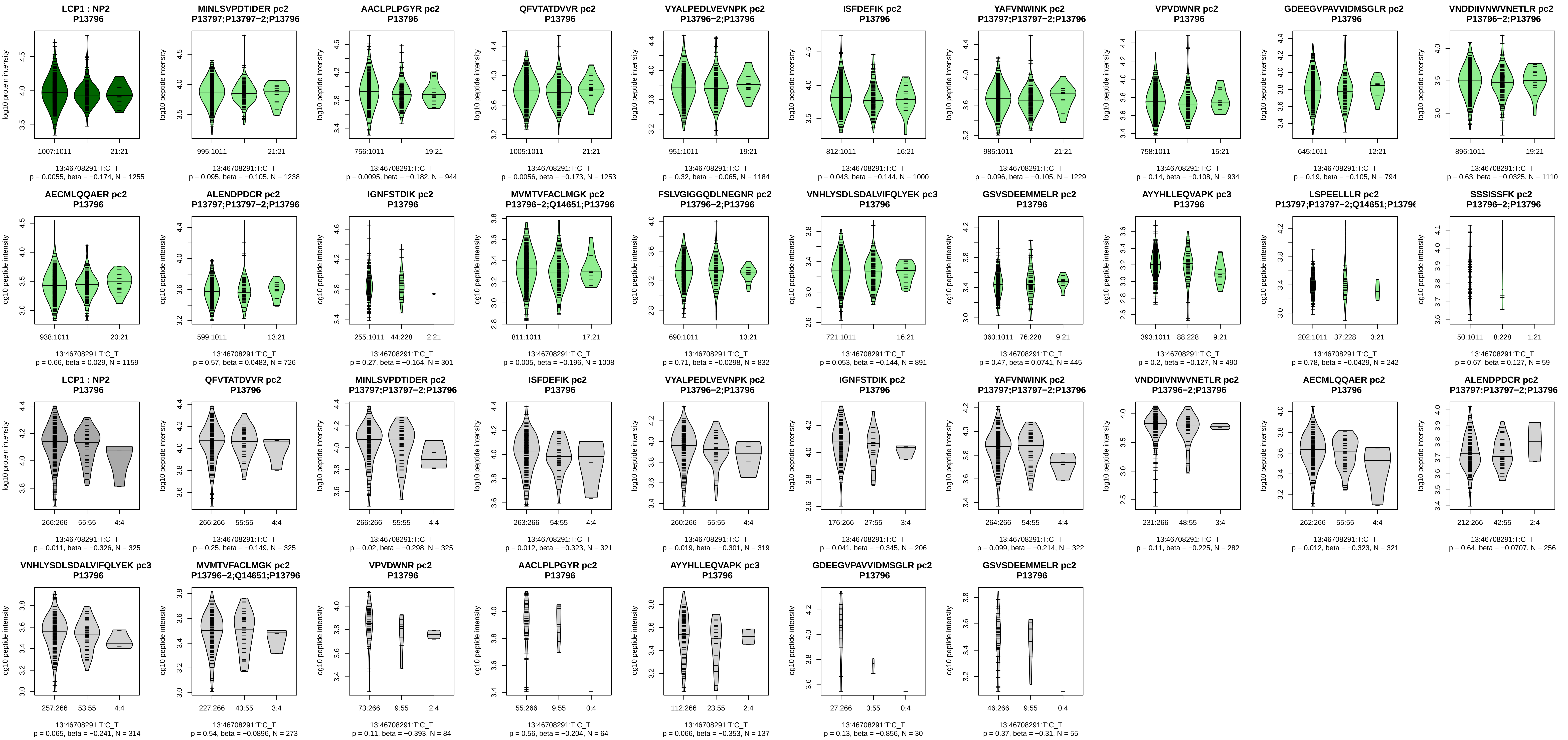

Assay Target: LCP1
UniProt: P13796
rsID: rs4941543
Target SNP: 13:46708291:T:C
Proxy SNP: 13:46708291:T:C
UKBPPP log10(p): 4209.4
UKBPPP BETA: −1.447
UKBPPP MSPA: 0.196
1238:944:1253:1184:1000:1229

13. CTSH (P09668)
UKBPPP MSPA=0.989

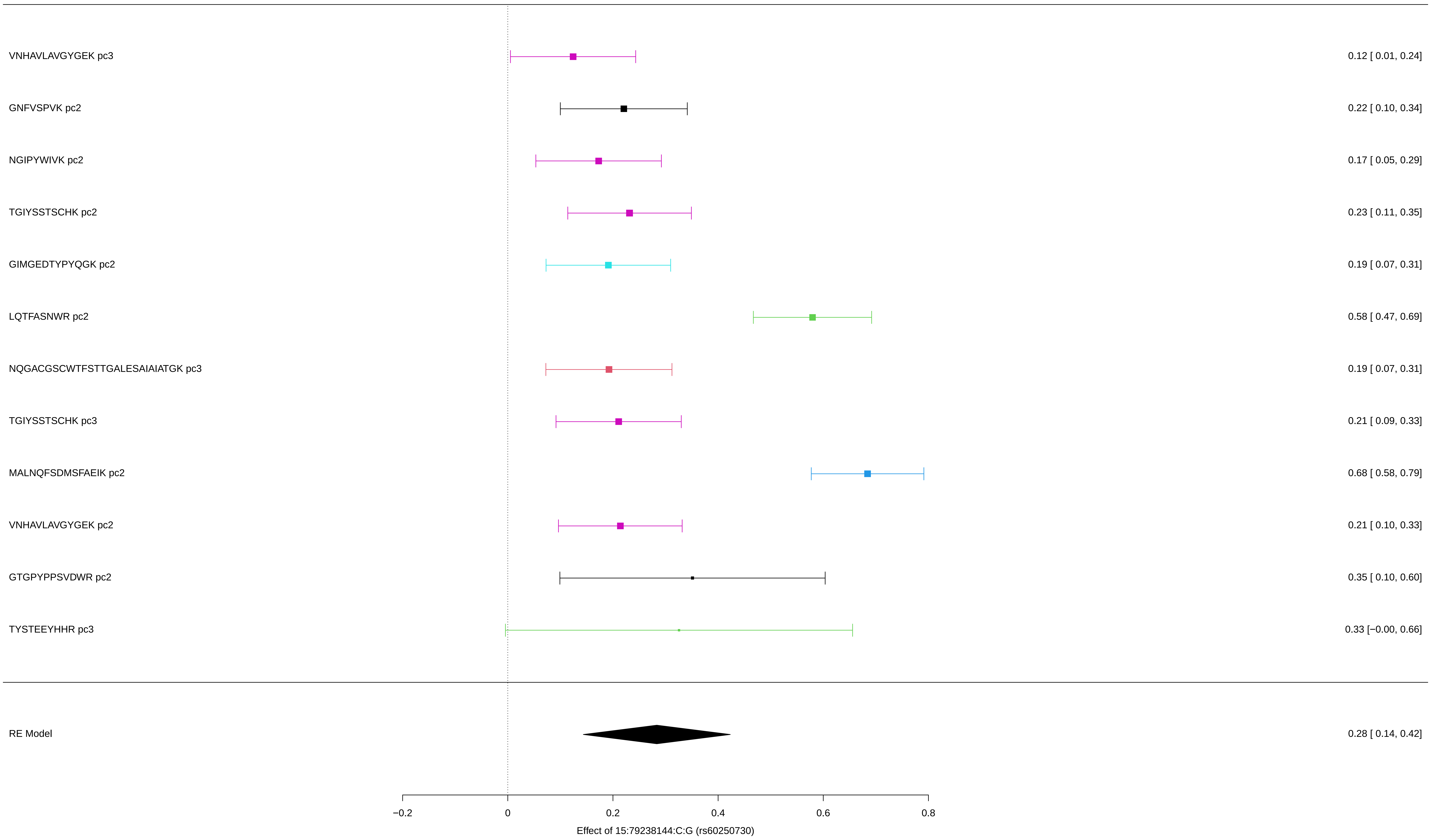

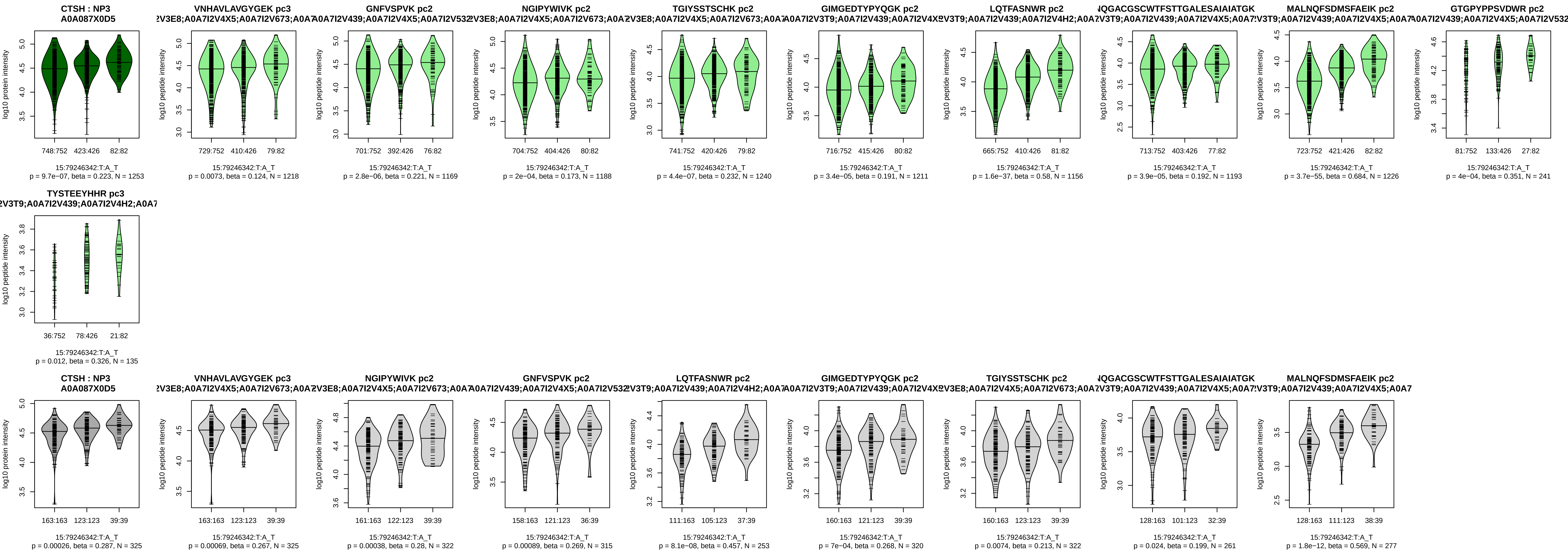

Assay Target: CTSH
UniProt: P09668
rsID: rs60250730
Target SNP: 15:79238144:C:G
Proxy SNP: 15:79246342:T:A
UKBPPP log10(p): 3936
UKBPPP BETA: 1.102
UKBPPP MSPA: 0.989
--*-*-*-*-*-*-*-*-
1218:1169:1188:1240:1211:115

14. IL7R (P16871)
UKBPPP MSPA=0.97

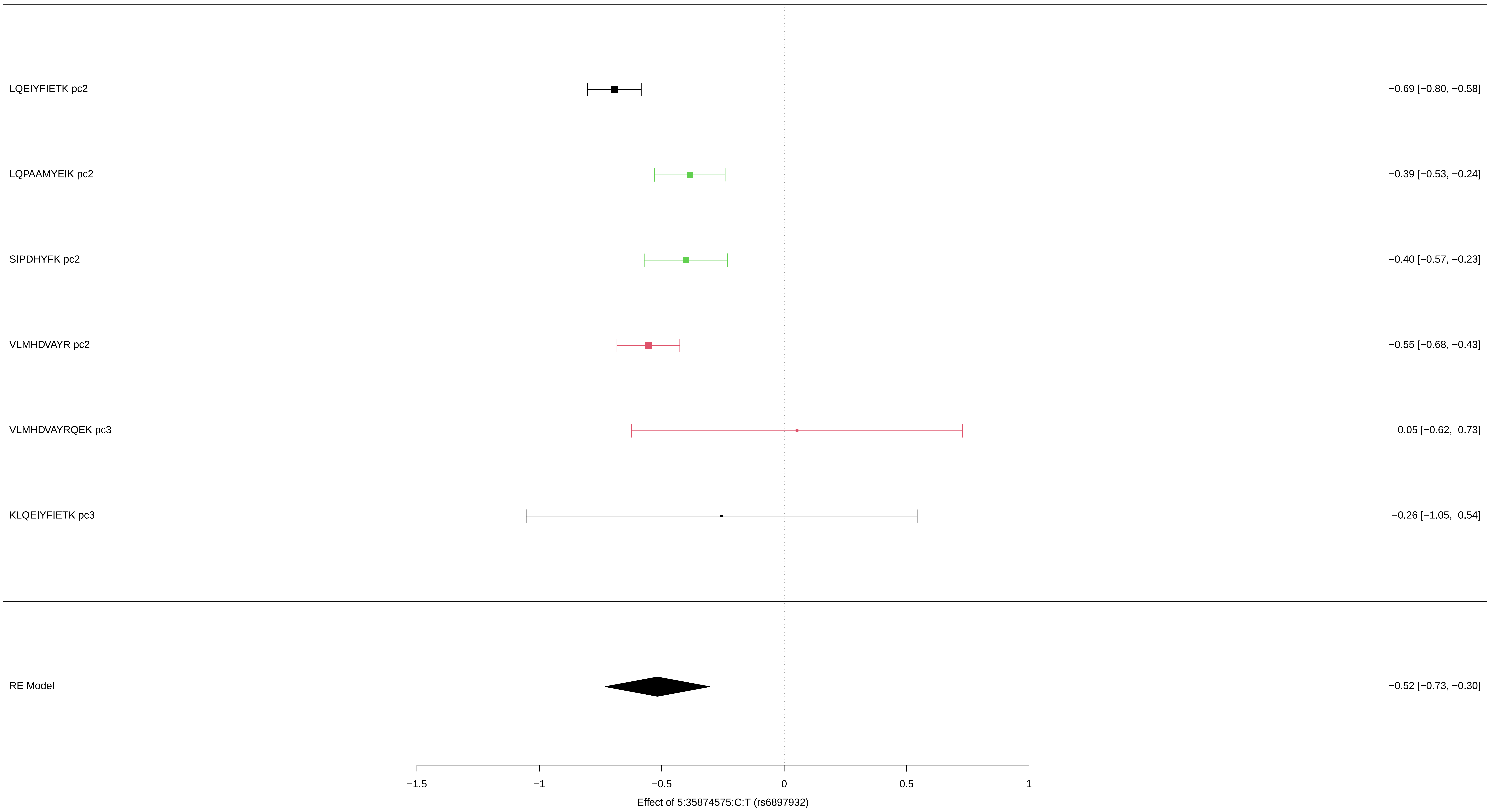

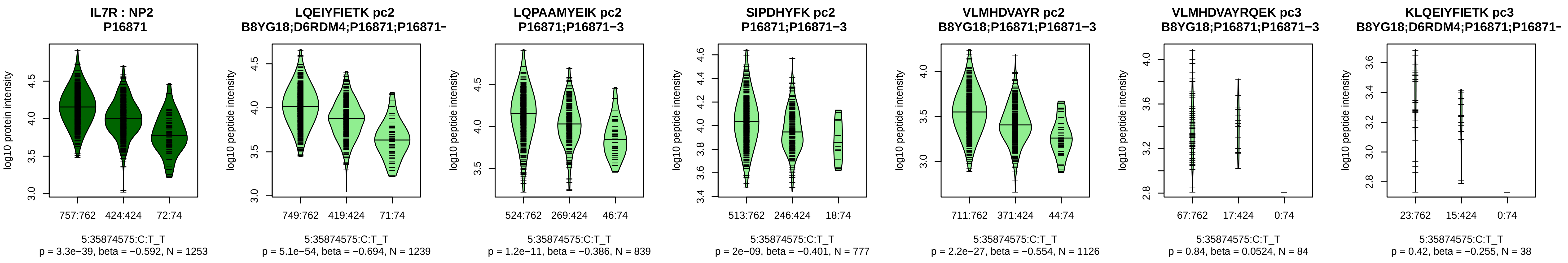

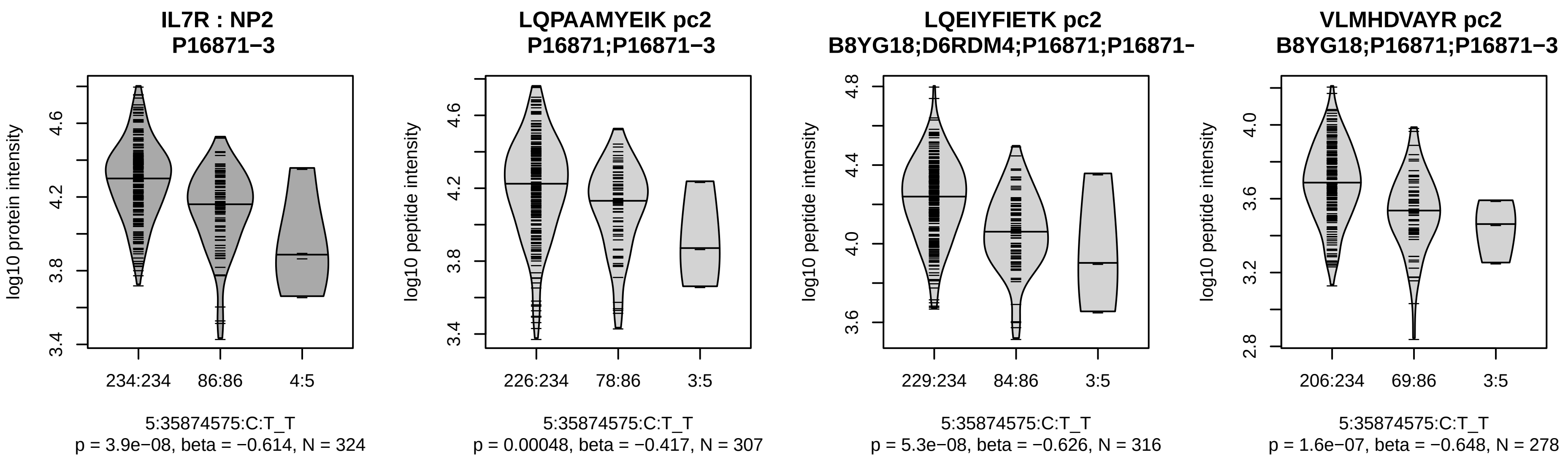

Assay Target: IL7R
UniProt: P16871
rsID: rs6897932
Target SNP: 5:35874575:C:T
Proxy SNP: 5:35874575:C:T
UKBPPP log10(p): 3931.5
UKBPPP BETA: −0.889
UKBPPP MSPA: 0.97
::*:*:–:–
1239:839:777:1126:84:38

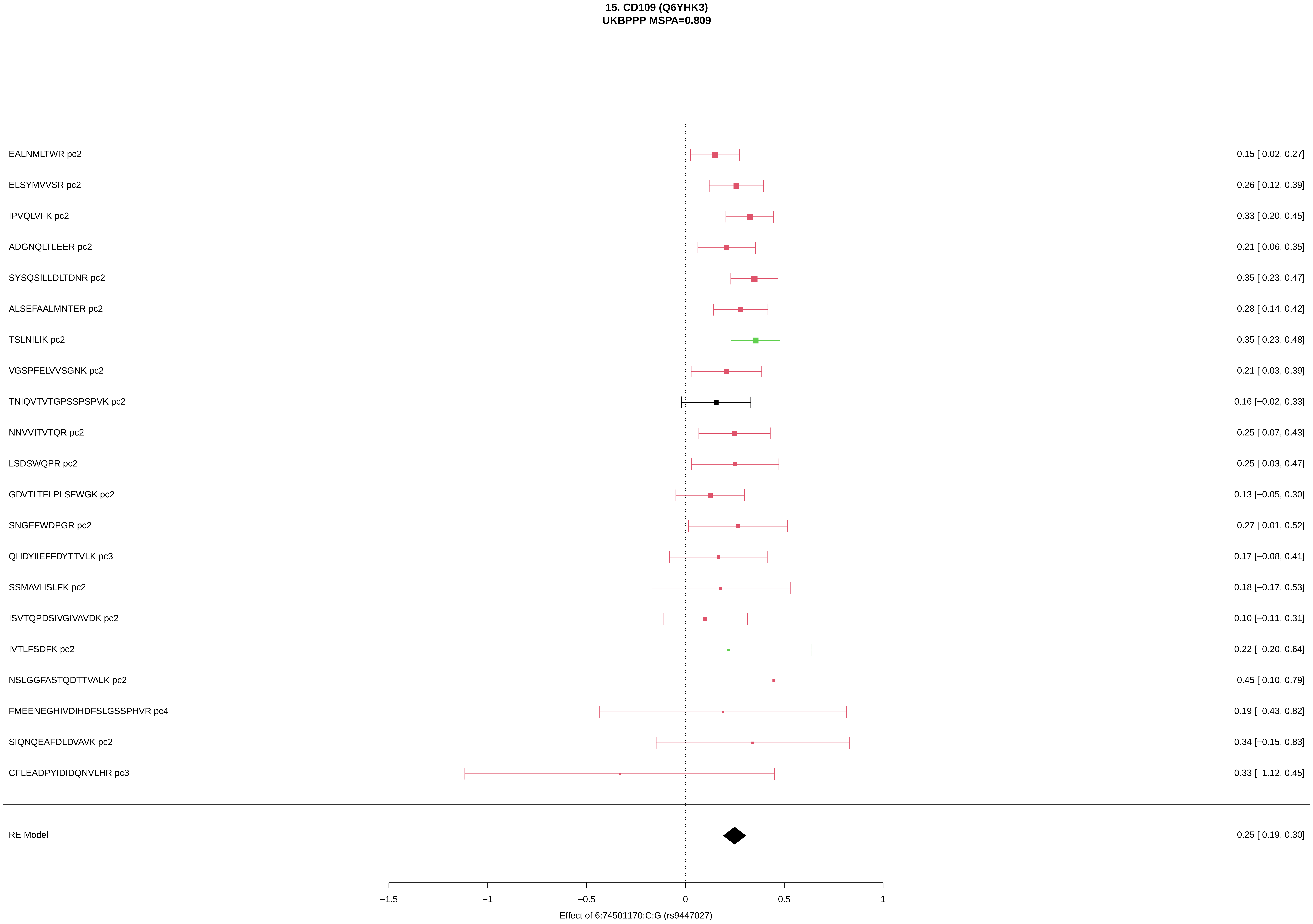

15. CD109 (Q6YHK3)
UKBPPP MSPA=0.809
EALNMLTWR pc2
0.15 [0.02, 0.27]
ELSYMVVSR pc2
0.26 [0.12, 0.39]
IPVQLVFK pc2
0.33 [0.20, 0.45]
ADGNQLTLEER pc2
0.21 [0.06, 0.35]
SYSQSILLDLTDNR pc2
0.35 [0.23, 0.47]
ALSEFAALMNTER pc2
0.28 [0.14, 0.42]
TSLNILIK pc2
0.35 [0.23, 0.48]
VGSPFELVVSGNK pc2
0.21 [0.03, 0.39]
TNIQVTVTGPSSPSPVK pc2
0.16 [−0.02, 0.33]
NNVVITVTQR pc2
0.25 [0.07, 0.43]
LSDSWQPR pc2
0.25 [0.03, 0.47]
GDVTLTFLPLSFWGK pc2
0.13 [−0.05, 0.30]
SNGEFWDPGR pc2
0.27 [0.01, 0.52]
QHDYIIEFFDYTTVLK pc3
0.17 [−0.08, 0.41]
SSMAVHSLFK pc2
0.18 [−0.17, 0.53]
ISVTQPDSIVGIVAVDK pc2
0.10 [−0.11, 0.31]
IVTLFSDFK pc2
0.22 [−0.20, 0.64]
NSLGGFASTQDTTVALK pc2
0.45 [0.10, 0.79]
FMEENEGHIVDIHDFSLGSSPHVR pc4
0.19 [−0.43, 0.82]
SIQNQEAFDLDVAVK pc2
0.34 [−0.15, 0.83]
CFLEADPYIDIDQNVLHR pc3
−0.33 [−1.12, 0.45]
RE Model
0.25 [0.19, 0.30]
−1.5
−1
−0.5
0
0.5
1
Effect of 6:74501170:C:G (rs9447027)

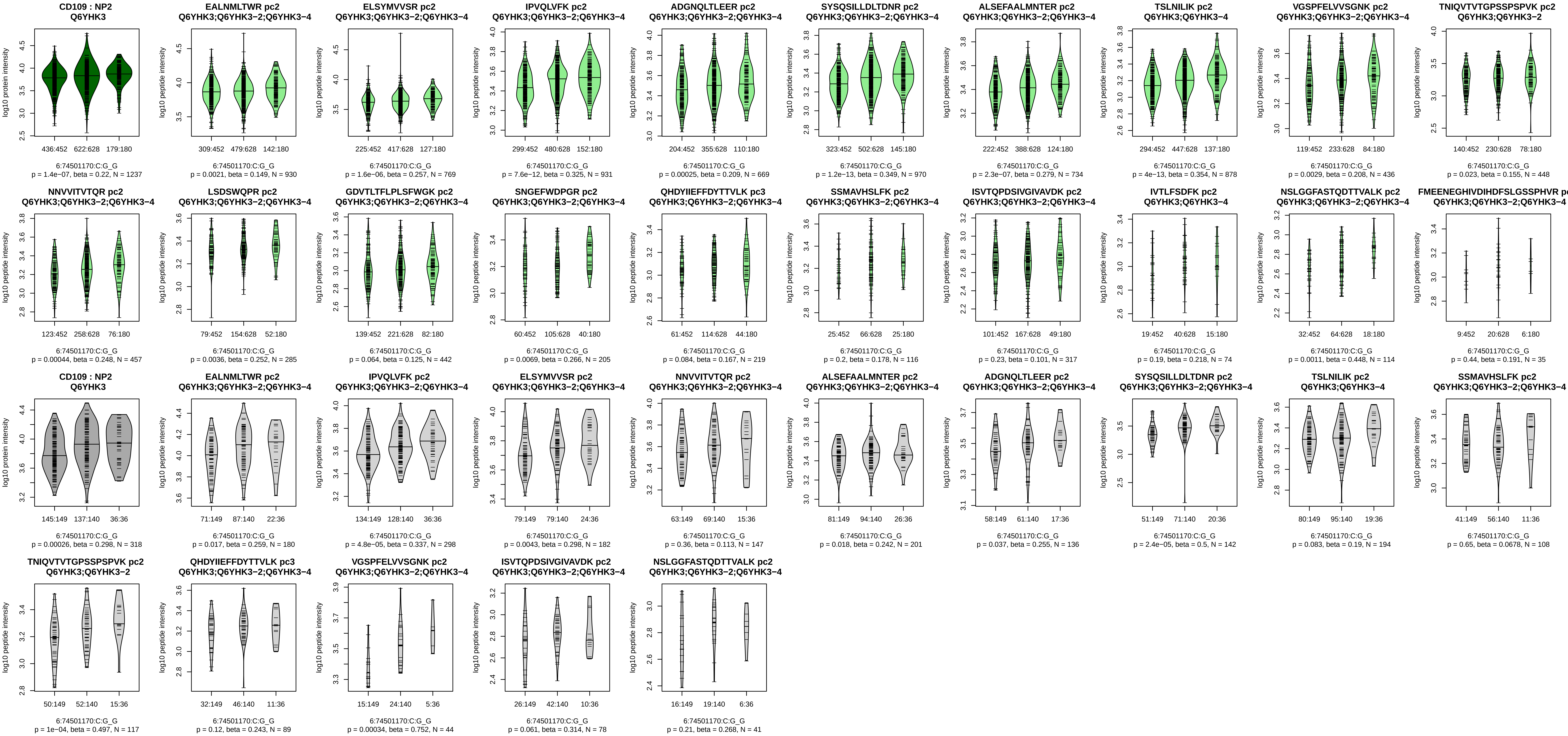

Assay Target: CD109
UniProt: Q6YHK3
rsID: rs9447027
Target SNP: 6:74501170:C:G
Proxy SNP: 6:74501170:C:G
UKBPPP log10(p): 3909.6
UKBPPP BETA: 0.845
UKBPPP MSPA: 0.809
930:769:931:669:970:734:878:4

16. TPSAB1 (Q15661)
UKBPPP MSPA=0

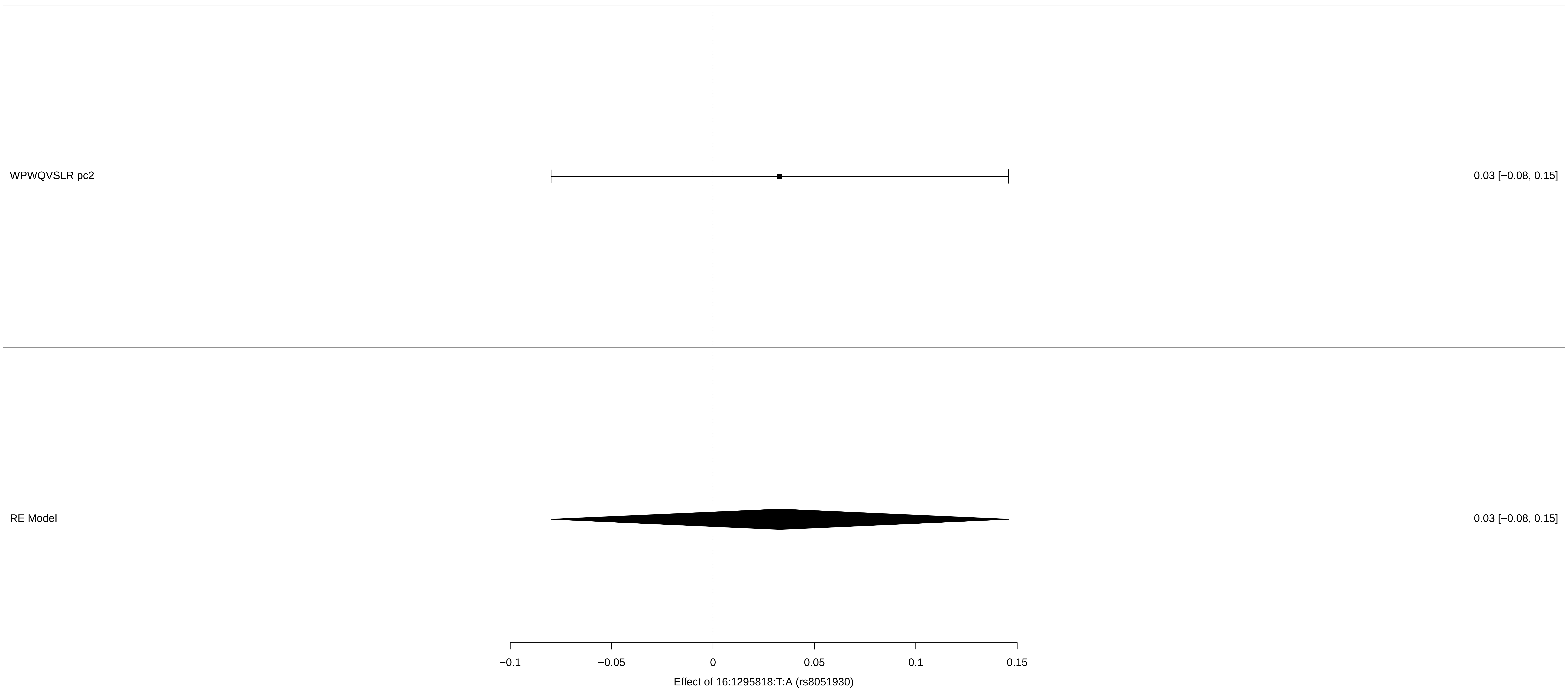

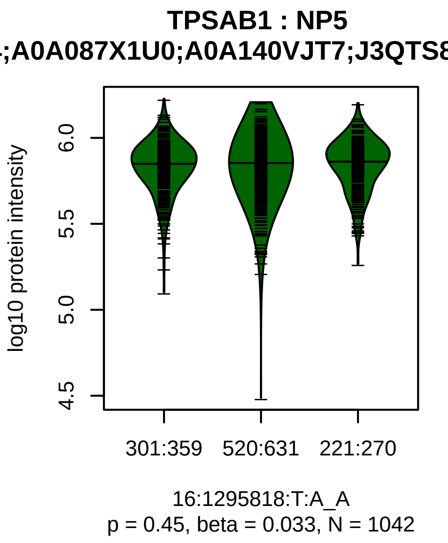

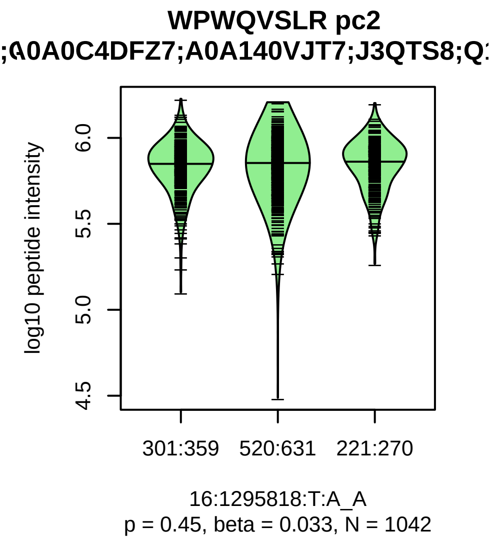

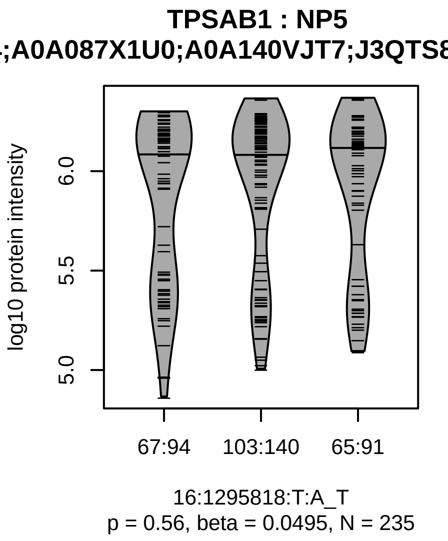

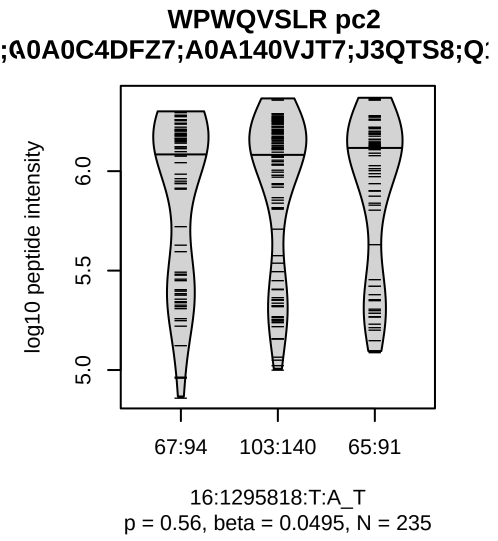

Assay Target: TPSAB1
UniProt: Q15661
rsID: rs8051930
Target SNP: 16:1295818:T:A
Proxy SNP: 16:1295818:T:A
UKBPPP log10(p): 3832.7
UKBPPP BETA: 0.814
UKBPPP MSPA: 0
–
1042

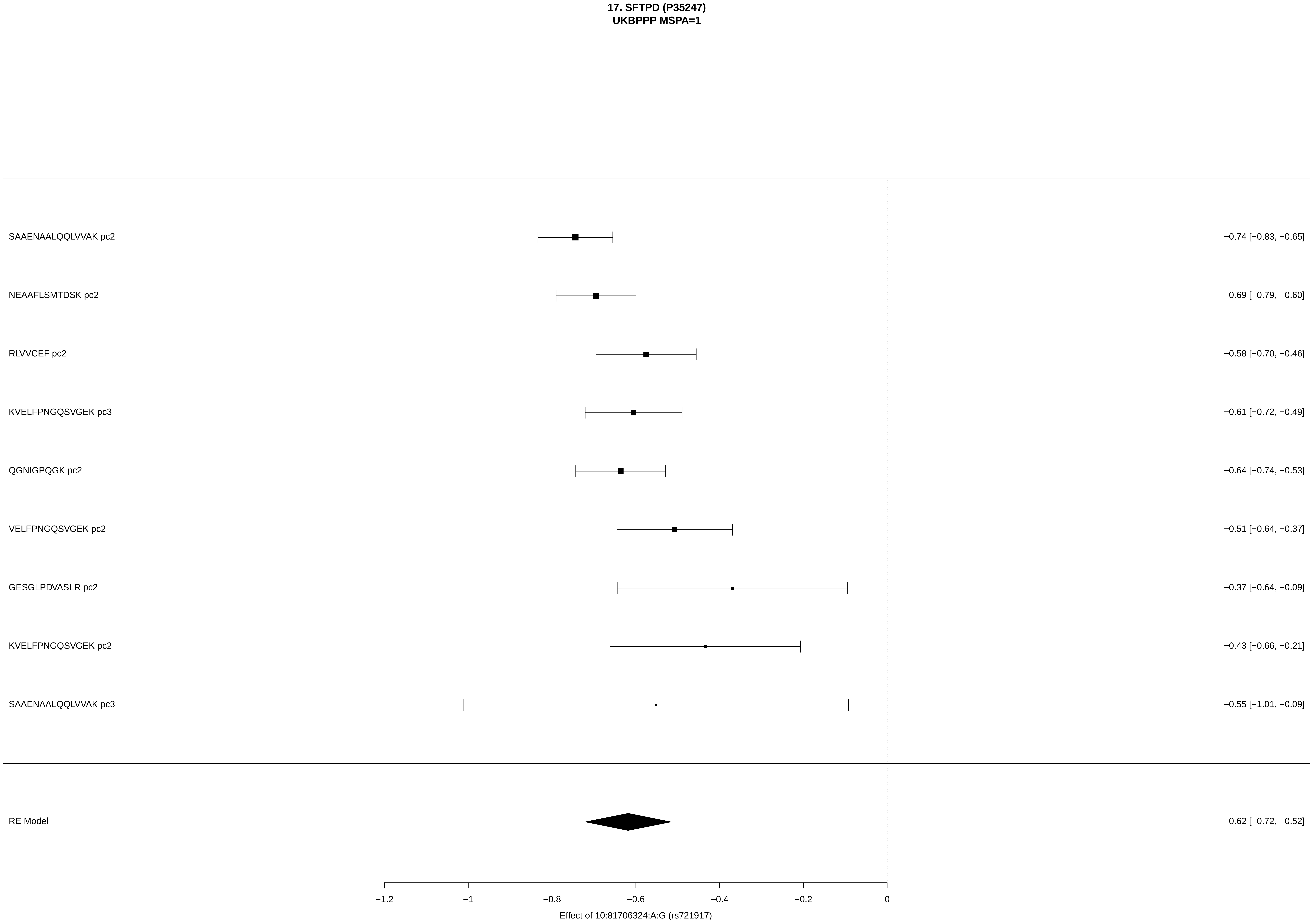
17. SFTPD (P35247)
UKBPPP MSPA=1
SAAENAALQQLVVAK pc2
−0.74 [−0.83, −0.65]
NEAAFLSMTDSK pc2
−0.69 [−0.79, −0.60]
RLVVCEF pc2
−0.58 [−0.70, −0.46]
KVELFPNGQSVGEK pc3
−0.61 [−0.72, −0.49]
QGNIGPQGK pc2
−0.64 [−0.74, −0.53]
VELFPNGQSVGEK pc2
−0.51 [−0.64, −0.37]
GESGLPDVASLR pc2
−0.37 [−0.64, −0.09]
KVELFPNGQSVGEK pc2
−0.43 [−0.66, −0.21]
SAAENAALQQLVVAK pc3
−0.55 [−1.01, −0.09]
RE Model
−0.62 [−0.72, −0.52]
−1.2
−1
−0.8
−0.6
−0.4
−0.2
0
Effect of 10:81706324:A:G (rs721917)

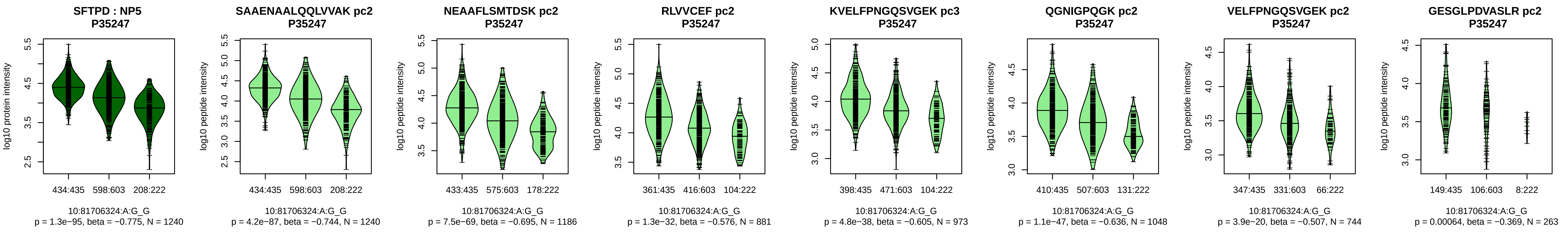

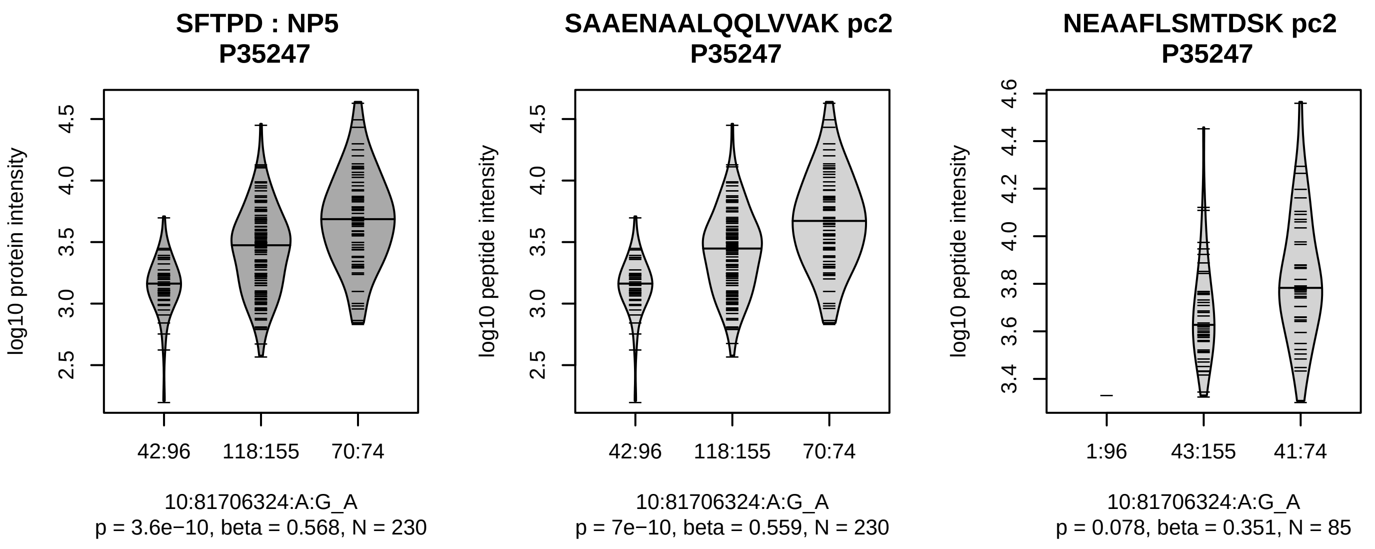

Assay Target: SFTPD
UniProt: P35247
rsID: rs721917
Target SNP: 10:81706324:A:G
Proxy SNP: 10:81706324:A:G
UKBPPP log10(p): 3560.7
UKBPPP BETA: −0.755
UKBPPP MSPA: 1
1240:1186:881:973:1048:744:26

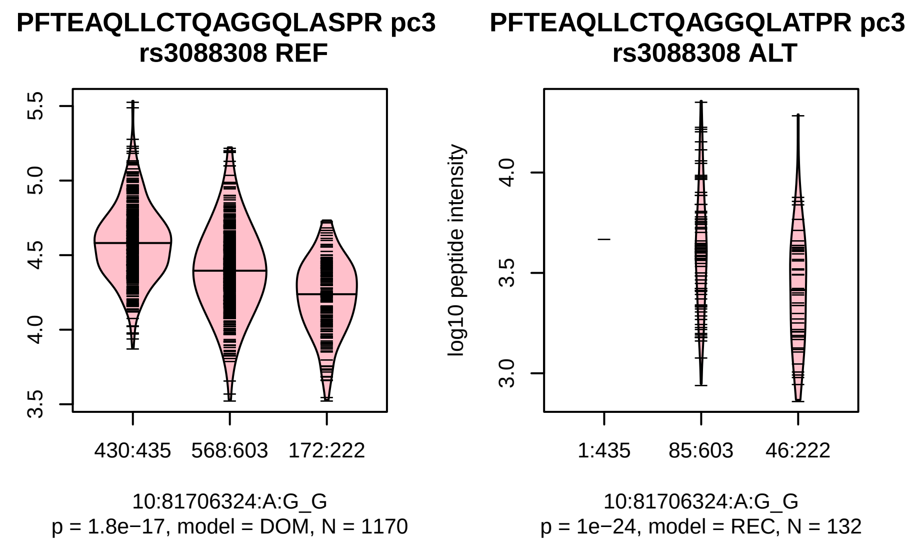

18. PON2 (Q15165)
UKBPPP MSPA=0.956

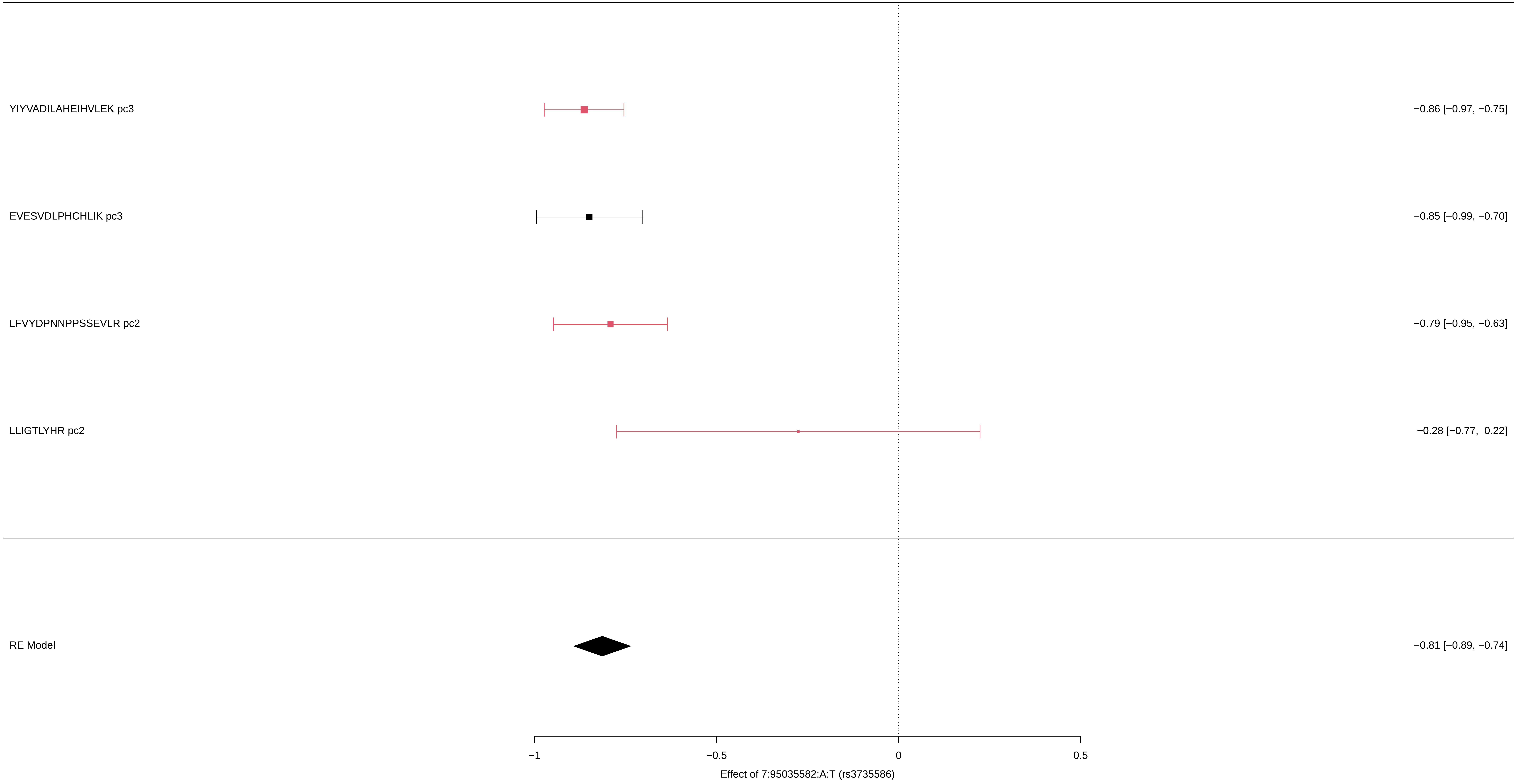

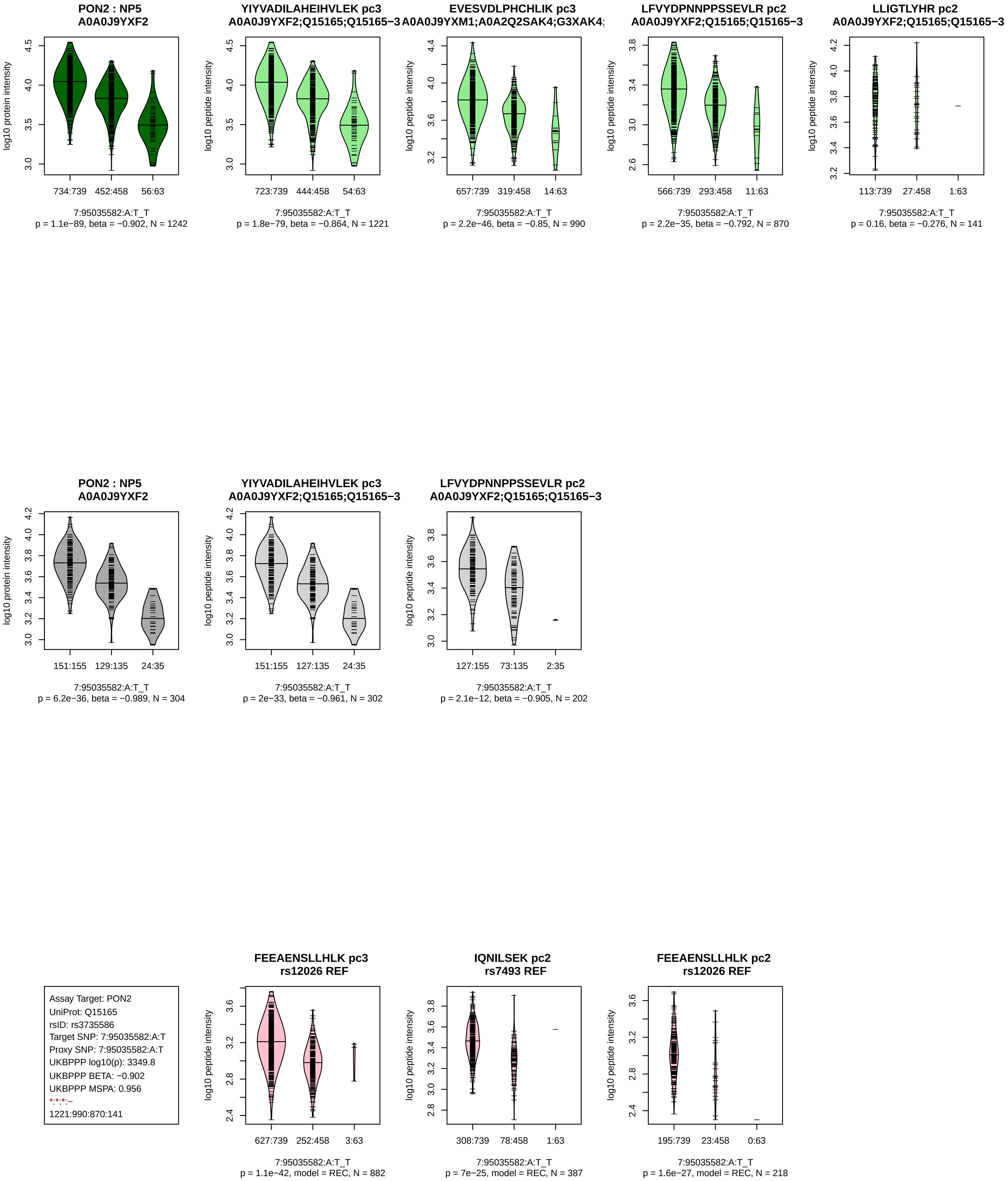
PON2 : NP5
A0A0J9YXF2
log10 protein intensity
734:739
452:458
56:63
7:95035582:A:T_T
p = 1.1e−89, beta = −0.902, N = 1242
YIYVADILAHEIHVLEK pc3
A0A0J9YXF2;Q15165;Q15165−3
log10 peptide intensity
723:739
444:458
54:63
7:95035582:A:T_T
p = 1.8e−79, beta = −0.864, N = 1221
EVESVDLPHCHLIK pc3
A0A0J9YXM1;A0A2Q2SAK4;G3XAK4;
657:739
319:458
14:63
7:95035582:A:T_T
p = 2.2e−46, beta = −0.85, N = 990
LFVYDPNNPPSSEVLR pc2
A0A0J9YXF2;Q15165;Q15165−3
566:739
293:458
11:63
7:95035582:A:T_T
p = 2.2e−35, beta = −0.792, N = 870
LLIGTLYHR pc2
A0A0J9YXF2;Q15165;Q15165−3
113:739
27:458
1:63
7:95035582:A:T_T
p = 0.16, beta = −0.276, N = 141
PON2 : NP5
A0A0J9YXF2
151:155
129:135
24:35
7:95035582:A:T_T
p = 6.2e−36, beta = −0.989, N = 304
YIYVADILAHEIHVLEK pc3
A0A0J9YXF2;Q15165;Q15165−3
151:155
127:135
24:35
7:95035582:A:T_T
p = 2e−33, beta = −0.961, N = 302
LFVYDPNNPPSSEVLR pc2
A0A0J9YXF2;Q15165;Q15165−3
127:155
73:135
2:35
7:95035582:A:T_T
p = 2.1e−12, beta = −0.905, N = 202
Assay Target: PON2
UniProt: Q15165
rsID: rs3735586
Target SNP: 7:95035582:A:T
Proxy SNP: 7:95035582:A:T
UKBPPP log10(p): 3349.8
UKBPPP BETA: −0.902
UKBPPP MSPA: 0.956
::*:–
1221:990:870:141
FEEAENSLLHLK pc3
rs12026 REF
627:739
252:458
3:63
7:95035582:A:T_T
p = 1.1e−42, model = REC, N = 882
IQNILSEK pc2
rs7493 REF
308:739
78:458
1:63
7:95035582:A:T_T
p = 7e−25, model = REC, N = 387
FEEAENSLLHLK pc2
rs12026 REF
195:739
23:458
0:63
7:95035582:A:T_T
p = 1.6e−27, model = REC, N = 218

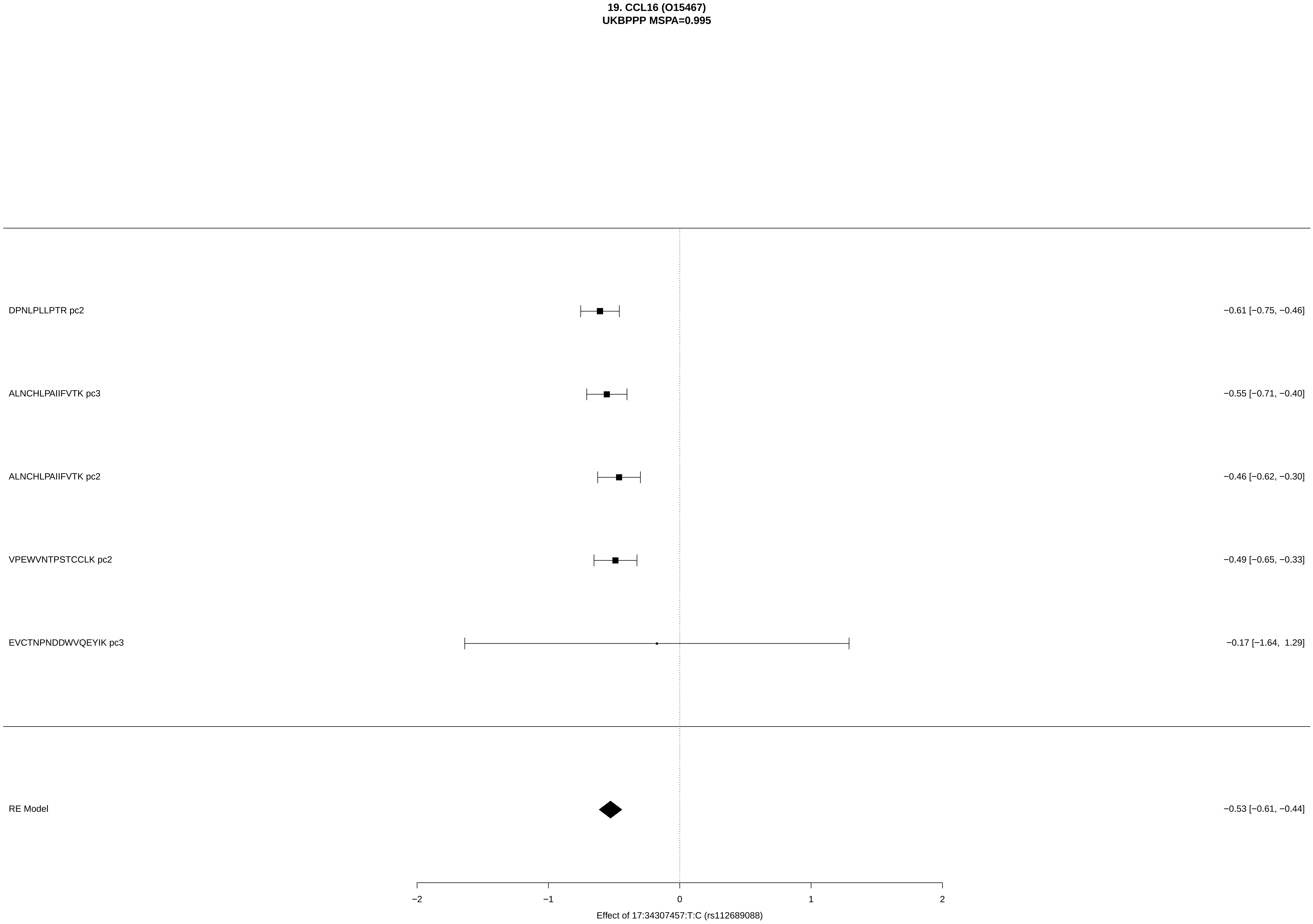
19. CCL16 (O15467)
UKBPPP MSPA=0.995
DPNLPLLPTR pc2
−0.61 [−0.75, −0.46]
ALNCHLPAIIFVTK pc3
−0.55 [−0.71, −0.40]
ALNCHLPAIIFVTK pc2
−0.46 [−0.62, −0.30]
VPEWVNTPSTCCLK pc2
−0.49 [−0.65, −0.33]
EVCTNPNDDWVQEYIK pc3
−0.17 [−1.64, 1.29]
RE Model
−0.53 [−0.61, −0.44]
−2
−1
0
1
2
Effect of 17:34307457:T:C (rs112689088)

CCL16 : NP3
O15467

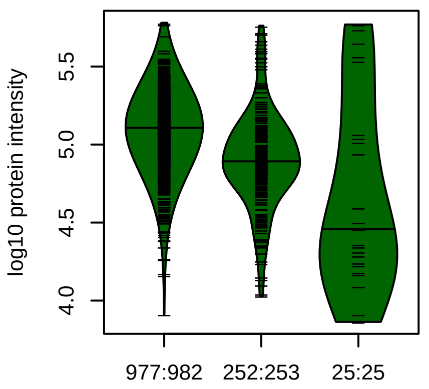

17:34307457:T:C_C
p = 1.6e−25, beta = −0.609, N = 1254

DPNLPLLPTR pc2
O15467

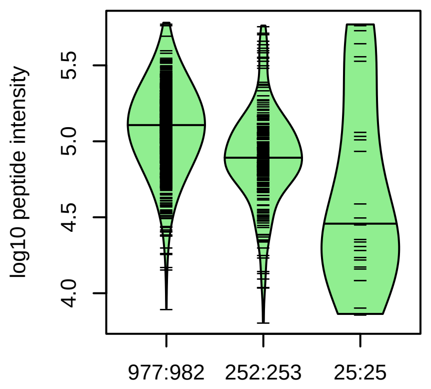

17:34307457:T:C_C
p = 2.3e−25, beta = −0.607, N = 1254

ALNCHLPAIIFVTK pc3
O15467

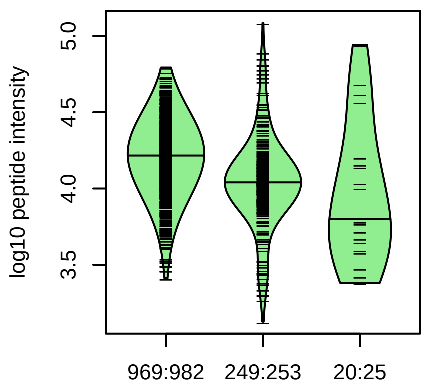

17:34307457:T:C_C
p = 5e−20, beta = −0.555, N = 1238

VPEWVNTPSTCCLK pc2
O15467

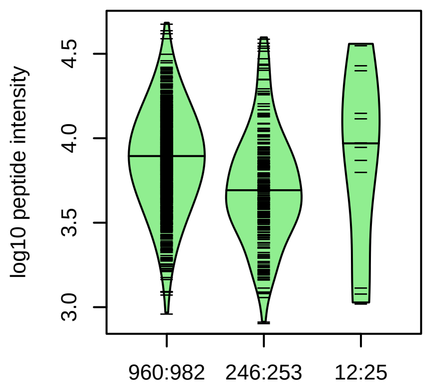

17:34307457:T:C_C
p = 2.8e−14, beta = −0.489, N = 1218

EVCTNPNDDWVQEYIK pc3
O15467

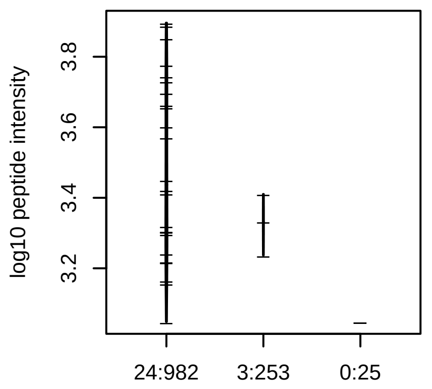

17:34307457:T:C_C
p = 0.76, beta = −0.174, N = 27

CCL16 : NP3
O15467

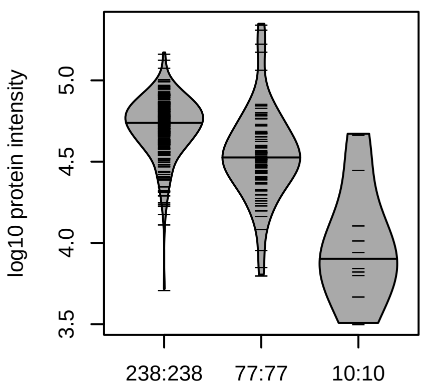

17:34307457:T:C_C
p = 6.8e−17, beta = −0.834, N = 325

ALNCHLPAIIFVTK pc3
O15467

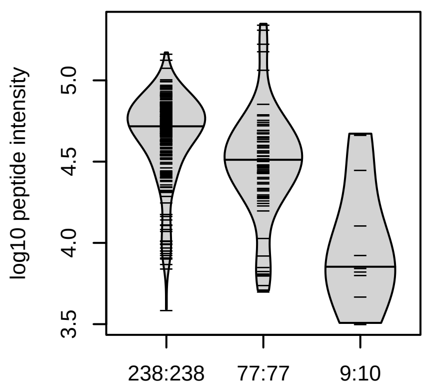

17:34307457:T:C_C
p = 6.6e−12, beta = −0.709, N = 324

DPNLPLLPTR pc2
O15467

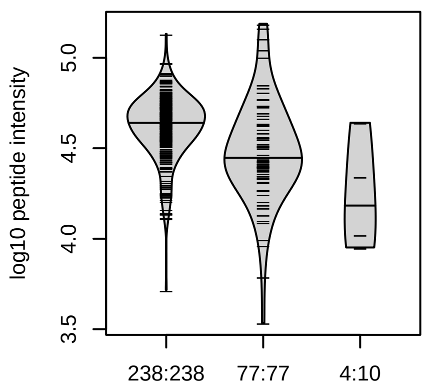

17:34307457:T:C_C
p = 2e−08, beta = −0.645, N = 319

VPEWVNTPSTCCLK pc2
O15467

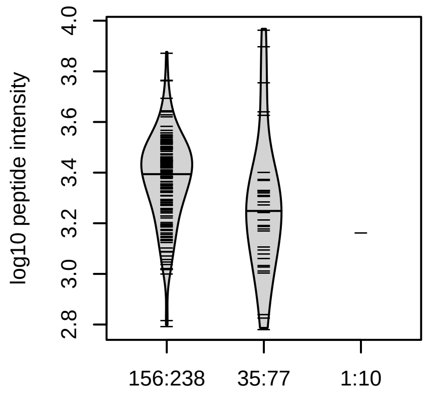

17:34307457:T:C_C
p = 0.00069, beta = −0.582, N = 192

Assay Target: CCL16
UniProt: O15467
rsID: rs112689088
Target SNP: 17:34307457:T:C
Proxy SNP: 17:34307457:T:C
UKBPPP log10(p): 3249.4
UKBPPP BETA: −1.203
UKBPPP MSPA: 0.995
::*:*:–
1254:1238:1219:1218:27

20. PON1 (P27169)
UKBPPP MSPA=0.945

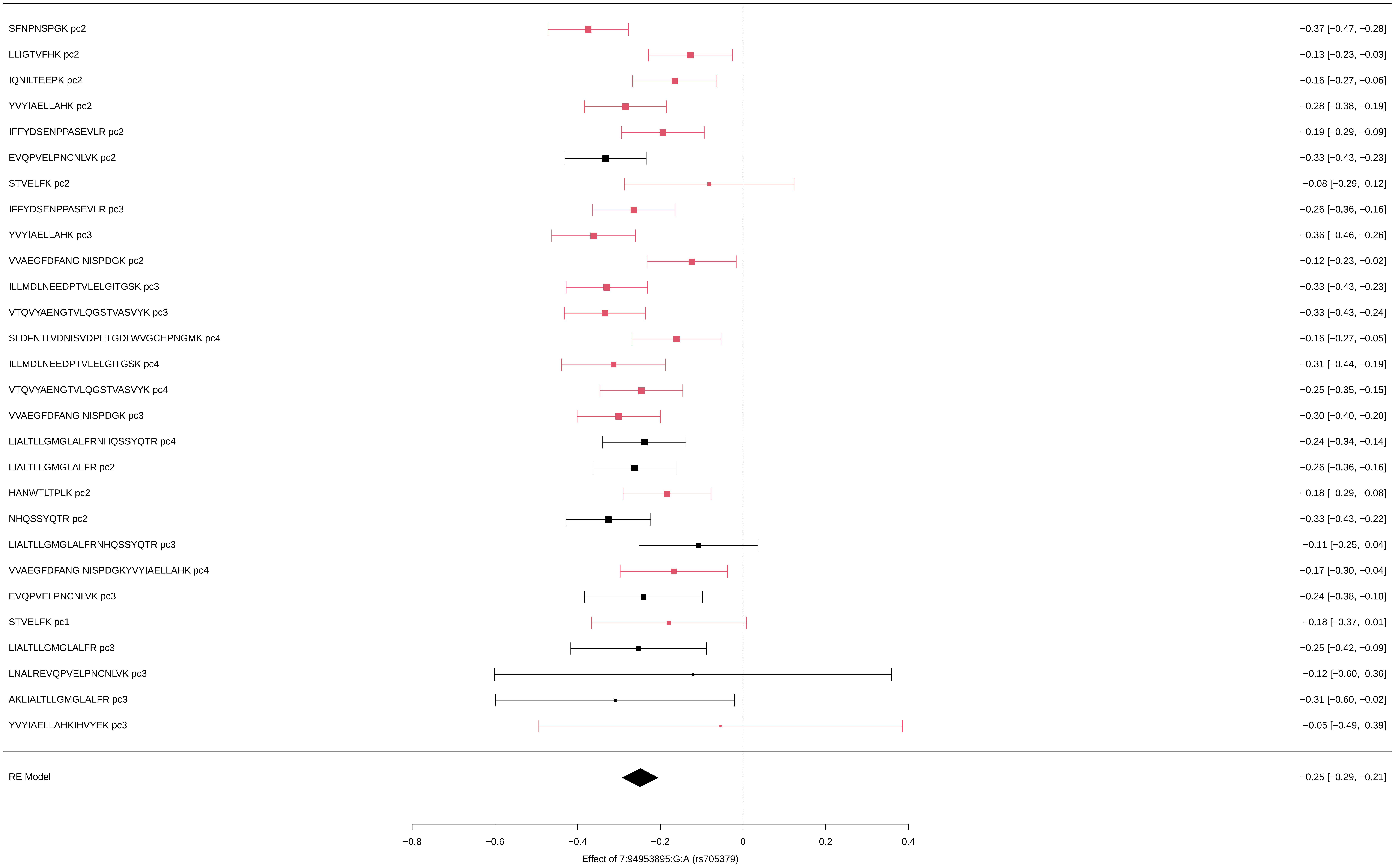

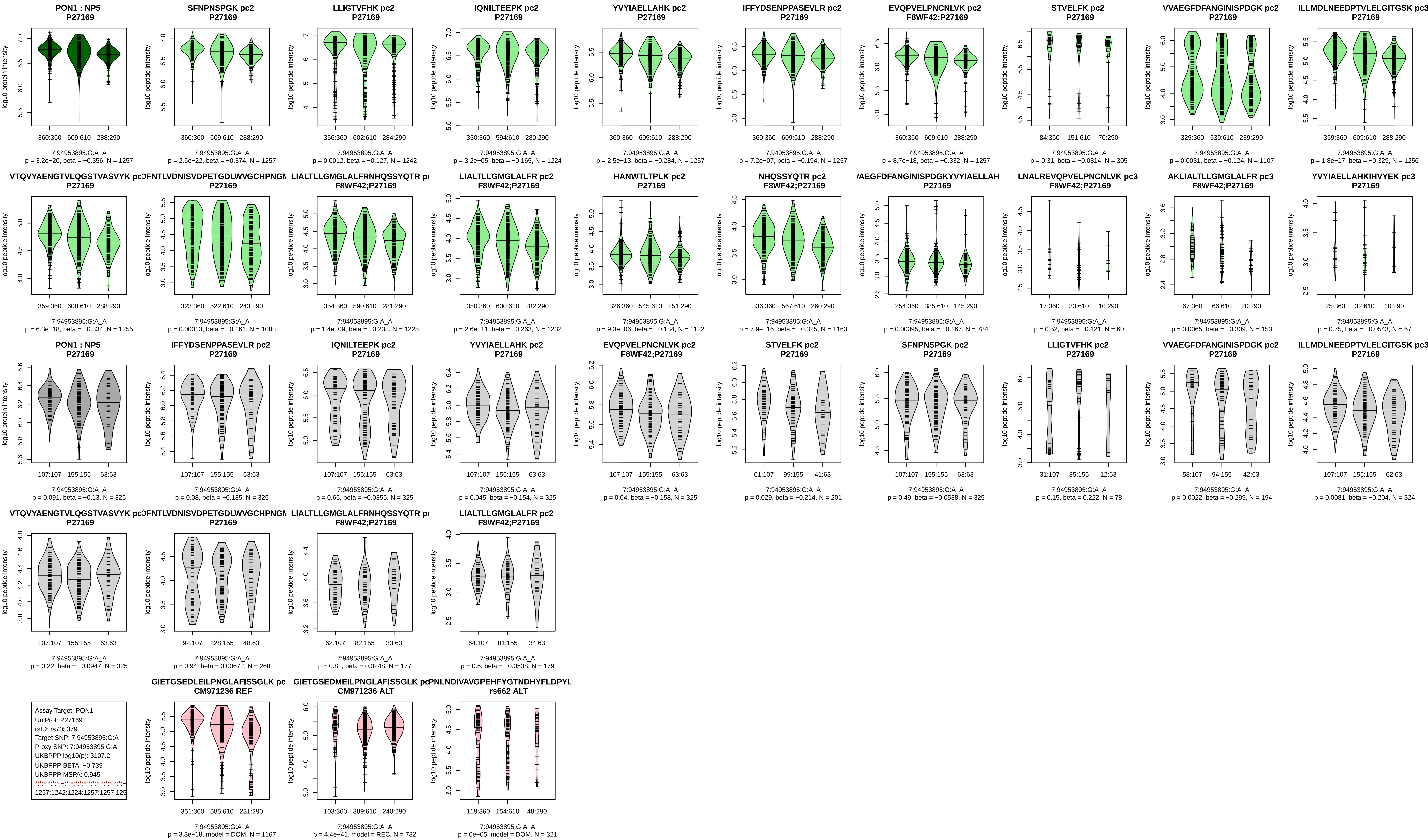

PON1 : NP5
P27169
360:360 609:610 288:290
7:94953895:G:A_A
p = 3.2e−20, beta = −0.356, N = 1257
SFNPNSPGK pc2
P27169
360:360 609:610 288:290
7:94953895:G:A_A
p = 2.6e−22, beta = −0.374, N = 1257
LLIGTVFHK pc2
P27169
356:360 602:610 284:290
7:94953895:G:A_A
p = 0.0012, beta = −0.127, N = 1242
IQNILTEEPK pc2
P27169
350:360 594:610 280:290
7:94953895:G:A_A
p = 3.2e−05, beta = −0.165, N = 1224
YVYIAELLAHK pc2
P27169
360:360 609:610 288:290
7:94953895:G:A_A
p = 2.5e−13, beta = −0.284, N = 1257
IFFYDSENPPASEVLR pc2
P27169
360:360 609:610 288:290
7:94953895:G:A_A
p = 7.2e−07, beta = −0.194, N = 1257
EVQPVELPNCNLVK pc2
F8WF42;P27169
360:360 609:610 288:290
7:94953895:G:A_A
p = 8.7e−18, beta = −0.332, N = 1257
STVELFK pc2
P27169
84:360 151:610 70:290
7:94953895:G:A_A
p = 0.31, beta = −0.0814, N = 305
VVAEGFDFANGINISPDGK pc2
P27169
329:360 539:610 239:290
7:94953895:G:A_A
p = 0.0031, beta = −0.124, N = 1107
ILLMDLNEEDPTVLELGITGSK pc3
P27169
359:360 609:610 288:290
7:94953895:G:A_A
p = 1.8e−17, beta = −0.329, N = 1256
VTQVYAENGTVLQGSTVASVYK pc
P27169
359:360 608:610 288:290
7:94953895:G:A_A
p = 6.3e−18, beta = −0.334, N = 1255
FNTLVDNISVDPETGDLWVGCHPNGM
P27169
323:360 522:610 243:290
7:94953895:G:A_A
p = 0.00013, beta = −0.161, N = 1088
LIALTLLGMGLALFRNHQSSYQTR p
F8WF42;P27169
354:360 590:610 281:290
7:94953895:G:A_A
p = 1.4e−09, beta = −0.238, N = 1225
LIALTLLGMGLALFR pc2
F8WF42;P27169
350:360 600:610 282:290
7:94953895:G:A_A
p = 2.6e−11, beta = −0.263, N = 1232
HANWTLTPLK pc2
P27169
326:360 545:610 251:290
7:94953895:G:A_A
p = 9.3e−06, beta = −0.184, N = 1122
NHQSSYQTR pc2
F8WF42;P27169
336:360 567:610 260:290
7:94953895:G:A_A
p = 7.9e−16, beta = −0.325, N = 1163
AEGFDFANGINISPDGKYVYIAELLAH
P27169
254:360 385:610 145:290
7:94953895:G:A_A
p = 0.00095, beta = −0.167, N = 784
LNALREVQPVELPNCNLVK pc3
F8WF42;P27169
17:360 33:610 10:290
7:94953895:G:A_A
p = 0.52, beta = −0.121, N = 60
AKLIALTLLGMGLALFR pc3
F8WF42;P27169
67:360 66:610 20:290
7:94953895:G:A_A
p = 0.0065, beta = −0.309, N = 153
YVYIAELLAHKIHVYEK pc3
P27169
25:360 32:610 10:290
7:94953895:G:A_A
p = 0.75, beta = −0.0543, N = 67
PON1 : NP5
P27169
107:107 155:155 63:63
7:94953895:G:A_A
p = 0.091, beta = −0.13, N = 325
IFFYDSENPPASEVLR pc2
P27169
107:107 155:155 63:63
7:94953895:G:A_A
p = 0.08, beta = −0.135, N = 325
IQNILTEEPK pc2
P27169
107:107 155:155 63:63
7:94953895:G:A_A
p = 0.65, beta = −0.0355, N = 325
YVYIAELLAHK pc2
P27169
107:107 155:155 63:63
7:94953895:G:A_A
p = 0.045, beta = −0.154, N = 325
EVQPVELPNCNLVK pc2
F8WF42;P27169
107:107 155:155 63:63
7:94953895:G:A_A
p = 0.04, beta = −0.158, N = 325
STVELFK pc2
P27169
61:107 99:155 41:63
7:94953895:G:A_A
p = 0.029, beta = −0.214, N = 201
SFNPNSPGK pc2
P27169
107:107 155:155 63:63
7:94953895:G:A_A
p = 0.49, beta = −0.0538, N = 325
LLIGTVFHK pc2
P27169
31:107 35:155 12:63
7:94953895:G:A_A
p = 0.15, beta = 0.222, N = 78
VVAEGFDFANGINISPDGK pc2
P27169
58:107 94:155 42:63
7:94953895:G:A_A
p = 0.0022, beta = −0.299, N = 194
ILLMDLNEEDPTVLELGITGSK pc3
P27169
107:107 155:155 62:63
7:94953895:G:A_A
p = 0.0081, beta = −0.204, N = 324
VTQVYAENGTVLQGSTVASVYK pc
P27169
107:107 155:155 63:63
7:94953895:G:A_A
p = 0.22, beta = −0.0947, N = 325
FNTLVDNISVDPETGDLWVGCHPNGM
P27169
92:107 128:155 48:63
7:94953895:G:A_A
p = 0.94, beta = 0.00672, N = 268
LIALTLLGMGLALFRNHQSSYQTR p
F8WF42;P27169
62:107 82:155 33:63
7:94953895:G:A_A
p = 0.81, beta = 0.0248, N = 177
LIALTLLGMGLALFR pc2
F8WF42;P27169
64:107 81:155 34:63
7:94953895:G:A_A
p = 0.6, beta = −0.0538, N = 179
Assay Target: PON1
UniProt: P27169
rsID: rs705379
Target SNP: 7:94953895:G:A
Proxy SNP: 7:94953895:G:A
UKBPPP log10(p): 3107.2
UKBPPP BETA: −0.739
UKBPPP MSPA: 0.945
1257:1242:1224:1257:1257:125
GIETGSEDLEILPNGLAFISSGLK pc
CM971236 REF
351:360 585:610 231:290
7:94953895:G:A_A
p = 3.3e−18, model = DOM, N = 1167
GIETGSEDMEILPNGLAFISSGLK pc
CM971236 ALT
103:360 389:610 240:290
7:94953895:G:A_A
p = 4.4e−41, model = REC, N = 732
PNLNDIVAVGPEHFYGTNDHYFLDPYL
rs662 ALT
119:360 154:610 48:290
7:94953895:G:A_A
p = 6e−05, model = DOM, N = 321
log10 protein intensity
log10 peptide intensity

21. APOE (P02649)
UKBPPP MSPA=0.421

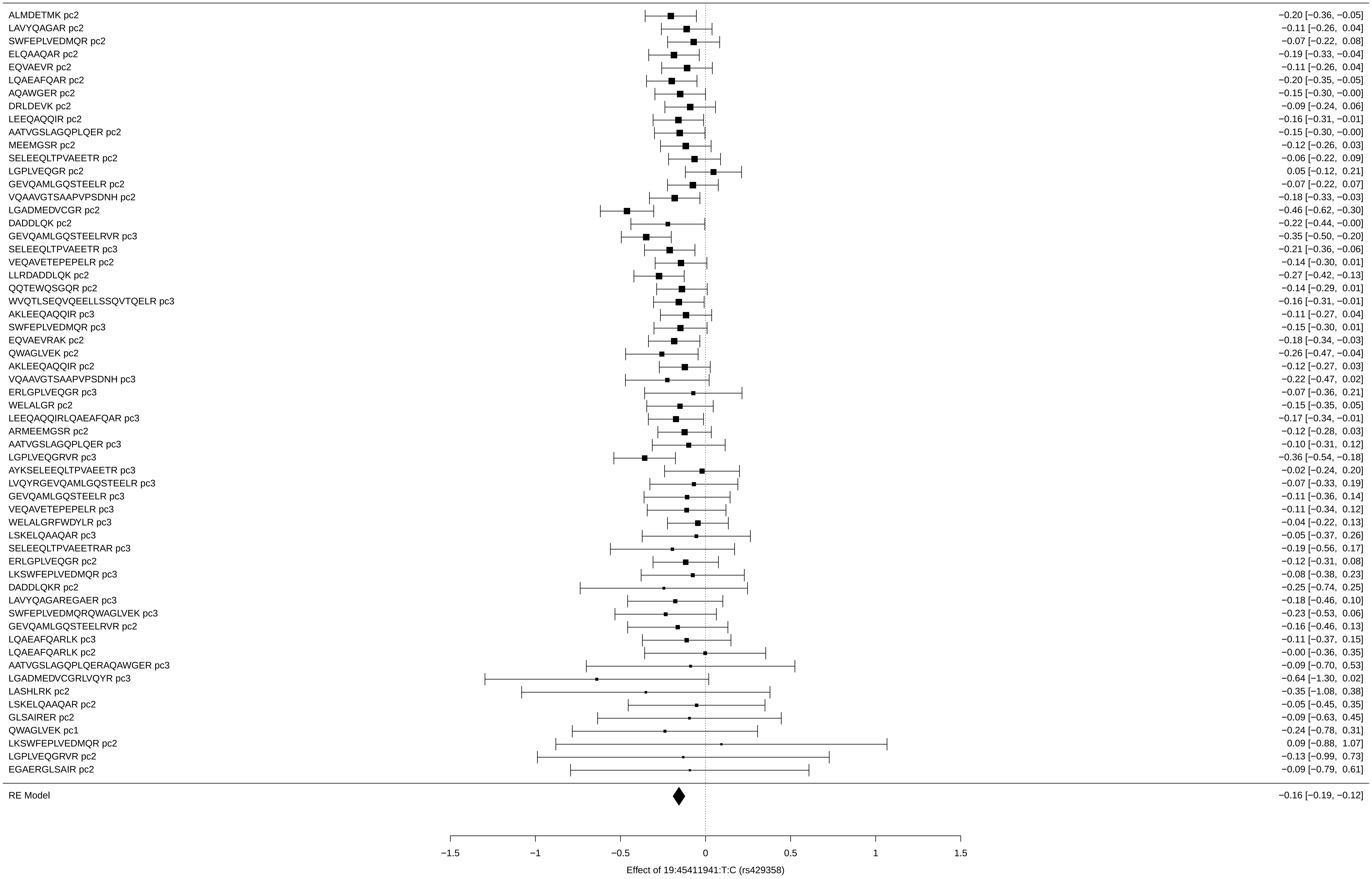

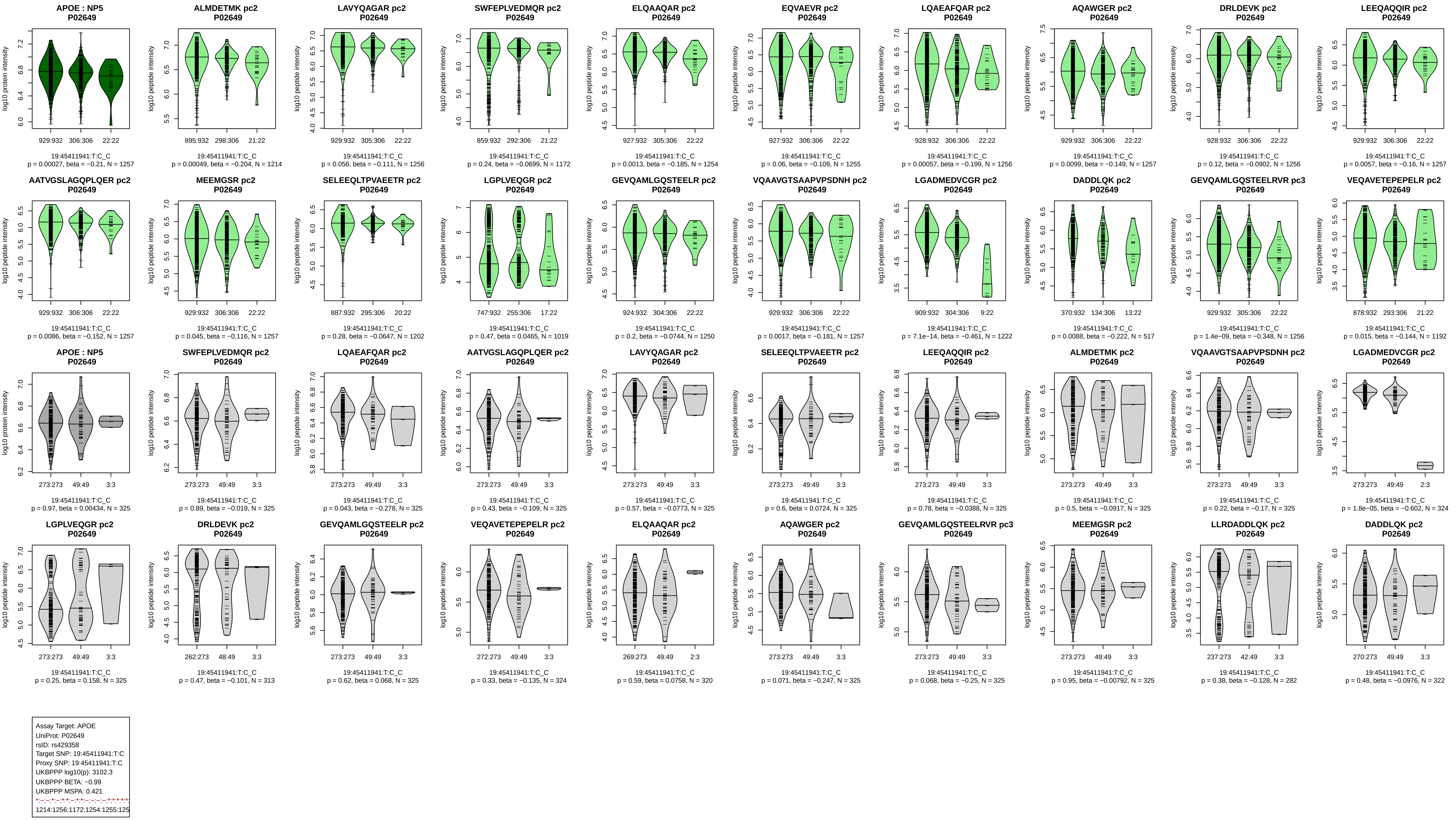
APOE : NP5 P02649
p = 0.00027, beta = −0.21, N = 1257
ALMDETMK pc2 P02649
p = 0.00049, beta = −0.204, N = 1214
LAVYQAGAR pc2 P02649
p = 0.056, beta = −0.111, N = 1256
SWFEPLVEDMQR pc2 P02649
p = 0.24, beta = −0.0699, N = 1172
ELQAAQAR pc2 P02649
p = 0.0013, beta = −0.185, N = 1254
EQVAEVR pc2 P02649
p = 0.06, beta = −0.109, N = 1255
LQAEAFQAR pc2 P02649
p = 0.00057, beta = −0.199, N = 1256
AQAWGER pc2 P02649
p = 0.0099, beta = −0.149, N = 1257
DRLDEVK pc2 P02649
p = 0.12, beta = −0.0902, N = 1256
LEEQAQQIR pc2 P02649
p = 0.0057, beta = −0.16, N = 1257
AATVGSLAGQPLQER pc2 P02649
p = 0.0086, beta = −0.152, N = 1257
MEEMGSR pc2 P02649
p = 0.045, beta = −0.116, N = 1257
SELEEQLTPVAEETR pc2 P02649
p = 0.28, beta = −0.0647, N = 1202
LGPLVEQGR pc2 P02649
p = 0.47, beta = −0.0465, N = 1019
GEVQAMLGQSTEELR pc2 P02649
p = 0.2, beta = −0.0744, N = 1250
VQAAVGTSAAPVPSDNH pc2 P02649
p = 0.0017, beta = −0.181, N = 1257
LGADMEDVCGR pc2 P02649
p = 7.1e−14, beta = −0.461, N = 1222
DADDLQK pc2 P02649
p = 0.0088, beta = −0.222, N = 517
GEVQAMLGQSTEELRVR pc3 P02649
p = 1.4e−09, beta = −0.348, N = 1256
VEQAVETEPEPELR pc2 P02649
p = 0.015, beta = −0.144, N = 1192
APOE : NP5 P02649
p = 0.97, beta = 0.00434, N = 325
SWFEPLVEDMQR pc2 P02649
p = 0.89, beta = −0.019, N = 325
LQAEAFQAR pc2 P02649
p = 0.043, beta = −0.278, N = 325
AATVGSLAGQPLQER pc2 P02649
p = 0.43, beta = −0.109, N = 325
LAVYQAGAR pc2 P02649
p = 0.57, beta = −0.0773, N = 325
SELEEQLTPVAEETR pc2 P02649
p = 0.6, beta = 0.0724, N = 325
LEEQAQQIR pc2 P02649
p = 0.78, beta = −0.0388, N = 325
ALMDETMK pc2 P02649
p = 0.5, beta = −0.0917, N = 325
VQAAVGTSAAPVPSDNH pc2 P02649
p = 0.22, beta = −0.17, N = 325
LGADMEDVCGR pc2 P02649
p = 1.8e−05, beta = −0.602, N = 324
LGPLVEQGR pc2 P02649
p = 0.25, beta = 0.158, N = 325
DRLDEVK pc2 P02649
p = 0.47, beta = −0.101, N = 313
GEVQAMLGQSTEELR pc2 P02649
p = 0.62, beta = 0.068, N = 325
VEQAVETEPEPELR pc2 P02649
p = 0.33, beta = −0.135, N = 324
ELQAAQAR pc2 P02649
p = 0.59, beta = 0.0758, N = 320
AQAWGER pc2 P02649
p = 0.071, beta = −0.247, N = 325
GEVQAMLGQSTEELRVR pc3 P02649
p = 0.068, beta = −0.25, N = 325
MEEMGSR pc2 P02649
p = 0.95, beta = −0.00792, N = 325
LLRDADDLQK pc2 P02649
p = 0.38, beta = −0.128, N = 282
DADDLQK pc2 P02649
p = 0.48, beta = −0.0976, N = 322
19:45411941:T:C_C
log10 protein intensity
log10 peptide intensity
Assay Target: APOE
UniProt: P02649
rsID: rs429358
Target SNP: 19:45411941:T:C
Proxy SNP: 19:45411941:T:C
UKBPPP log10(p): 3102.3
UKBPPP BETA: −0.99
UKBPPP MSPA: 0.421
1214:1256:1172:1254:1255:125

22. FUT8 (Q9BYC5)
UKBPPP MSPA=0.894

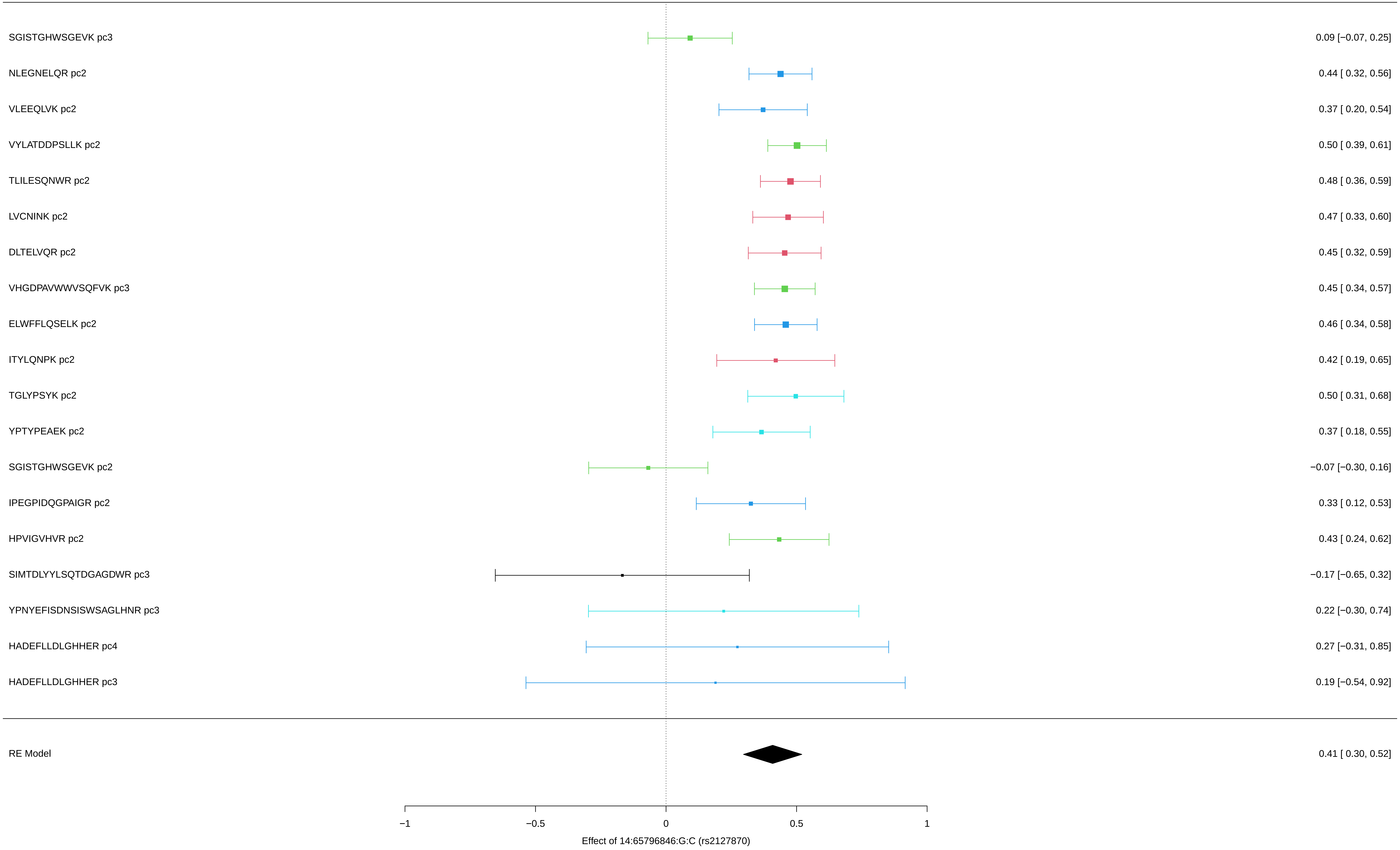

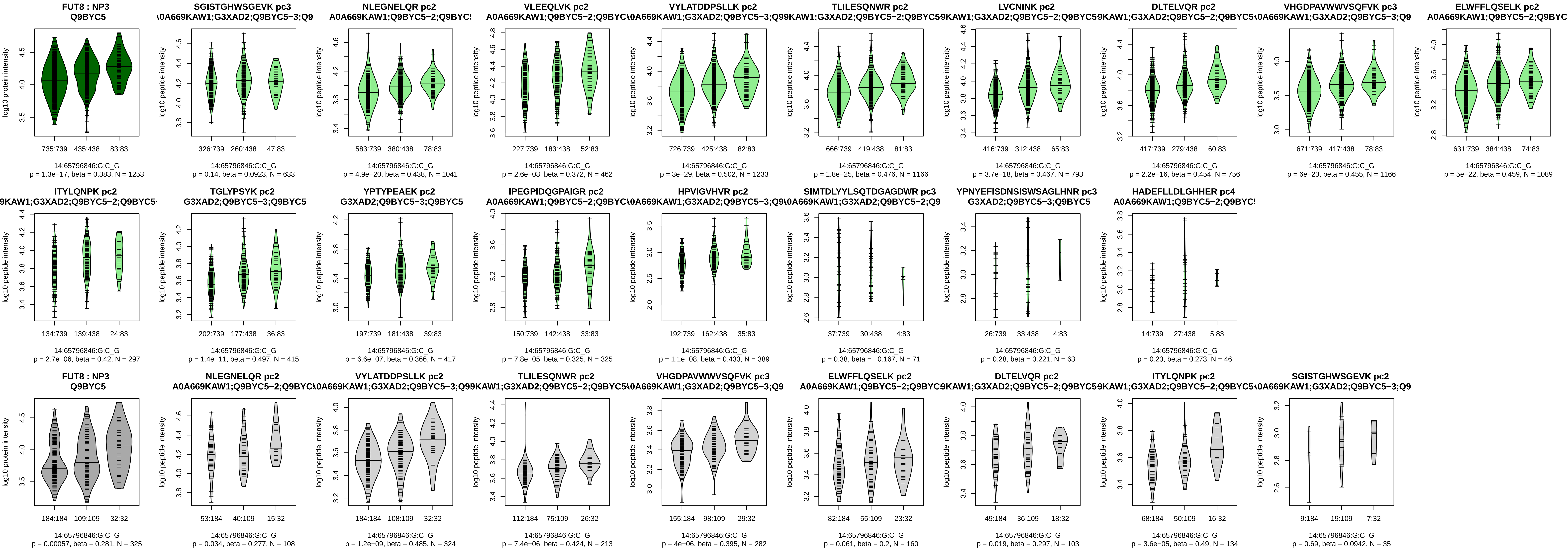

Assay Target: FUT8
UniProt: Q9BYC5
rsID: rs2127870
Target SNP: 14:65796846:G:C
Proxy SNP: 14:65796846:G:C
UKBPPP log10(p): 3097.8
UKBPPP BETA: −0.905
UKBPPP MSPA: 0.894
633:1041:462:1233:1166:793:75

23. VMO1 (Q7Z5L0)
UKBPPP MSPA=0.878

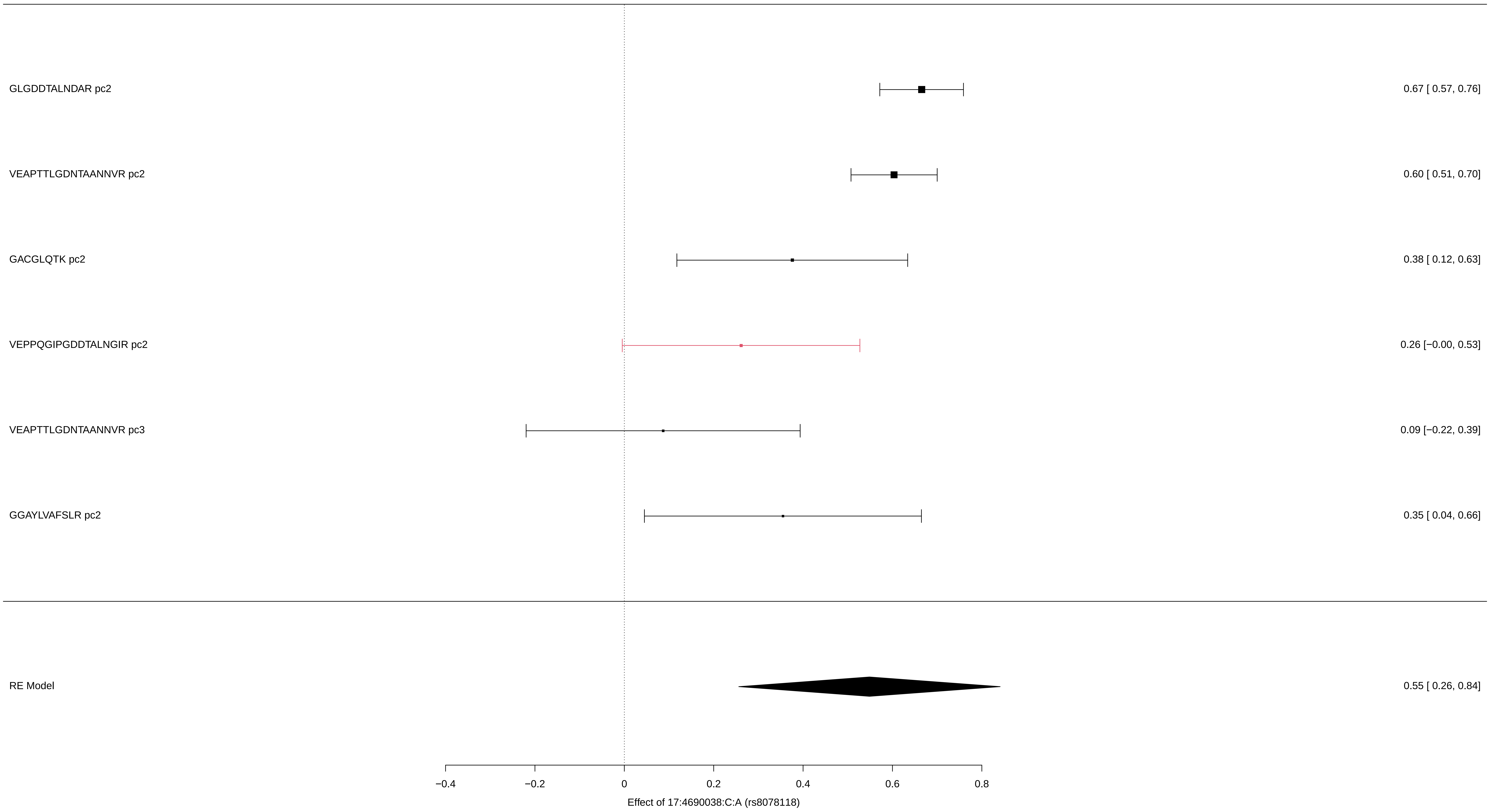

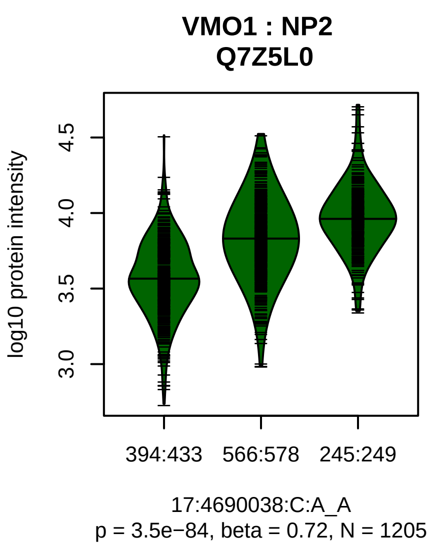

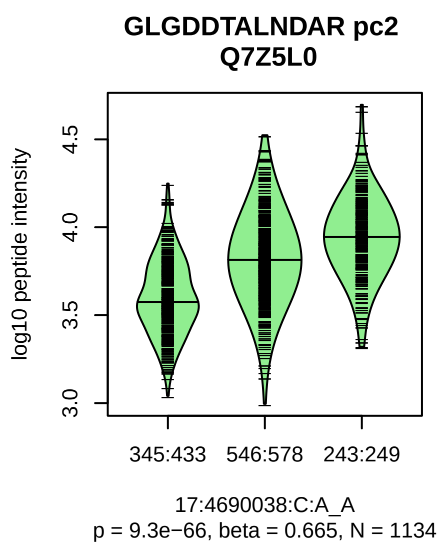

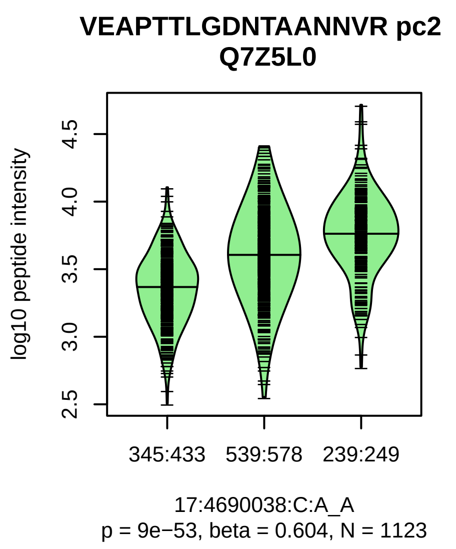

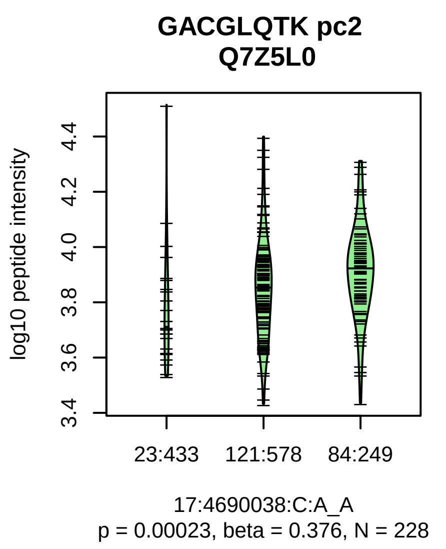

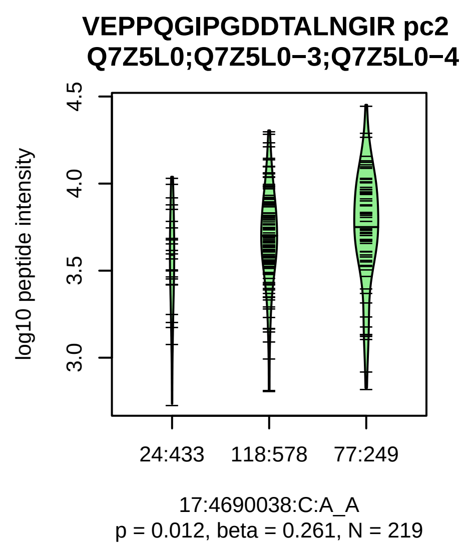

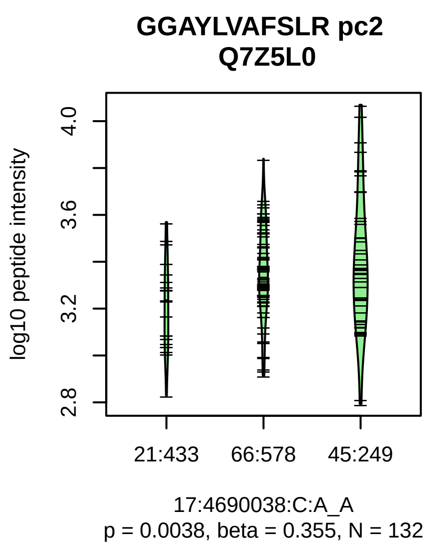

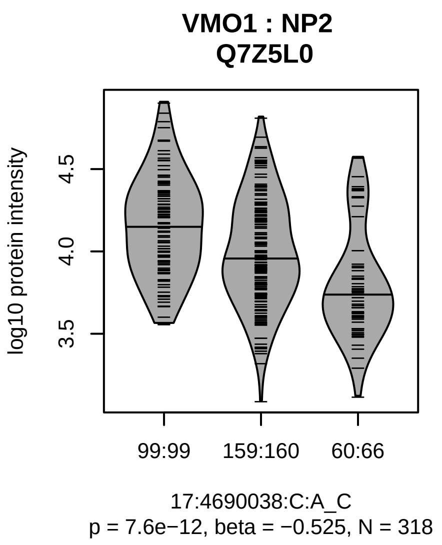

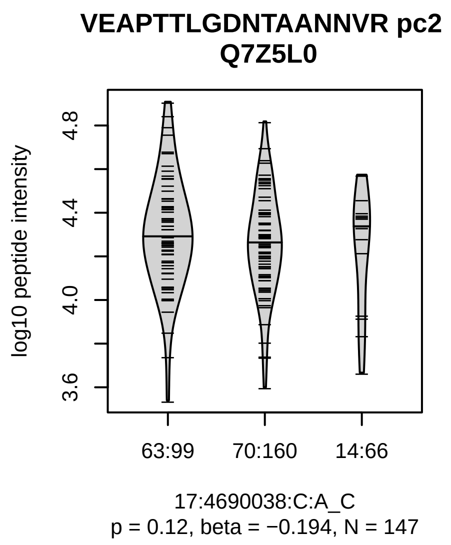

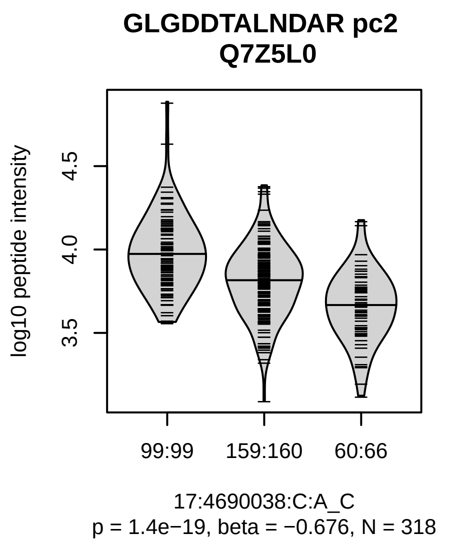

Assay Target: VMO1
UniProt: Q7Z5L0
rsID: rs8078118
Target SNP: 17:4690038:C:A
Proxy SNP: 17:4690038:C:A
UKBPPP log10(p): 3082.1
UKBPPP BETA: 0.732
UKBPPP MSPA: 0.878
::*:–:–:*
1134:1123:228:219:144:132

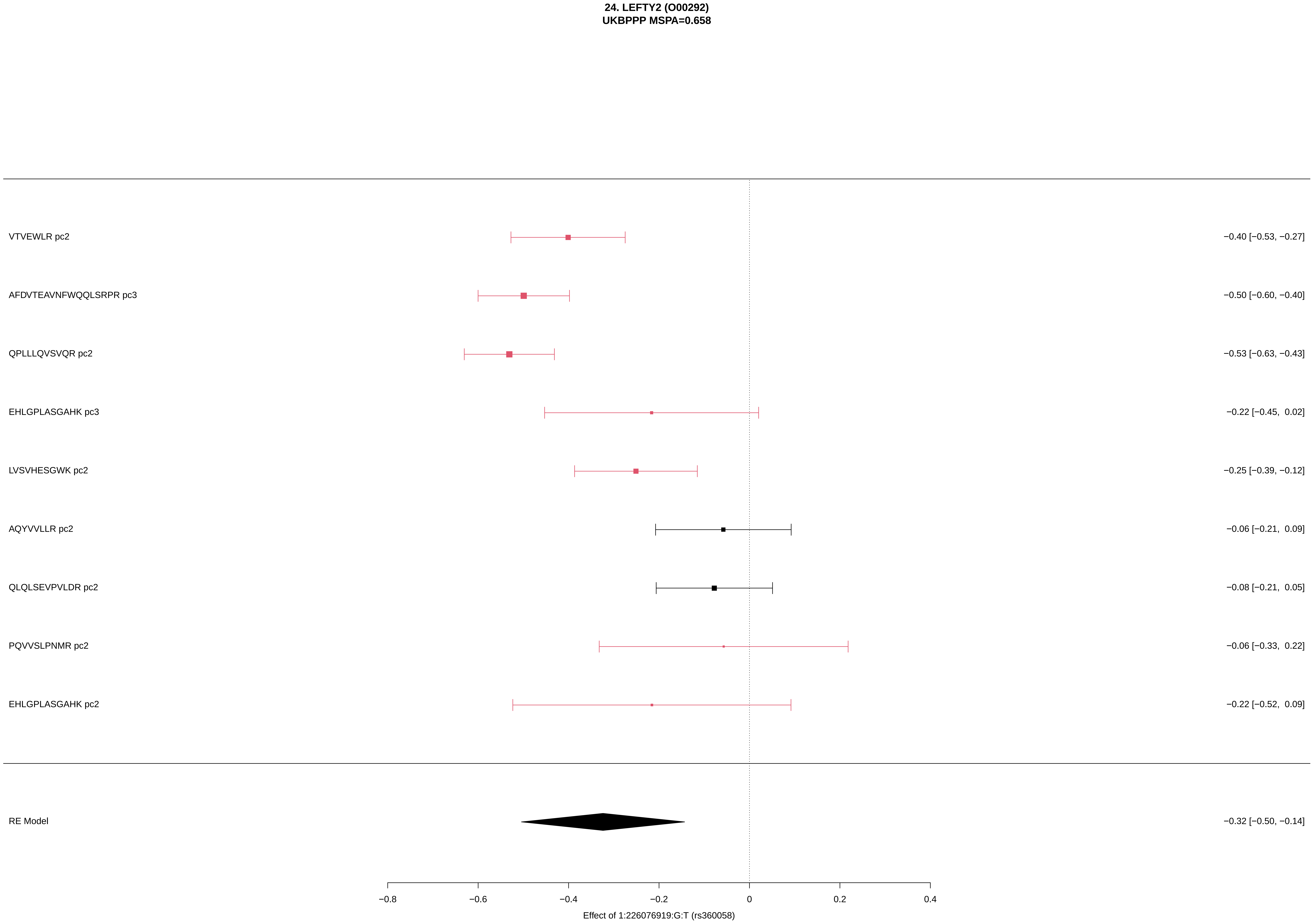

24. LEFTY2 (O00292)
UKBPPP MSPA=0.658
VTVEWLR pc2
−0.40 [−0.53, −0.27]
AFDVTEAVNFWQQLSRPR pc3
−0.50 [−0.60, −0.40]
QPLLLQVSVQR pc2
−0.53 [−0.63, −0.43]
EHLGPLASGAHK pc3
−0.22 [−0.45, 0.02]
LVSVHESGWK pc2
−0.25 [−0.39, −0.12]
AQYVVLLR pc2
−0.06 [−0.21, 0.09]
QLQLSEVPVLDR pc2
−0.08 [−0.21, 0.05]
PQVVSLPNMR pc2
−0.06 [−0.33, 0.22]
EHLGPLASGAHK pc2
−0.22 [−0.52, 0.09]
RE Model
−0.32 [−0.50, −0.14]
−0.8
−0.6
−0.4
−0.2
0
0.2
0.4
Effect of 1:226076919:G:T (rs360058)

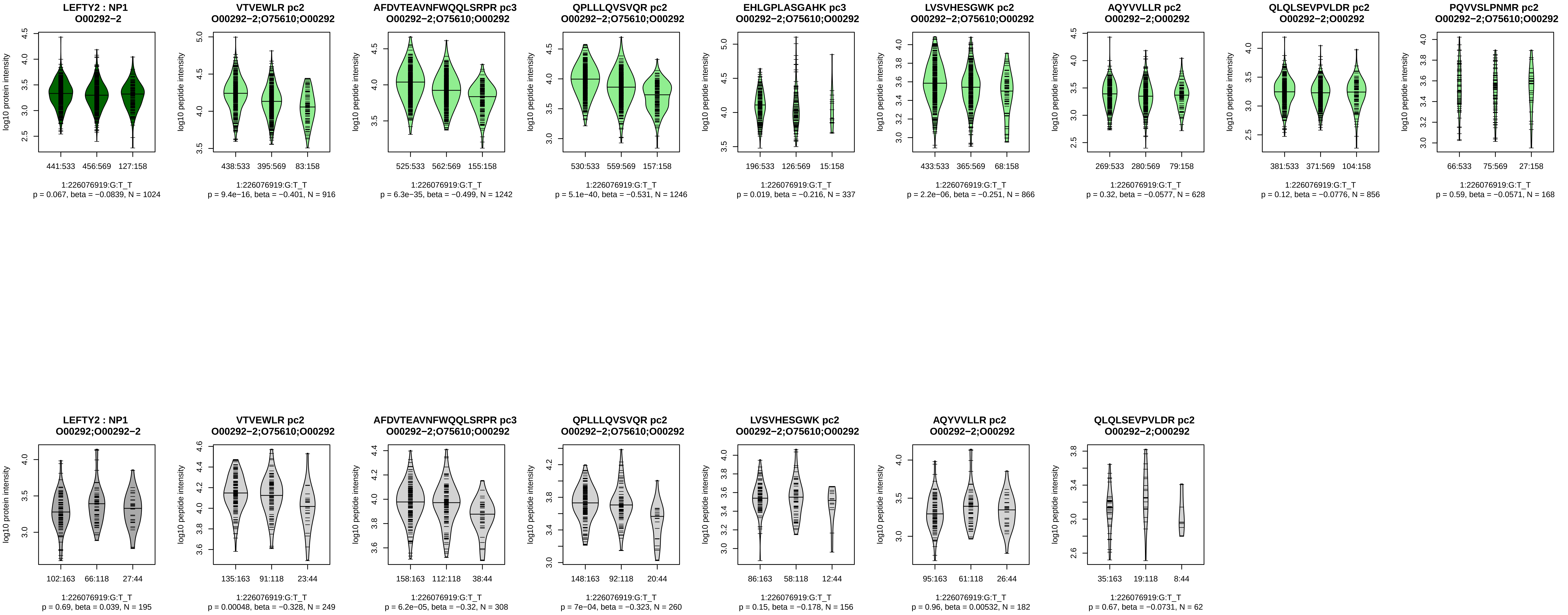

Assay Target: LEFTY2
UniProt: O00292
rsID: rs360058
Target SNP: 1:226076919:G:T
Proxy SNP: 1:226076919:G:T
UKBPPP log10(p): 2990.7
UKBPPP BETA: −0.709
UKBPPP MSPA: 0.658
::*:–:*:–:–:–:–
916:1242:1246:337:866:628:856

25. TGOLN2 (O43493)
UKBPPP MSPA=0

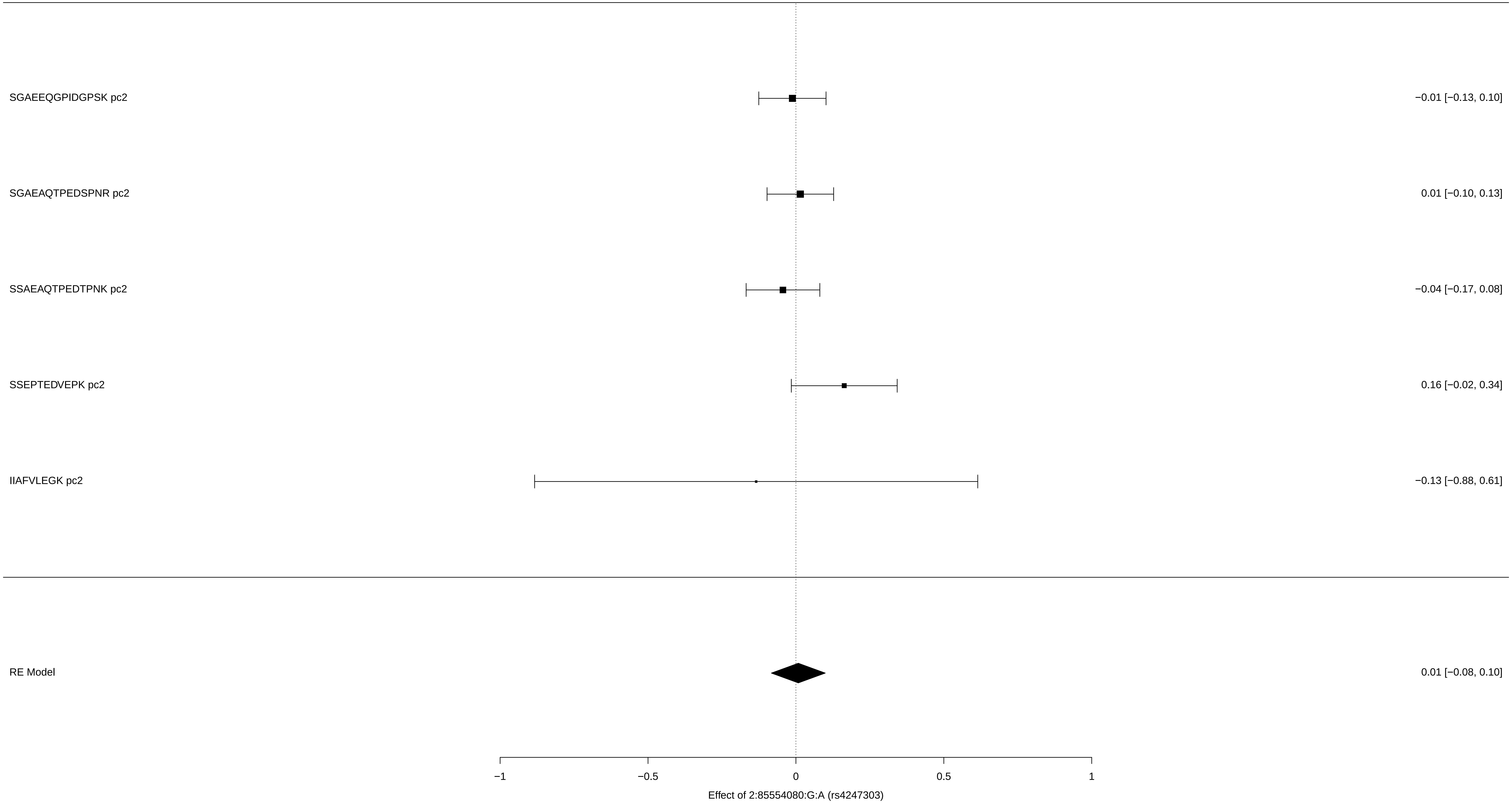

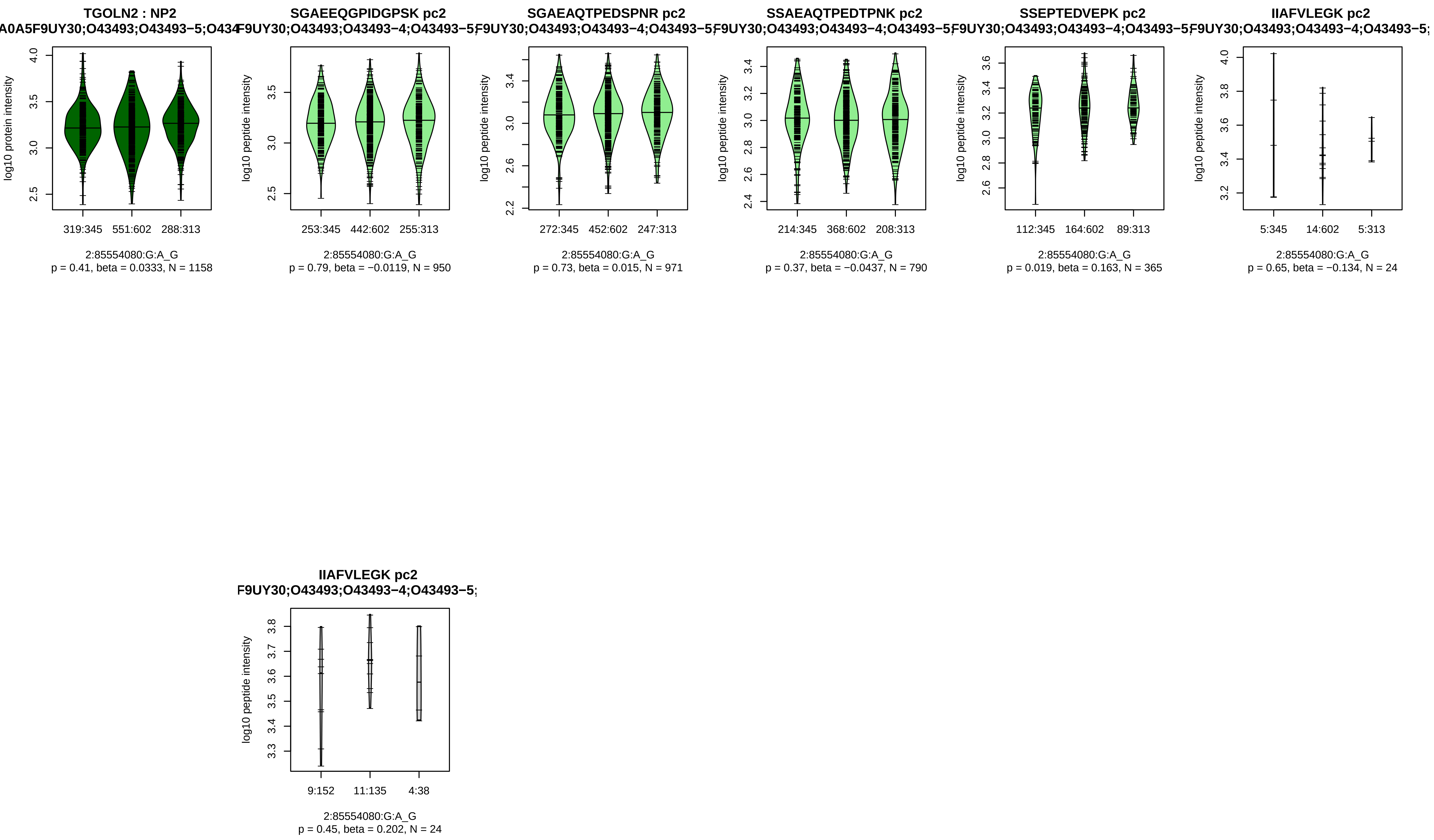

Assay Target: TGOLN2
UniProt: O43493
rsID: rs4247303
Target SNP: 2:85554080:G:A
Proxy SNP: 2:85554080:G:A
UKBPPP log10(p): 2870.9
UKBPPP BETA: −0.688
UKBPPP MSPA: 0
−:−:−:−:−:NA
950:971:790:365:24:12

26. CRTAC1 (Q9NQ79)
UKBPPP MSPA=0.169

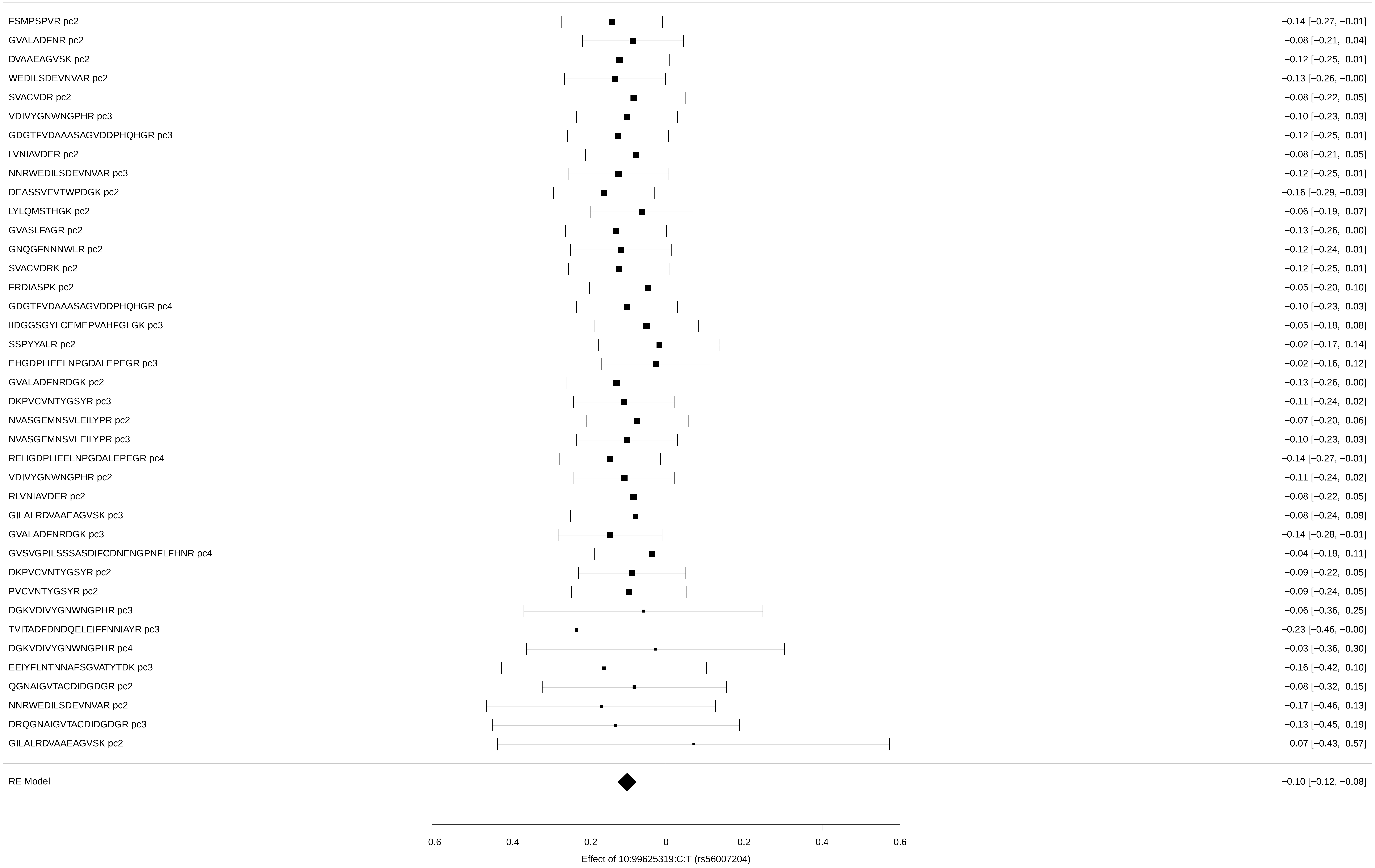

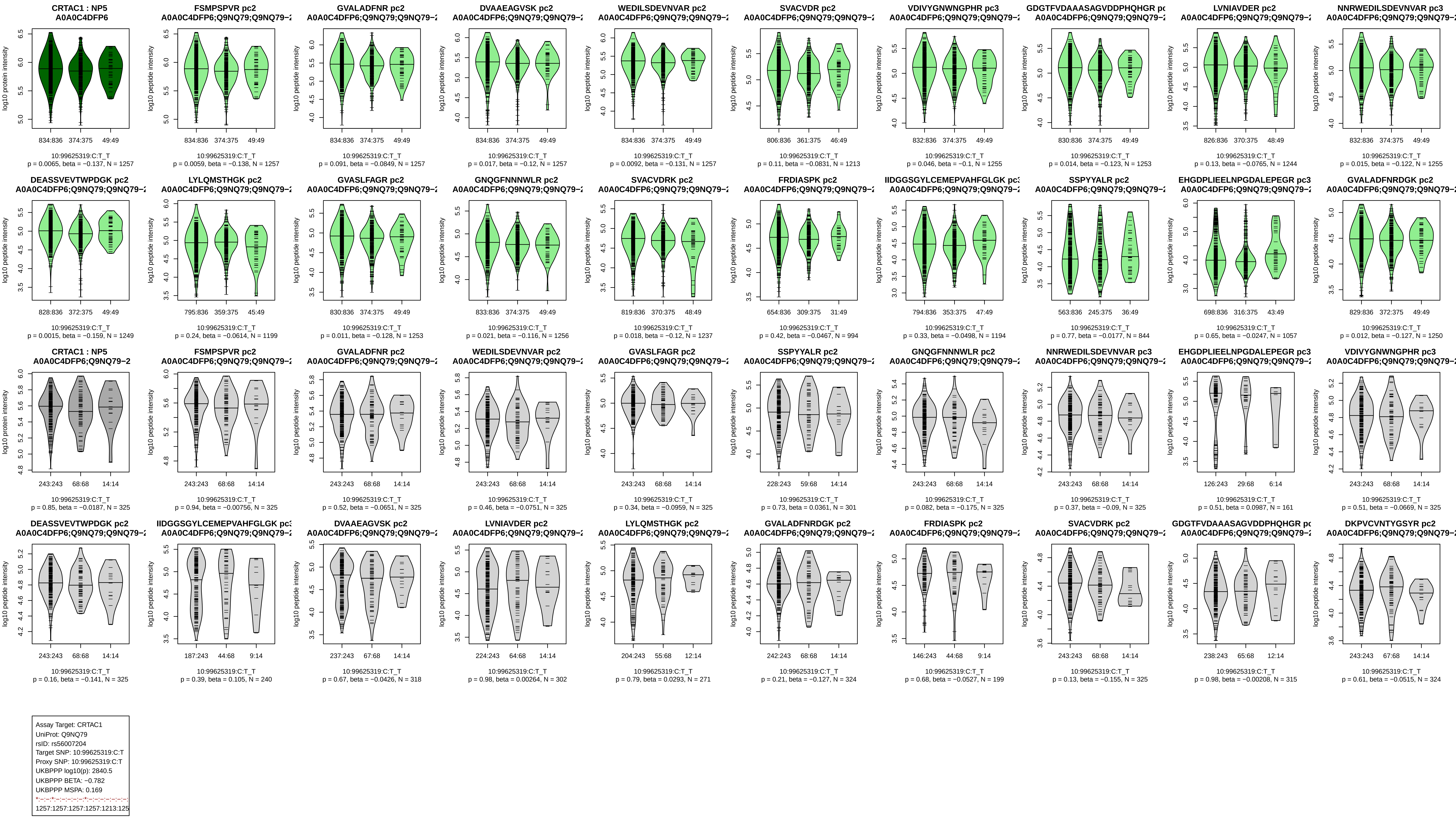
CRTAC1 : NP5
A0A0C4DFP6
p = 0.0065, beta = −0.137, N = 1257
FSMPSPVR pc2
p = 0.0059, beta = −0.138, N = 1257
GVALADFNR pc2
p = 0.091, beta = −0.0849, N = 1257
DVAAEAGVSK pc2
p = 0.017, beta = −0.12, N = 1257
WEDILSDEVNVAR pc2
p = 0.0092, beta = −0.131, N = 1257
SVACVDR pc2
p = 0.11, beta = −0.0831, N = 1213
VDIVYGNWNGPHR pc3
p = 0.046, beta = −0.1, N = 1255
GDGTFVDAAASAGVDDPHQHGR pc
p = 0.014, beta = −0.123, N = 1253
LVNIAVDER pc2
p = 0.13, beta = −0.0765, N = 1244
NNRWEDILSDEVNVAR pc3
p = 0.015, beta = −0.122, N = 1255
DEASSVEVTWPDGK pc2
p = 0.0015, beta = −0.159, N = 1249
LYLQMSTHGK pc2
p = 0.24, beta = −0.0614, N = 1199
GVASLFAGR pc2
p = 0.011, beta = −0.128, N = 1253
GNQGFNNNWLR pc2
p = 0.021, beta = −0.116, N = 1256
SVACVDRK pc2
p = 0.018, beta = −0.12, N = 1237
FRDIASPK pc2
p = 0.42, beta = −0.0467, N = 994
IIDGGSGYLCEMEPVAHFGLGK pc3
p = 0.33, beta = −0.0498, N = 1194
SSPYYALR pc2
p = 0.77, beta = −0.0177, N = 844
EHGDPLIEELNPGDALEPEGR pc3
p = 0.65, beta = −0.0247, N = 1057
GVALADFNRDGK pc2
p = 0.012, beta = −0.127, N = 1250
CRTAC1 : NP5
p = 0.85, beta = −0.0187, N = 325
FSMPSPVR pc2
p = 0.94, beta = −0.00756, N = 325
GVALADFNR pc2
p = 0.52, beta = −0.0651, N = 325
WEDILSDEVNVAR pc2
p = 0.46, beta = −0.0751, N = 325
GVASLFAGR pc2
p = 0.34, beta = −0.0959, N = 325
SSPYYALR pc2
p = 0.73, beta = 0.0361, N = 301
GNQGFNNNWLR pc2
p = 0.082, beta = −0.175, N = 325
NNRWEDILSDEVNVAR pc3
p = 0.37, beta = −0.09, N = 325
EHGDPLIEELNPGDALEPEGR pc3
p = 0.51, beta = 0.0987, N = 161
VDIVYGNWNGPHR pc3
p = 0.51, beta = −0.0669, N = 325
DEASSVEVTWPDGK pc2
p = 0.16, beta = −0.141, N = 325
IIDGGSGYLCEMEPVAHFGLGK pc3
p = 0.39, beta = 0.105, N = 240
DVAAEAGVSK pc2
p = 0.67, beta = −0.0426, N = 318
LVNIAVDER pc2
p = 0.98, beta = 0.00264, N = 302
LYLQMSTHGK pc2
p = 0.79, beta = 0.0293, N = 271
GVALADFNRDGK pc2
p = 0.21, beta = −0.127, N = 324
FRDIASPK pc2
p = 0.68, beta = −0.0527, N = 199
SVACVDRK pc2
p = 0.13, beta = −0.155, N = 325
GDGTFVDAAASAGVDDPHQHGR pc
p = 0.98, beta = −0.00208, N = 315
DKPVCVNTYGSYR pc2
p = 0.61, beta = −0.0515, N = 324
10:99625319:C:T_T
Assay Target: CRTAC1
UniProt: Q9NQ79
rsID: rs56007204
Target SNP: 10:99625319:C:T
Proxy SNP: 10:99625319:C:T
UKBPPP log10(p): 2840.5
UKBPPP BETA: −0.782
UKBPPP MSPA: 0.169
1257:1257:1257:1257:1213:125

27. CPVL (Q9H3G5)
UKBPPP MSPA=0

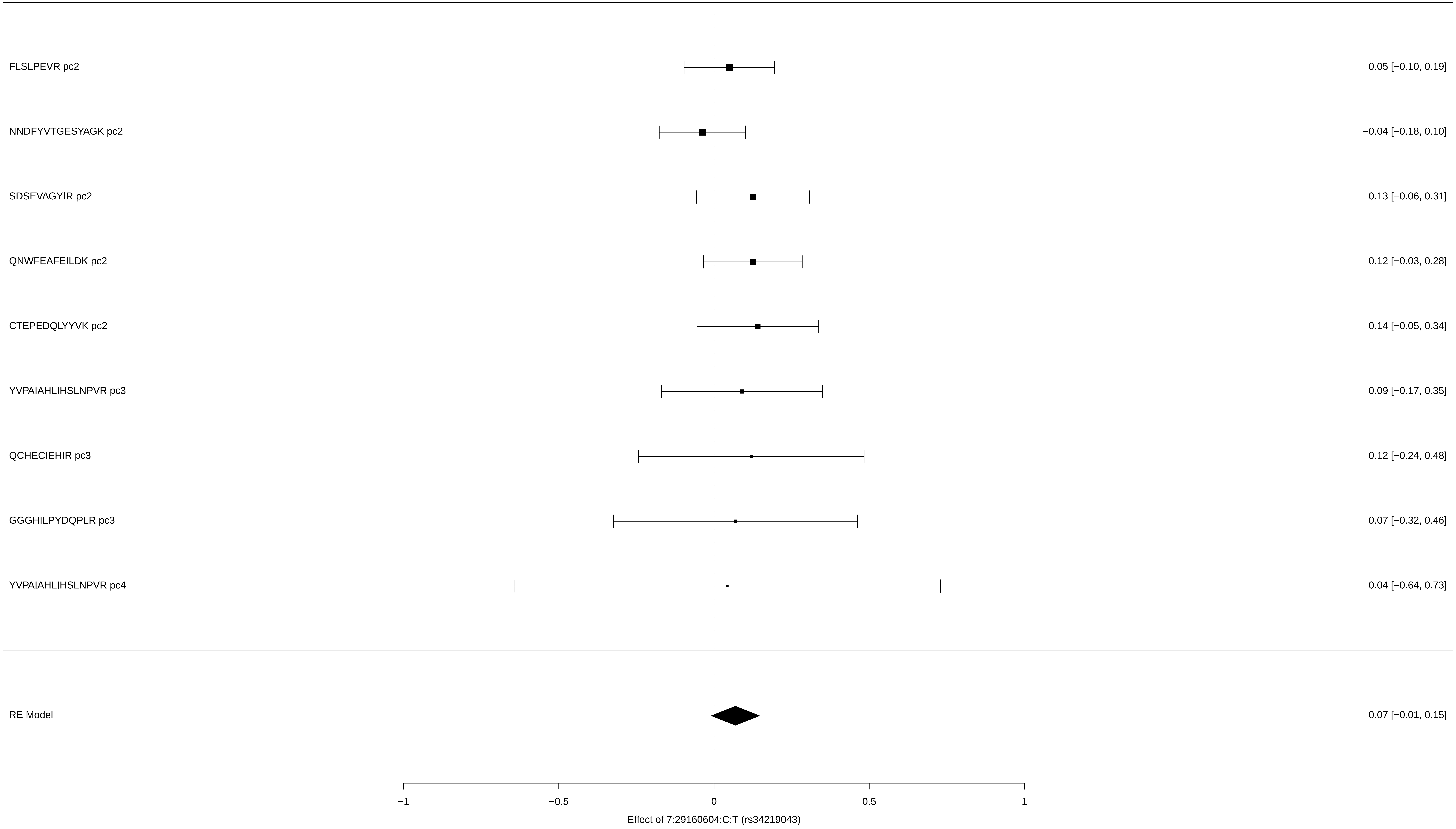

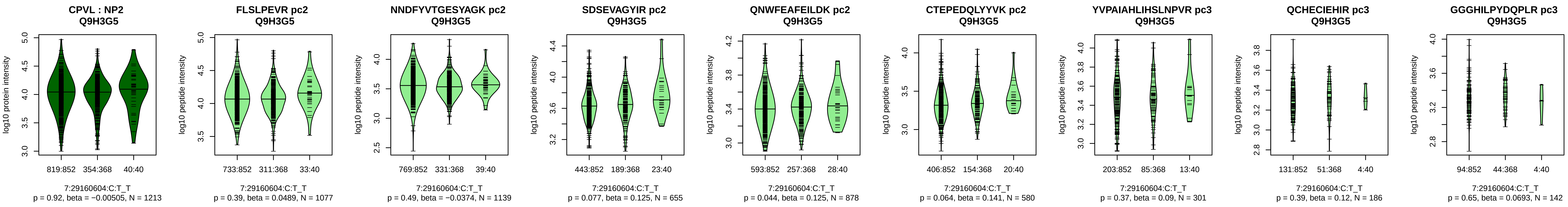

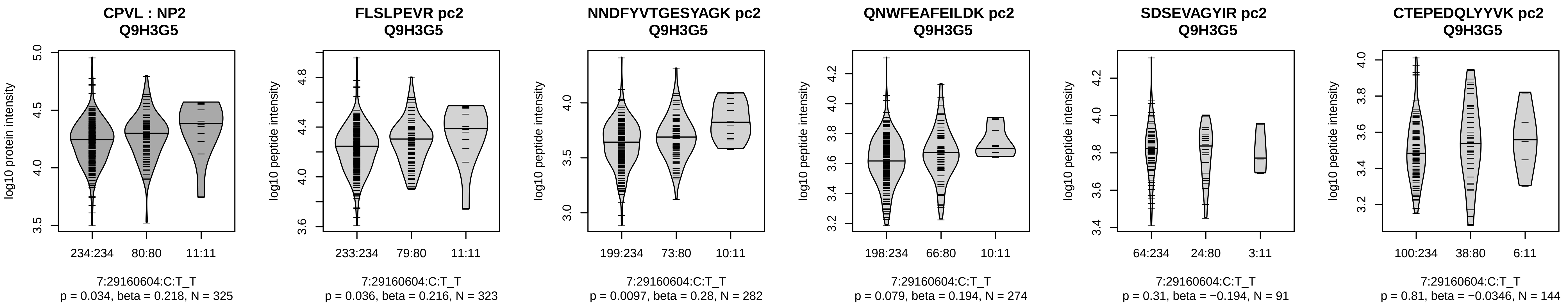

Assay Target: CPVL
UniProt: Q9H3G5
rsID: rs34219043
Target SNP: 7:29160604:C:T
Proxy SNP: 7:29160604:C:T
UKBPPP log10(p): 2728.2
UKBPPP BETA: −0.842
UKBPPP MSPA: 0
1077:1139:655:878:580:301:18[illegible]

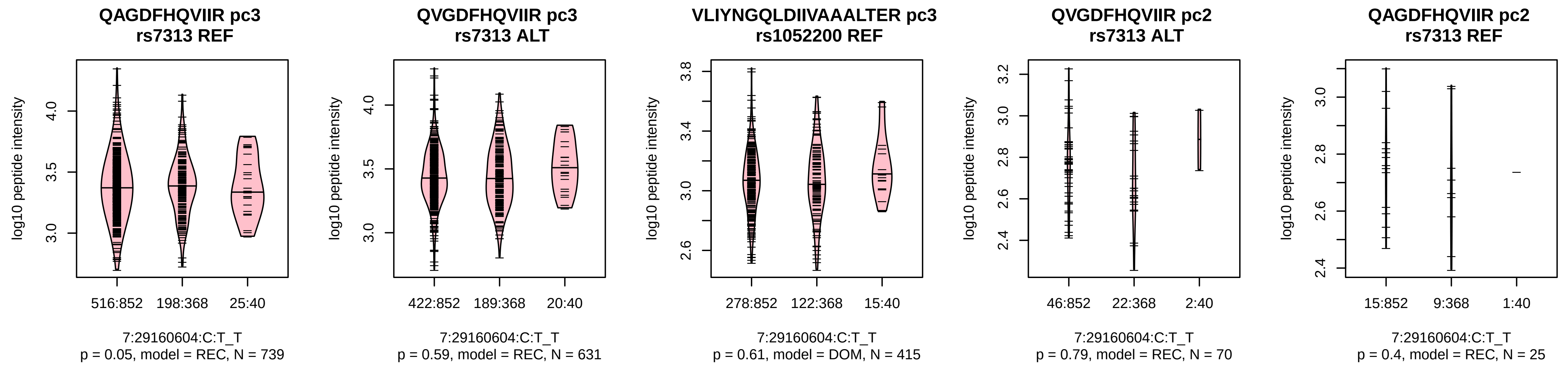

28. SERPINA11 (Q86U17)
UKBPPP MSPA=1

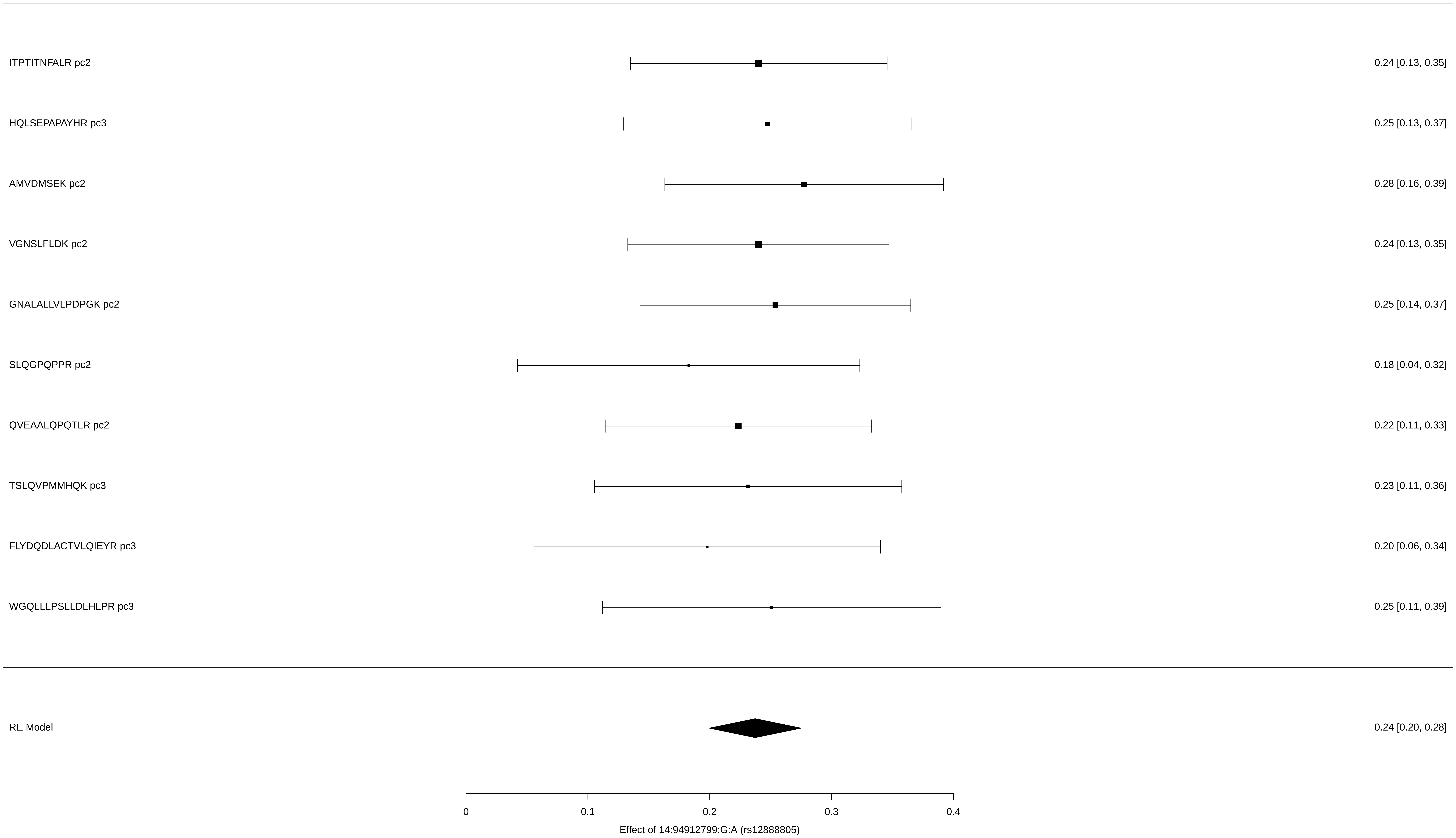

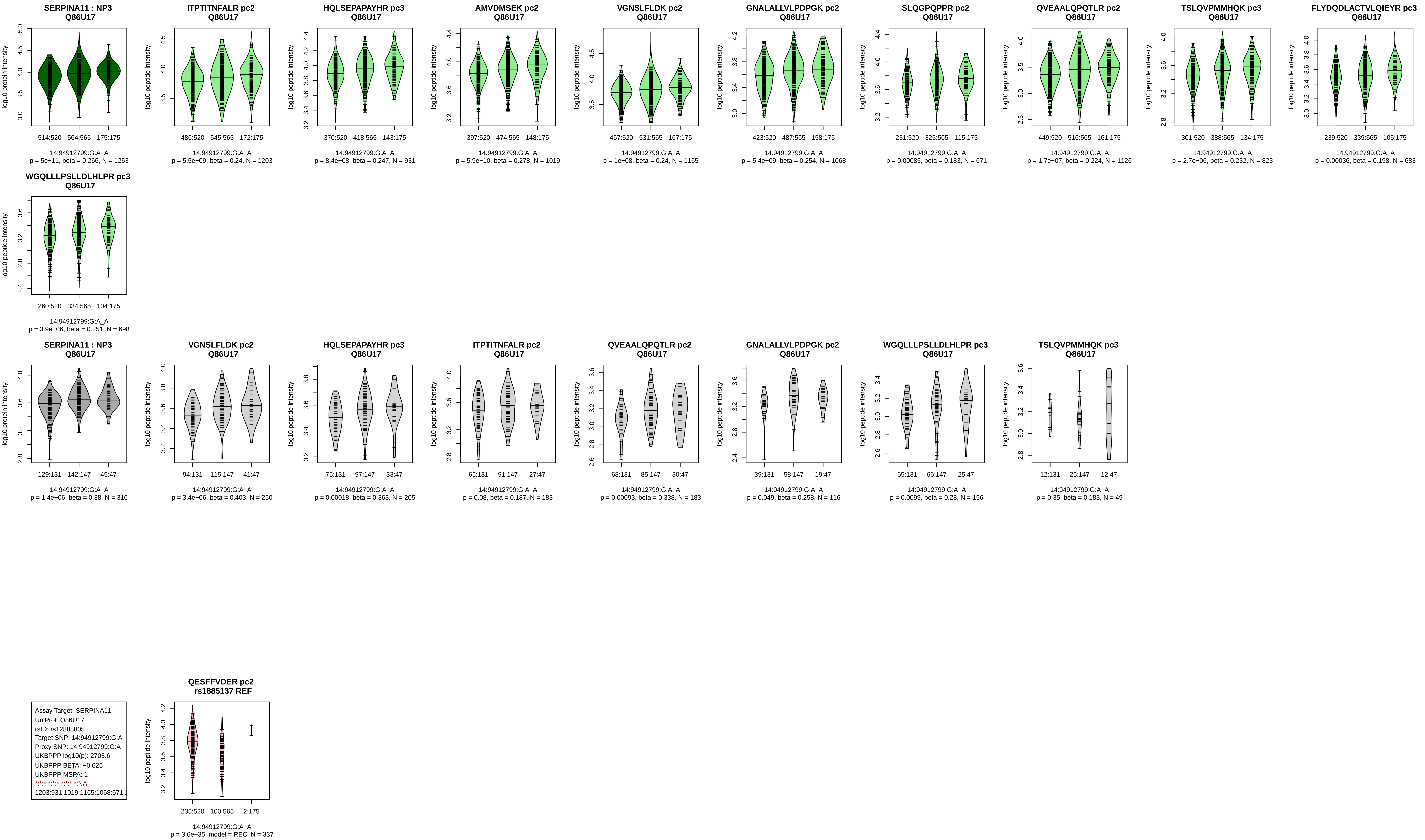

SERPINA11 : NP3 Q86U17
log10 protein intensity
514:520 564:565 175:175
14:94912799:G:A_A
p = 5e−11, beta = 0.266, N = 1253
ITPTITNFALR pc2 Q86U17
log10 peptide intensity
486:520 545:565 172:175
14:94912799:G:A_A
p = 5.5e−09, beta = 0.24, N = 1203
HQLSEPAPAYHR pc3 Q86U17
370:520 418:565 143:175
14:94912799:G:A_A
p = 8.4e−08, beta = 0.247, N = 931
AMVDMSEK pc2 Q86U17
397:520 474:565 148:175
14:94912799:G:A_A
p = 5.9e−10, beta = 0.278, N = 1019
VGNSLFLDK pc2 Q86U17
467:520 531:565 167:175
14:94912799:G:A_A
p = 1e−08, beta = 0.24, N = 1165
GNALALLVLPDPGK pc2 Q86U17
423:520 487:565 158:175
14:94912799:G:A_A
p = 5.4e−09, beta = 0.254, N = 1068
SLQGPQPPR pc2 Q86U17
231:520 325:565 115:175
14:94912799:G:A_A
p = 0.00085, beta = 0.183, N = 671
QVEAALQPQTLR pc2 Q86U17
449:520 516:565 161:175
14:94912799:G:A_A
p = 1.7e−07, beta = 0.224, N = 1126
TSLQVPMMHQK pc3 Q86U17
301:520 388:565 134:175
14:94912799:G:A_A
p = 2.7e−06, beta = 0.232, N = 823
FLYDQDLACTVLQIEYR pc3 Q86U17
239:520 339:565 105:175
14:94912799:G:A_A
p = 0.00036, beta = 0.198, N = 683
WGQLLLPSLLDLHLPR pc3 Q86U17
260:520 334:565 104:175
14:94912799:G:A_A
p = 3.9e−06, beta = 0.251, N = 698
SERPINA11 : NP3 Q86U17
129:131 142:147 45:47
14:94912799:G:A_A
p = 1.4e−06, beta = 0.38, N = 316
VGNSLFLDK pc2 Q86U17
94:131 115:147 41:47
14:94912799:G:A_A
p = 3.4e−06, beta = 0.403, N = 250
HQLSEPAPAYHR pc3 Q86U17
75:131 97:147 33:47
14:94912799:G:A_A
p = 0.00018, beta = 0.363, N = 205
ITPTITNFALR pc2 Q86U17
65:131 91:147 27:47
14:94912799:G:A_A
p = 0.08, beta = 0.187, N = 183
QVEAALQPQTLR pc2 Q86U17
68:131 85:147 30:47
14:94912799:G:A_A
p = 0.00093, beta = 0.338, N = 183
GNALALLVLPDPGK pc2 Q86U17
39:131 58:147 19:47
14:94912799:G:A_A
p = 0.049, beta = 0.258, N = 116
WGQLLLPSLLDLHLPR pc3 Q86U17
65:131 66:147 25:47
14:94912799:G:A_A
p = 0.0099, beta = 0.28, N = 156
TSLQVPMMHQK pc3 Q86U17
12:131 25:147 12:47
14:94912799:G:A_A
p = 0.35, beta = 0.183, N = 49
Assay Target: SERPINA11
UniProt: Q86U17
rsID: rs12888805
Target SNP: 14:94912799:G:A
Proxy SNP: 14:94912799:G:A
UKBPPP log10(p): 2705.6
UKBPPP BETA: −0.625
UKBPPP MSPA: 1
::*:*:*:*:*:*:*:*:NA
1203:931:1019:1165:1068:671:1
QESFFVDER pc2 rs1885137 REF
235:520 100:565 2:175
14:94912799:G:A_A
p = 3.6e−35, model = REC, N = 337

29. SPARCL1 (Q14515)
UKBPPP MSPA=0.932

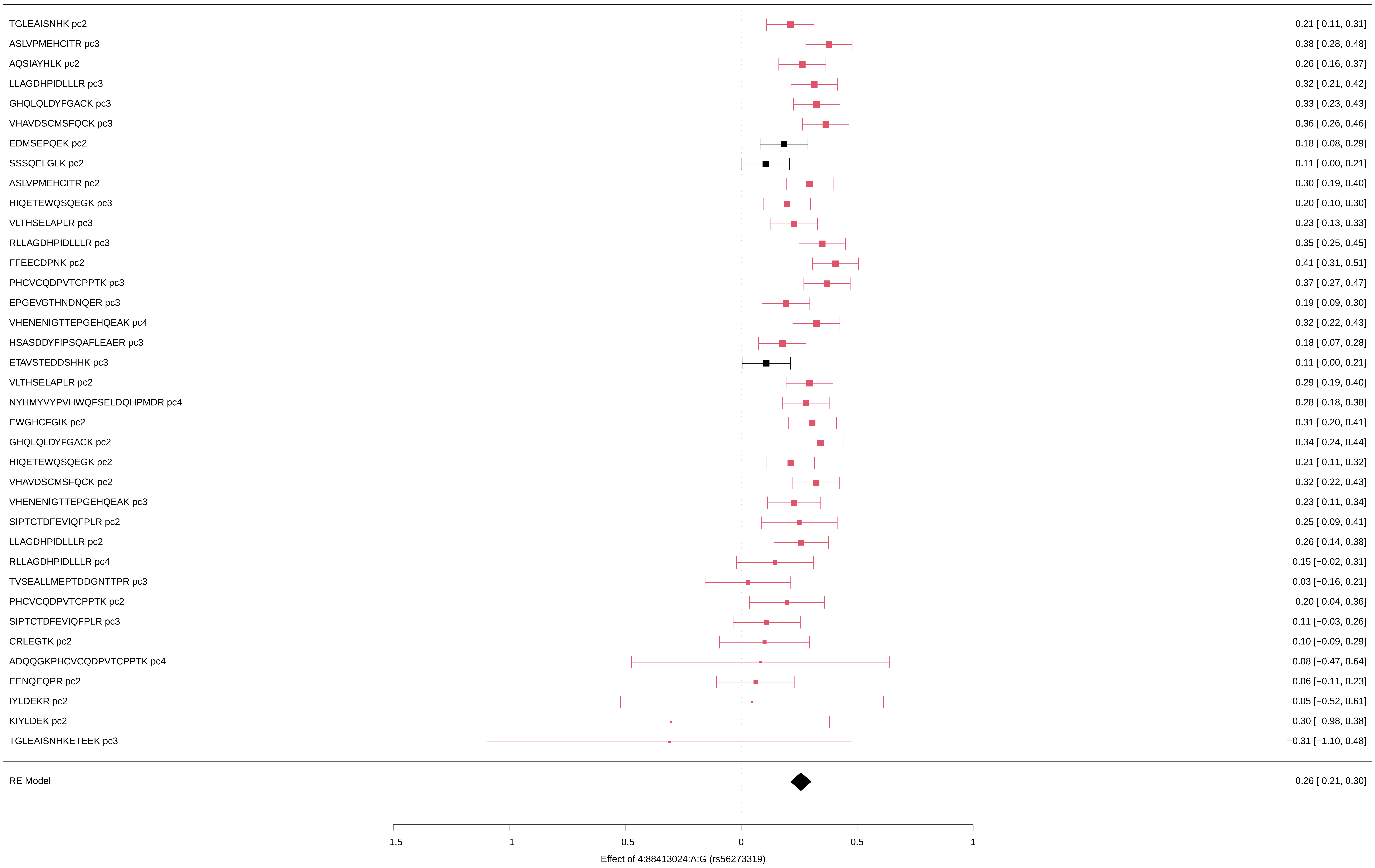

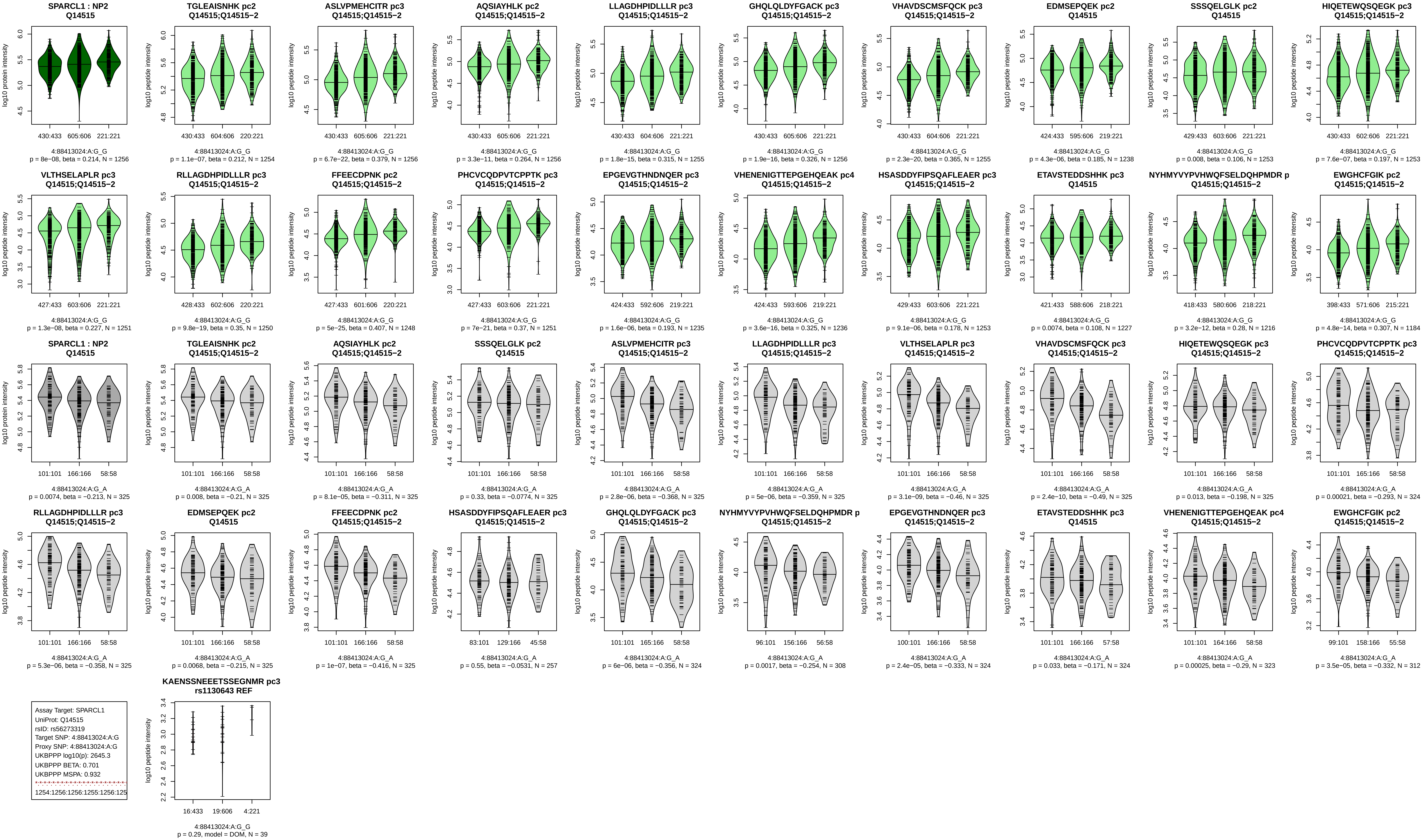
SPARCL1 : NP2
Q14515
log10 protein intensity
430:433 605:606 221:221
4:88413024:A:G_G
p = 8e−08, beta = 0.214, N = 1256
TGLEAISNHK pc2
Q14515;Q14515−2
log10 peptide intensity
430:433 604:606 220:221
4:88413024:A:G_G
p = 1.1e−07, beta = 0.212, N = 1254
ASLVPMEHCITR pc3
Q14515;Q14515−2
430:433 605:606 221:221
4:88413024:A:G_G
p = 6.7e−22, beta = 0.379, N = 1256
AQSIAYHLK pc2
Q14515;Q14515−2
430:433 605:606 221:221
4:88413024:A:G_G
p = 3.3e−11, beta = 0.264, N = 1256
LLAGDHPIDLLLR pc3
Q14515;Q14515−2
430:433 604:606 221:221
4:88413024:A:G_G
p = 1.8e−15, beta = 0.315, N = 1255
GHQLQLDYFGACK pc3
Q14515;Q14515−2
430:433 605:606 221:221
4:88413024:A:G_G
p = 1.9e−16, beta = 0.326, N = 1256
VHAVDSCMSFQCK pc3
Q14515;Q14515−2
430:433 604:606 221:221
4:88413024:A:G_G
p = 2.3e−20, beta = 0.365, N = 1255
EDMSEPQEK pc2
Q14515
424:433 595:606 219:221
4:88413024:A:G_G
p = 4.3e−06, beta = 0.185, N = 1238
SSSQELGLK pc2
Q14515
429:433 603:606 221:221
4:88413024:A:G_G
p = 0.008, beta = 0.106, N = 1253
HIQETEWQSQEGK pc3
Q14515;Q14515−2
430:433 602:606 221:221
4:88413024:A:G_G
p = 7.6e−07, beta = 0.197, N = 1253
VLTHSELAPLR pc3
Q14515;Q14515−2
427:433 603:606 221:221
4:88413024:A:G_G
p = 1.3e−08, beta = 0.227, N = 1251
RLLAGDHPIDLLLR pc3
Q14515;Q14515−2
428:433 602:606 220:221
4:88413024:A:G_G
p = 9.8e−19, beta = 0.35, N = 1250
FFEECDPNK pc2
Q14515;Q14515−2
427:433 601:606 220:221
4:88413024:A:G_G
p = 5e−25, beta = 0.407, N = 1248
PHCVCQDPVTCPPTK pc3
Q14515;Q14515−2
427:433 603:606 221:221
4:88413024:A:G_G
p = 7e−21, beta = 0.37, N = 1251
EPGEVGTHNDNQER pc3
Q14515;Q14515−2
424:433 592:606 219:221
4:88413024:A:G_G
p = 1.6e−06, beta = 0.193, N = 1235
VHENENIGTTEPGEHQEAK pc4
Q14515;Q14515−2
424:433 593:606 219:221
4:88413024:A:G_G
p = 3.6e−16, beta = 0.325, N = 1236
HSASDDYFIPSQAFLEAER pc3
Q14515;Q14515−2
429:433 603:606 221:221
4:88413024:A:G_G
p = 9.1e−06, beta = 0.178, N = 1253
ETAVSTEDDSHHK pc3
Q14515
421:433 588:606 218:221
4:88413024:A:G_G
p = 0.0074, beta = 0.108, N = 1227
NYHMYVYPVHWQFSELDQHPMDR p
Q14515;Q14515−2
418:433 580:606 218:221
4:88413024:A:G_G
p = 3.2e−12, beta = 0.28, N = 1216
EWGHCFGIK pc2
Q14515;Q14515−2
398:433 571:606 215:221
4:88413024:A:G_G
p = 4.8e−14, beta = 0.307, N = 1184
SPARCL1 : NP2
Q14515
101:101 166:166 58:58
4:88413024:A:G_A
p = 0.0074, beta = −0.213, N = 325
TGLEAISNHK pc2
Q14515;Q14515−2
101:101 166:166 58:58
4:88413024:A:G_A
p = 0.008, beta = −0.21, N = 325
AQSIAYHLK pc2
Q14515;Q14515−2
101:101 166:166 58:58
4:88413024:A:G_A
p = 8.1e−05, beta = −0.311, N = 325
SSSQELGLK pc2
Q14515
101:101 166:166 58:58
4:88413024:A:G_A
p = 0.33, beta = −0.0774, N = 325
ASLVPMEHCITR pc3
Q14515;Q14515−2
101:101 166:166 58:58
4:88413024:A:G_A
p = 2.8e−06, beta = −0.368, N = 325
LLAGDHPIDLLLR pc3
Q14515;Q14515−2
101:101 166:166 58:58
4:88413024:A:G_A
p = 5e−06, beta = −0.359, N = 325
VLTHSELAPLR pc3
Q14515;Q14515−2
101:101 166:166 58:58
4:88413024:A:G_A
p = 3.1e−09, beta = −0.46, N = 325
VHAVDSCMSFQCK pc3
Q14515;Q14515−2
101:101 166:166 58:58
4:88413024:A:G_A
p = 2.4e−10, beta = −0.49, N = 325
HIQETEWQSQEGK pc3
Q14515;Q14515−2
101:101 166:166 58:58
4:88413024:A:G_A
p = 0.013, beta = −0.198, N = 325
PHCVCQDPVTCPPTK pc3
Q14515;Q14515−2
101:101 165:166 58:58
4:88413024:A:G_A
p = 0.00021, beta = −0.293, N = 324
RLLAGDHPIDLLLR pc3
Q14515;Q14515−2
101:101 166:166 58:58
4:88413024:A:G_A
p = 5.3e−06, beta = −0.358, N = 325
EDMSEPQEK pc2
Q14515
101:101 166:166 58:58
4:88413024:A:G_A
p = 0.0068, beta = −0.215, N = 325
FFEECDPNK pc2
Q14515;Q14515−2
101:101 166:166 58:58
4:88413024:A:G_A
p = 1e−07, beta = −0.416, N = 325
HSASDDYFIPSQAFLEAER pc3
Q14515;Q14515−2
83:101 129:166 45:58
4:88413024:A:G_A
p = 0.55, beta = −0.0531, N = 257
GHQLQLDYFGACK pc3
Q14515;Q14515−2
101:101 165:166 58:58
4:88413024:A:G_A
p = 6e−06, beta = −0.356, N = 324
NYHMYVYPVHWQFSELDQHPMDR p
Q14515;Q14515−2
96:101 156:166 56:58
4:88413024:A:G_A
p = 0.0017, beta = −0.254, N = 308
EPGEVGTHNDNQER pc3
Q14515;Q14515−2
100:101 166:166 58:58
4:88413024:A:G_A
p = 2.4e−05, beta = −0.333, N = 324
ETAVSTEDDSHHK pc3
Q14515
101:101 166:166 57:58
4:88413024:A:G_A
p = 0.033, beta = −0.171, N = 324
VHENENIGTTEPGEHQEAK pc4
Q14515;Q14515−2
101:101 164:166 58:58
4:88413024:A:G_A
p = 0.00025, beta = −0.29, N = 323
EWGHCFGIK pc2
Q14515;Q14515−2
99:101 158:166 55:58
4:88413024:A:G_A
p = 3.5e−05, beta = −0.332, N = 312
Assay Target: SPARCL1
UniProt: Q14515
rsID: rs56273319
Target SNP: 4:88413024:A:G
Proxy SNP: 4:88413024:A:G
UKBPPP log10(p): 2645.3
UKBPPP BETA: 0.701
UKBPPP MSPA: 0.932
1254:1256:1256:1255:1256:125
KAENSSNEEETSSEGNMR pc3
rs1130643 REF
16:433 19:606 4:221
4:88413024:A:G_G
p = 0.29, model = DOM, N = 39

30. CFHR4 (Q92496)
UKBPPP MSPA=0.792

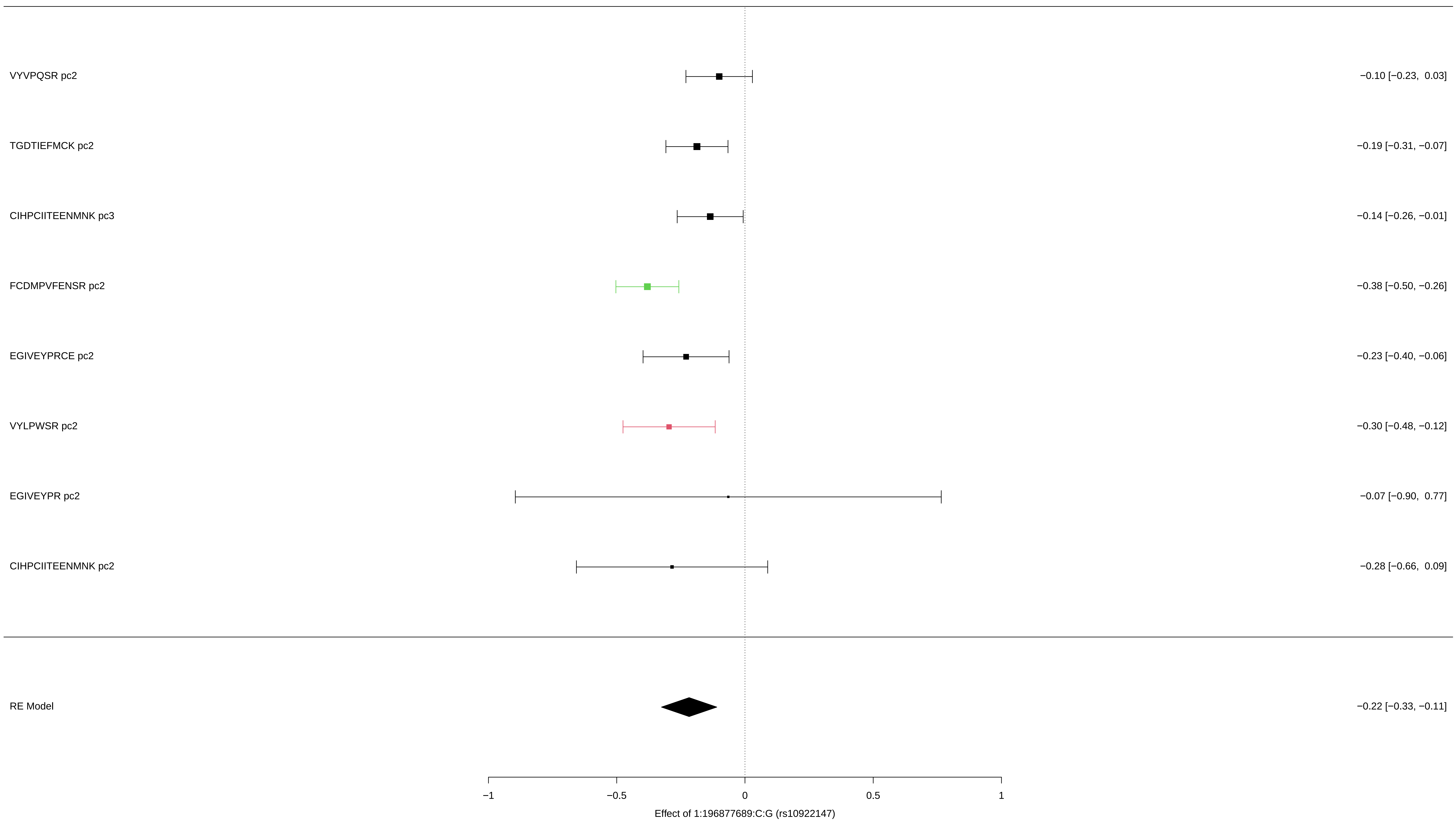

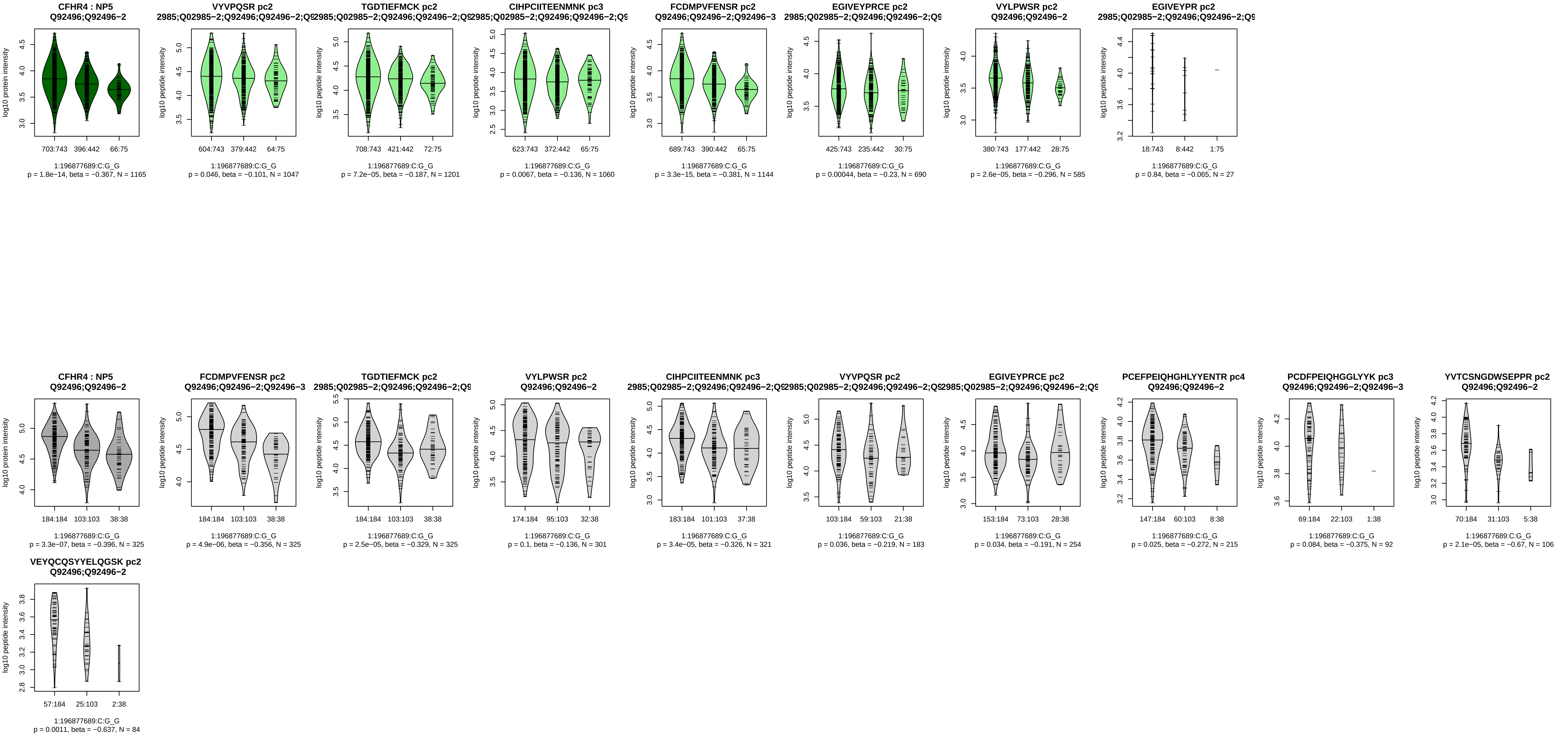

Assay Target: CFHR4
UniProt: Q92496
rsID: rs10922147
Target SNP: 1:196877689:C:G
Proxy SNP: 1:196877689:C:G
UKBPPP log10(p): 2512.3
UKBPPP BETA: −0.785
UKBPPP MSPA: 0.792
–;*;*;*;*;*;–;–
1047:1201:1060:1144:690:585:2

31. VCAN (P13611)
UKBPPP MSPA=0.351

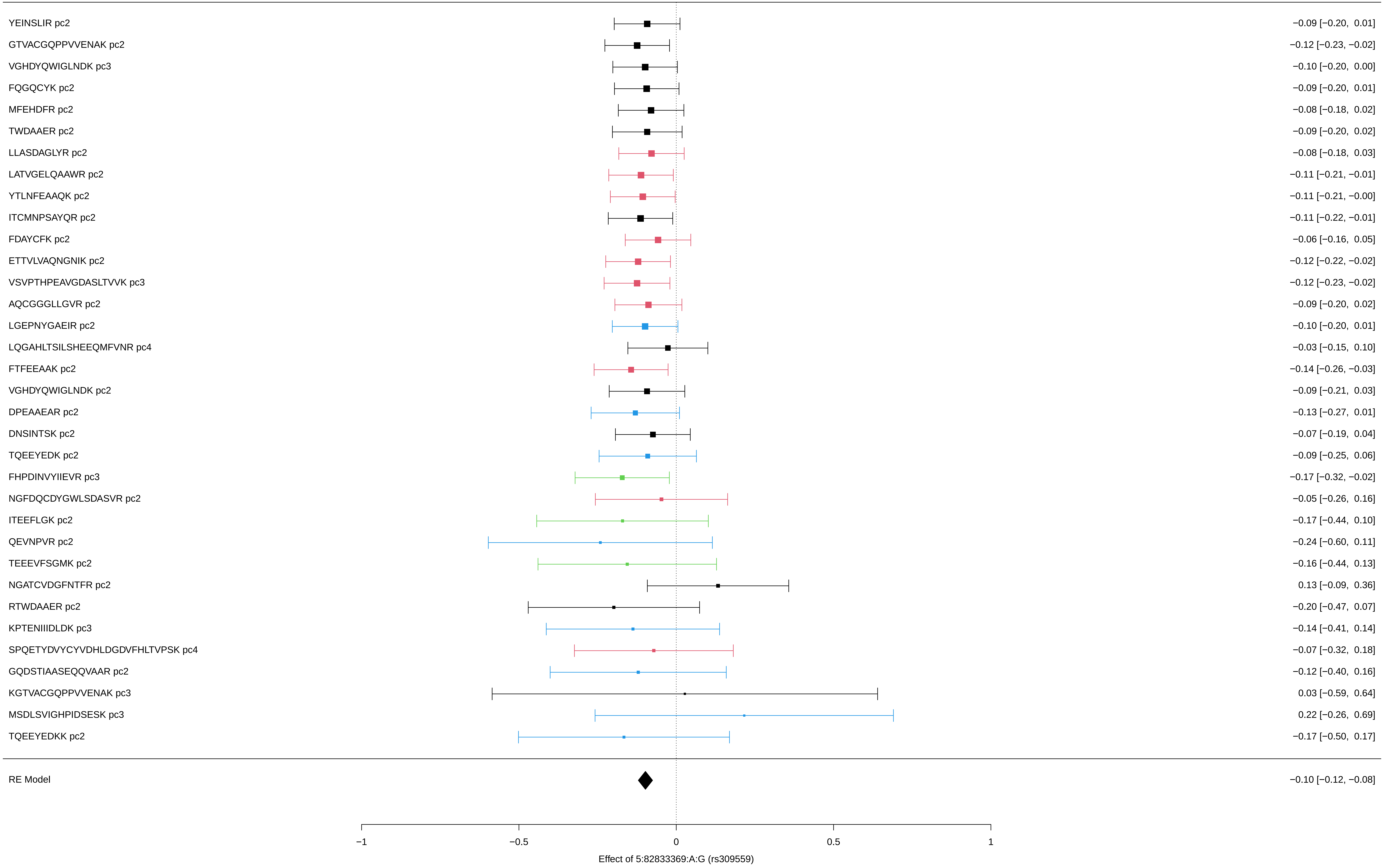

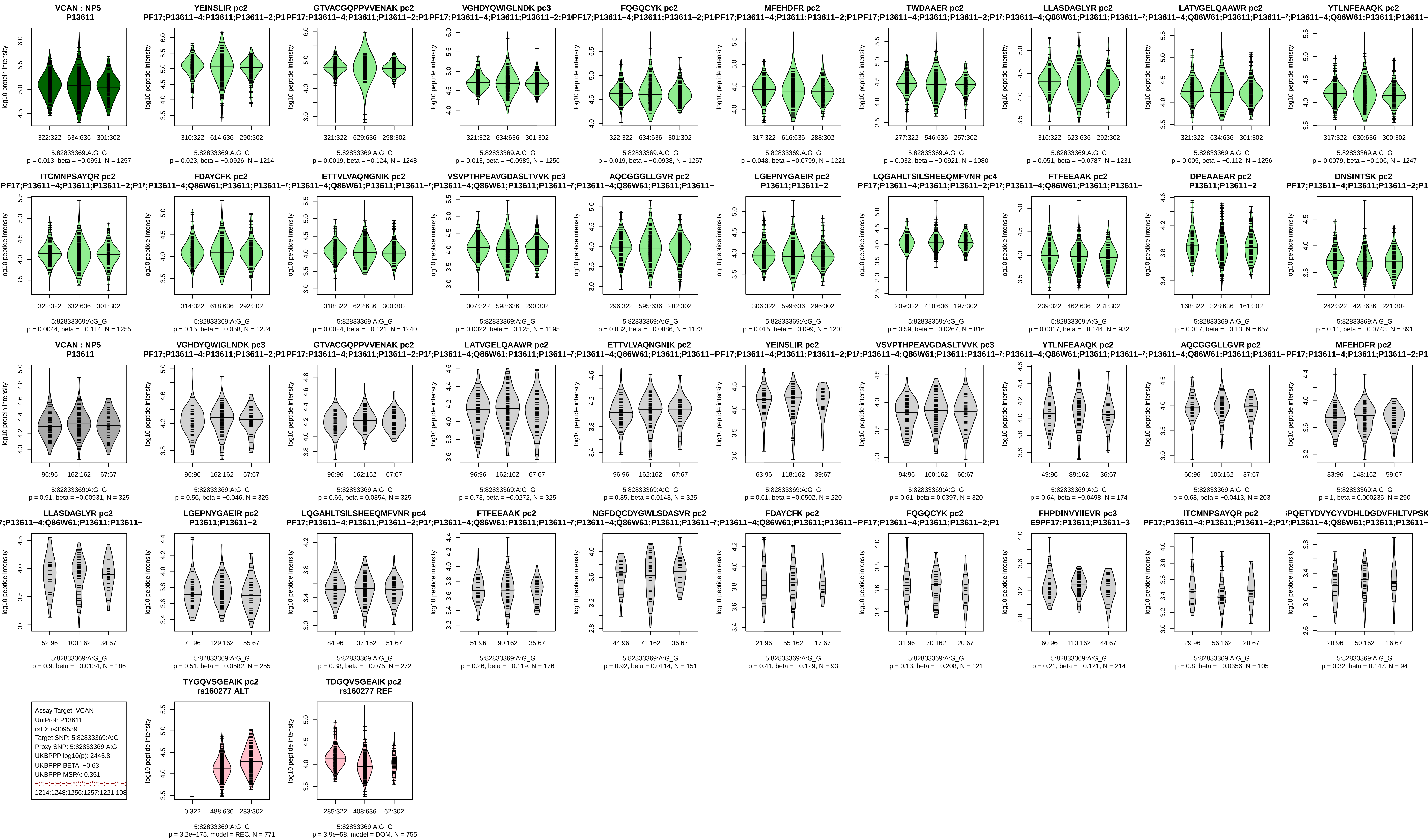
VCAN : NP5
P13611
log10 protein intensity
322:322 634:636 301:302
5:82833369:A:G_G
p = 0.013, beta = −0.0991, N = 1257
YEINSLIR pc2
log10 peptide intensity
310:322 614:636 290:302
5:82833369:A:G_G
p = 0.023, beta = −0.0926, N = 1214
GTVACGQPPVVENAK pc2
321:322 629:636 298:302
5:82833369:A:G_G
p = 0.0019, beta = −0.124, N = 1248
VGHDYQWIGLNDK pc3
321:322 634:636 301:302
5:82833369:A:G_G
p = 0.013, beta = −0.0989, N = 1256
FQGQCYK pc2
322:322 634:636 301:302
5:82833369:A:G_G
p = 0.019, beta = −0.0938, N = 1257
MFEHDFR pc2
317:322 616:636 288:302
5:82833369:A:G_G
p = 0.048, beta = −0.0799, N = 1221
TWDAAER pc2
277:322 546:636 257:302
5:82833369:A:G_G
p = 0.032, beta = −0.0921, N = 1080
LLASDAGLYR pc2
316:322 623:636 292:302
5:82833369:A:G_G
p = 0.051, beta = −0.0787, N = 1231
LATVGELQAAWR pc2
321:322 634:636 301:302
5:82833369:A:G_G
p = 0.005, beta = −0.112, N = 1256
YTLNFEAAQK pc2
317:322 630:636 300:302
5:82833369:A:G_G
p = 0.0079, beta = −0.106, N = 1247
ITCMNPSAYQR pc2
322:322 632:636 301:302
5:82833369:A:G_G
p = 0.0044, beta = −0.114, N = 1255
FDAYCFK pc2
314:322 618:636 292:302
5:82833369:A:G_G
p = 0.15, beta = −0.058, N = 1224
ETTVLVAQNGNIK pc2
318:322 622:636 300:302
5:82833369:A:G_G
p = 0.0024, beta = −0.121, N = 1240
VSVPTHPEAVGDASLTVVK pc3
307:322 598:636 290:302
5:82833369:A:G_G
p = 0.0022, beta = −0.125, N = 1195
AQCGGGLLGVR pc2
296:322 595:636 282:302
5:82833369:A:G_G
p = 0.032, beta = −0.0886, N = 1173
LGEPNYGAEIR pc2
P13611;P13611−2
306:322 599:636 296:302
5:82833369:A:G_G
p = 0.015, beta = −0.099, N = 1201
LQGAHLTSILSHEEQMFVNR pc4
209:322 410:636 197:302
5:82833369:A:G_G
p = 0.59, beta = −0.0267, N = 816
FTFEEAAK pc2
239:322 462:636 231:302
5:82833369:A:G_G
p = 0.0017, beta = −0.144, N = 932
DPEAAEAR pc2
P13611;P13611−2
168:322 328:636 161:302
5:82833369:A:G_G
p = 0.017, beta = −0.13, N = 657
DNSINTSK pc2
242:322 428:636 221:302
5:82833369:A:G_G
p = 0.11, beta = −0.0743, N = 891
VCAN : NP5
P13611
96:96 162:162 67:67
5:82833369:A:G_G
p = 0.91, beta = −0.00931, N = 325
VGHDYQWIGLNDK pc3
96:96 162:162 67:67
5:82833369:A:G_G
p = 0.56, beta = −0.046, N = 325
GTVACGQPPVVENAK pc2
96:96 162:162 67:67
5:82833369:A:G_G
p = 0.65, beta = 0.0354, N = 325
LATVGELQAAWR pc2
96:96 162:162 67:67
5:82833369:A:G_G
p = 0.73, beta = −0.0272, N = 325
ETTVLVAQNGNIK pc2
96:96 162:162 67:67
5:82833369:A:G_G
p = 0.85, beta = 0.0143, N = 325
YEINSLIR pc2
63:96 118:162 39:67
5:82833369:A:G_G
p = 0.61, beta = −0.0502, N = 220
VSVPTHPEAVGDASLTVVK pc3
94:96 160:162 66:67
5:82833369:A:G_G
p = 0.61, beta = 0.0397, N = 320
YTLNFEAAQK pc2
49:96 89:162 36:67
5:82833369:A:G_G
p = 0.64, beta = −0.0498, N = 174
AQCGGGLLGVR pc2
60:96 106:162 37:67
5:82833369:A:G_G
p = 0.68, beta = −0.0413, N = 203
MFEHDFR pc2
83:96 148:162 59:67
5:82833369:A:G_G
p = 1, beta = 0.000235, N = 290
LLASDAGLYR pc2
52:96 100:162 34:67
5:82833369:A:G_G
p = 0.9, beta = −0.0134, N = 186
LGEPNYGAEIR pc2
P13611;P13611−2
71:96 129:162 55:67
5:82833369:A:G_G
p = 0.51, beta = −0.0582, N = 255
LQGAHLTSILSHEEQMFVNR pc4
84:96 137:162 51:67
5:82833369:A:G_G
p = 0.38, beta = −0.075, N = 272
FTFEEAAK pc2
51:96 90:162 35:67
5:82833369:A:G_G
p = 0.26, beta = −0.119, N = 176
NGFDQCDYGWLSDASVR pc2
44:96 71:162 36:67
5:82833369:A:G_G
p = 0.92, beta = 0.0114, N = 151
FDAYCFK pc2
21:96 55:162 17:67
5:82833369:A:G_G
p = 0.41, beta = −0.129, N = 93
FQGQCYK pc2
31:96 70:162 20:67
5:82833369:A:G_G
p = 0.13, beta = −0.208, N = 121
FHPDINVYIIEVR pc3
E9PF17;P13611;P13611−3
60:96 110:162 44:67
5:82833369:A:G_G
p = 0.21, beta = −0.121, N = 214
ITCMNPSAYQR pc2
29:96 56:162 20:67
5:82833369:A:G_G
p = 0.8, beta = −0.0356, N = 105
28:96 50:162 16:67
5:82833369:A:G_G
p = 0.32, beta = 0.147, N = 94
Assay Target: VCAN
UniProt: P13611
rsID: rs309559
Target SNP: 5:82833369:A:G
Proxy SNP: 5:82833369:A:G
UKBPPP log10(p): 2445.8
UKBPPP BETA: −0.63
UKBPPP MSPA: 0.351
1214:1248:1256:1257:1221:108
TYGQVSGEAIK pc2
rs160277 ALT
0:322 488:636 283:302
5:82833369:A:G_G
p = 3.2e−175, model = REC, N = 771
TDGQVSGEAIK pc2
rs160277 REF
285:322 408:636 62:302
5:82833369:A:G_G
p = 3.9e−58, model = DOM, N = 755

32. LBP (P18428)
UKBPPP MSPA=0.88

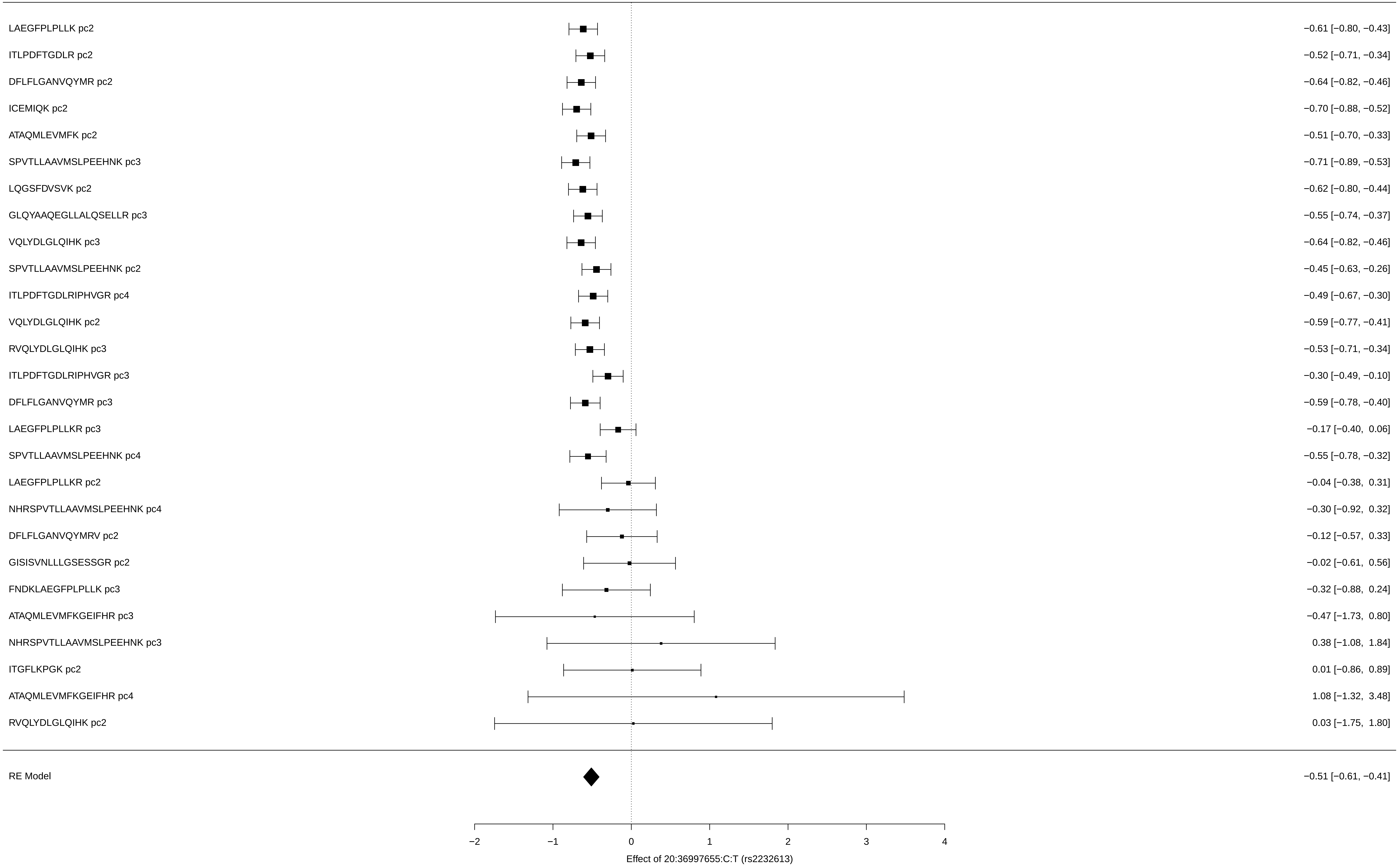

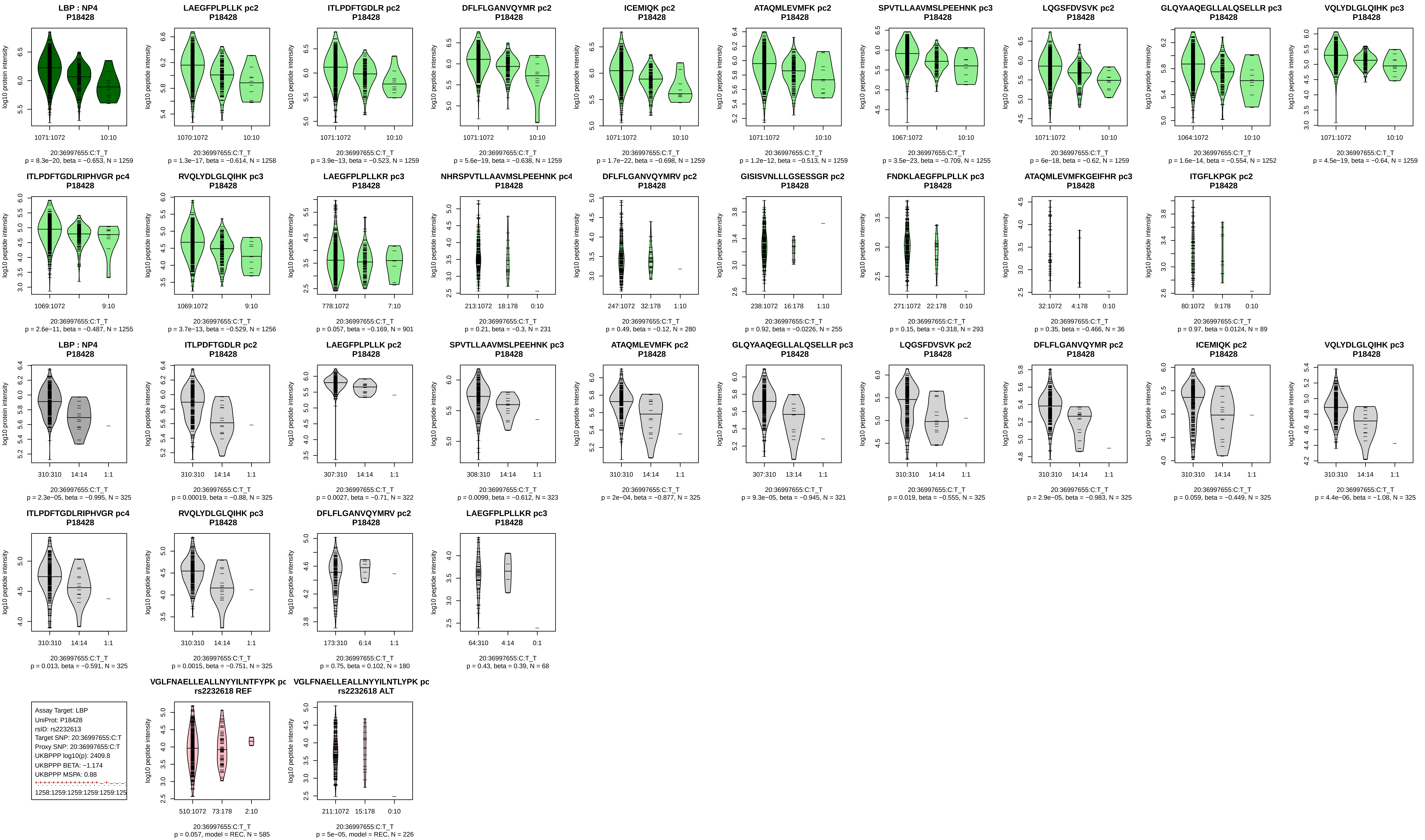
LBP : NP4
P18428
log10 protein intensity
1071:1072
10:10
20:36997655:C:T_T
p = 8.3e−20, beta = −0.653, N = 1259
LAEGFPLPLLK pc2
P18428
log10 peptide intensity
1070:1072
10:10
20:36997655:C:T_T
p = 1.3e−17, beta = −0.614, N = 1258
ITLPDFTGDLR pc2
P18428
1071:1072
10:10
20:36997655:C:T_T
p = 3.9e−13, beta = −0.523, N = 1259
DFLFLGANVQYMR pc2
P18428
1071:1072
10:10
20:36997655:C:T_T
p = 5.6e−19, beta = −0.638, N = 1259
ICEMIQK pc2
P18428
1071:1072
10:10
20:36997655:C:T_T
p = 1.7e−22, beta = −0.698, N = 1259
ATAQMLEVMFK pc2
P18428
1071:1072
10:10
20:36997655:C:T_T
p = 1.2e−12, beta = −0.513, N = 1259
SPVTLLAAVMSLPEEHNK pc3
P18428
1067:1072
10:10
20:36997655:C:T_T
p = 3.5e−23, beta = −0.709, N = 1255
LQGSFDVSVK pc2
P18428
1071:1072
10:10
20:36997655:C:T_T
p = 6e−18, beta = −0.62, N = 1259
GLQYAAQEGLLALQSELLR pc3
P18428
1064:1072
10:10
20:36997655:C:T_T
p = 1.6e−14, beta = −0.554, N = 1252
VQLYDLGLQIHK pc3
P18428
1071:1072
10:10
20:36997655:C:T_T
p = 4.5e−19, beta = −0.64, N = 1259
ITLPDFTGDLRIPHVGR pc4
P18428
1069:1072
9:10
20:36997655:C:T_T
p = 2.6e−11, beta = −0.487, N = 1255
RVQLYDLGLQIHK pc3
P18428
1069:1072
9:10
20:36997655:C:T_T
p = 3.7e−13, beta = −0.529, N = 1256
LAEGFPLPLLKR pc3
P18428
778:1072
7:10
20:36997655:C:T_T
p = 0.057, beta = −0.169, N = 901
NHRSPVTLLAAVMSLPEEHNK pc4
P18428
213:1072
18:178
0:10
20:36997655:C:T_T
p = 0.21, beta = −0.3, N = 231
DFLFLGANVQYMRV pc2
P18428
247:1072
32:178
1:10
20:36997655:C:T_T
p = 0.49, beta = −0.12, N = 280
GISISVNLLLGSESSGR pc2
P18428
238:1072
16:178
1:10
20:36997655:C:T_T
p = 0.92, beta = −0.0226, N = 255
FNDKLAEGFPLPLLK pc3
P18428
271:1072
22:178
0:10
20:36997655:C:T_T
p = 0.15, beta = −0.318, N = 293
ATAQMLEVMFKGEIFHR pc3
P18428
32:1072
4:178
0:10
20:36997655:C:T_T
p = 0.35, beta = −0.466, N = 36
ITGFLKPGK pc2
P18428
80:1072
9:178
0:10
20:36997655:C:T_T
p = 0.97, beta = 0.0124, N = 89
LBP : NP4
P18428
310:310
14:14
1:1
20:36997655:C:T_T
p = 2.3e−05, beta = −0.995, N = 325
ITLPDFTGDLR pc2
P18428
310:310
14:14
1:1
20:36997655:C:T_T
p = 0.00019, beta = −0.88, N = 325
LAEGFPLPLLK pc2
P18428
307:310
14:14
1:1
20:36997655:C:T_T
p = 0.0027, beta = −0.71, N = 322
SPVTLLAAVMSLPEEHNK pc3
P18428
308:310
14:14
1:1
20:36997655:C:T_T
p = 0.0099, beta = −0.612, N = 323
ATAQMLEVMFK pc2
P18428
310:310
14:14
1:1
20:36997655:C:T_T
p = 2e−04, beta = −0.877, N = 325
GLQYAAQEGLLALQSELLR pc3
P18428
307:310
13:14
1:1
20:36997655:C:T_T
p = 9.3e−05, beta = −0.945, N = 321
LQGSFDVSVK pc2
P18428
310:310
14:14
1:1
20:36997655:C:T_T
p = 0.019, beta = −0.555, N = 325
DFLFLGANVQYMR pc2
P18428
310:310
14:14
1:1
20:36997655:C:T_T
p = 2.9e−05, beta = −0.983, N = 325
ICEMIQK pc2
P18428
310:310
14:14
1:1
20:36997655:C:T_T
p = 0.059, beta = −0.449, N = 325
VQLYDLGLQIHK pc3
P18428
310:310
14:14
1:1
20:36997655:C:T_T
p = 4.4e−06, beta = −1.08, N = 325
ITLPDFTGDLRIPHVGR pc4
P18428
310:310
14:14
1:1
20:36997655:C:T_T
p = 0.013, beta = −0.591, N = 325
RVQLYDLGLQIHK pc3
P18428
310:310
14:14
1:1
20:36997655:C:T_T
p = 0.0015, beta = −0.751, N = 325
DFLFLGANVQYMRV pc2
P18428
173:310
6:14
1:1
20:36997655:C:T_T
p = 0.75, beta = 0.102, N = 180
LAEGFPLPLLKR pc3
P18428
64:310
4:14
0:1
20:36997655:C:T_T
p = 0.43, beta = 0.39, N = 68
Assay Target: LBP
UniProt: P18428
rsID: rs2232613
Target SNP: 20:36997655:C:T
Proxy SNP: 20:36997655:C:T
UKBPPP log10(p): 2409.8
UKBPPP BETA: −1.174
UKBPPP MSPA: 0.88
1258:1259:1259:1259:1259:125
VGLFNAELLEALLNYYILNTFYPK pc
rs2232618 REF
510:1072
73:178
2:10
20:36997655:C:T_T
p = 0.057, model = REC, N = 585
VGLFNAELLEALLNYYILNTLYPK pc
rs2232618 ALT
211:1072
15:178
0:10
20:36997655:C:T_T
p = 5e−05, model = REC, N = 226

33. FGL1 (Q08830)
UKBPPP MSPA=0.982

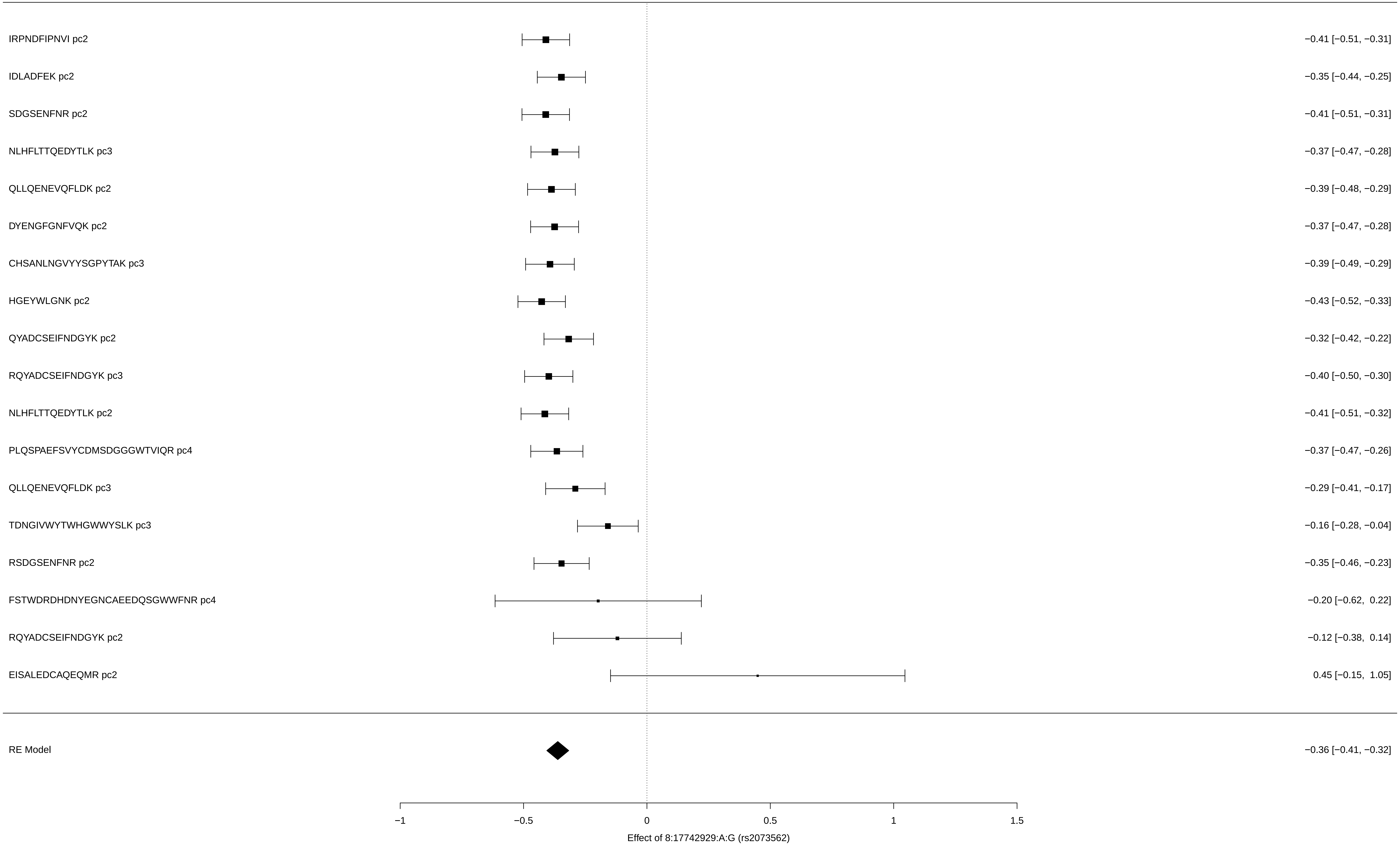

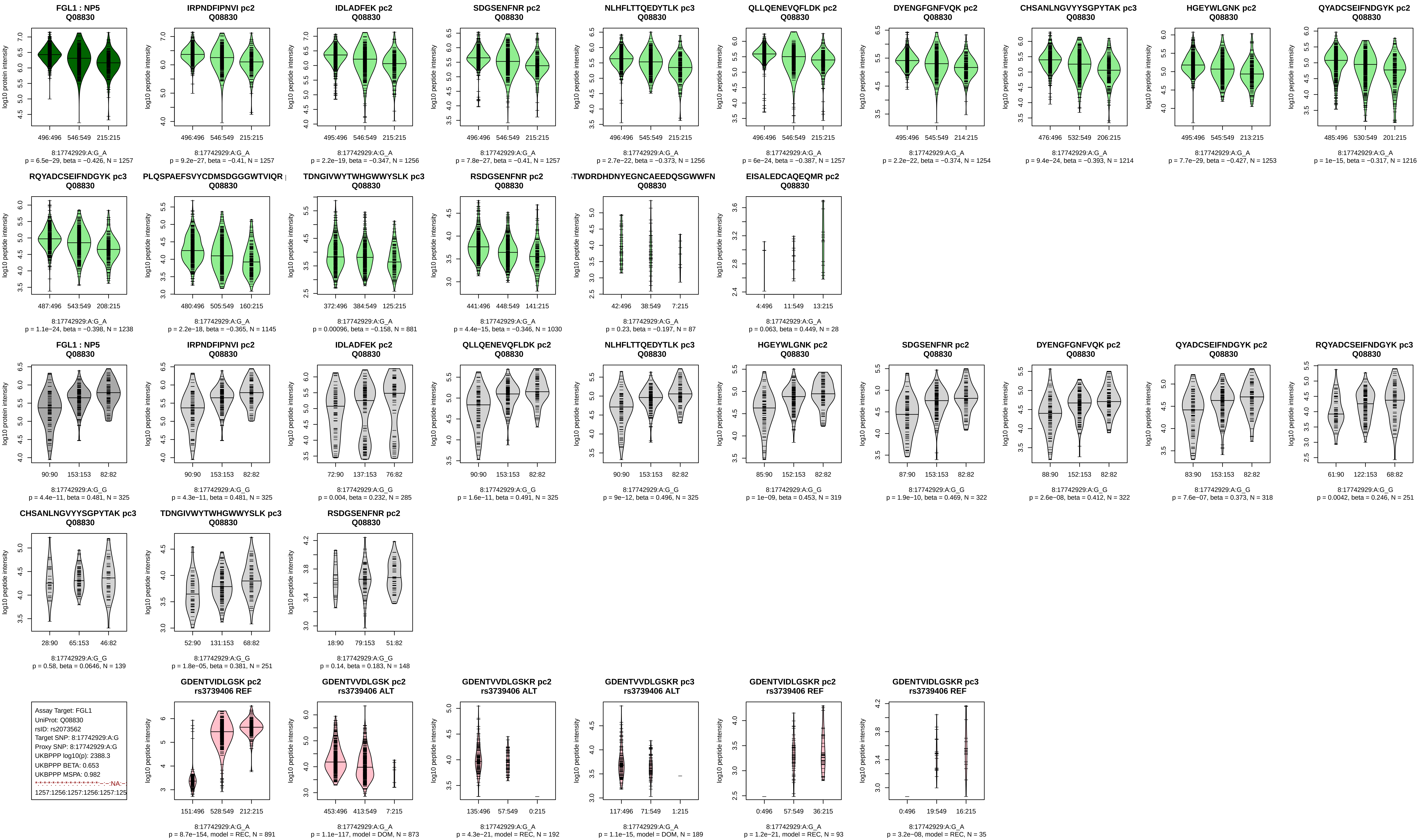
FGL1 : NP5
Q08830
log10 protein intensity
496:496 546:549 215:215
8:17742929:A:G_A
p = 6.5e−29, beta = −0.426, N = 1257
IRPNDFIPNVI pc2
Q08830
log10 peptide intensity
496:496 546:549 215:215
8:17742929:A:G_A
p = 9.2e−27, beta = −0.41, N = 1257
IDLADFEK pc2
Q08830
495:496 546:549 215:215
8:17742929:A:G_A
p = 2.2e−19, beta = −0.347, N = 1256
SDGSENFNR pc2
Q08830
496:496 546:549 215:215
8:17742929:A:G_A
p = 7.8e−27, beta = −0.41, N = 1257
NLHFLTTQEDYTLK pc3
Q08830
496:496 545:549 215:215
8:17742929:A:G_A
p = 2.7e−22, beta = −0.373, N = 1256
QLLQENEVQFLDK pc2
Q08830
496:496 546:549 215:215
8:17742929:A:G_A
p = 6e−24, beta = −0.387, N = 1257
DYENGFGNFVQK pc2
Q08830
495:496 545:549 214:215
8:17742929:A:G_A
p = 2.2e−22, beta = −0.374, N = 1254
CHSANLNGVYYSGPYTAK pc3
Q08830
476:496 532:549 206:215
8:17742929:A:G_A
p = 9.4e−24, beta = −0.393, N = 1214
HGEYWLGNK pc2
Q08830
495:496 545:549 213:215
8:17742929:A:G_A
p = 7.7e−29, beta = −0.427, N = 1253
QYADCSEIFNDGYK pc2
Q08830
485:496 530:549 201:215
8:17742929:A:G_A
p = 1e−15, beta = −0.317, N = 1216
RQYADCSEIFNDGYK pc3
Q08830
487:496 543:549 208:215
8:17742929:A:G_A
p = 1.1e−24, beta = −0.398, N = 1238
PLQSPAEFSVYCDMSDGGGWTVIQR
Q08830
480:496 505:549 160:215
8:17742929:A:G_A
p = 2.2e−18, beta = −0.365, N = 1145
TDNGIVWYTWHGWWYSLK pc3
Q08830
372:496 384:549 125:215
8:17742929:A:G_A
p = 0.00096, beta = −0.158, N = 881
RSDGSENFNR pc2
Q08830
441:496 448:549 141:215
8:17742929:A:G_A
p = 4.4e−15, beta = −0.346, N = 1030
TWDRDHDNYEGNCAEEDQSGWWFN
Q08830
42:496 38:549 7:215
8:17742929:A:G_A
p = 0.23, beta = −0.197, N = 87
EISALEDCAQEQMR pc2
Q08830
4:496 11:549 13:215
8:17742929:A:G_A
p = 0.063, beta = 0.449, N = 28
FGL1 : NP5
Q08830
90:90 153:153 82:82
8:17742929:A:G_G
p = 4.4e−11, beta = 0.481, N = 325
IRPNDFIPNVI pc2
Q08830
90:90 153:153 82:82
8:17742929:A:G_G
p = 4.3e−11, beta = 0.481, N = 325
IDLADFEK pc2
Q08830
72:90 137:153 76:82
8:17742929:A:G_G
p = 0.004, beta = 0.232, N = 285
QLLQENEVQFLDK pc2
Q08830
90:90 153:153 82:82
8:17742929:A:G_G
p = 1.6e−11, beta = 0.491, N = 325
NLHFLTTQEDYTLK pc3
Q08830
90:90 153:153 82:82
8:17742929:A:G_G
p = 9e−12, beta = 0.496, N = 325
HGEYWLGNK pc2
Q08830
85:90 152:153 82:82
8:17742929:A:G_G
p = 1e−09, beta = 0.453, N = 319
SDGSENFNR pc2
Q08830
87:90 153:153 82:82
8:17742929:A:G_G
p = 1.9e−10, beta = 0.469, N = 322
DYENGFGNFVQK pc2
Q08830
88:90 152:153 82:82
8:17742929:A:G_G
p = 2.6e−08, beta = 0.412, N = 322
QYADCSEIFNDGYK pc2
Q08830
83:90 153:153 82:82
8:17742929:A:G_G
p = 7.6e−07, beta = 0.373, N = 318
RQYADCSEIFNDGYK pc3
Q08830
61:90 122:153 68:82
8:17742929:A:G_G
p = 0.0042, beta = 0.246, N = 251
CHSANLNGVYYSGPYTAK pc3
Q08830
28:90 65:153 46:82
8:17742929:A:G_G
p = 0.58, beta = 0.0646, N = 139
TDNGIVWYTWHGWWYSLK pc3
Q08830
52:90 131:153 68:82
8:17742929:A:G_G
p = 1.8e−05, beta = 0.381, N = 251
RSDGSENFNR pc2
Q08830
18:90 79:153 51:82
8:17742929:A:G_G
p = 0.14, beta = 0.183, N = 148
Assay Target: FGL1
UniProt: Q08830
rsID: rs2073562
Target SNP: 8:17742929:A:G
Proxy SNP: 8:17742929:A:G
UKBPPP log10(p): 2388.3
UKBPPP BETA: 0.653
UKBPPP MSPA: 0.982
1257:1256:1257:1256:1257:125
GDENTVIDLGSK pc2
rs3739406 REF
151:496 528:549 212:215
8:17742929:A:G_A
p = 8.7e−154, model = REC, N = 891
GDENTVVDLGSK pc2
rs3739406 ALT
453:496 413:549 7:215
8:17742929:A:G_A
p = 1.1e−117, model = DOM, N = 873
GDENTVVDLGSKR pc2
rs3739406 ALT
135:496 57:549 0:215
8:17742929:A:G_A
p = 4.3e−21, model = REC, N = 192
GDENTVVDLGSKR pc3
rs3739406 ALT
117:496 71:549 1:215
8:17742929:A:G_A
p = 1.1e−15, model = DOM, N = 189
GDENTVIDLGSKR pc2
rs3739406 REF
0:496 57:549 36:215
8:17742929:A:G_A
p = 1.2e−21, model = REC, N = 93
GDENTVIDLGSKR pc3
rs3739406 REF
0:496 19:549 16:215
8:17742929:A:G_A
p = 3.2e−08, model = REC, N = 35

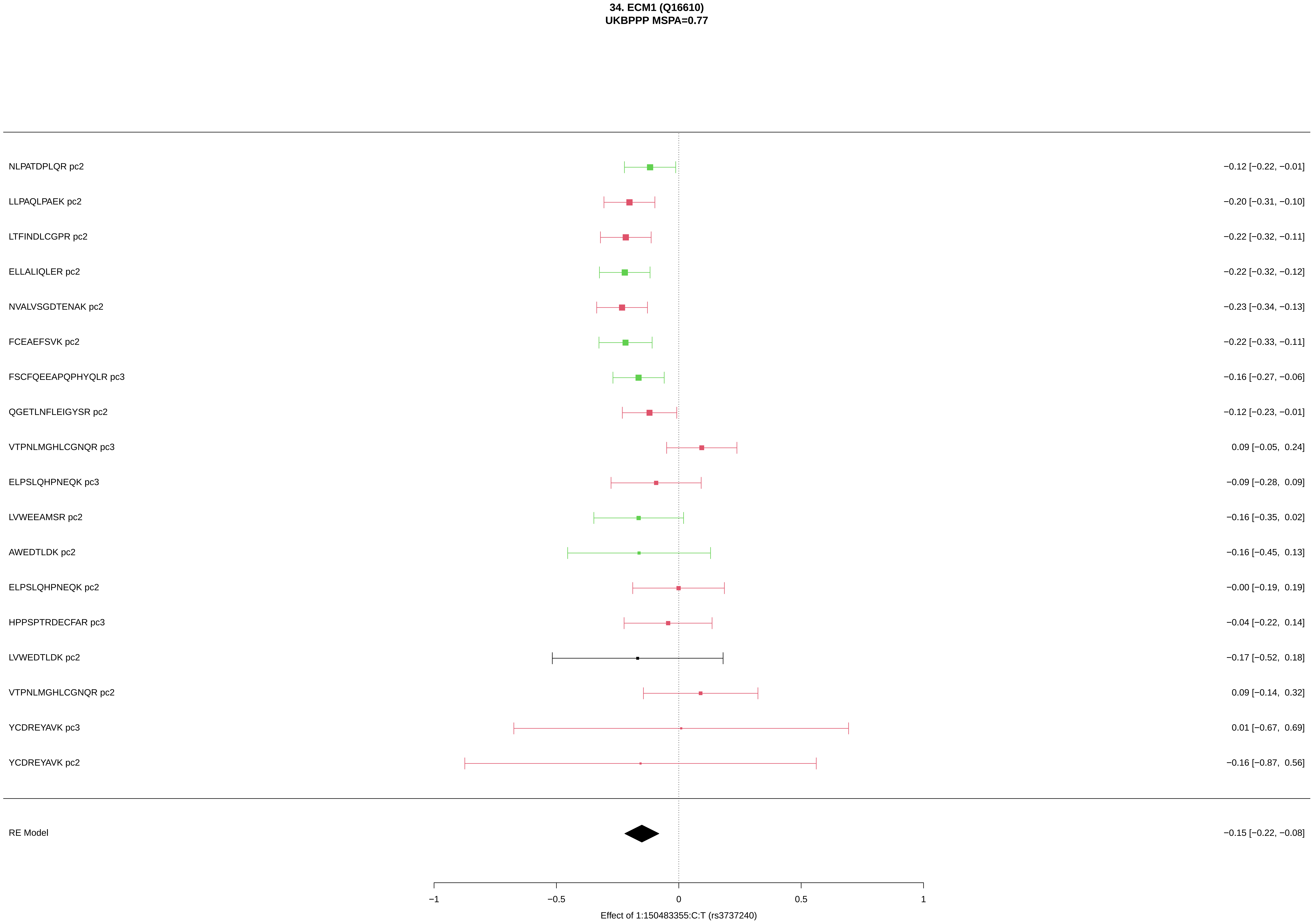
34. ECM1 (Q16610)
UKBPPP MSPA=0.77
NLPATDPLQR pc2
−0.12 [−0.22, −0.01]
LLPAQLPAEK pc2
−0.20 [−0.31, −0.10]
LTFINDLCGPR pc2
−0.22 [−0.32, −0.11]
ELLALIQLER pc2
−0.22 [−0.32, −0.12]
NVALVSGDTENAK pc2
−0.23 [−0.34, −0.13]
FCEAEFSVK pc2
−0.22 [−0.33, −0.11]
FSCFQEEAPQPHYQLR pc3
−0.16 [−0.27, −0.06]
QGETLNFLEIGYSR pc2
−0.12 [−0.23, −0.01]
VTPNLMGHLCGNQR pc3
0.09 [−0.05, 0.24]
ELPSLQHPNEQK pc3
−0.09 [−0.28, 0.09]
LVWEEAMSR pc2
−0.16 [−0.35, 0.02]
AWEDTLDK pc2
−0.16 [−0.45, 0.13]
ELPSLQHPNEQK pc2
−0.00 [−0.19, 0.19]
HPPSPTRDECFAR pc3
−0.04 [−0.22, 0.14]
LVWEDTLDK pc2
−0.17 [−0.52, 0.18]
VTPNLMGHLCGNQR pc2
0.09 [−0.14, 0.32]
YCDREYAVK pc3
0.01 [−0.67, 0.69]
YCDREYAVK pc2
−0.16 [−0.87, 0.56]
RE Model
−0.15 [−0.22, −0.08]
−1
−0.5
0
0.5
1
Effect of 1:150483355:C:T (rs3737240)

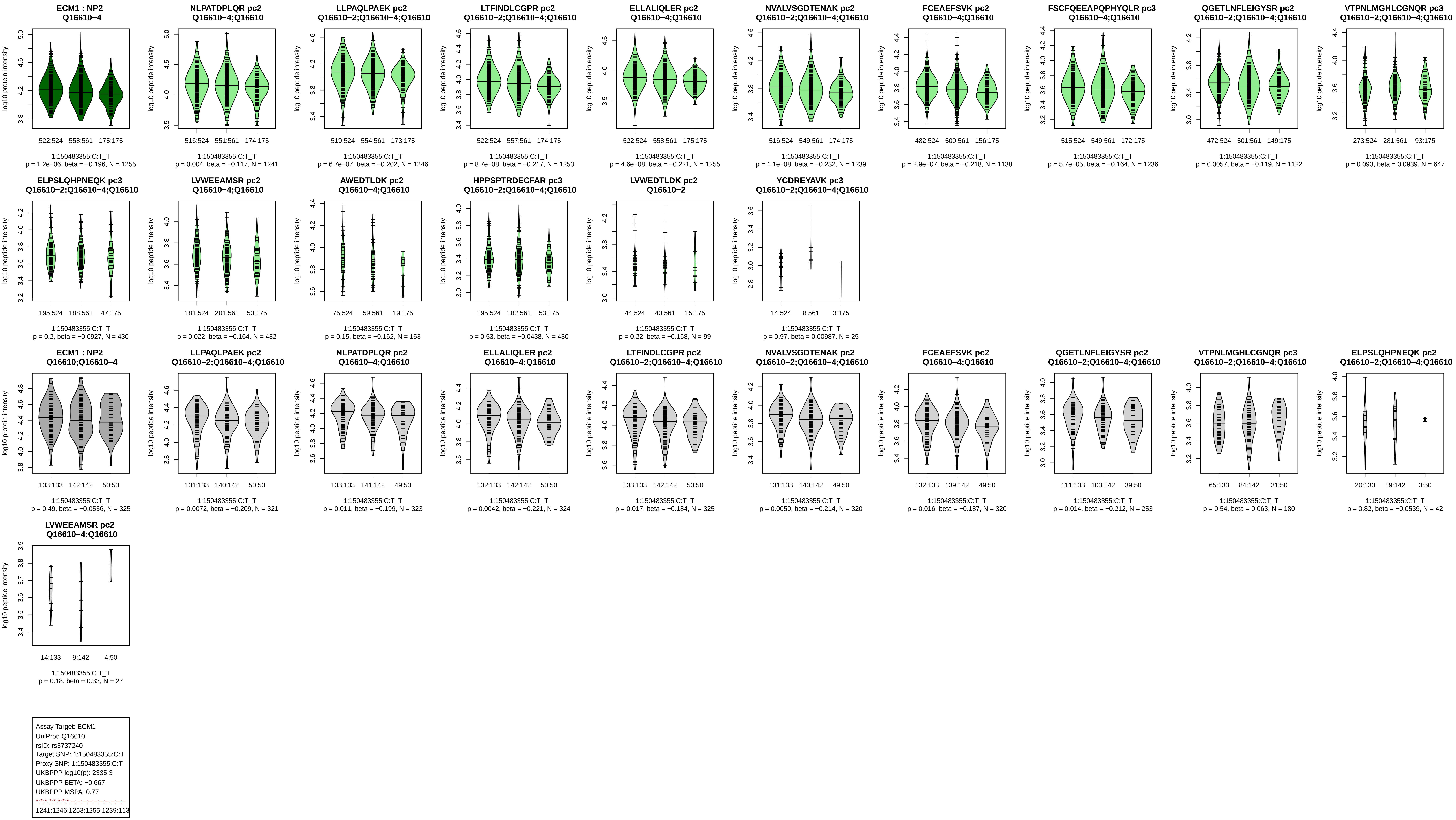
ECM1 : NP2
Q16610−4
log10 protein intensity
522:524 558:561 175:175
1:150483355:C:T_T
p = 1.2e−06, beta = −0.196, N = 1255
NLPATDPLQR pc2
Q16610−4;Q16610
log10 peptide intensity
516:524 551:561 174:175
1:150483355:C:T_T
p = 0.004, beta = −0.117, N = 1241
LLPAQLPAEK pc2
Q16610−2;Q16610−4;Q16610
519:524 554:561 173:175
1:150483355:C:T_T
p = 6.7e−07, beta = −0.202, N = 1246
LTFINDLCGPR pc2
Q16610−2;Q16610−4;Q16610
522:524 557:561 174:175
1:150483355:C:T_T
p = 8.7e−08, beta = −0.217, N = 1253
ELLALIQLER pc2
Q16610−4;Q16610
522:524 558:561 175:175
1:150483355:C:T_T
p = 4.6e−08, beta = −0.221, N = 1255
NVALVSGDTENAK pc2
Q16610−2;Q16610−4;Q16610
516:524 549:561 174:175
1:150483355:C:T_T
p = 1.1e−08, beta = −0.232, N = 1239
FCEAEFSVK pc2
Q16610−4;Q16610
482:524 500:561 156:175
1:150483355:C:T_T
p = 2.9e−07, beta = −0.218, N = 1138
FSCFQEEAPQPHYQLR pc3
Q16610−4;Q16610
515:524 549:561 172:175
1:150483355:C:T_T
p = 5.7e−05, beta = −0.164, N = 1236
QGETLNFLEIGYSR pc2
Q16610−2;Q16610−4;Q16610
472:524 501:561 149:175
1:150483355:C:T_T
p = 0.0057, beta = −0.119, N = 1122
VTPNLMGHLCGNQR pc3
Q16610−2;Q16610−4;Q16610
273:524 281:561 93:175
1:150483355:C:T_T
p = 0.093, beta = 0.0939, N = 647
ELPSLQHPNEQK pc3
Q16610−2;Q16610−4;Q16610
195:524 188:561 47:175
1:150483355:C:T_T
p = 0.2, beta = −0.0927, N = 430
LVWEEAMSR pc2
Q16610−4;Q16610
181:524 201:561 50:175
1:150483355:C:T_T
p = 0.022, beta = −0.164, N = 432
AWEDTLDK pc2
Q16610−4;Q16610
75:524 59:561 19:175
1:150483355:C:T_T
p = 0.15, beta = −0.162, N = 153
HPPSPTRDECFAR pc3
Q16610−2;Q16610−4;Q16610
195:524 182:561 53:175
1:150483355:C:T_T
p = 0.53, beta = −0.0438, N = 430
LVWEDTLDK pc2
Q16610−2
44:524 40:561 15:175
1:150483355:C:T_T
p = 0.22, beta = −0.168, N = 99
YCDREYAVK pc3
Q16610−2;Q16610−4;Q16610
14:524 8:561 3:175
1:150483355:C:T_T
p = 0.97, beta = 0.00987, N = 25
ECM1 : NP2
Q16610;Q16610−4
133:133 142:142 50:50
1:150483355:C:T_T
p = 0.49, beta = −0.0536, N = 325
LLPAQLPAEK pc2
Q16610−2;Q16610−4;Q16610
131:133 140:142 50:50
1:150483355:C:T_T
p = 0.0072, beta = −0.209, N = 321
NLPATDPLQR pc2
Q16610−4;Q16610
133:133 141:142 49:50
1:150483355:C:T_T
p = 0.011, beta = −0.199, N = 323
ELLALIQLER pc2
Q16610−4;Q16610
132:133 142:142 50:50
1:150483355:C:T_T
p = 0.0042, beta = −0.221, N = 324
LTFINDLCGPR pc2
Q16610−2;Q16610−4;Q16610
133:133 142:142 50:50
1:150483355:C:T_T
p = 0.017, beta = −0.184, N = 325
NVALVSGDTENAK pc2
Q16610−2;Q16610−4;Q16610
131:133 140:142 49:50
1:150483355:C:T_T
p = 0.0059, beta = −0.214, N = 320
FCEAEFSVK pc2
Q16610−4;Q16610
132:133 139:142 49:50
1:150483355:C:T_T
p = 0.016, beta = −0.187, N = 320
QGETLNFLEIGYSR pc2
Q16610−2;Q16610−4;Q16610
111:133 103:142 39:50
1:150483355:C:T_T
p = 0.014, beta = −0.212, N = 253
VTPNLMGHLCGNQR pc3
Q16610−2;Q16610−4;Q16610
65:133 84:142 31:50
1:150483355:C:T_T
p = 0.54, beta = 0.063, N = 180
ELPSLQHPNEQK pc2
Q16610−2;Q16610−4;Q16610
20:133 19:142 3:50
1:150483355:C:T_T
p = 0.82, beta = −0.0539, N = 42
LVWEEAMSR pc2
Q16610−4;Q16610
14:133 9:142 4:50
1:150483355:C:T_T
p = 0.18, beta = 0.33, N = 27
Assay Target: ECM1
UniProt: Q16610
rsID: rs3737240
Target SNP: 1:150483355:C:T
Proxy SNP: 1:150483355:C:T
UKBPPP log10(p): 2335.3
UKBPPP BETA: −0.667
UKBPPP MSPA: 0.77
1241:1246:1253:1255:1239:113

35. AHSG (P02765)
UKBPPP MSPA=0.155

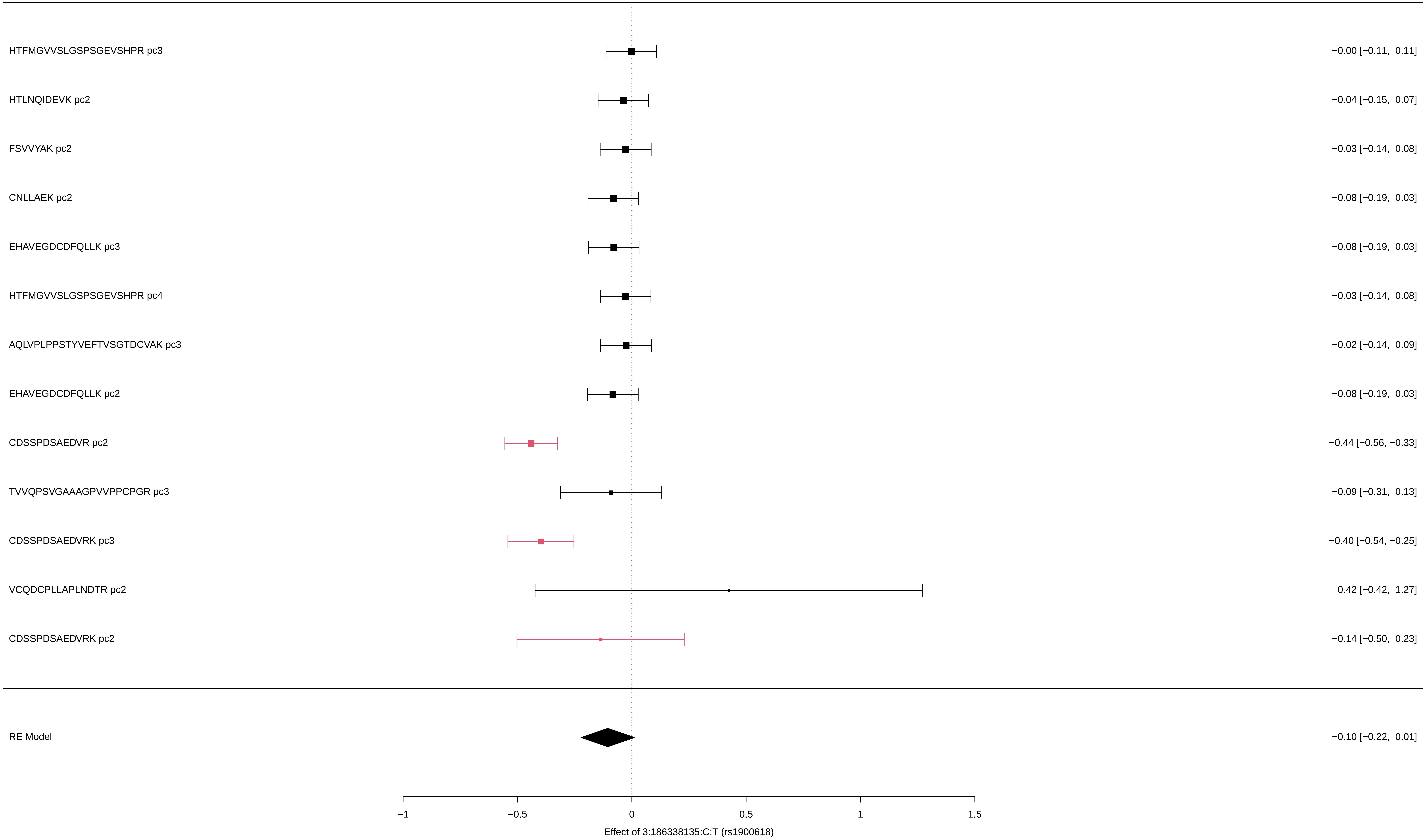

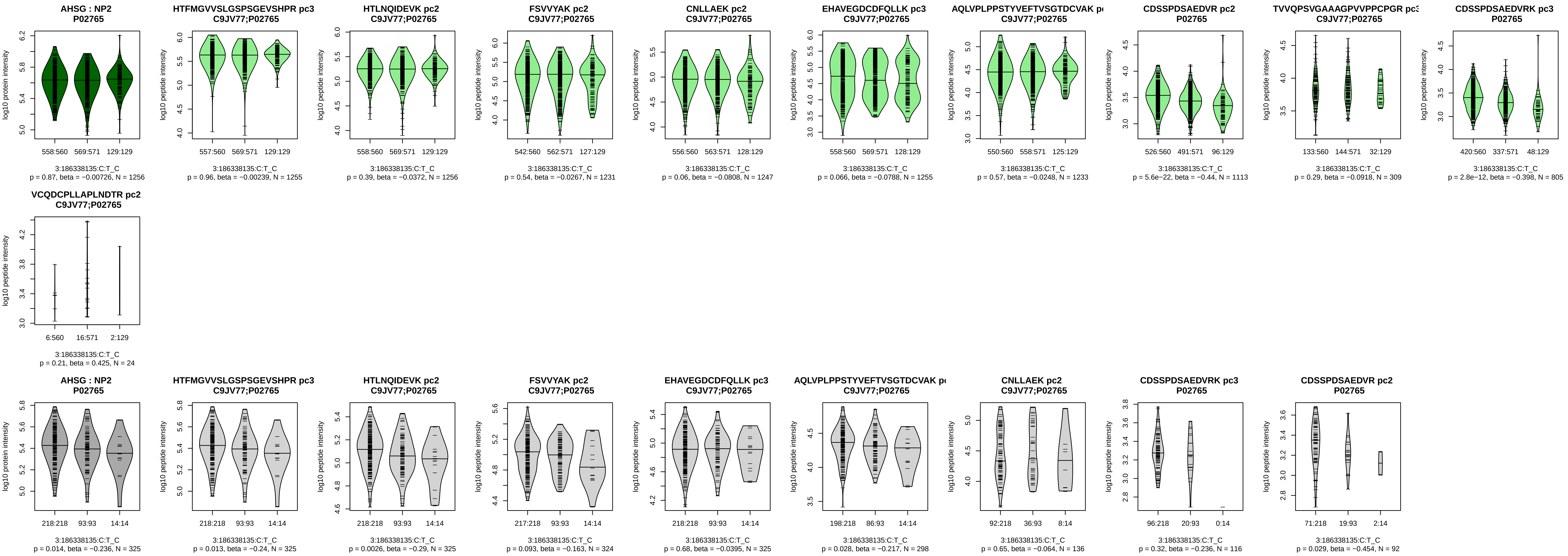

Assay Target: AHSG
UniProt: P02765
rsID: rs1900618
Target SNP: 3:186338135:C:T
Proxy SNP: 3:186338135:C:T
UKBPPP log10(p): 2299.5
UKBPPP BETA: 0.668
UKBPPP MSPA: 0.155
–:–:–:–:–:–:–:*:–:*:–:–
1255:1256:1231:1247:1255:125

36. FCN2 (Q15485)
UKBPPP MSPA=0.757

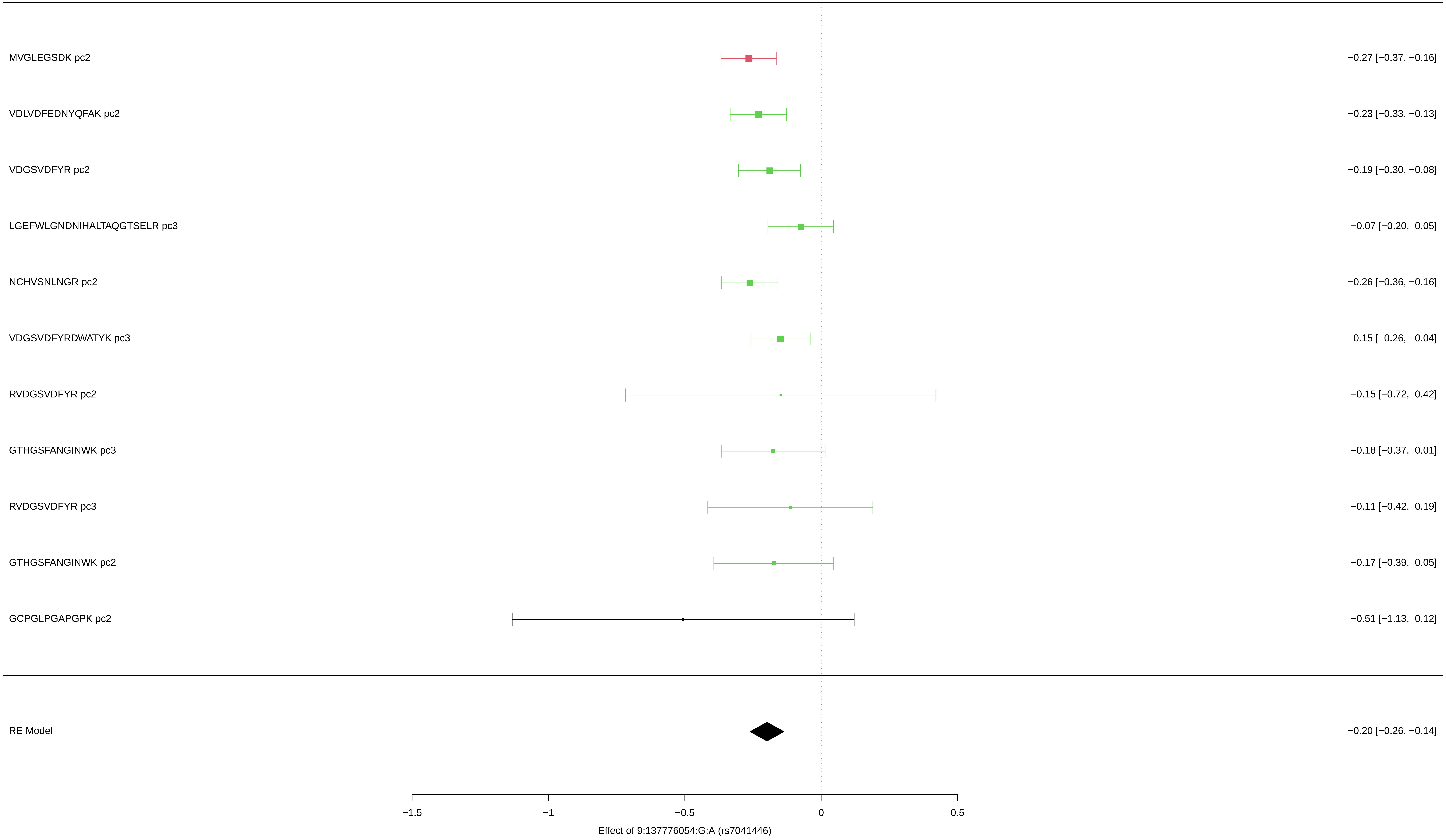

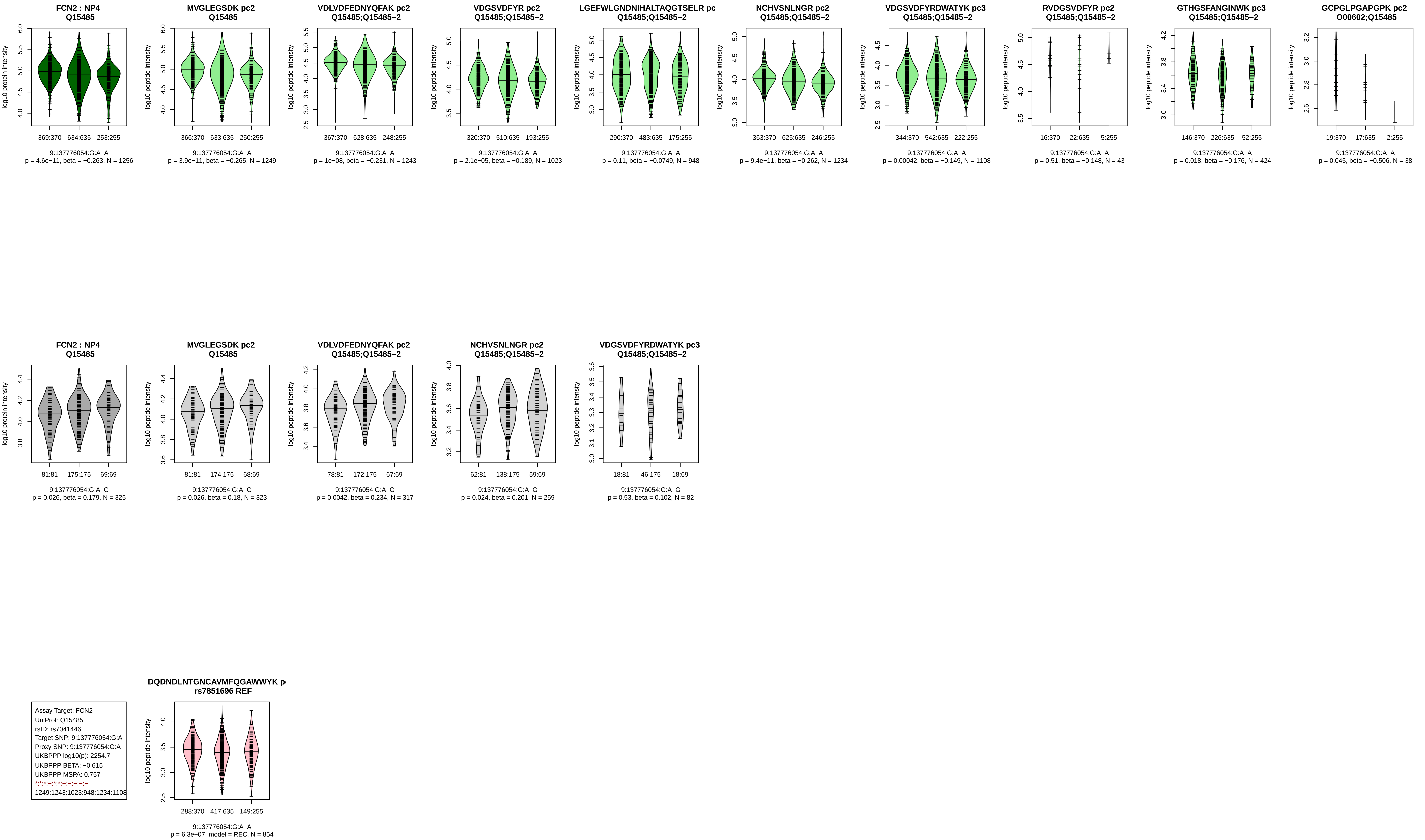
FCN2 : NP4
Q15485
log10 protein intensity
369:370 634:635 253:255
9:137776054:G:A_A
p = 4.6e−11, beta = −0.263, N = 1256
MVGLEGSDK pc2
Q15485
log10 peptide intensity
366:370 633:635 250:255
9:137776054:G:A_A
p = 3.9e−11, beta = −0.265, N = 1249
VDLVDFEDNYQFAK pc2
Q15485;Q15485−2
367:370 628:635 248:255
9:137776054:G:A_A
p = 1e−08, beta = −0.231, N = 1243
VDGSVDFYR pc2
Q15485;Q15485−2
320:370 510:635 193:255
9:137776054:G:A_A
p = 2.1e−05, beta = −0.189, N = 1023
LGEFWLGNDNIHALTAQGTSELR pc
Q15485;Q15485−2
290:370 483:635 175:255
9:137776054:G:A_A
p = 0.11, beta = −0.0749, N = 948
NCHVSNLNGR pc2
Q15485;Q15485−2
363:370 625:635 246:255
9:137776054:G:A_A
p = 9.4e−11, beta = −0.262, N = 1234
VDGSVDFYRDWATYK pc3
Q15485;Q15485−2
344:370 542:635 222:255
9:137776054:G:A_A
p = 0.00042, beta = −0.149, N = 1108
RVDGSVDFYR pc2
Q15485;Q15485−2
16:370 22:635 5:255
9:137776054:G:A_A
p = 0.51, beta = −0.148, N = 43
GTHGSFANGINWK pc3
Q15485;Q15485−2
146:370 226:635 52:255
9:137776054:G:A_A
p = 0.018, beta = −0.176, N = 424
GCPGLPGAPGPK pc2
O00602;Q15485
19:370 17:635 2:255
9:137776054:G:A_A
p = 0.045, beta = −0.506, N = 38
FCN2 : NP4
Q15485
81:81 175:175 69:69
9:137776054:G:A_G
p = 0.026, beta = 0.179, N = 325
MVGLEGSDK pc2
Q15485
81:81 174:175 68:69
9:137776054:G:A_G
p = 0.026, beta = 0.18, N = 323
VDLVDFEDNYQFAK pc2
Q15485;Q15485−2
78:81 172:175 67:69
9:137776054:G:A_G
p = 0.0042, beta = 0.234, N = 317
NCHVSNLNGR pc2
Q15485;Q15485−2
62:81 138:175 59:69
9:137776054:G:A_G
p = 0.024, beta = 0.201, N = 259
VDGSVDFYRDWATYK pc3
Q15485;Q15485−2
18:81 46:175 18:69
9:137776054:G:A_G
p = 0.53, beta = 0.102, N = 82
Assay Target: FCN2
UniProt: Q15485
rsID: rs7041446
Target SNP: 9:137776054:G:A
Proxy SNP: 9:137776054:G:A
UKBPPP log10(p): 2254.7
UKBPPP BETA: −0.615
UKBPPP MSPA: 0.757
1249:1243:1023:948:1234:1108
DQDNDLNTGNCAVMFQGAWWYK p
rs7851696 REF
288:370 417:635 149:255
9:137776054:G:A_A
p = 6.3e−07, model = REC, N = 854

37. LAMB1 (P07942)
UKBPPP MSPA=0

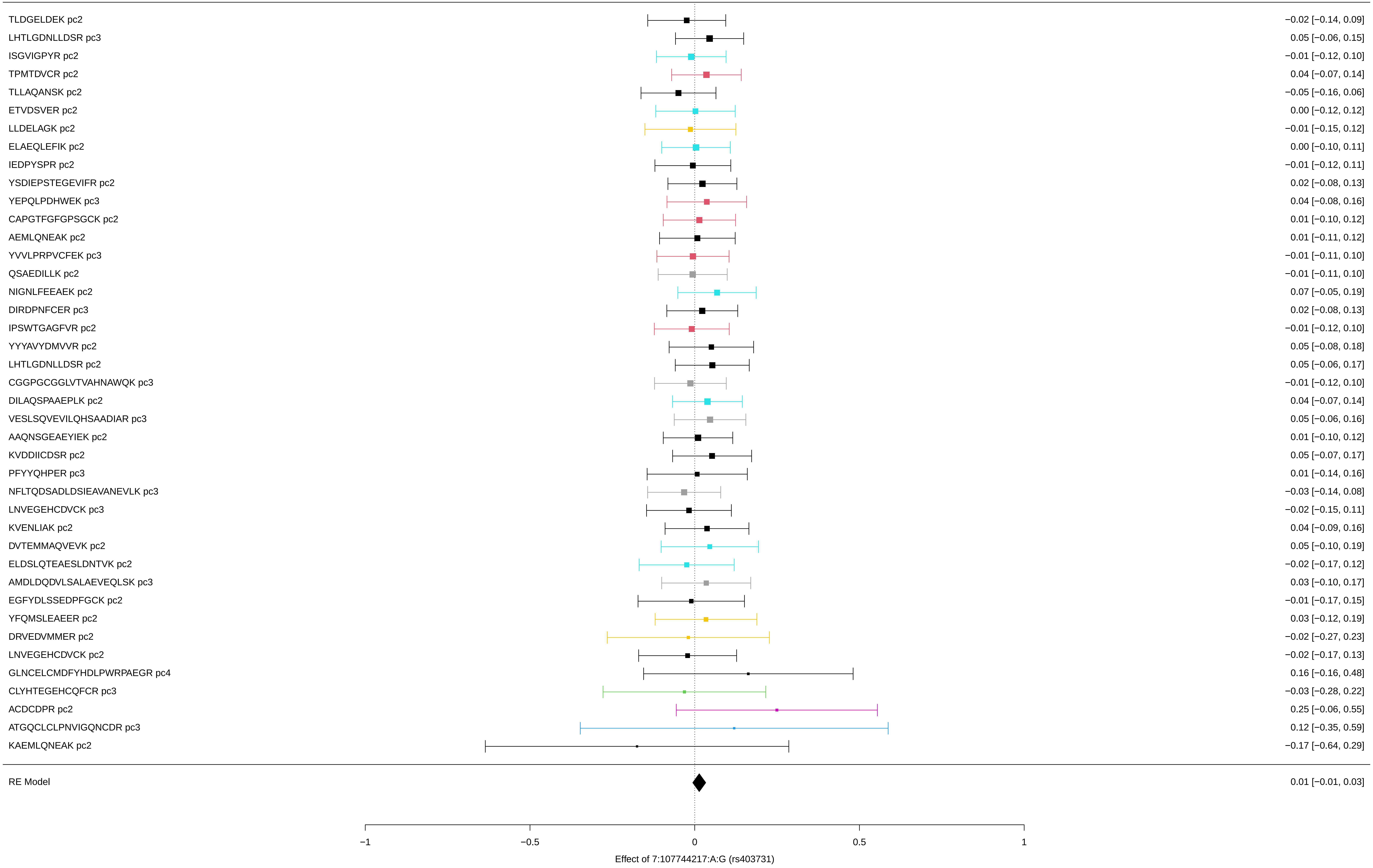

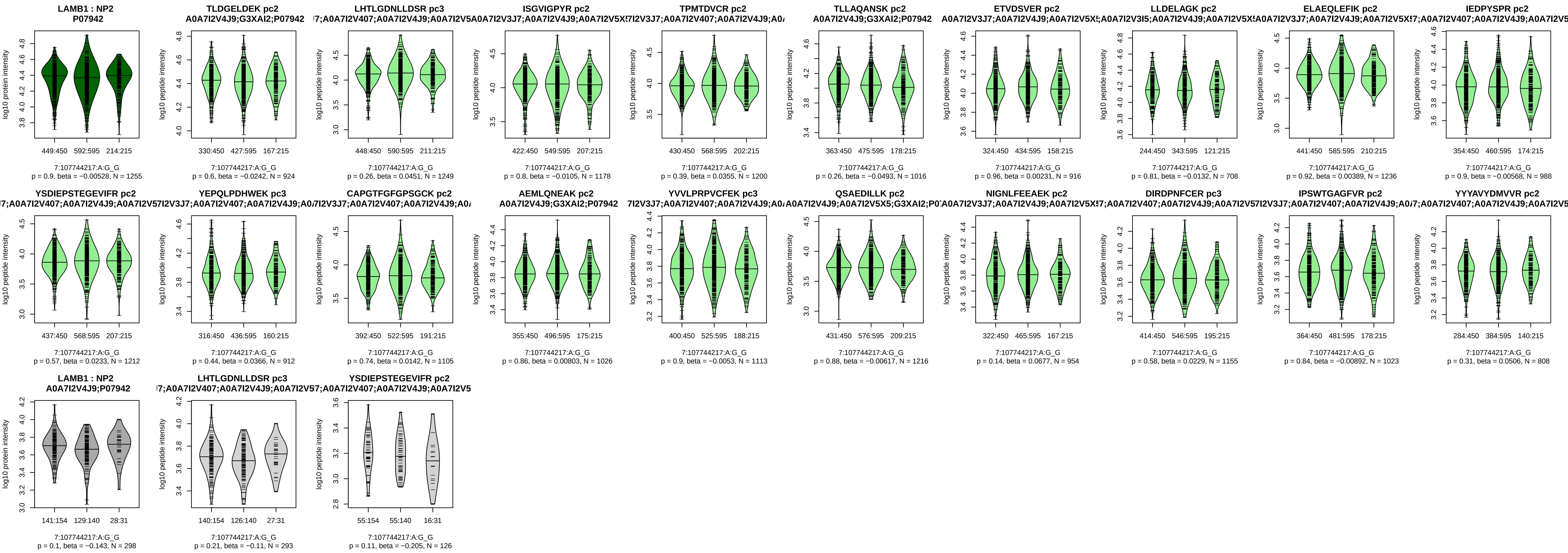
LAMB1 : NP2
P07942
p = 0.9, beta = −0.00528, N = 1255
TLDGELDEK pc2
A0A7I2V4J9;G3XAI2;P07942
p = 0.6, beta = −0.0242, N = 924
LHTLGDNLLDSR pc3
p = 0.26, beta = 0.0451, N = 1249
ISGVIGPYR pc2
p = 0.8, beta = −0.0105, N = 1178
TPMTDVCR pc2
p = 0.39, beta = 0.0355, N = 1200
TLLAQANSK pc2
A0A7I2V4J9;G3XAI2;P07942
p = 0.26, beta = −0.0493, N = 1016
ETVDSVER pc2
p = 0.96, beta = 0.00231, N = 916
LLDELAGK pc2
p = 0.81, beta = −0.0132, N = 708
ELAEQLEFIK pc2
p = 0.92, beta = 0.00389, N = 1236
IEDPYSPR pc2
p = 0.9, beta = −0.00568, N = 988
YSDIEPSTEGEVIFR pc2
p = 0.57, beta = 0.0233, N = 1212
YEPQLPDHWEK pc3
p = 0.44, beta = 0.0366, N = 912
CAPGTFGFGPSGCK pc2
p = 0.74, beta = 0.0142, N = 1105
AEMLQNEAK pc2
A0A7I2V4J9;G3XAI2;P07942
p = 0.86, beta = 0.00803, N = 1026
YVVLPRPVCFEK pc3
p = 0.9, beta = −0.0053, N = 1113
QSAEDILLK pc2
p = 0.88, beta = −0.00617, N = 1216
NIGNLFEEAEK pc2
p = 0.14, beta = 0.0677, N = 954
DIRDPNFCER pc3
p = 0.58, beta = 0.0229, N = 1155
IPSWTGAGFVR pc2
p = 0.84, beta = −0.00892, N = 1023
YYYAVYDMVVR pc2
p = 0.31, beta = 0.0506, N = 808
LAMB1 : NP2
A0A7I2V4J9;P07942
p = 0.1, beta = −0.143, N = 298
LHTLGDNLLDSR pc3
p = 0.21, beta = −0.11, N = 293
YSDIEPSTEGEVIFR pc2
p = 0.11, beta = −0.205, N = 126
7:107744217:A:G_G

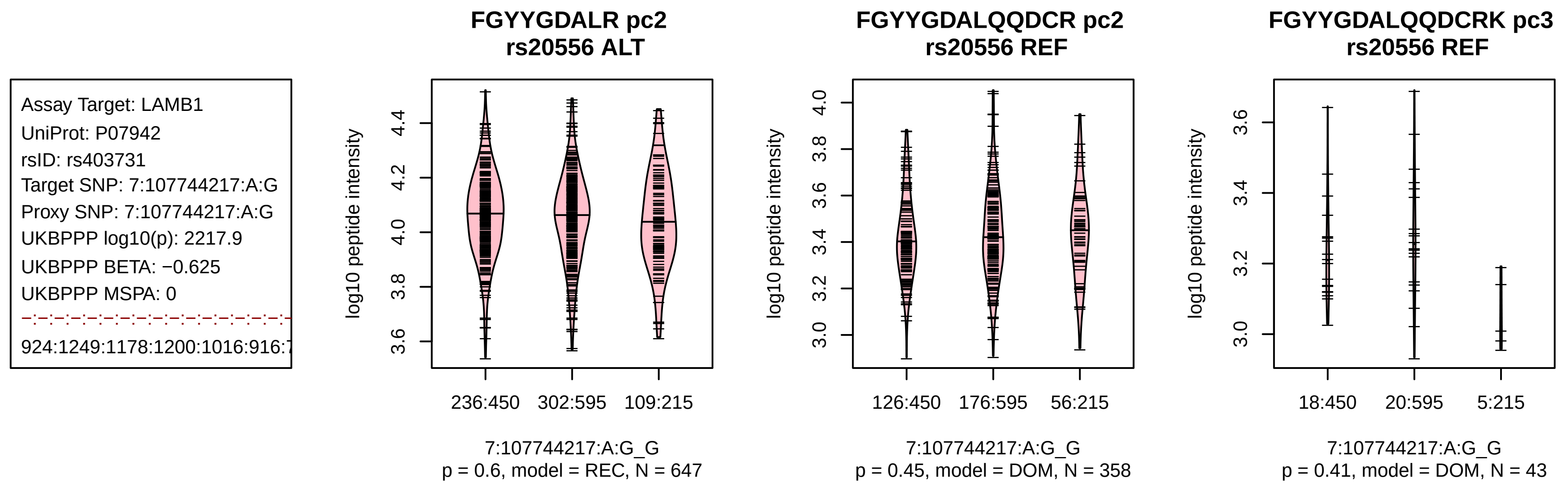
Assay Target: LAMB1
UniProt: P07942
rsID: rs403731
Target SNP: 7:107744217:A:G
Proxy SNP: 7:107744217:A:G
UKBPPP log10(p): 2217.9
UKBPPP BETA: −0.625
UKBPPP MSPA: 0
FGYYGDALR pc2
rs20556 ALT
p = 0.6, model = REC, N = 647
FGYYGDALQQDCR pc2
rs20556 REF
p = 0.45, model = DOM, N = 358
FGYYGDALQQDCRK pc3
rs20556 REF
p = 0.41, model = DOM, N = 43
7:107744217:A:G_G

38. FUCA1 (P04066)
UKBPPP MSPA=0

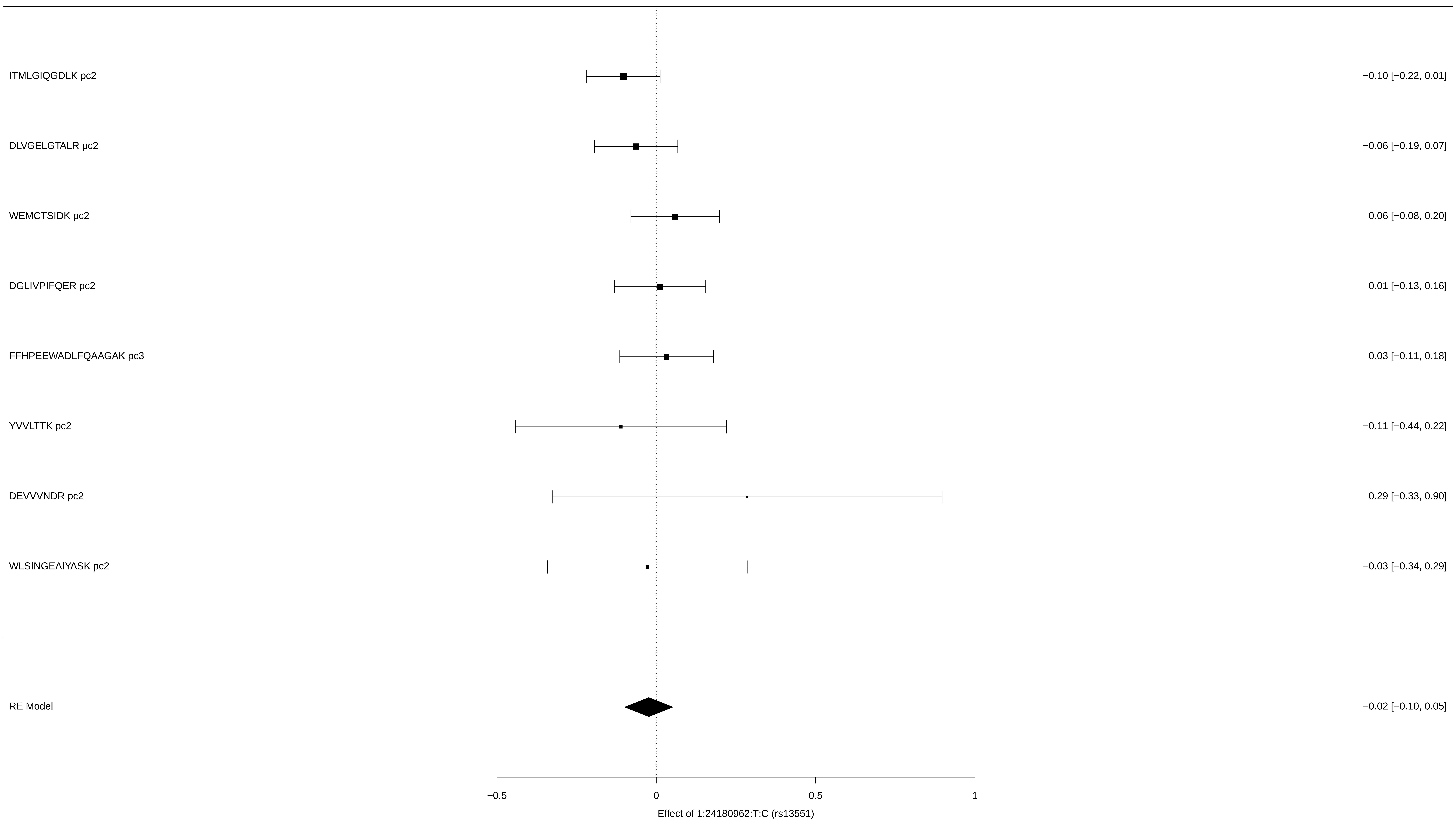

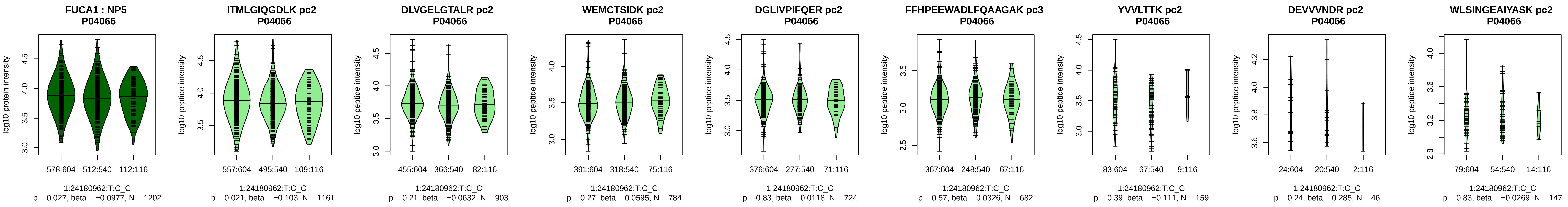

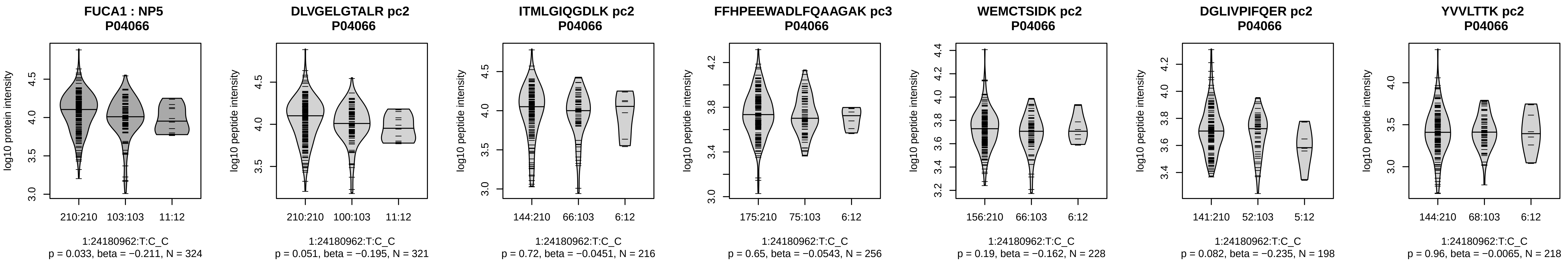

Assay Target: FUCA1
UniProt: P04066
rsID: rs13551
Target SNP: 1:24180962:T:C
Proxy SNP: 1:24180962:T:C
UKBPPP log10(p): 2197.8
UKBPPP BETA: −0.38
UKBPPP MSPA: 0
1161:903:784:724:682:159:46:1

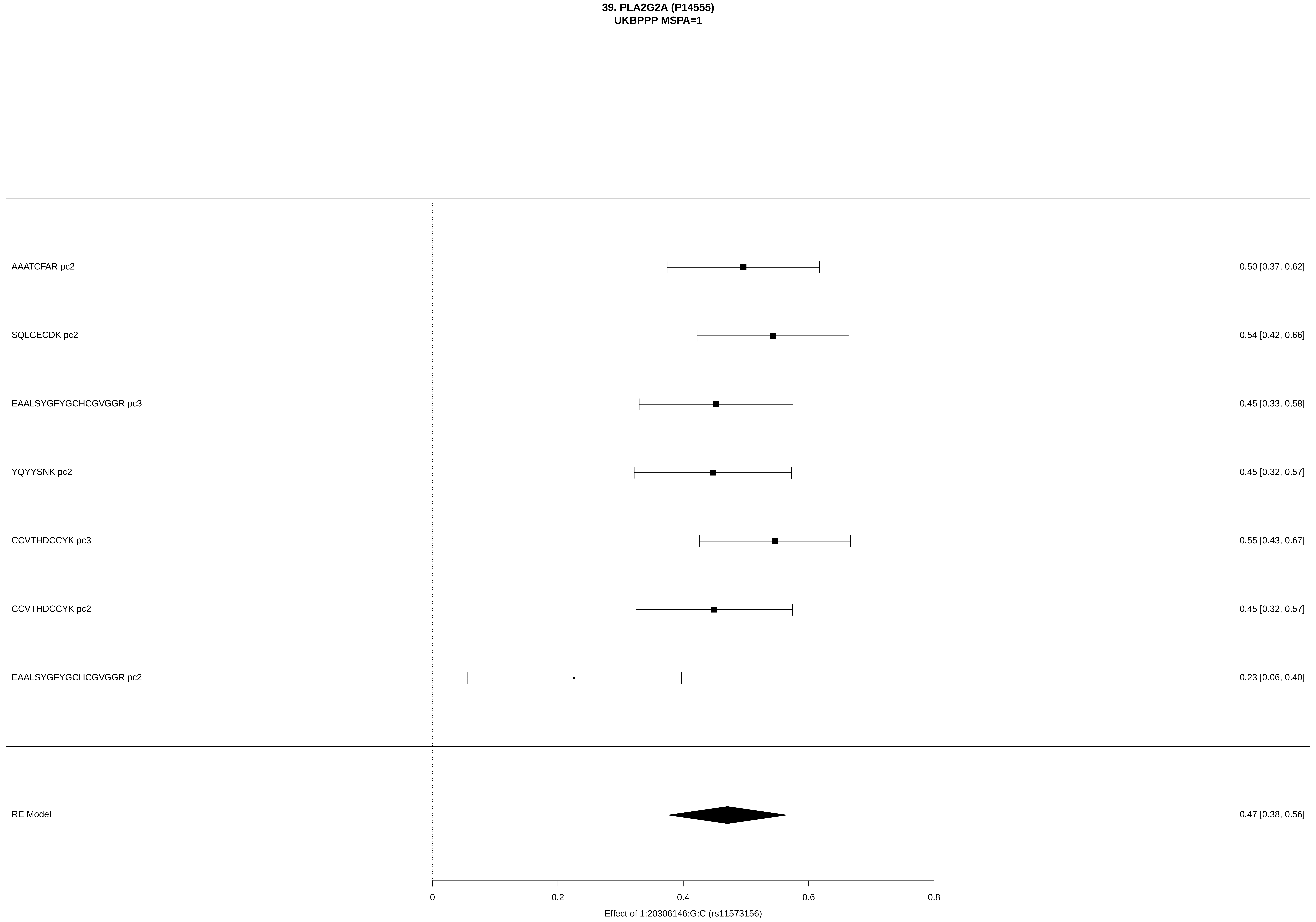
39. PLA2G2A (P14555)
UKBPPP MSPA=1
AAATCFAR pc2
0.50 [0.37, 0.62]
SQLCECDK pc2
0.54 [0.42, 0.66]
EAALSYGFYGCHCGVGGR pc3
0.45 [0.33, 0.58]
YQYYSNK pc2
0.45 [0.32, 0.57]
CCVTHDCCYK pc3
0.55 [0.43, 0.67]
CCVTHDCCYK pc2
0.45 [0.32, 0.57]
EAALSYGFYGCHCGVGGR pc2
0.23 [0.06, 0.40]
RE Model
0.47 [0.38, 0.56]
0
0.2
0.4
0.6
0.8
Effect of 1:20306146:G:C (rs11573156)

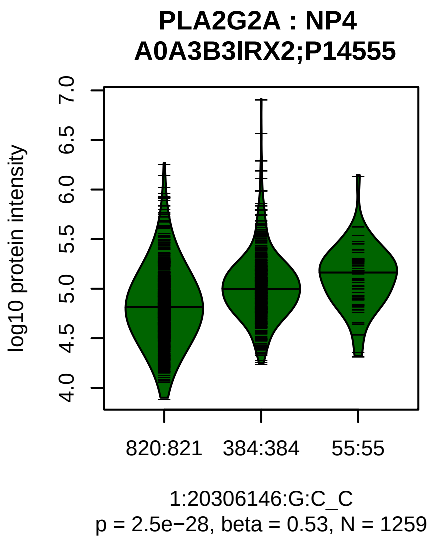

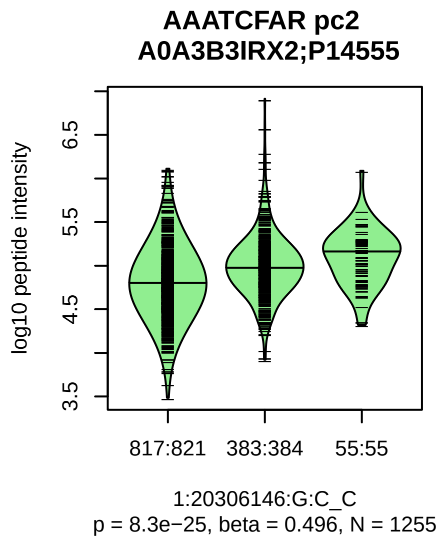

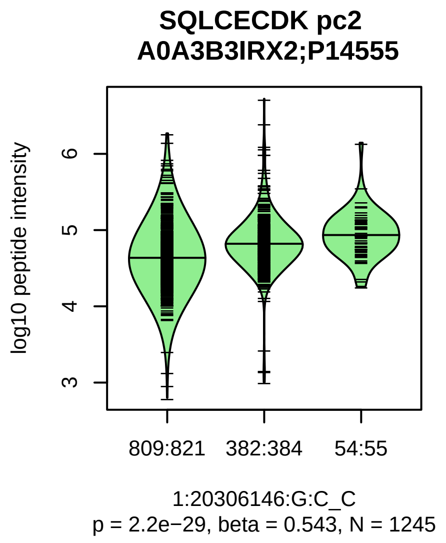

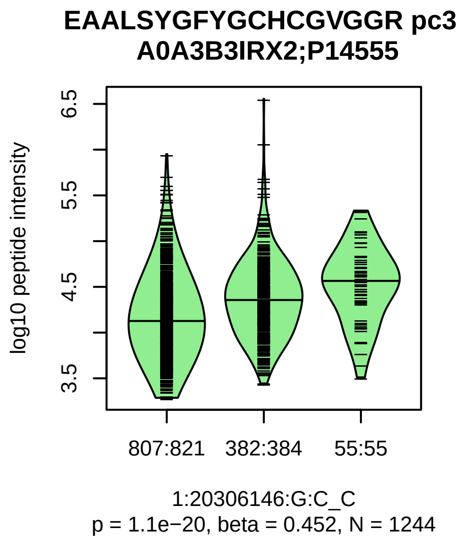

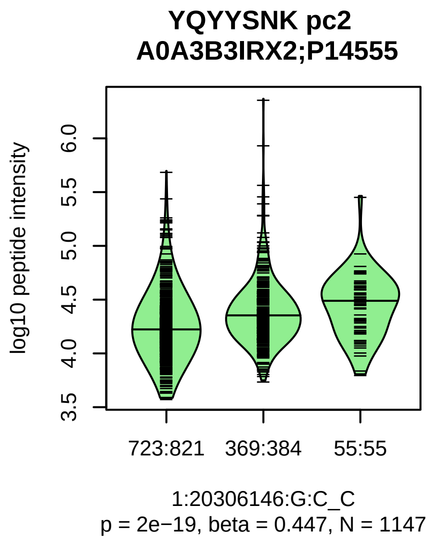

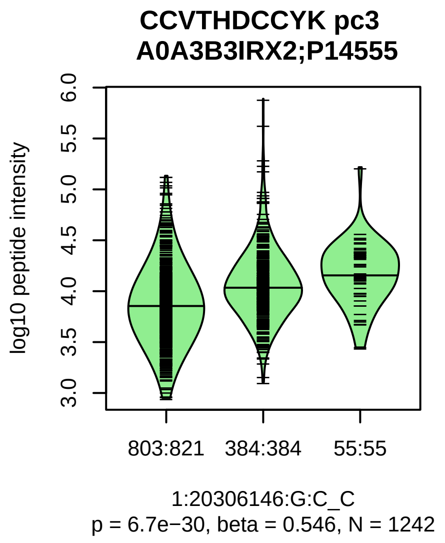

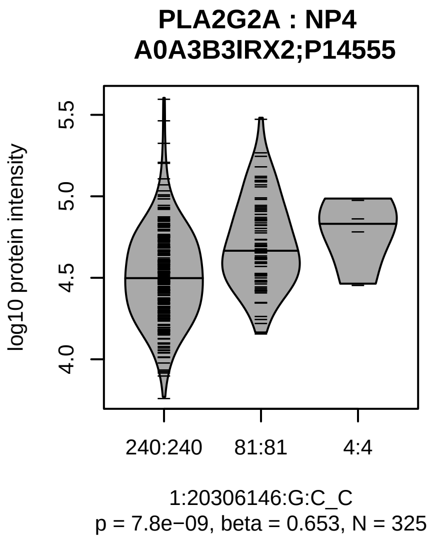

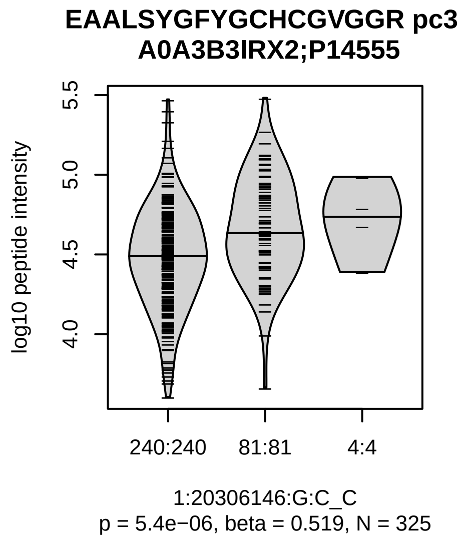

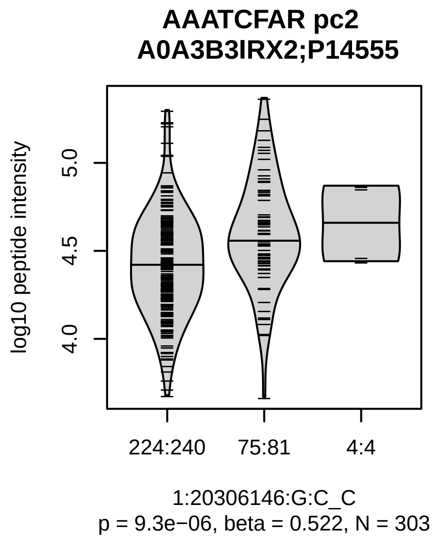

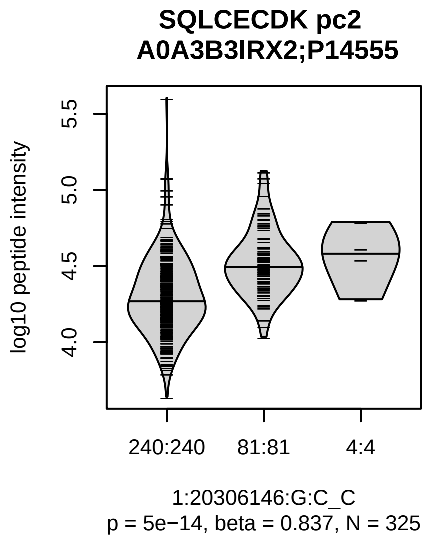

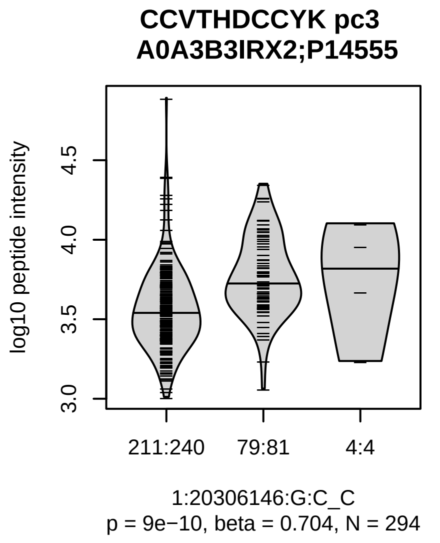

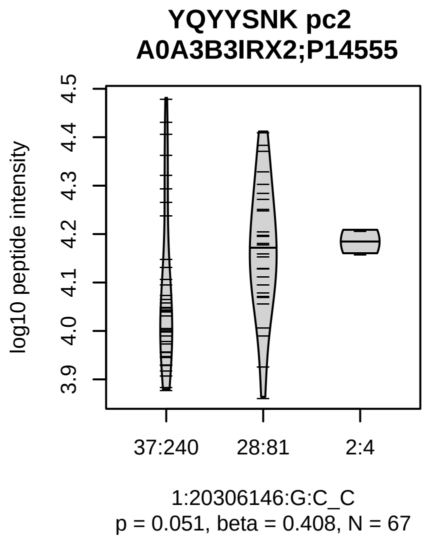

Assay Target: PLA2G2A
UniProt: P14555
rsID: rs11573156
Target SNP: 1:20306146:G:C
Proxy SNP: 1:20306146:G:C
UKBPPP log10(p): 2153.3
UKBPPP BETA: 0.721
UKBPPP MSPA: 1
1255:1245:1244:1147:1242:118

40. MMP3 (P08254)
UKBPPP MSPA=0.964

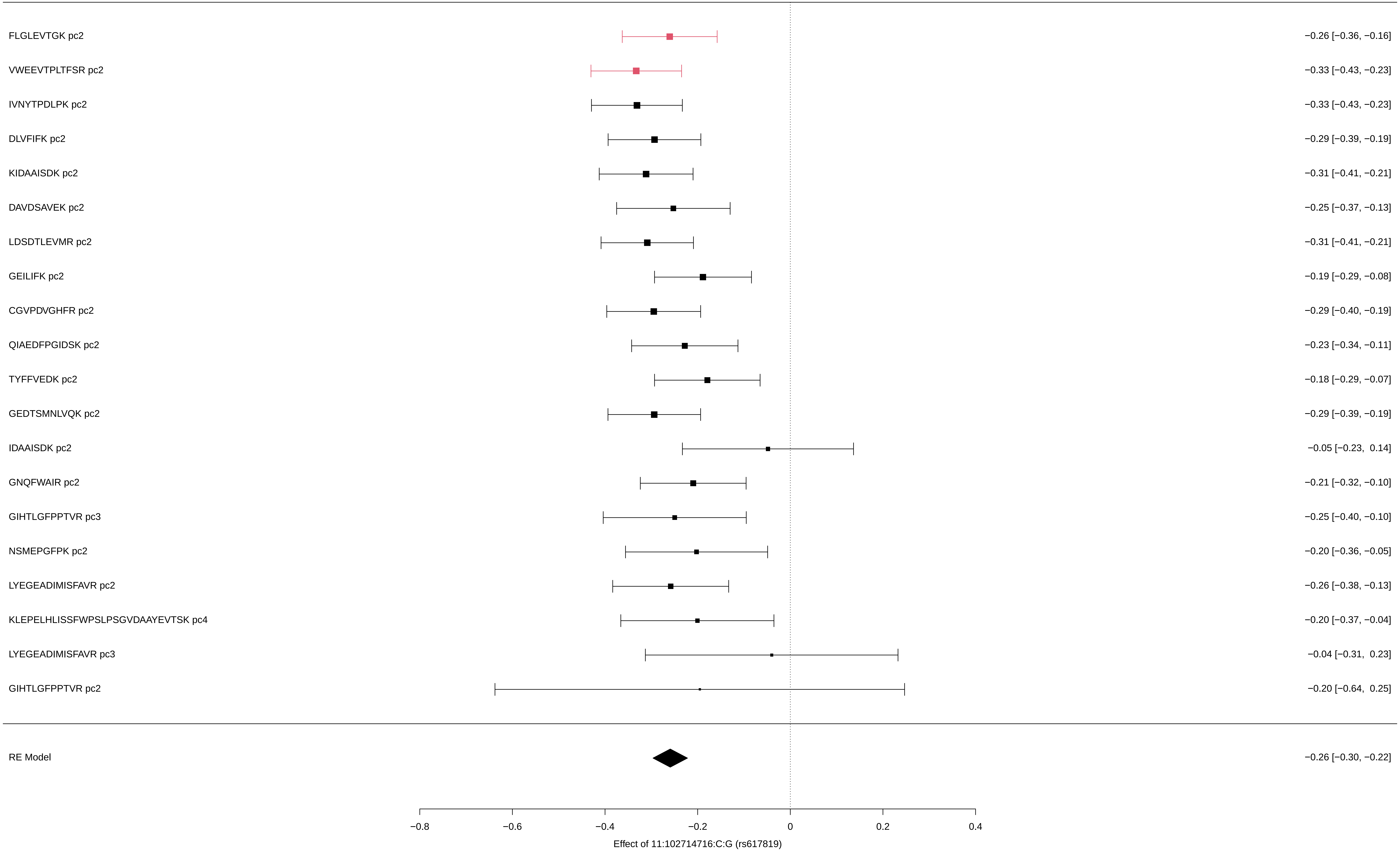

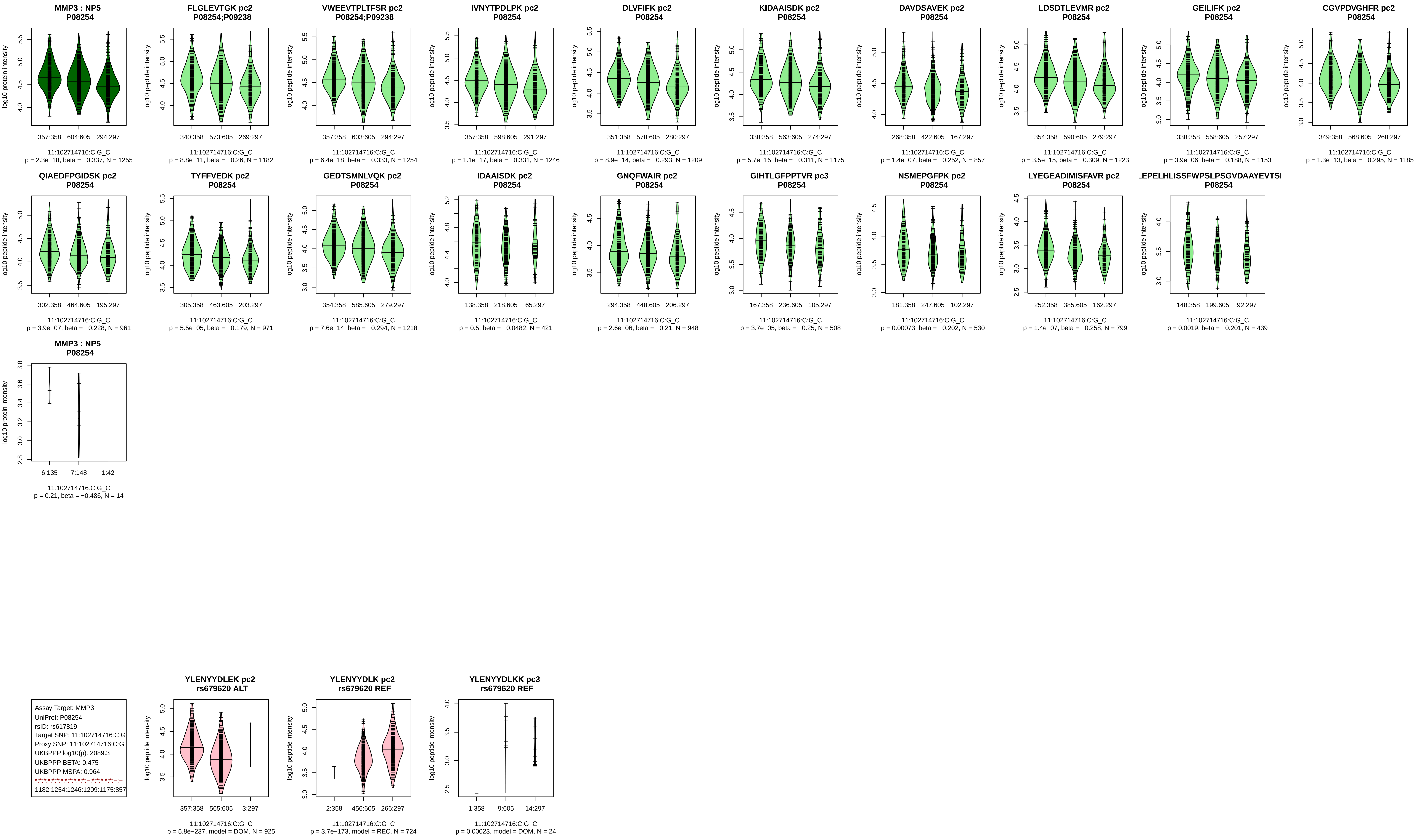

MMP3 : NP5 P08254
log10 protein intensity
357:358 604:605 294:297
11:102714716:C:G_C
p = 2.3e−18, beta = −0.337, N = 1255
FLGLEVTGK pc2 P08254;P09238
log10 peptide intensity
340:358 573:605 269:297
11:102714716:C:G_C
p = 8.8e−11, beta = −0.26, N = 1182
VWEEVTPLTFSR pc2 P08254;P09238
357:358 603:605 294:297
11:102714716:C:G_C
p = 6.4e−18, beta = −0.333, N = 1254
IVNYTPDLPK pc2 P08254
357:358 598:605 291:297
11:102714716:C:G_C
p = 1.1e−17, beta = −0.331, N = 1246
DLVFIFK pc2 P08254
351:358 578:605 280:297
11:102714716:C:G_C
p = 8.9e−14, beta = −0.293, N = 1209
KIDAAISDK pc2 P08254
338:358 563:605 274:297
11:102714716:C:G_C
p = 5.7e−15, beta = −0.311, N = 1175
DAVDSAVEK pc2 P08254
268:358 422:605 167:297
11:102714716:C:G_C
p = 1.4e−07, beta = −0.252, N = 857
LDSDTLEVMR pc2 P08254
354:358 590:605 279:297
11:102714716:C:G_C
p = 3.5e−15, beta = −0.309, N = 1223
GEILIFK pc2 P08254
338:358 558:605 257:297
11:102714716:C:G_C
p = 3.9e−06, beta = −0.188, N = 1153
CGVPDVGHFR pc2 P08254
349:358 568:605 268:297
11:102714716:C:G_C
p = 1.3e−13, beta = −0.295, N = 1185
QIAEDFPGIDSK pc2 P08254
302:358 464:605 195:297
11:102714716:C:G_C
p = 3.9e−07, beta = −0.228, N = 961
TYFFVEDK pc2 P08254
305:358 463:605 203:297
11:102714716:C:G_C
p = 5.5e−05, beta = −0.179, N = 971
GEDTSMNLVQK pc2 P08254
354:358 585:605 279:297
11:102714716:C:G_C
p = 7.6e−14, beta = −0.294, N = 1218
IDAAISDK pc2 P08254
138:358 218:605 65:297
11:102714716:C:G_C
p = 0.5, beta = −0.0482, N = 421
GNQFWAIR pc2 P08254
294:358 448:605 206:297
11:102714716:C:G_C
p = 2.6e−06, beta = −0.21, N = 948
GIHTLGFPPTVR pc3 P08254
167:358 236:605 105:297
11:102714716:C:G_C
p = 3.7e−05, beta = −0.25, N = 508
NSMEPGFPK pc2 P08254
181:358 247:605 102:297
11:102714716:C:G_C
p = 0.00073, beta = −0.202, N = 530
LYEGEADIMISFAVR pc2 P08254
252:358 385:605 162:297
11:102714716:C:G_C
p = 1.4e−07, beta = −0.258, N = 799
EPELHLISSFWPSLPSGVDAAYEVTS P08254
148:358 199:605 92:297
11:102714716:C:G_C
p = 0.0019, beta = −0.201, N = 439
MMP3 : NP5 P08254
log10 protein intensity
6:135 7:148 1:42
11:102714716:C:G_C
p = 0.21, beta = −0.486, N = 14
Assay Target: MMP3
UniProt: P08254
rsID: rs617819
Target SNP: 11:102714716:C:G
Proxy SNP: 11:102714716:C:G
UKBPPP log10(p): 2089.3
UKBPPP BETA: 0.475
UKBPPP MSPA: 0.964
1182:1254:1246:1209:1175:857
YLENYYDLEK pc2 rs679620 ALT
357:358 565:605 3:297
11:102714716:C:G_C
p = 5.8e−237, model = DOM, N = 925
YLENYYDLK pc2 rs679620 REF
2:358 456:605 266:297
11:102714716:C:G_C
p = 3.7e−173, model = REC, N = 724
YLENYYDLKK pc3 rs679620 REF
1:358 9:605 14:297
11:102714716:C:G_C
p = 0.00023, model = DOM, N = 24

41. GNLY (P22749)
UKBPPP MSPA=1

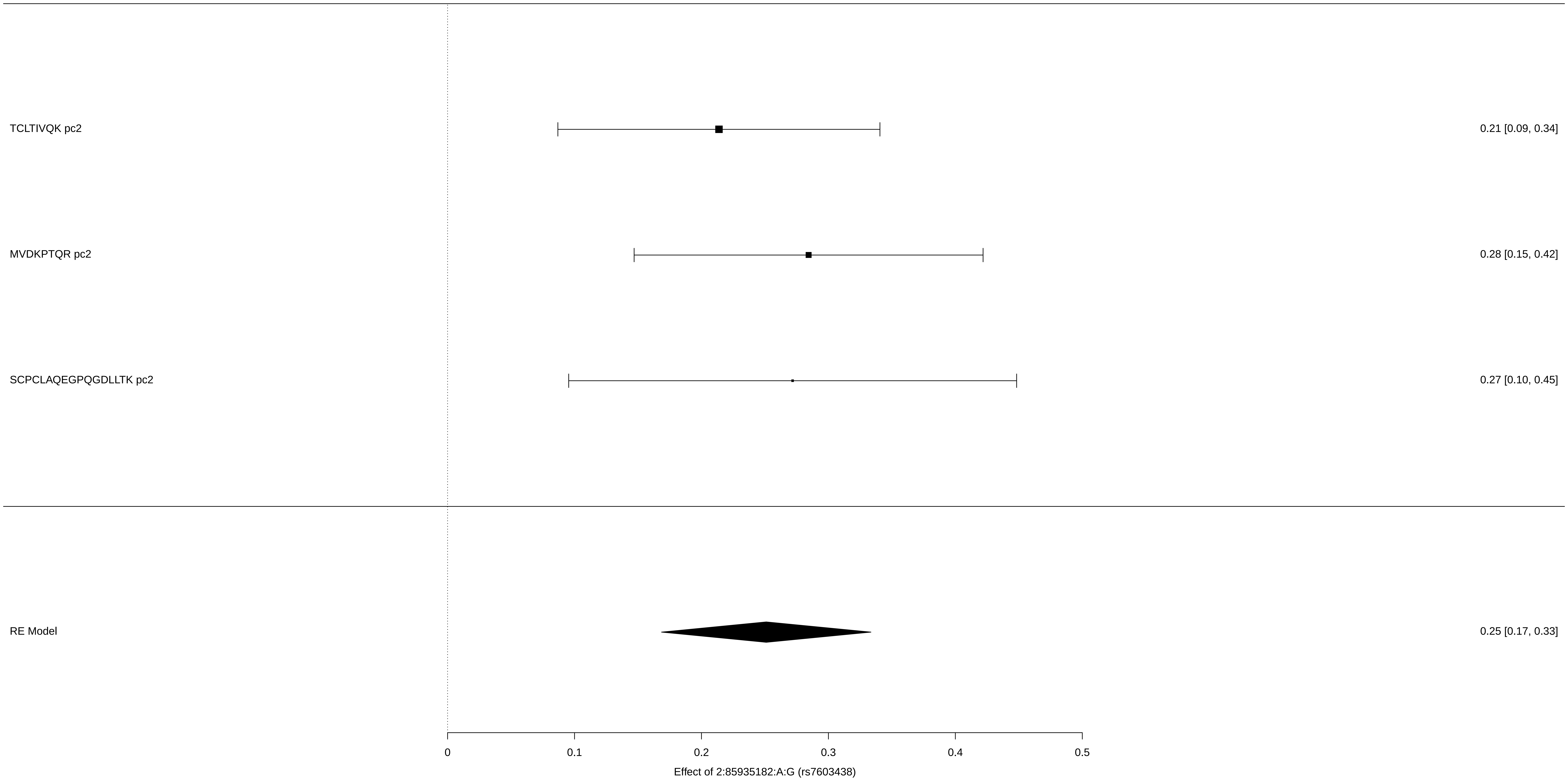

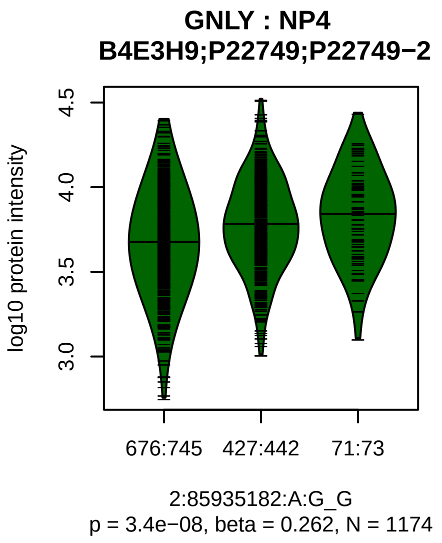

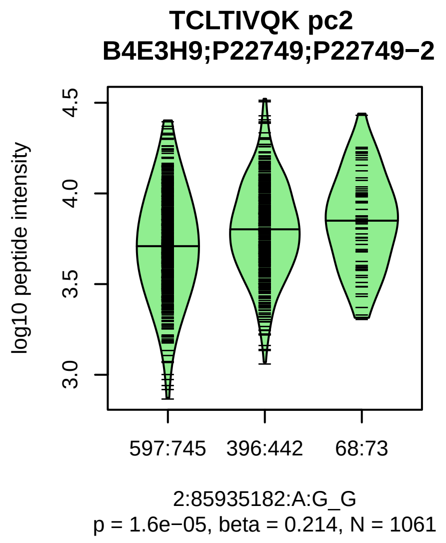

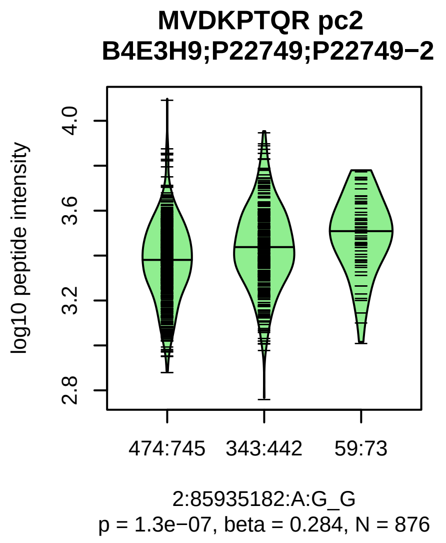

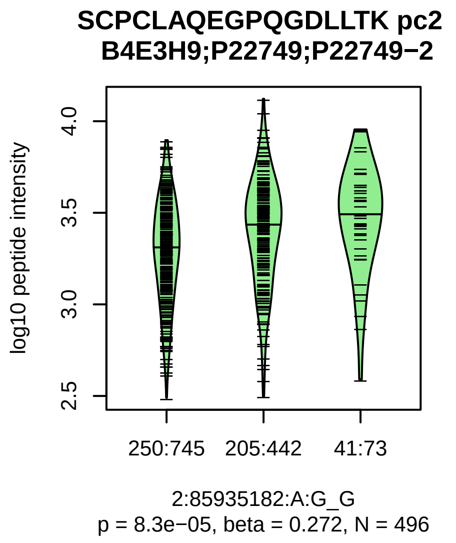

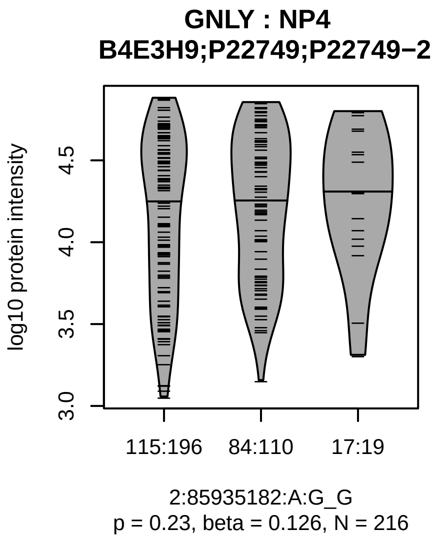

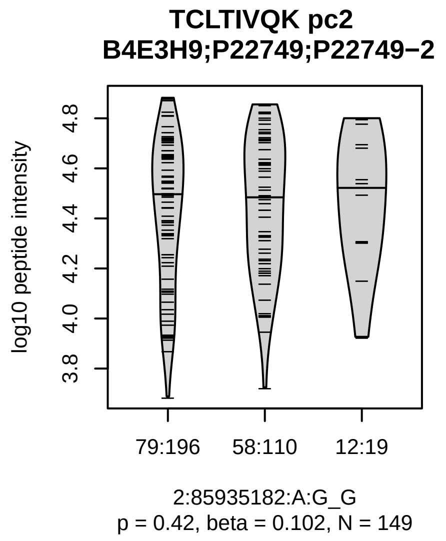

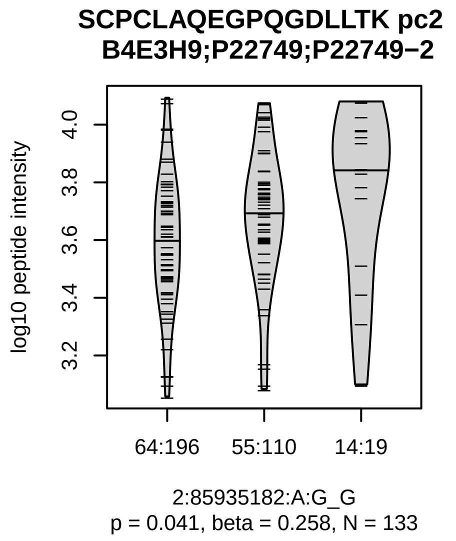

Assay Target: GNLY
UniProt: P22749
rsID: rs7603438
Target SNP: 2:85935182:A:G
Proxy SNP: 2:85935182:A:G
UKBPPP log10(p): 2087
UKBPPP BETA: 0.702
UKBPPP MSPA: 1
::*
1061:876:496

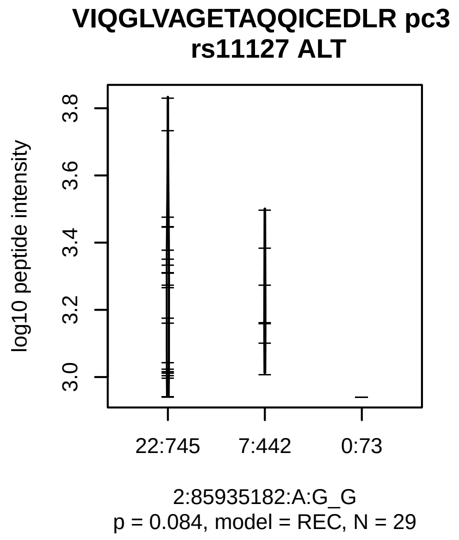

42. MSLN (Q13421)
UKBPPP MSPA=0.192

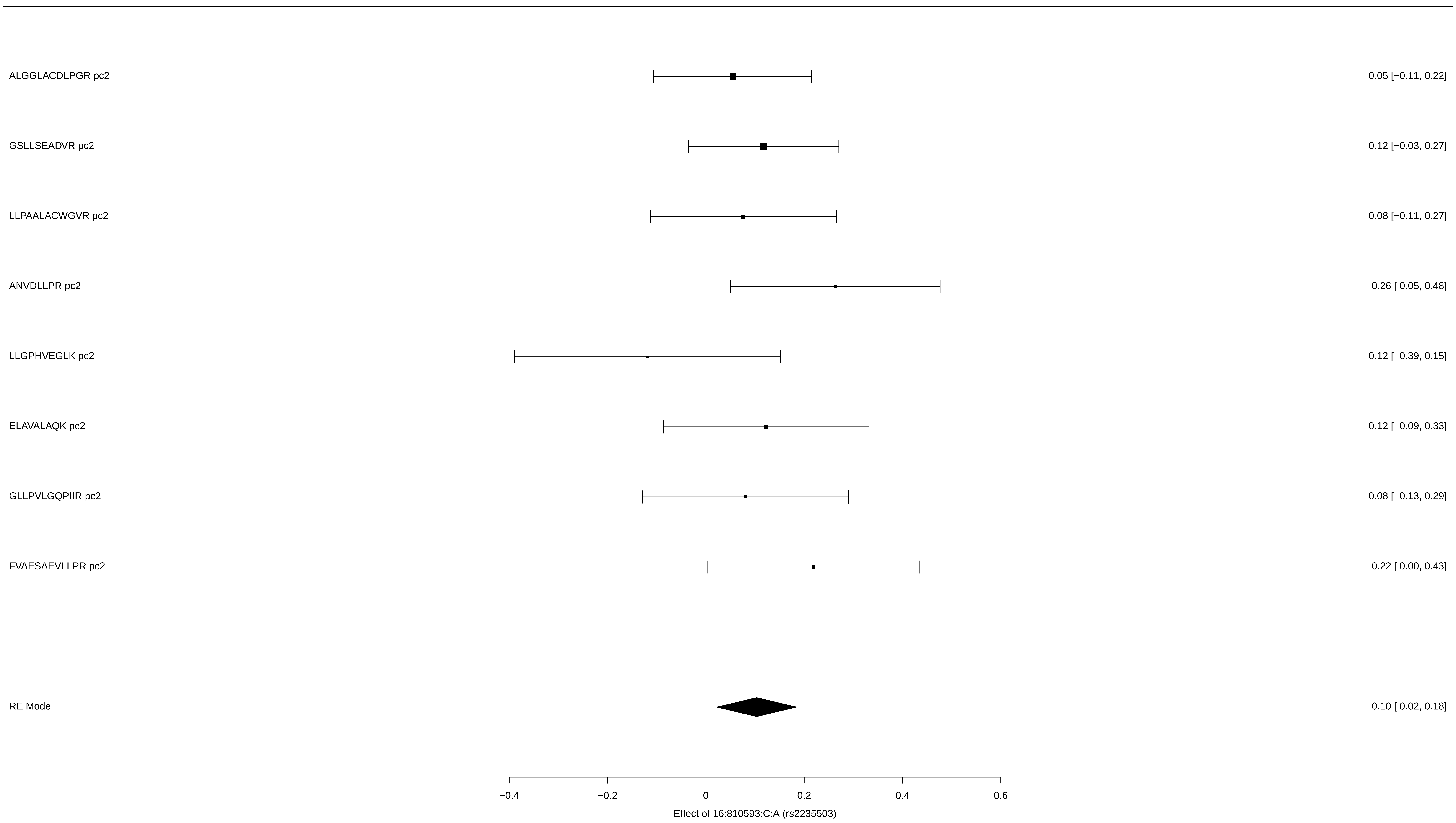

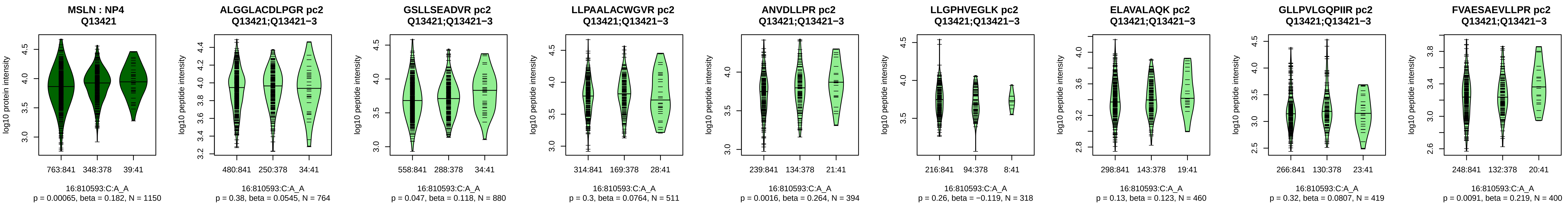

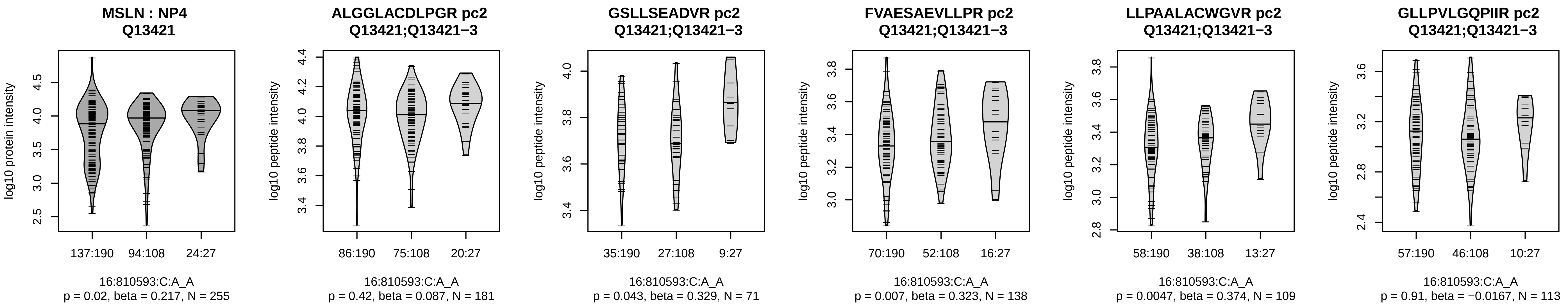

Assay Target: MSLN
UniProt: Q13421
rsID: rs2235503
Target SNP: 16:810593:C:A
Proxy SNP: 16:810593:C:A
UKBPPP log10(p): 2084.8
UKBPPP BETA: 0.737
UKBPPP MSPA: 0.192
−:−:−:*:−:−:−:*:NA
764:880:511:394:318:460:419:4

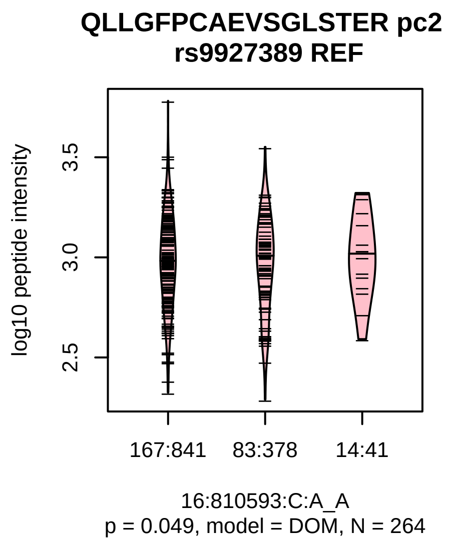

43. OGN (P20774)
UKBPPP MSPA=0.811

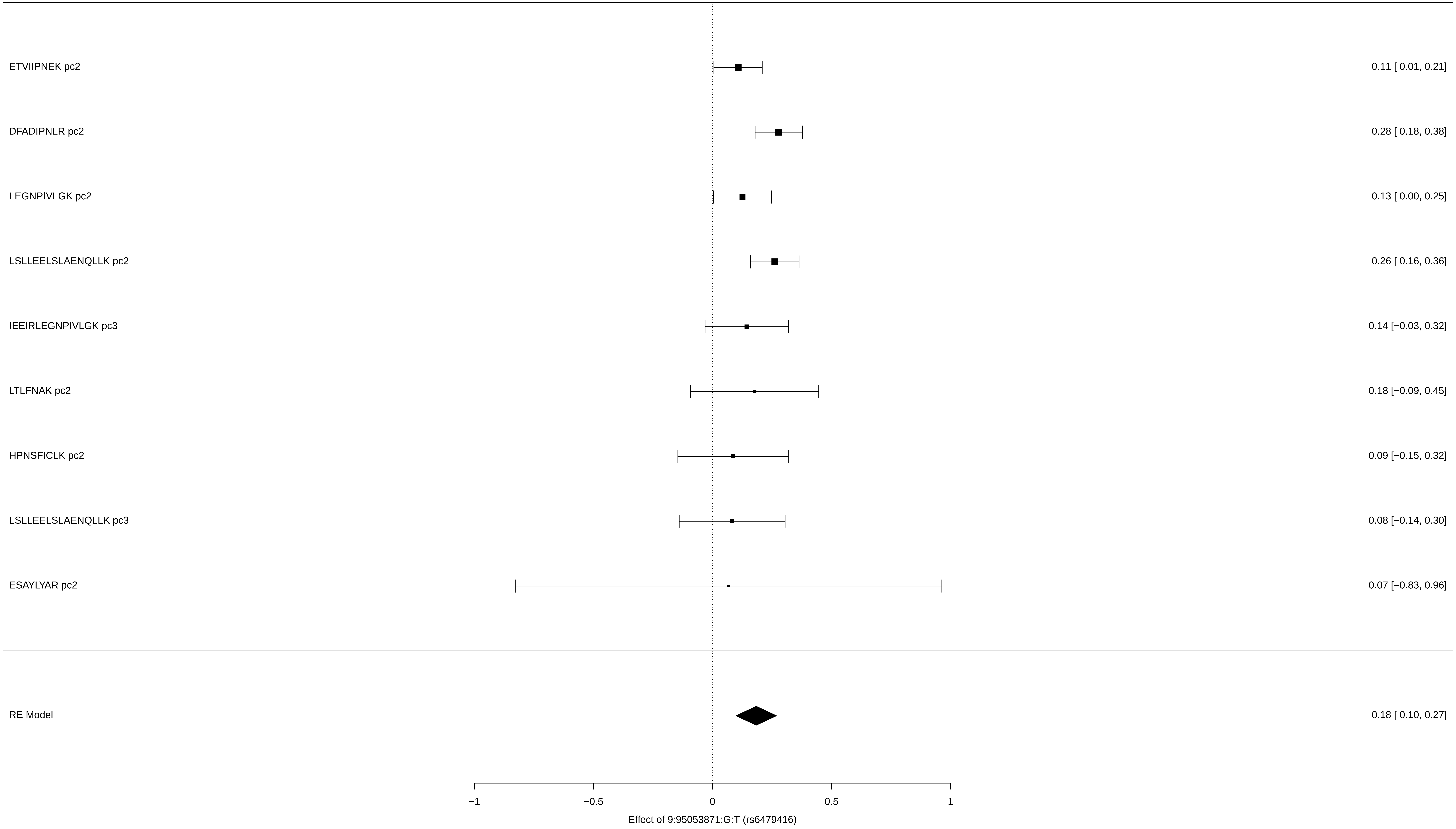

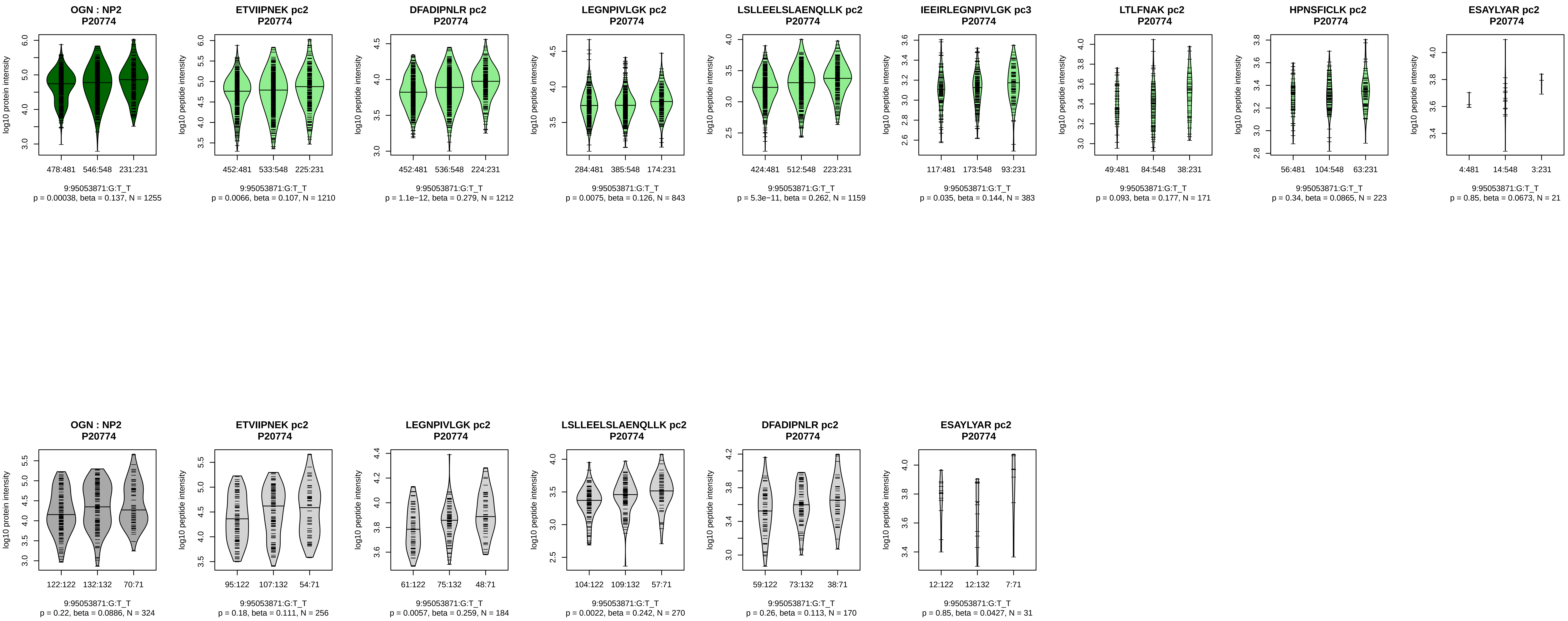

Assay Target: OGN
UniProt: P20774
rsID: rs6479416
Target SNP: 9:95053871:G:T
Proxy SNP: 9:95053871:G:T
UKBPPP log10(p): 2084.4
UKBPPP BETA: 0.579
UKBPPP MSPA: 0.811
::*:*:–:–:–:–:–
1210:1212:843:1159:383:171:22

44. QSOX1 (O00391)
UKBPPP MSPA=0.77 (3 conflicts)

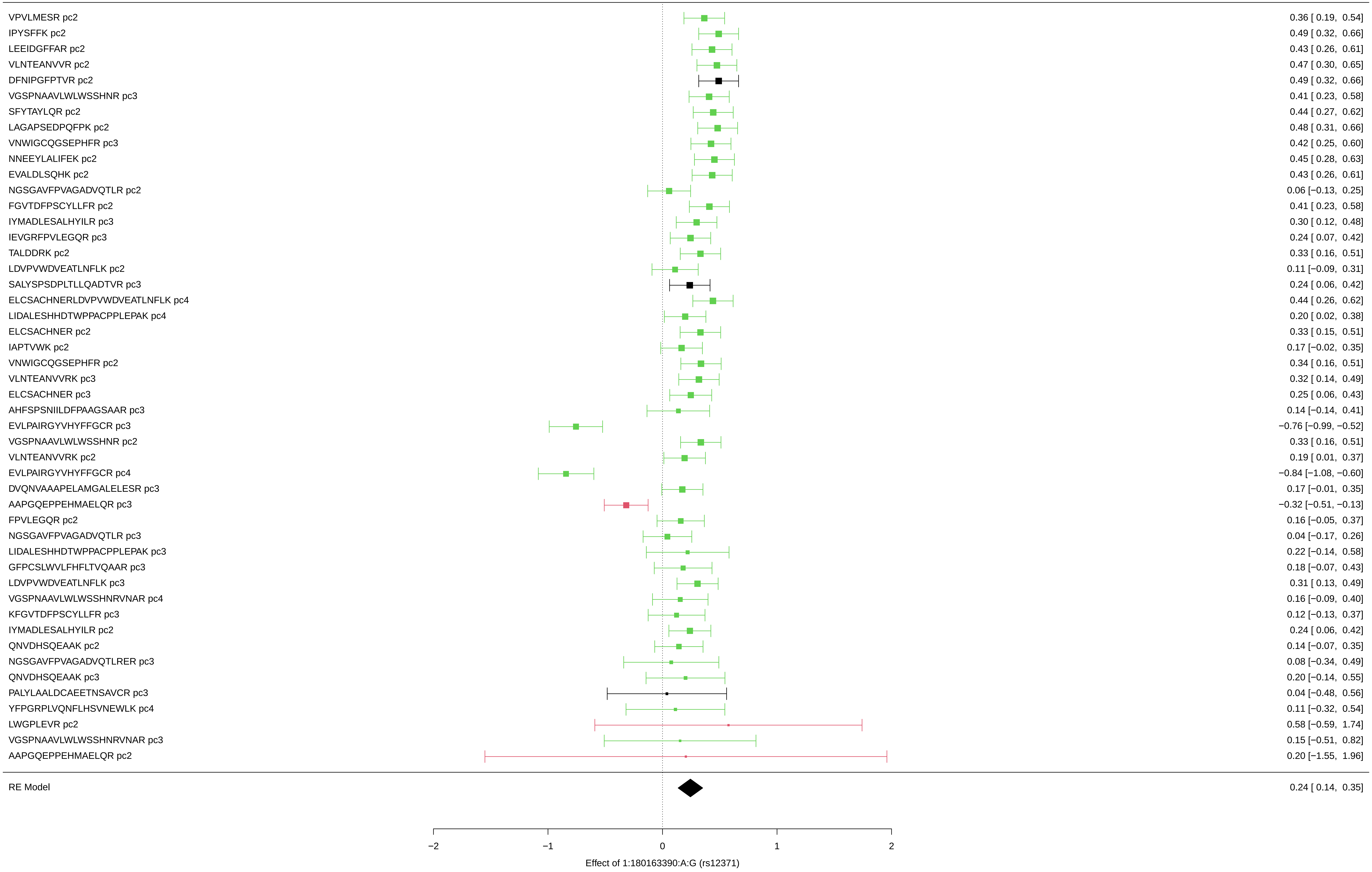

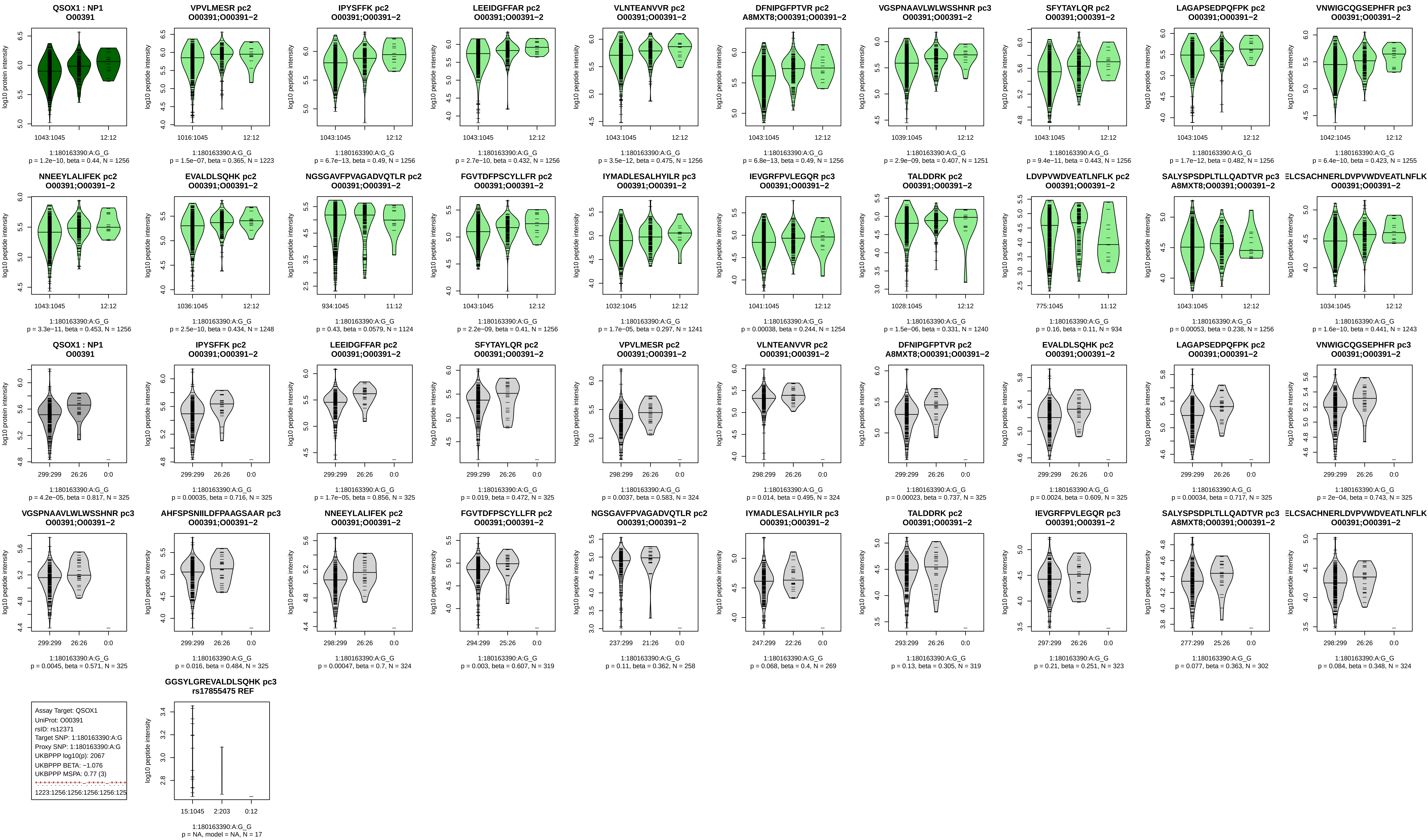
QSOX1 : NP1
O00391
1:180163390:A:G_G
p = 1.2e−10, beta = 0.44, N = 1256
VPVLMESR pc2
O00391;O00391−2
p = 1.5e−07, beta = 0.365, N = 1223
IPYSFFK pc2
O00391;O00391−2
p = 6.7e−13, beta = 0.49, N = 1256
LEEIDGFFAR pc2
O00391;O00391−2
p = 2.7e−10, beta = 0.432, N = 1256
VLNTEANVVR pc2
O00391;O00391−2
p = 3.5e−12, beta = 0.475, N = 1256
DFNIPGFPTVR pc2
A8MXT8;O00391;O00391−2
p = 6.8e−13, beta = 0.49, N = 1256
VGSPNAAVLWLWSSHNR pc3
O00391;O00391−2
p = 2.9e−09, beta = 0.407, N = 1251
SFYTAYLQR pc2
O00391;O00391−2
p = 9.4e−11, beta = 0.443, N = 1256
LAGAPSEDPQFPK pc2
O00391;O00391−2
p = 1.7e−12, beta = 0.482, N = 1256
VNWIGCQGSEPHFR pc3
O00391;O00391−2
p = 6.4e−10, beta = 0.423, N = 1255
NNEEYLALIFEK pc2
O00391;O00391−2
p = 3.3e−11, beta = 0.453, N = 1256
EVALDLSQHK pc2
O00391;O00391−2
p = 2.5e−10, beta = 0.434, N = 1248
NGSGAVFPVAGADVQTLR pc2
O00391;O00391−2
p = 0.43, beta = 0.0579, N = 1124
FGVTDFPSCYLLFR pc2
O00391;O00391−2
p = 2.2e−09, beta = 0.41, N = 1256
IYMADLESALHYILR pc3
O00391;O00391−2
p = 1.7e−05, beta = 0.297, N = 1241
IEVGRFPVLEGQR pc3
O00391;O00391−2
p = 0.00038, beta = 0.244, N = 1254
TALDDRK pc2
O00391;O00391−2
p = 1.5e−06, beta = 0.331, N = 1240
LDVPVWDVEATLNFLK pc2
O00391;O00391−2
p = 0.16, beta = 0.11, N = 934
SALYSPSDPLTLLQADTVR pc3
A8MXT8;O00391;O00391−2
p = 0.00053, beta = 0.238, N = 1256
ELCSACHNERLDVPVWDVEATLNFLK
O00391;O00391−2
p = 1.6e−10, beta = 0.441, N = 1243
QSOX1 : NP1
O00391
p = 4.2e−05, beta = 0.817, N = 325
IPYSFFK pc2
O00391;O00391−2
p = 0.00035, beta = 0.716, N = 325
LEEIDGFFAR pc2
O00391;O00391−2
p = 1.7e−05, beta = 0.856, N = 325
SFYTAYLQR pc2
O00391;O00391−2
p = 0.019, beta = 0.472, N = 325
VPVLMESR pc2
O00391;O00391−2
p = 0.0037, beta = 0.583, N = 324
VLNTEANVVR pc2
O00391;O00391−2
p = 0.014, beta = 0.495, N = 324
DFNIPGFPTVR pc2
A8MXT8;O00391;O00391−2
p = 0.00023, beta = 0.737, N = 325
EVALDLSQHK pc2
O00391;O00391−2
p = 0.0024, beta = 0.609, N = 325
LAGAPSEDPQFPK pc2
O00391;O00391−2
p = 0.00034, beta = 0.717, N = 325
VNWIGCQGSEPHFR pc3
O00391;O00391−2
p = 2e−04, beta = 0.743, N = 325
VGSPNAAVLWLWSSHNR pc3
O00391;O00391−2
p = 0.0045, beta = 0.571, N = 325
AHFSPSNIILDFPAAGSAAR pc3
O00391;O00391−2
p = 0.016, beta = 0.484, N = 325
NNEEYLALIFEK pc2
O00391;O00391−2
p = 0.00047, beta = 0.7, N = 324
FGVTDFPSCYLLFR pc2
O00391;O00391−2
p = 0.003, beta = 0.607, N = 319
NGSGAVFPVAGADVQTLR pc2
O00391;O00391−2
p = 0.11, beta = 0.362, N = 258
IYMADLESALHYILR pc3
O00391;O00391−2
p = 0.068, beta = 0.4, N = 269
TALDDRK pc2
O00391;O00391−2
p = 0.13, beta = 0.305, N = 319
IEVGRFPVLEGQR pc3
O00391;O00391−2
p = 0.21, beta = 0.251, N = 323
SALYSPSDPLTLLQADTVR pc3
A8MXT8;O00391;O00391−2
p = 0.077, beta = 0.363, N = 302
ELCSACHNERLDVPVWDVEATLNFLK
O00391;O00391−2
p = 0.084, beta = 0.348, N = 324
Assay Target: QSOX1
UniProt: O00391
rsID: rs12371
Target SNP: 1:180163390:A:G
Proxy SNP: 1:180163390:A:G
UKBPPP log10(p): 2067
UKBPPP BETA: −1.076
UKBPPP MSPA: 0.77 (3)
1223:1256:1256:1256:1256:125
GGSYLGREVALDLSQHK pc3
rs17855475 REF
1:180163390:A:G_G
p = NA, model = NA, N = 17

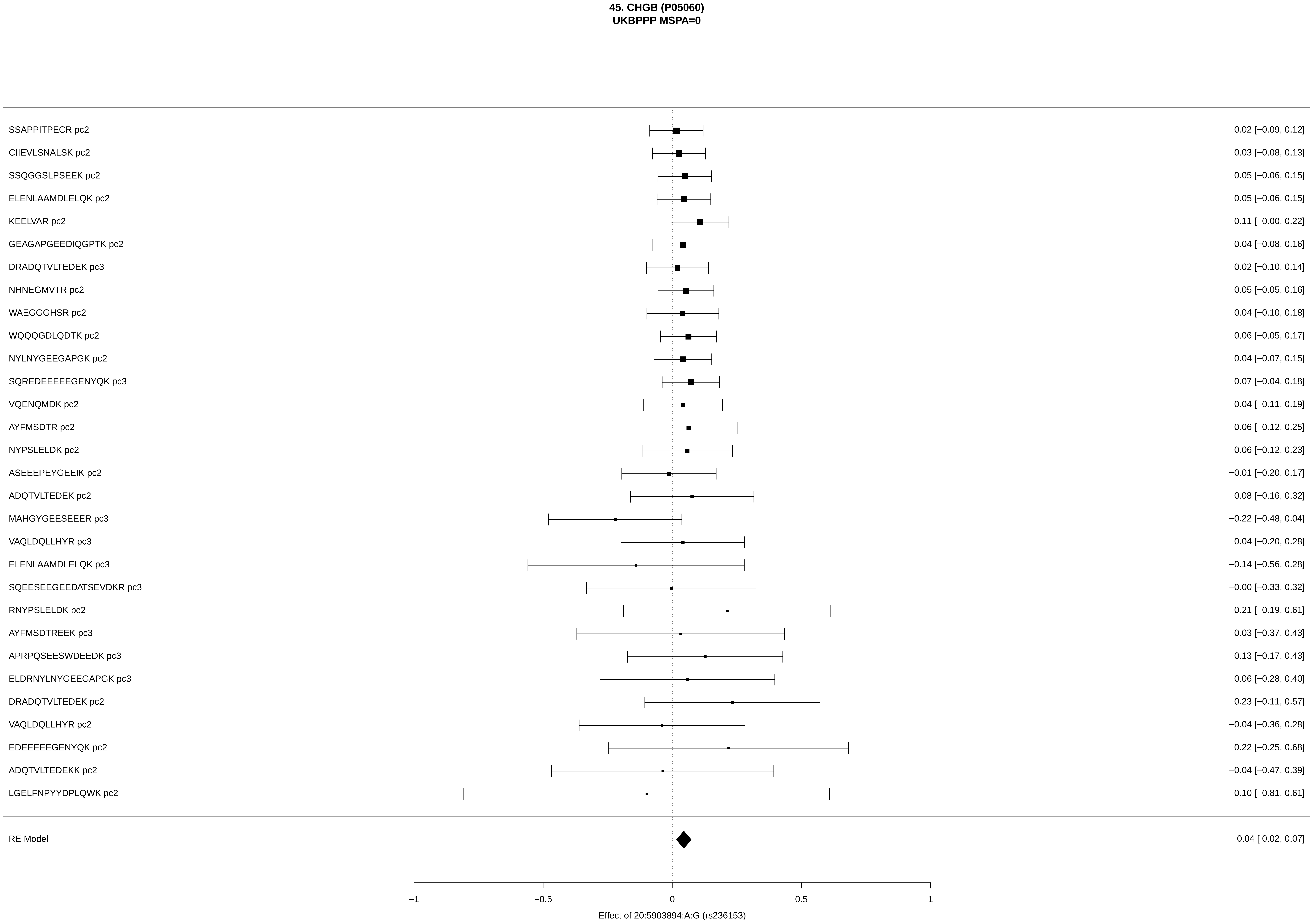
45. CHGB (P05060)
UKBPPP MSPA=0
SSAPPITPECR pc2
0.02 [−0.09, 0.12]
CIIEVLSNALSK pc2
0.03 [−0.08, 0.13]
SSQGGSLPSEEK pc2
0.05 [−0.06, 0.15]
ELENLAAMDLELQK pc2
0.05 [−0.06, 0.15]
KEELVAR pc2
0.11 [−0.00, 0.22]
GEAGAPGEEDIQGPTK pc2
0.04 [−0.08, 0.16]
DRADQTVLTEDEK pc3
0.02 [−0.10, 0.14]
NHNEGMVTR pc2
0.05 [−0.05, 0.16]
WAEGGGHSR pc2
0.04 [−0.10, 0.18]
WQQQGDLQDTK pc2
0.06 [−0.05, 0.17]
NYLNYGEEGAPGK pc2
0.04 [−0.07, 0.15]
SQREDEEEEEGENYQK pc3
0.07 [−0.04, 0.18]
VQENQMDK pc2
0.04 [−0.11, 0.19]
AYFMSDTR pc2
0.06 [−0.12, 0.25]
NYPSLELDK pc2
0.06 [−0.12, 0.23]
ASEEEPEYGEEIK pc2
−0.01 [−0.20, 0.17]
ADQTVLTEDEK pc2
0.08 [−0.16, 0.32]
MAHGYGEESEEER pc3
−0.22 [−0.48, 0.04]
VAQLDQLLHYR pc3
0.04 [−0.20, 0.28]
ELENLAAMDLELQK pc3
−0.14 [−0.56, 0.28]
SQEESEEGEEDATSEVDKR pc3
−0.00 [−0.33, 0.32]
RNYPSLELDK pc2
0.21 [−0.19, 0.61]
AYFMSDTREEK pc3
0.03 [−0.37, 0.43]
APRPQSEESWDEEDK pc3
0.13 [−0.17, 0.43]
ELDRNYLNYGEEGAPGK pc3
0.06 [−0.28, 0.40]
DRADQTVLTEDEK pc2
0.23 [−0.11, 0.57]
VAQLDQLLHYR pc2
−0.04 [−0.36, 0.28]
EDEEEEEGENYQK pc2
0.22 [−0.25, 0.68]
ADQTVLTEDEKK pc2
−0.04 [−0.47, 0.39]
LGELFNPYYDPLQWK pc2
−0.10 [−0.81, 0.61]
RE Model
0.04 [0.02, 0.07]
−1
−0.5
0
0.5
1
Effect of 20:5903894:A:G (rs236153)

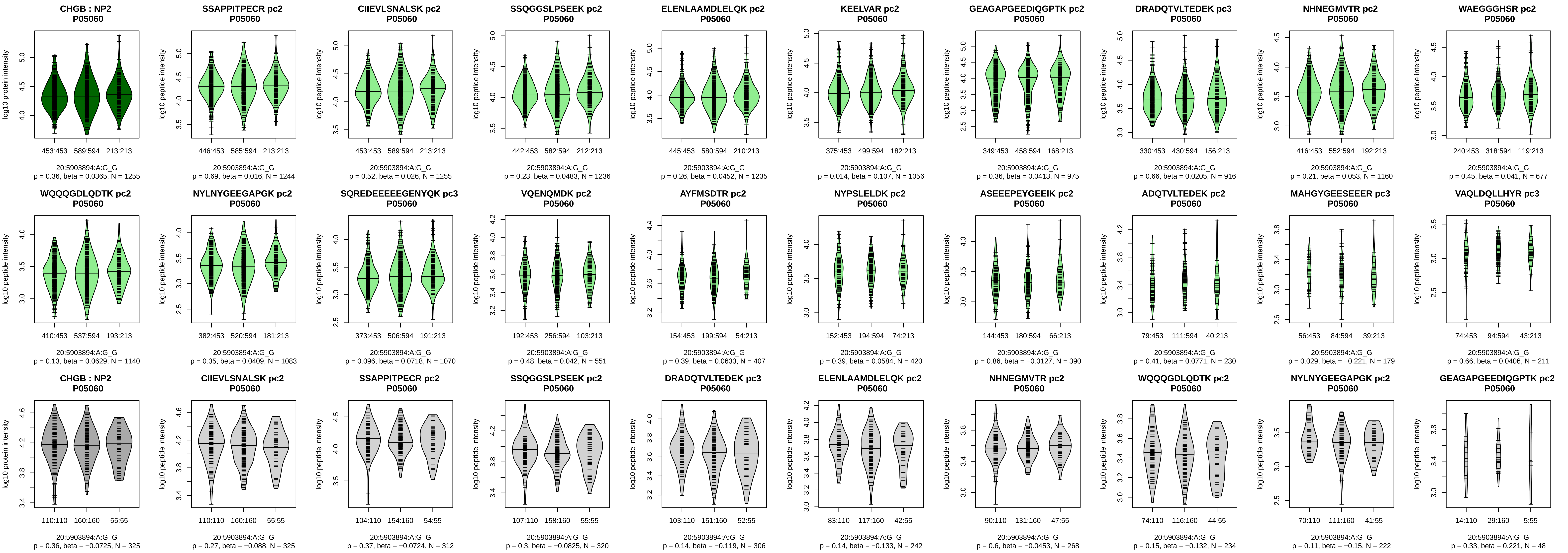
CHGB : NP2 P05060
20:5903894:A:G_G p = 0.36, beta = 0.0365, N = 1255
SSAPPITPECR pc2 P05060
20:5903894:A:G_G p = 0.69, beta = 0.016, N = 1244
CIIEVLSNALSK pc2 P05060
20:5903894:A:G_G p = 0.52, beta = 0.026, N = 1255
SSQGGSLPSEEK pc2 P05060
20:5903894:A:G_G p = 0.23, beta = 0.0483, N = 1236
ELENLAAMDLELQK pc2 P05060
20:5903894:A:G_G p = 0.26, beta = 0.0452, N = 1235
KEELVAR pc2 P05060
20:5903894:A:G_G p = 0.014, beta = 0.107, N = 1056
GEAGAPGEEDIQGPTK pc2 P05060
20:5903894:A:G_G p = 0.36, beta = 0.0413, N = 975
DRADQTVLTEDEK pc3 P05060
20:5903894:A:G_G p = 0.66, beta = 0.0205, N = 916
NHNEGMVTR pc2 P05060
20:5903894:A:G_G p = 0.21, beta = 0.053, N = 1160
WAEGGGHSR pc2 P05060
20:5903894:A:G_G p = 0.45, beta = 0.041, N = 677
WQQQGDLQDTK pc2 P05060
20:5903894:A:G_G p = 0.13, beta = 0.0629, N = 1140
NYLNYGEEGAPGK pc2 P05060
20:5903894:A:G_G p = 0.35, beta = 0.0409, N = 1083
SQREDEEEEEGENYQK pc3 P05060
20:5903894:A:G_G p = 0.096, beta = 0.0718, N = 1070
VQENQMDK pc2 P05060
20:5903894:A:G_G p = 0.48, beta = 0.042, N = 551
AYFMSDTR pc2 P05060
20:5903894:A:G_G p = 0.39, beta = 0.0633, N = 407
NYPSLELDK pc2 P05060
20:5903894:A:G_G p = 0.39, beta = 0.0584, N = 420
ASEEEPEYGEEIK pc2 P05060
20:5903894:A:G_G p = 0.86, beta = −0.0127, N = 390
ADQTVLTEDEK pc2 P05060
20:5903894:A:G_G p = 0.41, beta = 0.0771, N = 230
MAHGYGEESEEER pc3 P05060
20:5903894:A:G_G p = 0.029, beta = −0.221, N = 179
VAQLDQLLHYR pc3 P05060
20:5903894:A:G_G p = 0.66, beta = 0.0406, N = 211
CHGB : NP2 P05060
20:5903894:A:G_G p = 0.36, beta = −0.0725, N = 325
CIIEVLSNALSK pc2 P05060
20:5903894:A:G_G p = 0.27, beta = −0.088, N = 325
SSAPPITPECR pc2 P05060
20:5903894:A:G_G p = 0.37, beta = −0.0724, N = 312
SSQGGSLPSEEK pc2 P05060
20:5903894:A:G_G p = 0.3, beta = −0.0825, N = 320
DRADQTVLTEDEK pc3 P05060
20:5903894:A:G_G p = 0.14, beta = −0.119, N = 306
ELENLAAMDLELQK pc2 P05060
20:5903894:A:G_G p = 0.14, beta = −0.133, N = 242
NHNEGMVTR pc2 P05060
20:5903894:A:G_G p = 0.6, beta = −0.0453, N = 268
WQQQGDLQDTK pc2 P05060
20:5903894:A:G_G p = 0.15, beta = −0.132, N = 234
NYLNYGEEGAPGK pc2 P05060
20:5903894:A:G_G p = 0.11, beta = −0.15, N = 222
GEAGAPGEEDIQGPTK pc2 P05060
20:5903894:A:G_G p = 0.33, beta = 0.221, N = 48

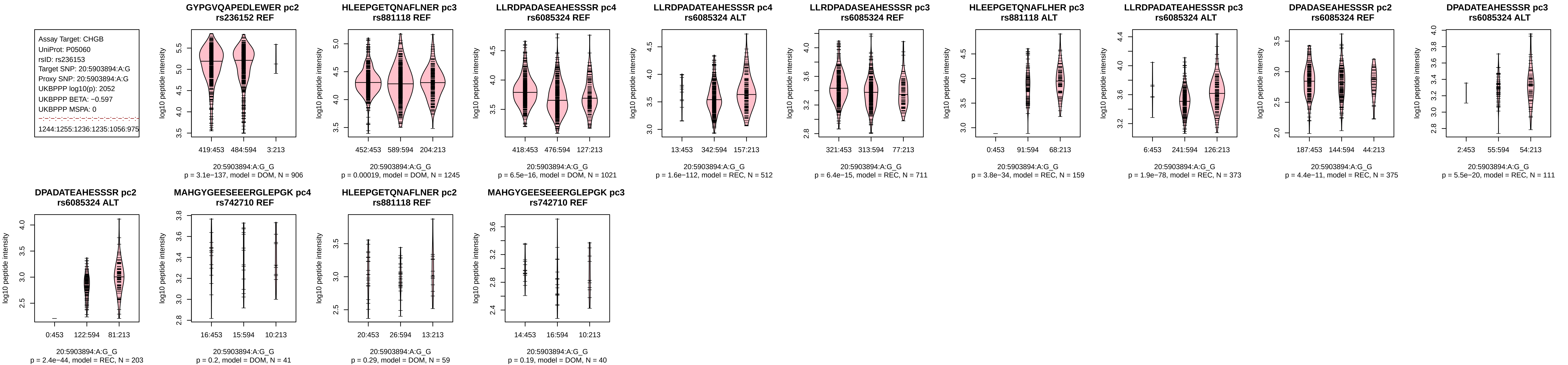
Assay Target: CHGB
UniProt: P05060
rsID: rs236153
Target SNP: 20:5903894:A:G
Proxy SNP: 20:5903894:A:G
UKBPPP log10(p): 2052
UKBPPP BETA: −0.597
UKBPPP MSPA: 0
1244:1255:1236:1235:1056:975
GYPGVQAPEDLEWER pc2 rs236152 REF
20:5903894:A:G_G p = 3.1e−137, model = DOM, N = 906
HLEEPGETQNAFLNER pc3 rs881118 REF
20:5903894:A:G_G p = 0.00019, model = DOM, N = 1245
LLRDPADASEAHESSSR pc4 rs6085324 REF
20:5903894:A:G_G p = 6.5e−16, model = DOM, N = 1021
LLRDPADATEAHESSSR pc4 rs6085324 ALT
20:5903894:A:G_G p = 1.6e−112, model = REC, N = 512
LLRDPADASEAHESSSR pc3 rs6085324 REF
20:5903894:A:G_G p = 6.4e−15, model = REC, N = 711
HLEEPGETQNAFLHER pc3 rs881118 ALT
20:5903894:A:G_G p = 3.8e−34, model = REC, N = 159
LLRDPADATEAHESSSR pc3 rs6085324 ALT
20:5903894:A:G_G p = 1.9e−78, model = REC, N = 373
DPADASEAHESSSR pc2 rs6085324 REF
20:5903894:A:G_G p = 4.4e−11, model = REC, N = 375
DPADATEAHESSSR pc3 rs6085324 ALT
20:5903894:A:G_G p = 5.5e−20, model = REC, N = 111
DPADATEAHESSSR pc2 rs6085324 ALT
20:5903894:A:G_G p = 2.4e−44, model = REC, N = 203
MAHGYGEESEEERGLEPGK pc4 rs742710 REF
20:5903894:A:G_G p = 0.2, model = DOM, N = 41
HLEEPGETQNAFLNER pc2 rs881118 REF
20:5903894:A:G_G p = 0.29, model = DOM, N = 59
MAHGYGEESEEERGLEPGK pc3 rs742710 REF
20:5903894:A:G_G p = 0.19, model = DOM, N = 40

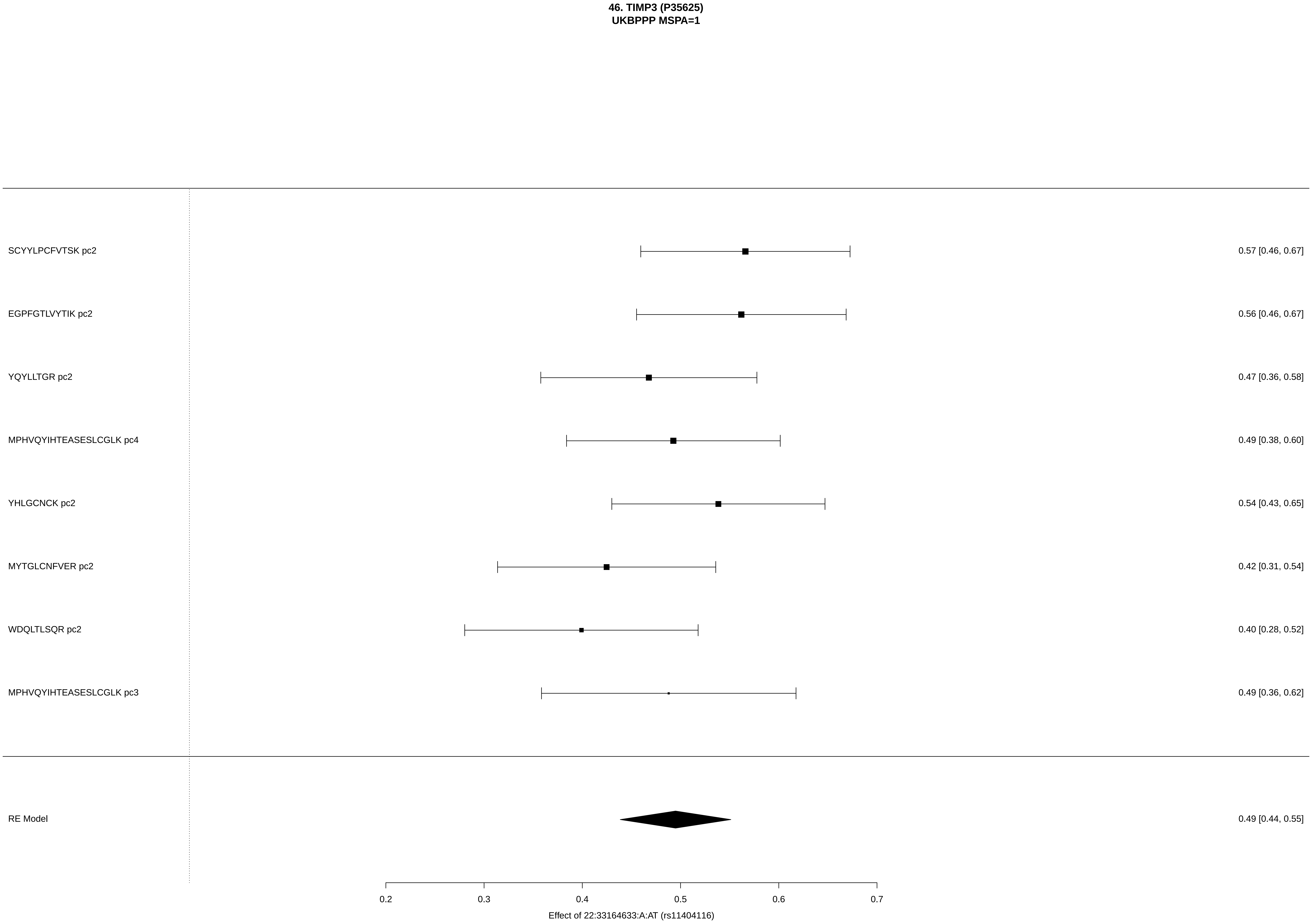
46. TIMP3 (P35625)
UKBPPP MSPA=1
SCYYLPCFVTSK pc2
0.57 [0.46, 0.67]
EGPFGTLVYTIK pc2
0.56 [0.46, 0.67]
YQYLLTGR pc2
0.47 [0.36, 0.58]
MPHVQYIHTEASESLCGLK pc4
0.49 [0.38, 0.60]
YHLGCNCK pc2
0.54 [0.43, 0.65]
MYTGLCNFVER pc2
0.42 [0.31, 0.54]
WDQLTLSQR pc2
0.40 [0.28, 0.52]
MPHVQYIHTEASESLCGLK pc3
0.49 [0.36, 0.62]
RE Model
0.49 [0.44, 0.55]
0.2
0.3
0.4
0.5
0.6
0.7
Effect of 22:33164633:A:AT (rs11404116)

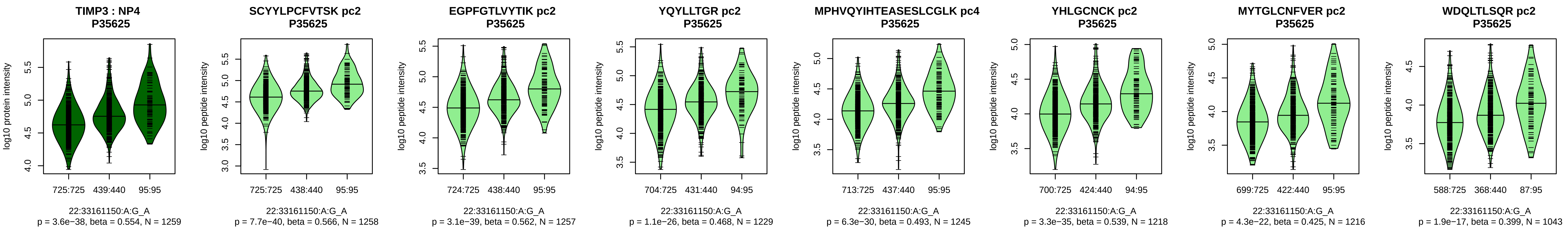

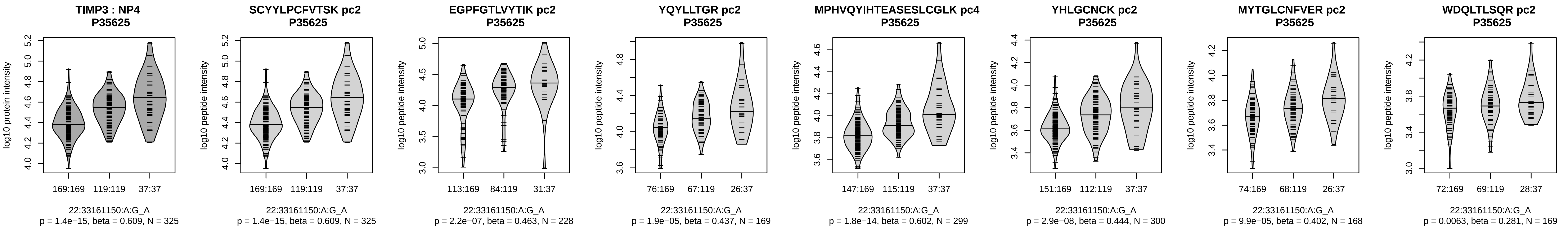

Assay Target: TIMP3
UniProt: P35625
rsID: rs11404116
Target SNP: 22:33164633:A:AT
Proxy SNP: 22:33161150:A:G
UKBPPP log10(p): 1997.5
UKBPPP BETA: −0.652
UKBPPP MSPA: 1
1258:1257:1229:1245:1218:121

47. CPB2 (Q96IY4)
UKBPPP MSPA=0.981

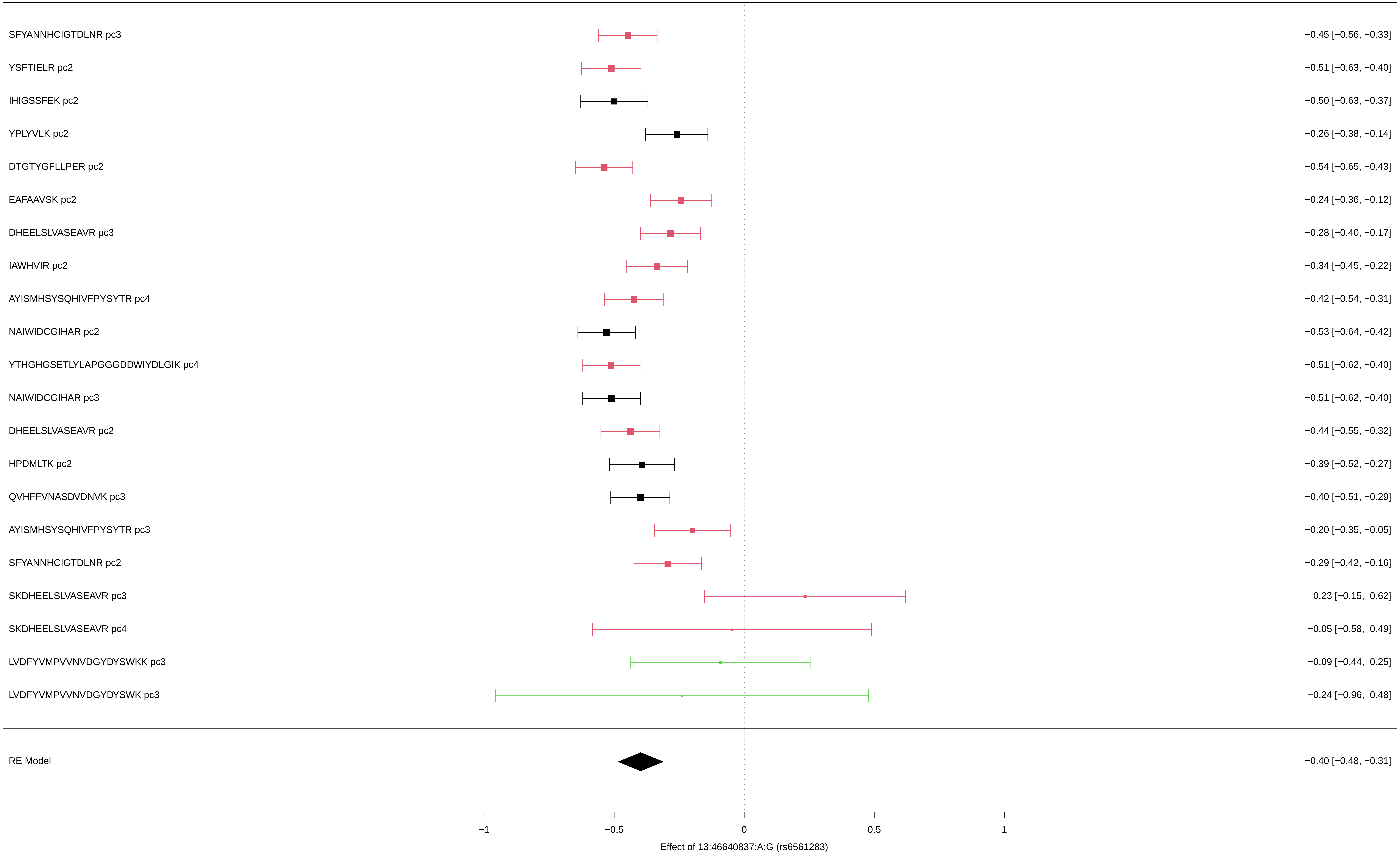

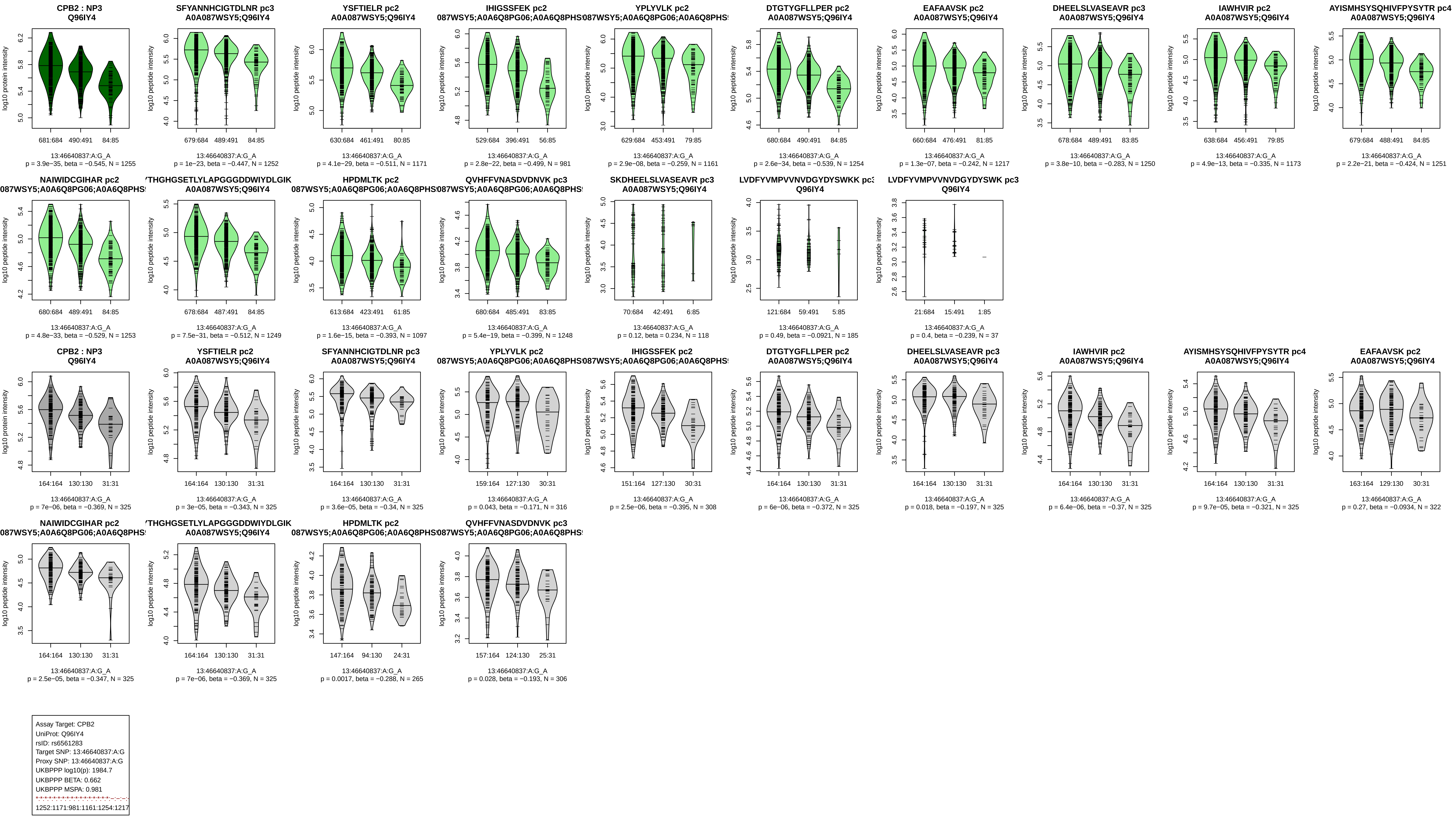
CPB2 : NP3
Q96IY4
log10 protein intensity
681:684 490:491 84:85
13:46640837:A:G_A
p = 3.9e−35, beta = −0.545, N = 1255
SFYANNHCIGTDLNR pc3
A0A087WSY5;Q96IY4
log10 peptide intensity
679:684 489:491 84:85
13:46640837:A:G_A
p = 1e−23, beta = −0.447, N = 1252
YSFTIELR pc2
A0A087WSY5;Q96IY4
630:684 461:491 80:85
13:46640837:A:G_A
p = 4.1e−29, beta = −0.511, N = 1171
IHIGSSFEK pc2
087WSY5;A0A6Q8PG06;A0A6Q8PHS
529:684 396:491 56:85
13:46640837:A:G_A
p = 2.8e−22, beta = −0.499, N = 981
YPLYVLK pc2
087WSY5;A0A6Q8PG06;A0A6Q8PHS
629:684 453:491 79:85
13:46640837:A:G_A
p = 2.9e−08, beta = −0.259, N = 1161
DTGTYGFLLPER pc2
A0A087WSY5;Q96IY4
680:684 490:491 84:85
13:46640837:A:G_A
p = 2.6e−34, beta = −0.539, N = 1254
EAFAAVSK pc2
A0A087WSY5;Q96IY4
660:684 476:491 81:85
13:46640837:A:G_A
p = 1.3e−07, beta = −0.242, N = 1217
DHEELSLVASEAVR pc3
A0A087WSY5;Q96IY4
678:684 489:491 83:85
13:46640837:A:G_A
p = 3.8e−10, beta = −0.283, N = 1250
IAWHVIR pc2
A0A087WSY5;Q96IY4
638:684 456:491 79:85
13:46640837:A:G_A
p = 4.9e−13, beta = −0.335, N = 1173
AYISMHSYSQHIVFPYSYTR pc4
A0A087WSY5;Q96IY4
679:684 488:491 84:85
13:46640837:A:G_A
p = 2.2e−21, beta = −0.424, N = 1251
NAIWIDCGIHAR pc2
087WSY5;A0A6Q8PG06;A0A6Q8PHS
680:684 489:491 84:85
13:46640837:A:G_A
p = 4.8e−33, beta = −0.529, N = 1253
THGHGSETLYLAPGGGDDWIYDLGIK
A0A087WSY5;Q96IY4
678:684 487:491 84:85
13:46640837:A:G_A
p = 7.5e−31, beta = −0.512, N = 1249
HPDMLTK pc2
087WSY5;A0A6Q8PG06;A0A6Q8PHS
613:684 423:491 61:85
13:46640837:A:G_A
p = 1.6e−15, beta = −0.393, N = 1097
QVHFFVNASDVDNVK pc3
087WSY5;A0A6Q8PG06;A0A6Q8PHS
680:684 485:491 83:85
13:46640837:A:G_A
p = 5.4e−19, beta = −0.399, N = 1248
SKDHEELSLVASEAVR pc3
A0A087WSY5;Q96IY4
70:684 42:491 6:85
13:46640837:A:G_A
p = 0.12, beta = 0.234, N = 118
LVDFYVMPVVNVDGYDYSWKK pc3
Q96IY4
121:684 59:491 5:85
13:46640837:A:G_A
p = 0.49, beta = −0.0921, N = 185
LVDFYVMPVVNVDGYDYSWK pc3
Q96IY4
21:684 15:491 1:85
13:46640837:A:G_A
p = 0.4, beta = −0.239, N = 37
CPB2 : NP3
Q96IY4
164:164 130:130 31:31
13:46640837:A:G_A
p = 7e−06, beta = −0.369, N = 325
YSFTIELR pc2
A0A087WSY5;Q96IY4
164:164 130:130 31:31
13:46640837:A:G_A
p = 3e−05, beta = −0.343, N = 325
SFYANNHCIGTDLNR pc3
A0A087WSY5;Q96IY4
164:164 130:130 31:31
13:46640837:A:G_A
p = 3.6e−05, beta = −0.34, N = 325
YPLYVLK pc2
087WSY5;A0A6Q8PG06;A0A6Q8PHS
159:164 127:130 30:31
13:46640837:A:G_A
p = 0.043, beta = −0.171, N = 316
IHIGSSFEK pc2
087WSY5;A0A6Q8PG06;A0A6Q8PHS
151:164 127:130 30:31
13:46640837:A:G_A
p = 2.5e−06, beta = −0.395, N = 308
DTGTYGFLLPER pc2
A0A087WSY5;Q96IY4
164:164 130:130 31:31
13:46640837:A:G_A
p = 6e−06, beta = −0.372, N = 325
DHEELSLVASEAVR pc3
A0A087WSY5;Q96IY4
164:164 130:130 31:31
13:46640837:A:G_A
p = 0.018, beta = −0.197, N = 325
IAWHVIR pc2
A0A087WSY5;Q96IY4
164:164 130:130 31:31
13:46640837:A:G_A
p = 6.4e−06, beta = −0.37, N = 325
AYISMHSYSQHIVFPYSYTR pc4
A0A087WSY5;Q96IY4
164:164 130:130 31:31
13:46640837:A:G_A
p = 9.7e−05, beta = −0.321, N = 325
EAFAAVSK pc2
A0A087WSY5;Q96IY4
163:164 129:130 30:31
13:46640837:A:G_A
p = 0.27, beta = −0.0934, N = 322
NAIWIDCGIHAR pc2
087WSY5;A0A6Q8PG06;A0A6Q8PHS
164:164 130:130 31:31
13:46640837:A:G_A
p = 2.5e−05, beta = −0.347, N = 325
THGHGSETLYLAPGGGDDWIYDLGIK
A0A087WSY5;Q96IY4
164:164 130:130 31:31
13:46640837:A:G_A
p = 7e−06, beta = −0.369, N = 325
HPDMLTK pc2
087WSY5;A0A6Q8PG06;A0A6Q8PHS
147:164 94:130 24:31
13:46640837:A:G_A
p = 0.0017, beta = −0.288, N = 265
QVHFFVNASDVDNVK pc3
087WSY5;A0A6Q8PG06;A0A6Q8PHS
157:164 124:130 25:31
13:46640837:A:G_A
p = 0.028, beta = −0.193, N = 306
Assay Target: CPB2
UniProt: Q96IY4
rsID: rs6561283
Target SNP: 13:46640837:A:G
Proxy SNP: 13:46640837:A:G
UKBPPP log10(p): 1984.7
UKBPPP BETA: 0.662
UKBPPP MSPA: 0.981
1252:1171:981:1161:1254:1217

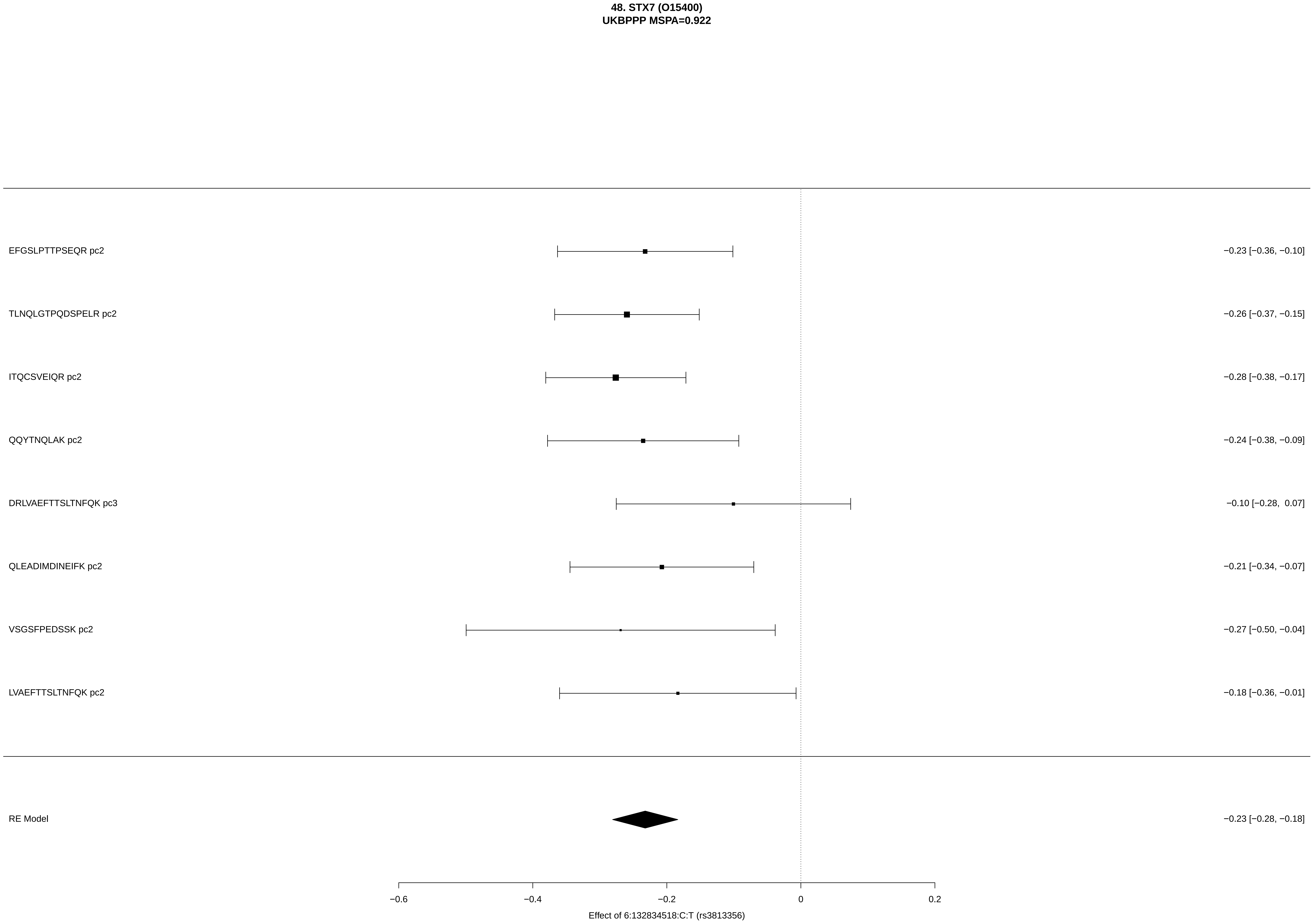
48. STX7 (O15400)
UKBPPP MSPA=0.922
EFGSLPTTPSEQR pc2
−0.23 [−0.36, −0.10]
TLNQLGTPQDSPELR pc2
−0.26 [−0.37, −0.15]
ITQCSVEIQR pc2
−0.28 [−0.38, −0.17]
QQYTNQLAK pc2
−0.24 [−0.38, −0.09]
DRLVAEFTTSLTNFQK pc3
−0.10 [−0.28, 0.07]
QLEADIMDINEIFK pc2
−0.21 [−0.34, −0.07]
VSGSFPEDSSK pc2
−0.27 [−0.50, −0.04]
LVAEFTTSLTNFQK pc2
−0.18 [−0.36, −0.01]
RE Model
−0.23 [−0.28, −0.18]
−0.6
−0.4
−0.2
0
0.2
Effect of 6:132834518:C:T (rs3813356)

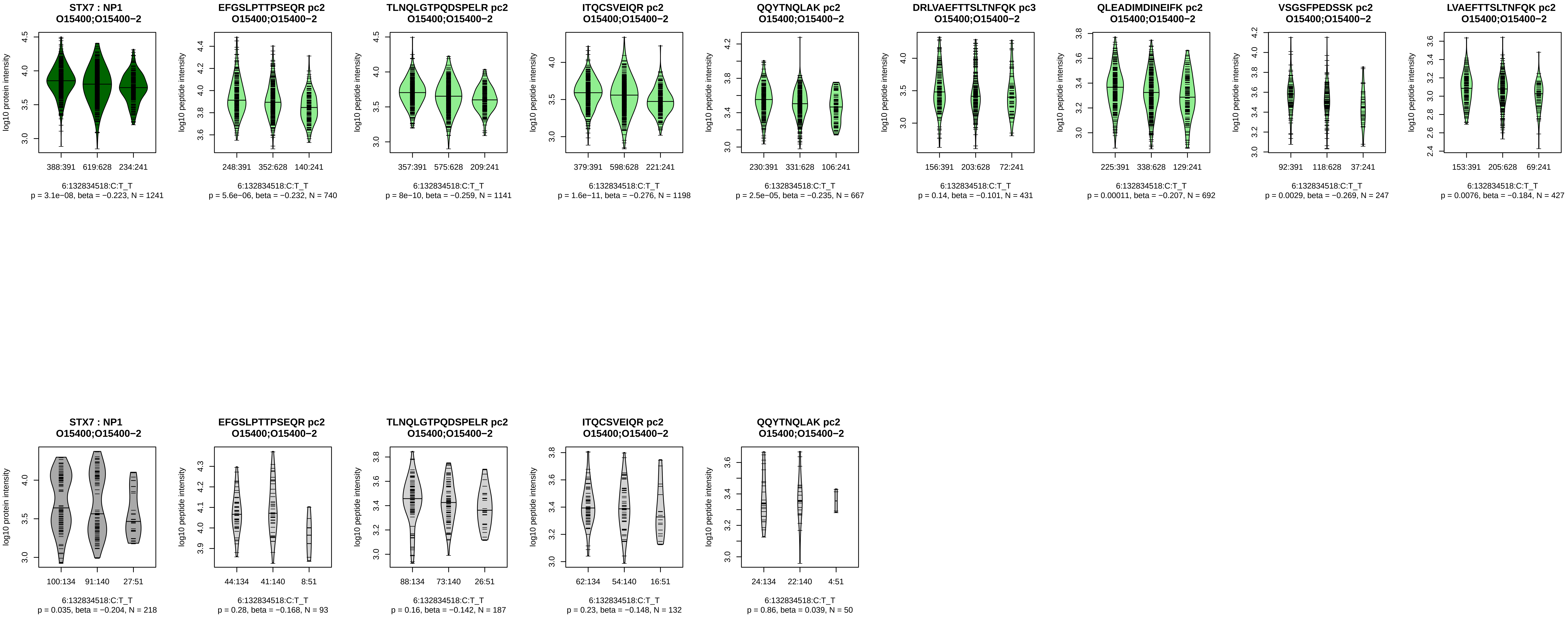

Assay Target: STX7
UniProt: O15400
rsID: rs3813356
Target SNP: 6:132834518:C:T
Proxy SNP: 6:132834518:C:T
UKBPPP log10(p): 1960.4
UKBPPP BETA: −0.582
UKBPPP MSPA: 0.922
::*:*:_:*:*
740:1141:1198:667:431:692:247

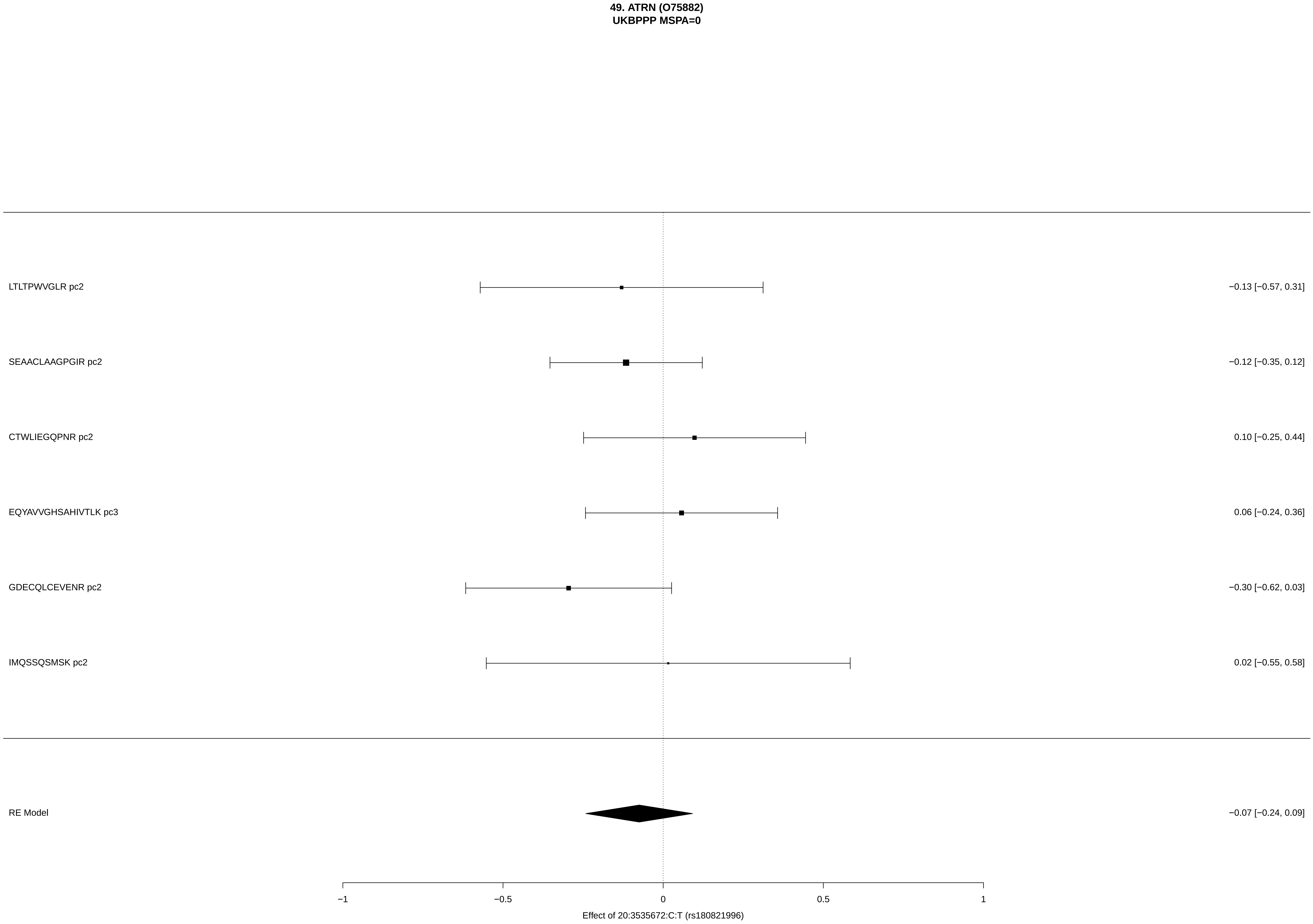

49. ATRN (O75882)
UKBPPP MSPA=0
LTLTPWVGLR pc2
SEAACLAAGPGIR pc2
CTWLIEGQPNR pc2
EQYAVVGHSAHIVTLK pc3
GDECQLCEVENR pc2
IMQSSQSMSK pc2
RE Model
−0.13 [−0.57, 0.31]
−0.12 [−0.35, 0.12]
0.10 [−0.25, 0.44]
0.06 [−0.24, 0.36]
−0.30 [−0.62, 0.03]
0.02 [−0.55, 0.58]
−0.07 [−0.24, 0.09]
−1
−0.5
0
0.5
1
Effect of 20:3535672:C:T (rs180821996)

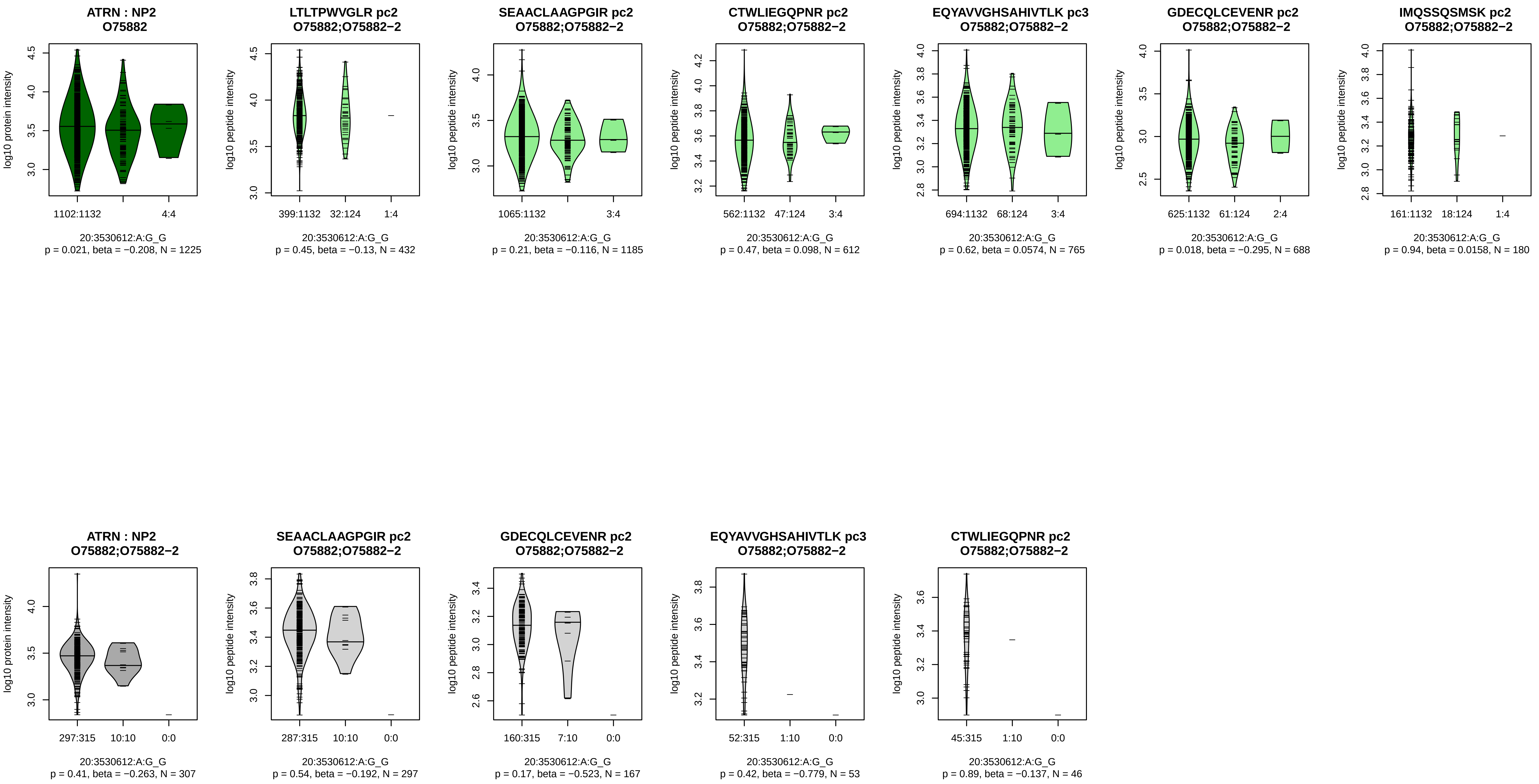

Assay Target: ATRN
UniProt: O75882
rsID: rs180821996
Target SNP: 20:3535672:C:T
Proxy SNP: 20:3530612:A:G
UKBPPP log10(p): 1939.9
UKBPPP BETA: −1.295
UKBPPP MSPA: 0
–:–:–:–:–:–
432:1185:612:765:688:180

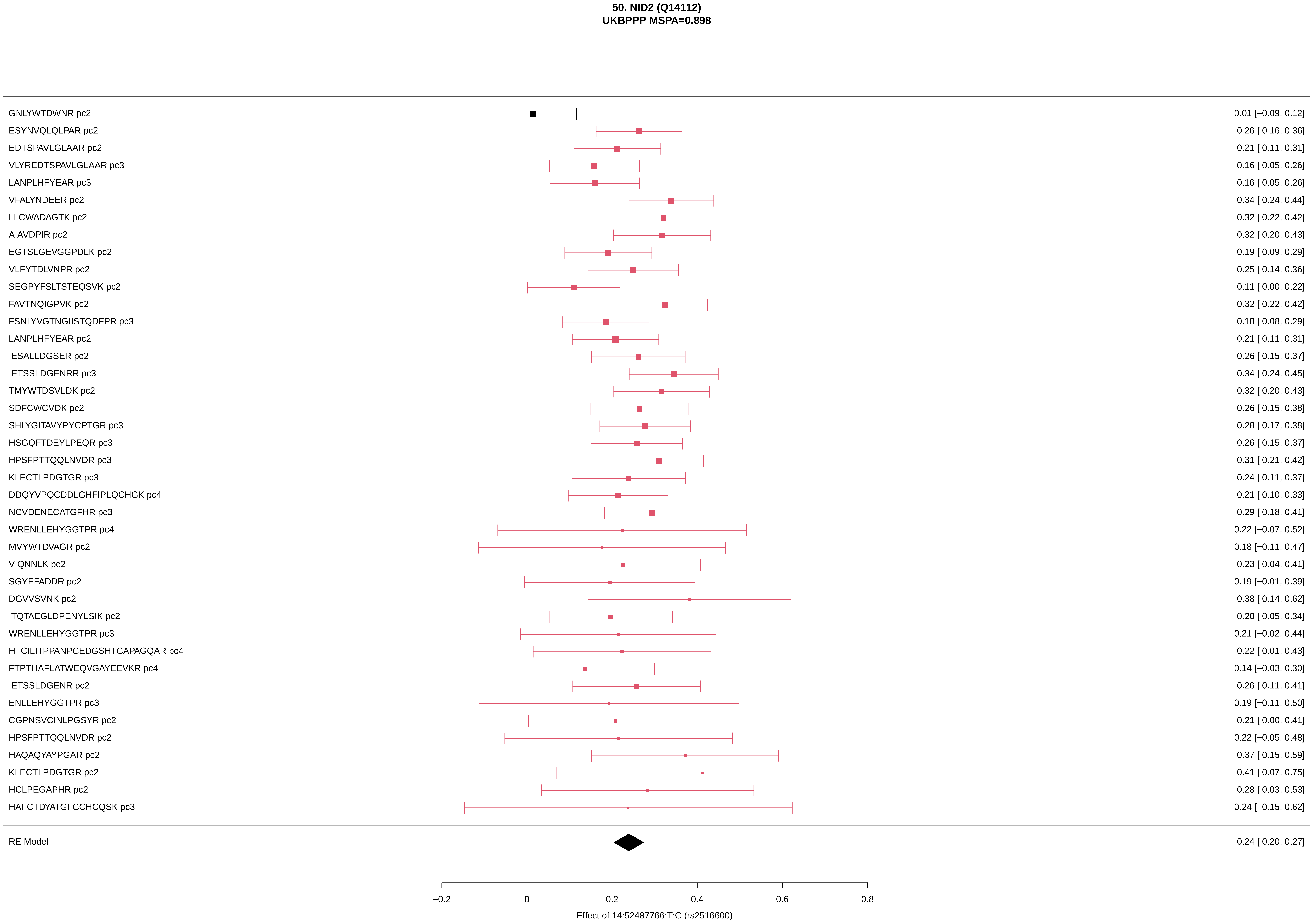
50. NID2 (Q14112)
UKBPPP MSPA=0.898
GNLYWTDWNR pc2 0.01 [−0.09, 0.12]
ESYNVQLQLPAR pc2 0.26 [0.16, 0.36]
EDTSPAVLGLAAR pc2 0.21 [0.11, 0.31]
VLYREDTSPAVLGLAAR pc3 0.16 [0.05, 0.26]
LANPLHFYEAR pc3 0.16 [0.05, 0.26]
VFALYNDEER pc2 0.34 [0.24, 0.44]
LLCWADAGTK pc2 0.32 [0.22, 0.42]
AIAVDPIR pc2 0.32 [0.20, 0.43]
EGTSLGEVGGPDLK pc2 0.19 [0.09, 0.29]
VLFYTDLVNPR pc2 0.25 [0.14, 0.36]
SEGPYFSLTSTEQSVK pc2 0.11 [0.00, 0.22]
FAVTNQIGPVK pc2 0.32 [0.22, 0.42]
FSNLYVGTNGIISTQDFPR pc3 0.18 [0.08, 0.29]
LANPLHFYEAR pc2 0.21 [0.11, 0.31]
IESALLDGSER pc2 0.26 [0.15, 0.37]
IETSSLDGENRR pc3 0.34 [0.24, 0.45]
TMYWTDSVLDK pc2 0.32 [0.20, 0.43]
SDFCWCVDK pc2 0.26 [0.15, 0.38]
SHLYGITAVYPYCPTGR pc3 0.28 [0.17, 0.38]
HSGQFTDEYLPEQR pc3 0.26 [0.15, 0.37]
HPSFPTTQQLNVDR pc3 0.31 [0.21, 0.42]
KLECTLPDGTGR pc3 0.24 [0.11, 0.37]
DDQYVPQCDDLGHFIPLQCHGK pc4 0.21 [0.10, 0.33]
NCVDENECATGFHR pc3 0.29 [0.18, 0.41]
WRENLLEHYGGTPR pc4 0.22 [−0.07, 0.52]
MVYWTDVAGR pc2 0.18 [−0.11, 0.47]
VIQNNLK pc2 0.23 [0.04, 0.41]
SGYEFADDR pc2 0.19 [−0.01, 0.39]
DGVVSVNK pc2 0.38 [0.14, 0.62]
ITQTAEGLDPENYLSIK pc2 0.20 [0.05, 0.34]
WRENLLEHYGGTPR pc3 0.21 [−0.02, 0.44]
HTCILITPPANPCEDGSHTCAPAGQAR pc4 0.22 [0.01, 0.43]
FTPTHAFLATWEQVGAYEEVKR pc4 0.14 [−0.03, 0.30]
IETSSLDGENR pc2 0.26 [0.11, 0.41]
ENLLEHYGGTPR pc3 0.19 [−0.11, 0.50]
CGPNSVCINLPGSYR pc2 0.21 [0.00, 0.41]
HPSFPTTQQLNVDR pc2 0.22 [−0.05, 0.48]
HAQAQYAYPGAR pc2 0.37 [0.15, 0.59]
KLECTLPDGTGR pc2 0.41 [0.07, 0.75]
HCLPEGAPHR pc2 0.28 [0.03, 0.53]
HAFCTDYATGFCCHCQSK pc3 0.24 [−0.15, 0.62]
RE Model 0.24 [0.20, 0.27]
−0.2
0
0.2
0.4
0.6
0.8
Effect of 14:52487766:T:C (rs2516600)

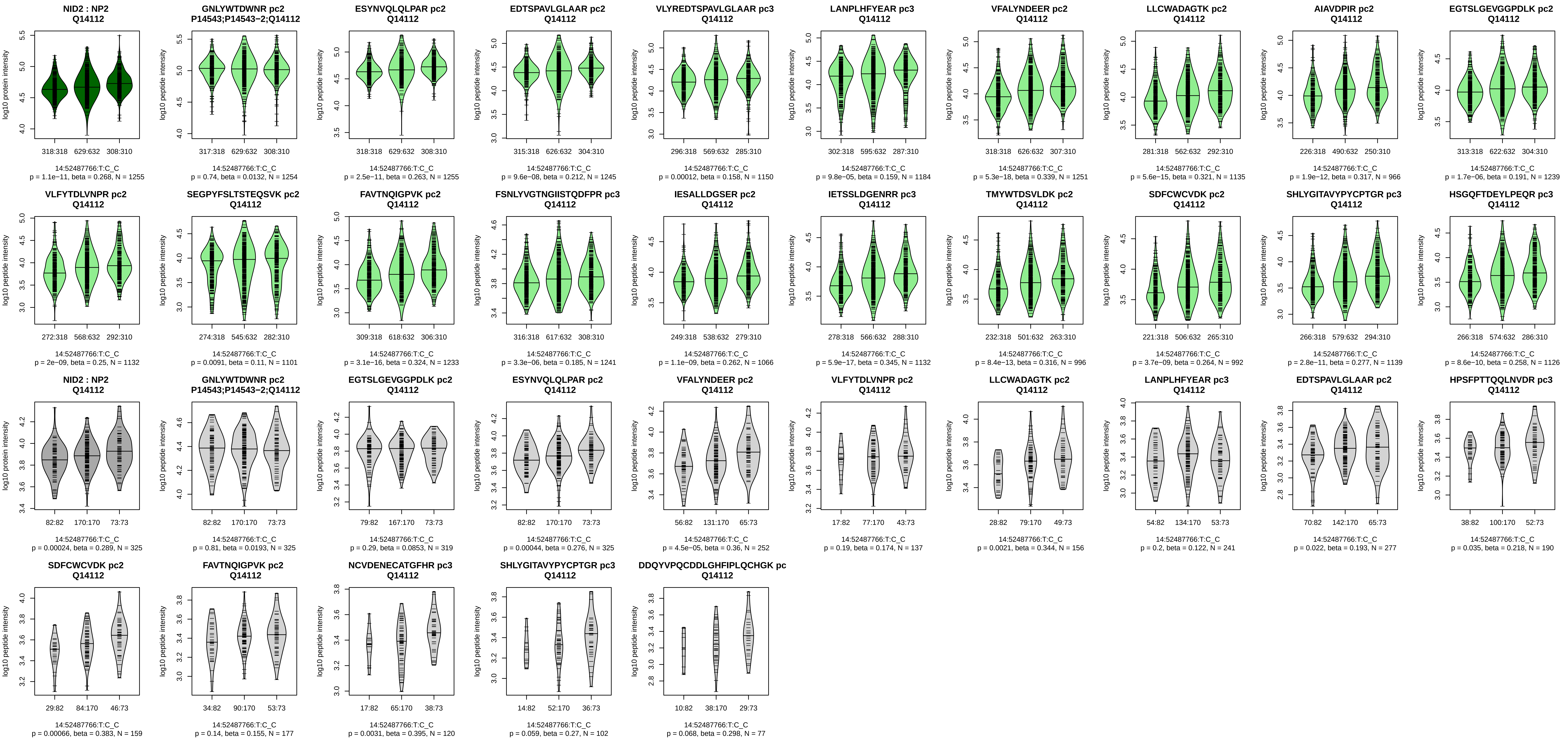

Assay Target: NID2
UniProt: Q14112
rsID: rs2516600
Target SNP: 14:52487766:T:C
Proxy SNP: 14:52487766:T:C
UKBPPP log10(p): 1914.1
UKBPPP BETA: 0.574
UKBPPP MSPA: 0.898
1254:1255:1245:1150:1184:125

51. CTSC (P53634)
UKBPPP MSPA=0.118

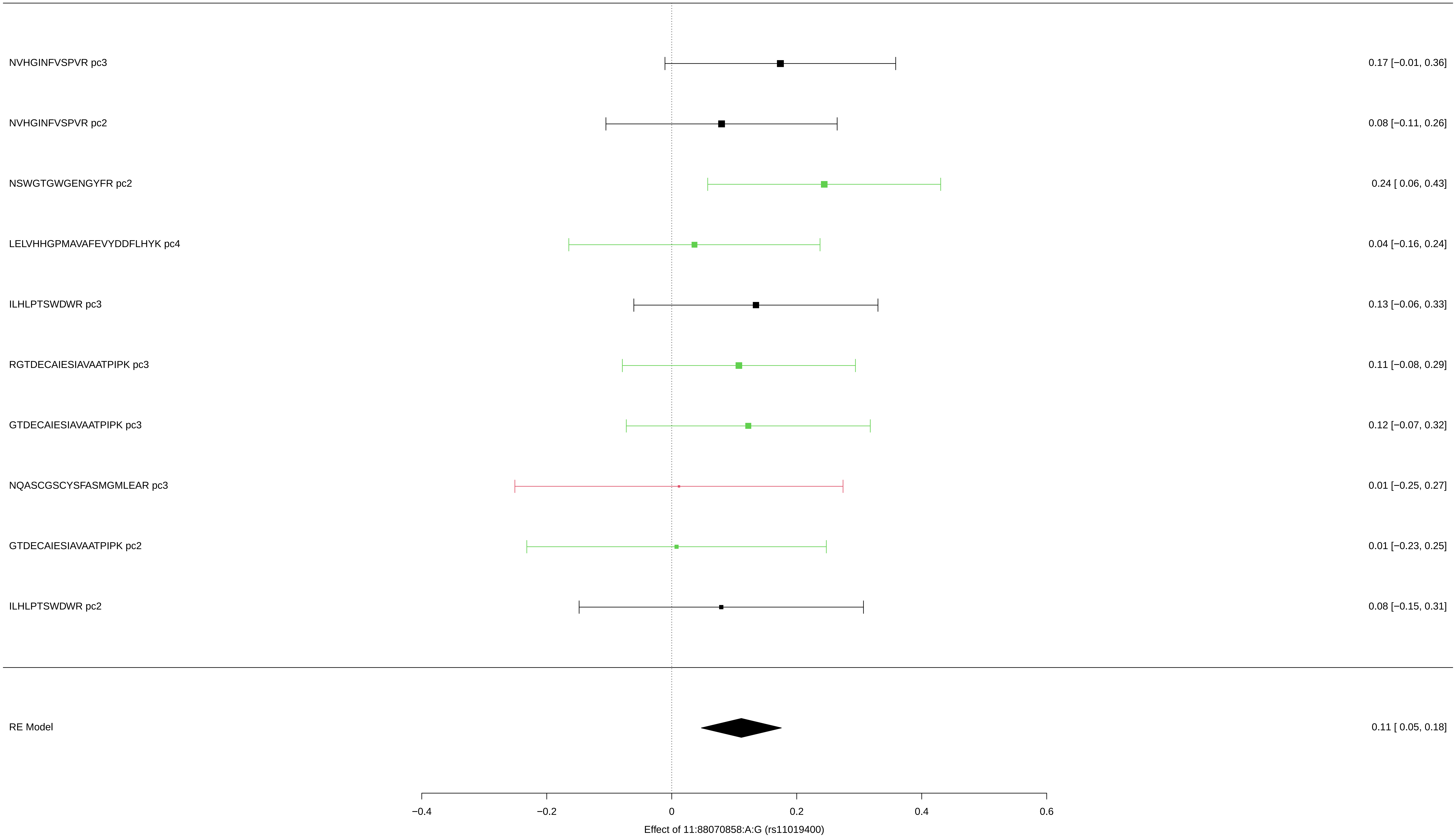

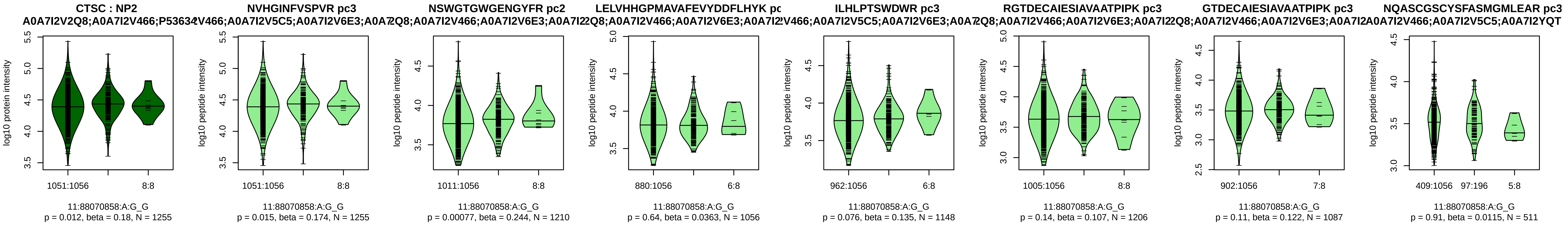

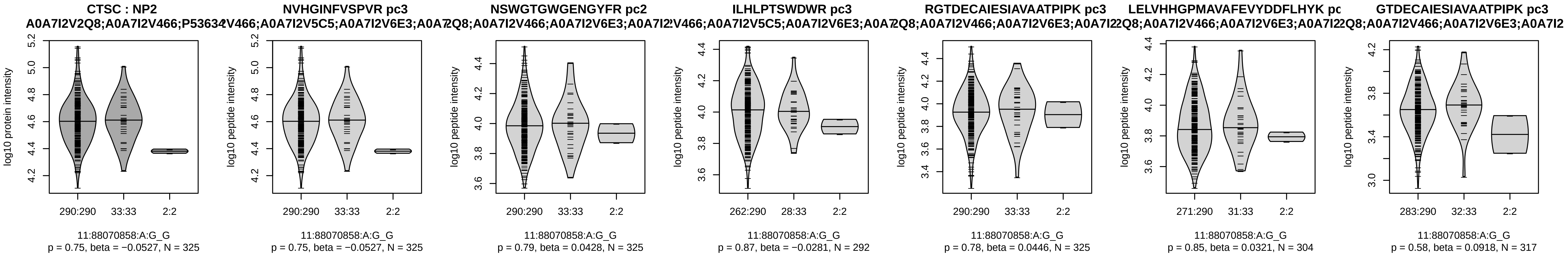

Assay Target: CTSC
UniProt: P53634
rsID: rs11019400
Target SNP: 11:88070858:A:G
Proxy SNP: 11:88070858:A:G
UKBPPP log10(p): 1890.8
UKBPPP BETA: 0.971
UKBPPP MSPA: 0.118
–:–:*:–:–:–:–:–:–:NA
1255:1249:1210:1056:1148:120

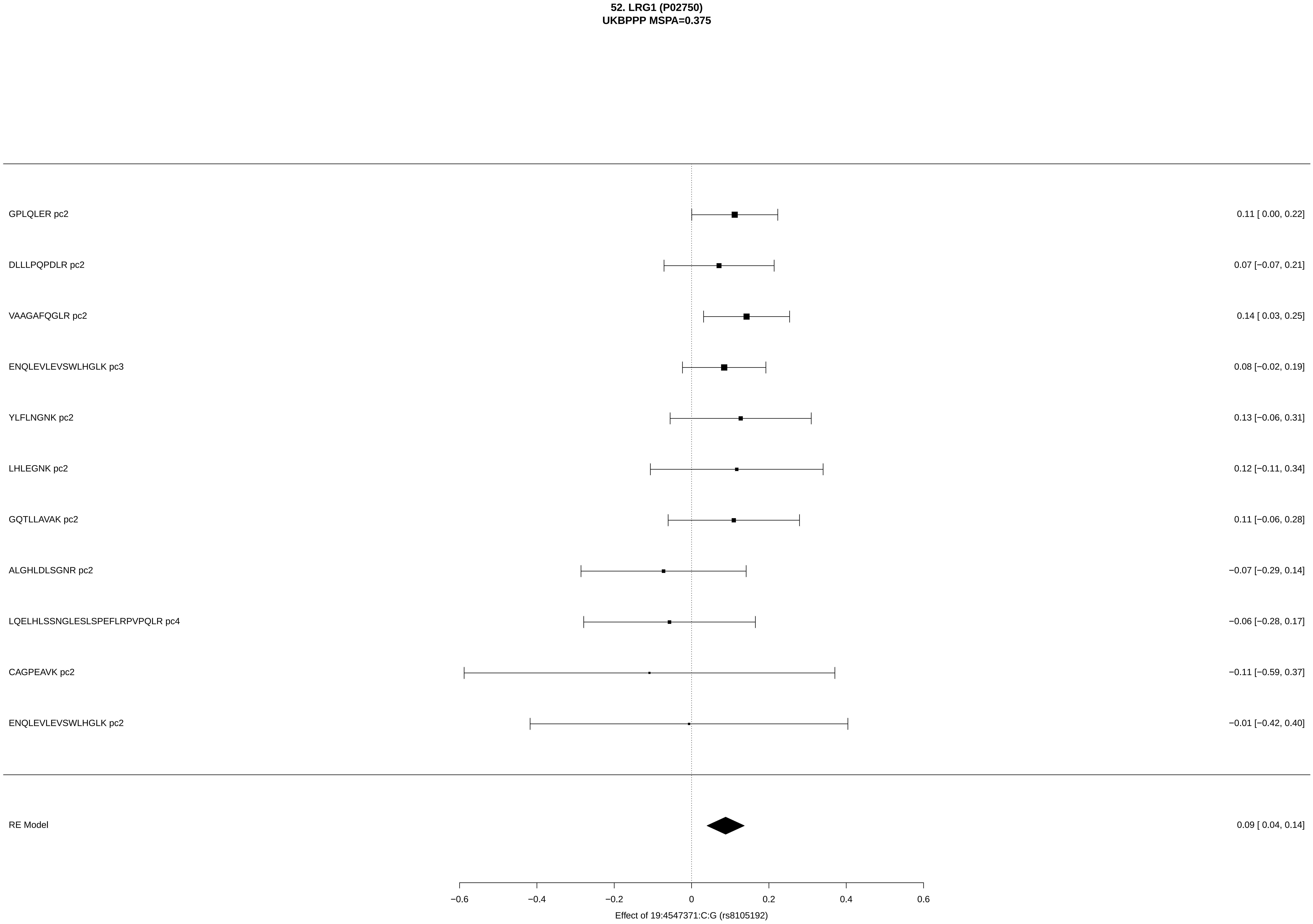
52. LRG1 (P02750)
UKBPPP MSPA=0.375
GPLQLER pc2
0.11 [0.00, 0.22]
DLLLPQPDLR pc2
0.07 [−0.07, 0.21]
VAAGAFQGLR pc2
0.14 [0.03, 0.25]
ENQLEVLEVSWLHGLK pc3
0.08 [−0.02, 0.19]
YLFLNGNK pc2
0.13 [−0.06, 0.31]
LHLEGNK pc2
0.12 [−0.11, 0.34]
GQTLLAVAK pc2
0.11 [−0.06, 0.28]
ALGHLDLSGNR pc2
−0.07 [−0.29, 0.14]
LQELHLSSNGLESLSPEFLRPVPQLR pc4
−0.06 [−0.28, 0.17]
CAGPEAVK pc2
−0.11 [−0.59, 0.37]
ENQLEVLEVSWLHGLK pc2
−0.01 [−0.42, 0.40]
RE Model
0.09 [0.04, 0.14]
−0.6
−0.4
−0.2
0
0.2
0.4
0.6
Effect of 19:4547371:C:G (rs8105192)

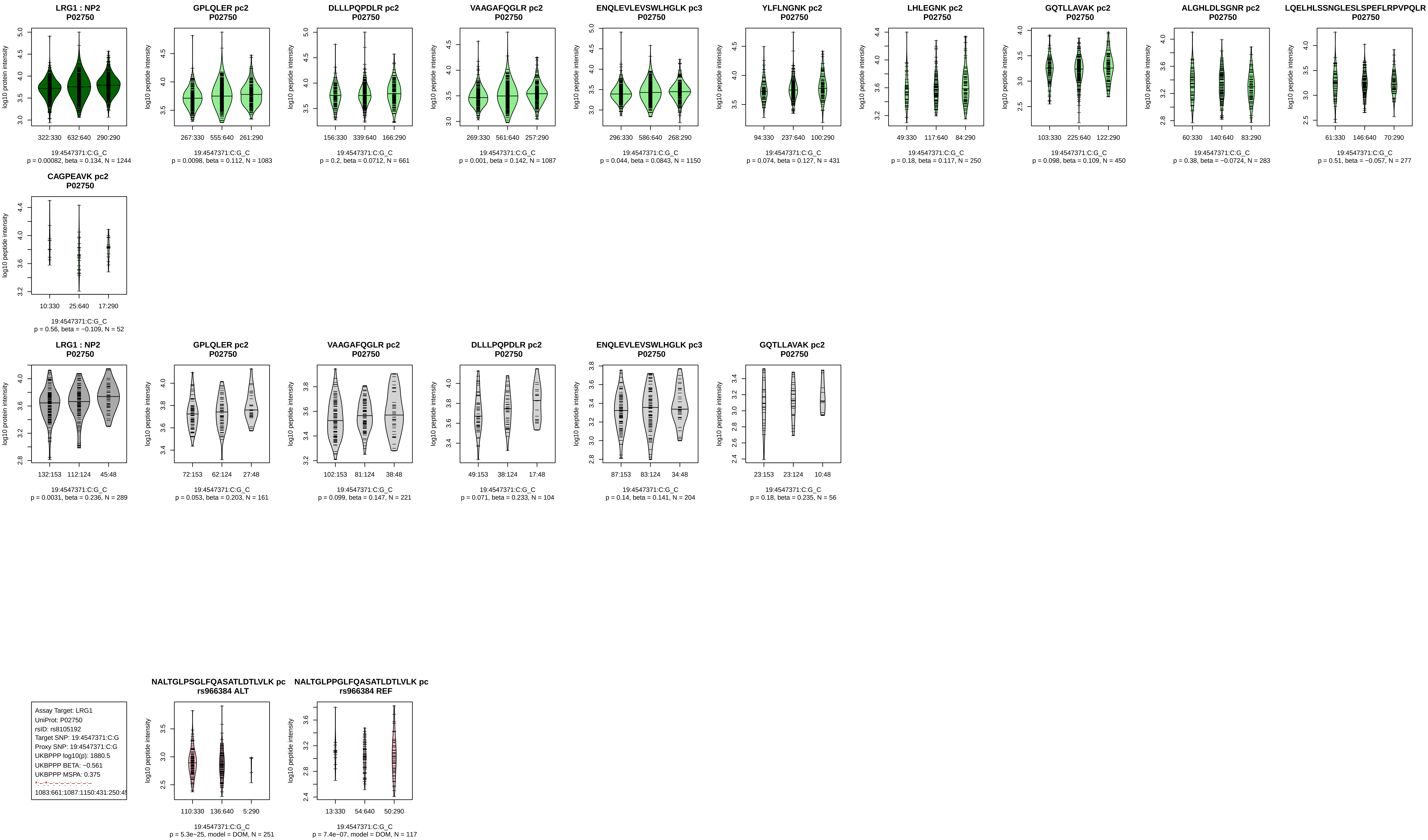
LRG1 : NP2
P02750
log10 protein intensity
322:330 632:640 290:290
19:4547371:C:G_C
p = 0.00082, beta = 0.134, N = 1244
GPLQLER pc2
P02750
log10 peptide intensity
267:330 555:640 261:290
19:4547371:C:G_C
p = 0.0098, beta = 0.112, N = 1083
DLLLPQPDLR pc2
P02750
156:330 339:640 166:290
19:4547371:C:G_C
p = 0.2, beta = 0.0712, N = 661
VAAGAFQGLR pc2
P02750
269:330 561:640 257:290
19:4547371:C:G_C
p = 0.001, beta = 0.142, N = 1087
ENQLEVLEVSWLHGLK pc3
P02750
296:330 586:640 268:290
19:4547371:C:G_C
p = 0.044, beta = 0.0843, N = 1150
YLFLNGNK pc2
P02750
94:330 237:640 100:290
19:4547371:C:G_C
p = 0.074, beta = 0.127, N = 431
LHLEGNK pc2
P02750
49:330 117:640 84:290
19:4547371:C:G_C
p = 0.18, beta = 0.117, N = 250
GQTLLAVAK pc2
P02750
103:330 225:640 122:290
19:4547371:C:G_C
p = 0.098, beta = 0.109, N = 450
ALGHLDLSGNR pc2
P02750
60:330 140:640 83:290
19:4547371:C:G_C
p = 0.38, beta = −0.0724, N = 283
LQELHLSSNGLESLSPEFLRPVPQLR
P02750
61:330 146:640 70:290
19:4547371:C:G_C
p = 0.51, beta = −0.057, N = 277
CAGPEAVK pc2
P02750
10:330 25:640 17:290
19:4547371:C:G_C
p = 0.56, beta = −0.109, N = 52
LRG1 : NP2
P02750
132:153 112:124 45:48
19:4547371:C:G_C
p = 0.0031, beta = 0.236, N = 289
GPLQLER pc2
P02750
72:153 62:124 27:48
19:4547371:C:G_C
p = 0.053, beta = 0.203, N = 161
VAAGAFQGLR pc2
P02750
102:153 81:124 38:48
19:4547371:C:G_C
p = 0.099, beta = 0.147, N = 221
DLLLPQPDLR pc2
P02750
49:153 38:124 17:48
19:4547371:C:G_C
p = 0.071, beta = 0.233, N = 104
ENQLEVLEVSWLHGLK pc3
P02750
87:153 83:124 34:48
19:4547371:C:G_C
p = 0.14, beta = 0.141, N = 204
GQTLLAVAK pc2
P02750
23:153 23:124 10:48
19:4547371:C:G_C
p = 0.18, beta = 0.235, N = 56
Assay Target: LRG1
UniProt: P02750
rsID: rs8105192
Target SNP: 19:4547371:C:G
Proxy SNP: 19:4547371:C:G
UKBPPP log10(p): 1880.5
UKBPPP BETA: −0.561
UKBPPP MSPA: 0.375
:–::–:–:–:–:–:–:–
1083:661:1087:1150:431:250:45
NALTGLPSGLFQASATLDTLVLK pc
rs966384 ALT
110:330 136:640 5:290
19:4547371:C:G_C
p = 5.3e−25, model = DOM, N = 251
NALTGLPPGLFQASATLDTLVLK pc
rs966384 REF
13:330 54:640 50:290
19:4547371:C:G_C
p = 7.4e−07, model = DOM, N = 117

53. ITGA2 (P17301)
UKBPPP MSPA=0

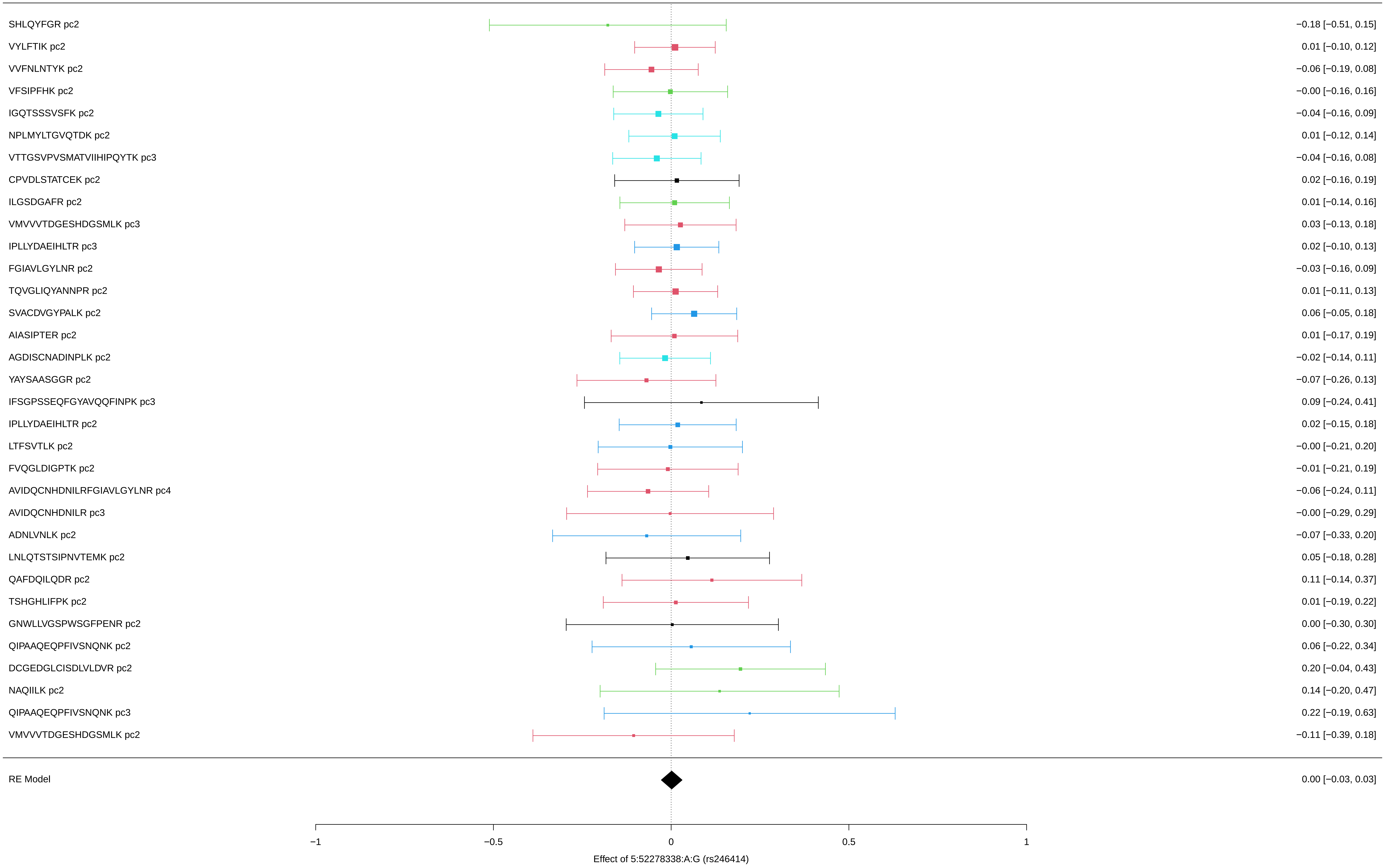

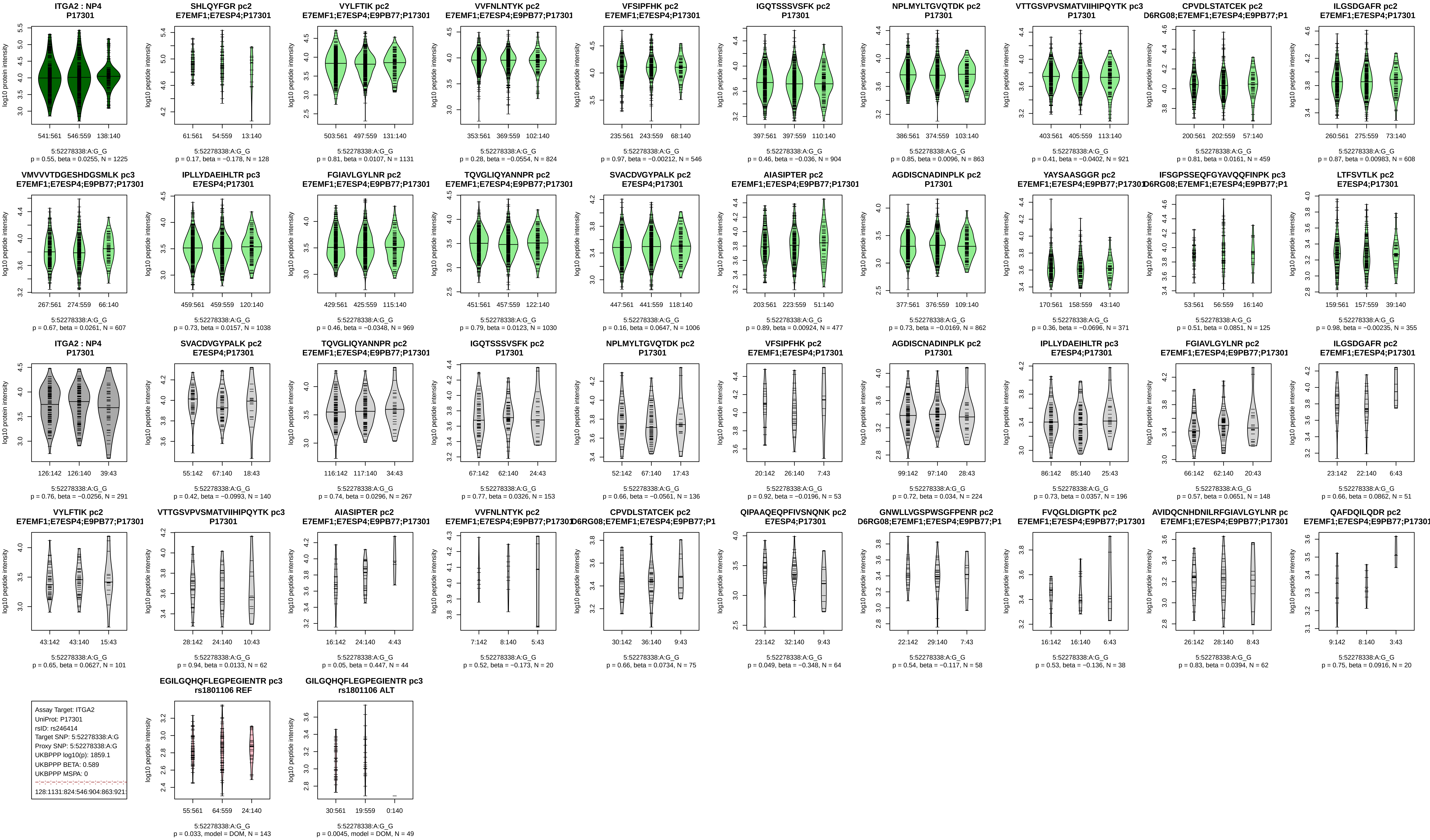
ITGA2 : NP4 P17301
541:561 546:559 138:140
5:52278338:A:G_G
p = 0.55, beta = 0.0255, N = 1225
SHLQYFGR pc2 E7EMF1;E7ESP4;P17301
61:561 54:559 13:140
5:52278338:A:G_G
p = 0.17, beta = −0.178, N = 128
VYLFTIK pc2 E7EMF1;E7ESP4;E9PB77;P17301
503:561 497:559 131:140
5:52278338:A:G_G
p = 0.81, beta = 0.0107, N = 1131
VVFNLNTYK pc2 E7EMF1;E7ESP4;E9PB77;P17301
353:561 369:559 102:140
5:52278338:A:G_G
p = 0.28, beta = −0.0554, N = 824
VFSIPFHK pc2 E7EMF1;E7ESP4;P17301
235:561 243:559 68:140
5:52278338:A:G_G
p = 0.97, beta = −0.00212, N = 546
IGQTSSSVSFK pc2 P17301
397:561 397:559 110:140
5:52278338:A:G_G
p = 0.46, beta = −0.036, N = 904
NPLMYLTGVQTDK pc2 P17301
386:561 374:559 103:140
5:52278338:A:G_G
p = 0.85, beta = 0.0096, N = 863
VTTGSVPVSMATVIIHIPQYTK pc3 P17301
403:561 405:559 113:140
5:52278338:A:G_G
p = 0.41, beta = −0.0402, N = 921
CPVDLSTATCEK pc2 D6RG08;E7EMF1;E7ESP4;E9PB77;P1
200:561 202:559 57:140
5:52278338:A:G_G
p = 0.81, beta = 0.0161, N = 459
ILGSDGAFR pc2 E7EMF1;E7ESP4;P17301
260:561 275:559 73:140
5:52278338:A:G_G
p = 0.87, beta = 0.00983, N = 608
VMVVVTDGESHDGSMLK pc3 E7EMF1;E7ESP4;E9PB77;P17301
267:561 274:559 66:140
5:52278338:A:G_G
p = 0.67, beta = 0.0261, N = 607
IPLLYDAEIHLTR pc3 E7ESP4;P17301
459:561 459:559 120:140
5:52278338:A:G_G
p = 0.73, beta = 0.0157, N = 1038
FGIAVLGYLNR pc2 E7EMF1;E7ESP4;E9PB77;P17301
429:561 425:559 115:140
5:52278338:A:G_G
p = 0.46, beta = −0.0348, N = 969
TQVGLIQYANNPR pc2 E7EMF1;E7ESP4;E9PB77;P17301
451:561 457:559 122:140
5:52278338:A:G_G
p = 0.79, beta = 0.0123, N = 1030
SVACDVGYPALK pc2 E7ESP4;P17301
447:561 441:559 118:140
5:52278338:A:G_G
p = 0.16, beta = 0.0647, N = 1006
AIASIPTER pc2 E7EMF1;E7ESP4;E9PB77;P17301
203:561 223:559 51:140
5:52278338:A:G_G
p = 0.89, beta = 0.00924, N = 477
AGDISCNADINPLK pc2 P17301
377:561 376:559 109:140
5:52278338:A:G_G
p = 0.73, beta = −0.0169, N = 862
YAYSAASGGR pc2 E7EMF1;E7ESP4;E9PB77;P17301
170:561 158:559 43:140
5:52278338:A:G_G
p = 0.36, beta = −0.0696, N = 371
IFSGPSSEQFGYAVQQFINPK pc3 D6RG08;E7EMF1;E7ESP4;E9PB77;P1
53:561 56:559 16:140
5:52278338:A:G_G
p = 0.51, beta = 0.0851, N = 125
LTFSVTLK pc2 E7ESP4;P17301
159:561 157:559 39:140
5:52278338:A:G_G
p = 0.98, beta = −0.00235, N = 355
ITGA2 : NP4 P17301
126:142 126:140 39:43
5:52278338:A:G_G
p = 0.76, beta = −0.0256, N = 291
SVACDVGYPALK pc2 E7ESP4;P17301
55:142 67:140 18:43
5:52278338:A:G_G
p = 0.42, beta = −0.0993, N = 140
TQVGLIQYANNPR pc2 E7EMF1;E7ESP4;E9PB77;P17301
116:142 117:140 34:43
5:52278338:A:G_G
p = 0.74, beta = 0.0296, N = 267
IGQTSSSVSFK pc2 P17301
67:142 62:140 24:43
5:52278338:A:G_G
p = 0.77, beta = 0.0326, N = 153
NPLMYLTGVQTDK pc2 P17301
52:142 67:140 17:43
5:52278338:A:G_G
p = 0.66, beta = −0.0561, N = 136
VFSIPFHK pc2 E7EMF1;E7ESP4;P17301
20:142 26:140 7:43
5:52278338:A:G_G
p = 0.92, beta = −0.0196, N = 53
AGDISCNADINPLK pc2 P17301
99:142 97:140 28:43
5:52278338:A:G_G
p = 0.72, beta = 0.034, N = 224
IPLLYDAEIHLTR pc3 E7ESP4;P17301
86:142 85:140 25:43
5:52278338:A:G_G
p = 0.73, beta = 0.0357, N = 196
FGIAVLGYLNR pc2 E7EMF1;E7ESP4;E9PB77;P17301
66:142 62:140 20:43
5:52278338:A:G_G
p = 0.57, beta = 0.0651, N = 148
ILGSDGAFR pc2 E7EMF1;E7ESP4;P17301
23:142 22:140 6:43
5:52278338:A:G_G
p = 0.66, beta = 0.0862, N = 51
VYLFTIK pc2 E7EMF1;E7ESP4;E9PB77;P17301
43:142 43:140 15:43
5:52278338:A:G_G
p = 0.65, beta = 0.0627, N = 101
VTTGSVPVSMATVIIHIPQYTK pc3 P17301
28:142 24:140 10:43
5:52278338:A:G_G
p = 0.94, beta = 0.0133, N = 62
AIASIPTER pc2 E7EMF1;E7ESP4;E9PB77;P17301
16:142 24:140 4:43
5:52278338:A:G_G
p = 0.05, beta = 0.447, N = 44
VVFNLNTYK pc2 E7EMF1;E7ESP4;E9PB77;P17301
7:142 8:140 5:43
5:52278338:A:G_G
p = 0.52, beta = −0.173, N = 20
CPVDLSTATCEK pc2 D6RG08;E7EMF1;E7ESP4;E9PB77;P1
30:142 36:140 9:43
5:52278338:A:G_G
p = 0.66, beta = 0.0734, N = 75
QIPAAQEQPFIVSNQNK pc2 E7ESP4;P17301
23:142 32:140 9:43
5:52278338:A:G_G
p = 0.049, beta = −0.348, N = 64
GNWLLVGSPWSGFPENR pc2 D6RG08;E7EMF1;E7ESP4;E9PB77;P1
22:142 29:140 7:43
5:52278338:A:G_G
p = 0.54, beta = −0.117, N = 58
FVQGLDIGPTK pc2 E7EMF1;E7ESP4;E9PB77;P17301
16:142 16:140 6:43
5:52278338:A:G_G
p = 0.53, beta = −0.136, N = 38
AVIDQCNHDNILRFGIAVLGYLNR pc E7EMF1;E7ESP4;E9PB77;P17301
26:142 28:140 8:43
5:52278338:A:G_G
p = 0.83, beta = 0.0394, N = 62
QAFDQILQDR pc2 E7EMF1;E7ESP4;E9PB77;P17301
9:142 8:140 3:43
5:52278338:A:G_G
p = 0.75, beta = 0.0916, N = 20
Assay Target: ITGA2
UniProt: P17301
rsID: rs246414
Target SNP: 5:52278338:A:G
Proxy SNP: 5:52278338:A:G
UKBPPP log10(p): 1859.1
UKBPPP BETA: 0.589
UKBPPP MSPA: 0
128:1131:824:546:904:863:921:
EGILGQHQFLEGPEGIENTR pc3 rs1801106 REF
55:561 64:559 24:140
5:52278338:A:G_G
p = 0.033, model = DOM, N = 143
GILGQHQFLEGPEGIENTR pc3 rs1801106 ALT
30:561 19:559 0:140
5:52278338:A:G_G
p = 0.0045, model = DOM, N = 49
log10 protein intensity
log10 peptide intensity

54. CHI3L1 (P36222)
UKBPPP MSPA=0.685

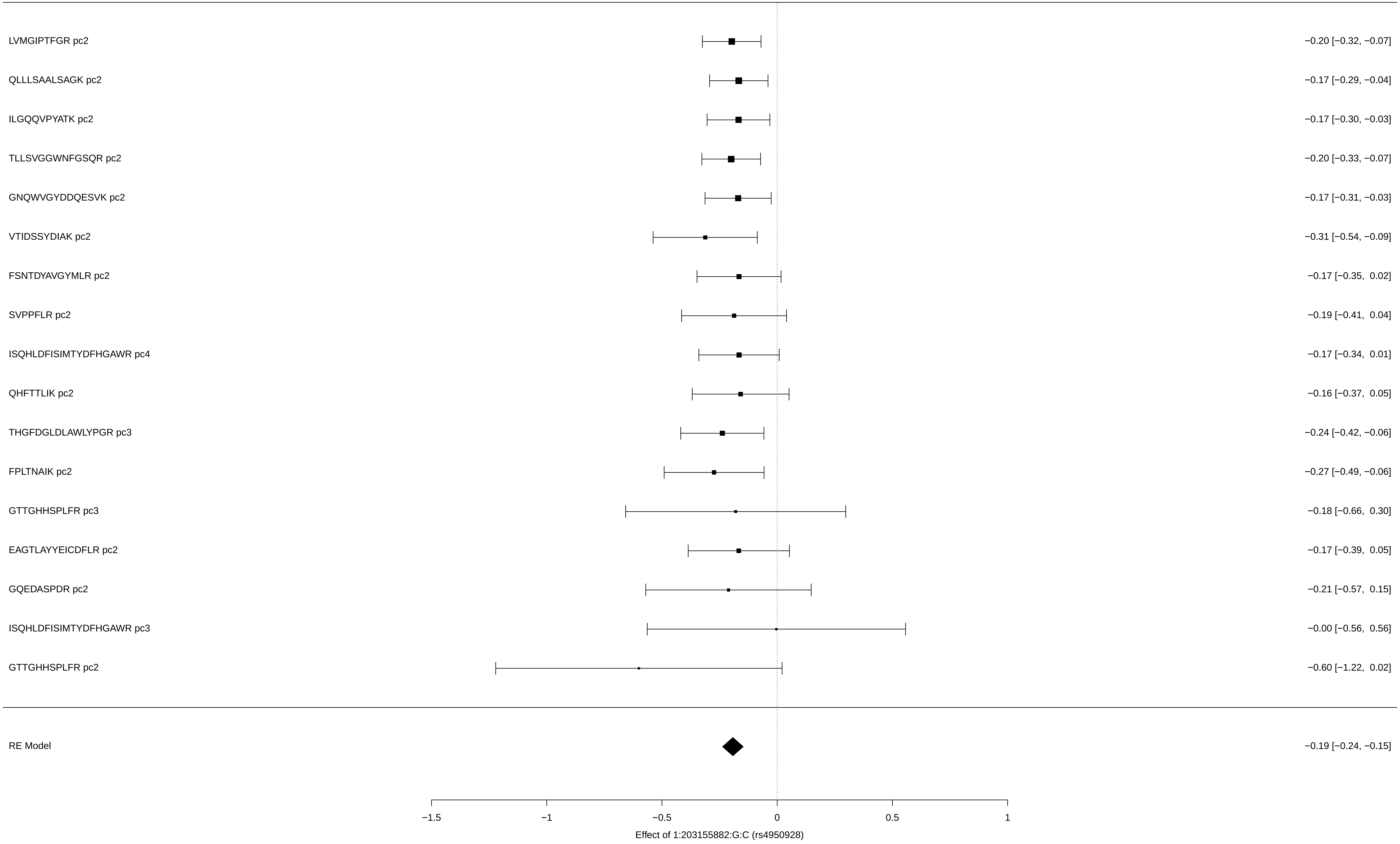

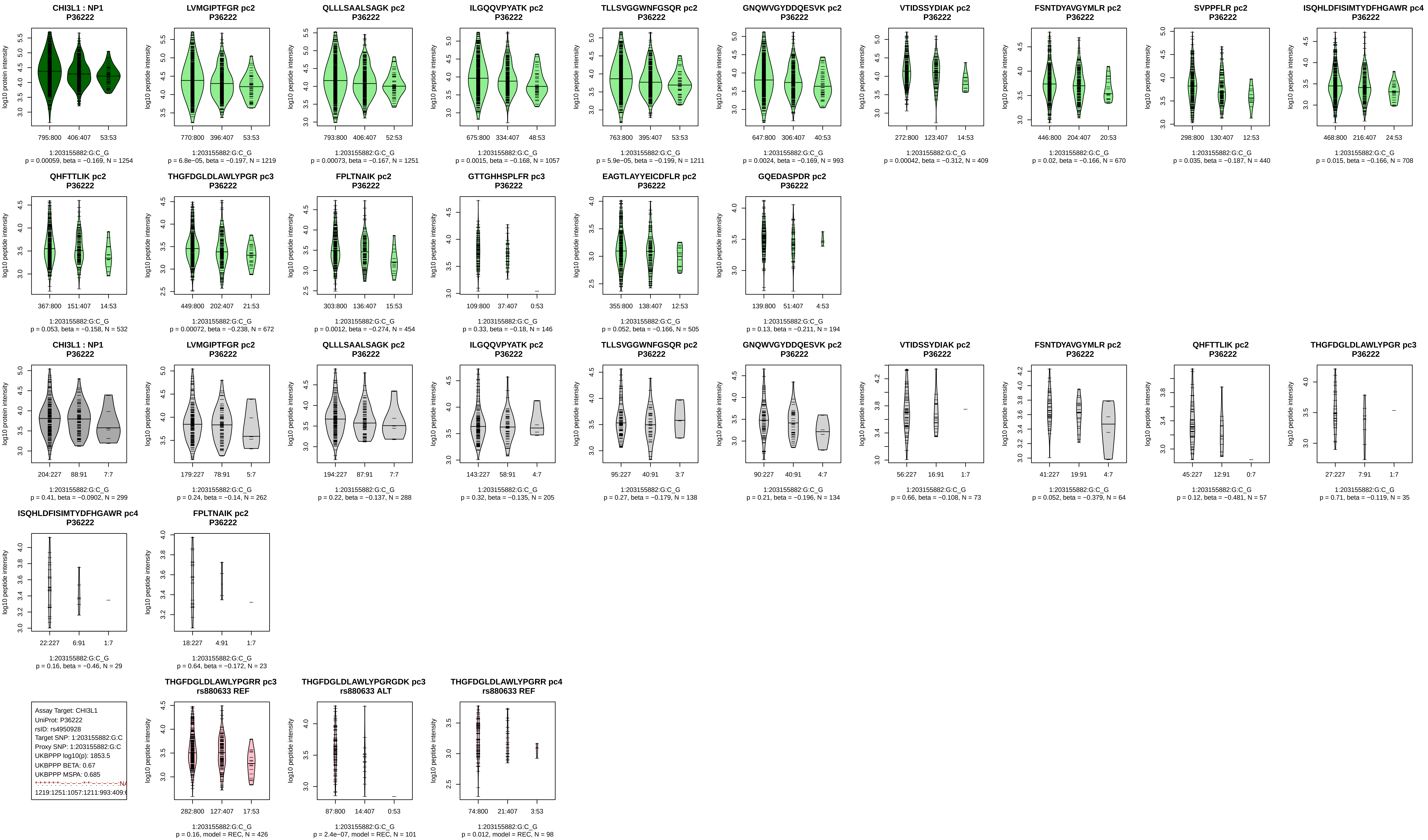

CHI3L1 : NP1 P36222
795:800 406:407 53:53
1:203155882:G:C_G
p = 0.00059, beta = −0.169, N = 1254
LVMGIPTFGR pc2 P36222
770:800 396:407 53:53
1:203155882:G:C_G
p = 6.8e−05, beta = −0.197, N = 1219
QLLLSAALSAGK pc2 P36222
793:800 406:407 52:53
1:203155882:G:C_G
p = 0.00073, beta = −0.167, N = 1251
ILGQQVPYATK pc2 P36222
675:800 334:407 48:53
1:203155882:G:C_G
p = 0.0015, beta = −0.168, N = 1057
TLLSVGGWNFGSQR pc2 P36222
763:800 395:407 53:53
1:203155882:G:C_G
p = 5.9e−05, beta = −0.199, N = 1211
GNQWVGYDDQESVK pc2 P36222
647:800 306:407 40:53
1:203155882:G:C_G
p = 0.0024, beta = −0.169, N = 993
VTIDSSYDIAK pc2 P36222
272:800 123:407 14:53
1:203155882:G:C_G
p = 0.00042, beta = −0.312, N = 409
FSNTDYAVGYMLR pc2 P36222
446:800 204:407 20:53
1:203155882:G:C_G
p = 0.02, beta = −0.166, N = 670
SVPPFLR pc2 P36222
298:800 130:407 12:53
1:203155882:G:C_G
p = 0.035, beta = −0.187, N = 440
ISQHLDFISIMTYDFHGAWR pc4 P36222
468:800 216:407 24:53
1:203155882:G:C_G
p = 0.015, beta = −0.166, N = 708
QHFTTLIK pc2 P36222
367:800 151:407 14:53
1:203155882:G:C_G
p = 0.053, beta = −0.158, N = 532
THGFDGLDLAWLYPGR pc3 P36222
449:800 202:407 21:53
1:203155882:G:C_G
p = 0.00072, beta = −0.238, N = 672
FPLTNAIK pc2 P36222
303:800 136:407 15:53
1:203155882:G:C_G
p = 0.0012, beta = −0.274, N = 454
GTTGHHSPLFR pc3 P36222
109:800 37:407 0:53
1:203155882:G:C_G
p = 0.33, beta = −0.18, N = 146
EAGTLAYYEICDFLR pc2 P36222
355:800 138:407 12:53
1:203155882:G:C_G
p = 0.052, beta = −0.166, N = 505
GQEDASPDR pc2 P36222
139:800 51:407 4:53
1:203155882:G:C_G
p = 0.13, beta = −0.211, N = 194
CHI3L1 : NP1 P36222
204:227 88:91 7:7
1:203155882:G:C_G
p = 0.41, beta = −0.0902, N = 299
LVMGIPTFGR pc2 P36222
179:227 78:91 5:7
1:203155882:G:C_G
p = 0.24, beta = −0.14, N = 262
QLLLSAALSAGK pc2 P36222
194:227 87:91 7:7
1:203155882:G:C_G
p = 0.22, beta = −0.137, N = 288
ILGQQVPYATK pc2 P36222
143:227 58:91 4:7
1:203155882:G:C_G
p = 0.32, beta = −0.135, N = 205
TLLSVGGWNFGSQR pc2 P36222
95:227 40:91 3:7
1:203155882:G:C_G
p = 0.27, beta = −0.179, N = 138
GNQWVGYDDQESVK pc2 P36222
90:227 40:91 4:7
1:203155882:G:C_G
p = 0.21, beta = −0.196, N = 134
VTIDSSYDIAK pc2 P36222
56:227 16:91 1:7
1:203155882:G:C_G
p = 0.66, beta = −0.108, N = 73
FSNTDYAVGYMLR pc2 P36222
41:227 19:91 4:7
1:203155882:G:C_G
p = 0.052, beta = −0.379, N = 64
QHFTTLIK pc2 P36222
45:227 12:91 0:7
1:203155882:G:C_G
p = 0.12, beta = −0.481, N = 57
THGFDGLDLAWLYPGR pc3 P36222
27:227 7:91 1:7
1:203155882:G:C_G
p = 0.71, beta = −0.119, N = 35
ISQHLDFISIMTYDFHGAWR pc4 P36222
22:227 6:91 1:7
1:203155882:G:C_G
p = 0.16, beta = −0.46, N = 29
FPLTNAIK pc2 P36222
18:227 4:91 1:7
1:203155882:G:C_G
p = 0.64, beta = −0.172, N = 23
Assay Target: CHI3L1
UniProt: P36222
rsID: rs4950928
Target SNP: 1:203155882:G:C
Proxy SNP: 1:203155882:G:C
UKBPPP log10(p): 1853.5
UKBPPP BETA: 0.67
UKBPPP MSPA: 0.685
THGFDGLDLAWLYPGRR pc3 rs880633 REF
282:800 127:407 17:53
1:203155882:G:C_G
p = 0.16, model = REC, N = 426
THGFDGLDLAWLYPGRGDK pc3 rs880633 ALT
87:800 14:407 0:53
1:203155882:G:C_G
p = 2.4e−07, model = REC, N = 101
THGFDGLDLAWLYPGRR pc4 rs880633 REF
74:800 21:407 3:53
1:203155882:G:C_G
p = 0.012, model = REC, N = 98
log10 protein intensity
log10 peptide intensity

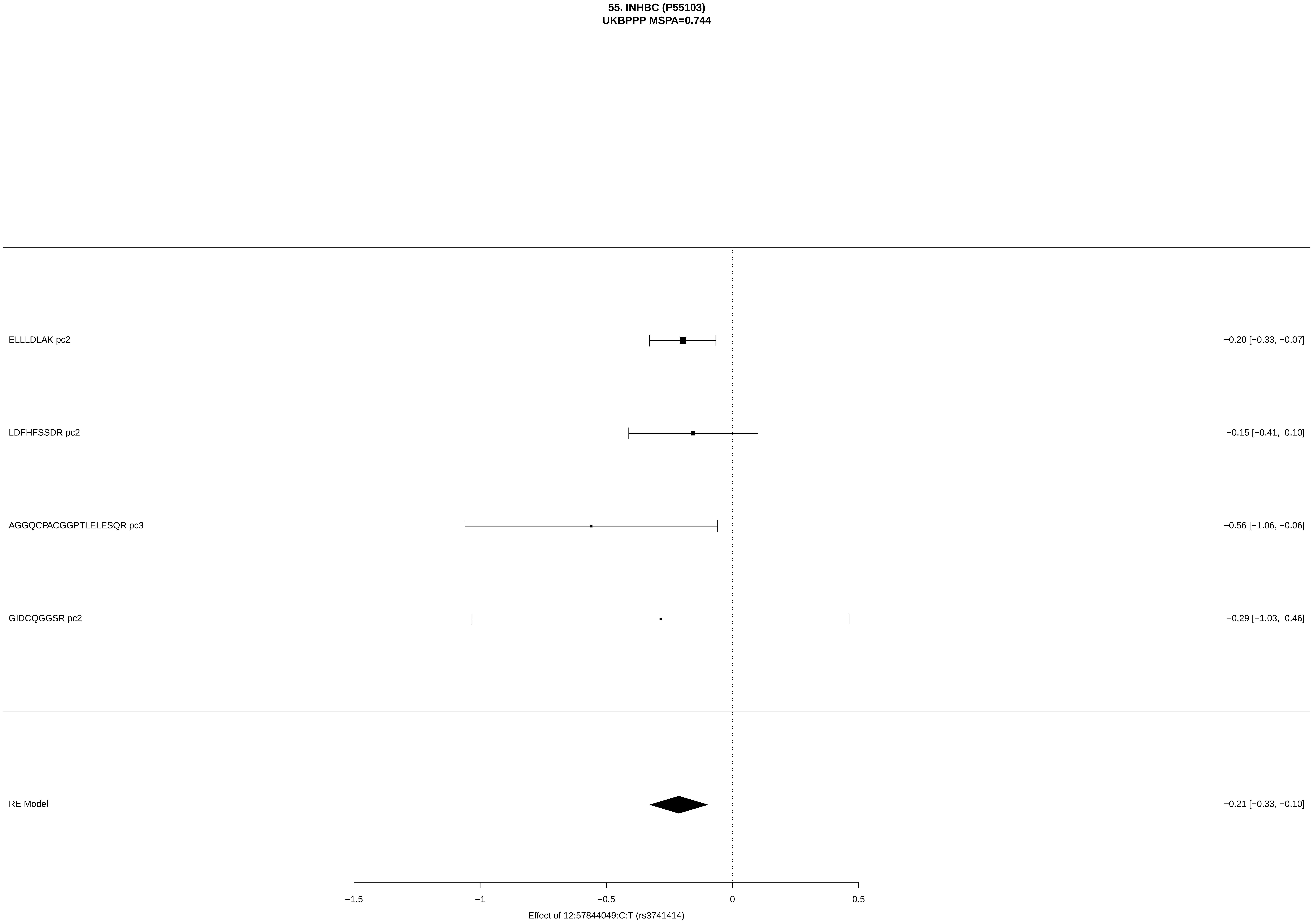
55. INHBC (P55103)
UKBPPP MSPA=0.744
ELLLDLAK pc2
−0.20 [−0.33, −0.07]
LDFHFSSDR pc2
−0.15 [−0.41, 0.10]
AGGQCPACGGPTLELESQR pc3
−0.56 [−1.06, −0.06]
GIDCQGGSR pc2
−0.29 [−1.03, 0.46]
RE Model
−0.21 [−0.33, −0.10]
−1.5
−1
−0.5
0
0.5
Effect of 12:57844049:C:T (rs3741414)

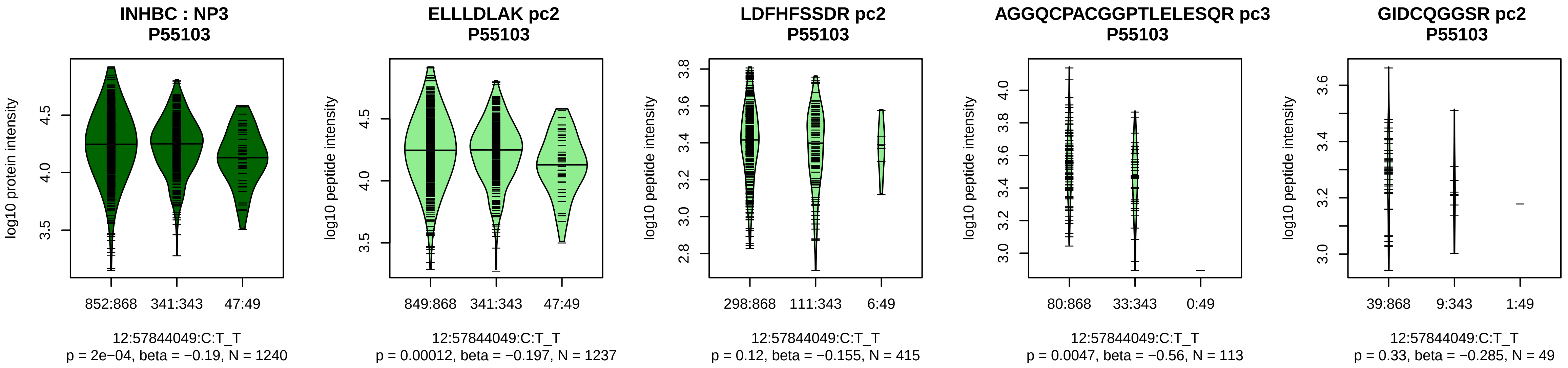

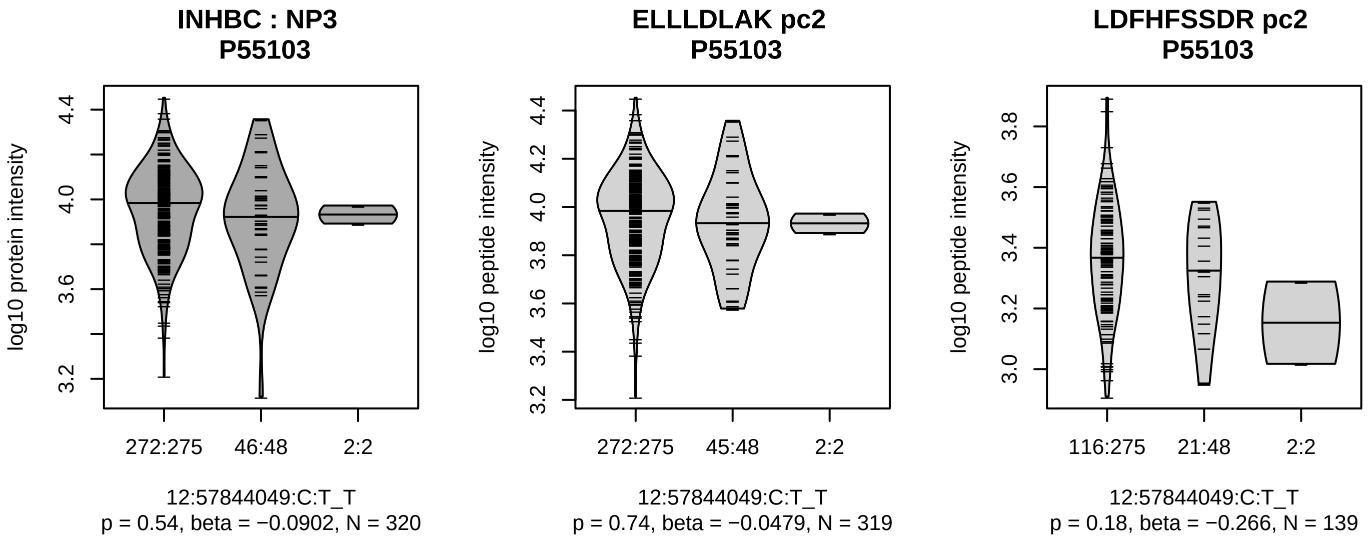

Assay Target: INHBC
UniProt: P55103
rsID: rs3741414
Target SNP: 12:57844049:C:T
Proxy SNP: 12:57844049:C:T
UKBPPP log10(p): 1817.4
UKBPPP BETA: −0.655
UKBPPP MSPA: 0.744
:−::−:NA
1237:415:113:49:1

56. CRHBP (P24387)
UKBPPP MSPA=0.496

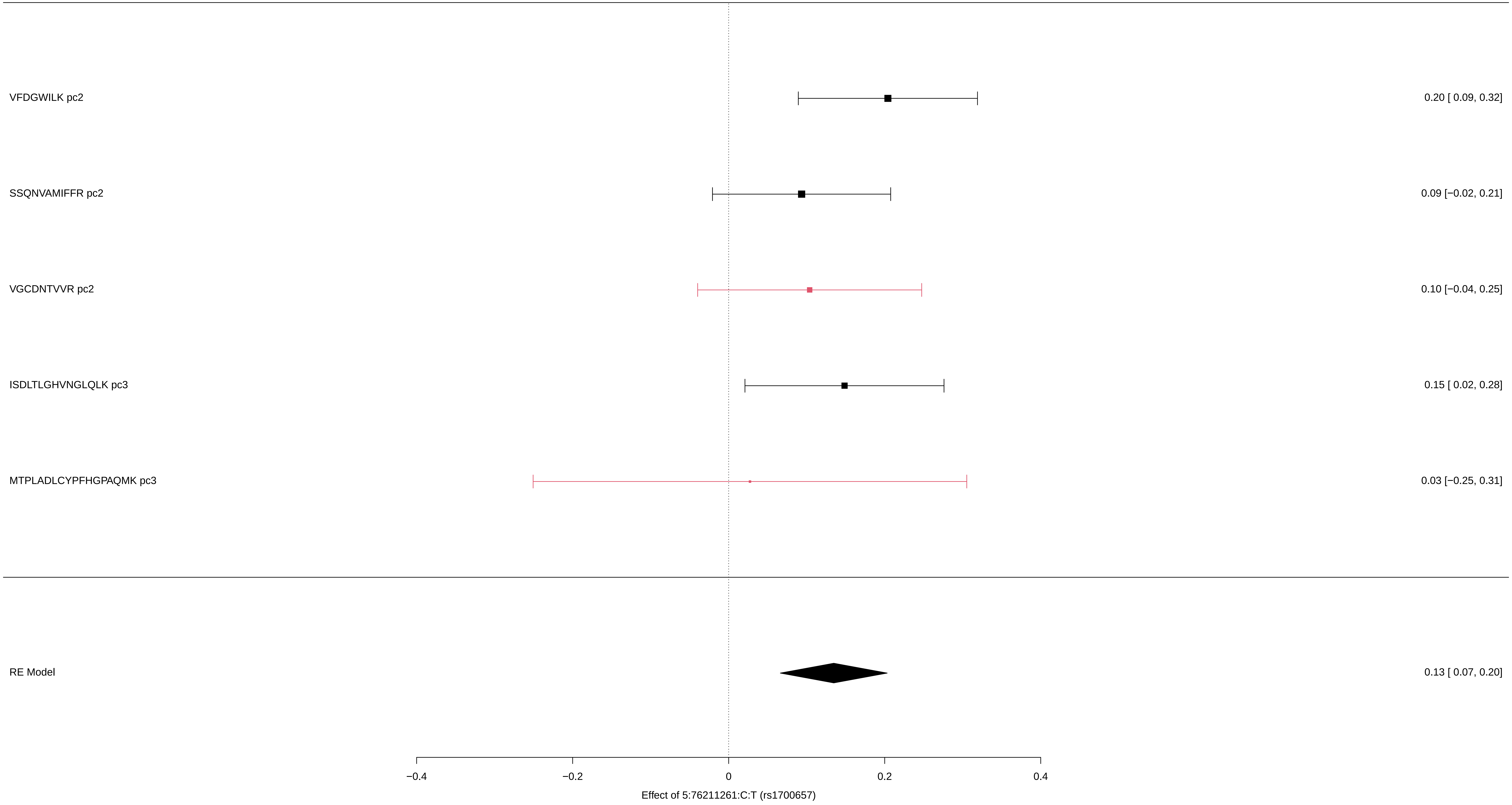

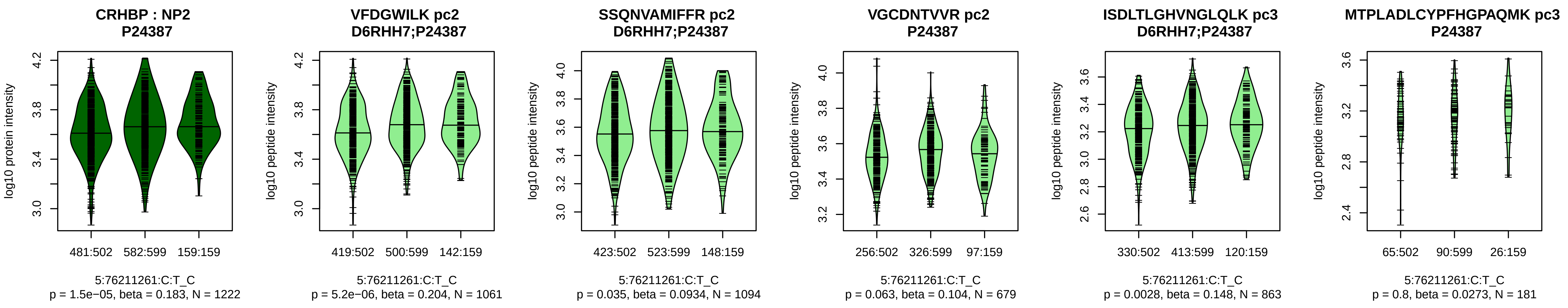

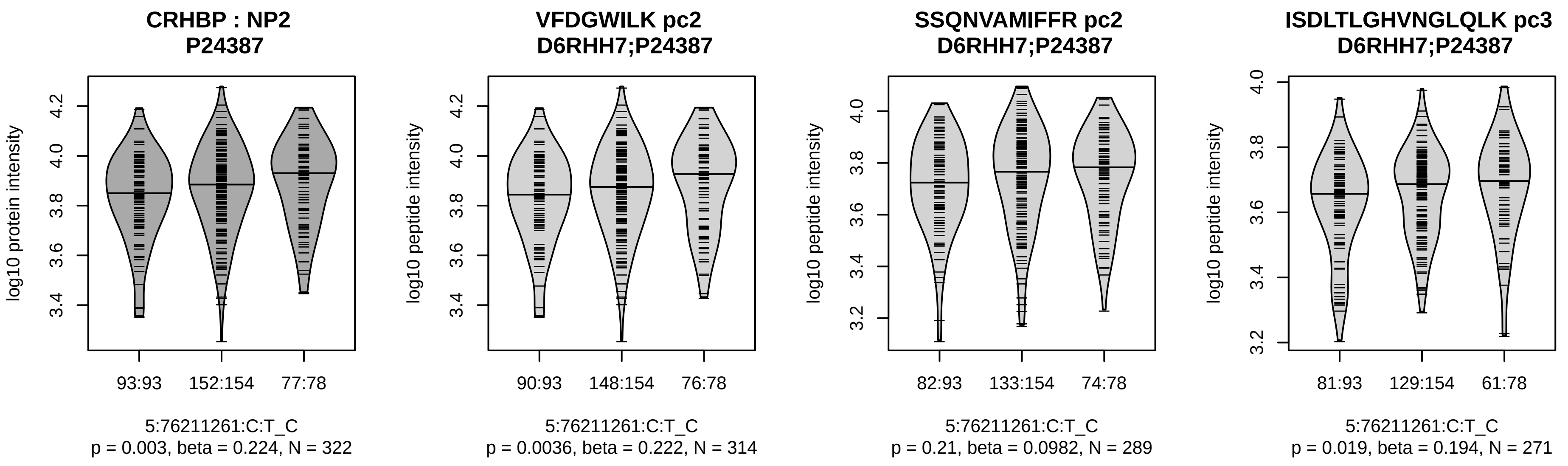

Assay Target: CRHBP
UniProt: P24387
rsID: rs1700657
Target SNP: 5:76211261:C:T
Proxy SNP: 5:76211261:C:T
UKBPPP log10(p): 1809.2
UKBPPP BETA: −0.569
UKBPPP MSPA: 0.496
:−:−::−
1061:1094:679:863:181

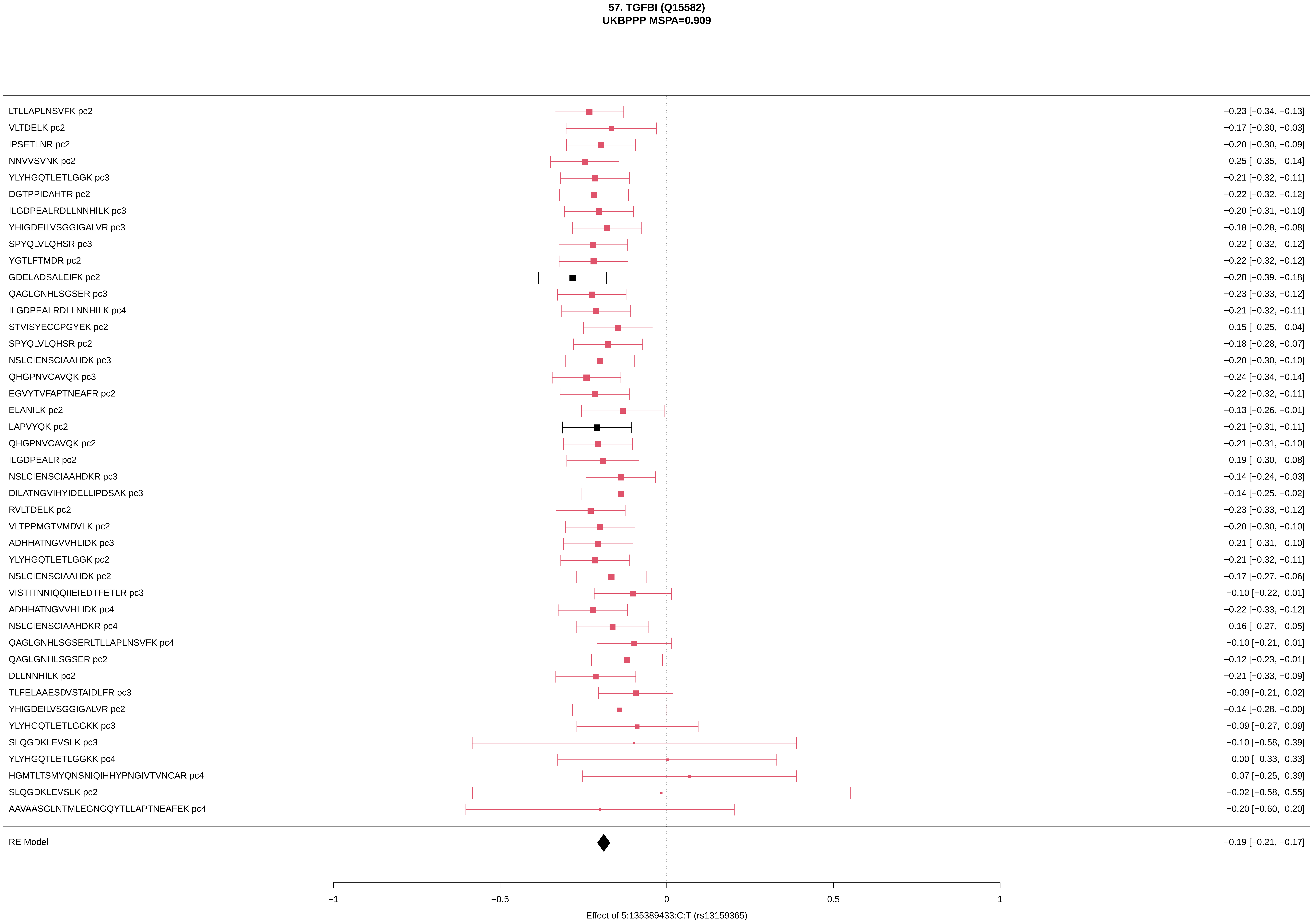
57. TGFBI (Q15582)
UKBPPP MSPA=0.909
LTLLAPLNSVFK pc2 −0.23 [−0.34, −0.13]
VLTDELK pc2 −0.17 [−0.30, −0.03]
IPSETLNR pc2 −0.20 [−0.30, −0.09]
NNVVSVNK pc2 −0.25 [−0.35, −0.14]
YLYHGQTLETLGGK pc3 −0.21 [−0.32, −0.11]
DGTPPIDAHTR pc2 −0.22 [−0.32, −0.12]
ILGDPEALRDLLNNHILK pc3 −0.20 [−0.31, −0.10]
YHIGDEILVSGGIGALVR pc3 −0.18 [−0.28, −0.08]
SPYQLVLQHSR pc3 −0.22 [−0.32, −0.12]
YGTLFTMDR pc2 −0.22 [−0.32, −0.12]
GDELADSALEIFK pc2 −0.28 [−0.39, −0.18]
QAGLGNHLSGSER pc3 −0.23 [−0.33, −0.12]
ILGDPEALRDLLNNHILK pc4 −0.21 [−0.32, −0.11]
STVISYECCPGYEK pc2 −0.15 [−0.25, −0.04]
SPYQLVLQHSR pc2 −0.18 [−0.28, −0.07]
NSLCIENSCIAAHDK pc3 −0.20 [−0.30, −0.10]
QHGPNVCAVQK pc3 −0.24 [−0.34, −0.14]
EGVYTVFAPTNEAFR pc2 −0.22 [−0.32, −0.11]
ELANILK pc2 −0.13 [−0.26, −0.01]
LAPVYQK pc2 −0.21 [−0.31, −0.11]
QHGPNVCAVQK pc2 −0.21 [−0.31, −0.10]
ILGDPEALR pc2 −0.19 [−0.30, −0.08]
NSLCIENSCIAAHDKR pc3 −0.14 [−0.24, −0.03]
DILATNGVIHYIDELLIPDSAK pc3 −0.14 [−0.25, −0.02]
RVLTDELK pc2 −0.23 [−0.33, −0.12]
VLTPPMGTVMDVLK pc2 −0.20 [−0.30, −0.10]
ADHHATNGVVHLIDK pc3 −0.21 [−0.31, −0.10]
YLYHGQTLETLGGK pc2 −0.21 [−0.32, −0.11]
NSLCIENSCIAAHDK pc2 −0.17 [−0.27, −0.06]
VISTITNNIQQIIEIEDTFETLR pc3 −0.10 [−0.22, 0.01]
ADHHATNGVVHLIDK pc4 −0.22 [−0.33, −0.12]
NSLCIENSCIAAHDKR pc4 −0.16 [−0.27, −0.05]
QAGLGNHLSGSERLTLLAPLNSVFK pc4 −0.10 [−0.21, 0.01]
QAGLGNHLSGSER pc2 −0.12 [−0.23, −0.01]
DLLNNHILK pc2 −0.21 [−0.33, −0.09]
TLFELAAESDVSTAIDLFR pc3 −0.09 [−0.21, 0.02]
YHIGDEILVSGGIGALVR pc2 −0.14 [−0.28, −0.00]
YLYHGQTLETLGGKK pc3 −0.09 [−0.27, 0.09]
SLQGDKLEVSLK pc3 −0.10 [−0.58, 0.39]
YLYHGQTLETLGGKK pc4 0.00 [−0.33, 0.33]
HGMTLTSMYQNSNIQIHHYPNGIVTVNCAR pc4 0.07 [−0.25, 0.39]
SLQGDKLEVSLK pc2 −0.02 [−0.58, 0.55]
AAVAASGLNTMLEGNGQYTLLAPTNEAFEK pc4 −0.20 [−0.60, 0.20]
RE Model −0.19 [−0.21, −0.17]
−1
−0.5
0
0.5
1
Effect of 5:135389433:C:T (rs13159365)

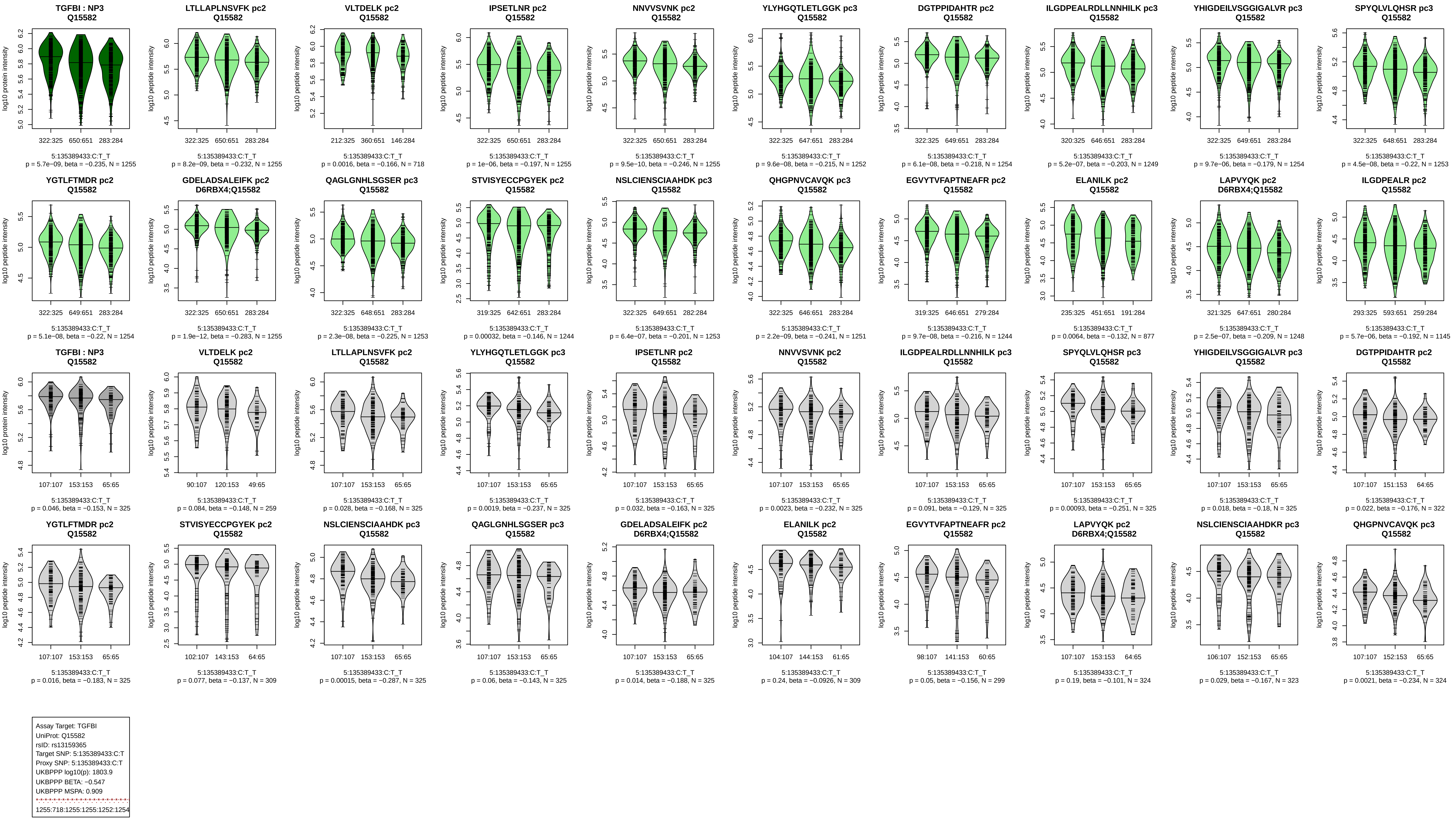

TGFBI : NP3 Q15582
5:135389433:C:T_T p = 5.7e−09, beta = −0.235, N = 1255
LTLLAPLNSVFK pc2 Q15582
5:135389433:C:T_T p = 8.2e−09, beta = −0.232, N = 1255
VLTDELK pc2 Q15582
5:135389433:C:T_T p = 0.0016, beta = −0.166, N = 718
IPSETLNR pc2 Q15582
5:135389433:C:T_T p = 1e−06, beta = −0.197, N = 1255
NNVVSVNK pc2 Q15582
5:135389433:C:T_T p = 9.5e−10, beta = −0.246, N = 1255
YLYHGQTLETLGGK pc3 Q15582
5:135389433:C:T_T p = 9.6e−08, beta = −0.215, N = 1252
DGTPPIDAHTR pc2 Q15582
5:135389433:C:T_T p = 6.1e−08, beta = −0.218, N = 1254
ILGDPEALRDLLNNHILK pc3 Q15582
5:135389433:C:T_T p = 5.2e−07, beta = −0.203, N = 1249
YHIGDEILVSGGIGALVR pc3 Q15582
5:135389433:C:T_T p = 9.7e−06, beta = −0.179, N = 1254
SPYQLVLQHSR pc3 Q15582
5:135389433:C:T_T p = 4.5e−08, beta = −0.22, N = 1253
YGTLFTMDR pc2 Q15582
5:135389433:C:T_T p = 5.1e−08, beta = −0.22, N = 1254
GDELADSALEIFK pc2 D6RBX4;Q15582
5:135389433:C:T_T p = 1.9e−12, beta = −0.283, N = 1255
QAGLGNHLSGSER pc3 Q15582
5:135389433:C:T_T p = 2.3e−08, beta = −0.225, N = 1253
STVISYECCPGYEK pc2 Q15582
5:135389433:C:T_T p = 0.00032, beta = −0.146, N = 1244
NSLCIENSCIAAHDK pc3 Q15582
5:135389433:C:T_T p = 6.4e−07, beta = −0.201, N = 1253
QHGPNVCAVQK pc3 Q15582
5:135389433:C:T_T p = 2.2e−09, beta = −0.241, N = 1251
EGVYTVFAPTNEAFR pc2 Q15582
5:135389433:C:T_T p = 9.7e−08, beta = −0.216, N = 1244
ELANILK pc2 Q15582
5:135389433:C:T_T p = 0.0064, beta = −0.132, N = 877
LAPVYQK pc2 D6RBX4;Q15582
5:135389433:C:T_T p = 2.5e−07, beta = −0.209, N = 1248
ILGDPEALR pc2 Q15582
5:135389433:C:T_T p = 5.7e−06, beta = −0.192, N = 1145
TGFBI : NP3 Q15582
5:135389433:C:T_T p = 0.046, beta = −0.153, N = 325
VLTDELK pc2 Q15582
5:135389433:C:T_T p = 0.084, beta = −0.148, N = 259
LTLLAPLNSVFK pc2 Q15582
5:135389433:C:T_T p = 0.028, beta = −0.168, N = 325
YLYHGQTLETLGGK pc3 Q15582
5:135389433:C:T_T p = 0.0019, beta = −0.237, N = 325
IPSETLNR pc2 Q15582
5:135389433:C:T_T p = 0.032, beta = −0.163, N = 325
NNVVSVNK pc2 Q15582
5:135389433:C:T_T p = 0.0023, beta = −0.232, N = 325
ILGDPEALRDLLNNHILK pc3 Q15582
5:135389433:C:T_T p = 0.091, beta = −0.129, N = 325
SPYQLVLQHSR pc3 Q15582
5:135389433:C:T_T p = 0.00093, beta = −0.251, N = 325
YHIGDEILVSGGIGALVR pc3 Q15582
5:135389433:C:T_T p = 0.018, beta = −0.18, N = 325
DGTPPIDAHTR pc2 Q15582
5:135389433:C:T_T p = 0.022, beta = −0.176, N = 322
YGTLFTMDR pc2 Q15582
5:135389433:C:T_T p = 0.016, beta = −0.183, N = 325
STVISYECCPGYEK pc2 Q15582
5:135389433:C:T_T p = 0.077, beta = −0.137, N = 309
NSLCIENSCIAAHDK pc3 Q15582
5:135389433:C:T_T p = 0.00015, beta = −0.287, N = 325
QAGLGNHLSGSER pc3 Q15582
5:135389433:C:T_T p = 0.06, beta = −0.143, N = 325
GDELADSALEIFK pc2 D6RBX4;Q15582
5:135389433:C:T_T p = 0.014, beta = −0.188, N = 325
ELANILK pc2 Q15582
5:135389433:C:T_T p = 0.24, beta = −0.0926, N = 309
EGVYTVFAPTNEAFR pc2 Q15582
5:135389433:C:T_T p = 0.05, beta = −0.156, N = 299
LAPVYQK pc2 D6RBX4;Q15582
5:135389433:C:T_T p = 0.19, beta = −0.101, N = 324
NSLCIENSCIAAHDKR pc3 Q15582
5:135389433:C:T_T p = 0.029, beta = −0.167, N = 323
QHGPNVCAVQK pc3 Q15582
5:135389433:C:T_T p = 0.0021, beta = −0.234, N = 324
Assay Target: TGFBI
UniProt: Q15582
rsID: rs13159365
Target SNP: 5:135389433:C:T
Proxy SNP: 5:135389433:C:T
UKBPPP log10(p): 1803.9
UKBPPP BETA: −0.547
UKBPPP MSPA: 0.909
1255:718:1255:1255:1252:1254

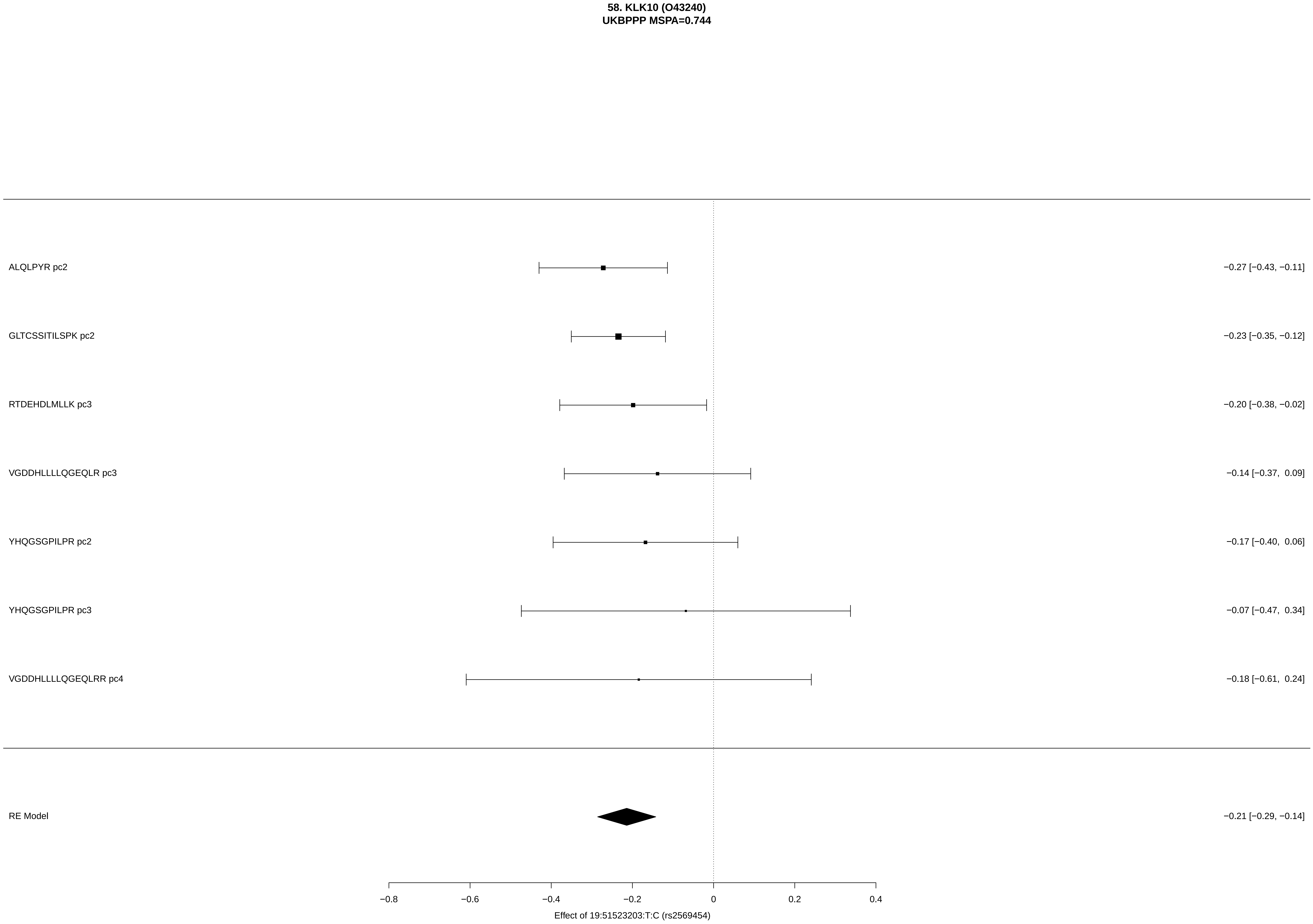

58. KLK10 (O43240)
UKBPPP MSPA=0.744
ALQLPYR pc2
−0.27 [−0.43, −0.11]
GLTCSSITILSPK pc2
−0.23 [−0.35, −0.12]
RTDEHDLMLLK pc3
−0.20 [−0.38, −0.02]
VGDDHLLLLQGEQLR pc3
−0.14 [−0.37, 0.09]
YHQGSGPILPR pc2
−0.17 [−0.40, 0.06]
YHQGSGPILPR pc3
−0.07 [−0.47, 0.34]
VGDDHLLLLQGEQLRR pc4
−0.18 [−0.61, 0.24]
RE Model
−0.21 [−0.29, −0.14]
−0.8
−0.6
−0.4
−0.2
0
0.2
0.4
Effect of 19:51523203:T:C (rs2569454)

KLK10 : NP3
O43240

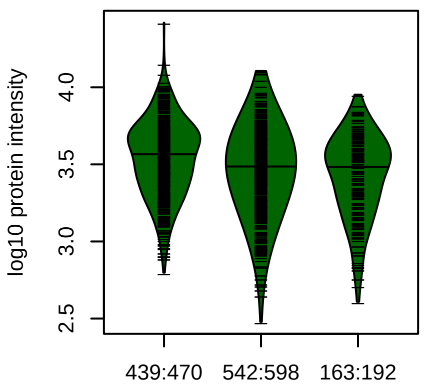

19:51523203:T:C_T
p = 6.1e−07, beta = −0.213, N = 1144

ALQLPYR pc2
O43240

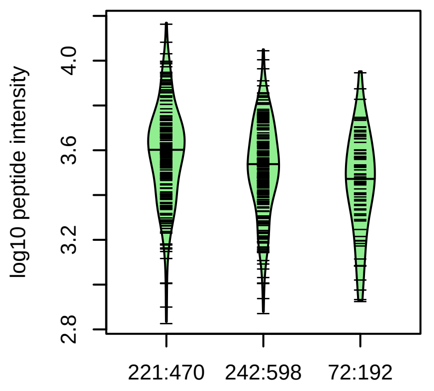

19:51523203:T:C_T
p = 1.2e−05, beta = −0.272, N = 535

GLTCSSITILSPK pc2
O43240

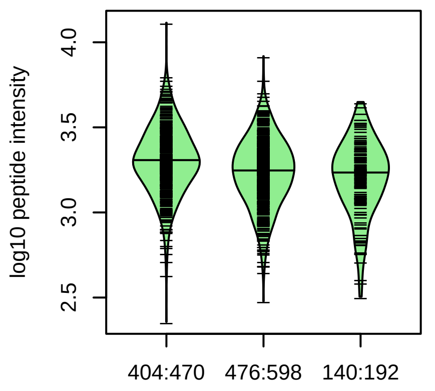

19:51523203:T:C_T
p = 2.3e−07, beta = −0.235, N = 1020

RTDEHDLMLLK pc3
O43240

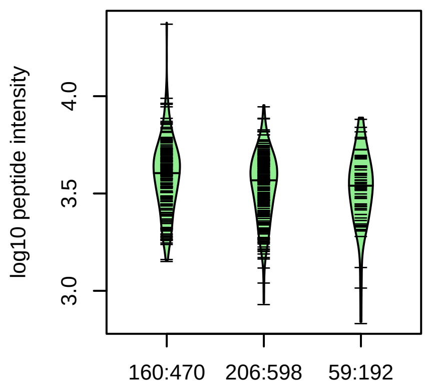

19:51523203:T:C_T
p = 0.005, beta = −0.198, N = 425

VGDDHLLLLQGEQLR pc3
O43240

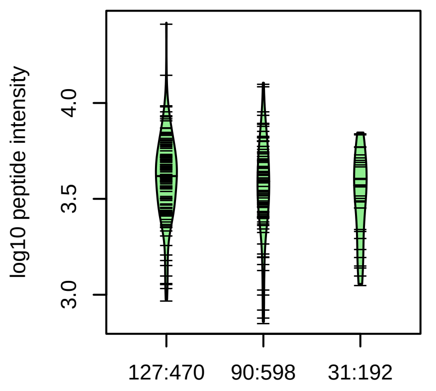

19:51523203:T:C_T
p = 0.12, beta = −0.138, N = 248

YHQGSGPILPR pc2
O43240

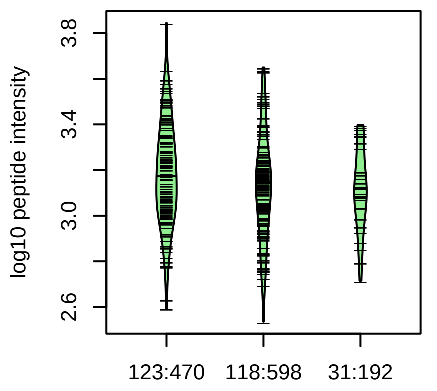

19:51523203:T:C_T
p = 0.059, beta = −0.168, N = 272

VGDDHLLLLQGEQLRR pc4
O43240

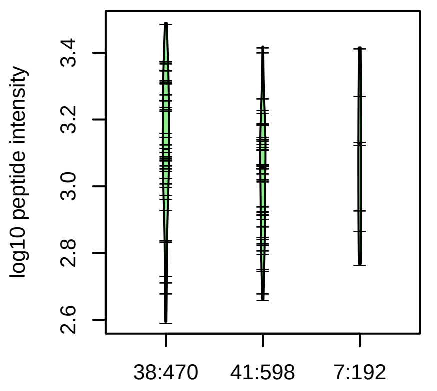

19:51523203:T:C_T
p = 0.27, beta = −0.184, N = 86

Assay Target: KLK10
UniProt: O43240
rsID: rs2569454
Target SNP: 19:51523203:T:C
Proxy SNP: 19:51523203:T:C
UKBPPP log10(p): 1762.7
UKBPPP BETA: 0.576
UKBPPP MSPA: 0.744
::*:−:−:−:−:NA:NA
535:1020:425:248:272:76:86:4:5

LDPEAYGAPCAR pc2
rs3745535 ALT

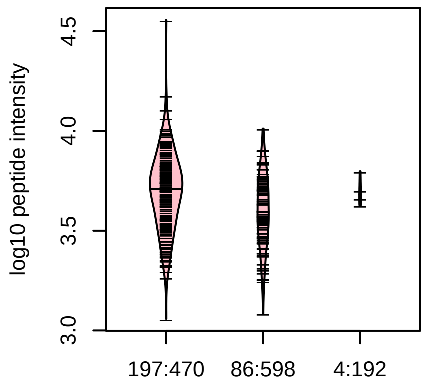

19:51523203:T:C_T
p = 6.1e−35, model = REC, N = 287

59. LGALS3 (P17931)
UKBPPP MSPA=0

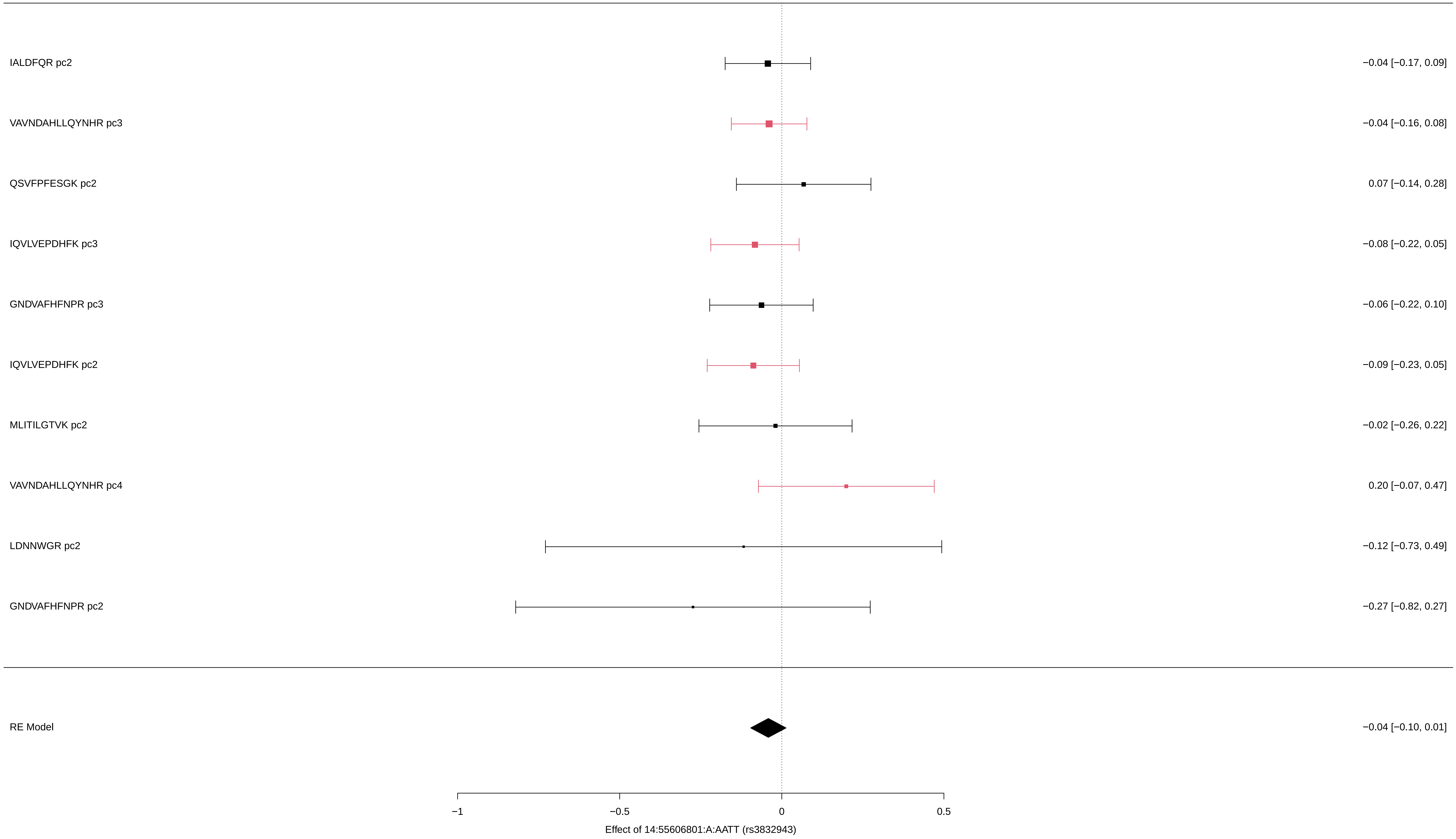

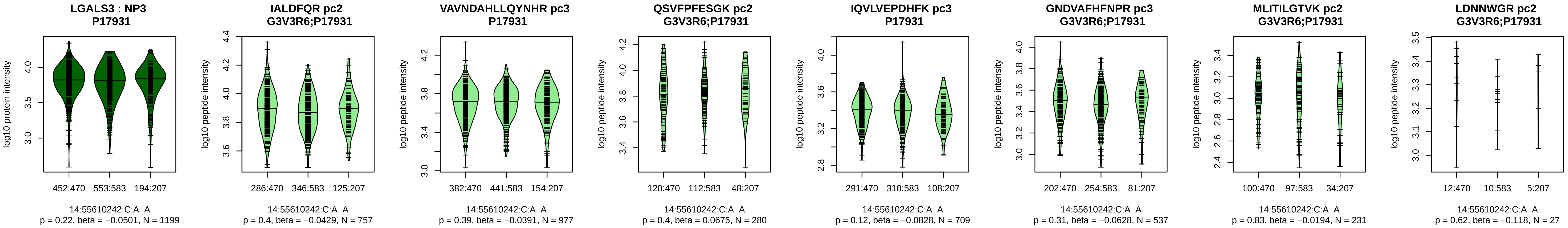

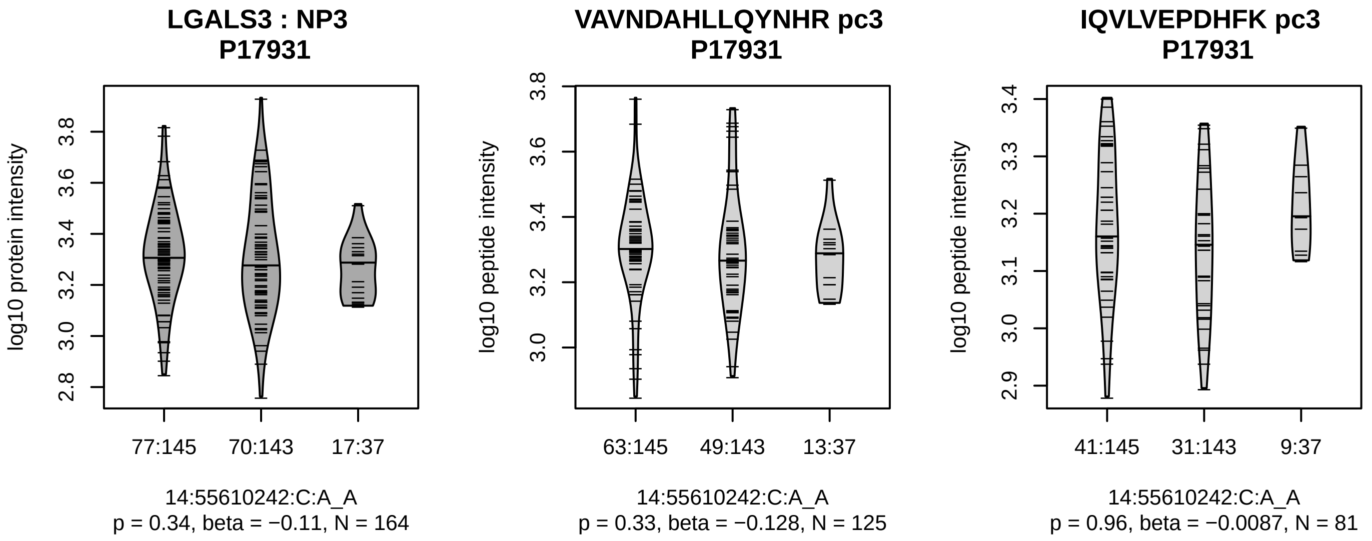

Assay Target: LGALS3
UniProt: P17931
rsID: rs3832943
Target SNP: 14:55606801:A:AA
Proxy SNP: 14:55610242:C:A
UKBPPP log10(p): 1725.9
UKBPPP BETA: −0.526
UKBPPP MSPA: 0
–:–:–:–:–:–:–:–
757:977:280:709:537:658:231:1

60. IL16 (Q14005)
UKBPPP MSPA=0

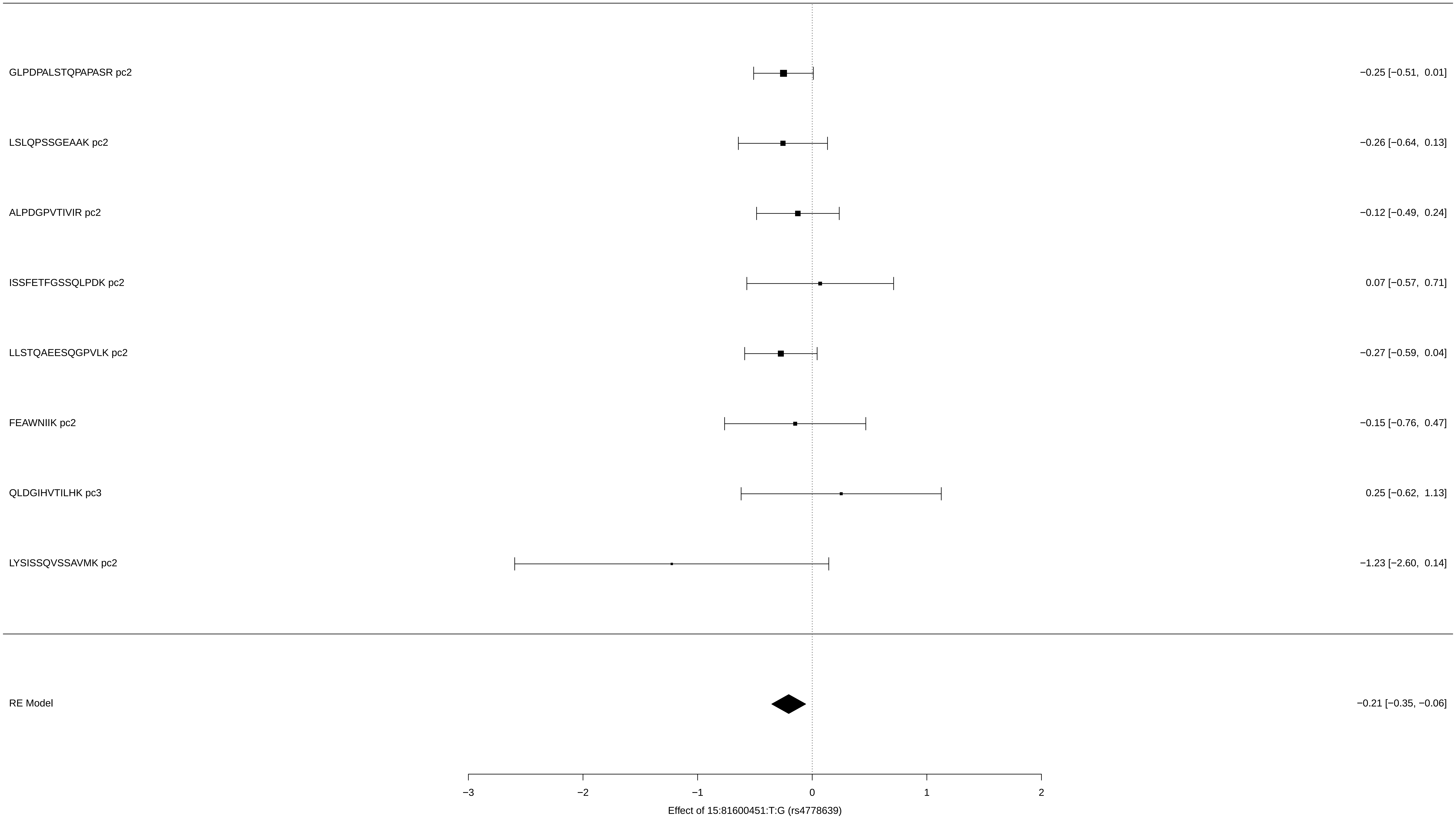

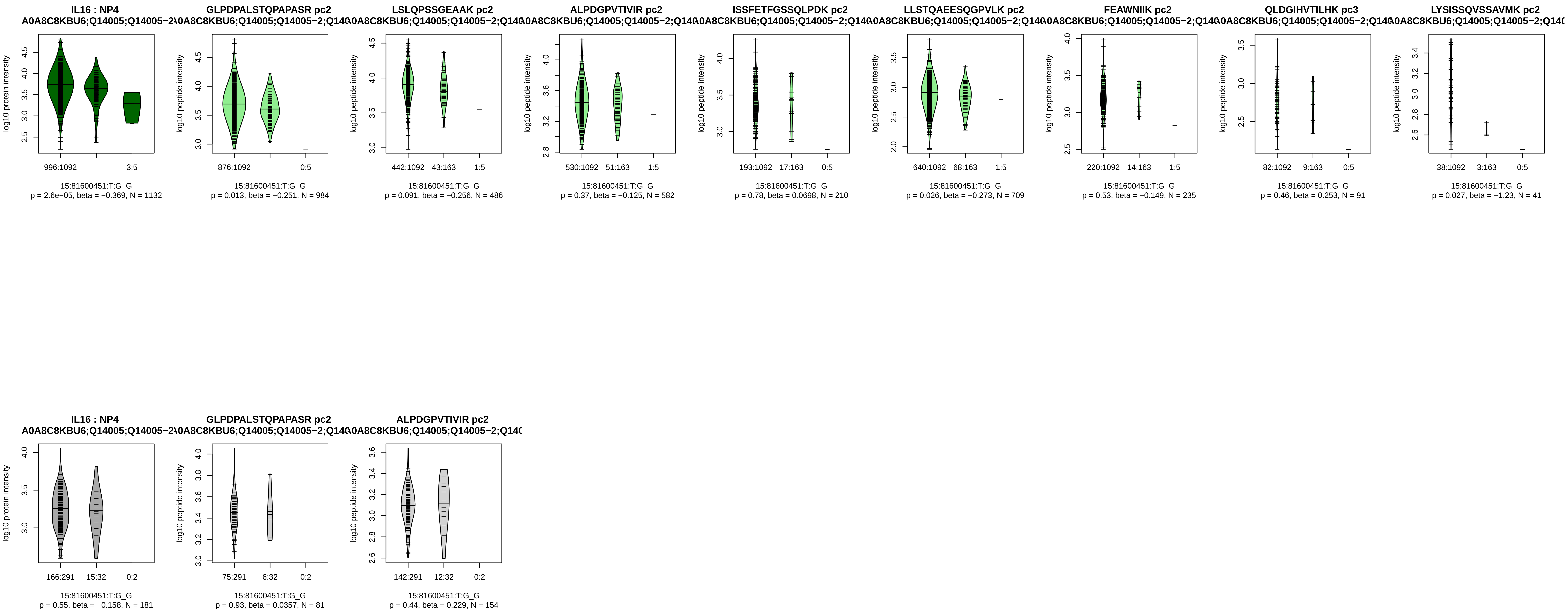

Assay Target: IL16
UniProt: Q14005
rsID: rs4778639
Target SNP: 15:81600451:T:G
Proxy SNP: 15:81600451:T:G
UKBPPP log10(p): 1708.9
UKBPPP BETA: −0.944
UKBPPP MSPA: 0
−:−:−:−:−:−:−:−
984:486:582:210:709:235:91:41

61. LPA (P08519)
UKBPPP MSPA=0.859

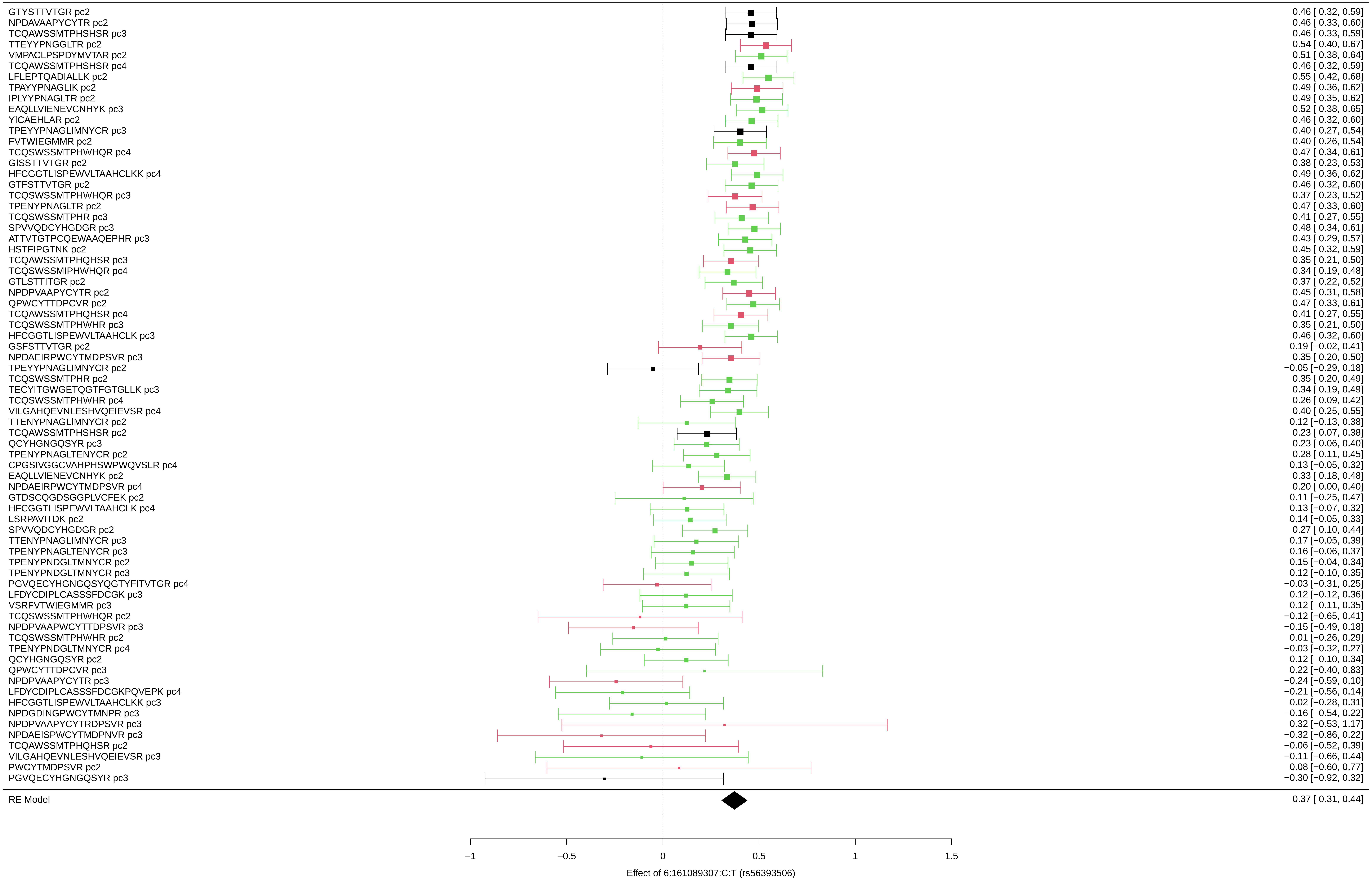

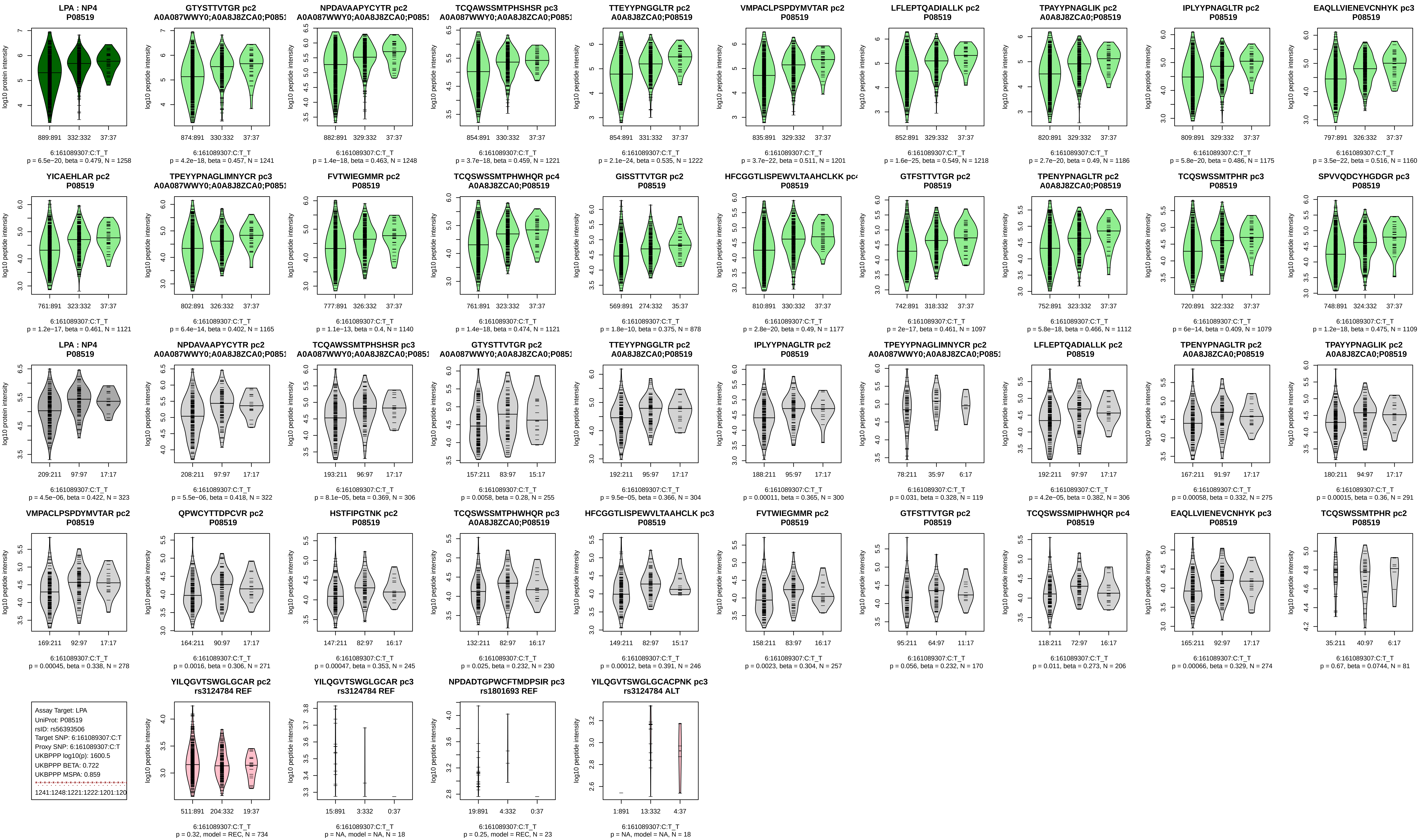

LPA : NP4 P08519
889:891 332:332 37:37
6:161089307:C:T_T
p = 6.5e−20, beta = 0.479, N = 1258
GTYSTTVTGR pc2 A0A087WWY0;A0A8J8ZCA0;P0851
874:891 330:332 37:37
6:161089307:C:T_T
p = 4.2e−18, beta = 0.457, N = 1241
NPDAVAAPYCYTR pc2 A0A087WWY0;A0A8J8ZCA0;P0851
882:891 329:332 37:37
6:161089307:C:T_T
p = 1.4e−18, beta = 0.463, N = 1248
TCQAWSSMTPHSHSR pc3 A0A087WWY0;A0A8J8ZCA0;P0851
854:891 330:332 37:37
6:161089307:C:T_T
p = 3.7e−18, beta = 0.459, N = 1221
TTEYYPNGGLTR pc2 A0A8J8ZCA0;P08519
854:891 331:332 37:37
6:161089307:C:T_T
p = 2.1e−24, beta = 0.535, N = 1222
VMPACLPSPDYMVTAR pc2 P08519
835:891 329:332 37:37
6:161089307:C:T_T
p = 3.7e−22, beta = 0.511, N = 1201
LFLEPTQADIALLK pc2 P08519
852:891 329:332 37:37
6:161089307:C:T_T
p = 1.6e−25, beta = 0.549, N = 1218
TPAYYPNAGLIK pc2 A0A8J8ZCA0;P08519
820:891 329:332 37:37
6:161089307:C:T_T
p = 2.7e−20, beta = 0.49, N = 1186
IPLYYPNAGLTR pc2 P08519
809:891 329:332 37:37
6:161089307:C:T_T
p = 5.8e−20, beta = 0.486, N = 1175
EAQLLVIENEVCNHYK pc3 P08519
797:891 326:332 37:37
6:161089307:C:T_T
p = 3.5e−22, beta = 0.516, N = 1160
YICAEHLAR pc2 P08519
761:891 323:332 37:37
6:161089307:C:T_T
p = 1.2e−17, beta = 0.461, N = 1121
TPEYYPNAGLIMNYCR pc3 A0A087WWY0;A0A8J8ZCA0;P0851
802:891 326:332 37:37
6:161089307:C:T_T
p = 6.4e−14, beta = 0.402, N = 1165
FVTWIEGMMR pc2 P08519
777:891 326:332 37:37
6:161089307:C:T_T
p = 1.1e−13, beta = 0.4, N = 1140
TCQSWSSMTPHWHQR pc4 A0A8J8ZCA0;P08519
761:891 323:332 37:37
6:161089307:C:T_T
p = 1.4e−18, beta = 0.474, N = 1121
GISSTTVTGR pc2 P08519
569:891 274:332 35:37
6:161089307:C:T_T
p = 1.8e−10, beta = 0.375, N = 878
HFCGGTLISPEWVLTAAHCLKK pc4 P08519
810:891 330:332 37:37
6:161089307:C:T_T
p = 2.8e−20, beta = 0.49, N = 1177
GTFSTTVTGR pc2 P08519
742:891 318:332 37:37
6:161089307:C:T_T
p = 2e−17, beta = 0.461, N = 1097
TPENYPNAGLTR pc2 A0A8J8ZCA0;P08519
752:891 323:332 37:37
6:161089307:C:T_T
p = 5.8e−18, beta = 0.466, N = 1112
TCQSWSSMTPHR pc3 P08519
720:891 322:332 37:37
6:161089307:C:T_T
p = 6e−14, beta = 0.409, N = 1079
SPVVQDCYHGDGR pc3 P08519
748:891 324:332 37:37
6:161089307:C:T_T
p = 1.2e−18, beta = 0.475, N = 1109
LPA : NP4 P08519
209:211 97:97 17:17
6:161089307:C:T_T
p = 4.5e−06, beta = 0.422, N = 323
NPDAVAAPYCYTR pc2 A0A087WWY0;A0A8J8ZCA0;P0851
208:211 97:97 17:17
6:161089307:C:T_T
p = 5.5e−06, beta = 0.418, N = 322
TCQAWSSMTPHSHSR pc3 A0A087WWY0;A0A8J8ZCA0;P0851
193:211 96:97 17:17
6:161089307:C:T_T
p = 8.1e−05, beta = 0.369, N = 306
GTYSTTVTGR pc2 A0A087WWY0;A0A8J8ZCA0;P0851
157:211 83:97 15:17
6:161089307:C:T_T
p = 0.0058, beta = 0.28, N = 255
TTEYYPNGGLTR pc2 A0A8J8ZCA0;P08519
192:211 95:97 17:17
6:161089307:C:T_T
p = 9.5e−05, beta = 0.366, N = 304
IPLYYPNAGLTR pc2 P08519
188:211 95:97 17:17
6:161089307:C:T_T
p = 0.00011, beta = 0.365, N = 300
TPEYYPNAGLIMNYCR pc2 A0A087WWY0;A0A8J8ZCA0;P0851
78:211 35:97 6:17
6:161089307:C:T_T
p = 0.031, beta = 0.328, N = 119
LFLEPTQADIALLK pc2 P08519
192:211 97:97 17:17
6:161089307:C:T_T
p = 4.2e−05, beta = 0.382, N = 306
TPENYPNAGLTR pc2 A0A8J8ZCA0;P08519
167:211 91:97 17:17
6:161089307:C:T_T
p = 0.00058, beta = 0.332, N = 275
TPAYYPNAGLIK pc2 A0A8J8ZCA0;P08519
180:211 94:97 17:17
6:161089307:C:T_T
p = 0.00015, beta = 0.36, N = 291
VMPACLPSPDYMVTAR pc2 P08519
169:211 92:97 17:17
6:161089307:C:T_T
p = 0.00045, beta = 0.338, N = 278
QPWCYTTDPCVR pc2 P08519
164:211 90:97 17:17
6:161089307:C:T_T
p = 0.0016, beta = 0.306, N = 271
HSTFIPGTNK pc2 P08519
147:211 82:97 16:17
6:161089307:C:T_T
p = 0.00047, beta = 0.353, N = 245
TCQSWSSMTPHWHQR pc3 A0A8J8ZCA0;P08519
132:211 82:97 16:17
6:161089307:C:T_T
p = 0.025, beta = 0.232, N = 230
HFCGGTLISPEWVLTAAHCLK pc3 P08519
149:211 82:97 15:17
6:161089307:C:T_T
p = 0.00012, beta = 0.391, N = 246
FVTWIEGMMR pc2 P08519
158:211 83:97 16:17
6:161089307:C:T_T
p = 0.0023, beta = 0.304, N = 257
GTFSTTVTGR pc2 P08519
95:211 64:97 11:17
6:161089307:C:T_T
p = 0.056, beta = 0.232, N = 170
TCQSWSSMIPHWHQR pc4 P08519
118:211 72:97 16:17
6:161089307:C:T_T
p = 0.011, beta = 0.273, N = 206
EAQLLVIENEVCNHYK pc3 P08519
165:211 92:97 17:17
6:161089307:C:T_T
p = 0.00066, beta = 0.329, N = 274
TCQSWSSMTPHR pc2 P08519
35:211 40:97 6:17
6:161089307:C:T_T
p = 0.67, beta = 0.0744, N = 81
Assay Target: LPA
UniProt: P08519
rsID: rs56393506
Target SNP: 6:161089307:C:T
Proxy SNP: 6:161089307:C:T
UKBPPP log10(p): 1600.5
UKBPPP BETA: 0.722
UKBPPP MSPA: 0.859
1241:1248:1221:1222:1201:120
YILQGVTSWGLGCAR pc2 rs3124784 REF
511:891 204:332 19:37
6:161089307:C:T_T
p = 0.32, model = REC, N = 734
YILQGVTSWGLGCAR pc3 rs3124784 REF
15:891 3:332 0:37
6:161089307:C:T_T
p = NA, model = NA, N = 18
NPDADTGPWCFTMDPSIR pc3 rs1801693 REF
19:891 4:332 0:37
6:161089307:C:T_T
p = 0.25, model = REC, N = 23
YILQGVTSWGLGCACPNK pc3 rs3124784 ALT
1:891 13:332 4:37
6:161089307:C:T_T
p = NA, model = NA, N = 18
log10 protein intensity
log10 peptide intensity

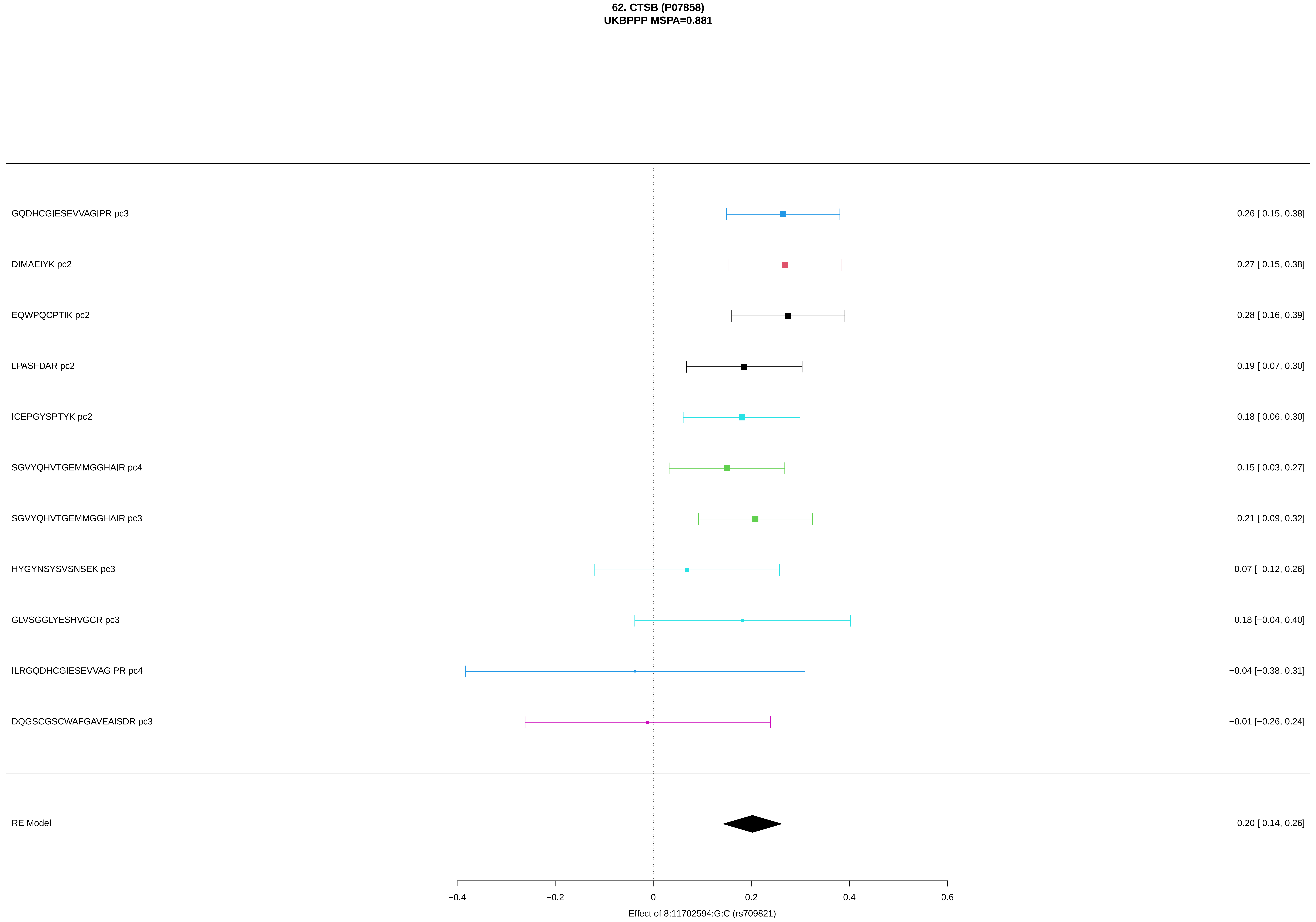
62. CTSB (P07858)
UKBPPP MSPA=0.881
GQDHCGIESEVVAGIPR pc3
0.26 [0.15, 0.38]
DIMAEIYK pc2
0.27 [0.15, 0.38]
EQWPQCPTIK pc2
0.28 [0.16, 0.39]
LPASFDAR pc2
0.19 [0.07, 0.30]
ICEPGYSPTYK pc2
0.18 [0.06, 0.30]
SGVYQHVTGEMMGGHAIR pc4
0.15 [0.03, 0.27]
SGVYQHVTGEMMGGHAIR pc3
0.21 [0.09, 0.32]
HYGYNSYSVSNSEK pc3
0.07 [−0.12, 0.26]
GLVSGGLYESHVGCR pc3
0.18 [−0.04, 0.40]
ILRGQDHCGIESEVVAGIPR pc4
−0.04 [−0.38, 0.31]
DQGSCGSCWAFGAVEAISDR pc3
−0.01 [−0.26, 0.24]
RE Model
0.20 [0.14, 0.26]
−0.4
−0.2
0
0.2
0.4
0.6
Effect of 8:11702594:G:C (rs709821)

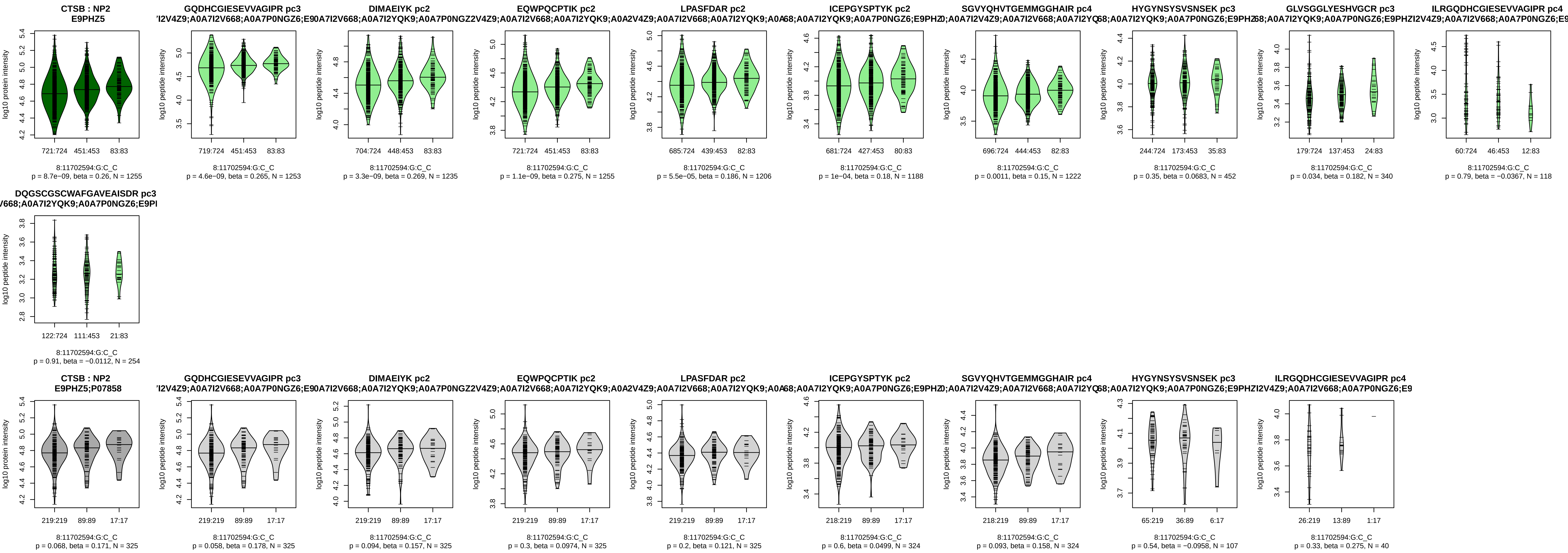

Assay Target: CTSB
UniProt: P07858
rsID: rs709821
Target SNP: 8:11702594:G:C
Proxy SNP: 8:11702594:G:C
UKBPPP log10(p): 1581.4
UKBPPP BETA: 0.591
UKBPPP MSPA: 0.881
::*:*:*:*:-:-:-:-:NA
1253:1235:1255:1206:1188:122

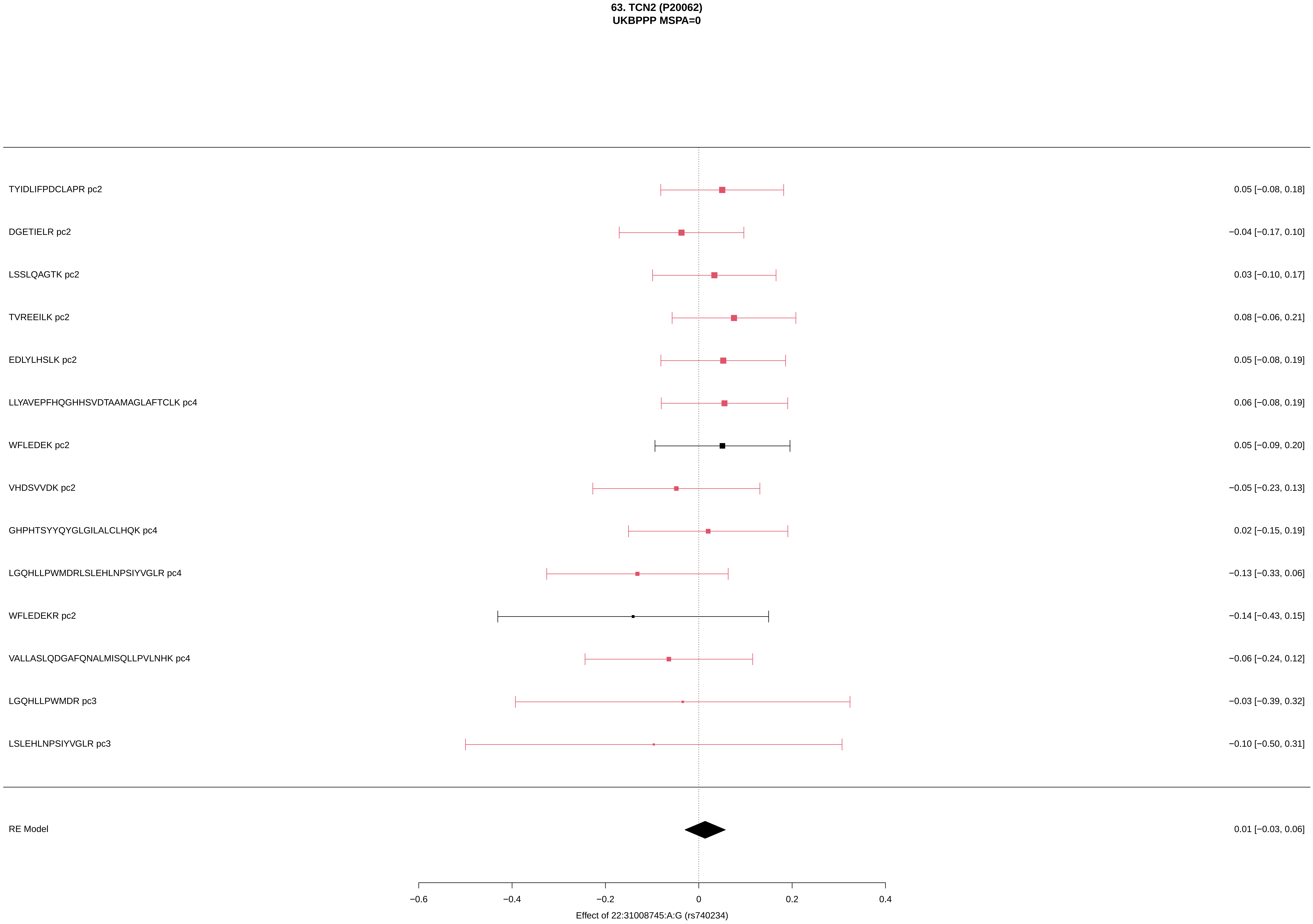

63. TCN2 (P20062)
UKBPPP MSPA=0
TYIDLIFPDCLAPR pc2
0.05 [−0.08, 0.18]
DGETIELR pc2
−0.04 [−0.17, 0.10]
LSSLQAGTK pc2
0.03 [−0.10, 0.17]
TVREEILK pc2
0.08 [−0.06, 0.21]
EDLYLHSLK pc2
0.05 [−0.08, 0.19]
LLYAVEPFHQGHHSVDTAAMAGLAFTCLK pc4
0.06 [−0.08, 0.19]
WFLEDEK pc2
0.05 [−0.09, 0.20]
VHDSVVDK pc2
−0.05 [−0.23, 0.13]
GHPHTSYYQYGLGILALCLHQK pc4
0.02 [−0.15, 0.19]
LGQHLLPWMDRLSLEHLNPSIYVGLR pc4
−0.13 [−0.33, 0.06]
WFLEDEKR pc2
−0.14 [−0.43, 0.15]
VALLASLQDGAFQNALMISQLLPVLNHK pc4
−0.06 [−0.24, 0.12]
LGQHLLPWMDR pc3
−0.03 [−0.39, 0.32]
LSLEHLNPSIYVGLR pc3
−0.10 [−0.50, 0.31]
RE Model
0.01 [−0.03, 0.06]
−0.6
−0.4
−0.2
0
0.2
0.4
Effect of 22:31008745:A:G (rs740234)

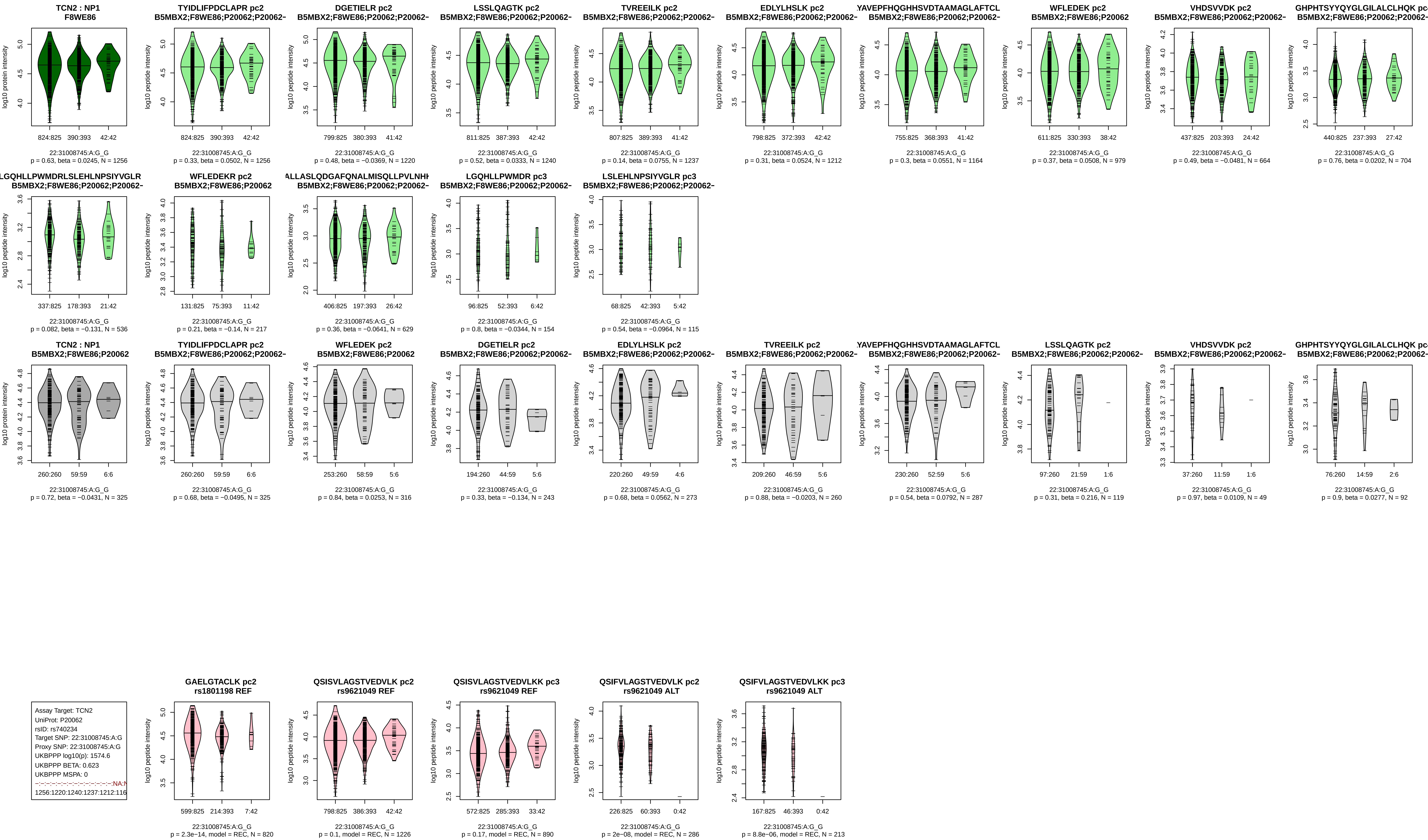
TCN2 : NP1
F8WE86
22:31008745:A:G_G
p = 0.63, beta = 0.0245, N = 1256
TYIDLIFPDCLAPR pc2
22:31008745:A:G_G
p = 0.33, beta = 0.0502, N = 1256
DGETIELR pc2
22:31008745:A:G_G
p = 0.48, beta = −0.0369, N = 1220
LSSLQAGTK pc2
22:31008745:A:G_G
p = 0.52, beta = 0.0333, N = 1240
TVREEILK pc2
22:31008745:A:G_G
p = 0.14, beta = 0.0755, N = 1237
EDLYLHSLK pc2
22:31008745:A:G_G
p = 0.31, beta = 0.0524, N = 1212
22:31008745:A:G_G
p = 0.3, beta = 0.0551, N = 1164
WFLEDEK pc2
B5MBX2;F8WE86;P20062
22:31008745:A:G_G
p = 0.37, beta = 0.0508, N = 979
VHDSVVDK pc2
22:31008745:A:G_G
p = 0.49, beta = −0.0481, N = 664
22:31008745:A:G_G
p = 0.76, beta = 0.0202, N = 704
22:31008745:A:G_G
p = 0.082, beta = −0.131, N = 536
WFLEDEKR pc2
B5MBX2;F8WE86;P20062
22:31008745:A:G_G
p = 0.21, beta = −0.14, N = 217
22:31008745:A:G_G
p = 0.36, beta = −0.0641, N = 629
LGQHLLPWMDR pc3
22:31008745:A:G_G
p = 0.8, beta = −0.0344, N = 154
LSLEHLNPSIYVGLR pc3
22:31008745:A:G_G
p = 0.54, beta = −0.0964, N = 115
TCN2 : NP1
B5MBX2;F8WE86;P20062
22:31008745:A:G_G
p = 0.72, beta = −0.0431, N = 325
TYIDLIFPDCLAPR pc2
22:31008745:A:G_G
p = 0.68, beta = −0.0495, N = 325
WFLEDEK pc2
B5MBX2;F8WE86;P20062
22:31008745:A:G_G
p = 0.84, beta = 0.0253, N = 316
DGETIELR pc2
22:31008745:A:G_G
p = 0.33, beta = −0.134, N = 243
EDLYLHSLK pc2
22:31008745:A:G_G
p = 0.68, beta = 0.0562, N = 273
TVREEILK pc2
22:31008745:A:G_G
p = 0.88, beta = −0.0203, N = 260
22:31008745:A:G_G
p = 0.54, beta = 0.0792, N = 287
LSSLQAGTK pc2
22:31008745:A:G_G
p = 0.31, beta = 0.216, N = 119
VHDSVVDK pc2
22:31008745:A:G_G
p = 0.97, beta = 0.0109, N = 49
22:31008745:A:G_G
p = 0.9, beta = 0.0277, N = 92
Assay Target: TCN2
UniProt: P20062
rsID: rs740234
Target SNP: 22:31008745:A:G
Proxy SNP: 22:31008745:A:G
UKBPPP log10(p): 1574.6
UKBPPP BETA: 0.623
UKBPPP MSPA: 0
GAELGTACLK pc2
rs1801198 REF
22:31008745:A:G_G
p = 2.3e−14, model = REC, N = 820
QSISVLAGSTVEDVLK pc2
rs9621049 REF
22:31008745:A:G_G
p = 0.1, model = REC, N = 1226
QSISVLAGSTVEDVLKK pc3
rs9621049 REF
22:31008745:A:G_G
p = 0.17, model = REC, N = 890
QSIFVLAGSTVEDVLK pc2
rs9621049 ALT
22:31008745:A:G_G
p = 2e−08, model = REC, N = 286
QSIFVLAGSTVEDVLKK pc3
rs9621049 ALT
22:31008745:A:G_G
p = 8.8e−06, model = REC, N = 213

64. TFRC (P02786)
UKBPPP MSPA=0.337

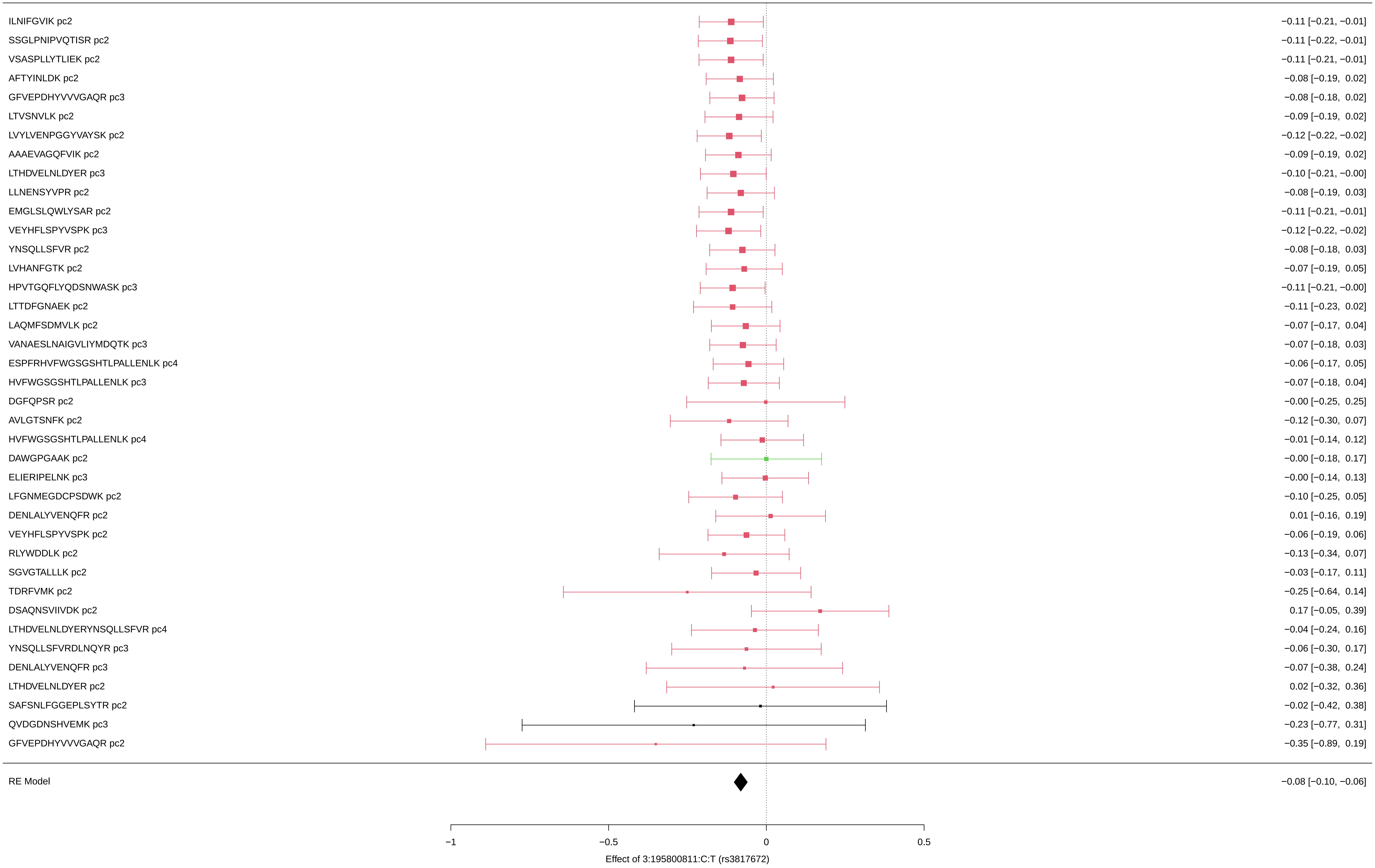

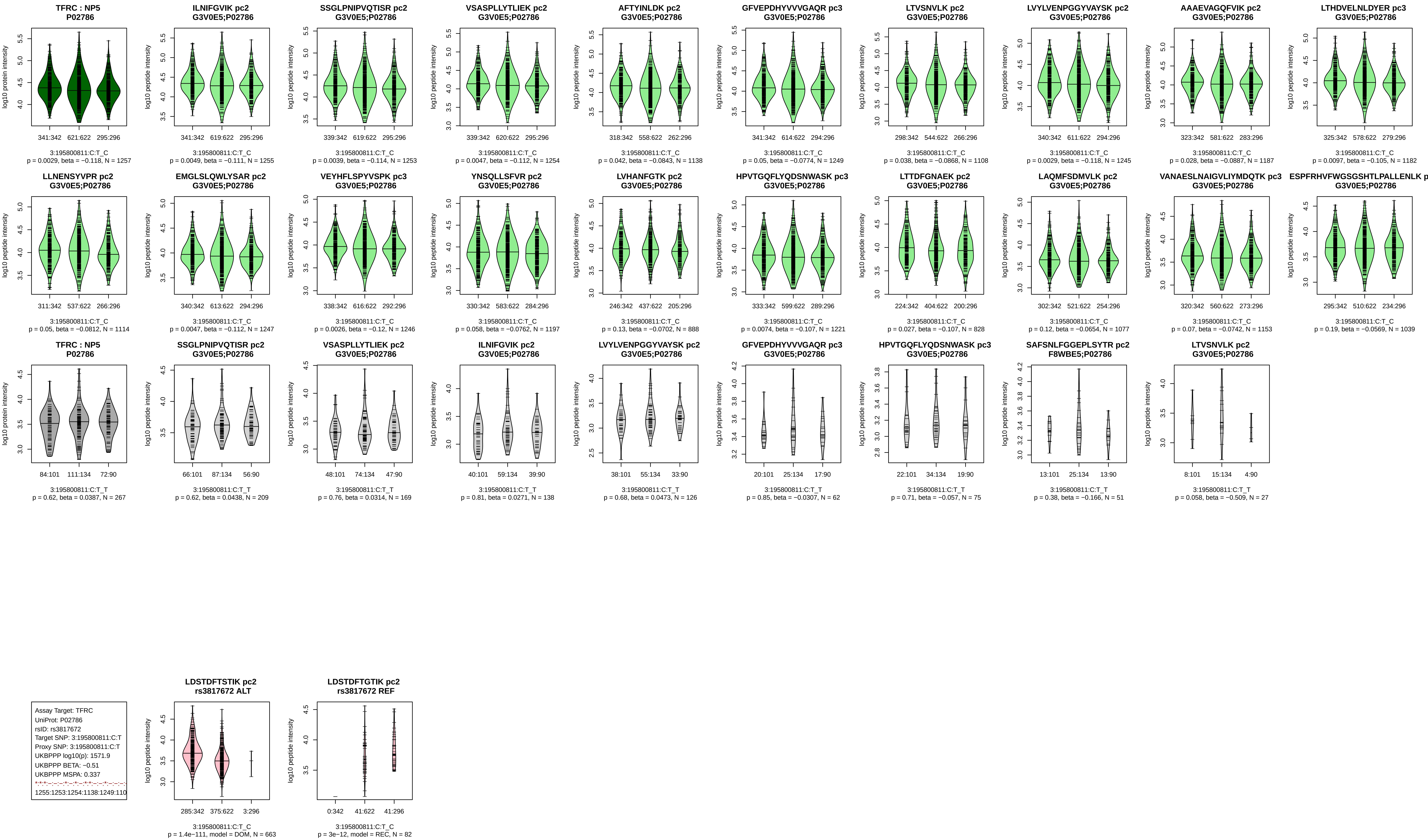
TFRC : NP5
P02786
341:342 621:622 295:296
3:195800811:C:T_C
p = 0.0029, beta = −0.118, N = 1257
ILNIFGVIK pc2
G3V0E5;P02786
341:342 619:622 295:296
3:195800811:C:T_C
p = 0.0049, beta = −0.111, N = 1255
SSGLPNIPVQTISR pc2
G3V0E5;P02786
339:342 619:622 295:296
3:195800811:C:T_C
p = 0.0039, beta = −0.114, N = 1253
VSASPLLYTLIEK pc2
G3V0E5;P02786
339:342 620:622 295:296
3:195800811:C:T_C
p = 0.0047, beta = −0.112, N = 1254
AFTYINLDK pc2
G3V0E5;P02786
318:342 558:622 262:296
3:195800811:C:T_C
p = 0.042, beta = −0.0843, N = 1138
GFVEPDHYVVVGAQR pc3
G3V0E5;P02786
341:342 614:622 294:296
3:195800811:C:T_C
p = 0.05, beta = −0.0774, N = 1249
LTVSNVLK pc2
G3V0E5;P02786
298:342 544:622 266:296
3:195800811:C:T_C
p = 0.038, beta = −0.0868, N = 1108
LVYLVENPGGYVAYSK pc2
G3V0E5;P02786
340:342 611:622 294:296
3:195800811:C:T_C
p = 0.0029, beta = −0.118, N = 1245
AAAEVAGQFVIK pc2
G3V0E5;P02786
323:342 581:622 283:296
3:195800811:C:T_C
p = 0.028, beta = −0.0887, N = 1187
LTHDVELNLDYER pc3
G3V0E5;P02786
325:342 578:622 279:296
3:195800811:C:T_C
p = 0.0097, beta = −0.105, N = 1182
LLNENSYVPR pc2
G3V0E5;P02786
311:342 537:622 266:296
3:195800811:C:T_C
p = 0.05, beta = −0.0812, N = 1114
EMGLSLQWLYSAR pc2
G3V0E5;P02786
340:342 613:622 294:296
3:195800811:C:T_C
p = 0.0047, beta = −0.112, N = 1247
VEYHFLSPYVSPK pc3
G3V0E5;P02786
338:342 616:622 292:296
3:195800811:C:T_C
p = 0.0026, beta = −0.12, N = 1246
YNSQLLSFVR pc2
G3V0E5;P02786
330:342 583:622 284:296
3:195800811:C:T_C
p = 0.058, beta = −0.0762, N = 1197
LVHANFGTK pc2
G3V0E5;P02786
246:342 437:622 205:296
3:195800811:C:T_C
p = 0.13, beta = −0.0702, N = 888
HPVTGQFLYQDSNWASK pc3
G3V0E5;P02786
333:342 599:622 289:296
3:195800811:C:T_C
p = 0.0074, beta = −0.107, N = 1221
LTTDFGNAEK pc2
G3V0E5;P02786
224:342 404:622 200:296
3:195800811:C:T_C
p = 0.027, beta = −0.107, N = 828
LAQMFSDMVLK pc2
G3V0E5;P02786
302:342 521:622 254:296
3:195800811:C:T_C
p = 0.12, beta = −0.0654, N = 1077
VANAESLNAIGVLIYMDQTK pc3
G3V0E5;P02786
320:342 560:622 273:296
3:195800811:C:T_C
p = 0.07, beta = −0.0742, N = 1153
ESPFRHVFWGSGSHTLPALLENLK p
G3V0E5;P02786
295:342 510:622 234:296
3:195800811:C:T_C
p = 0.19, beta = −0.0569, N = 1039
TFRC : NP5
P02786
84:101 111:134 72:90
3:195800811:C:T_T
p = 0.62, beta = 0.0387, N = 267
SSGLPNIPVQTISR pc2
G3V0E5;P02786
66:101 87:134 56:90
3:195800811:C:T_T
p = 0.62, beta = 0.0438, N = 209
VSASPLLYTLIEK pc2
G3V0E5;P02786
48:101 74:134 47:90
3:195800811:C:T_T
p = 0.76, beta = 0.0314, N = 169
ILNIFGVIK pc2
G3V0E5;P02786
40:101 59:134 39:90
3:195800811:C:T_T
p = 0.81, beta = 0.0271, N = 138
LVYLVENPGGYVAYSK pc2
G3V0E5;P02786
38:101 55:134 33:90
3:195800811:C:T_T
p = 0.68, beta = 0.0473, N = 126
GFVEPDHYVVVGAQR pc3
G3V0E5;P02786
20:101 25:134 17:90
3:195800811:C:T_T
p = 0.85, beta = −0.0307, N = 62
HPVTGQFLYQDSNWASK pc3
G3V0E5;P02786
22:101 34:134 19:90
3:195800811:C:T_T
p = 0.71, beta = −0.057, N = 75
SAFSNLFGGEPLSYTR pc2
F8WBE5;P02786
13:101 25:134 13:90
3:195800811:C:T_T
p = 0.38, beta = −0.166, N = 51
LTVSNVLK pc2
G3V0E5;P02786
8:101 15:134 4:90
3:195800811:C:T_T
p = 0.058, beta = −0.509, N = 27
Assay Target: TFRC
UniProt: P02786
rsID: rs3817672
Target SNP: 3:195800811:C:T
Proxy SNP: 3:195800811:C:T
UKBPPP log10(p): 1571.9
UKBPPP BETA: −0.51
UKBPPP MSPA: 0.337
1255:1253:1254:1138:1249:110
LDSTDFTSTIK pc2
rs3817672 ALT
285:342 375:622 3:296
3:195800811:C:T_C
p = 1.4e−111, model = DOM, N = 663
LDSTDFTGTIK pc2
rs3817672 REF
0:342 41:622 41:296
3:195800811:C:T_C
p = 3e−12, model = REC, N = 82

65. CES1 (P23141)
UKBPPP MSPA=0

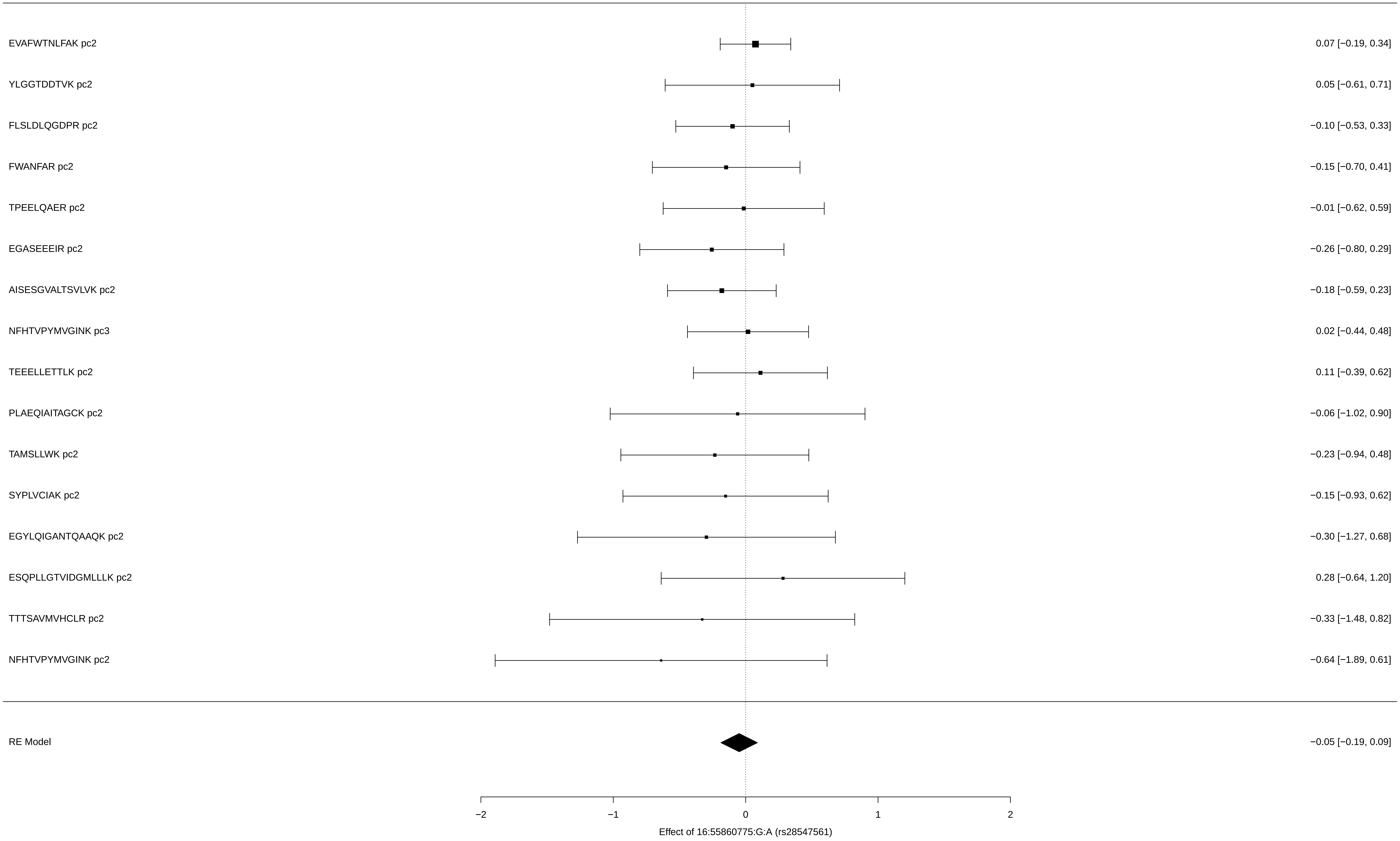

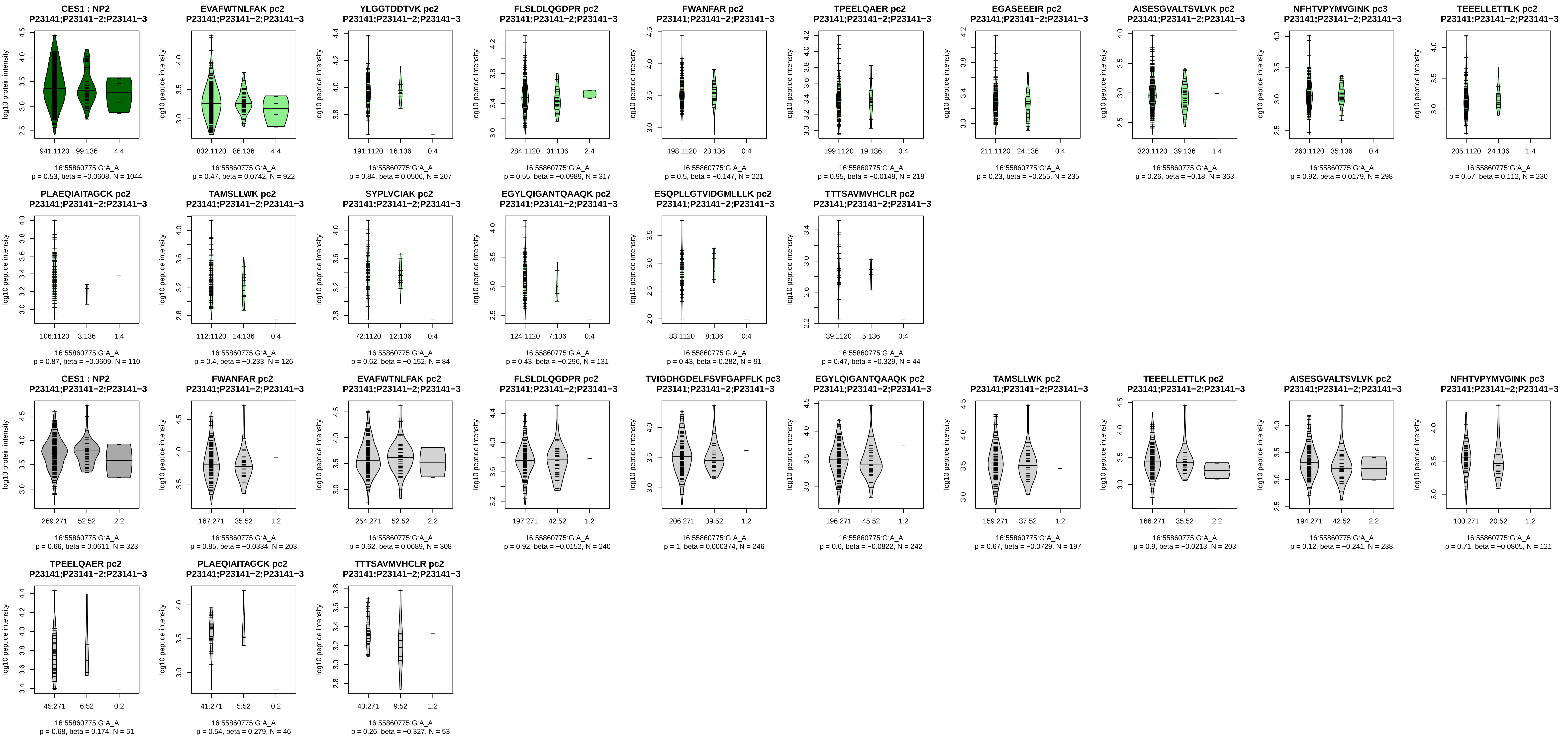

Assay Target: CES1
UniProt: P23141
rsID: rs28547561
Target SNP: 16:55860775:G:A
Proxy SNP: 16:55860775:G:A
UKBPPP log10(p): 1568
UKBPPP BETA: −1.136
UKBPPP MSPA: 0
922:207:317:221:218:235:363:2

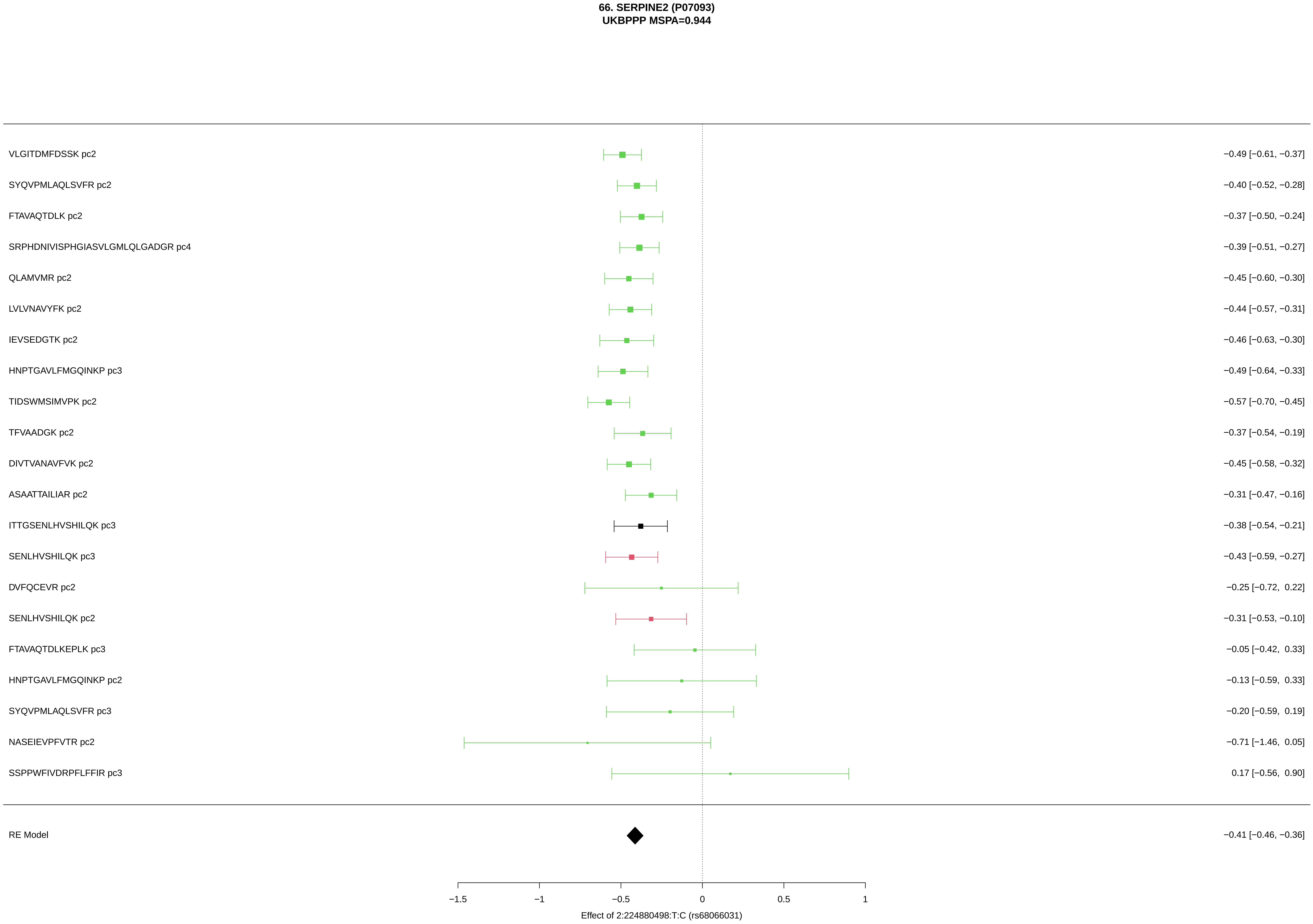
66. SERPINE2 (P07093)
UKBPPP MSPA=0.944
VLGITDMFDSSK pc2
−0.49 [−0.61, −0.37]
SYQVPMLAQLSVFR pc2
−0.40 [−0.52, −0.28]
FTAVAQTDLK pc2
−0.37 [−0.50, −0.24]
SRPHDNIVISPHGIASVLGMLQLGADGR pc4
−0.39 [−0.51, −0.27]
QLAMVMR pc2
−0.45 [−0.60, −0.30]
LVLVNAVYFK pc2
−0.44 [−0.57, −0.31]
IEVSEDGTK pc2
−0.46 [−0.63, −0.30]
HNPTGAVLFMGQINKP pc3
−0.49 [−0.64, −0.33]
TIDSWMSIMVPK pc2
−0.57 [−0.70, −0.45]
TFVAADGK pc2
−0.37 [−0.54, −0.19]
DIVTVANAVFVK pc2
−0.45 [−0.58, −0.32]
ASAATTAILIAR pc2
−0.31 [−0.47, −0.16]
ITTGSENLHVSHILQK pc3
−0.38 [−0.54, −0.21]
SENLHVSHILQK pc3
−0.43 [−0.59, −0.27]
DVFQCEVR pc2
−0.25 [−0.72, 0.22]
SENLHVSHILQK pc2
−0.31 [−0.53, −0.10]
FTAVAQTDLKEPLK pc3
−0.05 [−0.42, 0.33]
HNPTGAVLFMGQINKP pc2
−0.13 [−0.59, 0.33]
SYQVPMLAQLSVFR pc3
−0.20 [−0.59, 0.19]
NASEIEVPFVTR pc2
−0.71 [−1.46, 0.05]
SSPPWFIVDRPFLFFIR pc3
0.17 [−0.56, 0.90]
RE Model
−0.41 [−0.46, −0.36]
−1.5
−1
−0.5
0
0.5
1
Effect of 2:224880498:T:C (rs68066031)

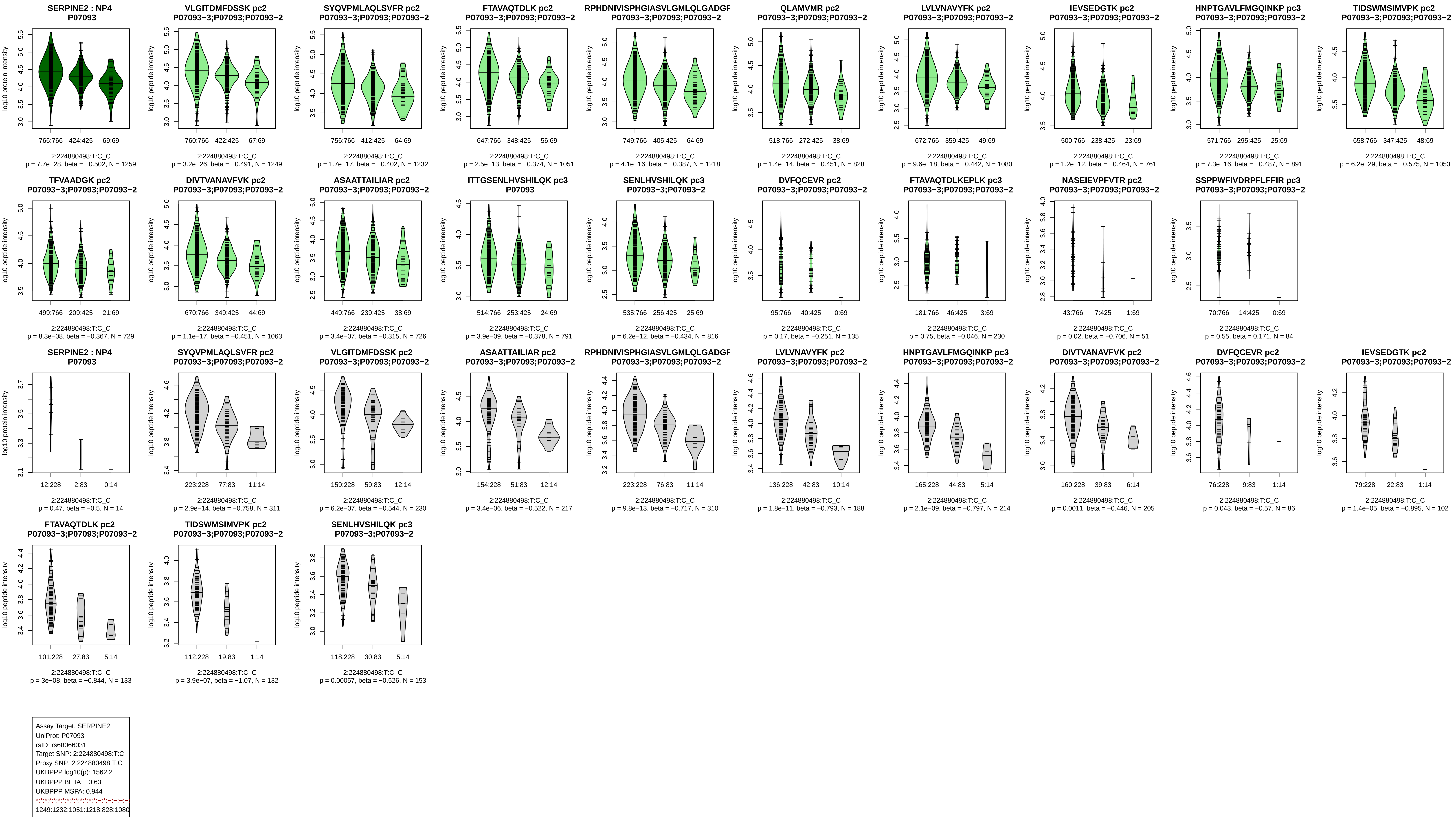
SERPINE2 : NP4
P07093
766:766 424:425 69:69
2:224880498:T:C_C
p = 7.7e−28, beta = −0.502, N = 1259
VLGITDMFDSSK pc2
P07093−3;P07093;P07093−2
760:766 422:425 67:69
2:224880498:T:C_C
p = 3.2e−26, beta = −0.491, N = 1249
SYQVPMLAQLSVFR pc2
P07093−3;P07093;P07093−2
756:766 412:425 64:69
2:224880498:T:C_C
p = 1.7e−17, beta = −0.402, N = 1232
FTAVAQTDLK pc2
P07093−3;P07093;P07093−2
647:766 348:425 56:69
2:224880498:T:C_C
p = 2.5e−13, beta = −0.374, N = 1051
RPHDNIVISPHGIASVLGMLQLGADGF
P07093−3;P07093;P07093−2
749:766 405:425 64:69
2:224880498:T:C_C
p = 4.1e−16, beta = −0.387, N = 1218
QLAMVMR pc2
P07093−3;P07093;P07093−2
518:766 272:425 38:69
2:224880498:T:C_C
p = 1.4e−14, beta = −0.451, N = 828
LVLVNAVYFK pc2
P07093−3;P07093;P07093−2
672:766 359:425 49:69
2:224880498:T:C_C
p = 9.6e−18, beta = −0.442, N = 1080
IEVSEDGTK pc2
P07093−3;P07093;P07093−2
500:766 238:425 23:69
2:224880498:T:C_C
p = 1.2e−12, beta = −0.464, N = 761
HNPTGAVLFMGQINKP pc3
P07093−3;P07093;P07093−2
571:766 295:425 25:69
2:224880498:T:C_C
p = 7.3e−16, beta = −0.487, N = 891
TIDSWMSIMVPK pc2
P07093−3;P07093;P07093−2
658:766 347:425 48:69
2:224880498:T:C_C
p = 6.2e−29, beta = −0.575, N = 1053
TFVAADGK pc2
P07093−3;P07093;P07093−2
499:766 209:425 21:69
2:224880498:T:C_C
p = 8.3e−08, beta = −0.367, N = 729
DIVTVANAVFVK pc2
P07093−3;P07093;P07093−2
670:766 349:425 44:69
2:224880498:T:C_C
p = 1.1e−17, beta = −0.451, N = 1063
ASAATTAILIAR pc2
P07093−3;P07093;P07093−2
449:766 239:425 38:69
2:224880498:T:C_C
p = 3.4e−07, beta = −0.315, N = 726
ITTGSENLHVSHILQK pc3
P07093
514:766 253:425 24:69
2:224880498:T:C_C
p = 3.9e−09, beta = −0.378, N = 791
SENLHVSHILQK pc3
P07093−3;P07093;P07093−2
535:766 256:425 25:69
2:224880498:T:C_C
p = 6.2e−12, beta = −0.434, N = 816
DVFQCEVR pc2
P07093−3;P07093;P07093−2
95:766 40:425 0:69
2:224880498:T:C_C
p = 0.17, beta = −0.251, N = 135
FTAVAQTDLKEPLK pc3
P07093−3;P07093;P07093−2
181:766 46:425 3:69
2:224880498:T:C_C
p = 0.75, beta = −0.046, N = 230
NASEIEVPFVTR pc2
P07093−3;P07093;P07093−2
43:766 7:425 1:69
2:224880498:T:C_C
p = 0.02, beta = −0.706, N = 51
SSPPWFIVDRPFLFFIR pc3
P07093−3;P07093;P07093−2
70:766 14:425 0:69
2:224880498:T:C_C
p = 0.55, beta = 0.171, N = 84
SERPINE2 : NP4
P07093
12:228 2:83 0:14
2:224880498:T:C_C
p = 0.47, beta = −0.5, N = 14
SYQVPMLAQLSVFR pc2
P07093−3;P07093;P07093−2
223:228 77:83 11:14
2:224880498:T:C_C
p = 2.9e−14, beta = −0.758, N = 311
VLGITDMFDSSK pc2
P07093−3;P07093;P07093−2
159:228 59:83 12:14
2:224880498:T:C_C
p = 6.2e−07, beta = −0.544, N = 230
ASAATTAILIAR pc2
P07093−3;P07093;P07093−2
154:228 51:83 12:14
2:224880498:T:C_C
p = 3.4e−06, beta = −0.522, N = 217
RPHDNIVISPHGIASVLGMLQLGADGF
P07093−3;P07093;P07093−2
223:228 76:83 11:14
2:224880498:T:C_C
p = 9.8e−13, beta = −0.717, N = 310
LVLVNAVYFK pc2
P07093−3;P07093;P07093−2
136:228 42:83 10:14
2:224880498:T:C_C
p = 1.8e−11, beta = −0.793, N = 188
HNPTGAVLFMGQINKP pc3
P07093−3;P07093;P07093−2
165:228 44:83 5:14
2:224880498:T:C_C
p = 2.1e−09, beta = −0.797, N = 214
DIVTVANAVFVK pc2
P07093−3;P07093;P07093−2
160:228 39:83 6:14
2:224880498:T:C_C
p = 0.0011, beta = −0.446, N = 205
DVFQCEVR pc2
P07093−3;P07093;P07093−2
76:228 9:83 1:14
2:224880498:T:C_C
p = 0.043, beta = −0.57, N = 86
IEVSEDGTK pc2
P07093−3;P07093;P07093−2
79:228 22:83 1:14
2:224880498:T:C_C
p = 1.4e−05, beta = −0.895, N = 102
FTAVAQTDLK pc2
P07093−3;P07093;P07093−2
101:228 27:83 5:14
2:224880498:T:C_C
p = 3e−08, beta = −0.844, N = 133
TIDSWMSIMVPK pc2
P07093−3;P07093;P07093−2
112:228 19:83 1:14
2:224880498:T:C_C
p = 3.9e−07, beta = −1.07, N = 132
SENLHVSHILQK pc3
P07093−3;P07093−2
118:228 30:83 5:14
2:224880498:T:C_C
p = 0.00057, beta = −0.526, N = 153
Assay Target: SERPINE2
UniProt: P07093
rsID: rs68066031
Target SNP: 2:224880498:T:C
Proxy SNP: 2:224880498:T:C
UKBPPP log10(p): 1562.2
UKBPPP BETA: −0.63
UKBPPP MSPA: 0.944
1249:1232:1051:1218:828:1080

67. SERPINA4 (P29622)
UKBPPP MSPA=0.9

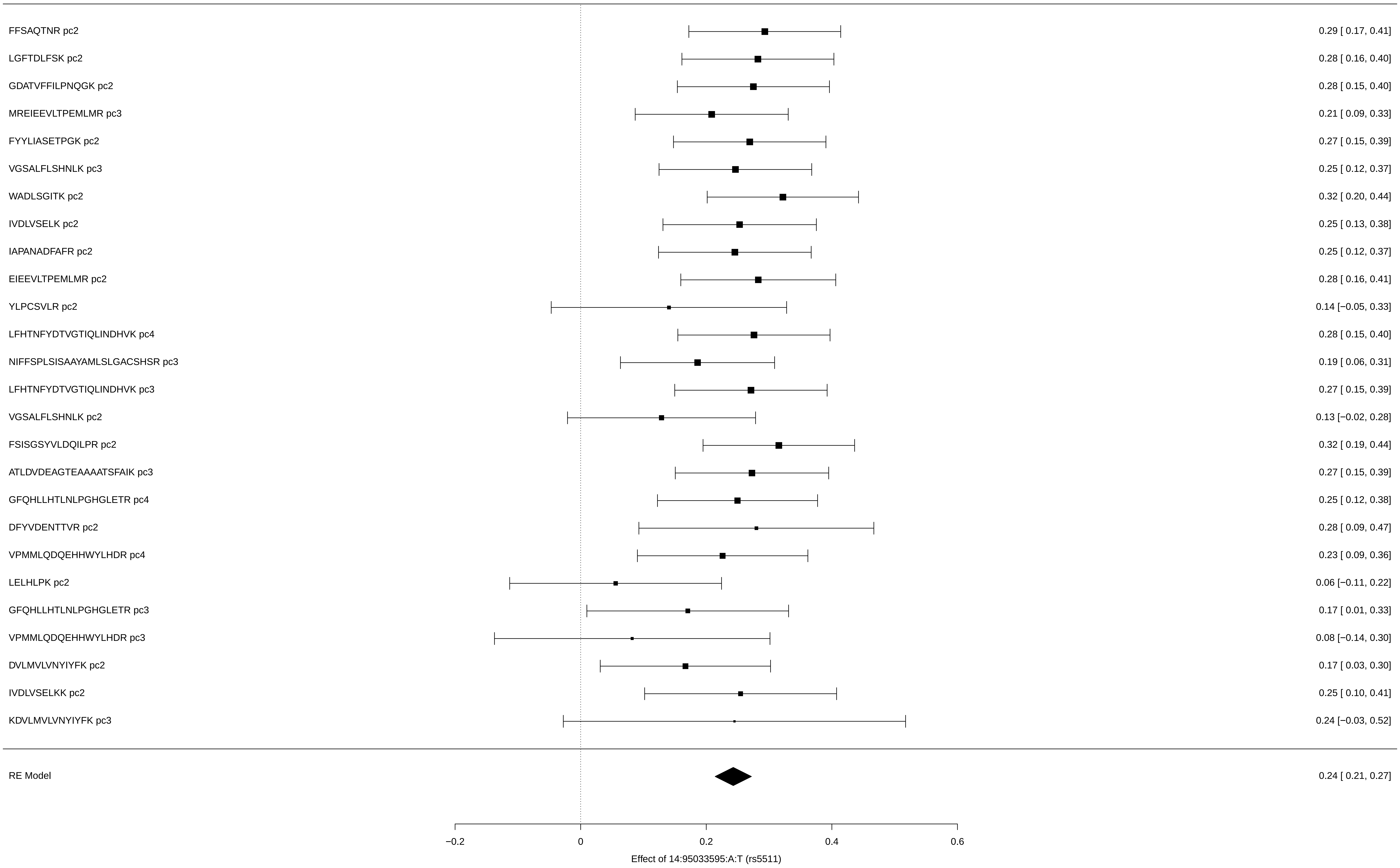

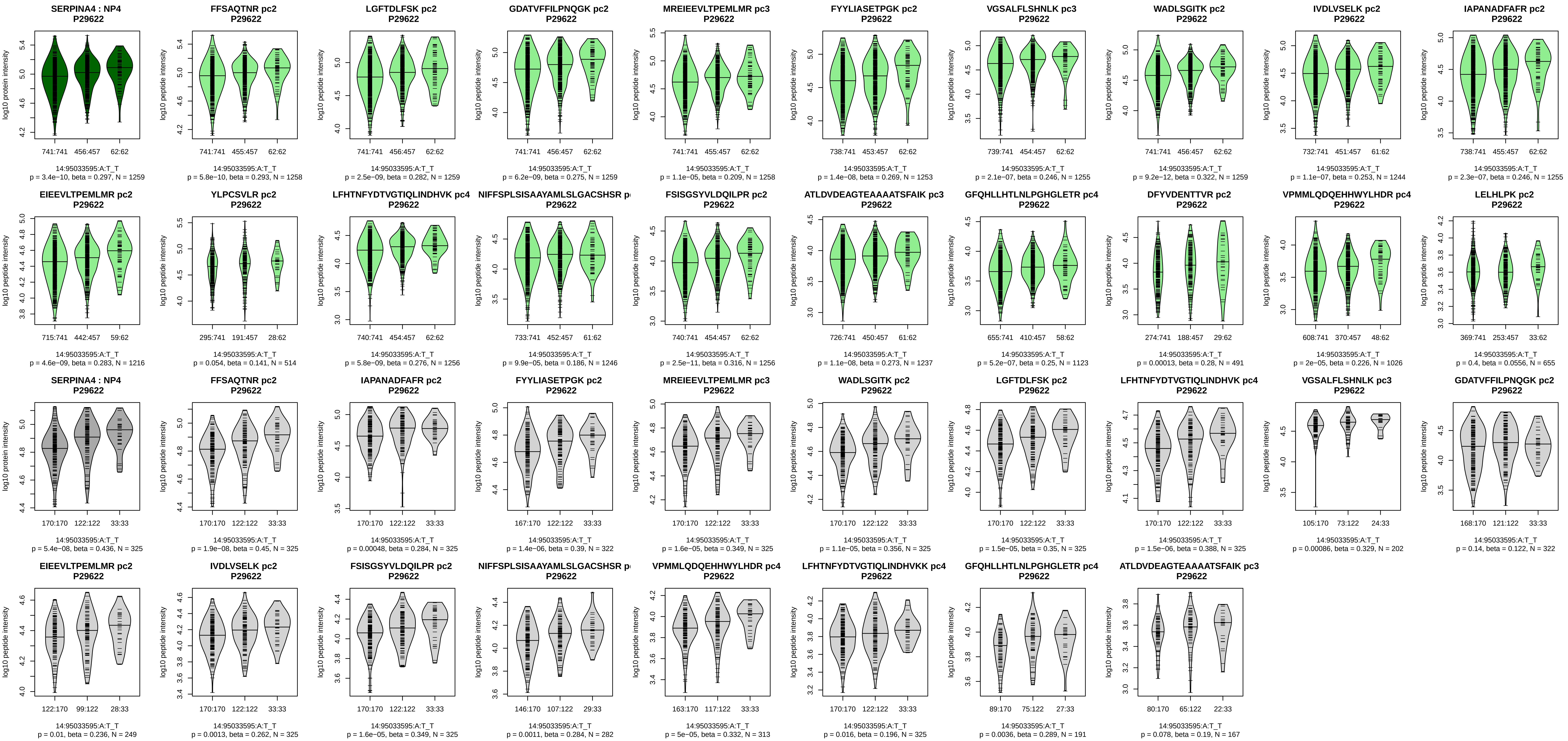

Assay Target: SERPINA4
UniProt: P29622
rsID: rs5511
Target SNP: 14:95033595:A:T
Proxy SNP: 14:95033595:A:T
UKBPPP log10(p): 1529.9
UKBPPP BETA: 0.612
UKBPPP MSPA: 0.9
1258:1259:1259:1258:1253:125

68. FAM3B (P58499)
UKBPPP MSPA=0.159

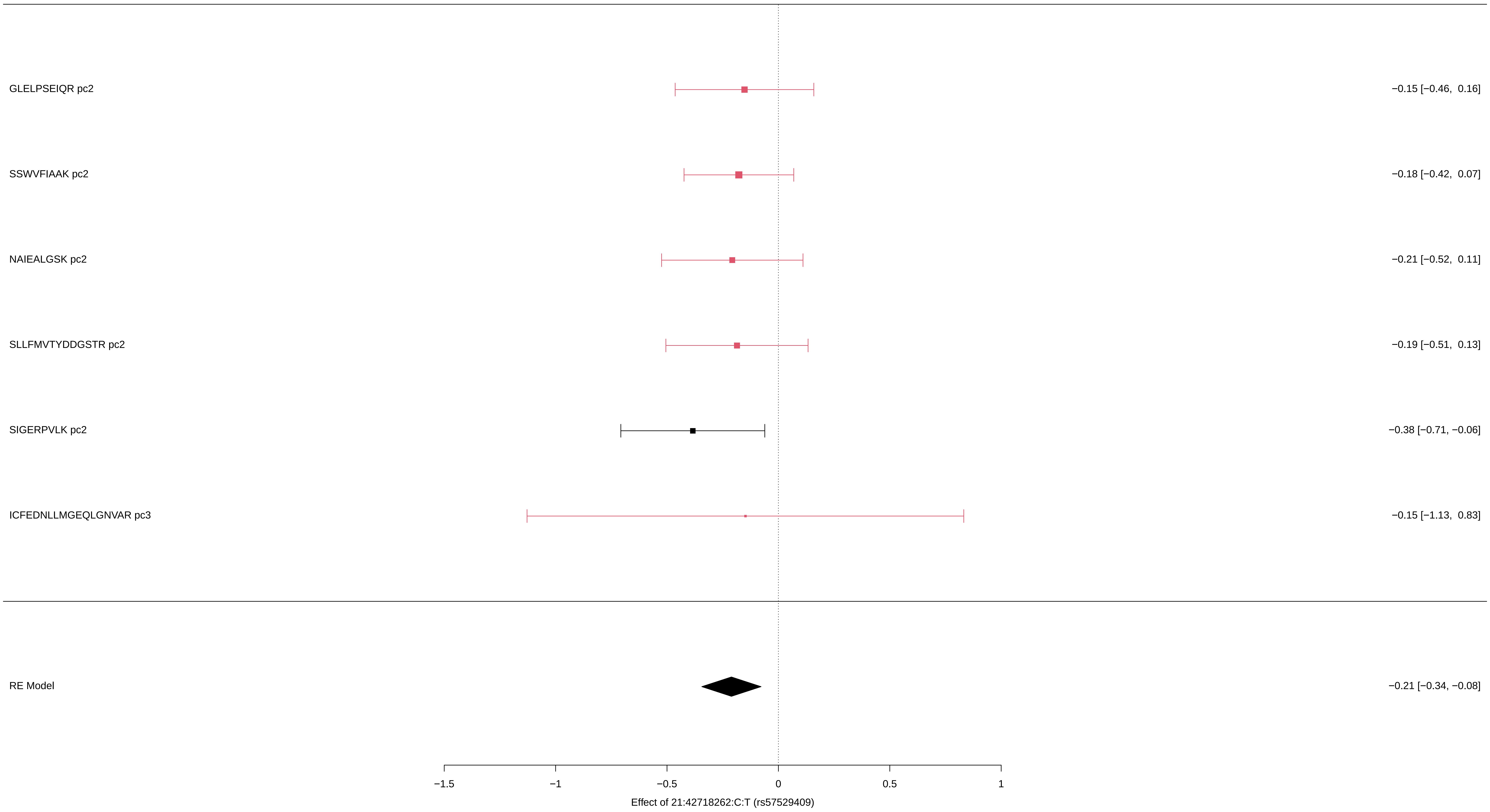

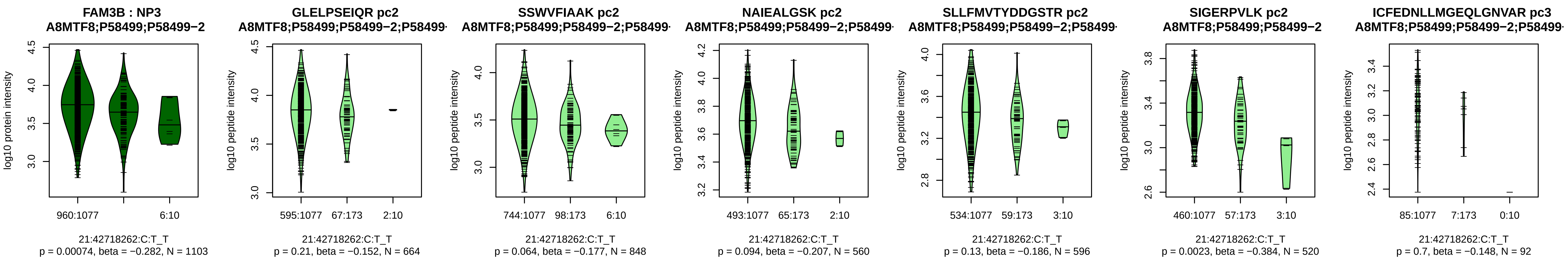

Assay Target: FAM3B
UniProt: P58499
rsID: rs57529409
Target SNP: 21:42718262:C:T
Proxy SNP: 21:42718262:C:T
UKBPPP log10(p): 1516.9
UKBPPP BETA: −1.087
UKBPPP MSPA: 0.159
–:–:–:–:*:–
664:848:560:596:520:92

69. CDH6 (P55285)
UKBPPP MSPA=0.698

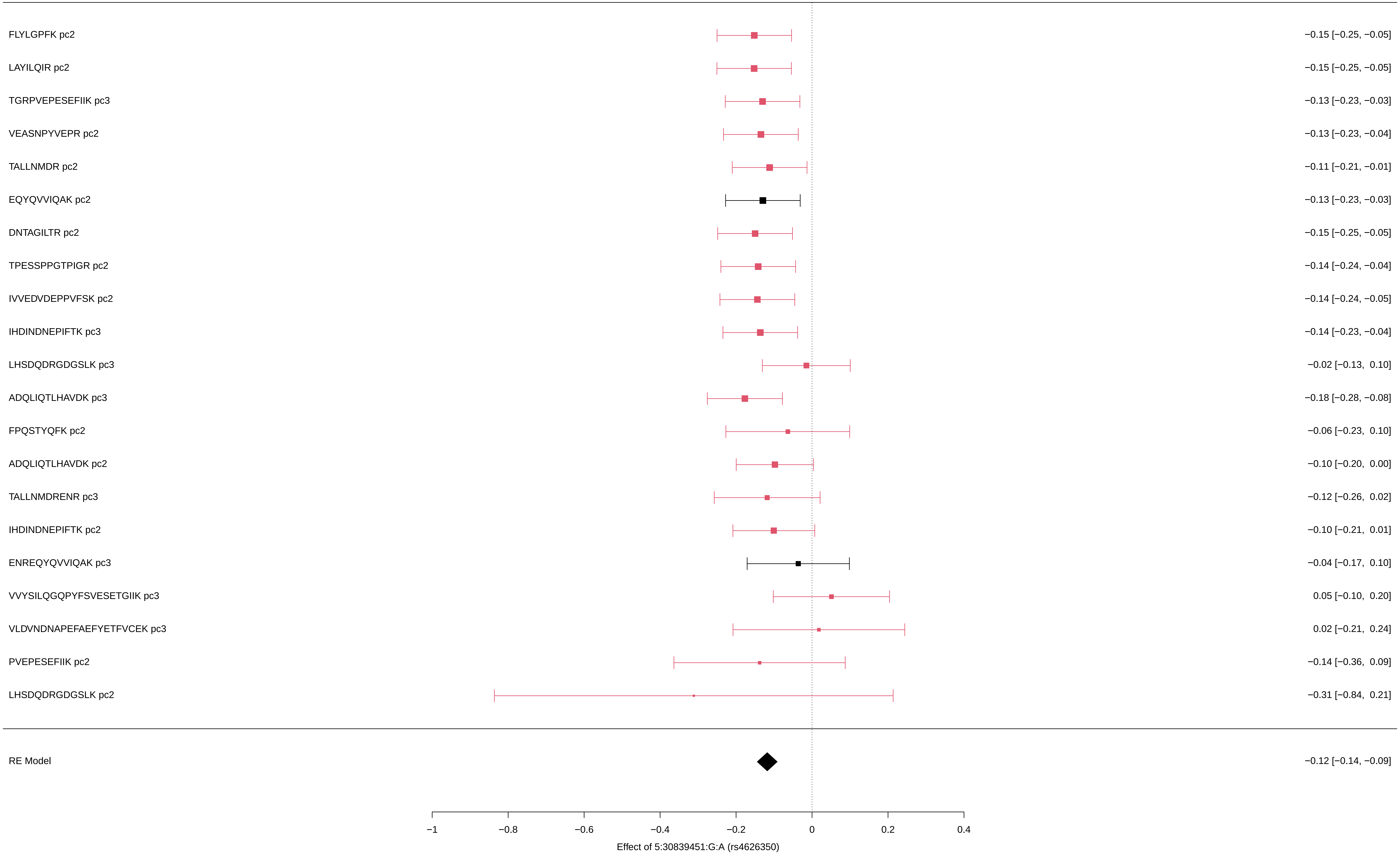

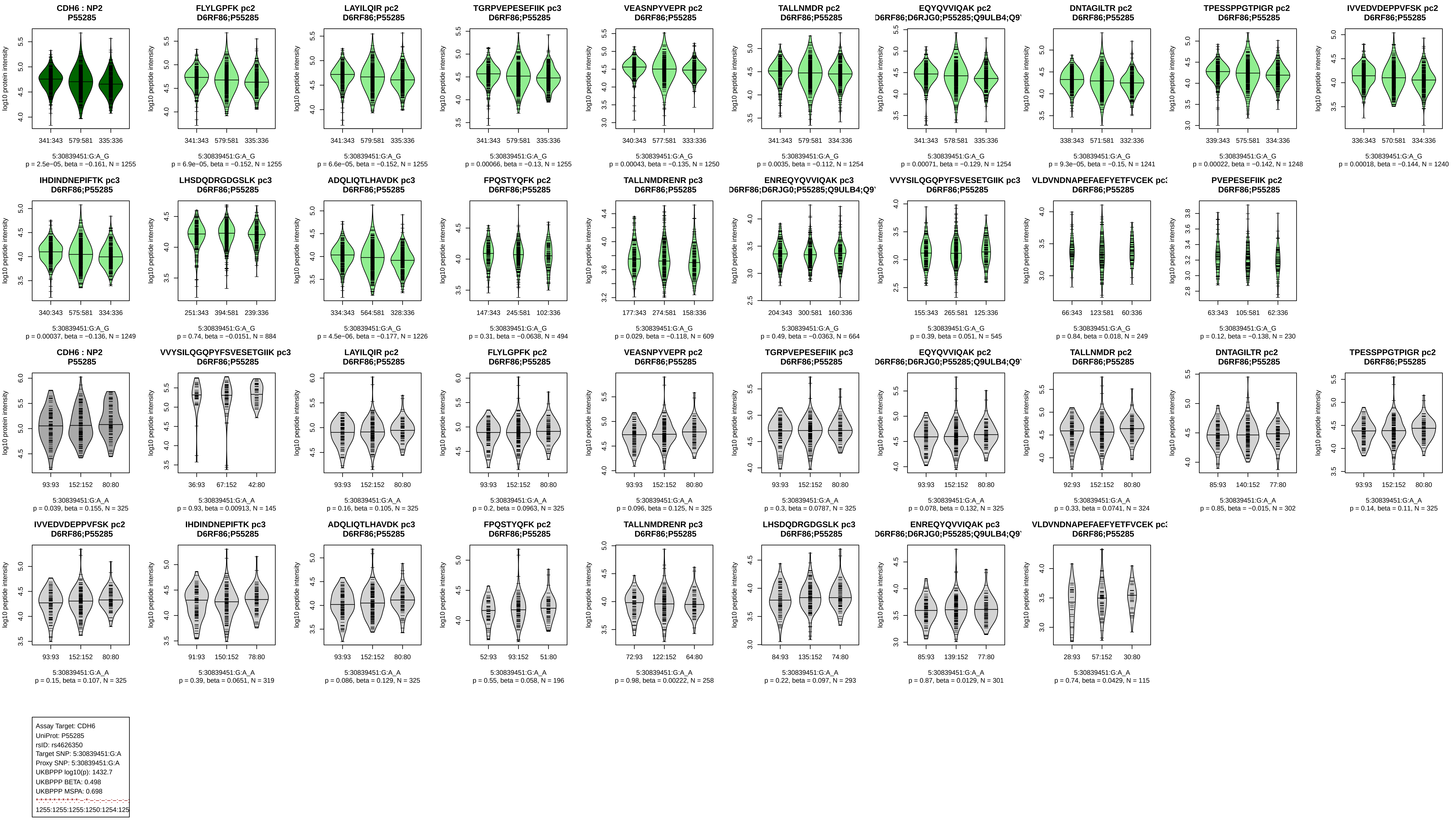
CDH6 : NP2
P55285
p = 2.5e−05, beta = −0.161, N = 1255
FLYLGPFK pc2
D6RF86;P55285
p = 6.9e−05, beta = −0.152, N = 1255
LAYILQIR pc2
D6RF86;P55285
p = 6.6e−05, beta = −0.152, N = 1255
TGRPVEPESEFIIK pc3
D6RF86;P55285
p = 0.00066, beta = −0.13, N = 1255
VEASNPYVEPR pc2
D6RF86;P55285
p = 0.00043, beta = −0.135, N = 1250
TALLNMDR pc2
D6RF86;P55285
p = 0.0035, beta = −0.112, N = 1254
EQYQVVIQAK pc2
p = 0.00071, beta = −0.129, N = 1254
DNTAGILTR pc2
D6RF86;P55285
p = 9.3e−05, beta = −0.15, N = 1241
TPESSPPGTPIGR pc2
D6RF86;P55285
p = 0.00022, beta = −0.142, N = 1248
IVVEDVDEPPVFSK pc2
D6RF86;P55285
p = 0.00018, beta = −0.144, N = 1240
IHDINDNEPIFTK pc3
D6RF86;P55285
p = 0.00037, beta = −0.136, N = 1249
LHSDQDRGDGSLK pc3
D6RF86;P55285
p = 0.74, beta = −0.0151, N = 884
ADQLIQTLHAVDK pc3
D6RF86;P55285
p = 4.5e−06, beta = −0.177, N = 1226
FPQSTYQFK pc2
D6RF86;P55285
p = 0.31, beta = −0.0638, N = 494
TALLNMDRENR pc3
D6RF86;P55285
p = 0.029, beta = −0.118, N = 609
ENREQYQVVIQAK pc3
p = 0.49, beta = −0.0363, N = 664
VVYSILQGQPYFSVESETGIIK pc3
D6RF86;P55285
p = 0.39, beta = 0.051, N = 545
D6RF86;P55285
p = 0.84, beta = 0.018, N = 249
PVEPESEFIIK pc2
D6RF86;P55285
p = 0.12, beta = −0.138, N = 230
CDH6 : NP2
P55285
p = 0.039, beta = 0.155, N = 325
VVYSILQGQPYFSVESETGIIK pc3
D6RF86;P55285
p = 0.93, beta = 0.00913, N = 145
LAYILQIR pc2
D6RF86;P55285
p = 0.16, beta = 0.105, N = 325
FLYLGPFK pc2
D6RF86;P55285
p = 0.2, beta = 0.0963, N = 325
VEASNPYVEPR pc2
D6RF86;P55285
p = 0.096, beta = 0.125, N = 325
TGRPVEPESEFIIK pc3
D6RF86;P55285
p = 0.3, beta = 0.0787, N = 325
EQYQVVIQAK pc2
p = 0.078, beta = 0.132, N = 325
TALLNMDR pc2
D6RF86;P55285
p = 0.33, beta = 0.0741, N = 324
DNTAGILTR pc2
D6RF86;P55285
p = 0.85, beta = −0.015, N = 302
TPESSPPGTPIGR pc2
D6RF86;P55285
p = 0.14, beta = 0.11, N = 325
IVVEDVDEPPVFSK pc2
D6RF86;P55285
p = 0.15, beta = 0.107, N = 325
IHDINDNEPIFTK pc3
D6RF86;P55285
p = 0.39, beta = 0.0651, N = 319
ADQLIQTLHAVDK pc3
D6RF86;P55285
p = 0.086, beta = 0.129, N = 325
FPQSTYQFK pc2
D6RF86;P55285
p = 0.55, beta = 0.058, N = 196
TALLNMDRENR pc3
D6RF86;P55285
p = 0.98, beta = 0.00222, N = 258
LHSDQDRGDGSLK pc3
D6RF86;P55285
p = 0.22, beta = 0.097, N = 293
ENREQYQVVIQAK pc3
p = 0.87, beta = 0.0129, N = 301
D6RF86;P55285
p = 0.74, beta = 0.0429, N = 115
Assay Target: CDH6
UniProt: P55285
rsID: rs4626350
Target SNP: 5:30839451:G:A
Proxy SNP: 5:30839451:G:A
UKBPPP log10(p): 1432.7
UKBPPP BETA: 0.498
UKBPPP MSPA: 0.698

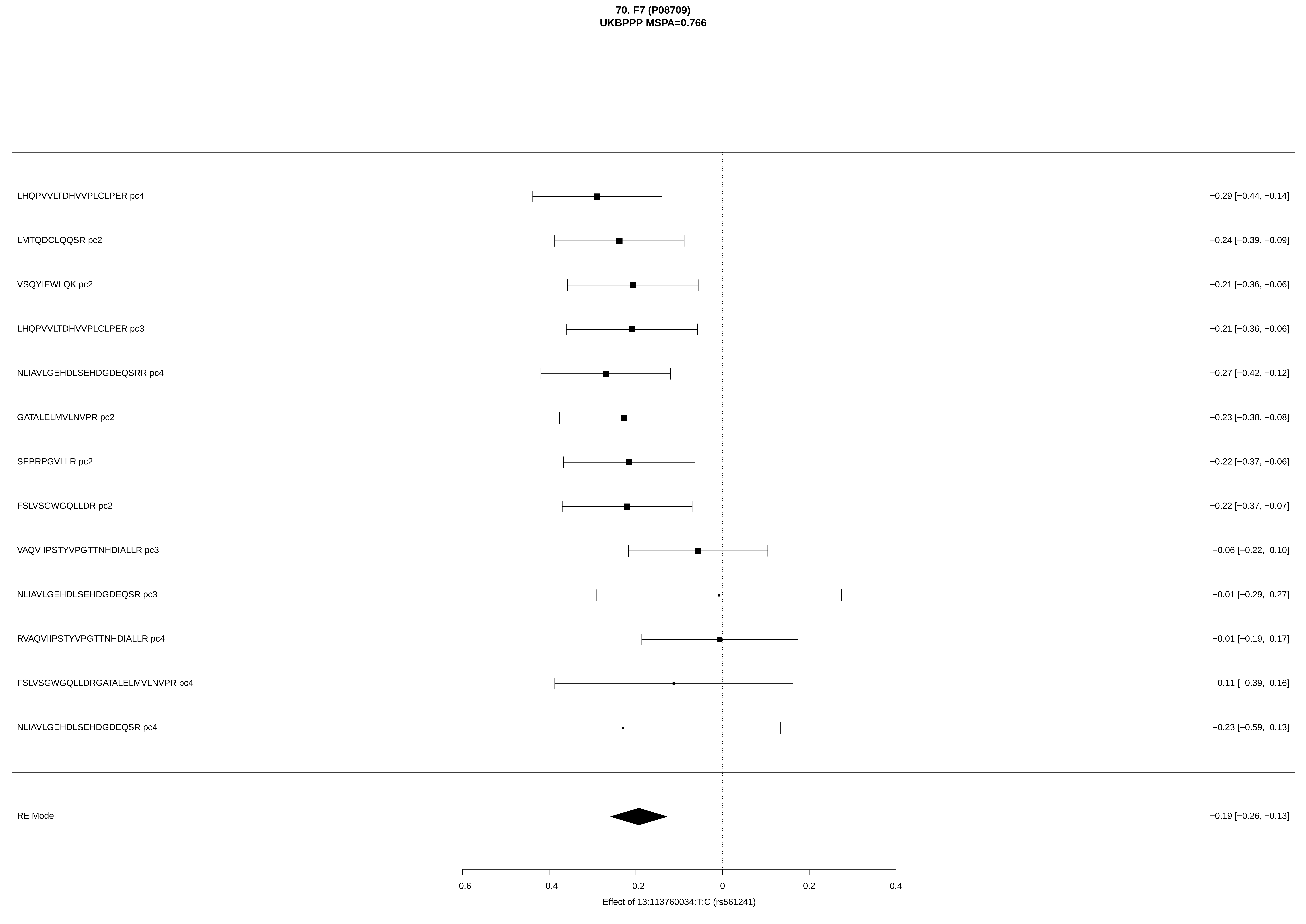

70. F7 (P08709)
UKBPPP MSPA=0.766
LHQPVVLTDHVVPLCLPER pc4
−0.29 [−0.44, −0.14]
LMTQDCLQQSR pc2
−0.24 [−0.39, −0.09]
VSQYIEWLQK pc2
−0.21 [−0.36, −0.06]
LHQPVVLTDHVVPLCLPER pc3
−0.21 [−0.36, −0.06]
NLIAVLGEHDLSEHDGDEQSRR pc4
−0.27 [−0.42, −0.12]
GATALELMVLNVPR pc2
−0.23 [−0.38, −0.08]
SEPRPGVLLR pc2
−0.22 [−0.37, −0.06]
FSLVSGWGQLLDR pc2
−0.22 [−0.37, −0.07]
VAQVIIPSTYVPGTTNHDIALLR pc3
−0.06 [−0.22, 0.10]
NLIAVLGEHDLSEHDGDEQSR pc3
−0.01 [−0.29, 0.27]
RVAQVIIPSTYVPGTTNHDIALLR pc4
−0.01 [−0.19, 0.17]
FSLVSGWGQLLDRGATALELMVLNVPR pc4
−0.11 [−0.39, 0.16]
NLIAVLGEHDLSEHDGDEQSR pc4
−0.23 [−0.59, 0.13]
RE Model
−0.19 [−0.26, −0.13]
−0.6
−0.4
−0.2
0
0.2
0.4
Effect of 13:113760034:T:C (rs561241)

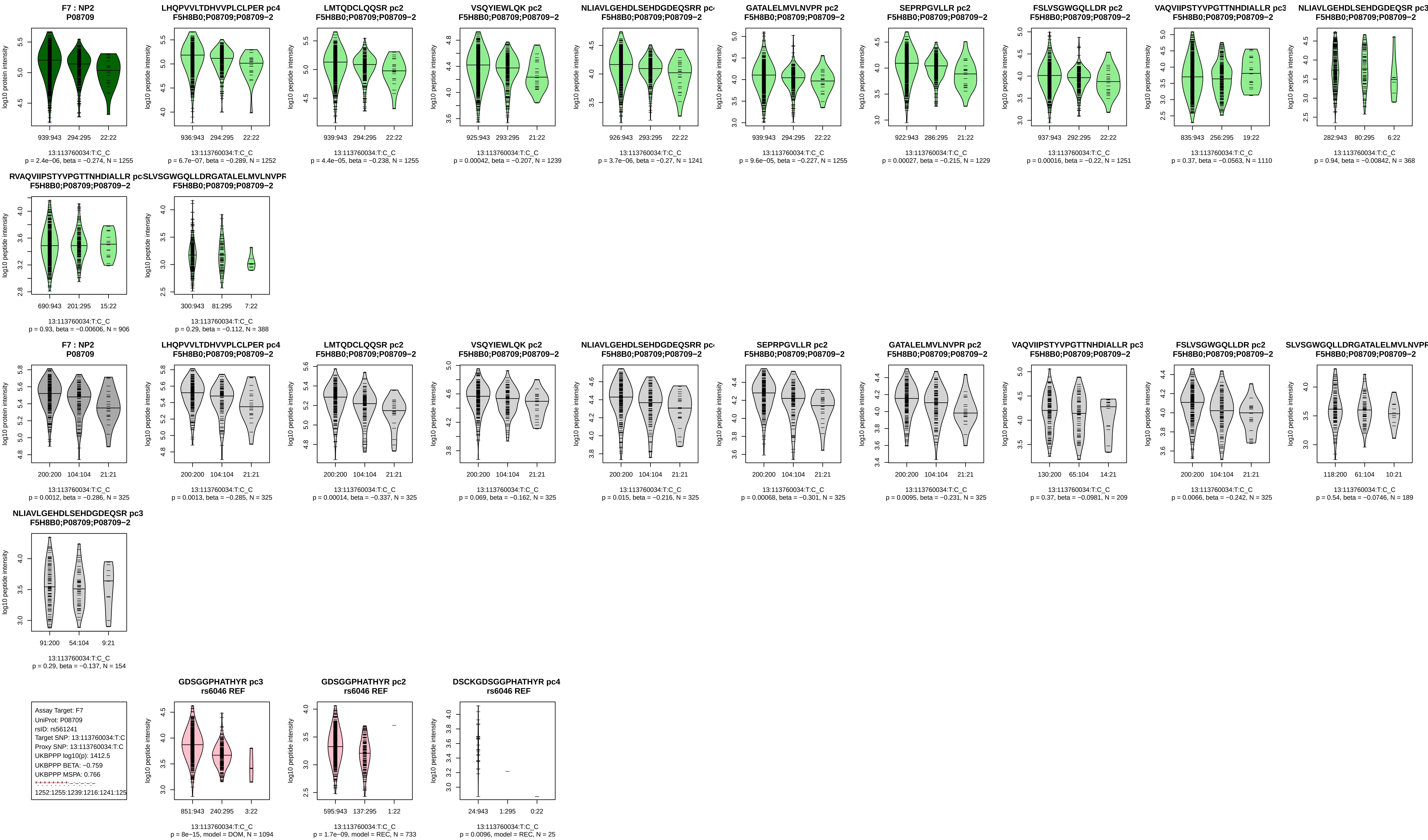

F7 : NP2
P08709
log10 protein intensity
939:943 294:295 22:22
13:113760034:T:C_C
p = 2.4e−06, beta = −0.274, N = 1255
LHQPVVLTDHVVPLCLPER pc4
F5H8B0;P08709;P08709−2
log10 peptide intensity
936:943 294:295 22:22
13:113760034:T:C_C
p = 6.7e−07, beta = −0.289, N = 1252
LMTQDCLQQSR pc2
F5H8B0;P08709;P08709−2
939:943 294:295 22:22
13:113760034:T:C_C
p = 4.4e−05, beta = −0.238, N = 1255
VSQYIEWLQK pc2
F5H8B0;P08709;P08709−2
925:943 293:295 21:22
13:113760034:T:C_C
p = 0.00042, beta = −0.207, N = 1239
NLIAVLGEHDLSEHDGDEQSRR pc
F5H8B0;P08709;P08709−2
926:943 293:295 22:22
13:113760034:T:C_C
p = 3.7e−06, beta = −0.27, N = 1241
GATALELMVLNVPR pc2
F5H8B0;P08709;P08709−2
939:943 294:295 22:22
13:113760034:T:C_C
p = 9.6e−05, beta = −0.227, N = 1255
SEPRPGVLLR pc2
F5H8B0;P08709;P08709−2
922:943 286:295 21:22
13:113760034:T:C_C
p = 0.00027, beta = −0.215, N = 1229
FSLVSGWGQLLDR pc2
F5H8B0;P08709;P08709−2
937:943 292:295 22:22
13:113760034:T:C_C
p = 0.00016, beta = −0.22, N = 1251
VAQVIIPSTYVPGTTNHDIALLR pc3
F5H8B0;P08709;P08709−2
835:943 256:295 19:22
13:113760034:T:C_C
p = 0.37, beta = −0.0563, N = 1110
NLIAVLGEHDLSEHDGDEQSR pc2
F5H8B0;P08709;P08709−2
282:943 80:295 6:22
13:113760034:T:C_C
p = 0.94, beta = −0.00842, N = 368
RVAQVIIPSTYVPGTTNHDIALLR pc
F5H8B0;P08709;P08709−2
690:943 201:295 15:22
13:113760034:T:C_C
p = 0.93, beta = −0.00606, N = 906
SLVSGWGQLLDRGATALELMVLNVPR
F5H8B0;P08709;P08709−2
300:943 81:295 7:22
13:113760034:T:C_C
p = 0.29, beta = −0.112, N = 388
F7 : NP2
P08709
200:200 104:104 21:21
13:113760034:T:C_C
p = 0.0012, beta = −0.286, N = 325
LHQPVVLTDHVVPLCLPER pc4
F5H8B0;P08709;P08709−2
200:200 104:104 21:21
13:113760034:T:C_C
p = 0.0013, beta = −0.285, N = 325
LMTQDCLQQSR pc2
F5H8B0;P08709;P08709−2
200:200 104:104 21:21
13:113760034:T:C_C
p = 0.00014, beta = −0.337, N = 325
VSQYIEWLQK pc2
F5H8B0;P08709;P08709−2
200:200 104:104 21:21
13:113760034:T:C_C
p = 0.069, beta = −0.162, N = 325
NLIAVLGEHDLSEHDGDEQSRR pc
F5H8B0;P08709;P08709−2
200:200 104:104 21:21
13:113760034:T:C_C
p = 0.015, beta = −0.216, N = 325
SEPRPGVLLR pc2
F5H8B0;P08709;P08709−2
200:200 104:104 21:21
13:113760034:T:C_C
p = 0.00068, beta = −0.301, N = 325
GATALELMVLNVPR pc2
F5H8B0;P08709;P08709−2
200:200 104:104 21:21
13:113760034:T:C_C
p = 0.0095, beta = −0.231, N = 325
VAQVIIPSTYVPGTTNHDIALLR pc3
F5H8B0;P08709;P08709−2
130:200 65:104 14:21
13:113760034:T:C_C
p = 0.37, beta = −0.0981, N = 209
FSLVSGWGQLLDR pc2
F5H8B0;P08709;P08709−2
200:200 104:104 21:21
13:113760034:T:C_C
p = 0.0066, beta = −0.242, N = 325
SLVSGWGQLLDRGATALELMVLNVPR
F5H8B0;P08709;P08709−2
118:200 61:104 10:21
13:113760034:T:C_C
p = 0.54, beta = −0.0746, N = 189
NLIAVLGEHDLSEHDGDEQSR pc3
F5H8B0;P08709;P08709−2
91:200 54:104 9:21
13:113760034:T:C_C
p = 0.29, beta = −0.137, N = 154
Assay Target: F7
UniProt: P08709
rsID: rs561241
Target SNP: 13:113760034:T:C
Proxy SNP: 13:113760034:T:C
UKBPPP log10(p): 1412.5
UKBPPP BETA: −0.759
UKBPPP MSPA: 0.766
1252:1255:1239:1216:1241:125
GDSGGPHATHYR pc3
rs6046 REF
851:943 240:295 3:22
13:113760034:T:C_C
p = 8e−15, model = DOM, N = 1094
GDSGGPHATHYR pc2
rs6046 REF
595:943 137:295 1:22
13:113760034:T:C_C
p = 1.7e−09, model = REC, N = 733
DSCKGDSGGPHATHYR pc4
rs6046 REF
24:943 1:295 0:22
13:113760034:T:C_C
p = 0.0096, model = REC, N = 25

71. SSC5D (A1L4H1)
UKBPPP MSPA=0.925

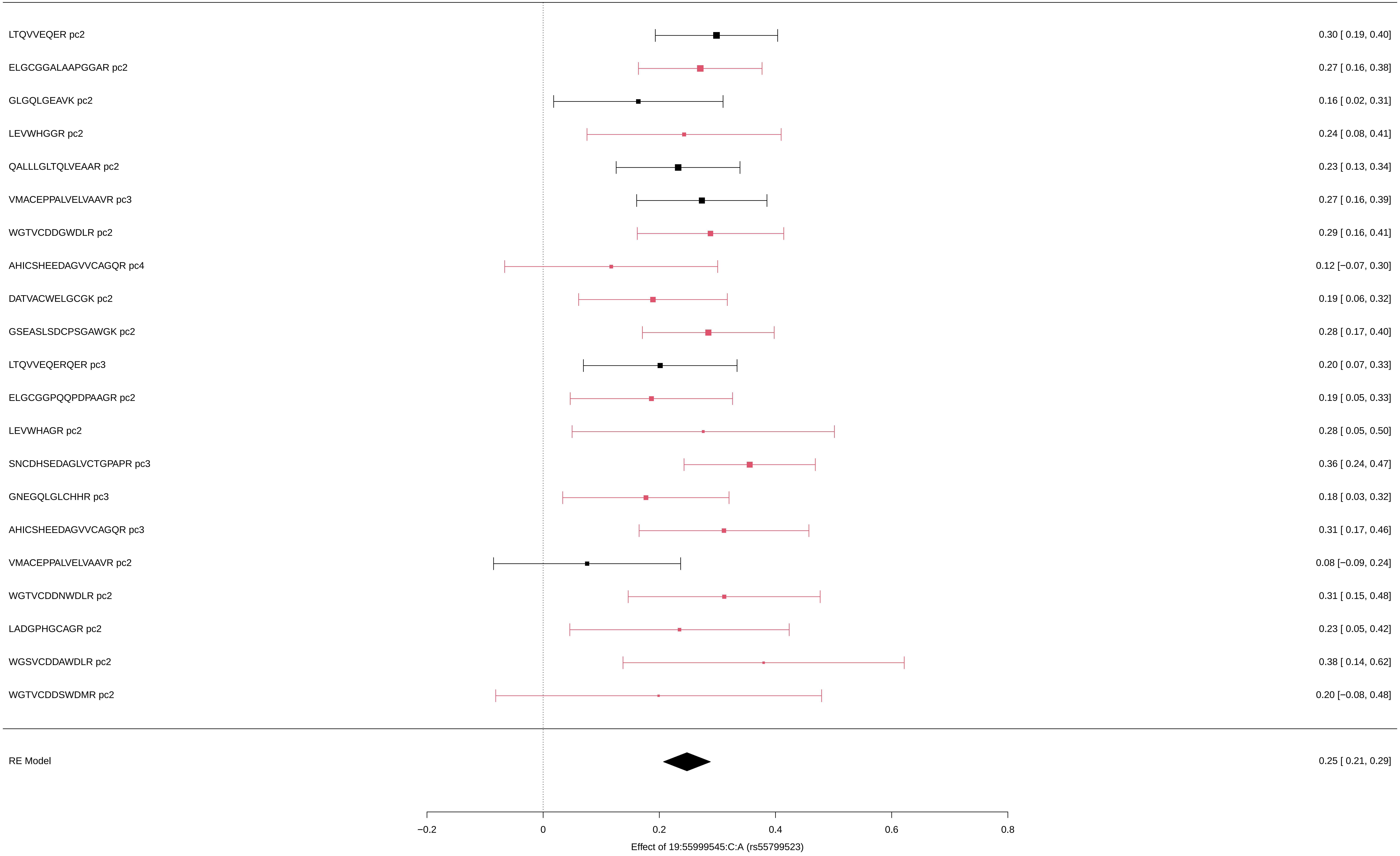

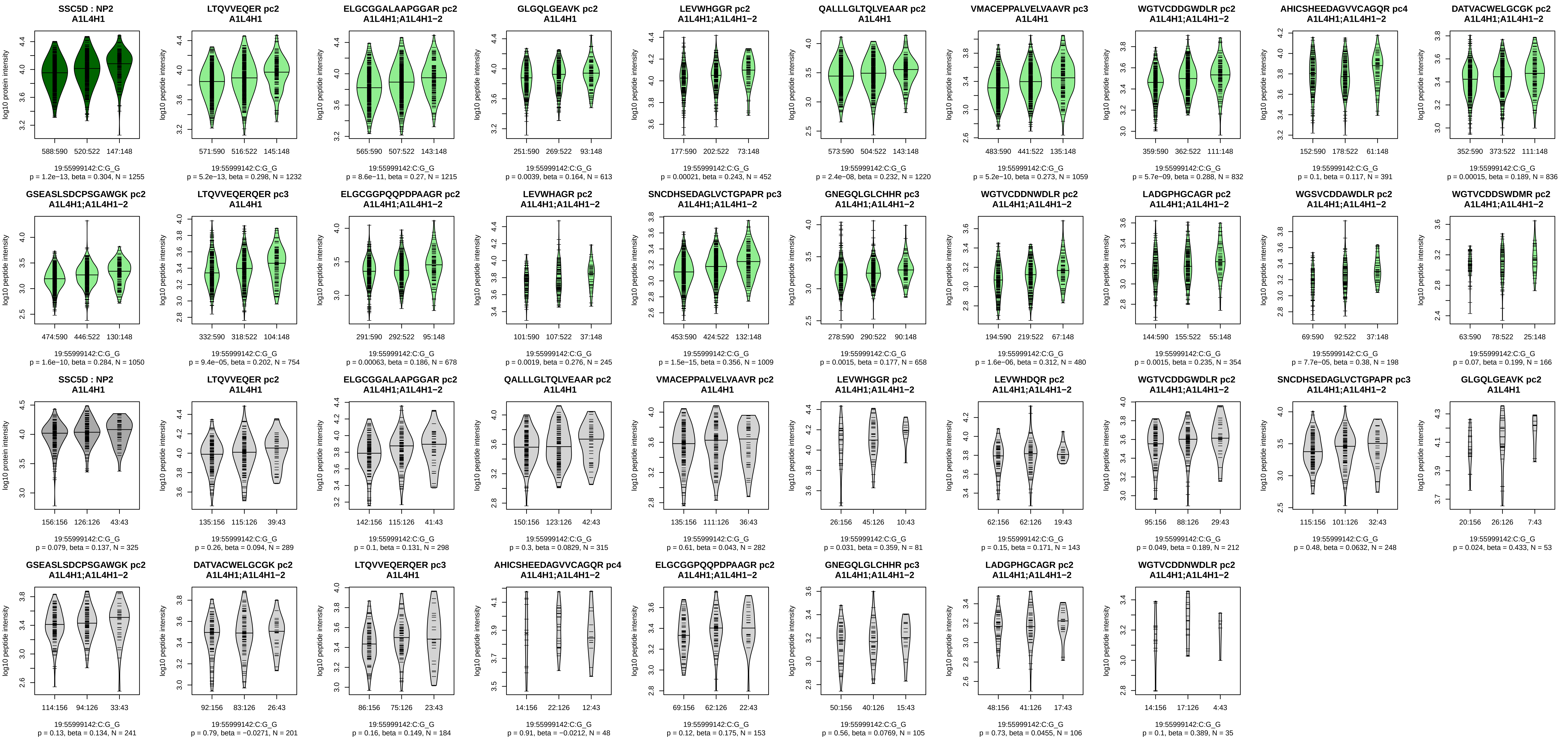

Assay Target: SSC5D
UniProt: A1L4H1
rsID: rs55799523
Target SNP: 19:55999545:C:A
Proxy SNP: 19:55999142:C:G
UKBPPP log10(p): 1347.2
UKBPPP BETA: 0.53
UKBPPP MSPA: 0.925
[illegible]
1232:1215:613:452:1220:1059:8

72. CCL18 (P55774)
UKBPPP MSPA=0.937

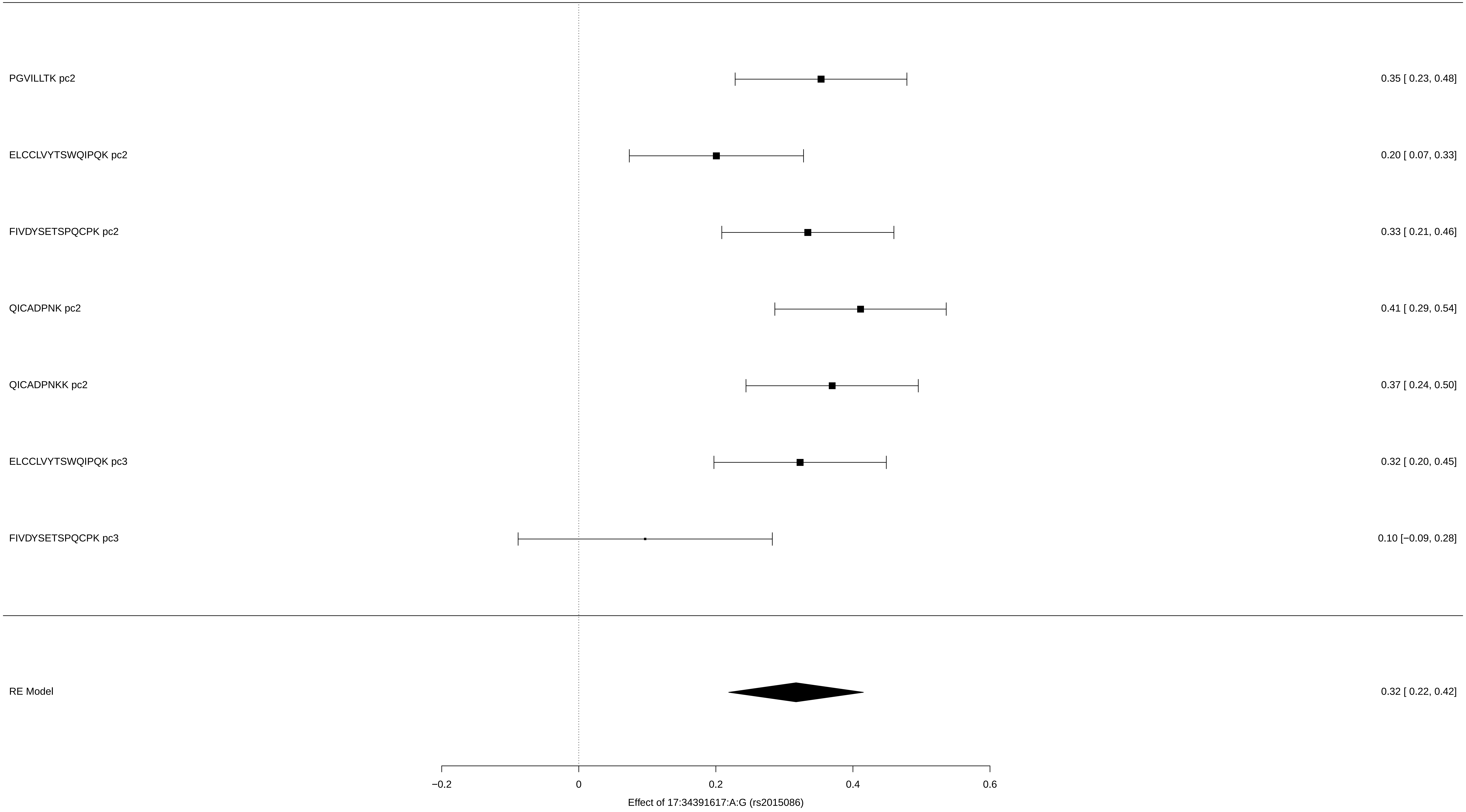

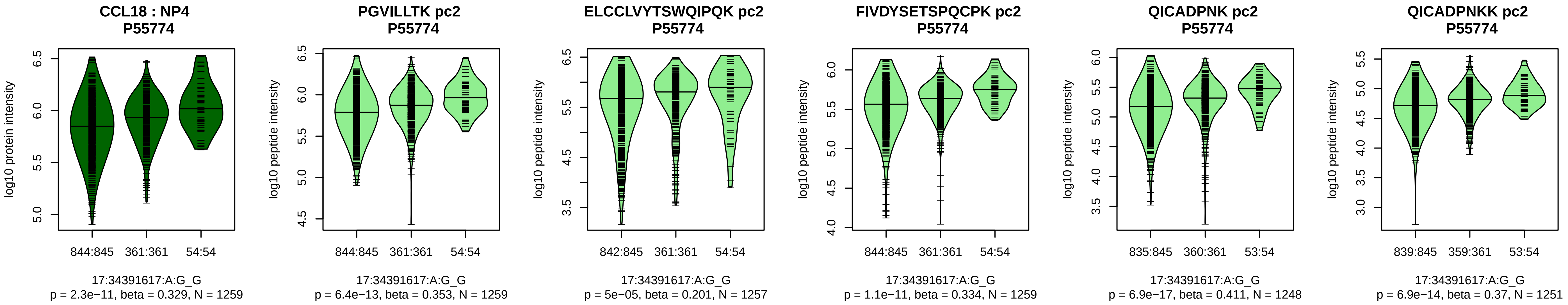

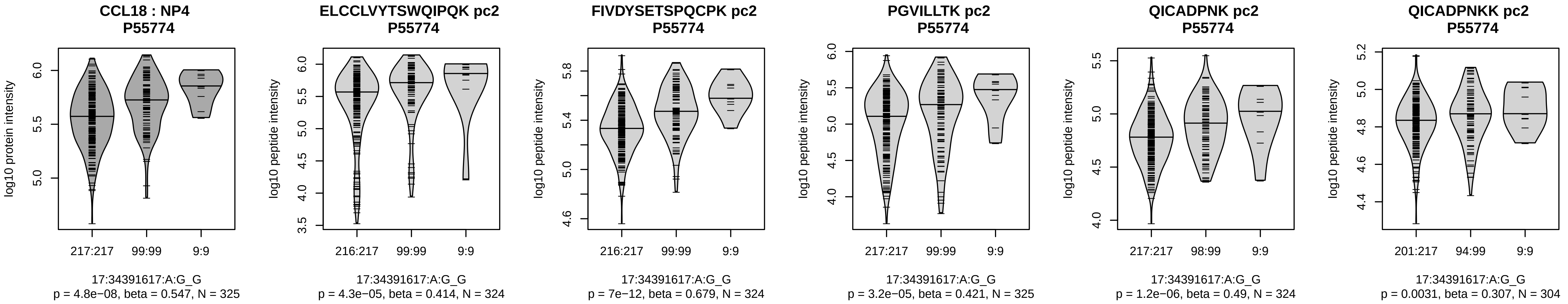

Assay Target: CCL18
UniProt: P55774
rsID: rs2015086
Target SNP: 17:34391617:A:G
Proxy SNP: 17:34391617:A:G
UKBPPP log10(p): 1335.8
UKBPPP BETA: 0.68
UKBPPP MSPA: 0.937
::*:*:*:*:–
1259:1257:1259:1248:1251:125

73. HSPB1 (P04792)
UKBPPP MSPA=0.919

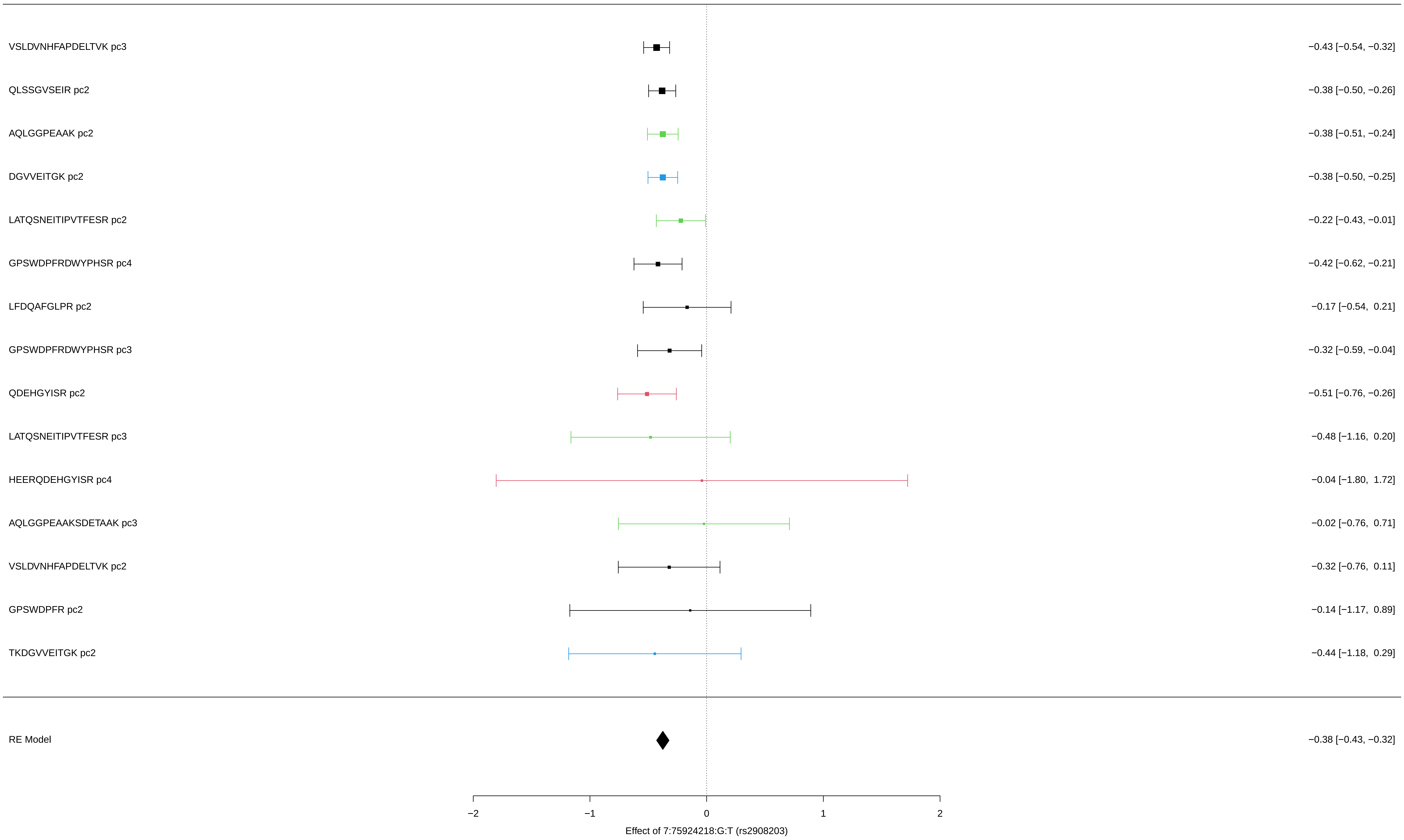

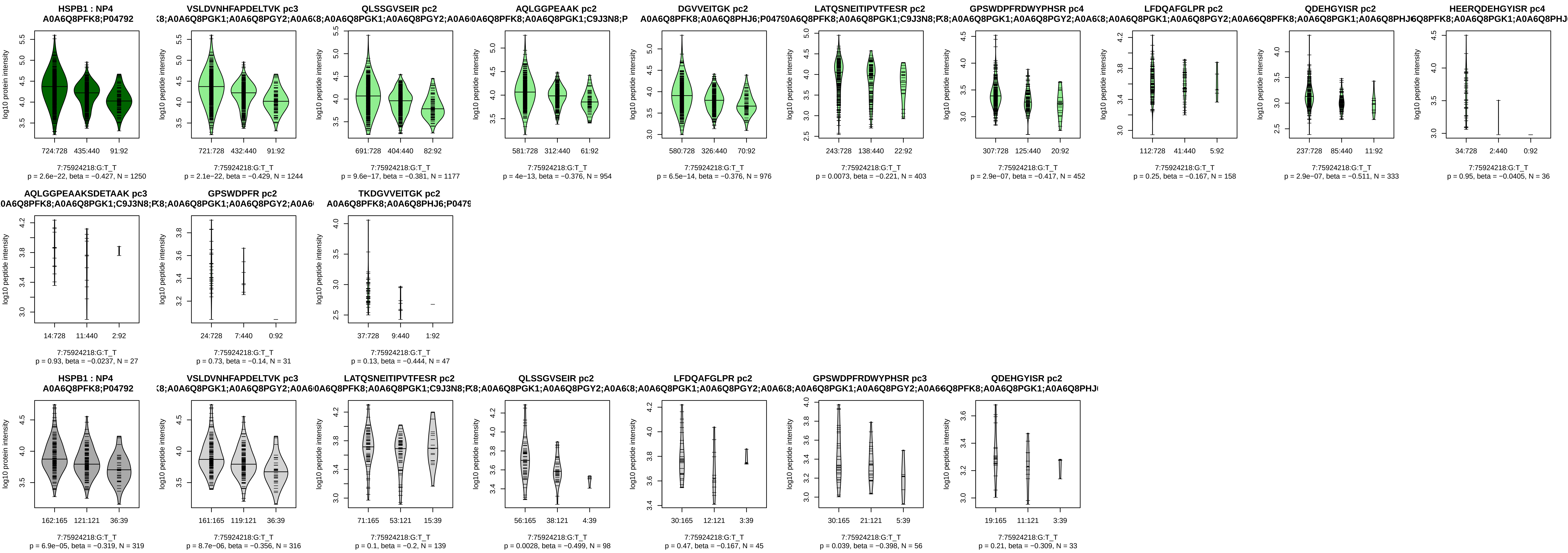

Assay Target: HSPB1
UniProt: P04792
rsID: rs2908203
Target SNP: 7:75924218:G:T
Proxy SNP: 7:75924218:G:T
UKBPPP log10(p): 1291.3
UKBPPP BETA: −0.554
UKBPPP MSPA: 0.919
::*:*:*:–:*:*:–:–:–:–:–:–:NA:NA
1244:1177:954:976:403:452:158

74. CCL14 (Q16627)
UKBPPP MSPA=0.86

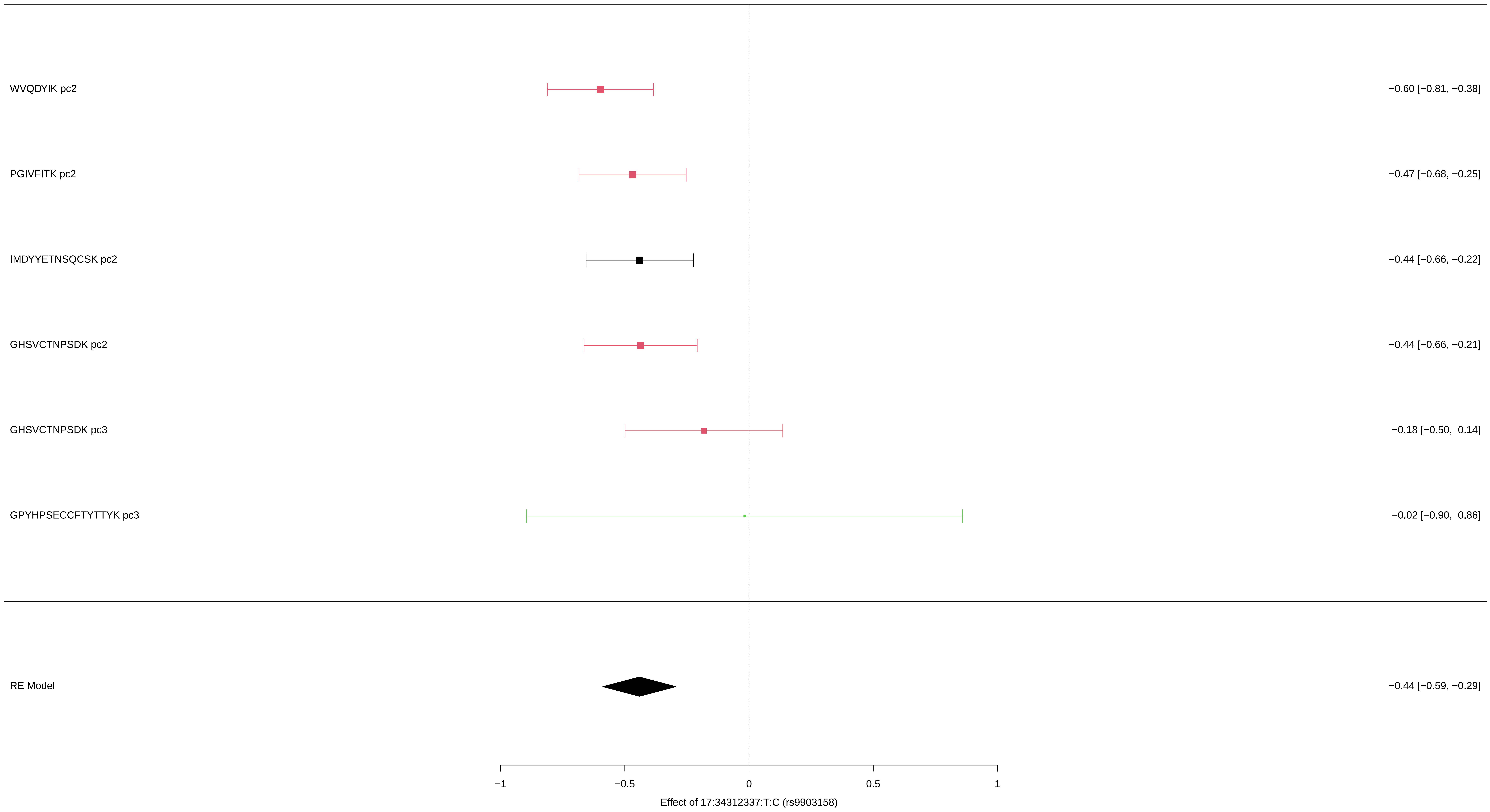

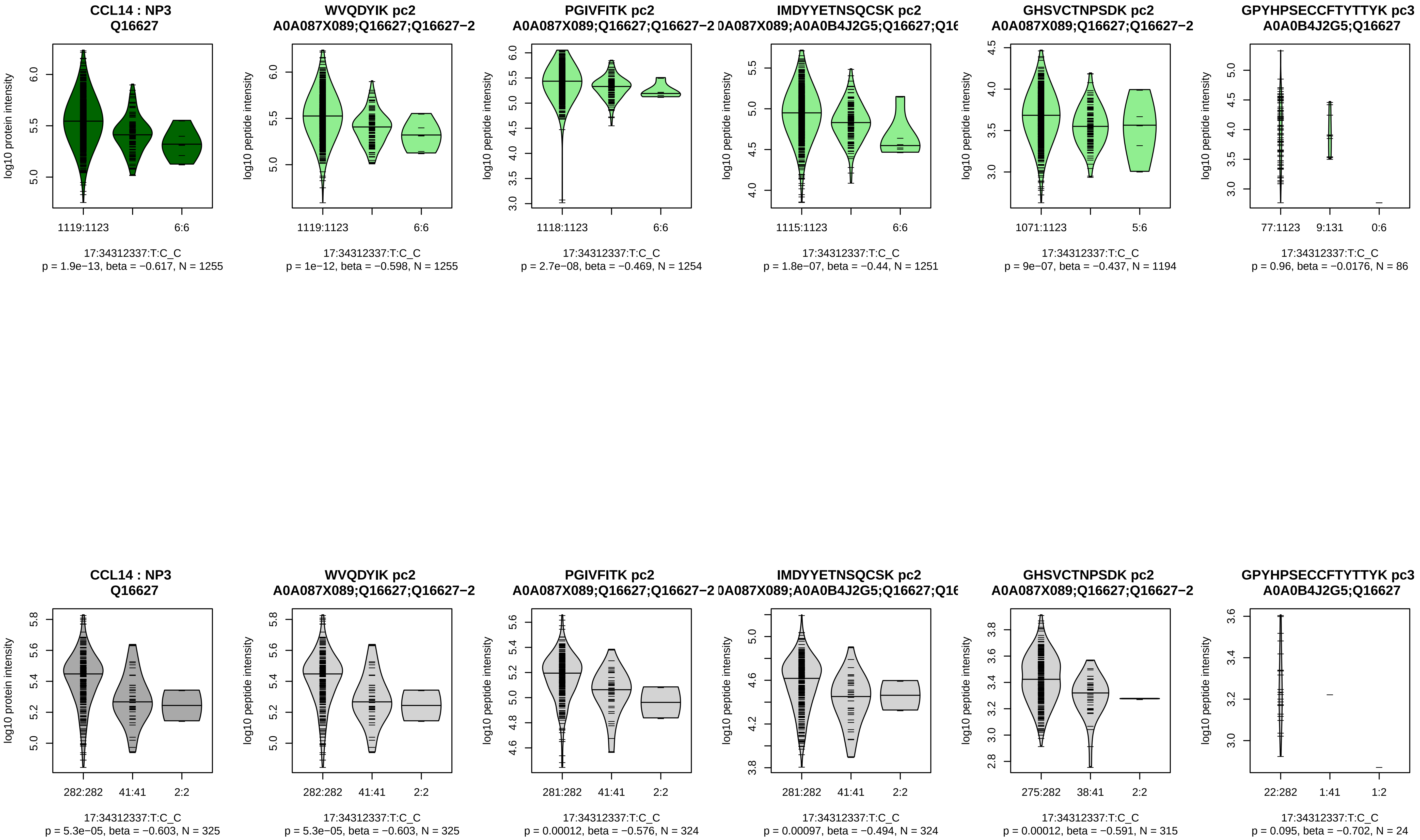

Assay Target: CCL14
UniProt: Q16627
rsID: rs9903158
Target SNP: 17:34312337:T:C
Proxy SNP: 17:34312337:T:C
UKBPPP log10(p): 1253.1
UKBPPP BETA: −0.997
UKBPPP MSPA: 0.86
;;*;*;–;–
1255:1254:1251:1194:722:86

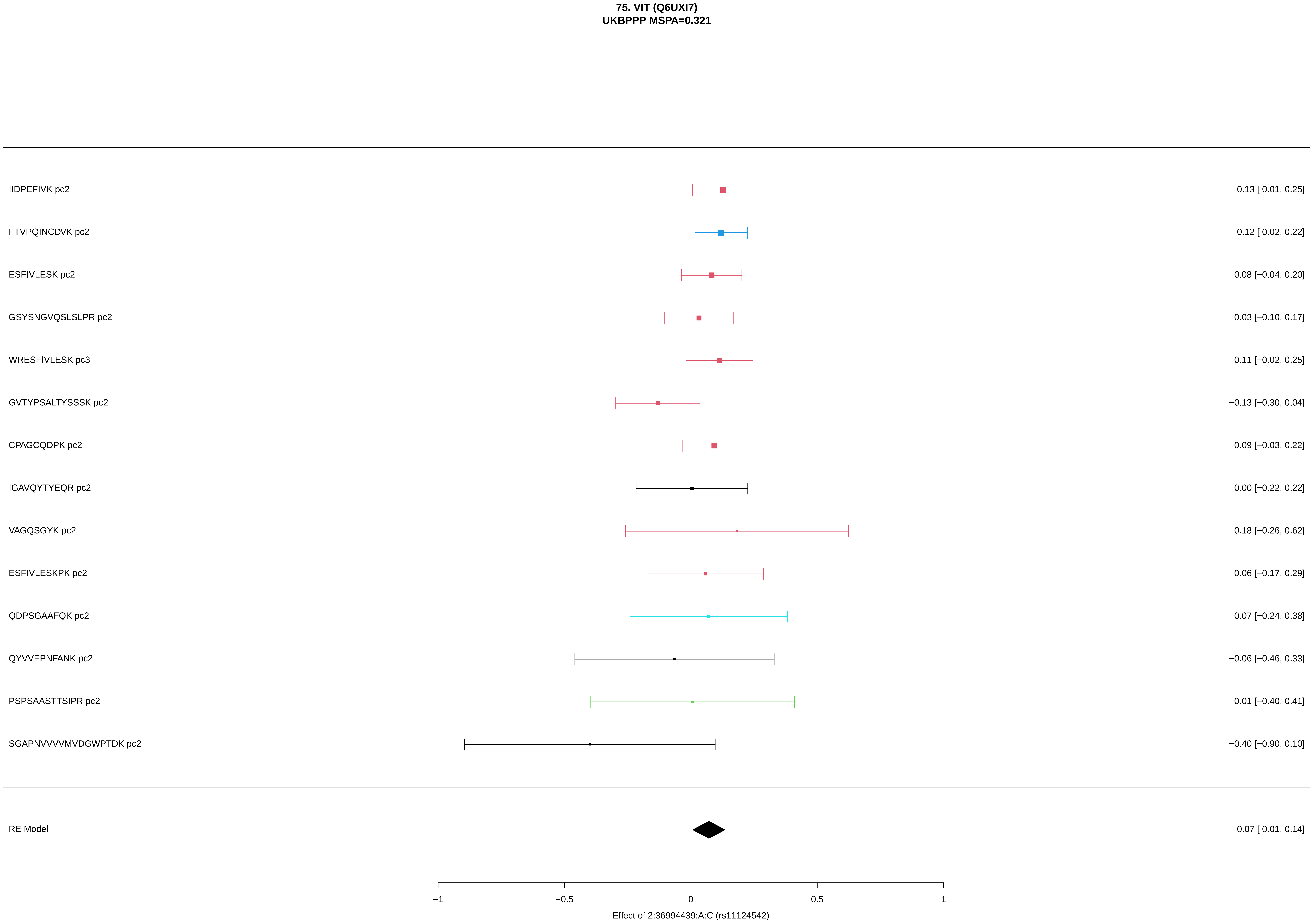
75. VIT (Q6UXI7)
UKBPPP MSPA=0.321
IIDPEFIVK pc2
0.13 [0.01, 0.25]
FTVPQINCDVK pc2
0.12 [0.02, 0.22]
ESFIVLESK pc2
0.08 [−0.04, 0.20]
GSYSNGVQSLSLPR pc2
0.03 [−0.10, 0.17]
WRESFIVLESK pc3
0.11 [−0.02, 0.25]
GVTYPSALTYSSSK pc2
−0.13 [−0.30, 0.04]
CPAGCQDPK pc2
0.09 [−0.03, 0.22]
IGAVQYTYEQR pc2
0.00 [−0.22, 0.22]
VAGQSGYK pc2
0.18 [−0.26, 0.62]
ESFIVLESKPK pc2
0.06 [−0.17, 0.29]
QDPSGAAFQK pc2
0.07 [−0.24, 0.38]
QYVVEPNFANK pc2
−0.06 [−0.46, 0.33]
PSPSAASTTSIPR pc2
0.01 [−0.40, 0.41]
SGAPNVVVVMVDGWPTDK pc2
−0.40 [−0.90, 0.10]
RE Model
0.07 [0.01, 0.14]
−1
−0.5
0
0.5
1
Effect of 2:36994439:A:C (rs11124542)

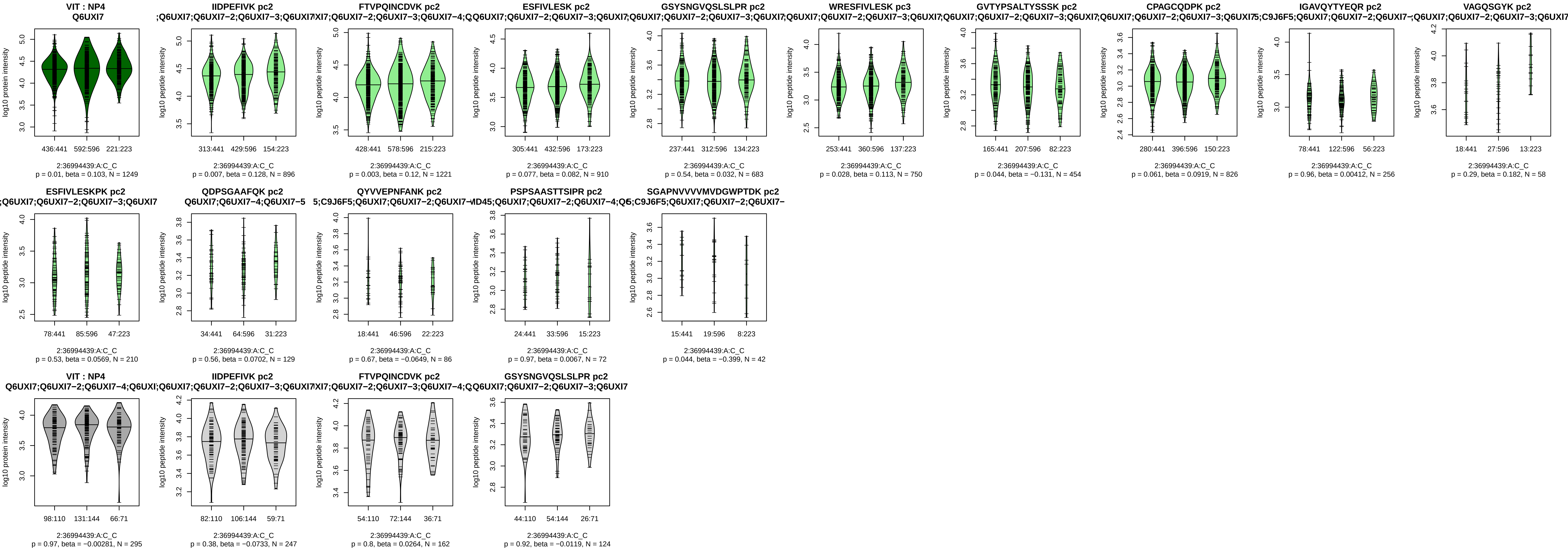

Assay Target: VIT
UniProt: Q6UXI7
rsID: rs11124542
Target SNP: 2:36994439:A:C
Proxy SNP: 2:36994439:A:C
UKBPPP log10(p): 1223.6
UKBPPP BETA: 0.413
UKBPPP MSPA: 0.321
::–:–:–:–:–:–:–:–:–:–:–
896:1221:910:683:750:454:826:

76. IGFBP3 (P17936)
UKBPPP MSPA=0

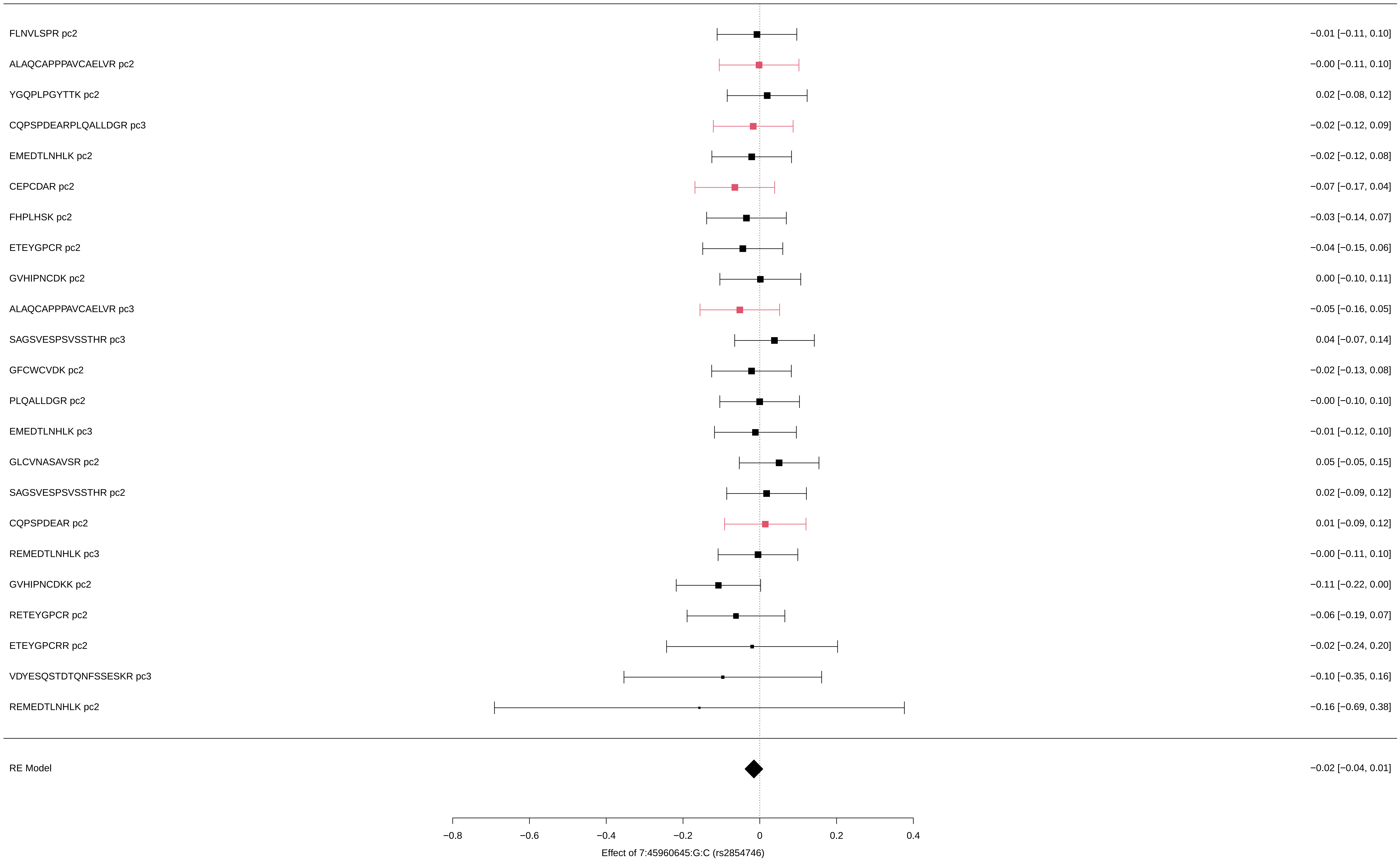

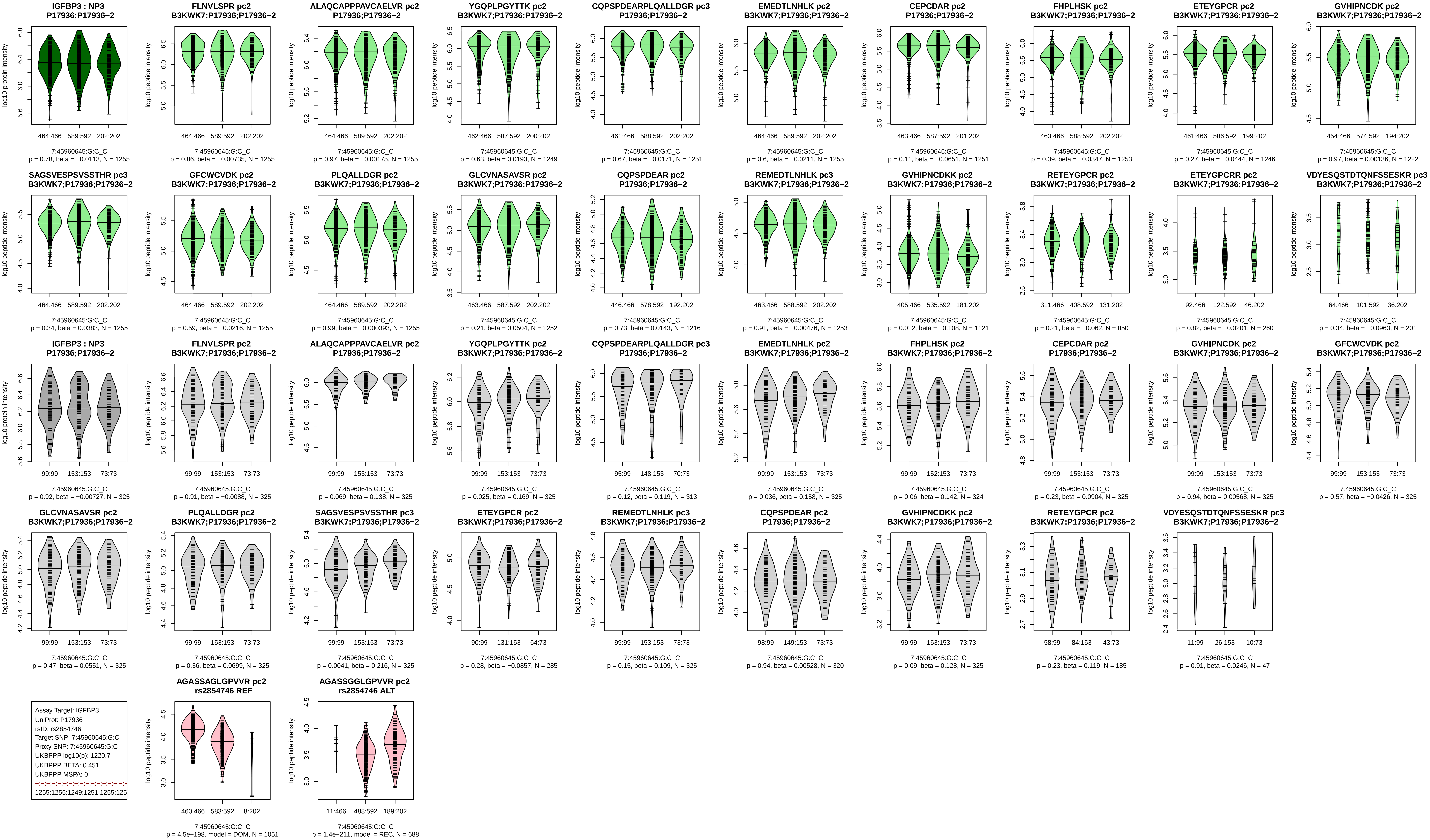

IGFBP3 : NP3 P17936;P17936−2
464:466 589:592 202:202
7:45960645:G:C_C
p = 0.78, beta = −0.0113, N = 1255
FLNVLSPR pc2 B3KWK7;P17936;P17936−2
464:466 589:592 202:202
7:45960645:G:C_C
p = 0.86, beta = −0.00735, N = 1255
ALAQCAPPPAVCAELVR pc2 P17936;P17936−2
464:466 589:592 202:202
7:45960645:G:C_C
p = 0.97, beta = −0.00175, N = 1255
YGQPLPGYTTK pc2 B3KWK7;P17936;P17936−2
462:466 587:592 200:202
7:45960645:G:C_C
p = 0.63, beta = 0.0193, N = 1249
CQPSPDEARPLQALLDGR pc3 P17936;P17936−2
461:466 588:592 202:202
7:45960645:G:C_C
p = 0.67, beta = −0.0171, N = 1251
EMEDTLNHLK pc2 B3KWK7;P17936;P17936−2
464:466 589:592 202:202
7:45960645:G:C_C
p = 0.6, beta = −0.0211, N = 1255
CEPCDAR pc2 P17936;P17936−2
463:466 587:592 201:202
7:45960645:G:C_C
p = 0.11, beta = −0.0651, N = 1251
FHPLHSK pc2 B3KWK7;P17936;P17936−2
463:466 588:592 202:202
7:45960645:G:C_C
p = 0.39, beta = −0.0347, N = 1253
ETEYGPCR pc2 B3KWK7;P17936;P17936−2
461:466 586:592 199:202
7:45960645:G:C_C
p = 0.27, beta = −0.0444, N = 1246
GVHIPNCDK pc2 B3KWK7;P17936;P17936−2
454:466 574:592 194:202
7:45960645:G:C_C
p = 0.97, beta = 0.00136, N = 1222
SAGSVESPSVSSTHR pc3 B3KWK7;P17936;P17936−2
464:466 589:592 202:202
7:45960645:G:C_C
p = 0.34, beta = 0.0383, N = 1255
GFCWCVDK pc2 B3KWK7;P17936;P17936−2
464:466 589:592 202:202
7:45960645:G:C_C
p = 0.59, beta = −0.0216, N = 1255
PLQALLDGR pc2 B3KWK7;P17936;P17936−2
464:466 589:592 202:202
7:45960645:G:C_C
p = 0.99, beta = −0.000393, N = 1255
GLCVNASAVSR pc2 B3KWK7;P17936;P17936−2
463:466 587:592 202:202
7:45960645:G:C_C
p = 0.21, beta = 0.0504, N = 1252
CQPSPDEAR pc2 P17936;P17936−2
446:466 578:592 192:202
7:45960645:G:C_C
p = 0.73, beta = 0.0143, N = 1216
REMEDTLNHLK pc3 B3KWK7;P17936;P17936−2
463:466 588:592 202:202
7:45960645:G:C_C
p = 0.91, beta = −0.00476, N = 1253
GVHIPNCDKK pc2 B3KWK7;P17936;P17936−2
405:466 535:592 181:202
7:45960645:G:C_C
p = 0.012, beta = −0.108, N = 1121
RETEYGPCR pc2 B3KWK7;P17936;P17936−2
311:466 408:592 131:202
7:45960645:G:C_C
p = 0.21, beta = −0.062, N = 850
ETEYGPCRR pc2 B3KWK7;P17936;P17936−2
92:466 122:592 46:202
7:45960645:G:C_C
p = 0.82, beta = −0.0201, N = 260
VDYESQSTDTQNFSSESKR pc3 B3KWK7;P17936;P17936−2
64:466 101:592 36:202
7:45960645:G:C_C
p = 0.34, beta = −0.0963, N = 201
IGFBP3 : NP3 P17936;P17936−2
99:99 153:153 73:73
7:45960645:G:C_C
p = 0.92, beta = −0.00727, N = 325
FLNVLSPR pc2 B3KWK7;P17936;P17936−2
99:99 153:153 73:73
7:45960645:G:C_C
p = 0.91, beta = −0.0088, N = 325
ALAQCAPPPAVCAELVR pc2 P17936;P17936−2
99:99 153:153 73:73
7:45960645:G:C_C
p = 0.069, beta = 0.138, N = 325
YGQPLPGYTTK pc2 B3KWK7;P17936;P17936−2
99:99 153:153 73:73
7:45960645:G:C_C
p = 0.025, beta = 0.169, N = 325
CQPSPDEARPLQALLDGR pc3 P17936;P17936−2
95:99 148:153 70:73
7:45960645:G:C_C
p = 0.12, beta = 0.119, N = 313
EMEDTLNHLK pc2 B3KWK7;P17936;P17936−2
99:99 153:153 73:73
7:45960645:G:C_C
p = 0.036, beta = 0.158, N = 325
FHPLHSK pc2 B3KWK7;P17936;P17936−2
99:99 152:153 73:73
7:45960645:G:C_C
p = 0.06, beta = 0.142, N = 324
CEPCDAR pc2 P17936;P17936−2
99:99 153:153 73:73
7:45960645:G:C_C
p = 0.23, beta = 0.0904, N = 325
GVHIPNCDK pc2 B3KWK7;P17936;P17936−2
99:99 153:153 73:73
7:45960645:G:C_C
p = 0.94, beta = 0.00568, N = 325
GFCWCVDK pc2 B3KWK7;P17936;P17936−2
99:99 153:153 73:73
7:45960645:G:C_C
p = 0.57, beta = −0.0426, N = 325
GLCVNASAVSR pc2 B3KWK7;P17936;P17936−2
99:99 153:153 73:73
7:45960645:G:C_C
p = 0.47, beta = 0.0551, N = 325
PLQALLDGR pc2 B3KWK7;P17936;P17936−2
99:99 153:153 73:73
7:45960645:G:C_C
p = 0.36, beta = 0.0699, N = 325
SAGSVESPSVSSTHR pc3 B3KWK7;P17936;P17936−2
99:99 153:153 73:73
7:45960645:G:C_C
p = 0.0041, beta = 0.216, N = 325
ETEYGPCR pc2 B3KWK7;P17936;P17936−2
90:99 131:153 64:73
7:45960645:G:C_C
p = 0.28, beta = −0.0857, N = 285
REMEDTLNHLK pc3 B3KWK7;P17936;P17936−2
99:99 153:153 73:73
7:45960645:G:C_C
p = 0.15, beta = 0.109, N = 325
CQPSPDEAR pc2 P17936;P17936−2
98:99 149:153 73:73
7:45960645:G:C_C
p = 0.94, beta = 0.00528, N = 320
GVHIPNCDKK pc2 B3KWK7;P17936;P17936−2
99:99 153:153 73:73
7:45960645:G:C_C
p = 0.09, beta = 0.128, N = 325
RETEYGPCR pc2 B3KWK7;P17936;P17936−2
58:99 84:153 43:73
7:45960645:G:C_C
p = 0.23, beta = 0.119, N = 185
VDYESQSTDTQNFSSESKR pc3 B3KWK7;P17936;P17936−2
11:99 26:153 10:73
7:45960645:G:C_C
p = 0.91, beta = 0.0246, N = 47
Assay Target: IGFBP3
UniProt: P17936
rsID: rs2854746
Target SNP: 7:45960645:G:C
Proxy SNP: 7:45960645:G:C
UKBPPP log10(p): 1220.7
UKBPPP BETA: 0.451
UKBPPP MSPA: 0
1255:1255:1249:1251:1255:125
AGASSAGLGPVVR pc2 rs2854746 REF
460:466 583:592 8:202
7:45960645:G:C_C
p = 4.5e−198, model = DOM, N = 1051
AGASSGGLGPVVR pc2 rs2854746 ALT
11:466 488:592 189:202
7:45960645:G:C_C
p = 1.4e−211, model = REC, N = 688
log10 protein intensity
log10 peptide intensity

77. CPXM2 (Q8N436)
UKBPPP MSPA=0.793

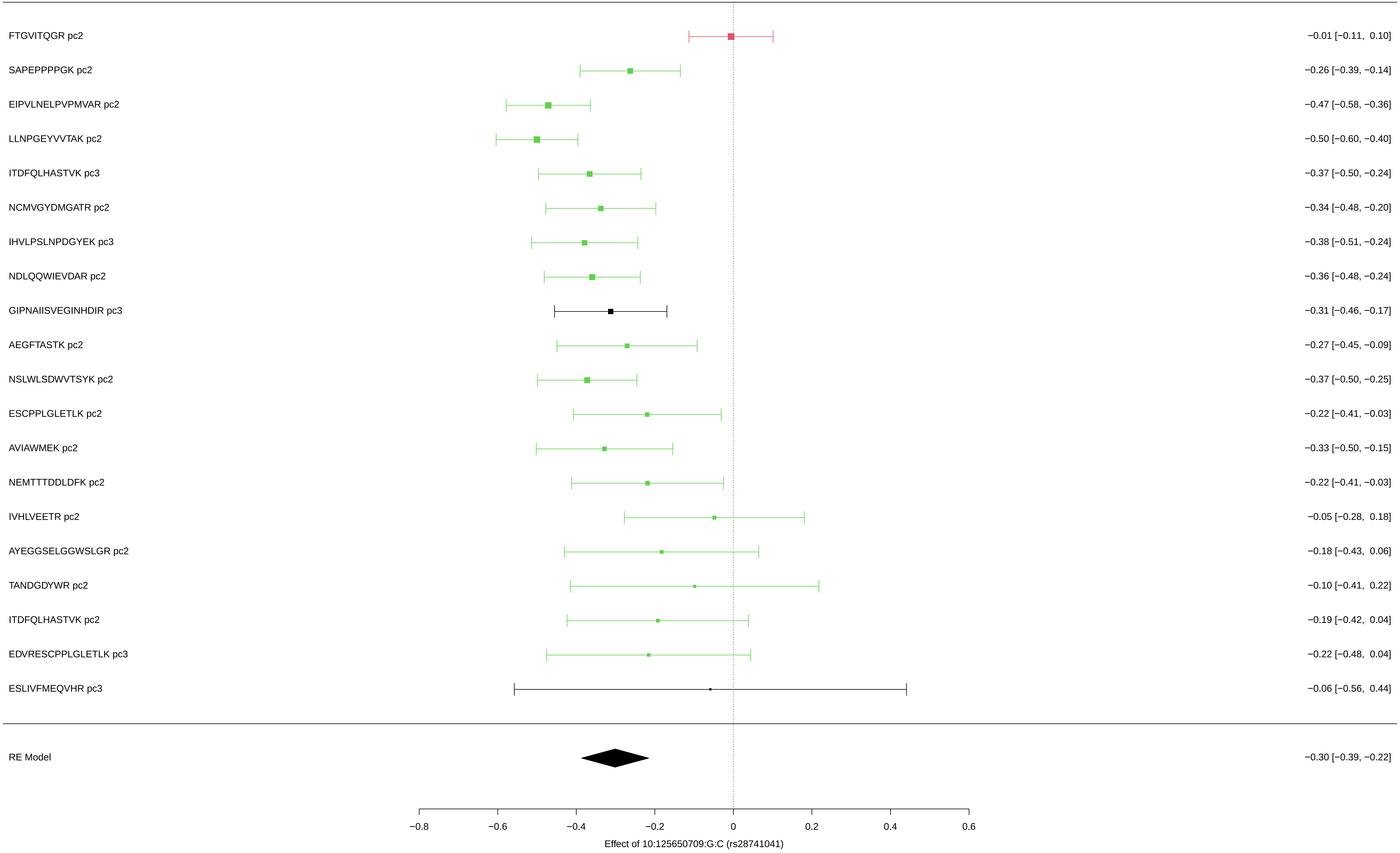

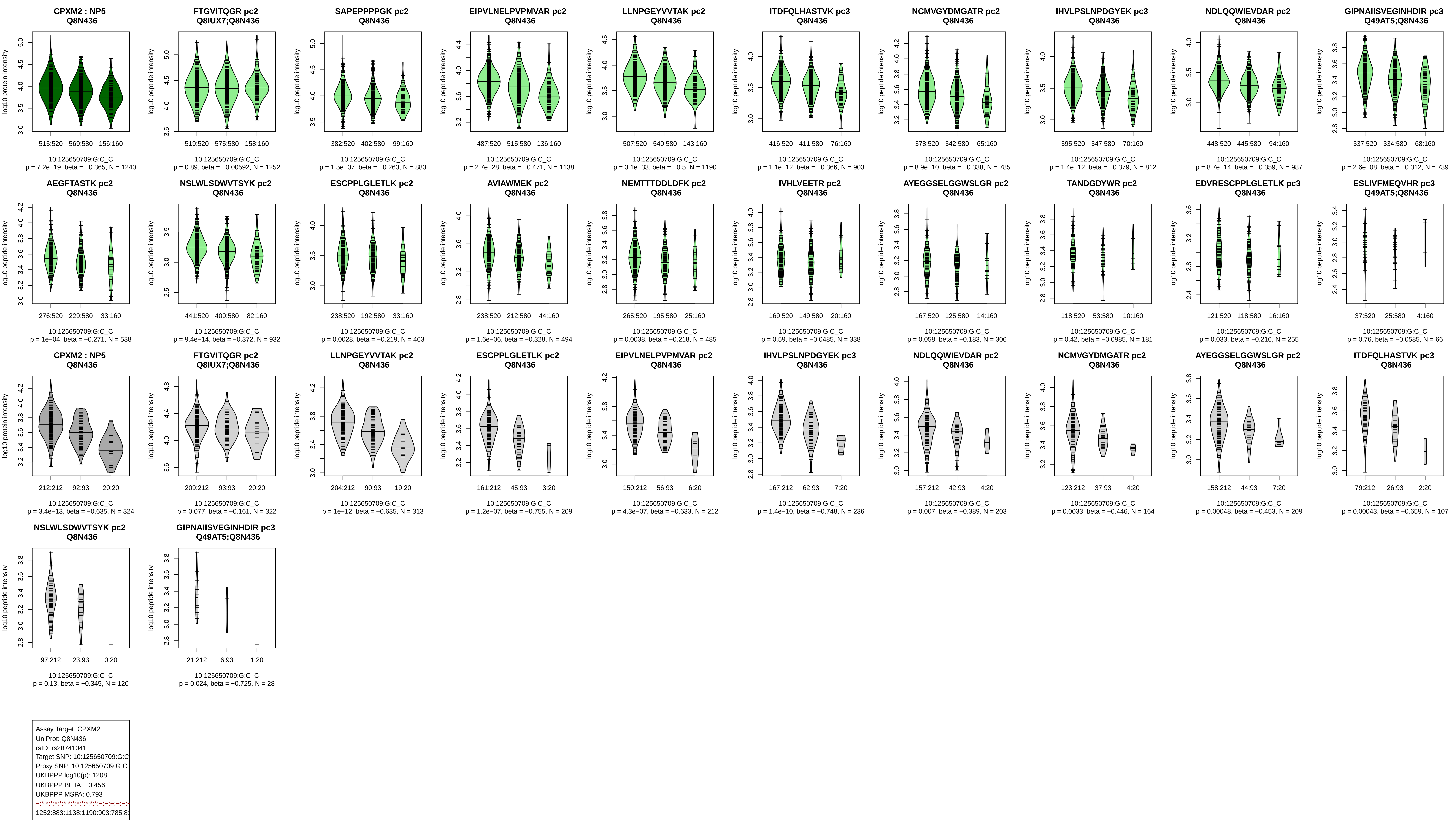
CPXM2 : NP5
Q8N436
515:520 569:580 156:160
10:125650709:G:C_C
p = 7.2e−19, beta = −0.365, N = 1240
FTGVITQGR pc2
Q8IUX7;Q8N436
519:520 575:580 158:160
10:125650709:G:C_C
p = 0.89, beta = −0.00592, N = 1252
SAPEPPPPGK pc2
Q8N436
382:520 402:580 99:160
10:125650709:G:C_C
p = 1.5e−07, beta = −0.263, N = 883
EIPVLNELPVPMVAR pc2
Q8N436
487:520 515:580 136:160
10:125650709:G:C_C
p = 2.7e−28, beta = −0.471, N = 1138
LLNPGEYVVTAK pc2
Q8N436
507:520 540:580 143:160
10:125650709:G:C_C
p = 3.1e−33, beta = −0.5, N = 1190
ITDFQLHASTVK pc3
Q8N436
416:520 411:580 76:160
10:125650709:G:C_C
p = 1.1e−12, beta = −0.366, N = 903
NCMVGYDMGATR pc2
Q8N436
378:520 342:580 65:160
10:125650709:G:C_C
p = 8.9e−10, beta = −0.338, N = 785
IHVLPSLNPDGYEK pc3
Q8N436
395:520 347:580 70:160
10:125650709:G:C_C
p = 1.4e−12, beta = −0.379, N = 812
NDLQQWIEVDAR pc2
Q8N436
448:520 445:580 94:160
10:125650709:G:C_C
p = 8.7e−14, beta = −0.359, N = 987
GIPNAIISVEGINHDIR pc3
Q49AT5;Q8N436
337:520 334:580 68:160
10:125650709:G:C_C
p = 2.6e−08, beta = −0.312, N = 739
AEGFTASTK pc2
Q8N436
276:520 229:580 33:160
10:125650709:G:C_C
p = 1e−04, beta = −0.271, N = 538
NSLWLSDWVTSYK pc2
Q8N436
441:520 409:580 82:160
10:125650709:G:C_C
p = 9.4e−14, beta = −0.372, N = 932
ESCPPLGLETLK pc2
Q8N436
238:520 192:580 33:160
10:125650709:G:C_C
p = 0.0028, beta = −0.219, N = 463
AVIAWMEK pc2
Q8N436
238:520 212:580 44:160
10:125650709:G:C_C
p = 1.6e−06, beta = −0.328, N = 494
NEMTTTDDLDFK pc2
Q8N436
265:520 195:580 25:160
10:125650709:G:C_C
p = 0.0038, beta = −0.218, N = 485
IVHLVEETR pc2
Q8N436
169:520 149:580 20:160
10:125650709:G:C_C
p = 0.59, beta = −0.0485, N = 338
AYEGGSELGGWSLGR pc2
Q8N436
167:520 125:580 14:160
10:125650709:G:C_C
p = 0.058, beta = −0.183, N = 306
TANDGDYWR pc2
Q8N436
118:520 53:580 10:160
10:125650709:G:C_C
p = 0.42, beta = −0.0985, N = 181
EDVRESCPPLGLETLK pc3
Q8N436
121:520 118:580 16:160
10:125650709:G:C_C
p = 0.033, beta = −0.216, N = 255
ESLIVFMEQVHR pc3
Q49AT5;Q8N436
37:520 25:580 4:160
10:125650709:G:C_C
p = 0.76, beta = −0.0585, N = 66
CPXM2 : NP5
Q8N436
212:212 92:93 20:20
10:125650709:G:C_C
p = 3.4e−13, beta = −0.635, N = 324
FTGVITQGR pc2
Q8IUX7;Q8N436
209:212 93:93 20:20
10:125650709:G:C_C
p = 0.077, beta = −0.161, N = 322
LLNPGEYVVTAK pc2
Q8N436
204:212 90:93 19:20
10:125650709:G:C_C
p = 1e−12, beta = −0.635, N = 313
ESCPPLGLETLK pc2
Q8N436
161:212 45:93 3:20
10:125650709:G:C_C
p = 1.2e−07, beta = −0.755, N = 209
EIPVLNELPVPMVAR pc2
Q8N436
150:212 56:93 6:20
10:125650709:G:C_C
p = 4.3e−07, beta = −0.633, N = 212
IHVLPSLNPDGYEK pc3
Q8N436
167:212 62:93 7:20
10:125650709:G:C_C
p = 1.4e−10, beta = −0.748, N = 236
NDLQQWIEVDAR pc2
Q8N436
157:212 42:93 4:20
10:125650709:G:C_C
p = 0.007, beta = −0.389, N = 203
NCMVGYDMGATR pc2
Q8N436
123:212 37:93 4:20
10:125650709:G:C_C
p = 0.0033, beta = −0.446, N = 164
AYEGGSELGGWSLGR pc2
Q8N436
158:212 44:93 7:20
10:125650709:G:C_C
p = 0.00048, beta = −0.453, N = 209
ITDFQLHASTVK pc3
Q8N436
79:212 26:93 2:20
10:125650709:G:C_C
p = 0.00043, beta = −0.659, N = 107
NSLWLSDWVTSYK pc2
Q8N436
97:212 23:93 0:20
10:125650709:G:C_C
p = 0.13, beta = −0.345, N = 120
GIPNAIISVEGINHDIR pc3
Q49AT5;Q8N436
21:212 6:93 1:20
10:125650709:G:C_C
p = 0.024, beta = −0.725, N = 28
log10 protein intensity
log10 peptide intensity
Assay Target: CPXM2
UniProt: Q8N436
rsID: rs28741041
Target SNP: 10:125650709:G:C
Proxy SNP: 10:125650709:G:C
UKBPPP log10(p): 1208
UKBPPP BETA: −0.456
UKBPPP MSPA: 0.793
1252:883:1138:1190:903:785:8

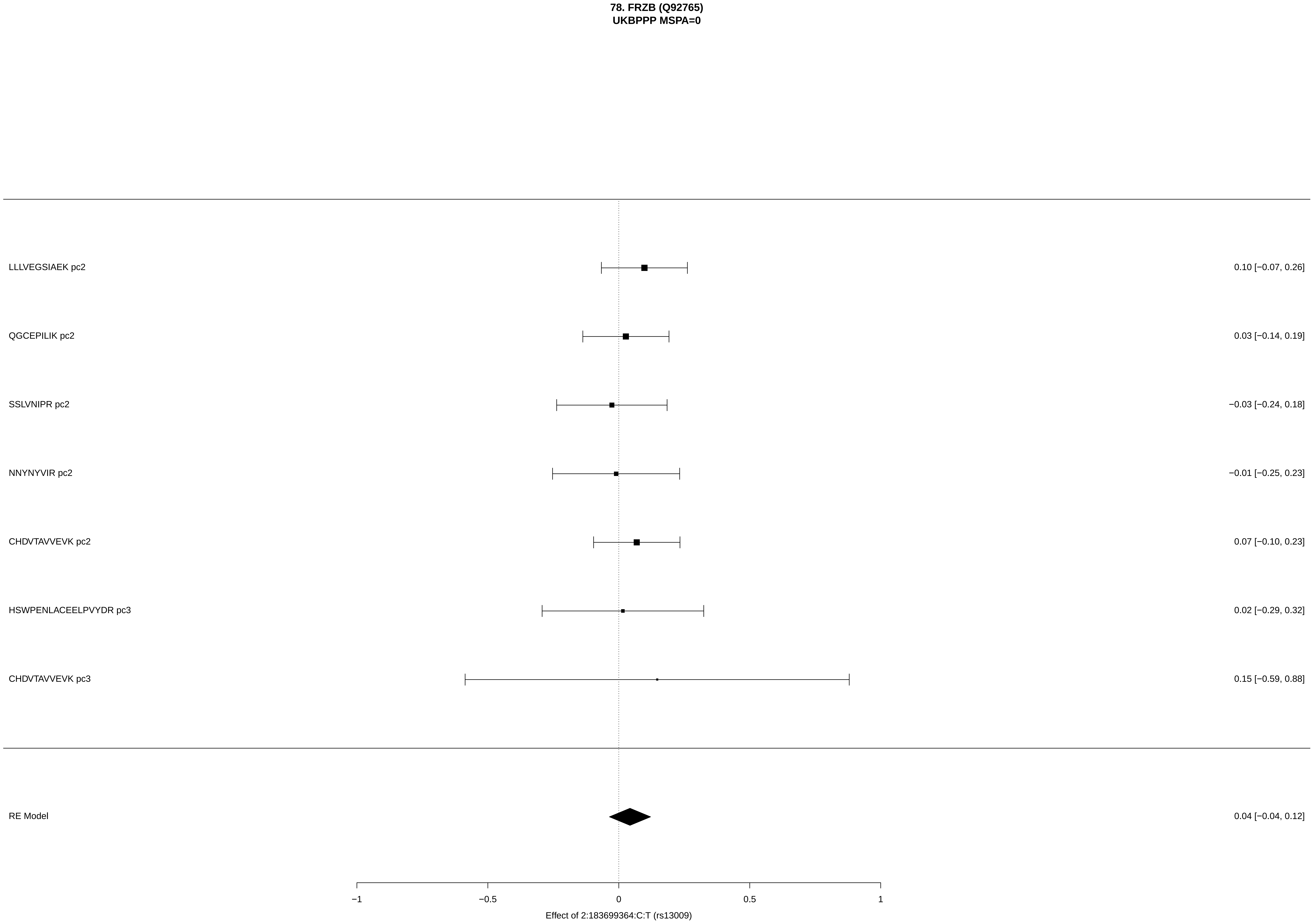
78. FRZB (Q92765)
UKBPPP MSPA=0
LLLVEGSIAEK pc2
0.10 [−0.07, 0.26]
QGCEPILIK pc2
0.03 [−0.14, 0.19]
SSLVNIPR pc2
−0.03 [−0.24, 0.18]
NNYNYVIR pc2
−0.01 [−0.25, 0.23]
CHDVTAVVEVK pc2
0.07 [−0.10, 0.23]
HSWPENLACEELPVYDR pc3
0.02 [−0.29, 0.32]
CHDVTAVVEVK pc3
0.15 [−0.59, 0.88]
RE Model
0.04 [−0.04, 0.12]
−1
−0.5
0
0.5
1
Effect of 2:183699364:C:T (rs13009)

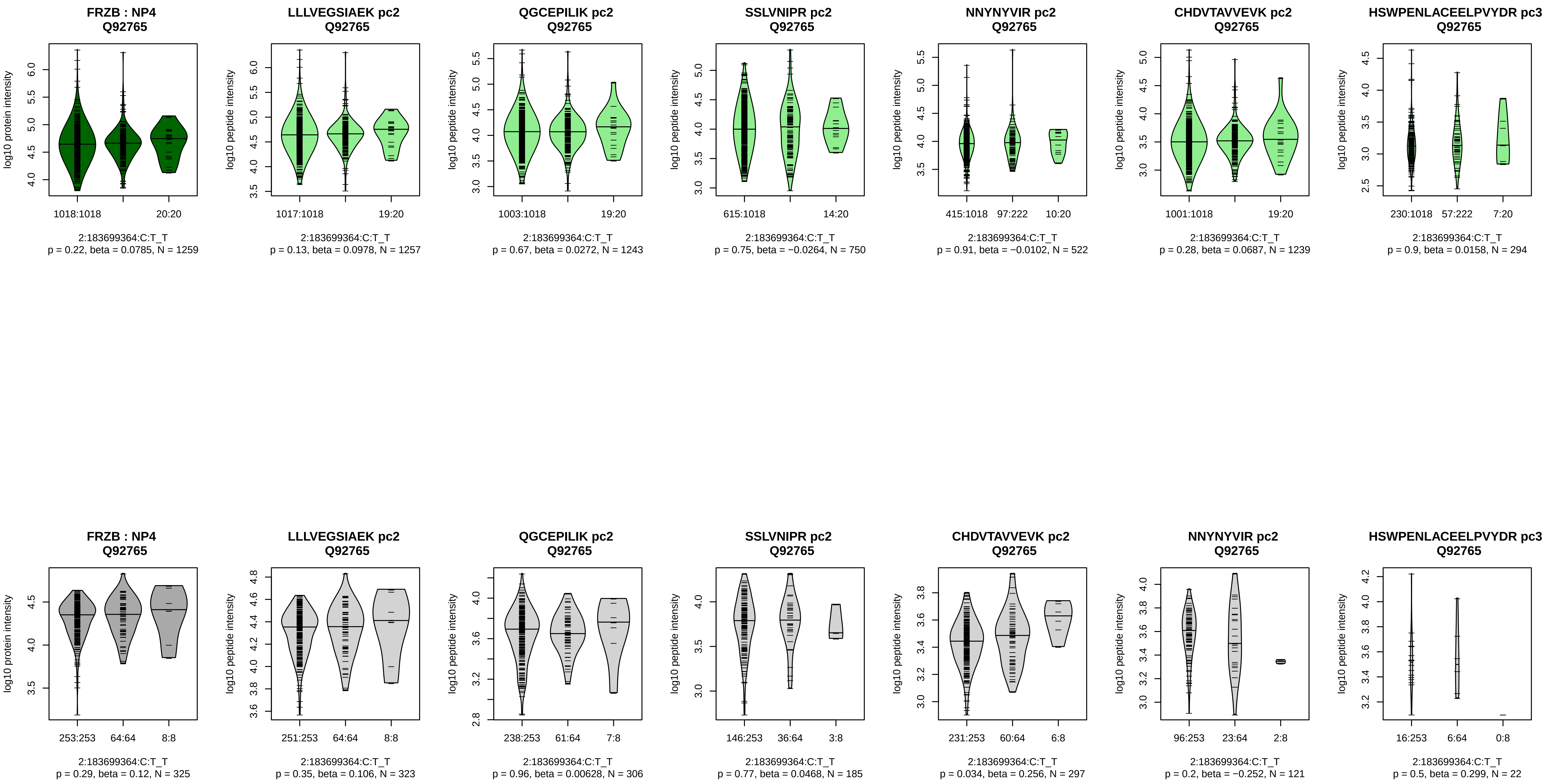

Assay Target: FRZB
UniProt: Q92765
rsID: rs13009
Target SNP: 2:183699364:C:T
Proxy SNP: 2:183699364:C:T
UKBPPP log10(p): 1192.8
UKBPPP BETA: −0.783
UKBPPP MSPA: 0
−:−:−:−:−:−:−:NA
1257:1243:750:522:1239:294:53

79. HGFAC (Q04756)
UKBPPP MSPA=0.084

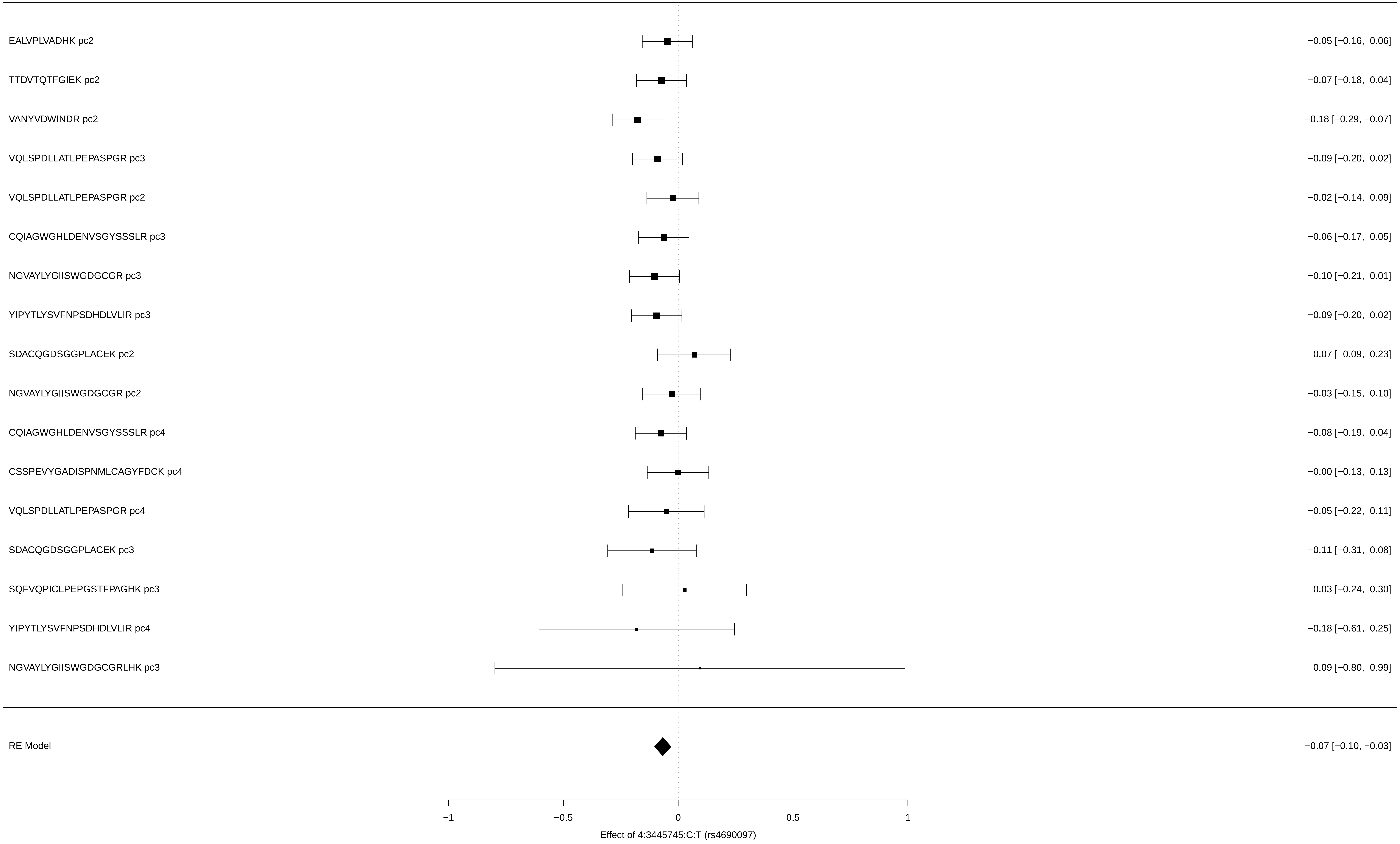

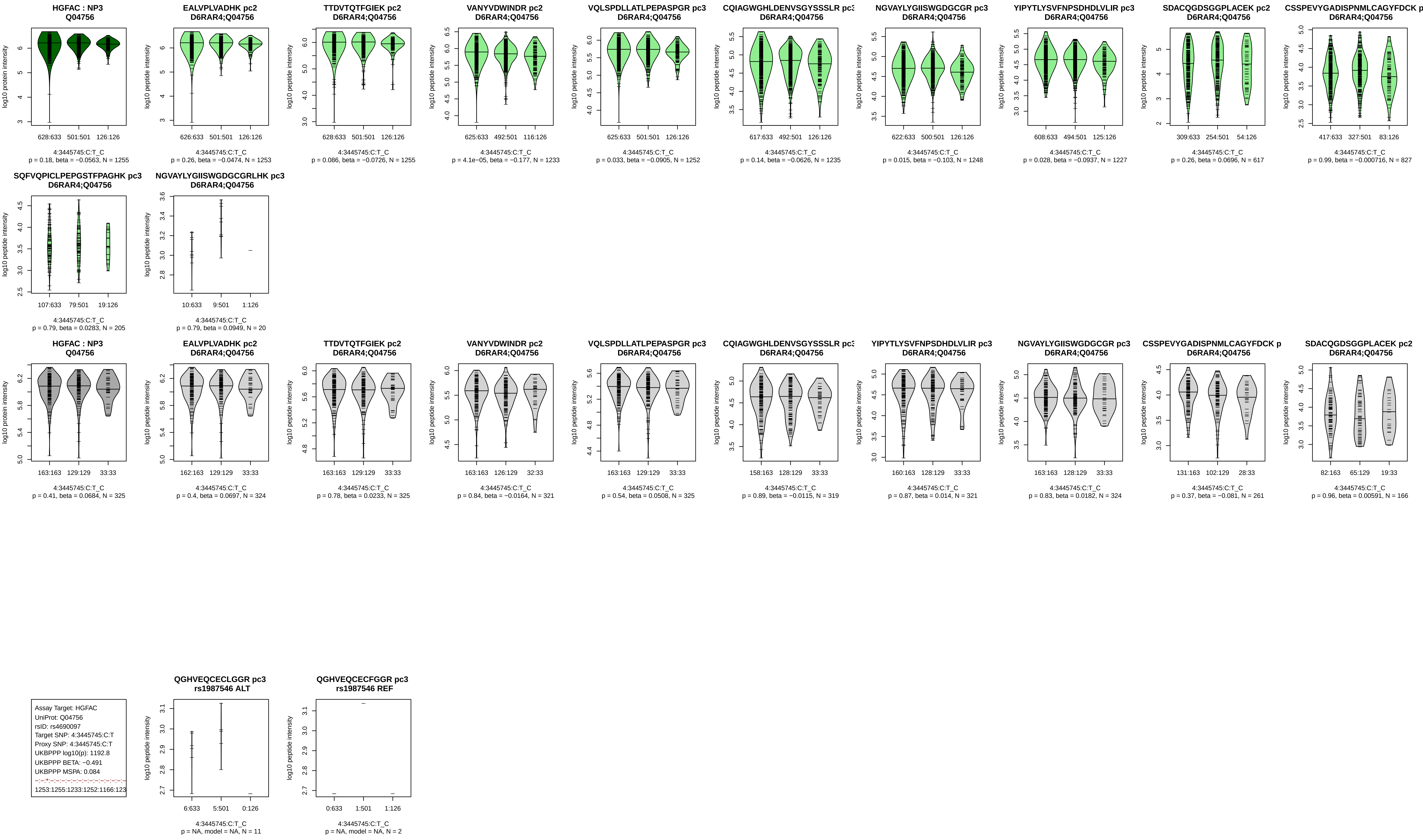
HGFAC : NP3 Q04756
4:3445745:C:T_C p = 0.18, beta = −0.0563, N = 1255
EALVPLVADHK pc2 D6RAR4;Q04756
4:3445745:C:T_C p = 0.26, beta = −0.0474, N = 1253
TTDVTQTFGIEK pc2 D6RAR4;Q04756
4:3445745:C:T_C p = 0.086, beta = −0.0726, N = 1255
VANYVDWINDR pc2 D6RAR4;Q04756
4:3445745:C:T_C p = 4.1e−05, beta = −0.177, N = 1233
VQLSPDLLATLPEPASPGR pc3 D6RAR4;Q04756
4:3445745:C:T_C p = 0.033, beta = −0.0905, N = 1252
CQIAGWGHLDENVSGYSSSLR pc3 D6RAR4;Q04756
4:3445745:C:T_C p = 0.14, beta = −0.0626, N = 1235
NGVAYLYGIISWGDGCGR pc3 D6RAR4;Q04756
4:3445745:C:T_C p = 0.015, beta = −0.103, N = 1248
YIPYTLYSVFNPSDHDLVLIR pc3 D6RAR4;Q04756
4:3445745:C:T_C p = 0.028, beta = −0.0937, N = 1227
SDACQGDSGGPLACEK pc2 D6RAR4;Q04756
4:3445745:C:T_C p = 0.26, beta = 0.0696, N = 617
CSSPEVYGADISPNMLCAGYFDCK p D6RAR4;Q04756
4:3445745:C:T_C p = 0.99, beta = −0.000716, N = 827
SQFVQPICLPEPGSTFPAGHK pc3 D6RAR4;Q04756
4:3445745:C:T_C p = 0.79, beta = 0.0283, N = 205
NGVAYLYGIISWGDGCGRLHK pc3 D6RAR4;Q04756
4:3445745:C:T_C p = 0.79, beta = 0.0949, N = 20
HGFAC : NP3 Q04756
4:3445745:C:T_C p = 0.41, beta = 0.0684, N = 325
EALVPLVADHK pc2 D6RAR4;Q04756
4:3445745:C:T_C p = 0.4, beta = 0.0697, N = 324
TTDVTQTFGIEK pc2 D6RAR4;Q04756
4:3445745:C:T_C p = 0.78, beta = 0.0233, N = 325
VANYVDWINDR pc2 D6RAR4;Q04756
4:3445745:C:T_C p = 0.84, beta = −0.0164, N = 321
VQLSPDLLATLPEPASPGR pc3 D6RAR4;Q04756
4:3445745:C:T_C p = 0.54, beta = 0.0508, N = 325
CQIAGWGHLDENVSGYSSSLR pc3 D6RAR4;Q04756
4:3445745:C:T_C p = 0.89, beta = −0.0115, N = 319
YIPYTLYSVFNPSDHDLVLIR pc3 D6RAR4;Q04756
4:3445745:C:T_C p = 0.87, beta = 0.014, N = 321
NGVAYLYGIISWGDGCGR pc3 D6RAR4;Q04756
4:3445745:C:T_C p = 0.83, beta = 0.0182, N = 324
CSSPEVYGADISPNMLCAGYFDCK p D6RAR4;Q04756
4:3445745:C:T_C p = 0.37, beta = −0.081, N = 261
SDACQGDSGGPLACEK pc2 D6RAR4;Q04756
4:3445745:C:T_C p = 0.96, beta = 0.00591, N = 166
Assay Target: HGFAC
UniProt: Q04756
rsID: rs4690097
Target SNP: 4:3445745:C:T
Proxy SNP: 4:3445745:C:T
UKBPPP log10(p): 1192.8
UKBPPP BETA: −0.491
UKBPPP MSPA: 0.084
1253:1255:1233:1252:1166:123
QGHVEQCECLGGR pc3 rs1987546 ALT
4:3445745:C:T_C p = NA, model = NA, N = 11
QGHVEQCECFGGR pc3 rs1987546 REF
4:3445745:C:T_C p = NA, model = NA, N = 2

80. LPL (P06858)
UKBPPP MSPA=0.079

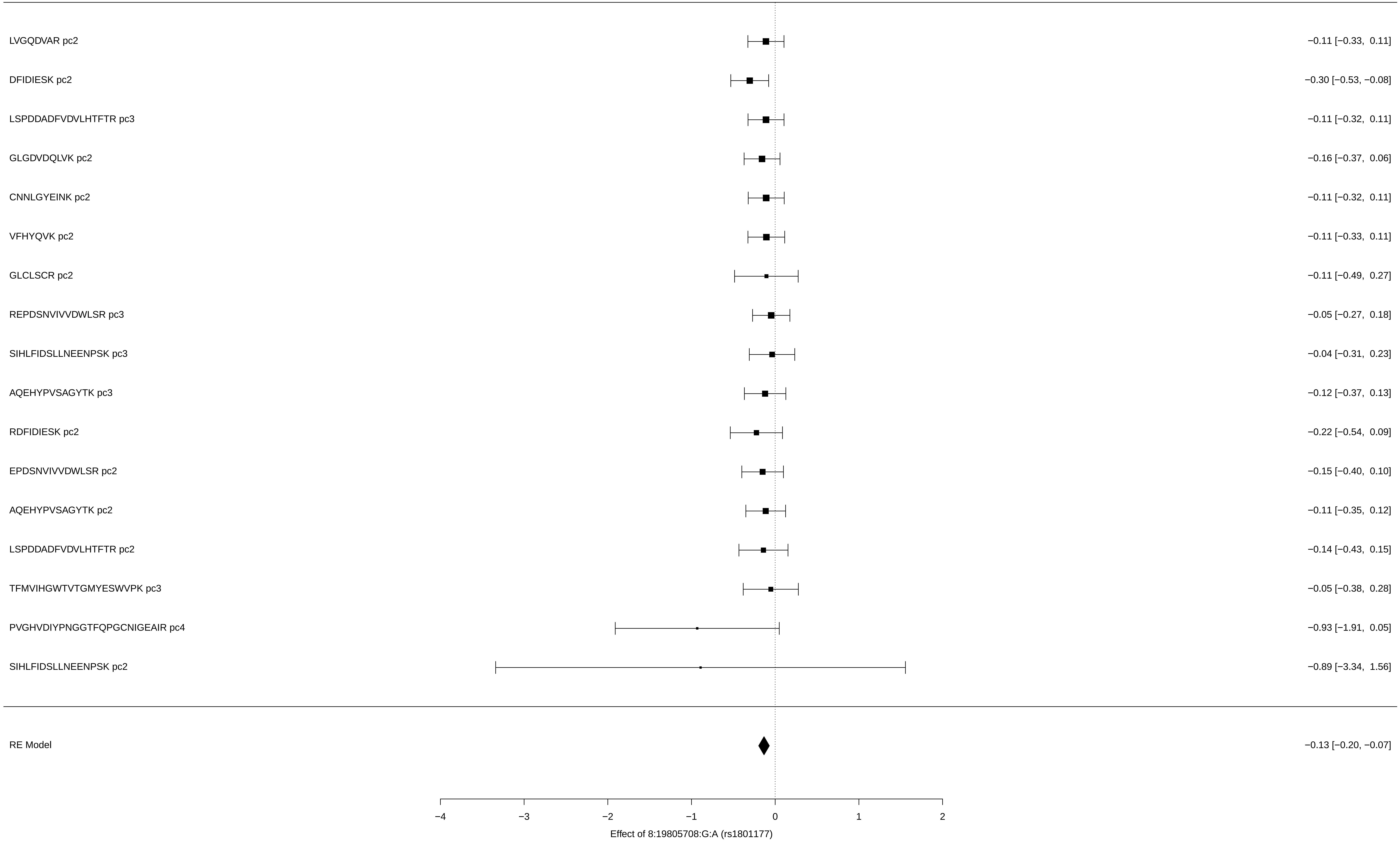

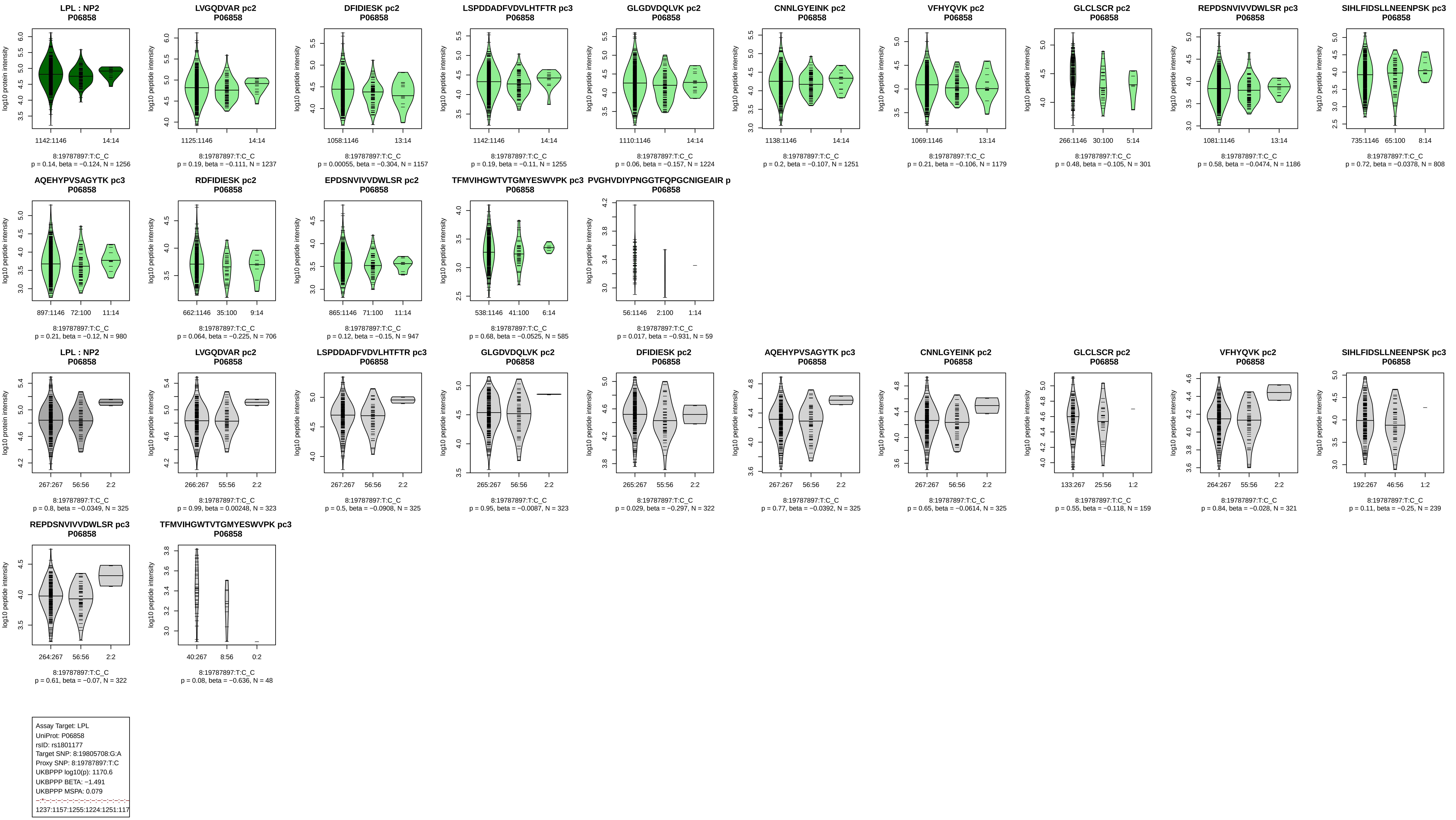
LPL : NP2 P06858
8:19787897:T:C_C
p = 0.14, beta = −0.124, N = 1256
LVGQDVAR pc2 P06858
8:19787897:T:C_C
p = 0.19, beta = −0.111, N = 1237
DFIDIESK pc2 P06858
8:19787897:T:C_C
p = 0.00055, beta = −0.304, N = 1157
LSPDDADFVDVLHTFTR pc3 P06858
8:19787897:T:C_C
p = 0.19, beta = −0.11, N = 1255
GLGDVDQLVK pc2 P06858
8:19787897:T:C_C
p = 0.06, beta = −0.157, N = 1224
CNNLGYEINK pc2 P06858
8:19787897:T:C_C
p = 0.2, beta = −0.107, N = 1251
VFHYQVK pc2 P06858
8:19787897:T:C_C
p = 0.21, beta = −0.106, N = 1179
GLCLSCR pc2 P06858
8:19787897:T:C_C
p = 0.48, beta = −0.105, N = 301
REPDSNVIVVDWLSR pc3 P06858
8:19787897:T:C_C
p = 0.58, beta = −0.0474, N = 1186
SIHLFIDSLLNEENPSK pc3 P06858
8:19787897:T:C_C
p = 0.72, beta = −0.0378, N = 808
AQEHYPVSAGYTK pc3 P06858
8:19787897:T:C_C
p = 0.21, beta = −0.12, N = 980
RDFIDIESK pc2 P06858
8:19787897:T:C_C
p = 0.064, beta = −0.225, N = 706
EPDSNVIVVDWLSR pc2 P06858
8:19787897:T:C_C
p = 0.12, beta = −0.15, N = 947
TFMVIHGWTVTGMYESWVPK pc3 P06858
8:19787897:T:C_C
p = 0.68, beta = −0.0525, N = 585
PVGHVDIYPNGGTFQPGCNIGEAIR p P06858
8:19787897:T:C_C
p = 0.017, beta = −0.931, N = 59
LPL : NP2 P06858
8:19787897:T:C_C
p = 0.8, beta = −0.0349, N = 325
LVGQDVAR pc2 P06858
8:19787897:T:C_C
p = 0.99, beta = 0.00248, N = 323
LSPDDADFVDVLHTFTR pc3 P06858
8:19787897:T:C_C
p = 0.5, beta = −0.0908, N = 325
GLGDVDQLVK pc2 P06858
8:19787897:T:C_C
p = 0.95, beta = −0.0087, N = 323
DFIDIESK pc2 P06858
8:19787897:T:C_C
p = 0.029, beta = −0.297, N = 322
AQEHYPVSAGYTK pc3 P06858
8:19787897:T:C_C
p = 0.77, beta = −0.0392, N = 325
CNNLGYEINK pc2 P06858
8:19787897:T:C_C
p = 0.65, beta = −0.0614, N = 325
GLCLSCR pc2 P06858
8:19787897:T:C_C
p = 0.55, beta = −0.118, N = 159
VFHYQVK pc2 P06858
8:19787897:T:C_C
p = 0.84, beta = −0.028, N = 321
SIHLFIDSLLNEENPSK pc3 P06858
8:19787897:T:C_C
p = 0.11, beta = −0.25, N = 239
REPDSNVIVVDWLSR pc3 P06858
8:19787897:T:C_C
p = 0.61, beta = −0.07, N = 322
TFMVIHGWTVTGMYESWVPK pc3 P06858
8:19787897:T:C_C
p = 0.08, beta = −0.636, N = 48
Assay Target: LPL
UniProt: P06858
rsID: rs1801177
Target SNP: 8:19805708:G:A
Proxy SNP: 8:19787897:T:C
UKBPPP log10(p): 1170.6
UKBPPP BETA: −1.491
UKBPPP MSPA: 0.079
1237:1157:1255:1224:1251:117

81. C1S (P09871)
UKBPPP MSPA=0.397 (2 conflicts)

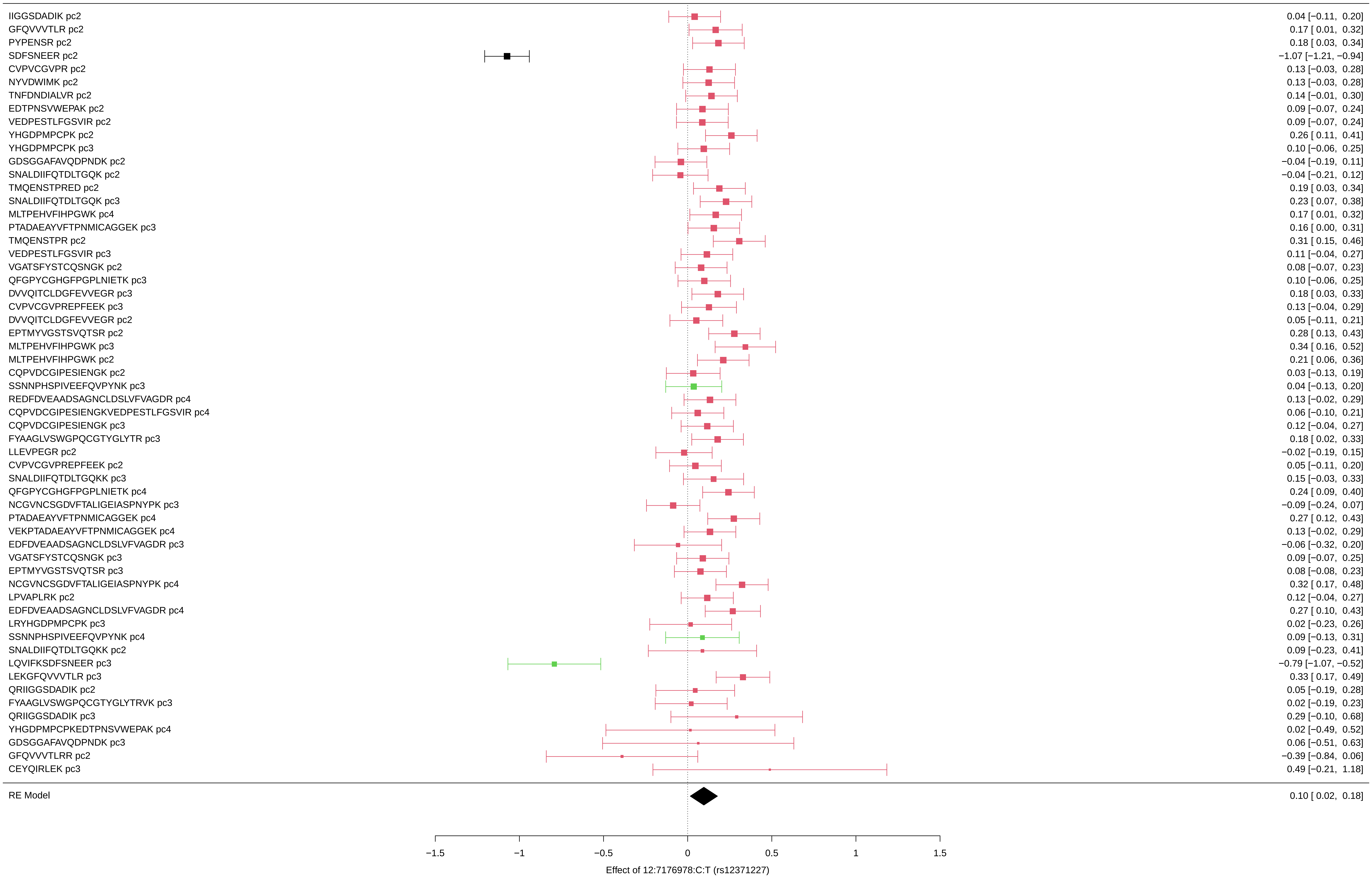

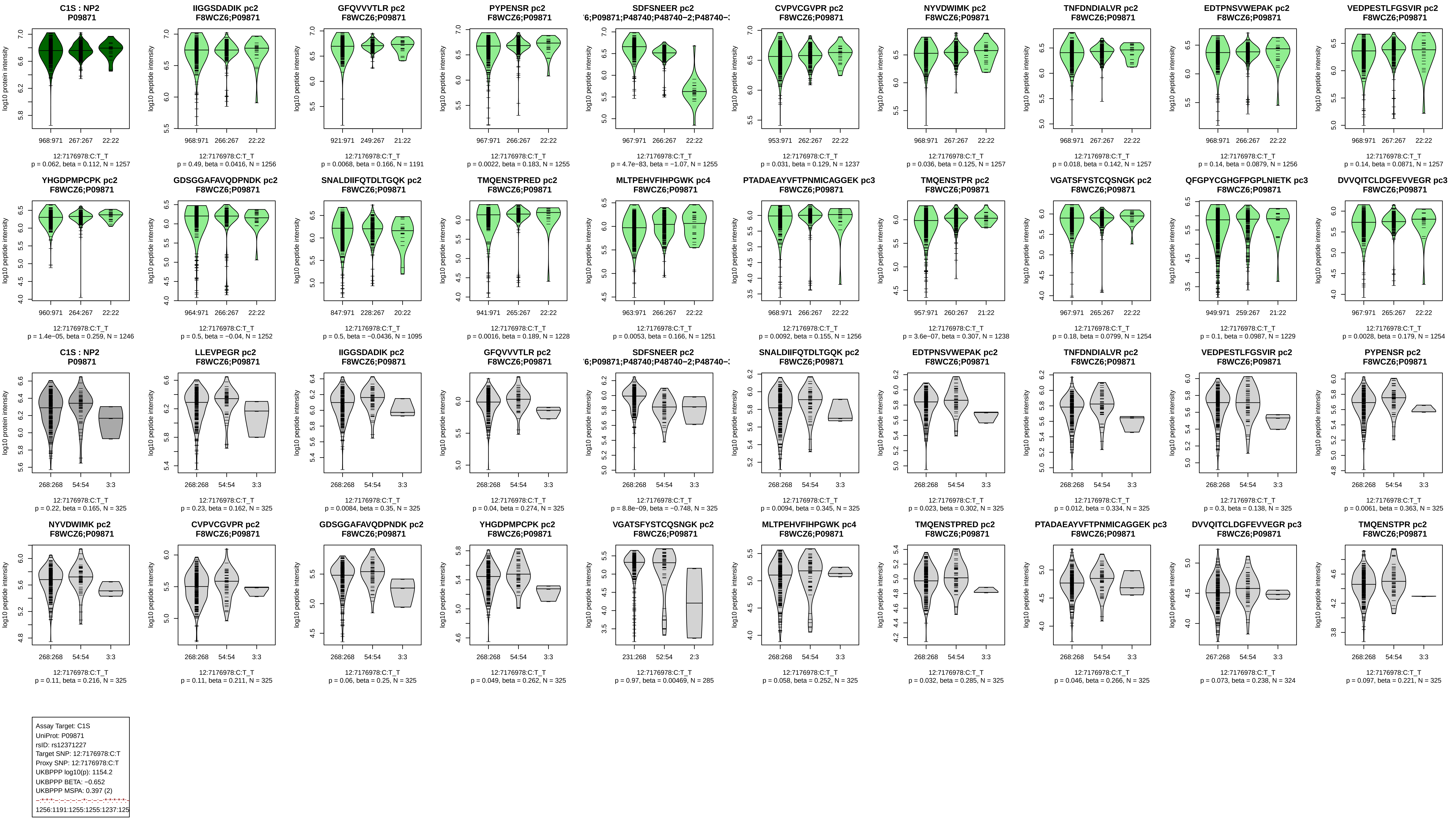
C1S : NP2
P09871
12:7176978:C:T_T
p = 0.062, beta = 0.112, N = 1257
IIGGSDADIK pc2
F8WCZ6;P09871
12:7176978:C:T_T
p = 0.49, beta = 0.0416, N = 1256
GFQVVVTLR pc2
F8WCZ6;P09871
12:7176978:C:T_T
p = 0.0068, beta = 0.166, N = 1191
PYPENSR pc2
F8WCZ6;P09871
12:7176978:C:T_T
p = 0.0022, beta = 0.183, N = 1255
SDFSNEER pc2
'6;P09871;P48740;P48740−2;P48740−
12:7176978:C:T_T
p = 4.7e−83, beta = −1.07, N = 1255
CVPVCGVPR pc2
F8WCZ6;P09871
12:7176978:C:T_T
p = 0.031, beta = 0.129, N = 1237
NYVDWIMK pc2
F8WCZ6;P09871
12:7176978:C:T_T
p = 0.036, beta = 0.125, N = 1257
TNFDNDIALVR pc2
F8WCZ6;P09871
12:7176978:C:T_T
p = 0.018, beta = 0.142, N = 1257
EDTPNSVWEPAK pc2
F8WCZ6;P09871
12:7176978:C:T_T
p = 0.14, beta = 0.0879, N = 1256
VEDPESTLFGSVIR pc2
F8WCZ6;P09871
12:7176978:C:T_T
p = 0.14, beta = 0.0871, N = 1257
YHGDPMPCPK pc2
F8WCZ6;P09871
12:7176978:C:T_T
p = 1.4e−05, beta = 0.259, N = 1246
GDSGGAFAVQDPNDK pc2
F8WCZ6;P09871
12:7176978:C:T_T
p = 0.5, beta = −0.04, N = 1252
SNALDIIFQTDLTGQK pc2
F8WCZ6;P09871
12:7176978:C:T_T
p = 0.5, beta = −0.0436, N = 1095
TMQENSTPRED pc2
F8WCZ6;P09871
12:7176978:C:T_T
p = 0.0016, beta = 0.189, N = 1228
MLTPEHVFIHPGWK pc4
F8WCZ6;P09871
12:7176978:C:T_T
p = 0.0053, beta = 0.166, N = 1251
PTADAEAYVFTPNMICAGGEK pc3
F8WCZ6;P09871
12:7176978:C:T_T
p = 0.0092, beta = 0.155, N = 1256
TMQENSTPR pc2
F8WCZ6;P09871
12:7176978:C:T_T
p = 3.6e−07, beta = 0.307, N = 1238
VGATSFYSTCQSNGK pc2
F8WCZ6;P09871
12:7176978:C:T_T
p = 0.18, beta = 0.0799, N = 1254
QFGPYCGHGFPGPLNIETK pc3
F8WCZ6;P09871
12:7176978:C:T_T
p = 0.1, beta = 0.0987, N = 1229
DVVQITCLDGFEVVEGR pc3
F8WCZ6;P09871
12:7176978:C:T_T
p = 0.0028, beta = 0.179, N = 1254
C1S : NP2
P09871
12:7176978:C:T_T
p = 0.22, beta = 0.165, N = 325
LLEVPEGR pc2
F8WCZ6;P09871
12:7176978:C:T_T
p = 0.23, beta = 0.162, N = 325
IIGGSDADIK pc2
F8WCZ6;P09871
12:7176978:C:T_T
p = 0.0084, beta = 0.35, N = 325
GFQVVVTLR pc2
F8WCZ6;P09871
12:7176978:C:T_T
p = 0.04, beta = 0.274, N = 325
SDFSNEER pc2
'6;P09871;P48740;P48740−2;P48740−
12:7176978:C:T_T
p = 8.8e−09, beta = −0.748, N = 325
SNALDIIFQTDLTGQK pc2
F8WCZ6;P09871
12:7176978:C:T_T
p = 0.0094, beta = 0.345, N = 325
EDTPNSVWEPAK pc2
F8WCZ6;P09871
12:7176978:C:T_T
p = 0.023, beta = 0.302, N = 325
TNFDNDIALVR pc2
F8WCZ6;P09871
12:7176978:C:T_T
p = 0.012, beta = 0.334, N = 325
VEDPESTLFGSVIR pc2
F8WCZ6;P09871
12:7176978:C:T_T
p = 0.3, beta = 0.138, N = 325
PYPENSR pc2
F8WCZ6;P09871
12:7176978:C:T_T
p = 0.0061, beta = 0.363, N = 325
NYVDWIMK pc2
F8WCZ6;P09871
12:7176978:C:T_T
p = 0.11, beta = 0.216, N = 325
CVPVCGVPR pc2
F8WCZ6;P09871
12:7176978:C:T_T
p = 0.11, beta = 0.211, N = 325
GDSGGAFAVQDPNDK pc2
F8WCZ6;P09871
12:7176978:C:T_T
p = 0.06, beta = 0.25, N = 325
YHGDPMPCPK pc2
F8WCZ6;P09871
12:7176978:C:T_T
p = 0.049, beta = 0.262, N = 325
VGATSFYSTCQSNGK pc2
F8WCZ6;P09871
12:7176978:C:T_T
p = 0.97, beta = 0.00469, N = 285
MLTPEHVFIHPGWK pc4
F8WCZ6;P09871
12:7176978:C:T_T
p = 0.058, beta = 0.252, N = 325
TMQENSTPRED pc2
F8WCZ6;P09871
12:7176978:C:T_T
p = 0.032, beta = 0.285, N = 325
PTADAEAYVFTPNMICAGGEK pc3
F8WCZ6;P09871
12:7176978:C:T_T
p = 0.046, beta = 0.266, N = 325
DVVQITCLDGFEVVEGR pc3
F8WCZ6;P09871
12:7176978:C:T_T
p = 0.073, beta = 0.238, N = 324
TMQENSTPR pc2
F8WCZ6;P09871
12:7176978:C:T_T
p = 0.097, beta = 0.221, N = 325
Assay Target: C1S
UniProt: P09871
rsID: rs12371227
Target SNP: 12:7176978:C:T
Proxy SNP: 12:7176978:C:T
UKBPPP log10(p): 1154.2
UKBPPP BETA: −0.652
UKBPPP MSPA: 0.397 (2)
1256:1191:1255:1255:1237:125

82. ANXA2 (P07355)
UKBPPP MSPA=0

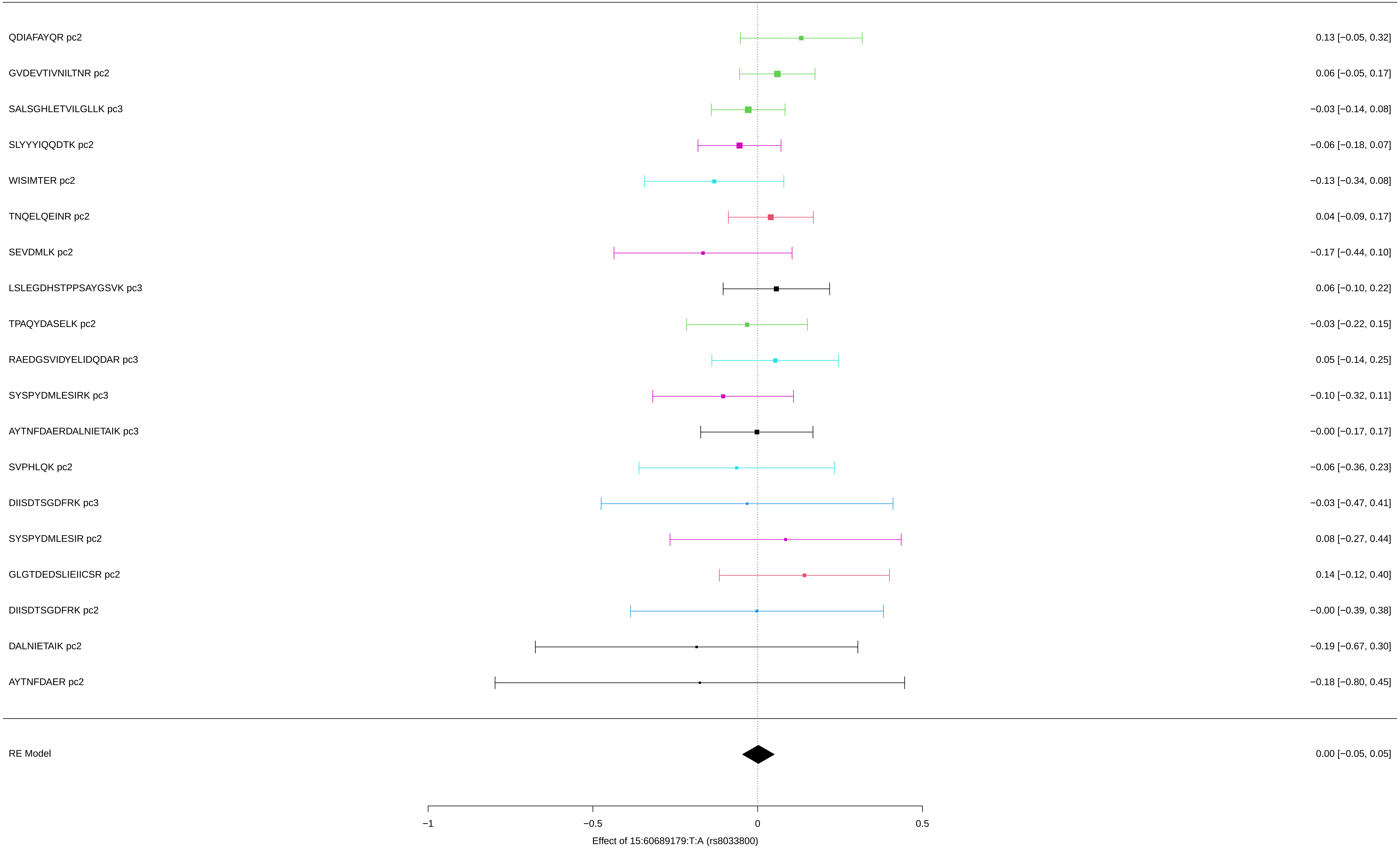

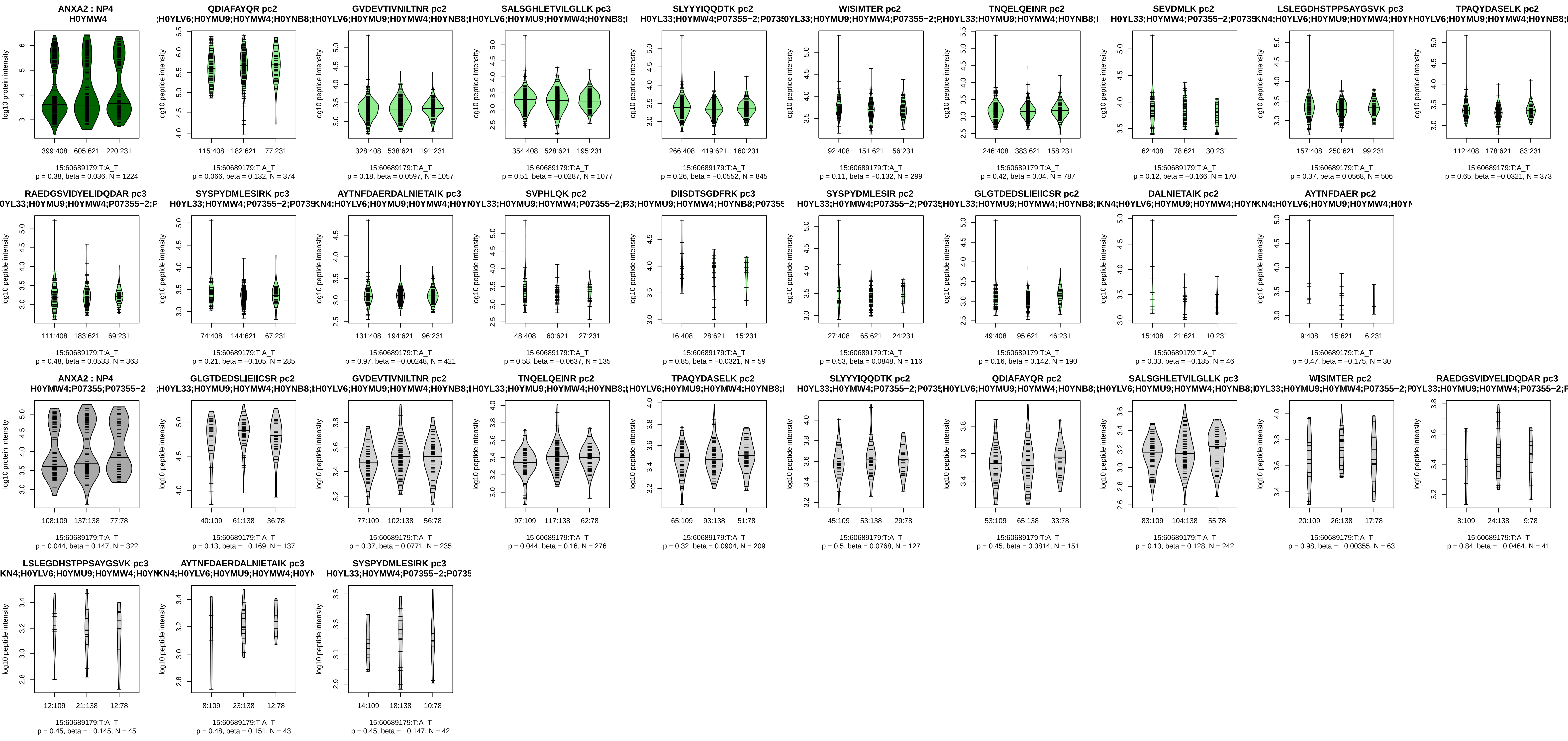

Assay Target: ANXA2
UniProt: P07355
rsID: rs8033800
Target SNP: 15:60689179:T:A
Proxy SNP: 15:60689179:T:A
UKBPPP log10(p): 1133.5
UKBPPP BETA: −0.454
UKBPPP MSPA: 0
374:1057:1077:845:299:787:17

83. HRG (P04196)
UKBPPP MSPA=0.359

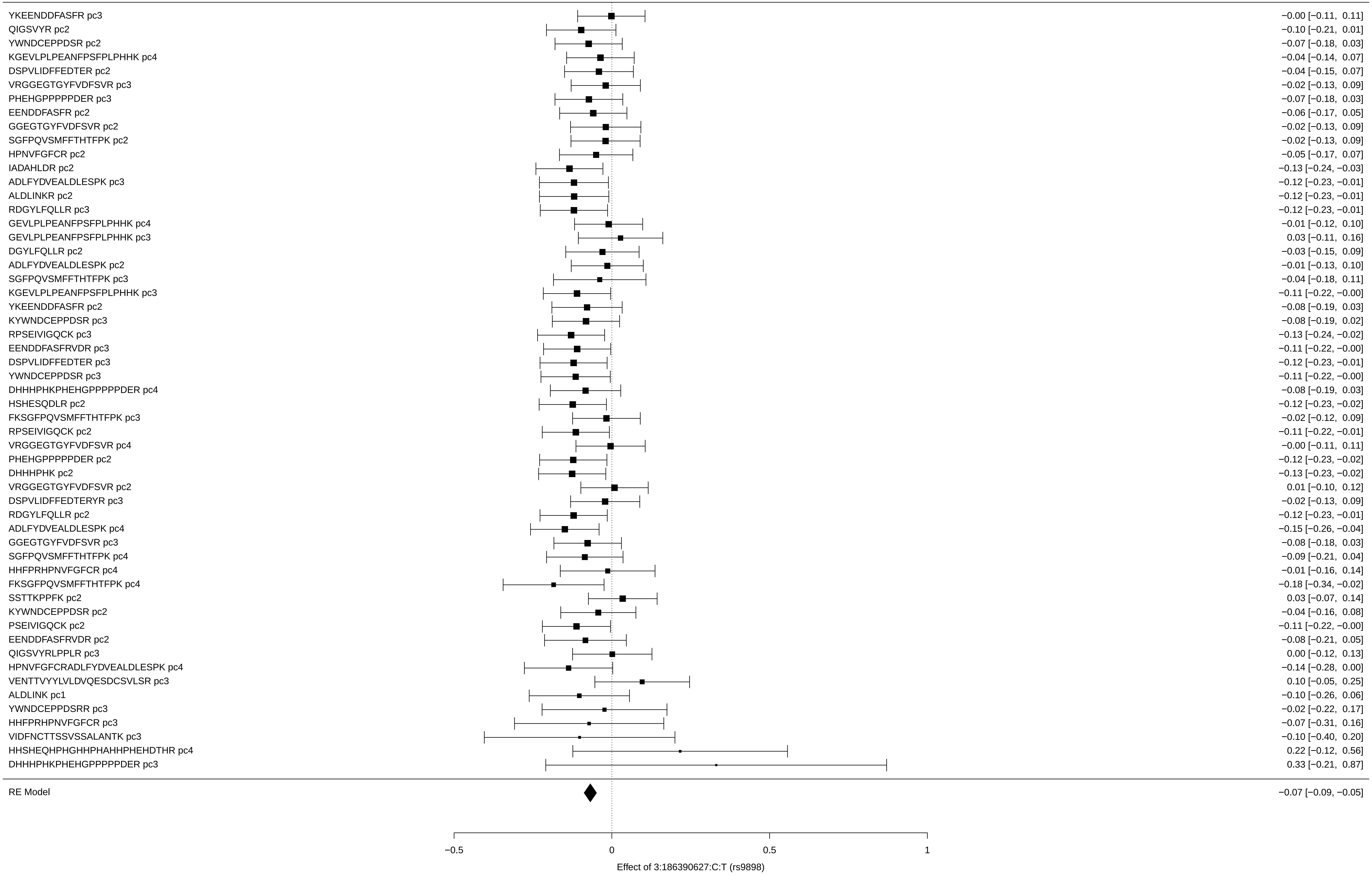

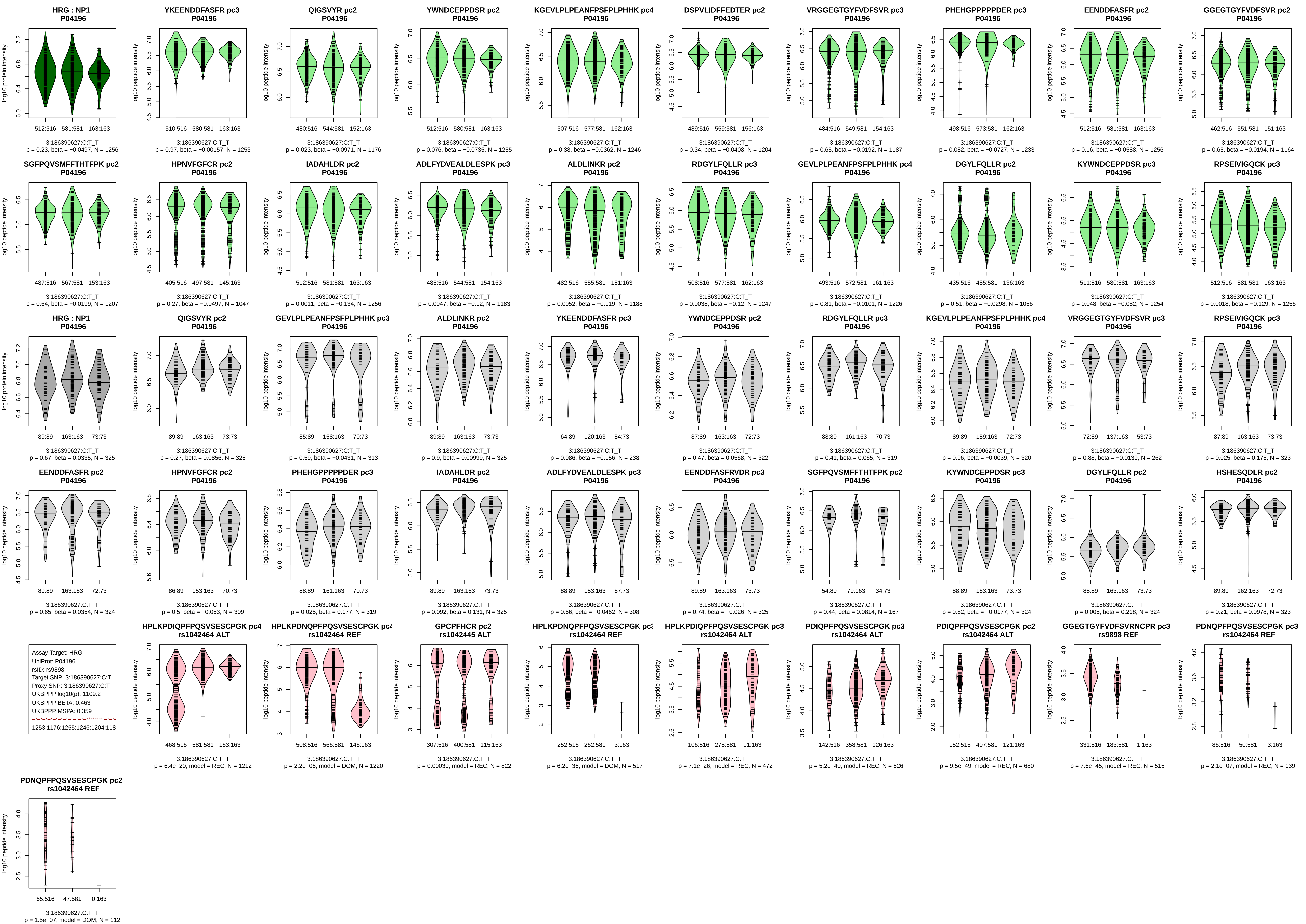
HRG : NP1 P04196
3:186390627:C:T_T p = 0.23, beta = −0.0497, N = 1256
YKEENDDFASFR pc3 P04196
3:186390627:C:T_T p = 0.97, beta = −0.00157, N = 1253
QIGSVYR pc2 P04196
3:186390627:C:T_T p = 0.023, beta = −0.0971, N = 1176
YWNDCEPPDSR pc2 P04196
3:186390627:C:T_T p = 0.076, beta = −0.0735, N = 1255
KGEVLPLPEANFPSFPLPHHK pc4 P04196
3:186390627:C:T_T p = 0.38, beta = −0.0362, N = 1246
DSPVLIDFFEDTER pc2 P04196
3:186390627:C:T_T p = 0.34, beta = −0.0408, N = 1204
VRGGEGTGYFVDFSVR pc3 P04196
3:186390627:C:T_T p = 0.65, beta = −0.0192, N = 1187
PHEHGPPPPPDER pc3 P04196
3:186390627:C:T_T p = 0.082, beta = −0.0727, N = 1233
EENDDFASFR pc2 P04196
3:186390627:C:T_T p = 0.16, beta = −0.0588, N = 1256
GGEGTGYFVDFSVR pc2 P04196
3:186390627:C:T_T p = 0.65, beta = −0.0194, N = 1164
SGFPQVSMFFTHTFPK pc2 P04196
3:186390627:C:T_T p = 0.64, beta = −0.0199, N = 1207
HPNVFGFCR pc2 P04196
3:186390627:C:T_T p = 0.27, beta = −0.0497, N = 1047
IADAHLDR pc2 P04196
3:186390627:C:T_T p = 0.0011, beta = −0.134, N = 1256
ADLFYDVEALDLESPK pc3 P04196
3:186390627:C:T_T p = 0.0047, beta = −0.12, N = 1183
ALDLINKR pc2 P04196
3:186390627:C:T_T p = 0.0052, beta = −0.119, N = 1188
RDGYLFQLLR pc3 P04196
3:186390627:C:T_T p = 0.0038, beta = −0.12, N = 1247
GEVLPLPEANFPSFPLPHHK pc4 P04196
3:186390627:C:T_T p = 0.81, beta = −0.0101, N = 1226
DGYLFQLLR pc2 P04196
3:186390627:C:T_T p = 0.51, beta = −0.0298, N = 1056
KYWNDCEPPDSR pc3 P04196
3:186390627:C:T_T p = 0.048, beta = −0.082, N = 1254
RPSEIVIGQCK pc3 P04196
3:186390627:C:T_T p = 0.0018, beta = −0.129, N = 1256
HRG : NP1 P04196
3:186390627:C:T_T p = 0.67, beta = 0.0335, N = 325
QIGSVYR pc2 P04196
3:186390627:C:T_T p = 0.27, beta = 0.0856, N = 325
GEVLPLPEANFPSFPLPHHK pc3 P04196
3:186390627:C:T_T p = 0.59, beta = −0.0431, N = 313
ALDLINKR pc2 P04196
3:186390627:C:T_T p = 0.9, beta = 0.00999, N = 325
YKEENDDFASFR pc3 P04196
3:186390627:C:T_T p = 0.086, beta = −0.156, N = 238
YWNDCEPPDSR pc2 P04196
3:186390627:C:T_T p = 0.47, beta = 0.0568, N = 322
RDGYLFQLLR pc3 P04196
3:186390627:C:T_T p = 0.41, beta = 0.065, N = 319
KGEVLPLPEANFPSFPLPHHK pc4 P04196
3:186390627:C:T_T p = 0.96, beta = −0.0039, N = 320
VRGGEGTGYFVDFSVR pc3 P04196
3:186390627:C:T_T p = 0.88, beta = −0.0139, N = 262
RPSEIVIGQCK pc3 P04196
3:186390627:C:T_T p = 0.025, beta = 0.175, N = 323
EENDDFASFR pc2 P04196
3:186390627:C:T_T p = 0.65, beta = 0.0354, N = 324
HPNVFGFCR pc2 P04196
3:186390627:C:T_T p = 0.5, beta = −0.053, N = 309
PHEHGPPPPPDER pc3 P04196
3:186390627:C:T_T p = 0.025, beta = 0.177, N = 319
IADAHLDR pc2 P04196
3:186390627:C:T_T p = 0.092, beta = 0.131, N = 325
ADLFYDVEALDLESPK pc3 P04196
3:186390627:C:T_T p = 0.56, beta = −0.0462, N = 308
EENDDFASFRVDR pc3 P04196
3:186390627:C:T_T p = 0.74, beta = −0.026, N = 325
SGFPQVSMFFTHTFPK pc2 P04196
3:186390627:C:T_T p = 0.44, beta = 0.0814, N = 167
KYWNDCEPPDSR pc3 P04196
3:186390627:C:T_T p = 0.82, beta = −0.0177, N = 324
DGYLFQLLR pc2 P04196
3:186390627:C:T_T p = 0.005, beta = 0.218, N = 324
HSHESQDLR pc2 P04196
3:186390627:C:T_T p = 0.21, beta = 0.0978, N = 323
Assay Target: HRG
UniProt: P04196
rsID: rs9898
Target SNP: 3:186390627:C:T
Proxy SNP: 3:186390627:C:T
UKBPPP log10(p): 1109.2
UKBPPP BETA: 0.463
UKBPPP MSPA: 0.359
1253:1176:1255:1246:1204:118
HPLKPDIQPFPQSVSESCPGK pc4 rs1042464 ALT
3:186390627:C:T_T p = 6.4e−20, model = REC, N = 1212
HPLKPDNQPFPQSVSESCPGK pc4 rs1042464 REF
3:186390627:C:T_T p = 2.2e−06, model = DOM, N = 1220
GPCPFHCR pc2 rs1042445 ALT
3:186390627:C:T_T p = 0.00039, model = REC, N = 822
HPLKPDNQPFPQSVSESCPGK pc3 rs1042464 REF
3:186390627:C:T_T p = 6.2e−36, model = DOM, N = 517
HPLKPDIQPFPQSVSESCPGK pc3 rs1042464 ALT
3:186390627:C:T_T p = 7.1e−26, model = REC, N = 472
PDIQPFPQSVSESCPGK pc3 rs1042464 ALT
3:186390627:C:T_T p = 5.2e−40, model = REC, N = 626
PDIQPFPQSVSESCPGK pc2 rs1042464 ALT
3:186390627:C:T_T p = 9.5e−49, model = REC, N = 680
GGEGTGYFVDFSVRNCPR pc3 rs9898 REF
3:186390627:C:T_T p = 7.6e−45, model = REC, N = 515
PDNQPFPQSVSESCPGK pc3 rs1042464 REF
3:186390627:C:T_T p = 2.1e−07, model = REC, N = 139
PDNQPFPQSVSESCPGK pc2 rs1042464 REF
3:186390627:C:T_T p = 1.5e−07, model = DOM, N = 112

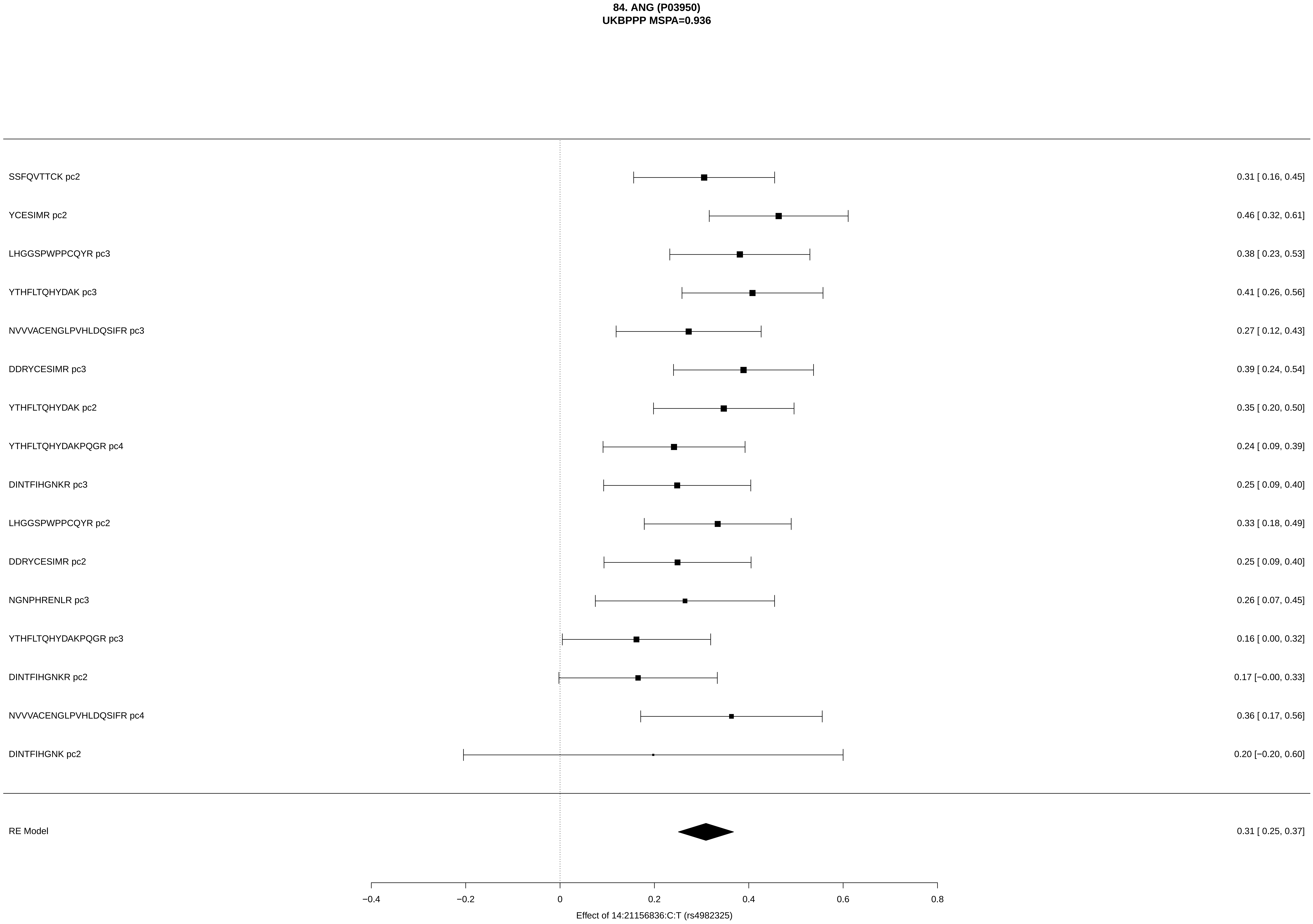
84. ANG (P03950)
UKBPPP MSPA=0.936
SSFQVTTCK pc2
0.31 [0.16, 0.45]
YCESIMR pc2
0.46 [0.32, 0.61]
LHGGSPWPPCQYR pc3
0.38 [0.23, 0.53]
YTHFLTQHYDAK pc3
0.41 [0.26, 0.56]
NVVVACENGLPVHLDQSIFR pc3
0.27 [0.12, 0.43]
DDRYCESIMR pc3
0.39 [0.24, 0.54]
YTHFLTQHYDAK pc2
0.35 [0.20, 0.50]
YTHFLTQHYDAKPQGR pc4
0.24 [0.09, 0.39]
DINTFIHGNKR pc3
0.25 [0.09, 0.40]
LHGGSPWPPCQYR pc2
0.33 [0.18, 0.49]
DDRYCESIMR pc2
0.25 [0.09, 0.40]
NGNPHRENLR pc3
0.26 [0.07, 0.45]
YTHFLTQHYDAKPQGR pc3
0.16 [0.00, 0.32]
DINTFIHGNKR pc2
0.17 [−0.00, 0.33]
NVVVACENGLPVHLDQSIFR pc4
0.36 [0.17, 0.56]
DINTFIHGNK pc2
0.20 [−0.20, 0.60]
RE Model
0.31 [0.25, 0.37]
−0.4
−0.2
0
0.2
0.4
0.6
0.8
Effect of 14:21156836:C:T (rs4982325)

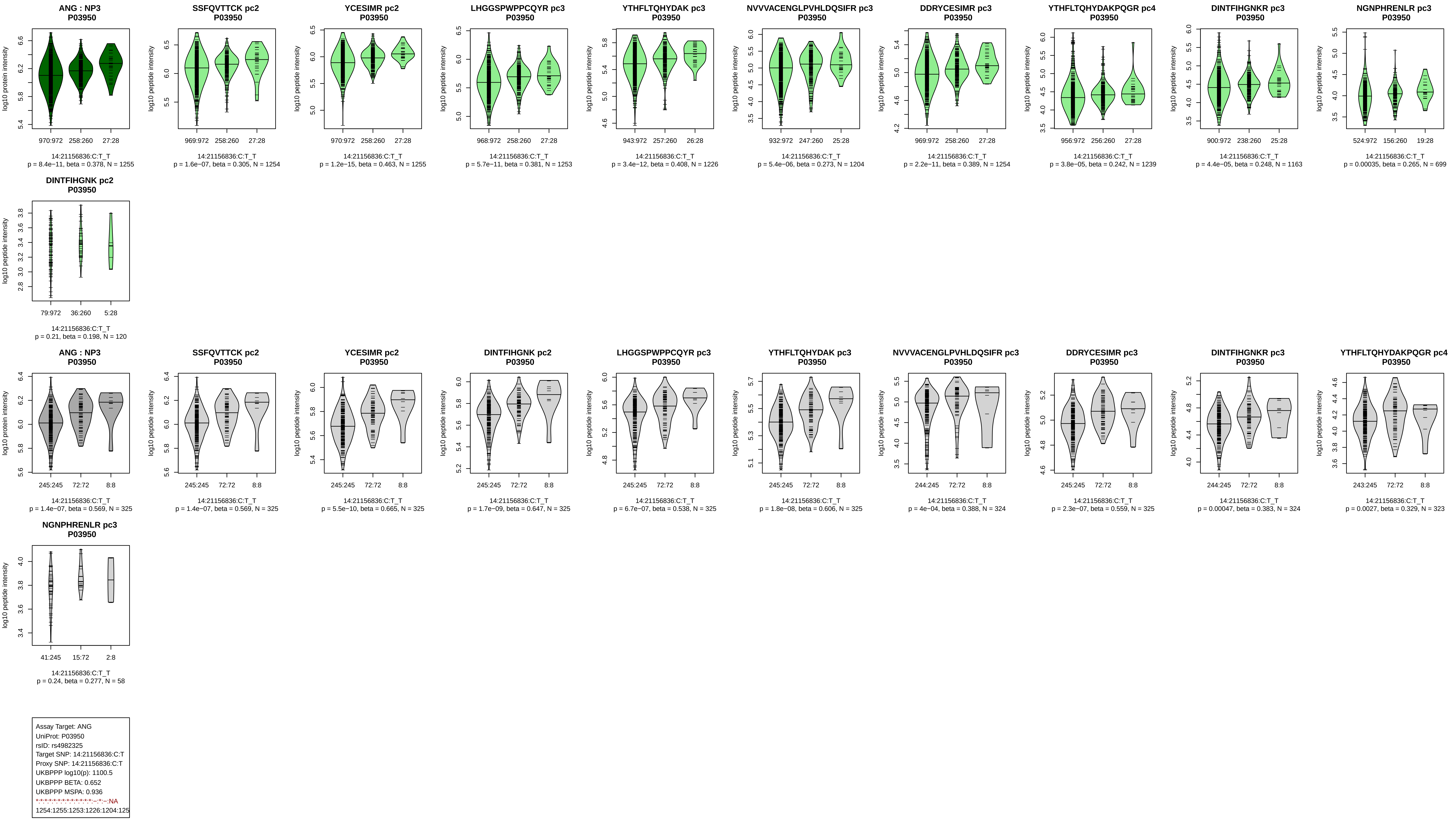
ANG : NP3
P03950
log10 protein intensity
970:972 258:260 27:28
14:21156836:C:T_T
p = 8.4e−11, beta = 0.378, N = 1255
SSFQVTTCK pc2
P03950
log10 peptide intensity
969:972 258:260 27:28
14:21156836:C:T_T
p = 1.6e−07, beta = 0.305, N = 1254
YCESIMR pc2
P03950
970:972 258:260 27:28
14:21156836:C:T_T
p = 1.2e−15, beta = 0.463, N = 1255
LHGGSPWPPCQYR pc3
P03950
968:972 258:260 27:28
14:21156836:C:T_T
p = 5.7e−11, beta = 0.381, N = 1253
YTHFLTQHYDAK pc3
P03950
943:972 257:260 26:28
14:21156836:C:T_T
p = 3.4e−12, beta = 0.408, N = 1226
NVVVACENGLPVHLDQSIFR pc3
P03950
932:972 247:260 25:28
14:21156836:C:T_T
p = 5.4e−06, beta = 0.273, N = 1204
DDRYCESIMR pc3
P03950
969:972 258:260 27:28
14:21156836:C:T_T
p = 2.2e−11, beta = 0.389, N = 1254
YTHFLTQHYDAKPQGR pc4
P03950
956:972 256:260 27:28
14:21156836:C:T_T
p = 3.8e−05, beta = 0.242, N = 1239
DINTFIHGNKR pc3
P03950
900:972 238:260 25:28
14:21156836:C:T_T
p = 4.4e−05, beta = 0.248, N = 1163
NGNPHRENLR pc3
P03950
524:972 156:260 19:28
14:21156836:C:T_T
p = 0.00035, beta = 0.265, N = 699
DINTFIHGNK pc2
P03950
79:972 36:260 5:28
14:21156836:C:T_T
p = 0.21, beta = 0.198, N = 120
ANG : NP3
P03950
log10 protein intensity
245:245 72:72 8:8
14:21156836:C:T_T
p = 1.4e−07, beta = 0.569, N = 325
SSFQVTTCK pc2
P03950
245:245 72:72 8:8
14:21156836:C:T_T
p = 1.4e−07, beta = 0.569, N = 325
YCESIMR pc2
P03950
245:245 72:72 8:8
14:21156836:C:T_T
p = 5.5e−10, beta = 0.665, N = 325
DINTFIHGNK pc2
P03950
245:245 72:72 8:8
14:21156836:C:T_T
p = 1.7e−09, beta = 0.647, N = 325
LHGGSPWPPCQYR pc3
P03950
245:245 72:72 8:8
14:21156836:C:T_T
p = 6.7e−07, beta = 0.538, N = 325
YTHFLTQHYDAK pc3
P03950
245:245 72:72 8:8
14:21156836:C:T_T
p = 1.8e−08, beta = 0.606, N = 325
NVVVACENGLPVHLDQSIFR pc3
P03950
244:245 72:72 8:8
14:21156836:C:T_T
p = 4e−04, beta = 0.388, N = 324
DDRYCESIMR pc3
P03950
245:245 72:72 8:8
14:21156836:C:T_T
p = 2.3e−07, beta = 0.559, N = 325
DINTFIHGNKR pc3
P03950
244:245 72:72 8:8
14:21156836:C:T_T
p = 0.00047, beta = 0.383, N = 324
YTHFLTQHYDAKPQGR pc4
P03950
243:245 72:72 8:8
14:21156836:C:T_T
p = 0.0027, beta = 0.329, N = 323
NGNPHRENLR pc3
P03950
41:245 15:72 2:8
14:21156836:C:T_T
p = 0.24, beta = 0.277, N = 58
Assay Target: ANG
UniProt: P03950
rsID: rs4982325
Target SNP: 14:21156836:C:T
Proxy SNP: 14:21156836:C:T
UKBPPP log10(p): 1100.5
UKBPPP BETA: 0.652
UKBPPP MSPA: 0.936
::*:*:*:*:*:*:*:*:*:*:*:−:*:−:NA
1254:1255:1253:1226:1204:125

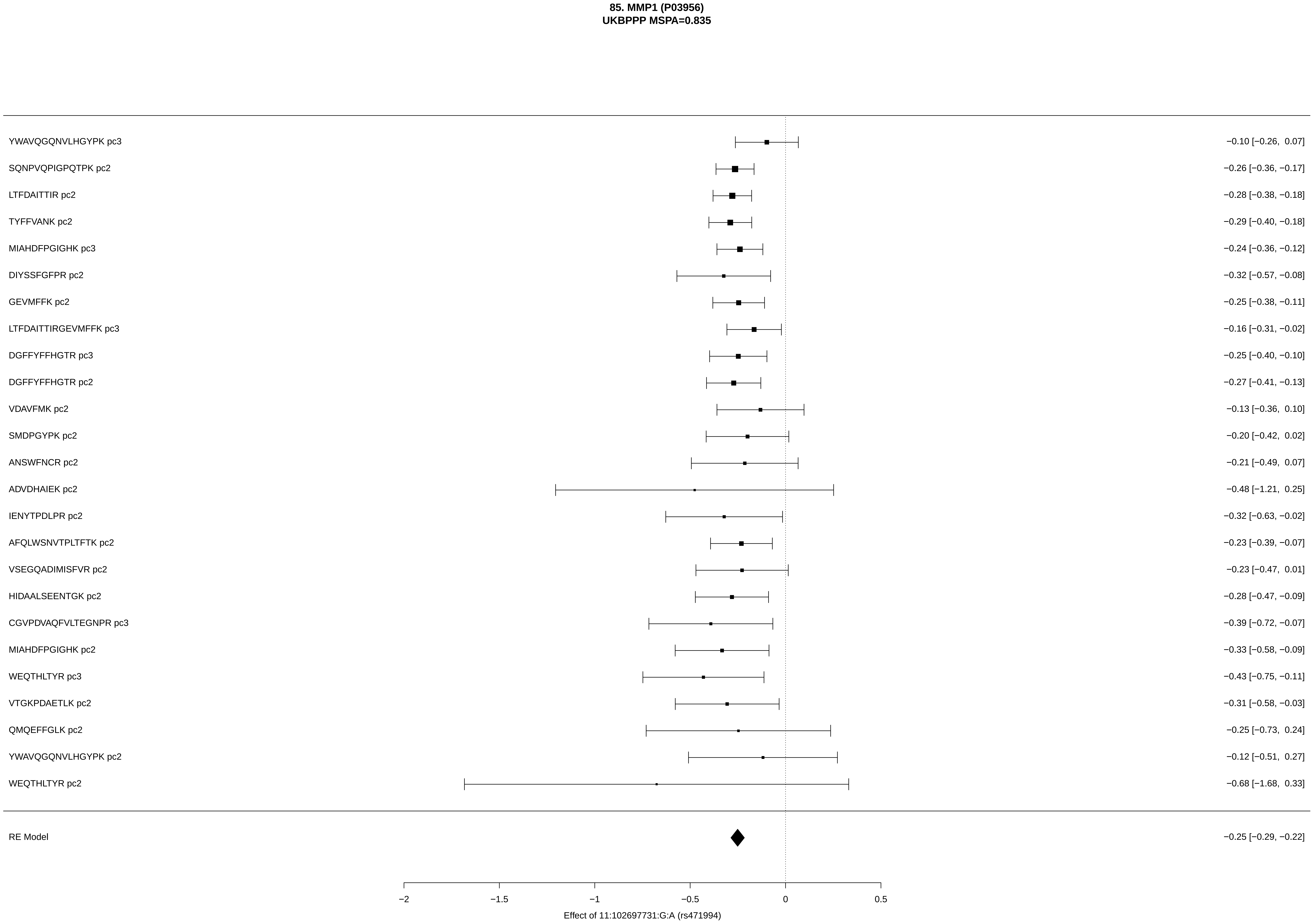
85. MMP1 (P03956)
UKBPPP MSPA=0.835
YWAVQGQNVLHGYPK pc3 −0.10 [−0.26, 0.07]
SQNPVQPIGPQTPK pc2 −0.26 [−0.36, −0.17]
LTFDAITTIR pc2 −0.28 [−0.38, −0.18]
TYFFVANK pc2 −0.29 [−0.40, −0.18]
MIAHDFPGIGHK pc3 −0.24 [−0.36, −0.12]
DIYSSFGFPR pc2 −0.32 [−0.57, −0.08]
GEVMFFK pc2 −0.25 [−0.38, −0.11]
LTFDAITTIRGEVMFFK pc3 −0.16 [−0.31, −0.02]
DGFFYFFHGTR pc3 −0.25 [−0.40, −0.10]
DGFFYFFHGTR pc2 −0.27 [−0.41, −0.13]
VDAVFMK pc2 −0.13 [−0.36, 0.10]
SMDPGYPK pc2 −0.20 [−0.42, 0.02]
ANSWFNCR pc2 −0.21 [−0.49, 0.07]
ADVDHAIEK pc2 −0.48 [−1.21, 0.25]
IENYTPDLPR pc2 −0.32 [−0.63, −0.02]
AFQLWSNVTPLTFTK pc2 −0.23 [−0.39, −0.07]
VSEGQADIMISFVR pc2 −0.23 [−0.47, 0.01]
HIDAALSEENTGK pc2 −0.28 [−0.47, −0.09]
CGVPDVAQFVLTEGNPR pc3 −0.39 [−0.72, −0.07]
MIAHDFPGIGHK pc2 −0.33 [−0.58, −0.09]
WEQTHLTYR pc3 −0.43 [−0.75, −0.11]
VTGKPDAETLK pc2 −0.31 [−0.58, −0.03]
QMQEFFGLK pc2 −0.25 [−0.73, 0.24]
YWAVQGQNVLHGYPK pc2 −0.12 [−0.51, 0.27]
WEQTHLTYR pc2 −0.68 [−1.68, 0.33]
RE Model −0.25 [−0.29, −0.22]
−2 −1.5 −1 −0.5 0 0.5
Effect of 11:102697731:G:A (rs471994)

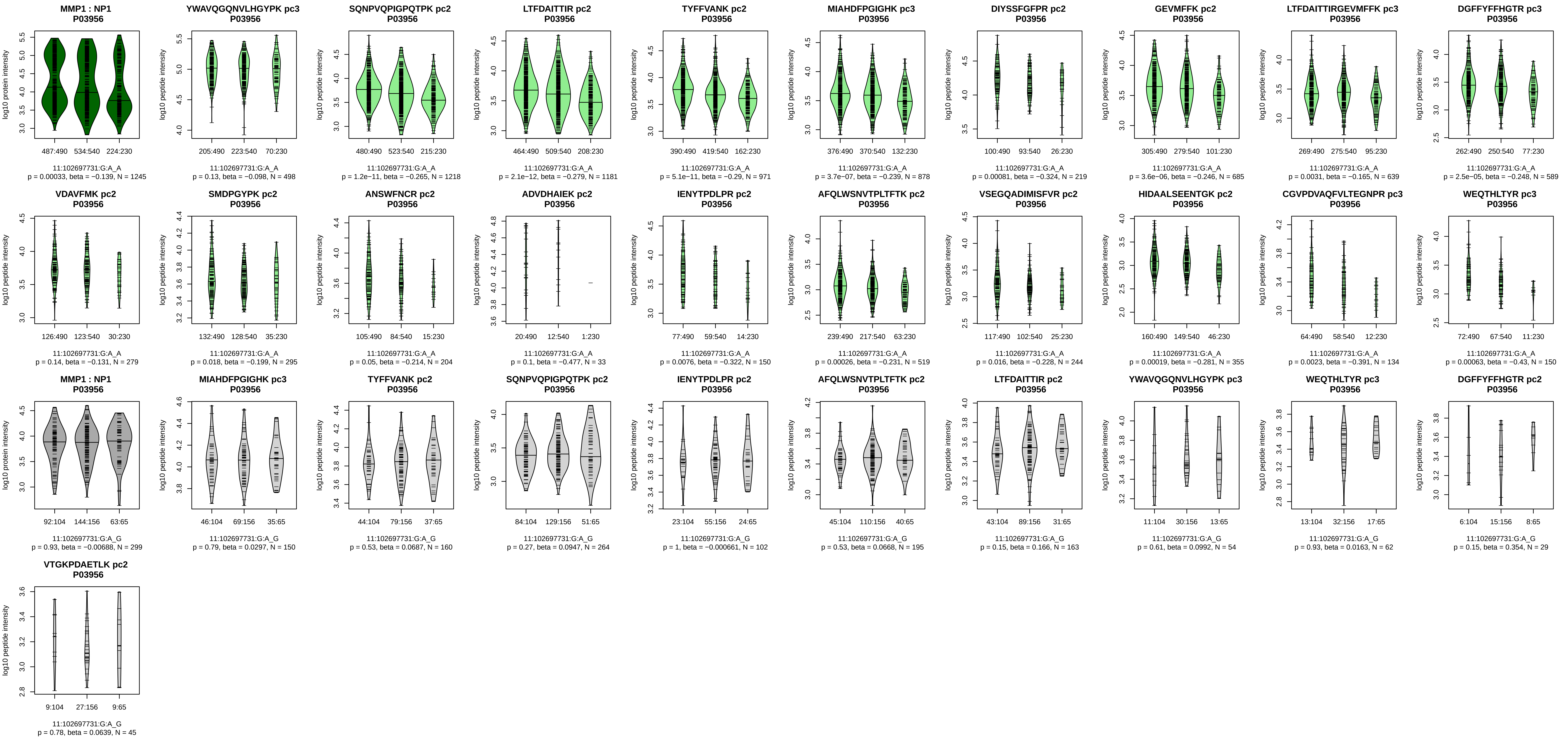

Assay Target: MMP1
UniProt: P03956
rsID: rs471994
Target SNP: 11:102697731:G:A
Proxy SNP: 11:102697731:G:A
UKBPPP log10(p): 1097.3
UKBPPP BETA: −0.457
UKBPPP MSPA: 0.835
498:1218:1181:971:878:219:685

86. BPIFB1 (Q8TDL5)
UKBPPP MSPA=0.834

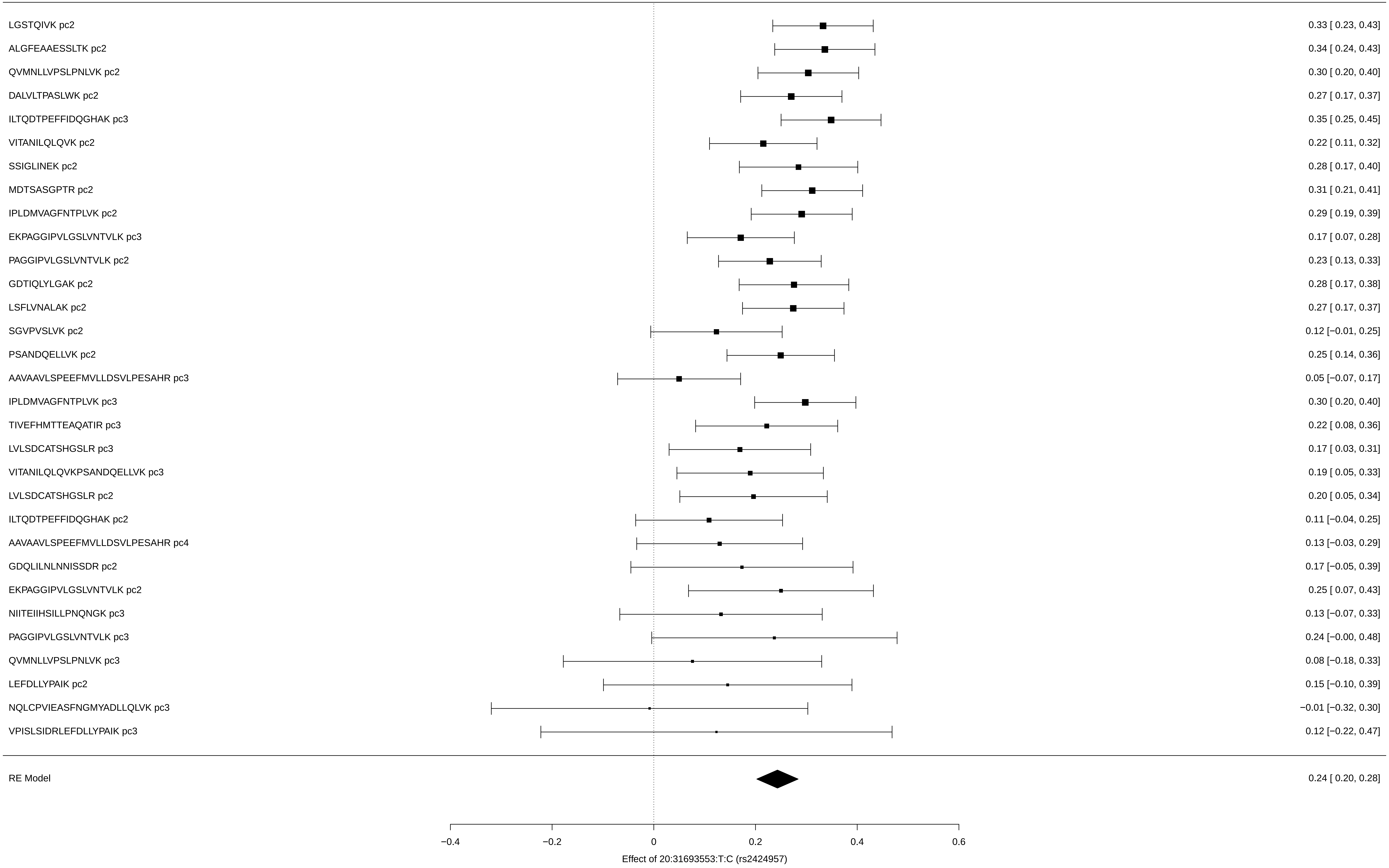

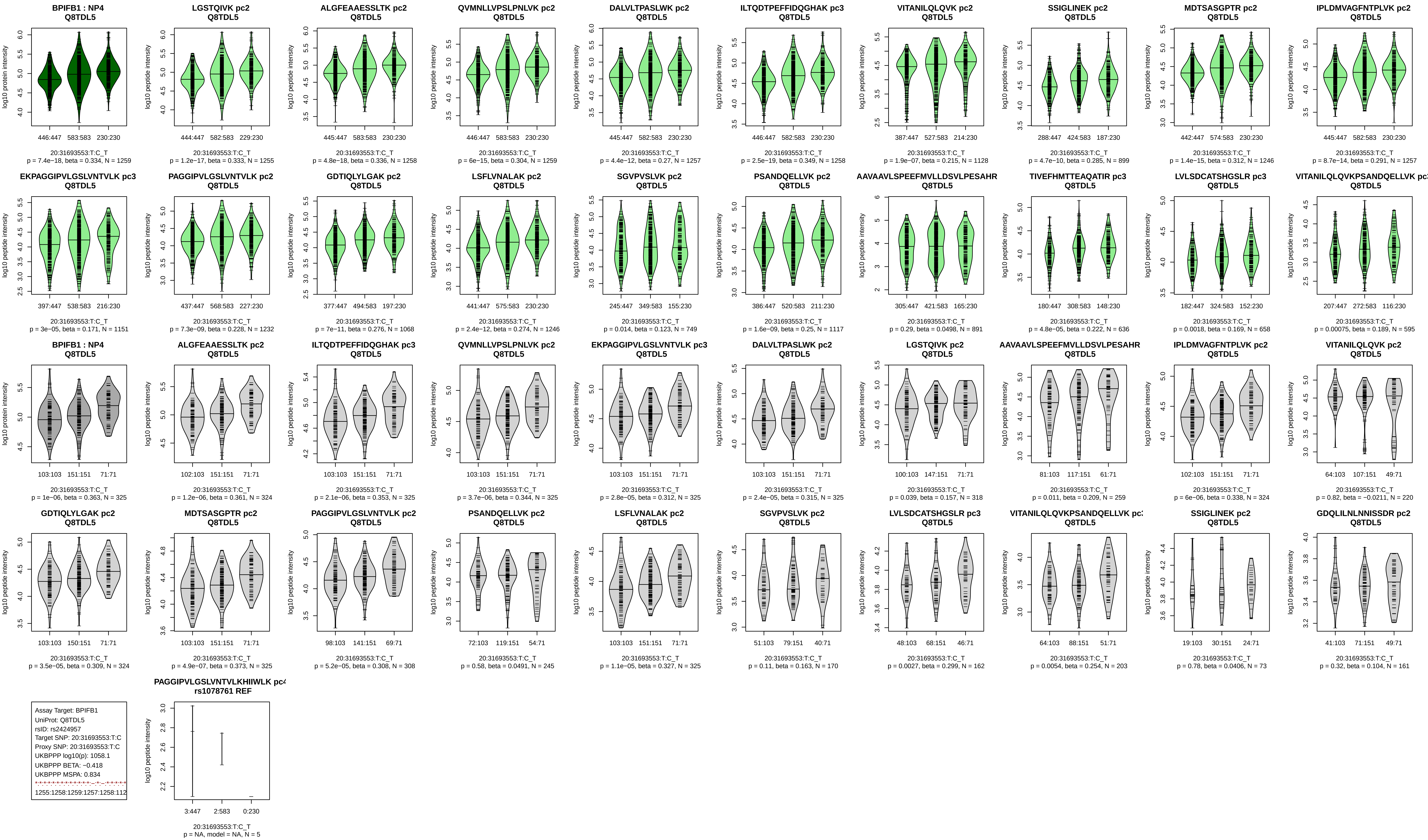

BPIFB1 : NP4 Q8TDL5
20:31693553:T:C_T p = 7.4e−18, beta = 0.334, N = 1259
LGSTQIVK pc2 Q8TDL5
20:31693553:T:C_T p = 1.2e−17, beta = 0.333, N = 1255
ALGFEAAESSLTK pc2 Q8TDL5
20:31693553:T:C_T p = 4.8e−18, beta = 0.336, N = 1258
QVMNLLVPSLPNLVK pc2 Q8TDL5
20:31693553:T:C_T p = 6e−15, beta = 0.304, N = 1259
DALVLTPASLWK pc2 Q8TDL5
20:31693553:T:C_T p = 4.4e−12, beta = 0.27, N = 1257
ILTQDTPEFFIDQGHAK pc3 Q8TDL5
20:31693553:T:C_T p = 2.5e−19, beta = 0.349, N = 1258
VITANILQLQVK pc2 Q8TDL5
20:31693553:T:C_T p = 1.9e−07, beta = 0.215, N = 1128
SSIGLINEK pc2 Q8TDL5
20:31693553:T:C_T p = 4.7e−10, beta = 0.285, N = 899
MDTSASGPTR pc2 Q8TDL5
20:31693553:T:C_T p = 1.4e−15, beta = 0.312, N = 1246
IPLDMVAGFNTPLVK pc2 Q8TDL5
20:31693553:T:C_T p = 8.7e−14, beta = 0.291, N = 1257
EKPAGGIPVLGSLVNTVLK pc3 Q8TDL5
20:31693553:T:C_T p = 3e−05, beta = 0.171, N = 1151
PAGGIPVLGSLVNTVLK pc2 Q8TDL5
20:31693553:T:C_T p = 7.3e−09, beta = 0.228, N = 1232
GDTIQLYLGAK pc2 Q8TDL5
20:31693553:T:C_T p = 7e−11, beta = 0.276, N = 1068
LSFLVNALAK pc2 Q8TDL5
20:31693553:T:C_T p = 2.4e−12, beta = 0.274, N = 1246
SGVPVSLVK pc2 Q8TDL5
20:31693553:T:C_T p = 0.014, beta = 0.123, N = 749
PSANDQELLVK pc2 Q8TDL5
20:31693553:T:C_T p = 1.6e−09, beta = 0.25, N = 1117
AAVAAVLSPEEFMVLLDSVLPESAHR Q8TDL5
20:31693553:T:C_T p = 0.29, beta = 0.0498, N = 891
TIVEFHMTTEAQATIR pc3 Q8TDL5
20:31693553:T:C_T p = 4.8e−05, beta = 0.222, N = 636
LVLSDCATSHGSLR pc3 Q8TDL5
20:31693553:T:C_T p = 0.0018, beta = 0.169, N = 658
VITANILQLQVKPSANDQELLVK pc3 Q8TDL5
20:31693553:T:C_T p = 0.00075, beta = 0.189, N = 595
BPIFB1 : NP4 Q8TDL5
20:31693553:T:C_T p = 1e−06, beta = 0.363, N = 325
ALGFEAAESSLTK pc2 Q8TDL5
20:31693553:T:C_T p = 1.2e−06, beta = 0.361, N = 324
ILTQDTPEFFIDQGHAK pc3 Q8TDL5
20:31693553:T:C_T p = 2.1e−06, beta = 0.353, N = 325
QVMNLLVPSLPNLVK pc2 Q8TDL5
20:31693553:T:C_T p = 3.7e−06, beta = 0.344, N = 325
EKPAGGIPVLGSLVNTVLK pc3 Q8TDL5
20:31693553:T:C_T p = 2.8e−05, beta = 0.312, N = 325
DALVLTPASLWK pc2 Q8TDL5
20:31693553:T:C_T p = 2.4e−05, beta = 0.315, N = 325
LGSTQIVK pc2 Q8TDL5
20:31693553:T:C_T p = 0.039, beta = 0.157, N = 318
AAVAAVLSPEEFMVLLDSVLPESAHR Q8TDL5
20:31693553:T:C_T p = 0.011, beta = 0.209, N = 259
IPLDMVAGFNTPLVK pc2 Q8TDL5
20:31693553:T:C_T p = 6e−06, beta = 0.338, N = 324
VITANILQLQVK pc2 Q8TDL5
20:31693553:T:C_T p = 0.82, beta = −0.0211, N = 220
GDTIQLYLGAK pc2 Q8TDL5
20:31693553:T:C_T p = 3.5e−05, beta = 0.309, N = 324
MDTSASGPTR pc2 Q8TDL5
20:31693553:T:C_T p = 4.9e−07, beta = 0.373, N = 325
PAGGIPVLGSLVNTVLK pc2 Q8TDL5
20:31693553:T:C_T p = 5.2e−05, beta = 0.308, N = 308
PSANDQELLVK pc2 Q8TDL5
20:31693553:T:C_T p = 0.58, beta = 0.0491, N = 245
LSFLVNALAK pc2 Q8TDL5
20:31693553:T:C_T p = 1.1e−05, beta = 0.327, N = 325
SGVPVSLVK pc2 Q8TDL5
20:31693553:T:C_T p = 0.11, beta = 0.163, N = 170
LVLSDCATSHGSLR pc3 Q8TDL5
20:31693553:T:C_T p = 0.0027, beta = 0.299, N = 162
VITANILQLQVKPSANDQELLVK pc3 Q8TDL5
20:31693553:T:C_T p = 0.0054, beta = 0.254, N = 203
SSIGLINEK pc2 Q8TDL5
20:31693553:T:C_T p = 0.78, beta = 0.0406, N = 73
GDQLILNLNNISSDR pc2 Q8TDL5
20:31693553:T:C_T p = 0.32, beta = 0.104, N = 161
Assay Target: BPIFB1
UniProt: Q8TDL5
rsID: rs2424957
Target SNP: 20:31693553:T:C
Proxy SNP: 20:31693553:T:C
UKBPPP log10(p): 1058.1
UKBPPP BETA: −0.418
UKBPPP MSPA: 0.834
1255:1258:1259:1257:1258:112
PAGGIPVLGSLVNTVLKHIIWLK pc4 rs1078761 REF
20:31693553:T:C_T p = NA, model = NA, N = 5

87. NOMO1 (Q15155)
UKBPPP MSPA=0

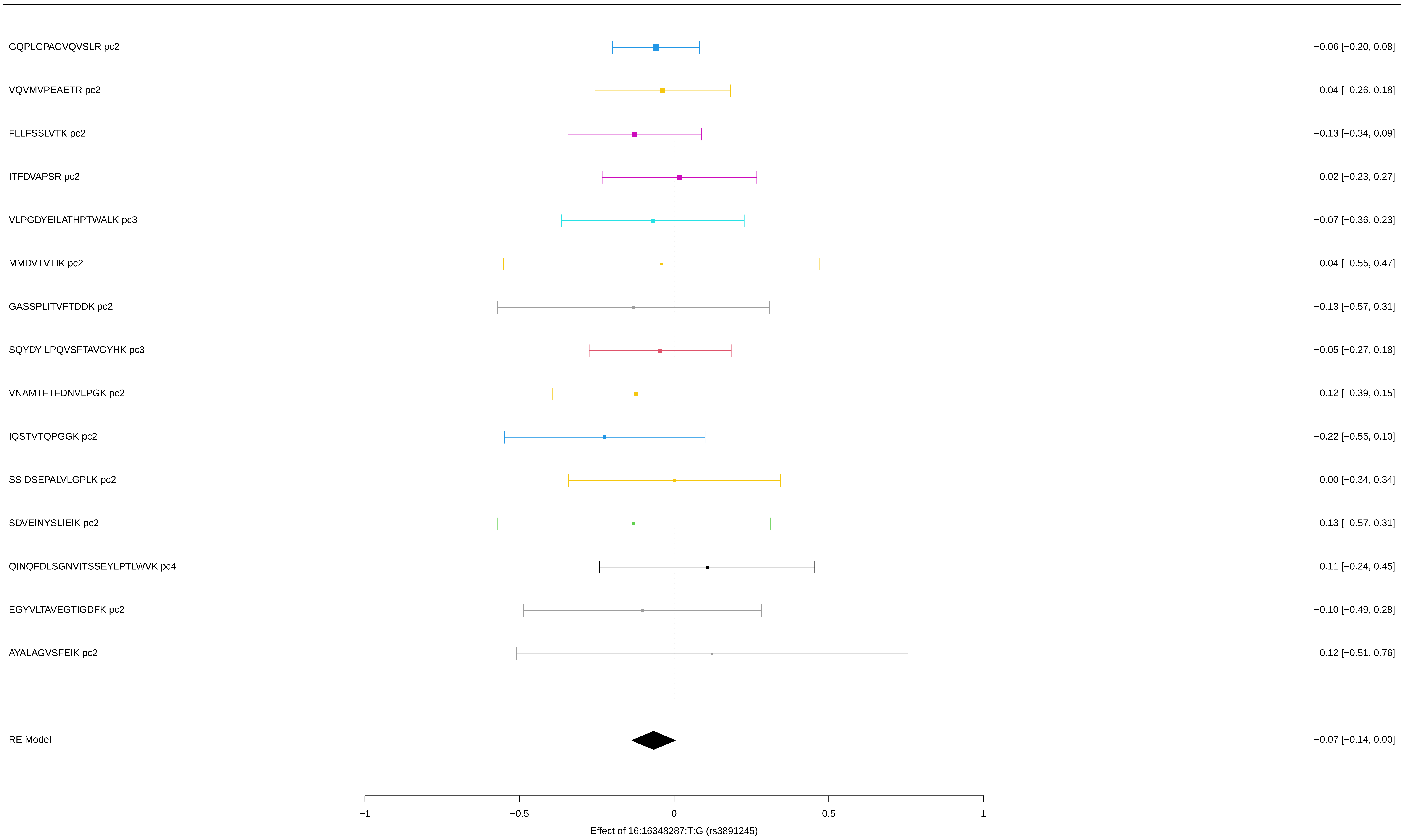

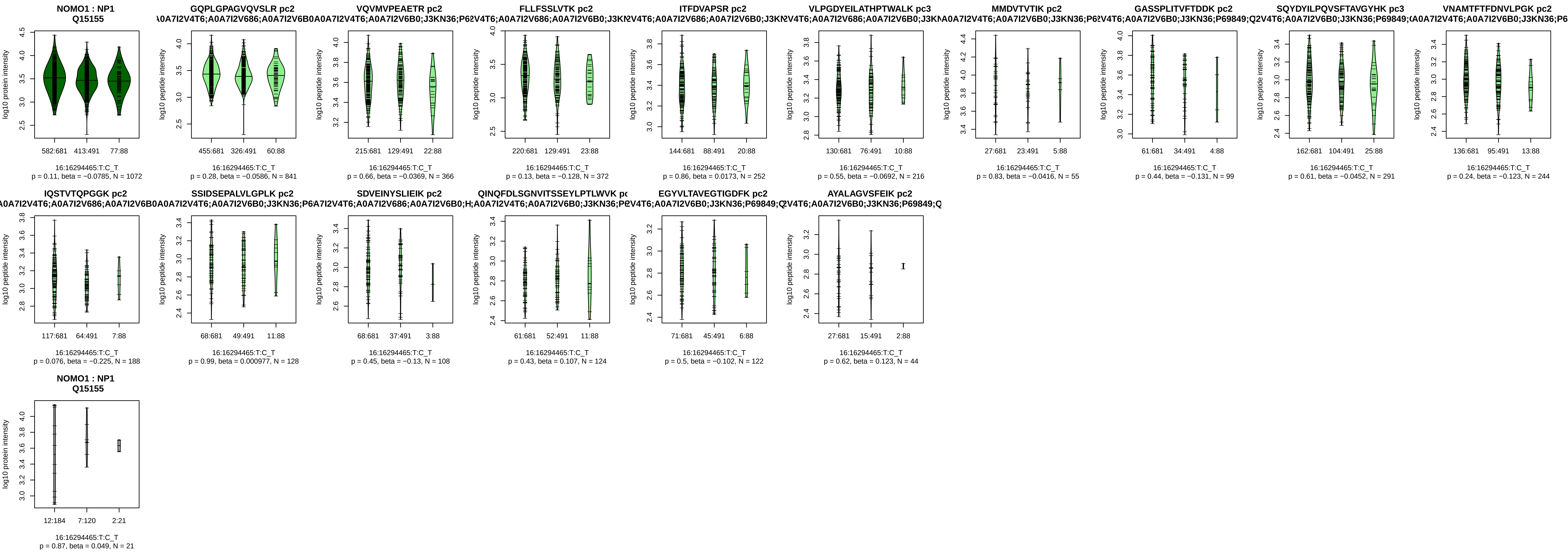

Assay Target: NOMO1
UniProt: Q15155
rsID: rs3891245
Target SNP: 16:16348287:T:G
Proxy SNP: 16:16294465:T:C
UKBPPP log10(p): 1051.8
UKBPPP BETA: −0.503
UKBPPP MSPA: 0
841:366:372:252:216:55:99:291

88. PLTP (P55058)
UKBPPP MSPA=0.804

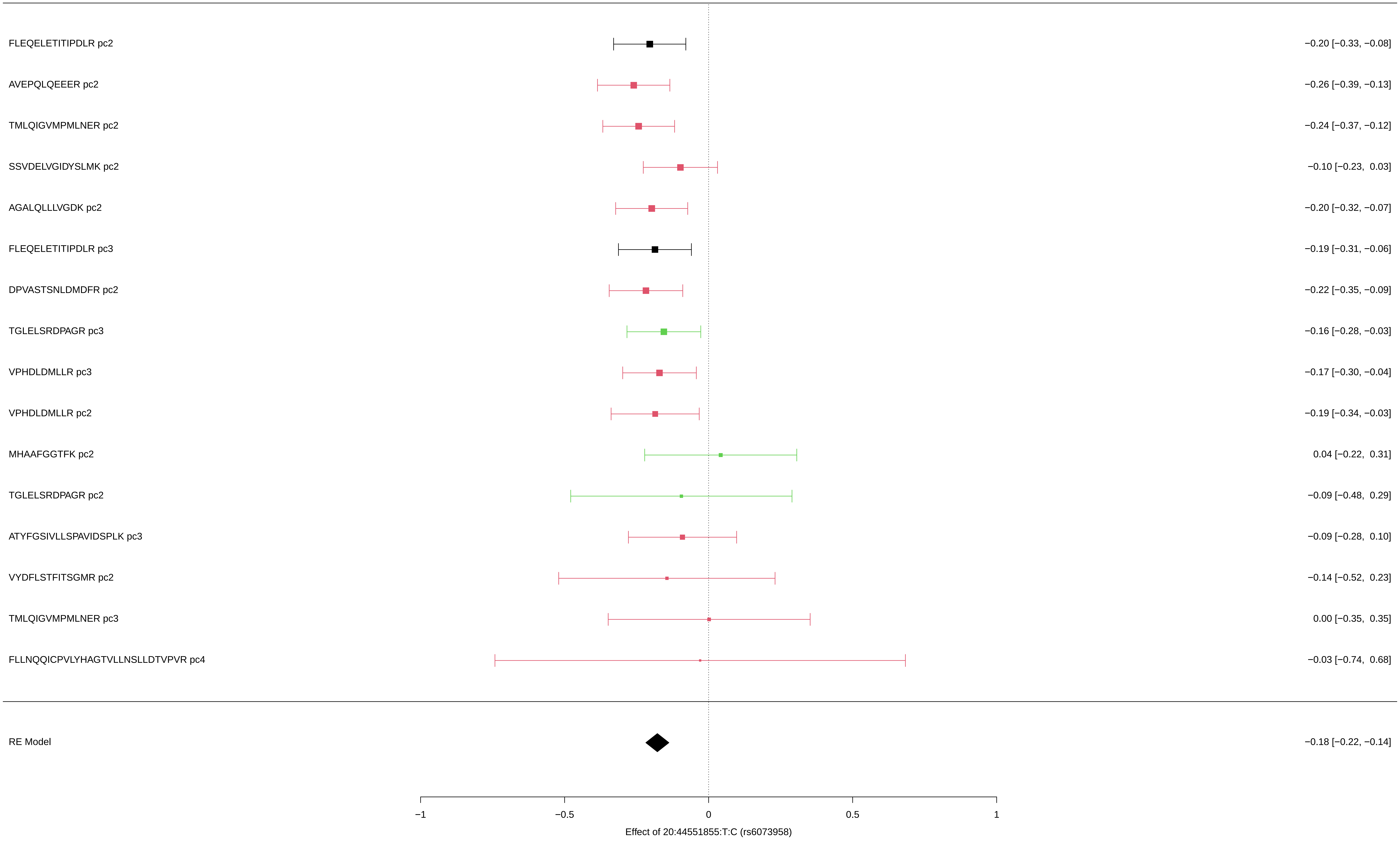

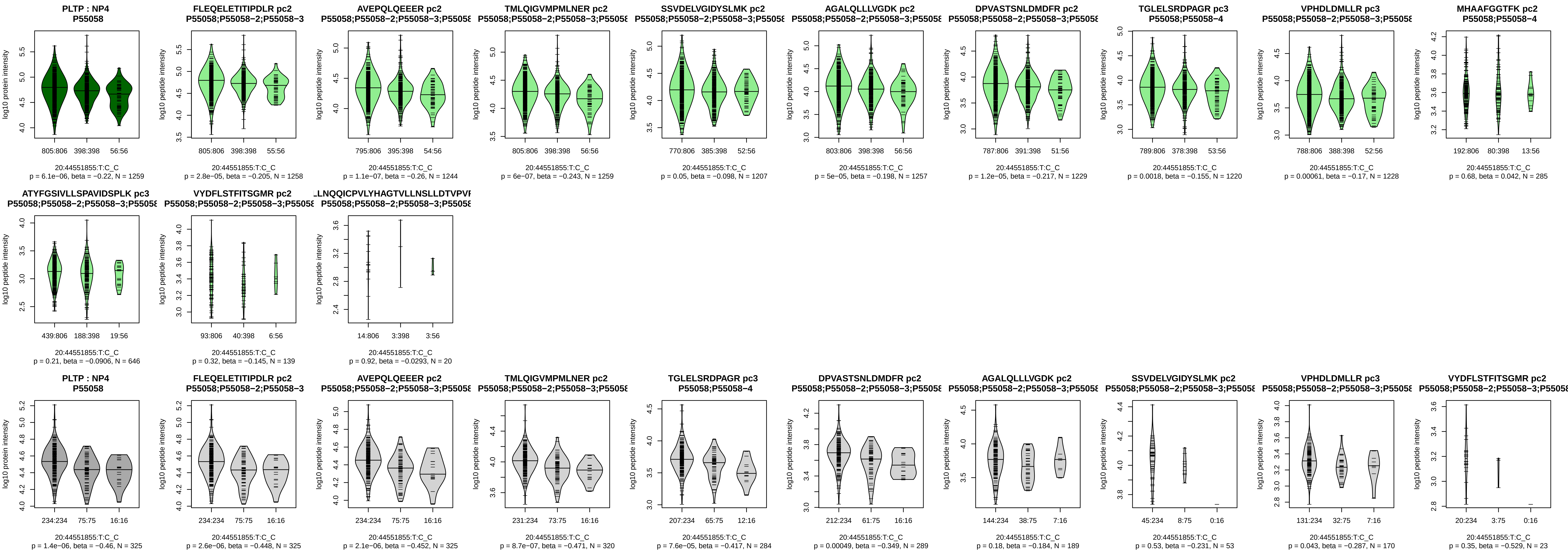

Assay Target: PLTP
UniProt: P55058
rsID: rs6073958
Target SNP: 20:44551855:T:C
Proxy SNP: 20:44551855:T:C
UKBPPP log10(p): 1006.5
UKBPPP BETA: −0.501
UKBPPP MSPA: 0.804
::*:–:*:*:*:*:*:*:–:–:–:–:–:–
1258:1244:1259:1207:1257:123

89. PZP (P20742)
UKBPPP MSPA=0.334

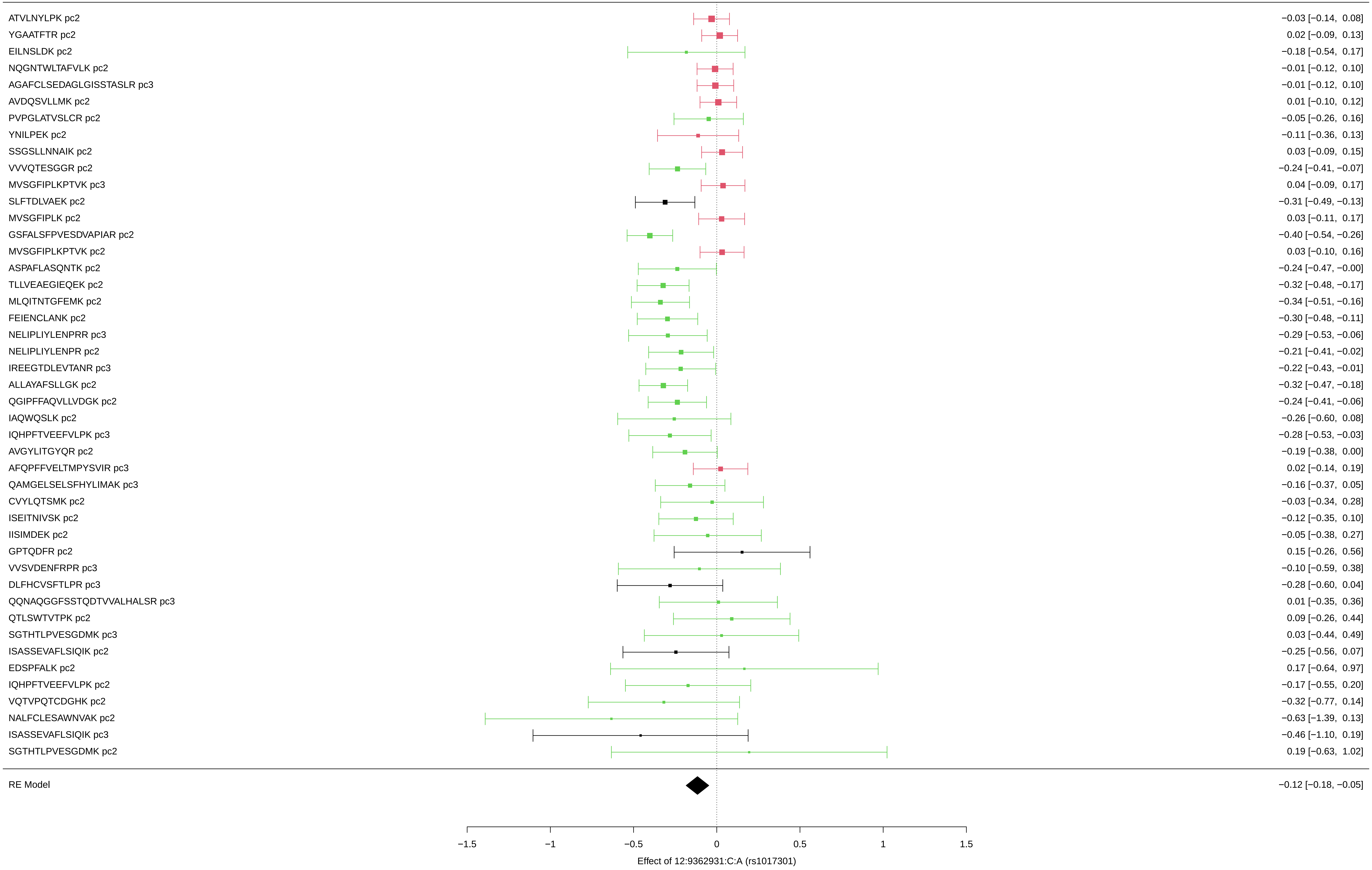

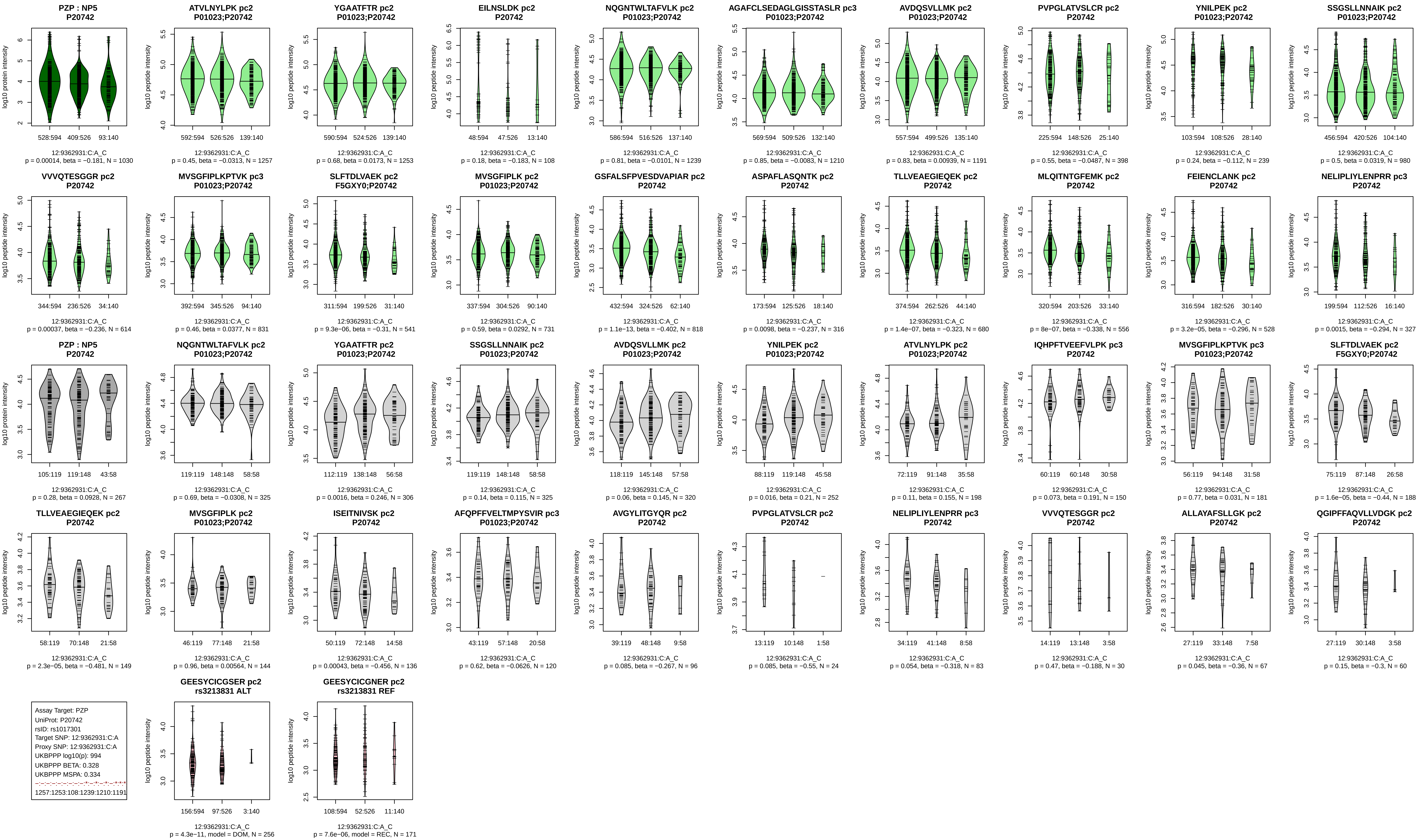
PZP : NP5
P20742
528:594 409:526 93:140
12:9362931:C:A_C
p = 0.00014, beta = −0.181, N = 1030
ATVLNYLPK pc2
P01023;P20742
592:594 526:526 139:140
12:9362931:C:A_C
p = 0.45, beta = −0.0313, N = 1257
YGAATFTR pc2
P01023;P20742
590:594 524:526 139:140
12:9362931:C:A_C
p = 0.68, beta = 0.0173, N = 1253
EILNSLDK pc2
P20742
48:594 47:526 13:140
12:9362931:C:A_C
p = 0.18, beta = −0.183, N = 108
NQGNTWLTAFVLK pc2
P01023;P20742
586:594 516:526 137:140
12:9362931:C:A_C
p = 0.81, beta = −0.0101, N = 1239
AGAFCLSEDAGLGISSTASLR pc3
P01023;P20742
569:594 509:526 132:140
12:9362931:C:A_C
p = 0.85, beta = −0.0083, N = 1210
AVDQSVLLMK pc2
P01023;P20742
557:594 499:526 135:140
12:9362931:C:A_C
p = 0.83, beta = 0.00939, N = 1191
PVPGLATVSLCR pc2
P20742
225:594 148:526 25:140
12:9362931:C:A_C
p = 0.55, beta = −0.0487, N = 398
YNILPEK pc2
P01023;P20742
103:594 108:526 28:140
12:9362931:C:A_C
p = 0.24, beta = −0.112, N = 239
SSGSLLNNAIK pc2
P01023;P20742
456:594 420:526 104:140
12:9362931:C:A_C
p = 0.5, beta = 0.0319, N = 980
VVVQTESGGR pc2
P20742
344:594 236:526 34:140
12:9362931:C:A_C
p = 0.00037, beta = −0.236, N = 614
MVSGFIPLKPTVK pc3
P01023;P20742
392:594 345:526 94:140
12:9362931:C:A_C
p = 0.46, beta = 0.0377, N = 831
SLFTDLVAEK pc2
F5GXY0;P20742
311:594 199:526 31:140
12:9362931:C:A_C
p = 9.3e−06, beta = −0.31, N = 541
MVSGFIPLK pc2
P01023;P20742
337:594 304:526 90:140
12:9362931:C:A_C
p = 0.59, beta = 0.0292, N = 731
GSFALSFPVESDVAPIAR pc2
P20742
432:594 324:526 62:140
12:9362931:C:A_C
p = 1.1e−13, beta = −0.402, N = 818
ASPAFLASQNTK pc2
P20742
173:594 125:526 18:140
12:9362931:C:A_C
p = 0.0098, beta = −0.237, N = 316
TLLVEAEGIEQEK pc2
P20742
374:594 262:526 44:140
12:9362931:C:A_C
p = 1.4e−07, beta = −0.323, N = 680
MLQITNTGFEMK pc2
P20742
320:594 203:526 33:140
12:9362931:C:A_C
p = 8e−07, beta = −0.338, N = 556
FEIENCLANK pc2
P20742
316:594 182:526 30:140
12:9362931:C:A_C
p = 3.2e−05, beta = −0.296, N = 528
NELIPLIYLENPRR pc3
P20742
199:594 112:526 16:140
12:9362931:C:A_C
p = 0.0015, beta = −0.294, N = 327
PZP : NP5
P20742
105:119 119:148 43:58
12:9362931:C:A_C
p = 0.28, beta = 0.0928, N = 267
NQGNTWLTAFVLK pc2
P01023;P20742
119:119 148:148 58:58
12:9362931:C:A_C
p = 0.69, beta = −0.0308, N = 325
YGAATFTR pc2
P01023;P20742
112:119 138:148 56:58
12:9362931:C:A_C
p = 0.0016, beta = 0.246, N = 306
SSGSLLNNAIK pc2
P01023;P20742
119:119 148:148 58:58
12:9362931:C:A_C
p = 0.14, beta = 0.115, N = 325
AVDQSVLLMK pc2
P01023;P20742
118:119 145:148 57:58
12:9362931:C:A_C
p = 0.06, beta = 0.145, N = 320
YNILPEK pc2
P01023;P20742
88:119 119:148 45:58
12:9362931:C:A_C
p = 0.016, beta = 0.21, N = 252
ATVLNYLPK pc2
P01023;P20742
72:119 91:148 35:58
12:9362931:C:A_C
p = 0.11, beta = 0.155, N = 198
IQHPFTVEEFVLPK pc3
P20742
60:119 60:148 30:58
12:9362931:C:A_C
p = 0.073, beta = 0.191, N = 150
MVSGFIPLKPTVK pc3
P01023;P20742
56:119 94:148 31:58
12:9362931:C:A_C
p = 0.77, beta = 0.031, N = 181
SLFTDLVAEK pc2
F5GXY0;P20742
75:119 87:148 26:58
12:9362931:C:A_C
p = 1.6e−05, beta = −0.44, N = 188
TLLVEAEGIEQEK pc2
P20742
58:119 70:148 21:58
12:9362931:C:A_C
p = 2.3e−05, beta = −0.481, N = 149
MVSGFIPLK pc2
P01023;P20742
46:119 77:148 21:58
12:9362931:C:A_C
p = 0.96, beta = 0.00564, N = 144
ISEITNIVSK pc2
P20742
50:119 72:148 14:58
12:9362931:C:A_C
p = 0.00043, beta = −0.456, N = 136
AFQPFFVELTMPYSVIR pc3
P01023;P20742
43:119 57:148 20:58
12:9362931:C:A_C
p = 0.62, beta = −0.0626, N = 120
AVGYLITGYQR pc2
P20742
39:119 48:148 9:58
12:9362931:C:A_C
p = 0.085, beta = −0.267, N = 96
PVPGLATVSLCR pc2
P20742
13:119 10:148 1:58
12:9362931:C:A_C
p = 0.085, beta = −0.55, N = 24
NELIPLIYLENPRR pc3
P20742
34:119 41:148 8:58
12:9362931:C:A_C
p = 0.054, beta = −0.318, N = 83
VVVQTESGGR pc2
P20742
14:119 13:148 3:58
12:9362931:C:A_C
p = 0.47, beta = −0.188, N = 30
ALLAYAFSLLGK pc2
P20742
27:119 33:148 7:58
12:9362931:C:A_C
p = 0.045, beta = −0.36, N = 67
QGIPFFAQVLLVDGK pc2
P20742
27:119 30:148 3:58
12:9362931:C:A_C
p = 0.15, beta = −0.3, N = 60
Assay Target: PZP
UniProt: P20742
rsID: rs1017301
Target SNP: 12:9362931:C:A
Proxy SNP: 12:9362931:C:A
UKBPPP log10(p): 994
UKBPPP BETA: 0.328
UKBPPP MSPA: 0.334
1257:1253:108:1239:1210:1191
GEESYCICGSER pc2
rs3213831 ALT
156:594 97:526 3:140
12:9362931:C:A_C
p = 4.3e−11, model = DOM, N = 256
GEESYCICGNER pc2
rs3213831 REF
108:594 52:526 11:140
12:9362931:C:A_C
p = 7.6e−06, model = REC, N = 171

90. SPINK5 (Q9NQ38)
UKBPPP MSPA=0.489 (2 conflicts)

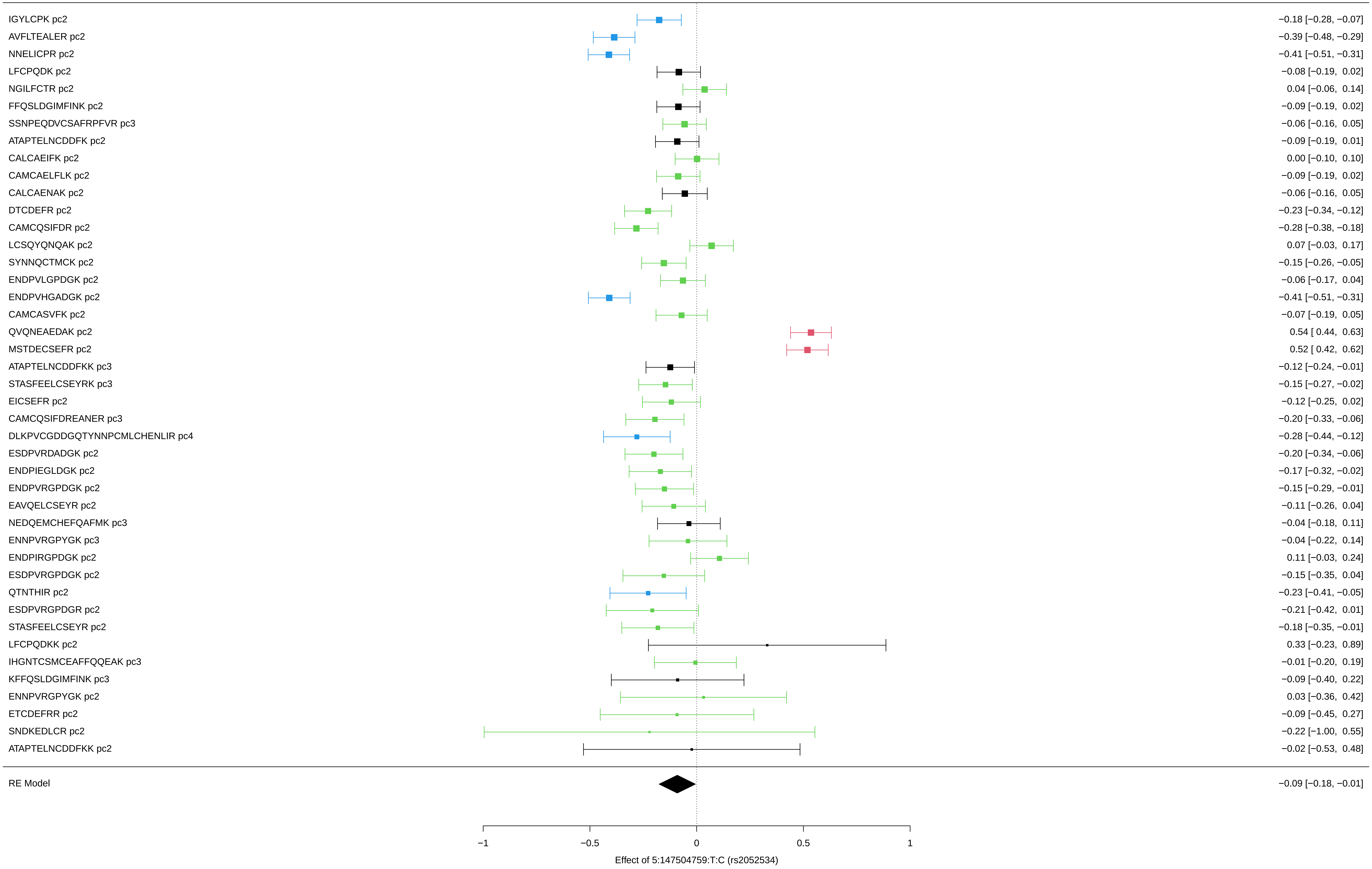

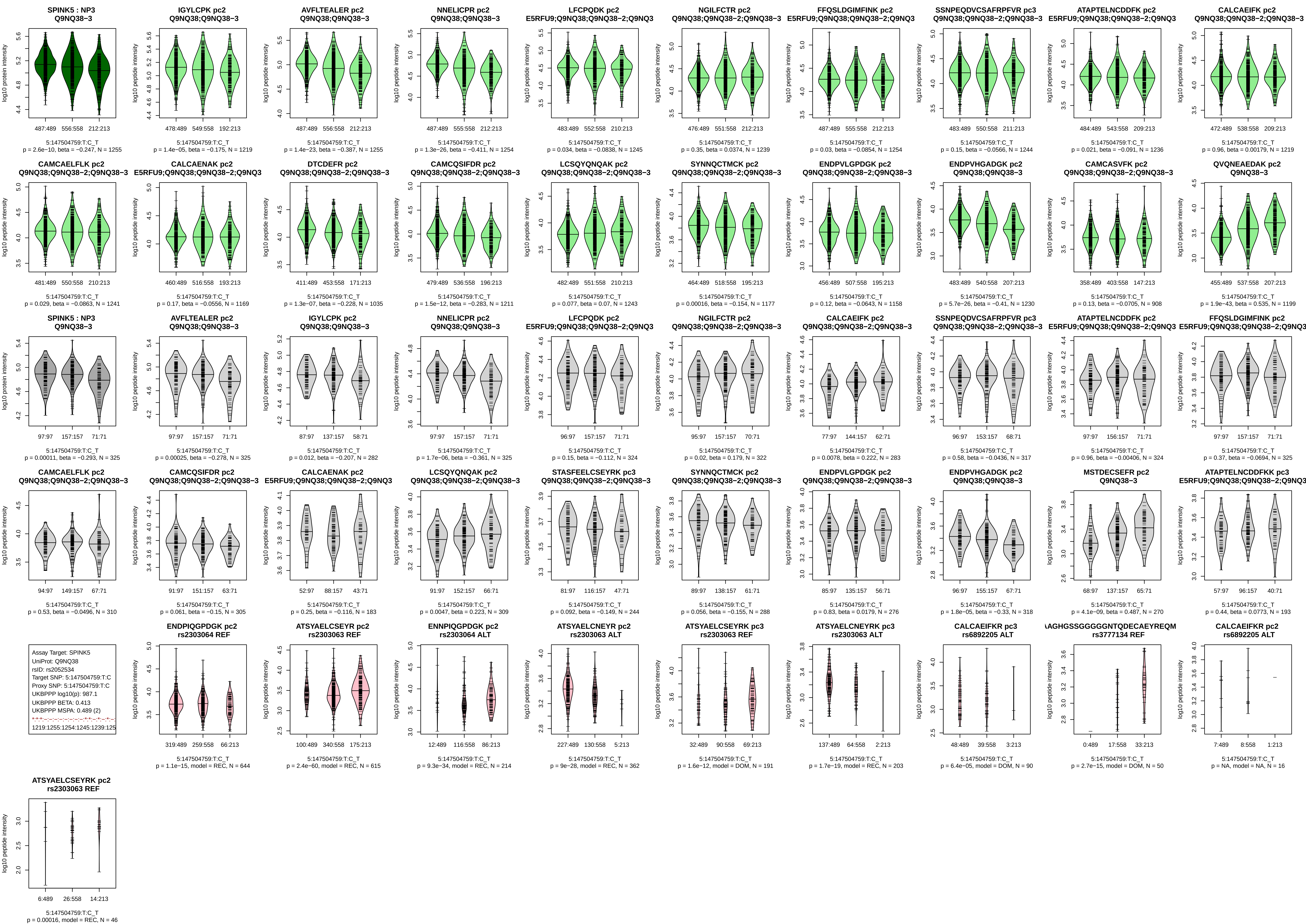
SPINK5 : NP3 Q9NQ38−3
log10 protein intensity
487:489 556:558 212:213
5:147504759:T:C_T
p = 2.6e−10, beta = −0.247, N = 1255
IGYLCPK pc2 Q9NQ38;Q9NQ38−3
478:489 549:558 192:213
p = 1.4e−05, beta = −0.175, N = 1219
AVFLTEALER pc2 Q9NQ38;Q9NQ38−3
487:489 556:558 212:213
p = 1.4e−23, beta = −0.387, N = 1255
NNELICPR pc2 Q9NQ38;Q9NQ38−3
487:489 555:558 212:213
p = 1.3e−26, beta = −0.411, N = 1254
LFCPQDK pc2 E5RFU9;Q9NQ38;Q9NQ38−2;Q9NQ3
483:489 552:558 210:213
p = 0.034, beta = −0.0838, N = 1245
NGILFCTR pc2 Q9NQ38;Q9NQ38−2;Q9NQ38−3
476:489 551:558 212:213
p = 0.35, beta = 0.0374, N = 1239
FFQSLDGIMFINK pc2 E5RFU9;Q9NQ38;Q9NQ38−2;Q9NQ3
487:489 555:558 212:213
p = 0.03, beta = −0.0854, N = 1254
SSNPEQDVCSAFRPFVR pc3 Q9NQ38;Q9NQ38−2;Q9NQ38−3
483:489 550:558 211:213
p = 0.15, beta = −0.0566, N = 1244
ATAPTELNCDDFK pc2 E5RFU9;Q9NQ38;Q9NQ38−2;Q9NQ3
484:489 543:558 209:213
p = 0.021, beta = −0.091, N = 1236
CALCAEIFK pc2 Q9NQ38;Q9NQ38−2;Q9NQ38−3
472:489 538:558 209:213
p = 0.96, beta = 0.00179, N = 1219
CAMCAELFLK pc2 Q9NQ38;Q9NQ38−2;Q9NQ38−3
481:489 550:558 210:213
p = 0.029, beta = −0.0863, N = 1241
CALCAENAK pc2 E5RFU9;Q9NQ38;Q9NQ38−2;Q9NQ3
460:489 516:558 193:213
p = 0.17, beta = −0.0556, N = 1169
DTCDEFR pc2 Q9NQ38;Q9NQ38−2;Q9NQ38−3
411:489 453:558 171:213
p = 1.3e−07, beta = −0.228, N = 1035
CAMCQSIFDR pc2 Q9NQ38;Q9NQ38−2;Q9NQ38−3
479:489 536:558 196:213
p = 1.5e−12, beta = −0.283, N = 1211
LCSQYQNQAK pc2 Q9NQ38;Q9NQ38−2;Q9NQ38−3
482:489 551:558 210:213
p = 0.077, beta = 0.07, N = 1243
SYNNQCTMCK pc2 Q9NQ38;Q9NQ38−2;Q9NQ38−3
464:489 518:558 195:213
p = 0.00016, beta = −0.154, N = 1177
ENDPVLGPDGK pc2 Q9NQ38;Q9NQ38−2;Q9NQ38−3
456:489 507:558 195:213
p = 0.12, beta = −0.0643, N = 1158
ENDPVHGADGK pc2 Q9NQ38;Q9NQ38−3
483:489 540:558 207:213
p = 5.7e−26, beta = −0.41, N = 1230
CAMCASVFK pc2 Q9NQ38;Q9NQ38−2;Q9NQ38−3
358:489 403:558 147:213
p = 0.13, beta = −0.0705, N = 908
QVQNEAEDAK pc2 Q9NQ38−3
455:489 537:558 207:213
p = 1.9e−43, beta = 0.535, N = 1199
SPINK5 : NP3 Q9NQ38−3
97:97 157:157 71:71
p = 0.00011, beta = −0.293, N = 325
AVFLTEALER pc2 Q9NQ38;Q9NQ38−3
97:97 157:157 71:71
p = 0.00025, beta = −0.278, N = 325
IGYLCPK pc2 Q9NQ38;Q9NQ38−3
87:97 137:157 58:71
p = 0.012, beta = −0.207, N = 282
NNELICPR pc2 Q9NQ38;Q9NQ38−3
97:97 157:157 71:71
p = 1.7e−06, beta = −0.361, N = 325
LFCPQDK pc2 E5RFU9;Q9NQ38;Q9NQ38−2;Q9NQ3
96:97 157:157 71:71
p = 0.15, beta = −0.112, N = 324
NGILFCTR pc2 Q9NQ38;Q9NQ38−2;Q9NQ38−3
95:97 157:157 70:71
p = 0.02, beta = 0.179, N = 322
CALCAEIFK pc2 Q9NQ38;Q9NQ38−2;Q9NQ38−3
77:97 144:157 62:71
p = 0.0078, beta = 0.222, N = 283
SSNPEQDVCSAFRPFVR pc3 Q9NQ38;Q9NQ38−2;Q9NQ38−3
96:97 153:157 68:71
p = 0.58, beta = −0.0436, N = 317
ATAPTELNCDDFK pc2 E5RFU9;Q9NQ38;Q9NQ38−2;Q9NQ3
97:97 156:157 71:71
p = 0.96, beta = −0.00406, N = 324
FFQSLDGIMFINK pc2 E5RFU9;Q9NQ38;Q9NQ38−2;Q9NQ3
97:97 157:157 71:71
p = 0.37, beta = −0.0694, N = 325
CAMCAELFLK pc2 Q9NQ38;Q9NQ38−2;Q9NQ38−3
94:97 149:157 67:71
p = 0.53, beta = −0.0496, N = 310
CAMCQSIFDR pc2 Q9NQ38;Q9NQ38−2;Q9NQ38−3
91:97 151:157 63:71
p = 0.061, beta = −0.15, N = 305
CALCAENAK pc2 E5RFU9;Q9NQ38;Q9NQ38−2;Q9NQ3
52:97 88:157 43:71
p = 0.25, beta = −0.116, N = 183
LCSQYQNQAK pc2 Q9NQ38;Q9NQ38−2;Q9NQ38−3
91:97 152:157 66:71
p = 0.0047, beta = 0.223, N = 309
STASFEELCSEYRK pc3 Q9NQ38;Q9NQ38−2;Q9NQ38−3
81:97 116:157 47:71
p = 0.092, beta = −0.149, N = 244
SYNNQCTMCK pc2 Q9NQ38;Q9NQ38−2;Q9NQ38−3
89:97 138:157 61:71
p = 0.056, beta = −0.155, N = 288
ENDPVLGPDGK pc2 Q9NQ38;Q9NQ38−2;Q9NQ38−3
85:97 135:157 56:71
p = 0.83, beta = 0.0179, N = 276
ENDPVHGADGK pc2 Q9NQ38;Q9NQ38−3
96:97 155:157 67:71
p = 1.8e−05, beta = −0.33, N = 318
MSTDECSEFR pc2 Q9NQ38−3
68:97 137:157 65:71
p = 4.1e−09, beta = 0.487, N = 270
ATAPTELNCDDFKK pc3 E5RFU9;Q9NQ38;Q9NQ38−2;Q9NQ3
57:97 96:157 40:71
p = 0.44, beta = 0.0773, N = 193
Assay Target: SPINK5
UniProt: Q9NQ38
rsID: rs2052534
Target SNP: 5:147504759:T:C
Proxy SNP: 5:147504759:T:C
UKBPPP log10(p): 987.1
UKBPPP BETA: 0.413
UKBPPP MSPA: 0.489 (2)
1219:1255:1254:1245:1239:125
ENDPIQGPDGK pc2 rs2303064 REF
319:489 259:558 66:213
p = 1.1e−15, model = REC, N = 644
ATSYAELCSEYR pc2 rs2303063 REF
100:489 340:558 175:213
p = 2.4e−60, model = REC, N = 615
ENNPIQGPDGK pc2 rs2303064 ALT
12:489 116:558 86:213
p = 9.3e−34, model = REC, N = 214
ATSYAELCNEYR pc2 rs2303063 ALT
227:489 130:558 5:213
p = 9e−28, model = REC, N = 362
ATSYAELCSEYRK pc3 rs2303063 REF
32:489 90:558 69:213
p = 1.6e−12, model = DOM, N = 191
ATSYAELCNEYRK pc3 rs2303063 ALT
137:489 64:558 2:213
p = 1.7e−19, model = REC, N = 203
CALCAEIFKR pc3 rs6892205 ALT
48:489 39:558 3:213
p = 6.4e−05, model = DOM, N = 90
AAGHGSSGGGGGNTQDECAEYREQM rs3777134 REF
0:489 17:558 33:213
p = 2.7e−15, model = DOM, N = 50
CALCAEIFKR pc2 rs6892205 ALT
7:489 8:558 1:213
p = NA, model = NA, N = 16
ATSYAELCSEYRK pc2 rs2303063 REF
6:489 26:558 14:213
p = 0.00016, model = REC, N = 46

91. F13B (P05160)
UKBPPP MSPA=0.055

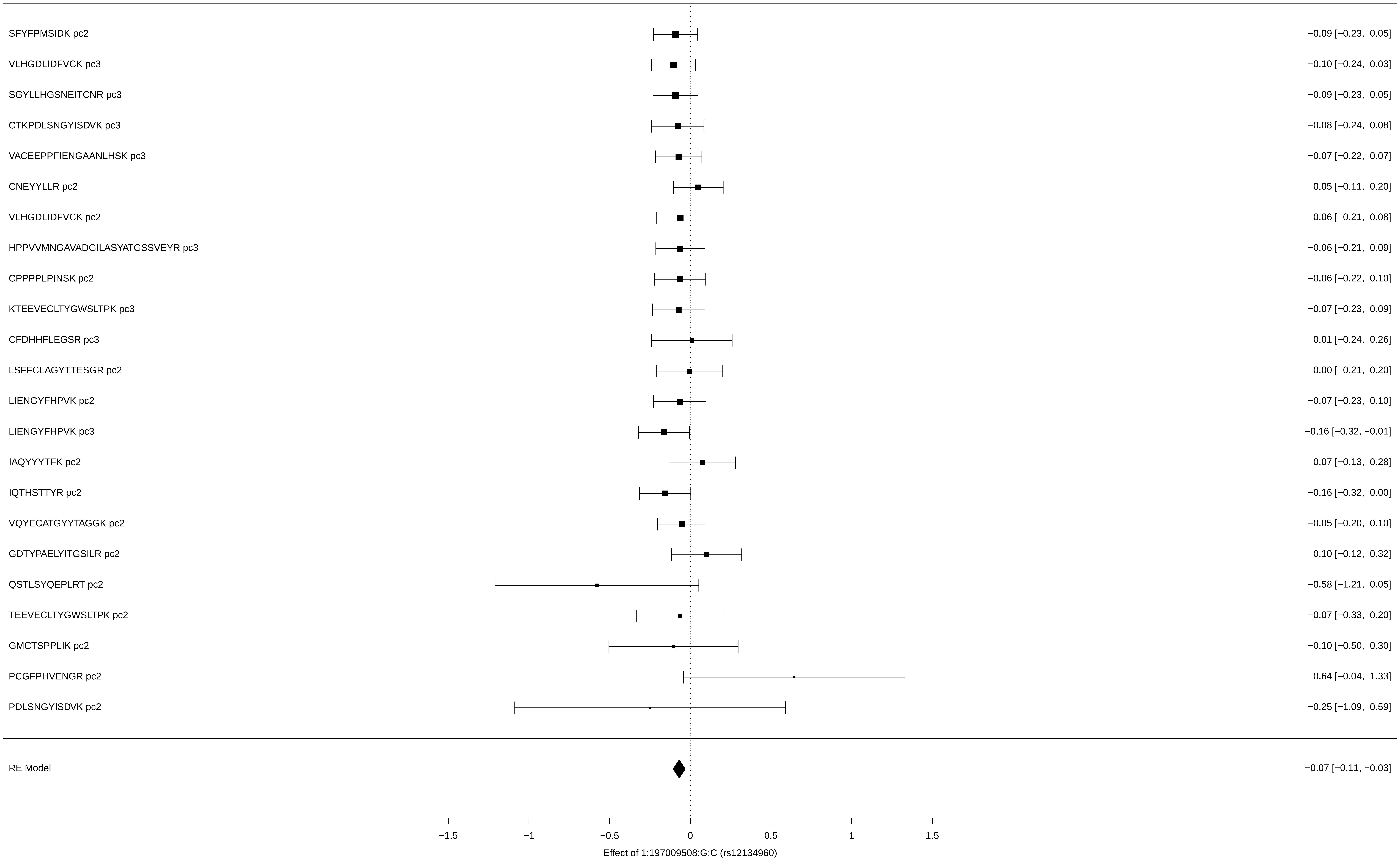

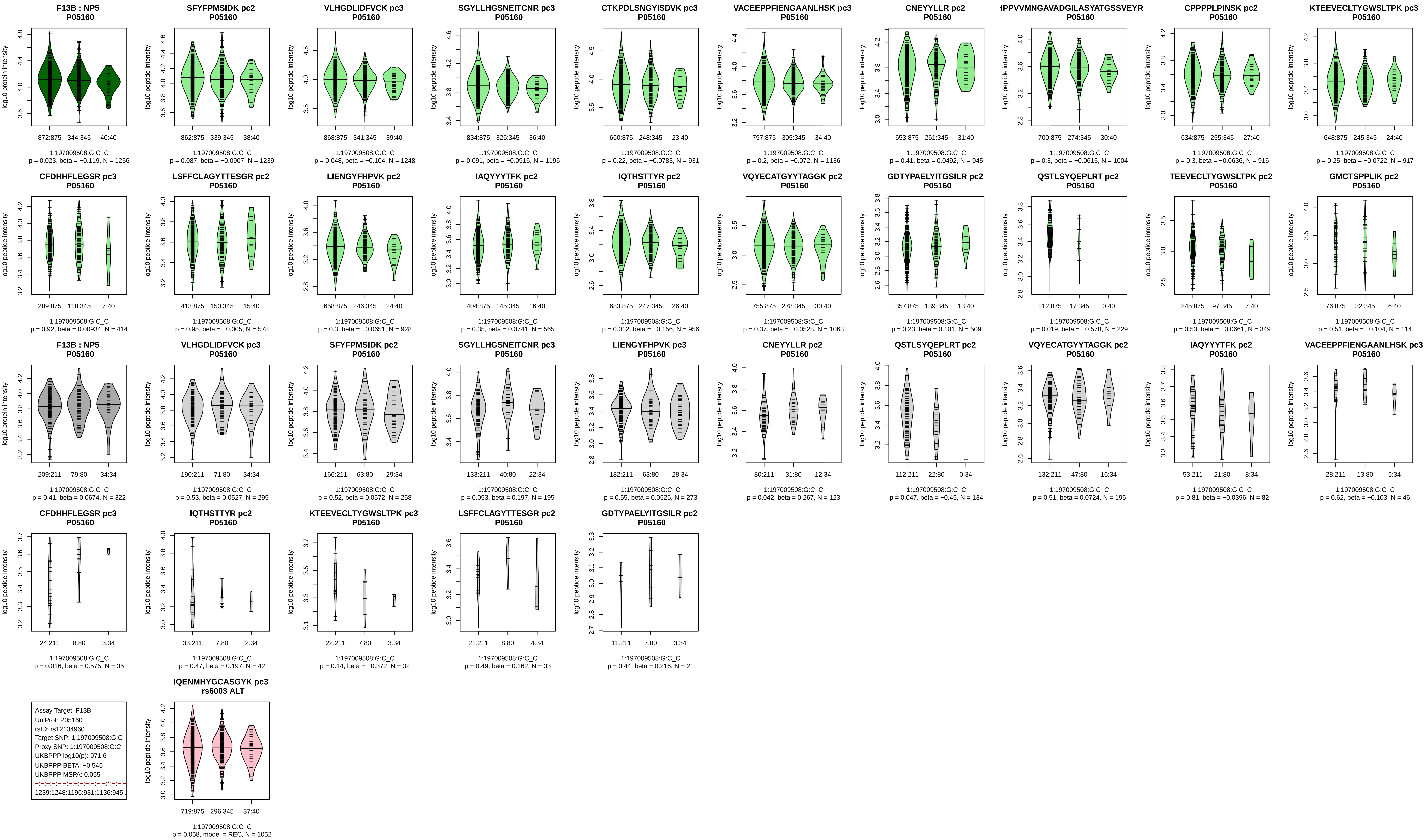
F13B : NP5 P05160
log10 protein intensity
872:875 344:345 40:40
1:197009508:G:C_C
p = 0.023, beta = −0.119, N = 1256
SFYFPMSIDK pc2 P05160
log10 peptide intensity
862:875 339:345 38:40
1:197009508:G:C_C
p = 0.087, beta = −0.0907, N = 1239
VLHGDLIDFVCK pc3 P05160
868:875 341:345 39:40
1:197009508:G:C_C
p = 0.048, beta = −0.104, N = 1248
SGYLLHGSNEITCNR pc3 P05160
834:875 326:345 36:40
1:197009508:G:C_C
p = 0.091, beta = −0.0916, N = 1196
CTKPDLSNGYISDVK pc3 P05160
660:875 248:345 23:40
1:197009508:G:C_C
p = 0.22, beta = −0.0783, N = 931
VACEEPPFIENGAANLHSK pc3 P05160
797:875 305:345 34:40
1:197009508:G:C_C
p = 0.2, beta = −0.072, N = 1136
CNEYYLLR pc2 P05160
653:875 261:345 31:40
1:197009508:G:C_C
p = 0.41, beta = 0.0492, N = 945
HPPVVMNGAVADGILASYATGSSVEYR P05160
700:875 274:345 30:40
1:197009508:G:C_C
p = 0.3, beta = −0.0615, N = 1004
CPPPPLPINSK pc2 P05160
634:875 255:345 27:40
1:197009508:G:C_C
p = 0.3, beta = −0.0636, N = 916
KTEEVECLTYGWSLTPK pc3 P05160
648:875 245:345 24:40
1:197009508:G:C_C
p = 0.25, beta = −0.0722, N = 917
CFDHHFLEGSR pc3 P05160
289:875 118:345 7:40
1:197009508:G:C_C
p = 0.92, beta = 0.00934, N = 414
LSFFCLAGYTTESGR pc2 P05160
413:875 150:345 15:40
1:197009508:G:C_C
p = 0.95, beta = −0.005, N = 578
LIENGYFHPVK pc2 P05160
658:875 246:345 24:40
1:197009508:G:C_C
p = 0.3, beta = −0.0651, N = 928
IAQYYYTFK pc2 P05160
404:875 145:345 16:40
1:197009508:G:C_C
p = 0.35, beta = 0.0741, N = 565
IQTHSTTYR pc2 P05160
683:875 247:345 26:40
1:197009508:G:C_C
p = 0.012, beta = −0.156, N = 956
VQYECATGYYTAGGK pc2 P05160
755:875 278:345 30:40
1:197009508:G:C_C
p = 0.37, beta = −0.0528, N = 1063
GDTYPAELYITGSILR pc2 P05160
357:875 139:345 13:40
1:197009508:G:C_C
p = 0.23, beta = 0.101, N = 509
QSTLSYQEPLRT pc2 P05160
212:875 17:345 0:40
1:197009508:G:C_C
p = 0.019, beta = −0.578, N = 229
TEEVECLTYGWSLTPK pc2 P05160
245:875 97:345 7:40
1:197009508:G:C_C
p = 0.53, beta = −0.0661, N = 349
GMCTSPPLIK pc2 P05160
76:875 32:345 6:40
1:197009508:G:C_C
p = 0.51, beta = −0.104, N = 114
F13B : NP5 P05160
209:211 79:80 34:34
1:197009508:G:C_C
p = 0.41, beta = 0.0674, N = 322
VLHGDLIDFVCK pc3 P05160
190:211 71:80 34:34
1:197009508:G:C_C
p = 0.53, beta = 0.0527, N = 295
SFYFPMSIDK pc2 P05160
166:211 63:80 29:34
1:197009508:G:C_C
p = 0.52, beta = 0.0572, N = 258
SGYLLHGSNEITCNR pc3 P05160
133:211 40:80 22:34
1:197009508:G:C_C
p = 0.053, beta = 0.197, N = 195
LIENGYFHPVK pc3 P05160
182:211 63:80 28:34
1:197009508:G:C_C
p = 0.55, beta = 0.0526, N = 273
CNEYYLLR pc2 P05160
80:211 31:80 12:34
1:197009508:G:C_C
p = 0.042, beta = 0.267, N = 123
QSTLSYQEPLRT pc2 P05160
112:211 22:80 0:34
1:197009508:G:C_C
p = 0.047, beta = −0.45, N = 134
VQYECATGYYTAGGK pc2 P05160
132:211 47:80 16:34
1:197009508:G:C_C
p = 0.51, beta = 0.0724, N = 195
IAQYYYTFK pc2 P05160
53:211 21:80 8:34
1:197009508:G:C_C
p = 0.81, beta = −0.0396, N = 82
VACEEPPFIENGAANLHSK pc3 P05160
28:211 13:80 5:34
1:197009508:G:C_C
p = 0.62, beta = −0.103, N = 46
CFDHHFLEGSR pc3 P05160
24:211 8:80 3:34
1:197009508:G:C_C
p = 0.016, beta = 0.575, N = 35
IQTHSTTYR pc2 P05160
33:211 7:80 2:34
1:197009508:G:C_C
p = 0.47, beta = 0.197, N = 42
KTEEVECLTYGWSLTPK pc3 P05160
22:211 7:80 3:34
1:197009508:G:C_C
p = 0.14, beta = −0.372, N = 32
LSFFCLAGYTTESGR pc2 P05160
21:211 8:80 4:34
1:197009508:G:C_C
p = 0.49, beta = 0.162, N = 33
GDTYPAELYITGSILR pc2 P05160
11:211 7:80 3:34
1:197009508:G:C_C
p = 0.44, beta = 0.216, N = 21
Assay Target: F13B
UniProt: P05160
rsID: rs12134960
Target SNP: 1:197009508:G:C
Proxy SNP: 1:197009508:G:C
UKBPPP log10(p): 971.6
UKBPPP BETA: −0.545
UKBPPP MSPA: 0.055
1239:1248:1196:931:1136:945:
IQENMHYGCASGYK pc3 rs6003 ALT
719:875 296:345 37:40
1:197009508:G:C_C
p = 0.058, model = REC, N = 1052

92. F11 (P03951)
UKBPPP MSPA=0.856

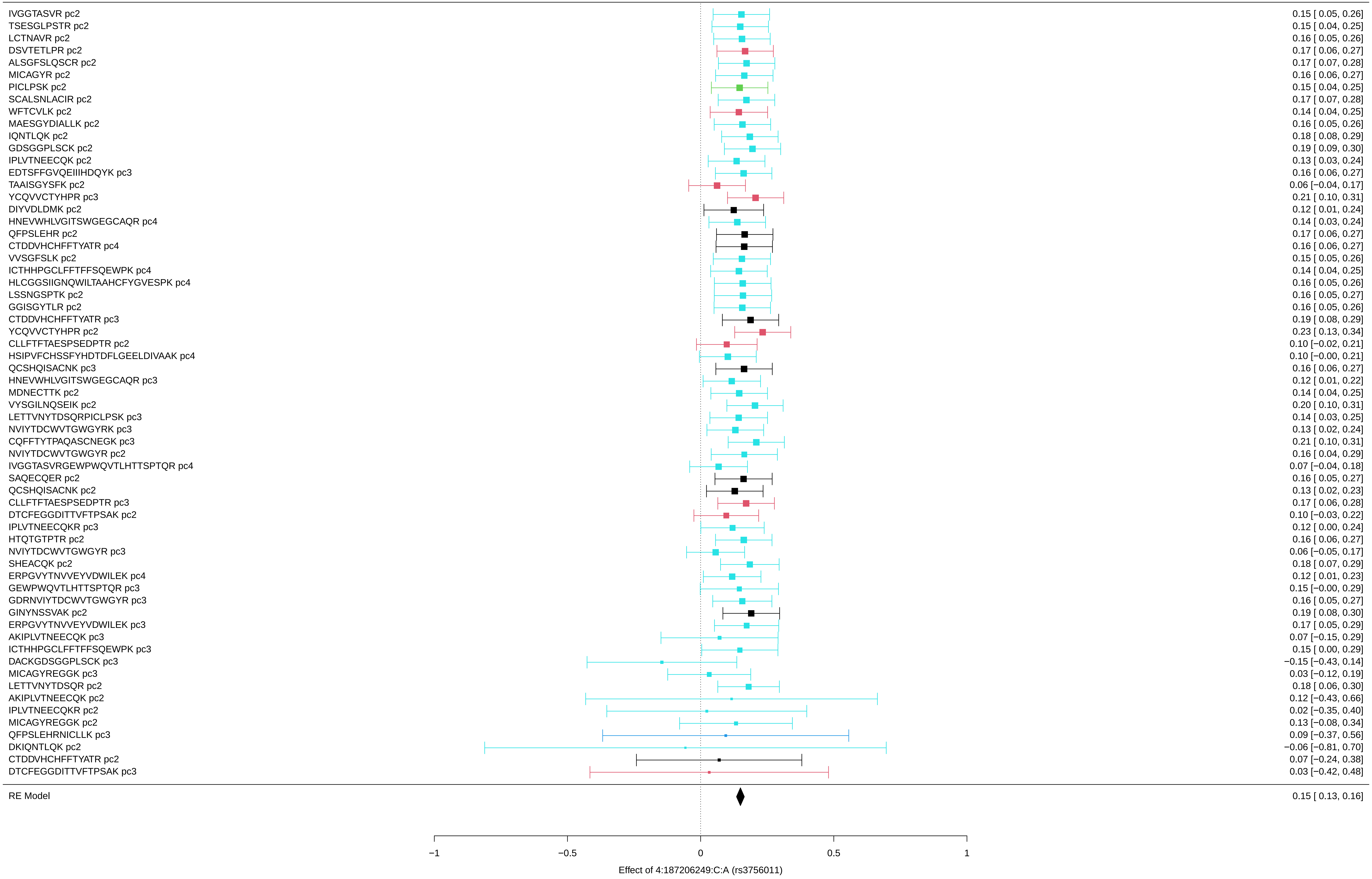

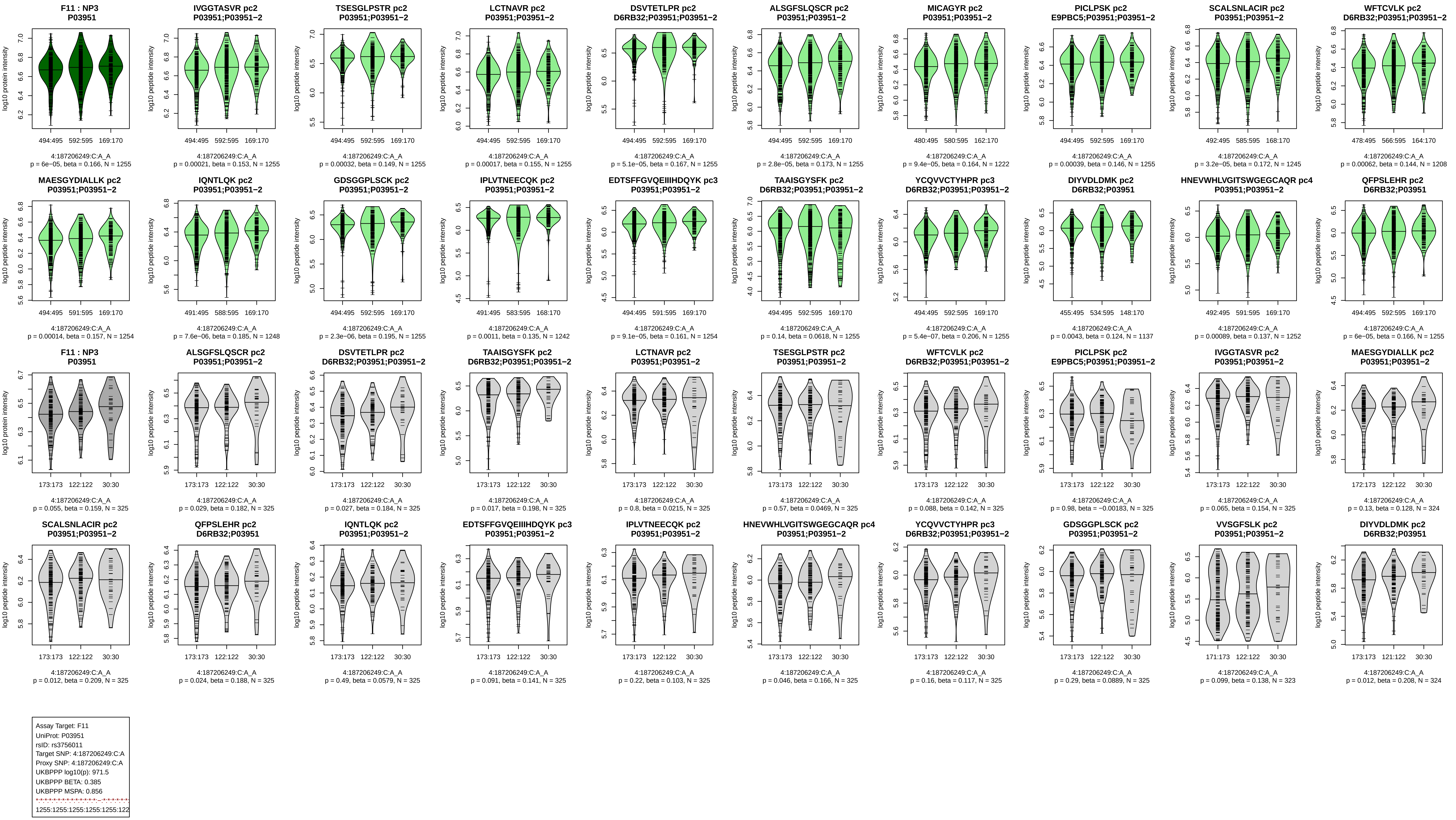
F11 : NP3
P03951
p = 6e−05, beta = 0.166, N = 1255
IVGGTASVR pc2
P03951;P03951−2
p = 0.00021, beta = 0.153, N = 1255
TSESGLPSTR pc2
P03951;P03951−2
p = 0.00032, beta = 0.149, N = 1255
LCTNAVR pc2
P03951;P03951−2
p = 0.00017, beta = 0.155, N = 1255
DSVTETLPR pc2
D6RB32;P03951;P03951−2
p = 5.1e−05, beta = 0.167, N = 1255
ALSGFSLQSCR pc2
P03951;P03951−2
p = 2.8e−05, beta = 0.173, N = 1255
MICAGYR pc2
P03951;P03951−2
p = 9.4e−05, beta = 0.164, N = 1222
PICLPSK pc2
E9PBC5;P03951;P03951−2
p = 0.00039, beta = 0.146, N = 1255
SCALSNLACIR pc2
P03951;P03951−2
p = 3.2e−05, beta = 0.172, N = 1245
WFTCVLK pc2
D6RB32;P03951;P03951−2
p = 0.00062, beta = 0.144, N = 1208
MAESGYDIALLK pc2
P03951;P03951−2
p = 0.00014, beta = 0.157, N = 1254
IQNTLQK pc2
P03951;P03951−2
p = 7.6e−06, beta = 0.185, N = 1248
GDSGGPLSCK pc2
P03951;P03951−2
p = 2.3e−06, beta = 0.195, N = 1255
IPLVTNEECQK pc2
P03951;P03951−2
p = 0.0011, beta = 0.135, N = 1242
EDTSFFGVQEIIHDQYK pc3
P03951;P03951−2
p = 9.1e−05, beta = 0.161, N = 1254
TAAISGYSFK pc2
D6RB32;P03951;P03951−2
p = 0.14, beta = 0.0618, N = 1255
YCQVVCTYHPR pc3
D6RB32;P03951;P03951−2
p = 5.4e−07, beta = 0.206, N = 1255
DIYVDLDMK pc2
D6RB32;P03951
p = 0.0043, beta = 0.124, N = 1137
HNEVWHLVGITSWGEGCAQR pc4
P03951;P03951−2
p = 0.00089, beta = 0.137, N = 1252
QFPSLEHR pc2
D6RB32;P03951
p = 6e−05, beta = 0.166, N = 1255
F11 : NP3
P03951
p = 0.055, beta = 0.159, N = 325
ALSGFSLQSCR pc2
P03951;P03951−2
p = 0.029, beta = 0.182, N = 325
DSVTETLPR pc2
D6RB32;P03951;P03951−2
p = 0.027, beta = 0.184, N = 325
TAAISGYSFK pc2
D6RB32;P03951;P03951−2
p = 0.017, beta = 0.198, N = 325
LCTNAVR pc2
P03951;P03951−2
p = 0.8, beta = 0.0215, N = 325
TSESGLPSTR pc2
P03951;P03951−2
p = 0.57, beta = 0.0469, N = 325
WFTCVLK pc2
D6RB32;P03951;P03951−2
p = 0.088, beta = 0.142, N = 325
PICLPSK pc2
E9PBC5;P03951;P03951−2
p = 0.98, beta = −0.00183, N = 325
IVGGTASVR pc2
P03951;P03951−2
p = 0.065, beta = 0.154, N = 325
MAESGYDIALLK pc2
P03951;P03951−2
p = 0.13, beta = 0.128, N = 324
SCALSNLACIR pc2
P03951;P03951−2
p = 0.012, beta = 0.209, N = 325
QFPSLEHR pc2
D6RB32;P03951
p = 0.024, beta = 0.188, N = 325
IQNTLQK pc2
P03951;P03951−2
p = 0.49, beta = 0.0579, N = 325
EDTSFFGVQEIIHDQYK pc3
P03951;P03951−2
p = 0.091, beta = 0.141, N = 325
IPLVTNEECQK pc2
P03951;P03951−2
p = 0.22, beta = 0.103, N = 325
HNEVWHLVGITSWGEGCAQR pc4
P03951;P03951−2
p = 0.046, beta = 0.166, N = 325
YCQVVCTYHPR pc3
D6RB32;P03951;P03951−2
p = 0.16, beta = 0.117, N = 325
GDSGGPLSCK pc2
P03951;P03951−2
p = 0.29, beta = 0.0889, N = 325
VVSGFSLK pc2
P03951;P03951−2
p = 0.099, beta = 0.138, N = 323
DIYVDLDMK pc2
D6RB32;P03951
p = 0.012, beta = 0.208, N = 324
4:187206249:C:A_A
log10 protein intensity
log10 peptide intensity
Assay Target: F11
UniProt: P03951
rsID: rs3756011
Target SNP: 4:187206249:C:A
Proxy SNP: 4:187206249:C:A
UKBPPP log10(p): 971.5
UKBPPP BETA: 0.385
UKBPPP MSPA: 0.856
1255:1255:1255:1255:1255:122

93. LTBP3 (Q9NS15)
UKBPPP MSPA=0.908

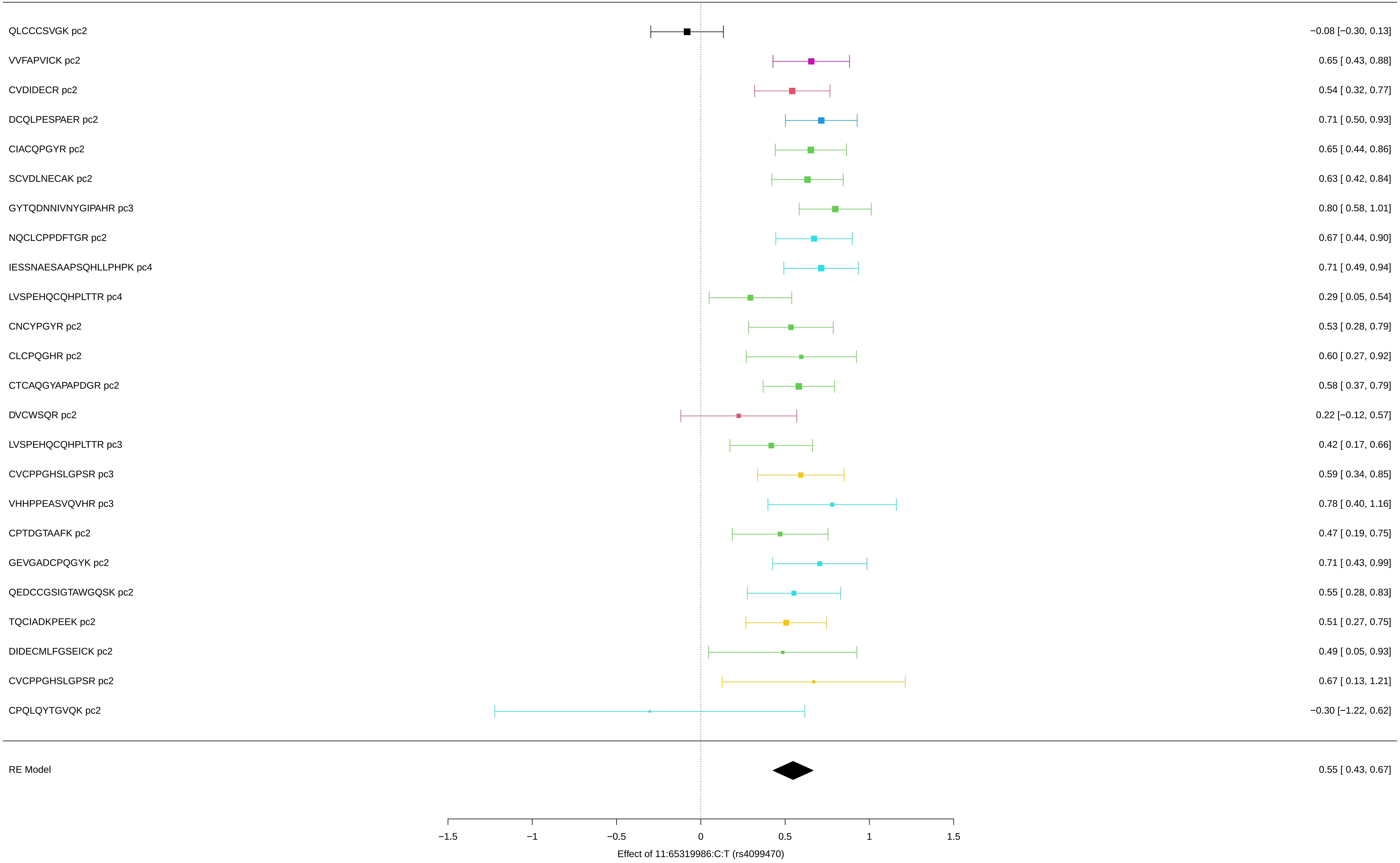

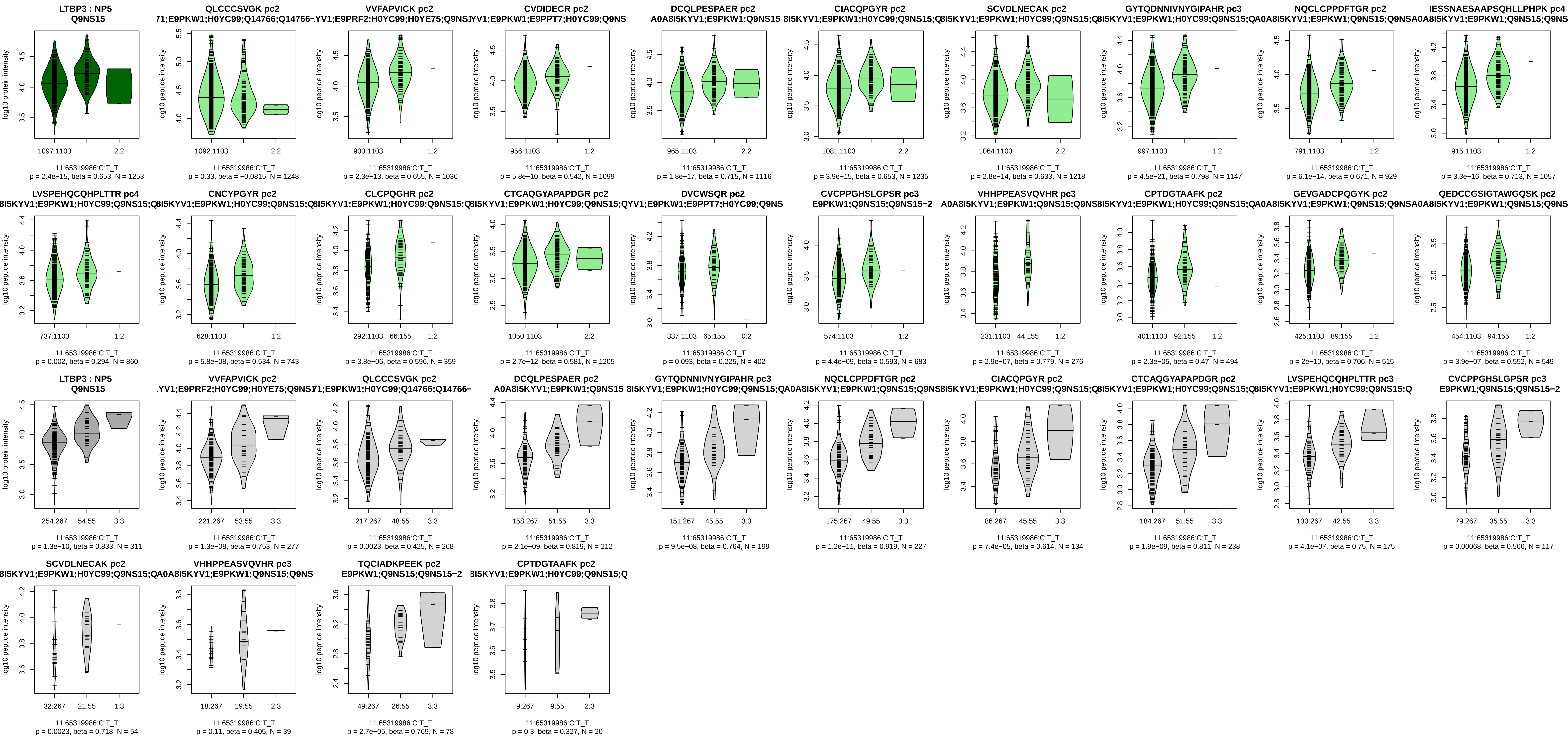

Assay Target: LTBP3
UniProt: Q9NS15
rsID: rs4099470
Target SNP: 11:65319986:C:T
Proxy SNP: 11:65319986:C:T
UKBPPP log10(p): 956.2
UKBPPP BETA: 0.857
UKBPPP MSPA: 0.908
1248:1036:1099:1116:1235:121

94. STAB2 (Q8WWQ8)
UKBPPP MSPA=0

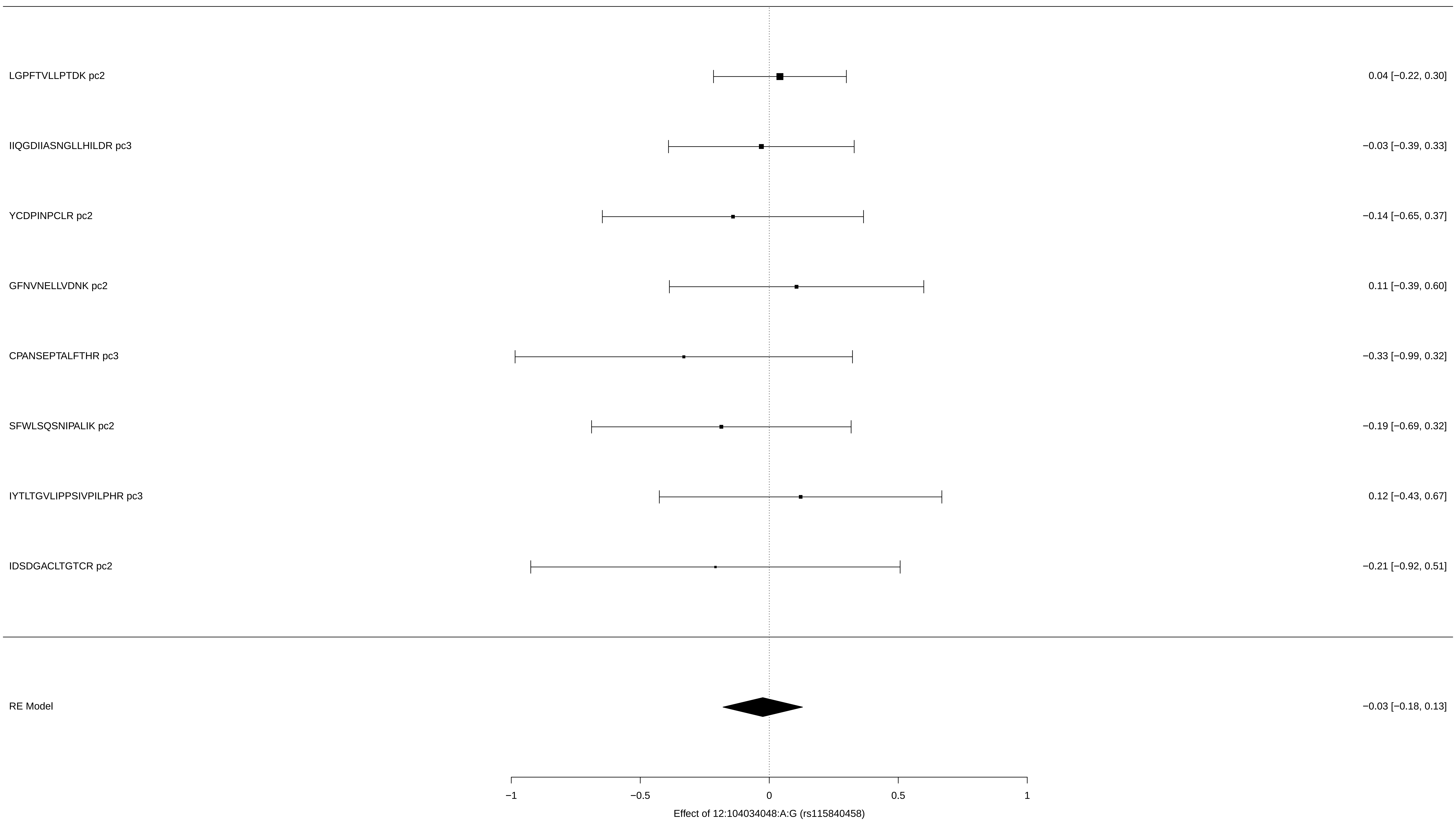

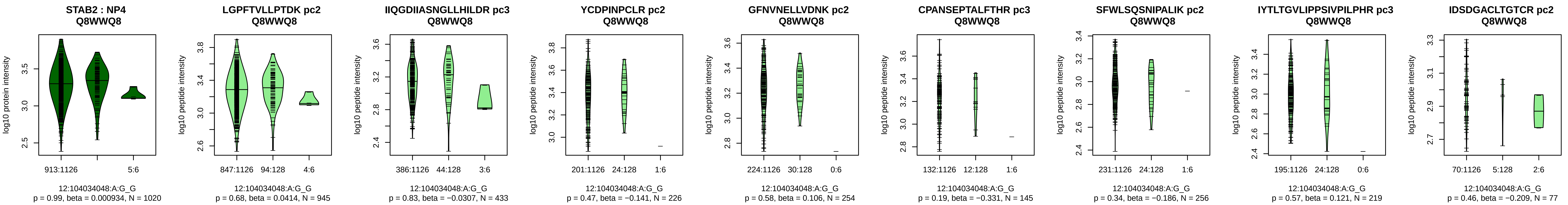

Assay Target: STAB2
UniProt: Q8WWQ8
rsID: rs115840458
Target SNP: 12:104034048:A:G
Proxy SNP: 12:104034048:A:G
UKBPPP log10(p): 952.3
UKBPPP BETA: 0.786
UKBPPP MSPA: 0
–:–:–:–:–:–:–:–
945:433:226:254:145:256:219:7

95. ICAM1 (P05362)
UKBPPP MSPA=0.143

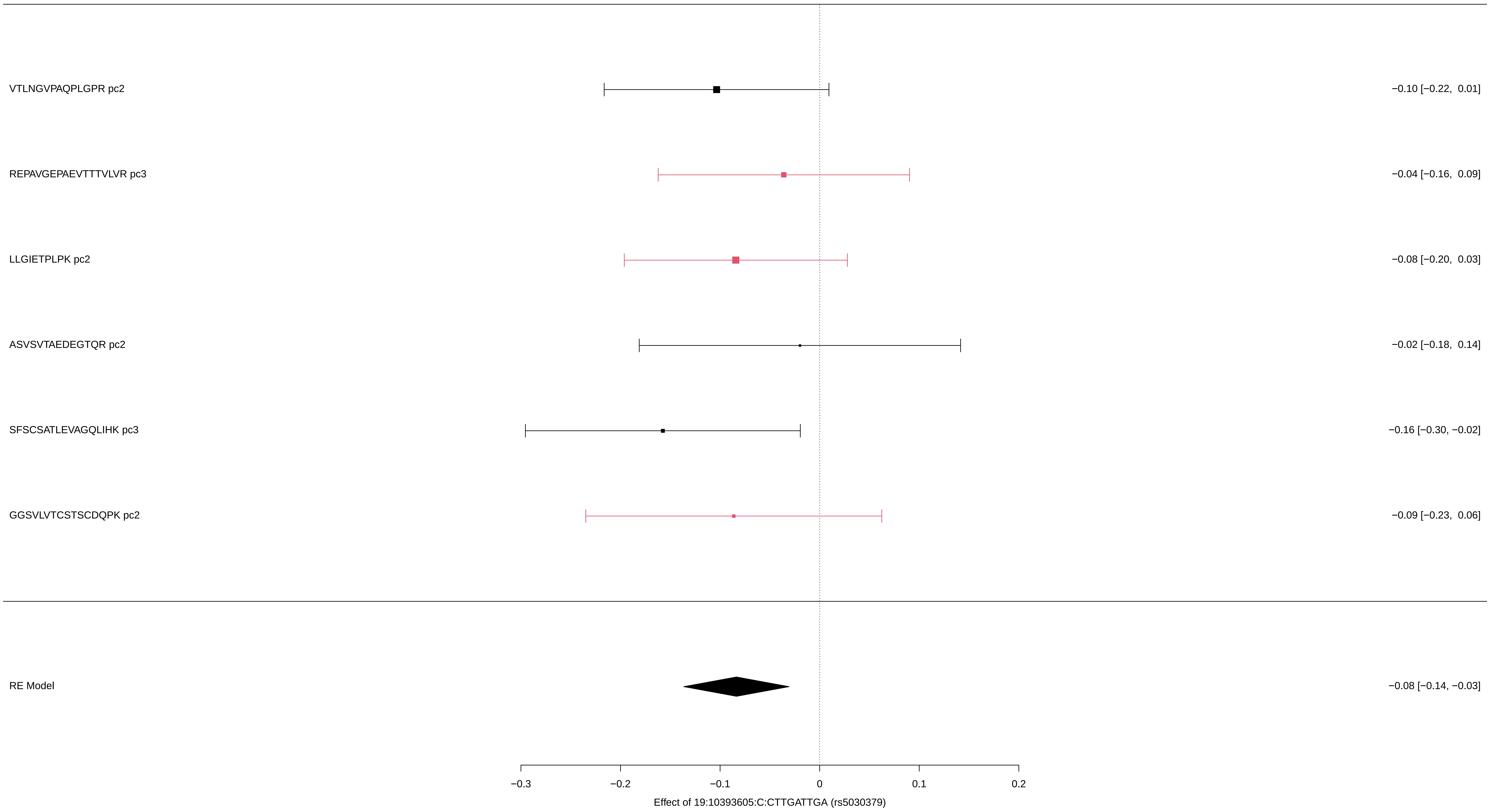

ICAM1 : NP4
P05362

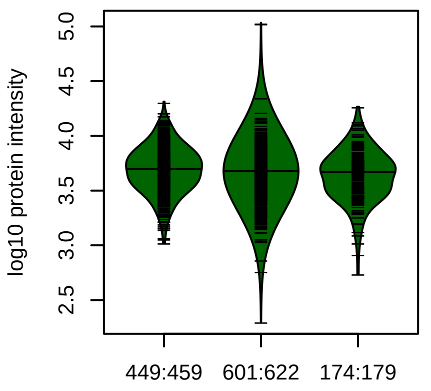

19:10393234:A:G_G
p = 0.0065, beta = −0.114, N = 1224

VTLNGVPAQPLGPR pc2
E7ESS4;P05362

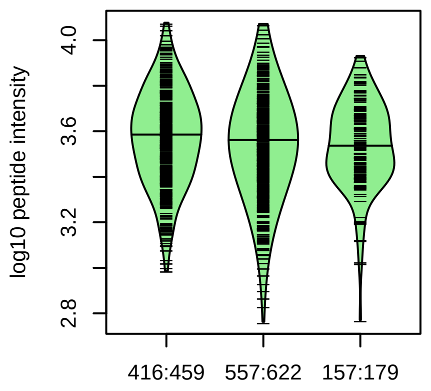

19:10393234:A:G_G
p = 0.018, beta = −0.104, N = 1130

REPAVGEPAEVTTTVLVR pc3
P05362

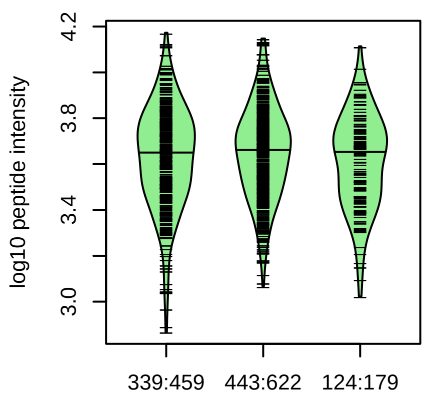

19:10393234:A:G_G
p = 0.46, beta = −0.036, N = 906

LLGIETPLPK pc2
P05362

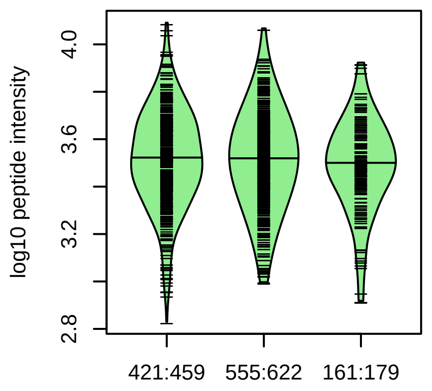

19:10393234:A:G_G
p = 0.053, beta = −0.0842, N = 1137

ASVSVTAEDEGTQR pc2
E7ESS4;P05362

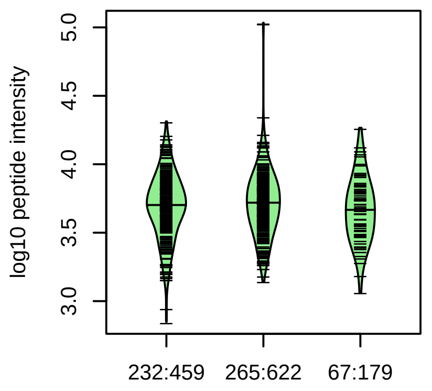

19:10393234:A:G_G
p = 0.75, beta = −0.0198, N = 564

SFSCSATLEVAGQLIHK pc3
E7ESS4;P05362

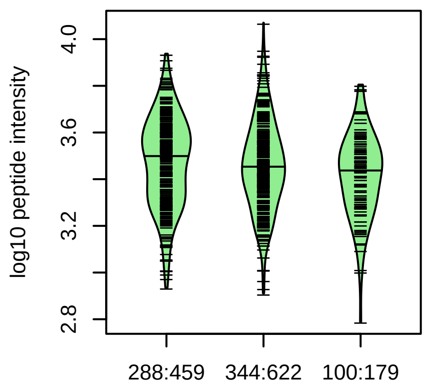

19:10393234:A:G_G
p = 0.0034, beta = −0.157, N = 732

GGSVLVTCSTSCDQPK pc2
P05362

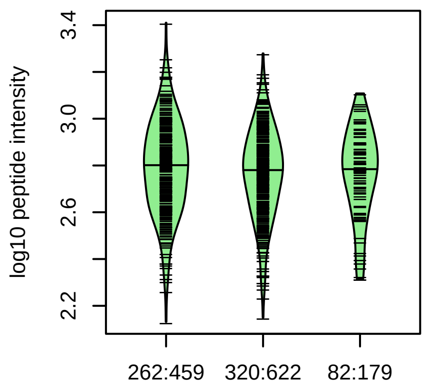

19:10393234:A:G_G
p = 0.14, beta = −0.0863, N = 664

ICAM1 : NP4
P05362

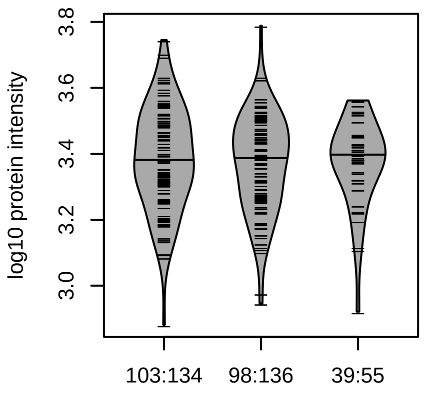

19:10393234:A:G_G
p = 0.72, beta = −0.0317, N = 240

VTLNGVPAQPLGPR pc2
E7ESS4;P05362

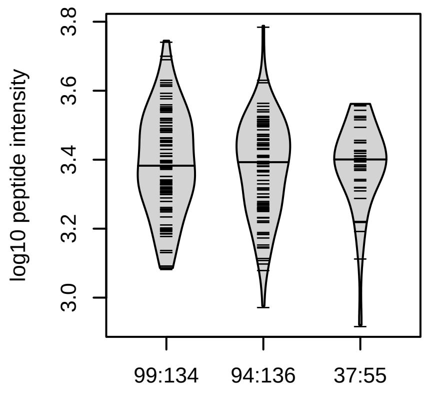

19:10393234:A:G_G
p = 0.74, beta = −0.0302, N = 230

LLGIETPLPK pc2
P05362

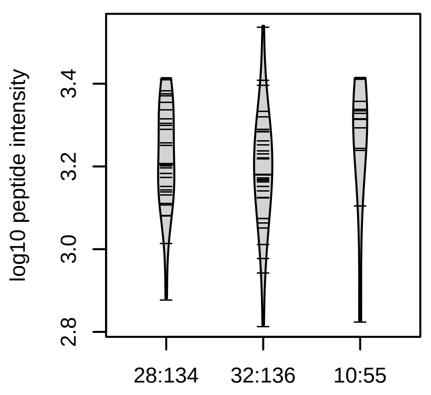

19:10393234:A:G_G
p = 0.8, beta = 0.0415, N = 70

Assay Target: ICAM1
UniProt: P05362
rsID: rs5030379
Target SNP: 19:10393605:C:CT
Proxy SNP: 19:10393234:A:G
UKBPPP log10(p): 945.1
UKBPPP BETA: 0.413
UKBPPP MSPA: 0.143
–:–:–:–:*:–
1130:906:1137:564:732:664

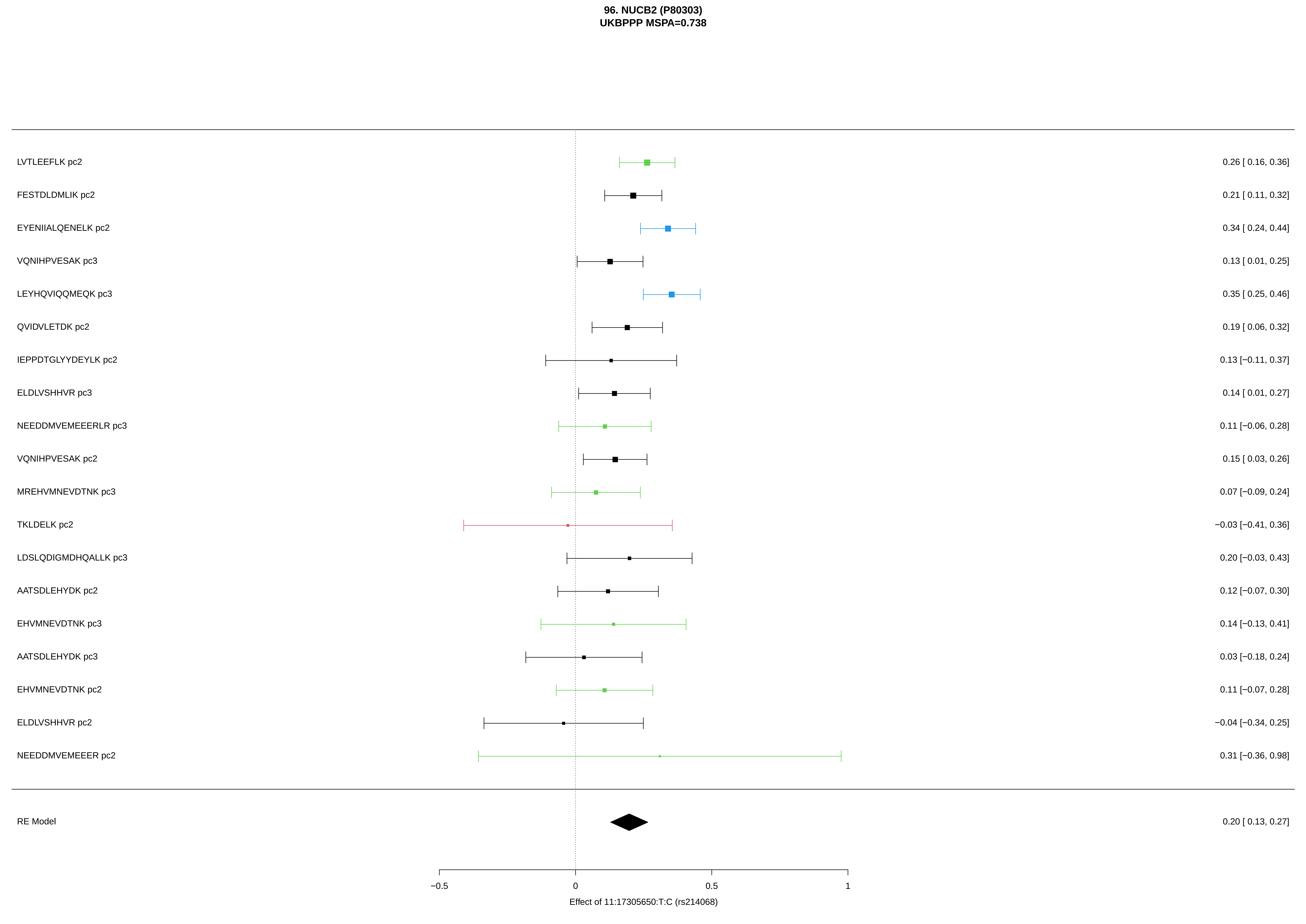
96. NUCB2 (P80303)
UKBPPP MSPA=0.738
LVTLEEFLK pc2
0.26 [0.16, 0.36]
FESTDLDMLIK pc2
0.21 [0.11, 0.32]
EYENIIALQENELK pc2
0.34 [0.24, 0.44]
VQNIHPVESAK pc3
0.13 [0.01, 0.25]
LEYHQVIQQMEQK pc3
0.35 [0.25, 0.46]
QVIDVLETDK pc2
0.19 [0.06, 0.32]
IEPPDTGLYYDEYLK pc2
0.13 [−0.11, 0.37]
ELDLVSHHVR pc3
0.14 [0.01, 0.27]
NEEDDMVEMEEERLR pc3
0.11 [−0.06, 0.28]
VQNIHPVESAK pc2
0.15 [0.03, 0.26]
MREHVMNEVDTNK pc3
0.07 [−0.09, 0.24]
TKLDELK pc2
−0.03 [−0.41, 0.36]
LDSLQDIGMDHQALLK pc3
0.20 [−0.03, 0.43]
AATSDLEHYDK pc2
0.12 [−0.07, 0.30]
EHVMNEVDTNK pc3
0.14 [−0.13, 0.41]
AATSDLEHYDK pc3
0.03 [−0.18, 0.24]
EHVMNEVDTNK pc2
0.11 [−0.07, 0.28]
ELDLVSHHVR pc2
−0.04 [−0.34, 0.25]
NEEDDMVEMEEER pc2
0.31 [−0.36, 0.98]
RE Model
0.20 [0.13, 0.27]
−0.5
0
0.5
1
Effect of 11:17305650:T:C (rs214068)

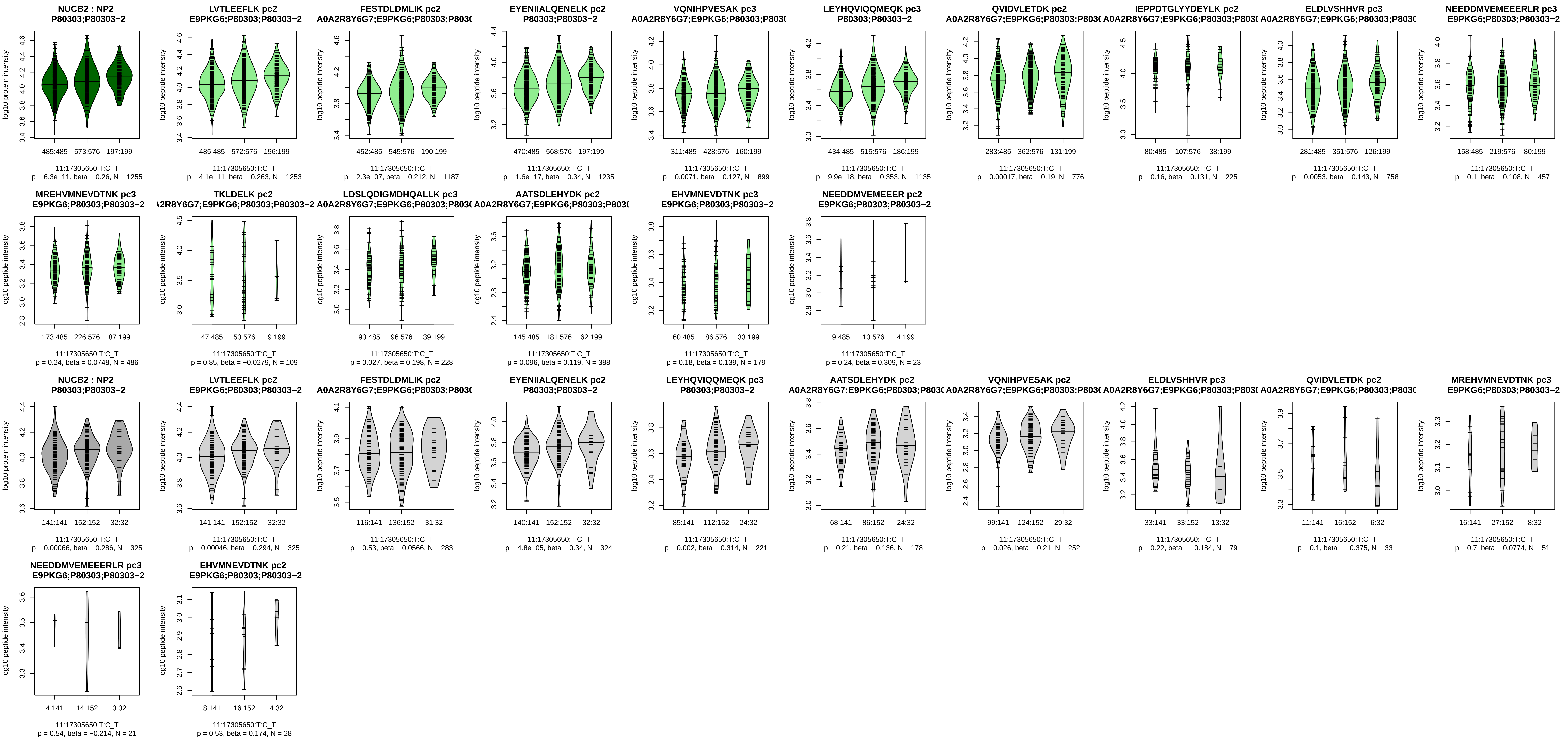

Assay Target: NUCB2
UniProt: P80303
rsID: rs214068
Target SNP: 11:17305650:T:C
Proxy SNP: 11:17305650:T:C
UKBPPP log10(p): 922.3
UKBPPP BETA: −0.402
UKBPPP MSPA: 0.738
1253:1187:1235:899:1135:776:2

97. FN1 (P02751)
UKBPPP MSPA=0.651

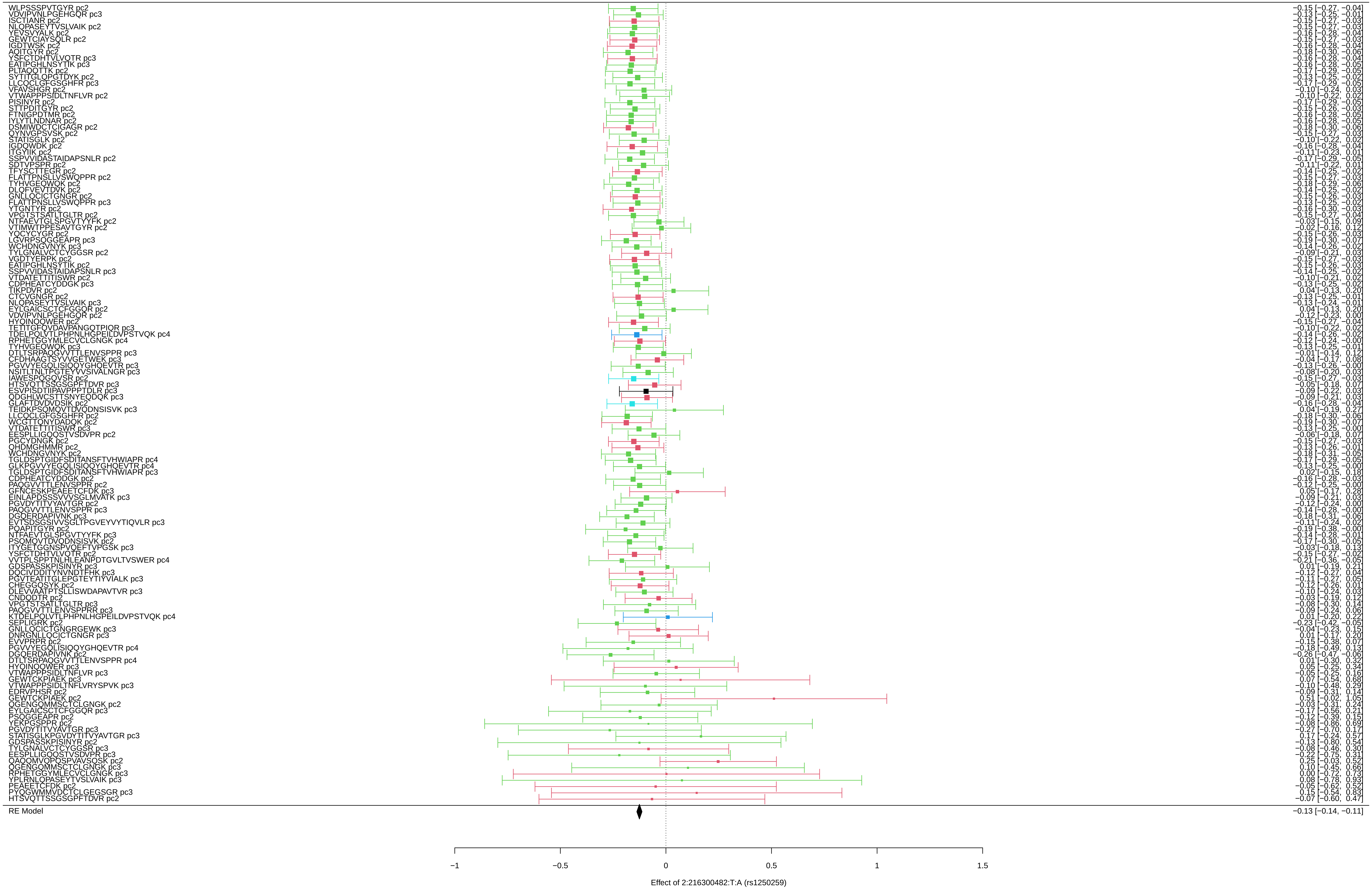

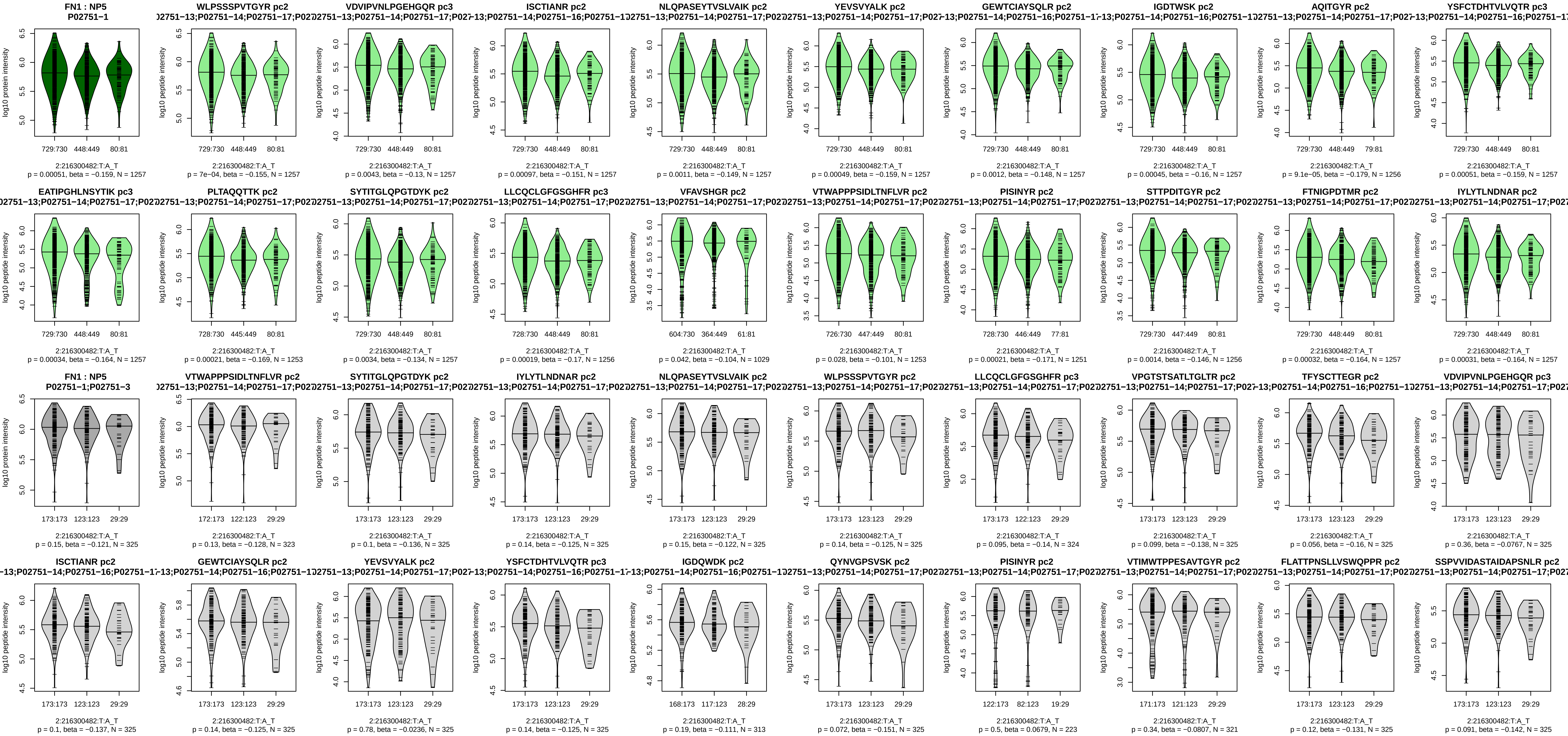

Assay Target: FN1
UniProt: P02751
rsID: rs1250259
Target SNP: 2:216300482:T:A
Proxy SNP: 2:216300482:T:A
UKBPPP log10(p): 919.4
UKBPPP BETA: 0.453
UKBPPP MSPA: 0.651
1257:1257:1257:1257:1257:125

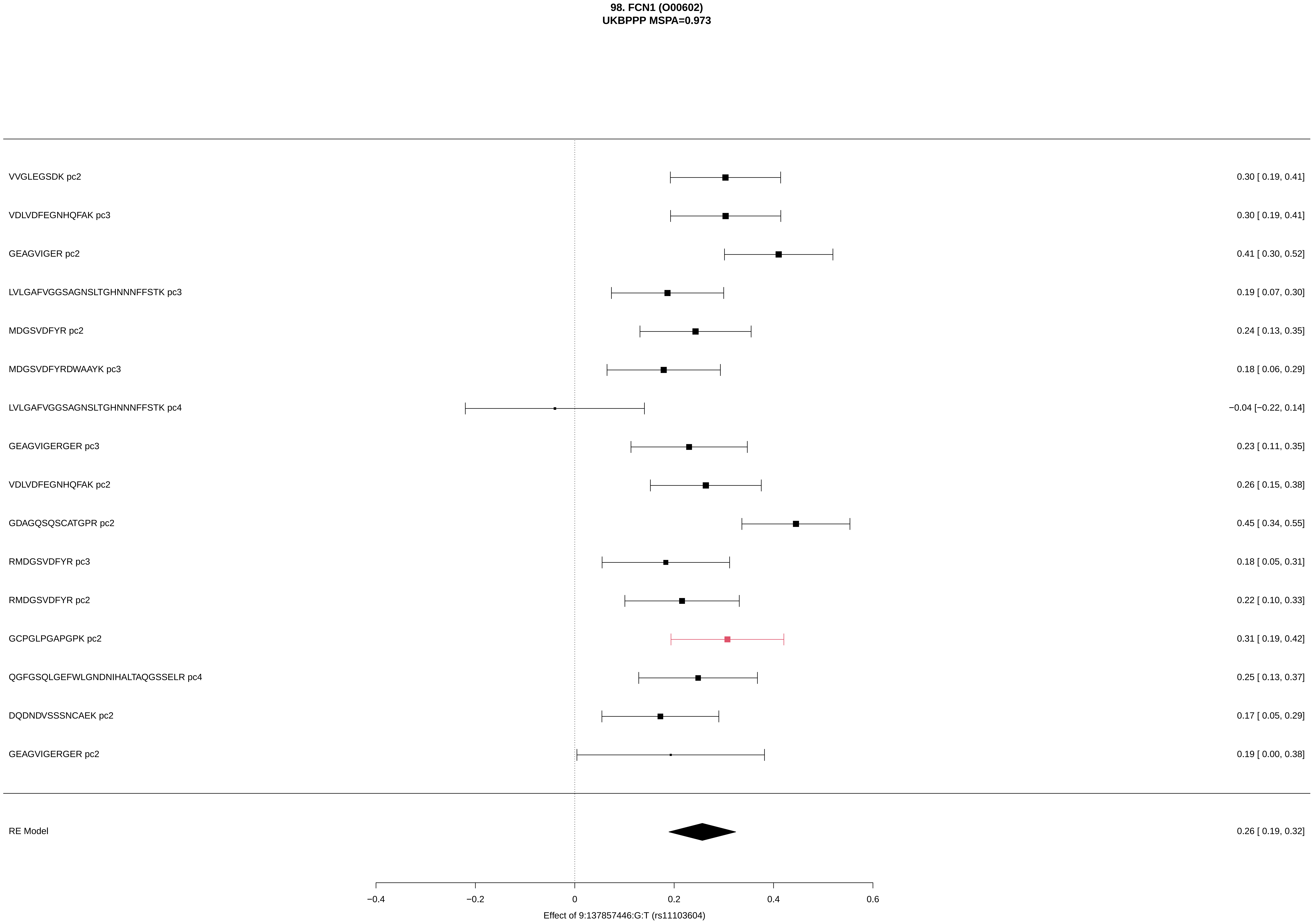
98. FCN1 (O00602)
UKBPPP MSPA=0.973
VVGLEGSDK pc2
0.30 [0.19, 0.41]
VDLVDFEGNHQFAK pc3
0.30 [0.19, 0.41]
GEAGVIGER pc2
0.41 [0.30, 0.52]
LVLGAFVGGSAGNSLTGHNNNFFSTK pc3
0.19 [0.07, 0.30]
MDGSVDFYR pc2
0.24 [0.13, 0.35]
MDGSVDFYRDWAAYK pc3
0.18 [0.06, 0.29]
LVLGAFVGGSAGNSLTGHNNNFFSTK pc4
−0.04 [−0.22, 0.14]
GEAGVIGERGER pc3
0.23 [0.11, 0.35]
VDLVDFEGNHQFAK pc2
0.26 [0.15, 0.38]
GDAGQSQSCATGPR pc2
0.45 [0.34, 0.55]
RMDGSVDFYR pc3
0.18 [0.05, 0.31]
RMDGSVDFYR pc2
0.22 [0.10, 0.33]
GCPGLPGAPGPK pc2
0.31 [0.19, 0.42]
QGFGSQLGEFWLGNDNIHALTAQGSSELR pc4
0.25 [0.13, 0.37]
DQDNDVSSSNCAEK pc2
0.17 [0.05, 0.29]
GEAGVIGERGER pc2
0.19 [0.00, 0.38]
RE Model
0.26 [0.19, 0.32]
−0.4
−0.2
0
0.2
0.4
0.6
Effect of 9:137857446:G:T (rs11103604)

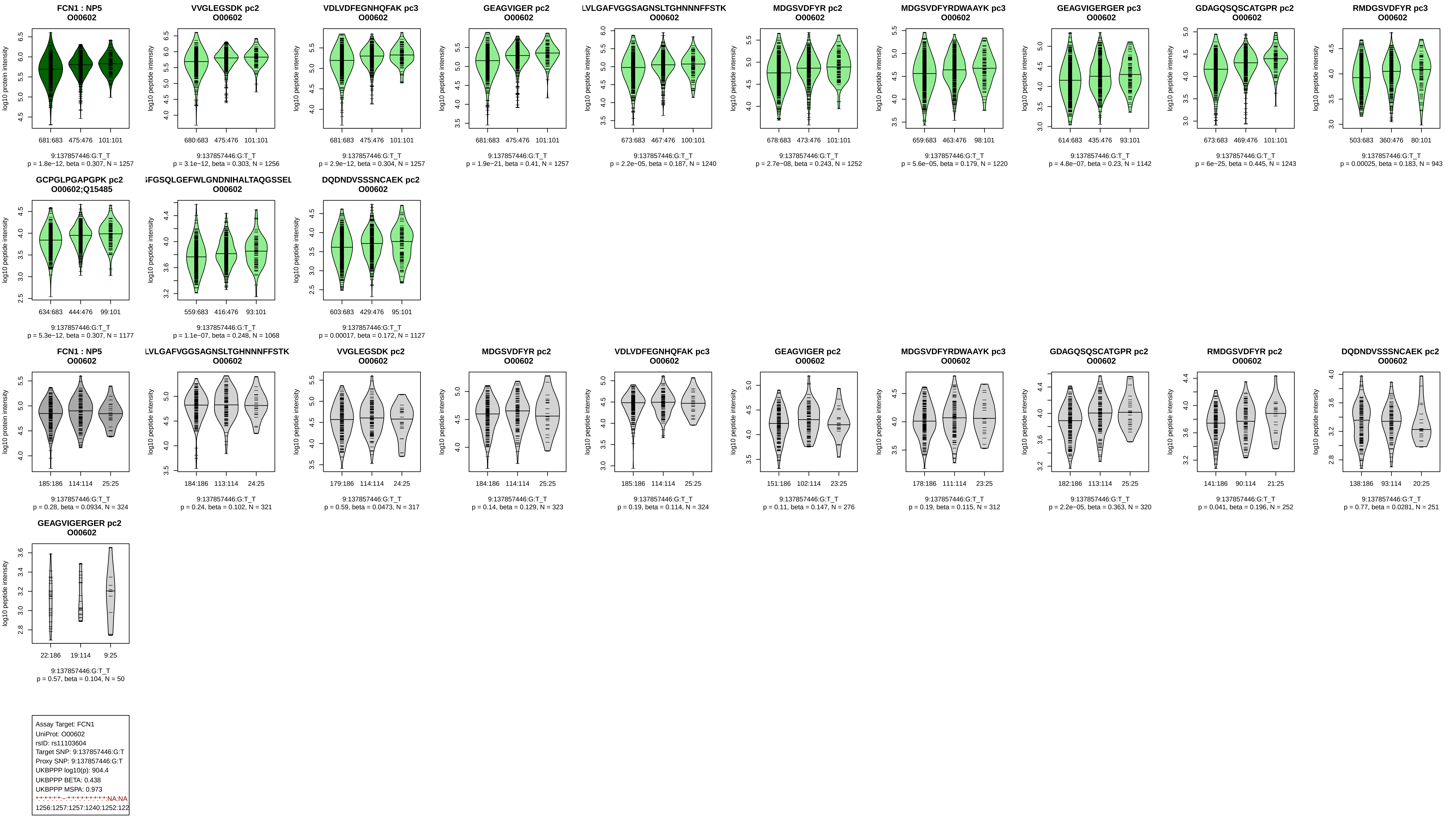
FCN1 : NP5
O00602
log10 protein intensity
681:683 475:476 101:101
9:137857446:G:T_T
p = 1.8e−12, beta = 0.307, N = 1257
VVGLEGSDK pc2
O00602
log10 peptide intensity
680:683 475:476 101:101
9:137857446:G:T_T
p = 3.1e−12, beta = 0.303, N = 1256
VDLVDFEGNHQFAK pc3
O00602
681:683 475:476 101:101
9:137857446:G:T_T
p = 2.9e−12, beta = 0.304, N = 1257
GEAGVIGER pc2
O00602
681:683 475:476 101:101
9:137857446:G:T_T
p = 1.9e−21, beta = 0.41, N = 1257
LVLGAFVGGSAGNSLTGHNNNFFSTK
O00602
673:683 467:476 100:101
9:137857446:G:T_T
p = 2.2e−05, beta = 0.187, N = 1240
MDGSVDFYR pc2
O00602
678:683 473:476 101:101
9:137857446:G:T_T
p = 2.7e−08, beta = 0.243, N = 1252
MDGSVDFYRDWAAYK pc3
O00602
659:683 463:476 98:101
9:137857446:G:T_T
p = 5.6e−05, beta = 0.179, N = 1220
GEAGVIGERGER pc3
O00602
614:683 435:476 93:101
9:137857446:G:T_T
p = 4.8e−07, beta = 0.23, N = 1142
GDAGQSQSCATGPR pc2
O00602
673:683 469:476 101:101
9:137857446:G:T_T
p = 6e−25, beta = 0.445, N = 1243
RMDGSVDFYR pc3
O00602
503:683 360:476 80:101
9:137857446:G:T_T
p = 0.00025, beta = 0.183, N = 943
GCPGLPGAPGPK pc2
O00602;Q15485
634:683 444:476 99:101
9:137857446:G:T_T
p = 5.3e−12, beta = 0.307, N = 1177
FGSQLGEFWLGNDNIHALTAQGSSEL
O00602
559:683 416:476 93:101
9:137857446:G:T_T
p = 1.1e−07, beta = 0.248, N = 1068
DQDNDVSSSNCAEK pc2
O00602
603:683 429:476 95:101
9:137857446:G:T_T
p = 0.00017, beta = 0.172, N = 1127
FCN1 : NP5
O00602
185:186 114:114 25:25
9:137857446:G:T_T
p = 0.28, beta = 0.0934, N = 324
LVLGAFVGGSAGNSLTGHNNNFFSTK
O00602
184:186 113:114 24:25
9:137857446:G:T_T
p = 0.24, beta = 0.102, N = 321
VVGLEGSDK pc2
O00602
179:186 114:114 24:25
9:137857446:G:T_T
p = 0.59, beta = 0.0473, N = 317
MDGSVDFYR pc2
O00602
184:186 114:114 25:25
9:137857446:G:T_T
p = 0.14, beta = 0.129, N = 323
VDLVDFEGNHQFAK pc3
O00602
185:186 114:114 25:25
9:137857446:G:T_T
p = 0.19, beta = 0.114, N = 324
GEAGVIGER pc2
O00602
151:186 102:114 23:25
9:137857446:G:T_T
p = 0.11, beta = 0.147, N = 276
MDGSVDFYRDWAAYK pc3
O00602
178:186 111:114 23:25
9:137857446:G:T_T
p = 0.19, beta = 0.115, N = 312
GDAGQSQSCATGPR pc2
O00602
182:186 113:114 25:25
9:137857446:G:T_T
p = 2.2e−05, beta = 0.363, N = 320
RMDGSVDFYR pc2
O00602
141:186 90:114 21:25
9:137857446:G:T_T
p = 0.041, beta = 0.196, N = 252
DQDNDVSSSNCAEK pc2
O00602
138:186 93:114 20:25
9:137857446:G:T_T
p = 0.77, beta = 0.0281, N = 251
GEAGVIGERGER pc2
O00602
22:186 19:114 9:25
9:137857446:G:T_T
p = 0.57, beta = 0.104, N = 50
Assay Target: FCN1
UniProt: O00602
rsID: rs11103604
Target SNP: 9:137857446:G:T
Proxy SNP: 9:137857446:G:T
UKBPPP log10(p): 904.4
UKBPPP BETA: 0.438
UKBPPP MSPA: 0.973
::*:*:*:*:–:*:*:*:*:*:*:*:*:*:NA:NA
1256:1257:1257:1240:1252:122

99. KLK7 (P49862)
UKBPPP MSPA=0.658

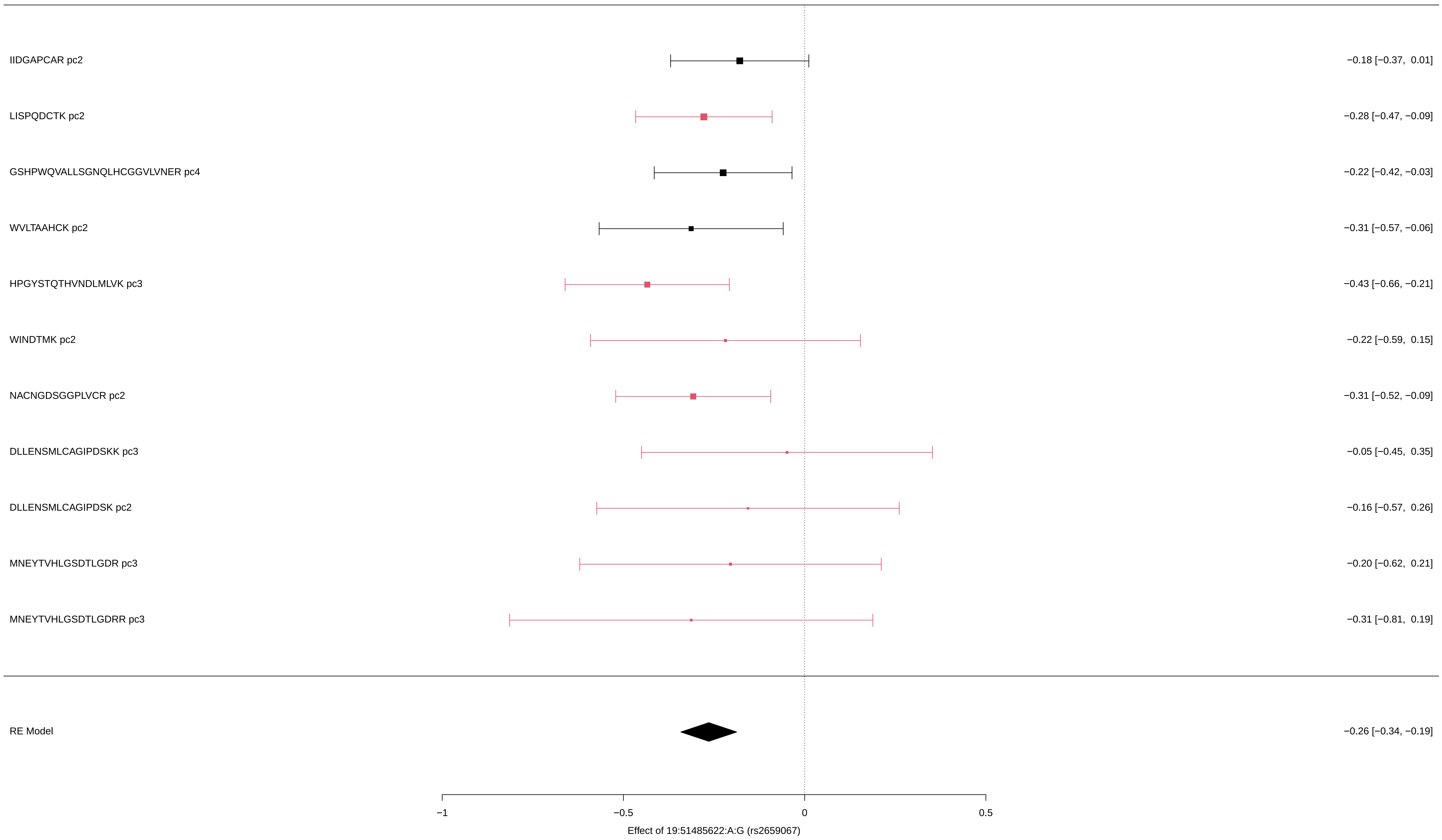

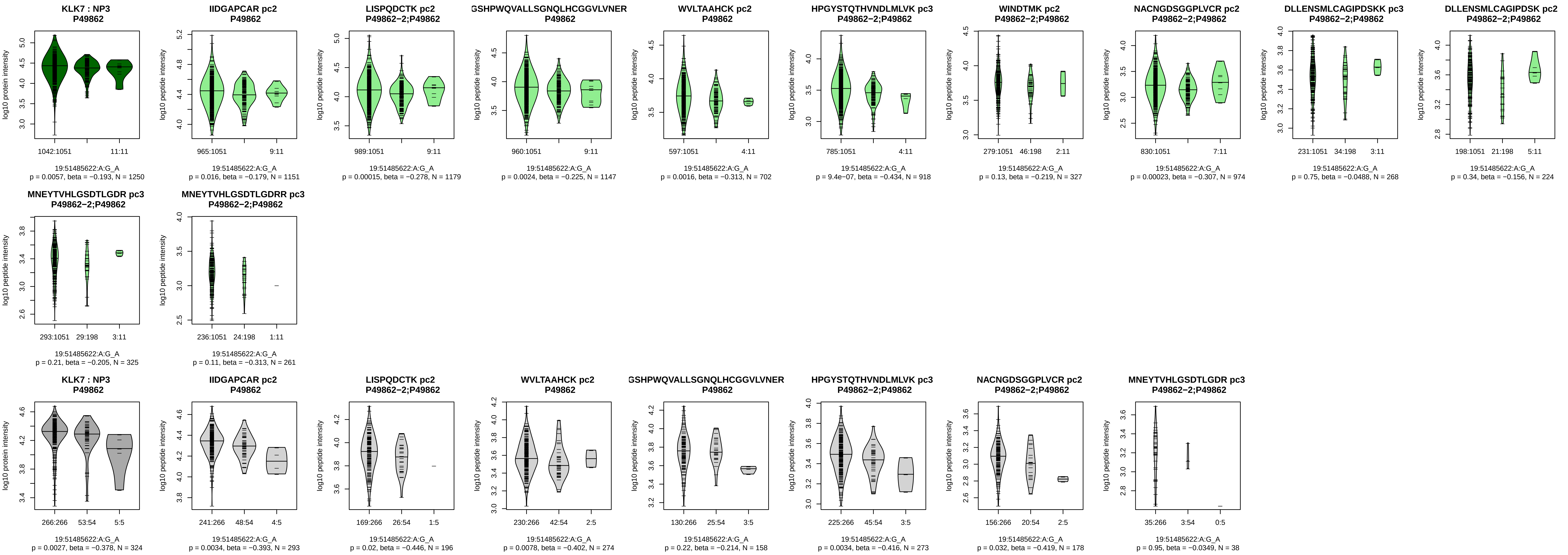

Assay Target: KLK7
UniProt: P49862
rsID: rs2659067
Target SNP: 19:51485622:A:G
Proxy SNP: 19:51485622:A:G
UKBPPP log10(p): 900.3
UKBPPP BETA: 0.681
UKBPPP MSPA: 0.658
–:*:*:*:*:–:*:–:–:–:–
1151:1179:1147:702:918:327:97

100. MATN2 (O00339)
UKBPPP MSPA=0

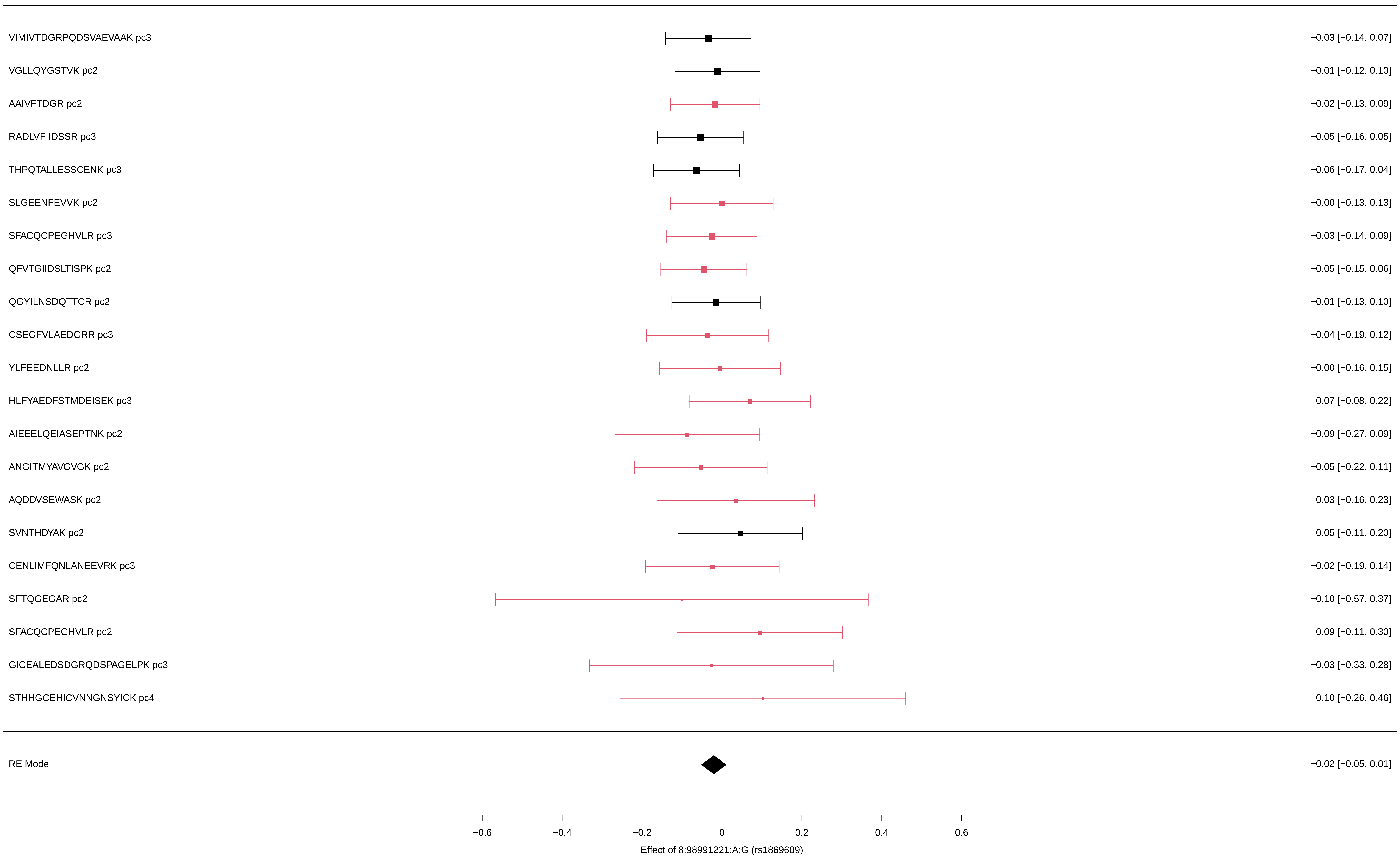

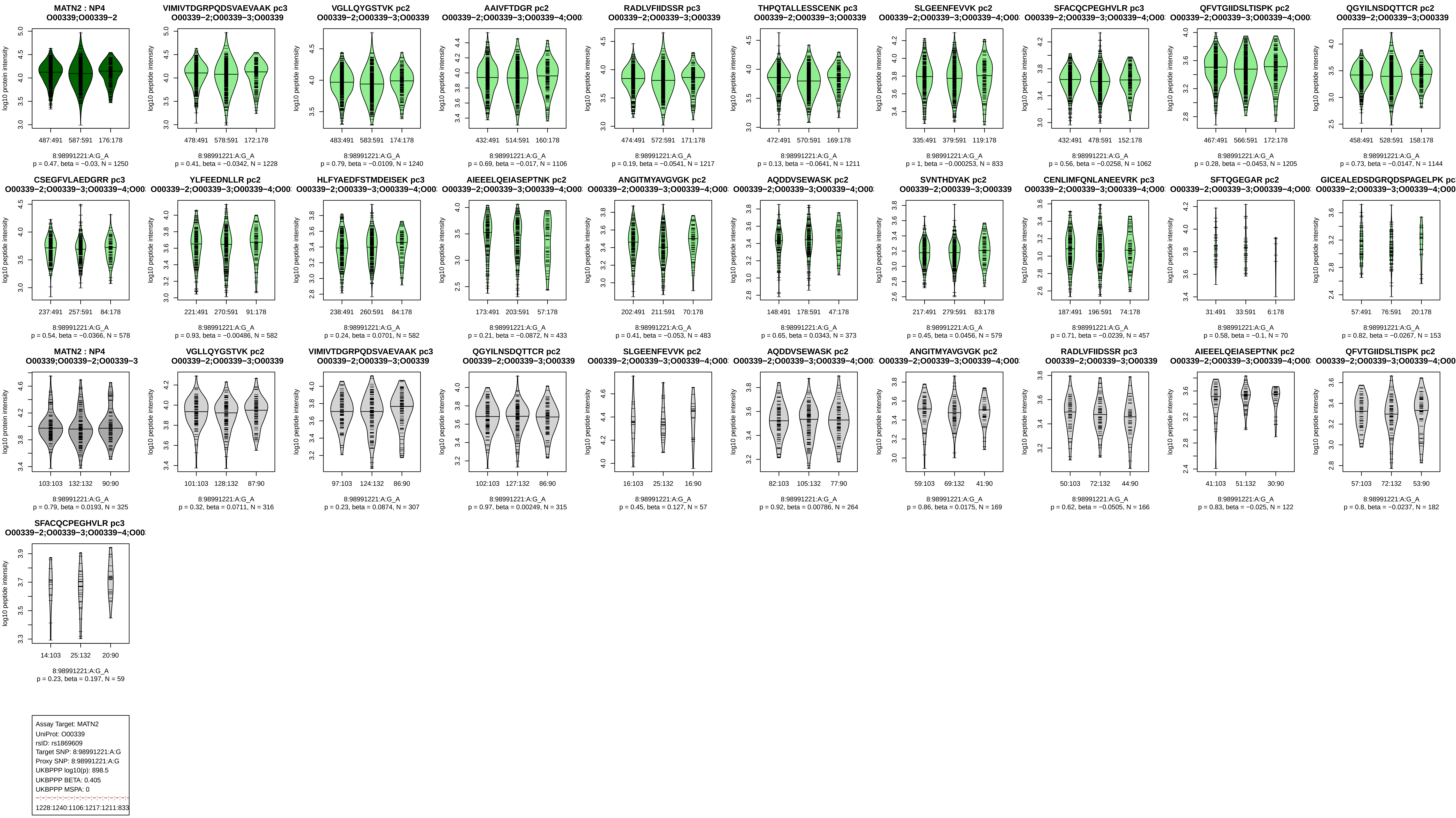

MATN2 : NP4
O00339;O00339−2
p = 0.47, beta = −0.03, N = 1250
VIMIVTDGRPQDSVAEVAAK pc3
p = 0.41, beta = −0.0342, N = 1228
VGLLQYGSTVK pc2
p = 0.79, beta = −0.0109, N = 1240
AAIVFTDGR pc2
p = 0.69, beta = −0.017, N = 1106
RADLVFIIDSSR pc3
p = 0.19, beta = −0.0541, N = 1217
THPQTALLESSCENK pc3
p = 0.13, beta = −0.0641, N = 1211
SLGEENFEVVK pc2
p = 1, beta = −0.000253, N = 833
SFACQCPEGHVLR pc3
p = 0.56, beta = −0.0258, N = 1062
QFVTGIIDSLTISPK pc2
p = 0.28, beta = −0.0453, N = 1205
QGYILNSDQTTCR pc2
p = 0.73, beta = −0.0147, N = 1144
CSEGFVLAEDGRR pc3
p = 0.54, beta = −0.0366, N = 578
YLFEEDNLLR pc2
p = 0.93, beta = −0.00486, N = 582
HLFYAEDFSTMDEISEK pc3
p = 0.24, beta = 0.0701, N = 582
AIEEELQEIASEPTNK pc2
p = 0.21, beta = −0.0872, N = 433
ANGITMYAVGVGK pc2
p = 0.41, beta = −0.053, N = 483
AQDDVSEWASK pc2
p = 0.65, beta = 0.0343, N = 373
SVNTHDYAK pc2
p = 0.45, beta = 0.0456, N = 579
CENLIMFQNLANEEVRK pc3
p = 0.71, beta = −0.0239, N = 457
SFTQGEGAR pc2
p = 0.58, beta = −0.1, N = 70
GICEALEDSDGRQDSPAGELPK pc
p = 0.82, beta = −0.0267, N = 153
MATN2 : NP4
O00339;O00339−2;O00339−3
p = 0.79, beta = 0.0193, N = 325
VGLLQYGSTVK pc2
p = 0.32, beta = 0.0711, N = 316
VIMIVTDGRPQDSVAEVAAK pc3
p = 0.23, beta = 0.0874, N = 307
QGYILNSDQTTCR pc2
p = 0.97, beta = 0.00249, N = 315
SLGEENFEVVK pc2
p = 0.45, beta = 0.127, N = 57
AQDDVSEWASK pc2
p = 0.92, beta = 0.00786, N = 264
ANGITMYAVGVGK pc2
p = 0.86, beta = 0.0175, N = 169
RADLVFIIDSSR pc3
p = 0.62, beta = −0.0505, N = 166
AIEEELQEIASEPTNK pc2
p = 0.83, beta = −0.025, N = 122
QFVTGIIDSLTISPK pc2
p = 0.8, beta = −0.0237, N = 182
SFACQCPEGHVLR pc3
p = 0.23, beta = 0.197, N = 59
8:98991221:A:G_A
Assay Target: MATN2
UniProt: O00339
rsID: rs1869609
Target SNP: 8:98991221:A:G
Proxy SNP: 8:98991221:A:G
UKBPPP log10(p): 898.5
UKBPPP BETA: 0.405
UKBPPP MSPA: 0
1228:1240:1106:1217:1211:833

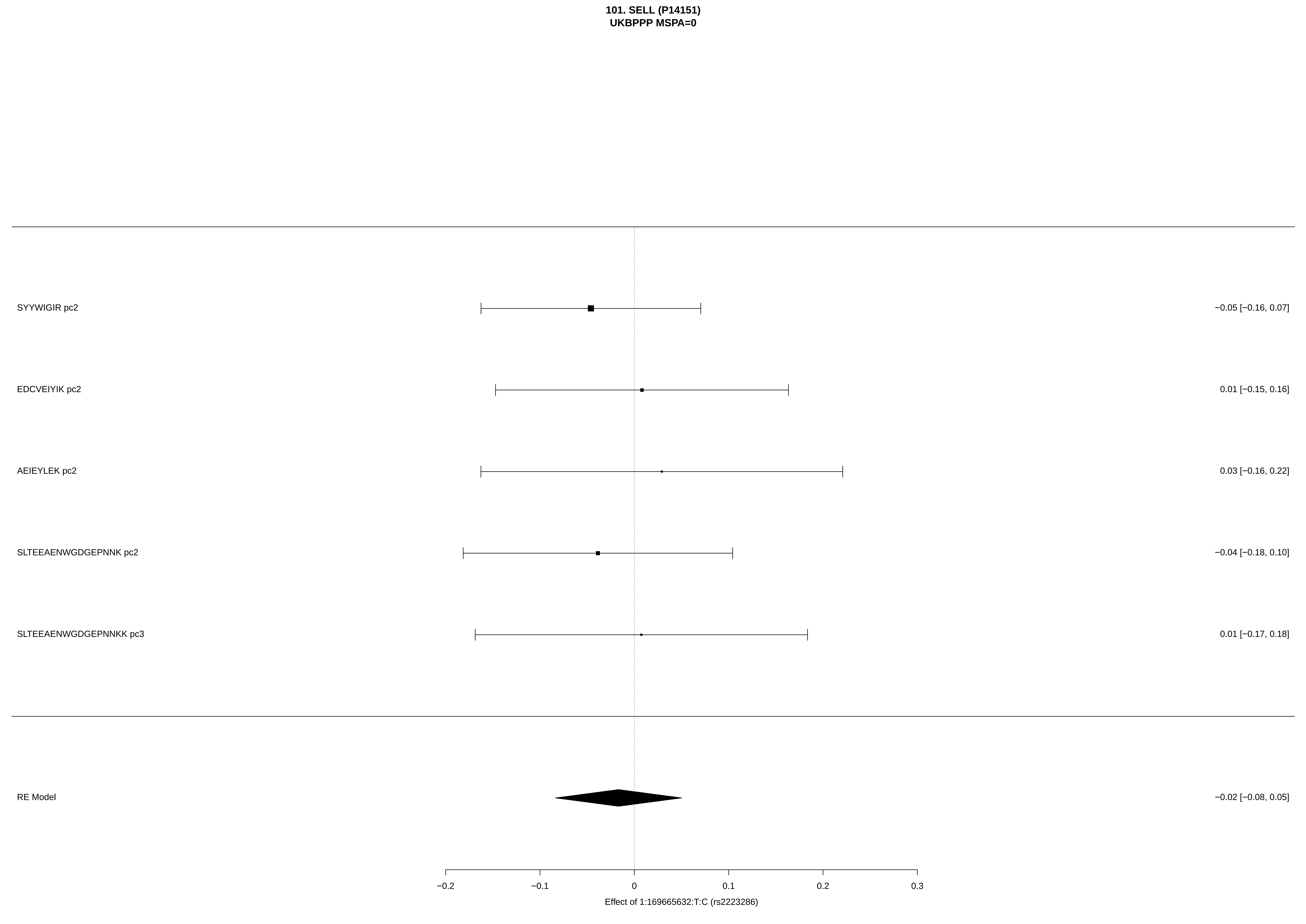
101. SELL (P14151)
UKBPPP MSPA=0
SYYWIGIR pc2
−0.05 [−0.16, 0.07]
EDCVEIYIK pc2
0.01 [−0.15, 0.16]
AEIEYLEK pc2
0.03 [−0.16, 0.22]
SLTEEAENWGDGEPNNK pc2
−0.04 [−0.18, 0.10]
SLTEEAENWGDGEPNNKK pc3
0.01 [−0.17, 0.18]
RE Model
−0.02 [−0.08, 0.05]
−0.2
−0.1
0
0.1
0.2
0.3
Effect of 1:169665632:T:C (rs2223286)

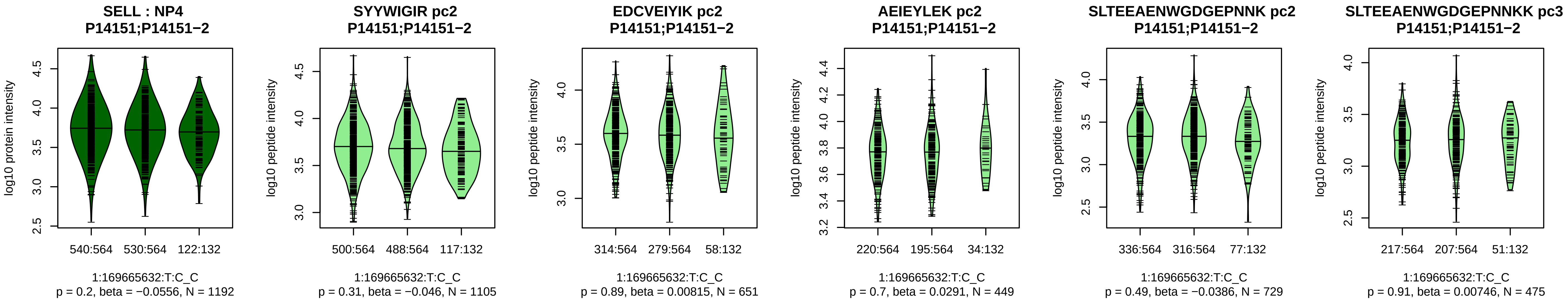

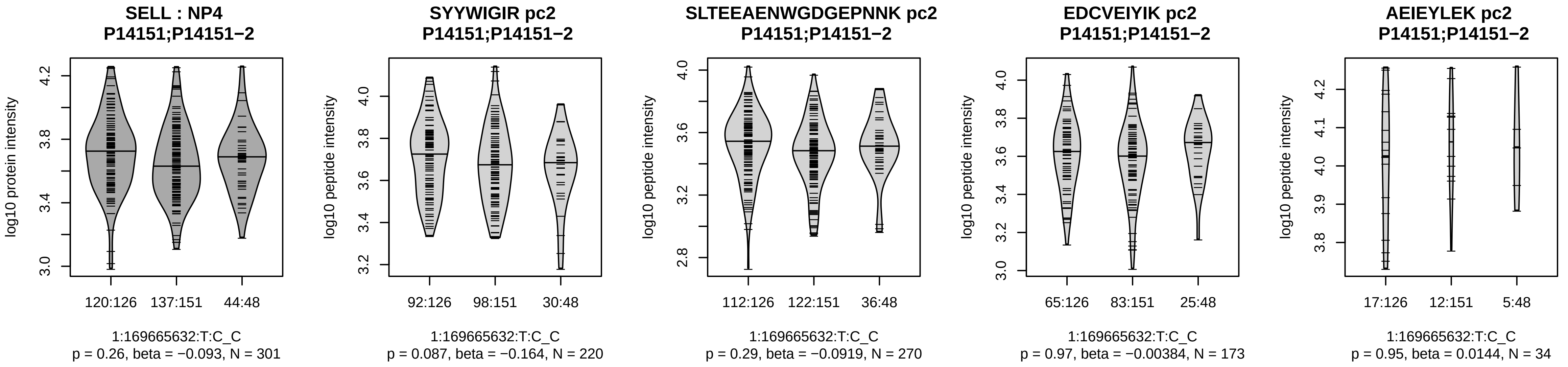

Assay Target: SELL
UniProt: P14151
rsID: rs2223286
Target SNP: 1:169665632:T:C
Proxy SNP: 1:169665632:T:C
UKBPPP log10(p): 888.4
UKBPPP BETA: −0.407
UKBPPP MSPA: 0
−:−:−:−:−
1105:651:449:729:475

102. EBI3_IL27 (Q14213)
UKBPPP MSPA=1

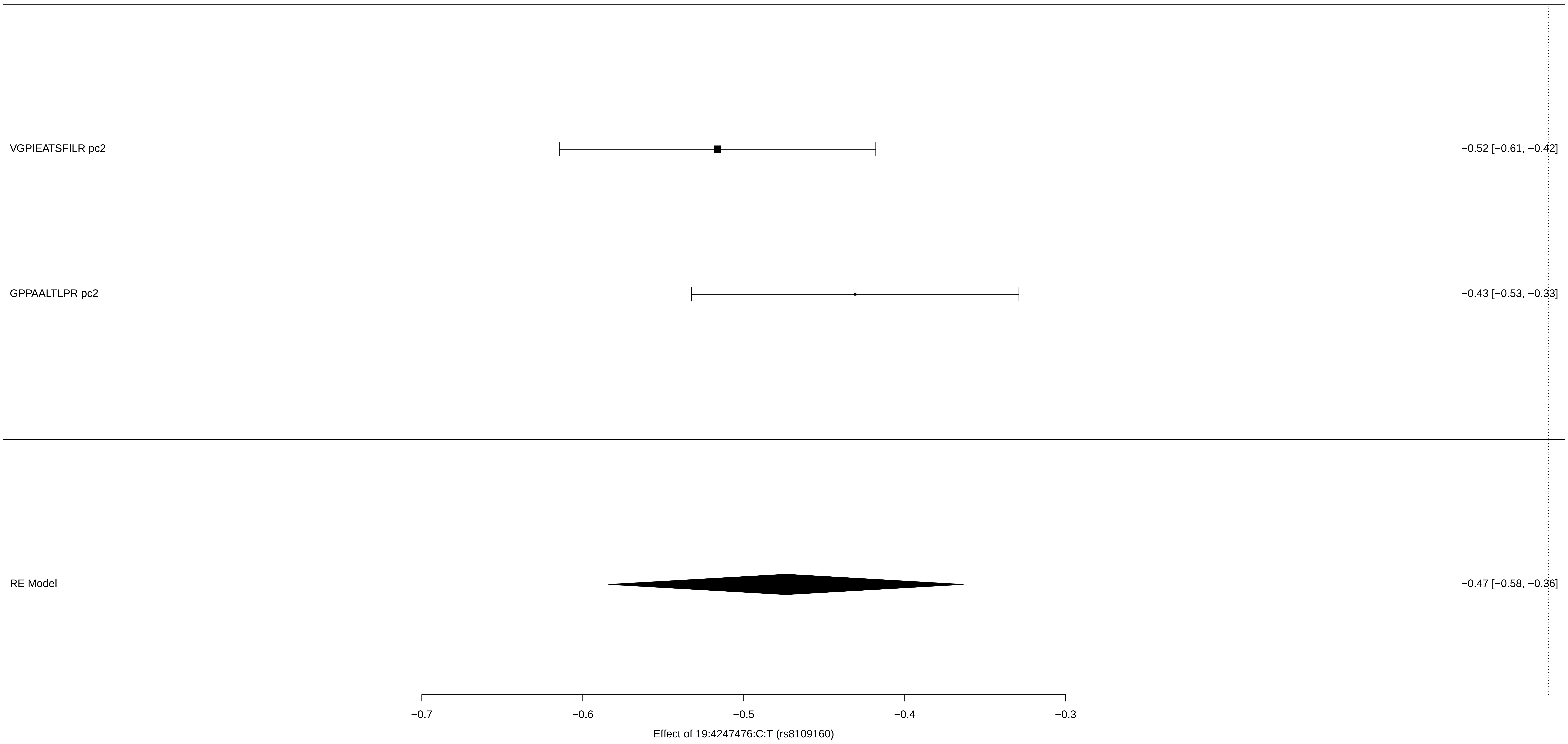

EBI3_IL27 : NP4
Q14213

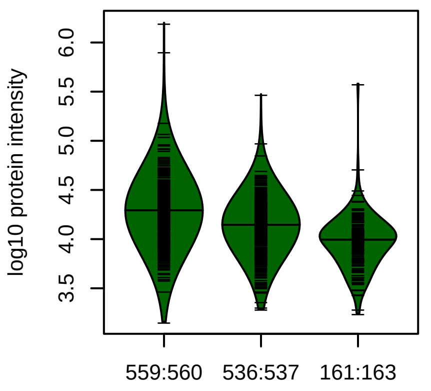

19:4247476:C:T_T
p = 7.6e−39, beta = −0.515, N = 1256

VGPIEATSFILR pc2
Q14213

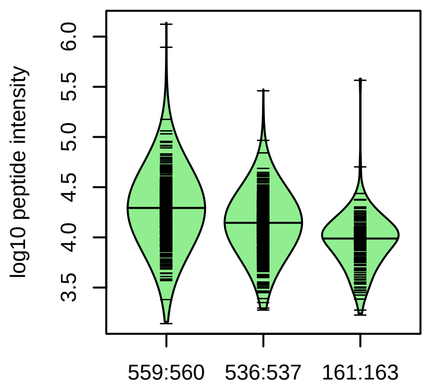

19:4247476:C:T_T
p = 5.3e−39, beta = −0.516, N = 1256

GPPAALTLPR pc2
Q14213

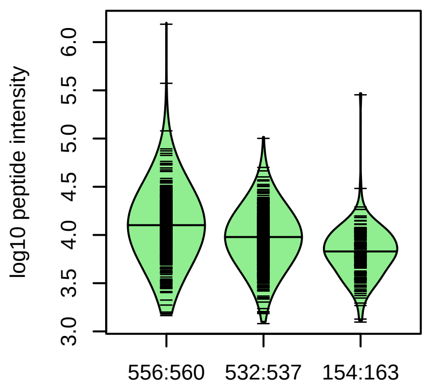

19:4247476:C:T_T
p = 1.7e−26, beta = −0.431, N = 1242

EBI3_IL27 : NP4
Q14213

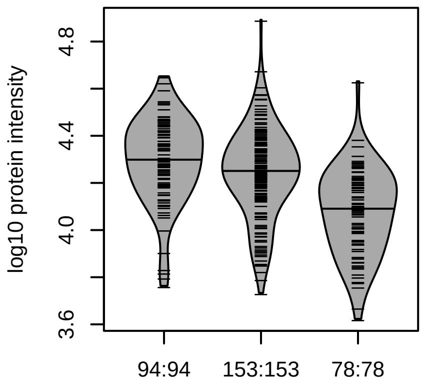

19:4247476:C:T_T
p = 7.6e−14, beta = −0.541, N = 325

VGPIEATSFILR pc2
Q14213

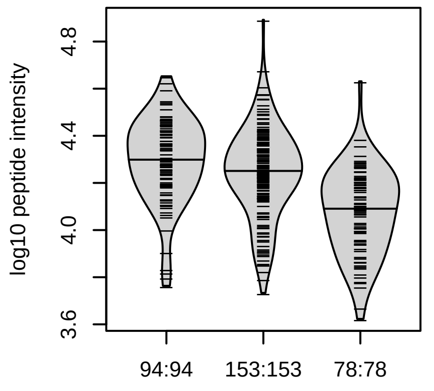

19:4247476:C:T_T
p = 8e−14, beta = −0.541, N = 325

GPPAALTLPR pc2
Q14213

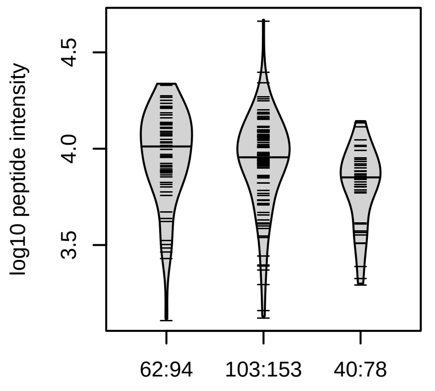

19:4247476:C:T_T
p = 0.00098, beta = −0.32, N = 205

Assay Target: EBI3_IL27
UniProt: Q14213
rsID: rs8109160
Target SNP: 19:4247476:C:T
Proxy SNP: 19:4247476:C:T
UKBPPP log10(p): 876.3
UKBPPP BETA: −0.401
UKBPPP MSPA: 1
:
1256:1242

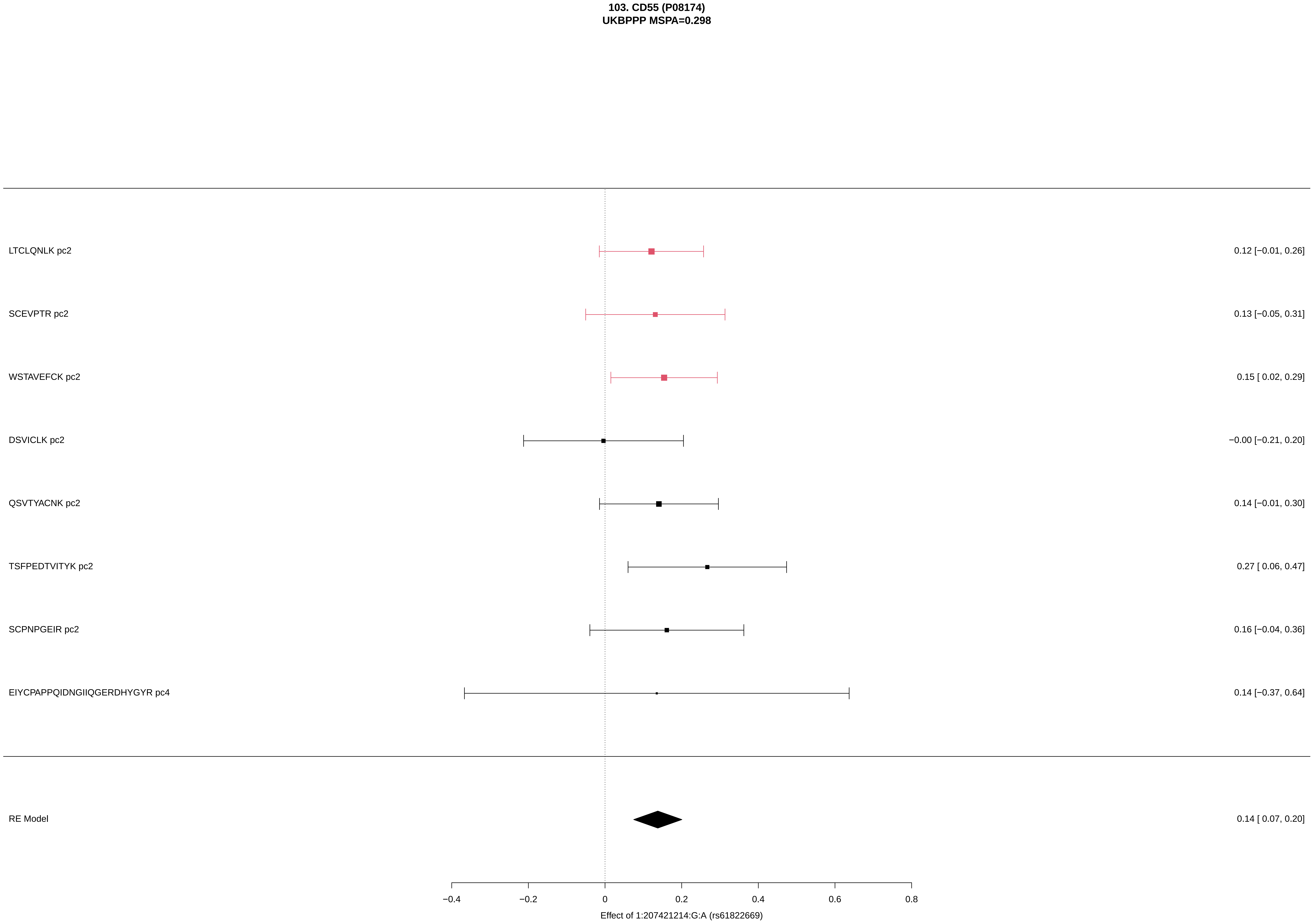

103. CD55 (P08174)
UKBPPP MSPA=0.298
LTCLQNLK pc2
0.12 [−0.01, 0.26]
SCEVPTR pc2
0.13 [−0.05, 0.31]
WSTAVEFCK pc2
0.15 [0.02, 0.29]
DSVICLK pc2
−0.00 [−0.21, 0.20]
QSVTYACNK pc2
0.14 [−0.01, 0.30]
TSFPEDTVITYK pc2
0.27 [0.06, 0.47]
SCPNPGEIR pc2
0.16 [−0.04, 0.36]
EIYCPAPPQIDNGIIQGERDHYGYR pc4
0.14 [−0.37, 0.64]
RE Model
0.14 [0.07, 0.20]
−0.4
−0.2
0
0.2
0.4
0.6
0.8
Effect of 1:207421214:G:A (rs61822669)

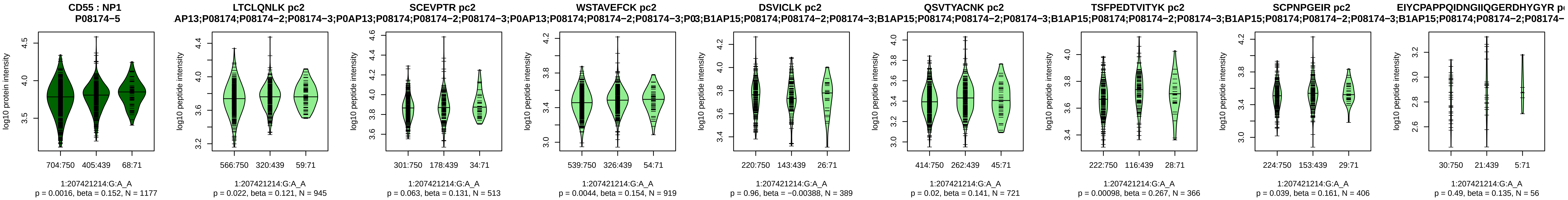

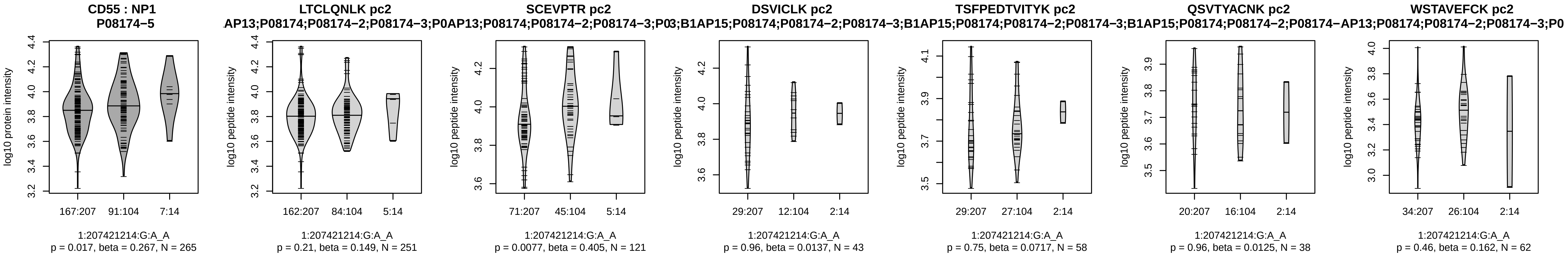

Assay Target: CD55
UniProt: P08174
rsID: rs61822669
Target SNP: 1:207421214:G:A
Proxy SNP: 1:207421214:G:A
UKBPPP log10(p): 863.1
UKBPPP BETA: −0.457
UKBPPP MSPA: 0.298
−:−:*:−:−:*:−:−
945:513:919:389:721:366:406:5

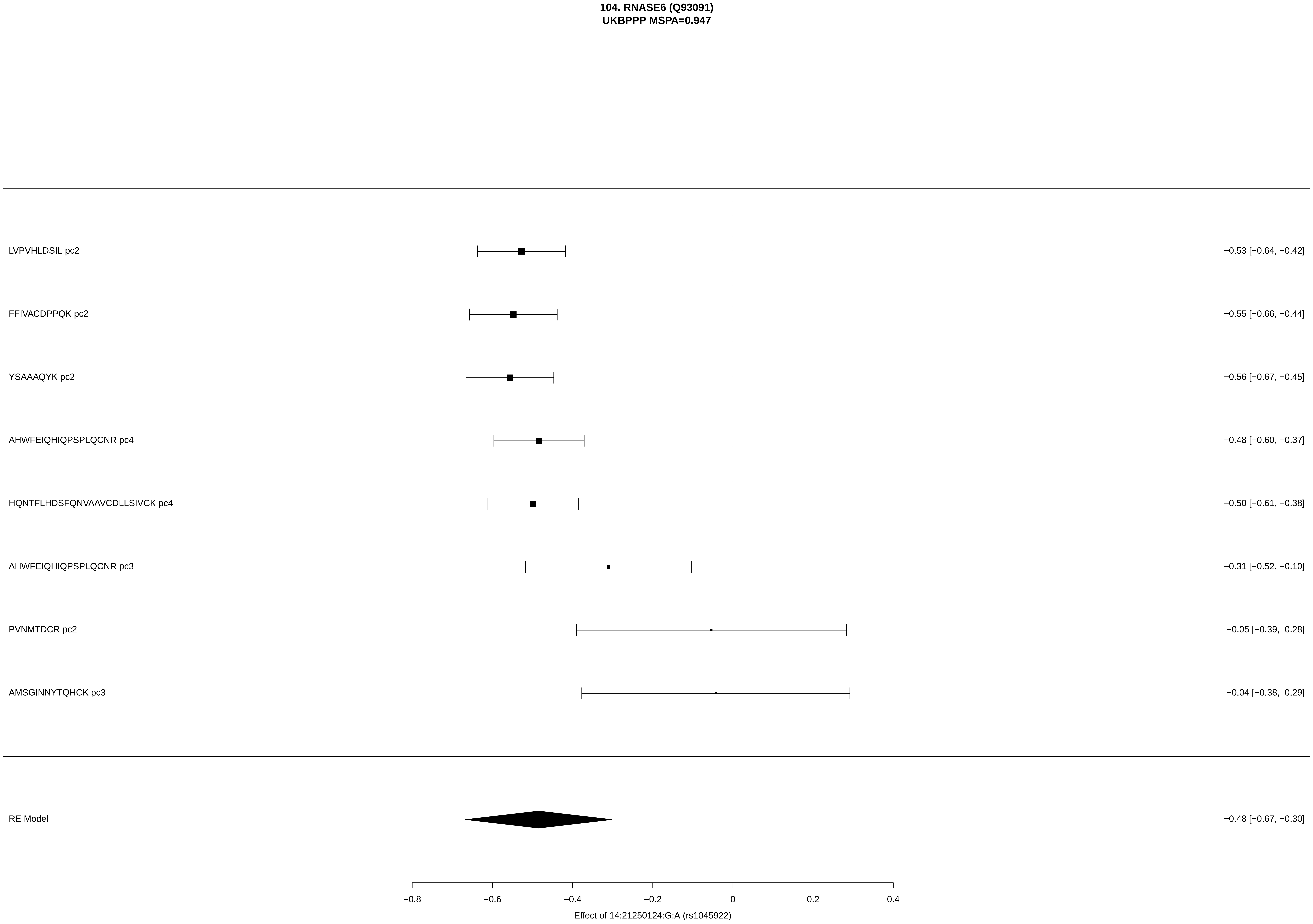
104. RNASE6 (Q93091)
UKBPPP MSPA=0.947
LVPVHLDSIL pc2
FFIVACDPPQK pc2
YSAAAQYK pc2
AHWFEIQHIQPSPLQCNR pc4
HQNTFLHDSFQNVAAVCDLLSIVCK pc4
AHWFEIQHIQPSPLQCNR pc3
PVNMTDCR pc2
AMSGINNYTQHCK pc3
RE Model
−0.53 [−0.64, −0.42]
−0.55 [−0.66, −0.44]
−0.56 [−0.67, −0.45]
−0.48 [−0.60, −0.37]
−0.50 [−0.61, −0.38]
−0.31 [−0.52, −0.10]
−0.05 [−0.39, 0.28]
−0.04 [−0.38, 0.29]
−0.48 [−0.67, −0.30]
−0.8
−0.6
−0.4
−0.2
0
0.2
0.4
Effect of 14:21250124:G:A (rs1045922)

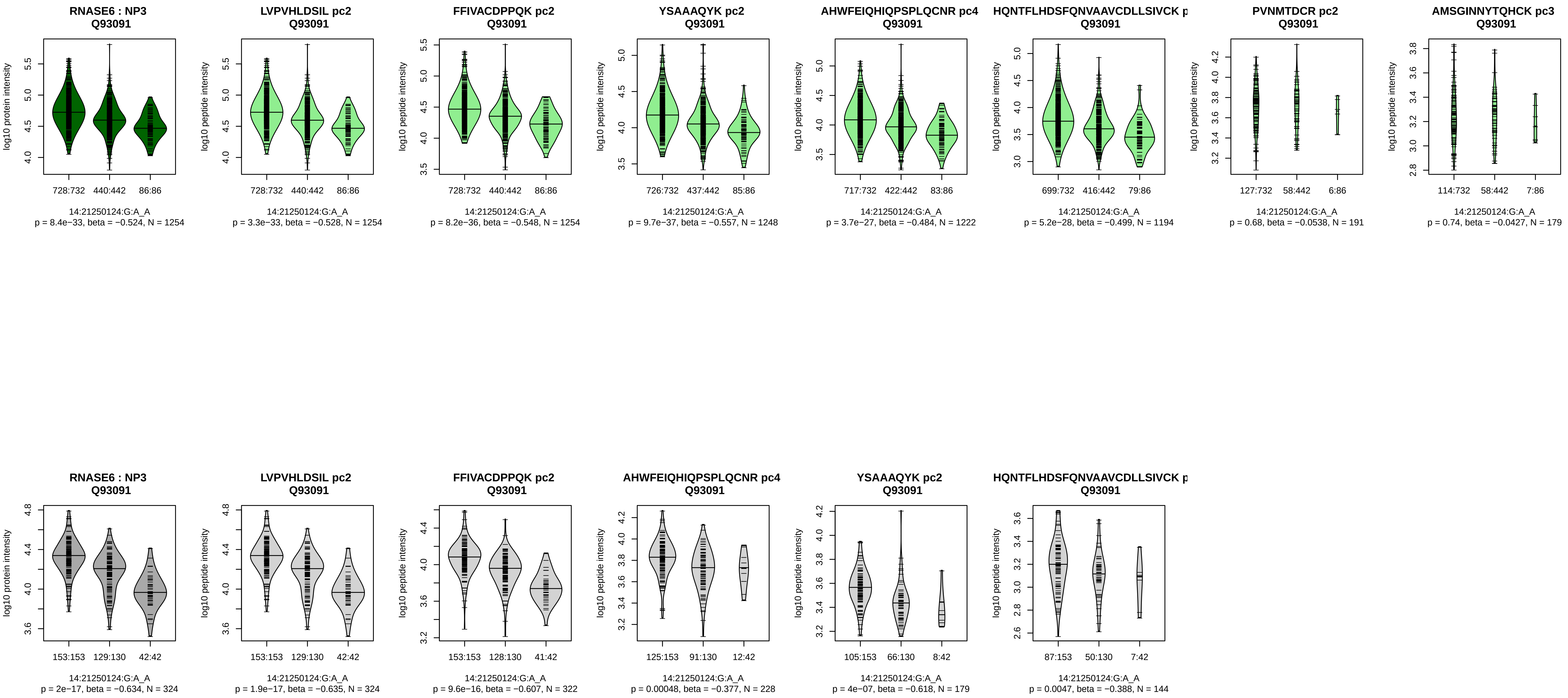

Assay Target: RNASE6
UniProt: Q93091
rsID: rs1045922
Target SNP: 14:21250124:G:A
Proxy SNP: 14:21250124:G:A
UKBPPP log10(p): 853.8
UKBPPP BETA: −0.427
UKBPPP MSPA: 0.947
::*:*:*:*:−:−:NA
1254:1254:1248:1222:1194:448

105. THBS2 (P35442)
UKBPPP MSPA=0.372

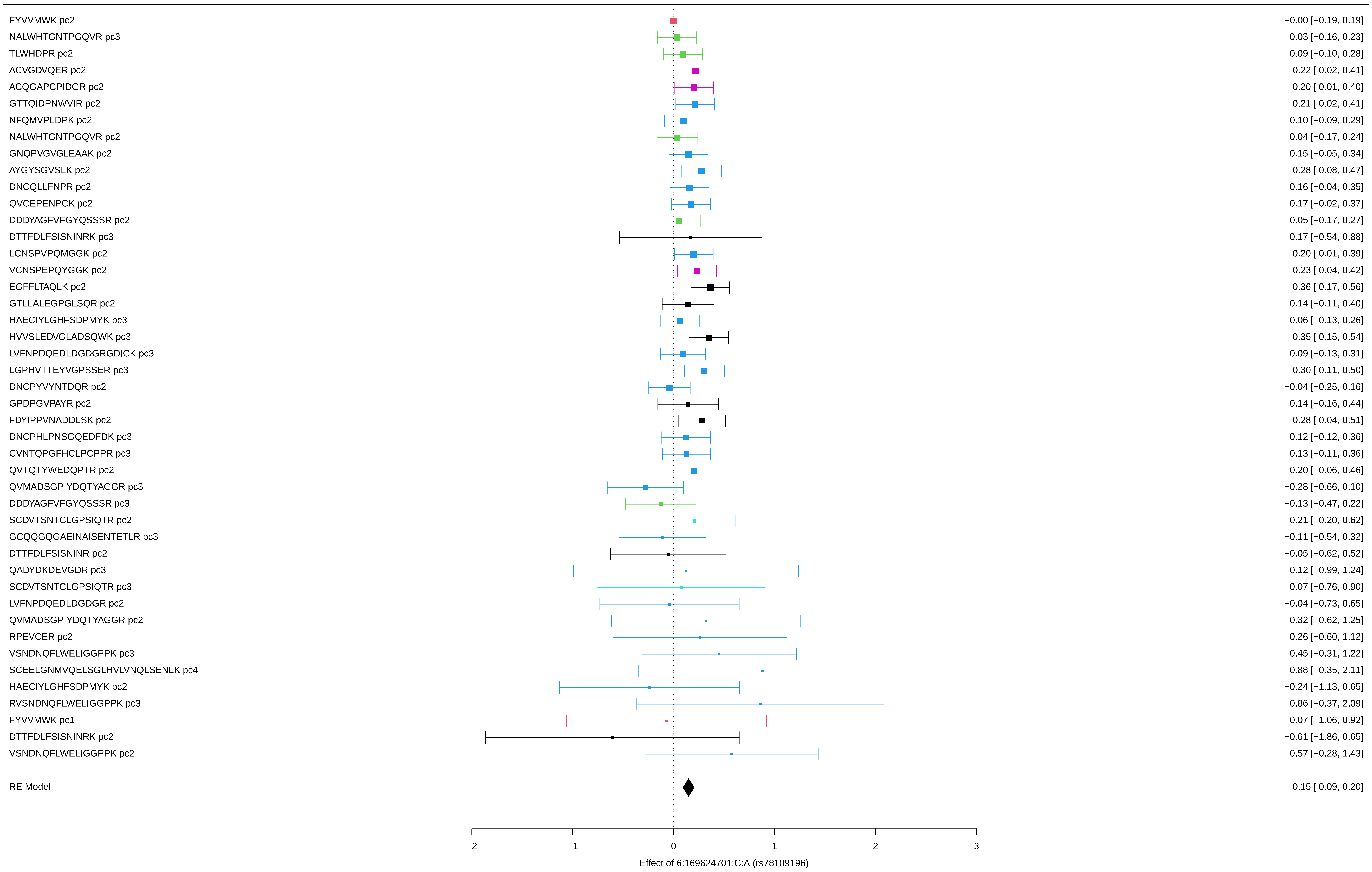

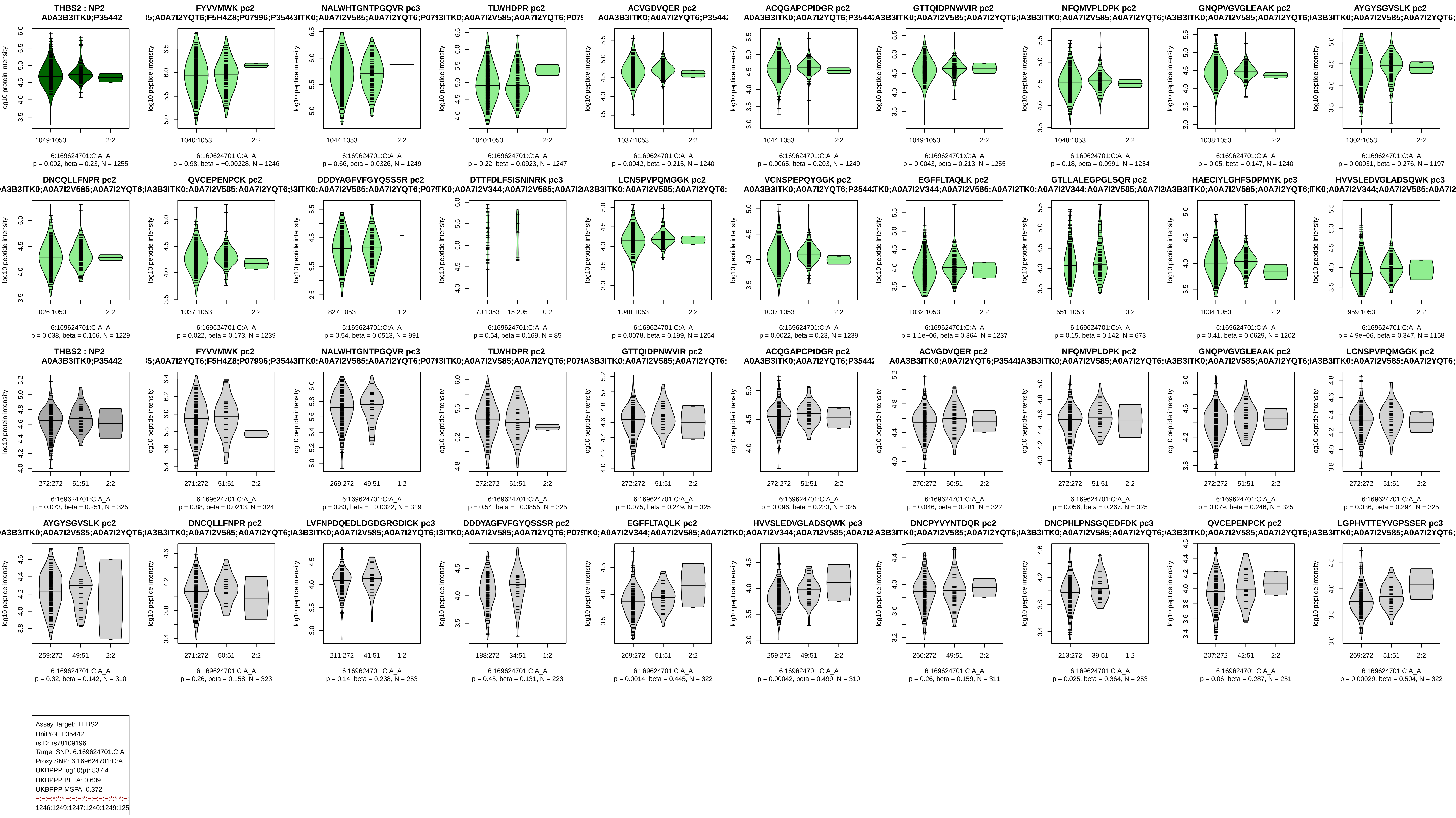
THBS2 : NP2
A0A3B3ITK0;P35442
6:169624701:C:A_A
p = 0.002, beta = 0.23, N = 1255
FYVVMWK pc2
p = 0.98, beta = −0.00228, N = 1246
NALWHTGNTPGQVR pc3
p = 0.66, beta = 0.0326, N = 1249
TLWHDPR pc2
p = 0.22, beta = 0.0923, N = 1247
ACVGDVQER pc2
p = 0.0042, beta = 0.215, N = 1240
ACQGAPCPIDGR pc2
p = 0.0065, beta = 0.203, N = 1249
GTTQIDPNWVIR pc2
p = 0.0043, beta = 0.213, N = 1255
NFQMVPLDPK pc2
p = 0.18, beta = 0.0991, N = 1254
GNQPVGVGLEAAK pc2
p = 0.05, beta = 0.147, N = 1240
AYGYSGVSLK pc2
p = 0.00031, beta = 0.276, N = 1197
DNCQLLFNPR pc2
p = 0.038, beta = 0.156, N = 1229
QVCEPENPCK pc2
p = 0.022, beta = 0.173, N = 1239
DDDYAGFVFGYQSSSR pc2
p = 0.54, beta = 0.0513, N = 991
DTTFDLFSISNINRK pc3
p = 0.54, beta = 0.169, N = 85
LCNSPVPQMGGK pc2
p = 0.0078, beta = 0.199, N = 1254
VCNSPEPQYGGK pc2
p = 0.0022, beta = 0.23, N = 1239
EGFFLTAQLK pc2
p = 1.1e−06, beta = 0.364, N = 1237
GTLLALEGPGLSQR pc2
p = 0.15, beta = 0.142, N = 673
HAECIYLGHFSDPMYK pc3
p = 0.41, beta = 0.0629, N = 1202
HVVSLEDVGLADSQWK pc3
p = 4.9e−06, beta = 0.347, N = 1158
THBS2 : NP2
p = 0.073, beta = 0.251, N = 325
FYVVMWK pc2
p = 0.88, beta = 0.0213, N = 324
NALWHTGNTPGQVR pc3
p = 0.83, beta = −0.0322, N = 319
TLWHDPR pc2
p = 0.54, beta = −0.0855, N = 325
GTTQIDPNWVIR pc2
p = 0.075, beta = 0.249, N = 325
ACQGAPCPIDGR pc2
p = 0.096, beta = 0.233, N = 325
ACVGDVQER pc2
p = 0.046, beta = 0.281, N = 322
NFQMVPLDPK pc2
p = 0.056, beta = 0.267, N = 325
GNQPVGVGLEAAK pc2
p = 0.079, beta = 0.246, N = 325
LCNSPVPQMGGK pc2
p = 0.036, beta = 0.294, N = 325
AYGYSGVSLK pc2
p = 0.32, beta = 0.142, N = 310
DNCQLLFNPR pc2
p = 0.26, beta = 0.158, N = 323
LVFNPDQEDLDGDGRGDICK pc3
p = 0.14, beta = 0.238, N = 253
DDDYAGFVFGYQSSSR pc2
p = 0.45, beta = 0.131, N = 223
EGFFLTAQLK pc2
p = 0.0014, beta = 0.445, N = 322
HVVSLEDVGLADSQWK pc3
p = 0.00042, beta = 0.499, N = 310
DNCPYVYNTDQR pc2
p = 0.26, beta = 0.159, N = 311
DNCPHLPNSGQEDFDK pc3
p = 0.025, beta = 0.364, N = 253
QVCEPENPCK pc2
p = 0.06, beta = 0.287, N = 251
LGPHVTTEYVGPSSER pc3
p = 0.00029, beta = 0.504, N = 322
Assay Target: THBS2
UniProt: P35442
rsID: rs78109196
Target SNP: 6:169624701:C:A
Proxy SNP: 6:169624701:C:A
UKBPPP log10(p): 837.4
UKBPPP BETA: 0.639
UKBPPP MSPA: 0.372

106. KLK14 (Q9P0G3)
UKBPPP MSPA=0

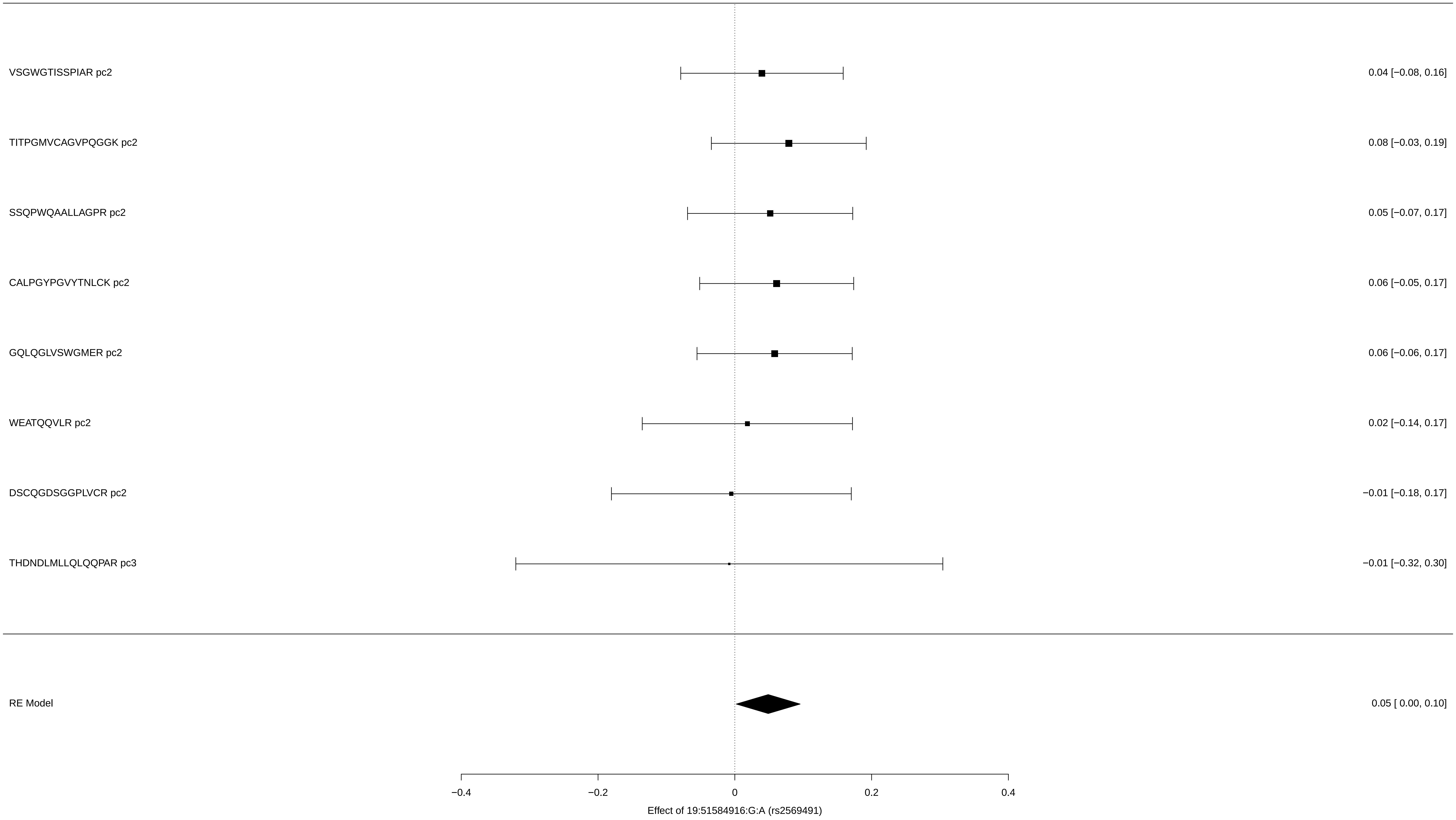

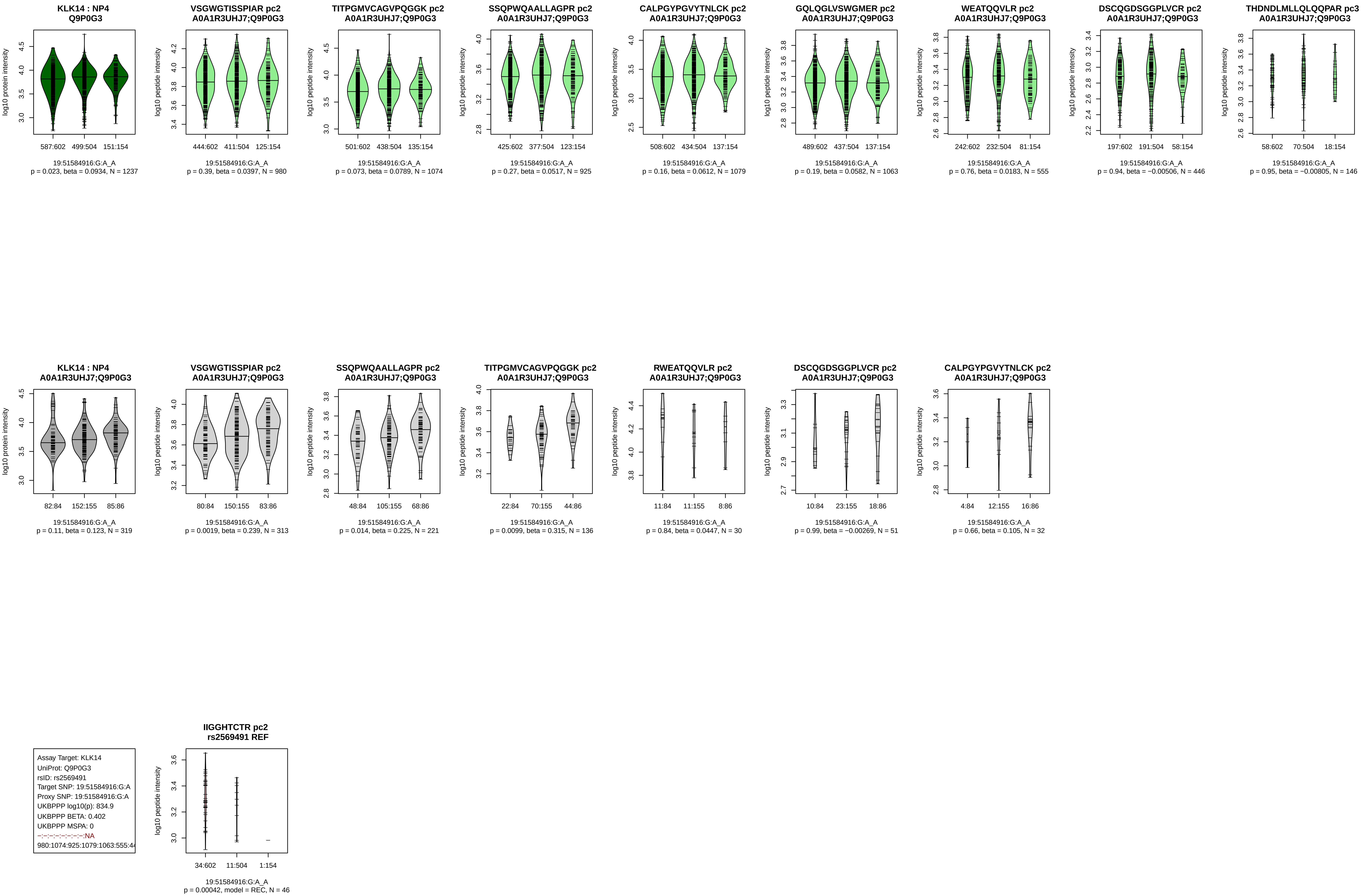
KLK14 : NP4
Q9P0G3
log10 protein intensity
587:602 499:504 151:154
19:51584916:G:A_A
p = 0.023, beta = 0.0934, N = 1237
VSGWGTISSPIAR pc2
A0A1R3UHJ7;Q9P0G3
444:602 411:504 125:154
p = 0.39, beta = 0.0397, N = 980
TITPGMVCAGVPQGGK pc2
501:602 438:504 135:154
p = 0.073, beta = 0.0789, N = 1074
SSQPWQAALLAGPR pc2
425:602 377:504 123:154
p = 0.27, beta = 0.0517, N = 925
CALPGYPGVYTNLCK pc2
508:602 434:504 137:154
p = 0.16, beta = 0.0612, N = 1079
GQLQGLVSWGMER pc2
489:602 437:504 137:154
p = 0.19, beta = 0.0582, N = 1063
WEATQQVLR pc2
242:602 232:504 81:154
p = 0.76, beta = 0.0183, N = 555
DSCQGDSGGPLVCR pc2
197:602 191:504 58:154
p = 0.94, beta = −0.00506, N = 446
THDNDLMLLQLQQPAR pc3
58:602 70:504 18:154
p = 0.95, beta = −0.00805, N = 146
KLK14 : NP4
A0A1R3UHJ7;Q9P0G3
82:84 152:155 85:86
p = 0.11, beta = 0.123, N = 319
VSGWGTISSPIAR pc2
80:84 150:155 83:86
p = 0.0019, beta = 0.239, N = 313
SSQPWQAALLAGPR pc2
48:84 105:155 68:86
p = 0.014, beta = 0.225, N = 221
TITPGMVCAGVPQGGK pc2
22:84 70:155 44:86
p = 0.0099, beta = 0.315, N = 136
RWEATQQVLR pc2
11:84 11:155 8:86
p = 0.84, beta = 0.0447, N = 30
DSCQGDSGGPLVCR pc2
10:84 23:155 18:86
p = 0.99, beta = −0.00269, N = 51
CALPGYPGVYTNLCK pc2
4:84 12:155 16:86
p = 0.66, beta = 0.105, N = 32
Assay Target: KLK14
UniProt: Q9P0G3
rsID: rs2569491
Target SNP: 19:51584916:G:A
Proxy SNP: 19:51584916:G:A
UKBPPP log10(p): 834.9
UKBPPP BETA: 0.402
UKBPPP MSPA: 0
−:−:−:−:−:−:−:−:NA
980:1074:925:1079:1063:555:44
IIGGHTCTR pc2
rs2569491 REF
log10 peptide intensity
34:602 11:504 1:154
p = 0.00042, model = REC, N = 46

107. CFB (P00751)
UKBPPP MSPA=0.94

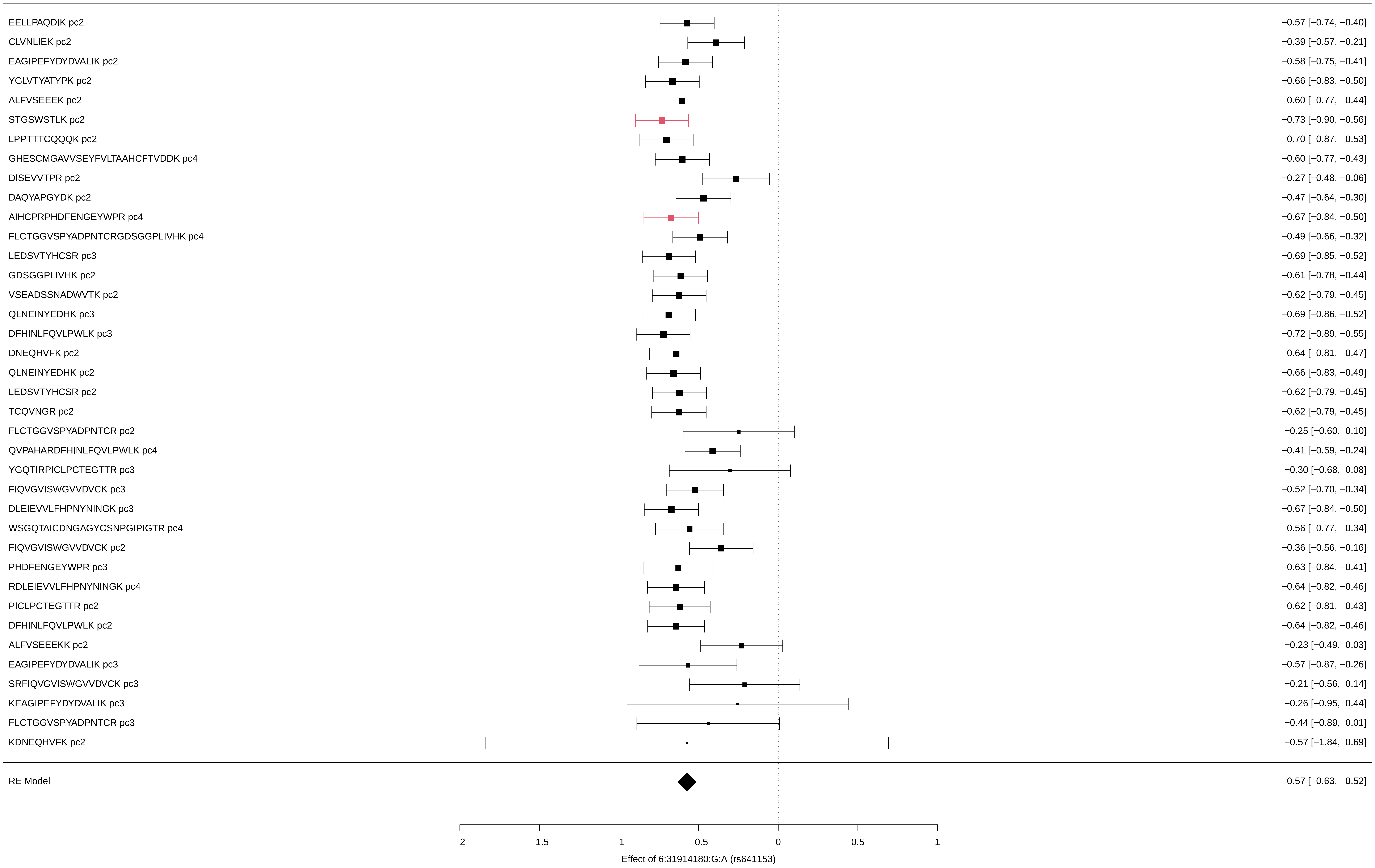

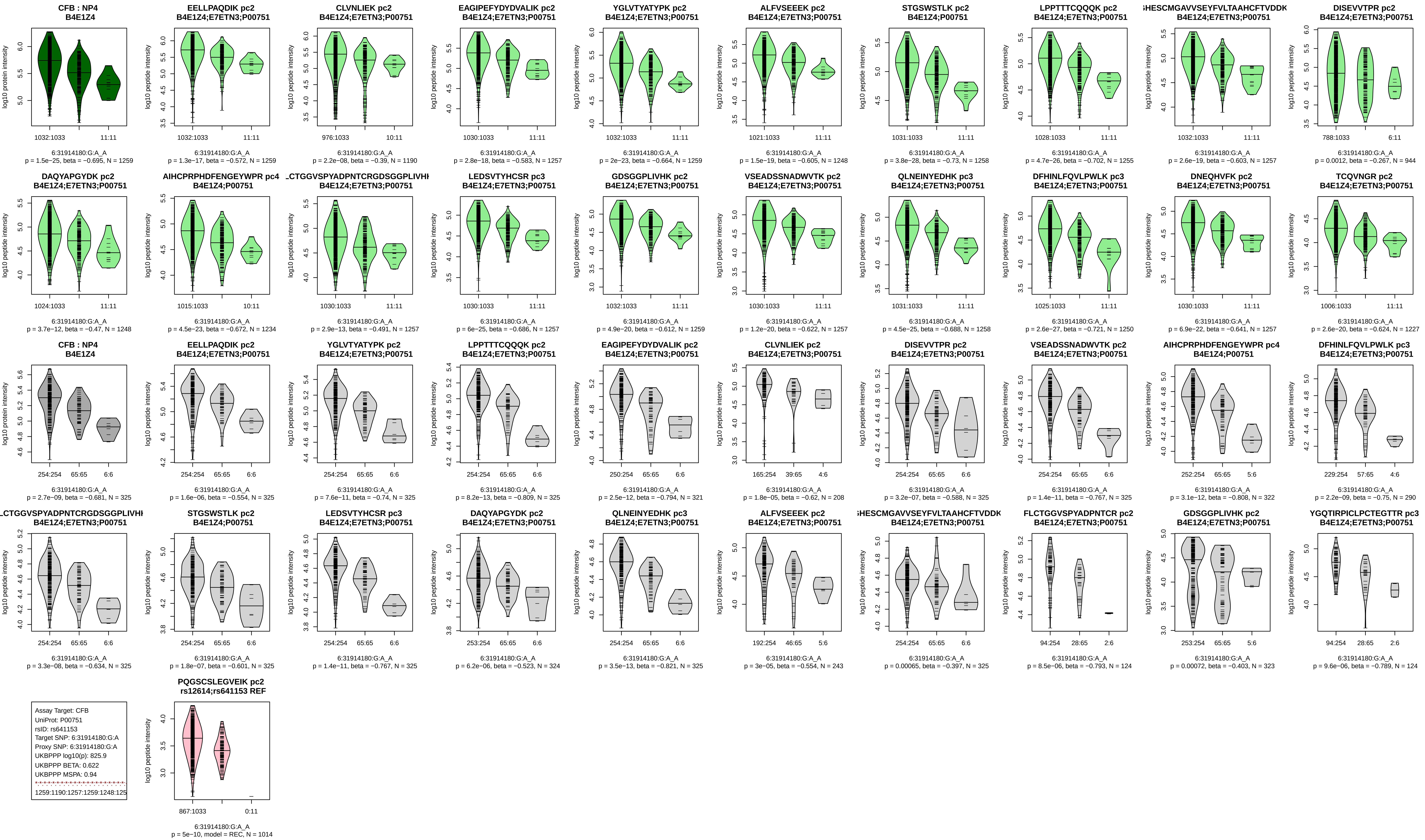
CFB : NP4
B4E1Z4
6:31914180:G:A_A
p = 1.5e−25, beta = −0.695, N = 1259
EELLPAQDIK pc2
B4E1Z4;E7ETN3;P00751
p = 1.3e−17, beta = −0.572, N = 1259
CLVNLIEK pc2
p = 2.2e−08, beta = −0.39, N = 1190
EAGIPEFYDYDVALIK pc2
p = 2.8e−18, beta = −0.583, N = 1257
YGLVTYATYPK pc2
p = 2e−23, beta = −0.664, N = 1259
ALFVSEEEK pc2
p = 1.5e−19, beta = −0.605, N = 1248
STGSWSTLK pc2
B4E1Z4;P00751
p = 3.8e−28, beta = −0.73, N = 1258
LPPTTTCQQQK pc2
p = 4.7e−26, beta = −0.702, N = 1255
p = 2.6e−19, beta = −0.603, N = 1257
DISEVVTPR pc2
p = 0.0012, beta = −0.267, N = 944
DAQYAPGYDK pc2
p = 3.7e−12, beta = −0.47, N = 1248
AIHCPRPHDFENGEYWPR pc4
p = 4.5e−23, beta = −0.672, N = 1234
p = 2.9e−13, beta = −0.491, N = 1257
LEDSVTYHCSR pc3
p = 6e−25, beta = −0.686, N = 1257
GDSGGPLIVHK pc2
p = 4.9e−20, beta = −0.612, N = 1259
VSEADSSNADWVTK pc2
p = 1.2e−20, beta = −0.622, N = 1257
QLNEINYEDHK pc3
p = 4.5e−25, beta = −0.688, N = 1258
DFHINLFQVLPWLK pc3
p = 2.6e−27, beta = −0.721, N = 1250
DNEQHVFK pc2
p = 6.9e−22, beta = −0.641, N = 1257
TCQVNGR pc2
p = 2.6e−20, beta = −0.624, N = 1227
p = 2.7e−09, beta = −0.681, N = 325
p = 1.6e−06, beta = −0.554, N = 325
p = 7.6e−11, beta = −0.74, N = 325
p = 8.2e−13, beta = −0.809, N = 325
p = 2.5e−12, beta = −0.794, N = 321
p = 1.8e−05, beta = −0.62, N = 208
p = 3.2e−07, beta = −0.588, N = 325
p = 1.4e−11, beta = −0.767, N = 325
p = 3.1e−12, beta = −0.808, N = 322
p = 2.2e−09, beta = −0.75, N = 290
p = 3.3e−08, beta = −0.634, N = 325
p = 1.8e−07, beta = −0.601, N = 325
p = 1.4e−11, beta = −0.767, N = 325
p = 6.2e−06, beta = −0.523, N = 324
p = 3.5e−13, beta = −0.821, N = 325
p = 3e−05, beta = −0.554, N = 243
p = 0.00065, beta = −0.397, N = 325
FLCTGGVSPYADPNTCR pc2
p = 8.5e−06, beta = −0.793, N = 124
p = 0.00072, beta = −0.403, N = 323
YGQTIRPICLPCTEGTTR pc3
p = 9.6e−06, beta = −0.789, N = 124
Assay Target: CFB
UniProt: P00751
rsID: rs641153
Target SNP: 6:31914180:G:A
Proxy SNP: 6:31914180:G:A
UKBPPP log10(p): 825.9
UKBPPP BETA: 0.622
UKBPPP MSPA: 0.94
PQGSCSLEGVEIK pc2
rs12614;rs641153 REF
p = 5e−10, model = REC, N = 1014

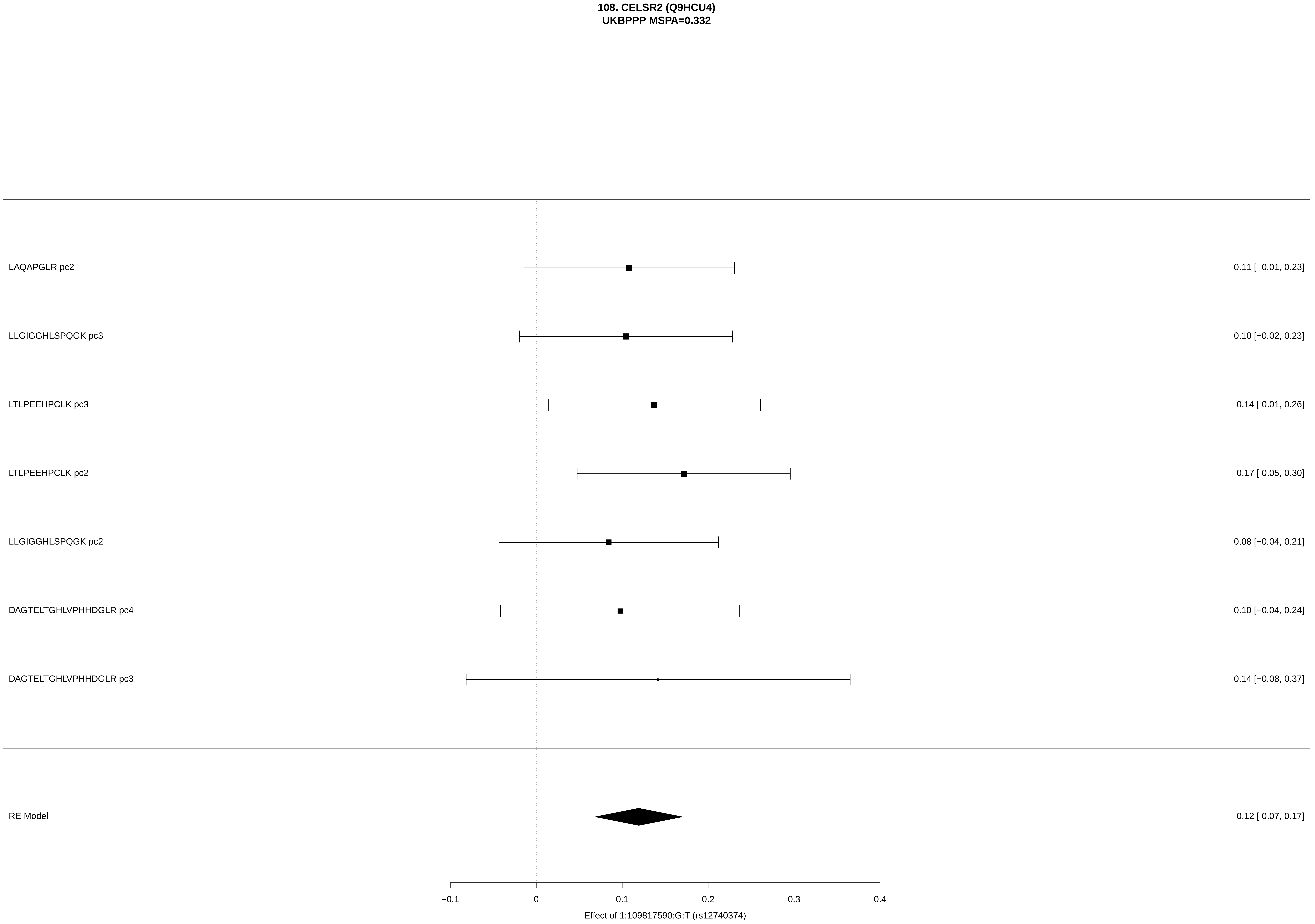
108. CELSR2 (Q9HCU4)
UKBPPP MSPA=0.332
LAQAPGLR pc2
0.11 [−0.01, 0.23]
LLGIGGHLSPQGK pc3
0.10 [−0.02, 0.23]
LTLPEEHPCLK pc3
0.14 [0.01, 0.26]
LTLPEEHPCLK pc2
0.17 [0.05, 0.30]
LLGIGGHLSPQGK pc2
0.08 [−0.04, 0.21]
DAGTELTGHLVPHHDGLR pc4
0.10 [−0.04, 0.24]
DAGTELTGHLVPHHDGLR pc3
0.14 [−0.08, 0.37]
RE Model
0.12 [0.07, 0.17]
−0.1
0
0.1
0.2
0.3
0.4
Effect of 1:109817590:G:T (rs12740374)

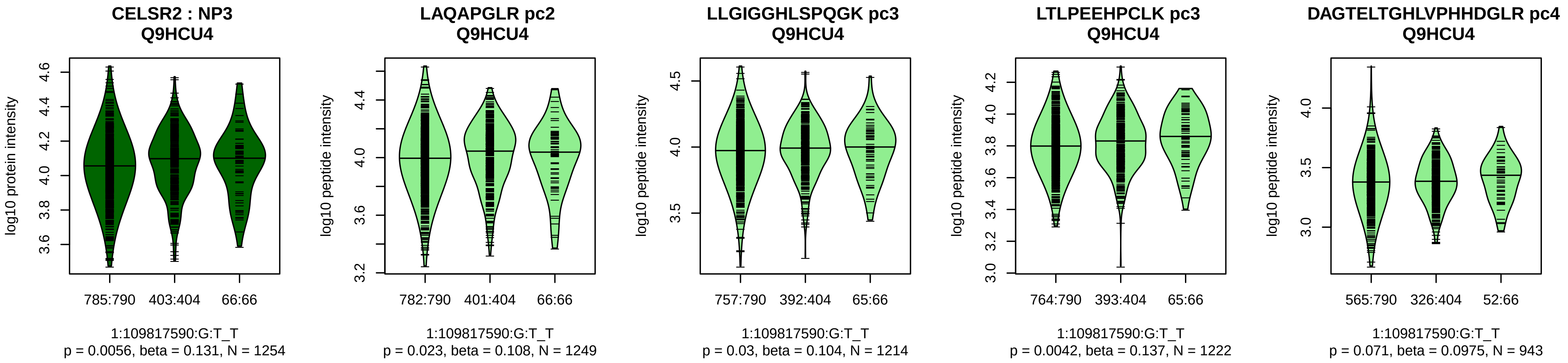

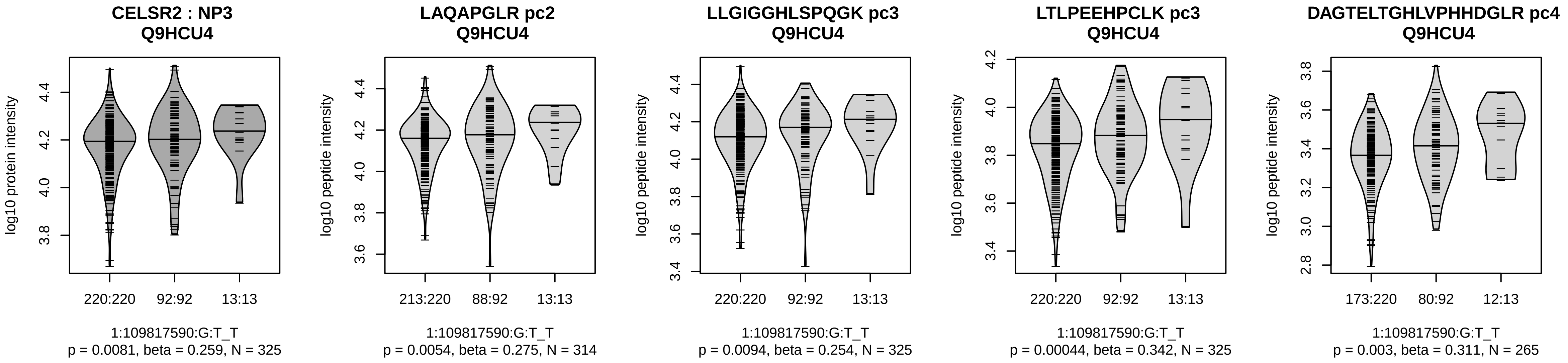

Assay Target: CELSR2
UniProt: Q9HCU4
rsID: rs12740374
Target SNP: 1:109817590:G:T
Proxy SNP: 1:109817590:G:T
UKBPPP log10(p): 813.5
UKBPPP BETA: 0.451
UKBPPP MSPA: 0.332
–;–;*;*;–;–;–
1249:1214:1222:1206:1153:943

109. SMOC2 (Q9H3U7)
UKBPPP MSPA=0.688

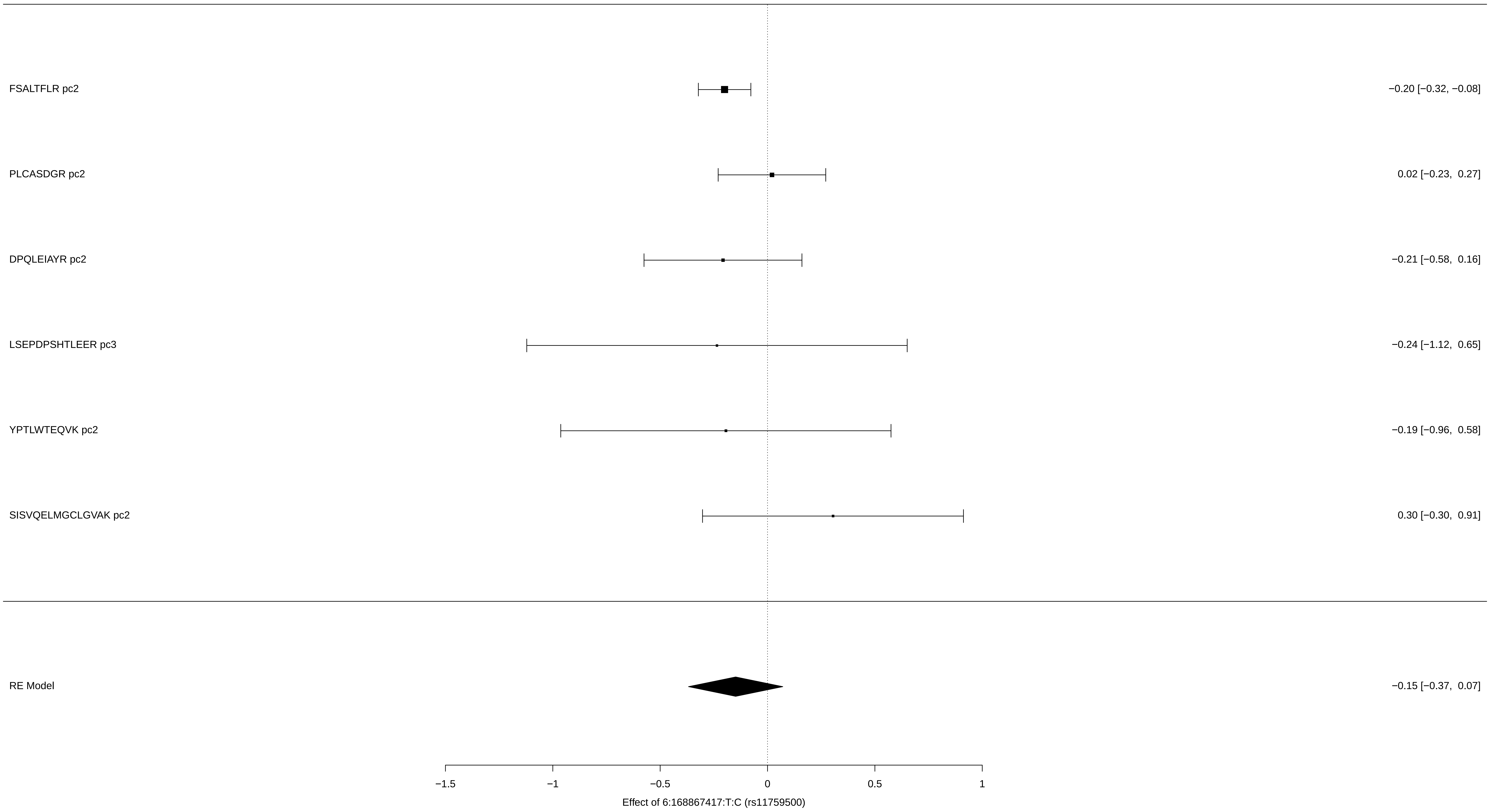

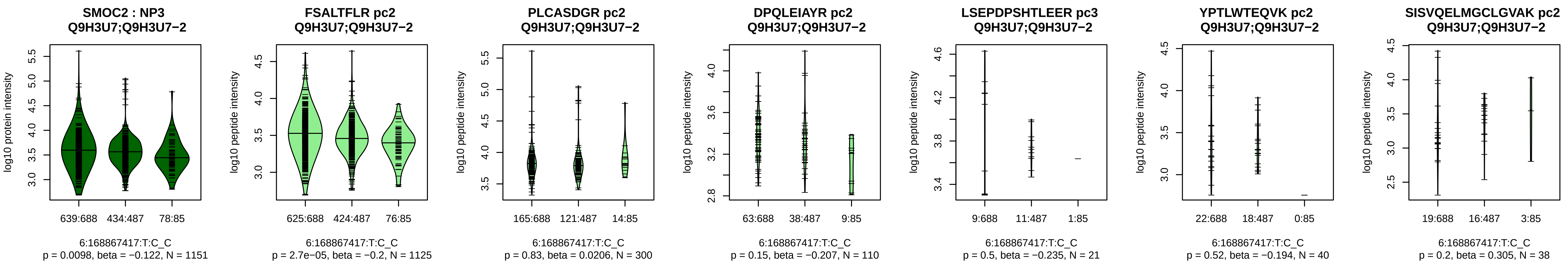

Assay Target: SMOC2
UniProt: Q9H3U7
rsID: rs11759500
Target SNP: 6:168867417:T:C
Proxy SNP: 6:168867417:T:C
UKBPPP log10(p): 784.1
UKBPPP BETA: −0.384
UKBPPP MSPA: 0.688
*:−:−:NA:−:−:−:NA:NA:NA:NA:N
1125:300:110:5:21:40:38:13:15:

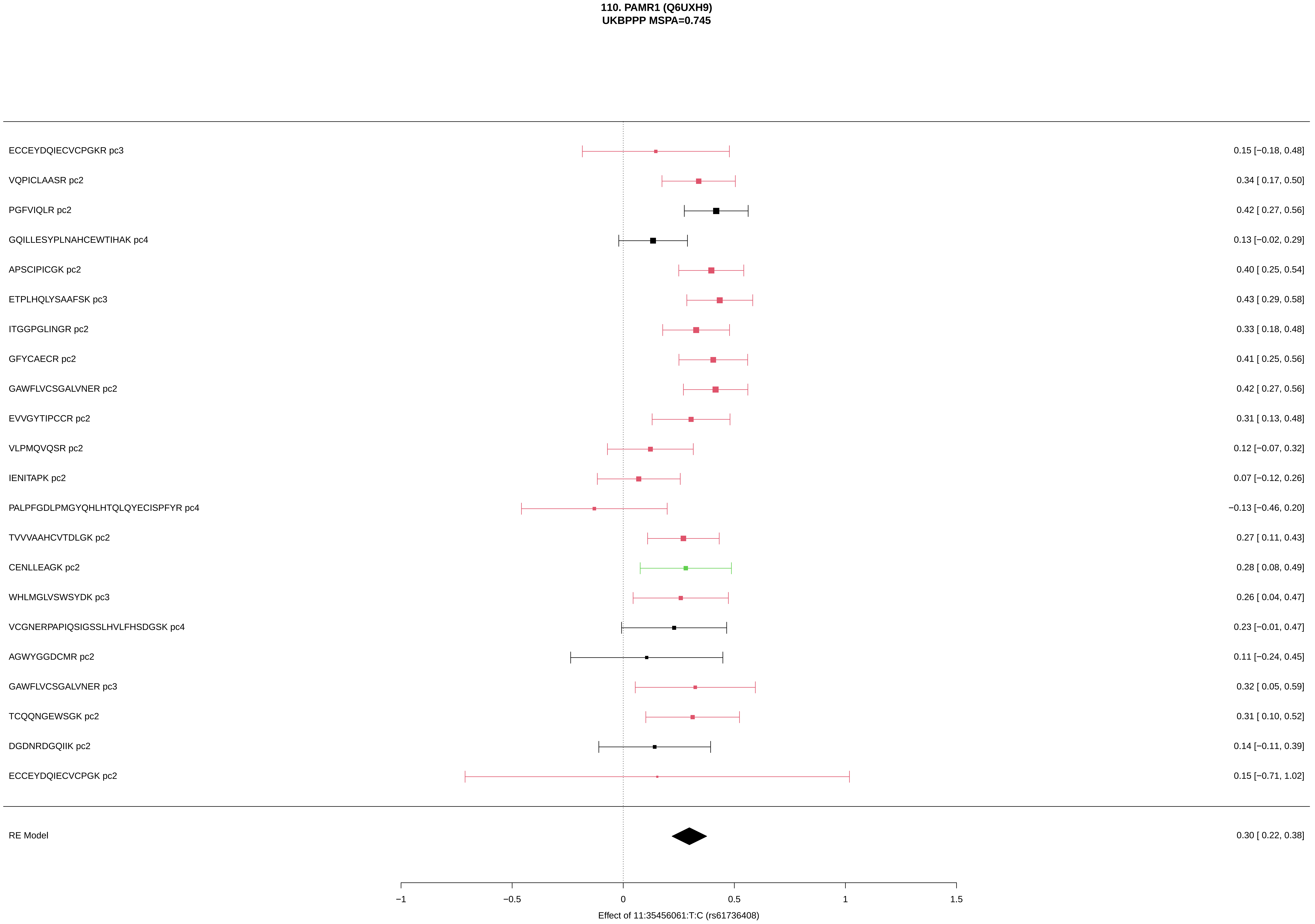

110. PAMR1 (Q6UXH9)
UKBPPP MSPA=0.745
ECCEYDQIECVCPGKR pc3
0.15 [−0.18, 0.48]
VQPICLAASR pc2
0.34 [0.17, 0.50]
PGFVIQLR pc2
0.42 [0.27, 0.56]
GQILLESYPLNAHCEWTIHAK pc4
0.13 [−0.02, 0.29]
APSCIPICGK pc2
0.40 [0.25, 0.54]
ETPLHQLYSAAFSK pc3
0.43 [0.29, 0.58]
ITGGPGLINGR pc2
0.33 [0.18, 0.48]
GFYCAECR pc2
0.41 [0.25, 0.56]
GAWFLVCSGALVNER pc2
0.42 [0.27, 0.56]
EVVGYTIPCCR pc2
0.31 [0.13, 0.48]
VLPMQVQSR pc2
0.12 [−0.07, 0.32]
IENITAPK pc2
0.07 [−0.12, 0.26]
PALPFGDLPMGYQHLHTQLQYECISPFYR pc4
−0.13 [−0.46, 0.20]
TVVVAAHCVTDLGK pc2
0.27 [0.11, 0.43]
CENLLEAGK pc2
0.28 [0.08, 0.49]
WHLMGLVSWSYDK pc3
0.26 [0.04, 0.47]
VCGNERPAPIQSIGSSLHVLFHSDGSK pc4
0.23 [−0.01, 0.47]
AGWYGGDCMR pc2
0.11 [−0.24, 0.45]
GAWFLVCSGALVNER pc3
0.32 [0.05, 0.59]
TCQQNGEWSGK pc2
0.31 [0.10, 0.52]
DGDNRDGQIIK pc2
0.14 [−0.11, 0.39]
ECCEYDQIECVCPGK pc2
0.15 [−0.71, 1.02]
RE Model
0.30 [0.22, 0.38]
−1
−0.5
0
0.5
1
1.5
Effect of 11:35456061:T:C (rs61736408)

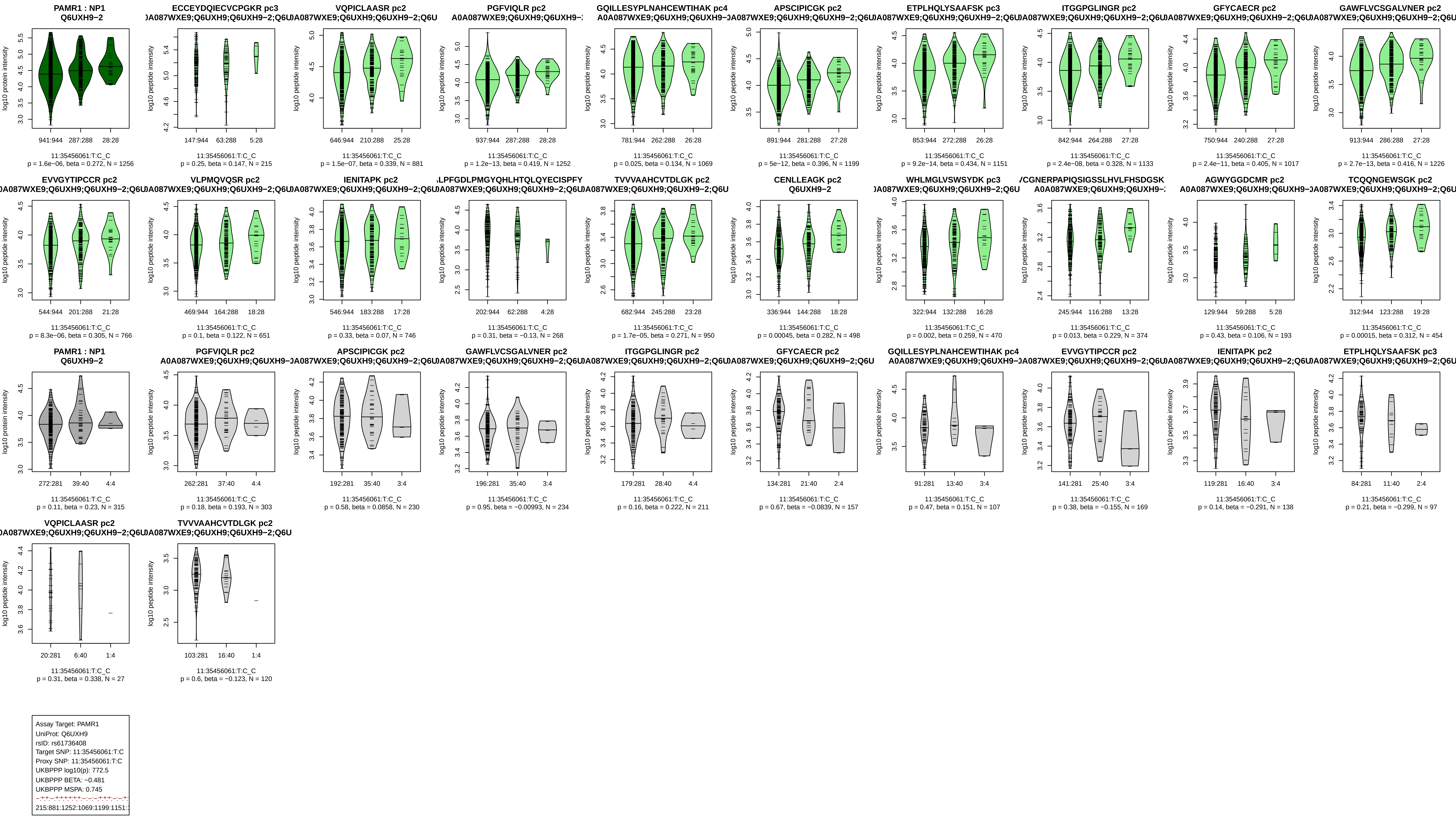
PAMR1 : NP1 Q6UXH9−2
log10 protein intensity
941:944 287:288 28:28
11:35456061:T:C_C
p = 1.6e−06, beta = 0.272, N = 1256
ECCEYDQIECVCPGKR pc3
log10 peptide intensity
147:944 63:288 5:28
p = 0.25, beta = 0.147, N = 215
VQPICLAASR pc2
646:944 210:288 25:28
p = 1.5e−07, beta = 0.339, N = 881
PGFVIQLR pc2
937:944 287:288 28:28
p = 1.2e−13, beta = 0.419, N = 1252
GQILLESYPLNAHCEWTIHAK pc4
781:944 262:288 26:28
p = 0.025, beta = 0.134, N = 1069
APSCIPICGK pc2
891:944 281:288 27:28
p = 5e−12, beta = 0.396, N = 1199
ETPLHQLYSAAFSK pc3
853:944 272:288 26:28
p = 9.2e−14, beta = 0.434, N = 1151
ITGGPGLINGR pc2
842:944 264:288 27:28
p = 2.4e−08, beta = 0.328, N = 1133
GFYCAECR pc2
750:944 240:288 27:28
p = 2.4e−11, beta = 0.405, N = 1017
GAWFLVCSGALVNER pc2
913:944 286:288 27:28
p = 2.7e−13, beta = 0.416, N = 1226
EVVGYTIPCCR pc2
544:944 201:288 21:28
p = 8.3e−06, beta = 0.305, N = 766
VLPMQVQSR pc2
469:944 164:288 18:28
p = 0.1, beta = 0.122, N = 651
IENITAPK pc2
546:944 183:288 17:28
p = 0.33, beta = 0.07, N = 746
LPFGDLPMGYQHLHTQLQYECISPFY
202:944 62:288 4:28
p = 0.31, beta = −0.13, N = 268
TVVVAAHCVTDLGK pc2
682:944 245:288 23:28
p = 1.7e−05, beta = 0.271, N = 950
CENLLEAGK pc2 Q6UXH9−2
336:944 144:288 18:28
p = 0.00045, beta = 0.282, N = 498
WHLMGLVSWSYDK pc3
322:944 132:288 16:28
p = 0.002, beta = 0.259, N = 470
VCGNERPAPIQSIGSSLHVLFHSDGSK
245:944 116:288 13:28
p = 0.013, beta = 0.229, N = 374
AGWYGGDCMR pc2
129:944 59:288 5:28
p = 0.43, beta = 0.106, N = 193
TCQQNGEWSGK pc2
312:944 123:288 19:28
p = 0.00015, beta = 0.312, N = 454
PAMR1 : NP1 Q6UXH9−2
272:281 39:40 4:4
p = 0.11, beta = 0.23, N = 315
PGFVIQLR pc2
262:281 37:40 4:4
p = 0.18, beta = 0.193, N = 303
APSCIPICGK pc2
192:281 35:40 3:4
p = 0.58, beta = 0.0858, N = 230
GAWFLVCSGALVNER pc2
196:281 35:40 3:4
p = 0.95, beta = −0.00993, N = 234
ITGGPGLINGR pc2
179:281 28:40 4:4
p = 0.16, beta = 0.222, N = 211
GFYCAECR pc2
134:281 21:40 2:4
p = 0.67, beta = −0.0839, N = 157
GQILLESYPLNAHCEWTIHAK pc4
91:281 13:40 3:4
p = 0.47, beta = 0.151, N = 107
EVVGYTIPCCR pc2
141:281 25:40 3:4
p = 0.38, beta = −0.155, N = 169
IENITAPK pc2
119:281 16:40 3:4
p = 0.14, beta = −0.291, N = 138
ETPLHQLYSAAFSK pc3
84:281 11:40 2:4
p = 0.21, beta = −0.299, N = 97
VQPICLAASR pc2
20:281 6:40 1:4
p = 0.31, beta = 0.338, N = 27
TVVVAAHCVTDLGK pc2
103:281 16:40 1:4
p = 0.6, beta = −0.123, N = 120
Assay Target: PAMR1
UniProt: Q6UXH9
rsID: rs61736408
Target SNP: 11:35456061:T:C
Proxy SNP: 11:35456061:T:C
UKBPPP log10(p): 772.5
UKBPPP BETA: −0.481
UKBPPP MSPA: 0.745
215:881:1252:1069:1199:1151:

111. ACP1 (P24666)
UKBPPP MSPA=0.85

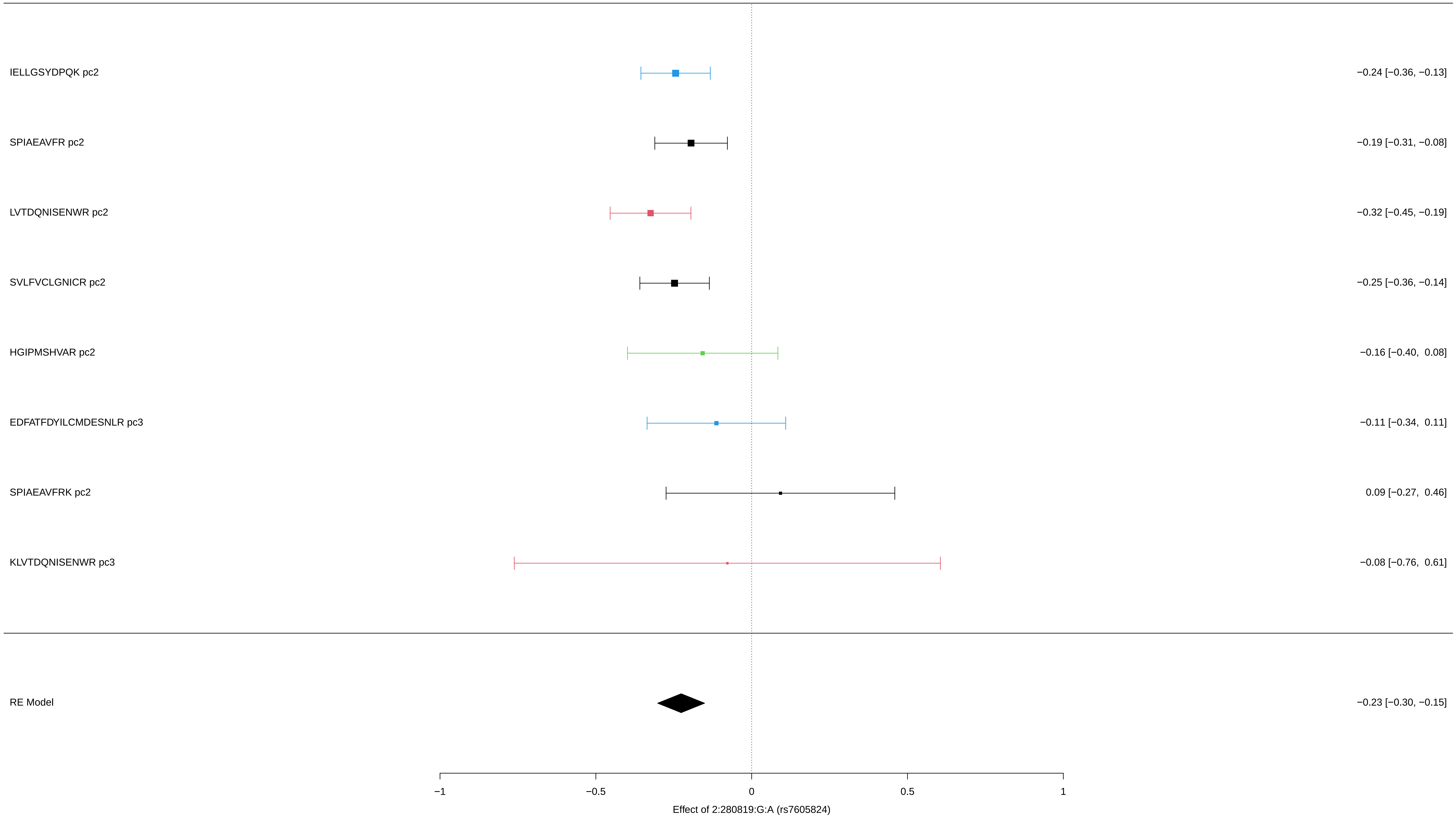

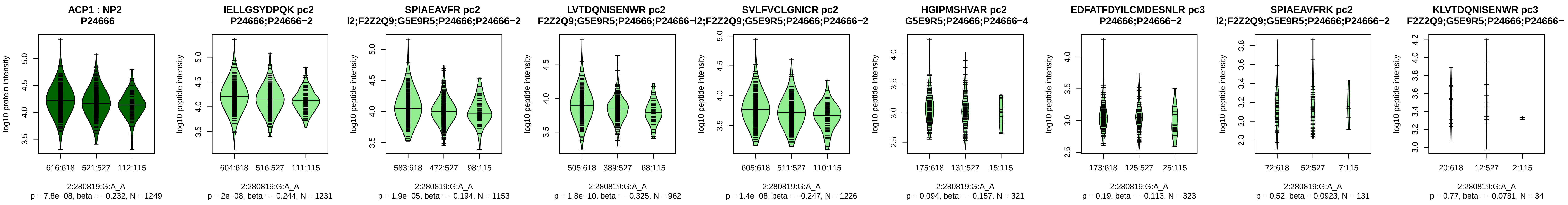

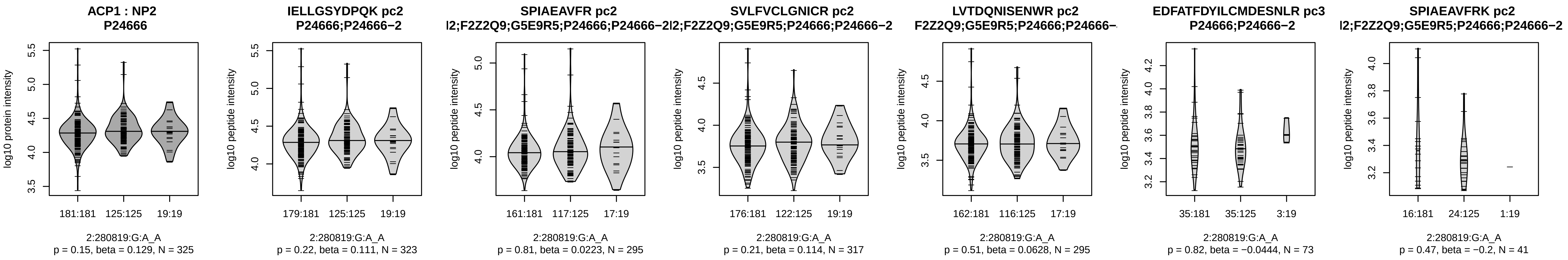

Assay Target: ACP1
UniProt: P24666
rsID: rs7605824
Target SNP: 2:280819:G:A
Proxy SNP: 2:280819:G:A
UKBPPP log10(p): 736.8
UKBPPP BETA: −0.376
UKBPPP MSPA: 0.85
::*:*:−:−:−:−:NA:NA:NA:NA
1231:1153:962:1226:321:323:1

112. INHBB (P09529)
UKBPPP MSPA=0.678

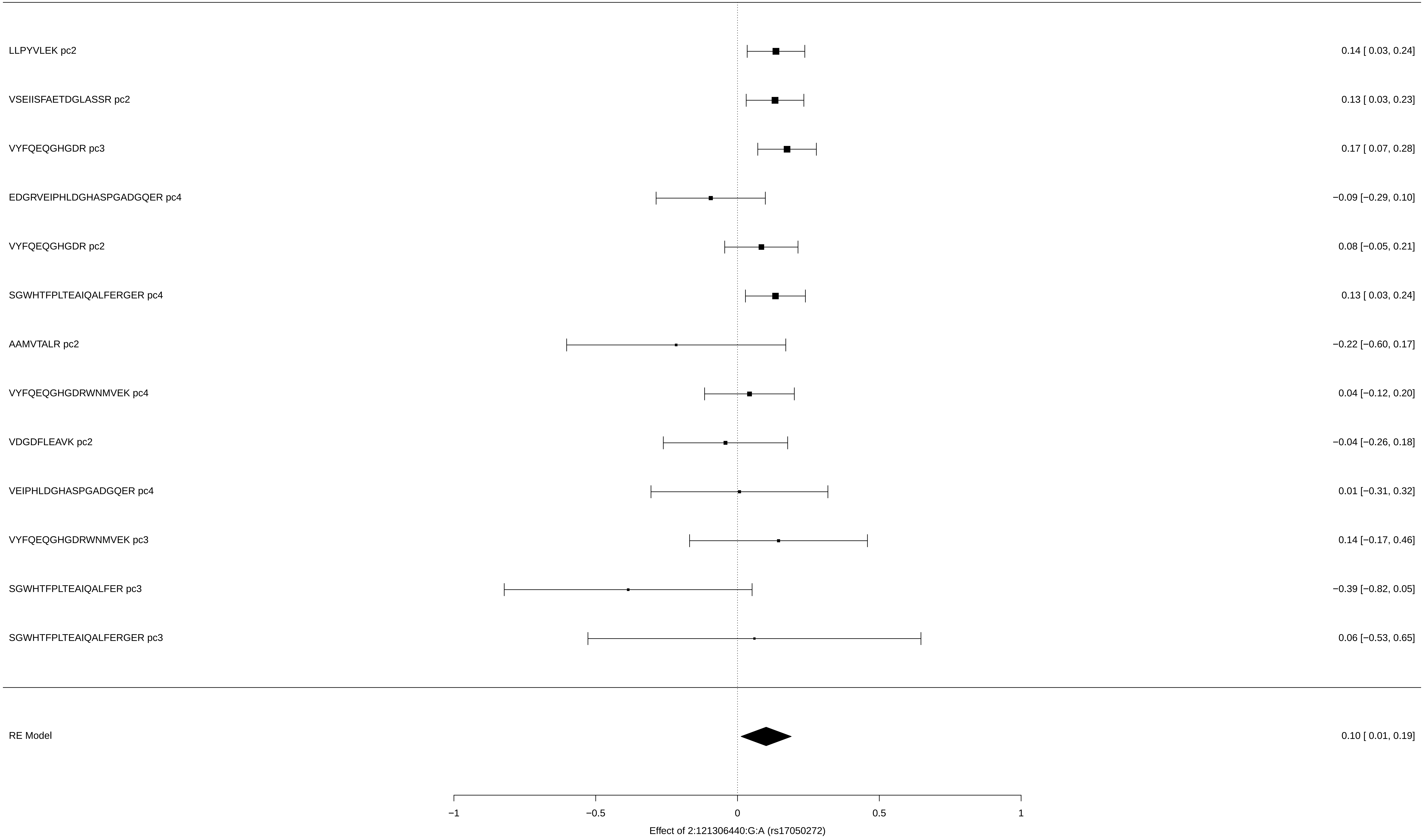

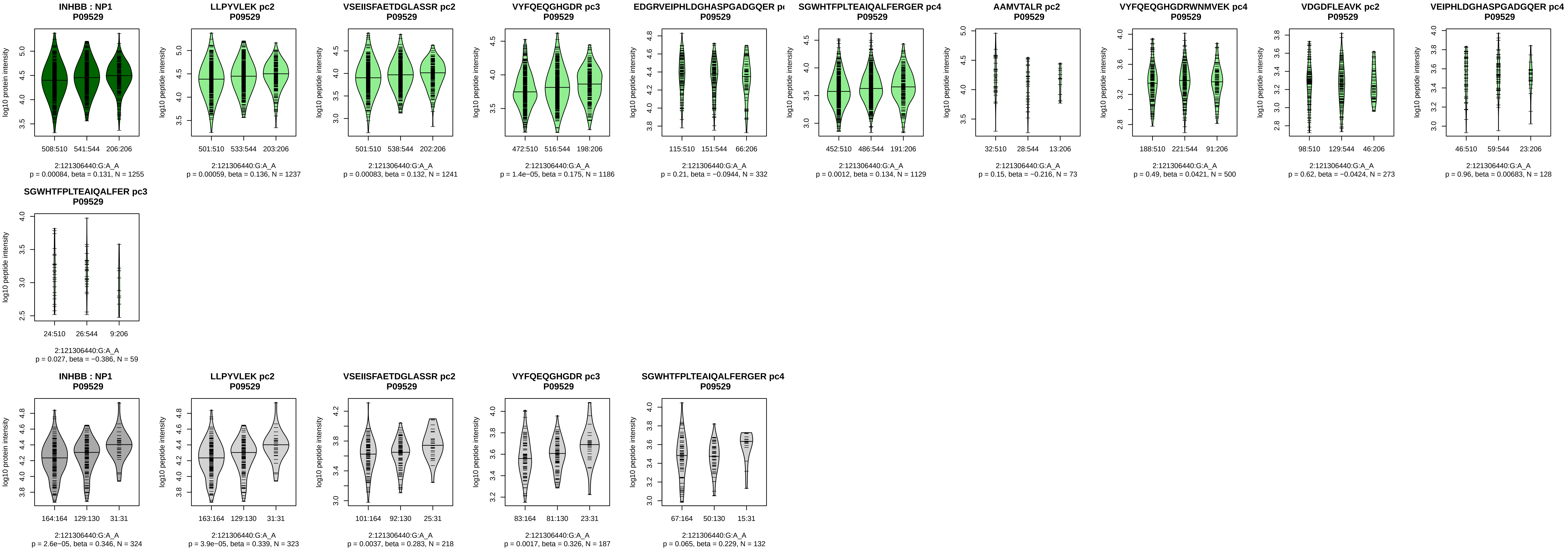

Assay Target: INHBB
UniProt: P09529
rsID: rs17050272
Target SNP: 2:121306440:G:A
Proxy SNP: 2:121306440:G:A
UKBPPP log10(p): 735
UKBPPP BETA: 0.355
UKBPPP MSPA: 0.678
::*:−:−:*:−:−:−:−:−:−:−:NA
1237:1241:1186:332:751:1129:7

113. CDH15 (P55291)
UKBPPP MSPA=0.9

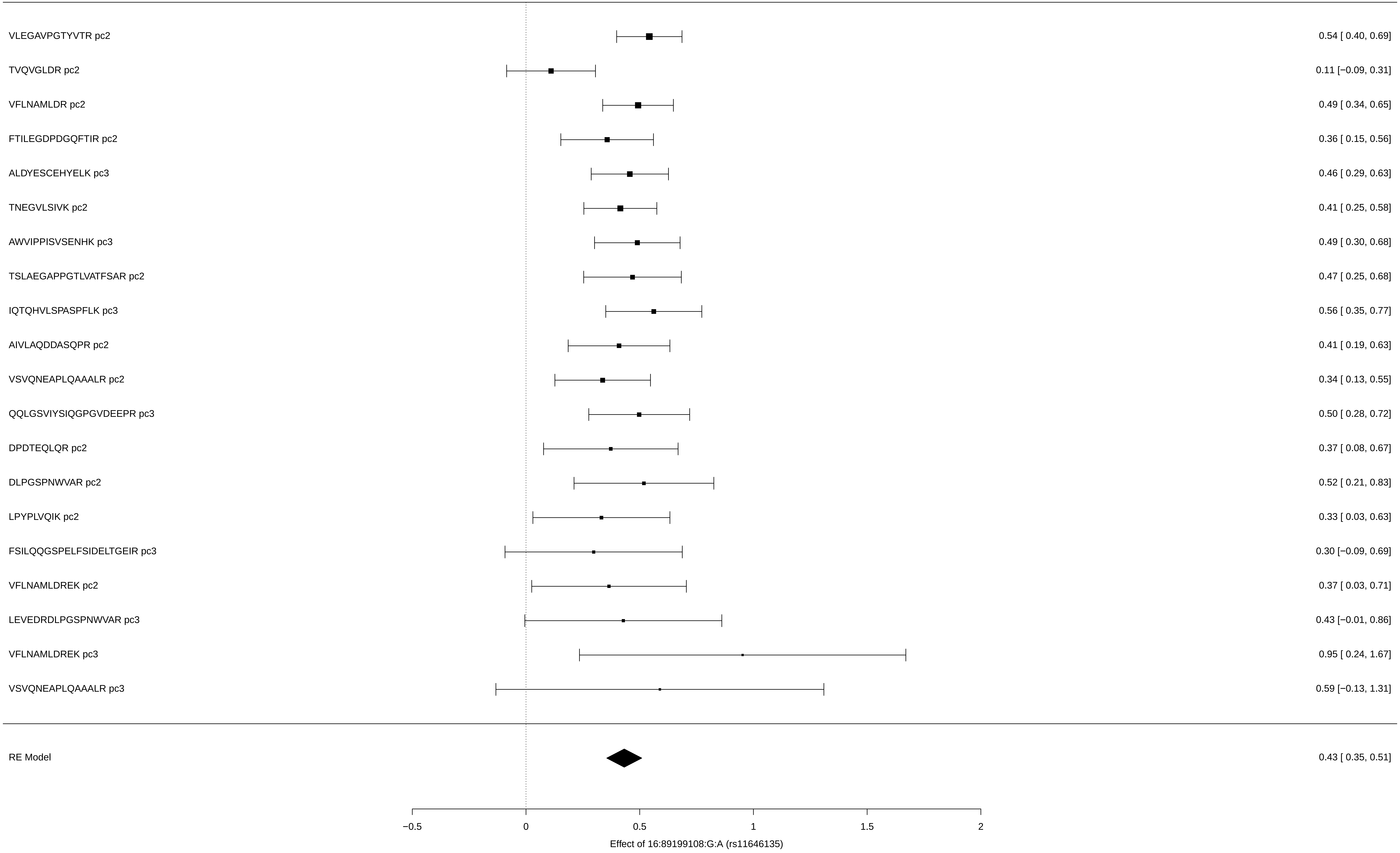

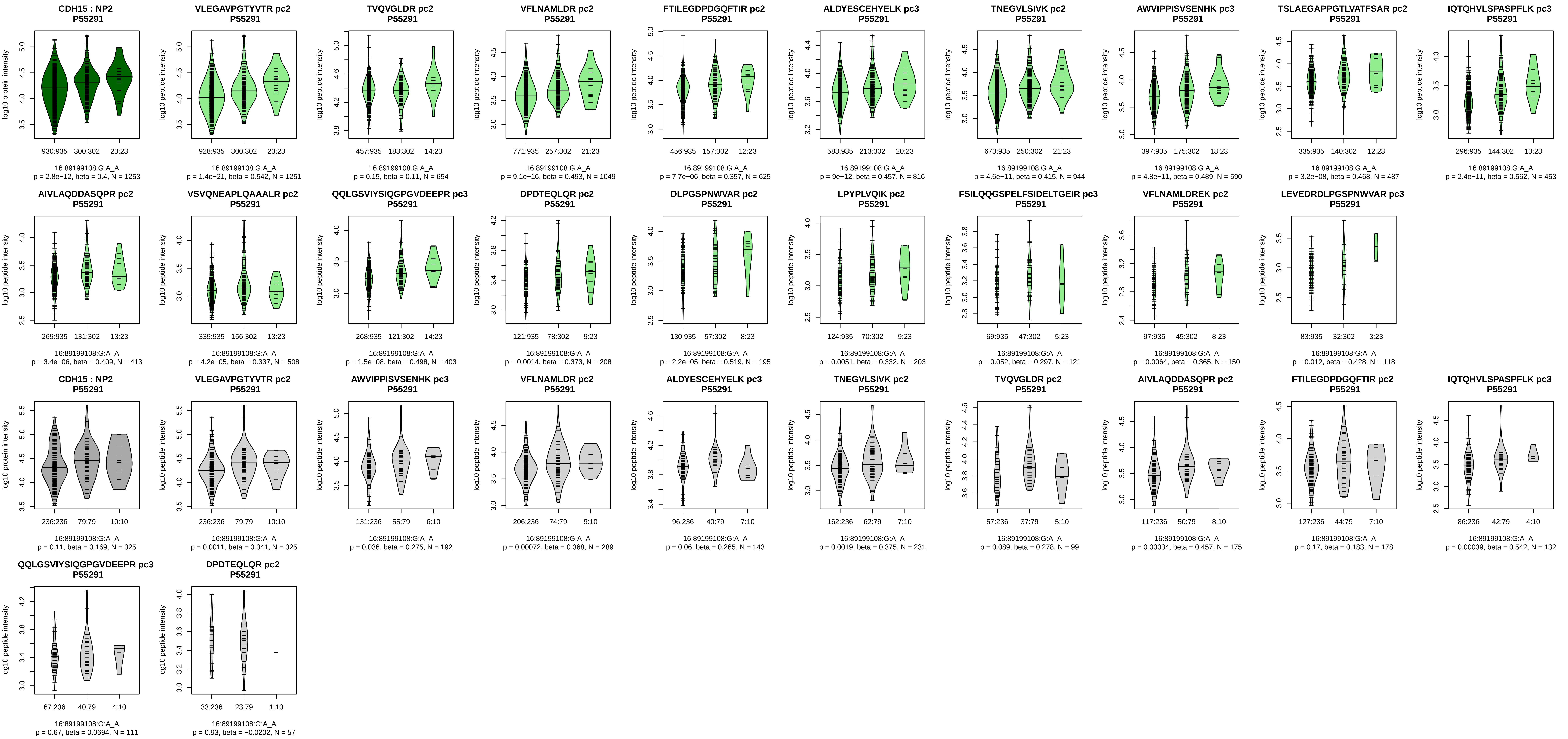

Assay Target: CDH15
UniProt: P55291
rsID: rs11646135
Target SNP: 16:89199108:G:A
Proxy SNP: 16:89199108:G:A
UKBPPP log10(p): 728.6
UKBPPP BETA: 0.432
UKBPPP MSPA: 0.9
[illegible]
1251:654:1049:625:816:944:590

114. AGRN (O00468)
UKBPPP MSPA=0.634

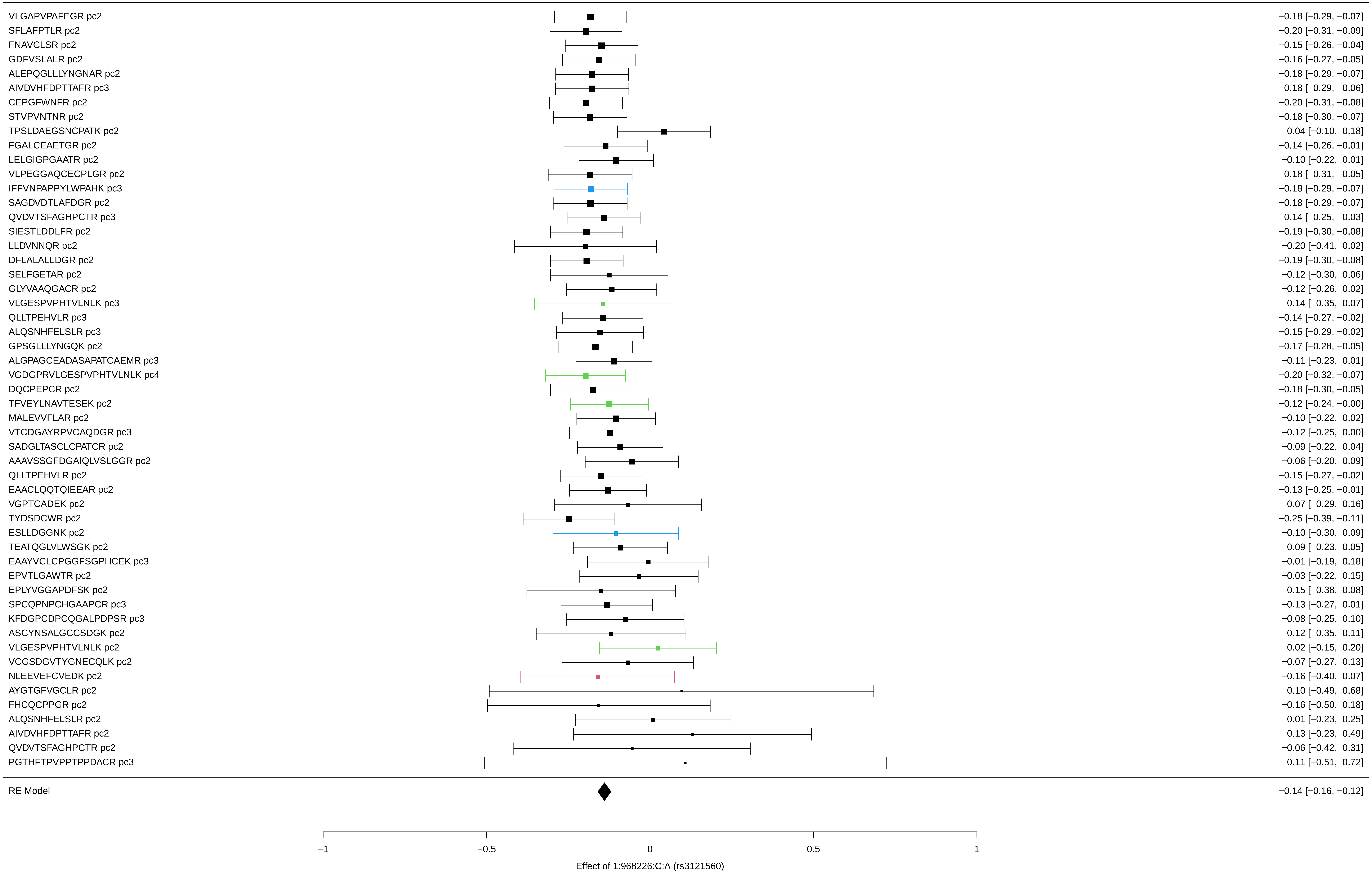

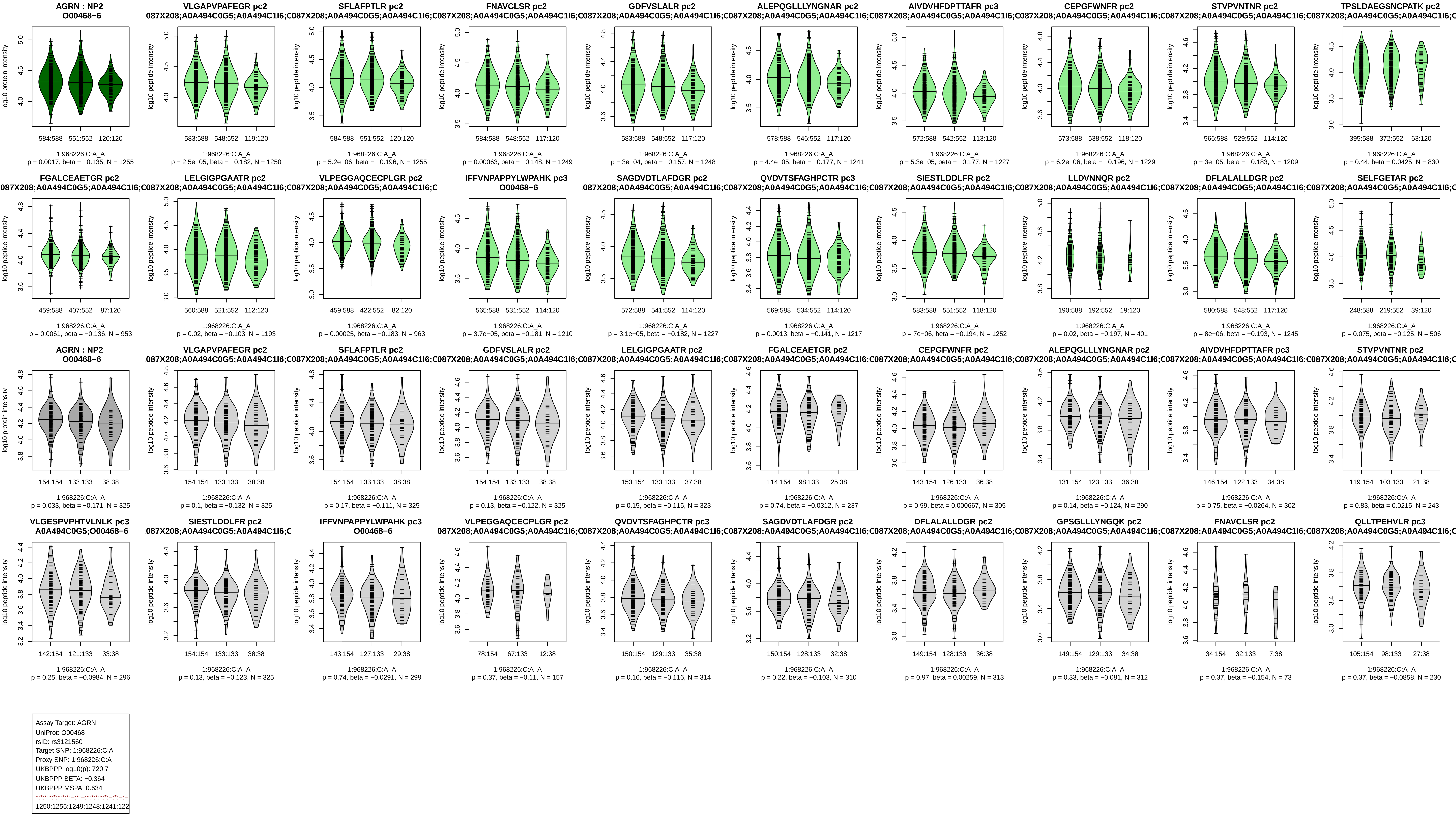
AGRN : NP2
O00468−6
584:588 551:552 120:120
1:968226:C:A_A
p = 0.0017, beta = −0.135, N = 1255
VLGAPVPAFEGR pc2
583:588 548:552 119:120
1:968226:C:A_A
p = 2.5e−05, beta = −0.182, N = 1250
SFLAFPTLR pc2
584:588 551:552 120:120
1:968226:C:A_A
p = 5.2e−06, beta = −0.196, N = 1255
FNAVCLSR pc2
584:588 548:552 117:120
1:968226:C:A_A
p = 0.00063, beta = −0.148, N = 1249
GDFVSLALR pc2
583:588 548:552 117:120
1:968226:C:A_A
p = 3e−04, beta = −0.157, N = 1248
ALEPQGLLLYNGNAR pc2
578:588 546:552 117:120
1:968226:C:A_A
p = 4.4e−05, beta = −0.177, N = 1241
AIVDVHFDPTTAFR pc3
572:588 542:552 113:120
1:968226:C:A_A
p = 5.3e−05, beta = −0.177, N = 1227
CEPGFWNFR pc2
573:588 538:552 118:120
1:968226:C:A_A
p = 6.2e−06, beta = −0.196, N = 1229
STVPVNTNR pc2
566:588 529:552 114:120
1:968226:C:A_A
p = 3e−05, beta = −0.183, N = 1209
TPSLDAEGSNCPATK pc2
395:588 372:552 63:120
1:968226:C:A_A
p = 0.44, beta = 0.0425, N = 830
FGALCEAETGR pc2
459:588 407:552 87:120
1:968226:C:A_A
p = 0.0061, beta = −0.136, N = 953
LELGIGPGAATR pc2
560:588 521:552 112:120
1:968226:C:A_A
p = 0.02, beta = −0.103, N = 1193
VLPEGGAQCECPLGR pc2
459:588 422:552 82:120
1:968226:C:A_A
p = 0.00025, beta = −0.183, N = 963
IFFVNPAPPYLWPAHK pc3
O00468−6
565:588 531:552 114:120
1:968226:C:A_A
p = 3.7e−05, beta = −0.181, N = 1210
SAGDVDTLAFDGR pc2
572:588 541:552 114:120
1:968226:C:A_A
p = 3.1e−05, beta = −0.182, N = 1227
QVDVTSFAGHPCTR pc3
569:588 534:552 114:120
1:968226:C:A_A
p = 0.0013, beta = −0.141, N = 1217
SIESTLDDLFR pc2
583:588 551:552 118:120
1:968226:C:A_A
p = 7e−06, beta = −0.194, N = 1252
LLDVNNQR pc2
190:588 192:552 19:120
1:968226:C:A_A
p = 0.02, beta = −0.197, N = 401
DFLALALLDGR pc2
580:588 548:552 117:120
1:968226:C:A_A
p = 8e−06, beta = −0.193, N = 1245
SELFGETAR pc2
248:588 219:552 39:120
1:968226:C:A_A
p = 0.075, beta = −0.125, N = 506
AGRN : NP2
O00468−6
154:154 133:133 38:38
1:968226:C:A_A
p = 0.033, beta = −0.171, N = 325
VLGAPVPAFEGR pc2
154:154 133:133 38:38
1:968226:C:A_A
p = 0.1, beta = −0.132, N = 325
SFLAFPTLR pc2
154:154 133:133 38:38
1:968226:C:A_A
p = 0.17, beta = −0.111, N = 325
GDFVSLALR pc2
154:154 133:133 38:38
1:968226:C:A_A
p = 0.13, beta = −0.122, N = 325
LELGIGPGAATR pc2
153:154 133:133 37:38
1:968226:C:A_A
p = 0.15, beta = −0.115, N = 323
FGALCEAETGR pc2
114:154 98:133 25:38
1:968226:C:A_A
p = 0.74, beta = −0.0312, N = 237
CEPGFWNFR pc2
143:154 126:133 36:38
1:968226:C:A_A
p = 0.99, beta = 0.000667, N = 305
ALEPQGLLLYNGNAR pc2
131:154 123:133 36:38
1:968226:C:A_A
p = 0.14, beta = −0.124, N = 290
AIVDVHFDPTTAFR pc3
146:154 122:133 34:38
1:968226:C:A_A
p = 0.75, beta = −0.0264, N = 302
STVPVNTNR pc2
119:154 103:133 21:38
1:968226:C:A_A
p = 0.83, beta = 0.0215, N = 243
VLGESPVPHTVLNLK pc3
A0A494C0G5;O00468−6
142:154 121:133 33:38
1:968226:C:A_A
p = 0.25, beta = −0.0984, N = 296
SIESTLDDLFR pc2
154:154 133:133 38:38
1:968226:C:A_A
p = 0.13, beta = −0.123, N = 325
IFFVNPAPPYLWPAHK pc3
O00468−6
143:154 127:133 29:38
1:968226:C:A_A
p = 0.74, beta = −0.0291, N = 299
VLPEGGAQCECPLGR pc2
78:154 67:133 12:38
1:968226:C:A_A
p = 0.37, beta = −0.11, N = 157
QVDVTSFAGHPCTR pc3
150:154 129:133 35:38
1:968226:C:A_A
p = 0.16, beta = −0.116, N = 314
SAGDVDTLAFDGR pc2
150:154 128:133 32:38
1:968226:C:A_A
p = 0.22, beta = −0.103, N = 310
DFLALALLDGR pc2
149:154 128:133 36:38
1:968226:C:A_A
p = 0.97, beta = 0.00259, N = 313
GPSGLLLYNGQK pc2
149:154 129:133 34:38
1:968226:C:A_A
p = 0.33, beta = −0.081, N = 312
FNAVCLSR pc2
34:154 32:133 7:38
1:968226:C:A_A
p = 0.37, beta = −0.154, N = 73
QLLTPEHVLR pc3
105:154 98:133 27:38
1:968226:C:A_A
p = 0.37, beta = −0.0858, N = 230
log10 protein intensity
log10 peptide intensity
Assay Target: AGRN
UniProt: O00468
rsID: rs3121560
Target SNP: 1:968226:C:A
Proxy SNP: 1:968226:C:A
UKBPPP log10(p): 720.7
UKBPPP BETA: −0.364
UKBPPP MSPA: 0.634
1250:1255:1249:1248:1241:122

115. MFGE8 (Q08431)
UKBPPP MSPA=0.195

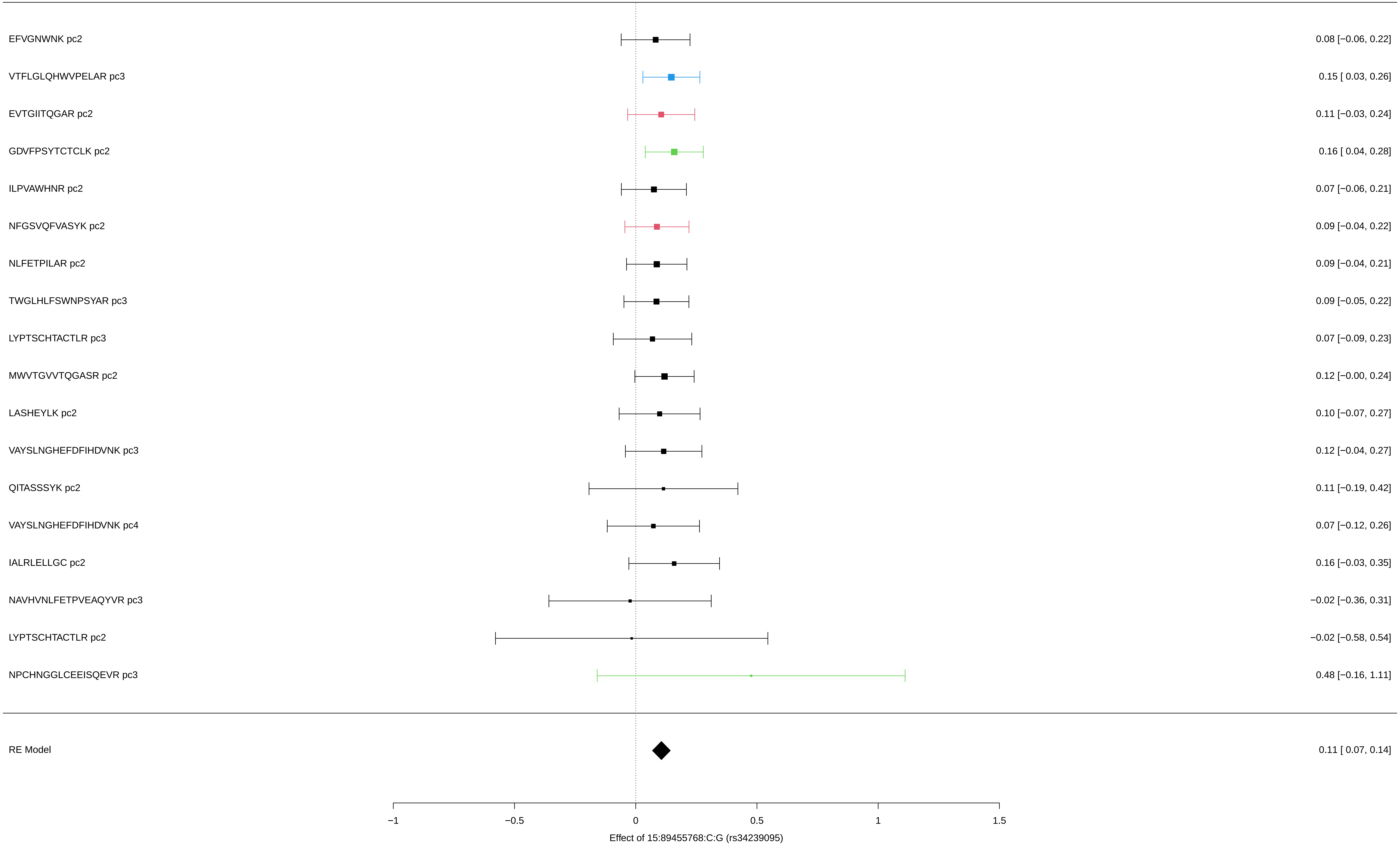

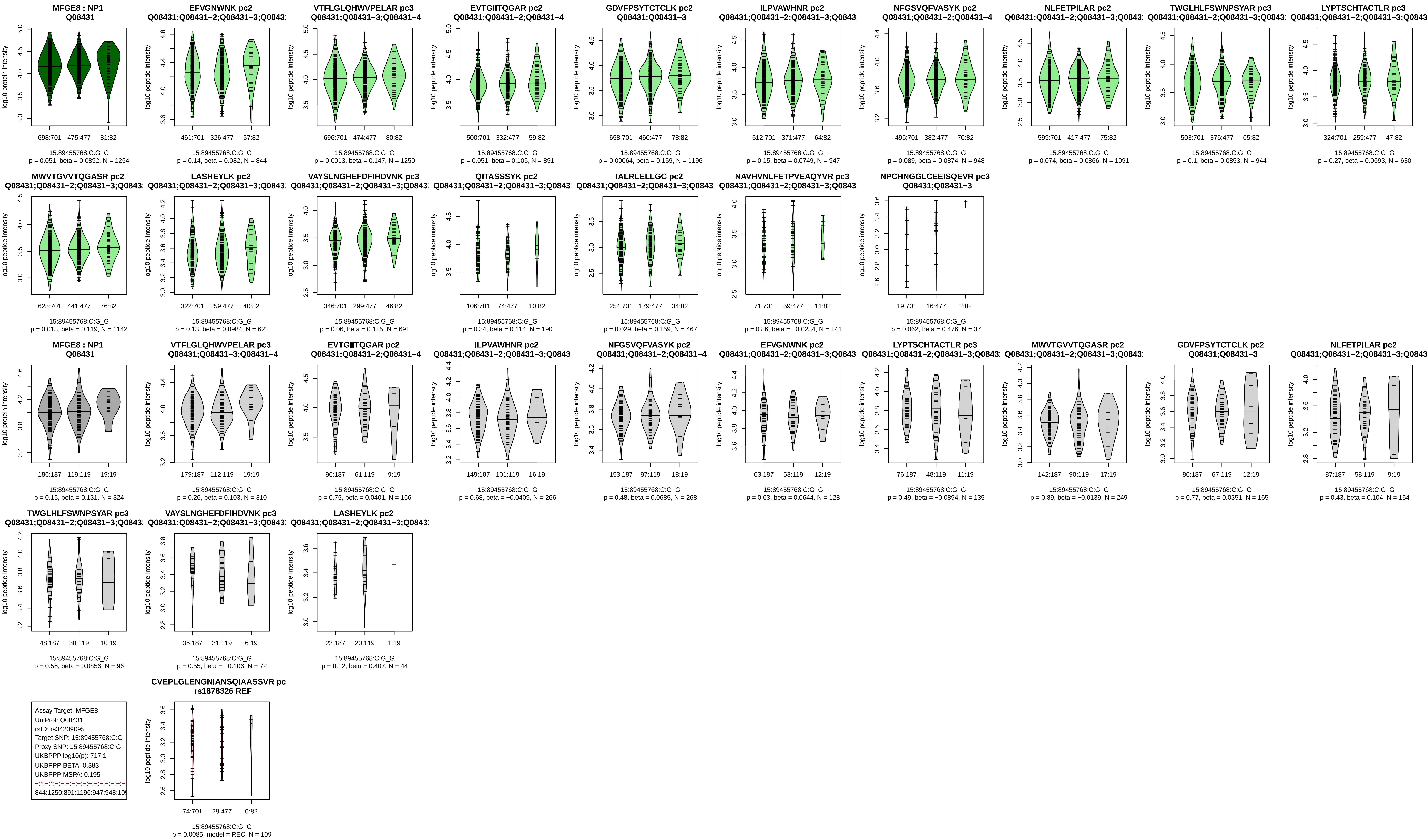

MFGE8 : NP1
Q08431
log10 protein intensity
698:701 475:477 81:82
15:89455768:C:G_G
p = 0.051, beta = 0.0892, N = 1254
EFVGNWNK pc2
Q08431;Q08431−2;Q08431−3;Q0843
log10 peptide intensity
461:701 326:477 57:82
15:89455768:C:G_G
p = 0.14, beta = 0.082, N = 844
VTFLGLQHWVPELAR pc3
Q08431;Q08431−3;Q08431−4
696:701 474:477 80:82
15:89455768:C:G_G
p = 0.0013, beta = 0.147, N = 1250
EVTGIITQGAR pc2
Q08431;Q08431−2;Q08431−4
500:701 332:477 59:82
15:89455768:C:G_G
p = 0.051, beta = 0.105, N = 891
GDVFPSYTCTCLK pc2
Q08431;Q08431−3
658:701 460:477 78:82
15:89455768:C:G_G
p = 0.00064, beta = 0.159, N = 1196
ILPVAWHNR pc2
Q08431;Q08431−2;Q08431−3;Q0843
512:701 371:477 64:82
15:89455768:C:G_G
p = 0.15, beta = 0.0749, N = 947
NFGSVQFVASYK pc2
Q08431;Q08431−2;Q08431−4
496:701 382:477 70:82
15:89455768:C:G_G
p = 0.089, beta = 0.0874, N = 948
NLFETPILAR pc2
Q08431;Q08431−2;Q08431−3;Q0843
599:701 417:477 75:82
15:89455768:C:G_G
p = 0.074, beta = 0.0866, N = 1091
TWGLHLFSWNPSYAR pc3
Q08431;Q08431−2;Q08431−3;Q0843
503:701 376:477 65:82
15:89455768:C:G_G
p = 0.1, beta = 0.0853, N = 944
LYPTSCHTACTLR pc3
Q08431;Q08431−2;Q08431−3;Q0843
324:701 259:477 47:82
15:89455768:C:G_G
p = 0.27, beta = 0.0693, N = 630
MWVTGVVTQGASR pc2
Q08431;Q08431−2;Q08431−3;Q0843
625:701 441:477 76:82
15:89455768:C:G_G
p = 0.013, beta = 0.119, N = 1142
LASHEYLK pc2
Q08431;Q08431−2;Q08431−3;Q0843
322:701 259:477 40:82
15:89455768:C:G_G
p = 0.13, beta = 0.0984, N = 621
VAYSLNGHEFDFIHDVNK pc3
Q08431;Q08431−2;Q08431−3;Q0843
346:701 299:477 46:82
15:89455768:C:G_G
p = 0.06, beta = 0.115, N = 691
QITASSSYK pc2
Q08431;Q08431−2;Q08431−3;Q0843
106:701 74:477 10:82
15:89455768:C:G_G
p = 0.34, beta = 0.114, N = 190
IALRLELLGC pc2
Q08431;Q08431−2;Q08431−3;Q0843
254:701 179:477 34:82
15:89455768:C:G_G
p = 0.029, beta = 0.159, N = 467
NAVHVNLFETPVEAQYVR pc3
Q08431;Q08431−2;Q08431−3;Q0843
71:701 59:477 11:82
15:89455768:C:G_G
p = 0.86, beta = −0.0234, N = 141
NPCHNGGLCEEISQEVR pc3
Q08431;Q08431−3
19:701 16:477 2:82
15:89455768:C:G_G
p = 0.062, beta = 0.476, N = 37
MFGE8 : NP1
Q08431
186:187 119:119 19:19
15:89455768:C:G_G
p = 0.15, beta = 0.131, N = 324
VTFLGLQHWVPELAR pc3
Q08431;Q08431−3;Q08431−4
179:187 112:119 19:19
15:89455768:C:G_G
p = 0.26, beta = 0.103, N = 310
EVTGIITQGAR pc2
Q08431;Q08431−2;Q08431−4
96:187 61:119 9:19
15:89455768:C:G_G
p = 0.75, beta = 0.0401, N = 166
ILPVAWHNR pc2
Q08431;Q08431−2;Q08431−3;Q0843
149:187 101:119 16:19
15:89455768:C:G_G
p = 0.68, beta = −0.0409, N = 266
NFGSVQFVASYK pc2
Q08431;Q08431−2;Q08431−4
153:187 97:119 18:19
15:89455768:C:G_G
p = 0.48, beta = 0.0685, N = 268
EFVGNWNK pc2
Q08431;Q08431−2;Q08431−3;Q0843
63:187 53:119 12:19
15:89455768:C:G_G
p = 0.63, beta = 0.0644, N = 128
LYPTSCHTACTLR pc3
Q08431;Q08431−2;Q08431−3;Q0843
76:187 48:119 11:19
15:89455768:C:G_G
p = 0.49, beta = −0.0894, N = 135
MWVTGVVTQGASR pc2
Q08431;Q08431−2;Q08431−3;Q0843
142:187 90:119 17:19
15:89455768:C:G_G
p = 0.89, beta = −0.0139, N = 249
GDVFPSYTCTCLK pc2
Q08431;Q08431−3
86:187 67:119 12:19
15:89455768:C:G_G
p = 0.77, beta = 0.0351, N = 165
NLFETPILAR pc2
Q08431;Q08431−2;Q08431−3;Q0843
87:187 58:119 9:19
15:89455768:C:G_G
p = 0.43, beta = 0.104, N = 154
TWGLHLFSWNPSYAR pc3
Q08431;Q08431−2;Q08431−3;Q0843
48:187 38:119 10:19
15:89455768:C:G_G
p = 0.56, beta = 0.0856, N = 96
VAYSLNGHEFDFIHDVNK pc3
Q08431;Q08431−2;Q08431−3;Q0843
35:187 31:119 6:19
15:89455768:C:G_G
p = 0.55, beta = −0.106, N = 72
LASHEYLK pc2
Q08431;Q08431−2;Q08431−3;Q0843
23:187 20:119 1:19
15:89455768:C:G_G
p = 0.12, beta = 0.407, N = 44
Assay Target: MFGE8
UniProt: Q08431
rsID: rs34239095
Target SNP: 15:89455768:C:G
Proxy SNP: 15:89455768:C:G
UKBPPP log10(p): 717.1
UKBPPP BETA: 0.383
UKBPPP MSPA: 0.195
844:1250:891:1196:947:948:10
CVEPLGLENGNIANSQIAASSVR pc
rs1878326 REF
74:701 29:477 6:82
15:89455768:C:G_G
p = 0.0085, model = REC, N = 109

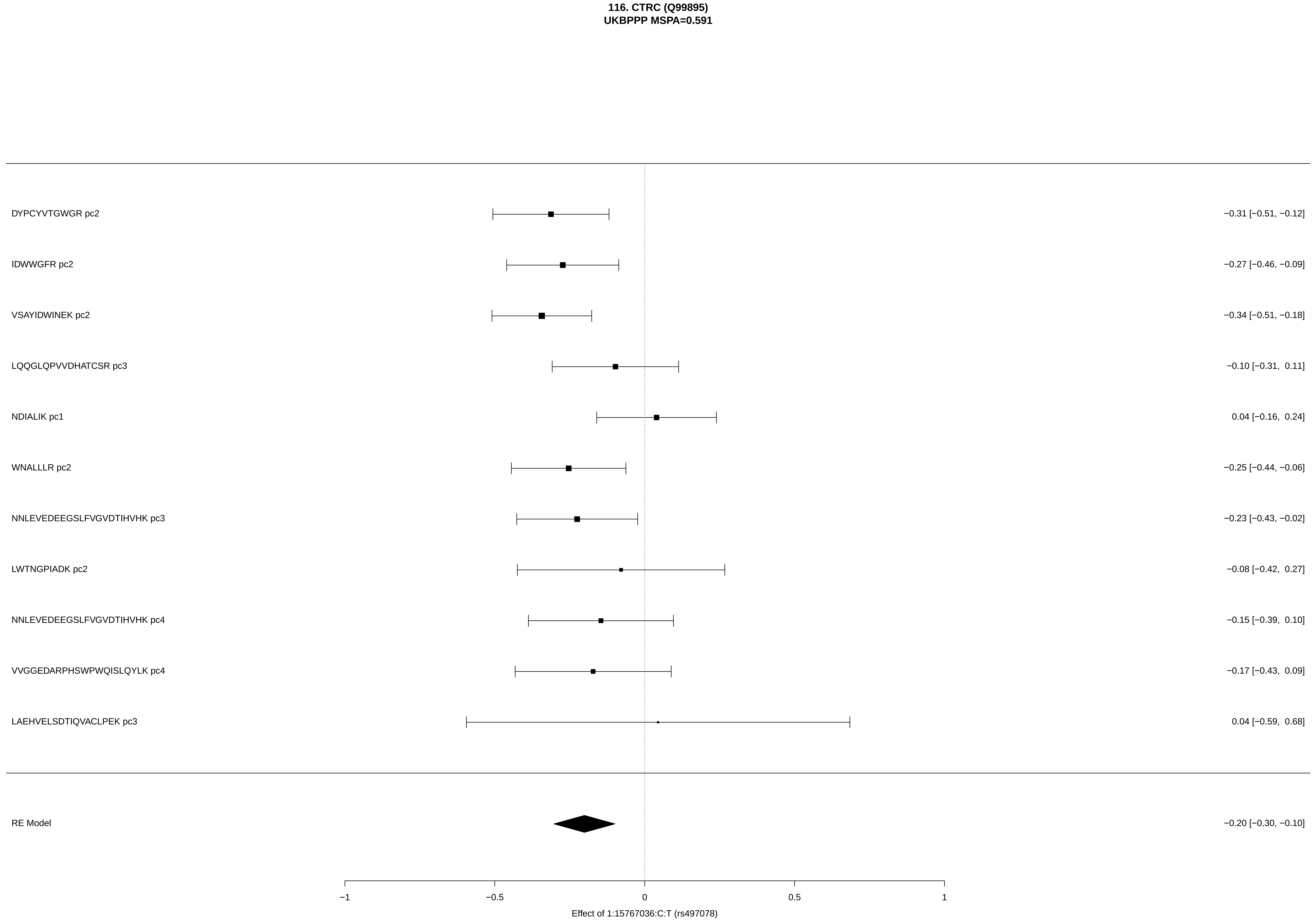

116. CTRC (Q99895)
UKBPPP MSPA=0.591
DYPCYVTGWGR pc2
−0.31 [−0.51, −0.12]
IDWWGFR pc2
−0.27 [−0.46, −0.09]
VSAYIDWINEK pc2
−0.34 [−0.51, −0.18]
LQQGLQPVVDHATCSR pc3
−0.10 [−0.31, 0.11]
NDIALIK pc1
0.04 [−0.16, 0.24]
WNALLLR pc2
−0.25 [−0.44, −0.06]
NNLEVEDEEGSLFVGVDTIHVHK pc3
−0.23 [−0.43, −0.02]
LWTNGPIADK pc2
−0.08 [−0.42, 0.27]
NNLEVEDEEGSLFVGVDTIHVHK pc4
−0.15 [−0.39, 0.10]
VVGGEDARPHSWPWQISLQYLK pc4
−0.17 [−0.43, 0.09]
LAEHVELSDTIQVACLPEK pc3
0.04 [−0.59, 0.68]
RE Model
−0.20 [−0.30, −0.10]
−1
−0.5
0
0.5
1
Effect of 1:15767036:C:T (rs497078)

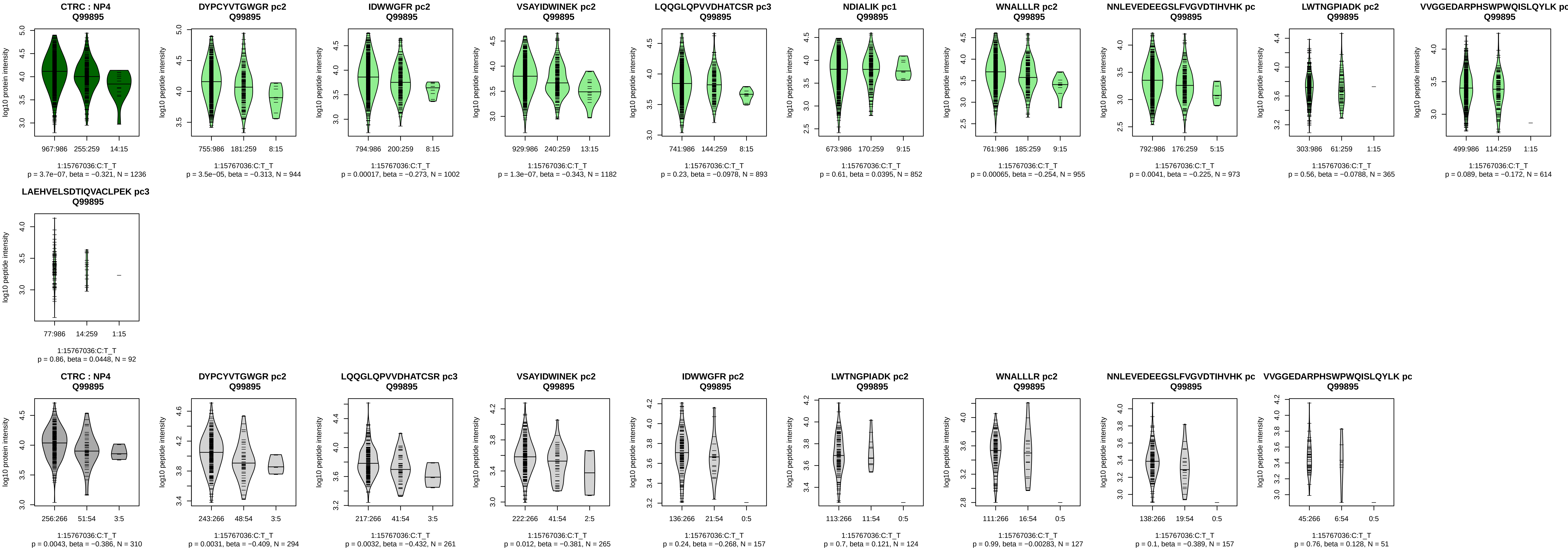

Assay Target: CTRC
UniProt: Q99895
rsID: rs497078
Target SNP: 1:15767036:C:T
Proxy SNP: 1:15767036:C:T
UKBPPP log10(p): 716.9
UKBPPP BETA: −0.552
UKBPPP MSPA: 0.591
::*:–:–:*:*:–:–:–:–:NA
944:1002:1182:893:852:955:973

117. DKK3 (Q9UBP4)
UKBPPP MSPA=0.903

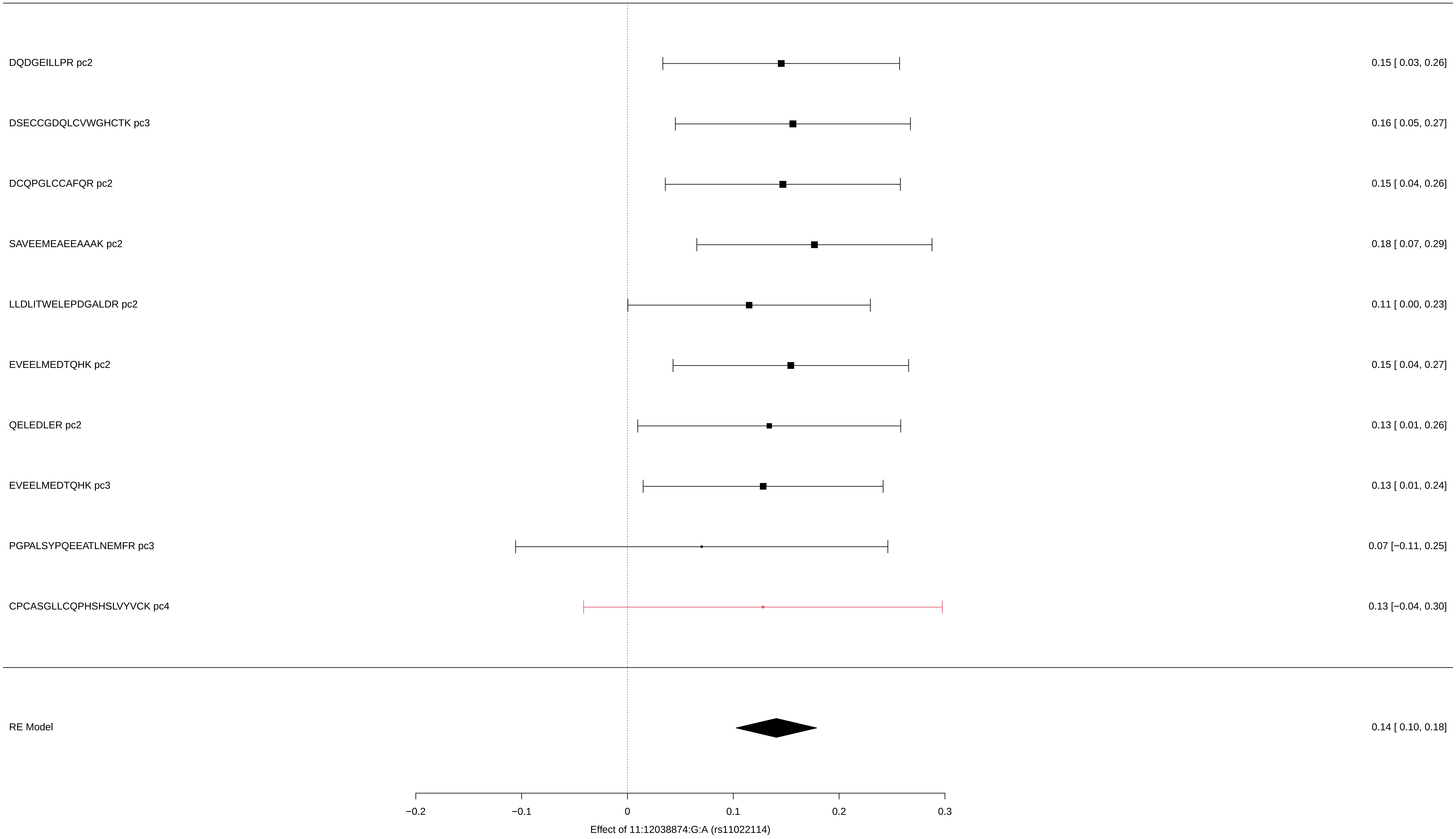

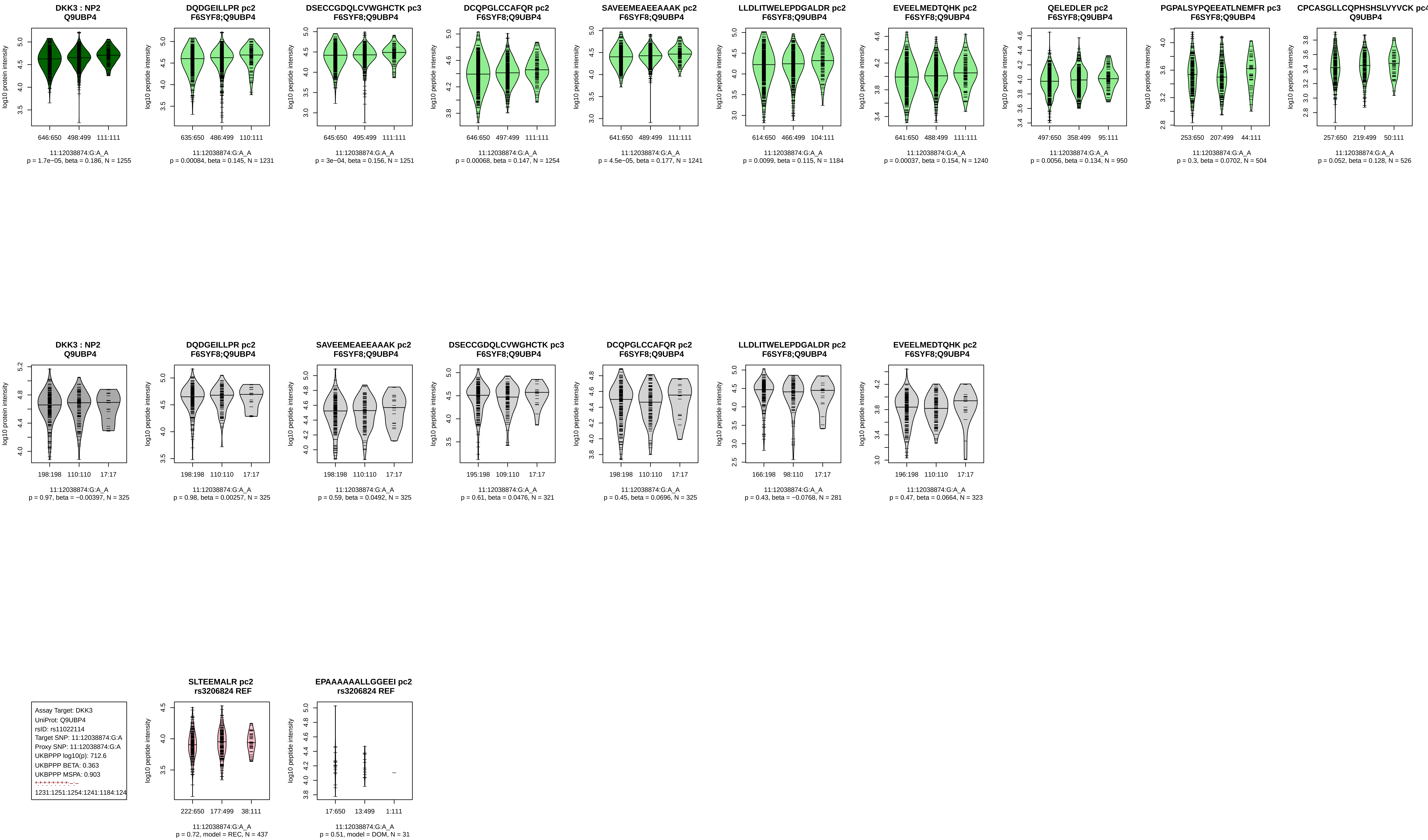
DKK3 : NP2
Q9UBP4
646:650 498:499 111:111
11:12038874:G:A_A
p = 1.7e−05, beta = 0.186, N = 1255
DQDGEILLPR pc2
F6SYF8;Q9UBP4
635:650 486:499 110:111
11:12038874:G:A_A
p = 0.00084, beta = 0.145, N = 1231
DSECCGDQLCVWGHCTK pc3
F6SYF8;Q9UBP4
645:650 495:499 111:111
11:12038874:G:A_A
p = 3e−04, beta = 0.156, N = 1251
DCQPGLCCAFQR pc2
F6SYF8;Q9UBP4
646:650 497:499 111:111
11:12038874:G:A_A
p = 0.00068, beta = 0.147, N = 1254
SAVEEMEAEEAAAK pc2
F6SYF8;Q9UBP4
641:650 489:499 111:111
11:12038874:G:A_A
p = 4.5e−05, beta = 0.177, N = 1241
LLDLITWELEPDGALDR pc2
F6SYF8;Q9UBP4
614:650 466:499 104:111
11:12038874:G:A_A
p = 0.0099, beta = 0.115, N = 1184
EVEELMEDTQHK pc2
F6SYF8;Q9UBP4
641:650 488:499 111:111
11:12038874:G:A_A
p = 0.00037, beta = 0.154, N = 1240
QELEDLER pc2
F6SYF8;Q9UBP4
497:650 358:499 95:111
11:12038874:G:A_A
p = 0.0056, beta = 0.134, N = 950
PGPALSYPQEEATLNEMFR pc3
F6SYF8;Q9UBP4
253:650 207:499 44:111
11:12038874:G:A_A
p = 0.3, beta = 0.0702, N = 504
CPCASGLLCQPHSHSLVYVCK pc4
Q9UBP4
257:650 219:499 50:111
11:12038874:G:A_A
p = 0.052, beta = 0.128, N = 526
DKK3 : NP2
Q9UBP4
198:198 110:110 17:17
11:12038874:G:A_A
p = 0.97, beta = −0.00397, N = 325
DQDGEILLPR pc2
F6SYF8;Q9UBP4
198:198 110:110 17:17
11:12038874:G:A_A
p = 0.98, beta = 0.00257, N = 325
SAVEEMEAEEAAAK pc2
F6SYF8;Q9UBP4
198:198 110:110 17:17
11:12038874:G:A_A
p = 0.59, beta = 0.0492, N = 325
DSECCGDQLCVWGHCTK pc3
F6SYF8;Q9UBP4
195:198 109:110 17:17
11:12038874:G:A_A
p = 0.61, beta = 0.0476, N = 321
DCQPGLCCAFQR pc2
F6SYF8;Q9UBP4
198:198 110:110 17:17
11:12038874:G:A_A
p = 0.45, beta = 0.0696, N = 325
LLDLITWELEPDGALDR pc2
F6SYF8;Q9UBP4
166:198 98:110 17:17
11:12038874:G:A_A
p = 0.43, beta = −0.0768, N = 281
EVEELMEDTQHK pc2
F6SYF8;Q9UBP4
196:198 110:110 17:17
11:12038874:G:A_A
p = 0.47, beta = 0.0664, N = 323
Assay Target: DKK3
UniProt: Q9UBP4
rsID: rs11022114
Target SNP: 11:12038874:G:A
Proxy SNP: 11:12038874:G:A
UKBPPP log10(p): 712.6
UKBPPP BETA: 0.363
UKBPPP MSPA: 0.903
1231:1251:1254:1241:1184:124
SLTEEMALR pc2
rs3206824 REF
222:650 177:499 38:111
11:12038874:G:A_A
p = 0.72, model = REC, N = 437
EPAAAAAALLGGEEI pc2
rs3206824 REF
17:650 13:499 1:111
11:12038874:G:A_A
p = 0.51, model = DOM, N = 31

118. DPT (Q07507)
UKBPPP MSPA=0.652

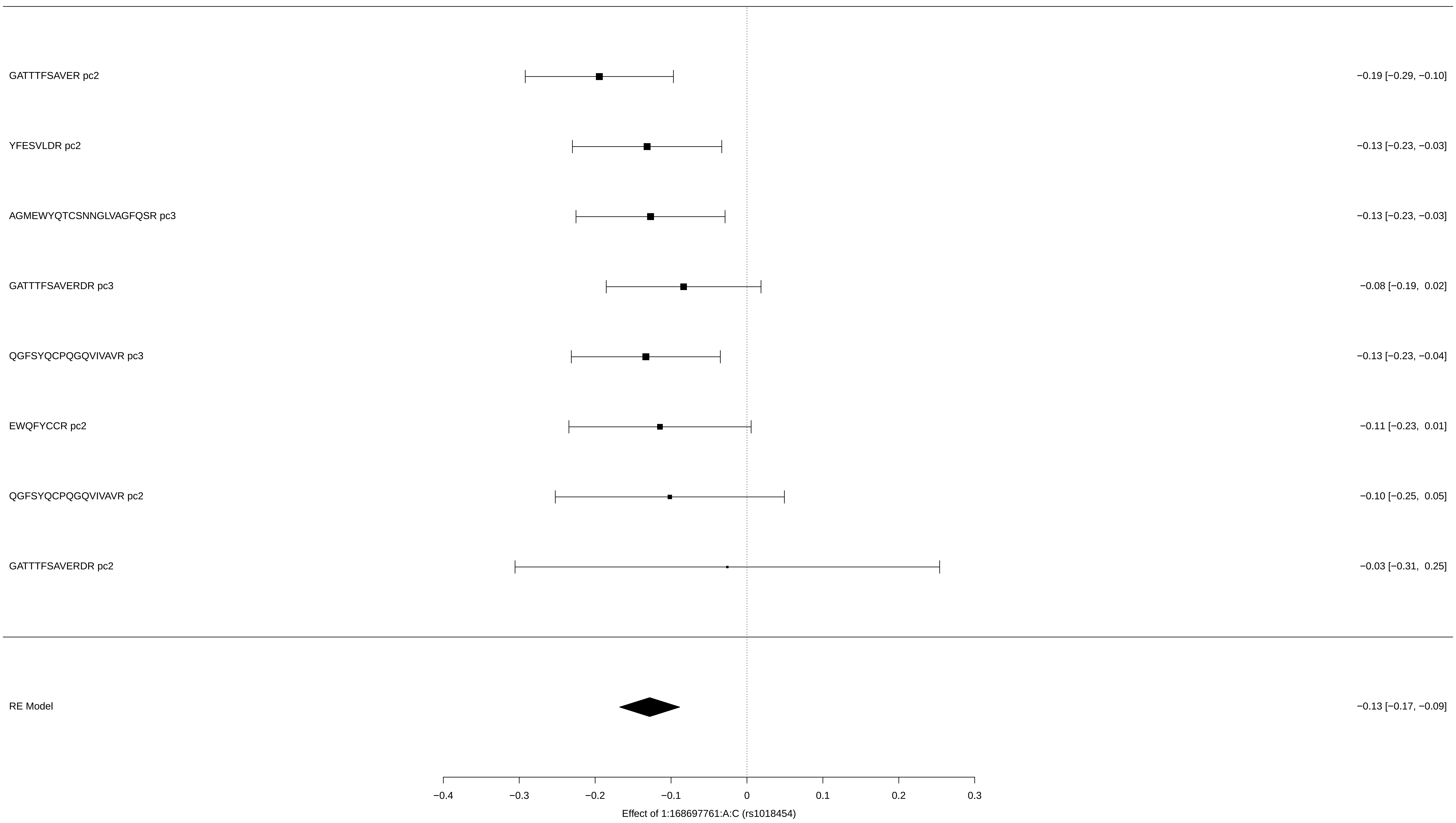

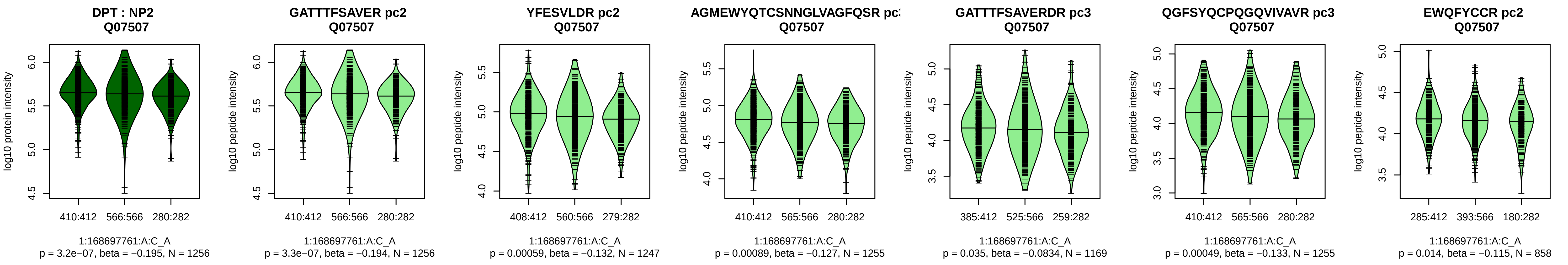

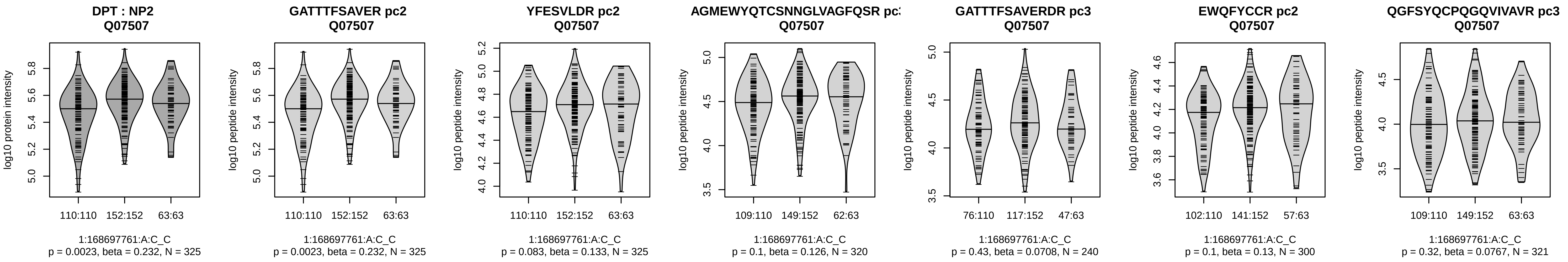

Assay Target: DPT
UniProt: Q07507
rsID: rs1018454
Target SNP: 1:168697761:A:C
Proxy SNP: 1:168697761:A:C
UKBPPP log10(p): 710.9
UKBPPP BETA: 0.323
UKBPPP MSPA: 0.652
::*:–:*:–:–:–
1256:1247:1255:1169:1255:858

119. ORM1 (P02763)
UKBPPP MSPA=0.703

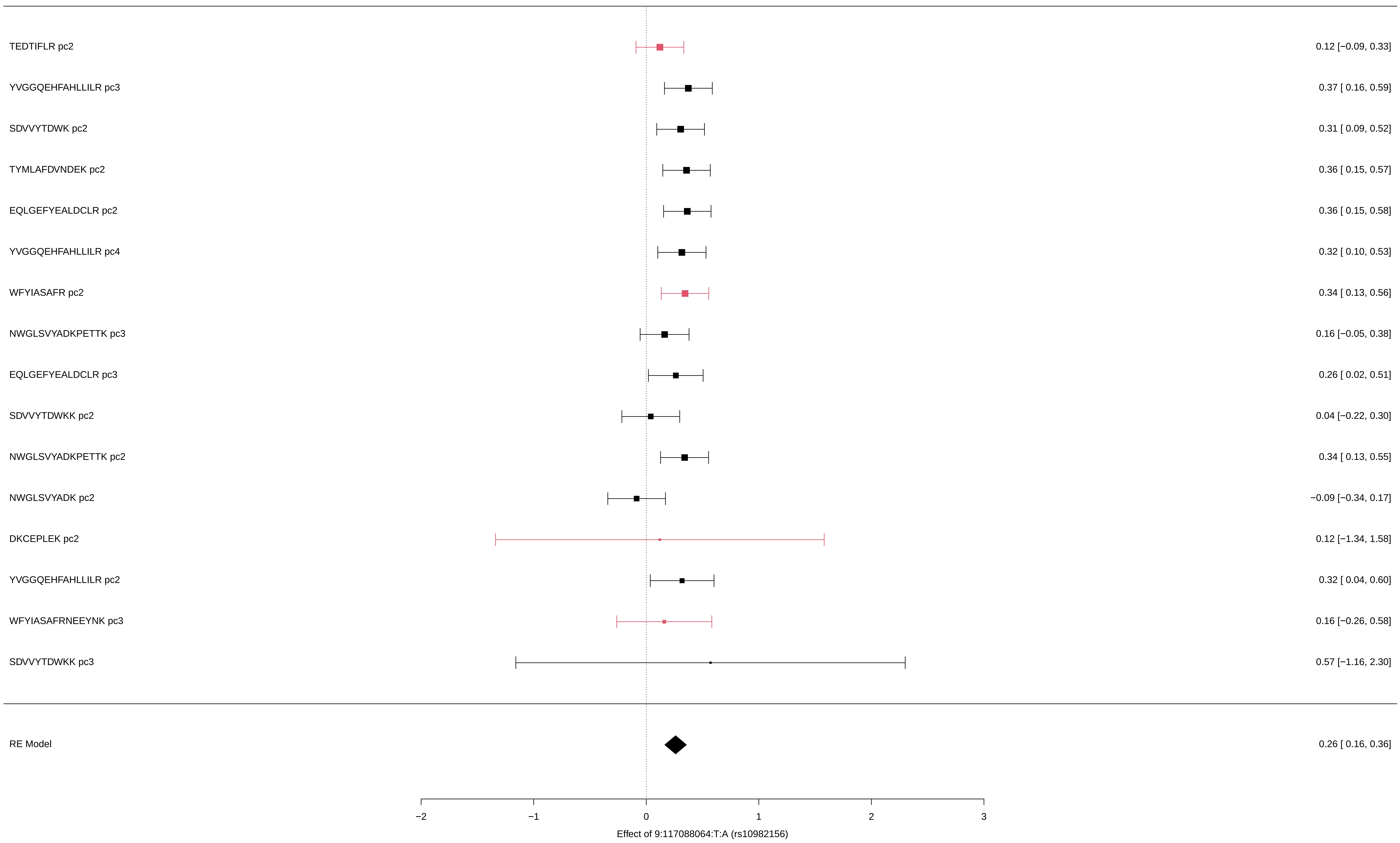

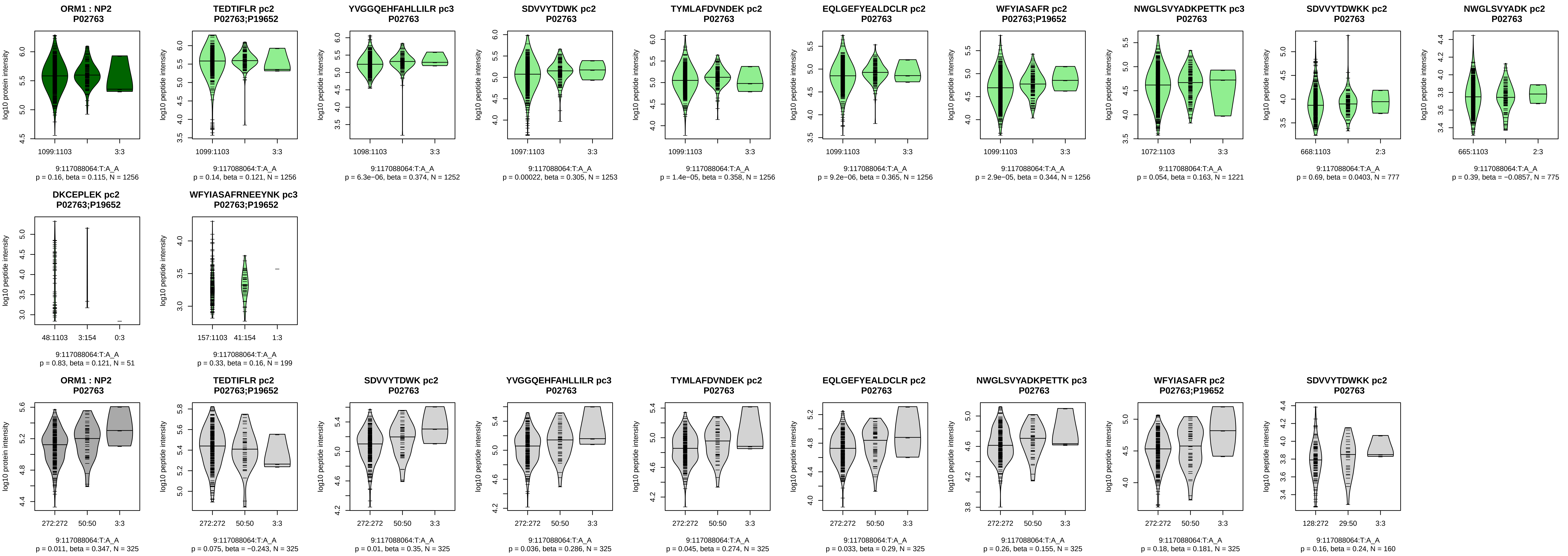

Assay Target: ORM1
UniProt: P02763
rsID: rs10982156
Target SNP: 9:117088064:T:A
Proxy SNP: 9:117088064:T:A
UKBPPP log10(p): 706.3
UKBPPP BETA: 0.721
UKBPPP MSPA: 0.703
–:*:*:*:*:*:*:–:*:–:*:–:–:*:–:–:NA
1256:1252:1253:1256:1256:125

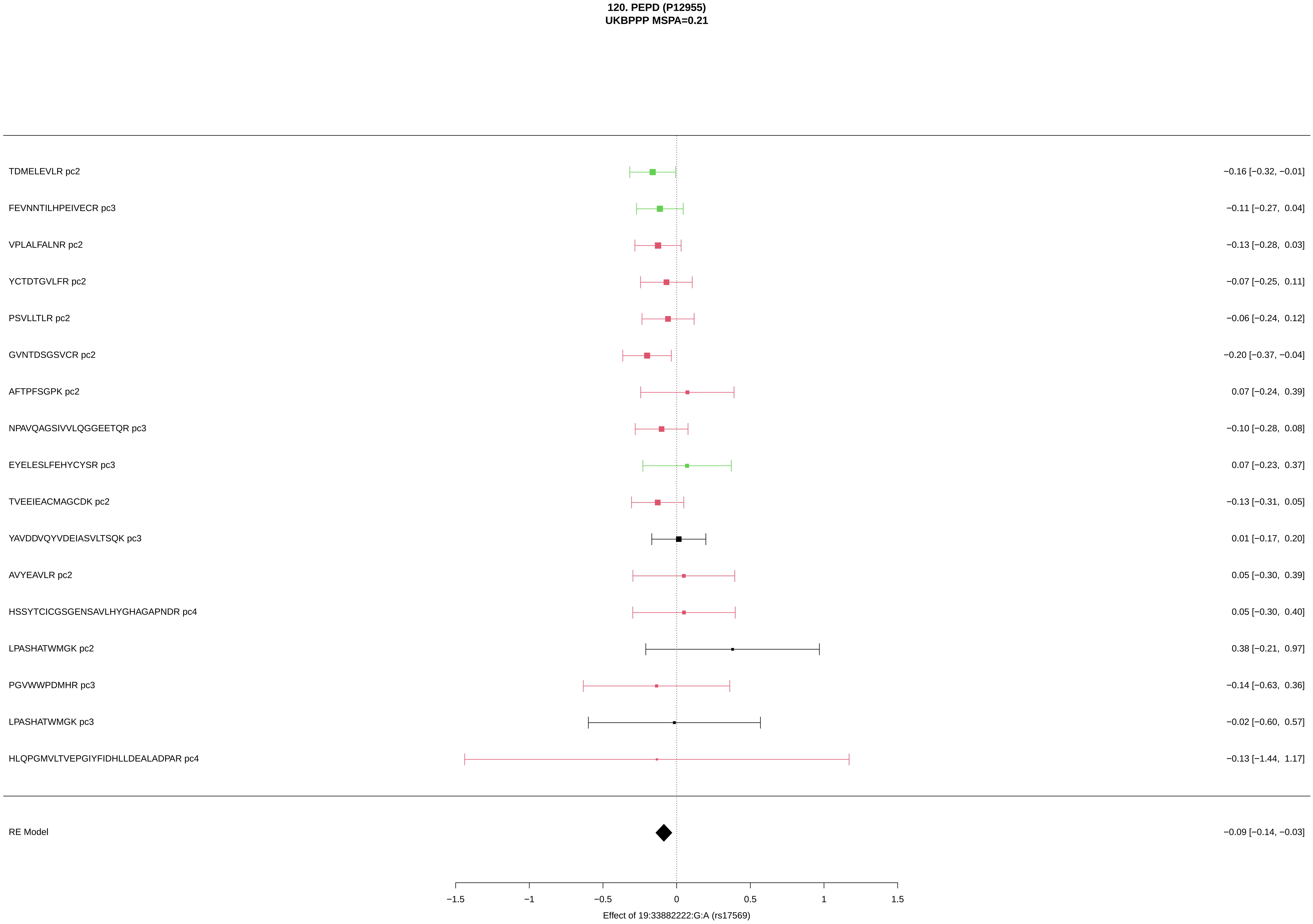

120. PEPD (P12955)
UKBPPP MSPA=0.21
TDMELEVLR pc2
−0.16 [−0.32, −0.01]
FEVNNTILHPEIVECR pc3
−0.11 [−0.27, 0.04]
VPLALFALNR pc2
−0.13 [−0.28, 0.03]
YCTDTGVLFR pc2
−0.07 [−0.25, 0.11]
PSVLLTLR pc2
−0.06 [−0.24, 0.12]
GVNTDSGSVCR pc2
−0.20 [−0.37, −0.04]
AFTPFSGPK pc2
0.07 [−0.24, 0.39]
NPAVQAGSIVVLQGGEETQR pc3
−0.10 [−0.28, 0.08]
EYELESLFEHYCYSR pc3
0.07 [−0.23, 0.37]
TVEEIEACMAGCDK pc2
−0.13 [−0.31, 0.05]
YAVDDVQYVDEIASVLTSQK pc3
0.01 [−0.17, 0.20]
AVYEAVLR pc2
0.05 [−0.30, 0.39]
HSSYTCICGSGENSAVLHYGHAGAPNDR pc4
0.05 [−0.30, 0.40]
LPASHATWMGK pc2
0.38 [−0.21, 0.97]
PGVWWPDMHR pc3
−0.14 [−0.63, 0.36]
LPASHATWMGK pc3
−0.02 [−0.60, 0.57]
HLQPGMVLTVEPGIYFIDHLLDEALADPAR pc4
−0.13 [−1.44, 1.17]
RE Model
−0.09 [−0.14, −0.03]
−1.5
−1
−0.5
0
0.5
1
1.5
Effect of 19:33882222:G:A (rs17569)

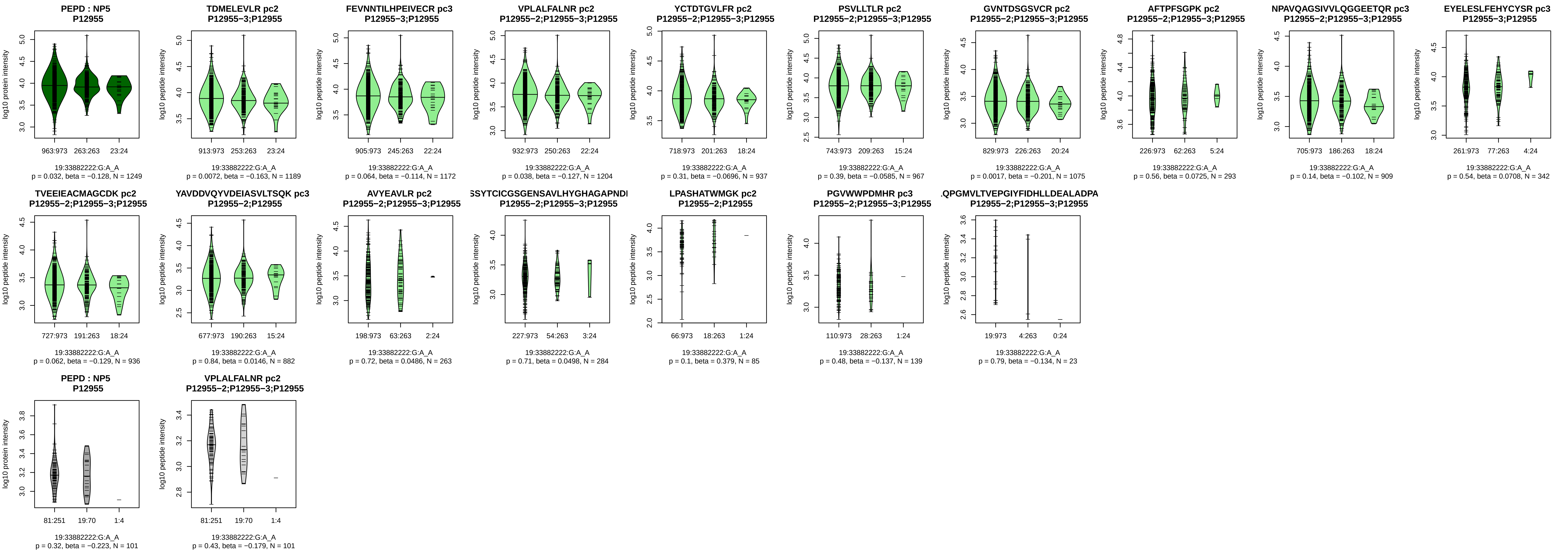

Assay Target: PEPD
UniProt: P12955
rsID: rs17569
Target SNP: 19:33882222:G:A
Proxy SNP: 19:33882222:G:A
UKBPPP log10(p): 697.8
UKBPPP BETA: −0.478
UKBPPP MSPA: 0.21
−:−:−:−:−:−:−:−:−:−:−:−:−:−:−
1189:1172:1204:937:967:1075:2

121. C1QA (P02745)
UKBPPP MSPA=0

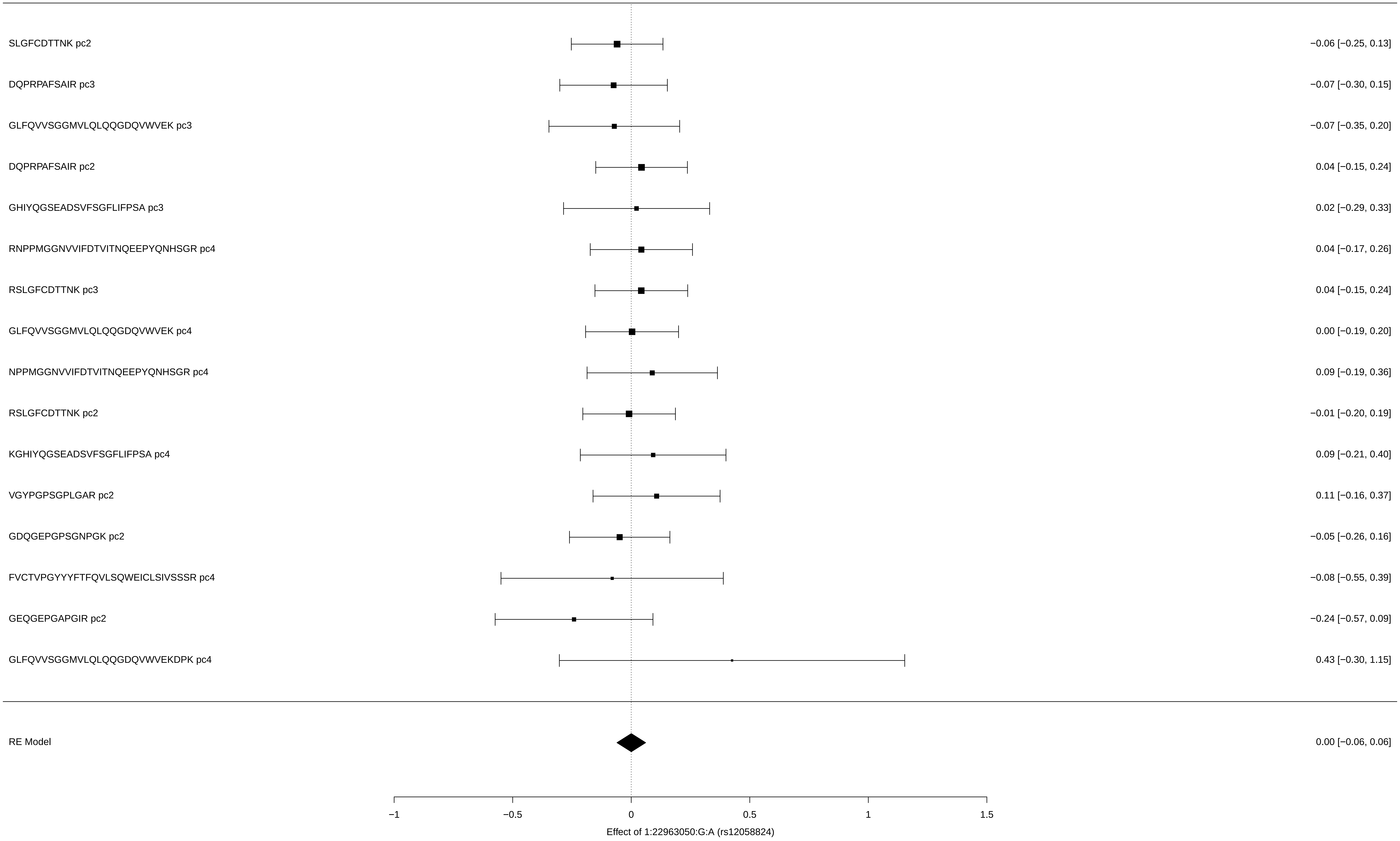

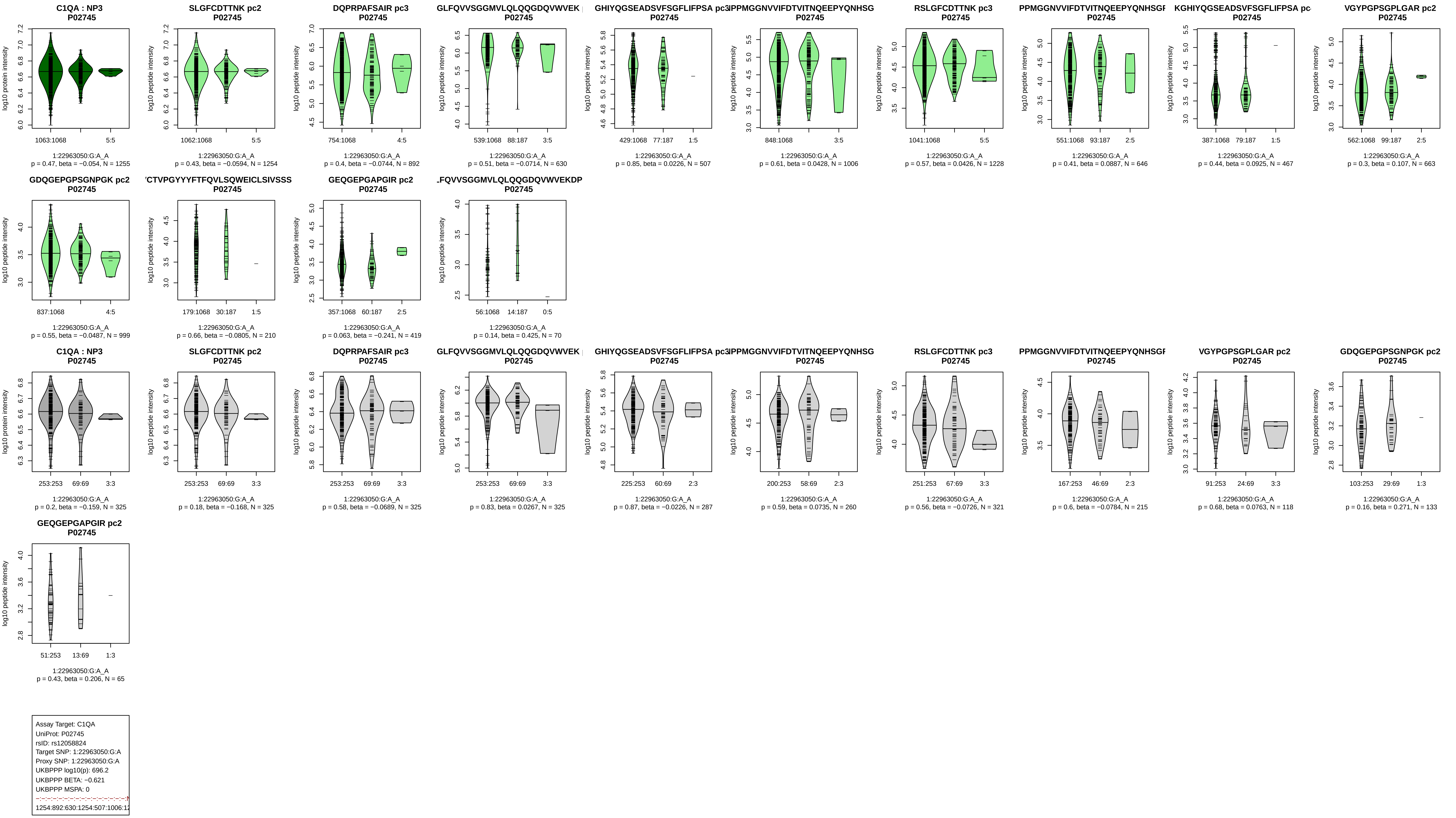
C1QA : NP3
P02745
1063:1068 5:5
1:22963050:G:A_A
p = 0.47, beta = −0.054, N = 1255
SLGFCDTTNK pc2
P02745
1062:1068 5:5
1:22963050:G:A_A
p = 0.43, beta = −0.0594, N = 1254
DQPRPAFSAIR pc3
P02745
754:1068 4:5
1:22963050:G:A_A
p = 0.4, beta = −0.0744, N = 892
GLFQVVSGGMVLQLQQGDQVWVEK
P02745
539:1068 88:187 3:5
1:22963050:G:A_A
p = 0.51, beta = −0.0714, N = 630
GHIYQGSEADSVFSGFLIFPSA pc3
P02745
429:1068 77:187 1:5
1:22963050:G:A_A
p = 0.85, beta = 0.0226, N = 507
IPPMGGNVVIFDTVITNQEEPYQNHSG
P02745
848:1068 3:5
1:22963050:G:A_A
p = 0.61, beta = 0.0428, N = 1006
RSLGFCDTTNK pc3
P02745
1041:1068 5:5
1:22963050:G:A_A
p = 0.57, beta = 0.0426, N = 1228
PPMGGNVVIFDTVITNQEEPYQNHSGF
P02745
551:1068 93:187 2:5
1:22963050:G:A_A
p = 0.41, beta = 0.0887, N = 646
KGHIYQGSEADSVFSGFLIFPSA pc
P02745
387:1068 79:187 1:5
1:22963050:G:A_A
p = 0.44, beta = 0.0925, N = 467
VGYPGPSGPLGAR pc2
P02745
562:1068 99:187 2:5
1:22963050:G:A_A
p = 0.3, beta = 0.107, N = 663
GDQGEPGPSGNPGK pc2
P02745
837:1068 4:5
1:22963050:G:A_A
p = 0.55, beta = −0.0487, N = 999
CTVPGYYYFTFQVLSQWEICLSIVSSS
P02745
179:1068 30:187 1:5
1:22963050:G:A_A
p = 0.66, beta = −0.0805, N = 210
GEQGEPGAPGIR pc2
P02745
357:1068 60:187 2:5
1:22963050:G:A_A
p = 0.063, beta = −0.241, N = 419
LFQVVSGGMVLQLQQGDQVWVEKDP
P02745
56:1068 14:187 0:5
1:22963050:G:A_A
p = 0.14, beta = 0.425, N = 70
C1QA : NP3
P02745
253:253 69:69 3:3
1:22963050:G:A_A
p = 0.2, beta = −0.159, N = 325
SLGFCDTTNK pc2
P02745
253:253 69:69 3:3
1:22963050:G:A_A
p = 0.18, beta = −0.168, N = 325
DQPRPAFSAIR pc3
P02745
253:253 69:69 3:3
1:22963050:G:A_A
p = 0.58, beta = −0.0689, N = 325
GLFQVVSGGMVLQLQQGDQVWVEK
P02745
253:253 69:69 3:3
1:22963050:G:A_A
p = 0.83, beta = 0.0267, N = 325
GHIYQGSEADSVFSGFLIFPSA pc3
P02745
225:253 60:69 2:3
1:22963050:G:A_A
p = 0.87, beta = −0.0226, N = 287
IPPMGGNVVIFDTVITNQEEPYQNHSG
P02745
200:253 58:69 2:3
1:22963050:G:A_A
p = 0.59, beta = 0.0735, N = 260
RSLGFCDTTNK pc3
P02745
251:253 67:69 3:3
1:22963050:G:A_A
p = 0.56, beta = −0.0726, N = 321
PPMGGNVVIFDTVITNQEEPYQNHSGF
P02745
167:253 46:69 2:3
1:22963050:G:A_A
p = 0.6, beta = −0.0784, N = 215
VGYPGPSGPLGAR pc2
P02745
91:253 24:69 3:3
1:22963050:G:A_A
p = 0.68, beta = 0.0763, N = 118
GDQGEPGPSGNPGK pc2
P02745
103:253 29:69 1:3
1:22963050:G:A_A
p = 0.16, beta = 0.271, N = 133
GEQGEPGAPGIR pc2
P02745
51:253 13:69 1:3
1:22963050:G:A_A
p = 0.43, beta = 0.206, N = 65
log10 protein intensity
log10 peptide intensity
Assay Target: C1QA
UniProt: P02745
rsID: rs12058824
Target SNP: 1:22963050:G:A
Proxy SNP: 1:22963050:G:A
UKBPPP log10(p): 696.2
UKBPPP BETA: −0.621
UKBPPP MSPA: 0
1254:892:630:1254:507:1006:1

122. PDCD5 (O14737)
UKBPPP MSPA=0.866

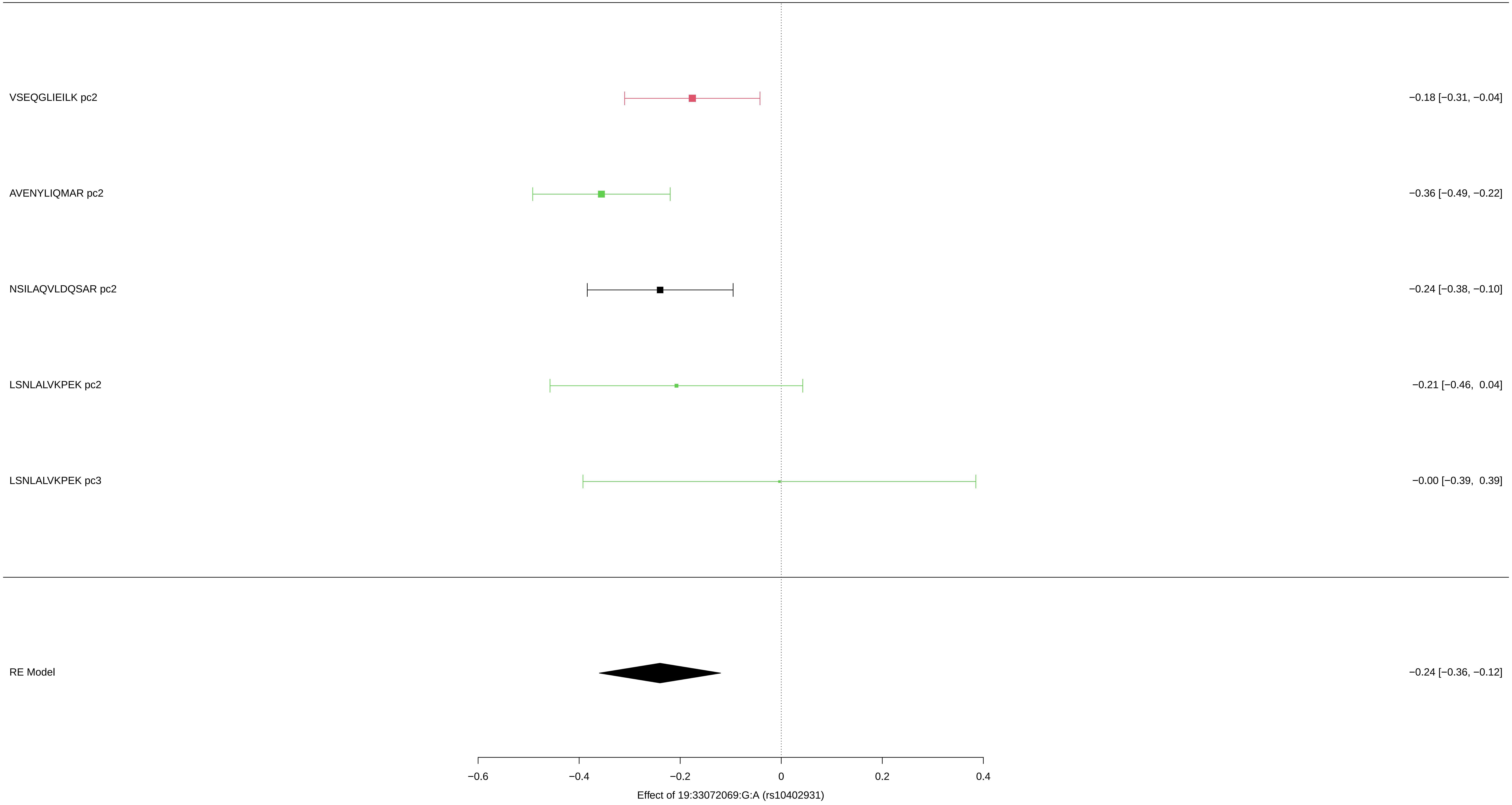

PDCD5 : NP2
O14737

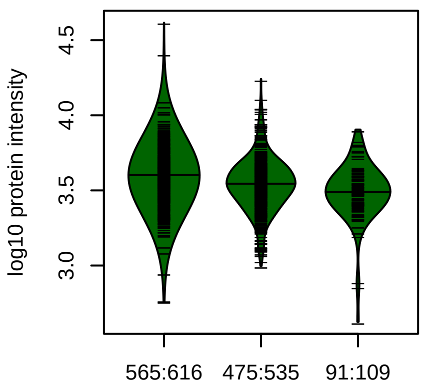

19:33072069:G:A_G
p = 1.4e−08, beta = −0.262, N = 1131

VSEQGLIEILK pc2
K7EQA1;O14737

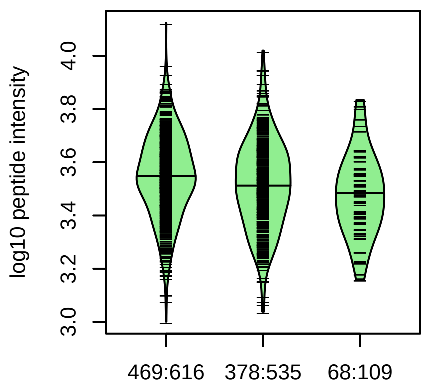

19:33072069:G:A_G
p = 0.00074, beta = −0.176, N = 915

AVENYLIQMAR pc2
K7EQA1;O14737;O14737−2

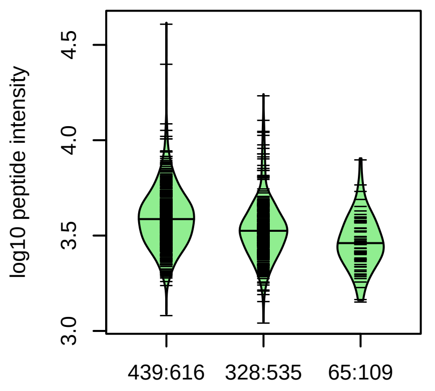

19:33072069:G:A_G
p = 3e−11, beta = −0.356, N = 832

NSILAQVLDQSAR pc2
K7EQA1;K7ESJ4;O14737;O14737-

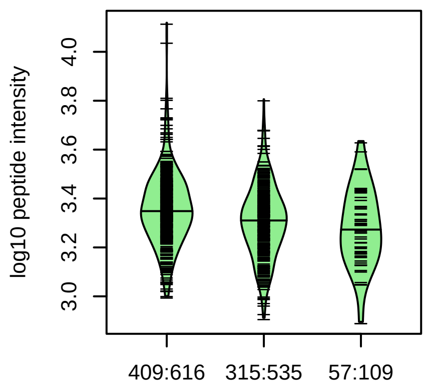

19:33072069:G:A_G
p = 2.1e−05, beta = −0.24, N = 781

LSNLALVKPEK pc2
K7EQA1;O14737;O14737−2

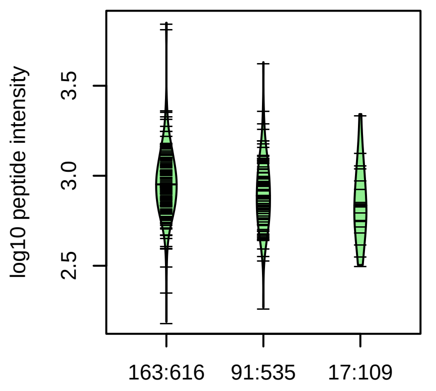

19:33072069:G:A_G
p = 0.034, beta = −0.208, N = 271

PDCD5 : NP2
O14737

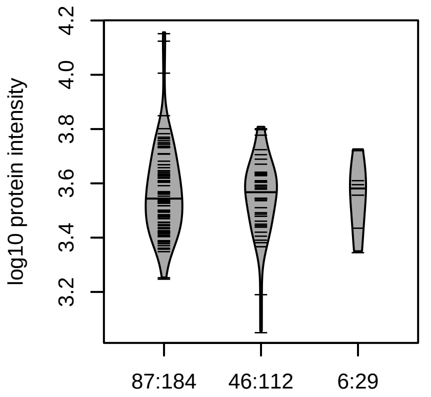

19:33072069:G:A_G
p = 0.64, beta = −0.0681, N = 139

AVENYLIQMAR pc2
K7EQA1;O14737;O14737−2

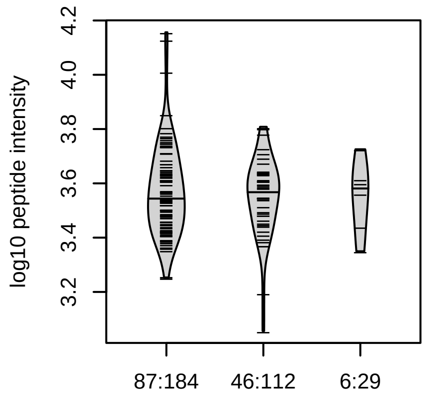

19:33072069:G:A_G
p = 0.64, beta = −0.0681, N = 139

Assay Target: PDCD5
UniProt: O14737
rsID: rs10402931
Target SNP: 19:33072069:G:A
Proxy SNP: 19:33072069:G:A
UKBPPP log10(p): 691.9
UKBPPP BETA: 0.376
UKBPPP MSPA: 0.866
::*:−:−:NA:NA
915:832:781:271:121:2:1

123. IGFBP7 (Q16270)
UKBPPP MSPA=0.458

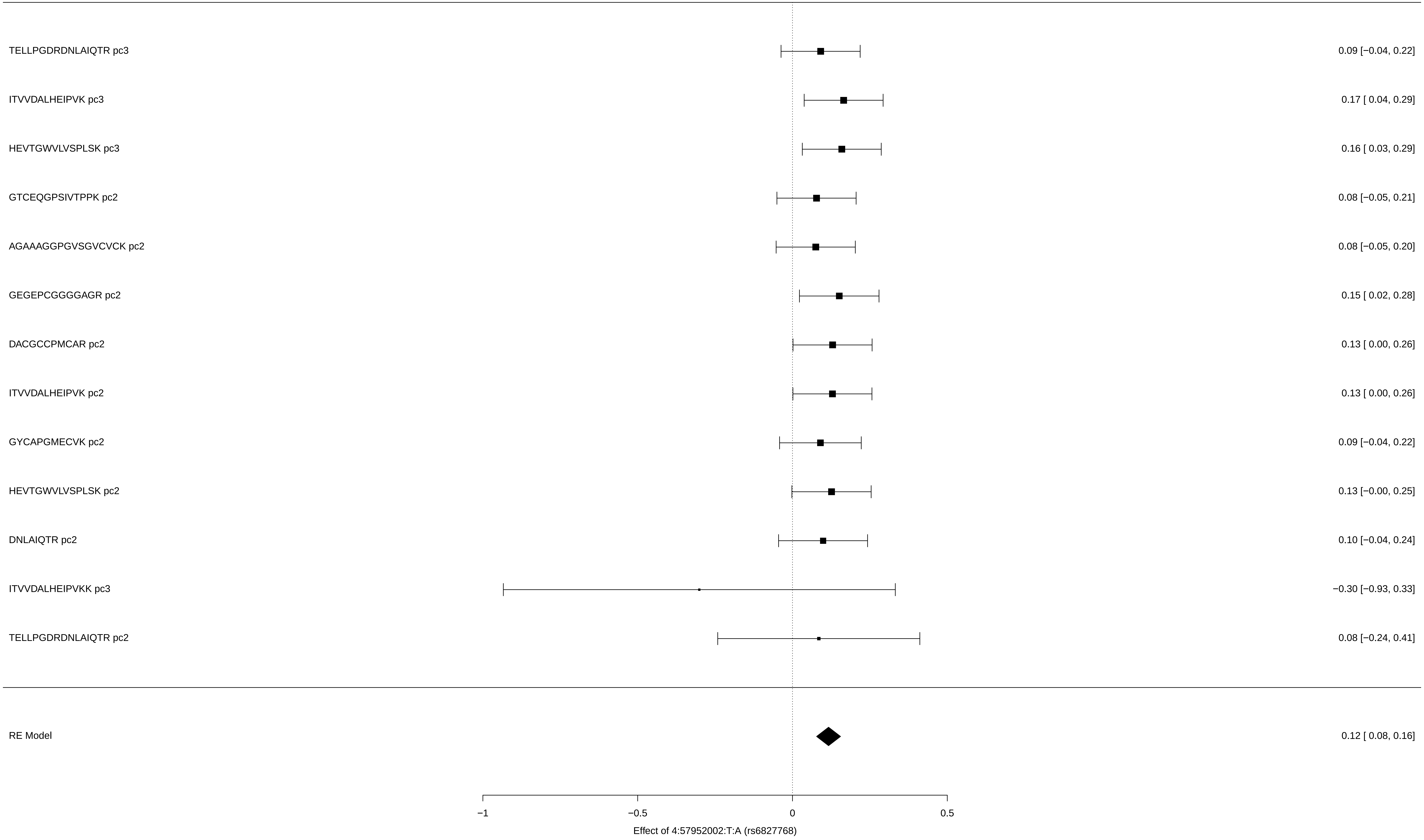

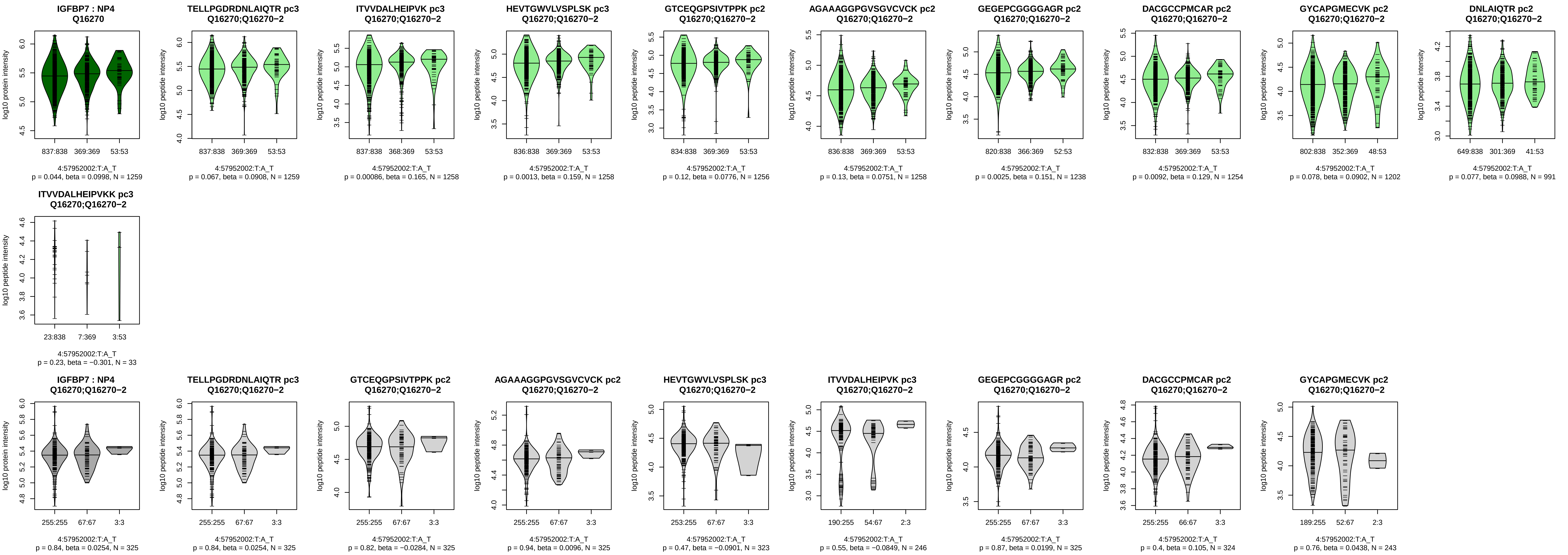

Assay Target: IGFBP7
UniProt: Q16270
rsID: rs6827768
Target SNP: 4:57952002:T:A
Proxy SNP: 4:57952002:T:A
UKBPPP log10(p): 684.7
UKBPPP BETA: −0.393
UKBPPP MSPA: 0.458
−:*:*:−:−:*:*:*:−:−:−:−:−:NA
1259:1258:1258:1256:1258:123

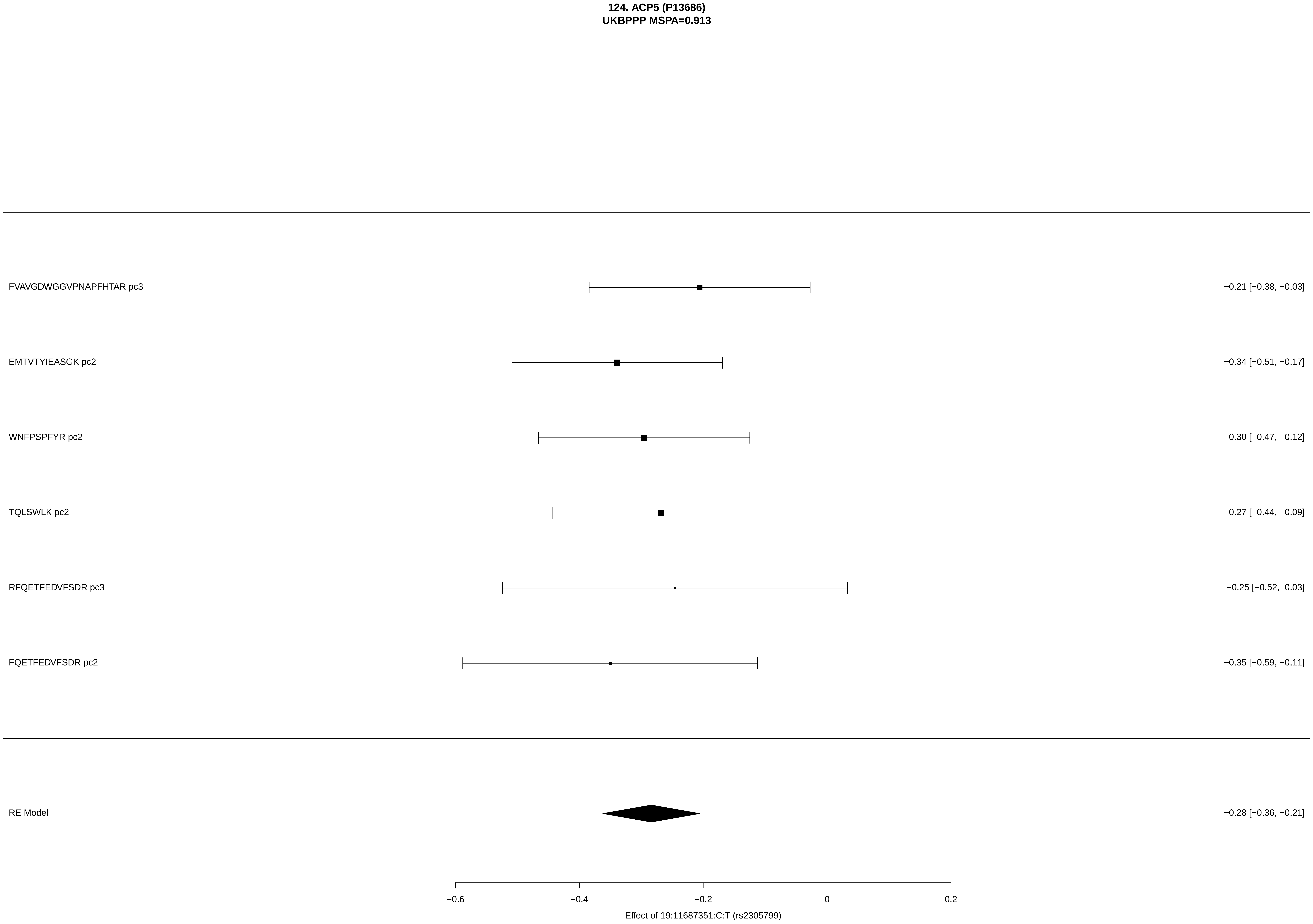
124. ACP5 (P13686)
UKBPPP MSPA=0.913
FVAVGDWGGVPNAPFHTAR pc3
−0.21 [−0.38, −0.03]
EMTVTYIEASGK pc2
−0.34 [−0.51, −0.17]
WNFPSPFYR pc2
−0.30 [−0.47, −0.12]
TQLSWLK pc2
−0.27 [−0.44, −0.09]
RFQETFEDVFSDR pc3
−0.25 [−0.52, 0.03]
FQETFEDVFSDR pc2
−0.35 [−0.59, −0.11]
RE Model
−0.28 [−0.36, −0.21]
−0.6
−0.4
−0.2
0
0.2
Effect of 19:11687351:C:T (rs2305799)

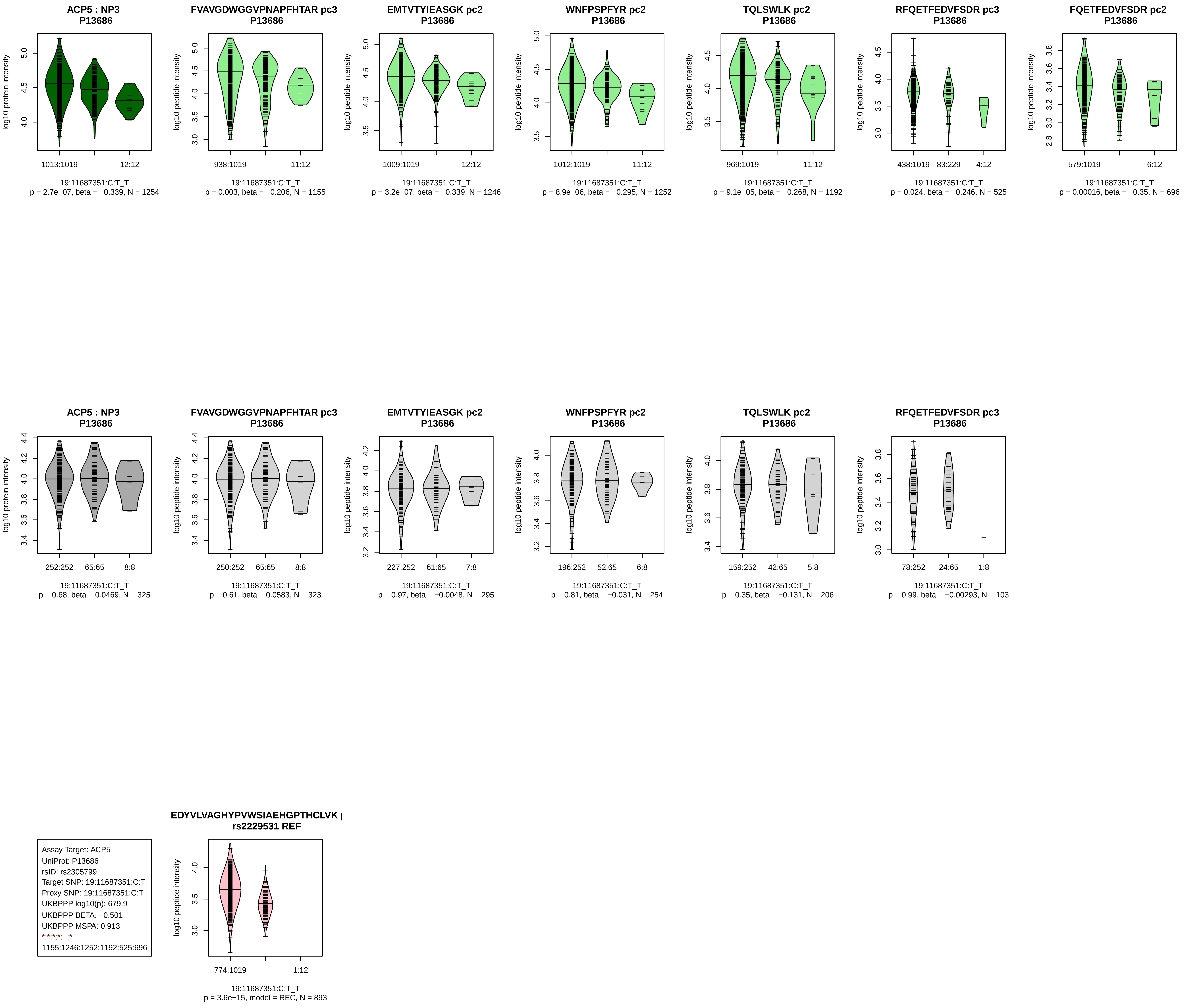
ACP5 : NP3
P13686
log10 protein intensity
1013:1019
12:12
19:11687351:C:T_T
p = 2.7e−07, beta = −0.339, N = 1254
FVAVGDWGGVPNAPFHTAR pc3
P13686
log10 peptide intensity
938:1019
11:12
19:11687351:C:T_T
p = 0.003, beta = −0.206, N = 1155
EMTVTYIEASGK pc2
P13686
1009:1019
12:12
19:11687351:C:T_T
p = 3.2e−07, beta = −0.339, N = 1246
WNFPSPFYR pc2
P13686
1012:1019
11:12
19:11687351:C:T_T
p = 8.9e−06, beta = −0.295, N = 1252
TQLSWLK pc2
P13686
969:1019
11:12
19:11687351:C:T_T
p = 9.1e−05, beta = −0.268, N = 1192
RFQETFEDVFSDR pc3
P13686
438:1019
83:229
4:12
19:11687351:C:T_T
p = 0.024, beta = −0.246, N = 525
FQETFEDVFSDR pc2
P13686
579:1019
6:12
19:11687351:C:T_T
p = 0.00016, beta = −0.35, N = 696
ACP5 : NP3
P13686
252:252
65:65
8:8
19:11687351:C:T_T
p = 0.68, beta = 0.0469, N = 325
FVAVGDWGGVPNAPFHTAR pc3
P13686
250:252
65:65
8:8
19:11687351:C:T_T
p = 0.61, beta = 0.0583, N = 323
EMTVTYIEASGK pc2
P13686
227:252
61:65
7:8
19:11687351:C:T_T
p = 0.97, beta = −0.0048, N = 295
WNFPSPFYR pc2
P13686
196:252
52:65
6:8
19:11687351:C:T_T
p = 0.81, beta = −0.031, N = 254
TQLSWLK pc2
P13686
159:252
42:65
5:8
19:11687351:C:T_T
p = 0.35, beta = −0.131, N = 206
RFQETFEDVFSDR pc3
P13686
78:252
24:65
1:8
19:11687351:C:T_T
p = 0.99, beta = −0.00293, N = 103
Assay Target: ACP5
UniProt: P13686
rsID: rs2305799
Target SNP: 19:11687351:C:T
Proxy SNP: 19:11687351:C:T
UKBPPP log10(p): 679.9
UKBPPP BETA: −0.501
UKBPPP MSPA: 0.913
::*:*:–:*
1155:1246:1252:1192:525:696
EDYVLVAGHYPVWSIAEHGPTHCLVK
rs2229531 REF
774:1019
1:12
19:11687351:C:T_T
p = 3.6e−15, model = REC, N = 893

125. PTPRB (P23467)
UKBPPP MSPA=0.041

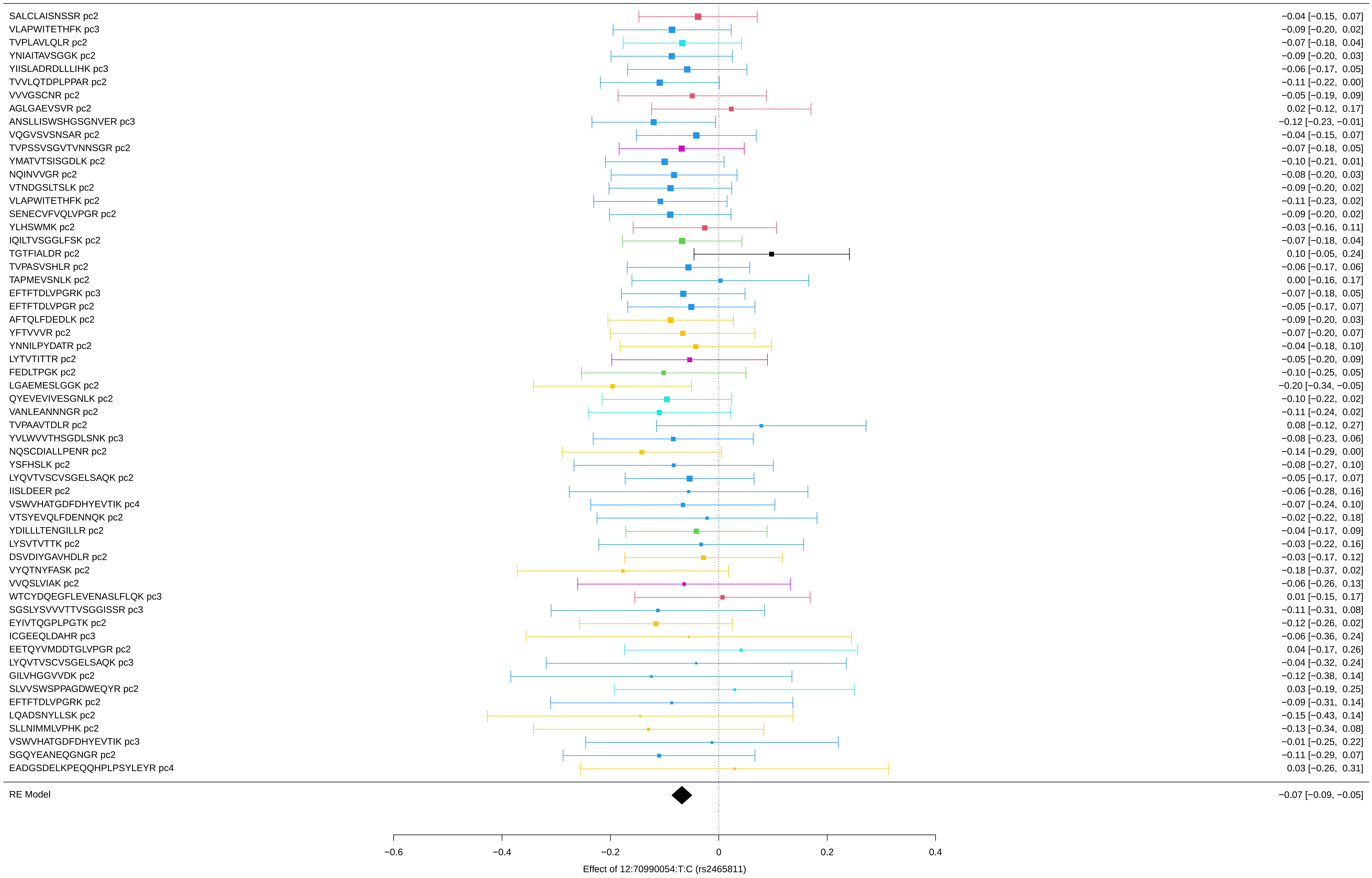

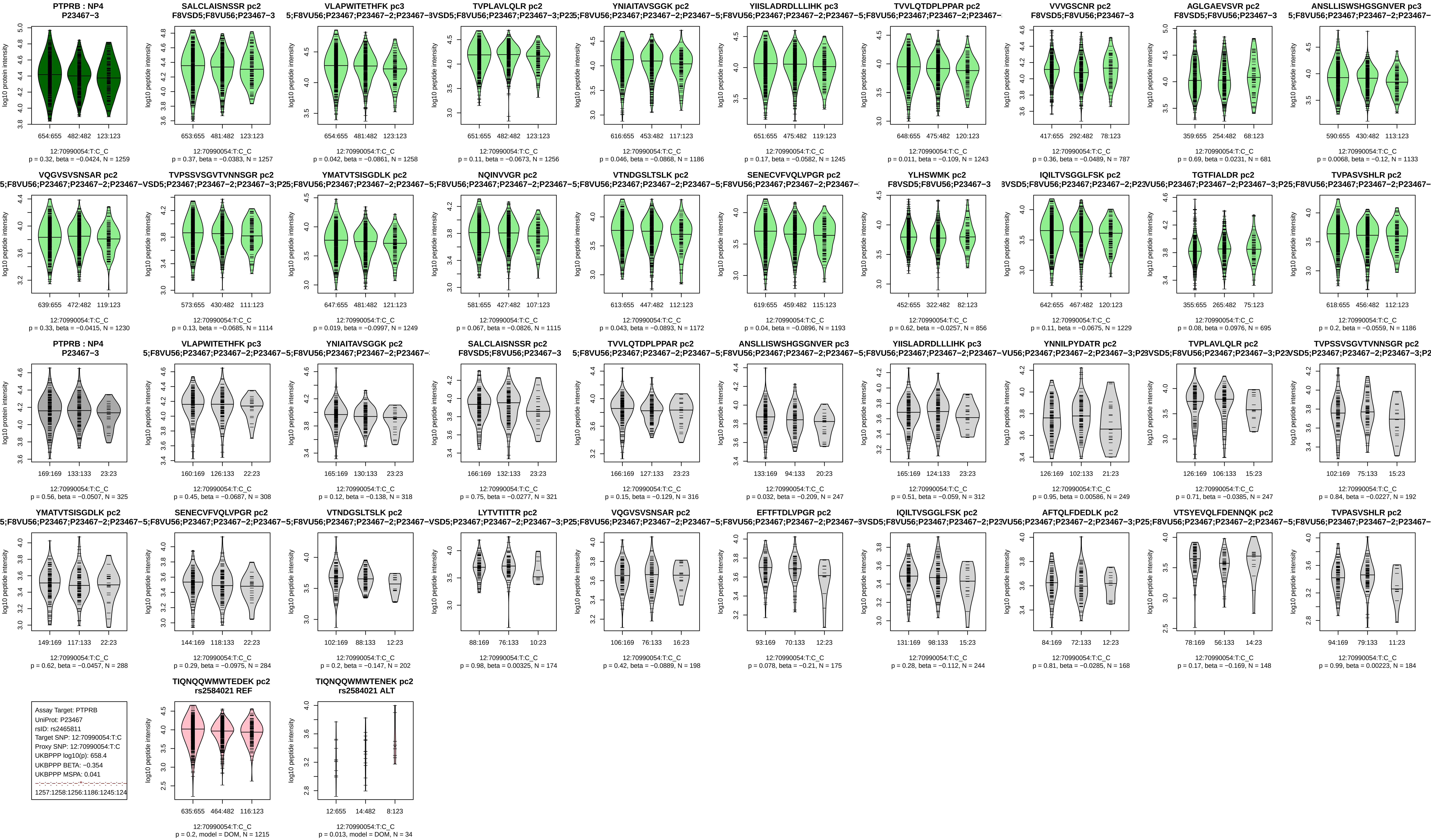
PTPRB : NP4
P23467−3
p = 0.32, beta = −0.0424, N = 1259
SALCLAISNSSR pc2
F8VSD5;F8VU56;P23467−3
p = 0.37, beta = −0.0383, N = 1257
VLAPWITETHFK pc3
p = 0.042, beta = −0.0861, N = 1258
TVPLAVLQLR pc2
p = 0.11, beta = −0.0673, N = 1256
YNIAITAVSGGK pc2
p = 0.046, beta = −0.0868, N = 1186
YIISLADRDLLLIHK pc3
p = 0.17, beta = −0.0582, N = 1245
TVVLQTDPLPPAR pc2
p = 0.011, beta = −0.109, N = 1243
VVVGSCNR pc2
F8VSD5;F8VU56;P23467−3
p = 0.36, beta = −0.0489, N = 787
AGLGAEVSVR pc2
F8VSD5;F8VU56;P23467−3
p = 0.69, beta = 0.0231, N = 681
ANSLLISWSHGSGNVER pc3
p = 0.0068, beta = −0.12, N = 1133
VQGVSVSNSAR pc2
p = 0.33, beta = −0.0415, N = 1230
TVPSSVSGVTVNNSGR pc2
p = 0.13, beta = −0.0685, N = 1114
YMATVTSISGDLK pc2
p = 0.019, beta = −0.0997, N = 1249
NQINVVGR pc2
p = 0.067, beta = −0.0826, N = 1115
VTNDGSLTSLK pc2
p = 0.043, beta = −0.0893, N = 1172
SENECVFVQLVPGR pc2
p = 0.04, beta = −0.0896, N = 1193
YLHSWMK pc2
F8VSD5;F8VU56;P23467−3
p = 0.62, beta = −0.0257, N = 856
IQILTVSGGLFSK pc2
p = 0.11, beta = −0.0675, N = 1229
TGTFIALDR pc2
p = 0.08, beta = 0.0976, N = 695
TVPASVSHLR pc2
p = 0.2, beta = −0.0559, N = 1186
PTPRB : NP4
P23467−3
p = 0.56, beta = −0.0507, N = 325
VLAPWITETHFK pc3
p = 0.45, beta = −0.0687, N = 308
YNIAITAVSGGK pc2
p = 0.12, beta = −0.138, N = 318
SALCLAISNSSR pc2
F8VSD5;F8VU56;P23467−3
p = 0.75, beta = −0.0277, N = 321
TVVLQTDPLPPAR pc2
p = 0.15, beta = −0.129, N = 316
ANSLLISWSHGSGNVER pc3
p = 0.032, beta = −0.209, N = 247
YIISLADRDLLLIHK pc3
p = 0.51, beta = −0.059, N = 312
YNNILPYDATR pc2
p = 0.95, beta = 0.00586, N = 249
TVPLAVLQLR pc2
p = 0.71, beta = −0.0385, N = 247
TVPSSVSGVTVNNSGR pc2
p = 0.84, beta = −0.0227, N = 192
YMATVTSISGDLK pc2
p = 0.62, beta = −0.0457, N = 288
SENECVFVQLVPGR pc2
p = 0.29, beta = −0.0975, N = 284
VTNDGSLTSLK pc2
p = 0.2, beta = −0.147, N = 202
LYTVTITTR pc2
p = 0.98, beta = 0.00325, N = 174
VQGVSVSNSAR pc2
p = 0.42, beta = −0.0889, N = 198
EFTFTDLVPGR pc2
p = 0.078, beta = −0.21, N = 175
IQILTVSGGLFSK pc2
p = 0.28, beta = −0.112, N = 244
AFTQLFDEDLK pc2
p = 0.81, beta = −0.0285, N = 168
VTSYEVQLFDENNQK pc2
p = 0.17, beta = −0.169, N = 148
TVPASVSHLR pc2
p = 0.99, beta = 0.00223, N = 184
Assay Target: PTPRB
UniProt: P23467
rsID: rs2465811
Target SNP: 12:70990054:T:C
Proxy SNP: 12:70990054:T:C
UKBPPP log10(p): 658.4
UKBPPP BETA: −0.354
UKBPPP MSPA: 0.041
TIQNQQWMWTEDEK pc2
rs2584021 REF
p = 0.2, model = DOM, N = 1215
TIQNQQWMWTENEK pc2
rs2584021 ALT
p = 0.013, model = DOM, N = 34

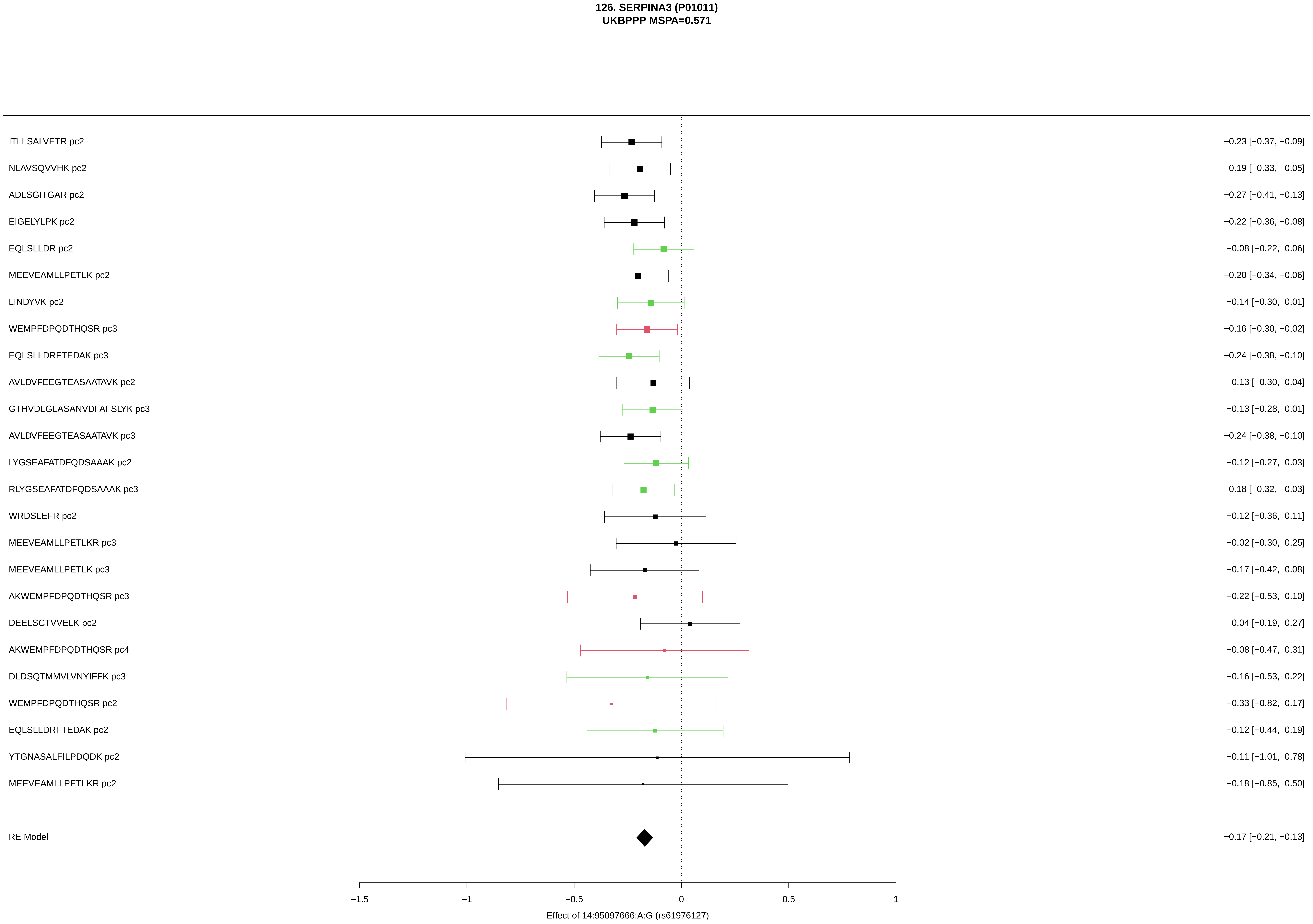
126. SERPINA3 (P01011)
UKBPPP MSPA=0.571
ITLLSALVETR pc2 −0.23 [−0.37, −0.09]
NLAVSQVVHK pc2 −0.19 [−0.33, −0.05]
ADLSGITGAR pc2 −0.27 [−0.41, −0.13]
EIGELYLPK pc2 −0.22 [−0.36, −0.08]
EQLSLLDR pc2 −0.08 [−0.22, 0.06]
MEEVEAMLLPETLK pc2 −0.20 [−0.34, −0.06]
LINDYVK pc2 −0.14 [−0.30, 0.01]
WEMPFDPQDTHQSR pc3 −0.16 [−0.30, −0.02]
EQLSLLDRFTEDAK pc3 −0.24 [−0.38, −0.10]
AVLDVFEEGTEASAATAVK pc2 −0.13 [−0.30, 0.04]
GTHVDLGLASANVDFAFSLYK pc3 −0.13 [−0.28, 0.01]
AVLDVFEEGTEASAATAVK pc3 −0.24 [−0.38, −0.10]
LYGSEAFATDFQDSAAAK pc2 −0.12 [−0.27, 0.03]
RLYGSEAFATDFQDSAAAK pc3 −0.18 [−0.32, −0.03]
WRDSLEFR pc2 −0.12 [−0.36, 0.11]
MEEVEAMLLPETLKR pc3 −0.02 [−0.30, 0.25]
MEEVEAMLLPETLK pc3 −0.17 [−0.42, 0.08]
AKWEMPFDPQDTHQSR pc3 −0.22 [−0.53, 0.10]
DEELSCTVVELK pc2 0.04 [−0.19, 0.27]
AKWEMPFDPQDTHQSR pc4 −0.08 [−0.47, 0.31]
DLDSQTMMVLVNYIFFK pc3 −0.16 [−0.53, 0.22]
WEMPFDPQDTHQSR pc2 −0.33 [−0.82, 0.17]
EQLSLLDRFTEDAK pc2 −0.12 [−0.44, 0.19]
YTGNASALFILPDQDK pc2 −0.11 [−1.01, 0.78]
MEEVEAMLLPETLKR pc2 −0.18 [−0.85, 0.50]
RE Model −0.17 [−0.21, −0.13]
−1.5
−1
−0.5
0
0.5
1
Effect of 14:95097666:A:G (rs61976127)

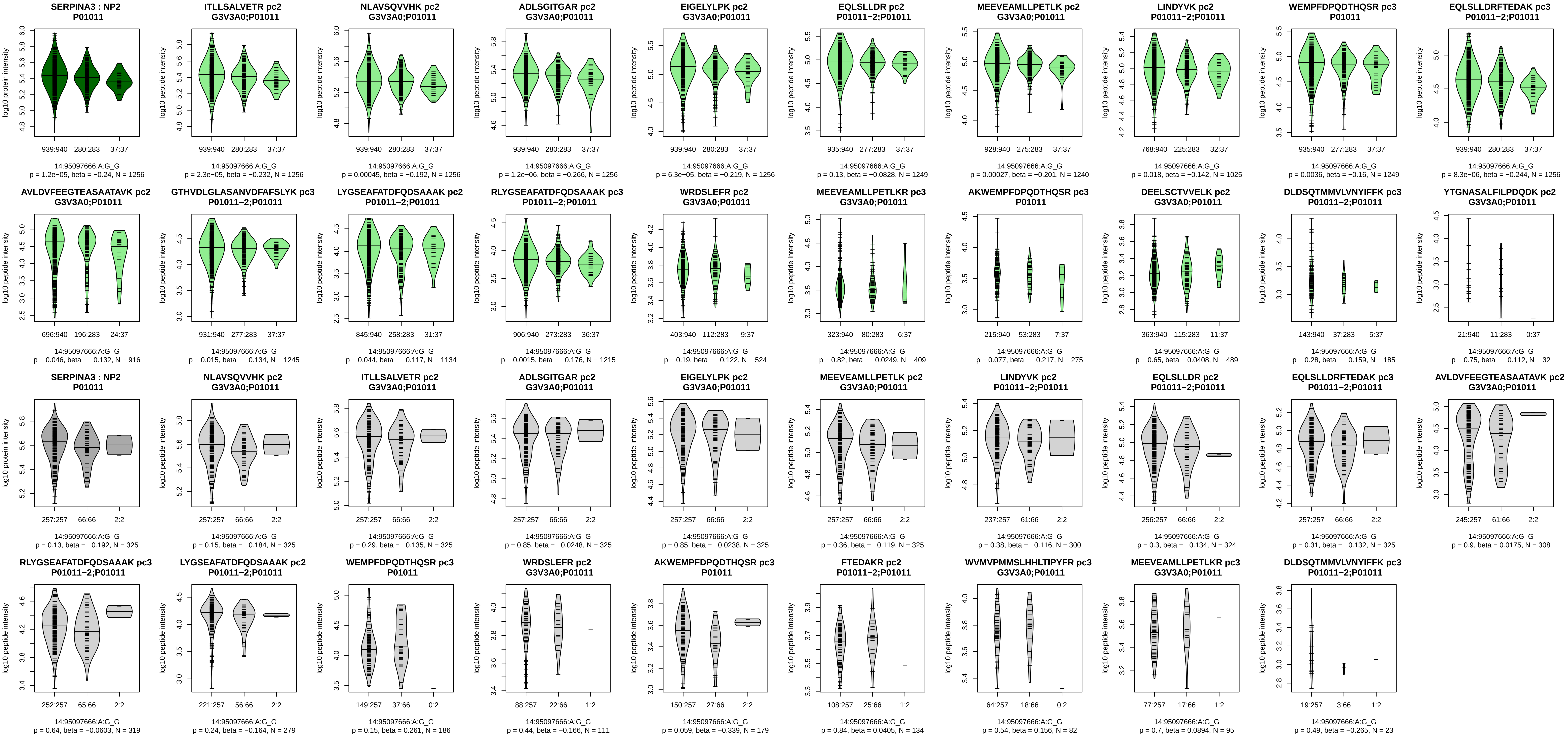

Assay Target: SERPINA3
UniProt: P01011
rsID: rs61976127
Target SNP: 14:95097666:A:G
Proxy SNP: 14:95097666:A:G
UKBPPP log10(p): 645.2
UKBPPP BETA: −0.445
UKBPPP MSPA: 0.571
1256:1256:1256:1256:1249:124

127. TFPI (P10646)
UKBPPP MSPA=0

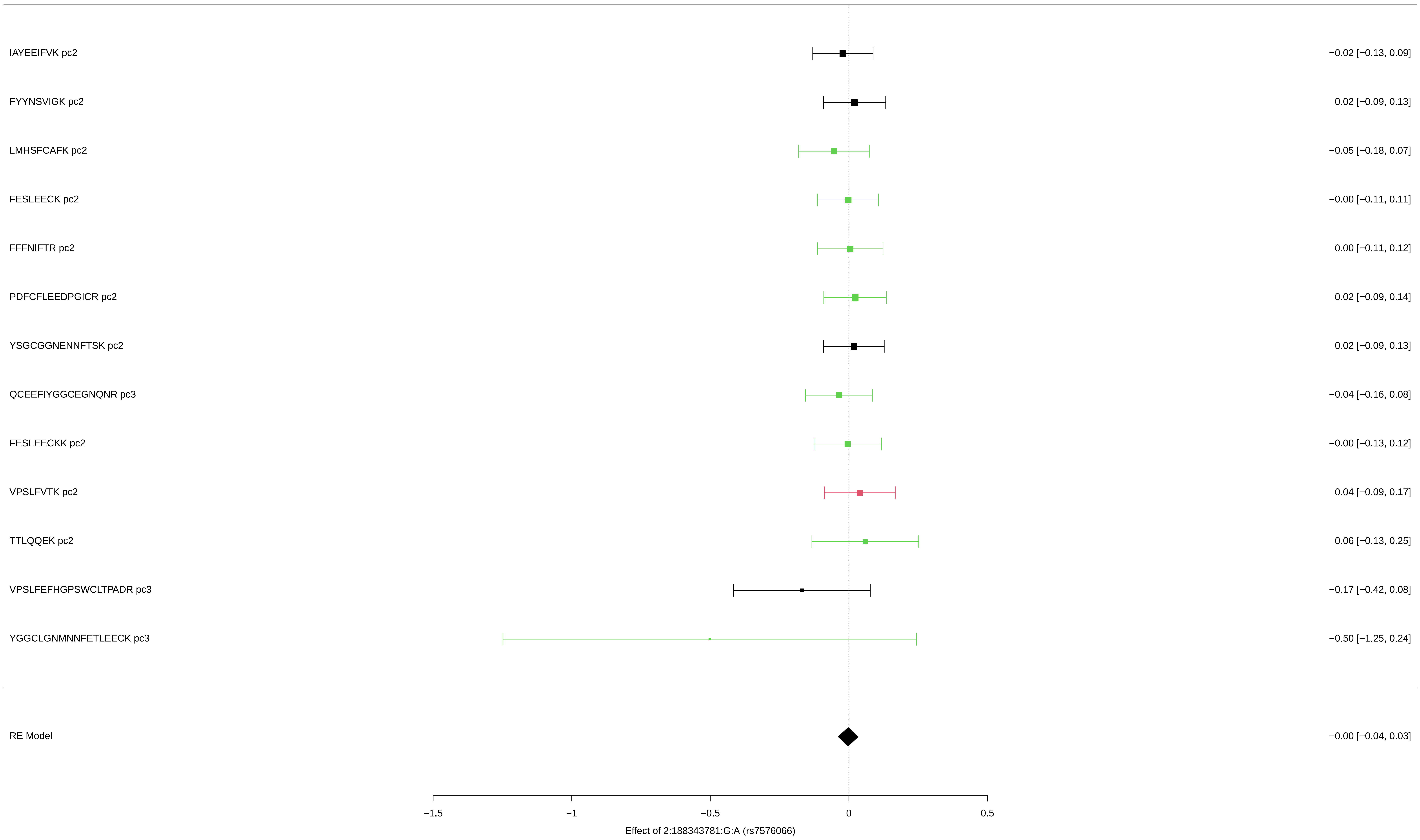

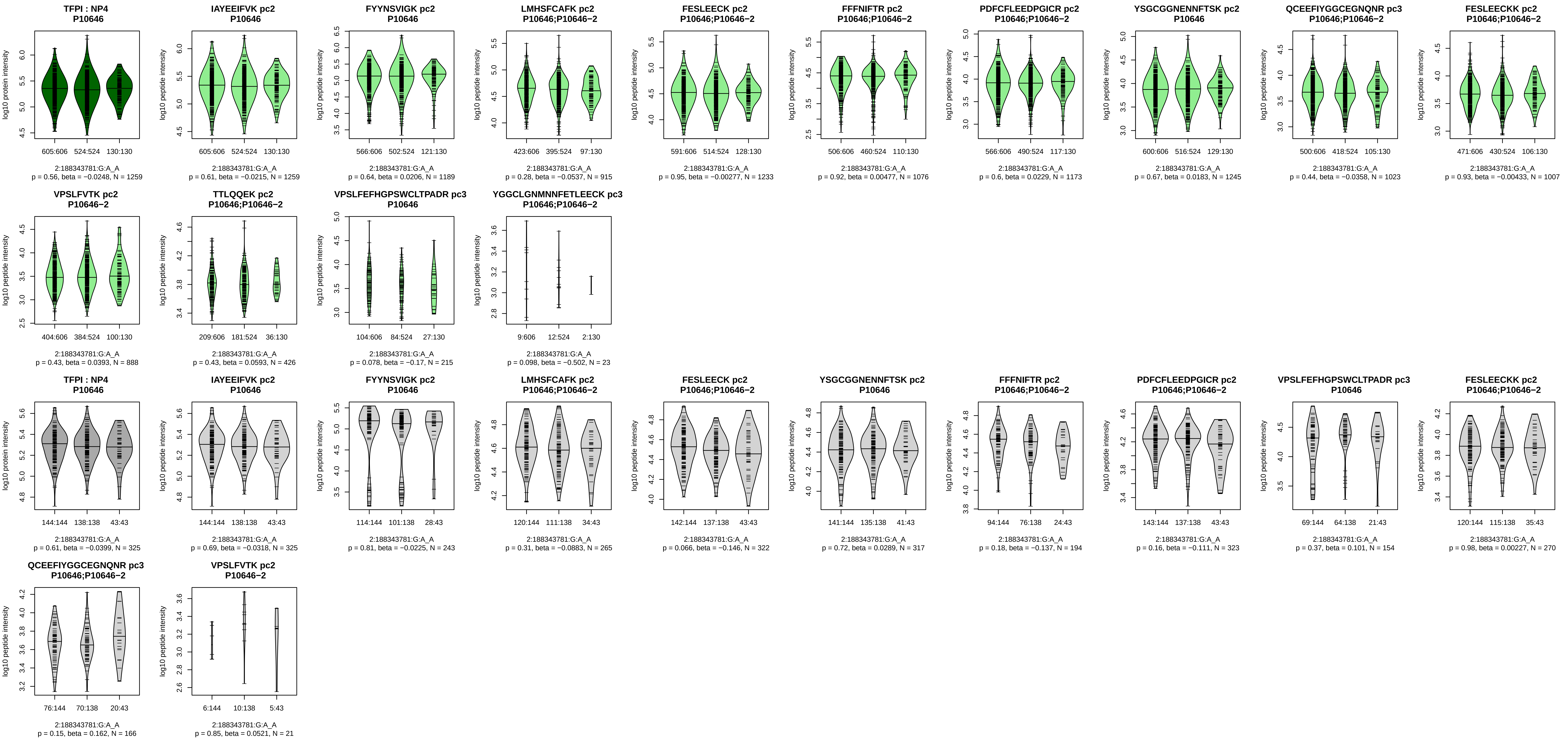

Assay Target: TFPI
UniProt: P10646
rsID: rs7576066
Target SNP: 2:188343781:G:A
Proxy SNP: 2:188343781:G:A
UKBPPP log10(p): 643.7
UKBPPP BETA: 0.35
UKBPPP MSPA: 0
−:−:−:−:−:−:−:−:−:−:−:−:−:NA
1259:1189:915:1233:1076:1173

128. APOC1 (P02654)
UKBPPP MSPA=0.71

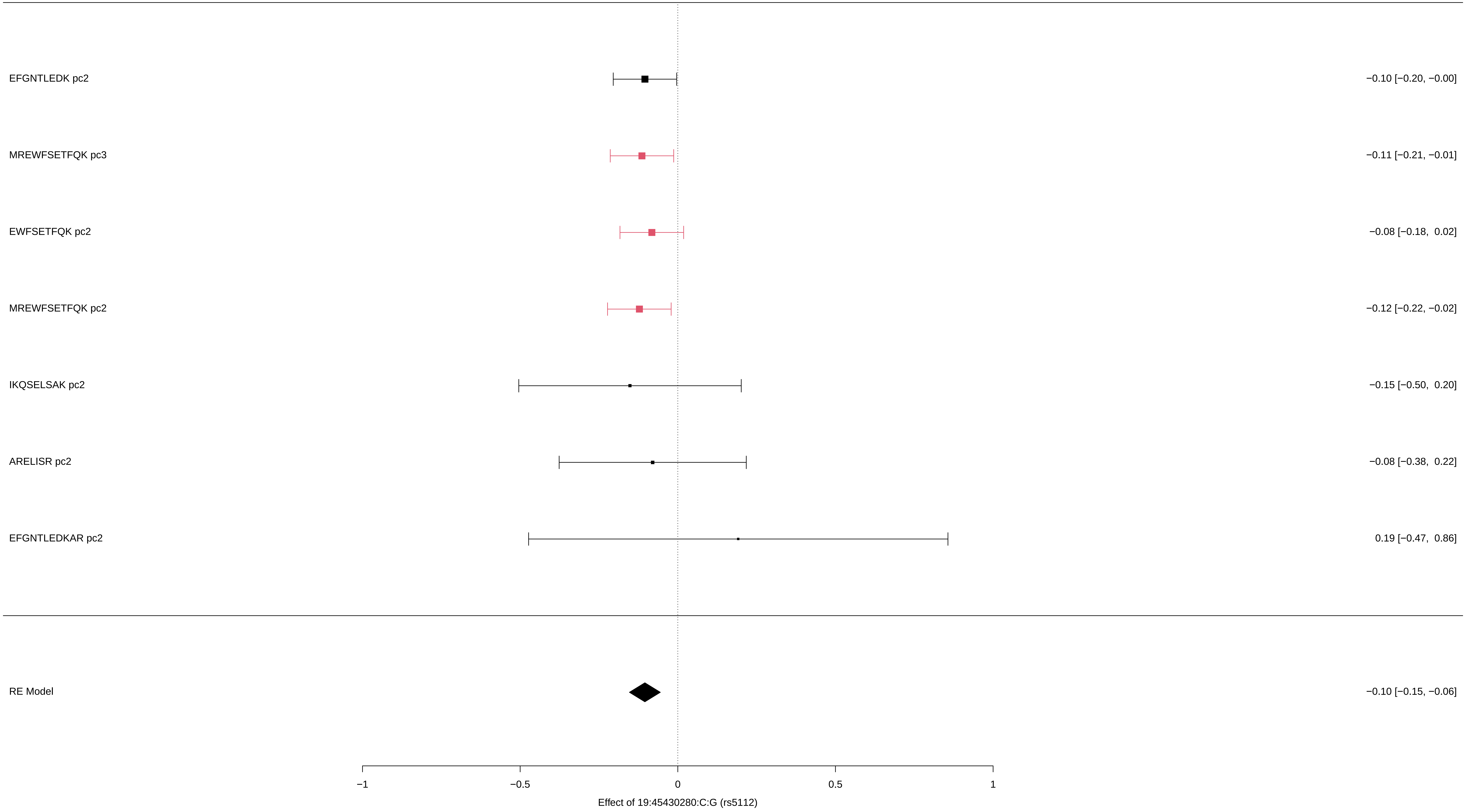

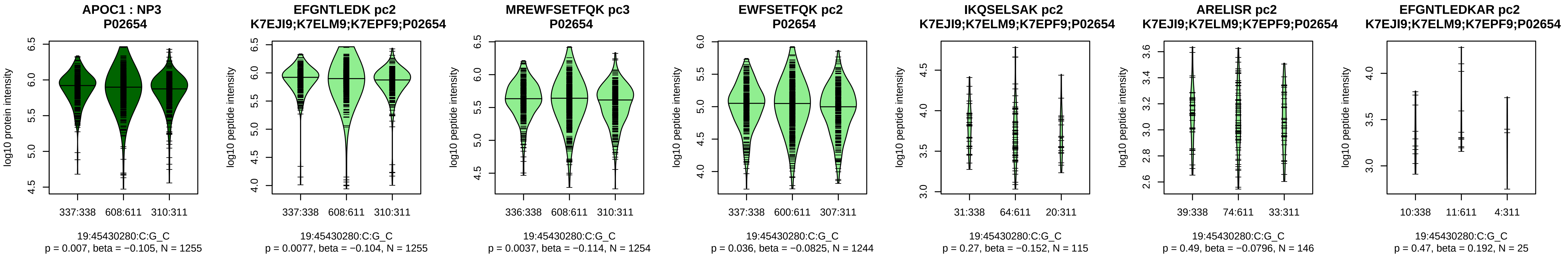

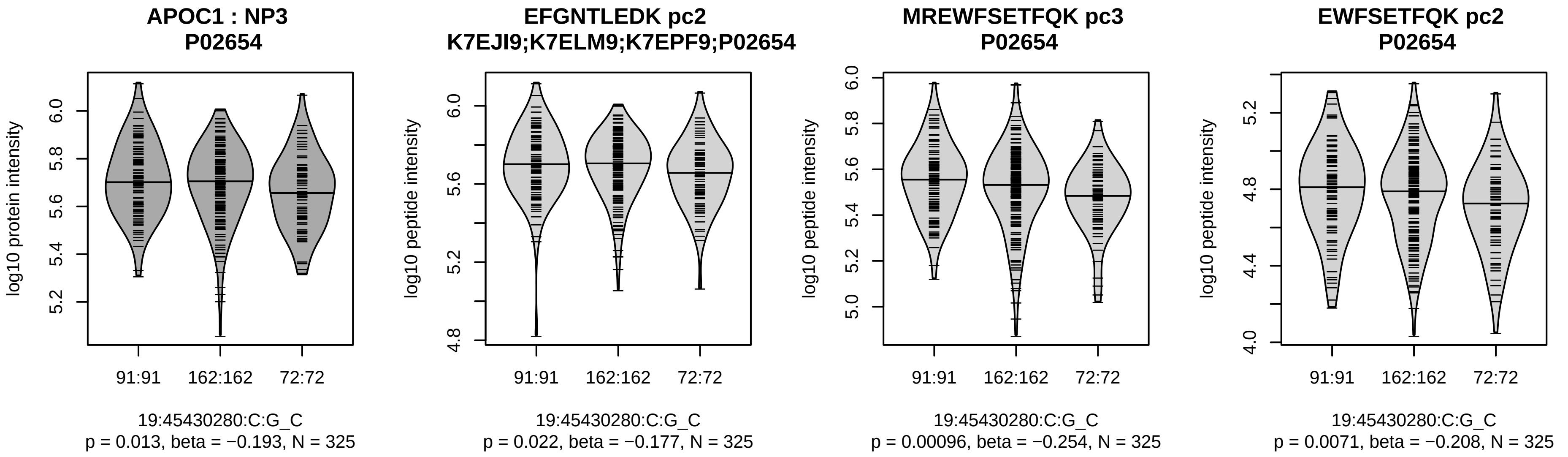

Assay Target: APOC1
UniProt: P02654
rsID: rs5112
Target SNP: 19:45430280:C:G
Proxy SNP: 19:45430280:C:G
UKBPPP log10(p): 624.8
UKBPPP BETA: 0.333
UKBPPP MSPA: 0.71
::−:*:−:−:NA:−
1255:1254:1244:1245:115:146:

129. GPC1 (P35052)
UKBPPP MSPA=0

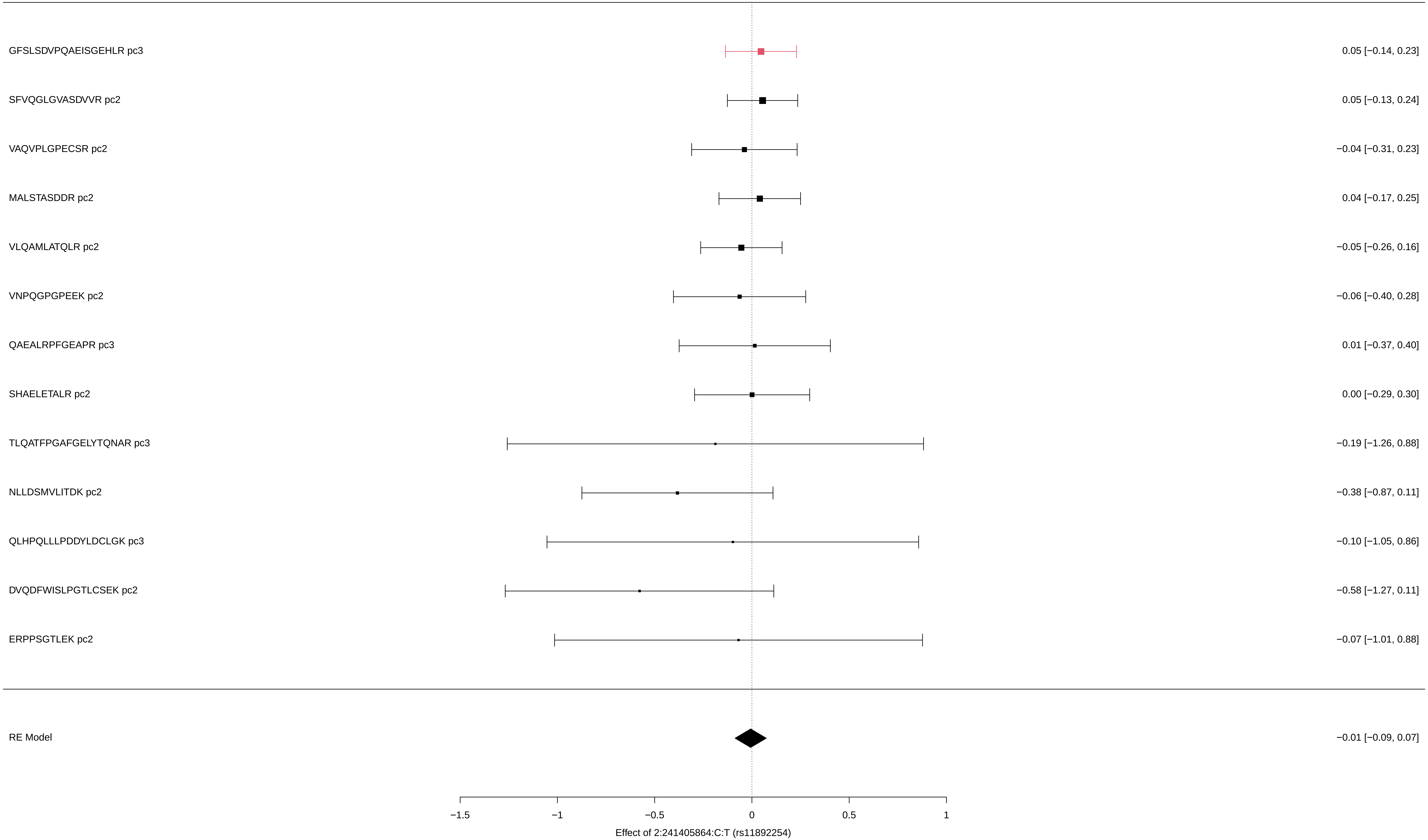

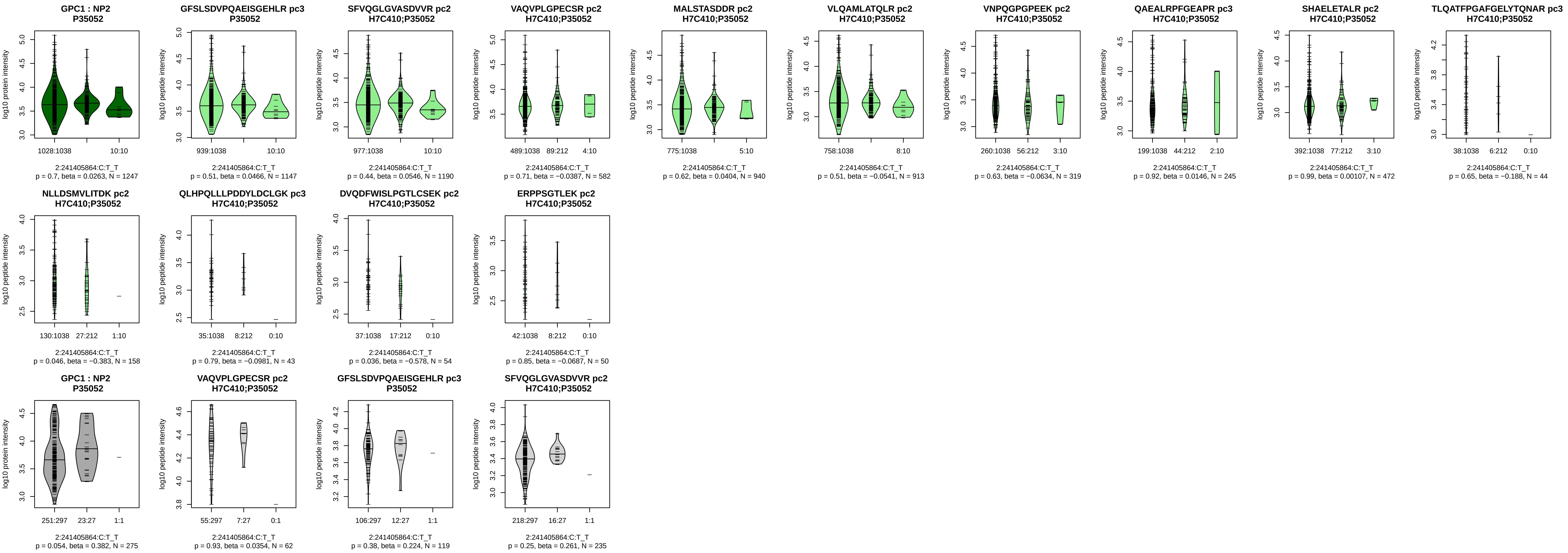

Assay Target: GPC1
UniProt: P35052
rsID: rs11892254
Target SNP: 2:241405864:C:T
Proxy SNP: 2:241405864:C:T
UKBPPP log10(p): 624.6
UKBPPP BETA: 0.5
UKBPPP MSPA: 0
–:–:–:–:–:–:–:–:–:–:–:–:–:NA:NA
1147:1190:582:940:913:319:245

130. GLO1 (Q04760)
UKBPPP MSPA=0.625

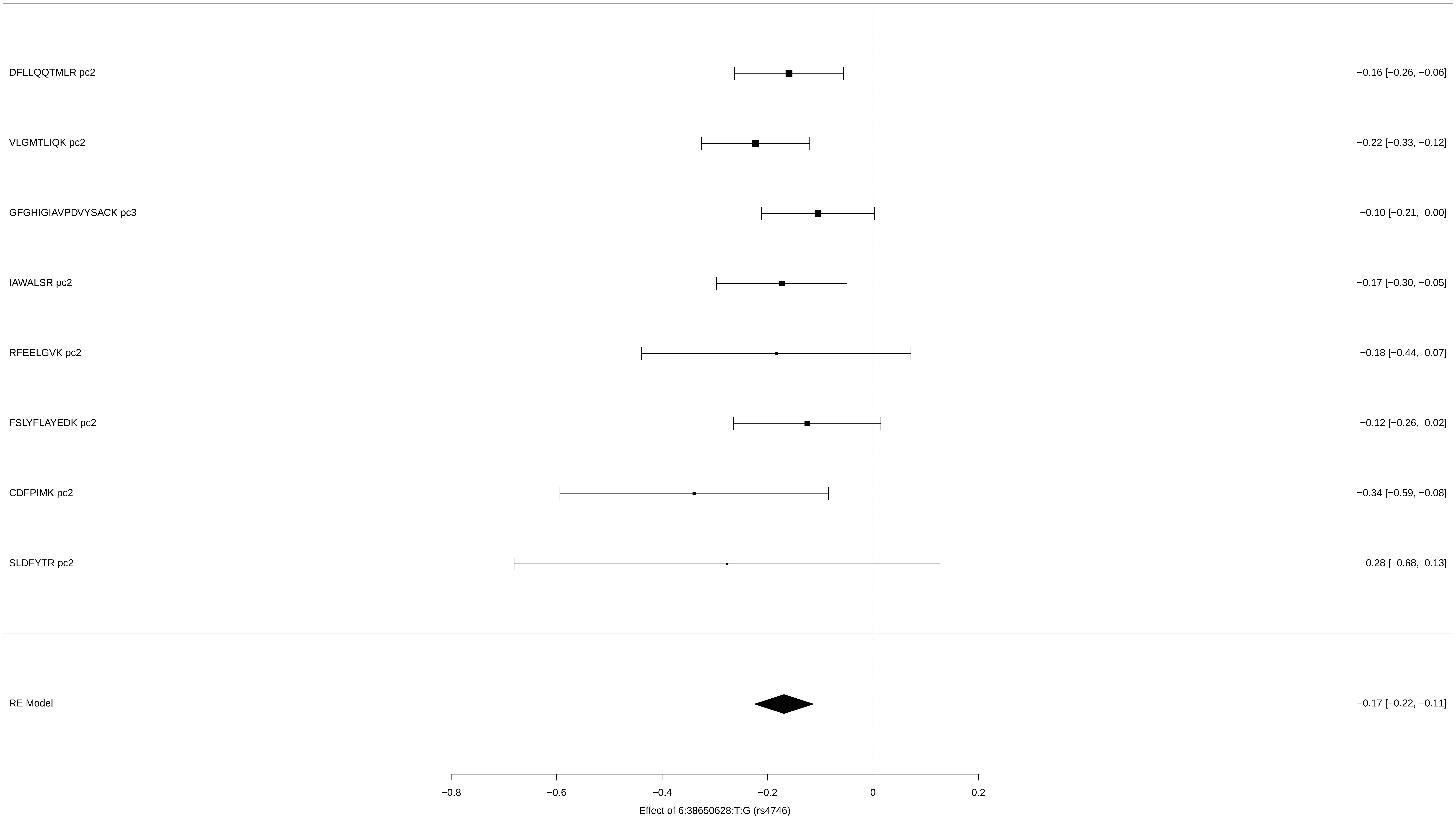

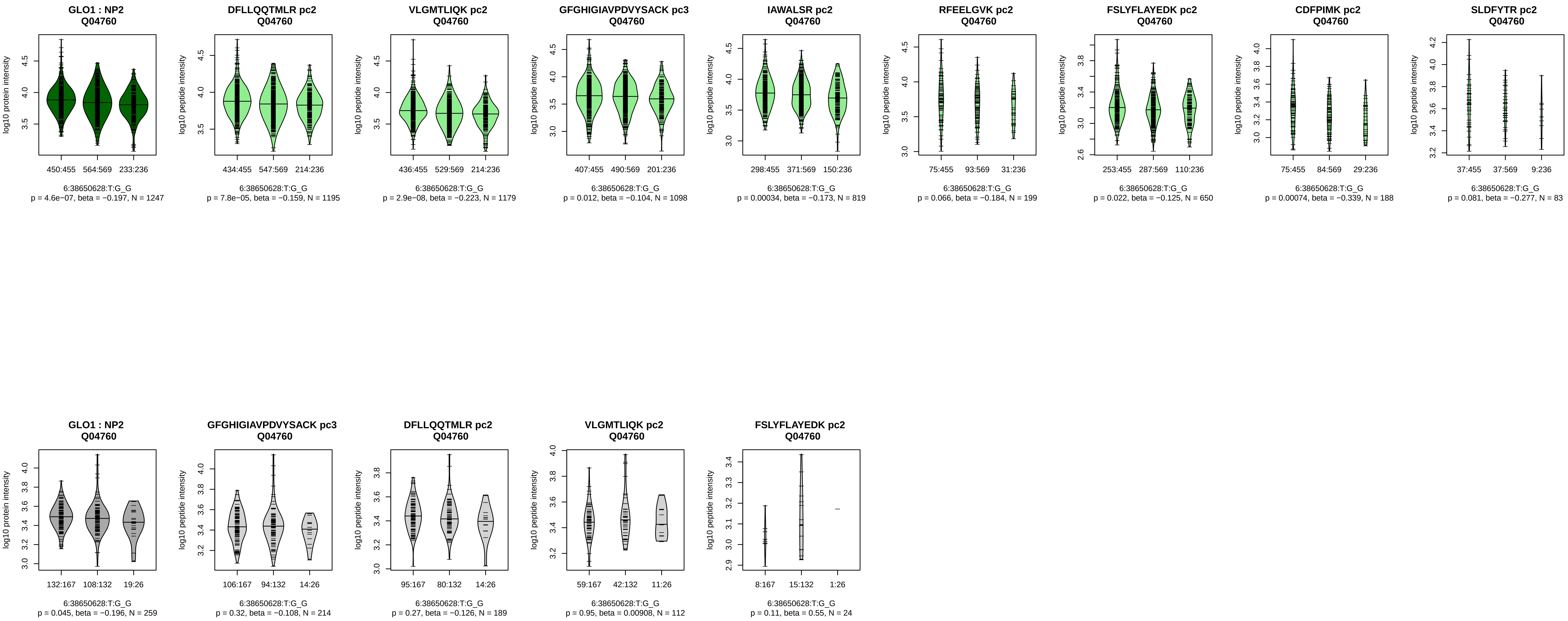

Assay Target: GLO1
UniProt: Q04760
rsID: rs4746
Target SNP: 6:38650628:T:G
Proxy SNP: 6:38650628:T:G
UKBPPP log10(p): 616.9
UKBPPP BETA: −0.326
UKBPPP MSPA: 0.625
::–:*:–:–:*:–
1195:1179:1098:819:199:650:18

131. GAS6 (Q14393)
UKBPPP MSPA=0.497

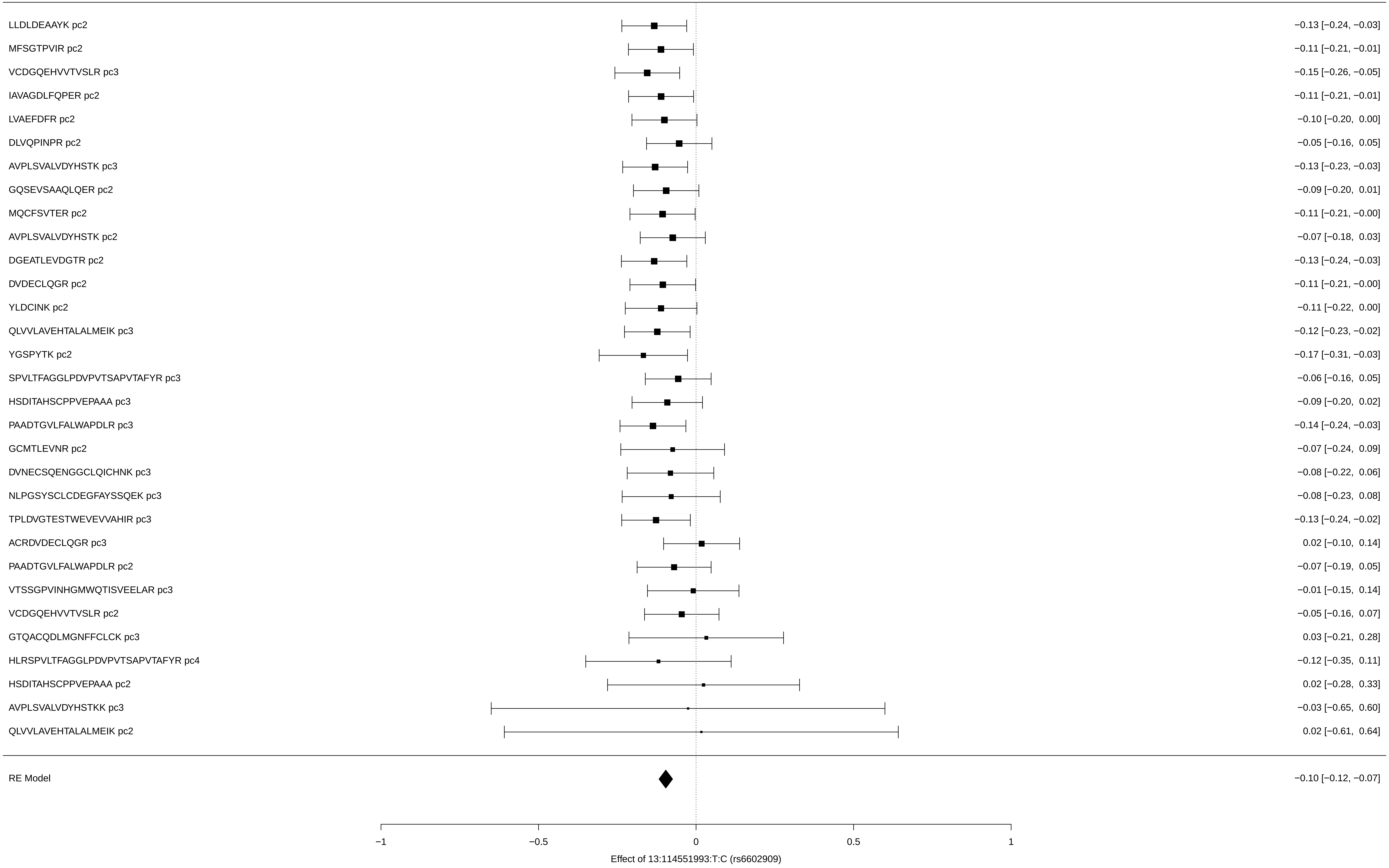

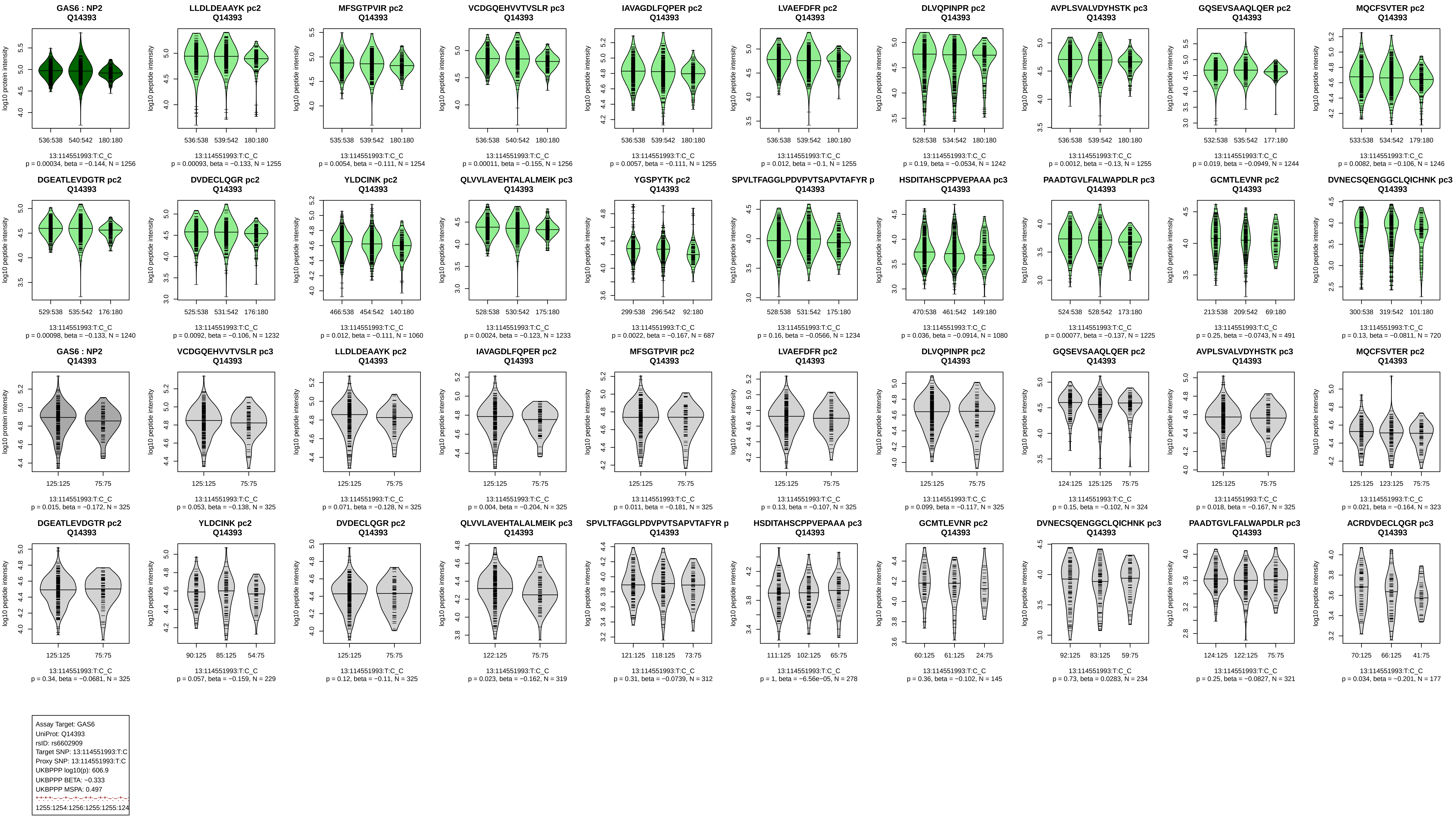
GAS6 : NP2 Q14393
13:114551993:T:C_C
p = 0.00034, beta = −0.144, N = 1256
LLDLDEAAYK pc2 Q14393
p = 0.00093, beta = −0.133, N = 1255
MFSGTPVIR pc2 Q14393
p = 0.0054, beta = −0.111, N = 1254
VCDGQEHVVTVSLR pc3 Q14393
p = 0.00011, beta = −0.155, N = 1256
IAVAGDLFQPER pc2 Q14393
p = 0.0057, beta = −0.111, N = 1255
LVAEFDFR pc2 Q14393
p = 0.012, beta = −0.1, N = 1255
DLVQPINPR pc2 Q14393
p = 0.19, beta = −0.0534, N = 1242
AVPLSVALVDYHSTK pc3 Q14393
p = 0.0012, beta = −0.13, N = 1255
GQSEVSAAQLQER pc2 Q14393
p = 0.019, beta = −0.0949, N = 1244
MQCFSVTER pc2 Q14393
p = 0.0082, beta = −0.106, N = 1246
DGEATLEVDGTR pc2 Q14393
p = 0.00098, beta = −0.133, N = 1240
DVDECLQGR pc2 Q14393
p = 0.0092, beta = −0.106, N = 1232
YLDCINK pc2 Q14393
p = 0.012, beta = −0.111, N = 1060
QLVVLAVEHTALALMEIK pc3 Q14393
p = 0.0024, beta = −0.123, N = 1233
YGSPYTK pc2 Q14393
p = 0.0022, beta = −0.167, N = 687
SPVLTFAGGLPDVPVTSAPVTAFYR p Q14393
p = 0.16, beta = −0.0566, N = 1234
HSDITAHSCPPVEPAAA pc3 Q14393
p = 0.036, beta = −0.0914, N = 1080
PAADTGVLFALWAPDLR pc3 Q14393
p = 0.00077, beta = −0.137, N = 1225
GCMTLEVNR pc2 Q14393
p = 0.25, beta = −0.0743, N = 491
DVNECSQENGGCLQICHNK pc3 Q14393
p = 0.13, beta = −0.0811, N = 720
GAS6 : NP2 Q14393
p = 0.015, beta = −0.172, N = 325
VCDGQEHVVTVSLR pc3 Q14393
p = 0.053, beta = −0.138, N = 325
LLDLDEAAYK pc2 Q14393
p = 0.071, beta = −0.128, N = 325
IAVAGDLFQPER pc2 Q14393
p = 0.004, beta = −0.204, N = 325
MFSGTPVIR pc2 Q14393
p = 0.011, beta = −0.181, N = 325
LVAEFDFR pc2 Q14393
p = 0.13, beta = −0.107, N = 325
DLVQPINPR pc2 Q14393
p = 0.099, beta = −0.117, N = 325
GQSEVSAAQLQER pc2 Q14393
p = 0.15, beta = −0.102, N = 324
AVPLSVALVDYHSTK pc3 Q14393
p = 0.018, beta = −0.167, N = 325
MQCFSVTER pc2 Q14393
p = 0.021, beta = −0.164, N = 323
DGEATLEVDGTR pc2 Q14393
p = 0.34, beta = −0.0681, N = 325
YLDCINK pc2 Q14393
p = 0.057, beta = −0.159, N = 229
DVDECLQGR pc2 Q14393
p = 0.12, beta = −0.11, N = 325
QLVVLAVEHTALALMEIK pc3 Q14393
p = 0.023, beta = −0.162, N = 319
SPVLTFAGGLPDVPVTSAPVTAFYR p Q14393
p = 0.31, beta = −0.0739, N = 312
HSDITAHSCPPVEPAAA pc3 Q14393
p = 1, beta = −6.56e−05, N = 278
GCMTLEVNR pc2 Q14393
p = 0.36, beta = −0.102, N = 145
DVNECSQENGGCLQICHNK pc3 Q14393
p = 0.73, beta = 0.0283, N = 234
PAADTGVLFALWAPDLR pc3 Q14393
p = 0.25, beta = −0.0827, N = 321
ACRDVDECLQGR pc3 Q14393
p = 0.034, beta = −0.201, N = 177
Assay Target: GAS6
UniProt: Q14393
rsID: rs6602909
Target SNP: 13:114551993:T:C
Proxy SNP: 13:114551993:T:C
UKBPPP log10(p): 606.9
UKBPPP BETA: −0.333
UKBPPP MSPA: 0.497
1255:1254:1256:1255:1255:124

132. PCDH12 (Q9NPG4)
UKBPPP MSPA=0

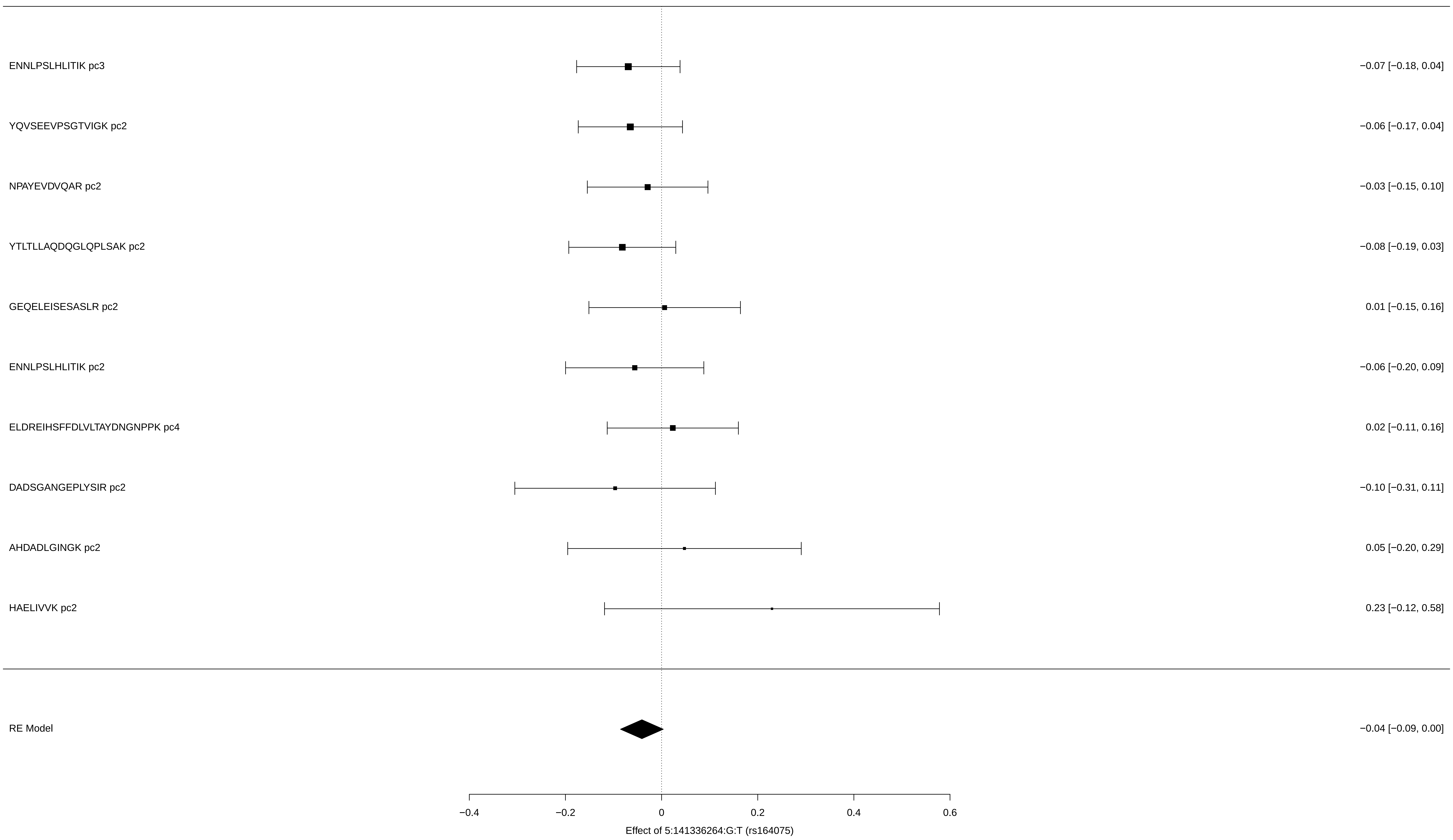

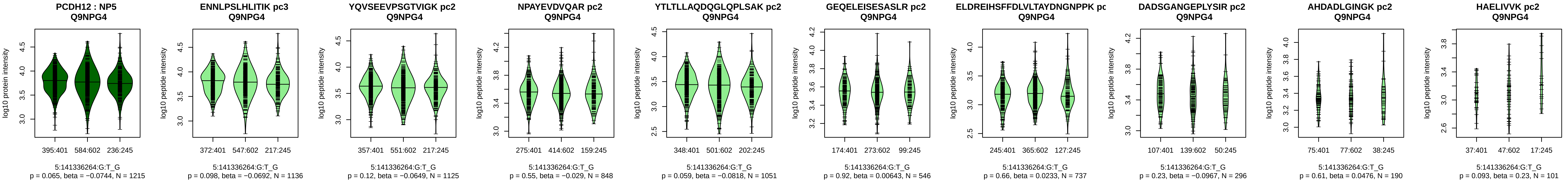

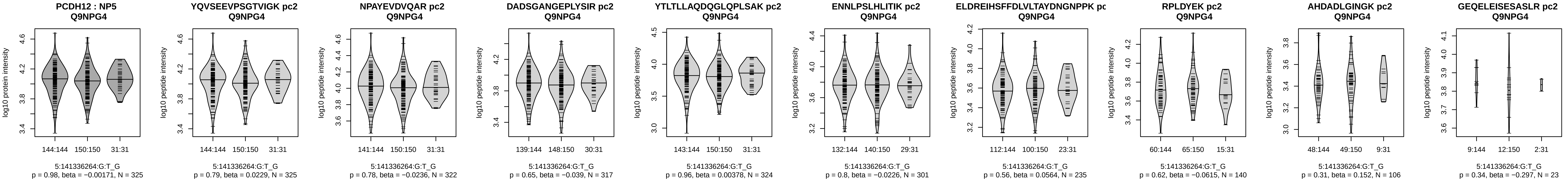

Assay Target: PCDH12
UniProt: Q9NPG4
rsID: rs164075
Target SNP: 5:141336264:G:T
Proxy SNP: 5:141336264:G:T
UKBPPP log10(p): 600.4
UKBPPP BETA: 0.325
UKBPPP MSPA: 0
–:–:–:–:–:–:–:–:–:NA
1136:1125:848:1051:546:618:73

133. GDF15 (Q99988)
UKBPPP MSPA=0

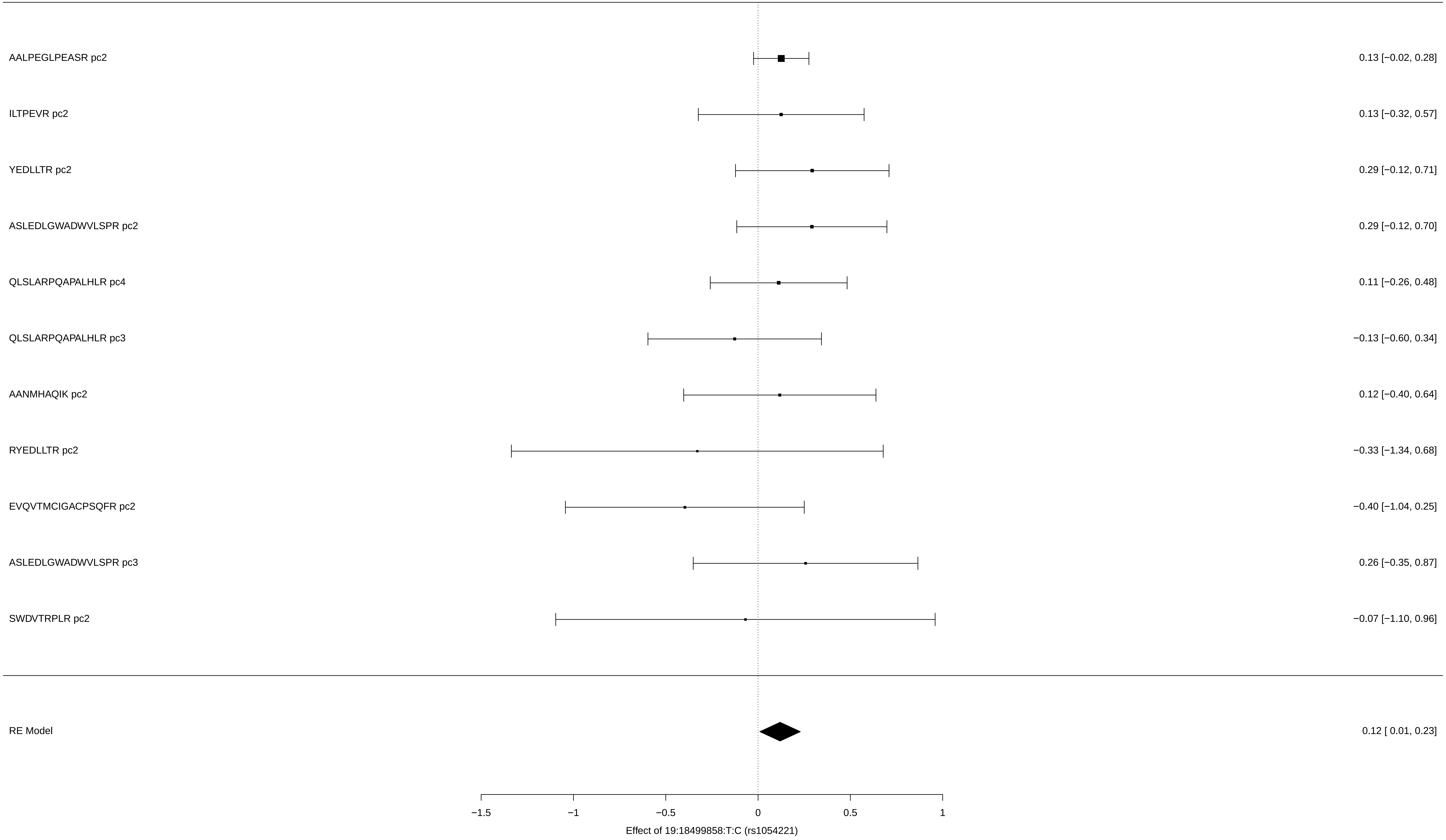

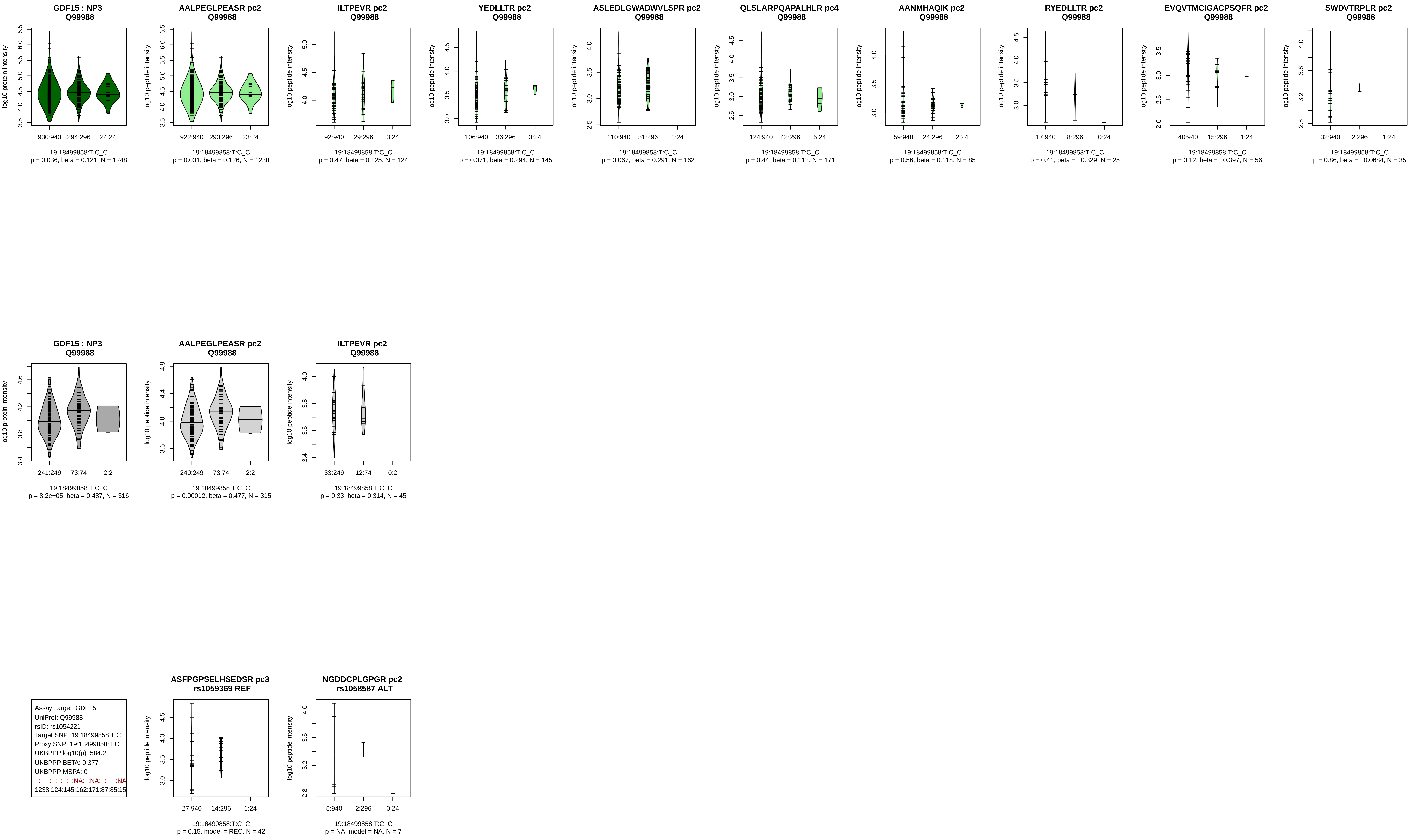
GDF15 : NP3
Q99988
log10 protein intensity
930:940
294:296
24:24
19:18499858:T:C_C
p = 0.036, beta = 0.121, N = 1248
AALPEGLPEASR pc2
Q99988
log10 peptide intensity
922:940
293:296
23:24
19:18499858:T:C_C
p = 0.031, beta = 0.126, N = 1238
ILTPEVR pc2
Q99988
92:940
29:296
3:24
19:18499858:T:C_C
p = 0.47, beta = 0.125, N = 124
YEDLLTR pc2
Q99988
106:940
36:296
3:24
19:18499858:T:C_C
p = 0.071, beta = 0.294, N = 145
ASLEDLGWADWVLSPR pc2
Q99988
110:940
51:296
1:24
19:18499858:T:C_C
p = 0.067, beta = 0.291, N = 162
QLSLARPQAPALHLR pc4
Q99988
124:940
42:296
5:24
19:18499858:T:C_C
p = 0.44, beta = 0.112, N = 171
AANMHAQIK pc2
Q99988
59:940
24:296
2:24
19:18499858:T:C_C
p = 0.56, beta = 0.118, N = 85
RYEDLLTR pc2
Q99988
17:940
8:296
0:24
19:18499858:T:C_C
p = 0.41, beta = −0.329, N = 25
EVQVTMCIGACPSQFR pc2
Q99988
40:940
15:296
1:24
19:18499858:T:C_C
p = 0.12, beta = −0.397, N = 56
SWDVTRPLR pc2
Q99988
32:940
2:296
1:24
19:18499858:T:C_C
p = 0.86, beta = −0.0684, N = 35
GDF15 : NP3
Q99988
log10 protein intensity
241:249
73:74
2:2
19:18499858:T:C_C
p = 8.2e−05, beta = 0.487, N = 316
AALPEGLPEASR pc2
Q99988
240:249
73:74
2:2
19:18499858:T:C_C
p = 0.00012, beta = 0.477, N = 315
ILTPEVR pc2
Q99988
33:249
12:74
0:2
19:18499858:T:C_C
p = 0.33, beta = 0.314, N = 45
Assay Target: GDF15
UniProt: Q99988
rsID: rs1054221
Target SNP: 19:18499858:T:C
Proxy SNP: 19:18499858:T:C
UKBPPP log10(p): 584.2
UKBPPP BETA: 0.377
UKBPPP MSPA: 0
−:−:−:−:−:−:−:NA:−:NA:−:−:−:NA
1238:124:145:162:171:87:85:15
ASFPGPSELHSEDSR pc3
rs1059369 REF
27:940
14:296
1:24
19:18499858:T:C_C
p = 0.15, model = REC, N = 42
NGDDCPLGPGR pc2
rs1058587 ALT
5:940
2:296
0:24
19:18499858:T:C_C
p = NA, model = NA, N = 7

134. TF (P02787)
UKBPPP MSPA=0.142 (2 conflicts)

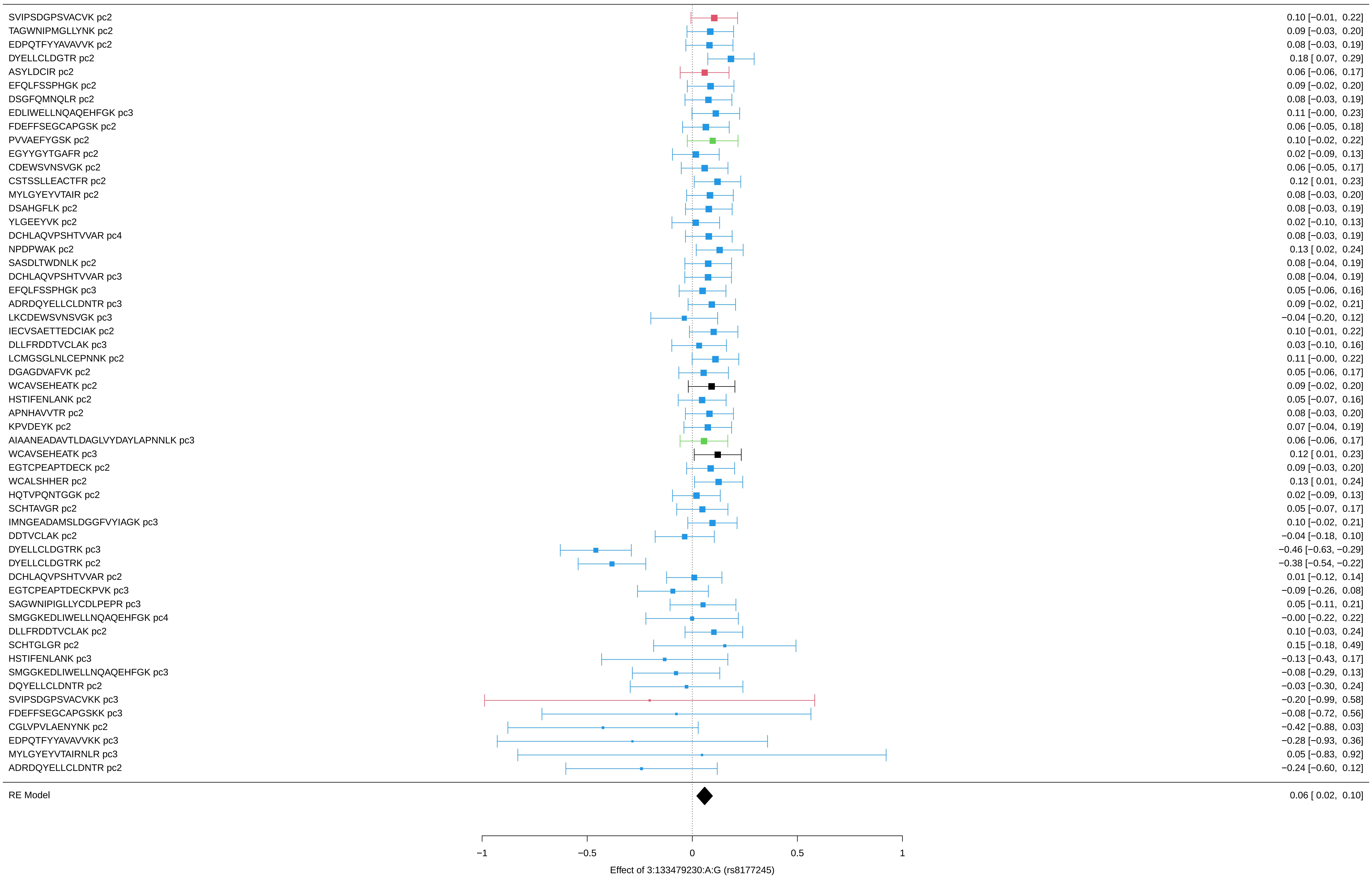

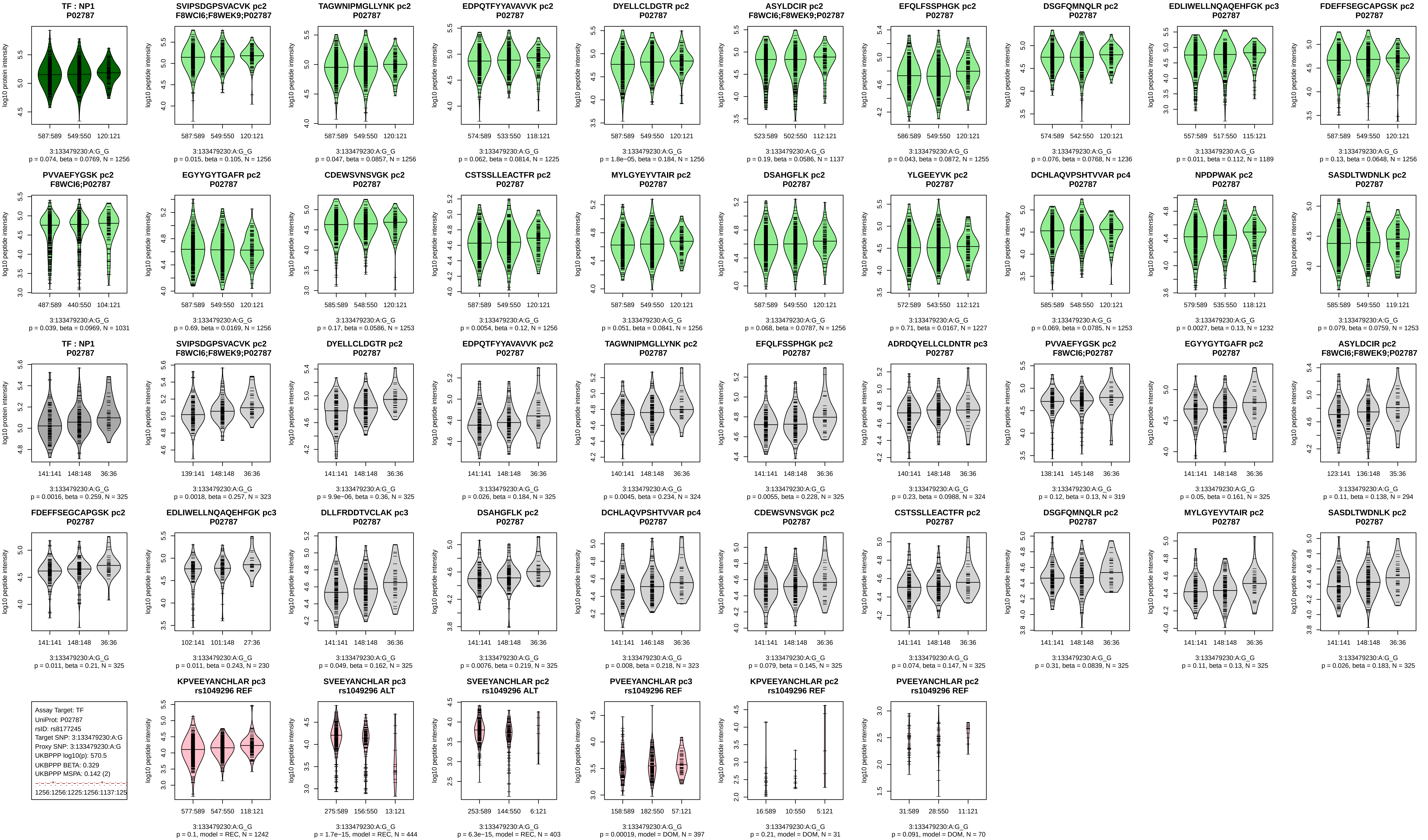

TF : NP1
P02787
log10 protein intensity
587:589 549:550 120:121
3:133479230:A:G_G
p = 0.074, beta = 0.0769, N = 1256
SVIPSDGPSVACVK pc2
F8WCI6;F8WEK9;P02787
log10 peptide intensity
587:589 549:550 120:121
3:133479230:A:G_G
p = 0.015, beta = 0.105, N = 1256
TAGWNIPMGLLYNK pc2
P02787
587:589 549:550 120:121
3:133479230:A:G_G
p = 0.047, beta = 0.0857, N = 1256
EDPQTFYYAVAVVK pc2
P02787
574:589 533:550 118:121
3:133479230:A:G_G
p = 0.062, beta = 0.0814, N = 1225
DYELLCLDGTR pc2
P02787
587:589 549:550 120:121
3:133479230:A:G_G
p = 1.8e−05, beta = 0.184, N = 1256
ASYLDCIR pc2
F8WCI6;F8WEK9;P02787
523:589 502:550 112:121
3:133479230:A:G_G
p = 0.19, beta = 0.0586, N = 1137
EFQLFSSPHGK pc2
P02787
586:589 549:550 120:121
3:133479230:A:G_G
p = 0.043, beta = 0.0872, N = 1255
DSGFQMNQLR pc2
P02787
574:589 542:550 120:121
3:133479230:A:G_G
p = 0.076, beta = 0.0768, N = 1236
EDLIWELLNQAQEHFGK pc3
P02787
557:589 517:550 115:121
3:133479230:A:G_G
p = 0.011, beta = 0.112, N = 1189
FDEFFSEGCAPGSK pc2
P02787
587:589 549:550 120:121
3:133479230:A:G_G
p = 0.13, beta = 0.0648, N = 1256
PVVAEFYGSK pc2
F8WCI6;P02787
487:589 440:550 104:121
3:133479230:A:G_G
p = 0.039, beta = 0.0969, N = 1031
EGYYGYTGAFR pc2
P02787
587:589 549:550 120:121
3:133479230:A:G_G
p = 0.69, beta = 0.0169, N = 1256
CDEWSVNSVGK pc2
P02787
585:589 548:550 120:121
3:133479230:A:G_G
p = 0.17, beta = 0.0586, N = 1253
CSTSSLLEACTFR pc2
P02787
587:589 549:550 120:121
3:133479230:A:G_G
p = 0.0054, beta = 0.12, N = 1256
MYLGYEYVTAIR pc2
P02787
587:589 549:550 120:121
3:133479230:A:G_G
p = 0.051, beta = 0.0841, N = 1256
DSAHGFLK pc2
P02787
587:589 549:550 120:121
3:133479230:A:G_G
p = 0.068, beta = 0.0787, N = 1256
YLGEEYVK pc2
P02787
572:589 543:550 112:121
3:133479230:A:G_G
p = 0.71, beta = 0.0167, N = 1227
DCHLAQVPSHTVVAR pc4
P02787
585:589 548:550 120:121
3:133479230:A:G_G
p = 0.069, beta = 0.0785, N = 1253
NPDPWAK pc2
P02787
579:589 535:550 118:121
3:133479230:A:G_G
p = 0.0027, beta = 0.13, N = 1232
SASDLTWDNLK pc2
P02787
585:589 549:550 119:121
3:133479230:A:G_G
p = 0.079, beta = 0.0759, N = 1253
TF : NP1
P02787
141:141 148:148 36:36
3:133479230:A:G_G
p = 0.0016, beta = 0.259, N = 325
SVIPSDGPSVACVK pc2
F8WCI6;F8WEK9;P02787
139:141 148:148 36:36
3:133479230:A:G_G
p = 0.0018, beta = 0.257, N = 323
DYELLCLDGTR pc2
P02787
141:141 148:148 36:36
3:133479230:A:G_G
p = 9.9e−06, beta = 0.36, N = 325
EDPQTFYYAVAVVK pc2
P02787
141:141 148:148 36:36
3:133479230:A:G_G
p = 0.026, beta = 0.184, N = 325
TAGWNIPMGLLYNK pc2
P02787
140:141 148:148 36:36
3:133479230:A:G_G
p = 0.0045, beta = 0.234, N = 324
EFQLFSSPHGK pc2
P02787
141:141 148:148 36:36
3:133479230:A:G_G
p = 0.0055, beta = 0.228, N = 325
ADRDQYELLCLDNTR pc3
P02787
140:141 148:148 36:36
3:133479230:A:G_G
p = 0.23, beta = 0.0988, N = 324
PVVAEFYGSK pc2
F8WCI6;P02787
138:141 145:148 36:36
3:133479230:A:G_G
p = 0.12, beta = 0.13, N = 319
EGYYGYTGAFR pc2
P02787
141:141 148:148 36:36
3:133479230:A:G_G
p = 0.05, beta = 0.161, N = 325
ASYLDCIR pc2
F8WCI6;F8WEK9;P02787
123:141 136:148 35:36
3:133479230:A:G_G
p = 0.11, beta = 0.138, N = 294
FDEFFSEGCAPGSK pc2
P02787
141:141 148:148 36:36
3:133479230:A:G_G
p = 0.011, beta = 0.21, N = 325
EDLIWELLNQAQEHFGK pc3
P02787
102:141 101:148 27:36
3:133479230:A:G_G
p = 0.011, beta = 0.243, N = 230
DLLFRDDTVCLAK pc3
P02787
141:141 148:148 36:36
3:133479230:A:G_G
p = 0.049, beta = 0.162, N = 325
DSAHGFLK pc2
P02787
141:141 148:148 36:36
3:133479230:A:G_G
p = 0.0076, beta = 0.219, N = 325
DCHLAQVPSHTVVAR pc4
P02787
141:141 146:148 36:36
3:133479230:A:G_G
p = 0.008, beta = 0.218, N = 323
CDEWSVNSVGK pc2
P02787
141:141 148:148 36:36
3:133479230:A:G_G
p = 0.079, beta = 0.145, N = 325
CSTSSLLEACTFR pc2
P02787
141:141 148:148 36:36
3:133479230:A:G_G
p = 0.074, beta = 0.147, N = 325
DSGFQMNQLR pc2
P02787
141:141 148:148 36:36
3:133479230:A:G_G
p = 0.31, beta = 0.0839, N = 325
MYLGYEYVTAIR pc2
P02787
141:141 148:148 36:36
3:133479230:A:G_G
p = 0.11, beta = 0.13, N = 325
SASDLTWDNLK pc2
P02787
141:141 148:148 36:36
3:133479230:A:G_G
p = 0.026, beta = 0.183, N = 325
Assay Target: TF
UniProt: P02787
rsID: rs8177245
Target SNP: 3:133479230:A:G
Proxy SNP: 3:133479230:A:G
UKBPPP log10(p): 570.5
UKBPPP BETA: 0.329
UKBPPP MSPA: 0.142 (2)
1256:1256:1225:1256:1137:125
KPVEEYANCHLAR pc3
rs1049296 REF
577:589 547:550 118:121
3:133479230:A:G_G
p = 0.1, model = REC, N = 1242
SVEEYANCHLAR pc3
rs1049296 ALT
275:589 156:550 13:121
3:133479230:A:G_G
p = 1.7e−15, model = REC, N = 444
SVEEYANCHLAR pc2
rs1049296 ALT
253:589 144:550 6:121
3:133479230:A:G_G
p = 6.3e−15, model = REC, N = 403
PVEEYANCHLAR pc3
rs1049296 REF
158:589 182:550 57:121
3:133479230:A:G_G
p = 0.00019, model = DOM, N = 397
KPVEEYANCHLAR pc2
rs1049296 REF
16:589 10:550 5:121
3:133479230:A:G_G
p = 0.21, model = DOM, N = 31
PVEEYANCHLAR pc2
rs1049296 REF
31:589 28:550 11:121
3:133479230:A:G_G
p = 0.091, model = DOM, N = 70

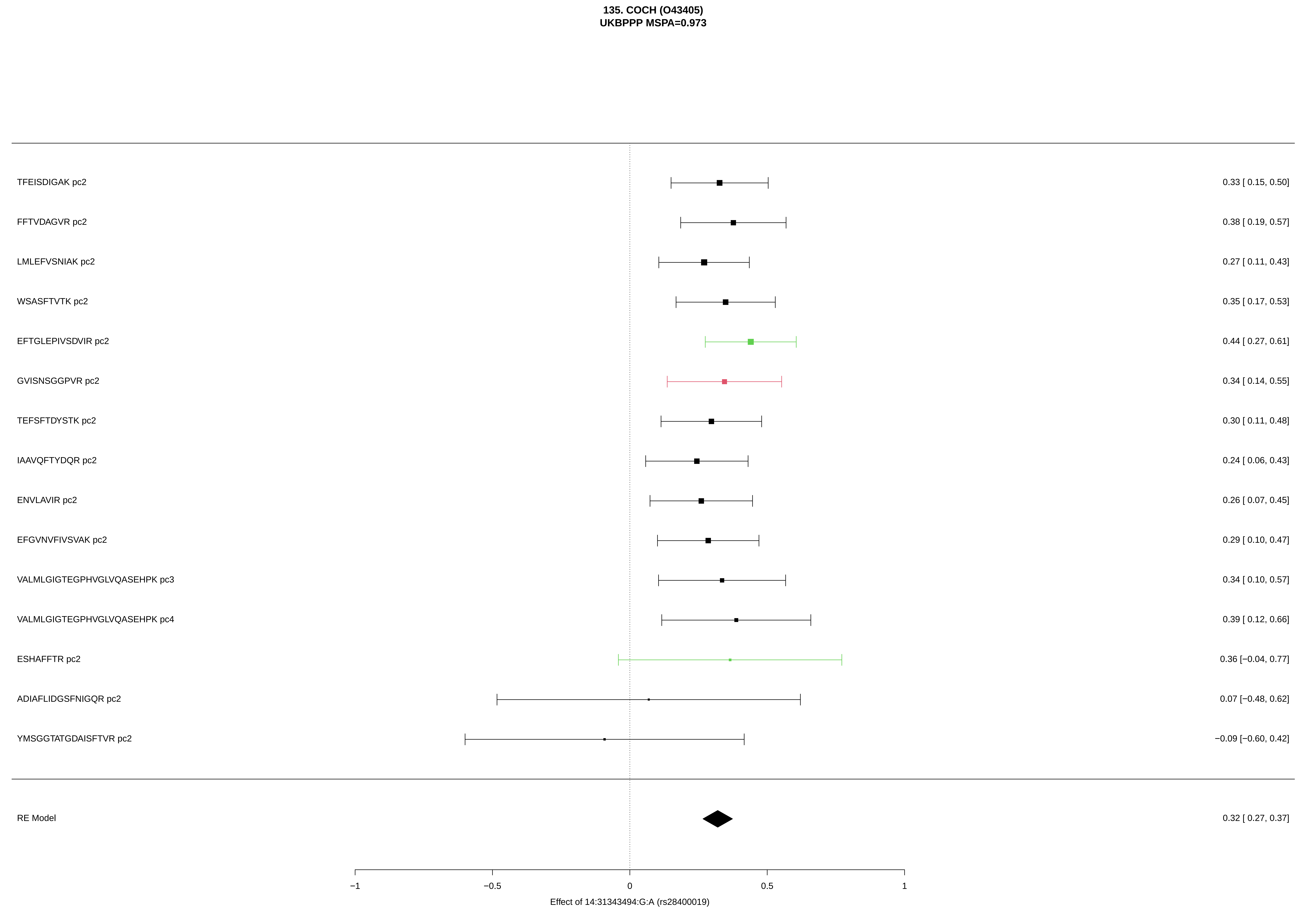
135. COCH (O43405)
UKBPPP MSPA=0.973
TFEISDIGAK pc2
0.33 [0.15, 0.50]
FFTVDAGVR pc2
0.38 [0.19, 0.57]
LMLEFVSNIAK pc2
0.27 [0.11, 0.43]
WSASFTVTK pc2
0.35 [0.17, 0.53]
EFTGLEPIVSDVIR pc2
0.44 [0.27, 0.61]
GVISNSGGPVR pc2
0.34 [0.14, 0.55]
TEFSFTDYSTK pc2
0.30 [0.11, 0.48]
IAAVQFTYDQR pc2
0.24 [0.06, 0.43]
ENVLAVIR pc2
0.26 [0.07, 0.45]
EFGVNVFIVSVAK pc2
0.29 [0.10, 0.47]
VALMLGIGTEGPHVGLVQASEHPK pc3
0.34 [0.10, 0.57]
VALMLGIGTEGPHVGLVQASEHPK pc4
0.39 [0.12, 0.66]
ESHAFFTR pc2
0.36 [−0.04, 0.77]
ADIAFLIDGSFNIGQR pc2
0.07 [−0.48, 0.62]
YMSGGTATGDAISFTVR pc2
−0.09 [−0.60, 0.42]
RE Model
0.32 [0.27, 0.37]
−1
−0.5
0
0.5
1
Effect of 14:31343494:G:A (rs28400019)

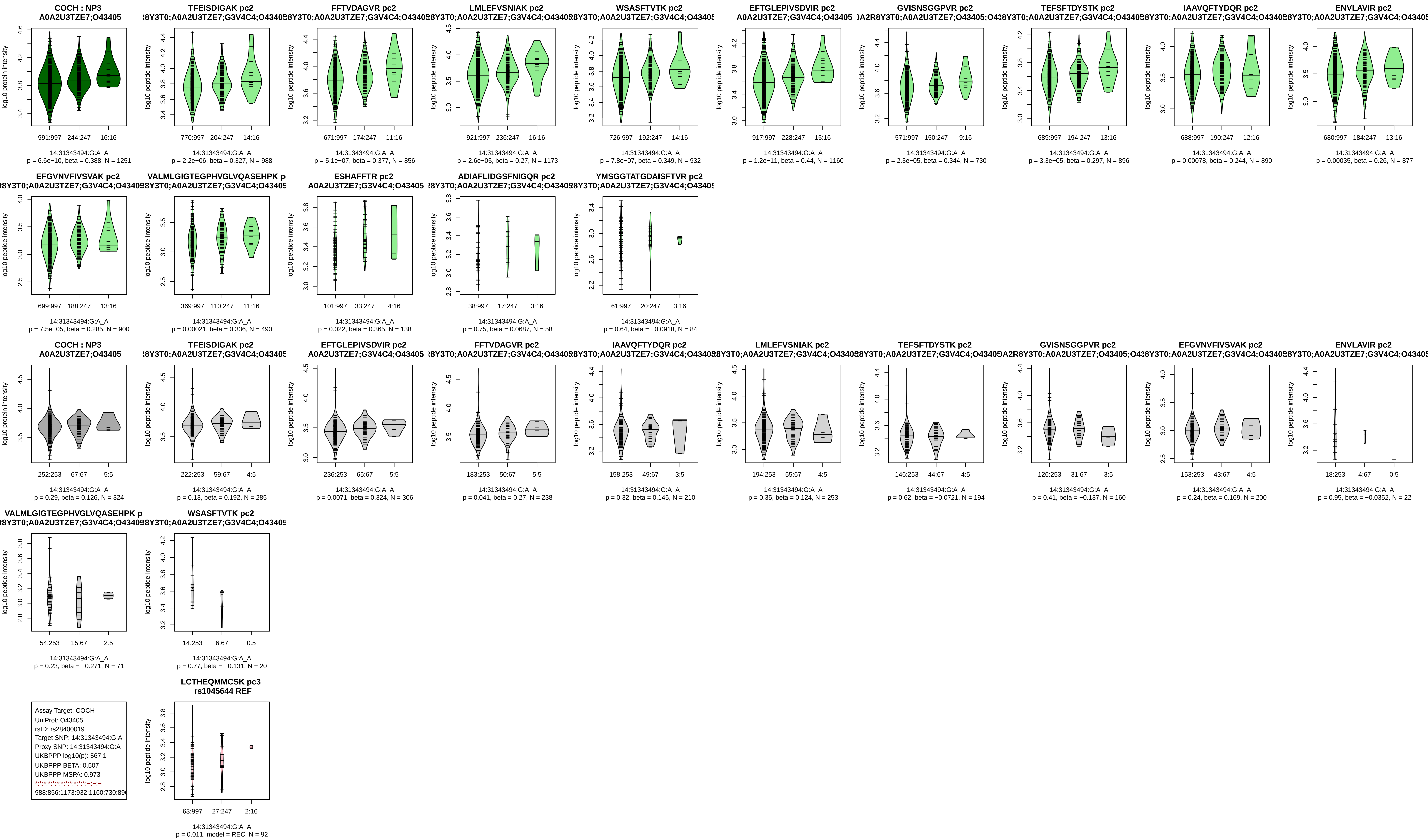
COCH : NP3
A0A2U3TZE7;O43405
log10 protein intensity
991:997 244:247 16:16
14:31343494:G:A_A
p = 6.6e−10, beta = 0.388, N = 1251
TFEISDIGAK pc2
log10 peptide intensity
770:997 204:247 14:16
14:31343494:G:A_A
p = 2.2e−06, beta = 0.327, N = 988
FFTVDAGVR pc2
671:997 174:247 11:16
14:31343494:G:A_A
p = 5.1e−07, beta = 0.377, N = 856
LMLEFVSNIAK pc2
921:997 236:247 16:16
14:31343494:G:A_A
p = 2.6e−05, beta = 0.27, N = 1173
WSASFTVTK pc2
726:997 192:247 14:16
14:31343494:G:A_A
p = 7.8e−07, beta = 0.349, N = 932
EFTGLEPIVSDVIR pc2
A0A2U3TZE7;G3V4C4;O43405
917:997 228:247 15:16
14:31343494:G:A_A
p = 1.2e−11, beta = 0.44, N = 1160
GVISNSGGPVR pc2
571:997 150:247 9:16
14:31343494:G:A_A
p = 2.3e−05, beta = 0.344, N = 730
TEFSFTDYSTK pc2
689:997 194:247 13:16
14:31343494:G:A_A
p = 3.3e−05, beta = 0.297, N = 896
IAAVQFTYDQR pc2
688:997 190:247 12:16
14:31343494:G:A_A
p = 0.00078, beta = 0.244, N = 890
ENVLAVIR pc2
680:997 184:247 13:16
14:31343494:G:A_A
p = 0.00035, beta = 0.26, N = 877
EFGVNVFIVSVAK pc2
699:997 188:247 13:16
14:31343494:G:A_A
p = 7.5e−05, beta = 0.285, N = 900
VALMLGIGTEGPHVGLVQASEHPK pc2
369:997 110:247 11:16
14:31343494:G:A_A
p = 0.00021, beta = 0.336, N = 490
ESHAFFTR pc2
A0A2U3TZE7;G3V4C4;O43405
101:997 33:247 4:16
14:31343494:G:A_A
p = 0.022, beta = 0.365, N = 138
ADIAFLIDGSFNIGQR pc2
38:997 17:247 3:16
14:31343494:G:A_A
p = 0.75, beta = 0.0687, N = 58
YMSGGTATGDAISFTVR pc2
61:997 20:247 3:16
14:31343494:G:A_A
p = 0.64, beta = −0.0918, N = 84
COCH : NP3
A0A2U3TZE7;O43405
252:253 67:67 5:5
14:31343494:G:A_A
p = 0.29, beta = 0.126, N = 324
TFEISDIGAK pc2
222:253 59:67 4:5
14:31343494:G:A_A
p = 0.13, beta = 0.192, N = 285
EFTGLEPIVSDVIR pc2
A0A2U3TZE7;G3V4C4;O43405
236:253 65:67 5:5
14:31343494:G:A_A
p = 0.0071, beta = 0.324, N = 306
FFTVDAGVR pc2
183:253 50:67 5:5
14:31343494:G:A_A
p = 0.041, beta = 0.27, N = 238
IAAVQFTYDQR pc2
158:253 49:67 3:5
14:31343494:G:A_A
p = 0.32, beta = 0.145, N = 210
LMLEFVSNIAK pc2
194:253 55:67 4:5
14:31343494:G:A_A
p = 0.35, beta = 0.124, N = 253
TEFSFTDYSTK pc2
146:253 44:67 4:5
14:31343494:G:A_A
p = 0.62, beta = −0.0721, N = 194
GVISNSGGPVR pc2
126:253 31:67 3:5
14:31343494:G:A_A
p = 0.41, beta = −0.137, N = 160
EFGVNVFIVSVAK pc2
153:253 43:67 4:5
14:31343494:G:A_A
p = 0.24, beta = 0.169, N = 200
ENVLAVIR pc2
18:253 4:67 0:5
14:31343494:G:A_A
p = 0.95, beta = −0.0352, N = 22
VALMLGIGTEGPHVGLVQASEHPK p
54:253 15:67 2:5
14:31343494:G:A_A
p = 0.23, beta = −0.271, N = 71
WSASFTVTK pc2
14:253 6:67 0:5
14:31343494:G:A_A
p = 0.77, beta = −0.131, N = 20
Assay Target: COCH
UniProt: O43405
rsID: rs28400019
Target SNP: 14:31343494:G:A
Proxy SNP: 14:31343494:G:A
UKBPPP log10(p): 567.1
UKBPPP BETA: 0.507
UKBPPP MSPA: 0.973
988:856:1173:932:1160:730:89
LCTHEQMMCSK pc3
rs1045644 REF
63:997 27:247 2:16
14:31343494:G:A_A
p = 0.011, model = REC, N = 92

136. SPON1 (Q9HCB6)
UKBPPP MSPA=0.389

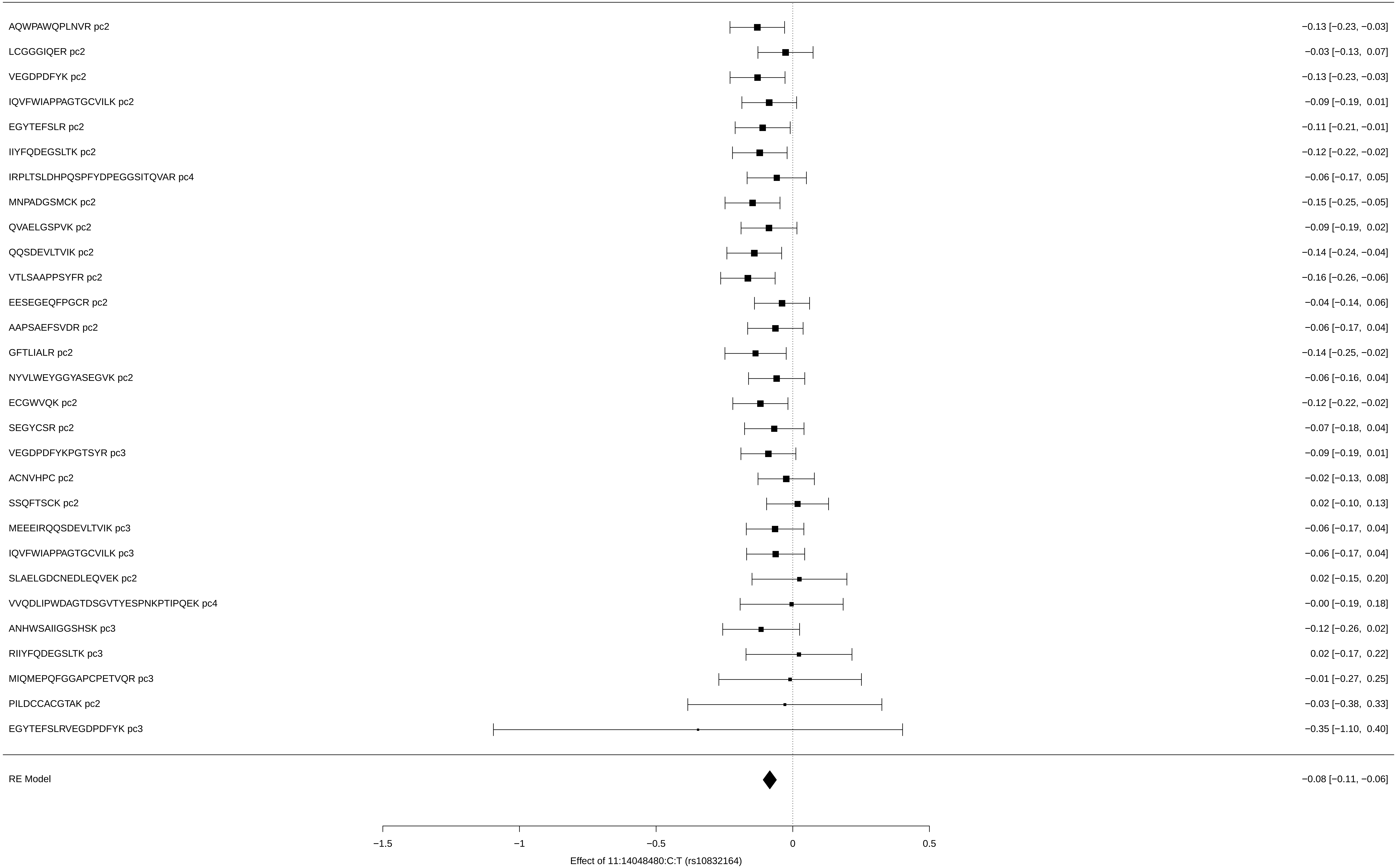

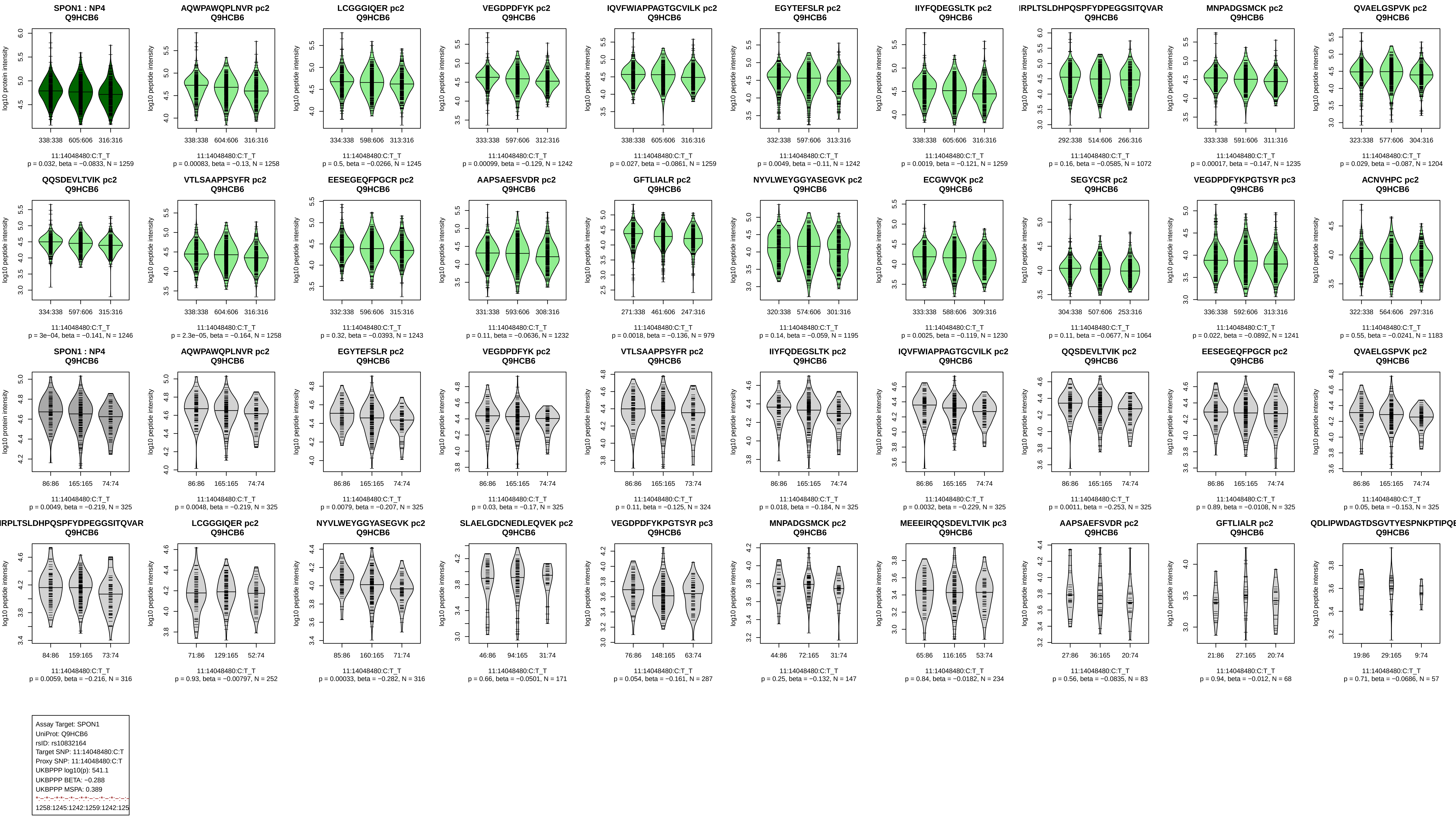
SPON1 : NP4
Q9HCB6
log10 protein intensity
338:338 605:606 316:316
11:14048480:C:T_T
p = 0.032, beta = −0.0833, N = 1259
AQWPAWQPLNVR pc2
Q9HCB6
log10 peptide intensity
338:338 604:606 316:316
11:14048480:C:T_T
p = 0.00083, beta = −0.13, N = 1258
LCGGGIQER pc2
Q9HCB6
334:338 598:606 313:316
11:14048480:C:T_T
p = 0.5, beta = −0.0266, N = 1245
VEGDPDFYK pc2
Q9HCB6
333:338 597:606 312:316
11:14048480:C:T_T
p = 0.00099, beta = −0.129, N = 1242
IQVFWIAPPAGTGCVILK pc2
Q9HCB6
338:338 605:606 316:316
11:14048480:C:T_T
p = 0.027, beta = −0.0861, N = 1259
EGYTEFSLR pc2
Q9HCB6
332:338 597:606 313:316
11:14048480:C:T_T
p = 0.0049, beta = −0.11, N = 1242
IIYFQDEGSLTK pc2
Q9HCB6
338:338 605:606 316:316
11:14048480:C:T_T
p = 0.0019, beta = −0.121, N = 1259
IRPLTSLDHPQSPFYDPEGGSITQVAR
Q9HCB6
292:338 514:606 266:316
11:14048480:C:T_T
p = 0.16, beta = −0.0585, N = 1072
MNPADGSMCK pc2
Q9HCB6
333:338 591:606 311:316
11:14048480:C:T_T
p = 0.00017, beta = −0.147, N = 1235
QVAELGSPVK pc2
Q9HCB6
323:338 577:606 304:316
11:14048480:C:T_T
p = 0.029, beta = −0.087, N = 1204
QQSDEVLTVIK pc2
Q9HCB6
334:338 597:606 315:316
11:14048480:C:T_T
p = 3e−04, beta = −0.141, N = 1246
VTLSAAPPSYFR pc2
Q9HCB6
338:338 604:606 316:316
11:14048480:C:T_T
p = 2.3e−05, beta = −0.164, N = 1258
EESEGEQFPGCR pc2
Q9HCB6
332:338 596:606 315:316
11:14048480:C:T_T
p = 0.32, beta = −0.0393, N = 1243
AAPSAEFSVDR pc2
Q9HCB6
331:338 593:606 308:316
11:14048480:C:T_T
p = 0.11, beta = −0.0636, N = 1232
GFTLIALR pc2
Q9HCB6
271:338 461:606 247:316
11:14048480:C:T_T
p = 0.0018, beta = −0.136, N = 979
NYVLWEYGGYASEGVK pc2
Q9HCB6
320:338 574:606 301:316
11:14048480:C:T_T
p = 0.14, beta = −0.059, N = 1195
ECGWVQK pc2
Q9HCB6
333:338 588:606 309:316
11:14048480:C:T_T
p = 0.0025, beta = −0.119, N = 1230
SEGYCSR pc2
Q9HCB6
304:338 507:606 253:316
11:14048480:C:T_T
p = 0.11, beta = −0.0677, N = 1064
VEGDPDFYKPGTSYR pc3
Q9HCB6
336:338 592:606 313:316
11:14048480:C:T_T
p = 0.022, beta = −0.0892, N = 1241
ACNVHPC pc2
Q9HCB6
322:338 564:606 297:316
11:14048480:C:T_T
p = 0.55, beta = −0.0241, N = 1183
SPON1 : NP4
Q9HCB6
86:86 165:165 74:74
11:14048480:C:T_T
p = 0.0049, beta = −0.219, N = 325
AQWPAWQPLNVR pc2
Q9HCB6
86:86 165:165 74:74
11:14048480:C:T_T
p = 0.0048, beta = −0.219, N = 325
EGYTEFSLR pc2
Q9HCB6
86:86 165:165 74:74
11:14048480:C:T_T
p = 0.0079, beta = −0.207, N = 325
VEGDPDFYK pc2
Q9HCB6
86:86 165:165 74:74
11:14048480:C:T_T
p = 0.03, beta = −0.17, N = 325
VTLSAAPPSYFR pc2
Q9HCB6
86:86 165:165 73:74
11:14048480:C:T_T
p = 0.11, beta = −0.125, N = 324
IIYFQDEGSLTK pc2
Q9HCB6
86:86 165:165 74:74
11:14048480:C:T_T
p = 0.018, beta = −0.184, N = 325
IQVFWIAPPAGTGCVILK pc2
Q9HCB6
86:86 165:165 74:74
11:14048480:C:T_T
p = 0.0032, beta = −0.229, N = 325
QQSDEVLTVIK pc2
Q9HCB6
86:86 165:165 74:74
11:14048480:C:T_T
p = 0.0011, beta = −0.253, N = 325
EESEGEQFPGCR pc2
Q9HCB6
86:86 165:165 74:74
11:14048480:C:T_T
p = 0.89, beta = −0.0108, N = 325
QVAELGSPVK pc2
Q9HCB6
86:86 165:165 74:74
11:14048480:C:T_T
p = 0.05, beta = −0.153, N = 325
IRPLTSLDHPQSPFYDPEGGSITQVAR
Q9HCB6
84:86 159:165 73:74
11:14048480:C:T_T
p = 0.0059, beta = −0.216, N = 316
LCGGGIQER pc2
Q9HCB6
71:86 129:165 52:74
11:14048480:C:T_T
p = 0.93, beta = −0.00797, N = 252
NYVLWEYGGYASEGVK pc2
Q9HCB6
85:86 160:165 71:74
11:14048480:C:T_T
p = 0.00033, beta = −0.282, N = 316
SLAELGDCNEDLEQVEK pc2
Q9HCB6
46:86 94:165 31:74
11:14048480:C:T_T
p = 0.66, beta = −0.0501, N = 171
VEGDPDFYKPGTSYR pc3
Q9HCB6
76:86 148:165 63:74
11:14048480:C:T_T
p = 0.054, beta = −0.161, N = 287
MNPADGSMCK pc2
Q9HCB6
44:86 72:165 31:74
11:14048480:C:T_T
p = 0.25, beta = −0.132, N = 147
MEEEIRQQSDEVLTVIK pc3
Q9HCB6
65:86 116:165 53:74
11:14048480:C:T_T
p = 0.84, beta = −0.0182, N = 234
AAPSAEFSVDR pc2
Q9HCB6
27:86 36:165 20:74
11:14048480:C:T_T
p = 0.56, beta = −0.0835, N = 83
GFTLIALR pc2
Q9HCB6
21:86 27:165 20:74
11:14048480:C:T_T
p = 0.94, beta = −0.012, N = 68
QDLIPWDAGTDSGVTYESPNKTPIPQE
Q9HCB6
19:86 29:165 9:74
11:14048480:C:T_T
p = 0.71, beta = −0.0686, N = 57
Assay Target: SPON1
UniProt: Q9HCB6
rsID: rs10832164
Target SNP: 11:14048480:C:T
Proxy SNP: 11:14048480:C:T
UKBPPP log10(p): 541.1
UKBPPP BETA: −0.288
UKBPPP MSPA: 0.389
1258:1245:1242:1259:1242:125

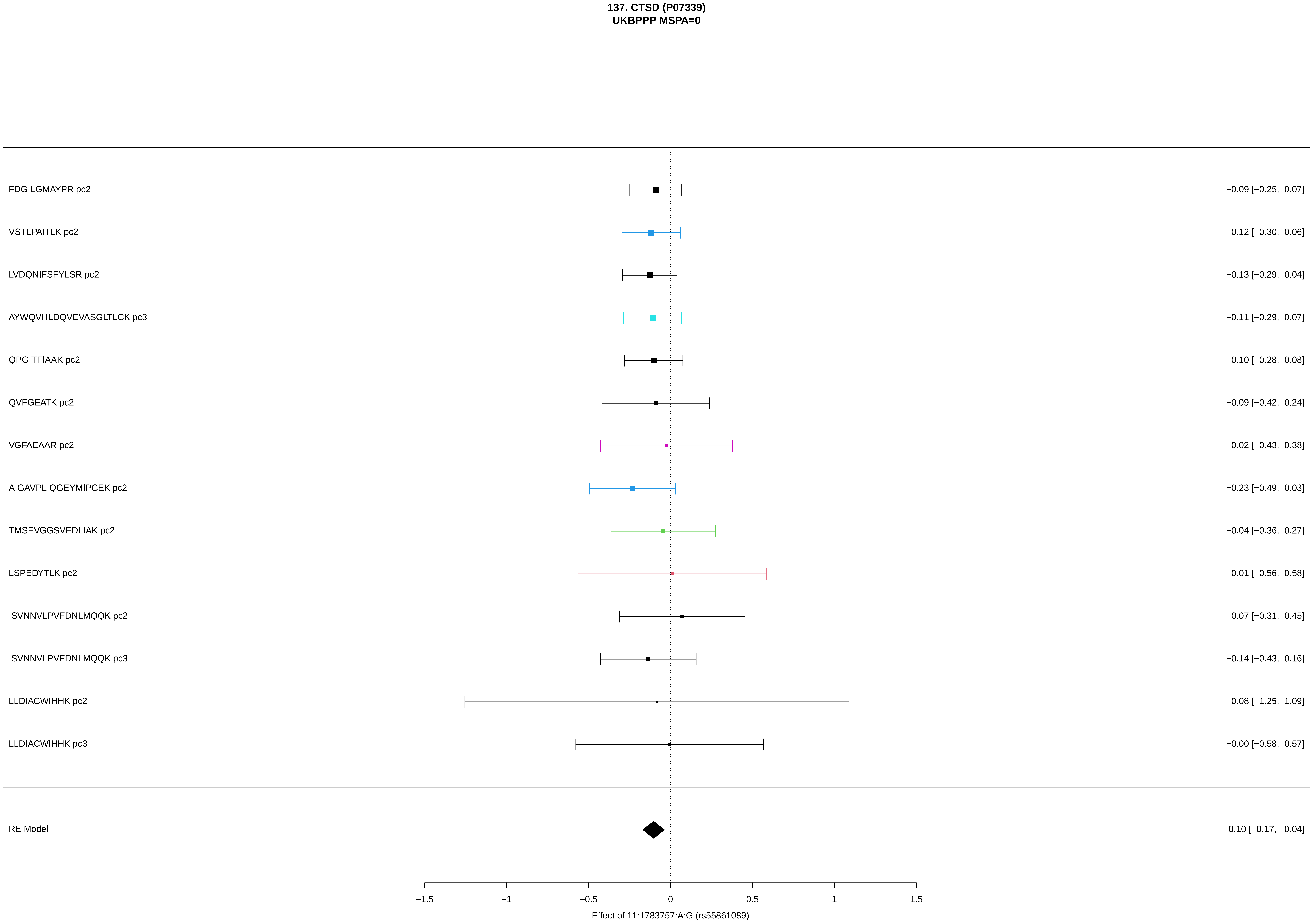

137. CTSD (P07339)
UKBPPP MSPA=0
FDGILGMAYPR pc2
−0.09 [−0.25, 0.07]
VSTLPAITLK pc2
−0.12 [−0.30, 0.06]
LVDQNIFSFYLSR pc2
−0.13 [−0.29, 0.04]
AYWQVHLDQVEVASGLTLCK pc3
−0.11 [−0.29, 0.07]
QPGITFIAAK pc2
−0.10 [−0.28, 0.08]
QVFGEATK pc2
−0.09 [−0.42, 0.24]
VGFAEAAR pc2
−0.02 [−0.43, 0.38]
AIGAVPLIQGEYMIPCEK pc2
−0.23 [−0.49, 0.03]
TMSEVGGSVEDLIAK pc2
−0.04 [−0.36, 0.27]
LSPEDYTLK pc2
0.01 [−0.56, 0.58]
ISVNNVLPVFDNLMQQK pc2
0.07 [−0.31, 0.45]
ISVNNVLPVFDNLMQQK pc3
−0.14 [−0.43, 0.16]
LLDIACWIHHK pc2
−0.08 [−1.25, 1.09]
LLDIACWIHHK pc3
−0.00 [−0.58, 0.57]
RE Model
−0.10 [−0.17, −0.04]
−1.5
−1
−0.5
0
0.5
1
1.5
Effect of 11:1783757:A:G (rs55861089)

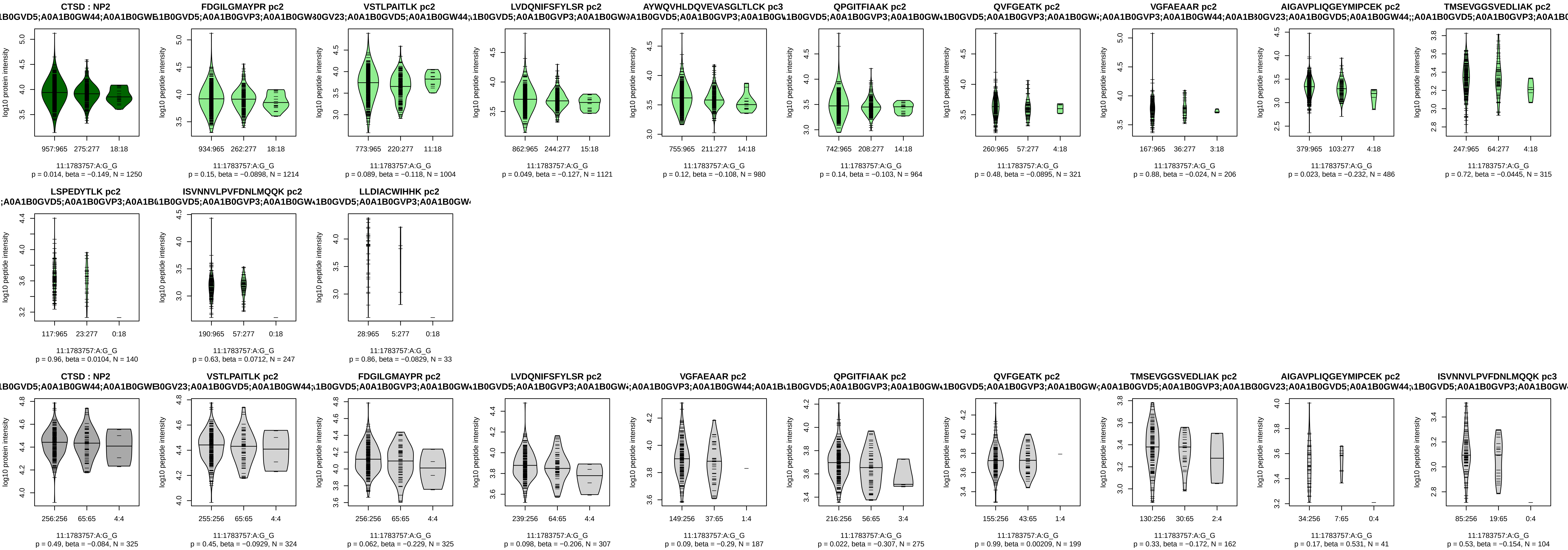

Assay Target: CTSD
UniProt: P07339
rsID: rs55861089
Target SNP: 11:1783757:A:G
Proxy SNP: 11:1783757:A:G
UKBPPP log10(p): 540.3
UKBPPP BETA: −0.492
UKBPPP MSPA: 0
−:−:−:−:−:−:−:−:−:−:−:−:NA
1214:1004:1121:980:964:321:2(

138. HS3ST3B1 (Q9Y662)
UKBPPP MSPA=0.395 (1 conflicts)

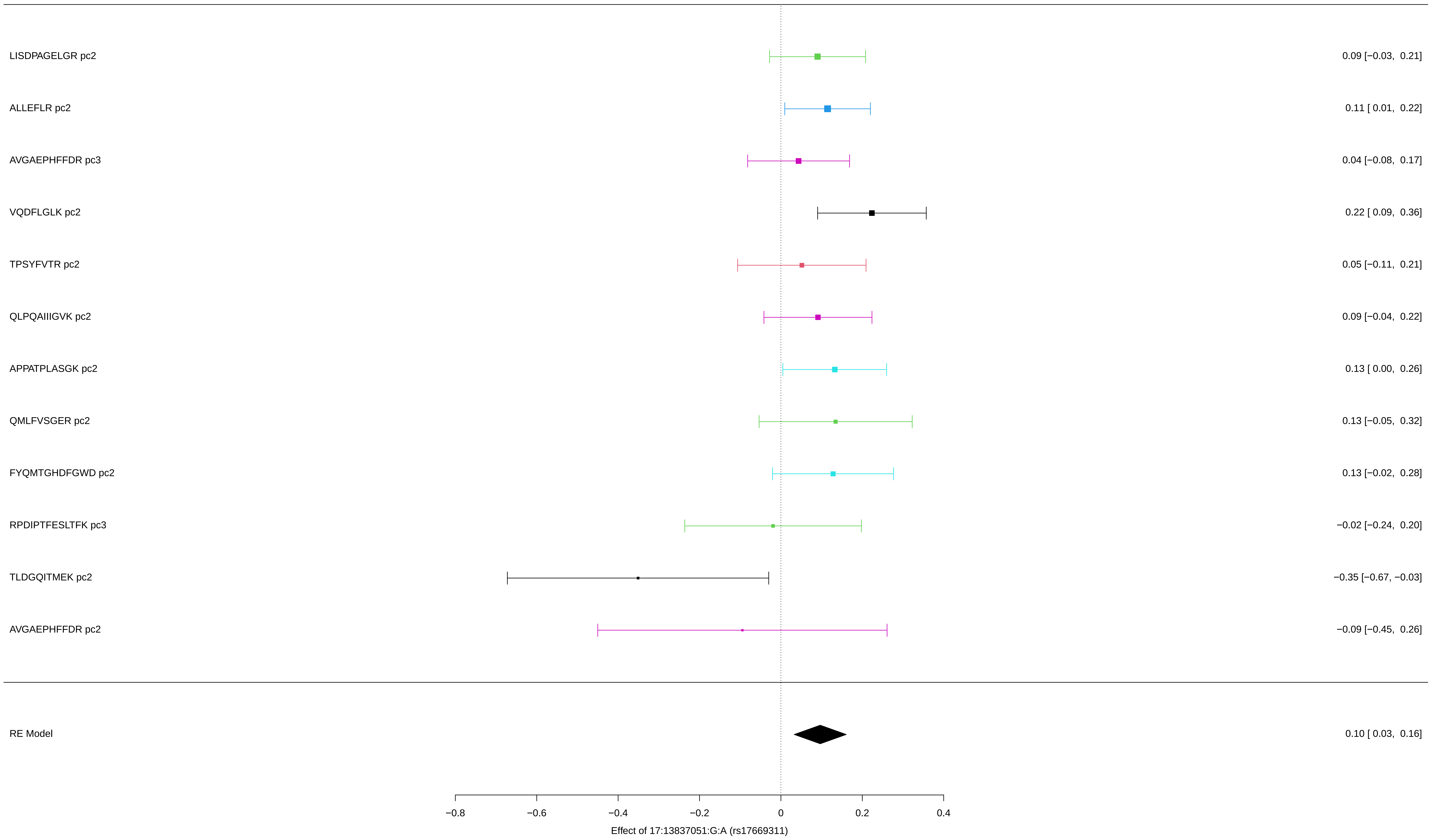

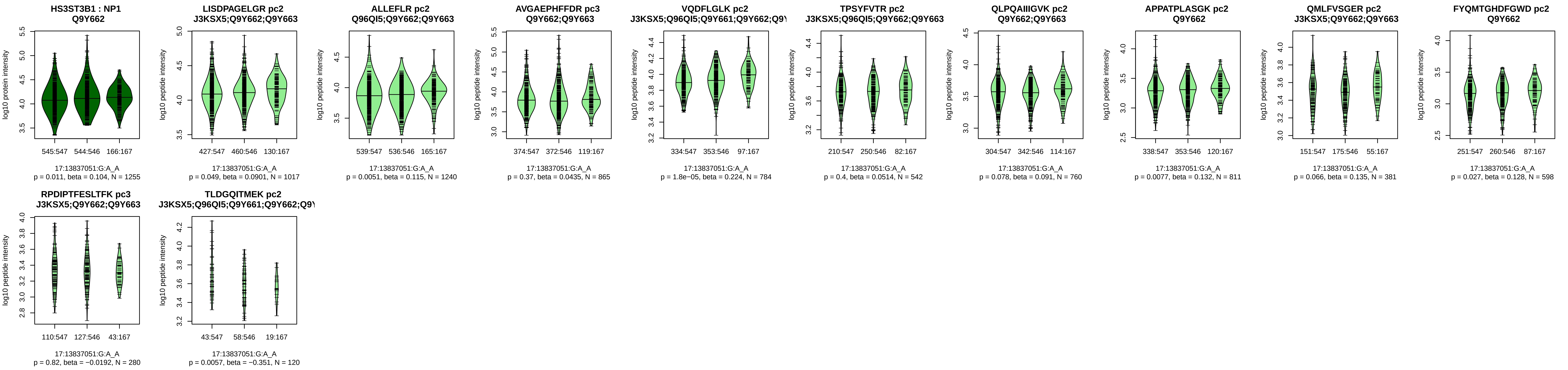

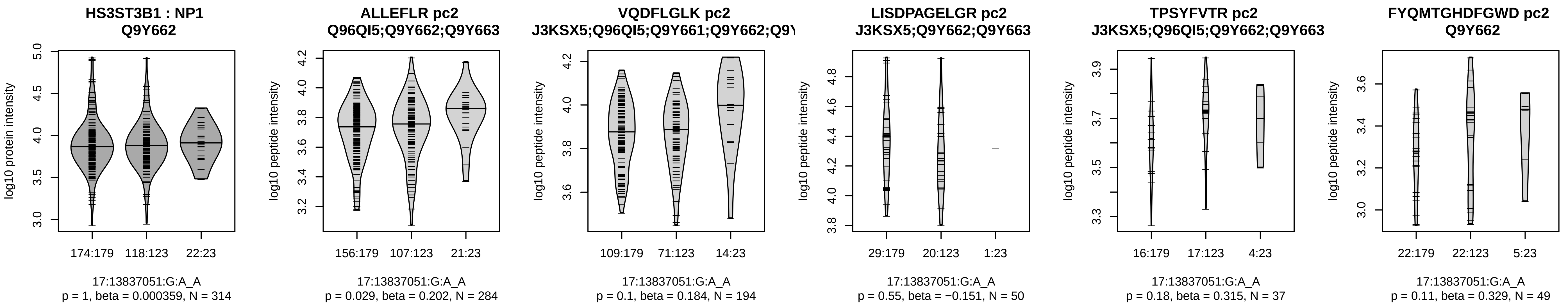

Assay Target: HS3ST3B1
UniProt: Q9Y662
rsID: rs17669311
Target SNP: 17:13837051:G:A
Proxy SNP: 17:13837051:G:A
UKBPPP log10(p): 538.7
UKBPPP BETA: 0.32
UKBPPP MSPA: 0.395 (1)
–·*·–·*·–·–·*·–·–·*·–
1017:1240:865:784:542:760:81

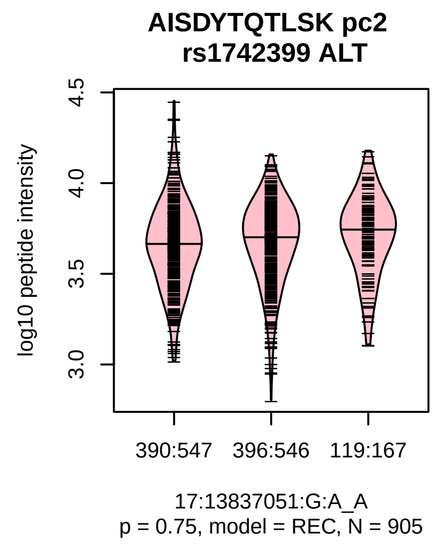

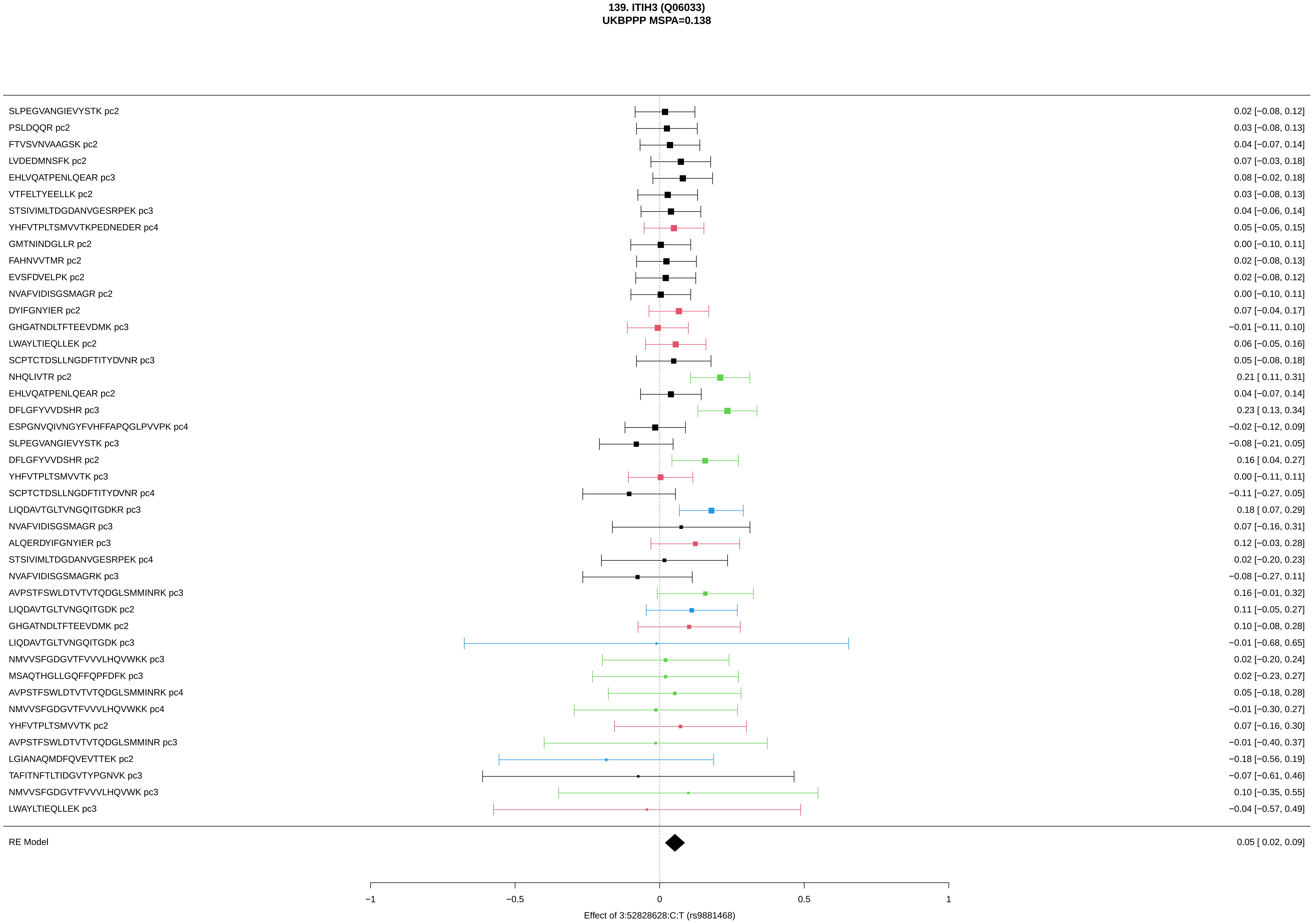

139. ITIH3 (Q06033)
UKBPPP MSPA=0.138
SLPEGVANGIEVYSTK pc2
PSLDQQR pc2
FTVSVNVAAGSK pc2
LVDEDMNSFK pc2
EHLVQATPENLQEAR pc3
VTFELTYEELLK pc2
STSIVIMLTDGDANVGESRPEK pc3
YHFVTPLTSMVVTKPEDNEDER pc4
GMTNINDGLLR pc2
FAHNVVTMR pc2
EVSFDVELPK pc2
NVAFVIDISGSMAGR pc2
DYIFGNYIER pc2
GHGATNDLTFTEEVDMK pc3
LWAYLTIEQLLEK pc2
SCPTCTDSLLNGDFTITYDVNR pc3
NHQLIVTR pc2
EHLVQATPENLQEAR pc2
DFLGFYVVDSHR pc3
ESPGNVQIVNGYFVHFFAPQGLPVVPK pc4
SLPEGVANGIEVYSTK pc3
DFLGFYVVDSHR pc2
YHFVTPLTSMVVTK pc3
SCPTCTDSLLNGDFTITYDVNR pc4
LIQDAVTGLTVNGQITGDKR pc3
NVAFVIDISGSMAGR pc3
ALQERDYIFGNYIER pc3
STSIVIMLTDGDANVGESRPEK pc4
NVAFVIDISGSMAGRK pc3
AVPSTFSWLDTVTVTQDGLSMMINRK pc3
LIQDAVTGLTVNGQITGDK pc2
GHGATNDLTFTEEVDMK pc2
LIQDAVTGLTVNGQITGDK pc3
NMVVSFGDGVTFVVVLHQVWKK pc3
MSAQTHGLLGQFFQPFDFK pc3
AVPSTFSWLDTVTVTQDGLSMMINRK pc4
NMVVSFGDGVTFVVVLHQVWKK pc4
YHFVTPLTSMVVTK pc2
AVPSTFSWLDTVTVTQDGLSMMINR pc3
LGIANAQMDFQVEVTTEK pc2
TAFITNFTLTIDGVTYPGNVK pc3
NMVVSFGDGVTFVVVLHQVWK pc3
LWAYLTIEQLLEK pc3
RE Model
0.02 [−0.08, 0.12]
0.03 [−0.08, 0.13]
0.04 [−0.07, 0.14]
0.07 [−0.03, 0.18]
0.08 [−0.02, 0.18]
0.03 [−0.08, 0.13]
0.04 [−0.06, 0.14]
0.05 [−0.05, 0.15]
0.00 [−0.10, 0.11]
0.02 [−0.08, 0.13]
0.02 [−0.08, 0.12]
0.00 [−0.10, 0.11]
0.07 [−0.04, 0.17]
−0.01 [−0.11, 0.10]
0.06 [−0.05, 0.16]
0.05 [−0.08, 0.18]
0.21 [0.11, 0.31]
0.04 [−0.07, 0.14]
0.23 [0.13, 0.34]
−0.02 [−0.12, 0.09]
−0.08 [−0.21, 0.05]
0.16 [0.04, 0.27]
0.00 [−0.11, 0.11]
−0.11 [−0.27, 0.05]
0.18 [0.07, 0.29]
0.07 [−0.16, 0.31]
0.12 [−0.03, 0.28]
0.02 [−0.20, 0.23]
−0.08 [−0.27, 0.11]
0.16 [−0.01, 0.32]
0.11 [−0.05, 0.27]
0.10 [−0.08, 0.28]
−0.01 [−0.68, 0.65]
0.02 [−0.20, 0.24]
0.02 [−0.23, 0.27]
0.05 [−0.18, 0.28]
−0.01 [−0.30, 0.27]
0.07 [−0.16, 0.30]
−0.01 [−0.40, 0.37]
−0.18 [−0.56, 0.19]
−0.07 [−0.61, 0.46]
0.10 [−0.35, 0.55]
−0.04 [−0.57, 0.49]
0.05 [0.02, 0.09]
−1
−0.5
0
0.5
1
Effect of 3:52828628:C:T (rs9881468)

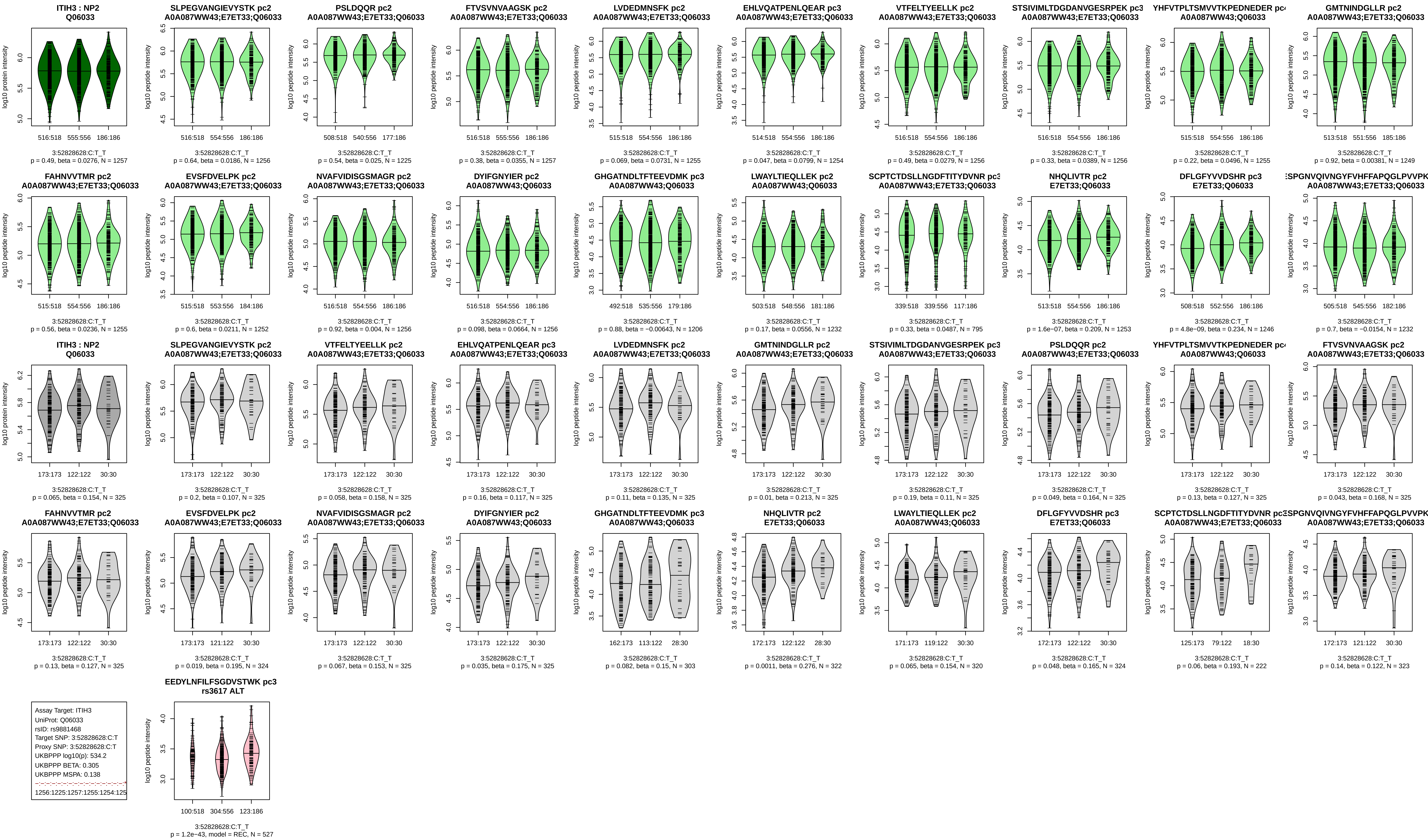
ITIH3 : NP2 Q06033
p = 0.49, beta = 0.0276, N = 1257
SLPEGVANGIEVYSTK pc2 A0A087WW43;E7ET33;Q06033
p = 0.64, beta = 0.0186, N = 1256
PSLDQQR pc2 A0A087WW43;E7ET33;Q06033
p = 0.54, beta = 0.025, N = 1225
FTVSVNVAAGSK pc2 A0A087WW43;E7ET33;Q06033
p = 0.38, beta = 0.0355, N = 1257
LVDEDMNSFK pc2 A0A087WW43;E7ET33;Q06033
p = 0.069, beta = 0.0731, N = 1255
EHLVQATPENLQEAR pc3 A0A087WW43;E7ET33;Q06033
p = 0.047, beta = 0.0799, N = 1254
VTFELTYEELLK pc2 A0A087WW43;E7ET33;Q06033
p = 0.49, beta = 0.0279, N = 1256
STSIVIMLTDGDANVGESRPEK pc3 A0A087WW43;E7ET33;Q06033
p = 0.33, beta = 0.0389, N = 1256
YHFVTPLTSMVVTKPEDNEDER pc A0A087WW43;Q06033
p = 0.22, beta = 0.0496, N = 1255
GMTNINDGLLR pc2 A0A087WW43;E7ET33;Q06033
p = 0.92, beta = 0.00381, N = 1249
FAHNVVTMR pc2 A0A087WW43;E7ET33;Q06033
p = 0.56, beta = 0.0236, N = 1255
EVSFDVELPK pc2 A0A087WW43;E7ET33;Q06033
p = 0.6, beta = 0.0211, N = 1252
NVAFVIDISGSMAGR pc2 A0A087WW43;E7ET33;Q06033
p = 0.92, beta = 0.004, N = 1256
DYIFGNYIER pc2 A0A087WW43;Q06033
p = 0.098, beta = 0.0664, N = 1256
GHGATNDLTFTEEVDMK pc3 A0A087WW43;Q06033
p = 0.88, beta = −0.00643, N = 1206
LWAYLTIEQLLEK pc2 A0A087WW43;Q06033
p = 0.17, beta = 0.0556, N = 1232
SCPTCTDSLLNGDFTITYDVNR pc3 A0A087WW43;E7ET33;Q06033
p = 0.33, beta = 0.0487, N = 795
NHQLIVTR pc2 E7ET33;Q06033
p = 1.6e−07, beta = 0.209, N = 1253
DFLGFYVVDSHR pc3 E7ET33;Q06033
p = 4.8e−09, beta = 0.234, N = 1246
SPGNVQIVNGYFVHFFAPQGLPVVPK A0A087WW43;E7ET33;Q06033
p = 0.7, beta = −0.0154, N = 1232
p = 0.065, beta = 0.154, N = 325
p = 0.2, beta = 0.107, N = 325
p = 0.058, beta = 0.158, N = 325
p = 0.16, beta = 0.117, N = 325
p = 0.11, beta = 0.135, N = 325
p = 0.01, beta = 0.213, N = 325
p = 0.19, beta = 0.11, N = 325
p = 0.049, beta = 0.164, N = 325
p = 0.13, beta = 0.127, N = 325
p = 0.043, beta = 0.168, N = 325
p = 0.13, beta = 0.127, N = 325
p = 0.019, beta = 0.195, N = 324
p = 0.067, beta = 0.153, N = 325
p = 0.035, beta = 0.175, N = 325
p = 0.082, beta = 0.15, N = 303
p = 0.0011, beta = 0.276, N = 322
p = 0.065, beta = 0.154, N = 320
p = 0.048, beta = 0.165, N = 324
p = 0.06, beta = 0.193, N = 222
p = 0.14, beta = 0.122, N = 323
3:52828628:C:T_T
Assay Target: ITIH3
UniProt: Q06033
rsID: rs9881468
Target SNP: 3:52828628:C:T
Proxy SNP: 3:52828628:C:T
UKBPPP log10(p): 534.2
UKBPPP BETA: 0.305
UKBPPP MSPA: 0.138
1256:1225:1257:1255:1254:125
EEDYLNFILFSGDVSTWK pc3 rs3617 ALT
p = 1.2e−43, model = REC, N = 527

140. NRP1 (O14786)
UKBPPP MSPA=0.71

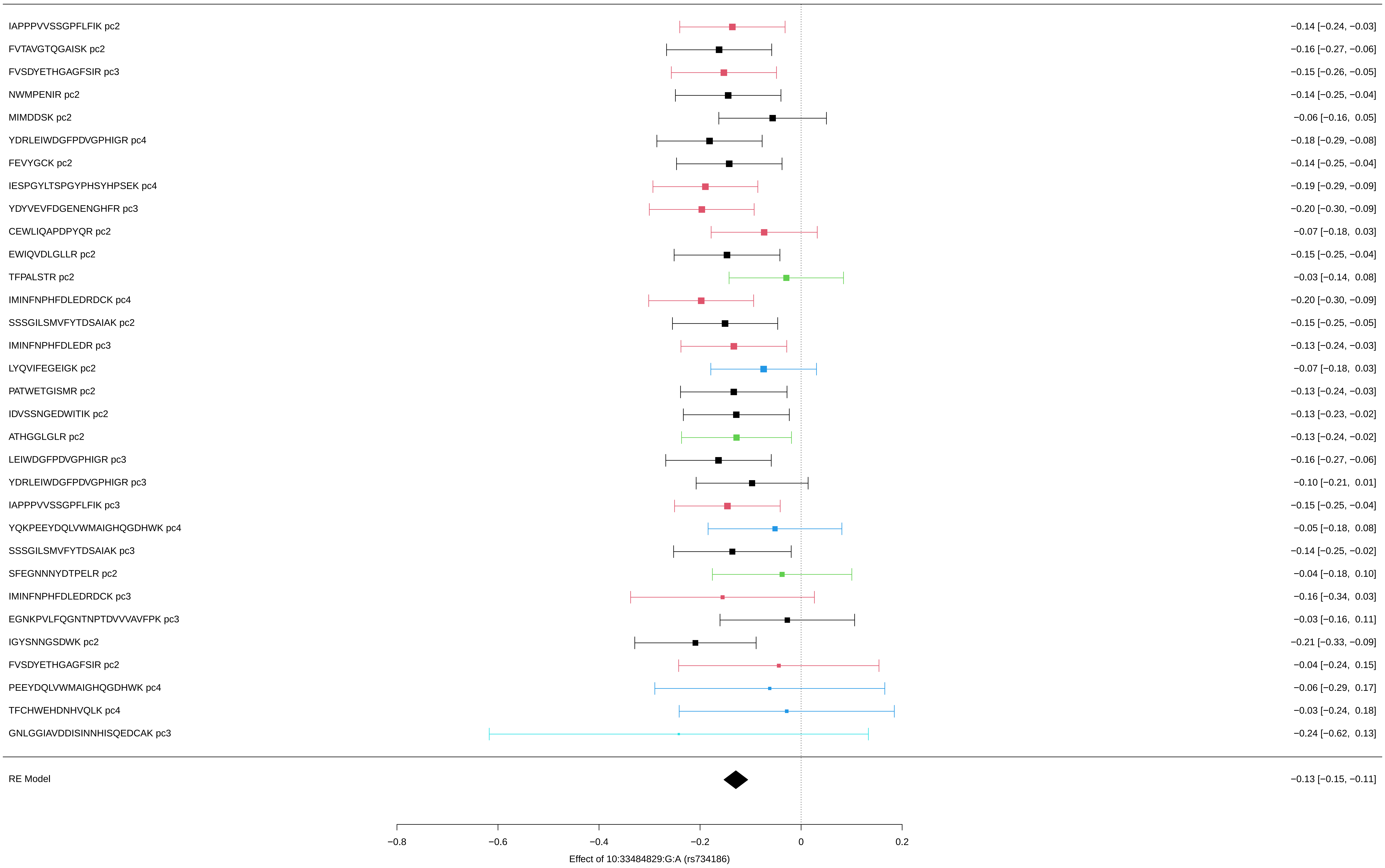

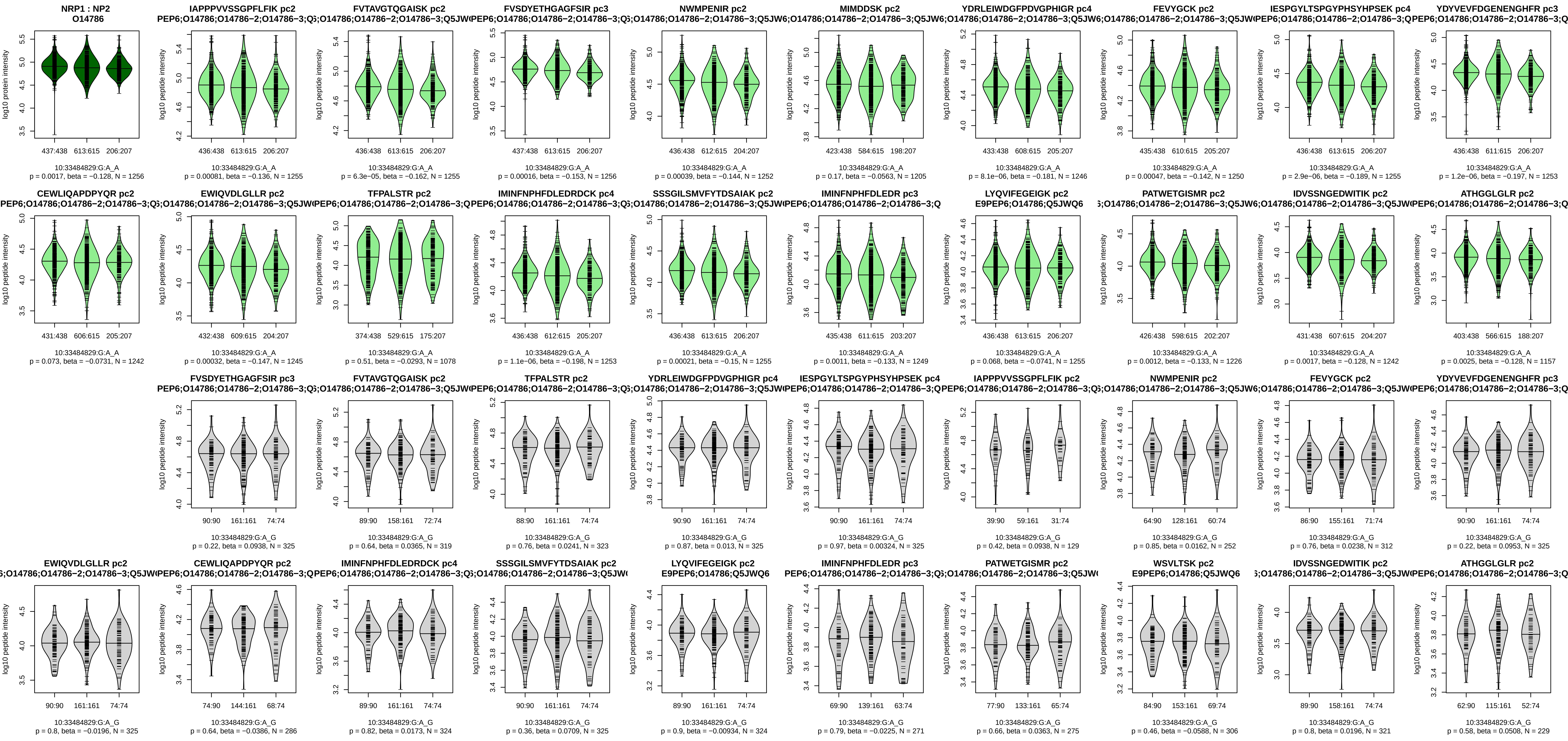

Assay Target: NRP1
UniProt: O14786
rsID: rs734186
Target SNP: 10:33484829:G:A
Proxy SNP: 10:33484829:G:A
UKBPPP log10(p): 521.2
UKBPPP BETA: −0.304
UKBPPP MSPA: 0.71
1255:1255:1256:1252:1205:124

141. SHBG (P04278)
UKBPPP MSPA=0.887

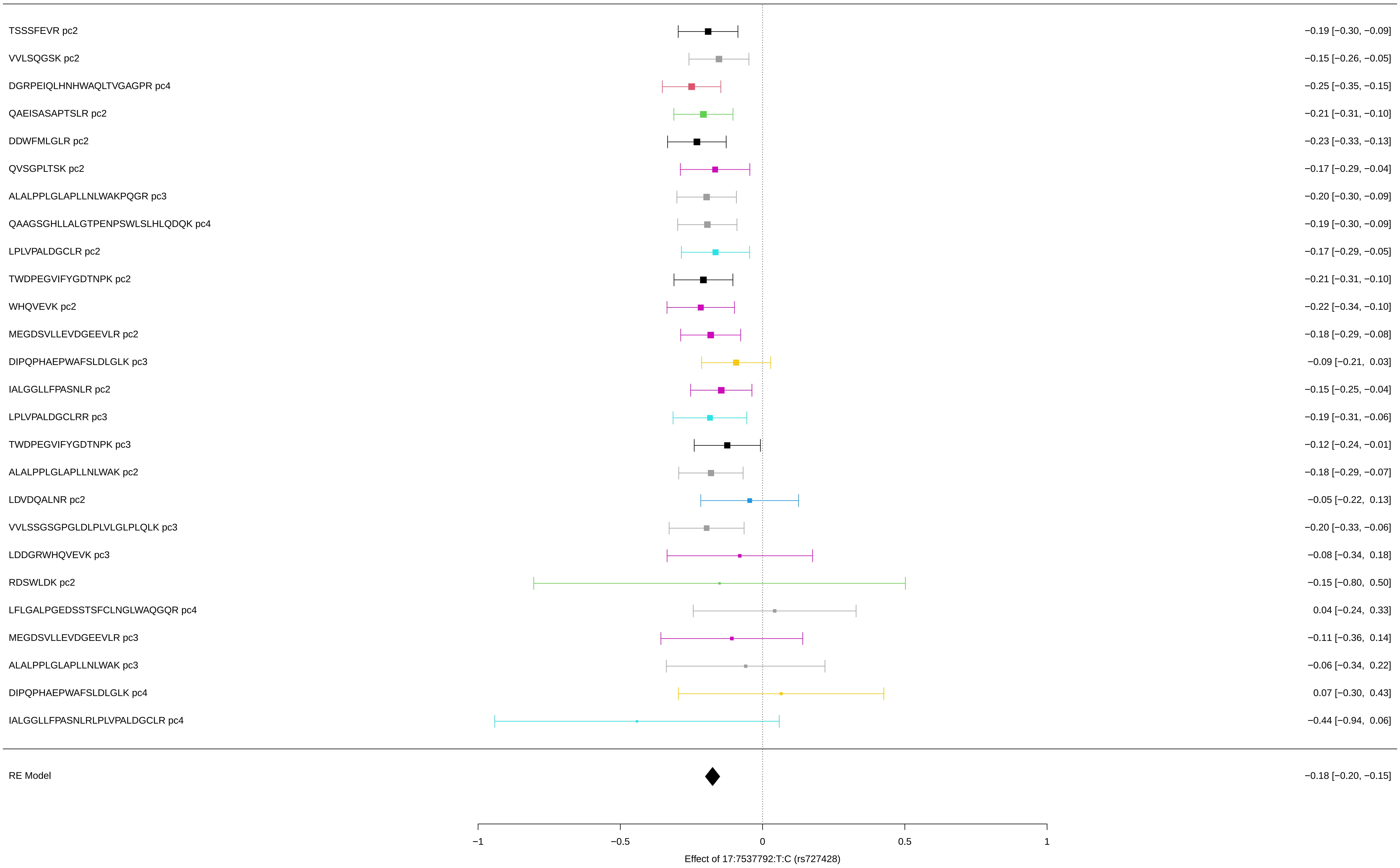

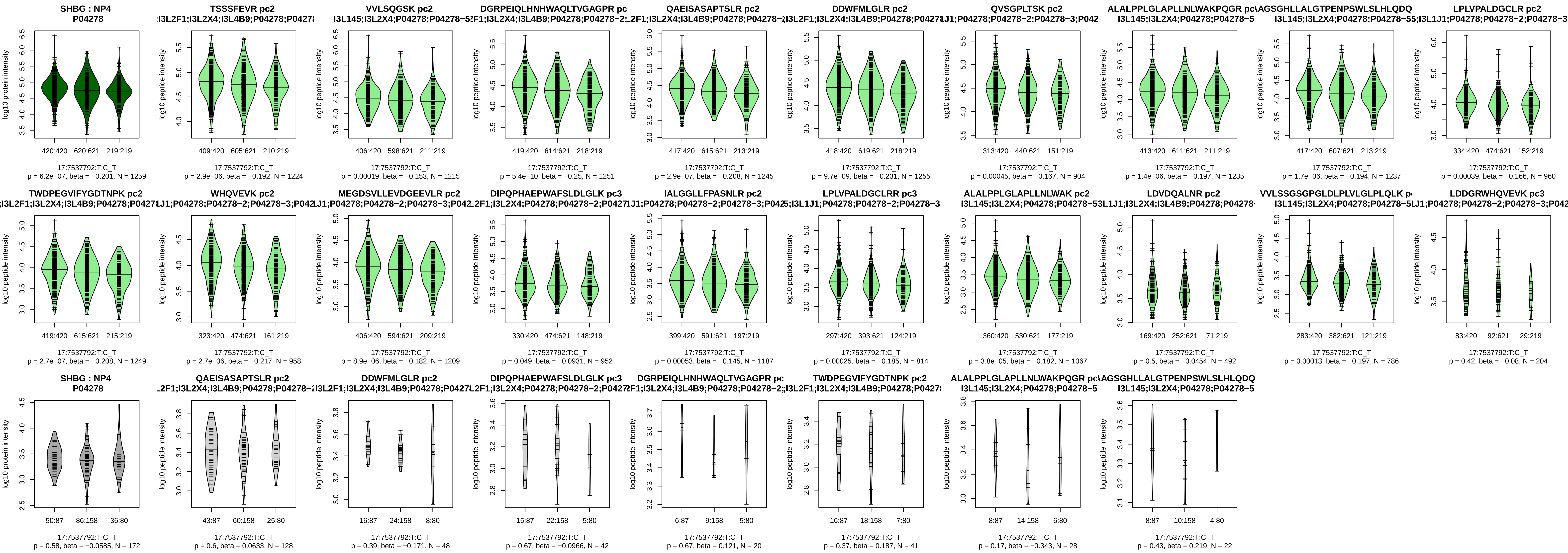

Assay Target: SHBG
UniProt: P04278
rsID: rs727428
Target SNP: 17:7537792:T:C
Proxy SNP: 17:7537792:T:C
UKBPPP log10(p): 514.8
UKBPPP BETA: 0.272
UKBPPP MSPA: 0.887
1224:1215:1251:1245:1255:904

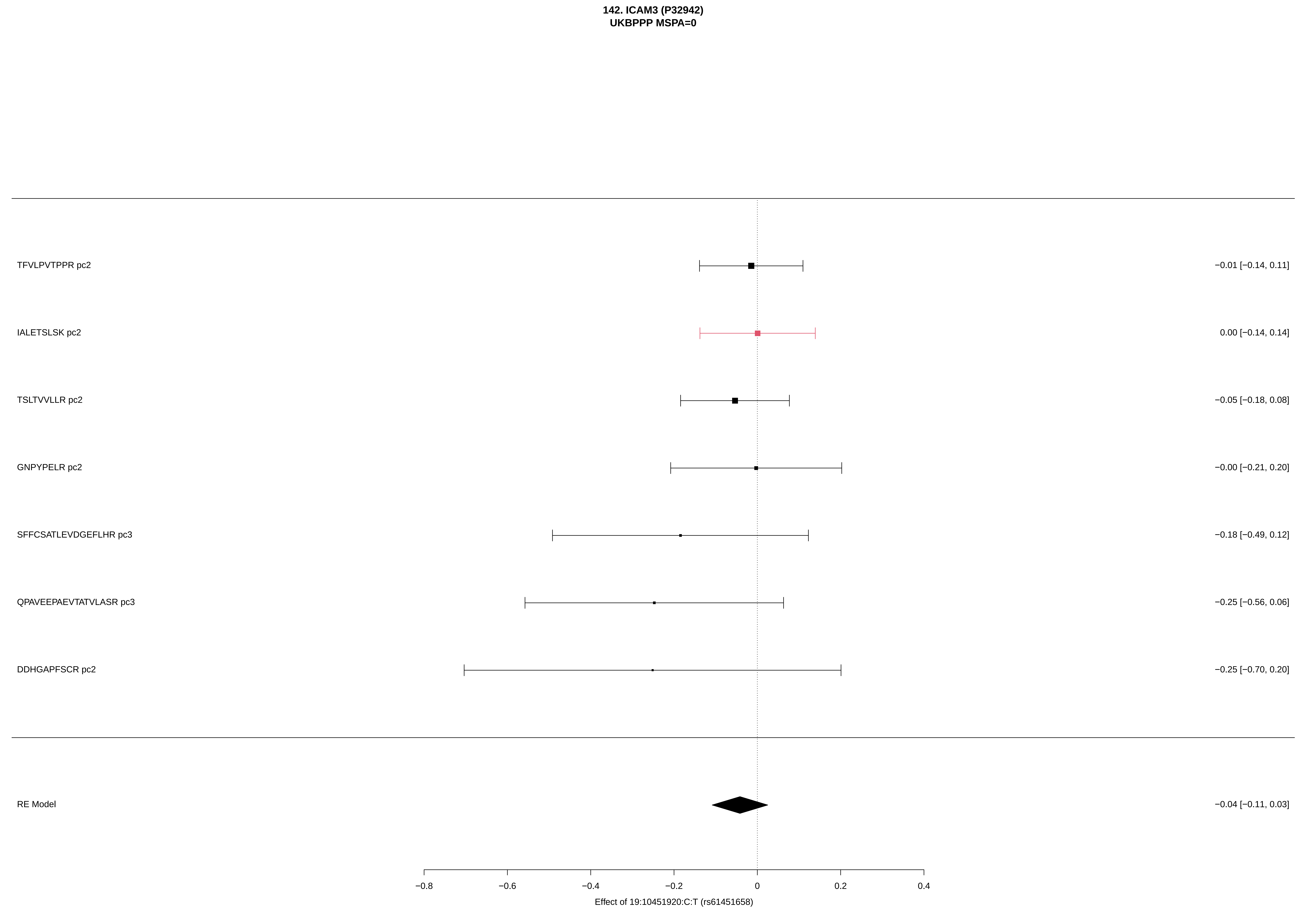

142. ICAM3 (P32942)
UKBPPP MSPA=0
TFVLPVTPPR pc2
−0.01 [−0.14, 0.11]
IALETSLSK pc2
0.00 [−0.14, 0.14]
TSLTVVLLR pc2
−0.05 [−0.18, 0.08]
GNPYPELR pc2
−0.00 [−0.21, 0.20]
SFFCSATLEVDGEFLHR pc3
−0.18 [−0.49, 0.12]
QPAVEEPAEVTATVLASR pc3
−0.25 [−0.56, 0.06]
DDHGAPFSCR pc2
−0.25 [−0.70, 0.20]
RE Model
−0.04 [−0.11, 0.03]
−0.8
−0.6
−0.4
−0.2
0
0.2
0.4
Effect of 19:10451920:C:T (rs61451658)

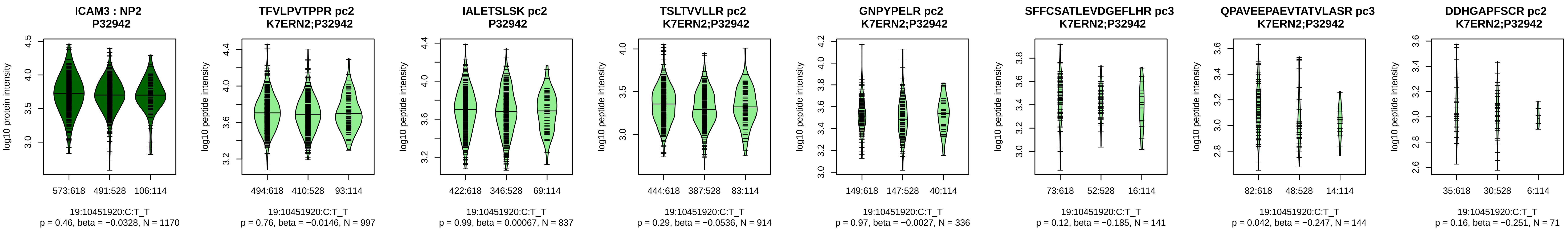

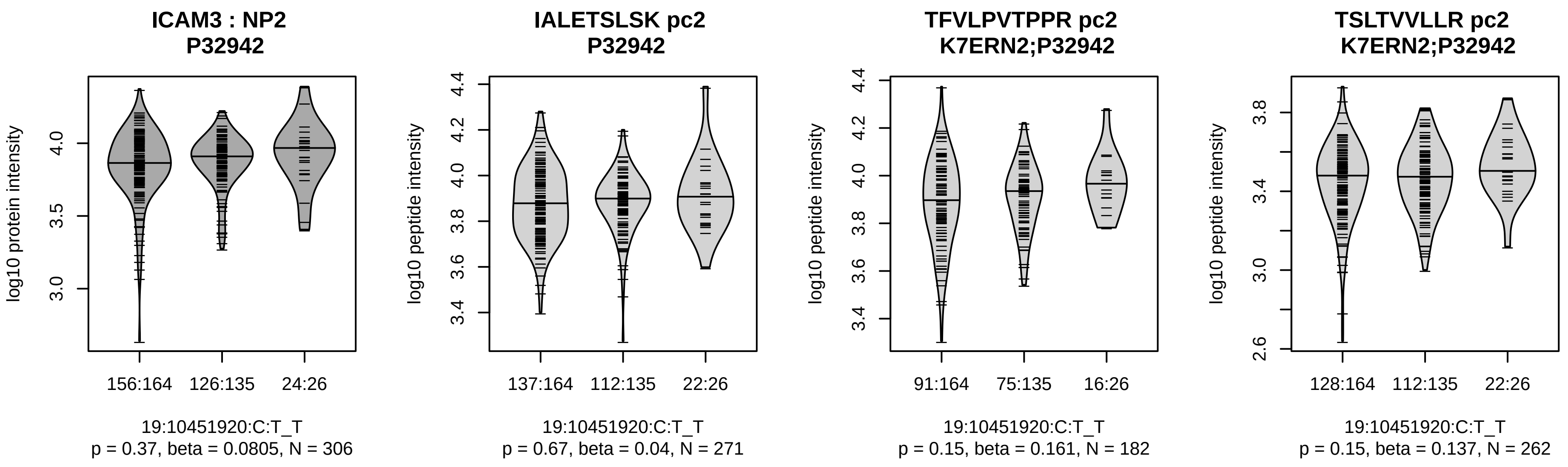

Assay Target: ICAM3
UniProt: P32942
rsID: rs61451658
Target SNP: 19:10451920:C:T
Proxy SNP: 19:10451920:C:T
UKBPPP log10(p): 510
UKBPPP BETA: 0.309
UKBPPP MSPA: 0
–:–:–:–:–:–:–
997:837:914:336:141:144:71

143. ANGPTL3 (Q9Y5C1)
UKBPPP MSPA=0.751

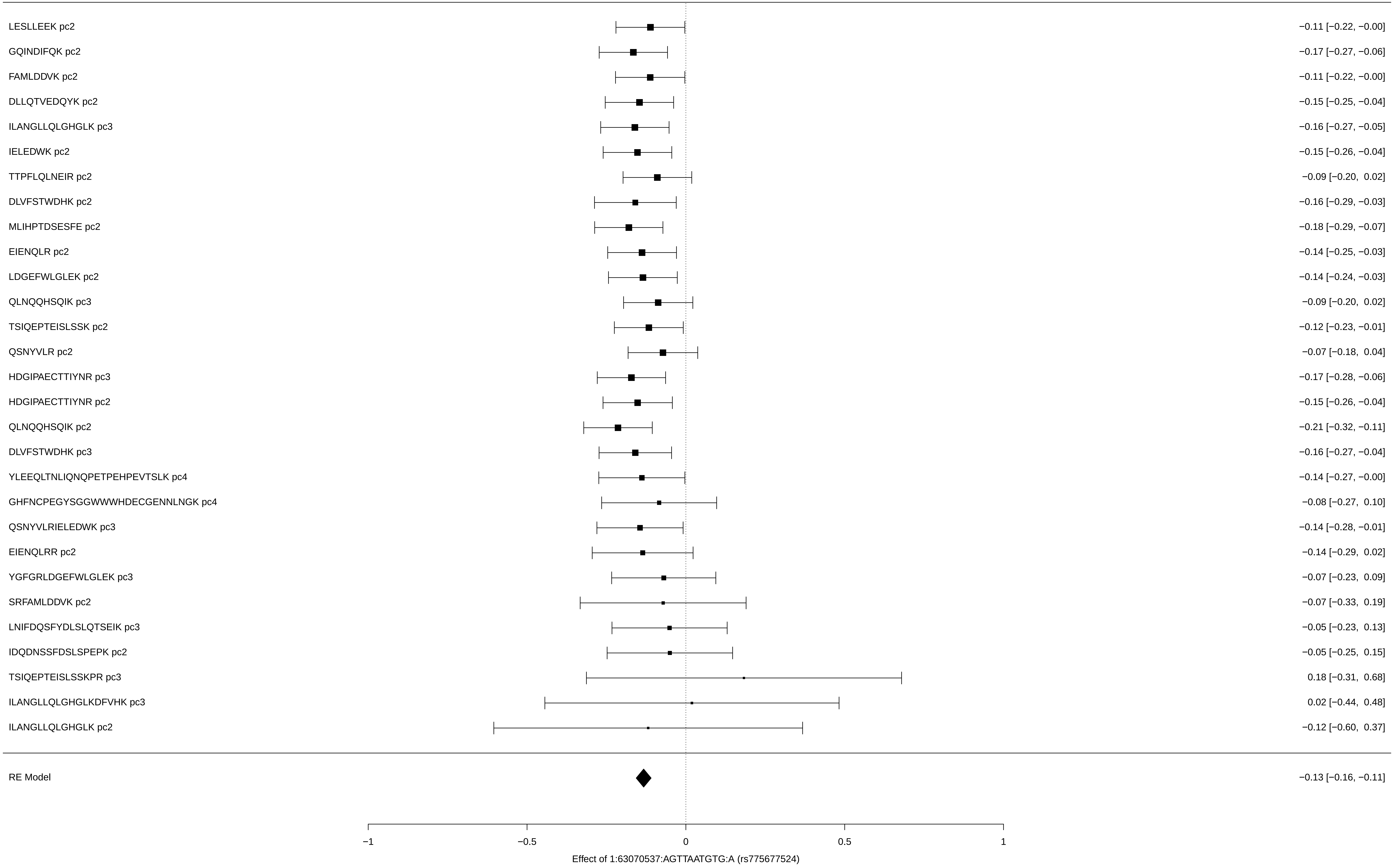

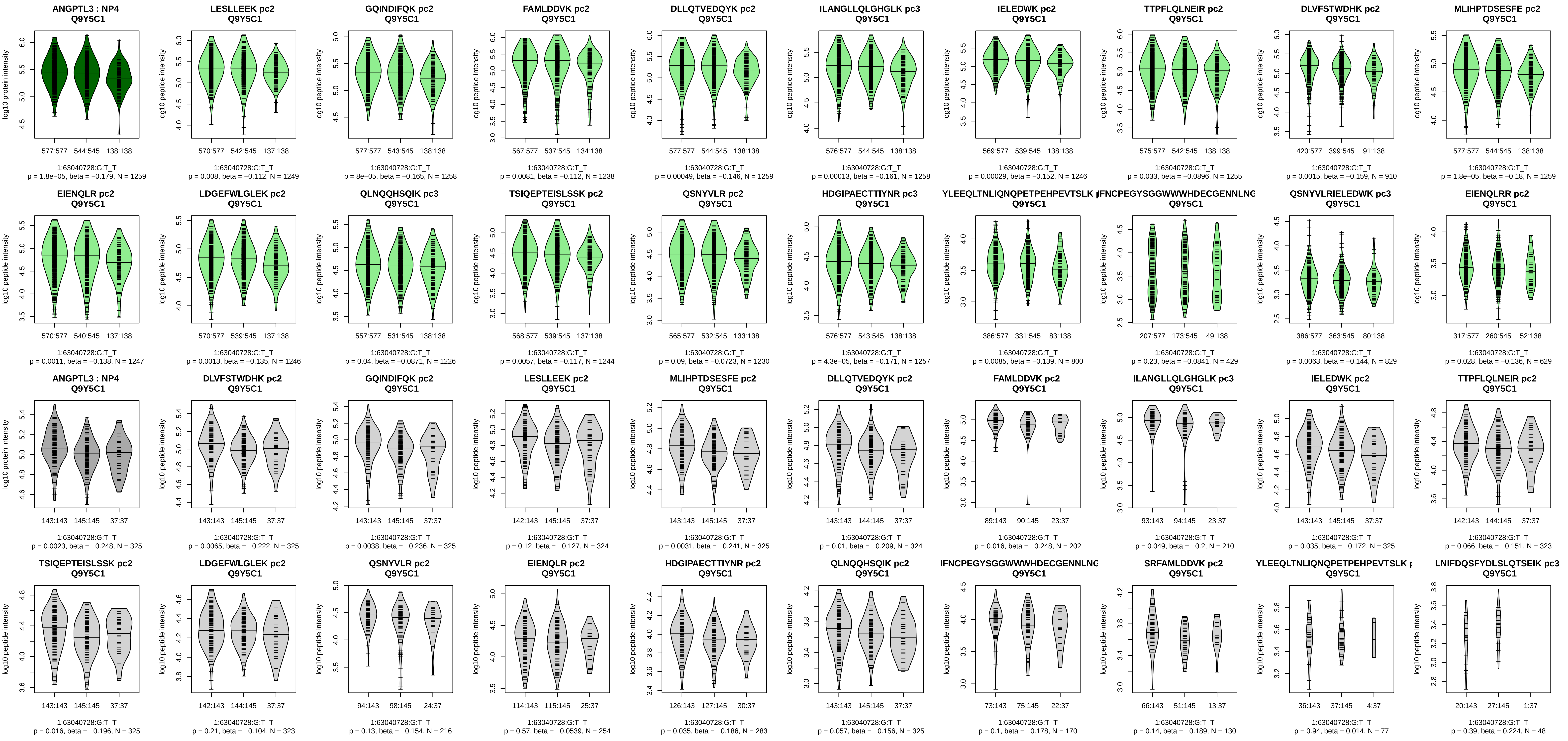

Assay Target: ANGPTL3
UniProt: Q9Y5C1
rsID: rs775677524
Target SNP: 1:63070537:AGTTA
Proxy SNP: 1:63040728:G:T
UKBPPP log10(p): 500.3
UKBPPP BETA: −0.282
UKBPPP MSPA: 0.751
1249:1258:1238:1259:1258:124

144. ASGR2 (P07307)
UKBPPP MSPA=0.375

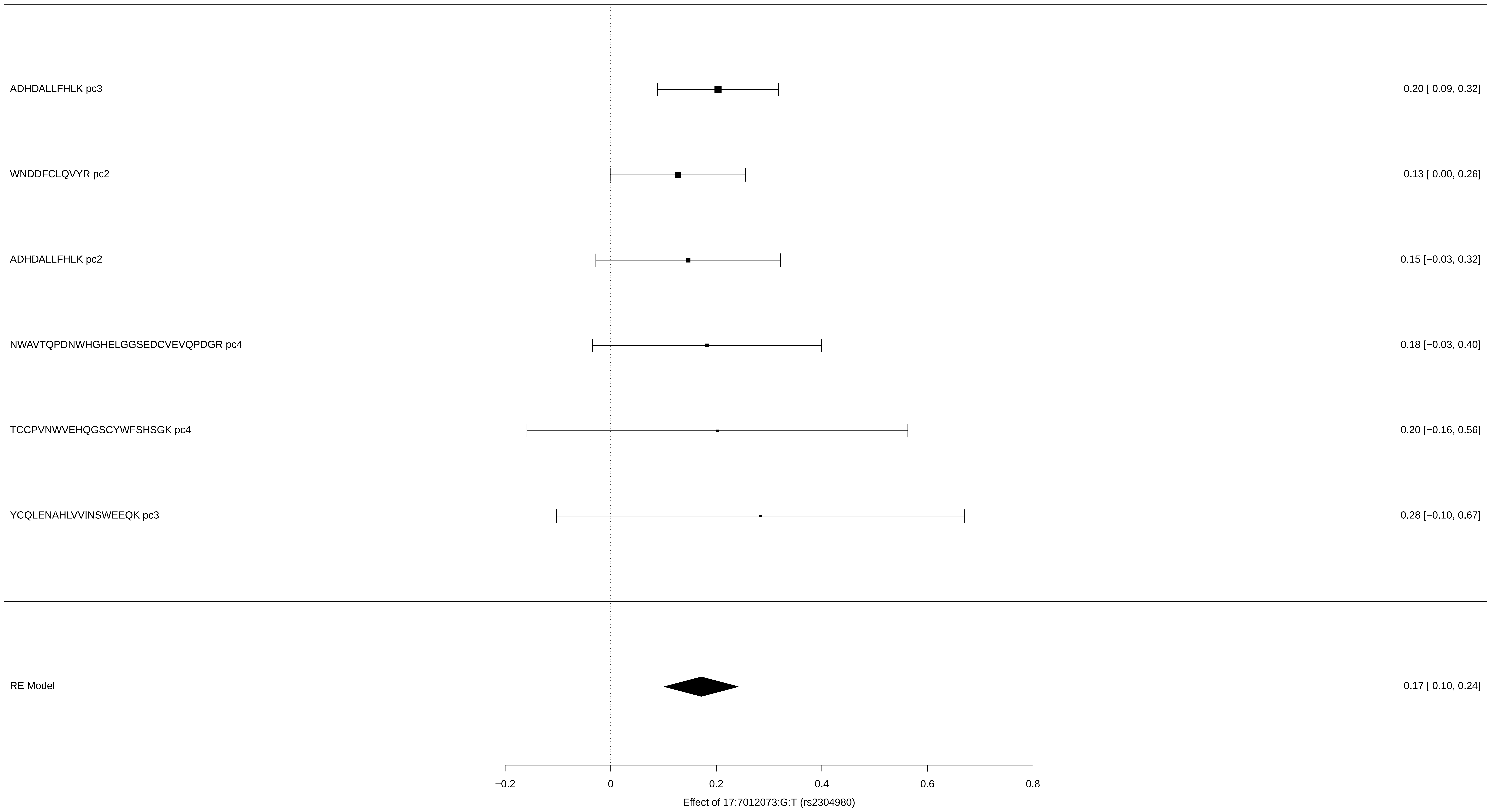

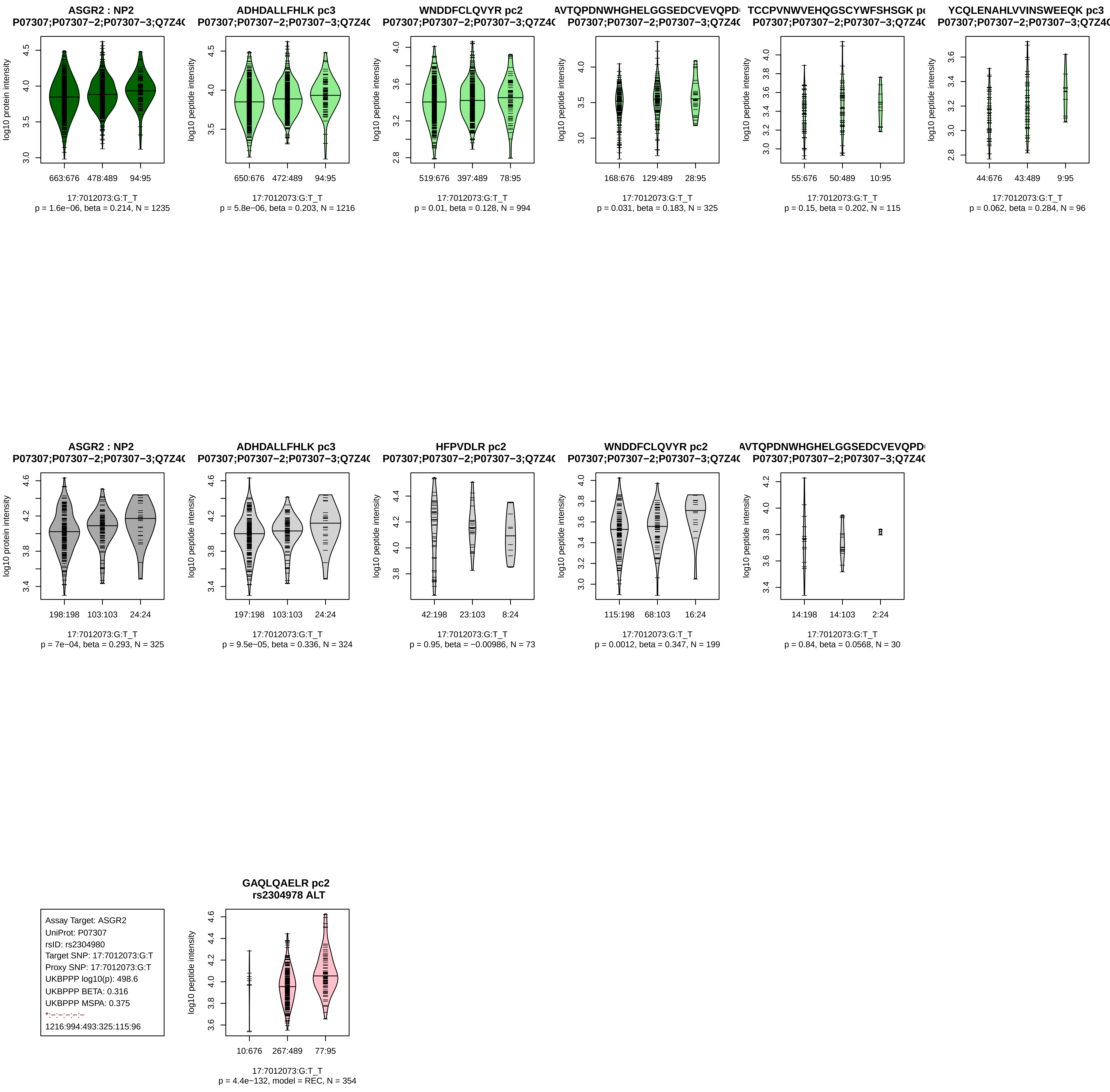
ASGR2 : NP2
P07307;P07307−2;P07307−3;Q7Z4(
log10 protein intensity
663:676 478:489 94:95
17:7012073:G:T_T
p = 1.6e−06, beta = 0.214, N = 1235
ADHDALLFHLK pc3
P07307;P07307−2;P07307−3;Q7Z4(
log10 peptide intensity
650:676 472:489 94:95
17:7012073:G:T_T
p = 5.8e−06, beta = 0.203, N = 1216
WNDDFCLQVYR pc2
P07307;P07307−2;P07307−3;Q7Z4(
log10 peptide intensity
519:676 397:489 78:95
17:7012073:G:T_T
p = 0.01, beta = 0.128, N = 994
AVTQPDNWHGHELGGSEDCVEVQPD(
P07307;P07307−2;P07307−3;Q7Z4(
log10 peptide intensity
168:676 129:489 28:95
17:7012073:G:T_T
p = 0.031, beta = 0.183, N = 325
TCCPVNWVEHQGSCYWFSHSGK p(
P07307;P07307−2;P07307−3;Q7Z4(
log10 peptide intensity
55:676 50:489 10:95
17:7012073:G:T_T
p = 0.15, beta = 0.202, N = 115
YCQLENAHLVVINSWEEQK pc3
P07307;P07307−2;P07307−3;Q7Z4(
log10 peptide intensity
44:676 43:489 9:95
17:7012073:G:T_T
p = 0.062, beta = 0.284, N = 96
ASGR2 : NP2
P07307;P07307−2;P07307−3;Q7Z4(
log10 protein intensity
198:198 103:103 24:24
17:7012073:G:T_T
p = 7e−04, beta = 0.293, N = 325
ADHDALLFHLK pc3
P07307;P07307−2;P07307−3;Q7Z4(
log10 peptide intensity
197:198 103:103 24:24
17:7012073:G:T_T
p = 9.5e−05, beta = 0.336, N = 324
HFPVDLR pc2
P07307;P07307−2;P07307−3;Q7Z4(
log10 peptide intensity
42:198 23:103 8:24
17:7012073:G:T_T
p = 0.95, beta = −0.00986, N = 73
WNDDFCLQVYR pc2
P07307;P07307−2;P07307−3;Q7Z4(
log10 peptide intensity
115:198 68:103 16:24
17:7012073:G:T_T
p = 0.0012, beta = 0.347, N = 199
AVTQPDNWHGHELGGSEDCVEVQPD(
P07307;P07307−2;P07307−3;Q7Z4(
log10 peptide intensity
14:198 14:103 2:24
17:7012073:G:T_T
p = 0.84, beta = 0.0568, N = 30
Assay Target: ASGR2
UniProt: P07307
rsID: rs2304980
Target SNP: 17:7012073:G:T
Proxy SNP: 17:7012073:G:T
UKBPPP log10(p): 498.6
UKBPPP BETA: 0.316
UKBPPP MSPA: 0.375
*:−:−:−:−:−
1216:994:493:325:115:96
GAQLQAELR pc2
rs2304978 ALT
log10 peptide intensity
10:676 267:489 77:95
17:7012073:G:T_T
p = 4.4e−132, model = REC, N = 354

145. FCER2 (P06734)
UKBPPP MSPA=0.353

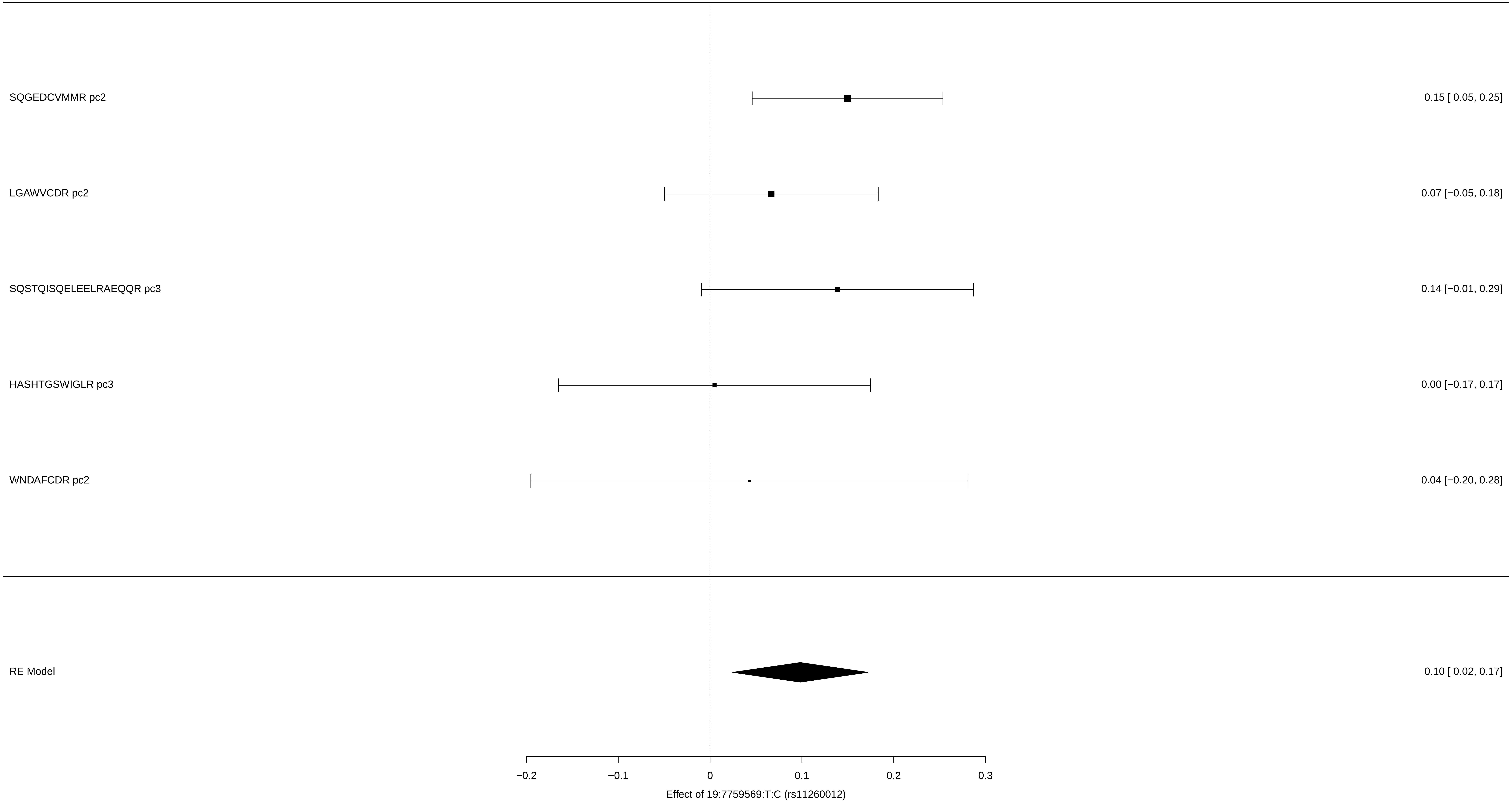

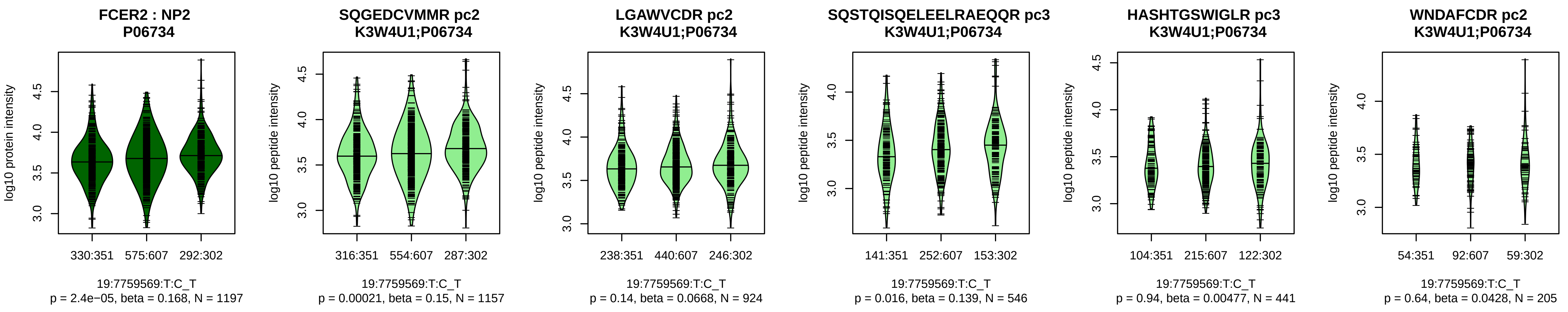

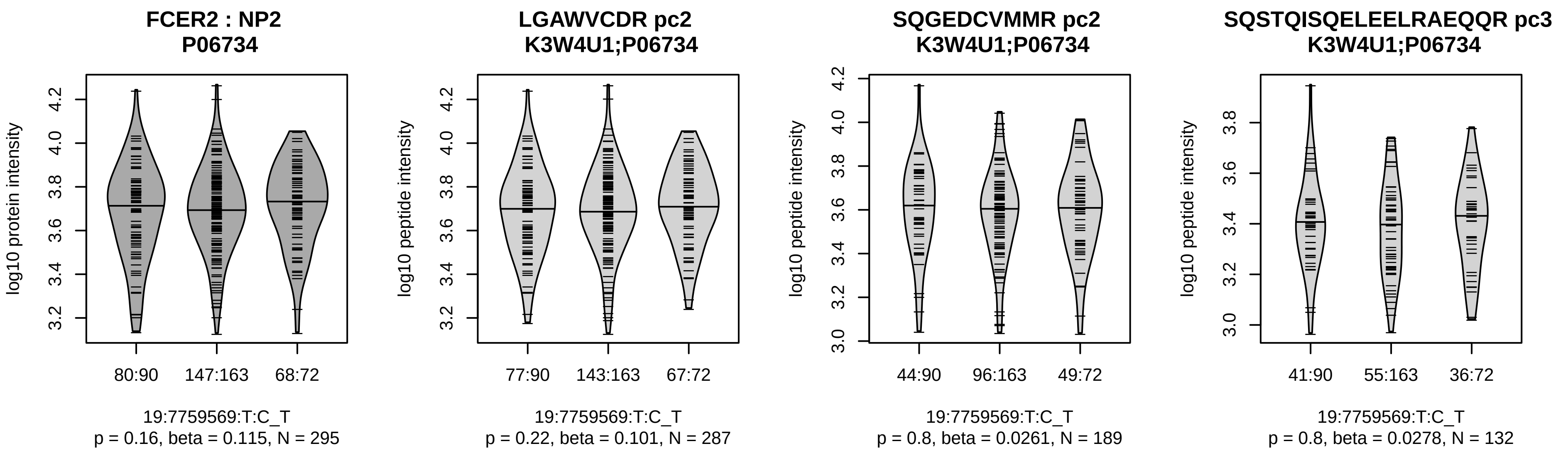

Assay Target: FCER2
UniProt: P06734
rsID: rs11260012
Target SNP: 19:7759569:T:C
Proxy SNP: 19:7759569:T:C
UKBPPP log10(p): 497.2
UKBPPP BETA: −0.289
UKBPPP MSPA: 0.353
*:−:−:−:−
1157:924:546:441:205

146. GALNT2 (Q10471)
UKBPPP MSPA=0.705

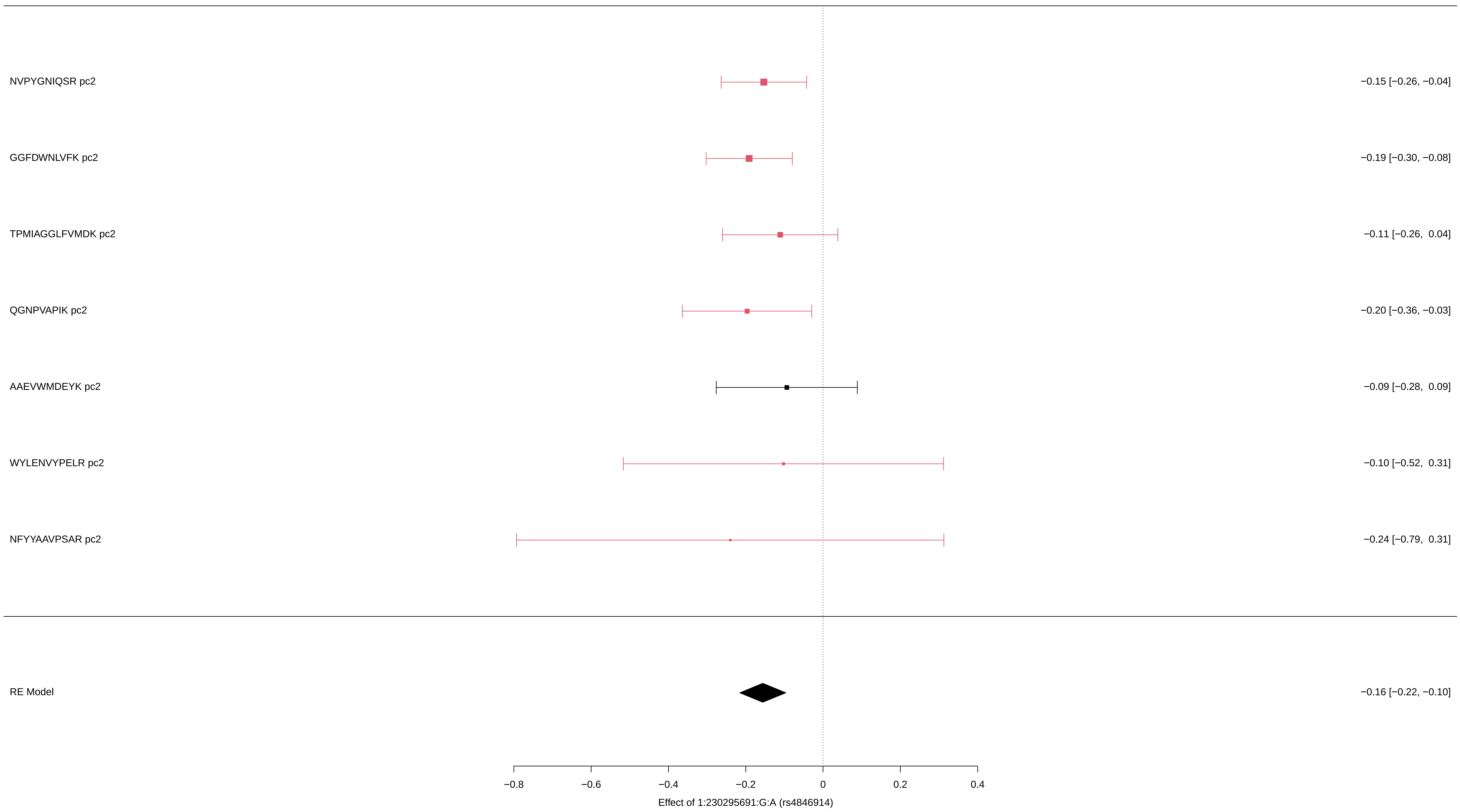

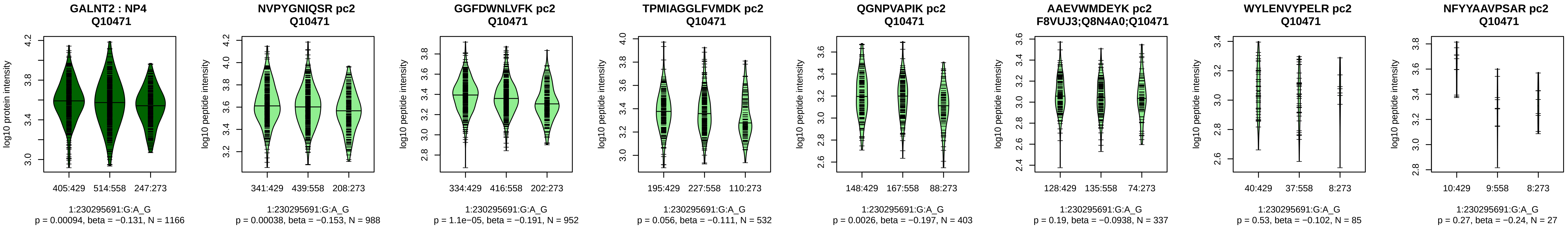

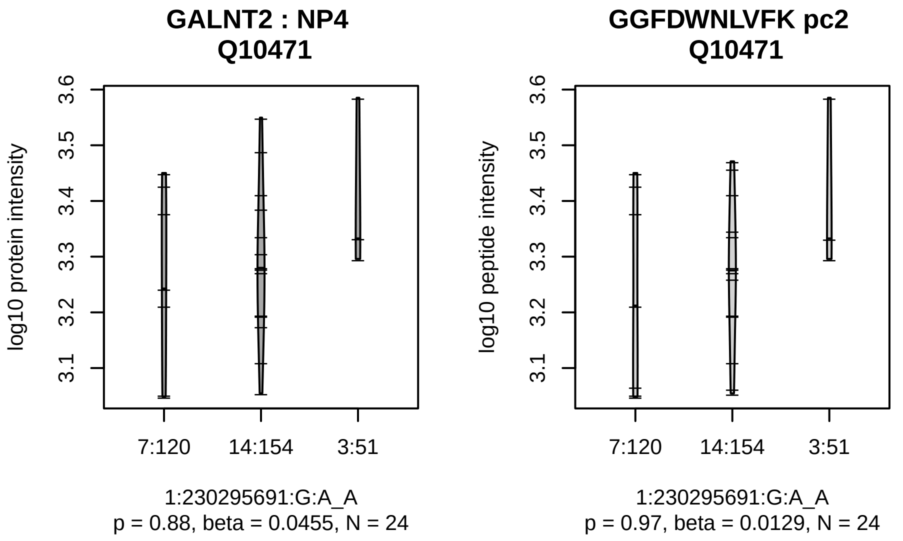

Assay Target: GALNT2
UniProt: Q10471
rsID: rs4846914
Target SNP: 1:230295691:G:A
Proxy SNP: 1:230295691:G:A
UKBPPP log10(p): 490
UKBPPP BETA: 0.287
UKBPPP MSPA: 0.705
::−:*:−:−:−:NA
988:952:532:403:337:85:27:14

147. CILP (O75339)
UKBPPP MSPA=0.555

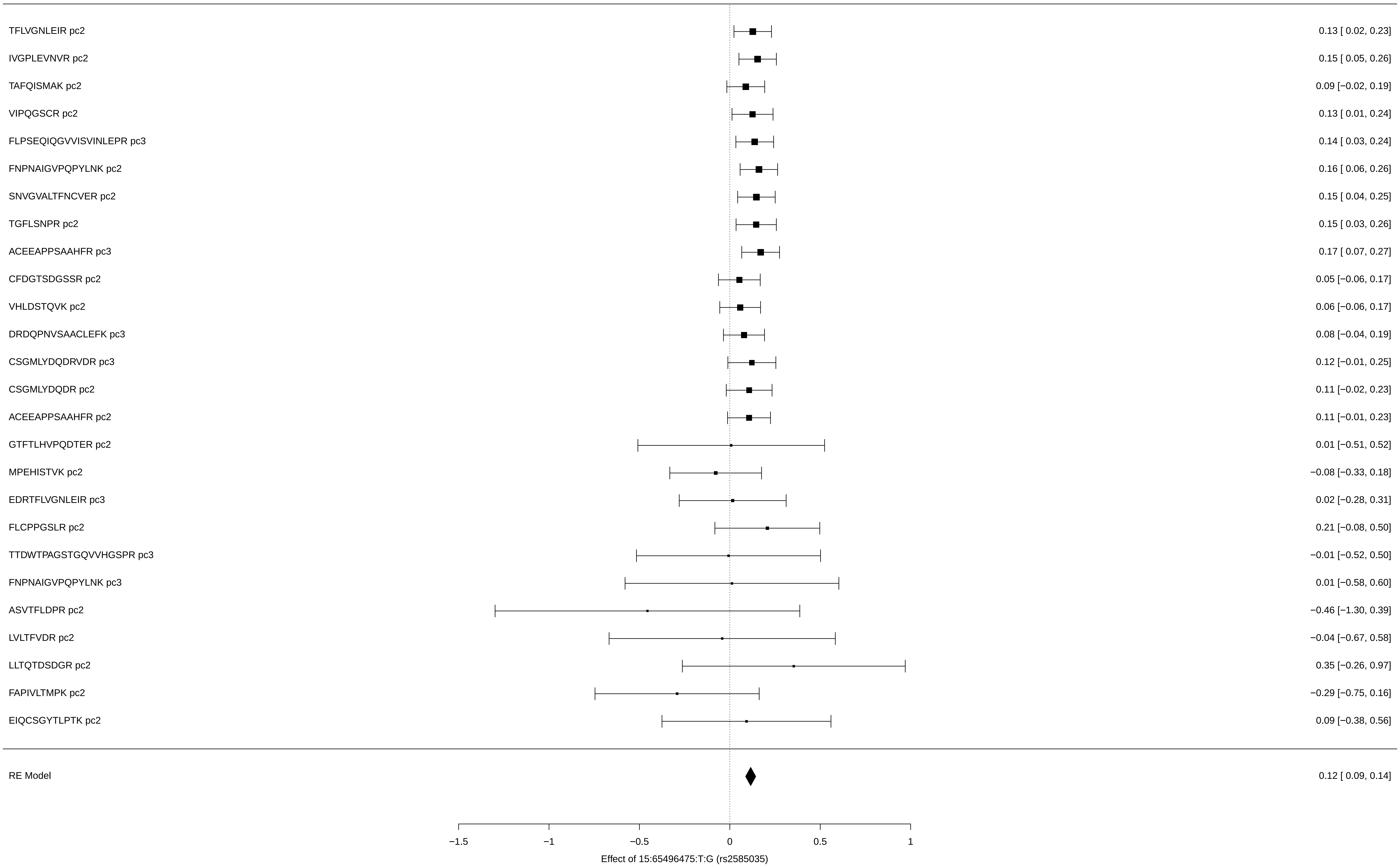

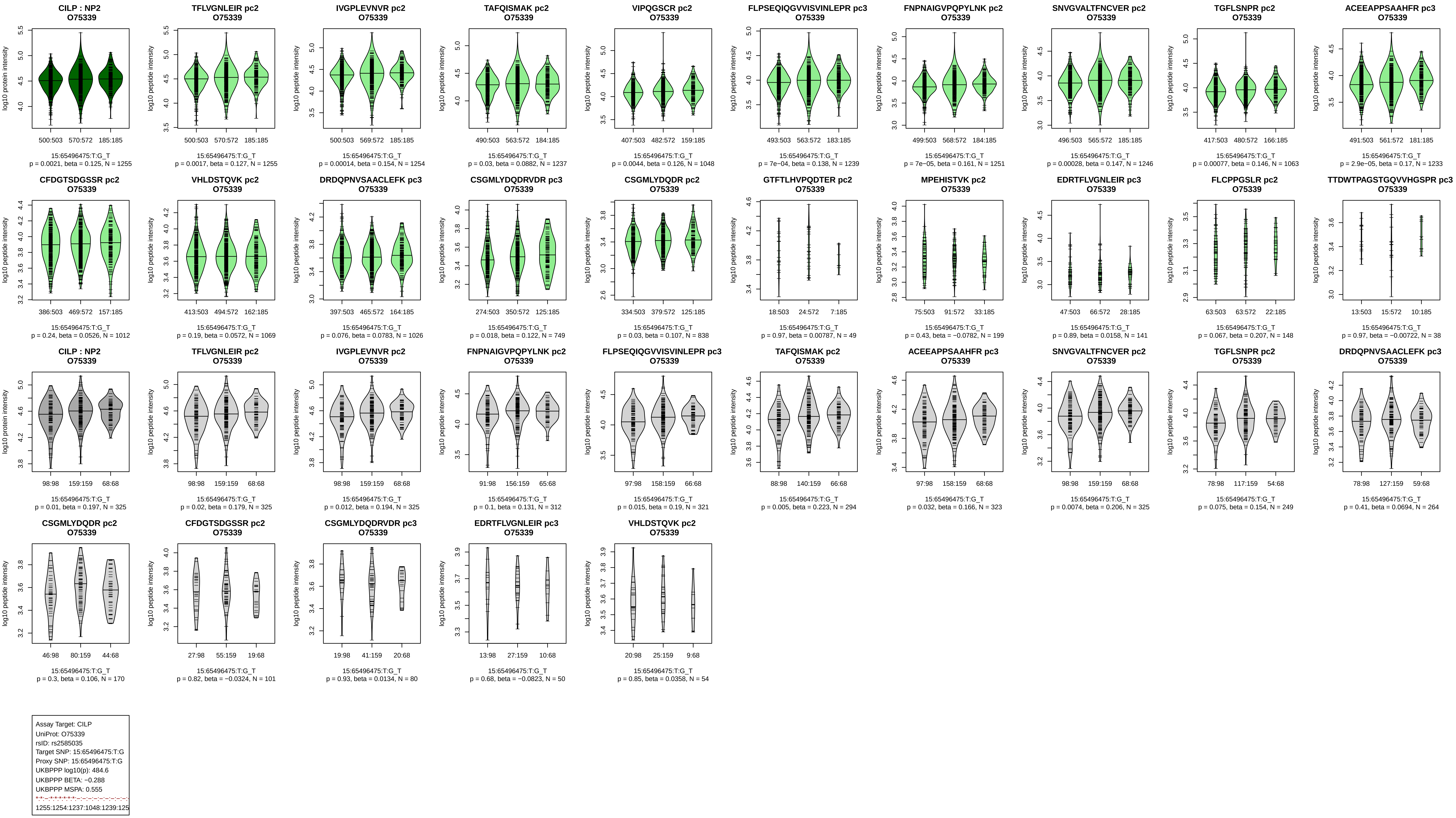
CILP : NP2
O75339
500:503 570:572 185:185
15:65496475:T:G_T
p = 0.0021, beta = 0.125, N = 1255
TFLVGNLEIR pc2
O75339
500:503 570:572 185:185
15:65496475:T:G_T
p = 0.0017, beta = 0.127, N = 1255
IVGPLEVNVR pc2
O75339
500:503 569:572 185:185
15:65496475:T:G_T
p = 0.00014, beta = 0.154, N = 1254
TAFQISMAK pc2
O75339
490:503 563:572 184:185
15:65496475:T:G_T
p = 0.03, beta = 0.0882, N = 1237
VIPQGSCR pc2
O75339
407:503 482:572 159:185
15:65496475:T:G_T
p = 0.0044, beta = 0.126, N = 1048
FLPSEQIQGVVISVINLEPR pc3
O75339
493:503 563:572 183:185
15:65496475:T:G_T
p = 7e−04, beta = 0.138, N = 1239
FNPNAIGVPQPYLNK pc2
O75339
499:503 568:572 184:185
15:65496475:T:G_T
p = 7e−05, beta = 0.161, N = 1251
SNVGVALTFNCVER pc2
O75339
496:503 565:572 185:185
15:65496475:T:G_T
p = 0.00028, beta = 0.147, N = 1246
TGFLSNPR pc2
O75339
417:503 480:572 166:185
15:65496475:T:G_T
p = 0.00077, beta = 0.146, N = 1063
ACEEAPPSAAHFR pc3
O75339
491:503 561:572 181:185
15:65496475:T:G_T
p = 2.9e−05, beta = 0.17, N = 1233
CFDGTSDGSSR pc2
O75339
386:503 469:572 157:185
15:65496475:T:G_T
p = 0.24, beta = 0.0526, N = 1012
VHLDSTQVK pc2
O75339
413:503 494:572 162:185
15:65496475:T:G_T
p = 0.19, beta = 0.0572, N = 1069
DRDQPNVSAACLEFK pc3
O75339
397:503 465:572 164:185
15:65496475:T:G_T
p = 0.076, beta = 0.0783, N = 1026
CSGMLYDQDRVDR pc3
O75339
274:503 350:572 125:185
15:65496475:T:G_T
p = 0.018, beta = 0.122, N = 749
CSGMLYDQDR pc2
O75339
334:503 379:572 125:185
15:65496475:T:G_T
p = 0.03, beta = 0.107, N = 838
GTFTLHVPQDTER pc2
O75339
18:503 24:572 7:185
15:65496475:T:G_T
p = 0.97, beta = 0.00787, N = 49
MPEHISTVK pc2
O75339
75:503 91:572 33:185
15:65496475:T:G_T
p = 0.43, beta = −0.0782, N = 199
EDRTFLVGNLEIR pc3
O75339
47:503 66:572 28:185
15:65496475:T:G_T
p = 0.89, beta = 0.0158, N = 141
FLCPPGSLR pc2
O75339
63:503 63:572 22:185
15:65496475:T:G_T
p = 0.067, beta = 0.207, N = 148
TTDWTPAGSTGQVVHGSPR pc3
O75339
13:503 15:572 10:185
15:65496475:T:G_T
p = 0.97, beta = −0.00722, N = 38
CILP : NP2
O75339
98:98 159:159 68:68
15:65496475:T:G_T
p = 0.01, beta = 0.197, N = 325
TFLVGNLEIR pc2
O75339
98:98 159:159 68:68
15:65496475:T:G_T
p = 0.02, beta = 0.179, N = 325
IVGPLEVNVR pc2
O75339
98:98 159:159 68:68
15:65496475:T:G_T
p = 0.012, beta = 0.194, N = 325
FNPNAIGVPQPYLNK pc2
O75339
91:98 156:159 65:68
15:65496475:T:G_T
p = 0.1, beta = 0.131, N = 312
FLPSEQIQGVVISVINLEPR pc3
O75339
97:98 158:159 66:68
15:65496475:T:G_T
p = 0.015, beta = 0.19, N = 321
TAFQISMAK pc2
O75339
88:98 140:159 66:68
15:65496475:T:G_T
p = 0.005, beta = 0.223, N = 294
ACEEAPPSAAHFR pc3
O75339
97:98 158:159 68:68
15:65496475:T:G_T
p = 0.032, beta = 0.166, N = 323
SNVGVALTFNCVER pc2
O75339
98:98 159:159 68:68
15:65496475:T:G_T
p = 0.0074, beta = 0.206, N = 325
TGFLSNPR pc2
O75339
78:98 117:159 54:68
15:65496475:T:G_T
p = 0.075, beta = 0.154, N = 249
DRDQPNVSAACLEFK pc3
O75339
78:98 127:159 59:68
15:65496475:T:G_T
p = 0.41, beta = 0.0694, N = 264
CSGMLYDQDR pc2
O75339
46:98 80:159 44:68
15:65496475:T:G_T
p = 0.3, beta = 0.106, N = 170
CFDGTSDGSSR pc2
O75339
27:98 55:159 19:68
15:65496475:T:G_T
p = 0.82, beta = −0.0324, N = 101
CSGMLYDQDRVDR pc3
O75339
19:98 41:159 20:68
15:65496475:T:G_T
p = 0.93, beta = 0.0134, N = 80
EDRTFLVGNLEIR pc3
O75339
13:98 27:159 10:68
15:65496475:T:G_T
p = 0.68, beta = −0.0823, N = 50
VHLDSTQVK pc2
O75339
20:98 25:159 9:68
15:65496475:T:G_T
p = 0.85, beta = 0.0358, N = 54
log10 protein intensity
log10 peptide intensity
Assay Target: CILP
UniProt: O75339
rsID: rs2585035
Target SNP: 15:65496475:T:G
Proxy SNP: 15:65496475:T:G
UKBPPP log10(p): 484.6
UKBPPP BETA: −0.288
UKBPPP MSPA: 0.555
1255:1254:1237:1048:1239:125

148. RNASE4 (P34096)
UKBPPP MSPA=0.456

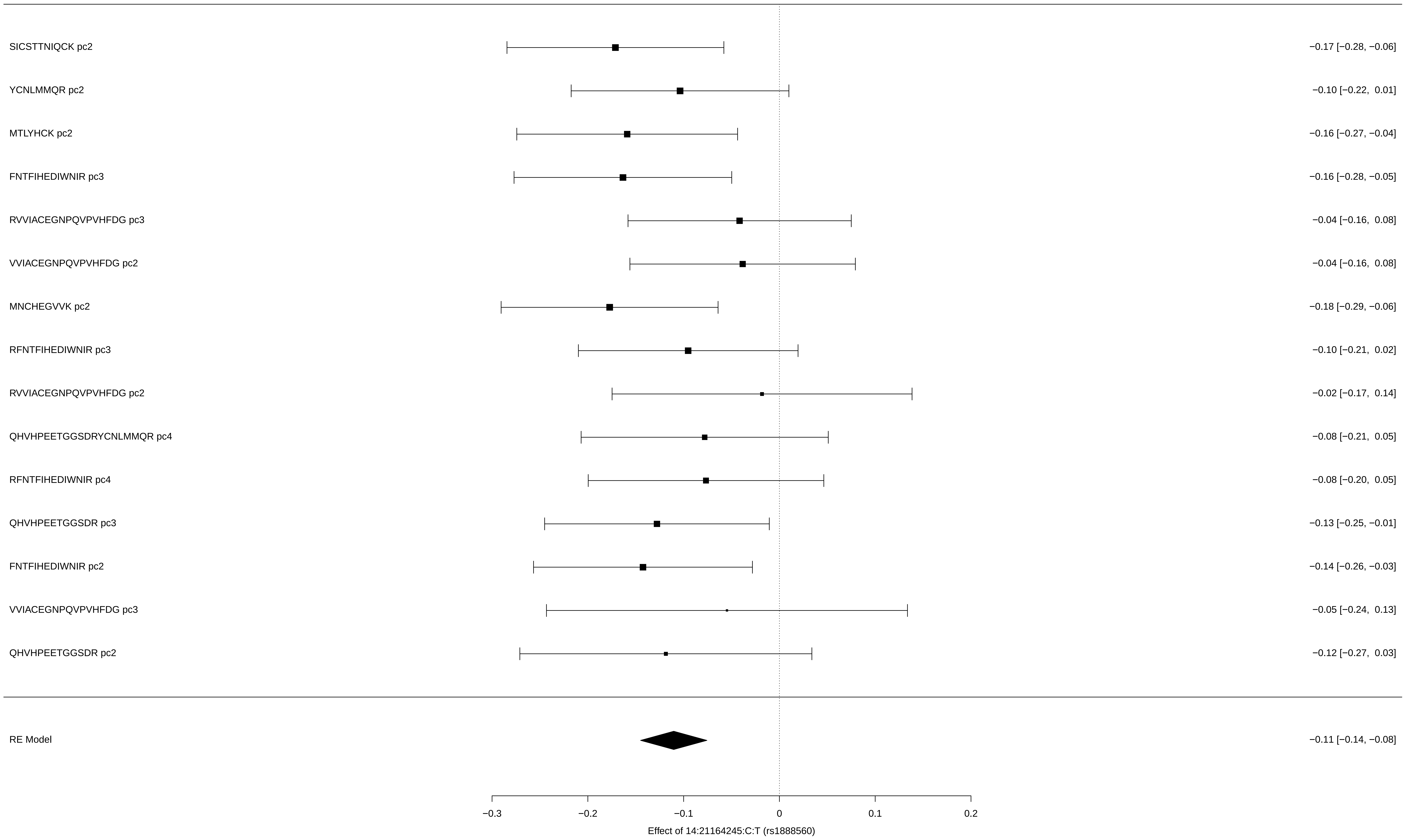

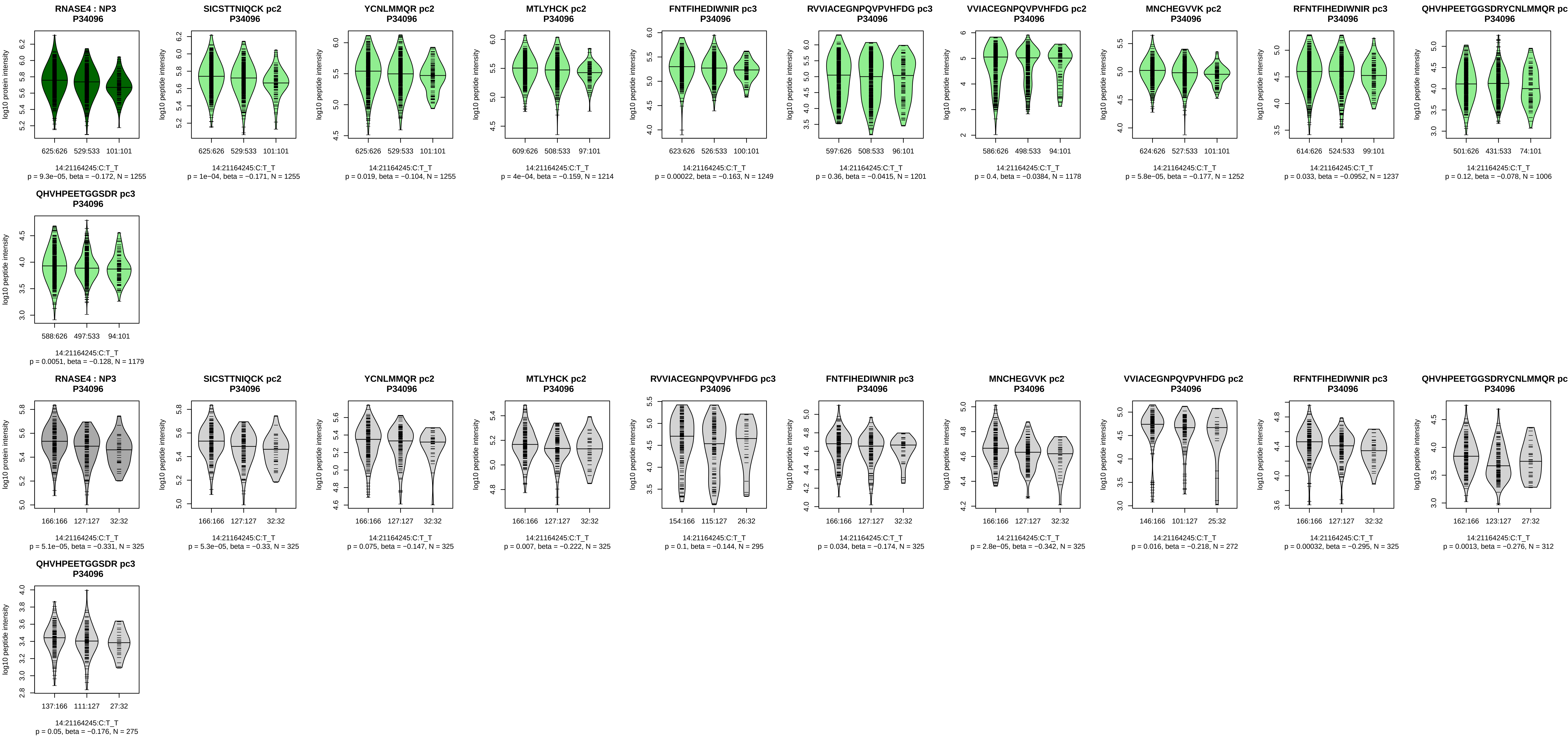

Assay Target: RNASE4
UniProt: P34096
rsID: rs1888560
Target SNP: 14:21164245:C:T
Proxy SNP: 14:21164245:C:T
UKBPPP log10(p): 484.2
UKBPPP BETA: −0.304
UKBPPP MSPA: 0.456
:–::*:–:–:*:–:–:–:–:*:*:–:–
1255:1255:1214:1249:1201:117

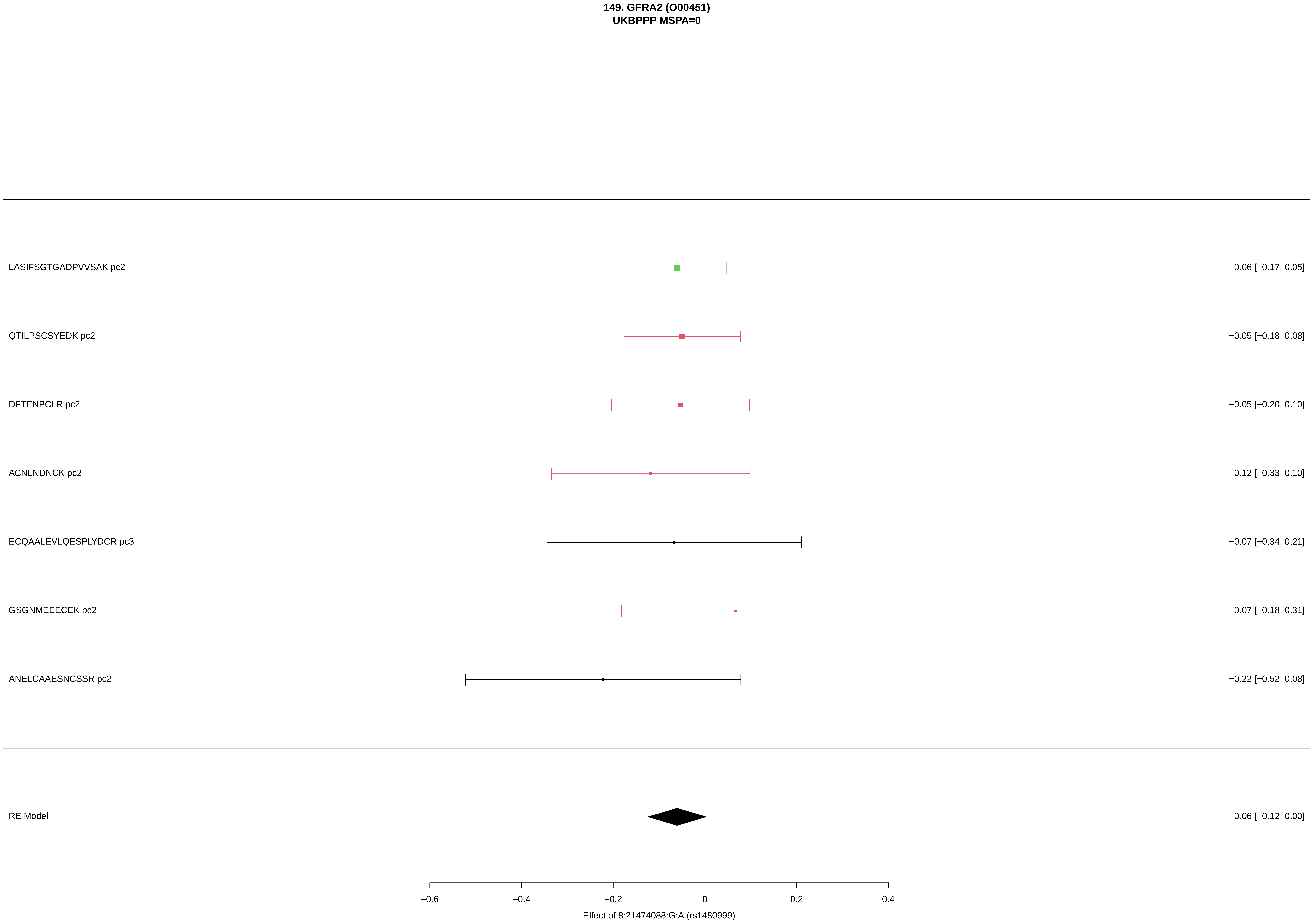
149. GFRA2 (O00451)
UKBPPP MSPA=0
LASIFSGTGADPVVSAK pc2
−0.06 [−0.17, 0.05]
QTILPSCSYEDK pc2
−0.05 [−0.18, 0.08]
DFTENPCLR pc2
−0.05 [−0.20, 0.10]
ACNLNDNCK pc2
−0.12 [−0.33, 0.10]
ECQAALEVLQESPLYDCR pc3
−0.07 [−0.34, 0.21]
GSGNMEEECEK pc2
0.07 [−0.18, 0.31]
ANELCAAESNCSSR pc2
−0.22 [−0.52, 0.08]
RE Model
−0.06 [−0.12, 0.00]
−0.6
−0.4
−0.2
0
0.2
0.4
Effect of 8:21474088:G:A (rs1480999)

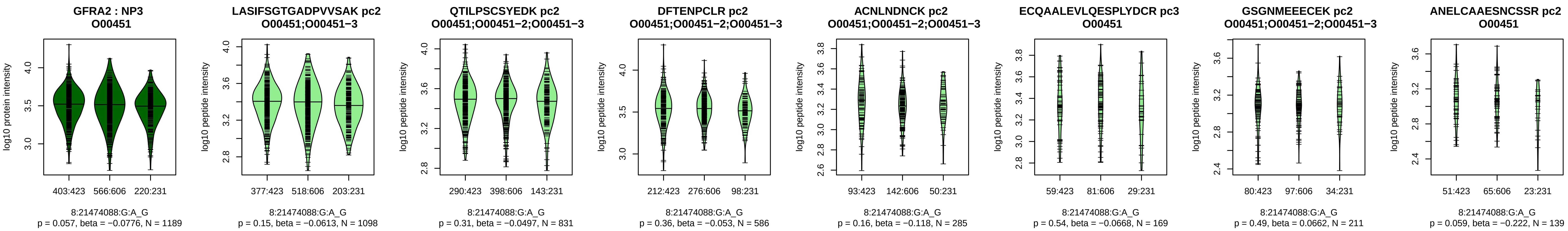

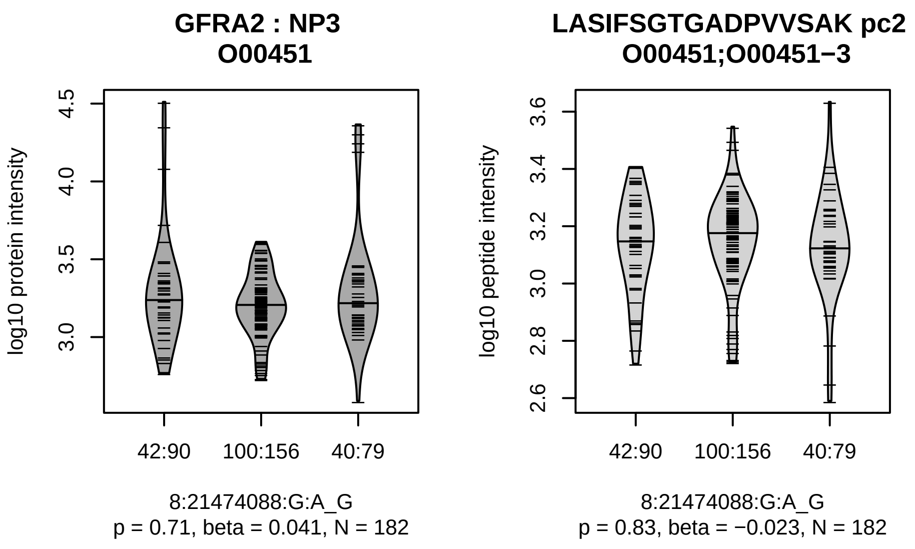

Assay Target: GFRA2
UniProt: O00451
rsID: rs1480999
Target SNP: 8:21474088:G:A
Proxy SNP: 8:21474088:G:A
UKBPPP log10(p): 474.1
UKBPPP BETA: 0.294
UKBPPP MSPA: 0
−:−:−:−:−:−
1098:831:586:285:169:211:139

150. CCN2 (P29279)
UKBPPP MSPA=0.615

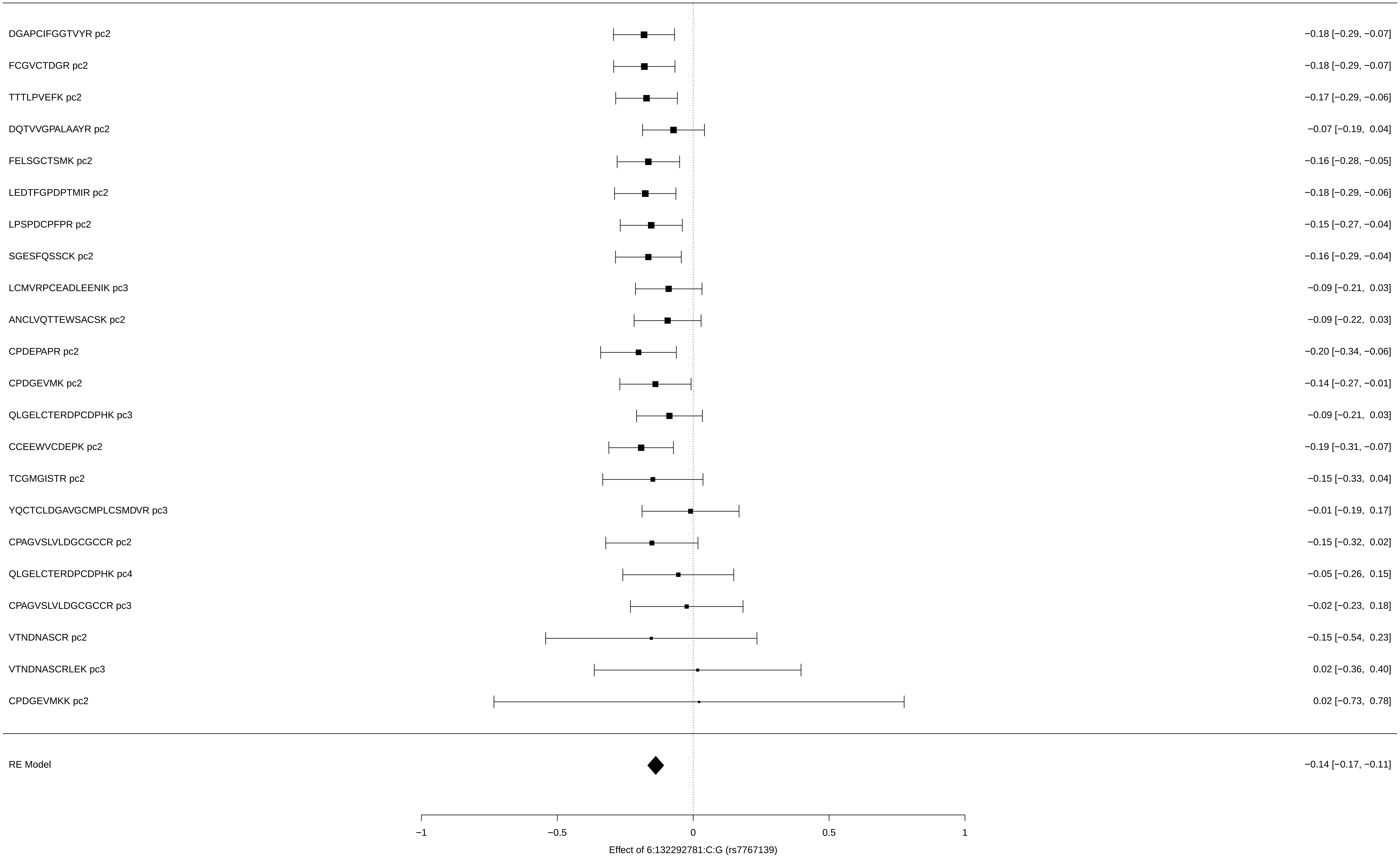

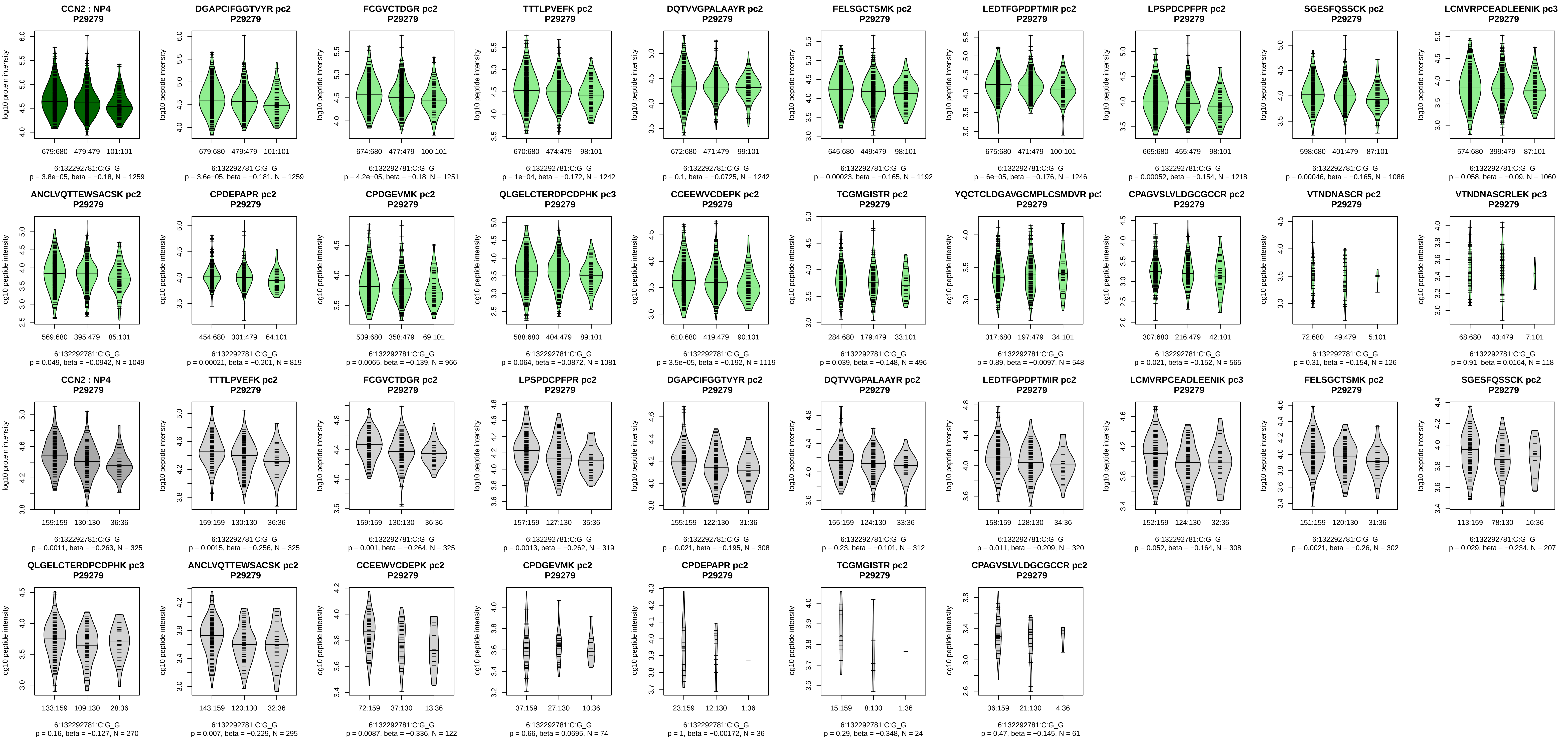

Assay Target: CCN2
UniProt: P29279
rsID: rs7767139
Target SNP: 6:132292781:C:G
Proxy SNP: 6:132292781:C:G
UKBPPP log10(p): 471.2
UKBPPP BETA: −0.343
UKBPPP MSPA: 0.615
1259:1251:1242:1242:1192:124

151. ENPP2 (Q13822)
UKBPPP MSPA=0

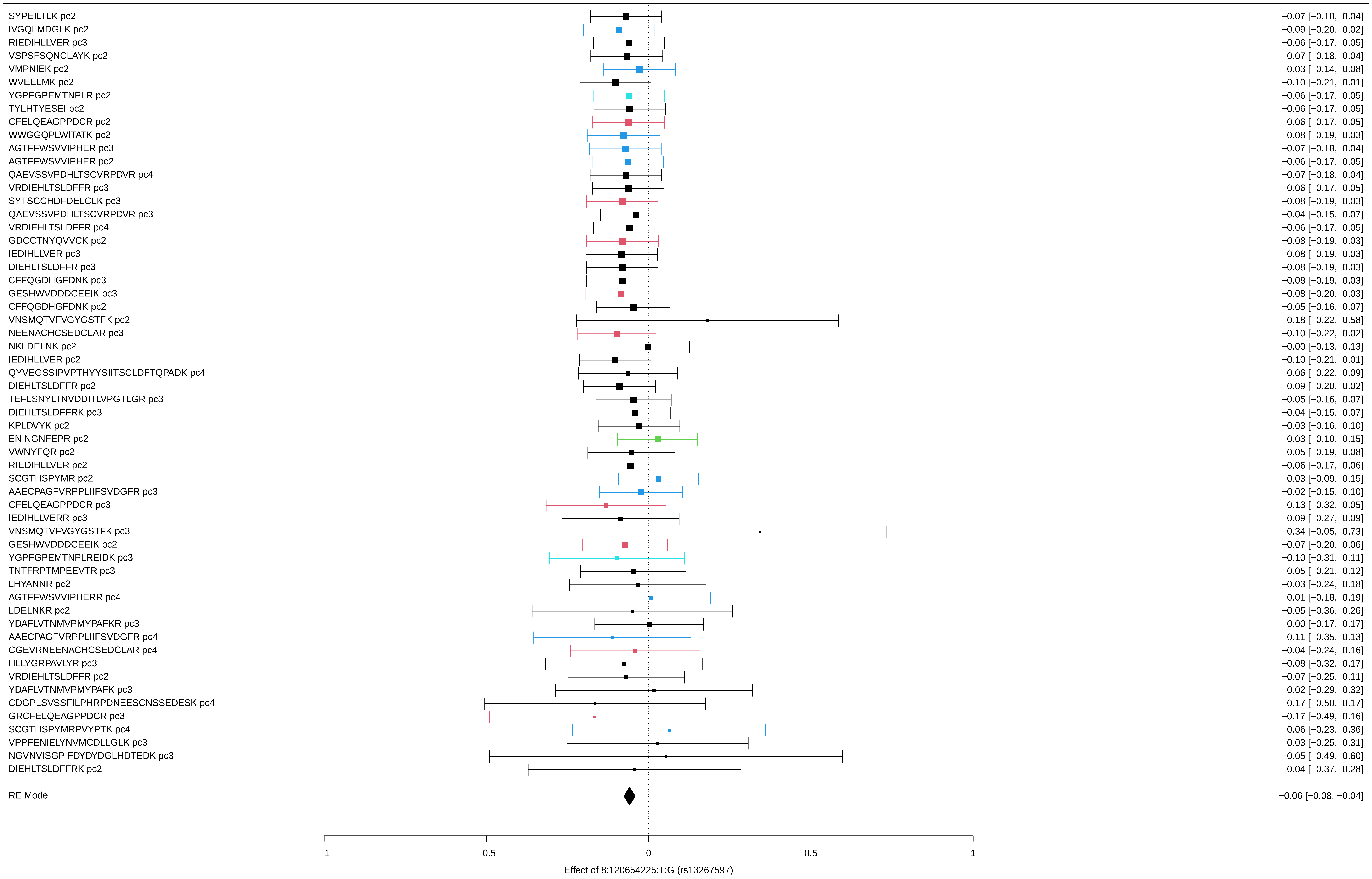

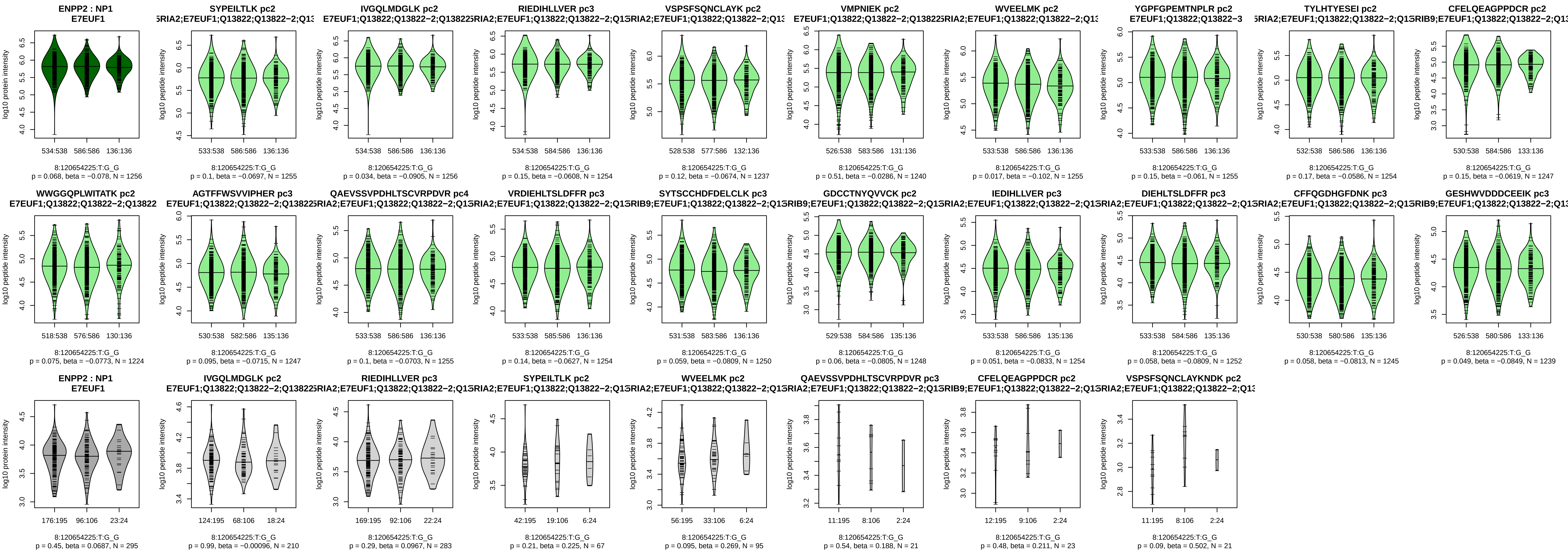

Assay Target: ENPP2
UniProt: Q13822
rsID: rs13267597
Target SNP: 8:120654225:T:G
Proxy SNP: 8:120654225:T:G
UKBPPP log10(p): 468.9
UKBPPP BETA: −0.25
UKBPPP MSPA: 0
1255:1256:1254:1237:1240:125

152. SMOC1 (Q9H4F8)
UKBPPP MSPA=0

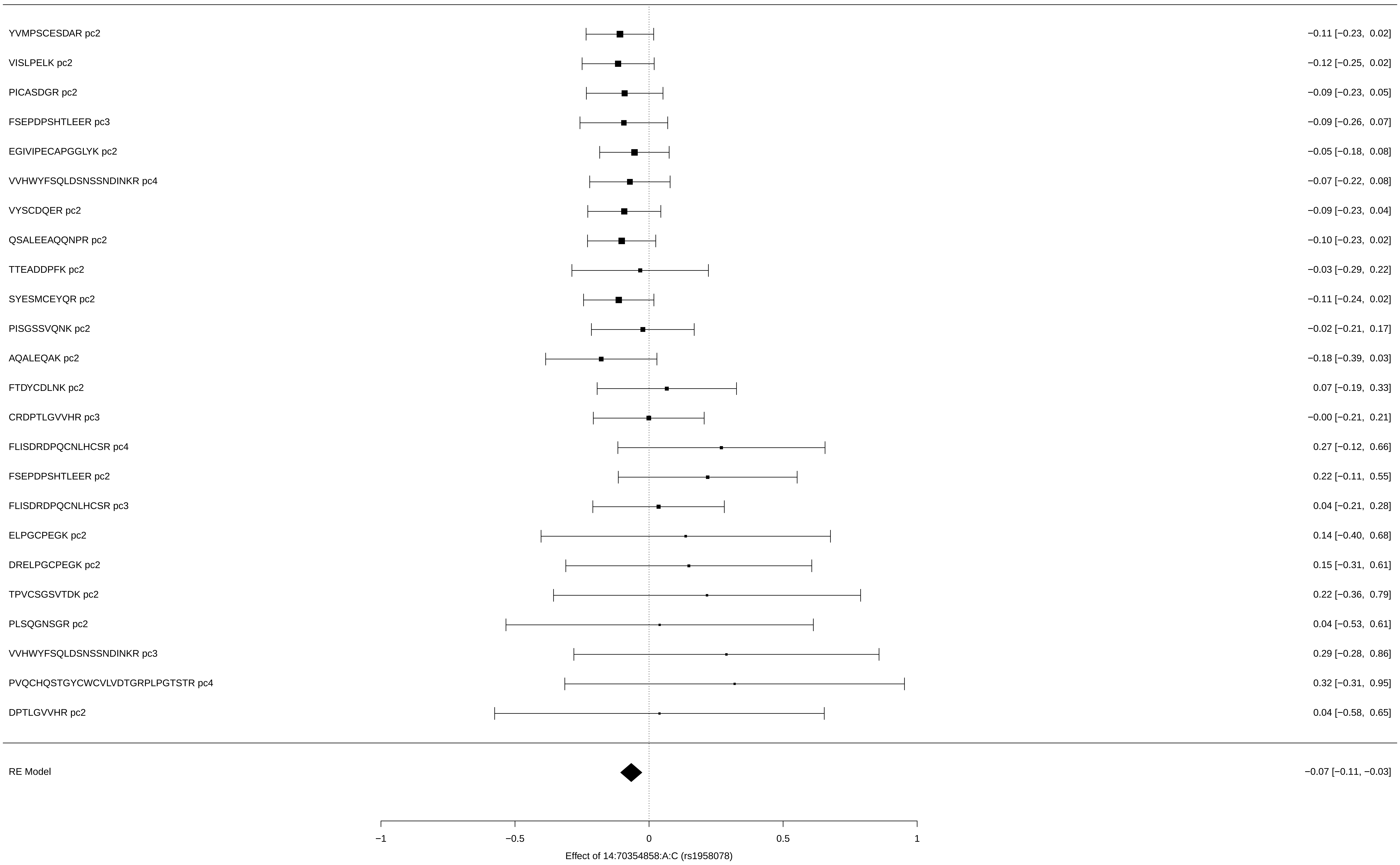

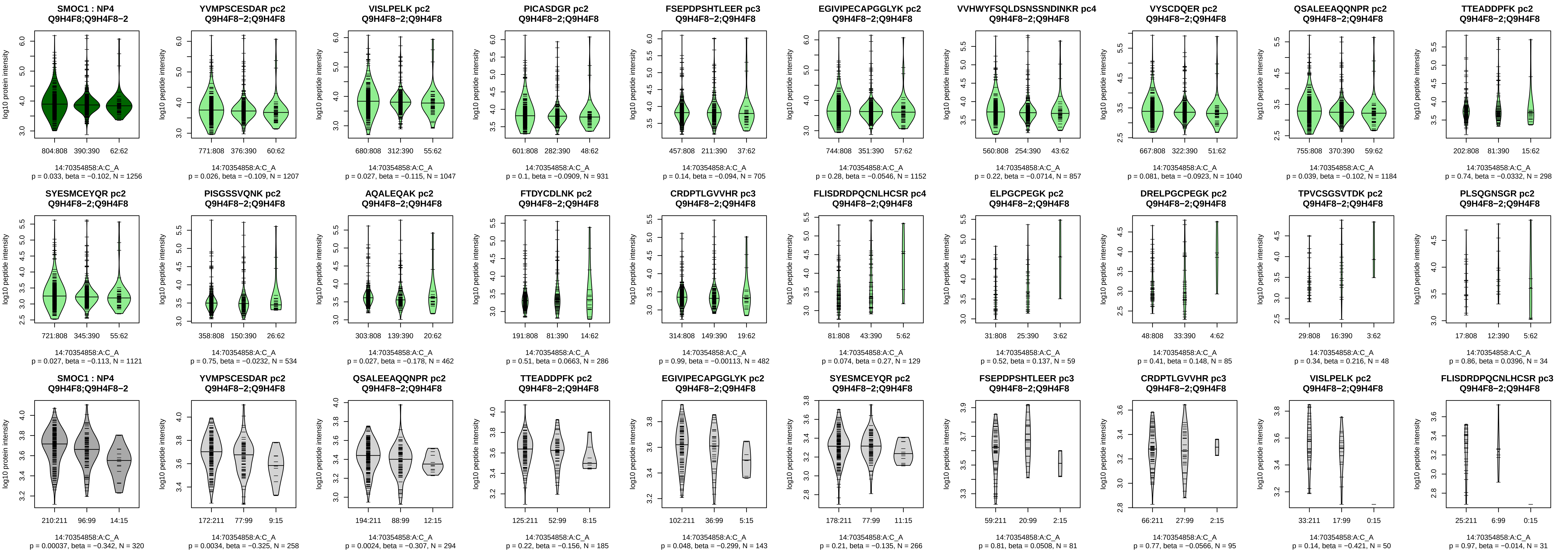

Assay Target: SMOC1
UniProt: Q9H4F8
rsID: rs1958078
Target SNP: 14:70354858:A:C
Proxy SNP: 14:70354858:A:C
UKBPPP log10(p): 455.1
UKBPPP BETA: 0.358
UKBPPP MSPA: 0
1207:1047:931:705:1152:857:1[illegible]

153. CD2AP (Q9Y5K6)
UKBPPP MSPA=0.625

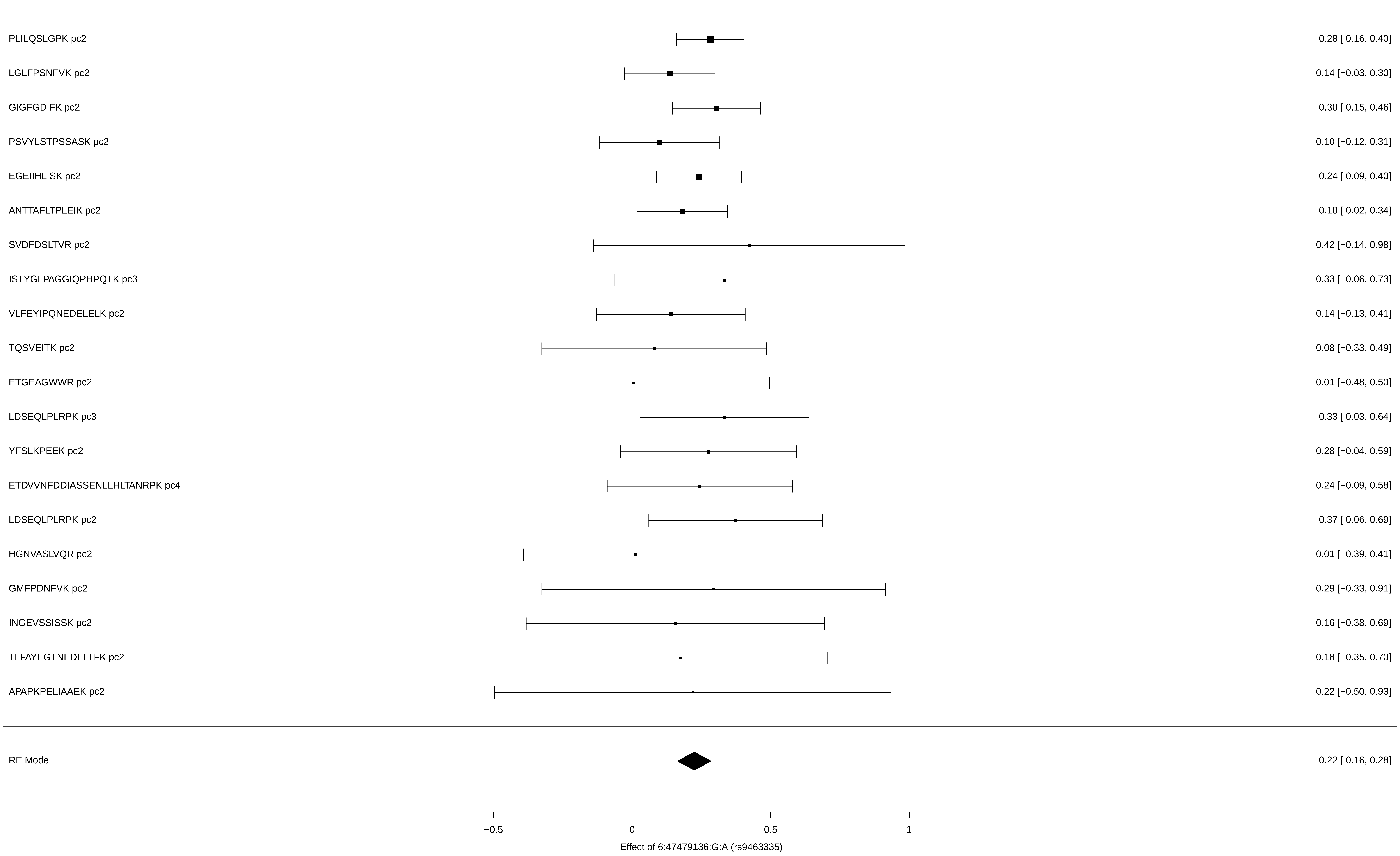

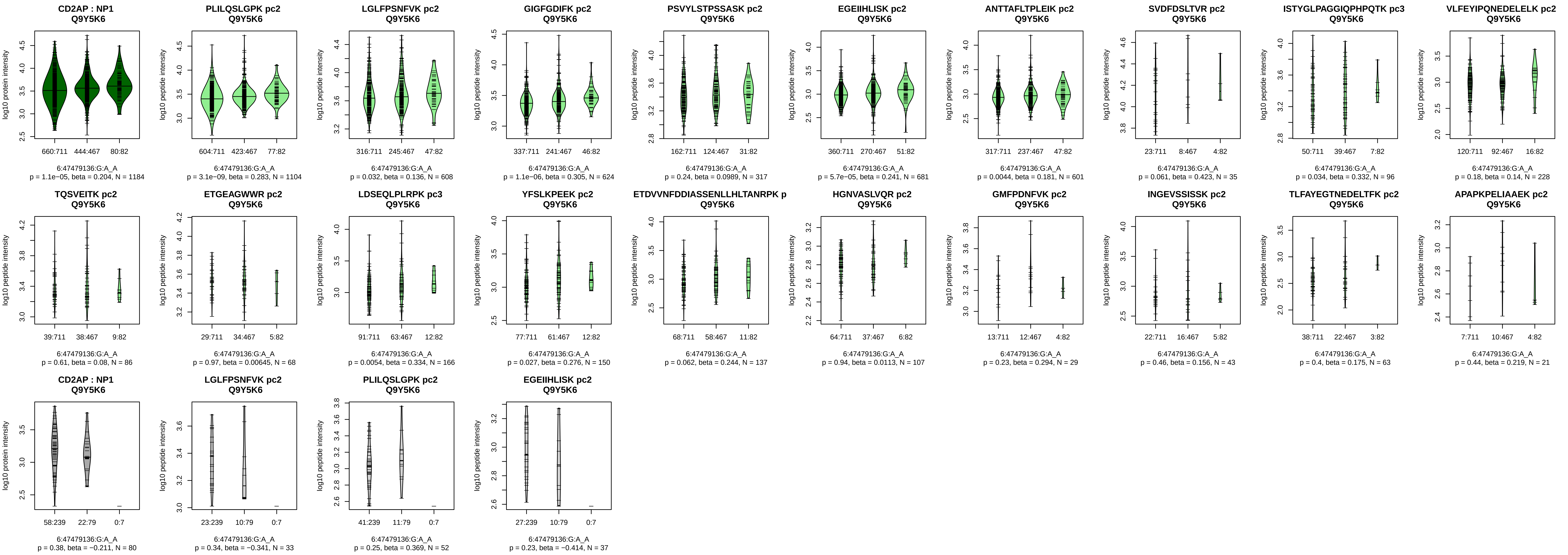

Assay Target: CD2AP
UniProt: Q9Y5K6
rsID: rs9463335
Target SNP: 6:47479136:G:A
Proxy SNP: 6:47479136:G:A
UKBPPP log10(p): 452.9
UKBPPP BETA: 0.306
UKBPPP MSPA: 0.625
:–::–:*:*:–:–:–:–:–:*:–:–:*:–:–:–:
1104:608:624:317:681:601:35:9

154. ITGB6 (P18564)
UKBPPP MSPA=0

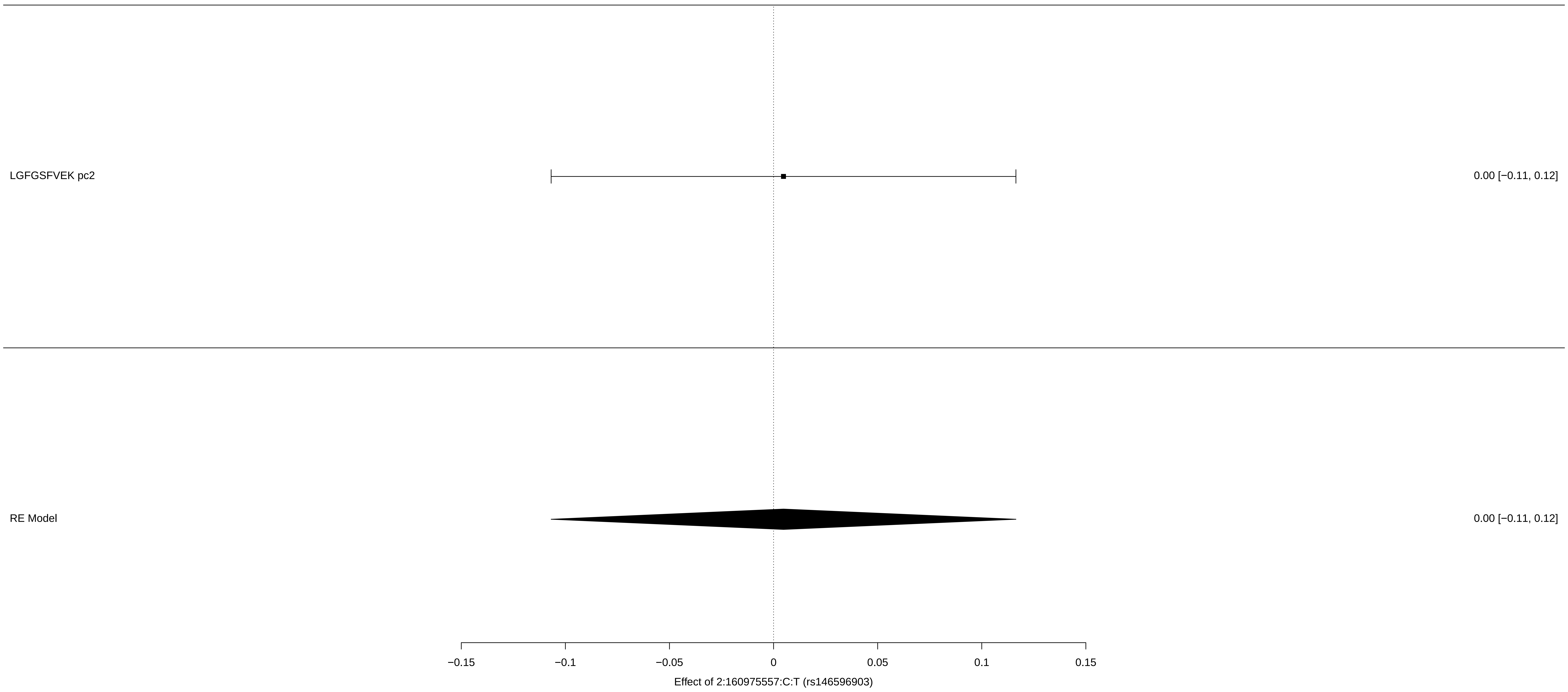

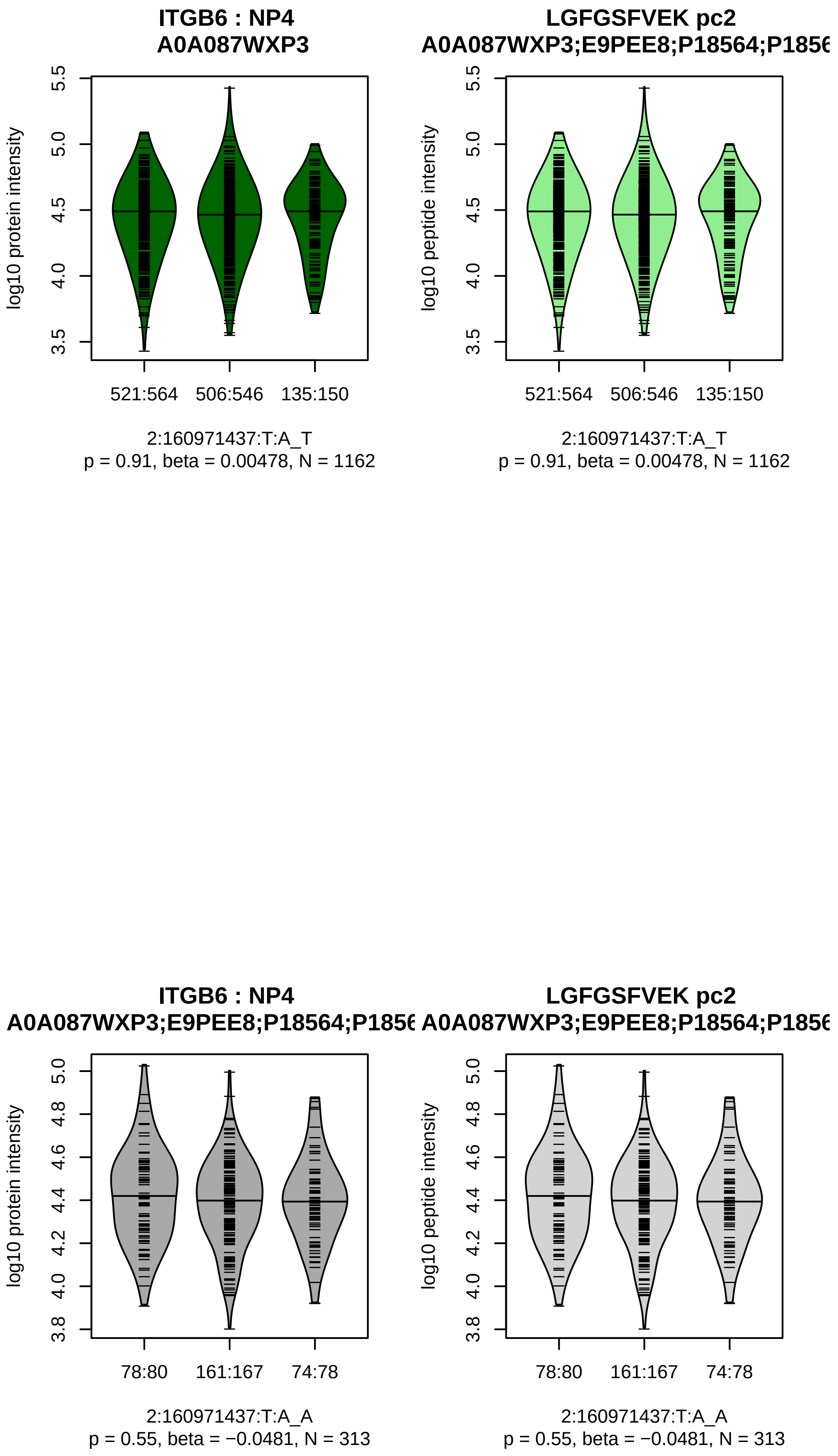

Assay Target: ITGB6
UniProt: P18564
rsID: rs146596903
Target SNP: 2:160975557:C:T
Proxy SNP: 2:160971437:T:A
UKBPPP log10(p): 451.3
UKBPPP BETA: 0.286
UKBPPP MSPA: 0
–
1162

155. PI3 (P19957)
UKBPPP MSPA=0.852

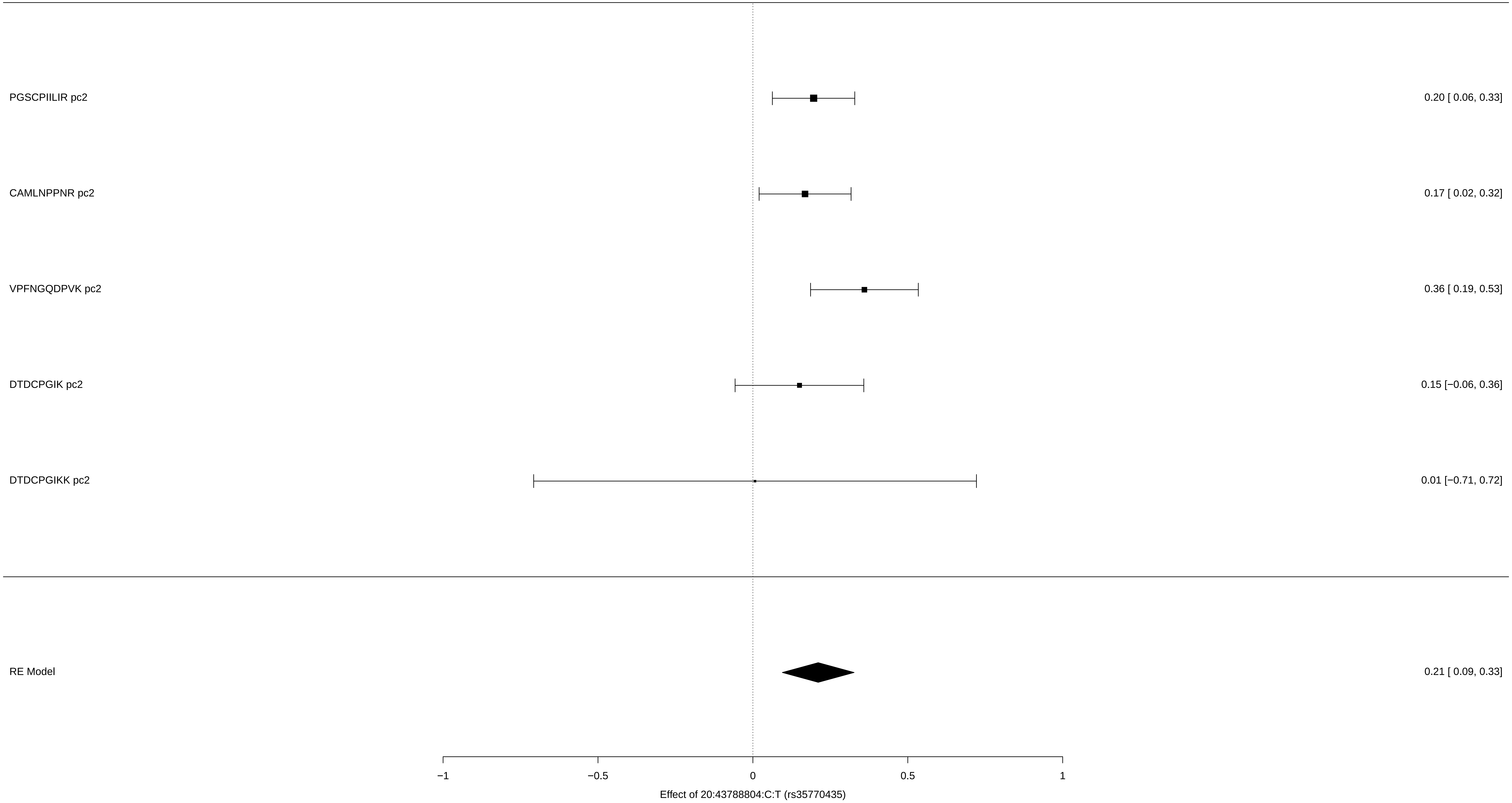

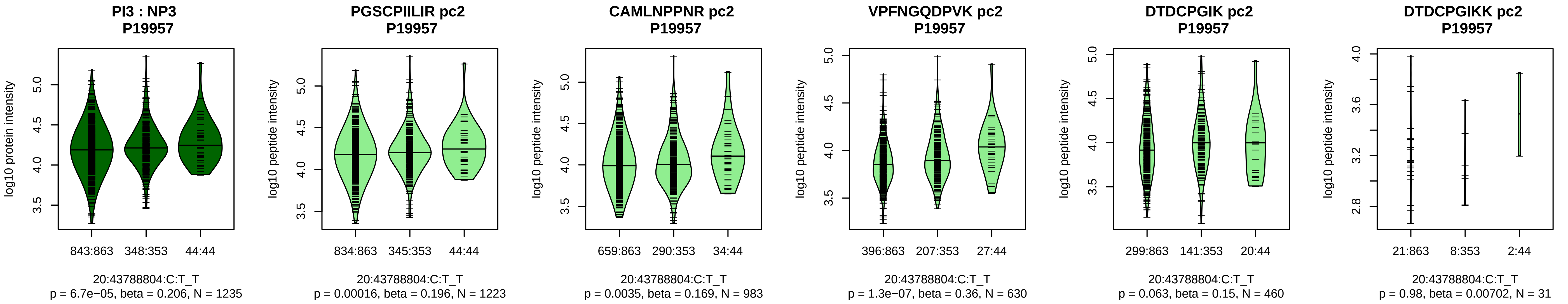

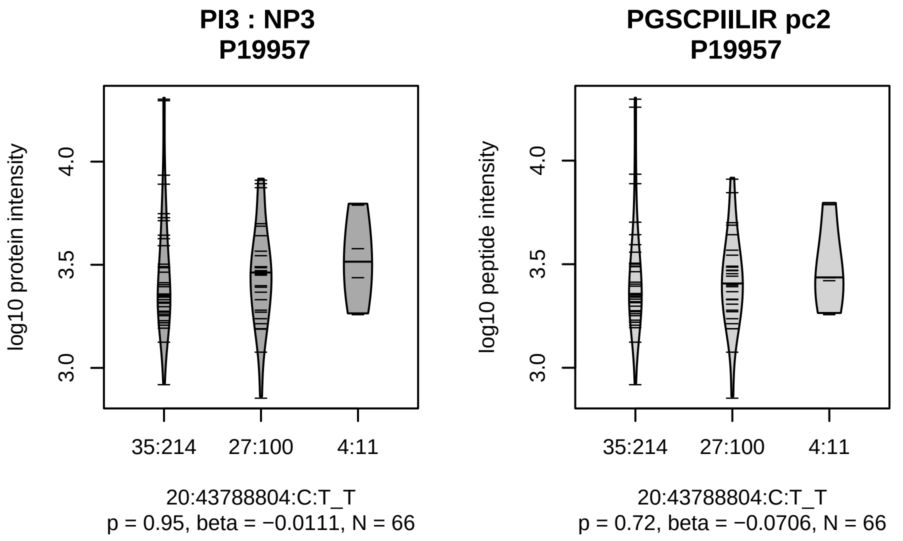

Assay Target: PI3
UniProt: P19957
rsID: rs35770435
Target SNP: 20:43788804:C:T
Proxy SNP: 20:43788804:C:T
UKBPPP log10(p): 450.3
UKBPPP BETA: 0.345
UKBPPP MSPA: 0.852
::*:–:–
1223:983:630:460:31

156. ITGBL1 (O95965)
UKBPPP MSPA=0.384

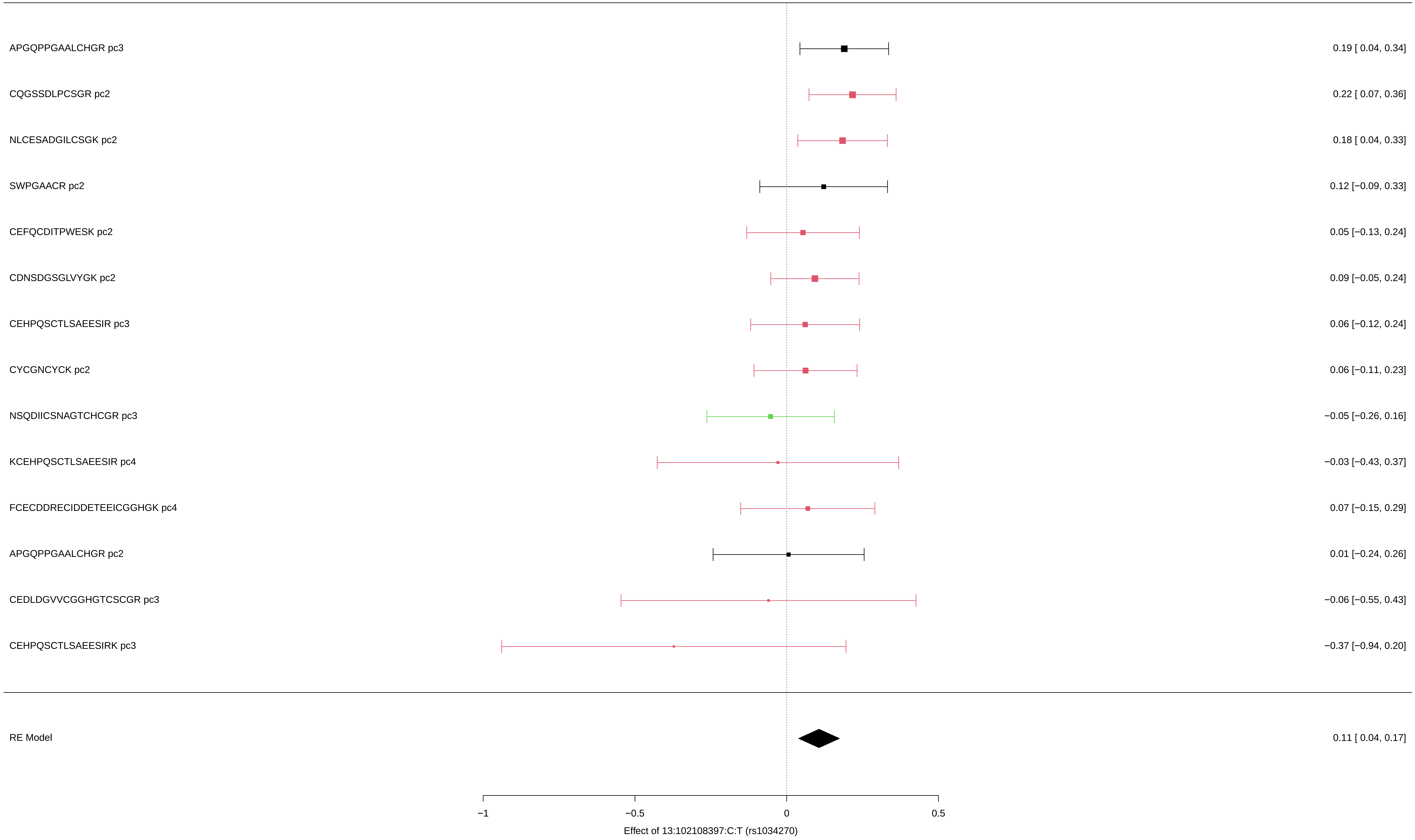

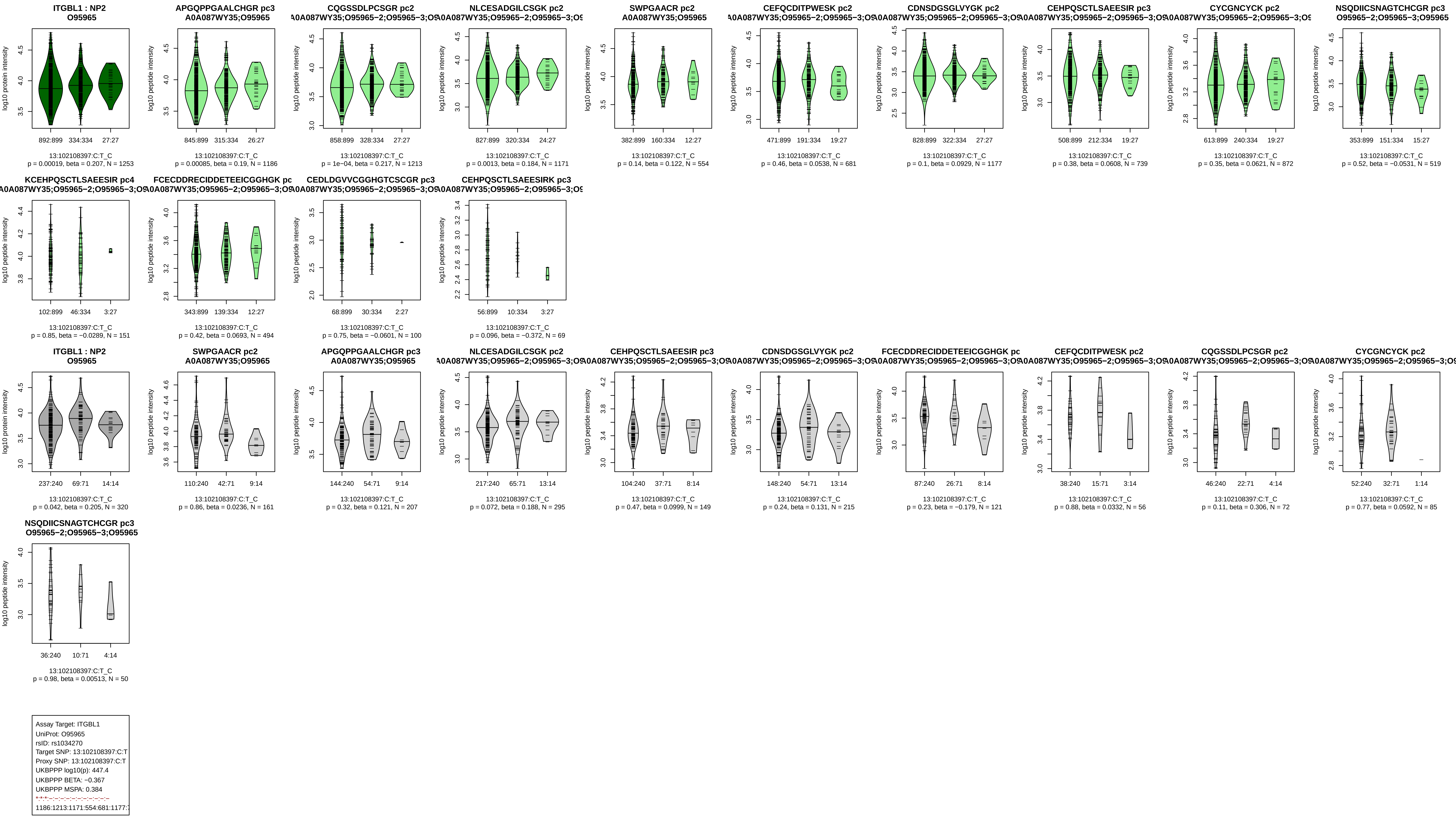
ITGBL1 : NP2
O95965
13:102108397:C:T_C
p = 0.00019, beta = 0.207, N = 1253
APGQPPGAALCHGR pc3
A0A087WY35;O95965
p = 0.00085, beta = 0.19, N = 1186
CQGSSDLPCSGR pc2
p = 1e−04, beta = 0.217, N = 1213
NLCESADGILCSGK pc2
p = 0.0013, beta = 0.184, N = 1171
SWPGAACR pc2
A0A087WY35;O95965
p = 0.14, beta = 0.122, N = 554
CEFQCDITPWESK pc2
p = 0.46, beta = 0.0538, N = 681
CDNSDGSGLVYGK pc2
p = 0.1, beta = 0.0929, N = 1177
CEHPQSCTLSAEESIR pc3
p = 0.38, beta = 0.0608, N = 739
CYCGNCYCK pc2
p = 0.35, beta = 0.0621, N = 872
NSQDIICSNAGTCHCGR pc3
O95965−2;O95965−3;O95965
p = 0.52, beta = −0.0531, N = 519
KCEHPQSCTLSAEESIR pc4
p = 0.85, beta = −0.0289, N = 151
FCECDDRECIDDETEEICGGHGK pc
p = 0.42, beta = 0.0693, N = 494
CEDLDGVVCGGHGTCSCGR pc3
p = 0.75, beta = −0.0601, N = 100
CEHPQSCTLSAEESIRK pc3
p = 0.096, beta = −0.372, N = 69
ITGBL1 : NP2
O95965
p = 0.042, beta = 0.205, N = 320
SWPGAACR pc2
p = 0.86, beta = 0.0236, N = 161
APGQPPGAALCHGR pc3
p = 0.32, beta = 0.121, N = 207
NLCESADGILCSGK pc2
p = 0.072, beta = 0.188, N = 295
CEHPQSCTLSAEESIR pc3
p = 0.47, beta = 0.0999, N = 149
CDNSDGSGLVYGK pc2
p = 0.24, beta = 0.131, N = 215
FCECDDRECIDDETEEICGGHGK pc
p = 0.23, beta = −0.179, N = 121
CEFQCDITPWESK pc2
p = 0.88, beta = 0.0332, N = 56
CQGSSDLPCSGR pc2
p = 0.11, beta = 0.306, N = 72
CYCGNCYCK pc2
p = 0.77, beta = 0.0592, N = 85
NSQDIICSNAGTCHCGR pc3
O95965−2;O95965−3;O95965
p = 0.98, beta = 0.00513, N = 50
log10 protein intensity
log10 peptide intensity
Assay Target: ITGBL1
UniProt: O95965
rsID: rs1034270
Target SNP: 13:102108397:C:T
Proxy SNP: 13:102108397:C:T
UKBPPP log10(p): 447.4
UKBPPP BETA: −0.367
UKBPPP MSPA: 0.384
1186:1213:1171:554:681:1177:

157. CST3 (P01034)
UKBPPP MSPA=0.69

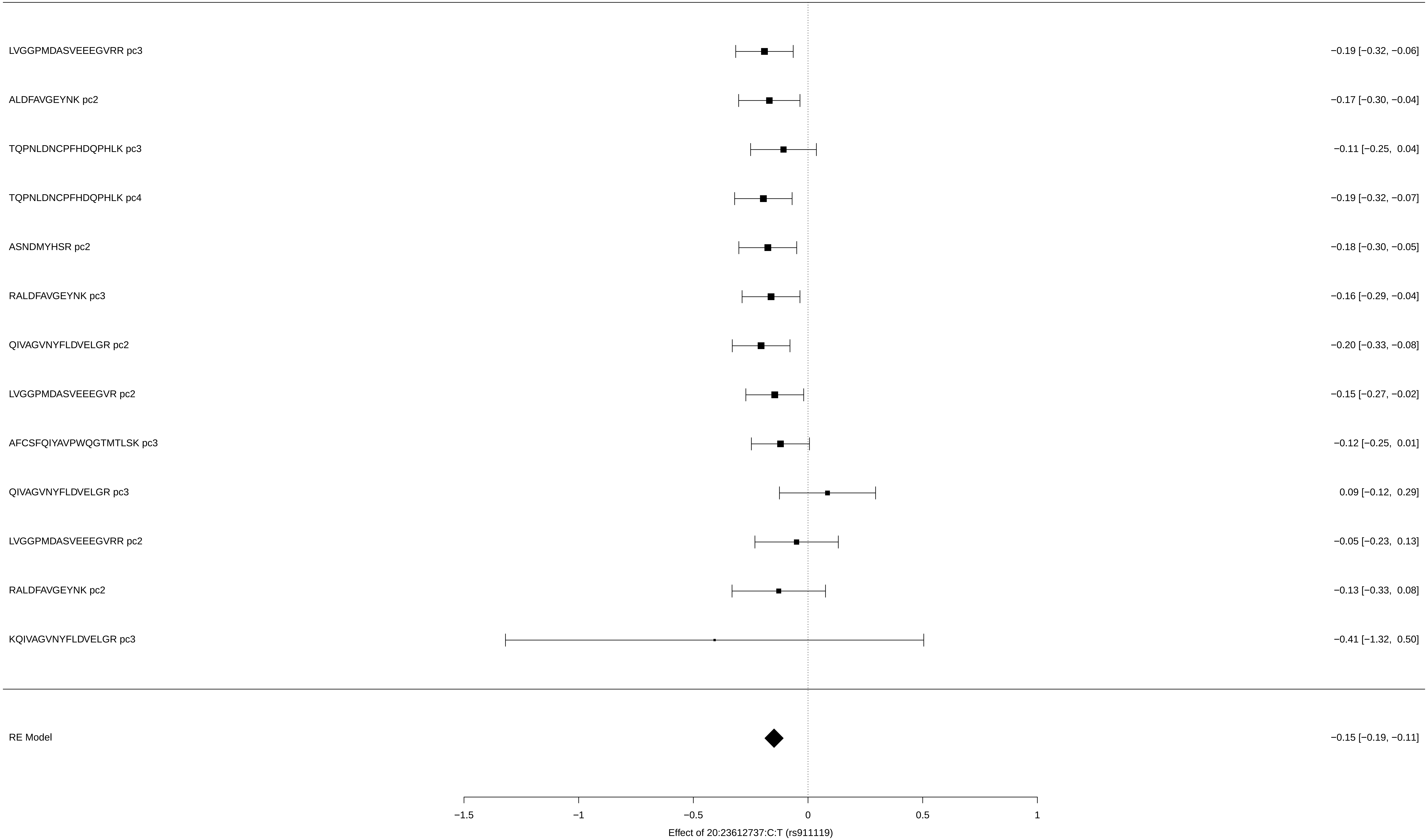

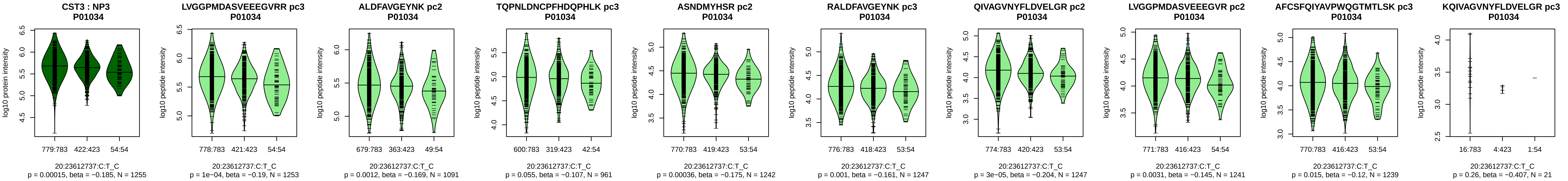

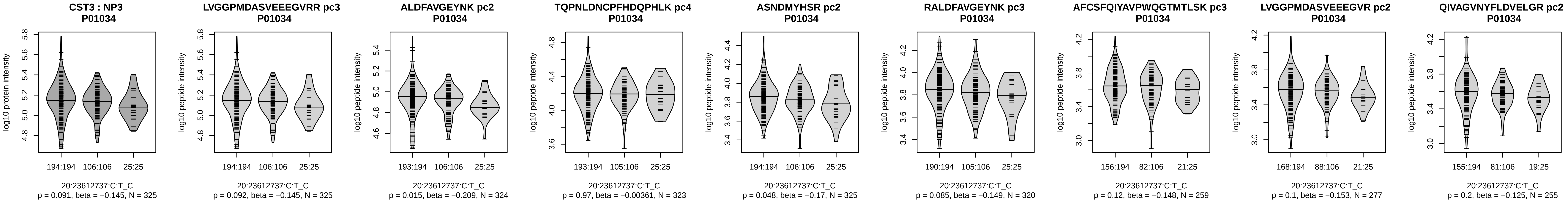

Assay Target: CST3
UniProt: P01034
rsID: rs911119
Target SNP: 20:23612737:C:T
Proxy SNP: 20:23612737:C:T
UKBPPP log10(p): 447.4
UKBPPP BETA: 0.307
UKBPPP MSPA: 0.69
::–:*:*:*:*:*:–:–:–:–:NA:–
1253:1091:961:1253:1242:1247

158. TNFSF13 (O75888)
UKBPPP MSPA=0

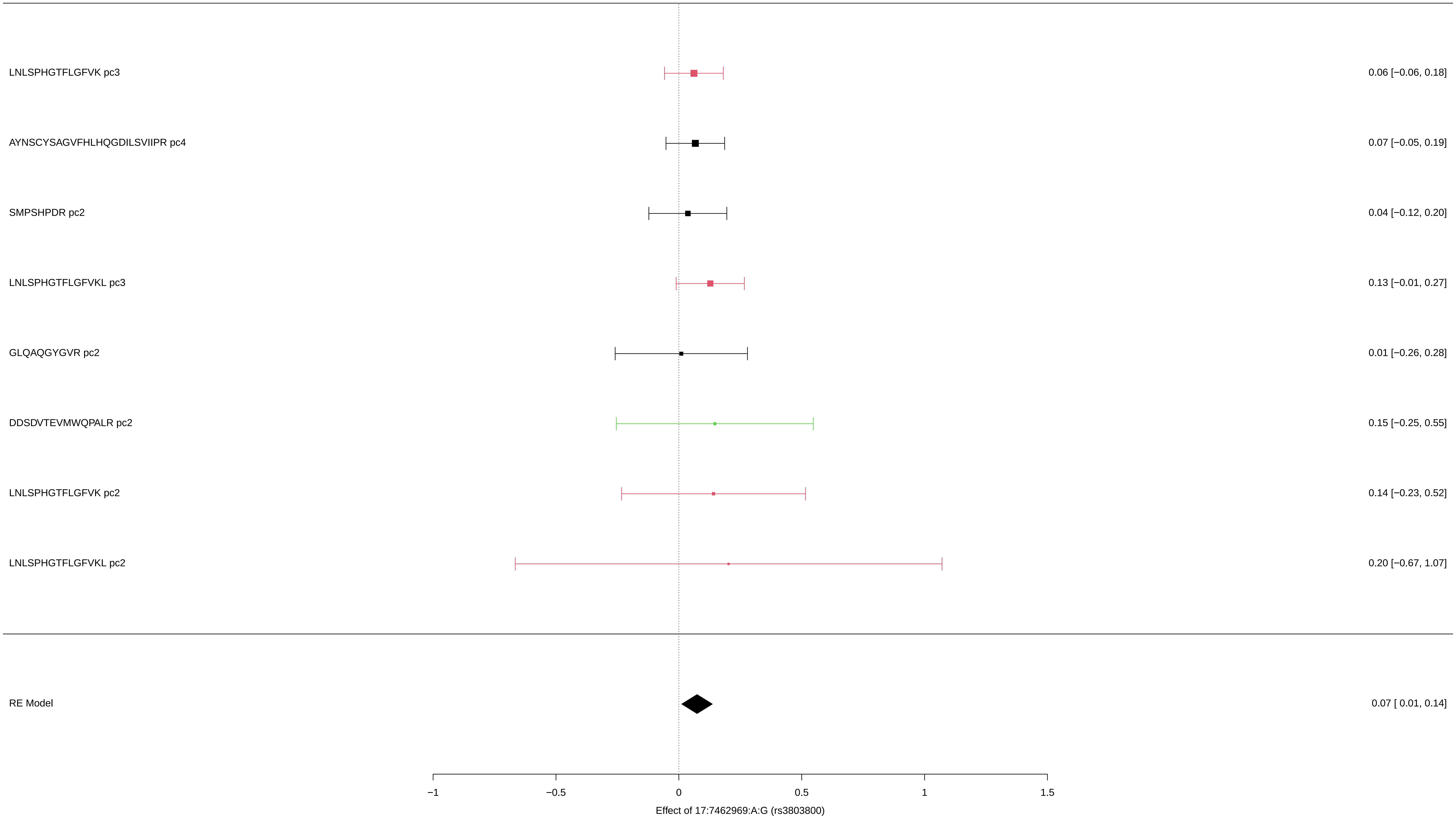

TNFSF13 : NP4
O75888;O75888−4;Q2QBA2

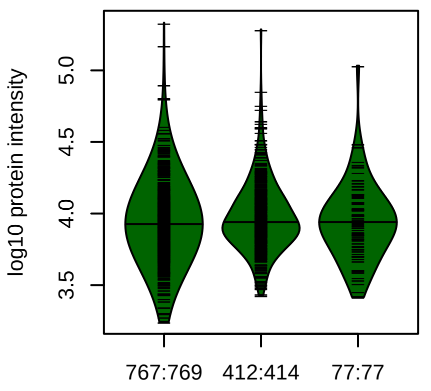

17:7462969:A:G_A
p = 0.2, beta = 0.0591, N = 1256

LNLSPHGTFLGFVK pc3
388;O75888−2;O75888−4;O75888−5;Q

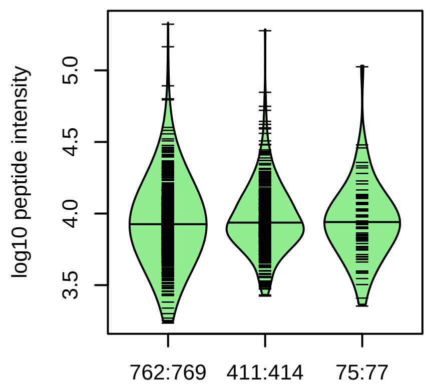

17:7462969:A:G_A
p = 0.19, beta = 0.0613, N = 1248

AYNSCYSAGVFHLHQGDILSVIIPR pc
Q75888−2;O75888−3;O75888−4;O7588

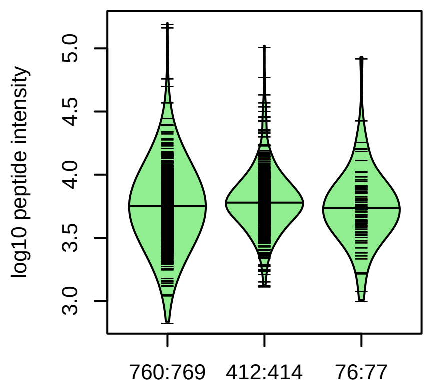

17:7462969:A:G_A
p = 0.15, beta = 0.0671, N = 1248

SMPSHPDR pc2
75888−2;O75888−3;O75888−4;O75888

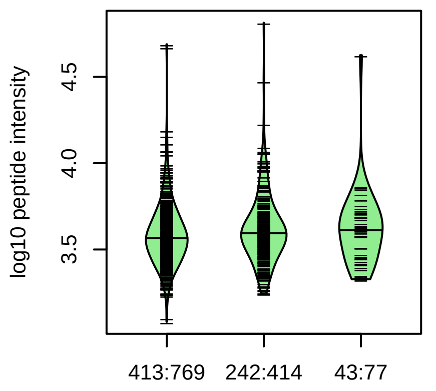

17:7462969:A:G_A
p = 0.55, beta = 0.0366, N = 698

LNLSPHGTFLGFVKL pc3
888;O75888−2;O75888−4;O75888−5;Q

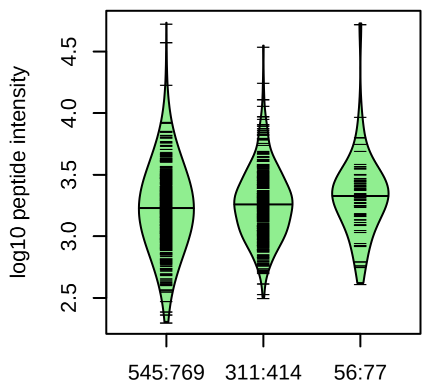

17:7462969:A:G_A
p = 0.018, beta = 0.128, N = 912

GLQAQGYGVR pc2
Q75888−2;O75888−3;O75888−4;O7588

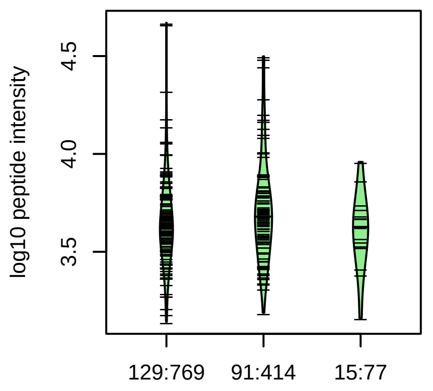

17:7462969:A:G_A
p = 0.92, beta = 0.0101, N = 235

DDSDVTEVMWQPALR pc2
O75888−3;O75888−4;O75888−5;Q2QB

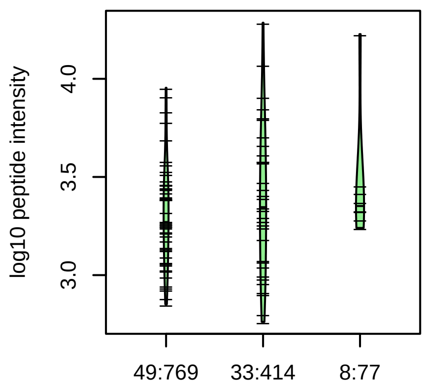

17:7462969:A:G_A
p = 0.35, beta = 0.146, N = 90

LNLSPHGTFLGFVK pc3
388;O75888−2;O75888−4;O75888−5;Q

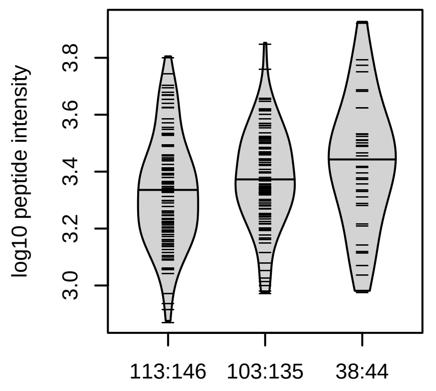

17:7462969:A:G_A
p = 0.0047, beta = 0.243, N = 254

GLQAQGYGVR pc2
Q75888−2;O75888−3;O75888−4;O7588

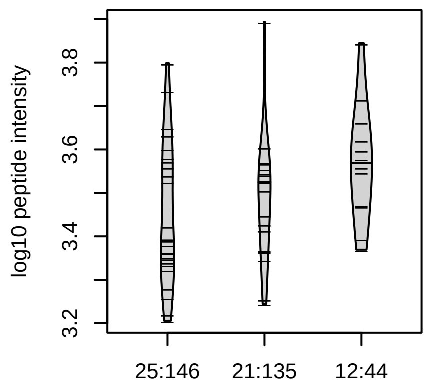

17:7462969:A:G_A
p = 0.022, beta = 0.369, N = 58

Assay Target: TNFSF13
UniProt: O75888
rsID: rs3803800
Target SNP: 17:7462969:A:G
Proxy SNP: 17:7462969:A:G
UKBPPP log10(p): 444
UKBPPP BETA: −0.313
UKBPPP MSPA: 0
−:−:−:−:−:−:−:NA:−
1248:1248:698:912:235:90:114:

159. SERPINF1 (P36955)
UKBPPP MSPA=0.467

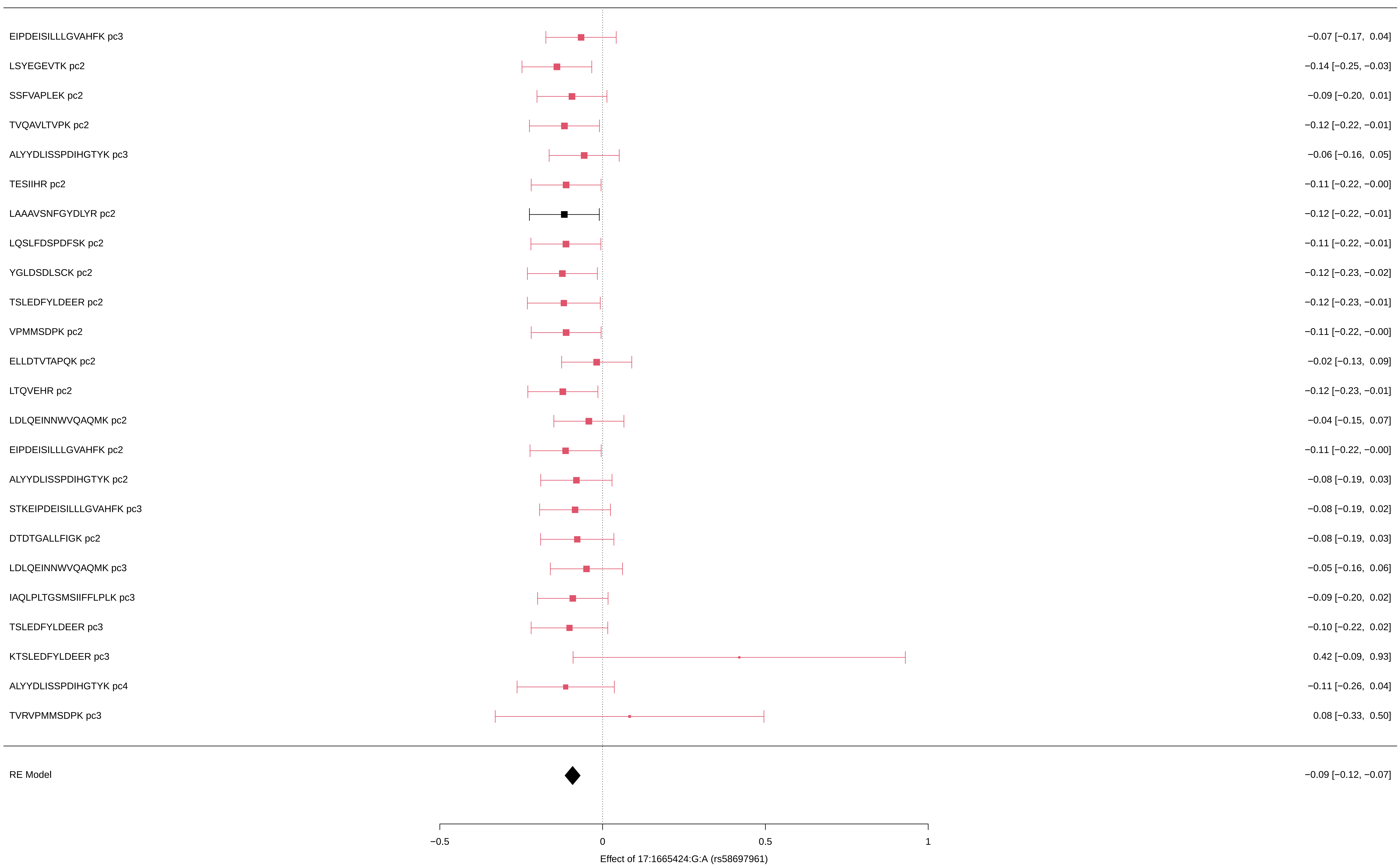

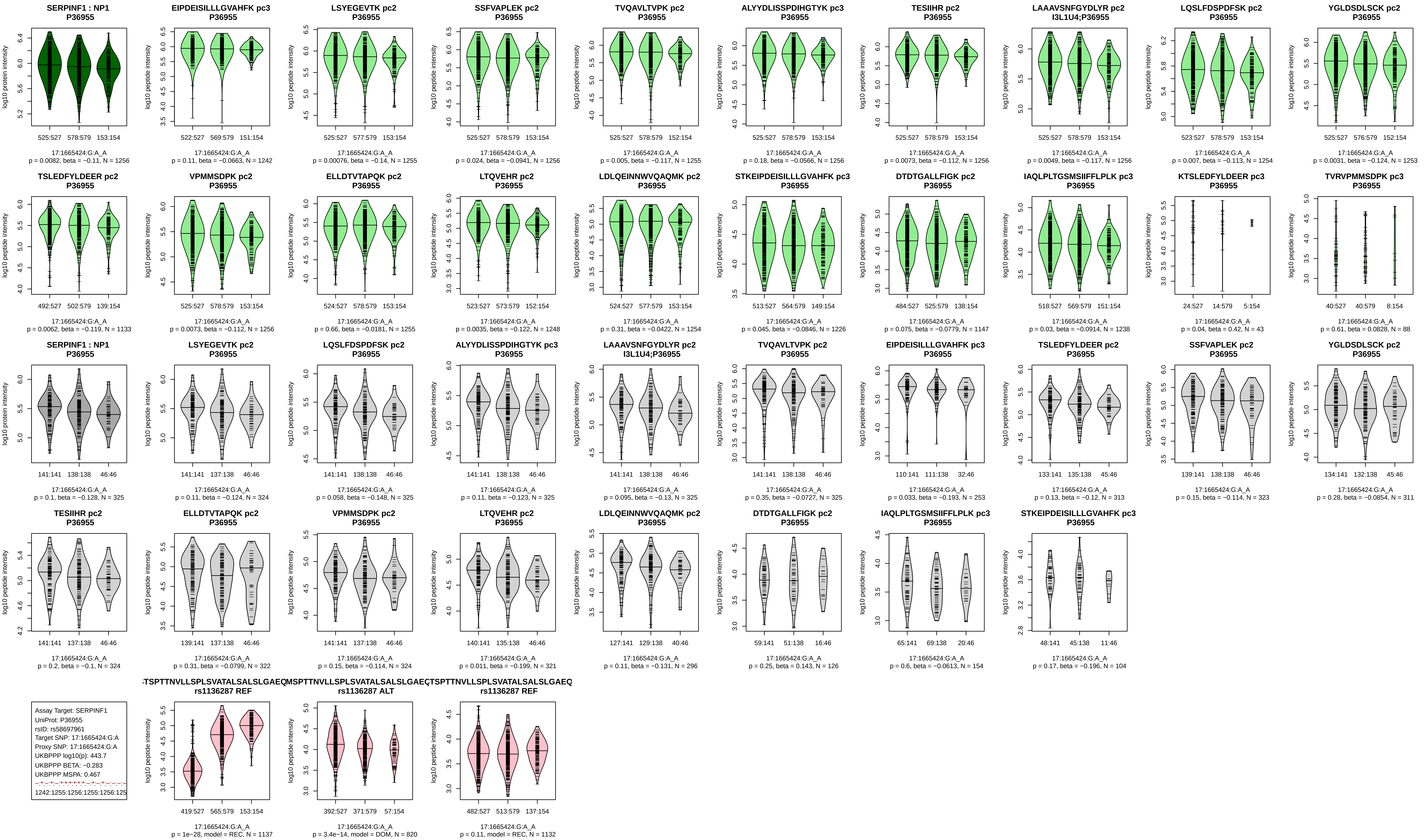

SERPINF1 : NP1
P36955
EIPDEISILLLGVAHFK pc3
LSYEGEVTK pc2
SSFVAPLEK pc2
TVQAVLTVPK pc2
ALYYDLISSPDIHGTYK pc3
TESIIHR pc2
LAAAVSNFGYDLYR pc2
I3L1U4;P36955
LQSLFDSPDFSK pc2
YGLDSDLSCK pc2
log10 protein intensity
log10 peptide intensity
17:1665424:G:A_A
p = 0.0082, beta = −0.11, N = 1256
p = 0.11, beta = −0.0663, N = 1242
p = 0.00076, beta = −0.14, N = 1255
p = 0.024, beta = −0.0941, N = 1256
p = 0.005, beta = −0.117, N = 1255
p = 0.18, beta = −0.0566, N = 1256
p = 0.0073, beta = −0.112, N = 1256
p = 0.0049, beta = −0.117, N = 1256
p = 0.007, beta = −0.113, N = 1254
p = 0.0031, beta = −0.124, N = 1253
TSLEDFYLDEER pc2
VPMMSDPK pc2
ELLDTVTAPQK pc2
LTQVEHR pc2
LDLQEINNWVQAQMK pc2
STKEIPDEISILLLGVAHFK pc3
DTDTGALLFIGK pc2
IAQLPLTGSMSIIFFLPLK pc3
KTSLEDFYLDEER pc3
TVRVPMMSDPK pc3
p = 0.0062, beta = −0.119, N = 1133
p = 0.0073, beta = −0.112, N = 1256
p = 0.66, beta = −0.0181, N = 1255
p = 0.0035, beta = −0.122, N = 1248
p = 0.31, beta = −0.0422, N = 1254
p = 0.045, beta = −0.0846, N = 1226
p = 0.075, beta = −0.0779, N = 1147
p = 0.03, beta = −0.0914, N = 1238
p = 0.04, beta = 0.42, N = 43
p = 0.61, beta = 0.0828, N = 88
p = 0.1, beta = −0.128, N = 325
p = 0.11, beta = −0.124, N = 324
p = 0.058, beta = −0.148, N = 325
p = 0.11, beta = −0.123, N = 325
p = 0.095, beta = −0.13, N = 325
p = 0.35, beta = −0.0727, N = 325
p = 0.033, beta = −0.193, N = 253
p = 0.13, beta = −0.12, N = 313
p = 0.15, beta = −0.114, N = 323
p = 0.28, beta = −0.0854, N = 311
p = 0.2, beta = −0.1, N = 324
p = 0.31, beta = −0.0799, N = 322
p = 0.15, beta = −0.114, N = 324
p = 0.011, beta = −0.199, N = 321
p = 0.11, beta = −0.131, N = 296
p = 0.25, beta = 0.143, N = 126
p = 0.6, beta = −0.0613, N = 154
p = 0.17, beta = −0.196, N = 104
Assay Target: SERPINF1
UniProt: P36955
rsID: rs58697961
Target SNP: 17:1665424:G:A
Proxy SNP: 17:1665424:G:A
UKBPPP log10(p): 443.7
UKBPPP BETA: −0.283
UKBPPP MSPA: 0.467
1242:1255:1256:1255:1256:125
rs1136287 REF
rs1136287 ALT
p = 1e−28, model = REC, N = 1137
p = 3.4e−14, model = DOM, N = 820
p = 0.11, model = REC, N = 1132

160. FBN2 (P35556)
UKBPPP MSPA=0.14

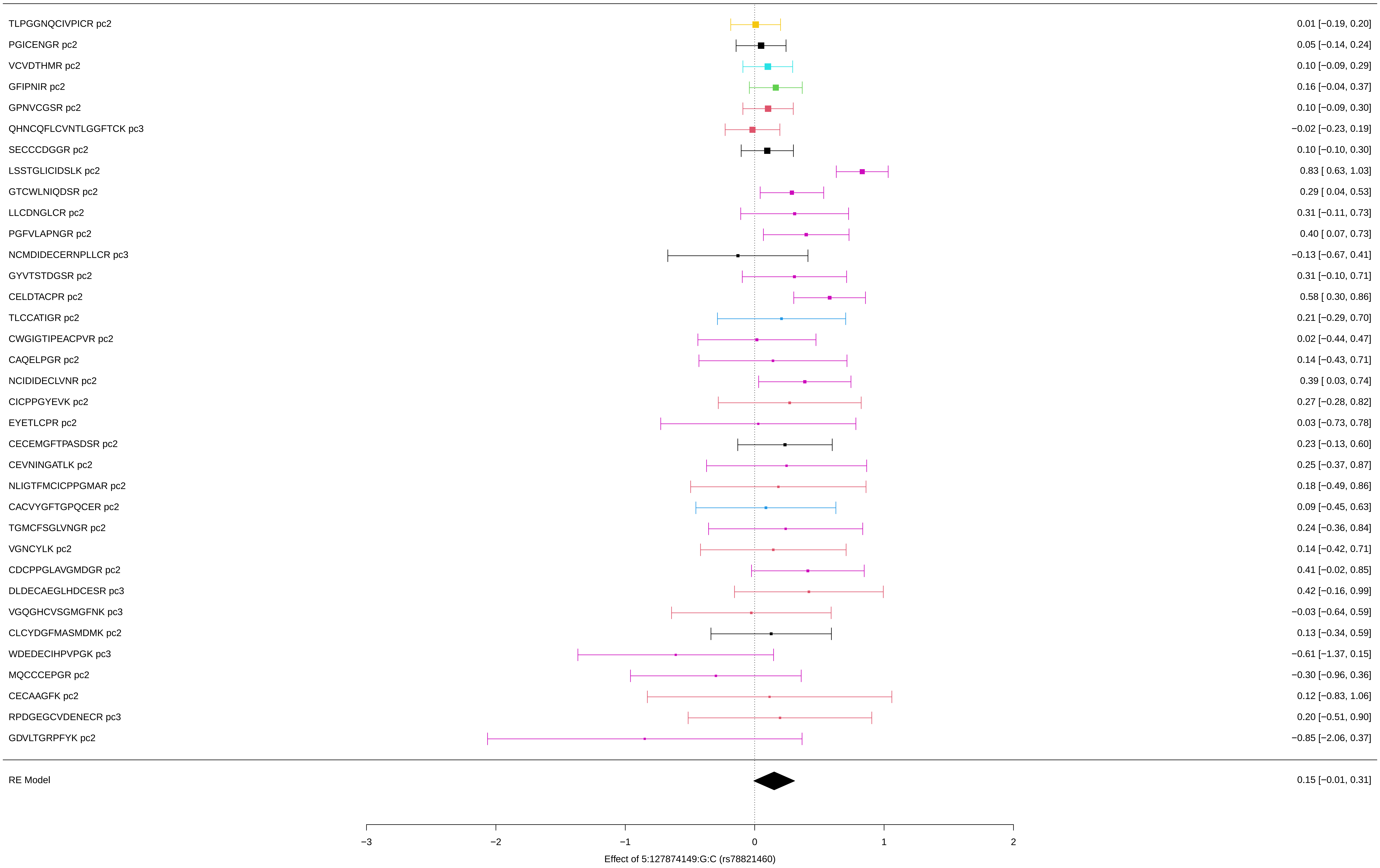

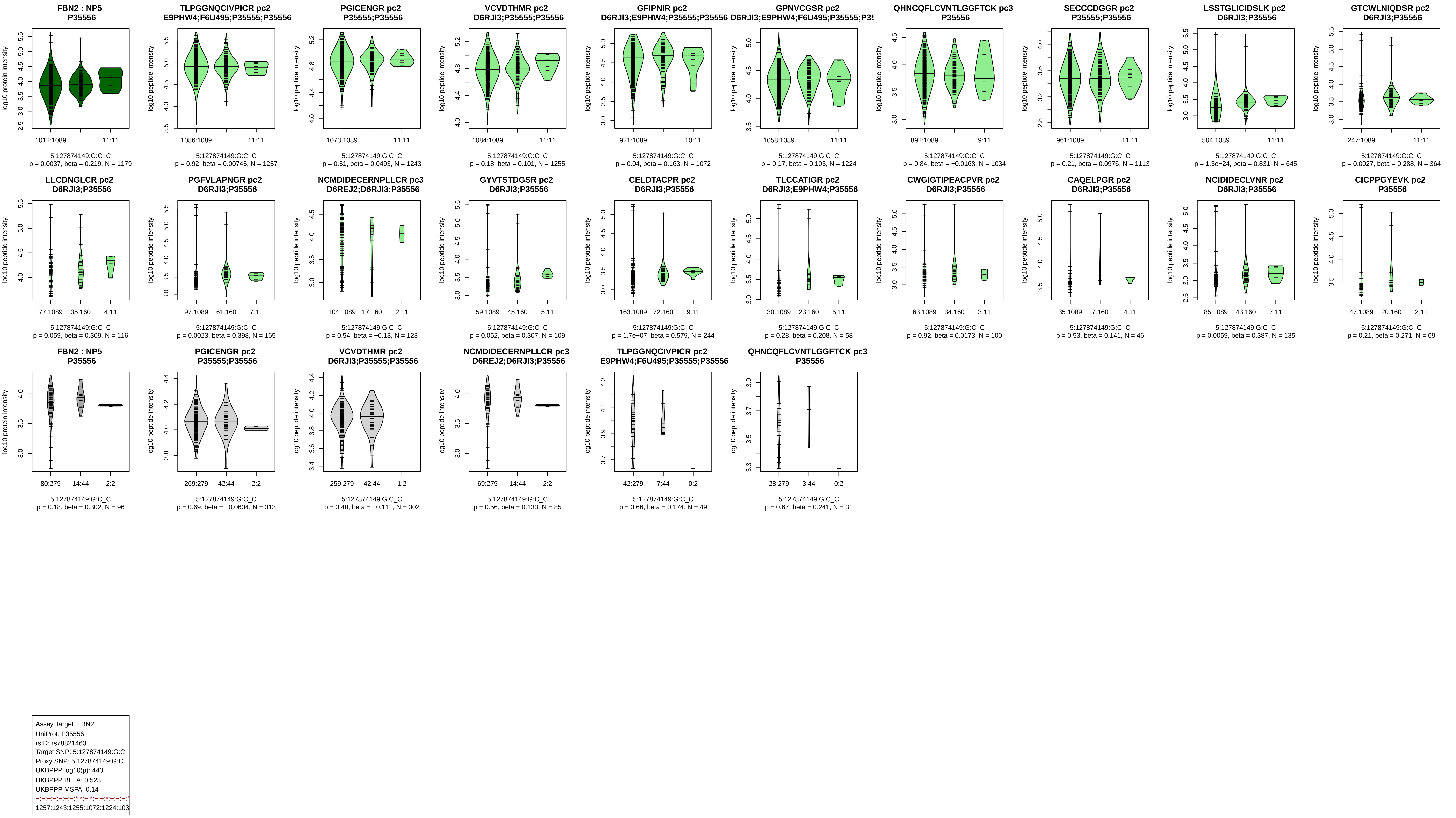
FBN2 : NP5
P35556
log10 protein intensity
1012:1089
11:11
5:127874149:G:C_C
p = 0.0037, beta = 0.219, N = 1179
TLPGGNQCIVPICR pc2
E9PHW4;F6U495;P35555;P35556
log10 peptide intensity
1086:1089
11:11
5:127874149:G:C_C
p = 0.92, beta = 0.00745, N = 1257
PGICENGR pc2
P35555;P35556
1073:1089
11:11
5:127874149:G:C_C
p = 0.51, beta = 0.0493, N = 1243
VCVDTHMR pc2
D6RJI3;P35555;P35556
1084:1089
11:11
5:127874149:G:C_C
p = 0.18, beta = 0.101, N = 1255
GFIPNIR pc2
D6RJI3;E9PHW4;P35555;P35556
921:1089
10:11
5:127874149:G:C_C
p = 0.04, beta = 0.163, N = 1072
GPNVCGSR pc2
D6RJI3;E9PHW4;F6U495;P35555;P35
1058:1089
11:11
5:127874149:G:C_C
p = 0.17, beta = 0.103, N = 1224
QHNCQFLCVNTLGGFTCK pc3
P35556
892:1089
9:11
5:127874149:G:C_C
p = 0.84, beta = −0.0168, N = 1034
SECCCDGGR pc2
P35555;P35556
961:1089
11:11
5:127874149:G:C_C
p = 0.21, beta = 0.0976, N = 1113
LSSTGLICIDSLK pc2
D6RJI3;P35556
504:1089
11:11
5:127874149:G:C_C
p = 1.3e−24, beta = 0.831, N = 645
GTCWLNIQDSR pc2
D6RJI3;P35556
247:1089
11:11
5:127874149:G:C_C
p = 0.0027, beta = 0.288, N = 364
LLCDNGLCR pc2
D6RJI3;P35556
77:1089
35:160
4:11
5:127874149:G:C_C
p = 0.059, beta = 0.309, N = 116
PGFVLAPNGR pc2
D6RJI3;P35556
97:1089
61:160
7:11
5:127874149:G:C_C
p = 0.0023, beta = 0.398, N = 165
NCMDIDECERNPLLCR pc3
D6REJ2;D6RJI3;P35556
104:1089
17:160
2:11
5:127874149:G:C_C
p = 0.54, beta = −0.13, N = 123
GYVTSTDGSR pc2
D6RJI3;P35556
59:1089
45:160
5:11
5:127874149:G:C_C
p = 0.052, beta = 0.307, N = 109
CELDTACPR pc2
D6RJI3;P35556
163:1089
72:160
9:11
5:127874149:G:C_C
p = 1.7e−07, beta = 0.579, N = 244
TLCCATIGR pc2
D6RJI3;E9PHW4;P35556
30:1089
23:160
5:11
5:127874149:G:C_C
p = 0.28, beta = 0.208, N = 58
CWGIGTIPEACPVR pc2
D6RJI3;P35556
63:1089
34:160
3:11
5:127874149:G:C_C
p = 0.92, beta = 0.0173, N = 100
CAQELPGR pc2
D6RJI3;P35556
35:1089
7:160
4:11
5:127874149:G:C_C
p = 0.53, beta = 0.141, N = 46
NCIDIDECLVNR pc2
D6RJI3;P35556
85:1089
43:160
7:11
5:127874149:G:C_C
p = 0.0059, beta = 0.387, N = 135
CICPPGYEVK pc2
P35556
47:1089
20:160
2:11
5:127874149:G:C_C
p = 0.21, beta = 0.271, N = 69
FBN2 : NP5
P35556
80:279
14:44
2:2
5:127874149:G:C_C
p = 0.18, beta = 0.302, N = 96
PGICENGR pc2
P35555;P35556
269:279
42:44
2:2
5:127874149:G:C_C
p = 0.69, beta = −0.0604, N = 313
VCVDTHMR pc2
D6RJI3;P35555;P35556
259:279
42:44
1:2
5:127874149:G:C_C
p = 0.48, beta = −0.111, N = 302
NCMDIDECERNPLLCR pc3
D6REJ2;D6RJI3;P35556
69:279
14:44
2:2
5:127874149:G:C_C
p = 0.56, beta = 0.133, N = 85
TLPGGNQCIVPICR pc2
E9PHW4;F6U495;P35555;P35556
42:279
7:44
0:2
5:127874149:G:C_C
p = 0.66, beta = 0.174, N = 49
QHNCQFLCVNTLGGFTCK pc3
P35556
28:279
3:44
0:2
5:127874149:G:C_C
p = 0.67, beta = 0.241, N = 31
Assay Target: FBN2
UniProt: P35556
rsID: rs78821460
Target SNP: 5:127874149:G:C
Proxy SNP: 5:127874149:G:C
UKBPPP log10(p): 443
UKBPPP BETA: 0.523
UKBPPP MSPA: 0.14
1257:1243:1255:1072:1224:103

161. CNDP1 (Q96KN2)
UKBPPP MSPA=0.811

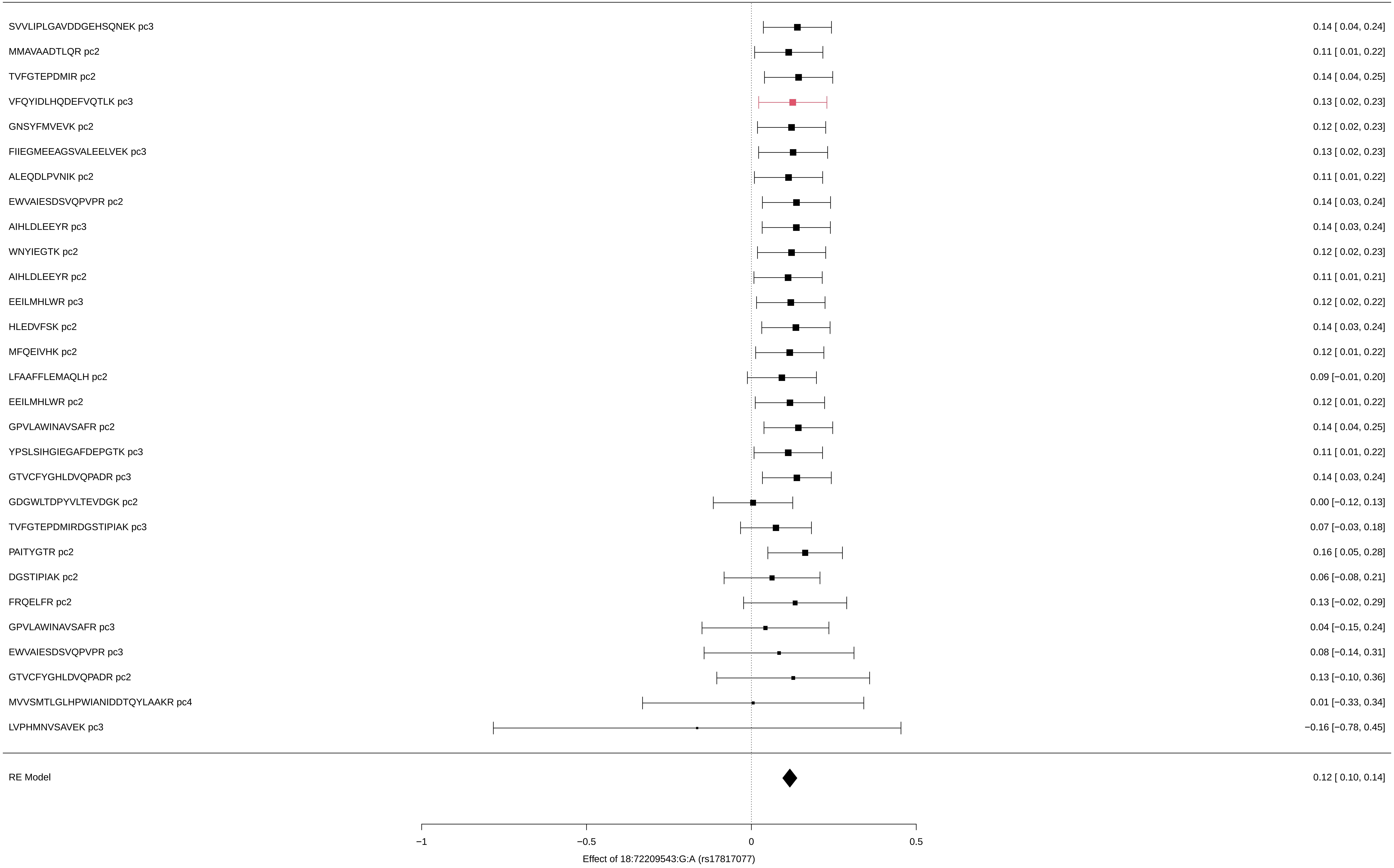

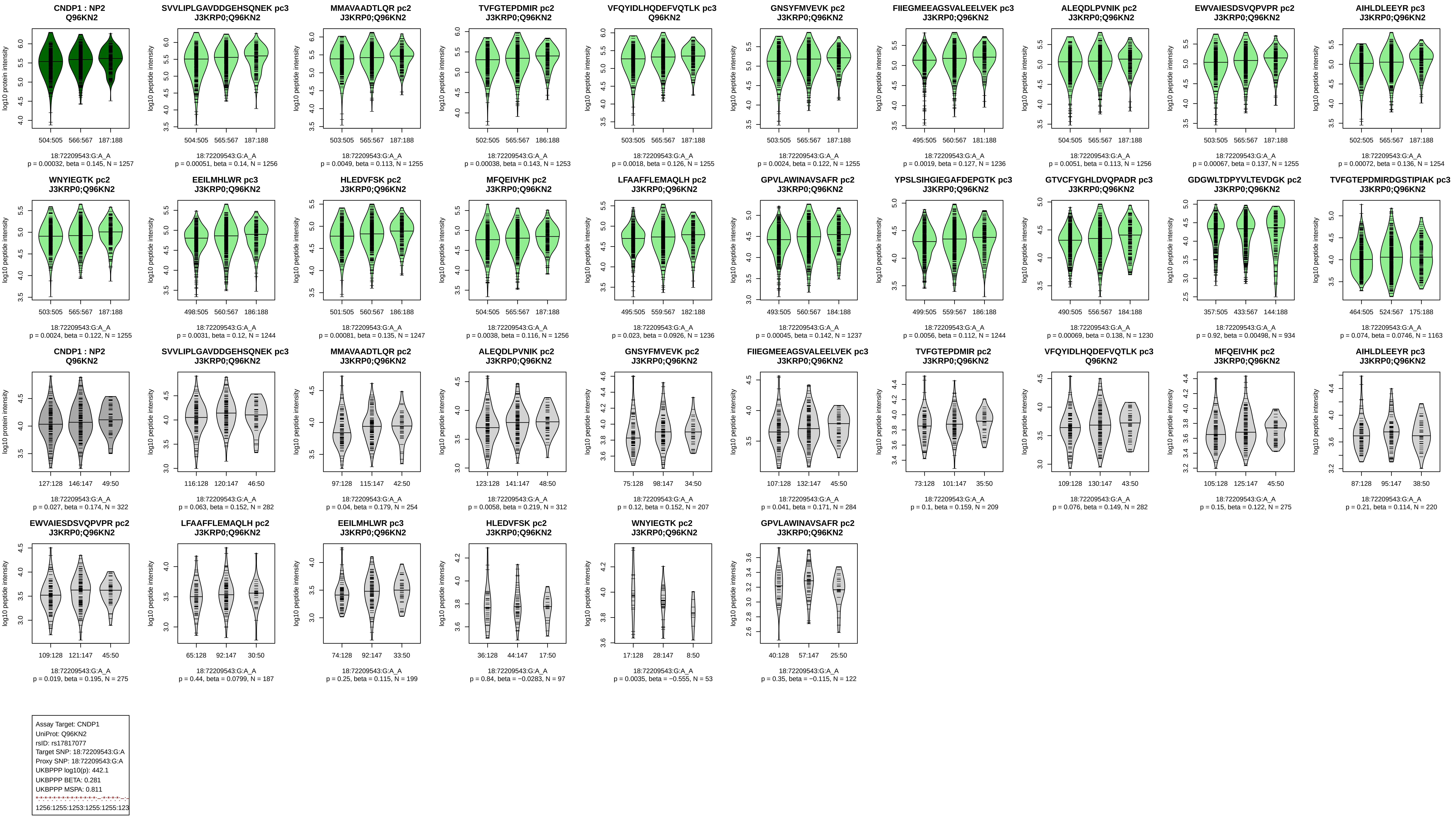
CNDP1 : NP2 Q96KN2
504:505 566:567 187:188
18:72209543:G:A_A
p = 0.00032, beta = 0.145, N = 1257
SVVLIPLGAVDDGEHSQNEK pc3 J3KRP0;Q96KN2
504:505 565:567 187:188
18:72209543:G:A_A
p = 0.00051, beta = 0.14, N = 1256
MMAVAADTLQR pc2 J3KRP0;Q96KN2
503:505 565:567 187:188
18:72209543:G:A_A
p = 0.0049, beta = 0.113, N = 1255
TVFGTEPDMIR pc2 J3KRP0;Q96KN2
502:505 565:567 186:188
18:72209543:G:A_A
p = 0.00038, beta = 0.143, N = 1253
VFQYIDLHQDEFVQTLK pc3 Q96KN2
503:505 565:567 187:188
18:72209543:G:A_A
p = 0.0018, beta = 0.126, N = 1255
GNSYFMVEVK pc2 J3KRP0;Q96KN2
503:505 565:567 187:188
18:72209543:G:A_A
p = 0.0024, beta = 0.122, N = 1255
FIIEGMEEAGSVALEELVEK pc3 J3KRP0;Q96KN2
495:505 560:567 181:188
18:72209543:G:A_A
p = 0.0019, beta = 0.127, N = 1236
ALEQDLPVNIK pc2 J3KRP0;Q96KN2
504:505 565:567 187:188
18:72209543:G:A_A
p = 0.0051, beta = 0.113, N = 1256
EWVAIESDSVQPVPR pc2 J3KRP0;Q96KN2
503:505 565:567 187:188
18:72209543:G:A_A
p = 0.00067, beta = 0.137, N = 1255
AIHLDLEEYR pc3 J3KRP0;Q96KN2
502:505 565:567 187:188
18:72209543:G:A_A
p = 0.00072, beta = 0.136, N = 1254
WNYIEGTK pc2 J3KRP0;Q96KN2
503:505 565:567 187:188
18:72209543:G:A_A
p = 0.0024, beta = 0.122, N = 1255
EEILMHLWR pc3 J3KRP0;Q96KN2
498:505 560:567 186:188
18:72209543:G:A_A
p = 0.0031, beta = 0.12, N = 1244
HLEDVFSK pc2 J3KRP0;Q96KN2
501:505 560:567 186:188
18:72209543:G:A_A
p = 0.00081, beta = 0.135, N = 1247
MFQEIVHK pc2 J3KRP0;Q96KN2
504:505 565:567 187:188
18:72209543:G:A_A
p = 0.0038, beta = 0.116, N = 1256
LFAAFFLEMAQLH pc2 J3KRP0;Q96KN2
495:505 559:567 182:188
18:72209543:G:A_A
p = 0.023, beta = 0.0926, N = 1236
GPVLAWINAVSAFR pc2 J3KRP0;Q96KN2
493:505 560:567 184:188
18:72209543:G:A_A
p = 0.00045, beta = 0.142, N = 1237
YPSLSIHGIEGAFDEPGTK pc3 J3KRP0;Q96KN2
499:505 559:567 186:188
18:72209543:G:A_A
p = 0.0056, beta = 0.112, N = 1244
GTVCFYGHLDVQPADR pc3 J3KRP0;Q96KN2
490:505 556:567 184:188
18:72209543:G:A_A
p = 0.00069, beta = 0.138, N = 1230
GDGWLTDPYVLTEVDGK pc2 J3KRP0;Q96KN2
357:505 433:567 144:188
18:72209543:G:A_A
p = 0.92, beta = 0.00498, N = 934
TVFGTEPDMIRDGSTIPIAK pc3 J3KRP0;Q96KN2
464:505 524:567 175:188
18:72209543:G:A_A
p = 0.074, beta = 0.0746, N = 1163
CNDP1 : NP2 Q96KN2
127:128 146:147 49:50
18:72209543:G:A_A
p = 0.027, beta = 0.174, N = 322
SVVLIPLGAVDDGEHSQNEK pc3 J3KRP0;Q96KN2
116:128 120:147 46:50
18:72209543:G:A_A
p = 0.063, beta = 0.152, N = 282
MMAVAADTLQR pc2 J3KRP0;Q96KN2
97:128 115:147 42:50
18:72209543:G:A_A
p = 0.04, beta = 0.179, N = 254
ALEQDLPVNIK pc2 J3KRP0;Q96KN2
123:128 141:147 48:50
18:72209543:G:A_A
p = 0.0058, beta = 0.219, N = 312
GNSYFMVEVK pc2 J3KRP0;Q96KN2
75:128 98:147 34:50
18:72209543:G:A_A
p = 0.12, beta = 0.152, N = 207
FIIEGMEEAGSVALEELVEK pc3 J3KRP0;Q96KN2
107:128 132:147 45:50
18:72209543:G:A_A
p = 0.041, beta = 0.171, N = 284
TVFGTEPDMIR pc2 J3KRP0;Q96KN2
73:128 101:147 35:50
18:72209543:G:A_A
p = 0.1, beta = 0.159, N = 209
VFQYIDLHQDEFVQTLK pc3 Q96KN2
109:128 130:147 43:50
18:72209543:G:A_A
p = 0.076, beta = 0.149, N = 282
MFQEIVHK pc2 J3KRP0;Q96KN2
105:128 125:147 45:50
18:72209543:G:A_A
p = 0.15, beta = 0.122, N = 275
AIHLDLEEYR pc3 J3KRP0;Q96KN2
87:128 95:147 38:50
18:72209543:G:A_A
p = 0.21, beta = 0.114, N = 220
EWVAIESDSVQPVPR pc2 J3KRP0;Q96KN2
109:128 121:147 45:50
18:72209543:G:A_A
p = 0.019, beta = 0.195, N = 275
LFAAFFLEMAQLH pc2 J3KRP0;Q96KN2
65:128 92:147 30:50
18:72209543:G:A_A
p = 0.44, beta = 0.0799, N = 187
EEILMHLWR pc3 J3KRP0;Q96KN2
74:128 92:147 33:50
18:72209543:G:A_A
p = 0.25, beta = 0.115, N = 199
HLEDVFSK pc2 J3KRP0;Q96KN2
36:128 44:147 17:50
18:72209543:G:A_A
p = 0.84, beta = −0.0283, N = 97
WNYIEGTK pc2 J3KRP0;Q96KN2
17:128 28:147 8:50
18:72209543:G:A_A
p = 0.0035, beta = −0.555, N = 53
GPVLAWINAVSAFR pc2 J3KRP0;Q96KN2
40:128 57:147 25:50
18:72209543:G:A_A
p = 0.35, beta = −0.115, N = 122
log10 protein intensity
log10 peptide intensity
Assay Target: CNDP1
UniProt: Q96KN2
rsID: rs17817077
Target SNP: 18:72209543:G:A
Proxy SNP: 18:72209543:G:A
UKBPPP log10(p): 442.1
UKBPPP BETA: 0.281
UKBPPP MSPA: 0.811
1256:1255:1253:1255:1255:123

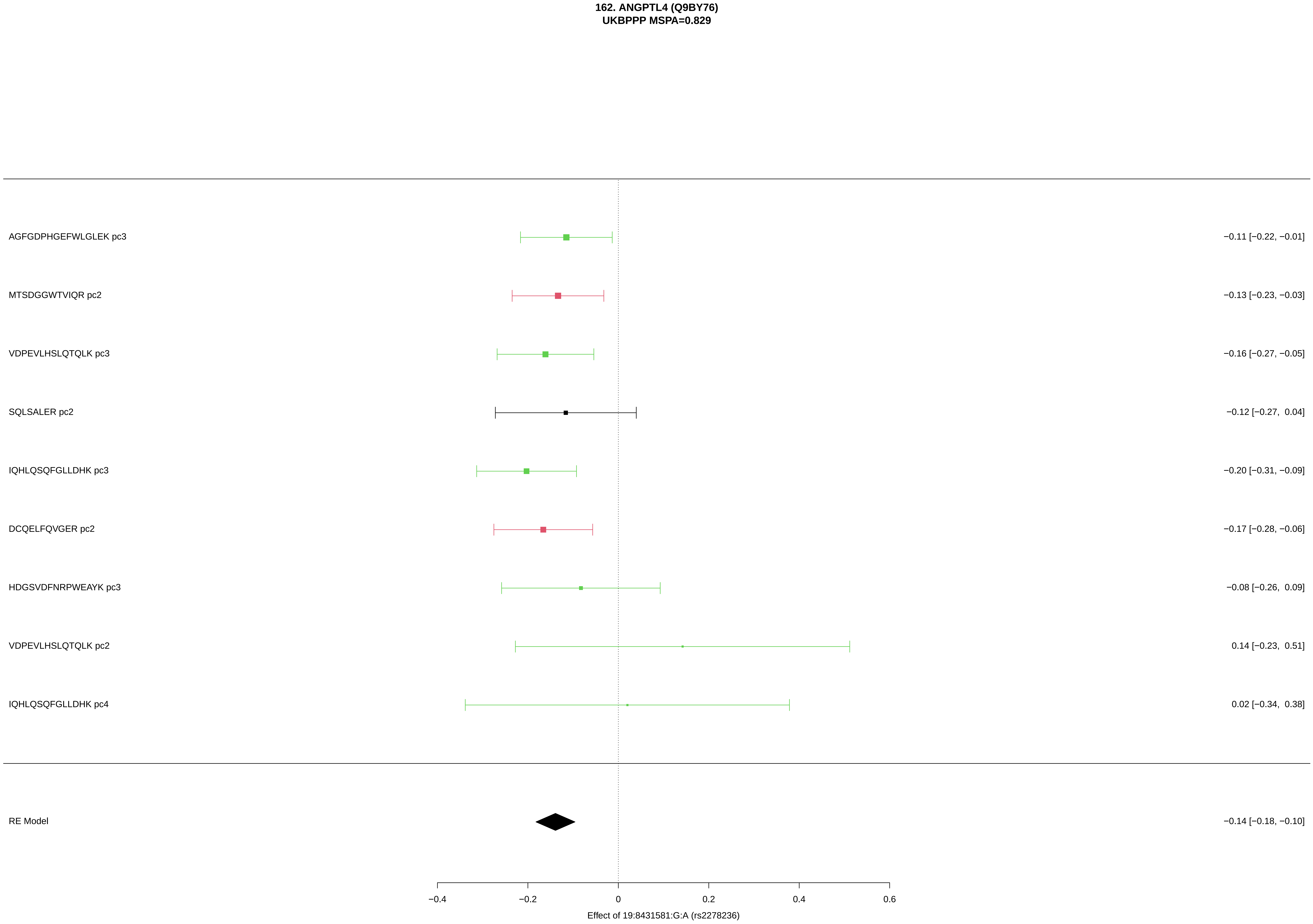
162. ANGPTL4 (Q9BY76)
UKBPPP MSPA=0.829
AGFGDPHGEFWLGLEK pc3
−0.11 [−0.22, −0.01]
MTSDGGWTVIQR pc2
−0.13 [−0.23, −0.03]
VDPEVLHSLQTQLK pc3
−0.16 [−0.27, −0.05]
SQLSALER pc2
−0.12 [−0.27, 0.04]
IQHLQSQFGLLDHK pc3
−0.20 [−0.31, −0.09]
DCQELFQVGER pc2
−0.17 [−0.28, −0.06]
HDGSVDFNRPWEAYK pc3
−0.08 [−0.26, 0.09]
VDPEVLHSLQTQLK pc2
0.14 [−0.23, 0.51]
IQHLQSQFGLLDHK pc4
0.02 [−0.34, 0.38]
RE Model
−0.14 [−0.18, −0.10]
−0.4
−0.2
0
0.2
0.4
0.6
Effect of 19:8431581:G:A (rs2278236)

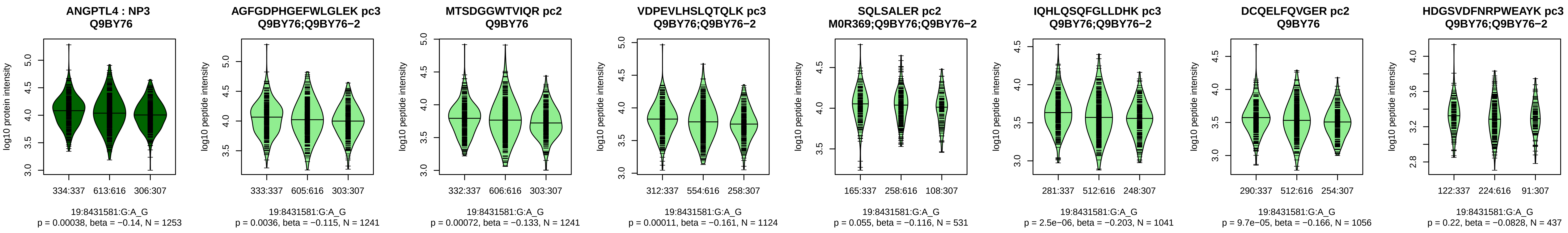

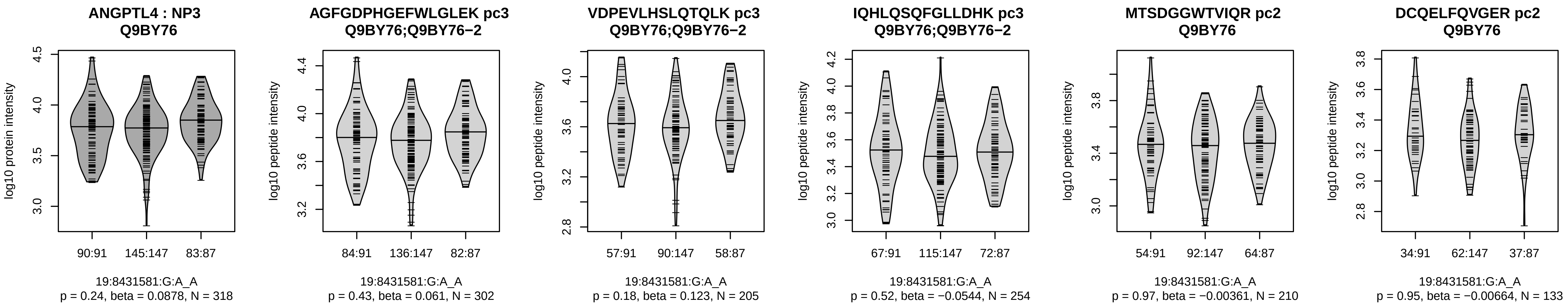

Assay Target: ANGPTL4
UniProt: Q9BY76
rsID: rs2278236
Target SNP: 19:8431581:G:A
Proxy SNP: 19:8431581:G:A
UKBPPP log10(p): 437
UKBPPP BETA: 0.271
UKBPPP MSPA: 0.829
::*:−:*:*:−:−:−:NA:NA
1241:1241:1124:531:1041:1056

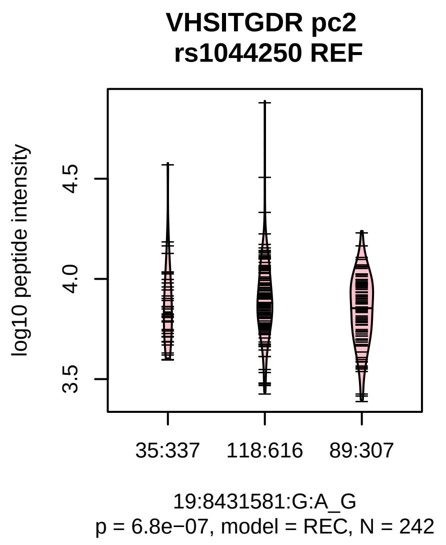

163. APOL1 (O14791)
UKBPPP MSPA=0.061

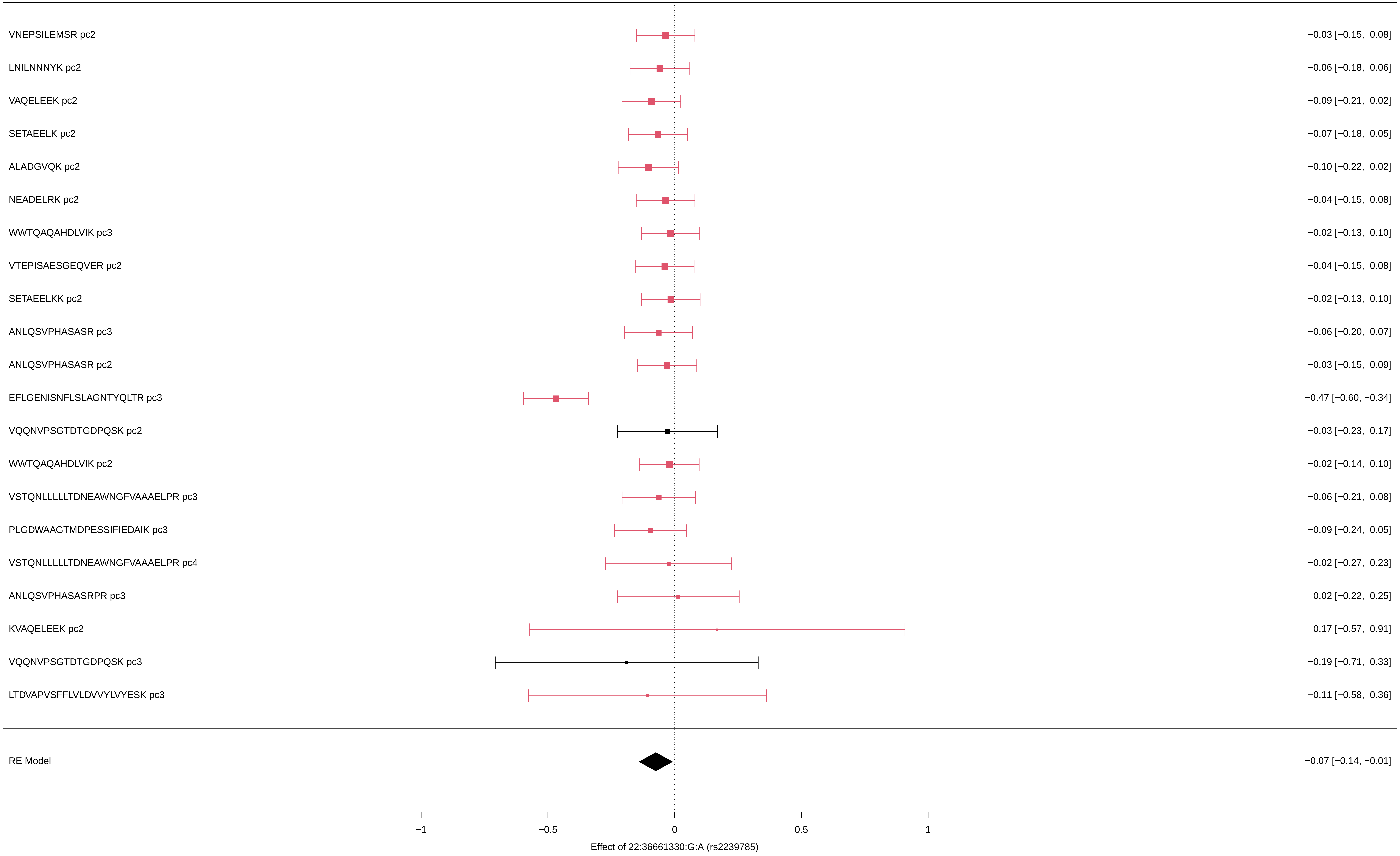

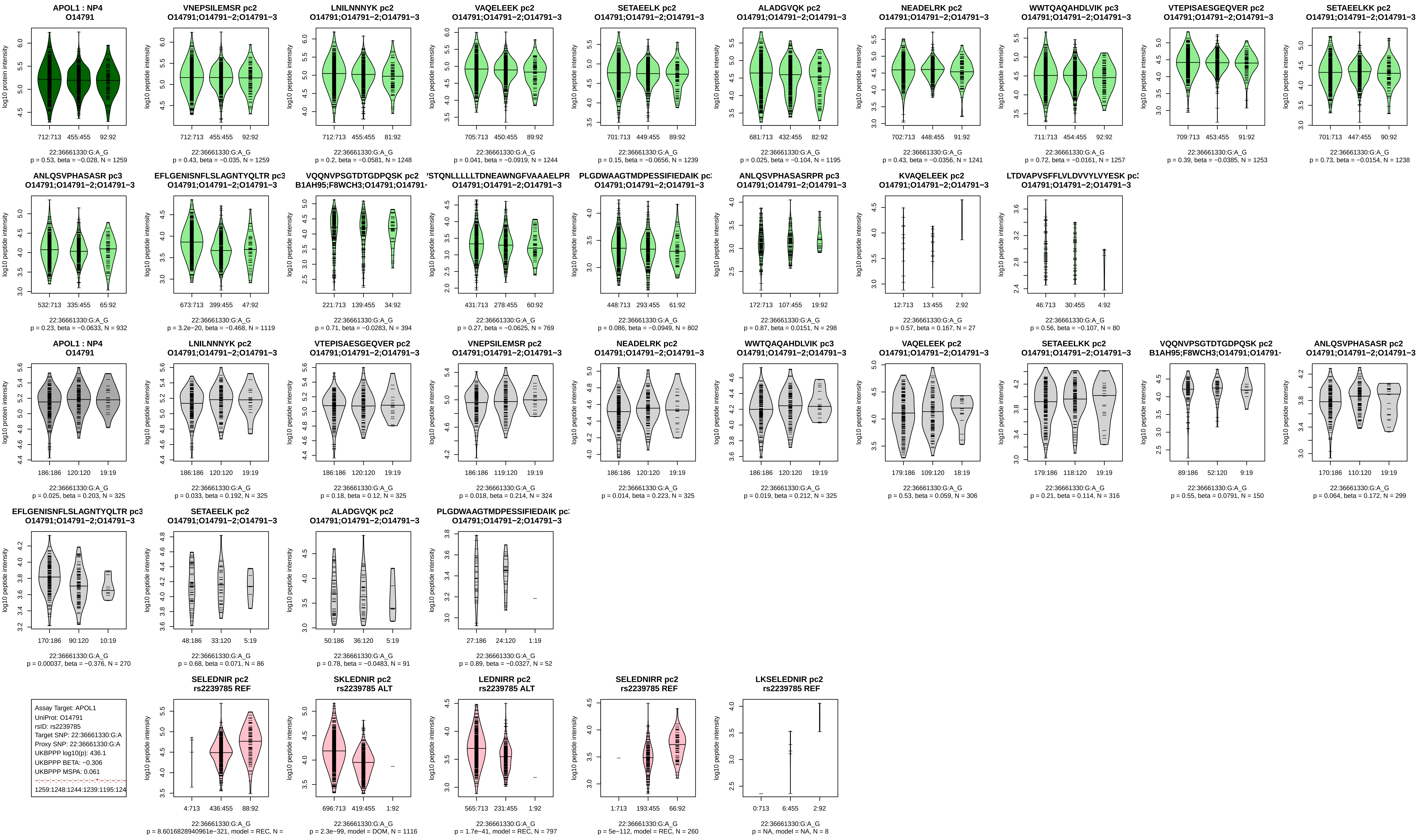
APOL1 : NP4
O14791
22:36661330:G:A_G
p = 0.53, beta = −0.028, N = 1259
VNEPSILEMSR pc2
O14791;O14791−2;O14791−3
p = 0.43, beta = −0.035, N = 1259
LNILNNNYK pc2
p = 0.2, beta = −0.0581, N = 1248
VAQELEEK pc2
p = 0.041, beta = −0.0919, N = 1244
SETAEELK pc2
p = 0.15, beta = −0.0656, N = 1239
ALADGVQK pc2
p = 0.025, beta = −0.104, N = 1195
NEADELRK pc2
p = 0.43, beta = −0.0356, N = 1241
WWTQAQAHDLVIK pc3
p = 0.72, beta = −0.0161, N = 1257
VTEPISAESGEQVER pc2
p = 0.39, beta = −0.0385, N = 1253
SETAEELKK pc2
p = 0.73, beta = −0.0154, N = 1238
ANLQSVPHASASR pc3
p = 0.23, beta = −0.0633, N = 932
EFLGENISNFLSLAGNTYQLTR pc3
p = 3.2e−20, beta = −0.468, N = 1119
VQQNVPSGTDTGDPQSK pc2
B1AH95;F8WCH3;O14791;O14791-
p = 0.71, beta = −0.0283, N = 394
STQNLLLLTDNEAWNGFVAAAELPR
p = 0.27, beta = −0.0625, N = 769
PLGDWAAGTMDPESSIFIEDAIK pc
p = 0.086, beta = −0.0949, N = 802
ANLQSVPHASASRPR pc3
p = 0.87, beta = 0.0151, N = 298
KVAQELEEK pc2
p = 0.57, beta = 0.167, N = 27
LTDVAPVSFFLVLDVVYLVYESK pc
p = 0.56, beta = −0.107, N = 80
APOL1 : NP4
p = 0.025, beta = 0.203, N = 325
LNILNNNYK pc2
p = 0.033, beta = 0.192, N = 325
VTEPISAESGEQVER pc2
p = 0.18, beta = 0.12, N = 325
VNEPSILEMSR pc2
p = 0.018, beta = 0.214, N = 324
NEADELRK pc2
p = 0.014, beta = 0.223, N = 325
WWTQAQAHDLVIK pc3
p = 0.019, beta = 0.212, N = 325
VAQELEEK pc2
p = 0.53, beta = 0.059, N = 306
SETAEELKK pc2
p = 0.21, beta = 0.114, N = 316
VQQNVPSGTDTGDPQSK pc2
p = 0.55, beta = 0.0791, N = 150
ANLQSVPHASASR pc2
p = 0.064, beta = 0.172, N = 299
EFLGENISNFLSLAGNTYQLTR pc3
p = 0.00037, beta = −0.376, N = 270
SETAEELK pc2
p = 0.68, beta = 0.071, N = 86
ALADGVQK pc2
p = 0.78, beta = −0.0483, N = 91
PLGDWAAGTMDPESSIFIEDAIK pc
p = 0.89, beta = −0.0327, N = 52
Assay Target: APOL1
UniProt: O14791
rsID: rs2239785
Target SNP: 22:36661330:G:A
Proxy SNP: 22:36661330:G:A
UKBPPP log10(p): 436.1
UKBPPP BETA: −0.306
UKBPPP MSPA: 0.061
1259:1248:1244:1239:1195:124
SELEDNIR pc2
rs2239785 REF
p = 8.6016828940961e−321, model = REC, N =
SKLEDNIR pc2
rs2239785 ALT
p = 2.3e−99, model = DOM, N = 1116
LEDNIRR pc2
rs2239785 ALT
p = 1.7e−41, model = REC, N = 797
SELEDNIRR pc2
rs2239785 REF
p = 5e−112, model = REC, N = 260
LKSELEDNIR pc2
rs2239785 REF
p = NA, model = NA, N = 8

164. NCAM1 (P13591)
UKBPPP MSPA=0

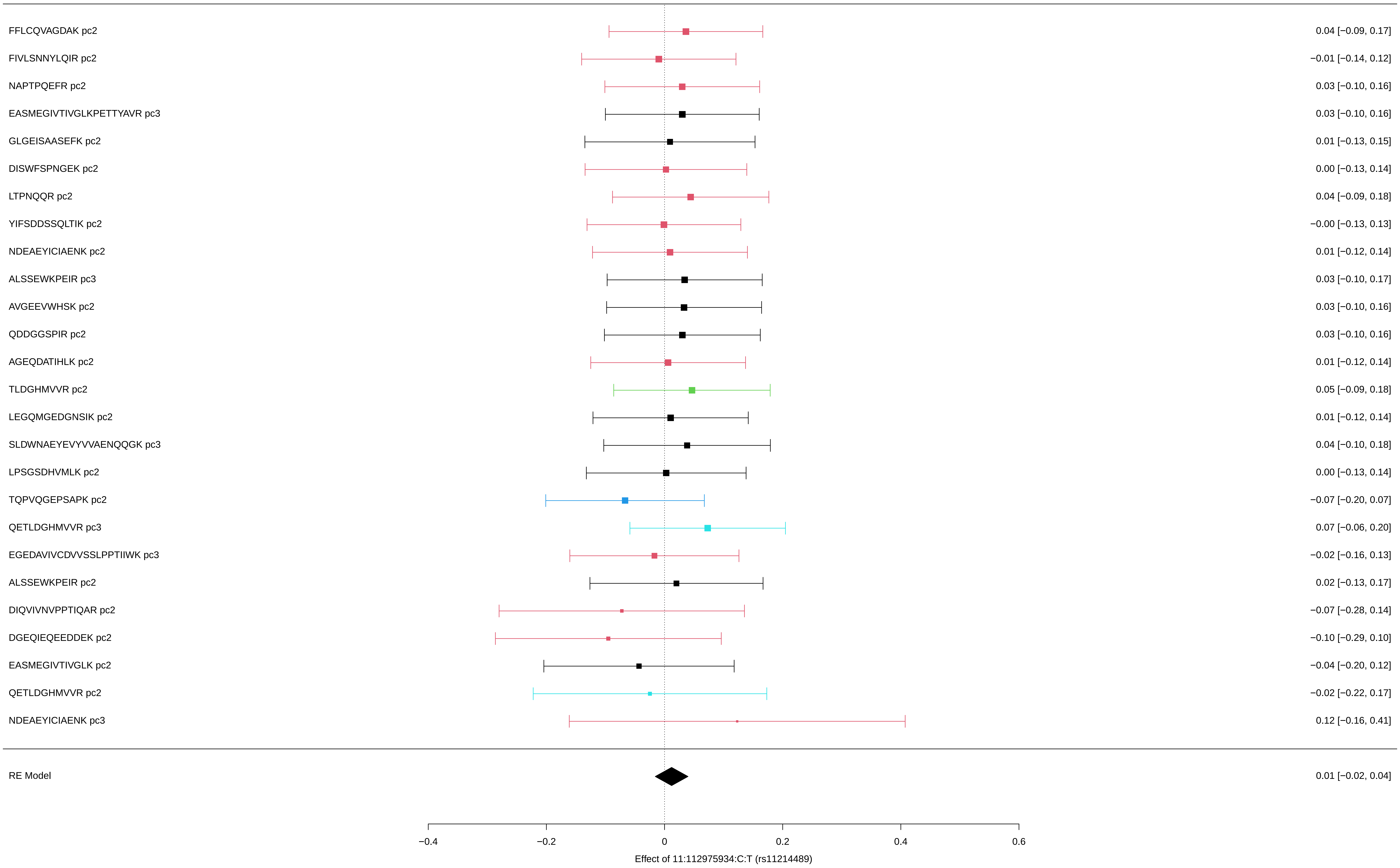

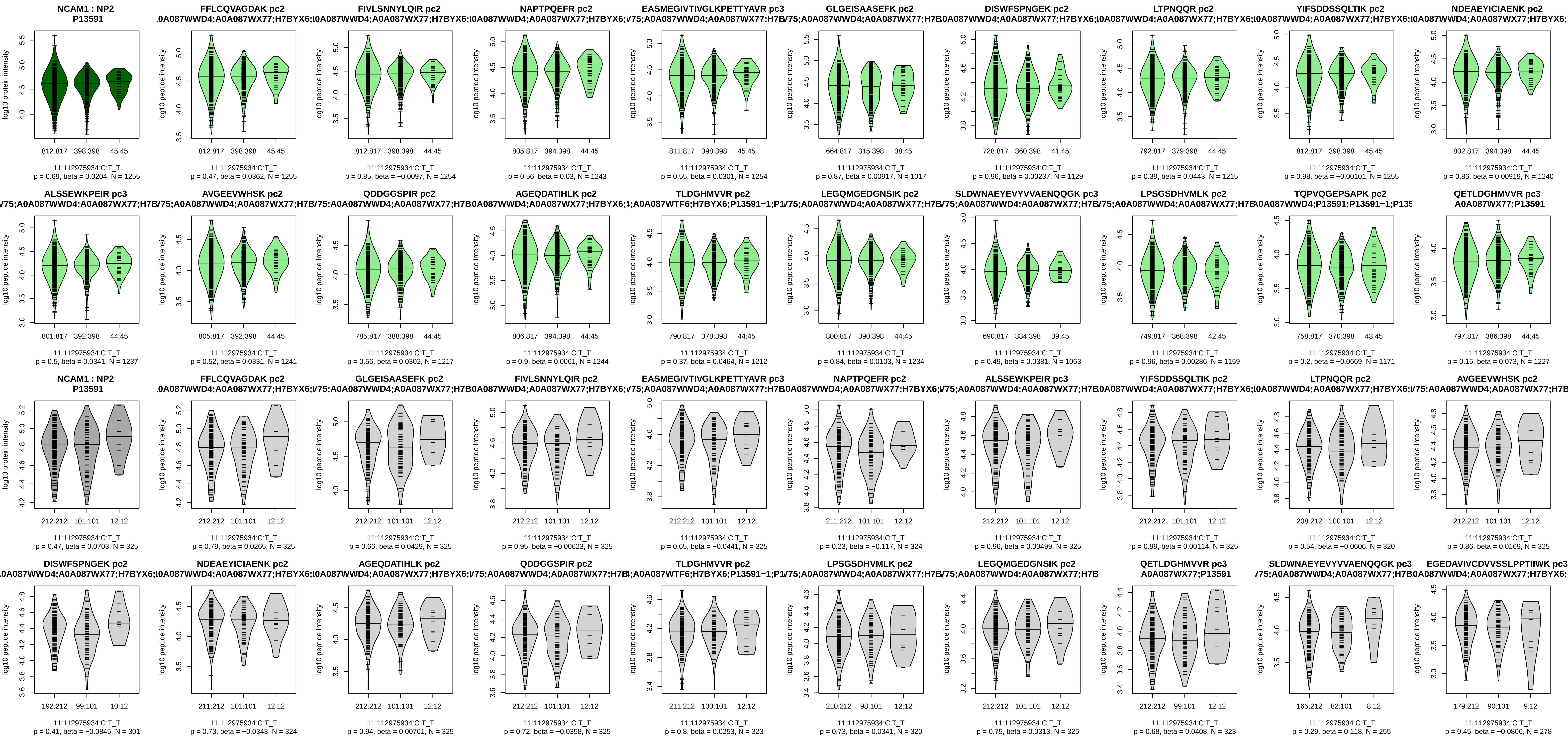

Assay Target: NCAM1
UniProt: P13591
rsID: rs11214489
Target SNP: 11:112975934:C:T
Proxy SNP: 11:112975934:C:T
UKBPPP log10(p): 432.7
UKBPPP BETA: −0.332
UKBPPP MSPA: 0
1255:1254:1243:1254:1017:112

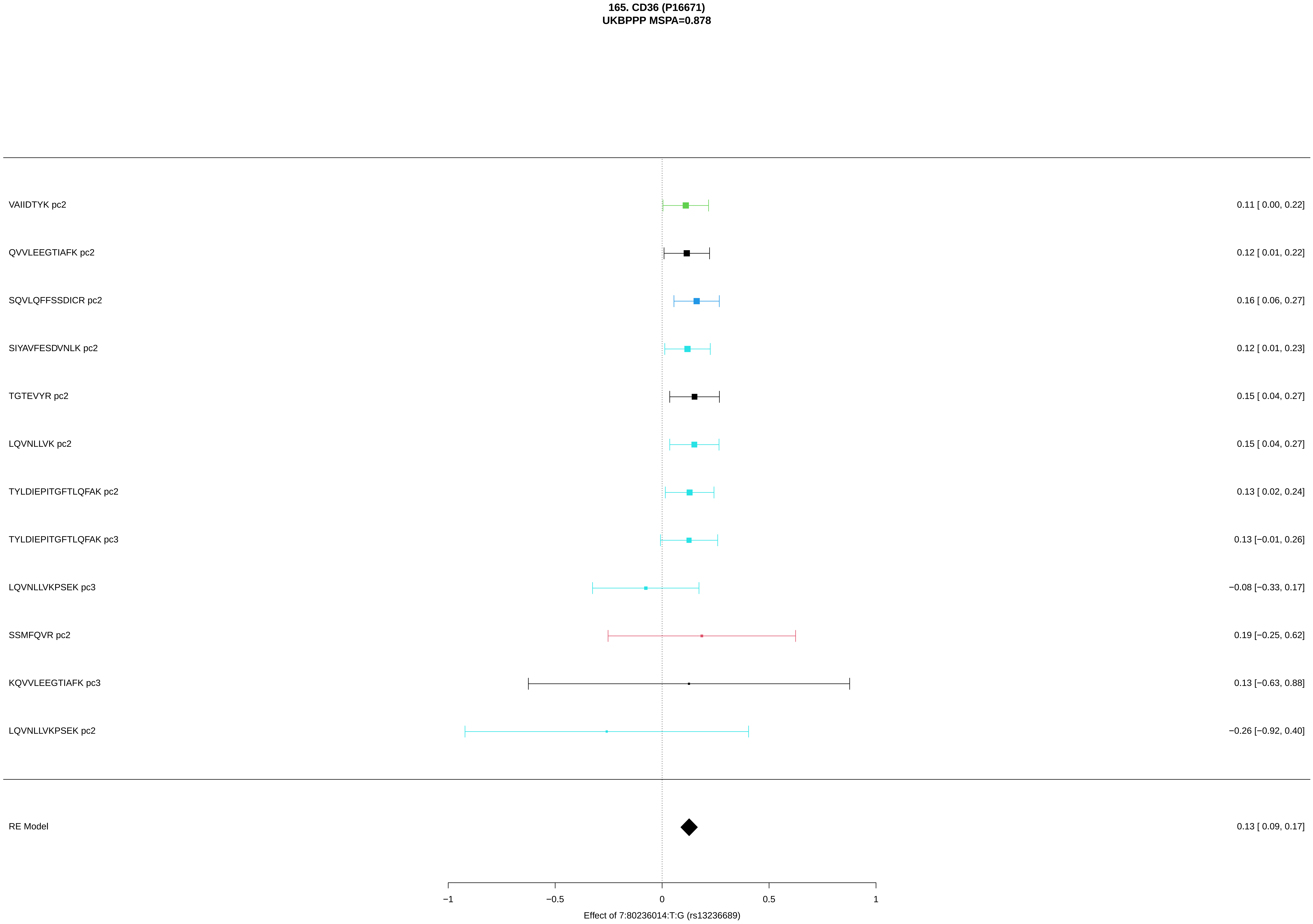
165. CD36 (P16671)
UKBPPP MSPA=0.878
VAIIDTYK pc2
QVVLEEGTIAFK pc2
SQVLQFFSSDICR pc2
SIYAVFESDVNLK pc2
TGTEVYR pc2
LQVNLLVK pc2
TYLDIEPITGFTLQFAK pc2
TYLDIEPITGFTLQFAK pc3
LQVNLLVKPSEK pc3
SSMFQVR pc2
KQVVLEEGTIAFK pc3
LQVNLLVKPSEK pc2
RE Model
0.11 [0.00, 0.22]
0.12 [0.01, 0.22]
0.16 [0.06, 0.27]
0.12 [0.01, 0.23]
0.15 [0.04, 0.27]
0.15 [0.04, 0.27]
0.13 [0.02, 0.24]
0.13 [−0.01, 0.26]
−0.08 [−0.33, 0.17]
0.19 [−0.25, 0.62]
0.13 [−0.63, 0.88]
−0.26 [−0.92, 0.40]
0.13 [0.09, 0.17]
−1
−0.5
0
0.5
1
Effect of 7:80236014:T:G (rs13236689)

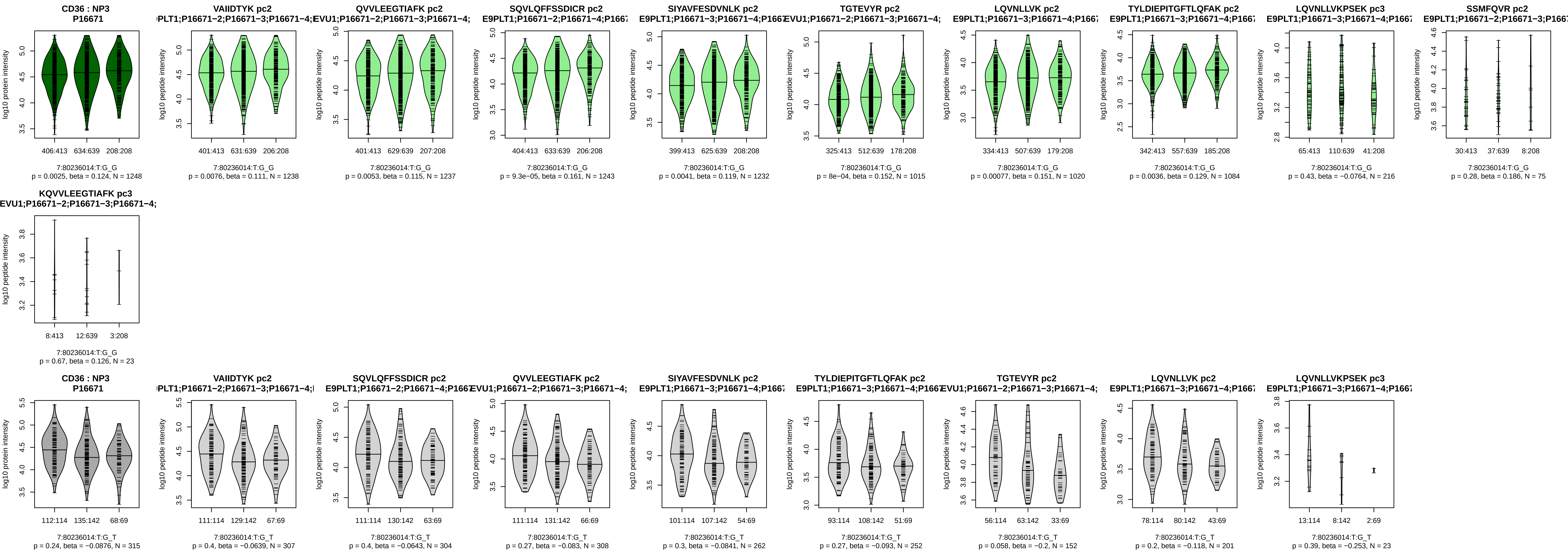

Assay Target: CD36
UniProt: P16671
rsID: rs13236689
Target SNP: 7:80236014:T:G
Proxy SNP: 7:80236014:T:G
UKBPPP log10(p): 413.8
UKBPPP BETA: 0.266
UKBPPP MSPA: 0.878
::*:*:*:*:*:–:–:–:–:–
1238:1237:1243:1232:1015:102

166. LGALS1 (P09382)
UKBPPP MSPA=0.123

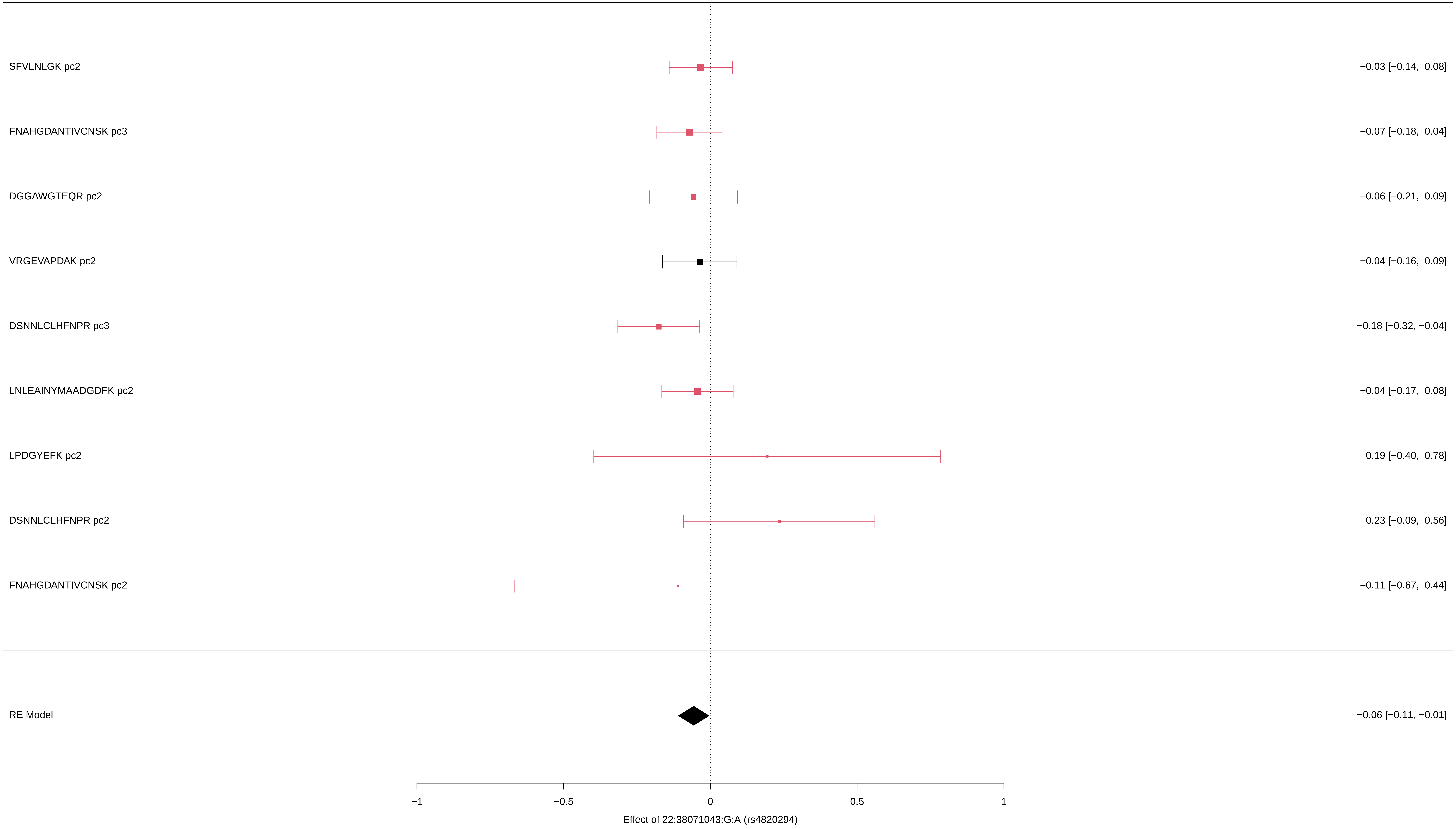

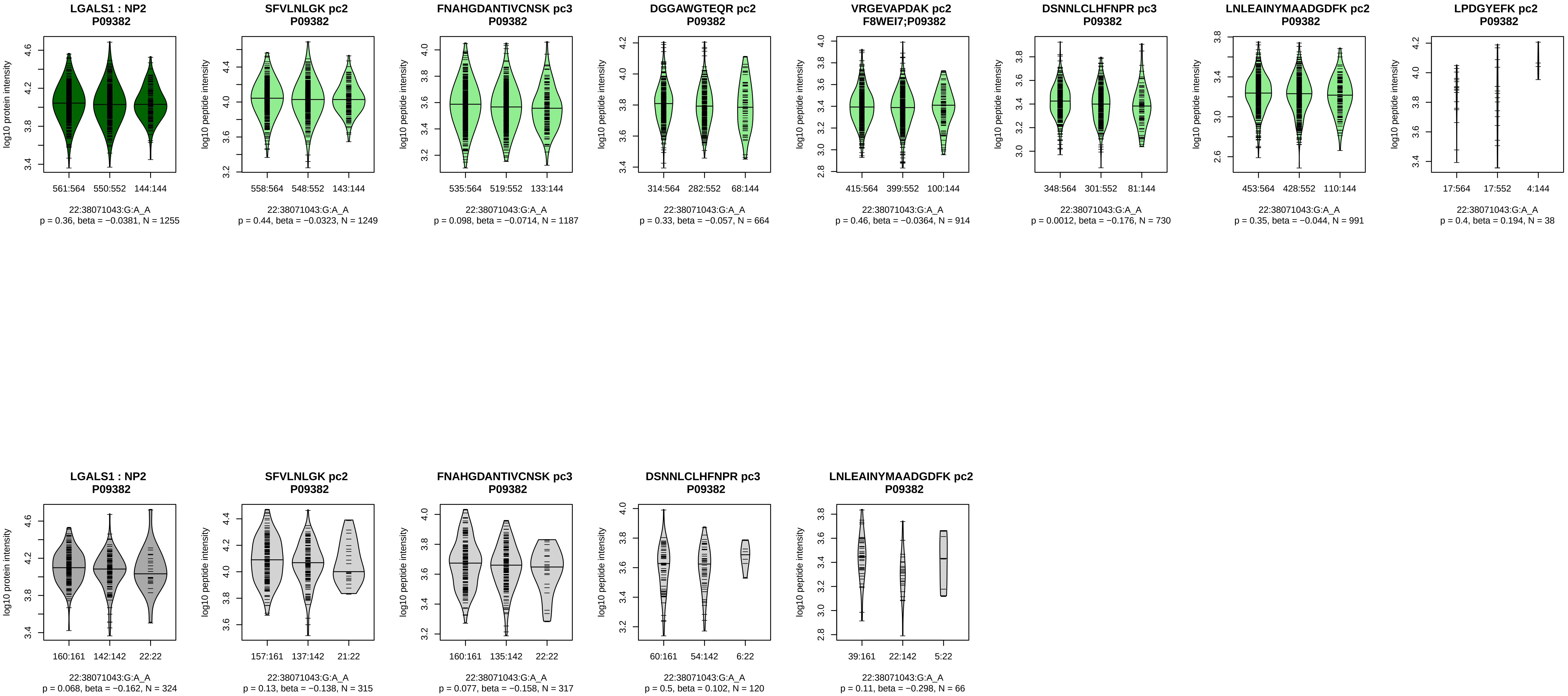

Assay Target: LGALS1
UniProt: P09382
rsID: rs4820294
Target SNP: 22:38071043:G:A
Proxy SNP: 22:38071043:G:A
UKBPPP log10(p): 413
UKBPPP BETA: −0.279
UKBPPP MSPA: 0.123
–:–:–:–:*:–:–:–:–
1249:1187:664:914:730:991:38:

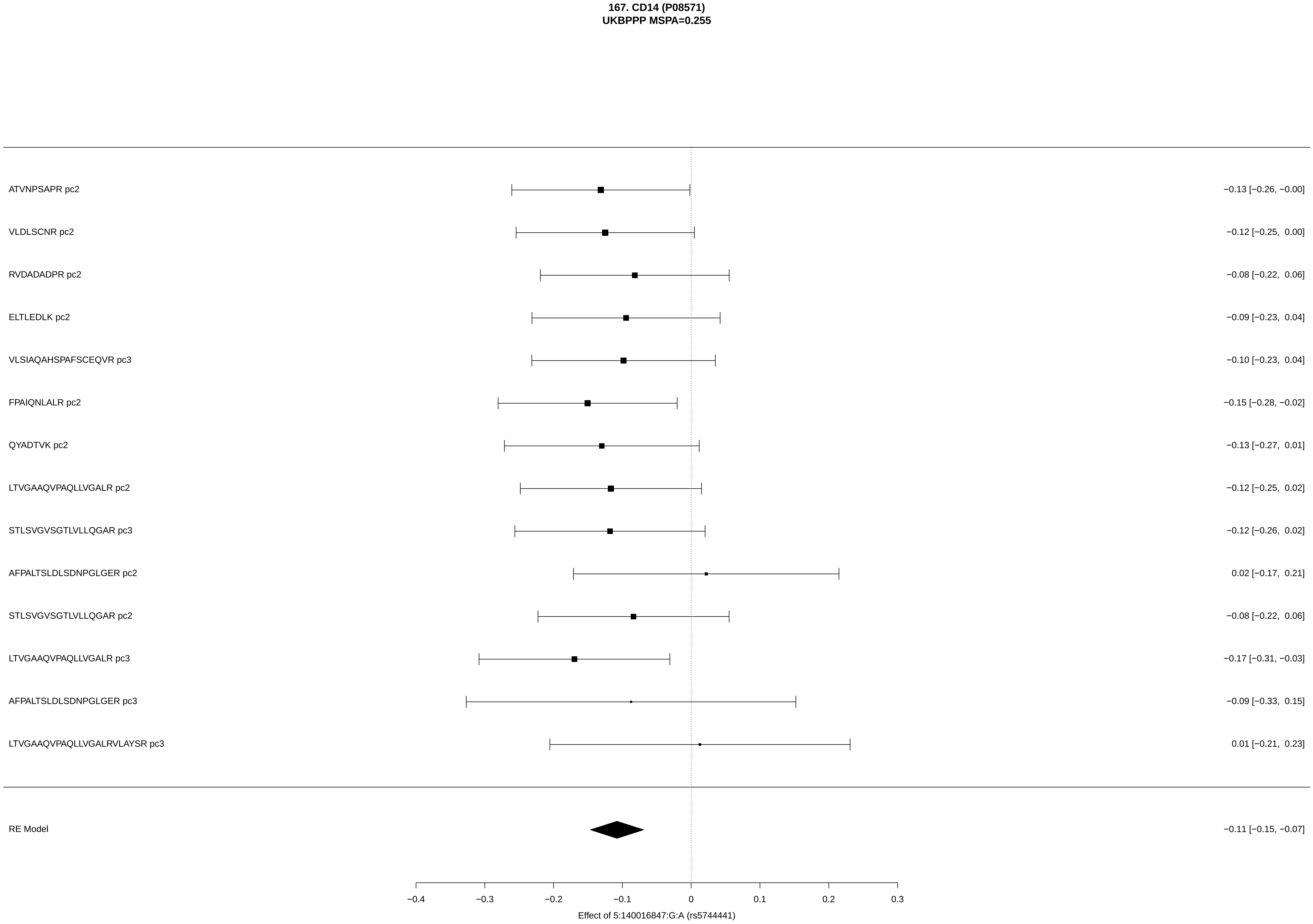
167. CD14 (P08571)
UKBPPP MSPA=0.255
ATVNPSAPR pc2
−0.13 [−0.26, −0.00]
VLDLSCNR pc2
−0.12 [−0.25, 0.00]
RVDADADPR pc2
−0.08 [−0.22, 0.06]
ELTLEDLK pc2
−0.09 [−0.23, 0.04]
VLSIAQAHSPAFSCEQVR pc3
−0.10 [−0.23, 0.04]
FPAIQNLALR pc2
−0.15 [−0.28, −0.02]
QYADTVK pc2
−0.13 [−0.27, 0.01]
LTVGAAQVPAQLLVGALR pc2
−0.12 [−0.25, 0.02]
STLSVGVSGTLVLLQGAR pc3
−0.12 [−0.26, 0.02]
AFPALTSLDLSDNPGLGER pc2
0.02 [−0.17, 0.21]
STLSVGVSGTLVLLQGAR pc2
−0.08 [−0.22, 0.06]
LTVGAAQVPAQLLVGALR pc3
−0.17 [−0.31, −0.03]
AFPALTSLDLSDNPGLGER pc3
−0.09 [−0.33, 0.15]
LTVGAAQVPAQLLVGALRVLAYSR pc3
0.01 [−0.21, 0.23]
RE Model
−0.11 [−0.15, −0.07]
−0.4
−0.3
−0.2
−0.1
0
0.1
0.2
0.3
Effect of 5:140016847:G:A (rs5744441)

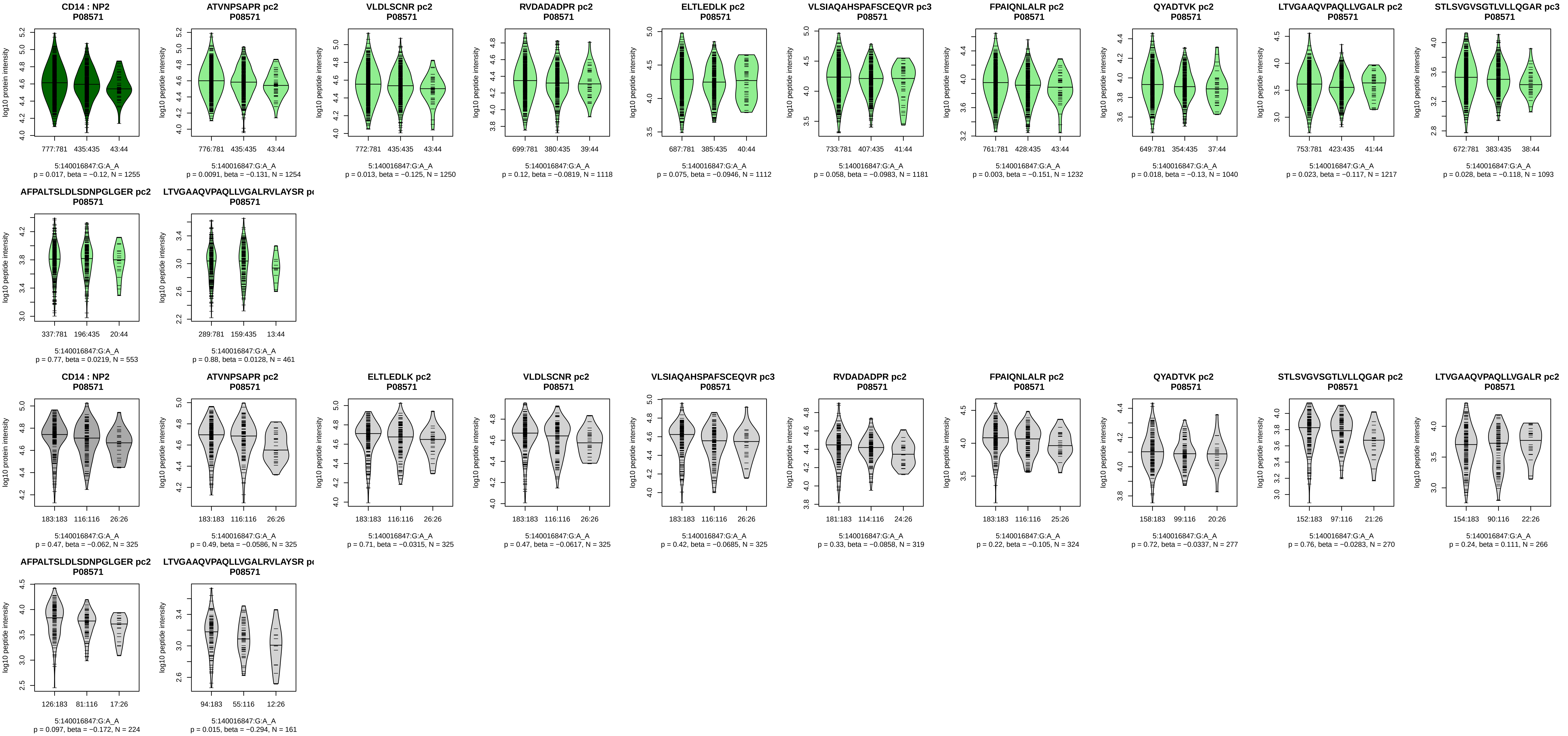

Assay Target: CD14
UniProt: P08571
rsID: rs5744441
Target SNP: 5:140016847:G:A
Proxy SNP: 5:140016847:G:A
UKBPPP log10(p): 411.3
UKBPPP BETA: −0.313
UKBPPP MSPA: 0.255
:–:–:–:–::–:–:–:–:–:*:–:–
1254:1250:1118:1112:1181:123

168. MYOC (Q99972)
UKBPPP MSPA=0.33

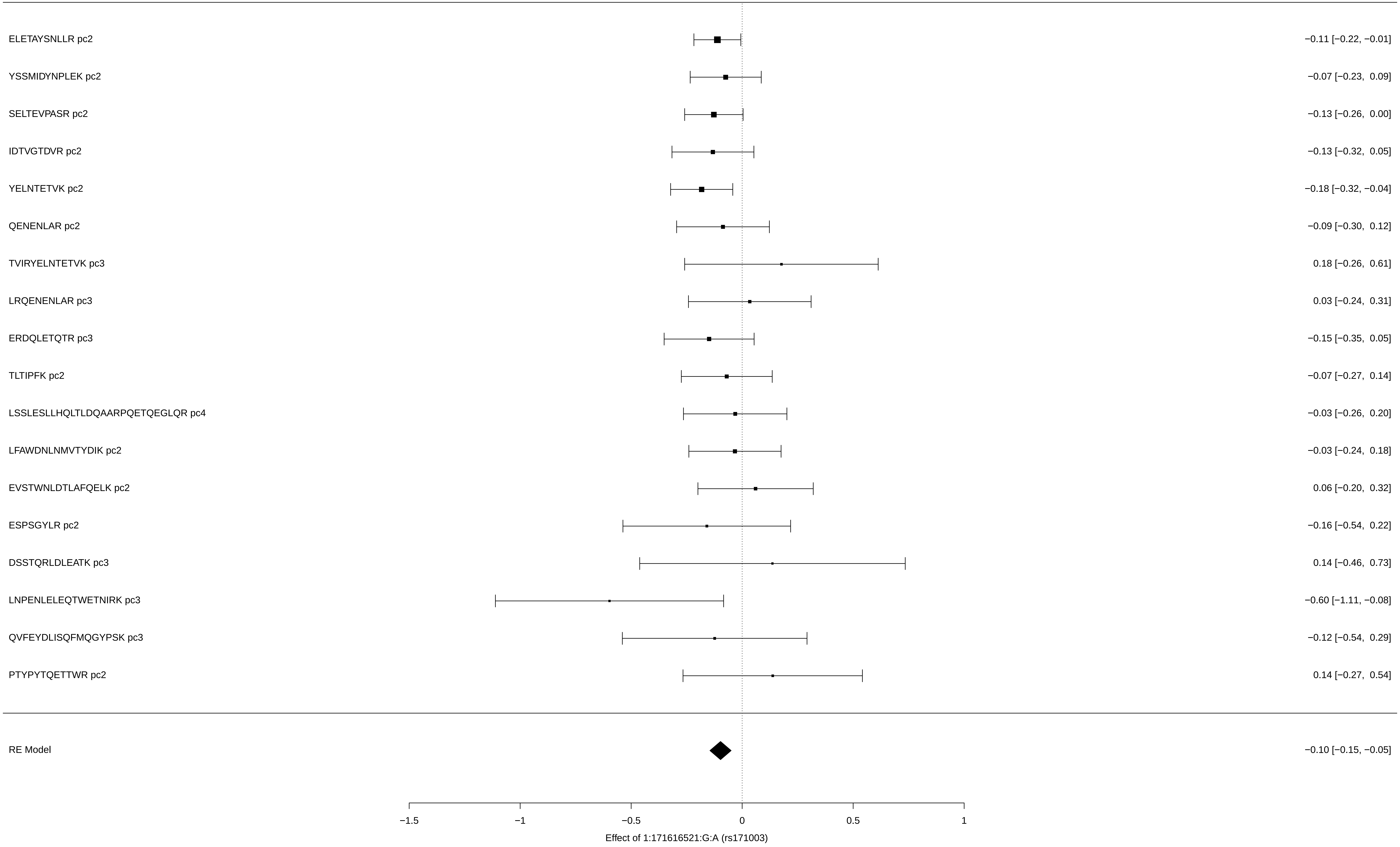

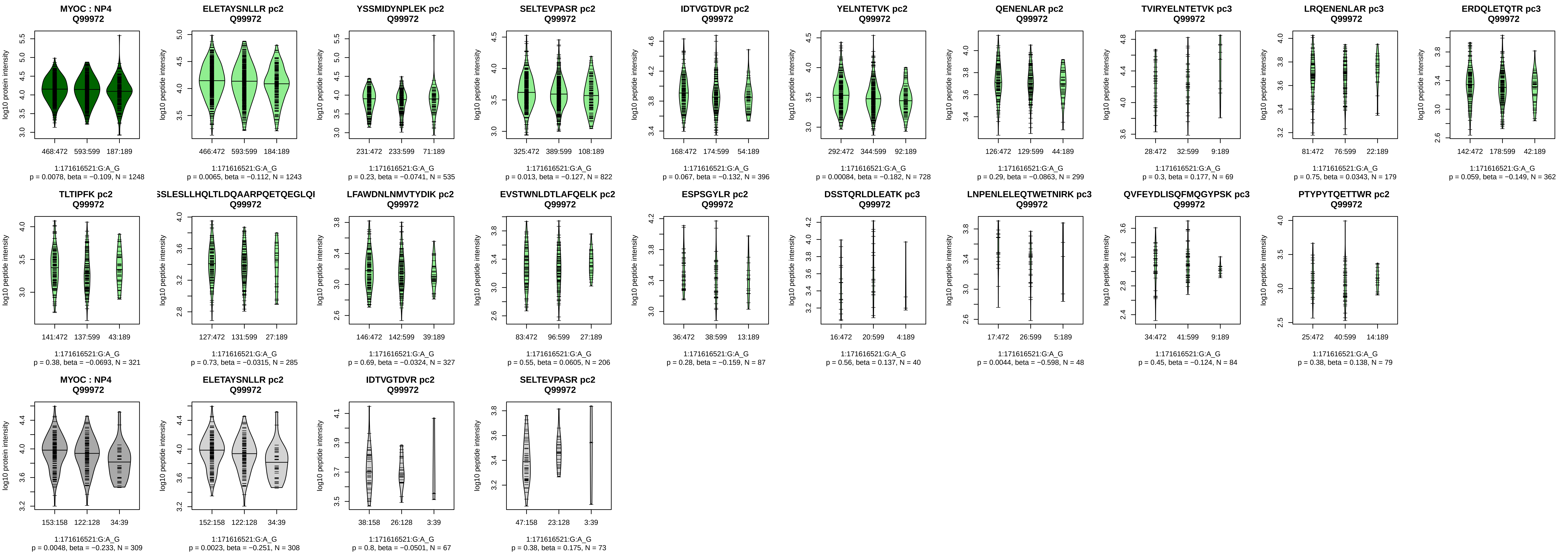

Assay Target: MYOC
UniProt: Q99972
rsID: rs171003
Target SNP: 1:171616521:G:A
Proxy SNP: 1:171616521:G:A
UKBPPP log10(p): 404.1
UKBPPP BETA: 0.255
UKBPPP MSPA: 0.33
-:-:-::-:-:-:-:-:-:-:-:-:*:-:
1243:535:822:396:728:299:69:1

169. FAS (P25445)
UKBPPP MSPA=0

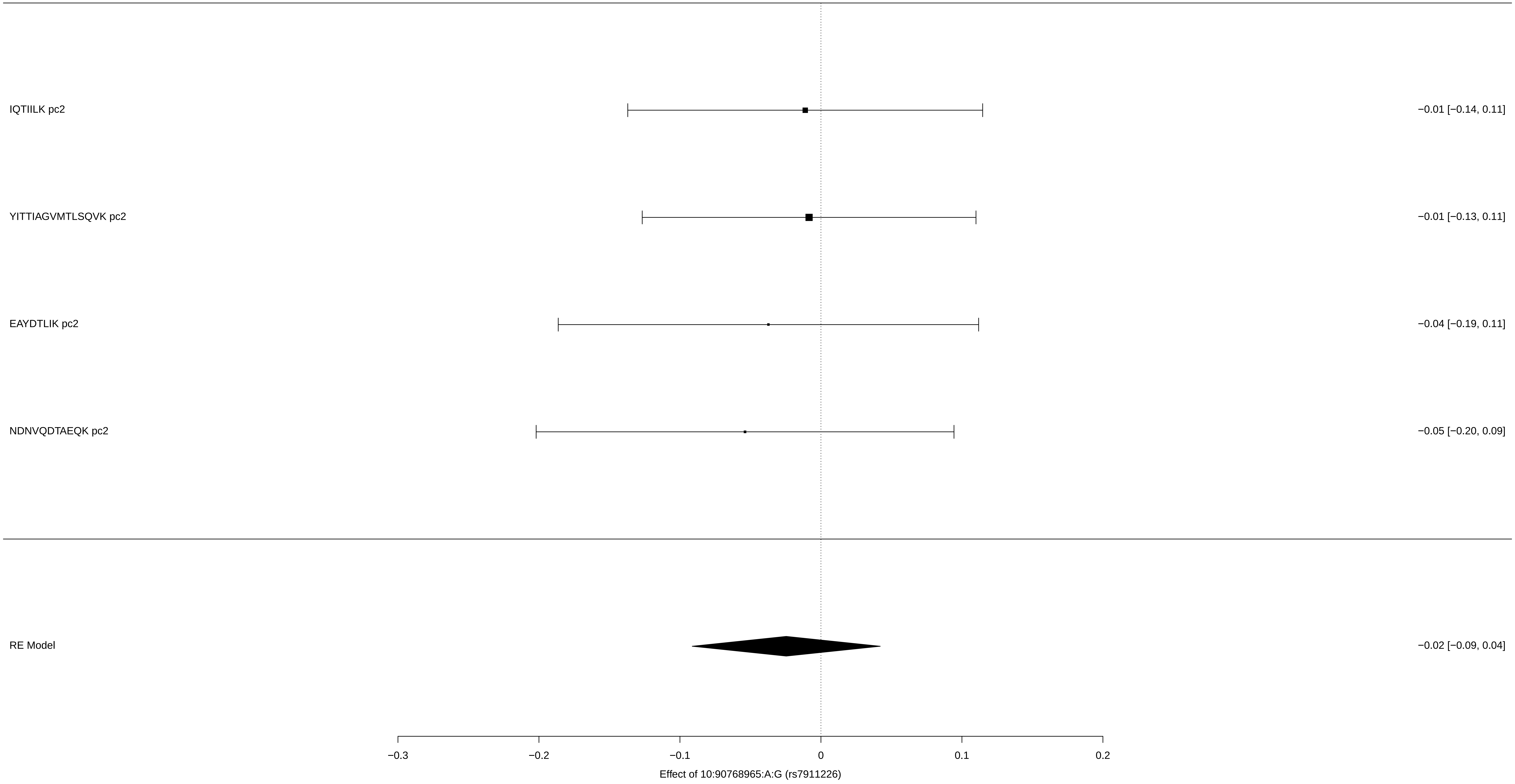

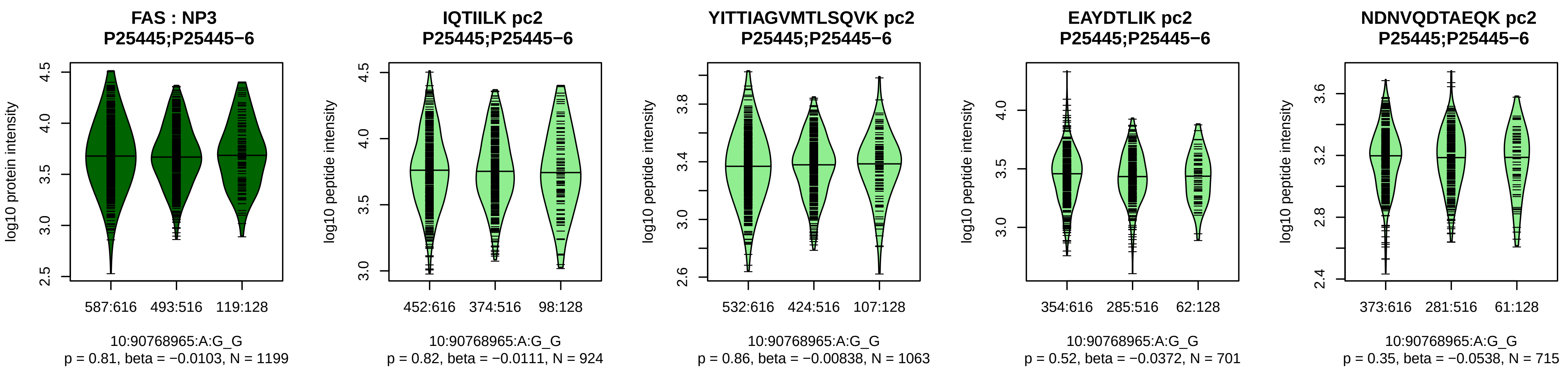

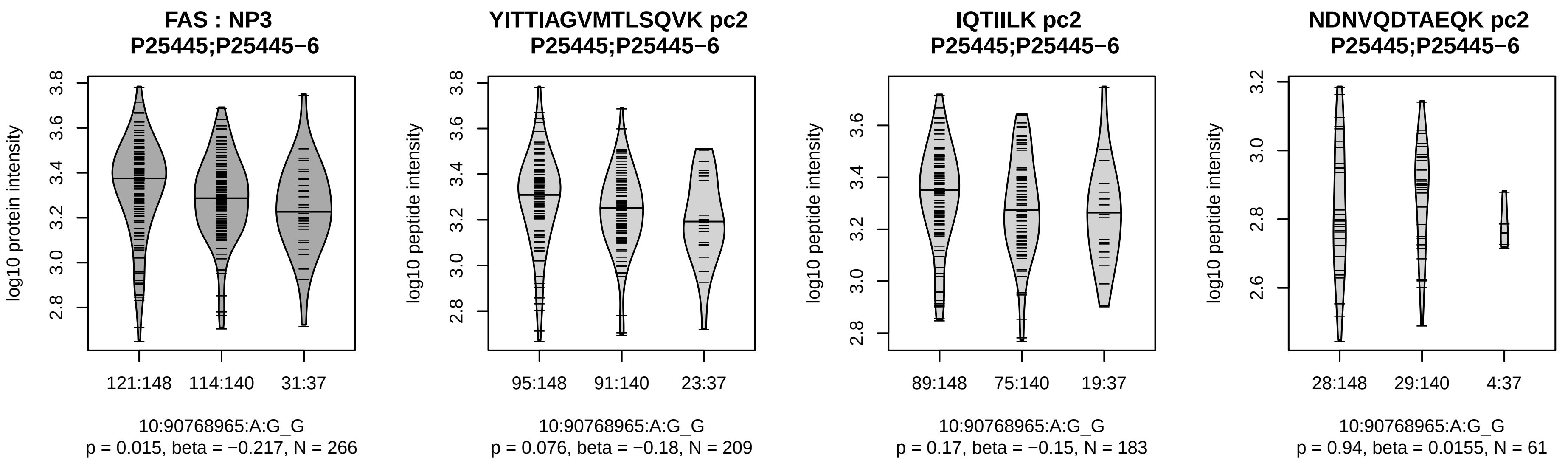

Assay Target: FAS
UniProt: P25445
rsID: rs7911226
Target SNP: 10:90768965:A:G
Proxy SNP: 10:90768965:A:G
UKBPPP log10(p): 399.8
UKBPPP BETA: −0.26
UKBPPP MSPA: 0
–:–:–:–
924:1063:701:715

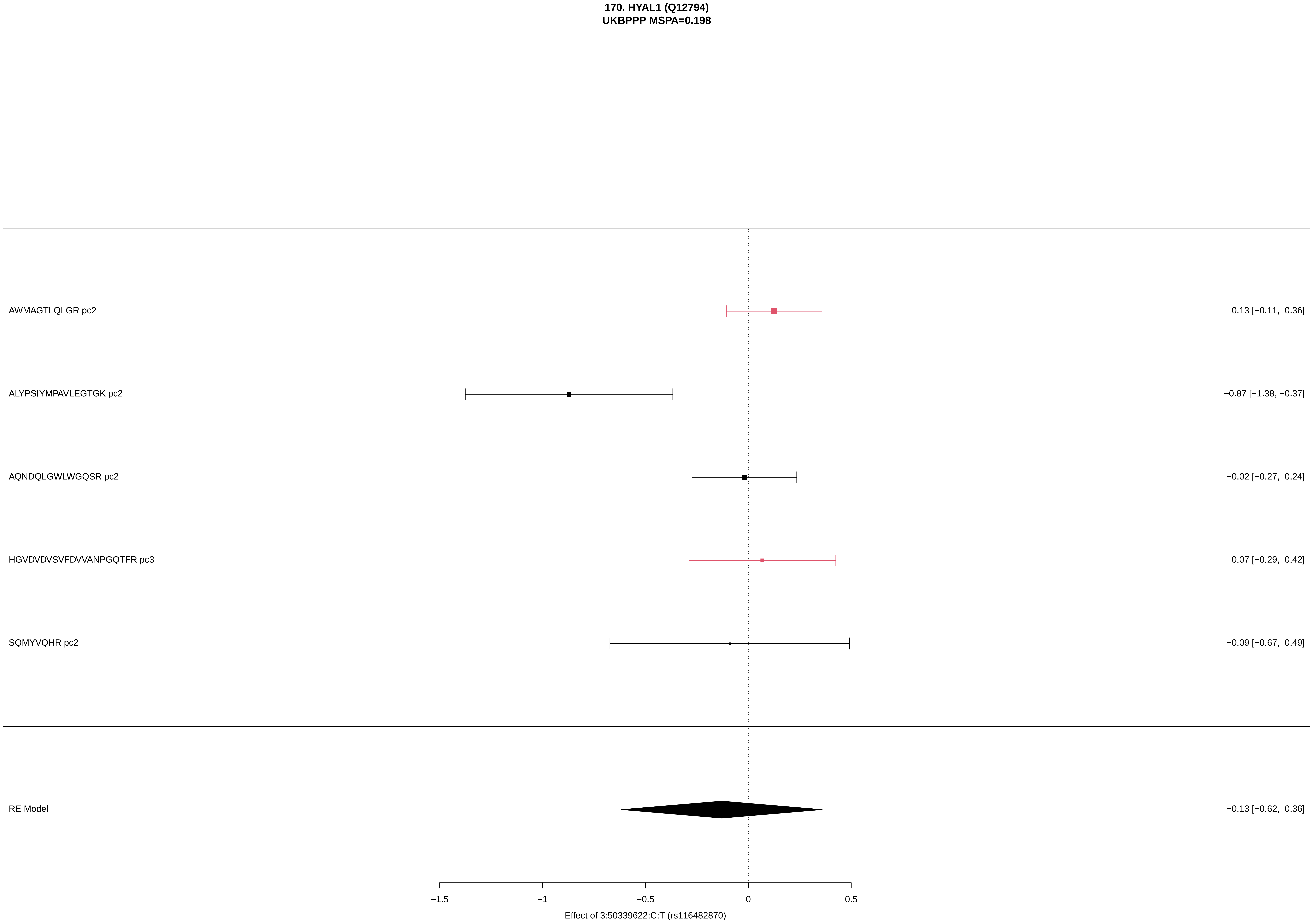
170. HYAL1 (Q12794)
UKBPPP MSPA=0.198
AWMAGTLQLGR pc2
0.13 [−0.11, 0.36]
ALYPSIYMPAVLEGTGK pc2
−0.87 [−1.38, −0.37]
AQNDQLGWLWGQSR pc2
−0.02 [−0.27, 0.24]
HGVDVDVSVFDVVANPGQTFR pc3
0.07 [−0.29, 0.42]
SQMYVQHR pc2
−0.09 [−0.67, 0.49]
RE Model
−0.13 [−0.62, 0.36]
−1.5
−1
−0.5
0
0.5
Effect of 3:50339622:C:T (rs116482870)

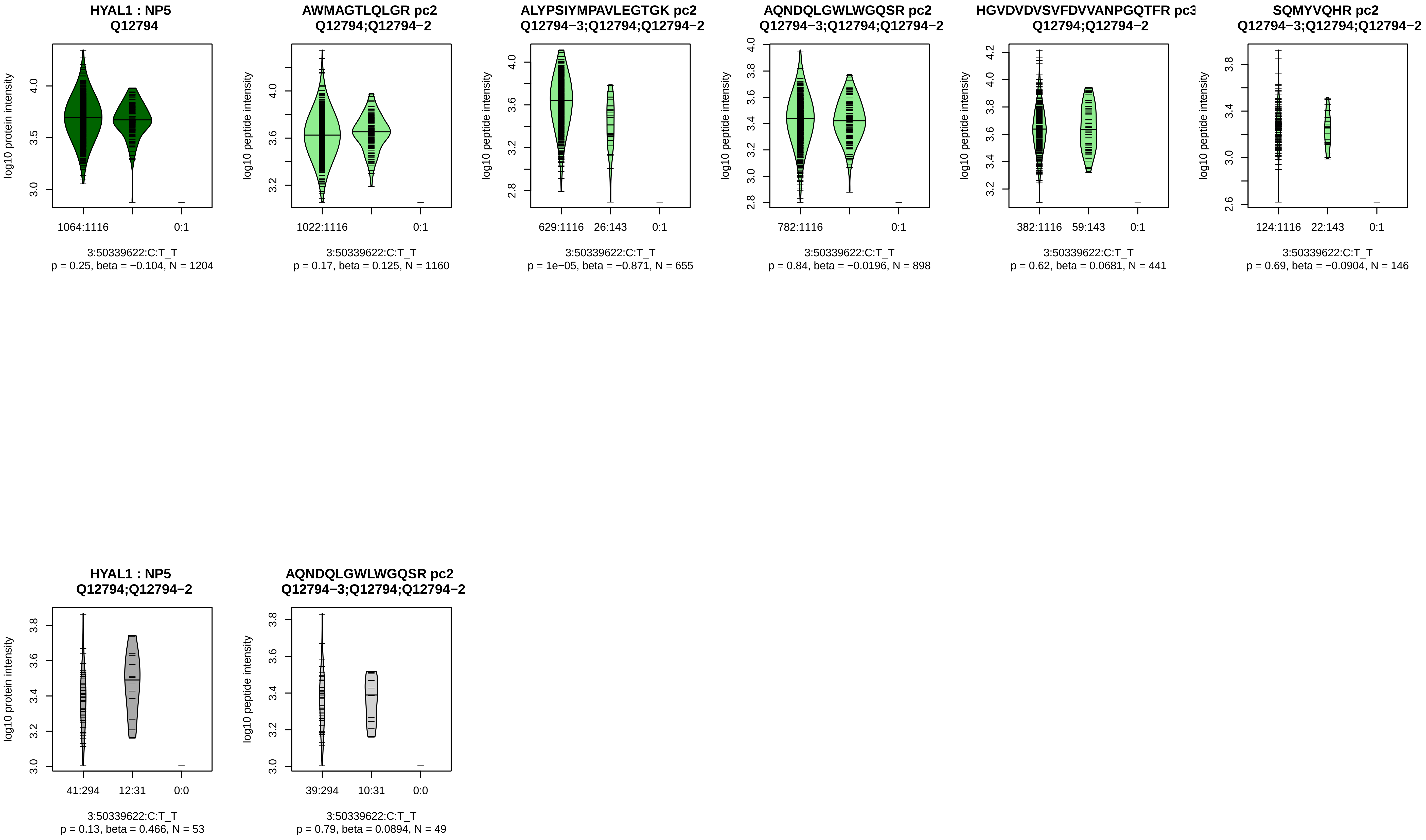

Assay Target: HYAL1
UniProt: Q12794
rsID: rs116482870
Target SNP: 3:50339622:C:T
Proxy SNP: 3:50339622:C:T
UKBPPP log10(p): 398.1
UKBPPP BETA: −0.542
UKBPPP MSPA: 0.198
–:*:–:–:–
1160:655:898:441:146

171. CD8A (P01732)
UKBPPP MSPA=0.818

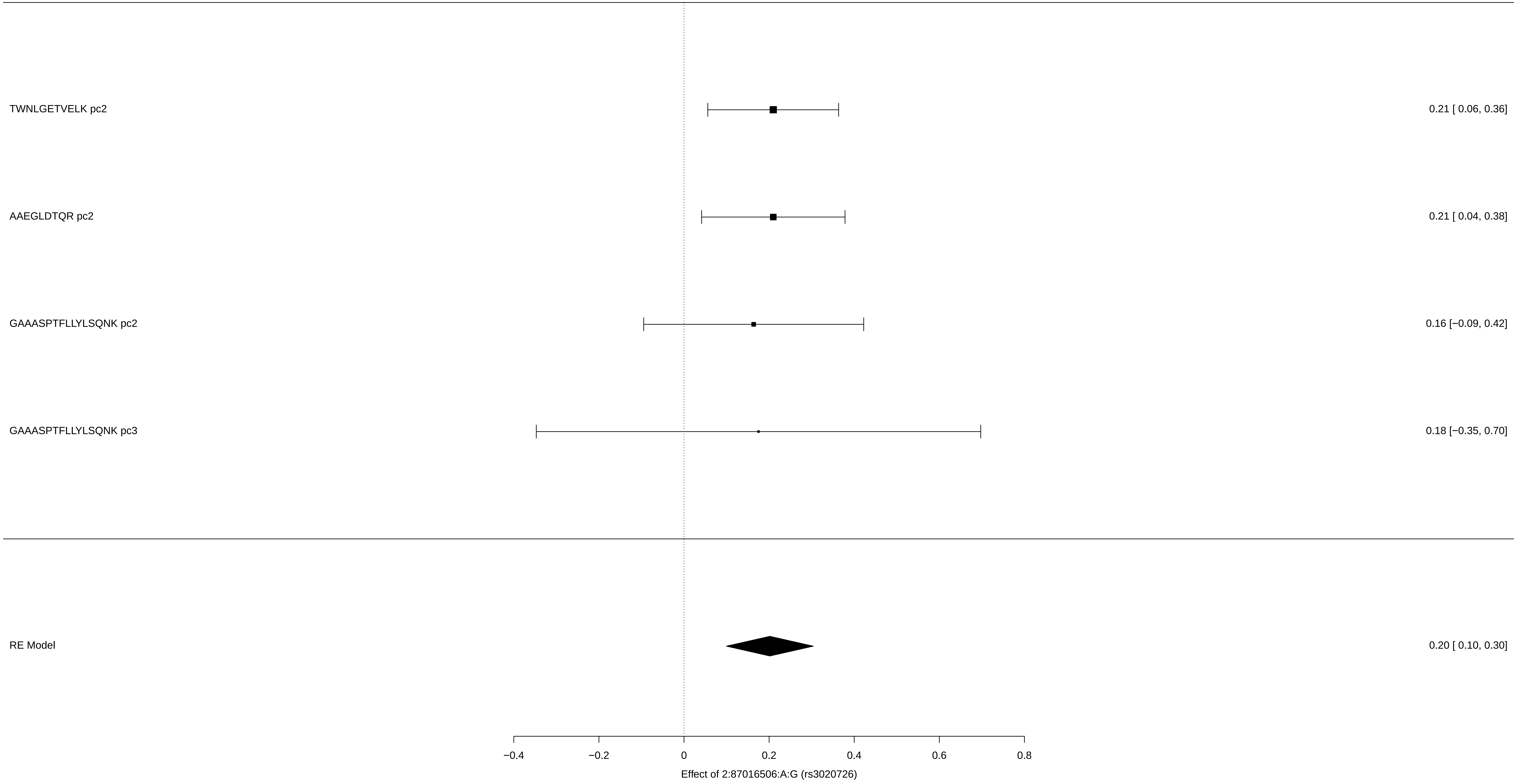

CD8A : NP3
B8ZZZ4;P01732;P01732−2

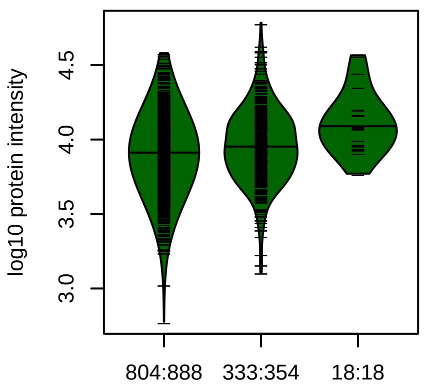

2:87016506:A:G_G
p = 3.9e−05, beta = 0.241, N = 1155

TWNLGETVELK pc2
B8ZZZ4;P01732;P01732−2

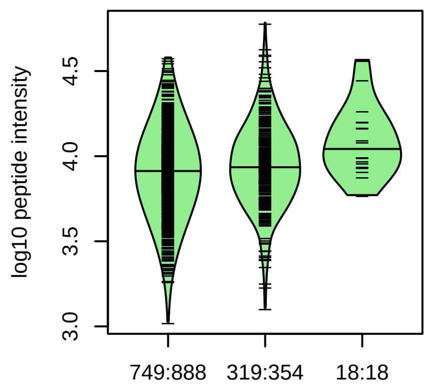

2:87016506:A:G_G
p = 0.00046, beta = 0.21, N = 1086

AAEGLDTQR pc2
B8ZZZ4;P01732;P01732−2

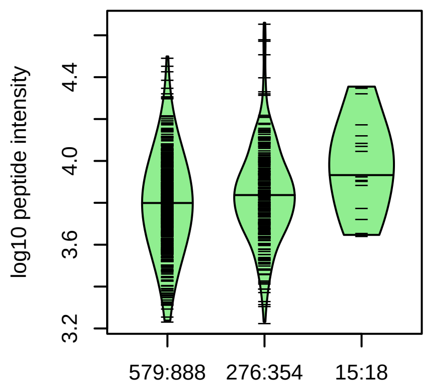

2:87016506:A:G_G
p = 0.0014, beta = 0.21, N = 870

GAAASPTFLLYLSQNK pc2
B8ZZZ4;P01732;P01732−2

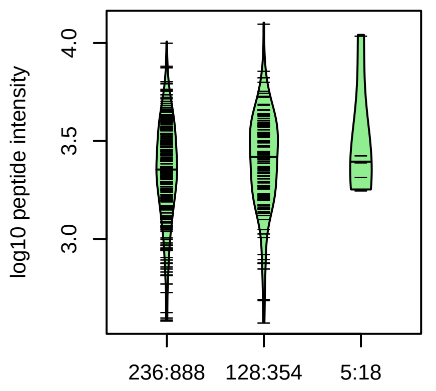

2:87016506:A:G_G
p = 0.1, beta = 0.164, N = 369

TWNLGETVELK pc2
B8ZZZ4;P01732;P01732−2

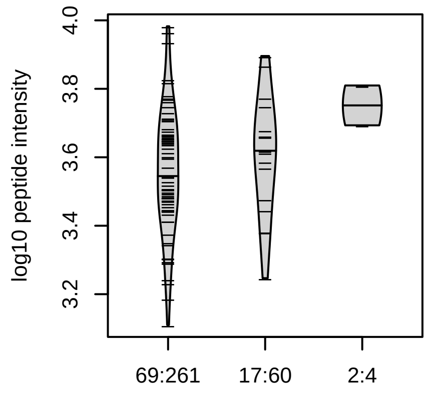

2:87016506:A:G_G
p = 0.17, beta = 0.297, N = 88

AAEGLDTQR pc2
B8ZZZ4;P01732;P01732−2

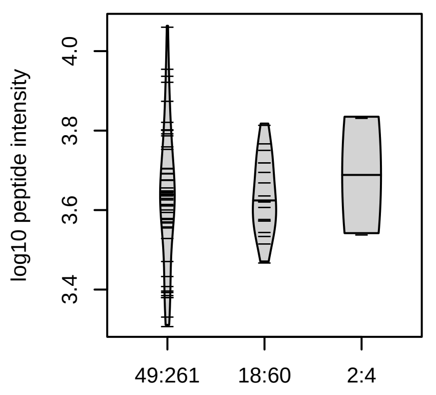

2:87016506:A:G_G
p = 0.91, beta = 0.0243, N = 69

Assay Target: CD8A
UniProt: P01732
rsID: rs3020726
Target SNP: 2:87016506:A:G
Proxy SNP: 2:87016506:A:G
UKBPPP log10(p): 383.5
UKBPPP BETA: 0.345
UKBPPP MSPA: 0.818
::–:–
1086:870:369:65

172. ITIH4 (Q14624)
UKBPPP MSPA=0.512

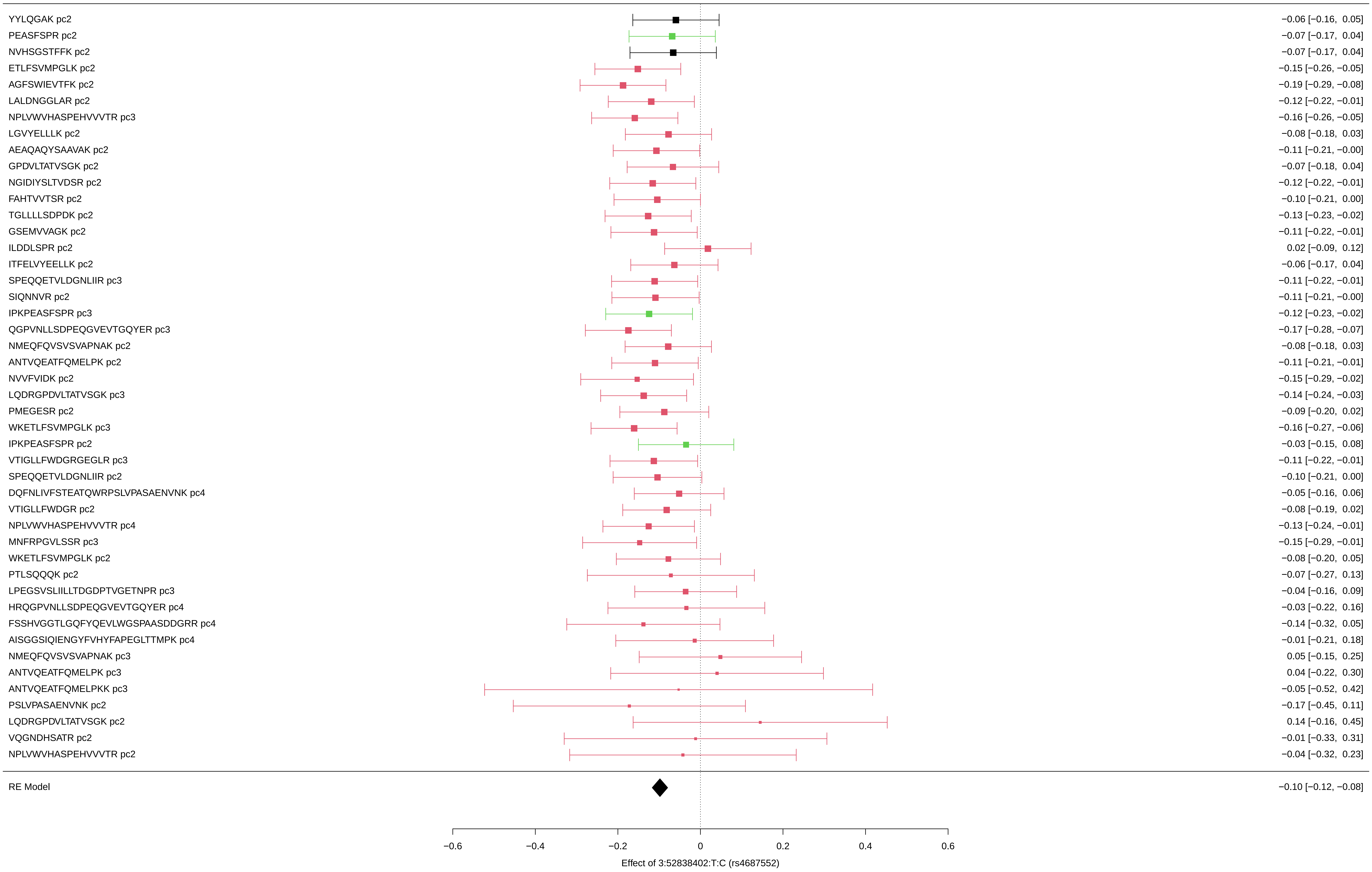

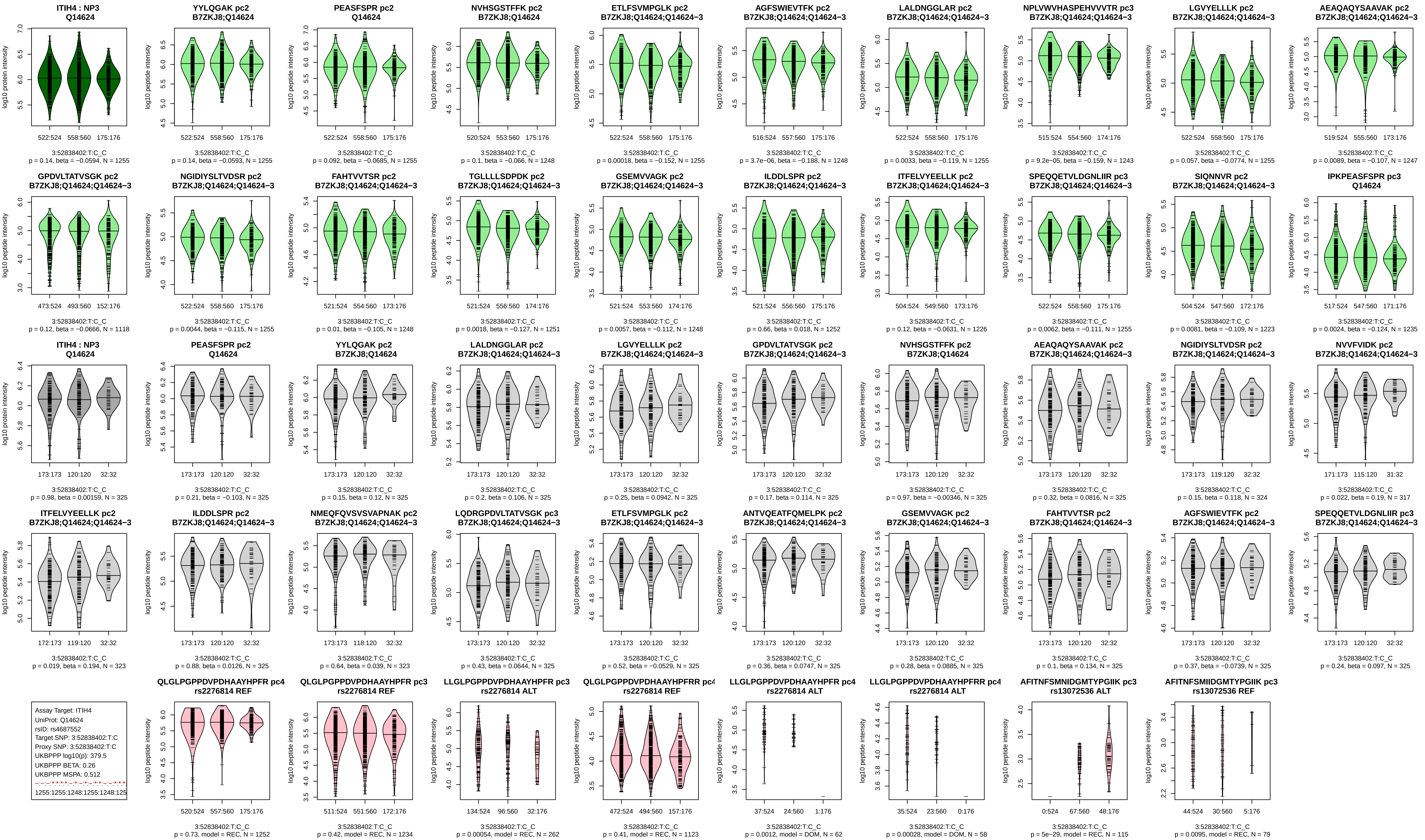
ITIH4 : NP3 Q14624
522:524 558:560 175:176
3:52838402:T:C_C
p = 0.14, beta = −0.0594, N = 1255
YYLQGAK pc2 B7ZKJ8;Q14624
522:524 558:560 175:176
3:52838402:T:C_C
p = 0.14, beta = −0.0593, N = 1255
PEASFSPR pc2 Q14624
522:524 558:560 175:176
3:52838402:T:C_C
p = 0.092, beta = −0.0685, N = 1255
NVHSGSTFFK pc2 B7ZKJ8;Q14624
520:524 553:560 175:176
3:52838402:T:C_C
p = 0.1, beta = −0.066, N = 1248
ETLFSVMPGLK pc2 B7ZKJ8;Q14624;Q14624−3
522:524 558:560 175:176
3:52838402:T:C_C
p = 0.00018, beta = −0.152, N = 1255
AGFSWIEVTFK pc2 B7ZKJ8;Q14624;Q14624−3
516:524 557:560 175:176
3:52838402:T:C_C
p = 3.7e−06, beta = −0.188, N = 1248
LALDNGGLAR pc2 B7ZKJ8;Q14624;Q14624−3
522:524 558:560 175:176
3:52838402:T:C_C
p = 0.0033, beta = −0.119, N = 1255
NPLVWVHASPEHVVVTR pc3 B7ZKJ8;Q14624;Q14624−3
515:524 554:560 174:176
3:52838402:T:C_C
p = 9.2e−05, beta = −0.159, N = 1243
LGVYELLLK pc2 B7ZKJ8;Q14624;Q14624−3
522:524 558:560 175:176
3:52838402:T:C_C
p = 0.057, beta = −0.0774, N = 1255
AEAQAQYSAAVAK pc2 B7ZKJ8;Q14624;Q14624−3
519:524 555:560 173:176
3:52838402:T:C_C
p = 0.0089, beta = −0.107, N = 1247
GPDVLTATVSGK pc2 B7ZKJ8;Q14624;Q14624−3
473:524 493:560 152:176
3:52838402:T:C_C
p = 0.12, beta = −0.0666, N = 1118
NGIDIYSLTVDSR pc2 B7ZKJ8;Q14624;Q14624−3
522:524 558:560 175:176
3:52838402:T:C_C
p = 0.0044, beta = −0.115, N = 1255
FAHTVVTSR pc2 B7ZKJ8;Q14624;Q14624−3
521:524 554:560 173:176
3:52838402:T:C_C
p = 0.01, beta = −0.105, N = 1248
TGLLLLSDPDK pc2 B7ZKJ8;Q14624;Q14624−3
521:524 556:560 174:176
3:52838402:T:C_C
p = 0.0018, beta = −0.127, N = 1251
GSEMVVAGK pc2 B7ZKJ8;Q14624;Q14624−3
521:524 553:560 174:176
3:52838402:T:C_C
p = 0.0057, beta = −0.112, N = 1248
ILDDLSPR pc2 B7ZKJ8;Q14624;Q14624−3
521:524 556:560 175:176
3:52838402:T:C_C
p = 0.66, beta = 0.018, N = 1252
ITFELVYEELLK pc2 B7ZKJ8;Q14624;Q14624−3
504:524 549:560 173:176
3:52838402:T:C_C
p = 0.12, beta = −0.0631, N = 1226
SPEQQETVLDGNLIIR pc3 B7ZKJ8;Q14624;Q14624−3
522:524 558:560 175:176
3:52838402:T:C_C
p = 0.0062, beta = −0.111, N = 1255
SIQNNVR pc2 B7ZKJ8;Q14624;Q14624−3
504:524 547:560 172:176
3:52838402:T:C_C
p = 0.0081, beta = −0.109, N = 1223
IPKPEASFSPR pc3 Q14624
517:524 547:560 171:176
3:52838402:T:C_C
p = 0.0024, beta = −0.124, N = 1235
ITIH4 : NP3 Q14624
173:173 120:120 32:32
3:52838402:T:C_C
p = 0.98, beta = 0.00159, N = 325
PEASFSPR pc2 Q14624
173:173 120:120 32:32
3:52838402:T:C_C
p = 0.21, beta = −0.103, N = 325
YYLQGAK pc2 B7ZKJ8;Q14624
173:173 120:120 32:32
3:52838402:T:C_C
p = 0.15, beta = 0.12, N = 325
LALDNGGLAR pc2 B7ZKJ8;Q14624;Q14624−3
173:173 120:120 32:32
3:52838402:T:C_C
p = 0.2, beta = 0.106, N = 325
LGVYELLLK pc2 B7ZKJ8;Q14624;Q14624−3
173:173 120:120 32:32
3:52838402:T:C_C
p = 0.25, beta = 0.0942, N = 325
GPDVLTATVSGK pc2 B7ZKJ8;Q14624;Q14624−3
173:173 120:120 32:32
3:52838402:T:C_C
p = 0.17, beta = 0.114, N = 325
NVHSGSTFFK pc2 B7ZKJ8;Q14624
173:173 120:120 32:32
3:52838402:T:C_C
p = 0.97, beta = −0.00346, N = 325
AEAQAQYSAAVAK pc2 B7ZKJ8;Q14624;Q14624−3
173:173 120:120 32:32
3:52838402:T:C_C
p = 0.32, beta = 0.0816, N = 325
NGIDIYSLTVDSR pc2 B7ZKJ8;Q14624;Q14624−3
173:173 119:120 32:32
3:52838402:T:C_C
p = 0.15, beta = 0.118, N = 324
NVVFVIDK pc2 B7ZKJ8;Q14624;Q14624−3
171:173 115:120 31:32
3:52838402:T:C_C
p = 0.022, beta = 0.19, N = 317
ITFELVYEELLK pc2 B7ZKJ8;Q14624;Q14624−3
172:173 119:120 32:32
3:52838402:T:C_C
p = 0.019, beta = 0.194, N = 323
ILDDLSPR pc2 B7ZKJ8;Q14624;Q14624−3
173:173 120:120 32:32
3:52838402:T:C_C
p = 0.88, beta = 0.0126, N = 325
NMEQFQVSVSVAPNAK pc2 B7ZKJ8;Q14624;Q14624−3
173:173 118:120 32:32
3:52838402:T:C_C
p = 0.64, beta = 0.039, N = 323
LQDRGPDVLTATVSGK pc3 B7ZKJ8;Q14624;Q14624−3
173:173 120:120 32:32
3:52838402:T:C_C
p = 0.43, beta = 0.0644, N = 325
ETLFSVMPGLK pc2 B7ZKJ8;Q14624;Q14624−3
173:173 120:120 32:32
3:52838402:T:C_C
p = 0.52, beta = −0.0529, N = 325
ANTVQEATFQMELPK pc2 B7ZKJ8;Q14624;Q14624−3
173:173 120:120 32:32
3:52838402:T:C_C
p = 0.36, beta = 0.0747, N = 325
GSEMVVAGK pc2 B7ZKJ8;Q14624;Q14624−3
173:173 120:120 32:32
3:52838402:T:C_C
p = 0.28, beta = 0.0885, N = 325
FAHTVVTSR pc2 B7ZKJ8;Q14624;Q14624−3
173:173 120:120 32:32
3:52838402:T:C_C
p = 0.1, beta = 0.134, N = 325
AGFSWIEVTFK pc2 B7ZKJ8;Q14624;Q14624−3
173:173 120:120 32:32
3:52838402:T:C_C
p = 0.37, beta = −0.0739, N = 325
SPEQQETVLDGNLIIR pc3 B7ZKJ8;Q14624;Q14624−3
173:173 120:120 32:32
3:52838402:T:C_C
p = 0.24, beta = 0.097, N = 325
Assay Target: ITIH4
UniProt: Q14624
rsID: rs4687552
Target SNP: 3:52838402:T:C
Proxy SNP: 3:52838402:T:C
UKBPPP log10(p): 379.5
UKBPPP BETA: 0.26
UKBPPP MSPA: 0.512
1255:1255:1248:1255:1248:125
QLGLPGPPDVPDHAAYHPFR pc4 rs2276814 REF
520:524 557:560 175:176
3:52838402:T:C_C
p = 0.73, model = REC, N = 1252
QLGLPGPPDVPDHAAYHPFR pc3 rs2276814 REF
511:524 551:560 172:176
3:52838402:T:C_C
p = 0.42, model = REC, N = 1234
LLGLPGPPDVPDHAAYHPFR pc3 rs2276814 ALT
134:524 96:560 32:176
3:52838402:T:C_C
p = 0.00054, model = REC, N = 262
QLGLPGPPDVPDHAAYHPFRR pc4 rs2276814 REF
472:524 494:560 157:176
3:52838402:T:C_C
p = 0.41, model = REC, N = 1123
LLGLPGPPDVPDHAAYHPFR pc4 rs2276814 ALT
37:524 24:560 1:176
3:52838402:T:C_C
p = 0.0012, model = DOM, N = 62
LLGLPGPPDVPDHAAYHPFRR pc4 rs2276814 ALT
35:524 23:560 0:176
3:52838402:T:C_C
p = 0.00028, model = DOM, N = 58
AFITNFSMNIDGMTYPGIIK pc3 rs13072536 ALT
0:524 67:560 48:176
3:52838402:T:C_C
p = 5e−29, model = REC, N = 115
AFITNFSMIIDGMTYPGIIK pc3 rs13072536 REF
44:524 30:560 5:176
3:52838402:T:C_C
p = 0.0095, model = REC, N = 79

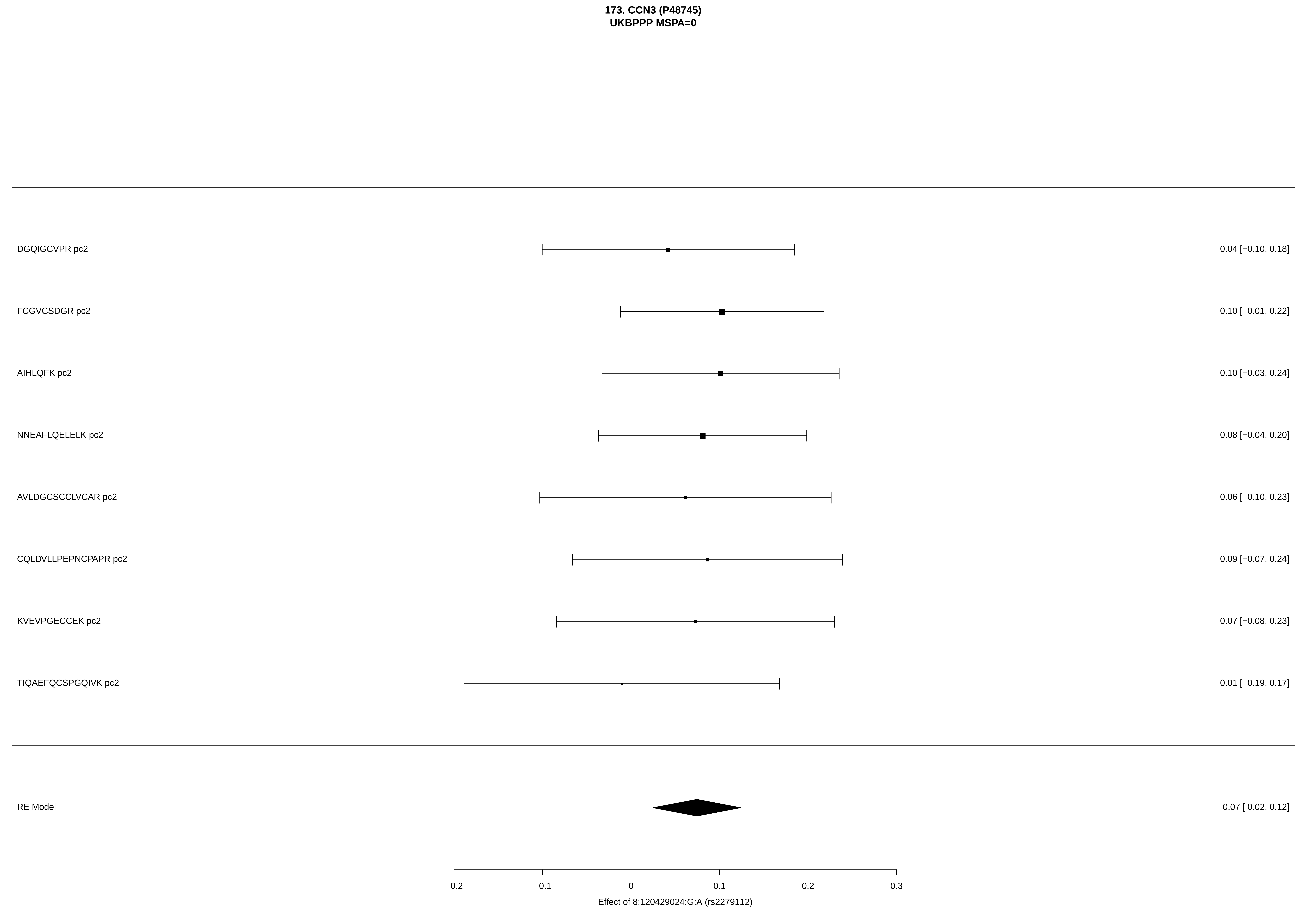
173. CCN3 (P48745)
UKBPPP MSPA=0
DGQIGCVPR pc2
0.04 [−0.10, 0.18]
FCGVCSDGR pc2
0.10 [−0.01, 0.22]
AIHLQFK pc2
0.10 [−0.03, 0.24]
NNEAFLQELELK pc2
0.08 [−0.04, 0.20]
AVLDGCSCCLVCAR pc2
0.06 [−0.10, 0.23]
CQLDVLLPEPNCPAPR pc2
0.09 [−0.07, 0.24]
KVEVPGECCEK pc2
0.07 [−0.08, 0.23]
TIQAEFQCSPGQIVK pc2
−0.01 [−0.19, 0.17]
RE Model
0.07 [0.02, 0.12]
−0.2
−0.1
0
0.1
0.2
0.3
Effect of 8:120429024:G:A (rs2279112)

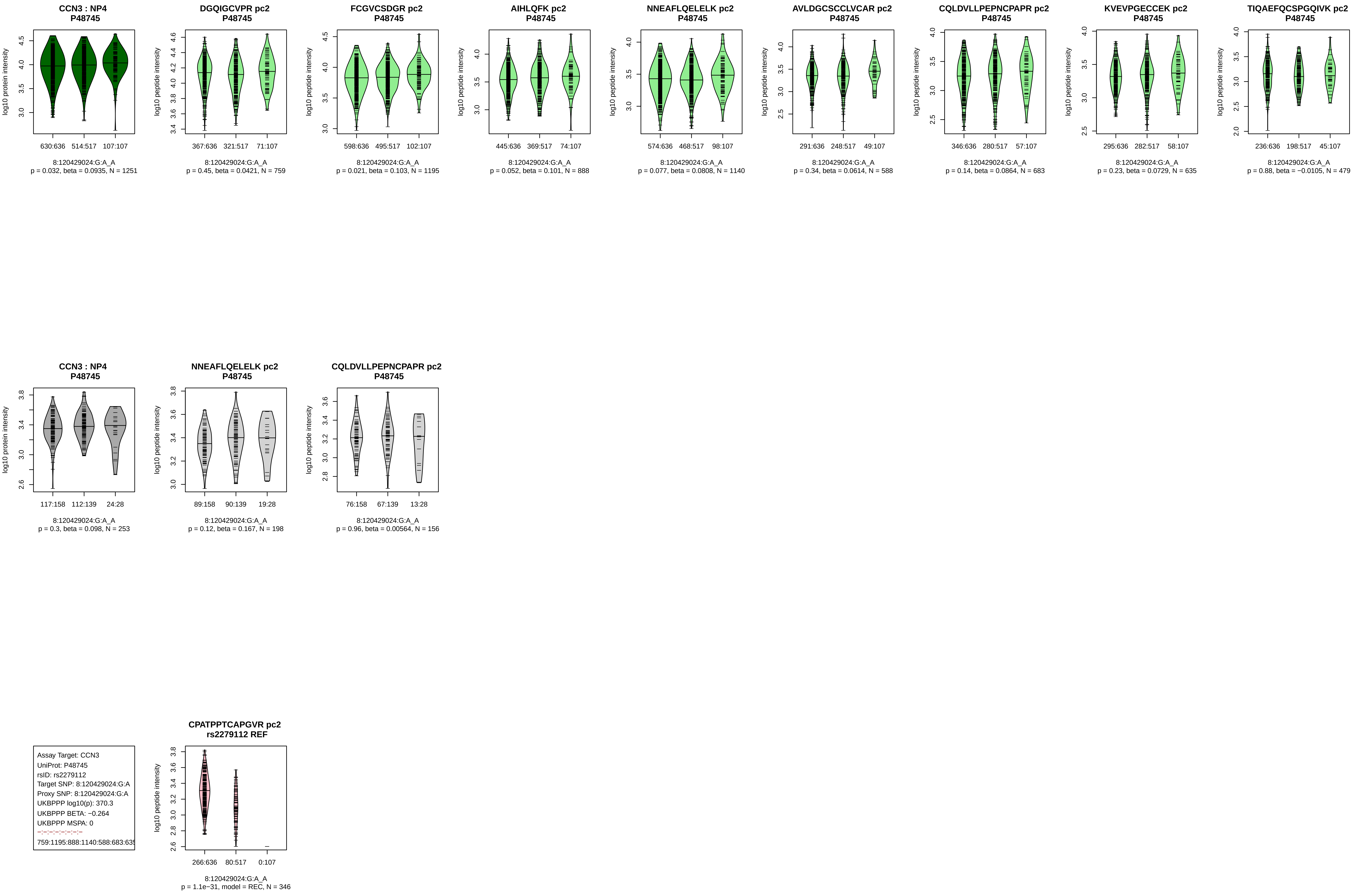
CCN3 : NP4
P48745
log10 protein intensity
630:636 514:517 107:107
8:120429024:G:A_A
p = 0.032, beta = 0.0935, N = 1251
DGQIGCVPR pc2
P48745
log10 peptide intensity
367:636 321:517 71:107
8:120429024:G:A_A
p = 0.45, beta = 0.0421, N = 759
FCGVCSDGR pc2
P48745
495:517 102:107 598:636
8:120429024:G:A_A
p = 0.021, beta = 0.103, N = 1195
AIHLQFK pc2
P48745
445:636 369:517 74:107
8:120429024:G:A_A
p = 0.052, beta = 0.101, N = 888
NNEAFLQELELK pc2
P48745
574:636 468:517 98:107
8:120429024:G:A_A
p = 0.077, beta = 0.0808, N = 1140
AVLDGCSCCLVCAR pc2
P48745
291:636 248:517 49:107
8:120429024:G:A_A
p = 0.34, beta = 0.0614, N = 588
CQLDVLLPEPNCPAPR pc2
P48745
346:636 280:517 57:107
8:120429024:G:A_A
p = 0.14, beta = 0.0864, N = 683
KVEVPGECCEK pc2
P48745
295:636 282:517 58:107
8:120429024:G:A_A
p = 0.23, beta = 0.0729, N = 635
TIQAEFQCSPGQIVK pc2
P48745
236:636 198:517 45:107
8:120429024:G:A_A
p = 0.88, beta = −0.0105, N = 479
CCN3 : NP4
P48745
117:158 112:139 24:28
8:120429024:G:A_A
p = 0.3, beta = 0.098, N = 253
NNEAFLQELELK pc2
P48745
89:158 90:139 19:28
8:120429024:G:A_A
p = 0.12, beta = 0.167, N = 198
CQLDVLLPEPNCPAPR pc2
P48745
76:158 67:139 13:28
8:120429024:G:A_A
p = 0.96, beta = 0.00564, N = 156
Assay Target: CCN3
UniProt: P48745
rsID: rs2279112
Target SNP: 8:120429024:G:A
Proxy SNP: 8:120429024:G:A
UKBPPP log10(p): 370.3
UKBPPP BETA: −0.264
UKBPPP MSPA: 0
759:1195:888:1140:588:683:635
CPATPPTCAPGVR pc2
rs2279112 REF
266:636 80:517 0:107
8:120429024:G:A_A
p = 1.1e−31, model = REC, N = 346

174. MARCO (Q9UEW3)
UKBPPP MSPA=0.655

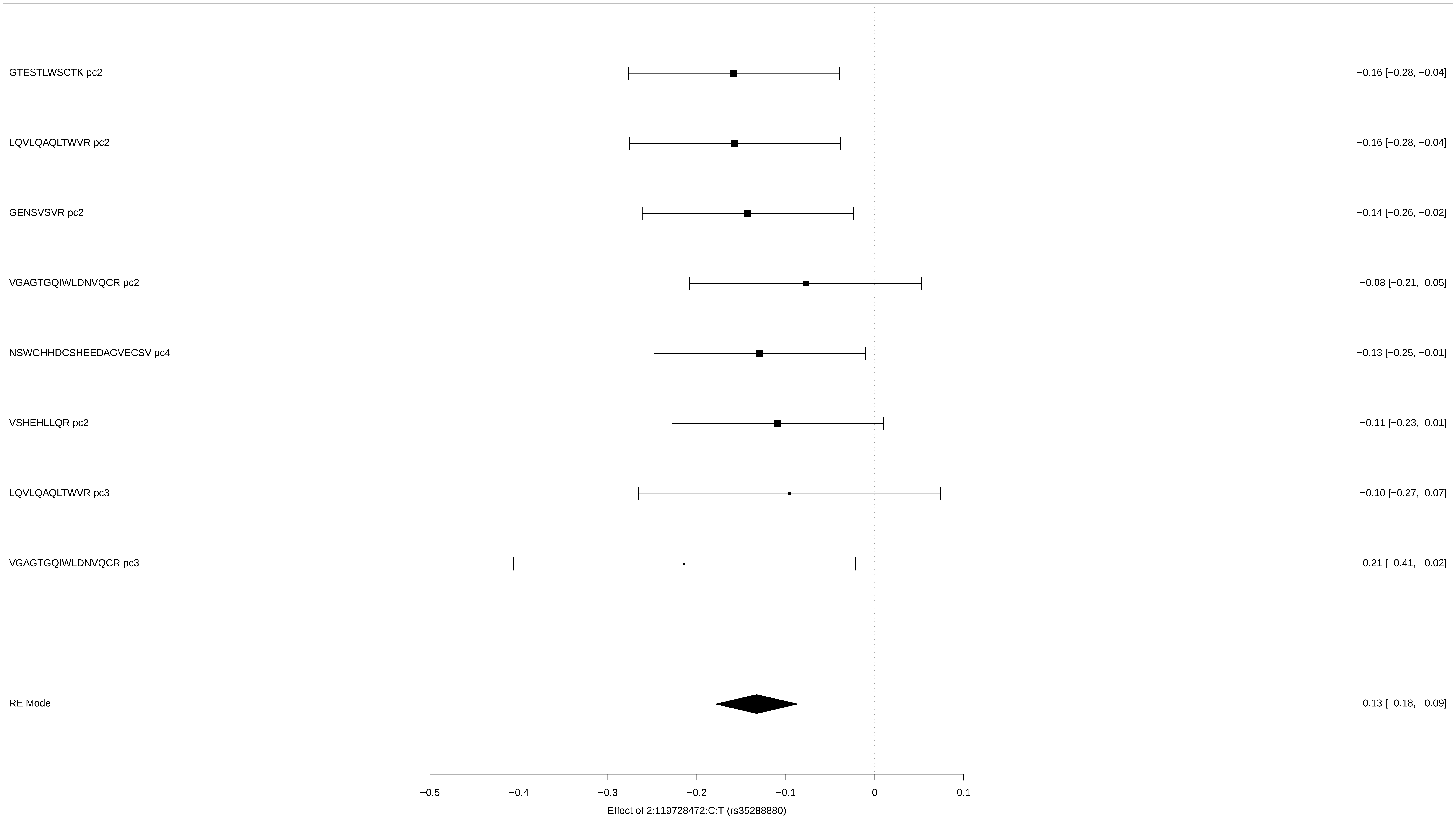

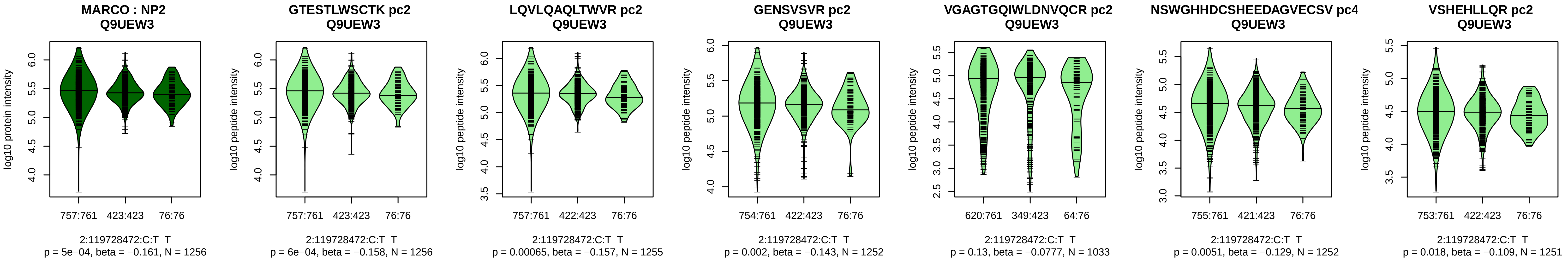

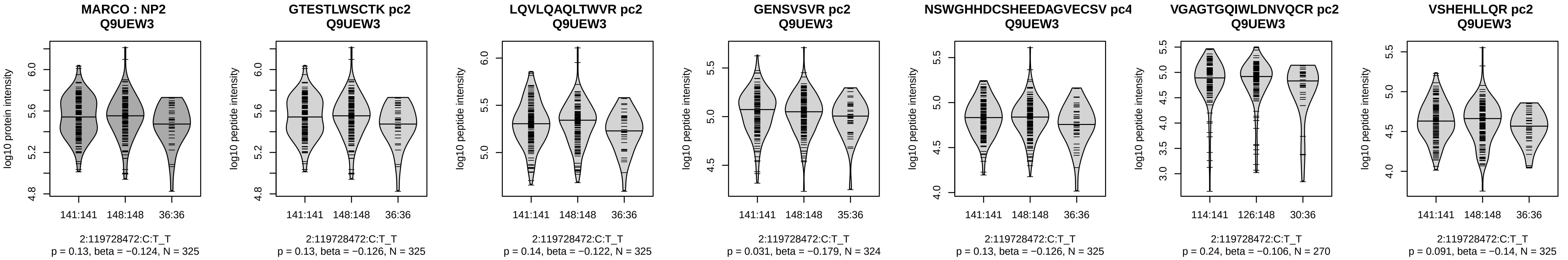

Assay Target: MARCO
UniProt: Q9UEW3
rsID: rs35288880
Target SNP: 2:119728472:C:T
Proxy SNP: 2:119728472:C:T
UKBPPP log10(p): 367.5
UKBPPP BETA: −0.324
UKBPPP MSPA: 0.655
::*:–:*:–:–:*
1256:1255:1252:1033:1252:125

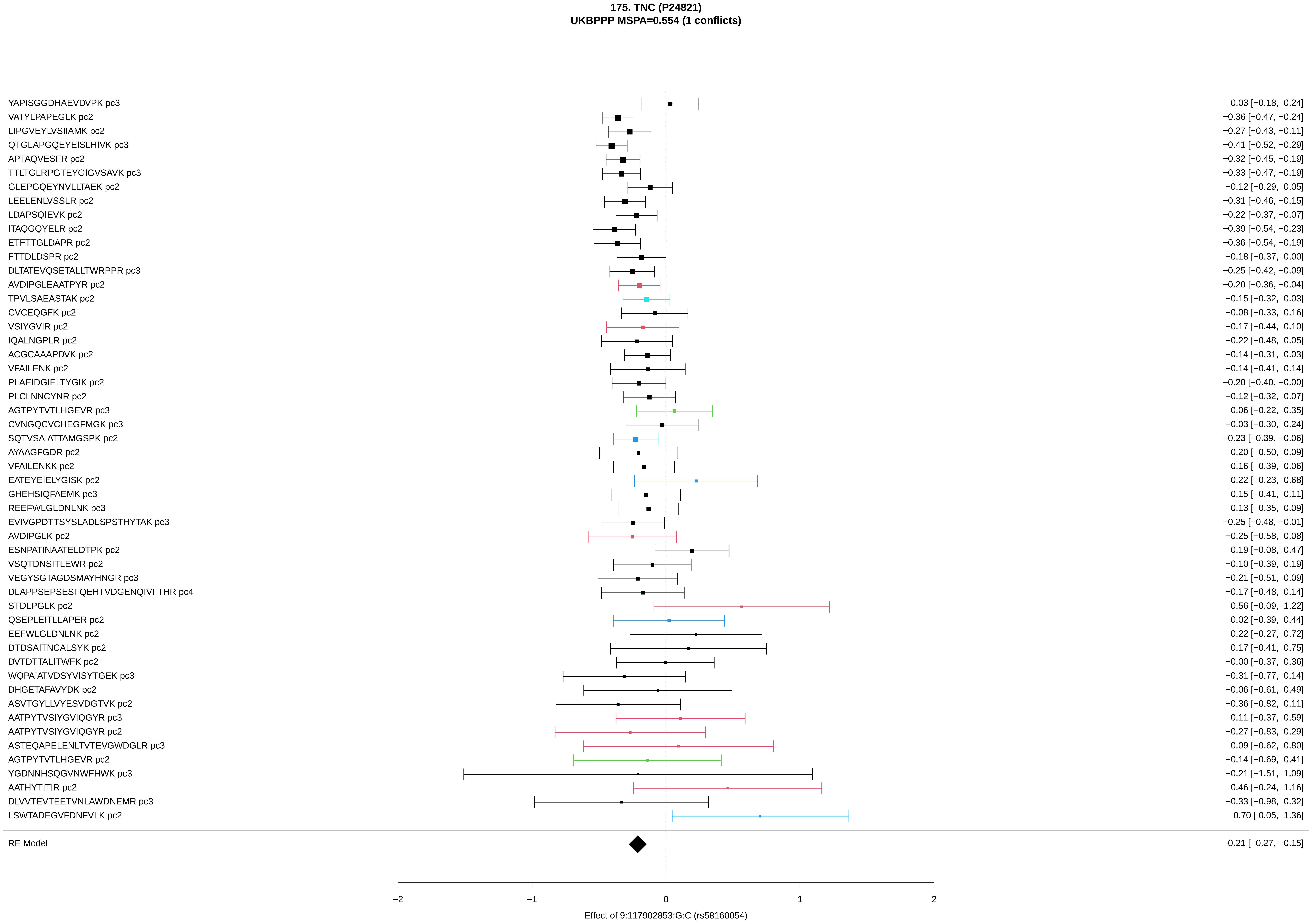
175. TNC (P24821)
UKBPPP MSPA=0.554 (1 conflicts)
YAPISGGDHAEVDVPK pc3 0.03 [−0.18, 0.24]
VATYLPAPEGLK pc2 −0.36 [−0.47, −0.24]
LIPGVEYLVSIIAMK pc2 −0.27 [−0.43, −0.11]
QTGLAPGQEYEISLHIVK pc3 −0.41 [−0.52, −0.29]
APTAQVESFR pc2 −0.32 [−0.45, −0.19]
TTLTGLRPGTEYGIGVSAVK pc3 −0.33 [−0.47, −0.19]
GLEPGQEYNVLLTAEK pc2 −0.12 [−0.29, 0.05]
LEELENLVSSLR pc2 −0.31 [−0.46, −0.15]
LDAPSQIEVK pc2 −0.22 [−0.37, −0.07]
ITAQGQYELR pc2 −0.39 [−0.54, −0.23]
ETFTTGLDAPR pc2 −0.36 [−0.54, −0.19]
FTTDLDSPR pc2 −0.18 [−0.37, 0.00]
DLTATEVQSETALLTWRPPR pc3 −0.25 [−0.42, −0.09]
AVDIPGLEAATPYR pc2 −0.20 [−0.36, −0.04]
TPVLSAEASTAK pc2 −0.15 [−0.32, 0.03]
CVCEQGFK pc2 −0.08 [−0.33, 0.16]
VSIYGVIR pc2 −0.17 [−0.44, 0.10]
IQALNGPLR pc2 −0.22 [−0.48, 0.05]
ACGCAAAPDVK pc2 −0.14 [−0.31, 0.03]
VFAILENK pc2 −0.14 [−0.41, 0.14]
PLAEIDGIELTYGIK pc2 −0.20 [−0.40, −0.00]
PLCLNNCYNR pc2 −0.12 [−0.32, 0.07]
AGTPYTVTLHGEVR pc3 0.06 [−0.22, 0.35]
CVNGQCVCHEGFMGK pc3 −0.03 [−0.30, 0.24]
SQTVSAIATTAMGSPK pc2 −0.23 [−0.39, −0.06]
AYAAGFGDR pc2 −0.20 [−0.50, 0.09]
VFAILENKK pc2 −0.16 [−0.39, 0.06]
EATEYEIELYGISK pc2 0.22 [−0.23, 0.68]
GHEHSIQFAEMK pc3 −0.15 [−0.41, 0.11]
REEFWLGLDNLNK pc3 −0.13 [−0.35, 0.09]
EVIVGPDTTSYSLADLSPSTHYTAK pc3 −0.25 [−0.48, −0.01]
AVDIPGLK pc2 −0.25 [−0.58, 0.08]
ESNPATINAATELDTPK pc2 0.19 [−0.08, 0.47]
VSQTDNSITLEWR pc2 −0.10 [−0.39, 0.19]
VEGYSGTAGDSMAYHNGR pc3 −0.21 [−0.51, 0.09]
DLAPPSEPSESFQEHTVDGENQIVFTHR pc4 −0.17 [−0.48, 0.14]
STDLPGLK pc2 0.56 [−0.09, 1.22]
QSEPLEITLLAPER pc2 0.02 [−0.39, 0.44]
EEFWLGLDNLNK pc2 0.22 [−0.27, 0.72]
DTDSAITNCALSYK pc2 0.17 [−0.41, 0.75]
DVTDTTALITWFK pc2 −0.00 [−0.37, 0.36]
WQPAIATVDSYVISYTGEK pc3 −0.31 [−0.77, 0.14]
DHGETAFAVYDK pc2 −0.06 [−0.61, 0.49]
ASVTGYLLVYESVDGTVK pc2 −0.36 [−0.82, 0.11]
AATPYTVSIYGVIQGYR pc3 0.11 [−0.37, 0.59]
AATPYTVSIYGVIQGYR pc2 −0.27 [−0.83, 0.29]
ASTEQAPELENLTVTEVGWDGLR pc3 0.09 [−0.62, 0.80]
AGTPYTVTLHGEVR pc2 −0.14 [−0.69, 0.41]
YGDNNHSQGVNWFHWK pc3 −0.21 [−1.51, 1.09]
AATHYTITIR pc2 0.46 [−0.24, 1.16]
DLVVTEVTEETVNLAWDNEMR pc3 −0.33 [−0.98, 0.32]
LSWTADEGVFDNFVLK pc2 0.70 [0.05, 1.36]
RE Model −0.21 [−0.27, −0.15]
−2
−1
0
1
2
Effect of 9:117902853:G:C (rs58160054)

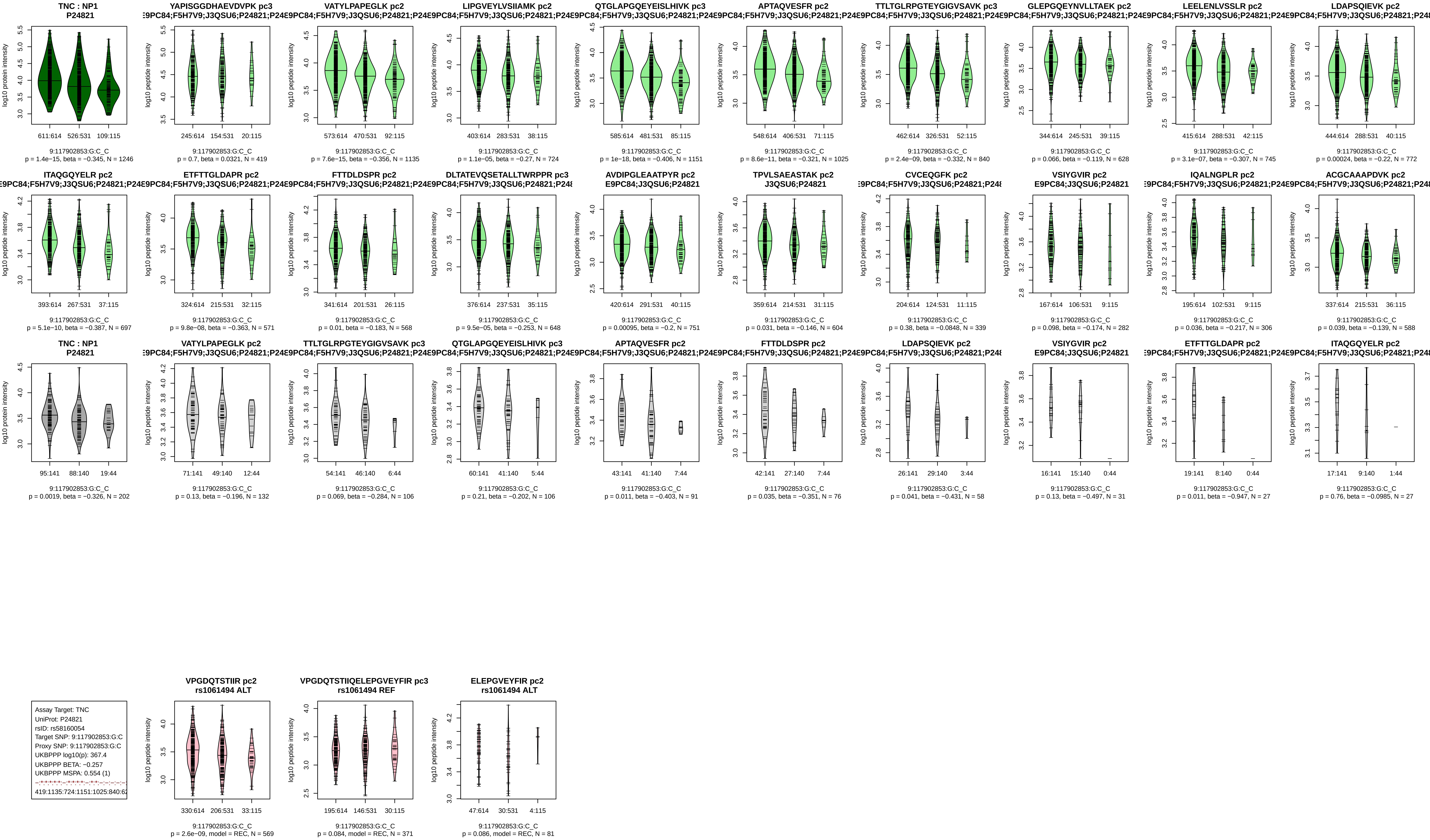
TNC : NP1
P24821
611:614 526:531 109:115
9:117902853:G:C_C
p = 1.4e−15, beta = −0.345, N = 1246
YAPISGGDHAEVDVPK pc3
245:614 154:531 20:115
9:117902853:G:C_C
p = 0.7, beta = 0.0321, N = 419
VATYLPAPEGLK pc2
573:614 470:531 92:115
9:117902853:G:C_C
p = 7.6e−15, beta = −0.356, N = 1135
LIPGVEYLVSIIAMK pc2
403:614 283:531 38:115
9:117902853:G:C_C
p = 1.1e−05, beta = −0.27, N = 724
QTGLAPGQEYEISLHIVK pc3
585:614 481:531 85:115
9:117902853:G:C_C
p = 1e−18, beta = −0.406, N = 1151
APTAQVESFR pc2
548:614 406:531 71:115
9:117902853:G:C_C
p = 8.6e−11, beta = −0.321, N = 1025
TTLTGLRPGTEYGIGVSAVK pc3
462:614 326:531 52:115
9:117902853:G:C_C
p = 2.4e−09, beta = −0.332, N = 840
GLEPGQEYNVLLTAEK pc2
344:614 245:531 39:115
9:117902853:G:C_C
p = 0.066, beta = −0.119, N = 628
LEELENLVSSLR pc2
415:614 288:531 42:115
9:117902853:G:C_C
p = 3.1e−07, beta = −0.307, N = 745
LDAPSQIEVK pc2
444:614 288:531 40:115
9:117902853:G:C_C
p = 0.00024, beta = −0.22, N = 772
ITAQGQYELR pc2
393:614 267:531 37:115
9:117902853:G:C_C
p = 5.1e−10, beta = −0.387, N = 697
ETFTTGLDAPR pc2
324:614 215:531 32:115
9:117902853:G:C_C
p = 9.8e−08, beta = −0.363, N = 571
FTTDLDSPR pc2
341:614 201:531 26:115
9:117902853:G:C_C
p = 0.01, beta = −0.183, N = 568
DLTATEVQSETALLTWRPPR pc3
376:614 237:531 35:115
9:117902853:G:C_C
p = 9.5e−05, beta = −0.253, N = 648
AVDIPGLEAATPYR pc2
E9PC84;J3QSU6;P24821
420:614 291:531 40:115
9:117902853:G:C_C
p = 0.00095, beta = −0.2, N = 751
TPVLSAEASTAK pc2
J3QSU6;P24821
359:614 214:531 31:115
9:117902853:G:C_C
p = 0.031, beta = −0.146, N = 604
CVCEQGFK pc2
204:614 124:531 11:115
9:117902853:G:C_C
p = 0.38, beta = −0.0848, N = 339
VSIYGVIR pc2
E9PC84;J3QSU6;P24821
167:614 106:531 9:115
9:117902853:G:C_C
p = 0.098, beta = −0.174, N = 282
IQALNGPLR pc2
195:614 102:531 9:115
9:117902853:G:C_C
p = 0.036, beta = −0.217, N = 306
ACGCAAAPDVK pc2
337:614 215:531 36:115
9:117902853:G:C_C
p = 0.039, beta = −0.139, N = 588
TNC : NP1
P24821
95:141 88:140 19:44
9:117902853:G:C_C
p = 0.0019, beta = −0.326, N = 202
VATYLPAPEGLK pc2
71:141 49:140 12:44
9:117902853:G:C_C
p = 0.13, beta = −0.196, N = 132
TTLTGLRPGTEYGIGVSAVK pc3
54:141 46:140 6:44
9:117902853:G:C_C
p = 0.069, beta = −0.284, N = 106
QTGLAPGQEYEISLHIVK pc3
60:141 41:140 5:44
9:117902853:G:C_C
p = 0.21, beta = −0.202, N = 106
APTAQVESFR pc2
43:141 41:140 7:44
9:117902853:G:C_C
p = 0.011, beta = −0.403, N = 91
FTTDLDSPR pc2
42:141 27:140 7:44
9:117902853:G:C_C
p = 0.035, beta = −0.351, N = 76
LDAPSQIEVK pc2
26:141 29:140 3:44
9:117902853:G:C_C
p = 0.041, beta = −0.431, N = 58
VSIYGVIR pc2
E9PC84;J3QSU6;P24821
16:141 15:140 0:44
9:117902853:G:C_C
p = 0.13, beta = −0.497, N = 31
ETFTTGLDAPR pc2
19:141 8:140 0:44
9:117902853:G:C_C
p = 0.011, beta = −0.947, N = 27
ITAQGQYELR pc2
17:141 9:140 1:44
9:117902853:G:C_C
p = 0.76, beta = −0.0985, N = 27
Assay Target: TNC
UniProt: P24821
rsID: rs58160054
Target SNP: 9:117902853:G:C
Proxy SNP: 9:117902853:G:C
UKBPPP log10(p): 367.4
UKBPPP BETA: −0.257
UKBPPP MSPA: 0.554 (1)
VPGDQTSTIIR pc2
rs1061494 ALT
330:614 206:531 33:115
9:117902853:G:C_C
p = 2.6e−09, model = REC, N = 569
VPGDQTSTIIQELEPGVEYFIR pc3
rs1061494 REF
195:614 146:531 30:115
9:117902853:G:C_C
p = 0.084, model = REC, N = 371
ELEPGVEYFIR pc2
rs1061494 ALT
47:614 30:531 4:115
9:117902853:G:C_C
p = 0.086, model = REC, N = 81

176. THOP1 (P52888)
UKBPPP MSPA=0.757

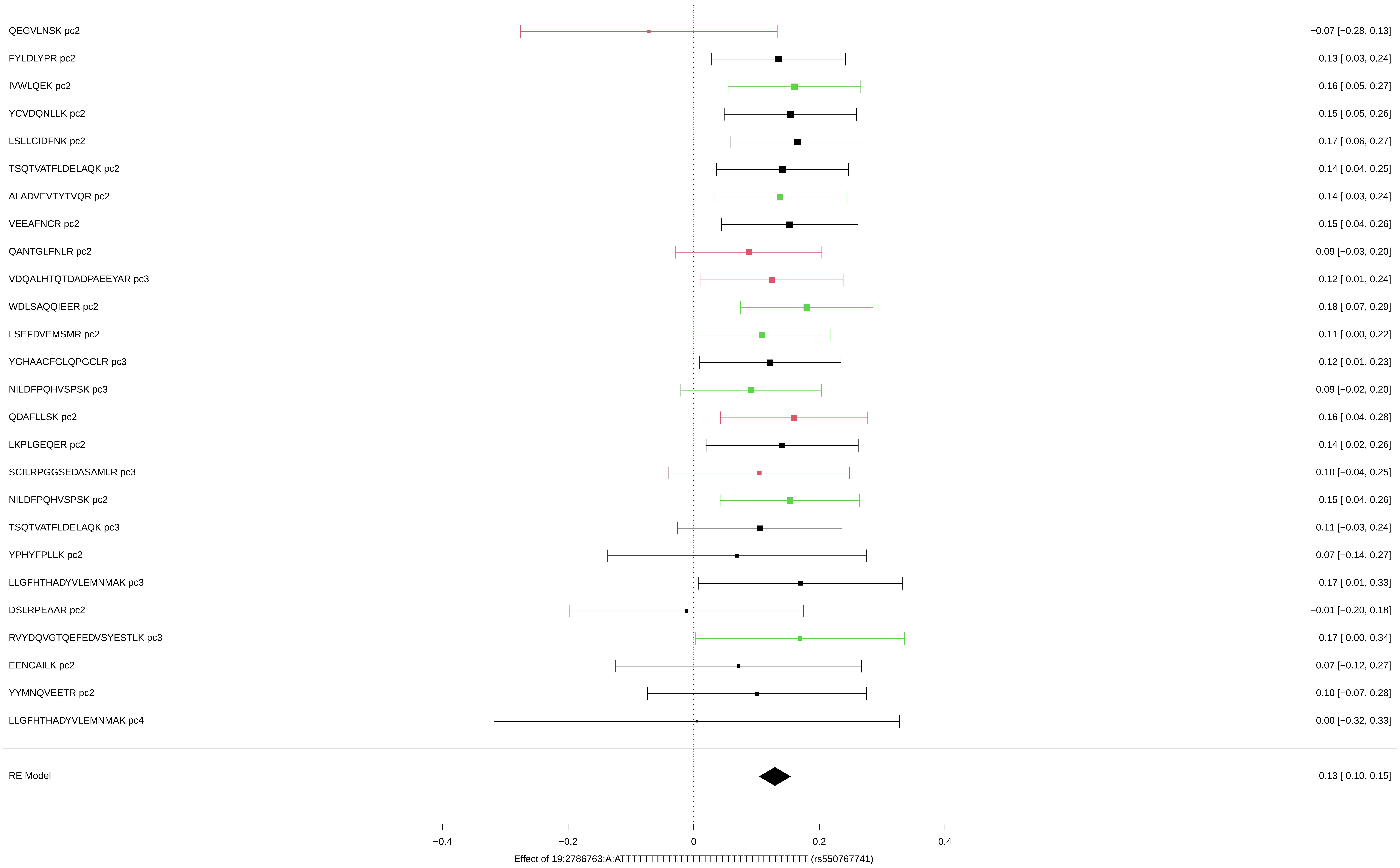

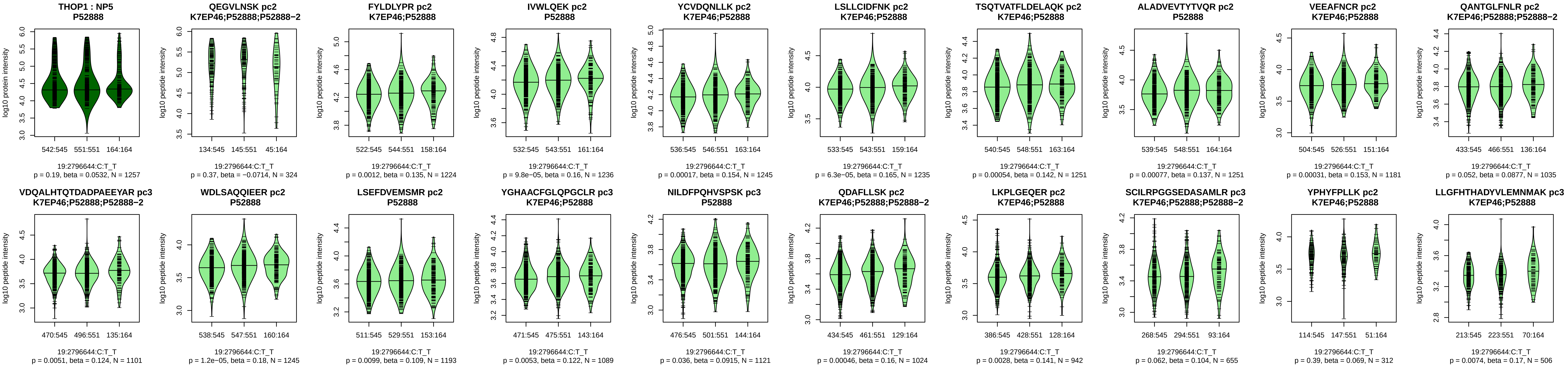

Assay Target: THOP1
UniProt: P52888
rsID: rs550767741
Target SNP: 19:2786763:A:ATT
Proxy SNP: 19:2796644:C:T
UKBPPP log10(p): 366.5
UKBPPP BETA: 0.266
UKBPPP MSPA: 0.757
-.*:*:*:*:*:*-.-:*:*:*-.-*:*-.-*:-.-
324:1224:1236:1245:1235:1251

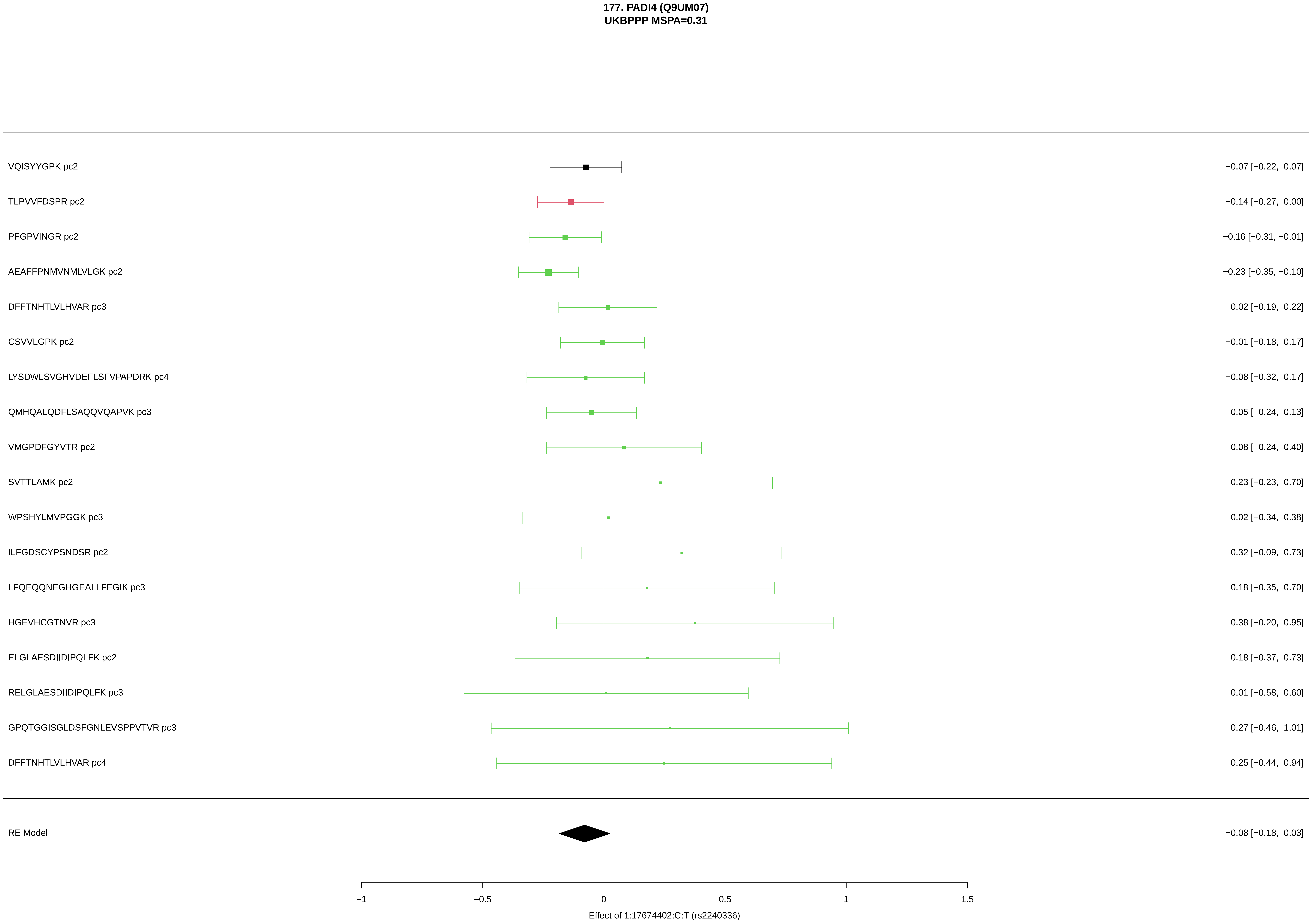
177. PADI4 (Q9UM07)
UKBPPP MSPA=0.31
VQISYYGPK pc2 −0.07 [−0.22, 0.07]
TLPVVFDSPR pc2 −0.14 [−0.27, 0.00]
PFGPVINGR pc2 −0.16 [−0.31, −0.01]
AEAFFPNMVNMLVLGK pc2 −0.23 [−0.35, −0.10]
DFFTNHTLVLHVAR pc3 0.02 [−0.19, 0.22]
CSVVLGPK pc2 −0.01 [−0.18, 0.17]
LYSDWLSVGHVDEFLSFVPAPDRK pc4 −0.08 [−0.32, 0.17]
QMHQALQDFLSAQQVQAPVK pc3 −0.05 [−0.24, 0.13]
VMGPDFGYVTR pc2 0.08 [−0.24, 0.40]
SVTTLAMK pc2 0.23 [−0.23, 0.70]
WPSHYLMVPGGK pc3 0.02 [−0.34, 0.38]
ILFGDSCYPSNDSR pc2 0.32 [−0.09, 0.73]
LFQEQQNEGHGEALLFEGIK pc3 0.18 [−0.35, 0.70]
HGEVHCGTNVR pc3 0.38 [−0.20, 0.95]
ELGLAESDIIDIPQLFK pc2 0.18 [−0.37, 0.73]
RELGLAESDIIDIPQLFK pc3 0.01 [−0.58, 0.60]
GPQTGGISGLDSFGNLEVSPPVTVR pc3 0.27 [−0.46, 1.01]
DFFTNHTLVLHVAR pc4 0.25 [−0.44, 0.94]
RE Model −0.08 [−0.18, 0.03]
−1
−0.5
0
0.5
1
1.5
Effect of 1:17674402:C:T (rs2240336)

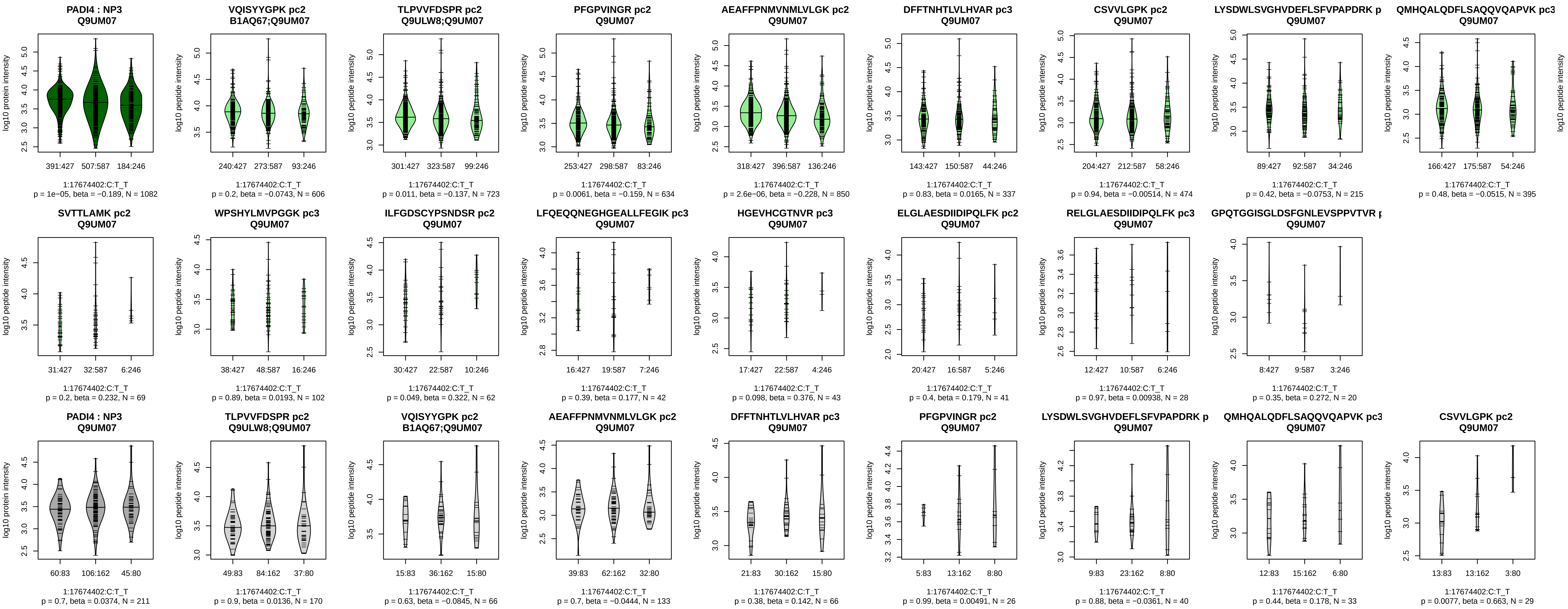

Assay Target: PADI4
UniProt: Q9UM07
rsID: rs2240336
Target SNP: 1:17674402:C:T
Proxy SNP: 1:17674402:C:T
UKBPPP log10(p): 366.3
UKBPPP BETA: −0.253
UKBPPP MSPA: 0.31
−:−:*:*:−:−:−:−:−:−:−:−:NA:−
606:723:634:850:337:474:215:3

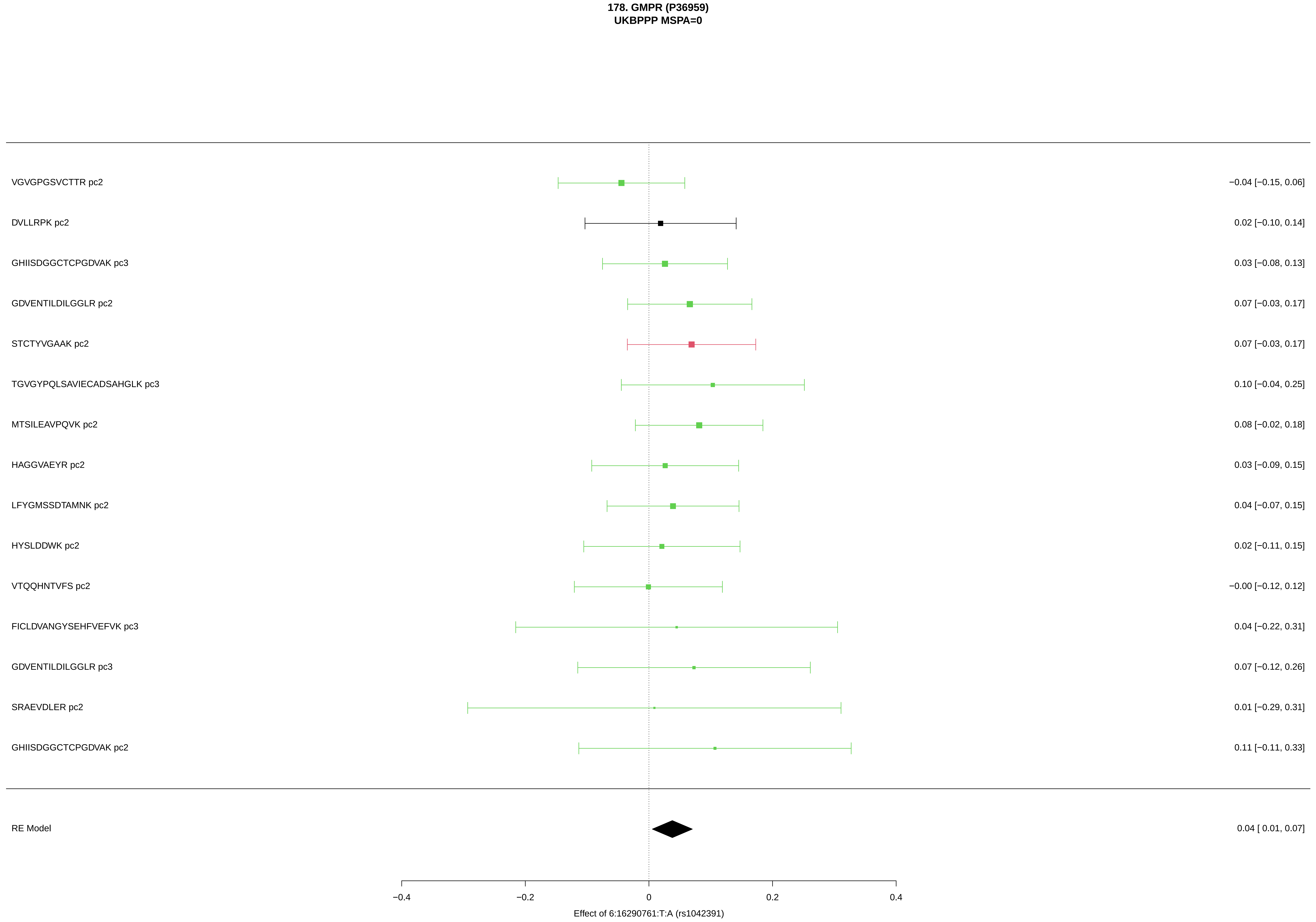
178. GMPR (P36959)
UKBPPP MSPA=0
VGVGPGSVCTTR pc2
−0.04 [−0.15, 0.06]
DVLLRPK pc2
0.02 [−0.10, 0.14]
GHIISDGGCTCPGDVAK pc3
0.03 [−0.08, 0.13]
GDVENTILDILGGLR pc2
0.07 [−0.03, 0.17]
STCTYVGAAK pc2
0.07 [−0.03, 0.17]
TGVGYPQLSAVIECADSAHGLK pc3
0.10 [−0.04, 0.25]
MTSILEAVPQVK pc2
0.08 [−0.02, 0.18]
HAGGVAEYR pc2
0.03 [−0.09, 0.15]
LFYGMSSDTAMNK pc2
0.04 [−0.07, 0.15]
HYSLDDWK pc2
0.02 [−0.11, 0.15]
VTQQHNTVFS pc2
−0.00 [−0.12, 0.12]
FICLDVANGYSEHFVEFVK pc3
0.04 [−0.22, 0.31]
GDVENTILDILGGLR pc3
0.07 [−0.12, 0.26]
SRAEVDLER pc2
0.01 [−0.29, 0.31]
GHIISDGGCTCPGDVAK pc2
0.11 [−0.11, 0.33]
RE Model
0.04 [0.01, 0.07]
−0.4
−0.2
0
0.2
0.4
Effect of 6:16290761:T:A (rs1042391)

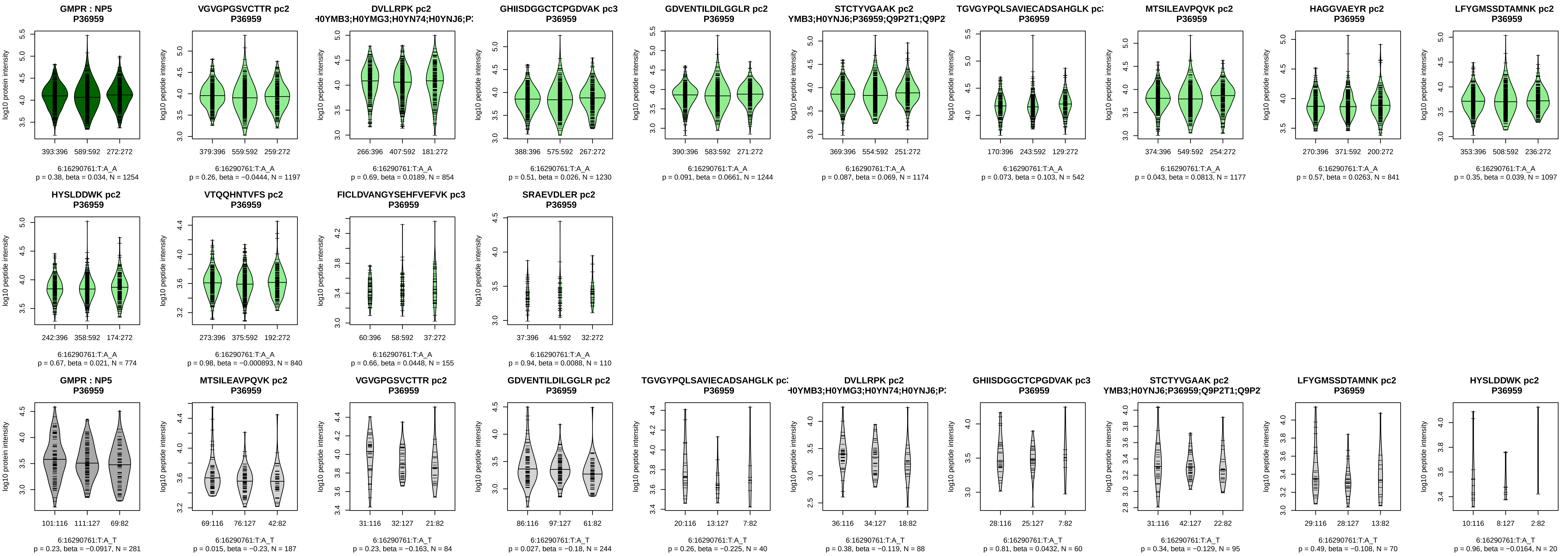

Assay Target: GMPR
UniProt: P36959
rsID: rs1042391
Target SNP: 6:16290761:T:A
Proxy SNP: 6:16290761:T:A
UKBPPP log10(p): 362.5
UKBPPP BETA: 0.252
UKBPPP MSPA: 0
–:–:–:–:–:–:–:–:–:–:–:–:–:–:NA
1197:854:1230:1244:1174:542:1

179. DNM1 (Q05193)
UKBPPP MSPA=0.661

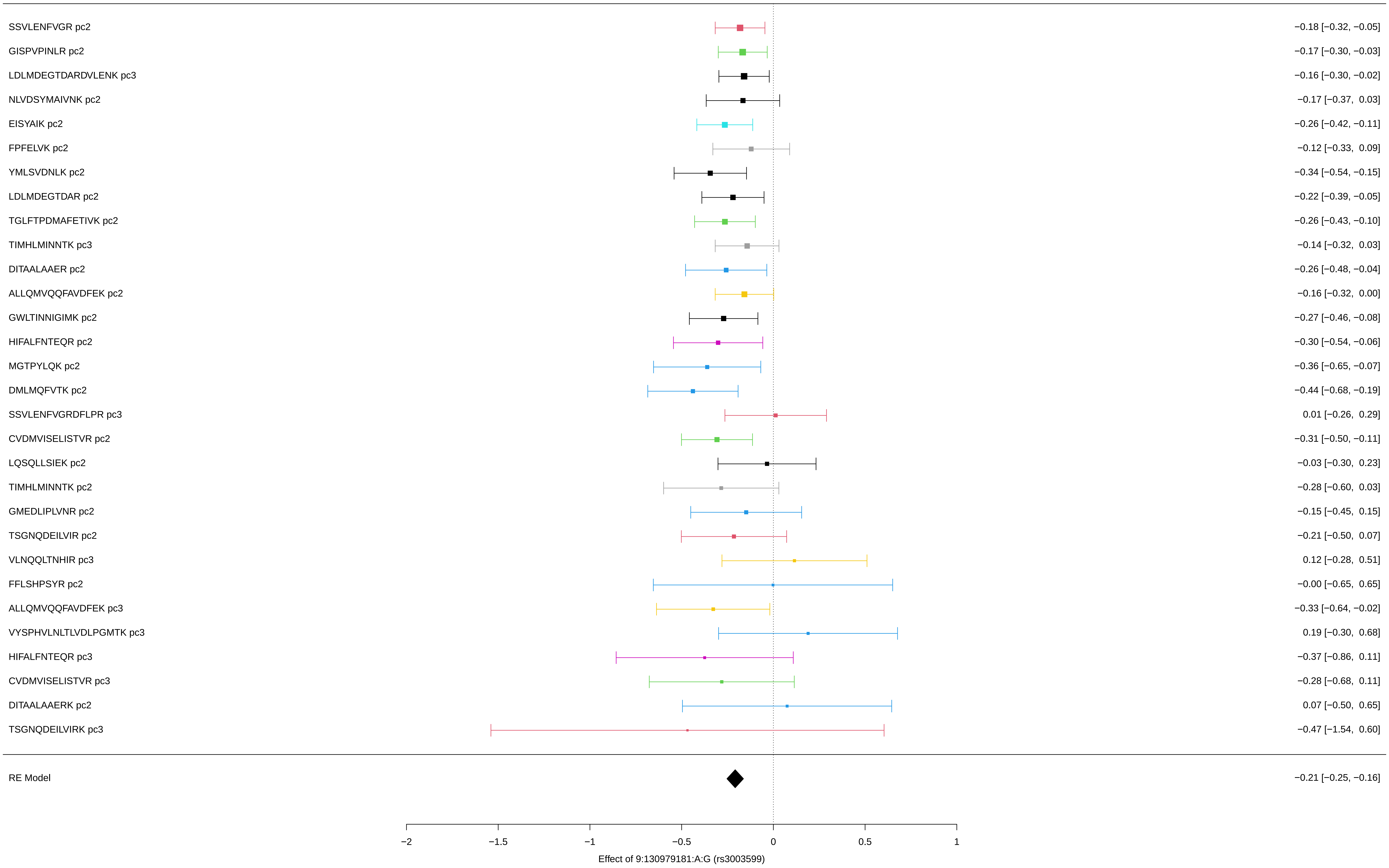

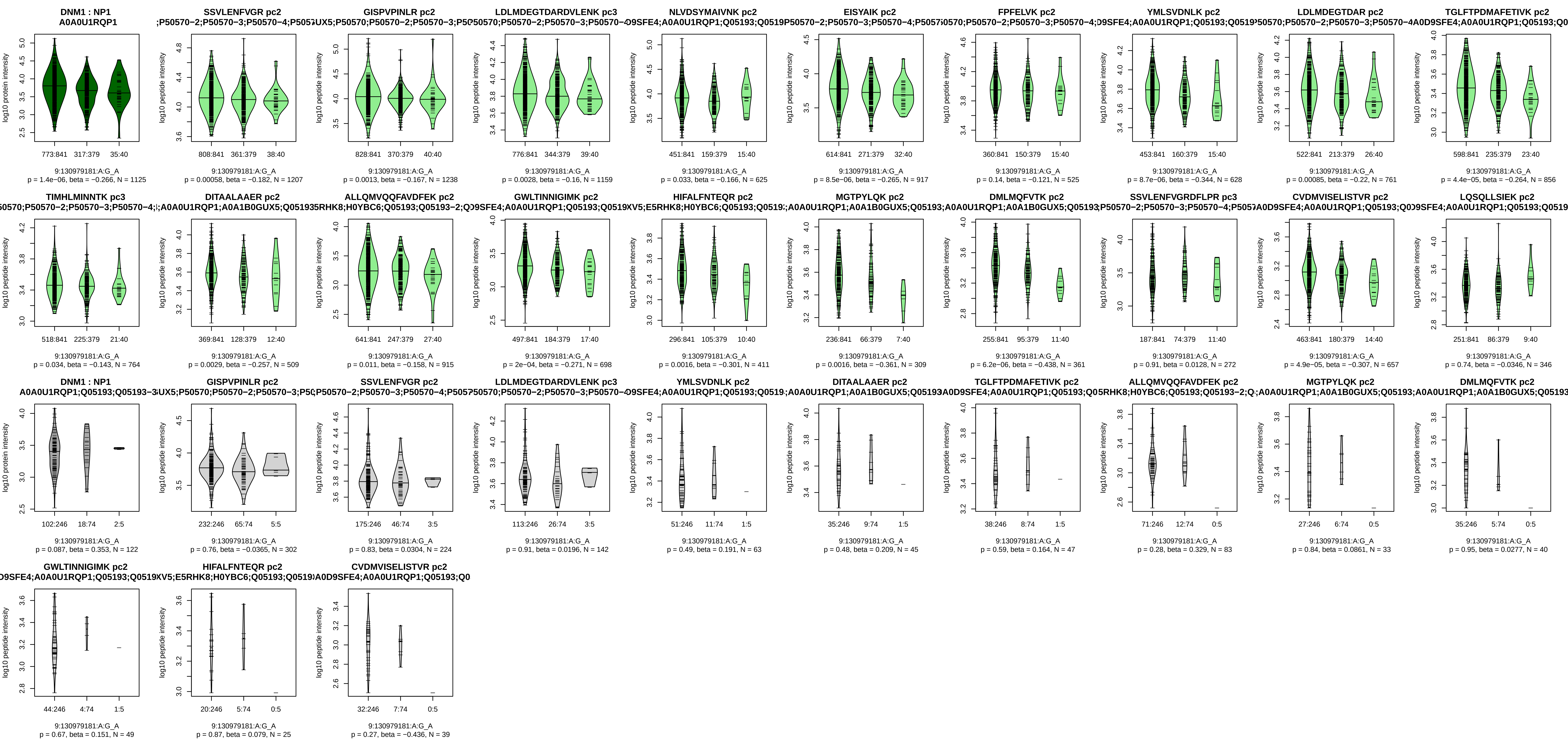

Assay Target: DNM1
UniProt: Q05193
rsID: rs3003599
Target SNP: 9:130979181:A:G
Proxy SNP: 9:130979181:A:G
UKBPPP log10(p): 355.2
UKBPPP BETA: 0.313
UKBPPP MSPA: 0.661
1207:1238:1159:625:917:525:62

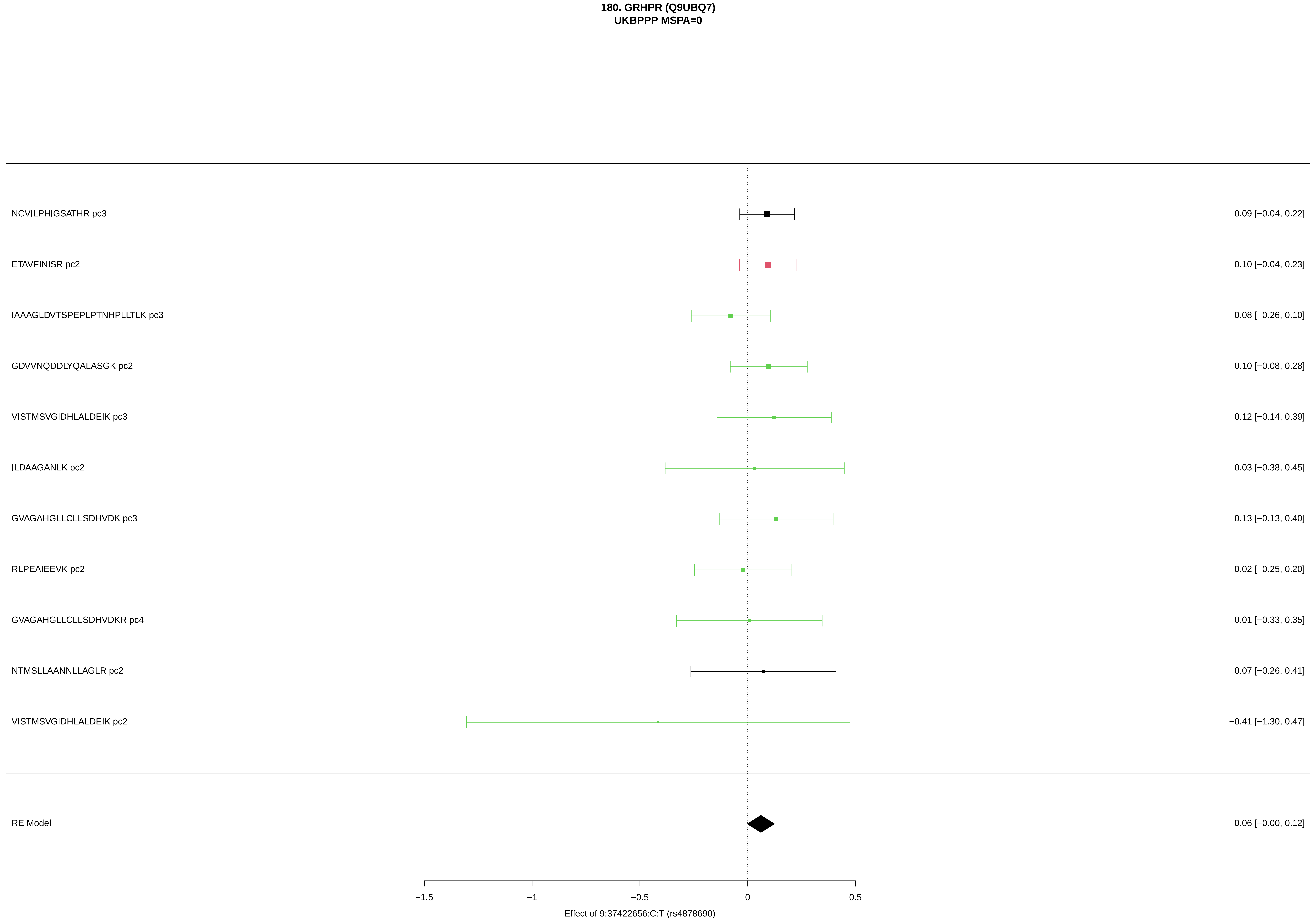

180. GRHPR (Q9UBQ7)
UKBPPP MSPA=0
NCVILPHIGSATHR pc3
0.09 [−0.04, 0.22]
ETAVFINISR pc2
0.10 [−0.04, 0.23]
IAAAGLDVTSPEPLPTNHPLLTLK pc3
−0.08 [−0.26, 0.10]
GDVVNQDDLYQALASGK pc2
0.10 [−0.08, 0.28]
VISTMSVGIDHLALDEIK pc3
0.12 [−0.14, 0.39]
ILDAAGANLK pc2
0.03 [−0.38, 0.45]
GVAGAHGLLCLLSDHVDK pc3
0.13 [−0.13, 0.40]
RLPEAIEEVK pc2
−0.02 [−0.25, 0.20]
GVAGAHGLLCLLSDHVDKR pc4
0.01 [−0.33, 0.35]
NTMSLLAANNLLAGLR pc2
0.07 [−0.26, 0.41]
VISTMSVGIDHLALDEIK pc2
−0.41 [−1.30, 0.47]
RE Model
0.06 [−0.00, 0.12]
−1.5
−1
−0.5
0
0.5
Effect of 9:37422656:C:T (rs4878690)

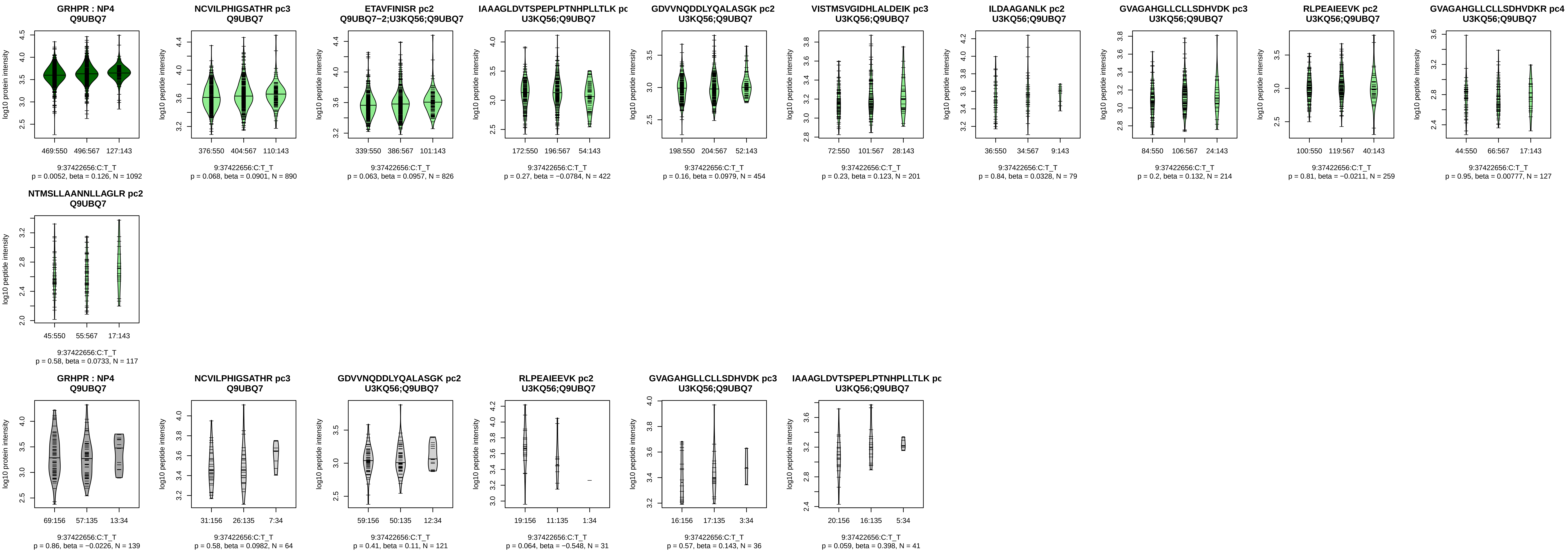

Assay Target: GRHPR
UniProt: Q9UBQ7
rsID: rs4878690
Target SNP: 9:37422656:C:T
Proxy SNP: 9:37422656:C:T
UKBPPP log10(p): 355.1
UKBPPP BETA: 0.264
UKBPPP MSPA: 0
−:−:−:−:−:−:−:−:NA:−
890:826:422:454:201:79:214:25

181. GRN (P28799)
UKBPPP MSPA=0

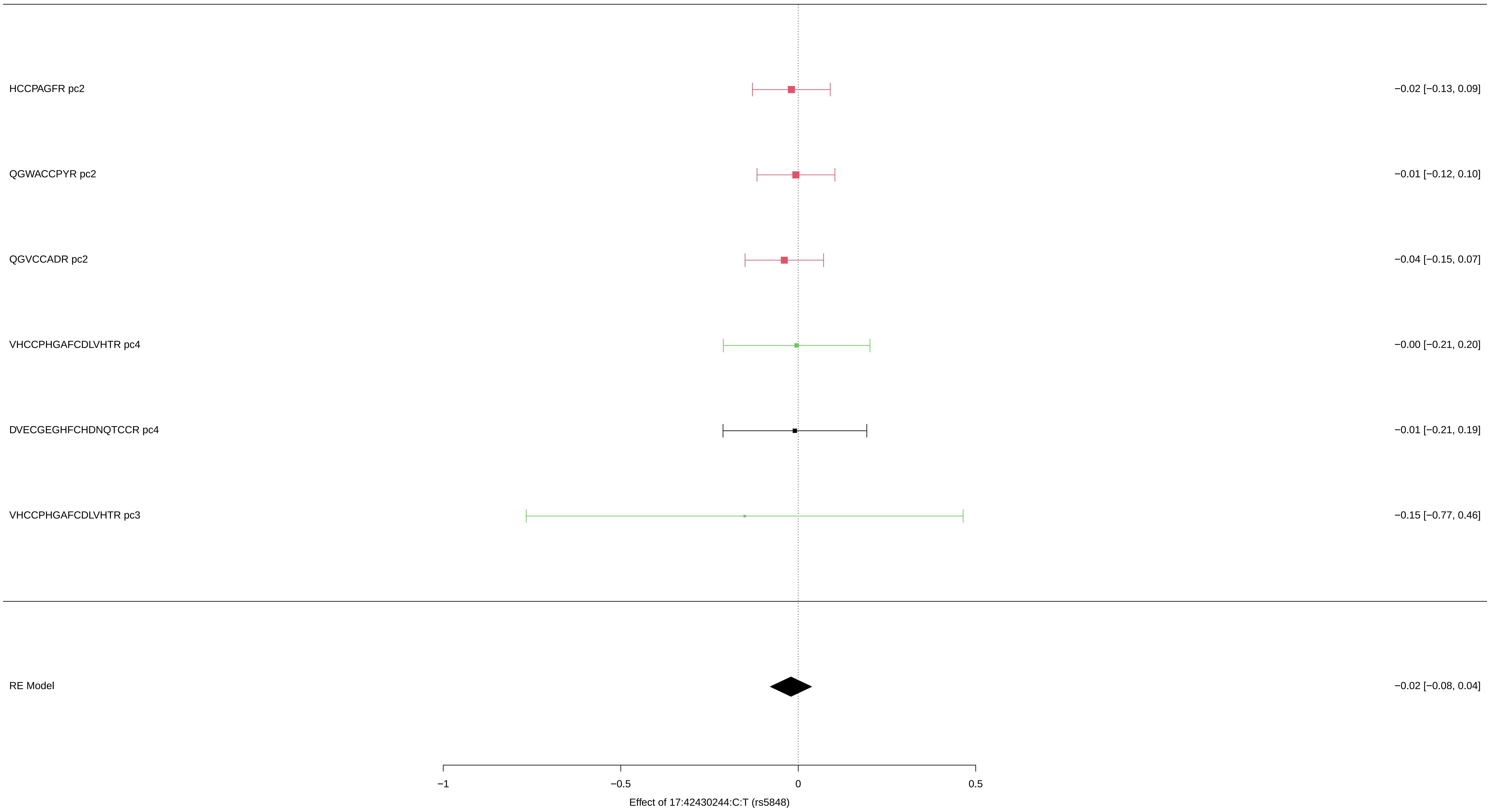

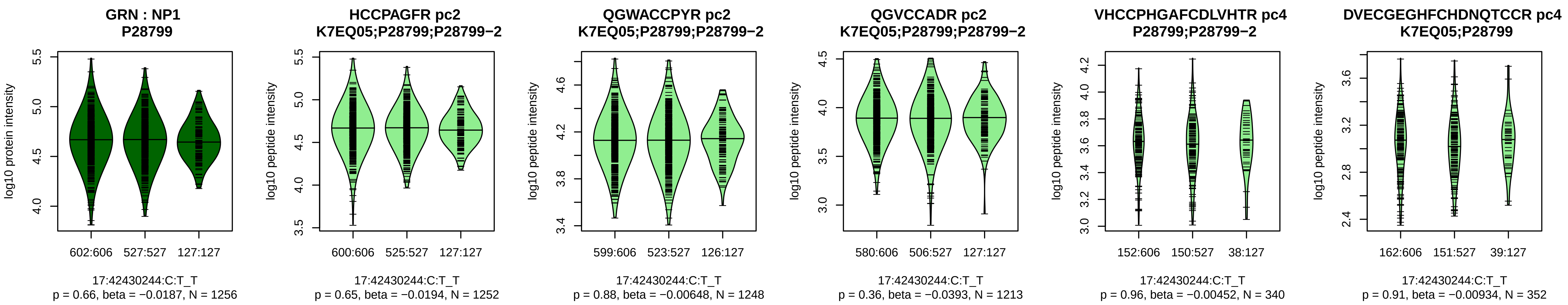

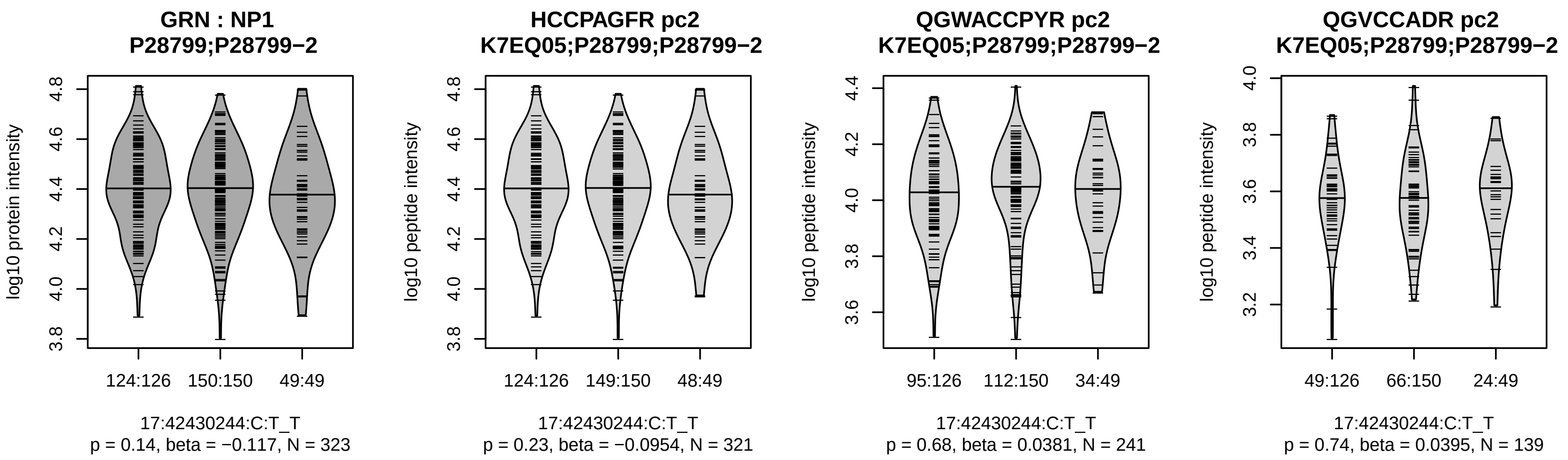

Assay Target: GRN
UniProt: P28799
rsID: rs5848
Target SNP: 17:42430244:C:T
Proxy SNP: 17:42430244:C:T
UKBPPP log10(p): 351.4
UKBPPP BETA: −0.25
UKBPPP MSPA: 0
−:−:−:−:−:−:NA
1252:1248:1213:340:352:38:5

182. PI16 (Q6UXB8)
UKBPPP MSPA=0.183

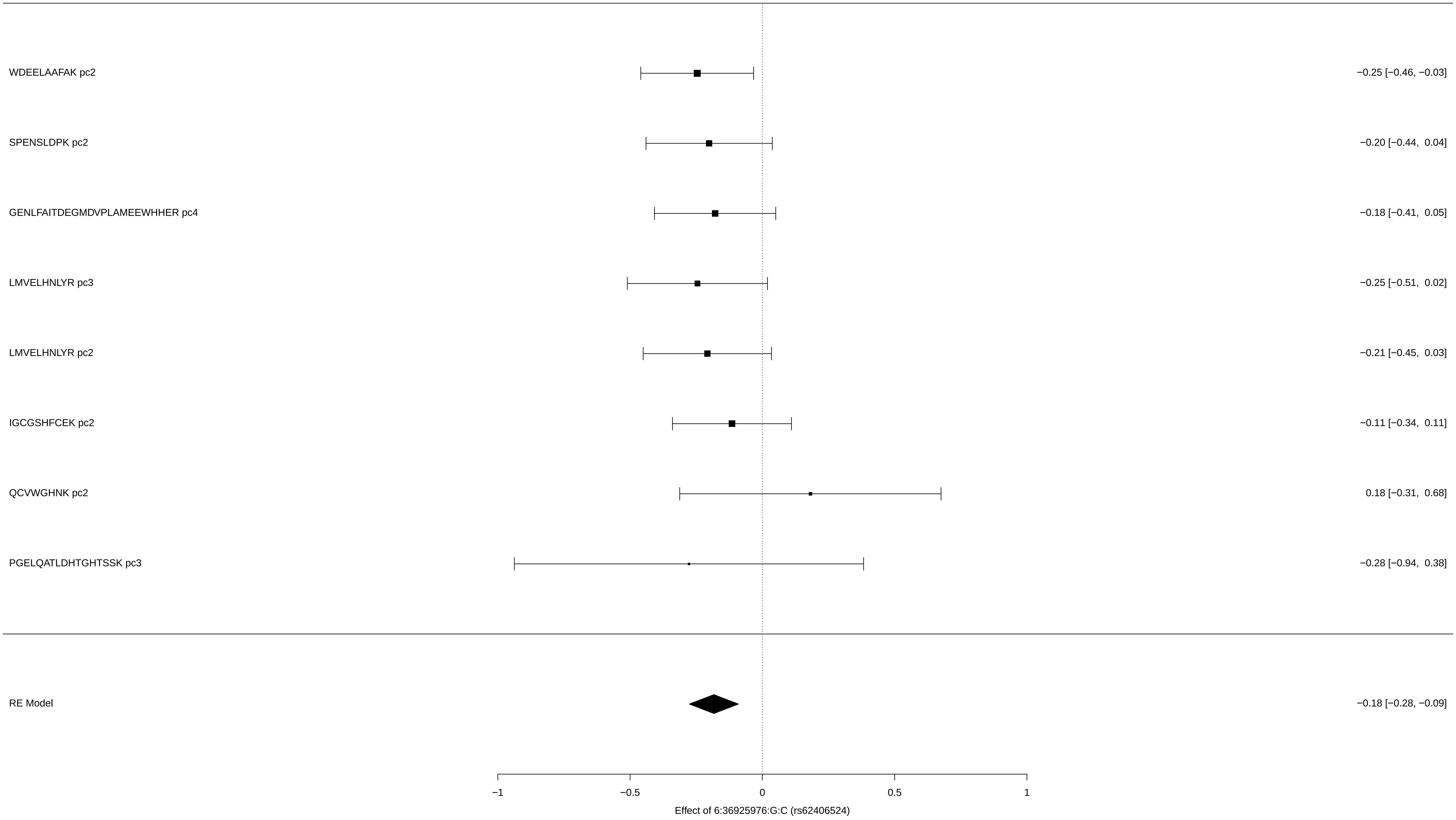

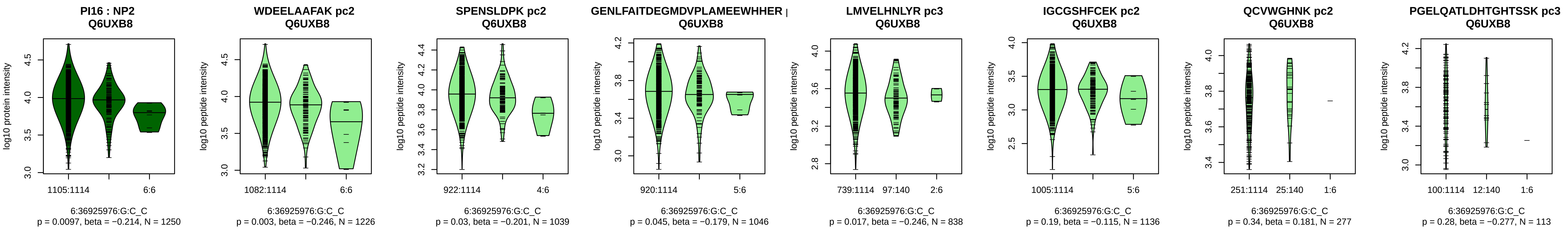

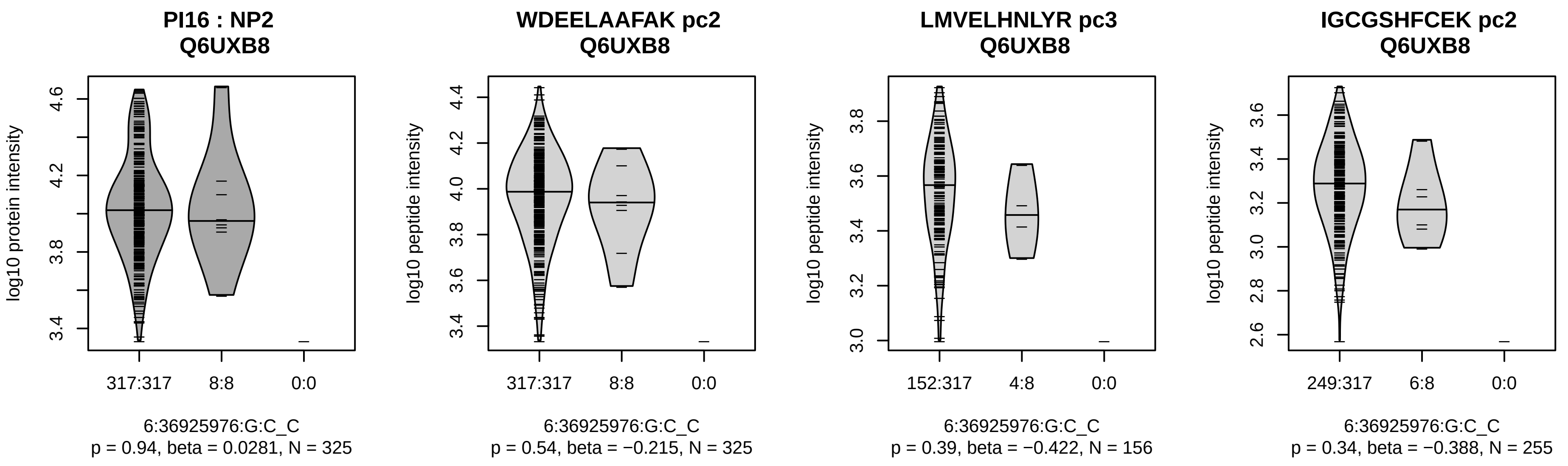

Assay Target: PI16
UniProt: Q6UXB8
rsID: rs62406524
Target SNP: 6:36925976:G:C
Proxy SNP: 6:36925976:G:C
UKBPPP log10(p): 350.5
UKBPPP BETA: −0.422
UKBPPP MSPA: 0.183
*:–:–:–:–:–:–:–
1226:1039:1046:838:1024:1136

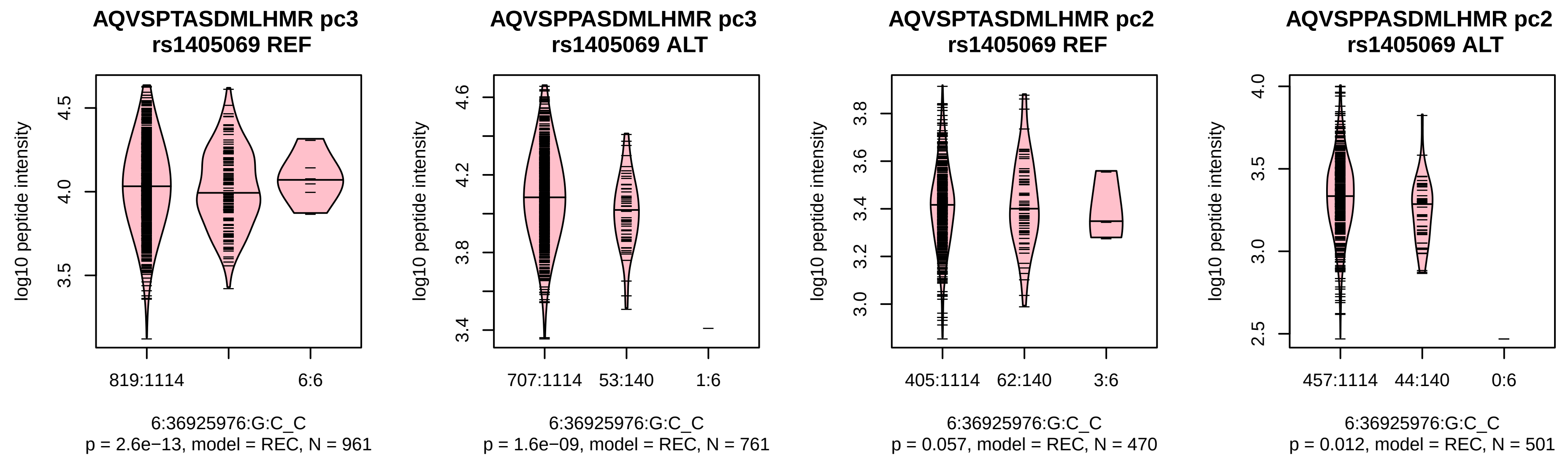

183. PODXL (O00592)
UKBPPP MSPA=0

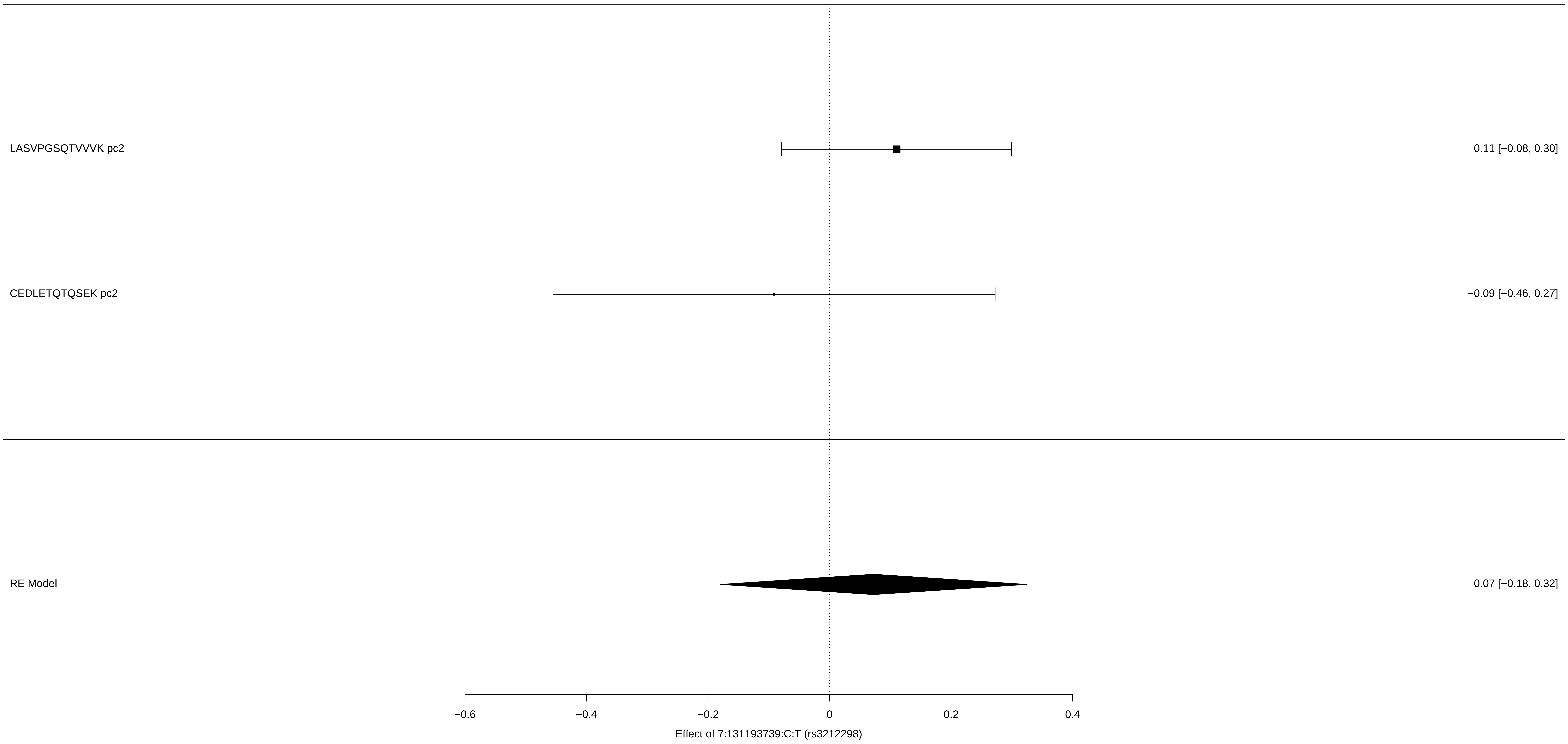

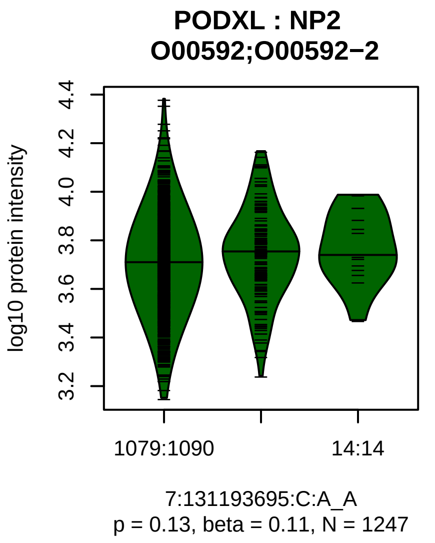

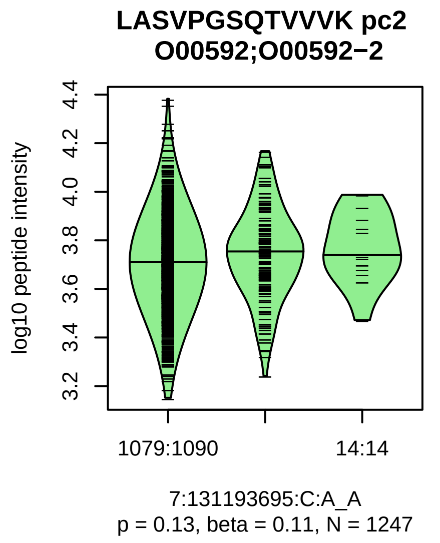

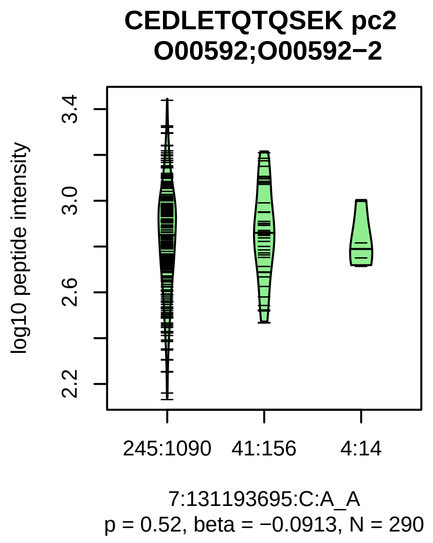

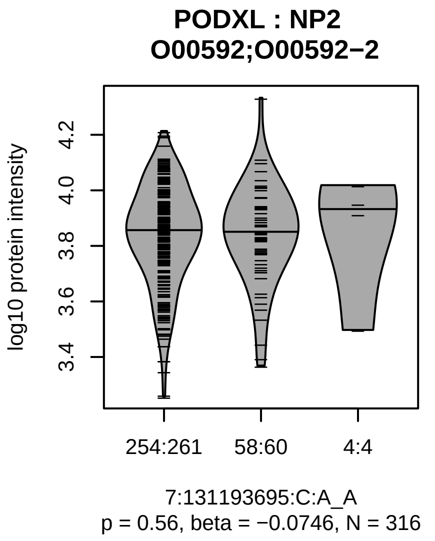

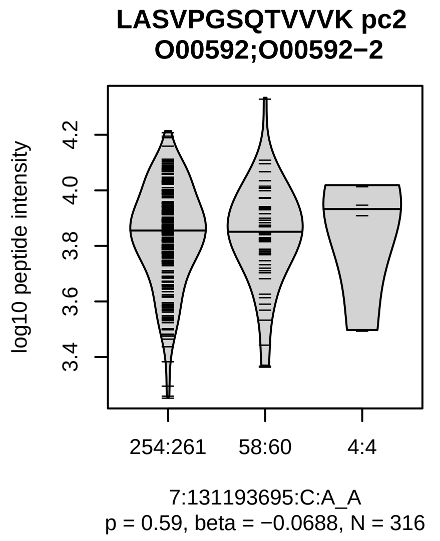

Assay Target: PODXL
UniProt: O00592
rsID: rs3212298
Target SNP: 7:131193739:C:T
Proxy SNP: 7:131193695:C:A
UKBPPP log10(p): 325.3
UKBPPP BETA: −0.555
UKBPPP MSPA: 0
–:–
1247:290

184. PDIA5 (Q14554)
UKBPPP MSPA=0.989

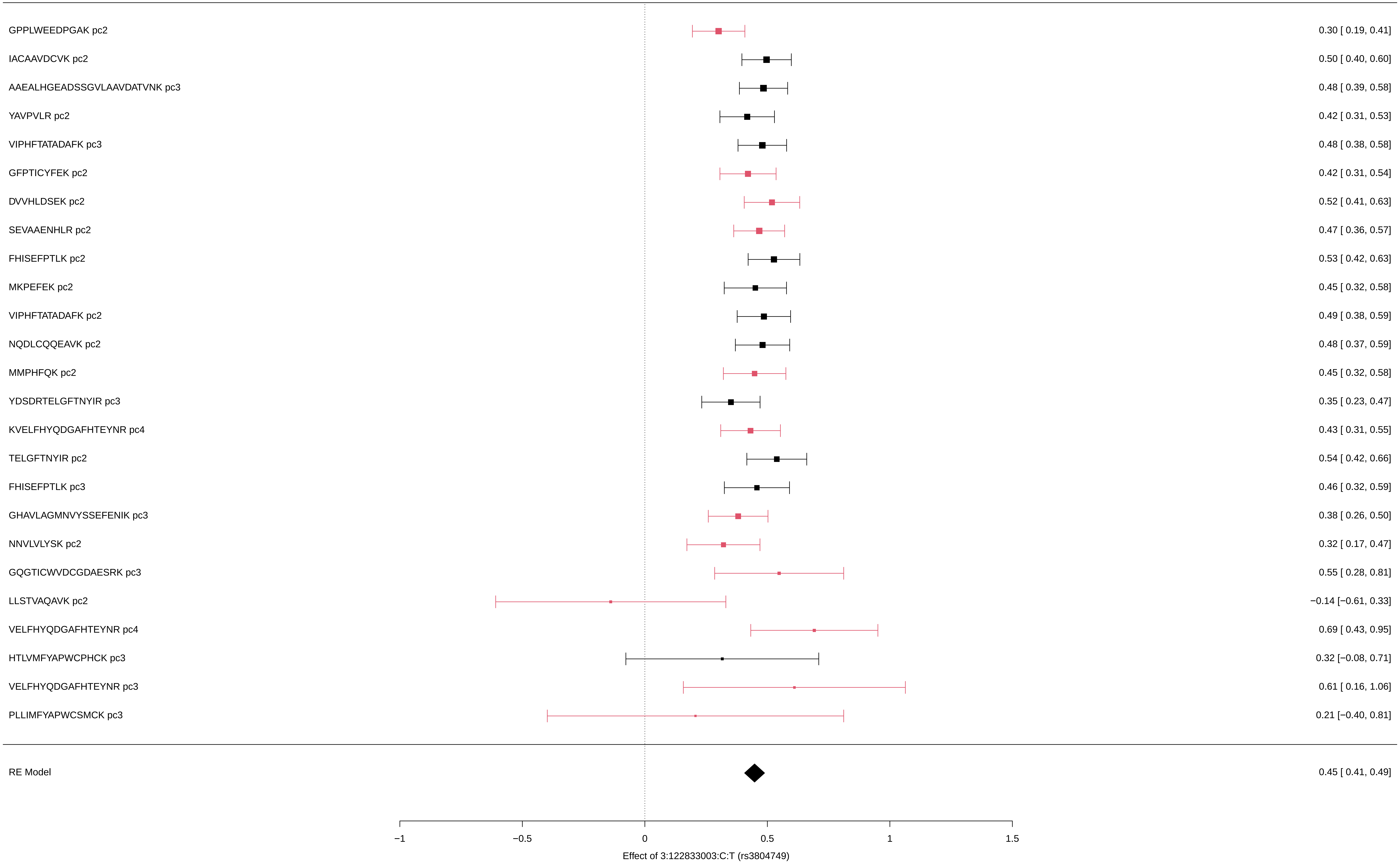

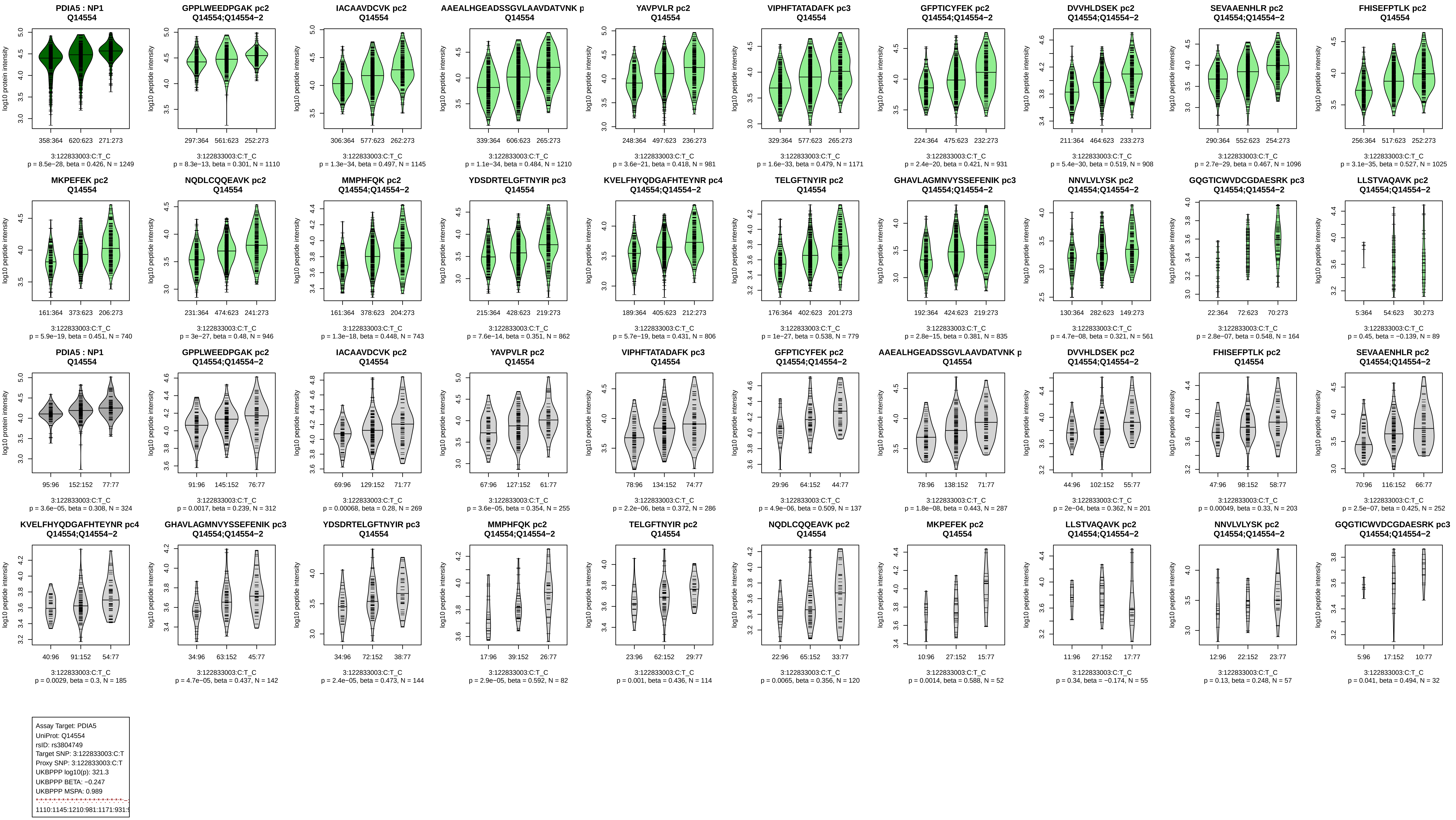
PDIA5 : NP1 Q14554
3:122833003:C:T_C
p = 8.5e−28, beta = 0.426, N = 1249
GPPLWEEDPGAK pc2 Q14554;Q14554−2
3:122833003:C:T_C
p = 8.3e−13, beta = 0.301, N = 1110
IACAAVDCVK pc2 Q14554
3:122833003:C:T_C
p = 1.3e−34, beta = 0.497, N = 1145
AAEALHGEADSSGVLAAVDATVNK p Q14554
3:122833003:C:T_C
p = 1.1e−34, beta = 0.484, N = 1210
YAVPVLR pc2 Q14554
3:122833003:C:T_C
p = 3.6e−21, beta = 0.418, N = 981
VIPHFTATADAFK pc3 Q14554
3:122833003:C:T_C
p = 1.6e−33, beta = 0.479, N = 1171
GFPTICYFEK pc2 Q14554;Q14554−2
3:122833003:C:T_C
p = 2.4e−20, beta = 0.421, N = 931
DVVHLDSEK pc2 Q14554;Q14554−2
3:122833003:C:T_C
p = 5.4e−30, beta = 0.519, N = 908
SEVAAENHLR pc2 Q14554;Q14554−2
3:122833003:C:T_C
p = 2.7e−29, beta = 0.467, N = 1096
FHISEFPTLK pc2 Q14554
3:122833003:C:T_C
p = 3.1e−35, beta = 0.527, N = 1025
MKPEFEK pc2 Q14554
3:122833003:C:T_C
p = 5.9e−19, beta = 0.451, N = 740
NQDLCQQEAVK pc2 Q14554
3:122833003:C:T_C
p = 3e−27, beta = 0.48, N = 946
MMPHFQK pc2 Q14554;Q14554−2
3:122833003:C:T_C
p = 1.3e−18, beta = 0.448, N = 743
YDSDRTELGFTNYIR pc3 Q14554
3:122833003:C:T_C
p = 7.6e−14, beta = 0.351, N = 862
KVELFHYQDGAFHTEYNR pc4 Q14554;Q14554−2
3:122833003:C:T_C
p = 5.7e−19, beta = 0.431, N = 806
TELGFTNYIR pc2 Q14554
3:122833003:C:T_C
p = 1e−27, beta = 0.538, N = 779
GHAVLAGMNVYSSEFENIK pc3 Q14554;Q14554−2
3:122833003:C:T_C
p = 2.8e−15, beta = 0.381, N = 835
NNVLVLYSK pc2 Q14554;Q14554−2
3:122833003:C:T_C
p = 4.7e−08, beta = 0.321, N = 561
GQGTICWVDCGDAESRK pc3 Q14554;Q14554−2
3:122833003:C:T_C
p = 2.8e−07, beta = 0.548, N = 164
LLSTVAQAVK pc2 Q14554;Q14554−2
3:122833003:C:T_C
p = 0.45, beta = −0.139, N = 89
PDIA5 : NP1 Q14554
3:122833003:C:T_C
p = 3.6e−05, beta = 0.308, N = 324
GPPLWEEDPGAK pc2 Q14554;Q14554−2
3:122833003:C:T_C
p = 0.0017, beta = 0.239, N = 312
IACAAVDCVK pc2 Q14554
3:122833003:C:T_C
p = 0.00068, beta = 0.28, N = 269
YAVPVLR pc2 Q14554
3:122833003:C:T_C
p = 3.6e−05, beta = 0.354, N = 255
VIPHFTATADAFK pc3 Q14554
3:122833003:C:T_C
p = 2.2e−06, beta = 0.372, N = 286
GFPTICYFEK pc2 Q14554;Q14554−2
3:122833003:C:T_C
p = 4.9e−06, beta = 0.509, N = 137
AAEALHGEADSSGVLAAVDATVNK p Q14554
3:122833003:C:T_C
p = 1.8e−08, beta = 0.443, N = 287
DVVHLDSEK pc2 Q14554;Q14554−2
3:122833003:C:T_C
p = 2e−04, beta = 0.362, N = 201
FHISEFPTLK pc2 Q14554
3:122833003:C:T_C
p = 0.00049, beta = 0.33, N = 203
SEVAAENHLR pc2 Q14554;Q14554−2
3:122833003:C:T_C
p = 2.5e−07, beta = 0.425, N = 252
KVELFHYQDGAFHTEYNR pc4 Q14554;Q14554−2
3:122833003:C:T_C
p = 0.0029, beta = 0.3, N = 185
GHAVLAGMNVYSSEFENIK pc3 Q14554;Q14554−2
3:122833003:C:T_C
p = 4.7e−05, beta = 0.437, N = 142
YDSDRTELGFTNYIR pc3 Q14554
3:122833003:C:T_C
p = 2.4e−05, beta = 0.473, N = 144
MMPHFQK pc2 Q14554;Q14554−2
3:122833003:C:T_C
p = 2.9e−05, beta = 0.592, N = 82
TELGFTNYIR pc2 Q14554
3:122833003:C:T_C
p = 0.001, beta = 0.436, N = 114
NQDLCQQEAVK pc2 Q14554
3:122833003:C:T_C
p = 0.0065, beta = 0.356, N = 120
MKPEFEK pc2 Q14554
3:122833003:C:T_C
p = 0.0014, beta = 0.588, N = 52
LLSTVAQAVK pc2 Q14554;Q14554−2
3:122833003:C:T_C
p = 0.34, beta = −0.174, N = 55
NNVLVLYSK pc2 Q14554;Q14554−2
3:122833003:C:T_C
p = 0.13, beta = 0.248, N = 57
GQGTICWVDCGDAESRK pc3 Q14554;Q14554−2
3:122833003:C:T_C
p = 0.041, beta = 0.494, N = 32
Assay Target: PDIA5
UniProt: Q14554
rsID: rs3804749
Target SNP: 3:122833003:C:T
Proxy SNP: 3:122833003:C:T
UKBPPP log10(p): 321.3
UKBPPP BETA: −0.247
UKBPPP MSPA: 0.989
1110:1145:1210:981:1171:931:9

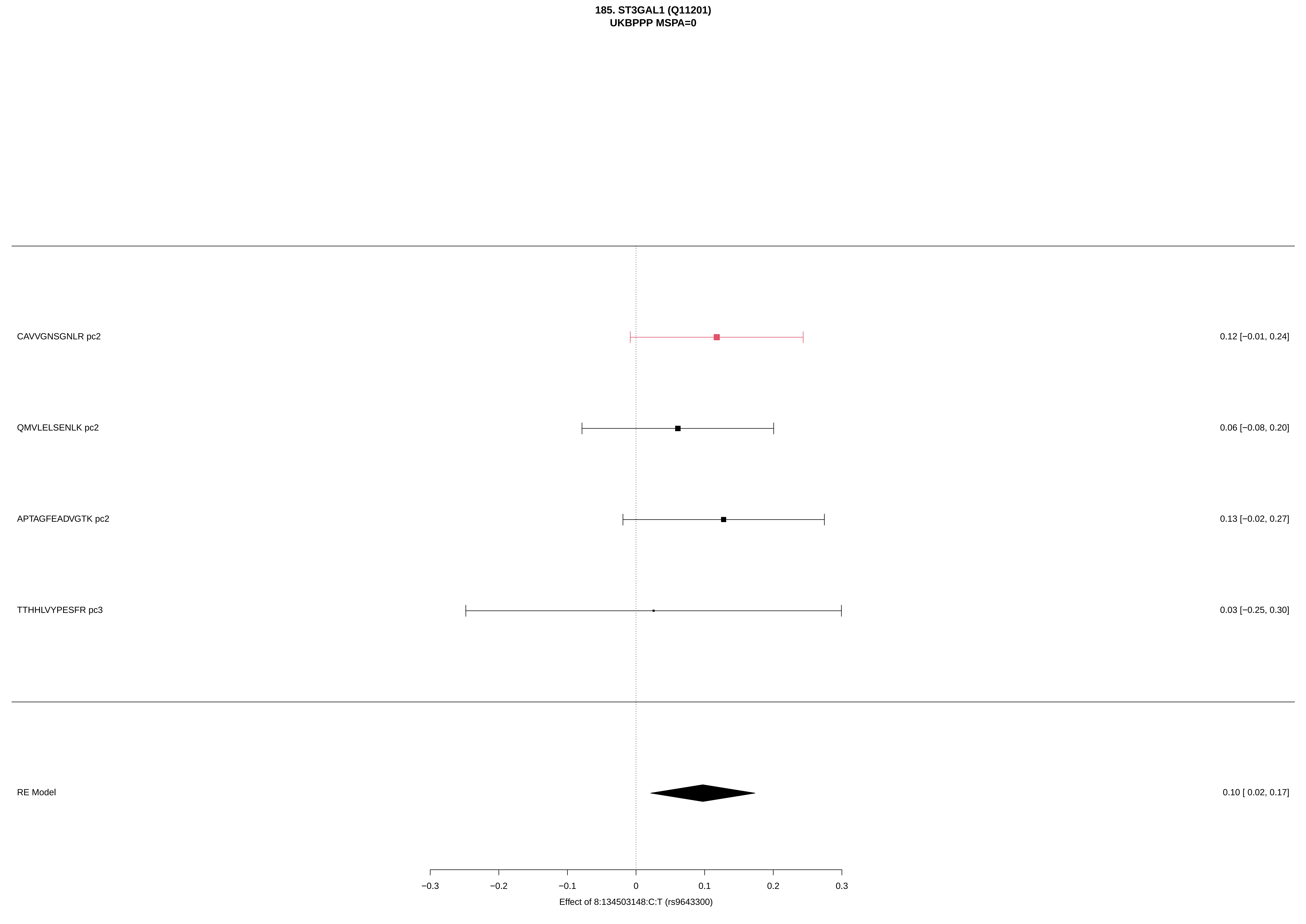
185. ST3GAL1 (Q11201)
UKBPPP MSPA=0
CAVVGNSGNLR pc2
0.12 [−0.01, 0.24]
QMVLELSENLK pc2
0.06 [−0.08, 0.20]
APTAGFEADVGTK pc2
0.13 [−0.02, 0.27]
TTHHLVYPESFR pc3
0.03 [−0.25, 0.30]
RE Model
0.10 [0.02, 0.17]
−0.3
−0.2
−0.1
0
0.1
0.2
0.3
Effect of 8:134503148:C:T (rs9643300)

ST3GAL1 : NP4
Q11201

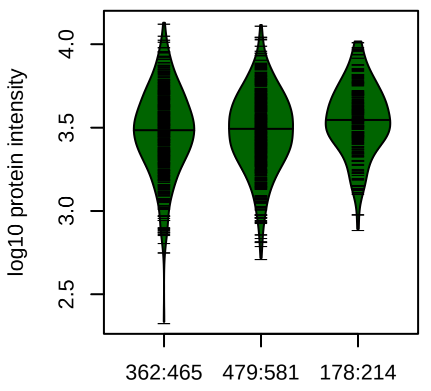

8:134503148:C:T_C
p = 0.0041, beta = 0.127, N = 1019

CAVVGNSGNLR pc2
Q11201;Q16842

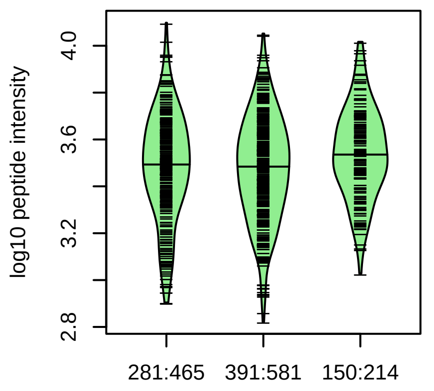

8:134503148:C:T_C
p = 0.016, beta = 0.118, N = 822

QMVLELSENLK pc2
Q11201

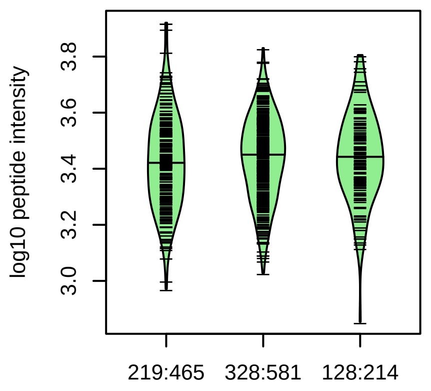

8:134503148:C:T_C
p = 0.26, beta = 0.0609, N = 675

APTAGFEADVGTK pc2
Q11201

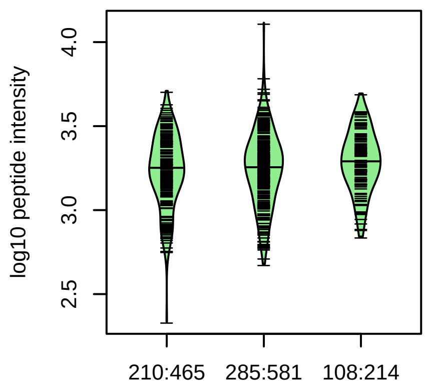

8:134503148:C:T_C
p = 0.025, beta = 0.128, N = 603

TTHHLVYPESFR pc3
Q11201

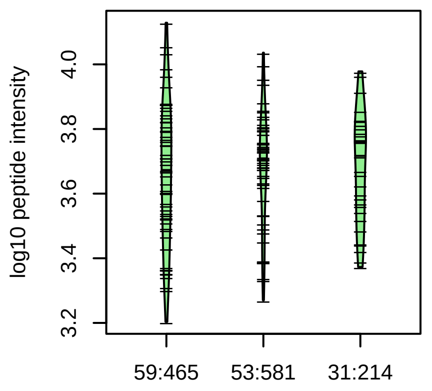

8:134503148:C:T_C
p = 0.81, beta = 0.0256, N = 143

ST3GAL1 : NP4
Q11201

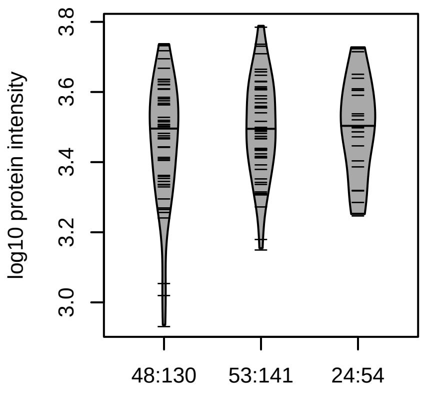

8:134503148:C:T_C
p = 0.76, beta = 0.0364, N = 125

CAVVGNSGNLR pc2
Q11201;Q16842

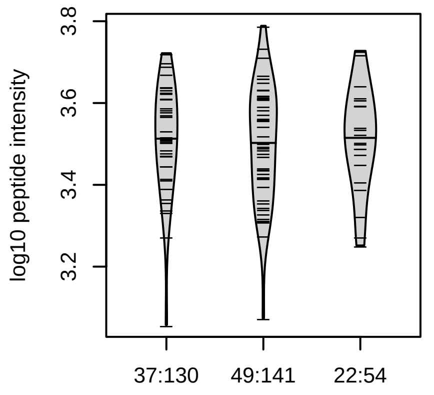

8:134503148:C:T_C
p = 0.37, beta = −0.116, N = 108

APTAGFEADVGTK pc2
Q11201

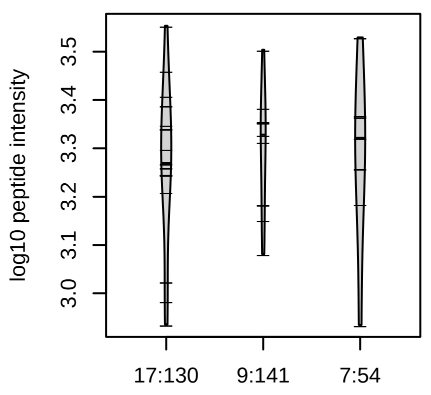

8:134503148:C:T_C
p = 0.87, beta = −0.0325, N = 33

Assay Target: ST3GAL1
UniProt: Q11201
rsID: rs9643300
Target SNP: 8:134503148:C:T
Proxy SNP: 8:134503148:C:T
UKBPPP log10(p): 312.8
UKBPPP BETA: −0.227
UKBPPP MSPA: 0
–:–:–:–
822:675:603:143

186. CD59 (P13987)
UKBPPP MSPA=0.551

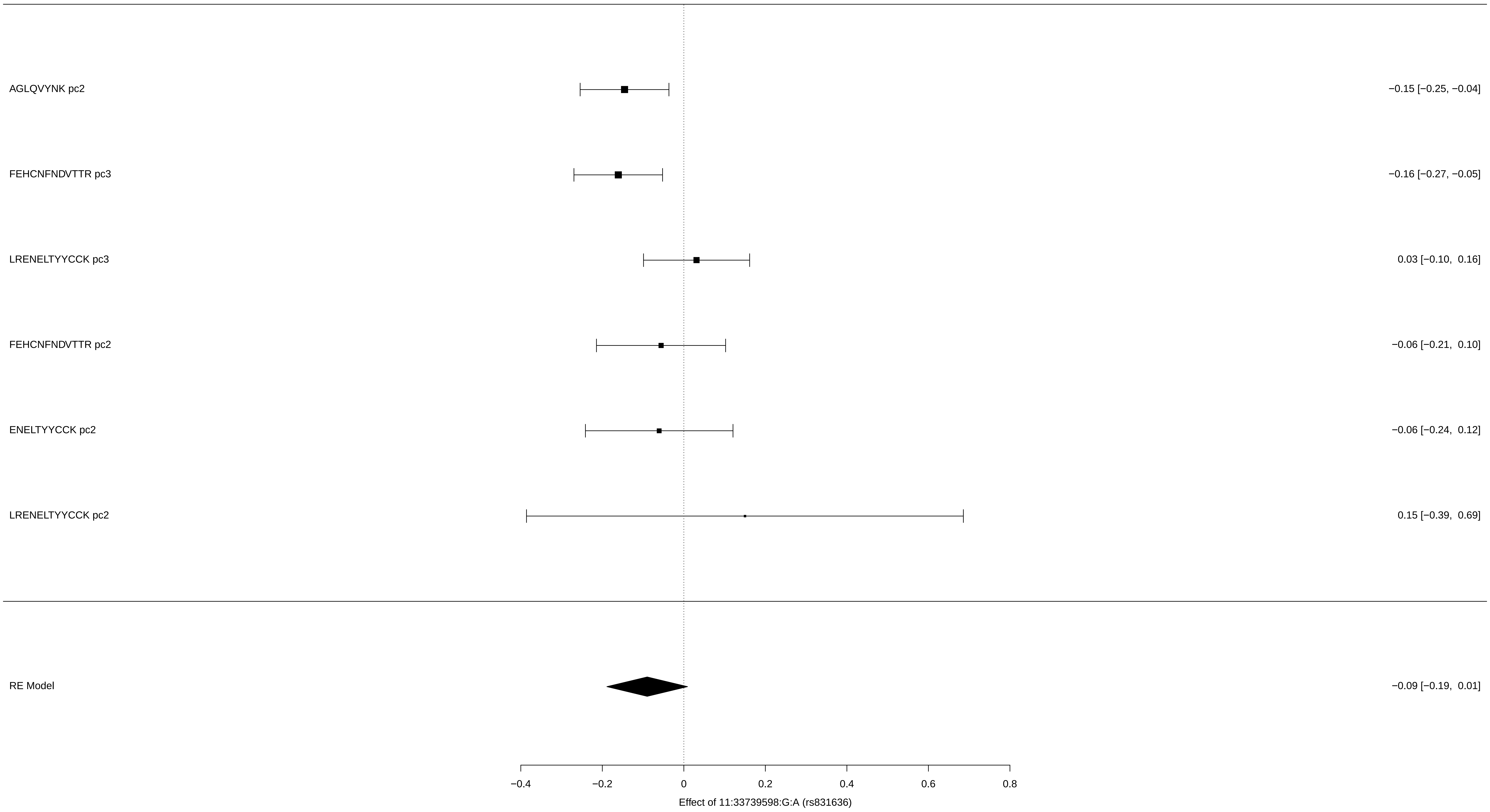

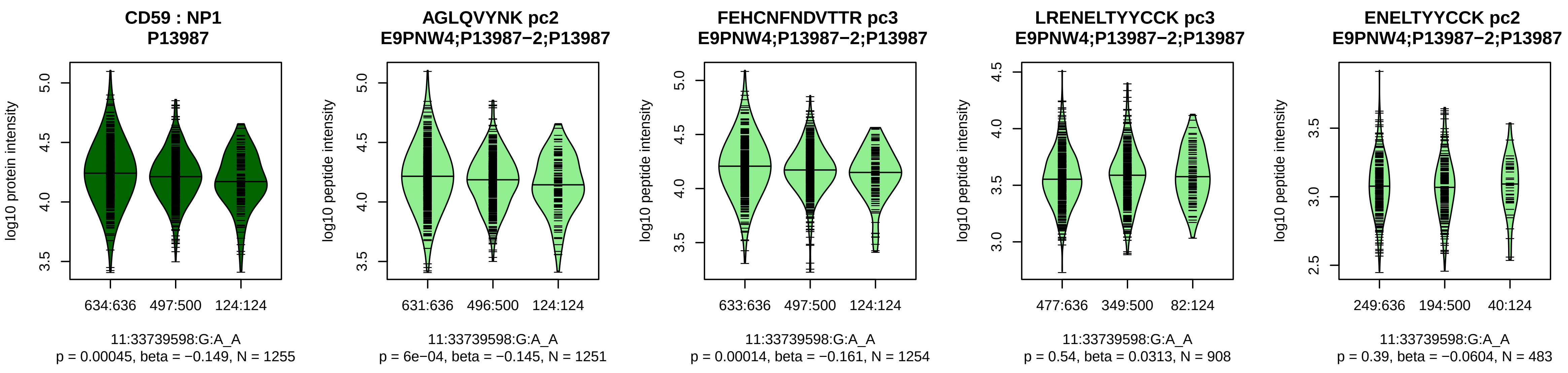

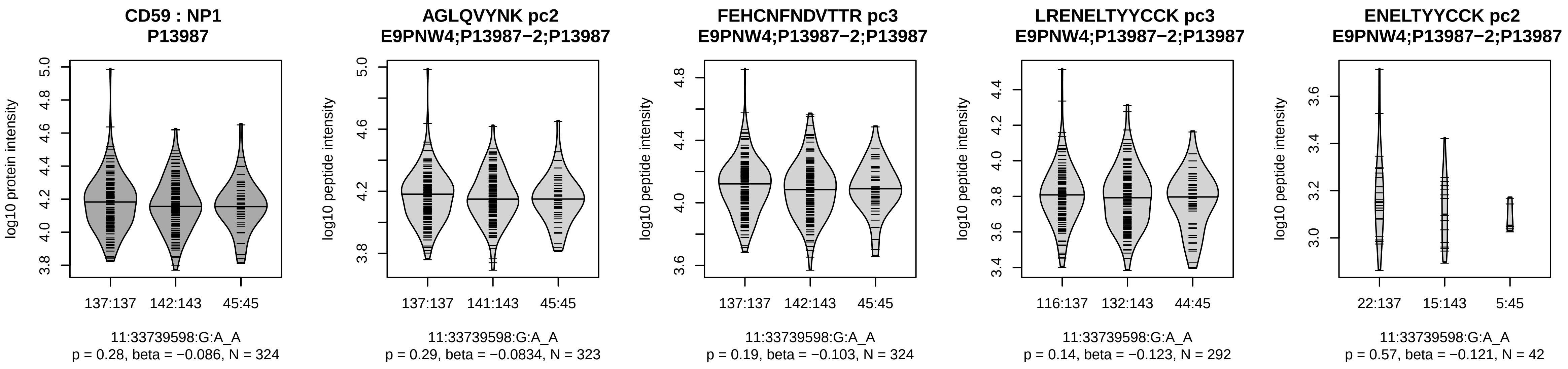

Assay Target: CD59
UniProt: P13987
rsID: rs831636
Target SNP: 11:33739598:G:A
Proxy SNP: 11:33739598:G:A
UKBPPP log10(p): 305.8
UKBPPP BETA: −0.229
UKBPPP MSPA: 0.551
::–:–:–:–
1251:1254:908:589:483:60

187. SDC4 (P31431)
UKBPPP MSPA=0.502

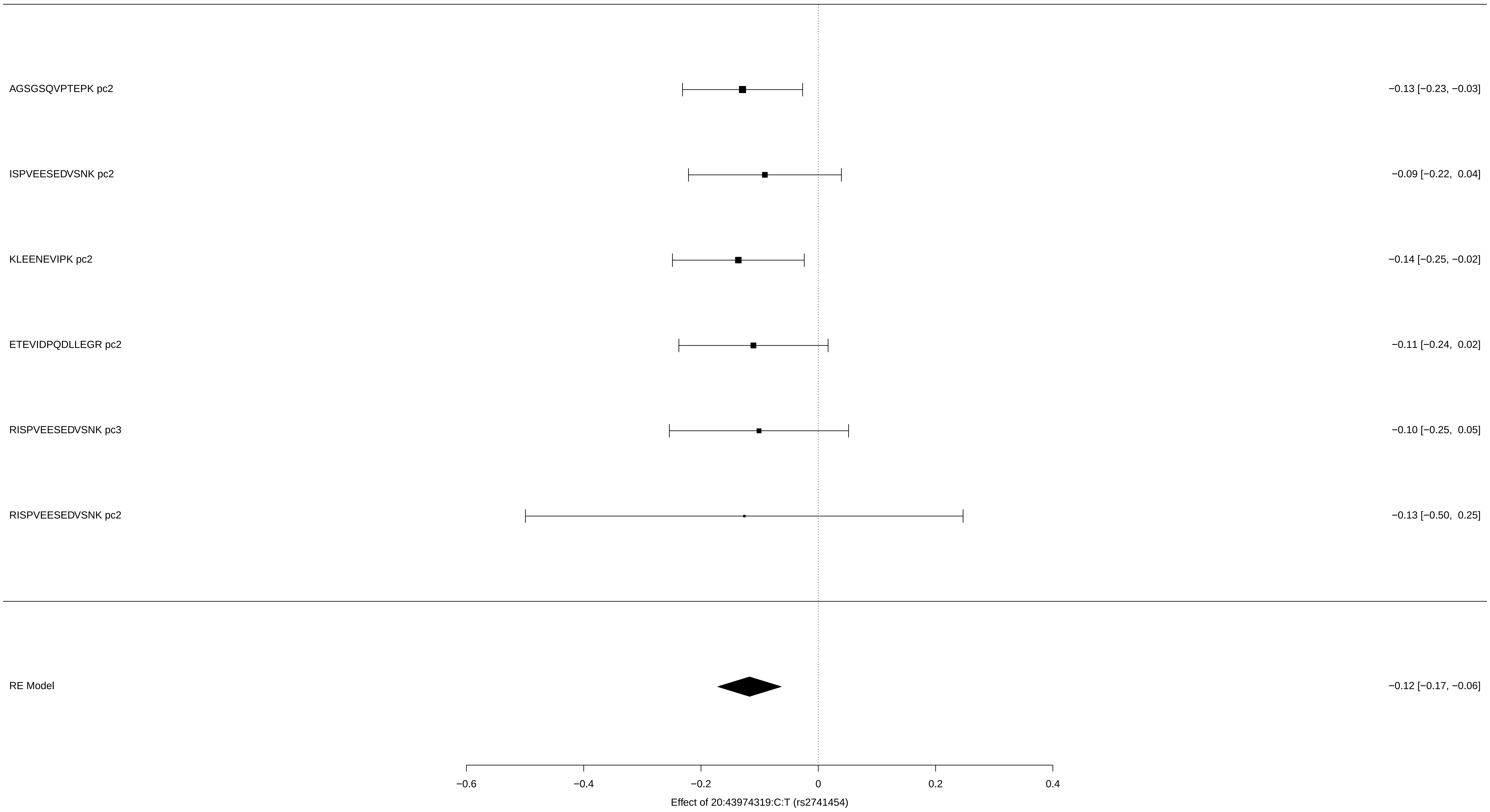

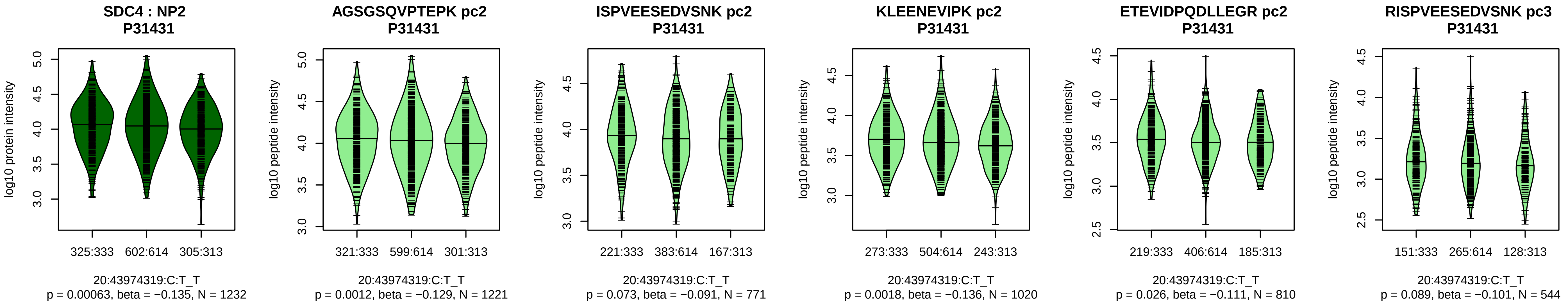

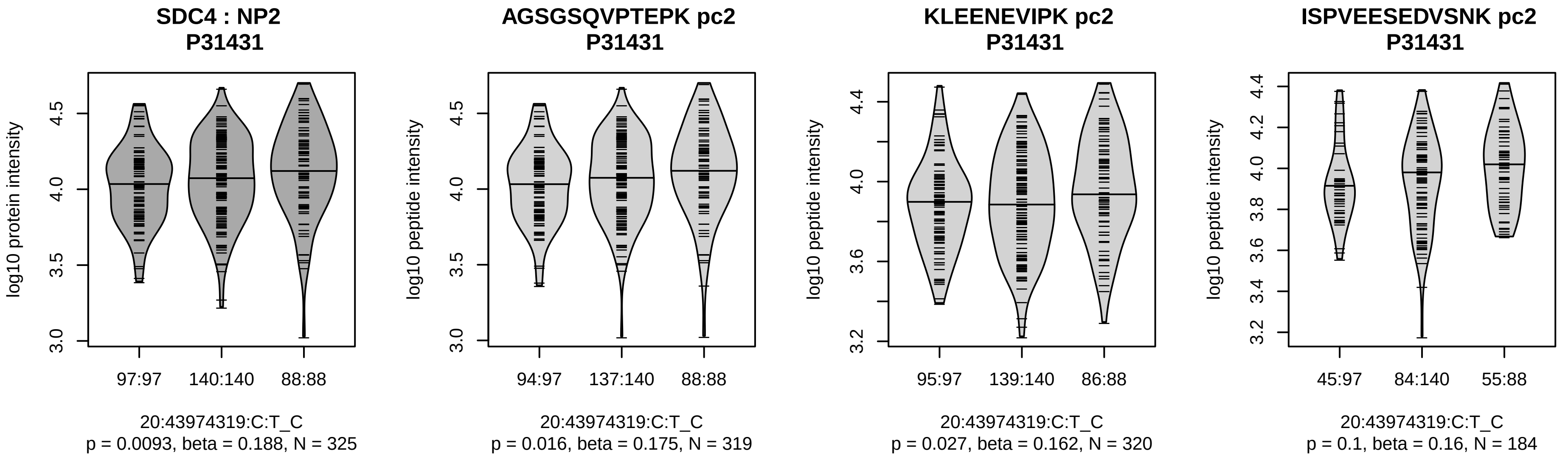

Assay Target: SDC4
UniProt: P31431
rsID: rs2741454
Target SNP: 20:43974319:C:T
Proxy SNP: 20:43974319:C:T
UKBPPP log10(p): 302.4
UKBPPP BETA: −0.226
UKBPPP MSPA: 0.502
:–::–:–:–
1221:771:1020:810:544:99

188. C1QTNF1 (Q9BXJ1)
UKBPPP MSPA=0.455

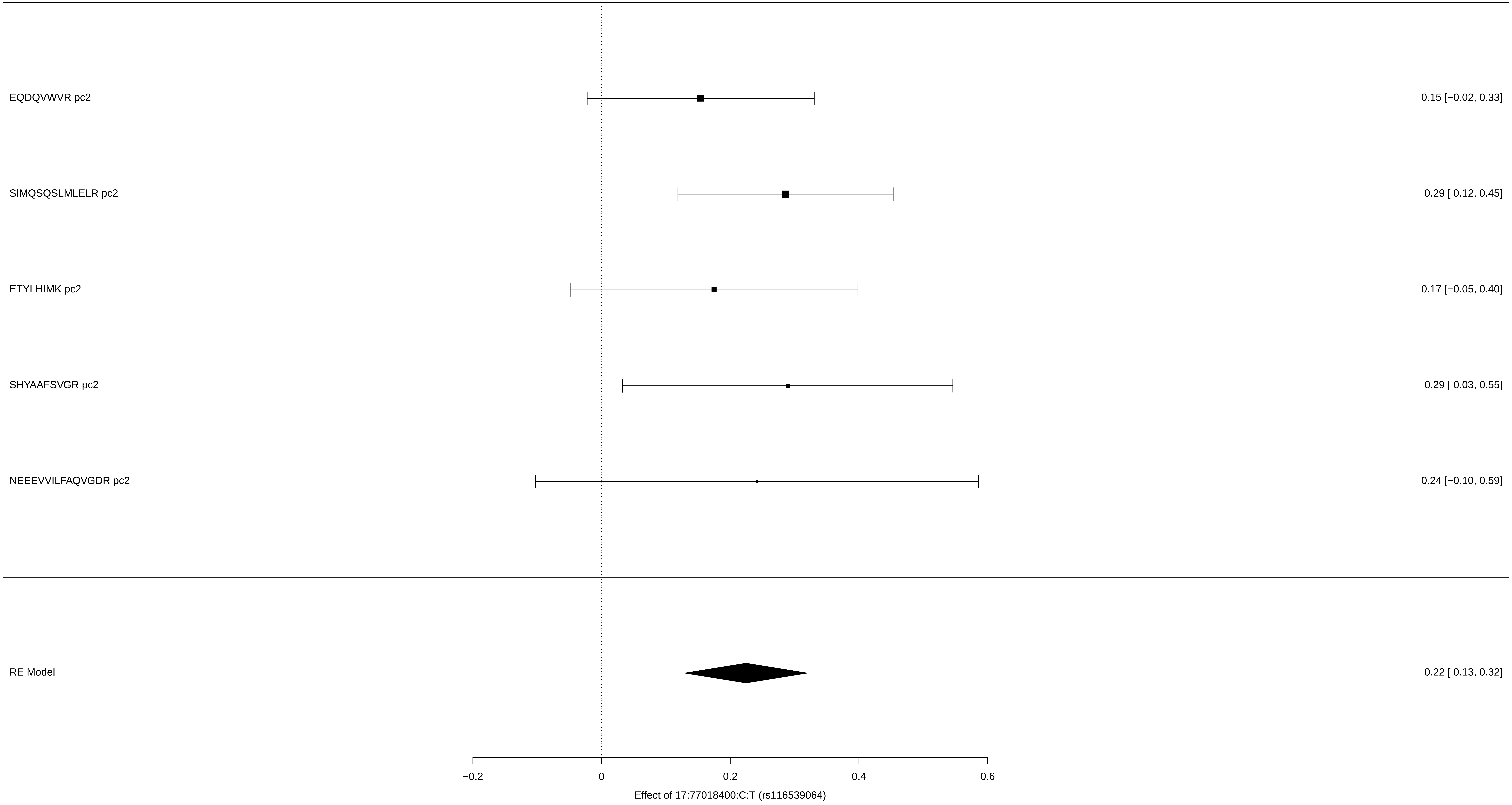

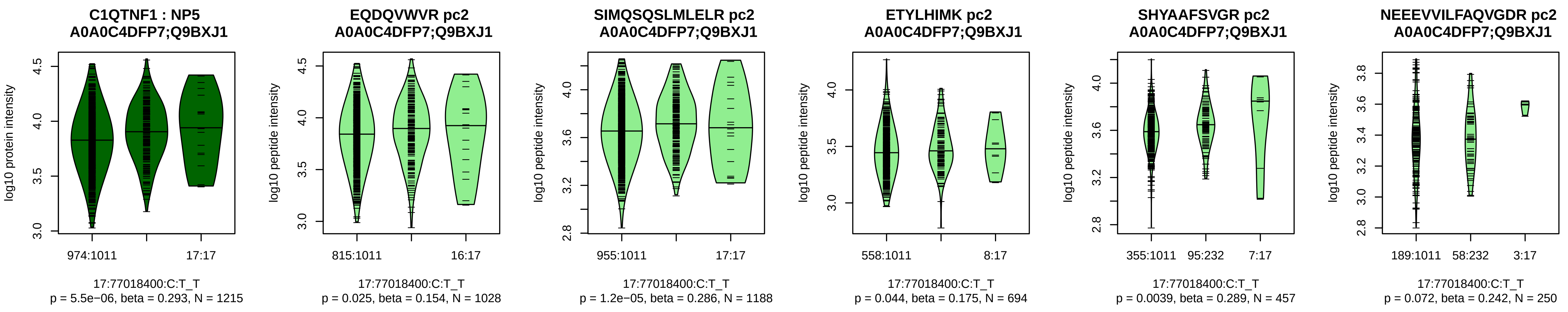

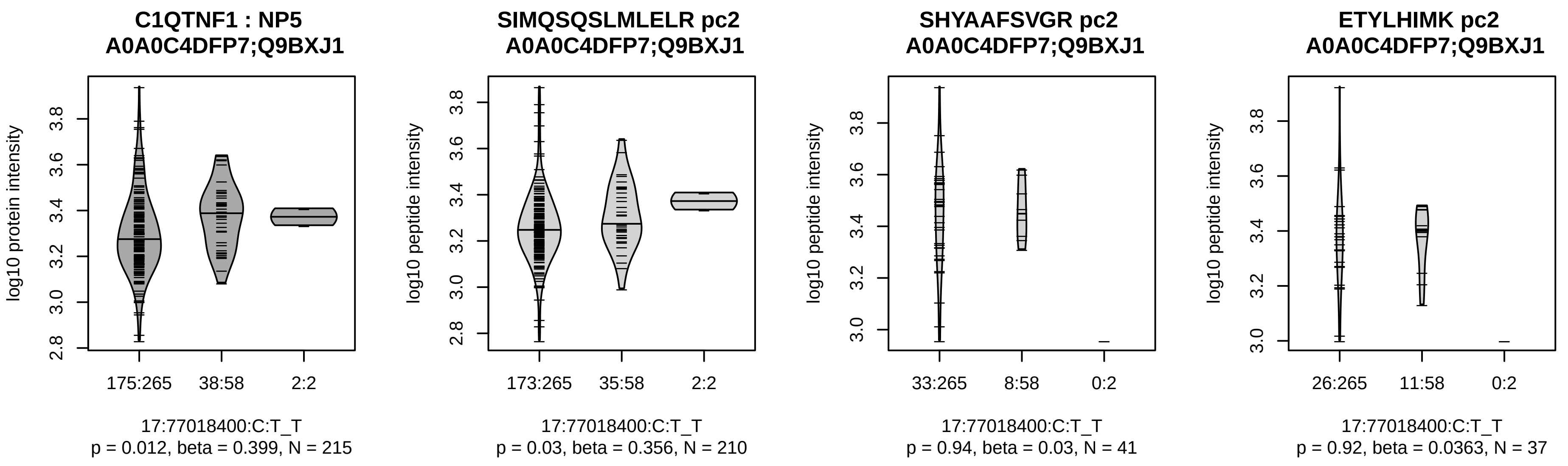

Assay Target: C1QTNF1
UniProt: Q9BXJ1
rsID: rs116539064
Target SNP: 17:77018400:C:T
Proxy SNP: 17:77018400:C:T
UKBPPP log10(p): 297.8
UKBPPP BETA: 0.387
UKBPPP MSPA: 0.455
−:*:−:*:−:NA
1028:1188:694:457:250:4

189. THBS4 (P35443)
UKBPPP MSPA=0.026

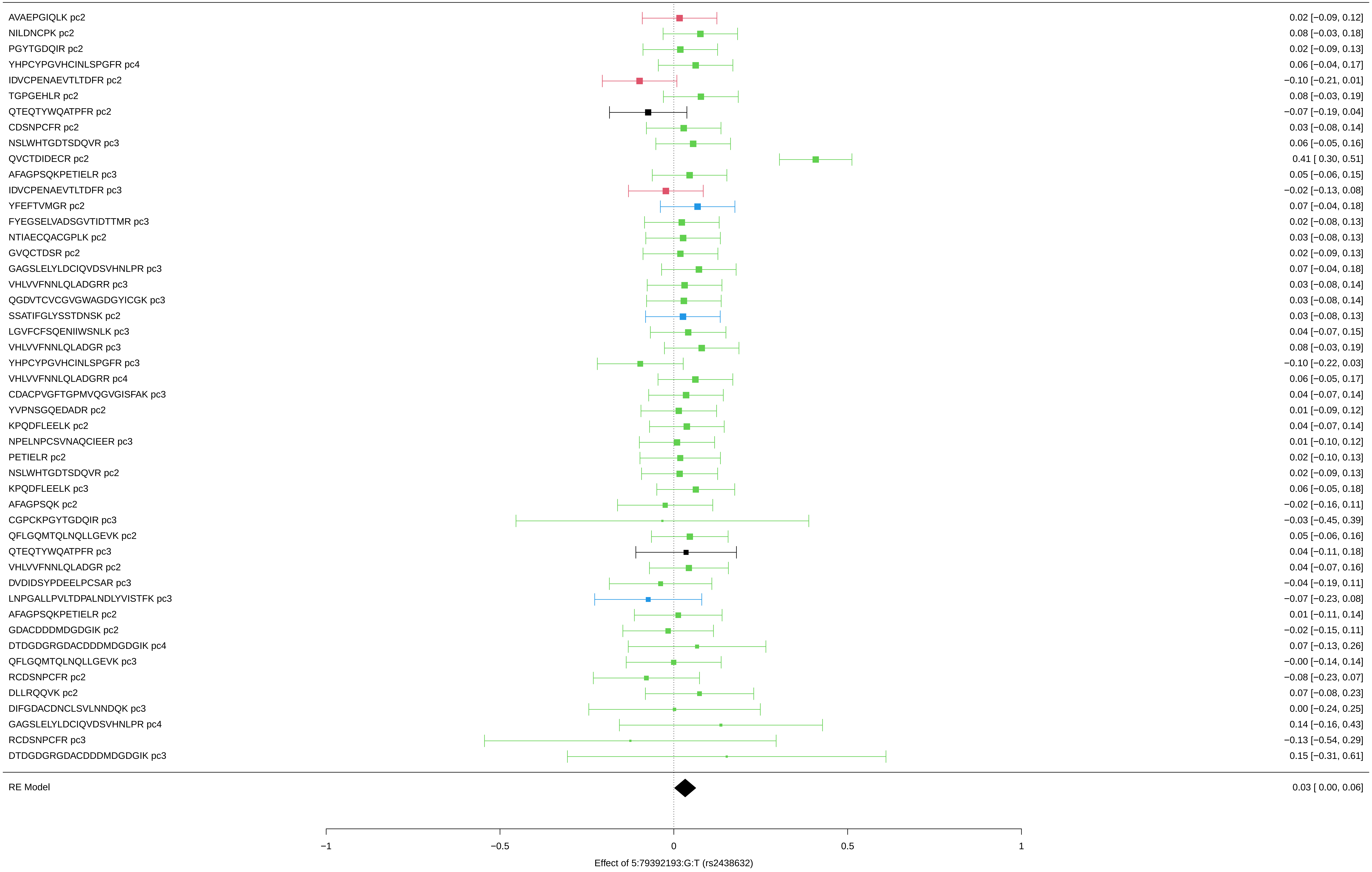

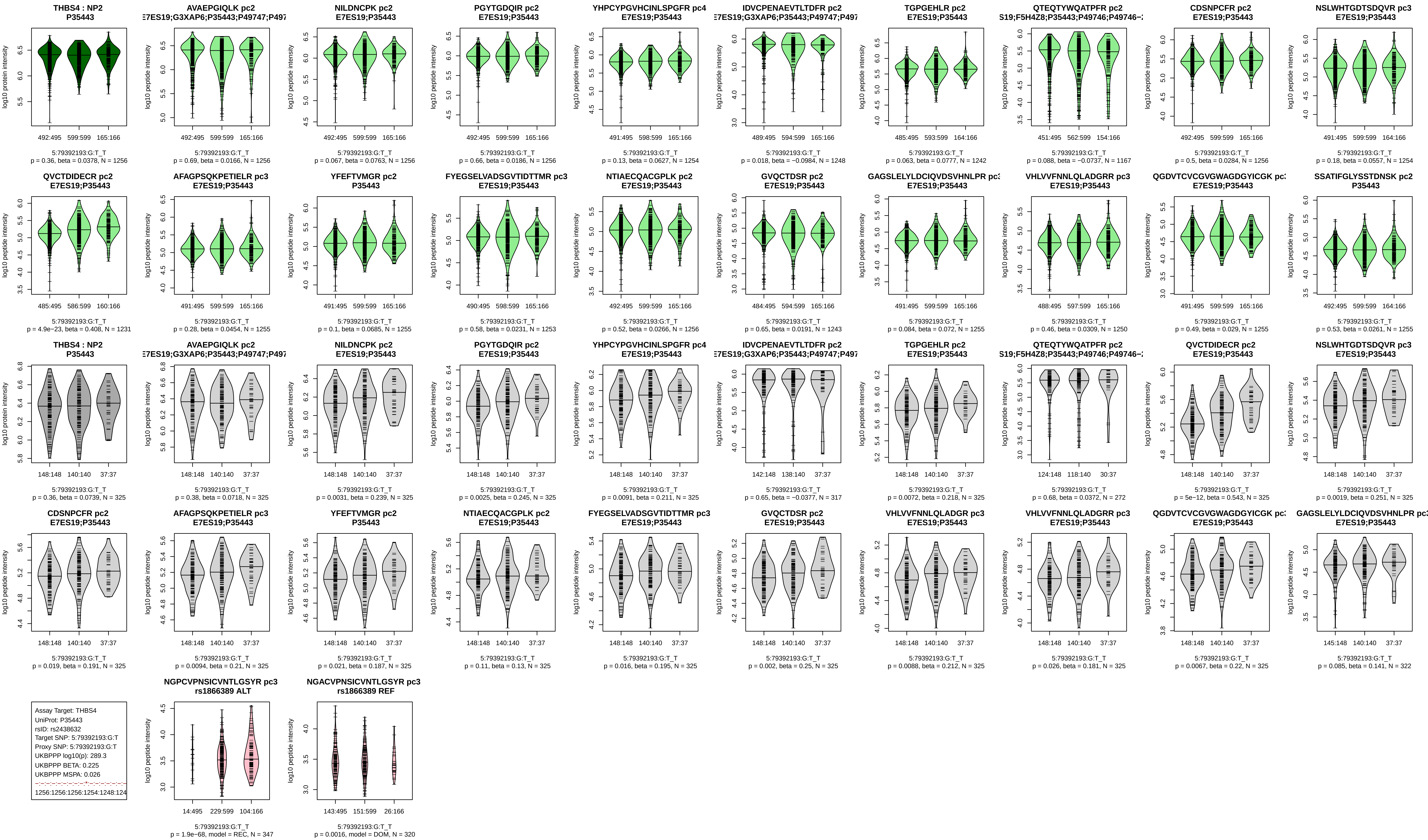

THBS4 : NP2
P35443
log10 protein intensity
492:495 599:599 165:166
5:79392193:G:T_T
p = 0.36, beta = 0.0378, N = 1256
AVAEPGIQLK pc2
E7ES19;G3XAP6;P35443;P49747;P497
log10 peptide intensity
492:495 599:599 165:166
5:79392193:G:T_T
p = 0.69, beta = 0.0166, N = 1256
NILDNCPK pc2
E7ES19;P35443
492:495 599:599 165:166
5:79392193:G:T_T
p = 0.067, beta = 0.0763, N = 1256
PGYTGDQIR pc2
E7ES19;P35443
492:495 599:599 165:166
5:79392193:G:T_T
p = 0.66, beta = 0.0186, N = 1256
YHPCYPGVHCINLSPGFR pc4
E7ES19;P35443
491:495 598:599 165:166
5:79392193:G:T_T
p = 0.13, beta = 0.0627, N = 1254
IDVCPENAEVTLTDFR pc2
E7ES19;G3XAP6;P35443;P49747;P497
489:495 594:599 165:166
5:79392193:G:T_T
p = 0.018, beta = −0.0984, N = 1248
TGPGEHLR pc2
E7ES19;P35443
485:495 593:599 164:166
5:79392193:G:T_T
p = 0.063, beta = 0.0777, N = 1242
QTEQTYWQATPFR pc2
S19;F5H4Z8;P35443;P49746;P49746−2
451:495 562:599 154:166
5:79392193:G:T_T
p = 0.088, beta = −0.0737, N = 1167
CDSNPCFR pc2
E7ES19;P35443
492:495 599:599 165:166
5:79392193:G:T_T
p = 0.5, beta = 0.0284, N = 1256
NSLWHTGDTSDQVR pc3
E7ES19;P35443
491:495 599:599 164:166
5:79392193:G:T_T
p = 0.18, beta = 0.0557, N = 1254
QVCTDIDECR pc2
E7ES19;P35443
485:495 586:599 160:166
5:79392193:G:T_T
p = 4.9e−23, beta = 0.408, N = 1231
AFAGPSQKPETIELR pc3
E7ES19;P35443
491:495 599:599 165:166
5:79392193:G:T_T
p = 0.28, beta = 0.0454, N = 1255
YFEFTVMGR pc2
P35443
491:495 599:599 165:166
5:79392193:G:T_T
p = 0.1, beta = 0.0685, N = 1255
FYEGSELVADSGVTIDTTMR pc3
E7ES19;P35443
490:495 598:599 165:166
5:79392193:G:T_T
p = 0.58, beta = 0.0231, N = 1253
NTIAECQACGPLK pc2
E7ES19;P35443
492:495 599:599 165:166
5:79392193:G:T_T
p = 0.52, beta = 0.0266, N = 1256
GVQCTDSR pc2
E7ES19;P35443
484:495 594:599 165:166
5:79392193:G:T_T
p = 0.65, beta = 0.0191, N = 1243
GAGSLELYLDCIQVDSVHNLPR pc3
E7ES19;P35443
491:495 599:599 165:166
5:79392193:G:T_T
p = 0.084, beta = 0.072, N = 1255
VHLVVFNNLQLADGRR pc3
E7ES19;P35443
488:495 597:599 165:166
5:79392193:G:T_T
p = 0.46, beta = 0.0309, N = 1250
QGDVTCVCGVGWAGDGYICGK pc3
E7ES19;P35443
491:495 599:599 165:166
5:79392193:G:T_T
p = 0.49, beta = 0.029, N = 1255
SSATIFGLYSSTDNSK pc2
P35443
492:495 599:599 164:166
5:79392193:G:T_T
p = 0.53, beta = 0.0261, N = 1255
THBS4 : NP2
P35443
148:148 140:140 37:37
5:79392193:G:T_T
p = 0.36, beta = 0.0739, N = 325
AVAEPGIQLK pc2
E7ES19;G3XAP6;P35443;P49747;P497
148:148 140:140 37:37
5:79392193:G:T_T
p = 0.38, beta = 0.0718, N = 325
NILDNCPK pc2
E7ES19;P35443
148:148 140:140 37:37
5:79392193:G:T_T
p = 0.0031, beta = 0.239, N = 325
PGYTGDQIR pc2
E7ES19;P35443
148:148 140:140 37:37
5:79392193:G:T_T
p = 0.0025, beta = 0.245, N = 325
YHPCYPGVHCINLSPGFR pc4
E7ES19;P35443
148:148 140:140 37:37
5:79392193:G:T_T
p = 0.0091, beta = 0.211, N = 325
IDVCPENAEVTLTDFR pc2
E7ES19;G3XAP6;P35443;P49747;P497
142:148 138:140 37:37
5:79392193:G:T_T
p = 0.65, beta = −0.0377, N = 317
TGPGEHLR pc2
E7ES19;P35443
148:148 140:140 37:37
5:79392193:G:T_T
p = 0.0072, beta = 0.218, N = 325
QTEQTYWQATPFR pc2
S19;F5H4Z8;P35443;P49746;P49746−2
124:148 118:140 30:37
5:79392193:G:T_T
p = 0.68, beta = 0.0372, N = 272
QVCTDIDECR pc2
E7ES19;P35443
148:148 140:140 37:37
5:79392193:G:T_T
p = 5e−12, beta = 0.543, N = 325
NSLWHTGDTSDQVR pc3
E7ES19;P35443
148:148 140:140 37:37
5:79392193:G:T_T
p = 0.0019, beta = 0.251, N = 325
CDSNPCFR pc2
E7ES19;P35443
148:148 140:140 37:37
5:79392193:G:T_T
p = 0.019, beta = 0.191, N = 325
AFAGPSQKPETIELR pc3
E7ES19;P35443
148:148 140:140 37:37
5:79392193:G:T_T
p = 0.0094, beta = 0.21, N = 325
YFEFTVMGR pc2
P35443
148:148 140:140 37:37
5:79392193:G:T_T
p = 0.021, beta = 0.187, N = 325
NTIAECQACGPLK pc2
E7ES19;P35443
148:148 140:140 37:37
5:79392193:G:T_T
p = 0.11, beta = 0.13, N = 325
FYEGSELVADSGVTIDTTMR pc3
E7ES19;P35443
148:148 140:140 37:37
5:79392193:G:T_T
p = 0.016, beta = 0.195, N = 325
GVQCTDSR pc2
E7ES19;P35443
148:148 140:140 37:37
5:79392193:G:T_T
p = 0.002, beta = 0.25, N = 325
VHLVVFNNLQLADGR pc3
E7ES19;P35443
148:148 140:140 37:37
5:79392193:G:T_T
p = 0.0088, beta = 0.212, N = 325
VHLVVFNNLQLADGRR pc3
E7ES19;P35443
148:148 140:140 37:37
5:79392193:G:T_T
p = 0.026, beta = 0.181, N = 325
QGDVTCVCGVGWAGDGYICGK pc3
E7ES19;P35443
148:148 140:140 37:37
5:79392193:G:T_T
p = 0.0067, beta = 0.22, N = 325
GAGSLELYLDCIQVDSVHNLPR pc3
E7ES19;P35443
145:148 140:140 37:37
5:79392193:G:T_T
p = 0.085, beta = 0.141, N = 322
Assay Target: THBS4
UniProt: P35443
rsID: rs2438632
Target SNP: 5:79392193:G:T
Proxy SNP: 5:79392193:G:T
UKBPPP log10(p): 289.3
UKBPPP BETA: 0.225
UKBPPP MSPA: 0.026
1256:1256:1256:1254:1248:124
NGPCVPNSICVNTLGSYR pc3
rs1866389 ALT
14:495 229:599 104:166
5:79392193:G:T_T
p = 1.9e−68, model = REC, N = 347
NGACVPNSICVNTLGSYR pc3
rs1866389 REF
143:495 151:599 26:166
5:79392193:G:T_T
p = 0.0016, model = DOM, N = 320

190. CPE (P16870)
UKBPPP MSPA=0.873

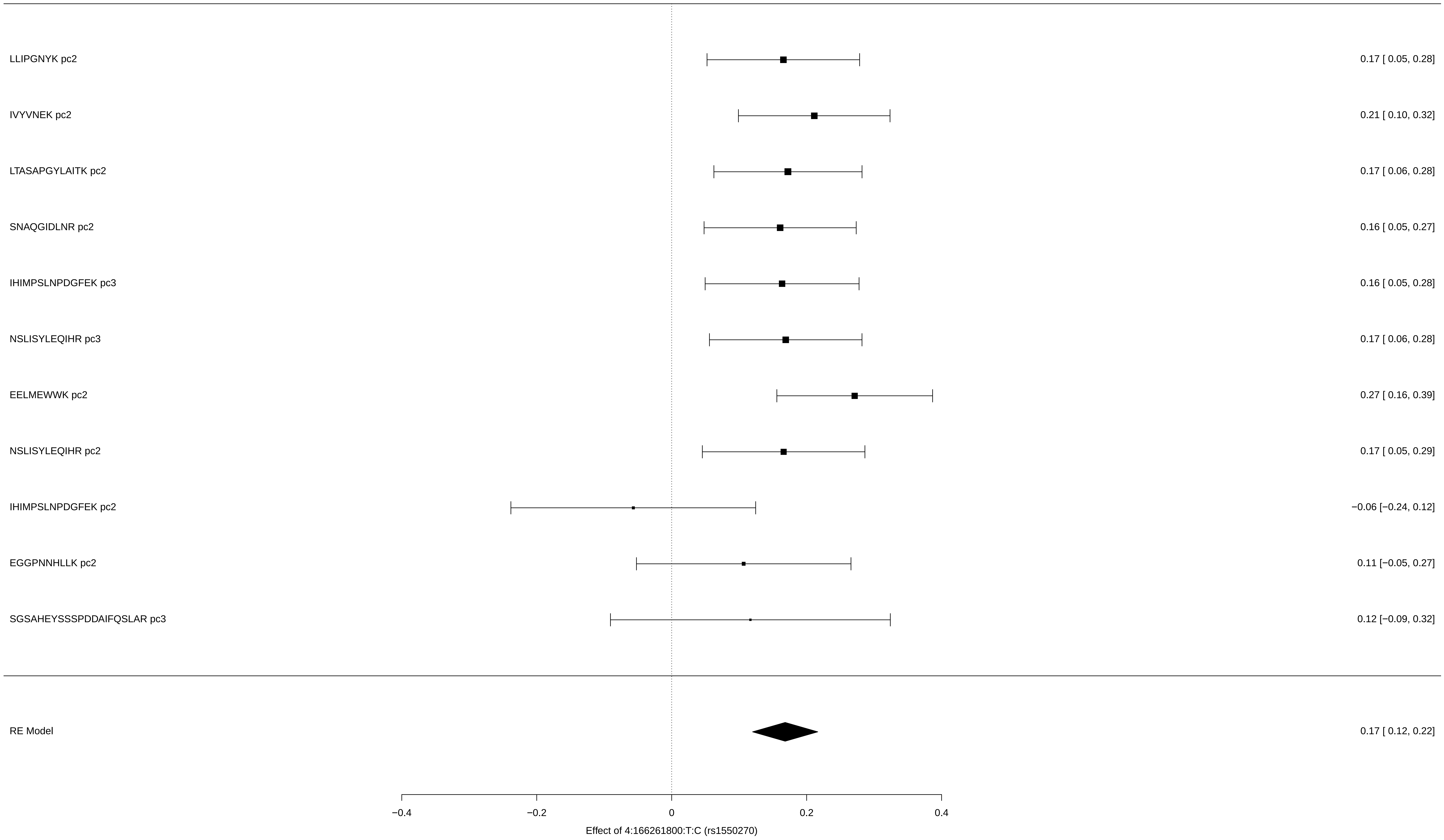

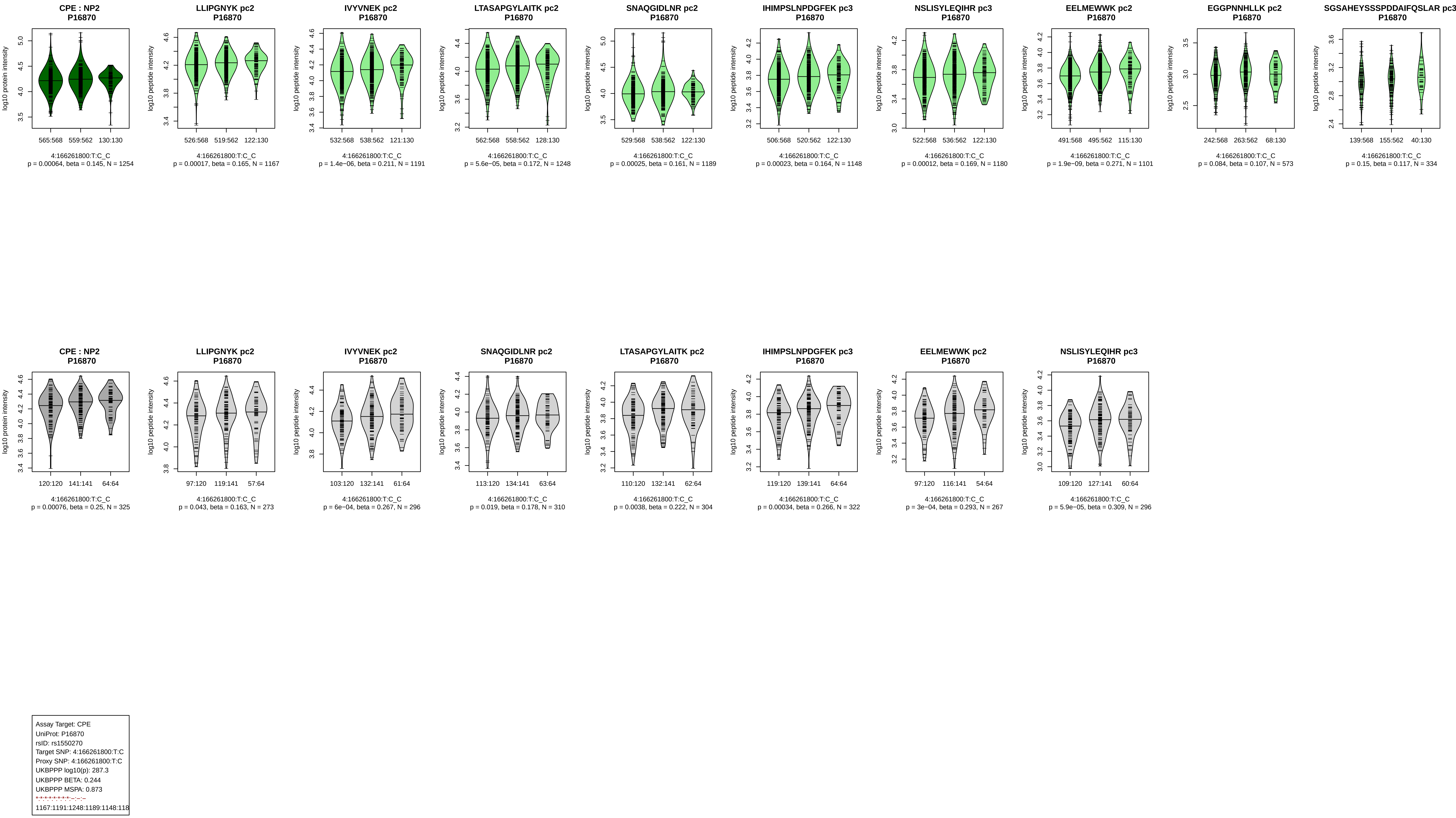
CPE : NP2
P16870
565:568 559:562 130:130
4:166261800:T:C_C
p = 0.00064, beta = 0.145, N = 1254
LLIPGNYK pc2
P16870
526:568 519:562 122:130
4:166261800:T:C_C
p = 0.00017, beta = 0.165, N = 1167
IVYVNEK pc2
P16870
532:568 538:562 121:130
4:166261800:T:C_C
p = 1.4e−06, beta = 0.211, N = 1191
LTASAPGYLAITK pc2
P16870
562:568 558:562 128:130
4:166261800:T:C_C
p = 5.6e−05, beta = 0.172, N = 1248
SNAQGIDLNR pc2
P16870
529:568 538:562 122:130
4:166261800:T:C_C
p = 0.00025, beta = 0.161, N = 1189
IHIMPSLNPDGFEK pc3
P16870
506:568 520:562 122:130
4:166261800:T:C_C
p = 0.00023, beta = 0.164, N = 1148
NSLISYLEQIHR pc3
P16870
522:568 536:562 122:130
4:166261800:T:C_C
p = 0.00012, beta = 0.169, N = 1180
EELMEWWK pc2
P16870
491:568 495:562 115:130
4:166261800:T:C_C
p = 1.9e−09, beta = 0.271, N = 1101
EGGPNNHLLK pc2
P16870
242:568 263:562 68:130
4:166261800:T:C_C
p = 0.084, beta = 0.107, N = 573
SGSAHEYSSSPDDAIFQSLAR pc3
P16870
139:568 155:562 40:130
4:166261800:T:C_C
p = 0.15, beta = 0.117, N = 334
log10 protein intensity
log10 peptide intensity
CPE : NP2
P16870
120:120 141:141 64:64
4:166261800:T:C_C
p = 0.00076, beta = 0.25, N = 325
LLIPGNYK pc2
P16870
97:120 119:141 57:64
4:166261800:T:C_C
p = 0.043, beta = 0.163, N = 273
IVYVNEK pc2
P16870
103:120 132:141 61:64
4:166261800:T:C_C
p = 6e−04, beta = 0.267, N = 296
SNAQGIDLNR pc2
P16870
113:120 134:141 63:64
4:166261800:T:C_C
p = 0.019, beta = 0.178, N = 310
LTASAPGYLAITK pc2
P16870
110:120 132:141 62:64
4:166261800:T:C_C
p = 0.0038, beta = 0.222, N = 304
IHIMPSLNPDGFEK pc3
P16870
119:120 139:141 64:64
4:166261800:T:C_C
p = 0.00034, beta = 0.266, N = 322
EELMEWWK pc2
P16870
97:120 116:141 54:64
4:166261800:T:C_C
p = 3e−04, beta = 0.293, N = 267
NSLISYLEQIHR pc3
P16870
109:120 127:141 60:64
4:166261800:T:C_C
p = 5.9e−05, beta = 0.309, N = 296
Assay Target: CPE
UniProt: P16870
rsID: rs1550270
Target SNP: 4:166261800:T:C
Proxy SNP: 4:166261800:T:C
UKBPPP log10(p): 287.3
UKBPPP BETA: 0.244
UKBPPP MSPA: 0.873
1167:1191:1248:1189:1148:118

191. DSG2 (Q14126)
UKBPPP MSPA=0.271

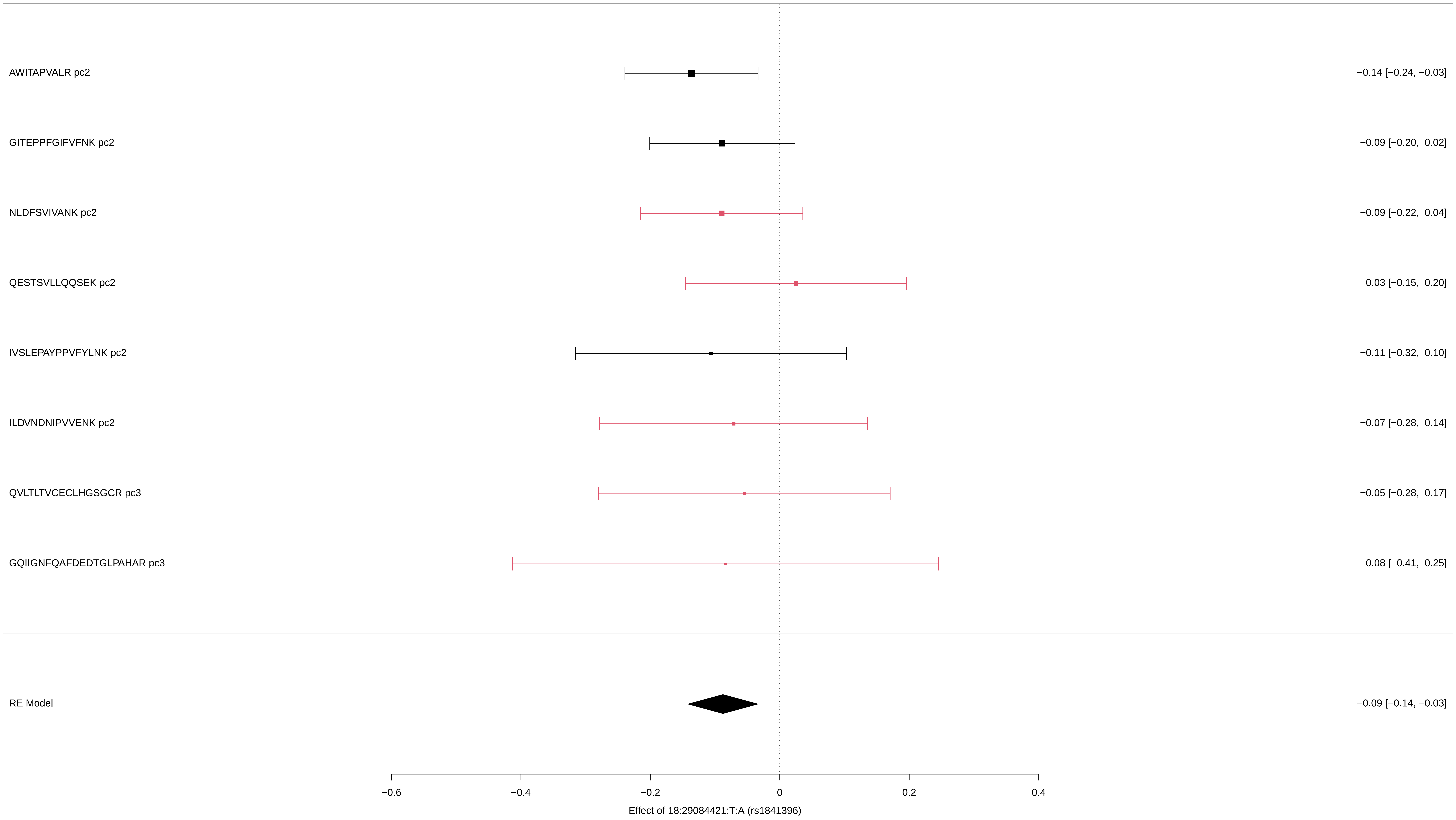

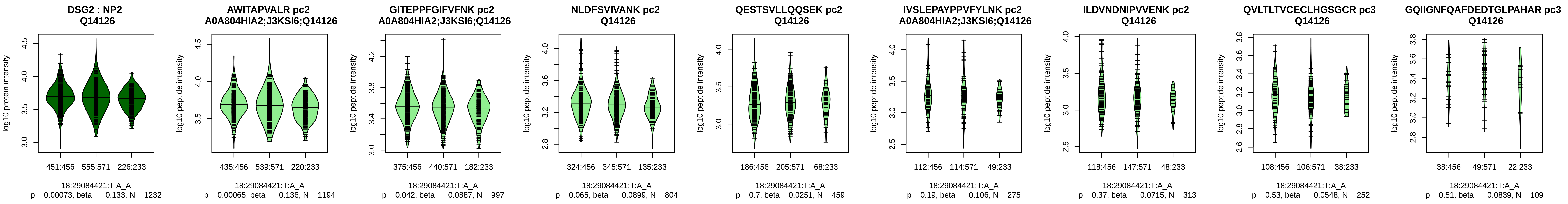

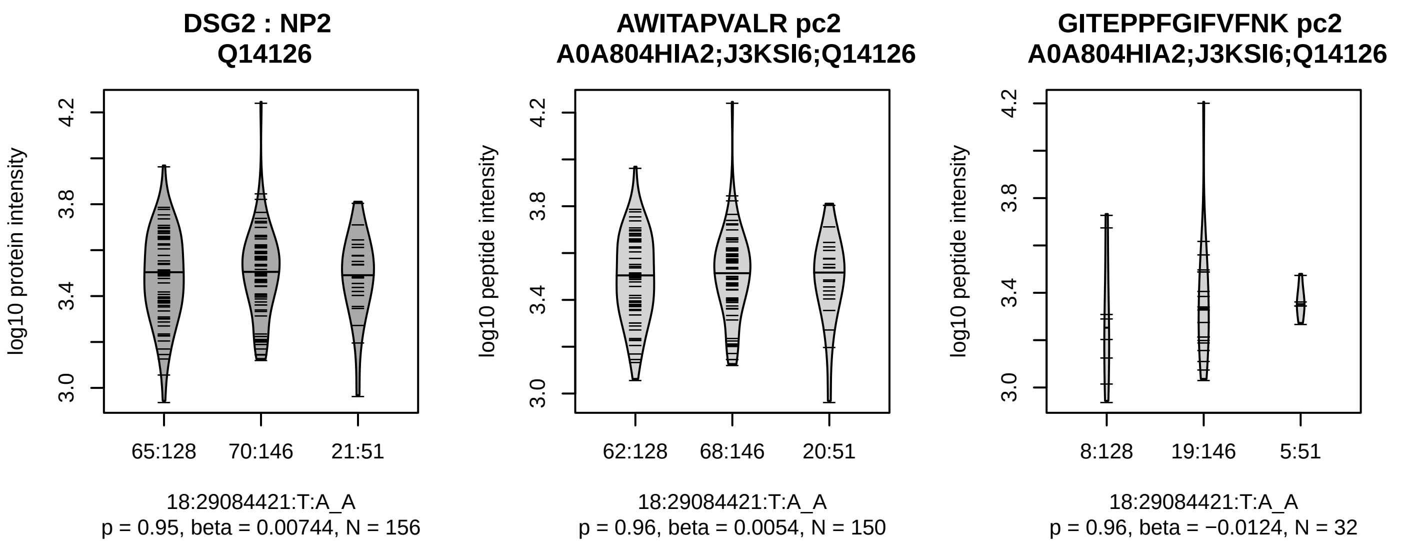

Assay Target: DSG2
UniProt: Q14126
rsID: rs1841396
Target SNP: 18:29084421:T:A
Proxy SNP: 18:29084421:T:A
UKBPPP log10(p): 274.8
UKBPPP BETA: −0.213
UKBPPP MSPA: 0.271
*:–:–:–:–:–:–:–
1194:997:804:459:275:313:252:

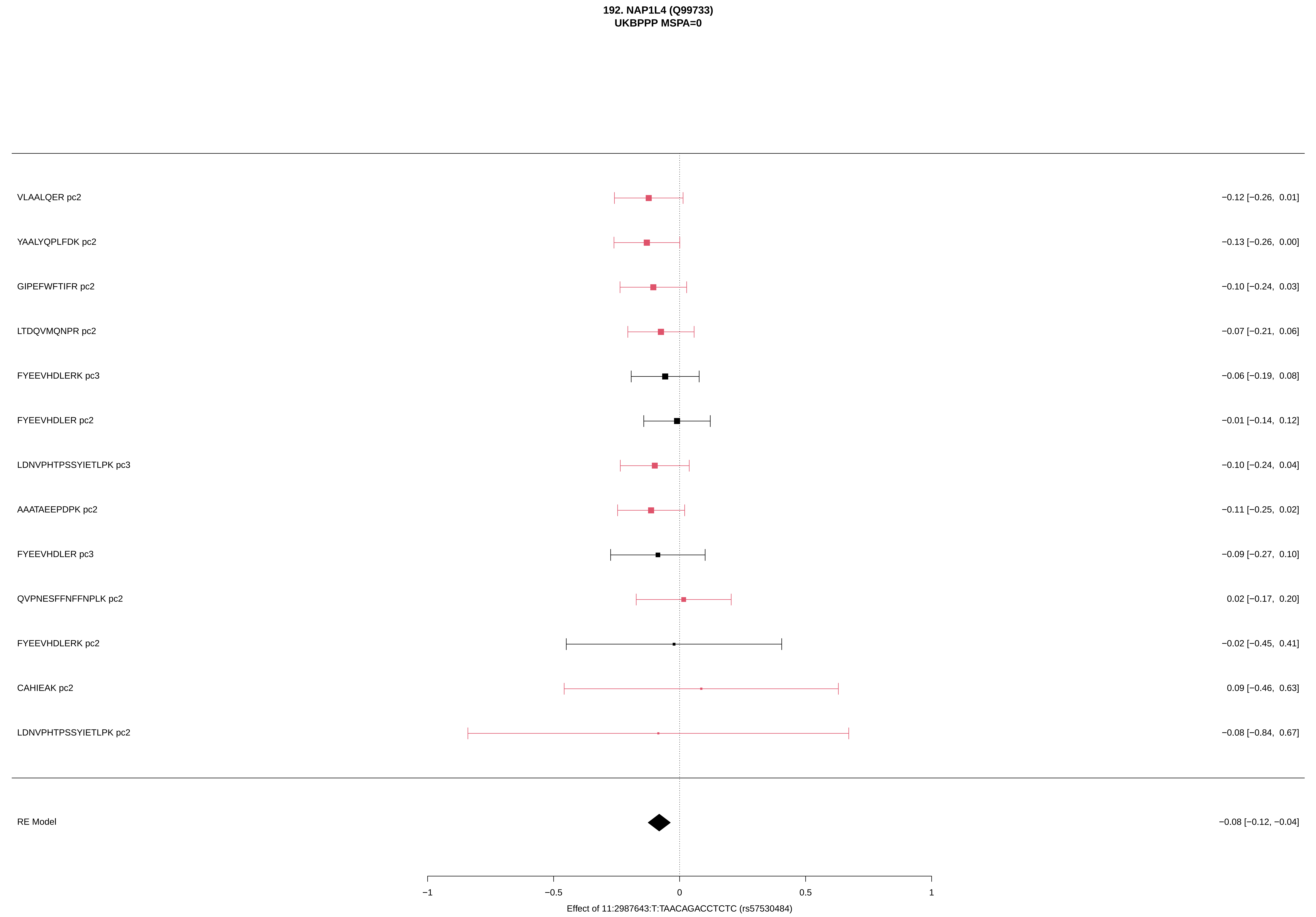
192. NAP1L4 (Q99733)
UKBPPP MSPA=0
VLAALQER pc2
YAALYQPLFDK pc2
GIPEFWFTIFR pc2
LTDQVMQNPR pc2
FYEEVHDLERK pc3
FYEEVHDLER pc2
LDNVPHTPSSYIETLPK pc3
AAATAEEPDPK pc2
FYEEVHDLER pc3
QVPNESFFNFFNPLK pc2
FYEEVHDLERK pc2
CAHIEAK pc2
LDNVPHTPSSYIETLPK pc2
RE Model
−0.12 [−0.26, 0.01]
−0.13 [−0.26, 0.00]
−0.10 [−0.24, 0.03]
−0.07 [−0.21, 0.06]
−0.06 [−0.19, 0.08]
−0.01 [−0.14, 0.12]
−0.10 [−0.24, 0.04]
−0.11 [−0.25, 0.02]
−0.09 [−0.27, 0.10]
0.02 [−0.17, 0.20]
−0.02 [−0.45, 0.41]
0.09 [−0.46, 0.63]
−0.08 [−0.84, 0.67]
−0.08 [−0.12, −0.04]
−1
−0.5
0
0.5
1
Effect of 11:2987643:T:TAACAGACCTCTC (rs57530484)

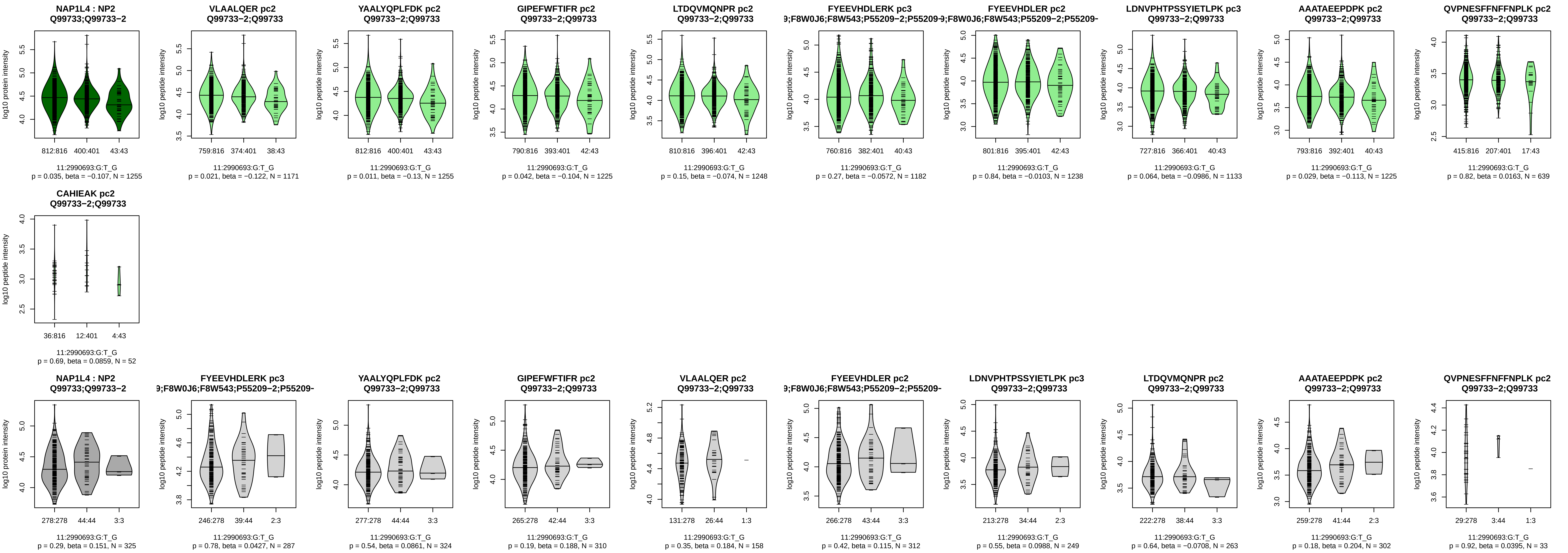

Assay Target: NAP1L4
UniProt: Q99733
rsID: rs57530484
Target SNP: 11:2987643:T:TAAC
Proxy SNP: 11:2990693:G:T
UKBPPP log10(p): 266.3
UKBPPP BETA: 0.251
UKBPPP MSPA: 0
−:−:−:−:−:−:−:−:−:−:−:−:NA
1171:1255:1225:1248:1182:123

193. APBB1IP (Q7Z5R6)
UKBPPP MSPA=0

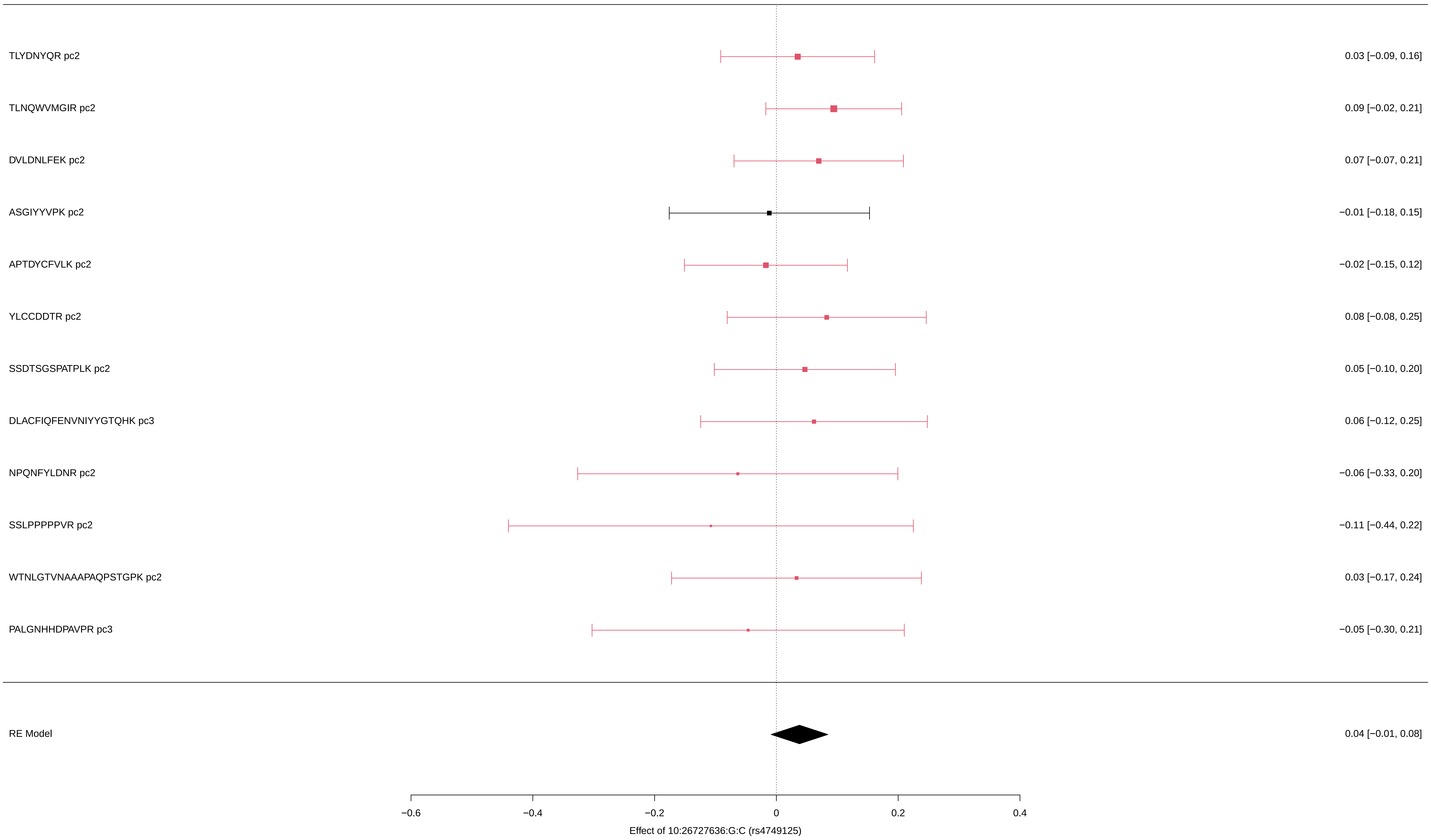

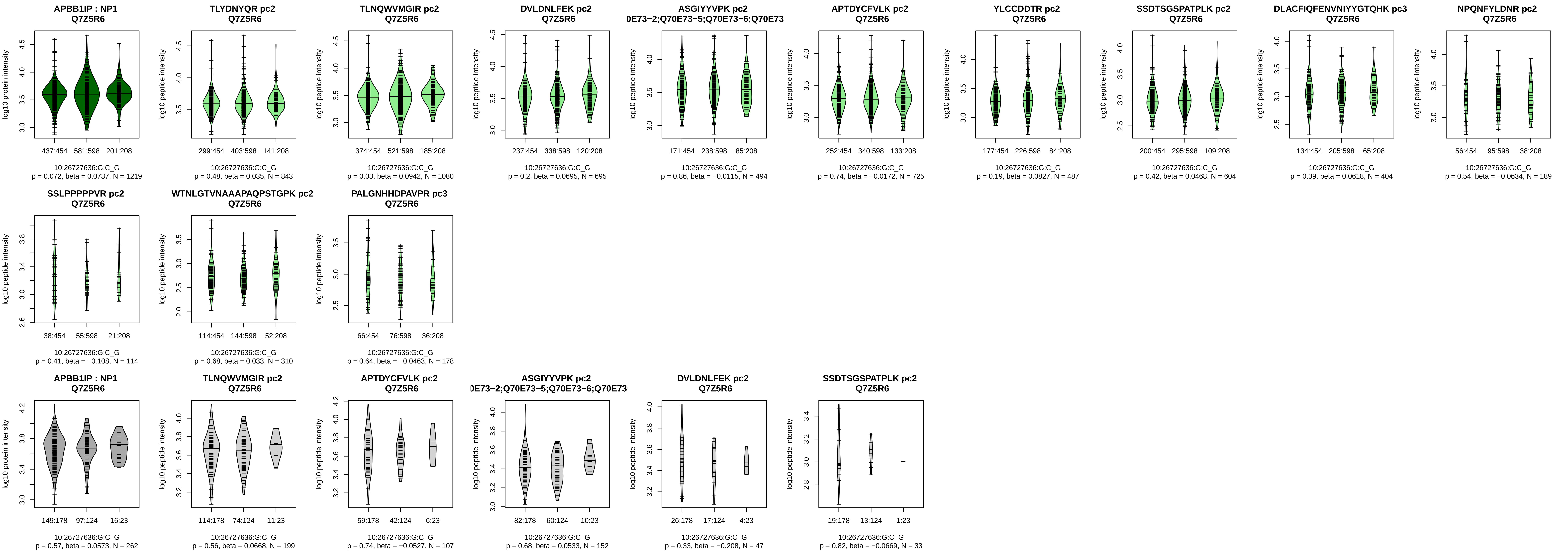

Assay Target: APBB1IP
UniProt: Q7Z5R6
rsID: rs4749125
Target SNP: 10:26727636:G:C
Proxy SNP: 10:26727636:G:C
UKBPPP log10(p): 264.2
UKBPPP BETA: −0.216
UKBPPP MSPA: 0
−:−:−:−:−:−:−:−:−:−:−:−:NA
843:1080:695:494:725:487:604:

194. S100A12 (P80511)
UKBPPP MSPA=0.833

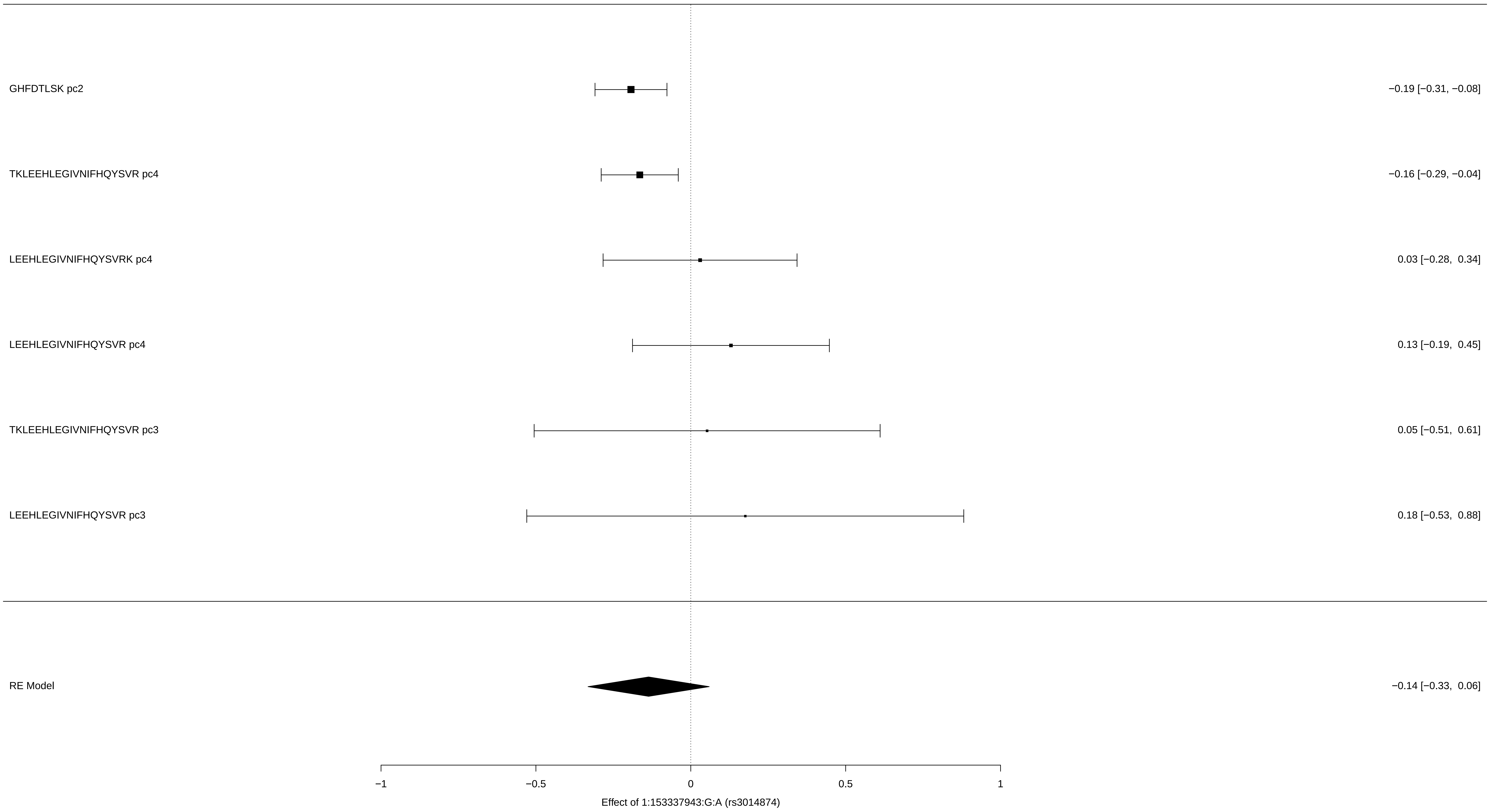

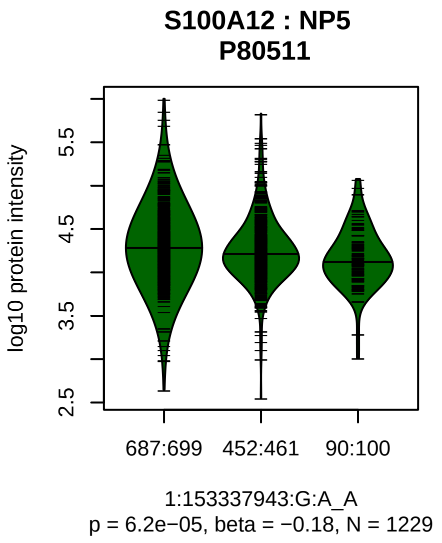

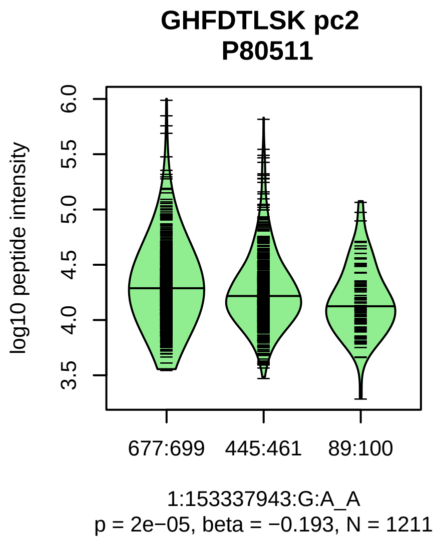

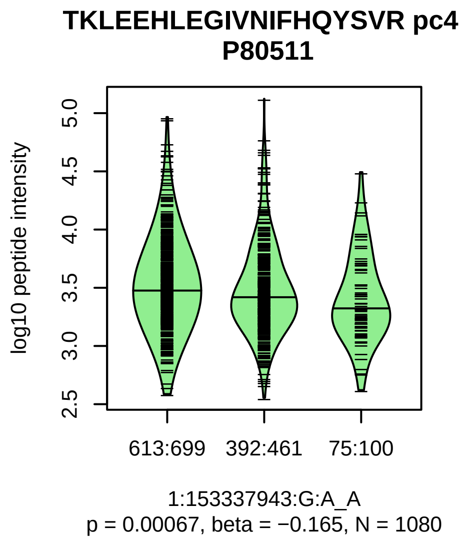

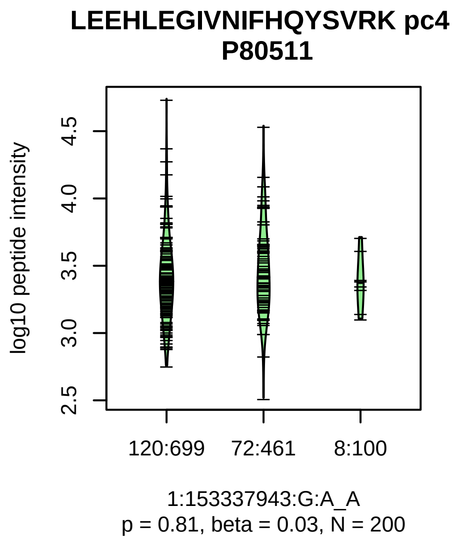

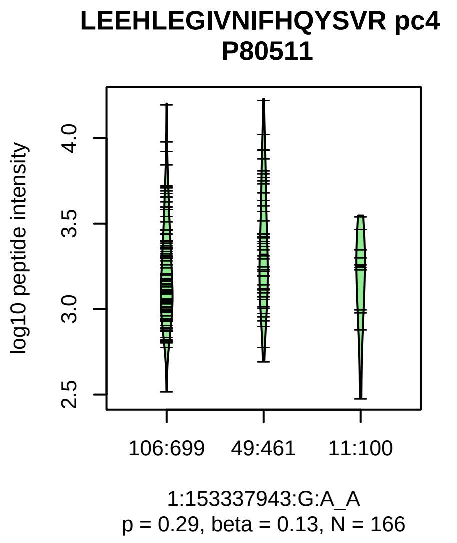

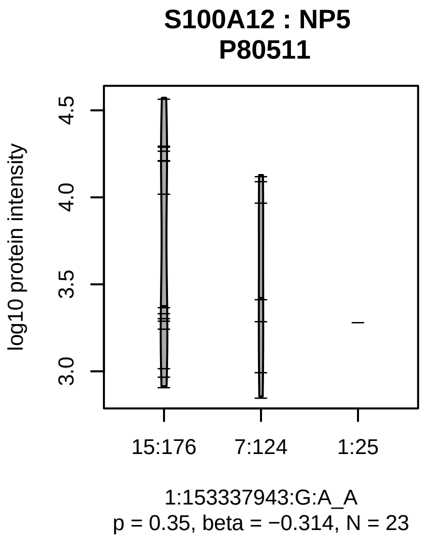

Assay Target: S100A12
UniProt: P80511
rsID: rs3014874
Target SNP: 1:153337943:G:A
Proxy SNP: 1:153337943:G:A
UKBPPP log10(p): 256.4
UKBPPP BETA: −0.235
UKBPPP MSPA: 0.833
::–:–:–:–
1211:1080:200:166:55:39

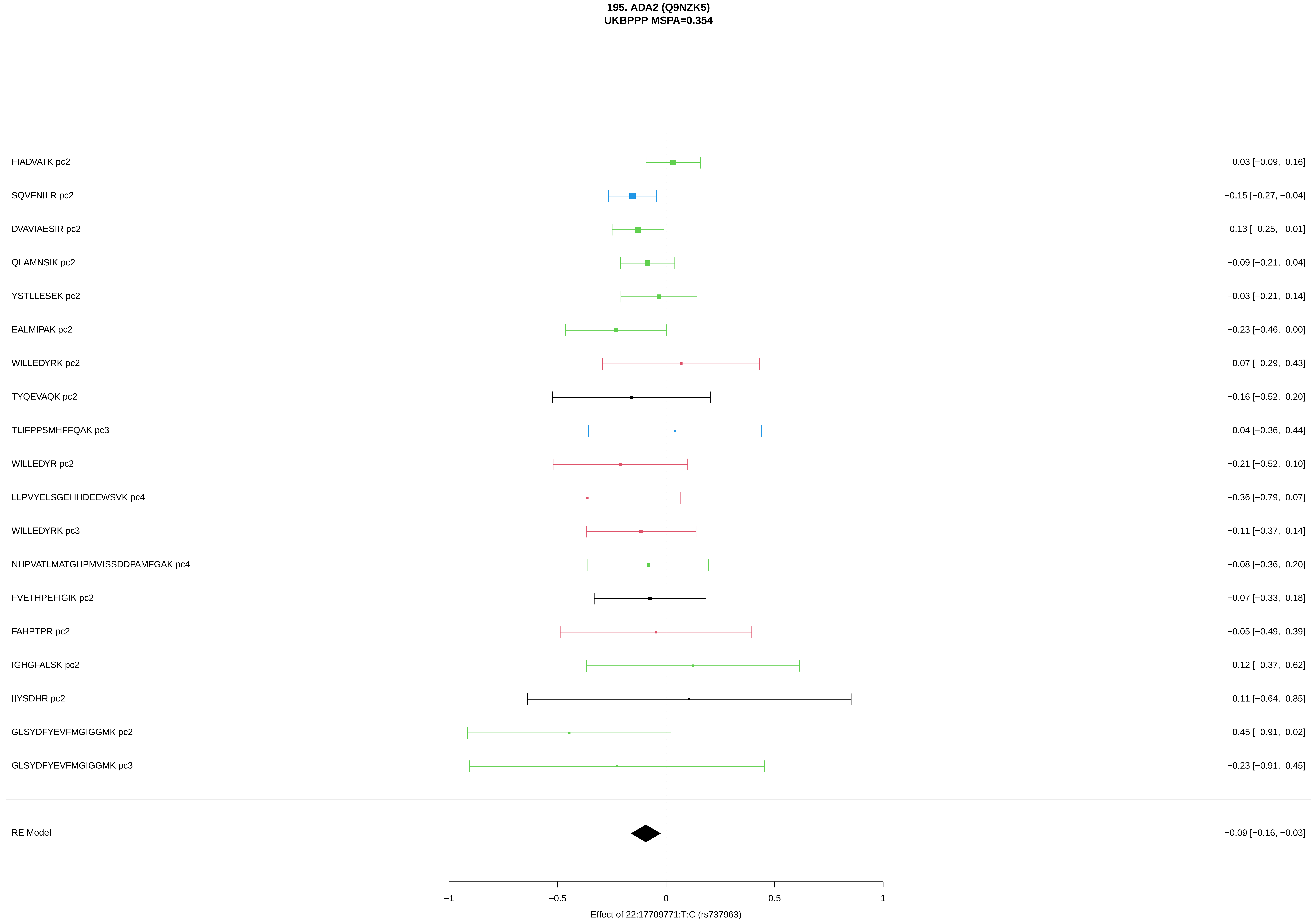
195. ADA2 (Q9NZK5)
UKBPPP MSPA=0.354
FIADVATK pc2
SQVFNILR pc2
DVAVIAESIR pc2
QLAMNSIK pc2
YSTLLESEK pc2
EALMIPAK pc2
WILLEDYRK pc2
TYQEVAQK pc2
TLIFPPSMHFFQAK pc3
WILLEDYR pc2
LLPVYELSGEHHDEEWSVK pc4
WILLEDYRK pc3
NHPVATLMATGHPMVISSDDPAMFGAK pc4
FVETHPEFIGIK pc2
FAHPTPR pc2
IGHGFALSK pc2
IIYSDHR pc2
GLSYDFYEVFMGIGGMK pc2
GLSYDFYEVFMGIGGMK pc3
RE Model
0.03 [−0.09, 0.16]
−0.15 [−0.27, −0.04]
−0.13 [−0.25, −0.01]
−0.09 [−0.21, 0.04]
−0.03 [−0.21, 0.14]
−0.23 [−0.46, 0.00]
0.07 [−0.29, 0.43]
−0.16 [−0.52, 0.20]
0.04 [−0.36, 0.44]
−0.21 [−0.52, 0.10]
−0.36 [−0.79, 0.07]
−0.11 [−0.37, 0.14]
−0.08 [−0.36, 0.20]
−0.07 [−0.33, 0.18]
−0.05 [−0.49, 0.39]
0.12 [−0.37, 0.62]
0.11 [−0.64, 0.85]
−0.45 [−0.91, 0.02]
−0.23 [−0.91, 0.45]
−0.09 [−0.16, −0.03]
−1
−0.5
0
0.5
1
Effect of 22:17709771:T:C (rs737963)

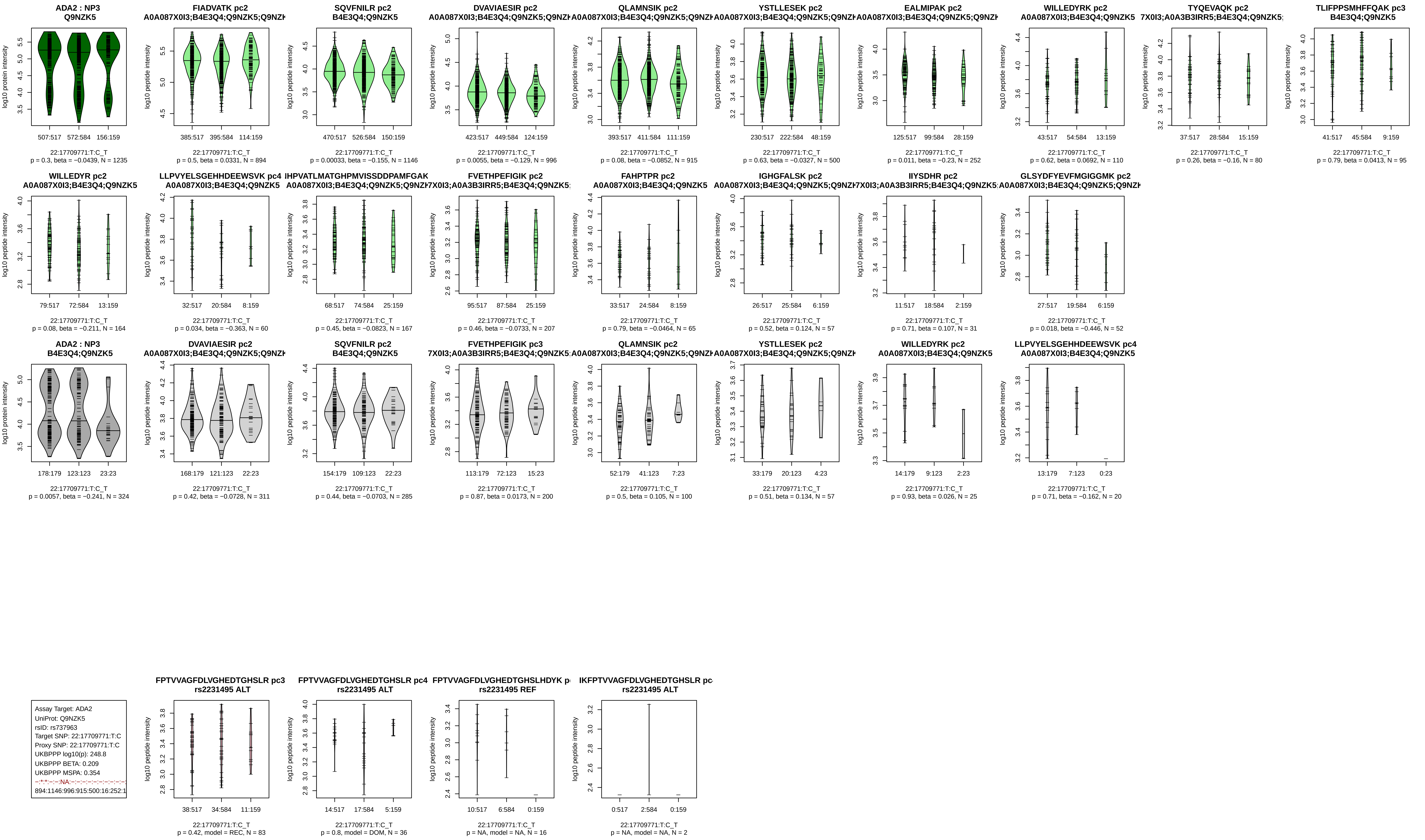

ADA2 : NP3 Q9NZK5
22:17709771:T:C_T
p = 0.3, beta = −0.0439, N = 1235
FIADVATK pc2
p = 0.5, beta = 0.0331, N = 894
SQVFNILR pc2 B4E3Q4;Q9NZK5
p = 0.00033, beta = −0.155, N = 1146
DVAVIAESIR pc2
p = 0.0055, beta = −0.129, N = 996
QLAMNSIK pc2
p = 0.08, beta = −0.0852, N = 915
YSTLLESEK pc2
p = 0.63, beta = −0.0327, N = 500
EALMIPAK pc2
p = 0.011, beta = −0.23, N = 252
WILLEDYRK pc2 A0A087X0I3;B4E3Q4;Q9NZK5
p = 0.62, beta = 0.0692, N = 110
TYQEVAQK pc2
p = 0.26, beta = −0.16, N = 80
TLIFPPSMHFFQAK pc3 B4E3Q4;Q9NZK5
p = 0.79, beta = 0.0413, N = 95
WILLEDYR pc2 A0A087X0I3;B4E3Q4;Q9NZK5
p = 0.08, beta = −0.211, N = 164
LLPVYELSGEHHDEEWSVK pc4 A0A087X0I3;B4E3Q4;Q9NZK5
p = 0.034, beta = −0.363, N = 60
IHPVATLMATGHPMVISSDDPAMFGAK
p = 0.45, beta = −0.0823, N = 167
FVETHPEFIGIK pc2
p = 0.46, beta = −0.0733, N = 207
FAHPTPR pc2 A0A087X0I3;B4E3Q4;Q9NZK5
p = 0.79, beta = −0.0464, N = 65
IGHGFALSK pc2
p = 0.52, beta = 0.124, N = 57
IIYSDHR pc2
p = 0.71, beta = 0.107, N = 31
GLSYDFYEVFMGIGGMK pc2
p = 0.018, beta = −0.446, N = 52
ADA2 : NP3 B4E3Q4;Q9NZK5
p = 0.0057, beta = −0.241, N = 324
DVAVIAESIR pc2
p = 0.42, beta = −0.0728, N = 311
SQVFNILR pc2 B4E3Q4;Q9NZK5
p = 0.44, beta = −0.0703, N = 285
FVETHPEFIGIK pc3
p = 0.87, beta = 0.0173, N = 200
QLAMNSIK pc2
p = 0.5, beta = 0.105, N = 100
YSTLLESEK pc2
p = 0.51, beta = 0.134, N = 57
WILLEDYRK pc2 A0A087X0I3;B4E3Q4;Q9NZK5
p = 0.93, beta = 0.026, N = 25
LLPVYELSGEHHDEEWSVK pc4 A0A087X0I3;B4E3Q4;Q9NZK5
p = 0.71, beta = −0.162, N = 20
Assay Target: ADA2
UniProt: Q9NZK5
rsID: rs737963
Target SNP: 22:17709771:T:C
Proxy SNP: 22:17709771:T:C
UKBPPP log10(p): 248.8
UKBPPP BETA: 0.209
UKBPPP MSPA: 0.354
894:1146:996:915:500:16:252:1
FPTVVAGFDLVGHEDTGHSLR pc3 rs2231495 ALT
p = 0.42, model = REC, N = 83
FPTVVAGFDLVGHEDTGHSLR pc4 rs2231495 ALT
p = 0.8, model = DOM, N = 36
FPTVVAGFDLVGHEDTGHSLHDYK rs2231495 REF
p = NA, model = NA, N = 16
IKFPTVVAGFDLVGHEDTGHSLR rs2231495 ALT
p = NA, model = NA, N = 2

196. ITGA6 (P23229)
UKBPPP MSPA=0.06

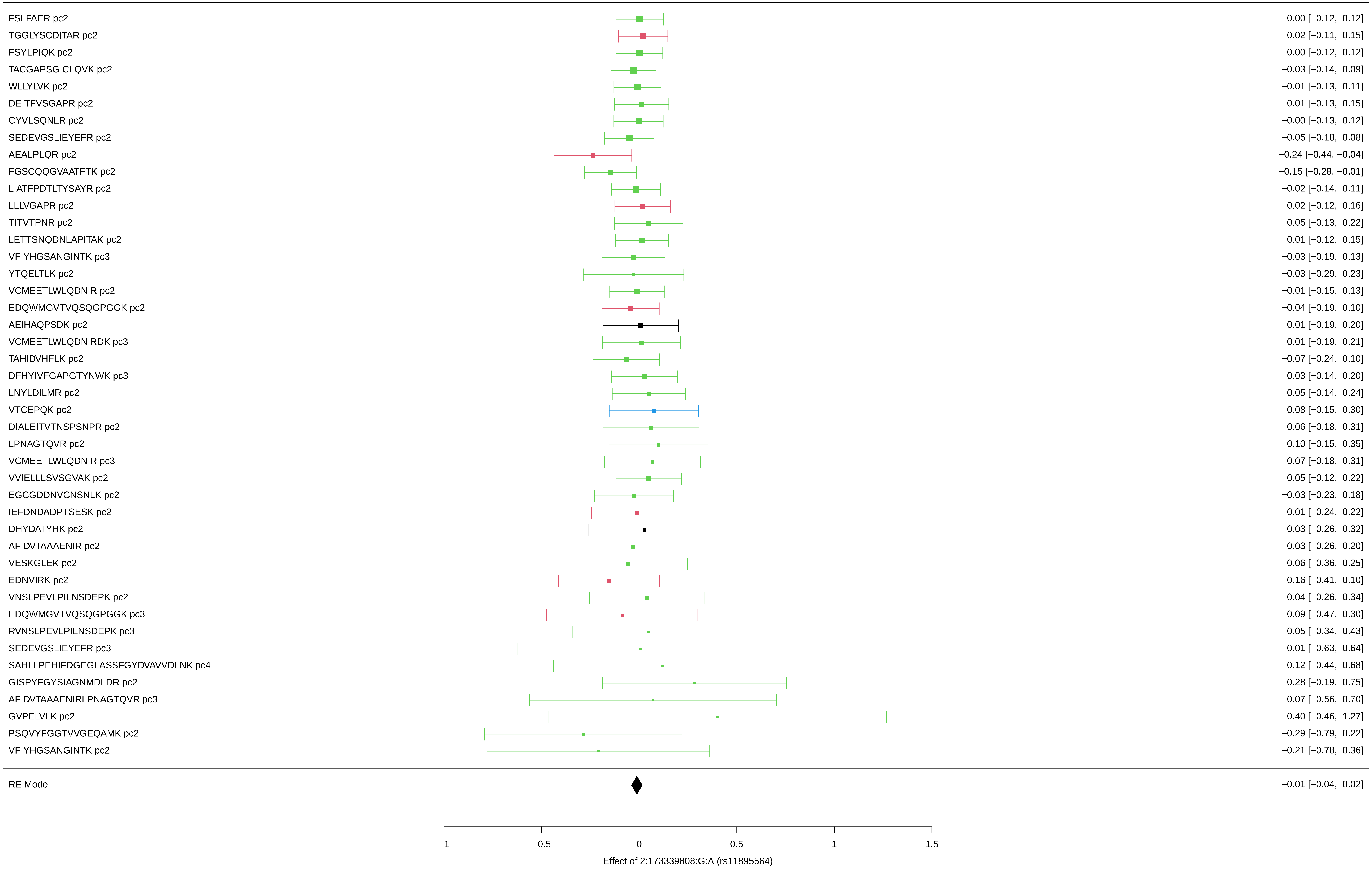

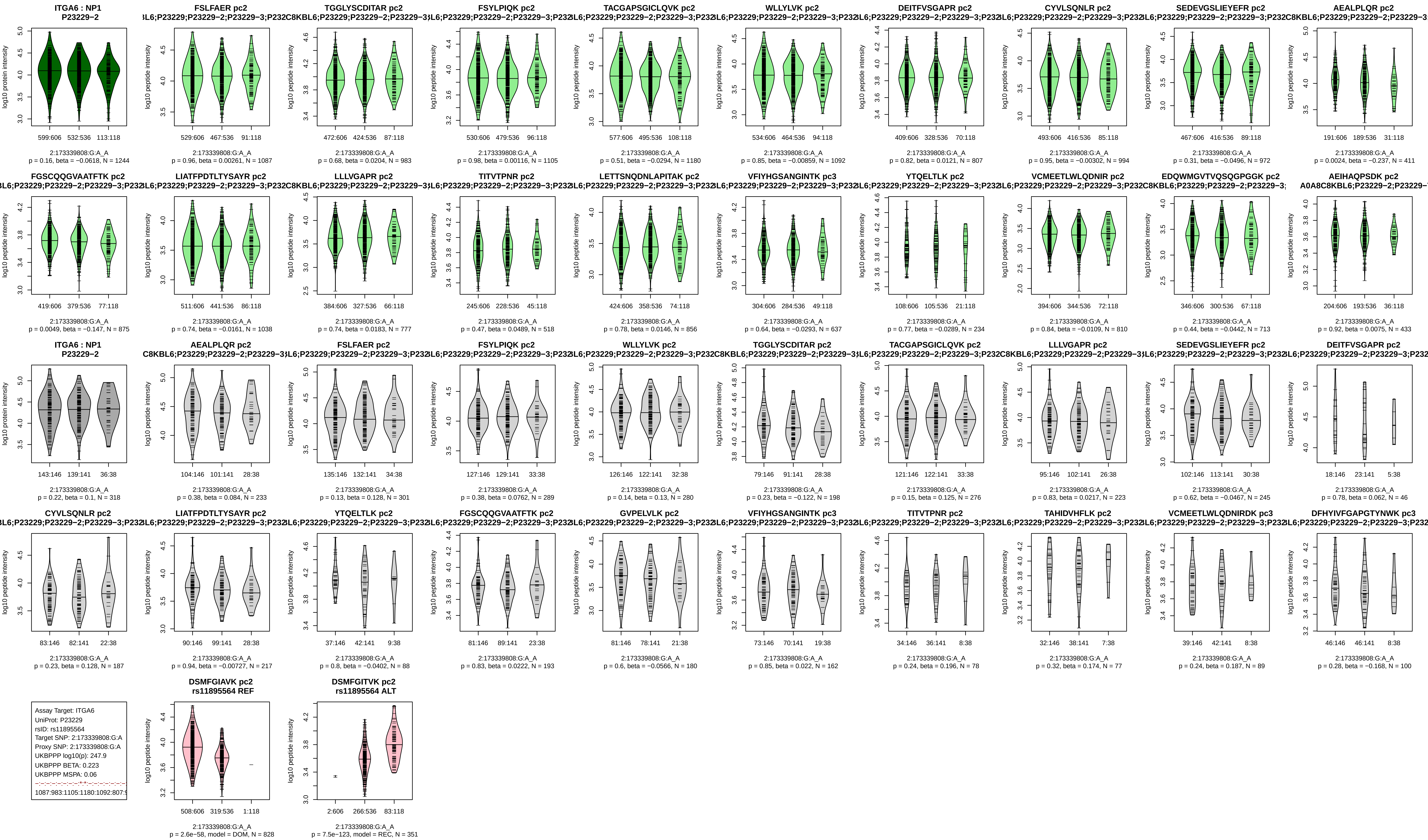
ITGA6 : NP1
P23229−2
599:606 532:536 113:118
2:173339808:G:A_A
p = 0.16, beta = −0.0618, N = 1244
FSLFAER pc2
529:606 467:536 91:118
2:173339808:G:A_A
p = 0.96, beta = 0.00261, N = 1087
TGGLYSCDITAR pc2
472:606 424:536 87:118
2:173339808:G:A_A
p = 0.68, beta = 0.0204, N = 983
FSYLPIQK pc2
530:606 479:536 96:118
2:173339808:G:A_A
p = 0.98, beta = 0.00116, N = 1105
TACGAPSGICLQVK pc2
577:606 495:536 108:118
2:173339808:G:A_A
p = 0.51, beta = −0.0294, N = 1180
WLLYLVK pc2
534:606 464:536 94:118
2:173339808:G:A_A
p = 0.85, beta = −0.00859, N = 1092
DEITFVSGAPR pc2
409:606 328:536 70:118
2:173339808:G:A_A
p = 0.82, beta = 0.0121, N = 807
CYVLSQNLR pc2
493:606 416:536 85:118
2:173339808:G:A_A
p = 0.95, beta = −0.00302, N = 994
SEDEVGSLIEYEFR pc2
467:606 416:536 89:118
2:173339808:G:A_A
p = 0.31, beta = −0.0496, N = 972
AEALPLQR pc2
191:606 189:536 31:118
2:173339808:G:A_A
p = 0.0024, beta = −0.237, N = 411
FGSCQQGVAATFTK pc2
419:606 379:536 77:118
2:173339808:G:A_A
p = 0.0049, beta = −0.147, N = 875
LIATFPDTLTYSAYR pc2
511:606 441:536 86:118
2:173339808:G:A_A
p = 0.74, beta = −0.0161, N = 1038
LLLVGAPR pc2
384:606 327:536 66:118
2:173339808:G:A_A
p = 0.74, beta = 0.0183, N = 777
TITVTPNR pc2
245:606 228:536 45:118
2:173339808:G:A_A
p = 0.47, beta = 0.0489, N = 518
LETTSNQDNLAPITAK pc2
424:606 358:536 74:118
2:173339808:G:A_A
p = 0.78, beta = 0.0146, N = 856
VFIYHGSANGINTK pc3
304:606 284:536 49:118
2:173339808:G:A_A
p = 0.64, beta = −0.0293, N = 637
YTQELTLK pc2
108:606 105:536 21:118
2:173339808:G:A_A
p = 0.77, beta = −0.0289, N = 234
VCMEETLWLQDNIR pc2
394:606 344:536 72:118
2:173339808:G:A_A
p = 0.84, beta = −0.0109, N = 810
EDQWMGVTVQSQGPGGK pc2
346:606 300:536 67:118
2:173339808:G:A_A
p = 0.44, beta = −0.0442, N = 713
AEIHAQPSDK pc2
204:606 193:536 36:118
2:173339808:G:A_A
p = 0.92, beta = 0.0075, N = 433
ITGA6 : NP1
P23229−2
143:146 139:141 36:38
2:173339808:G:A_A
p = 0.22, beta = 0.1, N = 318
AEALPLQR pc2
104:146 101:141 28:38
2:173339808:G:A_A
p = 0.38, beta = 0.084, N = 233
FSLFAER pc2
135:146 132:141 34:38
2:173339808:G:A_A
p = 0.13, beta = 0.128, N = 301
FSYLPIQK pc2
127:146 129:141 33:38
2:173339808:G:A_A
p = 0.38, beta = 0.0762, N = 289
WLLYLVK pc2
126:146 122:141 32:38
2:173339808:G:A_A
p = 0.14, beta = 0.13, N = 280
TGGLYSCDITAR pc2
79:146 91:141 28:38
2:173339808:G:A_A
p = 0.23, beta = −0.122, N = 198
TACGAPSGICLQVK pc2
121:146 122:141 33:38
2:173339808:G:A_A
p = 0.15, beta = 0.125, N = 276
LLLVGAPR pc2
95:146 102:141 26:38
2:173339808:G:A_A
p = 0.83, beta = 0.0217, N = 223
SEDEVGSLIEYEFR pc2
102:146 113:141 30:38
2:173339808:G:A_A
p = 0.62, beta = −0.0467, N = 245
DEITFVSGAPR pc2
18:146 23:141 5:38
2:173339808:G:A_A
p = 0.78, beta = 0.062, N = 46
CYVLSQNLR pc2
83:146 82:141 22:38
2:173339808:G:A_A
p = 0.23, beta = 0.128, N = 187
LIATFPDTLTYSAYR pc2
90:146 99:141 28:38
2:173339808:G:A_A
p = 0.94, beta = −0.00727, N = 217
YTQELTLK pc2
37:146 42:141 9:38
2:173339808:G:A_A
p = 0.8, beta = −0.0402, N = 88
FGSCQQGVAATFTK pc2
81:146 89:141 23:38
2:173339808:G:A_A
p = 0.83, beta = 0.0222, N = 193
GVPELVLK pc2
81:146 78:141 21:38
2:173339808:G:A_A
p = 0.6, beta = −0.0566, N = 180
VFIYHGSANGINTK pc3
73:146 70:141 19:38
2:173339808:G:A_A
p = 0.85, beta = 0.022, N = 162
TITVTPNR pc2
34:146 36:141 8:38
2:173339808:G:A_A
p = 0.24, beta = 0.196, N = 78
TAHIDVHFLK pc2
32:146 38:141 7:38
2:173339808:G:A_A
p = 0.32, beta = 0.174, N = 77
VCMEETLWLQDNIRDK pc3
39:146 42:141 8:38
2:173339808:G:A_A
p = 0.24, beta = 0.187, N = 89
DFHYIVFGAPGTYNWK pc3
46:146 46:141 8:38
2:173339808:G:A_A
p = 0.28, beta = −0.168, N = 100
Assay Target: ITGA6
UniProt: P23229
rsID: rs11895564
Target SNP: 2:173339808:G:A
Proxy SNP: 2:173339808:G:A
UKBPPP log10(p): 247.9
UKBPPP BETA: 0.223
UKBPPP MSPA: 0.06
DSMFGIAVK pc2
rs11895564 REF
508:606 319:536 1:118
2:173339808:G:A_A
p = 2.6e−58, model = DOM, N = 828
DSMFGITVK pc2
rs11895564 ALT
2:606 266:536 83:118
2:173339808:G:A_A
p = 7.5e−123, model = REC, N = 351

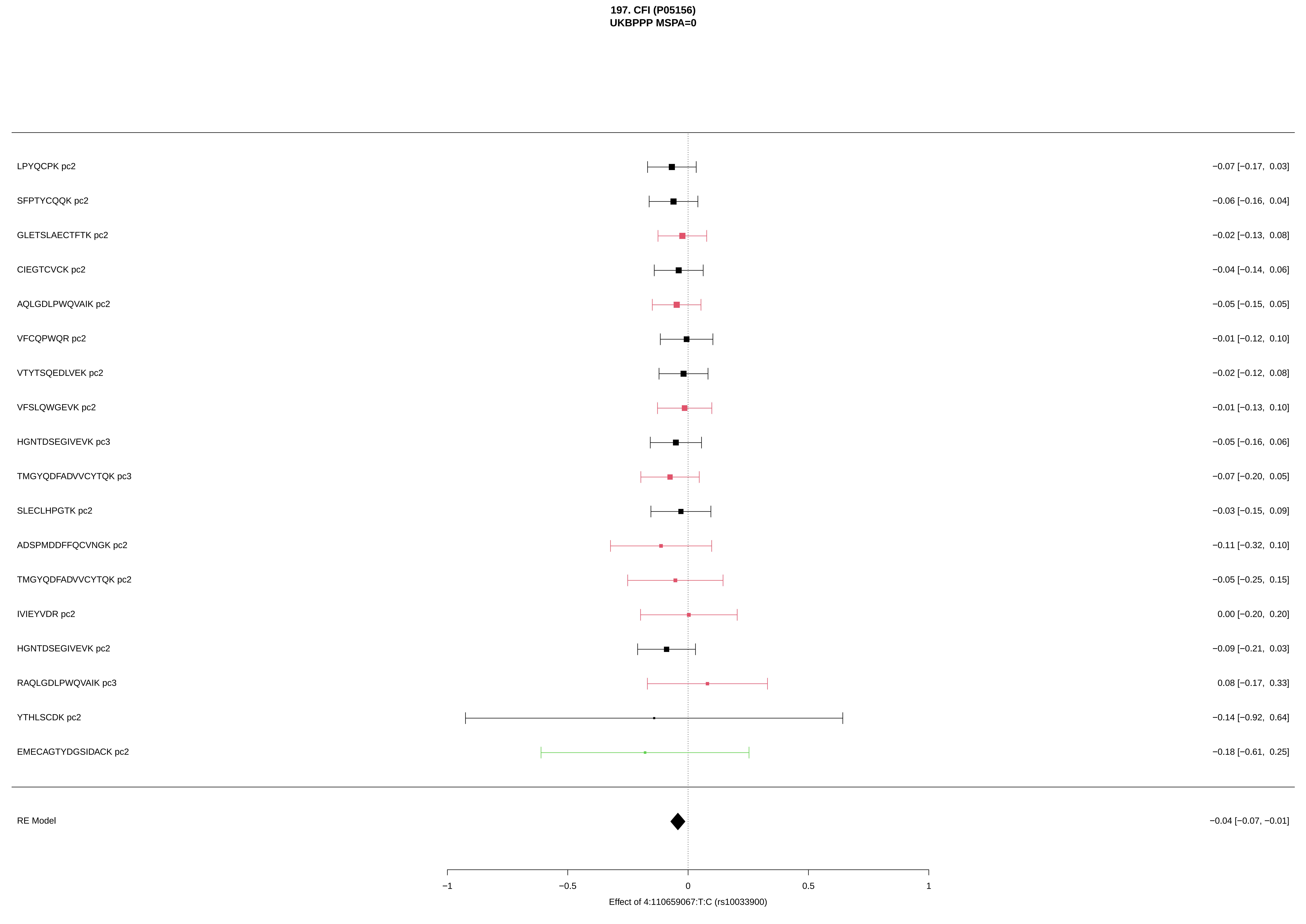

197. CFI (P05156)
UKBPPP MSPA=0
LPYQCPK pc2
SFPTYCQQK pc2
GLETSLAECTFTK pc2
CIEGTCVCK pc2
AQLGDLPWQVAIK pc2
VFCQPWQR pc2
VTYTSQEDLVEK pc2
VFSLQWGEVK pc2
HGNTDSEGIVEVK pc3
TMGYQDFADVVCYTQK pc3
SLECLHPGTK pc2
ADSPMDDFFQCVNGK pc2
TMGYQDFADVVCYTQK pc2
IVIEYVDR pc2
HGNTDSEGIVEVK pc2
RAQLGDLPWQVAIK pc3
YTHLSCDK pc2
EMECAGTYDGSIDACK pc2
RE Model
−0.07 [−0.17, 0.03]
−0.06 [−0.16, 0.04]
−0.02 [−0.13, 0.08]
−0.04 [−0.14, 0.06]
−0.05 [−0.15, 0.05]
−0.01 [−0.12, 0.10]
−0.02 [−0.12, 0.08]
−0.01 [−0.13, 0.10]
−0.05 [−0.16, 0.06]
−0.07 [−0.20, 0.05]
−0.03 [−0.15, 0.09]
−0.11 [−0.32, 0.10]
−0.05 [−0.25, 0.15]
0.00 [−0.20, 0.20]
−0.09 [−0.21, 0.03]
0.08 [−0.17, 0.33]
−0.14 [−0.92, 0.64]
−0.18 [−0.61, 0.25]
−0.04 [−0.07, −0.01]
−1
−0.5
0
0.5
1
Effect of 4:110659067:T:C (rs10033900)

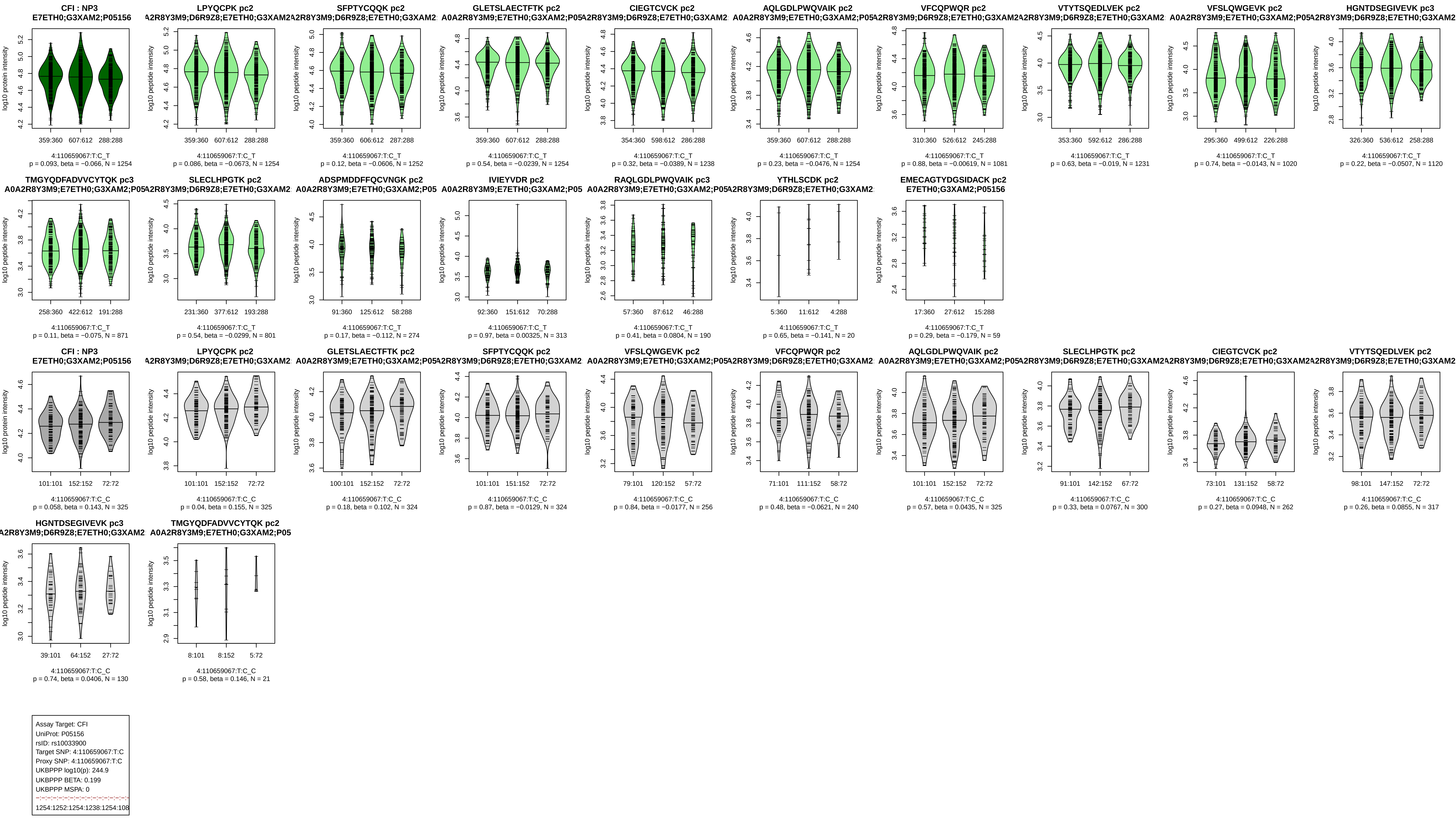
CFI : NP3
E7ETH0;G3XAM2;P05156
log10 protein intensity
4:110659067:T:C_T
p = 0.093, beta = −0.066, N = 1254
LPYQCPK pc2
p = 0.086, beta = −0.0673, N = 1254
SFPTYCQQK pc2
p = 0.12, beta = −0.0606, N = 1252
GLETSLAECTFTK pc2
p = 0.54, beta = −0.0239, N = 1254
CIEGTCVCK pc2
p = 0.32, beta = −0.0389, N = 1238
AQLGDLPWQVAIK pc2
p = 0.23, beta = −0.0476, N = 1254
VFCQPWQR pc2
p = 0.88, beta = −0.00619, N = 1081
VTYTSQEDLVEK pc2
p = 0.63, beta = −0.019, N = 1231
VFSLQWGEVK pc2
p = 0.74, beta = −0.0143, N = 1020
HGNTDSEGIVEVK pc3
p = 0.22, beta = −0.0507, N = 1120
TMGYQDFADVVCYTQK pc3
p = 0.11, beta = −0.075, N = 871
SLECLHPGTK pc2
p = 0.54, beta = −0.0299, N = 801
ADSPMDDFFQCVNGK pc2
p = 0.17, beta = −0.112, N = 274
IVIEYVDR pc2
p = 0.97, beta = 0.00325, N = 313
RAQLGDLPWQVAIK pc3
p = 0.41, beta = 0.0804, N = 190
YTHLSCDK pc2
p = 0.65, beta = −0.141, N = 20
EMECAGTYDGSIDACK pc2
p = 0.29, beta = −0.179, N = 59
log10 peptide intensity
CFI : NP3
4:110659067:T:C_C
p = 0.058, beta = 0.143, N = 325
LPYQCPK pc2
p = 0.04, beta = 0.155, N = 325
GLETSLAECTFTK pc2
p = 0.18, beta = 0.102, N = 324
SFPTYCQQK pc2
p = 0.87, beta = −0.0129, N = 324
VFSLQWGEVK pc2
p = 0.84, beta = −0.0177, N = 256
VFCQPWQR pc2
p = 0.48, beta = −0.0621, N = 240
AQLGDLPWQVAIK pc2
p = 0.57, beta = 0.0435, N = 325
SLECLHPGTK pc2
p = 0.33, beta = 0.0767, N = 300
CIEGTCVCK pc2
p = 0.27, beta = 0.0948, N = 262
VTYTSQEDLVEK pc2
p = 0.26, beta = 0.0855, N = 317
HGNTDSEGIVEVK pc3
p = 0.74, beta = 0.0406, N = 130
TMGYQDFADVVCYTQK pc2
p = 0.58, beta = 0.146, N = 21
Assay Target: CFI
UniProt: P05156
rsID: rs10033900
Target SNP: 4:110659067:T:C
Proxy SNP: 4:110659067:T:C
UKBPPP log10(p): 244.9
UKBPPP BETA: 0.199
UKBPPP MSPA: 0
1254:1252:1254:1238:1254:108

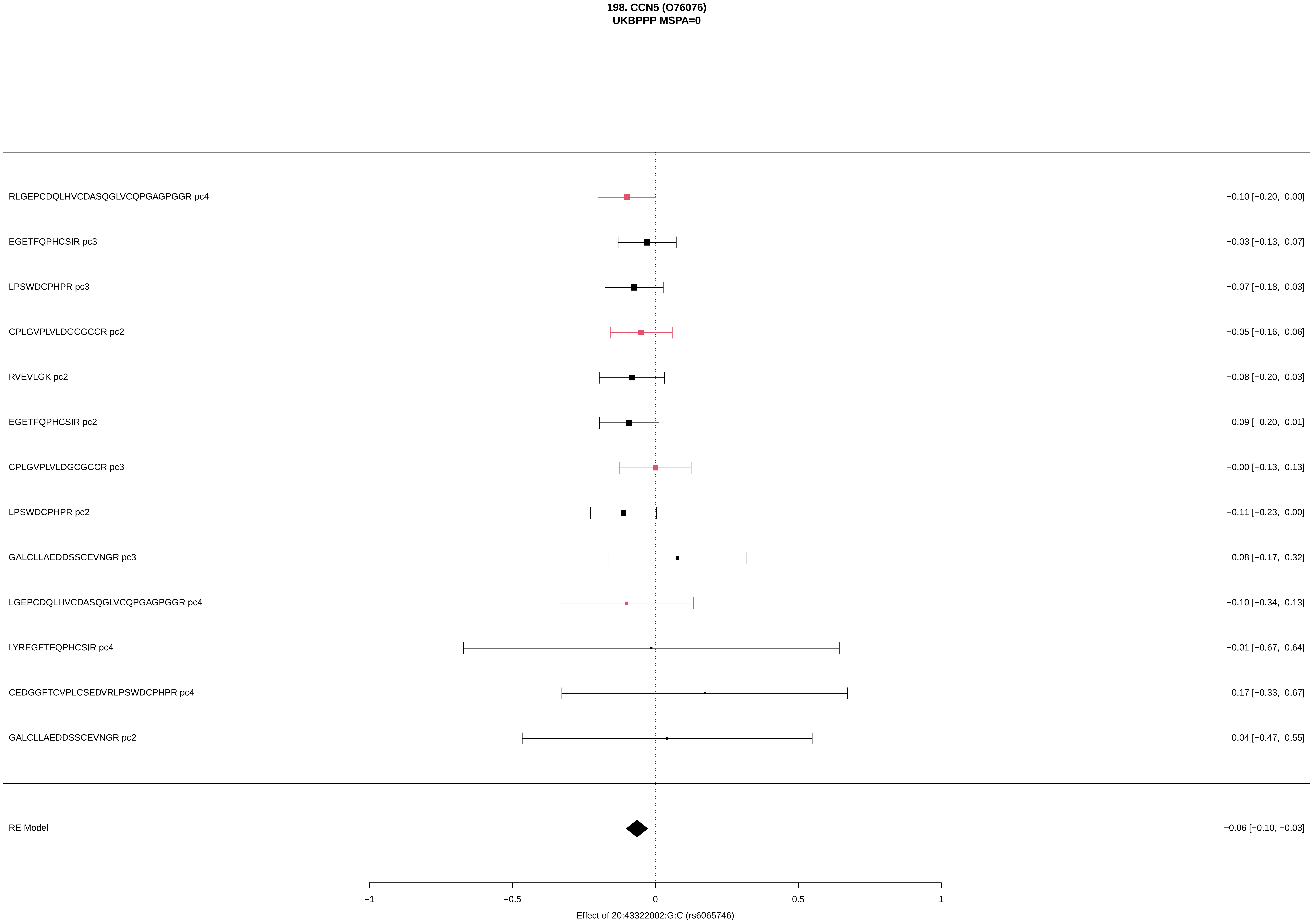
198. CCN5 (O76076)
UKBPPP MSPA=0
RLGEPCDQLHVCDASQGLVCQPGAGPGGR pc4
EGETFQPHCSIR pc3
LPSWDCPHPR pc3
CPLGVPLVLDGCGCCR pc2
RVEVLGK pc2
EGETFQPHCSIR pc2
CPLGVPLVLDGCGCCR pc3
LPSWDCPHPR pc2
GALCLLAEDDSSCEVNGR pc3
LGEPCDQLHVCDASQGLVCQPGAGPGGR pc4
LYREGETFQPHCSIR pc4
CEDGGFTCVPLCSEDVRLPSWDCPHPR pc4
GALCLLAEDDSSCEVNGR pc2
RE Model
−0.10 [−0.20, 0.00]
−0.03 [−0.13, 0.07]
−0.07 [−0.18, 0.03]
−0.05 [−0.16, 0.06]
−0.08 [−0.20, 0.03]
−0.09 [−0.20, 0.01]
−0.00 [−0.13, 0.13]
−0.11 [−0.23, 0.00]
0.08 [−0.17, 0.32]
−0.10 [−0.34, 0.13]
−0.01 [−0.67, 0.64]
0.17 [−0.33, 0.67]
0.04 [−0.47, 0.55]
−0.06 [−0.10, −0.03]
−1
−0.5
0
0.5
1
Effect of 20:43322002:G:C (rs6065746)

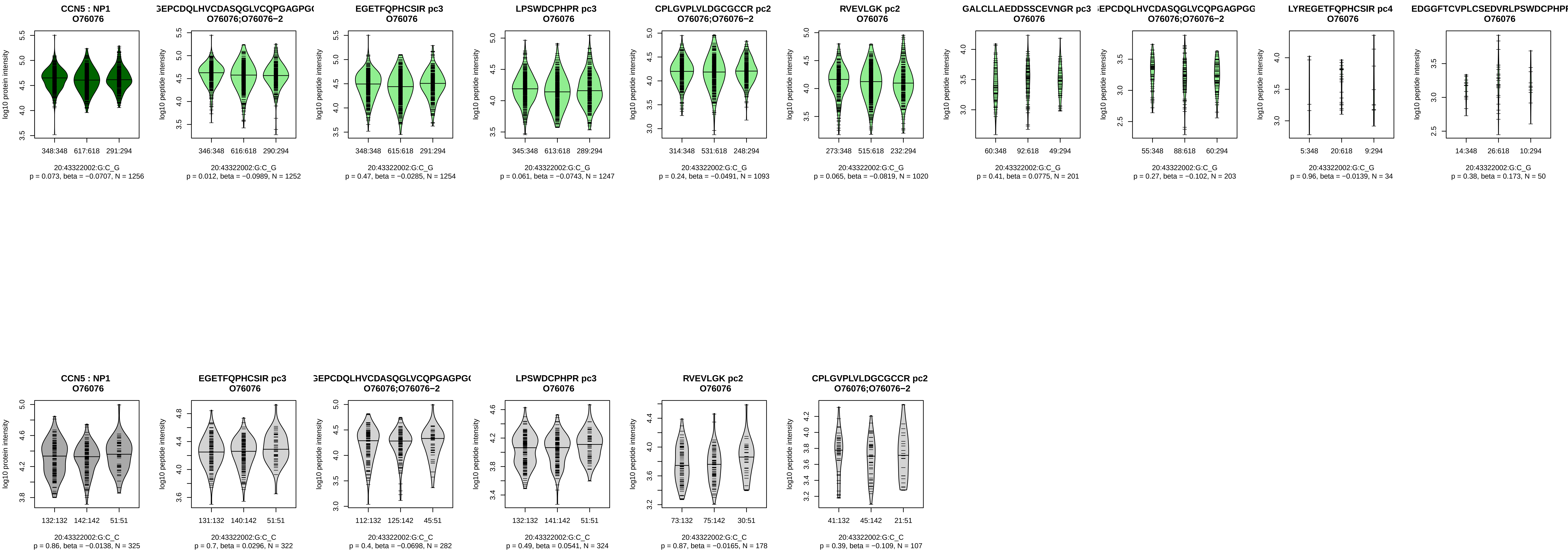

Assay Target: CCN5
UniProt: O76076
rsID: rs6065746
Target SNP: 20:43322002:G:C
Proxy SNP: 20:43322002:G:C
UKBPPP log10(p): 243.7
UKBPPP BETA: 0.189
UKBPPP MSPA: 0
−:−:−:−:−:−:−:−:−:−:−:−:−:−:NA
1252:1254:1247:1093:1020:119

199. PROC (P04070)
UKBPPP MSPA=0

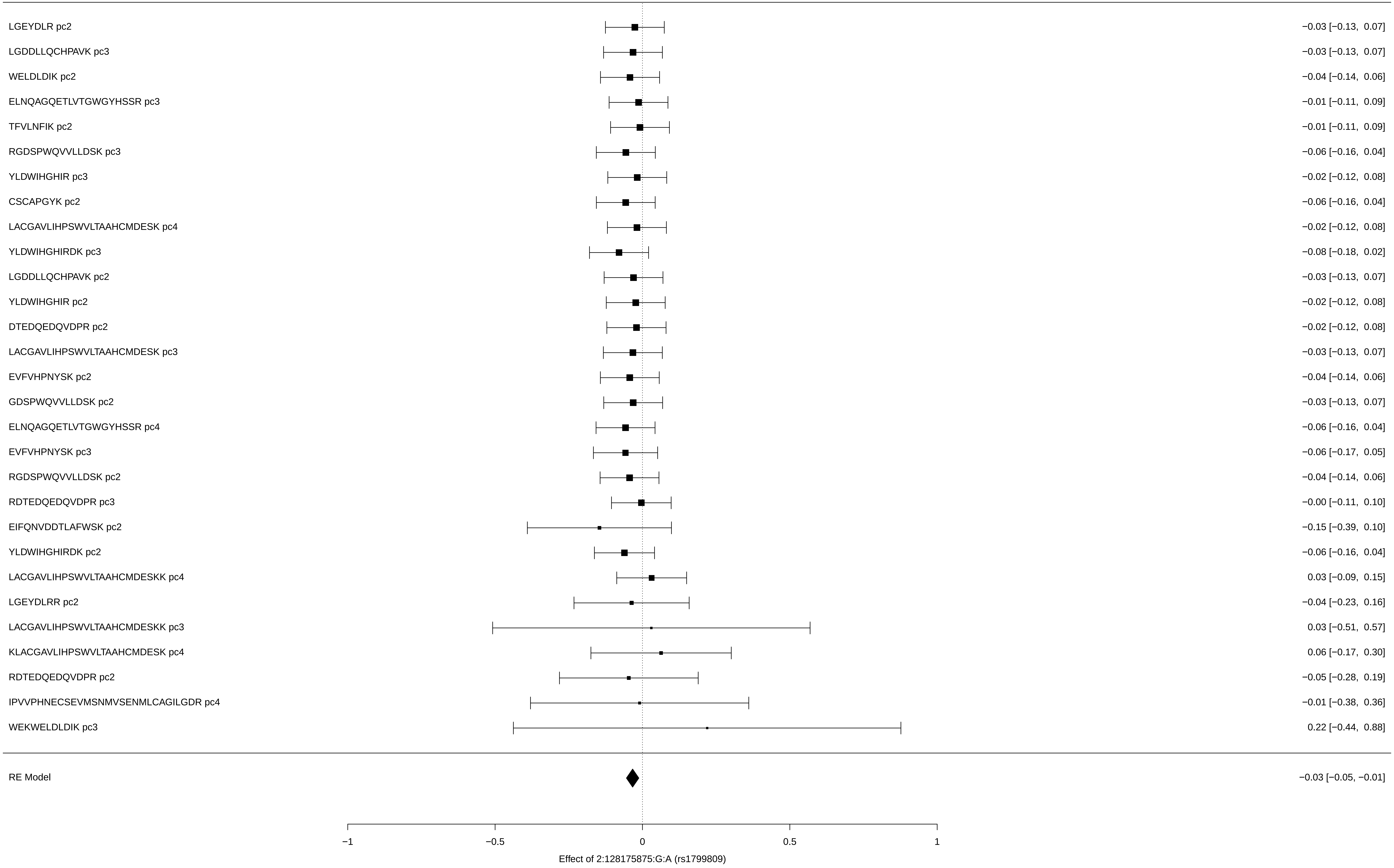

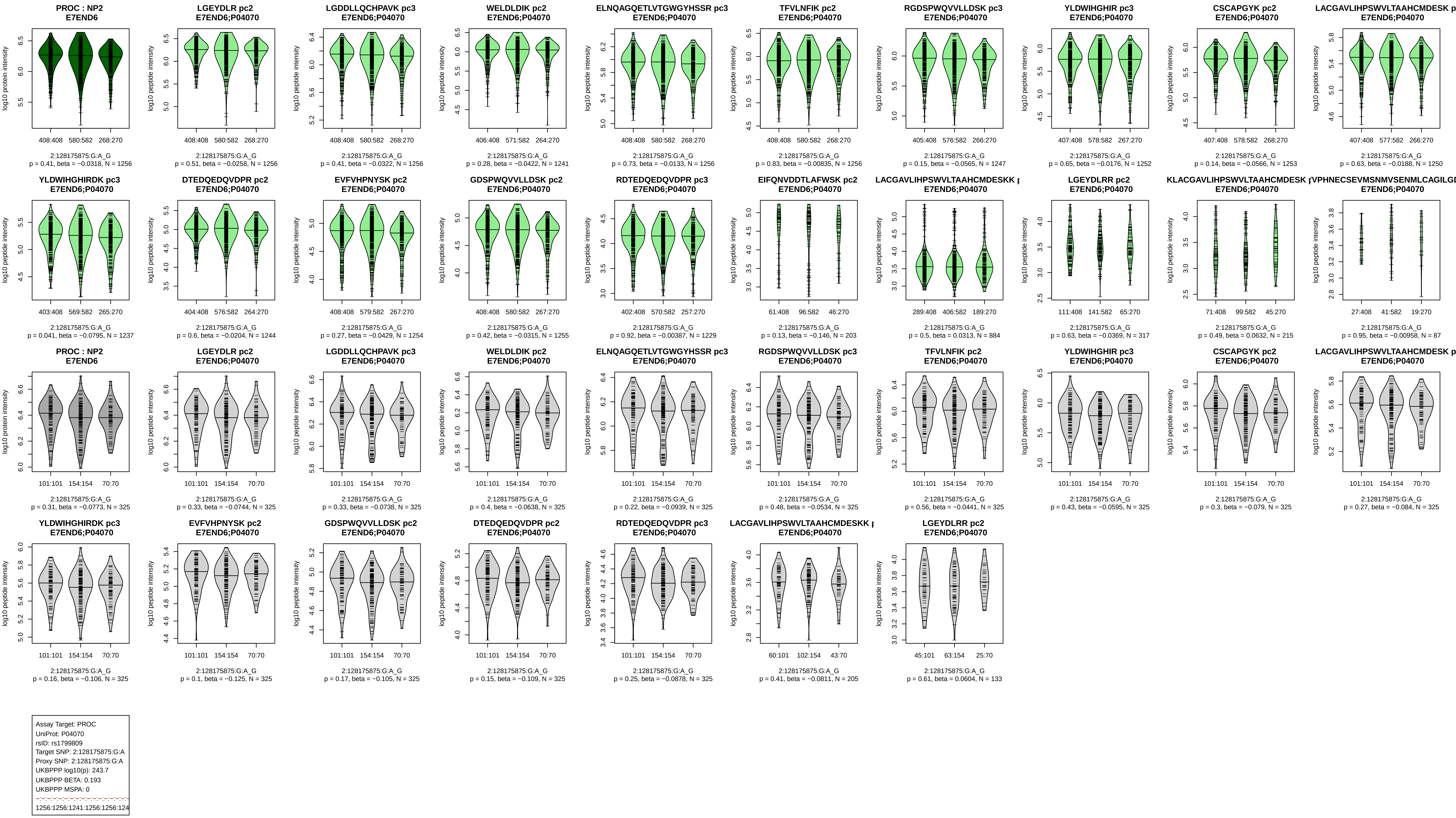

PROC : NP2 E7END6
p = 0.41, beta = −0.0318, N = 1256
LGEYDLR pc2 E7END6;P04070
p = 0.51, beta = −0.0258, N = 1256
LGDDLLQCHPAVK pc3 E7END6;P04070
p = 0.41, beta = −0.0322, N = 1256
WELDLDIK pc2 E7END6;P04070
p = 0.28, beta = −0.0422, N = 1241
ELNQAGQETLVTGWGYHSSR pc3 E7END6;P04070
p = 0.73, beta = −0.0133, N = 1256
TFVLNFIK pc2 E7END6;P04070
p = 0.83, beta = −0.00835, N = 1256
RGDSPWQVVLLDSK pc3 E7END6;P04070
p = 0.15, beta = −0.0565, N = 1247
YLDWIHGHIR pc3 E7END6;P04070
p = 0.65, beta = −0.0176, N = 1252
CSCAPGYK pc2 E7END6;P04070
p = 0.14, beta = −0.0566, N = 1253
LACGAVLIHPSWVLTAAHCMDESK p E7END6;P04070
p = 0.63, beta = −0.0188, N = 1250
YLDWIHGHIRDK pc3 E7END6;P04070
p = 0.041, beta = −0.0795, N = 1237
DTEDQEDQVDPR pc2 E7END6;P04070
p = 0.6, beta = −0.0204, N = 1244
EVFVHPNYSK pc2 E7END6;P04070
p = 0.27, beta = −0.0429, N = 1254
GDSPWQVVLLDSK pc2 E7END6;P04070
p = 0.42, beta = −0.0315, N = 1255
RDTEDQEDQVDPR pc3 E7END6;P04070
p = 0.92, beta = −0.00387, N = 1229
EIFQNVDDTLAFWSK pc2 E7END6;P04070
p = 0.13, beta = −0.146, N = 203
LACGAVLIHPSWVLTAAHCMDESKK p E7END6;P04070
p = 0.5, beta = −0.00315, N = 884
LGEYDLRR pc2 E7END6;P04070
p = 0.63, beta = −0.0369, N = 317
KLACGAVLIHPSWVLTAAHCMDESK p E7END6;P04070
p = 0.49, beta = 0.0632, N = 215
VPHNECSEVMSNMVSENMLCAGILGI E7END6;P04070
p = 0.95, beta = −0.00958, N = 87
PROC : NP2 E7END6
p = 0.31, beta = −0.0773, N = 325
LGEYDLR pc2 E7END6;P04070
p = 0.33, beta = −0.0744, N = 325
LGDDLLQCHPAVK pc3 E7END6;P04070
p = 0.33, beta = −0.0738, N = 325
WELDLDIK pc2 E7END6;P04070
p = 0.4, beta = −0.0638, N = 325
ELNQAGQETLVTGWGYHSSR pc3 E7END6;P04070
p = 0.22, beta = −0.0939, N = 325
RGDSPWQVVLLDSK pc3 E7END6;P04070
p = 0.48, beta = −0.0534, N = 325
TFVLNFIK pc2 E7END6;P04070
p = 0.56, beta = −0.0441, N = 325
YLDWIHGHIR pc3 E7END6;P04070
p = 0.43, beta = −0.0595, N = 325
CSCAPGYK pc2 E7END6;P04070
p = 0.3, beta = −0.079, N = 325
LACGAVLIHPSWVLTAAHCMDESK p E7END6;P04070
p = 0.27, beta = −0.084, N = 325
YLDWIHGHIRDK pc3 E7END6;P04070
p = 0.16, beta = −0.106, N = 325
EVFVHPNYSK pc2 E7END6;P04070
p = 0.1, beta = −0.125, N = 325
GDSPWQVVLLDSK pc2 E7END6;P04070
p = 0.17, beta = −0.105, N = 325
DTEDQEDQVDPR pc2 E7END6;P04070
p = 0.15, beta = −0.109, N = 325
RDTEDQEDQVDPR pc3 E7END6;P04070
p = 0.25, beta = −0.0878, N = 325
LACGAVLIHPSWVLTAAHCMDESKK p E7END6;P04070
p = 0.41, beta = −0.0811, N = 205
LGEYDLRR pc2 E7END6;P04070
p = 0.61, beta = 0.0604, N = 133
2:128175875:G:A_G
log10 protein intensity
log10 peptide intensity
Assay Target: PROC
UniProt: P04070
rsID: rs1799809
Target SNP: 2:128175875:G:A
Proxy SNP: 2:128175875:G:A
UKBPPP log10(p): 243.7
UKBPPP BETA: 0.193
UKBPPP MSPA: 0
1256:1256:1241:1256:1256:124

200. WARS (P23381)
UKBPPP MSPA=0.657

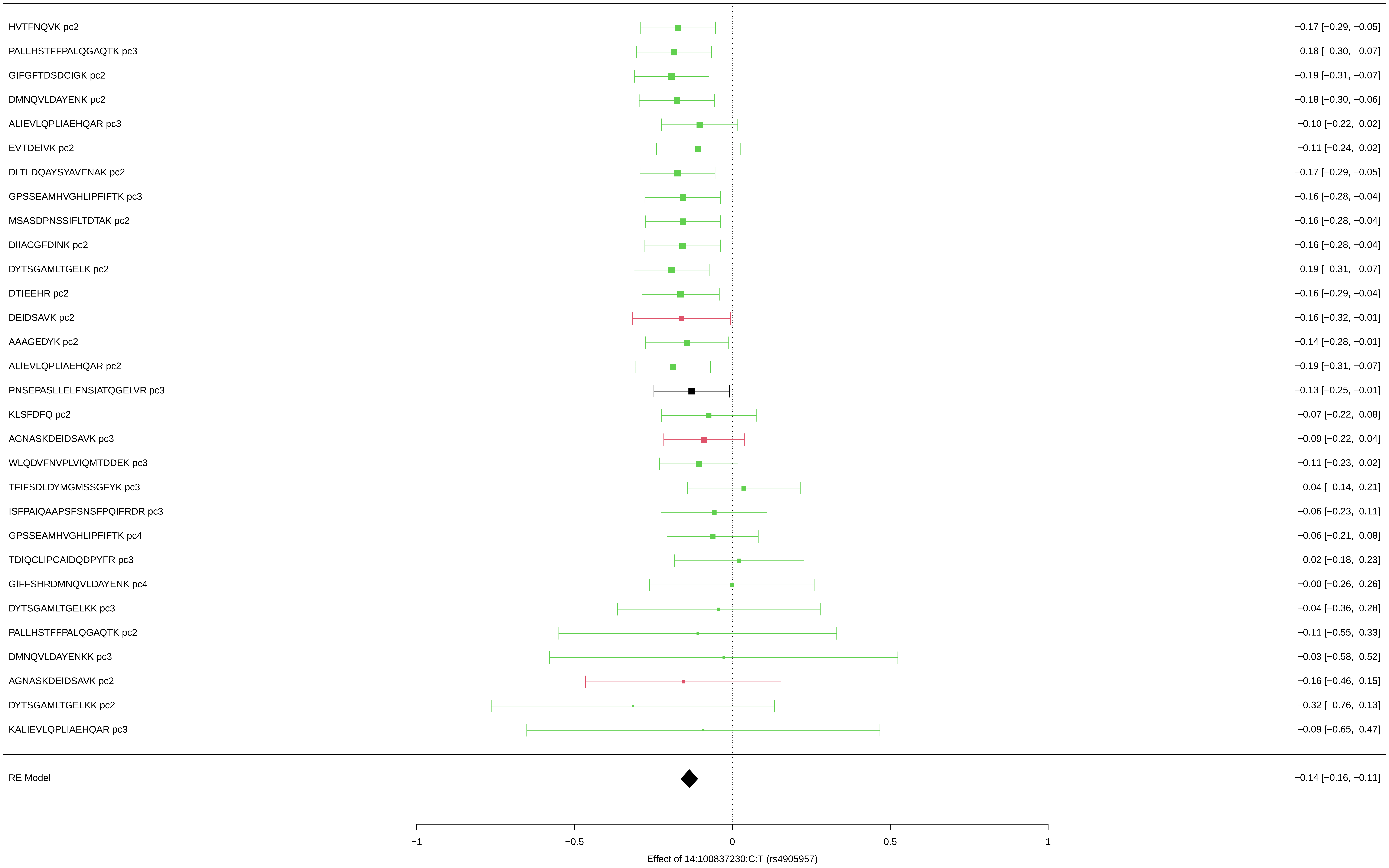

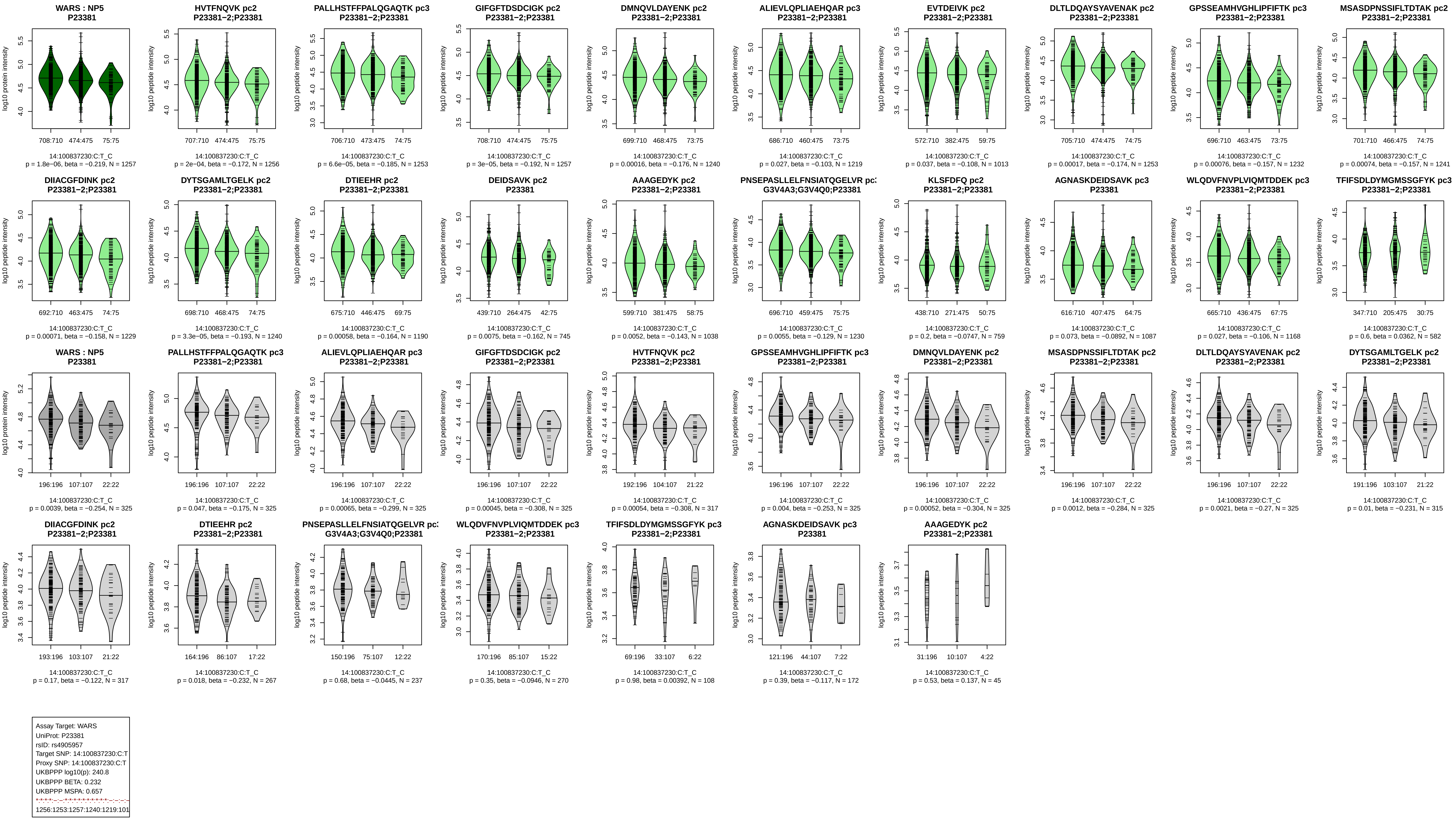
WARS : NP5
P23381
14:100837230:C:T_C
p = 1.8e−06, beta = −0.219, N = 1257
HVTFNQVK pc2
P23381−2;P23381
14:100837230:C:T_C
p = 2e−04, beta = −0.172, N = 1256
PALLHSTFFPALQGAQTK pc3
P23381−2;P23381
14:100837230:C:T_C
p = 6.6e−05, beta = −0.185, N = 1253
GIFGFTDSDCIGK pc2
P23381−2;P23381
14:100837230:C:T_C
p = 3e−05, beta = −0.192, N = 1257
DMNQVLDAYENK pc2
P23381−2;P23381
14:100837230:C:T_C
p = 0.00016, beta = −0.176, N = 1240
ALIEVLQPLIAEHQAR pc3
P23381−2;P23381
14:100837230:C:T_C
p = 0.027, beta = −0.103, N = 1219
EVTDEIVK pc2
P23381−2;P23381
14:100837230:C:T_C
p = 0.037, beta = −0.108, N = 1013
DLTLDQAYSYAVENAK pc2
P23381−2;P23381
14:100837230:C:T_C
p = 0.00017, beta = −0.174, N = 1253
GPSSEAMHVGHLIPFIFTK pc3
P23381−2;P23381
14:100837230:C:T_C
p = 0.00076, beta = −0.157, N = 1232
MSASDPNSSIFLTDTAK pc2
P23381−2;P23381
14:100837230:C:T_C
p = 0.00074, beta = −0.157, N = 1241
DIIACGFDINK pc2
P23381−2;P23381
14:100837230:C:T_C
p = 0.00071, beta = −0.158, N = 1229
DYTSGAMLTGELK pc2
P23381−2;P23381
14:100837230:C:T_C
p = 3.3e−05, beta = −0.193, N = 1240
DTIEEHR pc2
P23381−2;P23381
14:100837230:C:T_C
p = 0.00058, beta = −0.164, N = 1190
DEIDSAVK pc2
P23381
14:100837230:C:T_C
p = 0.0075, beta = −0.162, N = 745
AAAGEDYK pc2
P23381−2;P23381
14:100837230:C:T_C
p = 0.0052, beta = −0.143, N = 1038
G3V4A3;G3V4Q0;P23381
14:100837230:C:T_C
p = 0.0055, beta = −0.129, N = 1230
KLSFDFQ pc2
P23381−2;P23381
14:100837230:C:T_C
p = 0.2, beta = −0.0747, N = 759
AGNASKDEIDSAVK pc3
P23381
14:100837230:C:T_C
p = 0.073, beta = −0.0892, N = 1087
WLQDVFNVPLVIQMTDDEK pc3
P23381−2;P23381
14:100837230:C:T_C
p = 0.027, beta = −0.106, N = 1168
TFIFSDLDYMGMSSGFYK pc3
P23381−2;P23381
14:100837230:C:T_C
p = 0.6, beta = 0.0362, N = 582
WARS : NP5
P23381
14:100837230:C:T_C
p = 0.0039, beta = −0.254, N = 325
PALLHSTFFPALQGAQTK pc3
P23381−2;P23381
14:100837230:C:T_C
p = 0.047, beta = −0.175, N = 325
ALIEVLQPLIAEHQAR pc3
P23381−2;P23381
14:100837230:C:T_C
p = 0.00065, beta = −0.299, N = 325
GIFGFTDSDCIGK pc2
P23381−2;P23381
14:100837230:C:T_C
p = 0.00045, beta = −0.308, N = 325
HVTFNQVK pc2
P23381−2;P23381
14:100837230:C:T_C
p = 0.00054, beta = −0.308, N = 317
GPSSEAMHVGHLIPFIFTK pc3
P23381−2;P23381
14:100837230:C:T_C
p = 0.004, beta = −0.253, N = 325
DMNQVLDAYENK pc2
P23381−2;P23381
14:100837230:C:T_C
p = 0.00052, beta = −0.304, N = 325
MSASDPNSSIFLTDTAK pc2
P23381−2;P23381
14:100837230:C:T_C
p = 0.0012, beta = −0.284, N = 325
DLTLDQAYSYAVENAK pc2
P23381−2;P23381
14:100837230:C:T_C
p = 0.0021, beta = −0.27, N = 325
DYTSGAMLTGELK pc2
P23381−2;P23381
14:100837230:C:T_C
p = 0.01, beta = −0.231, N = 315
DIIACGFDINK pc2
P23381−2;P23381
14:100837230:C:T_C
p = 0.17, beta = −0.122, N = 317
DTIEEHR pc2
P23381−2;P23381
14:100837230:C:T_C
p = 0.018, beta = −0.232, N = 267
G3V4A3;G3V4Q0;P23381
14:100837230:C:T_C
p = 0.68, beta = −0.0445, N = 237
WLQDVFNVPLVIQMTDDEK pc3
P23381−2;P23381
14:100837230:C:T_C
p = 0.35, beta = −0.0946, N = 270
TFIFSDLDYMGMSSGFYK pc3
P23381−2;P23381
14:100837230:C:T_C
p = 0.98, beta = 0.00392, N = 108
AGNASKDEIDSAVK pc3
P23381
14:100837230:C:T_C
p = 0.39, beta = −0.117, N = 172
AAAGEDYK pc2
P23381−2;P23381
14:100837230:C:T_C
p = 0.53, beta = 0.137, N = 45
Assay Target: WARS
UniProt: P23381
rsID: rs4905957
Target SNP: 14:100837230:C:T
Proxy SNP: 14:100837230:C:T
UKBPPP log10(p): 240.8
UKBPPP BETA: 0.232
UKBPPP MSPA: 0.657

201. B4GALT1 (P15291)
UKBPPP MSPA=0

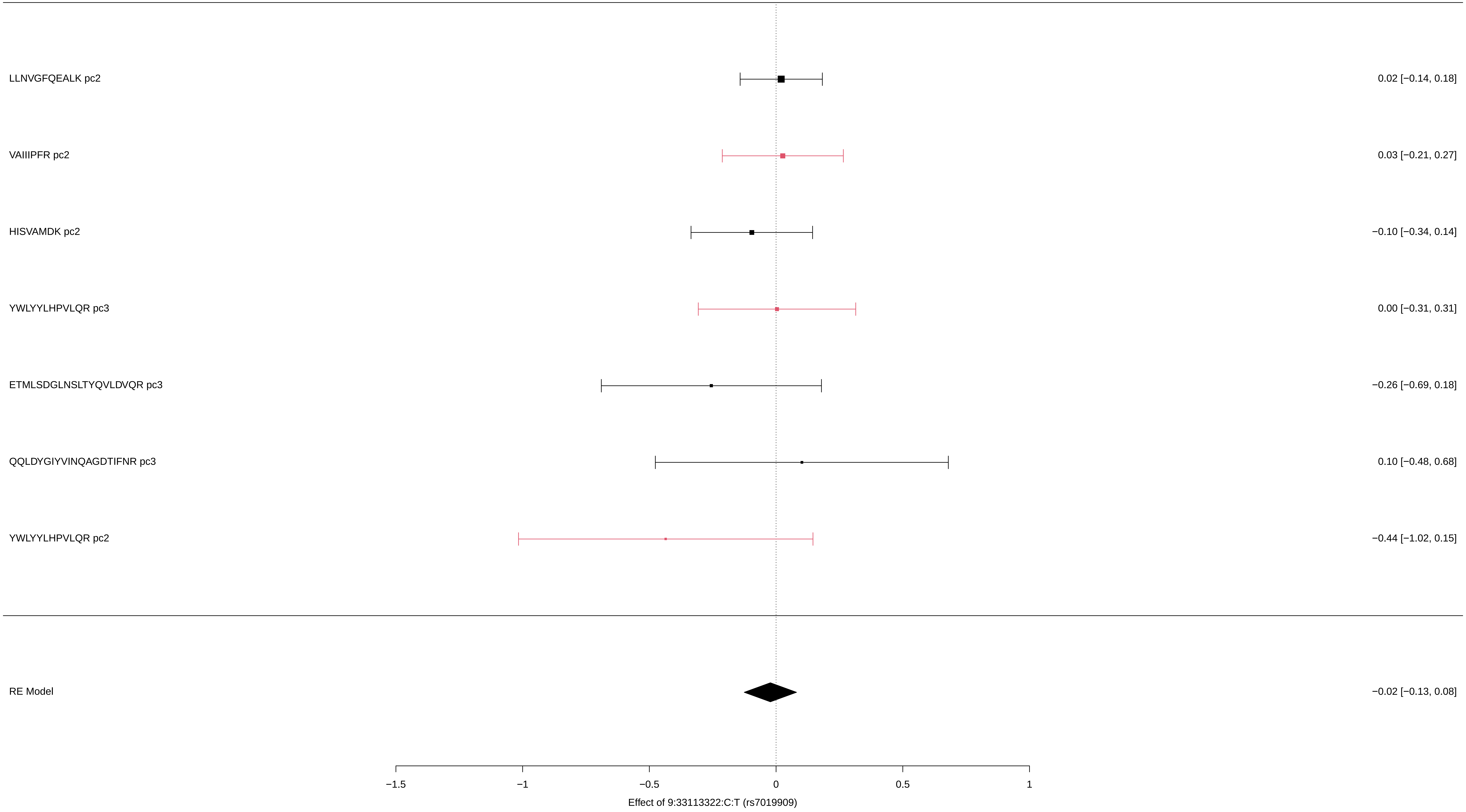

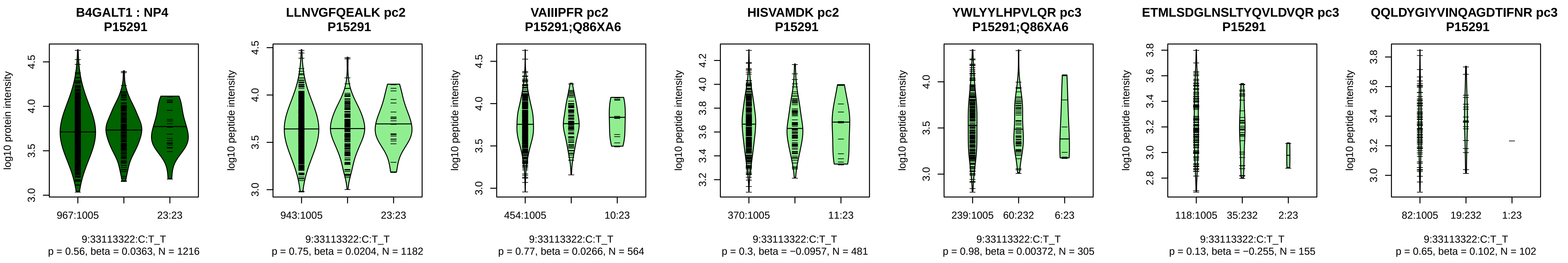

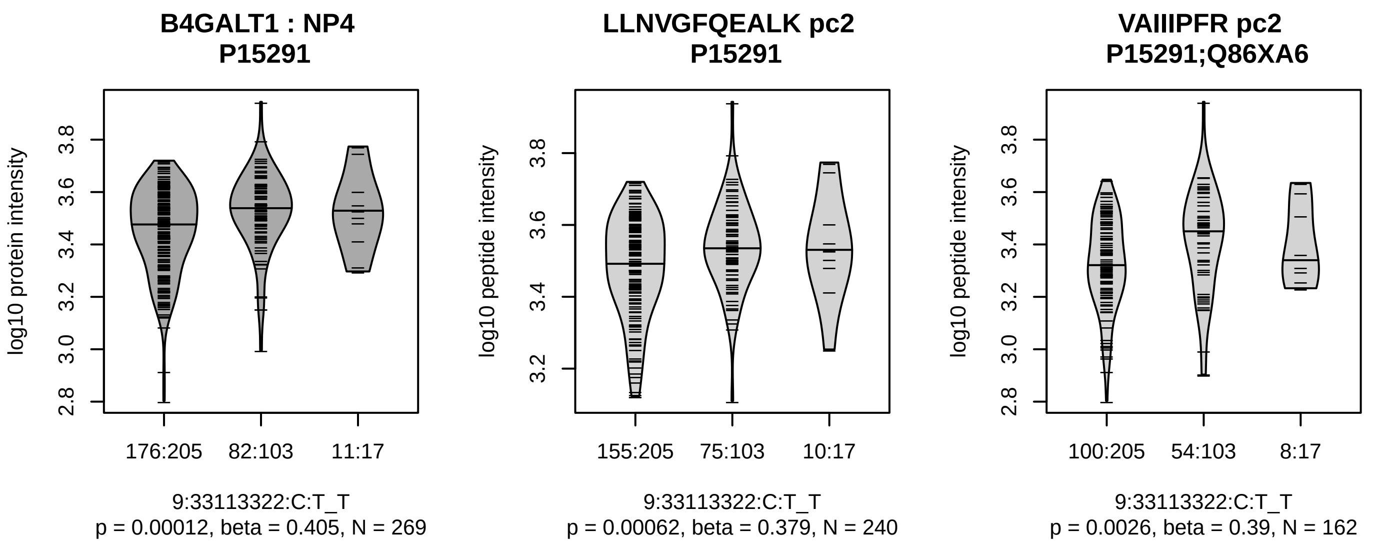

Assay Target: B4GALT1
UniProt: P15291
rsID: rs7019909
Target SNP: 9:33113322:C:T
Proxy SNP: 9:33113322:C:T
UKBPPP log10(p): 240.1
UKBPPP BETA: 0.314
UKBPPP MSPA: 0
–:–:–:–:–:–:–
1182:564:481:305:155:102:67

202. CELA3A (P09093)
UKBPPP MSPA=0.353

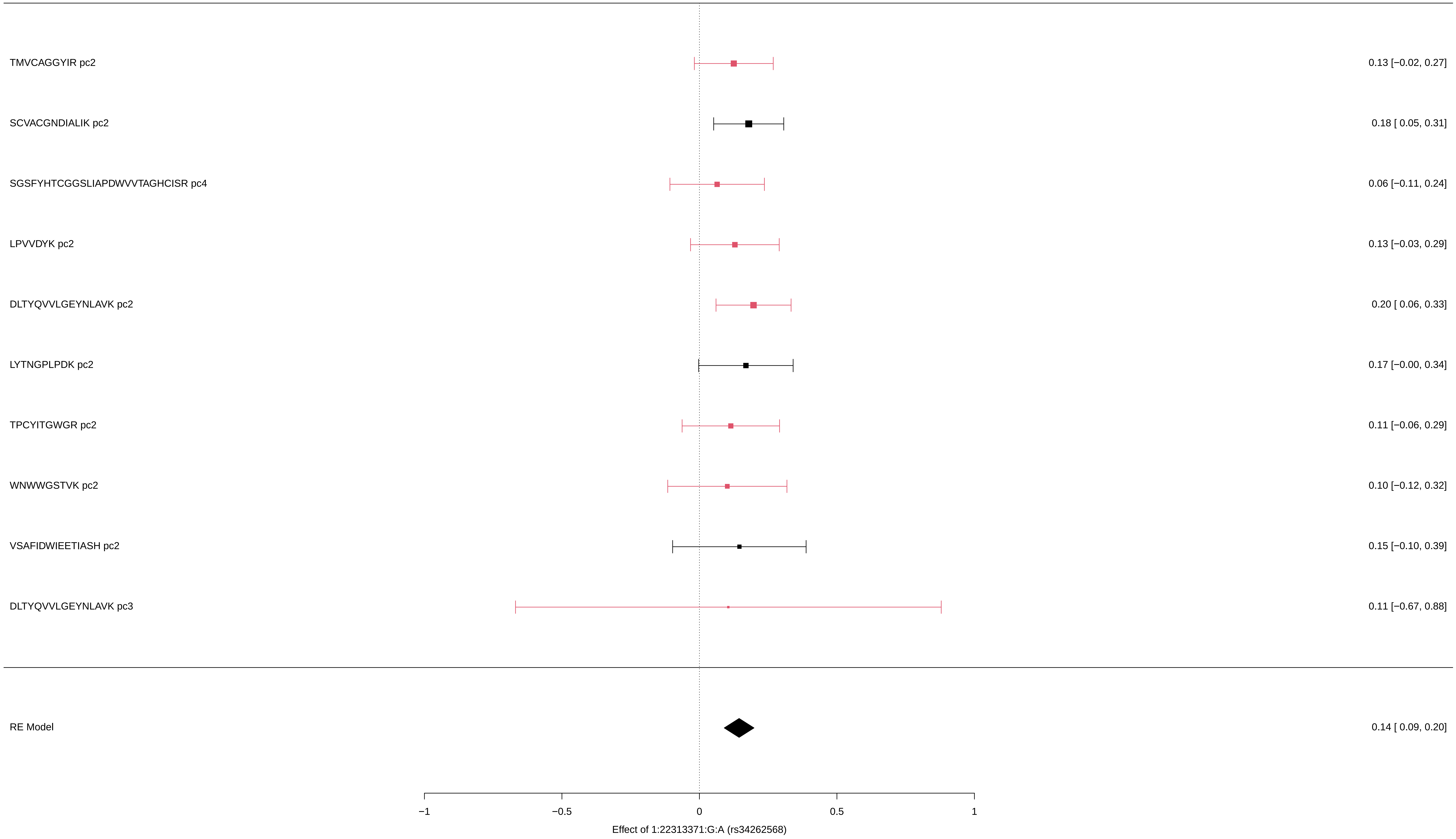

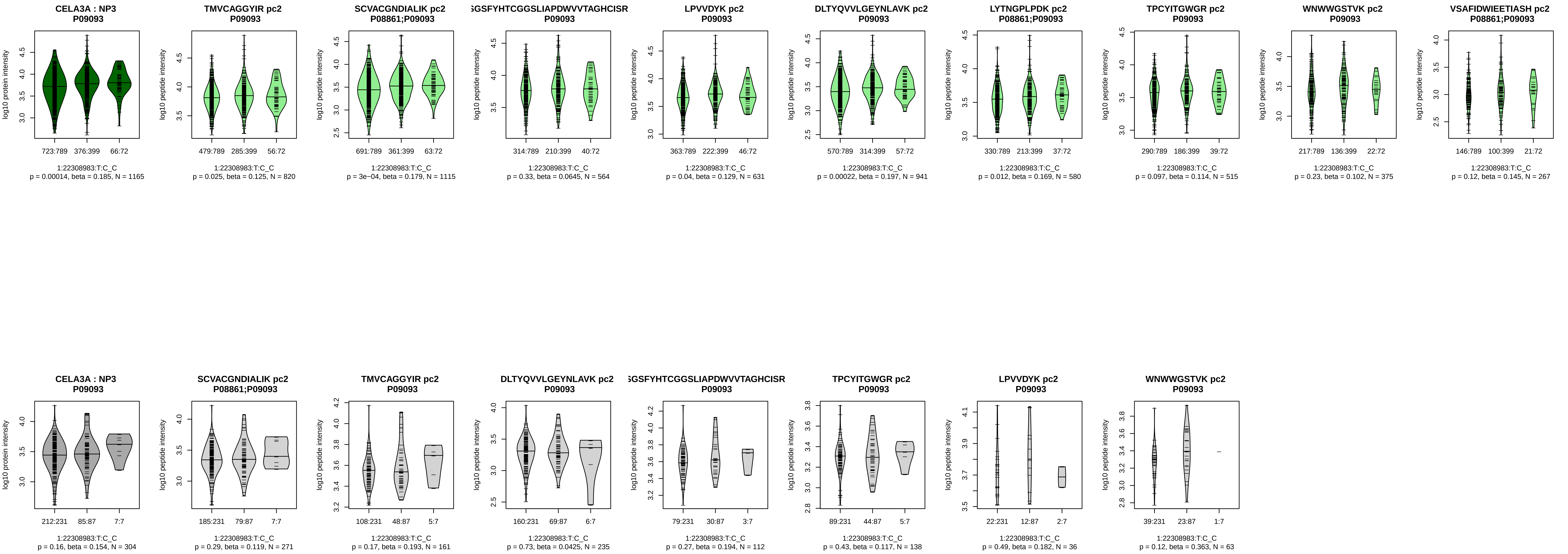

Assay Target: CELA3A
UniProt: P09093
rsID: rs34262568
Target SNP: 1:22313371:G:A
Proxy SNP: 1:22308983:T:C
UKBPPP log10(p): 233.1
UKBPPP BETA: 0.231
UKBPPP MSPA: 0.353
–:*:–:–:*:–:–:–:–:–
820:1115:564:631:941:580:515:

203. MAN1A2 (O60476)
UKBPPP MSPA=0

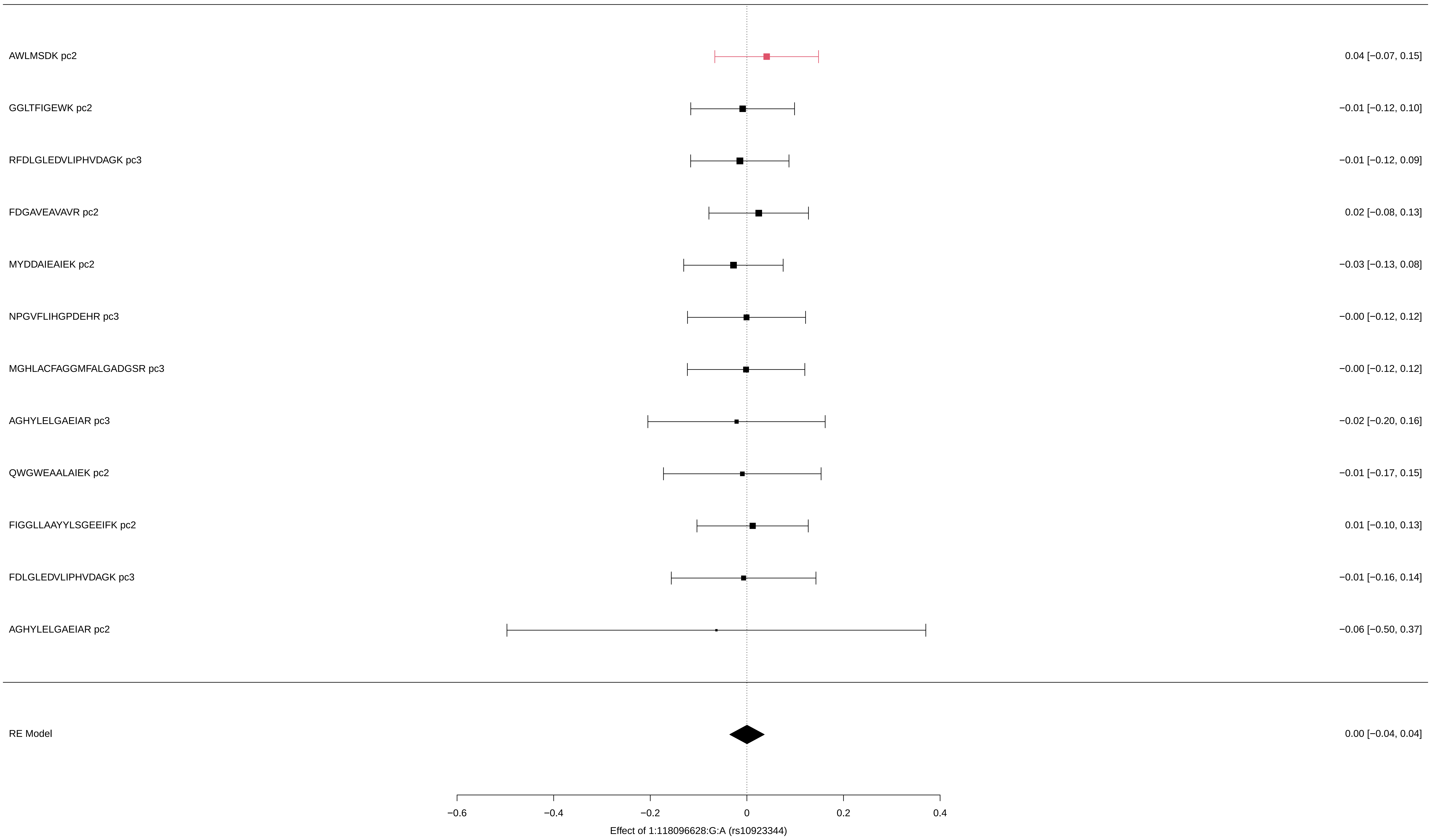

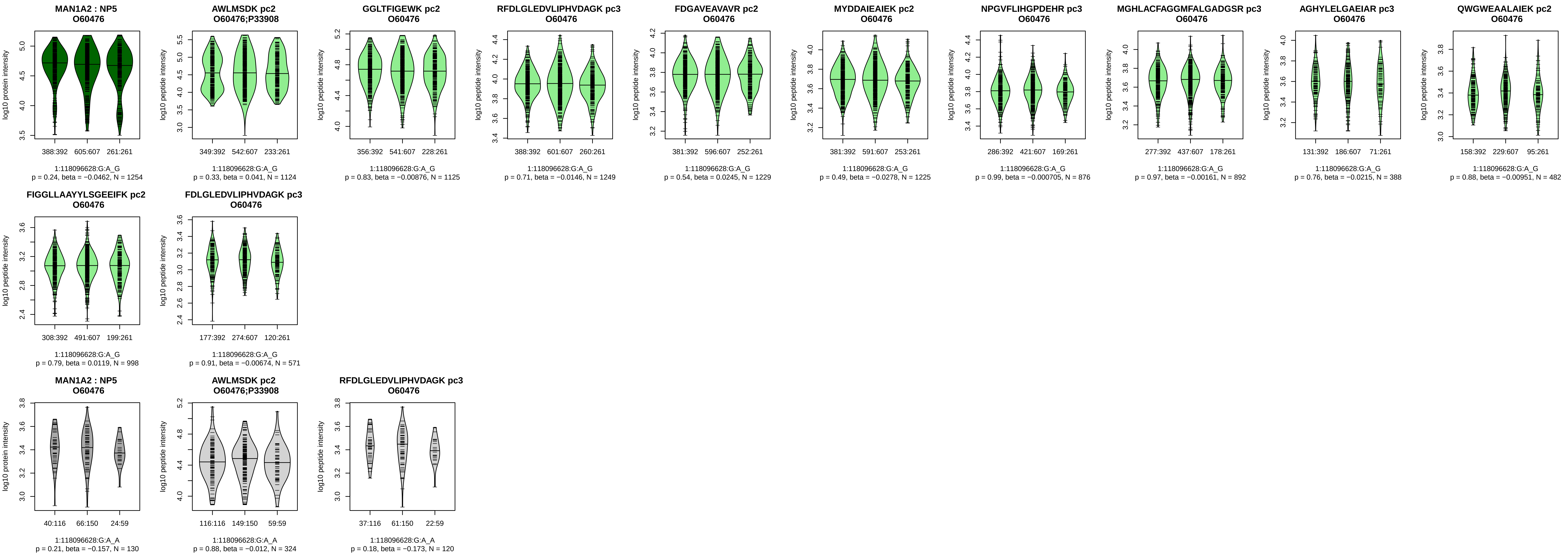

Assay Target: MAN1A2
UniProt: O60476
rsID: rs10923344
Target SNP: 1:118096628:G:A
Proxy SNP: 1:118096628:G:A
UKBPPP log10(p): 233.1
UKBPPP BETA: 0.195
UKBPPP MSPA: 0
1124:1125:1249:1229:1225:876

204. CDH5 (P33151)
UKBPPP MSPA=0.038

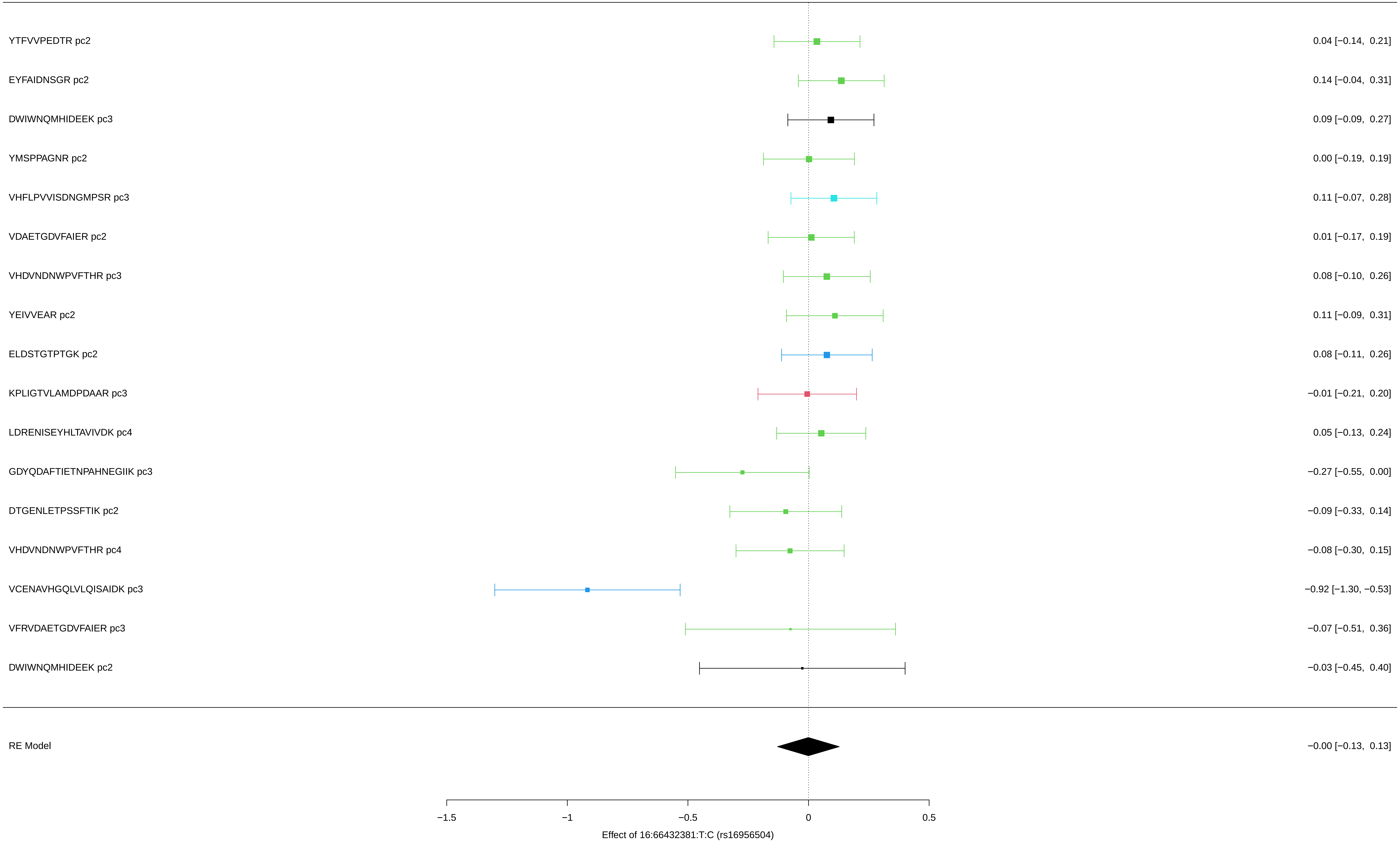

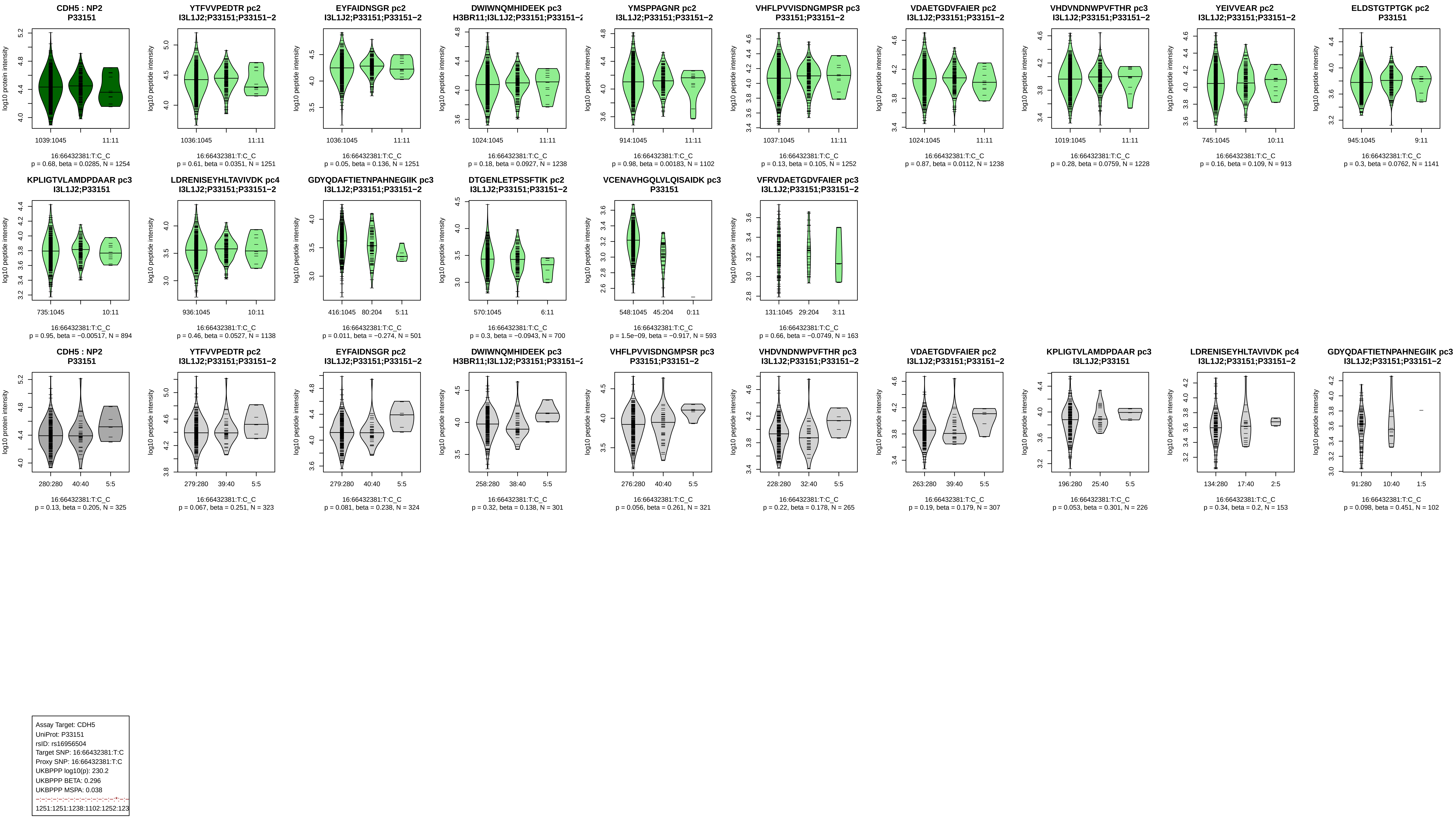
CDH5 : NP2
P33151
log10 protein intensity
1039:1045
11:11
16:66432381:T:C_C
p = 0.68, beta = 0.0285, N = 1254
YTFVVPEDTR pc2
I3L1J2;P33151;P33151−2
log10 peptide intensity
1036:1045
11:11
16:66432381:T:C_C
p = 0.61, beta = 0.0351, N = 1251
EYFAIDNSGR pc2
I3L1J2;P33151;P33151−2
1036:1045
11:11
16:66432381:T:C_C
p = 0.05, beta = 0.136, N = 1251
DWIWNQMHIDEEK pc3
H3BR11;I3L1J2;P33151;P33151−2
1024:1045
11:11
16:66432381:T:C_C
p = 0.18, beta = 0.0927, N = 1238
YMSPPAGNR pc2
I3L1J2;P33151;P33151−2
914:1045
11:11
16:66432381:T:C_C
p = 0.98, beta = 0.00183, N = 1102
VHFLPVVISDNGMPSR pc3
P33151;P33151−2
1037:1045
11:11
16:66432381:T:C_C
p = 0.13, beta = 0.105, N = 1252
VDAETGDVFAIER pc2
I3L1J2;P33151;P33151−2
1024:1045
11:11
16:66432381:T:C_C
p = 0.87, beta = 0.0112, N = 1238
VHDVNDNWPVFTHR pc3
I3L1J2;P33151;P33151−2
1019:1045
11:11
16:66432381:T:C_C
p = 0.28, beta = 0.0759, N = 1228
YEIVVEAR pc2
I3L1J2;P33151;P33151−2
745:1045
10:11
16:66432381:T:C_C
p = 0.16, beta = 0.109, N = 913
ELDSTGTPTGK pc2
P33151
945:1045
9:11
16:66432381:T:C_C
p = 0.3, beta = 0.0762, N = 1141
KPLIGTVLAMDPDAAR pc3
I3L1J2;P33151
735:1045
10:11
16:66432381:T:C_C
p = 0.95, beta = −0.00517, N = 894
LDRENISEYHLTAVIVDK pc4
I3L1J2;P33151;P33151−2
936:1045
10:11
16:66432381:T:C_C
p = 0.46, beta = 0.0527, N = 1138
GDYQDAFTIETNPAHNEGIIK pc3
I3L1J2;P33151;P33151−2
416:1045
80:204
5:11
16:66432381:T:C_C
p = 0.011, beta = −0.274, N = 501
DTGENLETPSSFTIK pc2
I3L1J2;P33151;P33151−2
570:1045
6:11
16:66432381:T:C_C
p = 0.3, beta = −0.0943, N = 700
VCENAVHGQLVLQISAIDK pc3
P33151
548:1045
45:204
0:11
16:66432381:T:C_C
p = 1.5e−09, beta = −0.917, N = 593
VFRVDAETGDVFAIER pc3
I3L1J2;P33151;P33151−2
131:1045
29:204
3:11
16:66432381:T:C_C
p = 0.66, beta = −0.0749, N = 163
CDH5 : NP2
P33151
280:280
40:40
5:5
16:66432381:T:C_C
p = 0.13, beta = 0.205, N = 325
YTFVVPEDTR pc2
I3L1J2;P33151;P33151−2
279:280
39:40
5:5
16:66432381:T:C_C
p = 0.067, beta = 0.251, N = 323
EYFAIDNSGR pc2
I3L1J2;P33151;P33151−2
279:280
40:40
5:5
16:66432381:T:C_C
p = 0.081, beta = 0.238, N = 324
DWIWNQMHIDEEK pc3
H3BR11;I3L1J2;P33151;P33151−2
258:280
38:40
5:5
16:66432381:T:C_C
p = 0.32, beta = 0.138, N = 301
VHFLPVVISDNGMPSR pc3
P33151;P33151−2
276:280
40:40
5:5
16:66432381:T:C_C
p = 0.056, beta = 0.261, N = 321
VHDVNDNWPVFTHR pc3
I3L1J2;P33151;P33151−2
228:280
32:40
5:5
16:66432381:T:C_C
p = 0.22, beta = 0.178, N = 265
VDAETGDVFAIER pc2
I3L1J2;P33151;P33151−2
263:280
39:40
5:5
16:66432381:T:C_C
p = 0.19, beta = 0.179, N = 307
KPLIGTVLAMDPDAAR pc3
I3L1J2;P33151
196:280
25:40
5:5
16:66432381:T:C_C
p = 0.053, beta = 0.301, N = 226
LDRENISEYHLTAVIVDK pc4
I3L1J2;P33151;P33151−2
134:280
17:40
2:5
16:66432381:T:C_C
p = 0.34, beta = 0.2, N = 153
GDYQDAFTIETNPAHNEGIIK pc3
I3L1J2;P33151;P33151−2
91:280
10:40
1:5
16:66432381:T:C_C
p = 0.098, beta = 0.451, N = 102
Assay Target: CDH5
UniProt: P33151
rsID: rs16956504
Target SNP: 16:66432381:T:C
Proxy SNP: 16:66432381:T:C
UKBPPP log10(p): 230.2
UKBPPP BETA: 0.296
UKBPPP MSPA: 0.038
1251:1251:1238:1102:1252:123

205. CLEC3B (P05452)
UKBPPP MSPA=0.074

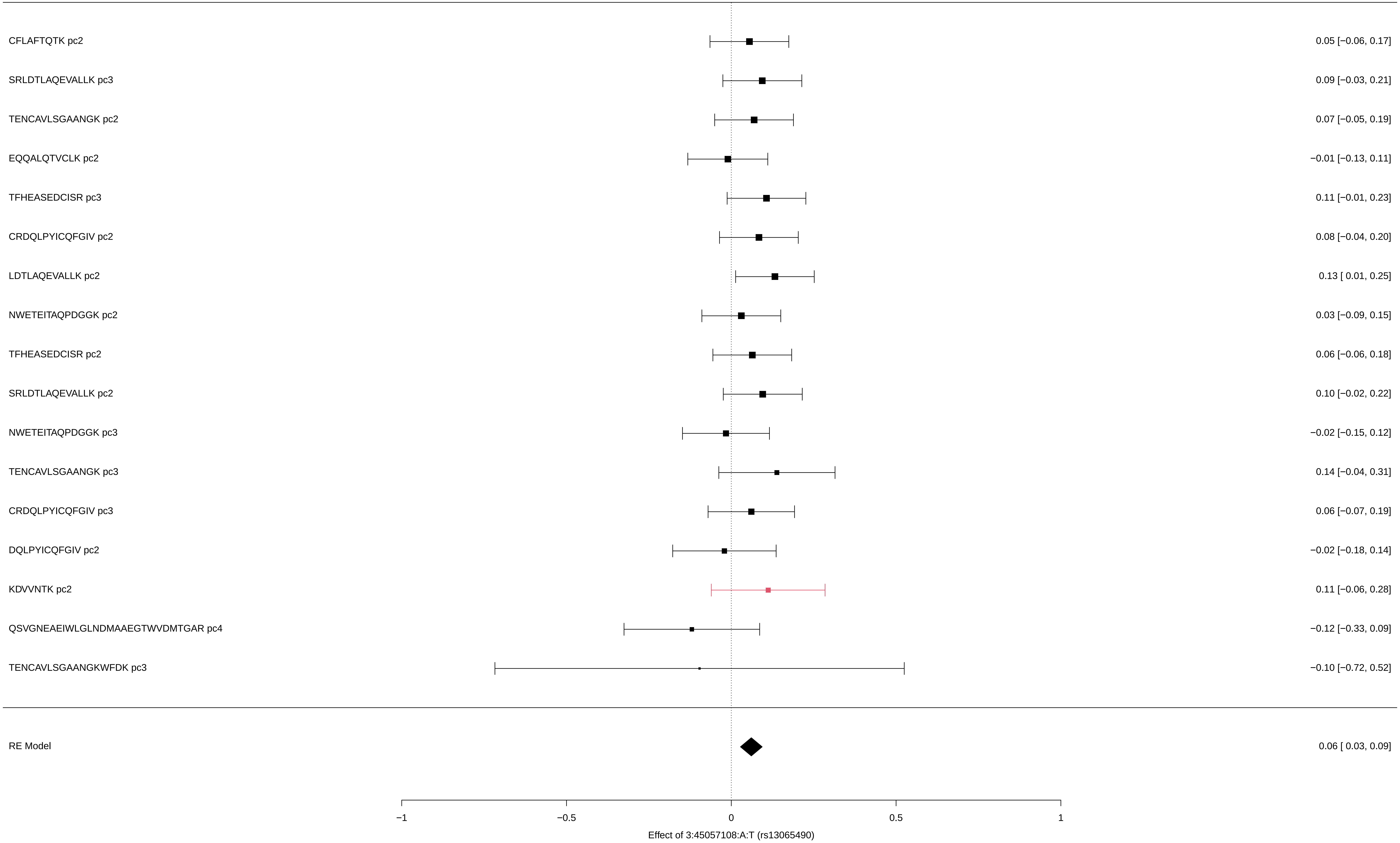

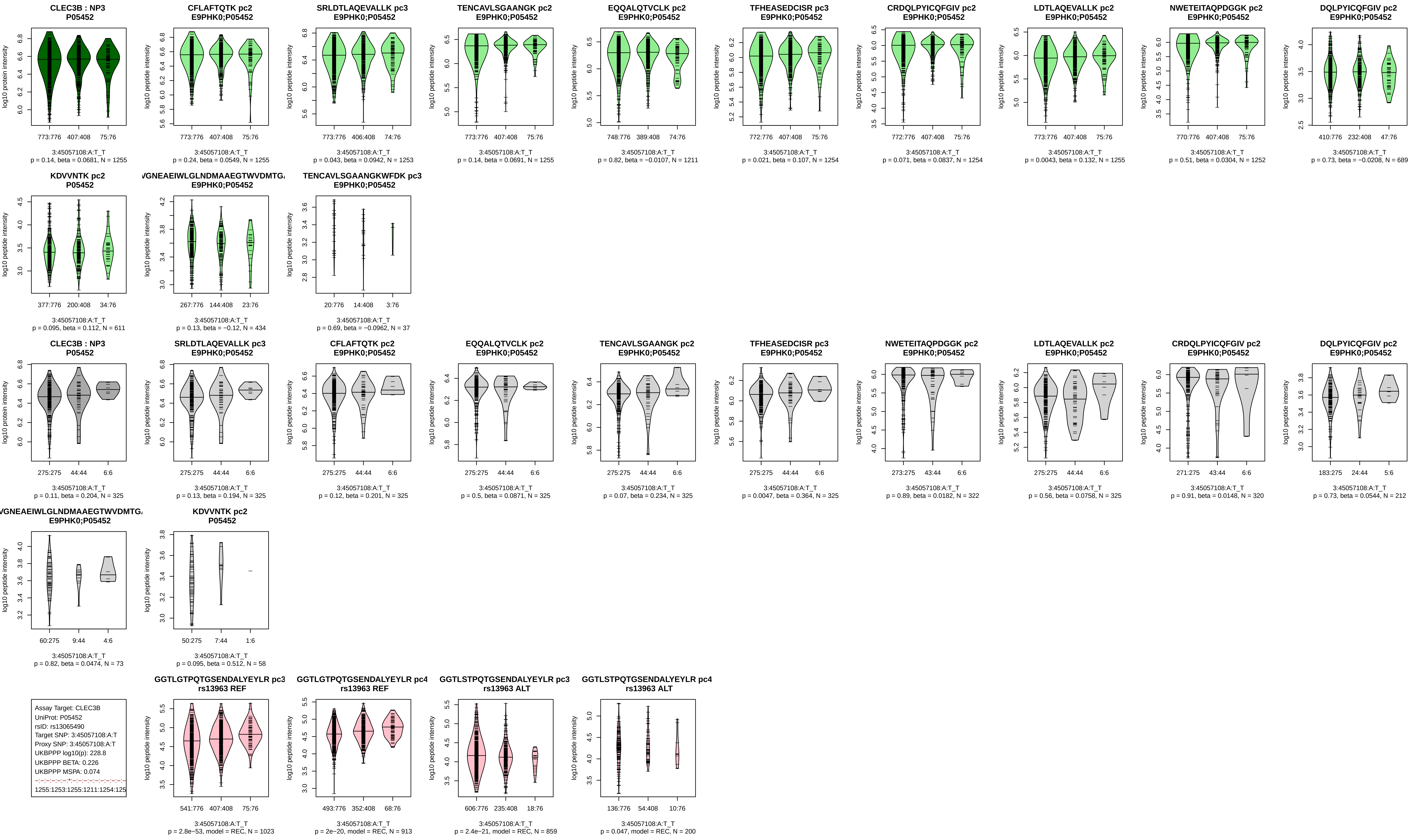
CLEC3B : NP3 P05452
CFLAFTQTK pc2 E9PHK0;P05452
SRLDTLAQEVALLK pc3 E9PHK0;P05452
TENCAVLSGAANGK pc2 E9PHK0;P05452
EQQALQTVCLK pc2 E9PHK0;P05452
TFHEASEDCISR pc3 E9PHK0;P05452
CRDQLPYICQFGIV pc2 E9PHK0;P05452
LDTLAQEVALLK pc2 E9PHK0;P05452
NWETEITAQPDGGK pc2 E9PHK0;P05452
DQLPYICQFGIV pc2 E9PHK0;P05452
log10 protein intensity
log10 peptide intensity
3:45057108:A:T_T
p = 0.14, beta = 0.0681, N = 1255
p = 0.24, beta = 0.0549, N = 1255
p = 0.043, beta = 0.0942, N = 1253
p = 0.14, beta = 0.0691, N = 1255
p = 0.82, beta = −0.0107, N = 1211
p = 0.021, beta = 0.107, N = 1254
p = 0.071, beta = 0.0837, N = 1254
p = 0.0043, beta = 0.132, N = 1255
p = 0.51, beta = 0.0304, N = 1252
p = 0.73, beta = −0.0208, N = 689
KDVVNTK pc2 P05452
VGNEAEIWLGLNDMAAEGTWVDMTG/ E9PHK0;P05452
TENCAVLSGAANGKWFDK pc3 E9PHK0;P05452
p = 0.095, beta = 0.112, N = 611
p = 0.13, beta = −0.12, N = 434
p = 0.69, beta = −0.0962, N = 37
CLEC3B : NP3 P05452
SRLDTLAQEVALLK pc3 E9PHK0;P05452
CFLAFTQTK pc2 E9PHK0;P05452
EQQALQTVCLK pc2 E9PHK0;P05452
TENCAVLSGAANGK pc2 E9PHK0;P05452
TFHEASEDCISR pc3 E9PHK0;P05452
NWETEITAQPDGGK pc2 E9PHK0;P05452
LDTLAQEVALLK pc2 E9PHK0;P05452
CRDQLPYICQFGIV pc2 E9PHK0;P05452
DQLPYICQFGIV pc2 E9PHK0;P05452
p = 0.11, beta = 0.204, N = 325
p = 0.13, beta = 0.194, N = 325
p = 0.12, beta = 0.201, N = 325
p = 0.5, beta = 0.0871, N = 325
p = 0.07, beta = 0.234, N = 325
p = 0.0047, beta = 0.364, N = 325
p = 0.89, beta = 0.0182, N = 322
p = 0.56, beta = 0.0758, N = 325
p = 0.91, beta = 0.0148, N = 320
p = 0.73, beta = 0.0544, N = 212
VGNEAEIWLGLNDMAAEGTWVDMTG/ E9PHK0;P05452
KDVVNTK pc2 P05452
p = 0.82, beta = 0.0474, N = 73
p = 0.095, beta = 0.512, N = 58
Assay Target: CLEC3B
UniProt: P05452
rsID: rs13065490
Target SNP: 3:45057108:A:T
Proxy SNP: 3:45057108:A:T
UKBPPP log10(p): 228.8
UKBPPP BETA: 0.226
UKBPPP MSPA: 0.074
1255:1253:1255:1211:1254:125
GGTLGTPQTGSENDALYEYLR pc3 rs13963 REF
GGTLGTPQTGSENDALYEYLR pc4 rs13963 REF
GGTLSTPQTGSENDALYEYLR pc3 rs13963 ALT
GGTLSTPQTGSENDALYEYLR pc4 rs13963 ALT
p = 2.8e−53, model = REC, N = 1023
p = 2e−20, model = REC, N = 913
p = 2.4e−21, model = REC, N = 859
p = 0.047, model = REC, N = 200

206. SERPINA5 (P05154)
UKBPPP MSPA=0

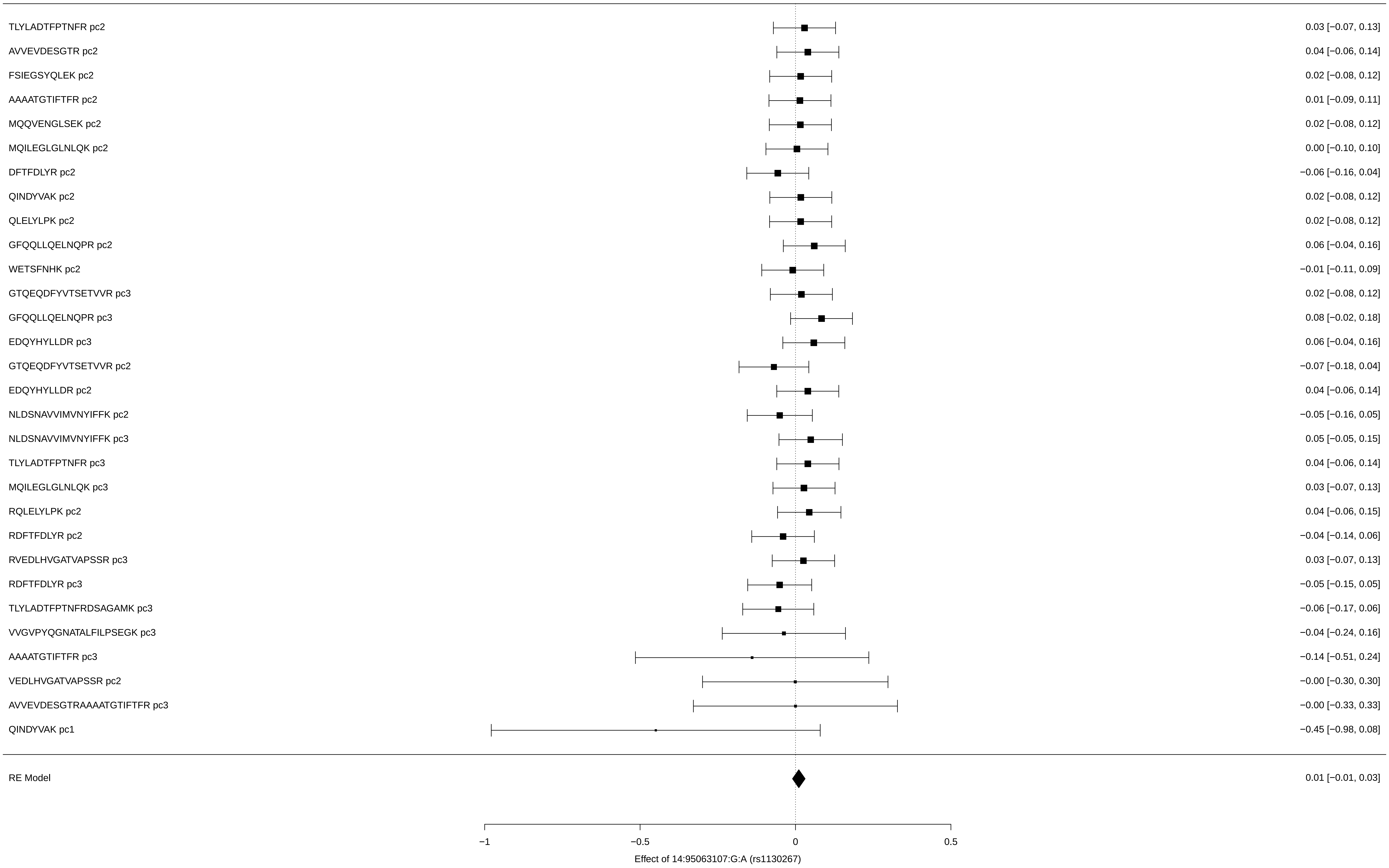

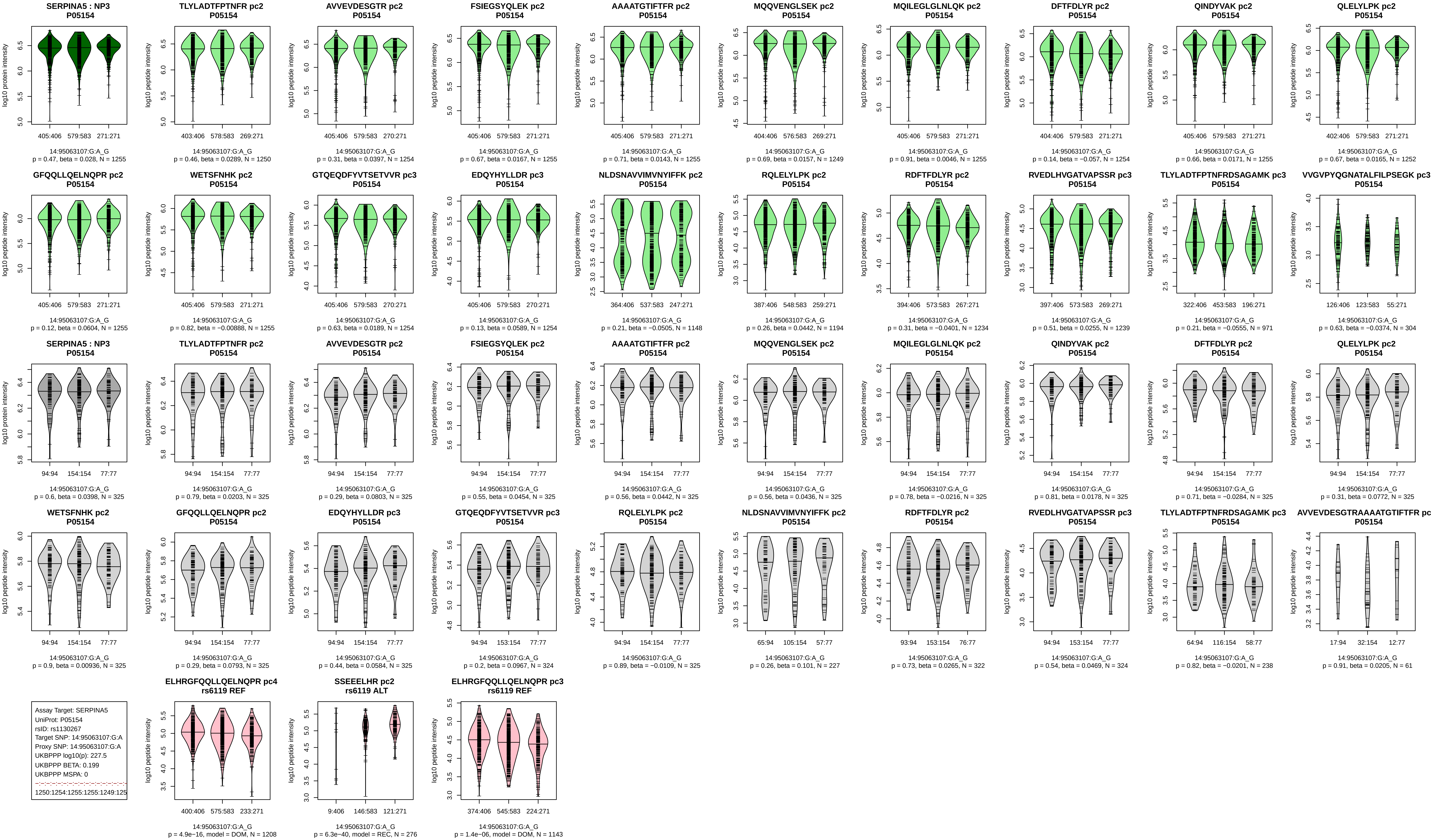
SERPINA5 : NP3
P05154
log10 protein intensity
405:406 579:583 271:271
14:95063107:G:A_G
p = 0.47, beta = 0.028, N = 1255
TLYLADTFPTNFR pc2
P05154
log10 peptide intensity
403:406 578:583 269:271
14:95063107:G:A_G
p = 0.46, beta = 0.0289, N = 1250
AVVEVDESGTR pc2
P05154
405:406 579:583 270:271
14:95063107:G:A_G
p = 0.31, beta = 0.0397, N = 1254
FSIEGSYQLEK pc2
P05154
405:406 579:583 271:271
14:95063107:G:A_G
p = 0.67, beta = 0.0167, N = 1255
AAAATGTIFTFR pc2
P05154
405:406 579:583 271:271
14:95063107:G:A_G
p = 0.71, beta = 0.0143, N = 1255
MQQVENGLSEK pc2
P05154
404:406 576:583 269:271
14:95063107:G:A_G
p = 0.69, beta = 0.0157, N = 1249
MQILEGLGLNLQK pc2
P05154
405:406 579:583 271:271
14:95063107:G:A_G
p = 0.91, beta = 0.0046, N = 1255
DFTFDLYR pc2
P05154
404:406 579:583 271:271
14:95063107:G:A_G
p = 0.14, beta = −0.057, N = 1254
QINDYVAK pc2
P05154
405:406 579:583 271:271
14:95063107:G:A_G
p = 0.66, beta = 0.0171, N = 1255
QLELYLPK pc2
P05154
402:406 579:583 271:271
14:95063107:G:A_G
p = 0.67, beta = 0.0165, N = 1252
GFQQLLQELNQPR pc2
P05154
405:406 579:583 271:271
14:95063107:G:A_G
p = 0.12, beta = 0.0604, N = 1255
WETSFNHK pc2
P05154
405:406 579:583 271:271
14:95063107:G:A_G
p = 0.82, beta = −0.00888, N = 1255
GTQEQDFYVTSETVVR pc3
P05154
405:406 579:583 270:271
14:95063107:G:A_G
p = 0.63, beta = 0.0189, N = 1254
EDQYHYLLDR pc3
P05154
405:406 579:583 270:271
14:95063107:G:A_G
p = 0.13, beta = 0.0589, N = 1254
NLDSNAVVIMVNYIFFK pc2
P05154
364:406 537:583 247:271
14:95063107:G:A_G
p = 0.21, beta = −0.0505, N = 1148
RQLELYLPK pc2
P05154
387:406 548:583 259:271
14:95063107:G:A_G
p = 0.26, beta = 0.0442, N = 1194
RDFTFDLYR pc2
P05154
394:406 573:583 267:271
14:95063107:G:A_G
p = 0.31, beta = −0.0401, N = 1234
RVEDLHVGATVAPSSR pc3
P05154
397:406 573:583 269:271
14:95063107:G:A_G
p = 0.51, beta = 0.0255, N = 1239
TLYLADTFPTNFRDSAGAMK pc3
P05154
322:406 453:583 196:271
14:95063107:G:A_G
p = 0.21, beta = −0.0555, N = 971
VVGVPYQGNATALFILPSEGK pc3
P05154
126:406 123:583 55:271
14:95063107:G:A_G
p = 0.63, beta = −0.0374, N = 304
SERPINA5 : NP3
P05154
94:94 154:154 77:77
14:95063107:G:A_G
p = 0.6, beta = 0.0398, N = 325
TLYLADTFPTNFR pc2
P05154
94:94 154:154 77:77
14:95063107:G:A_G
p = 0.79, beta = 0.0203, N = 325
AVVEVDESGTR pc2
P05154
94:94 154:154 77:77
14:95063107:G:A_G
p = 0.29, beta = 0.0803, N = 325
FSIEGSYQLEK pc2
P05154
94:94 154:154 77:77
14:95063107:G:A_G
p = 0.55, beta = 0.0454, N = 325
AAAATGTIFTFR pc2
P05154
94:94 154:154 77:77
14:95063107:G:A_G
p = 0.56, beta = 0.0442, N = 325
MQQVENGLSEK pc2
P05154
94:94 154:154 77:77
14:95063107:G:A_G
p = 0.56, beta = 0.0436, N = 325
MQILEGLGLNLQK pc2
P05154
94:94 154:154 77:77
14:95063107:G:A_G
p = 0.78, beta = −0.0216, N = 325
QINDYVAK pc2
P05154
94:94 154:154 77:77
14:95063107:G:A_G
p = 0.81, beta = 0.0178, N = 325
DFTFDLYR pc2
P05154
94:94 154:154 77:77
14:95063107:G:A_G
p = 0.71, beta = −0.0284, N = 325
QLELYLPK pc2
P05154
94:94 154:154 77:77
14:95063107:G:A_G
p = 0.31, beta = 0.0772, N = 325
WETSFNHK pc2
P05154
94:94 154:154 77:77
14:95063107:G:A_G
p = 0.9, beta = 0.00936, N = 325
GFQQLLQELNQPR pc2
P05154
94:94 154:154 77:77
14:95063107:G:A_G
p = 0.29, beta = 0.0793, N = 325
EDQYHYLLDR pc3
P05154
94:94 154:154 77:77
14:95063107:G:A_G
p = 0.44, beta = 0.0584, N = 325
GTQEQDFYVTSETVVR pc3
P05154
94:94 153:154 77:77
14:95063107:G:A_G
p = 0.2, beta = 0.0967, N = 324
RQLELYLPK pc2
P05154
94:94 154:154 77:77
14:95063107:G:A_G
p = 0.89, beta = −0.0109, N = 325
NLDSNAVVIMVNYIFFK pc2
P05154
65:94 105:154 57:77
14:95063107:G:A_G
p = 0.26, beta = 0.101, N = 227
RDFTFDLYR pc2
P05154
93:94 153:154 76:77
14:95063107:G:A_G
p = 0.73, beta = 0.0265, N = 322
RVEDLHVGATVAPSSR pc3
P05154
94:94 153:154 77:77
14:95063107:G:A_G
p = 0.54, beta = 0.0469, N = 324
TLYLADTFPTNFRDSAGAMK pc3
P05154
64:94 116:154 58:77
14:95063107:G:A_G
p = 0.82, beta = −0.0201, N = 238
AVVEVDESGTRAAAATGTIFTFR pc
P05154
17:94 32:154 12:77
14:95063107:G:A_G
p = 0.91, beta = 0.0205, N = 61
Assay Target: SERPINA5
UniProt: P05154
rsID: rs1130267
Target SNP: 14:95063107:G:A
Proxy SNP: 14:95063107:G:A
UKBPPP log10(p): 227.5
UKBPPP BETA: 0.199
UKBPPP MSPA: 0
1250:1254:1255:1255:1249:125
ELHRGFQQLLQELNQPR pc4
rs6119 REF
400:406 575:583 233:271
14:95063107:G:A_G
p = 4.9e−16, model = DOM, N = 1208
SSEEELHR pc2
rs6119 ALT
9:406 146:583 121:271
14:95063107:G:A_G
p = 6.3e−40, model = REC, N = 276
ELHRGFQQLLQELNQPR pc3
rs6119 REF
374:406 545:583 224:271
14:95063107:G:A_G
p = 1.4e−06, model = DOM, N = 1143

207. RNASE1 (P07998)
UKBPPP MSPA=0.141

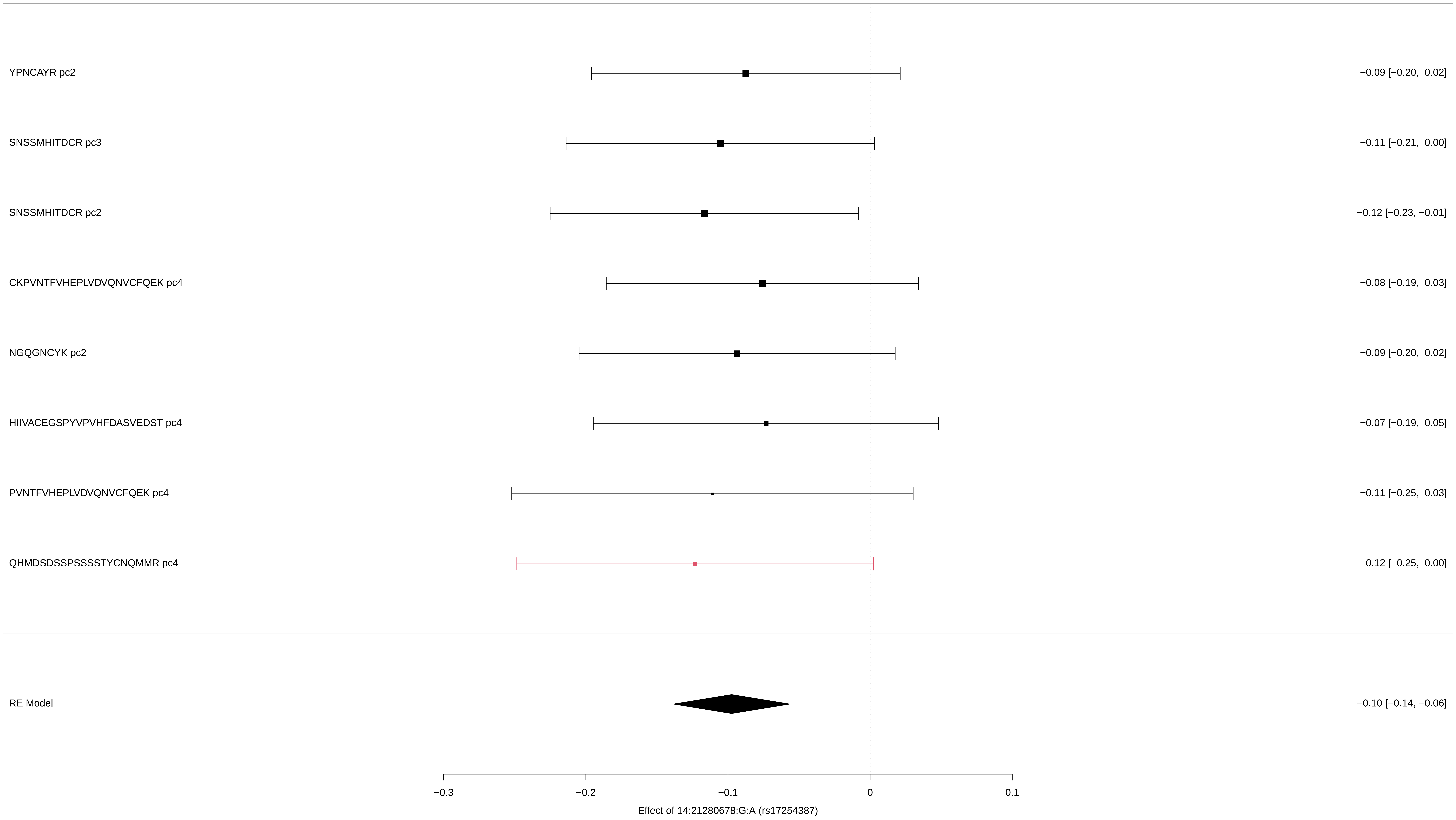

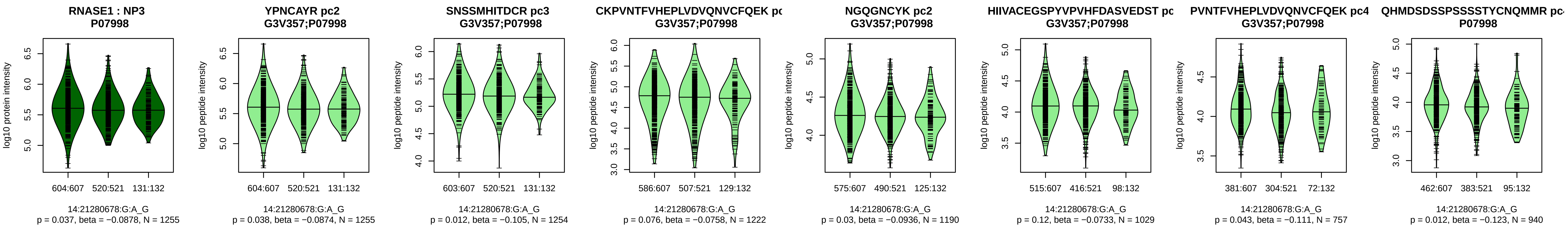

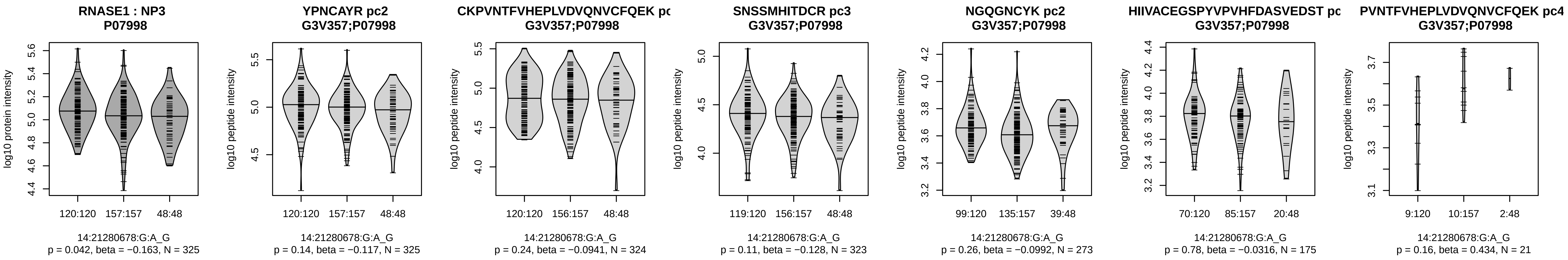

Assay Target: RNASE1
UniProt: P07998
rsID: rs17254387
Target SNP: 14:21280678:G:A
Proxy SNP: 14:21280678:G:A
UKBPPP log10(p): 226.5
UKBPPP BETA: 0.206
UKBPPP MSPA: 0.141
–:–:*:–:–:–:–:–
1255:1254:1254:1222:1190:102

208. CD5L (O43866)
UKBPPP MSPA=0

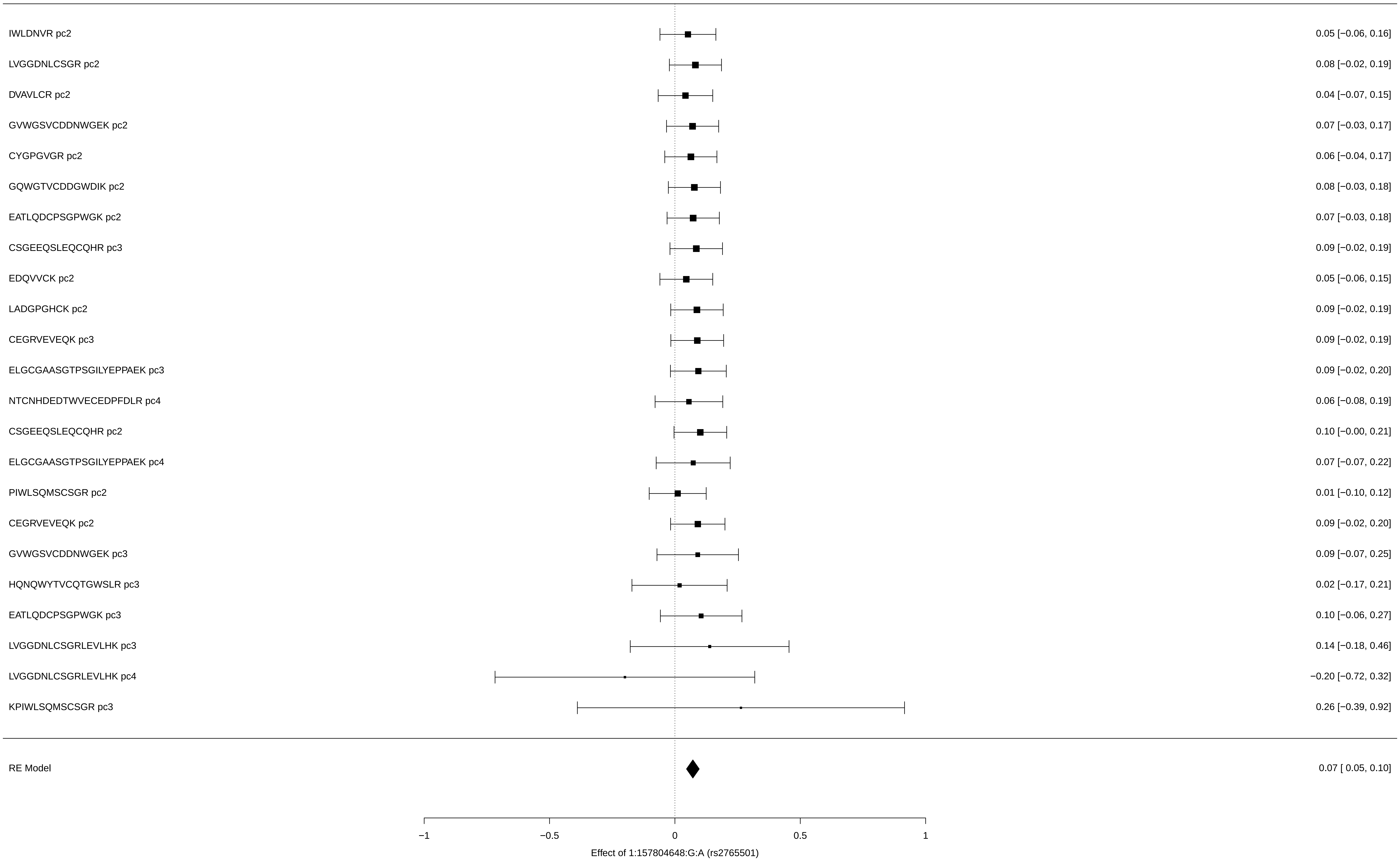

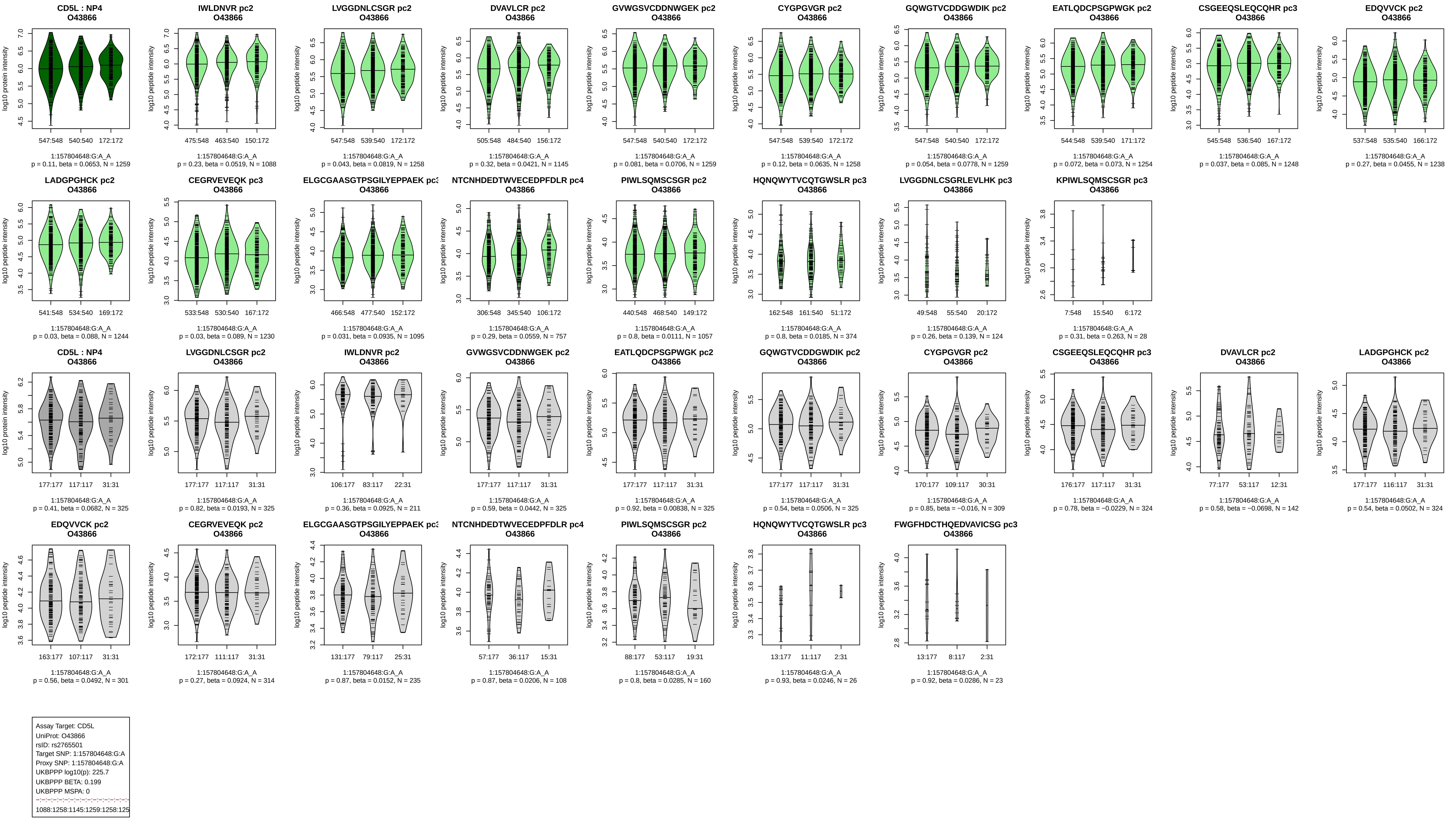
CD5L : NP4
O43866
log10 protein intensity
547:548 540:540 172:172
1:157804648:G:A_A
p = 0.11, beta = 0.0653, N = 1259
IWLDNVR pc2
O43866
log10 peptide intensity
475:548 463:540 150:172
1:157804648:G:A_A
p = 0.23, beta = 0.0519, N = 1088
LVGGDNLCSGR pc2
O43866
547:548 539:540 172:172
1:157804648:G:A_A
p = 0.043, beta = 0.0819, N = 1258
DVAVLCR pc2
O43866
505:548 484:540 156:172
1:157804648:G:A_A
p = 0.32, beta = 0.0421, N = 1145
GVWGSVCDDNWGEK pc2
O43866
547:548 540:540 172:172
1:157804648:G:A_A
p = 0.081, beta = 0.0706, N = 1259
CYGPGVGR pc2
O43866
547:548 539:540 172:172
1:157804648:G:A_A
p = 0.12, beta = 0.0635, N = 1258
GQWGTVCDDGWDIK pc2
O43866
547:548 540:540 172:172
1:157804648:G:A_A
p = 0.054, beta = 0.0778, N = 1259
EATLQDCPSGPWGK pc2
O43866
544:548 539:540 171:172
1:157804648:G:A_A
p = 0.072, beta = 0.073, N = 1254
CSGEEQSLEQCQHR pc3
O43866
545:548 536:540 167:172
1:157804648:G:A_A
p = 0.037, beta = 0.085, N = 1248
EDQVVCK pc2
O43866
537:548 535:540 166:172
1:157804648:G:A_A
p = 0.27, beta = 0.0455, N = 1238
LADGPGHCK pc2
O43866
541:548 534:540 169:172
1:157804648:G:A_A
p = 0.03, beta = 0.088, N = 1244
CEGRVEVEQK pc3
O43866
533:548 530:540 167:172
1:157804648:G:A_A
p = 0.03, beta = 0.089, N = 1230
ELGCGAASGTPSGILYEPPAEK pc3
O43866
466:548 477:540 152:172
1:157804648:G:A_A
p = 0.031, beta = 0.0935, N = 1095
NTCNHDEDTWVECEDPFDLR pc4
O43866
306:548 345:540 106:172
1:157804648:G:A_A
p = 0.29, beta = 0.0559, N = 757
PIWLSQMSCSGR pc2
O43866
440:548 468:540 149:172
1:157804648:G:A_A
p = 0.8, beta = 0.0111, N = 1057
HQNQWYTVCQTGWSLR pc3
O43866
162:548 161:540 51:172
1:157804648:G:A_A
p = 0.8, beta = 0.0185, N = 374
LVGGDNLCSGRLEVLHK pc3
O43866
49:548 55:540 20:172
1:157804648:G:A_A
p = 0.26, beta = 0.139, N = 124
KPIWLSQMSCSGR pc3
O43866
7:548 15:540 6:172
1:157804648:G:A_A
p = 0.31, beta = 0.263, N = 28
CD5L : NP4
O43866
177:177 117:117 31:31
1:157804648:G:A_A
p = 0.41, beta = 0.0682, N = 325
LVGGDNLCSGR pc2
O43866
177:177 117:117 31:31
1:157804648:G:A_A
p = 0.82, beta = 0.0193, N = 325
IWLDNVR pc2
O43866
106:177 83:117 22:31
1:157804648:G:A_A
p = 0.36, beta = 0.0925, N = 211
GVWGSVCDDNWGEK pc2
O43866
177:177 117:117 31:31
1:157804648:G:A_A
p = 0.59, beta = 0.0442, N = 325
EATLQDCPSGPWGK pc2
O43866
177:177 117:117 31:31
1:157804648:G:A_A
p = 0.92, beta = 0.00838, N = 325
GQWGTVCDDGWDIK pc2
O43866
177:177 117:117 31:31
1:157804648:G:A_A
p = 0.54, beta = 0.0506, N = 325
CYGPGVGR pc2
O43866
170:177 109:117 30:31
1:157804648:G:A_A
p = 0.85, beta = −0.016, N = 309
CSGEEQSLEQCQHR pc3
O43866
176:177 117:117 31:31
1:157804648:G:A_A
p = 0.78, beta = −0.0229, N = 324
DVAVLCR pc2
O43866
77:177 53:117 12:31
1:157804648:G:A_A
p = 0.58, beta = −0.0698, N = 142
LADGPGHCK pc2
O43866
177:177 116:117 31:31
1:157804648:G:A_A
p = 0.54, beta = 0.0502, N = 324
EDQVVCK pc2
O43866
163:177 107:117 31:31
1:157804648:G:A_A
p = 0.56, beta = 0.0492, N = 301
CEGRVEVEQK pc2
O43866
172:177 111:117 31:31
1:157804648:G:A_A
p = 0.27, beta = 0.0924, N = 314
ELGCGAASGTPSGILYEPPAEK pc3
O43866
131:177 79:117 25:31
1:157804648:G:A_A
p = 0.87, beta = 0.0152, N = 235
NTCNHDEDTWVECEDPFDLR pc4
O43866
57:177 36:117 15:31
1:157804648:G:A_A
p = 0.87, beta = 0.0206, N = 108
PIWLSQMSCSGR pc2
O43866
88:177 53:117 19:31
1:157804648:G:A_A
p = 0.8, beta = 0.0285, N = 160
HQNQWYTVCQTGWSLR pc3
O43866
13:177 11:117 2:31
1:157804648:G:A_A
p = 0.93, beta = 0.0246, N = 26
FWGFHDCTHQEDVAVICSG pc3
O43866
13:177 8:117 2:31
1:157804648:G:A_A
p = 0.92, beta = 0.0286, N = 23
Assay Target: CD5L
UniProt: O43866
rsID: rs2765501
Target SNP: 1:157804648:G:A
Proxy SNP: 1:157804648:G:A
UKBPPP log10(p): 225.7
UKBPPP BETA: 0.199
UKBPPP MSPA: 0
1088:1258:1145:1259:1258:125

209. COL5A1 (P20908)
UKBPPP MSPA=0

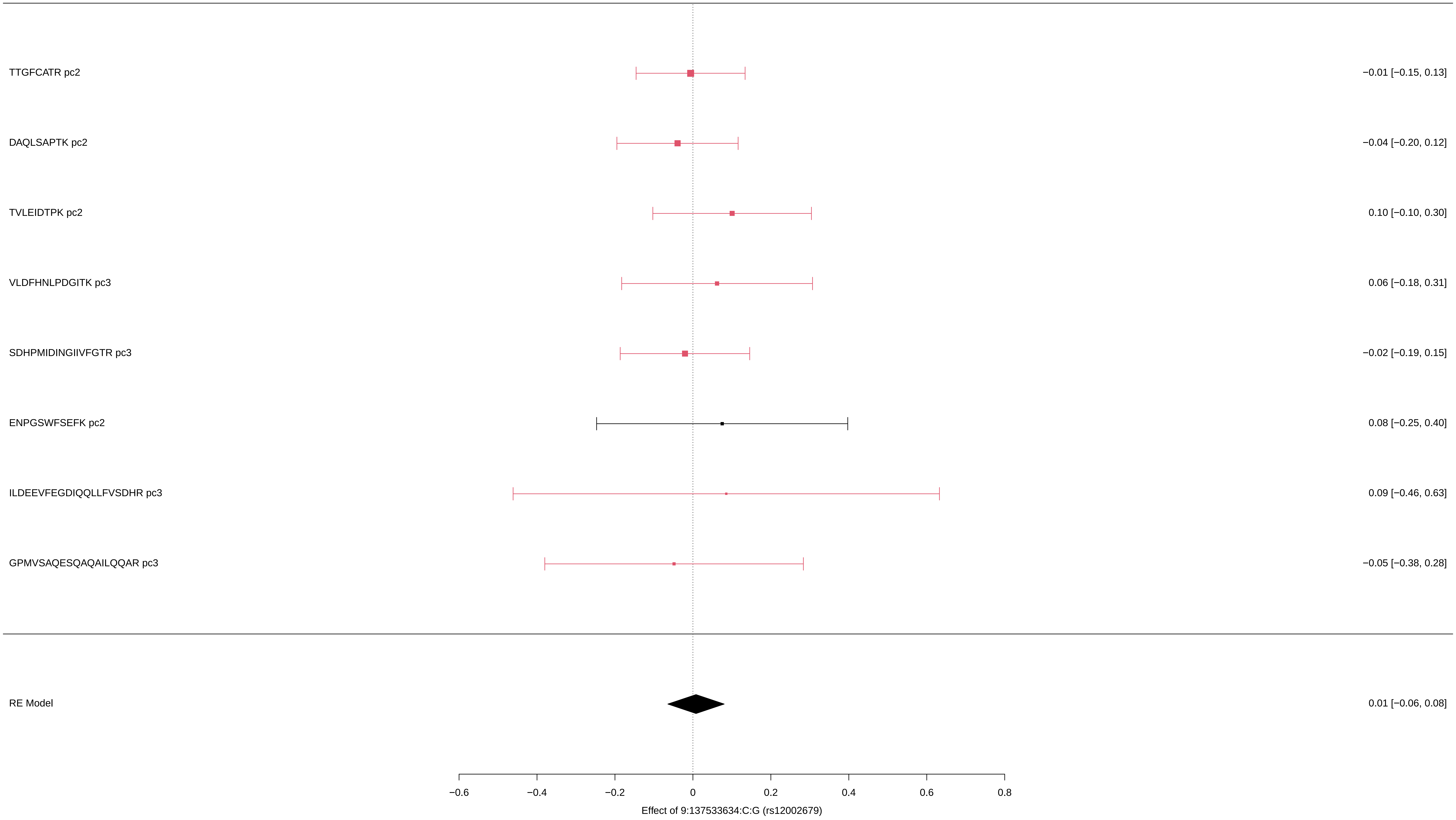

COL5A1 : NP2
P20908

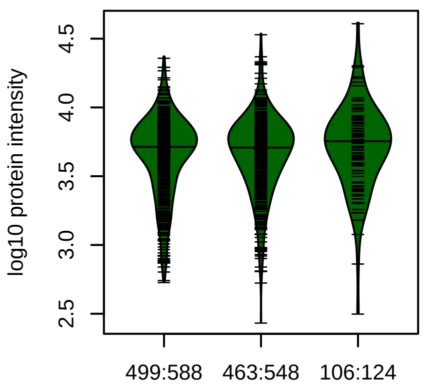

9:137533634:C:G_G
p = 0.38, beta = 0.0405, N = 1068

TTGFCATR pc2
P20908−2;P20908

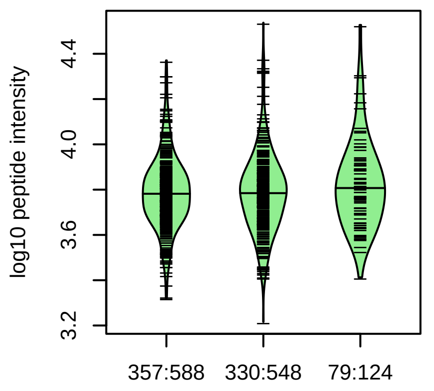

9:137533634:C:G_G
p = 0.91, beta = −0.00591, N = 766

DAQLSAPTK pc2
P20908−2;P20908

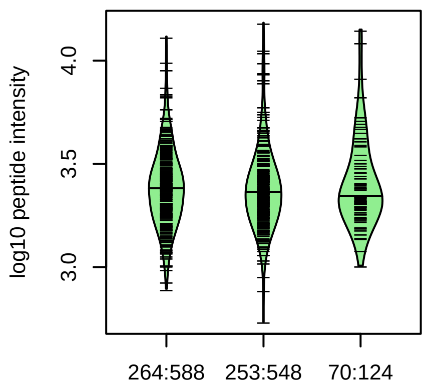

9:137533634:C:G_G
p = 0.51, beta = −0.0395, N = 587

TVLEIDTPK pc2
P20908−2;P20908

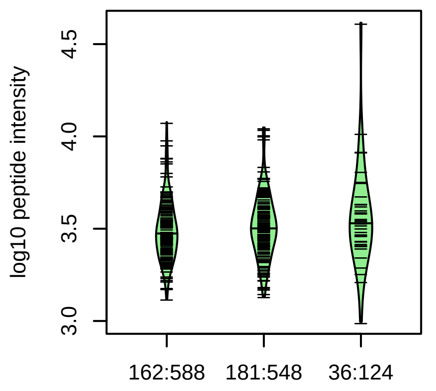

9:137533634:C:G_G
p = 0.2, beta = 0.101, N = 379

VLDFHNLPDGITK pc3
P20908−2;P20908

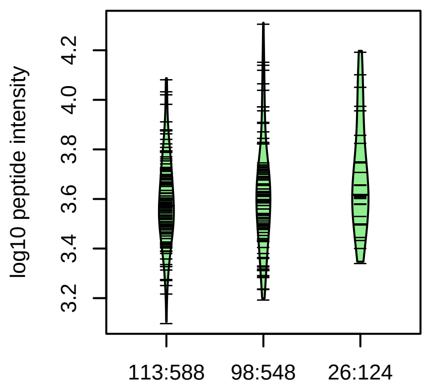

9:137533634:C:G_G
p = 0.51, beta = 0.0621, N = 237

SDHPMIDINGIIVFGTR pc3
P20908−2;P20908

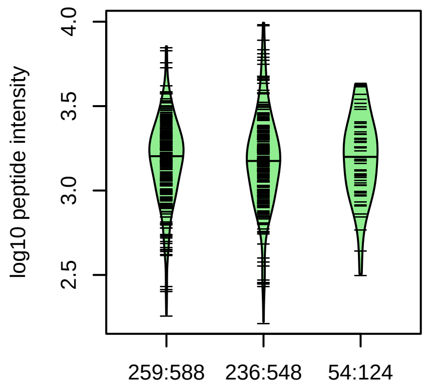

9:137533634:C:G_G
p = 0.75, beta = −0.0204, N = 549

ENPGSWFSEFK pc2
P20908

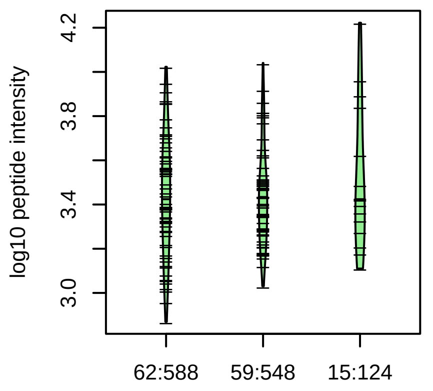

9:137533634:C:G_G
p = 0.55, beta = 0.0751, N = 136

ILDEEVFEGDIQQLLFVSDHR pc3
P20908−2;P20908

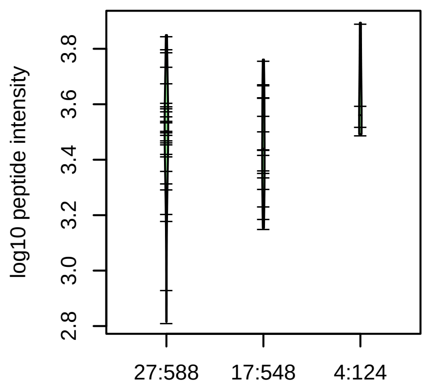

9:137533634:C:G_G
p = 0.69, beta = 0.0856, N = 48

GPMVSAQESQAQAILQQAR pc3
P20908−2;P20908

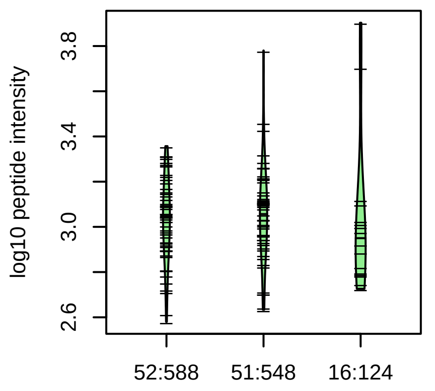

9:137533634:C:G_G
p = 0.71, beta = −0.0483, N = 119

COL5A1 : NP2
P20908

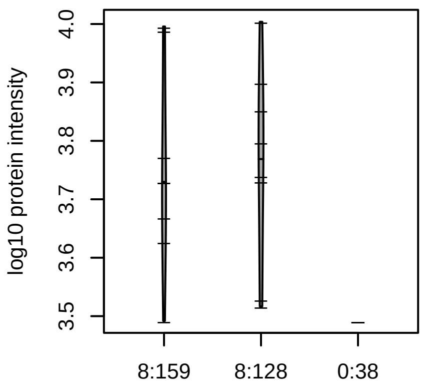

9:137533634:C:G_G
p = 0.55, beta = −0.274, N = 16

Assay Target: COL5A1
UniProt: P20908
rsID: rs12002679
Target SNP: 9:137533634:C:G
Proxy SNP: 9:137533634:C:G
UKBPPP log10(p): 223.2
UKBPPP BETA: 0.221
UKBPPP MSPA: 0
–:–:–:–:–:–:–:–
766:587:379:237:549:136:48:11

210. MAMDC2 (Q7Z304)
UKBPPP MSPA=0.257

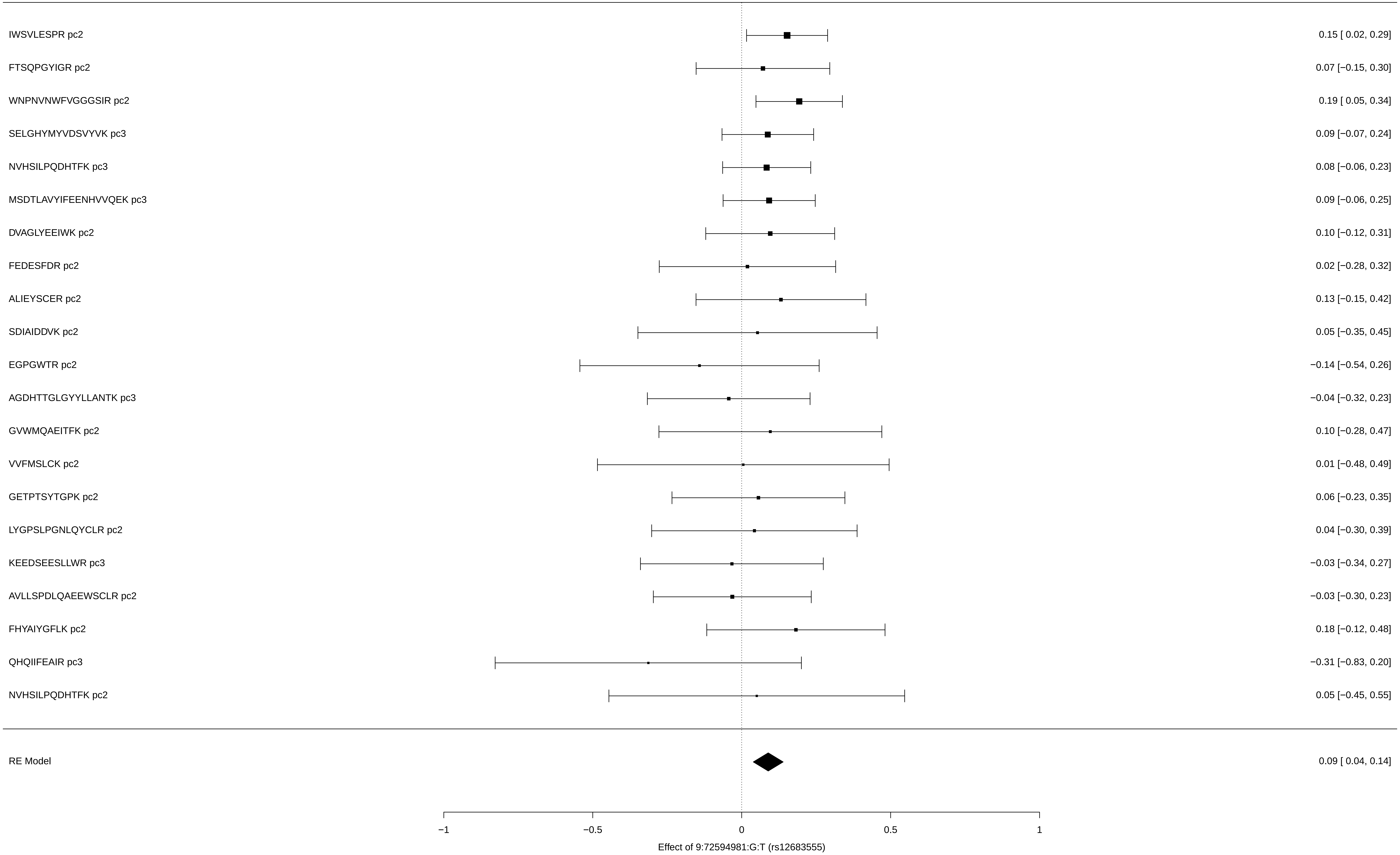

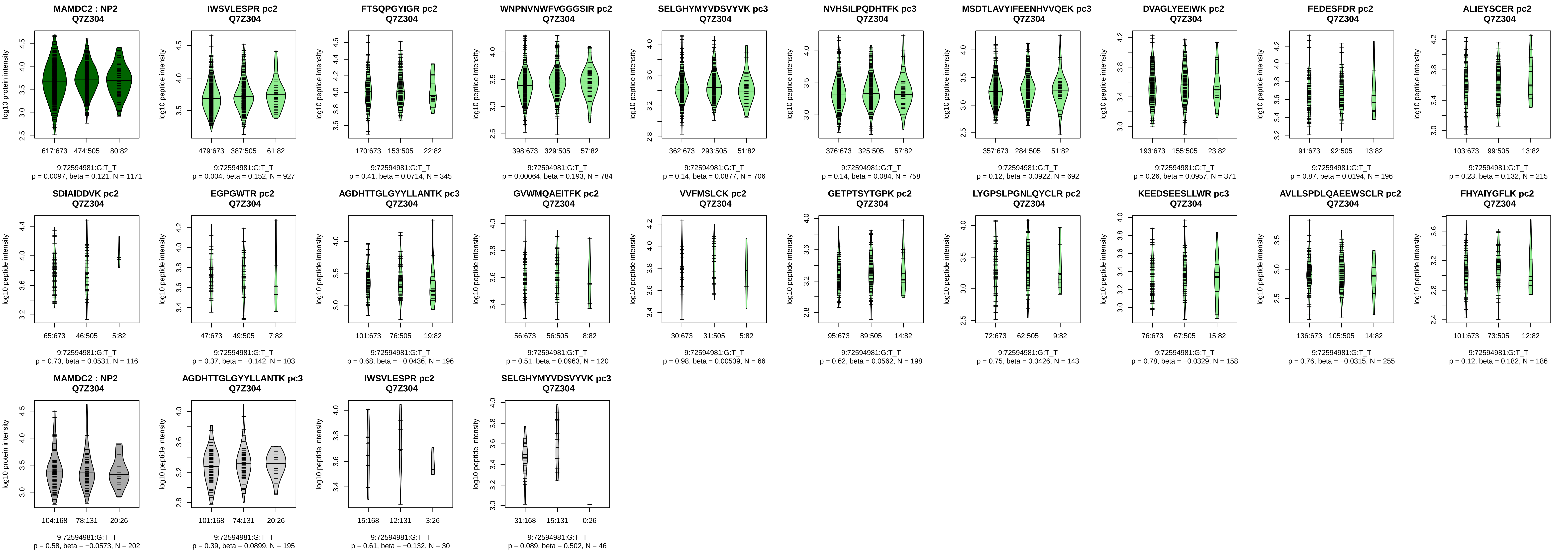

Assay Target: MAMDC2
UniProt: Q7Z304
rsID: rs12683555
Target SNP: 9:72594981:G:T
Proxy SNP: 9:72594981:G:T
UKBPPP log10(p): 222
UKBPPP BETA: 0.213
UKBPPP MSPA: 0.257
:–::–:–:–:–:–:–:–:–:–:–:–:–:–:–:–
927:345:784:706:758:692:371:1

211. SPON2 (Q9BUD6)
UKBPPP MSPA=0.965

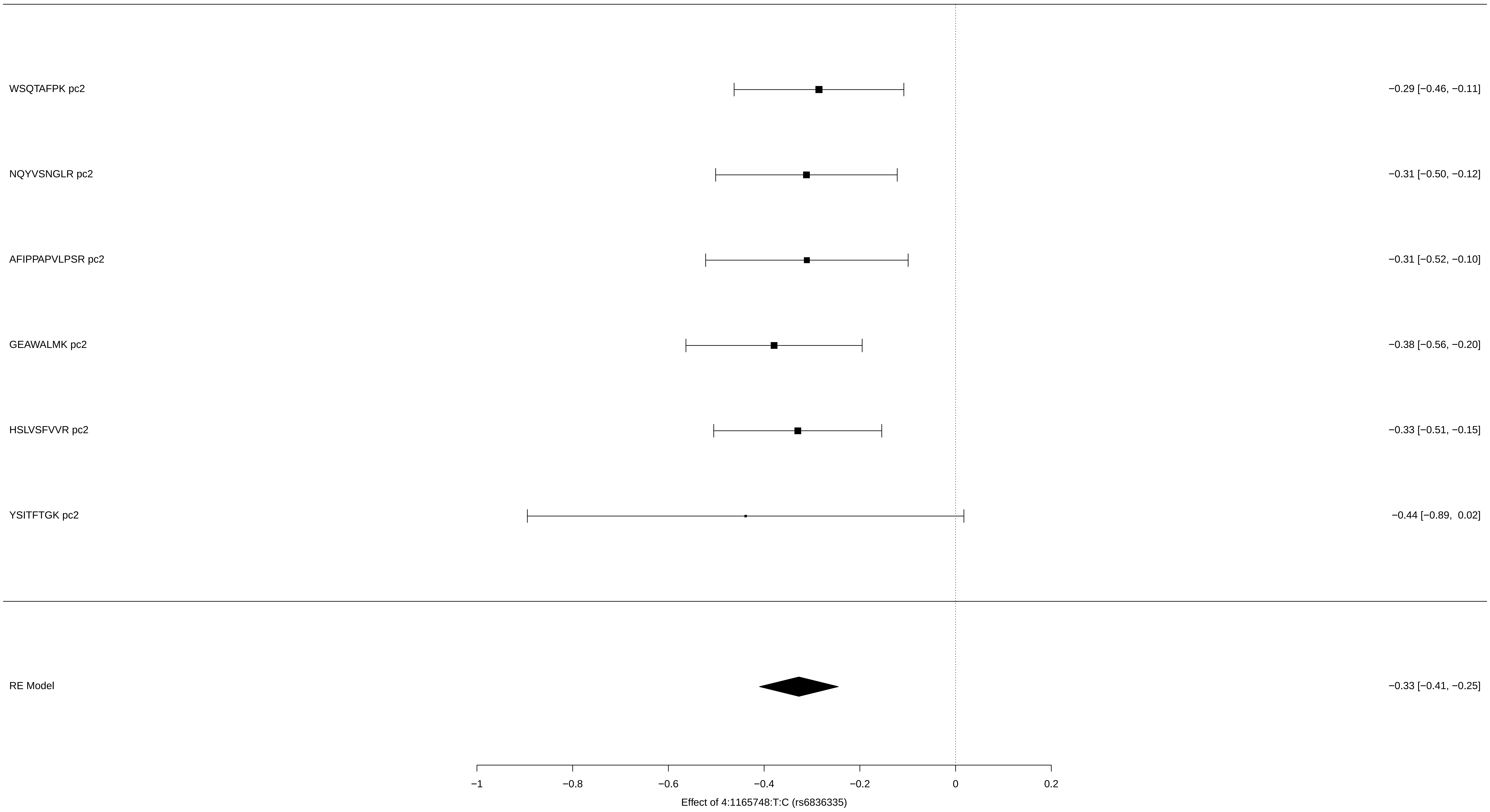

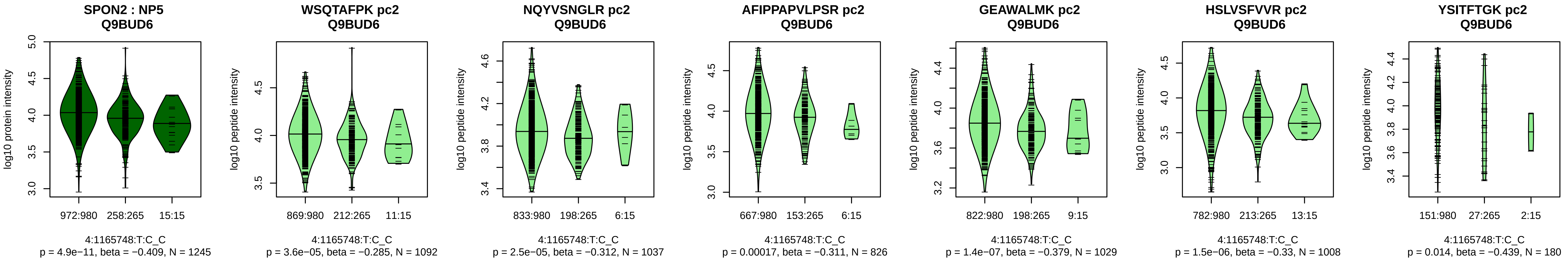

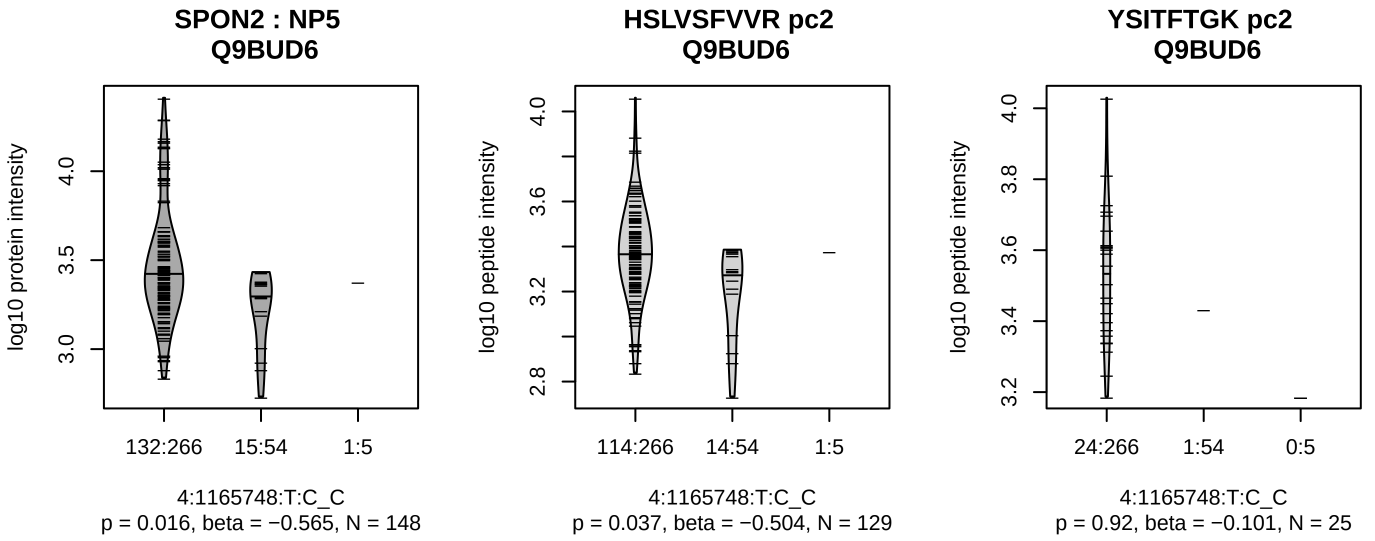

Assay Target: SPON2
UniProt: Q9BUD6
rsID: rs6836335
Target SNP: 4:1165748:T:C
Proxy SNP: 4:1165748:T:C
UKBPPP log10(p): 221.2
UKBPPP BETA: 0.293
UKBPPP MSPA: 0.965
::*:*:*:–
1092:1037:826:1029:1008:180

212. MASP1 (P48740)
UKBPPP MSPA=0.535

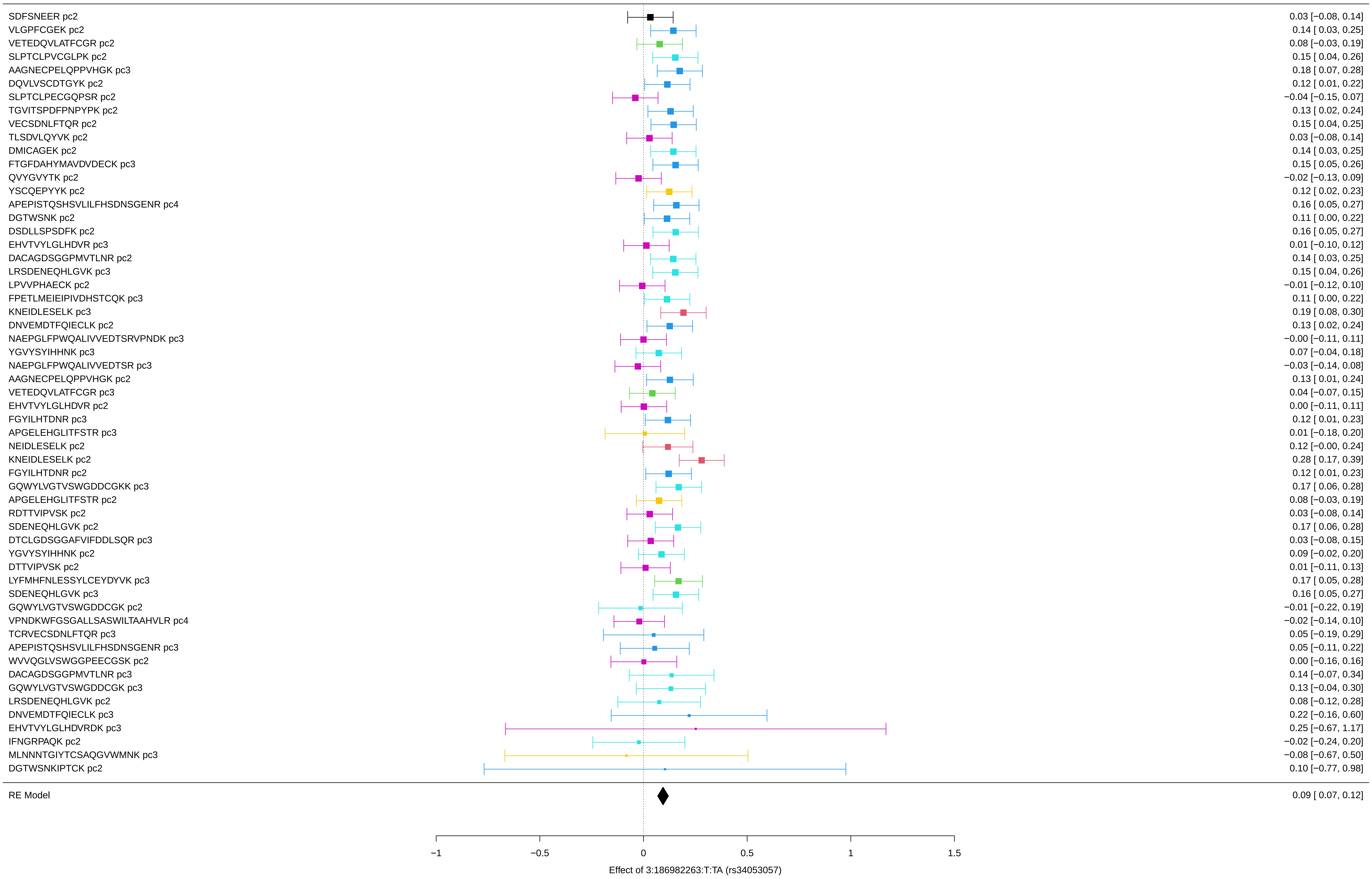

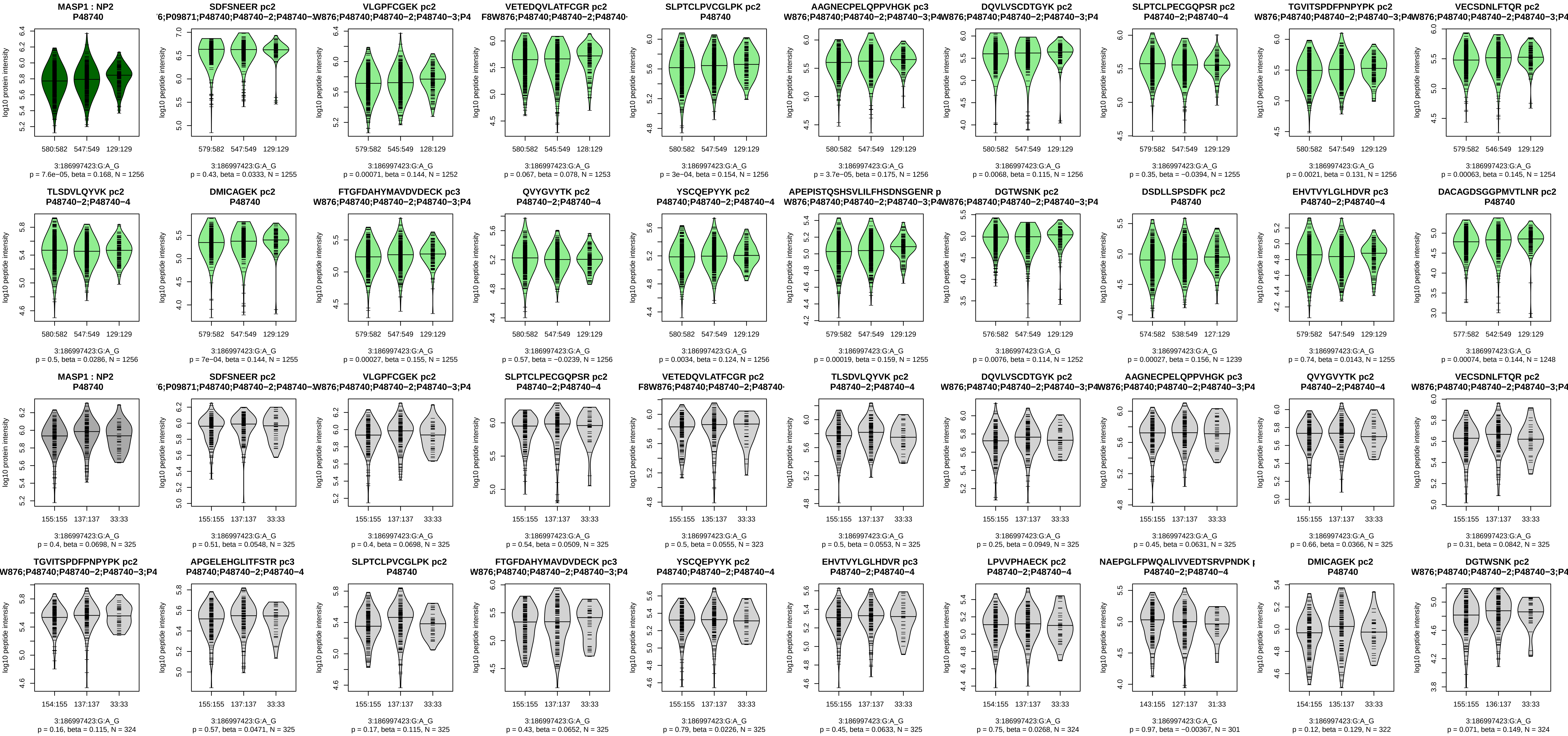

Assay Target: MASP1
UniProt: P48740
rsID: rs34053057
Target SNP: 3:186982263:T:TA
Proxy SNP: 3:186997423:G:A
UKBPPP log10(p): 219.9
UKBPPP BETA: −0.205
UKBPPP MSPA: 0.535
1255:1252:1253:1256:1256:125

213. RARRES2 (Q99969)
UKBPPP MSPA=0.772

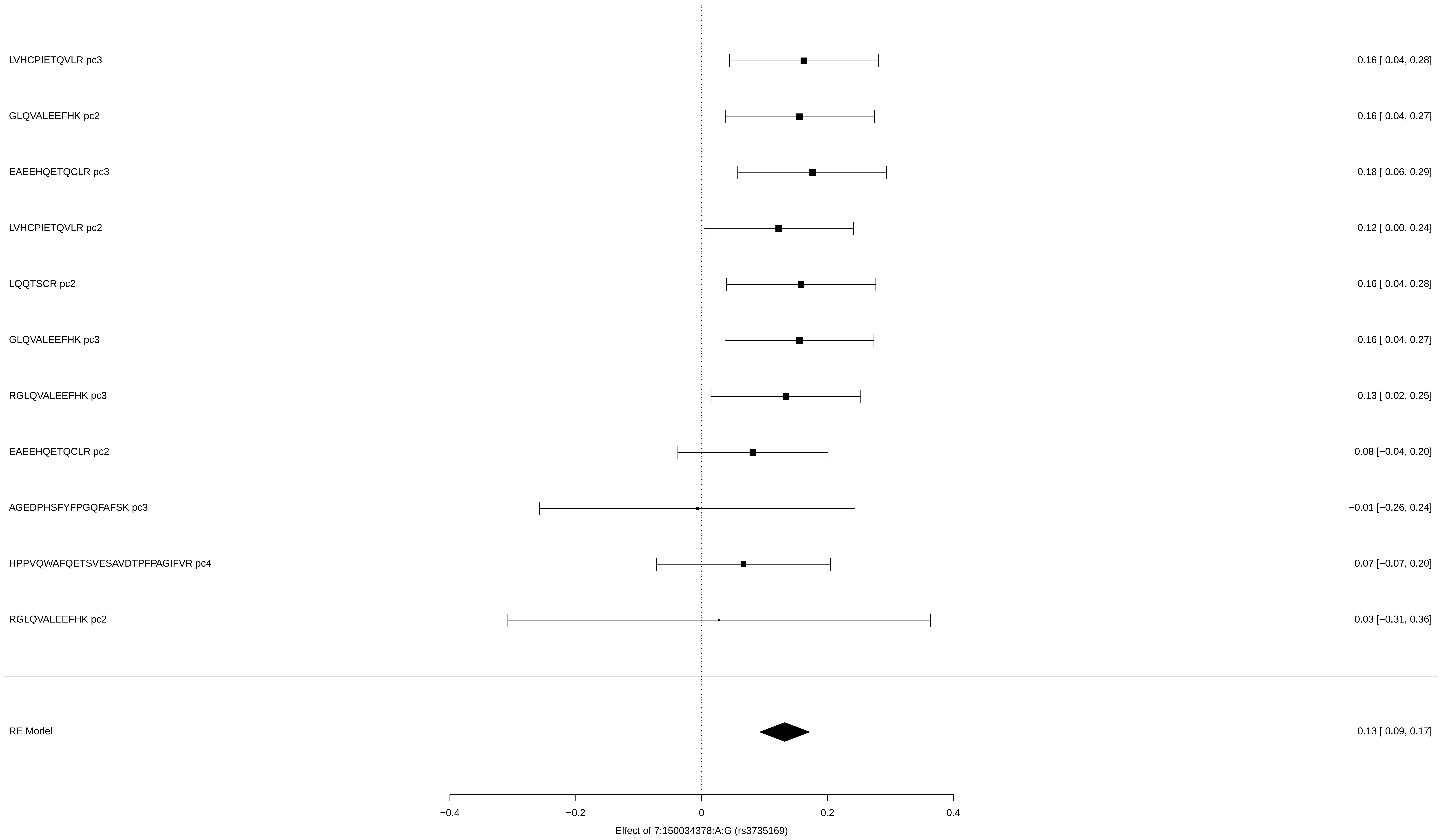

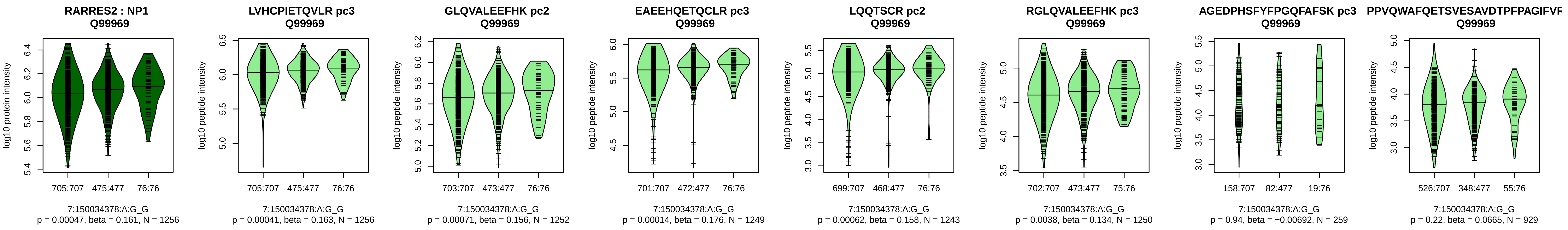

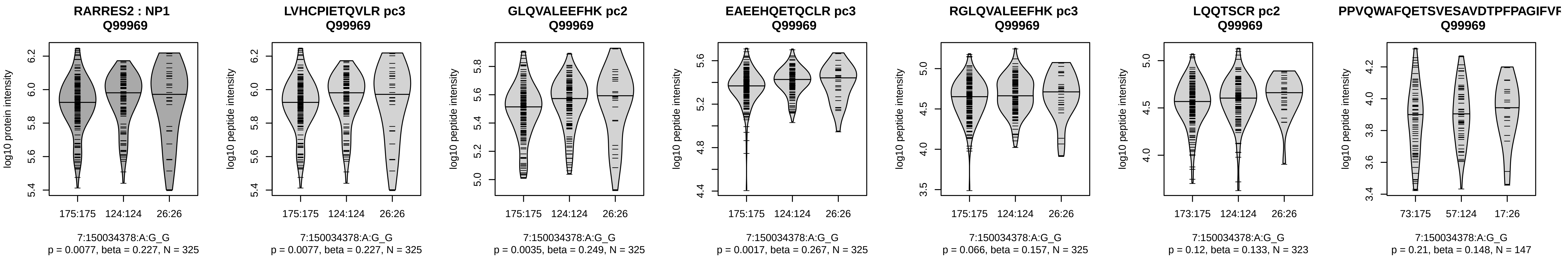

Assay Target: RARRES2
UniProt: Q99969
rsID: rs3735169
Target SNP: 7:150034378:A:G
Proxy SNP: 7:150034378:A:G
UKBPPP log10(p): 217.9
UKBPPP BETA: 0.217
UKBPPP MSPA: 0.772
::*:*:*:*:*:–:–:–:–:NA
1256:1252:1249:1253:1243:125

214. REG3A (Q06141)
UKBPPP MSPA=0

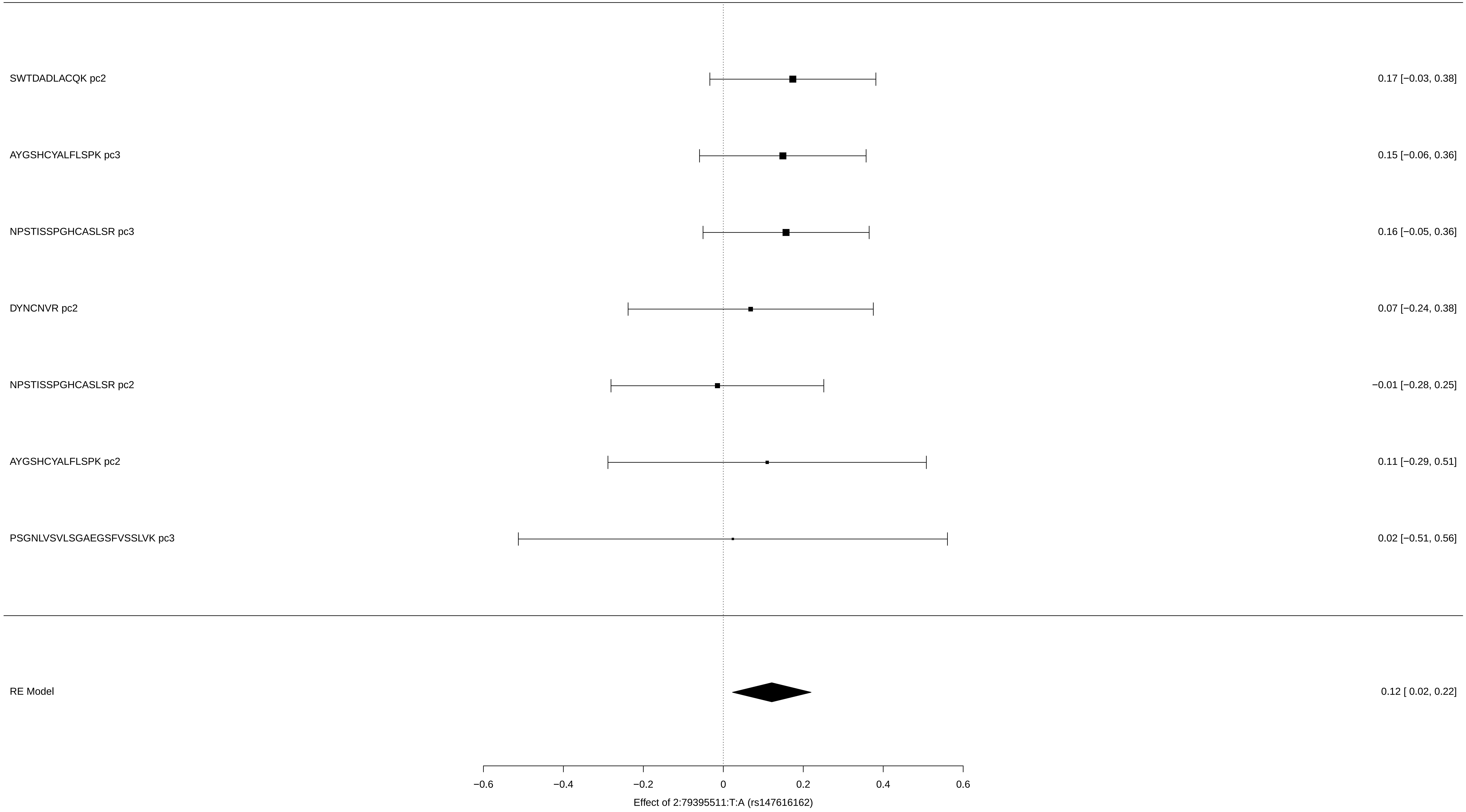

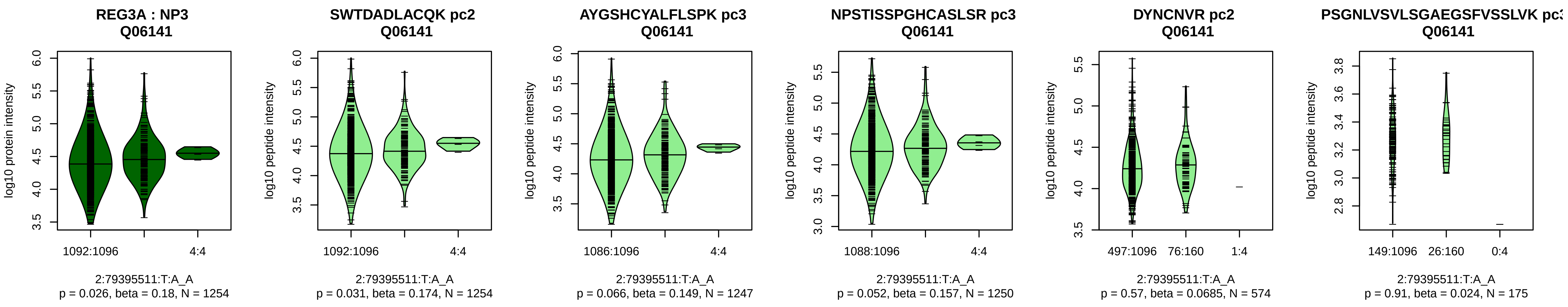

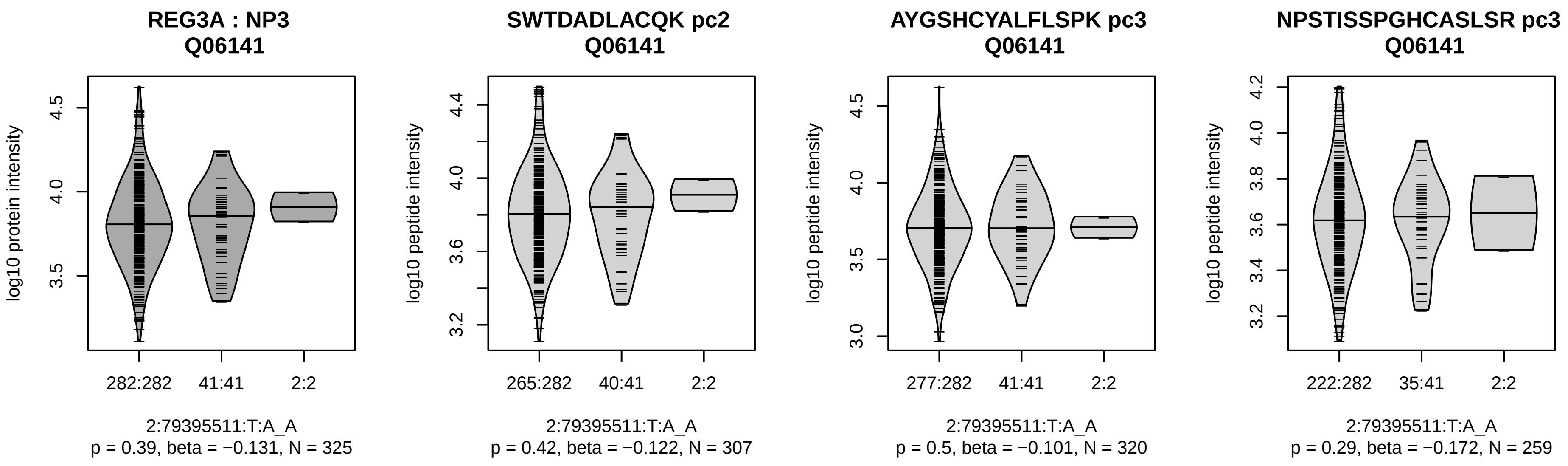

Assay Target: REG3A
UniProt: Q06141
rsID: rs147616162
Target SNP: 2:79395511:T:A
Proxy SNP: 2:79395511:T:A
UKBPPP log10(p): 215.7
UKBPPP BETA: 0.396
UKBPPP MSPA: 0
–:–:–:–:–:–:–
1254:1247:1250:574:694:324:1

215. CTSF (Q9UBX1)
UKBPPP MSPA=0

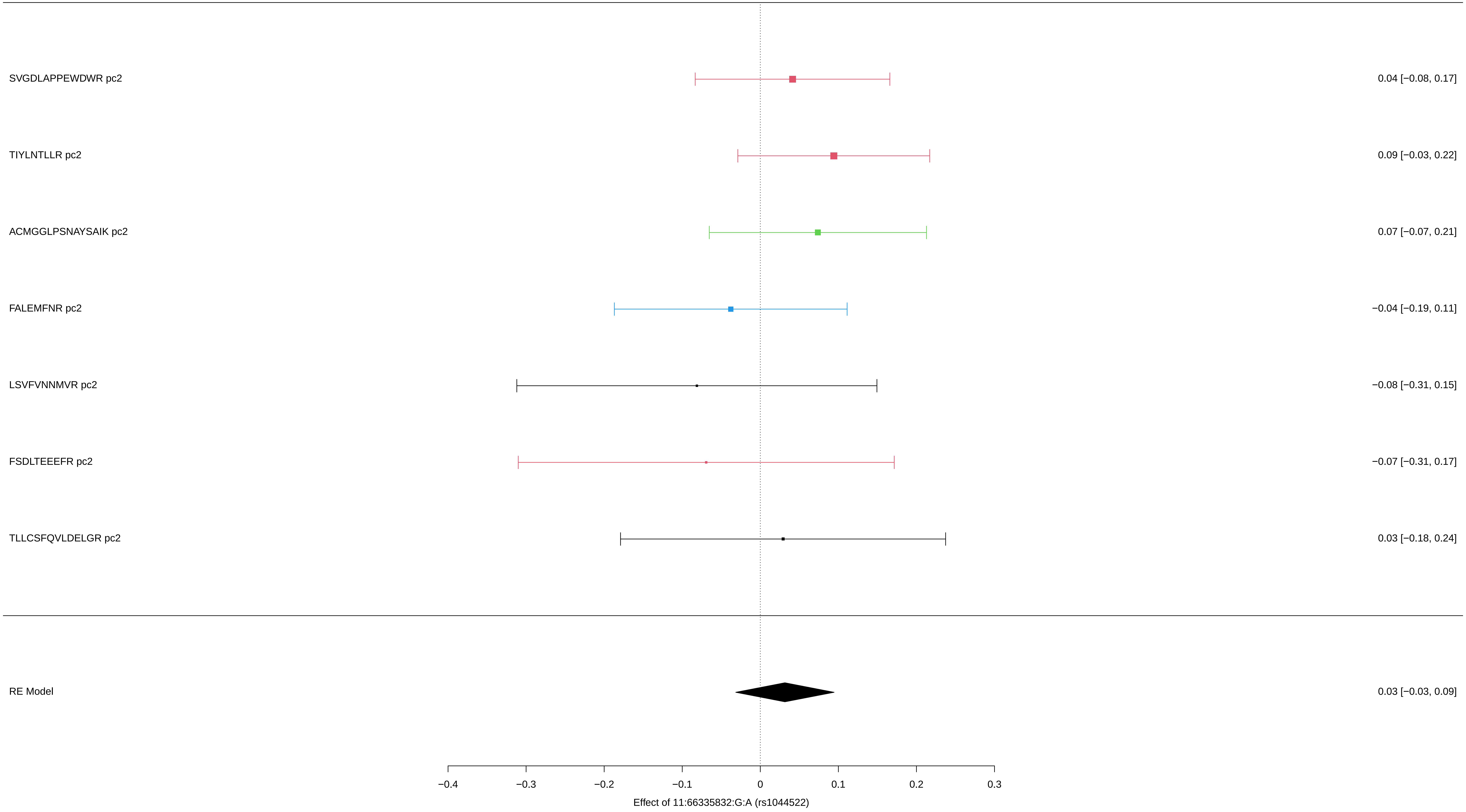

CTSF : NP4
A0A7I2V313

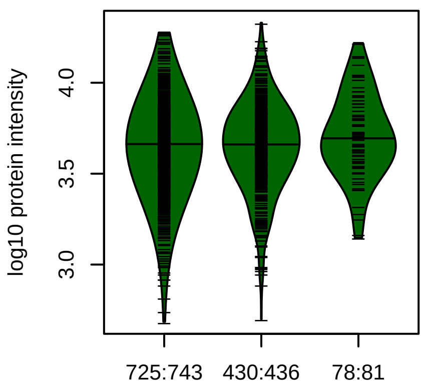

11:66335832:G:A_A
p = 0.16, beta = 0.0651, N = 1233

SVGDLAPPEWDWR pc2
A0A7I2V3X1;A0A7I2V411;A0A7I2V4A8

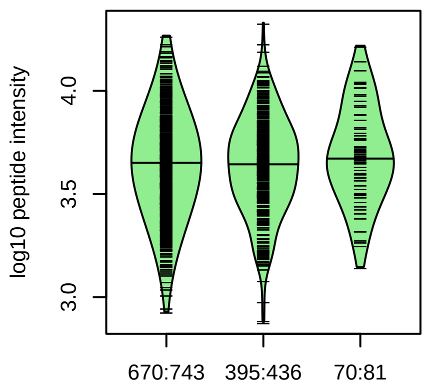

11:66335832:G:A_A
p = 0.39, beta = 0.0413, N = 1135

TIYLNTLLR pc2
A0A7I2V3X1;A0A7I2V411;A0A7I2V4A8

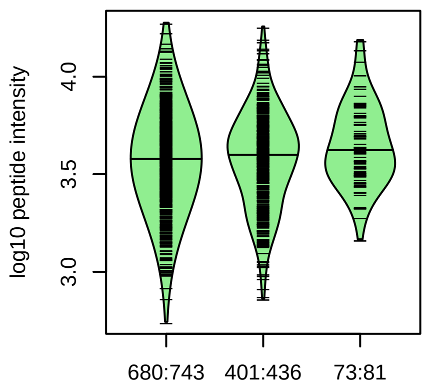

11:66335832:G:A_A
p = 0.049, beta = 0.0941, N = 1154

ACMGGLPSNAYSAIK pc2
A0A7I2V3X1;A0A7I2V411;A0A7I2V4A8

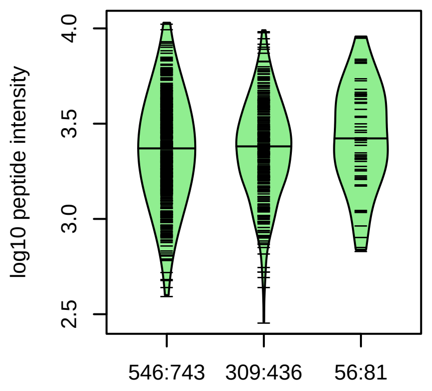

11:66335832:G:A_A
p = 0.17, beta = 0.0737, N = 911

FALEMFNR pc2
A0A7I2V4A8;A0A7I2V4V5;A0A7I2V5B

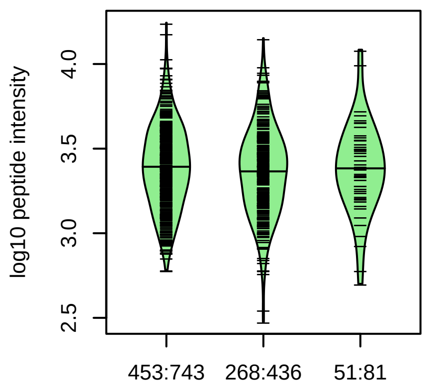

11:66335832:G:A_A
p = 0.51, beta = −0.0379, N = 772

LSVFVNNMVR pc2
I2V3X1;A0A7I2V411;A0A7I2V4A8;A0A

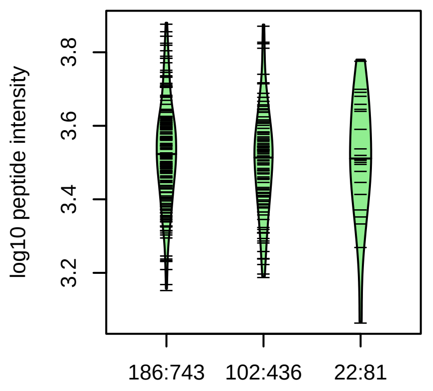

11:66335832:G:A_A
p = 0.36, beta = −0.0813, N = 310

FSDLTEEEFR pc2
A0A7I2V3X1;A0A7I2V411;A0A7I2V4A8

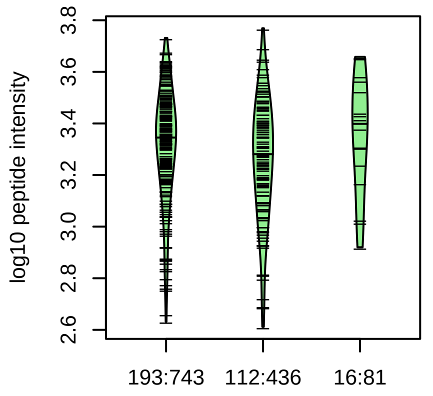

11:66335832:G:A_A
p = 0.46, beta = −0.0693, N = 321

TLLCSFQVLDELGR pc2
I2V3X1;A0A7I2V411;A0A7I2V4A8;A0A

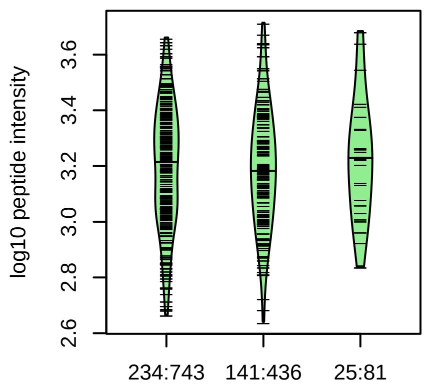

11:66335832:G:A_A
p = 0.72, beta = 0.0292, N = 400

TIYLNTLLR pc2
A0A7I2V3X1;A0A7I2V411;A0A7I2V4A8

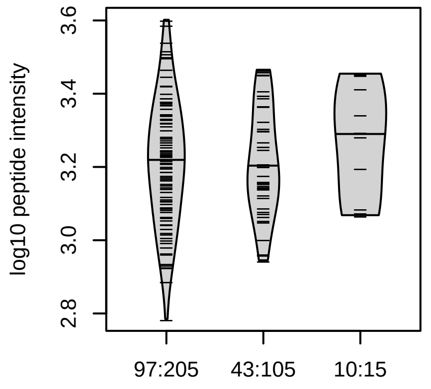

11:66335832:G:A_A
p = 0.73, beta = 0.0457, N = 150

SVGDLAPPEWDWR pc2
A0A7I2V3X1;A0A7I2V411;A0A7I2V4A8

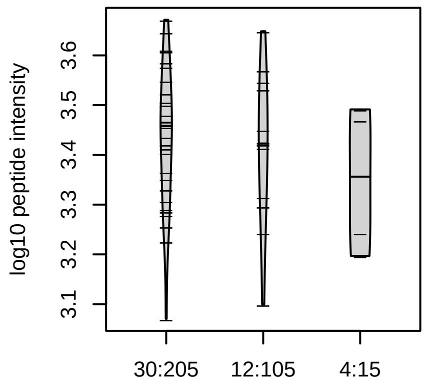

11:66335832:G:A_A
p = 0.22, beta = −0.266, N = 46

Assay Target: CTSF
UniProt: Q9UBX1
rsID: rs1044522
Target SNP: 11:66335832:G:A
Proxy SNP: 11:66335832:G:A
UKBPPP log10(p): 215
UKBPPP BETA: 0.215
UKBPPP MSPA: 0
--:--:--:--:--:--:--
1135:1154:911:772:310:321:400

216. PPP1R14A (Q96A00)
UKBPPP MSPA=0.77

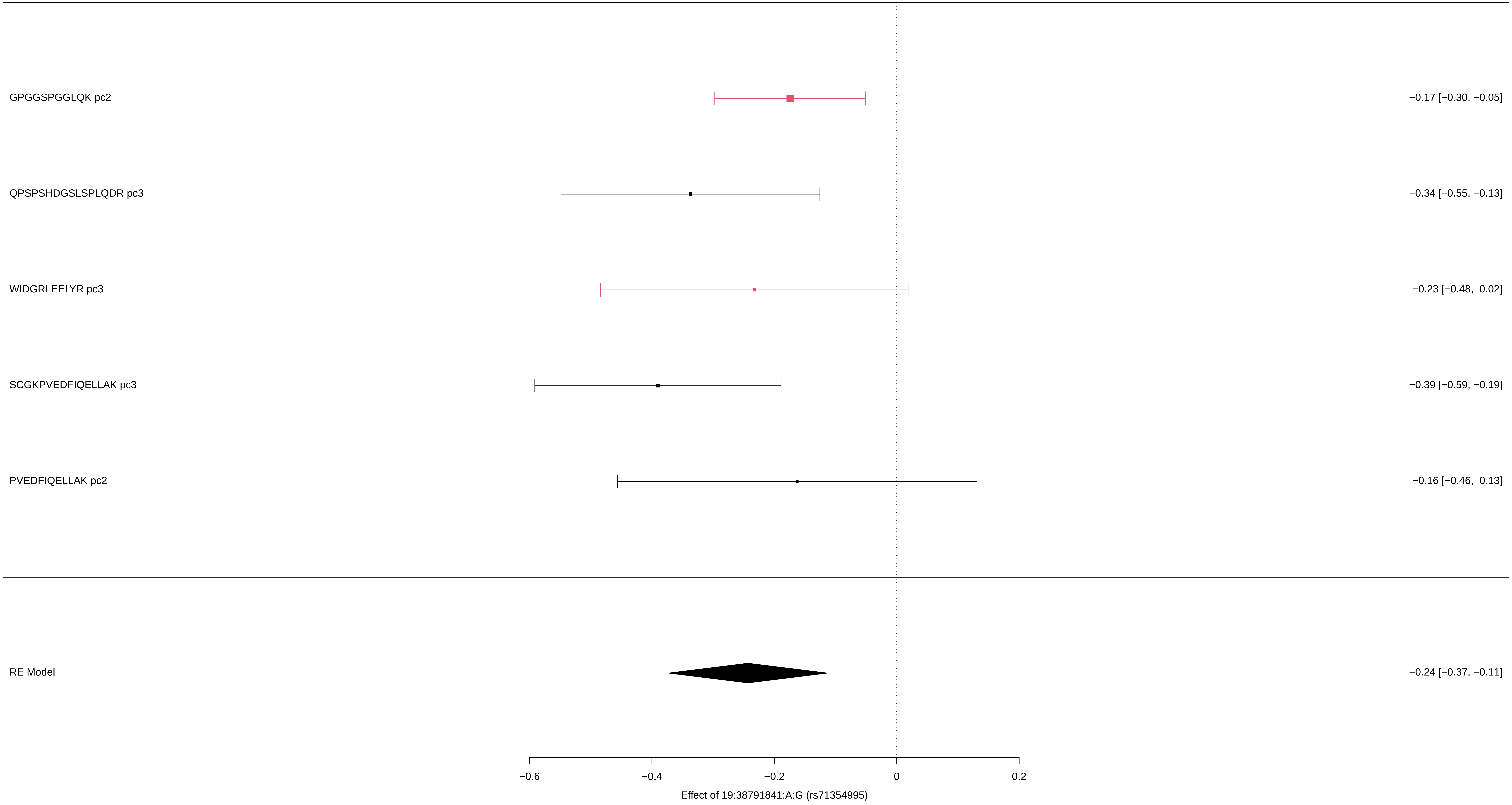

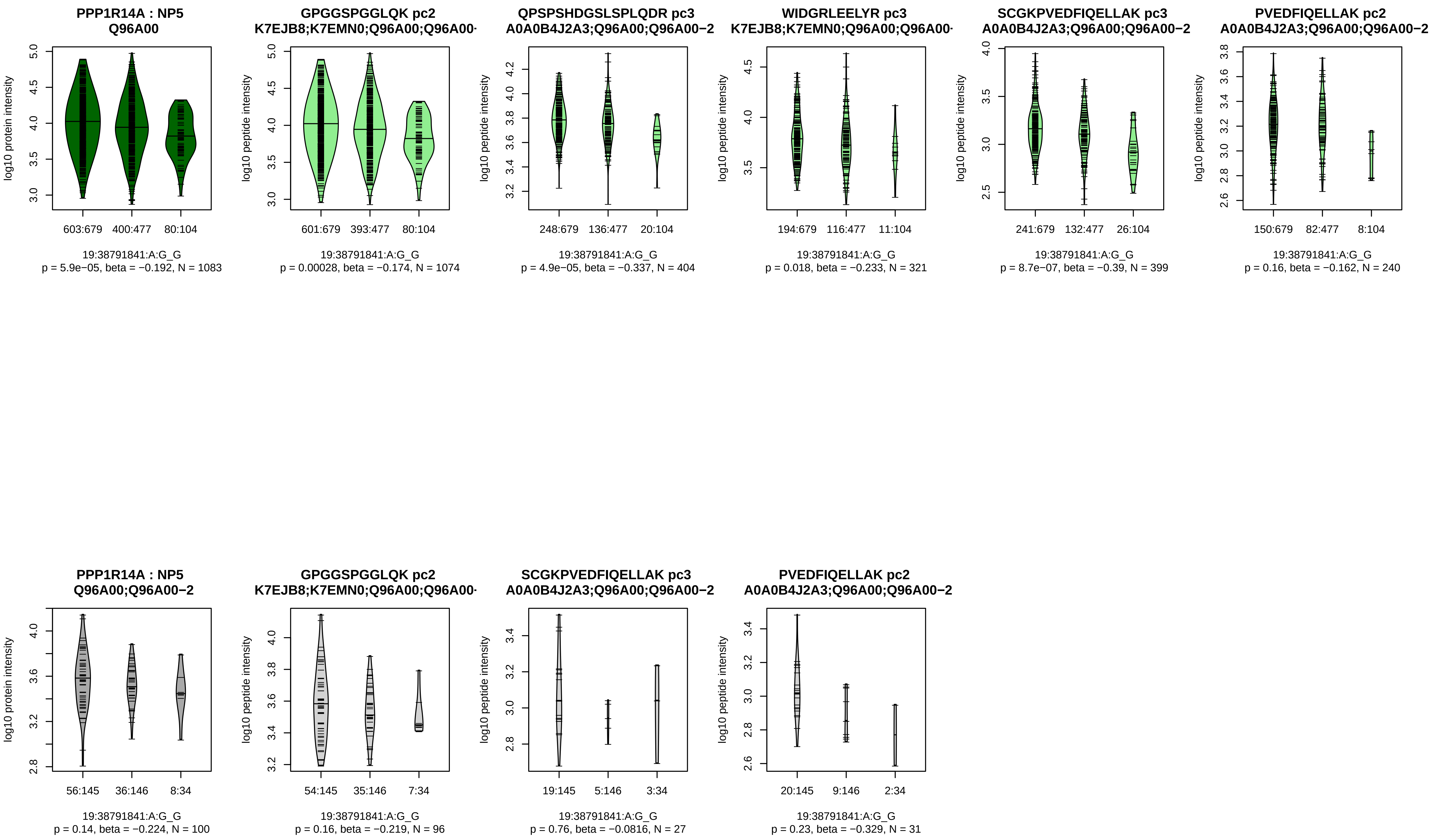

Assay Target: PPP1R14A
UniProt: Q96A00
rsID: rs71354995
Target SNP: 19:38791841:A:G
Proxy SNP: 19:38791841:A:G
UKBPPP log10(p): 215
UKBPPP BETA: −0.221
UKBPPP MSPA: 0.77
::–:*:–
1074:404:321:399:240

217. PTPRZ1 (P23471)
UKBPPP MSPA=0

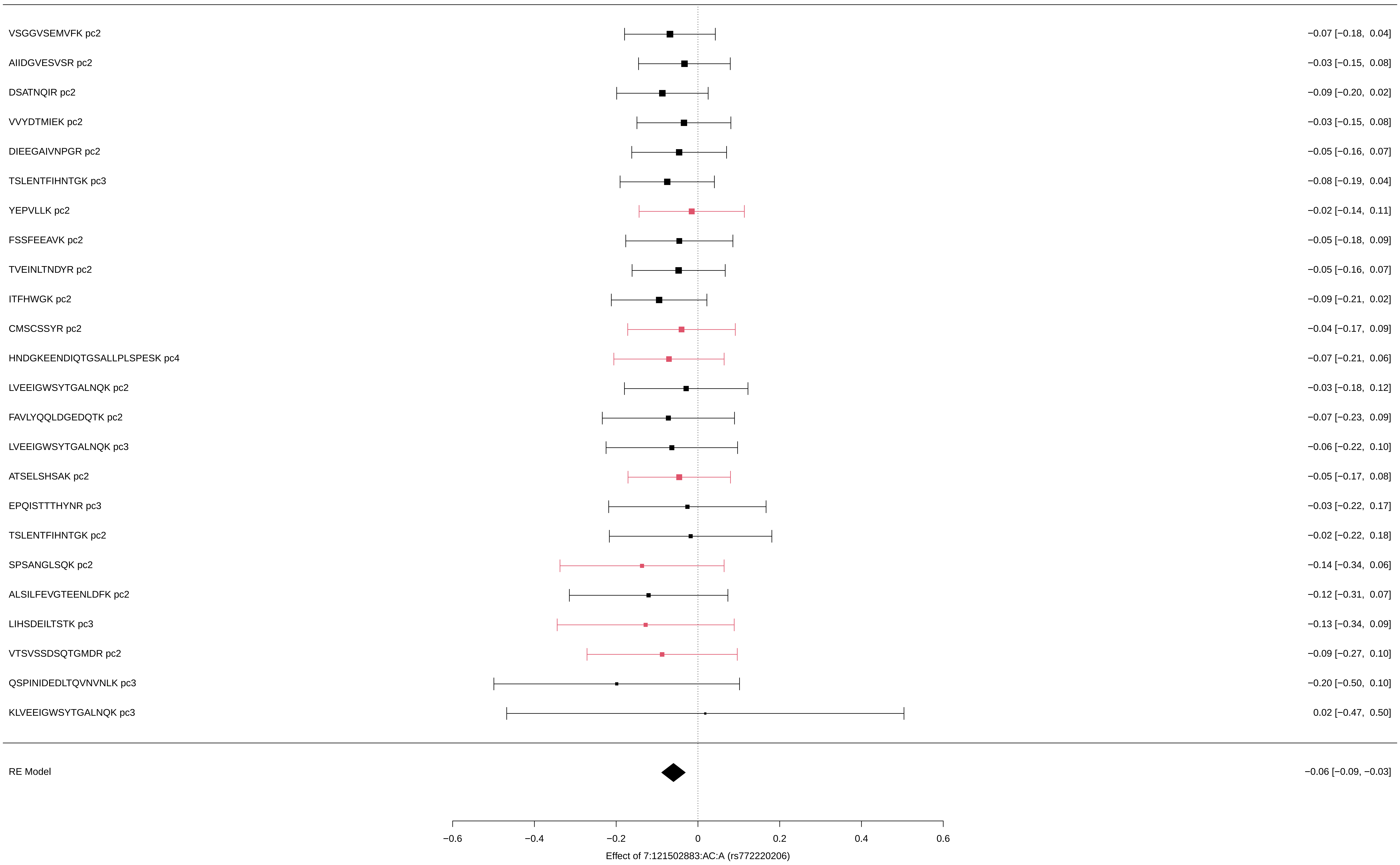

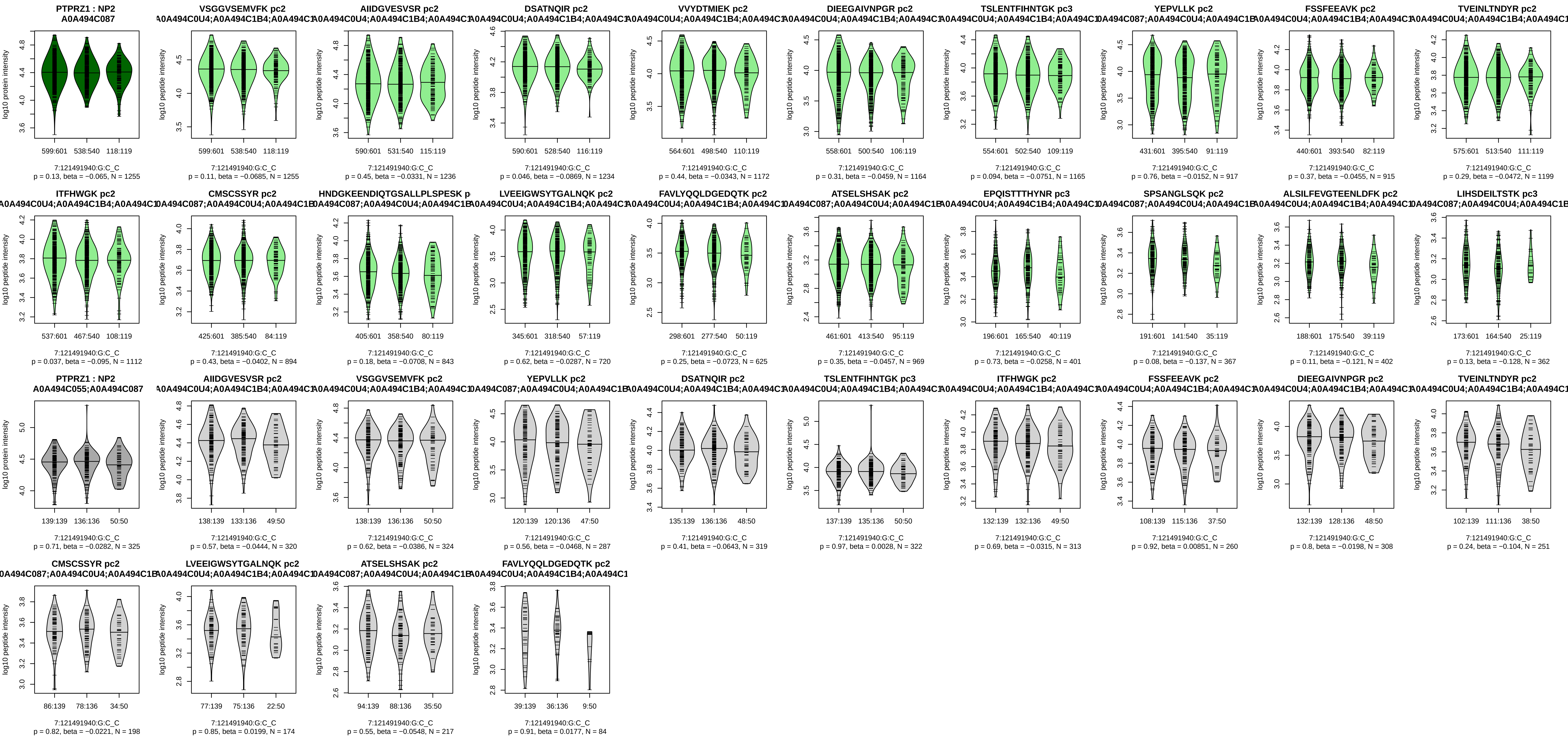

Assay Target: PTPRZ1
UniProt: P23471
rsID: rs772220206
Target SNP: 7:121502883:AC:A
Proxy SNP: 7:121491940:G:C
UKBPPP log10(p): 214.6
UKBPPP BETA: −0.183
UKBPPP MSPA: 0

1255:1236:1234:1172:1164:116

218. EFEMP1 (Q12805)
UKBPPP MSPA=0

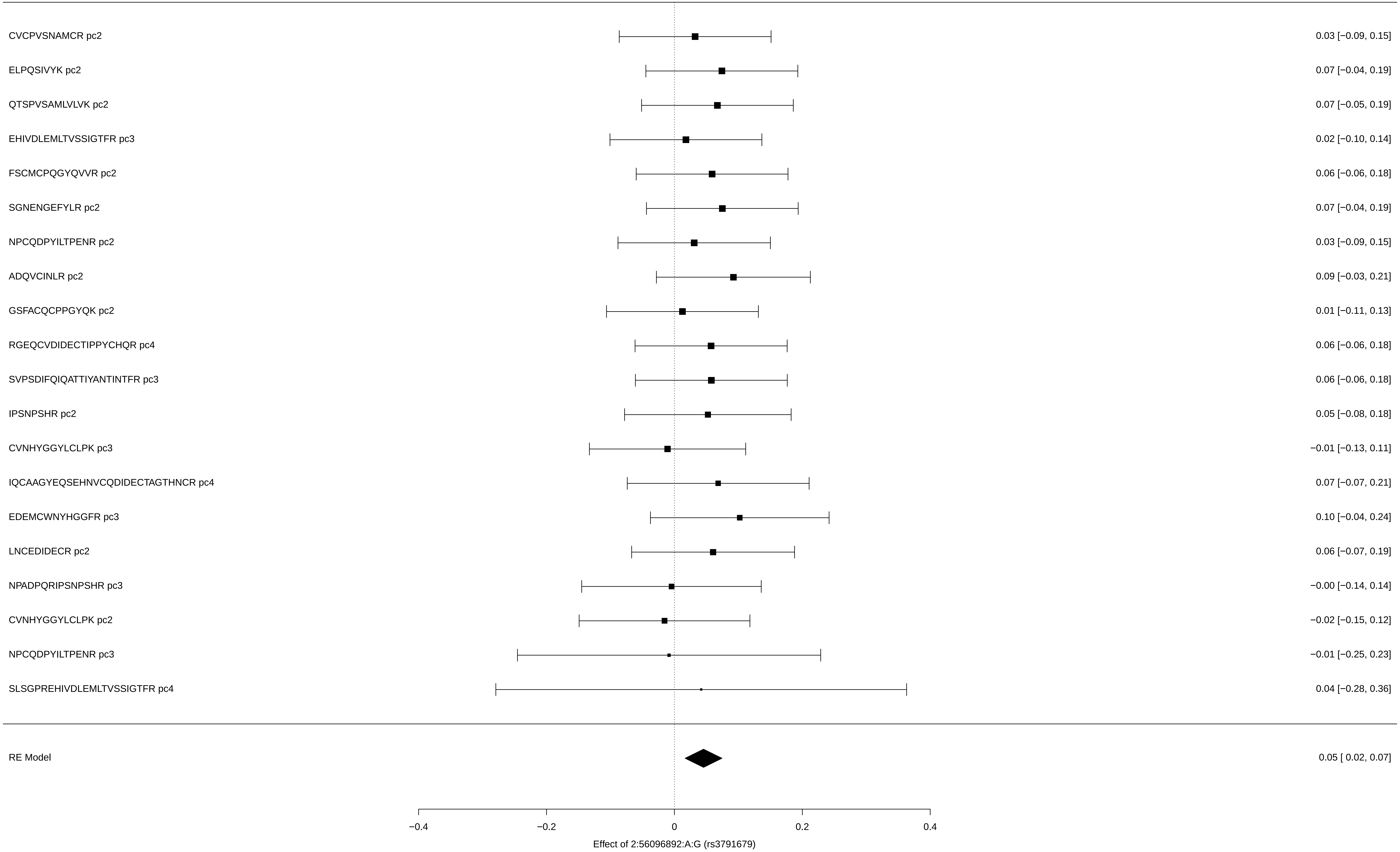

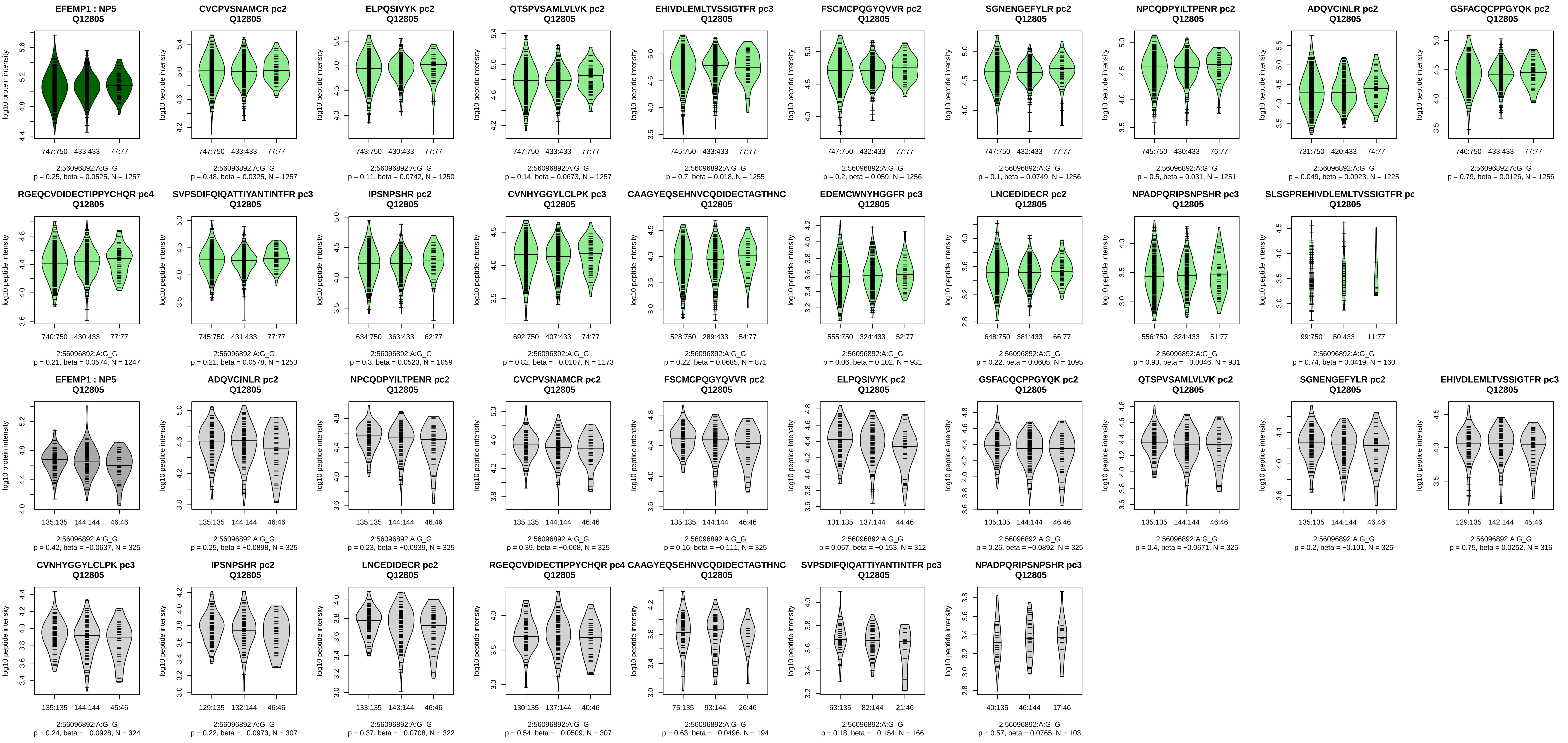

Assay Target: EFEMP1
UniProt: Q12805
rsID: rs3791679
Target SNP: 2:56096892:A:G
Proxy SNP: 2:56096892:A:G
UKBPPP log10(p): 208.1
UKBPPP BETA: 0.205
UKBPPP MSPA: 0
1257:1250:1257:1255:1256:125

219. SFRP1 (Q8N474)
UKBPPP MSPA=0

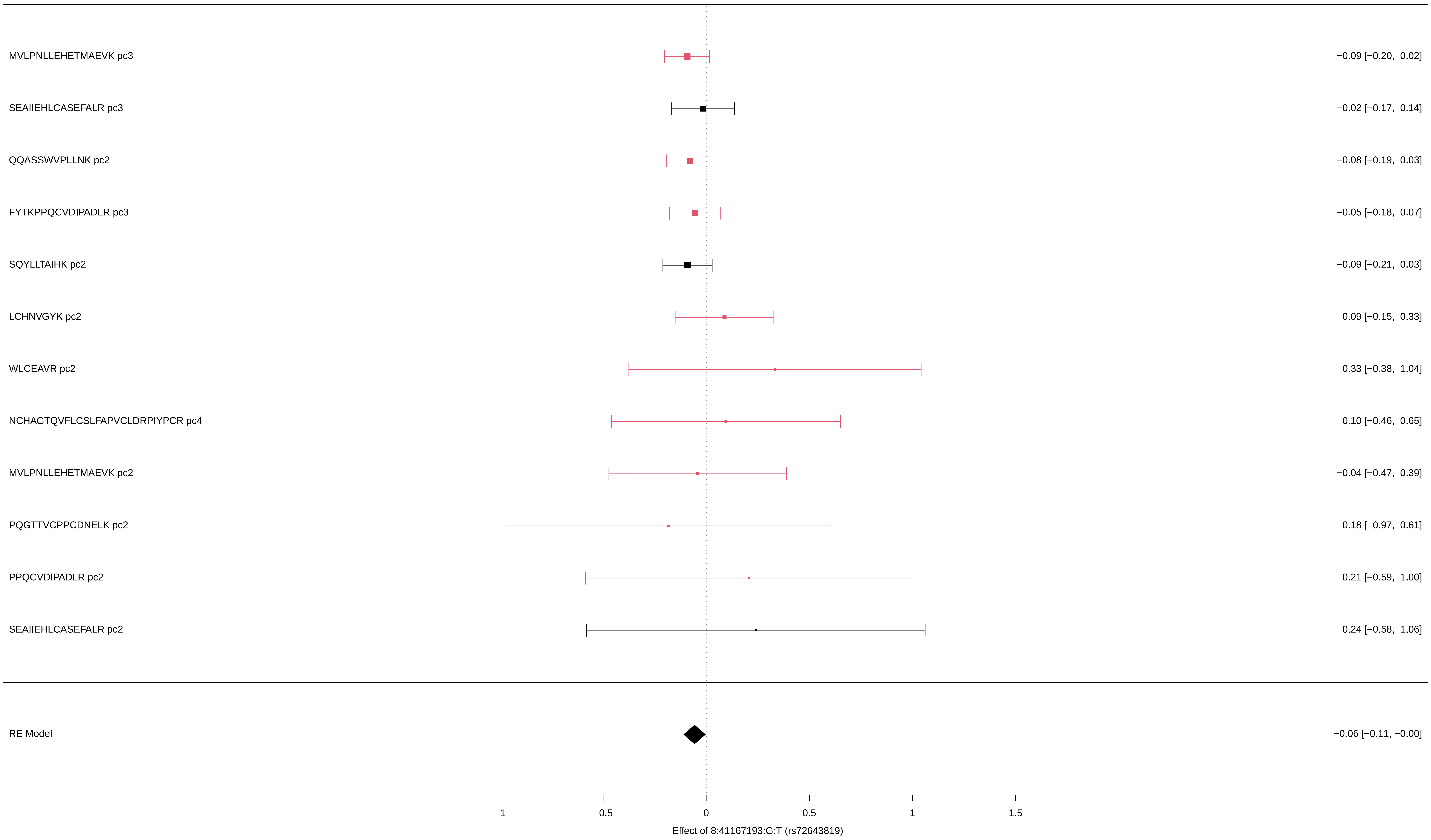

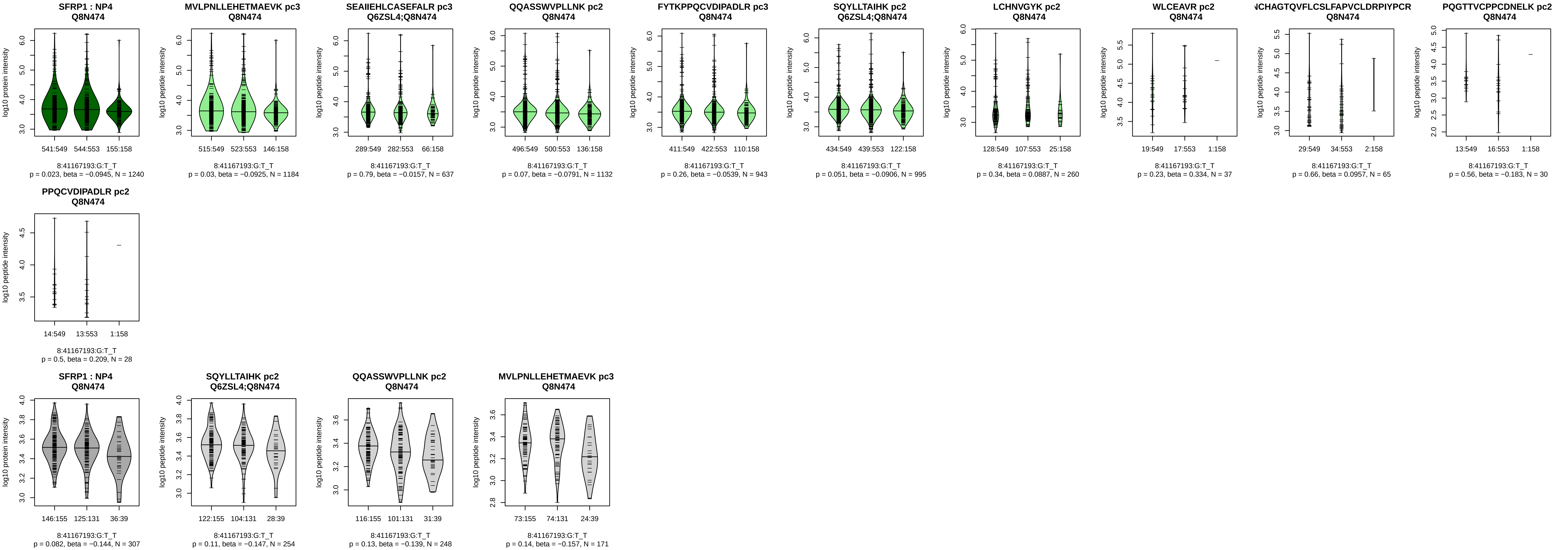

Assay Target: SFRP1
UniProt: Q8N474
rsID: rs72643819
Target SNP: 8:41167193:G:T
Proxy SNP: 8:41167193:G:T
UKBPPP log10(p): 206.1
UKBPPP BETA: −0.188
UKBPPP MSPA: 0
−:−:−:−:−:−:−:−:−:−:−:−:NA:NA
1184:637:1132:943:995:260:37:

220. GSTP1 (P09211)
UKBPPP MSPA=0.393

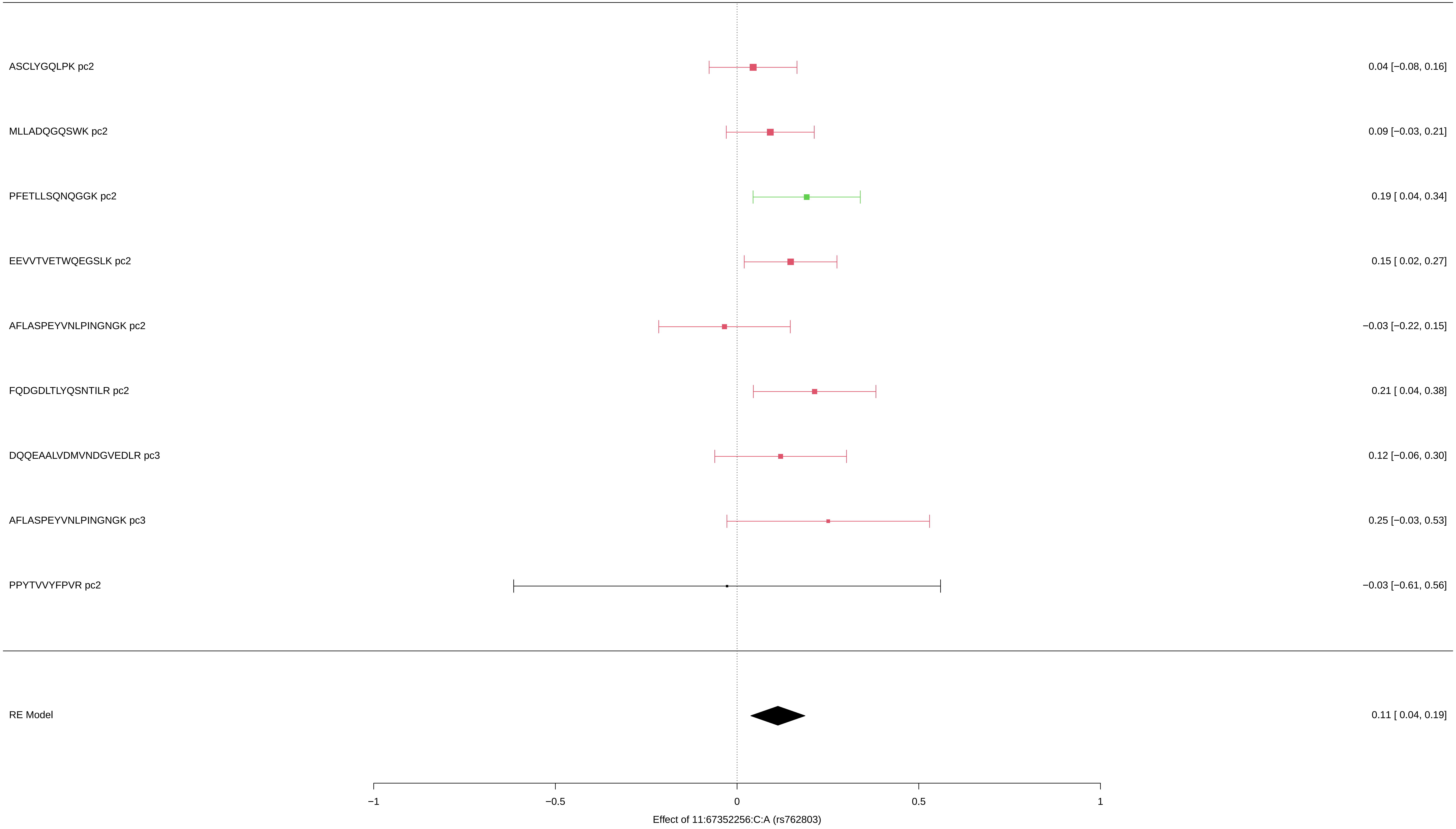

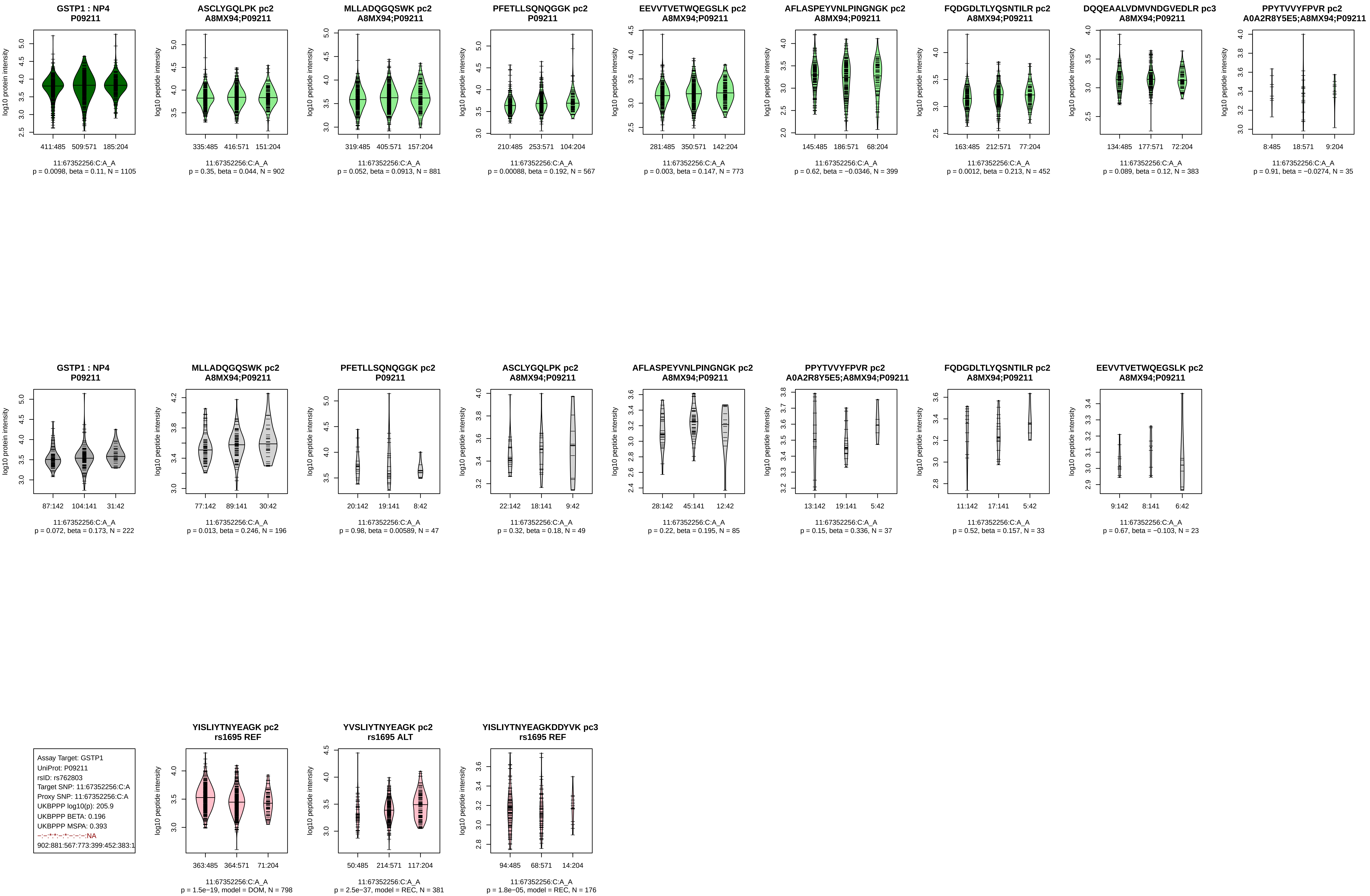
GSTP1 : NP4
P09211
411:485 509:571 185:204
11:67352256:C:A_A
p = 0.0098, beta = 0.11, N = 1105
ASCLYGQLPK pc2
A8MX94;P09211
335:485 416:571 151:204
11:67352256:C:A_A
p = 0.35, beta = 0.044, N = 902
MLLADQGQSWK pc2
A8MX94;P09211
319:485 405:571 157:204
11:67352256:C:A_A
p = 0.052, beta = 0.0913, N = 881
PFETLLSQNQGGK pc2
P09211
210:485 253:571 104:204
11:67352256:C:A_A
p = 0.00088, beta = 0.192, N = 567
EEVVTVETWQEGSLK pc2
A8MX94;P09211
281:485 350:571 142:204
11:67352256:C:A_A
p = 0.003, beta = 0.147, N = 773
AFLASPEYVNLPINGNGK pc2
A8MX94;P09211
145:485 186:571 68:204
11:67352256:C:A_A
p = 0.62, beta = −0.0346, N = 399
FQDGDLTLYQSNTILR pc2
A8MX94;P09211
163:485 212:571 77:204
11:67352256:C:A_A
p = 0.0012, beta = 0.213, N = 452
DQQEAALVDMVNDGVEDLR pc3
A8MX94;P09211
134:485 177:571 72:204
11:67352256:C:A_A
p = 0.089, beta = 0.12, N = 383
PPYTVVYFPVR pc2
A0A2R8Y5E5;A8MX94;P09211
8:485 18:571 9:204
11:67352256:C:A_A
p = 0.91, beta = −0.0274, N = 35
GSTP1 : NP4
P09211
87:142 104:141 31:42
11:67352256:C:A_A
p = 0.072, beta = 0.173, N = 222
MLLADQGQSWK pc2
A8MX94;P09211
77:142 89:141 30:42
11:67352256:C:A_A
p = 0.013, beta = 0.246, N = 196
PFETLLSQNQGGK pc2
P09211
20:142 19:141 8:42
11:67352256:C:A_A
p = 0.98, beta = 0.00589, N = 47
ASCLYGQLPK pc2
A8MX94;P09211
22:142 18:141 9:42
11:67352256:C:A_A
p = 0.32, beta = 0.18, N = 49
AFLASPEYVNLPINGNGK pc2
A8MX94;P09211
28:142 45:141 12:42
11:67352256:C:A_A
p = 0.22, beta = 0.195, N = 85
PPYTVVYFPVR pc2
A0A2R8Y5E5;A8MX94;P09211
13:142 19:141 5:42
11:67352256:C:A_A
p = 0.15, beta = 0.336, N = 37
FQDGDLTLYQSNTILR pc2
A8MX94;P09211
11:142 17:141 5:42
11:67352256:C:A_A
p = 0.52, beta = 0.157, N = 33
EEVVTVETWQEGSLK pc2
A8MX94;P09211
9:142 8:141 6:42
11:67352256:C:A_A
p = 0.67, beta = −0.103, N = 23
Assay Target: GSTP1
UniProt: P09211
rsID: rs762803
Target SNP: 11:67352256:C:A
Proxy SNP: 11:67352256:C:A
UKBPPP log10(p): 205.9
UKBPPP BETA: 0.196
UKBPPP MSPA: 0.393
−:−:*:*:−:*:−:−:−:NA
902:881:567:773:399:452:383:1
YISLIYTNYEAGK pc2
rs1695 REF
363:485 364:571 71:204
11:67352256:C:A_A
p = 1.5e−19, model = DOM, N = 798
YVSLIYTNYEAGK pc2
rs1695 ALT
50:485 214:571 117:204
11:67352256:C:A_A
p = 2.5e−37, model = REC, N = 381
YISLIYTNYEAGKDDYVK pc3
rs1695 REF
94:485 68:571 14:204
11:67352256:C:A_A
p = 1.8e−05, model = REC, N = 176
log10 protein intensity
log10 peptide intensity

221. PSMD9 (O00233)
UKBPPP MSPA=0.505

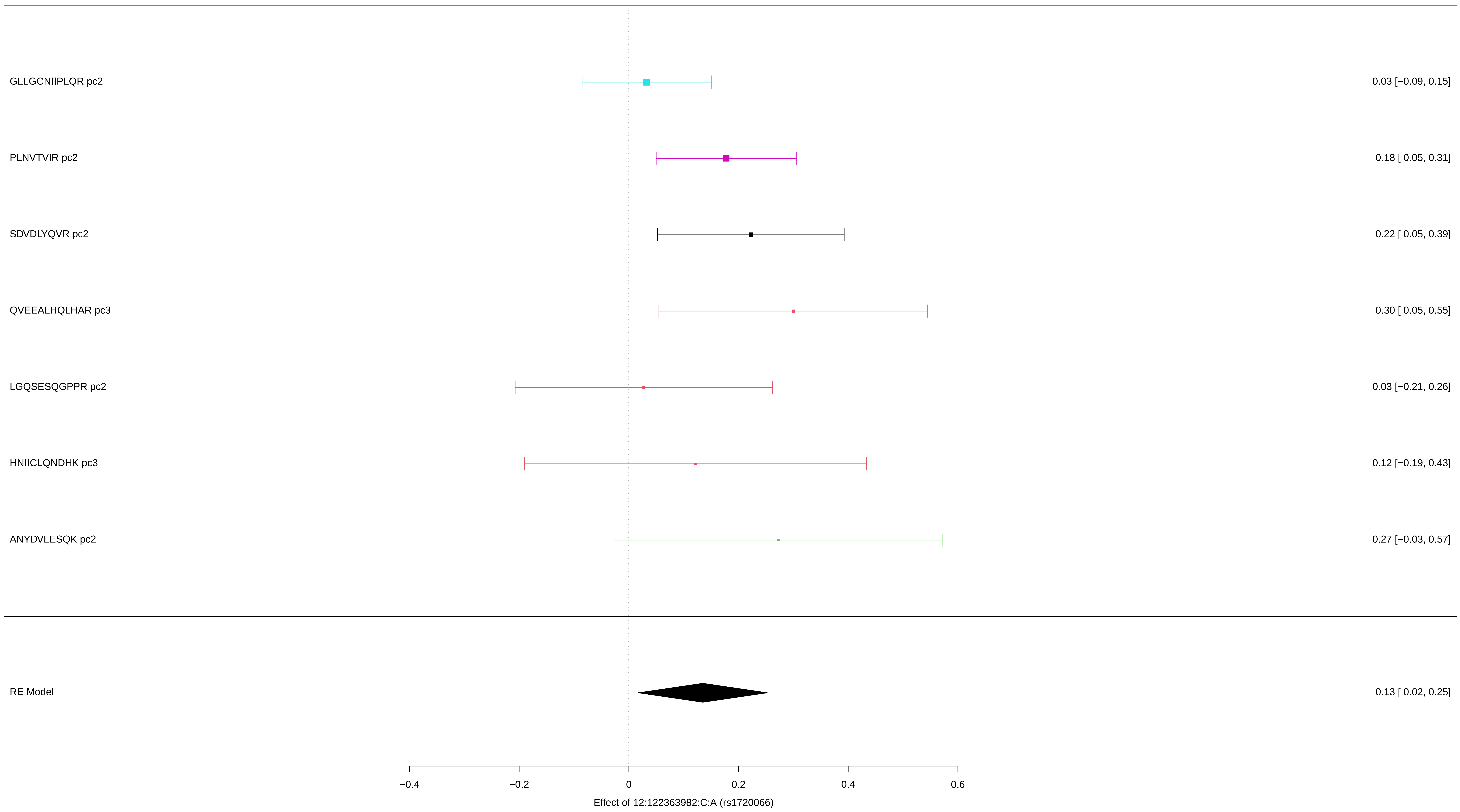

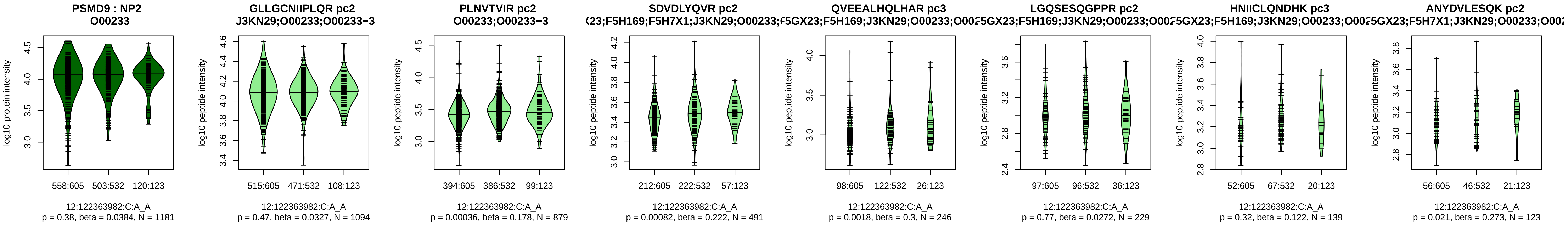

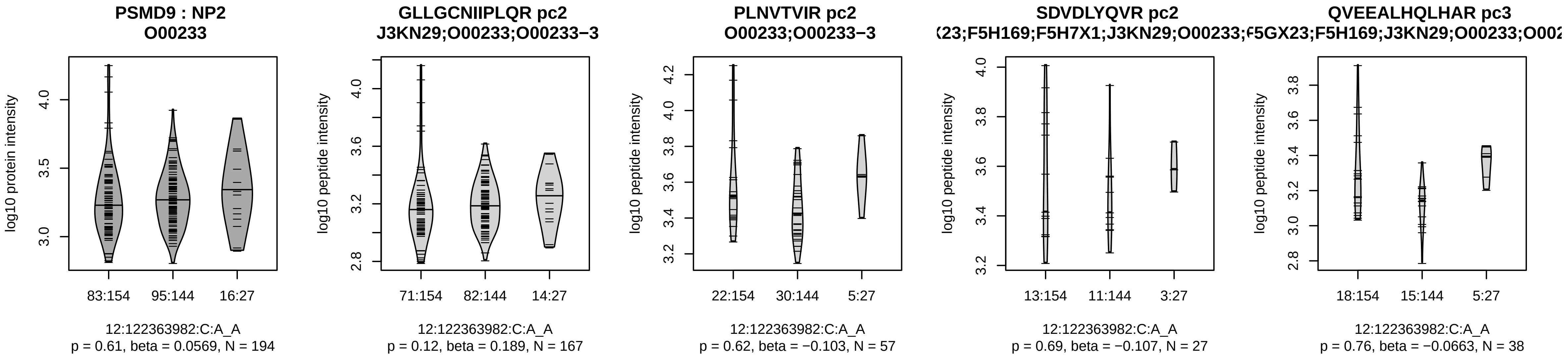

Assay Target: PSMD9
UniProt: O00233
rsID: rs1720066
Target SNP: 12:122363982:C:A
Proxy SNP: 12:122363982:C:A
UKBPPP log10(p): 203.8
UKBPPP BETA: −0.199
UKBPPP MSPA: 0.505
−:*:*:*:−:−:−:NA:NA:NA:NA
1094:879:491:246:229:139:123:

222. SFRP4 (Q6FHJ7)
UKBPPP MSPA=0.72

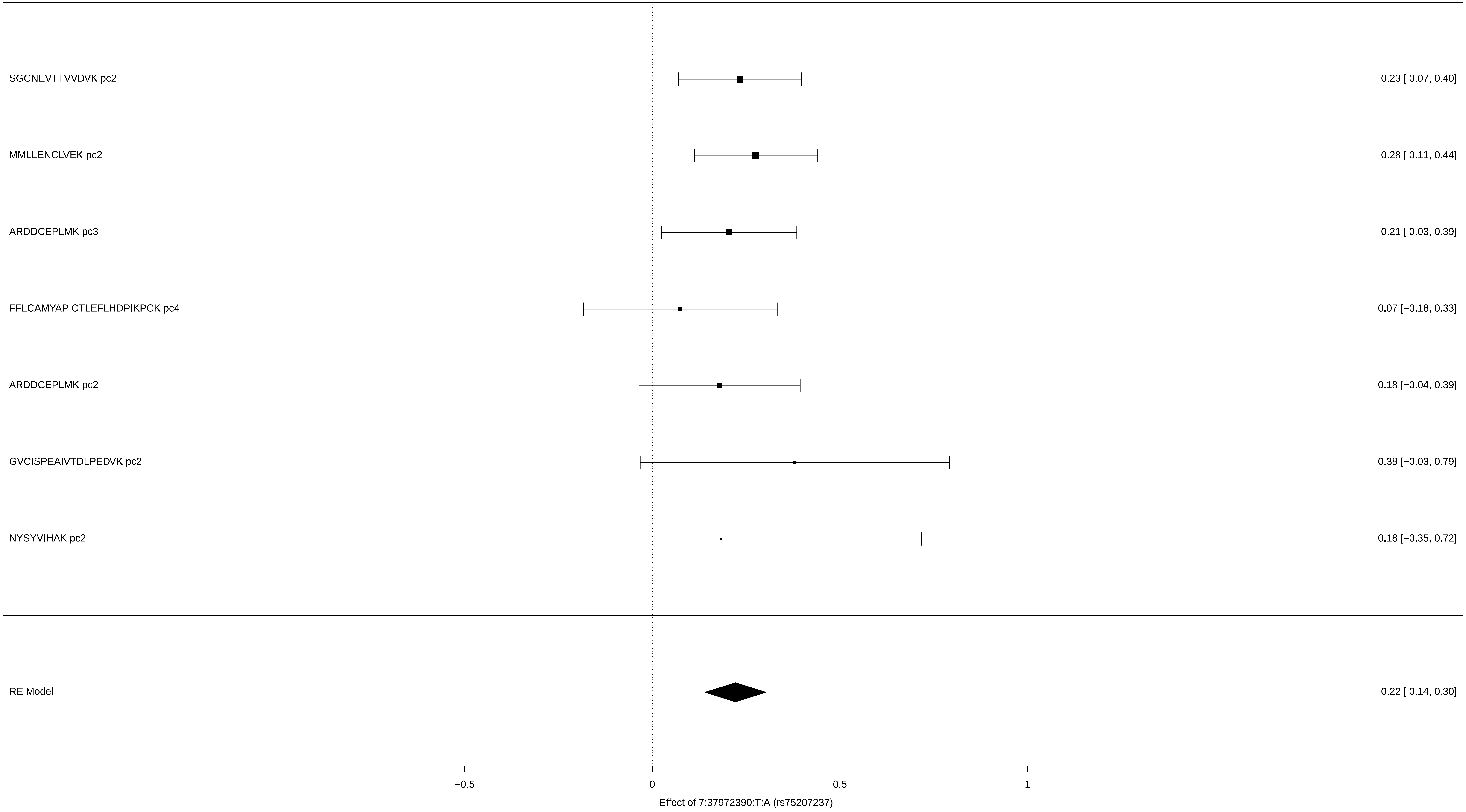

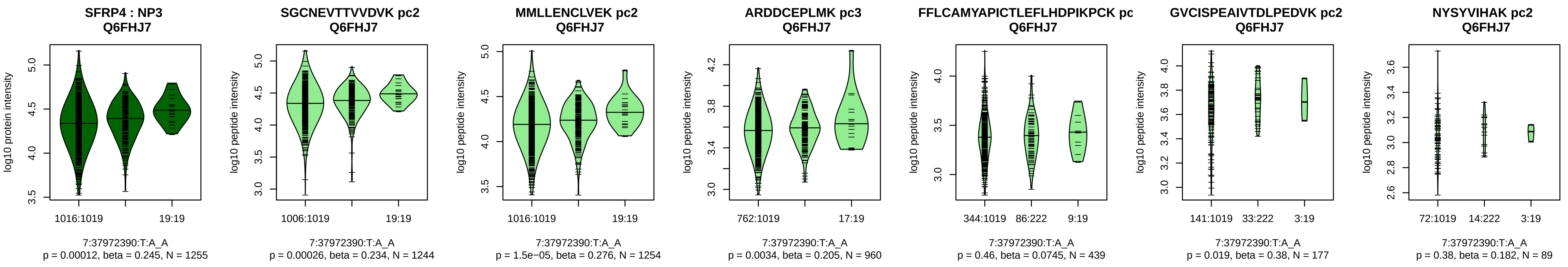

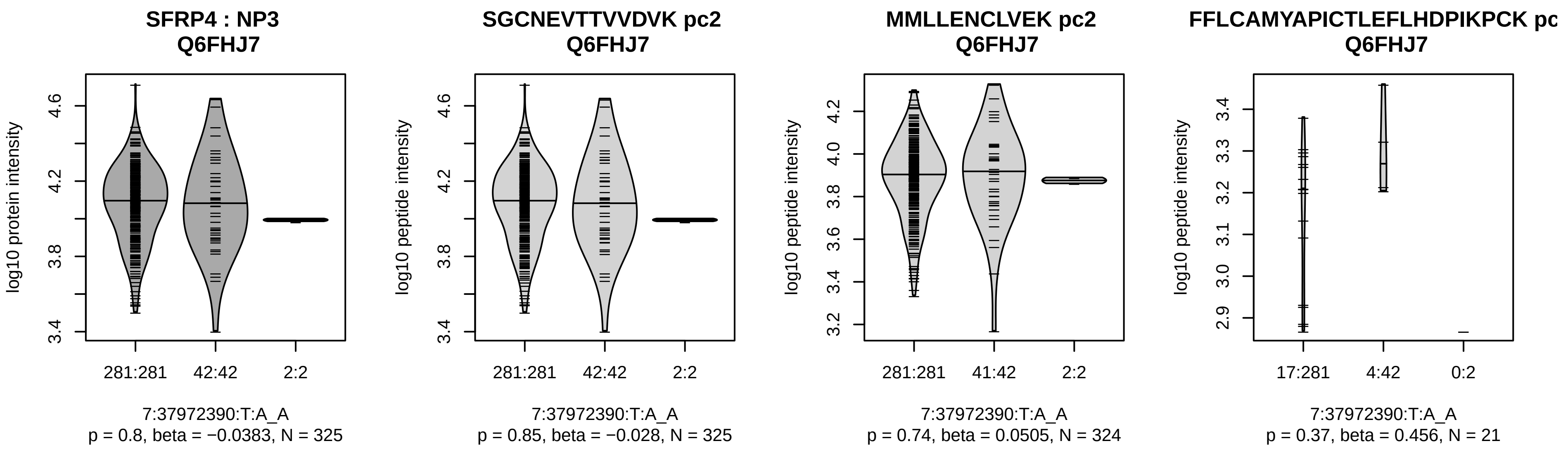

Assay Target: SFRP4
UniProt: Q6FHJ7
rsID: rs75207237
Target SNP: 7:37972390:T:A
Proxy SNP: 7:37972390:T:A
UKBPPP log10(p): 201
UKBPPP BETA: 0.28
UKBPPP MSPA: 0.72
::*:–:–:–:–
1244:1254:960:439:638:177:89

223. CCL5 (P13501)
UKBPPP MSPA=0.722

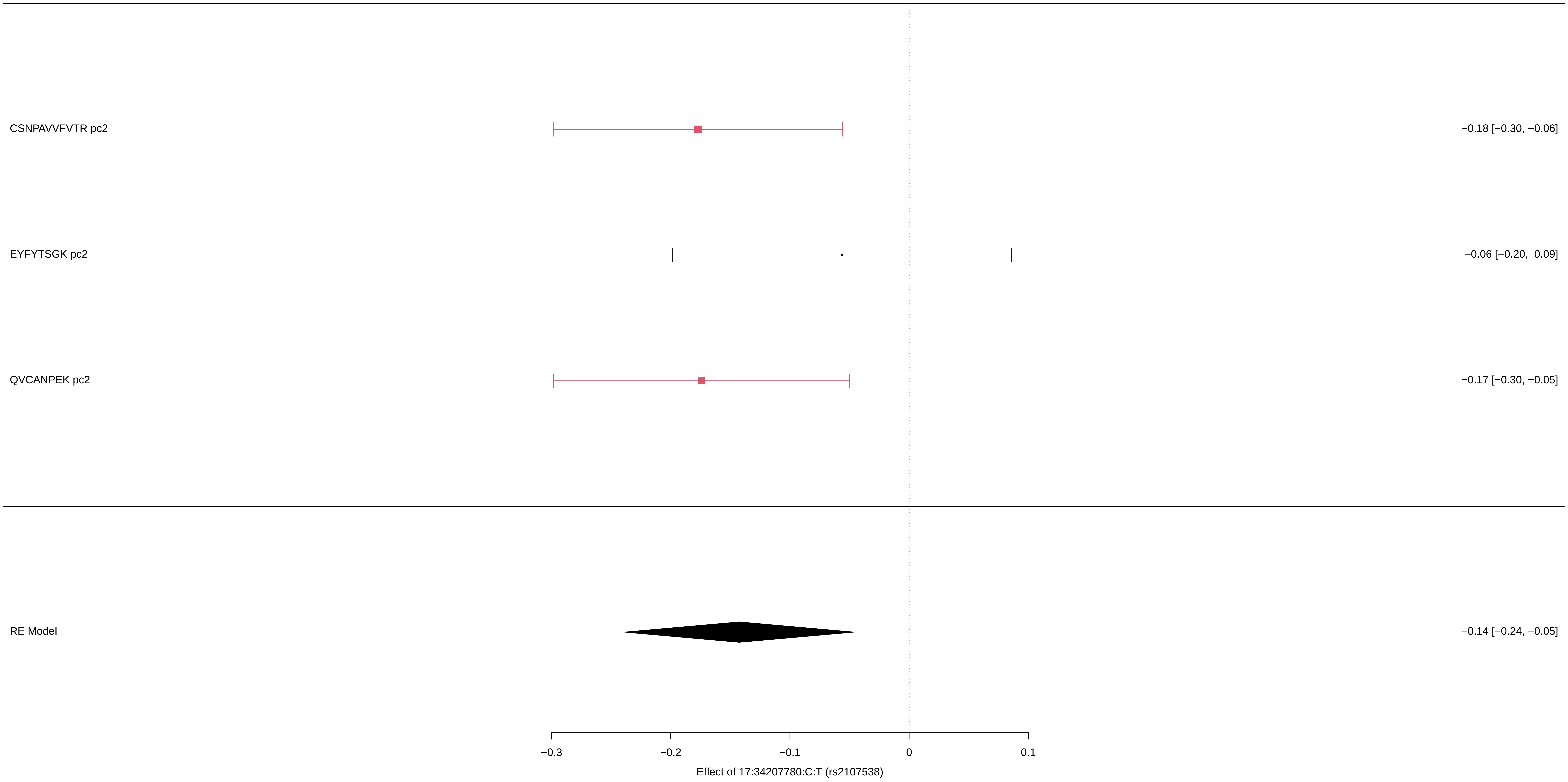

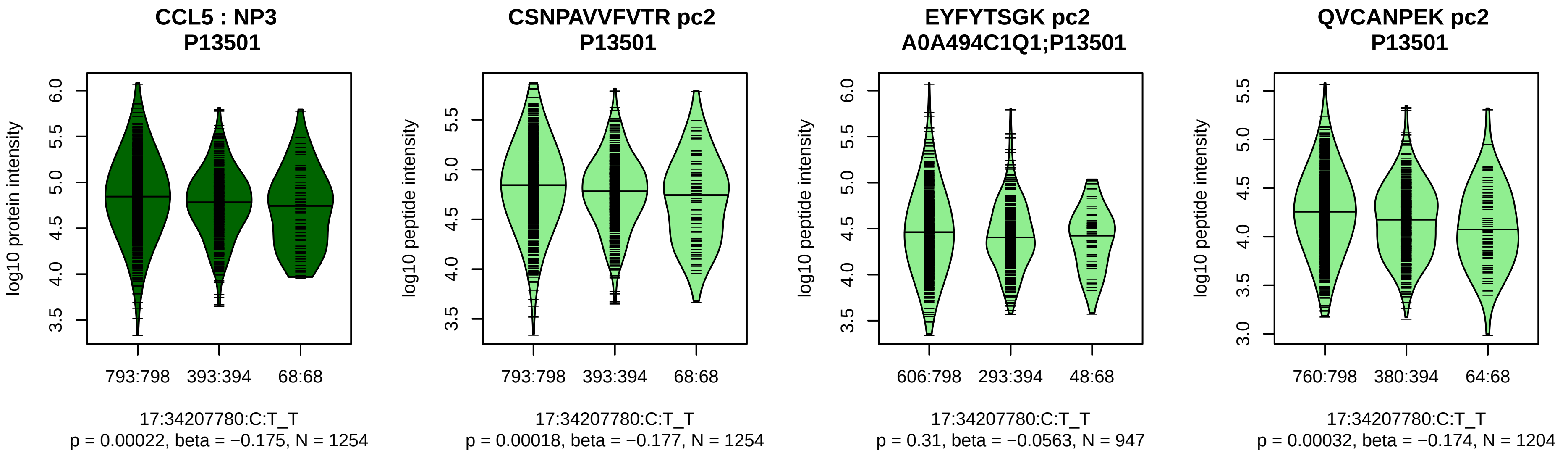

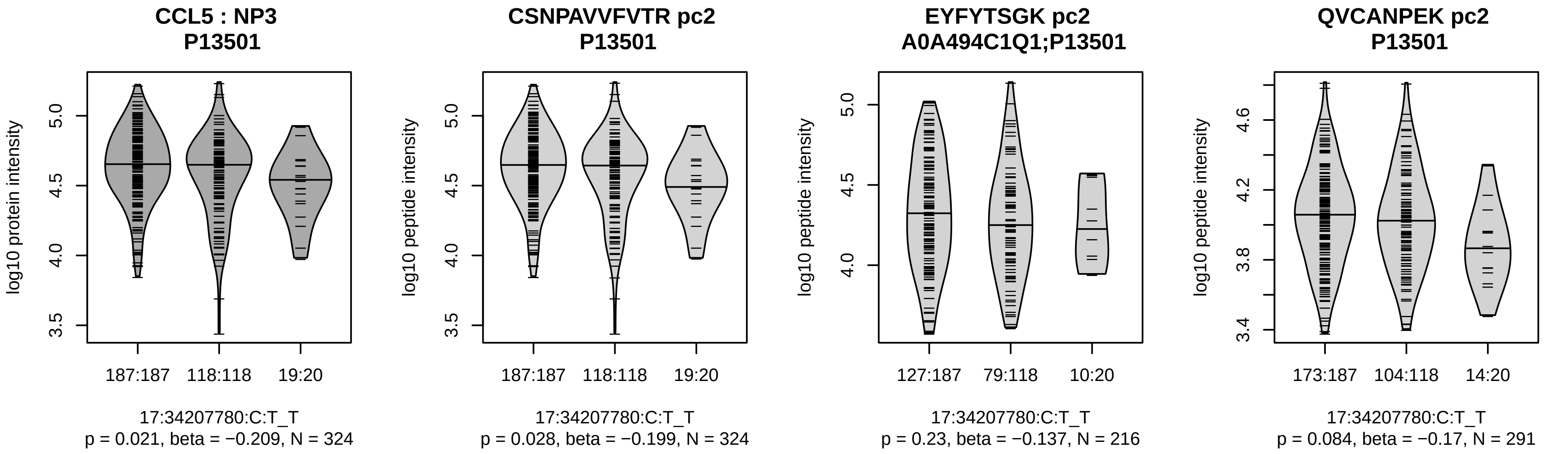

Assay Target: CCL5
UniProt: P13501
rsID: rs2107538
Target SNP: 17:34207780:C:T
Proxy SNP: 17:34207780:C:T
UKBPPP log10(p): 196.7
UKBPPP BETA: −0.231
UKBPPP MSPA: 0.722
:–:
1254:947:1204

224. PRKG1 (Q13976)
UKBPPP MSPA=0.191

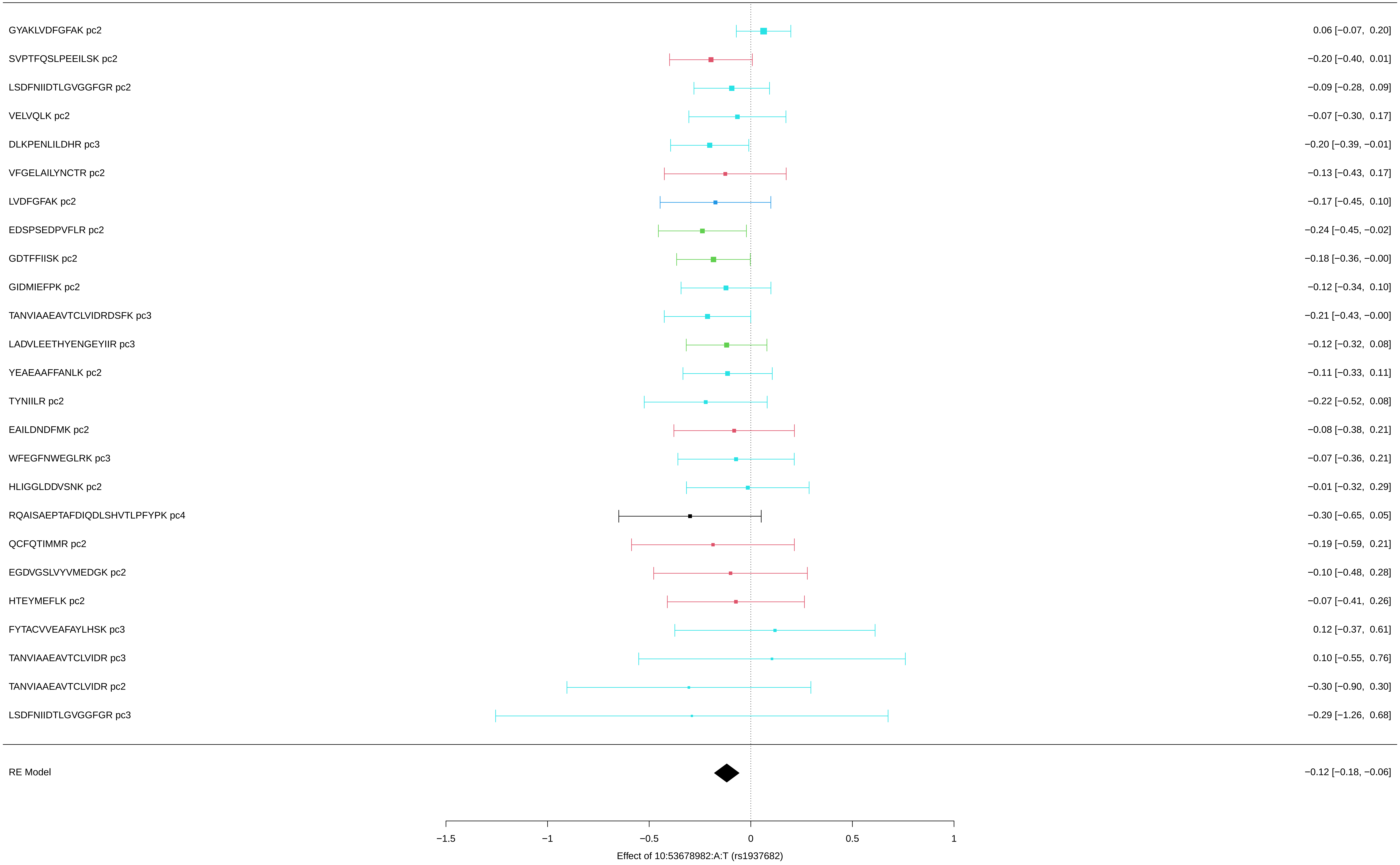

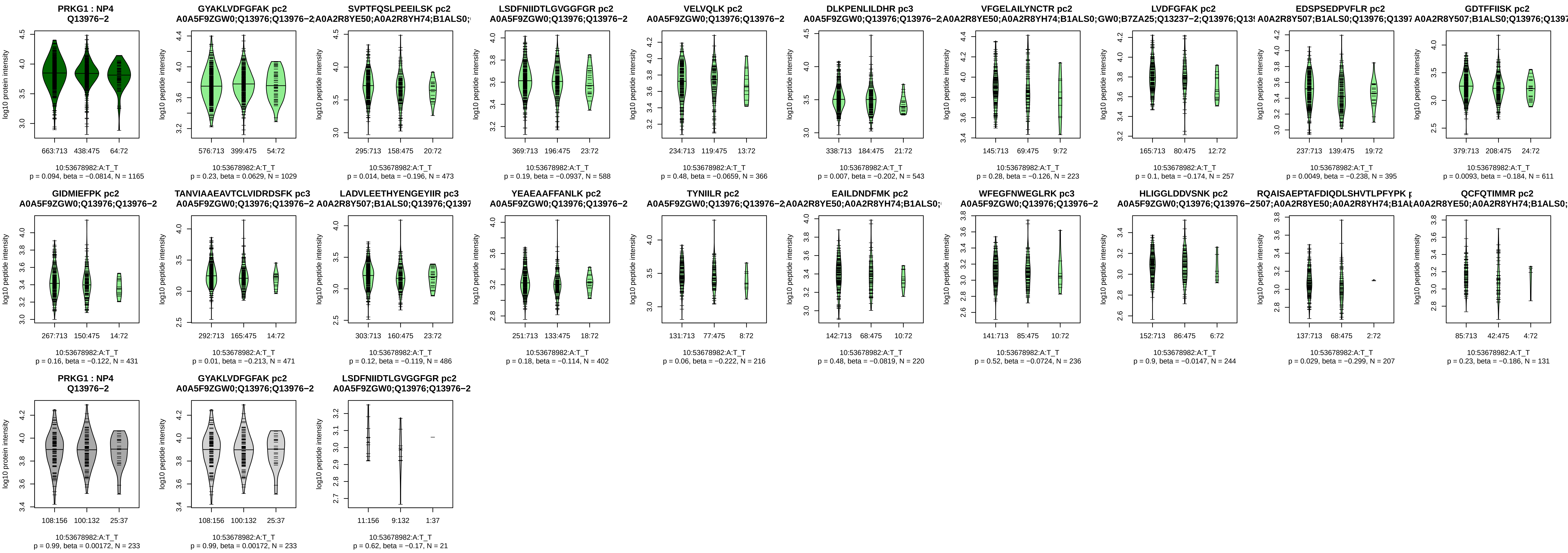

Assay Target: PRKG1
UniProt: Q13976
rsID: rs1937682
Target SNP: 10:53678982:A:T
Proxy SNP: 10:53678982:A:T
UKBPPP log10(p): 192.3
UKBPPP BETA: −0.214
UKBPPP MSPA: 0.191
[illegible]
1029:473:588:366:543:223:257:

225. PIGR (P01833)
UKBPPP MSPA=0

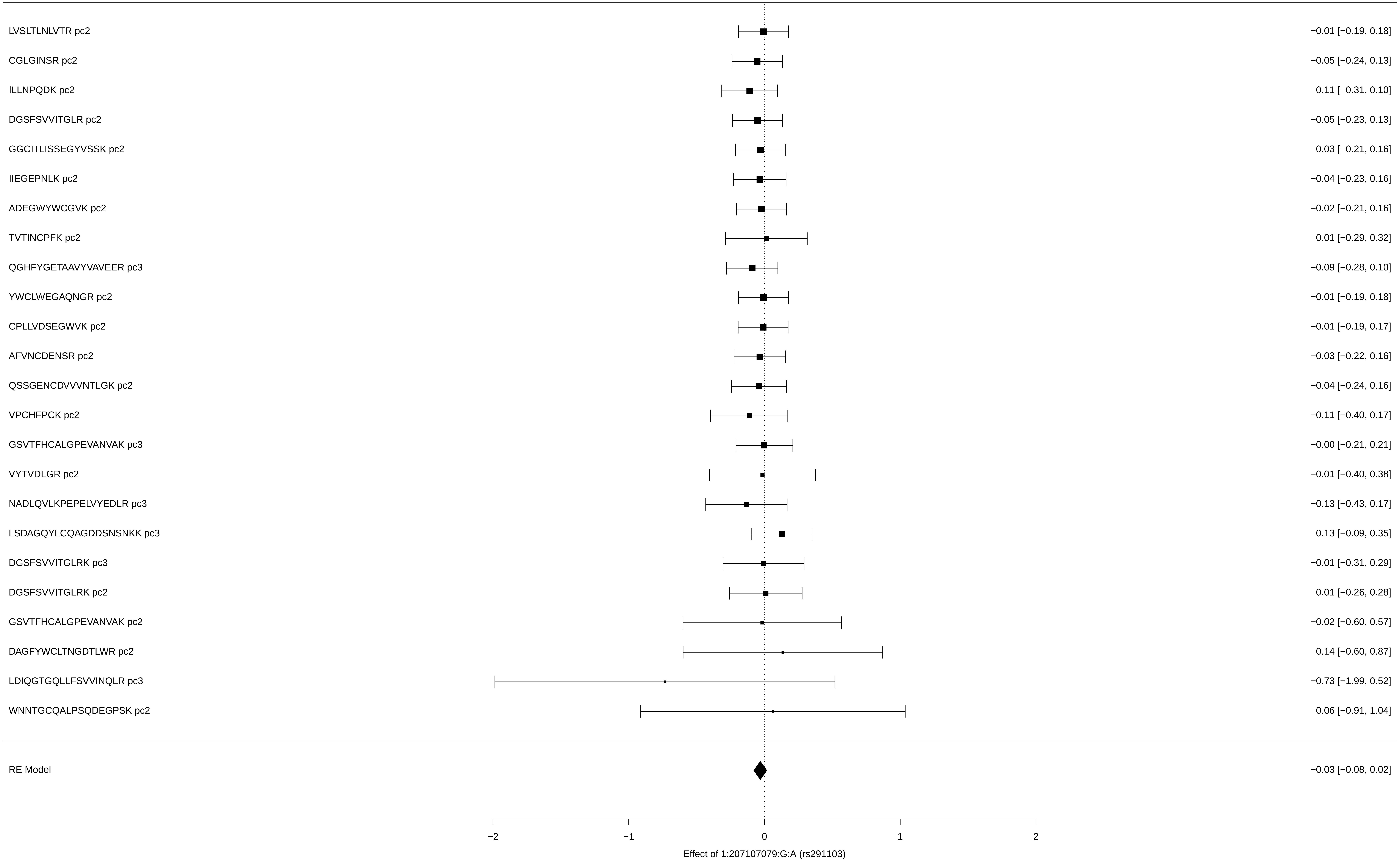

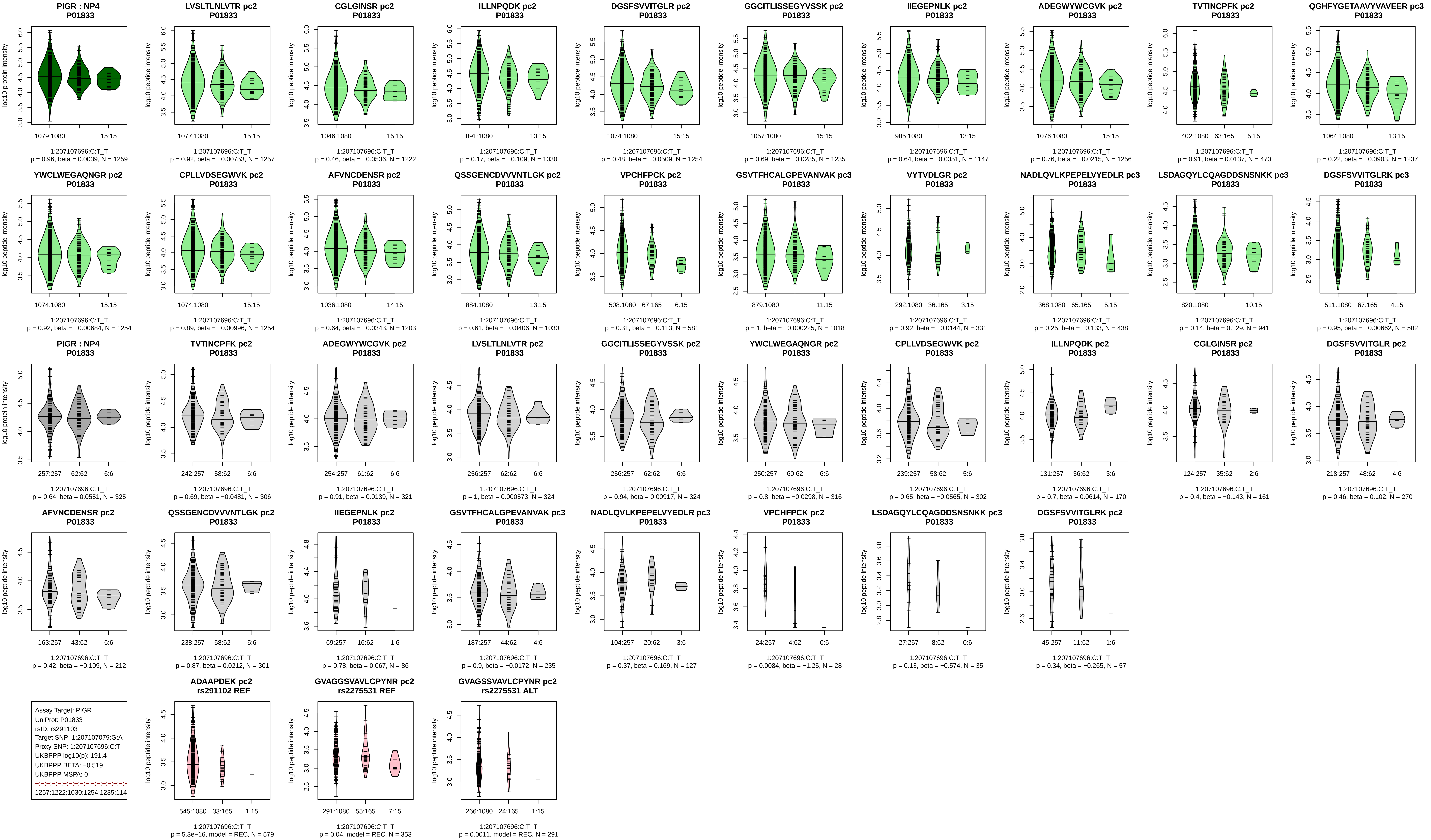

PIGR : NP4 P01833
1:207107696:C:T_T p = 0.96, beta = 0.0039, N = 1259
LVSLTLNLVTR pc2 P01833
1:207107696:C:T_T p = 0.92, beta = −0.00753, N = 1257
CGLGINSR pc2 P01833
1:207107696:C:T_T p = 0.46, beta = −0.0536, N = 1222
ILLNPQDK pc2 P01833
1:207107696:C:T_T p = 0.17, beta = −0.109, N = 1030
DGSFSVVITGLR pc2 P01833
1:207107696:C:T_T p = 0.48, beta = −0.0509, N = 1254
GGCITLISSEGYVSSK pc2 P01833
1:207107696:C:T_T p = 0.69, beta = −0.0285, N = 1235
IIEGEPNLK pc2 P01833
1:207107696:C:T_T p = 0.64, beta = −0.0351, N = 1147
ADEGWYWCGVK pc2 P01833
1:207107696:C:T_T p = 0.76, beta = −0.0215, N = 1256
TVTINCPFK pc2 P01833
1:207107696:C:T_T p = 0.91, beta = 0.0137, N = 470
QGHFYGETAAVYVAVEER pc3 P01833
1:207107696:C:T_T p = 0.22, beta = −0.0903, N = 1237
YWCLWEGAQNGR pc2 P01833
1:207107696:C:T_T p = 0.92, beta = −0.00684, N = 1254
CPLLVDSEGWVK pc2 P01833
1:207107696:C:T_T p = 0.89, beta = −0.00996, N = 1254
AFVNCDENSR pc2 P01833
1:207107696:C:T_T p = 0.64, beta = −0.0343, N = 1203
QSSGENCDVVVNTLGK pc2 P01833
1:207107696:C:T_T p = 0.61, beta = −0.0406, N = 1030
VPCHFPCK pc2 P01833
1:207107696:C:T_T p = 0.31, beta = −0.113, N = 581
GSVTFHCALGPEVANVAK pc3 P01833
1:207107696:C:T_T p = 1, beta = −0.000225, N = 1018
VYTVDLGR pc2 P01833
1:207107696:C:T_T p = 0.92, beta = −0.0144, N = 331
NADLQVLKPEPELVYEDLR pc3 P01833
1:207107696:C:T_T p = 0.25, beta = −0.133, N = 438
LSDAGQYLCQAGDDSNSNKK pc3 P01833
1:207107696:C:T_T p = 0.14, beta = 0.129, N = 941
DGSFSVVITGLRK pc3 P01833
1:207107696:C:T_T p = 0.95, beta = −0.00662, N = 582
PIGR : NP4 P01833
1:207107696:C:T_T p = 0.64, beta = 0.0551, N = 325
TVTINCPFK pc2 P01833
1:207107696:C:T_T p = 0.69, beta = −0.0481, N = 306
ADEGWYWCGVK pc2 P01833
1:207107696:C:T_T p = 0.91, beta = 0.0139, N = 321
LVSLTLNLVTR pc2 P01833
1:207107696:C:T_T p = 1, beta = 0.000573, N = 324
GGCITLISSEGYVSSK pc2 P01833
1:207107696:C:T_T p = 0.94, beta = 0.00917, N = 324
YWCLWEGAQNGR pc2 P01833
1:207107696:C:T_T p = 0.8, beta = −0.0298, N = 316
CPLLVDSEGWVK pc2 P01833
1:207107696:C:T_T p = 0.65, beta = −0.0565, N = 302
ILLNPQDK pc2 P01833
1:207107696:C:T_T p = 0.7, beta = 0.0614, N = 170
CGLGINSR pc2 P01833
1:207107696:C:T_T p = 0.4, beta = −0.143, N = 161
DGSFSVVITGLR pc2 P01833
1:207107696:C:T_T p = 0.46, beta = 0.102, N = 270
AFVNCDENSR pc2 P01833
1:207107696:C:T_T p = 0.42, beta = −0.109, N = 212
QSSGENCDVVVNTLGK pc2 P01833
1:207107696:C:T_T p = 0.87, beta = 0.0212, N = 301
IIEGEPNLK pc2 P01833
1:207107696:C:T_T p = 0.78, beta = 0.067, N = 86
GSVTFHCALGPEVANVAK pc3 P01833
1:207107696:C:T_T p = 0.9, beta = −0.0172, N = 235
NADLQVLKPEPELVYEDLR pc3 P01833
1:207107696:C:T_T p = 0.37, beta = 0.169, N = 127
VPCHFPCK pc2 P01833
1:207107696:C:T_T p = 0.0084, beta = −1.25, N = 28
LSDAGQYLCQAGDDSNSNKK pc3 P01833
1:207107696:C:T_T p = 0.13, beta = −0.574, N = 35
DGSFSVVITGLRK pc2 P01833
1:207107696:C:T_T p = 0.34, beta = −0.265, N = 57
Assay Target: PIGR
UniProt: P01833
rsID: rs291103
Target SNP: 1:207107079:G:A
Proxy SNP: 1:207107696:C:T
UKBPPP log10(p): 191.4
UKBPPP BETA: −0.519
UKBPPP MSPA: 0
1257:1222:1030:1254:1235:114
ADAAPDEK pc2 rs291102 REF
1:207107696:C:T_T p = 5.3e−16, model = REC, N = 579
GVAGGSVAVLCPYNR pc2 rs2275531 REF
1:207107696:C:T_T p = 0.04, model = REC, N = 353
GVAGSSVAVLCPYNR pc2 rs2275531 ALT
1:207107696:C:T_T p = 0.0011, model = REC, N = 291

226. PKLR (P30613)
UKBPPP MSPA=0.244

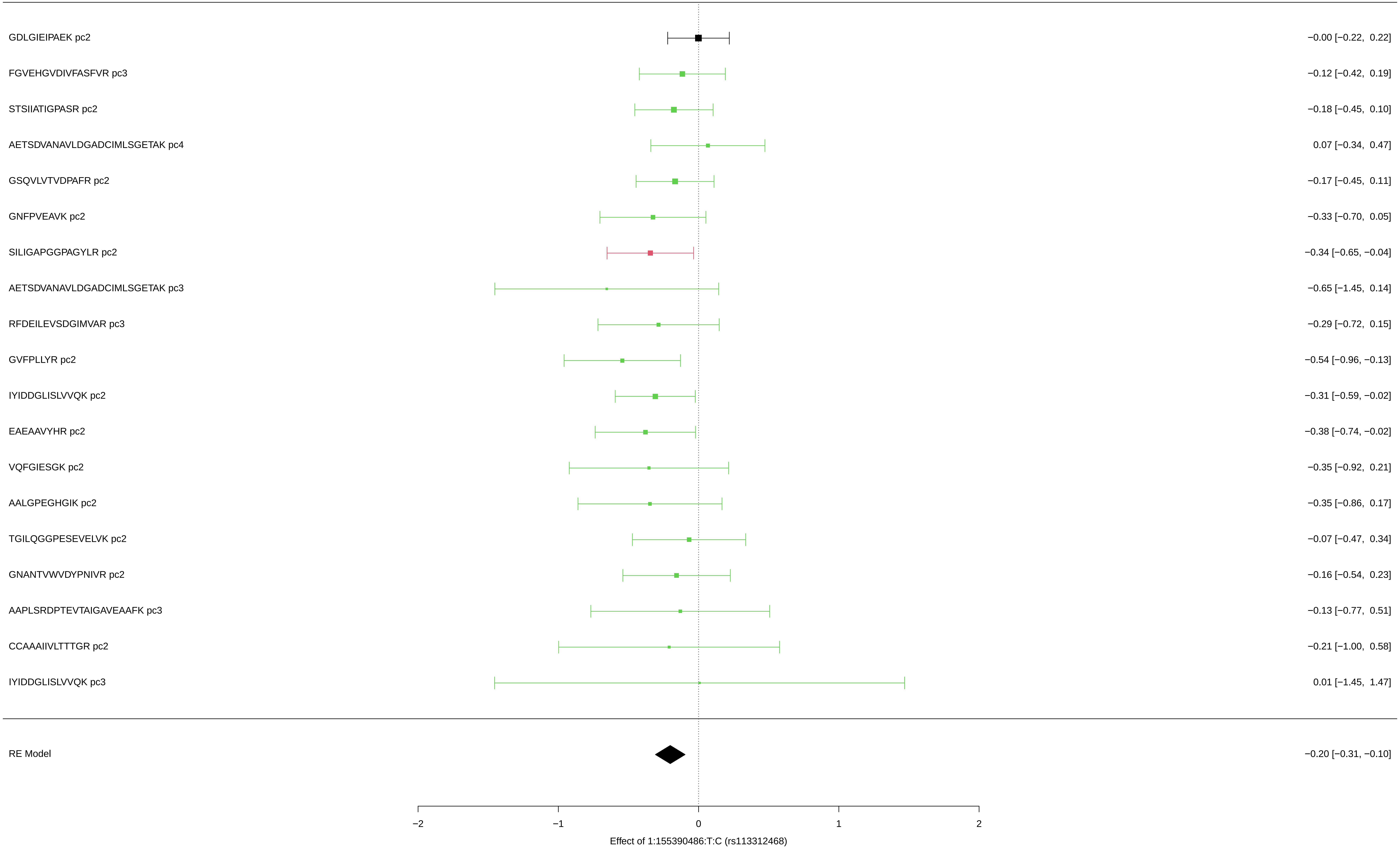

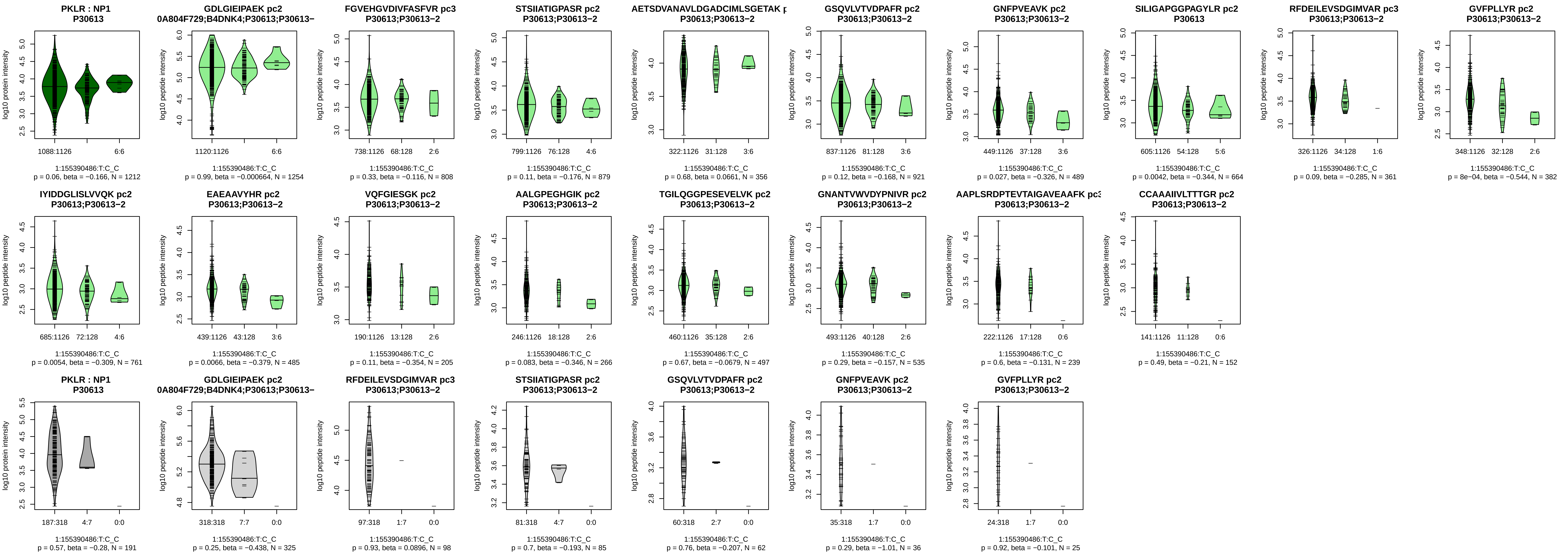

Assay Target: PKLR
UniProt: P30613
rsID: rs113312468
Target SNP: 1:155390486:T:C
Proxy SNP: 1:155390486:T:C
UKBPPP log10(p): 191.3
UKBPPP BETA: −0.366
UKBPPP MSPA: 0.244
1254:808:879:356:921:489:664:

227. AHNAK (Q09666)
UKBPPP MSPA=0.017

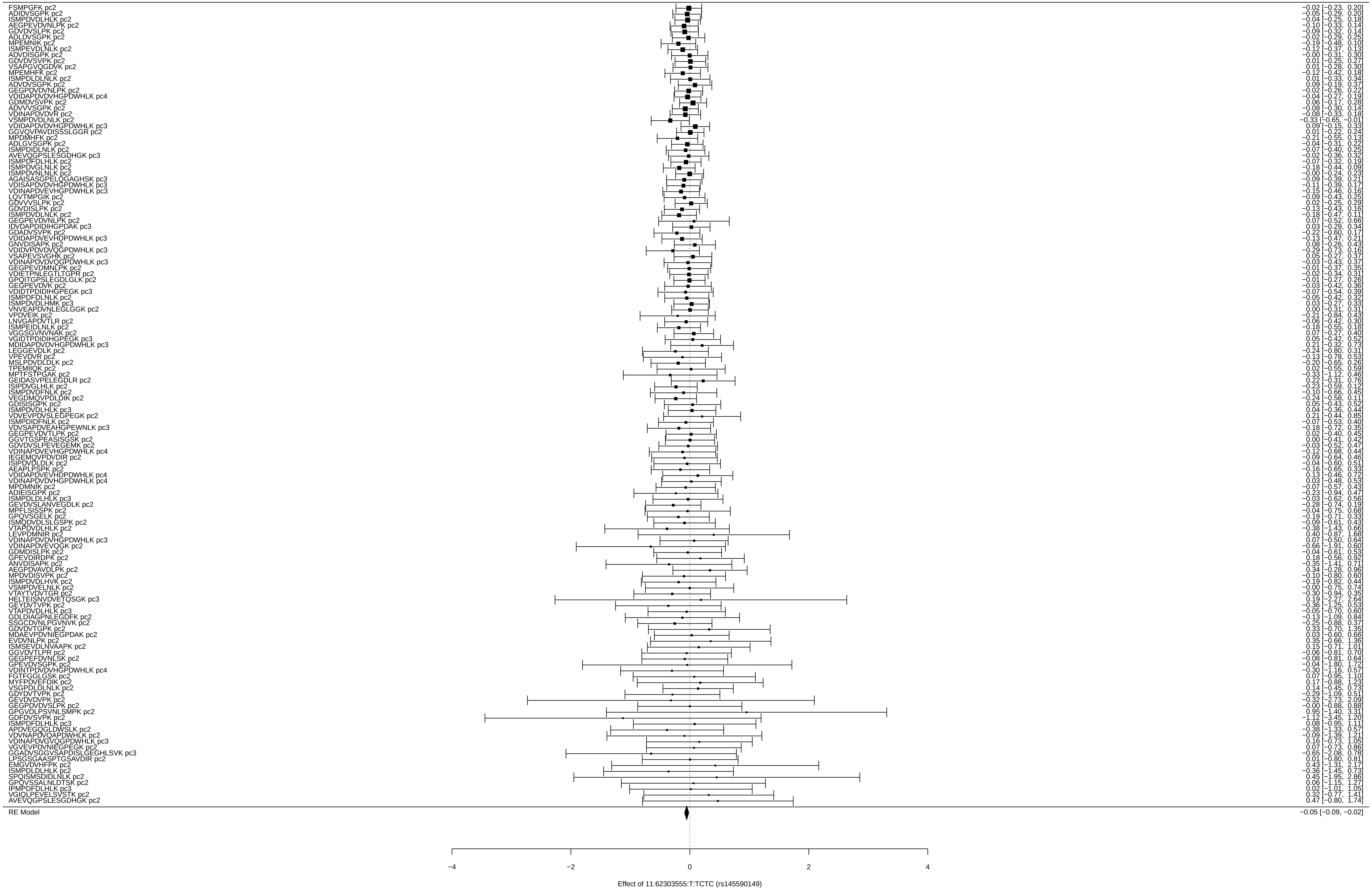

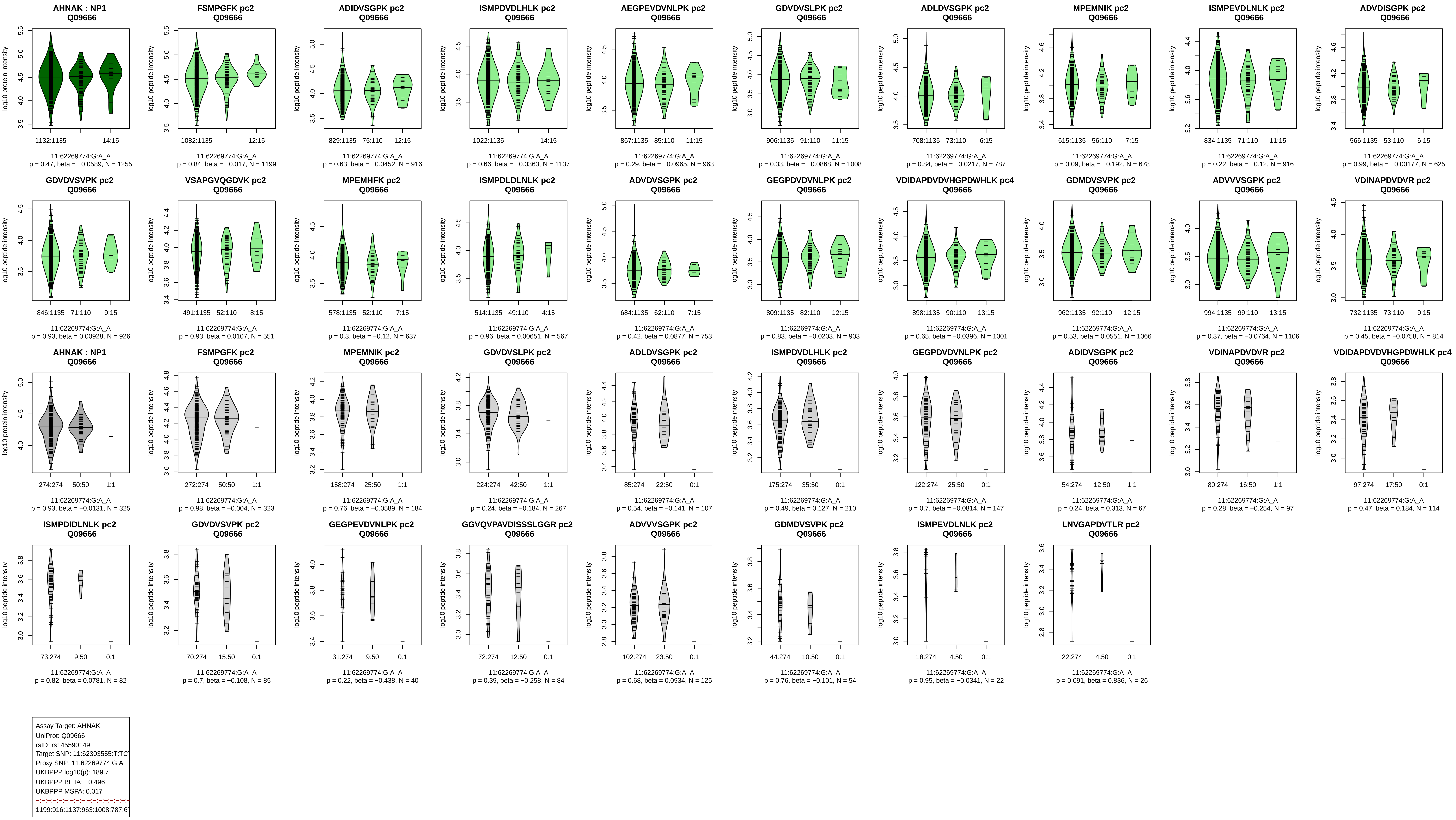
AHNAK : NP1 Q09666
1132:1135 14:15
11:62269774:G:A_A
p = 0.47, beta = −0.0589, N = 1255
FSMPGFK pc2 Q09666
1082:1135 12:15
11:62269774:G:A_A
p = 0.84, beta = −0.017, N = 1199
ADIDVSGPK pc2 Q09666
829:1135 75:110 12:15
11:62269774:G:A_A
p = 0.63, beta = −0.0452, N = 916
ISMPDVDLHLK pc2 Q09666
1022:1135 14:15
11:62269774:G:A_A
p = 0.66, beta = −0.0363, N = 1137
AEGPEVDVNLPK pc2 Q09666
867:1135 85:110 11:15
11:62269774:G:A_A
p = 0.29, beta = −0.0965, N = 963
GDVDVSLPK pc2 Q09666
906:1135 91:110 11:15
11:62269774:G:A_A
p = 0.33, beta = −0.0868, N = 1008
ADLDVSGPK pc2 Q09666
708:1135 73:110 6:15
11:62269774:G:A_A
p = 0.84, beta = −0.0217, N = 787
MPEMNIK pc2 Q09666
615:1135 56:110 7:15
11:62269774:G:A_A
p = 0.09, beta = −0.192, N = 678
ISMPEVDLNLK pc2 Q09666
834:1135 71:110 11:15
11:62269774:G:A_A
p = 0.22, beta = −0.12, N = 916
ADVDISGPK pc2 Q09666
566:1135 53:110 6:15
11:62269774:G:A_A
p = 0.99, beta = −0.00177, N = 625
GDVDVSVPK pc2 Q09666
846:1135 71:110 9:15
11:62269774:G:A_A
p = 0.93, beta = 0.00928, N = 926
VSAPGVQGDVK pc2 Q09666
491:1135 52:110 8:15
11:62269774:G:A_A
p = 0.93, beta = 0.0107, N = 551
MPEMHFK pc2 Q09666
578:1135 52:110 7:15
11:62269774:G:A_A
p = 0.3, beta = −0.12, N = 637
ISMPDLDLNLK pc2 Q09666
514:1135 49:110 4:15
11:62269774:G:A_A
p = 0.96, beta = 0.00651, N = 567
ADVDVSGPK pc2 Q09666
684:1135 62:110 7:15
11:62269774:G:A_A
p = 0.42, beta = 0.0877, N = 753
GEGPDVDVNLPK pc2 Q09666
809:1135 82:110 12:15
11:62269774:G:A_A
p = 0.83, beta = −0.0203, N = 903
VDIDAPDVDVHGPDWHLK pc4 Q09666
898:1135 90:110 13:15
11:62269774:G:A_A
p = 0.65, beta = −0.0396, N = 1001
GDMDVSVPK pc2 Q09666
962:1135 92:110 12:15
11:62269774:G:A_A
p = 0.53, beta = 0.0551, N = 1066
ADVVVSGPK pc2 Q09666
994:1135 99:110 13:15
11:62269774:G:A_A
p = 0.37, beta = −0.0764, N = 1106
VDINAPDVDVR pc2 Q09666
732:1135 73:110 9:15
11:62269774:G:A_A
p = 0.45, beta = −0.0758, N = 814
AHNAK : NP1 Q09666
274:274 50:50 1:1
11:62269774:G:A_A
p = 0.93, beta = −0.0131, N = 325
FSMPGFK pc2 Q09666
272:274 50:50 1:1
11:62269774:G:A_A
p = 0.98, beta = −0.004, N = 323
MPEMNIK pc2 Q09666
158:274 25:50 0:1
11:62269774:G:A_A
p = 0.76, beta = −0.0589, N = 184
GDVDVSLPK pc2 Q09666
224:274 42:50 1:1
11:62269774:G:A_A
p = 0.24, beta = −0.184, N = 267
ADLDVSGPK pc2 Q09666
85:274 22:50 0:1
11:62269774:G:A_A
p = 0.54, beta = −0.141, N = 107
ISMPDVDLHLK pc2 Q09666
175:274 35:50 0:1
11:62269774:G:A_A
p = 0.49, beta = 0.127, N = 210
GEGPDVDVNLPK pc2 Q09666
122:274 25:50 0:1
11:62269774:G:A_A
p = 0.7, beta = −0.0814, N = 147
ADIDVSGPK pc2 Q09666
54:274 12:50 1:1
11:62269774:G:A_A
p = 0.24, beta = 0.313, N = 67
VDINAPDVDVR pc2 Q09666
80:274 16:50 1:1
11:62269774:G:A_A
p = 0.28, beta = −0.254, N = 97
VDIDAPDVDVHGPDWHLK pc4 Q09666
97:274 17:50 0:1
11:62269774:G:A_A
p = 0.47, beta = 0.184, N = 114
ISMPDIDLNLK pc2 Q09666
73:274 9:50 0:1
11:62269774:G:A_A
p = 0.82, beta = 0.0781, N = 82
GDVDVSVPK pc2 Q09666
70:274 15:50 0:1
11:62269774:G:A_A
p = 0.7, beta = −0.108, N = 85
GEGPEVDVNLPK pc2 Q09666
31:274 9:50 0:1
11:62269774:G:A_A
p = 0.22, beta = −0.438, N = 40
GGVQVPAVDISSSLGGR pc2 Q09666
72:274 12:50 0:1
11:62269774:G:A_A
p = 0.39, beta = −0.258, N = 84
ADVVVSGPK pc2 Q09666
102:274 23:50 0:1
11:62269774:G:A_A
p = 0.68, beta = 0.0934, N = 125
GDMDVSVPK pc2 Q09666
44:274 10:50 0:1
11:62269774:G:A_A
p = 0.76, beta = −0.101, N = 54
ISMPEVDLNLK pc2 Q09666
18:274 4:50 0:1
11:62269774:G:A_A
p = 0.95, beta = −0.0341, N = 22
LNVGAPDVTLR pc2 Q09666
22:274 4:50 0:1
11:62269774:G:A_A
p = 0.091, beta = 0.836, N = 26
log10 protein intensity
log10 peptide intensity
Assay Target: AHNAK
UniProt: Q09666
rsID: rs145590149
Target SNP: 11:62303555:T:TC
Proxy SNP: 11:62269774:G:A
UKBPPP log10(p): 189.7
UKBPPP BETA: −0.496
UKBPPP MSPA: 0.017
1199:916:1137:963:1008:787:6

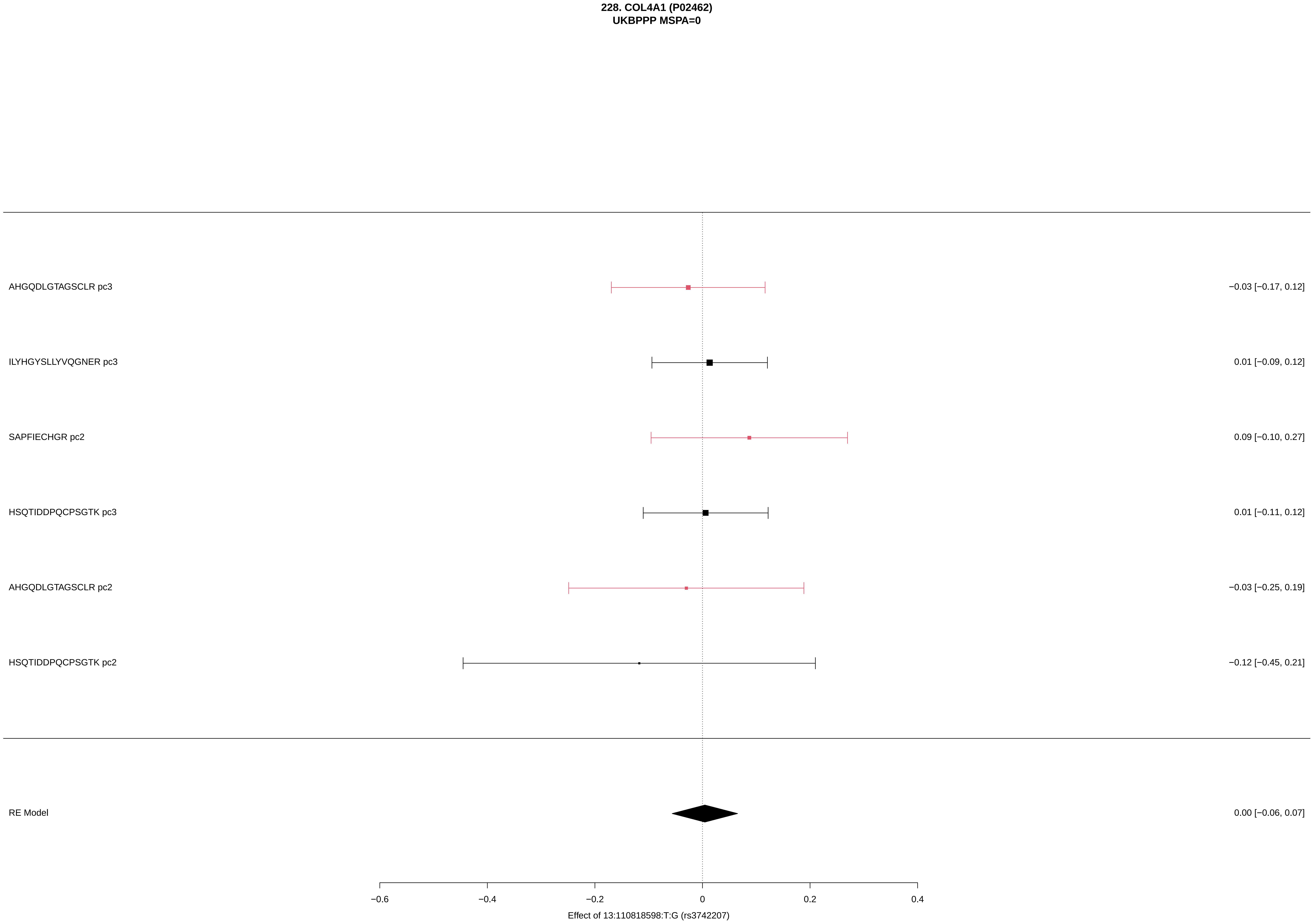
228. COL4A1 (P02462)
UKBPPP MSPA=0
AHGQDLGTAGSCLR pc3
−0.03 [−0.17, 0.12]
ILYHGYSLLYVQGNER pc3
0.01 [−0.09, 0.12]
SAPFIECHGR pc2
0.09 [−0.10, 0.27]
HSQTIDDPQCPSGTK pc3
0.01 [−0.11, 0.12]
AHGQDLGTAGSCLR pc2
−0.03 [−0.25, 0.19]
HSQTIDDPQCPSGTK pc2
−0.12 [−0.45, 0.21]
RE Model
0.00 [−0.06, 0.07]
−0.6
−0.4
−0.2
0
0.2
0.4
Effect of 13:110818598:T:G (rs3742207)

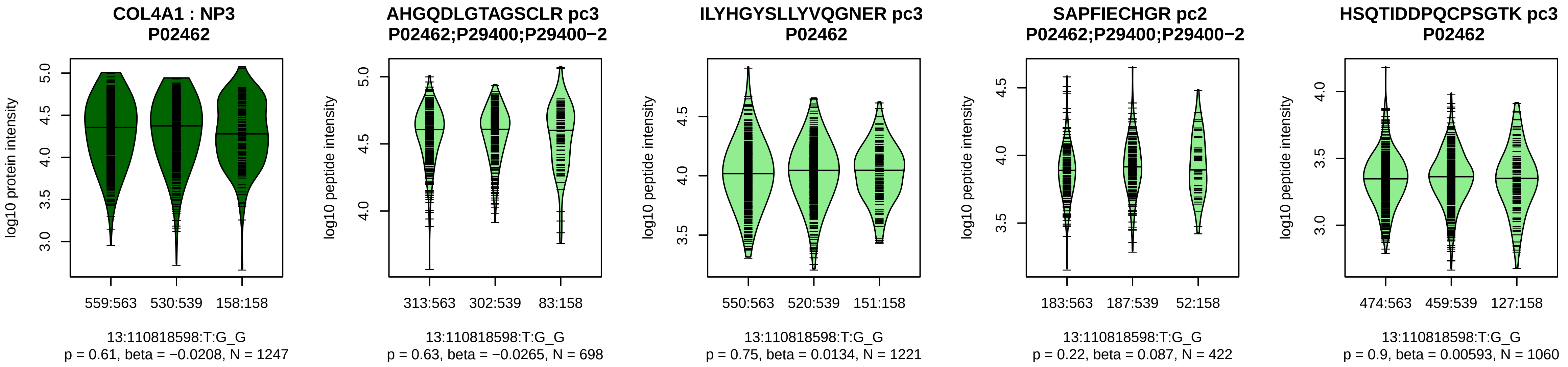

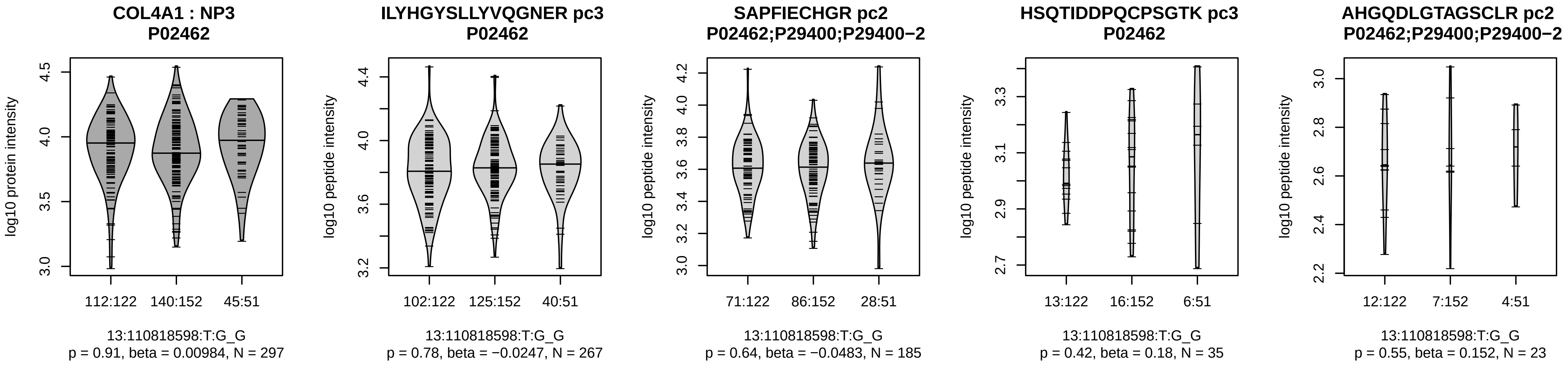

Assay Target: COL4A1
UniProt: P02462
rsID: rs3742207
Target SNP: 13:110818598:T:G
Proxy SNP: 13:110818598:T:G
UKBPPP log10(p): 186.6
UKBPPP BETA: −0.184
UKBPPP MSPA: 0
−:−:−:−:−:−:NA:NA
698:1221:422:1060:283:130:14

229. CSF1 (P09603)
UKBPPP MSPA=0.073

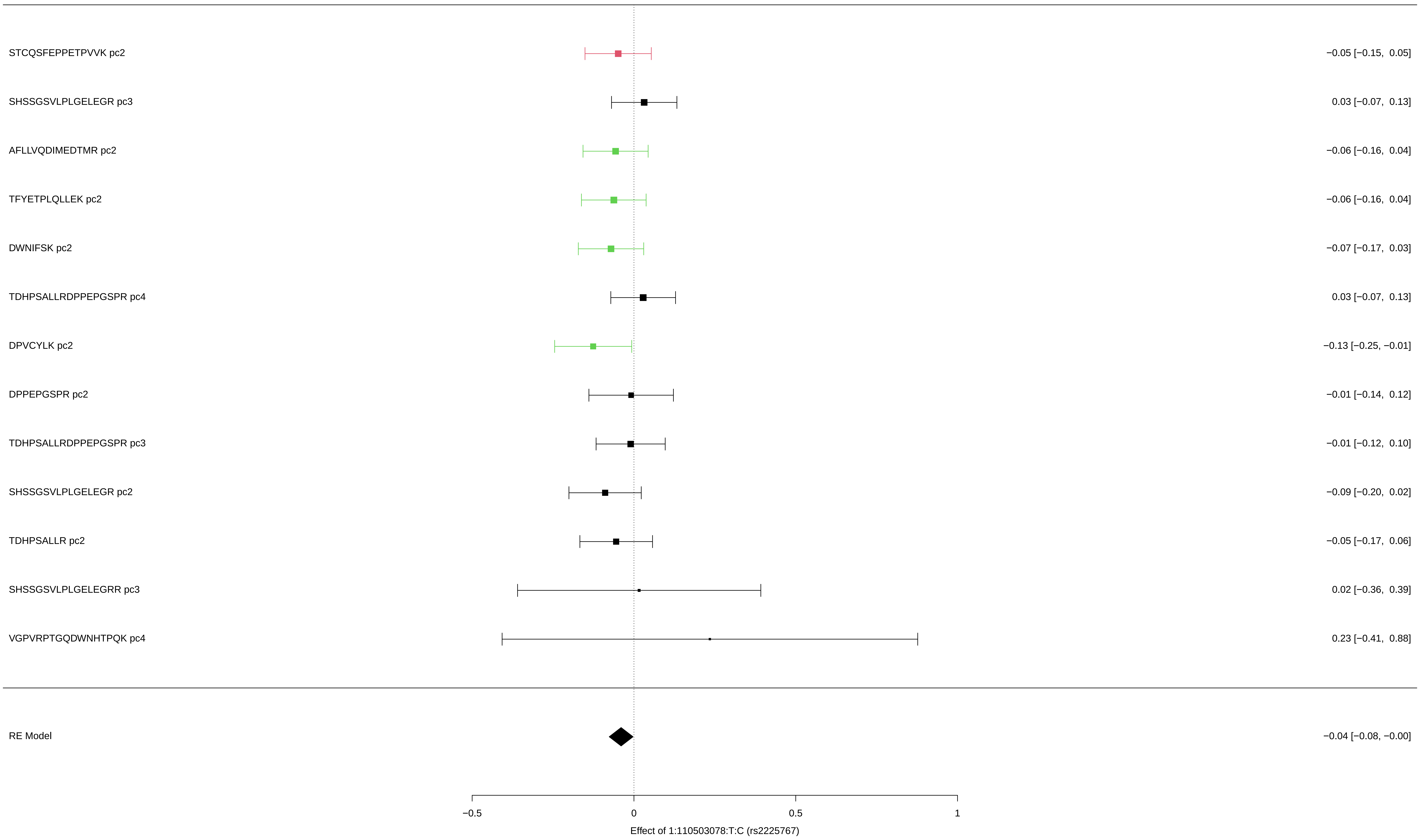

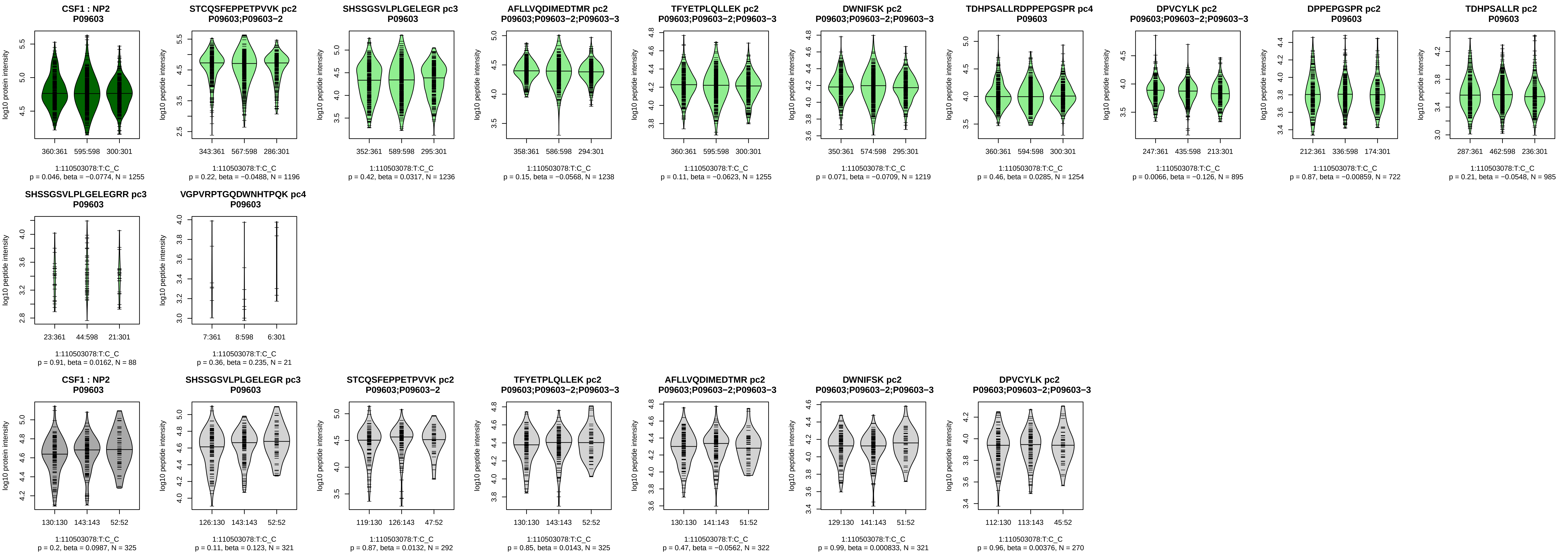

Assay Target: CSF1
UniProt: P09603
rsID: rs2225767
Target SNP: 1:110503078:T:C
Proxy SNP: 1:110503078:T:C
UKBPPP log10(p): 186.2
UKBPPP BETA: −0.177
UKBPPP MSPA: 0.073
–:–:–:–:–:–:*:–:–:–:–:–
1196:1236:1238:1255:1219:125

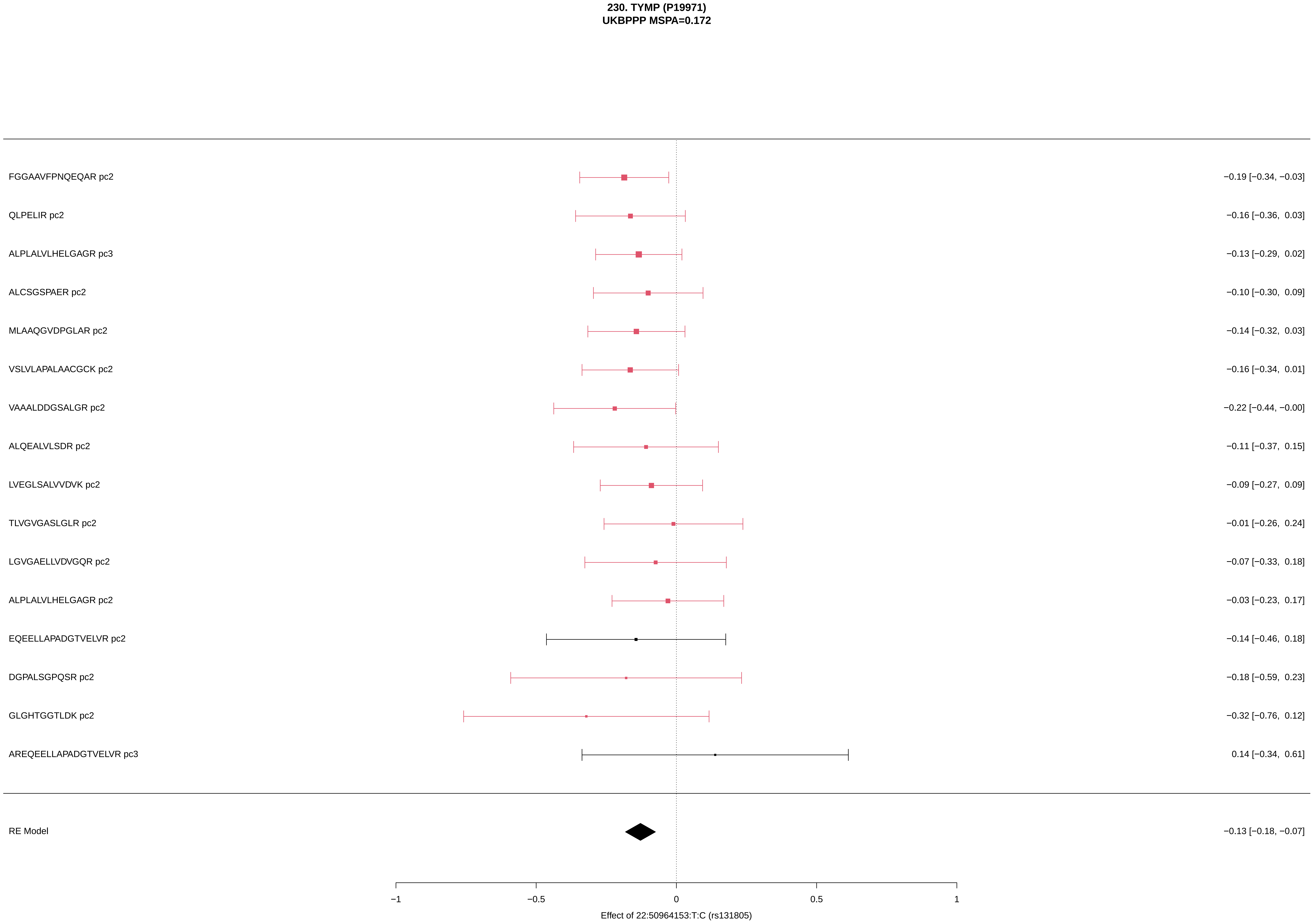

230. TYMP (P19971)
UKBPPP MSPA=0.172
FGGAAVFPNQEQAR pc2
QLPELIR pc2
ALPLALVLHELGAGR pc3
ALCSGSPAER pc2
MLAAQGVDPGLAR pc2
VSLVLAPALAACGCK pc2
VAAALDDGSALGR pc2
ALQEALVLSDR pc2
LVEGLSALVVDVK pc2
TLVGVGASLGLR pc2
LGVGAELLVDVGQR pc2
ALPLALVLHELGAGR pc2
EQEELLAPADGTVELVR pc2
DGPALSGPQSR pc2
GLGHTGGTLDK pc2
AREQEELLAPADGTVELVR pc3
RE Model
−0.19 [−0.34, −0.03]
−0.16 [−0.36, 0.03]
−0.13 [−0.29, 0.02]
−0.10 [−0.30, 0.09]
−0.14 [−0.32, 0.03]
−0.16 [−0.34, 0.01]
−0.22 [−0.44, −0.00]
−0.11 [−0.37, 0.15]
−0.09 [−0.27, 0.09]
−0.01 [−0.26, 0.24]
−0.07 [−0.33, 0.18]
−0.03 [−0.23, 0.17]
−0.14 [−0.46, 0.18]
−0.18 [−0.59, 0.23]
−0.32 [−0.76, 0.12]
0.14 [−0.34, 0.61]
−0.13 [−0.18, −0.07]
−1
−0.5
0
0.5
1
Effect of 22:50964153:T:C (rs131805)

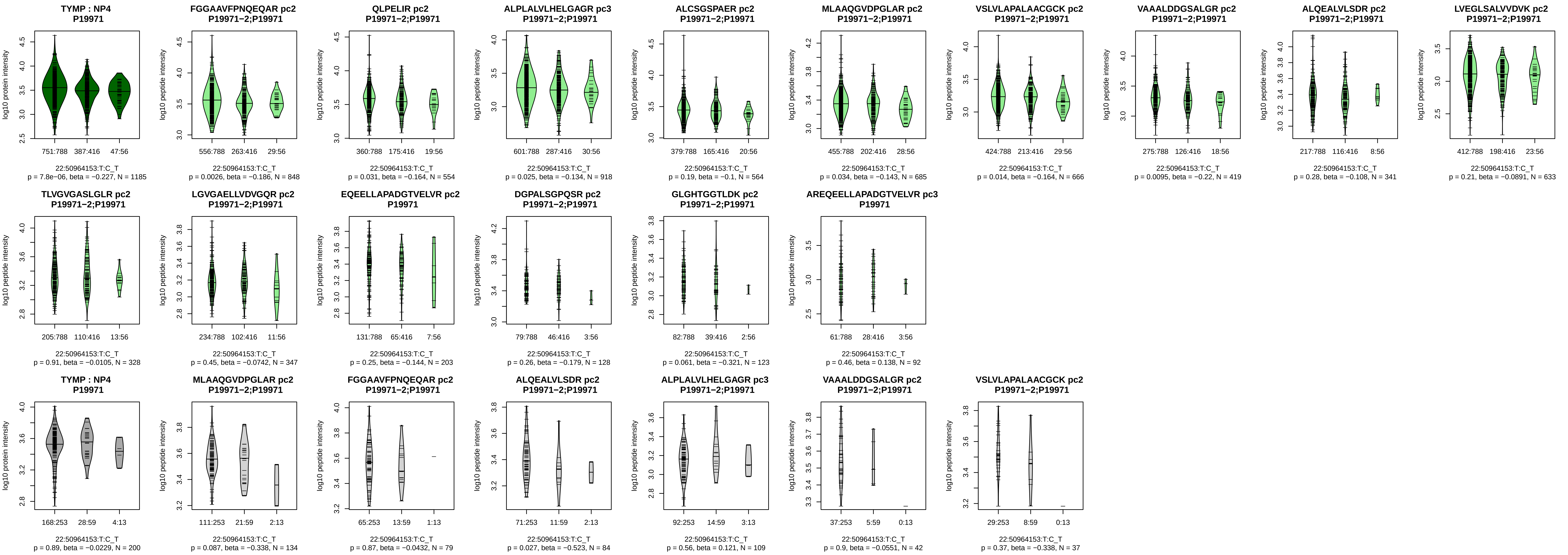

Assay Target: TYMP
UniProt: P19971
rsID: rs131805
Target SNP: 22:50964153:T:C
Proxy SNP: 22:50964153:T:C
UKBPPP log10(p): 185.4
UKBPPP BETA: 0.22
UKBPPP MSPA: 0.172
:–:–:–:–:–::–:–:–:–:–:–:–:–:–:N
848:554:918:564:685:666:419:3

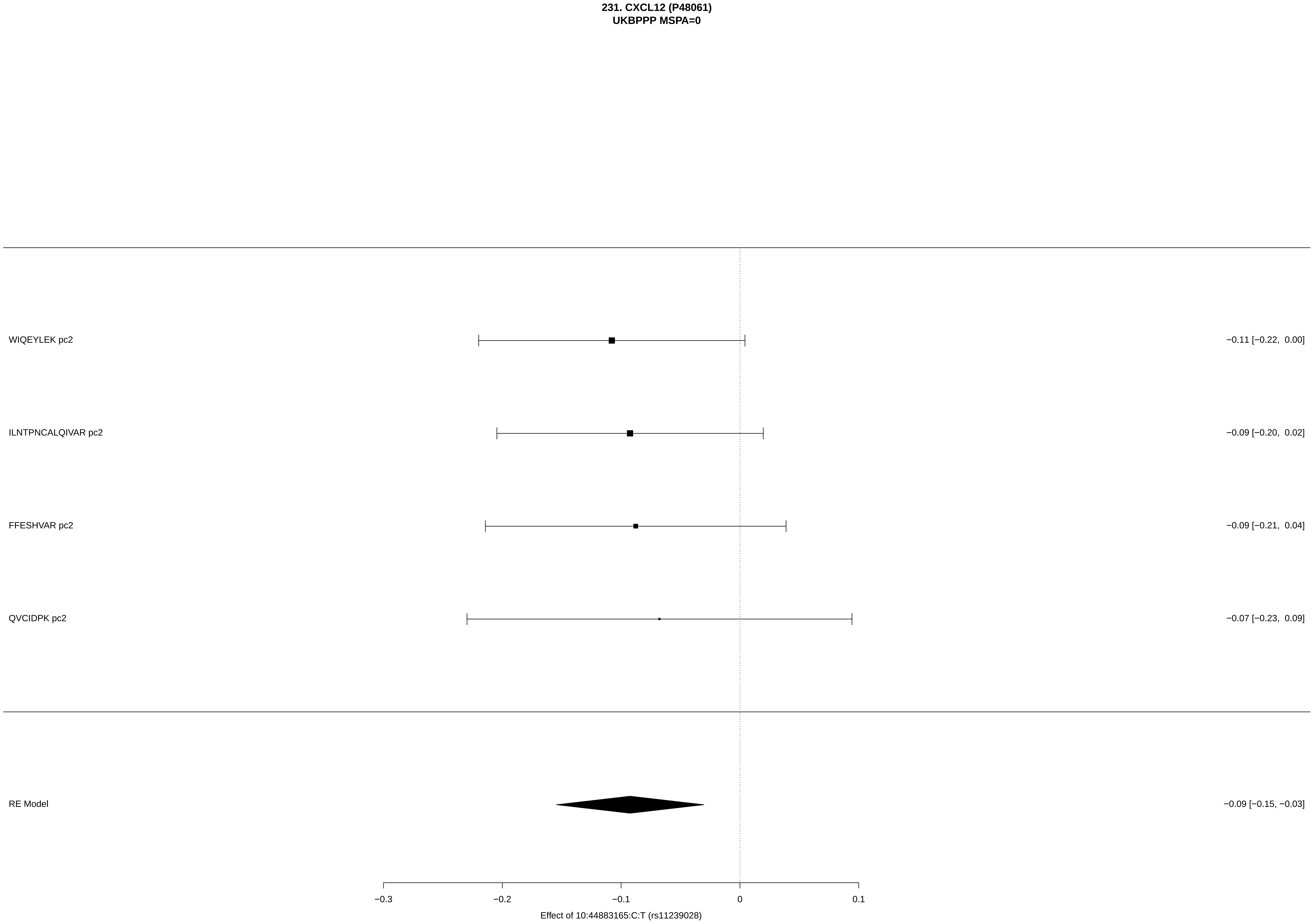
231. CXCL12 (P48061)
UKBPPP MSPA=0
WIQEYLEK pc2
−0.11 [−0.22, 0.00]
ILNTPNCALQIVAR pc2
−0.09 [−0.20, 0.02]
FFESHVAR pc2
−0.09 [−0.21, 0.04]
QVCIDPK pc2
−0.07 [−0.23, 0.09]
RE Model
−0.09 [−0.15, −0.03]
−0.3
−0.2
−0.1
0
0.1
Effect of 10:44883165:C:T (rs11239028)

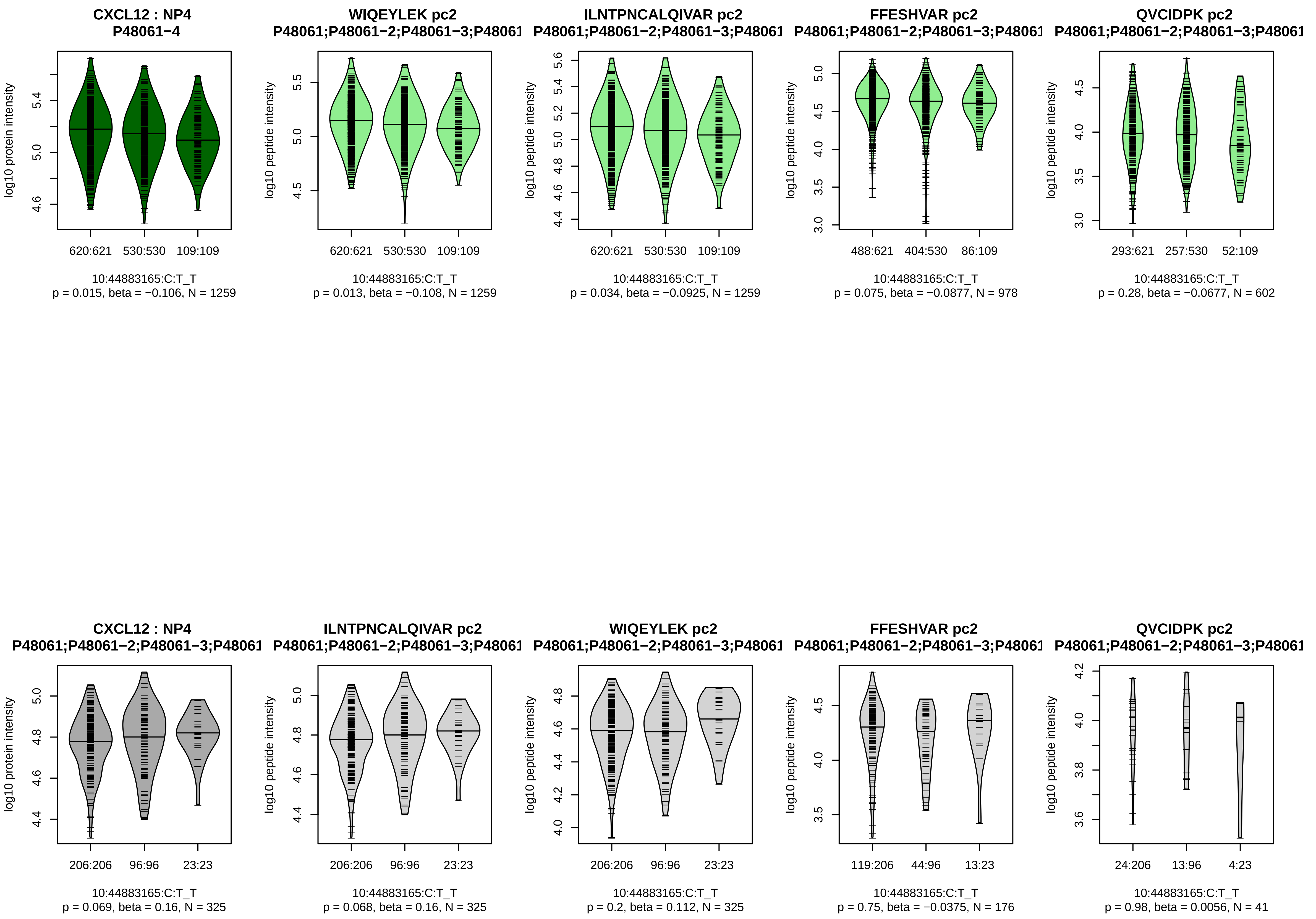

Assay Target: CXCL12
UniProt: P48061
rsID: rs11239028
Target SNP: 10:44883165:C:T
Proxy SNP: 10:44883165:C:T
UKBPPP log10(p): 184.6
UKBPPP BETA: 0.191
UKBPPP MSPA: 0
-:-:-:-
1259:1259:978:602

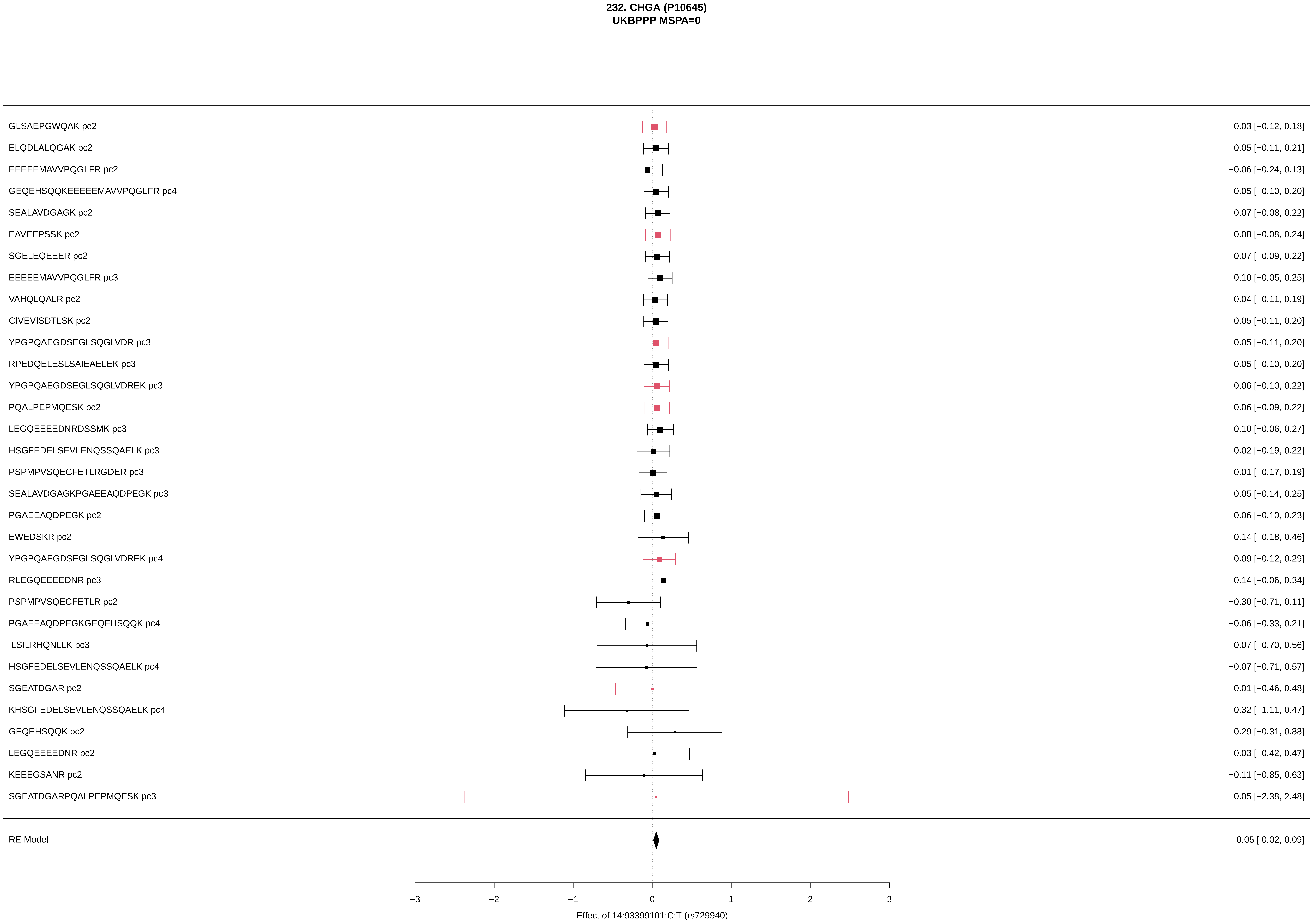

232. CHGA (P10645)
UKBPPP MSPA=0
GLSAEPGWQAK pc2
0.03 [−0.12, 0.18]
ELQDLALQGAK pc2
0.05 [−0.11, 0.21]
EEEEEMAVVPQGLFR pc2
−0.06 [−0.24, 0.13]
GEQEHSQQKEEEEEMAVVPQGLFR pc4
0.05 [−0.10, 0.20]
SEALAVDGAGK pc2
0.07 [−0.08, 0.22]
EAVEEPSSK pc2
0.08 [−0.08, 0.24]
SGELEQEEER pc2
0.07 [−0.09, 0.22]
EEEEEMAVVPQGLFR pc3
0.10 [−0.05, 0.25]
VAHQLQALR pc2
0.04 [−0.11, 0.19]
CIVEVISDTLSK pc2
0.05 [−0.11, 0.20]
YPGPQAEGDSEGLSQGLVDR pc3
0.05 [−0.11, 0.20]
RPEDQELESLSAIEAELEK pc3
0.05 [−0.10, 0.20]
YPGPQAEGDSEGLSQGLVDREK pc3
0.06 [−0.10, 0.22]
PQALPEPMQESK pc2
0.06 [−0.09, 0.22]
LEGQEEEEDNRDSSMK pc3
0.10 [−0.06, 0.27]
HSGFEDELSEVLENQSSQAELK pc3
0.02 [−0.19, 0.22]
PSPMPVSQECFETLRGDER pc3
0.01 [−0.17, 0.19]
SEALAVDGAGKPGAEEAQDPEGK pc3
0.05 [−0.14, 0.25]
PGAEEAQDPEGK pc2
0.06 [−0.10, 0.23]
EWEDSKR pc2
0.14 [−0.18, 0.46]
YPGPQAEGDSEGLSQGLVDREK pc4
0.09 [−0.12, 0.29]
RLEGQEEEEDNR pc3
0.14 [−0.06, 0.34]
PSPMPVSQECFETLR pc2
−0.30 [−0.71, 0.11]
PGAEEAQDPEGKGEQEHSQQK pc4
−0.06 [−0.33, 0.21]
ILSILRHQNLLK pc3
−0.07 [−0.70, 0.56]
HSGFEDELSEVLENQSSQAELK pc4
−0.07 [−0.71, 0.57]
SGEATDGAR pc2
0.01 [−0.46, 0.48]
KHSGFEDELSEVLENQSSQAELK pc4
−0.32 [−1.11, 0.47]
GEQEHSQQK pc2
0.29 [−0.31, 0.88]
LEGQEEEEDNR pc2
0.03 [−0.42, 0.47]
KEEEGSANR pc2
−0.11 [−0.85, 0.63]
SGEATDGARPQALPEPMQESK pc3
0.05 [−2.38, 2.48]
RE Model
0.05 [0.02, 0.09]
−3
−2
−1
0
1
2
3
Effect of 14:93399101:C:T (rs729940)

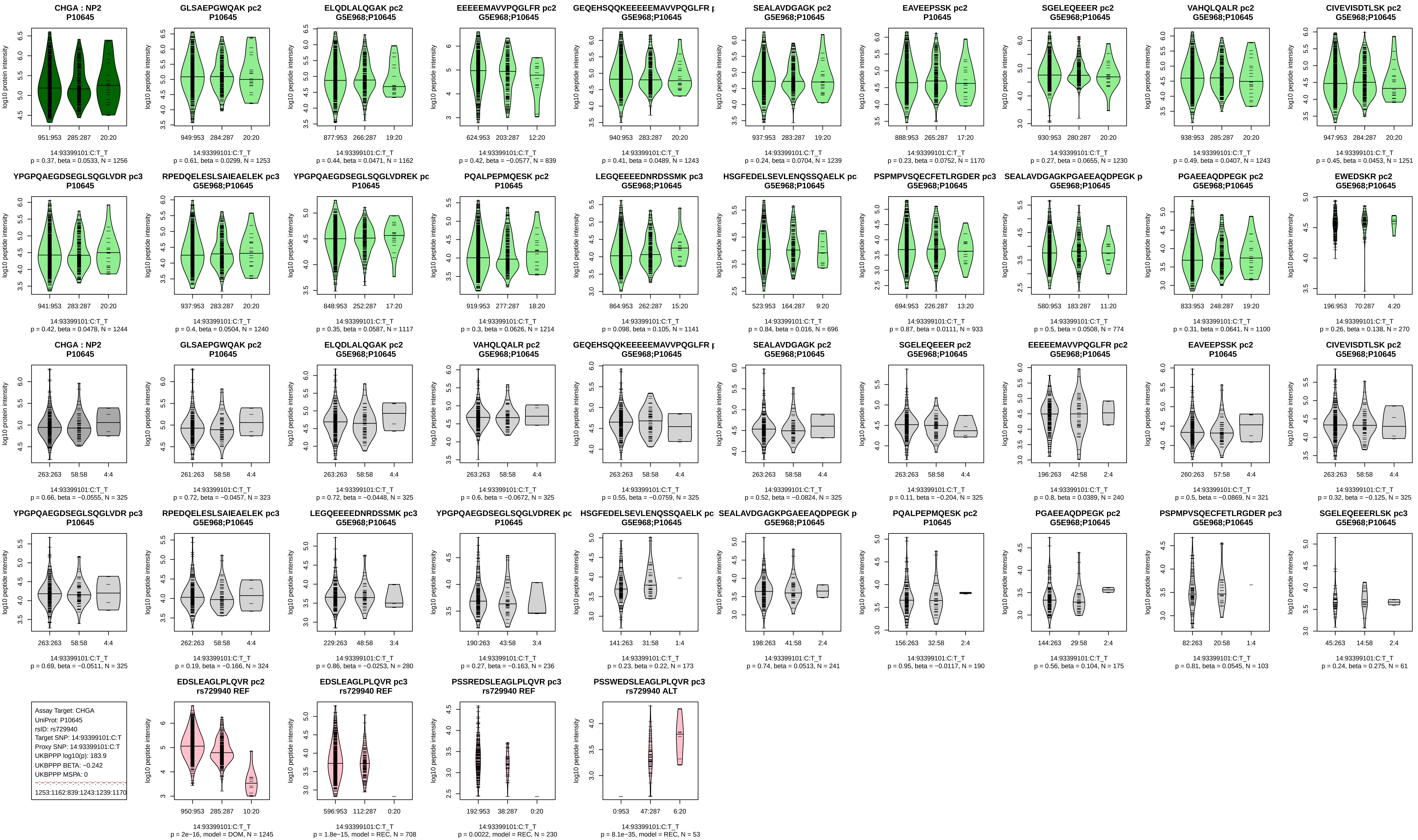

Assay Target: CHGA
UniProt: P10645
rsID: rs729940
Target SNP: 14:93399101:C:T
Proxy SNP: 14:93399101:C:T
UKBPPP log10(p): 183.9
UKBPPP BETA: −0.242
UKBPPP MSPA: 0
1253:1162:839:1243:1239:1170
CHGA : NP2 P10645
p = 0.37, beta = 0.0533, N = 1256
GLSAEPGWQAK pc2 P10645
p = 0.61, beta = 0.0299, N = 1253
ELQDLALQGAK pc2 G5E968;P10645
p = 0.44, beta = 0.0471, N = 1162
EEEEEMAVVPQGLFR pc2 G5E968;P10645
p = 0.42, beta = −0.0577, N = 839
SEALAVDGAGK pc2 G5E968;P10645
p = 0.24, beta = 0.0704, N = 1239
EAVEEPSSK pc2 P10645
p = 0.23, beta = 0.0752, N = 1170
SGELEQEEER pc2 G5E968;P10645
p = 0.27, beta = 0.0655, N = 1230
VAHQLQALR pc2 G5E968;P10645
p = 0.49, beta = 0.0407, N = 1243
CIVEVISDTLSK pc2 G5E968;P10645
p = 0.45, beta = 0.0453, N = 1251
EDSLEAGLPLQVR pc2 rs729940 REF
p = 2e−16, model = DOM, N = 1245
EDSLEAGLPLQVR pc3 rs729940 REF
p = 1.8e−15, model = REC, N = 708
PSSREDSLEAGLPLQVR pc3 rs729940 REF
p = 0.0022, model = REC, N = 230
PSSWEDSLEAGLPLQVR pc3 rs729940 ALT
p = 8.1e−35, model = REC, N = 53

233. COL15A1 (P39059)
UKBPPP MSPA=0.482

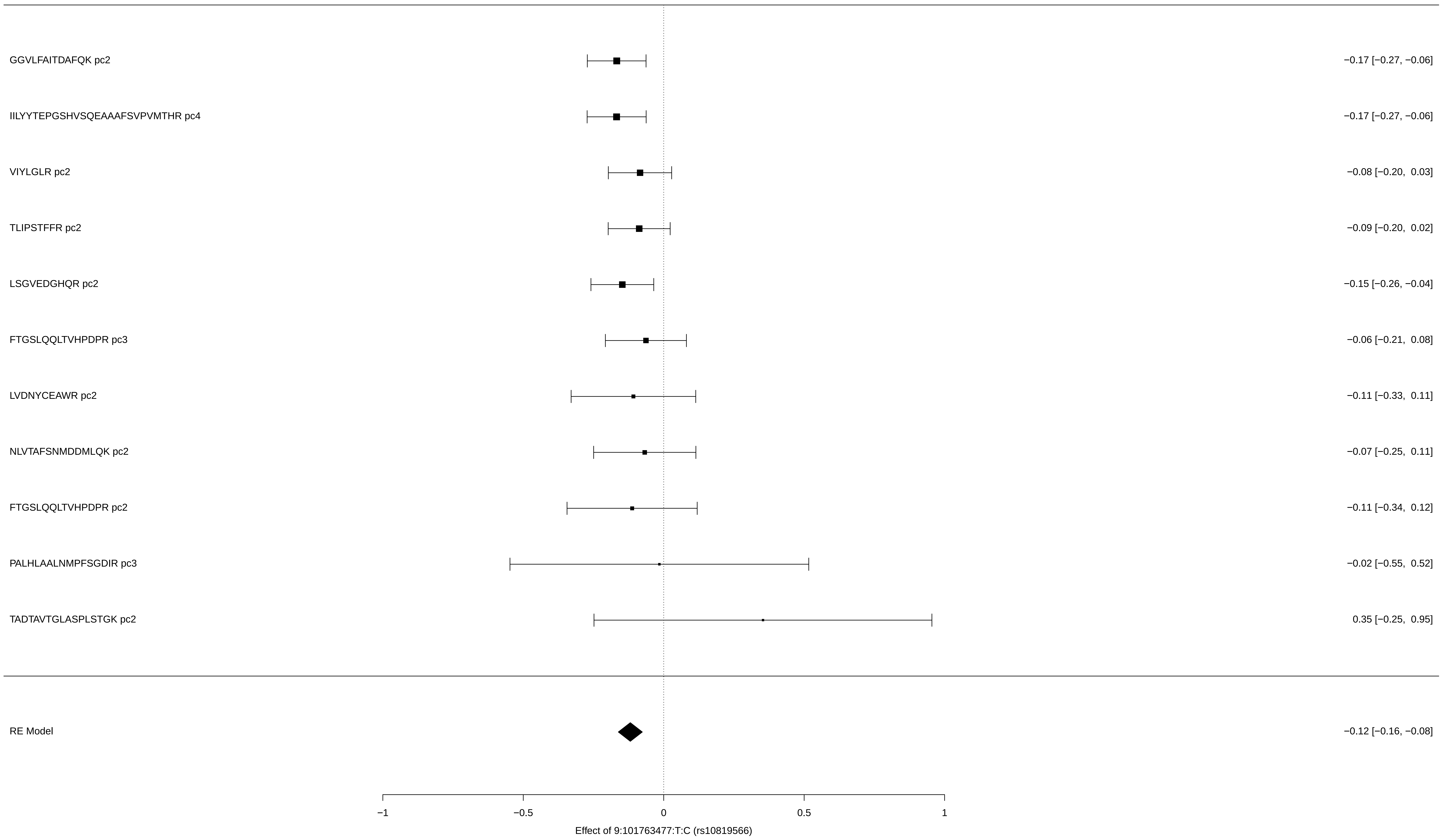

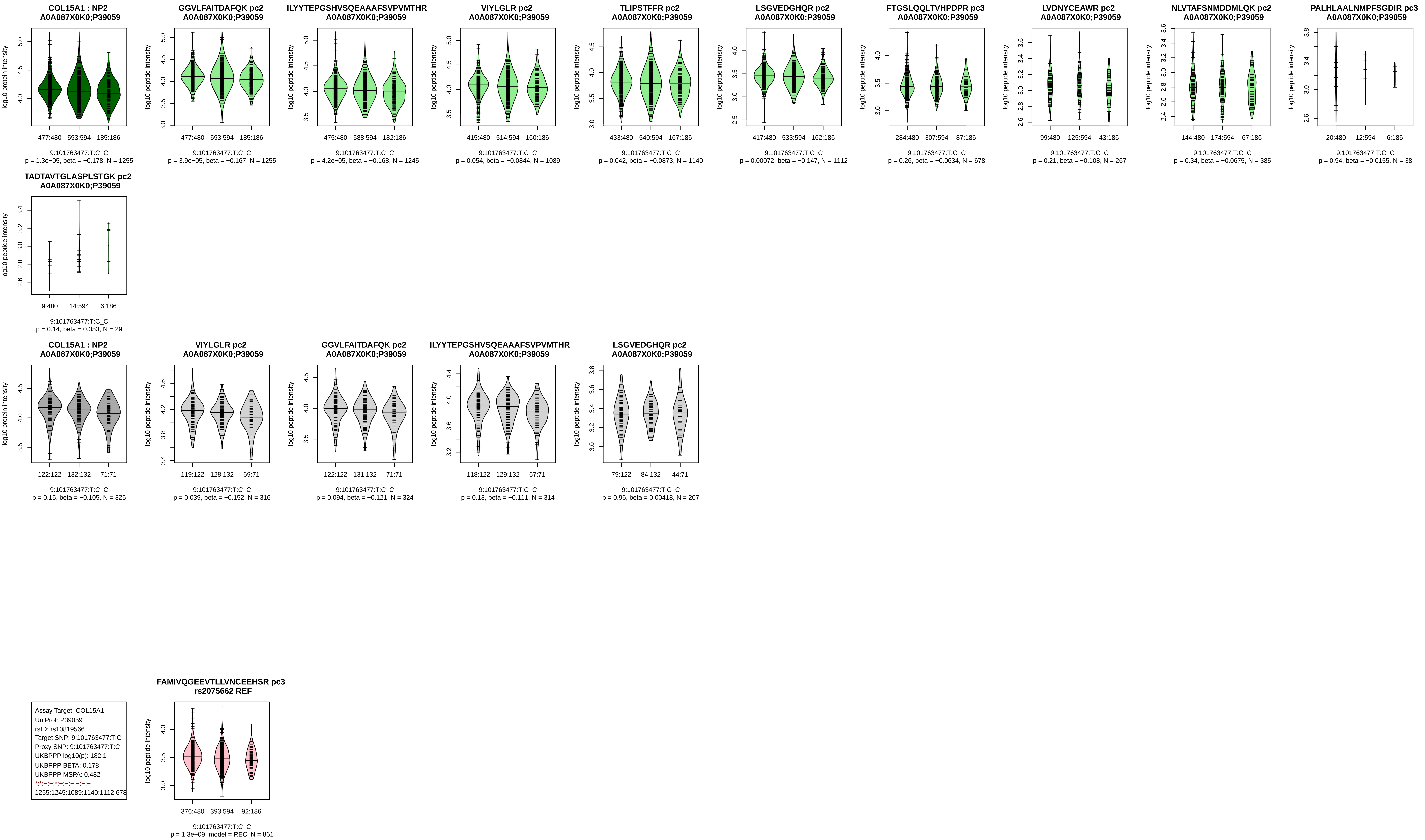
COL15A1 : NP2
A0A087X0K0;P39059
log10 protein intensity
477:480 593:594 185:186
9:101763477:T:C_C
p = 1.3e−05, beta = −0.178, N = 1255
GGVLFAITDAFQK pc2
A0A087X0K0;P39059
log10 peptide intensity
477:480 593:594 185:186
9:101763477:T:C_C
p = 3.9e−05, beta = −0.167, N = 1255
ILYYTEPGSHVSQEAAAFSVPVMTHR
A0A087X0K0;P39059
475:480 588:594 182:186
9:101763477:T:C_C
p = 4.2e−05, beta = −0.168, N = 1245
VIYLGLR pc2
A0A087X0K0;P39059
415:480 514:594 160:186
9:101763477:T:C_C
p = 0.054, beta = −0.0844, N = 1089
TLIPSTFFR pc2
A0A087X0K0;P39059
433:480 540:594 167:186
9:101763477:T:C_C
p = 0.042, beta = −0.0873, N = 1140
LSGVEDGHQR pc2
A0A087X0K0;P39059
417:480 533:594 162:186
9:101763477:T:C_C
p = 0.00072, beta = −0.147, N = 1112
FTGSLQQLTVHPDPR pc3
A0A087X0K0;P39059
284:480 307:594 87:186
9:101763477:T:C_C
p = 0.26, beta = −0.0634, N = 678
LVDNYCEAWR pc2
A0A087X0K0;P39059
99:480 125:594 43:186
9:101763477:T:C_C
p = 0.21, beta = −0.108, N = 267
NLVTAFSNMDDMLQK pc2
A0A087X0K0;P39059
144:480 174:594 67:186
9:101763477:T:C_C
p = 0.34, beta = −0.0675, N = 385
PALHLAALNMPFSGDIR pc3
A0A087X0K0;P39059
20:480 12:594 6:186
9:101763477:T:C_C
p = 0.94, beta = −0.0155, N = 38
TADTAVTGLASPLSTGK pc2
A0A087X0K0;P39059
9:480 14:594 6:186
9:101763477:T:C_C
p = 0.14, beta = 0.353, N = 29
COL15A1 : NP2
A0A087X0K0;P39059
122:122 132:132 71:71
9:101763477:T:C_C
p = 0.15, beta = −0.105, N = 325
VIYLGLR pc2
A0A087X0K0;P39059
119:122 128:132 69:71
9:101763477:T:C_C
p = 0.039, beta = −0.152, N = 316
GGVLFAITDAFQK pc2
A0A087X0K0;P39059
122:122 131:132 71:71
9:101763477:T:C_C
p = 0.094, beta = −0.121, N = 324
ILYYTEPGSHVSQEAAAFSVPVMTHR
A0A087X0K0;P39059
118:122 129:132 67:71
9:101763477:T:C_C
p = 0.13, beta = −0.111, N = 314
LSGVEDGHQR pc2
A0A087X0K0;P39059
79:122 84:132 44:71
9:101763477:T:C_C
p = 0.96, beta = 0.00418, N = 207
Assay Target: COL15A1
UniProt: P39059
rsID: rs10819566
Target SNP: 9:101763477:T:C
Proxy SNP: 9:101763477:T:C
UKBPPP log10(p): 182.1
UKBPPP BETA: 0.178
UKBPPP MSPA: 0.482
1255:1245:1089:1140:1112:678
FAMIVQGEEVTLLVNCEEHSR pc3
rs2075662 REF
376:480 393:594 92:186
9:101763477:T:C_C
p = 1.3e−09, model = REC, N = 861

234. S100A4 (P26447)
UKBPPP MSPA=0.745

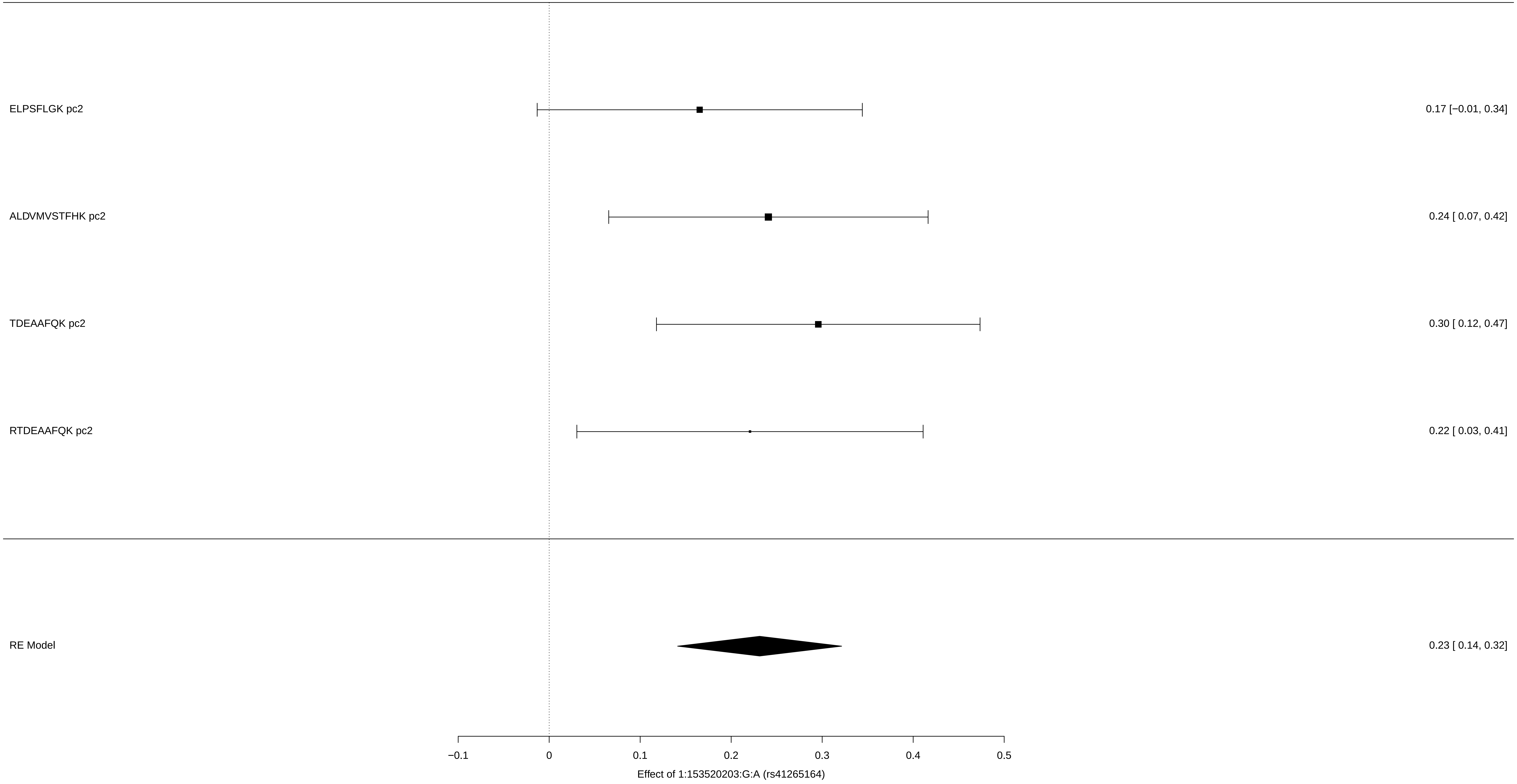

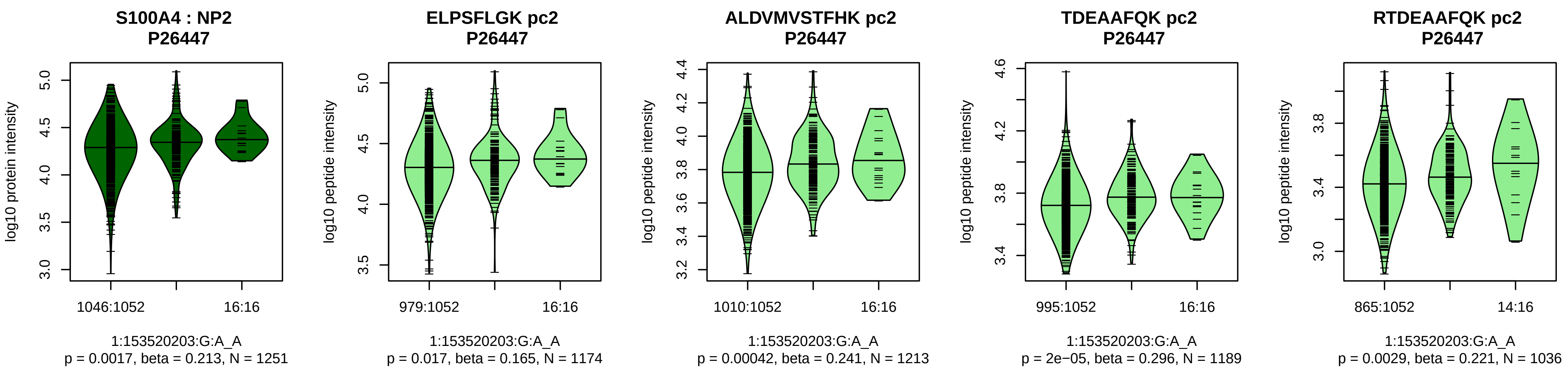

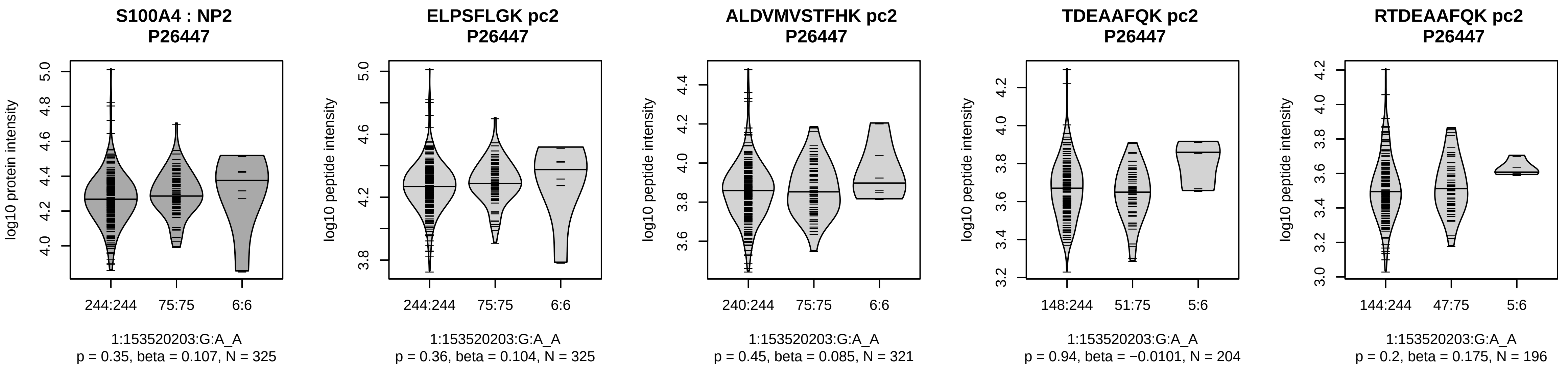

Assay Target: S100A4
UniProt: P26447
rsID: rs41265164
Target SNP: 1:153520203:G:A
Proxy SNP: 1:153520203:G:A
UKBPPP log10(p): 178.9
UKBPPP BETA: 0.373
UKBPPP MSPA: 0.745
−:*:*:*:NA
1174:1213:1189:1036:17

235. CTHRC1 (Q96CG8)
UKBPPP MSPA=0

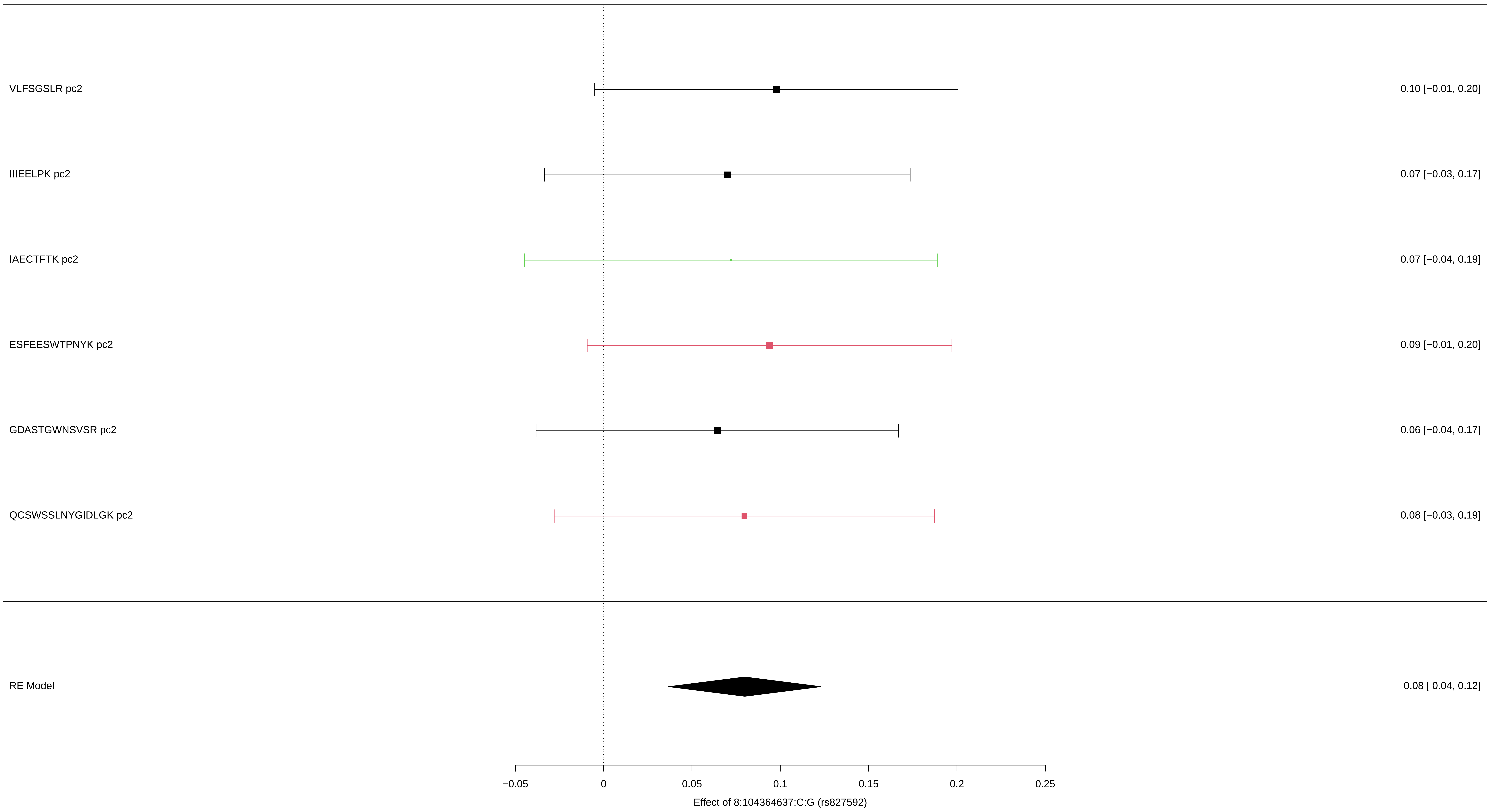

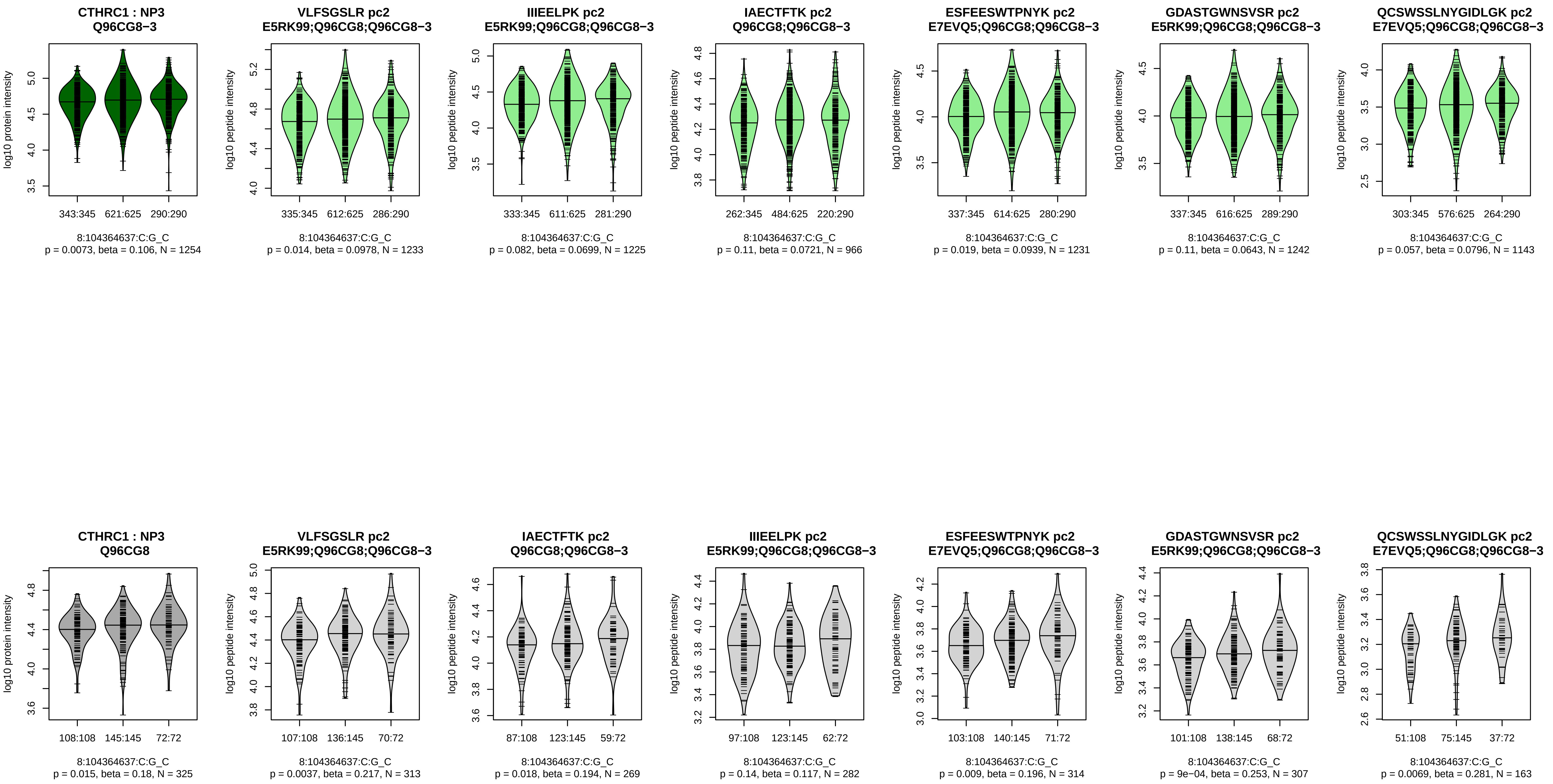

Assay Target: CTHRC1
UniProt: Q96CG8
rsID: rs827592
Target SNP: 8:104364637:C:G
Proxy SNP: 8:104364637:C:G
UKBPPP log10(p): 177.8
UKBPPP BETA: −0.157
UKBPPP MSPA: 0
–:–:–:–:–:–
1233:1225:966:1231:1242:1143

236. HYOU1 (Q9Y4L1)
UKBPPP MSPA=0.136

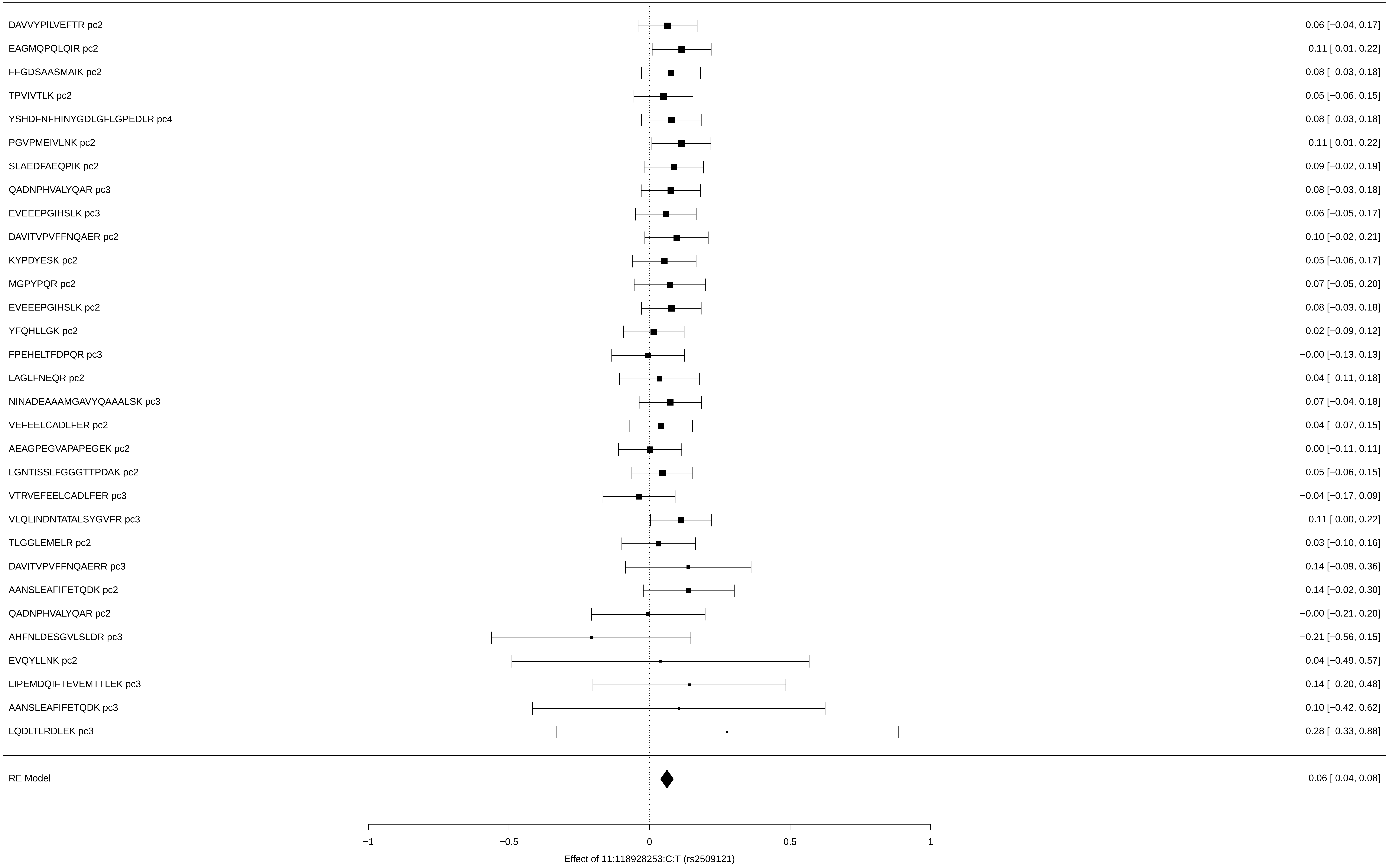

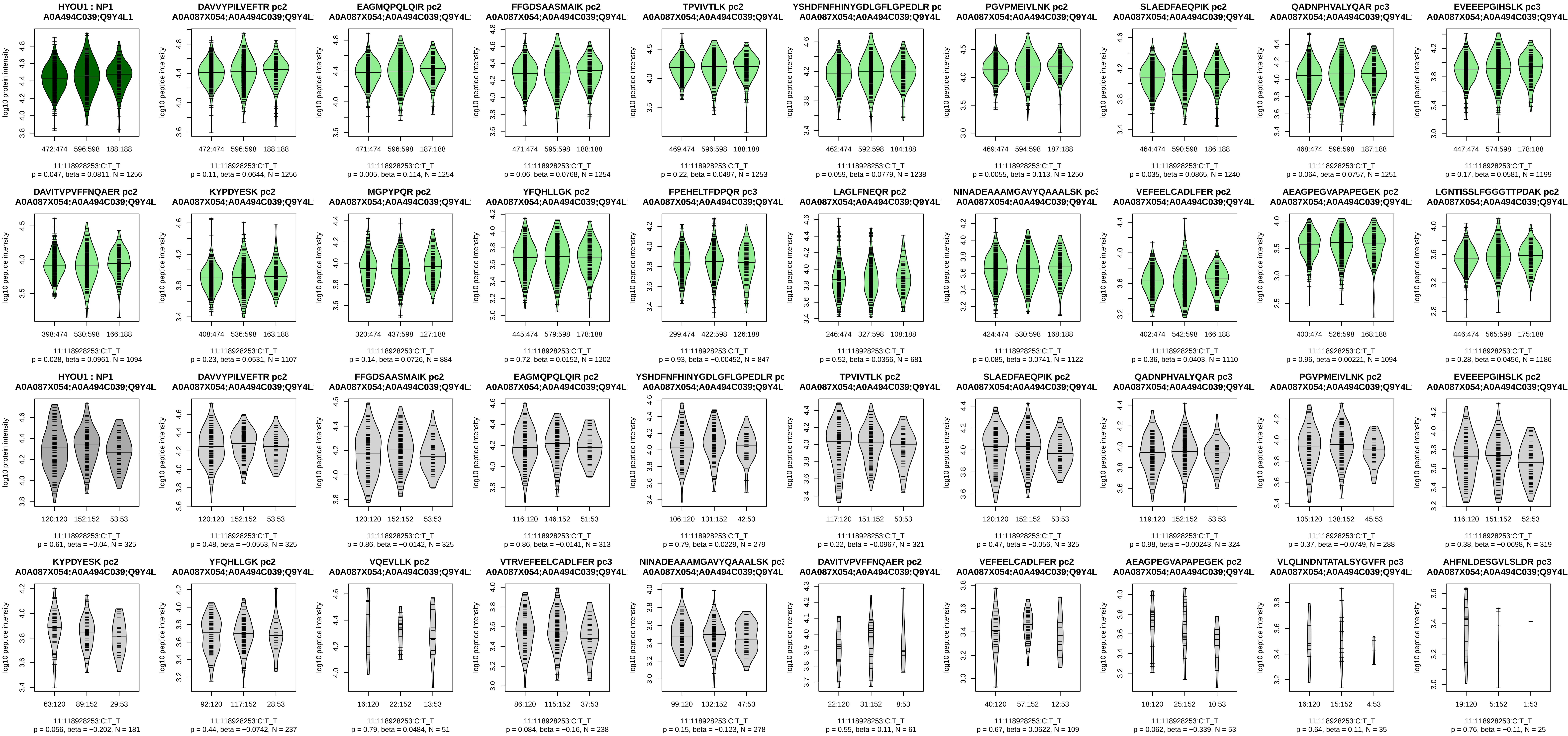

Assay Target: HYOU1
UniProt: Q9Y4L1
rsID: rs2509121
Target SNP: 11:118928253:C:T
Proxy SNP: 11:118928253:C:T
UKBPPP log10(p): 175.7
UKBPPP BETA: 0.173
UKBPPP MSPA: 0.136
1256:1254:1254:1253:1238:125

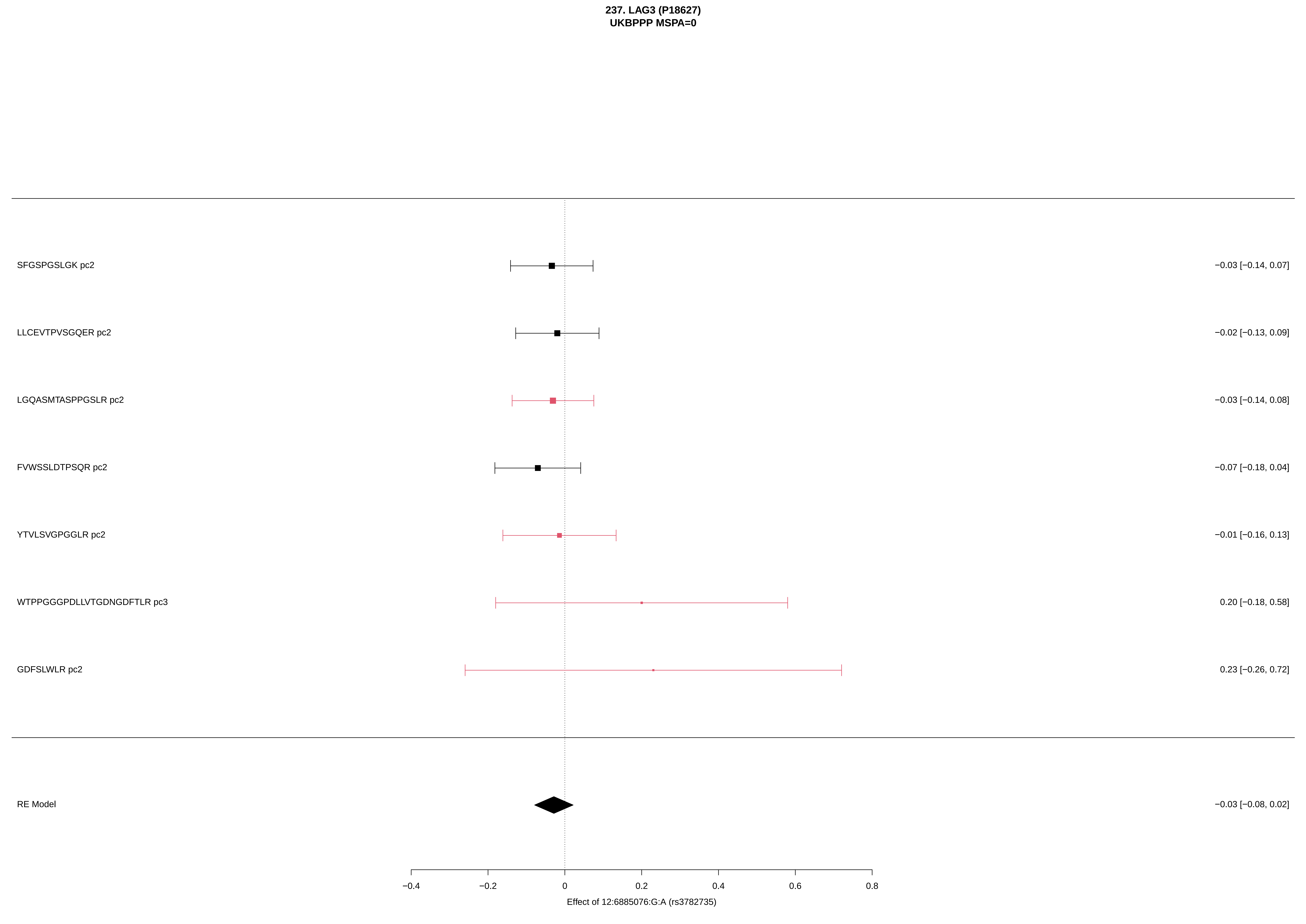
237. LAG3 (P18627)
UKBPPP MSPA=0
SFGSPGSLGK pc2
−0.03 [−0.14, 0.07]
LLCEVTPVSGQER pc2
−0.02 [−0.13, 0.09]
LGQASMTASPPGSLR pc2
−0.03 [−0.14, 0.08]
FVWSSLDTPSQR pc2
−0.07 [−0.18, 0.04]
YTVLSVGPGGLR pc2
−0.01 [−0.16, 0.13]
WTPPGGGPDLLVTGDNGDFTLR pc3
0.20 [−0.18, 0.58]
GDFSLWLR pc2
0.23 [−0.26, 0.72]
RE Model
−0.03 [−0.08, 0.02]
−0.4
−0.2
0
0.2
0.4
0.6
0.8
Effect of 12:6885076:G:A (rs3782735)

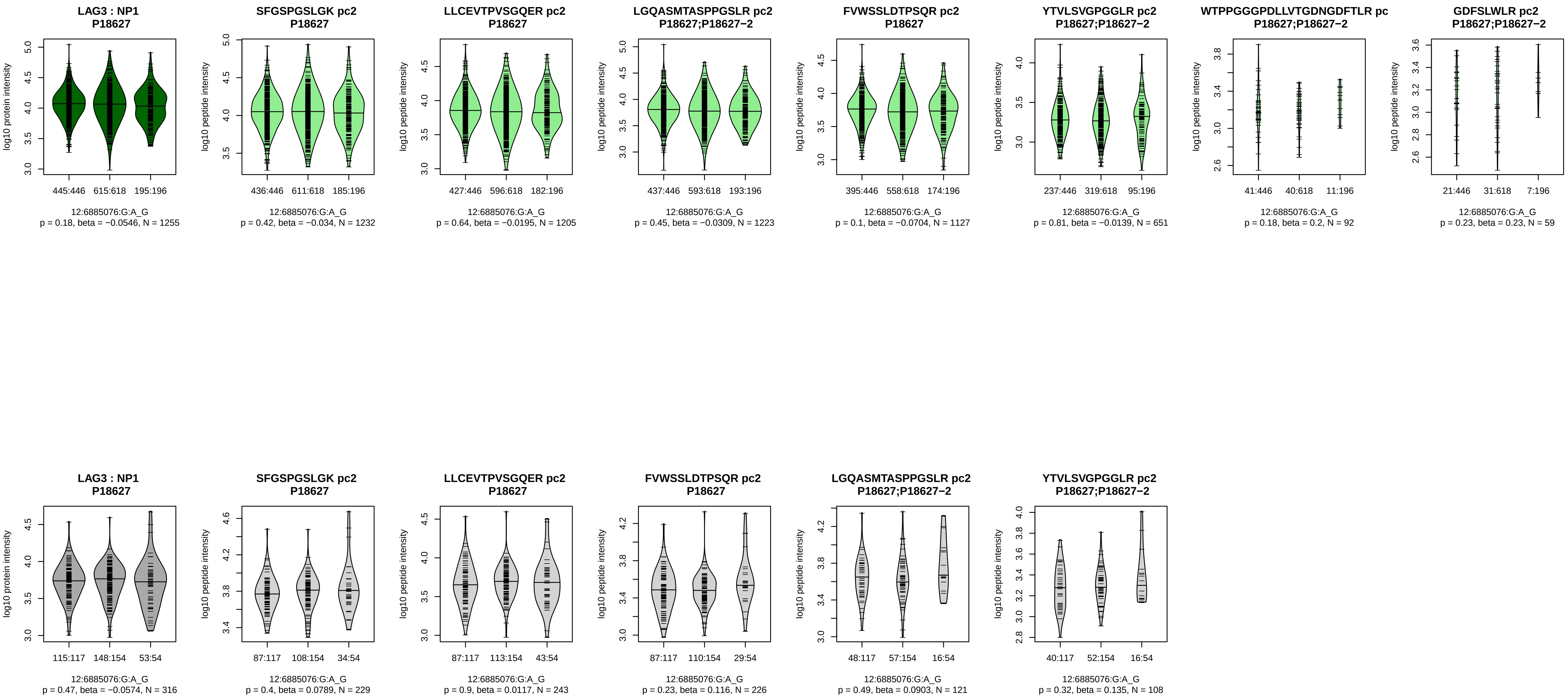

Assay Target: LAG3
UniProt: P18627
rsID: rs3782735
Target SNP: 12:6885076:G:A
Proxy SNP: 12:6885076:G:A
UKBPPP log10(p): 173.6
UKBPPP BETA: 0.174
UKBPPP MSPA: 0
–:–:–:–:–:–:–
1232:1205:1223:1127:651:92:5[illegible]

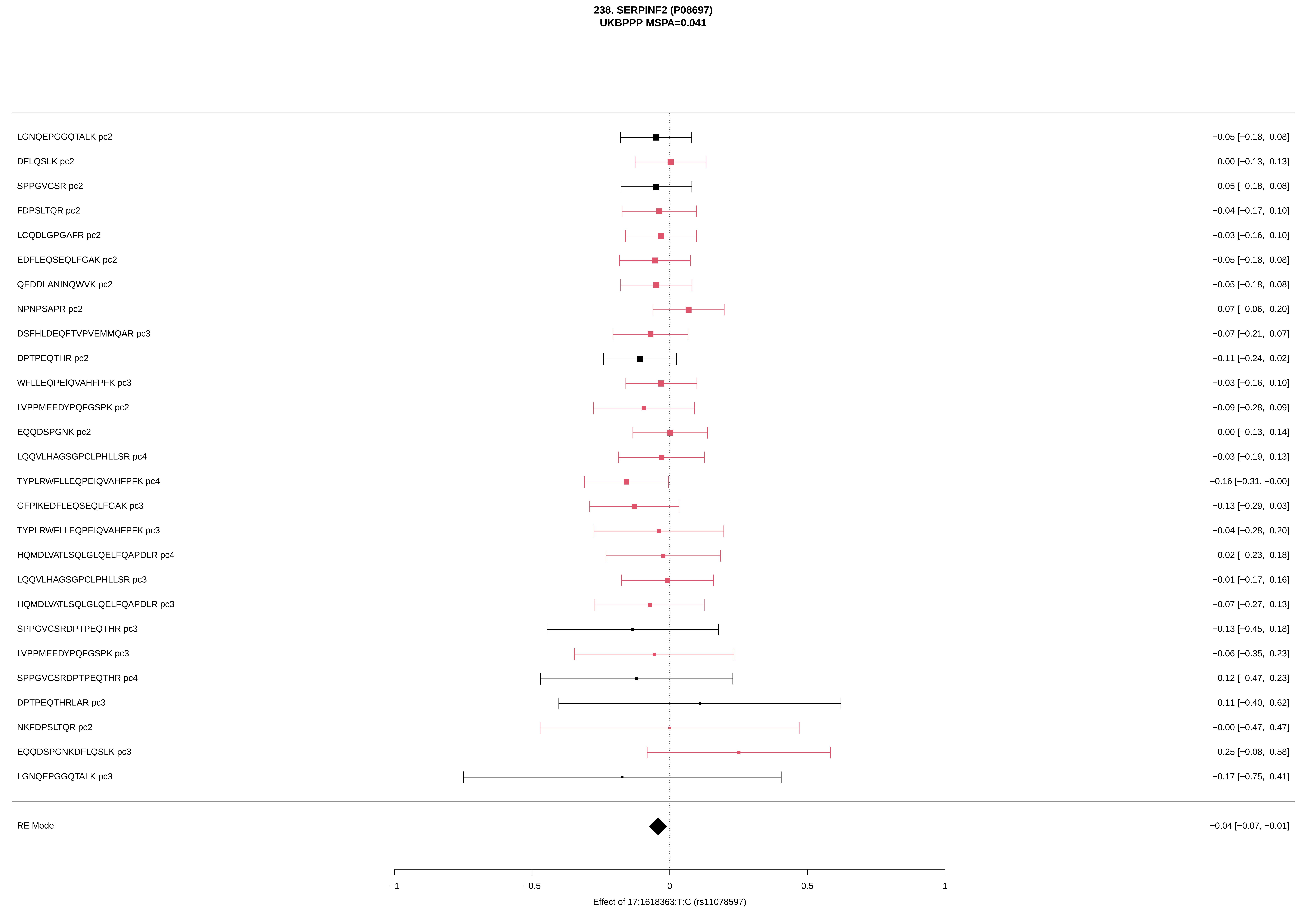

238. SERPINF2 (P08697)
UKBPPP MSPA=0.041
LGNQEPGGQTALK pc2 −0.05 [−0.18, 0.08]
DFLQSLK pc2 0.00 [−0.13, 0.13]
SPPGVCSR pc2 −0.05 [−0.18, 0.08]
FDPSLTQR pc2 −0.04 [−0.17, 0.10]
LCQDLGPGAFR pc2 −0.03 [−0.16, 0.10]
EDFLEQSEQLFGAK pc2 −0.05 [−0.18, 0.08]
QEDDLANINQWVK pc2 −0.05 [−0.18, 0.08]
NPNPSAPR pc2 0.07 [−0.06, 0.20]
DSFHLDEQFTVPVEMMQAR pc3 −0.07 [−0.21, 0.07]
DPTPEQTHR pc2 −0.11 [−0.24, 0.02]
WFLLEQPEIQVAHFPFK pc3 −0.03 [−0.16, 0.10]
LVPPMEEDYPQFGSPK pc2 −0.09 [−0.28, 0.09]
EQQDSPGNK pc2 0.00 [−0.13, 0.14]
LQQVLHAGSGPCLPHLLSR pc4 −0.03 [−0.19, 0.13]
TYPLRWFLLEQPEIQVAHFPFK pc4 −0.16 [−0.31, −0.00]
GFPIKEDFLEQSEQLFGAK pc3 −0.13 [−0.29, 0.03]
TYPLRWFLLEQPEIQVAHFPFK pc3 −0.04 [−0.28, 0.20]
HQMDLVATLSQLGLQELFQAPDLR pc4 −0.02 [−0.23, 0.18]
LQQVLHAGSGPCLPHLLSR pc3 −0.01 [−0.17, 0.16]
HQMDLVATLSQLGLQELFQAPDLR pc3 −0.07 [−0.27, 0.13]
SPPGVCSRDPTPEQTHR pc3 −0.13 [−0.45, 0.18]
LVPPMEEDYPQFGSPK pc3 −0.06 [−0.35, 0.23]
SPPGVCSRDPTPEQTHR pc4 −0.12 [−0.47, 0.23]
DPTPEQTHRLAR pc3 0.11 [−0.40, 0.62]
NKFDPSLTQR pc2 −0.00 [−0.47, 0.47]
EQQDSPGNKDFLQSLK pc3 0.25 [−0.08, 0.58]
LGNQEPGGQTALK pc3 −0.17 [−0.75, 0.41]
RE Model −0.04 [−0.07, −0.01]
−1
−0.5
0
0.5
1
Effect of 17:1618363:T:C (rs11078597)

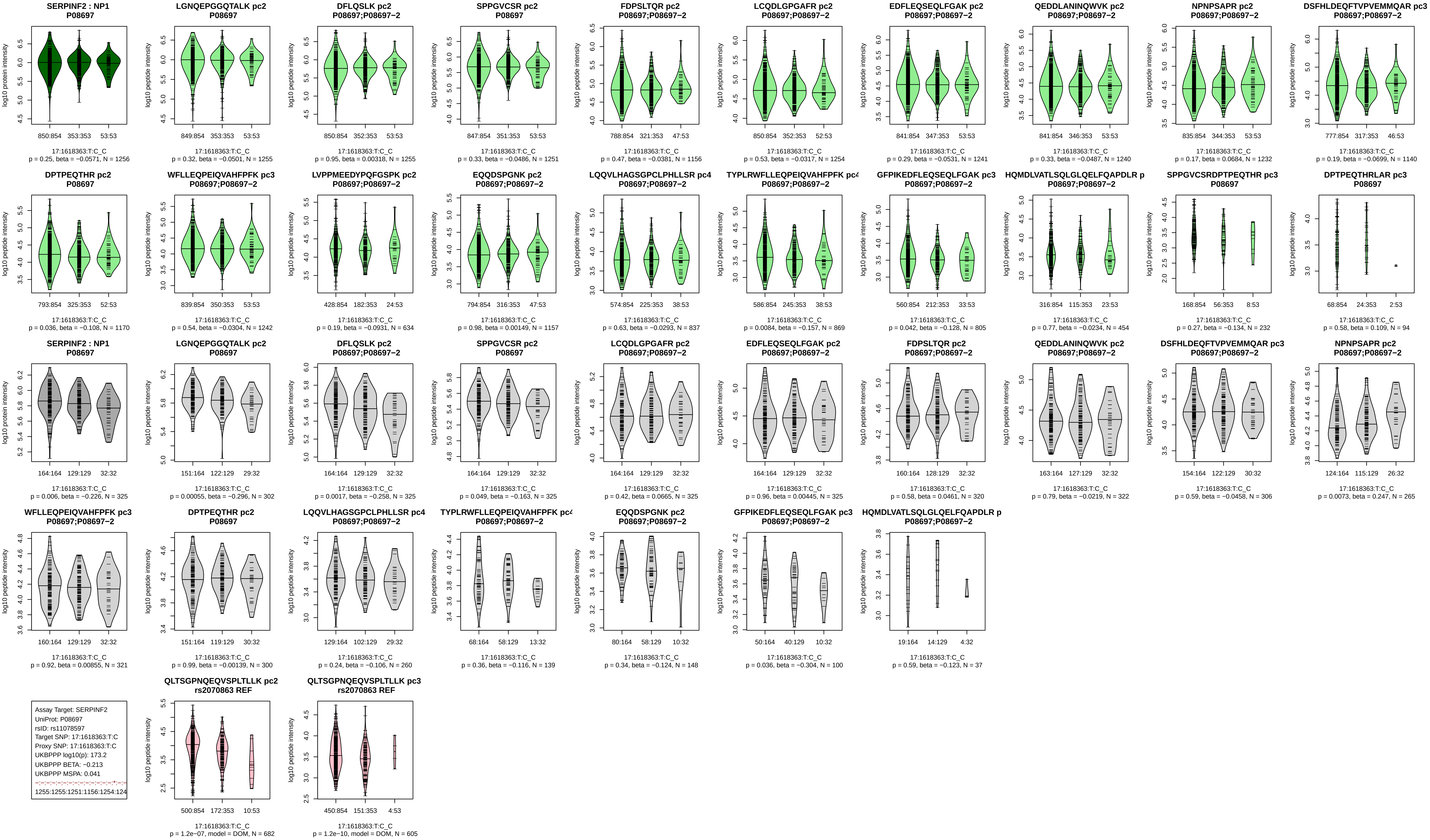
SERPINF2 : NP1
P08697
850:854 353:353 53:53
17:1618363:T:C_C
p = 0.25, beta = −0.0571, N = 1256
LGNQEPGGQTALK pc2
P08697
849:854 353:353 53:53
17:1618363:T:C_C
p = 0.32, beta = −0.0501, N = 1255
DFLQSLK pc2
P08697;P08697−2
850:854 352:353 53:53
17:1618363:T:C_C
p = 0.95, beta = 0.00318, N = 1255
SPPGVCSR pc2
P08697
847:854 351:353 53:53
17:1618363:T:C_C
p = 0.33, beta = −0.0486, N = 1251
FDPSLTQR pc2
P08697;P08697−2
788:854 321:353 47:53
17:1618363:T:C_C
p = 0.47, beta = −0.0381, N = 1156
LCQDLGPGAFR pc2
P08697;P08697−2
850:854 352:353 52:53
17:1618363:T:C_C
p = 0.53, beta = −0.0317, N = 1254
EDFLEQSEQLFGAK pc2
P08697;P08697−2
841:854 347:353 53:53
17:1618363:T:C_C
p = 0.29, beta = −0.0531, N = 1241
QEDDLANINQWVK pc2
P08697;P08697−2
841:854 346:353 53:53
17:1618363:T:C_C
p = 0.33, beta = −0.0487, N = 1240
NPNPSAPR pc2
P08697;P08697−2
835:854 344:353 53:53
17:1618363:T:C_C
p = 0.17, beta = 0.0684, N = 1232
DSFHLDEQFTVPVEMMQAR pc3
P08697;P08697−2
777:854 317:353 46:53
17:1618363:T:C_C
p = 0.19, beta = −0.0699, N = 1140
DPTPEQTHR pc2
P08697
793:854 325:353 52:53
17:1618363:T:C_C
p = 0.036, beta = −0.108, N = 1170
WFLLEQPEIQVAHFPFK pc3
P08697;P08697−2
839:854 350:353 53:53
17:1618363:T:C_C
p = 0.54, beta = −0.0304, N = 1242
LVPPMEEDYPQFGSPK pc2
P08697;P08697−2
428:854 182:353 24:53
17:1618363:T:C_C
p = 0.19, beta = −0.0931, N = 634
EQQDSPGNK pc2
P08697;P08697−2
794:854 316:353 47:53
17:1618363:T:C_C
p = 0.98, beta = 0.00149, N = 1157
LQQVLHAGSGPCLPHLLSR pc4
P08697;P08697−2
574:854 225:353 38:53
17:1618363:T:C_C
p = 0.63, beta = −0.0293, N = 837
TYPLRWFLLEQPEIQVAHFPFK pc4
P08697;P08697−2
586:854 245:353 38:53
17:1618363:T:C_C
p = 0.0084, beta = −0.157, N = 869
GFPIKEDFLEQSEQLFGAK pc3
P08697;P08697−2
560:854 212:353 33:53
17:1618363:T:C_C
p = 0.042, beta = −0.128, N = 805
HQMDLVATLSQLGLQELFQAPDLR p
P08697;P08697−2
316:854 115:353 23:53
17:1618363:T:C_C
p = 0.77, beta = −0.0234, N = 454
SPPGVCSRDPTPEQTHR pc3
P08697
168:854 56:353 8:53
17:1618363:T:C_C
p = 0.27, beta = −0.134, N = 232
DPTPEQTHRLAR pc3
P08697
68:854 24:353 2:53
17:1618363:T:C_C
p = 0.58, beta = 0.109, N = 94
SERPINF2 : NP1
P08697
164:164 129:129 32:32
17:1618363:T:C_C
p = 0.006, beta = −0.226, N = 325
LGNQEPGGQTALK pc2
P08697
151:164 122:129 29:32
17:1618363:T:C_C
p = 0.00055, beta = −0.296, N = 302
DFLQSLK pc2
P08697;P08697−2
164:164 129:129 32:32
17:1618363:T:C_C
p = 0.0017, beta = −0.258, N = 325
SPPGVCSR pc2
P08697
164:164 129:129 32:32
17:1618363:T:C_C
p = 0.049, beta = −0.163, N = 325
LCQDLGPGAFR pc2
P08697;P08697−2
164:164 129:129 32:32
17:1618363:T:C_C
p = 0.42, beta = 0.0665, N = 325
EDFLEQSEQLFGAK pc2
P08697;P08697−2
164:164 129:129 32:32
17:1618363:T:C_C
p = 0.96, beta = 0.00445, N = 325
FDPSLTQR pc2
P08697;P08697−2
160:164 128:129 32:32
17:1618363:T:C_C
p = 0.58, beta = 0.0461, N = 320
QEDDLANINQWVK pc2
P08697;P08697−2
163:164 127:129 32:32
17:1618363:T:C_C
p = 0.79, beta = −0.0219, N = 322
DSFHLDEQFTVPVEMMQAR pc3
P08697;P08697−2
154:164 122:129 30:32
17:1618363:T:C_C
p = 0.59, beta = −0.0458, N = 306
NPNPSAPR pc2
P08697;P08697−2
124:164 115:129 26:32
17:1618363:T:C_C
p = 0.0073, beta = 0.247, N = 265
WFLLEQPEIQVAHFPFK pc3
P08697;P08697−2
160:164 129:129 32:32
17:1618363:T:C_C
p = 0.92, beta = 0.00855, N = 321
DPTPEQTHR pc2
P08697
151:164 119:129 30:32
17:1618363:T:C_C
p = 0.99, beta = −0.00139, N = 300
LQQVLHAGSGPCLPHLLSR pc4
P08697;P08697−2
129:164 102:129 29:32
17:1618363:T:C_C
p = 0.24, beta = −0.106, N = 260
TYPLRWFLLEQPEIQVAHFPFK pc4
P08697;P08697−2
68:164 58:129 13:32
17:1618363:T:C_C
p = 0.36, beta = −0.116, N = 139
EQQDSPGNK pc2
P08697;P08697−2
80:164 58:129 10:32
17:1618363:T:C_C
p = 0.34, beta = −0.124, N = 148
GFPIKEDFLEQSEQLFGAK pc3
P08697;P08697−2
50:164 40:129 10:32
17:1618363:T:C_C
p = 0.036, beta = −0.304, N = 100
HQMDLVATLSQLGLQELFQAPDLR p
P08697;P08697−2
19:164 14:129 4:32
17:1618363:T:C_C
p = 0.59, beta = −0.123, N = 37
Assay Target: SERPINF2
UniProt: P08697
rsID: rs11078597
Target SNP: 17:1618363:T:C
Proxy SNP: 17:1618363:T:C
UKBPPP log10(p): 173.2
UKBPPP BETA: −0.213
UKBPPP MSPA: 0.041
1255:1255:1251:1156:1254:124
QLTSGPNQEQVSPLTLLK pc2
rs2070863 REF
500:854 172:353 10:53
17:1618363:T:C_C
p = 1.2e−07, model = DOM, N = 682
QLTSGPNQEQVSPLTLLK pc3
rs2070863 REF
450:854 151:353 4:53
17:1618363:T:C_C
p = 1.2e−10, model = DOM, N = 605
log10 protein intensity
log10 peptide intensity

239. ANGPT1 (Q15389)
UKBPPP MSPA=0

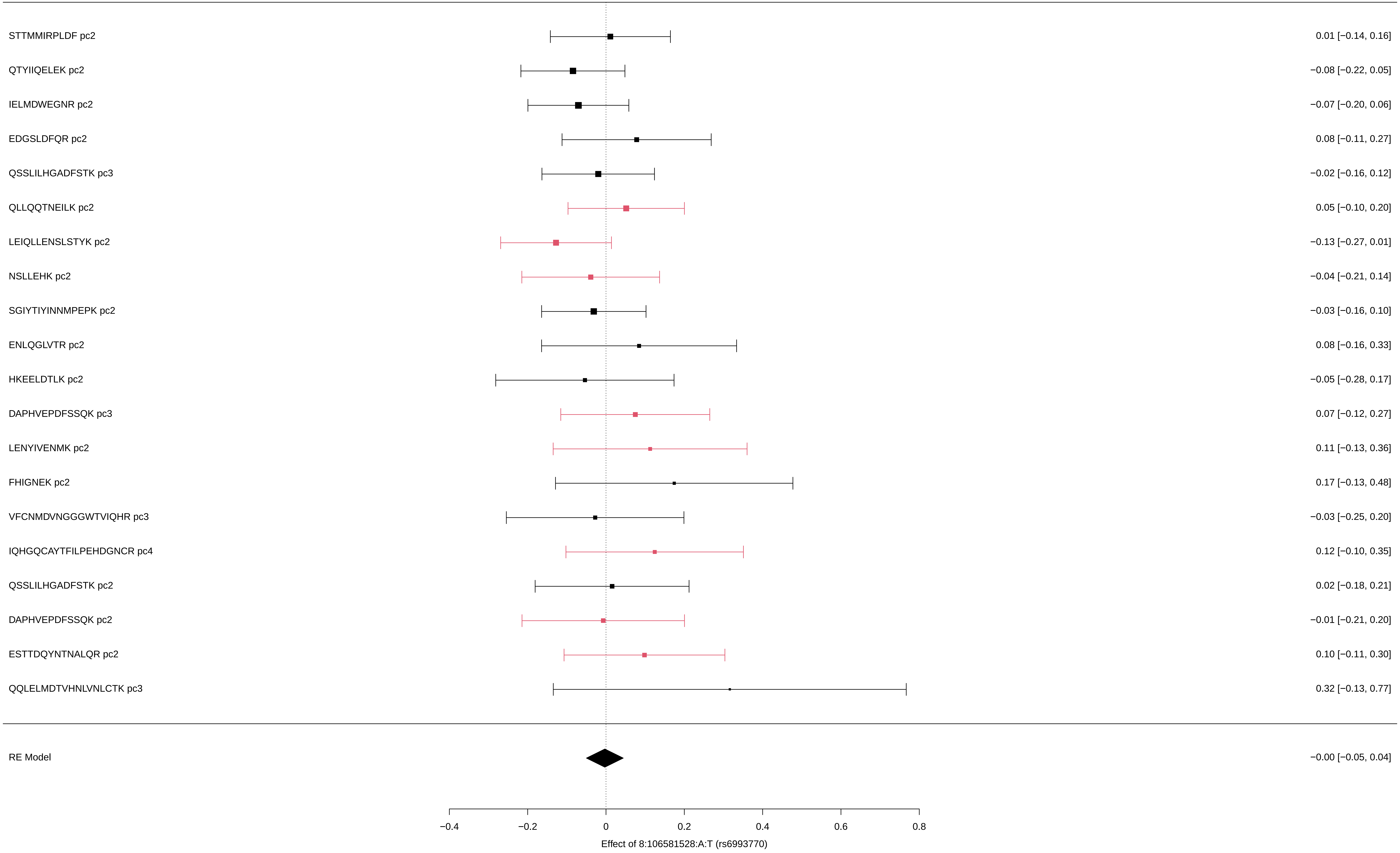

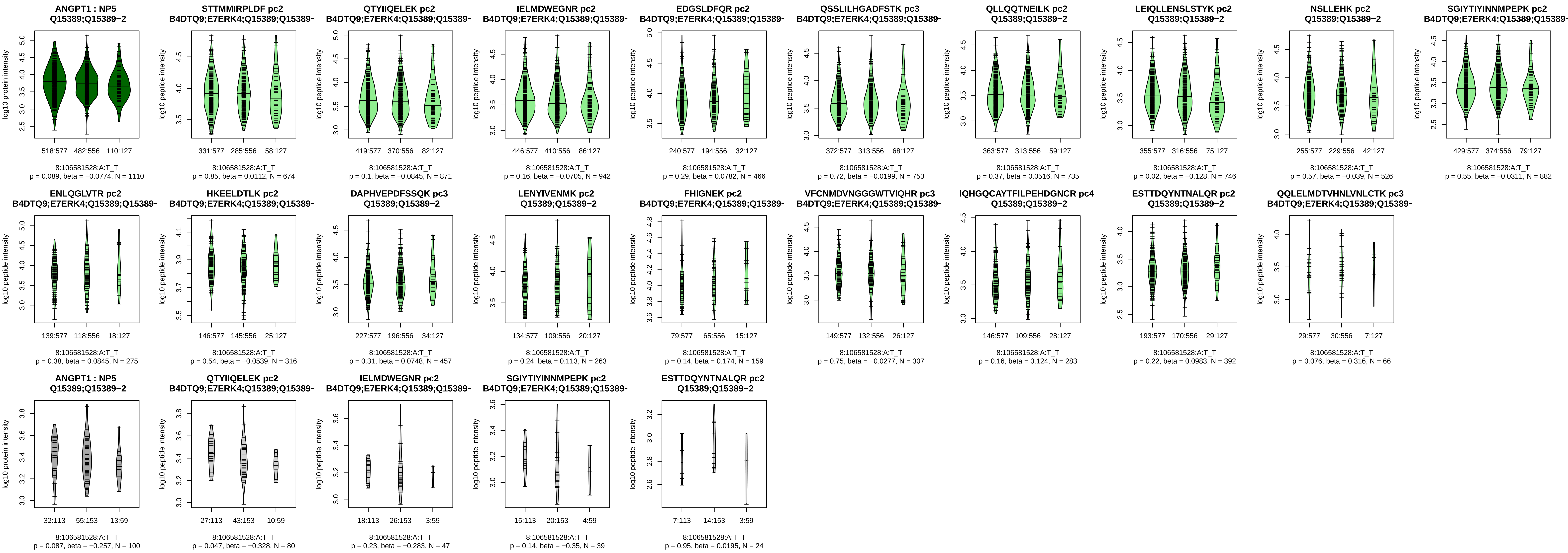

Assay Target: ANGPT1
UniProt: Q15389
rsID: rs6993770
Target SNP: 8:106581528:A:T
Proxy SNP: 8:106581528:A:T
UKBPPP log10(p): 172.5
UKBPPP BETA: −0.187
UKBPPP MSPA: 0

674:871:942:466:753:735:746:5

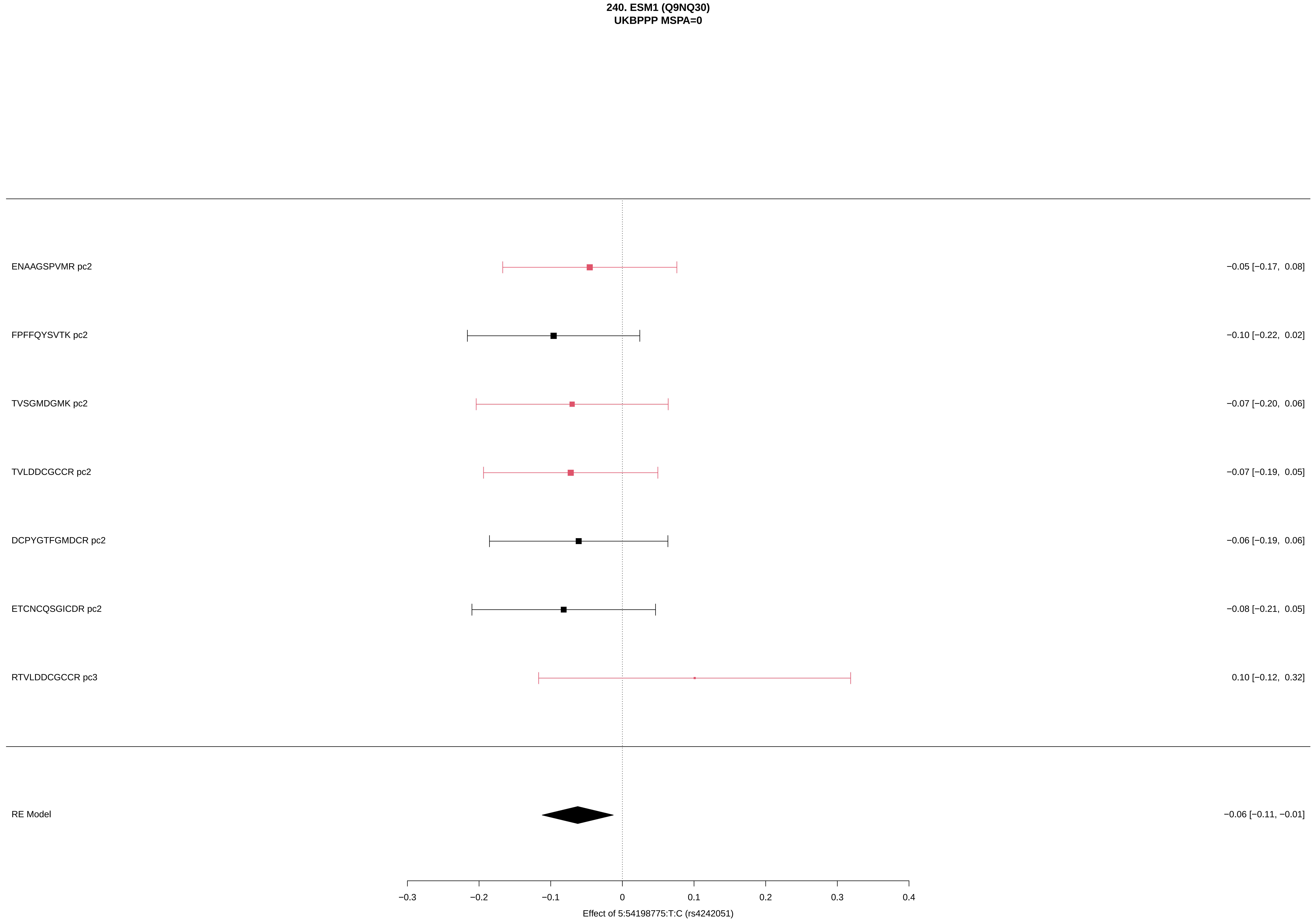
240. ESM1 (Q9NQ30)
UKBPPP MSPA=0
ENAAGSPVMR pc2
−0.05 [−0.17, 0.08]
FPFFQYSVTK pc2
−0.10 [−0.22, 0.02]
TVSGMDGMK pc2
−0.07 [−0.20, 0.06]
TVLDDCGCCR pc2
−0.07 [−0.19, 0.05]
DCPYGTFGMDCR pc2
−0.06 [−0.19, 0.06]
ETCNCQSGICDR pc2
−0.08 [−0.21, 0.05]
RTVLDDCGCCR pc3
0.10 [−0.12, 0.32]
RE Model
−0.06 [−0.11, −0.01]
−0.3
−0.2
−0.1
0
0.1
0.2
0.3
0.4
Effect of 5:54198775:T:C (rs4242051)

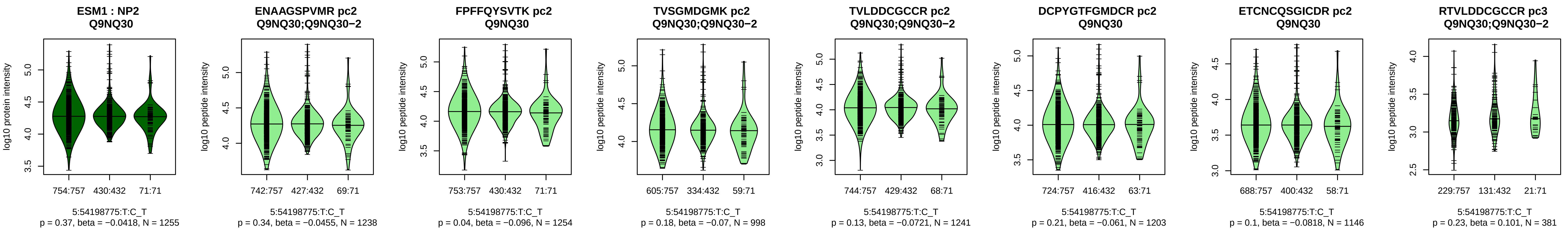

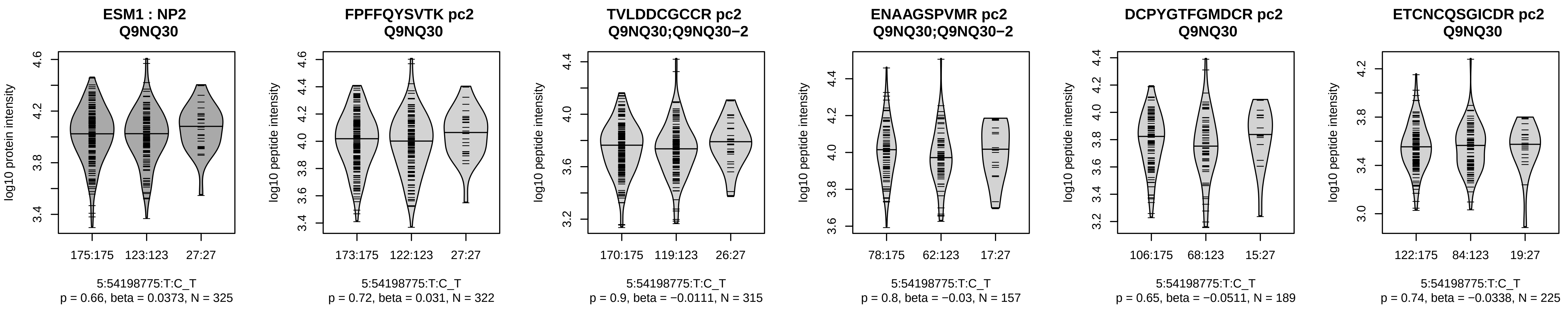

Assay Target: ESM1
UniProt: Q9NQ30
rsID: rs4242051
Target SNP: 5:54198775:T:C
Proxy SNP: 5:54198775:T:C
UKBPPP log10(p): 166.8
UKBPPP BETA: 0.191
UKBPPP MSPA: 0
−:−:−:−:−:−
1238:1254:998:1241:1203:1146

241. F10 (P00742)
UKBPPP MSPA=0.038

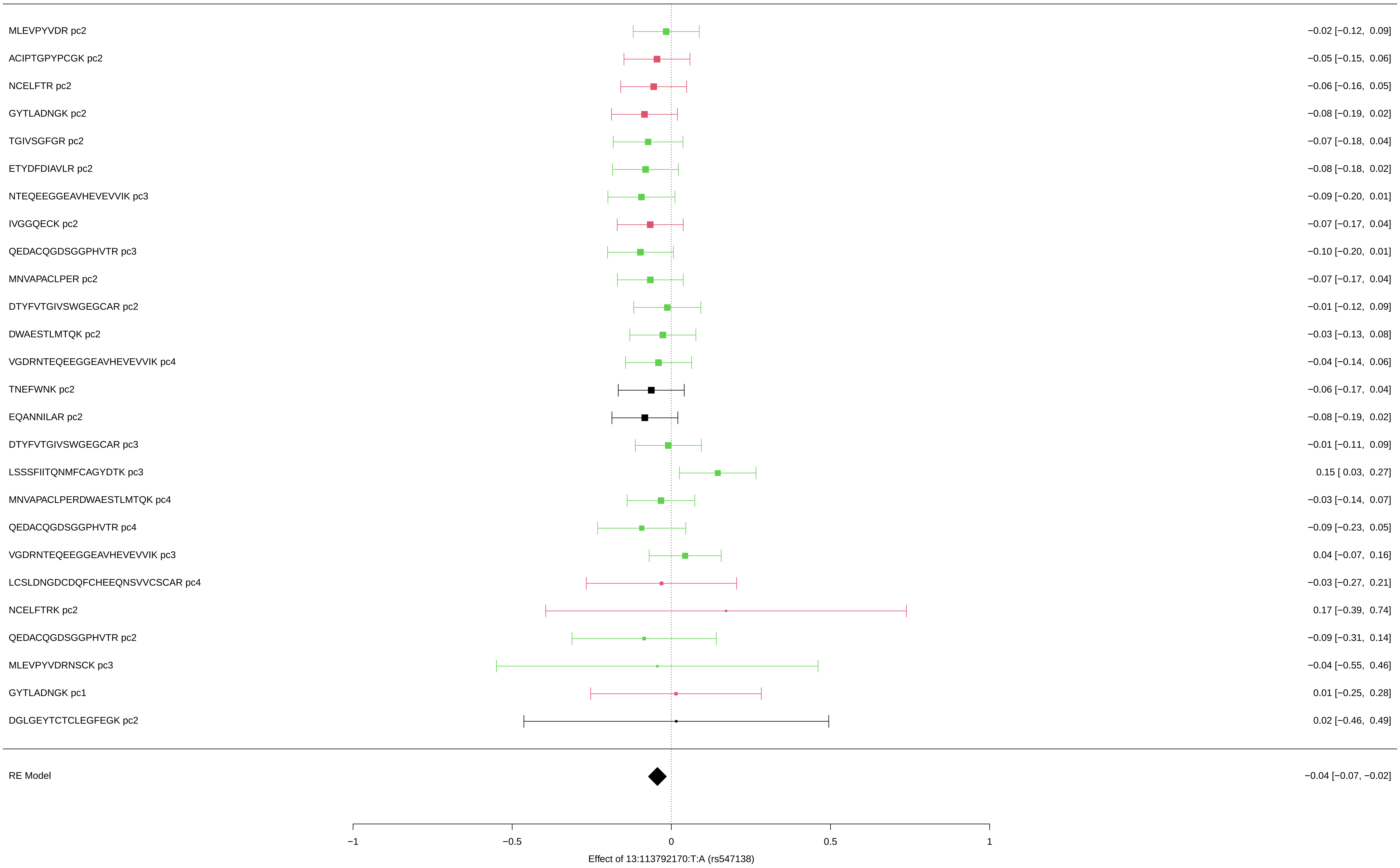

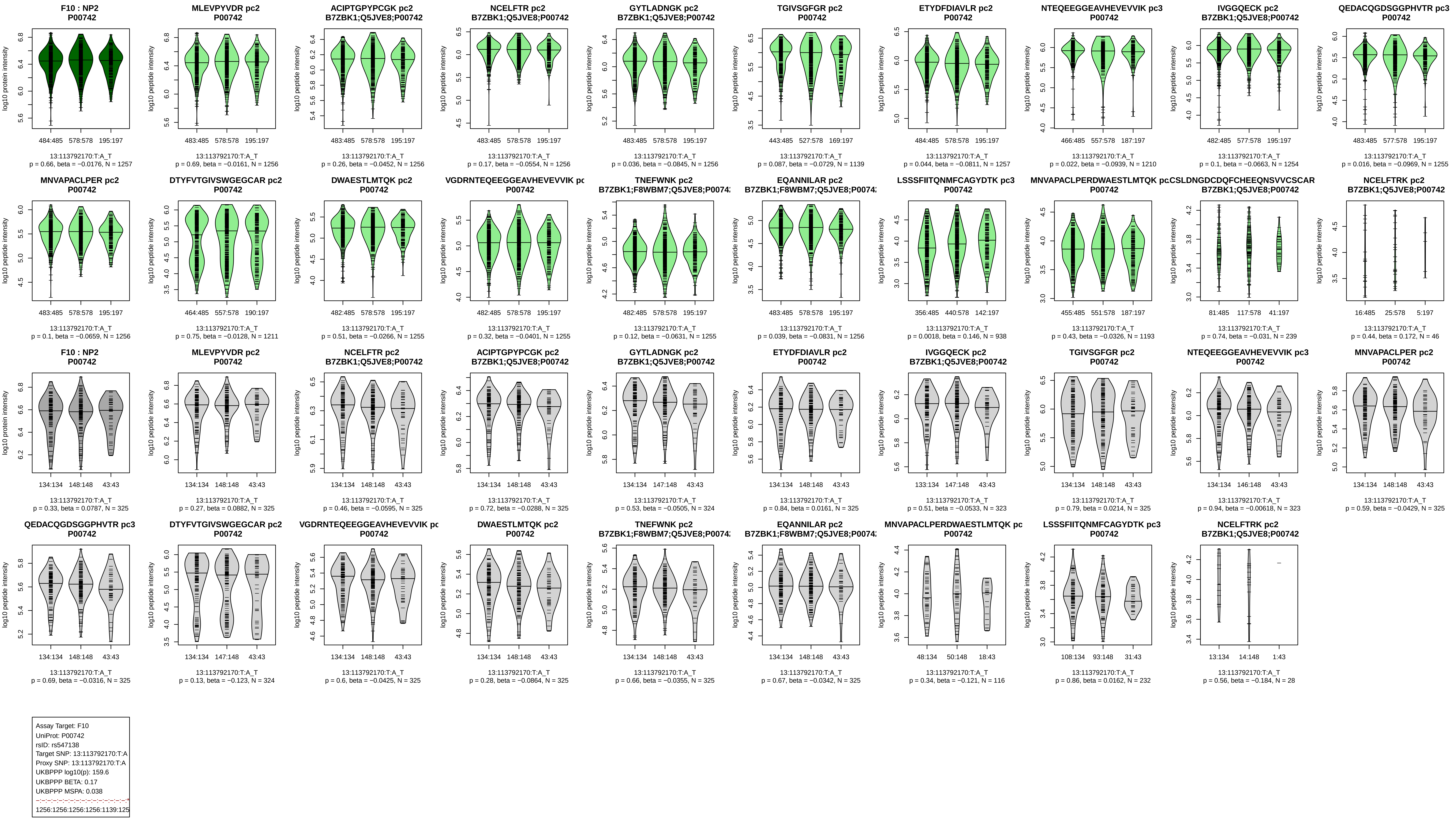

F10 : NP2
P00742
log10 protein intensity
484:485 578:578 195:197
13:113792170:T:A_T
p = 0.66, beta = −0.0176, N = 1257
MLEVPYVDR pc2
P00742
log10 peptide intensity
483:485 578:578 195:197
13:113792170:T:A_T
p = 0.69, beta = −0.0161, N = 1256
ACIPTGPYPCGK pc2
B7ZBK1;Q5JVE8;P00742
483:485 578:578 195:197
13:113792170:T:A_T
p = 0.26, beta = −0.0452, N = 1256
NCELFTR pc2
B7ZBK1;Q5JVE8;P00742
483:485 578:578 195:197
13:113792170:T:A_T
p = 0.17, beta = −0.0554, N = 1256
GYTLADNGK pc2
B7ZBK1;Q5JVE8;P00742
483:485 578:578 195:197
13:113792170:T:A_T
p = 0.036, beta = −0.0845, N = 1256
TGIVSGFGR pc2
P00742
443:485 527:578 169:197
13:113792170:T:A_T
p = 0.087, beta = −0.0729, N = 1139
ETYDFDIAVLR pc2
P00742
484:485 578:578 195:197
13:113792170:T:A_T
p = 0.044, beta = −0.0811, N = 1257
NTEQEEGGEAVHEVEVVIK pc3
P00742
466:485 557:578 187:197
13:113792170:T:A_T
p = 0.022, beta = −0.0939, N = 1210
IVGGQECK pc2
B7ZBK1;Q5JVE8;P00742
482:485 577:578 195:197
13:113792170:T:A_T
p = 0.1, beta = −0.0663, N = 1254
QEDACQGDSGGPHVTR pc3
P00742
483:485 577:578 195:197
13:113792170:T:A_T
p = 0.016, beta = −0.0969, N = 1255
MNVAPACLPER pc2
P00742
483:485 578:578 195:197
13:113792170:T:A_T
p = 0.1, beta = −0.0659, N = 1256
DTYFVTGIVSWGEGCAR pc2
P00742
464:485 557:578 190:197
13:113792170:T:A_T
p = 0.75, beta = −0.0128, N = 1211
DWAESTLMTQK pc2
P00742
482:485 578:578 195:197
13:113792170:T:A_T
p = 0.51, beta = −0.0266, N = 1255
VGDRNTEQEEGGEAVHEVEVVIK pc
P00742
482:485 578:578 195:197
13:113792170:T:A_T
p = 0.32, beta = −0.0401, N = 1255
TNEFWNK pc2
B7ZBK1;F8WBM7;Q5JVE8;P0074
482:485 578:578 195:197
13:113792170:T:A_T
p = 0.12, beta = −0.0631, N = 1255
EQANNILAR pc2
B7ZBK1;F8WBM7;Q5JVE8;P0074
483:485 578:578 195:197
13:113792170:T:A_T
p = 0.039, beta = −0.0831, N = 1256
LSSSFIITQNMFCAGYDTK pc3
P00742
356:485 440:578 142:197
13:113792170:T:A_T
p = 0.0018, beta = 0.146, N = 938
MNVAPACLPERDWAESTLMTQK pc
P00742
455:485 551:578 187:197
13:113792170:T:A_T
p = 0.43, beta = −0.0326, N = 1193
CSLDNGDCDQFCHEEQNSVVCSCAR
B7ZBK1;Q5JVE8;P00742
81:485 117:578 41:197
13:113792170:T:A_T
p = 0.74, beta = −0.031, N = 239
NCELFTRK pc2
B7ZBK1;Q5JVE8;P00742
16:485 25:578 5:197
13:113792170:T:A_T
p = 0.44, beta = 0.172, N = 46
F10 : NP2
P00742
134:134 148:148 43:43
13:113792170:T:A_T
p = 0.33, beta = 0.0787, N = 325
MLEVPYVDR pc2
P00742
134:134 148:148 43:43
13:113792170:T:A_T
p = 0.27, beta = 0.0882, N = 325
NCELFTR pc2
B7ZBK1;Q5JVE8;P00742
134:134 148:148 43:43
13:113792170:T:A_T
p = 0.46, beta = −0.0595, N = 325
ACIPTGPYPCGK pc2
B7ZBK1;Q5JVE8;P00742
134:134 148:148 43:43
13:113792170:T:A_T
p = 0.72, beta = −0.0288, N = 325
GYTLADNGK pc2
B7ZBK1;Q5JVE8;P00742
134:134 147:148 43:43
13:113792170:T:A_T
p = 0.53, beta = −0.0505, N = 324
ETYDFDIAVLR pc2
P00742
134:134 148:148 43:43
13:113792170:T:A_T
p = 0.84, beta = 0.0161, N = 325
IVGGQECK pc2
B7ZBK1;Q5JVE8;P00742
133:134 147:148 43:43
13:113792170:T:A_T
p = 0.51, beta = −0.0533, N = 323
TGIVSGFGR pc2
P00742
134:134 148:148 43:43
13:113792170:T:A_T
p = 0.79, beta = 0.0214, N = 325
NTEQEEGGEAVHEVEVVIK pc3
P00742
134:134 146:148 43:43
13:113792170:T:A_T
p = 0.94, beta = −0.00618, N = 323
MNVAPACLPER pc2
P00742
134:134 148:148 43:43
13:113792170:T:A_T
p = 0.59, beta = −0.0429, N = 325
QEDACQGDSGGPHVTR pc3
P00742
134:134 148:148 43:43
13:113792170:T:A_T
p = 0.69, beta = −0.0316, N = 325
DTYFVTGIVSWGEGCAR pc2
P00742
134:134 147:148 43:43
13:113792170:T:A_T
p = 0.13, beta = −0.123, N = 324
VGDRNTEQEEGGEAVHEVEVVIK pc
P00742
134:134 148:148 43:43
13:113792170:T:A_T
p = 0.6, beta = −0.0425, N = 325
DWAESTLMTQK pc2
P00742
134:134 148:148 43:43
13:113792170:T:A_T
p = 0.28, beta = −0.0864, N = 325
TNEFWNK pc2
B7ZBK1;F8WBM7;Q5JVE8;P0074
134:134 148:148 43:43
13:113792170:T:A_T
p = 0.66, beta = −0.0355, N = 325
EQANNILAR pc2
B7ZBK1;F8WBM7;Q5JVE8;P0074
134:134 148:148 43:43
13:113792170:T:A_T
p = 0.67, beta = −0.0342, N = 325
MNVAPACLPERDWAESTLMTQK pc
P00742
48:134 50:148 18:43
13:113792170:T:A_T
p = 0.34, beta = −0.121, N = 116
LSSSFIITQNMFCAGYDTK pc3
P00742
108:134 93:148 31:43
13:113792170:T:A_T
p = 0.86, beta = 0.0162, N = 232
NCELFTRK pc2
B7ZBK1;Q5JVE8;P00742
13:134 14:148 1:43
13:113792170:T:A_T
p = 0.56, beta = −0.184, N = 28
Assay Target: F10
UniProt: P00742
rsID: rs547138
Target SNP: 13:113792170:T:A
Proxy SNP: 13:113792170:T:A
UKBPPP log10(p): 159.6
UKBPPP BETA: 0.17
UKBPPP MSPA: 0.038
1256:1256:1256:1256:1139:125

242. EHD3 (Q9NZN3)
UKBPPP MSPA=0.668

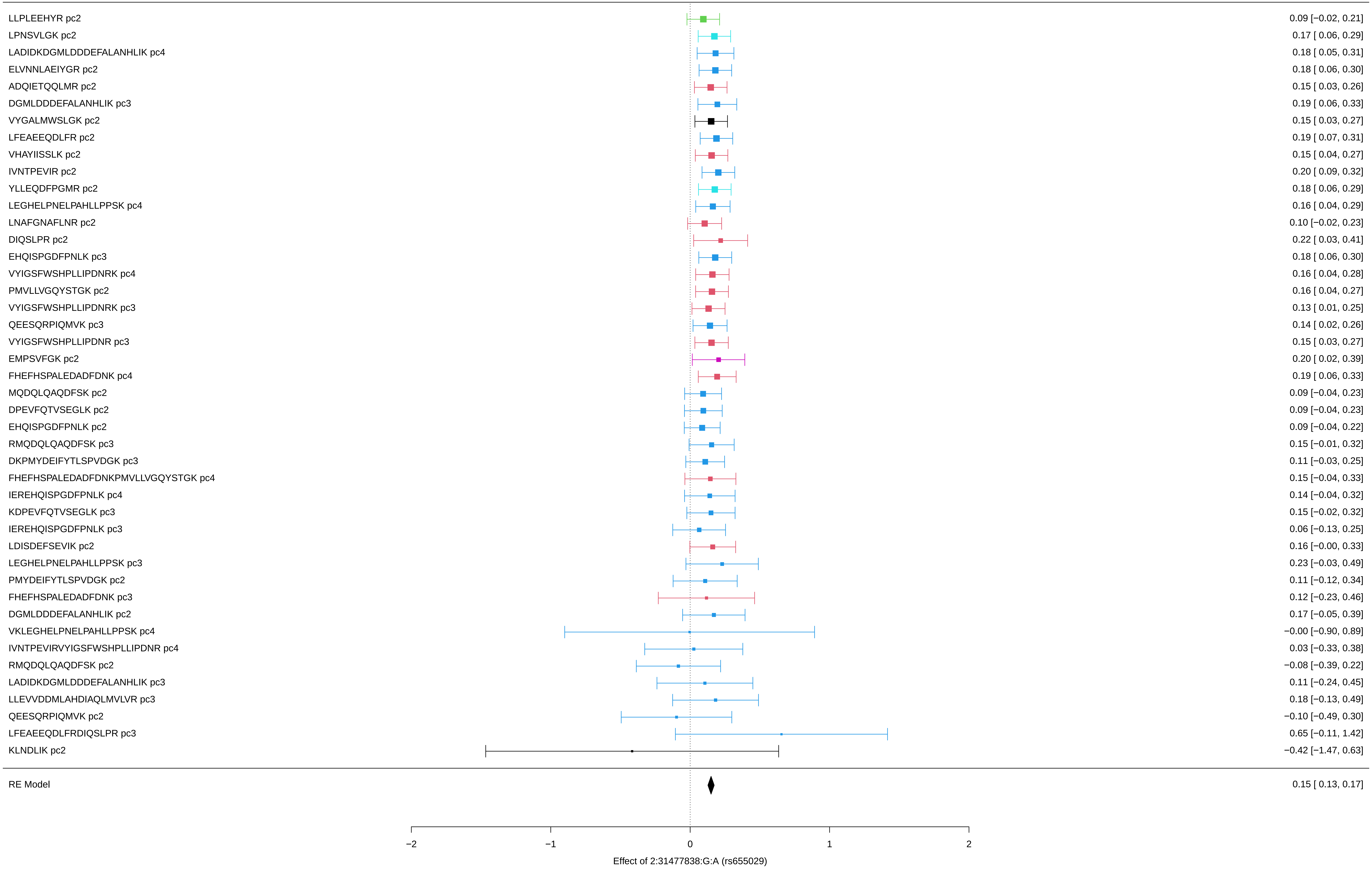

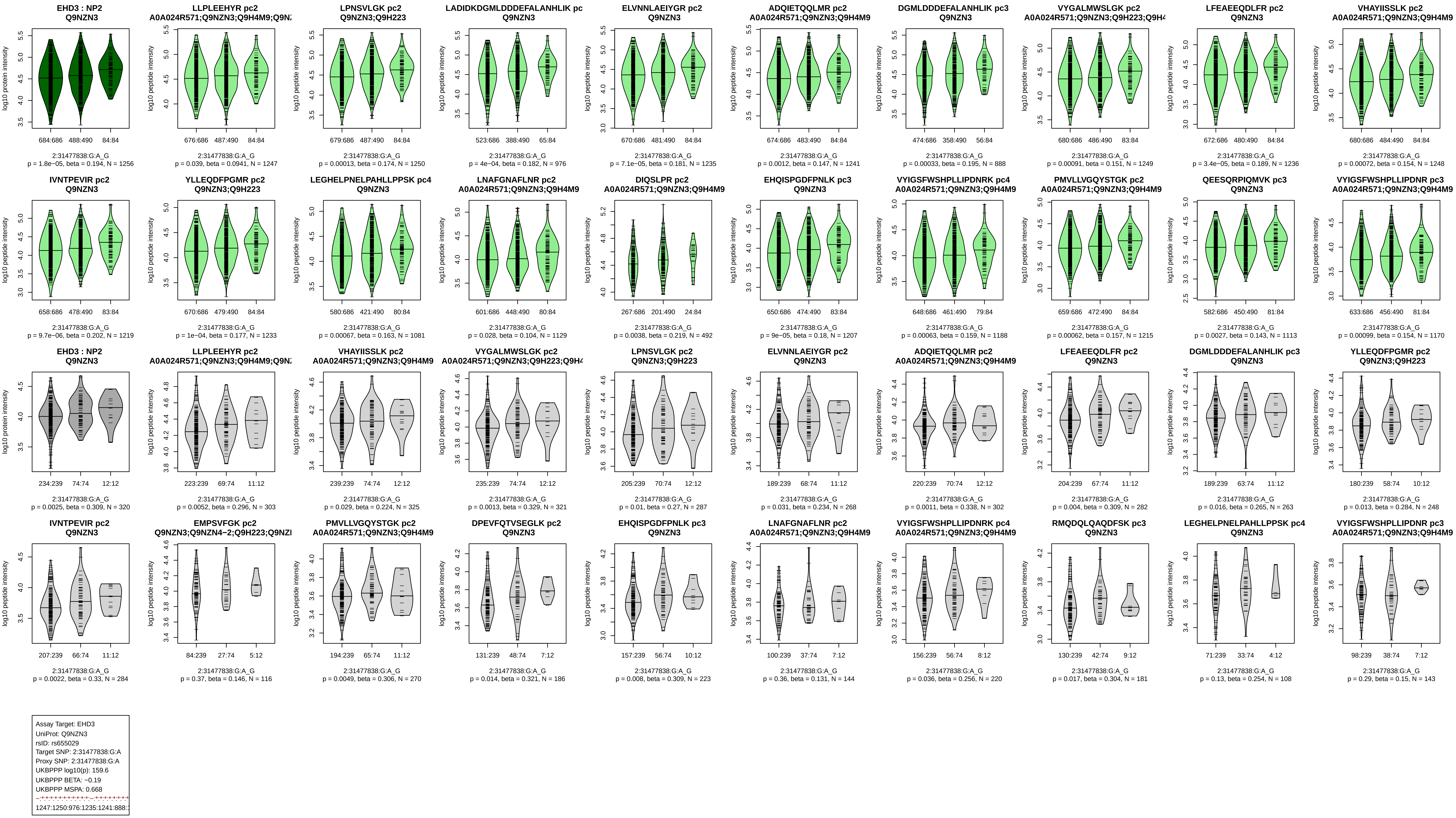
EHD3 : NP2
Q9NZN3
2:31477838:G:A_G
p = 1.8e−05, beta = 0.194, N = 1256
LLPLEEHYR pc2
A0A024R571;Q9NZN3;Q9H4M9;Q9N
p = 0.039, beta = 0.0941, N = 1247
LPNSVLGK pc2
Q9NZN3;Q9H223
p = 0.00013, beta = 0.174, N = 1250
LADIDKDGMLDDDEFALANHLIK pc
Q9NZN3
p = 4e−04, beta = 0.182, N = 976
ELVNNLAEIYGR pc2
Q9NZN3
p = 7.1e−05, beta = 0.181, N = 1235
ADQIETQQLMR pc2
A0A024R571;Q9NZN3;Q9H4M9
p = 0.0012, beta = 0.147, N = 1241
DGMLDDDEFALANHLIK pc3
Q9NZN3
p = 0.00033, beta = 0.195, N = 888
VYGALMWSLGK pc2
A0A024R571;Q9NZN3;Q9H223;Q9H4
p = 0.00091, beta = 0.151, N = 1249
LFEAEEQDLFR pc2
Q9NZN3
p = 3.4e−05, beta = 0.189, N = 1236
VHAYIISSLK pc2
A0A024R571;Q9NZN3;Q9H4M9
p = 0.00072, beta = 0.154, N = 1248
IVNTPEVIR pc2
Q9NZN3
p = 9.7e−06, beta = 0.202, N = 1219
YLLEQDFPGMR pc2
Q9NZN3;Q9H223
p = 1e−04, beta = 0.177, N = 1233
LEGHELPNELPAHLLPPSK pc4
Q9NZN3
p = 0.00067, beta = 0.163, N = 1081
LNAFGNAFLNR pc2
A0A024R571;Q9NZN3;Q9H4M9
p = 0.028, beta = 0.104, N = 1129
DIQSLPR pc2
A0A024R571;Q9NZN3;Q9H4M9
p = 0.0038, beta = 0.219, N = 492
EHQISPGDFPNLK pc3
Q9NZN3
p = 9e−05, beta = 0.18, N = 1207
VYIGSFWSHPLLIPDNRK pc4
A0A024R571;Q9NZN3;Q9H4M9
p = 0.00063, beta = 0.159, N = 1188
PMVLLVGQYSTGK pc2
A0A024R571;Q9NZN3;Q9H4M9
p = 0.00062, beta = 0.157, N = 1215
QEESQRPIQMVK pc3
Q9NZN3
p = 0.0027, beta = 0.143, N = 1113
VYIGSFWSHPLLIPDNR pc3
A0A024R571;Q9NZN3;Q9H4M9
p = 0.00099, beta = 0.154, N = 1170
EHD3 : NP2
Q9NZN3
p = 0.0025, beta = 0.309, N = 320
LLPLEEHYR pc2
A0A024R571;Q9NZN3;Q9H4M9;Q9N
p = 0.0052, beta = 0.296, N = 303
VHAYIISSLK pc2
A0A024R571;Q9NZN3;Q9H4M9
p = 0.029, beta = 0.224, N = 325
VYGALMWSLGK pc2
A0A024R571;Q9NZN3;Q9H223;Q9H4
p = 0.0013, beta = 0.329, N = 321
LPNSVLGK pc2
Q9NZN3;Q9H223
p = 0.01, beta = 0.27, N = 287
ELVNNLAEIYGR pc2
Q9NZN3
p = 0.031, beta = 0.234, N = 268
ADQIETQQLMR pc2
A0A024R571;Q9NZN3;Q9H4M9
p = 0.0011, beta = 0.338, N = 302
LFEAEEQDLFR pc2
Q9NZN3
p = 0.004, beta = 0.309, N = 282
DGMLDDDEFALANHLIK pc3
Q9NZN3
p = 0.016, beta = 0.265, N = 263
YLLEQDFPGMR pc2
Q9NZN3;Q9H223
p = 0.013, beta = 0.284, N = 248
IVNTPEVIR pc2
Q9NZN3
p = 0.0022, beta = 0.33, N = 284
EMPSVFGK pc2
Q9NZN3;Q9NZN4−2;Q9H223;Q9NZ
p = 0.37, beta = 0.146, N = 116
PMVLLVGQYSTGK pc2
A0A024R571;Q9NZN3;Q9H4M9
p = 0.0049, beta = 0.306, N = 270
DPEVFQTVSEGLK pc2
Q9NZN3
p = 0.014, beta = 0.321, N = 186
EHQISPGDFPNLK pc3
Q9NZN3
p = 0.008, beta = 0.309, N = 223
LNAFGNAFLNR pc2
A0A024R571;Q9NZN3;Q9H4M9
p = 0.36, beta = 0.131, N = 144
VYIGSFWSHPLLIPDNRK pc4
A0A024R571;Q9NZN3;Q9H4M9
p = 0.036, beta = 0.256, N = 220
RMQDQLQAQDFSK pc3
Q9NZN3
p = 0.017, beta = 0.304, N = 181
LEGHELPNELPAHLLPPSK pc4
Q9NZN3
p = 0.13, beta = 0.254, N = 108
VYIGSFWSHPLLIPDNR pc3
A0A024R571;Q9NZN3;Q9H4M9
p = 0.29, beta = 0.15, N = 143
Assay Target: EHD3
UniProt: Q9NZN3
rsID: rs655029
Target SNP: 2:31477838:G:A
Proxy SNP: 2:31477838:G:A
UKBPPP log10(p): 159.6
UKBPPP BETA: −0.19
UKBPPP MSPA: 0.668
1247:1250:976:1235:1241:888:

243. AXL (P30530)
UKBPPP MSPA=0.301

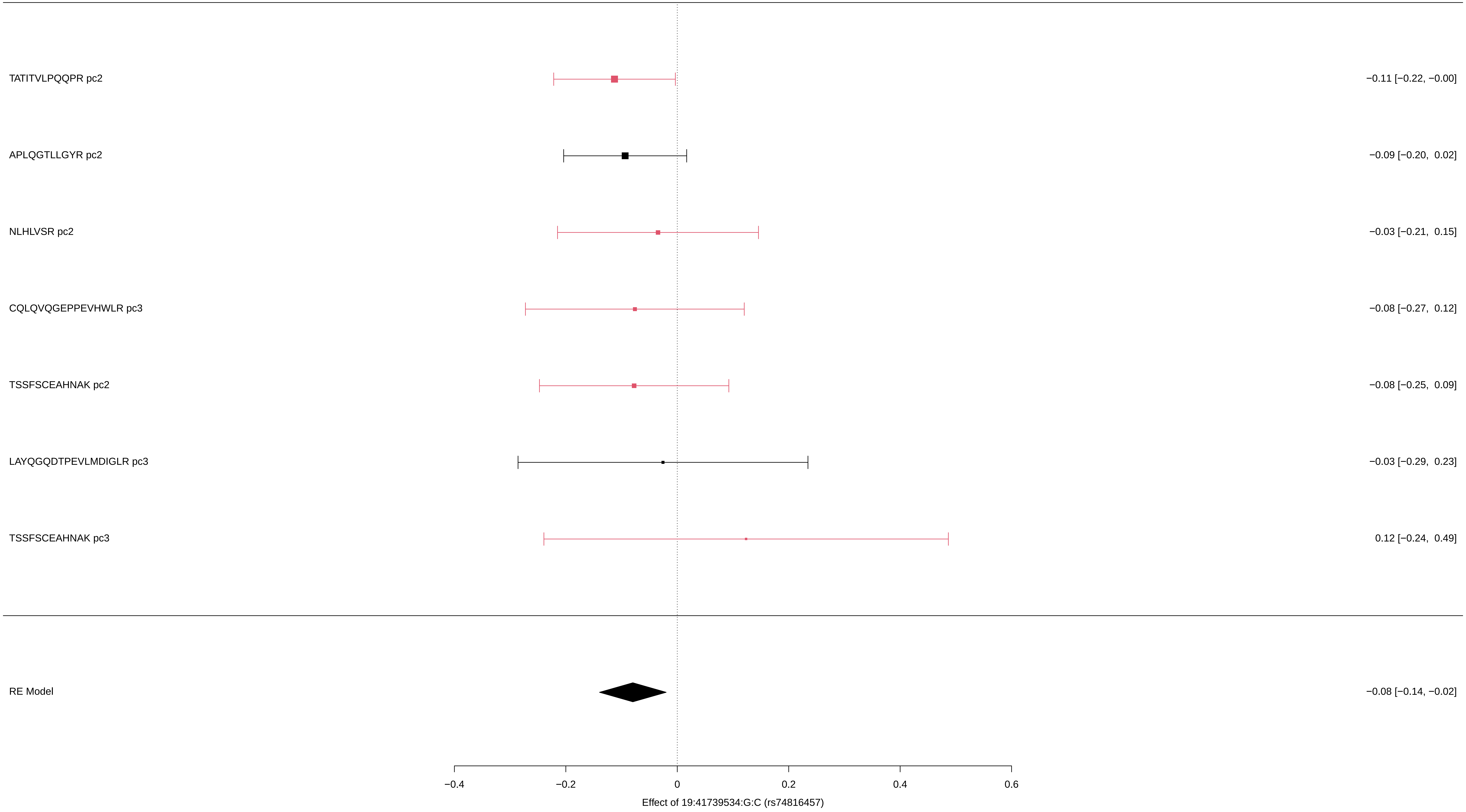

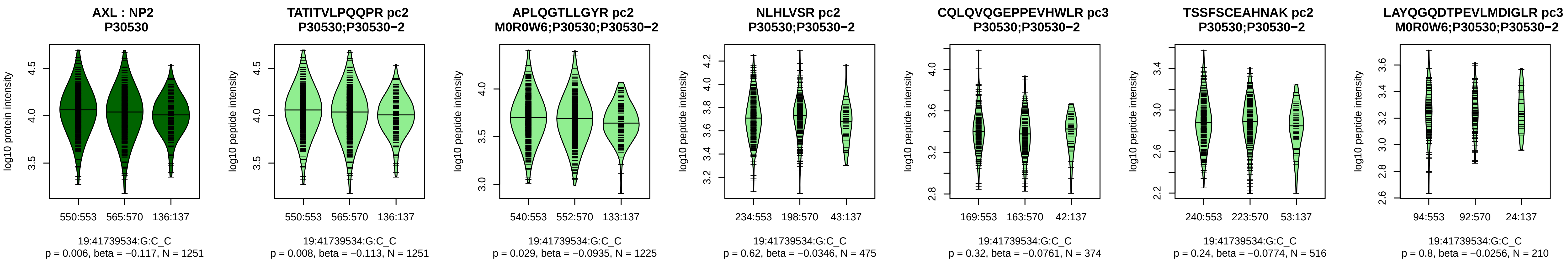

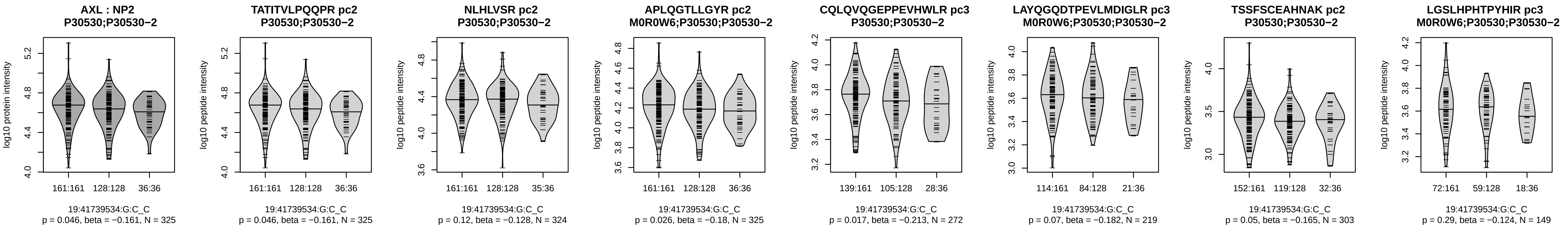

Assay Target: AXL
UniProt: P30530
rsID: rs74816457
Target SNP: 19:41739534:G:C
Proxy SNP: 19:41739534:G:C
UKBPPP log10(p): 159.4
UKBPPP BETA: −0.166
UKBPPP MSPA: 0.301
*:–:–:–:–:–:–
1251:1225:475:374:516:210:11[illegible]

244. NRP2 (O60462)
UKBPPP MSPA=0

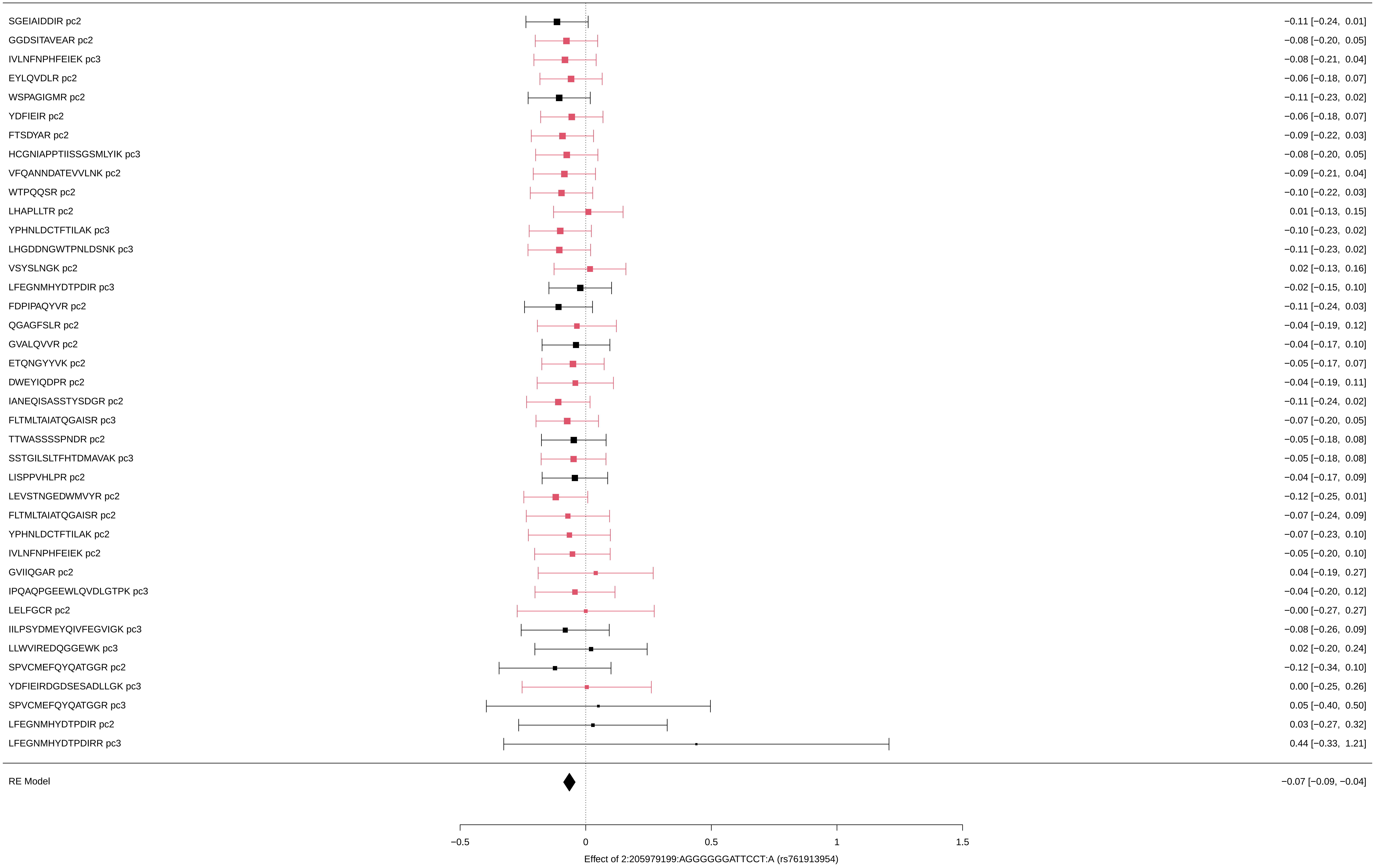

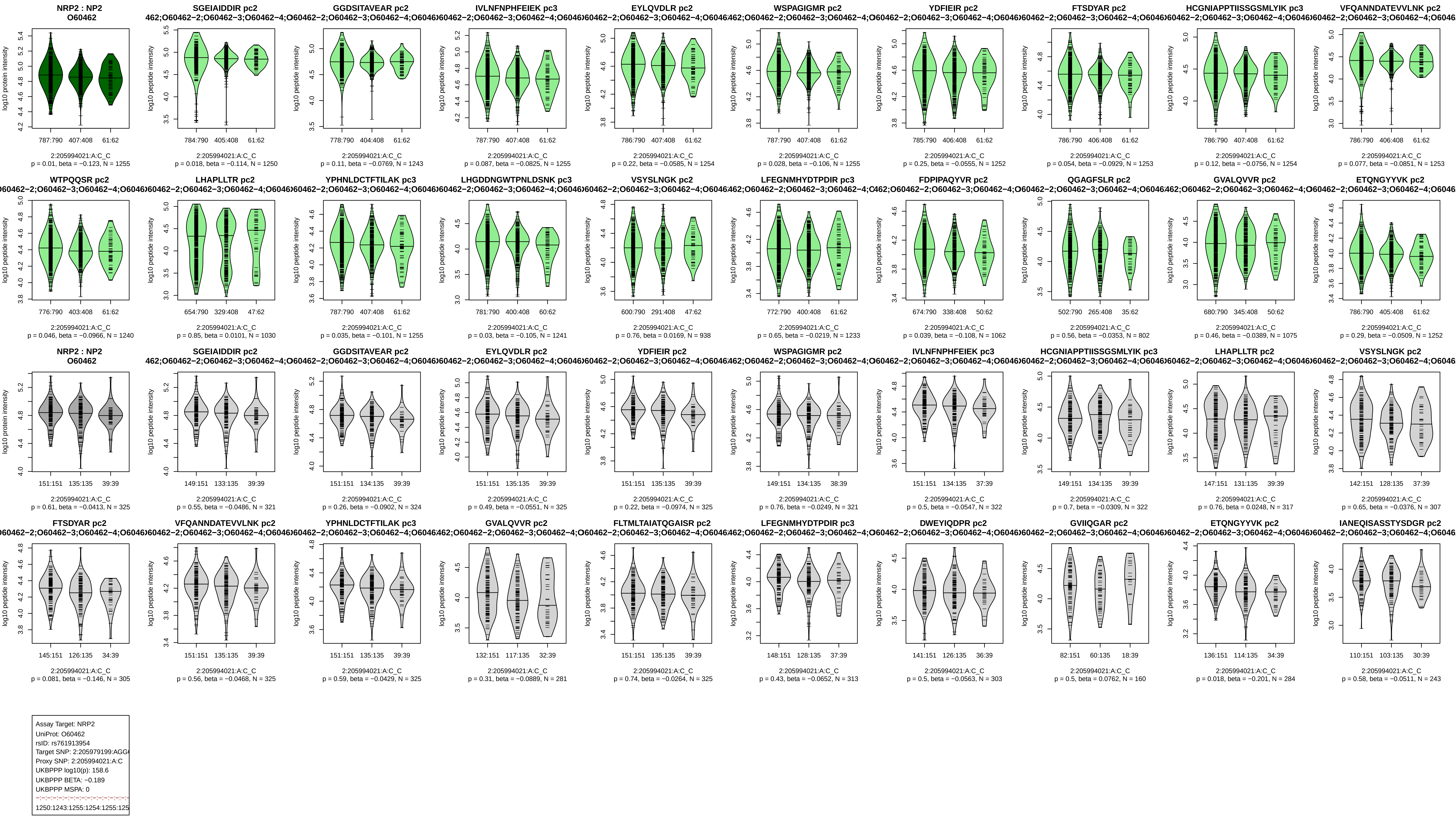
NRP2 : NP2 O60462
2:205994021:A:C_C
p = 0.01, beta = −0.123, N = 1255
SGEIAIDDIR pc2
p = 0.018, beta = −0.114, N = 1250
GGDSITAVEAR pc2
p = 0.11, beta = −0.0769, N = 1243
IVLNFNPHFEIEK pc3
p = 0.087, beta = −0.0825, N = 1255
EYLQVDLR pc2
p = 0.22, beta = −0.0585, N = 1254
WSPAGIGMR pc2
p = 0.028, beta = −0.106, N = 1255
YDFIEIR pc2
p = 0.25, beta = −0.0555, N = 1252
FTSDYAR pc2
p = 0.054, beta = −0.0929, N = 1253
HCGNIAPPTIISSGSMLYIK pc3
p = 0.12, beta = −0.0756, N = 1254
VFQANNDATEVVLNK pc2
p = 0.077, beta = −0.0851, N = 1253
WTPQQSR pc2
p = 0.046, beta = −0.0966, N = 1240
LHAPLLTR pc2
p = 0.85, beta = 0.0101, N = 1030
YPHNLDCTFTILAK pc3
p = 0.035, beta = −0.101, N = 1255
LHGDDNGWTPNLDSNK pc3
p = 0.03, beta = −0.105, N = 1241
VSYSLNGK pc2
p = 0.76, beta = 0.0169, N = 938
LFEGNMHYDTPDIR pc3
p = 0.65, beta = −0.0219, N = 1233
FDPIPAQYVR pc2
p = 0.039, beta = −0.108, N = 1062
QGAGFSLR pc2
p = 0.56, beta = −0.0353, N = 802
GVALQVVR pc2
p = 0.46, beta = −0.0389, N = 1075
ETQNGYYVK pc2
p = 0.29, beta = −0.0509, N = 1252
NRP2 : NP2 O60462
p = 0.61, beta = −0.0413, N = 325
SGEIAIDDIR pc2
p = 0.55, beta = −0.0486, N = 321
GGDSITAVEAR pc2
p = 0.26, beta = −0.0902, N = 324
EYLQVDLR pc2
p = 0.49, beta = −0.0551, N = 325
YDFIEIR pc2
p = 0.22, beta = −0.0974, N = 325
WSPAGIGMR pc2
p = 0.76, beta = −0.0249, N = 321
IVLNFNPHFEIEK pc3
p = 0.5, beta = −0.0547, N = 322
HCGNIAPPTIISSGSMLYIK pc3
p = 0.7, beta = −0.0309, N = 322
LHAPLLTR pc2
p = 0.76, beta = 0.0248, N = 317
VSYSLNGK pc2
p = 0.65, beta = −0.0376, N = 307
FTSDYAR pc2
p = 0.081, beta = −0.146, N = 305
VFQANNDATEVVLNK pc2
p = 0.56, beta = −0.0468, N = 325
YPHNLDCTFTILAK pc3
p = 0.59, beta = −0.0429, N = 325
GVALQVVR pc2
p = 0.31, beta = −0.0889, N = 281
FLTMLTAIATQGAISR pc2
p = 0.74, beta = −0.0264, N = 325
LFEGNMHYDTPDIR pc3
p = 0.43, beta = −0.0652, N = 313
DWEYIQDPR pc2
p = 0.5, beta = −0.0563, N = 303
GVIIQGAR pc2
p = 0.5, beta = 0.0762, N = 160
ETQNGYYVK pc2
p = 0.018, beta = −0.201, N = 284
IANEQISASSTYSDGR pc2
p = 0.58, beta = −0.0511, N = 243
Assay Target: NRP2
UniProt: O60462
rsID: rs761913954
Target SNP: 2:205979199:AGG
Proxy SNP: 2:205994021:A:C
UKBPPP log10(p): 158.6
UKBPPP BETA: −0.189
UKBPPP MSPA: 0
1250:1243:1255:1254:1255:125

245. C4BPB (P20851)
UKBPPP MSPA=0

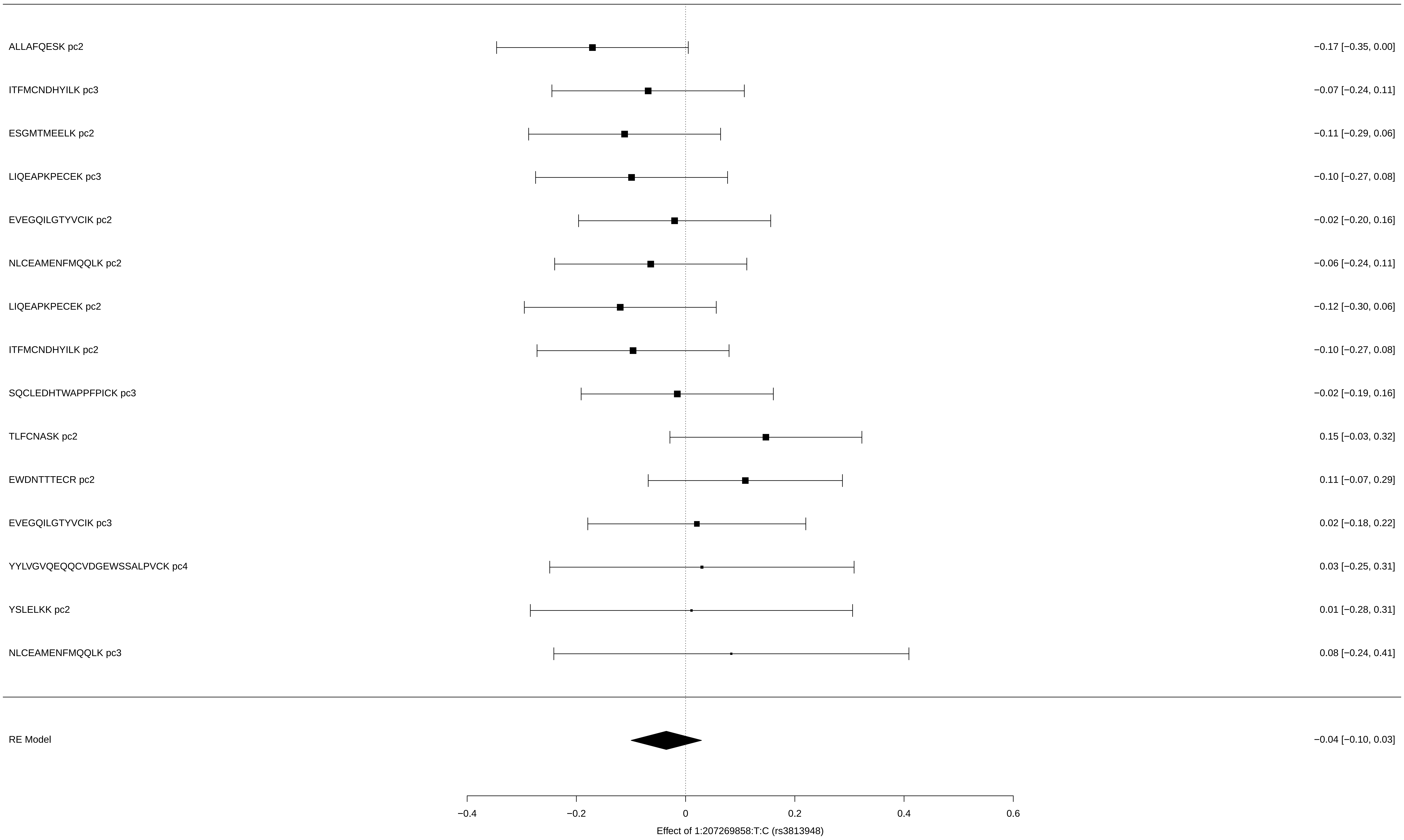

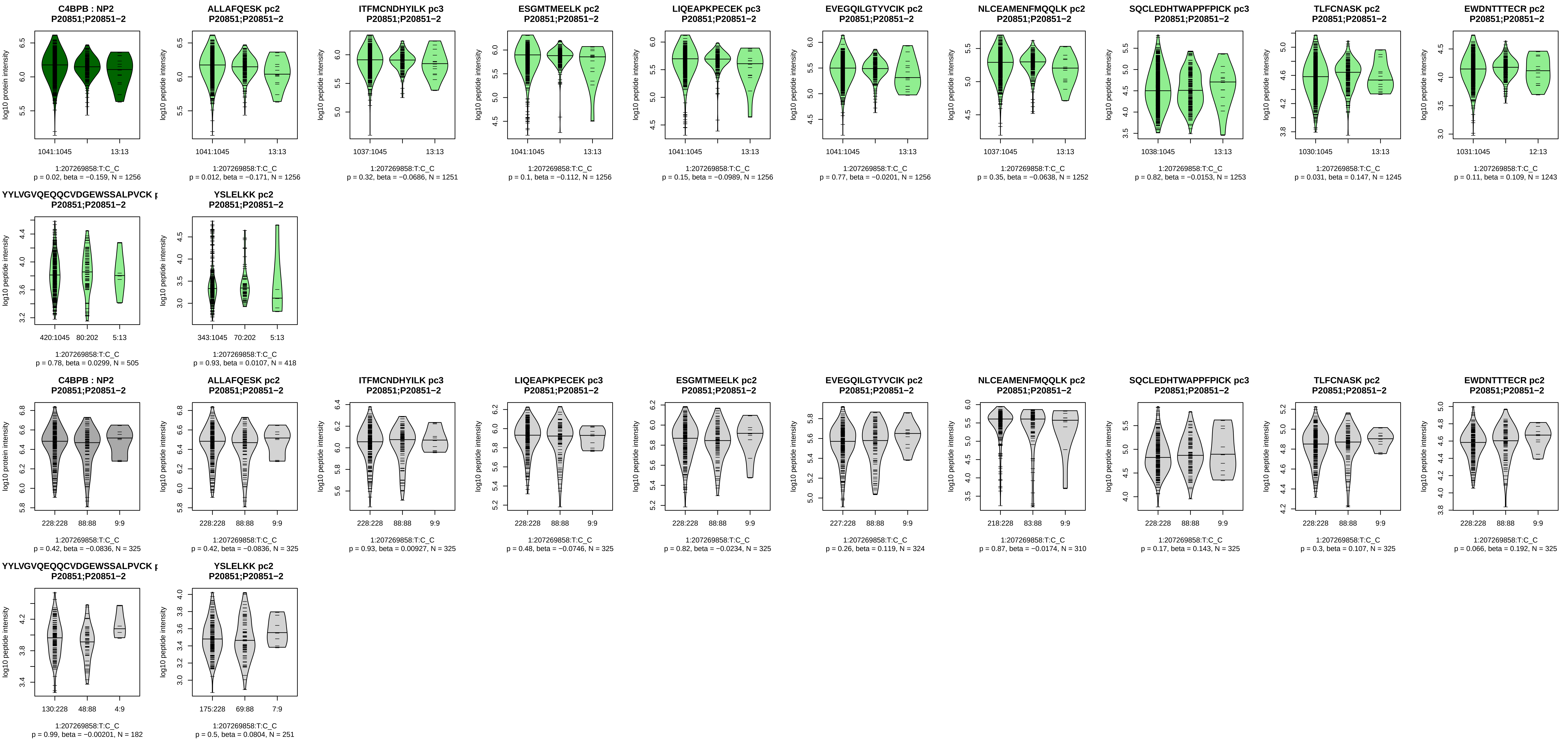

Assay Target: C4BPB
UniProt: P20851
rsID: rs3813948
Target SNP: 1:207269858:T:C
Proxy SNP: 1:207269858:T:C
UKBPPP log10(p): 158
UKBPPP BETA: −0.295
UKBPPP MSPA: 0
1256:1251:1256:1256:1256:125

246. FIS1 (Q9Y3D6)
UKBPPP MSPA=0.338

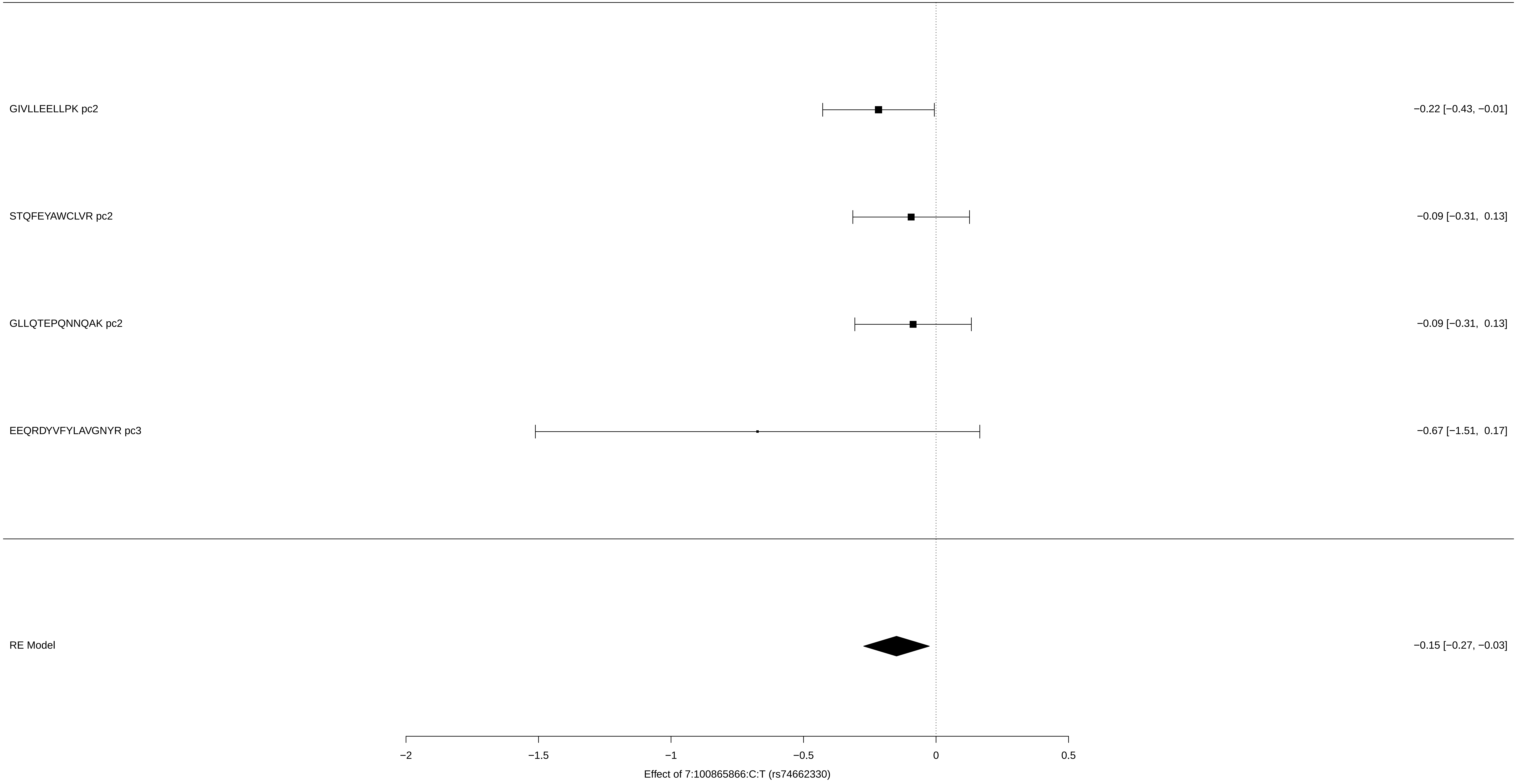

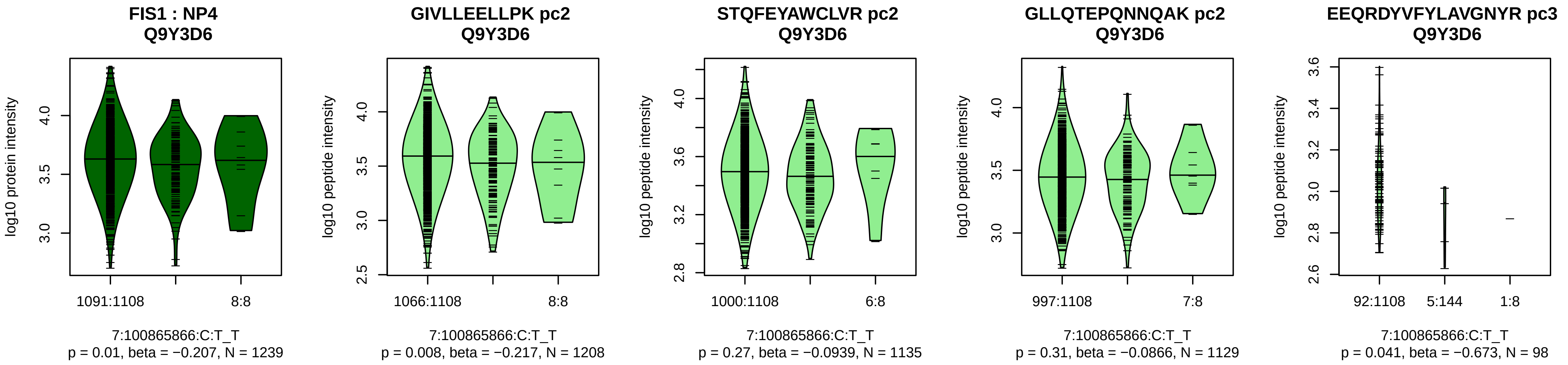

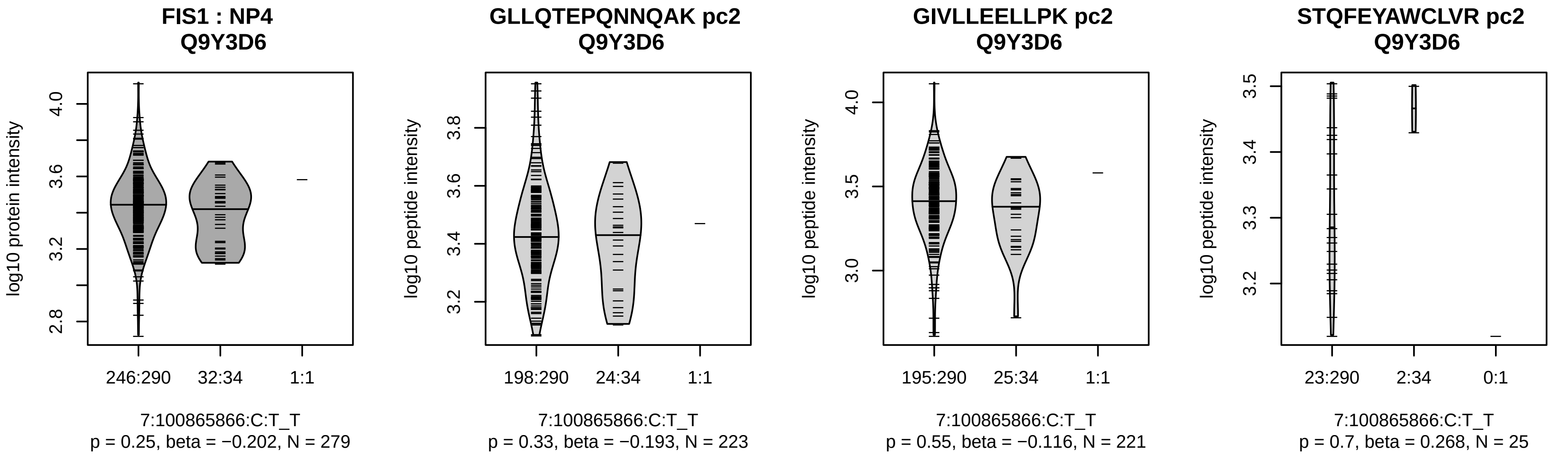

Assay Target: FIS1
UniProt: Q9Y3D6
rsID: rs74662330
Target SNP: 7:100865866:C:T
Proxy SNP: 7:100865866:C:T
UKBPPP log10(p): 155.2
UKBPPP BETA: −0.353
UKBPPP MSPA: 0.338
*:−:−:−
1208:1135:1129:98

247. NPY (P01303)
UKBPPP MSPA=0

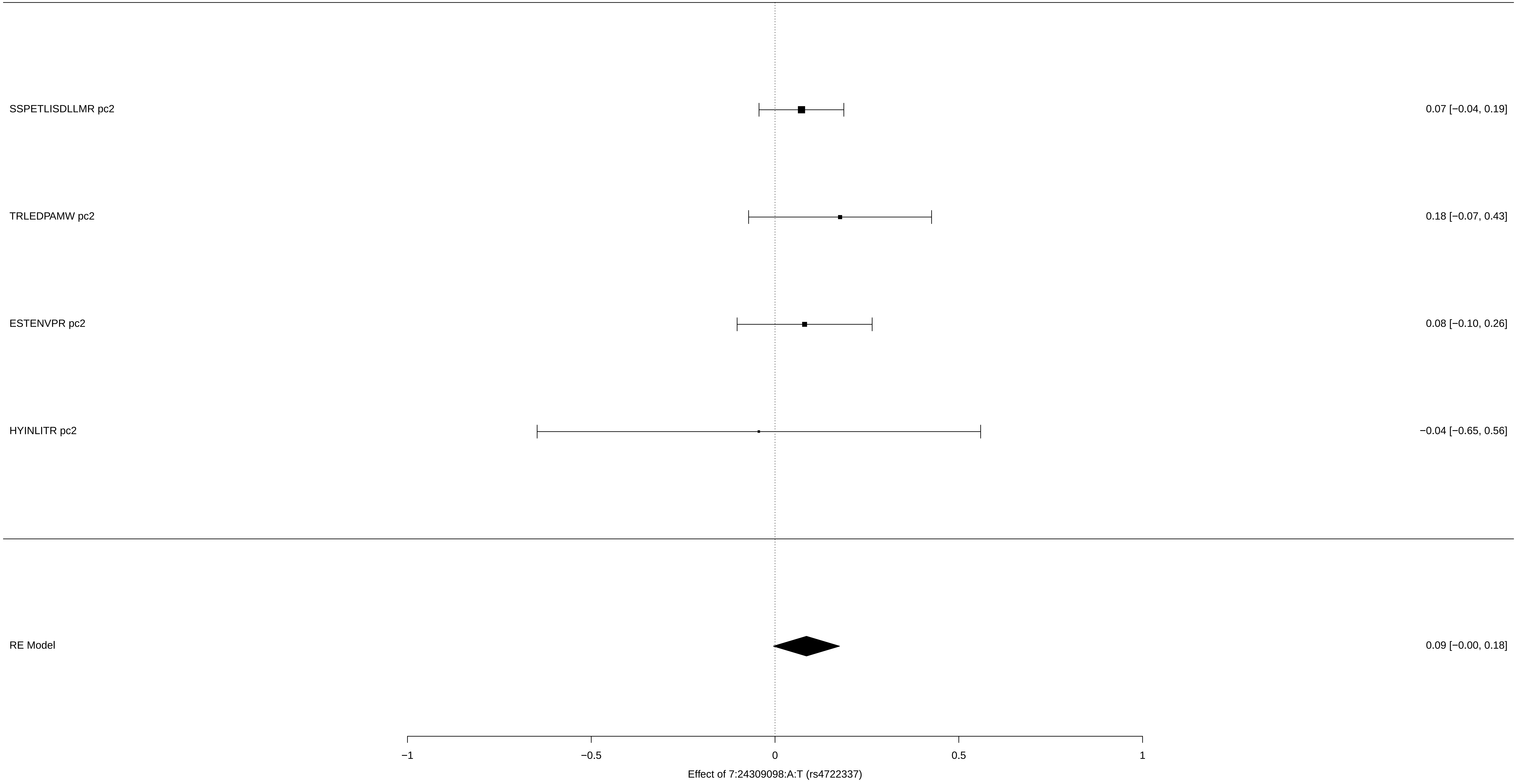

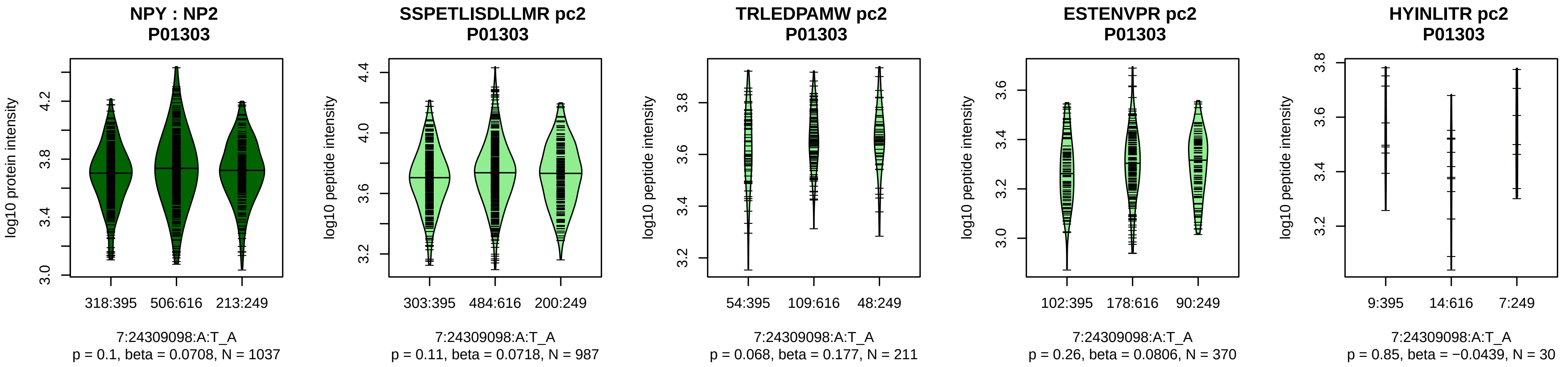

Assay Target: NPY
UniProt: P01303
rsID: rs4722337
Target SNP: 7:24309098:A:T
Proxy SNP: 7:24309098:A:T
UKBPPP log10(p): 152.7
UKBPPP BETA: −0.157
UKBPPP MSPA: 0
–:–:–:–
987:211:370:30

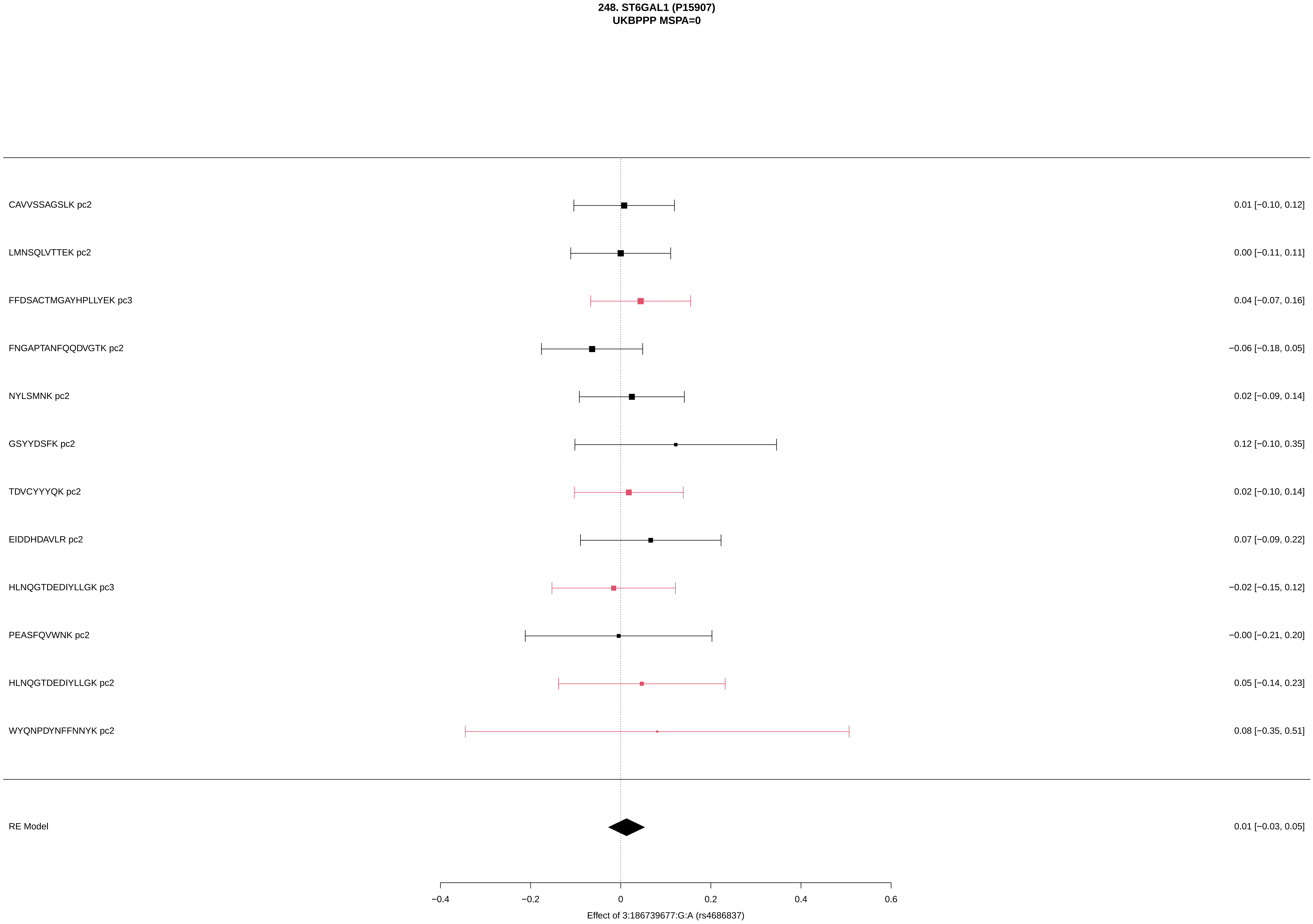
248. ST6GAL1 (P15907)
UKBPPP MSPA=0
CAVVSSAGSLK pc2
0.01 [−0.10, 0.12]
LMNSQLVTTEK pc2
0.00 [−0.11, 0.11]
FFDSACTMGAYHPLLYEK pc3
0.04 [−0.07, 0.16]
FNGAPTANFQQDVGTK pc2
−0.06 [−0.18, 0.05]
NYLSMNK pc2
0.02 [−0.09, 0.14]
GSYYDSFK pc2
0.12 [−0.10, 0.35]
TDVCYYYQK pc2
0.02 [−0.10, 0.14]
EIDDHDAVLR pc2
0.07 [−0.09, 0.22]
HLNQGTDEDIYLLGK pc3
−0.02 [−0.15, 0.12]
PEASFQVWNK pc2
−0.00 [−0.21, 0.20]
HLNQGTDEDIYLLGK pc2
0.05 [−0.14, 0.23]
WYQNPDYNFFNNYK pc2
0.08 [−0.35, 0.51]
RE Model
0.01 [−0.03, 0.05]
−0.4
−0.2
0
0.2
0.4
0.6
Effect of 3:186739677:G:A (rs4686837)

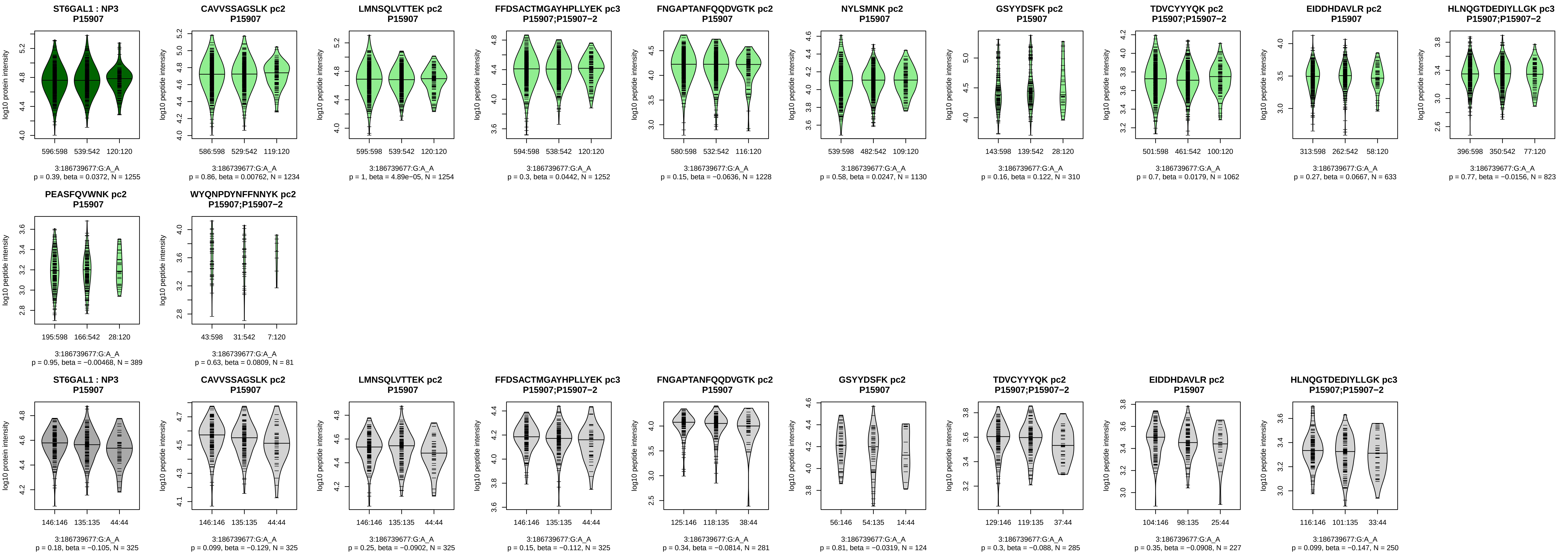

Assay Target: ST6GAL1
UniProt: P15907
rsID: rs4686837
Target SNP: 3:186739677:G:A
Proxy SNP: 3:186739677:G:A
UKBPPP log10(p): 152.6
UKBPPP BETA: −0.17
UKBPPP MSPA: 0
−:−:−:−:−:−:−:−:−:−:−:NA:NA
1234:1254:1252:1228:1130:310

249. HSPG2 (P98160)
UKBPPP MSPA=0.011

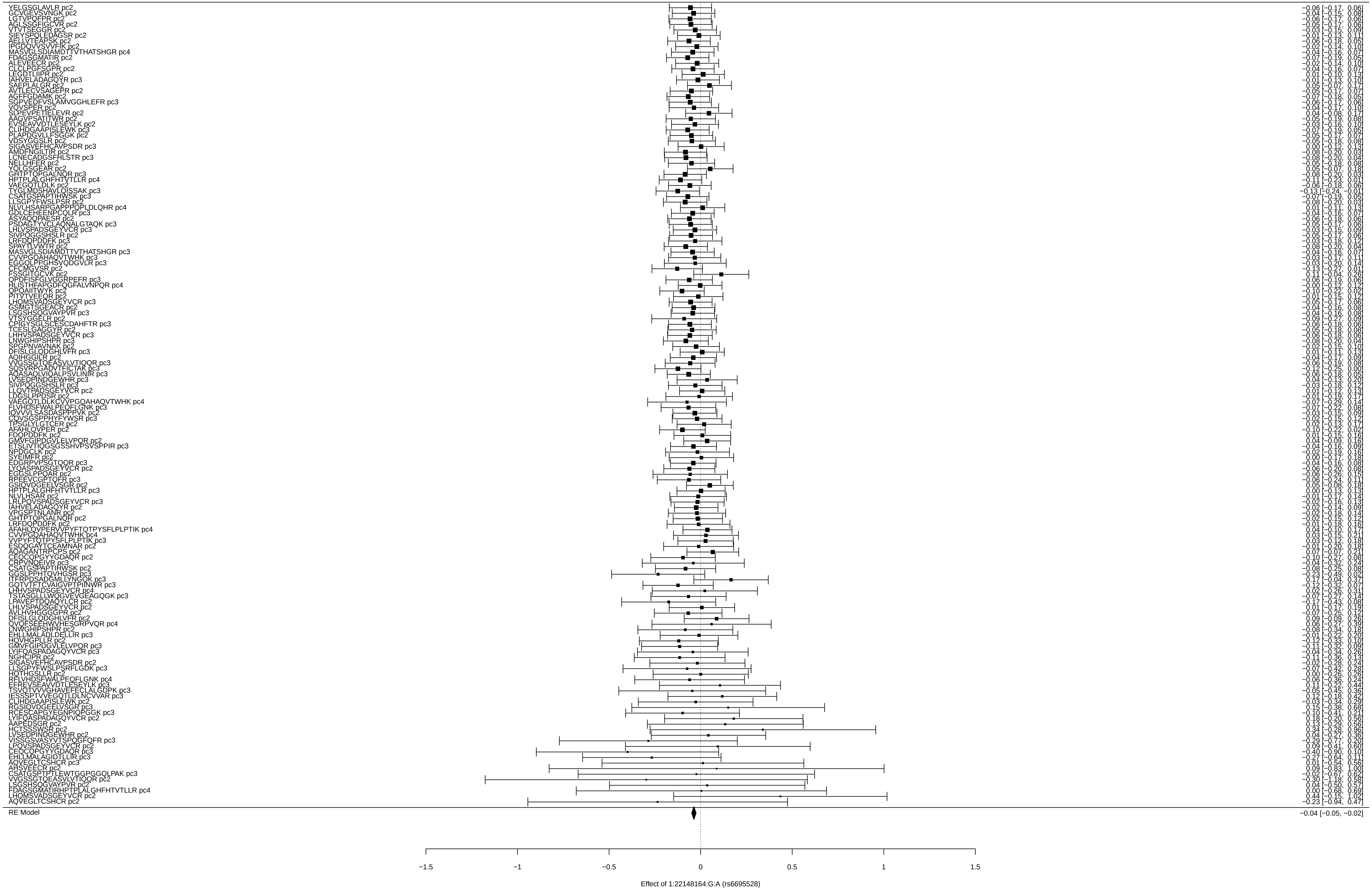

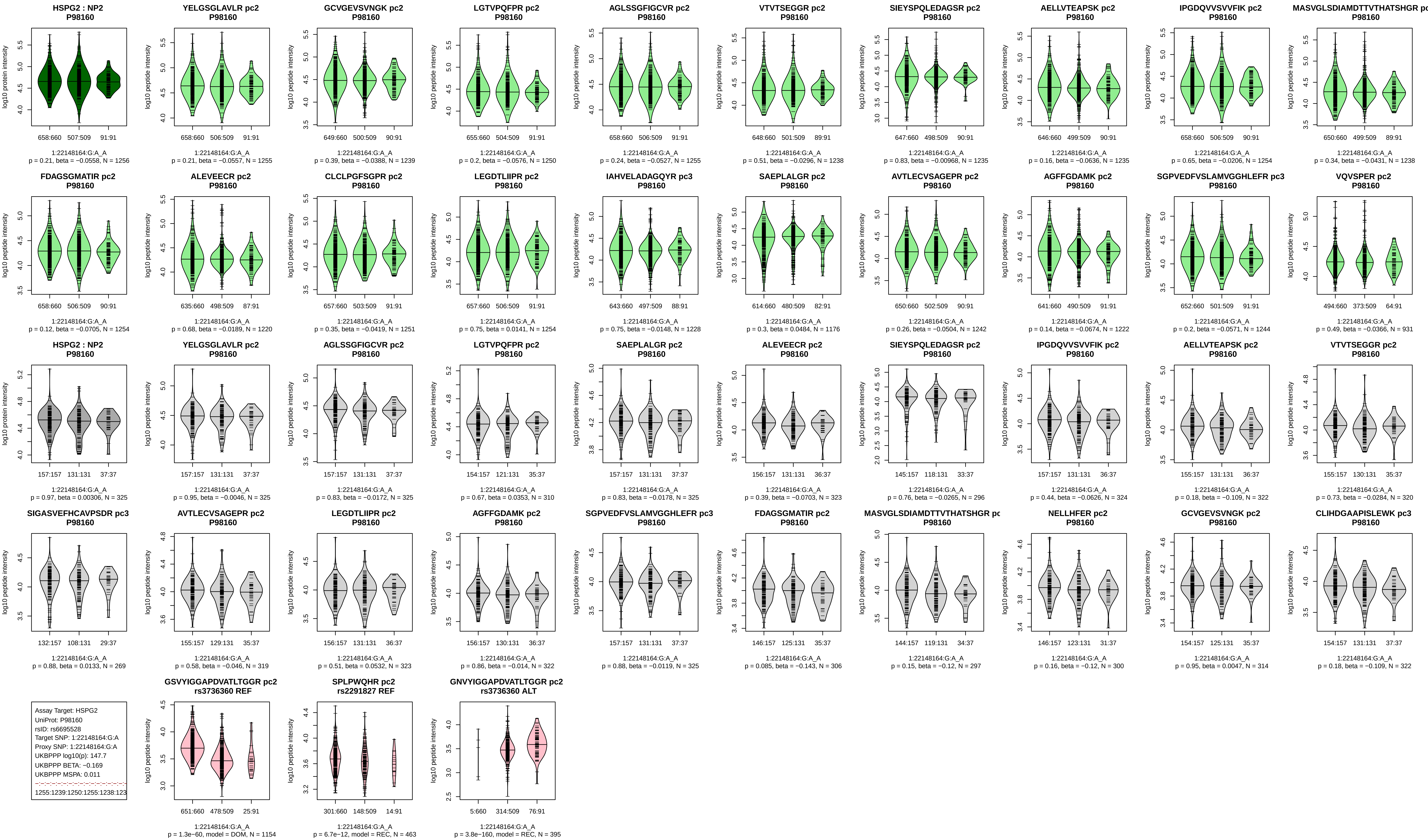

HSPG2 : NP2 P98160
1:22148164:G:A_A
p = 0.21, beta = −0.0558, N = 1256
YELGSGLAVLR pc2 P98160
p = 0.21, beta = −0.0557, N = 1255
GCVGEVSVNGK pc2 P98160
p = 0.39, beta = −0.0388, N = 1239
LGTVPQFPR pc2 P98160
p = 0.2, beta = −0.0576, N = 1250
AGLSSGFIGCVR pc2 P98160
p = 0.24, beta = −0.0527, N = 1255
VTVTSEGGR pc2 P98160
p = 0.51, beta = −0.0296, N = 1238
SIEYSPQLEDAGSR pc2 P98160
p = 0.83, beta = −0.00968, N = 1235
AELLVTEAPSK pc2 P98160
p = 0.16, beta = −0.0636, N = 1235
IPGDQVVSVVFIK pc2 P98160
p = 0.65, beta = −0.0206, N = 1254
MASVGLSDIAMDTTVTHATSHGR pc P98160
p = 0.34, beta = −0.0431, N = 1238
FDAGSGMATIR pc2 P98160
p = 0.12, beta = −0.0705, N = 1254
ALEVEECR pc2 P98160
p = 0.68, beta = −0.0189, N = 1220
CLCLPGFSGPR pc2 P98160
p = 0.35, beta = −0.0419, N = 1251
LEGDTLIIPR pc2 P98160
p = 0.75, beta = 0.0141, N = 1254
IAHVELADAGQYR pc3 P98160
p = 0.75, beta = −0.0148, N = 1228
SAEPLALGR pc2 P98160
p = 0.3, beta = 0.0484, N = 1176
AVTLECVSAGEPR pc2 P98160
p = 0.26, beta = −0.0504, N = 1242
AGFFGDAMK pc2 P98160
p = 0.14, beta = −0.0674, N = 1222
SGPVEDFVSLAMVGGHLEFR pc3 P98160
p = 0.2, beta = −0.0571, N = 1244
VQVSPER pc2 P98160
p = 0.49, beta = −0.0366, N = 931
HSPG2 : NP2 P98160
p = 0.97, beta = 0.00306, N = 325
YELGSGLAVLR pc2 P98160
p = 0.95, beta = −0.0046, N = 325
AGLSSGFIGCVR pc2 P98160
p = 0.83, beta = −0.0172, N = 325
LGTVPQFPR pc2 P98160
p = 0.67, beta = 0.0353, N = 310
SAEPLALGR pc2 P98160
p = 0.83, beta = −0.0178, N = 325
ALEVEECR pc2 P98160
p = 0.39, beta = −0.0703, N = 323
SIEYSPQLEDAGSR pc2 P98160
p = 0.76, beta = −0.0265, N = 296
IPGDQVVSVVFIK pc2 P98160
p = 0.44, beta = −0.0626, N = 324
AELLVTEAPSK pc2 P98160
p = 0.18, beta = −0.109, N = 322
VTVTSEGGR pc2 P98160
p = 0.73, beta = −0.0284, N = 320
SIGASVEFHCAVPSDR pc3 P98160
p = 0.88, beta = 0.0133, N = 269
AVTLECVSAGEPR pc2 P98160
p = 0.58, beta = −0.046, N = 319
LEGDTLIIPR pc2 P98160
p = 0.51, beta = 0.0532, N = 323
AGFFGDAMK pc2 P98160
p = 0.86, beta = −0.014, N = 322
SGPVEDFVSLAMVGGHLEFR pc3 P98160
p = 0.88, beta = −0.0119, N = 325
FDAGSGMATIR pc2 P98160
p = 0.085, beta = −0.143, N = 306
MASVGLSDIAMDTTVTHATSHGR pc P98160
p = 0.15, beta = −0.12, N = 297
NELLHFER pc2 P98160
p = 0.16, beta = −0.12, N = 300
GCVGEVSVNGK pc2 P98160
p = 0.95, beta = 0.0047, N = 314
CLIHDGAAPISLEWK pc3 P98160
p = 0.18, beta = −0.109, N = 322
Assay Target: HSPG2
UniProt: P98160
rsID: rs6695528
Target SNP: 1:22148164:G:A
Proxy SNP: 1:22148164:G:A
UKBPPP log10(p): 147.7
UKBPPP BETA: −0.169
UKBPPP MSPA: 0.011
1255:1239:1250:1255:1238:123
GSVYIGGAPDVATLTGGR pc2 rs3736360 REF
p = 1.3e−60, model = DOM, N = 1154
SPLPWQHR pc2 rs2291827 REF
p = 6.7e−12, model = REC, N = 463
GNVYIGGAPDVATLTGGR pc2 rs3736360 ALT
p = 3.8e−160, model = REC, N = 395

250. CAT (P04040)
UKBPPP MSPA=0

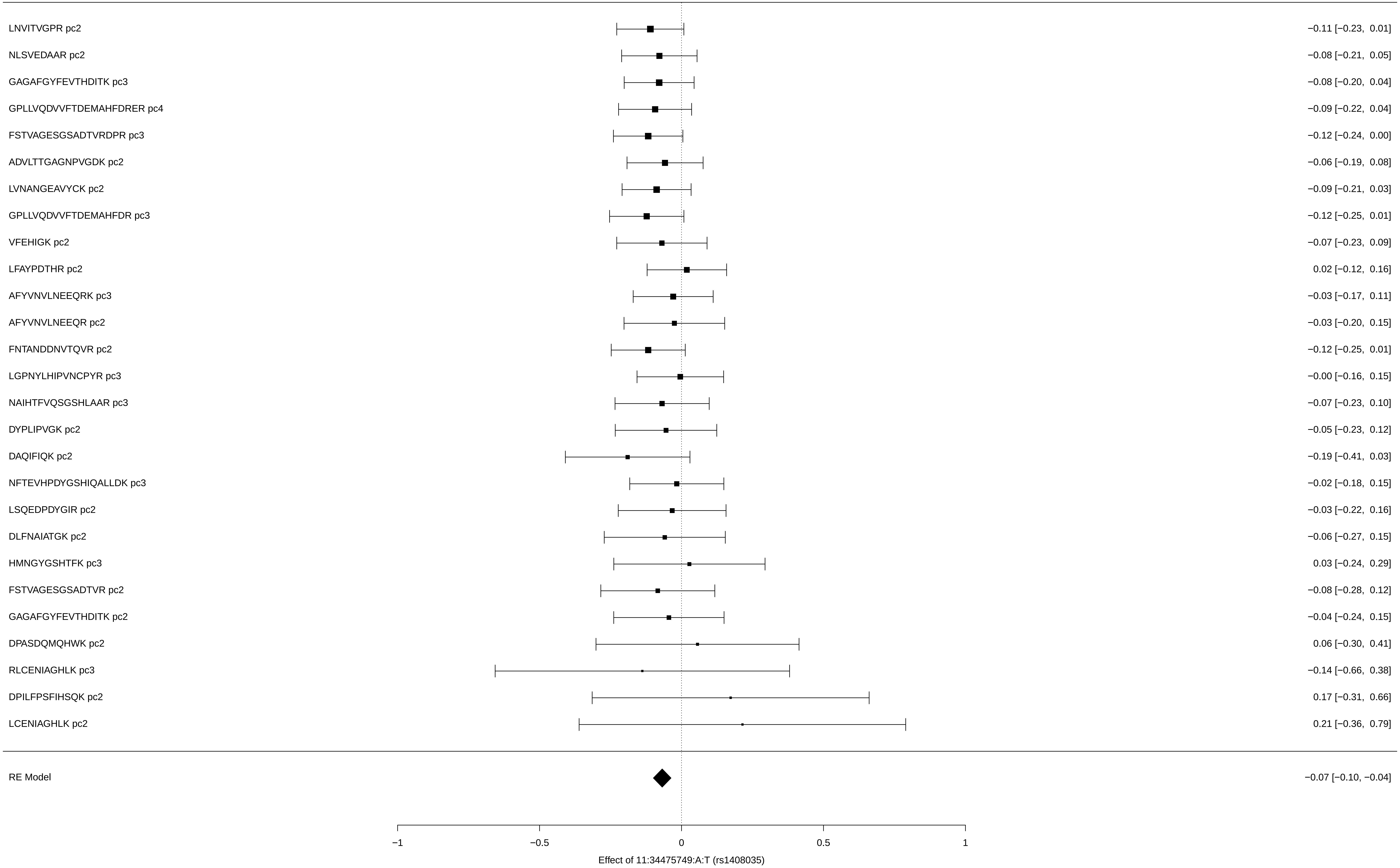

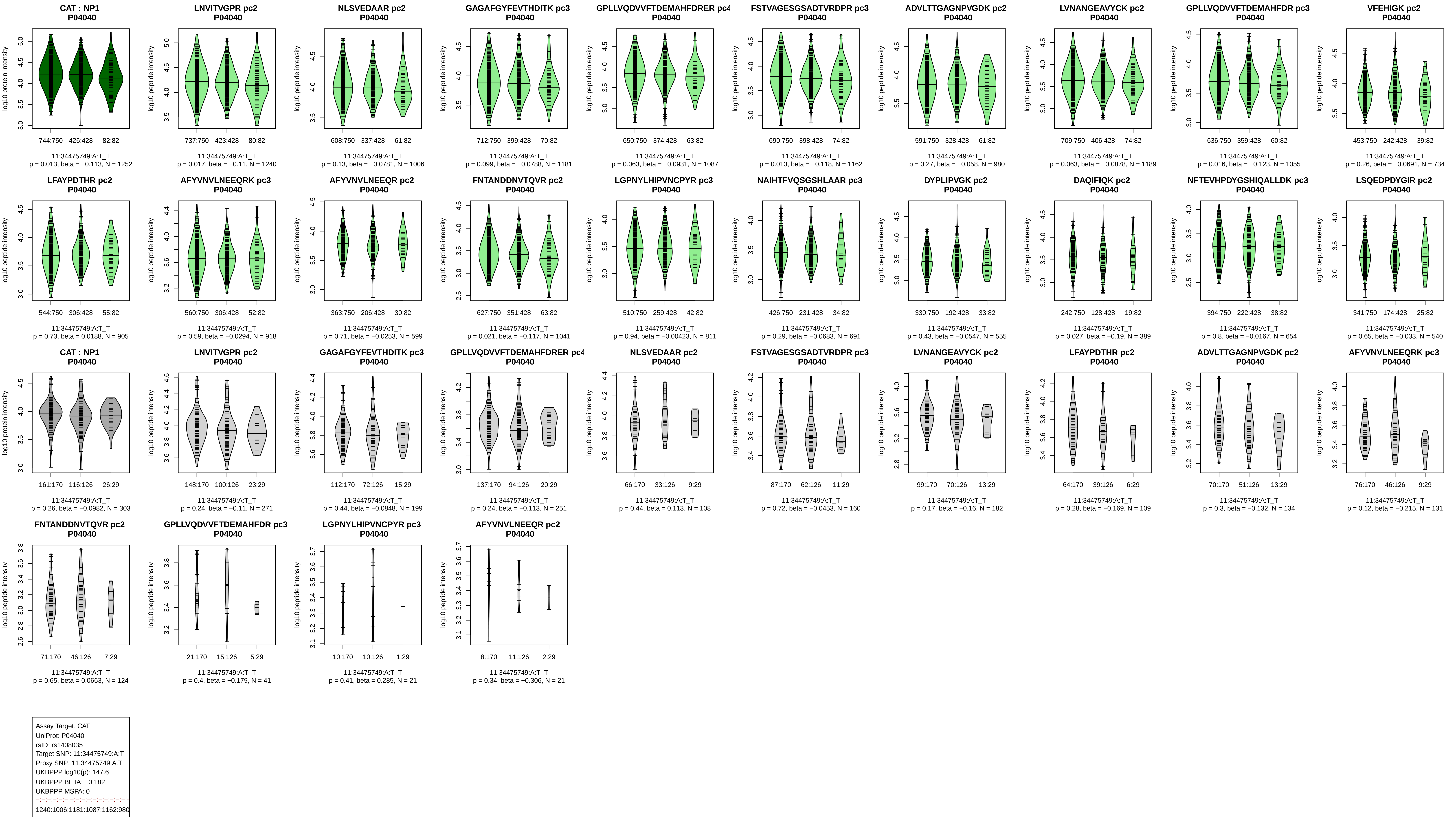
CAT : NP1
P04040
log10 protein intensity
744:750 426:428 82:82
11:34475749:A:T_T
p = 0.013, beta = −0.113, N = 1252
LNVITVGPR pc2
P04040
log10 peptide intensity
737:750 423:428 80:82
11:34475749:A:T_T
p = 0.017, beta = −0.11, N = 1240
NLSVEDAAR pc2
P04040
608:750 337:428 61:82
11:34475749:A:T_T
p = 0.13, beta = −0.0781, N = 1006
GAGAFGYFEVTHDITK pc3
P04040
712:750 399:428 70:82
11:34475749:A:T_T
p = 0.099, beta = −0.0788, N = 1181
GPLLVQDVVFTDEMAHFDRER pc4
P04040
650:750 374:428 63:82
11:34475749:A:T_T
p = 0.063, beta = −0.0931, N = 1087
FSTVAGESGSADTVRDPR pc3
P04040
690:750 398:428 74:82
11:34475749:A:T_T
p = 0.013, beta = −0.118, N = 1162
ADVLTTGAGNPVGDK pc2
P04040
591:750 328:428 61:82
11:34475749:A:T_T
p = 0.27, beta = −0.058, N = 980
LVNANGEAVYCK pc2
P04040
709:750 406:428 74:82
11:34475749:A:T_T
p = 0.063, beta = −0.0878, N = 1189
GPLLVQDVVFTDEMAHFDR pc3
P04040
636:750 359:428 60:82
11:34475749:A:T_T
p = 0.016, beta = −0.123, N = 1055
VFEHIGK pc2
P04040
453:750 242:428 39:82
11:34475749:A:T_T
p = 0.26, beta = −0.0691, N = 734
LFAYPDTHR pc2
P04040
544:750 306:428 55:82
11:34475749:A:T_T
p = 0.73, beta = 0.0188, N = 905
AFYVNVLNEEQRK pc3
P04040
560:750 306:428 52:82
11:34475749:A:T_T
p = 0.59, beta = −0.0294, N = 918
AFYVNVLNEEQR pc2
P04040
363:750 206:428 30:82
11:34475749:A:T_T
p = 0.71, beta = −0.0253, N = 599
FNTANDDNVTQVR pc2
P04040
627:750 351:428 63:82
11:34475749:A:T_T
p = 0.021, beta = −0.117, N = 1041
LGPNYLHIPVNCPYR pc3
P04040
510:750 259:428 42:82
11:34475749:A:T_T
p = 0.94, beta = −0.00423, N = 811
NAIHTFVQSGSHLAAR pc3
P04040
426:750 231:428 34:82
11:34475749:A:T_T
p = 0.29, beta = −0.0683, N = 691
DYPLIPVGK pc2
P04040
330:750 192:428 33:82
11:34475749:A:T_T
p = 0.43, beta = −0.0547, N = 555
DAQIFIQK pc2
P04040
242:750 128:428 19:82
11:34475749:A:T_T
p = 0.027, beta = −0.19, N = 389
NFTEVHPDYGSHIQALLDK pc3
P04040
394:750 222:428 38:82
11:34475749:A:T_T
p = 0.8, beta = −0.0167, N = 654
LSQEDPDYGIR pc2
P04040
341:750 174:428 25:82
11:34475749:A:T_T
p = 0.65, beta = −0.033, N = 540
CAT : NP1
P04040
161:170 116:126 26:29
11:34475749:A:T_T
p = 0.26, beta = −0.0982, N = 303
LNVITVGPR pc2
P04040
148:170 100:126 23:29
11:34475749:A:T_T
p = 0.24, beta = −0.11, N = 271
GAGAFGYFEVTHDITK pc3
P04040
112:170 72:126 15:29
11:34475749:A:T_T
p = 0.44, beta = −0.0848, N = 199
GPLLVQDVVFTDEMAHFDRER pc4
P04040
137:170 94:126 20:29
11:34475749:A:T_T
p = 0.24, beta = −0.113, N = 251
NLSVEDAAR pc2
P04040
66:170 33:126 9:29
11:34475749:A:T_T
p = 0.44, beta = 0.113, N = 108
FSTVAGESGSADTVRDPR pc3
P04040
87:170 62:126 11:29
11:34475749:A:T_T
p = 0.72, beta = −0.0453, N = 160
LVNANGEAVYCK pc2
P04040
99:170 70:126 13:29
11:34475749:A:T_T
p = 0.17, beta = −0.16, N = 182
LFAYPDTHR pc2
P04040
64:170 39:126 6:29
11:34475749:A:T_T
p = 0.28, beta = −0.169, N = 109
ADVLTTGAGNPVGDK pc2
P04040
70:170 51:126 13:29
11:34475749:A:T_T
p = 0.3, beta = −0.132, N = 134
AFYVNVLNEEQRK pc3
P04040
76:170 46:126 9:29
11:34475749:A:T_T
p = 0.12, beta = −0.215, N = 131
FNTANDDNVTQVR pc2
P04040
71:170 46:126 7:29
11:34475749:A:T_T
p = 0.65, beta = 0.0663, N = 124
GPLLVQDVVFTDEMAHFDR pc3
P04040
21:170 15:126 5:29
11:34475749:A:T_T
p = 0.4, beta = −0.179, N = 41
LGPNYLHIPVNCPYR pc3
P04040
10:170 10:126 1:29
11:34475749:A:T_T
p = 0.41, beta = 0.285, N = 21
AFYVNVLNEEQR pc2
P04040
8:170 11:126 2:29
11:34475749:A:T_T
p = 0.34, beta = −0.306, N = 21
Assay Target: CAT
UniProt: P04040
rsID: rs1408035
Target SNP: 11:34475749:A:T
Proxy SNP: 11:34475749:A:T
UKBPPP log10(p): 147.6
UKBPPP BETA: −0.182
UKBPPP MSPA: 0
1240:1006:1181:1087:1162:980

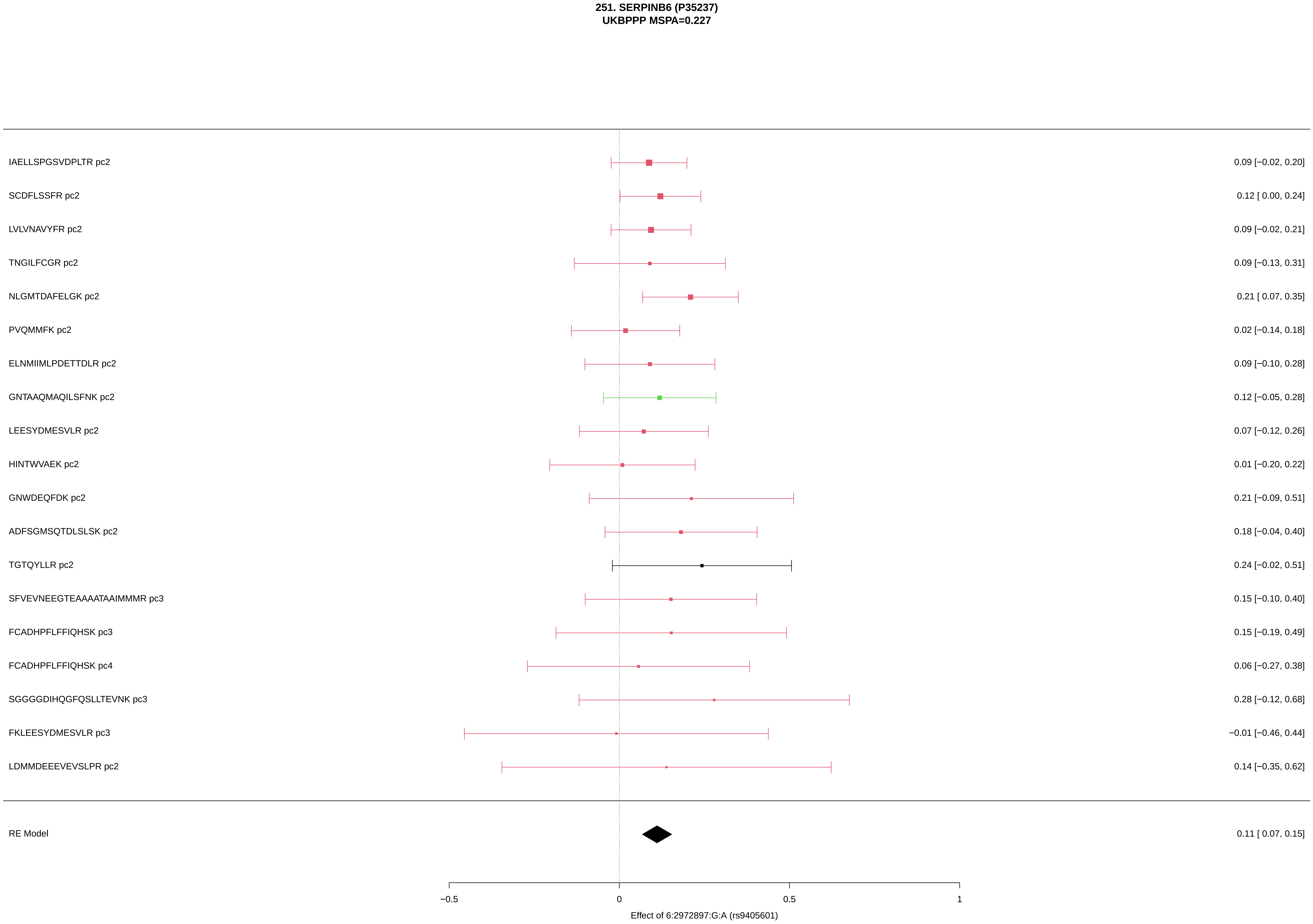
251. SERPINB6 (P35237)
UKBPPP MSPA=0.227
IAELLSPGSVDPLTR pc2
0.09 [−0.02, 0.20]
SCDFLSSFR pc2
0.12 [0.00, 0.24]
LVLVNAVYFR pc2
0.09 [−0.02, 0.21]
TNGILFCGR pc2
0.09 [−0.13, 0.31]
NLGMTDAFELGK pc2
0.21 [0.07, 0.35]
PVQMMFK pc2
0.02 [−0.14, 0.18]
ELNMIIMLPDETTDLR pc2
0.09 [−0.10, 0.28]
GNTAAQMAQILSFNK pc2
0.12 [−0.05, 0.28]
LEESYDMESVLR pc2
0.07 [−0.12, 0.26]
HINTWVAEK pc2
0.01 [−0.20, 0.22]
GNWDEQFDK pc2
0.21 [−0.09, 0.51]
ADFSGMSQTDLSLSK pc2
0.18 [−0.04, 0.40]
TGTQYLLR pc2
0.24 [−0.02, 0.51]
SFVEVNEEGTEAAAATAAIMMMR pc3
0.15 [−0.10, 0.40]
FCADHPFLFFIQHSK pc3
0.15 [−0.19, 0.49]
FCADHPFLFFIQHSK pc4
0.06 [−0.27, 0.38]
SGGGGDIHQGFQSLLTEVNK pc3
0.28 [−0.12, 0.68]
FKLEESYDMESVLR pc3
−0.01 [−0.46, 0.44]
LDMMDEEEVEVSLPR pc2
0.14 [−0.35, 0.62]
RE Model
0.11 [0.07, 0.15]
−0.5
0
0.5
1
Effect of 6:2972897:G:A (rs9405601)

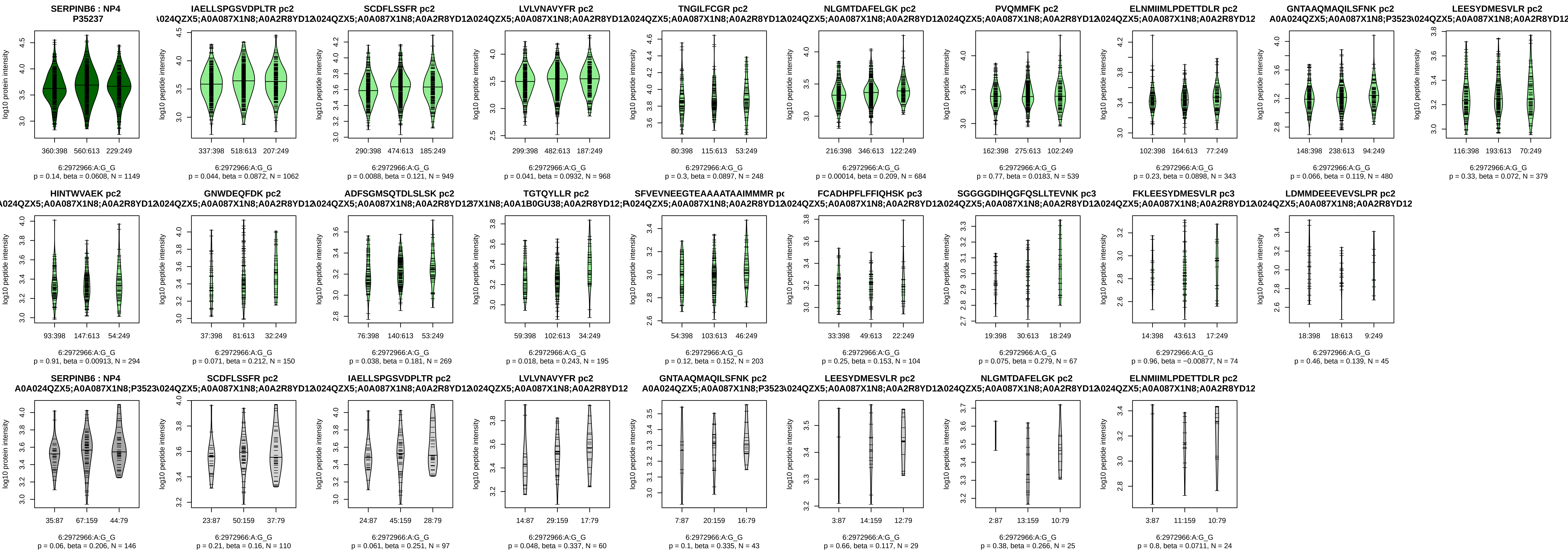

Assay Target: SERPINB6
UniProt: P35237
rsID: rs9405601
Target SNP: 6:2972897:G:A
Proxy SNP: 6:2972966:A:G
UKBPPP log10(p): 147.3
UKBPPP BETA: 0.159
UKBPPP MSPA: 0.227
1062:949:968:248:684:539:343:

252. LAMA4 (Q16363)
UKBPPP MSPA=NANA

Meta−analysis failed

c(0, 1)

Effect of 6:112453123:C:T (rs9320394)

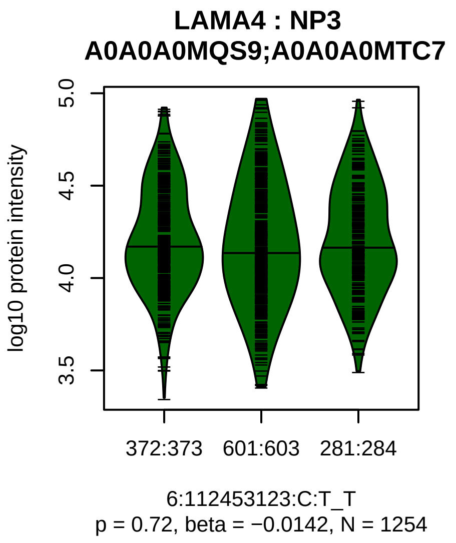

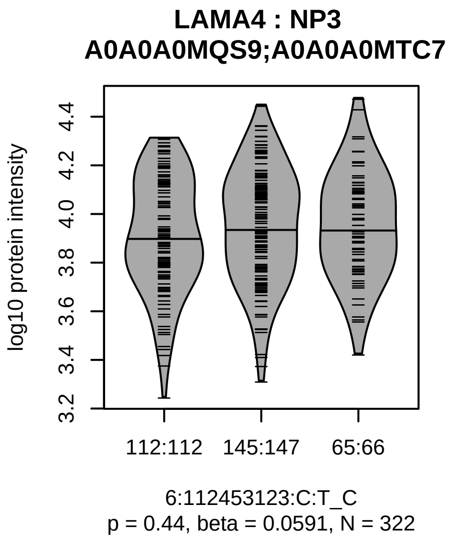

Assay Target: LAMA4
UniProt: Q16363
rsID: rs9320394
Target SNP: 6:112453123:C:T
Proxy SNP: 6:112453123:C:T
UKBPPP log10(p): 145.9
UKBPPP BETA: 0.158
UKBPPP MSPA: NANA
NA
2

253. AGER (Q15109)
UKBPPP MSPA=0.03

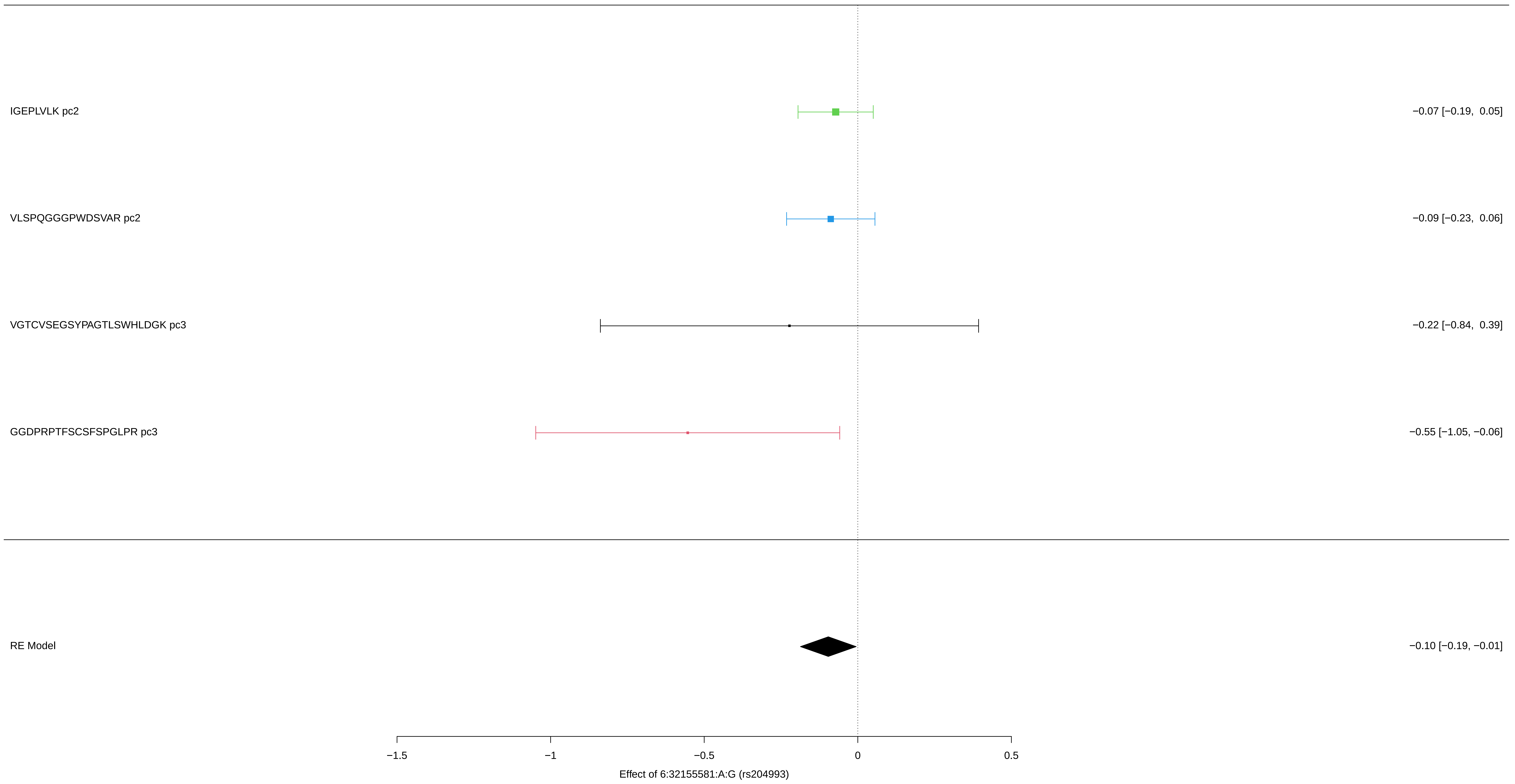

AGER : NP3
Q15109−3

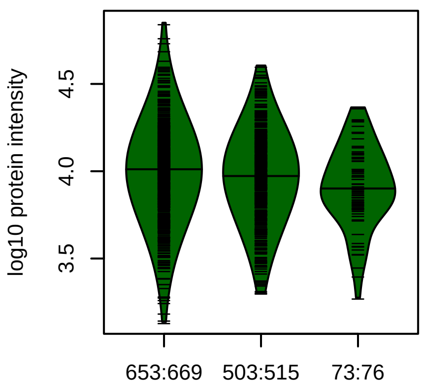

6:32155581:A:G_G
p = 0.025, beta = −0.105, N = 1229

IGEPLVLK pc2
Q15109−2;Q15109−3;Q15109−4;Q151A982;

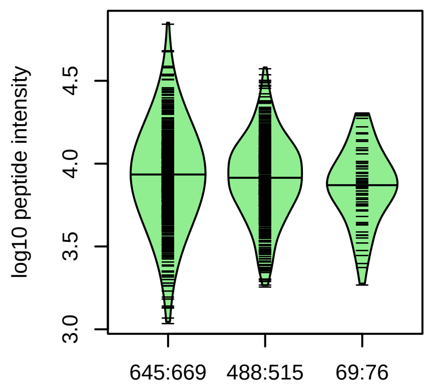

6:32155581:A:G_G
p = 0.13, beta = −0.0722, N = 1202

VLSPQGGGPWDSVAR pc2
Q15109−3;Q15109−4;Q15109−6;

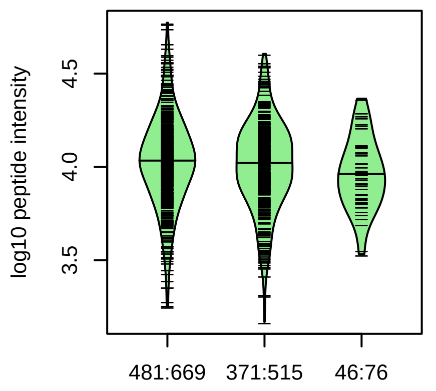

6:32155581:A:G_G
p = 0.12, beta = −0.088, N = 898

VGTCVSEGSYPAGTLSWHLDGK pc
A8MS87;Q15109−2;Q15109−3;Q151

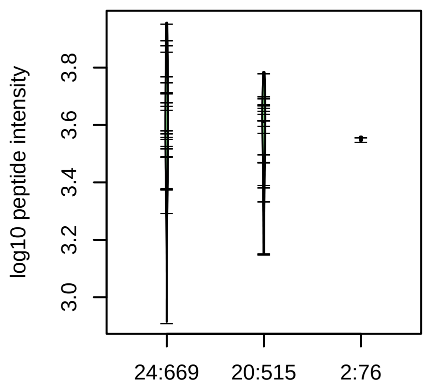

6:32155581:A:G_G
p = 0.36, beta = −0.222, N = 46

GGDPRPTFSCSFSPGLPR pc3
Q15109−2;Q15109−3;Q15109−4;Q151

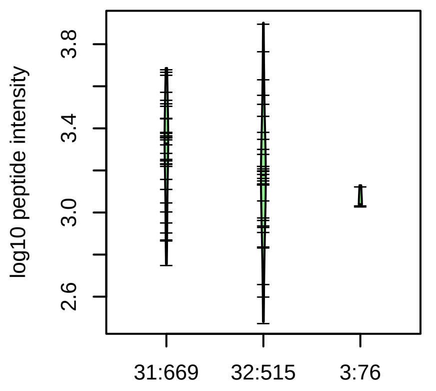

6:32155581:A:G_G
p = 0.0054, beta = −0.554, N = 66

AGER : NP3
Q15109−3;Q15109−4

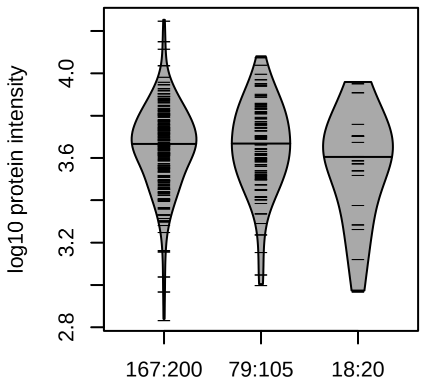

6:32155581:A:G_G
p = 0.82, beta = −0.0228, N = 264

IGEPLVLK pc2
Q15109−2;Q15109−3;Q15109−4;Q151A982;

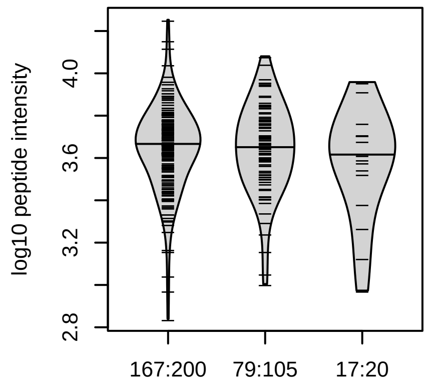

6:32155581:A:G_G
p = 0.86, beta = −0.0176, N = 263

VLSPQGGGPWDSVAR pc2
Q15109−3;Q15109−4;Q15109−6;

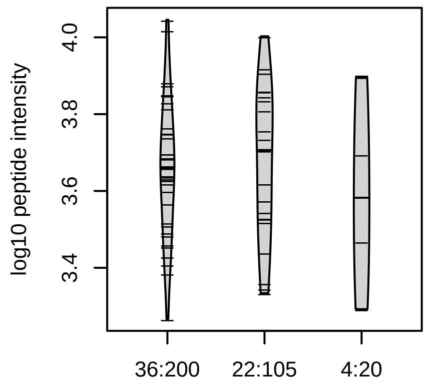

6:32155581:A:G_G
p = 0.88, beta = 0.0311, N = 62

Assay Target: AGER
UniProt: Q15109
rsID: rs204993
Target SNP: 6:32155581:A:G
Proxy SNP: 6:32155581:A:G
UKBPPP log10(p): 142.4
UKBPPP BETA: −0.16
UKBPPP MSPA: 0.03
−:−:−:*
1202:898:46:66

254. PARK7 (Q99497)
UKBPPP MSPA=0

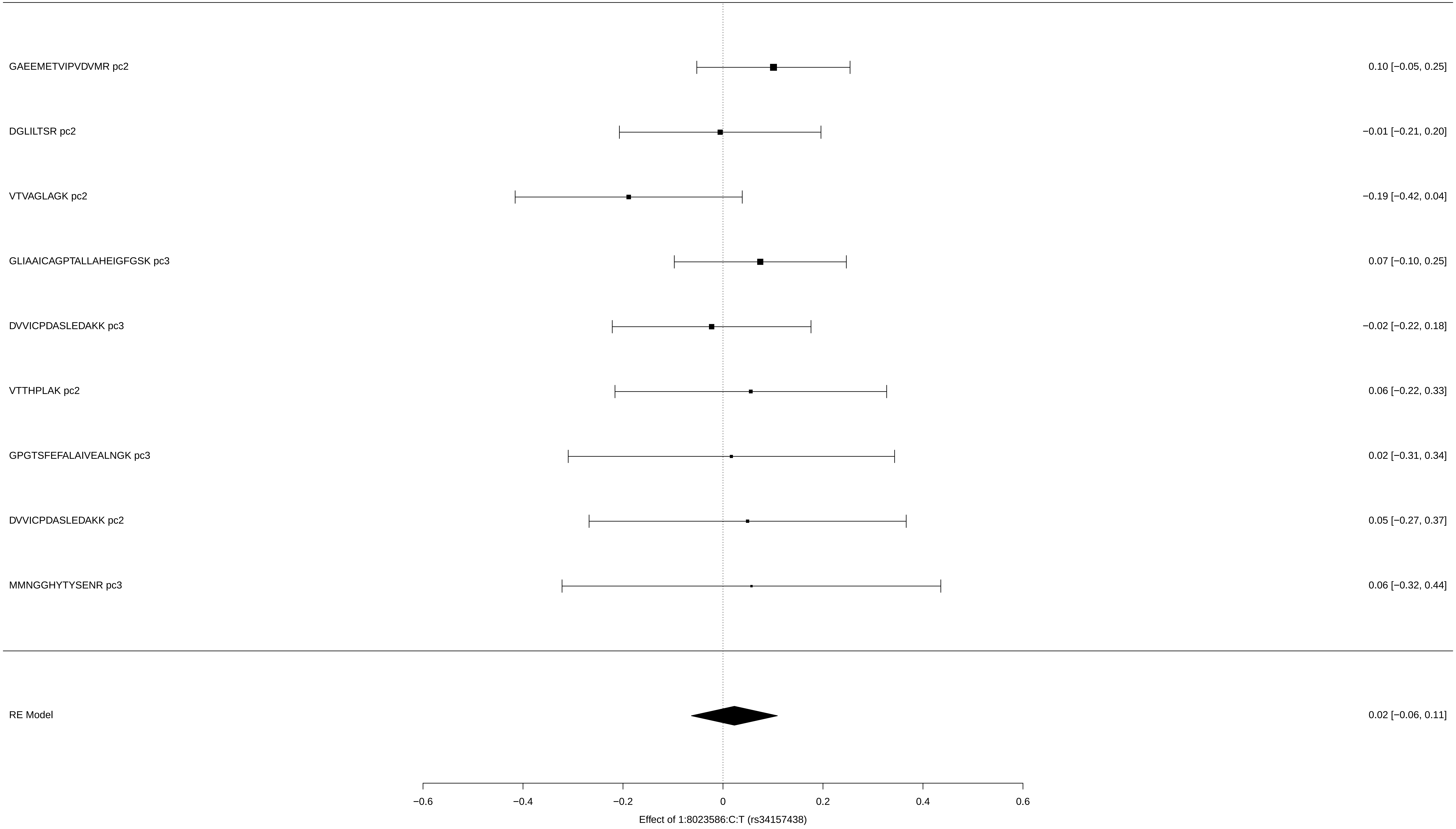

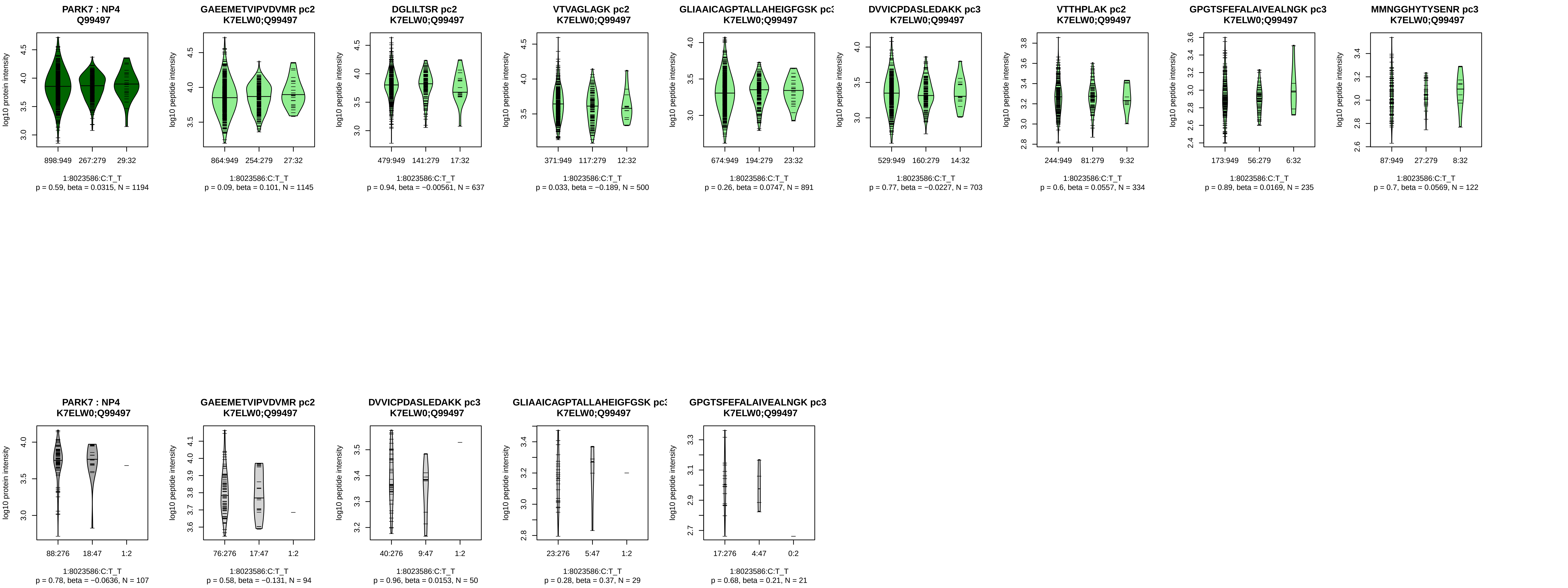

Assay Target: PARK7
UniProt: Q99497
rsID: rs34157438
Target SNP: 1:8023586:C:T
Proxy SNP: 1:8023586:C:T
UKBPPP log10(p): 141.4
UKBPPP BETA: 0.206
UKBPPP MSPA: 0
–:–:–:–:–:–:–:–:–:NA
1145:637:500:891:703:334:235:

255. COL18A1 (P39060)
UKBPPP MSPA=0.059

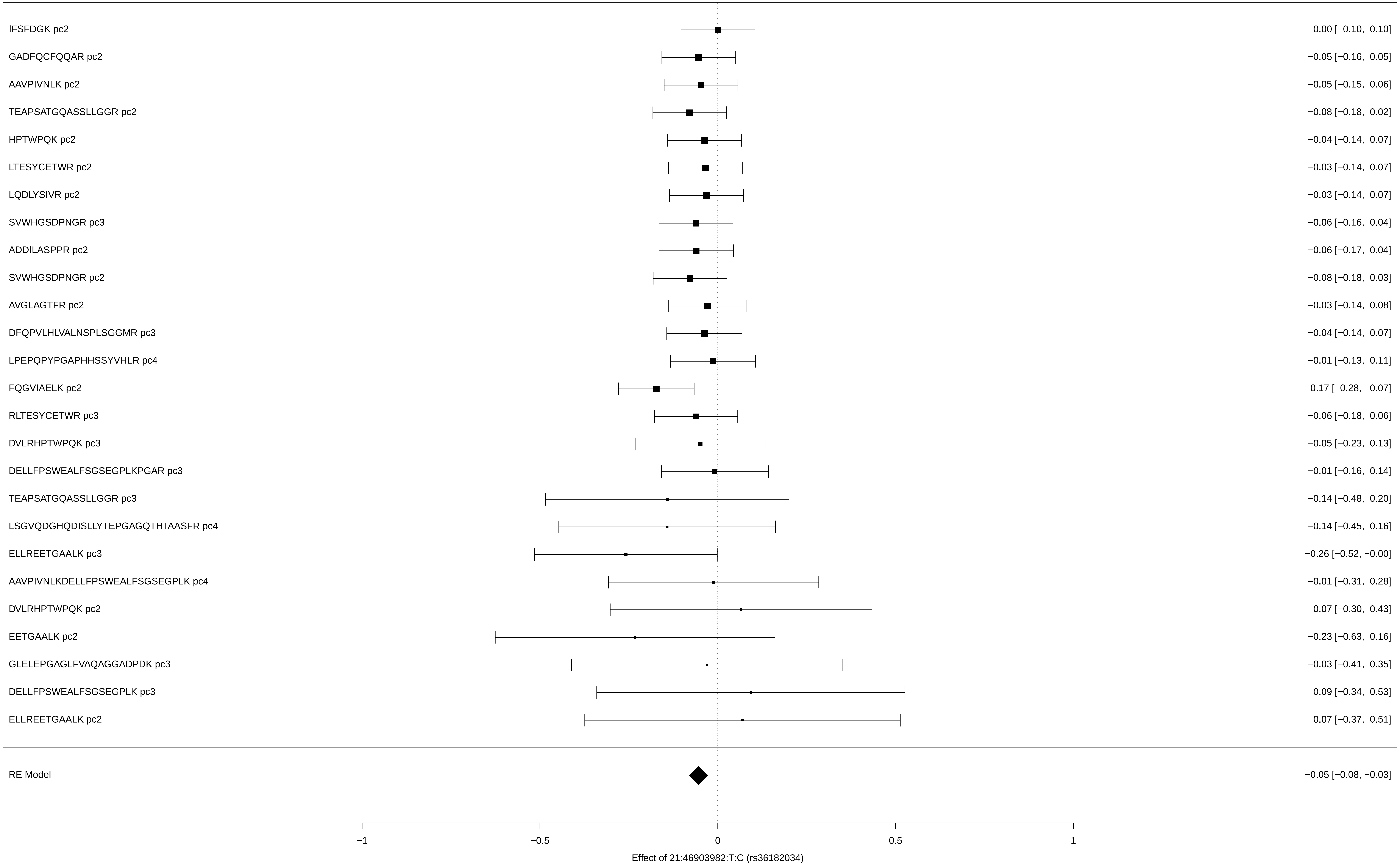

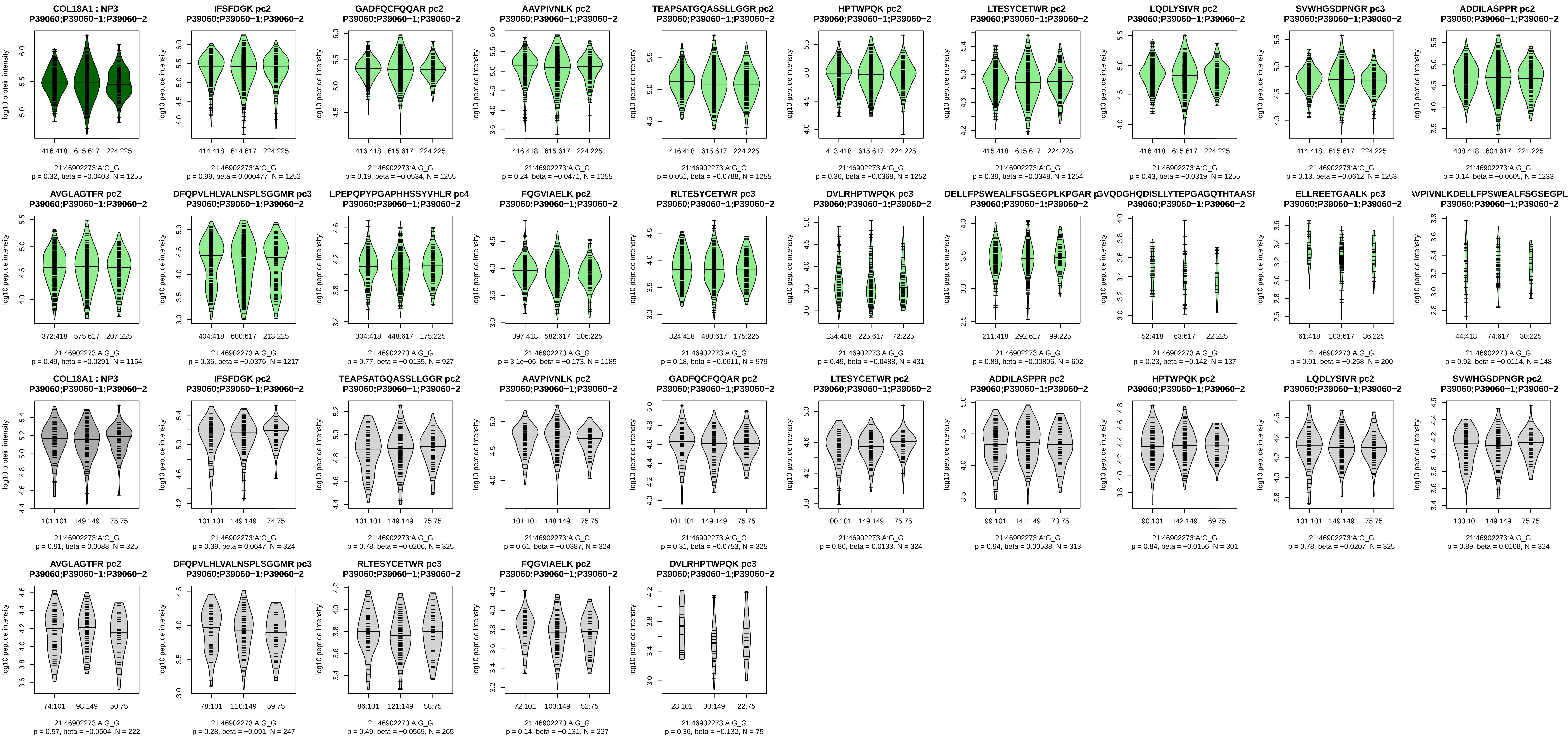

Assay Target: COL18A1
UniProt: P39060
rsID: rs36182034
Target SNP: 21:46903982:T:C
Proxy SNP: 21:46902273:A:G
UKBPPP log10(p): 140.7
UKBPPP BETA: −0.153
UKBPPP MSPA: 0.059
1252:1255:1255:1255:1252:125

256. CHMP6 (Q96FZ7)
UKBPPP MSPA=0.49

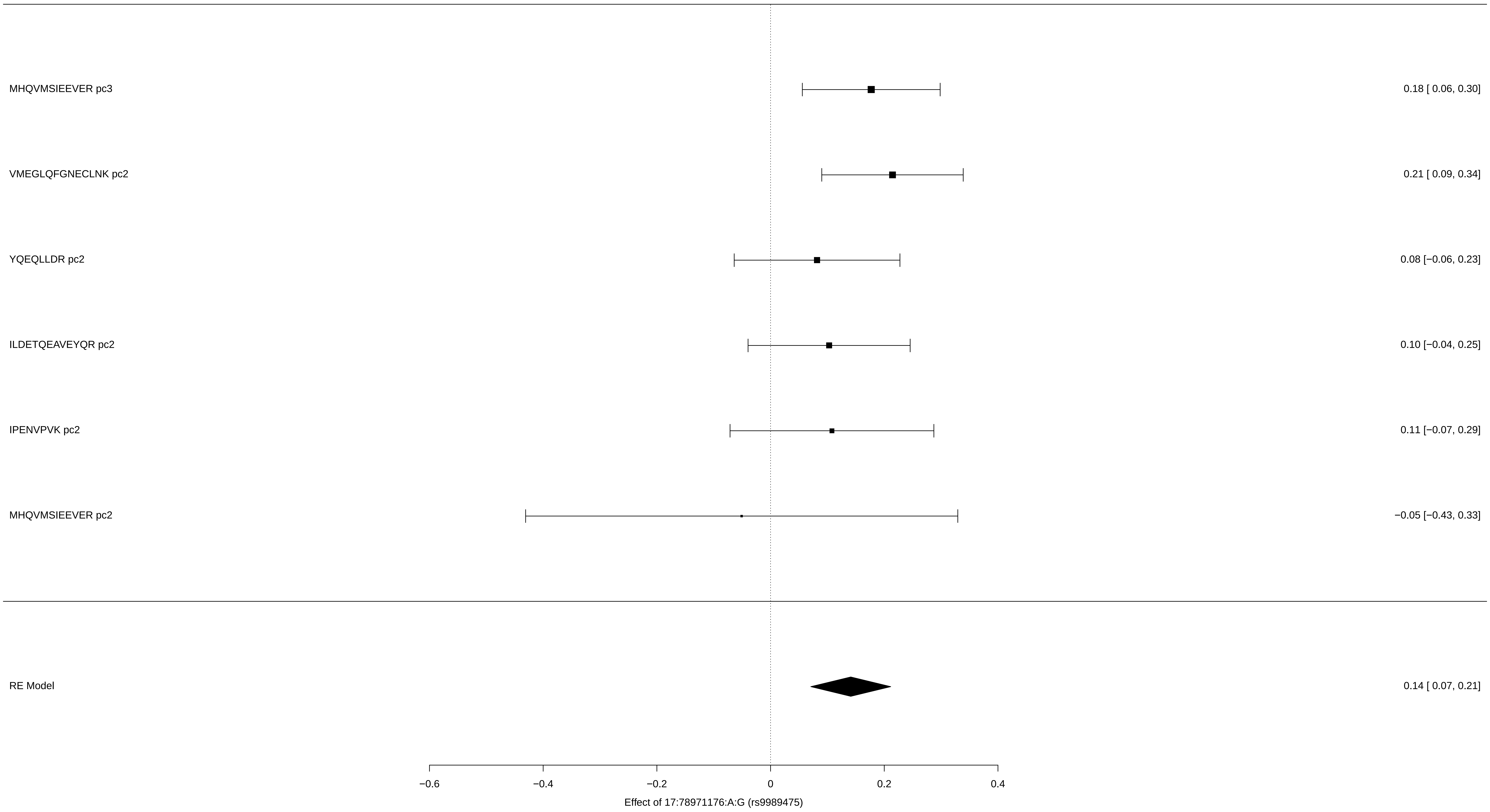

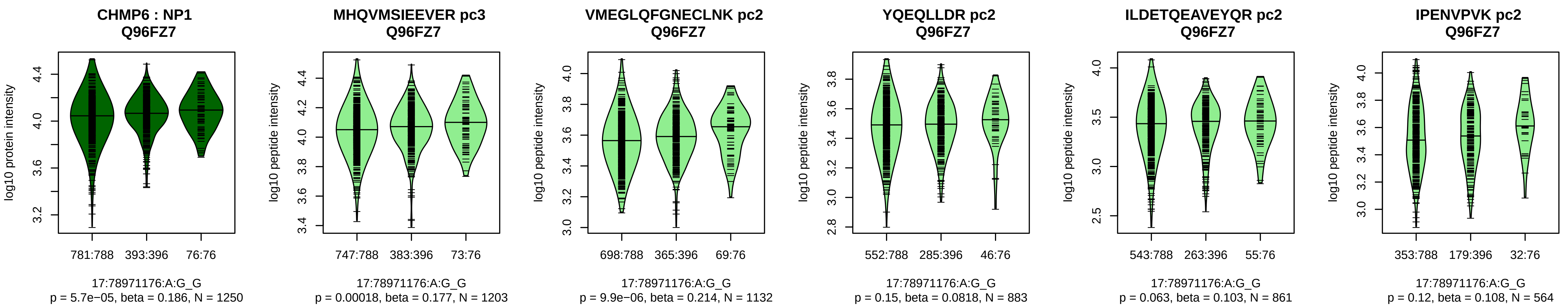

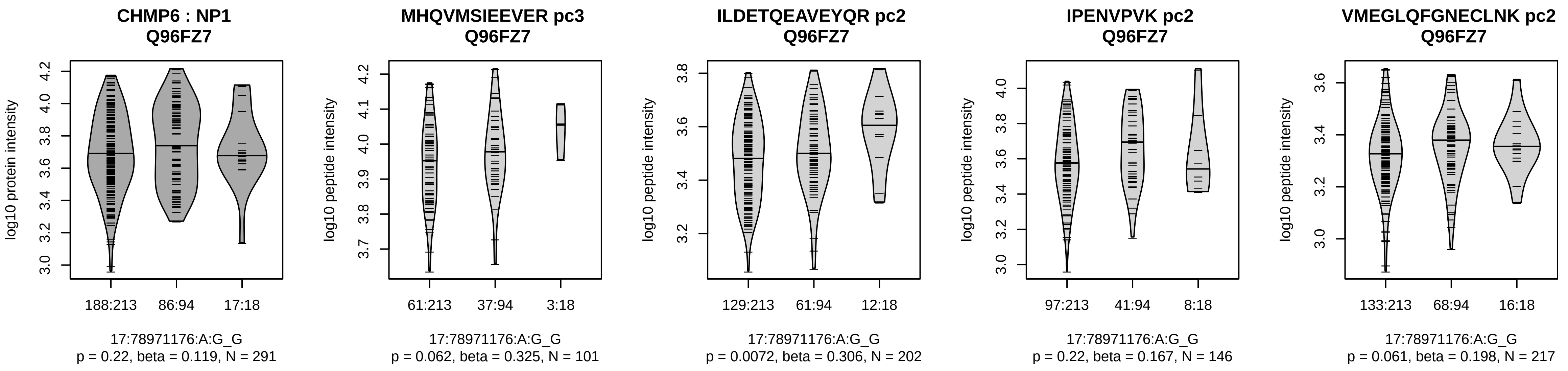

Assay Target: CHMP6
UniProt: Q96FZ7
rsID: rs9989475
Target SNP: 17:78971176:A:G
Proxy SNP: 17:78971176:A:G
UKBPPP log10(p): 139.3
UKBPPP BETA: 0.201
UKBPPP MSPA: 0.49
::–:–:–:–
1203:1132:883:861:564:118

257. PDE5A (O76074)
UKBPPP MSPA=0.604

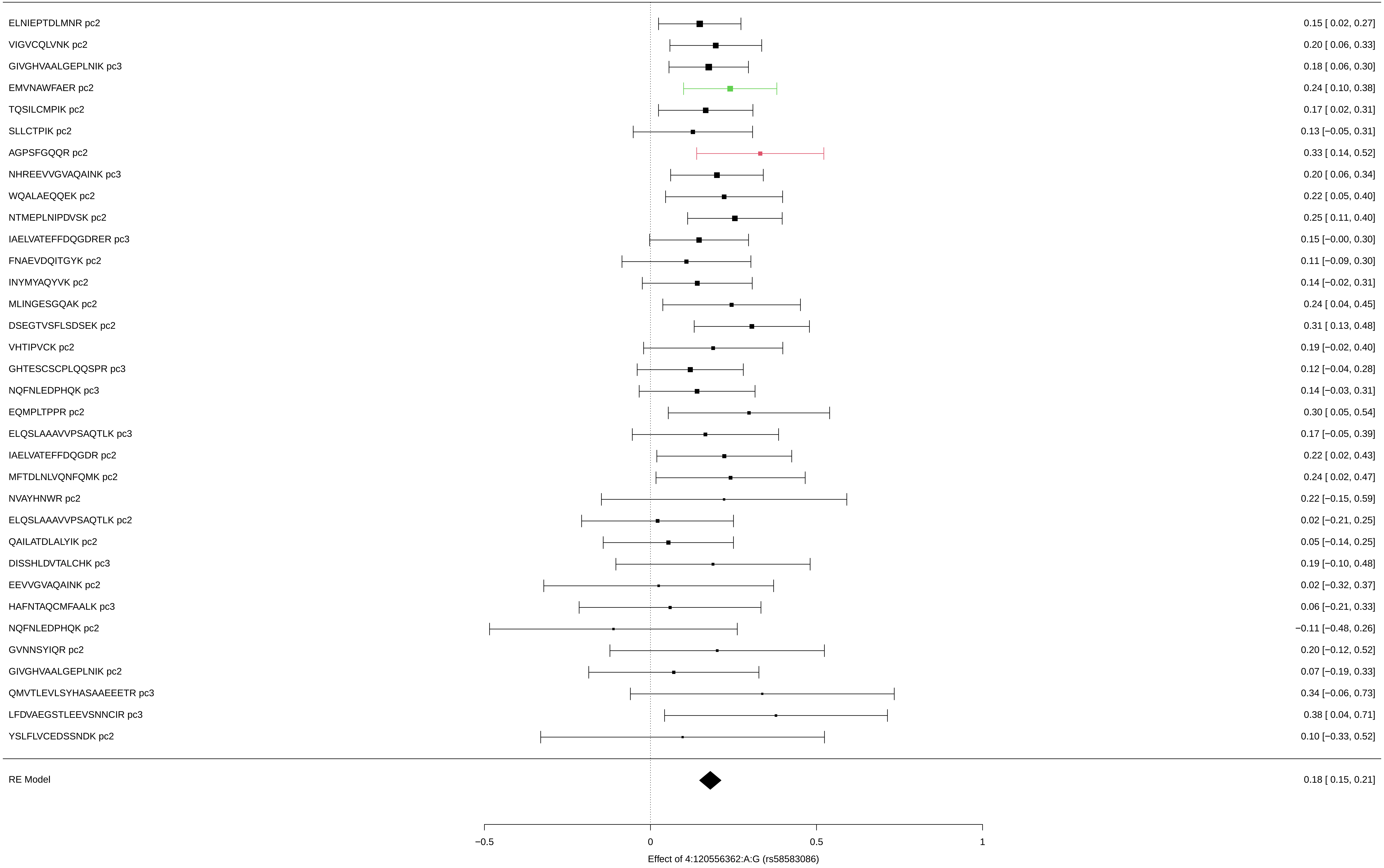

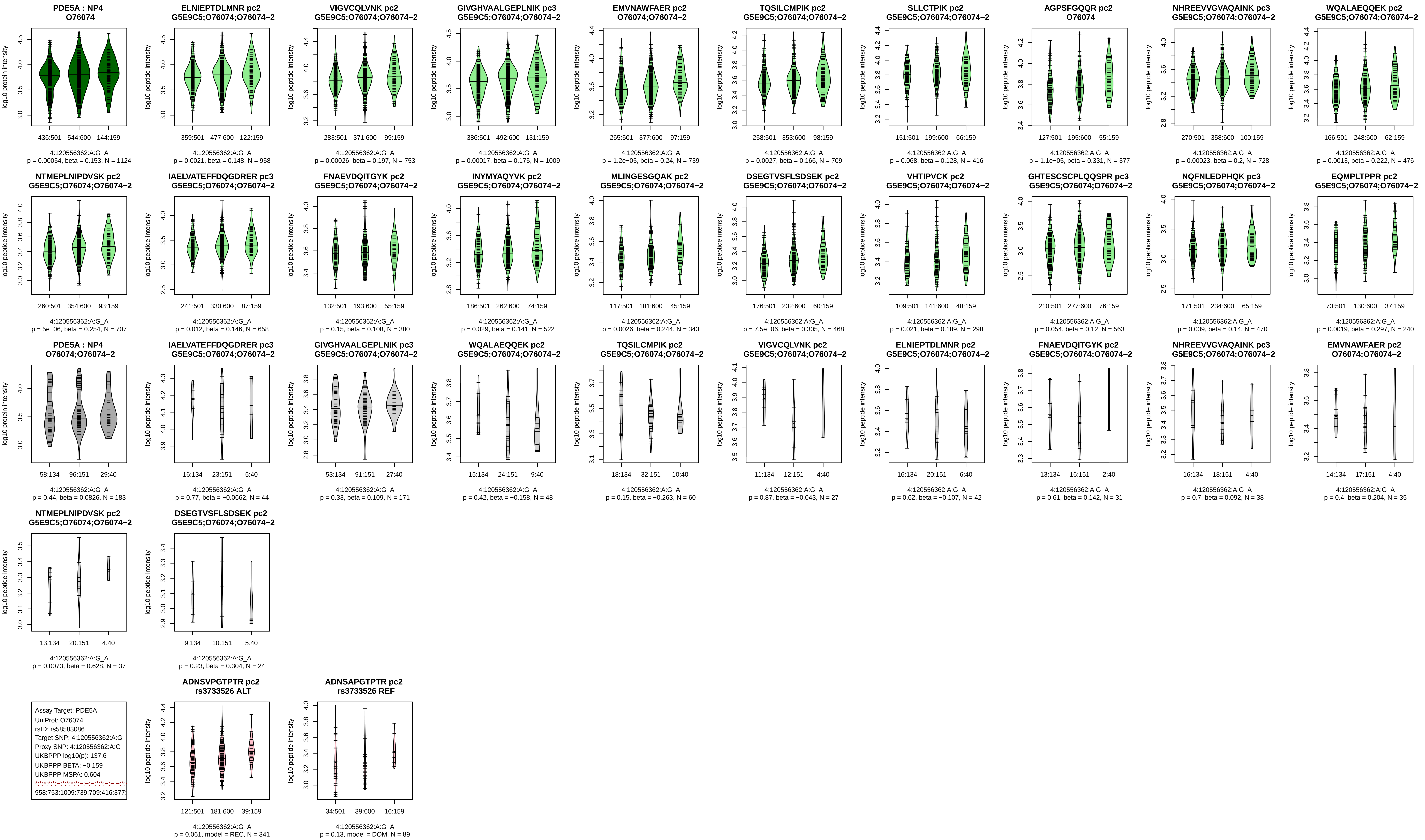
PDE5A : NP4
O76074
436:501 544:600 144:159
4:120556362:A:G_A
p = 0.00054, beta = 0.153, N = 1124
ELNIEPTDLMNR pc2
G5E9C5;O76074;O76074−2
359:501 477:600 122:159
4:120556362:A:G_A
p = 0.0021, beta = 0.148, N = 958
VIGVCQLVNK pc2
G5E9C5;O76074;O76074−2
283:501 371:600 99:159
4:120556362:A:G_A
p = 0.00026, beta = 0.197, N = 753
GIVGHVAALGEPLNIK pc3
G5E9C5;O76074;O76074−2
386:501 492:600 131:159
4:120556362:A:G_A
p = 0.00017, beta = 0.175, N = 1009
EMVNAWFAER pc2
O76074;O76074−2
265:501 377:600 97:159
4:120556362:A:G_A
p = 1.2e−05, beta = 0.24, N = 739
TQSILCMPIK pc2
G5E9C5;O76074;O76074−2
258:501 353:600 98:159
4:120556362:A:G_A
p = 0.0027, beta = 0.166, N = 709
SLLCTPIK pc2
G5E9C5;O76074;O76074−2
151:501 199:600 66:159
4:120556362:A:G_A
p = 0.068, beta = 0.128, N = 416
AGPSFGQQR pc2
O76074
127:501 195:600 55:159
4:120556362:A:G_A
p = 1.1e−05, beta = 0.331, N = 377
NHREEVVGVAQAINK pc3
G5E9C5;O76074;O76074−2
270:501 358:600 100:159
4:120556362:A:G_A
p = 0.00023, beta = 0.2, N = 728
WQALAEQQEK pc2
G5E9C5;O76074;O76074−2
166:501 248:600 62:159
4:120556362:A:G_A
p = 0.0013, beta = 0.222, N = 476
NTMEPLNIPDVSK pc2
G5E9C5;O76074;O76074−2
260:501 354:600 93:159
4:120556362:A:G_A
p = 5e−06, beta = 0.254, N = 707
IAELVATEFFDQGDRER pc3
G5E9C5;O76074;O76074−2
241:501 330:600 87:159
4:120556362:A:G_A
p = 0.012, beta = 0.146, N = 658
FNAEVDQITGYK pc2
G5E9C5;O76074;O76074−2
132:501 193:600 55:159
4:120556362:A:G_A
p = 0.15, beta = 0.108, N = 380
INYMYAQYVK pc2
G5E9C5;O76074;O76074−2
186:501 262:600 74:159
4:120556362:A:G_A
p = 0.029, beta = 0.141, N = 522
MLINGESGQAK pc2
G5E9C5;O76074;O76074−2
117:501 181:600 45:159
4:120556362:A:G_A
p = 0.0026, beta = 0.244, N = 343
DSEGTVSFLSDSEK pc2
G5E9C5;O76074;O76074−2
176:501 232:600 60:159
4:120556362:A:G_A
p = 7.5e−06, beta = 0.305, N = 468
VHTIPVCK pc2
G5E9C5;O76074;O76074−2
109:501 141:600 48:159
4:120556362:A:G_A
p = 0.021, beta = 0.189, N = 298
GHTESCSCPLQQSPR pc3
G5E9C5;O76074;O76074−2
210:501 277:600 76:159
4:120556362:A:G_A
p = 0.054, beta = 0.12, N = 563
NQFNLEDPHQK pc3
G5E9C5;O76074;O76074−2
171:501 234:600 65:159
4:120556362:A:G_A
p = 0.039, beta = 0.14, N = 470
EQMPLTPPR pc2
G5E9C5;O76074;O76074−2
73:501 130:600 37:159
4:120556362:A:G_A
p = 0.0019, beta = 0.297, N = 240
PDE5A : NP4
O76074;O76074−2
58:134 96:151 29:40
4:120556362:A:G_A
p = 0.44, beta = 0.0826, N = 183
IAELVATEFFDQGDRER pc3
G5E9C5;O76074;O76074−2
16:134 23:151 5:40
4:120556362:A:G_A
p = 0.77, beta = −0.0662, N = 44
GIVGHVAALGEPLNIK pc3
G5E9C5;O76074;O76074−2
53:134 91:151 27:40
4:120556362:A:G_A
p = 0.33, beta = 0.109, N = 171
WQALAEQQEK pc2
G5E9C5;O76074;O76074−2
15:134 24:151 9:40
4:120556362:A:G_A
p = 0.42, beta = −0.158, N = 48
TQSILCMPIK pc2
G5E9C5;O76074;O76074−2
18:134 32:151 10:40
4:120556362:A:G_A
p = 0.15, beta = −0.263, N = 60
VIGVCQLVNK pc2
G5E9C5;O76074;O76074−2
11:134 12:151 4:40
4:120556362:A:G_A
p = 0.87, beta = −0.043, N = 27
ELNIEPTDLMNR pc2
G5E9C5;O76074;O76074−2
16:134 20:151 6:40
4:120556362:A:G_A
p = 0.62, beta = −0.107, N = 42
FNAEVDQITGYK pc2
G5E9C5;O76074;O76074−2
13:134 16:151 2:40
4:120556362:A:G_A
p = 0.61, beta = 0.142, N = 31
NHREEVVGVAQAINK pc3
G5E9C5;O76074;O76074−2
16:134 18:151 4:40
4:120556362:A:G_A
p = 0.7, beta = 0.092, N = 38
EMVNAWFAER pc2
O76074;O76074−2
14:134 17:151 4:40
4:120556362:A:G_A
p = 0.4, beta = 0.204, N = 35
NTMEPLNIPDVSK pc2
G5E9C5;O76074;O76074−2
13:134 20:151 4:40
4:120556362:A:G_A
p = 0.0073, beta = 0.628, N = 37
DSEGTVSFLSDSEK pc2
G5E9C5;O76074;O76074−2
9:134 10:151 5:40
4:120556362:A:G_A
p = 0.23, beta = 0.304, N = 24
Assay Target: PDE5A
UniProt: O76074
rsID: rs58583086
Target SNP: 4:120556362:A:G
Proxy SNP: 4:120556362:A:G
UKBPPP log10(p): 137.6
UKBPPP BETA: −0.159
UKBPPP MSPA: 0.604
958:753:1009:739:709:416:377:
ADNSVPGTPTR pc2
rs3733526 ALT
121:501 181:600 39:159
4:120556362:A:G_A
p = 0.061, model = REC, N = 341
ADNSAPGTPTR pc2
rs3733526 REF
34:501 39:600 16:159
4:120556362:A:G_A
p = 0.13, model = DOM, N = 89

258. HPCAL1 (P37235)
UKBPPP MSPA=0.616

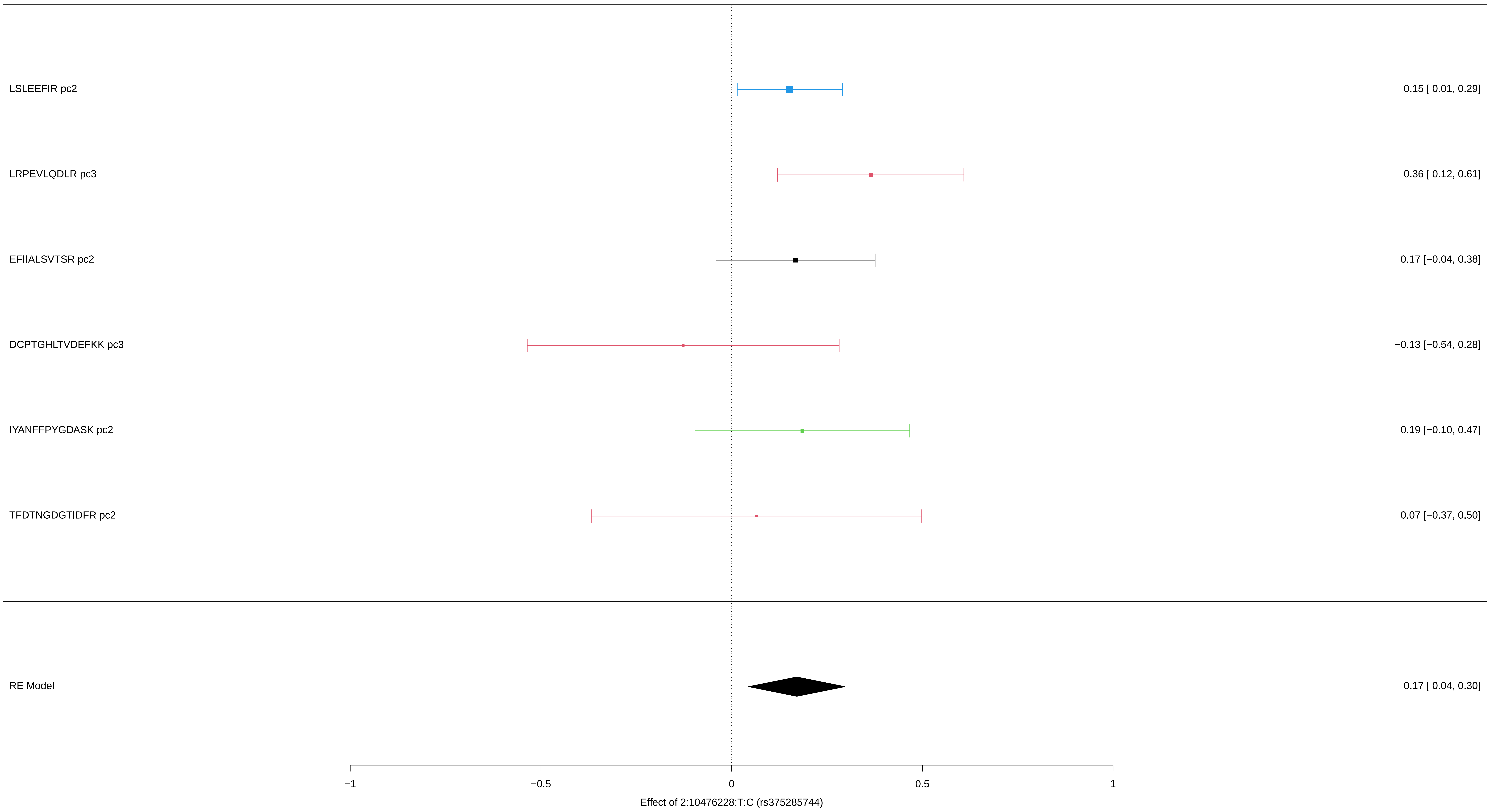

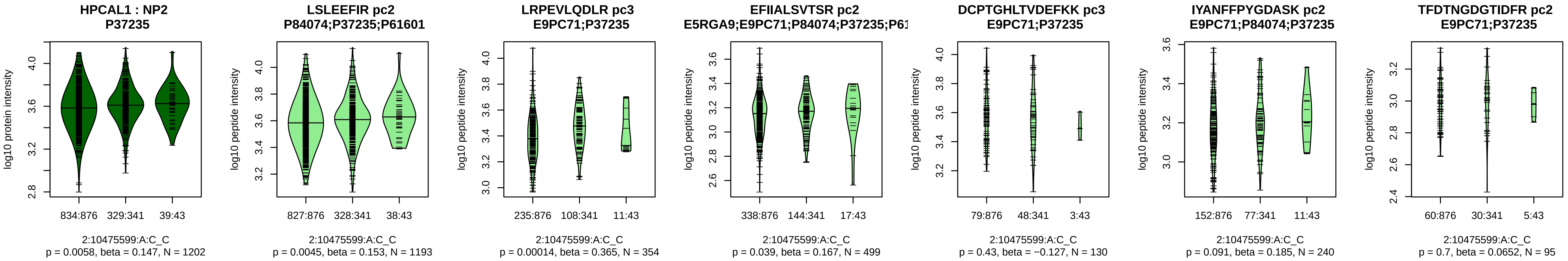

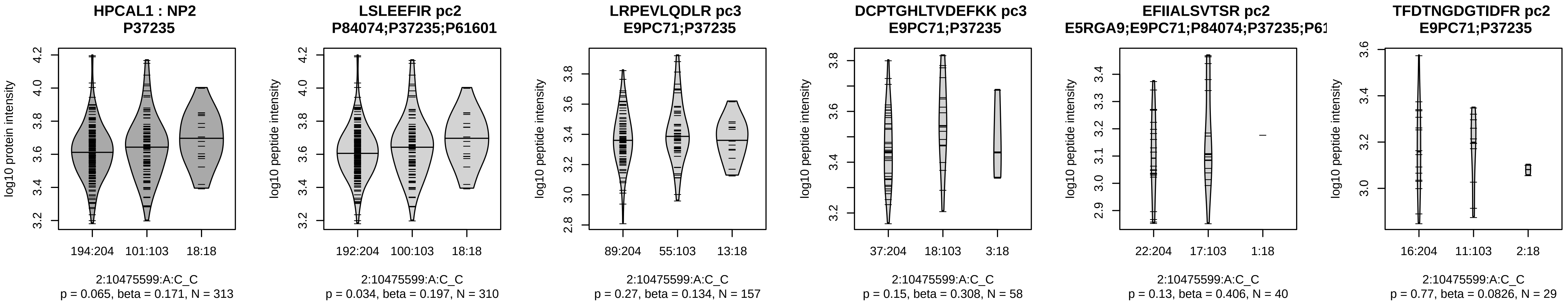

Assay Target: HPCAL1
UniProt: P37235
rsID: rs375285744
Target SNP: 2:10476228:T:C
Proxy SNP: 2:10475599:A:C
UKBPPP log10(p): 137
UKBPPP BETA: 0.213
UKBPPP MSPA: 0.616
::–:–:–:–:NA
1193:354:499:130:240:95:2

259. NCAN (O14594)
UKBPPP MSPA=0.149

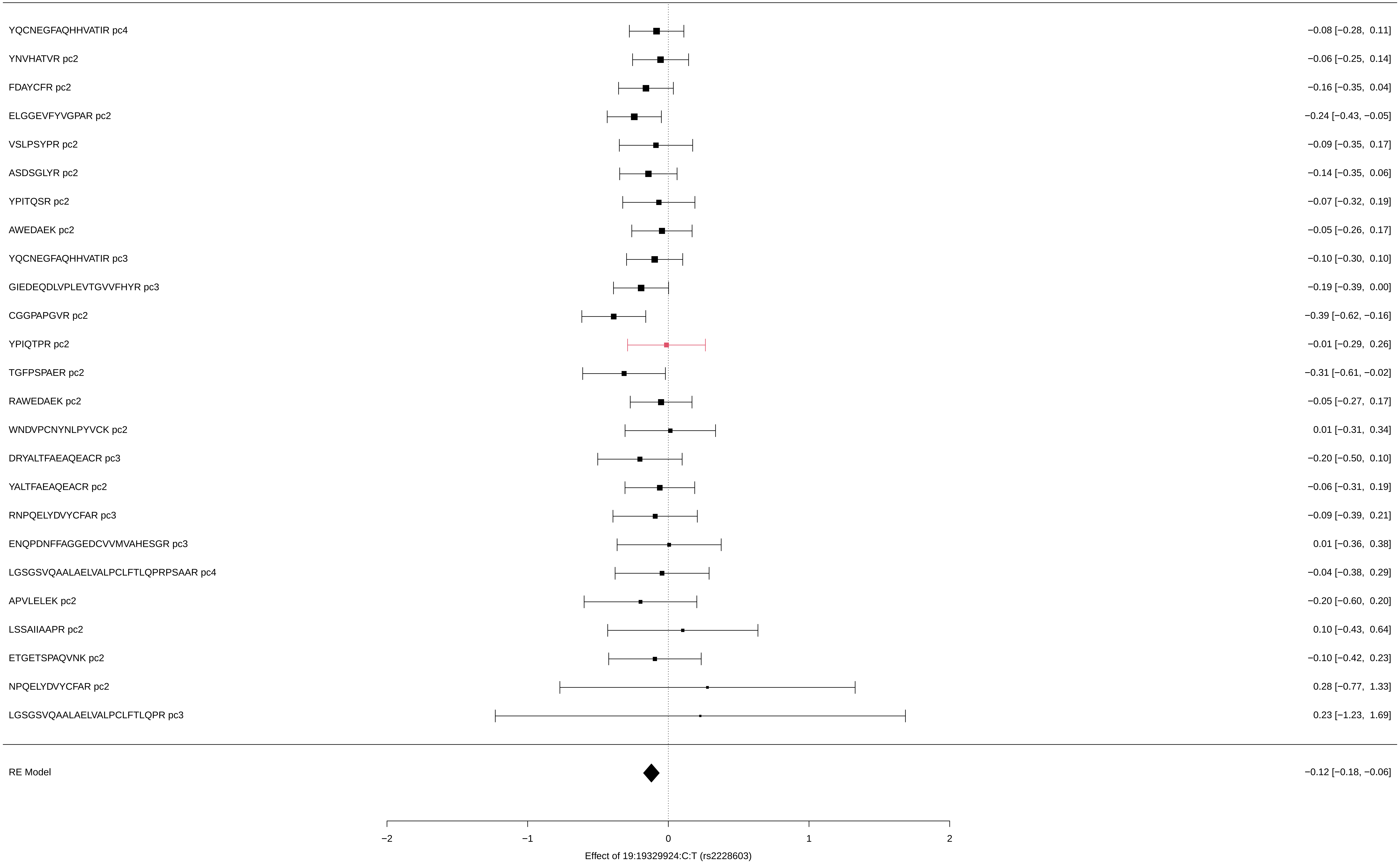

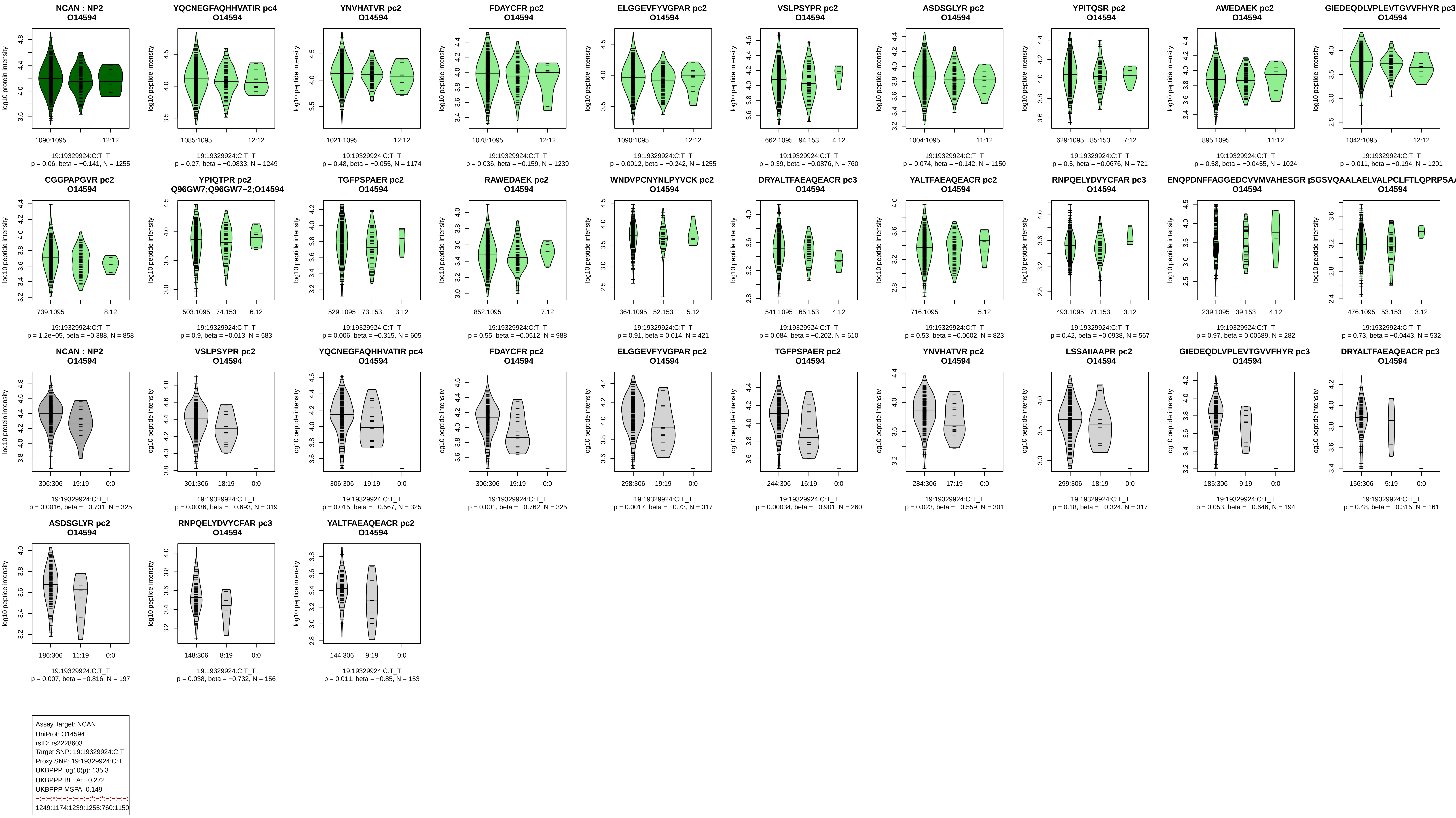
NCAN : NP2
O14594
log10 protein intensity
1090:1095
12:12
19:19329924:C:T_T
p = 0.06, beta = −0.141, N = 1255
YQCNEGFAQHHVATIR pc4
O14594
log10 peptide intensity
1085:1095
12:12
19:19329924:C:T_T
p = 0.27, beta = −0.0833, N = 1249
YNVHATVR pc2
O14594
1021:1095
12:12
19:19329924:C:T_T
p = 0.48, beta = −0.055, N = 1174
FDAYCFR pc2
O14594
1078:1095
12:12
19:19329924:C:T_T
p = 0.036, beta = −0.159, N = 1239
ELGGEVFYVGPAR pc2
O14594
1090:1095
12:12
19:19329924:C:T_T
p = 0.0012, beta = −0.242, N = 1255
VSLPSYPR pc2
O14594
662:1095
94:153
4:12
19:19329924:C:T_T
p = 0.39, beta = −0.0876, N = 760
ASDSGLYR pc2
O14594
1004:1095
11:12
19:19329924:C:T_T
p = 0.074, beta = −0.142, N = 1150
YPITQSR pc2
O14594
629:1095
85:153
7:12
19:19329924:C:T_T
p = 0.5, beta = −0.0676, N = 721
AWEDAEK pc2
O14594
895:1095
11:12
19:19329924:C:T_T
p = 0.58, beta = −0.0455, N = 1024
GIEDEQDLVPLEVTGVVFHYR pc3
O14594
1042:1095
12:12
19:19329924:C:T_T
p = 0.011, beta = −0.194, N = 1201
CGGPAPGVR pc2
O14594
739:1095
8:12
19:19329924:C:T_T
p = 1.2e−05, beta = −0.388, N = 858
YPIQTPR pc2
Q96GW7;Q96GW7−2;O14594
503:1095
74:153
6:12
19:19329924:C:T_T
p = 0.9, beta = −0.013, N = 583
TGFPSPAER pc2
O14594
529:1095
73:153
3:12
19:19329924:C:T_T
p = 0.006, beta = −0.315, N = 605
RAWEDAEK pc2
O14594
852:1095
7:12
19:19329924:C:T_T
p = 0.55, beta = −0.0512, N = 988
WNDVPCNYNLPYVCK pc2
O14594
364:1095
52:153
5:12
19:19329924:C:T_T
p = 0.91, beta = 0.014, N = 421
DRYALTFAEAQEACR pc3
O14594
541:1095
65:153
4:12
19:19329924:C:T_T
p = 0.084, beta = −0.202, N = 610
YALTFAEAQEACR pc2
O14594
716:1095
5:12
19:19329924:C:T_T
p = 0.53, beta = −0.0602, N = 823
RNPQELYDVYCFAR pc3
O14594
493:1095
71:153
3:12
19:19329924:C:T_T
p = 0.42, beta = −0.0938, N = 567
ENQPDNFFAGGEDCVVMVAHESGR
O14594
239:1095
39:153
4:12
19:19329924:C:T_T
p = 0.97, beta = 0.00589, N = 282
SGSVQAALAELVALPCLFTLQPRPSA
O14594
476:1095
53:153
3:12
19:19329924:C:T_T
p = 0.73, beta = −0.0443, N = 532
NCAN : NP2
O14594
306:306
19:19
0:0
19:19329924:C:T_T
p = 0.0016, beta = −0.731, N = 325
VSLPSYPR pc2
O14594
301:306
18:19
0:0
19:19329924:C:T_T
p = 0.0036, beta = −0.693, N = 319
YQCNEGFAQHHVATIR pc4
O14594
306:306
19:19
0:0
19:19329924:C:T_T
p = 0.015, beta = −0.567, N = 325
FDAYCFR pc2
O14594
306:306
19:19
0:0
19:19329924:C:T_T
p = 0.001, beta = −0.762, N = 325
ELGGEVFYVGPAR pc2
O14594
298:306
19:19
0:0
19:19329924:C:T_T
p = 0.0017, beta = −0.73, N = 317
TGFPSPAER pc2
O14594
244:306
16:19
0:0
19:19329924:C:T_T
p = 0.00034, beta = −0.901, N = 260
YNVHATVR pc2
O14594
284:306
17:19
0:0
19:19329924:C:T_T
p = 0.023, beta = −0.559, N = 301
LSSAIIAAPR pc2
O14594
299:306
18:19
0:0
19:19329924:C:T_T
p = 0.18, beta = −0.324, N = 317
GIEDEQDLVPLEVTGVVFHYR pc3
O14594
185:306
9:19
0:0
19:19329924:C:T_T
p = 0.053, beta = −0.646, N = 194
DRYALTFAEAQEACR pc3
O14594
156:306
5:19
0:0
19:19329924:C:T_T
p = 0.48, beta = −0.315, N = 161
ASDSGLYR pc2
O14594
186:306
11:19
0:0
19:19329924:C:T_T
p = 0.007, beta = −0.816, N = 197
RNPQELYDVYCFAR pc3
O14594
148:306
8:19
0:0
19:19329924:C:T_T
p = 0.038, beta = −0.732, N = 156
YALTFAEAQEACR pc2
O14594
144:306
9:19
0:0
19:19329924:C:T_T
p = 0.011, beta = −0.85, N = 153
Assay Target: NCAN
UniProt: O14594
rsID: rs2228603
Target SNP: 19:19329924:C:T
Proxy SNP: 19:19329924:C:T
UKBPPP log10(p): 135.3
UKBPPP BETA: −0.272
UKBPPP MSPA: 0.149
1249:1174:1239:1255:760:1150

260. CTSL (P07711)
UKBPPP MSPA=0

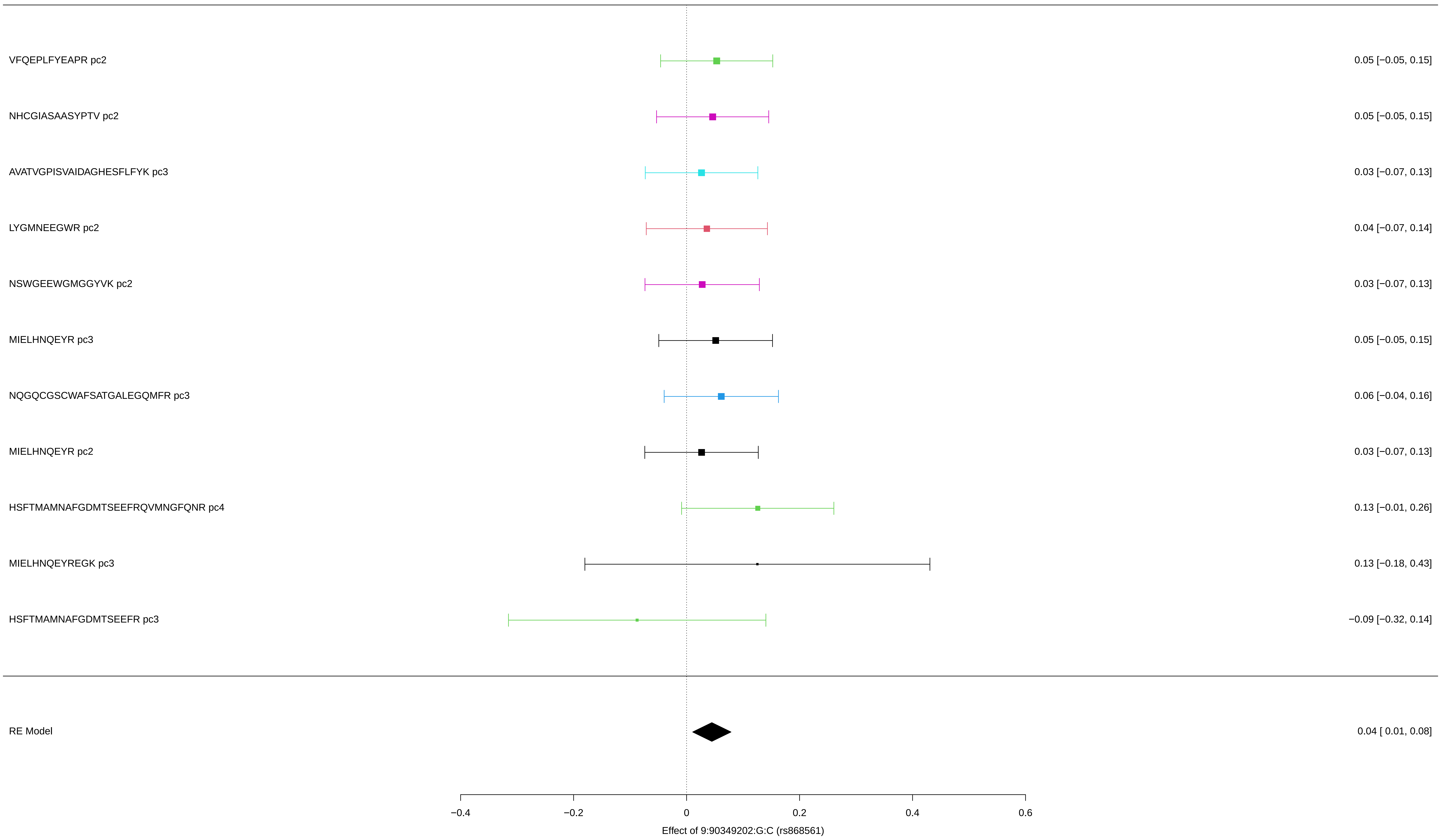

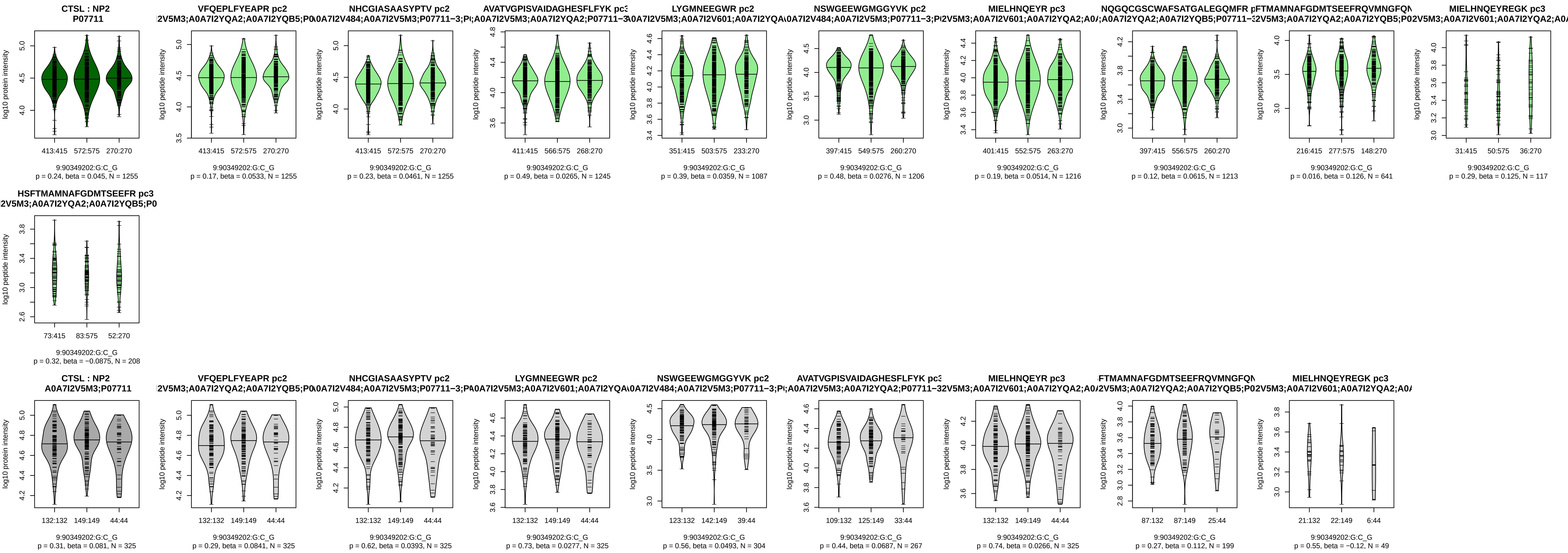

Assay Target: CTSL
UniProt: P07711
rsID: rs868561
Target SNP: 9:90349202:G:C
Proxy SNP: 9:90349202:G:C
UKBPPP log10(p): 133
UKBPPP BETA: −0.145
UKBPPP MSPA: 0
−:−:−:−:−:−:−:−:−:−:NA
1255:1255:1245:1087:1206:121

261. ASGR1 (P07306)
UKBPPP MSPA=0.045

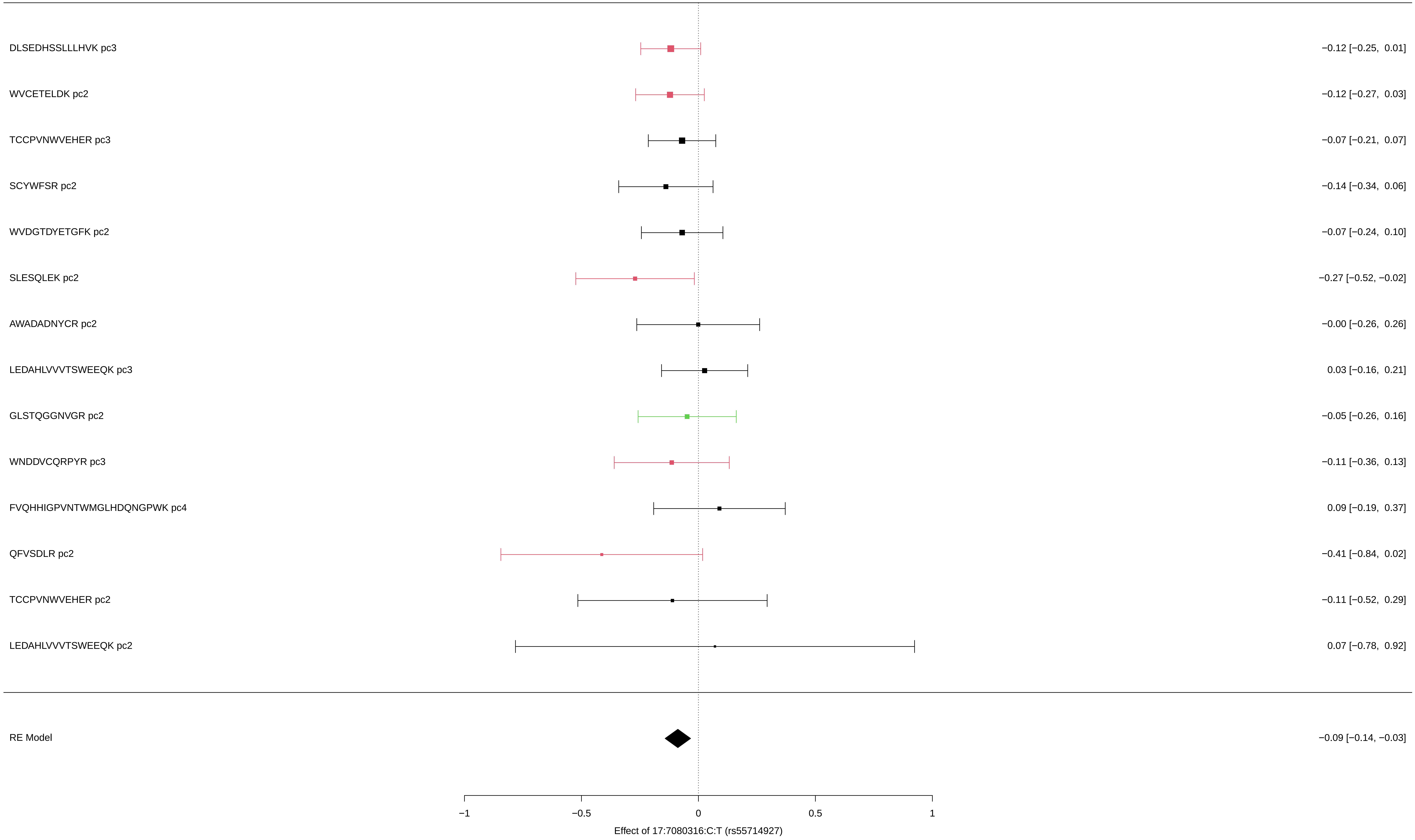

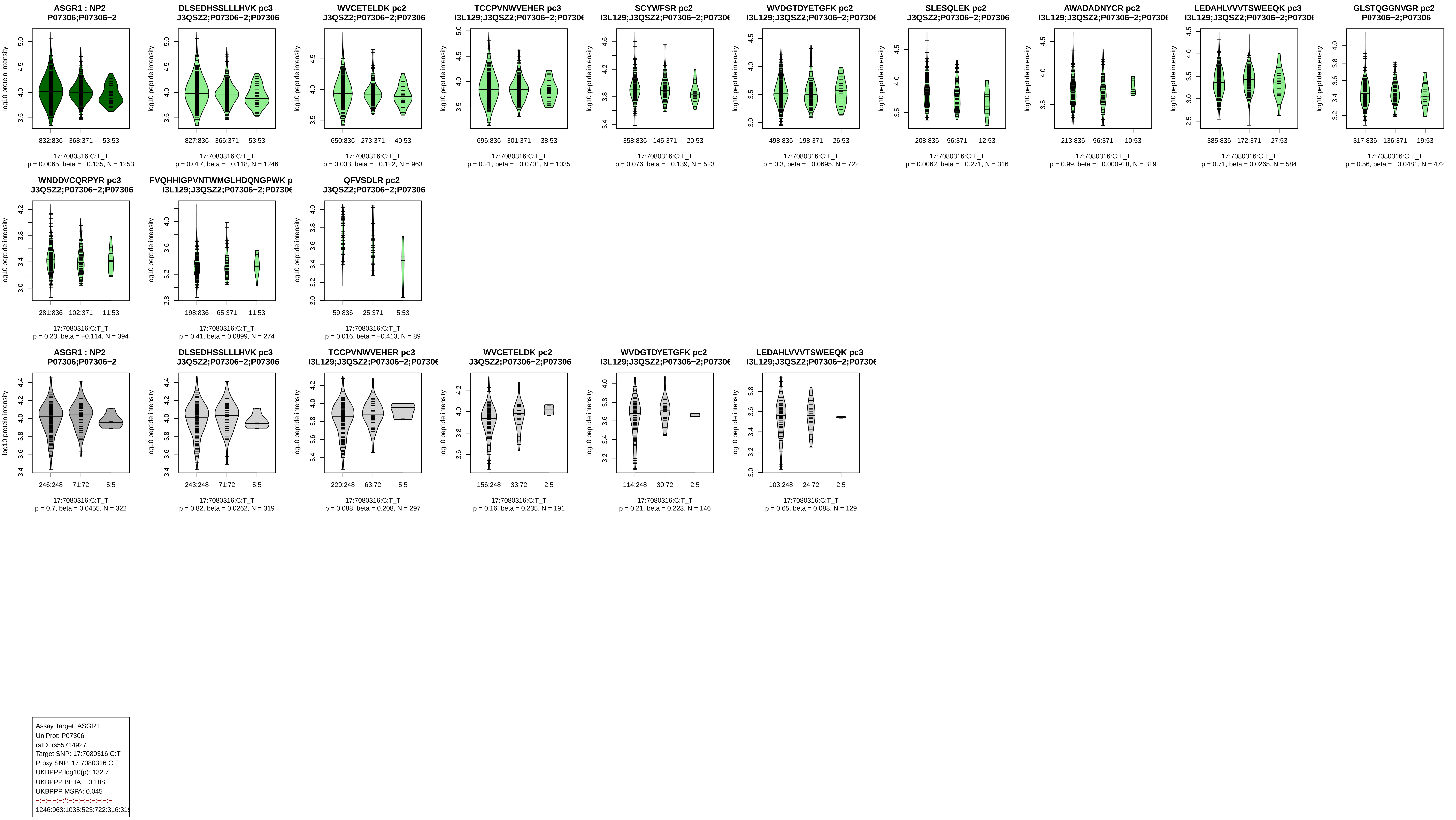

ASGR1 : NP2
P07306;P07306−2
832:836 368:371 53:53
17:7080316:C:T_T
p = 0.0065, beta = −0.135, N = 1253
DLSEDHSSLLLHVK pc3
J3QSZ2;P07306−2;P07306
827:836 366:371 53:53
17:7080316:C:T_T
p = 0.017, beta = −0.118, N = 1246
WVCETELDK pc2
J3QSZ2;P07306−2;P07306
650:836 273:371 40:53
17:7080316:C:T_T
p = 0.033, beta = −0.122, N = 963
TCCPVNWVEHER pc3
I3L129;J3QSZ2;P07306−2;P07306
696:836 301:371 38:53
17:7080316:C:T_T
p = 0.21, beta = −0.0701, N = 1035
SCYWFSR pc2
I3L129;J3QSZ2;P07306−2;P07306
358:836 145:371 20:53
17:7080316:C:T_T
p = 0.076, beta = −0.139, N = 523
WVDGTDYETGFK pc2
I3L129;J3QSZ2;P07306−2;P07306
498:836 198:371 26:53
17:7080316:C:T_T
p = 0.3, beta = −0.0695, N = 722
SLESQLEK pc2
J3QSZ2;P07306−2;P07306
208:836 96:371 12:53
17:7080316:C:T_T
p = 0.0062, beta = −0.271, N = 316
AWADADNYCR pc2
I3L129;J3QSZ2;P07306−2;P07306
213:836 96:371 10:53
17:7080316:C:T_T
p = 0.99, beta = −0.000918, N = 319
LEDAHLVVVTSWEEQK pc3
I3L129;J3QSZ2;P07306−2;P07306
385:836 172:371 27:53
17:7080316:C:T_T
p = 0.71, beta = 0.0265, N = 584
GLSTQGGNVGR pc2
P07306−2;P07306
317:836 136:371 19:53
17:7080316:C:T_T
p = 0.56, beta = −0.0481, N = 472
WNDDVCQRPYR pc3
J3QSZ2;P07306−2;P07306
281:836 102:371 11:53
17:7080316:C:T_T
p = 0.23, beta = −0.114, N = 394
FVQHHIGPVNTWMGLHDQNGPWK p
I3L129;J3QSZ2;P07306−2;P07306
198:836 65:371 11:53
17:7080316:C:T_T
p = 0.41, beta = 0.0899, N = 274
QFVSDLR pc2
J3QSZ2;P07306−2;P07306
59:836 25:371 5:53
17:7080316:C:T_T
p = 0.016, beta = −0.413, N = 89
ASGR1 : NP2
P07306;P07306−2
246:248 71:72 5:5
17:7080316:C:T_T
p = 0.7, beta = 0.0455, N = 322
DLSEDHSSLLLHVK pc3
J3QSZ2;P07306−2;P07306
243:248 71:72 5:5
17:7080316:C:T_T
p = 0.82, beta = 0.0262, N = 319
TCCPVNWVEHER pc3
I3L129;J3QSZ2;P07306−2;P07306
229:248 63:72 5:5
17:7080316:C:T_T
p = 0.088, beta = 0.208, N = 297
WVCETELDK pc2
J3QSZ2;P07306−2;P07306
156:248 33:72 2:5
17:7080316:C:T_T
p = 0.16, beta = 0.235, N = 191
WVDGTDYETGFK pc2
I3L129;J3QSZ2;P07306−2;P07306
114:248 30:72 2:5
17:7080316:C:T_T
p = 0.21, beta = 0.223, N = 146
LEDAHLVVVTSWEEQK pc3
I3L129;J3QSZ2;P07306−2;P07306
103:248 24:72 2:5
17:7080316:C:T_T
p = 0.65, beta = 0.088, N = 129
log10 protein intensity
log10 peptide intensity
Assay Target: ASGR1
UniProt: P07306
rsID: rs55714927
Target SNP: 17:7080316:C:T
Proxy SNP: 17:7080316:C:T
UKBPPP log10(p): 132.7
UKBPPP BETA: −0.188
UKBPPP MSPA: 0.045
1246:963:1035:523:722:316:31

262. CFD (P00746)
UKBPPP MSPA=0

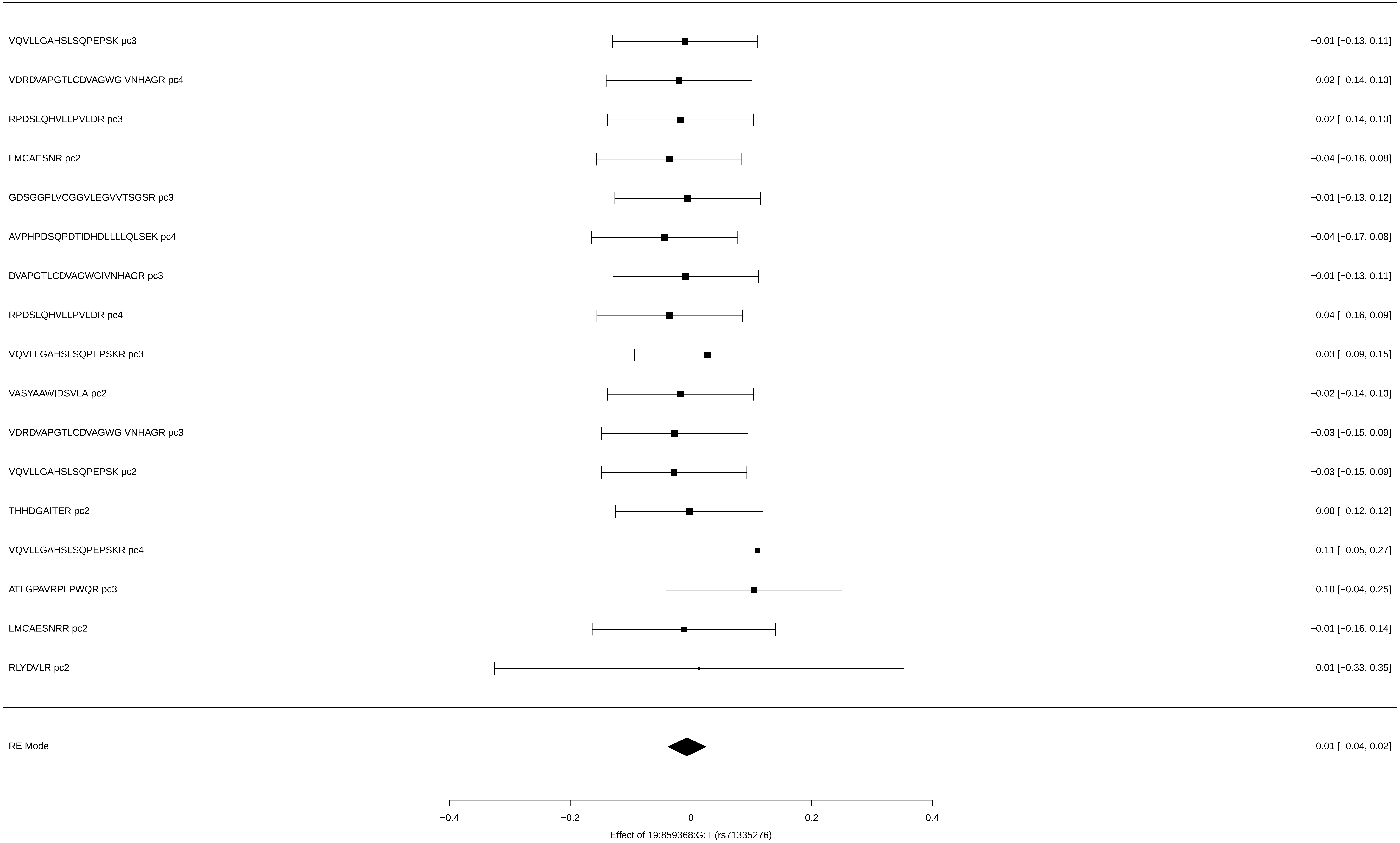

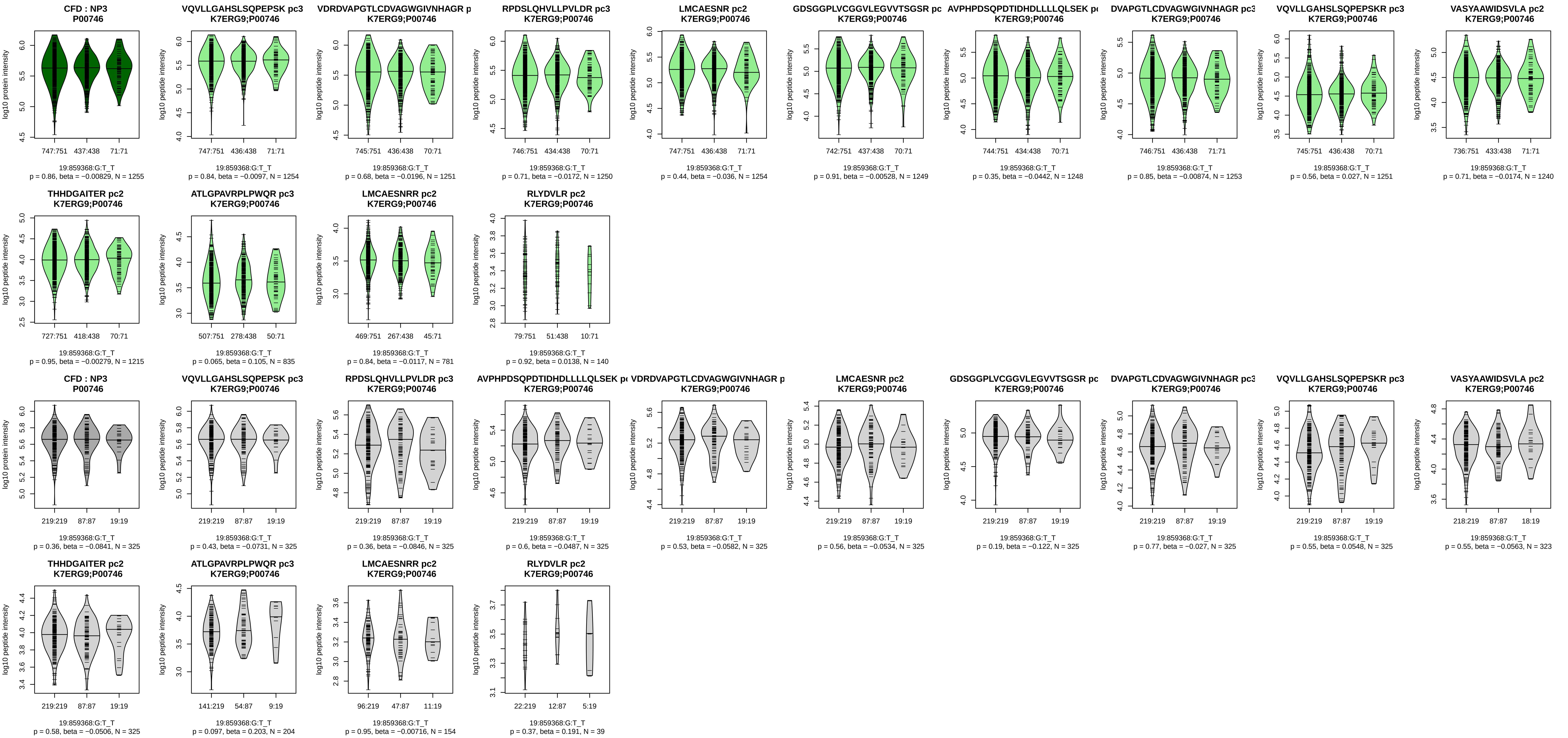

Assay Target: CFD
UniProt: P00746
rsID: rs71335276
Target SNP: 19:859368:G:T
Proxy SNP: 19:859368:G:T
UKBPPP log10(p): 131.4
UKBPPP BETA: −0.181
UKBPPP MSPA: 0
1254:1251:1250:1254:1249:124

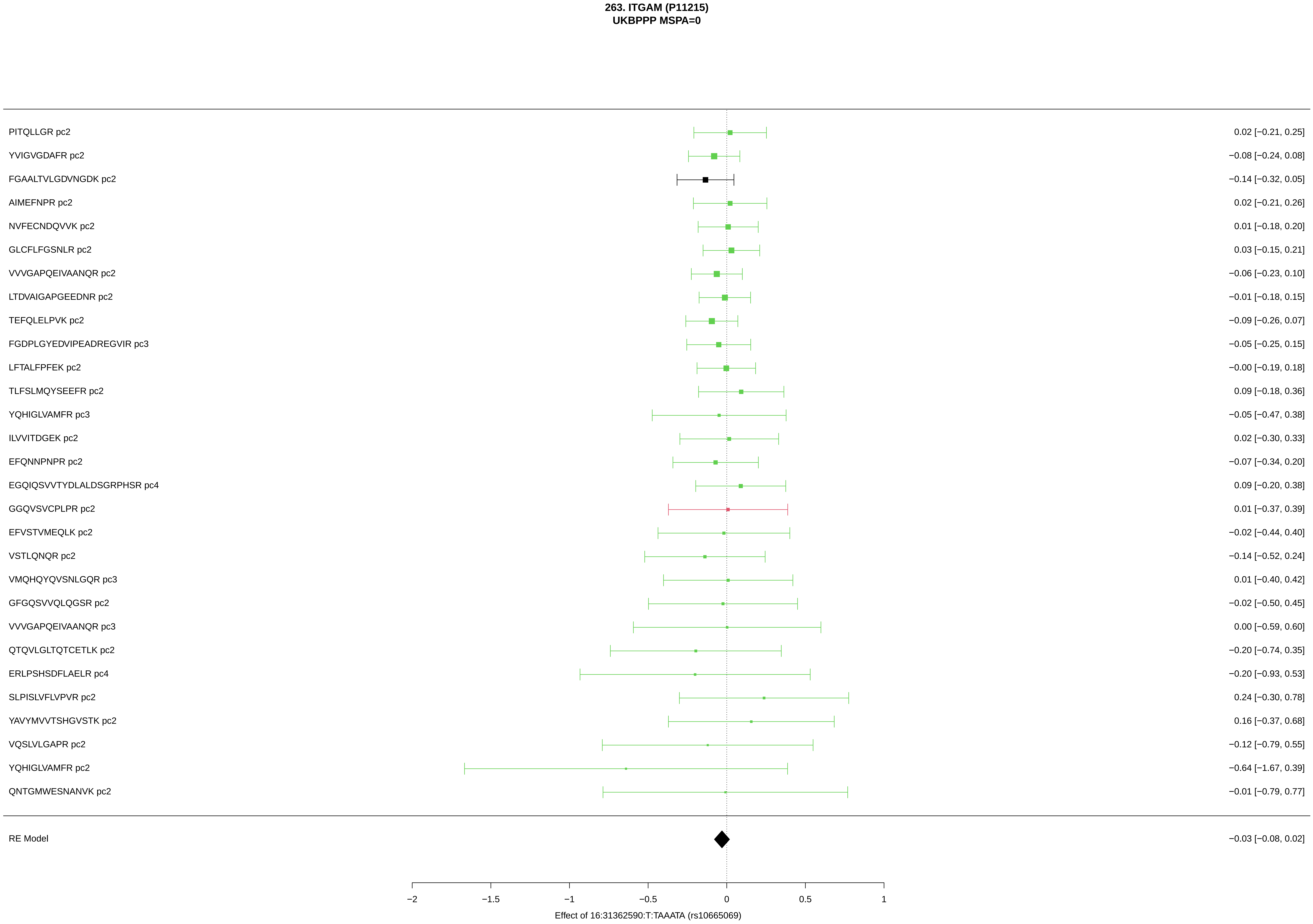
263. ITGAM (P11215)
UKBPPP MSPA=0
PITQLLGR pc2
YVIGVGDAFR pc2
FGAALTVLGDVNGDK pc2
AIMEFNPR pc2
NVFECNDQVVK pc2
GLCFLFGSNLR pc2
VVVGAPQEIVAANQR pc2
LTDVAIGAPGEEDNR pc2
TEFQLELPVK pc2
FGDPLGYEDVIPEADREGVIR pc3
LFTALFPFEK pc2
TLFSLMQYSEEFR pc2
YQHIGLVAMFR pc3
ILVVITDGEK pc2
EFQNNPNPR pc2
EGQIQSVVTYDLALDSGRPHSR pc4
GGQVSVCPLPR pc2
EFVSTVMEQLK pc2
VSTLQNQR pc2
VMQHQYQVSNLGQR pc3
GFGQSVVQLQGSR pc2
VVVGAPQEIVAANQR pc3
QTQVLGLTQTCETLK pc2
ERLPSHSDFLAELR pc4
SLPISLVFLVPVR pc2
YAVYMVVTSHGVSTK pc2
VQSLVLGAPR pc2
YQHIGLVAMFR pc2
QNTGMWESNANVK pc2
RE Model
0.02 [−0.21, 0.25]
−0.08 [−0.24, 0.08]
−0.14 [−0.32, 0.05]
0.02 [−0.21, 0.26]
0.01 [−0.18, 0.20]
0.03 [−0.15, 0.21]
−0.06 [−0.23, 0.10]
−0.01 [−0.18, 0.15]
−0.09 [−0.26, 0.07]
−0.05 [−0.25, 0.15]
−0.00 [−0.19, 0.18]
0.09 [−0.18, 0.36]
−0.05 [−0.47, 0.38]
0.02 [−0.30, 0.33]
−0.07 [−0.34, 0.20]
0.09 [−0.20, 0.38]
0.01 [−0.37, 0.39]
−0.02 [−0.44, 0.40]
−0.14 [−0.52, 0.24]
0.01 [−0.40, 0.42]
−0.02 [−0.50, 0.45]
0.00 [−0.59, 0.60]
−0.20 [−0.74, 0.35]
−0.20 [−0.93, 0.53]
0.24 [−0.30, 0.78]
0.16 [−0.37, 0.68]
−0.12 [−0.79, 0.55]
−0.64 [−1.67, 0.39]
−0.01 [−0.79, 0.77]
−0.03 [−0.08, 0.02]
−2
−1.5
−1
−0.5
0
0.5
1
Effect of 16:31362590:T:TAAATA (rs10665069)

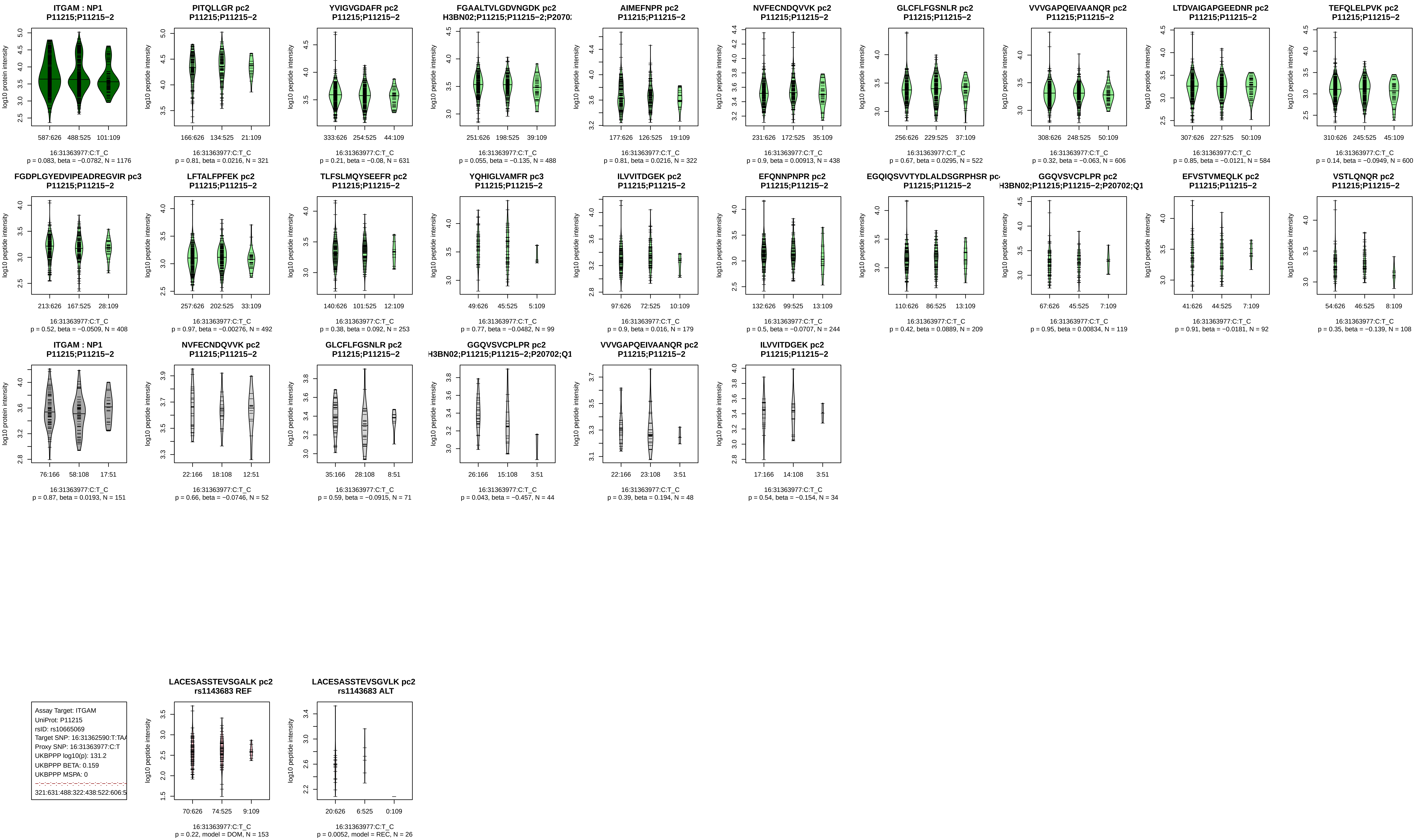

ITGAM : NP1 P11215;P11215−2
16:31363977:C:T_C p = 0.083, beta = −0.0782, N = 1176
PITQLLGR pc2 P11215;P11215−2
16:31363977:C:T_C p = 0.81, beta = 0.0216, N = 321
YVIGVGDAFR pc2 P11215;P11215−2
16:31363977:C:T_C p = 0.21, beta = −0.08, N = 631
FGAALTVLGDVNGDK pc2 H3BN02;P11215;P11215−2;P2070
16:31363977:C:T_C p = 0.055, beta = −0.135, N = 488
AIMEFNPR pc2 P11215;P11215−2
16:31363977:C:T_C p = 0.81, beta = 0.0216, N = 322
NVFECNDQVVK pc2 P11215;P11215−2
16:31363977:C:T_C p = 0.9, beta = 0.00913, N = 438
GLCFLFGSNLR pc2 P11215;P11215−2
16:31363977:C:T_C p = 0.67, beta = 0.0295, N = 522
VVVGAPQEIVAANQR pc2 P11215;P11215−2
16:31363977:C:T_C p = 0.32, beta = −0.063, N = 606
LTDVAIGAPGEEDNR pc2 P11215;P11215−2
16:31363977:C:T_C p = 0.85, beta = −0.0121, N = 584
TEFQLELPVK pc2 P11215;P11215−2
16:31363977:C:T_C p = 0.14, beta = −0.0949, N = 600
FGDPLGYEDVIPEADREGVIR pc3 P11215;P11215−2
16:31363977:C:T_C p = 0.52, beta = −0.0509, N = 408
LFTALFPFEK pc2 P11215;P11215−2
16:31363977:C:T_C p = 0.97, beta = −0.00276, N = 492
TLFSLMQYSEEFR pc2 P11215;P11215−2
16:31363977:C:T_C p = 0.38, beta = 0.092, N = 253
YQHIGLVAMFR pc3 P11215;P11215−2
16:31363977:C:T_C p = 0.77, beta = −0.0482, N = 99
ILVVITDGEK pc2 P11215;P11215−2
16:31363977:C:T_C p = 0.9, beta = 0.016, N = 179
EFQNNPNPR pc2 P11215;P11215−2
16:31363977:C:T_C p = 0.5, beta = −0.0707, N = 244
EGQIQSVVTYDLALDSGRPHSR pc P11215;P11215−2
16:31363977:C:T_C p = 0.42, beta = 0.0889, N = 209
GGQVSVCPLPR pc2 H3BN02;P11215;P11215−2;P20702;Q1
16:31363977:C:T_C p = 0.95, beta = 0.00834, N = 119
EFVSTVMEQLK pc2 P11215;P11215−2
16:31363977:C:T_C p = 0.91, beta = −0.0181, N = 92
VSTLQNQR pc2 P11215;P11215−2
16:31363977:C:T_C p = 0.35, beta = −0.139, N = 108
ITGAM : NP1 P11215;P11215−2
16:31363977:C:T_C p = 0.87, beta = 0.0193, N = 151
NVFECNDQVVK pc2 P11215;P11215−2
16:31363977:C:T_C p = 0.66, beta = −0.0746, N = 52
GLCFLFGSNLR pc2 P11215;P11215−2
16:31363977:C:T_C p = 0.59, beta = −0.0915, N = 71
GGQVSVCPLPR pc2 H3BN02;P11215;P11215−2;P20702;Q1
16:31363977:C:T_C p = 0.043, beta = −0.457, N = 44
VVVGAPQEIVAANQR pc2 P11215;P11215−2
16:31363977:C:T_C p = 0.39, beta = 0.194, N = 48
ILVVITDGEK pc2 P11215;P11215−2
16:31363977:C:T_C p = 0.54, beta = −0.154, N = 34
Assay Target: ITGAM
UniProt: P11215
rsID: rs10665069
Target SNP: 16:31362590:T:TA
Proxy SNP: 16:31363977:C:T
UKBPPP log10(p): 131.2
UKBPPP BETA: 0.159
UKBPPP MSPA: 0
321:631:488:322:438:522:606:5
LACESASSTEVSGALK pc2 rs1143683 REF
16:31363977:C:T_C p = 0.22, model = DOM, N = 153
LACESASSTEVSGVLK pc2 rs1143683 ALT
16:31363977:C:T_C p = 0.0052, model = REC, N = 26

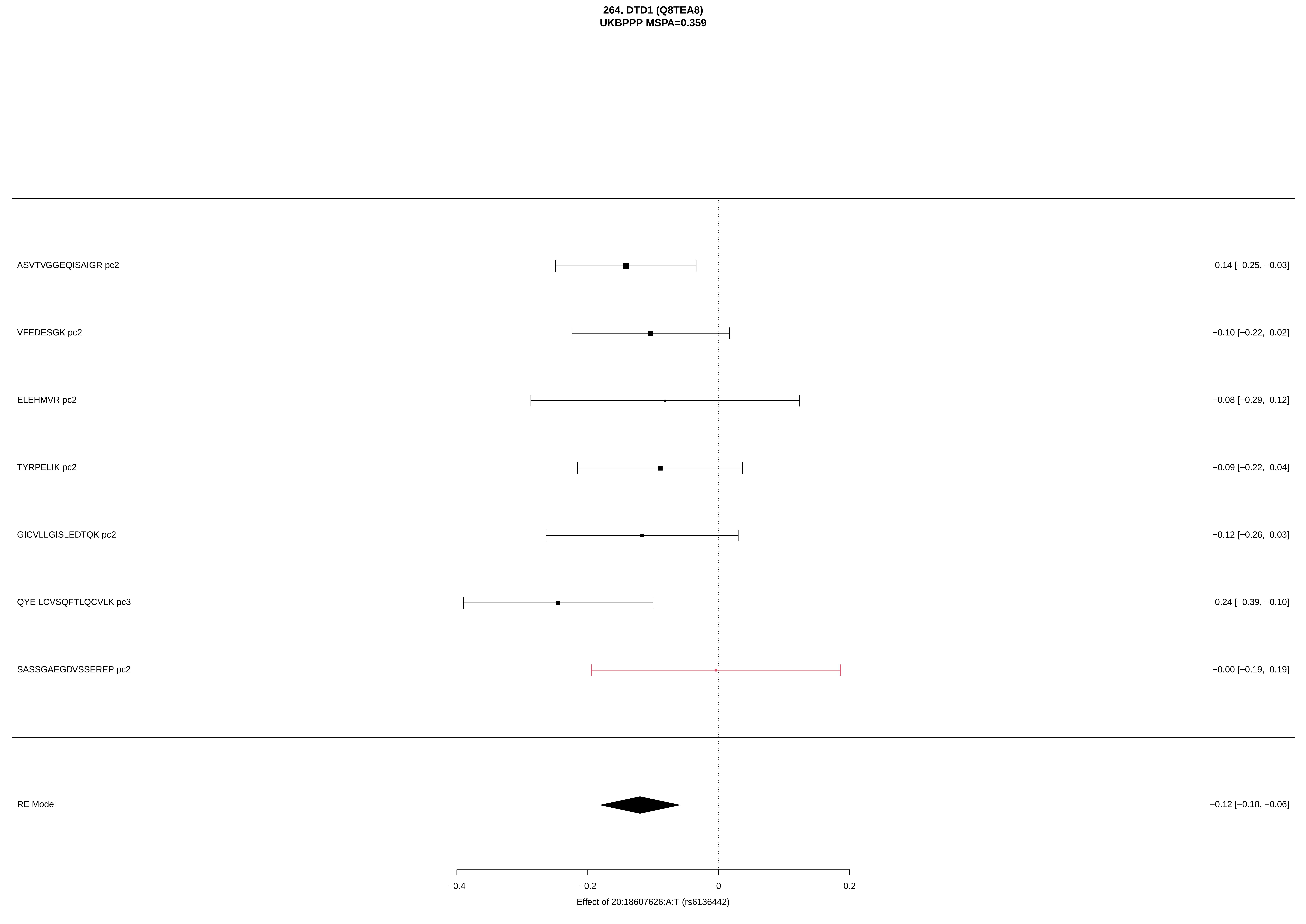
264. DTD1 (Q8TEA8)
UKBPPP MSPA=0.359
ASVTVGGEQISAIGR pc2
−0.14 [−0.25, −0.03]
VFEDESGK pc2
−0.10 [−0.22, 0.02]
ELEHMVR pc2
−0.08 [−0.29, 0.12]
TYRPELIK pc2
−0.09 [−0.22, 0.04]
GICVLLGISLEDTQK pc2
−0.12 [−0.26, 0.03]
QYEILCVSQFTLQCVLK pc3
−0.24 [−0.39, −0.10]
SASSGAEGDVSSEREP pc2
−0.00 [−0.19, 0.19]
RE Model
−0.12 [−0.18, −0.06]
−0.4
−0.2
0
0.2
Effect of 20:18607626:A:T (rs6136442)

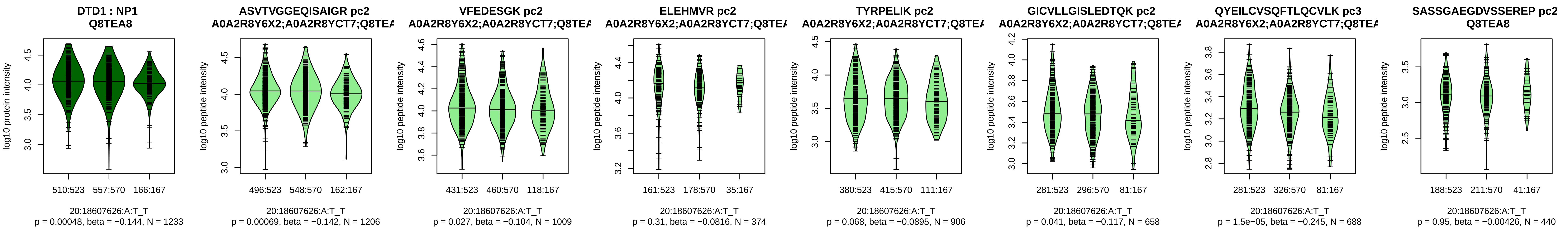

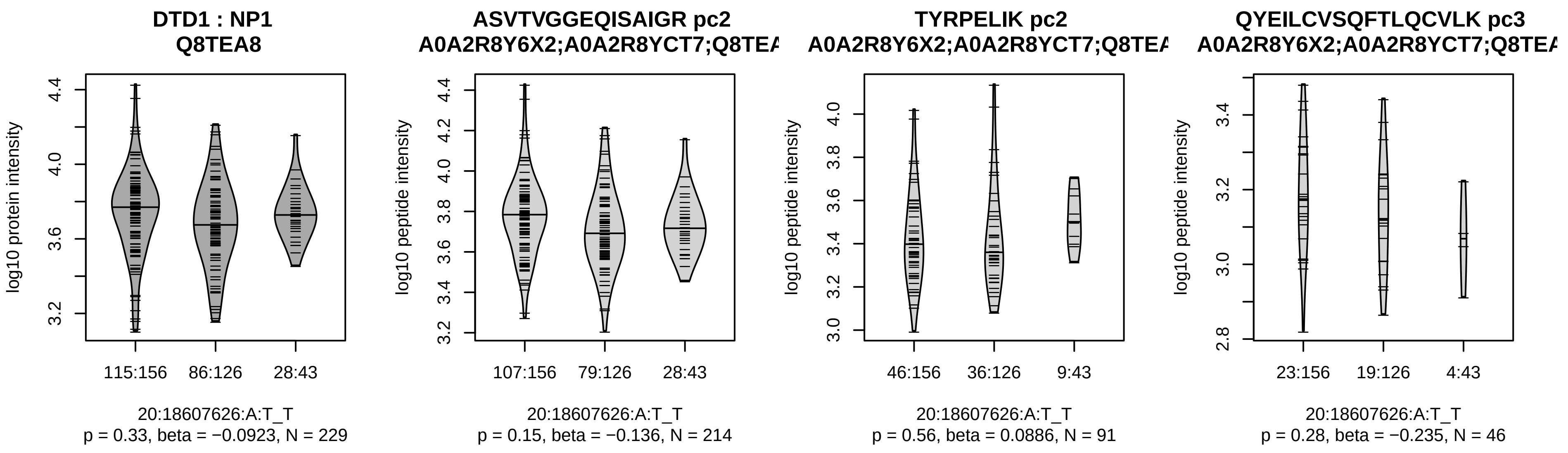

Assay Target: DTD1
UniProt: Q8TEA8
rsID: rs6136442
Target SNP: 20:18607626:A:T
Proxy SNP: 20:18607626:A:T
UKBPPP log10(p): 131
UKBPPP BETA: −0.156
UKBPPP MSPA: 0.359
:−:−:−:−::−
1206:1009:374:906:658:688:440

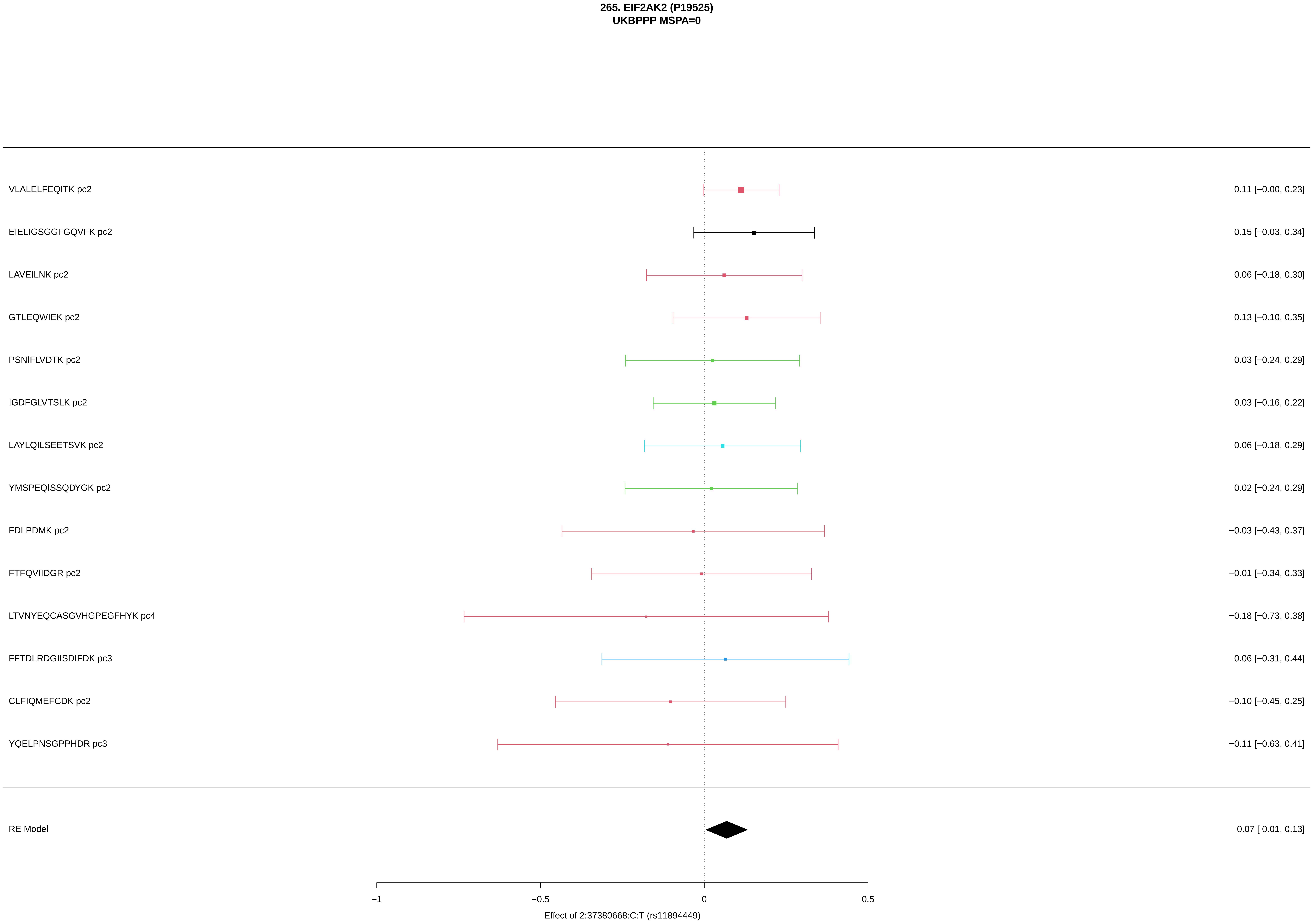
265. EIF2AK2 (P19525)
UKBPPP MSPA=0
VLALELFEQITK pc2
0.11 [−0.00, 0.23]
EIELIGSGGFGQVFK pc2
0.15 [−0.03, 0.34]
LAVEILNK pc2
0.06 [−0.18, 0.30]
GTLEQWIEK pc2
0.13 [−0.10, 0.35]
PSNIFLVDTK pc2
0.03 [−0.24, 0.29]
IGDFGLVTSLK pc2
0.03 [−0.16, 0.22]
LAYLQILSEETSVK pc2
0.06 [−0.18, 0.29]
YMSPEQISSQDYGK pc2
0.02 [−0.24, 0.29]
FDLPDMK pc2
−0.03 [−0.43, 0.37]
FTFQVIIDGR pc2
−0.01 [−0.34, 0.33]
LTVNYEQCASGVHGPEGFHYK pc4
−0.18 [−0.73, 0.38]
FFTDLRDGIISDIFDK pc3
0.06 [−0.31, 0.44]
CLFIQMEFCDK pc2
−0.10 [−0.45, 0.25]
YQELPNSGPPHDR pc3
−0.11 [−0.63, 0.41]
RE Model
0.07 [0.01, 0.13]
−1
−0.5
0
0.5
Effect of 2:37380668:C:T (rs11894449)

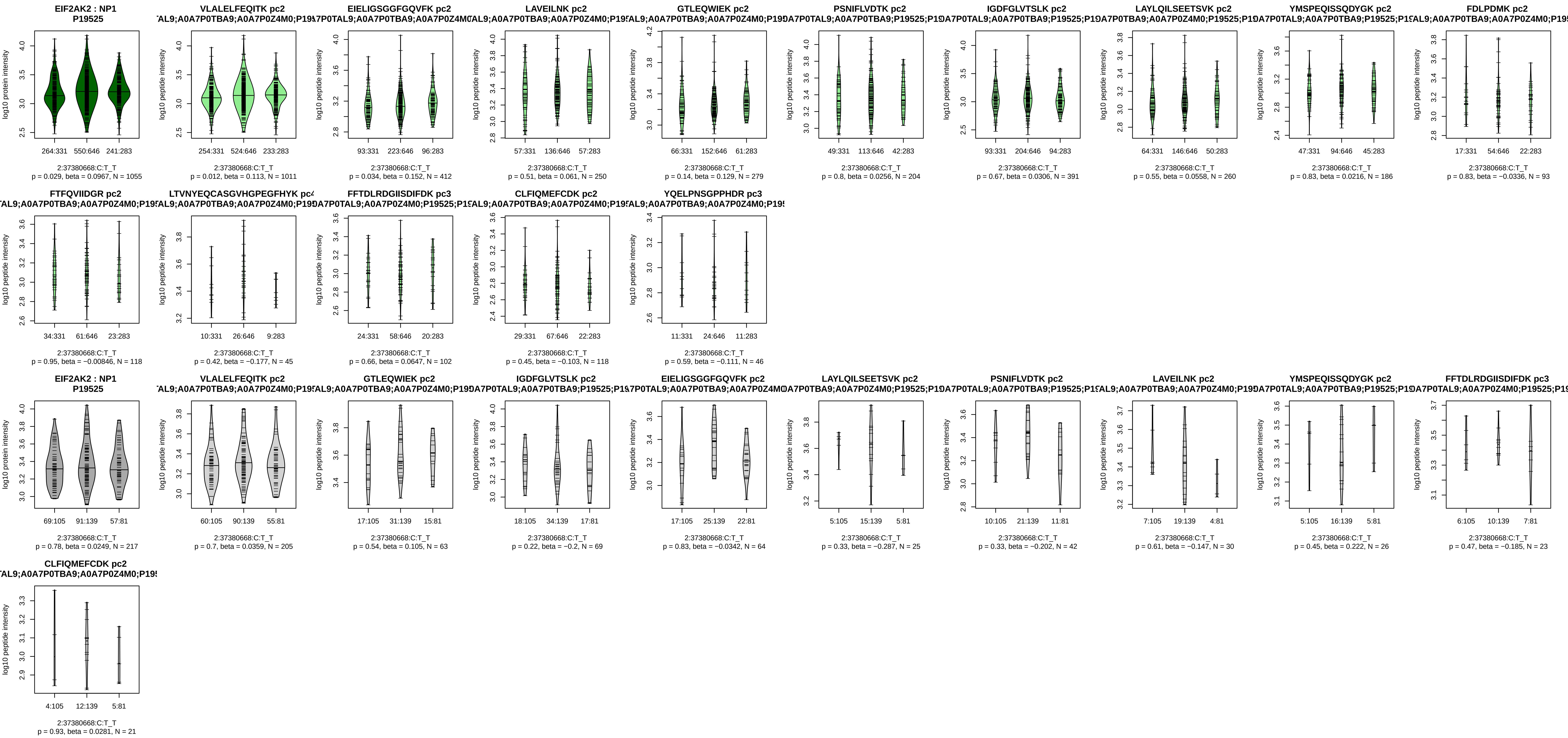

Assay Target: EIF2AK2
UniProt: P19525
rsID: rs11894449
Target SNP: 2:37380668:C:T
Proxy SNP: 2:37380668:C:T
UKBPPP log10(p): 130.4
UKBPPP BETA: 0.148
UKBPPP MSPA: 0
1011:412:250:279:204:391:260:

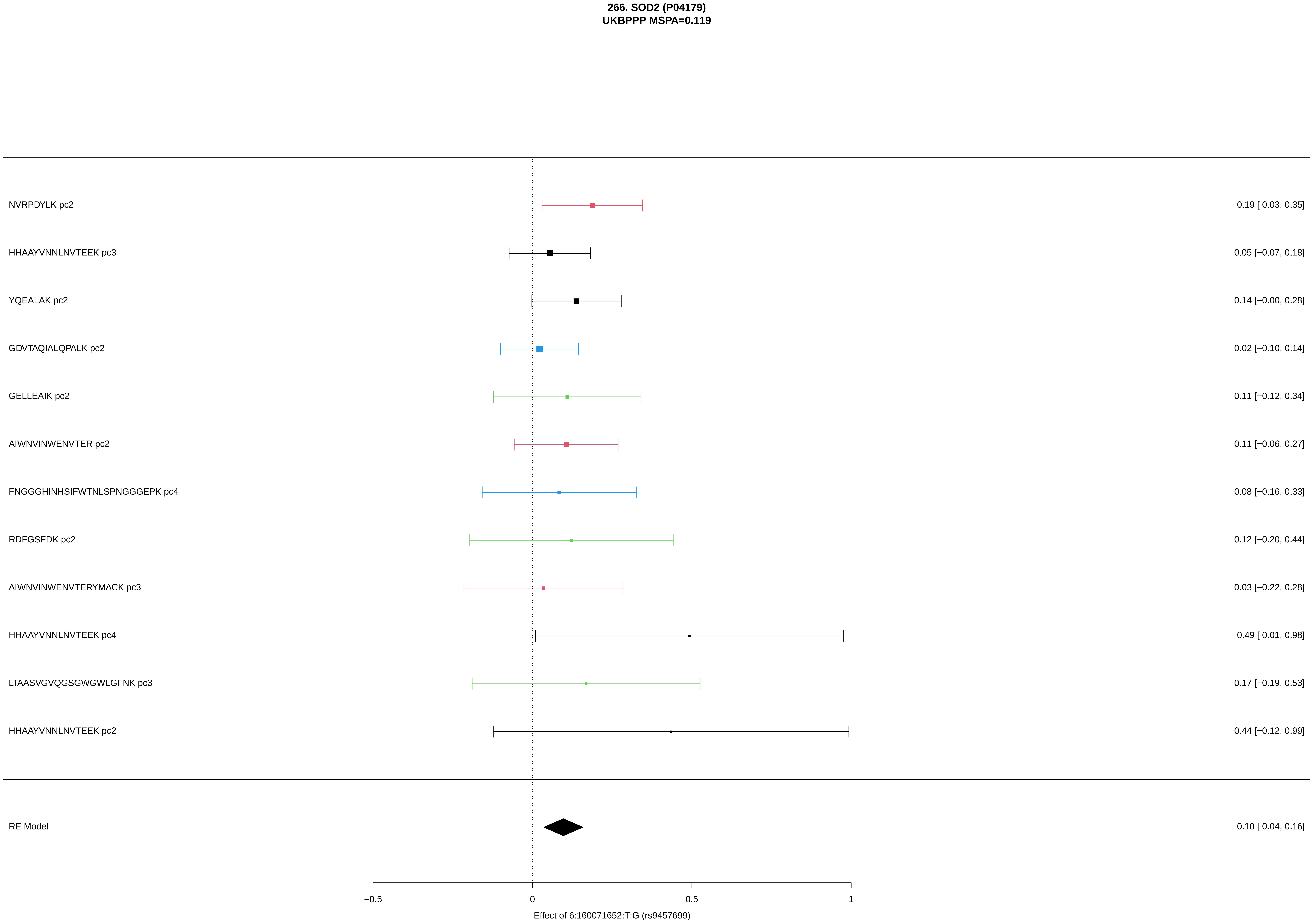
266. SOD2 (P04179)
UKBPPP MSPA=0.119
NVRPDYLK pc2
0.19 [0.03, 0.35]
HHAAYVNNLNVTEEK pc3
0.05 [−0.07, 0.18]
YQEALAK pc2
0.14 [−0.00, 0.28]
GDVTAQIALQPALK pc2
0.02 [−0.10, 0.14]
GELLEAIK pc2
0.11 [−0.12, 0.34]
AIWNVINWENVTER pc2
0.11 [−0.06, 0.27]
FNGGGHINHSIFWTNLSPNGGGEPK pc4
0.08 [−0.16, 0.33]
RDFGSFDK pc2
0.12 [−0.20, 0.44]
AIWNVINWENVTERYMACK pc3
0.03 [−0.22, 0.28]
HHAAYVNNLNVTEEK pc4
0.49 [0.01, 0.98]
LTAASVGVQGSGWGWLGFNK pc3
0.17 [−0.19, 0.53]
HHAAYVNNLNVTEEK pc2
0.44 [−0.12, 0.99]
RE Model
0.10 [0.04, 0.16]
−0.5
0
0.5
1
Effect of 6:160071652:T:G (rs9457699)

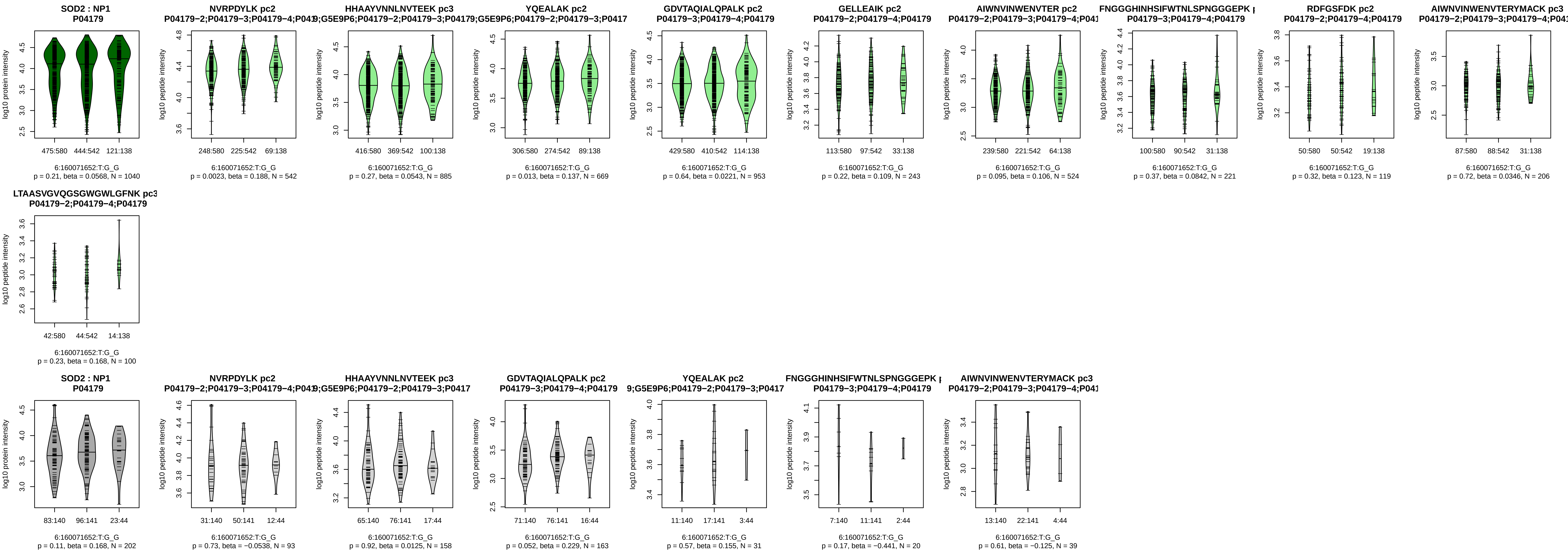

Assay Target: SOD2
UniProt: P04179
rsID: rs9457699
Target SNP: 6:160071652:T:G
Proxy SNP: 6:160071652:T:G
UKBPPP log10(p): 130.2
UKBPPP BETA: 0.157
UKBPPP MSPA: 0.119
*|−:−:−:−:−:−:−:−:−
542:885:669:953:243:524:221:1

267. PSTPIP2 (Q9H939)
UKBPPP MSPA=0.015

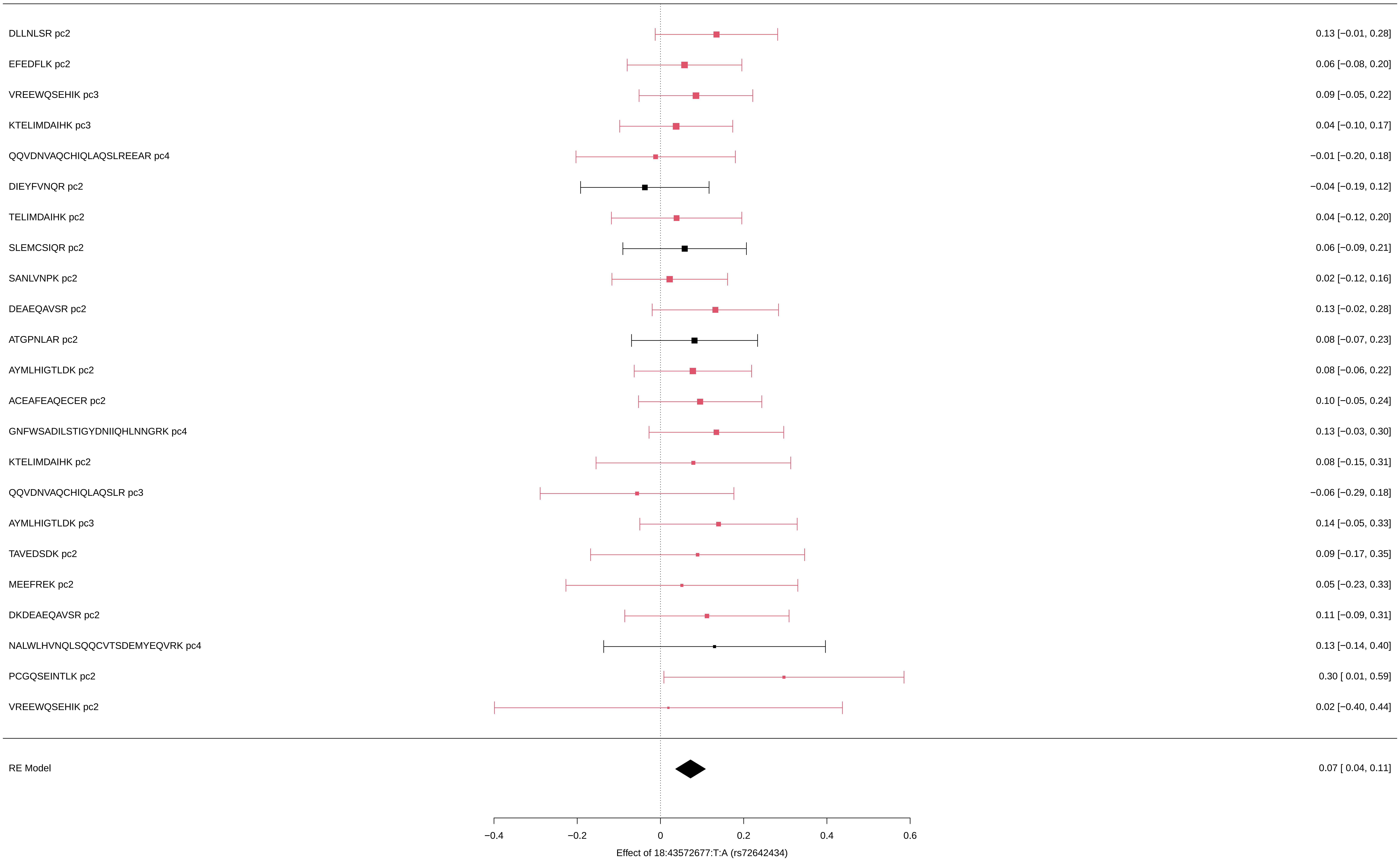

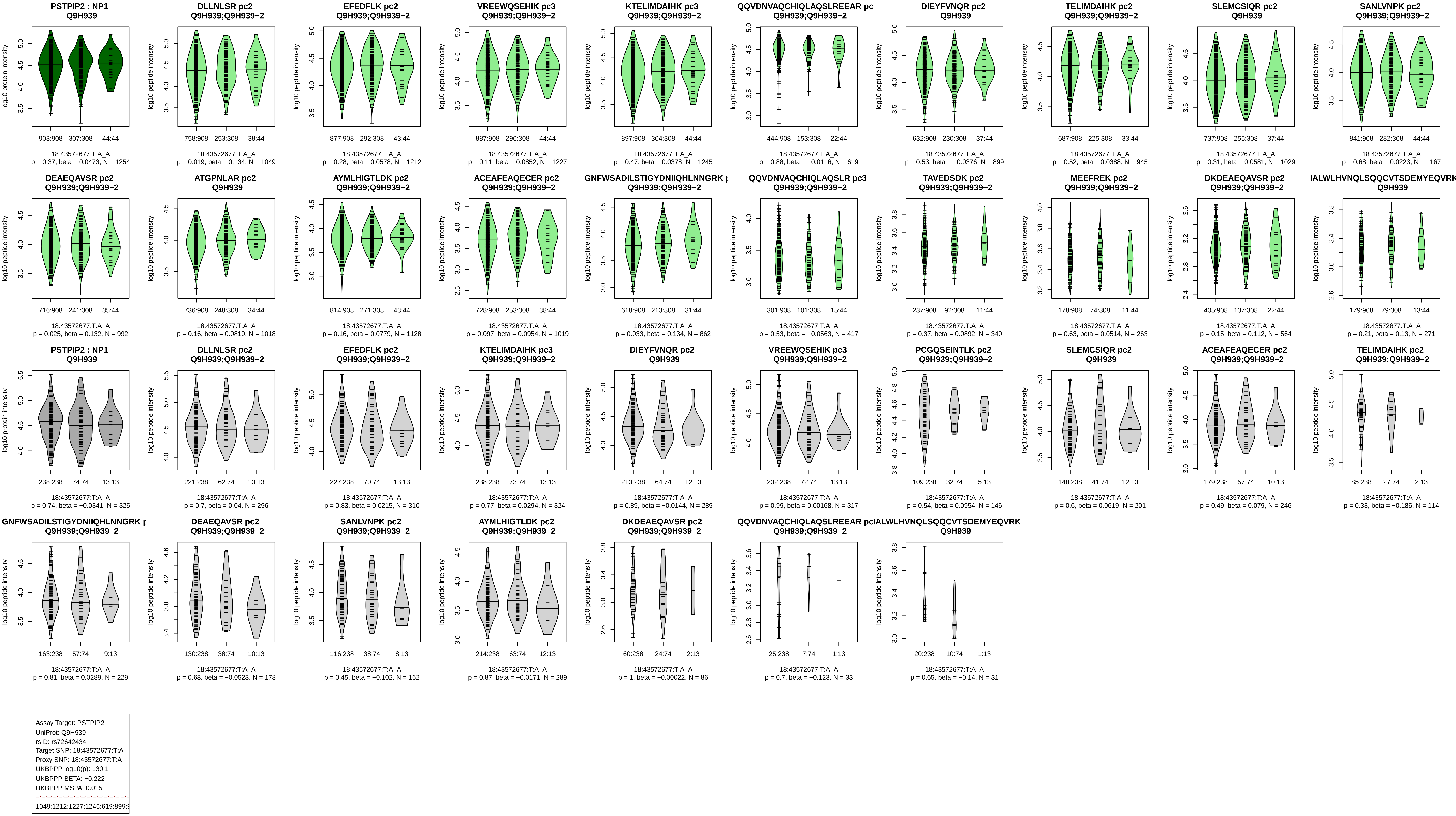
PSTPIP2 : NP1 Q9H939
903:908 307:308 44:44
18:43572677:T:A_A
p = 0.37, beta = 0.0473, N = 1254
DLLNLSR pc2 Q9H939;Q9H939−2
758:908 253:308 38:44
18:43572677:T:A_A
p = 0.019, beta = 0.134, N = 1049
EFEDFLK pc2 Q9H939;Q9H939−2
877:908 292:308 43:44
18:43572677:T:A_A
p = 0.28, beta = 0.0578, N = 1212
VREEWQSEHIK pc3 Q9H939;Q9H939−2
887:908 296:308 44:44
18:43572677:T:A_A
p = 0.11, beta = 0.0852, N = 1227
KTELIMDAIHK pc3 Q9H939;Q9H939−2
897:908 304:308 44:44
18:43572677:T:A_A
p = 0.47, beta = 0.0378, N = 1245
QQVDNVAQCHIQLAQSLREEAR pc Q9H939;Q9H939−2
444:908 153:308 22:44
18:43572677:T:A_A
p = 0.88, beta = −0.0116, N = 619
DIEYFVNQR pc2 Q9H939
632:908 230:308 37:44
18:43572677:T:A_A
p = 0.53, beta = −0.0376, N = 899
TELIMDAIHK pc2 Q9H939;Q9H939−2
687:908 225:308 33:44
18:43572677:T:A_A
p = 0.52, beta = 0.0388, N = 945
SLEMCSIQR pc2 Q9H939
737:908 255:308 37:44
18:43572677:T:A_A
p = 0.31, beta = 0.0581, N = 1029
SANLVNPK pc2 Q9H939;Q9H939−2
841:908 282:308 44:44
18:43572677:T:A_A
p = 0.68, beta = 0.0223, N = 1167
DEAEQAVSR pc2 Q9H939;Q9H939−2
716:908 241:308 35:44
18:43572677:T:A_A
p = 0.025, beta = 0.132, N = 992
ATGPNLAR pc2 Q9H939
736:908 248:308 34:44
18:43572677:T:A_A
p = 0.16, beta = 0.0819, N = 1018
AYMLHIGTLDK pc2 Q9H939;Q9H939−2
814:908 271:308 43:44
18:43572677:T:A_A
p = 0.16, beta = 0.0779, N = 1128
ACEAFEAQECER pc2 Q9H939;Q9H939−2
728:908 253:308 38:44
18:43572677:T:A_A
p = 0.097, beta = 0.0954, N = 1019
GNFWSADILSTIGYDNIIQHLNNGRK p Q9H939;Q9H939−2
618:908 213:308 31:44
18:43572677:T:A_A
p = 0.033, beta = 0.134, N = 862
QQVDNVAQCHIQLAQSLR pc3 Q9H939;Q9H939−2
301:908 101:308 15:44
18:43572677:T:A_A
p = 0.53, beta = −0.0563, N = 417
TAVEDSDK pc2 Q9H939;Q9H939−2
237:908 92:308 11:44
18:43572677:T:A_A
p = 0.37, beta = 0.0892, N = 340
MEEFREK pc2 Q9H939;Q9H939−2
178:908 74:308 11:44
18:43572677:T:A_A
p = 0.63, beta = 0.0514, N = 263
DKDEAEQAVSR pc2 Q9H939;Q9H939−2
405:908 137:308 22:44
18:43572677:T:A_A
p = 0.15, beta = 0.112, N = 564
IALWLHVNQLSQQCVTSDEMYEQVRK Q9H939
179:908 79:308 13:44
18:43572677:T:A_A
p = 0.21, beta = 0.13, N = 271
PSTPIP2 : NP1 Q9H939
238:238 74:74 13:13
18:43572677:T:A_A
p = 0.74, beta = −0.0341, N = 325
DLLNLSR pc2 Q9H939;Q9H939−2
221:238 62:74 13:13
18:43572677:T:A_A
p = 0.7, beta = 0.04, N = 296
EFEDFLK pc2 Q9H939;Q9H939−2
227:238 70:74 13:13
18:43572677:T:A_A
p = 0.83, beta = 0.0215, N = 310
KTELIMDAIHK pc3 Q9H939;Q9H939−2
238:238 73:74 13:13
18:43572677:T:A_A
p = 0.77, beta = 0.0294, N = 324
DIEYFVNQR pc2 Q9H939
213:238 64:74 12:13
18:43572677:T:A_A
p = 0.89, beta = −0.0144, N = 289
VREEWQSEHIK pc3 Q9H939;Q9H939−2
232:238 72:74 13:13
18:43572677:T:A_A
p = 0.99, beta = 0.00168, N = 317
PCGQSEINTLK pc2 Q9H939;Q9H939−2
109:238 32:74 5:13
18:43572677:T:A_A
p = 0.54, beta = 0.0954, N = 146
SLEMCSIQR pc2 Q9H939
148:238 41:74 12:13
18:43572677:T:A_A
p = 0.6, beta = 0.0619, N = 201
ACEAFEAQECER pc2 Q9H939;Q9H939−2
179:238 57:74 10:13
18:43572677:T:A_A
p = 0.49, beta = 0.079, N = 246
TELIMDAIHK pc2 Q9H939;Q9H939−2
85:238 27:74 2:13
18:43572677:T:A_A
p = 0.33, beta = −0.186, N = 114
GNFWSADILSTIGYDNIIQHLNNGRK p Q9H939;Q9H939−2
163:238 57:74 9:13
18:43572677:T:A_A
p = 0.81, beta = 0.0289, N = 229
DEAEQAVSR pc2 Q9H939;Q9H939−2
130:238 38:74 10:13
18:43572677:T:A_A
p = 0.68, beta = −0.0523, N = 178
SANLVNPK pc2 Q9H939;Q9H939−2
116:238 38:74 8:13
18:43572677:T:A_A
p = 0.45, beta = −0.102, N = 162
AYMLHIGTLDK pc2 Q9H939;Q9H939−2
214:238 63:74 12:13
18:43572677:T:A_A
p = 0.87, beta = −0.0171, N = 289
DKDEAEQAVSR pc2 Q9H939;Q9H939−2
60:238 24:74 2:13
18:43572677:T:A_A
p = 1, beta = −0.00022, N = 86
QQVDNVAQCHIQLAQSLREEAR pc Q9H939;Q9H939−2
25:238 7:74 1:13
18:43572677:T:A_A
p = 0.7, beta = −0.123, N = 33
IALWLHVNQLSQQCVTSDEMYEQVRK Q9H939
20:238 10:74 1:13
18:43572677:T:A_A
p = 0.65, beta = −0.14, N = 31
log10 protein intensity
log10 peptide intensity
Assay Target: PSTPIP2
UniProt: Q9H939
rsID: rs72642434
Target SNP: 18:43572677:T:A
Proxy SNP: 18:43572677:T:A
UKBPPP log10(p): 130.1
UKBPPP BETA: −0.222
UKBPPP MSPA: 0.015
1049:1212:1227:1245:619:899:9

268. ITGAV (P06756)
UKBPPP MSPA=0

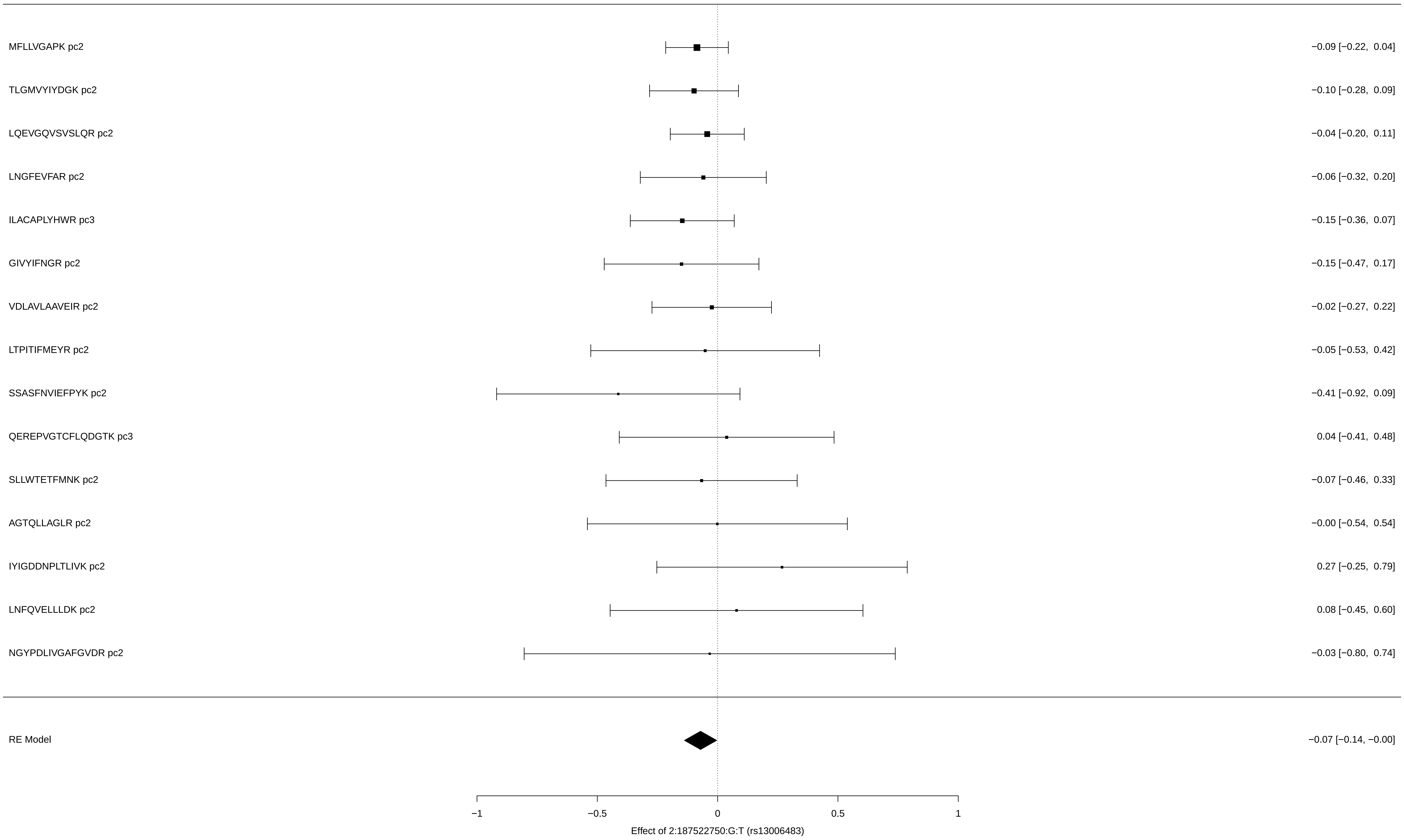

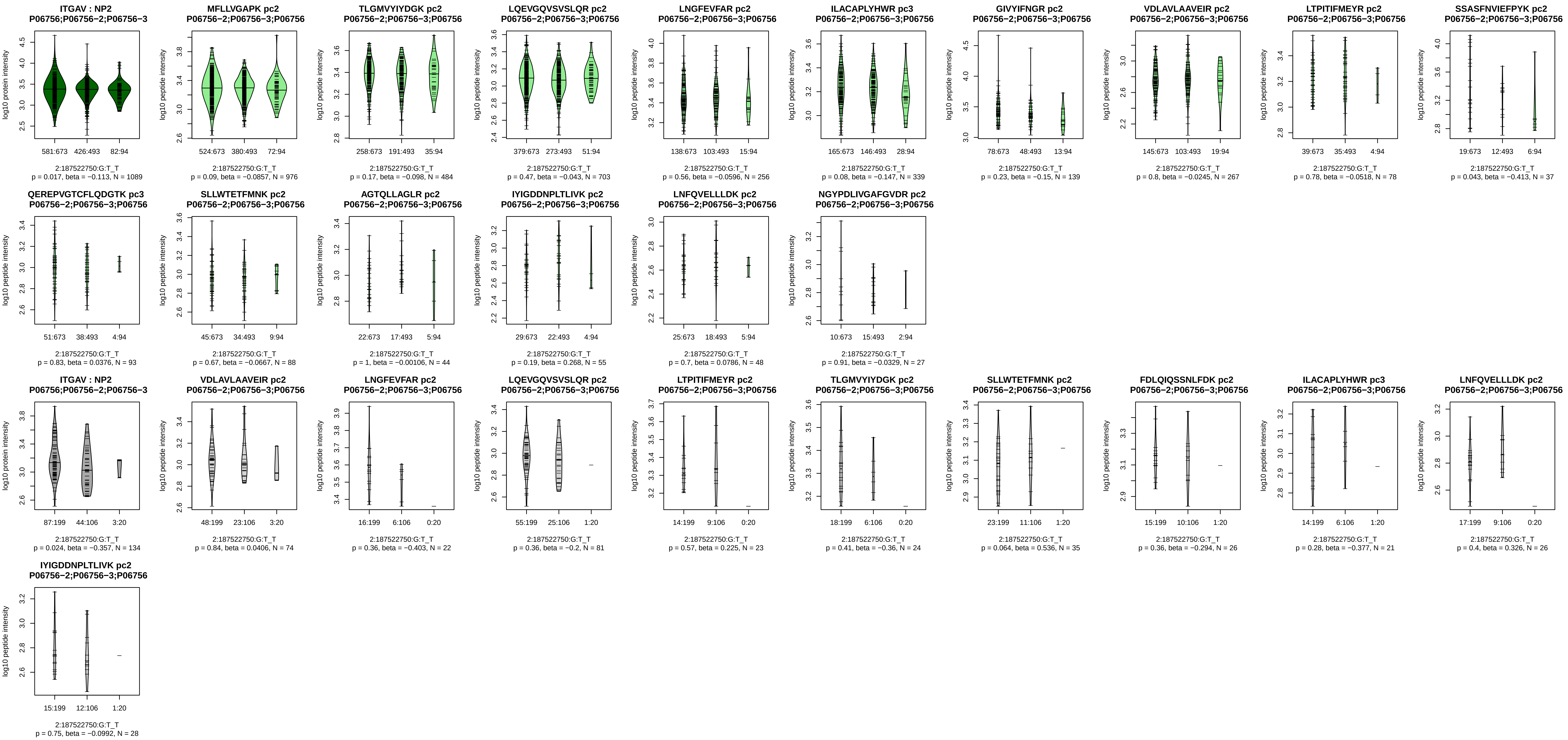

Assay Target: ITGAV
UniProt: P06756
rsID: rs13006483
Target SNP: 2:187522750:G:T
Proxy SNP: 2:187522750:G:T
UKBPPP log10(p): 124.5
UKBPPP BETA: −0.156
UKBPPP MSPA: 0
−:−:−:−:−:−:−:−:−:−:−:−:−:NA:−:-
976:484:703:256:339:139:267:7

269. ADD1 (P35611)
UKBPPP MSPA=0.012

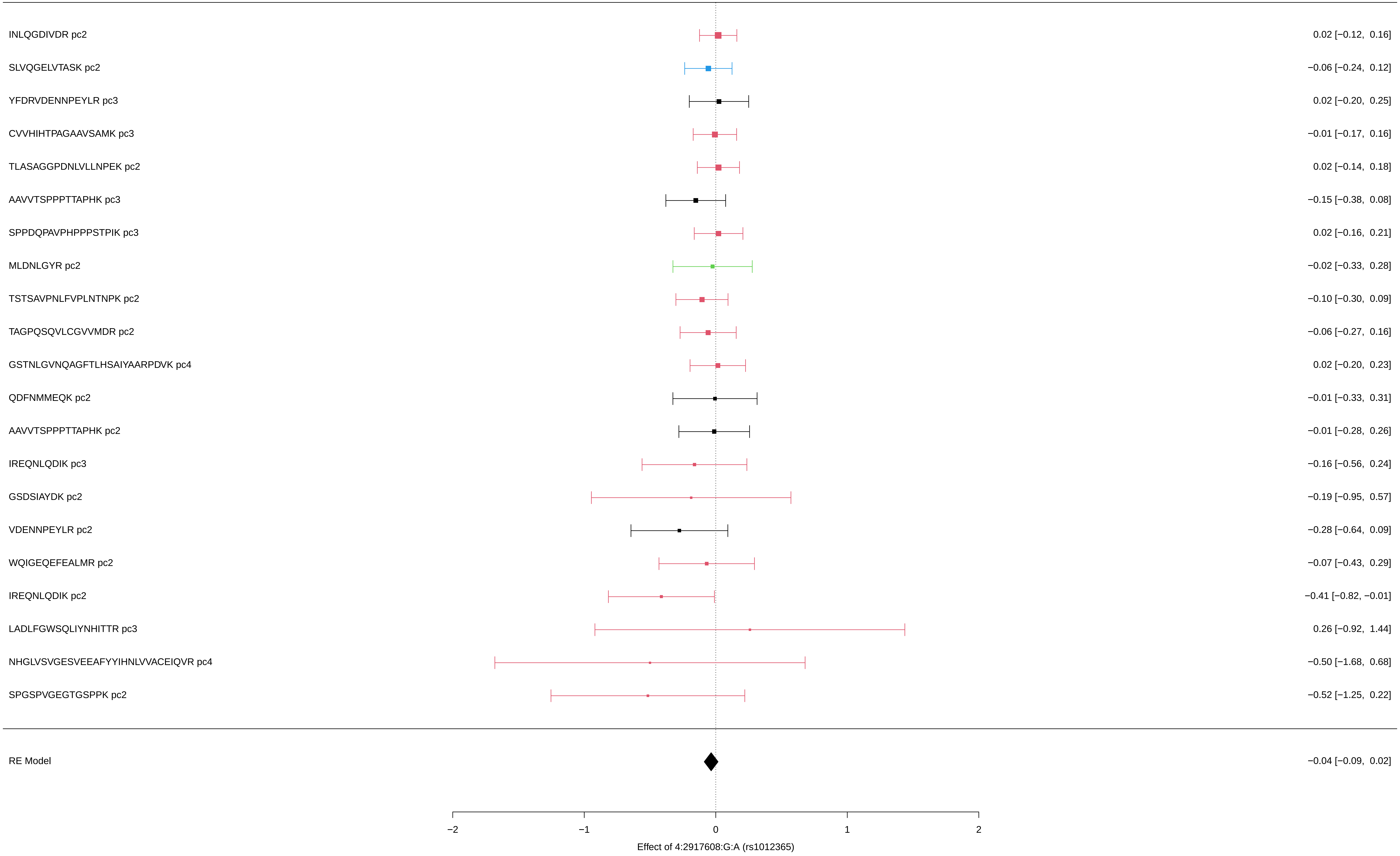

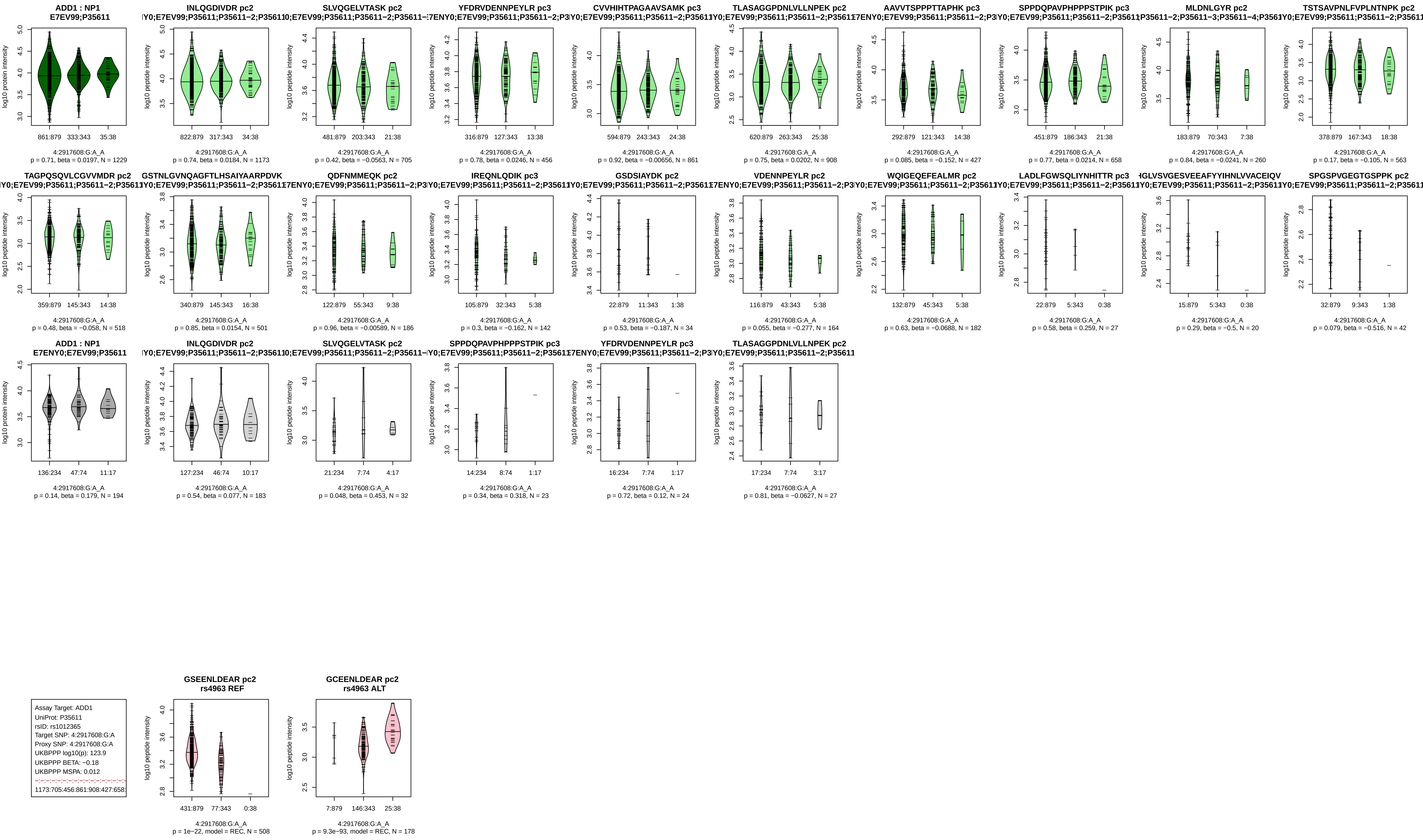
ADD1 : NP1
E7EV99;P35611
861:879 333:343 35:38
4:2917608:G:A_A
p = 0.71, beta = 0.0197, N = 1229
INLQGDIVDR pc2
822:879 317:343 34:38
p = 0.74, beta = 0.0184, N = 1173
SLVQGELVTASK pc2
481:879 203:343 21:38
p = 0.42, beta = −0.0563, N = 705
YFDRVDENNPEYLR pc3
316:879 127:343 13:38
p = 0.78, beta = 0.0246, N = 456
CVVHIHTPAGAAVSAMK pc3
594:879 243:343 24:38
p = 0.92, beta = −0.00656, N = 861
TLASAGGPDNLVLLNPEK pc2
620:879 263:343 25:38
p = 0.75, beta = 0.0202, N = 908
AAVVTSPPPTTAPHK pc3
292:879 121:343 14:38
p = 0.085, beta = −0.152, N = 427
SPPDQPAVPHPPPSTPIK pc3
451:879 186:343 21:38
p = 0.77, beta = 0.0214, N = 658
MLDNLGYR pc2
183:879 70:343 7:38
p = 0.84, beta = −0.0241, N = 260
TSTSAVPNLFVPLNTNPK pc2
378:879 167:343 18:38
p = 0.17, beta = −0.105, N = 563
TAGPQSQVLCGVVMDR pc2
359:879 145:343 14:38
p = 0.48, beta = −0.058, N = 518
GSTNLGVNQAGFTLHSAIYAARPDVK
340:879 145:343 16:38
p = 0.85, beta = 0.0154, N = 501
QDFNMMEQK pc2
122:879 55:343 9:38
p = 0.96, beta = −0.00589, N = 186
IREQNLQDIK pc3
105:879 32:343 5:38
p = 0.3, beta = −0.162, N = 142
GSDSIAYDK pc2
22:879 11:343 1:38
p = 0.53, beta = −0.187, N = 34
VDENNPEYLR pc2
116:879 43:343 5:38
p = 0.055, beta = −0.277, N = 164
WQIGEQEFEALMR pc2
132:879 45:343 5:38
p = 0.63, beta = −0.0688, N = 182
LADLFGWSQLIYNHITTR pc3
22:879 5:343 0:38
p = 0.58, beta = 0.259, N = 27
GLVSVGESVEEAFYYIHNLVVACEIQV
15:879 5:343 0:38
p = 0.29, beta = −0.5, N = 20
SPGSPVGEGTGSPPK pc2
32:879 9:343 1:38
p = 0.079, beta = −0.516, N = 42
ADD1 : NP1
E7ENY0;E7EV99;P35611
136:234 47:74 11:17
p = 0.14, beta = 0.179, N = 194
INLQGDIVDR pc2
127:234 46:74 10:17
p = 0.54, beta = 0.077, N = 183
SLVQGELVTASK pc2
21:234 7:74 4:17
p = 0.048, beta = 0.453, N = 32
SPPDQPAVPHPPPSTPIK pc3
14:234 8:74 1:17
p = 0.34, beta = 0.318, N = 23
YFDRVDENNPEYLR pc3
16:234 7:74 1:17
p = 0.72, beta = 0.12, N = 24
TLASAGGPDNLVLLNPEK pc2
17:234 7:74 3:17
p = 0.81, beta = −0.0627, N = 27
Assay Target: ADD1
UniProt: P35611
rsID: rs1012365
Target SNP: 4:2917608:G:A
Proxy SNP: 4:2917608:G:A
UKBPPP log10(p): 123.9
UKBPPP BETA: −0.18
UKBPPP MSPA: 0.012
GSEENLDEAR pc2
rs4963 REF
431:879 77:343 0:38
p = 1e−22, model = REC, N = 508
GCEENLDEAR pc2
rs4963 ALT
7:879 146:343 25:38
p = 9.3e−93, model = REC, N = 178

270. ITGAL (P20701)
UKBPPP MSPA=0

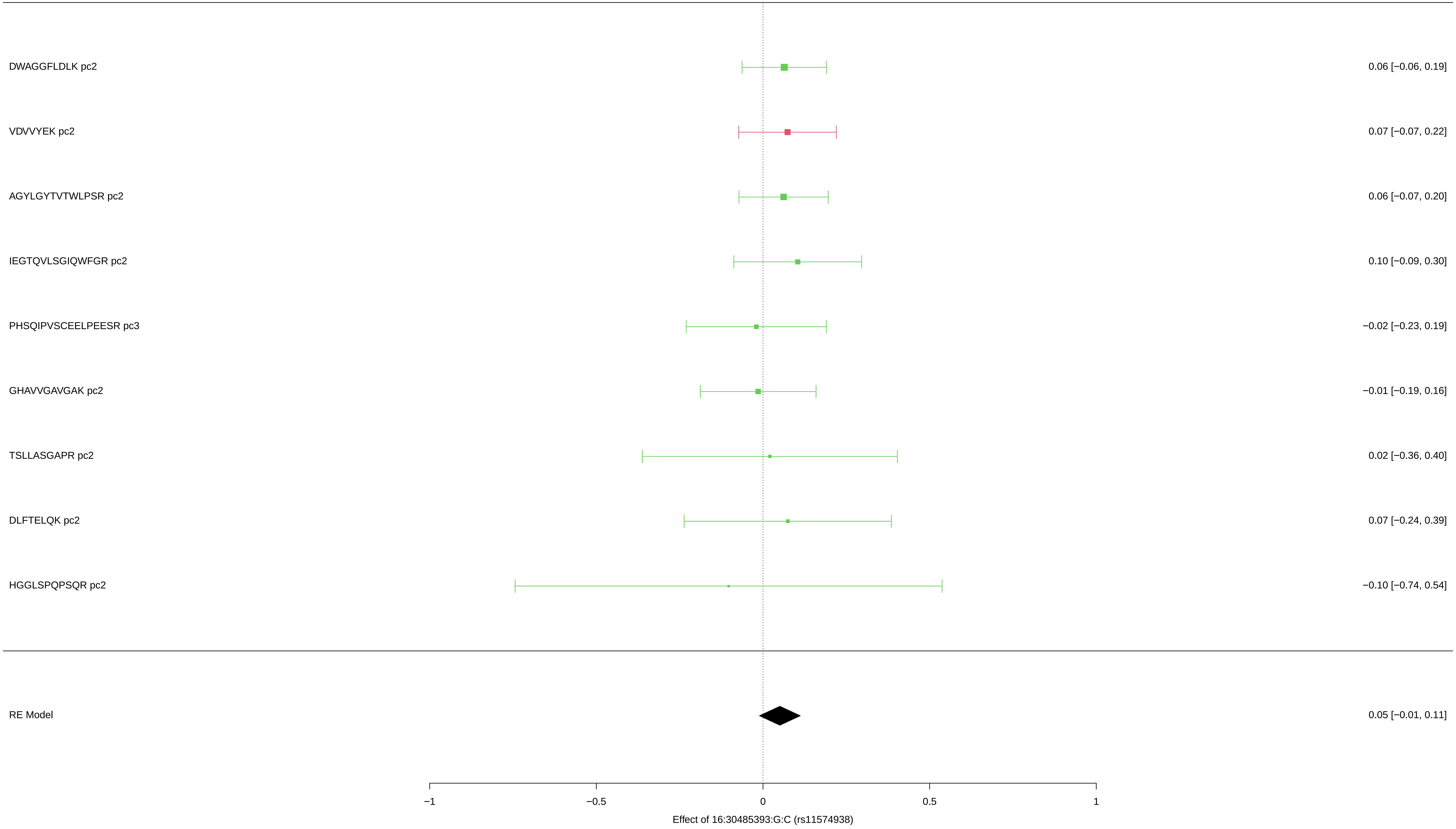

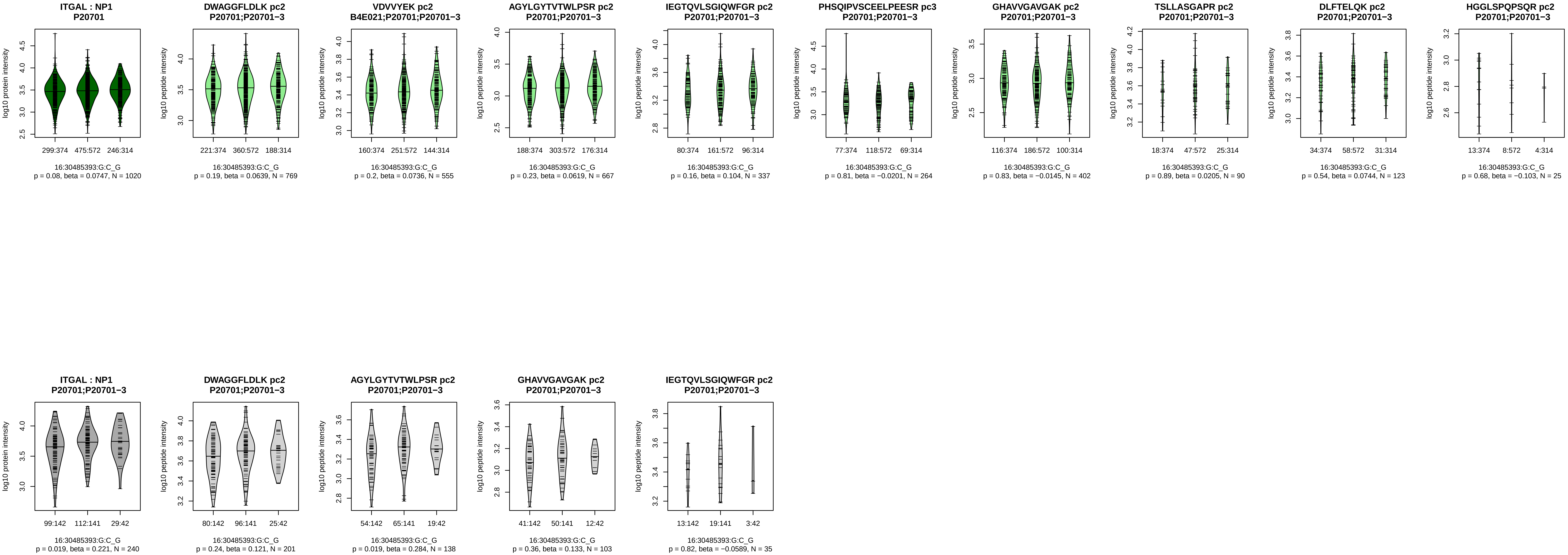

Assay Target: ITGAL
UniProt: P20701
rsID: rs11574938
Target SNP: 16:30485393:G:C
Proxy SNP: 16:30485393:G:C
UKBPPP log10(p): 123.7
UKBPPP BETA: −0.149
UKBPPP MSPA: 0
−:−:−:−:−:−:−:−:−:NA
769:555:667:337:264:402:90:12

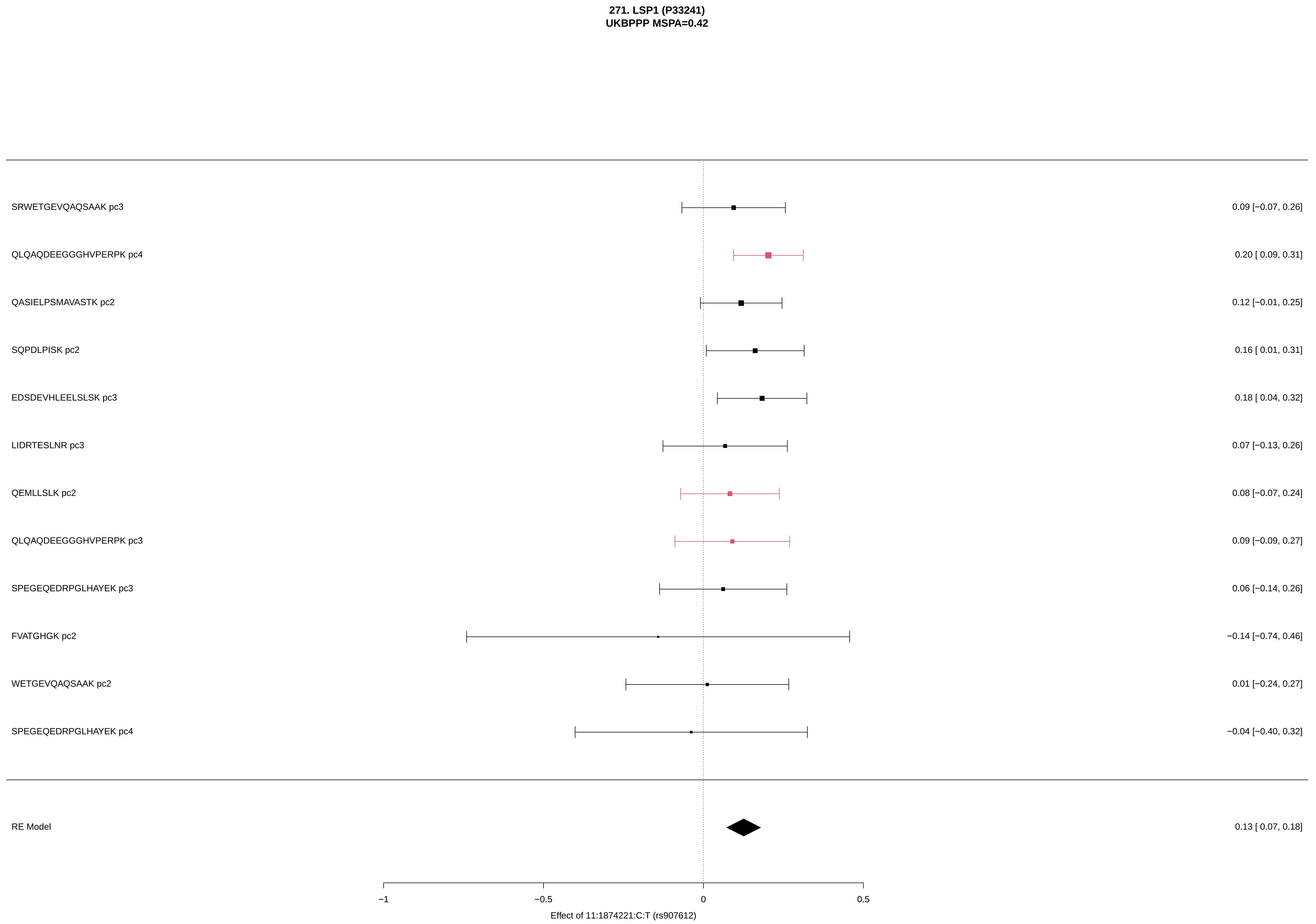
271. LSP1 (P33241)
UKBPPP MSPA=0.42
SRWETGEVQAQSAAK pc3
0.09 [−0.07, 0.26]
QLQAQDEEGGGHVPERPK pc4
0.20 [0.09, 0.31]
QASIELPSMAVASTK pc2
0.12 [−0.01, 0.25]
SQPDLPISK pc2
0.16 [0.01, 0.31]
EDSDEVHLEELSLSK pc3
0.18 [0.04, 0.32]
LIDRTESLNR pc3
0.07 [−0.13, 0.26]
QEMLLSLK pc2
0.08 [−0.07, 0.24]
QLQAQDEEGGGHVPERPK pc3
0.09 [−0.09, 0.27]
SPEGEQEDRPGLHAYEK pc3
0.06 [−0.14, 0.26]
FVATGHGK pc2
−0.14 [−0.74, 0.46]
WETGEVQAQSAAK pc2
0.01 [−0.24, 0.27]
SPEGEQEDRPGLHAYEK pc4
−0.04 [−0.40, 0.32]
RE Model
0.13 [0.07, 0.18]
−1
−0.5
0
0.5
Effect of 11:1874221:C:T (rs907612)

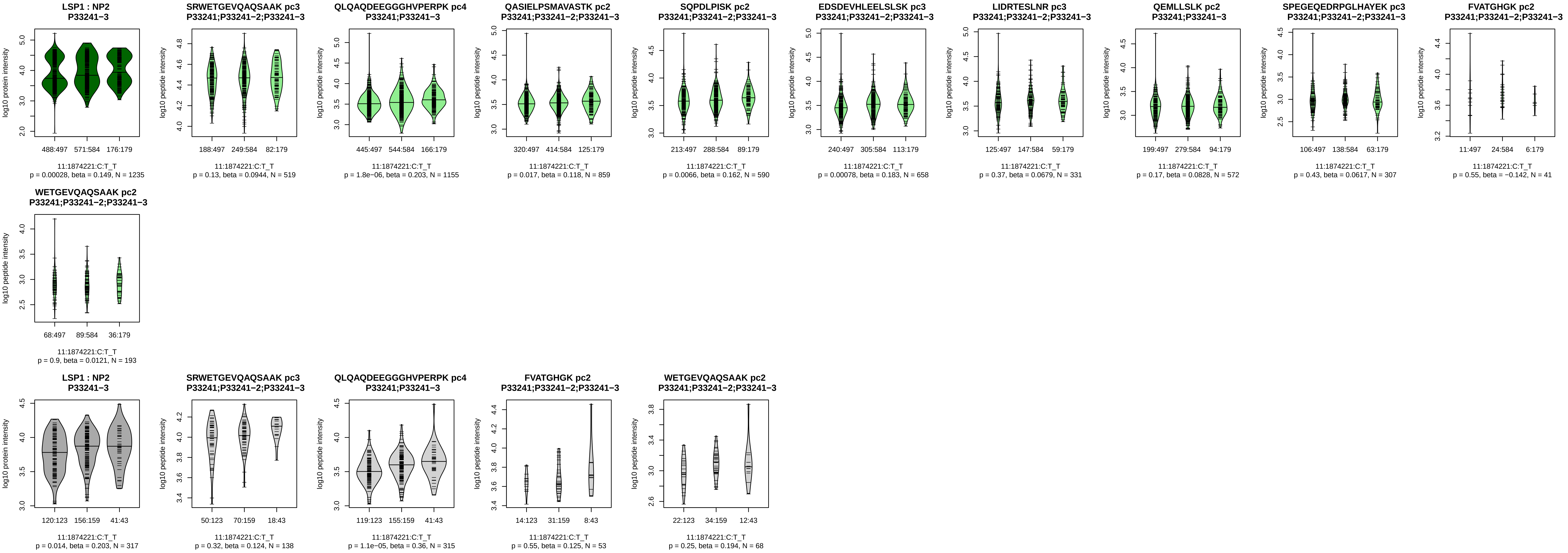

Assay Target: LSP1
UniProt: P33241
rsID: rs907612
Target SNP: 11:1874221:C:T
Proxy SNP: 11:1874221:C:T
UKBPPP log10(p): 121.7
UKBPPP BETA: 0.146
UKBPPP MSPA: 0.42
−:*:−:*:*:−:−:−:−:−:−:−:NA:NA
519:1155:859:590:658:331:572:

272. MMP9 (P14780)
UKBPPP MSPA=0

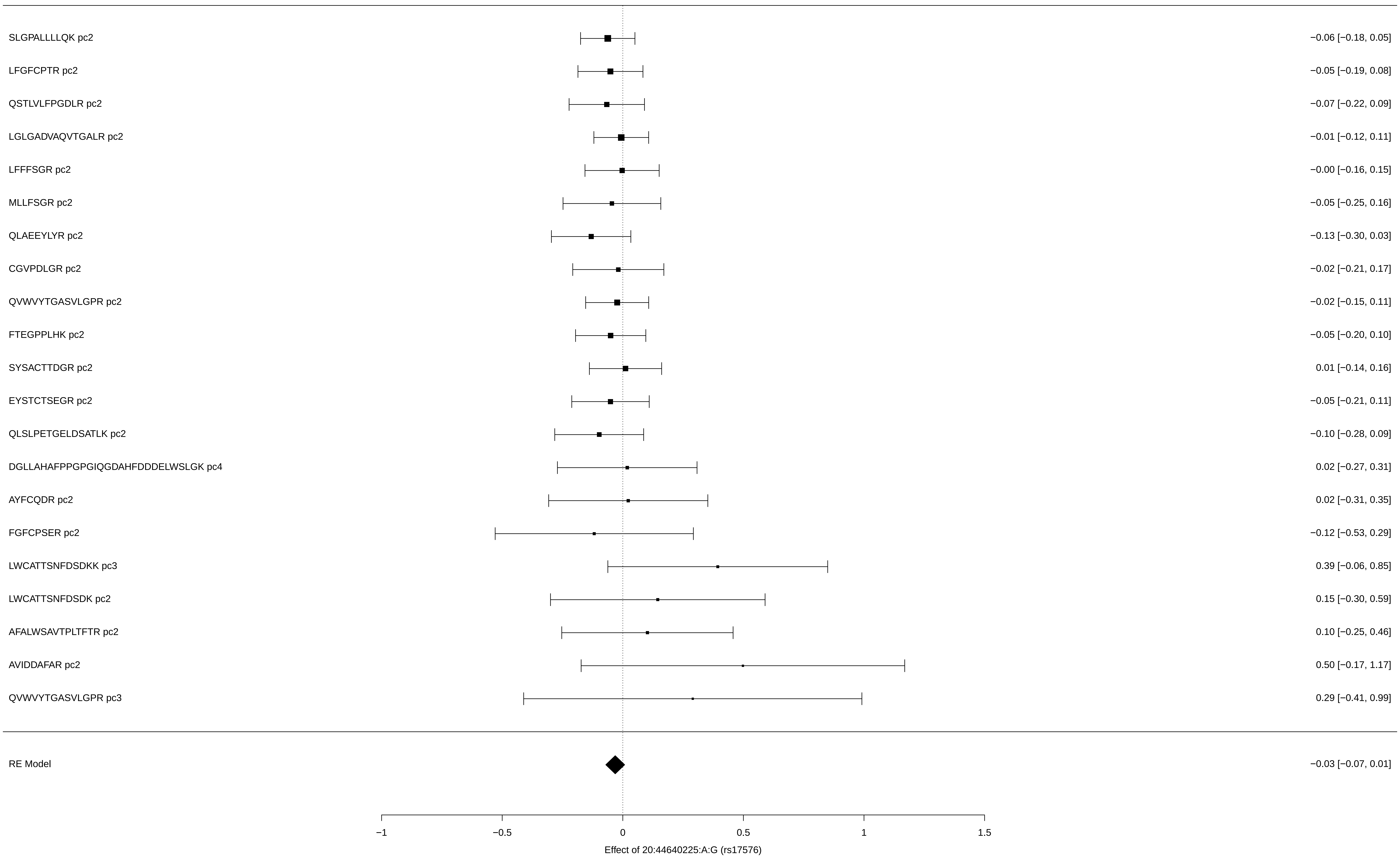

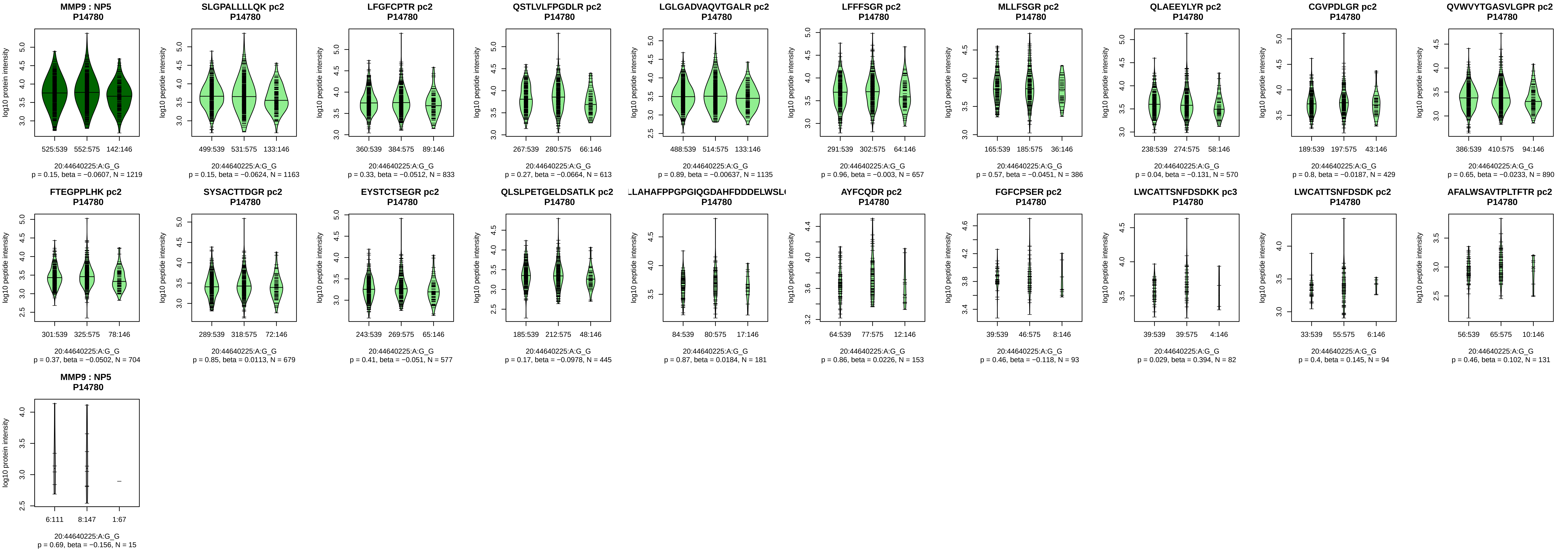

Assay Target: MMP9
UniProt: P14780
rsID: rs17576
Target SNP: 20:44640225:A:G
Proxy SNP: 20:44640225:A:G
UKBPPP log10(p): 116.2
UKBPPP BETA: 0.144
UKBPPP MSPA: 0
1163:833:613:1135:657:386:570

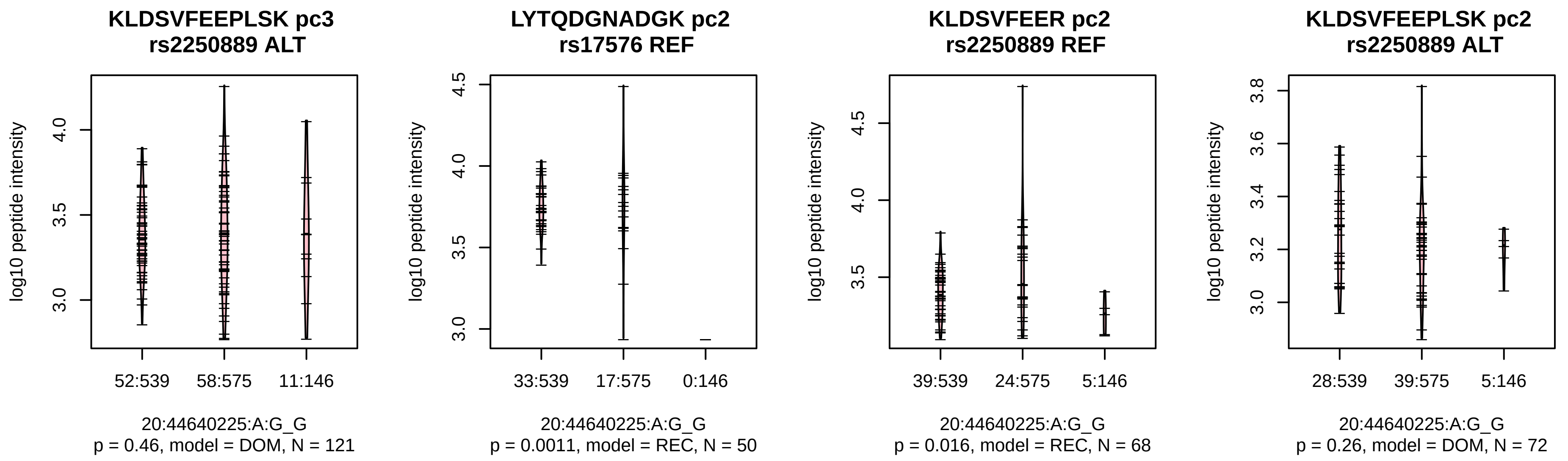

273. C5 (P01031)
UKBPPP MSPA=0.021

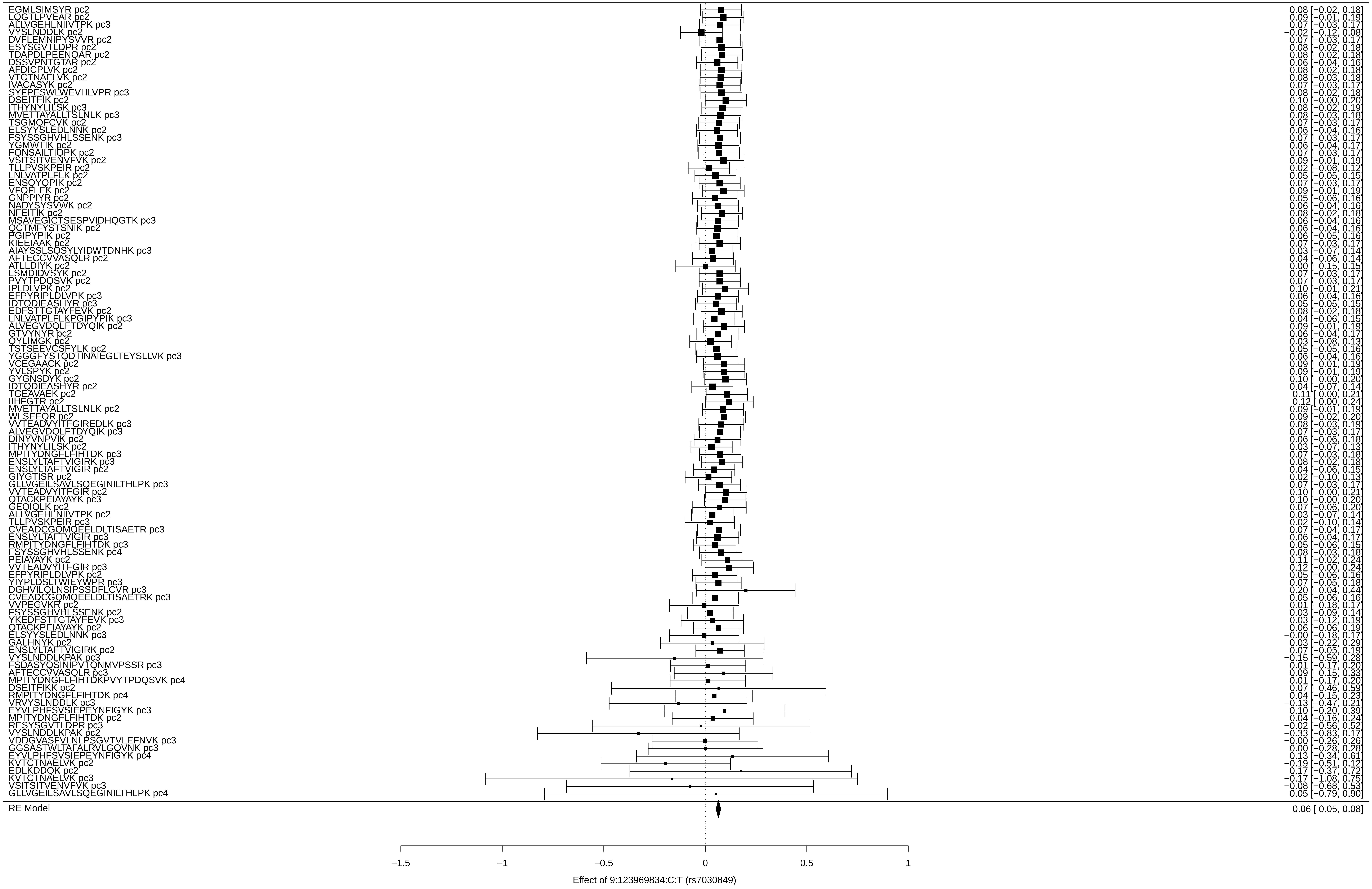

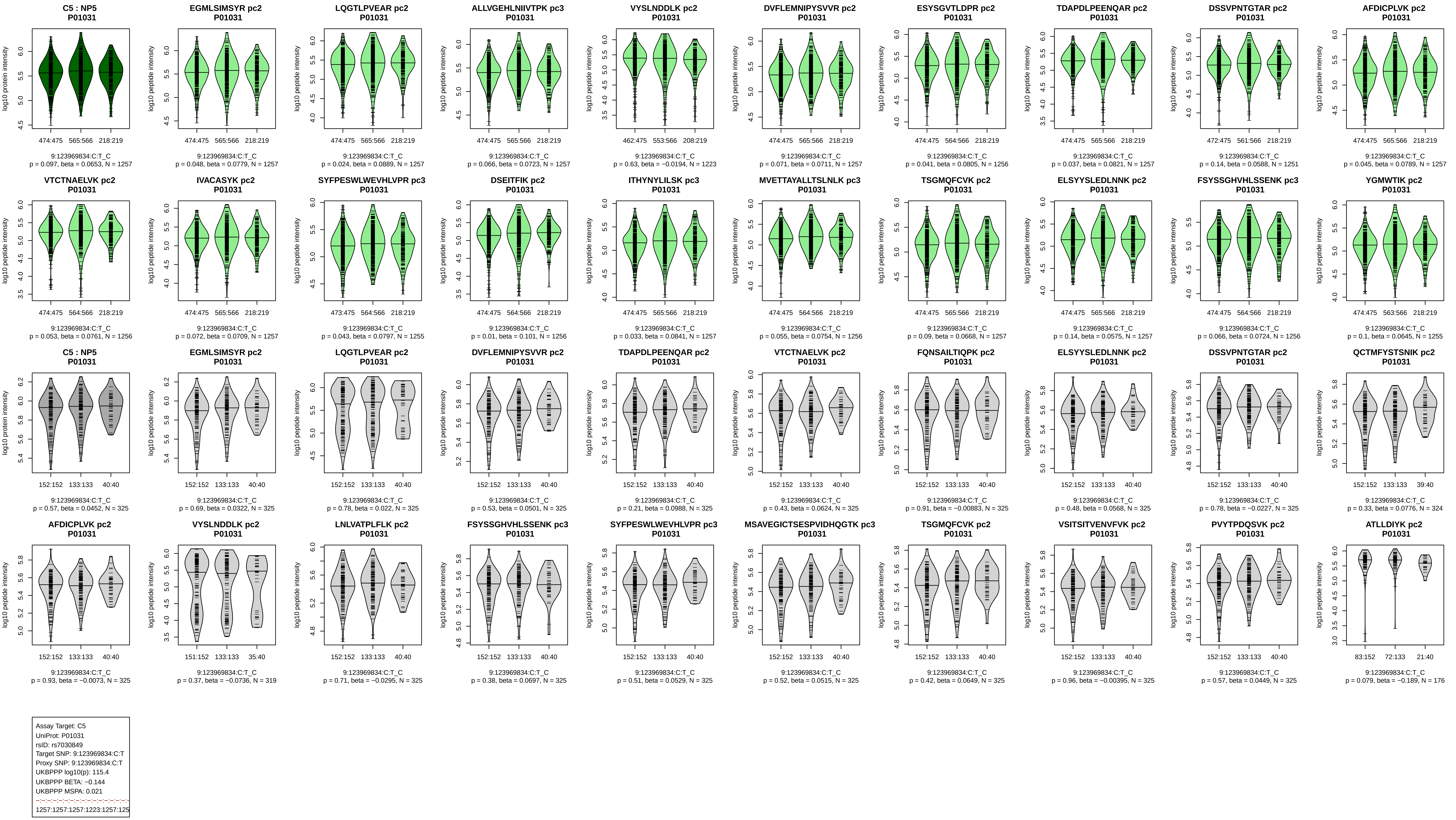
C5 : NP5 P01031
p = 0.097, beta = 0.0653, N = 1257
EGMLSIMSYR pc2 P01031
p = 0.048, beta = 0.0779, N = 1257
LQGTLPVEAR pc2 P01031
p = 0.024, beta = 0.0889, N = 1257
ALLVGEHLNIIVTPK pc3 P01031
p = 0.066, beta = 0.0723, N = 1257
VYSLNDDLK pc2 P01031
p = 0.63, beta = −0.0194, N = 1223
DVFLEMNIPYSVVR pc2 P01031
p = 0.071, beta = 0.0711, N = 1257
ESYSGVTLDPR pc2 P01031
p = 0.041, beta = 0.0805, N = 1256
TDAPDLPEENQAR pc2 P01031
p = 0.037, beta = 0.0821, N = 1257
DSSVPNTGTAR pc2 P01031
p = 0.14, beta = 0.0588, N = 1251
AFDICPLVK pc2 P01031
p = 0.045, beta = 0.0789, N = 1257
VTCTNAELVK pc2 P01031
p = 0.053, beta = 0.0761, N = 1256
IVACASYK pc2 P01031
p = 0.072, beta = 0.0709, N = 1257
SYFPESWLWEVHLVPR pc3 P01031
p = 0.043, beta = 0.0797, N = 1255
DSEITFIK pc2 P01031
p = 0.01, beta = 0.101, N = 1256
ITHYNYLILSK pc3 P01031
p = 0.033, beta = 0.0841, N = 1257
MVETTAYALLTSLNLK pc3 P01031
p = 0.055, beta = 0.0754, N = 1256
TSGMQFCVK pc2 P01031
p = 0.09, beta = 0.0668, N = 1257
ELSYYSLEDLNNK pc2 P01031
p = 0.14, beta = 0.0575, N = 1257
FSYSSGHVHLSSENK pc3 P01031
p = 0.066, beta = 0.0724, N = 1256
YGMWTIK pc2 P01031
p = 0.1, beta = 0.0645, N = 1255
C5 : NP5 P01031
p = 0.57, beta = 0.0452, N = 325
EGMLSIMSYR pc2 P01031
p = 0.69, beta = 0.0322, N = 325
LQGTLPVEAR pc2 P01031
p = 0.78, beta = 0.022, N = 325
DVFLEMNIPYSVVR pc2 P01031
p = 0.53, beta = 0.0501, N = 325
TDAPDLPEENQAR pc2 P01031
p = 0.21, beta = 0.0988, N = 325
VTCTNAELVK pc2 P01031
p = 0.43, beta = 0.0624, N = 325
FQNSAILTIQPK pc2 P01031
p = 0.91, beta = −0.00883, N = 325
ELSYYSLEDLNNK pc2 P01031
p = 0.48, beta = 0.0568, N = 325
DSSVPNTGTAR pc2 P01031
p = 0.78, beta = −0.0227, N = 325
QCTMFYSTSNIK pc2 P01031
p = 0.33, beta = 0.0776, N = 324
AFDICPLVK pc2 P01031
p = 0.93, beta = −0.0073, N = 325
VYSLNDDLK pc2 P01031
p = 0.37, beta = −0.0736, N = 319
LNLVATPLFLK pc2 P01031
p = 0.71, beta = −0.0295, N = 325
FSYSSGHVHLSSENK pc3 P01031
p = 0.38, beta = 0.0697, N = 325
SYFPESWLWEVHLVPR pc3 P01031
p = 0.51, beta = 0.0529, N = 325
MSAVEGICTSESPVIDHQGTK pc3 P01031
p = 0.52, beta = 0.0515, N = 325
TSGMQFCVK pc2 P01031
p = 0.42, beta = 0.0649, N = 325
VSITSITVENVFVK pc2 P01031
p = 0.96, beta = −0.00395, N = 325
PVYTPDQSVK pc2 P01031
p = 0.57, beta = 0.0449, N = 325
ATLLDIYK pc2 P01031
p = 0.079, beta = −0.189, N = 176
9:123969834:C:T_C
Assay Target: C5
UniProt: P01031
rsID: rs7030849
Target SNP: 9:123969834:C:T
Proxy SNP: 9:123969834:C:T
UKBPPP log10(p): 115.4
UKBPPP BETA: −0.144
UKBPPP MSPA: 0.021
1257:1257:1257:1223:1257:125

274. FSTL1 (Q12841)
UKBPPP MSPA=0.741

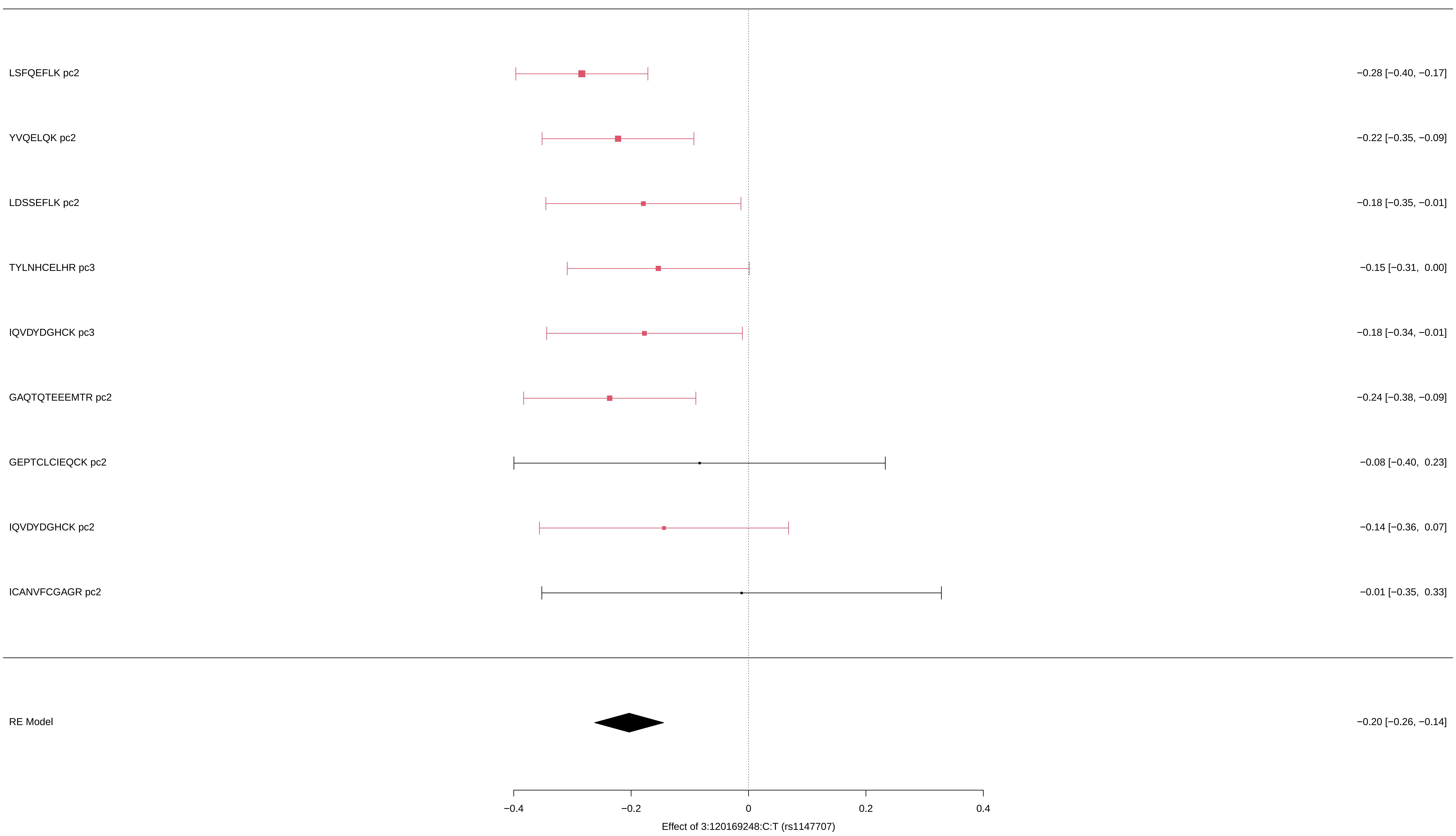

FSTL1 : NP2 Q12841 | **LSFQEFLK pc2** Q12841;Q12841−2 | **YVQELQK pc2** Q12841;Q12841−2 | **LDSSEFLK pc2** Q12841;Q12841−2 | **TYLNHCELHR pc3** Q12841;Q12841−2 | **IQVDYDGHCK pc3** Q12841;Q12841−2 | **GAQTQTEEEMTR pc2** Q12841;Q12841−2 | **GEPTCLCIEQCK pc2** Q12841 | **ICANVFCGAGR pc2** Q12841

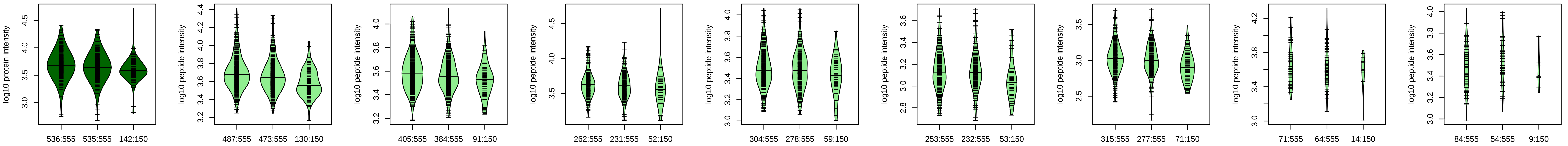

3:120169248:C:T_T
p = 1.8e−08, beta = −0.238, N = 1213

3:120169248:C:T_T
p = 1.2e−10, beta = −0.284, N = 1090

3:120169248:C:T_T
p = 1.1e−05, beta = −0.222, N = 880

3:120169248:C:T_T
p = 0.0057, beta = −0.179, N = 545

3:120169248:C:T_T
p = 0.011, beta = −0.154, N = 641

3:120169248:C:T_T
p = 0.0064, beta = −0.177, N = 538

3:120169248:C:T_T
p = 3.7e−05, beta = −0.236, N = 663

3:120169248:C:T_T
p = 0.5, beta = −0.0834, N = 149

3:120169248:C:T_T
p = 0.93, beta = −0.0118, N = 147

FSTL1 : NP2 Q12841 | **LSFQEFLK pc2** Q12841;Q12841−2

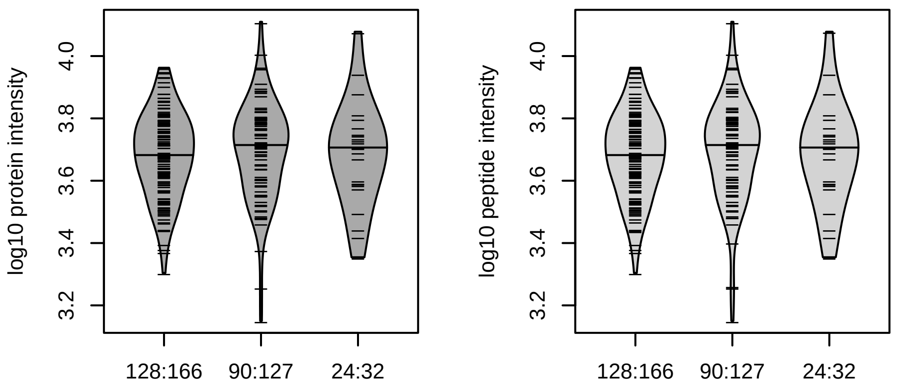

3:120169248:C:T_T
p = 0.66, beta = 0.0412, N = 242

3:120169248:C:T_T
p = 0.69, beta = 0.0383, N = 242

Assay Target: FSTL1
UniProt: Q12841
rsID: rs1147707
Target SNP: 3:120169248:C:T
Proxy SNP: 3:120169248:C:T
UKBPPP log10(p): 114.4
UKBPPP BETA: −0.147
UKBPPP MSPA: 0.741
::*:–:*:*:–:–:–
1090:880:545:641:538:663:149:

275. CRIP2 (P52943)
UKBPPP MSPA=0

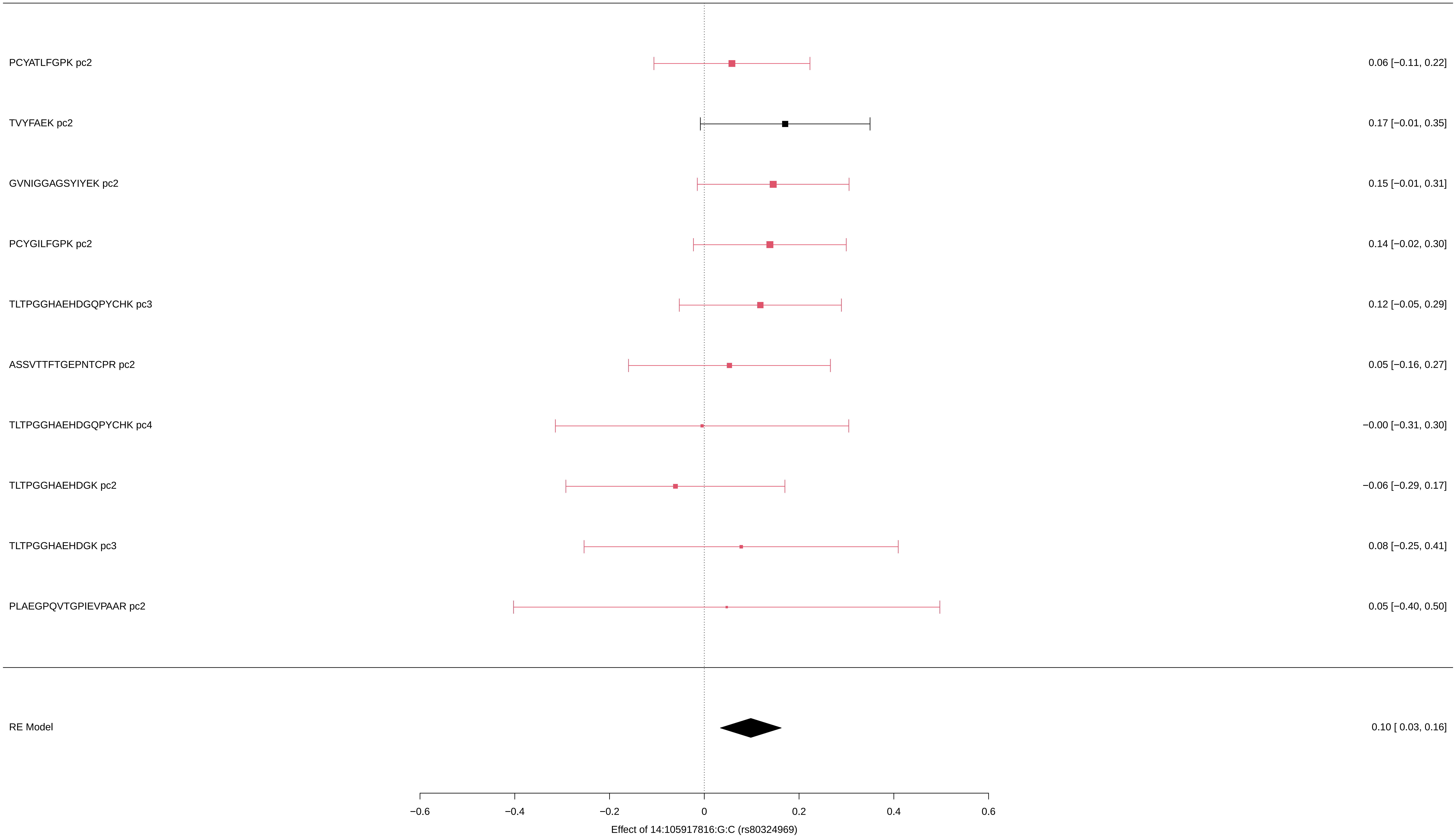

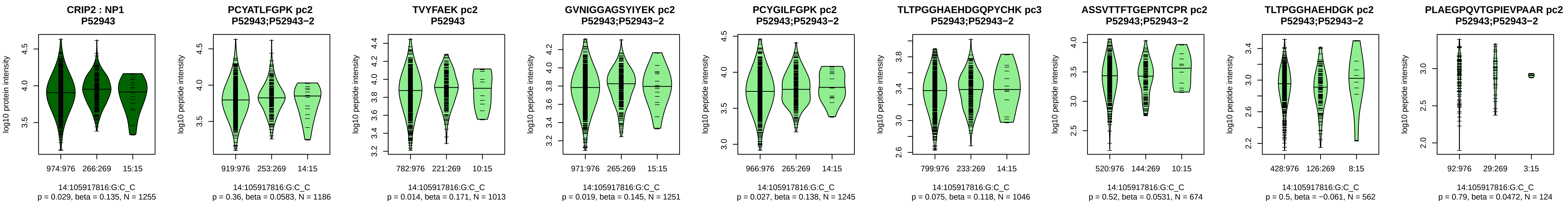

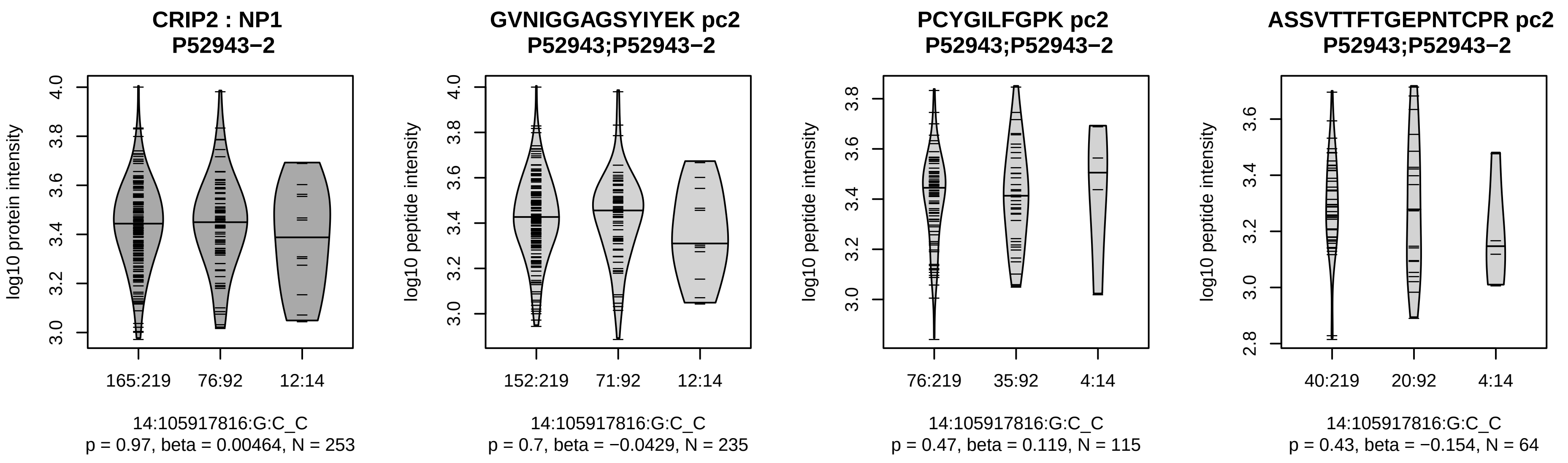

Assay Target: CRIP2
UniProt: P52943
rsID: rs80324969
Target SNP: 14:105917816:G:C
Proxy SNP: 14:105917816:G:C
UKBPPP log10(p): 112.5
UKBPPP BETA: 0.185
UKBPPP MSPA: 0
1186:1013:1251:1245:1046:674

276. PRSS2 (P07478)
UKBPPP MSPA=0

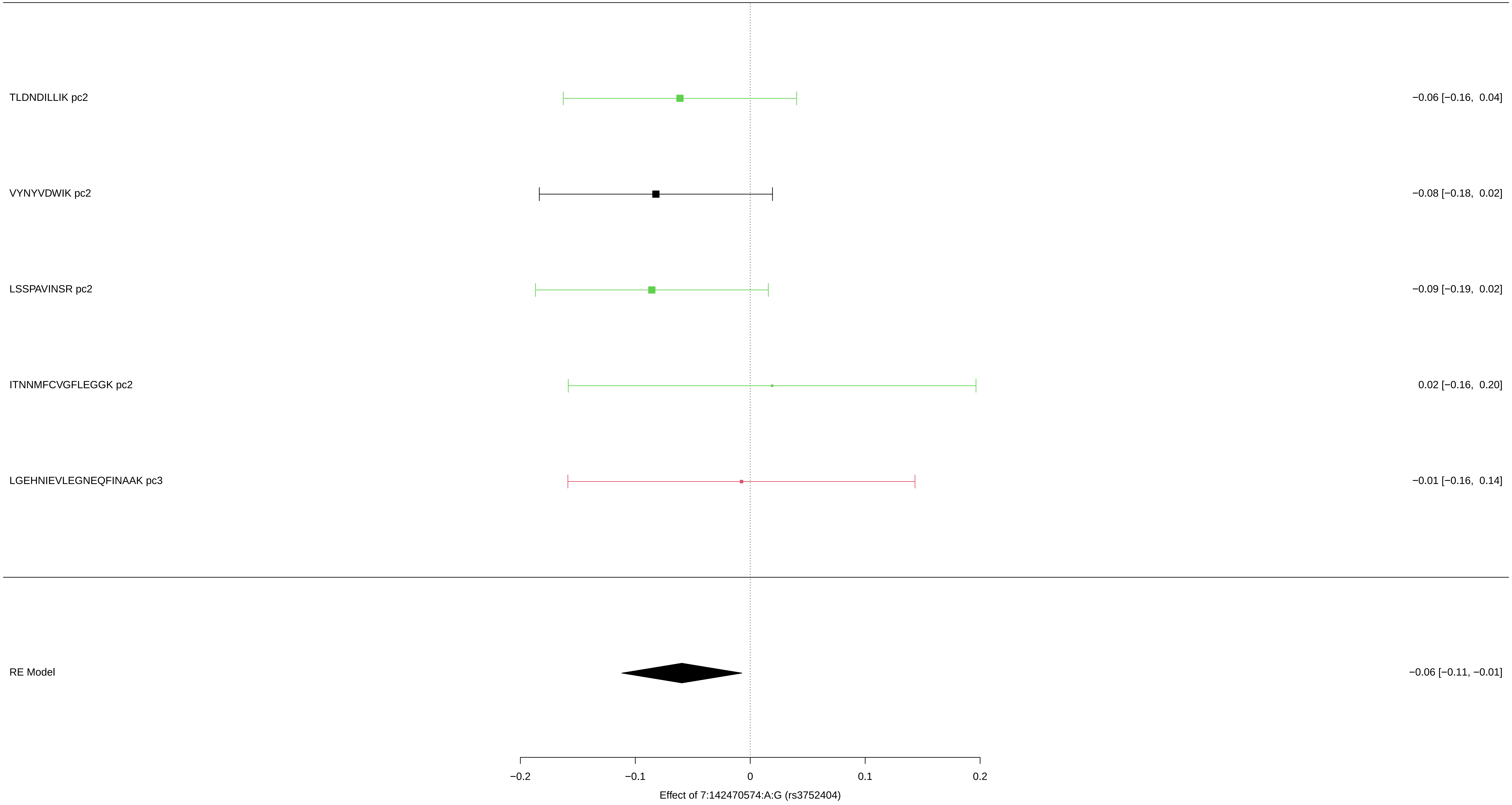

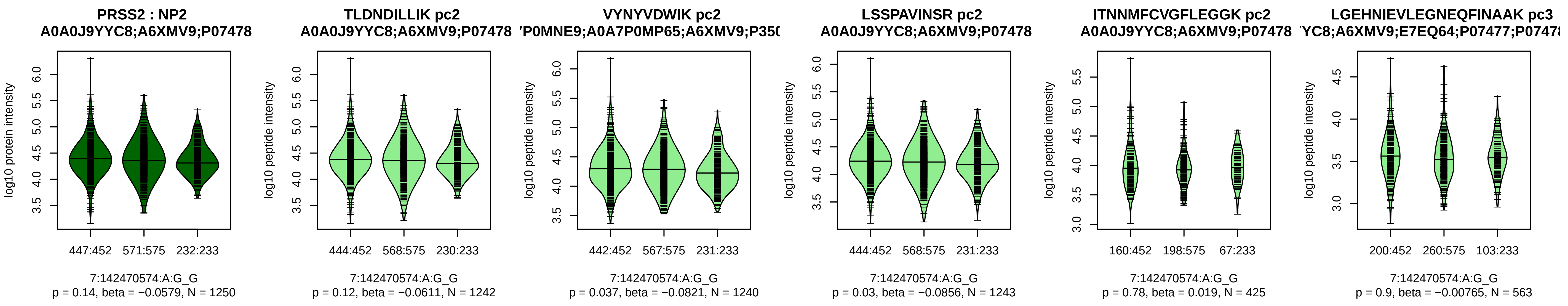

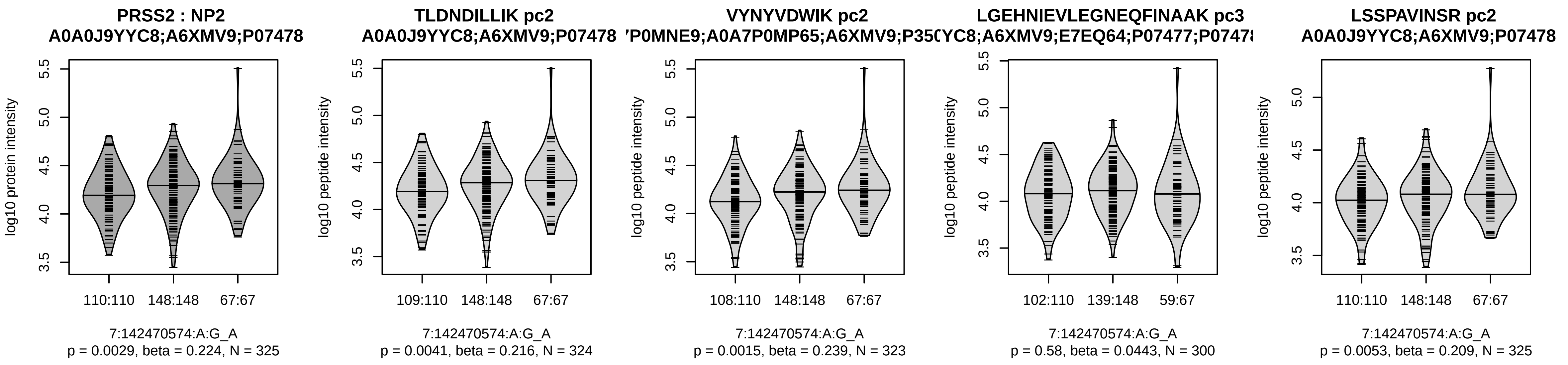

Assay Target: PRSS2
UniProt: P07478
rsID: rs3752404
Target SNP: 7:142470574:A:G
Proxy SNP: 7:142470574:A:G
UKBPPP log10(p): 110
UKBPPP BETA: −0.137
UKBPPP MSPA: 0
–:–:–:–:–
1242:1240:1243:425:563

277. VAT1 (Q99536)
UKBPPP MSPA=0

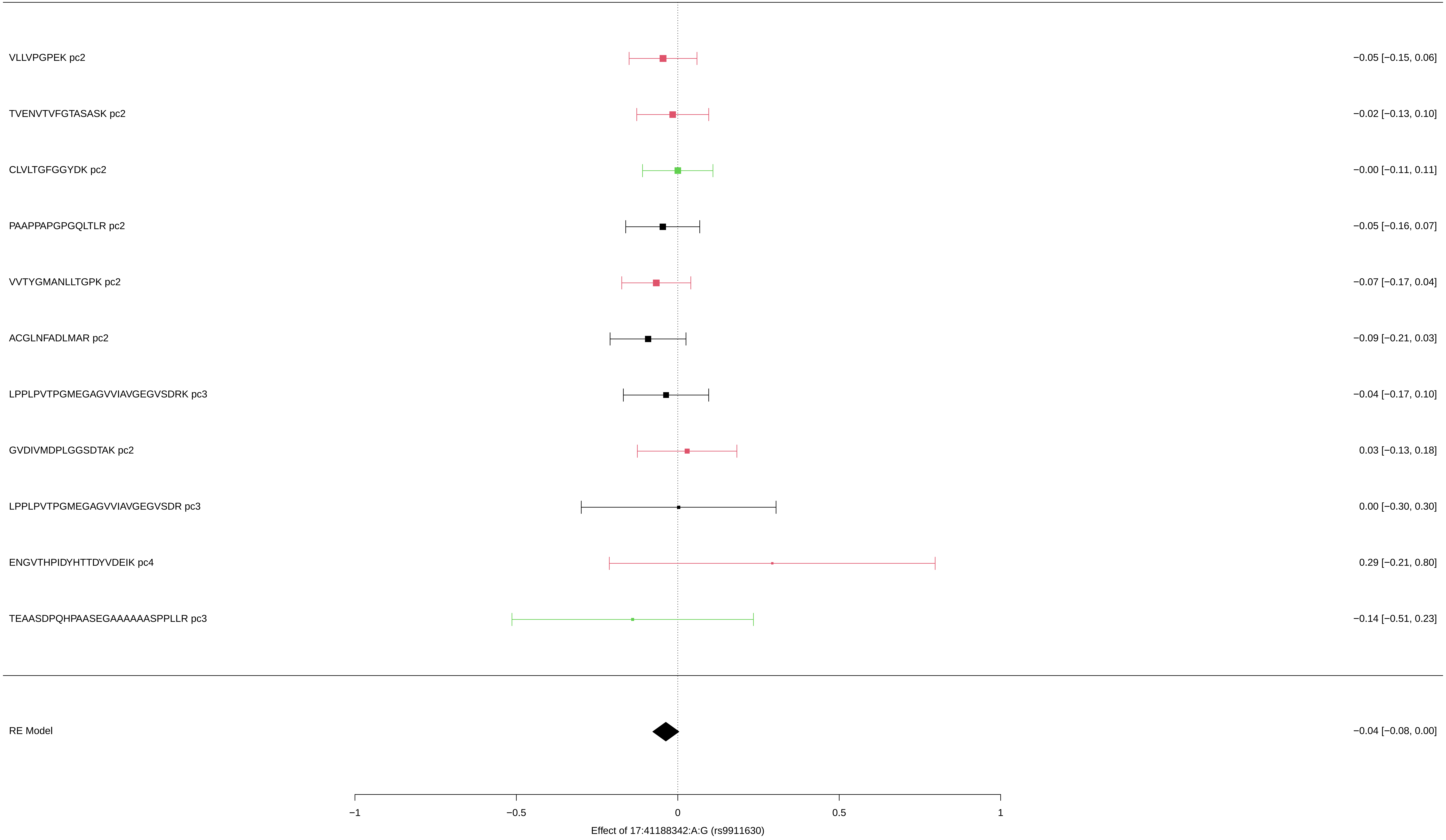

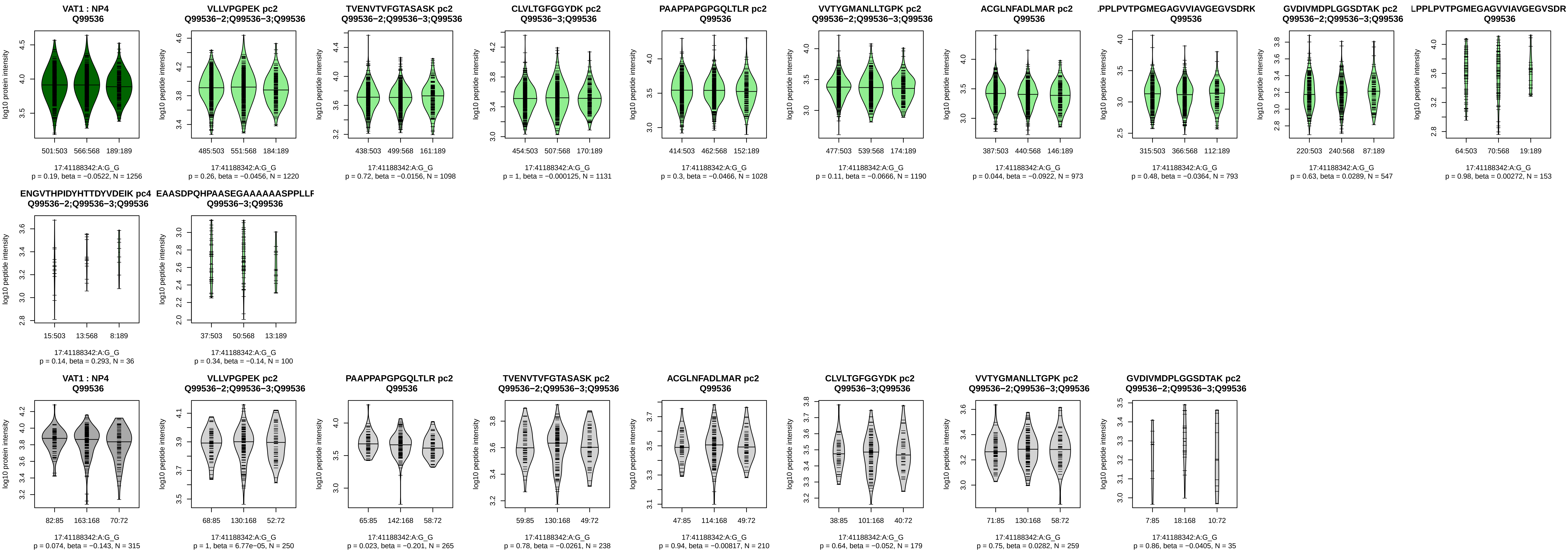

Assay Target: VAT1
UniProt: Q99536
rsID: rs9911630
Target SNP: 17:41188342:A:G
Proxy SNP: 17:41188342:A:G
UKBPPP log10(p): 109.3
UKBPPP BETA: −0.145
UKBPPP MSPA: 0
−:−:−:−:−:−:−:−:−
1220:1098:1131:1028:1190:973

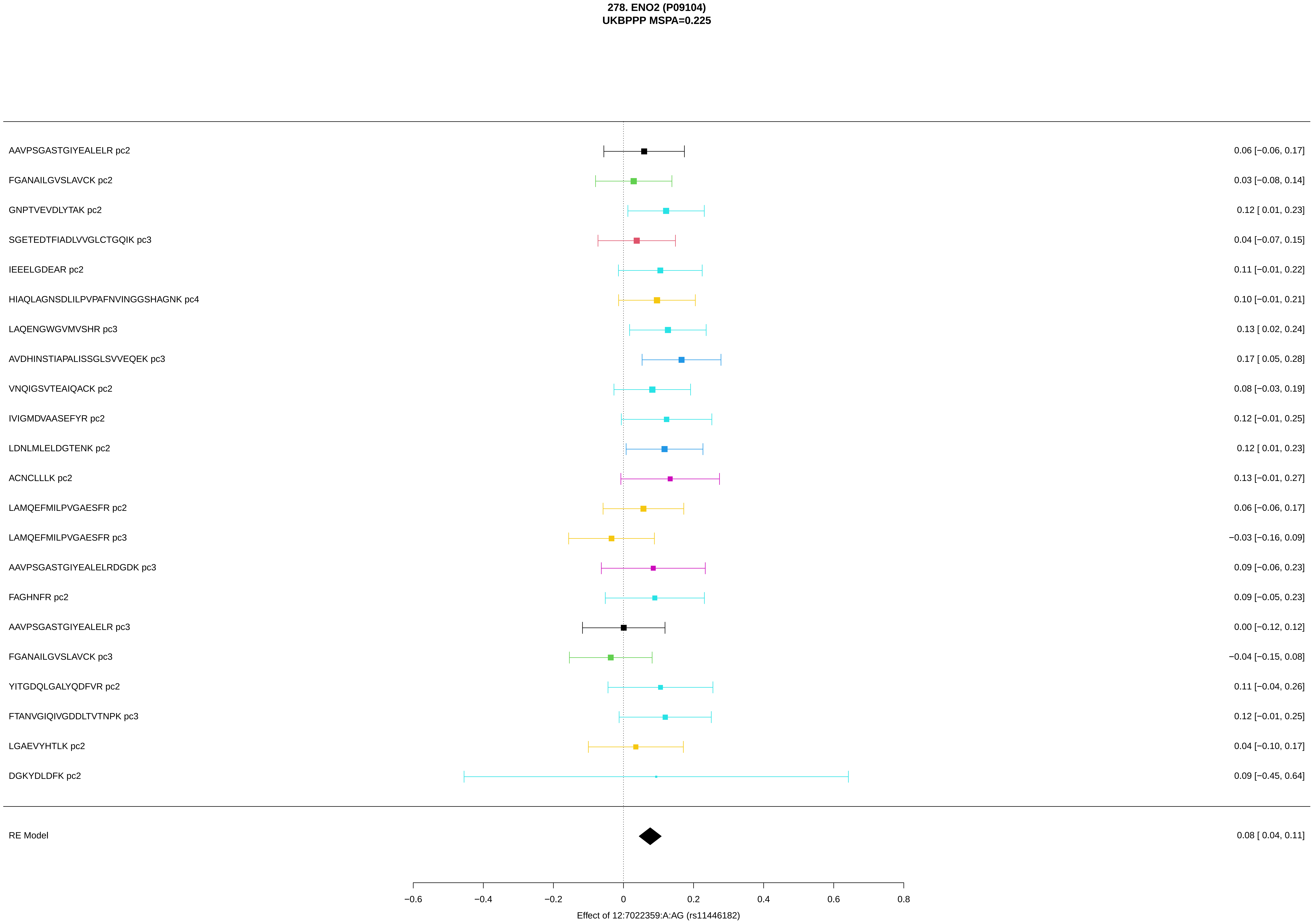

278. ENO2 (P09104)
UKBPPP MSPA=0.225
AAVPSGASTGIYEALELR pc2
0.06 [−0.06, 0.17]
FGANAILGVSLAVCK pc2
0.03 [−0.08, 0.14]
GNPTVEVDLYTAK pc2
0.12 [0.01, 0.23]
SGETEDTFIADLVVGLCTGQIK pc3
0.04 [−0.07, 0.15]
IEEELGDEAR pc2
0.11 [−0.01, 0.22]
HIAQLAGNSDLILPVPAFNVINGGSHAGNK pc4
0.10 [−0.01, 0.21]
LAQENGWGVMVSHR pc3
0.13 [0.02, 0.24]
AVDHINSTIAPALISSGLSVVEQEK pc3
0.17 [0.05, 0.28]
VNQIGSVTEAIQACK pc2
0.08 [−0.03, 0.19]
IVIGMDVAASEFYR pc2
0.12 [−0.01, 0.25]
LDNLMLELDGTENK pc2
0.12 [0.01, 0.23]
ACNCLLLK pc2
0.13 [−0.01, 0.27]
LAMQEFMILPVGAESFR pc2
0.06 [−0.06, 0.17]
LAMQEFMILPVGAESFR pc3
−0.03 [−0.16, 0.09]
AAVPSGASTGIYEALELRDGDK pc3
0.09 [−0.06, 0.23]
FAGHNFR pc2
0.09 [−0.05, 0.23]
AAVPSGASTGIYEALELR pc3
0.00 [−0.12, 0.12]
FGANAILGVSLAVCK pc3
−0.04 [−0.15, 0.08]
YITGDQLGALYQDFVR pc2
0.11 [−0.04, 0.26]
FTANVGIQIVGDDLTVTNPK pc3
0.12 [−0.01, 0.25]
LGAEVYHTLK pc2
0.04 [−0.10, 0.17]
DGKYDLDFK pc2
0.09 [−0.45, 0.64]
RE Model
0.08 [0.04, 0.11]
−0.6
−0.4
−0.2
0
0.2
0.4
0.6
0.8
Effect of 12:7022359:A:AG (rs11446182)

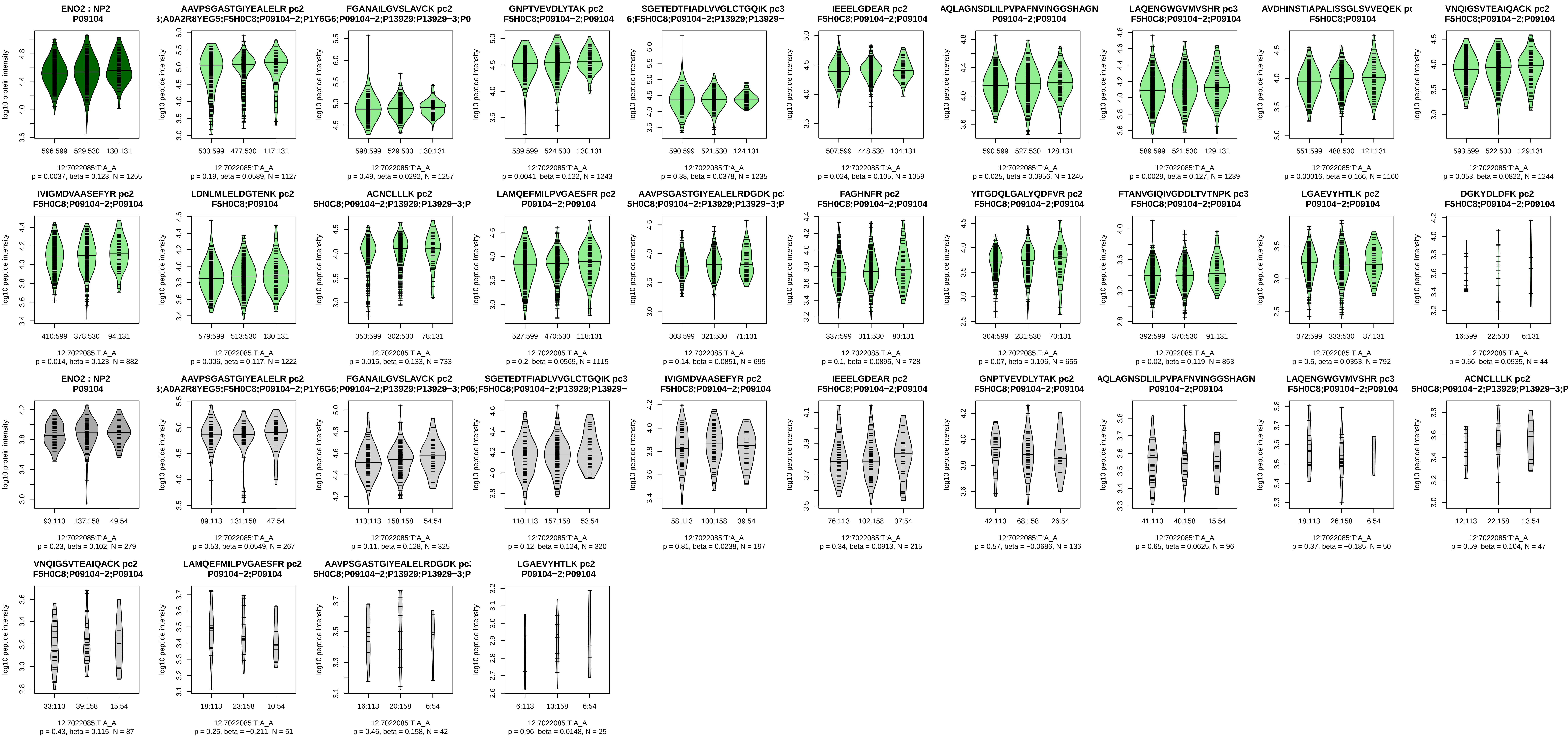

Assay Target: ENO2
UniProt: P09104
rsID: rs11446182
Target SNP: 12:7022359:A:AG
Proxy SNP: 12:7022085:T:A
UKBPPP log10(p): 108.9
UKBPPP BETA: 0.15
UKBPPP MSPA: 0.225
1127:1257:1243:1235:1059:124

279. APOA4 (P06727)
UKBPPP MSPA=0.048

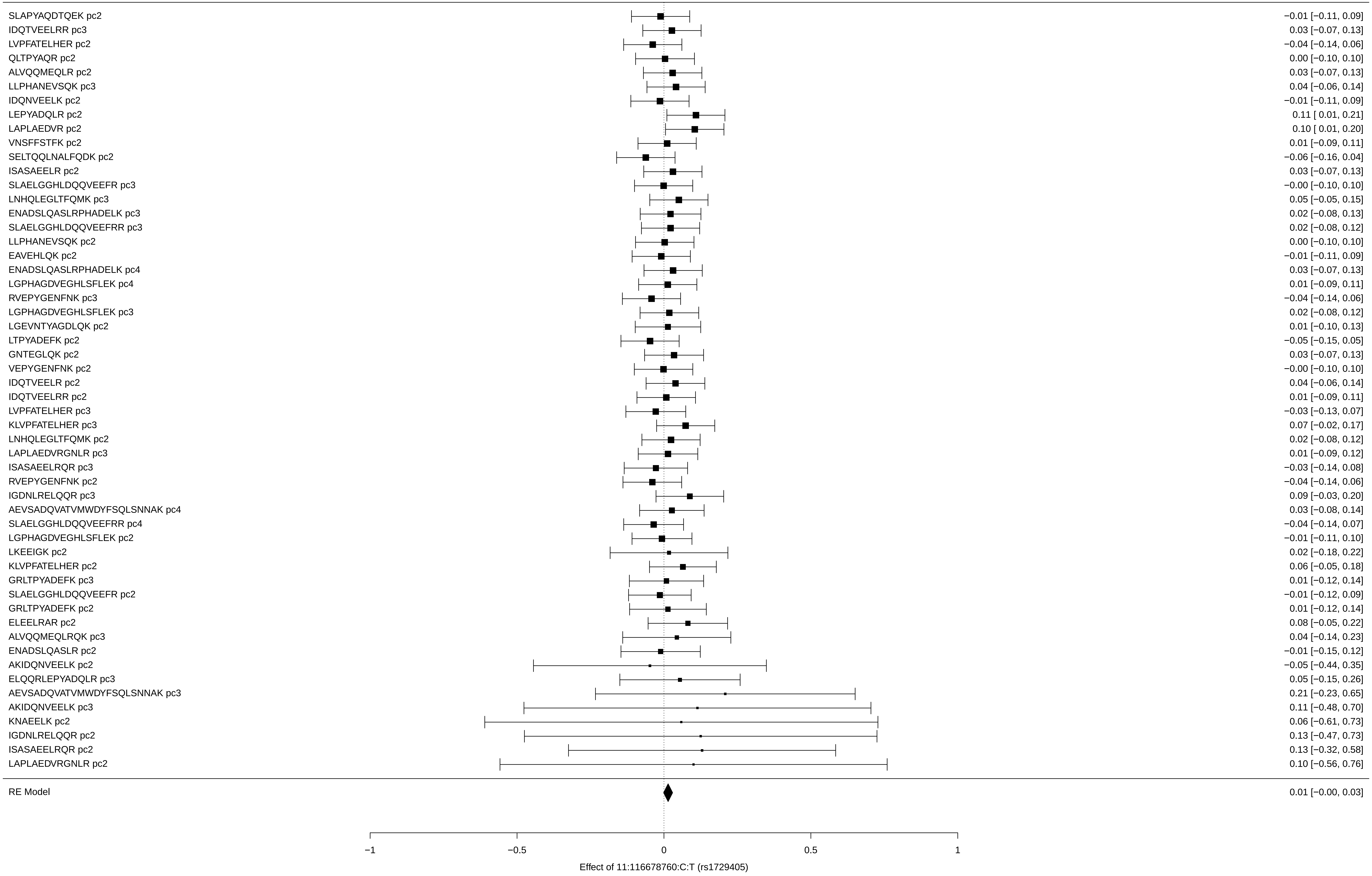

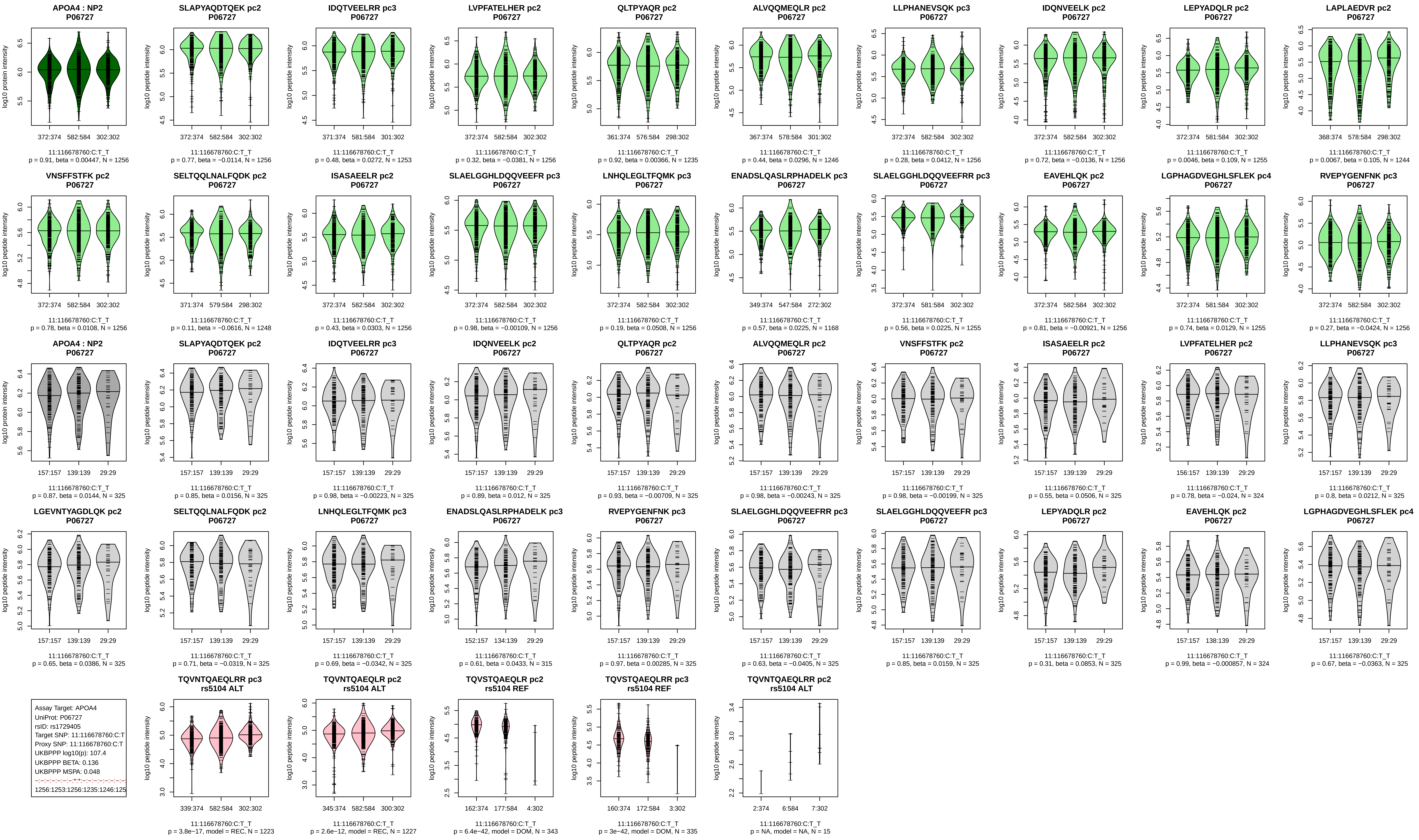
APOA4 : NP2 P06727
372:374 582:584 302:302
11:116678760:C:T_T p = 0.91, beta = 0.00447, N = 1256
SLAPYAQDTQEK pc2 P06727
372:374 582:584 302:302
11:116678760:C:T_T p = 0.77, beta = −0.0114, N = 1256
IDQTVEELRR pc3 P06727
371:374 581:584 301:302
11:116678760:C:T_T p = 0.48, beta = 0.0272, N = 1253
LVPFATELHER pc2 P06727
372:374 582:584 302:302
11:116678760:C:T_T p = 0.32, beta = −0.0381, N = 1256
QLTPYAQR pc2 P06727
361:374 576:584 298:302
11:116678760:C:T_T p = 0.92, beta = 0.00366, N = 1235
ALVQQMEQLR pc2 P06727
367:374 578:584 301:302
11:116678760:C:T_T p = 0.44, beta = 0.0296, N = 1246
LLPHANEVSQK pc3 P06727
372:374 582:584 302:302
11:116678760:C:T_T p = 0.28, beta = 0.0412, N = 1256
IDQNVEELK pc2 P06727
372:374 582:584 302:302
11:116678760:C:T_T p = 0.72, beta = −0.0136, N = 1256
LEPYADQLR pc2 P06727
372:374 581:584 302:302
11:116678760:C:T_T p = 0.0046, beta = 0.109, N = 1255
LAPLAEDVR pc2 P06727
368:374 578:584 298:302
11:116678760:C:T_T p = 0.0067, beta = 0.105, N = 1244
VNSFFSTFK pc2 P06727
372:374 582:584 302:302
11:116678760:C:T_T p = 0.78, beta = 0.0108, N = 1256
SELTQQLNALFQDK pc2 P06727
371:374 579:584 298:302
11:116678760:C:T_T p = 0.11, beta = −0.0616, N = 1248
ISASAEELR pc2 P06727
372:374 582:584 302:302
11:116678760:C:T_T p = 0.43, beta = 0.0303, N = 1256
SLAELGGHLDQQVEEFR pc3 P06727
372:374 582:584 302:302
11:116678760:C:T_T p = 0.98, beta = −0.00109, N = 1256
LNHQLEGLTFQMK pc3 P06727
372:374 582:584 302:302
11:116678760:C:T_T p = 0.19, beta = 0.0508, N = 1256
ENADSLQASLRPHADELK pc3 P06727
349:374 547:584 272:302
11:116678760:C:T_T p = 0.57, beta = 0.0225, N = 1168
SLAELGGHLDQQVEEFRR pc3 P06727
372:374 581:584 302:302
11:116678760:C:T_T p = 0.56, beta = 0.0225, N = 1255
EAVEHLQK pc2 P06727
372:374 582:584 302:302
11:116678760:C:T_T p = 0.81, beta = −0.00921, N = 1256
LGPHAGDVEGHLSFLEK pc4 P06727
372:374 581:584 302:302
11:116678760:C:T_T p = 0.74, beta = 0.0129, N = 1255
RVEPYGENFNK pc3 P06727
372:374 582:584 302:302
11:116678760:C:T_T p = 0.27, beta = −0.0424, N = 1256
APOA4 : NP2 P06727
157:157 139:139 29:29
11:116678760:C:T_T p = 0.87, beta = 0.0144, N = 325
SLAPYAQDTQEK pc2 P06727
157:157 139:139 29:29
11:116678760:C:T_T p = 0.85, beta = 0.0156, N = 325
IDQTVEELRR pc3 P06727
157:157 139:139 29:29
11:116678760:C:T_T p = 0.98, beta = −0.00223, N = 325
IDQNVEELK pc2 P06727
157:157 139:139 29:29
11:116678760:C:T_T p = 0.89, beta = 0.012, N = 325
QLTPYAQR pc2 P06727
157:157 139:139 29:29
11:116678760:C:T_T p = 0.93, beta = −0.00709, N = 325
ALVQQMEQLR pc2 P06727
157:157 139:139 29:29
11:116678760:C:T_T p = 0.98, beta = −0.00243, N = 325
VNSFFSTFK pc2 P06727
157:157 139:139 29:29
11:116678760:C:T_T p = 0.98, beta = −0.00199, N = 325
ISASAEELR pc2 P06727
157:157 139:139 29:29
11:116678760:C:T_T p = 0.55, beta = 0.0506, N = 325
LVPFATELHER pc2 P06727
156:157 139:139 29:29
11:116678760:C:T_T p = 0.78, beta = −0.024, N = 324
LLPHANEVSQK pc3 P06727
157:157 139:139 29:29
11:116678760:C:T_T p = 0.8, beta = 0.0212, N = 325
LGEVNTYAGDLQK pc2 P06727
157:157 139:139 29:29
11:116678760:C:T_T p = 0.65, beta = 0.0386, N = 325
SELTQQLNALFQDK pc2 P06727
157:157 139:139 29:29
11:116678760:C:T_T p = 0.71, beta = −0.0319, N = 325
LNHQLEGLTFQMK pc3 P06727
157:157 139:139 29:29
11:116678760:C:T_T p = 0.69, beta = −0.0342, N = 325
ENADSLQASLRPHADELK pc3 P06727
152:157 134:139 29:29
11:116678760:C:T_T p = 0.61, beta = 0.0433, N = 315
RVEPYGENFNK pc3 P06727
157:157 139:139 29:29
11:116678760:C:T_T p = 0.97, beta = 0.00285, N = 325
SLAELGGHLDQQVEEFRR pc3 P06727
157:157 139:139 29:29
11:116678760:C:T_T p = 0.63, beta = −0.0405, N = 325
SLAELGGHLDQQVEEFR pc3 P06727
157:157 139:139 29:29
11:116678760:C:T_T p = 0.85, beta = 0.0159, N = 325
LEPYADQLR pc2 P06727
157:157 139:139 29:29
11:116678760:C:T_T p = 0.31, beta = 0.0853, N = 325
EAVEHLQK pc2 P06727
157:157 138:139 29:29
11:116678760:C:T_T p = 0.99, beta = −0.000857, N = 324
LGPHAGDVEGHLSFLEK pc4 P06727
157:157 139:139 29:29
11:116678760:C:T_T p = 0.67, beta = −0.0363, N = 325
Assay Target: APOA4
UniProt: P06727
rsID: rs1729405
Target SNP: 11:116678760:C:T
Proxy SNP: 11:116678760:C:T
UKBPPP log10(p): 107.4
UKBPPP BETA: 0.136
UKBPPP MSPA: 0.048
1256:1253:1256:1235:1246:125
TQVNTQAEQLRR pc3 rs5104 ALT
339:374 582:584 302:302
11:116678760:C:T_T p = 3.8e−17, model = REC, N = 1223
TQVNTQAEQLR pc2 rs5104 ALT
345:374 582:584 300:302
11:116678760:C:T_T p = 2.6e−12, model = REC, N = 1227
TQVSTQAEQLR pc2 rs5104 REF
162:374 177:584 4:302
11:116678760:C:T_T p = 6.4e−42, model = DOM, N = 343
TQVSTQAEQLRR pc3 rs5104 REF
160:374 172:584 3:302
11:116678760:C:T_T p = 3e−42, model = DOM, N = 335
TQVNTQAEQLRR pc2 rs5104 ALT
2:374 6:584 7:302
11:116678760:C:T_T p = NA, model = NA, N = 15

280. S100A11 (P31949)
UKBPPP MSPA=0

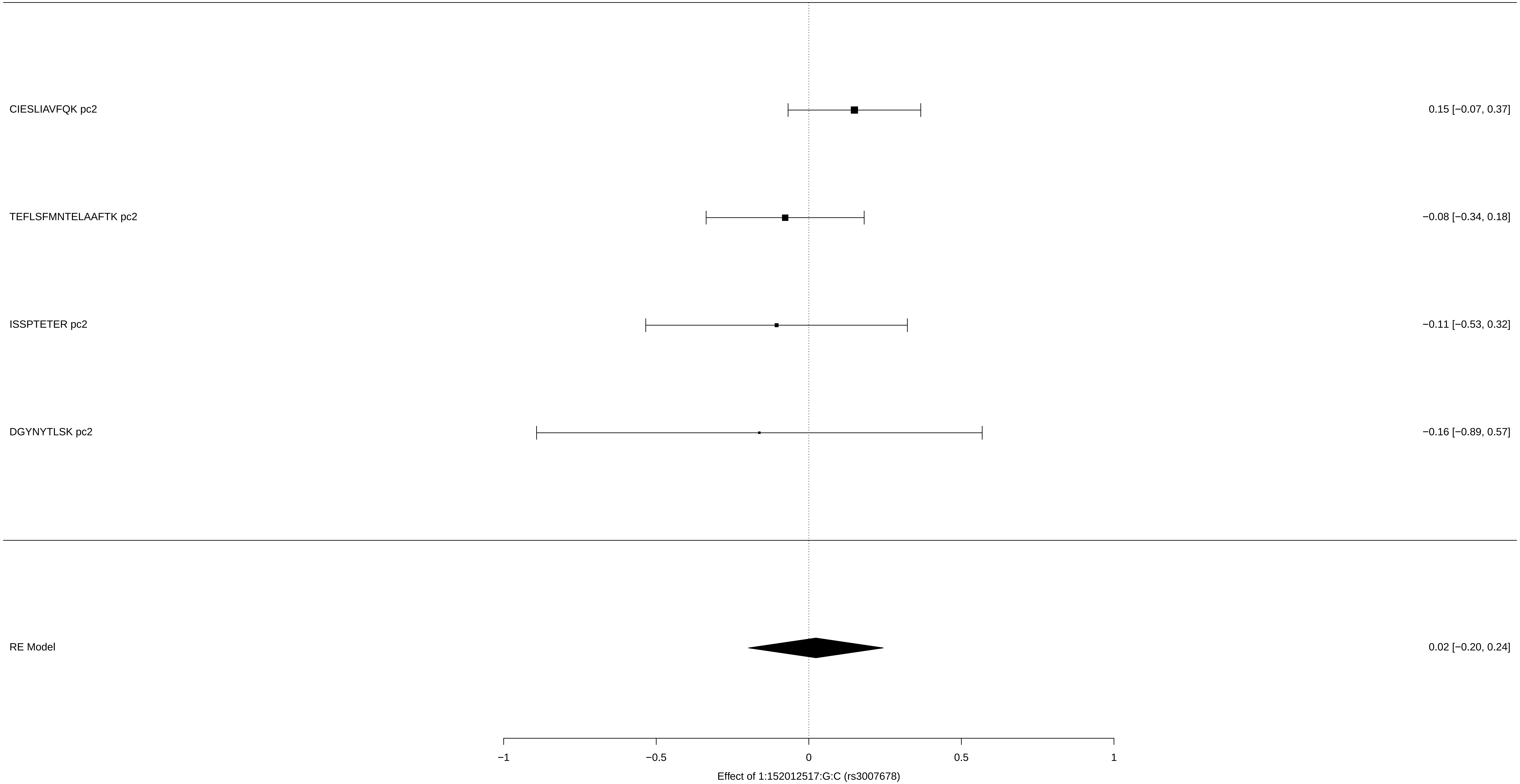

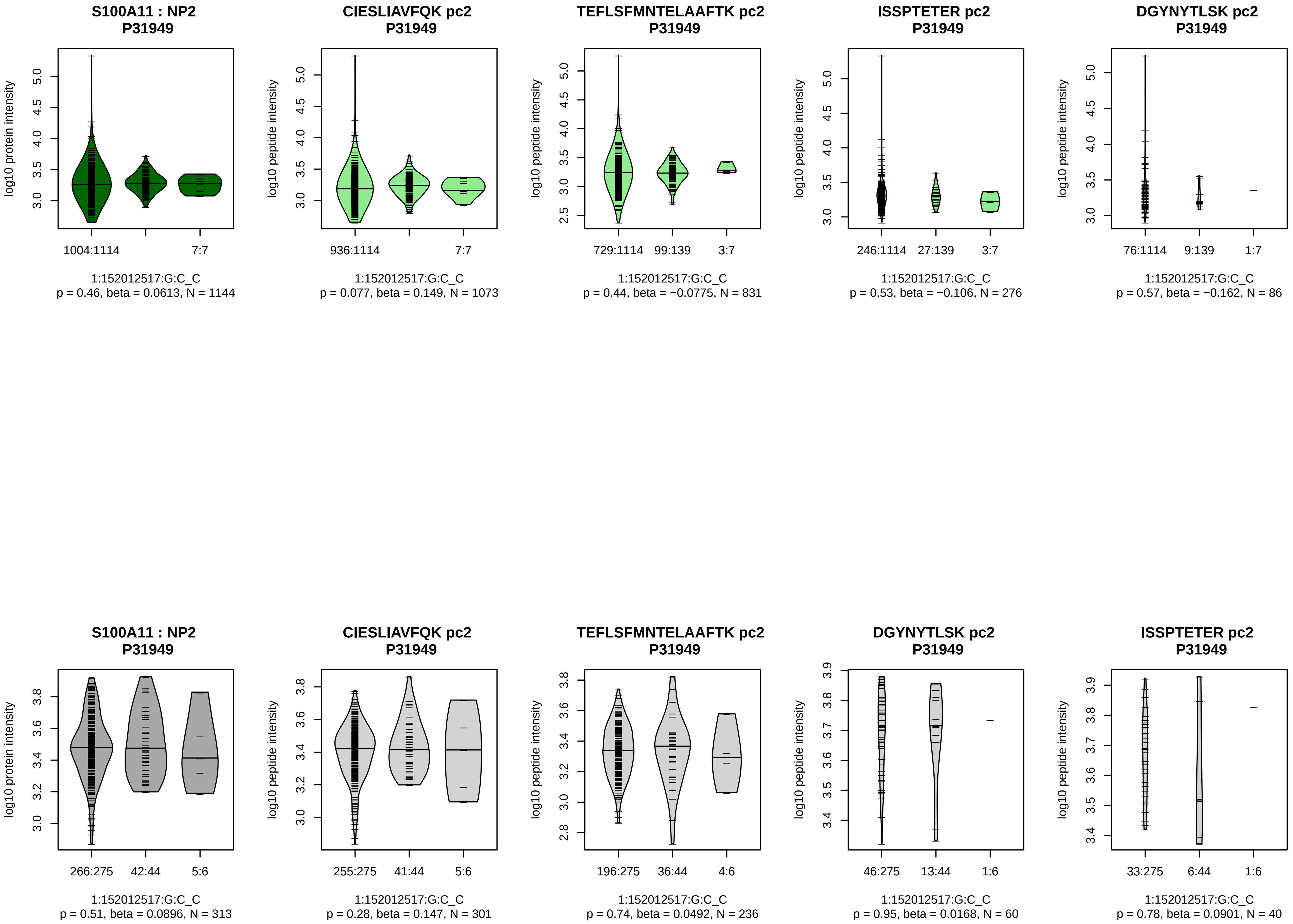

Assay Target: S100A11
UniProt: P31949
rsID: rs3007678
Target SNP: 1:152012517:G:C
Proxy SNP: 1:152012517:G:C
UKBPPP log10(p): 106.8
UKBPPP BETA: 0.31
UKBPPP MSPA: 0
–:–:–:–
1073:831:276:86

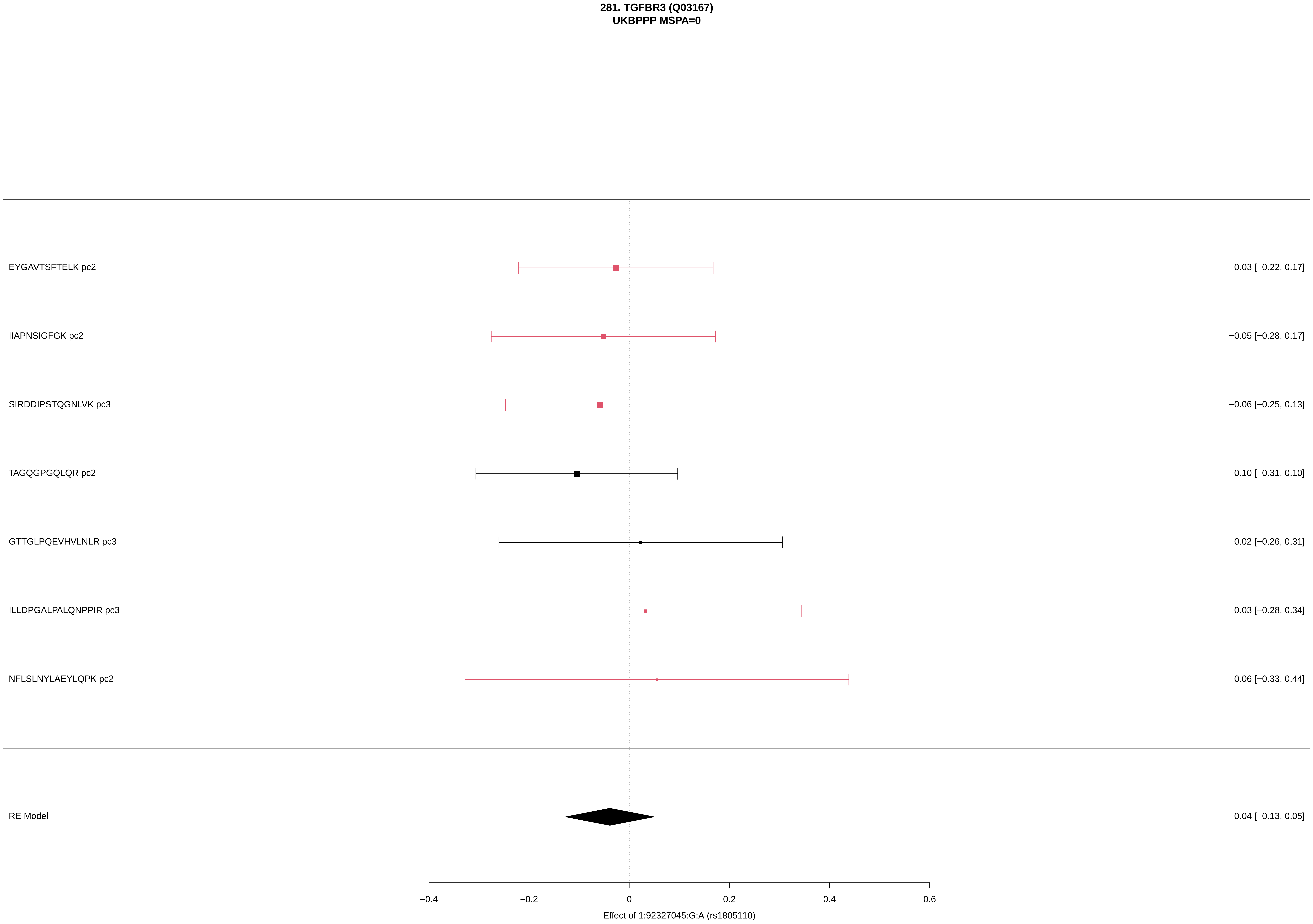
281. TGFBR3 (Q03167)
UKBPPP MSPA=0
EYGAVTSFTELK pc2
−0.03 [−0.22, 0.17]
IIAPNSIGFGK pc2
−0.05 [−0.28, 0.17]
SIRDDIPSTQGNLVK pc3
−0.06 [−0.25, 0.13]
TAGQGPGQLQR pc2
−0.10 [−0.31, 0.10]
GTTGLPQEVHVLNLR pc3
0.02 [−0.26, 0.31]
ILLDPGALPALQNPPIR pc3
0.03 [−0.28, 0.34]
NFLSLNYLAEYLQPK pc2
0.06 [−0.33, 0.44]
RE Model
−0.04 [−0.13, 0.05]
−0.4
−0.2
0
0.2
0.4
0.6
Effect of 1:92327045:G:A (rs1805110)

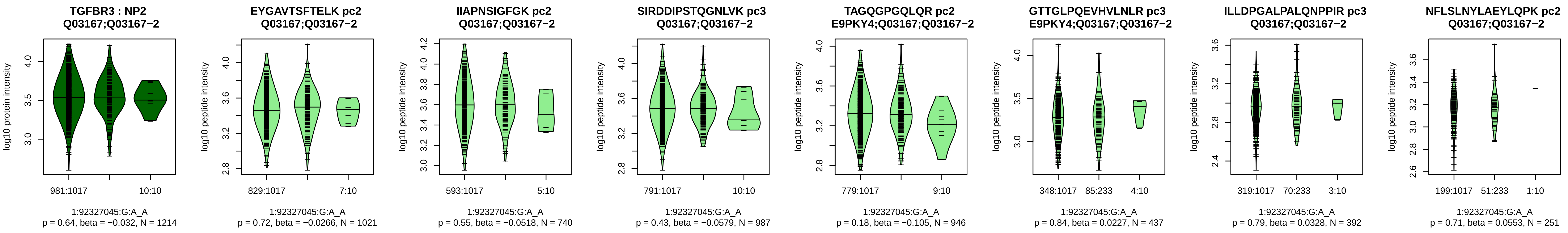

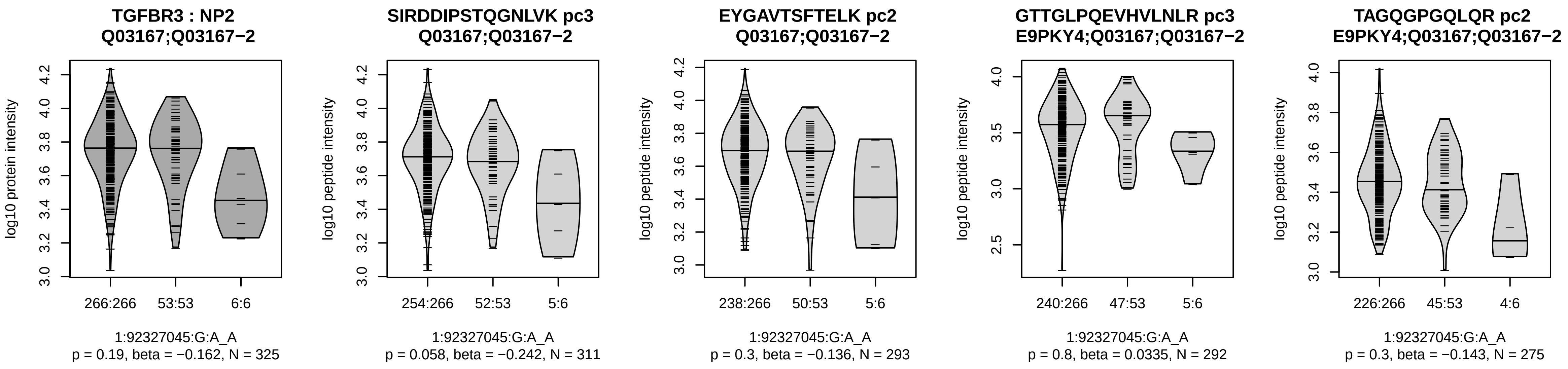

Assay Target: TGFBR3
UniProt: Q03167
rsID: rs1805110
Target SNP: 1:92327045:G:A
Proxy SNP: 1:92327045:G:A
UKBPPP log10(p): 106.6
UKBPPP BETA: −0.234
UKBPPP MSPA: 0
−:−:−:−:−:−:−
1021:740:987:946:437:392:251

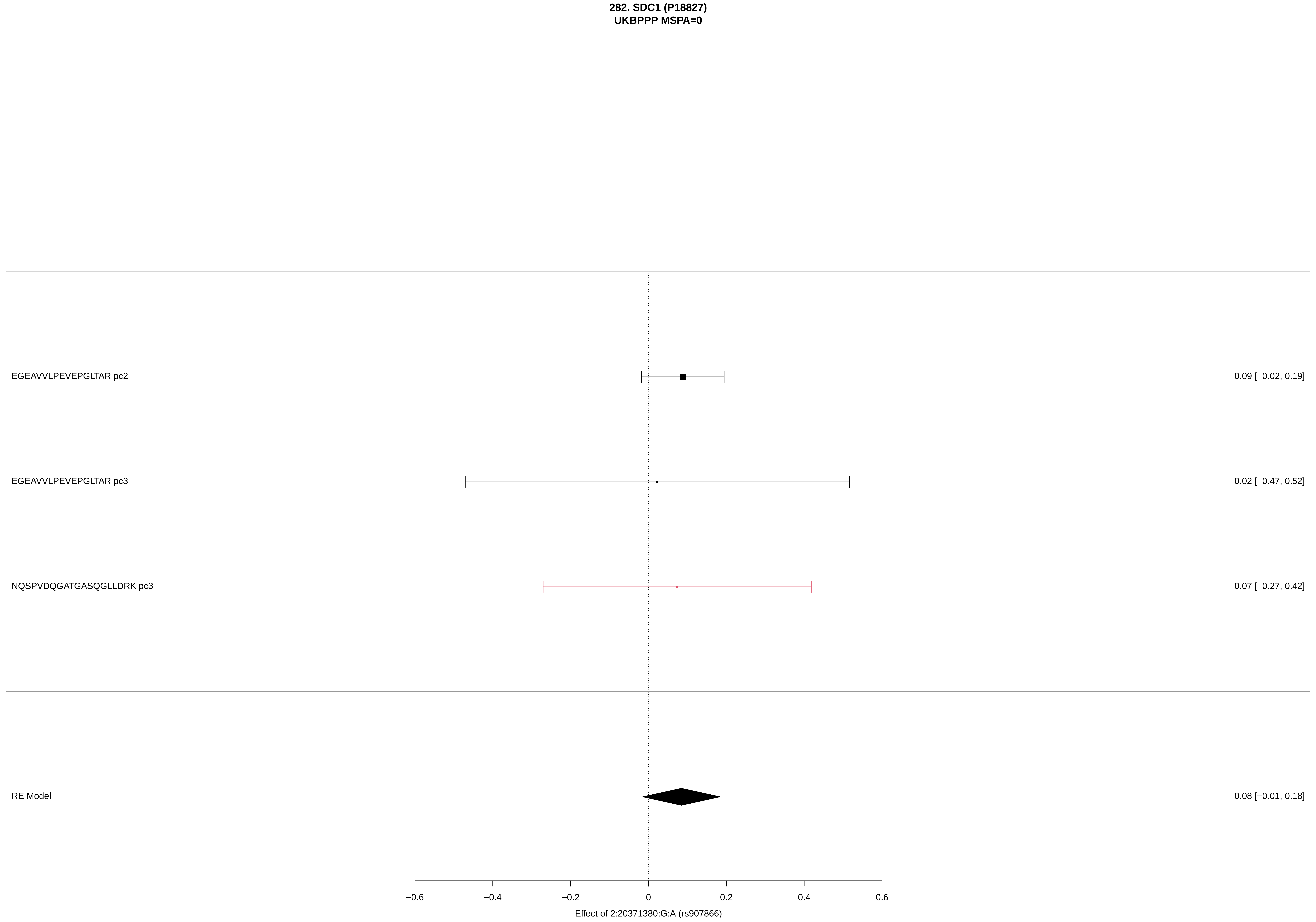

282. SDC1 (P18827)
UKBPPP MSPA=0
EGEAVVLPEVEPGLTAR pc2
0.09 [−0.02, 0.19]
EGEAVVLPEVEPGLTAR pc3
0.02 [−0.47, 0.52]
NQSPVDQGATGASQGLLDRK pc3
0.07 [−0.27, 0.42]
RE Model
0.08 [−0.01, 0.18]
−0.6
−0.4
−0.2
0
0.2
0.4
0.6
Effect of 2:20371380:G:A (rs907866)

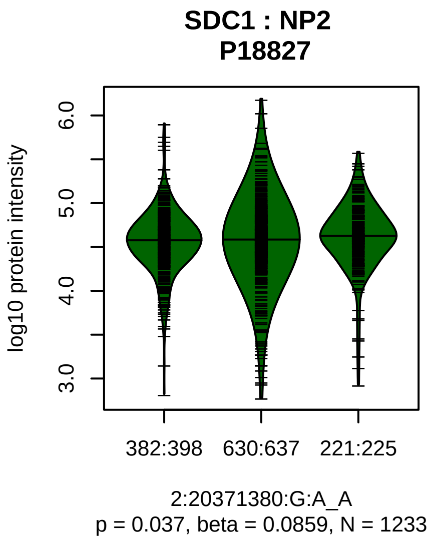

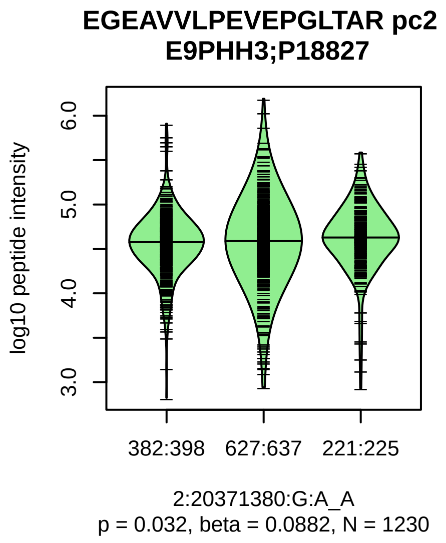

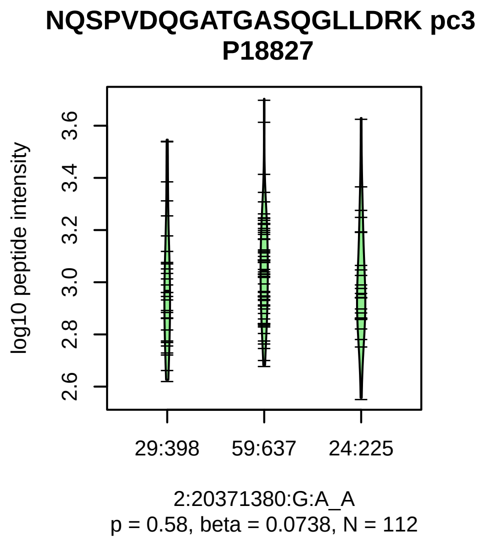

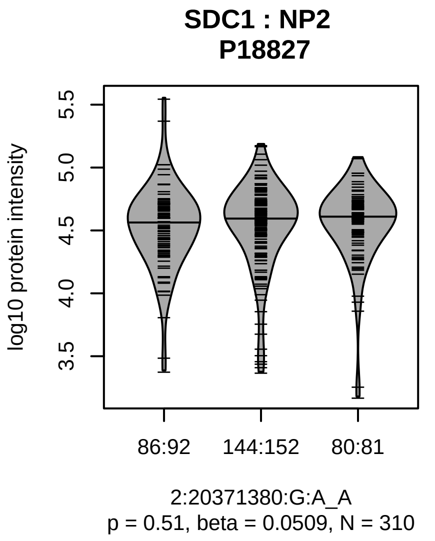

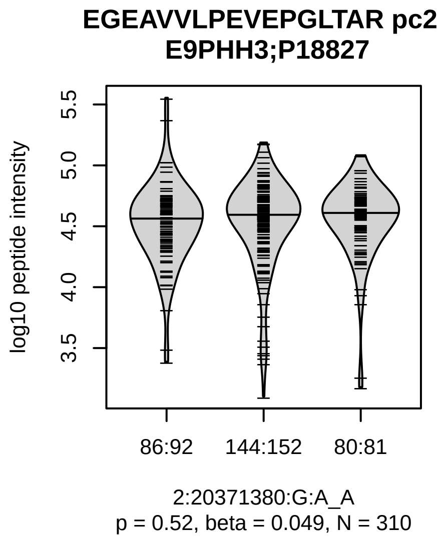

Assay Target: SDC1
UniProt: P18827
rsID: rs907866
Target SNP: 2:20371380:G:A
Proxy SNP: 2:20371380:G:A
UKBPPP log10(p): 106.1
UKBPPP BETA: 0.131
UKBPPP MSPA: 0
–:–:–
1230:53:112

283. IQGAP2 (Q13576)
UKBPPP MSPA=0.026

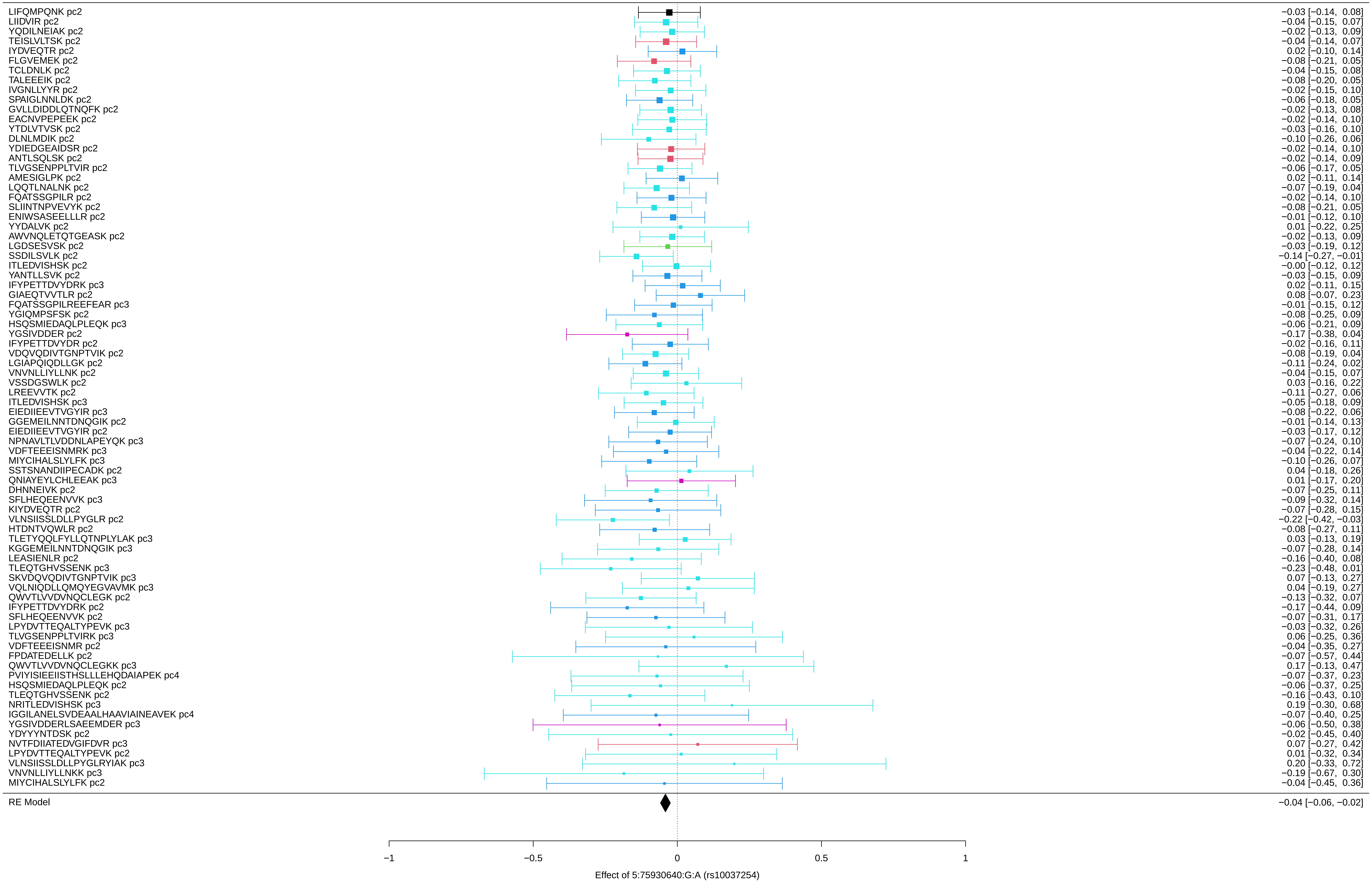

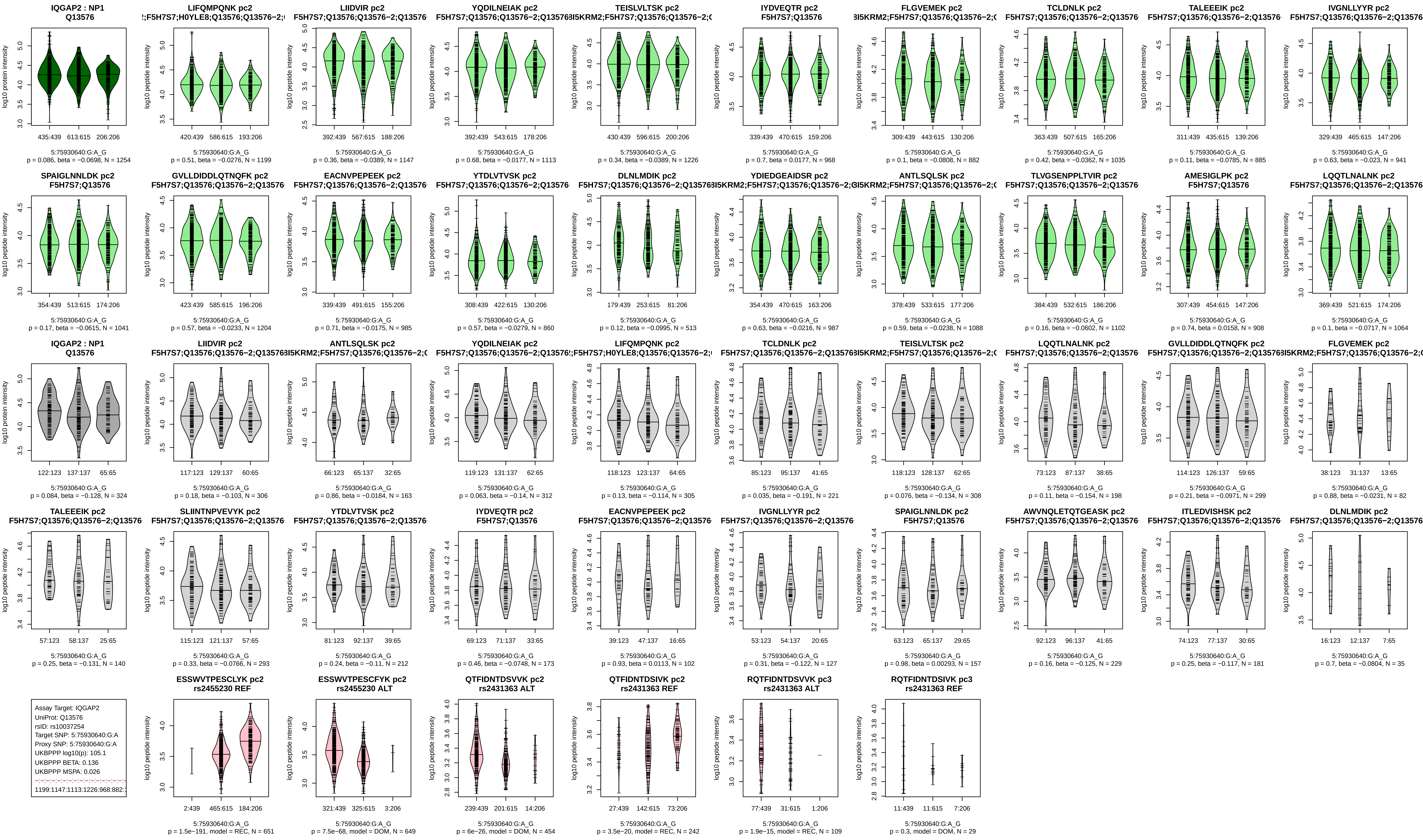

284. PRDX6 (P30041)
UKBPPP MSPA=0.726

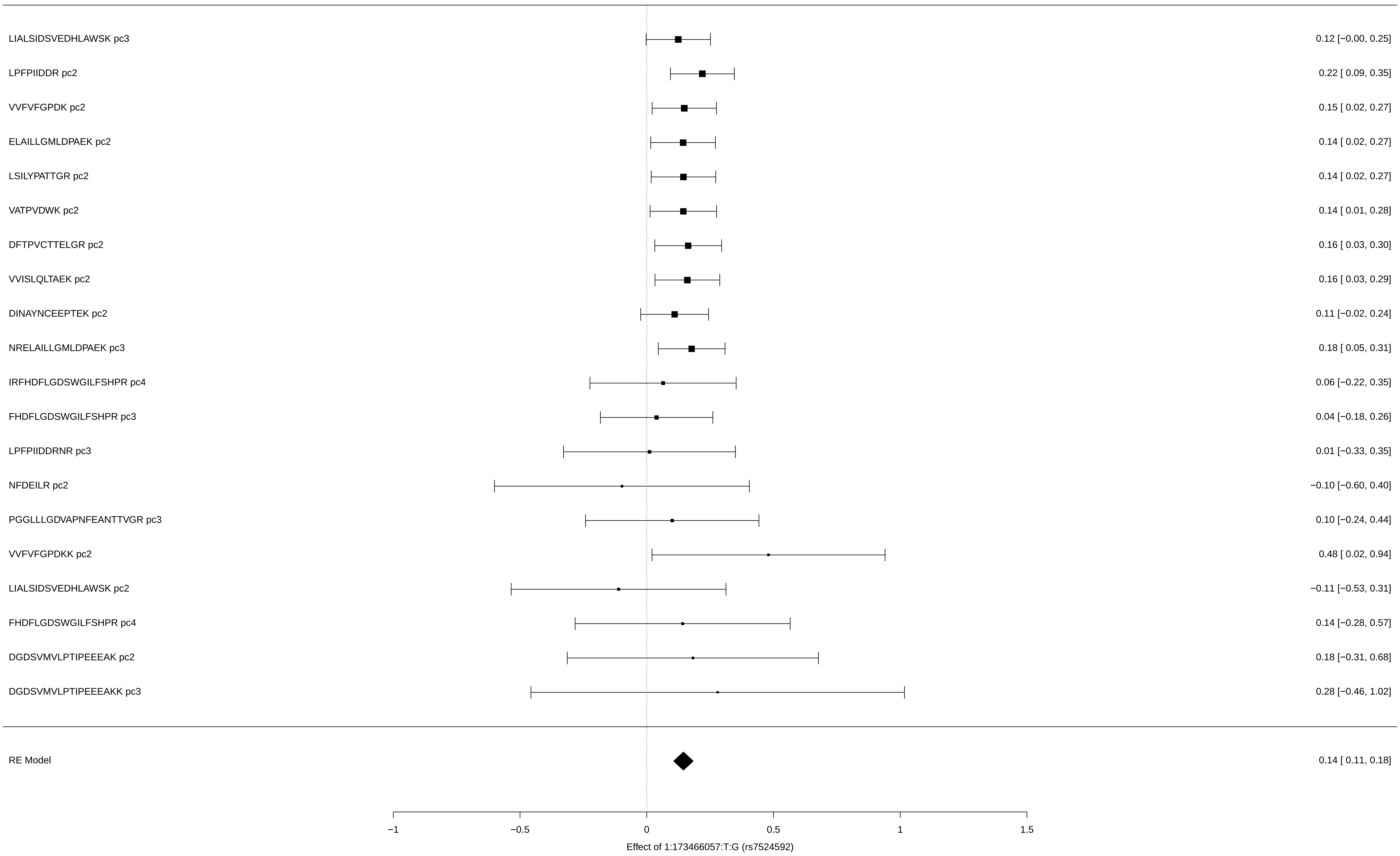

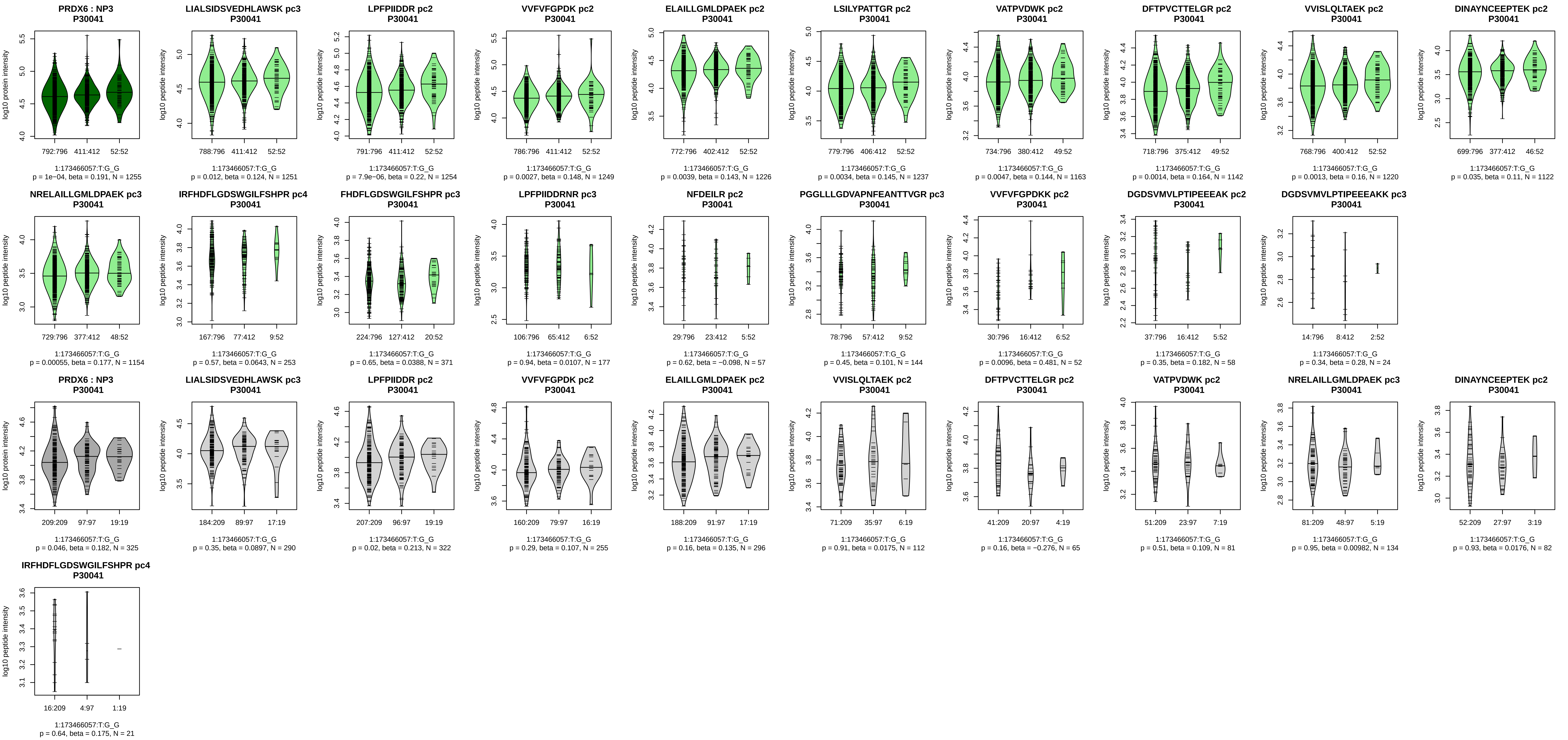

Assay Target: PRDX6
UniProt: P30041
rsID: rs7524592
Target SNP: 1:173466057:T:G
Proxy SNP: 1:173466057:T:G
UKBPPP log10(p): 104.6
UKBPPP BETA: 0.169
UKBPPP MSPA: 0.726
1251:1254:1249:1226:1237:116

285. ADH4 (P08319)
UKBPPP MSPA=0.779

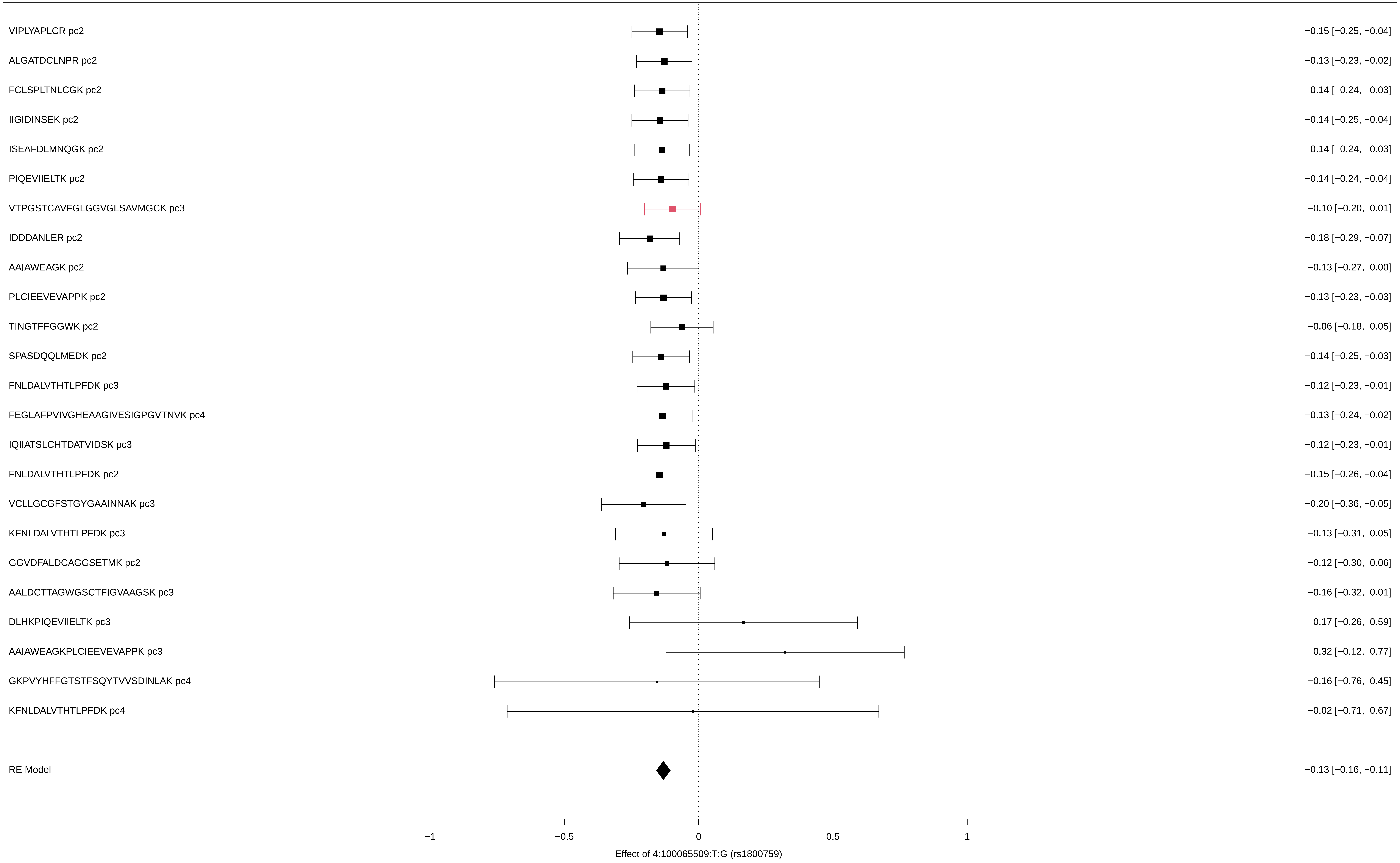

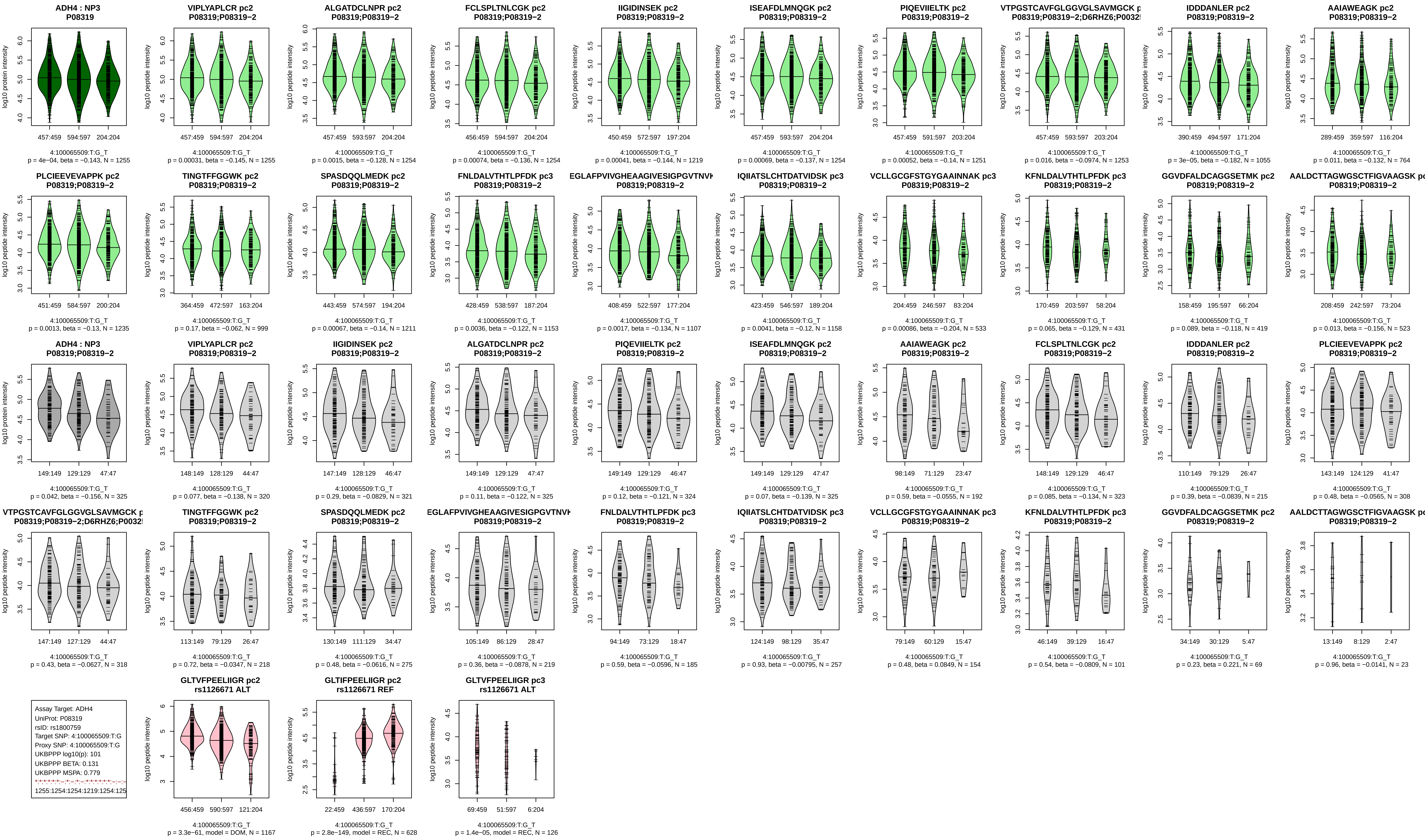
ADH4 : NP3
P08319
log10 protein intensity
457:459 594:597 204:204
4:100065509:T:G_T
p = 4e−04, beta = −0.143, N = 1255
VIPLYAPLCR pc2
P08319;P08319−2
log10 peptide intensity
457:459 594:597 204:204
4:100065509:T:G_T
p = 0.00031, beta = −0.145, N = 1255
ALGATDCLNPR pc2
P08319;P08319−2
457:459 593:597 204:204
4:100065509:T:G_T
p = 0.0015, beta = −0.128, N = 1254
FCLSPLTNLCGK pc2
P08319;P08319−2
456:459 594:597 204:204
4:100065509:T:G_T
p = 0.00074, beta = −0.136, N = 1254
IIGIDINSEK pc2
P08319;P08319−2
450:459 572:597 197:204
4:100065509:T:G_T
p = 0.00041, beta = −0.144, N = 1219
ISEAFDLMNQGK pc2
P08319;P08319−2
457:459 593:597 204:204
4:100065509:T:G_T
p = 0.00069, beta = −0.137, N = 1254
PIQEVIIELTK pc2
P08319;P08319−2
457:459 591:597 203:204
4:100065509:T:G_T
p = 0.00052, beta = −0.14, N = 1251
VTPGSTCAVFGLGGVGLSAVMGCK p
P08319;P08319−2;D6RHZ6;P0032
457:459 593:597 203:204
4:100065509:T:G_T
p = 0.016, beta = −0.0974, N = 1253
IDDDANLER pc2
P08319;P08319−2
390:459 494:597 171:204
4:100065509:T:G_T
p = 3e−05, beta = −0.182, N = 1055
AAIAWEAGK pc2
P08319;P08319−2
289:459 359:597 116:204
4:100065509:T:G_T
p = 0.011, beta = −0.132, N = 764
PLCIEEVEVAPPK pc2
P08319;P08319−2
451:459 584:597 200:204
4:100065509:T:G_T
p = 0.0013, beta = −0.13, N = 1235
TINGTFFGGWK pc2
P08319;P08319−2
364:459 472:597 163:204
4:100065509:T:G_T
p = 0.17, beta = −0.062, N = 999
SPASDQQLMEDK pc2
P08319;P08319−2
443:459 574:597 194:204
4:100065509:T:G_T
p = 0.00067, beta = −0.14, N = 1211
FNLDALVTHTLPFDK pc3
P08319;P08319−2
428:459 538:597 187:204
4:100065509:T:G_T
p = 0.0036, beta = −0.122, N = 1153
EGLAFPVIVGHEAAGIVESIGPGVTNVK
P08319;P08319−2
408:459 522:597 177:204
4:100065509:T:G_T
p = 0.0017, beta = −0.134, N = 1107
IQIIATSLCHTDATVIDSK pc3
P08319;P08319−2
423:459 546:597 189:204
4:100065509:T:G_T
p = 0.0041, beta = −0.12, N = 1158
VCLLGCGFSTGYGAAINNAK pc3
P08319;P08319−2
204:459 246:597 83:204
4:100065509:T:G_T
p = 0.00086, beta = −0.204, N = 533
KFNLDALVTHTLPFDK pc3
P08319;P08319−2
170:459 203:597 58:204
4:100065509:T:G_T
p = 0.065, beta = −0.129, N = 431
GGVDFALDCAGGSETMK pc2
P08319;P08319−2
158:459 195:597 66:204
4:100065509:T:G_T
p = 0.089, beta = −0.118, N = 419
AALDCTTAGWGSCTFIGVAAGSK pc
P08319;P08319−2
208:459 242:597 73:204
4:100065509:T:G_T
p = 0.013, beta = −0.156, N = 523
ADH4 : NP3
P08319;P08319−2
149:149 129:129 47:47
4:100065509:T:G_T
p = 0.042, beta = −0.156, N = 325
VIPLYAPLCR pc2
P08319;P08319−2
148:149 128:129 44:47
4:100065509:T:G_T
p = 0.077, beta = −0.138, N = 320
IIGIDINSEK pc2
P08319;P08319−2
147:149 128:129 46:47
4:100065509:T:G_T
p = 0.29, beta = −0.0829, N = 321
ALGATDCLNPR pc2
P08319;P08319−2
149:149 129:129 47:47
4:100065509:T:G_T
p = 0.11, beta = −0.122, N = 325
PIQEVIIELTK pc2
P08319;P08319−2
149:149 129:129 46:47
4:100065509:T:G_T
p = 0.12, beta = −0.121, N = 324
ISEAFDLMNQGK pc2
P08319;P08319−2
149:149 129:129 47:47
4:100065509:T:G_T
p = 0.07, beta = −0.139, N = 325
AAIAWEAGK pc2
P08319;P08319−2
98:149 71:129 23:47
4:100065509:T:G_T
p = 0.59, beta = −0.0555, N = 192
FCLSPLTNLCGK pc2
P08319;P08319−2
148:149 129:129 46:47
4:100065509:T:G_T
p = 0.085, beta = −0.134, N = 323
IDDDANLER pc2
P08319;P08319−2
110:149 79:129 26:47
4:100065509:T:G_T
p = 0.39, beta = −0.0839, N = 215
PLCIEEVEVAPPK pc2
P08319;P08319−2
143:149 124:129 41:47
4:100065509:T:G_T
p = 0.48, beta = −0.0565, N = 308
VTPGSTCAVFGLGGVGLSAVMGCK p
P08319;P08319−2;D6RHZ6;P0032
147:149 127:129 44:47
4:100065509:T:G_T
p = 0.43, beta = −0.0627, N = 318
TINGTFFGGWK pc2
P08319;P08319−2
113:149 79:129 26:47
4:100065509:T:G_T
p = 0.72, beta = −0.0347, N = 218
SPASDQQLMEDK pc2
P08319;P08319−2
130:149 111:129 34:47
4:100065509:T:G_T
p = 0.48, beta = −0.0616, N = 275
EGLAFPVIVGHEAAGIVESIGPGVTNVK
P08319;P08319−2
105:149 86:129 28:47
4:100065509:T:G_T
p = 0.36, beta = −0.0878, N = 219
FNLDALVTHTLPFDK pc3
P08319;P08319−2
94:149 73:129 18:47
4:100065509:T:G_T
p = 0.59, beta = −0.0596, N = 185
IQIIATSLCHTDATVIDSK pc3
P08319;P08319−2
124:149 98:129 35:47
4:100065509:T:G_T
p = 0.93, beta = −0.00795, N = 257
VCLLGCGFSTGYGAAINNAK pc3
P08319;P08319−2
79:149 60:129 15:47
4:100065509:T:G_T
p = 0.48, beta = 0.0849, N = 154
KFNLDALVTHTLPFDK pc3
P08319;P08319−2
46:149 39:129 16:47
4:100065509:T:G_T
p = 0.54, beta = −0.0809, N = 101
GGVDFALDCAGGSETMK pc2
P08319;P08319−2
34:149 30:129 5:47
4:100065509:T:G_T
p = 0.23, beta = 0.221, N = 69
AALDCTTAGWGSCTFIGVAAGSK pc
P08319;P08319−2
13:149 8:129 2:47
4:100065509:T:G_T
p = 0.96, beta = −0.0141, N = 23
Assay Target: ADH4
UniProt: P08319
rsID: rs1800759
Target SNP: 4:100065509:T:G
Proxy SNP: 4:100065509:T:G
UKBPPP log10(p): 101
UKBPPP BETA: 0.131
UKBPPP MSPA: 0.779
1255:1254:1254:1219:1254:125
GLTVFPEELIIGR pc2
rs1126671 ALT
456:459 590:597 121:204
4:100065509:T:G_T
p = 3.3e−61, model = DOM, N = 1167
GLTIFPEELIIGR pc2
rs1126671 REF
22:459 436:597 170:204
4:100065509:T:G_T
p = 2.8e−149, model = REC, N = 628
GLTVFPEELIIGR pc3
rs1126671 ALT
69:459 51:597 6:204
4:100065509:T:G_T
p = 1.4e−05, model = REC, N = 126

286. BSG (P35613)
UKBPPP MSPA=0

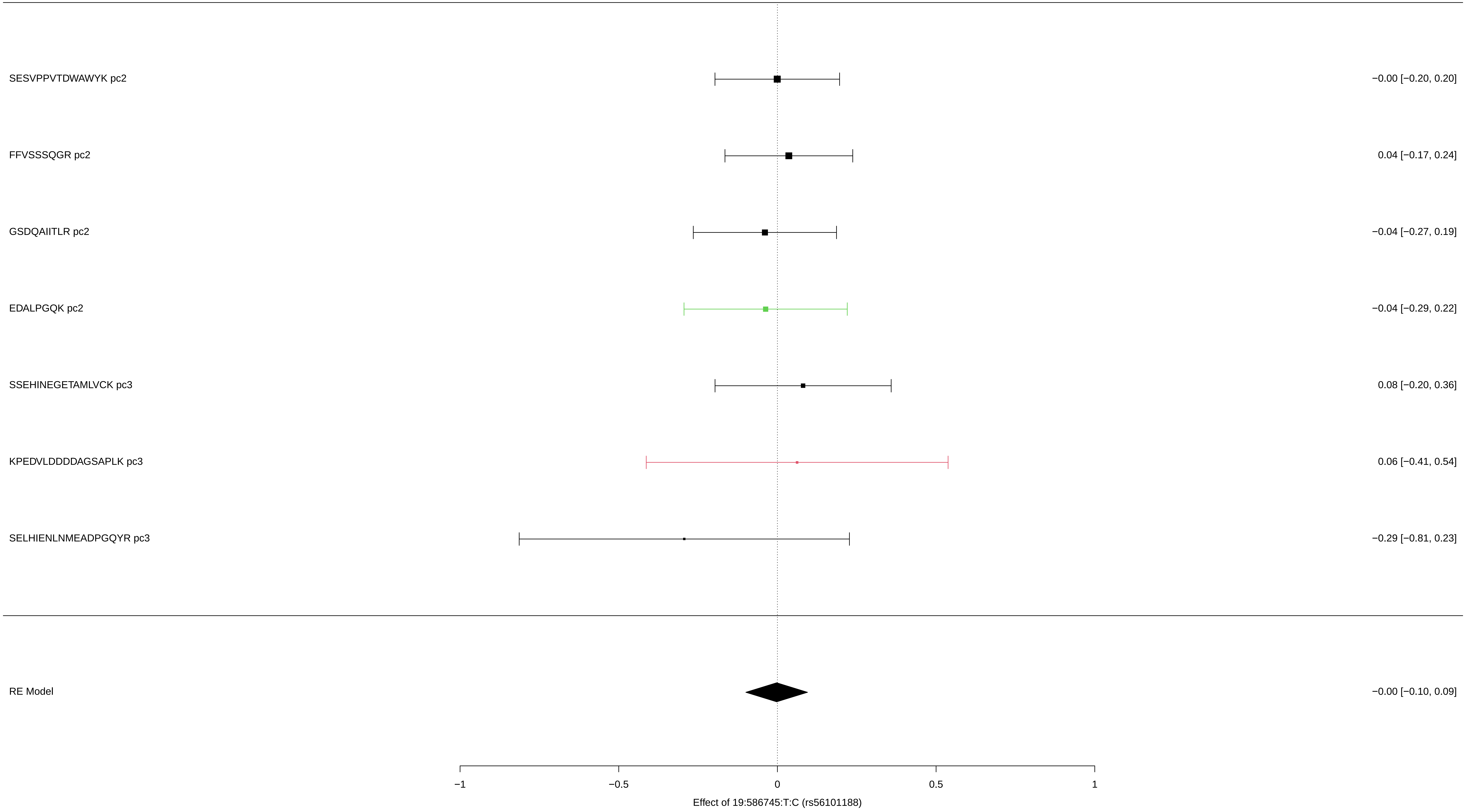

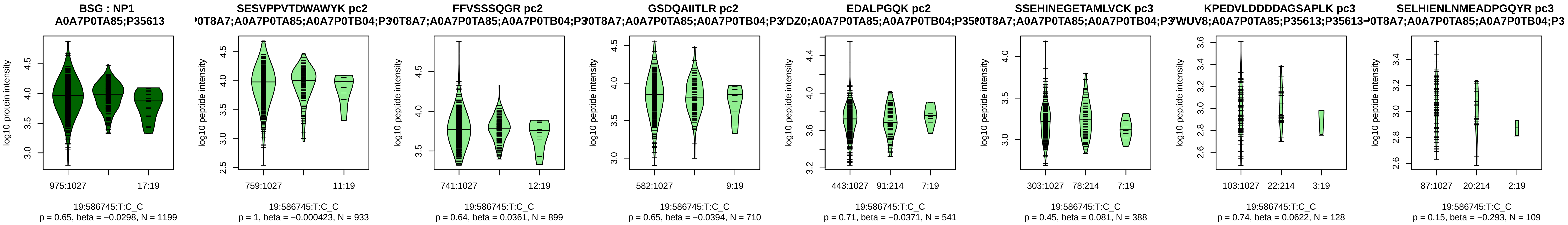

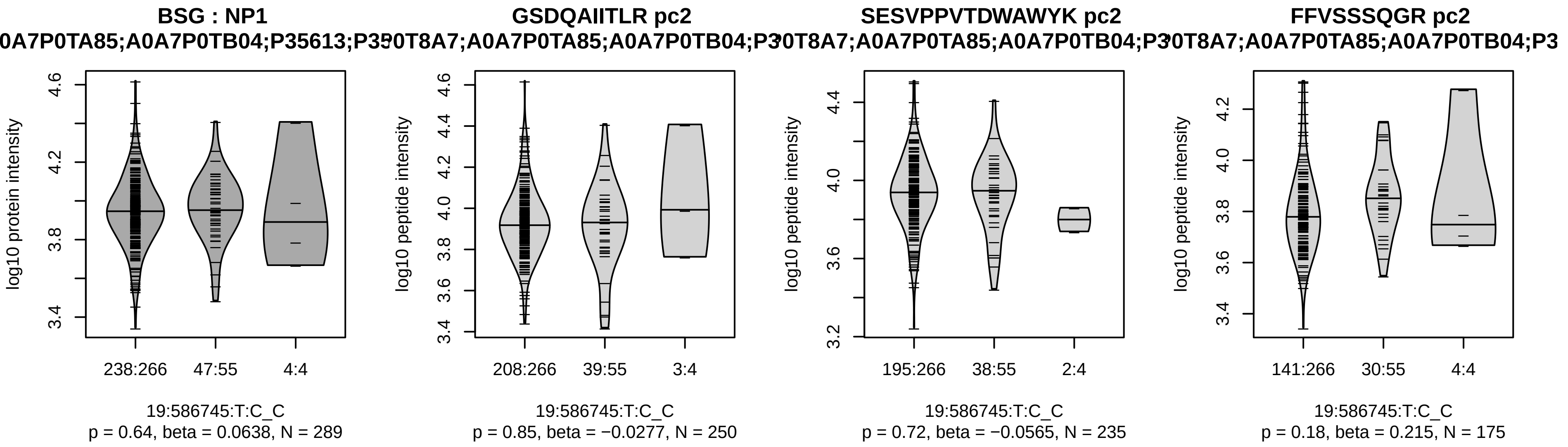

Assay Target: BSG
UniProt: P35613
rsID: rs56101188
Target SNP: 19:586745:T:C
Proxy SNP: 19:586745:T:C
UKBPPP log10(p): 100.8
UKBPPP BETA: 0.218
UKBPPP MSPA: 0
–:–:–:–:–:–:–
933:899:710:541:388:128:109

287. MCFD2 (Q8NI22)
UKBPPP MSPA=0

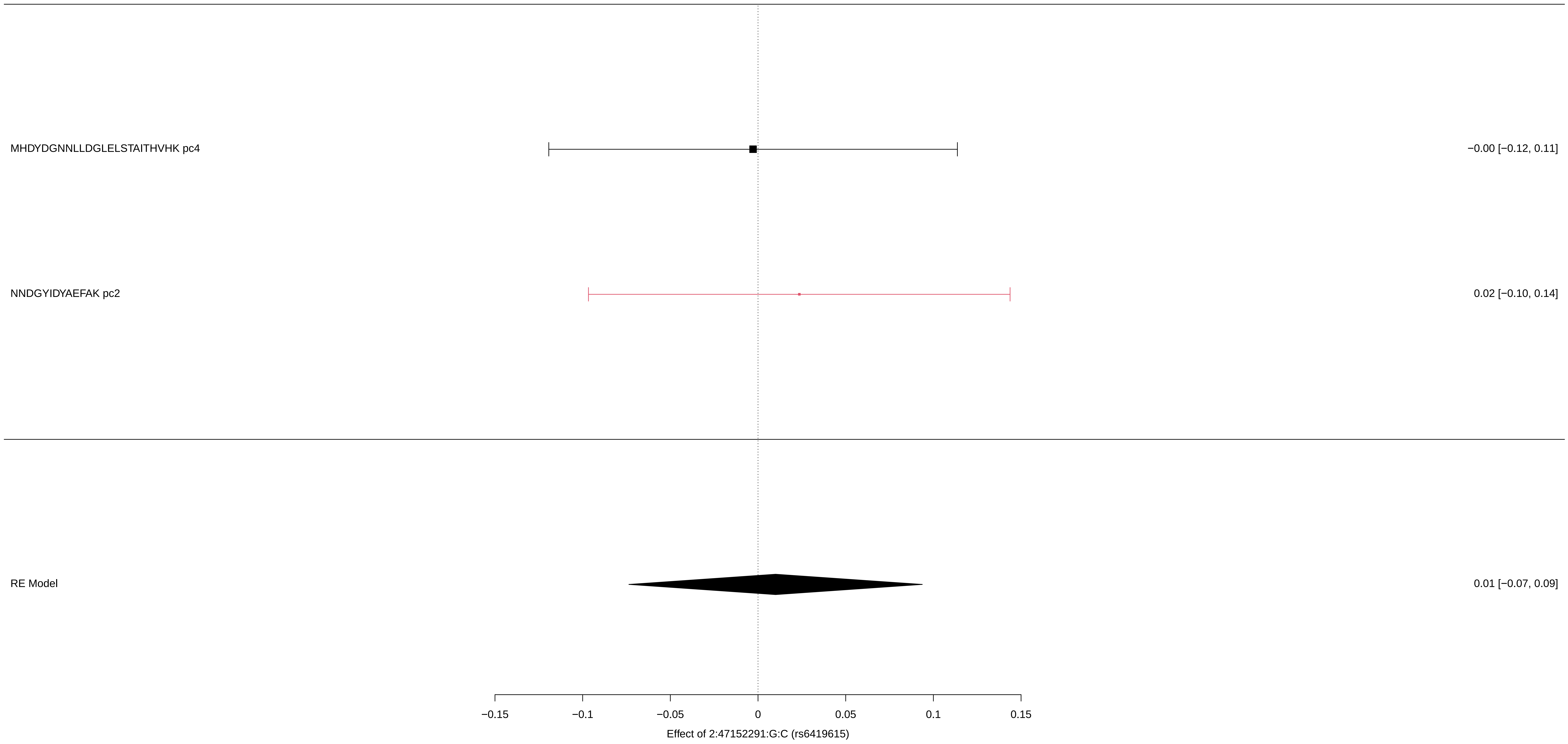

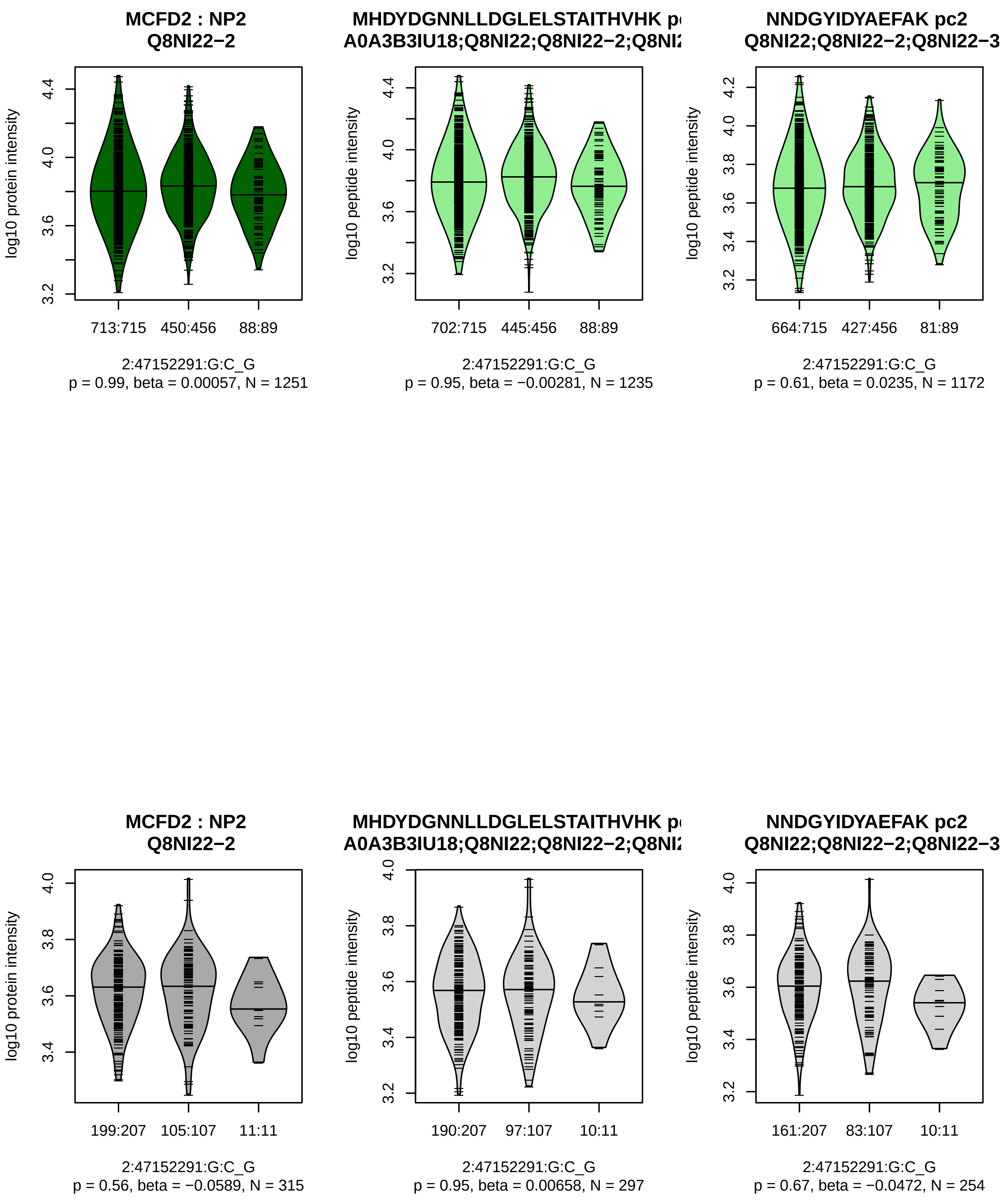

Assay Target: MCFD2
UniProt: Q8NI22
rsID: rs6419615
Target SNP: 2:47152291:G:C
Proxy SNP: 2:47152291:G:C
UKBPPP log10(p): 99.3
UKBPPP BETA: −0.146
UKBPPP MSPA: 0
−:−
1235:1172

288. CDH23 (Q9H251)
UKBPPP MSPA=0.027

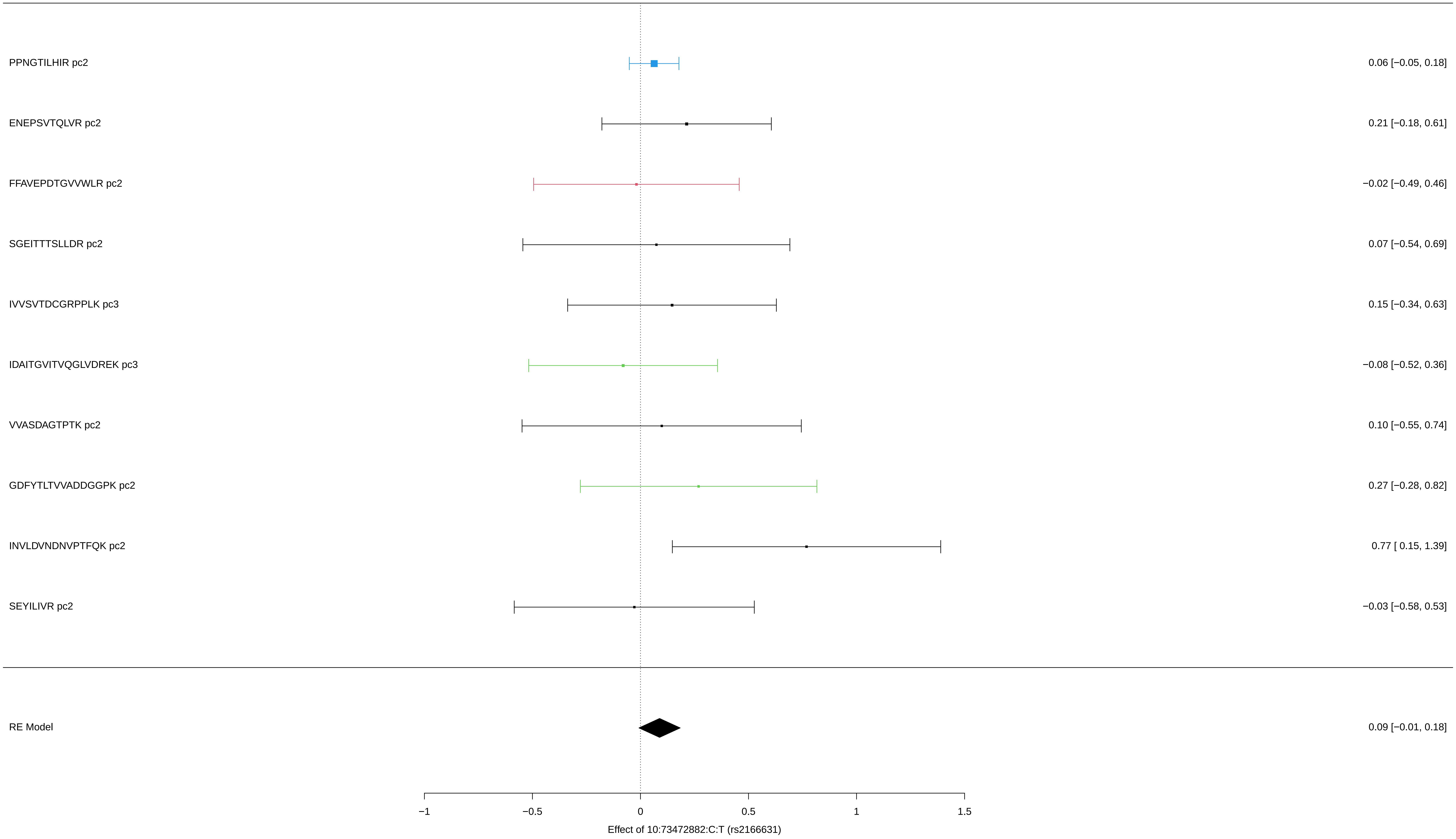

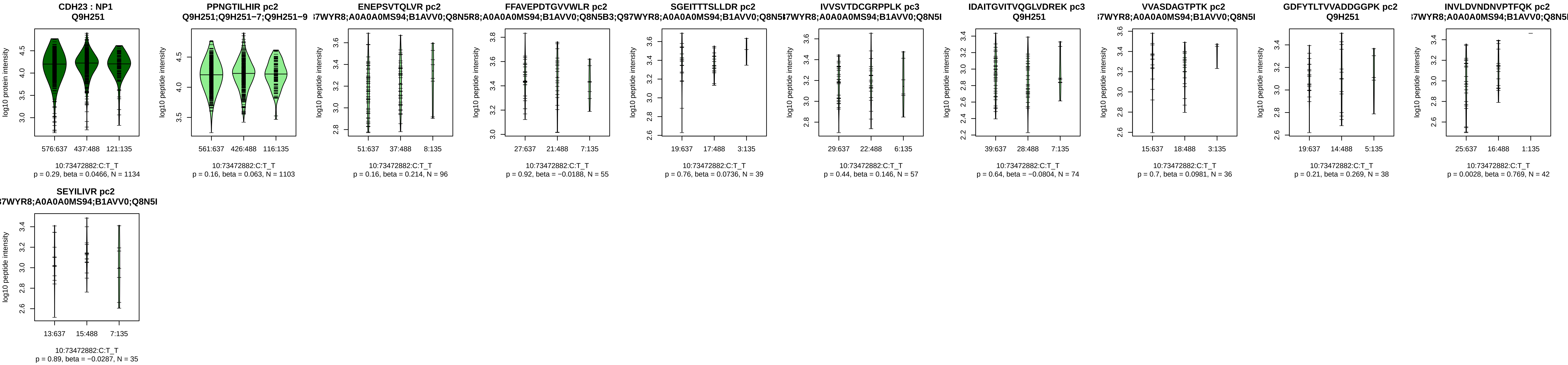

Assay Target: CDH23
UniProt: Q9H251
rsID: rs2166631
Target SNP: 10:73472882:C:T
Proxy SNP: 10:73472882:C:T
UKBPPP log10(p): 96.8
UKBPPP BETA: −0.157
UKBPPP MSPA: 0.027
−:−:−:−:−:−:−:−:*:−
1103:96:55:39:57:74:36:38:42:3

289. SEMA3F (Q13275)
UKBPPP MSPA=0

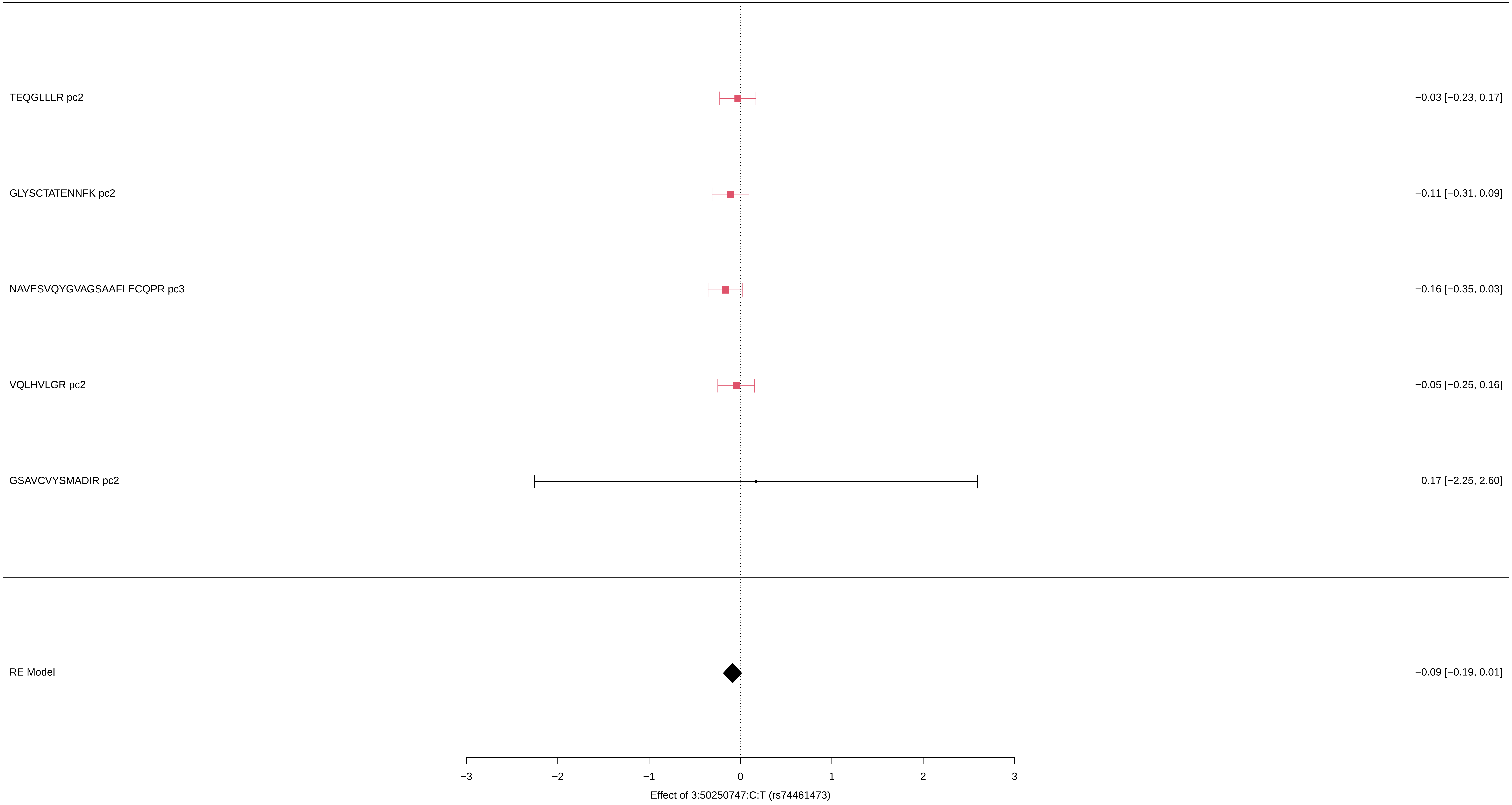

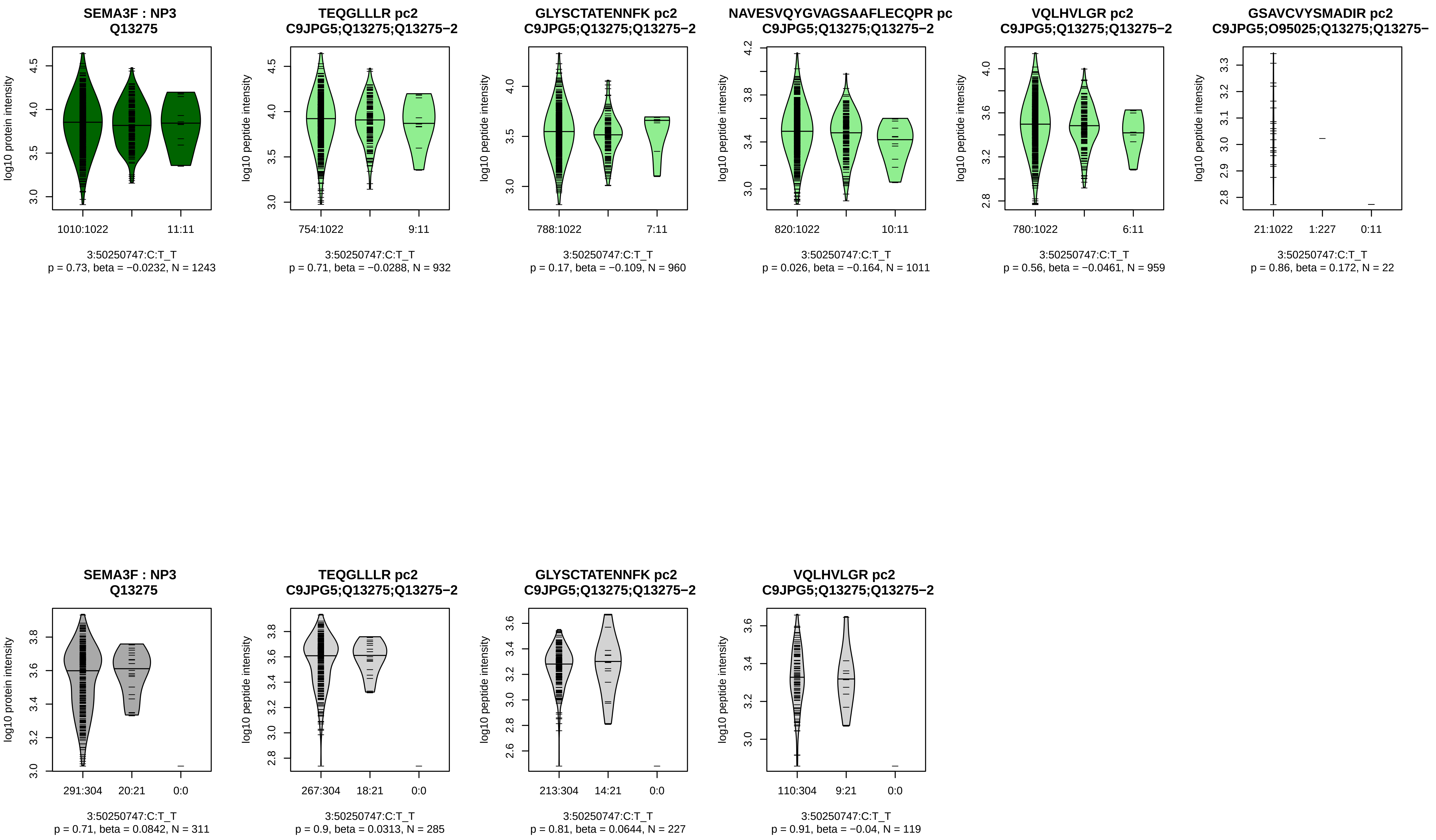

Assay Target: SEMA3F
UniProt: Q13275
rsID: rs74461473
Target SNP: 3:50250747:C:T
Proxy SNP: 3:50250747:C:T
UKBPPP log10(p): 95.6
UKBPPP BETA: −0.201
UKBPPP MSPA: 0
−:−:−:−:−
932:960:1011:959:22

290. ITIH1 (P19827)
UKBPPP MSPA=0.099

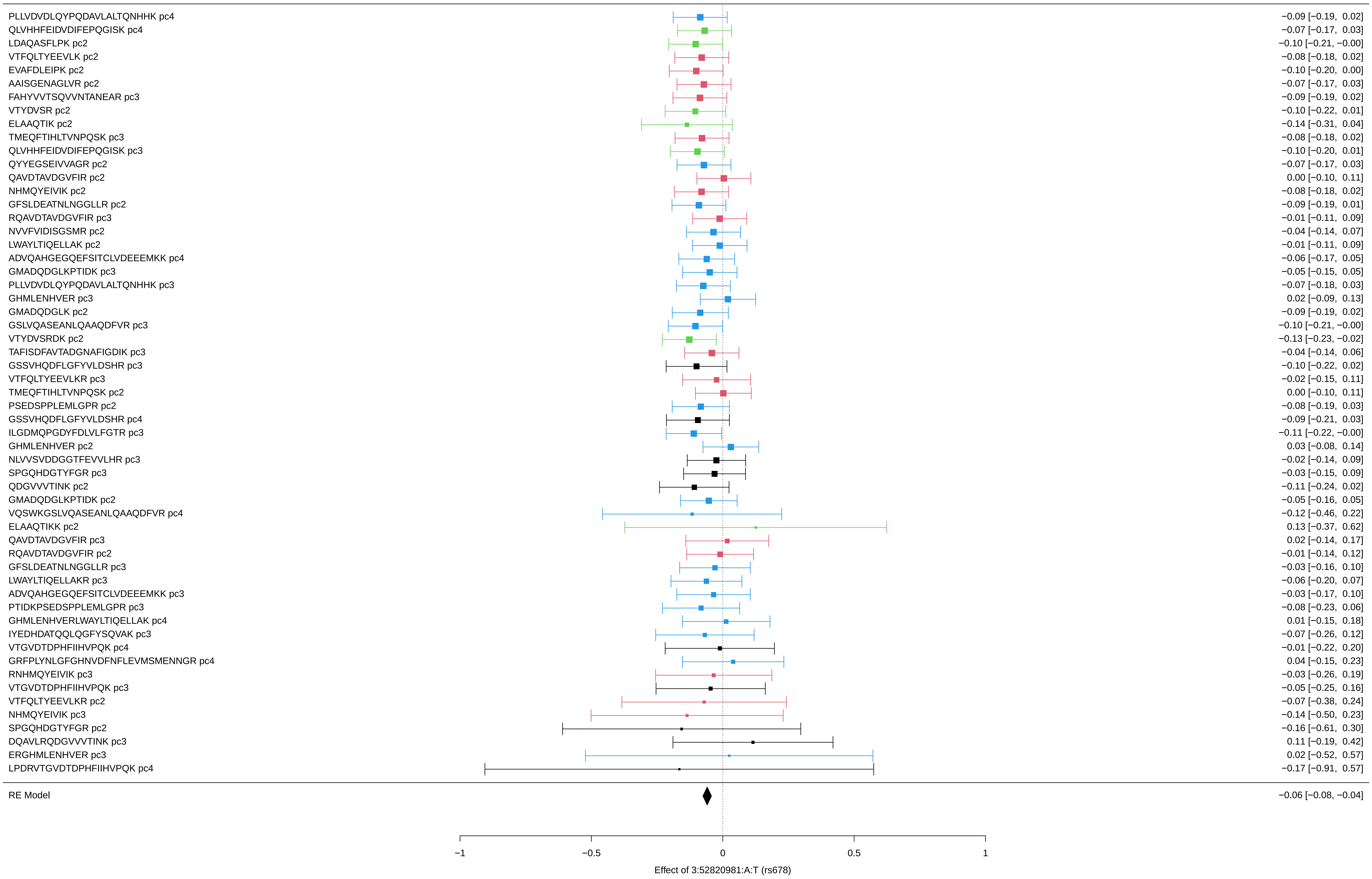

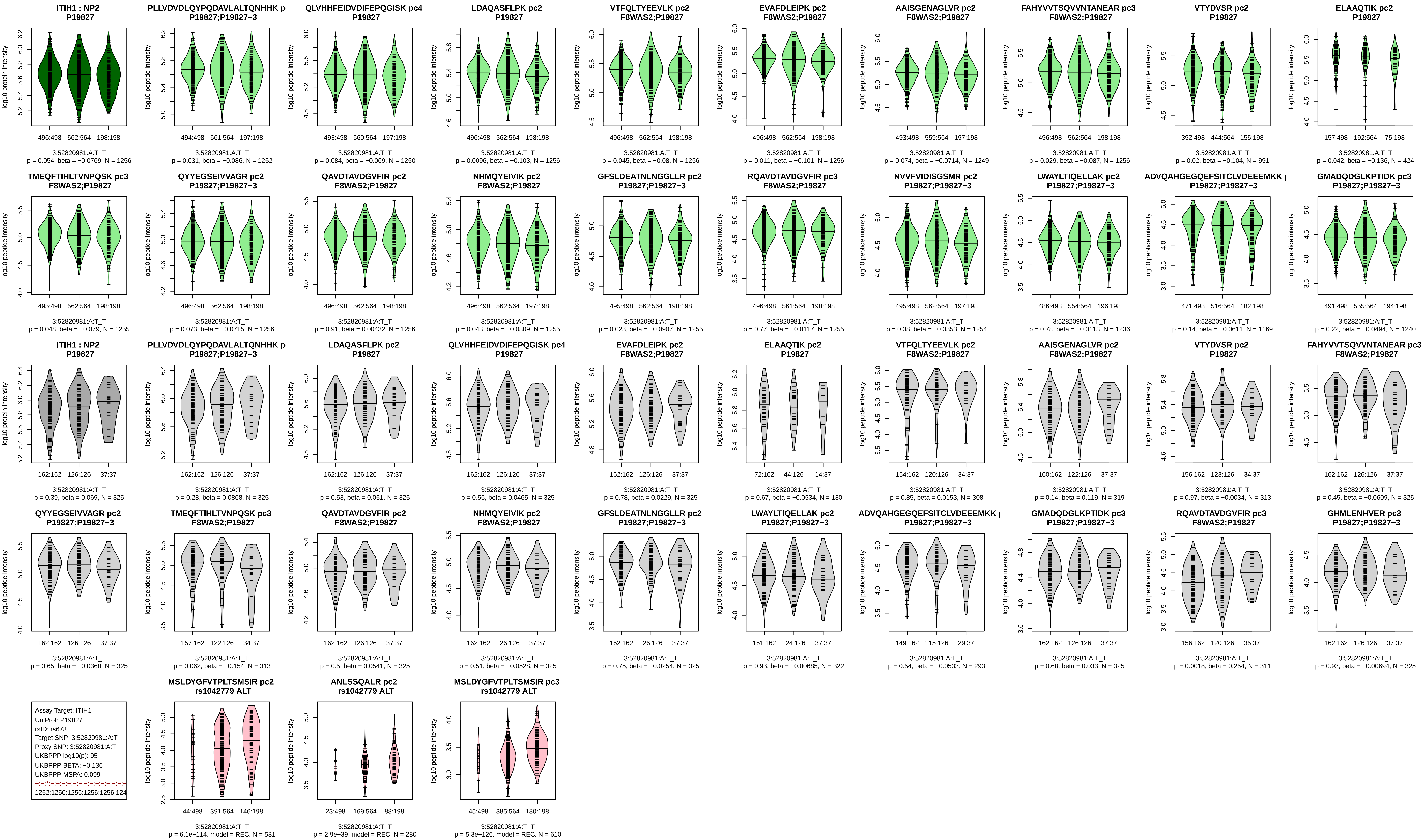
ITIH1 : NP2 P19827
PLLVDVDLQYPQDAVLALTQNHHK pc P19827;P19827−3
QLVHHFEIDVDIFEPQGISK pc4 P19827
LDAQASFLPK pc2 P19827
VTFQLTYEEVLK pc2 F8WAS2;P19827
EVAFDLEIPK pc2 F8WAS2;P19827
AAISGENAGLVR pc2 F8WAS2;P19827
FAHYVVTSQVVNTANEAR pc3 F8WAS2;P19827
VTYDVSR pc2 P19827
ELAAQTIK pc2 P19827
p = 0.054, beta = −0.0769, N = 1256
p = 0.031, beta = −0.086, N = 1252
p = 0.084, beta = −0.069, N = 1250
p = 0.0096, beta = −0.103, N = 1256
p = 0.045, beta = −0.08, N = 1256
p = 0.011, beta = −0.101, N = 1256
p = 0.074, beta = −0.0714, N = 1249
p = 0.029, beta = −0.087, N = 1256
p = 0.02, beta = −0.104, N = 991
p = 0.042, beta = −0.136, N = 424
TMEQFTIHLTVNPQSK pc3 F8WAS2;P19827
QYYEGSEIVVAGR pc2 P19827;P19827−3
QAVDTAVDGVFIR pc2 F8WAS2;P19827
NHMQYEIVIK pc2 F8WAS2;P19827
GFSLDEATNLNGGLLR pc2 P19827;P19827−3
RQAVDTAVDGVFIR pc3 F8WAS2;P19827
NVVFVIDISGSMR pc2 P19827;P19827−3
LWAYLTIQELLAK pc2 P19827;P19827−3
ADVQAHGEGQEFSITCLVDEEEMKK P19827;P19827−3
GMADQDGLKPTIDK pc3 P19827;P19827−3
p = 0.048, beta = −0.079, N = 1255
p = 0.073, beta = −0.0715, N = 1256
p = 0.91, beta = 0.00432, N = 1256
p = 0.043, beta = −0.0809, N = 1255
p = 0.023, beta = −0.0907, N = 1255
p = 0.77, beta = −0.0117, N = 1255
p = 0.38, beta = −0.0353, N = 1254
p = 0.78, beta = −0.0113, N = 1236
p = 0.14, beta = −0.0611, N = 1169
p = 0.22, beta = −0.0494, N = 1240
p = 0.39, beta = 0.069, N = 325
p = 0.28, beta = 0.0868, N = 325
p = 0.53, beta = 0.051, N = 325
p = 0.56, beta = 0.0465, N = 325
p = 0.78, beta = 0.0229, N = 325
p = 0.67, beta = −0.0534, N = 130
p = 0.85, beta = 0.0153, N = 308
p = 0.14, beta = 0.119, N = 319
p = 0.97, beta = −0.0034, N = 313
p = 0.45, beta = −0.0609, N = 325
GHMLENHVER pc3 P19827;P19827−3
p = 0.65, beta = −0.0368, N = 325
p = 0.062, beta = −0.154, N = 313
p = 0.5, beta = 0.0541, N = 325
p = 0.51, beta = −0.0528, N = 325
p = 0.75, beta = −0.0254, N = 325
p = 0.93, beta = −0.00685, N = 322
p = 0.54, beta = −0.0533, N = 293
p = 0.68, beta = 0.033, N = 325
p = 0.0018, beta = 0.254, N = 311
p = 0.93, beta = −0.00694, N = 325
Assay Target: ITIH1
UniProt: P19827
rsID: rs678
Target SNP: 3:52820981:A:T
Proxy SNP: 3:52820981:A:T
UKBPPP log10(p): 95
UKBPPP BETA: −0.136
UKBPPP MSPA: 0.099
1252:1250:1256:1256:1256:124
MSLDYGFVTPLTSMSIR pc2 rs1042779 ALT
ANLSSQALR pc2 rs1042779 ALT
MSLDYGFVTPLTSMSIR pc3 rs1042779 ALT
3:52820981:A:T_T
p = 6.1e−114, model = REC, N = 581
p = 2.9e−39, model = REC, N = 280
p = 5.3e−126, model = REC, N = 610

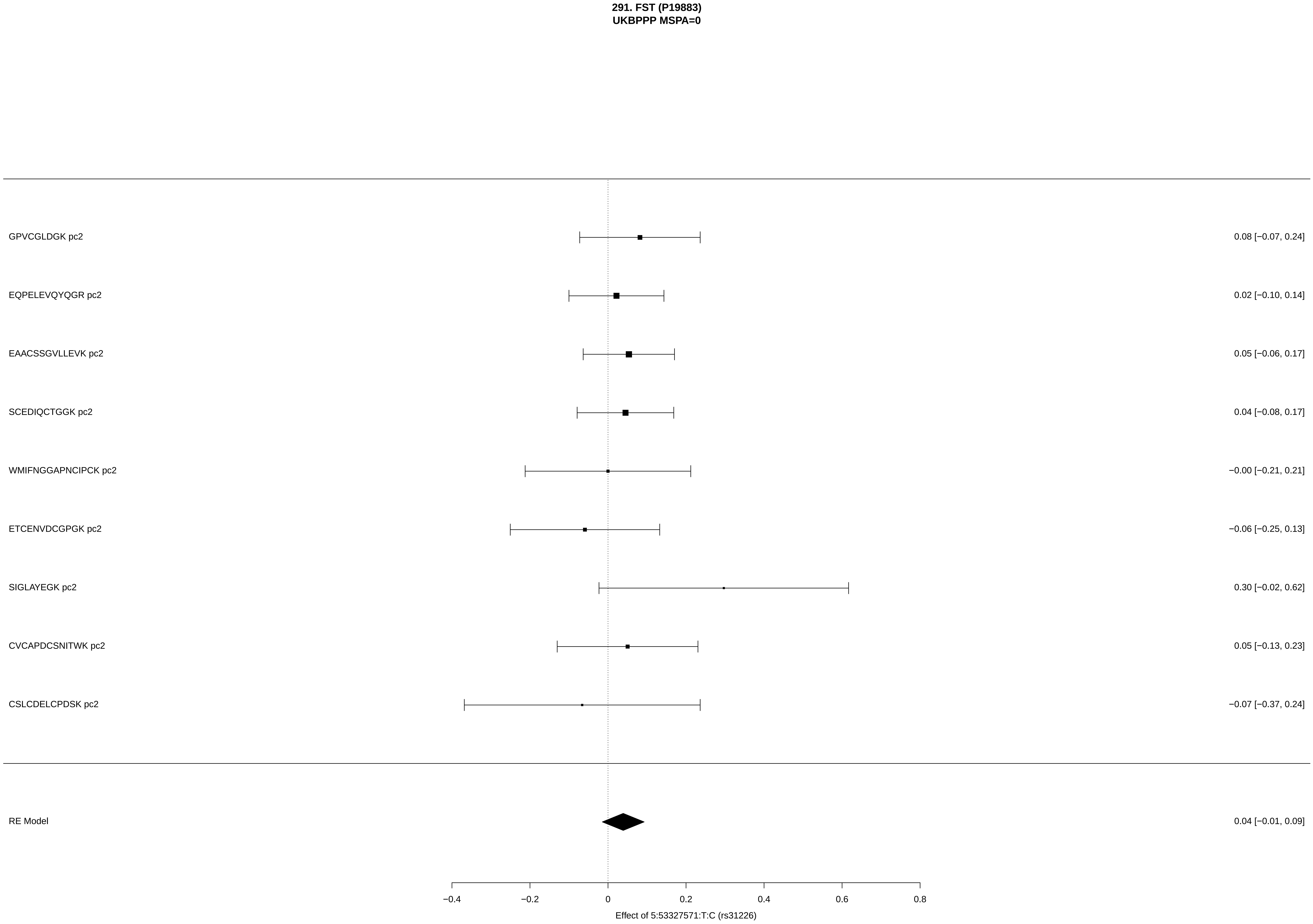

291. FST (P19883)
UKBPPP MSPA=0
GPVCGLDGK pc2
EQPELEVQYQGR pc2
EAACSSGVLLEVK pc2
SCEDIQCTGGK pc2
WMIFNGGAPNCIPCK pc2
ETCENVDCGPGK pc2
SIGLAYEGK pc2
CVCAPDCSNITWK pc2
CSLCDELCPDSK pc2
RE Model
0.08 [−0.07, 0.24]
0.02 [−0.10, 0.14]
0.05 [−0.06, 0.17]
0.04 [−0.08, 0.17]
−0.00 [−0.21, 0.21]
−0.06 [−0.25, 0.13]
0.30 [−0.02, 0.62]
0.05 [−0.13, 0.23]
−0.07 [−0.37, 0.24]
0.04 [−0.01, 0.09]
−0.4
−0.2
0
0.2
0.4
0.6
0.8
Effect of 5:53327571:T:C (rs31226)

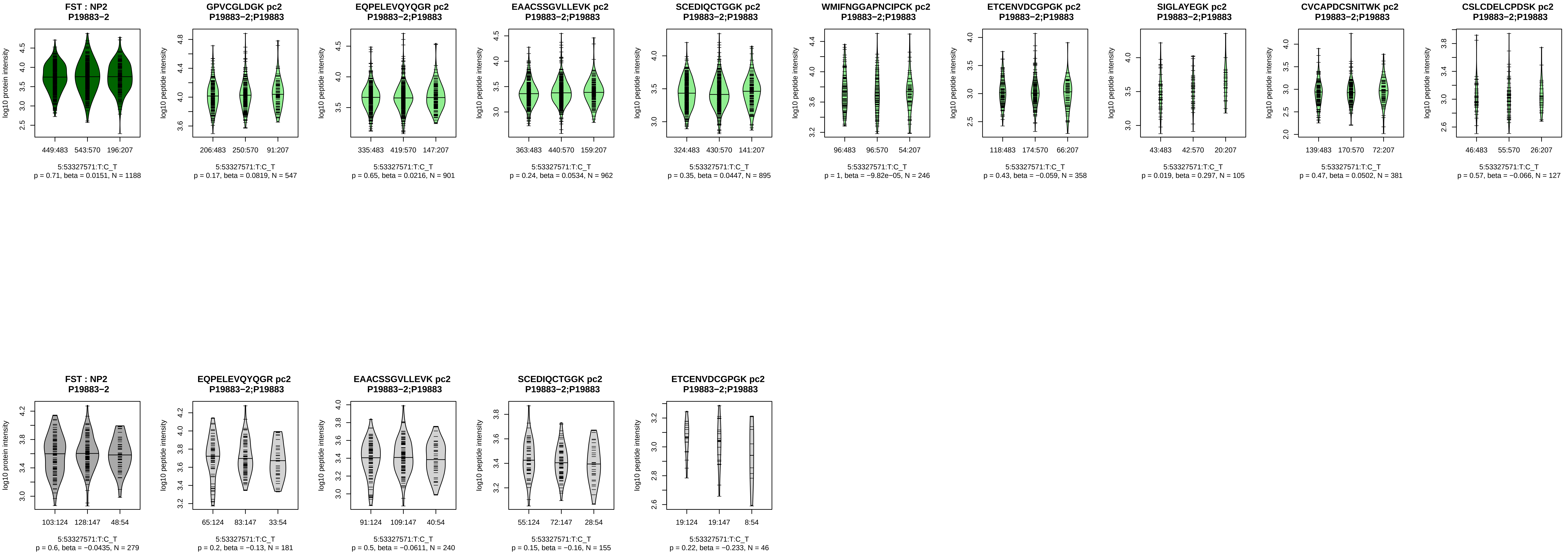

Assay Target: FST
UniProt: P19883
rsID: rs31226
Target SNP: 5:53327571:T:C
Proxy SNP: 5:53327571:T:C
UKBPPP log10(p): 94.3
UKBPPP BETA: −0.128
UKBPPP MSPA: 0
−:−:−:−:−:−:−:−:−:NA
547:901:962:895:246:358:105:3

292. TTR (P02766)
UKBPPP MSPA=0

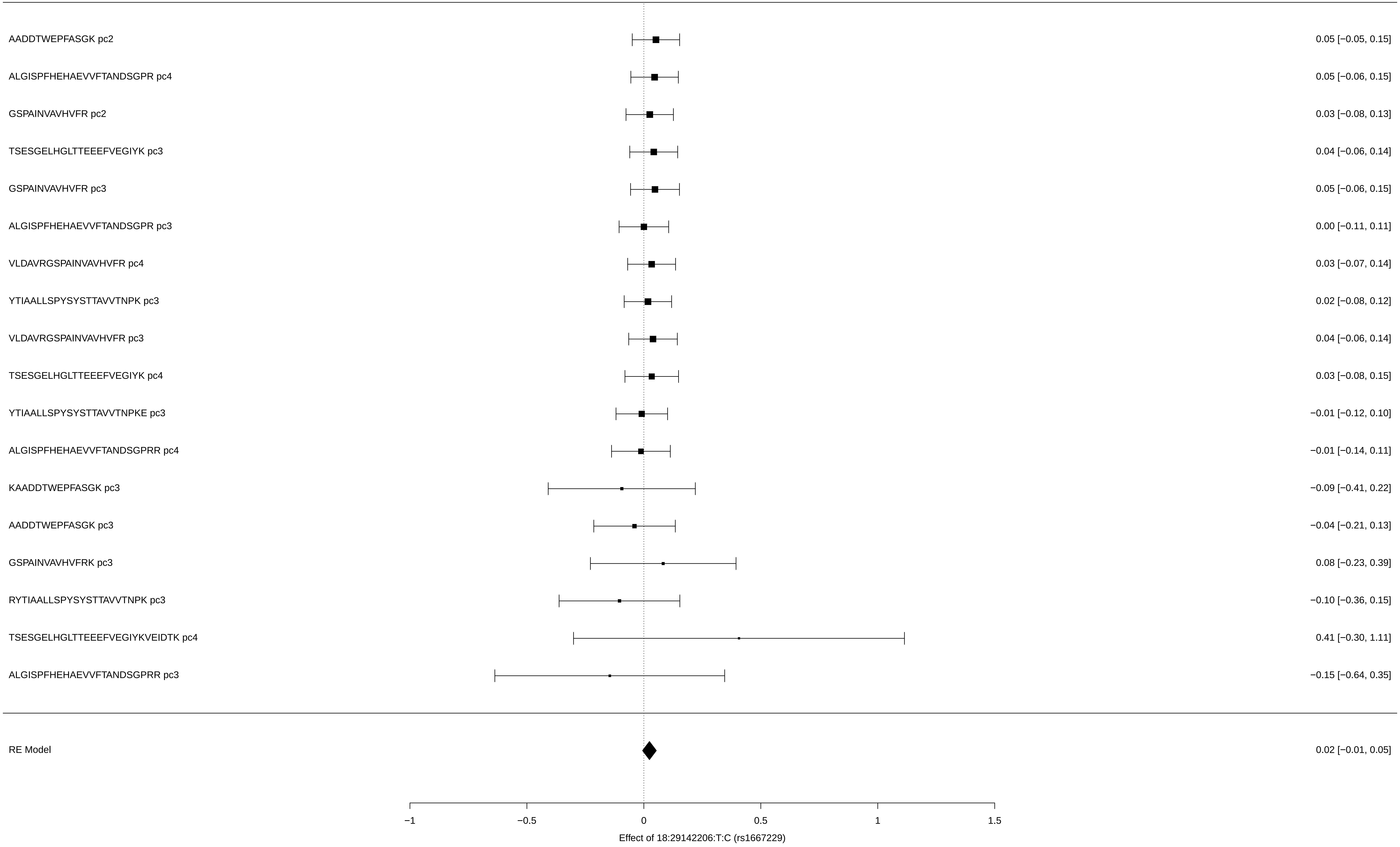

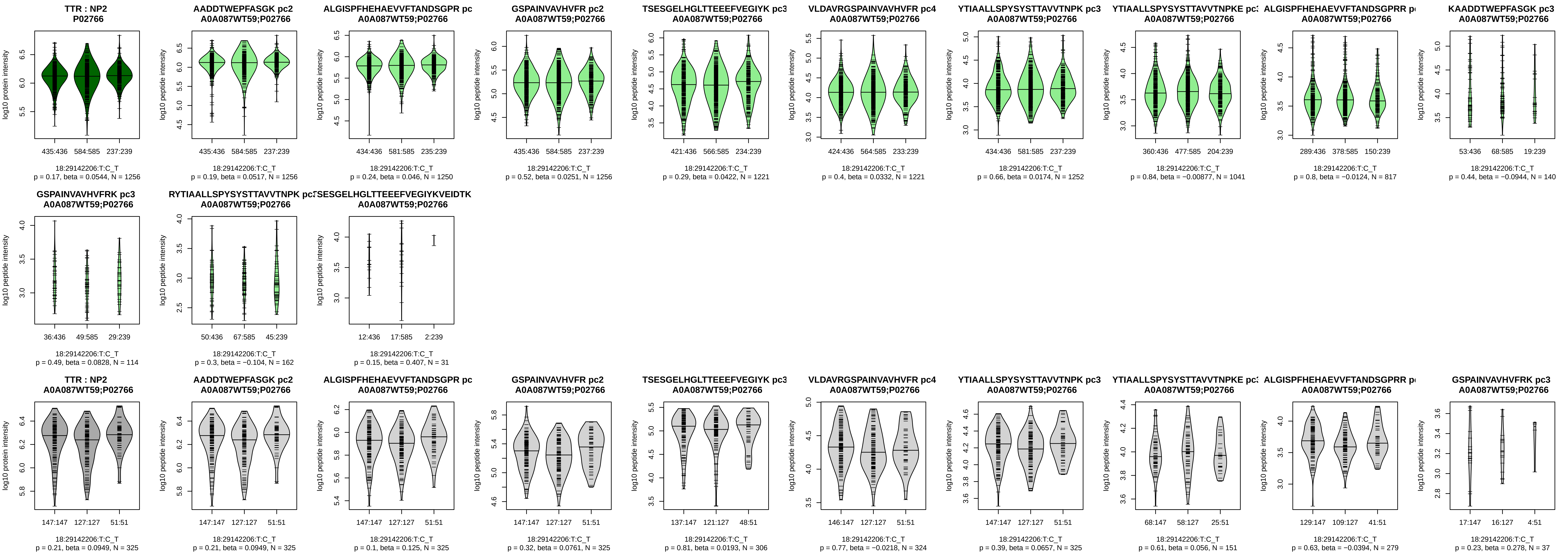

Assay Target: TTR
UniProt: P02766
rsID: rs1667229
Target SNP: 18:29142206:T:C
Proxy SNP: 18:29142206:T:C
UKBPPP log10(p): 94.2
UKBPPP BETA: 0.121
UKBPPP MSPA: 0
1256:1250:1256:1221:1189:114

293. TBCA (O75347)
UKBPPP MSPA=0

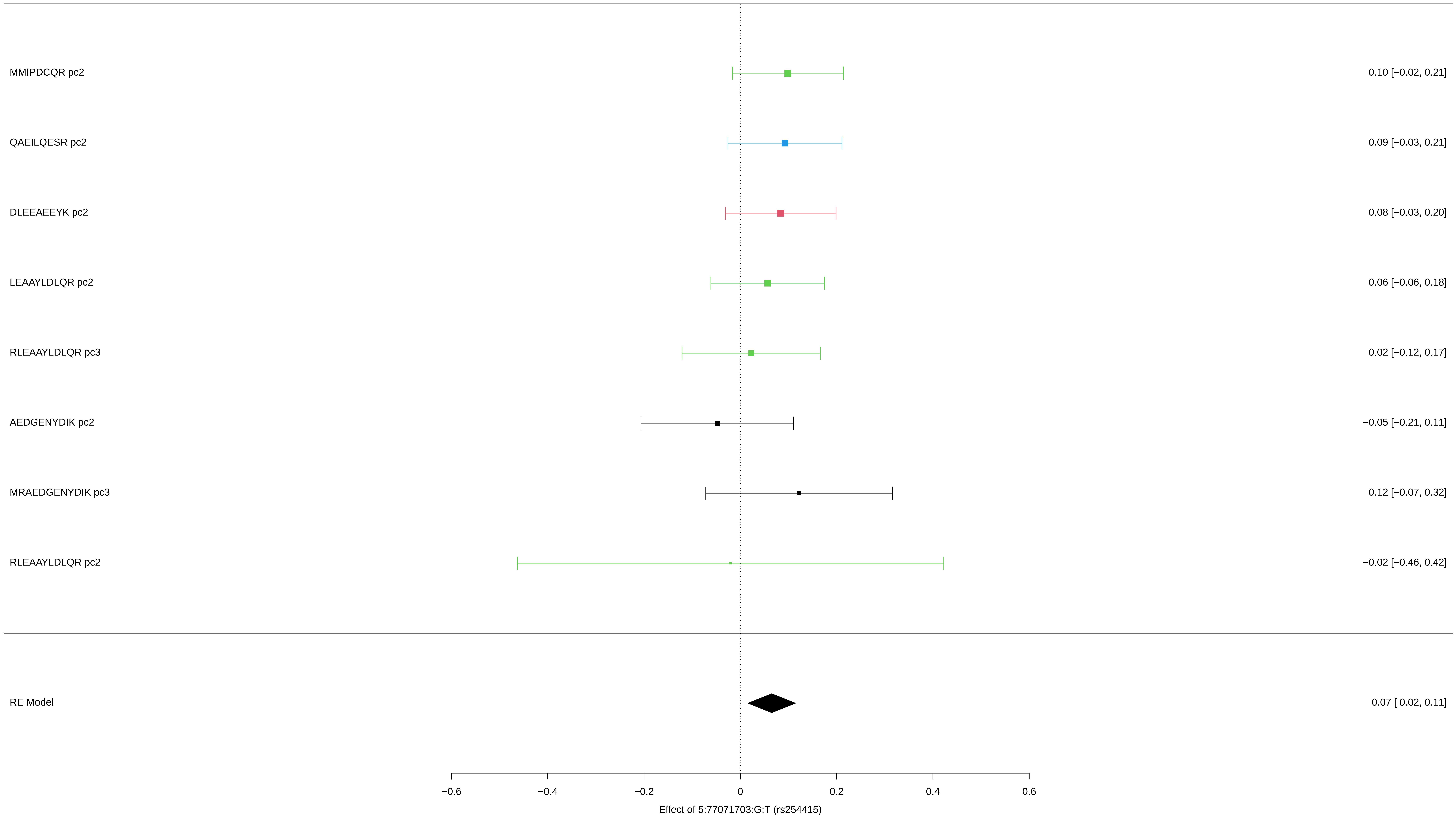

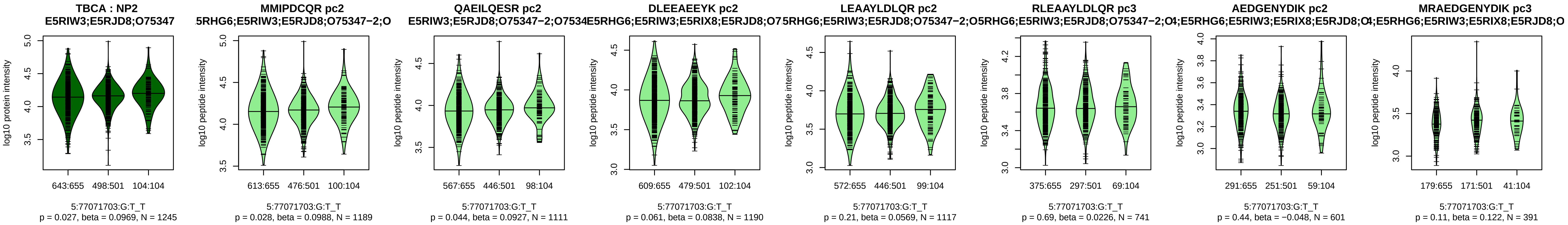

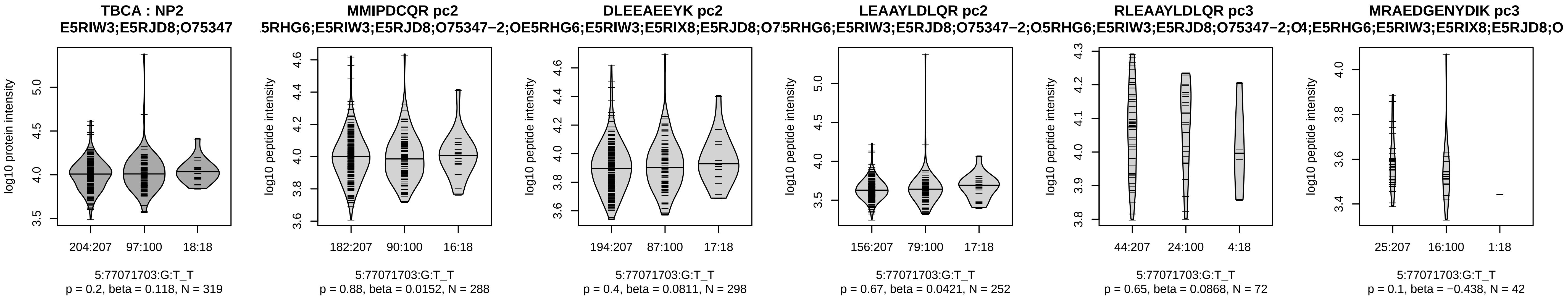

Assay Target: TBCA
UniProt: O75347
rsID: rs254415
Target SNP: 5:77071703:G:T
Proxy SNP: 5:77071703:G:T
UKBPPP log10(p): 94.1
UKBPPP BETA: 0.14
UKBPPP MSPA: 0
−:−:−:−:−:−:−:−:NA
1189:1111:1190:1117:741:601:3

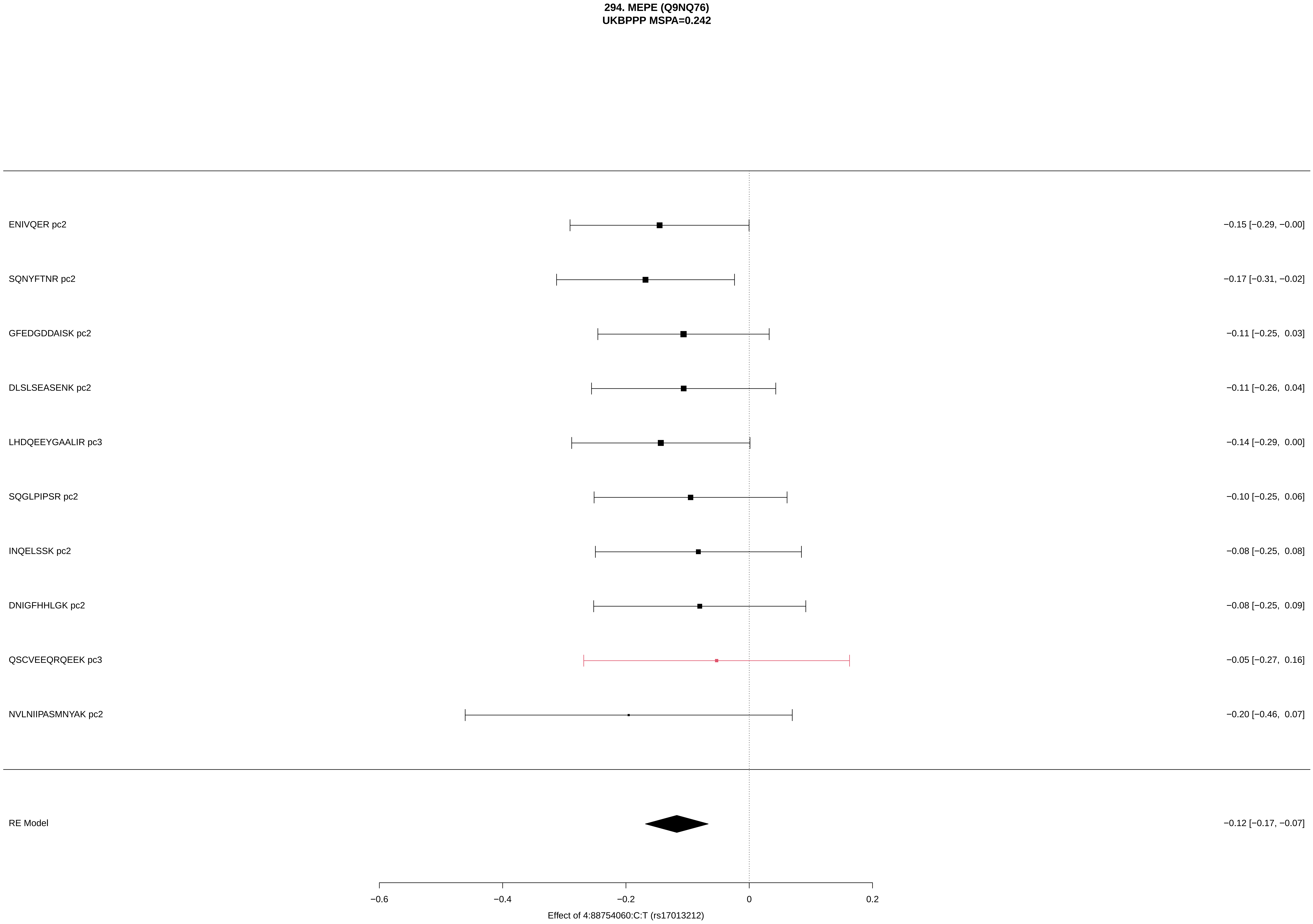

294. MEPE (Q9NQ76)
UKBPPP MSPA=0.242
ENIVQER pc2
−0.15 [−0.29, −0.00]
SQNYFTNR pc2
−0.17 [−0.31, −0.02]
GFEDGDDAISK pc2
−0.11 [−0.25, 0.03]
DLSLSEASENK pc2
−0.11 [−0.26, 0.04]
LHDQEEYGAALIR pc3
−0.14 [−0.29, 0.00]
SQGLPIPSR pc2
−0.10 [−0.25, 0.06]
INQELSSK pc2
−0.08 [−0.25, 0.08]
DNIGFHHLGK pc2
−0.08 [−0.25, 0.09]
QSCVEEQRQEEK pc3
−0.05 [−0.27, 0.16]
NVLNIIPASMNYAK pc2
−0.20 [−0.46, 0.07]
RE Model
−0.12 [−0.17, −0.07]
−0.6
−0.4
−0.2
0
0.2
Effect of 4:88754060:C:T (rs17013212)

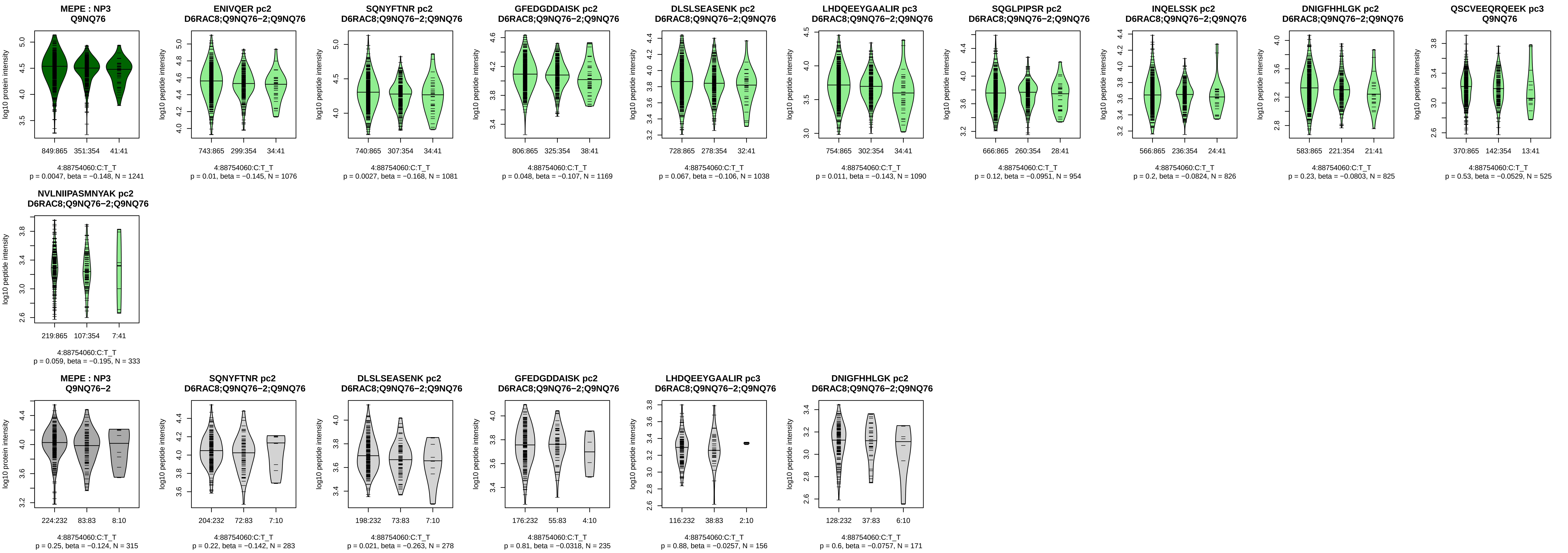

Assay Target: MEPE
UniProt: Q9NQ76
rsID: rs17013212
Target SNP: 4:88754060:C:T
Proxy SNP: 4:88754060:C:T
UKBPPP log10(p): 92.2
UKBPPP BETA: −0.168
UKBPPP MSPA: 0.242
::–:–:–:–:–:–:–:–:NA:NA
1076:1081:1169:1038:1090:954

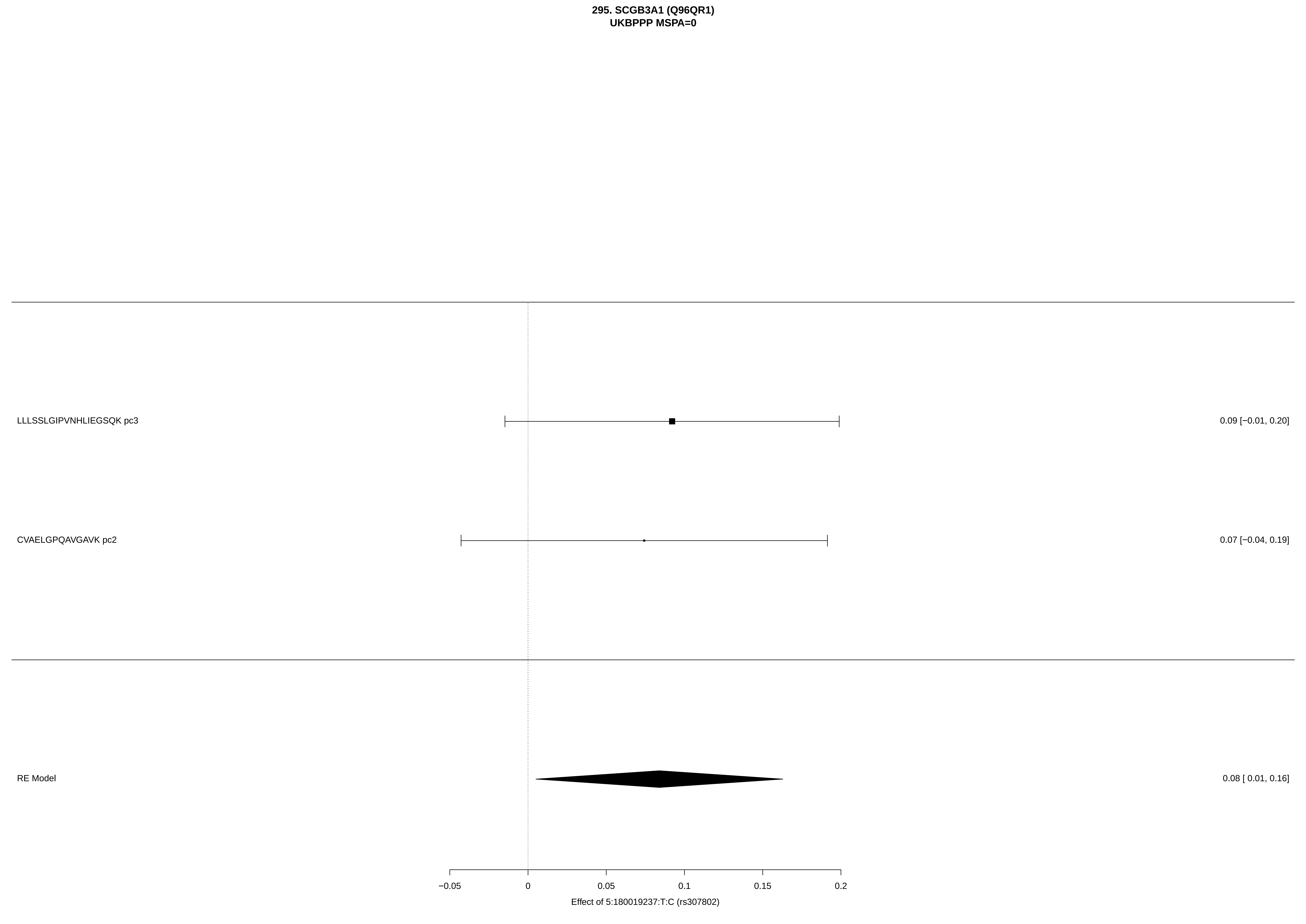
295. SCGB3A1 (Q96QR1)
UKBPPP MSPA=0
LLLSSLGIPVNHLIEGSQK pc3
0.09 [−0.01, 0.20]
CVAELGPQAVGAVK pc2
0.07 [−0.04, 0.19]
RE Model
0.08 [0.01, 0.16]
−0.05
0
0.05
0.1
0.15
0.2
Effect of 5:180019237:T:C (rs307802)

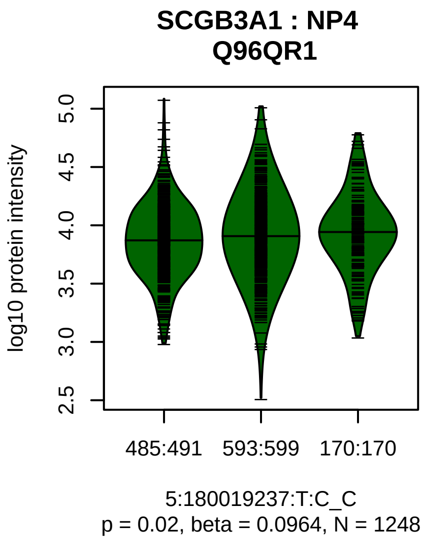

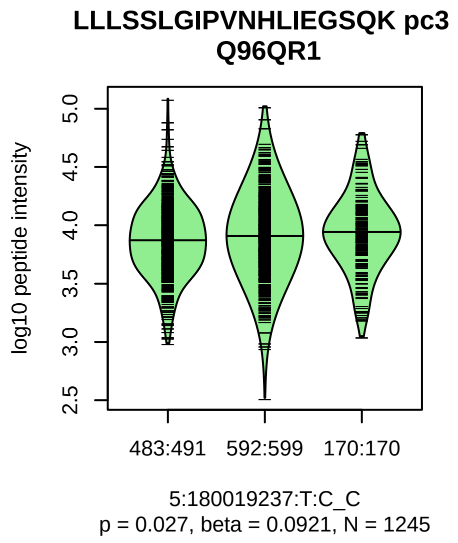

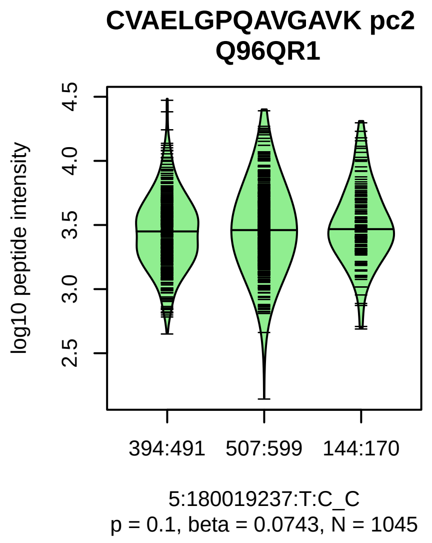

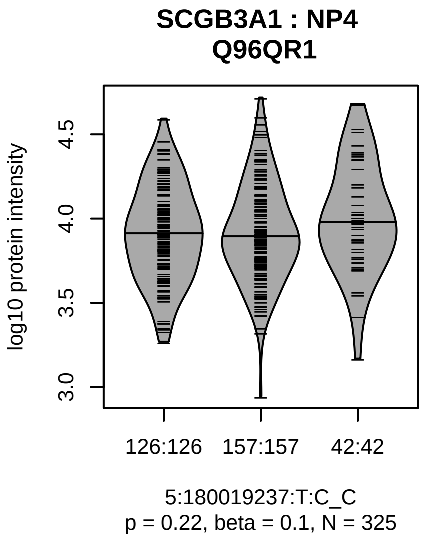

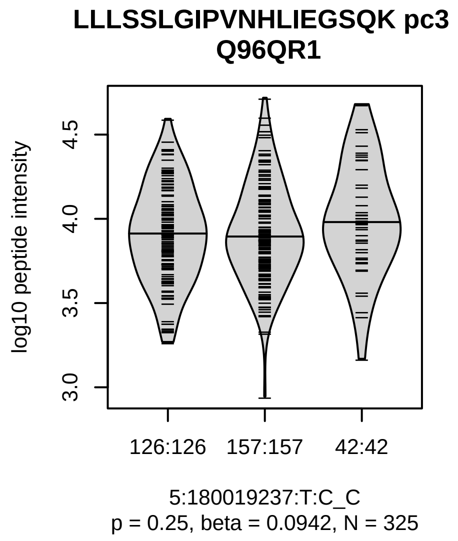

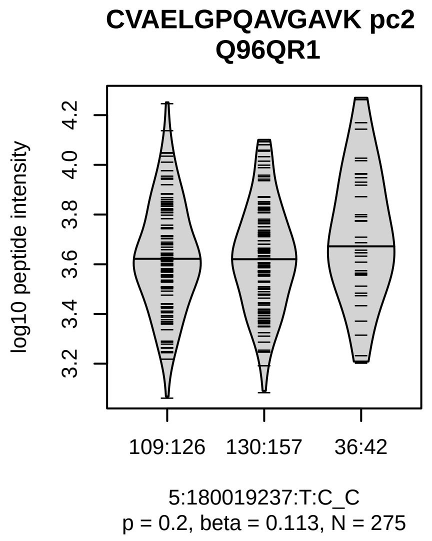

Assay Target: SCGB3A1
UniProt: Q96QR1
rsID: rs307802
Target SNP: 5:180019237:T:C
Proxy SNP: 5:180019237:T:C
UKBPPP log10(p): 91.5
UKBPPP BETA: 0.123
UKBPPP MSPA: 0
--:--
1245:1045

296. CD84 (Q9UIB8)
UKBPPP MSPA=0

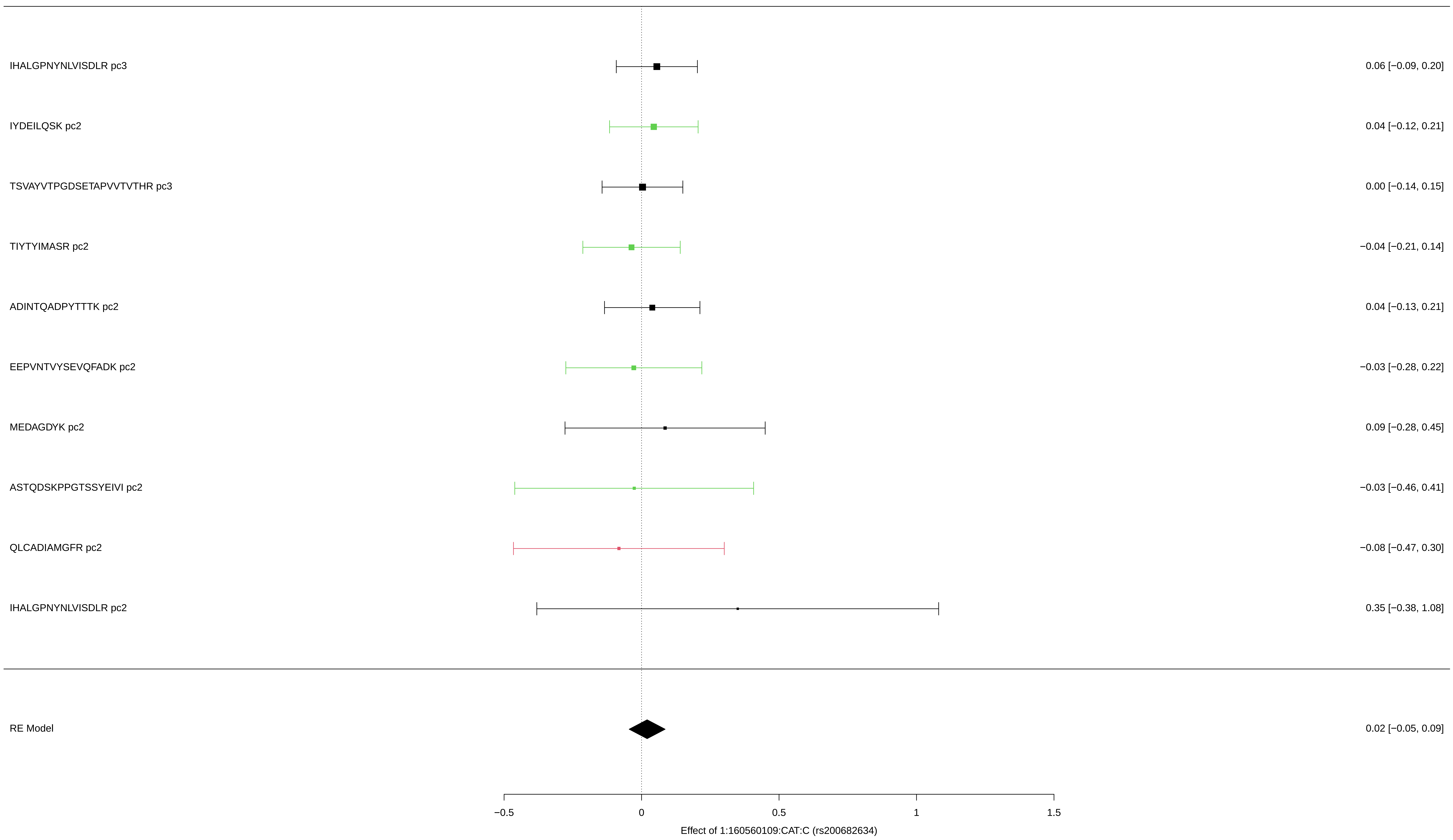

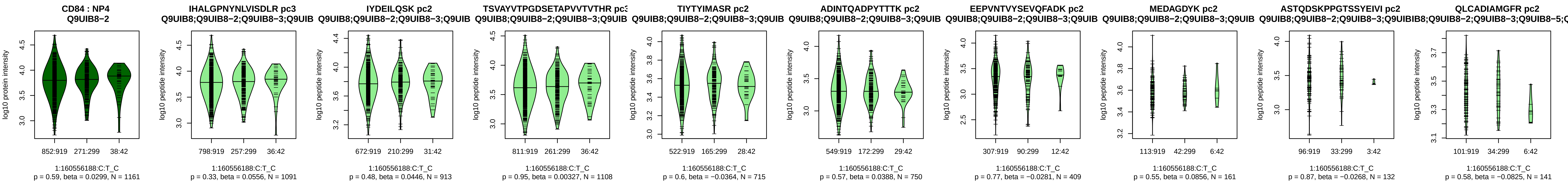

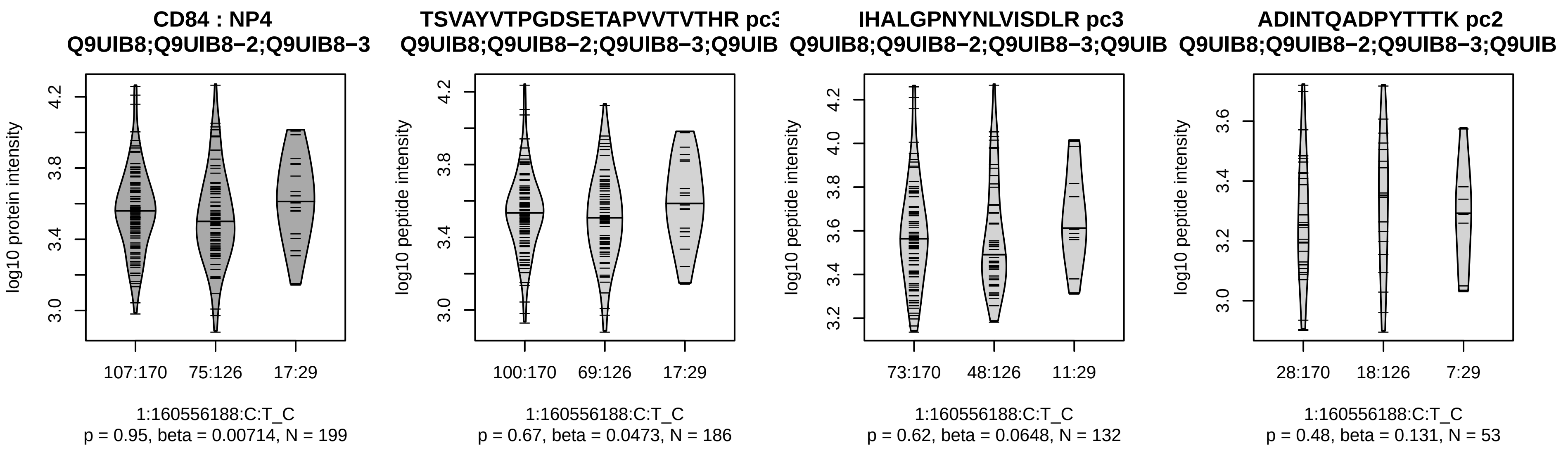

Assay Target: CD84
UniProt: Q9UIB8
rsID: rs200682634
Target SNP: 1:160560109:CAT:
Proxy SNP: 1:160556188:C:T
UKBPPP log10(p): 88.6
UKBPPP BETA: −0.211
UKBPPP MSPA: 0
1091:913:1108:715:750:409:16

297. NCS1 (P62166)
UKBPPP MSPA=0

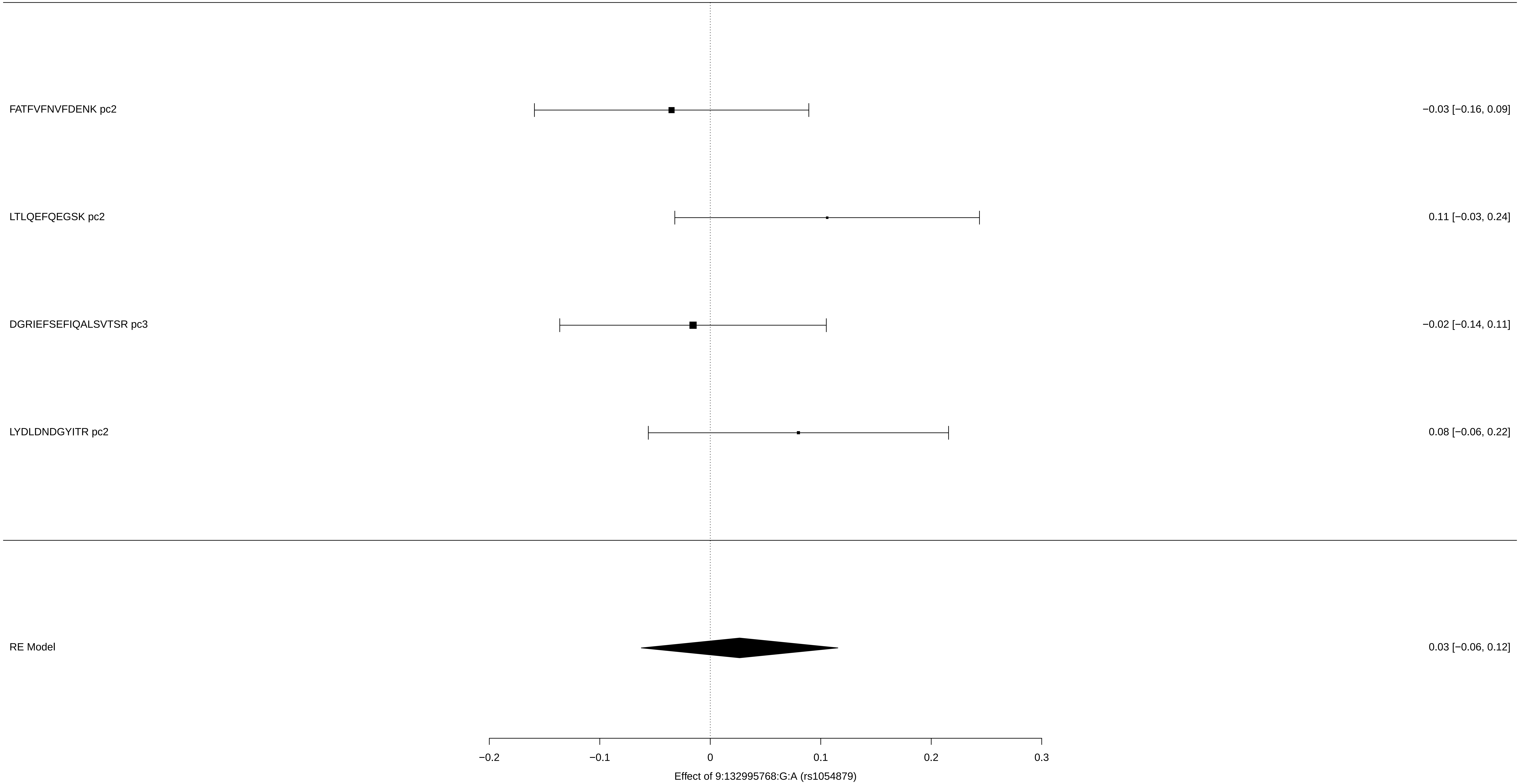

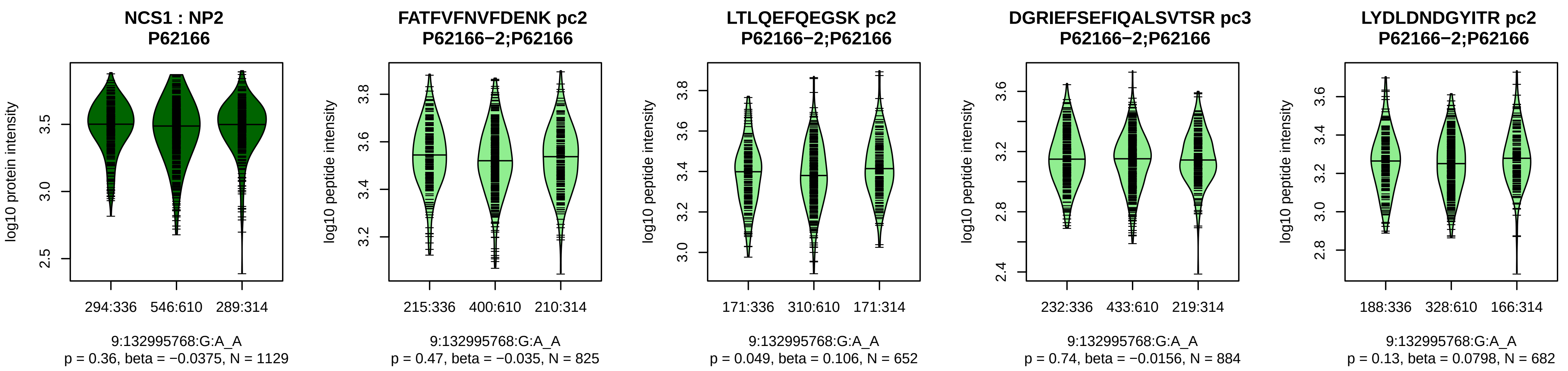

Assay Target: NCS1
UniProt: P62166
rsID: rs1054879
Target SNP: 9:132995768:G:A
Proxy SNP: 9:132995768:G:A
UKBPPP log10(p): 88
UKBPPP BETA: 0.11
UKBPPP MSPA: 0
−:−:−:−
825:652:884:682

298. LTBP2 (Q14767)
UKBPPP MSPA=0

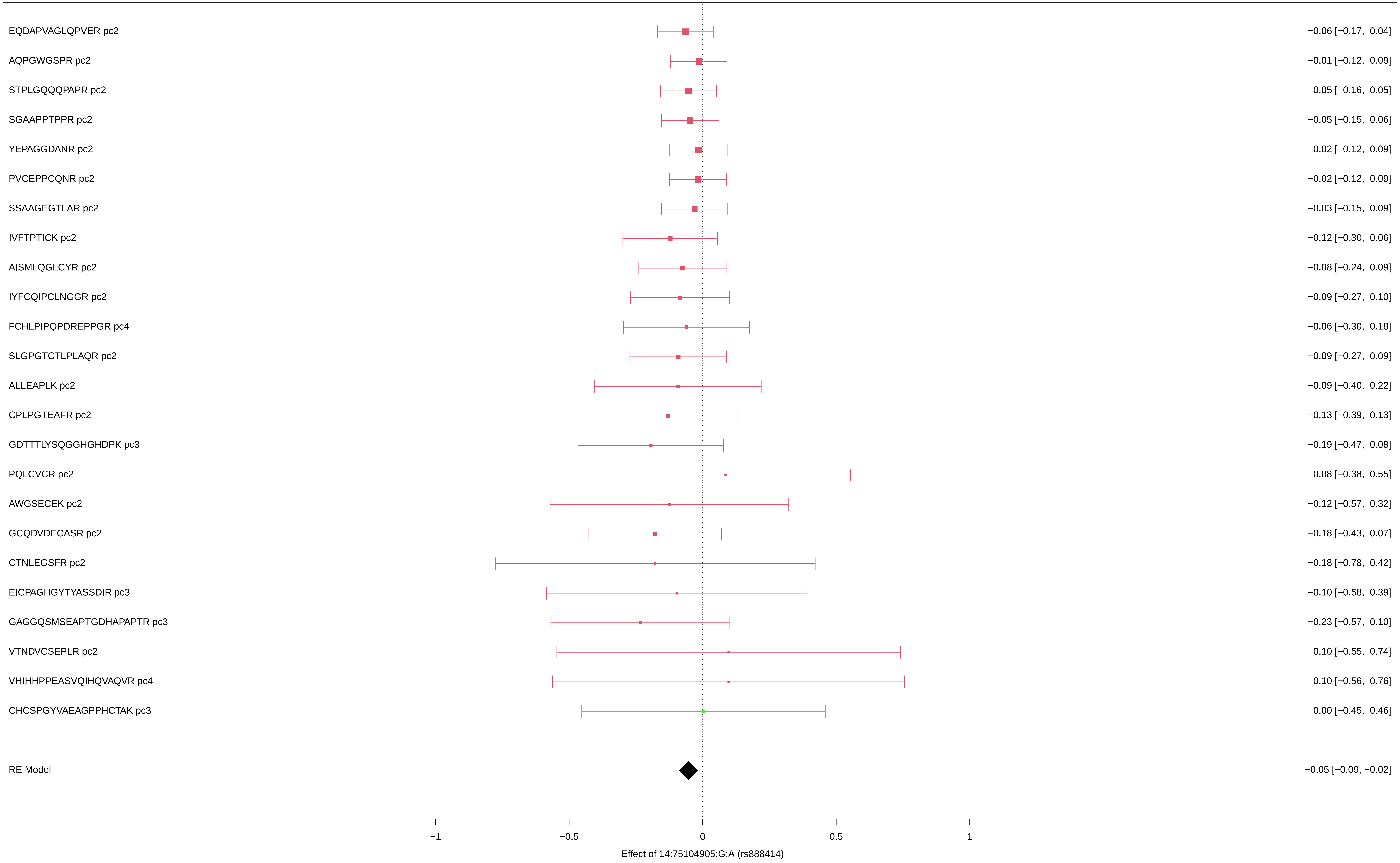

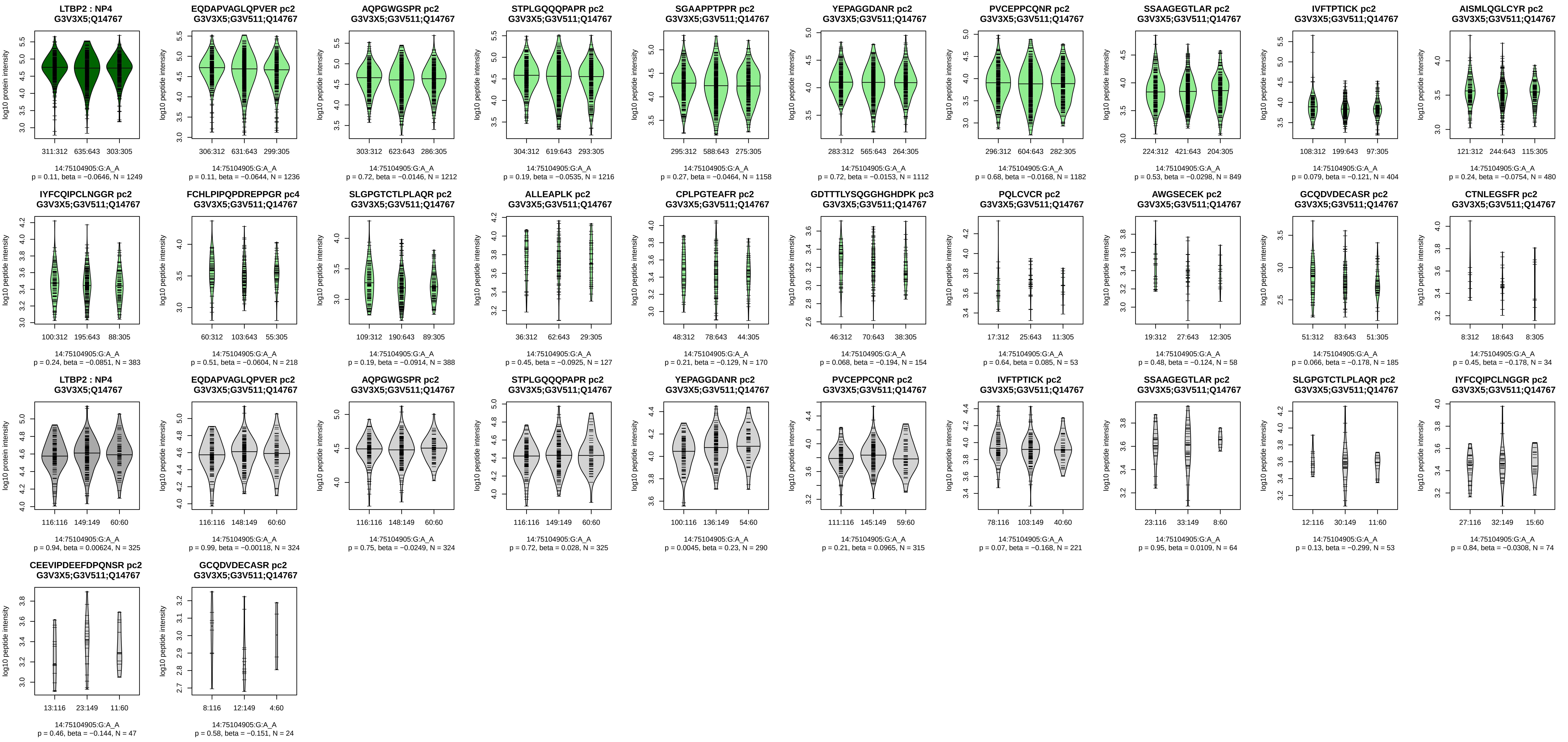

Assay Target: LTBP2
UniProt: Q14767
rsID: rs888414
Target SNP: 14:75104905:G:A
Proxy SNP: 14:75104905:G:A
UKBPPP log10(p): 86.9
UKBPPP BETA: −0.103
UKBPPP MSPA: 0
1236:1212:1216:1158:1112:118

299. DNAJA4 (Q8WW22)
UKBPPP MSPA=0

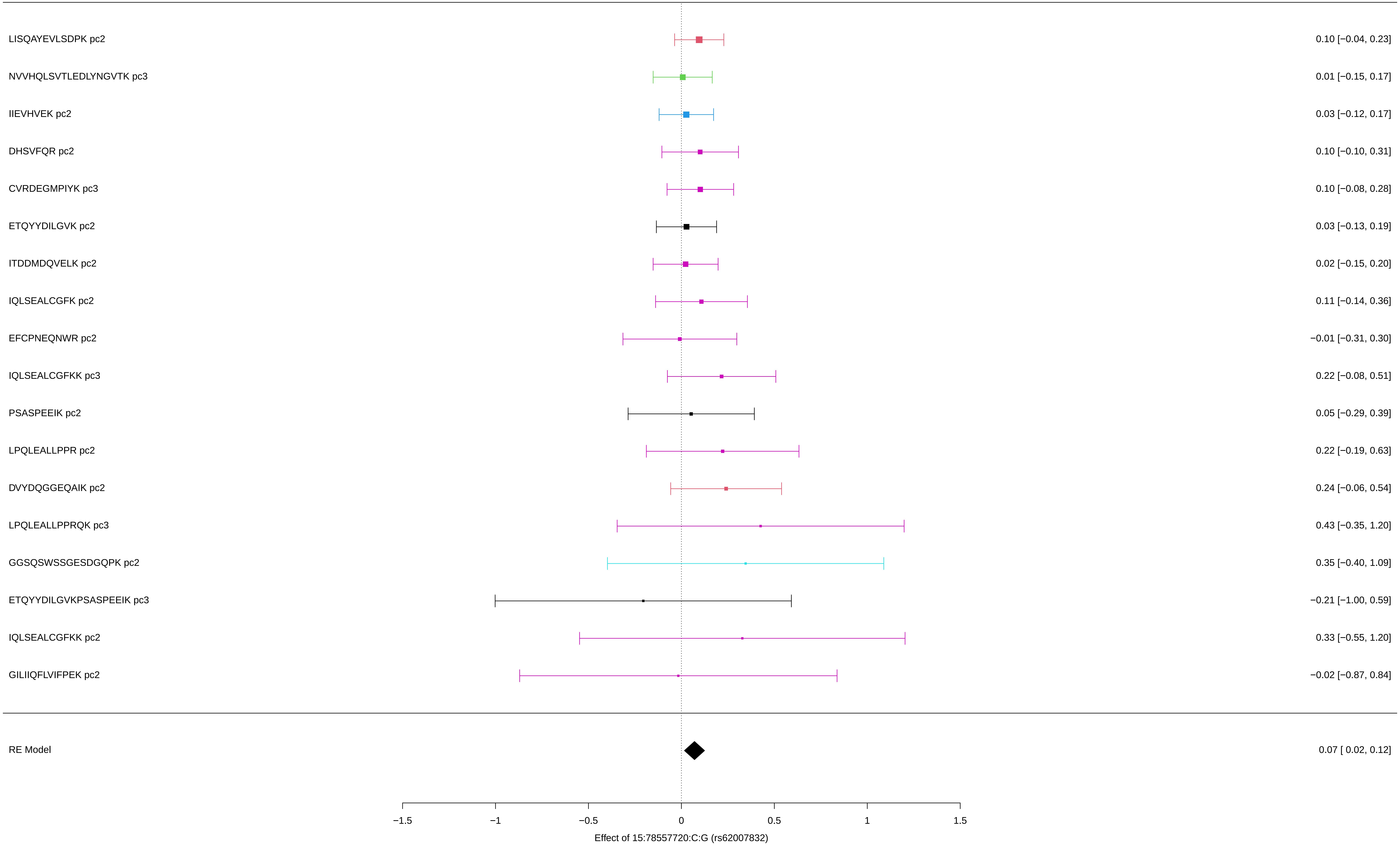

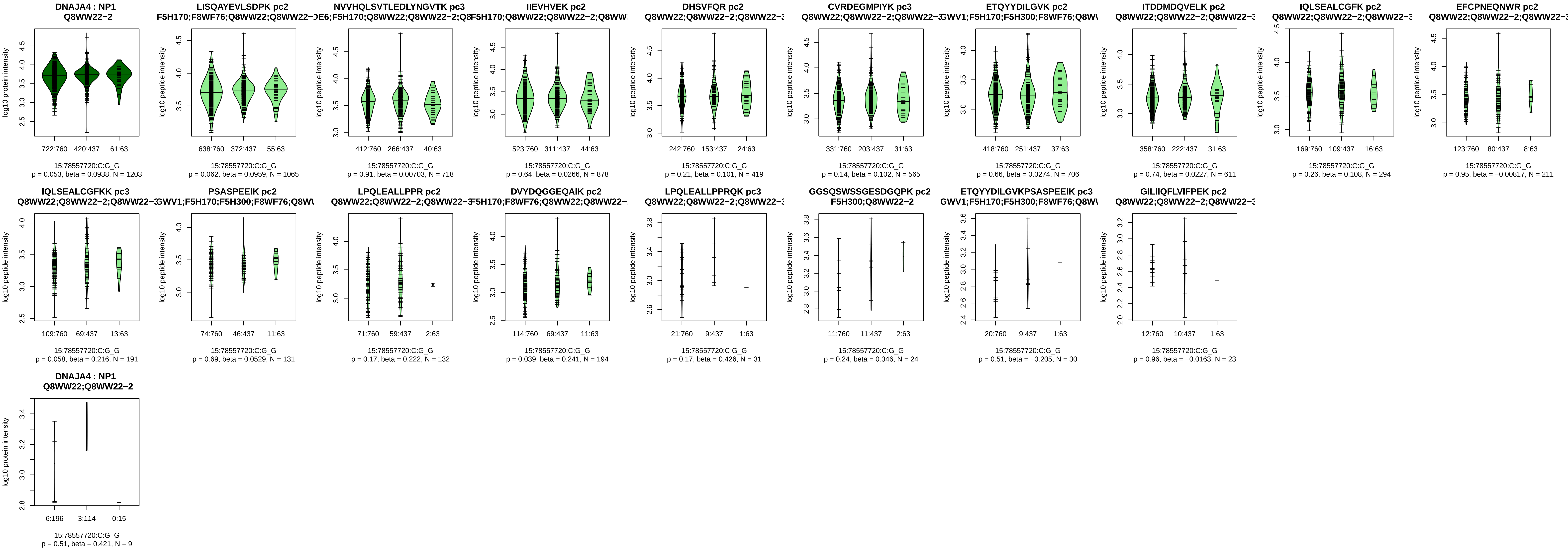

Assay Target: DNAJA4
UniProt: Q8WW22
rsID: rs62007832
Target SNP: 15:78557720:C:G
Proxy SNP: 15:78557720:C:G
UKBPPP log10(p): 86
UKBPPP BETA: 0.143
UKBPPP MSPA: 0
1065:718:878:419:565:706:611:

300. GP5 (P40197)
UKBPPP MSPA=0

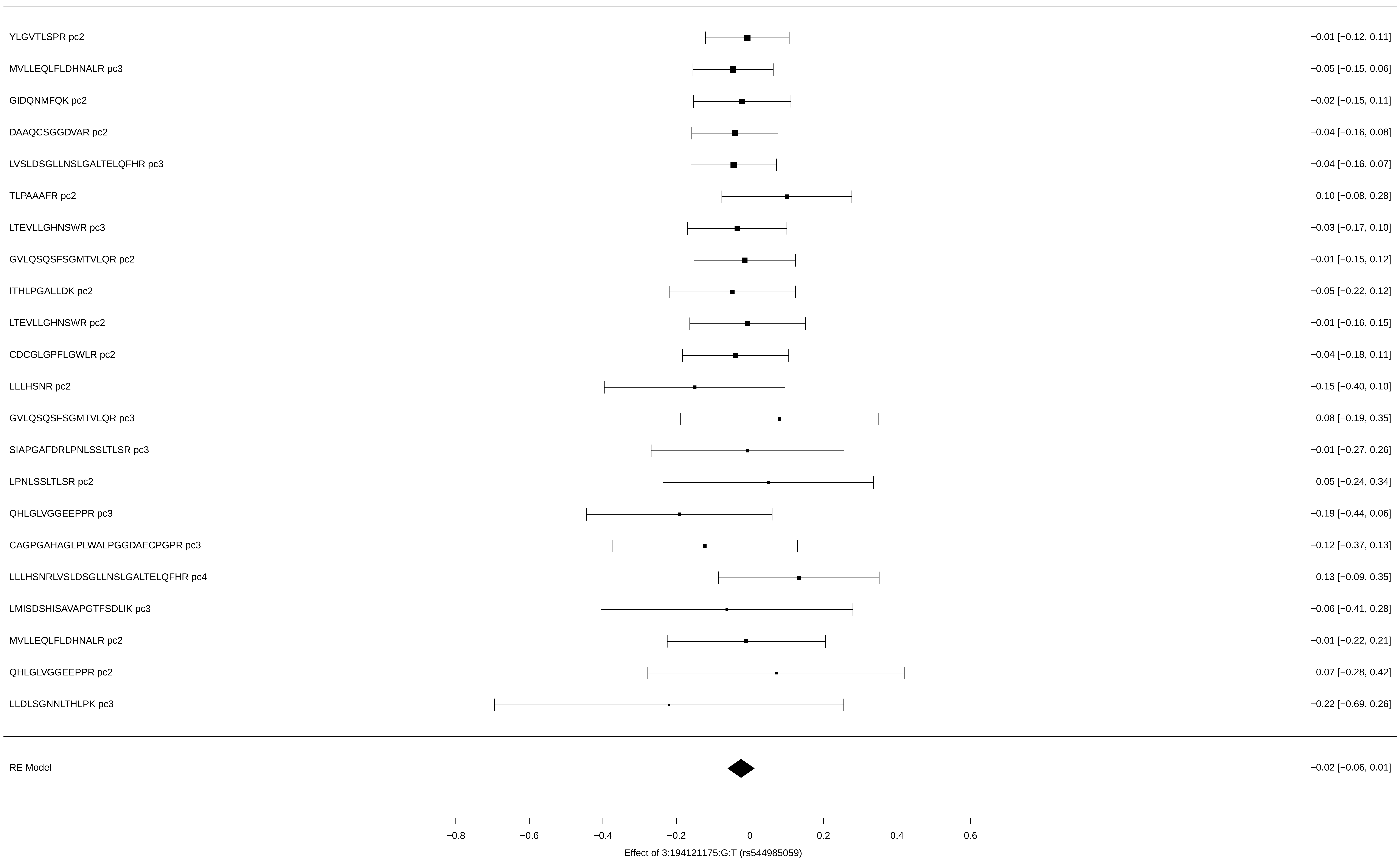

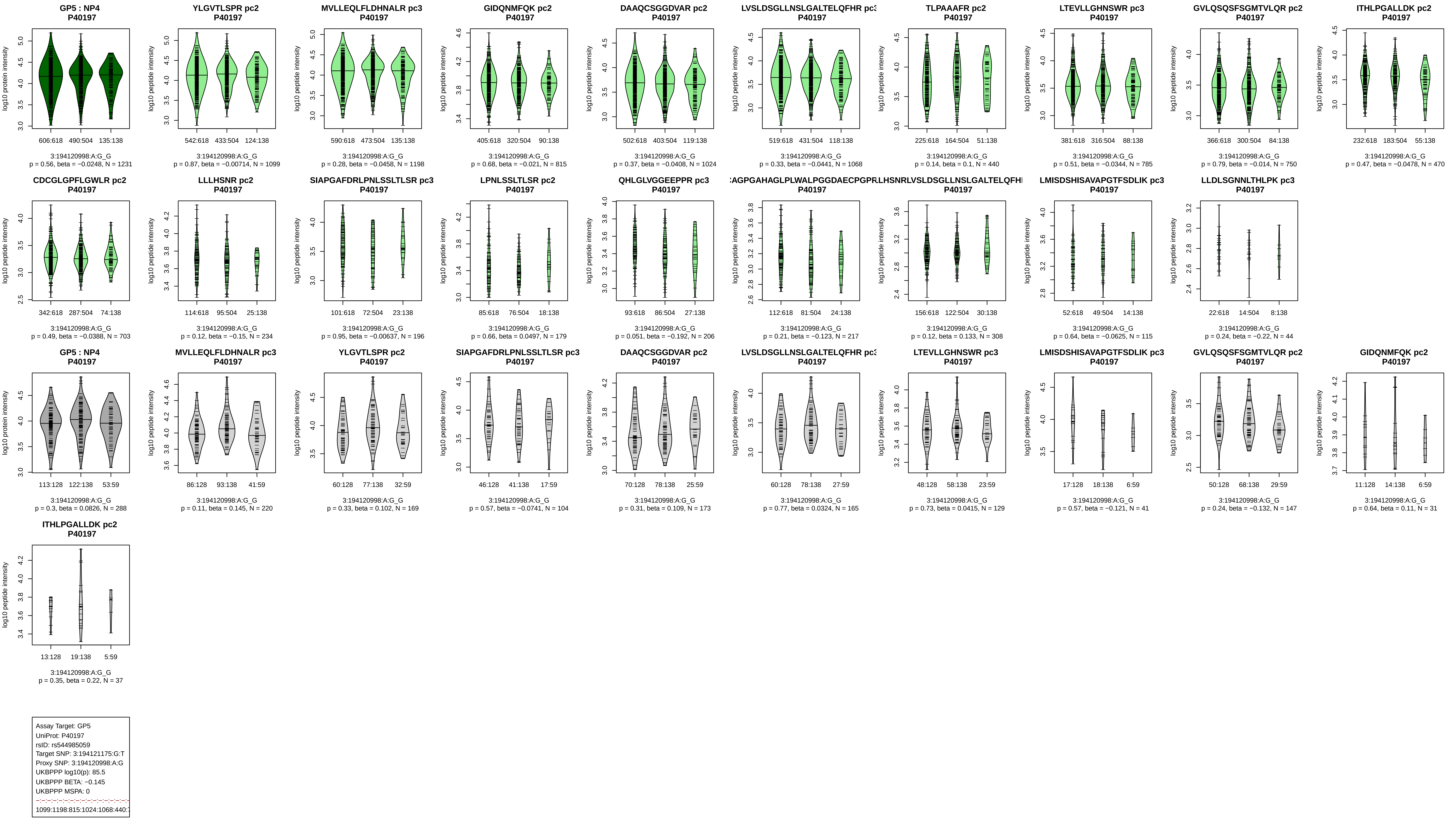
GP5 : NP4
P40197
log10 protein intensity
606:618 490:504 135:138
3:194120998:A:G_G
p = 0.56, beta = −0.0248, N = 1231
YLGVTLSPR pc2
P40197
log10 peptide intensity
542:618 433:504 124:138
3:194120998:A:G_G
p = 0.87, beta = −0.00714, N = 1099
MVLLEQLFLDHNALR pc3
P40197
590:618 473:504 135:138
3:194120998:A:G_G
p = 0.28, beta = −0.0458, N = 1198
GIDQNMFQK pc2
P40197
405:618 320:504 90:138
3:194120998:A:G_G
p = 0.68, beta = −0.021, N = 815
DAAQCSGGDVAR pc2
P40197
502:618 403:504 119:138
3:194120998:A:G_G
p = 0.37, beta = −0.0408, N = 1024
LVSLDSGLLNSLGALTELQFHR pc3
P40197
519:618 431:504 118:138
3:194120998:A:G_G
p = 0.33, beta = −0.0441, N = 1068
TLPAAAFR pc2
P40197
225:618 164:504 51:138
3:194120998:A:G_G
p = 0.14, beta = 0.1, N = 440
LTEVLLGHNSWR pc3
P40197
381:618 316:504 88:138
3:194120998:A:G_G
p = 0.51, beta = −0.0344, N = 785
GVLQSQSFSGMTVLQR pc2
P40197
366:618 300:504 84:138
3:194120998:A:G_G
p = 0.79, beta = −0.014, N = 750
ITHLPGALLDK pc2
P40197
232:618 183:504 55:138
3:194120998:A:G_G
p = 0.47, beta = −0.0478, N = 470
CDCGLGPFLGWLR pc2
P40197
342:618 287:504 74:138
3:194120998:A:G_G
p = 0.49, beta = −0.0388, N = 703
LLLHSNR pc2
P40197
114:618 95:504 25:138
3:194120998:A:G_G
p = 0.12, beta = −0.15, N = 234
SIAPGAFDRLPNLSSLTLSR pc3
P40197
101:618 72:504 23:138
3:194120998:A:G_G
p = 0.95, beta = −0.00637, N = 196
LPNLSSLTLSR pc2
P40197
85:618 76:504 18:138
3:194120998:A:G_G
p = 0.66, beta = 0.0497, N = 179
QHLGLVGGEEPPR pc3
P40197
93:618 86:504 27:138
3:194120998:A:G_G
p = 0.051, beta = −0.192, N = 206
AGPGAHAGLPLWALPGGDAECPGPR
P40197
112:618 81:504 24:138
3:194120998:A:G_G
p = 0.21, beta = −0.123, N = 217
LHSNRLVSLDSGLLNSLGALTELQFH
P40197
156:618 122:504 30:138
3:194120998:A:G_G
p = 0.12, beta = 0.133, N = 308
LMISDSHISAVAPGTFSDLIK pc3
P40197
52:618 49:504 14:138
3:194120998:A:G_G
p = 0.64, beta = −0.0625, N = 115
LLDLSGNNLTHLPK pc3
P40197
22:618 14:504 8:138
3:194120998:A:G_G
p = 0.24, beta = −0.22, N = 44
GP5 : NP4
P40197
113:128 122:138 53:59
3:194120998:A:G_G
p = 0.3, beta = 0.0826, N = 288
MVLLEQLFLDHNALR pc3
P40197
86:128 93:138 41:59
3:194120998:A:G_G
p = 0.11, beta = 0.145, N = 220
YLGVTLSPR pc2
P40197
60:128 77:138 32:59
3:194120998:A:G_G
p = 0.33, beta = 0.102, N = 169
SIAPGAFDRLPNLSSLTLSR pc3
P40197
46:128 41:138 17:59
3:194120998:A:G_G
p = 0.57, beta = −0.0741, N = 104
DAAQCSGGDVAR pc2
P40197
70:128 78:138 25:59
3:194120998:A:G_G
p = 0.31, beta = 0.109, N = 173
LVSLDSGLLNSLGALTELQFHR pc3
P40197
60:128 78:138 27:59
3:194120998:A:G_G
p = 0.77, beta = 0.0324, N = 165
LTEVLLGHNSWR pc3
P40197
48:128 58:138 23:59
3:194120998:A:G_G
p = 0.73, beta = 0.0415, N = 129
LMISDSHISAVAPGTFSDLIK pc3
P40197
17:128 18:138 6:59
3:194120998:A:G_G
p = 0.57, beta = −0.121, N = 41
GVLQSQSFSGMTVLQR pc2
P40197
50:128 68:138 29:59
3:194120998:A:G_G
p = 0.24, beta = −0.132, N = 147
GIDQNMFQK pc2
P40197
11:128 14:138 6:59
3:194120998:A:G_G
p = 0.64, beta = 0.11, N = 31
ITHLPGALLDK pc2
P40197
13:128 19:138 5:59
3:194120998:A:G_G
p = 0.35, beta = 0.22, N = 37
Assay Target: GP5
UniProt: P40197
rsID: rs544985059
Target SNP: 3:194121175:G:T
Proxy SNP: 3:194120998:A:G
UKBPPP log10(p): 85.5
UKBPPP BETA: −0.145
UKBPPP MSPA: 0
1099:1198:815:1024:1068:440:

301. CDHR2 (Q9BYE9)
UKBPPP MSPA=0

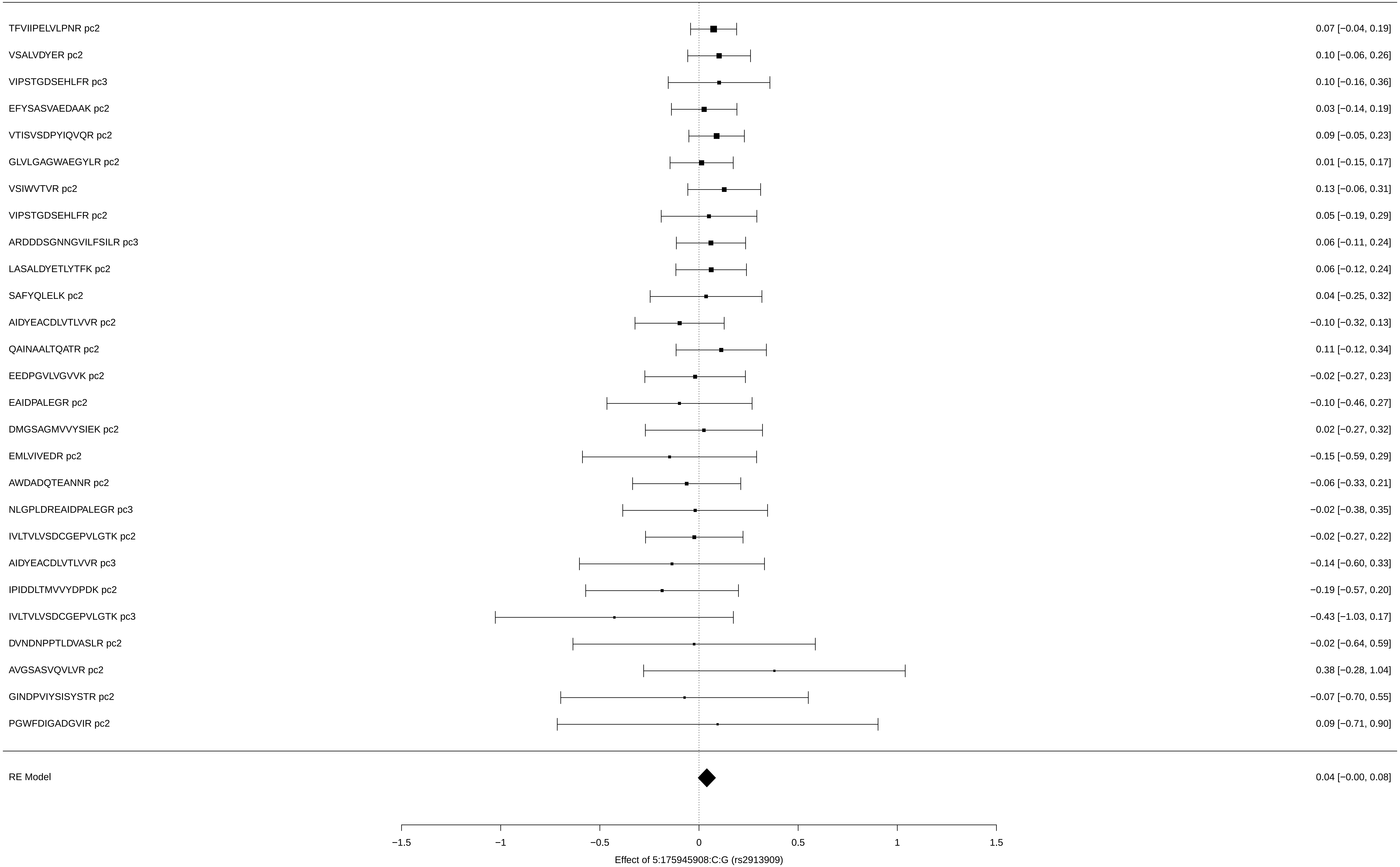

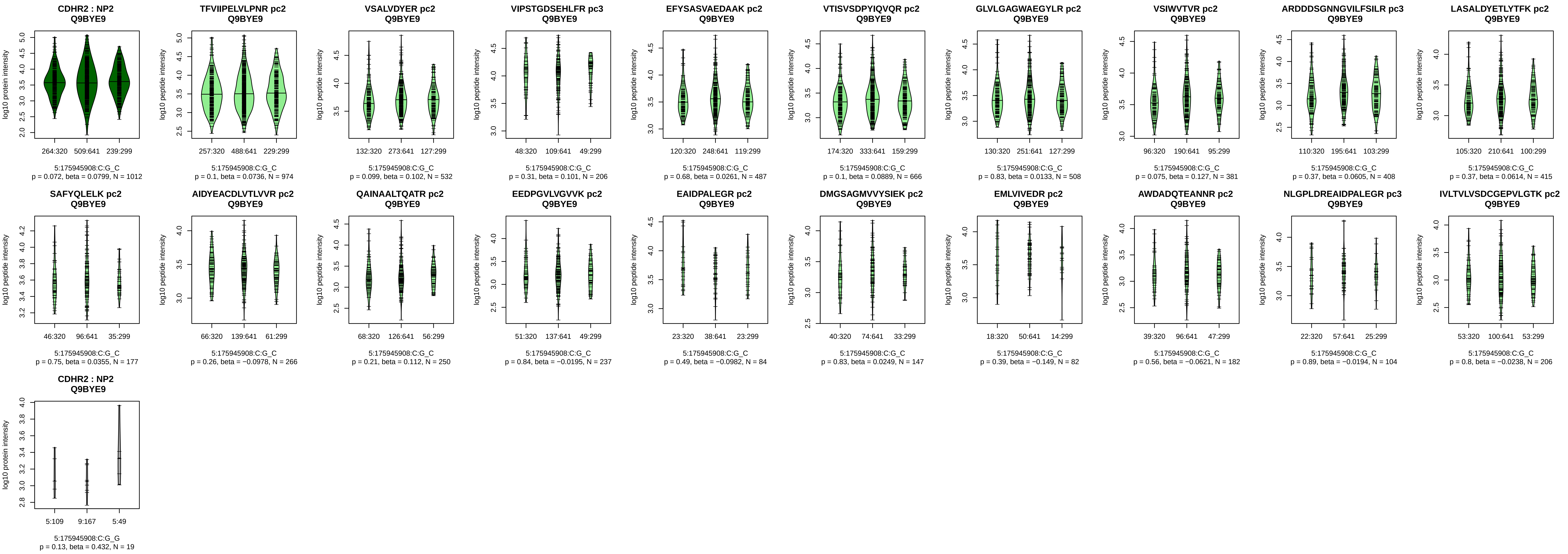

Assay Target: CDHR2
UniProt: Q9BYE9
rsID: rs2913909
Target SNP: 5:175945908:C:G
Proxy SNP: 5:175945908:C:G
UKBPPP log10(p): 81.7
UKBPPP BETA: −0.1
UKBPPP MSPA: 0

974:532:206:487:666:508:381:2

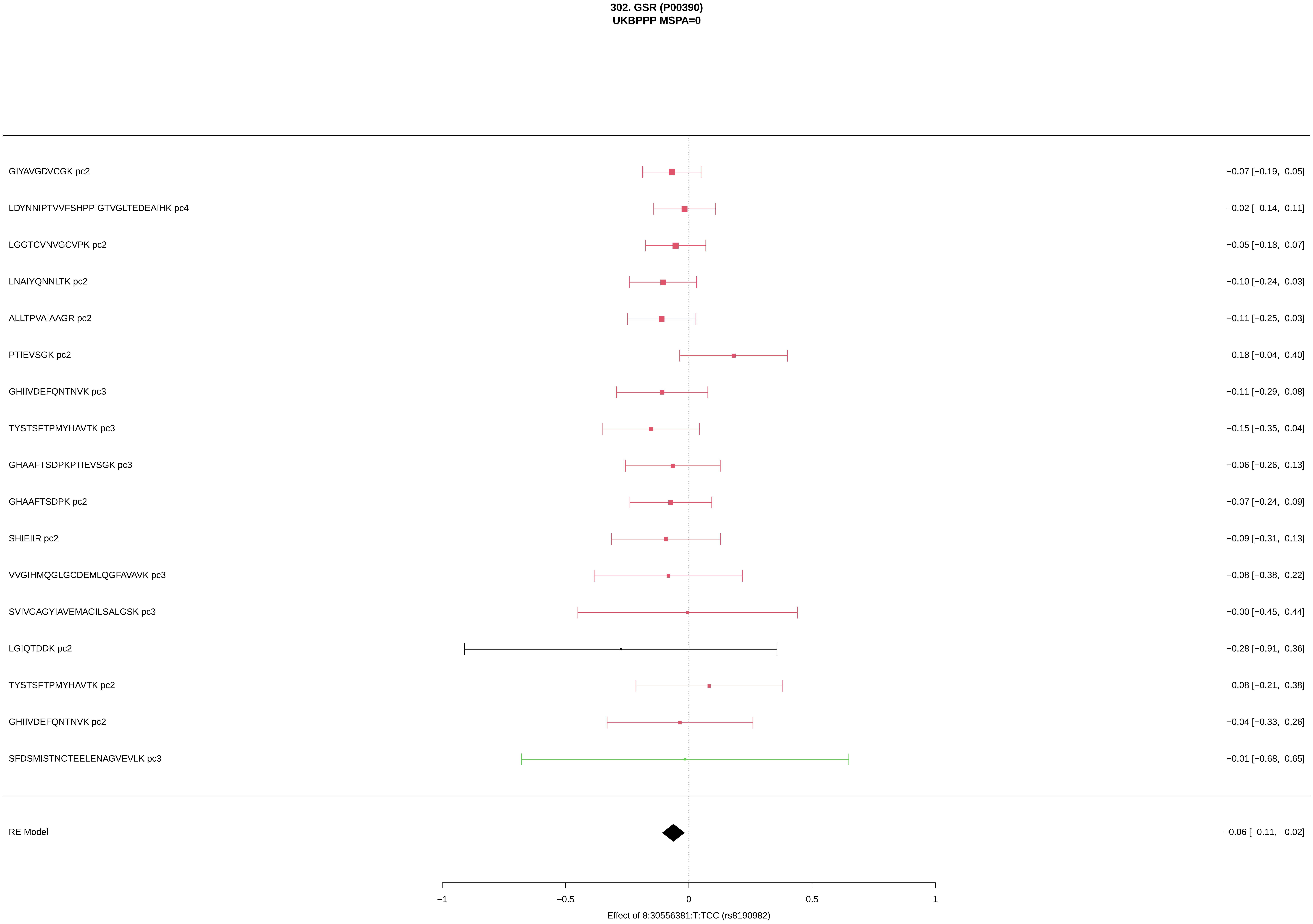

302. GSR (P00390)
UKBPPP MSPA=0
GIYAVGDVCGK pc2
−0.07 [−0.19, 0.05]
LDYNNIPTVVFSHPPIGTVGLTEDEAIHK pc4
−0.02 [−0.14, 0.11]
LGGTCVNVGCVPK pc2
−0.05 [−0.18, 0.07]
LNAIYQNNLTK pc2
−0.10 [−0.24, 0.03]
ALLTPVAIAAGR pc2
−0.11 [−0.25, 0.03]
PTIEVSGK pc2
0.18 [−0.04, 0.40]
GHIIVDEFQNTNVK pc3
−0.11 [−0.29, 0.08]
TYSTSFTPMYHAVTK pc3
−0.15 [−0.35, 0.04]
GHAAFTSDPKPTIEVSGK pc3
−0.06 [−0.26, 0.13]
GHAAFTSDPK pc2
−0.07 [−0.24, 0.09]
SHIEIIR pc2
−0.09 [−0.31, 0.13]
VVGIHMQGLGCDEMLQGFAVAVK pc3
−0.08 [−0.38, 0.22]
SVIVGAGYIAVEMAGILSALGSK pc3
−0.00 [−0.45, 0.44]
LGIQTDDK pc2
−0.28 [−0.91, 0.36]
TYSTSFTPMYHAVTK pc2
0.08 [−0.21, 0.38]
GHIIVDEFQNTNVK pc2
−0.04 [−0.33, 0.26]
SFDSMISTNCTEELENAGVEVLK pc3
−0.01 [−0.68, 0.65]
RE Model
−0.06 [−0.11, −0.02]
−1
−0.5
0
0.5
1
Effect of 8:30556381:T:TCC (rs8190982)

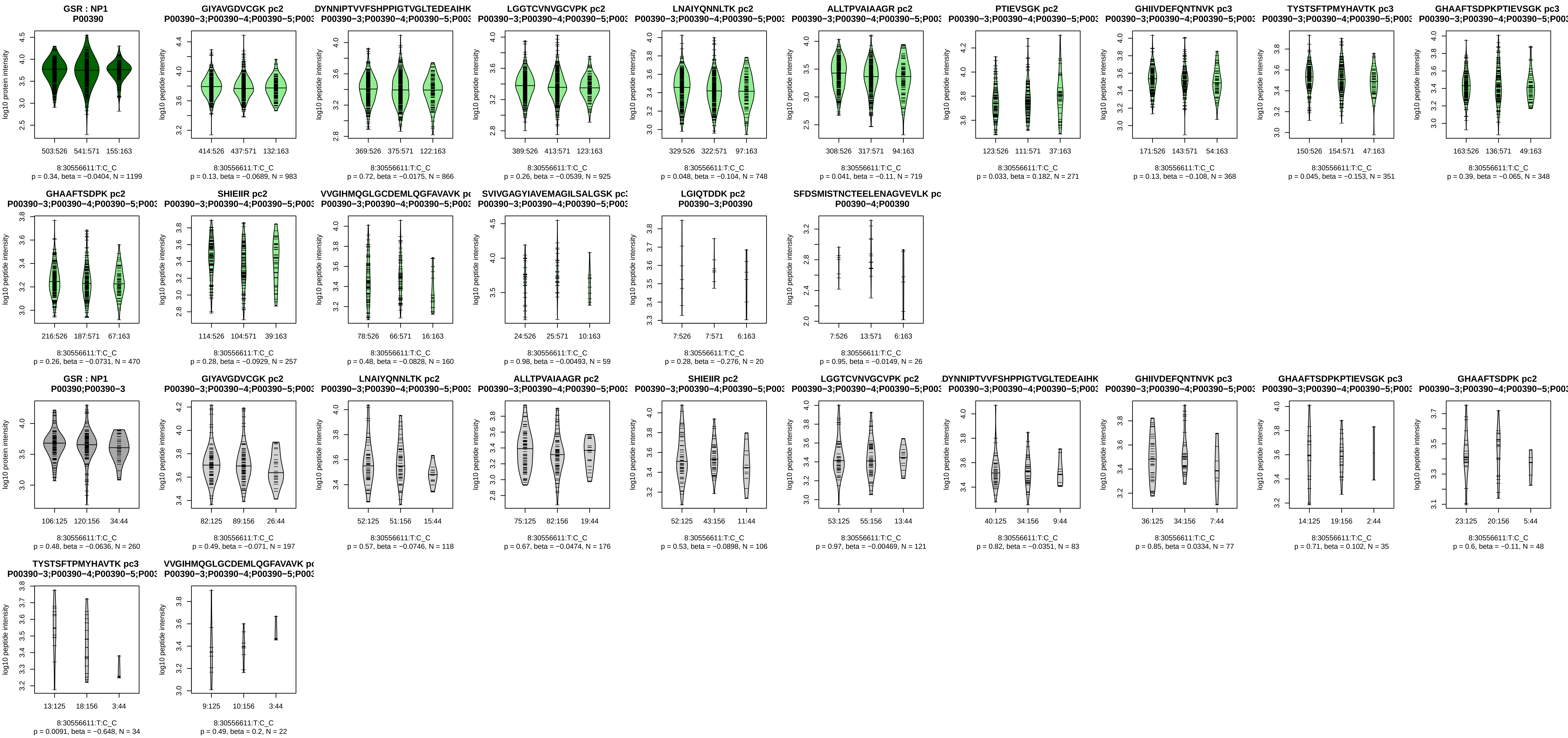

Assay Target: GSR
UniProt: P00390
rsID: rs8190982
Target SNP: 8:30556381:T:TCC
Proxy SNP: 8:30556611:T:C
UKBPPP log10(p): 81.7
UKBPPP BETA: −0.126
UKBPPP MSPA: 0
983:866:925:748:719:271:368:3

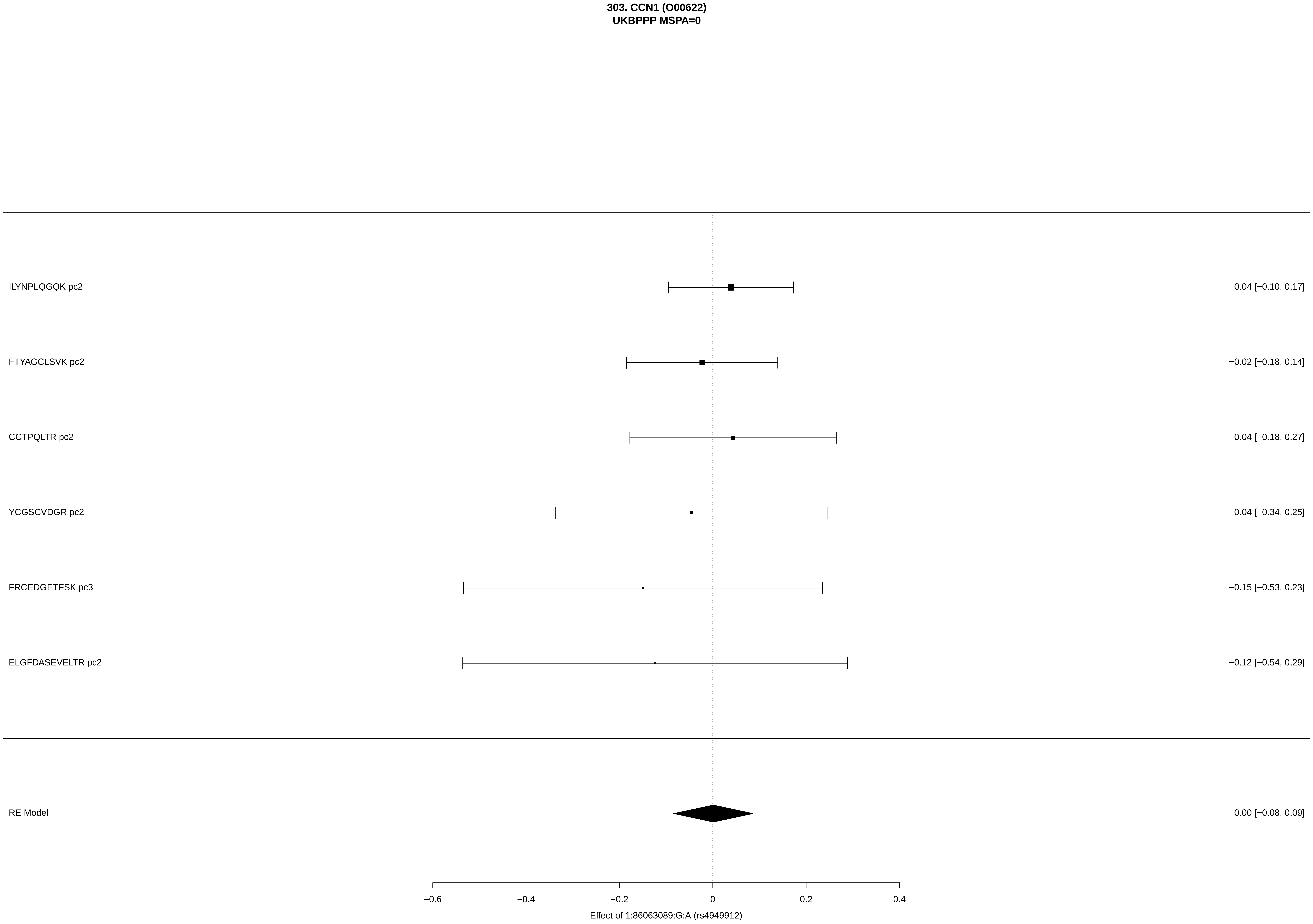
303. CCN1 (O00622)
UKBPPP MSPA=0
ILYNPLQGQK pc2
0.04 [−0.10, 0.17]
FTYAGCLSVK pc2
−0.02 [−0.18, 0.14]
CCTPQLTR pc2
0.04 [−0.18, 0.27]
YCGSCVDGR pc2
−0.04 [−0.34, 0.25]
FRCEDGETFSK pc3
−0.15 [−0.53, 0.23]
ELGFDASEVELTR pc2
−0.12 [−0.54, 0.29]
RE Model
0.00 [−0.08, 0.09]
−0.6
−0.4
−0.2
0
0.2
0.4
Effect of 1:86063089:G:A (rs4949912)

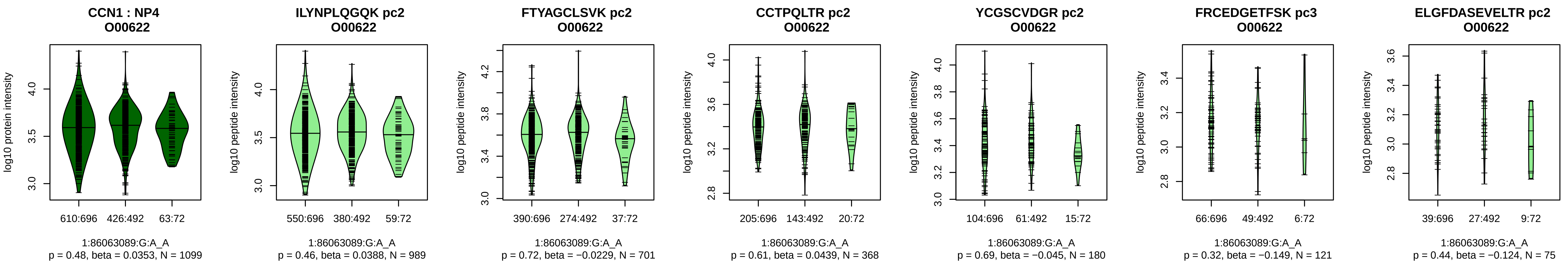

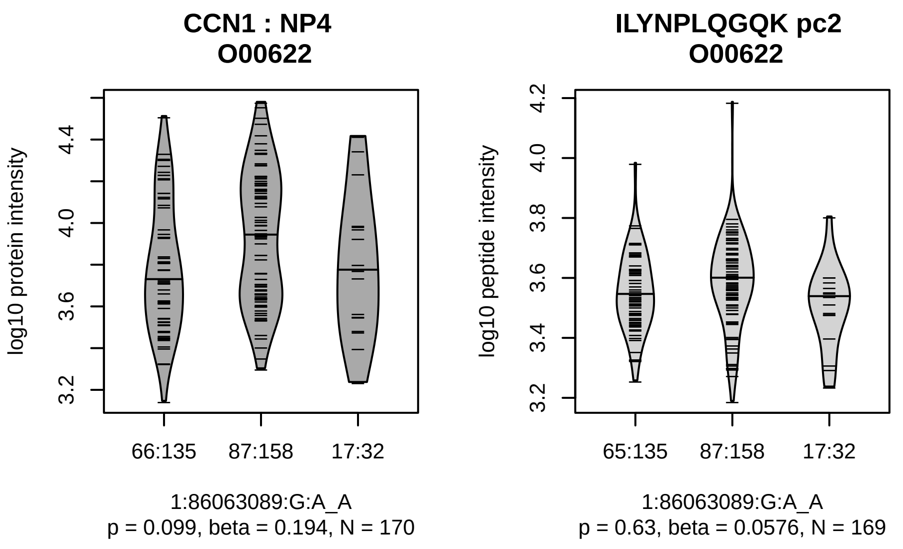

Assay Target: CCN1
UniProt: O00622
rsID: rs4949912
Target SNP: 1:86063089:G:A
Proxy SNP: 1:86063089:G:A
UKBPPP log10(p): 81
UKBPPP BETA: −0.13
UKBPPP MSPA: 0
–:–:–:–:–:–
989:701:368:180:121:75

304. LCAT (P04180)
UKBPPP MSPA=0

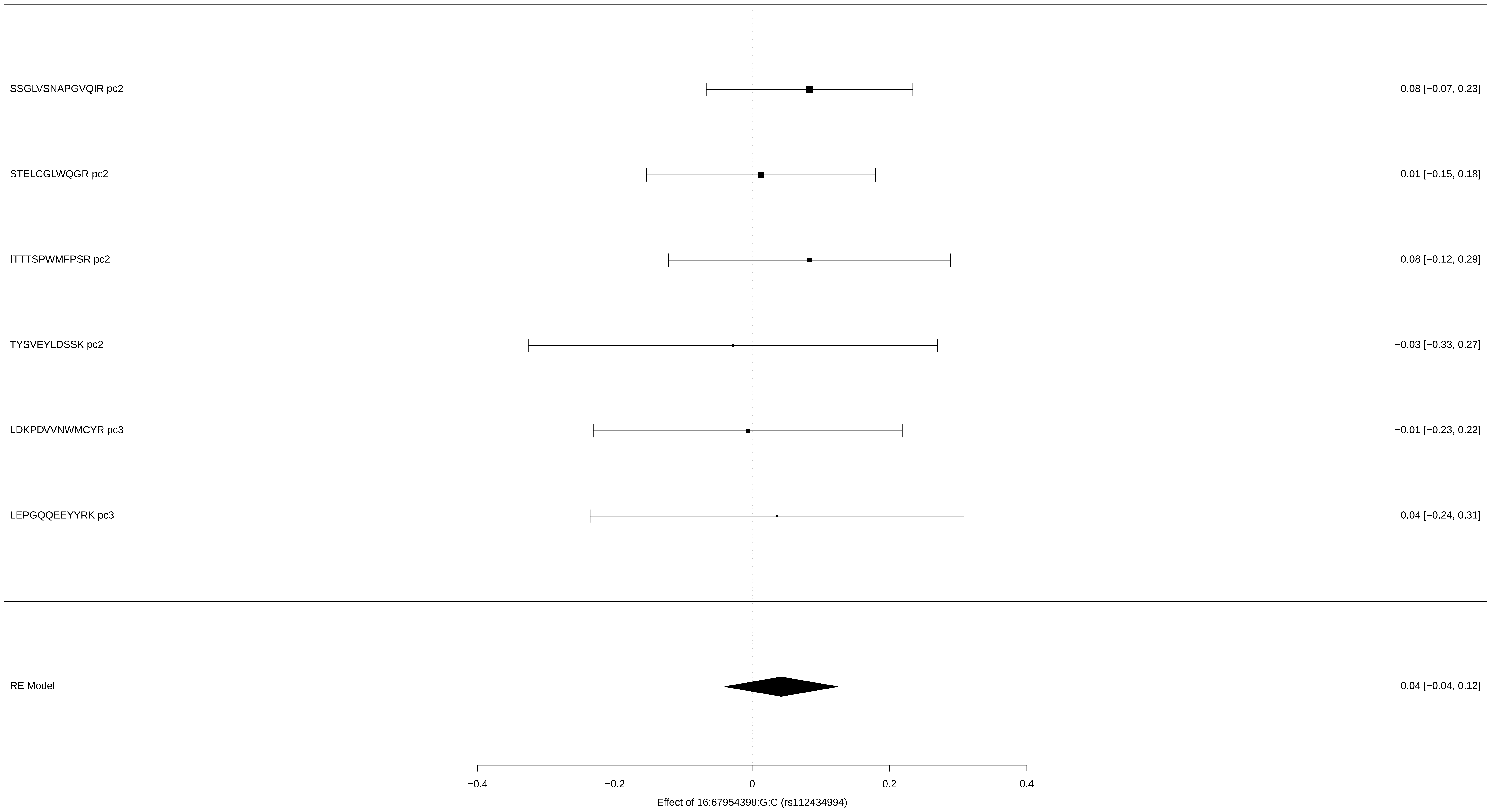

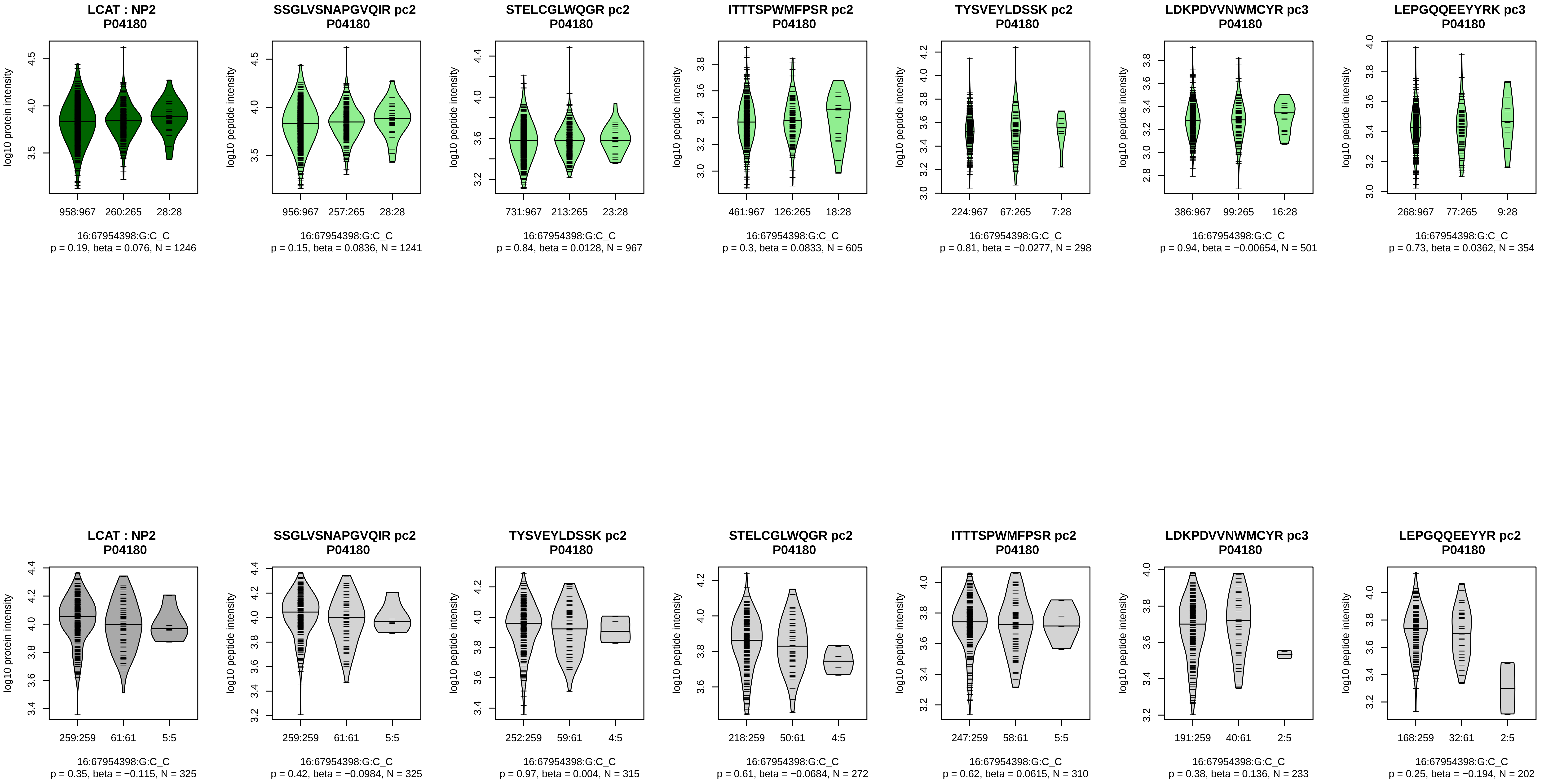

Assay Target: LCAT
UniProt: P04180
rsID: rs112434994
Target SNP: 16:67954398:G:C
Proxy SNP: 16:67954398:G:C
UKBPPP log10(p): 79
UKBPPP BETA: 0.175
UKBPPP MSPA: 0
–:–:–:–:–:–
1241:967:605:298:501:354

305. CACYBP (Q9HB71)
UKBPPP MSPA=0.134

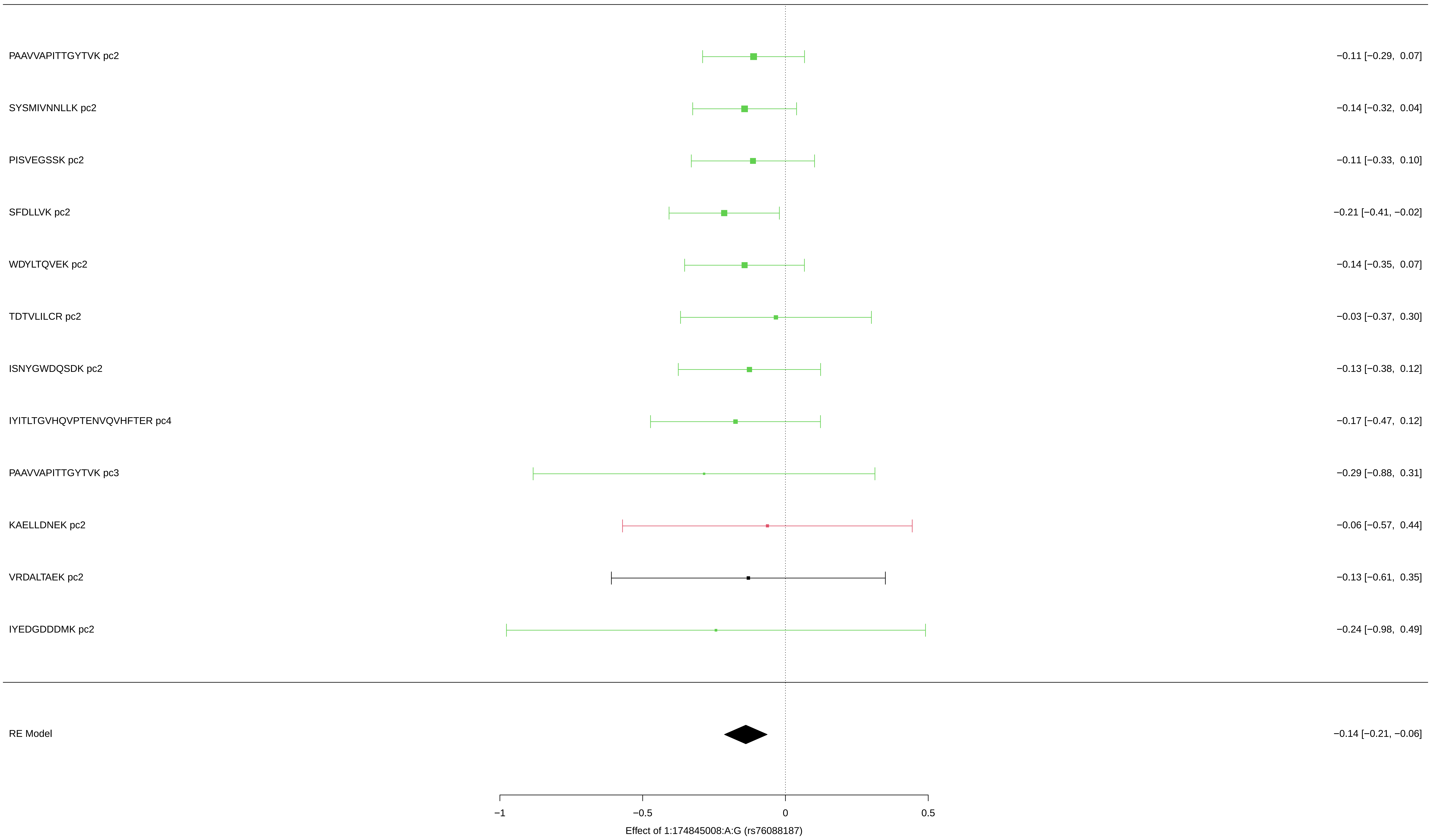

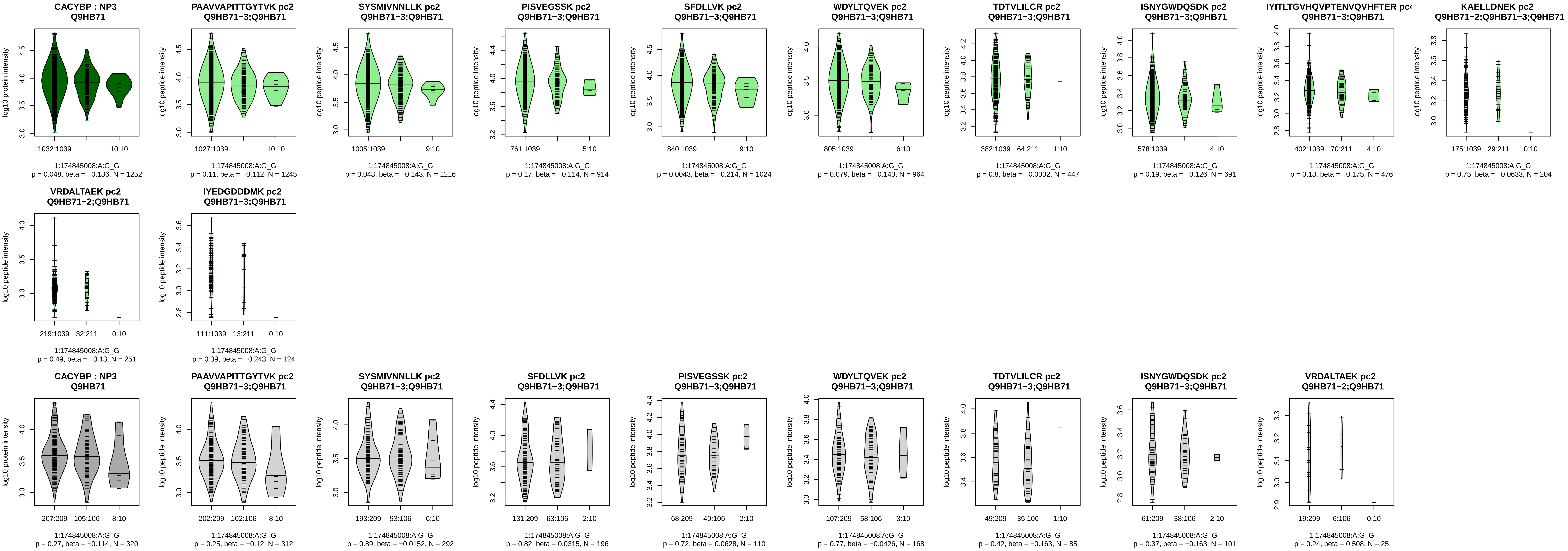

Assay Target: CACYBP
UniProt: Q9HB71
rsID: rs76088187
Target SNP: 1:174845008:A:G
Proxy SNP: 1:174845008:A:G
UKBPPP log10(p): 74.4
UKBPPP BETA: −0.177
UKBPPP MSPA: 0.134
−:−:−:*:−:−:−:−:−:−:−:NA
1245:1216:914:1024:964:447:69

306. SNED1 (Q8TER0)
UKBPPP MSPA=0.123

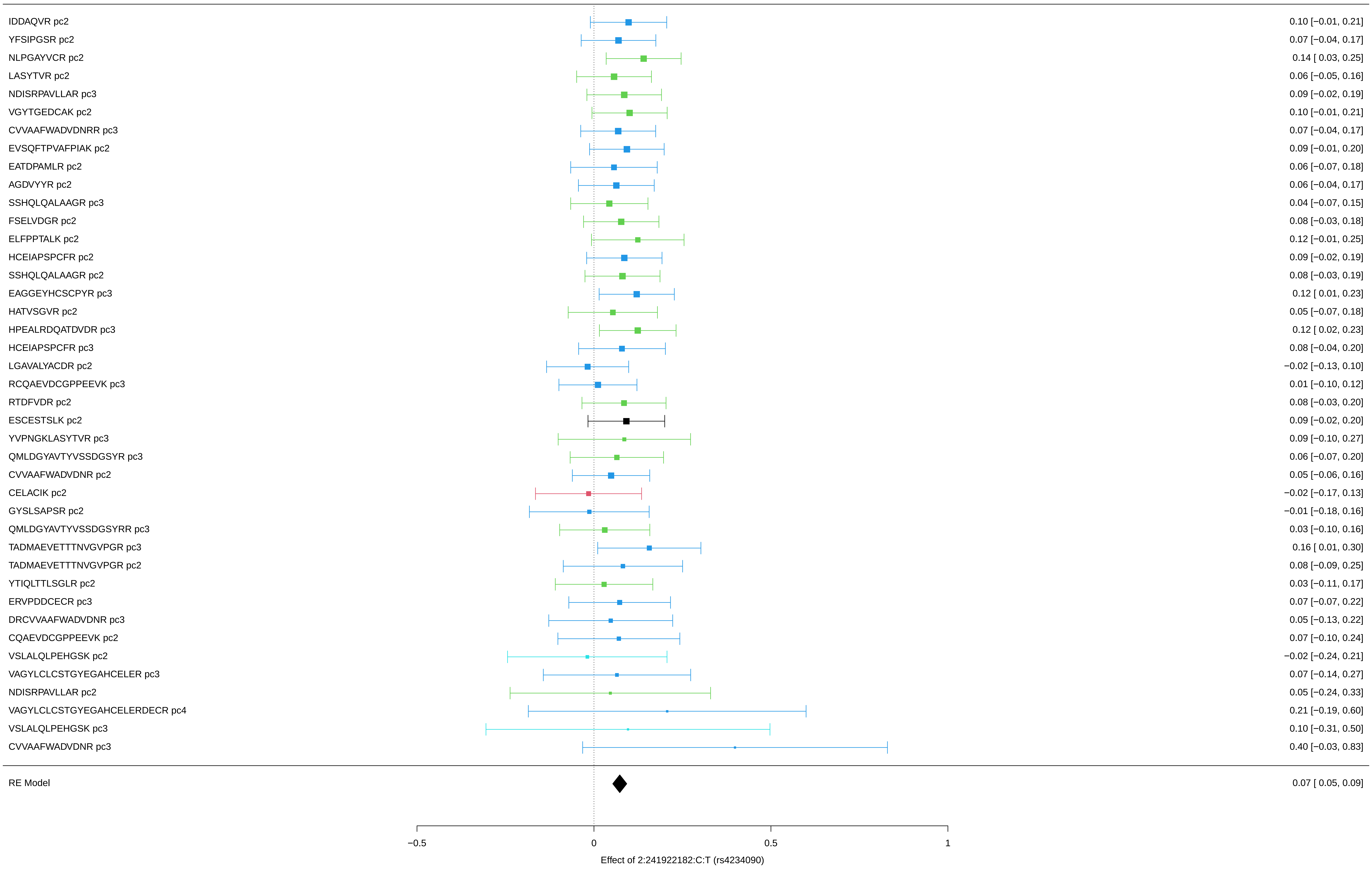

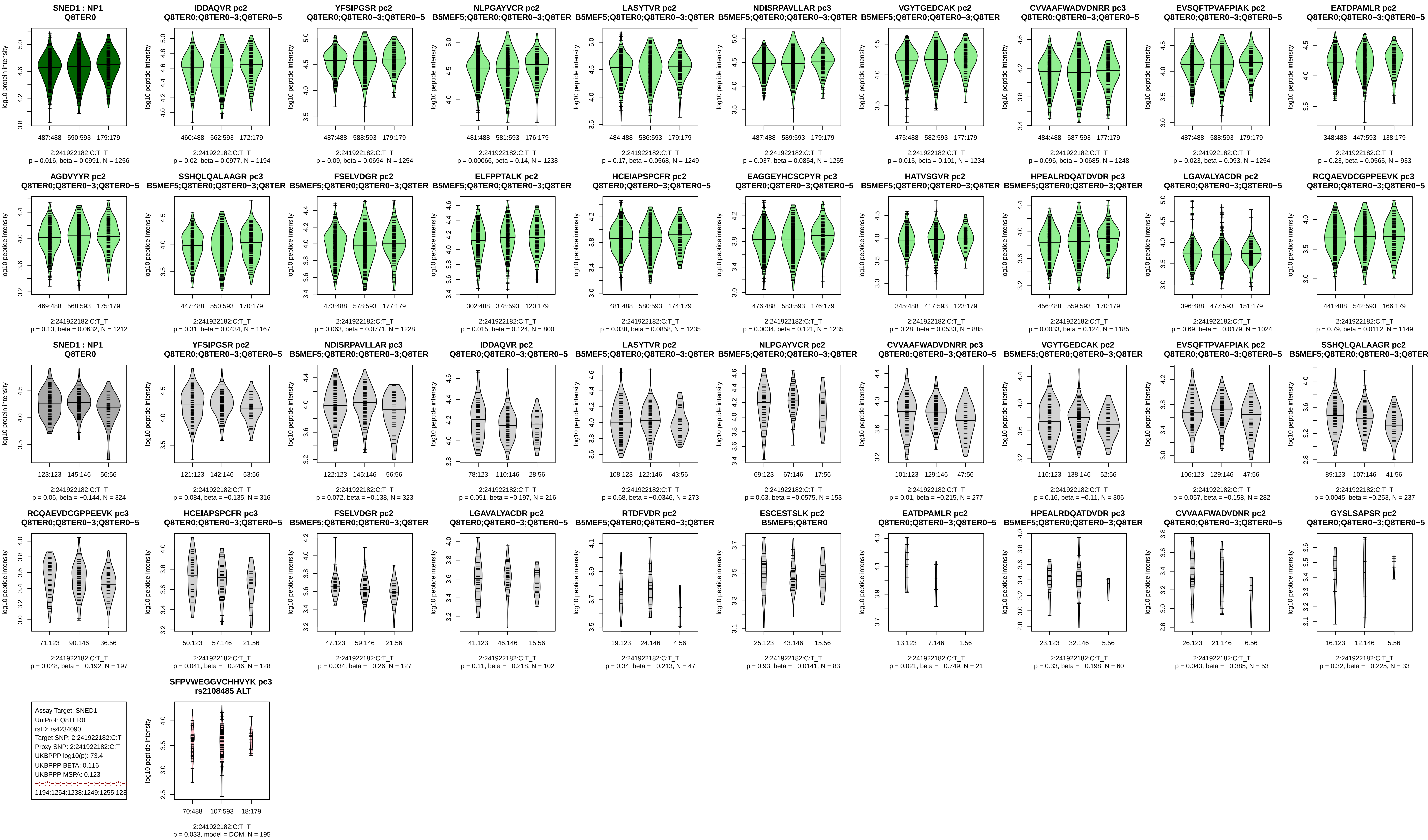

SNED1 : NP1 Q8TER0
487:488 590:593 179:179
2:241922182:C:T_T
p = 0.016, beta = 0.0991, N = 1256
IDDAQVR pc2 Q8TER0;Q8TER0−3;Q8TER0−5
460:488 562:593 172:179
p = 0.02, beta = 0.0977, N = 1194
YFSIPGSR pc2 Q8TER0;Q8TER0−3;Q8TER0−5
487:488 588:593 179:179
p = 0.09, beta = 0.0694, N = 1254
NLPGAYVCR pc2 B5MEF5;Q8TER0;Q8TER0−3;Q8TER
481:488 581:593 176:179
p = 0.00066, beta = 0.14, N = 1238
LASYTVR pc2 B5MEF5;Q8TER0;Q8TER0−3;Q8TER
484:488 586:593 179:179
p = 0.17, beta = 0.0568, N = 1249
NDISRPAVLLAR pc3 B5MEF5;Q8TER0;Q8TER0−3;Q8TER
487:488 589:593 179:179
p = 0.037, beta = 0.0854, N = 1255
VGYTGEDCAK pc2 B5MEF5;Q8TER0;Q8TER0−3;Q8TER
475:488 582:593 177:179
p = 0.015, beta = 0.101, N = 1234
CVVAAFWADVDNRR pc3 Q8TER0;Q8TER0−3;Q8TER0−5
484:488 587:593 177:179
p = 0.096, beta = 0.0685, N = 1248
EVSQFTPVAFPIAK pc2 Q8TER0;Q8TER0−3;Q8TER0−5
487:488 588:593 179:179
p = 0.023, beta = 0.093, N = 1254
EATDPAMLR pc2 Q8TER0;Q8TER0−3;Q8TER0−5
348:488 447:593 138:179
p = 0.23, beta = 0.0565, N = 933
AGDVYYR pc2 Q8TER0;Q8TER0−3;Q8TER0−5
469:488 568:593 175:179
p = 0.13, beta = 0.0632, N = 1212
SSHQLQALAAGR pc3 B5MEF5;Q8TER0;Q8TER0−3;Q8TER
447:488 550:593 170:179
p = 0.31, beta = 0.0434, N = 1167
FSELVDGR pc2 B5MEF5;Q8TER0;Q8TER0−3;Q8TER
473:488 578:593 177:179
p = 0.063, beta = 0.0771, N = 1228
ELFPPTALK pc2 B5MEF5;Q8TER0;Q8TER0−3;Q8TER
302:488 378:593 120:179
p = 0.015, beta = 0.124, N = 800
HCEIAPSPCFR pc2 Q8TER0;Q8TER0−3;Q8TER0−5
481:488 580:593 174:179
p = 0.038, beta = 0.0858, N = 1235
EAGGEYHCSCPYR pc3 Q8TER0;Q8TER0−3;Q8TER0−5
476:488 583:593 176:179
p = 0.0034, beta = 0.121, N = 1235
HATVSGVR pc2 B5MEF5;Q8TER0;Q8TER0−3;Q8TER
345:488 417:593 123:179
p = 0.28, beta = 0.0533, N = 885
HPEALRDQATDVDR pc3 B5MEF5;Q8TER0;Q8TER0−3;Q8TER
456:488 559:593 170:179
p = 0.0033, beta = 0.124, N = 1185
LGAVALYACDR pc2 Q8TER0;Q8TER0−3;Q8TER0−5
396:488 477:593 151:179
p = 0.69, beta = −0.0179, N = 1024
RCQAEVDCGPPEEVK pc3 Q8TER0;Q8TER0−3;Q8TER0−5
441:488 542:593 166:179
p = 0.79, beta = 0.0112, N = 1149
SNED1 : NP1 Q8TER0
123:123 145:146 56:56
p = 0.06, beta = −0.144, N = 324
YFSIPGSR pc2 Q8TER0;Q8TER0−3;Q8TER0−5
121:123 142:146 53:56
p = 0.084, beta = −0.135, N = 316
NDISRPAVLLAR pc3 B5MEF5;Q8TER0;Q8TER0−3;Q8TER
122:123 145:146 56:56
p = 0.072, beta = −0.138, N = 323
IDDAQVR pc2 Q8TER0;Q8TER0−3;Q8TER0−5
78:123 110:146 28:56
p = 0.051, beta = −0.197, N = 216
LASYTVR pc2 B5MEF5;Q8TER0;Q8TER0−3;Q8TER
108:123 122:146 43:56
p = 0.68, beta = −0.0346, N = 273
NLPGAYVCR pc2 B5MEF5;Q8TER0;Q8TER0−3;Q8TER
69:123 67:146 17:56
p = 0.63, beta = −0.0575, N = 153
CVVAAFWADVDNRR pc3 Q8TER0;Q8TER0−3;Q8TER0−5
101:123 129:146 47:56
p = 0.01, beta = −0.215, N = 277
VGYTGEDCAK pc2 B5MEF5;Q8TER0;Q8TER0−3;Q8TER
116:123 138:146 52:56
p = 0.16, beta = −0.11, N = 306
EVSQFTPVAFPIAK pc2 Q8TER0;Q8TER0−3;Q8TER0−5
106:123 129:146 47:56
p = 0.057, beta = −0.158, N = 282
SSHQLQALAAGR pc2 B5MEF5;Q8TER0;Q8TER0−3;Q8TER
89:123 107:146 41:56
p = 0.0045, beta = −0.253, N = 237
RCQAEVDCGPPEEVK pc3 Q8TER0;Q8TER0−3;Q8TER0−5
71:123 90:146 36:56
p = 0.048, beta = −0.192, N = 197
HCEIAPSPCFR pc3 Q8TER0;Q8TER0−3;Q8TER0−5
50:123 57:146 21:56
p = 0.041, beta = −0.246, N = 128
FSELVDGR pc2 B5MEF5;Q8TER0;Q8TER0−3;Q8TER
47:123 59:146 21:56
p = 0.034, beta = −0.26, N = 127
LGAVALYACDR pc2 Q8TER0;Q8TER0−3;Q8TER0−5
41:123 46:146 15:56
p = 0.11, beta = −0.218, N = 102
RTDFVDR pc2 B5MEF5;Q8TER0;Q8TER0−3;Q8TER
19:123 24:146 4:56
p = 0.34, beta = −0.213, N = 47
ESCESTSLK pc2 B5MEF5;Q8TER0
25:123 43:146 15:56
p = 0.93, beta = −0.0141, N = 83
EATDPAMLR pc2 Q8TER0;Q8TER0−3;Q8TER0−5
13:123 7:146 1:56
p = 0.021, beta = −0.749, N = 21
HPEALRDQATDVDR pc3 B5MEF5;Q8TER0;Q8TER0−3;Q8TER
23:123 32:146 5:56
p = 0.33, beta = −0.198, N = 60
CVVAAFWADVDNR pc2 Q8TER0;Q8TER0−3;Q8TER0−5
26:123 21:146 6:56
p = 0.043, beta = −0.385, N = 53
GYSLSAPSR pc2 Q8TER0;Q8TER0−3;Q8TER0−5
16:123 12:146 5:56
p = 0.32, beta = −0.225, N = 33
Assay Target: SNED1
UniProt: Q8TER0
rsID: rs4234090
Target SNP: 2:241922182:C:T
Proxy SNP: 2:241922182:C:T
UKBPPP log10(p): 73.4
UKBPPP BETA: 0.116
UKBPPP MSPA: 0.123
1194:1254:1238:1249:1255:123
SFPVWEGGVCHHVYK pc3 rs2108485 ALT
70:488 107:593 18:179
2:241922182:C:T_T
p = 0.033, model = DOM, N = 195
log10 protein intensity
log10 peptide intensity

307. ACAN (P16112)
UKBPPP MSPA=0.129

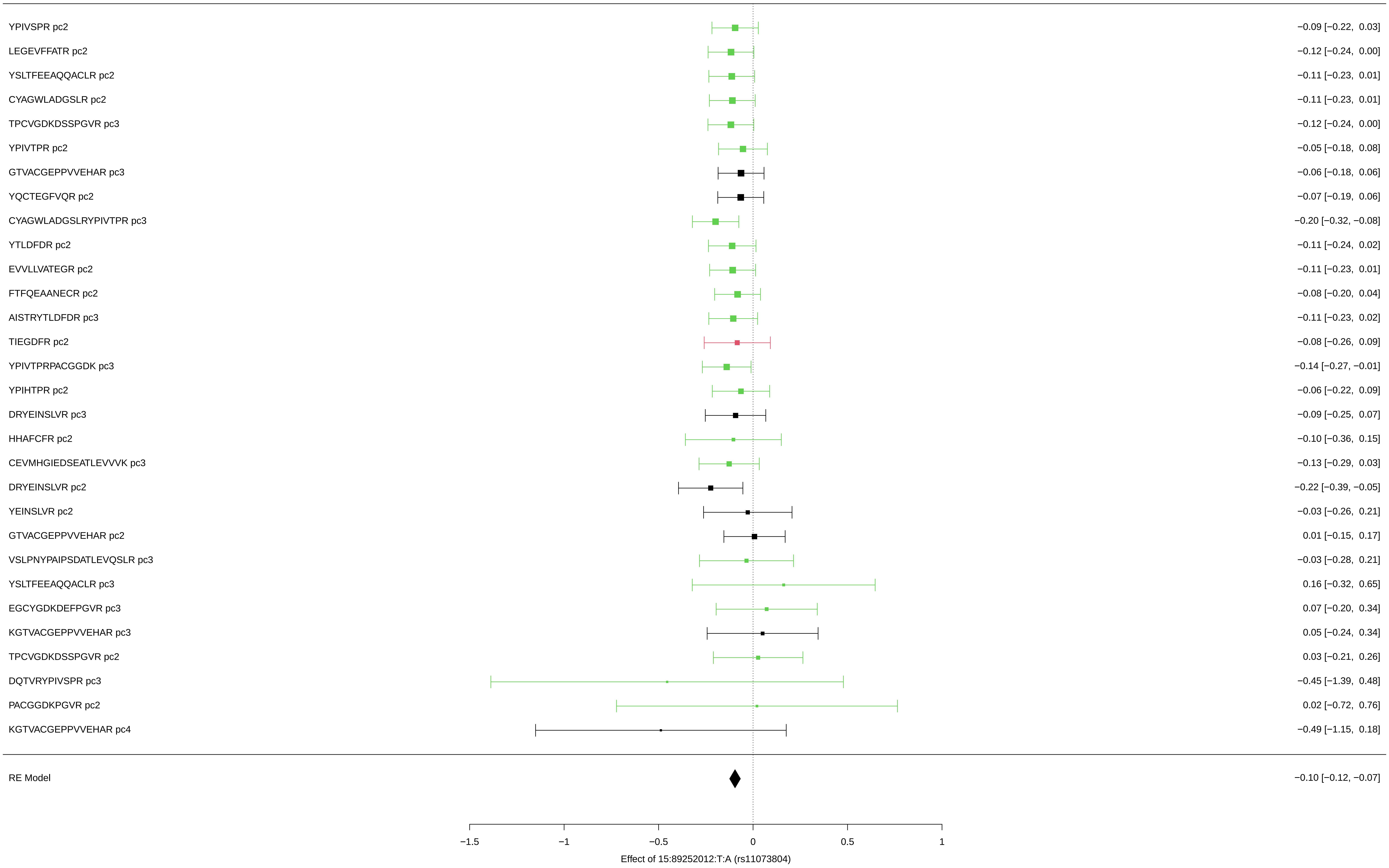

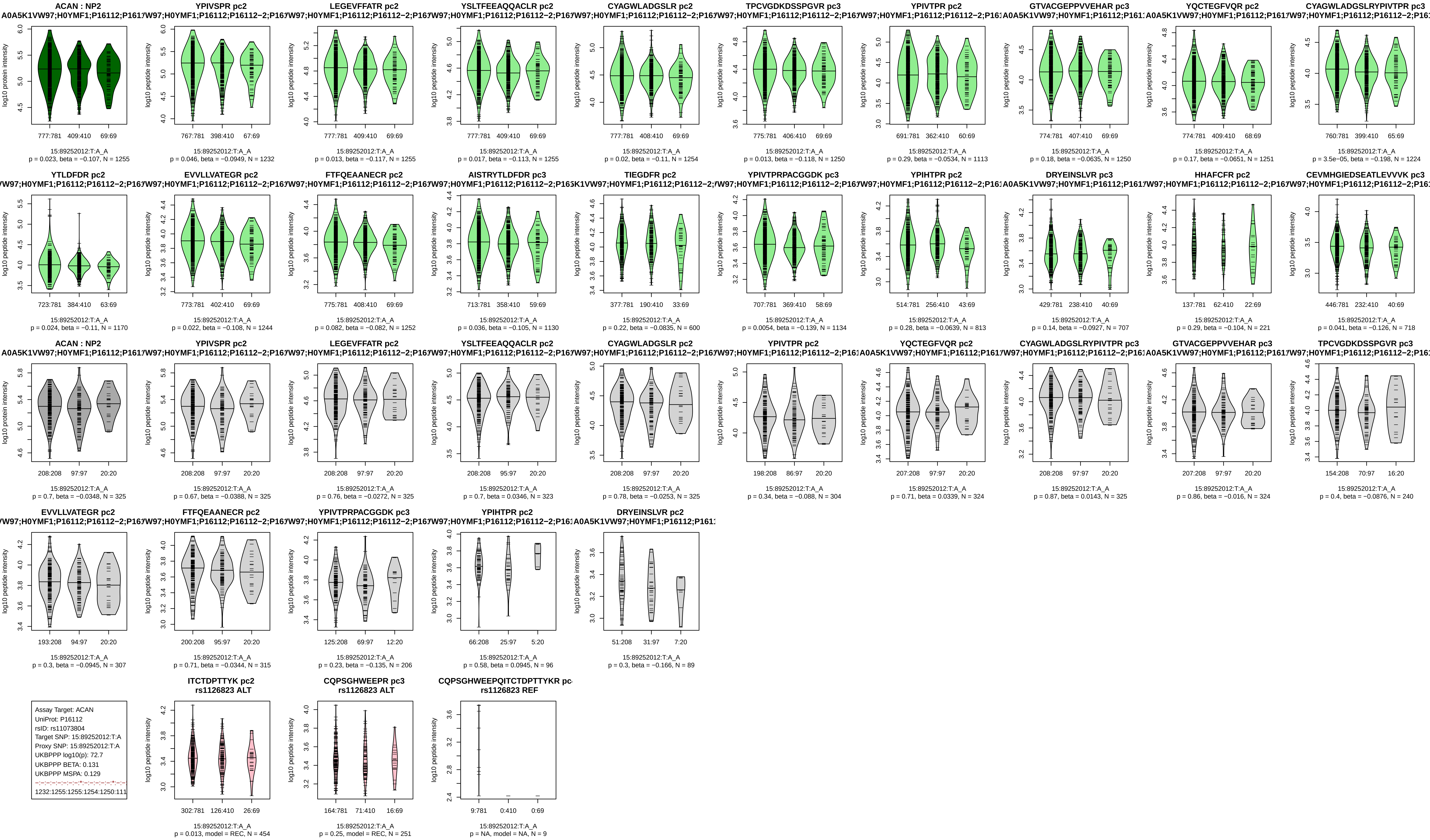

ACAN : NP2
777:781 409:410 69:69
15:89252012:T:A_A
p = 0.023, beta = −0.107, N = 1255
YPIVSPR pc2
767:781 398:410 67:69
15:89252012:T:A_A
p = 0.046, beta = −0.0949, N = 1232
LEGEVFFATR pc2
777:781 409:410 69:69
15:89252012:T:A_A
p = 0.013, beta = −0.117, N = 1255
YSLTFEEAQQACLR pc2
777:781 409:410 69:69
15:89252012:T:A_A
p = 0.017, beta = −0.113, N = 1255
CYAGWLADGSLR pc2
777:781 408:410 69:69
15:89252012:T:A_A
p = 0.02, beta = −0.11, N = 1254
TPCVGDKDSSPGVR pc3
775:781 406:410 69:69
15:89252012:T:A_A
p = 0.013, beta = −0.118, N = 1250
YPIVTPR pc2
691:781 362:410 60:69
15:89252012:T:A_A
p = 0.29, beta = −0.0534, N = 1113
GTVACGEPPVVEHAR pc3
774:781 407:410 69:69
15:89252012:T:A_A
p = 0.18, beta = −0.0635, N = 1250
YQCTEGFVQR pc2
774:781 409:410 68:69
15:89252012:T:A_A
p = 0.17, beta = −0.0651, N = 1251
CYAGWLADGSLRYPIVTPR pc3
760:781 399:410 65:69
15:89252012:T:A_A
p = 3.5e−05, beta = −0.198, N = 1224
YTLDFDR pc2
723:781 384:410 63:69
15:89252012:T:A_A
p = 0.024, beta = −0.11, N = 1170
EVVLLVATEGR pc2
773:781 402:410 69:69
15:89252012:T:A_A
p = 0.022, beta = −0.108, N = 1244
FTFQEAANECR pc2
775:781 408:410 69:69
15:89252012:T:A_A
p = 0.082, beta = −0.082, N = 1252
AISTRYTLDFDR pc3
713:781 358:410 59:69
15:89252012:T:A_A
p = 0.036, beta = −0.105, N = 1130
TIEGDFR pc2
377:781 190:410 33:69
15:89252012:T:A_A
p = 0.22, beta = −0.0835, N = 600
YPIVTPRPACGGDK pc3
707:781 369:410 58:69
15:89252012:T:A_A
p = 0.0054, beta = −0.139, N = 1134
YPIHTPR pc2
514:781 256:410 43:69
15:89252012:T:A_A
p = 0.28, beta = −0.0639, N = 813
DRYEINSLVR pc3
429:781 238:410 40:69
15:89252012:T:A_A
p = 0.14, beta = −0.0927, N = 707
HHAFCFR pc2
137:781 62:410 22:69
15:89252012:T:A_A
p = 0.29, beta = −0.104, N = 221
CEVMHGIEDSEATLEVVVK pc3
446:781 232:410 40:69
15:89252012:T:A_A
p = 0.041, beta = −0.126, N = 718
ACAN : NP2
208:208 97:97 20:20
15:89252012:T:A_A
p = 0.7, beta = −0.0348, N = 325
YPIVSPR pc2
208:208 97:97 20:20
15:89252012:T:A_A
p = 0.67, beta = −0.0388, N = 325
LEGEVFFATR pc2
208:208 97:97 20:20
15:89252012:T:A_A
p = 0.76, beta = −0.0272, N = 325
YSLTFEEAQQACLR pc2
208:208 95:97 20:20
15:89252012:T:A_A
p = 0.7, beta = 0.0346, N = 323
CYAGWLADGSLR pc2
208:208 97:97 20:20
15:89252012:T:A_A
p = 0.78, beta = −0.0253, N = 325
YPIVTPR pc2
198:208 86:97 20:20
15:89252012:T:A_A
p = 0.34, beta = −0.088, N = 304
YQCTEGFVQR pc2
207:208 97:97 20:20
15:89252012:T:A_A
p = 0.71, beta = 0.0339, N = 324
CYAGWLADGSLRYPIVTPR pc3
208:208 97:97 20:20
15:89252012:T:A_A
p = 0.87, beta = 0.0143, N = 325
GTVACGEPPVVEHAR pc3
207:208 97:97 20:20
15:89252012:T:A_A
p = 0.86, beta = −0.016, N = 324
TPCVGDKDSSPGVR pc3
154:208 70:97 16:20
15:89252012:T:A_A
p = 0.4, beta = −0.0876, N = 240
EVVLLVATEGR pc2
193:208 94:97 20:20
15:89252012:T:A_A
p = 0.3, beta = −0.0945, N = 307
FTFQEAANECR pc2
200:208 95:97 20:20
15:89252012:T:A_A
p = 0.71, beta = −0.0344, N = 315
YPIVTPRPACGGDK pc3
125:208 69:97 12:20
15:89252012:T:A_A
p = 0.23, beta = −0.135, N = 206
YPIHTPR pc2
66:208 25:97 5:20
15:89252012:T:A_A
p = 0.58, beta = 0.0945, N = 96
DRYEINSLVR pc2
51:208 31:97 7:20
15:89252012:T:A_A
p = 0.3, beta = −0.166, N = 89
Assay Target: ACAN
UniProt: P16112
rsID: rs11073804
Target SNP: 15:89252012:T:A
Proxy SNP: 15:89252012:T:A
UKBPPP log10(p): 72.7
UKBPPP BETA: 0.131
UKBPPP MSPA: 0.129
1232:1255:1255:1254:1250:111
ITCTDPTTYK pc2
rs1126823 ALT
302:781 126:410 26:69
15:89252012:T:A_A
p = 0.013, model = REC, N = 454
CQPSGHWEEPR pc3
rs1126823 ALT
164:781 71:410 16:69
15:89252012:T:A_A
p = 0.25, model = REC, N = 251
rs1126823 REF
9:781 0:410 0:69
15:89252012:T:A_A
p = NA, model = NA, N = 9
log10 protein intensity
log10 peptide intensity

308. MIF (P14174)
UKBPPP MSPA=0.617

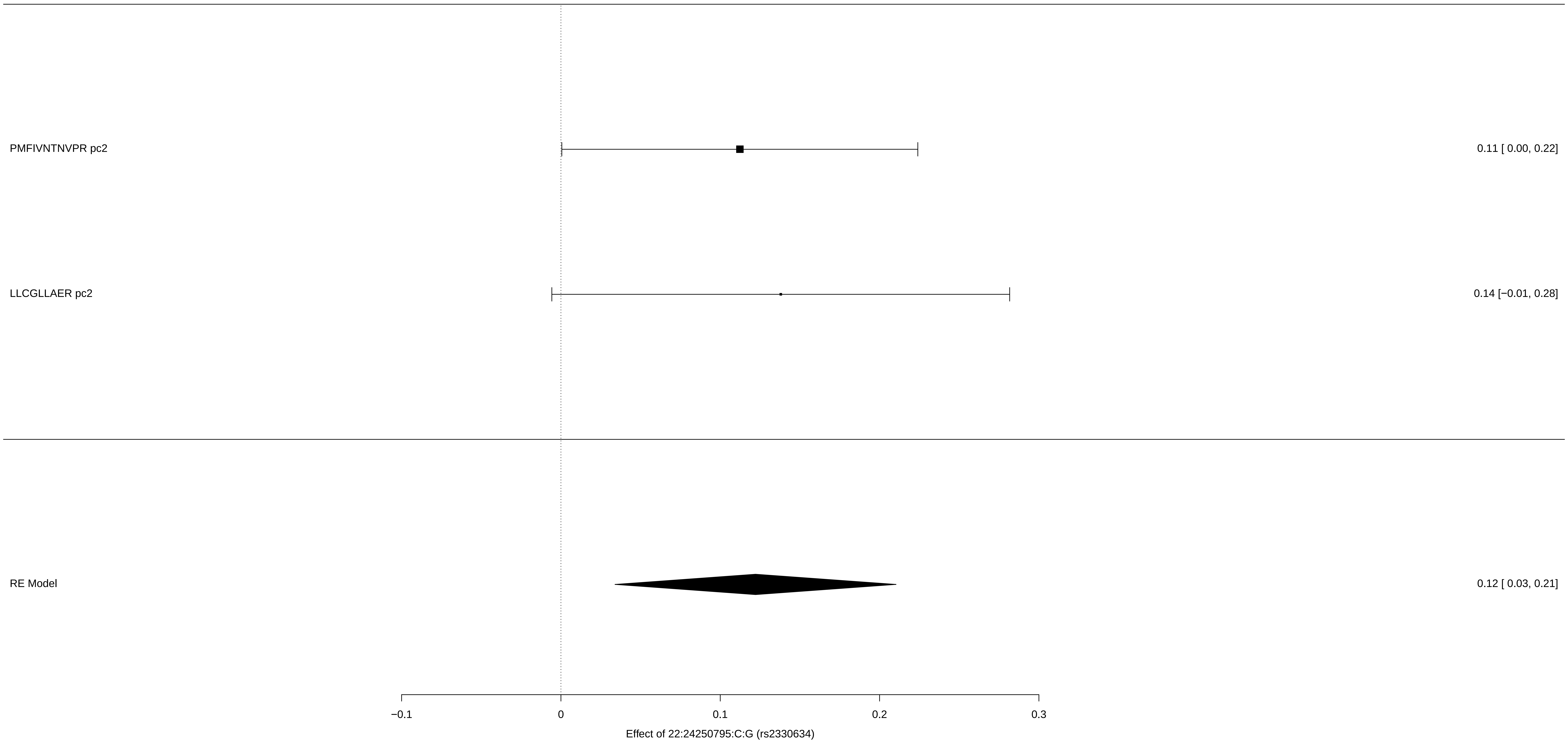

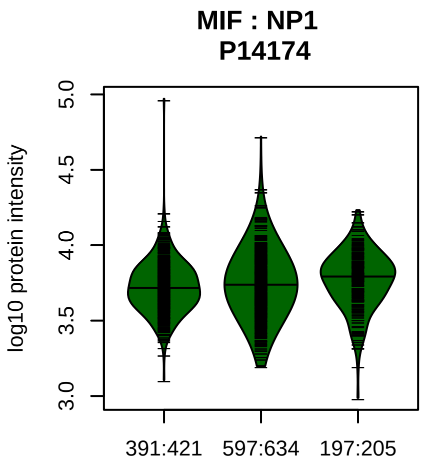

22:24250795:C:G_C
p = 0.0052, beta = 0.118, N = 1185

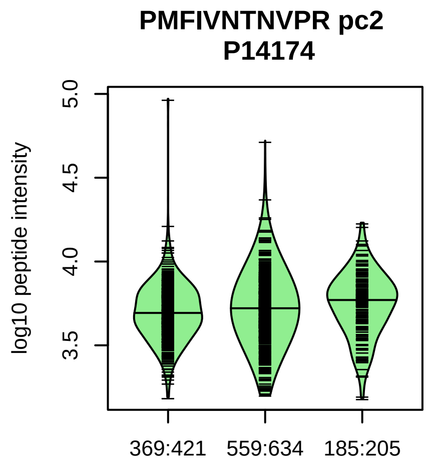

22:24250795:C:G_C
p = 0.0098, beta = 0.112, N = 1113

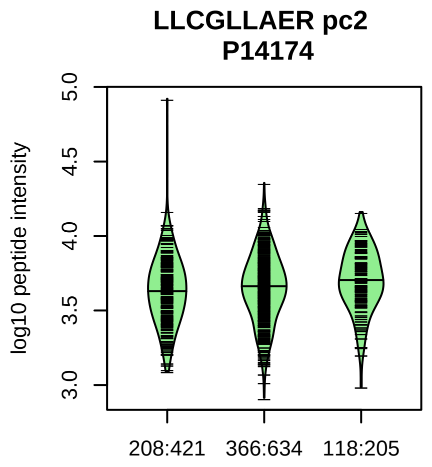

22:24250795:C:G_C
p = 0.014, beta = 0.138, N = 692

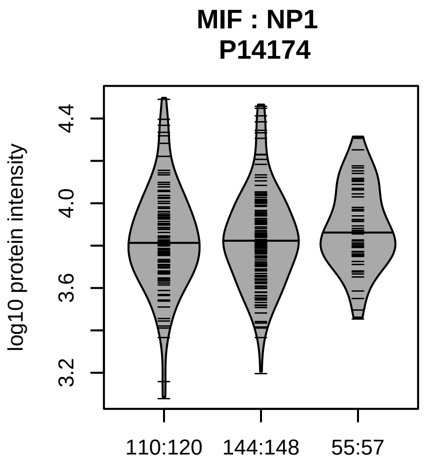

22:24250795:C:G_C
p = 0.14, beta = 0.115, N = 309

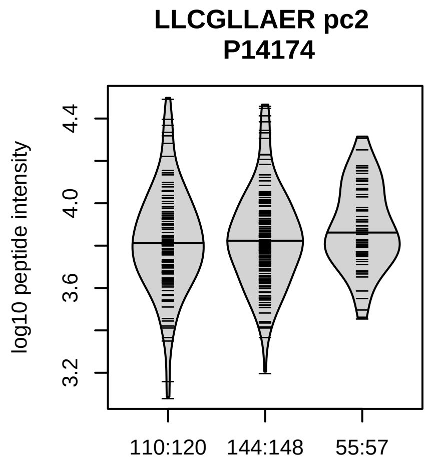

22:24250795:C:G_C
p = 0.12, beta = 0.123, N = 309

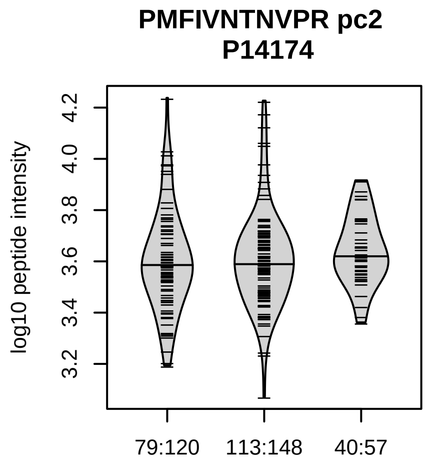

22:24250795:C:G_C
p = 0.074, beta = 0.165, N = 232

Assay Target: MIF
UniProt: P14174
rsID: rs2330634
Target SNP: 22:24250795:C:G
Proxy SNP: 22:24250795:C:G
UKBPPP log10(p): 71.1
UKBPPP BETA: −0.112
UKBPPP MSPA: 0.617
*:–
1113:692

309. HEG1 (Q9ULI3)
UKBPPP MSPA=0.591

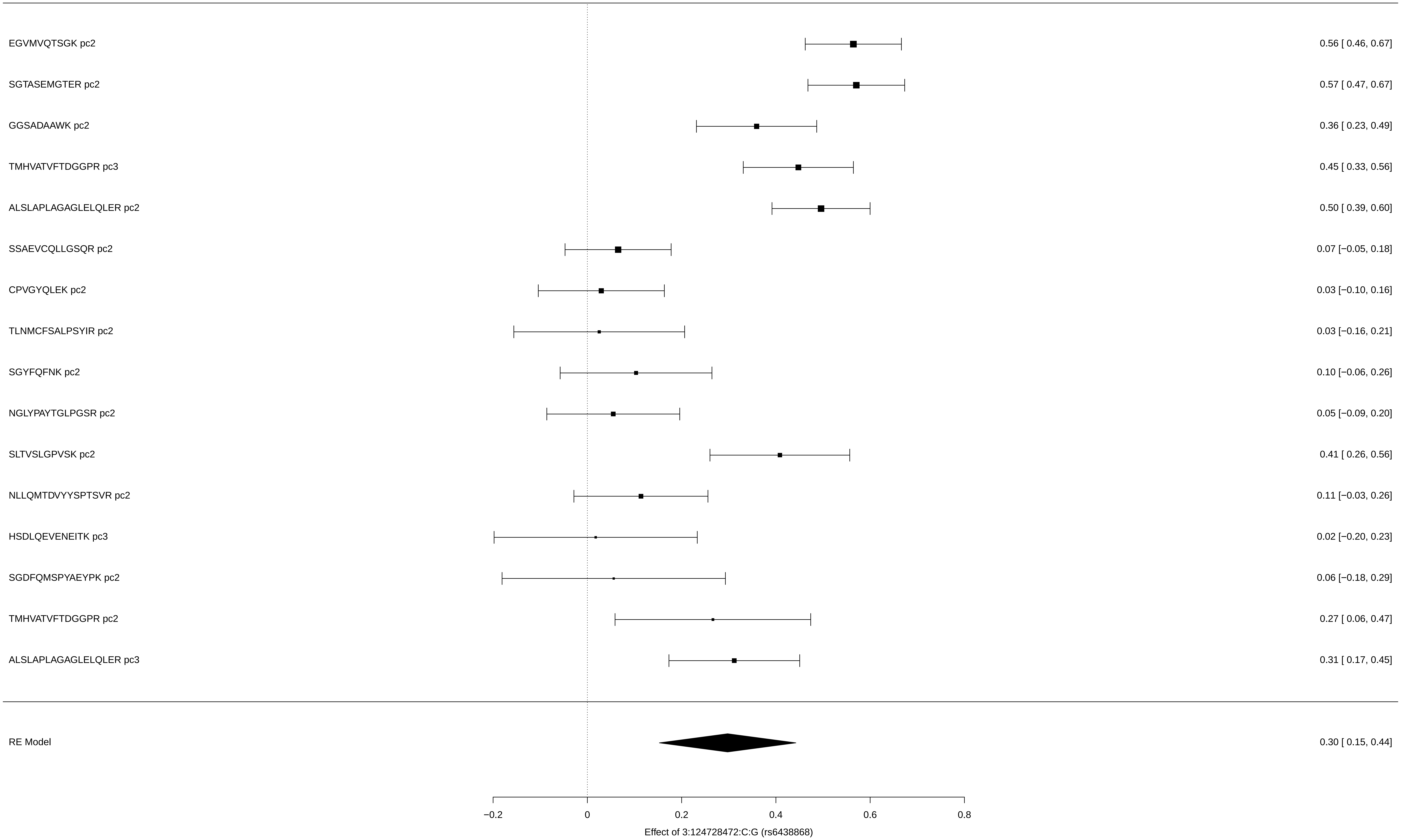

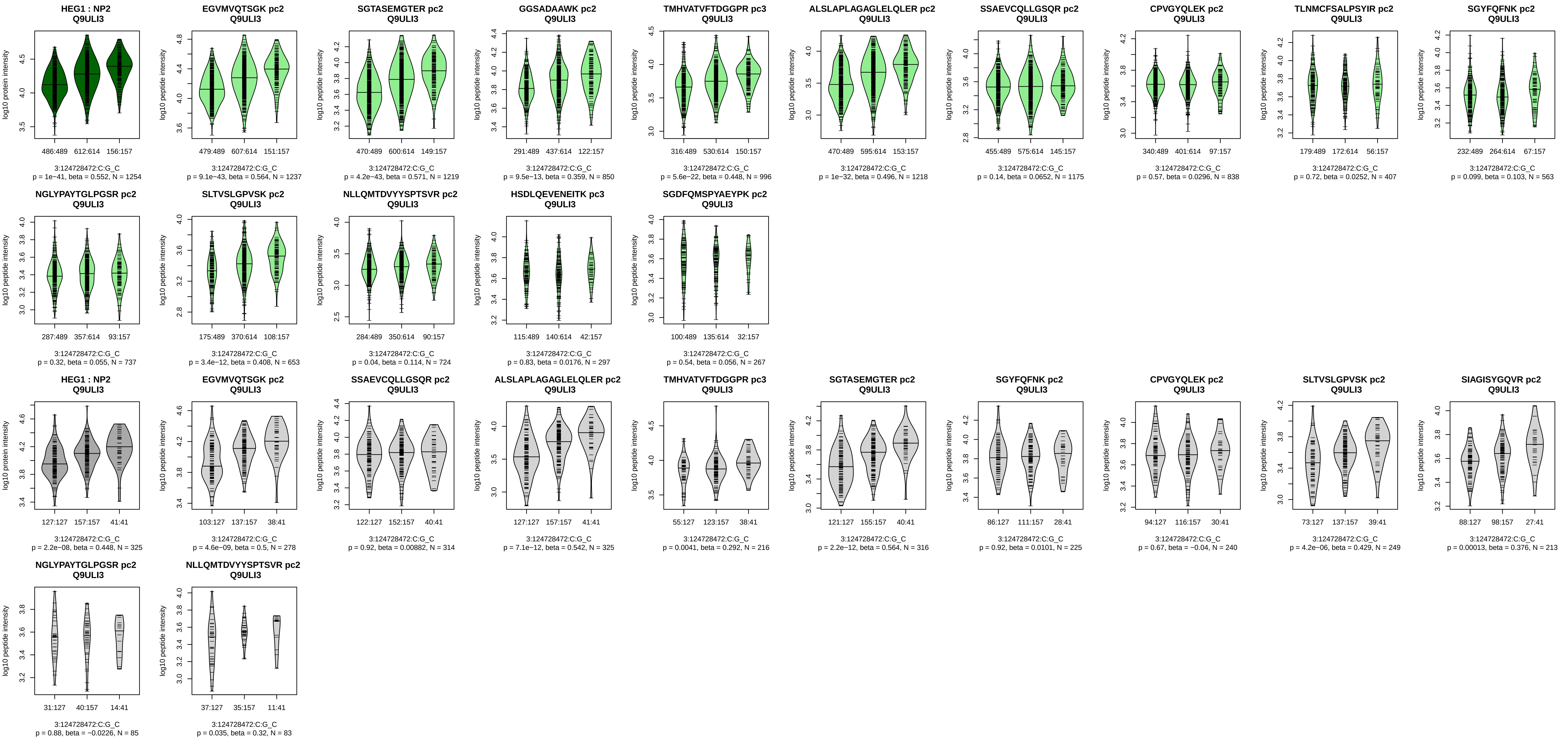

Assay Target: HEG1
UniProt: Q9ULI3
rsID: rs6438868
Target SNP: 3:124728472:C:G
Proxy SNP: 3:124728472:C:G
UKBPPP log10(p): 71.1
UKBPPP BETA: 0.104
UKBPPP MSPA: 0.591
::*:*:*:–:–:–:–:–:*:–:–:–:*:*:NA
1237:1219:850:996:1218:1175:8

310. YOD1 (Q5VVQ6)
UKBPPP MSPA=0

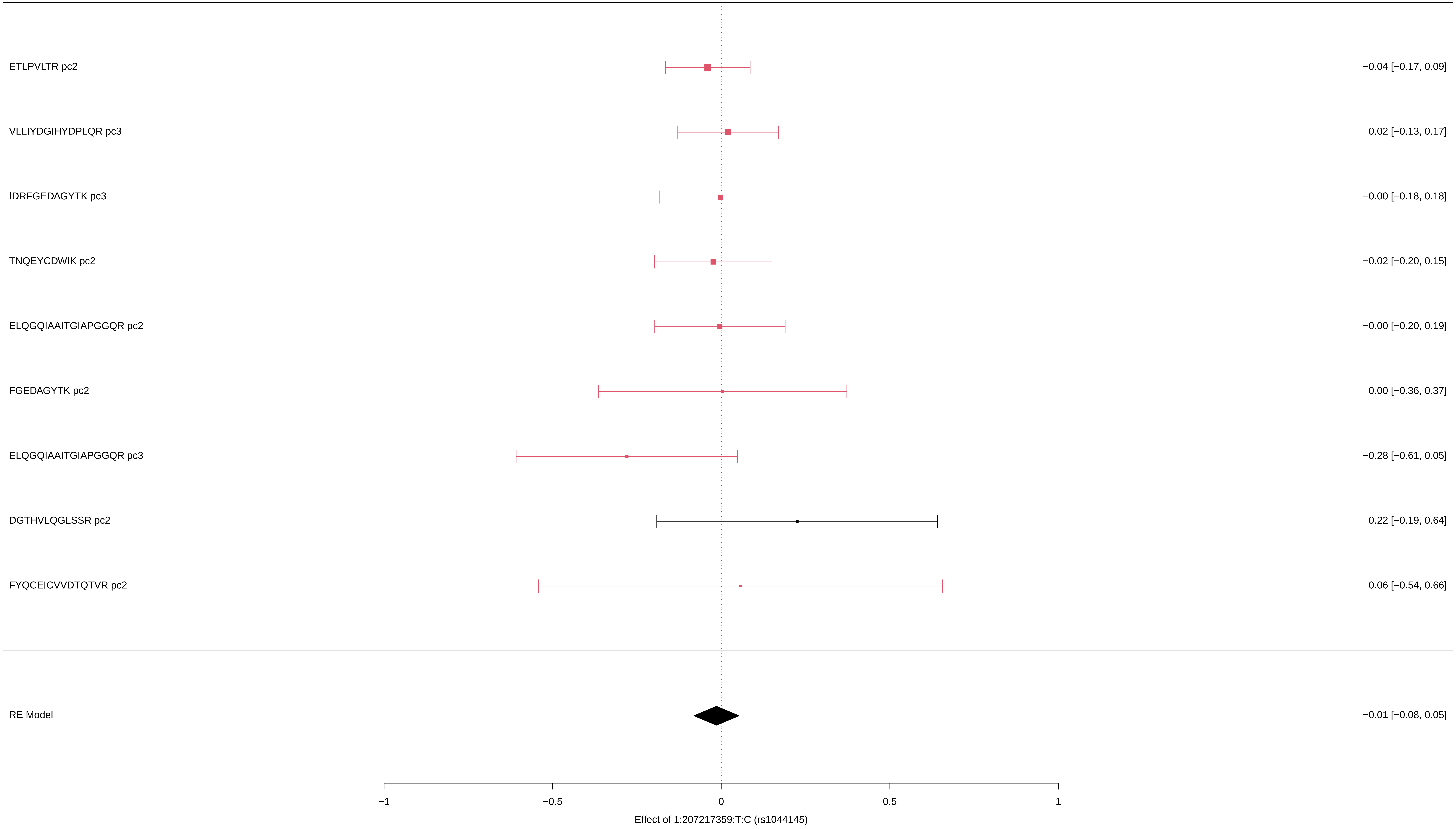

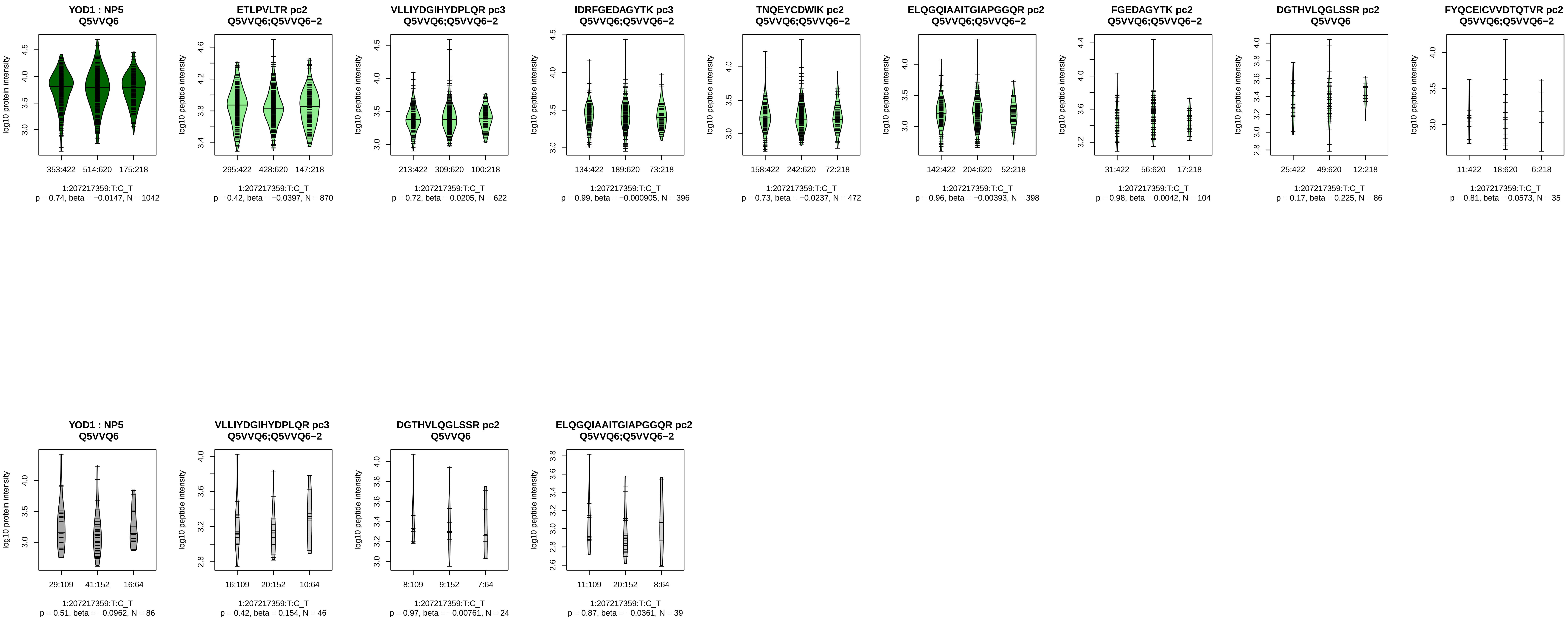

Assay Target: YOD1
UniProt: Q5VVQ6
rsID: rs1044145
Target SNP: 1:207217359:T:C
Proxy SNP: 1:207217359:T:C
UKBPPP log10(p): 70.2
UKBPPP BETA: 0.108
UKBPPP MSPA: 0
−:−:−:−:−:−:−:−:NA:NA:NA:NA
870:622:396:472:398:104:110:8

311. CSPG4 (Q6UVK1)
UKBPPP MSPA=0

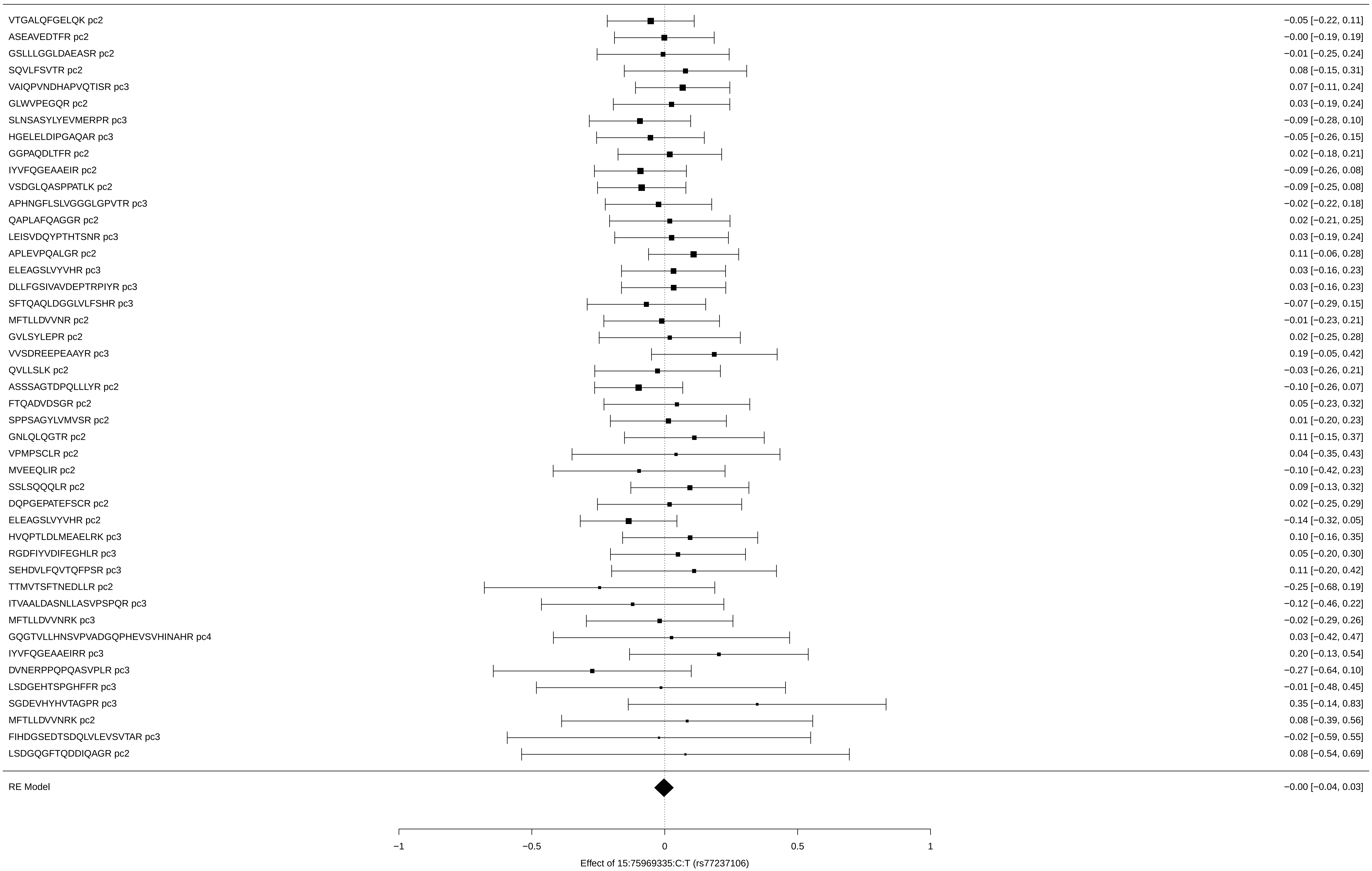

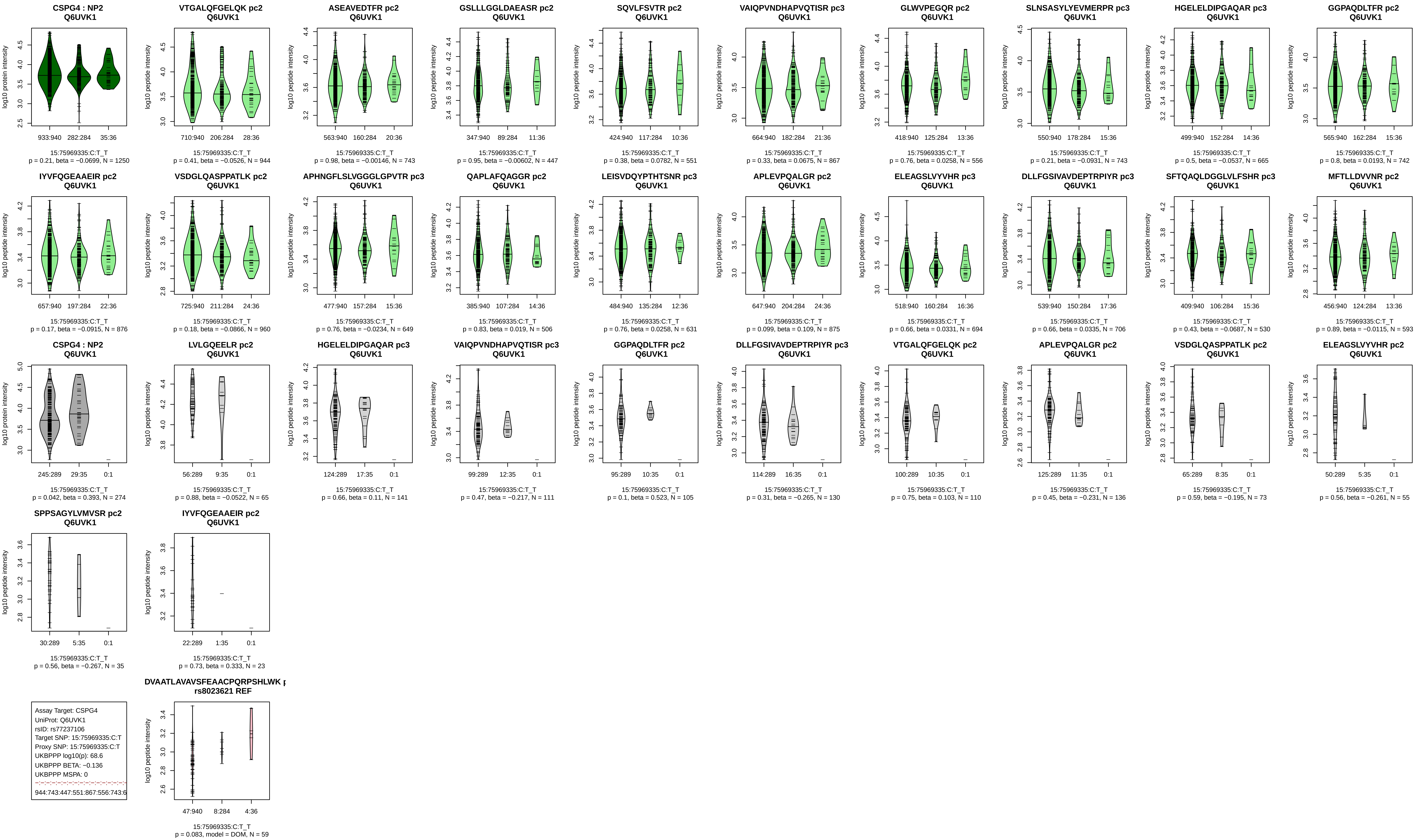
CSPG4 : NP2 Q6UVK1
933:940 282:284 35:36
15:75969335:C:T_T
p = 0.21, beta = −0.0699, N = 1250
VTGALQFGELQK pc2 Q6UVK1
710:940 206:284 28:36
15:75969335:C:T_T
p = 0.41, beta = −0.0526, N = 944
ASEAVEDTFR pc2 Q6UVK1
563:940 160:284 20:36
15:75969335:C:T_T
p = 0.98, beta = −0.00146, N = 743
GSLLLGGLDAEASR pc2 Q6UVK1
347:940 89:284 11:36
15:75969335:C:T_T
p = 0.95, beta = −0.00602, N = 447
SQVLFSVTR pc2 Q6UVK1
424:940 117:284 10:36
15:75969335:C:T_T
p = 0.38, beta = 0.0782, N = 551
VAIQPVNDHAPVQTISR pc3 Q6UVK1
664:940 182:284 21:36
15:75969335:C:T_T
p = 0.33, beta = 0.0675, N = 867
GLWVPEGQR pc2 Q6UVK1
418:940 125:284 13:36
15:75969335:C:T_T
p = 0.76, beta = 0.0258, N = 556
SLNSASYLYEVMERPR pc3 Q6UVK1
550:940 178:284 15:36
15:75969335:C:T_T
p = 0.21, beta = −0.0931, N = 743
HGELELDIPGAQAR pc3 Q6UVK1
499:940 152:284 14:36
15:75969335:C:T_T
p = 0.5, beta = −0.0537, N = 665
GGPAQDLTFR pc2 Q6UVK1
565:940 162:284 13:36
15:75969335:C:T_T
p = 0.8, beta = 0.0193, N = 742
IYVFQGEAAEIR pc2 Q6UVK1
657:940 197:284 22:36
15:75969335:C:T_T
p = 0.17, beta = −0.0915, N = 876
VSDGLQASPPATLK pc2 Q6UVK1
725:940 211:284 24:36
15:75969335:C:T_T
p = 0.18, beta = −0.0866, N = 960
APHNGFLSLVGGGLGPVTR pc3 Q6UVK1
477:940 157:284 15:36
15:75969335:C:T_T
p = 0.76, beta = −0.0234, N = 649
QAPLAFQAGGR pc2 Q6UVK1
385:940 107:284 14:36
15:75969335:C:T_T
p = 0.83, beta = 0.019, N = 506
LEISVDQYPTHTSNR pc3 Q6UVK1
484:940 135:284 12:36
15:75969335:C:T_T
p = 0.76, beta = 0.0258, N = 631
APLEVPQALGR pc2 Q6UVK1
647:940 204:284 24:36
15:75969335:C:T_T
p = 0.099, beta = 0.109, N = 875
ELEAGSLVYVHR pc3 Q6UVK1
518:940 160:284 16:36
15:75969335:C:T_T
p = 0.66, beta = 0.0331, N = 694
DLLFGSIVAVDEPTRPIYR pc3 Q6UVK1
539:940 150:284 17:36
15:75969335:C:T_T
p = 0.66, beta = 0.0335, N = 706
SFTQAQLDGGLVLFSHR pc3 Q6UVK1
409:940 106:284 15:36
15:75969335:C:T_T
p = 0.43, beta = −0.0687, N = 530
MFTLLDVVNR pc2 Q6UVK1
456:940 124:284 13:36
15:75969335:C:T_T
p = 0.89, beta = −0.0115, N = 593
CSPG4 : NP2 Q6UVK1
245:289 29:35 0:1
15:75969335:C:T_T
p = 0.042, beta = 0.393, N = 274
LVLGQEELR pc2 Q6UVK1
56:289 9:35 0:1
15:75969335:C:T_T
p = 0.88, beta = −0.0522, N = 65
HGELELDIPGAQAR pc3 Q6UVK1
124:289 17:35 0:1
15:75969335:C:T_T
p = 0.66, beta = 0.11, N = 141
VAIQPVNDHAPVQTISR pc3 Q6UVK1
99:289 12:35 0:1
15:75969335:C:T_T
p = 0.47, beta = −0.217, N = 111
GGPAQDLTFR pc2 Q6UVK1
95:289 10:35 0:1
15:75969335:C:T_T
p = 0.1, beta = 0.523, N = 105
DLLFGSIVAVDEPTRPIYR pc3 Q6UVK1
114:289 16:35 0:1
15:75969335:C:T_T
p = 0.31, beta = −0.265, N = 130
VTGALQFGELQK pc2 Q6UVK1
100:289 10:35 0:1
15:75969335:C:T_T
p = 0.75, beta = 0.103, N = 110
APLEVPQALGR pc2 Q6UVK1
125:289 11:35 0:1
15:75969335:C:T_T
p = 0.45, beta = −0.231, N = 136
VSDGLQASPPATLK pc2 Q6UVK1
65:289 8:35 0:1
15:75969335:C:T_T
p = 0.59, beta = −0.195, N = 73
ELEAGSLVYVHR pc2 Q6UVK1
50:289 5:35 0:1
15:75969335:C:T_T
p = 0.56, beta = −0.261, N = 55
SPPSAGYLVMVSR pc2 Q6UVK1
30:289 5:35 0:1
15:75969335:C:T_T
p = 0.56, beta = −0.267, N = 35
IYVFQGEAAEIR pc2 Q6UVK1
22:289 1:35 0:1
15:75969335:C:T_T
p = 0.73, beta = 0.333, N = 23
Assay Target: CSPG4
UniProt: Q6UVK1
rsID: rs77237106
Target SNP: 15:75969335:C:T
Proxy SNP: 15:75969335:C:T
UKBPPP log10(p): 68.6
UKBPPP BETA: −0.136
UKBPPP MSPA: 0
944:743:447:551:867:556:743:6
DVAATLAVAVSFEAACPQRPSHLWK p
rs8023621 REF
47:940 8:284 4:36
15:75969335:C:T_T
p = 0.083, model = DOM, N = 59
log10 protein intensity
log10 peptide intensity

312. ELN (P15502)
UKBPPP MSPA=0

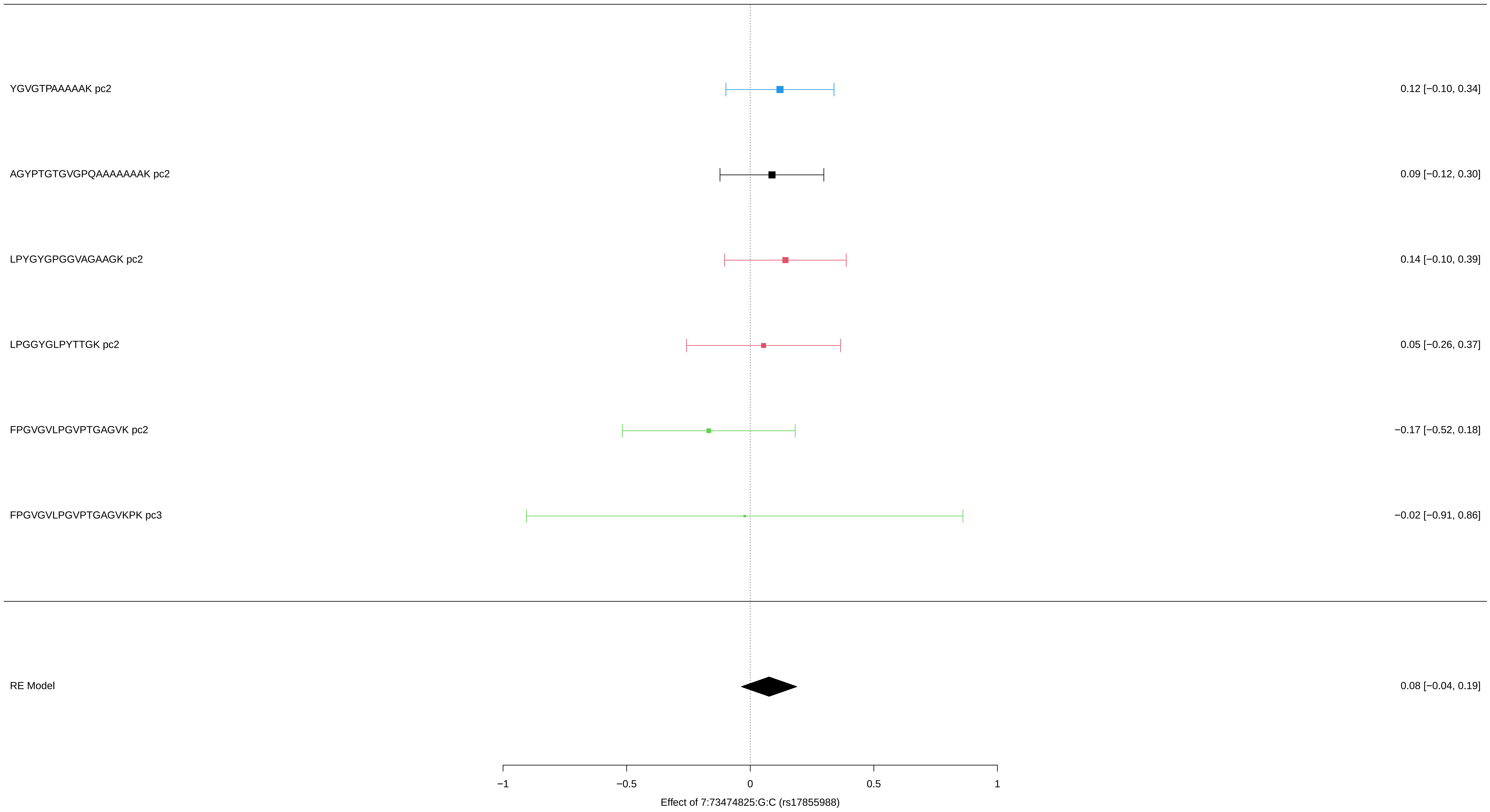

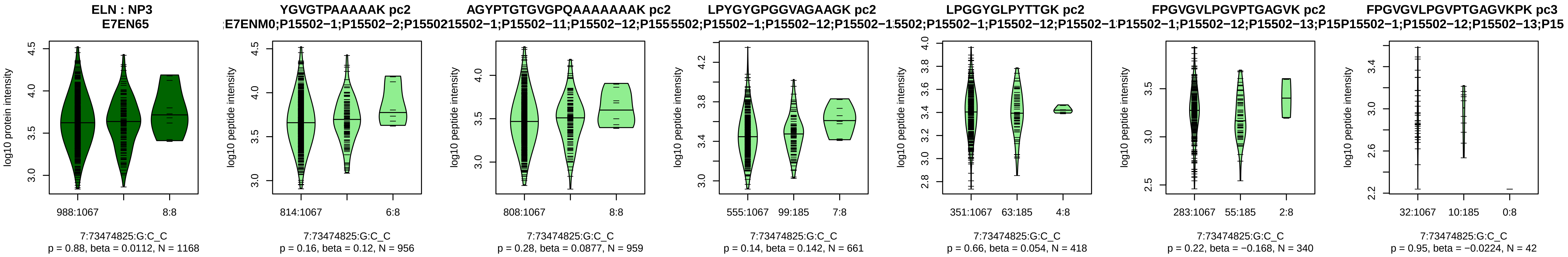

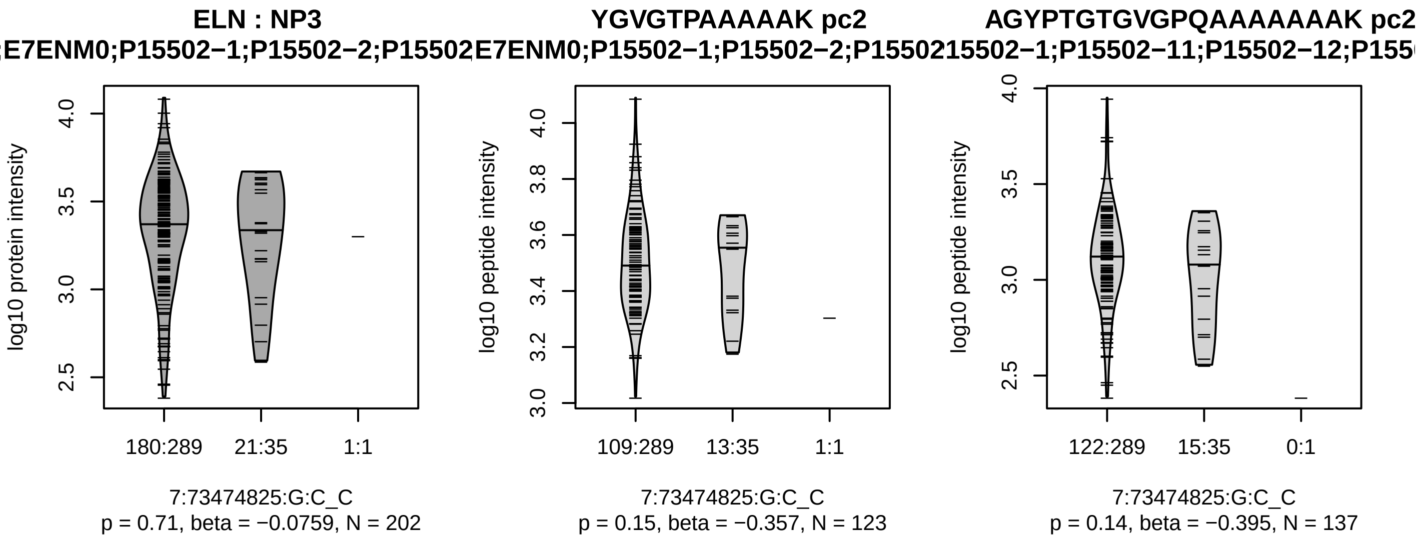

Assay Target: ELN
UniProt: P15502
rsID: rs17855988
Target SNP: 7:73474825:G:C
Proxy SNP: 7:73474825:G:C
UKBPPP log10(p): 68.2
UKBPPP BETA: 0.136
UKBPPP MSPA: 0
–:–:–:–:–:–
956:959:661:418:340:42

313. RALB (P11234)
UKBPPP MSPA=0.912

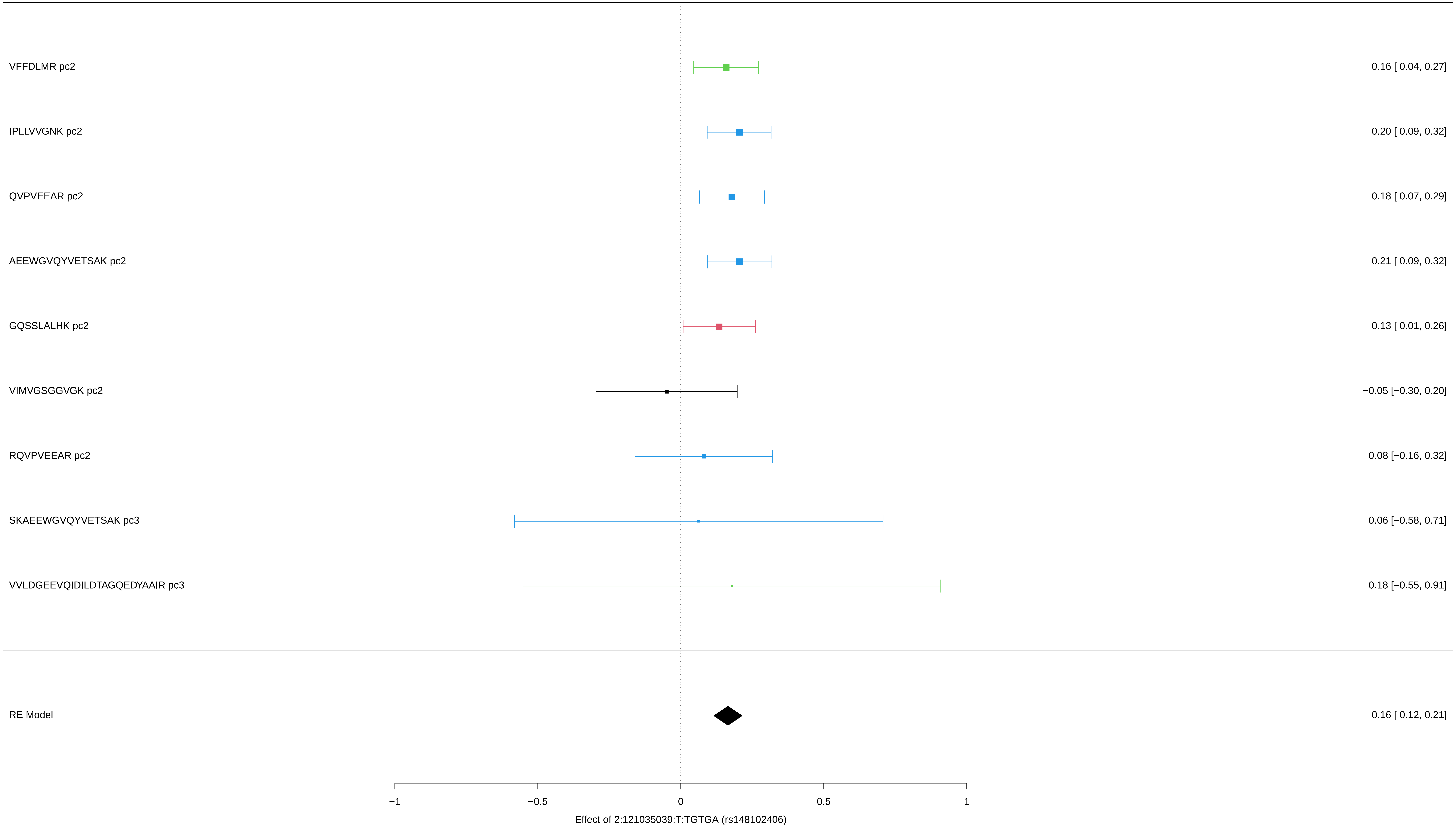

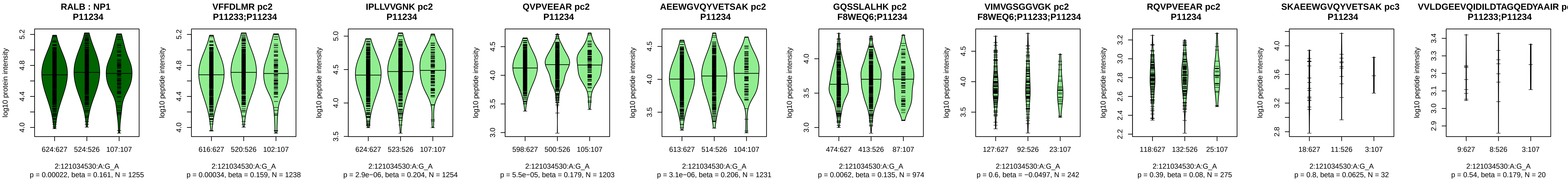

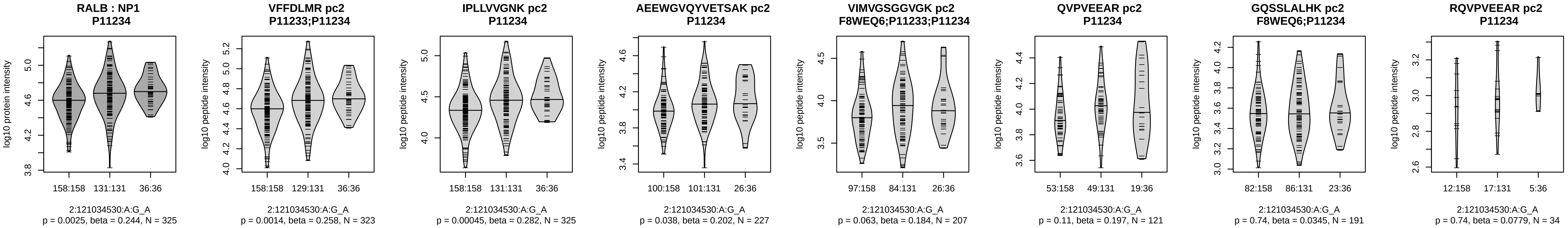

Assay Target: RALB
UniProt: P11234
rsID: rs148102406
Target SNP: 2:121035039:T:TG
Proxy SNP: 2:121034530:A:G
UKBPPP log10(p): 67.7
UKBPPP BETA: −0.119
UKBPPP MSPA: 0.912
::*:*:*:−:−:−:NA:−
1238:1254:1203:1231:974:242:2

314. C3 (P01024)
UKBPPP MSPA=0.03

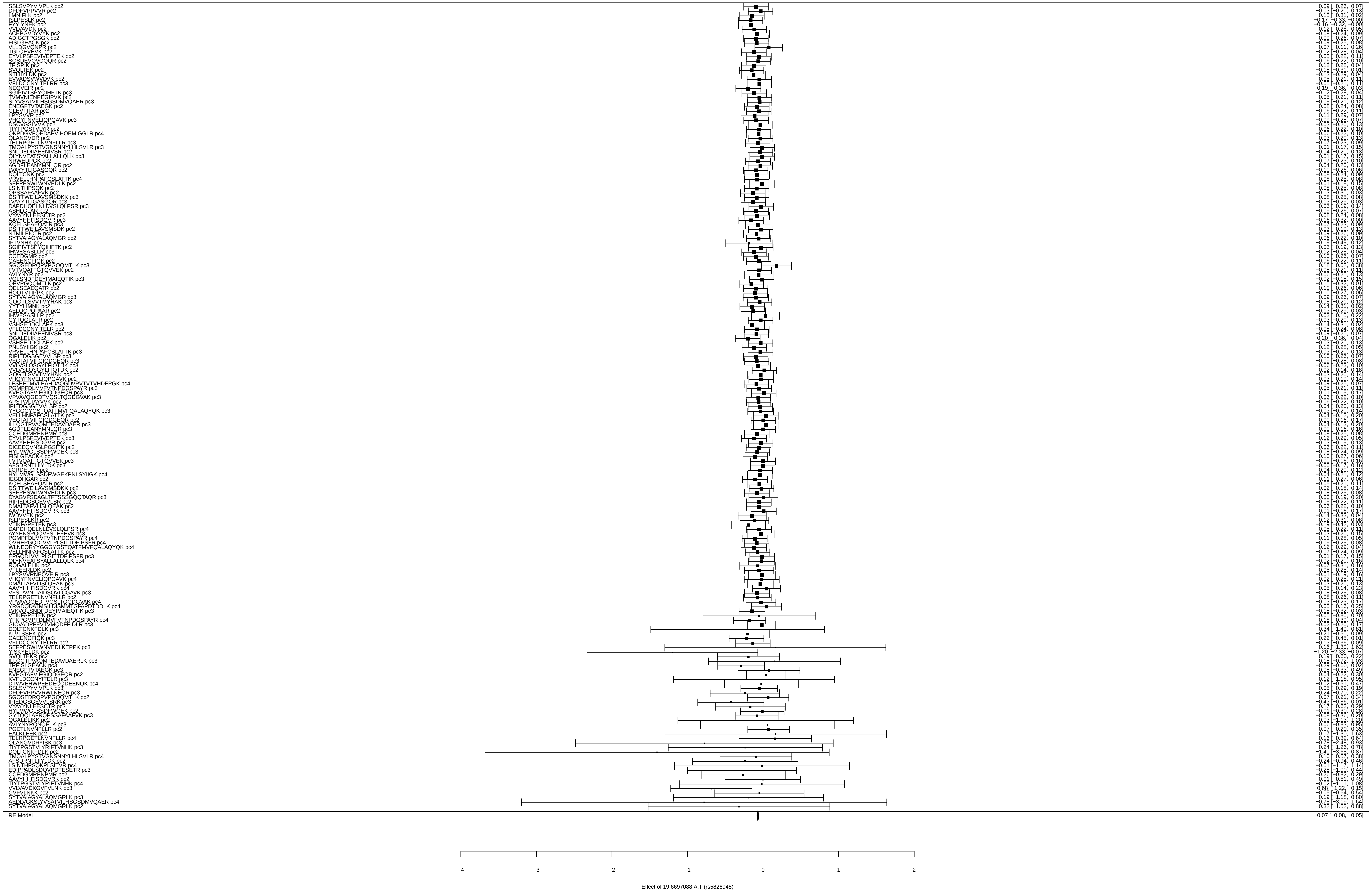

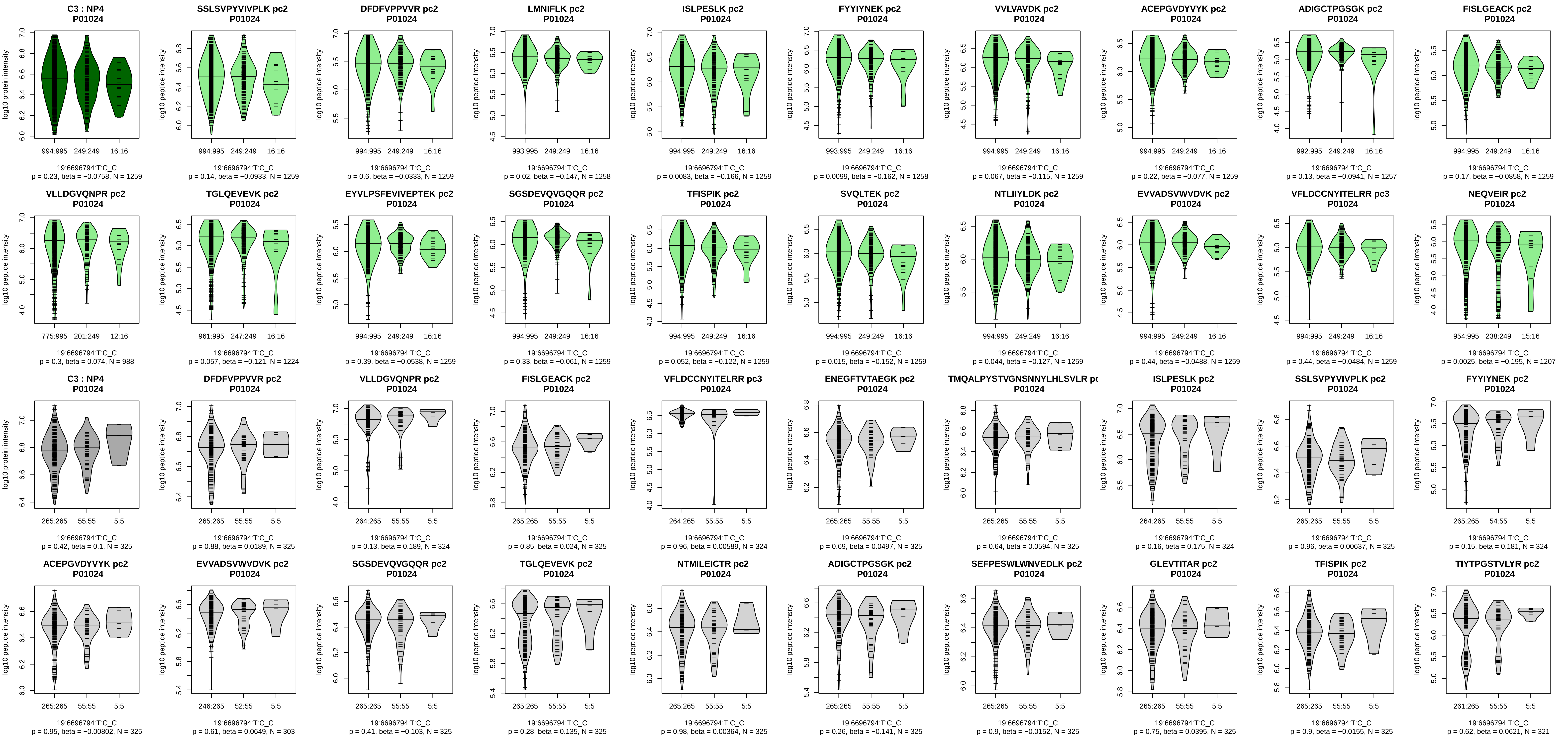

Assay Target: C3
UniProt: P01024
rsID: rs5826945
Target SNP: 19:6697088:A:T
Proxy SNP: 19:6696794:T:C
UKBPPP log10(p): 66.9
UKBPPP BETA: −0.157
UKBPPP MSPA: 0.03
1259:1259:1258:1259:1258:125

315. PCOLCE (Q15113)
UKBPPP MSPA=0.694

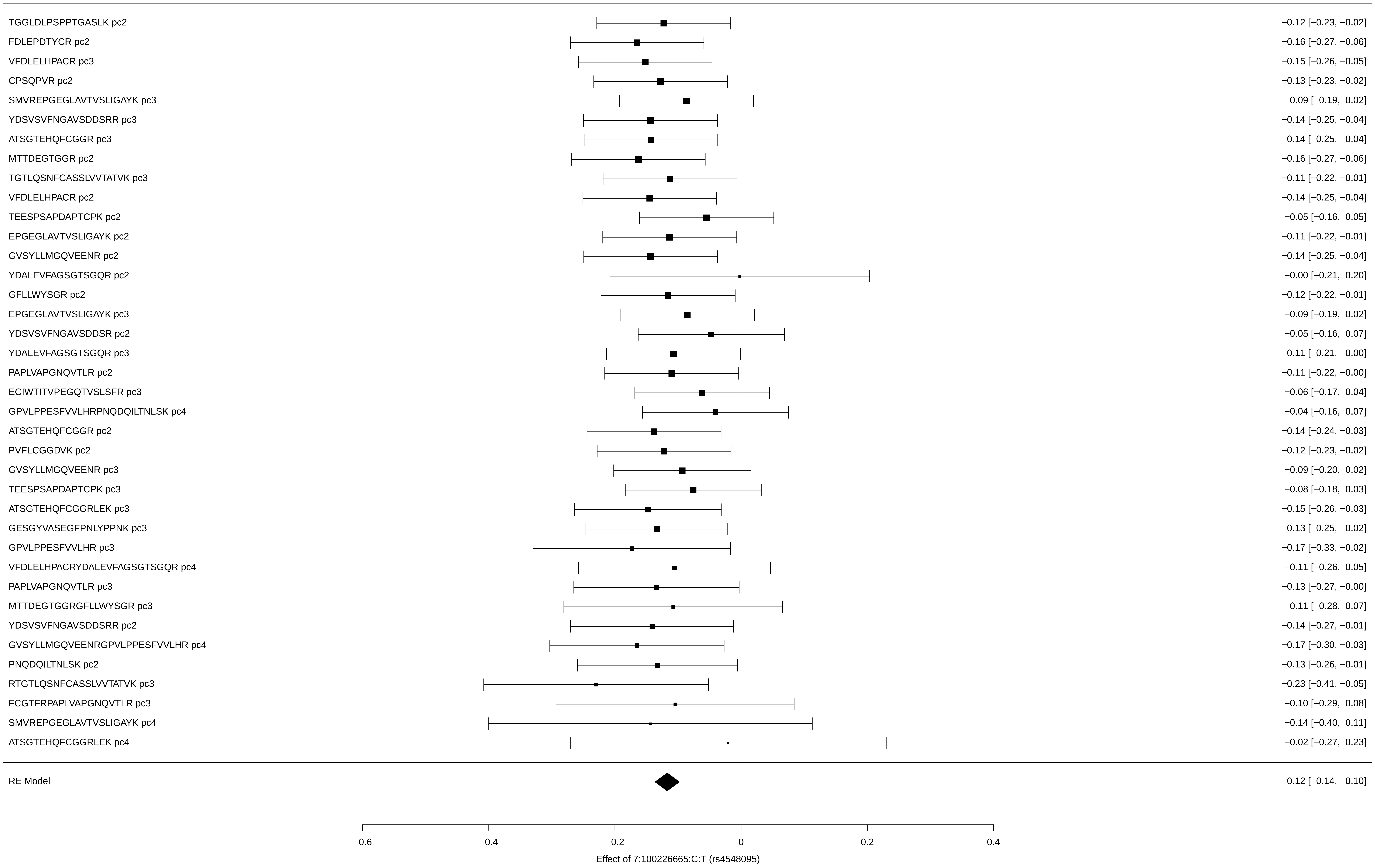

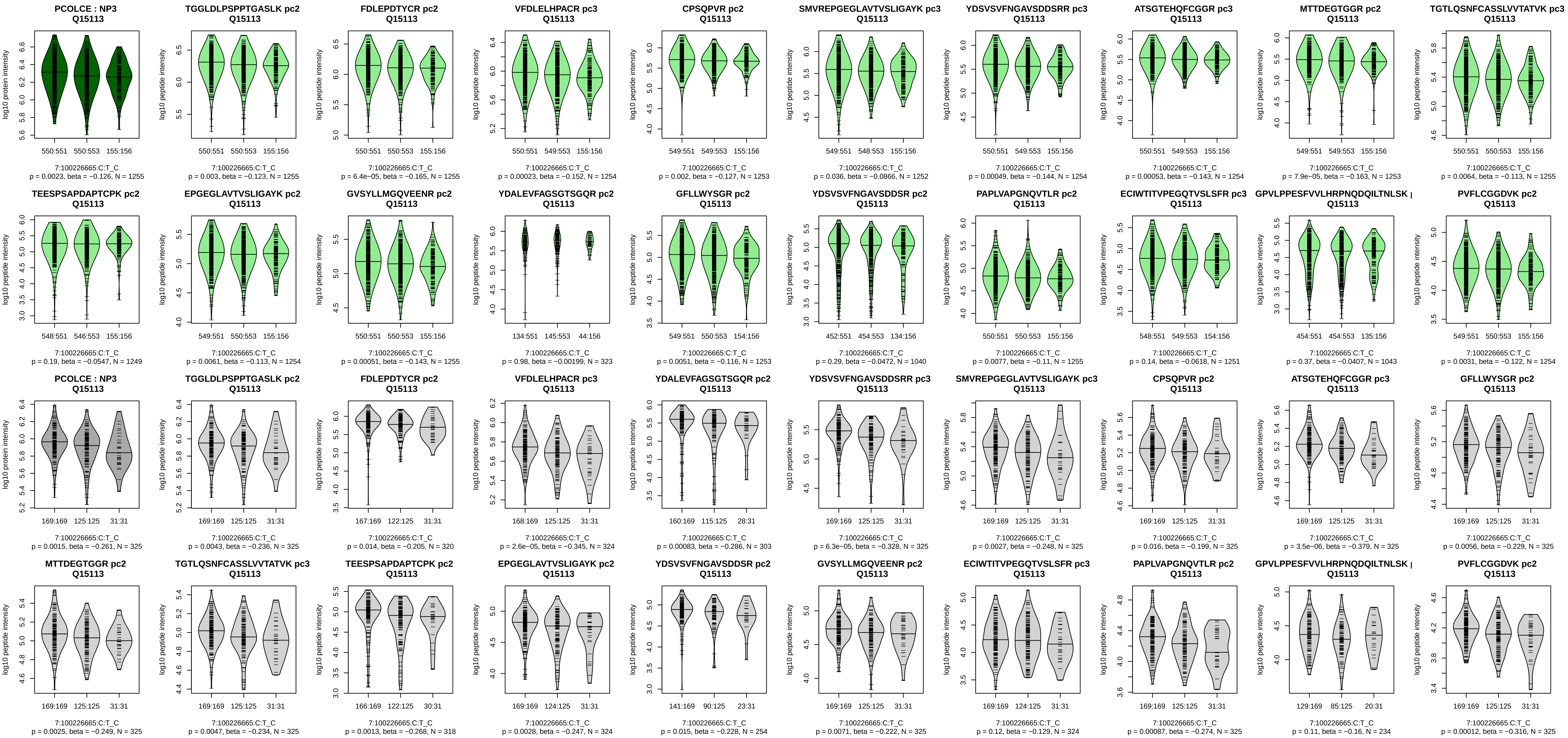

Assay Target: PCOLCE
UniProt: Q15113
rsID: rs4548095
Target SNP: 7:100226665:C:T
Proxy SNP: 7:100226665:C:T
UKBPPP log10(p): 63.7
UKBPPP BETA: 0.169
UKBPPP MSPA: 0.694
1255:1255:1254:1253:1252:125

316. PRG2 (P13727)
UKBPPP MSPA=0.192

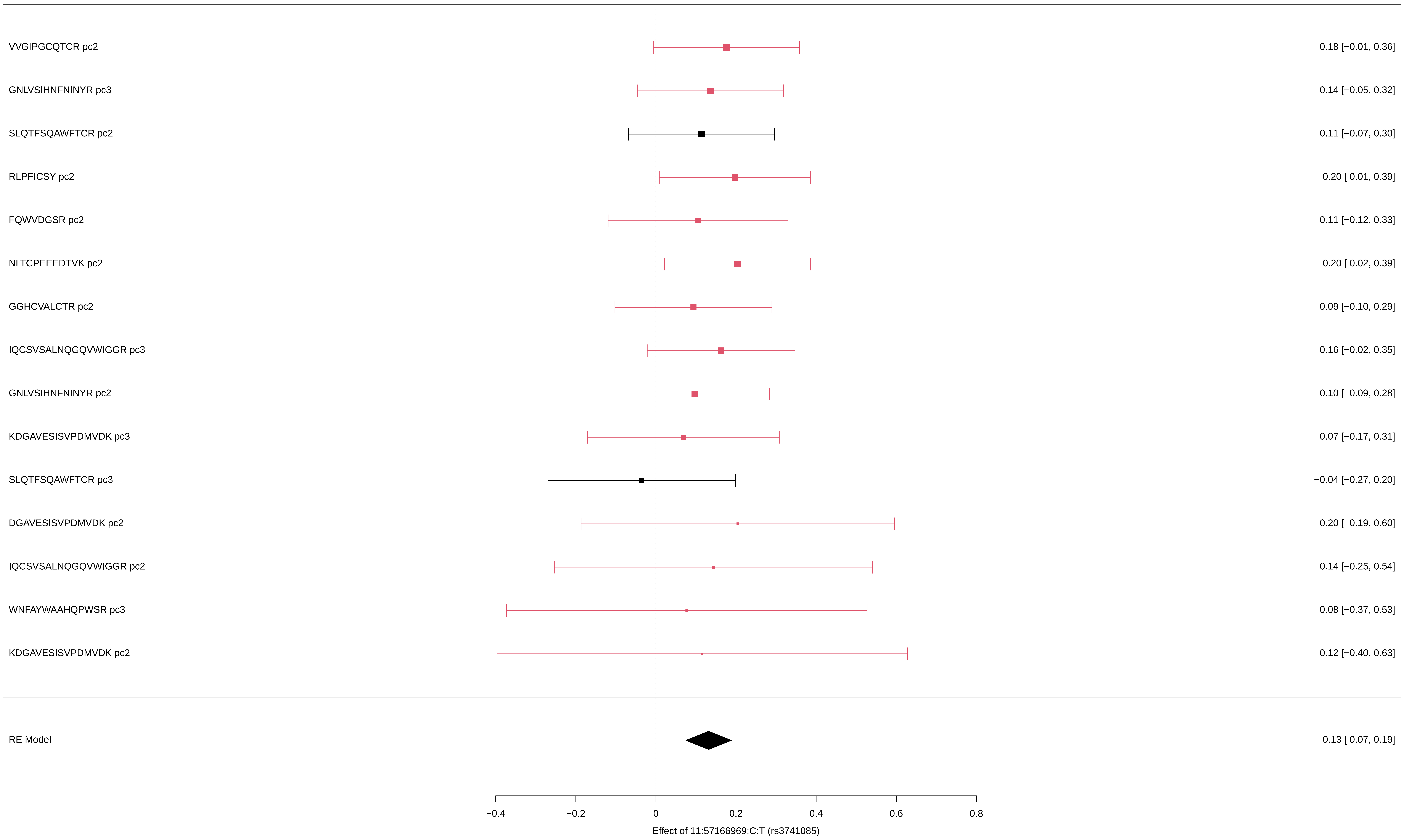

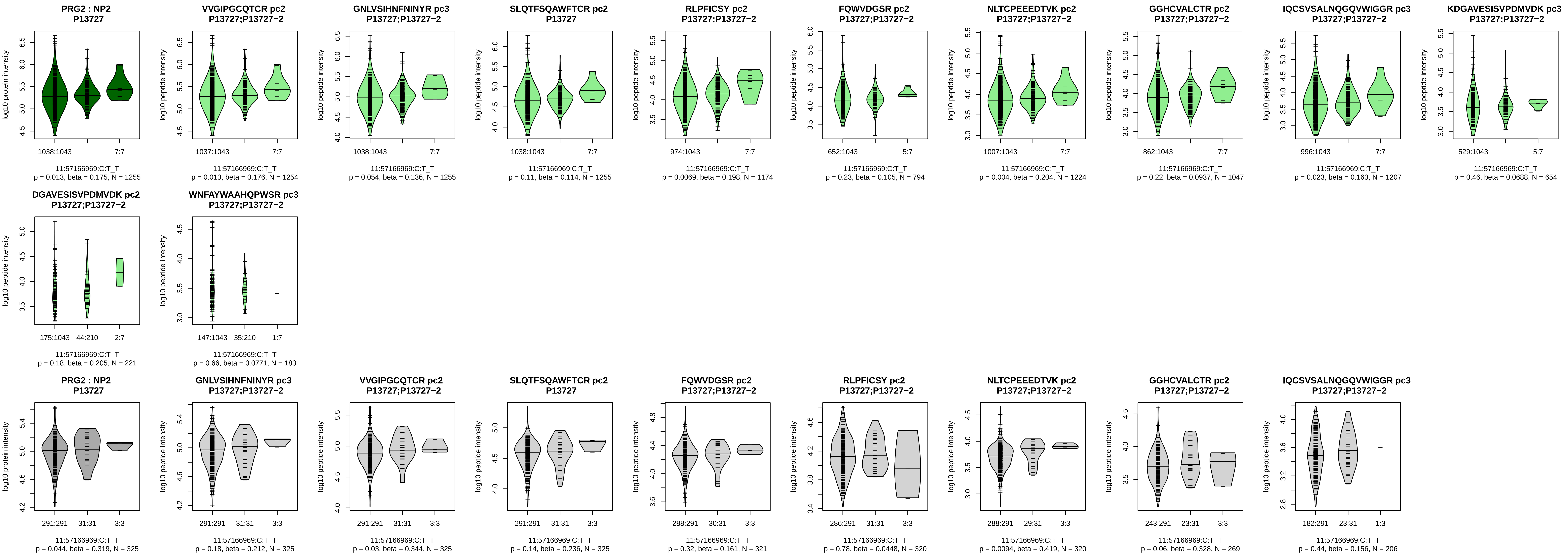

Assay Target: PRG2
UniProt: P13727
rsID: rs3741085
Target SNP: 11:57166969:C:T
Proxy SNP: 11:57166969:C:T
UKBPPP log10(p): 62.4
UKBPPP BETA: 0.15
UKBPPP MSPA: 0.192
–:–:–:*:–:*:–:–:–:–:–:–:–
1254:1255:1255:1174:794:1224

317. PSME2 (Q9UL46)
UKBPPP MSPA=0

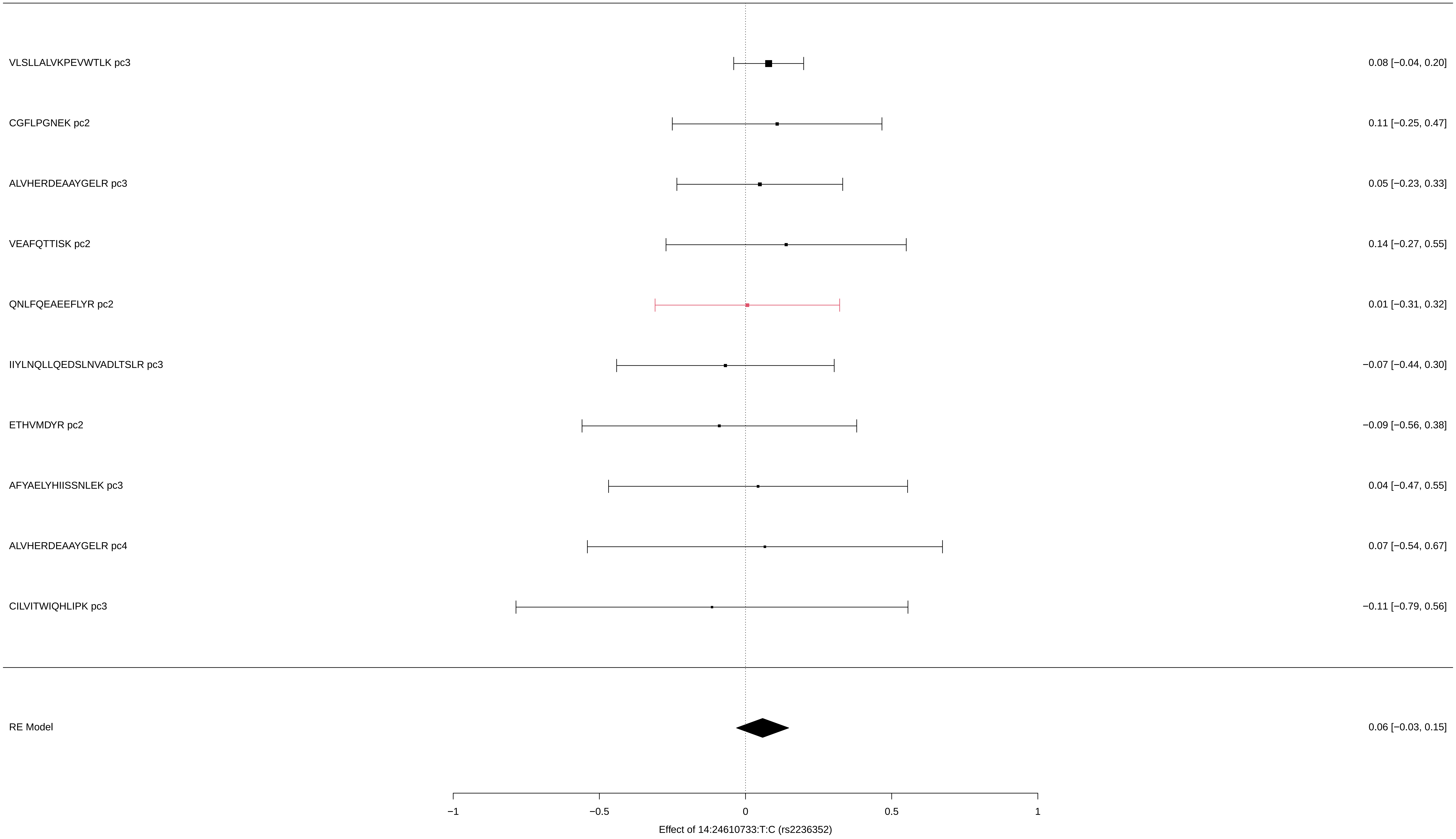

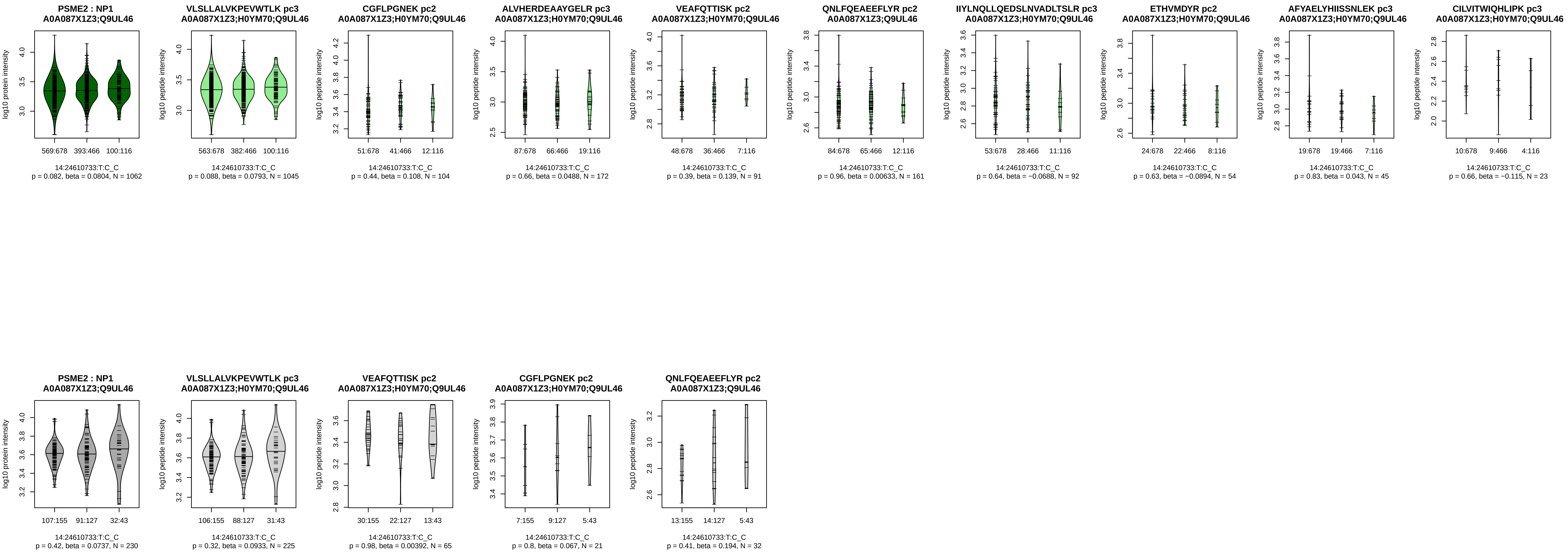

Assay Target: PSME2
UniProt: Q9UL46
rsID: rs2236352
Target SNP: 14:24610733:T:C
Proxy SNP: 14:24610733:T:C
UKBPPP log10(p): 58.8
UKBPPP BETA: 0.111
UKBPPP MSPA: 0
–:–:–:–:–:–:–:–:–:–
1045:104:172:91:161:92:54:45:[illegible]

318. CA2 (P00918)
UKBPPP MSPA=0.463

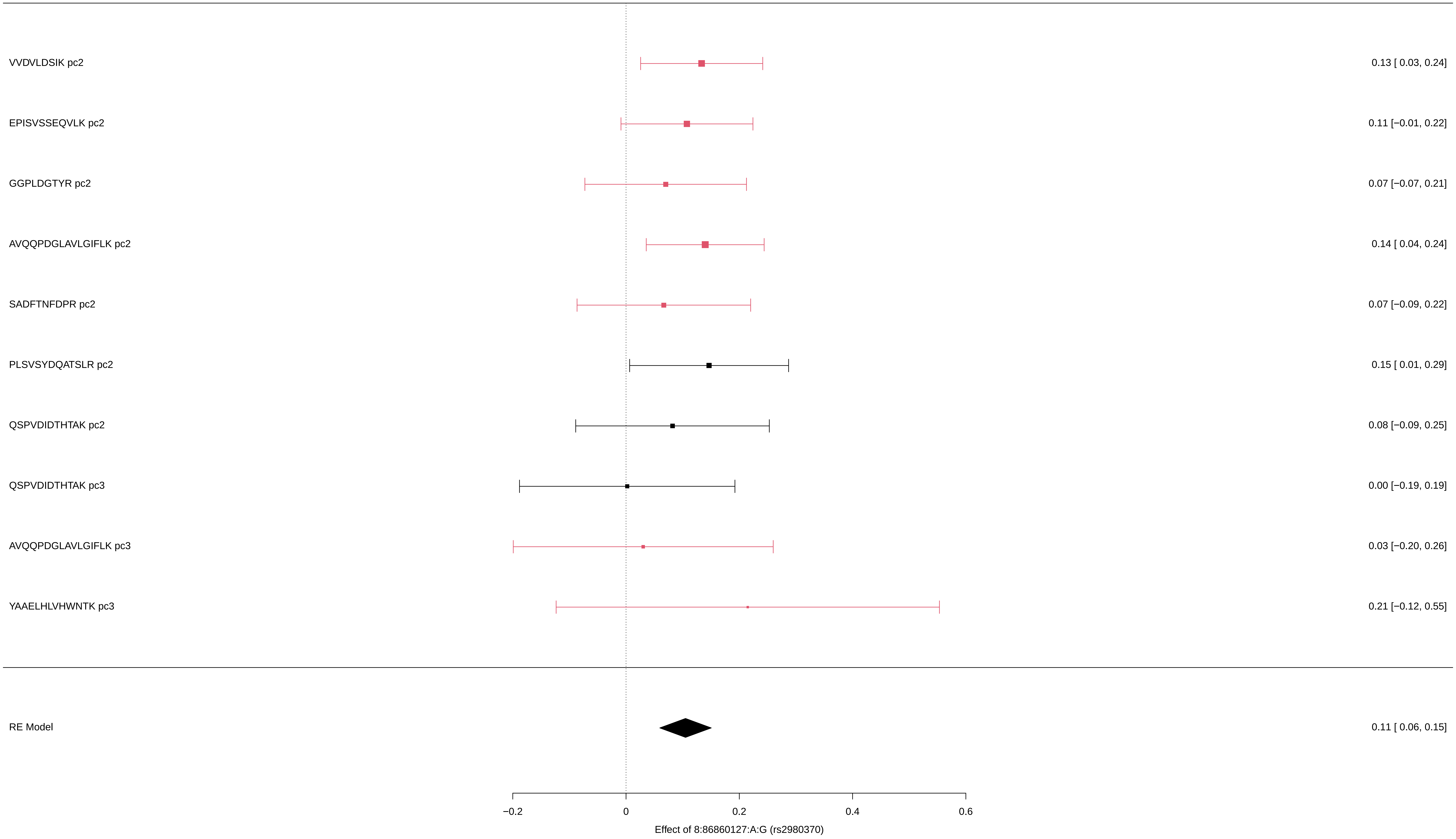

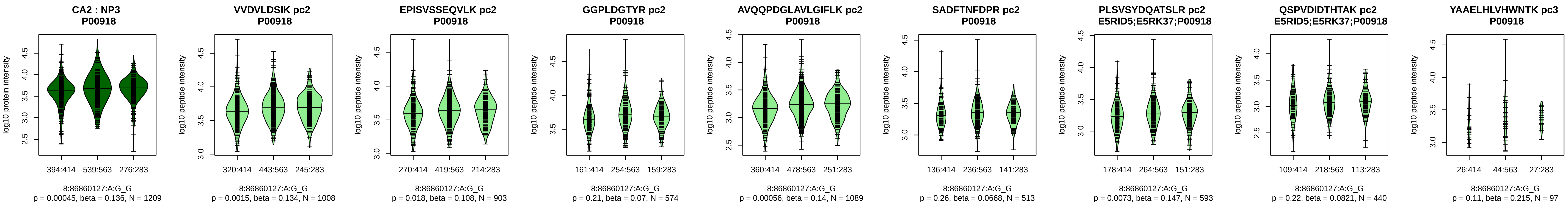

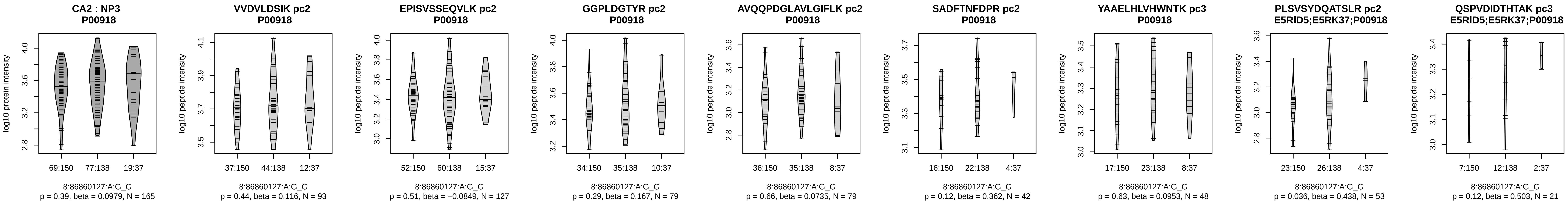

Assay Target: CA2
UniProt: P00918
rsID: rs2980370
Target SNP: 8:86860127:A:G
Proxy SNP: 8:86860127:A:G
UKBPPP log10(p): 58.8
UKBPPP BETA: 0.097
UKBPPP MSPA: 0.463
:−:−::−:*:−:−:−:−:NA:NA
1008:903:574:1089:513:593:44

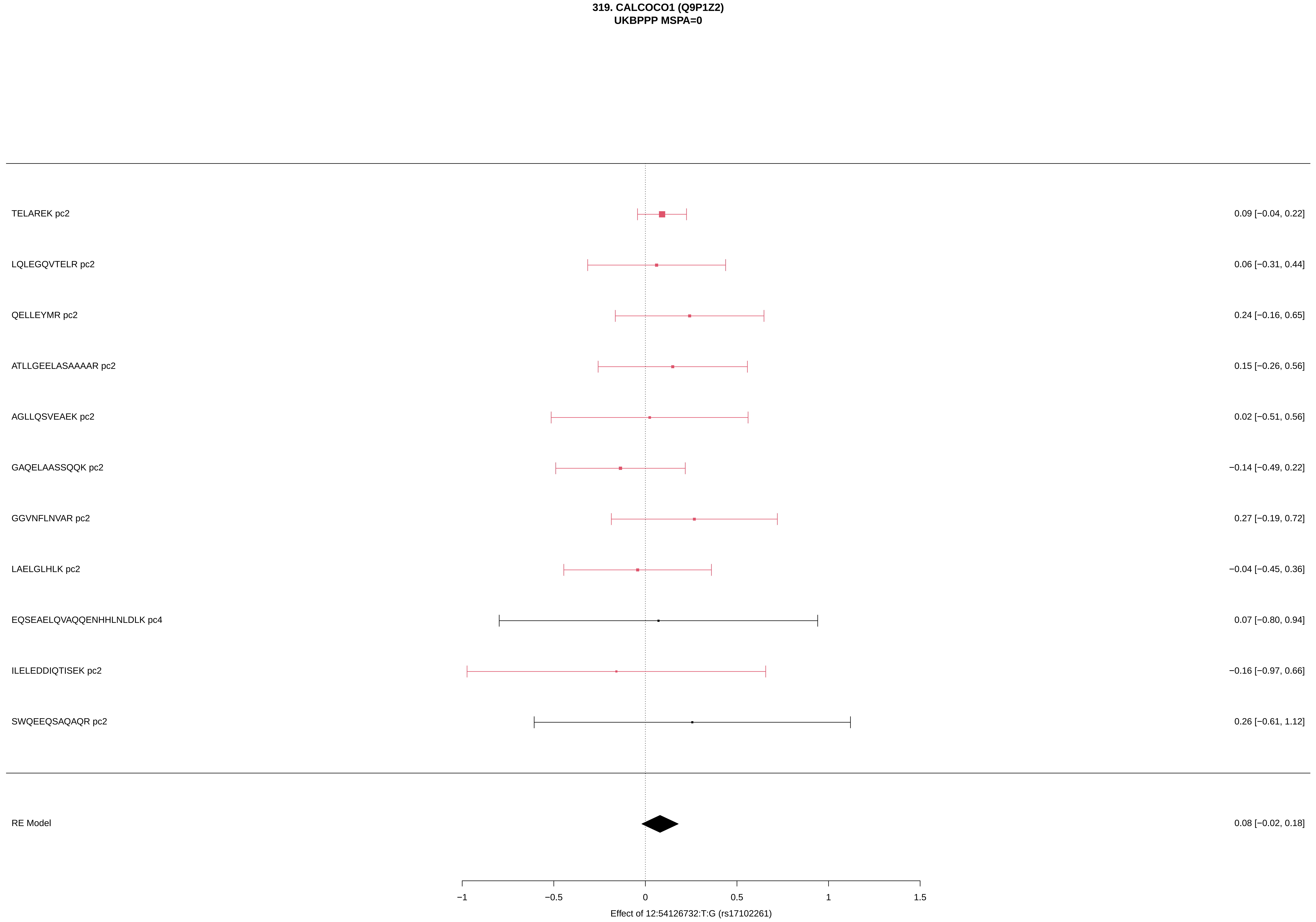
319. CALCOCO1 (Q9P1Z2)
UKBPPP MSPA=0
TELAREK pc2
0.09 [−0.04, 0.22]
LQLEGQVTELR pc2
0.06 [−0.31, 0.44]
QELLEYMR pc2
0.24 [−0.16, 0.65]
ATLLGEELASAAAAR pc2
0.15 [−0.26, 0.56]
AGLLQSVEAEK pc2
0.02 [−0.51, 0.56]
GAQELAASSQQK pc2
−0.14 [−0.49, 0.22]
GGVNFLNVAR pc2
0.27 [−0.19, 0.72]
LAELGLHLK pc2
−0.04 [−0.45, 0.36]
EQSEAELQVAQQENHHLNLDLK pc4
0.07 [−0.80, 0.94]
ILELEDDIQTISEK pc2
−0.16 [−0.97, 0.66]
SWQEEQSAQAQR pc2
0.26 [−0.61, 1.12]
RE Model
0.08 [−0.02, 0.18]
−1
−0.5
0
0.5
1
1.5
Effect of 12:54126732:T:G (rs17102261)

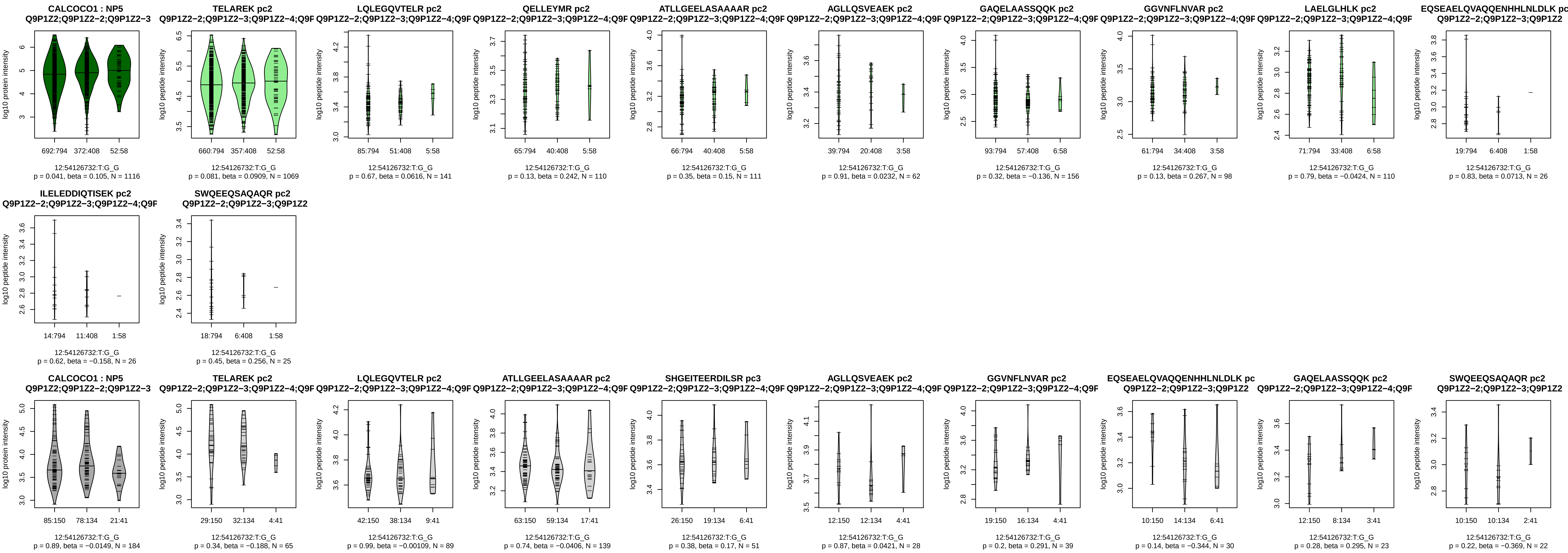

Assay Target: CALCOCO1
UniProt: Q9P1Z2
rsID: rs17102261
Target SNP: 12:54126732:T:G
Proxy SNP: 12:54126732:T:G
UKBPPP log10(p): 58.7
UKBPPP BETA: 0.133
UKBPPP MSPA: 0
−:−:−:−:−:−:−:−:−:−:NA:NA:NA
1069:141:110:111:62:156:98:11

320. CCL21 (O00585)
UKBPPP MSPA=0

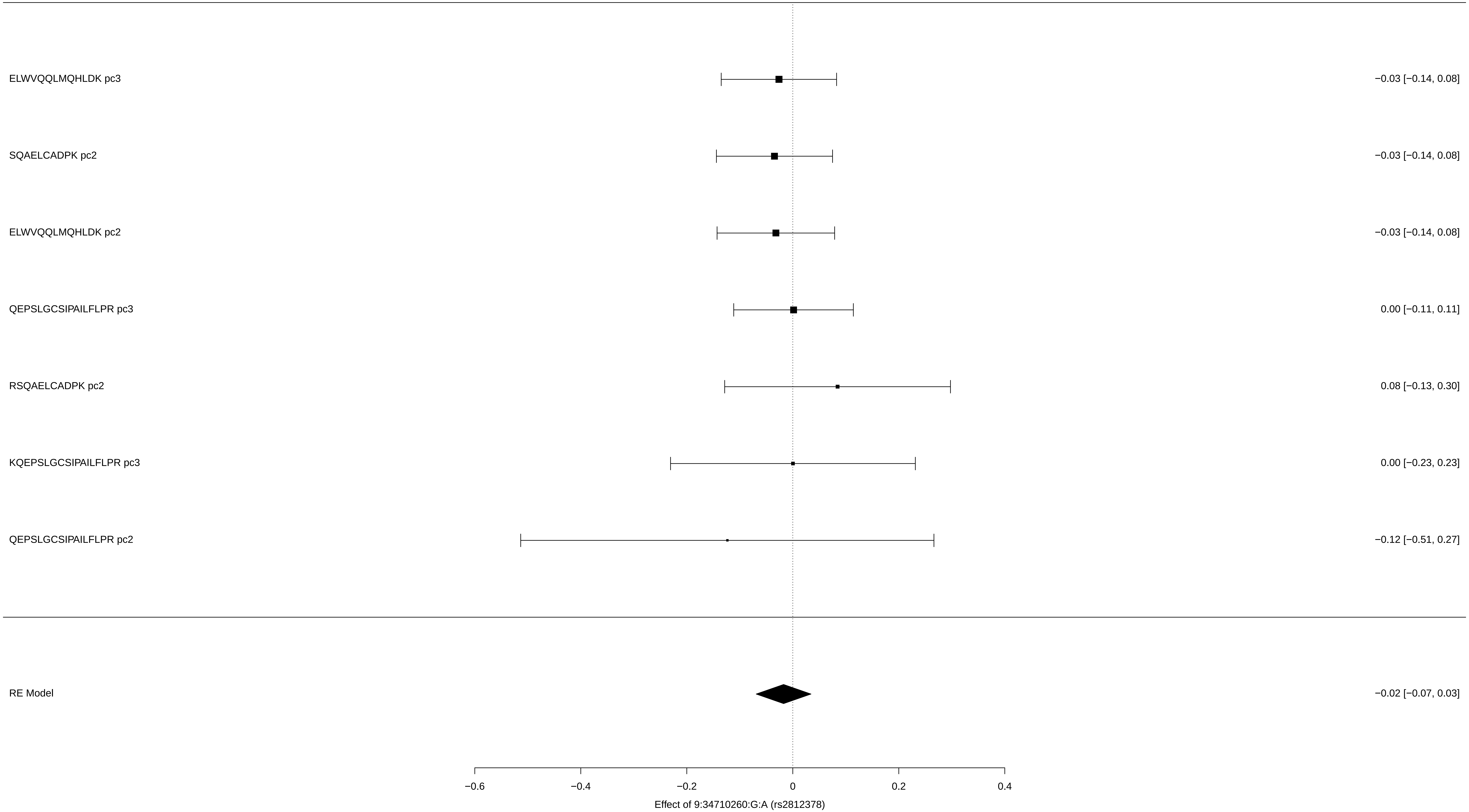

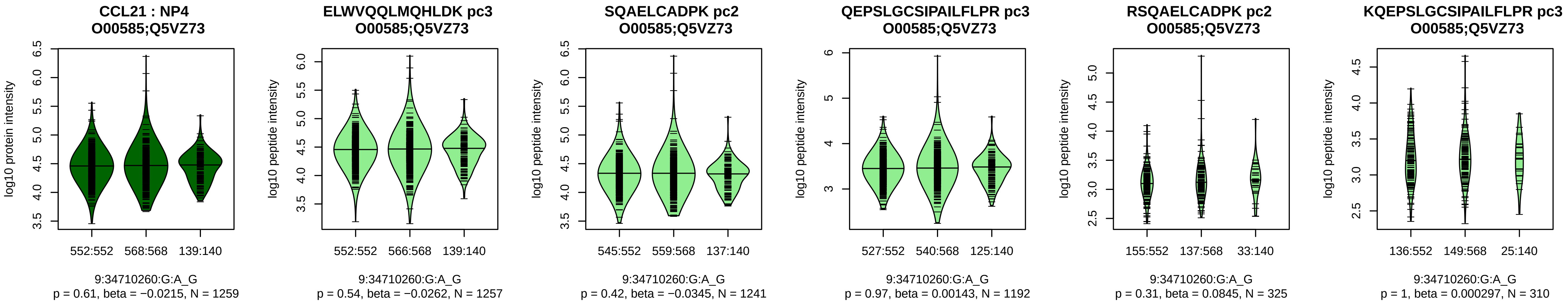

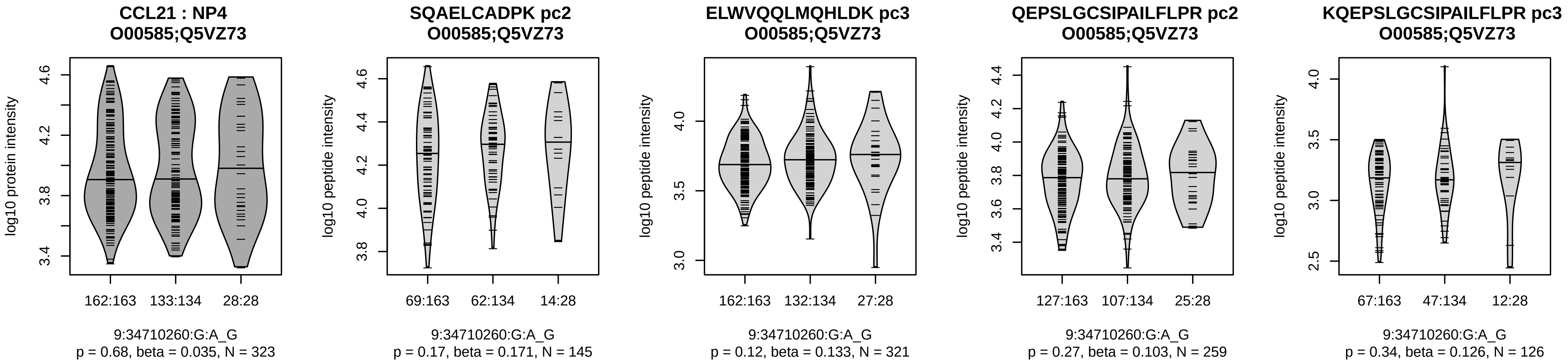

Assay Target: CCL21
UniProt: O00585
rsID: rs2812378
Target SNP: 9:34710260:G:A
Proxy SNP: 9:34710260:G:A
UKBPPP log10(p): 57.3
UKBPPP BETA: 0.101
UKBPPP MSPA: 0
−:−:−:−:−:−:−:NA:NA
1257:1241:1208:1192:325:310:1

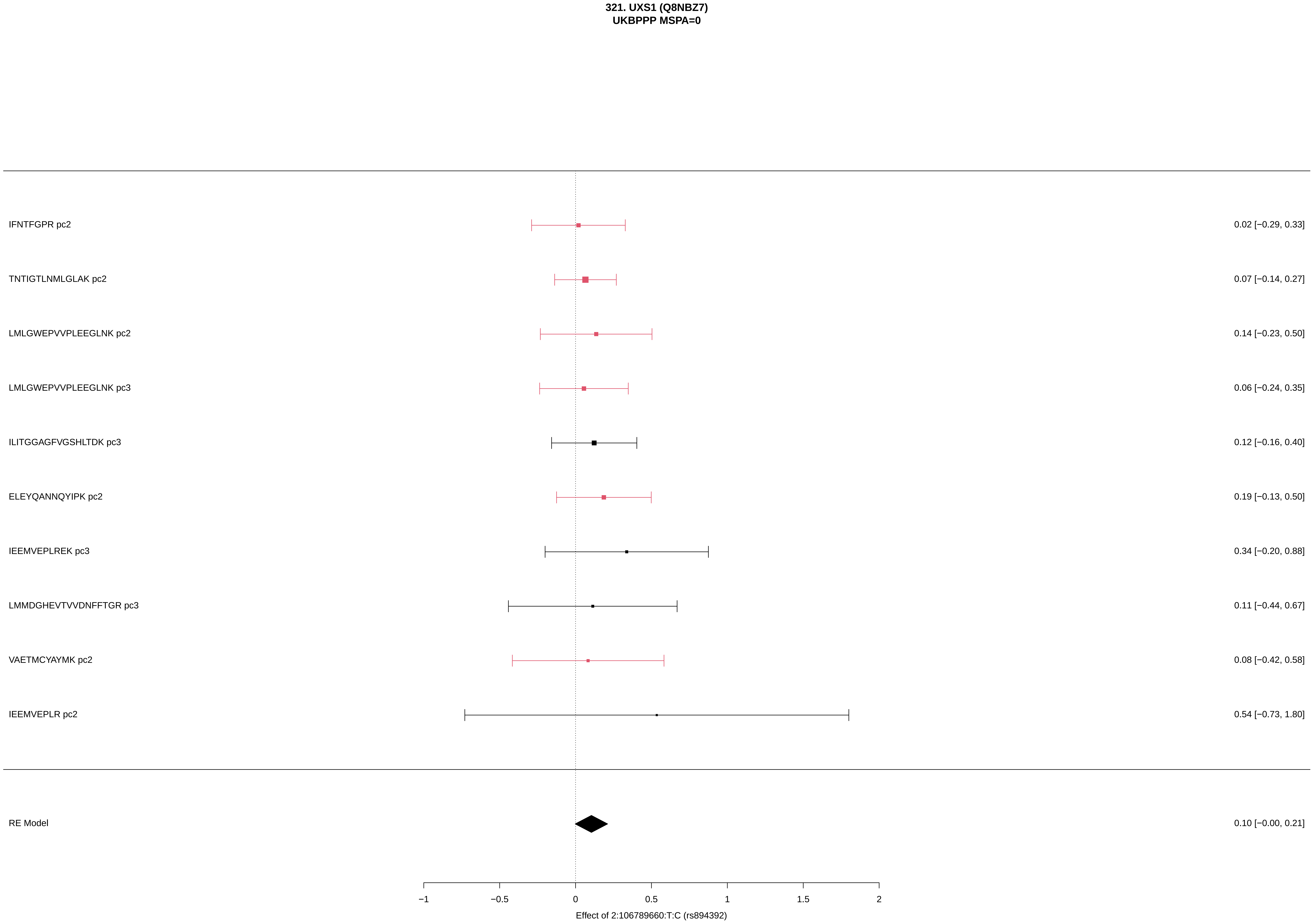
321. UXS1 (Q8NBZ7)
UKBPPP MSPA=0
IFNTFGPR pc2
0.02 [−0.29, 0.33]
TNTIGTLNMLGLAK pc2
0.07 [−0.14, 0.27]
LMLGWEPVVPLEEGLNK pc2
0.14 [−0.23, 0.50]
LMLGWEPVVPLEEGLNK pc3
0.06 [−0.24, 0.35]
ILITGGAGFVGSHLTDK pc3
0.12 [−0.16, 0.40]
ELEYQANNQYIPK pc2
0.19 [−0.13, 0.50]
IEEMVEPLREK pc3
0.34 [−0.20, 0.88]
LMMDGHEVTVVDNFFTGR pc3
0.11 [−0.44, 0.67]
VAETMCYAYMK pc2
0.08 [−0.42, 0.58]
IEEMVEPLR pc2
0.54 [−0.73, 1.80]
RE Model
0.10 [−0.00, 0.21]
−1
−0.5
0
0.5
1
1.5
2
Effect of 2:106789660:T:C (rs894392)

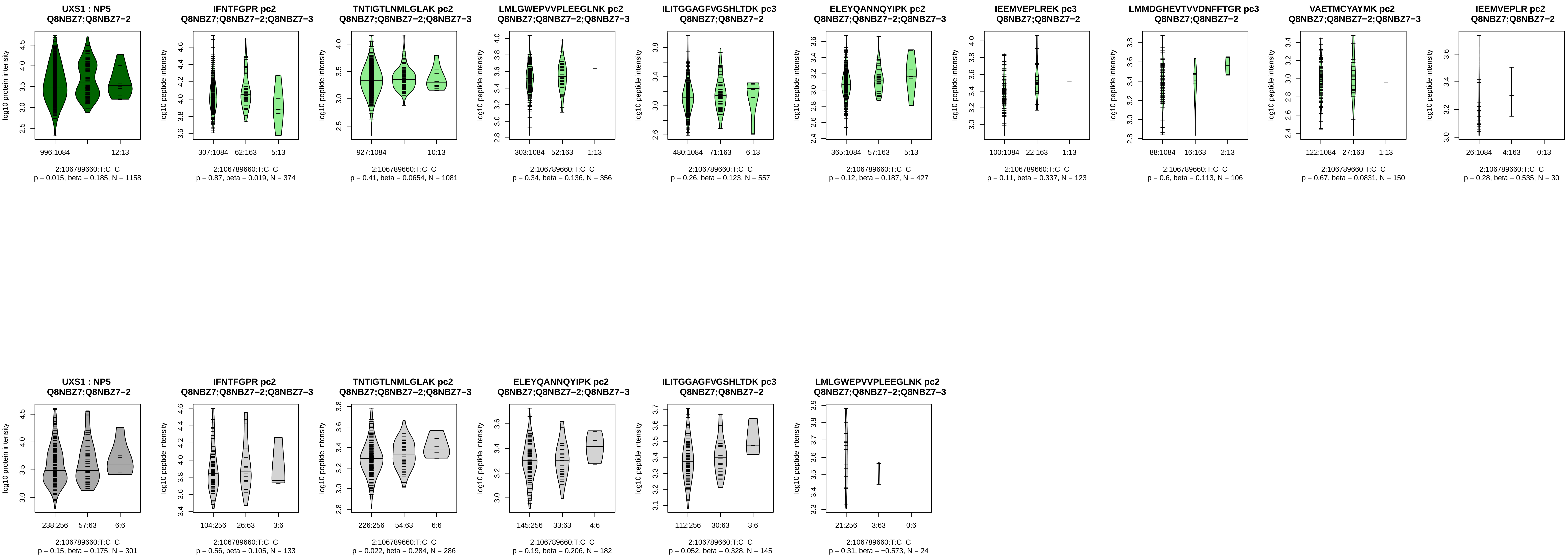

Assay Target: UXS1
UniProt: Q8NBZ7
rsID: rs894392
Target SNP: 2:106789660:T:C
Proxy SNP: 2:106789660:T:C
UKBPPP log10(p): 56.8
UKBPPP BETA: 0.182
UKBPPP MSPA: 0
−:−:−:−:−:−:−:−:−:NA:−
374:1081:356:444:557:427:123:

322. NBL1 (P41271)
UKBPPP MSPA=0

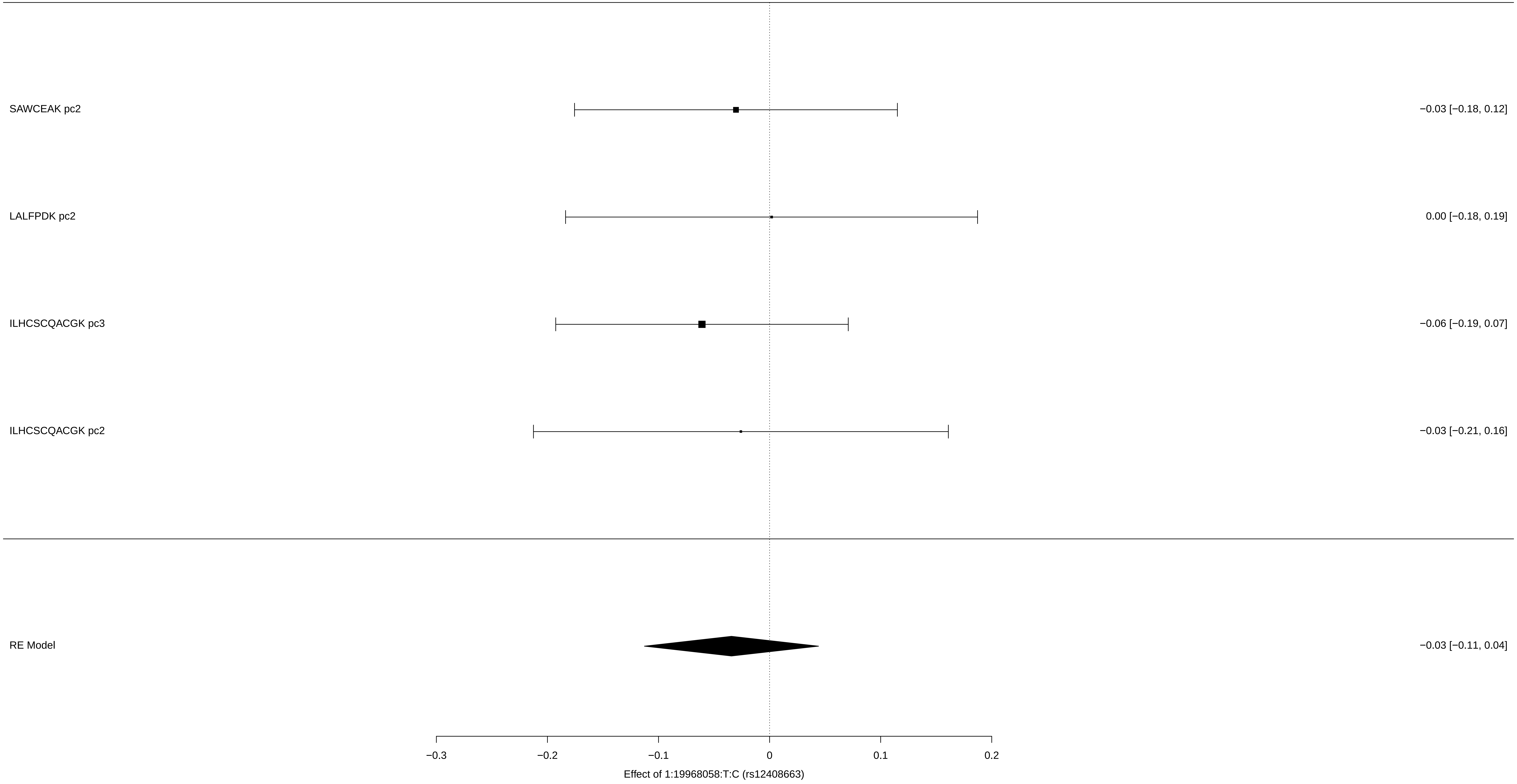

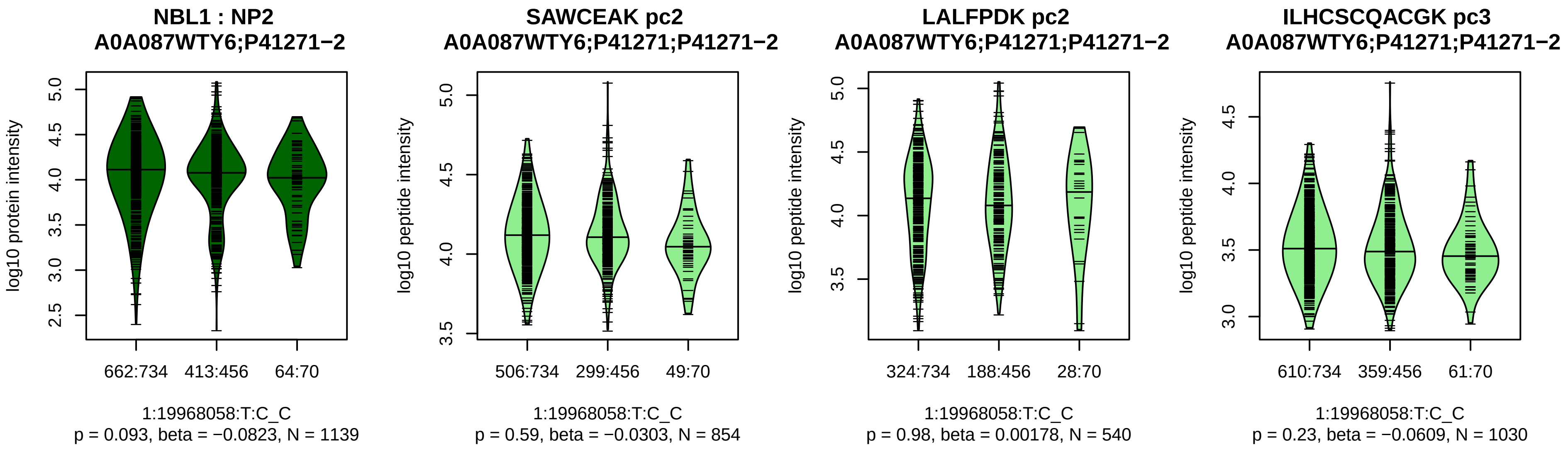

Assay Target: NBL1
UniProt: P41271
rsID: rs12408663
Target SNP: 1:19968058:T:C
Proxy SNP: 1:19968058:T:C
UKBPPP log10(p): 55.9
UKBPPP BETA: −0.114
UKBPPP MSPA: 0
−:−:−:−
854:540:1030:517

323. ANXA5 (P08758)
UKBPPP MSPA=0.883

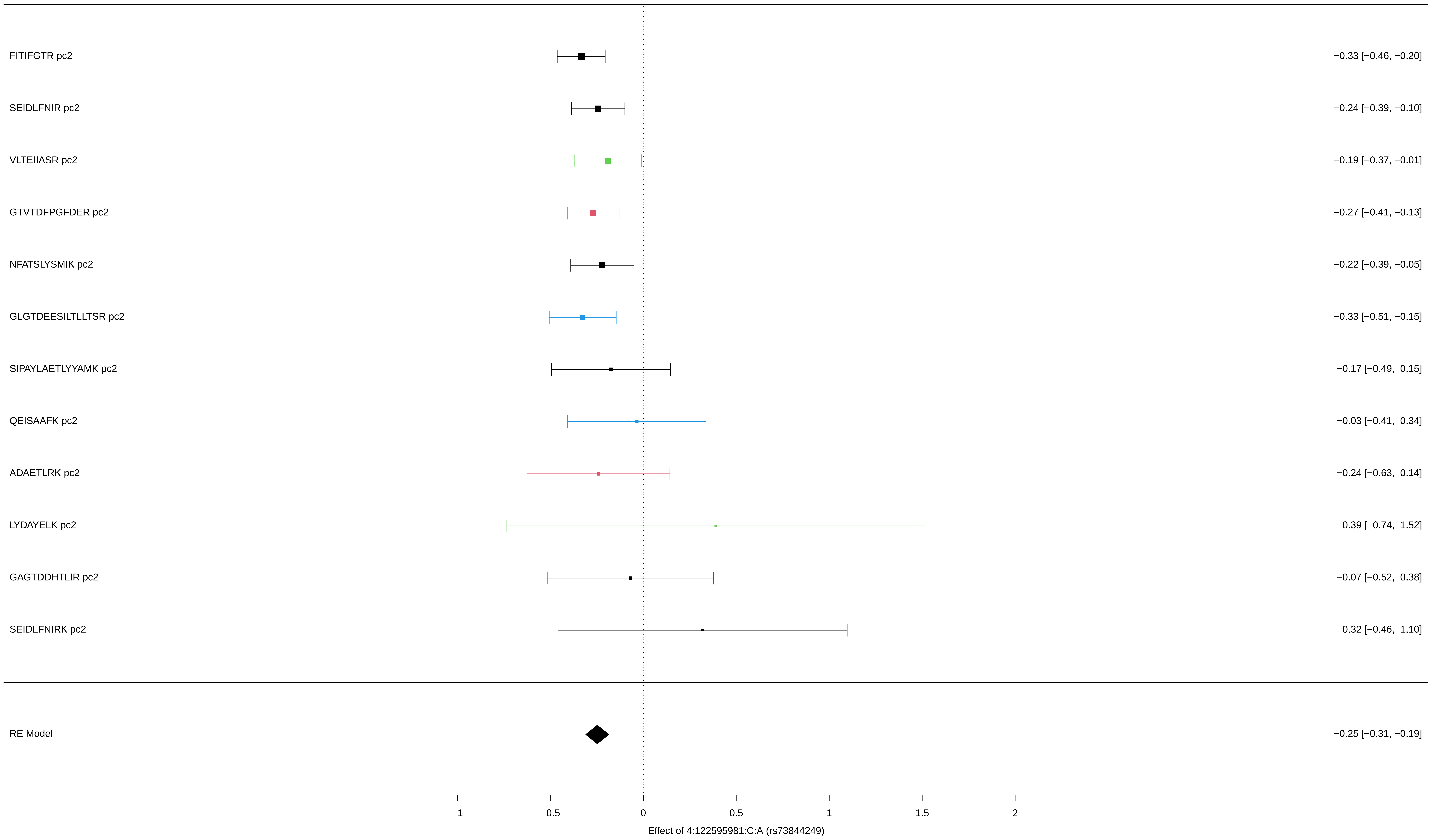

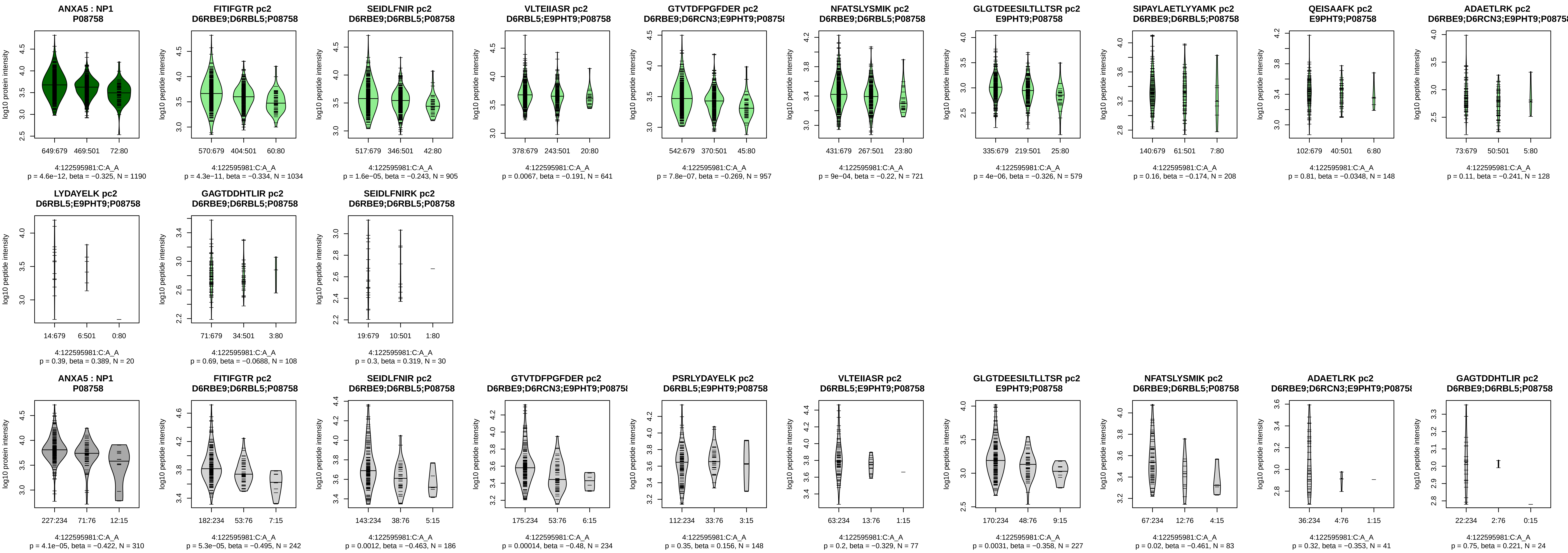

Assay Target: ANXA5
UniProt: P08758
rsID: rs73844249
Target SNP: 4:122595981:C:A
Proxy SNP: 4:122595981:C:A
UKBPPP log10(p): 55.5
UKBPPP BETA: −0.11
UKBPPP MSPA: 0.883
::*:*:*:*:–:–:–:–:–:–:NA
1034:905:641:957:721:579:208:

324. CDH2 (P19022)
UKBPPP MSPA=0

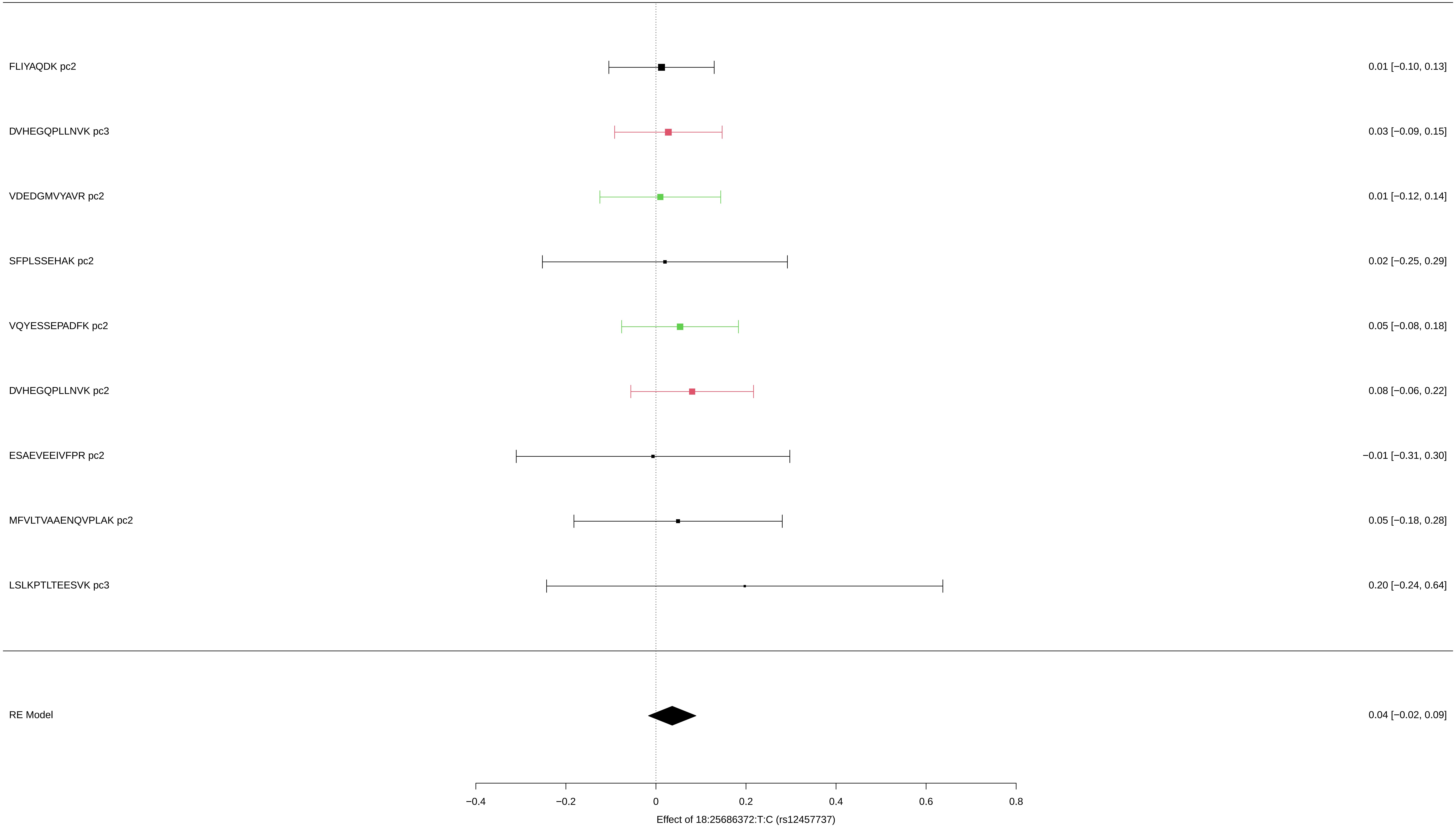

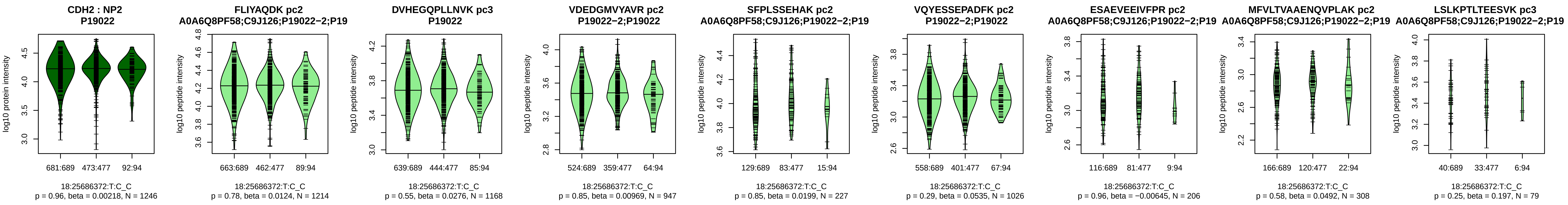

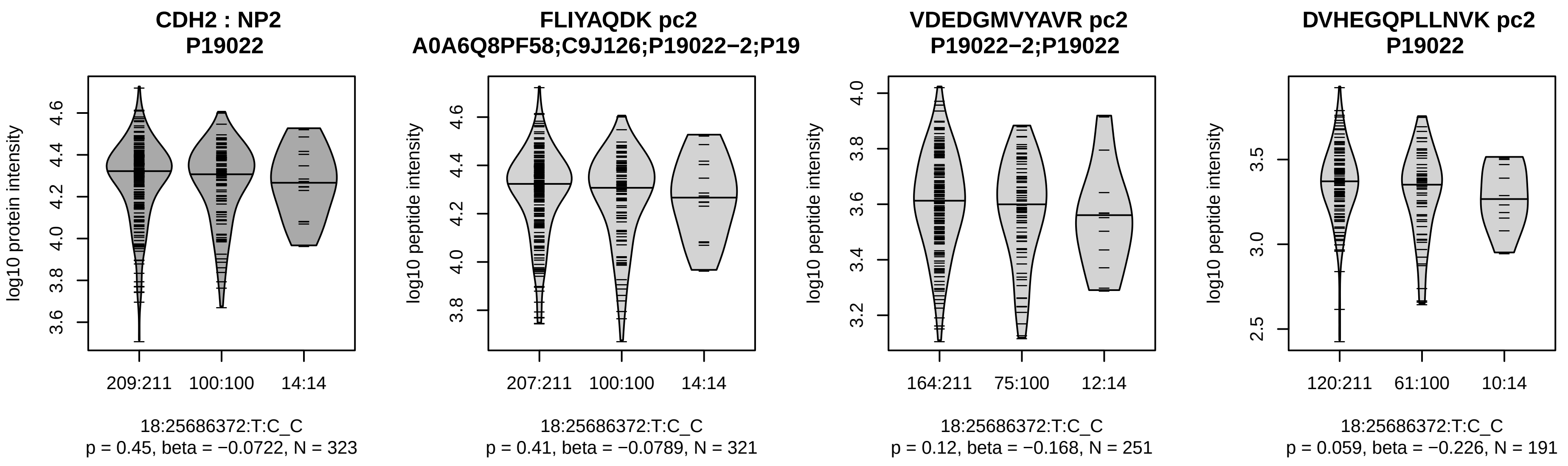

Assay Target: CDH2
UniProt: P19022
rsID: rs12457737
Target SNP: 18:25686372:T:C
Proxy SNP: 18:25686372:T:C
UKBPPP log10(p): 55.1
UKBPPP BETA: −0.096
UKBPPP MSPA: 0
−:−:−:−:−:−:−:−
1214:1168:947:227:1026:940:20

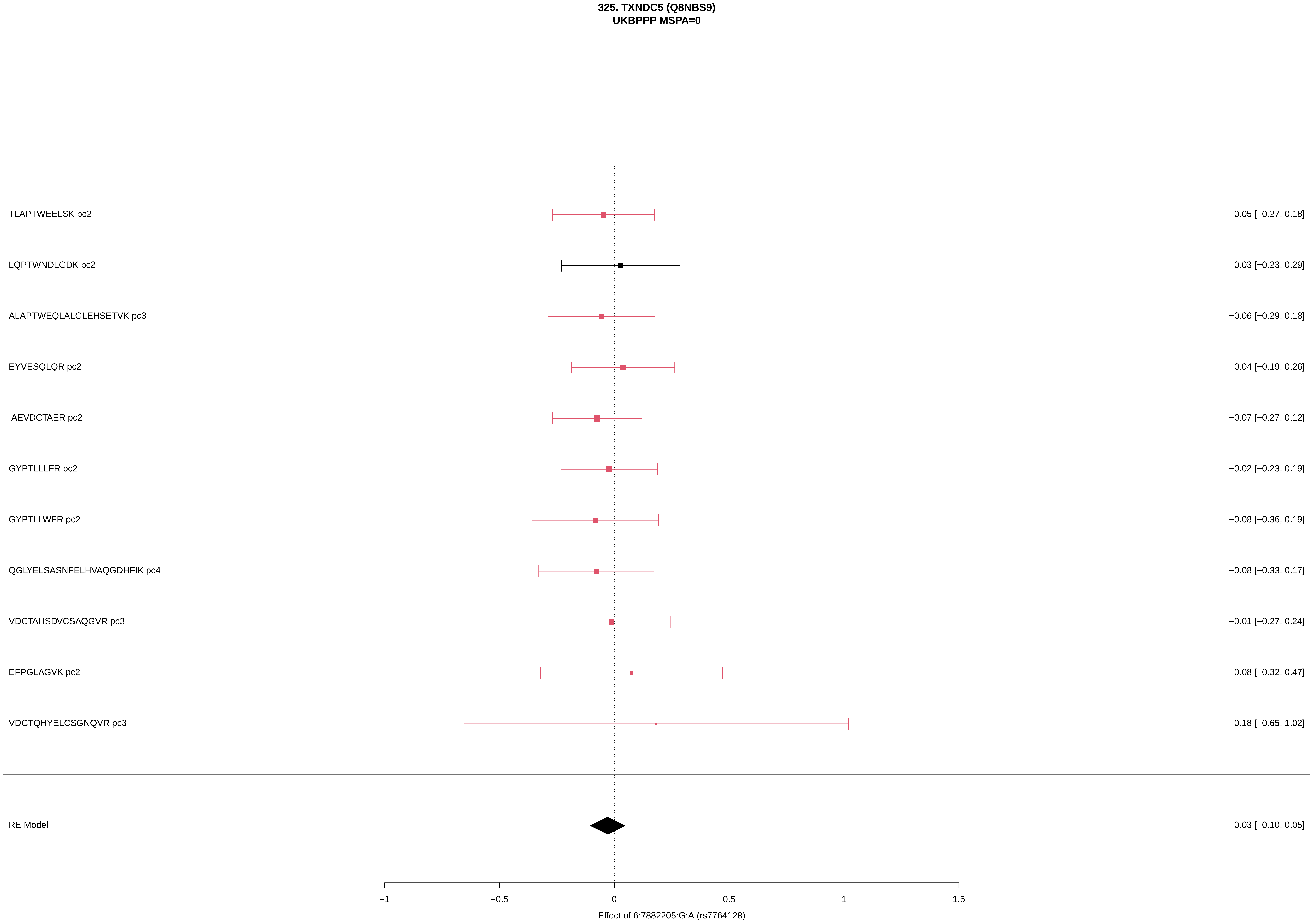

325. TXNDC5 (Q8NBS9)
UKBPPP MSPA=0
TLAPTWEELSK pc2
−0.05 [−0.27, 0.18]
LQPTWNDLGDK pc2
0.03 [−0.23, 0.29]
ALAPTWEQLALGLEHSETVK pc3
−0.06 [−0.29, 0.18]
EYVESQLQR pc2
0.04 [−0.19, 0.26]
IAEVDCTAER pc2
−0.07 [−0.27, 0.12]
GYPTLLLFR pc2
−0.02 [−0.23, 0.19]
GYPTLLWFR pc2
−0.08 [−0.36, 0.19]
QGLYELSASNFELHVAQGDHFIK pc4
−0.08 [−0.33, 0.17]
VDCTAHSDVCSAQGVR pc3
−0.01 [−0.27, 0.24]
EFPGLAGVK pc2
0.08 [−0.32, 0.47]
VDCTQHYELCSGNQVR pc3
0.18 [−0.65, 1.02]
RE Model
−0.03 [−0.10, 0.05]
−1
−0.5
0
0.5
1
1.5
Effect of 6:7882205:G:A (rs7764128)

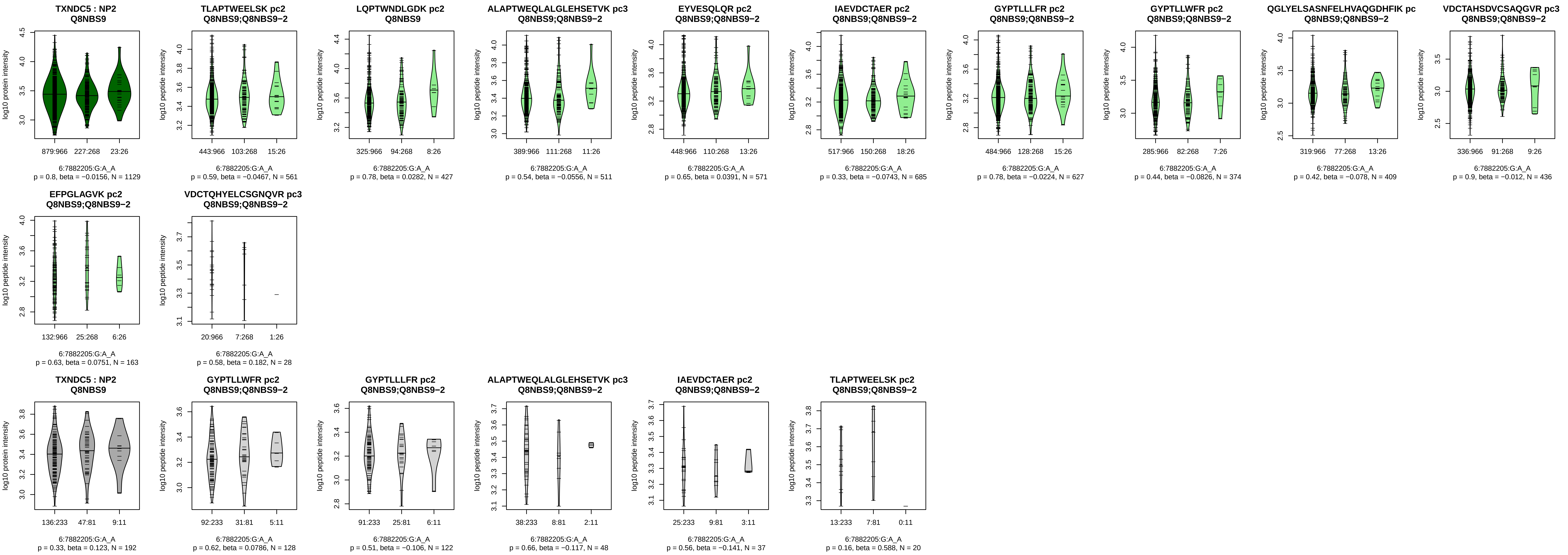

Assay Target: TXNDC5
UniProt: Q8NBS9
rsID: rs7764128
Target SNP: 6:7882205:G:A
Proxy SNP: 6:7882205:G:A
UKBPPP log10(p): 53.8
UKBPPP BETA: −0.148
UKBPPP MSPA: 0
−:−:−:−:−:−:−:−:−:−:−:NA:NA
561:427:511:571:685:627:374:4

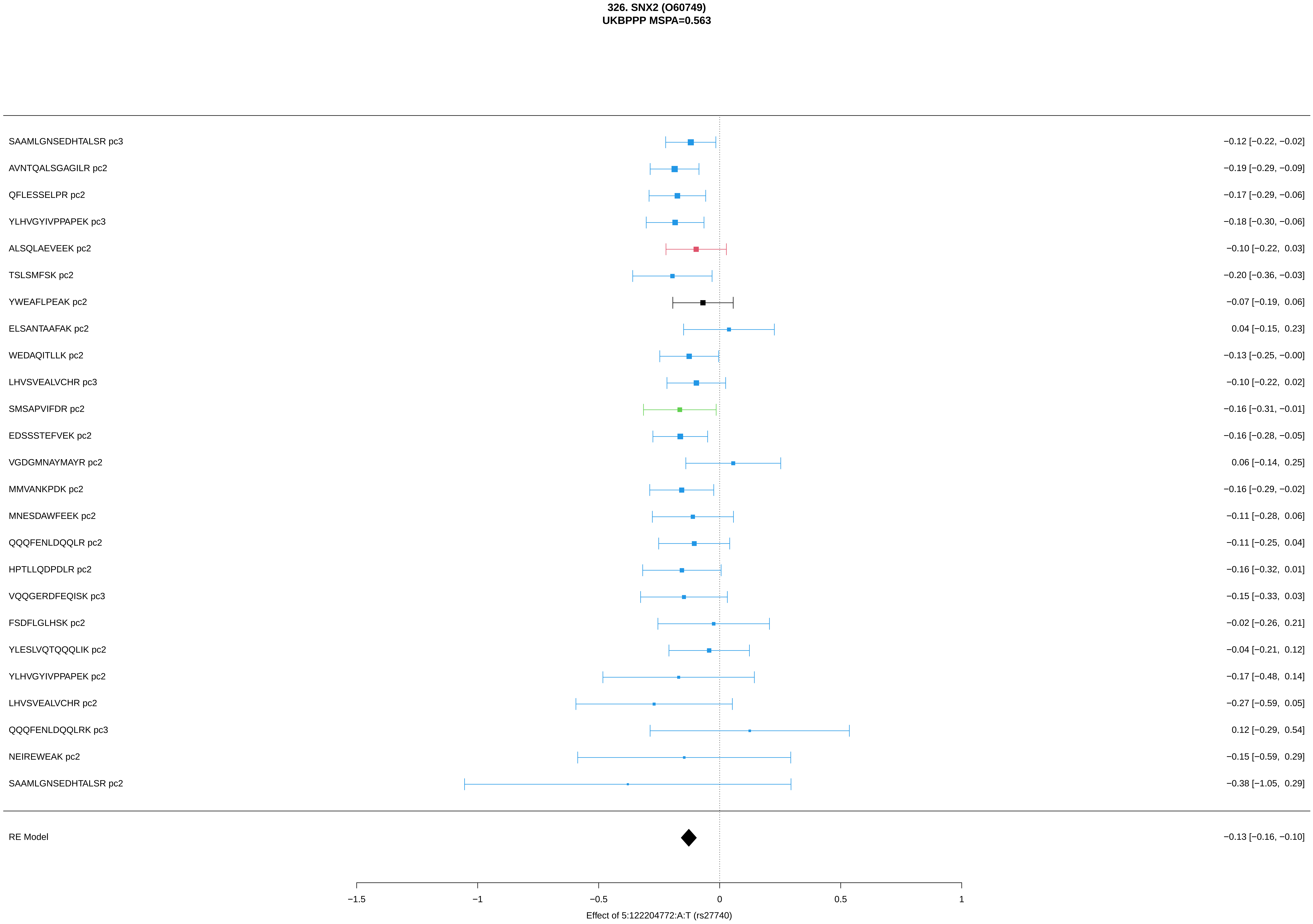
326. SNX2 (O60749)
UKBPPP MSPA=0.563
SAAMLGNSEDHTALSR pc3 −0.12 [−0.22, −0.02]
AVNTQALSGAGILR pc2 −0.19 [−0.29, −0.09]
QFLESSELPR pc2 −0.17 [−0.29, −0.06]
YLHVGYIVPPAPEK pc3 −0.18 [−0.30, −0.06]
ALSQLAEVEEK pc2 −0.10 [−0.22, 0.03]
TSLSMFSK pc2 −0.20 [−0.36, −0.03]
YWEAFLPEAK pc2 −0.07 [−0.19, 0.06]
ELSANTAAFAK pc2 0.04 [−0.15, 0.23]
WEDAQITLLK pc2 −0.13 [−0.25, −0.00]
LHVSVEALVCHR pc3 −0.10 [−0.22, 0.02]
SMSAPVIFDR pc2 −0.16 [−0.31, −0.01]
EDSSSTEFVEK pc2 −0.16 [−0.28, −0.05]
VGDGMNAYMAYR pc2 0.06 [−0.14, 0.25]
MMVANKPDK pc2 −0.16 [−0.29, −0.02]
MNESDAWFEEK pc2 −0.11 [−0.28, 0.06]
QQQFENLDQQLR pc2 −0.11 [−0.25, 0.04]
HPTLLQDPDLR pc2 −0.16 [−0.32, 0.01]
VQQGERDFEQISK pc3 −0.15 [−0.33, 0.03]
FSDFLGLHSK pc2 −0.02 [−0.26, 0.21]
YLESLVQTQQQLIK pc2 −0.04 [−0.21, 0.12]
YLHVGYIVPPAPEK pc2 −0.17 [−0.48, 0.14]
LHVSVEALVCHR pc2 −0.27 [−0.59, 0.05]
QQQFENLDQQLRK pc3 0.12 [−0.29, 0.54]
NEIREWEAK pc2 −0.15 [−0.59, 0.29]
SAAMLGNSEDHTALSR pc2 −0.38 [−1.05, 0.29]
RE Model −0.13 [−0.16, −0.10]
−1.5
−1
−0.5
0
0.5
1
Effect of 5:122204772:A:T (rs27740)

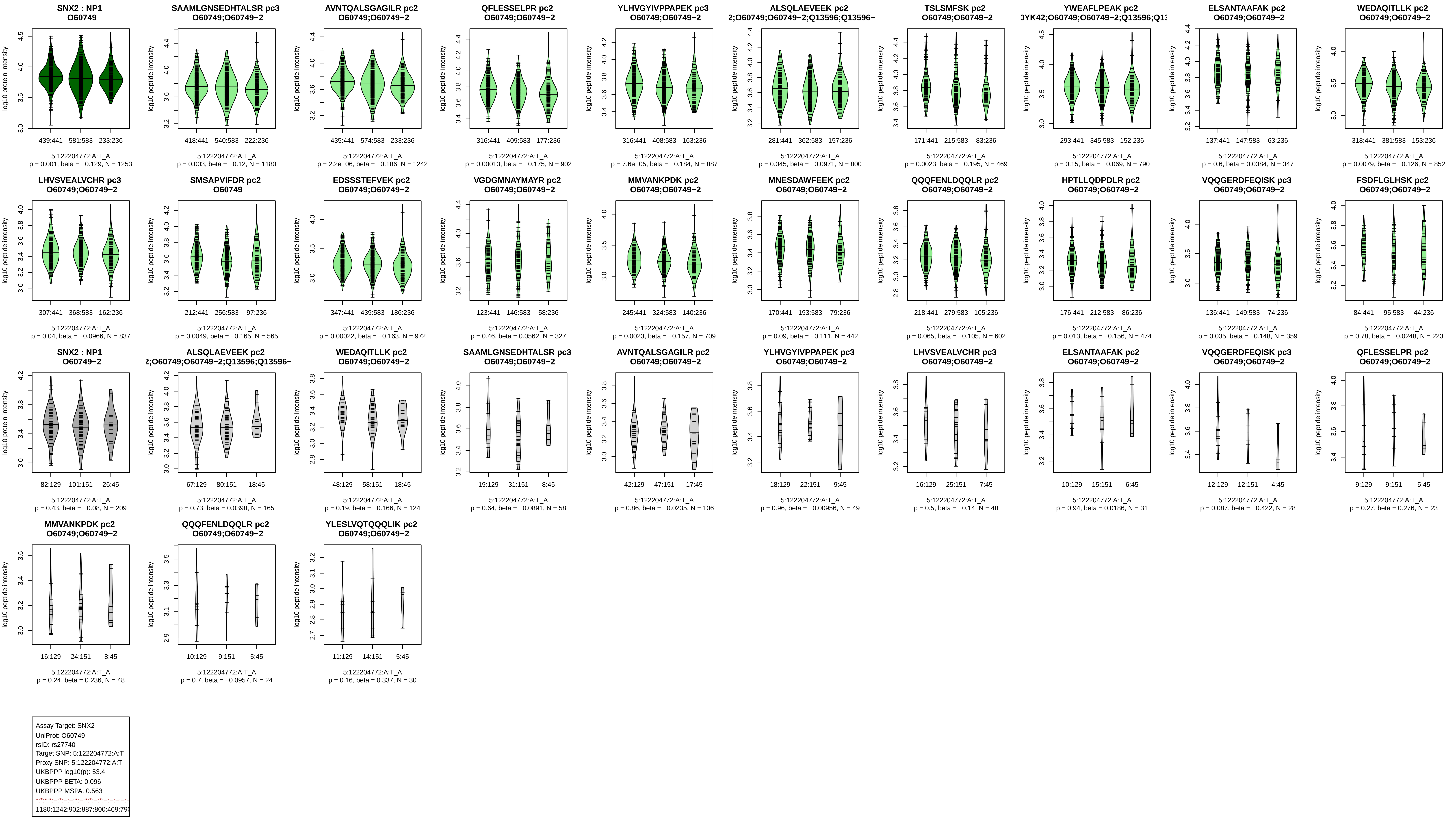
SNX2 : NP1
O60749
log10 protein intensity
439:441 581:583 233:236
5:122204772:A:T_A
p = 0.001, beta = −0.129, N = 1253
SAAMLGNSEDHTALSR pc3
O60749;O60749−2
log10 peptide intensity
418:441 540:583 222:236
5:122204772:A:T_A
p = 0.003, beta = −0.12, N = 1180
AVNTQALSGAGILR pc2
O60749;O60749−2
435:441 574:583 233:236
5:122204772:A:T_A
p = 2.2e−06, beta = −0.186, N = 1242
QFLESSELPR pc2
O60749;O60749−2
316:441 409:583 177:236
5:122204772:A:T_A
p = 0.00013, beta = −0.175, N = 902
YLHVGYIVPPAPEK pc3
O60749;O60749−2
316:441 408:583 163:236
5:122204772:A:T_A
p = 7.6e−05, beta = −0.184, N = 887
ALSQLAEVEEK pc2
2;O60749;O60749−2;Q13596;Q13596−
281:441 362:583 157:236
5:122204772:A:T_A
p = 0.045, beta = −0.0971, N = 800
TSLSMFSK pc2
O60749;O60749−2
171:441 215:583 83:236
5:122204772:A:T_A
p = 0.0023, beta = −0.195, N = 469
YWEAFLPEAK pc2
0YK42;O60749;O60749−2;Q13596;Q13
293:441 345:583 152:236
5:122204772:A:T_A
p = 0.15, beta = −0.069, N = 790
ELSANTAAFAK pc2
O60749;O60749−2
137:441 147:583 63:236
5:122204772:A:T_A
p = 0.6, beta = 0.0384, N = 347
WEDAQITLLK pc2
O60749;O60749−2
318:441 381:583 153:236
5:122204772:A:T_A
p = 0.0079, beta = −0.126, N = 852
LHVSVEALVCHR pc3
O60749;O60749−2
307:441 368:583 162:236
5:122204772:A:T_A
p = 0.04, beta = −0.0966, N = 837
SMSAPVIFDR pc2
O60749
212:441 256:583 97:236
5:122204772:A:T_A
p = 0.0049, beta = −0.165, N = 565
EDSSSTEFVEK pc2
O60749;O60749−2
347:441 439:583 186:236
5:122204772:A:T_A
p = 0.00022, beta = −0.163, N = 972
VGDGMNAYMAYR pc2
O60749;O60749−2
123:441 146:583 58:236
5:122204772:A:T_A
p = 0.46, beta = 0.0562, N = 327
MMVANKPDK pc2
O60749;O60749−2
245:441 324:583 140:236
5:122204772:A:T_A
p = 0.0023, beta = −0.157, N = 709
MNESDAWFEEK pc2
O60749;O60749−2
170:441 193:583 79:236
5:122204772:A:T_A
p = 0.09, beta = −0.111, N = 442
QQQFENLDQQLR pc2
O60749;O60749−2
218:441 279:583 105:236
5:122204772:A:T_A
p = 0.065, beta = −0.105, N = 602
HPTLLQDPDLR pc2
O60749;O60749−2
176:441 212:583 86:236
5:122204772:A:T_A
p = 0.013, beta = −0.156, N = 474
VQQGERDFEQISK pc3
O60749;O60749−2
136:441 149:583 74:236
5:122204772:A:T_A
p = 0.035, beta = −0.148, N = 359
FSDFLGLHSK pc2
O60749;O60749−2
84:441 95:583 44:236
5:122204772:A:T_A
p = 0.78, beta = −0.0248, N = 223
SNX2 : NP1
O60749−2
82:129 101:151 26:45
5:122204772:A:T_A
p = 0.43, beta = −0.08, N = 209
ALSQLAEVEEK pc2
2;O60749;O60749−2;Q13596;Q13596−
67:129 80:151 18:45
5:122204772:A:T_A
p = 0.73, beta = 0.0398, N = 165
WEDAQITLLK pc2
O60749;O60749−2
48:129 58:151 18:45
5:122204772:A:T_A
p = 0.19, beta = −0.166, N = 124
SAAMLGNSEDHTALSR pc3
O60749;O60749−2
19:129 31:151 8:45
5:122204772:A:T_A
p = 0.64, beta = −0.0891, N = 58
AVNTQALSGAGILR pc2
O60749;O60749−2
42:129 47:151 17:45
5:122204772:A:T_A
p = 0.86, beta = −0.0235, N = 106
YLHVGYIVPPAPEK pc3
O60749;O60749−2
18:129 22:151 9:45
5:122204772:A:T_A
p = 0.96, beta = −0.00956, N = 49
LHVSVEALVCHR pc3
O60749;O60749−2
16:129 25:151 7:45
5:122204772:A:T_A
p = 0.5, beta = −0.14, N = 48
ELSANTAAFAK pc2
O60749;O60749−2
10:129 15:151 6:45
5:122204772:A:T_A
p = 0.94, beta = 0.0186, N = 31
VQQGERDFEQISK pc3
O60749;O60749−2
12:129 12:151 4:45
5:122204772:A:T_A
p = 0.087, beta = −0.422, N = 28
QFLESSELPR pc2
O60749;O60749−2
9:129 9:151 5:45
5:122204772:A:T_A
p = 0.27, beta = 0.276, N = 23
MMVANKPDK pc2
O60749;O60749−2
16:129 24:151 8:45
5:122204772:A:T_A
p = 0.24, beta = 0.236, N = 48
QQQFENLDQQLR pc2
O60749;O60749−2
10:129 9:151 5:45
5:122204772:A:T_A
p = 0.7, beta = −0.0957, N = 24
YLESLVQTQQQLIK pc2
O60749;O60749−2
11:129 14:151 5:45
5:122204772:A:T_A
p = 0.16, beta = 0.337, N = 30
Assay Target: SNX2
UniProt: O60749
rsID: rs27740
Target SNP: 5:122204772:A:T
Proxy SNP: 5:122204772:A:T
UKBPPP log10(p): 53.4
UKBPPP BETA: 0.096
UKBPPP MSPA: 0.563
1180:1242:902:887:800:469:79(

327. ADAMTSL2 (Q86TH1)
UKBPPP MSPA=0

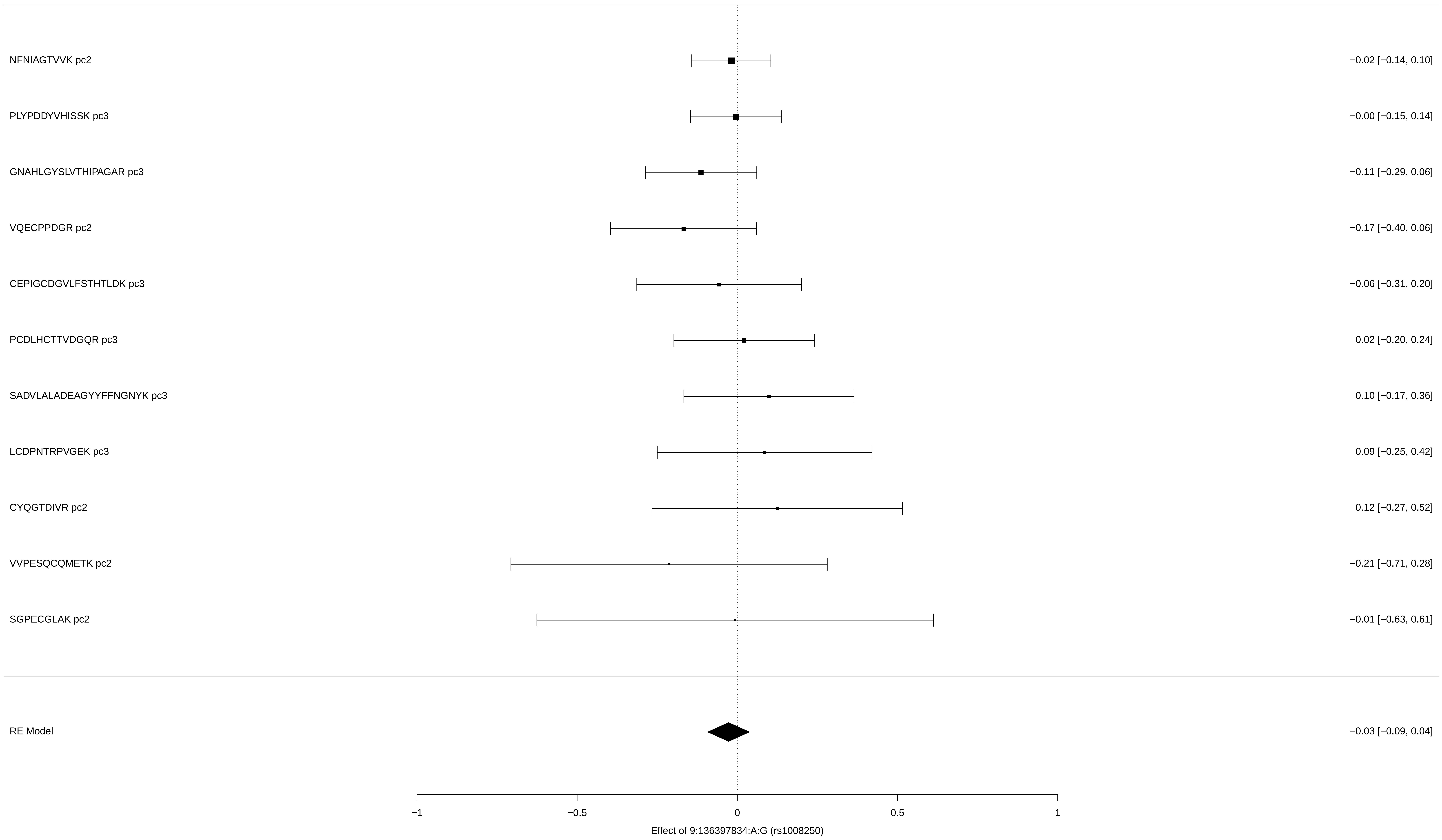

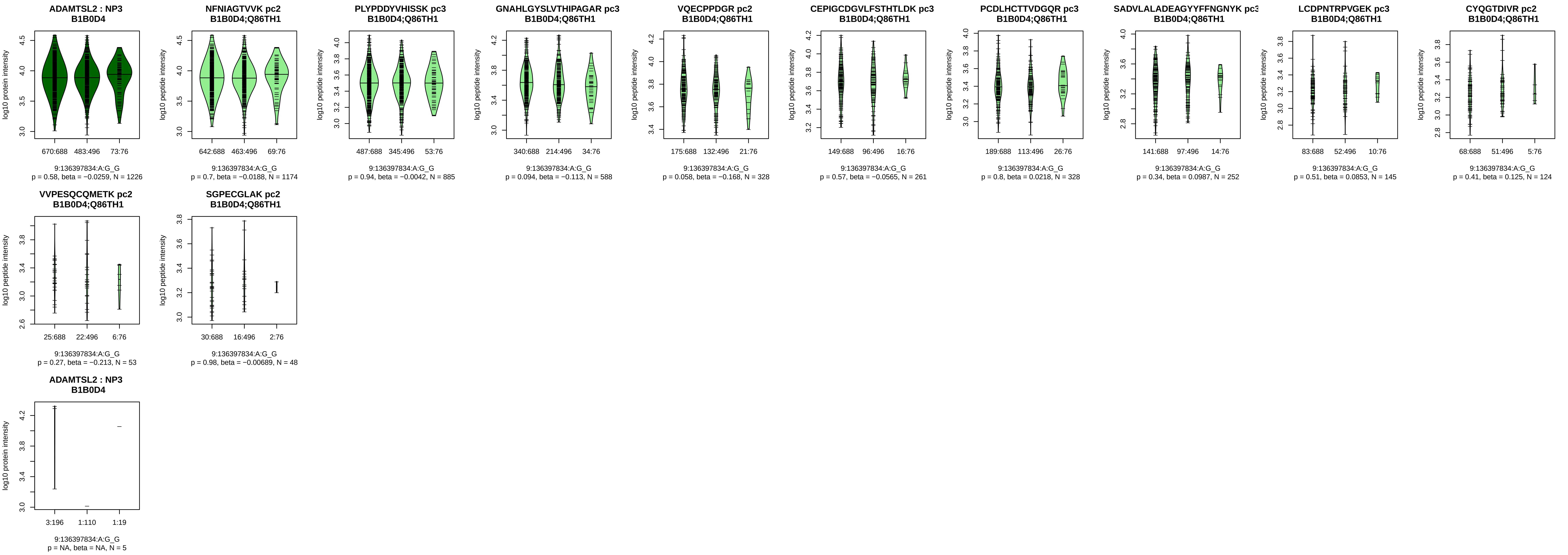

Assay Target: ADAMTSL2
UniProt: Q86TH1
rsID: rs1008250
Target SNP: 9:136397834:A:G
Proxy SNP: 9:136397834:A:G
UKBPPP log10(p): 53
UKBPPP BETA: 0.107
UKBPPP MSPA: 0
−:−:−:−:−:−:−:−:−:NA:−:NA:NA
1174:885:588:328:261:328:252:

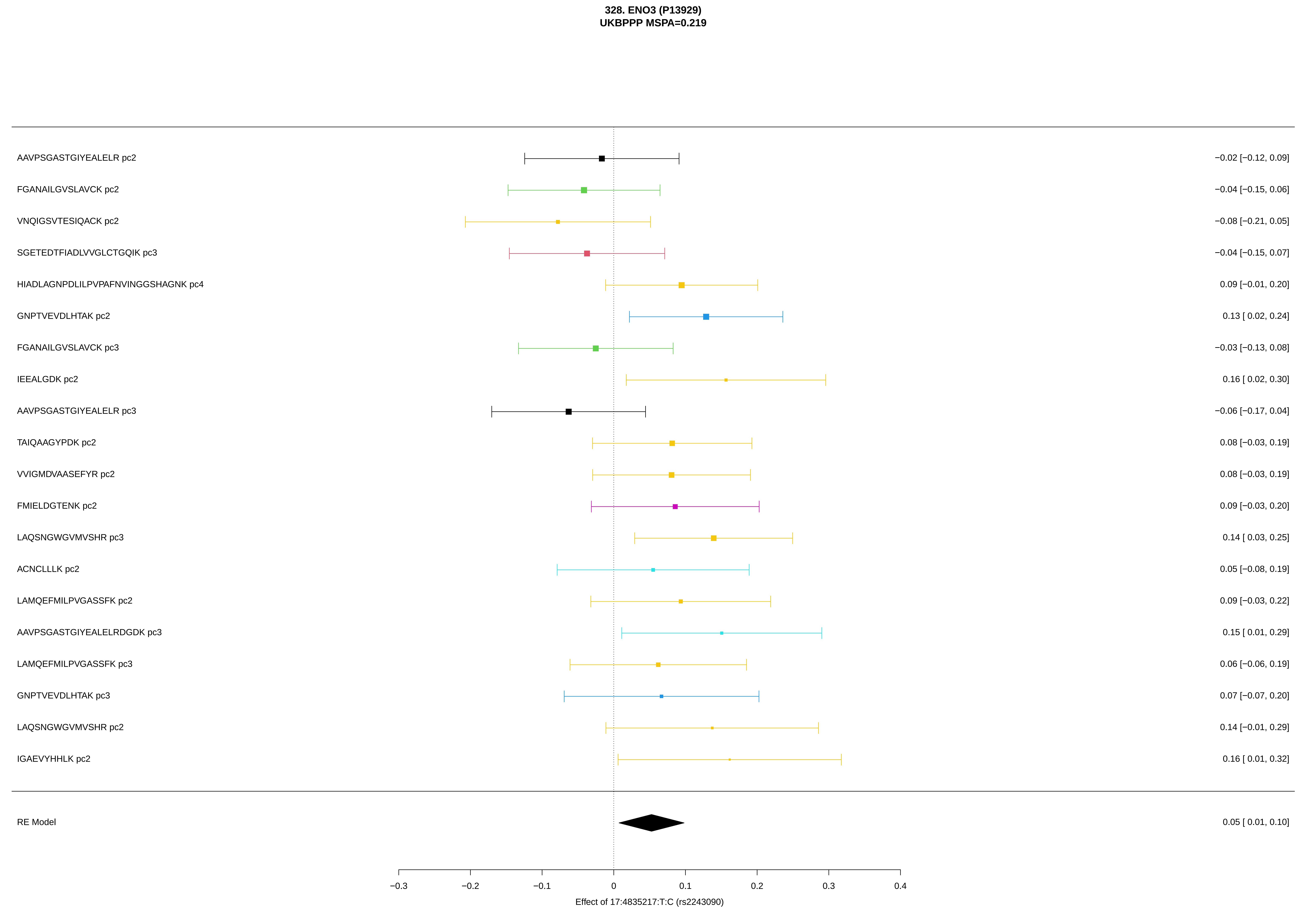
328. ENO3 (P13929)
UKBPPP MSPA=0.219
AAVPSGASTGIYEALELR pc2
FGANAILGVSLAVCK pc2
VNQIGSVTESIQACK pc2
SGETEDTFIADLVVGLCTGQIK pc3
HIADLAGNPDLILPVPAFNVINGGSHAGNK pc4
GNPTVEVDLHTAK pc2
FGANAILGVSLAVCK pc3
IEEALGDK pc2
AAVPSGASTGIYEALELR pc3
TAIQAAGYPDK pc2
VVIGMDVAASEFYR pc2
FMIELDGTENK pc2
LAQSNGWGVMVSHR pc3
ACNCLLLK pc2
LAMQEFMILPVGASSFK pc2
AAVPSGASTGIYEALELRDGDK pc3
LAMQEFMILPVGASSFK pc3
GNPTVEVDLHTAK pc3
LAQSNGWGVMVSHR pc2
IGAEVYHHLK pc2
RE Model
−0.02 [−0.12, 0.09]
−0.04 [−0.15, 0.06]
−0.08 [−0.21, 0.05]
−0.04 [−0.15, 0.07]
0.09 [−0.01, 0.20]
0.13 [0.02, 0.24]
−0.03 [−0.13, 0.08]
0.16 [0.02, 0.30]
−0.06 [−0.17, 0.04]
0.08 [−0.03, 0.19]
0.08 [−0.03, 0.19]
0.09 [−0.03, 0.20]
0.14 [0.03, 0.25]
0.05 [−0.08, 0.19]
0.09 [−0.03, 0.22]
0.15 [0.01, 0.29]
0.06 [−0.06, 0.19]
0.07 [−0.07, 0.20]
0.14 [−0.01, 0.29]
0.16 [0.01, 0.32]
0.05 [0.01, 0.10]
−0.3
−0.2
−0.1
0
0.1
0.2
0.3
0.4
Effect of 17:4835217:T:C (rs2243090)

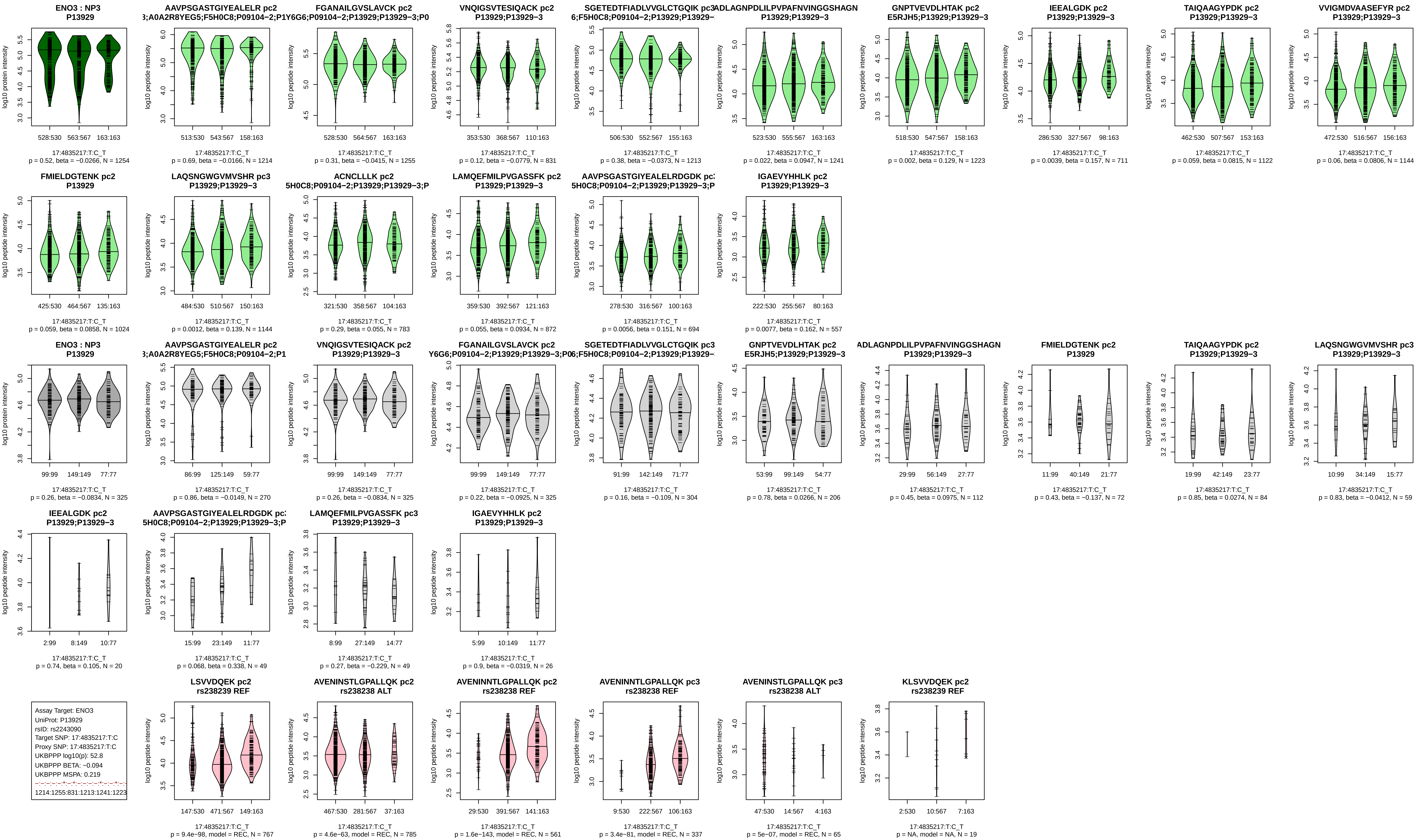
ENO3 : NP3
P13929
528:530 563:567 163:163
17:4835217:T:C_T
p = 0.52, beta = −0.0266, N = 1254
AAVPSGASTGIYEALELR pc2
3;A0A2R8YEG5;F5H0C8;P09104−2;P1Y6G6;P09104−2;P13929;P13929−3;P0
513:530 543:567 158:163
17:4835217:T:C_T
p = 0.69, beta = −0.0166, N = 1214
FGANAILGVSLAVCK pc2
528:530 564:567 163:163
17:4835217:T:C_T
p = 0.31, beta = −0.0415, N = 1255
VNQIGSVTESIQACK pc2
P13929;P13929−3
353:530 368:567 110:163
17:4835217:T:C_T
p = 0.12, beta = −0.0779, N = 831
SGETEDTFIADLVVGLCTGQIK pc3
6;F5H0C8;P09104−2;P13929;P13929−
506:530 552:567 155:163
17:4835217:T:C_T
p = 0.38, beta = −0.0373, N = 1213
ADLAGNPDLILPVPAFNVINGGSHAGN
P13929;P13929−3
523:530 555:567 163:163
17:4835217:T:C_T
p = 0.022, beta = 0.0947, N = 1241
GNPTVEVDLHTAK pc2
E5RJH5;P13929;P13929−3
518:530 547:567 158:163
17:4835217:T:C_T
p = 0.002, beta = 0.129, N = 1223
IEEALGDK pc2
P13929;P13929−3
286:530 327:567 98:163
17:4835217:T:C_T
p = 0.0039, beta = 0.157, N = 711
TAIQAAGYPDK pc2
P13929;P13929−3
462:530 507:567 153:163
17:4835217:T:C_T
p = 0.059, beta = 0.0815, N = 1122
VVIGMDVAASEFYR pc2
P13929;P13929−3
472:530 516:567 156:163
17:4835217:T:C_T
p = 0.06, beta = 0.0806, N = 1144
FMIELDGTENK pc2
P13929
425:530 464:567 135:163
17:4835217:T:C_T
p = 0.059, beta = 0.0858, N = 1024
LAQSNGWGVMVSHR pc3
P13929;P13929−3
484:530 510:567 150:163
17:4835217:T:C_T
p = 0.0012, beta = 0.139, N = 1144
ACNCLLLK pc2
5H0C8;P09104−2;P13929;P13929−3;P
321:530 358:567 104:163
17:4835217:T:C_T
p = 0.29, beta = 0.055, N = 783
LAMQEFMILPVGASSFK pc2
P13929;P13929−3
359:530 392:567 121:163
17:4835217:T:C_T
p = 0.055, beta = 0.0934, N = 872
AAVPSGASTGIYEALELRDGDK pc3
5H0C8;P09104−2;P13929;P13929−3;P
278:530 316:567 100:163
17:4835217:T:C_T
p = 0.0056, beta = 0.151, N = 694
IGAEVYHHLK pc2
P13929;P13929−3
222:530 255:567 80:163
17:4835217:T:C_T
p = 0.0077, beta = 0.162, N = 557
ENO3 : NP3
P13929
99:99 149:149 77:77
17:4835217:T:C_T
p = 0.26, beta = −0.0834, N = 325
AAVPSGASTGIYEALELR pc2
3;A0A2R8YEG5;F5H0C8;P09104−2;P1
86:99 125:149 59:77
17:4835217:T:C_T
p = 0.86, beta = −0.0149, N = 270
VNQIGSVTESIQACK pc2
P13929;P13929−3
99:99 149:149 77:77
17:4835217:T:C_T
p = 0.26, beta = −0.0834, N = 325
FGANAILGVSLAVCK pc2
Y6G6;P09104−2;P13929;P13929−3;P0
99:99 149:149 77:77
17:4835217:T:C_T
p = 0.22, beta = −0.0925, N = 325
SGETEDTFIADLVVGLCTGQIK pc3
6;F5H0C8;P09104−2;P13929;P13929−
91:99 142:149 71:77
17:4835217:T:C_T
p = 0.16, beta = −0.109, N = 304
GNPTVEVDLHTAK pc2
E5RJH5;P13929;P13929−3
53:99 99:149 54:77
17:4835217:T:C_T
p = 0.78, beta = 0.0266, N = 206
ADLAGNPDLILPVPAFNVINGGSHAGN
P13929;P13929−3
29:99 56:149 27:77
17:4835217:T:C_T
p = 0.45, beta = 0.0975, N = 112
FMIELDGTENK pc2
P13929
11:99 40:149 21:77
17:4835217:T:C_T
p = 0.43, beta = −0.137, N = 72
TAIQAAGYPDK pc2
P13929;P13929−3
19:99 42:149 23:77
17:4835217:T:C_T
p = 0.85, beta = 0.0274, N = 84
LAQSNGWGVMVSHR pc3
P13929;P13929−3
10:99 34:149 15:77
17:4835217:T:C_T
p = 0.83, beta = −0.0412, N = 59
IEEALGDK pc2
P13929;P13929−3
2:99 8:149 10:77
17:4835217:T:C_T
p = 0.74, beta = 0.105, N = 20
AAVPSGASTGIYEALELRDGDK pc3
5H0C8;P09104−2;P13929;P13929−3;P
15:99 23:149 11:77
17:4835217:T:C_T
p = 0.068, beta = 0.338, N = 49
LAMQEFMILPVGASSFK pc3
P13929;P13929−3
8:99 27:149 14:77
17:4835217:T:C_T
p = 0.27, beta = −0.229, N = 49
IGAEVYHHLK pc2
P13929;P13929−3
5:99 10:149 11:77
17:4835217:T:C_T
p = 0.9, beta = −0.0319, N = 26
Assay Target: ENO3
UniProt: P13929
rsID: rs2243090
Target SNP: 17:4835217:T:C
Proxy SNP: 17:4835217:T:C
UKBPPP log10(p): 52.8
UKBPPP BETA: −0.094
UKBPPP MSPA: 0.219
1214:1255:831:1213:1241:1223
LSVVDQEK pc2
rs238239 REF
147:530 471:567 149:163
17:4835217:T:C_T
p = 9.4e−98, model = REC, N = 767
AVENINSTLGPALLQK pc2
rs238238 ALT
467:530 281:567 37:163
17:4835217:T:C_T
p = 4.6e−63, model = REC, N = 785
AVENINNTLGPALLQK pc2
rs238238 REF
29:530 391:567 141:163
17:4835217:T:C_T
p = 1.6e−143, model = REC, N = 561
AVENINNTLGPALLQK pc3
rs238238 REF
9:530 222:567 106:163
17:4835217:T:C_T
p = 3.4e−81, model = REC, N = 337
AVENINSTLGPALLQK pc3
rs238238 ALT
47:530 14:567 4:163
17:4835217:T:C_T
p = 5e−07, model = REC, N = 65
KLSVVDQEK pc2
rs238239 REF
2:530 10:567 7:163
17:4835217:T:C_T
p = NA, model = NA, N = 19
log10 protein intensity
log10 peptide intensity

329. NOTCH2 (Q04721)
UKBPPP MSPA=0

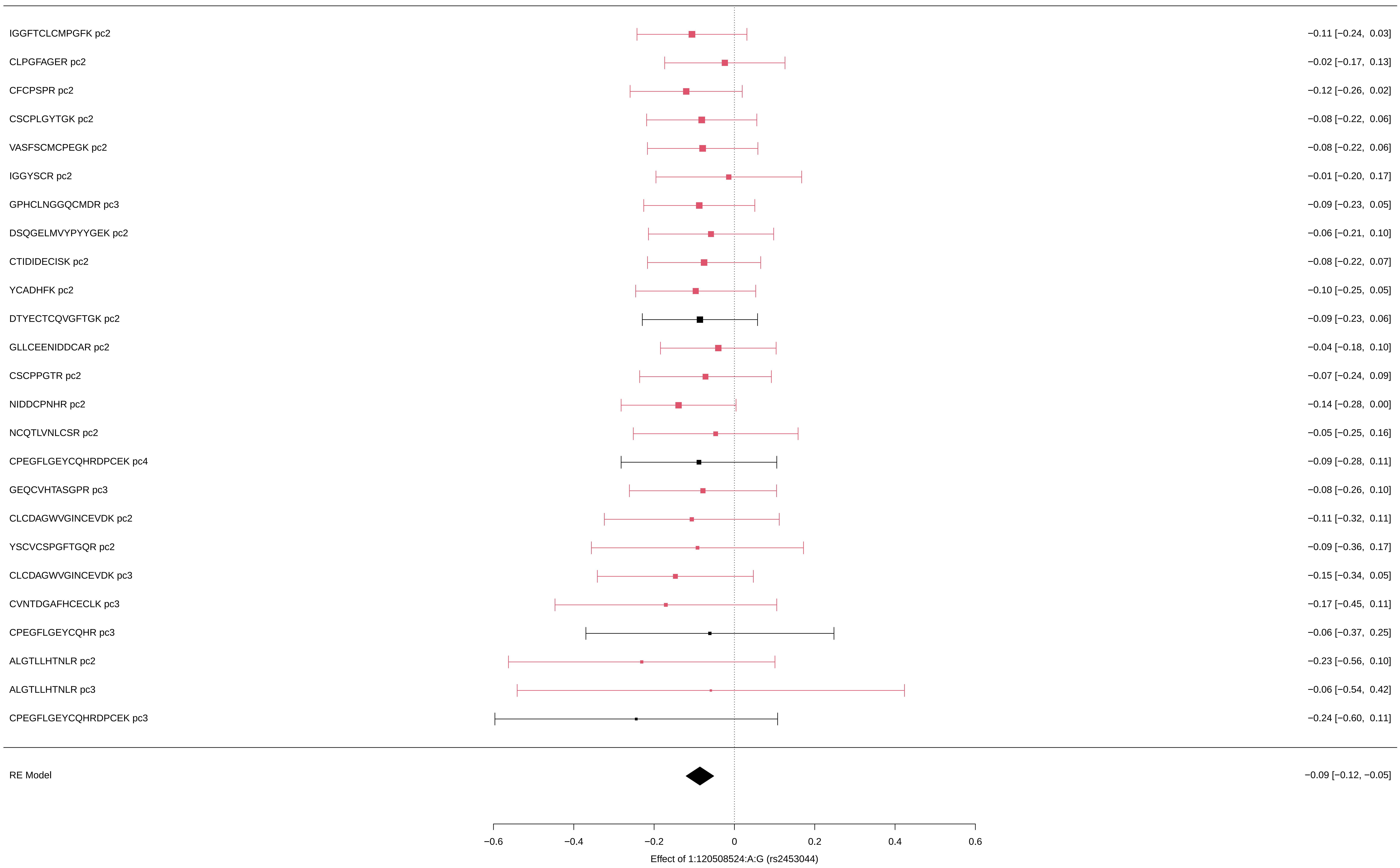

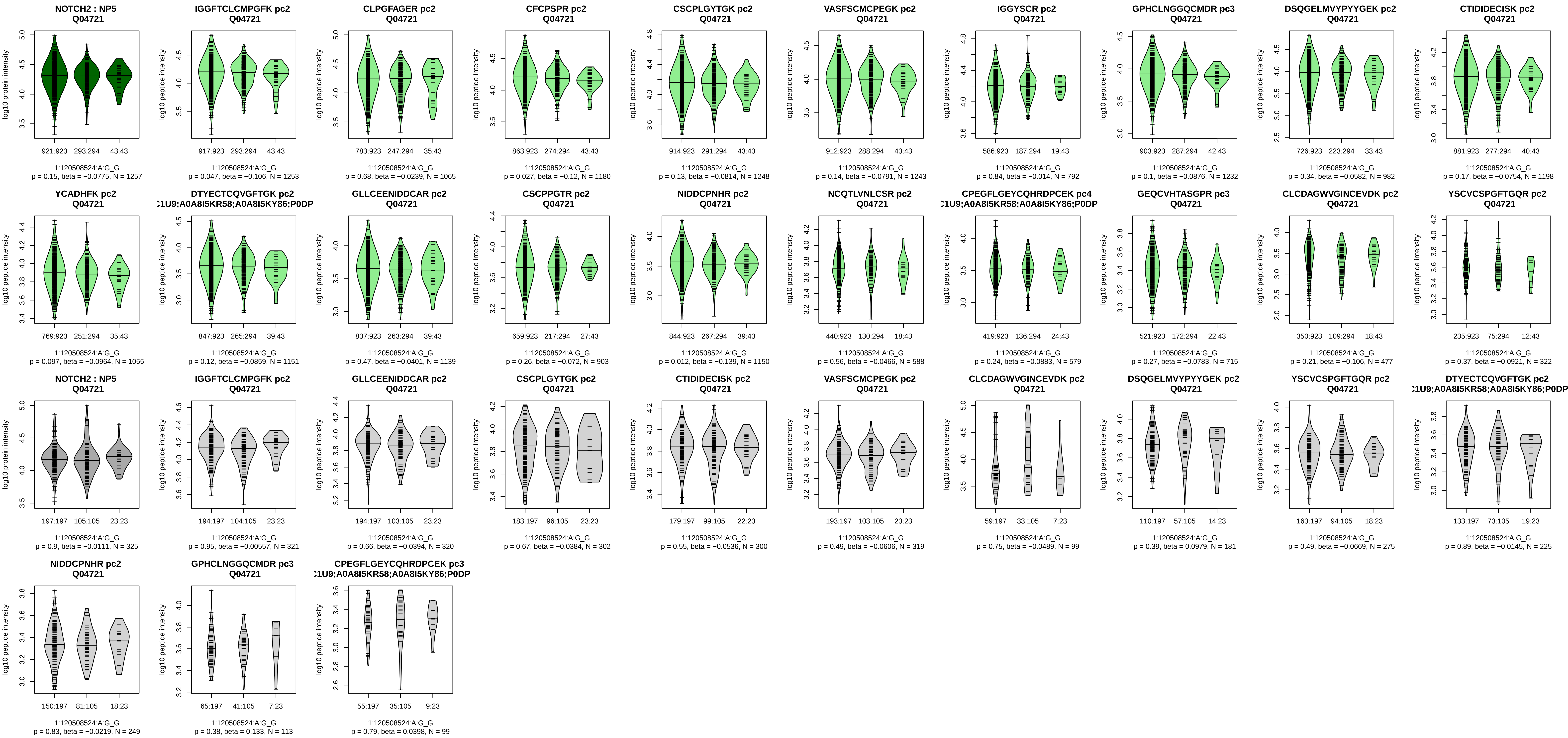

Assay Target: NOTCH2
UniProt: Q04721
rsID: rs2453044
Target SNP: 1:120508524:A:G
Proxy SNP: 1:120508524:A:G
UKBPPP log10(p): 50.4
UKBPPP BETA: −0.131
UKBPPP MSPA: 0

1253:1065:1180:1248:1243:792

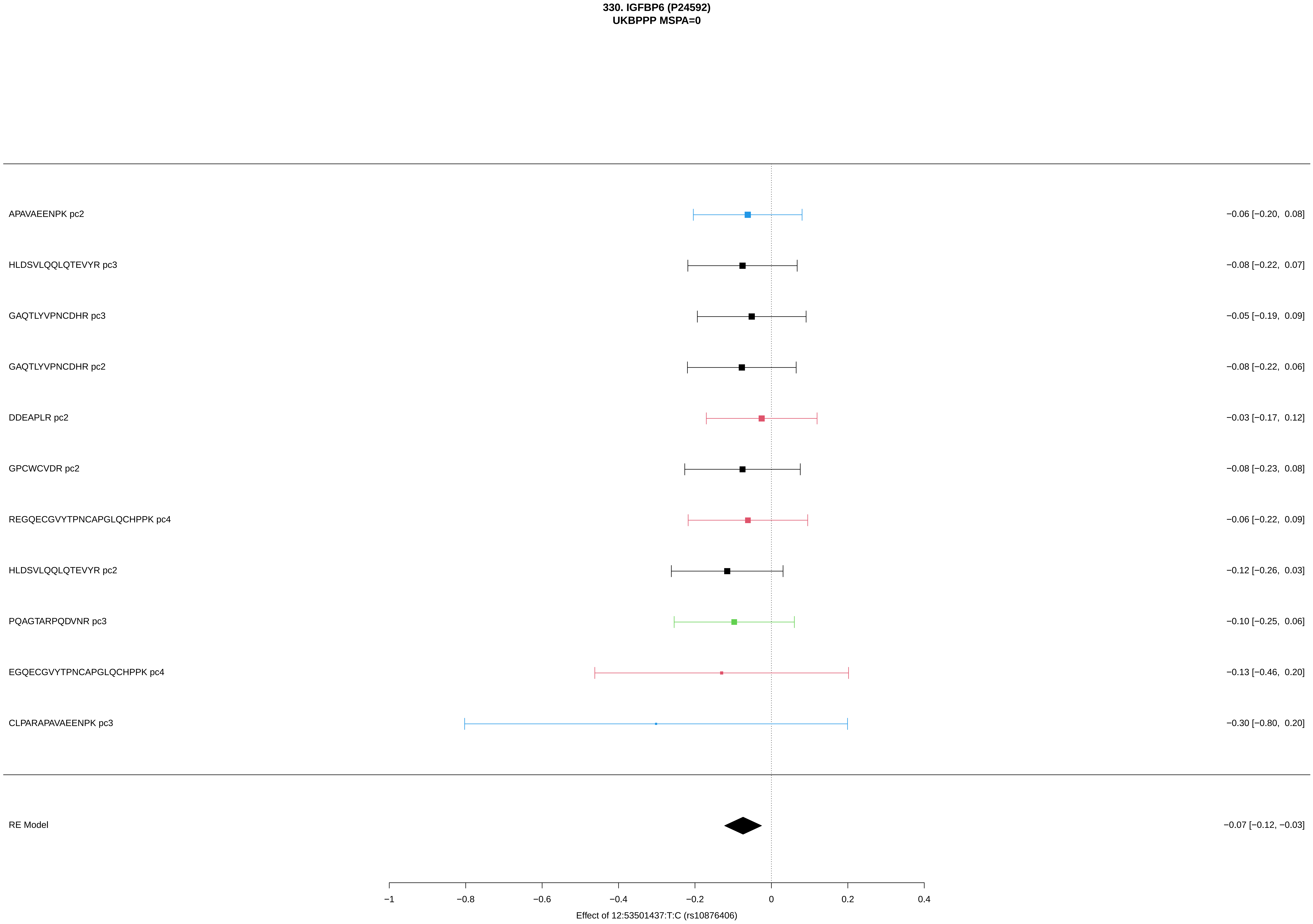
330. IGFBP6 (P24592)
UKBPPP MSPA=0
APAVAEENPK pc2
−0.06 [−0.20, 0.08]
HLDSVLQQLQTEVYR pc3
−0.08 [−0.22, 0.07]
GAQTLYVPNCDHR pc3
−0.05 [−0.19, 0.09]
GAQTLYVPNCDHR pc2
−0.08 [−0.22, 0.06]
DDEAPLR pc2
−0.03 [−0.17, 0.12]
GPCWCVDR pc2
−0.08 [−0.23, 0.08]
REGQECGVYTPNCAPGLQCHPPK pc4
−0.06 [−0.22, 0.09]
HLDSVLQQLQTEVYR pc2
−0.12 [−0.26, 0.03]
PQAGTARPQDVNR pc3
−0.10 [−0.25, 0.06]
EGQECGVYTPNCAPGLQCHPPK pc4
−0.13 [−0.46, 0.20]
CLPARAPAVAEENPK pc3
−0.30 [−0.80, 0.20]
RE Model
−0.07 [−0.12, −0.03]
−1
−0.8
−0.6
−0.4
−0.2
0
0.2
0.4
Effect of 12:53501437:T:C (rs10876406)

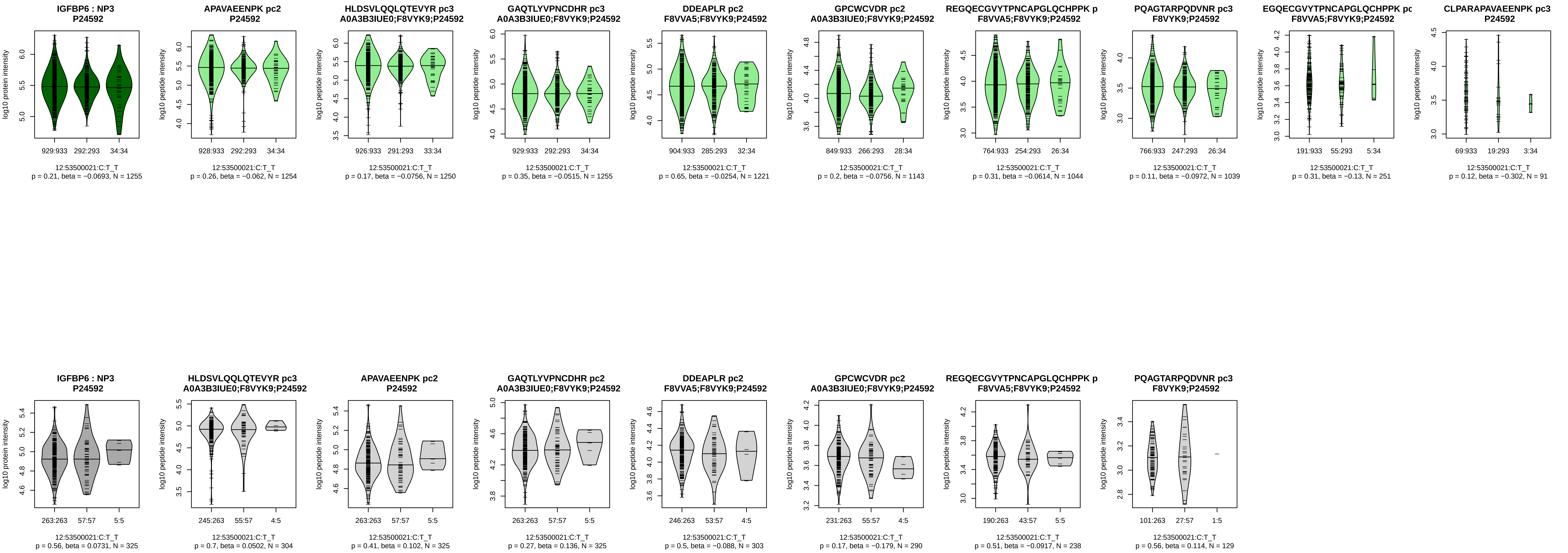

Assay Target: IGFBP6
UniProt: P24592
rsID: rs10876406
Target SNP: 12:53501437:T:C
Proxy SNP: 12:53500021:C:T
UKBPPP log10(p): 50
UKBPPP BETA: −0.131
UKBPPP MSPA: 0
1254:1250:1255:1254:1221:114

331. FABP4 (P15090)
UKBPPP MSPA=0.071

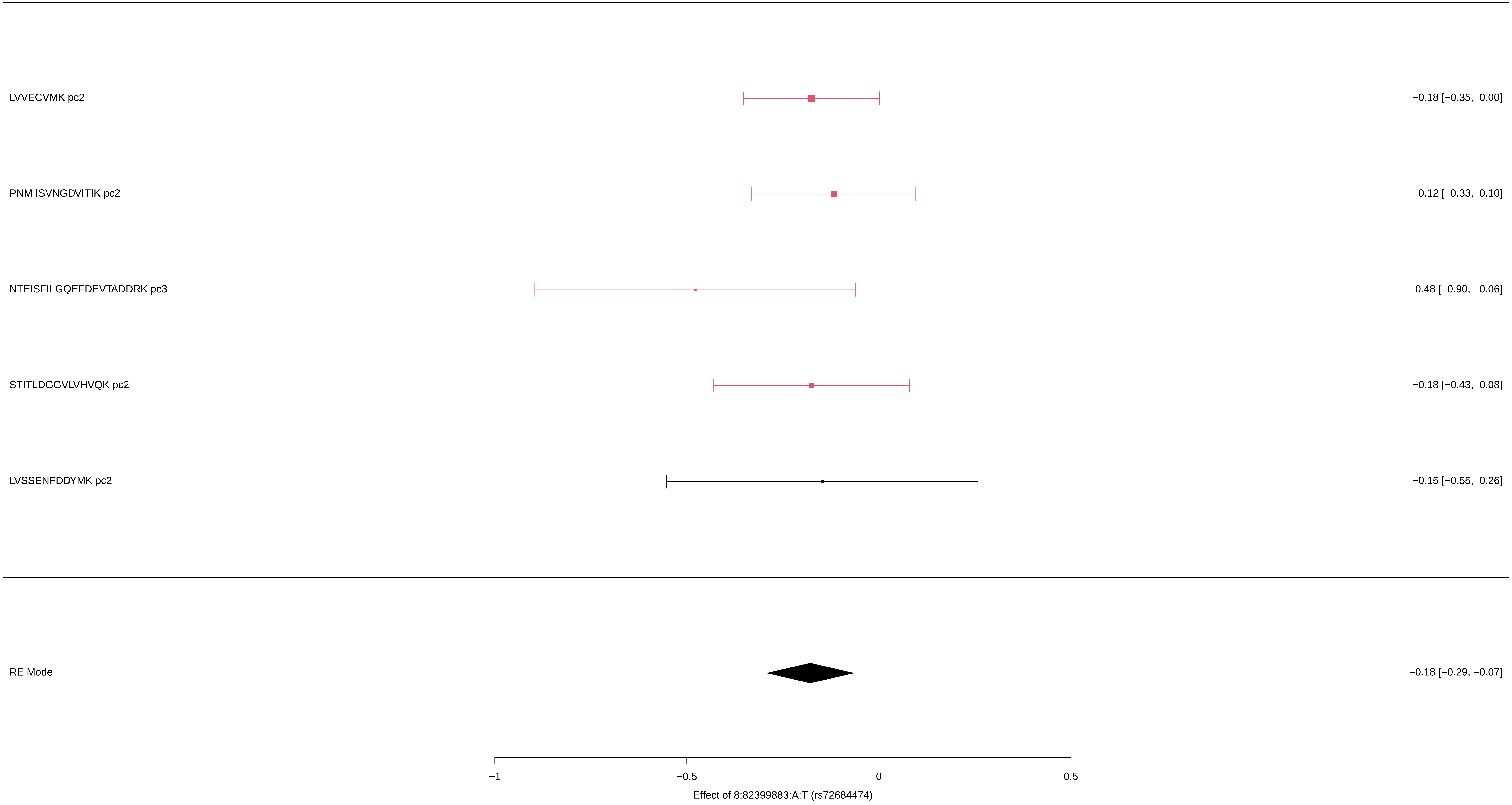

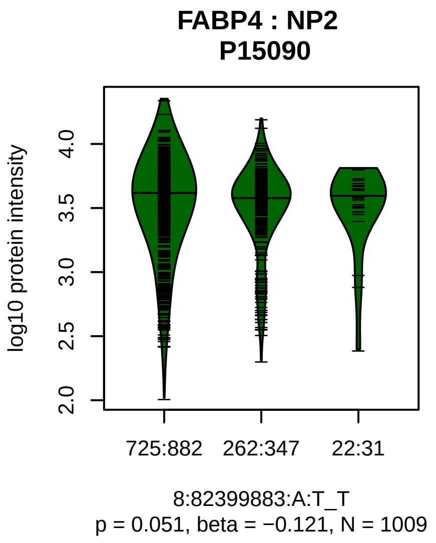

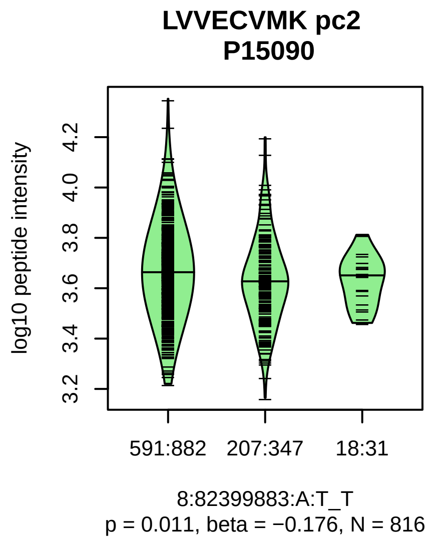

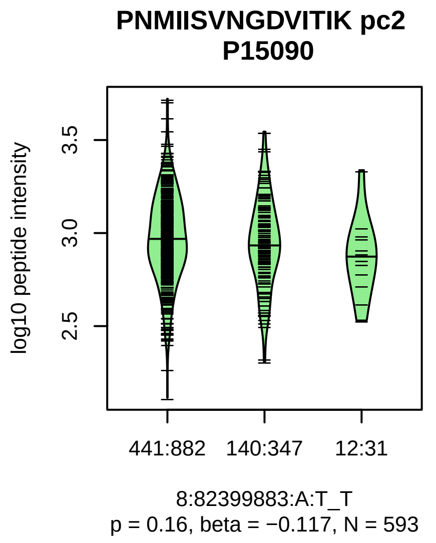

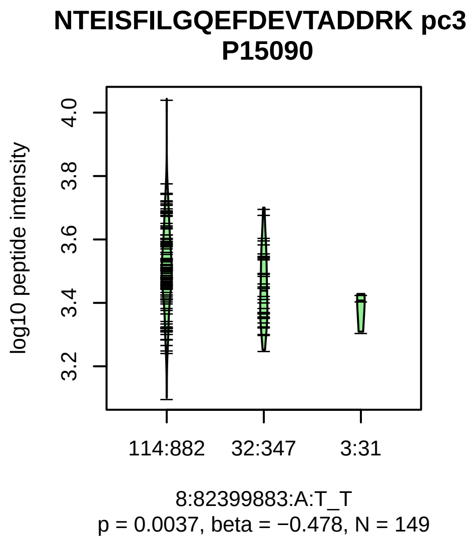

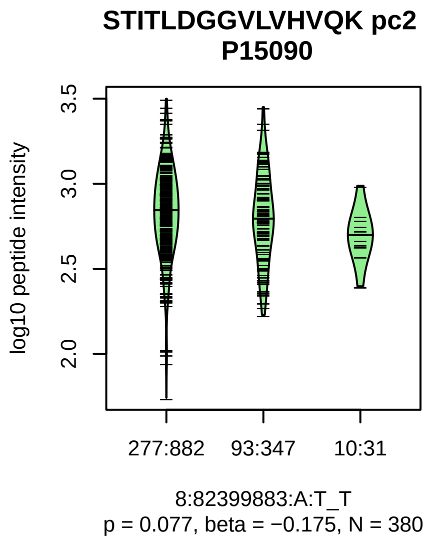

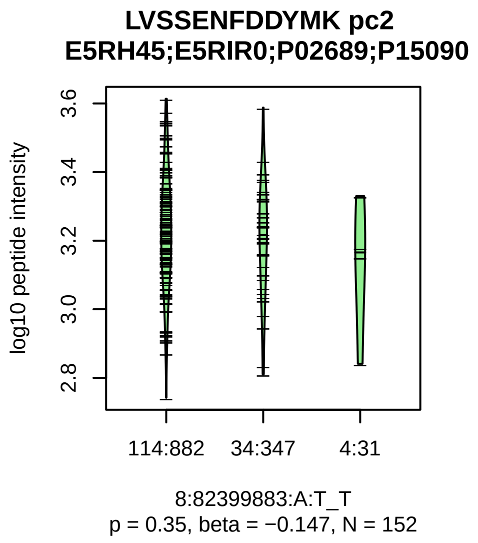

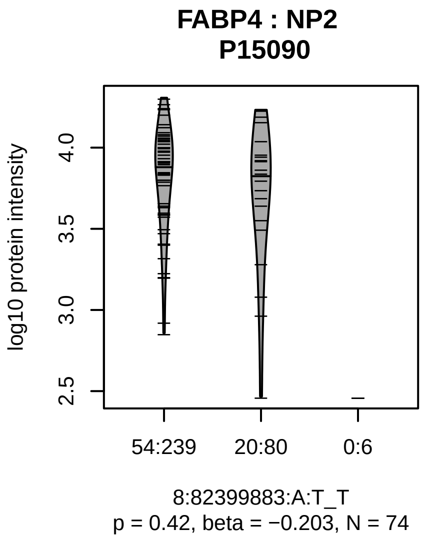

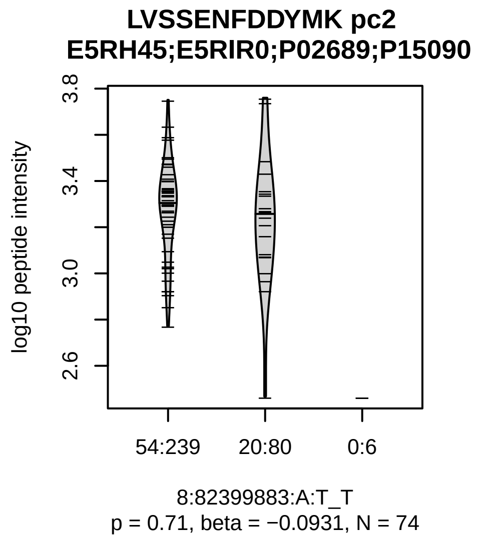

Assay Target: FABP4
UniProt: P15090
rsID: rs72684474
Target SNP: 8:82399883:A:T
Proxy SNP: 8:82399883:A:T
UKBPPP log10(p): 50
UKBPPP BETA: −0.115
UKBPPP MSPA: 0.071
–:–:*:–:–
816:593:149:380:152

332. ACAA1 (P09110)
UKBPPP MSPA=0

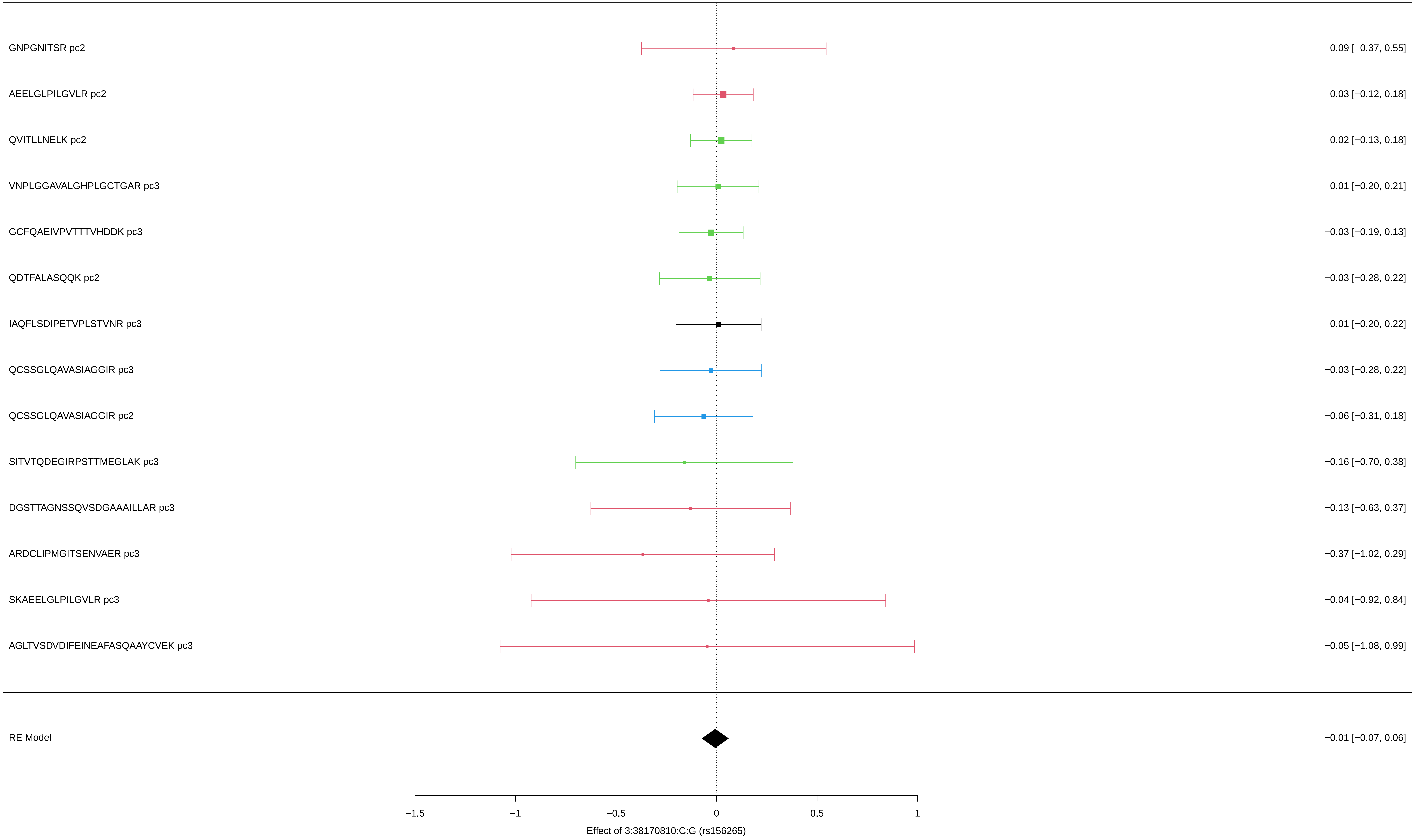

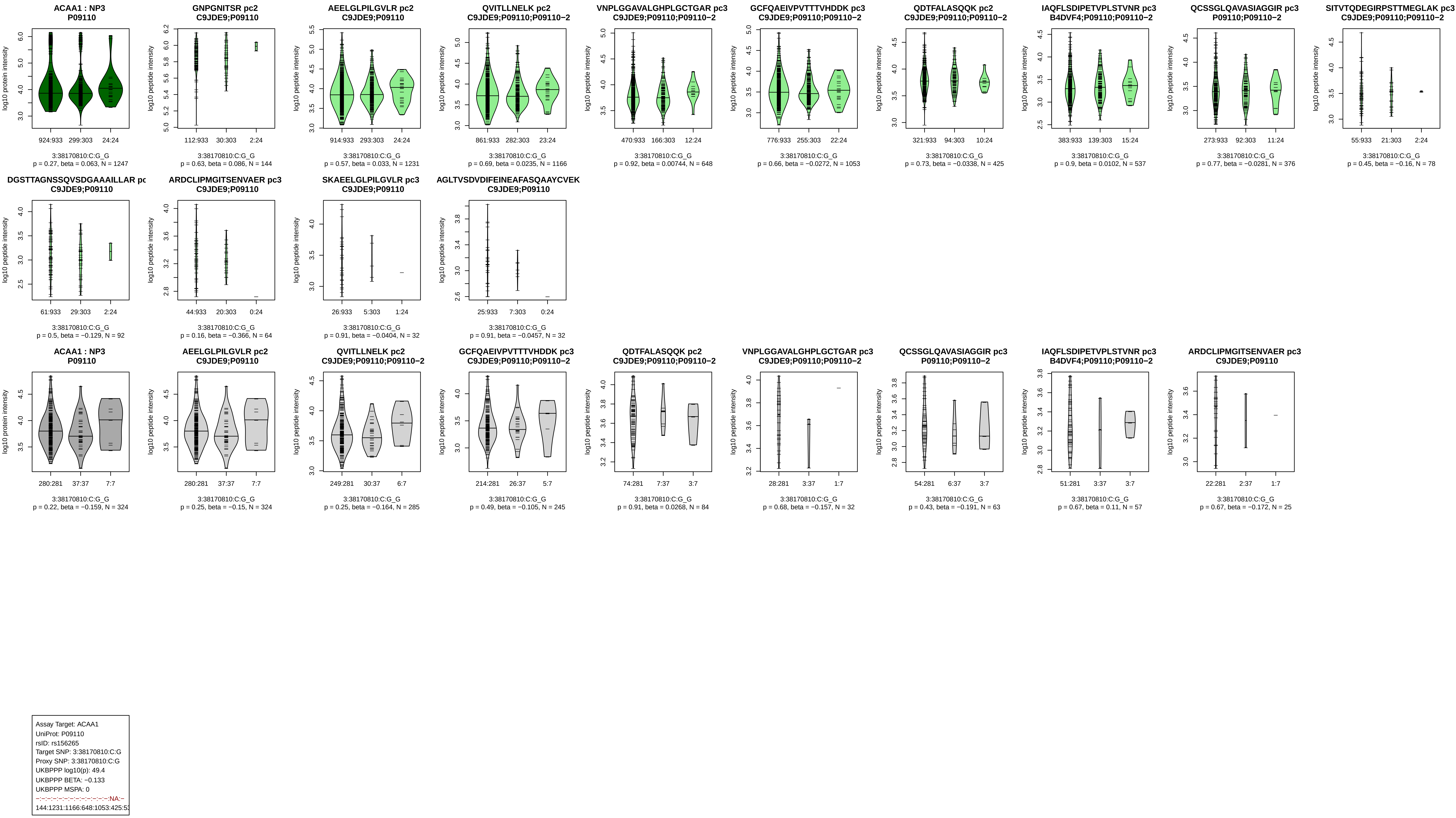
ACAA1 : NP3
P09110
3:38170810:C:G_G
p = 0.27, beta = 0.063, N = 1247
GNPGNITSR pc2
C9JDE9;P09110
p = 0.63, beta = 0.086, N = 144
AEELGLPILGVLR pc2
C9JDE9;P09110
p = 0.57, beta = 0.033, N = 1231
QVITLLNELK pc2
C9JDE9;P09110;P09110−2
p = 0.69, beta = 0.0235, N = 1166
VNPLGGAVALGHPLGCTGAR pc3
C9JDE9;P09110;P09110−2
p = 0.92, beta = 0.00744, N = 648
GCFQAEIVPVTTTVHDDK pc3
C9JDE9;P09110;P09110−2
p = 0.66, beta = −0.0272, N = 1053
QDTFALASQQK pc2
C9JDE9;P09110;P09110−2
p = 0.73, beta = −0.0338, N = 425
IAQFLSDIPETVPLSTVNR pc3
B4DVF4;P09110;P09110−2
p = 0.9, beta = 0.0102, N = 537
QCSSGLQAVASIAGGIR pc3
P09110;P09110−2
p = 0.77, beta = −0.0281, N = 376
SITVTQDEGIRPSTTMEGLAK pc3
C9JDE9;P09110;P09110−2
p = 0.45, beta = −0.16, N = 78
DGSTTAGNSSQVSDGAAAILLAR pc
C9JDE9;P09110
p = 0.5, beta = −0.129, N = 92
ARDCLIPMGITSENVAER pc3
C9JDE9;P09110
p = 0.16, beta = −0.366, N = 64
SKAEELGLPILGVLR pc3
C9JDE9;P09110
p = 0.91, beta = −0.0404, N = 32
AGLTVSDVDIFEINEAFASQAAYCVEK
C9JDE9;P09110
p = 0.91, beta = −0.0457, N = 32
ACAA1 : NP3
P09110
p = 0.22, beta = −0.159, N = 324
AEELGLPILGVLR pc2
C9JDE9;P09110
p = 0.25, beta = −0.15, N = 324
QVITLLNELK pc2
C9JDE9;P09110;P09110−2
p = 0.25, beta = −0.164, N = 285
GCFQAEIVPVTTTVHDDK pc3
C9JDE9;P09110;P09110−2
p = 0.49, beta = −0.105, N = 245
QDTFALASQQK pc2
C9JDE9;P09110;P09110−2
p = 0.91, beta = 0.0268, N = 84
VNPLGGAVALGHPLGCTGAR pc3
C9JDE9;P09110;P09110−2
p = 0.68, beta = −0.157, N = 32
QCSSGLQAVASIAGGIR pc3
P09110;P09110−2
p = 0.43, beta = −0.191, N = 63
IAQFLSDIPETVPLSTVNR pc3
B4DVF4;P09110;P09110−2
p = 0.67, beta = 0.11, N = 57
ARDCLIPMGITSENVAER pc3
C9JDE9;P09110
p = 0.67, beta = −0.172, N = 25
Assay Target: ACAA1
UniProt: P09110
rsID: rs156265
Target SNP: 3:38170810:C:G
Proxy SNP: 3:38170810:C:G
UKBPPP log10(p): 49.4
UKBPPP BETA: −0.133
UKBPPP MSPA: 0
144:1231:1166:648:1053:425:53

333. TST (Q16762)
UKBPPP MSPA=0

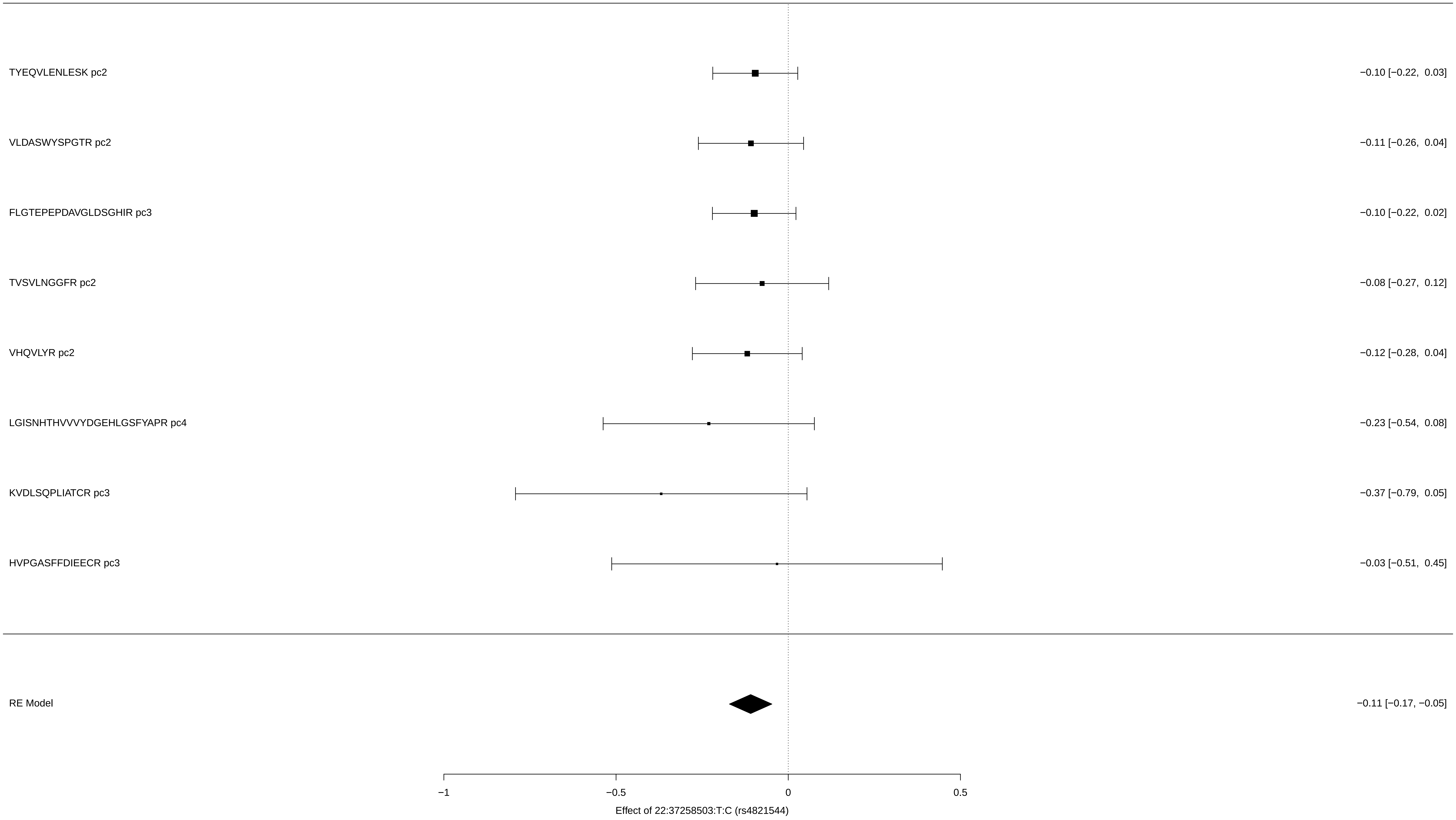

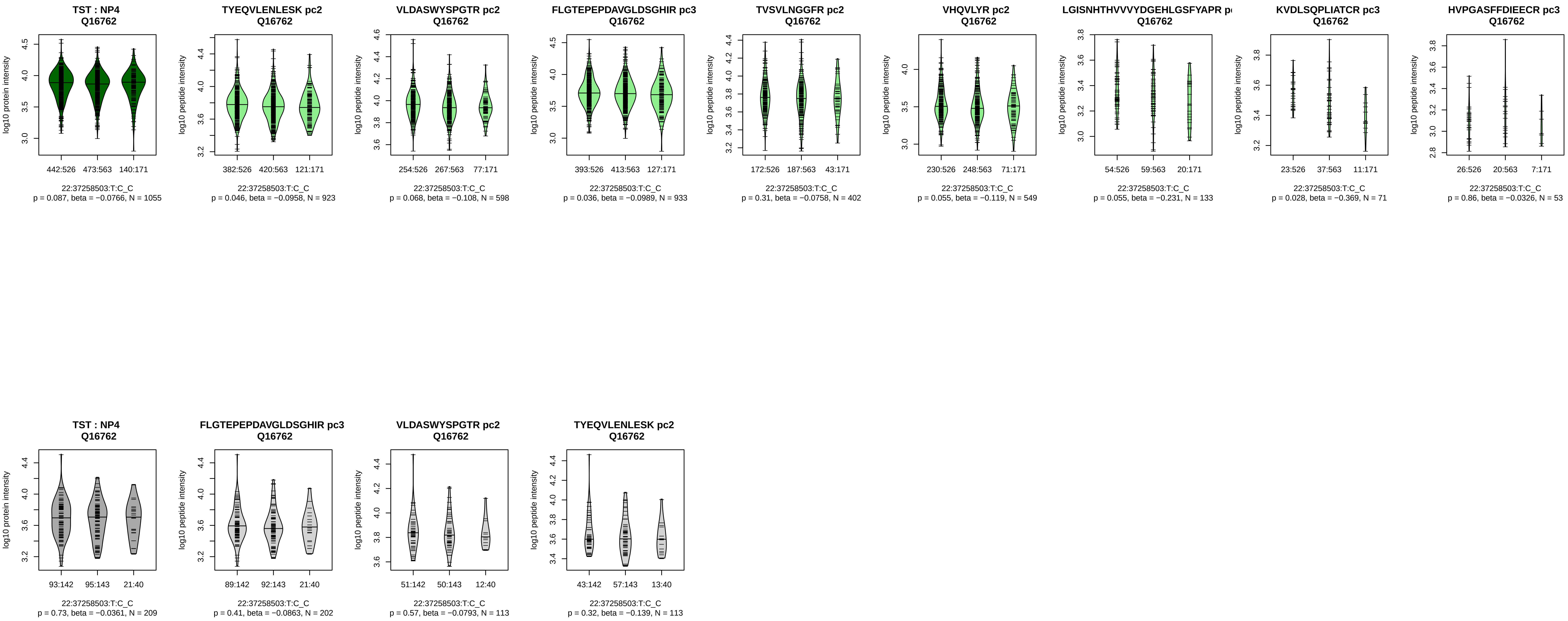

Assay Target: TST
UniProt: Q16762
rsID: rs4821544
Target SNP: 22:37258503:T:C
Proxy SNP: 22:37258503:T:C
UKBPPP log10(p): 49.3
UKBPPP BETA: −0.101
UKBPPP MSPA: 0
–:–:–:–:–:–:–:–
923:598:933:402:549:133:71:53

334. VWF (P04275)
UKBPPP MSPA=0.015 (1 conflicts)

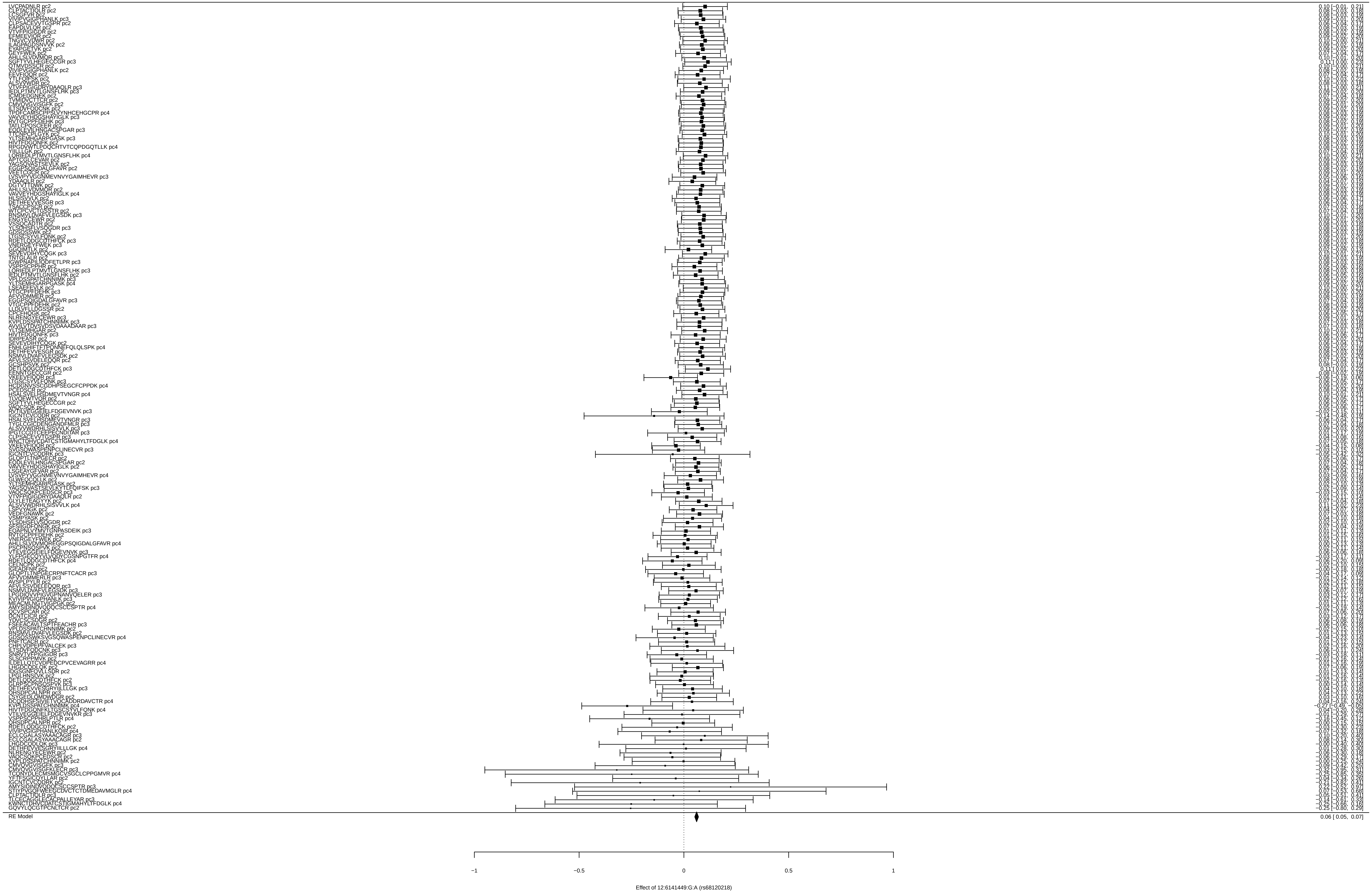

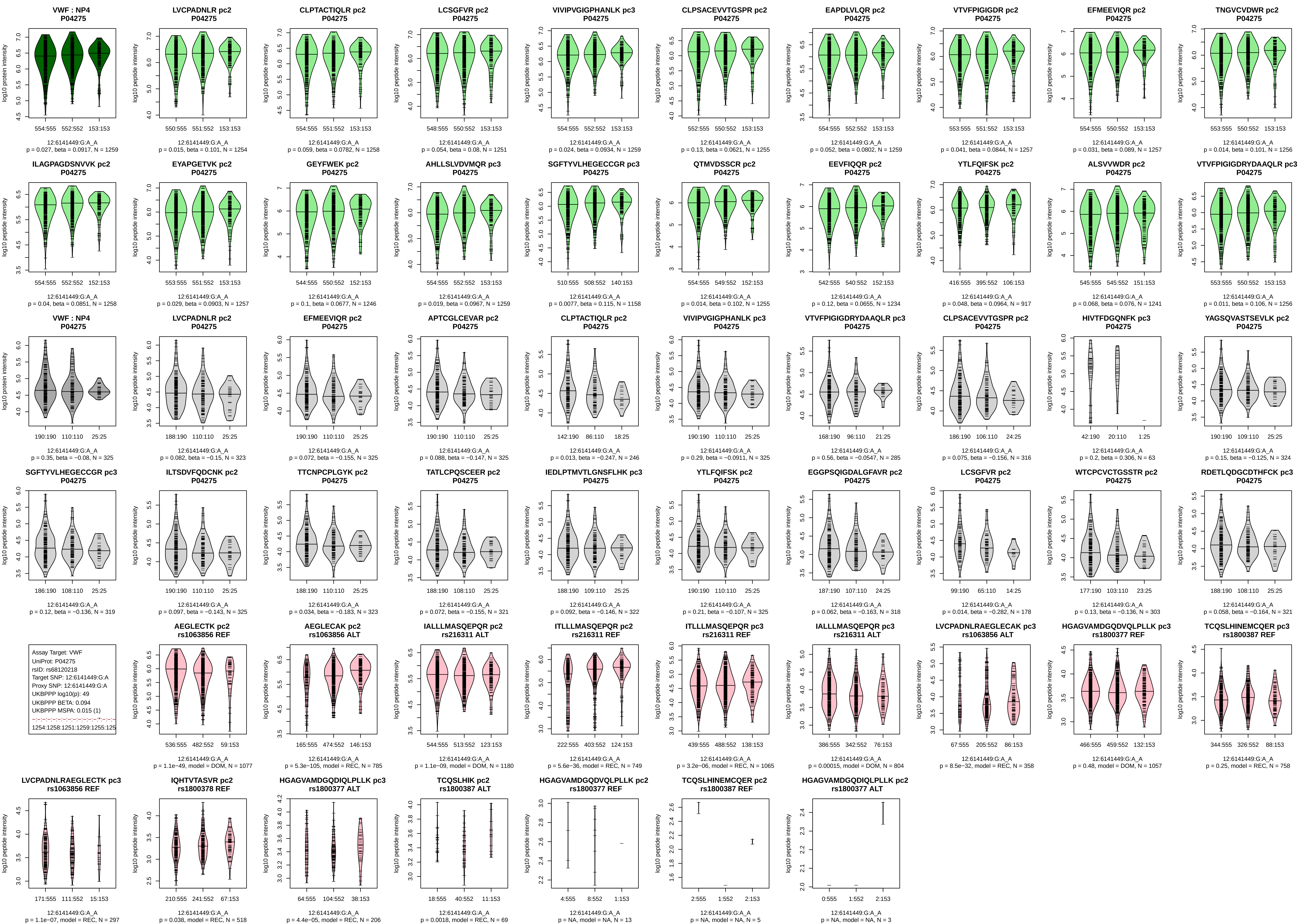
VWF : NP4
P04275
554:555 552:552 153:153
12:6141449:G:A_A
p = 0.027, beta = 0.0917, N = 1259
LVCPADNLR pc2
P04275
550:555 551:552 153:153
12:6141449:G:A_A
p = 0.015, beta = 0.101, N = 1254
CLPTACTIQLR pc2
P04275
554:555 551:552 153:153
12:6141449:G:A_A
p = 0.059, beta = 0.0782, N = 1258
LCSGFVR pc2
P04275
548:555 550:552 153:153
12:6141449:G:A_A
p = 0.054, beta = 0.08, N = 1251
VIVIPVGIGPHANLK pc3
P04275
554:555 552:552 153:153
12:6141449:G:A_A
p = 0.024, beta = 0.0934, N = 1259
CLPSACEVVTGSPR pc2
P04275
552:555 550:552 153:153
12:6141449:G:A_A
p = 0.13, beta = 0.0621, N = 1255
EAPDLVLQR pc2
P04275
554:555 552:552 153:153
12:6141449:G:A_A
p = 0.052, beta = 0.0802, N = 1259
VTVFPIGIGDR pc2
P04275
553:555 551:552 153:153
12:6141449:G:A_A
p = 0.041, beta = 0.0844, N = 1257
EFMEEVIQR pc2
P04275
554:555 550:552 153:153
12:6141449:G:A_A
p = 0.031, beta = 0.089, N = 1257
TNGVCVDWR pc2
P04275
553:555 550:552 153:153
12:6141449:G:A_A
p = 0.014, beta = 0.101, N = 1256
ILAGPAGDSNVVK pc2
P04275
554:555 552:552 152:153
12:6141449:G:A_A
p = 0.04, beta = 0.0851, N = 1258
EYAPGETVK pc2
P04275
553:555 551:552 153:153
12:6141449:G:A_A
p = 0.029, beta = 0.0903, N = 1257
GEYFWEK pc2
P04275
544:555 550:552 152:153
12:6141449:G:A_A
p = 0.1, beta = 0.0677, N = 1246
AHLLSLVDVMQR pc3
P04275
554:555 552:552 153:153
12:6141449:G:A_A
p = 0.019, beta = 0.0967, N = 1259
SGFTYVLHEGECCGR pc3
P04275
510:555 508:552 140:153
12:6141449:G:A_A
p = 0.0077, beta = 0.115, N = 1158
QTMVDSSCR pc2
P04275
554:555 549:552 152:153
12:6141449:G:A_A
p = 0.014, beta = 0.102, N = 1255
EEVFIQQR pc2
P04275
542:555 540:552 152:153
12:6141449:G:A_A
p = 0.12, beta = 0.0655, N = 1234
YTLFQIFSK pc2
P04275
416:555 395:552 106:153
12:6141449:G:A_A
p = 0.048, beta = 0.0964, N = 917
ALSVVWDR pc2
P04275
545:555 545:552 151:153
12:6141449:G:A_A
p = 0.068, beta = 0.076, N = 1241
VTVFPIGIGDRYDAAQLR pc3
P04275
553:555 550:552 153:153
12:6141449:G:A_A
p = 0.011, beta = 0.106, N = 1256
VWF : NP4
P04275
190:190 110:110 25:25
12:6141449:G:A_A
p = 0.35, beta = −0.08, N = 325
LVCPADNLR pc2
P04275
188:190 110:110 25:25
12:6141449:G:A_A
p = 0.082, beta = −0.15, N = 323
EFMEEVIQR pc2
P04275
190:190 110:110 25:25
12:6141449:G:A_A
p = 0.072, beta = −0.155, N = 325
APTCGLCEVAR pc2
P04275
190:190 110:110 25:25
12:6141449:G:A_A
p = 0.088, beta = −0.147, N = 325
CLPTACTIQLR pc2
P04275
142:190 86:110 18:25
12:6141449:G:A_A
p = 0.013, beta = −0.247, N = 246
VIVIPVGIGPHANLK pc3
P04275
190:190 110:110 25:25
12:6141449:G:A_A
p = 0.29, beta = −0.0911, N = 325
VTVFPIGIGDRYDAAQLR pc3
P04275
168:190 96:110 21:25
12:6141449:G:A_A
p = 0.56, beta = −0.0547, N = 285
CLPSACEVVTGSPR pc2
P04275
186:190 106:110 24:25
12:6141449:G:A_A
p = 0.075, beta = −0.156, N = 316
HIVTFDGQNFK pc3
P04275
42:190 20:110 1:25
12:6141449:G:A_A
p = 0.2, beta = 0.306, N = 63
YAGSQVASTSEVLK pc2
P04275
190:190 109:110 25:25
12:6141449:G:A_A
p = 0.15, beta = −0.125, N = 324
SGFTYVLHEGECCGR pc3
P04275
186:190 108:110 25:25
12:6141449:G:A_A
p = 0.12, beta = −0.136, N = 319
ILTSDVFQDCNK pc2
P04275
190:190 110:110 25:25
12:6141449:G:A_A
p = 0.097, beta = −0.143, N = 325
TTCNPCPLGYK pc2
P04275
188:190 110:110 25:25
12:6141449:G:A_A
p = 0.034, beta = −0.183, N = 323
TATLCPQSCEER pc2
P04275
188:190 108:110 25:25
12:6141449:G:A_A
p = 0.072, beta = −0.155, N = 321
IEDLPTMVTLGNSFLHK pc3
P04275
188:190 109:110 25:25
12:6141449:G:A_A
p = 0.092, beta = −0.146, N = 322
YTLFQIFSK pc2
P04275
190:190 110:110 25:25
12:6141449:G:A_A
p = 0.21, beta = −0.107, N = 325
EGGPSQIGDALGFAVR pc2
P04275
187:190 107:110 24:25
12:6141449:G:A_A
p = 0.062, beta = −0.163, N = 318
LCSGFVR pc2
P04275
99:190 65:110 14:25
12:6141449:G:A_A
p = 0.014, beta = −0.282, N = 178
WTCPCVCTGSSTR pc2
P04275
177:190 103:110 23:25
12:6141449:G:A_A
p = 0.13, beta = −0.136, N = 303
RDETLQDGCDTHFCK pc3
P04275
188:190 108:110 25:25
12:6141449:G:A_A
p = 0.058, beta = −0.164, N = 321
Assay Target: VWF
UniProt: P04275
rsID: rs68120218
Target SNP: 12:6141449:G:A
Proxy SNP: 12:6141449:G:A
UKBPPP log10(p): 49
UKBPPP BETA: 0.094
UKBPPP MSPA: 0.015 (1)
1254:1258:1251:1259:1255:125
AEGLECTK pc2
rs1063856 REF
536:555 482:552 59:153
12:6141449:G:A_A
p = 1.1e−49, model = DOM, N = 1077
AEGLECAK pc2
rs1063856 ALT
165:555 474:552 146:153
12:6141449:G:A_A
p = 5.3e−105, model = REC, N = 785
IALLLMASQEPQR pc2
rs216311 ALT
544:555 513:552 123:153
12:6141449:G:A_A
p = 1.1e−09, model = DOM, N = 1180
ITLLLMASQEPQR pc2
rs216311 REF
222:555 403:552 124:153
12:6141449:G:A_A
p = 5.6e−36, model = REC, N = 749
ITLLLMASQEPQR pc3
rs216311 REF
439:555 488:552 138:153
12:6141449:G:A_A
p = 3.2e−06, model = REC, N = 1065
IALLLMASQEPQR pc3
rs216311 ALT
386:555 342:552 76:153
12:6141449:G:A_A
p = 0.00015, model = DOM, N = 804
LVCPADNLRAEGLECAK pc3
rs1063856 ALT
67:555 205:552 86:153
12:6141449:G:A_A
p = 8.5e−32, model = REC, N = 358
HGAGVAMDGQDVQLPLLK pc3
rs1800377 REF
466:555 459:552 132:153
12:6141449:G:A_A
p = 0.48, model = DOM, N = 1057
TCQSLHINEMCQER pc3
rs1800387 REF
344:555 326:552 88:153
12:6141449:G:A_A
p = 0.25, model = REC, N = 758
LVCPADNLRAEGLECTK pc3
rs1063856 REF
171:555 111:552 15:153
12:6141449:G:A_A
p = 1.1e−07, model = REC, N = 297
IQHTVTASVR pc2
rs1800378 REF
210:555 241:552 67:153
12:6141449:G:A_A
p = 0.038, model = REC, N = 518
HGAGVAMDGQDIQLPLLK pc3
rs1800377 ALT
64:555 104:552 38:153
12:6141449:G:A_A
p = 4.4e−05, model = REC, N = 206
TCQSLHIK pc2
rs1800387 ALT
18:555 40:552 11:153
12:6141449:G:A_A
p = 0.0018, model = REC, N = 69
HGAGVAMDGQDVQLPLLK pc2
rs1800377 REF
4:555 8:552 1:153
12:6141449:G:A_A
p = NA, model = NA, N = 13
TCQSLHINEMCQER pc2
rs1800387 REF
2:555 1:552 2:153
12:6141449:G:A_A
p = NA, model = NA, N = 5
HGAGVAMDGQDIQLPLLK pc2
rs1800377 ALT
0:555 1:552 2:153
12:6141449:G:A_A
p = NA, model = NA, N = 3
log10 protein intensity
log10 peptide intensity

335. SLC9A3R1 (O14745)
UKBPPP MSPA=0

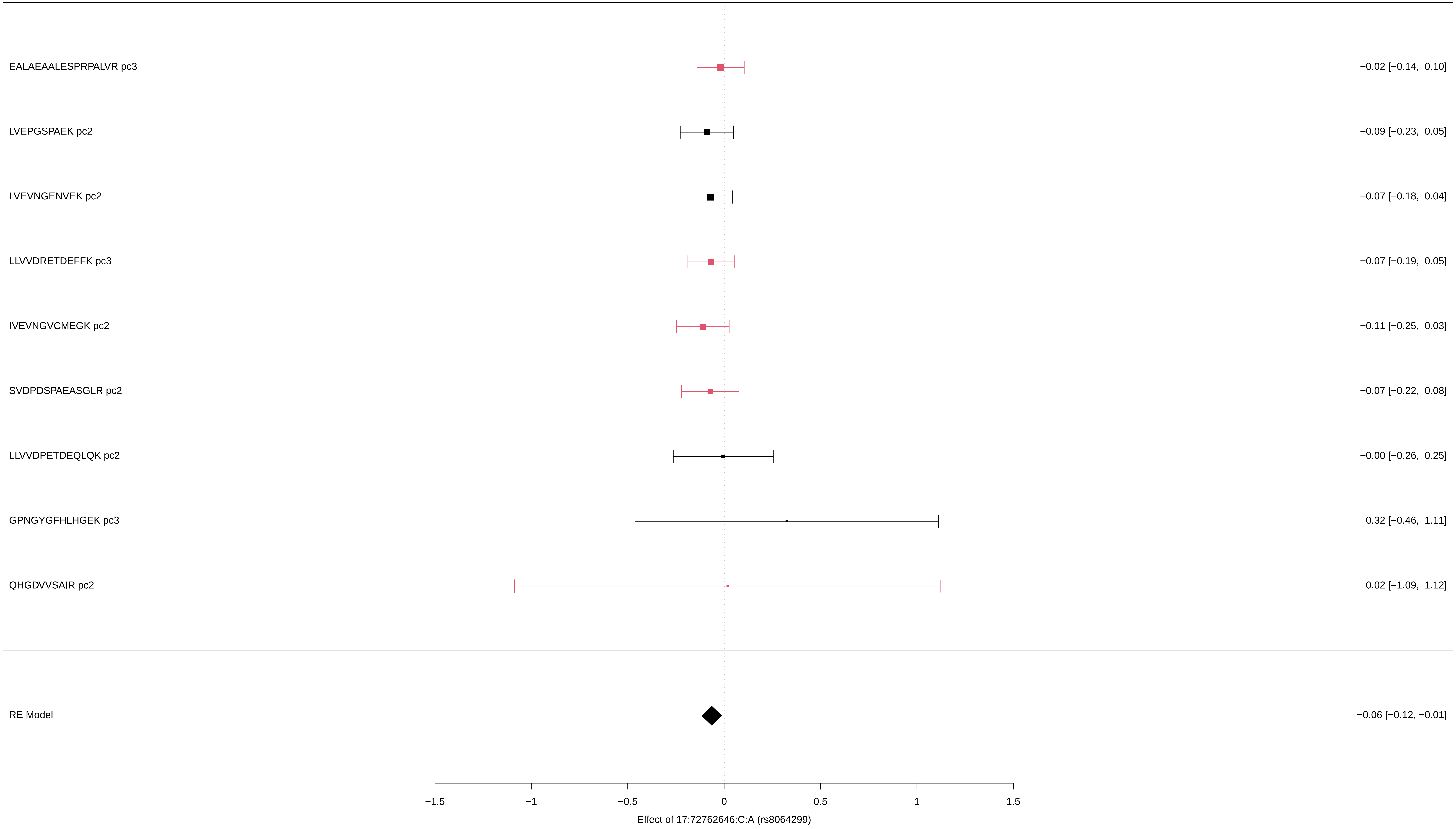

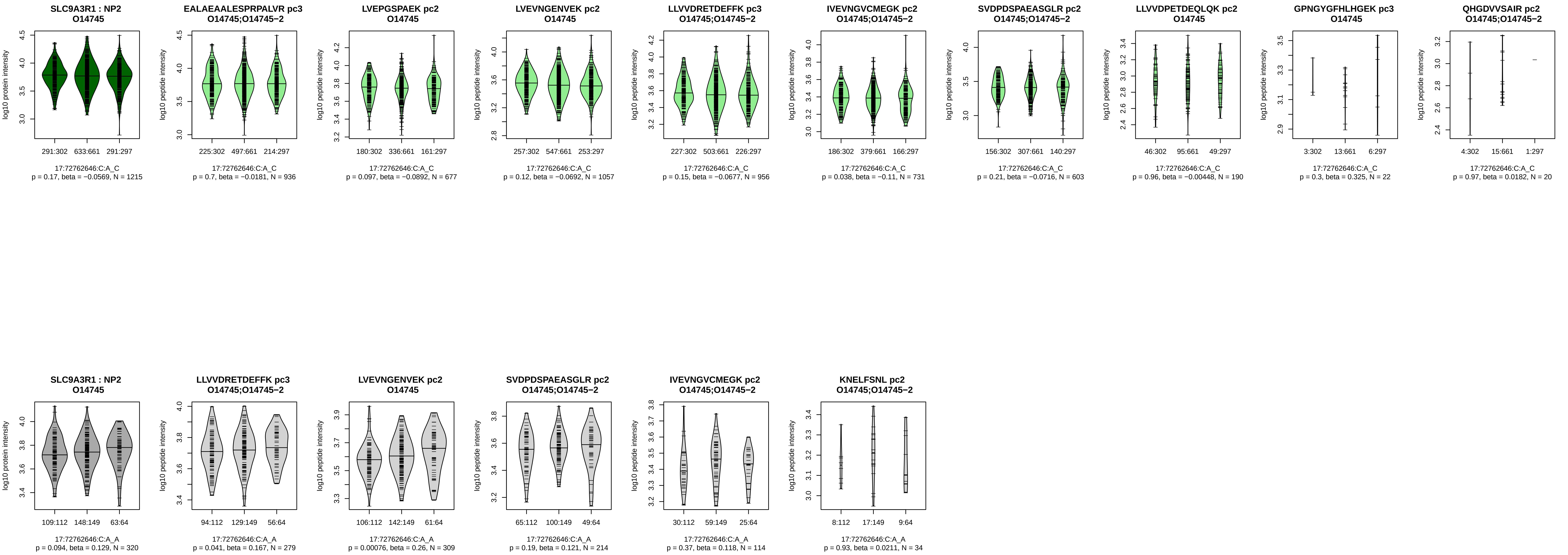

Assay Target: SLC9A3R1
UniProt: O14745
rsID: rs8064299
Target SNP: 17:72762646:C:A
Proxy SNP: 17:72762646:C:A
UKBPPP log10(p): 48.8
UKBPPP BETA: 0.092
UKBPPP MSPA: 0
–:–:–:–:–:–:–:–
936:677:1057:956:731:603:190:

336. GAL (P22466)
UKBPPP MSPA=0

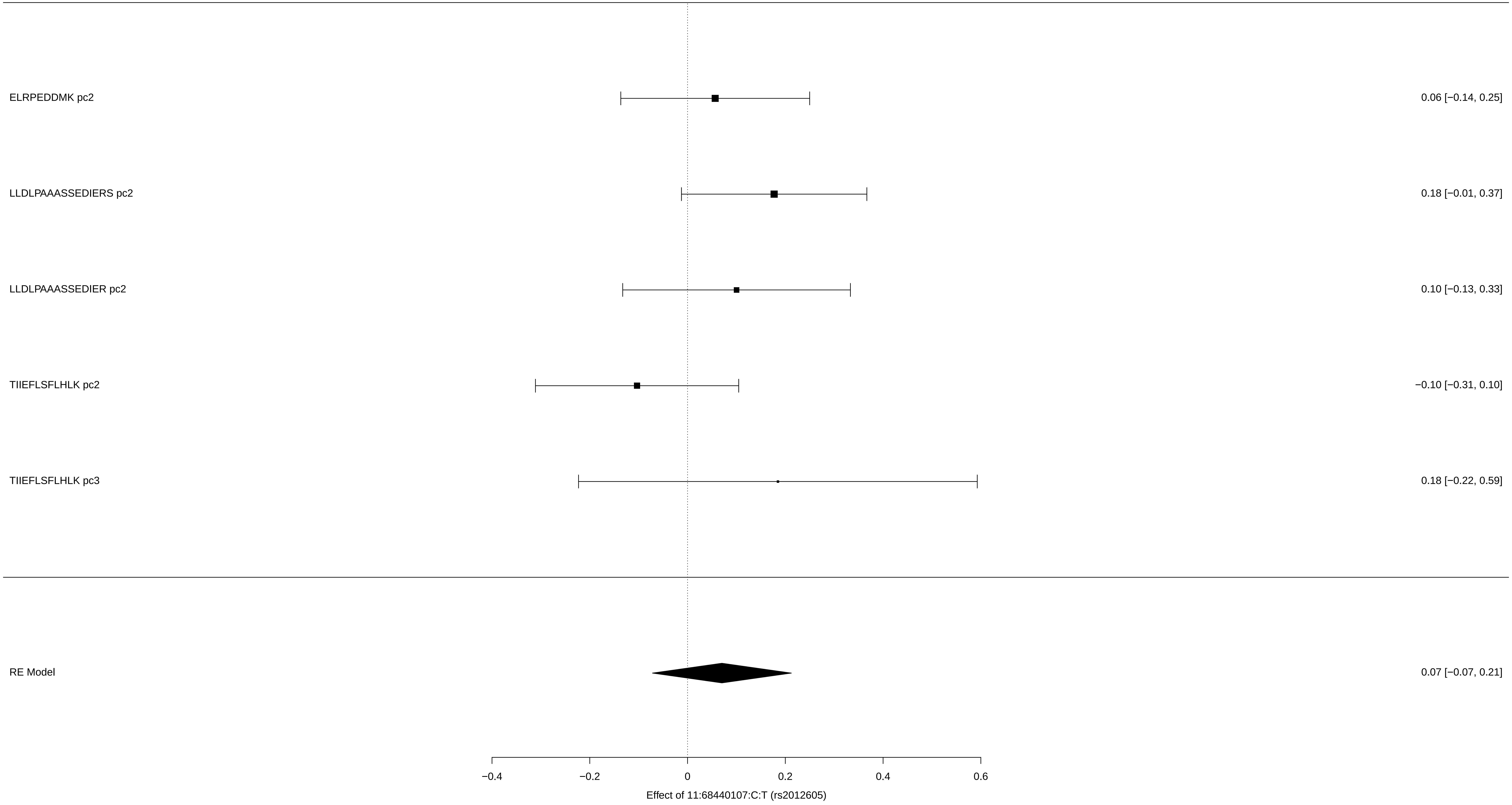

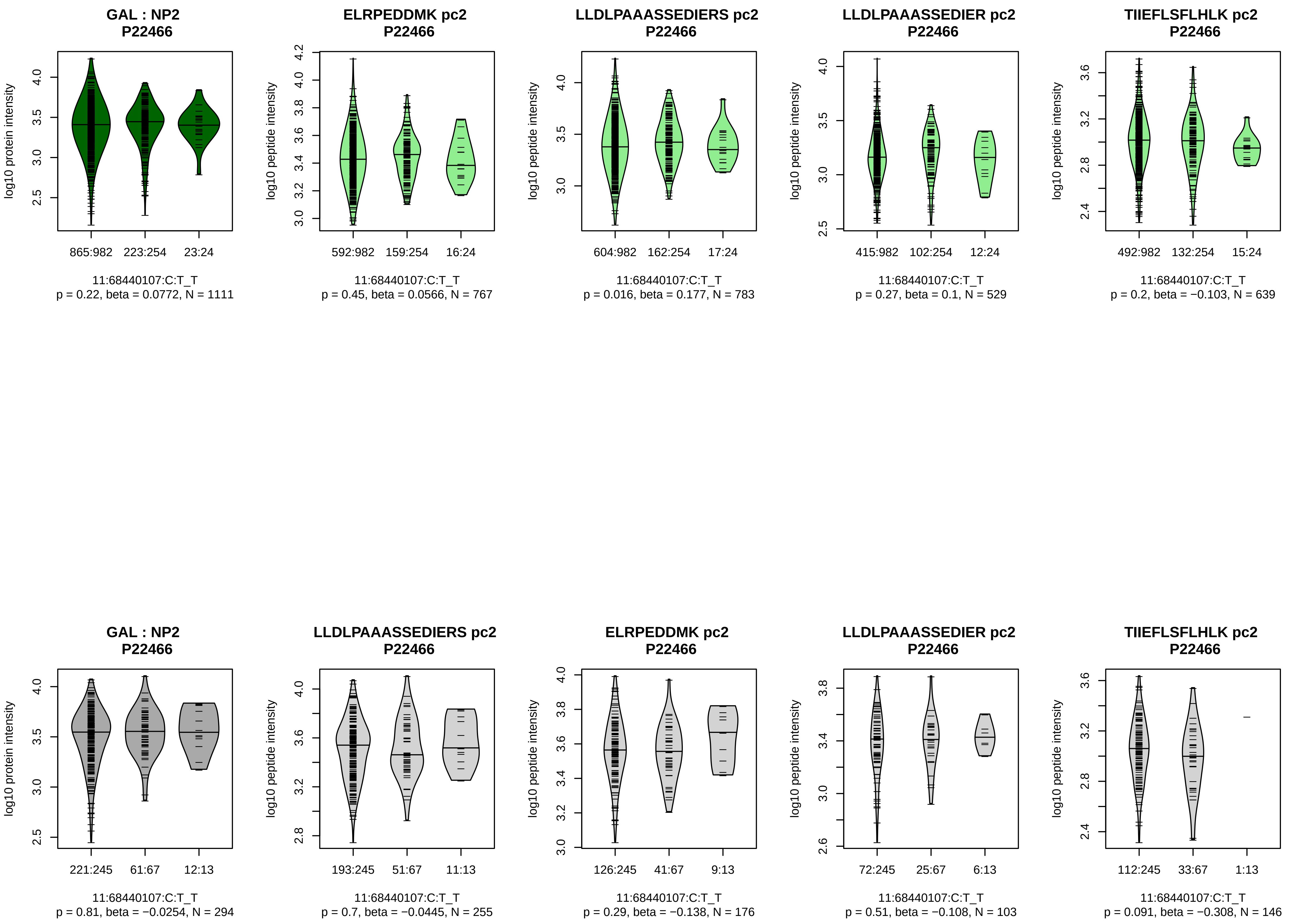

Assay Target: GAL
UniProt: P22466
rsID: rs2012605
Target SNP: 11:68440107:C:T
Proxy SNP: 11:68440107:C:T
UKBPPP log10(p): 47.9
UKBPPP BETA: 0.148
UKBPPP MSPA: 0
–:–:–:–:–
767:783:529:639:190

337. COL6A3 (P12111)
UKBPPP MSPA=0.017

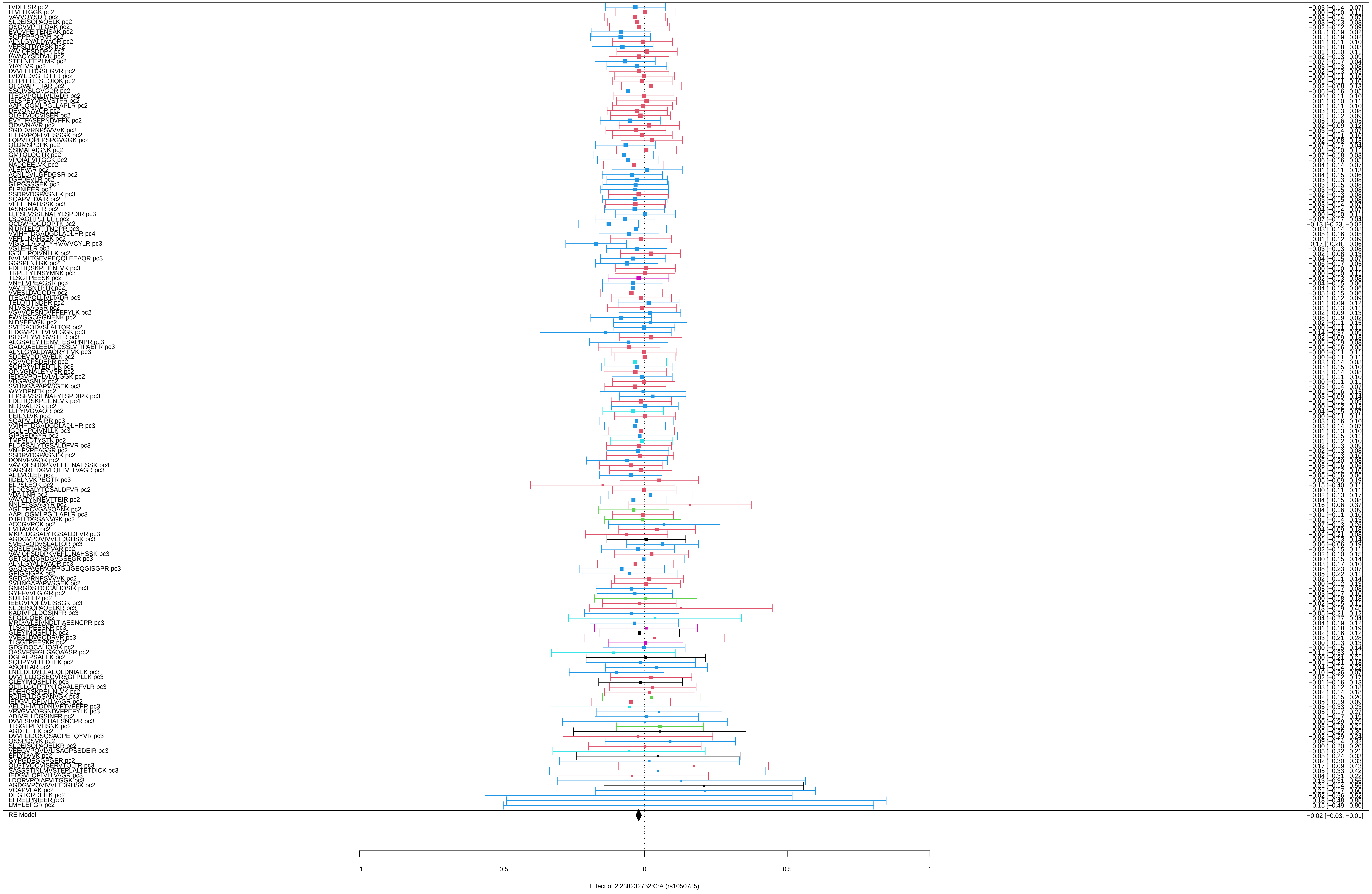

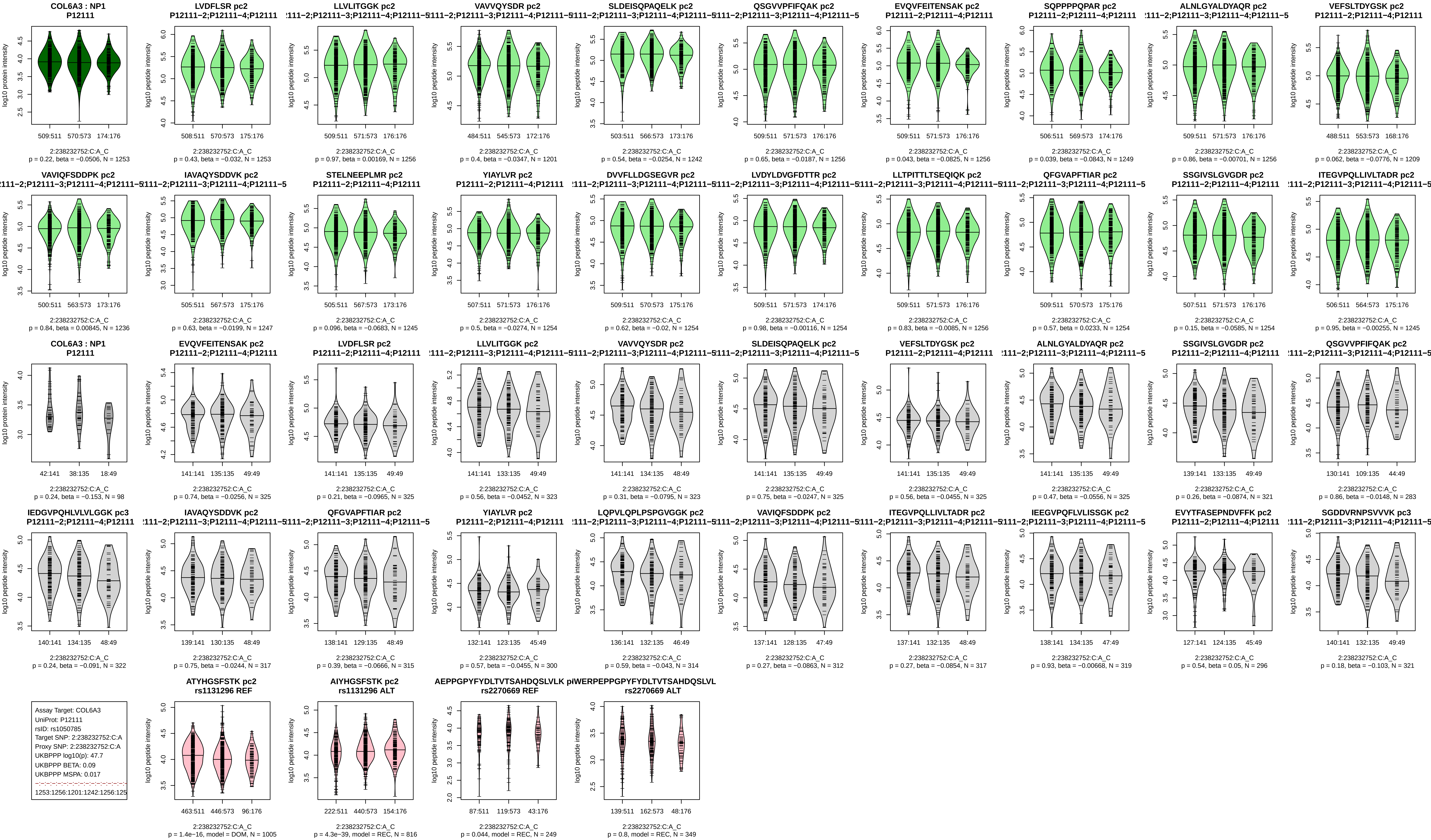
COL6A3 : NP1
P12111
log10 protein intensity
509:511 570:573 174:176
2:238232752:C:A_C
p = 0.22, beta = −0.0506, N = 1253
LVDFLSR pc2
P12111−2;P12111−4;P12111
log10 peptide intensity
508:511 570:573 175:176
2:238232752:C:A_C
p = 0.43, beta = −0.032, N = 1253
LLVLITGGK pc2
111−2;P12111−3;P12111−4;P12111−5
log10 peptide intensity
509:511 571:573 176:176
2:238232752:C:A_C
p = 0.97, beta = 0.00169, N = 1256
VAVVQYSDR pc2
111−2;P12111−3;P12111−4;P12111−5
log10 peptide intensity
484:511 545:573 172:176
2:238232752:C:A_C
p = 0.4, beta = −0.0347, N = 1201
SLDEISQPAQELK pc2
111−2;P12111−3;P12111−4;P12111−5
log10 peptide intensity
503:511 566:573 173:176
2:238232752:C:A_C
p = 0.54, beta = −0.0254, N = 1242
QSGVVPFIFQAK pc2
111−2;P12111−3;P12111−4;P12111−5
log10 peptide intensity
509:511 571:573 176:176
2:238232752:C:A_C
p = 0.65, beta = −0.0187, N = 1256
EVQVFEITENSAK pc2
P12111−2;P12111−4;P12111
log10 peptide intensity
509:511 571:573 176:176
2:238232752:C:A_C
p = 0.043, beta = −0.0825, N = 1256
SQPPPPQPAR pc2
P12111−2;P12111−4;P12111
log10 peptide intensity
506:511 569:573 174:176
2:238232752:C:A_C
p = 0.039, beta = −0.0843, N = 1249
ALNLGYALDYAQR pc2
111−2;P12111−3;P12111−4;P12111−5
log10 peptide intensity
509:511 571:573 176:176
2:238232752:C:A_C
p = 0.86, beta = −0.00701, N = 1256
VEFSLTDYGSK pc2
P12111−2;P12111−4;P12111
log10 peptide intensity
488:511 553:573 168:176
2:238232752:C:A_C
p = 0.062, beta = −0.0776, N = 1209
VAVIQFSDDPK pc2
111−2;P12111−3;P12111−4;P12111−5
log10 peptide intensity
500:511 563:573 173:176
2:238232752:C:A_C
p = 0.84, beta = 0.00845, N = 1236
IAVAQYSDDVK pc2
111−2;P12111−3;P12111−4;P12111−5
log10 peptide intensity
505:511 567:573 175:176
2:238232752:C:A_C
p = 0.63, beta = −0.0199, N = 1247
STELNEEPLMR pc2
P12111−2;P12111−4;P12111
log10 peptide intensity
505:511 567:573 173:176
2:238232752:C:A_C
p = 0.096, beta = −0.0683, N = 1245
YIAYLVR pc2
P12111−2;P12111−4;P12111
log10 peptide intensity
507:511 571:573 176:176
2:238232752:C:A_C
p = 0.5, beta = −0.0274, N = 1254
DVVFLLDGSEGVR pc2
111−2;P12111−3;P12111−4;P12111−5
log10 peptide intensity
509:511 570:573 175:176
2:238232752:C:A_C
p = 0.62, beta = −0.02, N = 1254
LVDYLDVGFDTTR pc2
111−2;P12111−3;P12111−4;P12111−5
log10 peptide intensity
509:511 571:573 174:176
2:238232752:C:A_C
p = 0.98, beta = −0.00116, N = 1254
LLTPITTLTSEQIQK pc2
111−2;P12111−3;P12111−4;P12111−5
log10 peptide intensity
509:511 571:573 176:176
2:238232752:C:A_C
p = 0.83, beta = −0.0585, N = 1256
QFGVAPFTIAR pc2
111−2;P12111−3;P12111−4;P12111−5
log10 peptide intensity
509:511 570:573 175:176
2:238232752:C:A_C
p = 0.57, beta = 0.0233, N = 1254
SSGIVSLGVGDR pc2
P12111−2;P12111−4;P12111
log10 peptide intensity
507:511 571:573 176:176
2:238232752:C:A_C
p = 0.15, beta = −0.0585, N = 1254
ITEGVPQLLIVLTADR pc2
111−2;P12111−3;P12111−4;P12111−5
log10 peptide intensity
506:511 564:573 175:176
2:238232752:C:A_C
p = 0.95, beta = −0.00255, N = 1245
COL6A3 : NP1
P12111
log10 protein intensity
42:141 38:135 18:49
2:238232752:C:A_C
p = 0.24, beta = −0.153, N = 98
EVQVFEITENSAK pc2
P12111−2;P12111−4;P12111
log10 peptide intensity
141:141 135:135 49:49
2:238232752:C:A_C
p = 0.74, beta = −0.0256, N = 325
LVDFLSR pc2
P12111−2;P12111−4;P12111
log10 peptide intensity
141:141 135:135 49:49
2:238232752:C:A_C
p = 0.21, beta = −0.0965, N = 325
LLVLITGGK pc2
111−2;P12111−3;P12111−4;P12111−5
log10 peptide intensity
141:141 133:135 49:49
2:238232752:C:A_C
p = 0.56, beta = −0.0452, N = 323
VAVVQYSDR pc2
111−2;P12111−3;P12111−4;P12111−5
log10 peptide intensity
141:141 134:135 48:49
2:238232752:C:A_C
p = 0.31, beta = −0.0795, N = 323
SLDEISQPAQELK pc2
111−2;P12111−3;P12111−4;P12111−5
log10 peptide intensity
141:141 135:135 49:49
2:238232752:C:A_C
p = 0.75, beta = −0.0247, N = 325
VEFSLTDYGSK pc2
P12111−2;P12111−4;P12111
log10 peptide intensity
141:141 135:135 49:49
2:238232752:C:A_C
p = 0.56, beta = −0.0455, N = 325
ALNLGYALDYAQR pc2
111−2;P12111−3;P12111−4;P12111−5
log10 peptide intensity
141:141 135:135 49:49
2:238232752:C:A_C
p = 0.47, beta = −0.0556, N = 325
SSGIVSLGVGDR pc2
P12111−2;P12111−4;P12111
log10 peptide intensity
139:141 133:135 49:49
2:238232752:C:A_C
p = 0.26, beta = −0.0874, N = 321
QSGVVPFIFQAK pc2
111−2;P12111−3;P12111−4;P12111−5
log10 peptide intensity
130:141 109:135 44:49
2:238232752:C:A_C
p = 0.86, beta = −0.0148, N = 283
IEDGVPQHLVLVLGGK pc3
P12111−2;P12111−4;P12111
log10 peptide intensity
140:141 134:135 48:49
2:238232752:C:A_C
p = 0.24, beta = −0.091, N = 322
IAVAQYSDDVK pc2
111−2;P12111−3;P12111−4;P12111−5
log10 peptide intensity
139:141 130:135 48:49
2:238232752:C:A_C
p = 0.75, beta = −0.0244, N = 317
QFGVAPFTIAR pc2
111−2;P12111−3;P12111−4;P12111−5
log10 peptide intensity
138:141 129:135 48:49
2:238232752:C:A_C
p = 0.39, beta = −0.0666, N = 315
YIAYLVR pc2
P12111−2;P12111−4;P12111
log10 peptide intensity
132:141 123:135 45:49
2:238232752:C:A_C
p = 0.57, beta = −0.0455, N = 300
LQPVLQPLPSPGVGGK pc2
111−2;P12111−3;P12111−4;P12111−5
log10 peptide intensity
136:141 132:135 46:49
2:238232752:C:A_C
p = 0.59, beta = −0.043, N = 314
VAVIQFSDDPK pc2
111−2;P12111−3;P12111−4;P12111−5
log10 peptide intensity
137:141 128:135 47:49
2:238232752:C:A_C
p = 0.27, beta = −0.0863, N = 312
ITEGVPQLLIVLTADR pc2
111−2;P12111−3;P12111−4;P12111−5
log10 peptide intensity
137:141 132:135 48:49
2:238232752:C:A_C
p = 0.27, beta = −0.0854, N = 317
IEEGVPQFLVLISSGK pc2
111−2;P12111−3;P12111−4;P12111−5
log10 peptide intensity
138:141 134:135 47:49
2:238232752:C:A_C
p = 0.93, beta = −0.00668, N = 319
EVYTFASEPNDVFFK pc2
P12111−2;P12111−4;P12111
log10 peptide intensity
127:141 124:135 45:49
2:238232752:C:A_C
p = 0.54, beta = 0.05, N = 296
SGDDVRNPSVVVK pc3
111−2;P12111−3;P12111−4;P12111−5
log10 peptide intensity
140:141 132:135 49:49
2:238232752:C:A_C
p = 0.18, beta = −0.103, N = 321
Assay Target: COL6A3
UniProt: P12111
rsID: rs1050785
Target SNP: 2:238232752:C:A
Proxy SNP: 2:238232752:C:A
UKBPPP log10(p): 47.7
UKBPPP BETA: 0.09
UKBPPP MSPA: 0.017
1253:1256:1201:1242:1256:125
ATYHGSFSTK pc2
rs1131296 REF
log10 peptide intensity
463:511 446:573 96:176
2:238232752:C:A_C
p = 1.4e−16, model = DOM, N = 1005
AIYHGSFSTK pc2
rs1131296 ALT
log10 peptide intensity
222:511 440:573 154:176
2:238232752:C:A_C
p = 4.3e−39, model = REC, N = 816
AEPPGPYFYDLTVTSAHDQSLVLK p
rs2270669 REF
log10 peptide intensity
87:511 119:573 43:176
2:238232752:C:A_C
p = 0.044, model = REC, N = 249
IWERPEPPGPYFYDLTVTSAHDQSLVL
rs2270669 ALT
log10 peptide intensity
139:511 162:573 48:176
2:238232752:C:A_C
p = 0.8, model = REC, N = 349

338. SERPINE1 (P05121)
UKBPPP MSPA=0

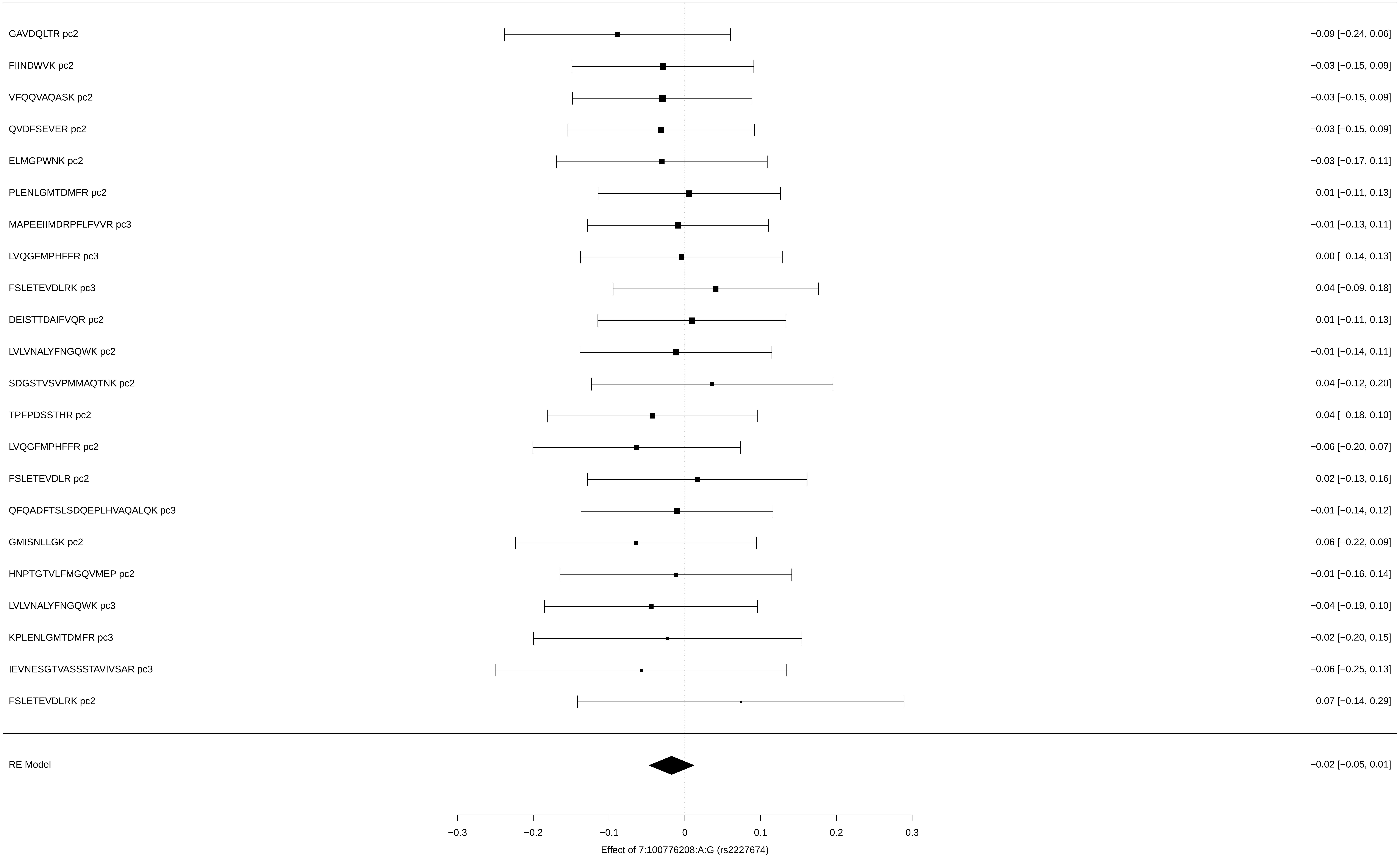

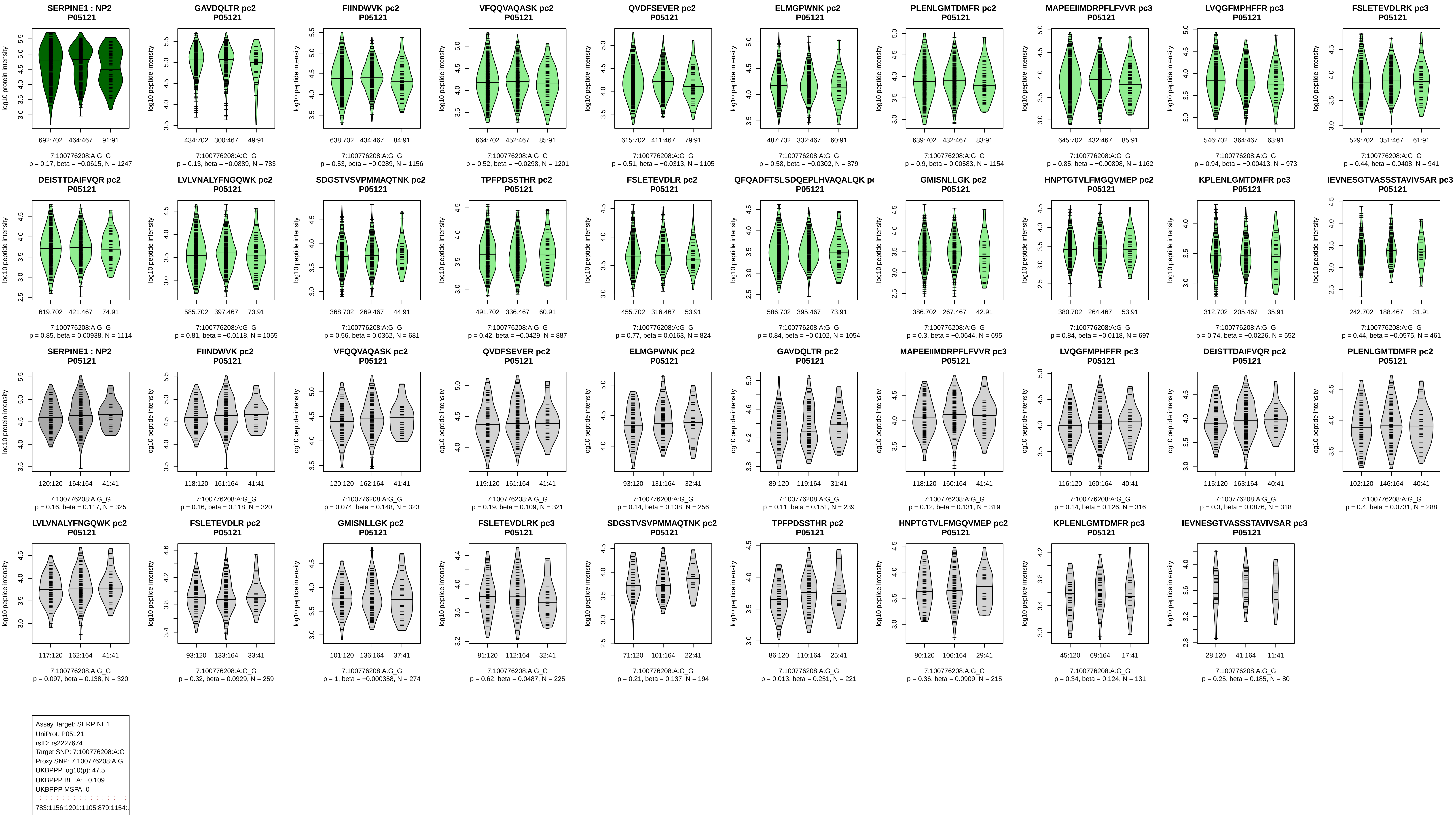
SERPINE1 : NP2
P05121
692:702 464:467 91:91
7:100776208:A:G_G
p = 0.17, beta = −0.0615, N = 1247
GAVDQLTR pc2
P05121
434:702 300:467 49:91
7:100776208:A:G_G
p = 0.13, beta = −0.0889, N = 783
FIINDWVK pc2
P05121
638:702 434:467 84:91
7:100776208:A:G_G
p = 0.53, beta = −0.0289, N = 1156
VFQQVAQASK pc2
P05121
664:702 452:467 85:91
7:100776208:A:G_G
p = 0.52, beta = −0.0298, N = 1201
QVDFSEVER pc2
P05121
615:702 411:467 79:91
7:100776208:A:G_G
p = 0.51, beta = −0.0313, N = 1105
ELMGPWNK pc2
P05121
487:702 332:467 60:91
7:100776208:A:G_G
p = 0.58, beta = −0.0302, N = 879
PLENLGMTDMFR pc2
P05121
639:702 432:467 83:91
7:100776208:A:G_G
p = 0.9, beta = 0.00583, N = 1154
MAPEEIIMDRPFLFVVR pc3
P05121
645:702 432:467 85:91
7:100776208:A:G_G
p = 0.85, beta = −0.00898, N = 1162
LVQGFMPHFFR pc3
P05121
546:702 364:467 63:91
7:100776208:A:G_G
p = 0.94, beta = −0.00413, N = 973
FSLETEVDLRK pc3
P05121
529:702 351:467 61:91
7:100776208:A:G_G
p = 0.44, beta = 0.0408, N = 941
DEISTTDAIFVQR pc2
P05121
619:702 421:467 74:91
7:100776208:A:G_G
p = 0.85, beta = 0.00938, N = 1114
LVLVNALYFNGQWK pc2
P05121
585:702 397:467 73:91
7:100776208:A:G_G
p = 0.81, beta = −0.0118, N = 1055
SDGSTVSVPMMAQTNK pc2
P05121
368:702 269:467 44:91
7:100776208:A:G_G
p = 0.56, beta = 0.0362, N = 681
TPFPDSSTHR pc2
P05121
491:702 336:467 60:91
7:100776208:A:G_G
p = 0.42, beta = −0.0429, N = 887
FSLETEVDLR pc2
P05121
455:702 316:467 53:91
7:100776208:A:G_G
p = 0.77, beta = −0.0163, N = 824
QFQADFTSLSDQEPLHVAQALQK pc
P05121
586:702 395:467 73:91
7:100776208:A:G_G
p = 0.84, beta = −0.0102, N = 1054
GMISNLLGK pc2
P05121
386:702 267:467 42:91
7:100776208:A:G_G
p = 0.3, beta = −0.0644, N = 695
HNPTGTVLFMGQVMEP pc2
P05121
380:702 264:467 53:91
7:100776208:A:G_G
p = 0.84, beta = −0.0118, N = 697
KPLENLGMTDMFR pc3
P05121
312:702 205:467 35:91
7:100776208:A:G_G
p = 0.74, beta = −0.0226, N = 552
IEVNESGTVASSSTAVIVSAR pc3
P05121
242:702 188:467 31:91
7:100776208:A:G_G
p = 0.44, beta = −0.0575, N = 461
SERPINE1 : NP2
P05121
120:120 164:164 41:41
7:100776208:A:G_G
p = 0.16, beta = 0.117, N = 325
FIINDWVK pc2
P05121
118:120 161:164 41:41
7:100776208:A:G_G
p = 0.16, beta = 0.118, N = 320
VFQQVAQASK pc2
P05121
120:120 162:164 41:41
7:100776208:A:G_G
p = 0.074, beta = 0.148, N = 323
QVDFSEVER pc2
P05121
119:120 161:164 41:41
7:100776208:A:G_G
p = 0.19, beta = 0.109, N = 321
ELMGPWNK pc2
P05121
93:120 131:164 32:41
7:100776208:A:G_G
p = 0.14, beta = 0.138, N = 256
GAVDQLTR pc2
P05121
89:120 119:164 31:41
7:100776208:A:G_G
p = 0.11, beta = 0.151, N = 239
MAPEEIIMDRPFLFVVR pc3
P05121
118:120 160:164 41:41
7:100776208:A:G_G
p = 0.12, beta = 0.131, N = 319
LVQGFMPHFFR pc3
P05121
116:120 160:164 40:41
7:100776208:A:G_G
p = 0.14, beta = 0.126, N = 316
DEISTTDAIFVQR pc2
P05121
115:120 163:164 40:41
7:100776208:A:G_G
p = 0.3, beta = 0.0876, N = 318
PLENLGMTDMFR pc2
P05121
102:120 146:164 40:41
7:100776208:A:G_G
p = 0.4, beta = 0.0731, N = 288
LVLVNALYFNGQWK pc2
P05121
117:120 162:164 41:41
7:100776208:A:G_G
p = 0.097, beta = 0.138, N = 320
FSLETEVDLR pc2
P05121
93:120 133:164 33:41
7:100776208:A:G_G
p = 0.32, beta = 0.0929, N = 259
GMISNLLGK pc2
P05121
101:120 136:164 37:41
7:100776208:A:G_G
p = 1, beta = −0.000358, N = 274
FSLETEVDLRK pc3
P05121
81:120 112:164 32:41
7:100776208:A:G_G
p = 0.62, beta = 0.0487, N = 225
SDGSTVSVPMMAQTNK pc2
P05121
71:120 101:164 22:41
7:100776208:A:G_G
p = 0.21, beta = 0.137, N = 194
TPFPDSSTHR pc2
P05121
86:120 110:164 25:41
7:100776208:A:G_G
p = 0.013, beta = 0.251, N = 221
HNPTGTVLFMGQVMEP pc2
P05121
80:120 106:164 29:41
7:100776208:A:G_G
p = 0.36, beta = 0.0909, N = 215
KPLENLGMTDMFR pc3
P05121
45:120 69:164 17:41
7:100776208:A:G_G
p = 0.34, beta = 0.124, N = 131
IEVNESGTVASSSTAVIVSAR pc3
P05121
28:120 41:164 11:41
7:100776208:A:G_G
p = 0.25, beta = 0.185, N = 80
log10 protein intensity
log10 peptide intensity
Assay Target: SERPINE1
UniProt: P05121
rsID: rs2227674
Target SNP: 7:100776208:A:G
Proxy SNP: 7:100776208:A:G
UKBPPP log10(p): 47.5
UKBPPP BETA: −0.109
UKBPPP MSPA: 0
783:1156:1201:1105:879:1154:

339. PSME1 (Q06323)
UKBPPP MSPA=0.085

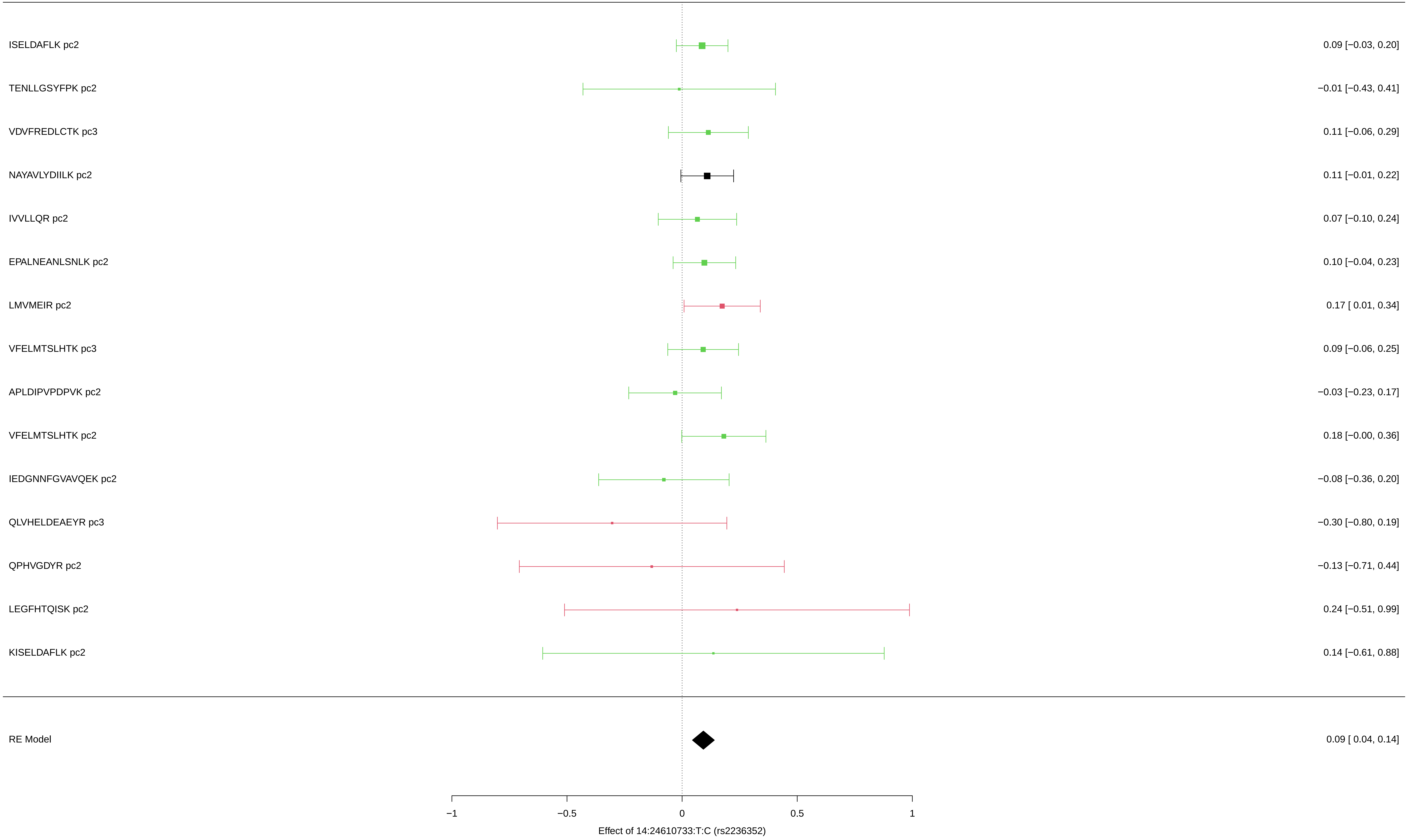

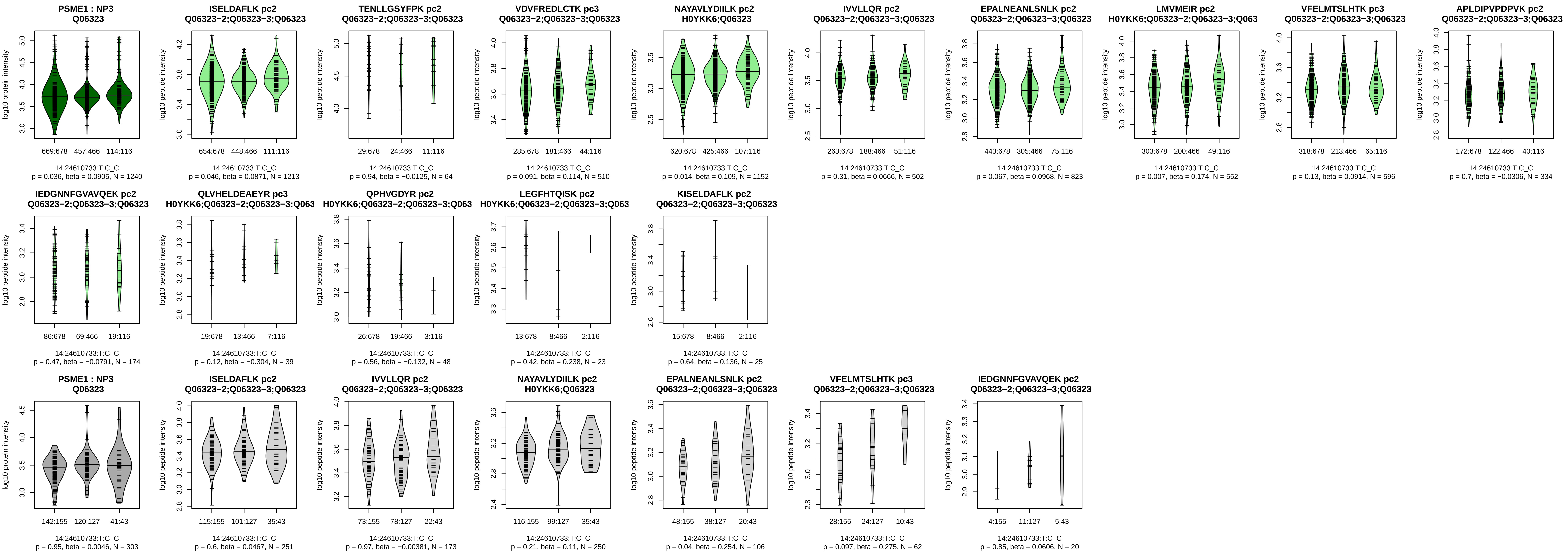

Assay Target: PSME1
UniProt: Q06323
rsID: rs2236352
Target SNP: 14:24610733:T:C
Proxy SNP: 14:24610733:T:C
UKBPPP log10(p): 47.5
UKBPPP BETA: 0.1
UKBPPP MSPA: 0.085
–:–:–:–:–:–:*:–:–:–:–:–:–:–:–:NA
1213:64:510:1152:502:823:552:

340. TIMP2 (P16035)
UKBPPP MSPA=0

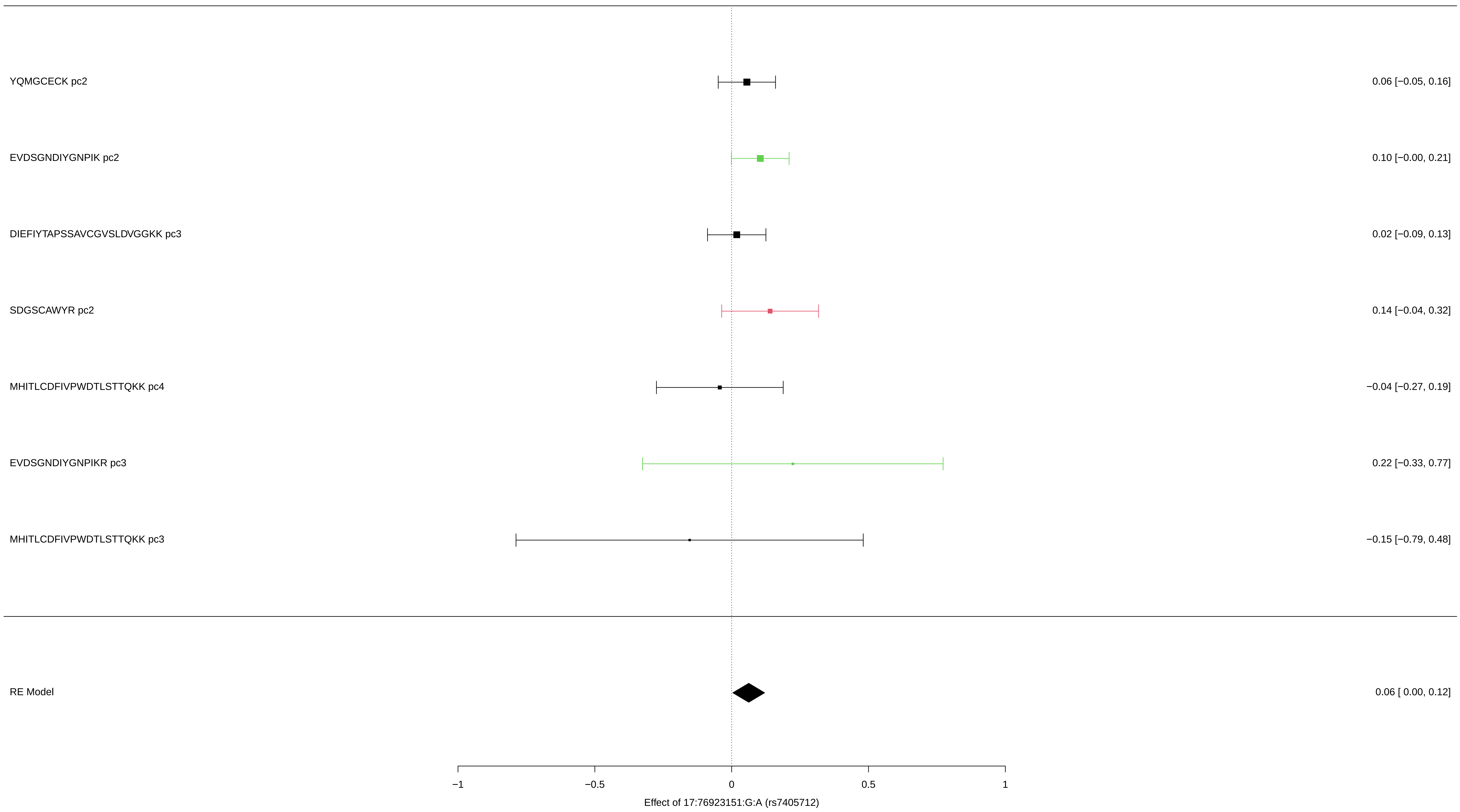

TIMP2 : NP5
P16035

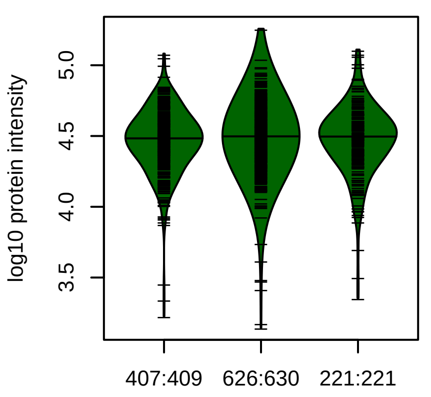

17:76923151:G:A_A
p = 0.25, beta = 0.0464, N = 1254

YQMGCECK pc2
B4DFW2;K7EIX4;P16035

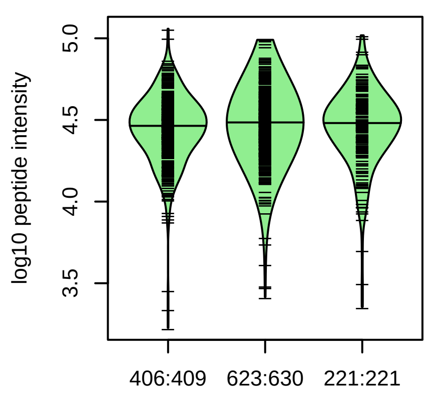

17:76923151:G:A_A
p = 0.17, beta = 0.0556, N = 1250

EVDSGNDIYGNPIK pc2
P16035

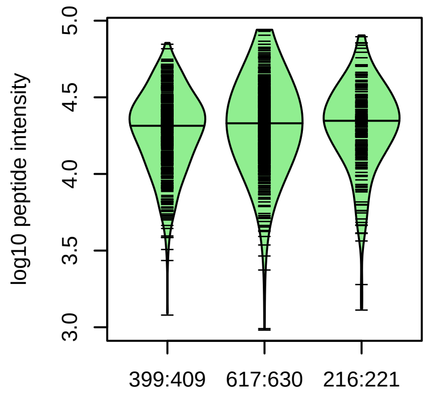

17:76923151:G:A_A
p = 0.011, beta = 0.104, N = 1232

DIEFIYTAPSSAVCGVSLDVGGKK pc
B4DFW2;K7EIX4;P16035

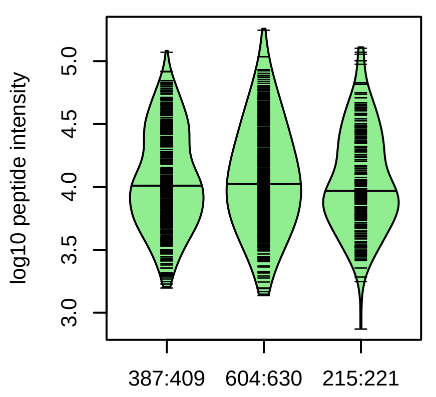

17:76923151:G:A_A
p = 0.66, beta = 0.0184, N = 1206

SDGSCAWYR pc2
B4DFW2;P16035

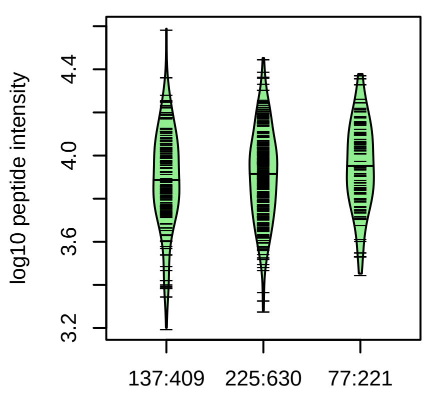

17:76923151:G:A_A
p = 0.041, beta = 0.141, N = 439

MHITLCDFIVPWDTLSTTQKK pc4
B4DFW2;K7EIX4;P16035

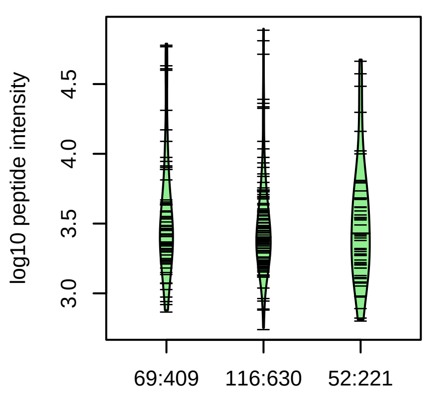

17:76923151:G:A_A
p = 0.63, beta = −0.0433, N = 237

EVDSGNDIYGNPIKR pc3
P16035

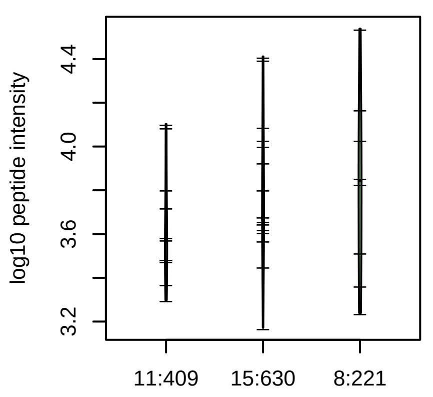

17:76923151:G:A_A
p = 0.3, beta = 0.223, N = 34

TIMP2 : NP5
P16035

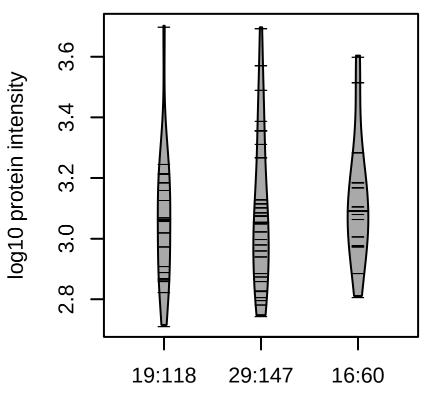

17:76923151:G:A_A
p = 0.58, beta = 0.0911, N = 64

EVDSGNDIYGNPIK pc2
P16035

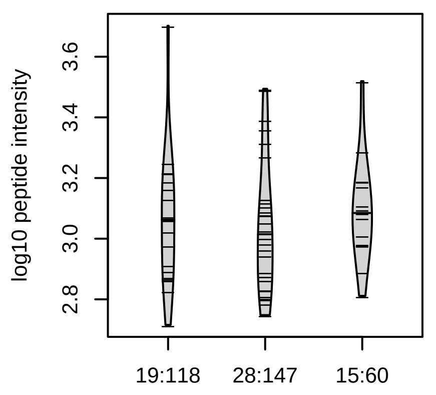

17:76923151:G:A_A
p = 0.64, beta = 0.0774, N = 62

Assay Target: TIMP2
UniProt: P16035
rsID: rs7405712
Target SNP: 17:76923151:G:A
Proxy SNP: 17:76923151:G:A
UKBPPP log10(p): 46.6
UKBPPP BETA: 0.089
UKBPPP MSPA: 0
–:–:–:–:–:–:NA:–
1250:1232:1206:439:237:34:1[illegible]

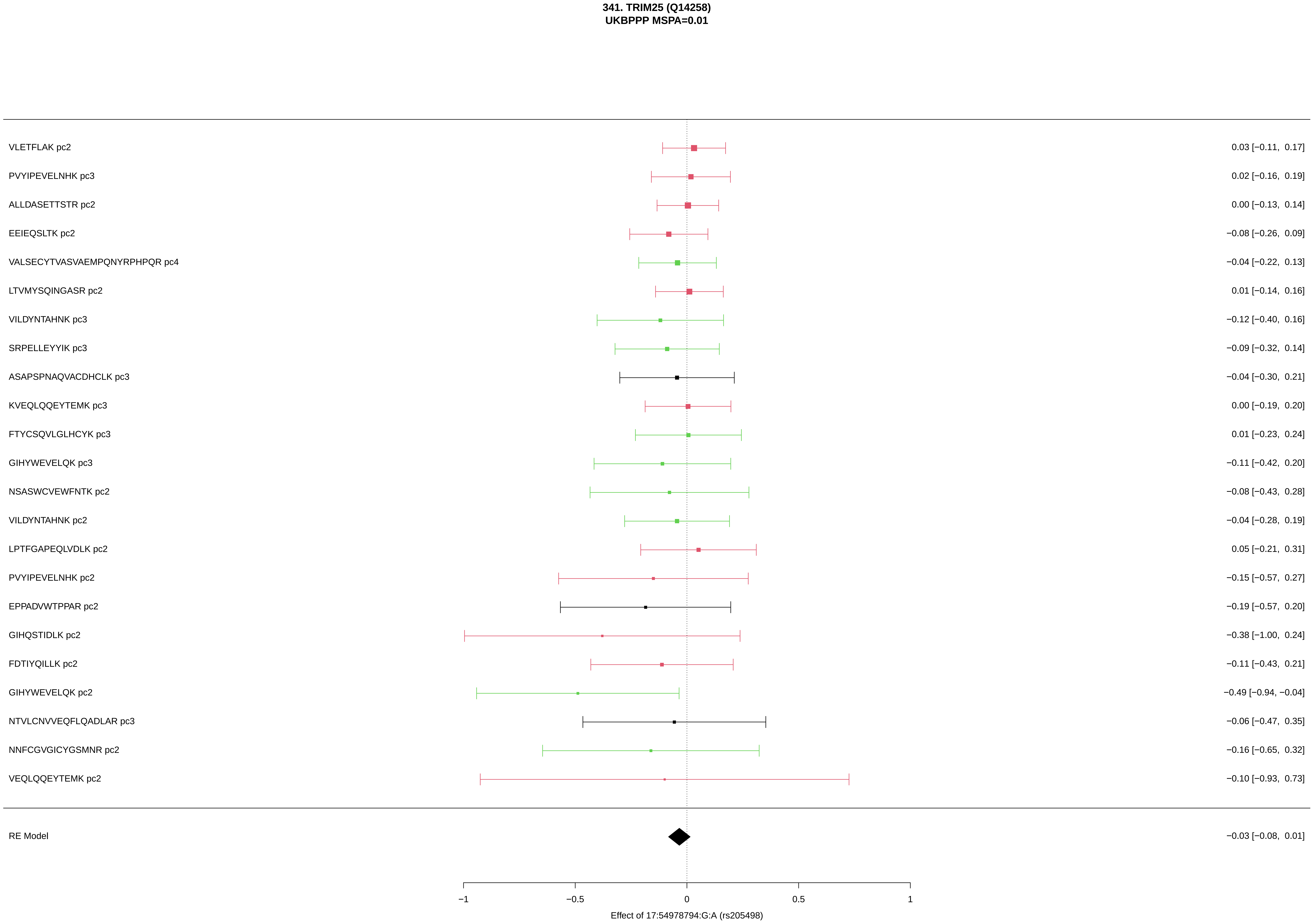
341. TRIM25 (Q14258)
UKBPPP MSPA=0.01
VLETFLAK pc2 0.03 [−0.11, 0.17]
PVYIPEVELNHK pc3 0.02 [−0.16, 0.19]
ALLDASETTSTR pc2 0.00 [−0.13, 0.14]
EEIEQSLTK pc2 −0.08 [−0.26, 0.09]
VALSECYTVASVAEMPQNYRPHPQR pc4 −0.04 [−0.22, 0.13]
LTVMYSQINGASR pc2 0.01 [−0.14, 0.16]
VILDYNTAHNK pc3 −0.12 [−0.40, 0.16]
SRPELLEYYIK pc3 −0.09 [−0.32, 0.14]
ASAPSPNAQVACDHCLK pc3 −0.04 [−0.30, 0.21]
KVEQLQQEYTEMK pc3 0.00 [−0.19, 0.20]
FTYCSQVLGLHCYK pc3 0.01 [−0.23, 0.24]
GIHYWEVELQK pc3 −0.11 [−0.42, 0.20]
NSASWCVEWFNTK pc2 −0.08 [−0.43, 0.28]
VILDYNTAHNK pc2 −0.04 [−0.28, 0.19]
LPTFGAPEQLVDLK pc2 0.05 [−0.21, 0.31]
PVYIPEVELNHK pc2 −0.15 [−0.57, 0.27]
EPPADVWTPPAR pc2 −0.19 [−0.57, 0.20]
GIHQSTIDLK pc2 −0.38 [−1.00, 0.24]
FDTIYQILLK pc2 −0.11 [−0.43, 0.21]
GIHYWEVELQK pc2 −0.49 [−0.94, −0.04]
NTVLCNVVEQFLQADLAR pc3 −0.06 [−0.47, 0.35]
NNFCGVGICYGSMNR pc2 −0.16 [−0.65, 0.32]
VEQLQQEYTEMK pc2 −0.10 [−0.93, 0.73]
RE Model −0.03 [−0.08, 0.01]
−1 −0.5 0 0.5 1
Effect of 17:54978794:G:A (rs205498)

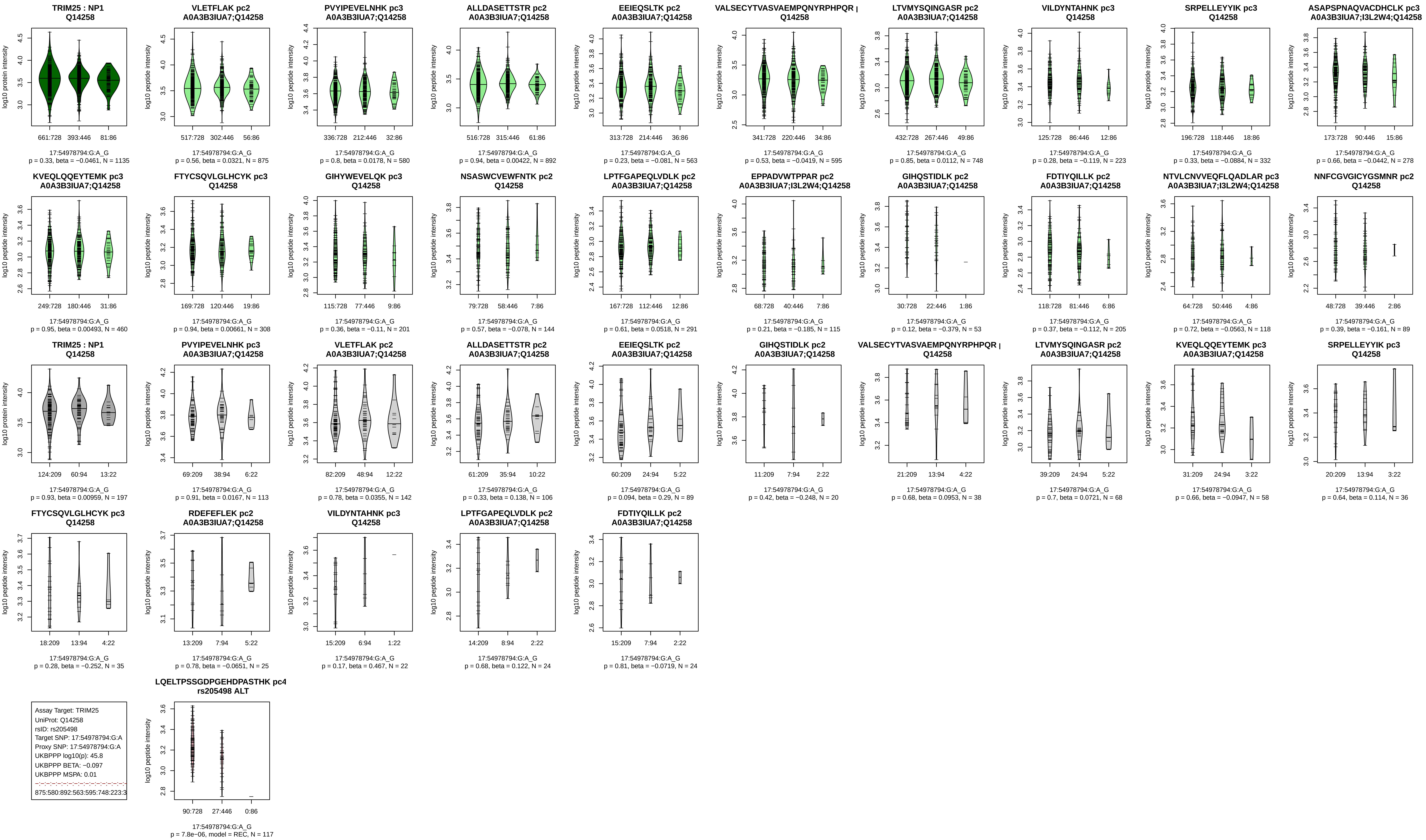
TRIM25 : NP1
Q14258
log10 protein intensity
661:728
393:446
81:86
17:54978794:G:A_G
p = 0.33, beta = −0.0461, N = 1135
VLETFLAK pc2
A0A3B3IUA7;Q14258
log10 peptide intensity
517:728
302:446
56:86
17:54978794:G:A_G
p = 0.56, beta = 0.0321, N = 875
PVYIPEVELNHK pc3
A0A3B3IUA7;Q14258
log10 peptide intensity
336:728
212:446
32:86
17:54978794:G:A_G
p = 0.8, beta = 0.0178, N = 580
ALLDASETTSTR pc2
A0A3B3IUA7;Q14258
log10 peptide intensity
516:728
315:446
61:86
17:54978794:G:A_G
p = 0.94, beta = 0.00422, N = 892
EEIEQSLTK pc2
A0A3B3IUA7;Q14258
log10 peptide intensity
313:728
214:446
36:86
17:54978794:G:A_G
p = 0.23, beta = −0.081, N = 563
VALSECYTVASVAEMPQNYRPHPQR |
Q14258
log10 peptide intensity
341:728
220:446
34:86
17:54978794:G:A_G
p = 0.53, beta = −0.0419, N = 595
LTVMYSQINGASR pc2
A0A3B3IUA7;Q14258
log10 peptide intensity
432:728
267:446
49:86
17:54978794:G:A_G
p = 0.85, beta = 0.0112, N = 748
VILDYNTAHNK pc3
Q14258
log10 peptide intensity
125:728
86:446
12:86
17:54978794:G:A_G
p = 0.28, beta = −0.119, N = 223
SRPELLEYYIK pc3
Q14258
log10 peptide intensity
196:728
118:446
18:86
17:54978794:G:A_G
p = 0.33, beta = −0.0884, N = 332
ASAPSPNAQVACDHCLK pc3
A0A3B3IUA7;I3L2W4;Q14258
log10 peptide intensity
173:728
90:446
15:86
17:54978794:G:A_G
p = 0.66, beta = −0.0442, N = 278
KVEQLQQEYTEMK pc3
A0A3B3IUA7;Q14258
log10 peptide intensity
249:728
180:446
31:86
17:54978794:G:A_G
p = 0.95, beta = 0.00493, N = 460
FTYCSQVLGLHCYK pc3
Q14258
log10 peptide intensity
169:728
120:446
19:86
17:54978794:G:A_G
p = 0.94, beta = 0.00661, N = 308
GIHYWEVELQK pc3
Q14258
log10 peptide intensity
115:728
77:446
9:86
17:54978794:G:A_G
p = 0.36, beta = −0.11, N = 201
NSASWCVEWFNTK pc2
Q14258
log10 peptide intensity
79:728
58:446
7:86
17:54978794:G:A_G
p = 0.57, beta = −0.078, N = 144
LPTFGAPEQLVDLK pc2
A0A3B3IUA7;Q14258
log10 peptide intensity
167:728
112:446
12:86
17:54978794:G:A_G
p = 0.61, beta = 0.0518, N = 291
EPPADVWTPPAR pc2
A0A3B3IUA7;I3L2W4;Q14258
log10 peptide intensity
68:728
40:446
7:86
17:54978794:G:A_G
p = 0.21, beta = −0.185, N = 115
GIHQSTIDLK pc2
A0A3B3IUA7;Q14258
log10 peptide intensity
30:728
22:446
1:86
17:54978794:G:A_G
p = 0.12, beta = −0.379, N = 53
FDTIYQILLK pc2
A0A3B3IUA7;Q14258
log10 peptide intensity
118:728
81:446
6:86
17:54978794:G:A_G
p = 0.37, beta = −0.112, N = 205
NTVLCNVVEQFLQADLAR pc3
A0A3B3IUA7;I3L2W4;Q14258
log10 peptide intensity
64:728
50:446
4:86
17:54978794:G:A_G
p = 0.72, beta = −0.0563, N = 118
NNFCGVGICYGSMNR pc2
Q14258
log10 peptide intensity
48:728
39:446
2:86
17:54978794:G:A_G
p = 0.39, beta = −0.161, N = 89
TRIM25 : NP1
Q14258
log10 protein intensity
124:209
60:94
13:22
17:54978794:G:A_G
p = 0.93, beta = 0.00959, N = 197
PVYIPEVELNHK pc3
A0A3B3IUA7;Q14258
log10 peptide intensity
69:209
38:94
6:22
17:54978794:G:A_G
p = 0.91, beta = 0.0167, N = 113
VLETFLAK pc2
A0A3B3IUA7;Q14258
log10 peptide intensity
82:209
48:94
12:22
17:54978794:G:A_G
p = 0.78, beta = 0.0355, N = 142
ALLDASETTSTR pc2
A0A3B3IUA7;Q14258
log10 peptide intensity
61:209
35:94
10:22
17:54978794:G:A_G
p = 0.33, beta = 0.138, N = 106
EEIEQSLTK pc2
A0A3B3IUA7;Q14258
log10 peptide intensity
60:209
24:94
5:22
17:54978794:G:A_G
p = 0.094, beta = 0.29, N = 89
GIHQSTIDLK pc2
A0A3B3IUA7;Q14258
log10 peptide intensity
11:209
7:94
2:22
17:54978794:G:A_G
p = 0.42, beta = −0.248, N = 20
VALSECYTVASVAEMPQNYRPHPQR |
Q14258
log10 peptide intensity
21:209
13:94
4:22
17:54978794:G:A_G
p = 0.68, beta = 0.0953, N = 38
LTVMYSQINGASR pc2
A0A3B3IUA7;Q14258
log10 peptide intensity
39:209
24:94
5:22
17:54978794:G:A_G
p = 0.7, beta = 0.0721, N = 68
KVEQLQQEYTEMK pc3
A0A3B3IUA7;Q14258
log10 peptide intensity
31:209
24:94
3:22
17:54978794:G:A_G
p = 0.66, beta = −0.0947, N = 58
SRPELLEYYIK pc3
Q14258
log10 peptide intensity
20:209
13:94
3:22
17:54978794:G:A_G
p = 0.64, beta = 0.114, N = 36
FTYCSQVLGLHCYK pc3
Q14258
log10 peptide intensity
18:209
13:94
4:22
17:54978794:G:A_G
p = 0.28, beta = −0.252, N = 35
RDEFEFLEK pc2
A0A3B3IUA7;Q14258
log10 peptide intensity
13:209
7:94
5:22
17:54978794:G:A_G
p = 0.78, beta = −0.0651, N = 25
VILDYNTAHNK pc3
Q14258
log10 peptide intensity
15:209
6:94
1:22
17:54978794:G:A_G
p = 0.17, beta = 0.467, N = 22
LPTFGAPEQLVDLK pc2
A0A3B3IUA7;Q14258
log10 peptide intensity
14:209
8:94
2:22
17:54978794:G:A_G
p = 0.68, beta = 0.122, N = 24
FDTIYQILLK pc2
A0A3B3IUA7;Q14258
log10 peptide intensity
15:209
7:94
2:22
17:54978794:G:A_G
p = 0.81, beta = −0.0719, N = 24
Assay Target: TRIM25
UniProt: Q14258
rsID: rs205498
Target SNP: 17:54978794:G:A
Proxy SNP: 17:54978794:G:A
UKBPPP log10(p): 45.8
UKBPPP BETA: −0.097
UKBPPP MSPA: 0.01
875:580:892:563:595:748:223:3
LQELTPSSGDPGEHDPASTHK pc4
rs205498 ALT
log10 peptide intensity
90:728
27:446
0:86
17:54978794:G:A_G
p = 7.8e−06, model = REC, N = 117

342. LTA4H (P09960)
UKBPPP MSPA=0

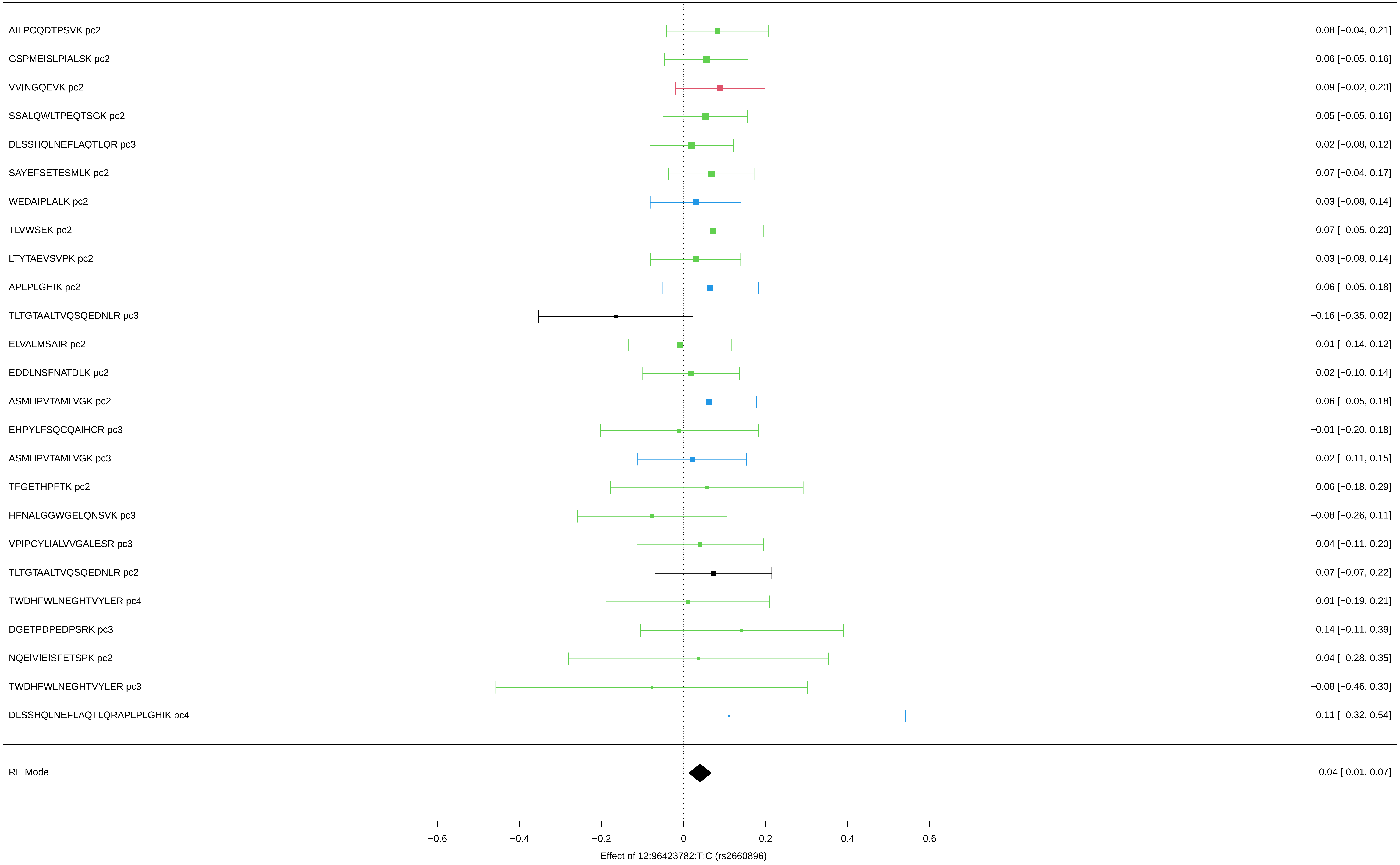

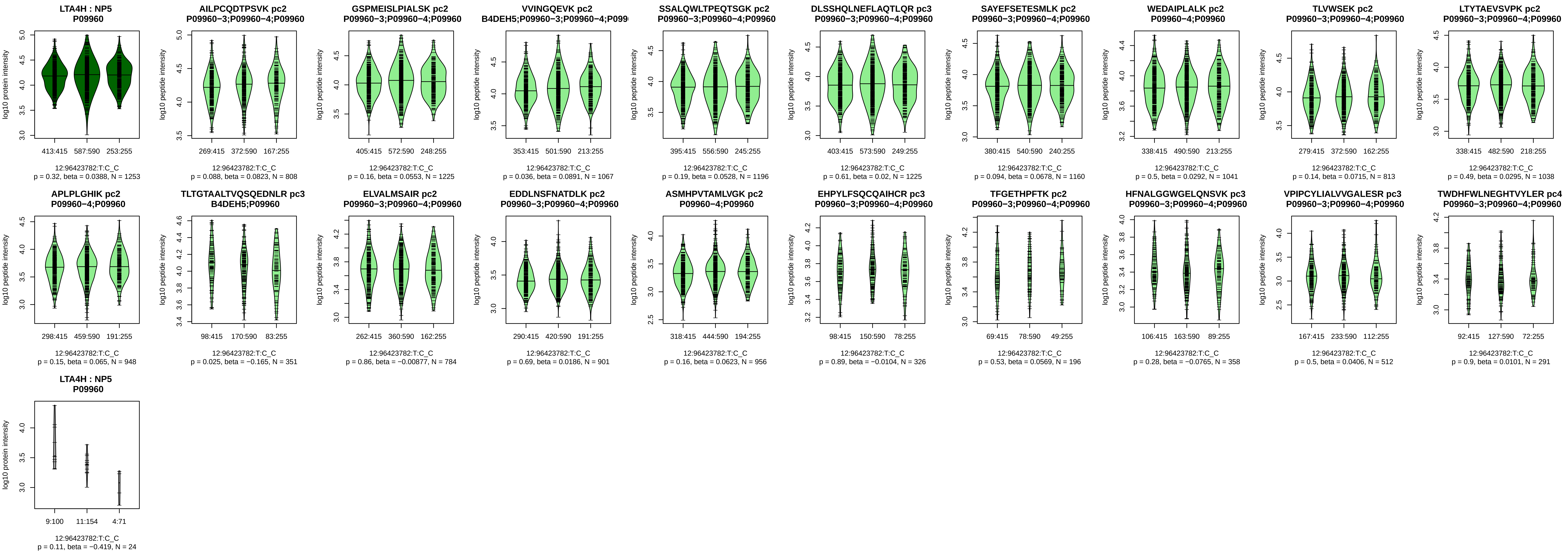

Assay Target: LTA4H
UniProt: P09960
rsID: rs2660896
Target SNP: 12:96423782:T:C
Proxy SNP: 12:96423782:T:C
UKBPPP log10(p): 45.7
UKBPPP BETA: 0.087
UKBPPP MSPA: 0

808:1225:1067:1196:1225:1160

343. PTPRK (Q15262)
UKBPPP MSPA=0

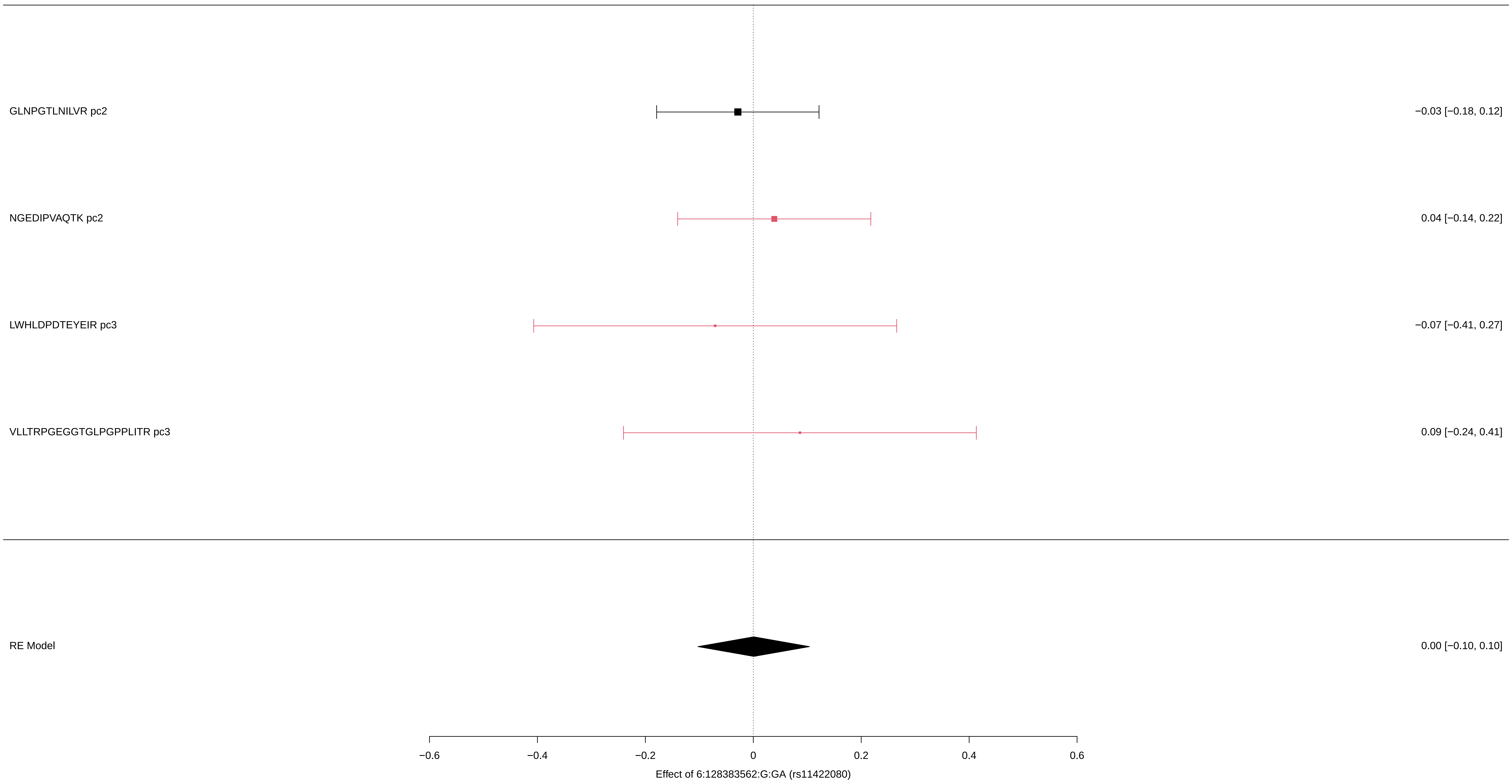

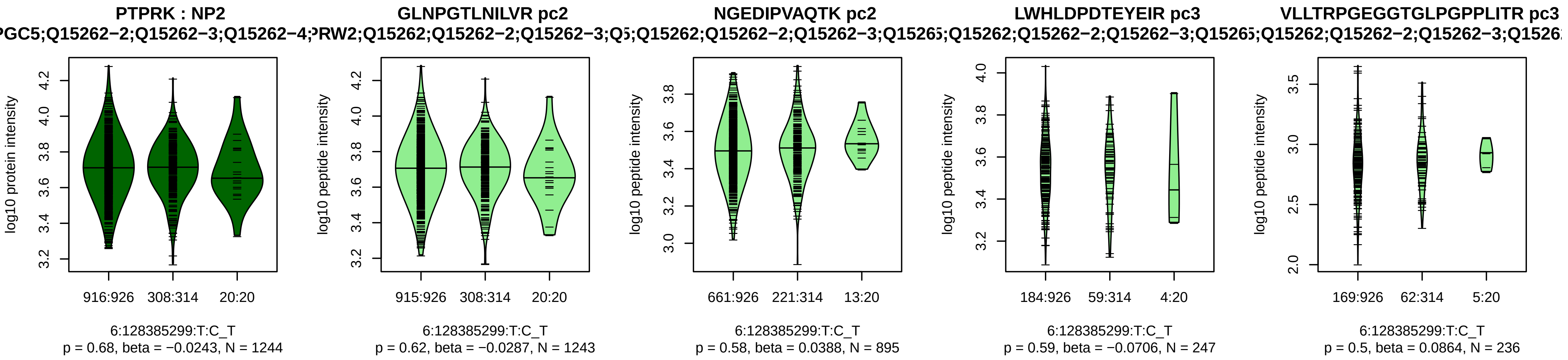

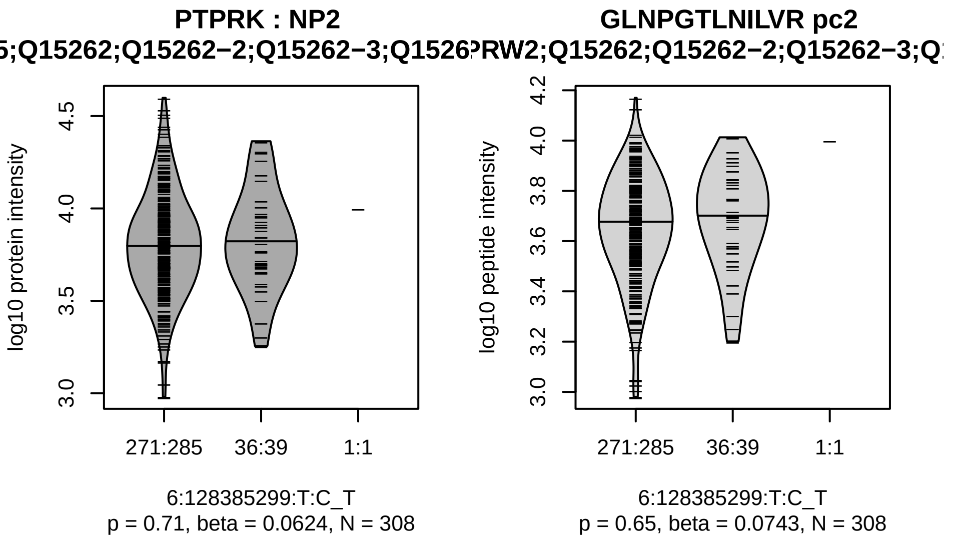

Assay Target: PTPRK
UniProt: Q15262
rsID: rs11422080
Target SNP: 6:128383562:G:GA
Proxy SNP: 6:128385299:T:C
UKBPPP log10(p): 44.7
UKBPPP BETA: −0.12
UKBPPP MSPA: 0
−:−:−:−
1243:895:247:236

344. SERPINB1 (P30740)
UKBPPP MSPA=0

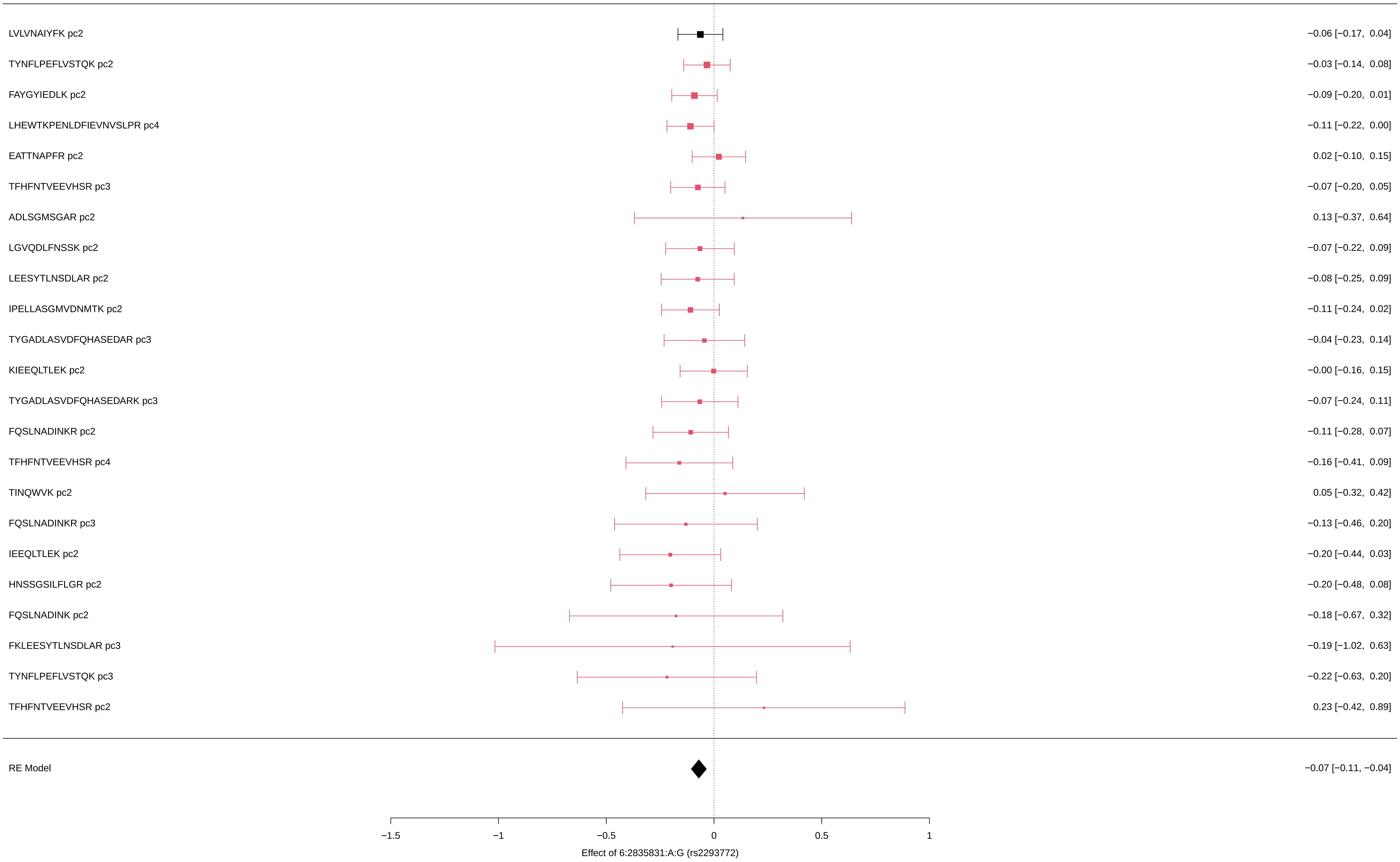

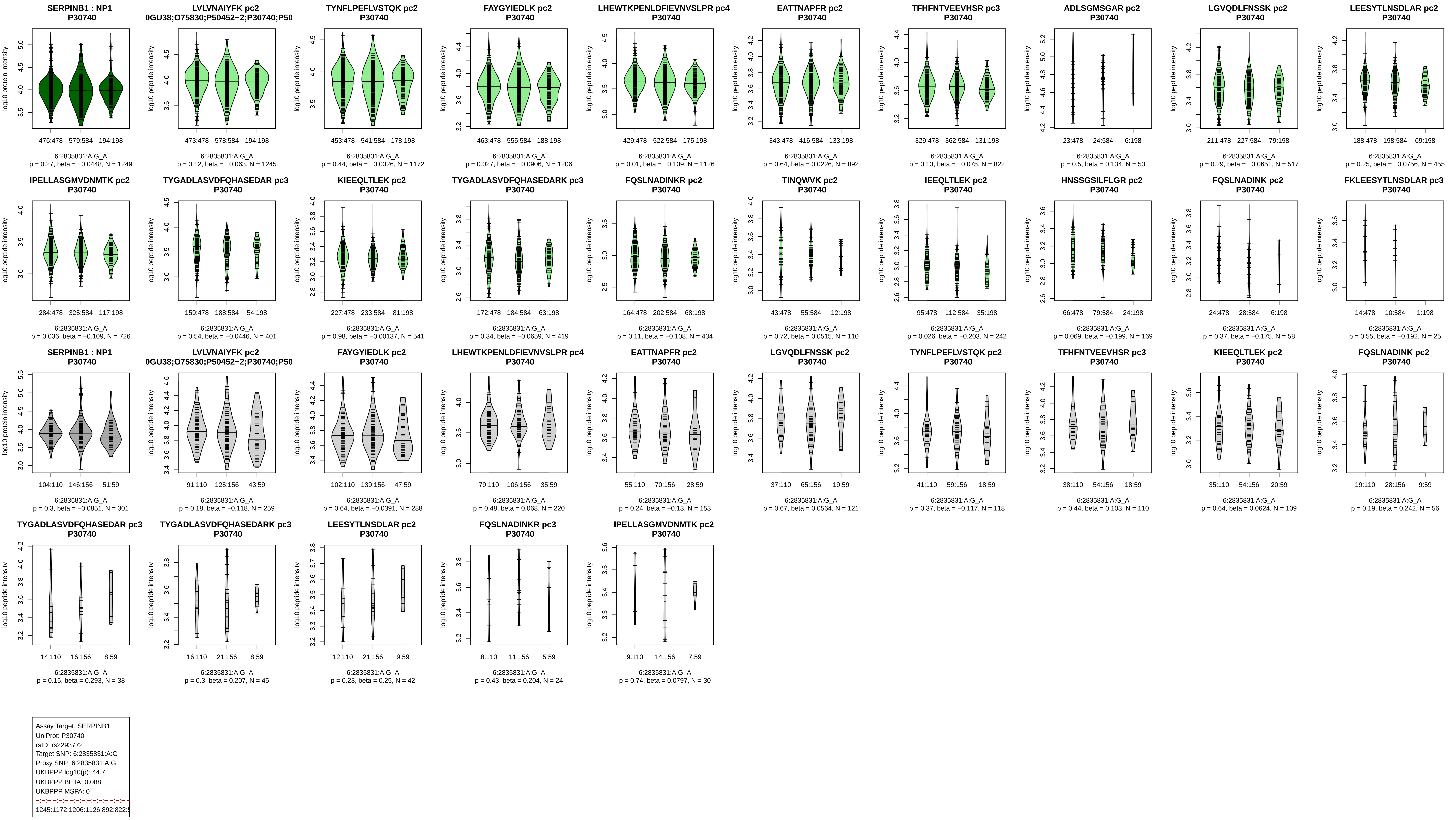
SERPINB1 : NP1 P30740
p = 0.27, beta = −0.0448, N = 1249
LVLVNAIYFK pc2 0GU38;O75830;P50452−2;P30740;P50
p = 0.12, beta = −0.063, N = 1245
TYNFLPEFLVSTQK pc2 P30740
p = 0.44, beta = −0.0326, N = 1172
FAYGYIEDLK pc2 P30740
p = 0.027, beta = −0.0906, N = 1206
LHEWTKPENLDFIEVNVSLPR pc4 P30740
p = 0.01, beta = −0.109, N = 1126
EATTNAPFR pc2 P30740
p = 0.64, beta = 0.0226, N = 892
TFHFNTVEEVHSR pc3 P30740
p = 0.13, beta = −0.075, N = 822
ADLSGMSGAR pc2 P30740
p = 0.5, beta = 0.134, N = 53
LGVQDLFNSSK pc2 P30740
p = 0.29, beta = −0.0651, N = 517
LEESYTLNSDLAR pc2 P30740
p = 0.25, beta = −0.0756, N = 455
IPELLASGMVDNMTK pc2 P30740
p = 0.036, beta = −0.109, N = 726
TYGADLASVDFQHASEDAR pc3 P30740
p = 0.54, beta = −0.0446, N = 401
KIEEQLTLEK pc2 P30740
p = 0.98, beta = −0.00137, N = 541
TYGADLASVDFQHASEDARK pc3 P30740
p = 0.34, beta = −0.0659, N = 419
FQSLNADINKR pc2 P30740
p = 0.11, beta = −0.108, N = 434
TINQWVK pc2 P30740
p = 0.72, beta = 0.0515, N = 110
IEEQLTLEK pc2 P30740
p = 0.026, beta = −0.203, N = 242
HNSSGSILFLGR pc2 P30740
p = 0.069, beta = −0.199, N = 169
FQSLNADINK pc2 P30740
p = 0.37, beta = −0.175, N = 58
FKLEESYTLNSDLAR pc3 P30740
p = 0.55, beta = −0.192, N = 25
SERPINB1 : NP1 P30740
p = 0.3, beta = −0.0851, N = 301
LVLVNAIYFK pc2 0GU38;O75830;P50452−2;P30740;P50
p = 0.18, beta = −0.118, N = 259
FAYGYIEDLK pc2 P30740
p = 0.64, beta = −0.0391, N = 288
LHEWTKPENLDFIEVNVSLPR pc4 P30740
p = 0.48, beta = 0.068, N = 220
EATTNAPFR pc2 P30740
p = 0.24, beta = −0.13, N = 153
LGVQDLFNSSK pc2 P30740
p = 0.67, beta = 0.0564, N = 121
TYNFLPEFLVSTQK pc2 P30740
p = 0.37, beta = −0.117, N = 118
TFHFNTVEEVHSR pc3 P30740
p = 0.44, beta = 0.103, N = 110
KIEEQLTLEK pc2 P30740
p = 0.64, beta = 0.0624, N = 109
FQSLNADINK pc2 P30740
p = 0.19, beta = 0.242, N = 56
TYGADLASVDFQHASEDAR pc3 P30740
p = 0.15, beta = 0.293, N = 38
TYGADLASVDFQHASEDARK pc3 P30740
p = 0.3, beta = 0.207, N = 45
LEESYTLNSDLAR pc2 P30740
p = 0.23, beta = 0.25, N = 42
FQSLNADINKR pc3 P30740
p = 0.43, beta = 0.204, N = 24
IPELLASGMVDNMTK pc2 P30740
p = 0.74, beta = 0.0797, N = 30
6:2835831:A:G_A
Assay Target: SERPINB1
UniProt: P30740
rsID: rs2293772
Target SNP: 6:2835831:A:G
Proxy SNP: 6:2835831:A:G
UKBPPP log10(p): 44.7
UKBPPP BETA: 0.088
UKBPPP MSPA: 0
1245:1172:1206:1126:892:822:5

345. PDIA4 (P13667)
UKBPPP MSPA=0

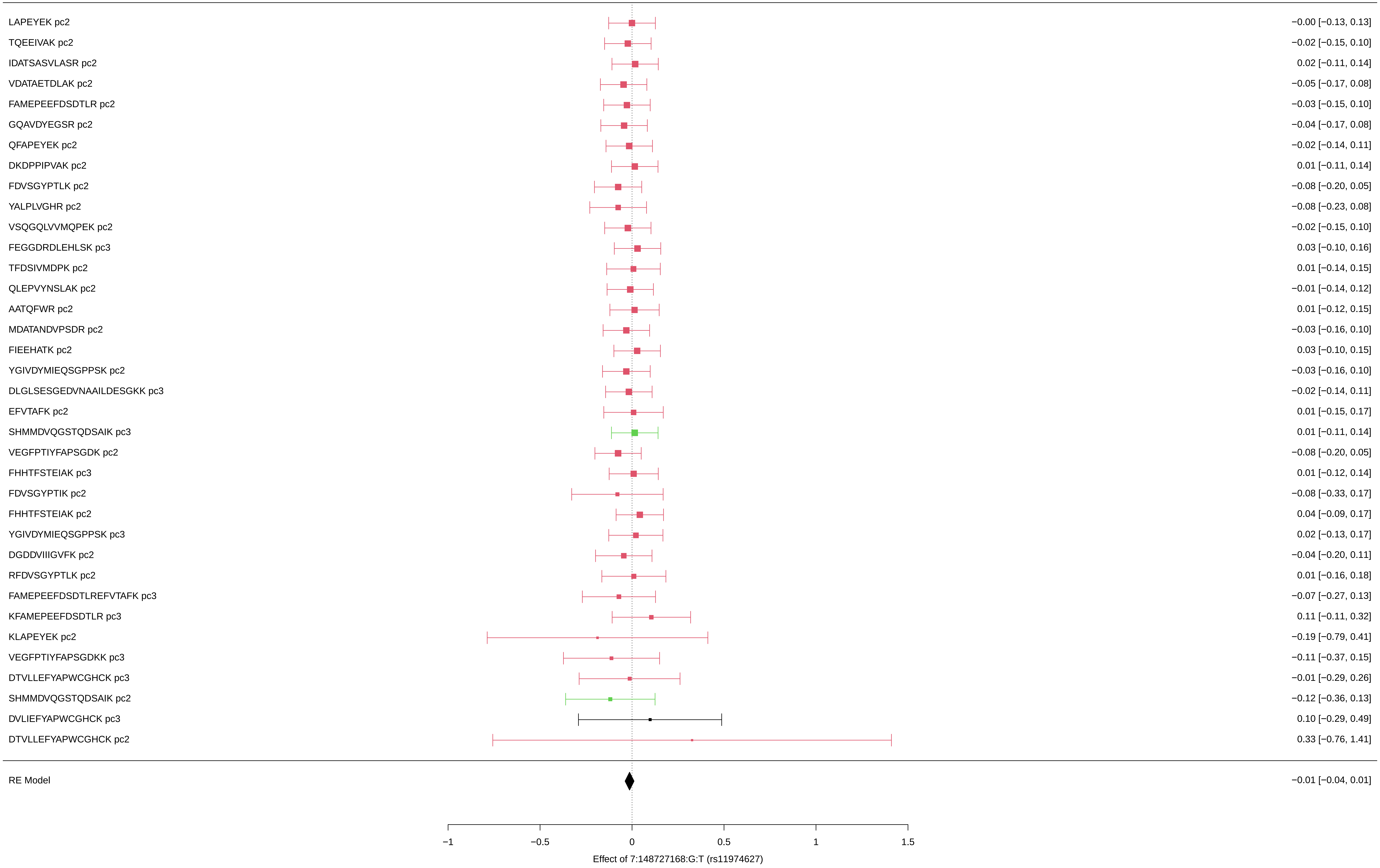

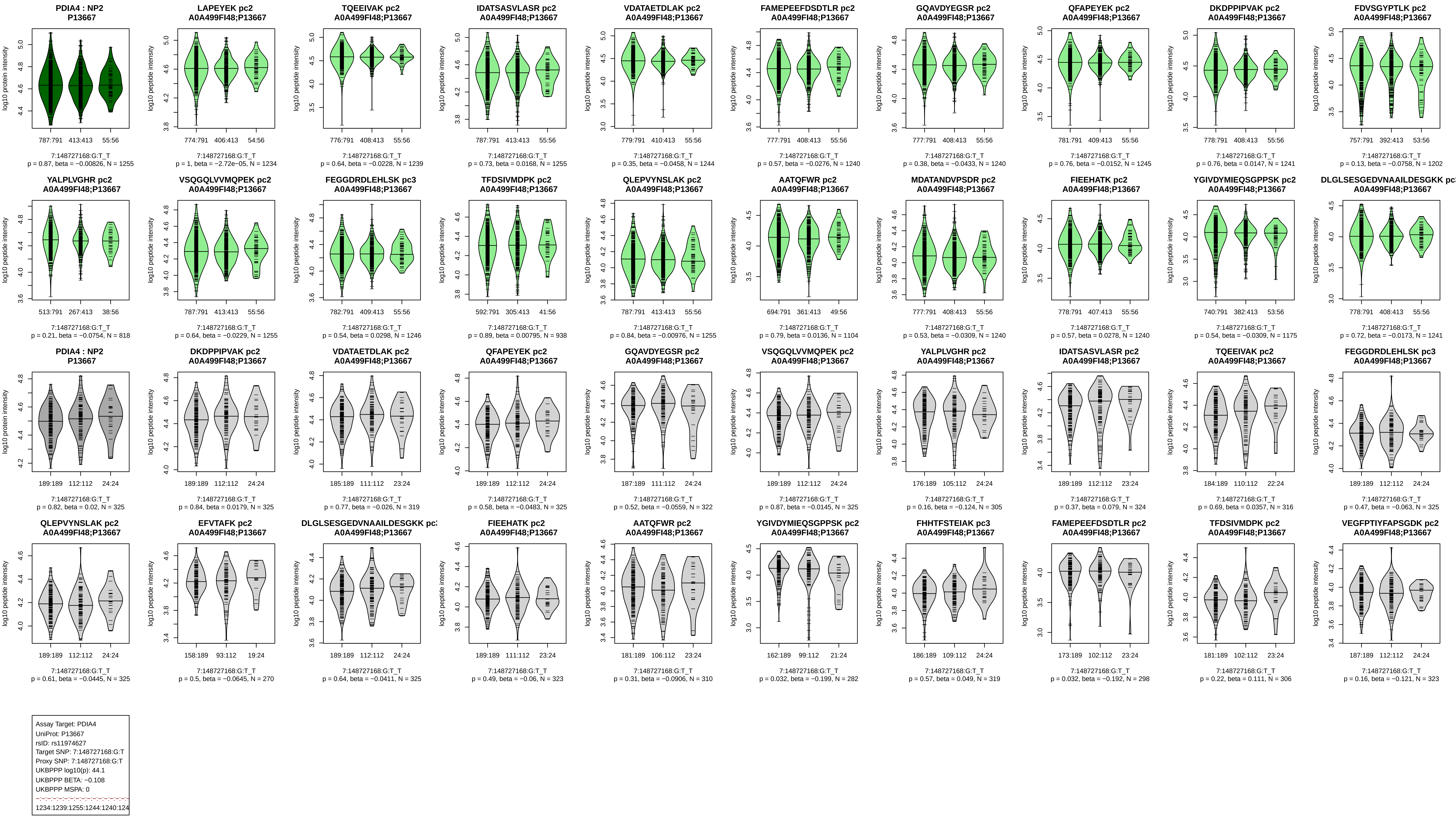
PDIA4 : NP2
P13667
7:148727168:G:T_T
p = 0.87, beta = −0.00826, N = 1255
LAPEYEK pc2
A0A499FI48;P13667
p = 1, beta = −2.72e−05, N = 1234
TQEEIVAK pc2
A0A499FI48;P13667
p = 0.64, beta = −0.0228, N = 1239
IDATSASVLASR pc2
A0A499FI48;P13667
p = 0.73, beta = 0.0168, N = 1255
VDATAETDLAK pc2
A0A499FI48;P13667
p = 0.35, beta = −0.0458, N = 1244
FAMEPEEFDSDTLR pc2
A0A499FI48;P13667
p = 0.57, beta = −0.0276, N = 1240
GQAVDYEGSR pc2
A0A499FI48;P13667
p = 0.38, beta = −0.0433, N = 1240
QFAPEYEK pc2
A0A499FI48;P13667
p = 0.76, beta = −0.0152, N = 1245
DKDPPIPVAK pc2
A0A499FI48;P13667
p = 0.76, beta = 0.0147, N = 1241
FDVSGYPTLK pc2
A0A499FI48;P13667
p = 0.13, beta = −0.0758, N = 1202
YALPLVGHR pc2
A0A499FI48;P13667
p = 0.21, beta = −0.0754, N = 818
VSQGQLVVMQPEK pc2
A0A499FI48;P13667
p = 0.64, beta = −0.0229, N = 1255
FEGGDRDLEHLSK pc3
A0A499FI48;P13667
p = 0.54, beta = 0.0298, N = 1246
TFDSIVMDPK pc2
A0A499FI48;P13667
p = 0.89, beta = 0.00795, N = 938
QLEPVYNSLAK pc2
A0A499FI48;P13667
p = 0.84, beta = −0.00976, N = 1255
AATQFWR pc2
A0A499FI48;P13667
p = 0.79, beta = 0.0136, N = 1104
MDATANDVPSDR pc2
A0A499FI48;P13667
p = 0.53, beta = −0.0309, N = 1240
FIEEHATK pc2
A0A499FI48;P13667
p = 0.57, beta = 0.0278, N = 1240
YGIVDYMIEQSGPPSK pc2
A0A499FI48;P13667
p = 0.54, beta = −0.0309, N = 1175
DLGLSESGEDVNAAILDESGKK pc
A0A499FI48;P13667
p = 0.72, beta = −0.0173, N = 1241
PDIA4 : NP2
P13667
p = 0.82, beta = 0.02, N = 325
DKDPPIPVAK pc2
A0A499FI48;P13667
p = 0.84, beta = 0.0179, N = 325
VDATAETDLAK pc2
A0A499FI48;P13667
p = 0.77, beta = −0.026, N = 319
QFAPEYEK pc2
A0A499FI48;P13667
p = 0.58, beta = −0.0483, N = 325
GQAVDYEGSR pc2
A0A499FI48;P13667
p = 0.52, beta = −0.0559, N = 322
VSQGQLVVMQPEK pc2
A0A499FI48;P13667
p = 0.87, beta = −0.0145, N = 325
YALPLVGHR pc2
A0A499FI48;P13667
p = 0.16, beta = −0.124, N = 305
IDATSASVLASR pc2
A0A499FI48;P13667
p = 0.37, beta = 0.079, N = 324
TQEEIVAK pc2
A0A499FI48;P13667
p = 0.69, beta = 0.0357, N = 316
FEGGDRDLEHLSK pc3
A0A499FI48;P13667
p = 0.47, beta = −0.063, N = 325
QLEPVYNSLAK pc2
A0A499FI48;P13667
p = 0.61, beta = −0.0445, N = 325
EFVTAFK pc2
A0A499FI48;P13667
p = 0.5, beta = −0.0645, N = 270
DLGLSESGEDVNAAILDESGKK pc
A0A499FI48;P13667
p = 0.64, beta = −0.0411, N = 325
FIEEHATK pc2
A0A499FI48;P13667
p = 0.49, beta = −0.06, N = 323
AATQFWR pc2
A0A499FI48;P13667
p = 0.31, beta = −0.0906, N = 310
YGIVDYMIEQSGPPSK pc2
A0A499FI48;P13667
p = 0.032, beta = −0.199, N = 282
FHHTFSTEIAK pc3
A0A499FI48;P13667
p = 0.57, beta = 0.049, N = 319
FAMEPEEFDSDTLR pc2
A0A499FI48;P13667
p = 0.032, beta = −0.192, N = 298
TFDSIVMDPK pc2
A0A499FI48;P13667
p = 0.22, beta = 0.111, N = 306
VEGFPTIYFAPSGDK pc2
A0A499FI48;P13667
p = 0.16, beta = −0.121, N = 323
Assay Target: PDIA4
UniProt: P13667
rsID: rs11974627
Target SNP: 7:148727168:G:T
Proxy SNP: 7:148727168:G:T
UKBPPP log10(p): 44.1
UKBPPP BETA: −0.108
UKBPPP MSPA: 0
1234:1239:1255:1244:1240:124

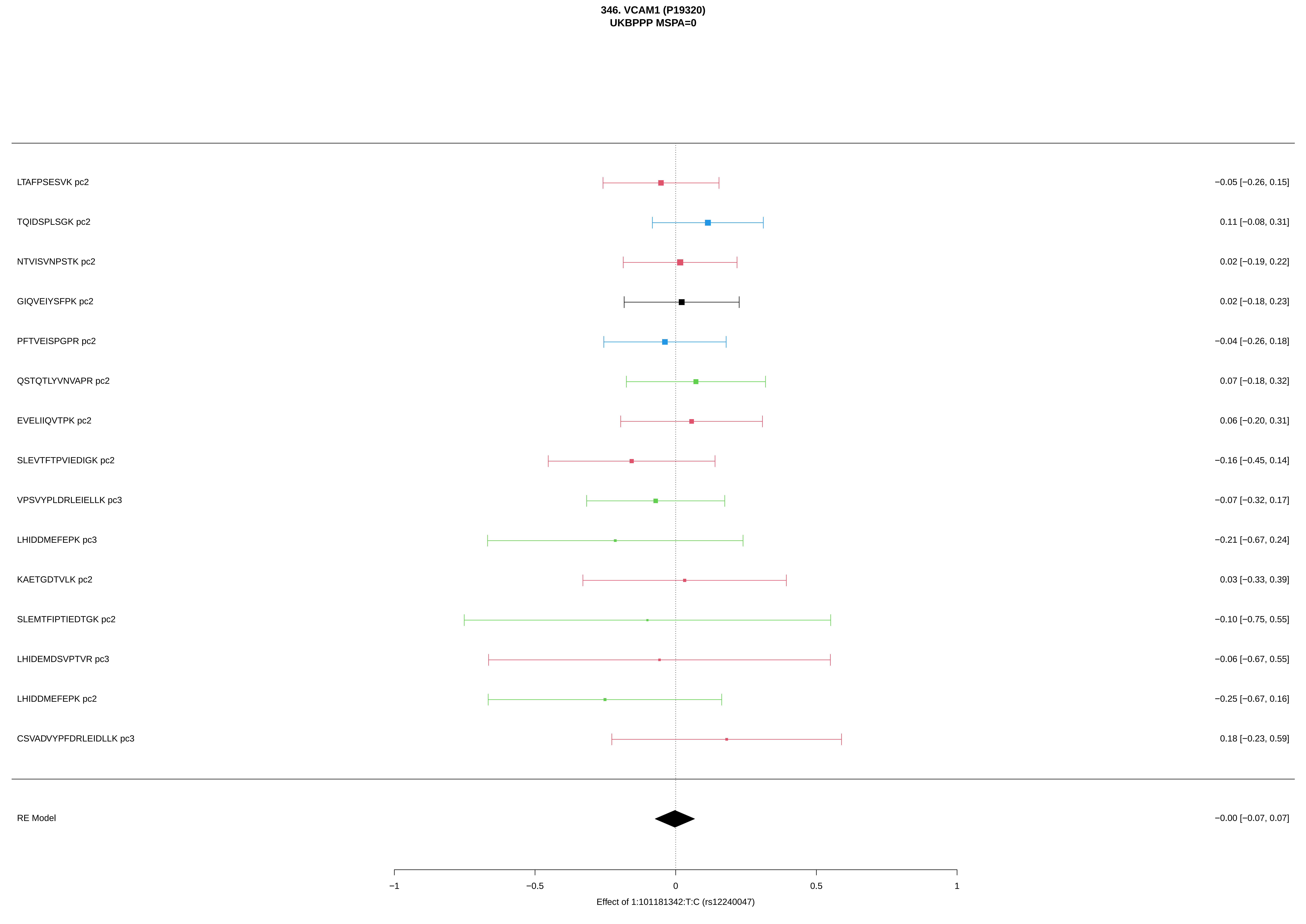

346. VCAM1 (P19320)
UKBPPP MSPA=0
LTAFPSESVK pc2
−0.05 [−0.26, 0.15]
TQIDSPLSGK pc2
0.11 [−0.08, 0.31]
NTVISVNPSTK pc2
0.02 [−0.19, 0.22]
GIQVEIYSFPK pc2
0.02 [−0.18, 0.23]
PFTVEISPGPR pc2
−0.04 [−0.26, 0.18]
QSTQTLYVNVAPR pc2
0.07 [−0.18, 0.32]
EVELIIQVTPK pc2
0.06 [−0.20, 0.31]
SLEVTFTPVIEDIGK pc2
−0.16 [−0.45, 0.14]
VPSVYPLDRLEIELLK pc3
−0.07 [−0.32, 0.17]
LHIDDMEFEPK pc3
−0.21 [−0.67, 0.24]
KAETGDTVLK pc2
0.03 [−0.33, 0.39]
SLEMTFIPTIEDTGK pc2
−0.10 [−0.75, 0.55]
LHIDEMDSVPTVR pc3
−0.06 [−0.67, 0.55]
LHIDDMEFEPK pc2
−0.25 [−0.67, 0.16]
CSVADVYPFDRLEIDLLK pc3
0.18 [−0.23, 0.59]
RE Model
−0.00 [−0.07, 0.07]
−1
−0.5
0
0.5
1
Effect of 1:101181342:T:C (rs12240047)

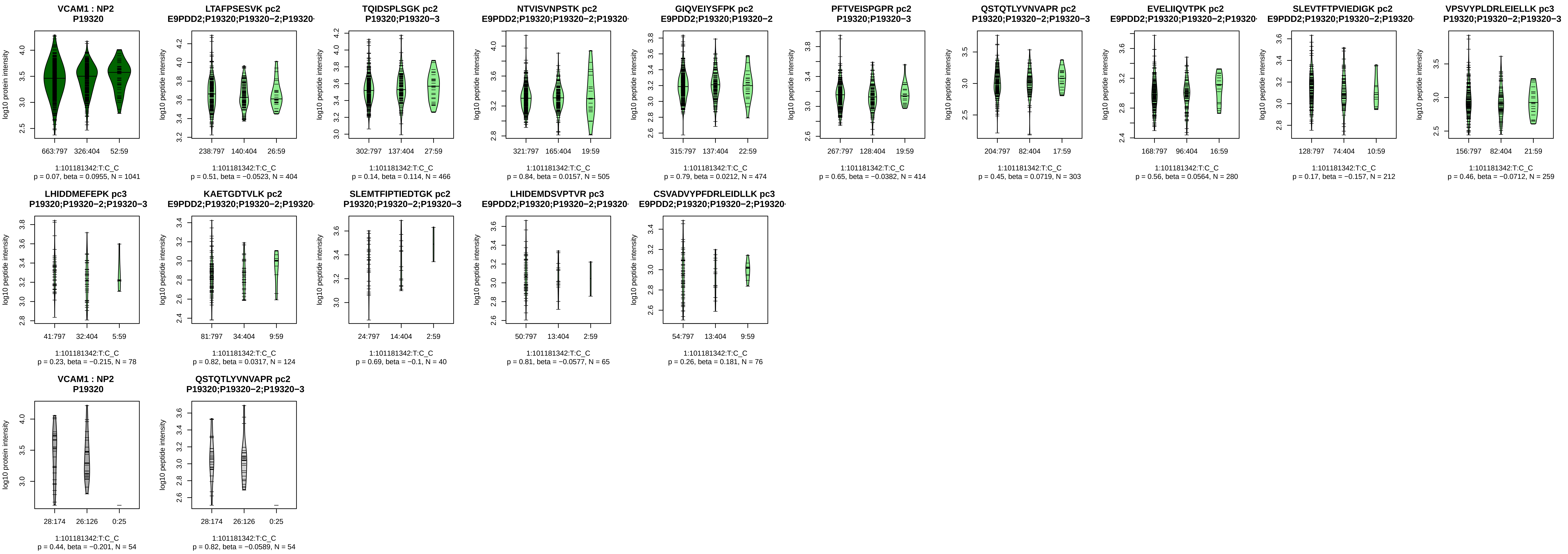

Assay Target: VCAM1
UniProt: P19320
rsID: rs12240047
Target SNP: 1:101181342:T:C
Proxy SNP: 1:101181342:T:C
UKBPPP log10(p): 42.7
UKBPPP BETA: −0.102
UKBPPP MSPA: 0
-:-:-:-:-:-:-:-:-:-:-:-:-
404:466:505:474:414:303:280:2

347. CA1 (P00915)
UKBPPP MSPA=0

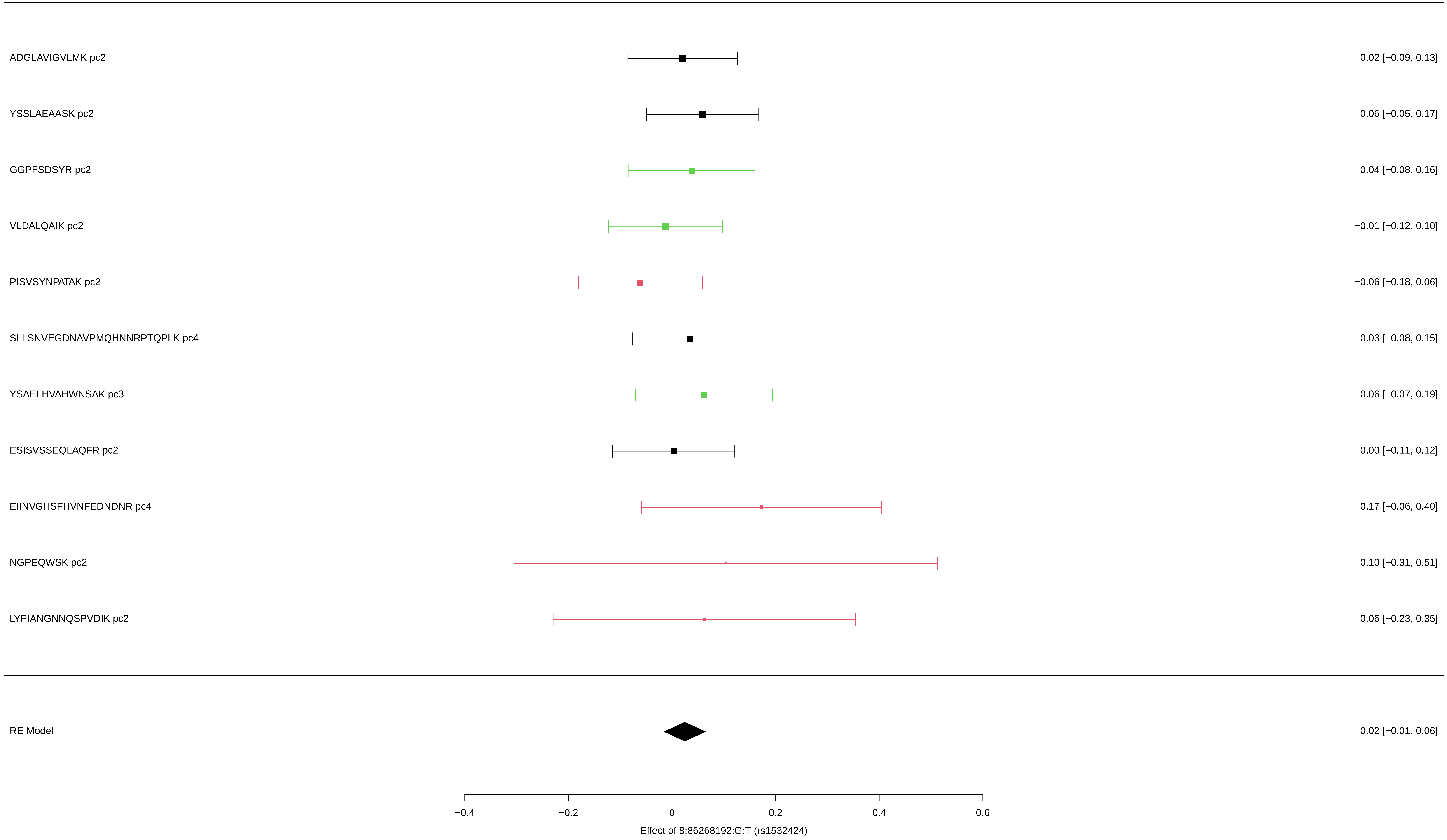

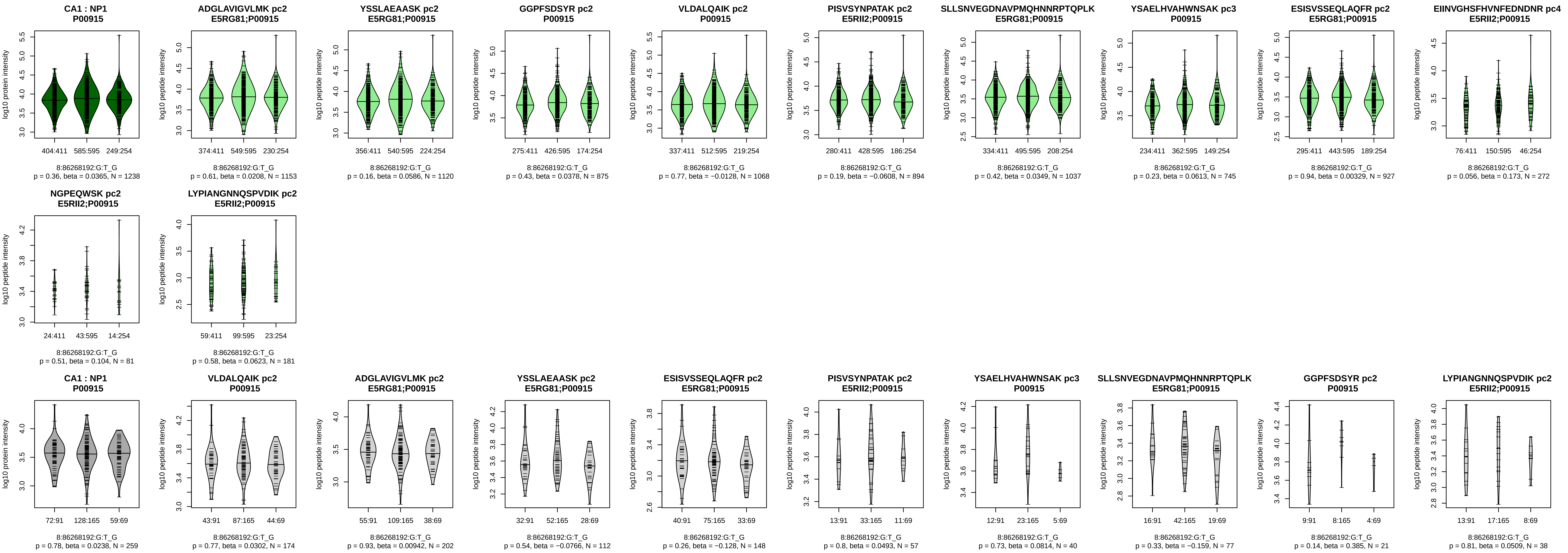

Assay Target: CA1
UniProt: P00915
rsID: rs1532424
Target SNP: 8:86268192:G:T
Proxy SNP: 8:86268192:G:T
UKBPPP log10(p): 41.7
UKBPPP BETA: −0.081
UKBPPP MSPA: 0
–:–:–:–:–:–:–:–:–:–:–:NA:NA:NA
1153:1120:875:1068:894:1037:7

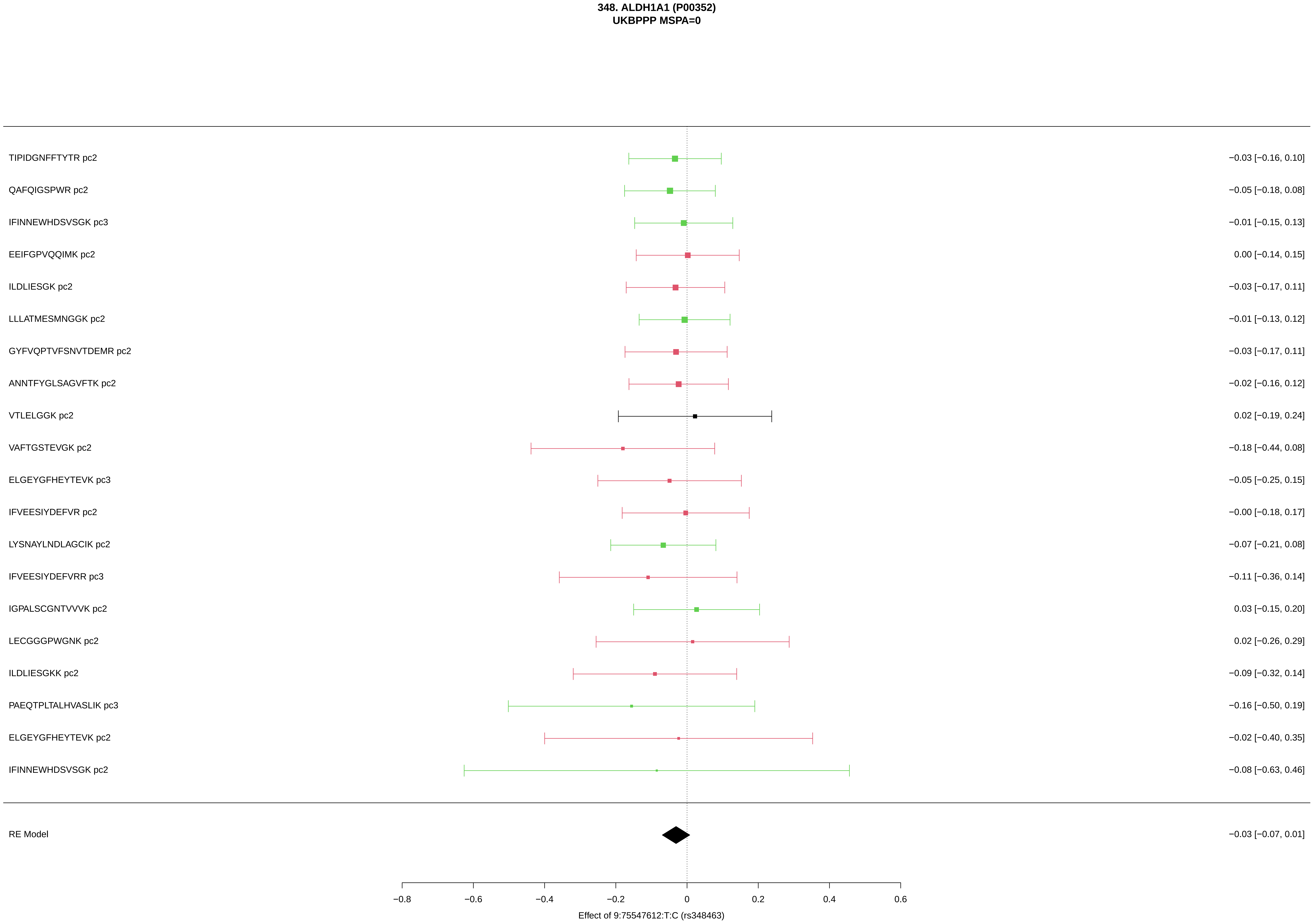
348. ALDH1A1 (P00352)
UKBPPP MSPA=0
TIPIDGNFFTYTR pc2
QAFQIGSPWR pc2
IFINNEWHDSVSGK pc3
EEIFGPVQQIMK pc2
ILDLIESGK pc2
LLLATMESMNGGK pc2
GYFVQPTVFSNVTDEMR pc2
ANNTFYGLSAGVFTK pc2
VTLELGGK pc2
VAFTGSTEVGK pc2
ELGEYGFHEYTEVK pc3
IFVEESIYDEFVR pc2
LYSNAYLNDLAGCIK pc2
IFVEESIYDEFVRR pc3
IGPALSCGNTVVVK pc2
LECGGGPWGNK pc2
ILDLIESGKK pc2
PAEQTPLTALHVASLIK pc3
ELGEYGFHEYTEVK pc2
IFINNEWHDSVSGK pc2
RE Model
−0.03 [−0.16, 0.10]
−0.05 [−0.18, 0.08]
−0.01 [−0.15, 0.13]
0.00 [−0.14, 0.15]
−0.03 [−0.17, 0.11]
−0.01 [−0.13, 0.12]
−0.03 [−0.17, 0.11]
−0.02 [−0.16, 0.12]
0.02 [−0.19, 0.24]
−0.18 [−0.44, 0.08]
−0.05 [−0.25, 0.15]
−0.00 [−0.18, 0.17]
−0.07 [−0.21, 0.08]
−0.11 [−0.36, 0.14]
0.03 [−0.15, 0.20]
0.02 [−0.26, 0.29]
−0.09 [−0.32, 0.14]
−0.16 [−0.50, 0.19]
−0.02 [−0.40, 0.35]
−0.08 [−0.63, 0.46]
−0.03 [−0.07, 0.01]
−0.8
−0.6
−0.4
−0.2
0
0.2
0.4
0.6
Effect of 9:75547612:T:C (rs348463)

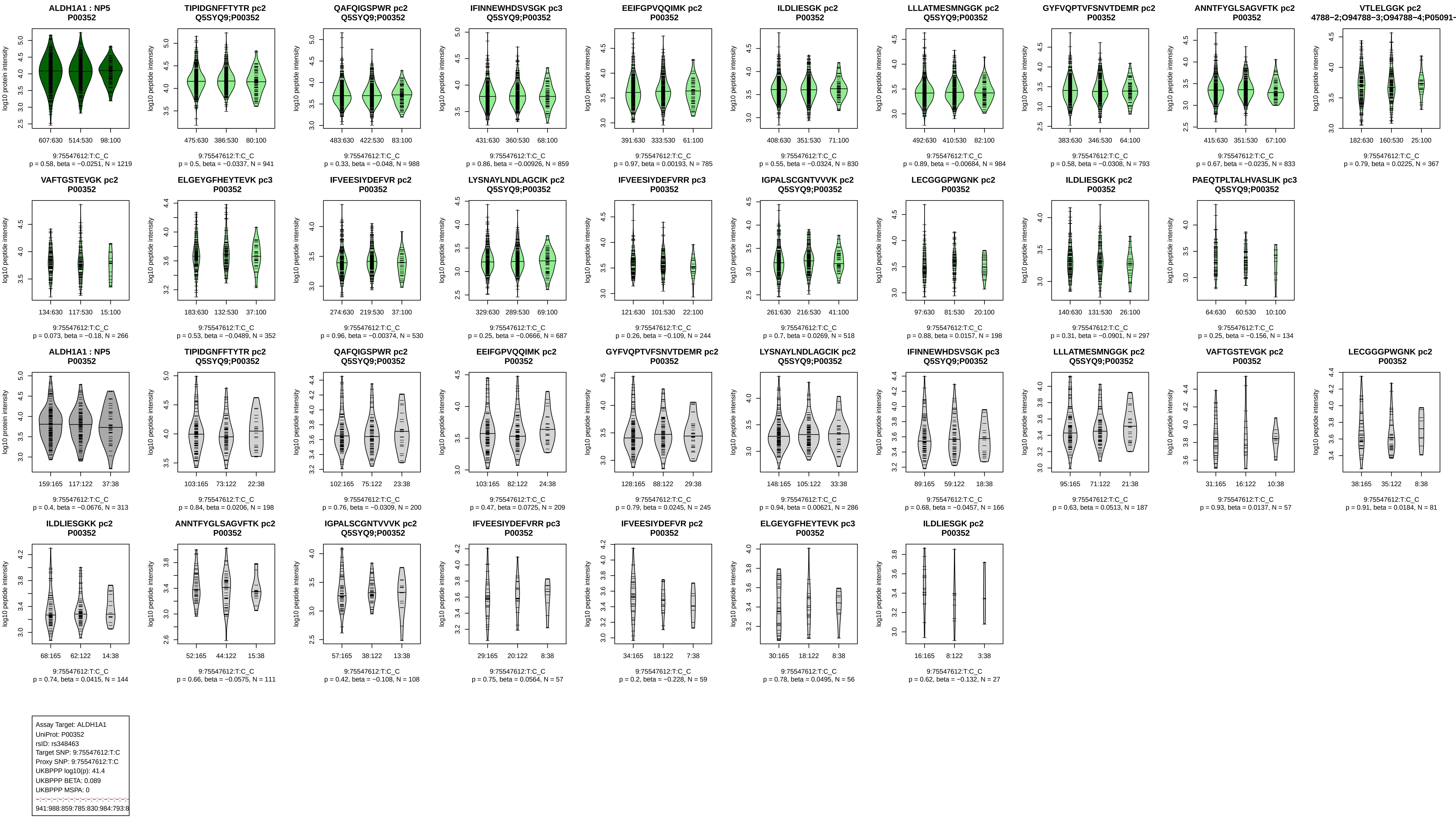
ALDH1A1 : NP5
P00352
607:630 514:530 98:100
9:75547612:T:C_C
p = 0.58, beta = −0.0251, N = 1219
TIPIDGNFFTYTR pc2
Q5SYQ9;P00352
475:630 386:530 80:100
9:75547612:T:C_C
p = 0.5, beta = −0.0337, N = 941
QAFQIGSPWR pc2
Q5SYQ9;P00352
483:630 422:530 83:100
9:75547612:T:C_C
p = 0.33, beta = −0.048, N = 988
IFINNEWHDSVSGK pc3
Q5SYQ9;P00352
431:630 360:530 68:100
9:75547612:T:C_C
p = 0.86, beta = −0.00926, N = 859
EEIFGPVQQIMK pc2
P00352
391:630 333:530 61:100
9:75547612:T:C_C
p = 0.97, beta = 0.00193, N = 785
ILDLIESGK pc2
P00352
408:630 351:530 71:100
9:75547612:T:C_C
p = 0.55, beta = −0.0324, N = 830
LLLATMESMNGGK pc2
Q5SYQ9;P00352
492:630 410:530 82:100
9:75547612:T:C_C
p = 0.89, beta = −0.00684, N = 984
GYFVQPTVFSNVTDEMR pc2
P00352
383:630 346:530 64:100
9:75547612:T:C_C
p = 0.58, beta = −0.0308, N = 793
ANNTFYGLSAGVFTK pc2
P00352
415:630 351:530 67:100
9:75547612:T:C_C
p = 0.67, beta = −0.0235, N = 833
VTLELGGK pc2
4788−2;O94788−3;O94788−4;P05091−
182:630 160:530 25:100
9:75547612:T:C_C
p = 0.79, beta = 0.0225, N = 367
VAFTGSTEVGK pc2
P00352
134:630 117:530 15:100
9:75547612:T:C_C
p = 0.073, beta = −0.18, N = 266
ELGEYGFHEYTEVK pc3
P00352
183:630 132:530 37:100
9:75547612:T:C_C
p = 0.53, beta = −0.0489, N = 352
IFVEESIYDEFVR pc2
P00352
274:630 219:530 37:100
9:75547612:T:C_C
p = 0.96, beta = −0.00374, N = 530
LYSNAYLNDLAGCIK pc2
Q5SYQ9;P00352
329:630 289:530 69:100
9:75547612:T:C_C
p = 0.25, beta = −0.0666, N = 687
IFVEESIYDEFVRR pc3
P00352
121:630 101:530 22:100
9:75547612:T:C_C
p = 0.26, beta = −0.109, N = 244
IGPALSCGNTVVVK pc2
Q5SYQ9;P00352
261:630 216:530 41:100
9:75547612:T:C_C
p = 0.7, beta = 0.0269, N = 518
LECGGGPWGNK pc2
P00352
97:630 81:530 20:100
9:75547612:T:C_C
p = 0.88, beta = 0.0157, N = 198
ILDLIESGKK pc2
P00352
140:630 131:530 26:100
9:75547612:T:C_C
p = 0.31, beta = −0.0901, N = 297
PAEQTPLTALHVASLIK pc3
Q5SYQ9;P00352
64:630 60:530 10:100
9:75547612:T:C_C
p = 0.25, beta = −0.156, N = 134
ALDH1A1 : NP5
P00352
159:165 117:122 37:38
9:75547612:T:C_C
p = 0.4, beta = −0.0676, N = 313
TIPIDGNFFTYTR pc2
Q5SYQ9;P00352
103:165 73:122 22:38
9:75547612:T:C_C
p = 0.84, beta = 0.0206, N = 198
QAFQIGSPWR pc2
Q5SYQ9;P00352
102:165 75:122 23:38
9:75547612:T:C_C
p = 0.76, beta = −0.0309, N = 200
EEIFGPVQQIMK pc2
P00352
103:165 82:122 24:38
9:75547612:T:C_C
p = 0.47, beta = 0.0725, N = 209
GYFVQPTVFSNVTDEMR pc2
P00352
128:165 88:122 29:38
9:75547612:T:C_C
p = 0.79, beta = 0.0245, N = 245
LYSNAYLNDLAGCIK pc2
Q5SYQ9;P00352
148:165 105:122 33:38
9:75547612:T:C_C
p = 0.94, beta = 0.00621, N = 286
IFINNEWHDSVSGK pc3
Q5SYQ9;P00352
89:165 59:122 18:38
9:75547612:T:C_C
p = 0.68, beta = −0.0457, N = 166
LLLATMESMNGGK pc2
Q5SYQ9;P00352
95:165 71:122 21:38
9:75547612:T:C_C
p = 0.63, beta = 0.0513, N = 187
VAFTGSTEVGK pc2
P00352
31:165 16:122 10:38
9:75547612:T:C_C
p = 0.93, beta = 0.0137, N = 57
LECGGGPWGNK pc2
P00352
38:165 35:122 8:38
9:75547612:T:C_C
p = 0.91, beta = 0.0184, N = 81
ILDLIESGKK pc2
P00352
68:165 62:122 14:38
9:75547612:T:C_C
p = 0.74, beta = 0.0415, N = 144
ANNTFYGLSAGVFTK pc2
P00352
52:165 44:122 15:38
9:75547612:T:C_C
p = 0.66, beta = −0.0575, N = 111
IGPALSCGNTVVVK pc2
Q5SYQ9;P00352
57:165 38:122 13:38
9:75547612:T:C_C
p = 0.42, beta = −0.108, N = 108
IFVEESIYDEFVRR pc3
P00352
29:165 20:122 8:38
9:75547612:T:C_C
p = 0.75, beta = 0.0564, N = 57
IFVEESIYDEFVR pc2
P00352
34:165 18:122 7:38
9:75547612:T:C_C
p = 0.2, beta = −0.228, N = 59
ELGEYGFHEYTEVK pc3
P00352
30:165 18:122 8:38
9:75547612:T:C_C
p = 0.78, beta = 0.0495, N = 56
ILDLIESGK pc2
P00352
16:165 8:122 3:38
9:75547612:T:C_C
p = 0.62, beta = −0.132, N = 27
log10 protein intensity
log10 peptide intensity
Assay Target: ALDH1A1
UniProt: P00352
rsID: rs348463
Target SNP: 9:75547612:T:C
Proxy SNP: 9:75547612:T:C
UKBPPP log10(p): 41.4
UKBPPP BETA: 0.089
UKBPPP MSPA: 0
941:988:859:785:830:984:793:8

349. MDH1 (P40925)
UKBPPP MSPA=0

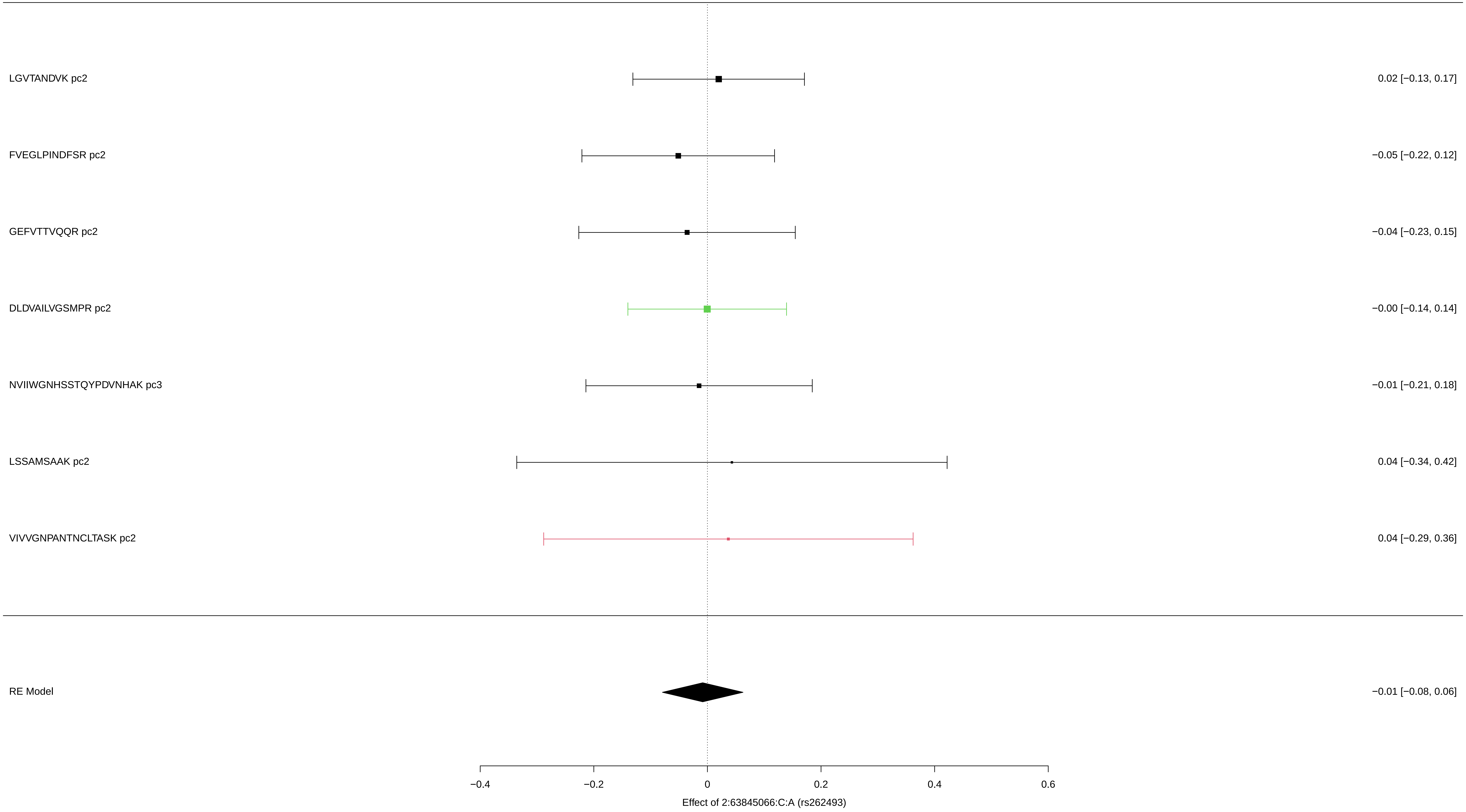

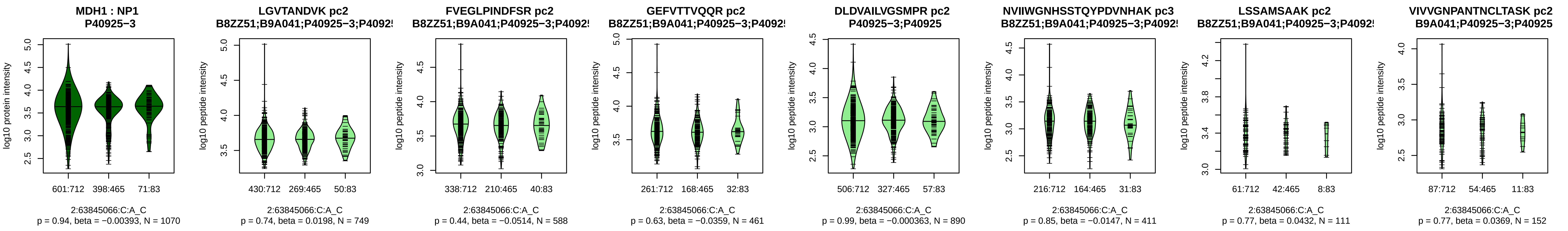

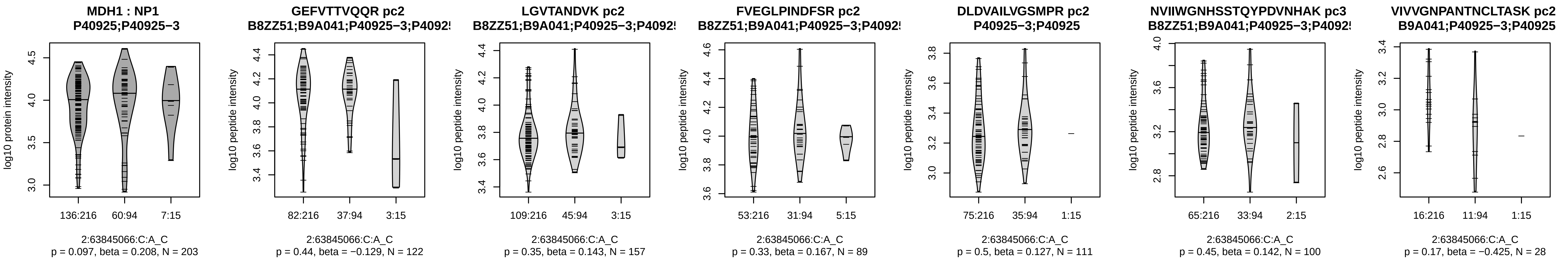

Assay Target: MDH1
UniProt: P40925
rsID: rs262493
Target SNP: 2:63845066:C:A
Proxy SNP: 2:63845066:C:A
UKBPPP log10(p): 41.3
UKBPPP BETA: −0.092
UKBPPP MSPA: 0
−:−:−:−:−:−:−
749:588:461:890:411:111:152

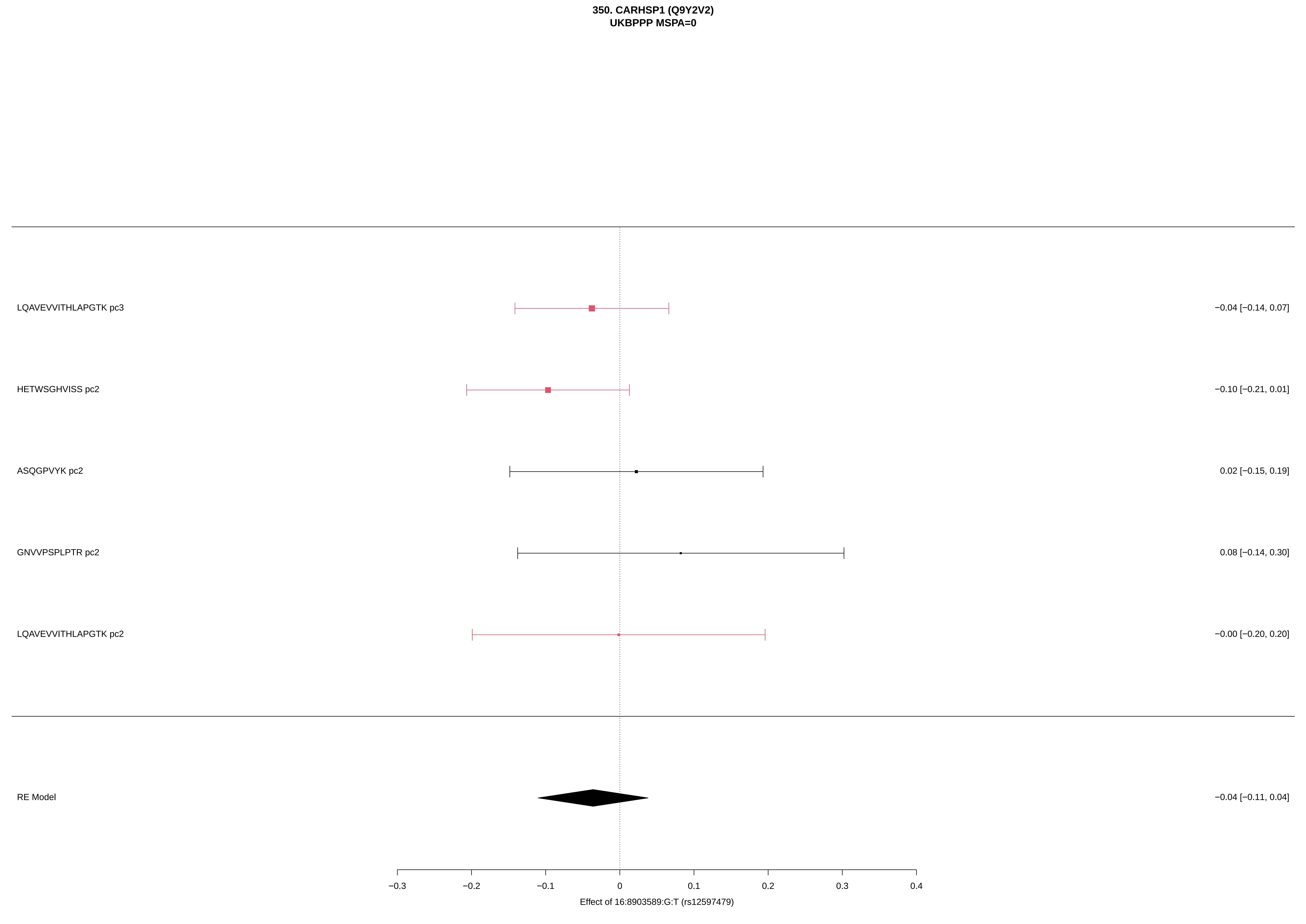
350. CARHSP1 (Q9Y2V2)
UKBPPP MSPA=0
LQAVEVVITHLAPGTK pc3
−0.04 [−0.14, 0.07]
HETWSGHVISS pc2
−0.10 [−0.21, 0.01]
ASQGPVYK pc2
0.02 [−0.15, 0.19]
GNVVPSPLPTR pc2
0.08 [−0.14, 0.30]
LQAVEVVITHLAPGTK pc2
−0.00 [−0.20, 0.20]
RE Model
−0.04 [−0.11, 0.04]
−0.3
−0.2
−0.1
0
0.1
0.2
0.3
0.4
Effect of 16:8903589:G:T (rs12597479)

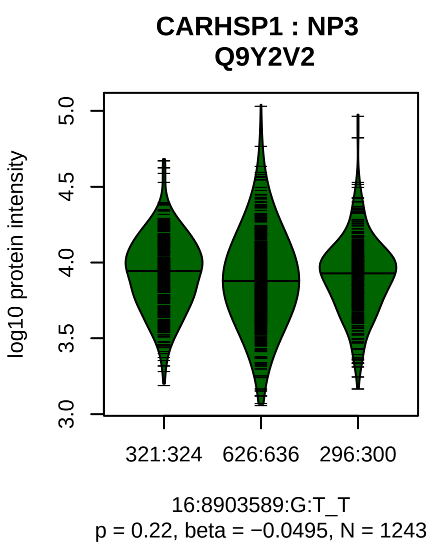

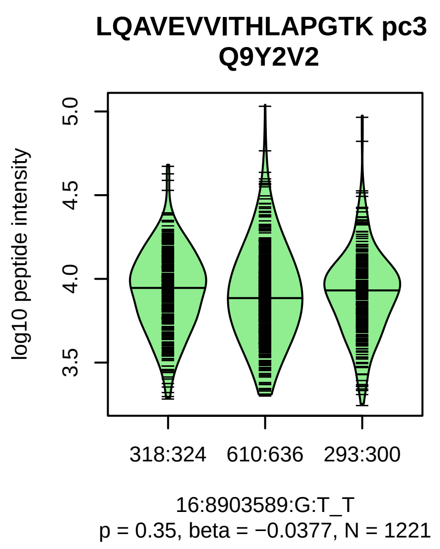

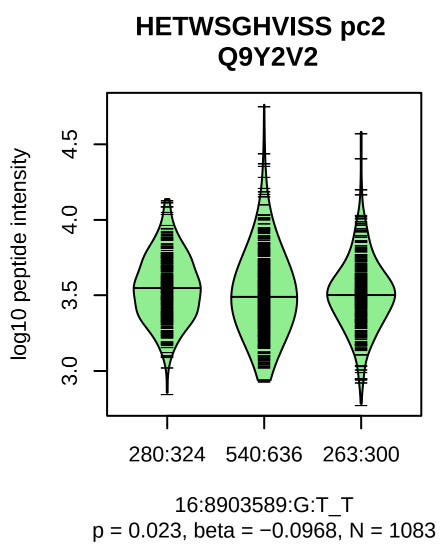

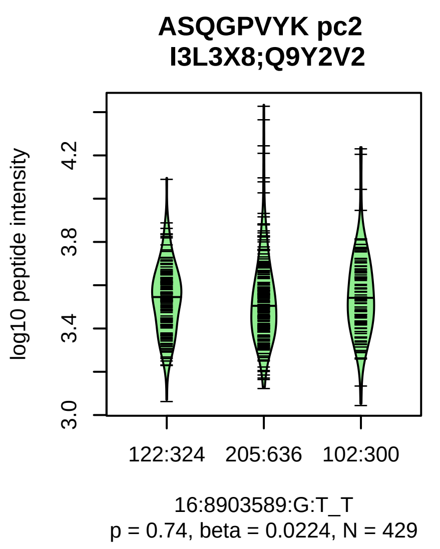

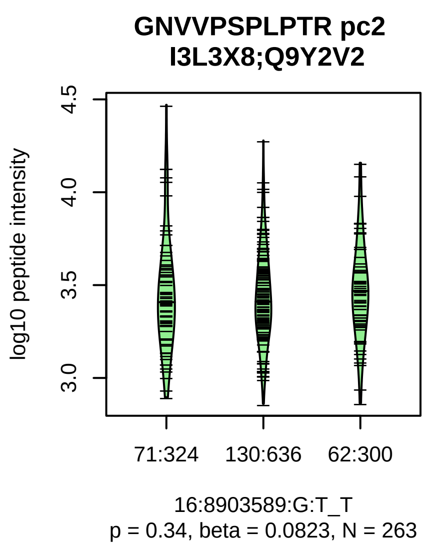

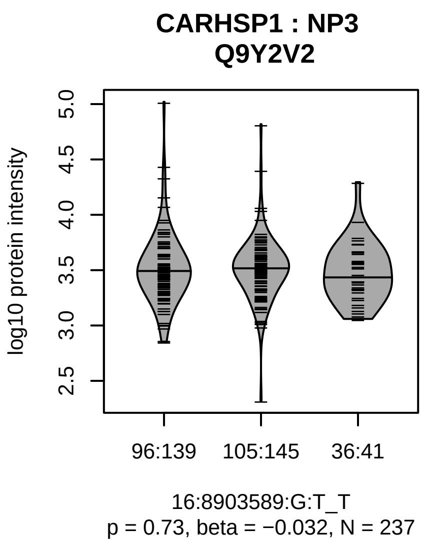

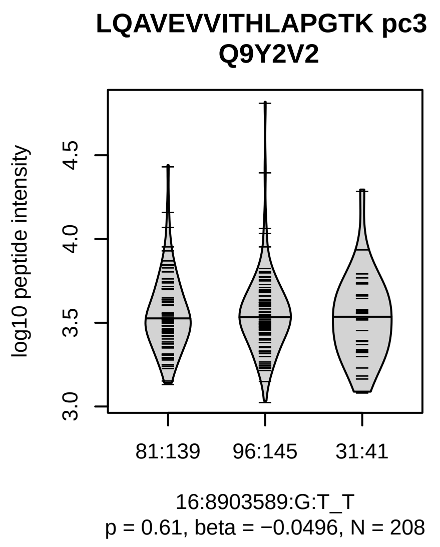

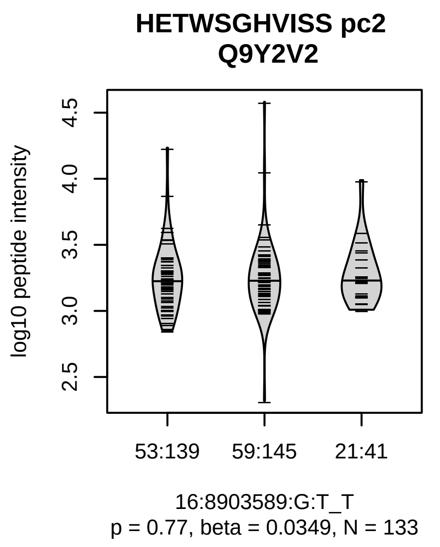

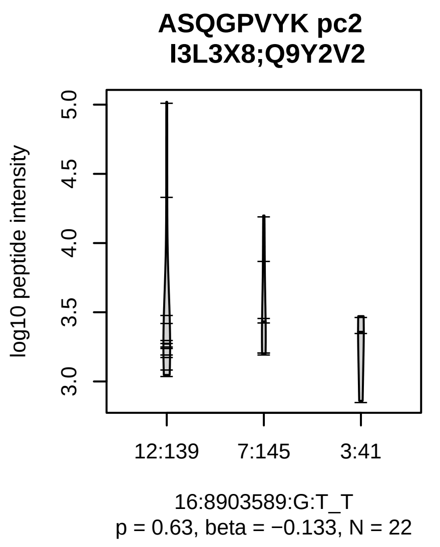

Assay Target: CARHSP1
UniProt: Q9Y2V2
rsID: rs12597479
Target SNP: 16:8903589:G:T
Proxy SNP: 16:8903589:G:T
UKBPPP log10(p): 40.5
UKBPPP BETA: −0.081
UKBPPP MSPA: 0
–:–:–:–:–
1221:1083:429:263:338

351. NSFL1C (Q9UNZ2)
UKBPPP MSPA=0

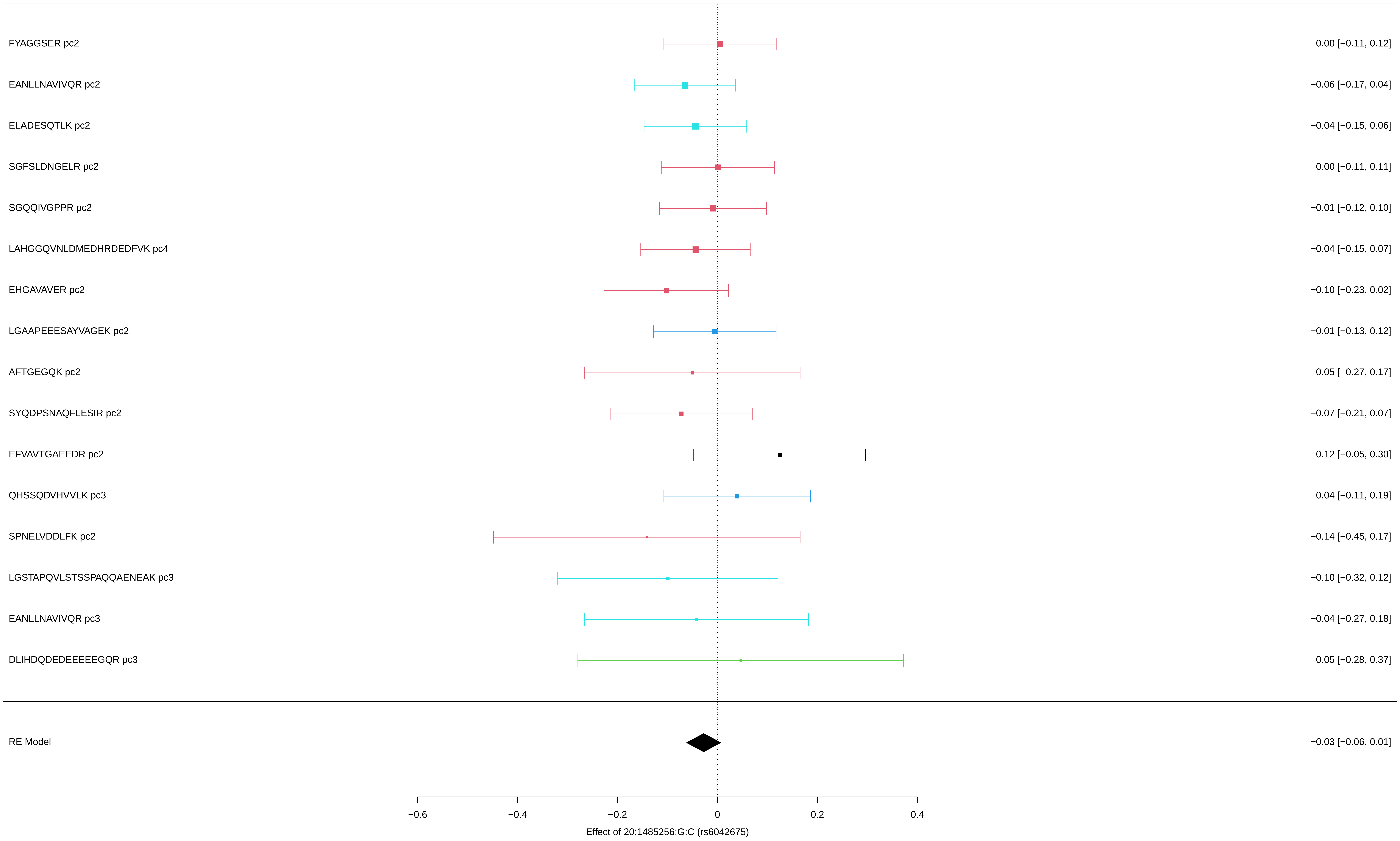

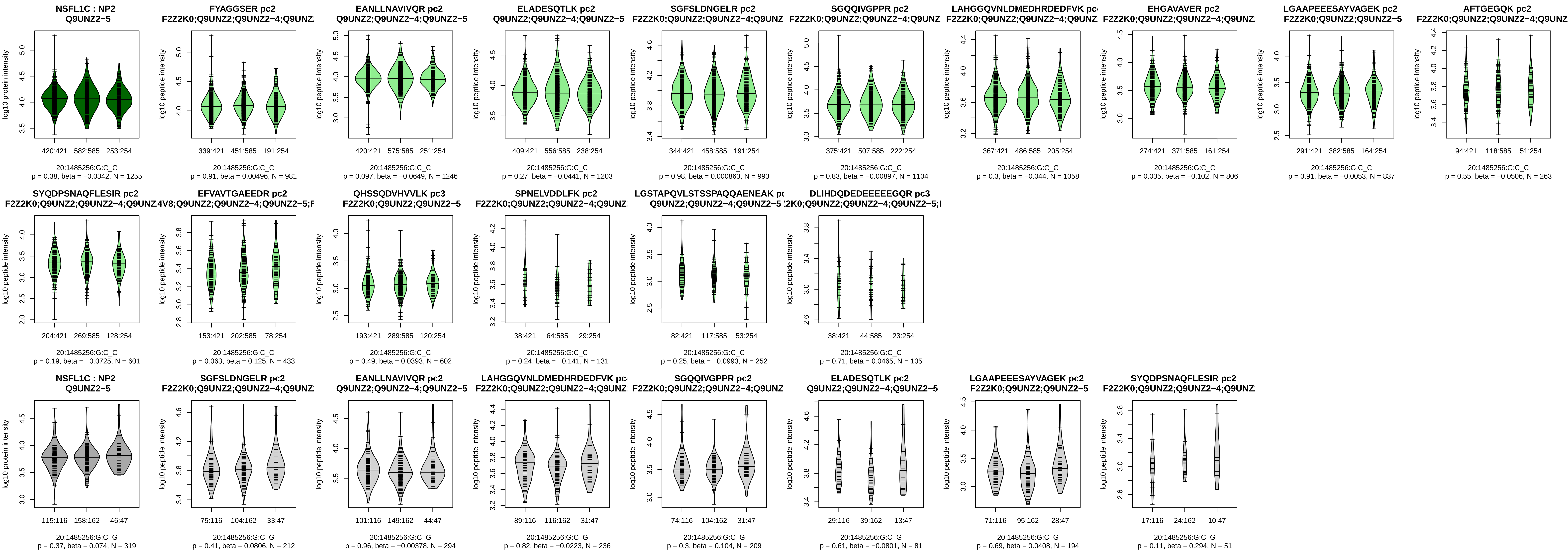

Assay Target: NSFL1C
UniProt: Q9UNZ2
rsID: rs6042675
Target SNP: 20:1485256:G:C
Proxy SNP: 20:1485256:G:C
UKBPPP log10(p): 38.3
UKBPPP BETA: −0.08
UKBPPP MSPA: 0
981:1246:1203:993:1104:1058:8

352. TINAGL1 (Q9GZM7)
UKBPPP MSPA=0

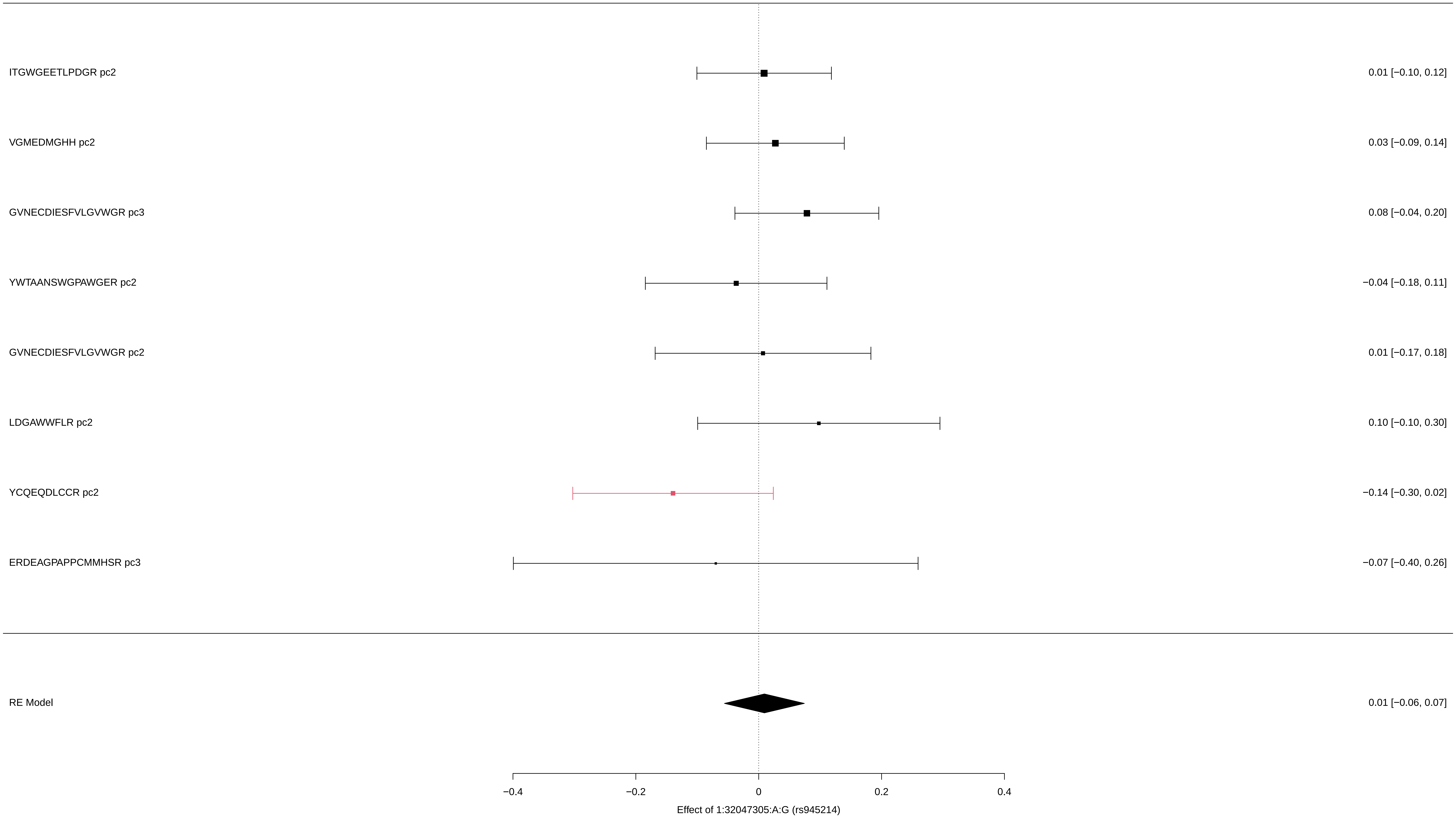

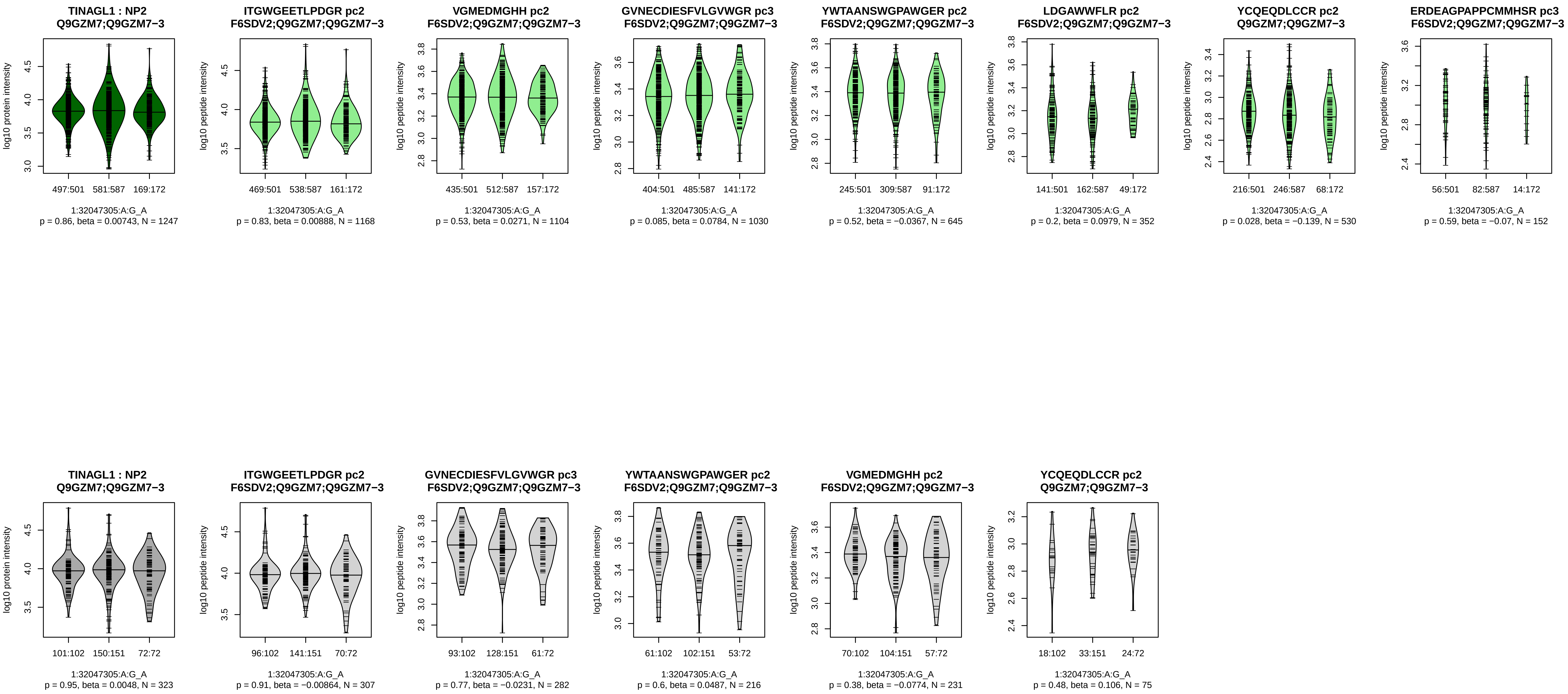

Assay Target: TINAGL1
UniProt: Q9GZM7
rsID: rs945214
Target SNP: 1:32047305:A:G
Proxy SNP: 1:32047305:A:G
UKBPPP log10(p): 38
UKBPPP BETA: −0.082
UKBPPP MSPA: 0
–:–:–:–:–:–:–:–
1168:1104:1030:645:427:352:53

353. PCDH1 (Q08174)
UKBPPP MSPA=0

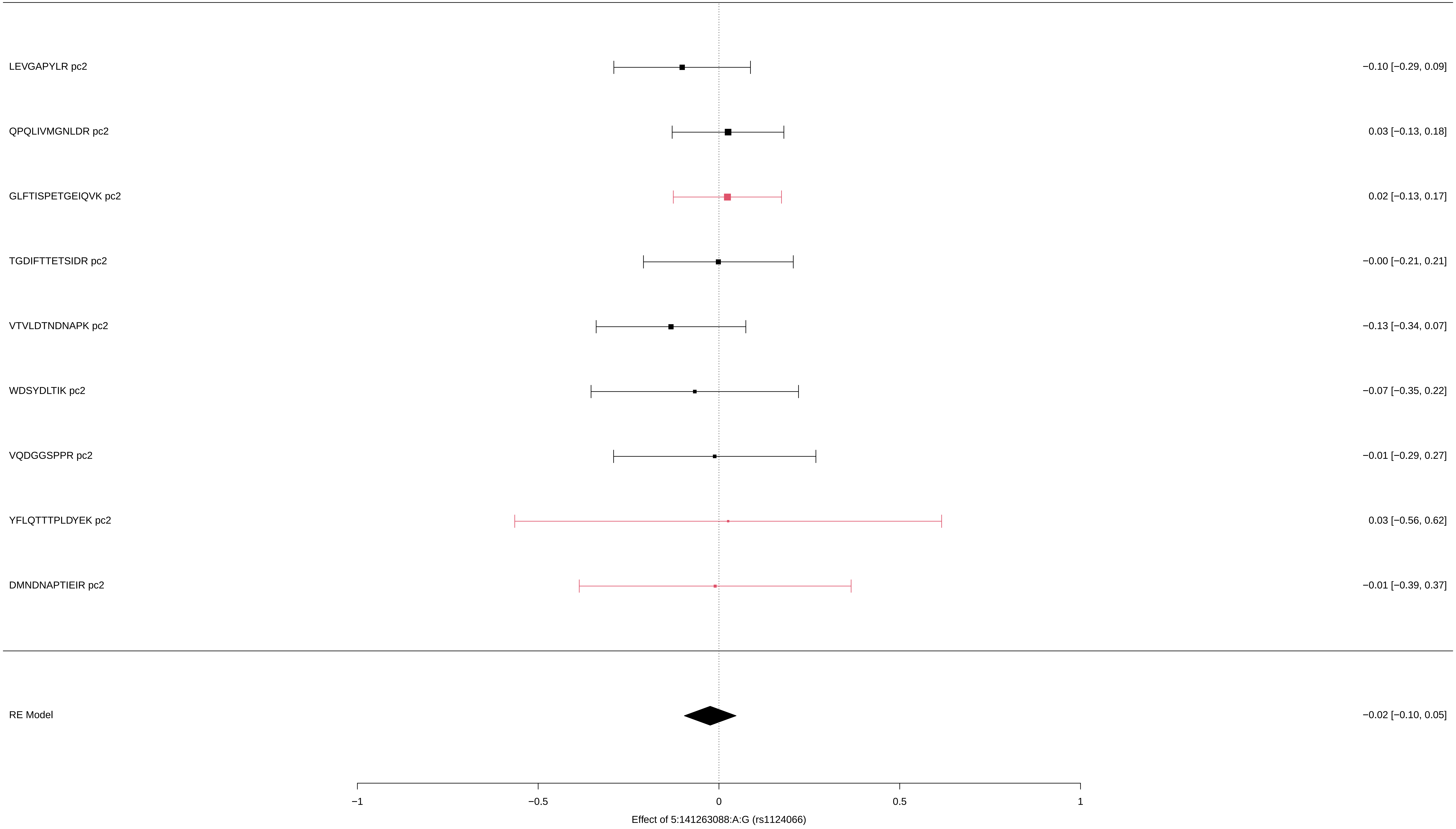

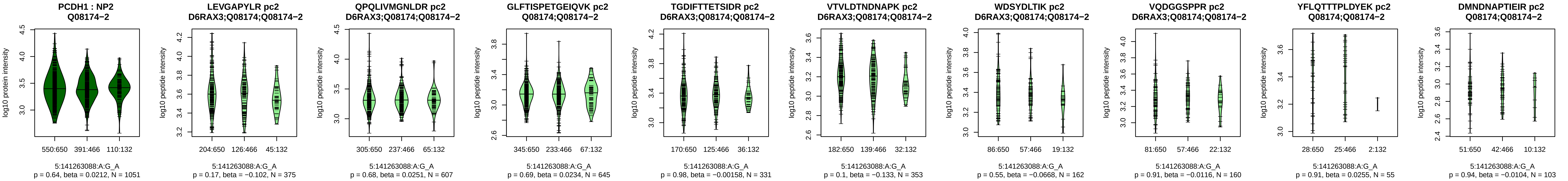

Assay Target: PCDH1
UniProt: Q08174
rsID: rs1124066
Target SNP: 5:141263088:A:G
Proxy SNP: 5:141263088:A:G
UKBPPP log10(p): 37
UKBPPP BETA: 0.078
UKBPPP MSPA: 0
−:−:−:−:−:−:−:−:−:NA:NA
375:607:645:331:353:162:160:5

354. PDGFC (Q9NRA1)
UKBPPP MSPA=0

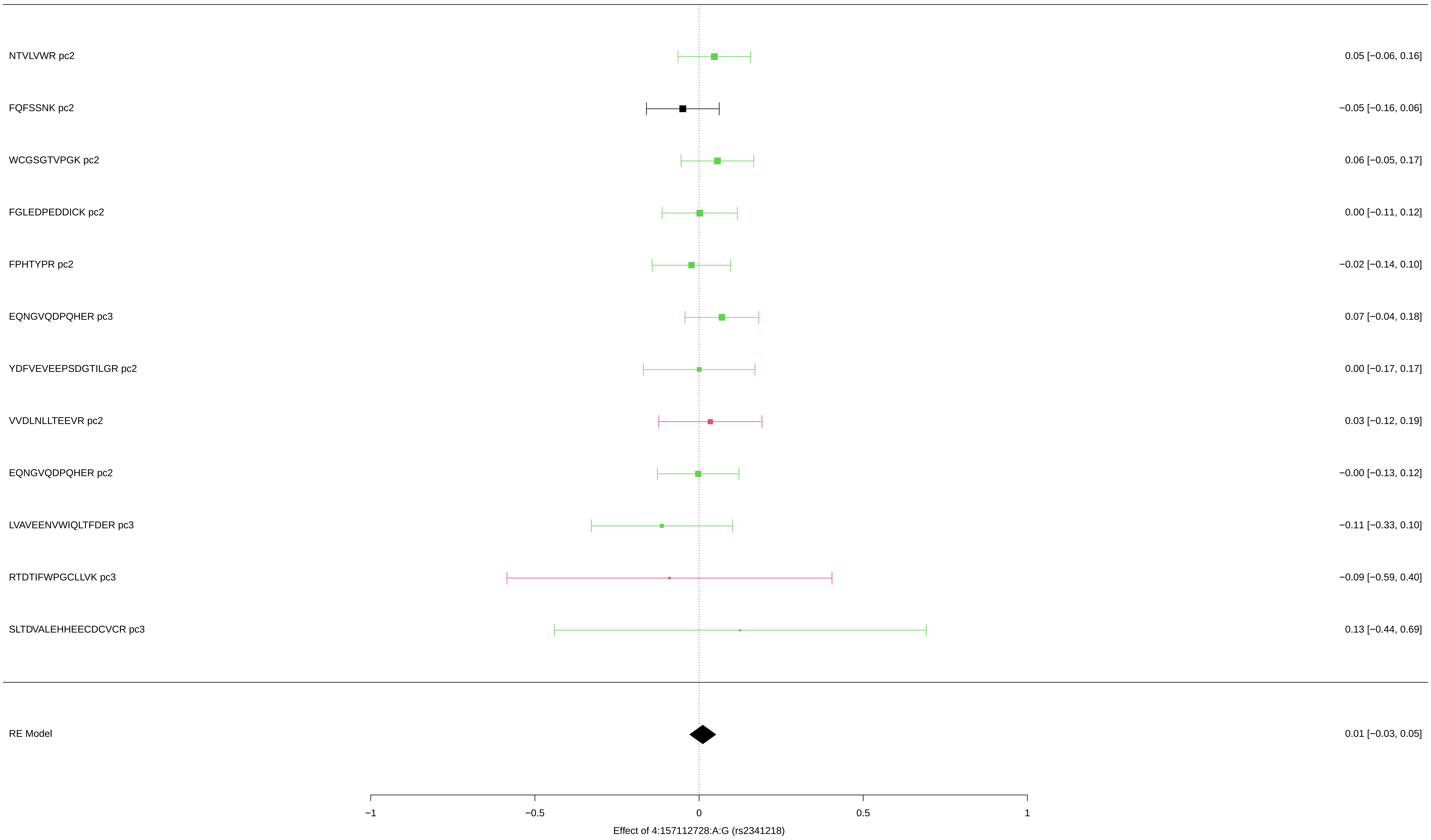

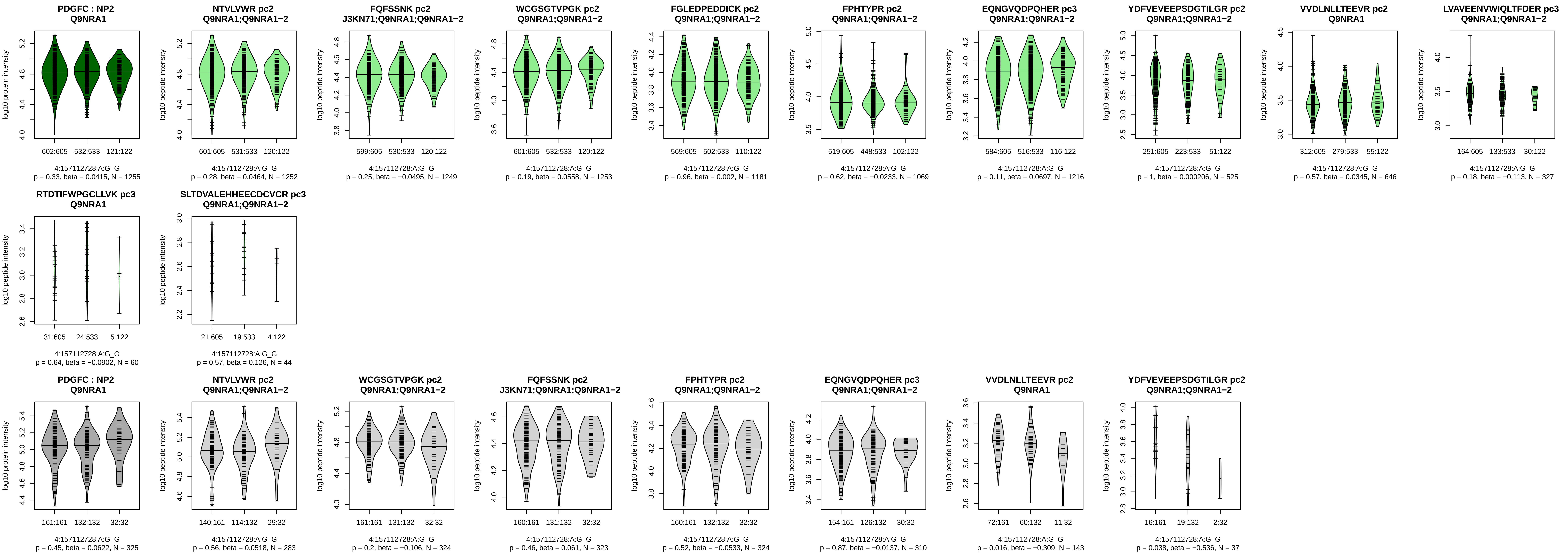

Assay Target: PDGFC
UniProt: Q9NRA1
rsID: rs2341218
Target SNP: 4:157112728:A:G
Proxy SNP: 4:157112728:A:G
UKBPPP log10(p): 36.6
UKBPPP BETA: −0.086
UKBPPP MSPA: 0
−:−:−:−:−:−:−:−:−:−:NA:NA:N
1252:1249:1253:1181:1069:121

355. NEXN (Q0ZGT2)
UKBPPP MSPA=0

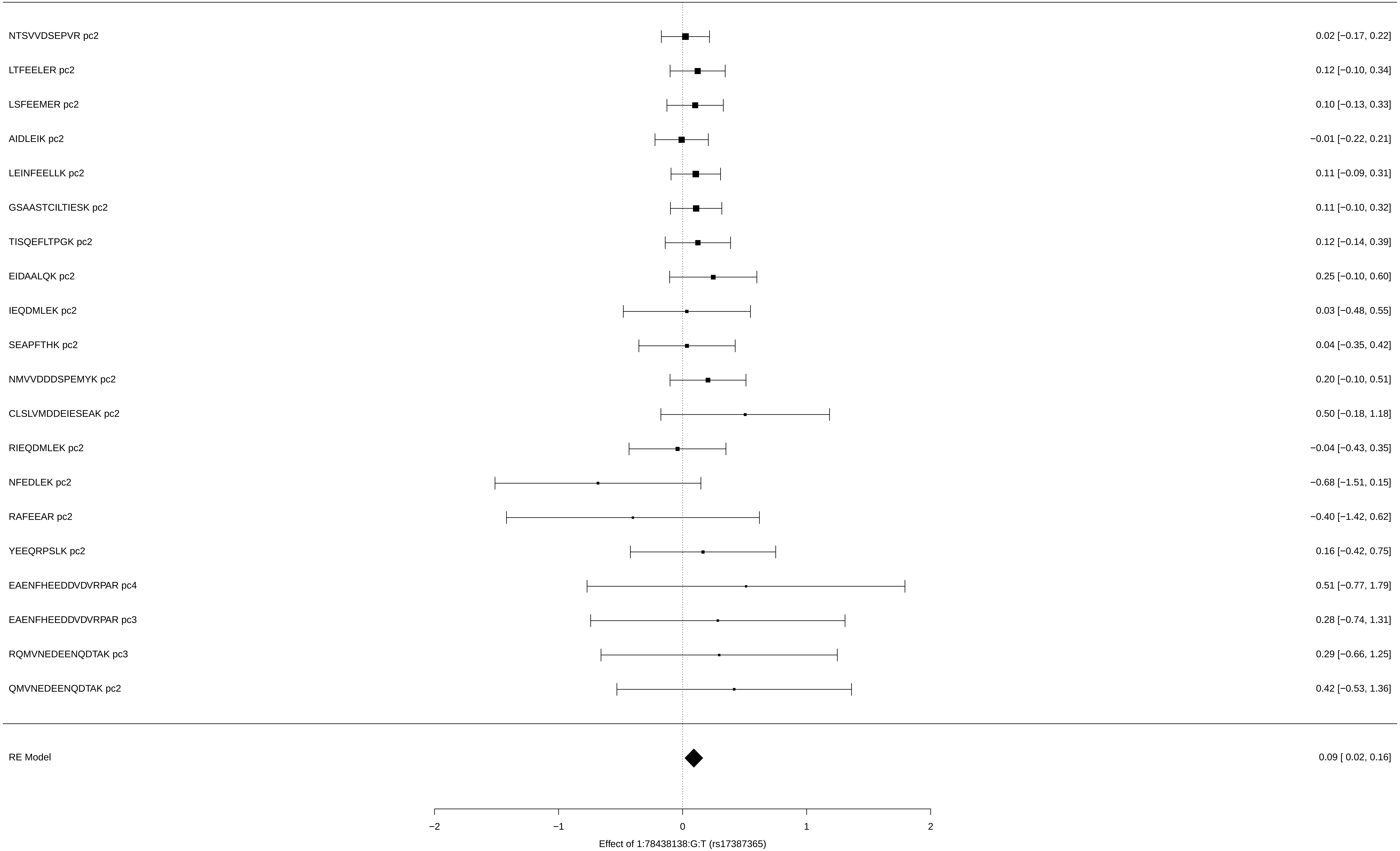

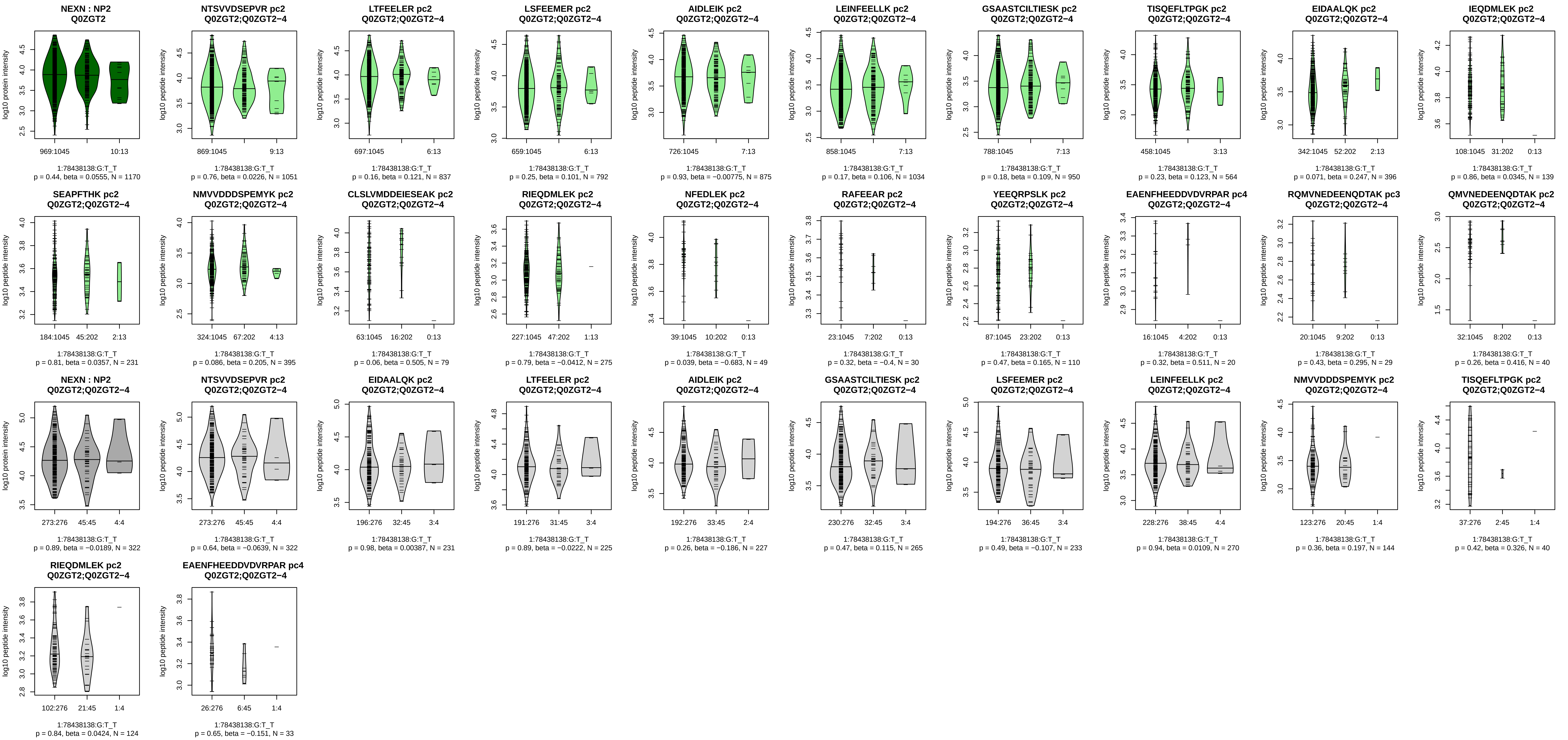

Assay Target: NEXN
UniProt: Q0ZGT2
rsID: rs17387365
Target SNP: 1:78438138:G:T
Proxy SNP: 1:78438138:G:T
UKBPPP log10(p): 35.4
UKBPPP BETA: −0.143
UKBPPP MSPA: 0

1051:837:792:875:1034:950:564

356. FSTL3 (O95633)
UKBPPP MSPA=0

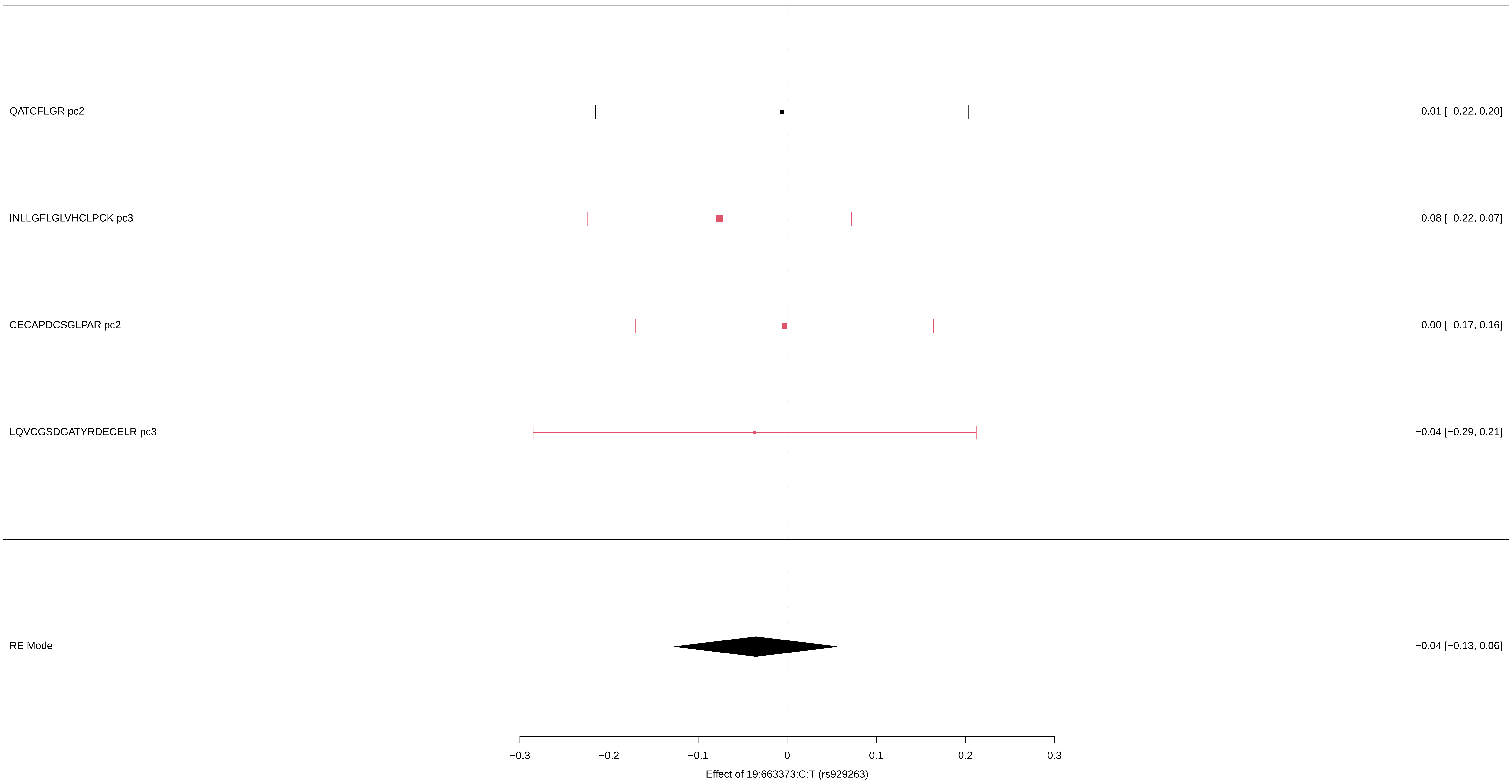

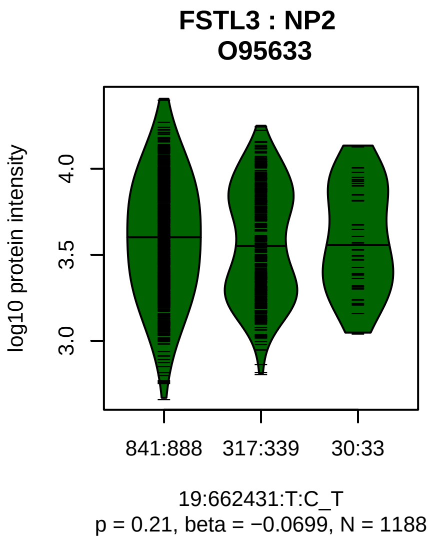

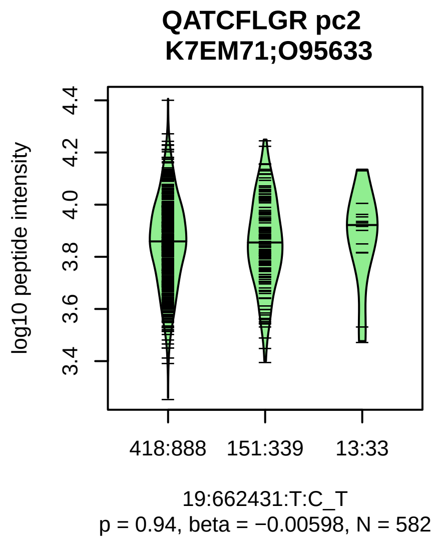

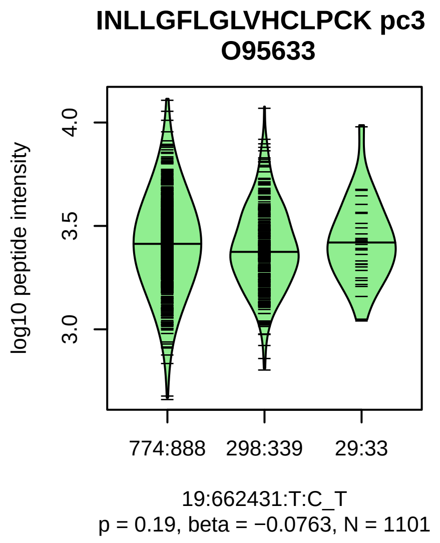

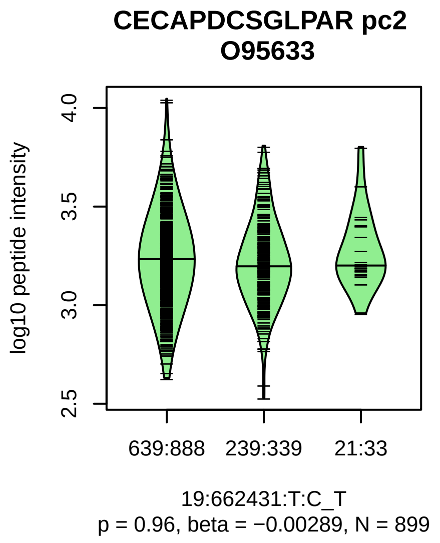

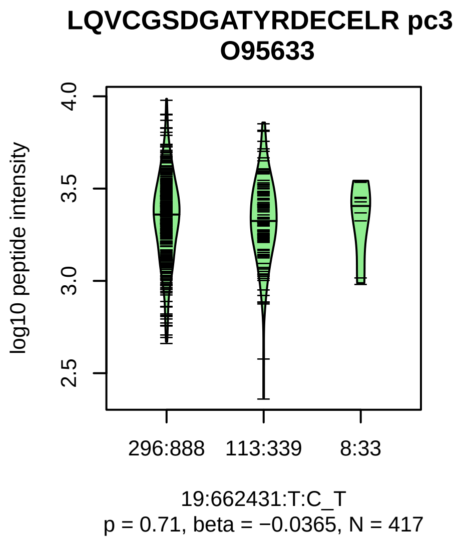

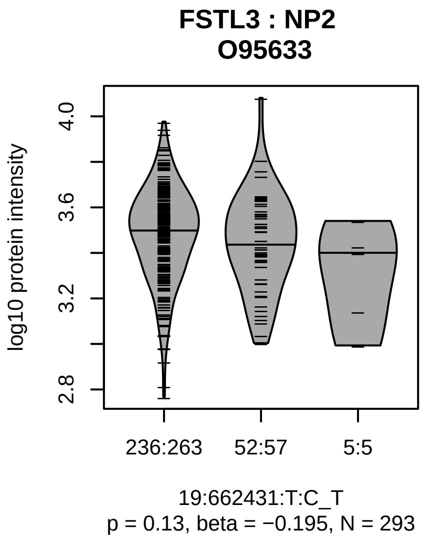

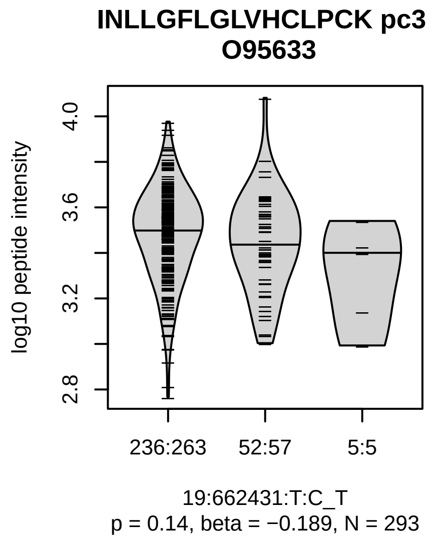

Assay Target: FSTL3
UniProt: O95633
rsID: rs929263
Target SNP: 19:663373:C:T
Proxy SNP: 19:662431:T:C
UKBPPP log10(p): 34.9
UKBPPP BETA: 0.099
UKBPPP MSPA: 0
–:–:–:–
582:1101:899:417

357. HCLS1 (P14317)
UKBPPP MSPA=0

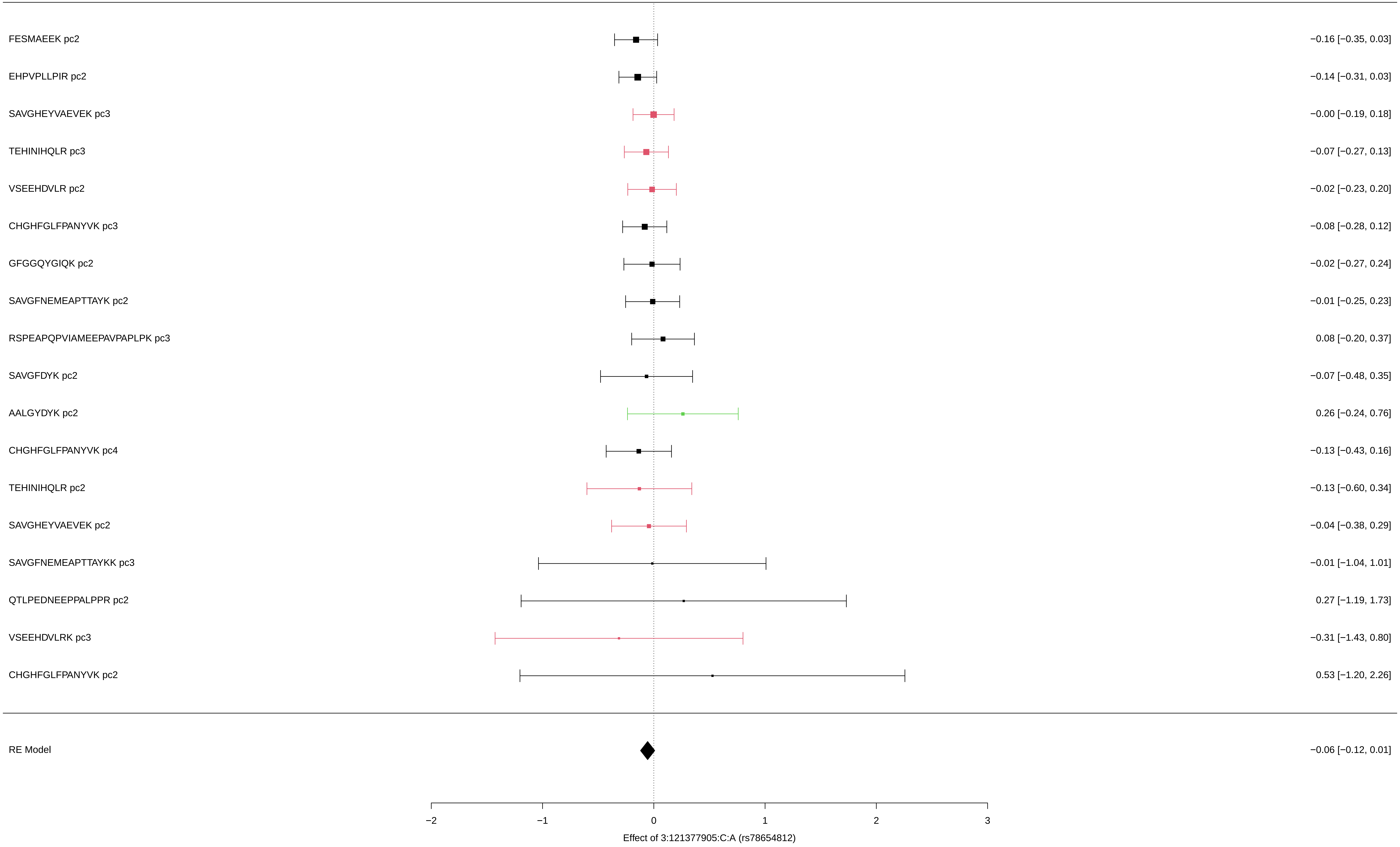

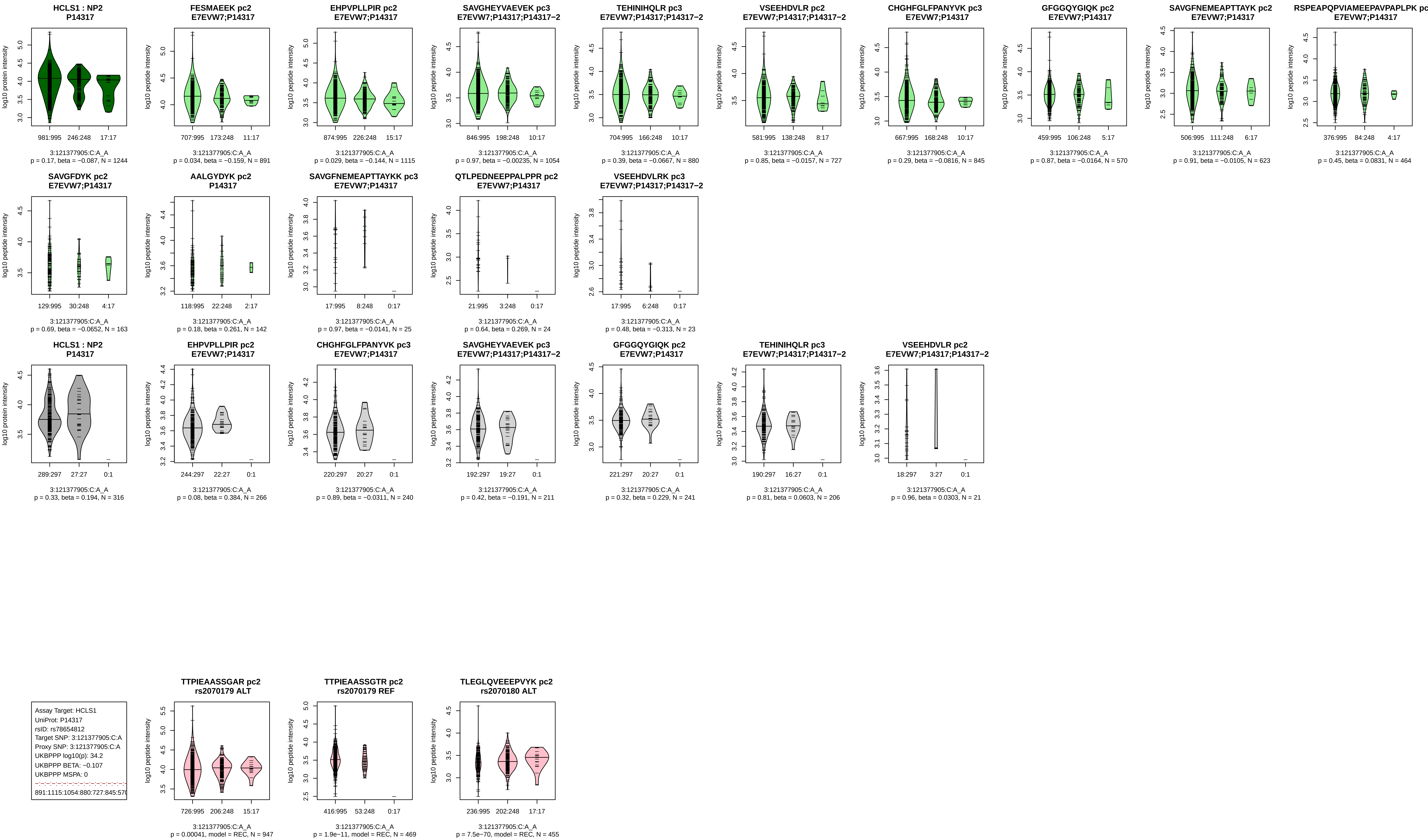
HCLS1 : NP2
P14317
981:995 246:248 17:17
3:121377905:C:A_A
p = 0.17, beta = −0.087, N = 1244
FESMAEEK pc2
E7EVW7;P14317
707:995 173:248 11:17
3:121377905:C:A_A
p = 0.034, beta = −0.159, N = 891
EHPVPLLPIR pc2
E7EVW7;P14317
874:995 226:248 15:17
3:121377905:C:A_A
p = 0.029, beta = −0.144, N = 1115
SAVGHEYVAEVEK pc3
E7EVW7;P14317;P14317−2
846:995 198:248 10:17
3:121377905:C:A_A
p = 0.97, beta = −0.00235, N = 1054
TEHINIHQLR pc3
E7EVW7;P14317;P14317−2
704:995 166:248 10:17
3:121377905:C:A_A
p = 0.39, beta = −0.0667, N = 880
VSEEHDVLR pc2
E7EVW7;P14317;P14317−2
581:995 138:248 8:17
3:121377905:C:A_A
p = 0.85, beta = −0.0157, N = 727
CHGHFGLFPANYVK pc3
E7EVW7;P14317
667:995 168:248 10:17
3:121377905:C:A_A
p = 0.29, beta = −0.0816, N = 845
GFGGQYGIQK pc2
E7EVW7;P14317
459:995 106:248 5:17
3:121377905:C:A_A
p = 0.87, beta = −0.0164, N = 570
SAVGFNEMEAPTTAYK pc2
E7EVW7;P14317
506:995 111:248 6:17
3:121377905:C:A_A
p = 0.91, beta = −0.0105, N = 623
RSPEAPQPVIAMEEPAVPAPLPK pc
E7EVW7;P14317
376:995 84:248 4:17
3:121377905:C:A_A
p = 0.45, beta = 0.0831, N = 464
SAVGFDYK pc2
E7EVW7;P14317
129:995 30:248 4:17
3:121377905:C:A_A
p = 0.69, beta = −0.0652, N = 163
AALGYDYK pc2
P14317
118:995 22:248 2:17
3:121377905:C:A_A
p = 0.18, beta = 0.261, N = 142
SAVGFNEMEAPTTAYKK pc3
E7EVW7;P14317
17:995 8:248 0:17
3:121377905:C:A_A
p = 0.97, beta = −0.0141, N = 25
QTLPEDNEEPPALPPR pc2
E7EVW7;P14317
21:995 3:248 0:17
3:121377905:C:A_A
p = 0.64, beta = 0.269, N = 24
VSEEHDVLRK pc3
E7EVW7;P14317;P14317−2
17:995 6:248 0:17
3:121377905:C:A_A
p = 0.48, beta = −0.313, N = 23
HCLS1 : NP2
P14317
289:297 27:27 0:1
3:121377905:C:A_A
p = 0.33, beta = 0.194, N = 316
EHPVPLLPIR pc2
E7EVW7;P14317
244:297 22:27 0:1
3:121377905:C:A_A
p = 0.08, beta = 0.384, N = 266
CHGHFGLFPANYVK pc3
E7EVW7;P14317
220:297 20:27 0:1
3:121377905:C:A_A
p = 0.89, beta = −0.0311, N = 240
SAVGHEYVAEVEK pc3
E7EVW7;P14317;P14317−2
192:297 19:27 0:1
3:121377905:C:A_A
p = 0.42, beta = −0.191, N = 211
GFGGQYGIQK pc2
E7EVW7;P14317
221:297 20:27 0:1
3:121377905:C:A_A
p = 0.32, beta = 0.229, N = 241
TEHINIHQLR pc3
E7EVW7;P14317;P14317−2
190:297 16:27 0:1
3:121377905:C:A_A
p = 0.81, beta = 0.0603, N = 206
VSEEHDVLR pc2
E7EVW7;P14317;P14317−2
18:297 3:27 0:1
3:121377905:C:A_A
p = 0.96, beta = 0.0303, N = 21
Assay Target: HCLS1
UniProt: P14317
rsID: rs78654812
Target SNP: 3:121377905:C:A
Proxy SNP: 3:121377905:C:A
UKBPPP log10(p): 34.2
UKBPPP BETA: −0.107
UKBPPP MSPA: 0
891:1115:1054:880:727:845:57
TTPIEAASSGAR pc2
rs2070179 ALT
726:995 206:248 15:17
3:121377905:C:A_A
p = 0.00041, model = REC, N = 947
TTPIEAASSGTR pc2
rs2070179 REF
416:995 53:248 0:17
3:121377905:C:A_A
p = 1.9e−11, model = REC, N = 469
TLEGLQVEEEPVYK pc2
rs2070180 ALT
236:995 202:248 17:17
3:121377905:C:A_A
p = 7.5e−70, model = REC, N = 455

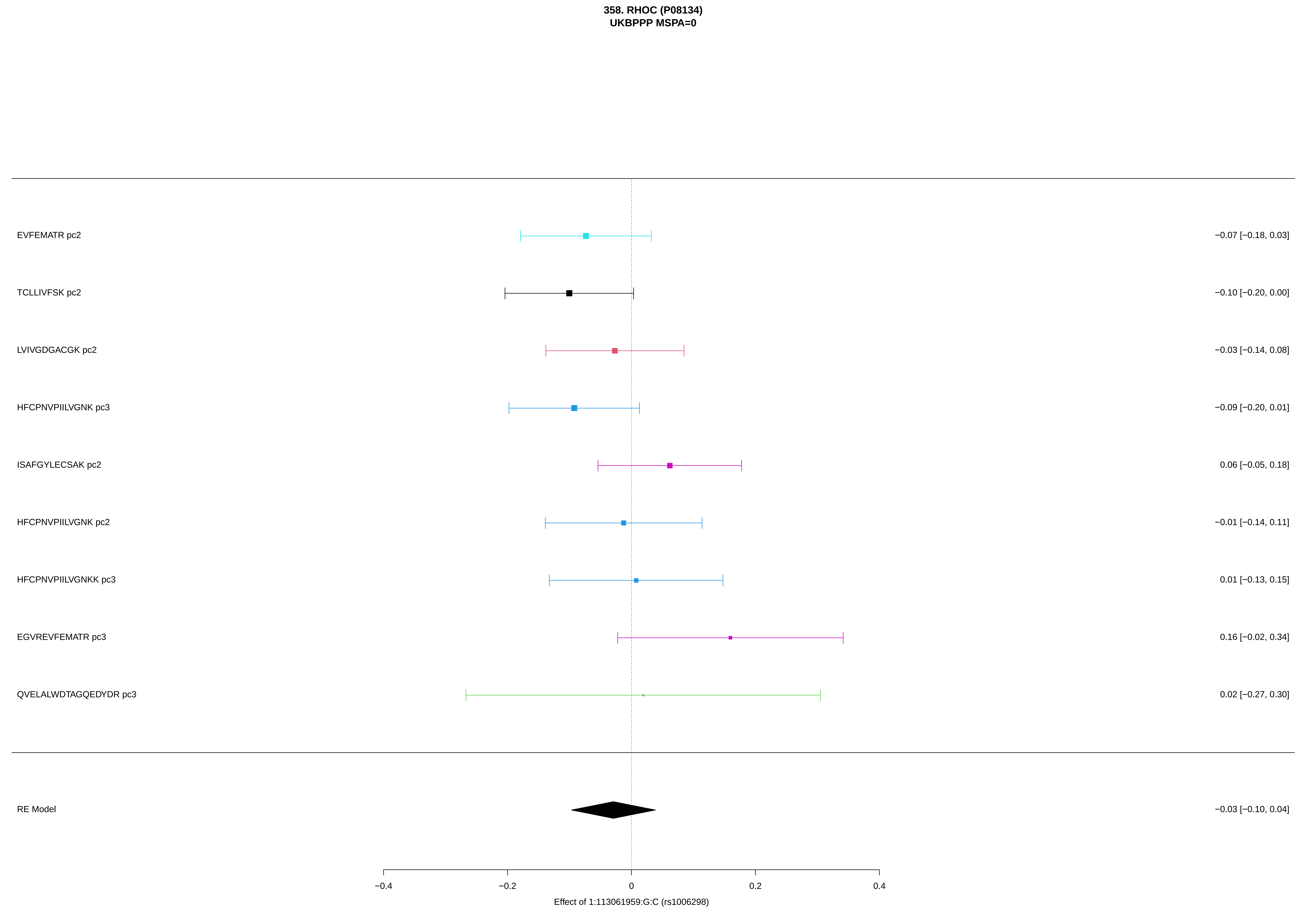
358. RHOC (P08134)
UKBPPP MSPA=0
EVFEMATR pc2
−0.07 [−0.18, 0.03]
TCLLIVFSK pc2
−0.10 [−0.20, 0.00]
LVIVGDGACGK pc2
−0.03 [−0.14, 0.08]
HFCPNVPIILVGNK pc3
−0.09 [−0.20, 0.01]
ISAFGYLECSAK pc2
0.06 [−0.05, 0.18]
HFCPNVPIILVGNK pc2
−0.01 [−0.14, 0.11]
HFCPNVPIILVGNKK pc3
0.01 [−0.13, 0.15]
EGVREVFEMATR pc3
0.16 [−0.02, 0.34]
QVELALWDTAGQEDYDR pc3
0.02 [−0.27, 0.30]
RE Model
−0.03 [−0.10, 0.04]
−0.4
−0.2
0
0.2
0.4
Effect of 1:113061959:G:C (rs1006298)

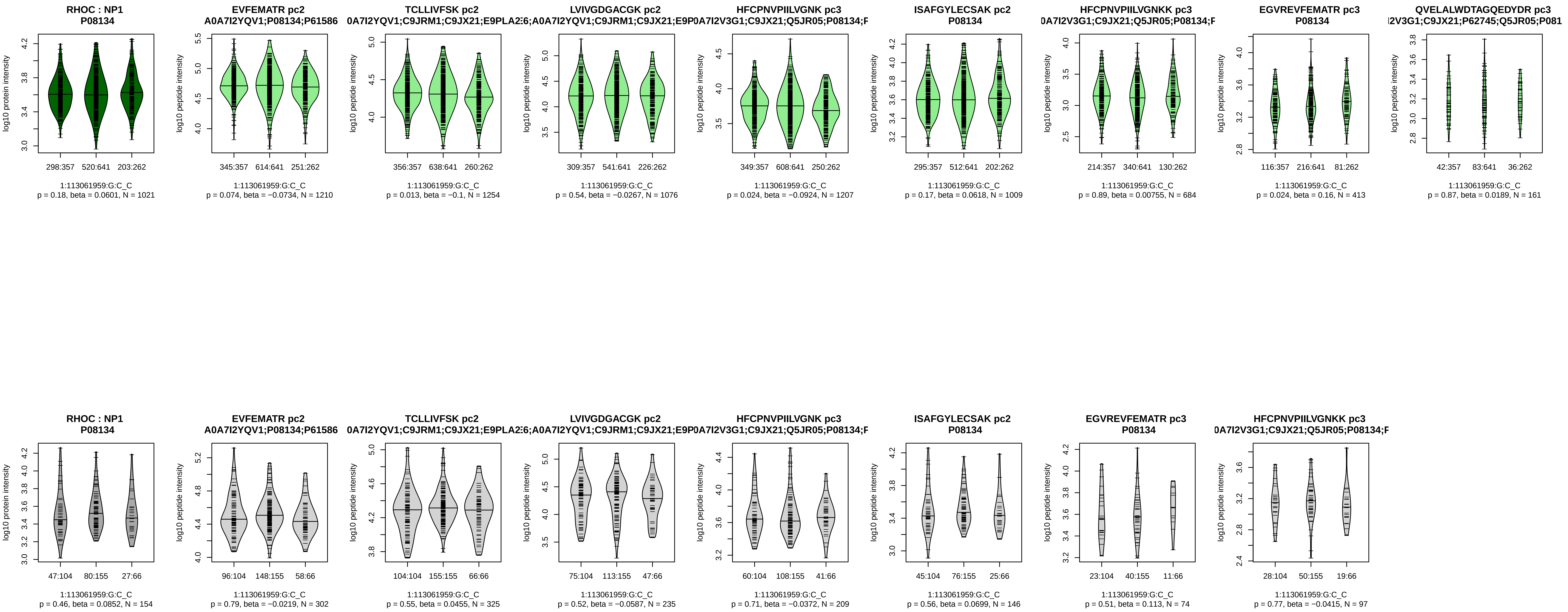

Assay Target: RHOC
UniProt: P08134
rsID: rs1006298
Target SNP: 1:113061959:G:C
Proxy SNP: 1:113061959:G:C
UKBPPP log10(p): 33.9
UKBPPP BETA: 0.076
UKBPPP MSPA: 0
–:–:–:–:–:–:–
1210:1254:1076:1207:1009:842

359. GNAS (O95467)
UKBPPP MSPA=NANA

Meta−analysis failed

c(0, 1)

Effect of 20:57274151:G:C (rs2296528)

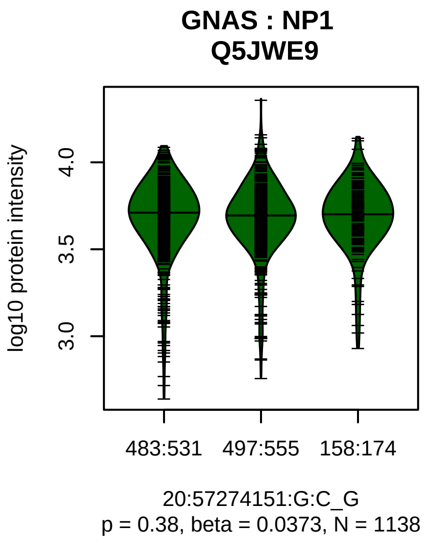

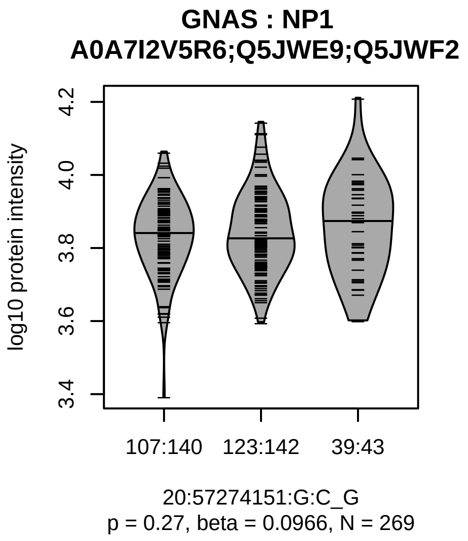

Assay Target: GNAS
UniProt: O95467
rsID: rs2296528
Target SNP: 20:57274151:G:C
Proxy SNP: 20:57274151:G:C
UKBPPP log10(p): 32
UKBPPP BETA: 0.075
UKBPPP MSPA: NANA
NA:NA
3:1

360. PLIN3 (O60664)
UKBPPP MSPA=0

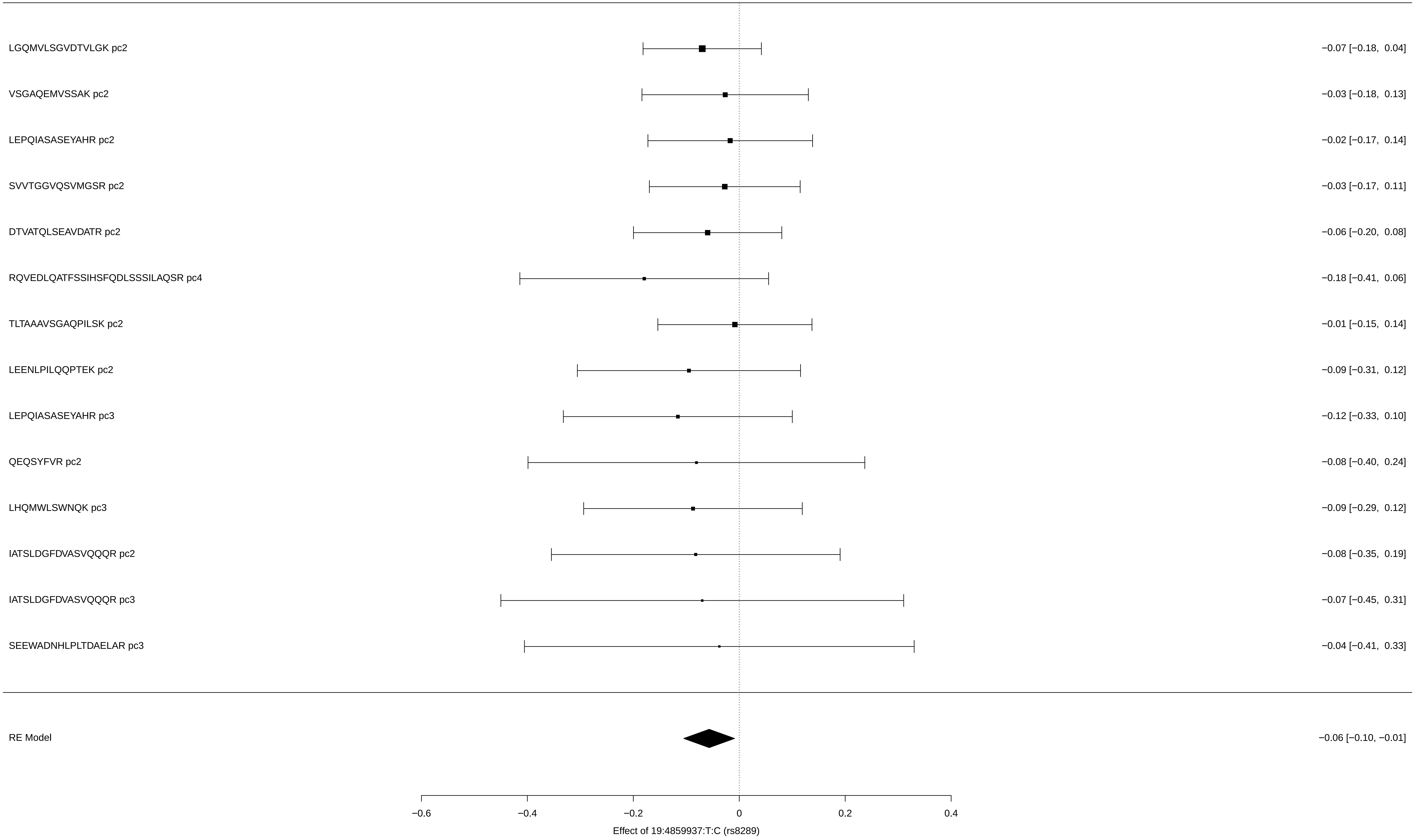

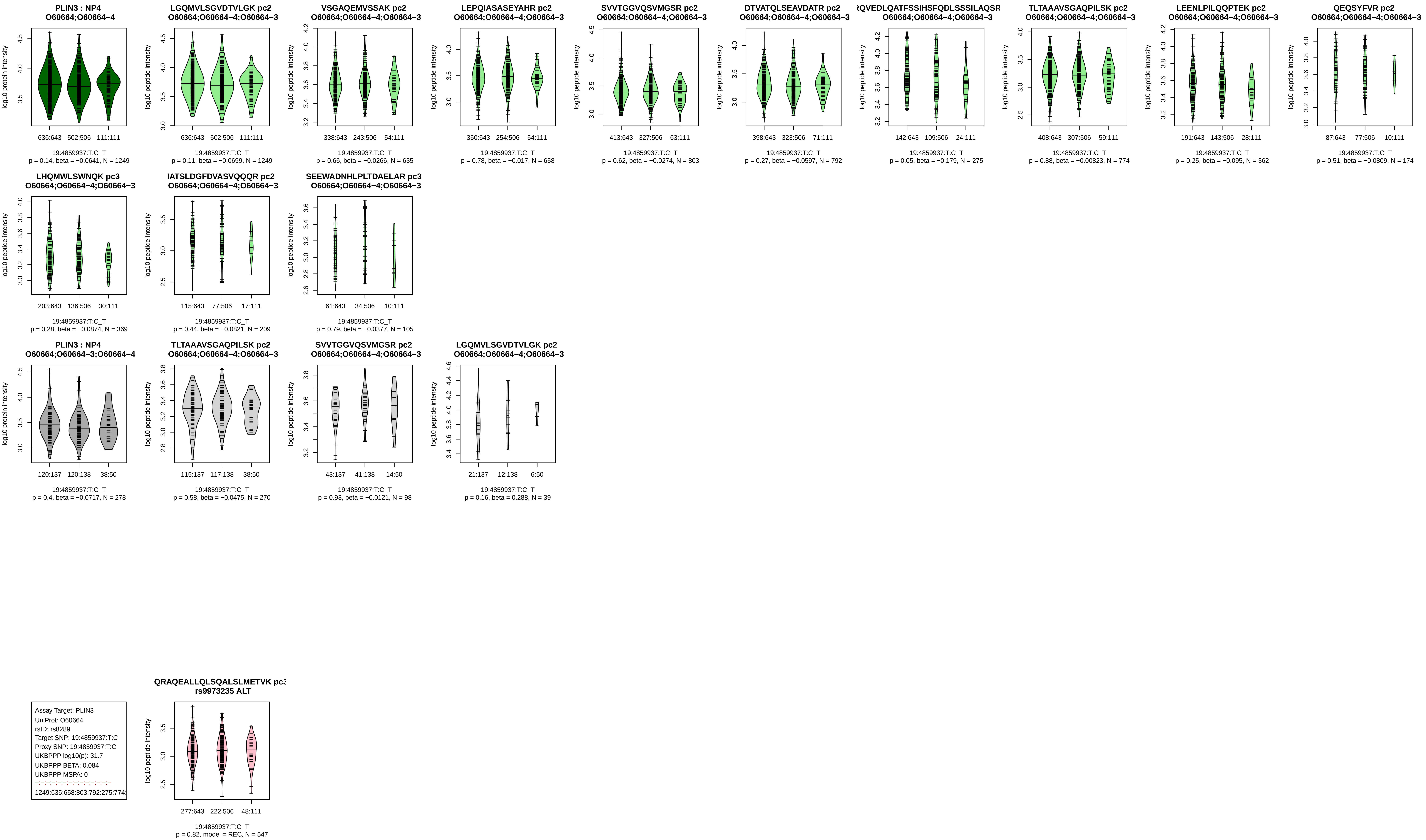
PLIN3 : NP4
O60664;O60664−4
log10 protein intensity
636:643 502:506 111:111
19:4859937:T:C_T
p = 0.14, beta = −0.0641, N = 1249
LGQMVLSGVDTVLGK pc2
O60664;O60664−4;O60664−3
log10 peptide intensity
636:643 502:506 111:111
19:4859937:T:C_T
p = 0.11, beta = −0.0699, N = 1249
VSGAQEMVSSAK pc2
O60664;O60664−4;O60664−3
338:643 243:506 54:111
19:4859937:T:C_T
p = 0.66, beta = −0.0266, N = 635
LEPQIASASEYAHR pc2
O60664;O60664−4;O60664−3
350:643 254:506 54:111
19:4859937:T:C_T
p = 0.78, beta = −0.017, N = 658
SVVTGGVQSVMGSR pc2
O60664;O60664−4;O60664−3
413:643 327:506 63:111
19:4859937:T:C_T
p = 0.62, beta = −0.0274, N = 803
DTVATQLSEAVDATR pc2
O60664;O60664−4;O60664−3
398:643 323:506 71:111
19:4859937:T:C_T
p = 0.27, beta = −0.0597, N = 792
QVEDLQATFSSIHSFQDLSSSILAQSR
O60664;O60664−4;O60664−3
142:643 109:506 24:111
19:4859937:T:C_T
p = 0.05, beta = −0.179, N = 275
TLTAAAVSGAQPILSK pc2
O60664;O60664−4;O60664−3
408:643 307:506 59:111
19:4859937:T:C_T
p = 0.88, beta = −0.00823, N = 774
LEENLPILQQPTEK pc2
O60664;O60664−4;O60664−3
191:643 143:506 28:111
19:4859937:T:C_T
p = 0.25, beta = −0.095, N = 362
QEQSYFVR pc2
O60664;O60664−4;O60664−3
87:643 77:506 10:111
19:4859937:T:C_T
p = 0.51, beta = −0.0809, N = 174
LHQMWLSWNQK pc3
O60664;O60664−4;O60664−3
203:643 136:506 30:111
19:4859937:T:C_T
p = 0.28, beta = −0.0874, N = 369
IATSLDGFDVASVQQQR pc2
O60664;O60664−4;O60664−3
115:643 77:506 17:111
19:4859937:T:C_T
p = 0.44, beta = −0.0821, N = 209
SEEWADNHLPLTDAELAR pc3
O60664;O60664−4;O60664−3
61:643 34:506 10:111
19:4859937:T:C_T
p = 0.79, beta = −0.0377, N = 105
PLIN3 : NP4
O60664;O60664−3;O60664−4
120:137 120:138 38:50
19:4859937:T:C_T
p = 0.4, beta = −0.0717, N = 278
TLTAAAVSGAQPILSK pc2
O60664;O60664−4;O60664−3
115:137 117:138 38:50
19:4859937:T:C_T
p = 0.58, beta = −0.0475, N = 270
SVVTGGVQSVMGSR pc2
O60664;O60664−4;O60664−3
43:137 41:138 14:50
19:4859937:T:C_T
p = 0.93, beta = −0.0121, N = 98
LGQMVLSGVDTVLGK pc2
O60664;O60664−4;O60664−3
21:137 12:138 6:50
19:4859937:T:C_T
p = 0.16, beta = 0.288, N = 39
Assay Target: PLIN3
UniProt: O60664
rsID: rs8289
Target SNP: 19:4859937:T:C
Proxy SNP: 19:4859937:T:C
UKBPPP log10(p): 31.7
UKBPPP BETA: 0.084
UKBPPP MSPA: 0
1249:635:658:803:792:275:774:
QRAQEALLQLSQALSLMETVK pc3
rs9973235 ALT
277:643 222:506 48:111
19:4859937:T:C_T
p = 0.82, model = REC, N = 547

361. CD34 (P28906)
UKBPPP MSPA=0

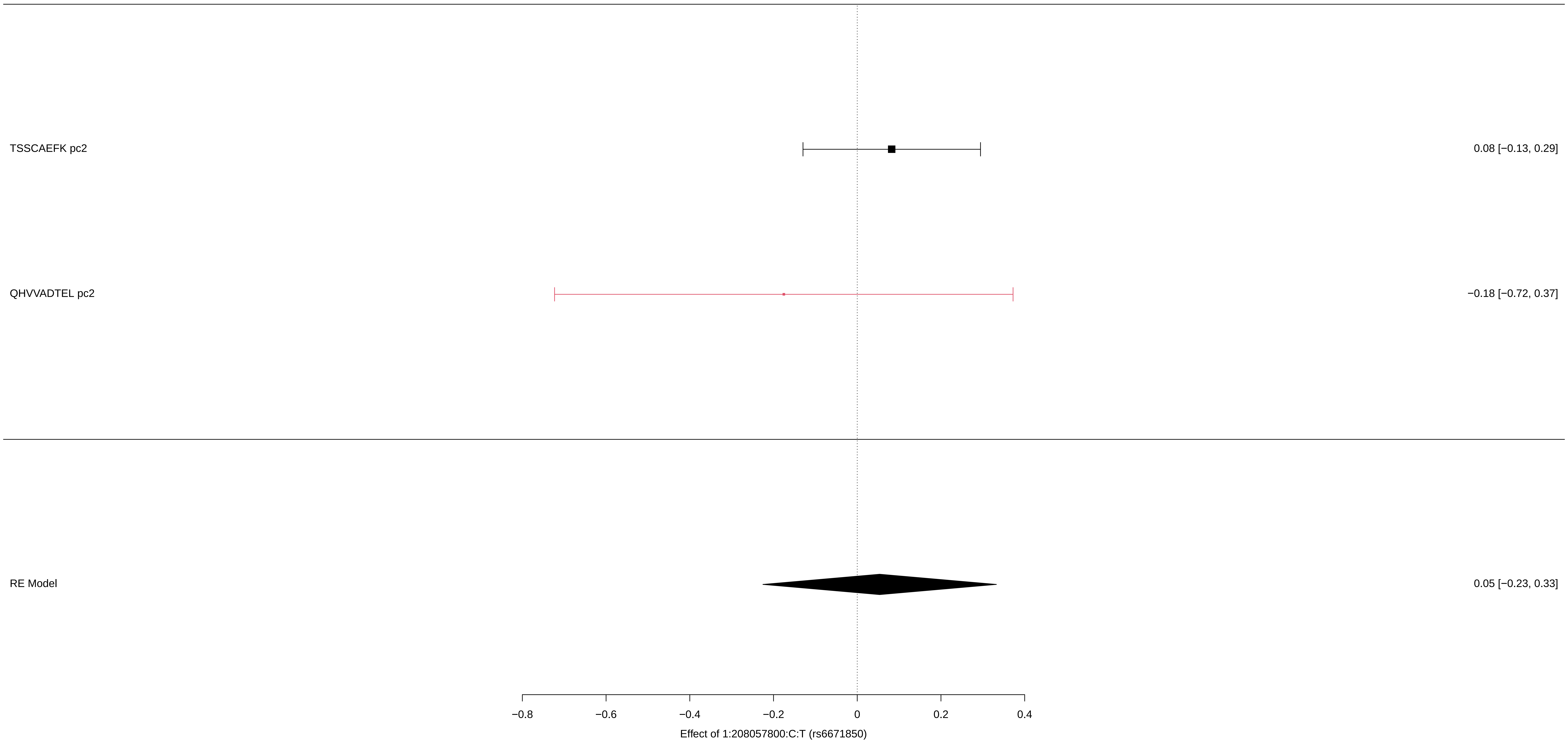

CD34 : NP2
P28906

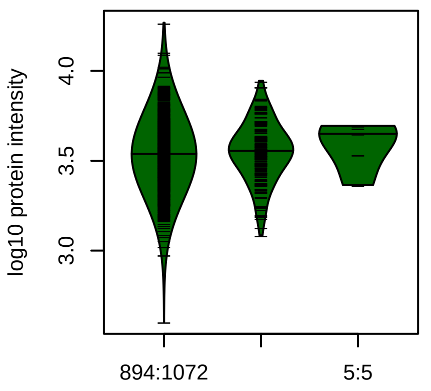

1:208057800:C:T_T
p = 0.35, beta = 0.0772, N = 1051

TSSCAEFK pc2
P28906;P28906−2

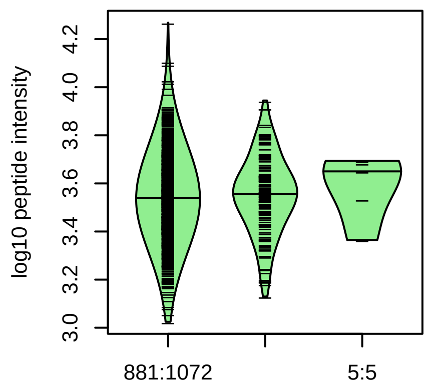

1:208057800:C:T_T
p = 0.32, beta = 0.0824, N = 1037

QHVVADTEL pc2
P28906;Q5JTA5

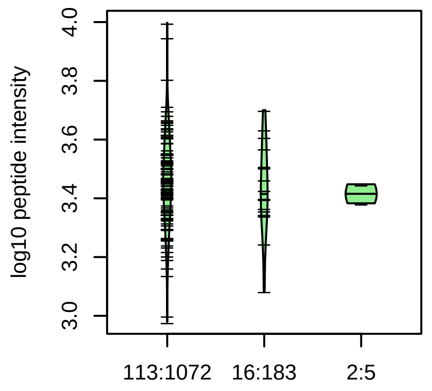

1:208057800:C:T_T
p = 0.41, beta = −0.175, N = 131

CD34 : NP2
P28906

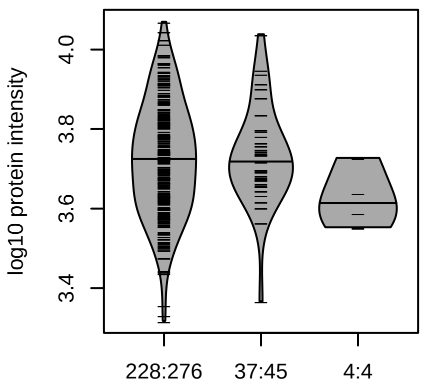

1:208057800:C:T_T
p = 0.87, beta = −0.0249, N = 269

TSSCAEFK pc2
P28906;P28906−2

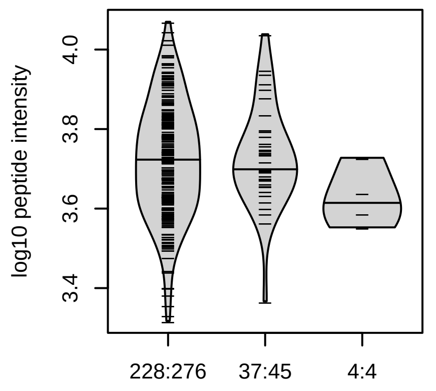

1:208057800:C:T_T
p = 0.77, beta = −0.0428, N = 269

Assay Target: CD34
UniProt: P28906
rsID: rs6671850
Target SNP: 1:208057800:C:T
Proxy SNP: 1:208057800:C:T
UKBPPP log10(p): 30.4
UKBPPP BETA: 0.14
UKBPPP MSPA: 0
−:−:NA
1037:131:9

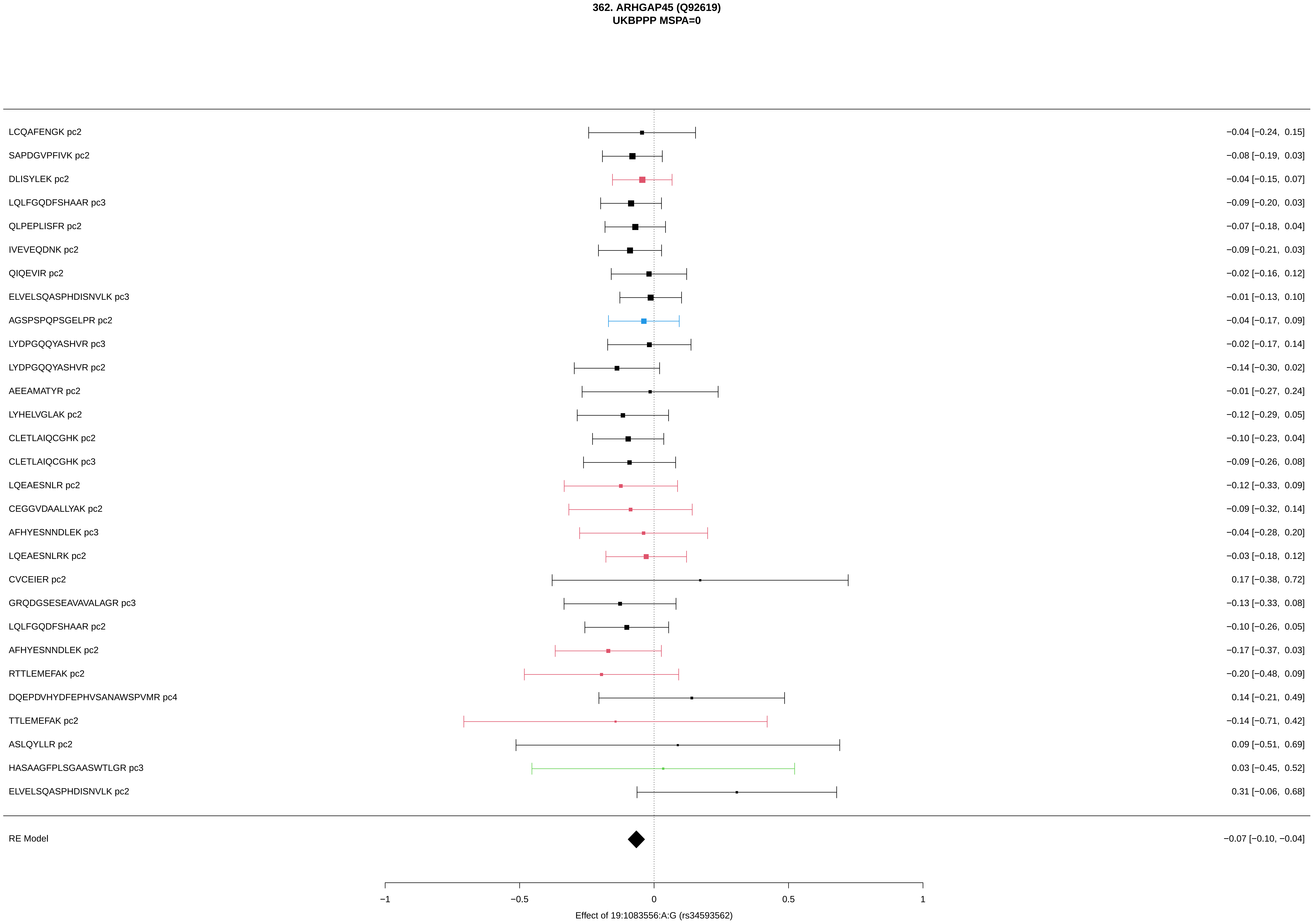
362. ARHGAP45 (Q92619)
UKBPPP MSPA=0
LCQAFENGK pc2 −0.04 [−0.24, 0.15]
SAPDGVPFIVK pc2 −0.08 [−0.19, 0.03]
DLISYLEK pc2 −0.04 [−0.15, 0.07]
LQLFGQDFSHAAR pc3 −0.09 [−0.20, 0.03]
QLPEPLISFR pc2 −0.07 [−0.18, 0.04]
IVEVEQDNK pc2 −0.09 [−0.21, 0.03]
QIQEVIR pc2 −0.02 [−0.16, 0.12]
ELVELSQASPHDISNVLK pc3 −0.01 [−0.13, 0.10]
AGSPSPQPSGELPR pc2 −0.04 [−0.17, 0.09]
LYDPGQQYASHVR pc3 −0.02 [−0.17, 0.14]
LYDPGQQYASHVR pc2 −0.14 [−0.30, 0.02]
AEEAMATYR pc2 −0.01 [−0.27, 0.24]
LYHELVGLAK pc2 −0.12 [−0.29, 0.05]
CLETLAIQCGHK pc2 −0.10 [−0.23, 0.04]
CLETLAIQCGHK pc3 −0.09 [−0.26, 0.08]
LQEAESNLR pc2 −0.12 [−0.33, 0.09]
CEGGVDAALLYAK pc2 −0.09 [−0.32, 0.14]
AFHYESNNDLEK pc3 −0.04 [−0.28, 0.20]
LQEAESNLRK pc2 −0.03 [−0.18, 0.12]
CVCEIER pc2 0.17 [−0.38, 0.72]
GRQDGSESEAVAVALAGR pc3 −0.13 [−0.33, 0.08]
LQLFGQDFSHAAR pc2 −0.10 [−0.26, 0.05]
AFHYESNNDLEK pc2 −0.17 [−0.37, 0.03]
RTTLEMEFAK pc2 −0.20 [−0.48, 0.09]
DQEPDVHYDFEPHVSANAWSPVMR pc4 0.14 [−0.21, 0.49]
TTLEMEFAK pc2 −0.14 [−0.71, 0.42]
ASLQYLLR pc2 0.09 [−0.51, 0.69]
HASAAGFPLSGAASWTLGR pc3 0.03 [−0.45, 0.52]
ELVELSQASPHDISNVLK pc2 0.31 [−0.06, 0.68]
RE Model −0.07 [−0.10, −0.04]
−1
−0.5
0
0.5
1
Effect of 19:1083556:A:G (rs34593562)

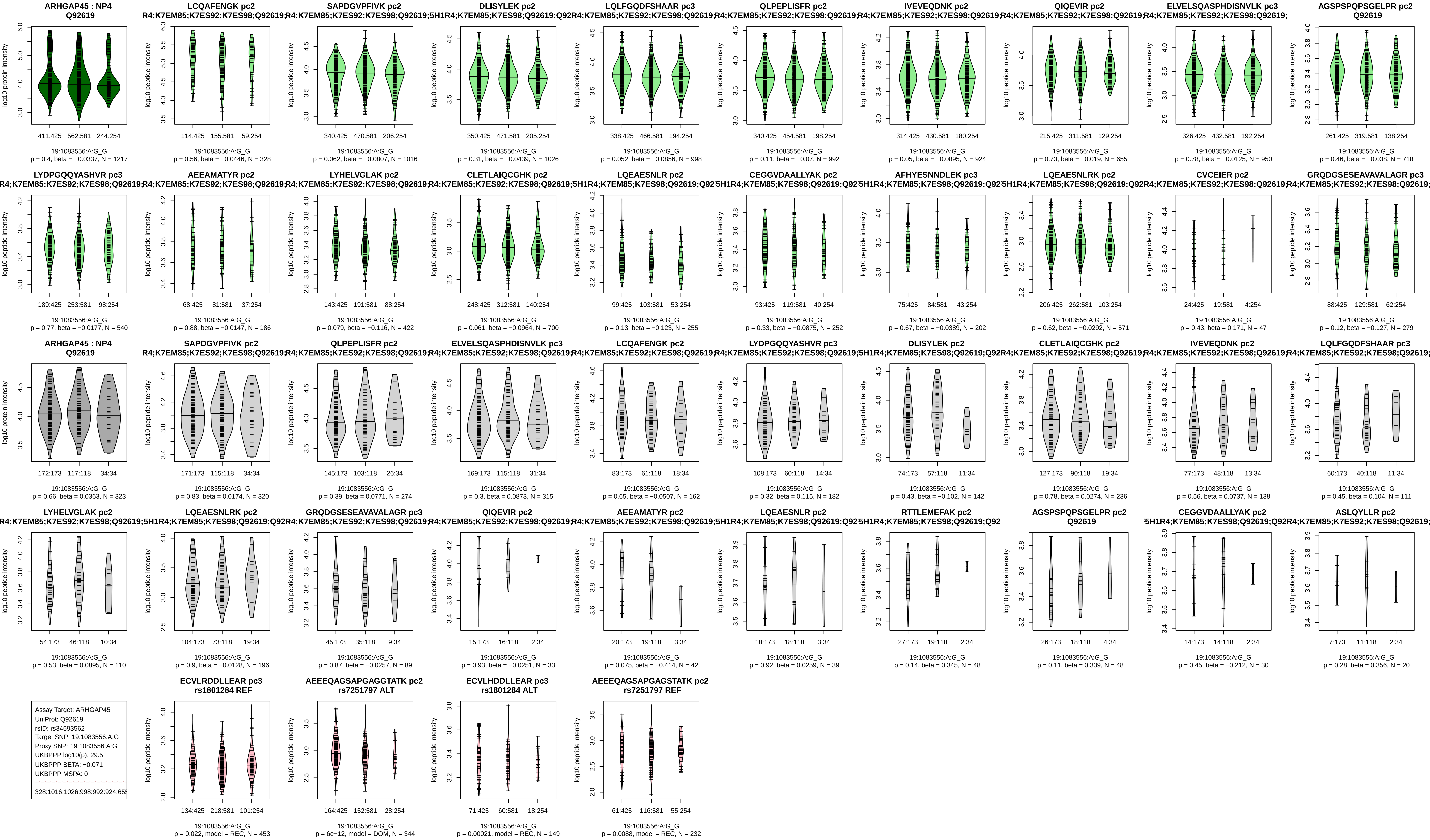

ARHGAP45 : NP4
Q92619
p = 0.4, beta = −0.0337, N = 1217
LCQAFENGK pc2
p = 0.56, beta = −0.0446, N = 328
SAPDGVPFIVK pc2
p = 0.062, beta = −0.0807, N = 1016
DLISYLEK pc2
p = 0.31, beta = −0.0439, N = 1026
LQLFGQDFSHAAR pc3
p = 0.052, beta = −0.0856, N = 998
QLPEPLISFR pc2
p = 0.11, beta = −0.07, N = 992
IVEVEQDNK pc2
p = 0.05, beta = −0.0895, N = 924
QIQEVIR pc2
p = 0.73, beta = −0.019, N = 655
ELVELSQASPHDISNVLK pc3
p = 0.78, beta = −0.0125, N = 950
AGSPSPQPSGELPR pc2
Q92619
p = 0.46, beta = −0.038, N = 718
LYDPGQQYASHVR pc3
p = 0.77, beta = −0.0177, N = 540
AEEAMATYR pc2
p = 0.88, beta = −0.0147, N = 186
LYHELVGLAK pc2
p = 0.079, beta = −0.116, N = 422
CLETLAIQCGHK pc2
p = 0.061, beta = −0.0964, N = 700
LQEAESNLR pc2
p = 0.13, beta = −0.123, N = 255
CEGGVDAALLYAK pc2
p = 0.33, beta = −0.0875, N = 252
AFHYESNNDLEK pc3
p = 0.67, beta = −0.0389, N = 202
LQEAESNLRK pc2
p = 0.62, beta = −0.0292, N = 571
CVCEIER pc2
p = 0.43, beta = 0.171, N = 47
GRQDGSESEAVAVALAGR pc3
p = 0.12, beta = −0.127, N = 279
ARHGAP45 : NP4
Q92619
p = 0.66, beta = 0.0363, N = 323
SAPDGVPFIVK pc2
p = 0.83, beta = 0.0174, N = 320
QLPEPLISFR pc2
p = 0.39, beta = 0.0771, N = 274
ELVELSQASPHDISNVLK pc3
p = 0.3, beta = 0.0873, N = 315
LCQAFENGK pc2
p = 0.65, beta = −0.0507, N = 162
LYDPGQQYASHVR pc3
p = 0.32, beta = 0.115, N = 182
DLISYLEK pc2
p = 0.43, beta = −0.102, N = 142
CLETLAIQCGHK pc2
p = 0.78, beta = 0.0274, N = 236
IVEVEQDNK pc2
p = 0.56, beta = 0.0737, N = 138
LQLFGQDFSHAAR pc3
p = 0.45, beta = 0.104, N = 111
LYHELVGLAK pc2
p = 0.53, beta = 0.0895, N = 110
LQEAESNLRK pc2
p = 0.9, beta = −0.0128, N = 196
GRQDGSESEAVAVALAGR pc3
p = 0.87, beta = −0.0257, N = 89
QIQEVIR pc2
p = 0.93, beta = −0.0251, N = 33
AEEAMATYR pc2
p = 0.075, beta = −0.414, N = 42
LQEAESNLR pc2
p = 0.92, beta = 0.0259, N = 39
RTTLEMEFAK pc2
p = 0.14, beta = 0.345, N = 48
AGSPSPQPSGELPR pc2
Q92619
p = 0.11, beta = 0.339, N = 48
CEGGVDAALLYAK pc2
p = 0.45, beta = −0.212, N = 30
ASLQYLLR pc2
p = 0.28, beta = 0.356, N = 20
Assay Target: ARHGAP45
UniProt: Q92619
rsID: rs34593562
Target SNP: 19:1083556:A:G
Proxy SNP: 19:1083556:A:G
UKBPPP log10(p): 29.5
UKBPPP BETA: −0.071
UKBPPP MSPA: 0
ECVLRDDLLEAR pc3
rs1801284 REF
p = 0.022, model = REC, N = 453
AEEEQAGSAPGAGGTATK pc2
rs7251797 ALT
p = 6e−12, model = DOM, N = 344
ECVLHDDLLEAR pc3
rs1801284 ALT
p = 0.00021, model = REC, N = 149
AEEEQAGSAPGAGSTATK pc2
rs7251797 REF
p = 0.0088, model = REC, N = 232
19:1083556:A:G_G
log10 protein intensity
log10 peptide intensity

363. P4HB (P07237)
UKBPPP MSPA=0.13

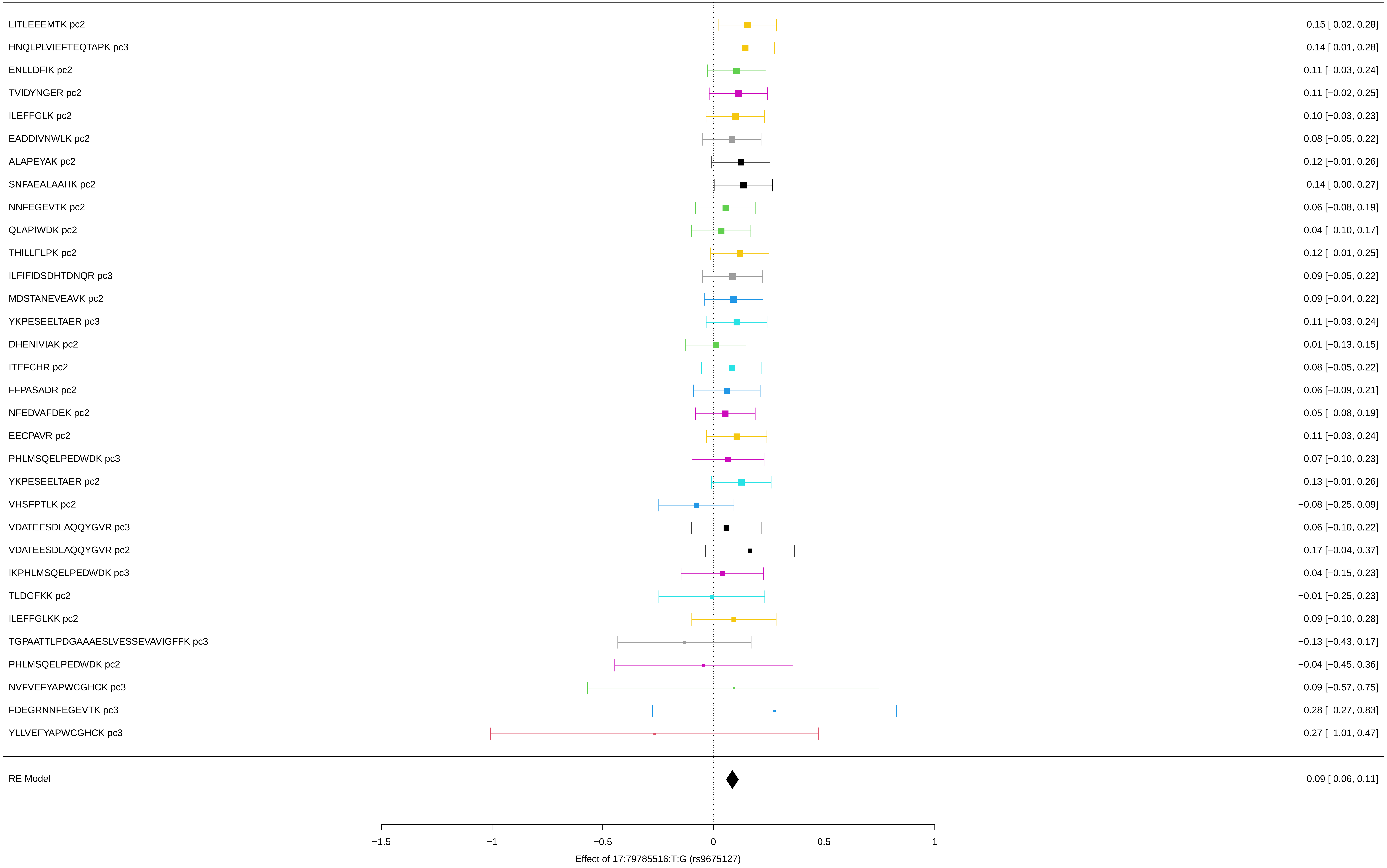

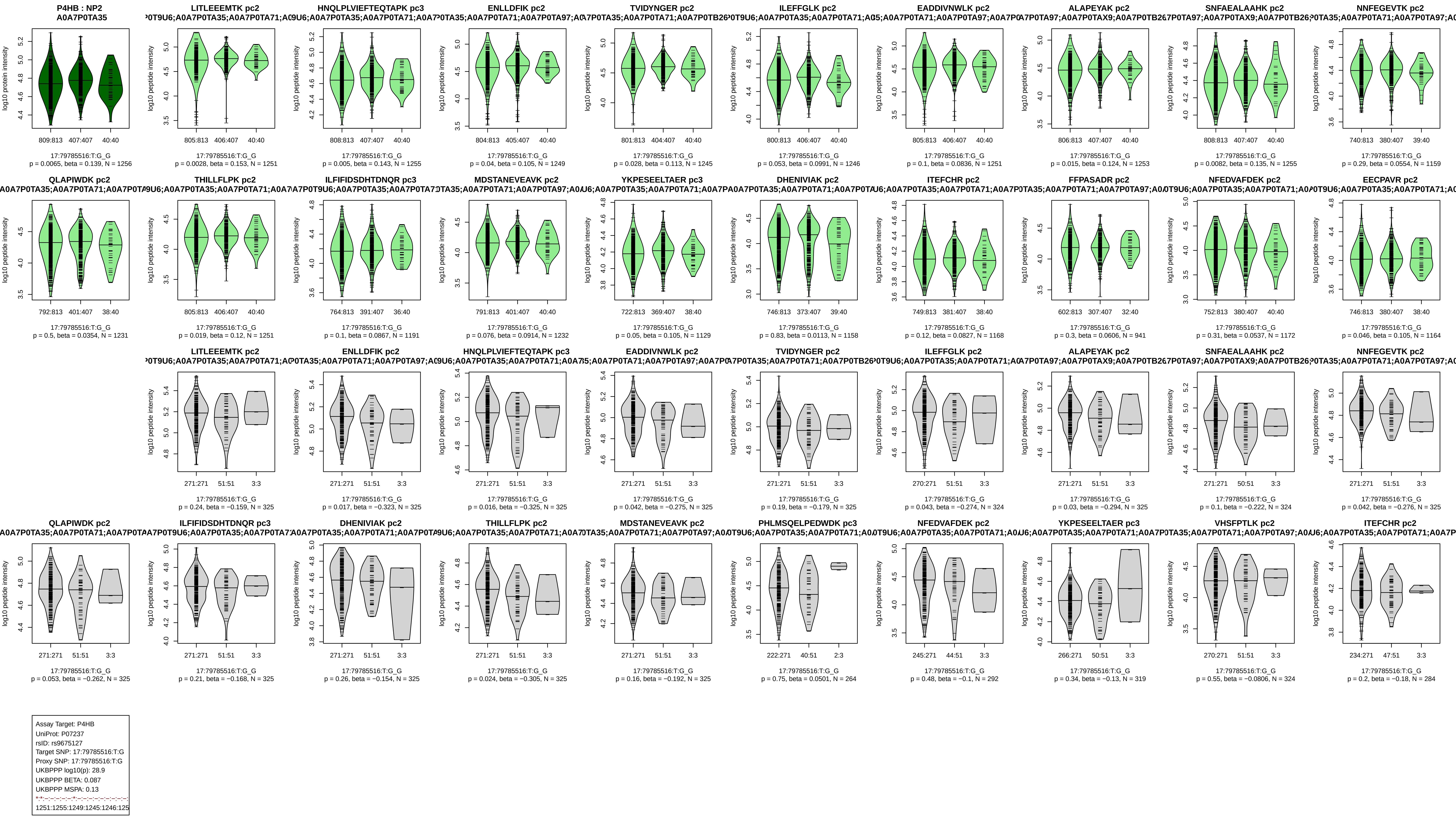
P4HB : NP2
A0A7P0TA35
p = 0.0065, beta = 0.139, N = 1256
LITLEEEMTK pc2
p = 0.0028, beta = 0.153, N = 1251
HNQLPLVIEFTEQTAPK pc3
p = 0.005, beta = 0.143, N = 1255
ENLLDFIK pc2
p = 0.04, beta = 0.105, N = 1249
TVIDYNGER pc2
p = 0.028, beta = 0.113, N = 1245
ILEFFGLK pc2
p = 0.053, beta = 0.0991, N = 1246
EADDIVNWLK pc2
p = 0.1, beta = 0.0836, N = 1251
ALAPEYAK pc2
p = 0.015, beta = 0.124, N = 1253
SNFAEALAAHK pc2
p = 0.0082, beta = 0.135, N = 1255
NNFEGEVTK pc2
p = 0.29, beta = 0.0554, N = 1159
QLAPIWDK pc2
p = 0.5, beta = 0.0354, N = 1231
THILLFLPK pc2
p = 0.019, beta = 0.12, N = 1251
ILFIFIDSDHTDNQR pc3
p = 0.1, beta = 0.0867, N = 1191
MDSTANEVEAVK pc2
p = 0.076, beta = 0.0914, N = 1232
YKPESEELTAER pc3
p = 0.05, beta = 0.105, N = 1129
DHENIVIAK pc2
p = 0.83, beta = 0.0113, N = 1158
ITEFCHR pc2
p = 0.12, beta = 0.0827, N = 1168
FFPASADR pc2
p = 0.3, beta = 0.0606, N = 941
NFEDVAFDEK pc2
p = 0.31, beta = 0.0537, N = 1172
EECPAVR pc2
p = 0.046, beta = 0.105, N = 1164
LITLEEEMTK pc2
p = 0.24, beta = −0.159, N = 325
ENLLDFIK pc2
p = 0.017, beta = −0.323, N = 325
HNQLPLVIEFTEQTAPK pc3
p = 0.016, beta = −0.325, N = 325
EADDIVNWLK pc2
p = 0.042, beta = −0.275, N = 325
TVIDYNGER pc2
p = 0.19, beta = −0.179, N = 325
ILEFFGLK pc2
p = 0.043, beta = −0.274, N = 324
ALAPEYAK pc2
p = 0.03, beta = −0.294, N = 325
SNFAEALAAHK pc2
p = 0.1, beta = −0.222, N = 324
NNFEGEVTK pc2
p = 0.042, beta = −0.276, N = 325
QLAPIWDK pc2
p = 0.053, beta = −0.262, N = 325
ILFIFIDSDHTDNQR pc3
p = 0.21, beta = −0.168, N = 325
DHENIVIAK pc2
p = 0.26, beta = −0.154, N = 325
THILLFLPK pc2
p = 0.024, beta = −0.305, N = 325
MDSTANEVEAVK pc2
p = 0.16, beta = −0.192, N = 325
PHLMSQELPEDWDK pc3
p = 0.75, beta = 0.0501, N = 264
NFEDVAFDEK pc2
p = 0.48, beta = −0.1, N = 292
YKPESEELTAER pc3
p = 0.34, beta = −0.13, N = 319
VHSFPTLK pc2
p = 0.55, beta = −0.0806, N = 324
ITEFCHR pc2
p = 0.2, beta = −0.18, N = 284
17:79785516:T:G_G
log10 protein intensity
log10 peptide intensity
Assay Target: P4HB
UniProt: P07237
rsID: rs9675127
Target SNP: 17:79785516:T:G
Proxy SNP: 17:79785516:T:G
UKBPPP log10(p): 28.9
UKBPPP BETA: 0.087
UKBPPP MSPA: 0.13
1251:1255:1249:1245:1246:125

364. CCDC80 (Q76M96)
UKBPPP MSPA=0

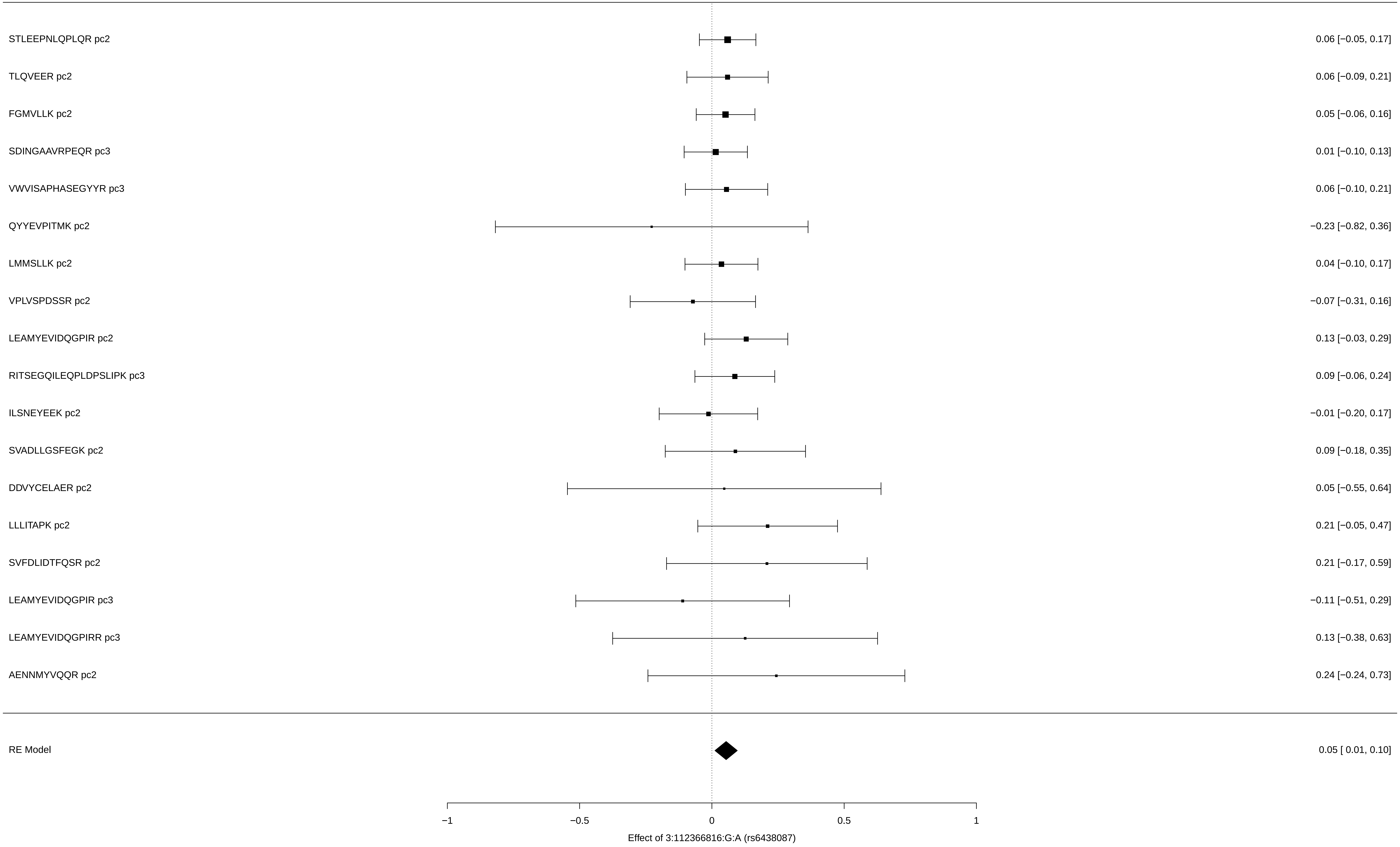

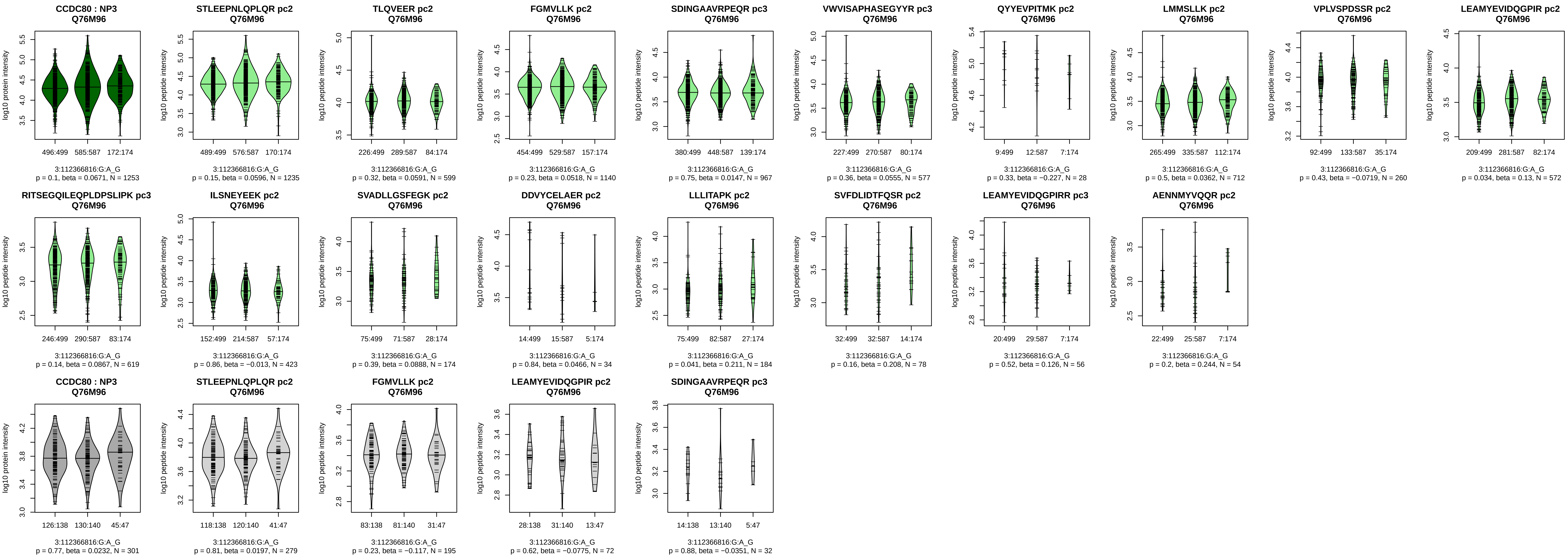

Assay Target: CCDC80
UniProt: Q76M96
rsID: rs6438087
Target SNP: 3:112366816:G:A
Proxy SNP: 3:112366816:G:A
UKBPPP log10(p): 28.2
UKBPPP BETA: −0.064
UKBPPP MSPA: 0

1235:599:1140:967:577:28:712:

365. CXCL14 (O95715)
UKBPPP MSPA=0

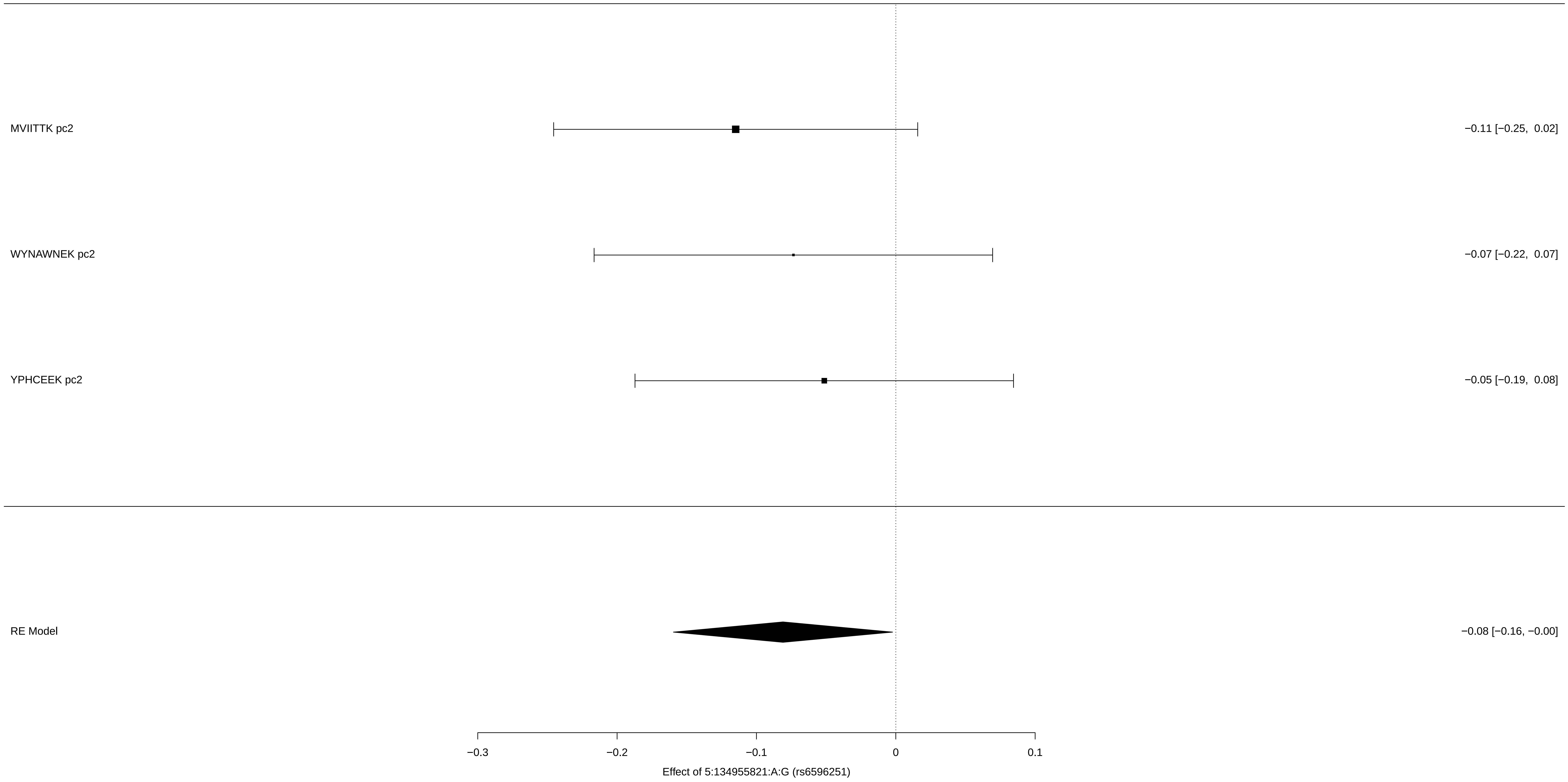

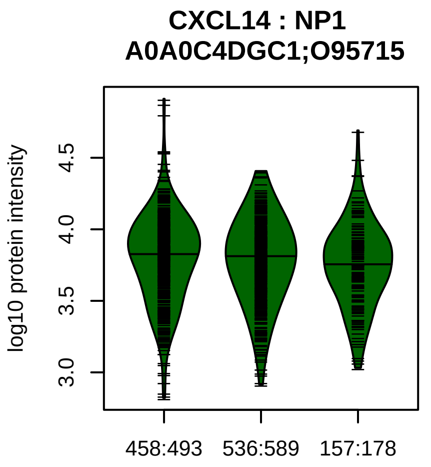

5:134955821:A:G_G
p = 0.064, beta = −0.0796, N = 1151

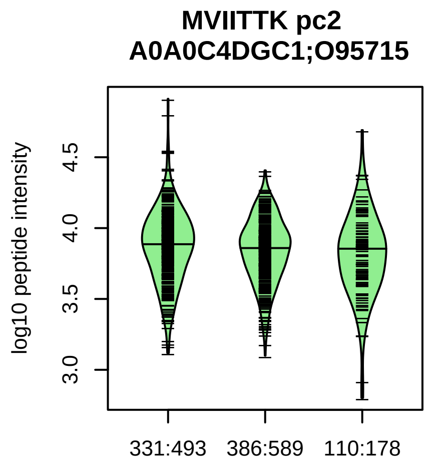

5:134955821:A:G_G
p = 0.024, beta = −0.115, N = 827

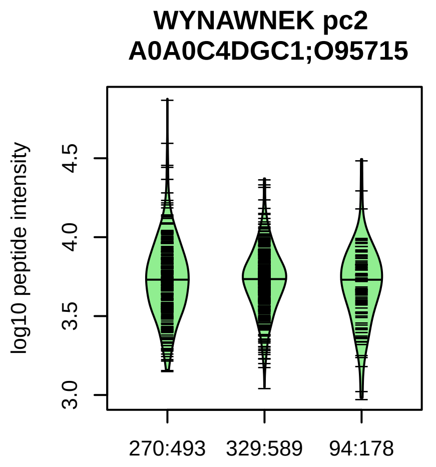

5:134955821:A:G_G
p = 0.19, beta = −0.0735, N = 693

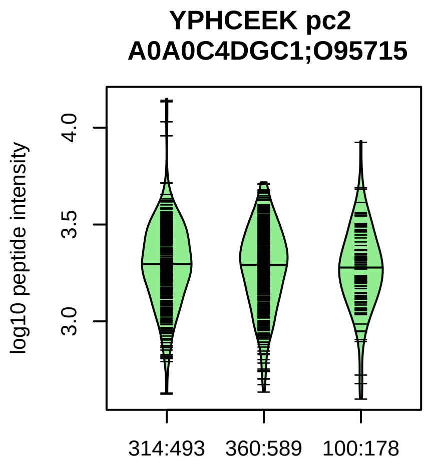

5:134955821:A:G_G
p = 0.33, beta = −0.0513, N = 774

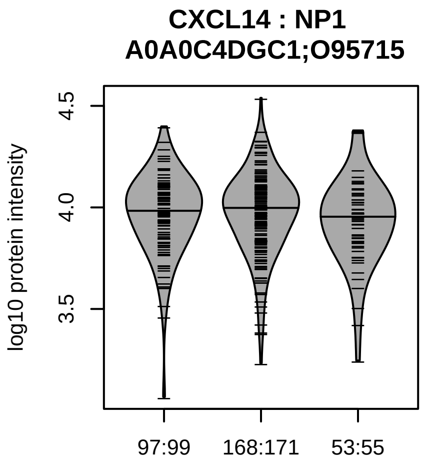

5:134955821:A:G_G
p = 0.39, beta = −0.0711, N = 318

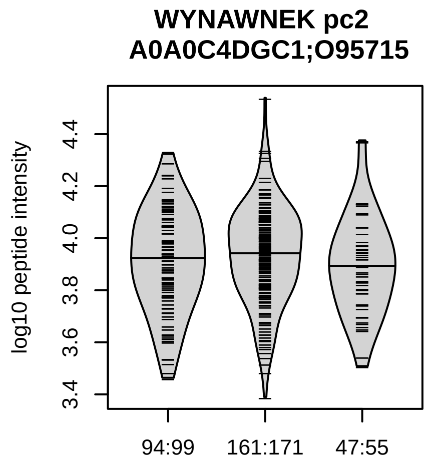

5:134955821:A:G_G
p = 0.8, beta = −0.0214, N = 302

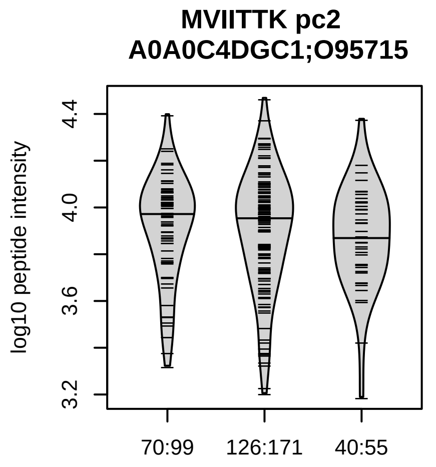

5:134955821:A:G_G
p = 0.35, beta = −0.0889, N = 236

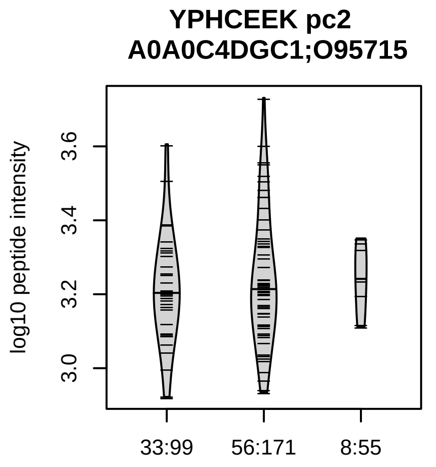

5:134955821:A:G_G
p = 0.48, beta = 0.118, N = 97

Assay Target: CXCL14
UniProt: O95715
rsID: rs6596251
Target SNP: 5:134955821:A:G
Proxy SNP: 5:134955821:A:G
UKBPPP log10(p): 27.1
UKBPPP BETA: −0.063
UKBPPP MSPA: 0
−:−:−
827:693:774

366. APOA1 (P02647)
UKBPPP MSPA=0.007

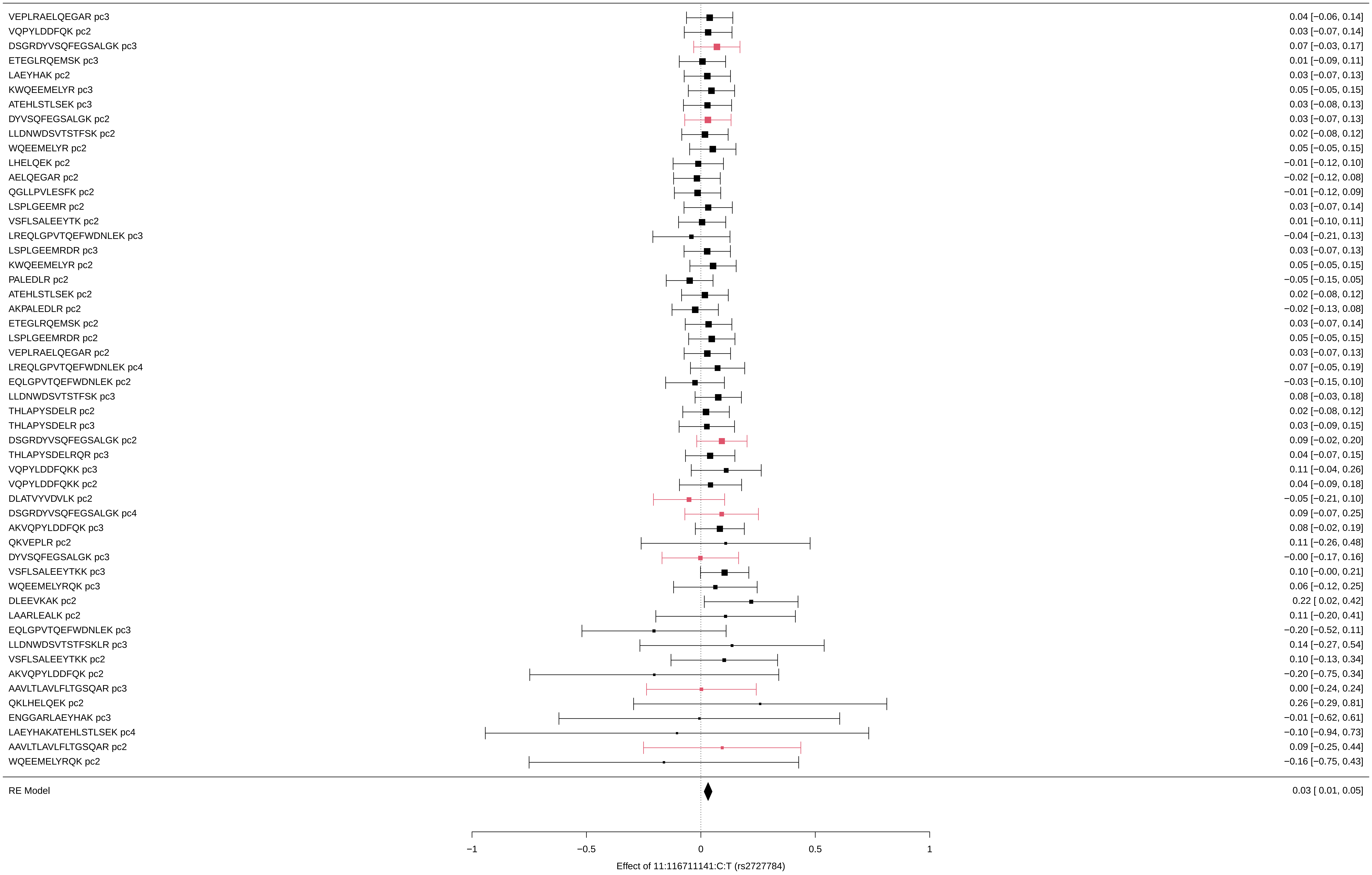

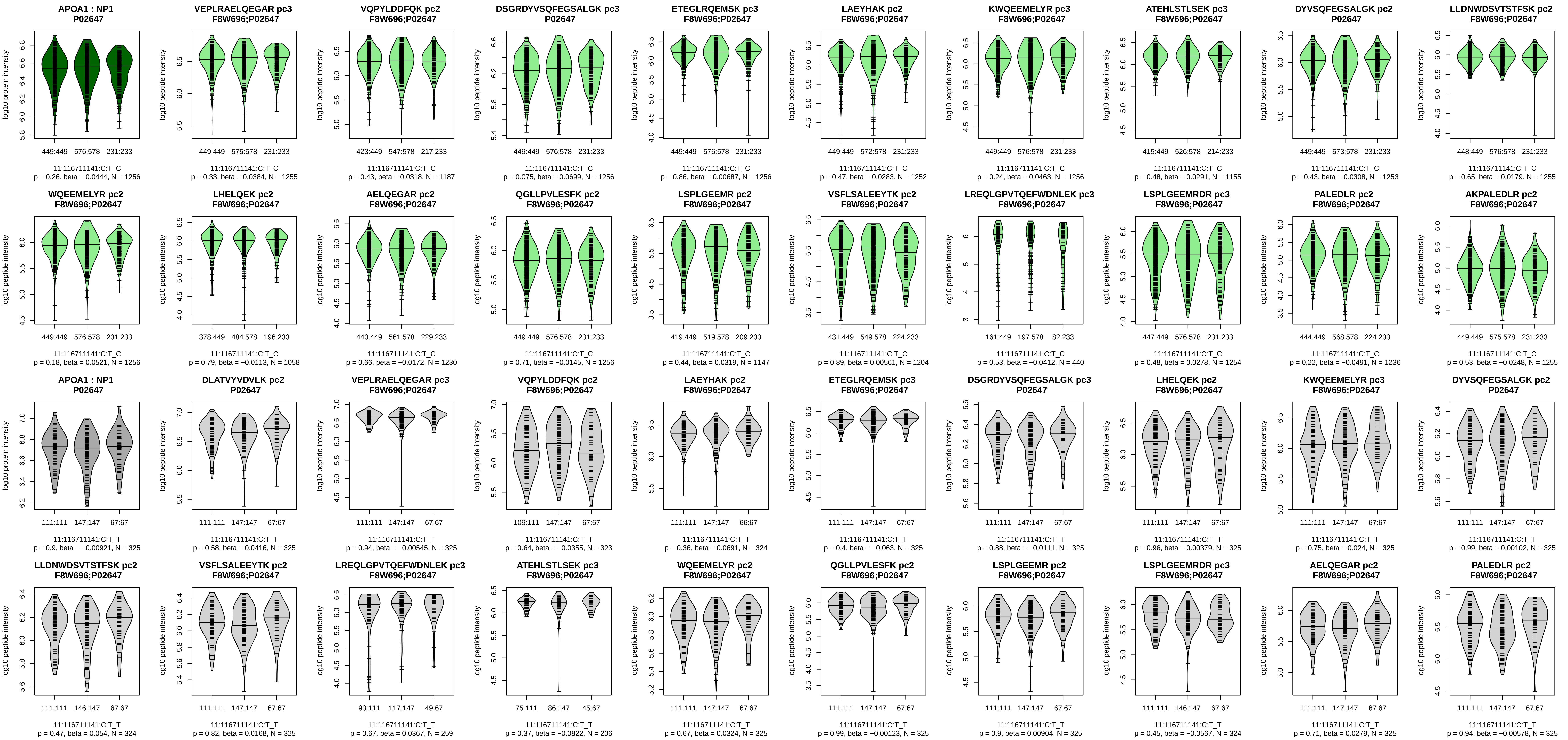

Assay Target: APOA1
UniProt: P02647
rsID: rs2727784
Target SNP: 11:116711141:C:T
Proxy SNP: 11:116711141:C:T
UKBPPP log10(p): 26.5
UKBPPP BETA: −0.066
UKBPPP MSPA: 0.007
1255:1187:1256:1256:1252:125

367. APP (P05067)
UKBPPP MSPA=0

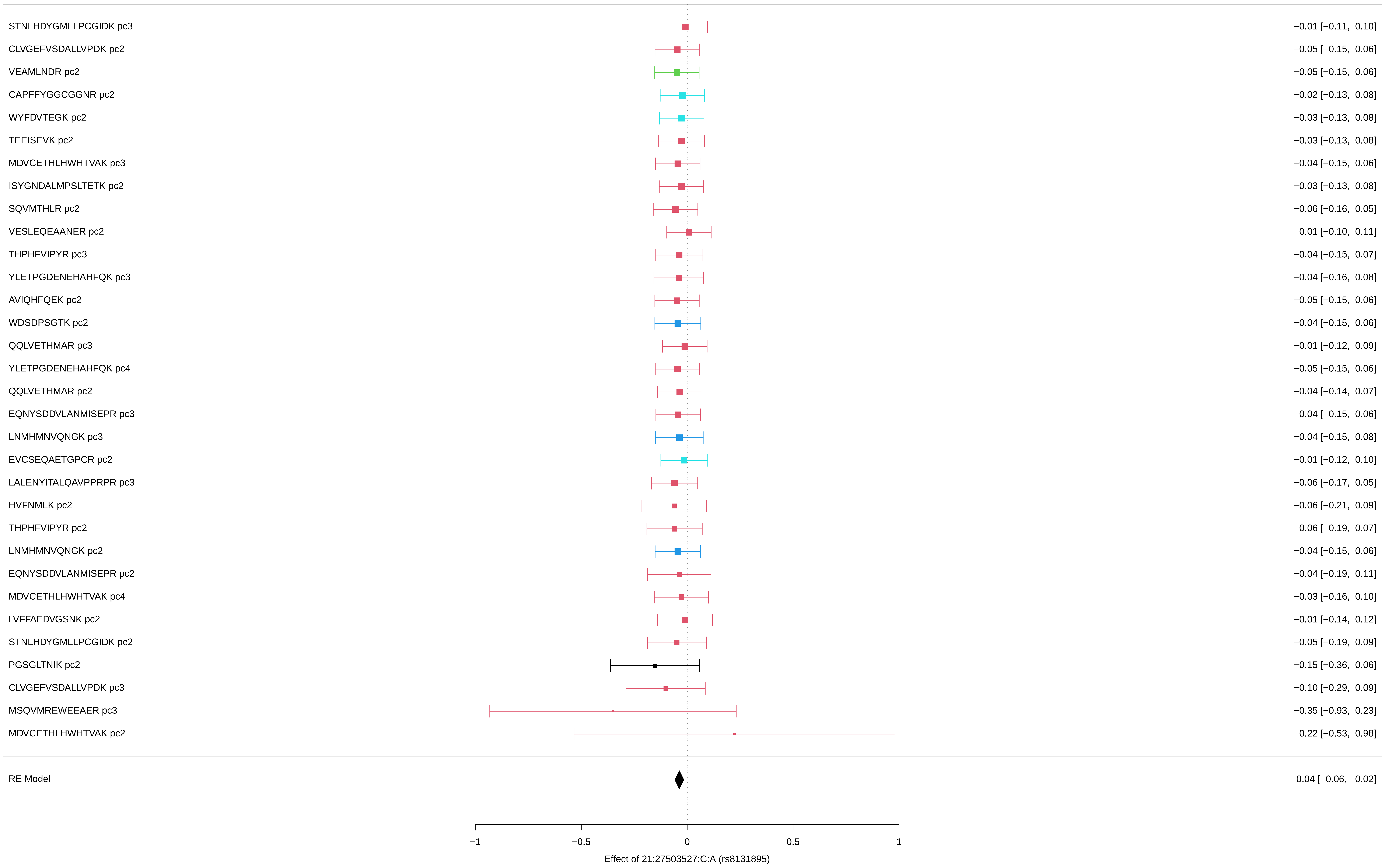

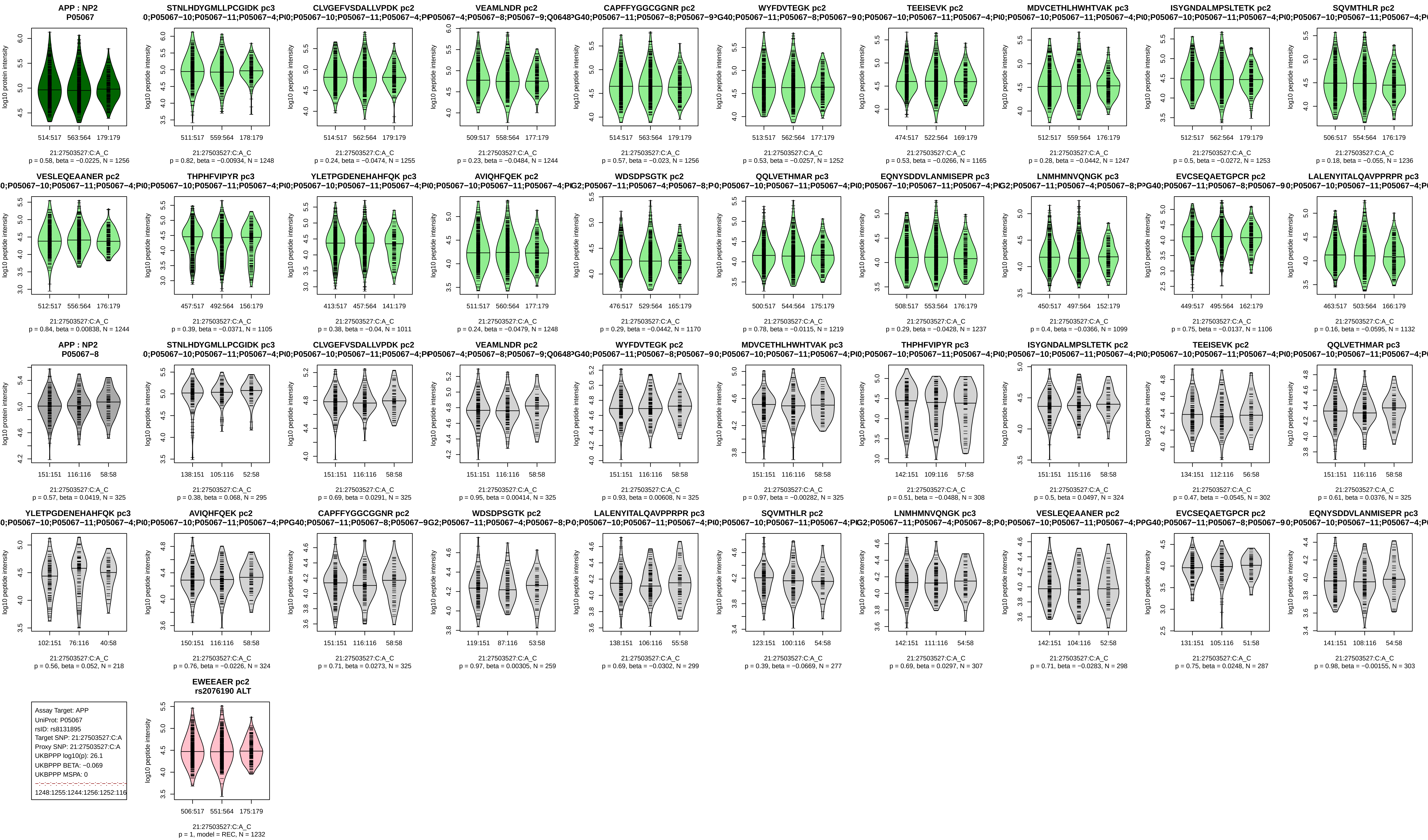

APP : NP2 P05067
p = 0.58, beta = −0.0225, N = 1256
STNLHDYGMLLPCGIDK pc3
p = 0.82, beta = −0.00934, N = 1248
CLVGEFVSDALLVPDK pc2
p = 0.24, beta = −0.0474, N = 1255
VEAMLNDR pc2
p = 0.23, beta = −0.0484, N = 1244
CAPFFYGGCGGNR pc2
p = 0.57, beta = −0.023, N = 1256
WYFDVTEGK pc2
p = 0.53, beta = −0.0257, N = 1252
TEEISEVK pc2
p = 0.53, beta = −0.0266, N = 1165
MDVCETHLHWHTVAK pc3
p = 0.28, beta = −0.0442, N = 1247
ISYGNDALMPSLTETK pc2
p = 0.5, beta = −0.0272, N = 1253
SQVMTHLR pc2
p = 0.18, beta = −0.055, N = 1236
VESLEQEAANER pc2
p = 0.84, beta = 0.00838, N = 1244
THPHFVIPYR pc3
p = 0.39, beta = −0.0371, N = 1105
YLETPGDENEHAHFQK pc3
p = 0.38, beta = −0.04, N = 1011
AVIQHFQEK pc2
p = 0.24, beta = −0.0479, N = 1248
WDSDPSGTK pc2
p = 0.29, beta = −0.0442, N = 1170
QQLVETHMAR pc3
p = 0.78, beta = −0.0115, N = 1219
EQNYSDDVLANMISEPR pc3
p = 0.29, beta = −0.0428, N = 1237
LNMHMNVQNGK pc3
p = 0.4, beta = −0.0366, N = 1099
EVCSEQAETGPCR pc2
p = 0.75, beta = −0.0137, N = 1106
LALENYITALQAVPPRPR pc3
p = 0.16, beta = −0.0595, N = 1132
APP : NP2 P05067−8
p = 0.57, beta = 0.0419, N = 325
STNLHDYGMLLPCGIDK pc3
p = 0.38, beta = 0.068, N = 295
CLVGEFVSDALLVPDK pc2
p = 0.69, beta = 0.0291, N = 325
VEAMLNDR pc2
p = 0.95, beta = 0.00414, N = 325
WYFDVTEGK pc2
p = 0.93, beta = 0.00608, N = 325
MDVCETHLHWHTVAK pc3
p = 0.97, beta = −0.00282, N = 325
THPHFVIPYR pc3
p = 0.51, beta = −0.0488, N = 308
ISYGNDALMPSLTETK pc2
p = 0.5, beta = 0.0497, N = 324
TEEISEVK pc2
p = 0.47, beta = −0.0545, N = 302
QQLVETHMAR pc3
p = 0.61, beta = 0.0376, N = 325
YLETPGDENEHAHFQK pc3
p = 0.56, beta = 0.052, N = 218
AVIQHFQEK pc2
p = 0.76, beta = −0.0226, N = 324
CAPFFYGGCGGNR pc2
p = 0.71, beta = 0.0273, N = 325
WDSDPSGTK pc2
p = 0.97, beta = 0.00305, N = 259
LALENYITALQAVPPRPR pc3
p = 0.69, beta = −0.0302, N = 299
SQVMTHLR pc2
p = 0.39, beta = −0.0669, N = 277
LNMHMNVQNGK pc3
p = 0.69, beta = 0.0297, N = 307
VESLEQEAANER pc2
p = 0.71, beta = −0.0283, N = 298
EVCSEQAETGPCR pc2
p = 0.75, beta = 0.0248, N = 287
EQNYSDDVLANMISEPR pc3
p = 0.98, beta = −0.00155, N = 303
Assay Target: APP
UniProt: P05067
rsID: rs8131895
Target SNP: 21:27503527:C:A
Proxy SNP: 21:27503527:C:A
UKBPPP log10(p): 26.1
UKBPPP BETA: −0.069
UKBPPP MSPA: 0
1248:1255:1244:1256:1252:116
EWEEAER pc2
rs2076190 ALT
p = 1, model = REC, N = 1232

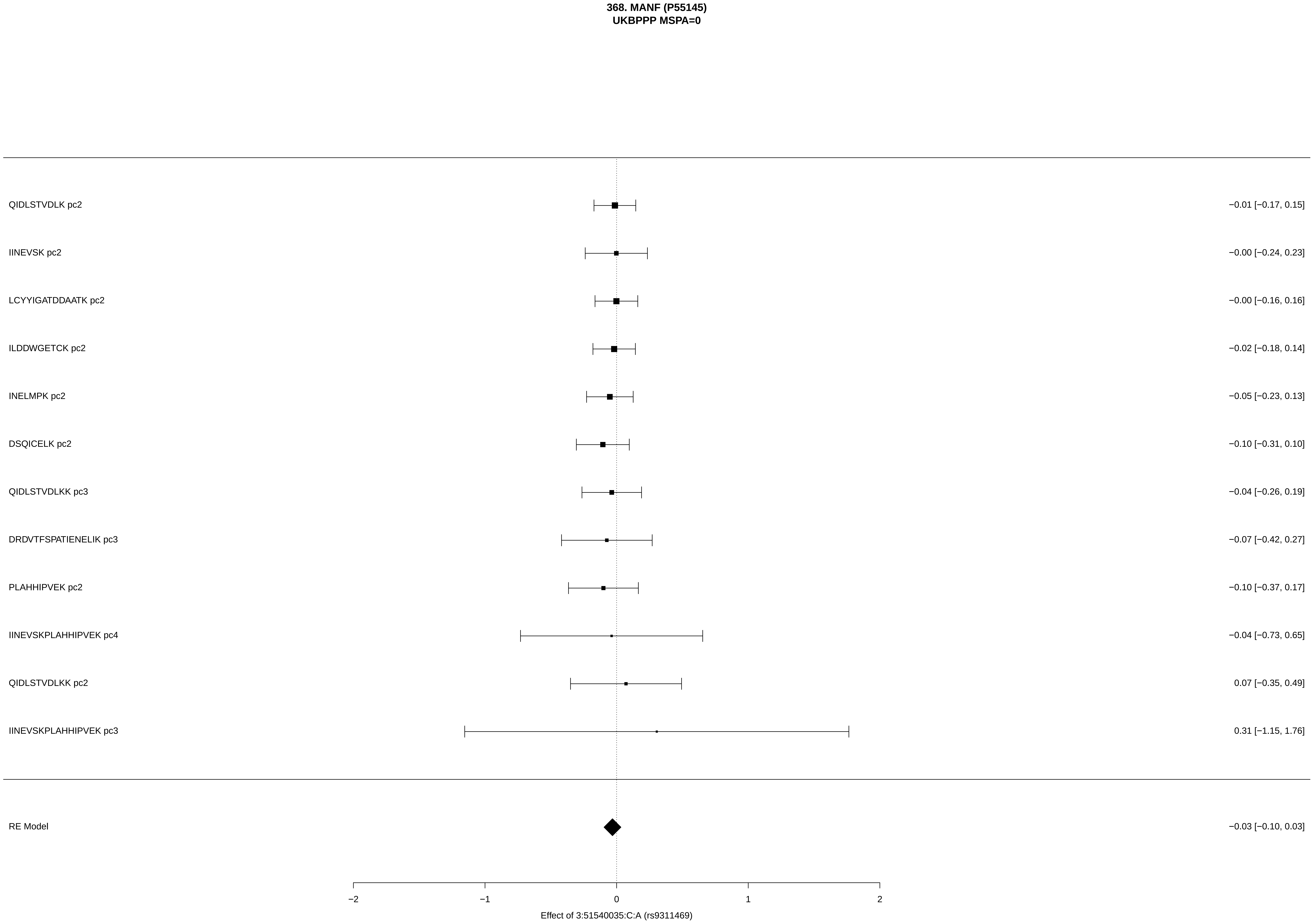
368. MANF (P55145)
UKBPPP MSPA=0
QIDLSTVDLK pc2
−0.01 [−0.17, 0.15]
IINEVSK pc2
−0.00 [−0.24, 0.23]
LCYYIGATDDAATK pc2
−0.00 [−0.16, 0.16]
ILDDWGETCK pc2
−0.02 [−0.18, 0.14]
INELMPK pc2
−0.05 [−0.23, 0.13]
DSQICELK pc2
−0.10 [−0.31, 0.10]
QIDLSTVDLKK pc3
−0.04 [−0.26, 0.19]
DRDVTFSPATIENELIK pc3
−0.07 [−0.42, 0.27]
PLAHHIPVEK pc2
−0.10 [−0.37, 0.17]
IINEVSKPLAHHIPVEK pc4
−0.04 [−0.73, 0.65]
QIDLSTVDLKK pc2
0.07 [−0.35, 0.49]
IINEVSKPLAHHIPVEK pc3
0.31 [−1.15, 1.76]
RE Model
−0.03 [−0.10, 0.03]
−2
−1
0
1
2
Effect of 3:51540035:C:A (rs9311469)

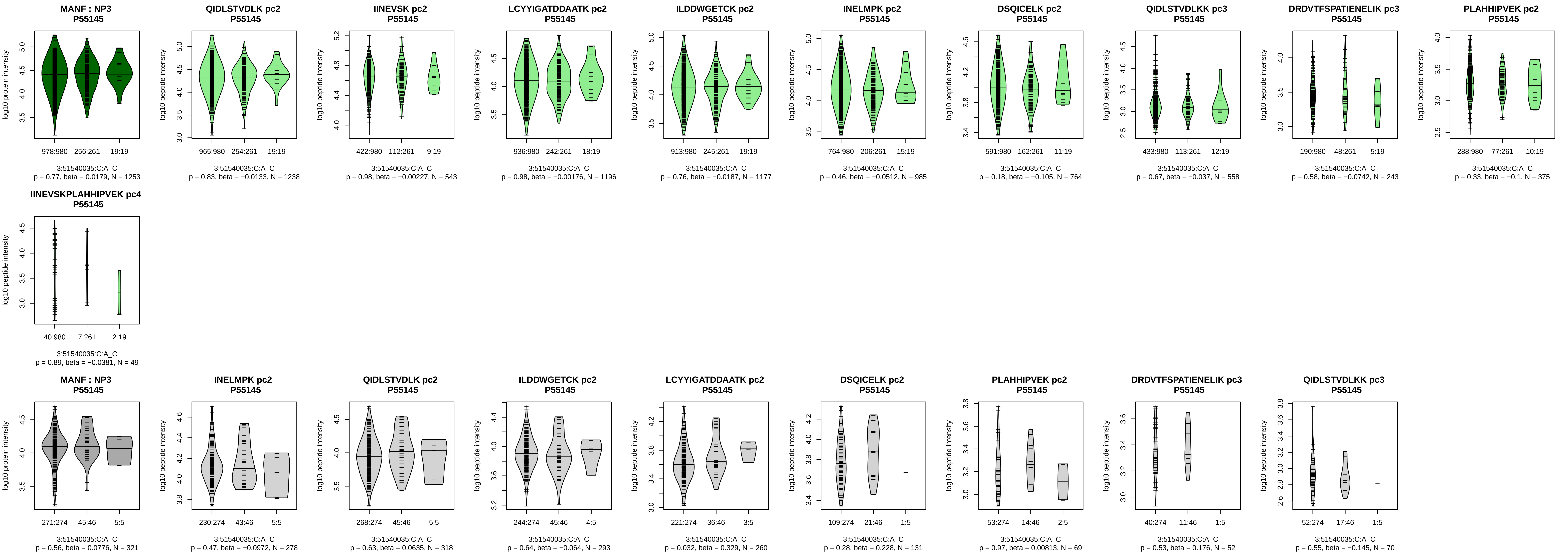

Assay Target: MANF
UniProt: P55145
rsID: rs9311469
Target SNP: 3:51540035:C:A
Proxy SNP: 3:51540035:C:A
UKBPPP log10(p): 26.1
UKBPPP BETA: 0.097
UKBPPP MSPA: 0
–:–:–:–:–:–:–:–:–:–:–:NA
1238:543:1196:1177:985:764:55

369. APOA2 (P02652)
UKBPPP MSPA=0

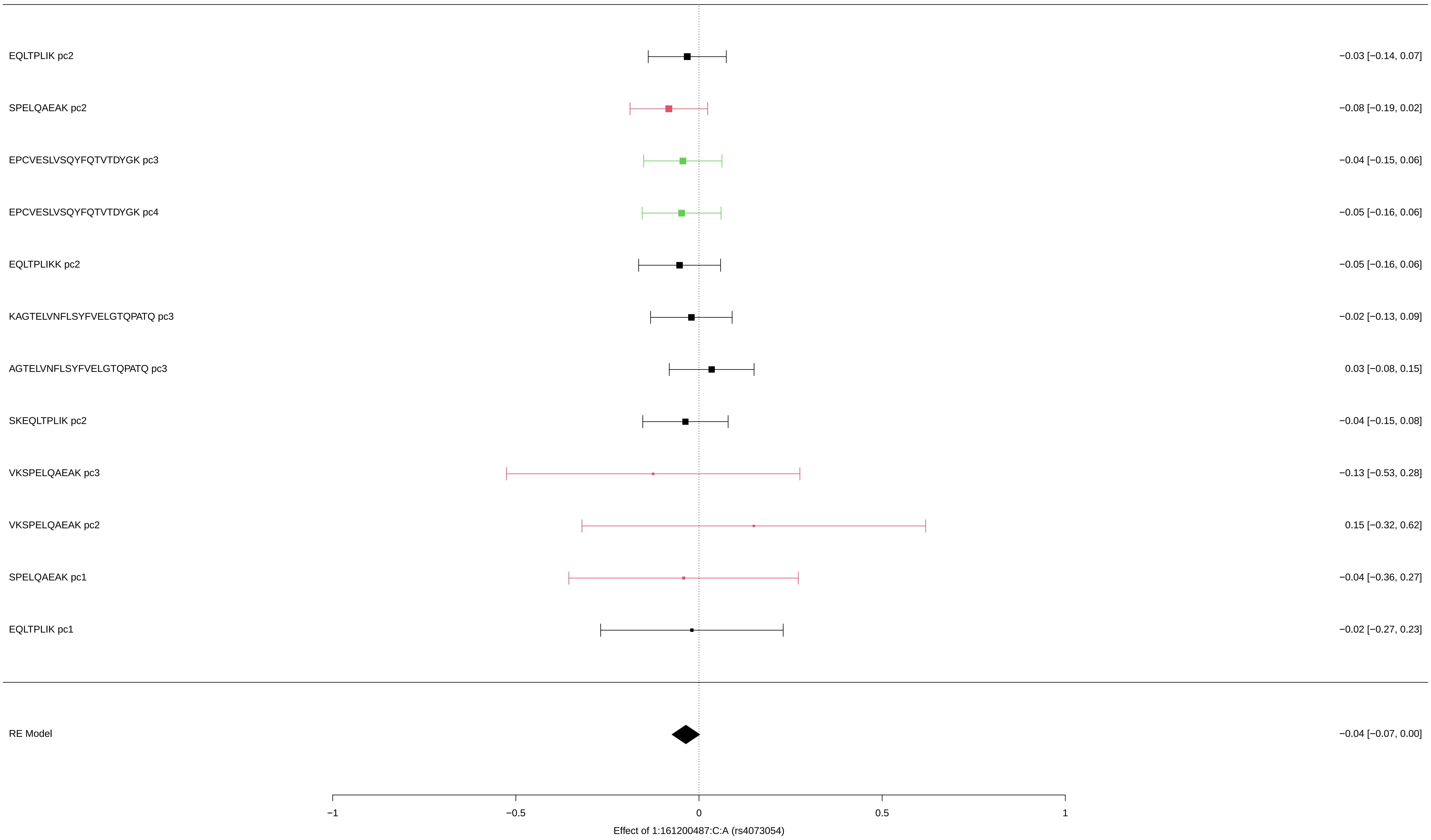

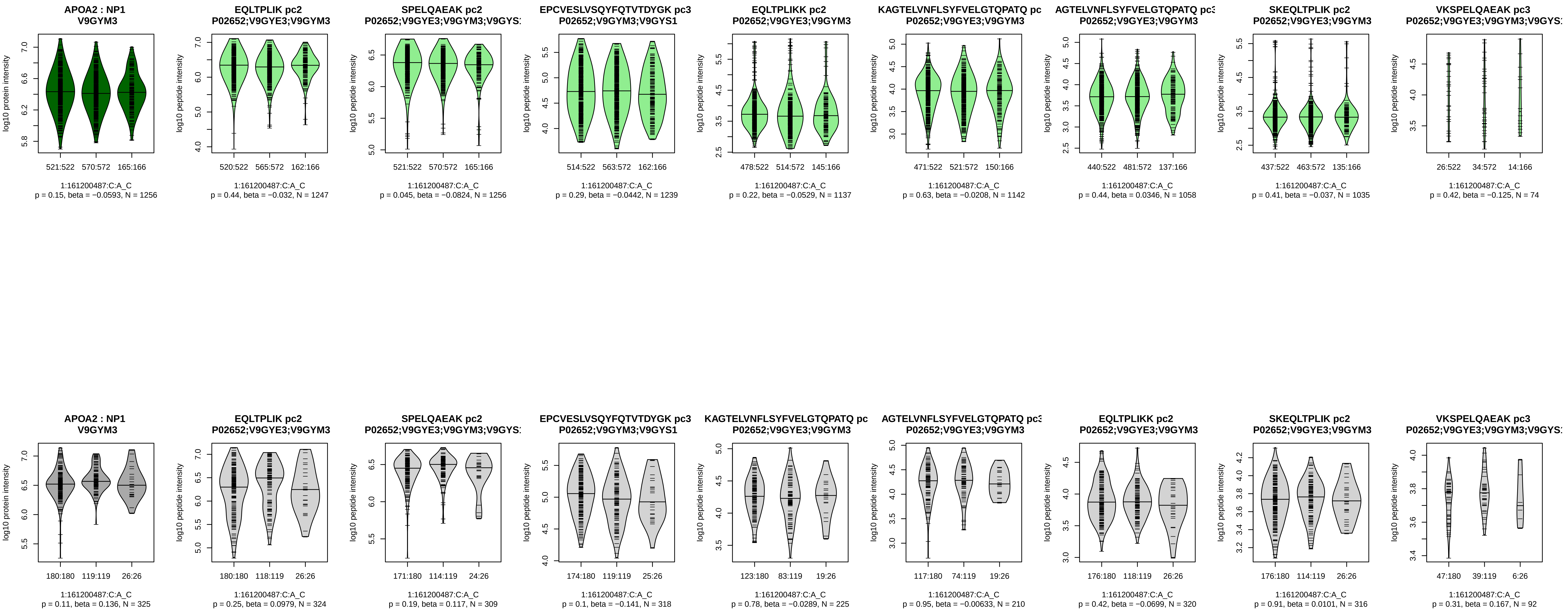

Assay Target: APOA2
UniProt: P02652
rsID: rs4073054
Target SNP: 1:161200487:C:A
Proxy SNP: 1:161200487:C:A
UKBPPP log10(p): 25.2
UKBPPP BETA: 0.065
UKBPPP MSPA: 0
1247:1256:1239:1221:1137:114

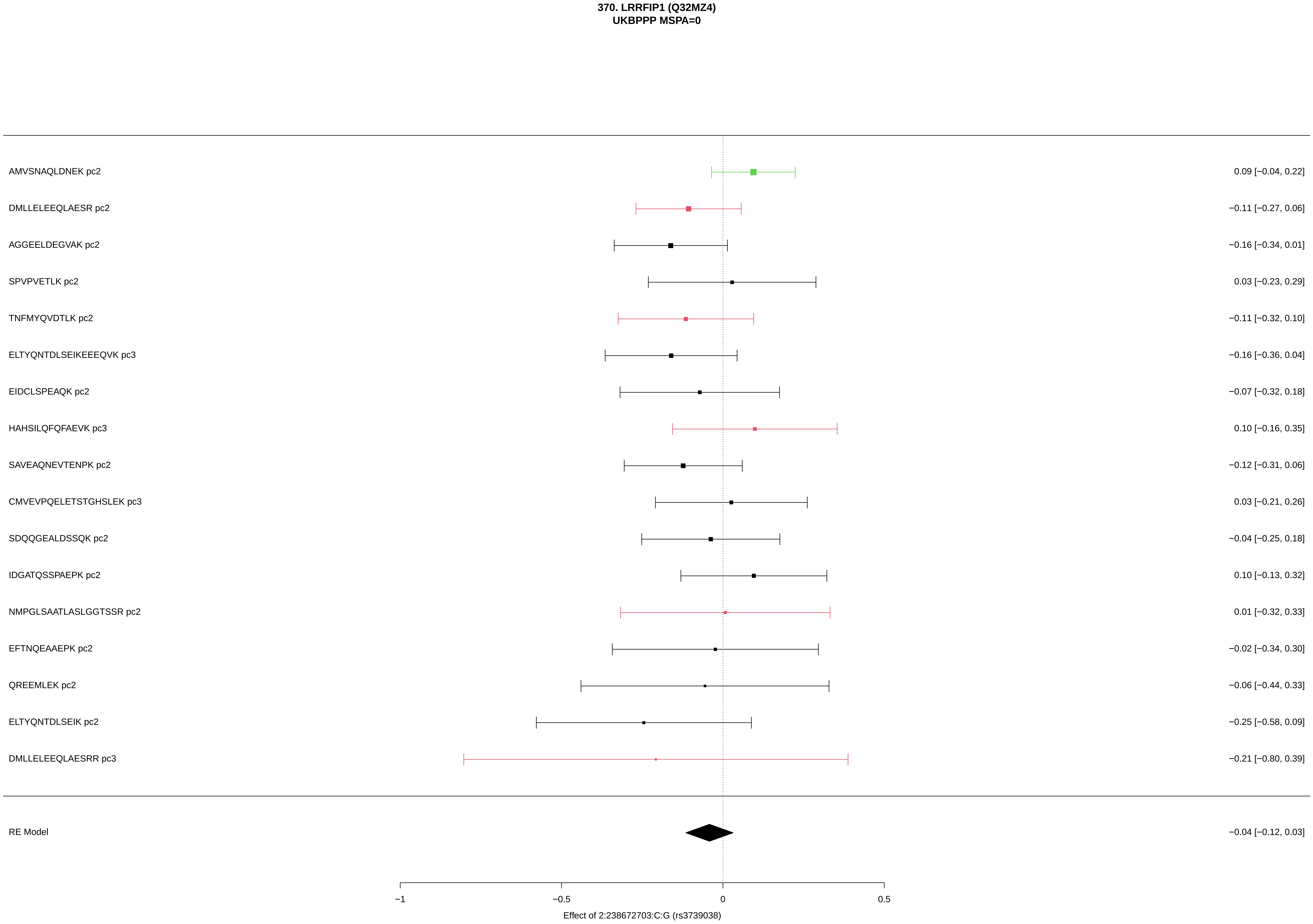
370. LRRFIP1 (Q32MZ4)
UKBPPP MSPA=0
AMVSNAQLDNEK pc2
0.09 [−0.04, 0.22]
DMLLELEEQLAESR pc2
−0.11 [−0.27, 0.06]
AGGEELDEGVAK pc2
−0.16 [−0.34, 0.01]
SPVPVETLK pc2
0.03 [−0.23, 0.29]
TNFMYQVDTLK pc2
−0.11 [−0.32, 0.10]
ELTYQNTDLSEIKEEEQVK pc3
−0.16 [−0.36, 0.04]
EIDCLSPEAQK pc2
−0.07 [−0.32, 0.18]
HAHSILQFQFAEVK pc3
0.10 [−0.16, 0.35]
SAVEAQNEVTENPK pc2
−0.12 [−0.31, 0.06]
CMVEVPQELETSTGHSLEK pc3
0.03 [−0.21, 0.26]
SDQQGEALDSSQK pc2
−0.04 [−0.25, 0.18]
IDGATQSSPAEPK pc2
0.10 [−0.13, 0.32]
NMPGLSAATLASLGGTSSR pc2
0.01 [−0.32, 0.33]
EFTNQEAAEPK pc2
−0.02 [−0.34, 0.30]
QREEMLEK pc2
−0.06 [−0.44, 0.33]
ELTYQNTDLSEIK pc2
−0.25 [−0.58, 0.09]
DMLLELEEQLAESRR pc3
−0.21 [−0.80, 0.39]
RE Model
−0.04 [−0.12, 0.03]
−1
−0.5
0
0.5
Effect of 2:238672703:C:G (rs3739038)

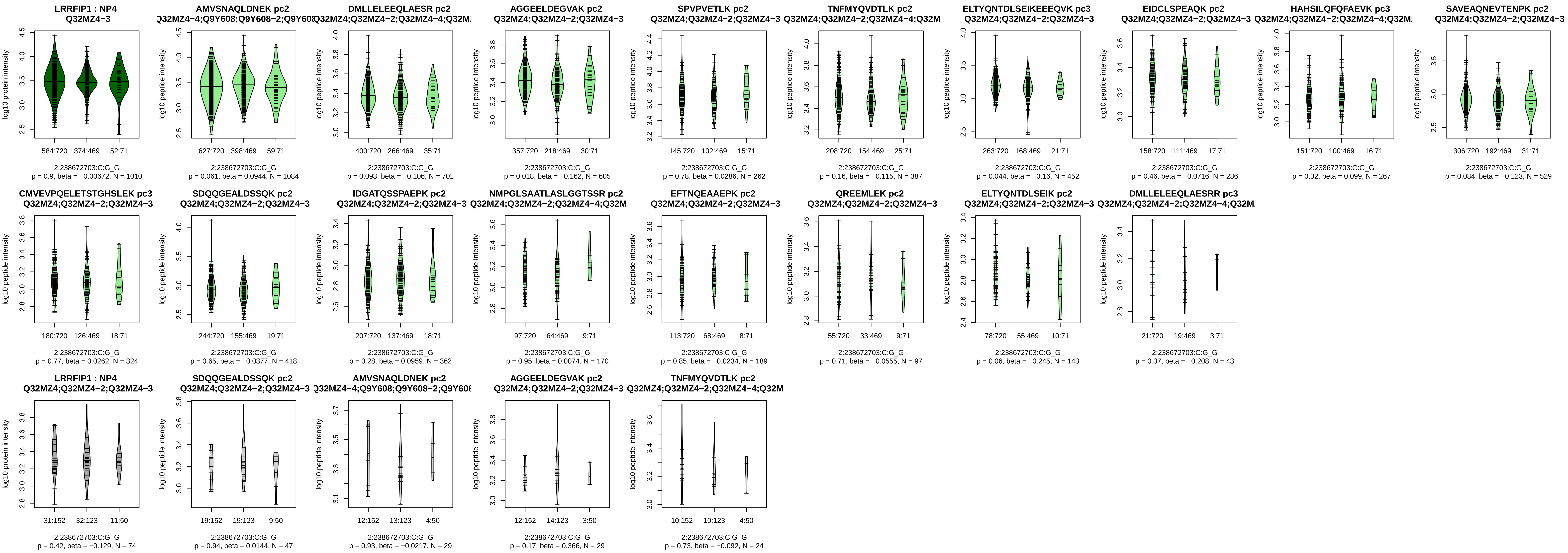

Assay Target: LRRFIP1
UniProt: Q32MZ4
rsID: rs3739038
Target SNP: 2:238672703:C:G
Proxy SNP: 2:238672703:C:G
UKBPPP log10(p): 25.2
UKBPPP BETA: −0.074
UKBPPP MSPA: 0
1084:701:605:262:387:452:286:

371. PLXDC2 (Q6UX71)
UKBPPP MSPA=0.085

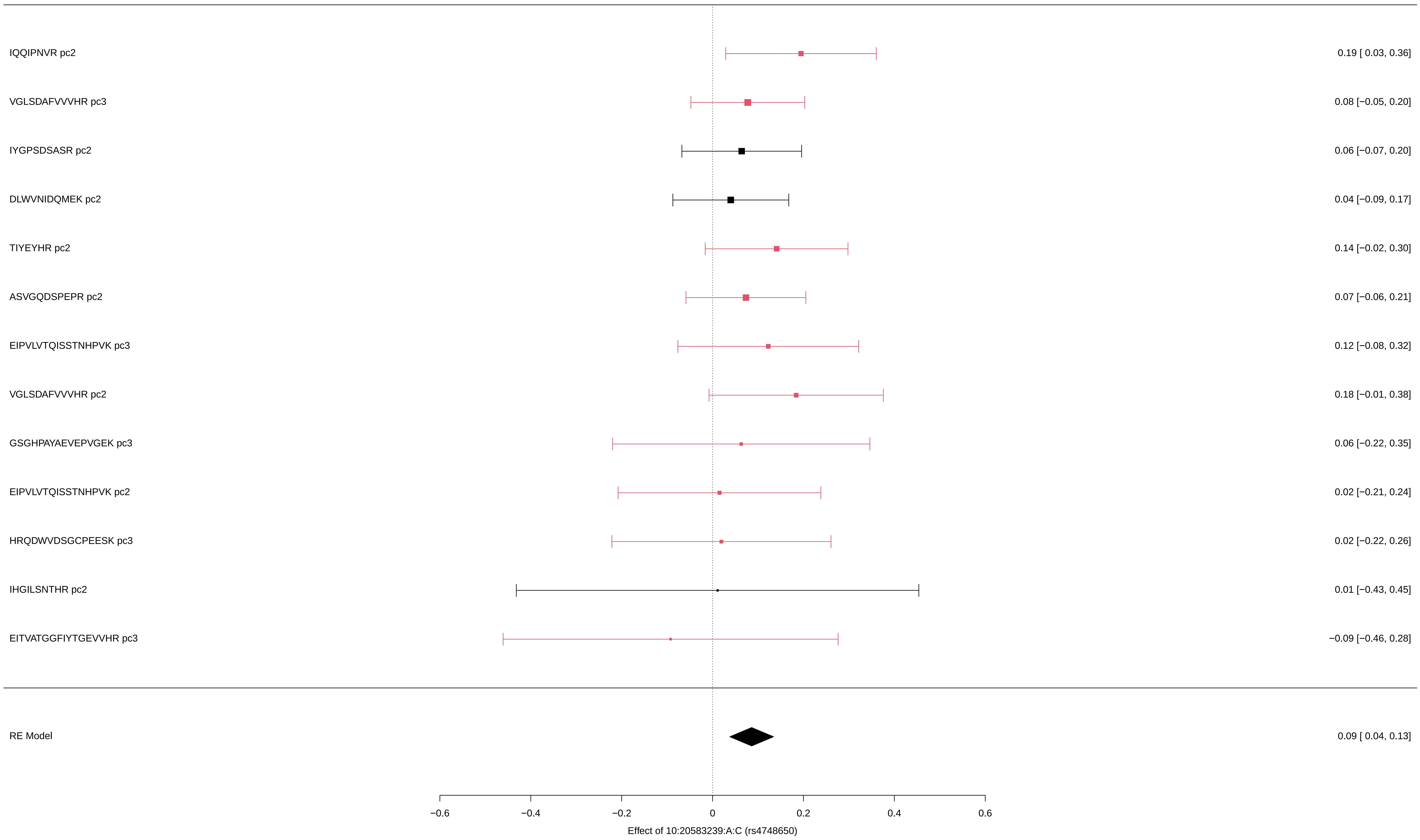

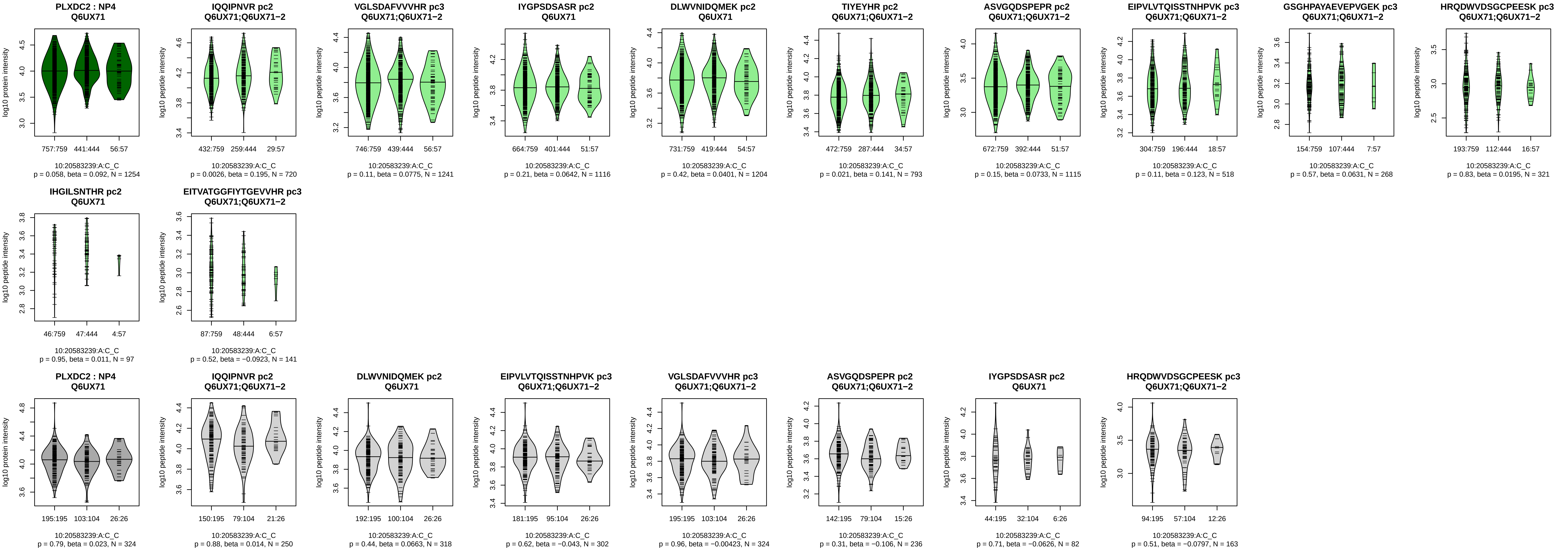

Assay Target: PLXDC2
UniProt: Q6UX71
rsID: rs4748650
Target SNP: 10:20583239:A:C
Proxy SNP: 10:20583239:A:C
UKBPPP log10(p): 25
UKBPPP BETA: 0.078
UKBPPP MSPA: 0.085
*-:-:-:-:-:-:-:-:-:-:-
720:1241:1116:1204:793:1115:5

372. DBNL (Q9UJU6)
UKBPPP MSPA=0

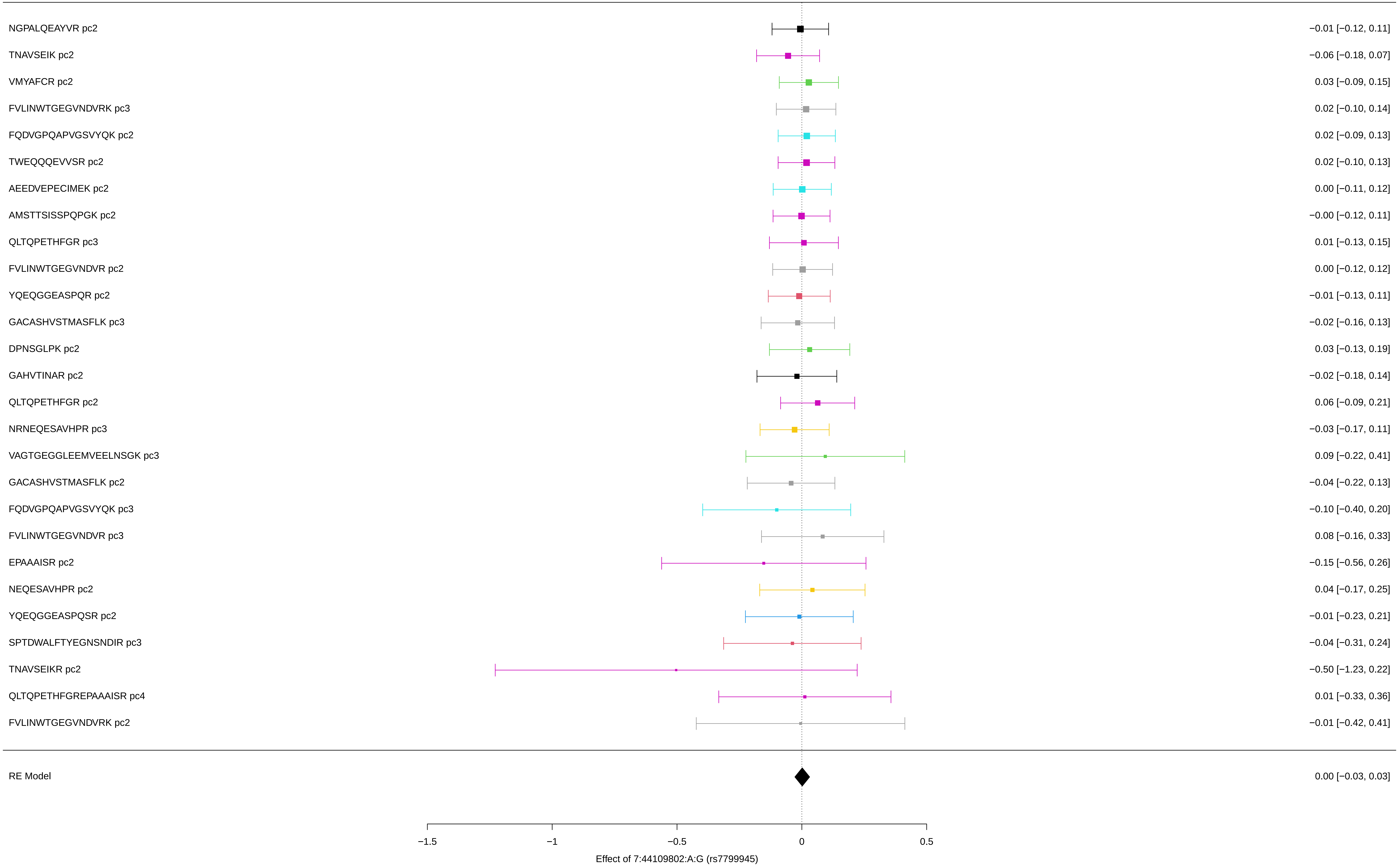

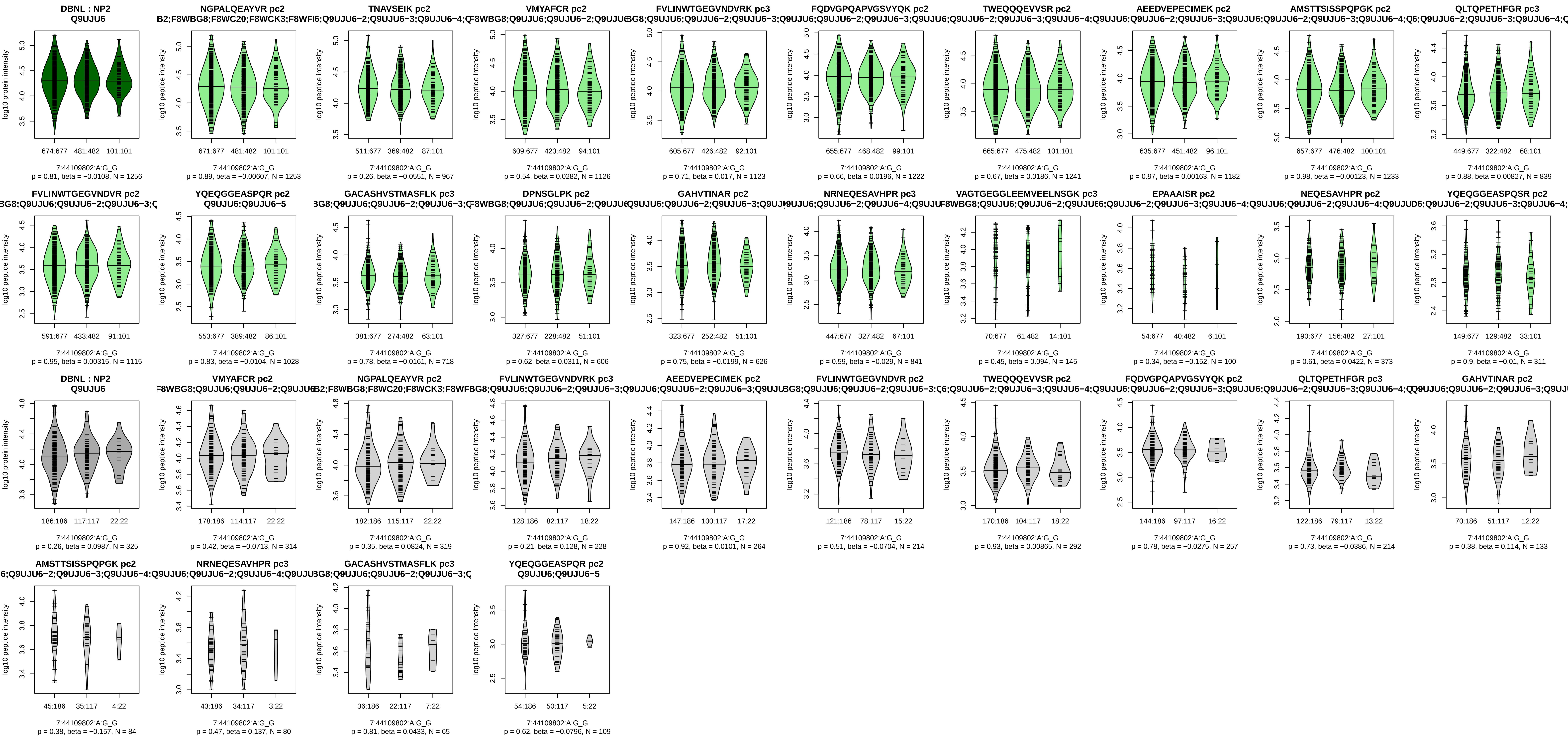

Assay Target: DBNL
UniProt: Q9UJU6
rsID: rs7799945
Target SNP: 7:44109802:A:G
Proxy SNP: 7:44109802:A:G
UKBPPP log10(p): 24.9
UKBPPP BETA: 0.068
UKBPPP MSPA: 0
1253:967:1126:1123:1222:1241

373. TWF2 (Q6IBS0)
UKBPPP MSPA=0

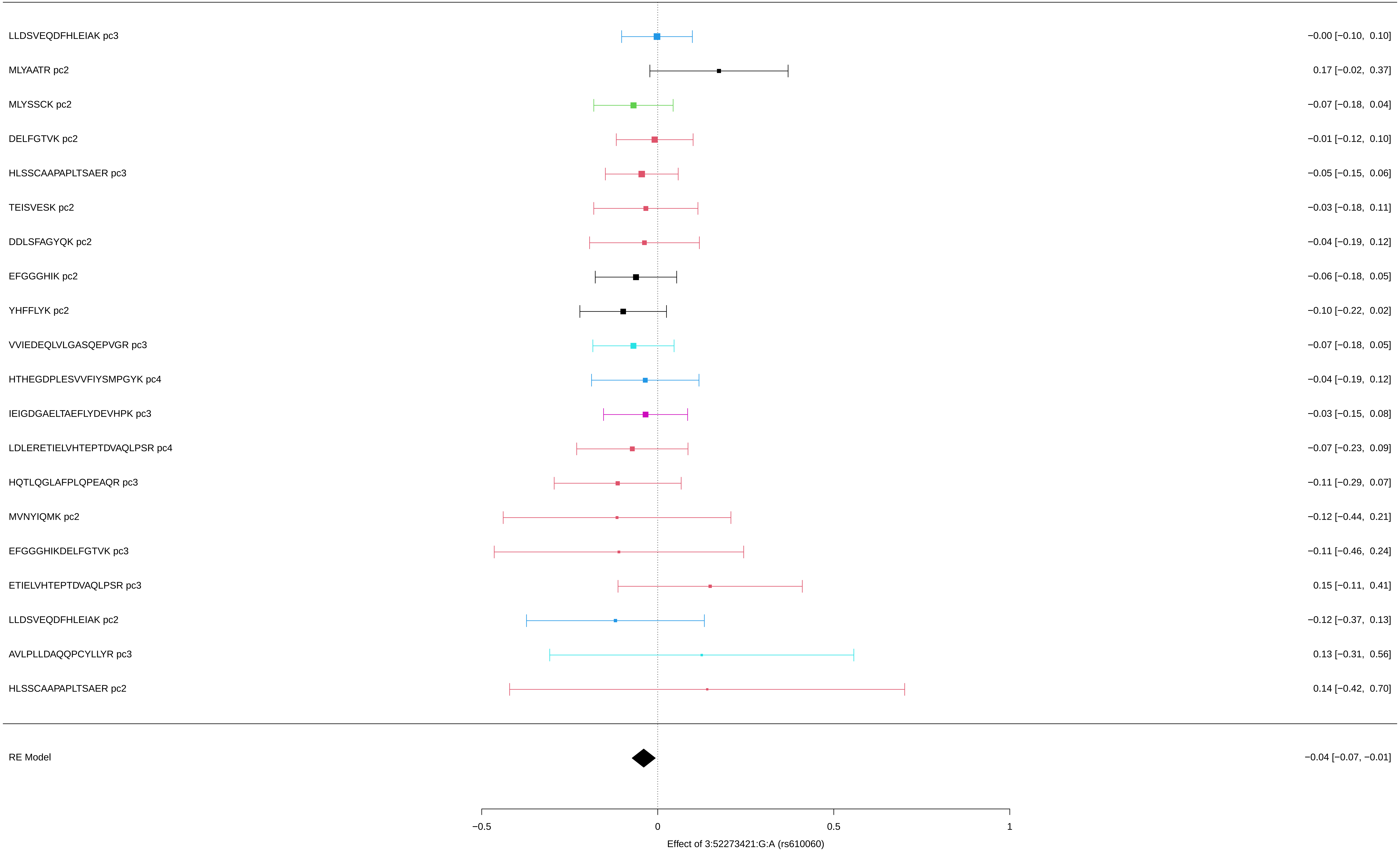

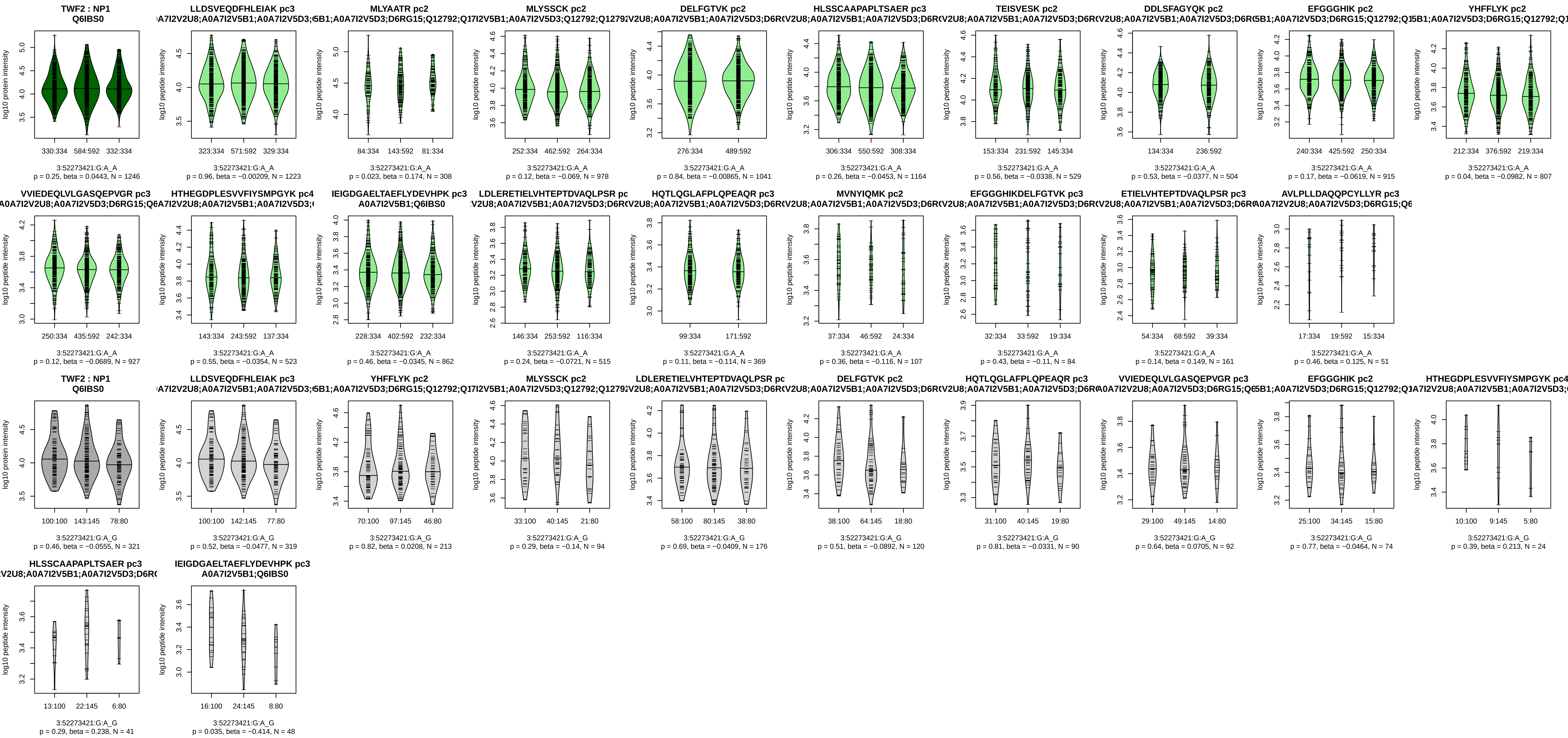

Assay Target: TWF2
UniProt: Q6IBS0
rsID: rs610060
Target SNP: 3:52273421:G:A
Proxy SNP: 3:52273421:G:A
UKBPPP log10(p): 24.8
UKBPPP BETA: −0.064
UKBPPP MSPA: 0

1223:308:978:1041:1164:529:50

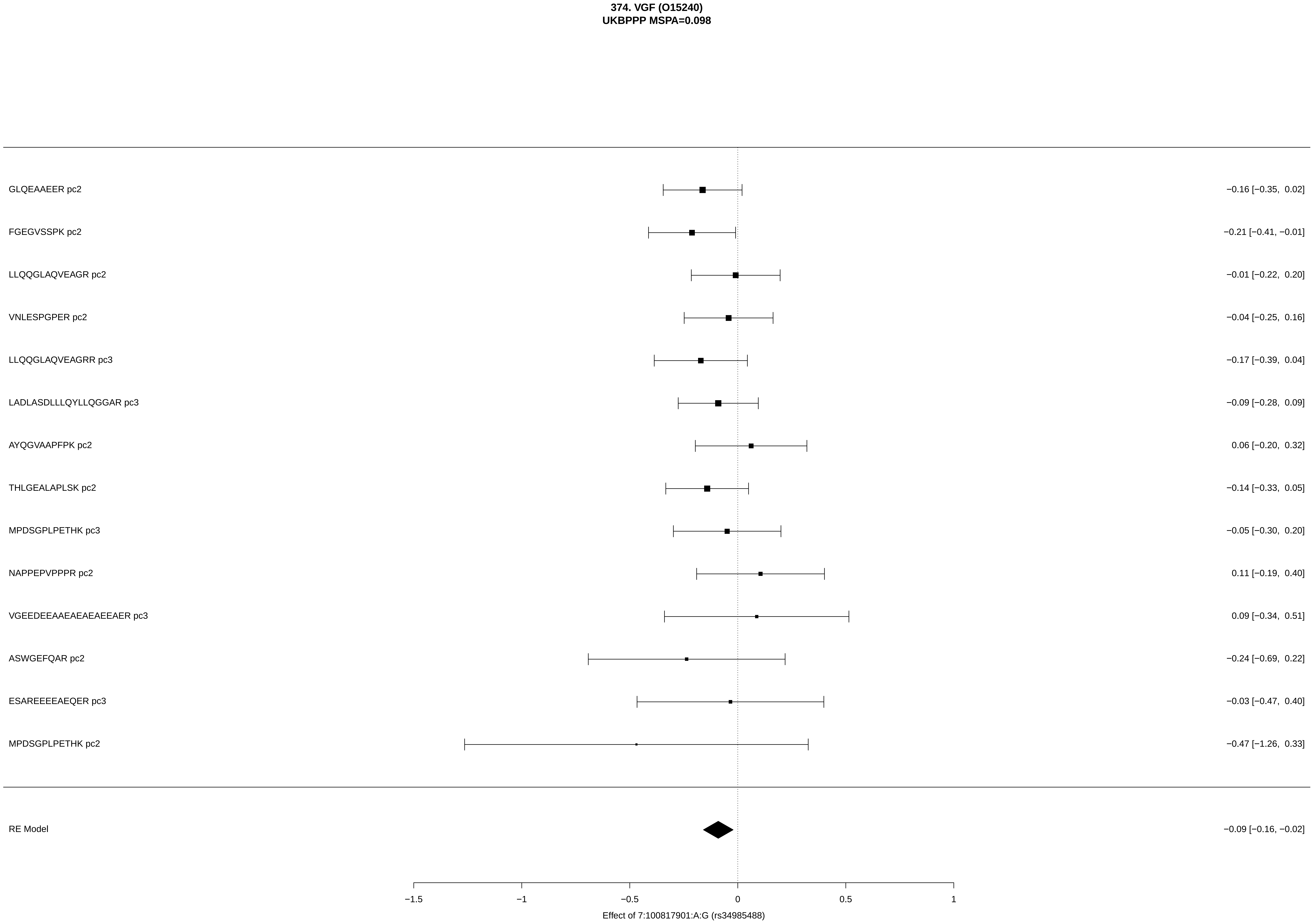

374. VGF (O15240)
UKBPPP MSPA=0.098
GLQEAAEER pc2
FGEGVSSPK pc2
LLQQGLAQVEAGR pc2
VNLESPGPER pc2
LLQQGLAQVEAGRR pc3
LADLASDLLLQYLLQGGAR pc3
AYQGVAAPFPK pc2
THLGEALAPLSK pc2
MPDSGPLPETHK pc3
NAPPEPVPPPR pc2
VGEEDEEAAEAEAEAEEAER pc3
ASWGEFQAR pc2
ESAREEEEAEQER pc3
MPDSGPLPETHK pc2
RE Model
−0.16 [−0.35, 0.02]
−0.21 [−0.41, −0.01]
−0.01 [−0.22, 0.20]
−0.04 [−0.25, 0.16]
−0.17 [−0.39, 0.04]
−0.09 [−0.28, 0.09]
0.06 [−0.20, 0.32]
−0.14 [−0.33, 0.05]
−0.05 [−0.30, 0.20]
0.11 [−0.19, 0.40]
0.09 [−0.34, 0.51]
−0.24 [−0.69, 0.22]
−0.03 [−0.47, 0.40]
−0.47 [−1.26, 0.33]
−0.09 [−0.16, −0.02]
−1.5
−1
−0.5
0
0.5
1
Effect of 7:100817901:A:G (rs34985488)

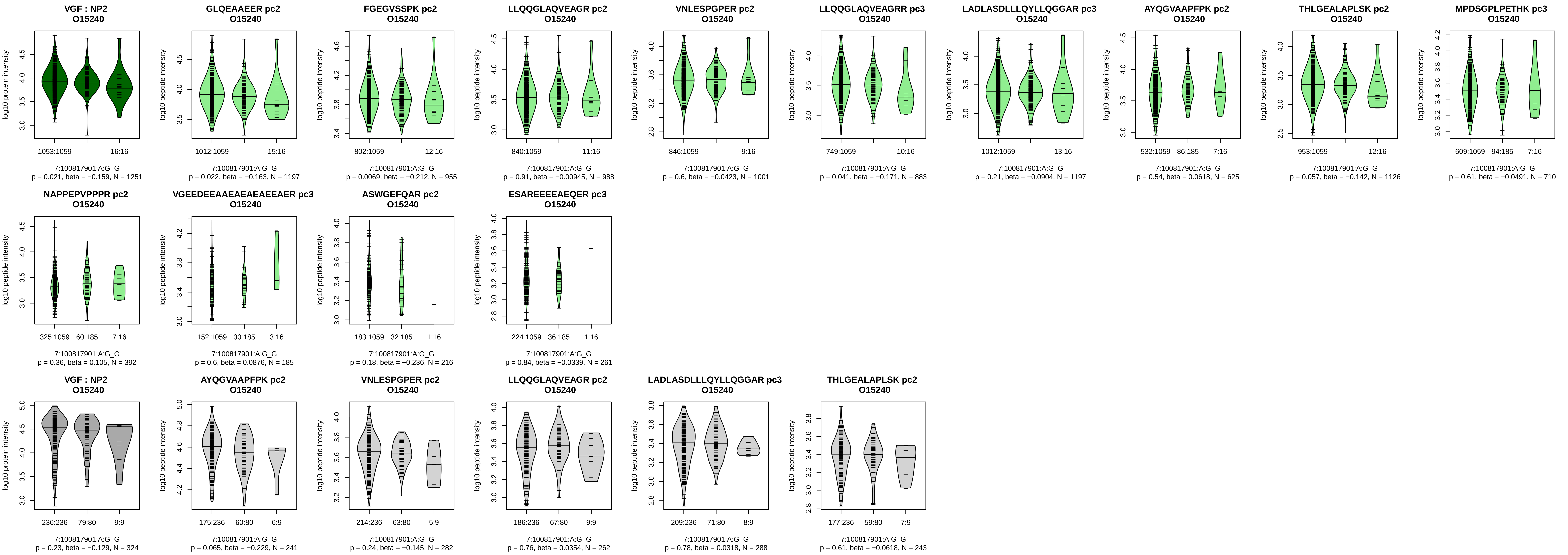

Assay Target: VGF
UniProt: O15240
rsID: rs34985488
Target SNP: 7:100817901:A:G
Proxy SNP: 7:100817901:A:G
UKBPPP log10(p): 24.5
UKBPPP BETA: −0.115
UKBPPP MSPA: 0.098
–*–:–:–:–:–:–:–:–:–:–
1197:955:988:1001:883:1197:6[illegible]

375. PRDX1 (Q06830)
UKBPPP MSPA=0.136

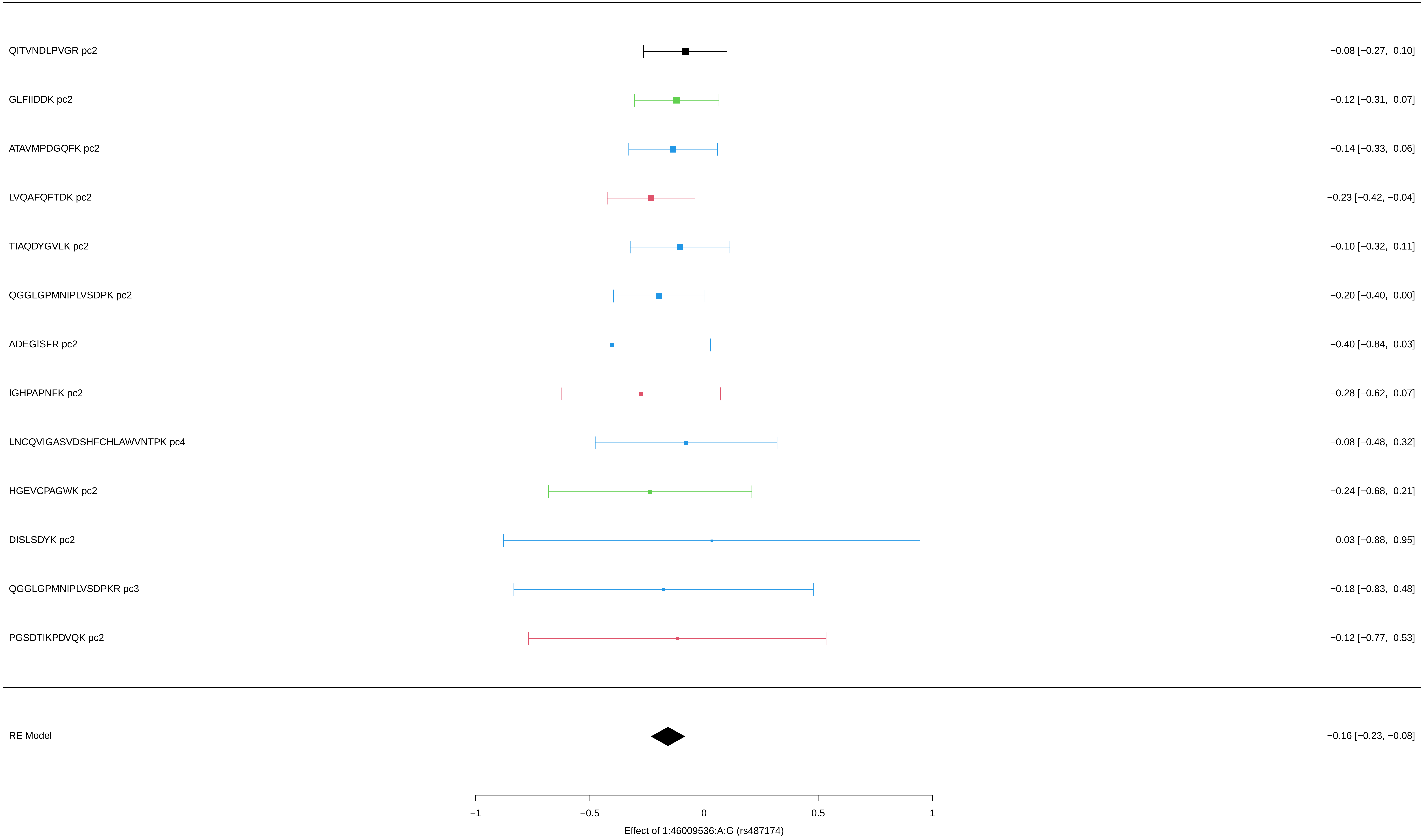

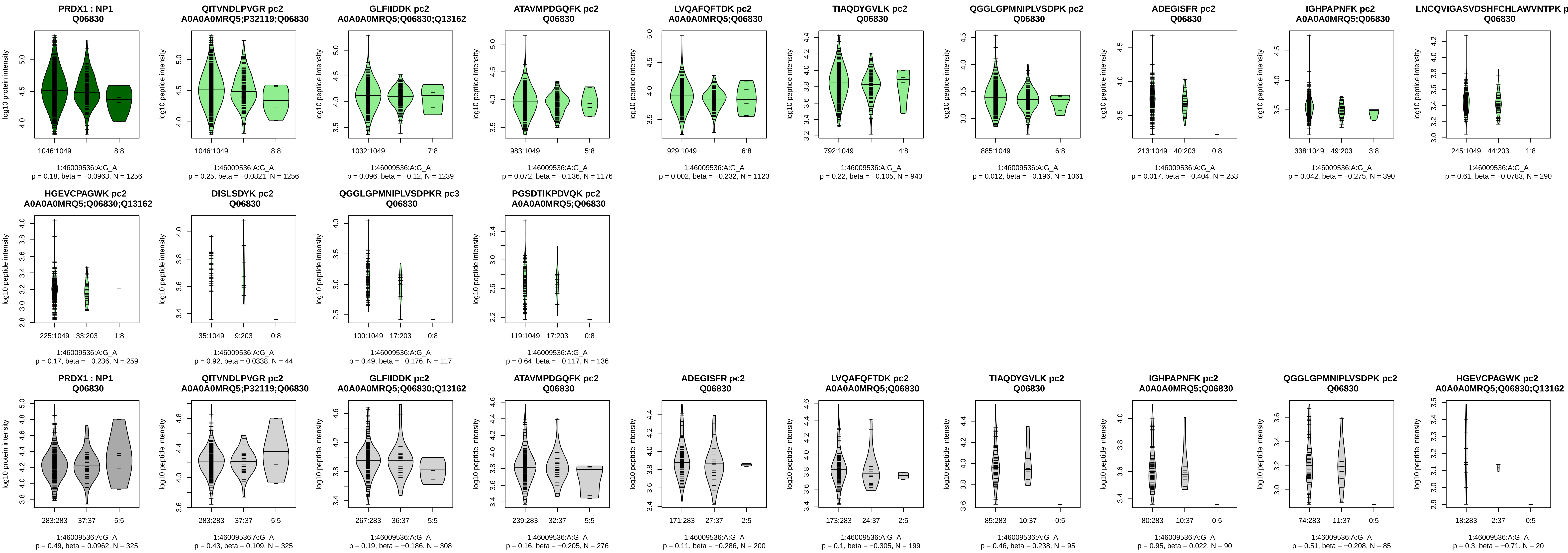

Assay Target: PRDX1
UniProt: Q06830
rsID: rs487174
Target SNP: 1:46009536:A:G
Proxy SNP: 1:46009536:A:G
UKBPPP log10(p): 22.1
UKBPPP BETA: 0.102
UKBPPP MSPA: 0.136
–:–:–:*:–:–:–:–:–:–:–:–
1256:1239:1176:1123:943:1061

376. SLC4A1 (P02730)
UKBPPP MSPA=0

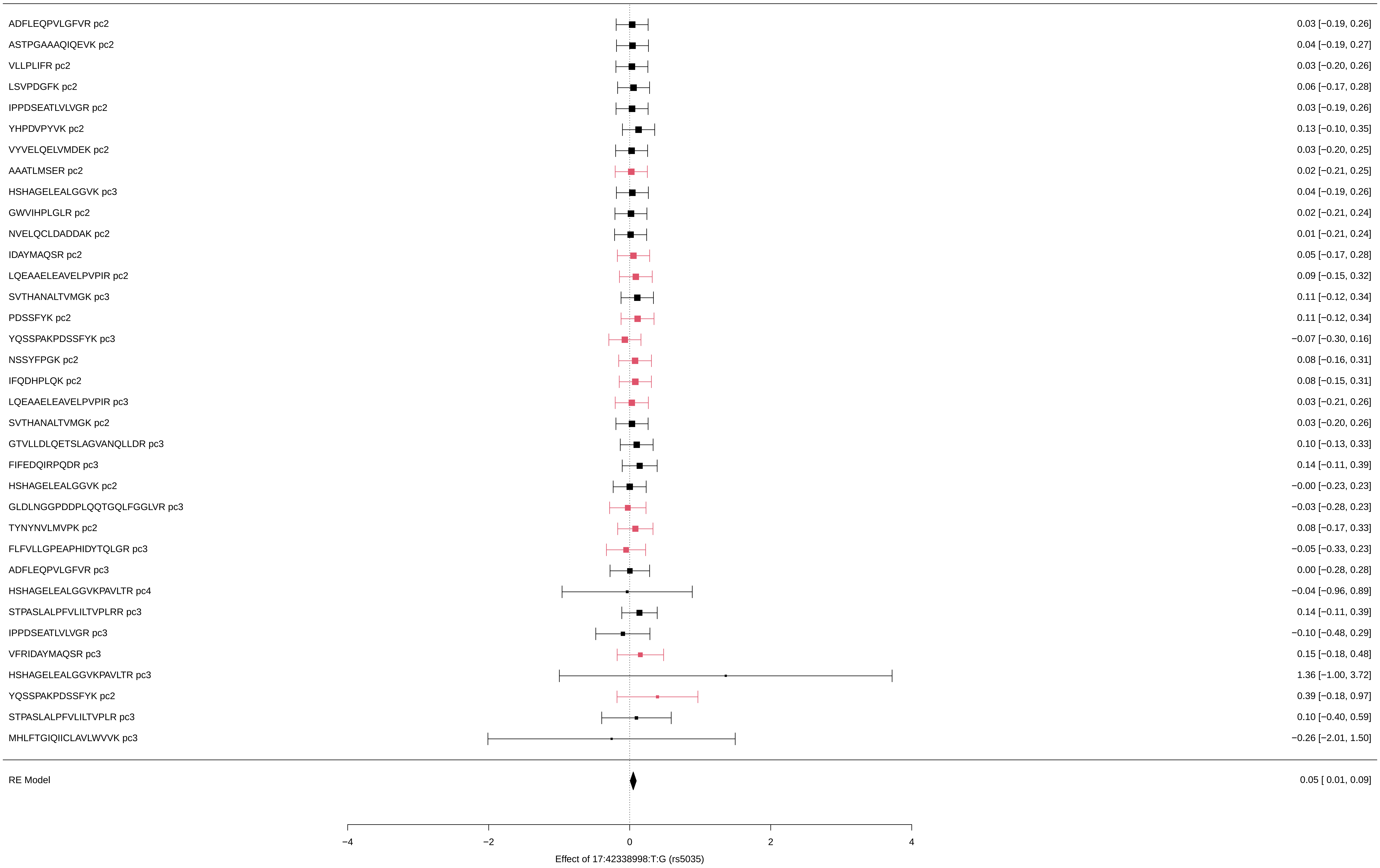

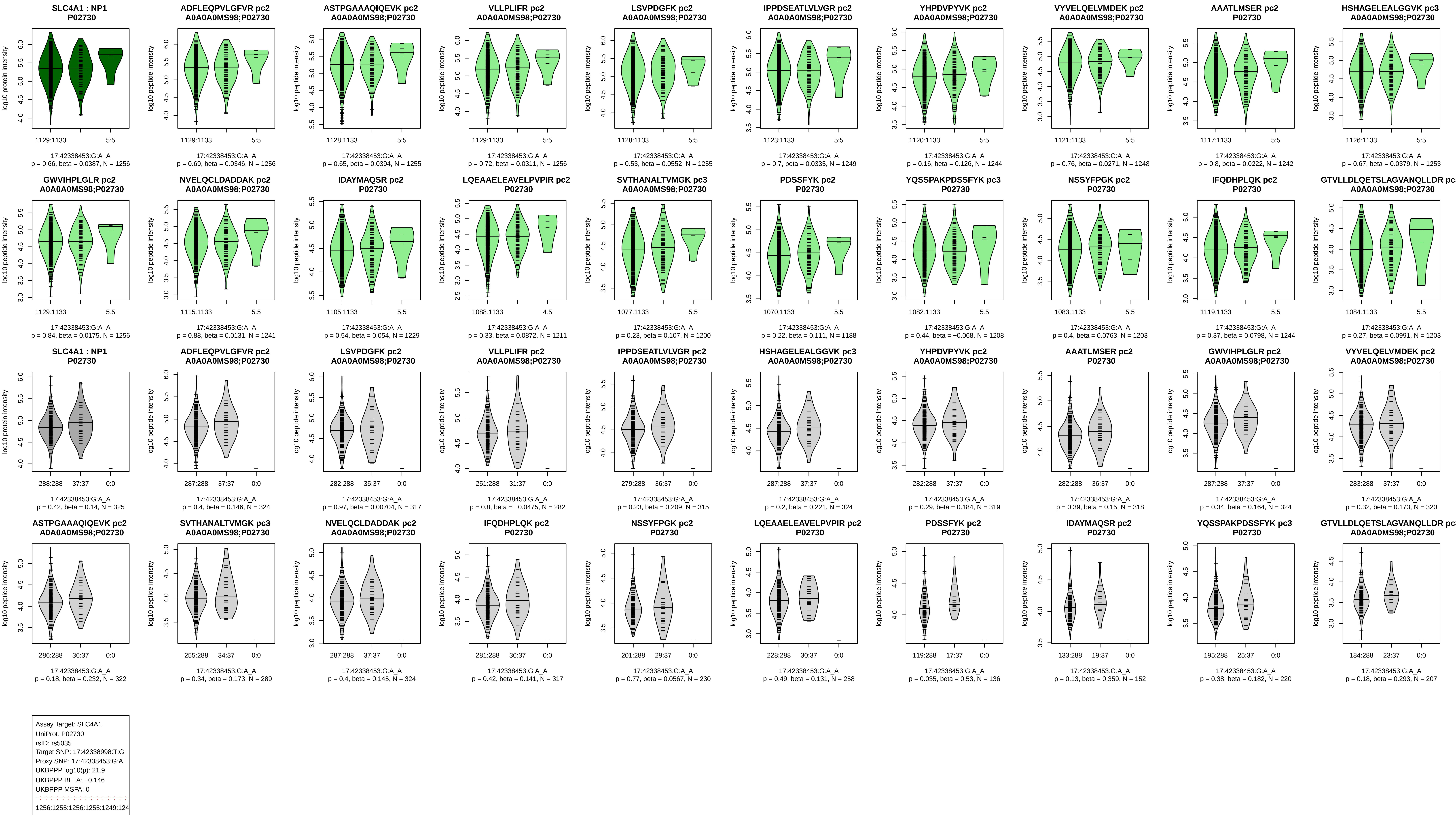

SLC4A1 : NP1
P02730
log10 protein intensity
1129:1133 5:5
17:42338453:G:A_A
p = 0.66, beta = 0.0387, N = 1256
ADFLEQPVLGFVR pc2
A0A0A0MS98;P02730
log10 peptide intensity
1129:1133 5:5
17:42338453:G:A_A
p = 0.69, beta = 0.0346, N = 1256
ASTPGAAAQIQEVK pc2
A0A0A0MS98;P02730
1128:1133 5:5
17:42338453:G:A_A
p = 0.65, beta = 0.0394, N = 1255
VLLPLIFR pc2
A0A0A0MS98;P02730
1129:1133 5:5
17:42338453:G:A_A
p = 0.72, beta = 0.0311, N = 1256
LSVPDGFK pc2
A0A0A0MS98;P02730
1128:1133 5:5
17:42338453:G:A_A
p = 0.53, beta = 0.0552, N = 1255
IPPDSEATLVLVGR pc2
A0A0A0MS98;P02730
1123:1133 5:5
17:42338453:G:A_A
p = 0.7, beta = 0.0335, N = 1249
YHPDVPYVK pc2
A0A0A0MS98;P02730
1120:1133 5:5
17:42338453:G:A_A
p = 0.16, beta = 0.126, N = 1244
VYVELQELVMDEK pc2
A0A0A0MS98;P02730
1121:1133 5:5
17:42338453:G:A_A
p = 0.76, beta = 0.0271, N = 1248
AAATLMSER pc2
P02730
1117:1133 5:5
17:42338453:G:A_A
p = 0.8, beta = 0.0222, N = 1242
HSHAGELEALGGVK pc3
A0A0A0MS98;P02730
1126:1133 5:5
17:42338453:G:A_A
p = 0.67, beta = 0.0379, N = 1253
GWVIHPLGLR pc2
A0A0A0MS98;P02730
1129:1133 5:5
17:42338453:G:A_A
p = 0.84, beta = 0.0175, N = 1256
NVELQCLDADDAK pc2
A0A0A0MS98;P02730
1115:1133 5:5
17:42338453:G:A_A
p = 0.88, beta = 0.0131, N = 1241
IDAYMAQSR pc2
P02730
1105:1133 5:5
17:42338453:G:A_A
p = 0.54, beta = 0.054, N = 1229
LQEAAELEAVELPVPIR pc2
P02730
1088:1133 4:5
17:42338453:G:A_A
p = 0.33, beta = 0.0872, N = 1211
SVTHANALTVMGK pc3
A0A0A0MS98;P02730
1077:1133 5:5
17:42338453:G:A_A
p = 0.23, beta = 0.107, N = 1200
PDSSFYK pc2
P02730
1070:1133 5:5
17:42338453:G:A_A
p = 0.22, beta = 0.111, N = 1188
YQSSPAKPDSSFYK pc3
P02730
1082:1133 5:5
17:42338453:G:A_A
p = 0.44, beta = −0.068, N = 1208
NSSYFPGK pc2
P02730
1083:1133 5:5
17:42338453:G:A_A
p = 0.4, beta = 0.0763, N = 1203
IFQDHPLQK pc2
P02730
1119:1133 5:5
17:42338453:G:A_A
p = 0.37, beta = 0.0798, N = 1244
GTVLLDLQETSLAGVANQLLDR pc
A0A0A0MS98;P02730
1084:1133 5:5
17:42338453:G:A_A
p = 0.27, beta = 0.0991, N = 1203
SLC4A1 : NP1
P02730
288:288 37:37 0:0
17:42338453:G:A_A
p = 0.42, beta = 0.14, N = 325
ADFLEQPVLGFVR pc2
A0A0A0MS98;P02730
287:288 37:37 0:0
17:42338453:G:A_A
p = 0.4, beta = 0.146, N = 324
LSVPDGFK pc2
A0A0A0MS98;P02730
282:288 35:37 0:0
17:42338453:G:A_A
p = 0.97, beta = 0.00704, N = 317
VLLPLIFR pc2
A0A0A0MS98;P02730
251:288 31:37 0:0
17:42338453:G:A_A
p = 0.8, beta = −0.0475, N = 282
IPPDSEATLVLVGR pc2
A0A0A0MS98;P02730
279:288 36:37 0:0
17:42338453:G:A_A
p = 0.23, beta = 0.209, N = 315
HSHAGELEALGGVK pc3
A0A0A0MS98;P02730
287:288 37:37 0:0
17:42338453:G:A_A
p = 0.2, beta = 0.221, N = 324
YHPDVPYVK pc2
A0A0A0MS98;P02730
282:288 37:37 0:0
17:42338453:G:A_A
p = 0.29, beta = 0.184, N = 319
AAATLMSER pc2
P02730
282:288 36:37 0:0
17:42338453:G:A_A
p = 0.39, beta = 0.15, N = 318
GWVIHPLGLR pc2
A0A0A0MS98;P02730
287:288 37:37 0:0
17:42338453:G:A_A
p = 0.34, beta = 0.164, N = 324
VYVELQELVMDEK pc2
A0A0A0MS98;P02730
283:288 37:37 0:0
17:42338453:G:A_A
p = 0.32, beta = 0.173, N = 320
ASTPGAAAQIQEVK pc2
A0A0A0MS98;P02730
286:288 36:37 0:0
17:42338453:G:A_A
p = 0.18, beta = 0.232, N = 322
SVTHANALTVMGK pc3
A0A0A0MS98;P02730
255:288 34:37 0:0
17:42338453:G:A_A
p = 0.34, beta = 0.173, N = 289
NVELQCLDADDAK pc2
A0A0A0MS98;P02730
287:288 37:37 0:0
17:42338453:G:A_A
p = 0.4, beta = 0.145, N = 324
IFQDHPLQK pc2
P02730
281:288 36:37 0:0
17:42338453:G:A_A
p = 0.42, beta = 0.141, N = 317
NSSYFPGK pc2
P02730
201:288 29:37 0:0
17:42338453:G:A_A
p = 0.77, beta = 0.0567, N = 230
LQEAAELEAVELPVPIR pc2
P02730
228:288 30:37 0:0
17:42338453:G:A_A
p = 0.49, beta = 0.131, N = 258
PDSSFYK pc2
P02730
119:288 17:37 0:0
17:42338453:G:A_A
p = 0.035, beta = 0.53, N = 136
IDAYMAQSR pc2
P02730
133:288 19:37 0:0
17:42338453:G:A_A
p = 0.13, beta = 0.359, N = 152
YQSSPAKPDSSFYK pc3
P02730
195:288 25:37 0:0
17:42338453:G:A_A
p = 0.38, beta = 0.182, N = 220
GTVLLDLQETSLAGVANQLLDR pc
A0A0A0MS98;P02730
184:288 23:37 0:0
17:42338453:G:A_A
p = 0.18, beta = 0.293, N = 207
Assay Target: SLC4A1
UniProt: P02730
rsID: rs5035
Target SNP: 17:42338998:T:G
Proxy SNP: 17:42338453:G:A
UKBPPP log10(p): 21.9
UKBPPP BETA: −0.146
UKBPPP MSPA: 0
1256:1255:1256:1255:1249:124

377. ANXA11 (P50995)
UKBPPP MSPA=0

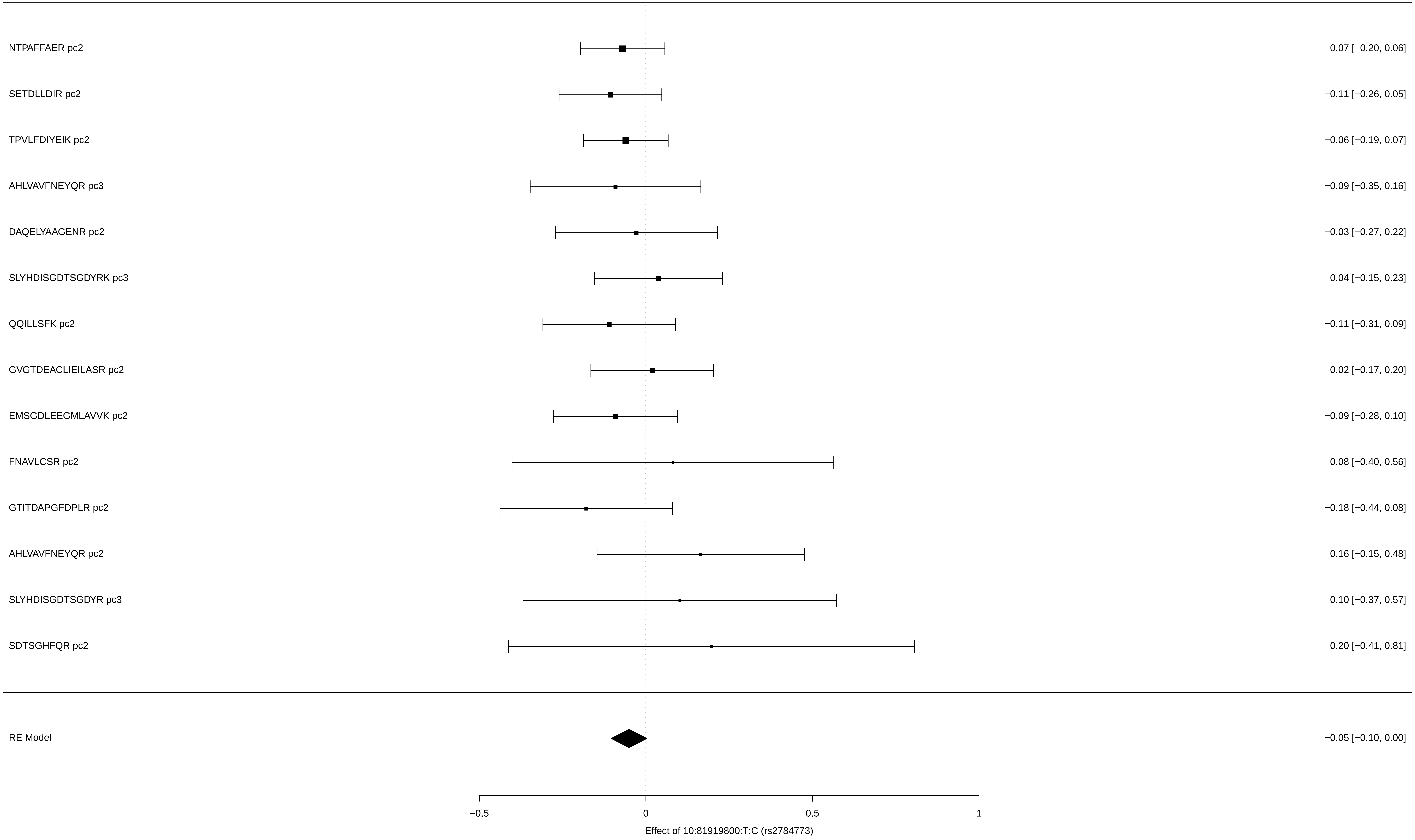

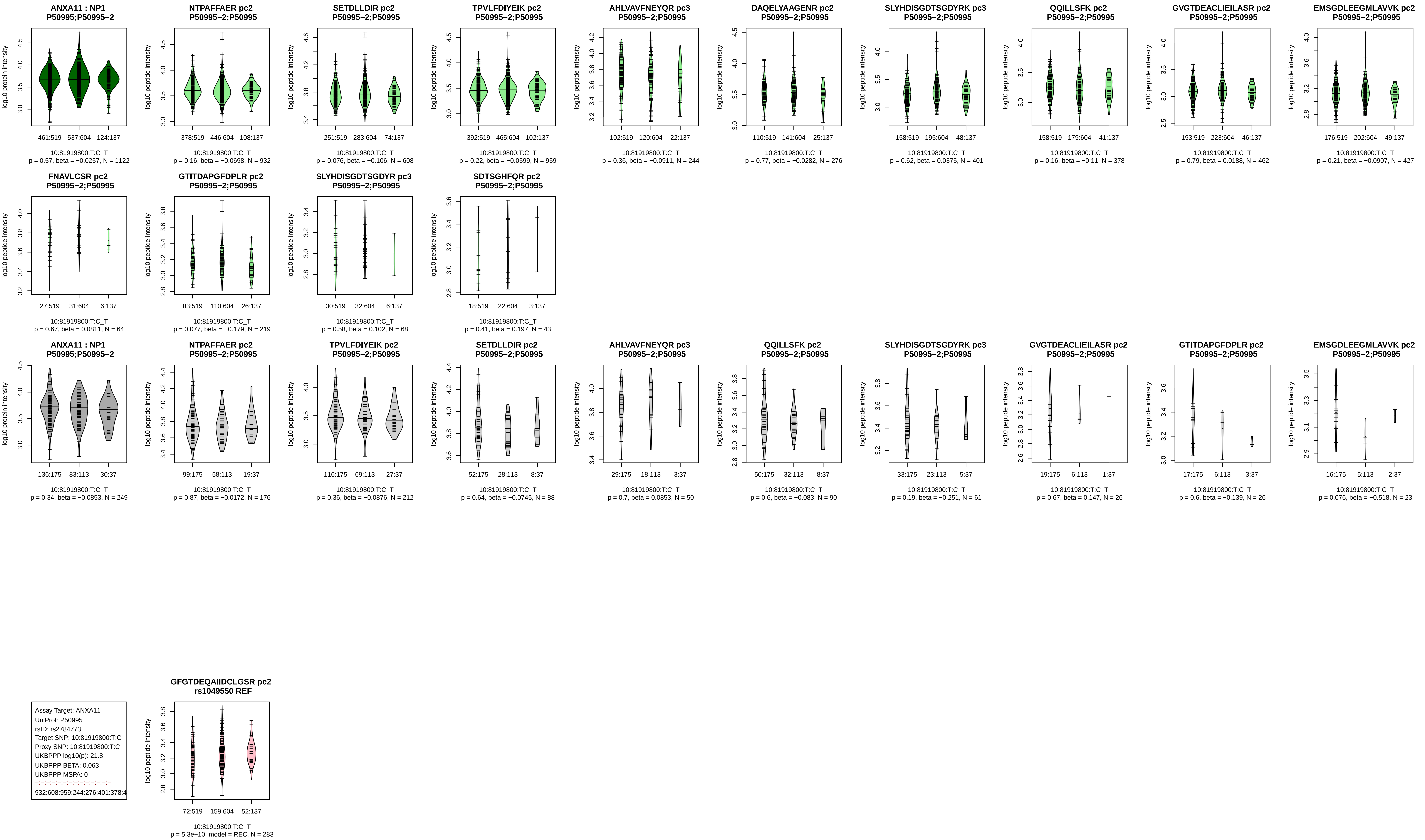
ANXA11 : NP1
P50995;P50995−2
461:519 537:604 124:137
10:81919800:T:C_T
p = 0.57, beta = −0.0257, N = 1122
NTPAFFAER pc2
P50995−2;P50995
378:519 446:604 108:137
10:81919800:T:C_T
p = 0.16, beta = −0.0698, N = 932
SETDLLDIR pc2
P50995−2;P50995
251:519 283:604 74:137
10:81919800:T:C_T
p = 0.076, beta = −0.106, N = 608
TPVLFDIYEIK pc2
P50995−2;P50995
392:519 465:604 102:137
10:81919800:T:C_T
p = 0.22, beta = −0.0599, N = 959
AHLVAVFNEYQR pc3
P50995−2;P50995
102:519 120:604 22:137
10:81919800:T:C_T
p = 0.36, beta = −0.0911, N = 244
DAQELYAAGENR pc2
P50995−2;P50995
110:519 141:604 25:137
10:81919800:T:C_T
p = 0.77, beta = −0.0282, N = 276
SLYHDISGDTSGDYRK pc3
P50995−2;P50995
158:519 195:604 48:137
10:81919800:T:C_T
p = 0.62, beta = 0.0375, N = 401
QQILLSFK pc2
P50995−2;P50995
158:519 179:604 41:137
10:81919800:T:C_T
p = 0.16, beta = −0.11, N = 378
GVGTDEACLIEILASR pc2
P50995−2;P50995
193:519 223:604 46:137
10:81919800:T:C_T
p = 0.79, beta = 0.0188, N = 462
EMSGDLEEGMLAVVK pc2
P50995−2;P50995
176:519 202:604 49:137
10:81919800:T:C_T
p = 0.21, beta = −0.0907, N = 427
FNAVLCSR pc2
P50995−2;P50995
27:519 31:604 6:137
10:81919800:T:C_T
p = 0.67, beta = 0.0811, N = 64
GTITDAPGFDPLR pc2
P50995−2;P50995
83:519 110:604 26:137
10:81919800:T:C_T
p = 0.077, beta = −0.179, N = 219
SLYHDISGDTSGDYR pc3
P50995−2;P50995
30:519 32:604 6:137
10:81919800:T:C_T
p = 0.58, beta = 0.102, N = 68
SDTSGHFQR pc2
P50995−2;P50995
18:519 22:604 3:137
10:81919800:T:C_T
p = 0.41, beta = 0.197, N = 43
ANXA11 : NP1
P50995;P50995−2
136:175 83:113 30:37
10:81919800:T:C_T
p = 0.34, beta = −0.0853, N = 249
NTPAFFAER pc2
P50995−2;P50995
99:175 58:113 19:37
10:81919800:T:C_T
p = 0.87, beta = −0.0172, N = 176
TPVLFDIYEIK pc2
P50995−2;P50995
116:175 69:113 27:37
10:81919800:T:C_T
p = 0.36, beta = −0.0876, N = 212
SETDLLDIR pc2
P50995−2;P50995
52:175 28:113 8:37
10:81919800:T:C_T
p = 0.64, beta = −0.0745, N = 88
AHLVAVFNEYQR pc3
P50995−2;P50995
29:175 18:113 3:37
10:81919800:T:C_T
p = 0.7, beta = 0.0853, N = 50
QQILLSFK pc2
P50995−2;P50995
50:175 32:113 8:37
10:81919800:T:C_T
p = 0.6, beta = −0.083, N = 90
SLYHDISGDTSGDYRK pc3
P50995−2;P50995
33:175 23:113 5:37
10:81919800:T:C_T
p = 0.19, beta = −0.251, N = 61
GVGTDEACLIEILASR pc2
P50995−2;P50995
19:175 6:113 1:37
10:81919800:T:C_T
p = 0.67, beta = 0.147, N = 26
GTITDAPGFDPLR pc2
P50995−2;P50995
17:175 6:113 3:37
10:81919800:T:C_T
p = 0.6, beta = −0.139, N = 26
EMSGDLEEGMLAVVK pc2
P50995−2;P50995
16:175 5:113 2:37
10:81919800:T:C_T
p = 0.076, beta = −0.518, N = 23
Assay Target: ANXA11
UniProt: P50995
rsID: rs2784773
Target SNP: 10:81919800:T:C
Proxy SNP: 10:81919800:T:C
UKBPPP log10(p): 21.8
UKBPPP BETA: 0.063
UKBPPP MSPA: 0
932:608:959:244:276:401:378:4
GFGTDEQAIIDCLGSR pc2
rs1049550 REF
72:519 159:604 52:137
10:81919800:T:C_T
p = 5.3e−10, model = REC, N = 283

378. PRDX3 (P30048)
UKBPPP MSPA=0.236

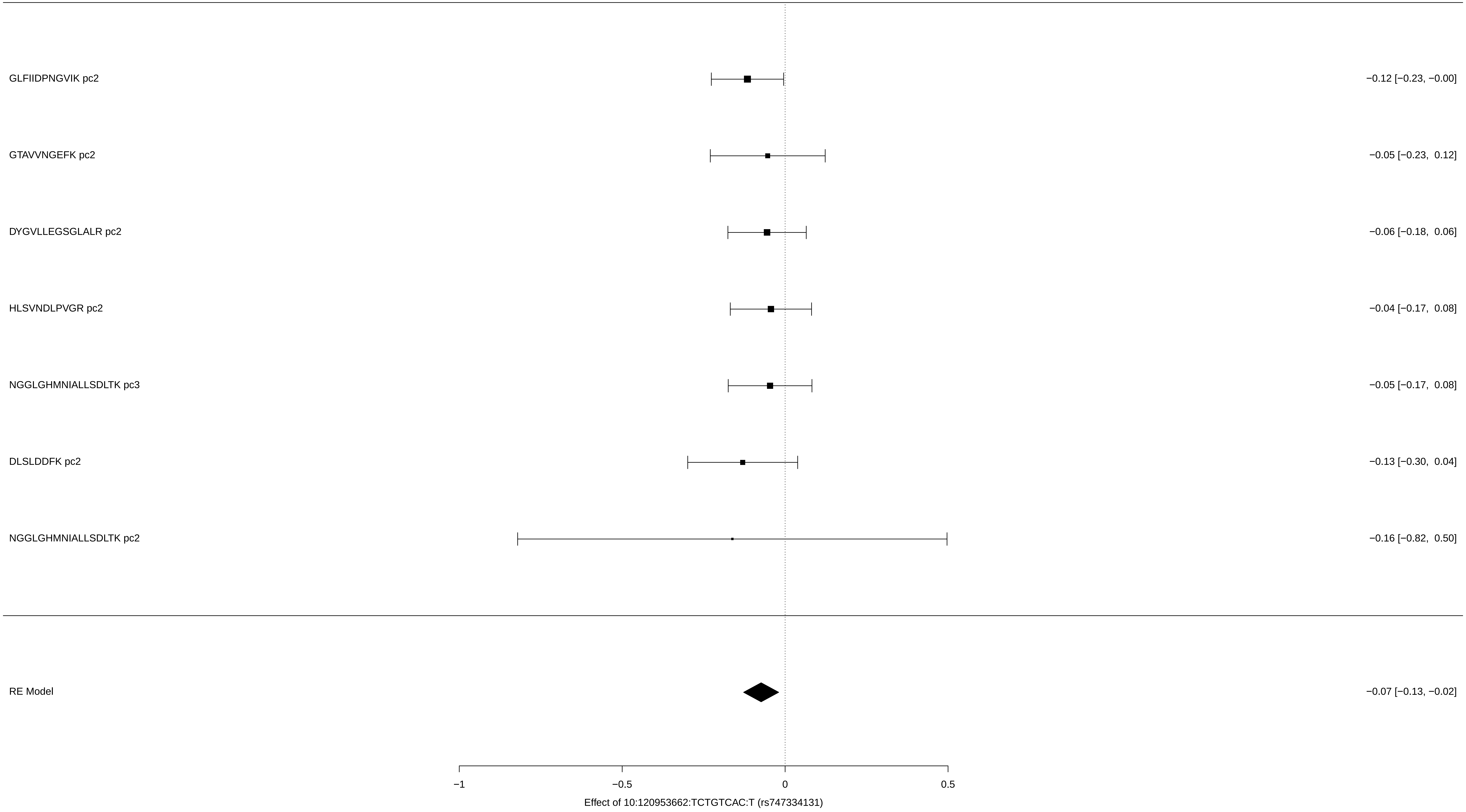

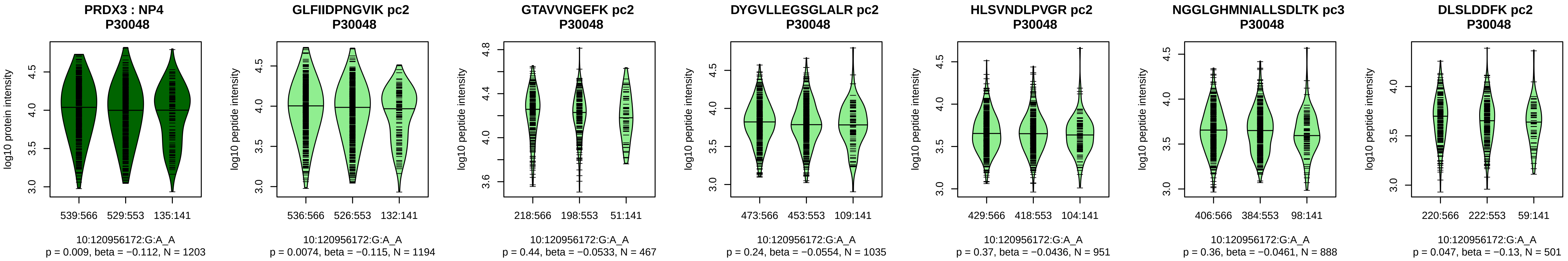

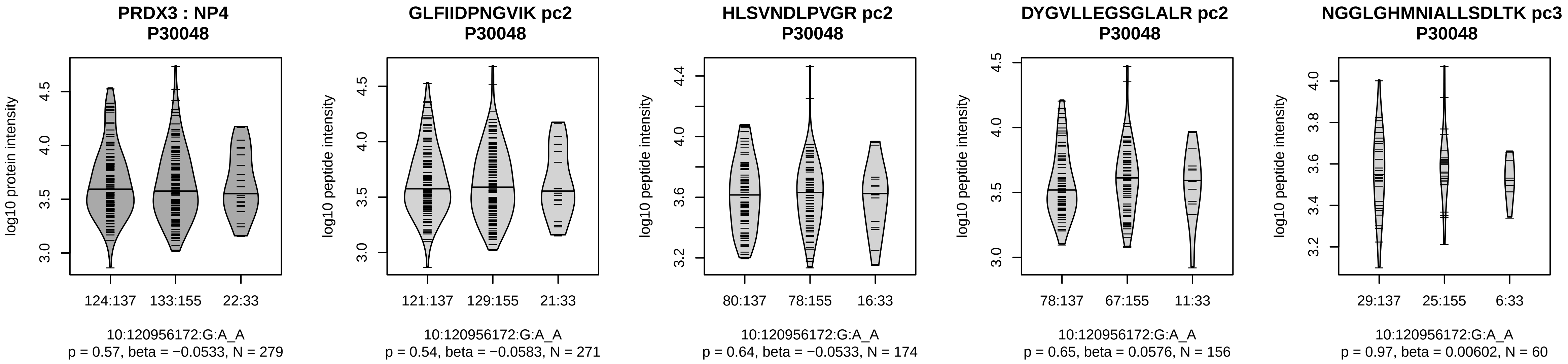

Assay Target: PRDX3
UniProt: P30048
rsID: rs747334131
Target SNP: 10:120953662:TCT
Proxy SNP: 10:120956172:G:A
UKBPPP log10(p): 21.5
UKBPPP BETA: −0.065
UKBPPP MSPA: 0.236
*:−:−:−:−:−:−
1194:467:1035:951:888:501:32

379. IGFBP2 (P18065)
UKBPPP MSPA=0

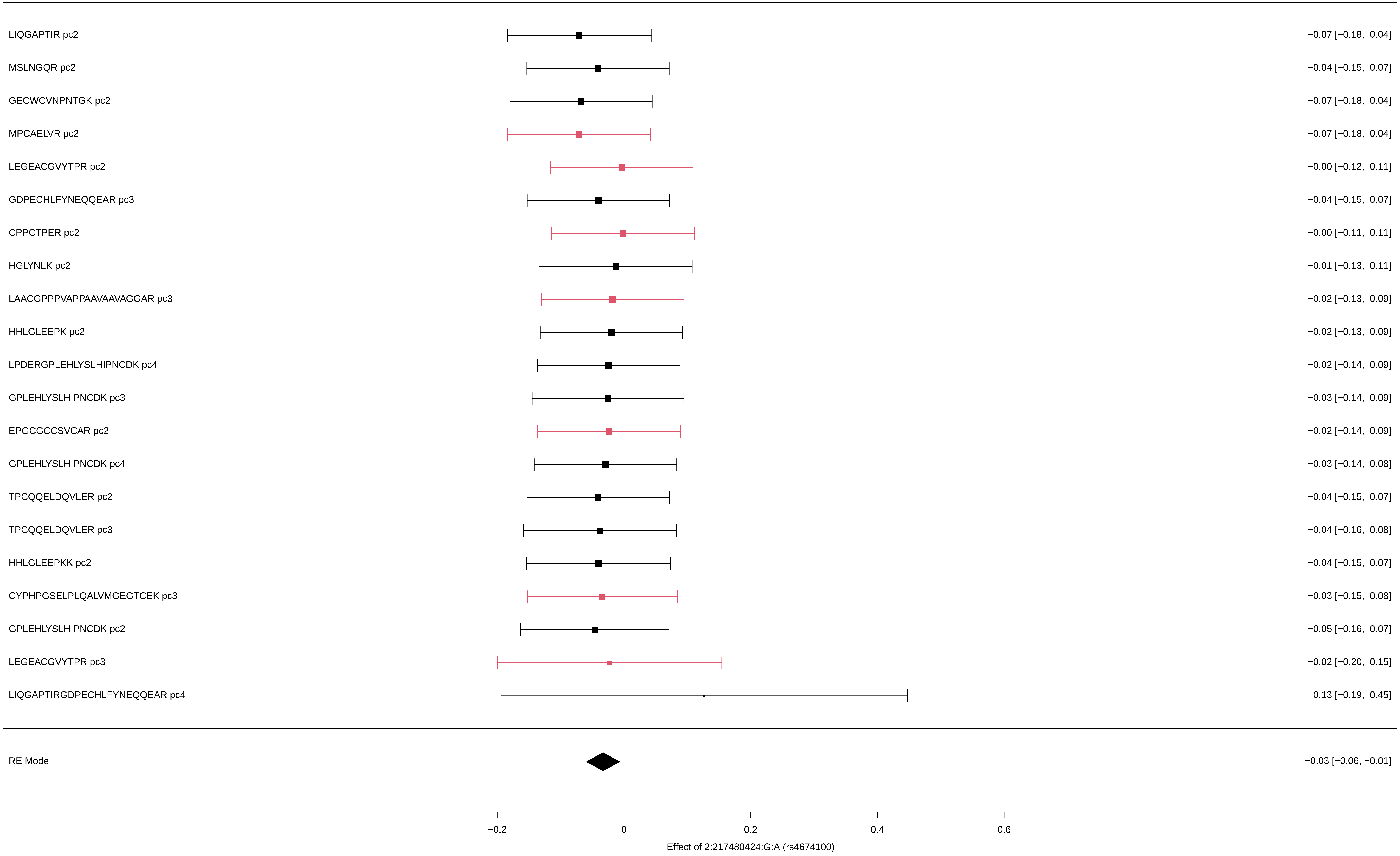

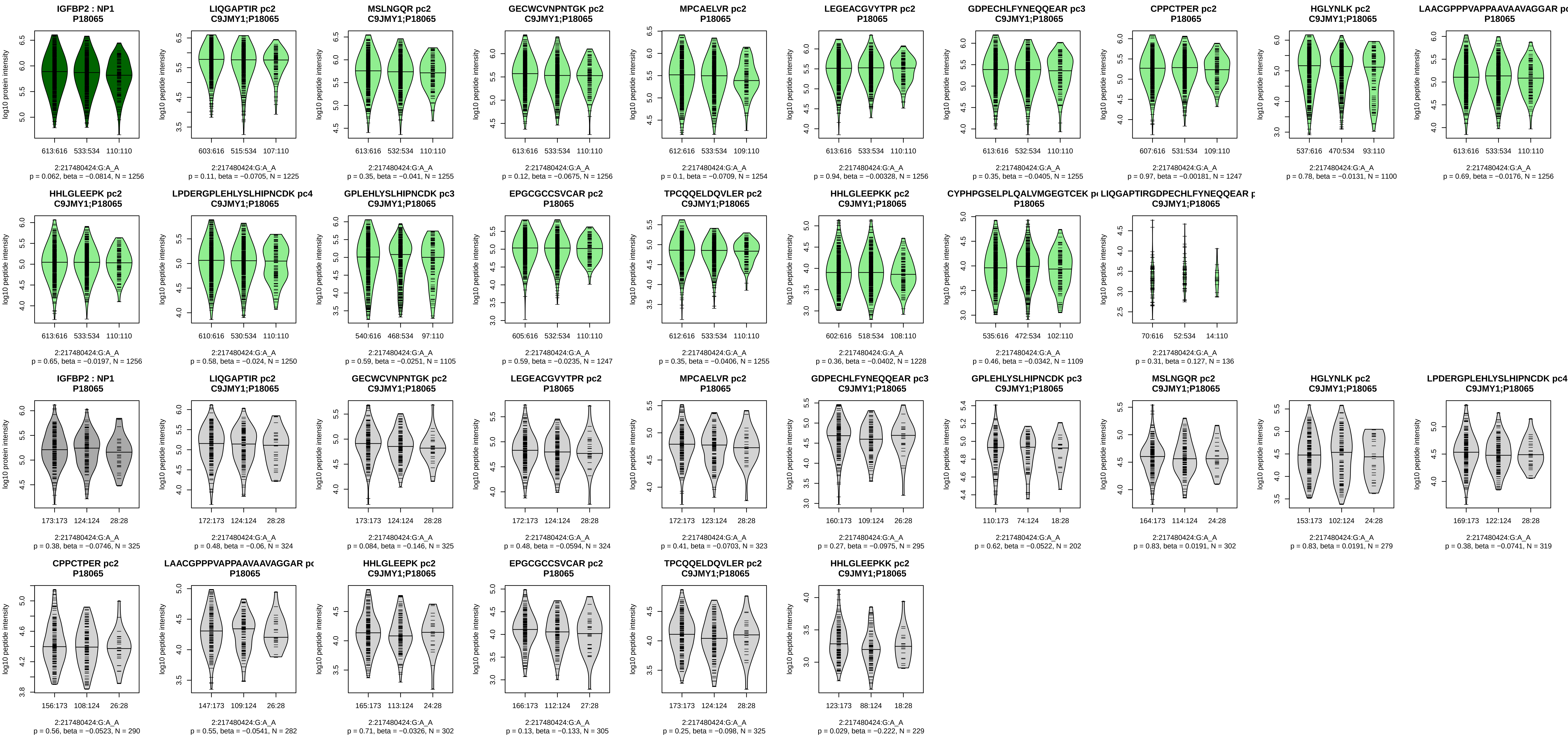

Assay Target: IGFBP2
UniProt: P18065
rsID: rs4674100
Target SNP: 2:217480424:G:A
Proxy SNP: 2:217480424:G:A
UKBPPP log10(p): 21.2
UKBPPP BETA: −0.066
UKBPPP MSPA: 0
1225:1255:1256:1254:1256:125

380. PGA4 (P0DJD7)
UKBPPP MSPA=0

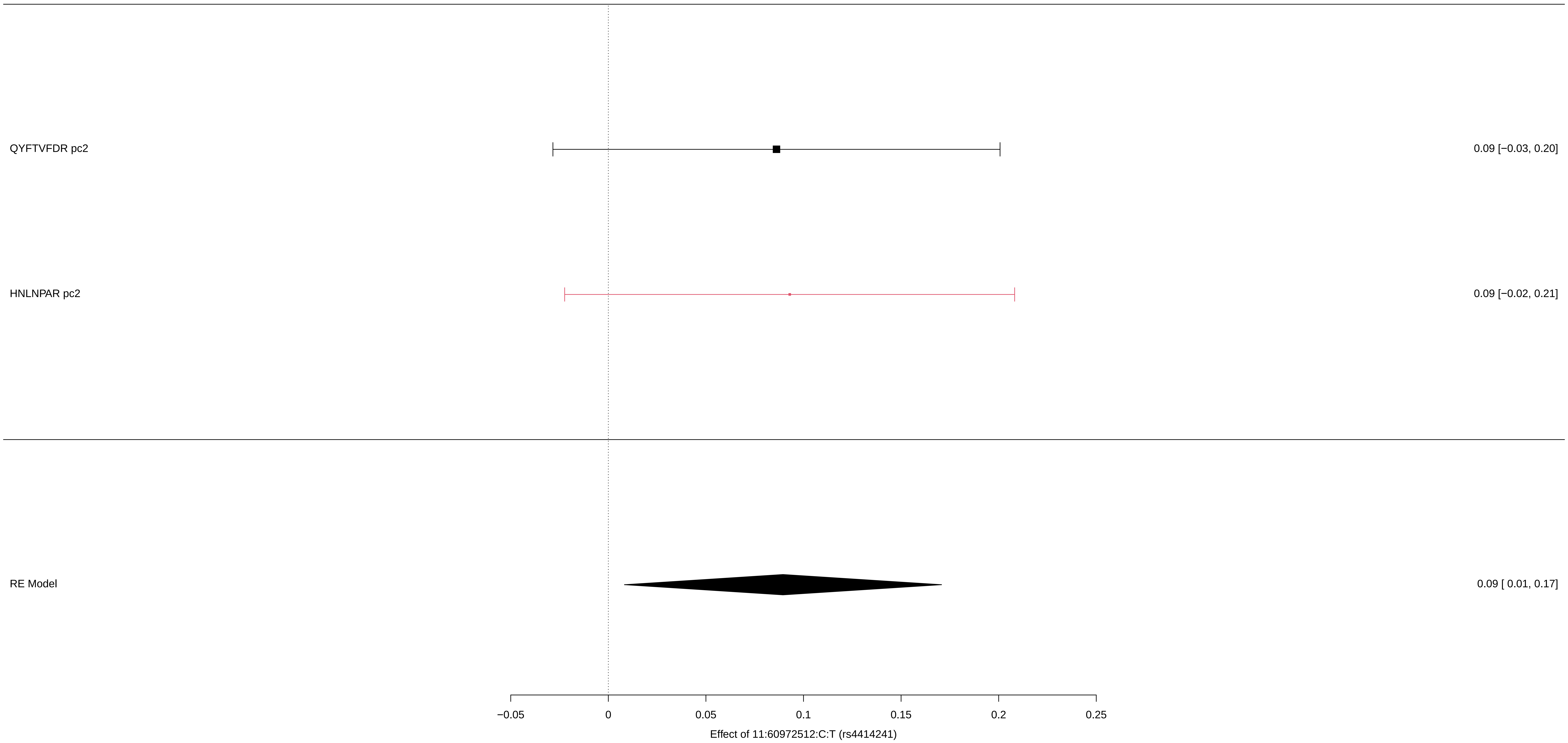

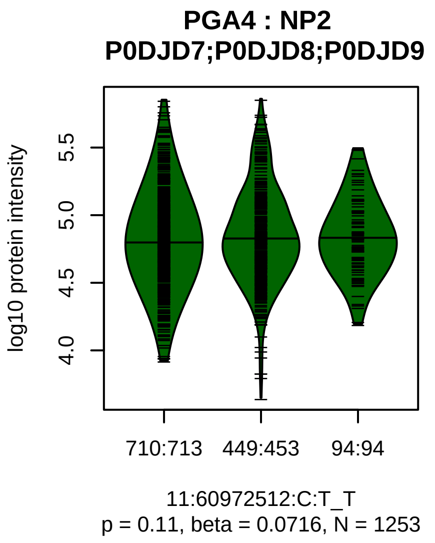

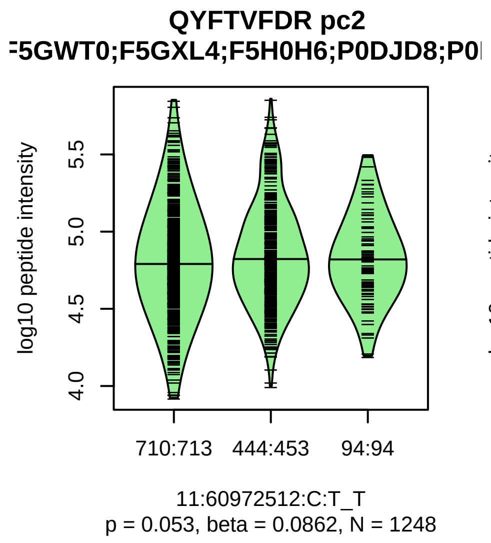

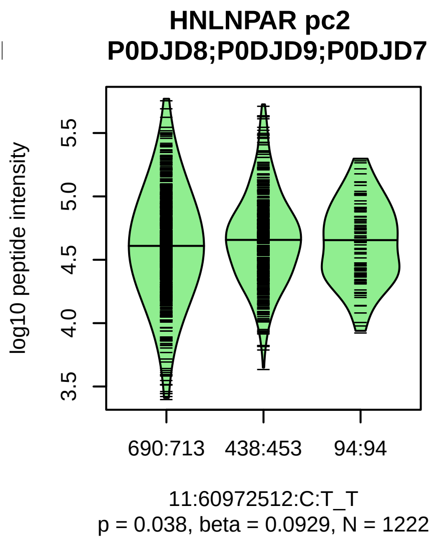

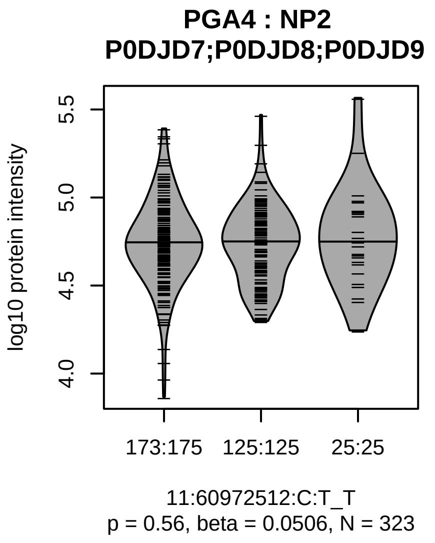

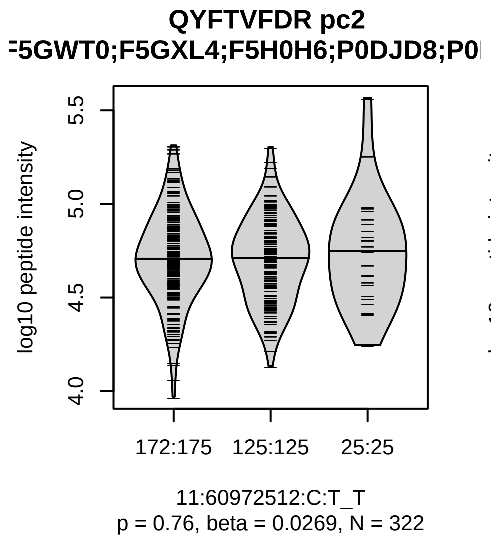

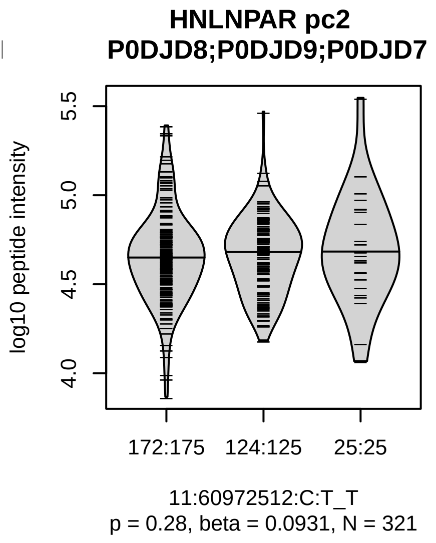

Assay Target: PGA4
UniProt: P0DJD7
rsID: rs4414241
Target SNP: 11:60972512:C:T
Proxy SNP: 11:60972512:C:T
UKBPPP log10(p): 21
UKBPPP BETA: 0.075
UKBPPP MSPA: 0
--:--
1248:1222

381. DAG1 (Q14118)
UKBPPP MSPA=0.451

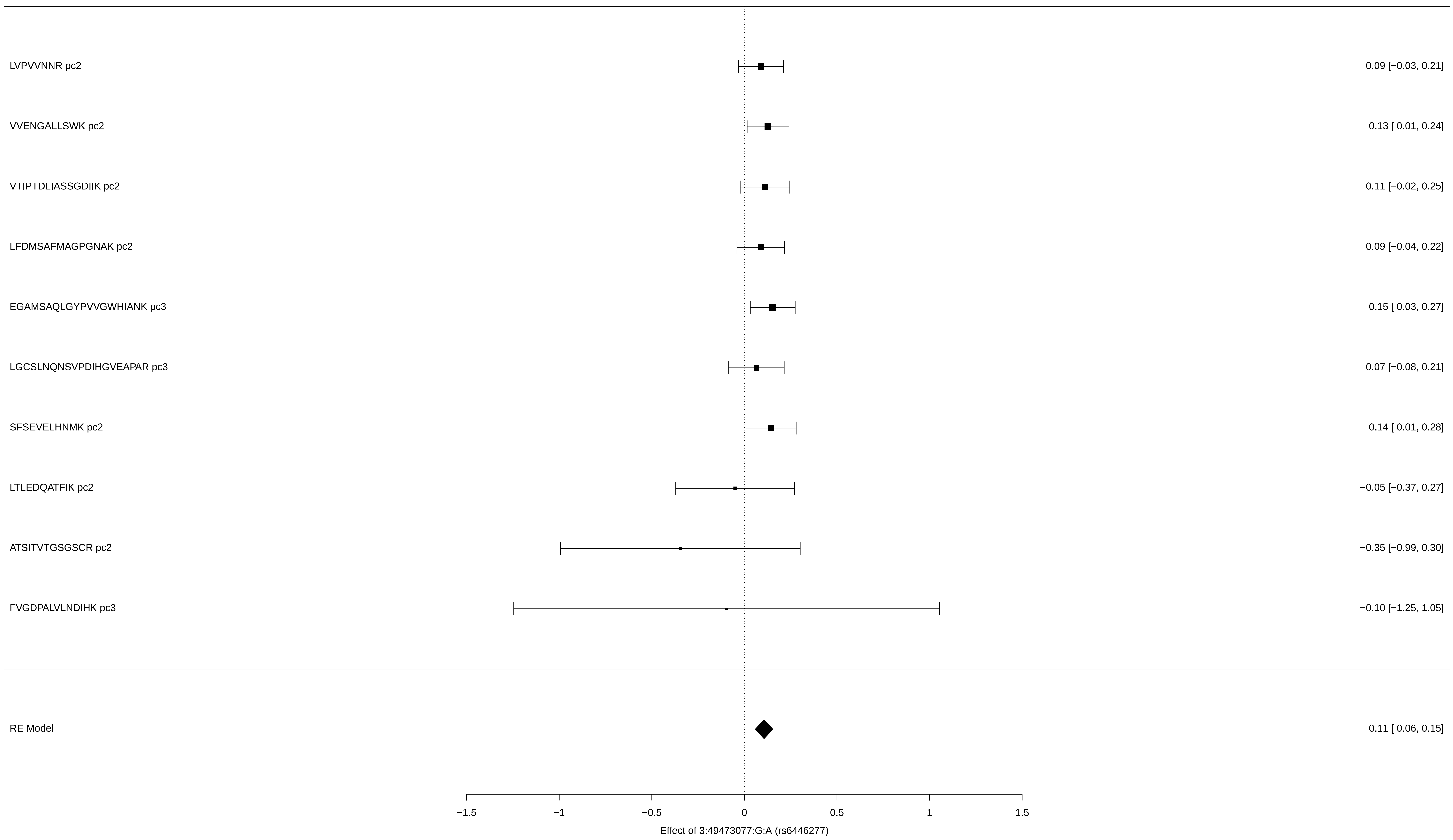

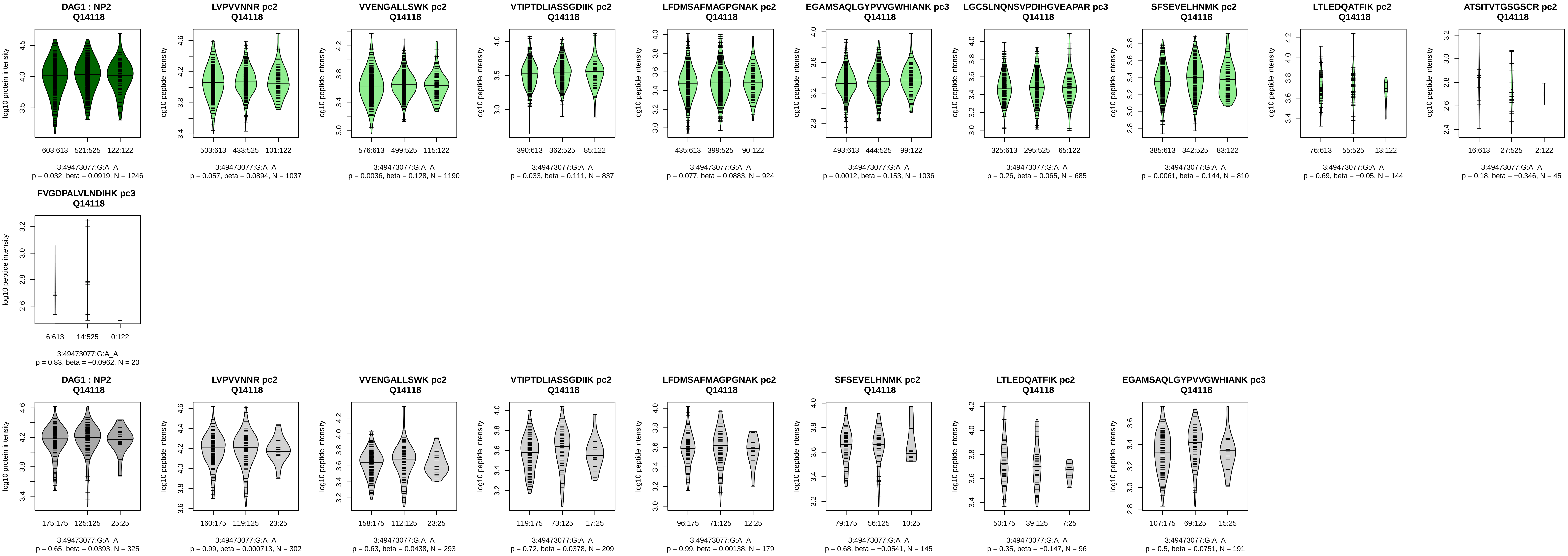

Assay Target: DAG1
UniProt: Q14118
rsID: rs6446277
Target SNP: 3:49473077:G:A
Proxy SNP: 3:49473077:G:A
UKBPPP log10(p): 21
UKBPPP BETA: 0.064
UKBPPP MSPA: 0.451
–:*:–:–:*:–:*:–:–:–
1037:1190:837:924:1036:685:8

382. CD74 (P04233)
UKBPPP MSPA=0

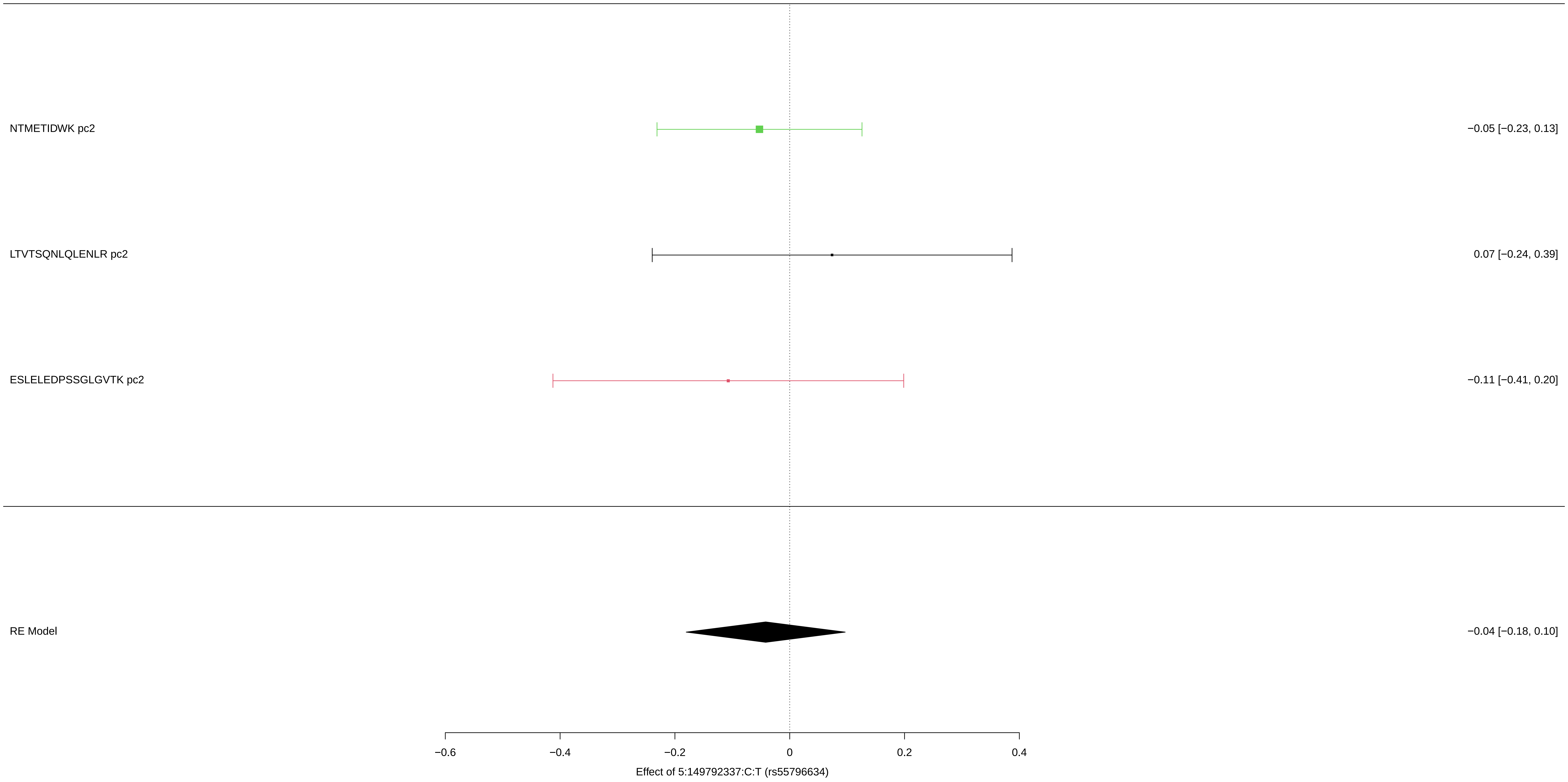

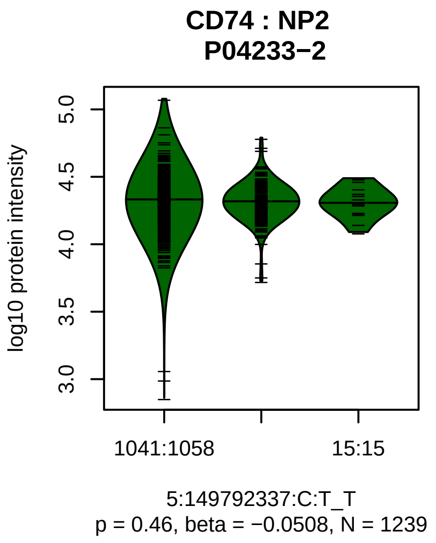

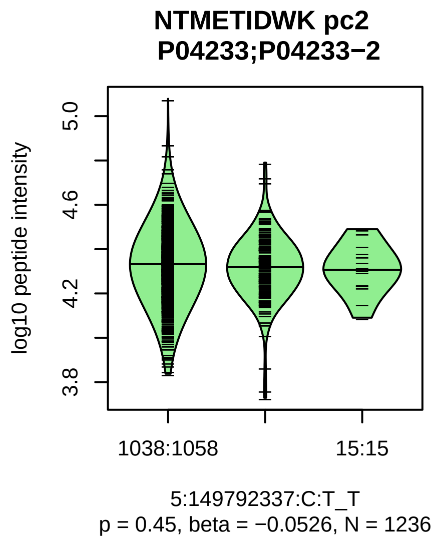

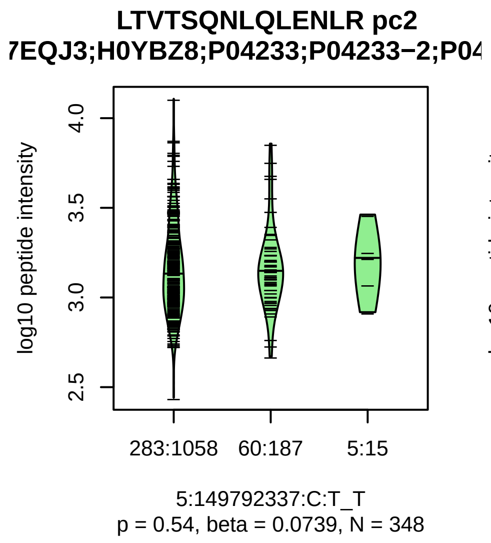

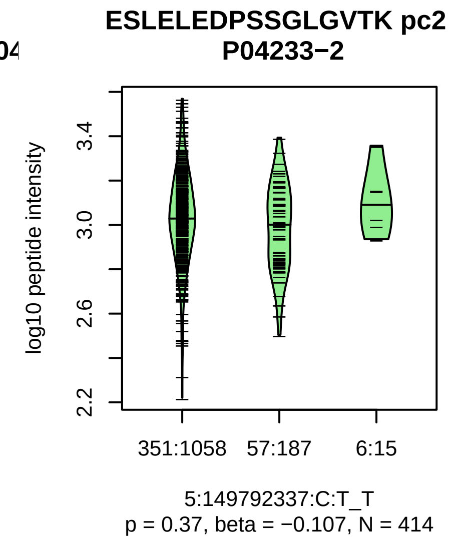

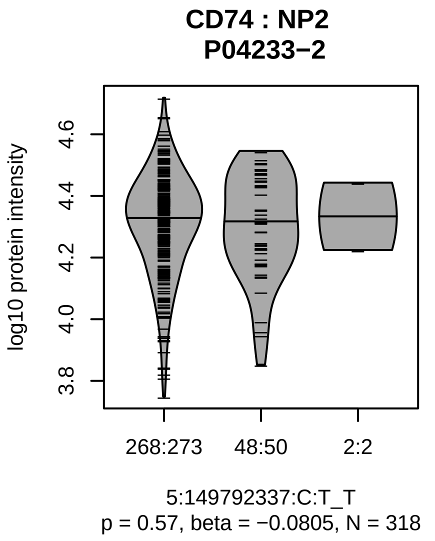

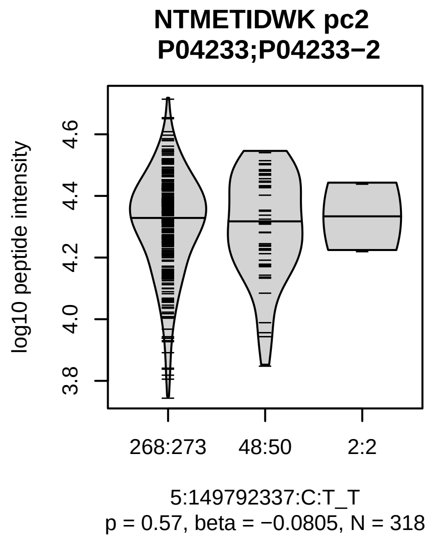

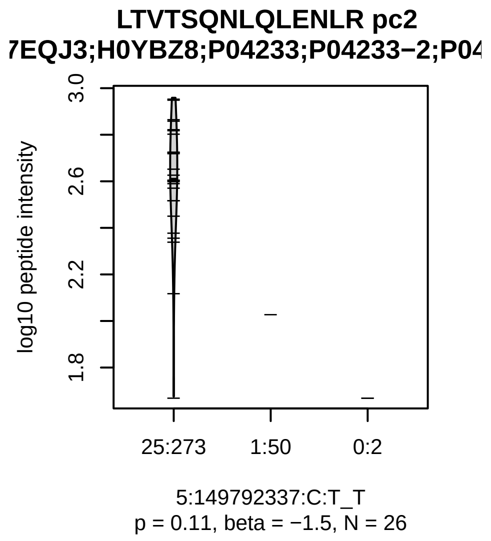

Assay Target: CD74
UniProt: P04233
rsID: rs55796634
Target SNP: 5:149792337:C:T
Proxy SNP: 5:149792337:C:T
UKBPPP log10(p): 20.1
UKBPPP BETA: −0.087
UKBPPP MSPA: 0
–:–:–
1236:348:414

383. SOST (Q9BQB4)
UKBPPP MSPA=0

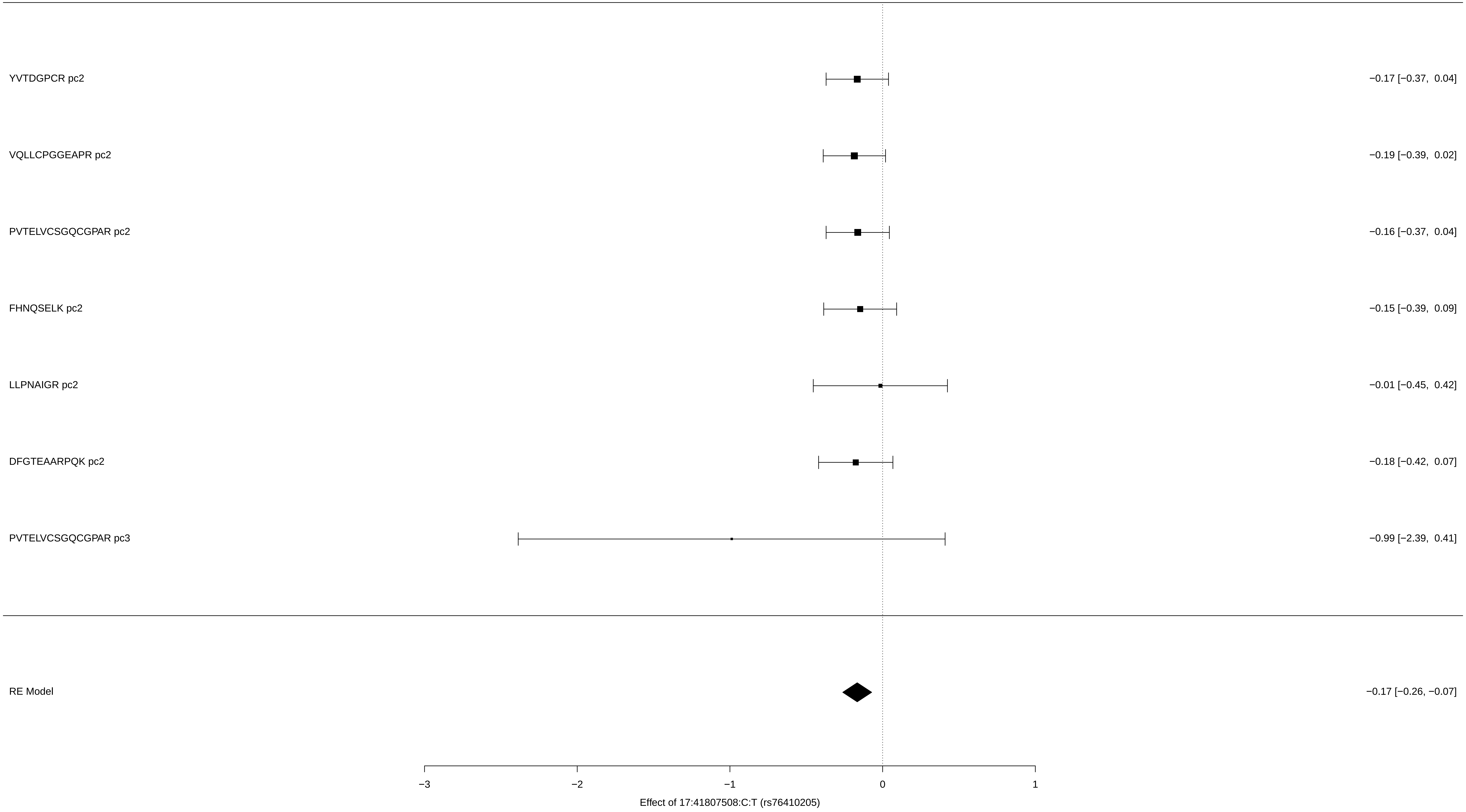

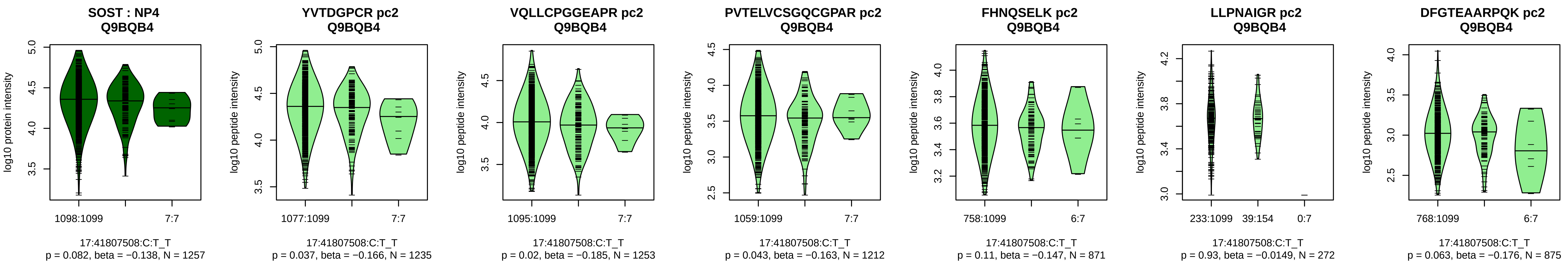

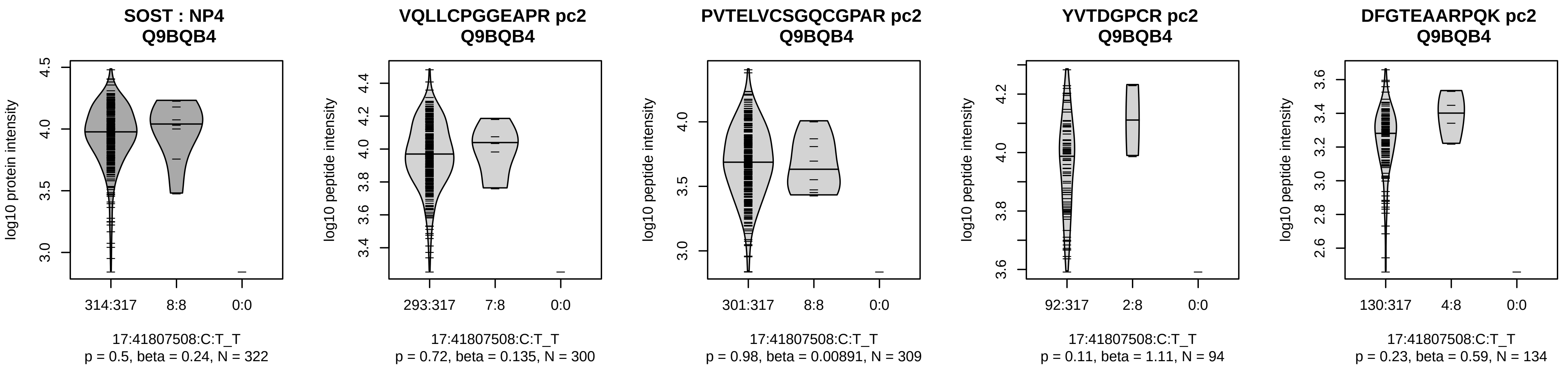

Assay Target: SOST
UniProt: Q9BQB4
rsID: rs76410205
Target SNP: 17:41807508:C:T
Proxy SNP: 17:41807508:C:T
UKBPPP log10(p): 19.7
UKBPPP BETA: −0.097
UKBPPP MSPA: 0
–:–:–:–:–:–:–
1235:1253:1212:871:272:875:3[illegible]

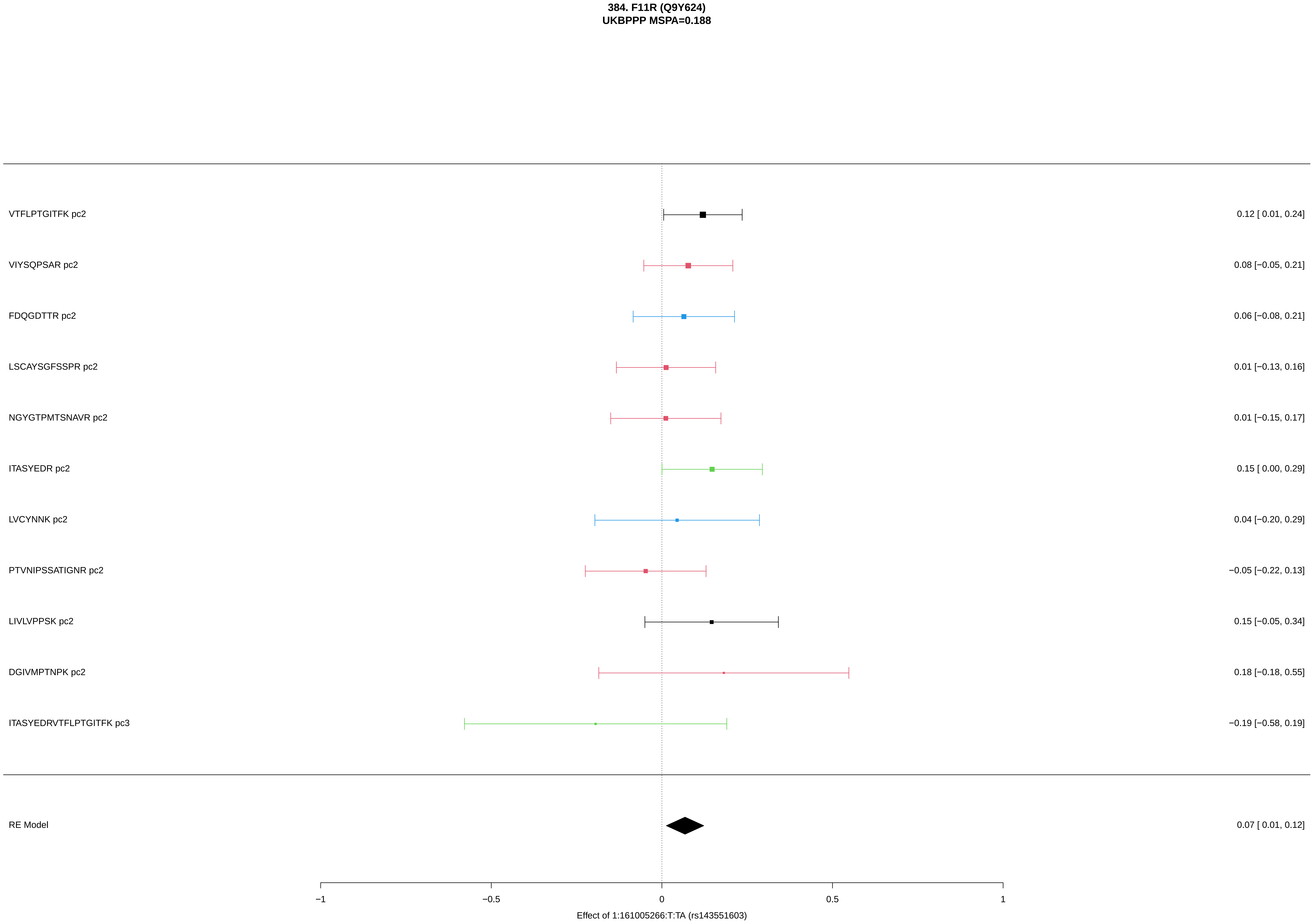
384. F11R (Q9Y624)
UKBPPP MSPA=0.188
VTFLPTGITFK pc2
0.12 [0.01, 0.24]
VIYSQPSAR pc2
0.08 [−0.05, 0.21]
FDQGDTTR pc2
0.06 [−0.08, 0.21]
LSCAYSGFSSPR pc2
0.01 [−0.13, 0.16]
NGYGTPMTSNAVR pc2
0.01 [−0.15, 0.17]
ITASYEDR pc2
0.15 [0.00, 0.29]
LVCYNNK pc2
0.04 [−0.20, 0.29]
PTVNIPSSATIGNR pc2
−0.05 [−0.22, 0.13]
LIVLVPPSK pc2
0.15 [−0.05, 0.34]
DGIVMPTNPK pc2
0.18 [−0.18, 0.55]
ITASYEDRVTFLPTGITFK pc3
−0.19 [−0.58, 0.19]
RE Model
0.07 [0.01, 0.12]
−1
−0.5
0
0.5
1
Effect of 1:161005266:T:TA (rs143551603)

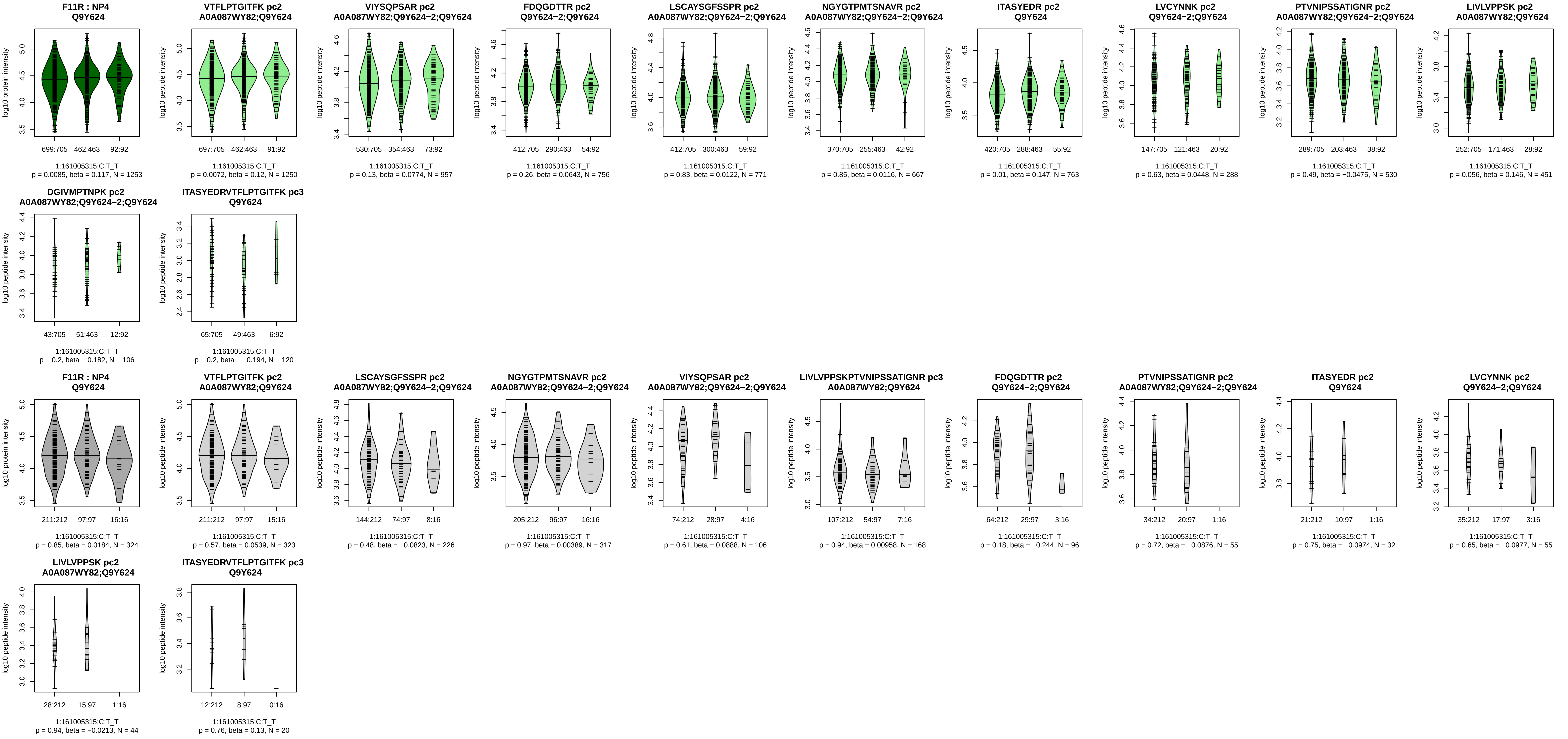

Assay Target: F11R
UniProt: Q9Y624
rsID: rs143551603
Target SNP: 1:161005266:T:TA
Proxy SNP: 1:161005315:C:T
UKBPPP log10(p): 18.7
UKBPPP BETA: 0.067
UKBPPP MSPA: 0.188
*–:–:–:–:–:–:–:–:–
1250:957:756:771:667:763:288:

385. SIL1 (Q9H173)
UKBPPP MSPA=0

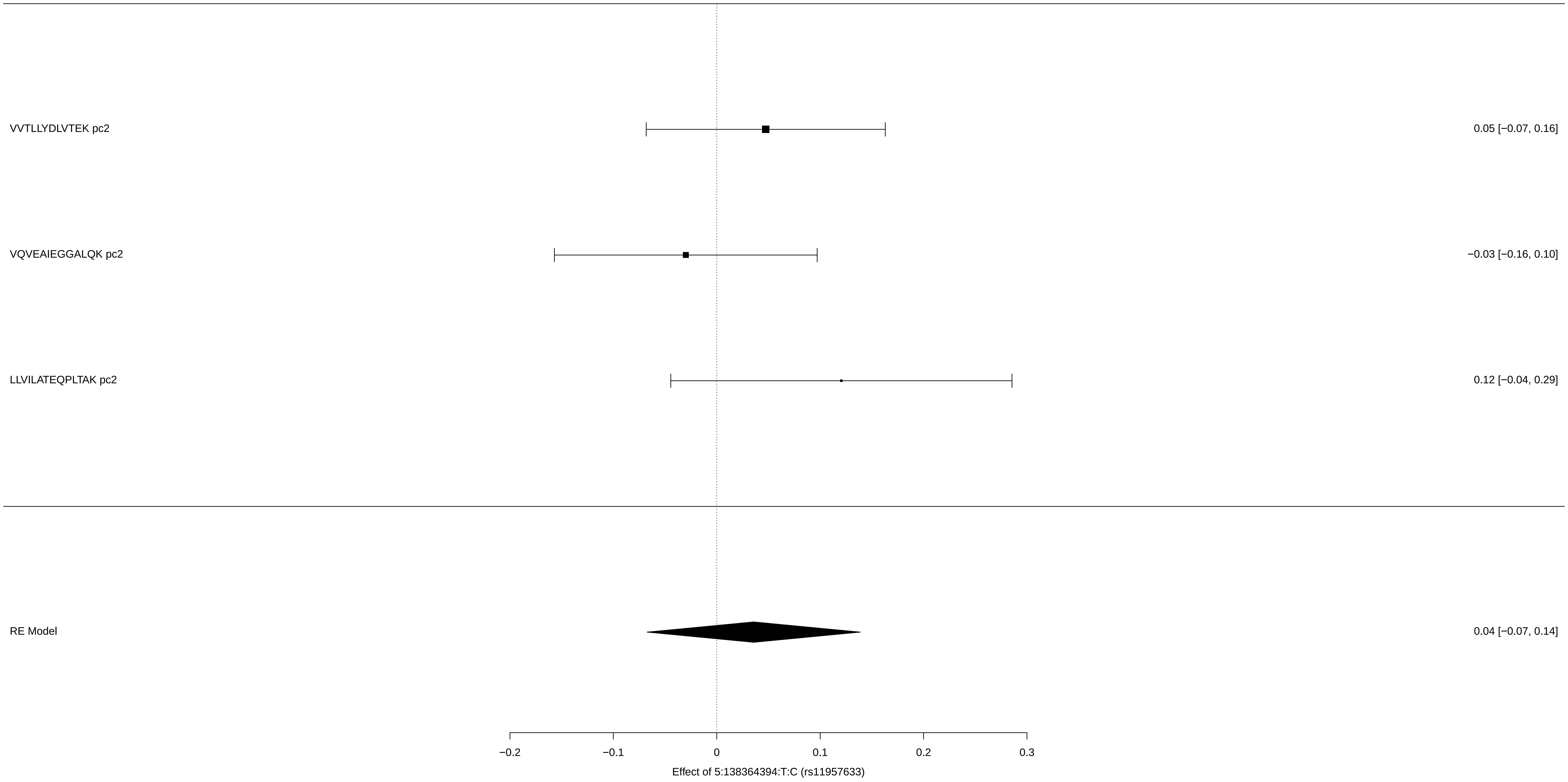

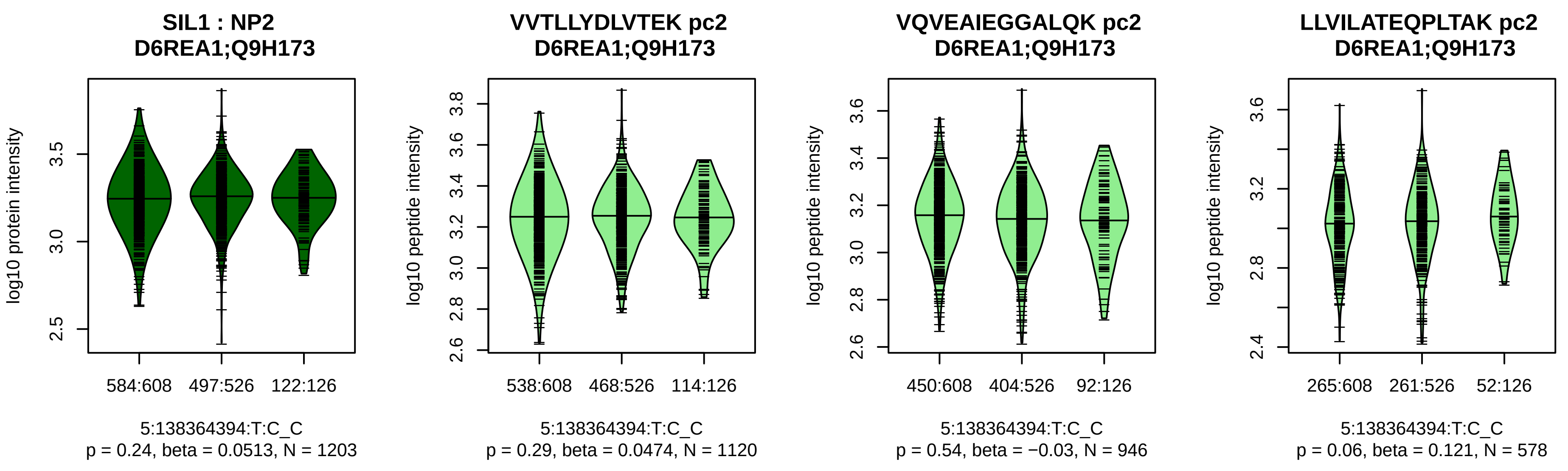

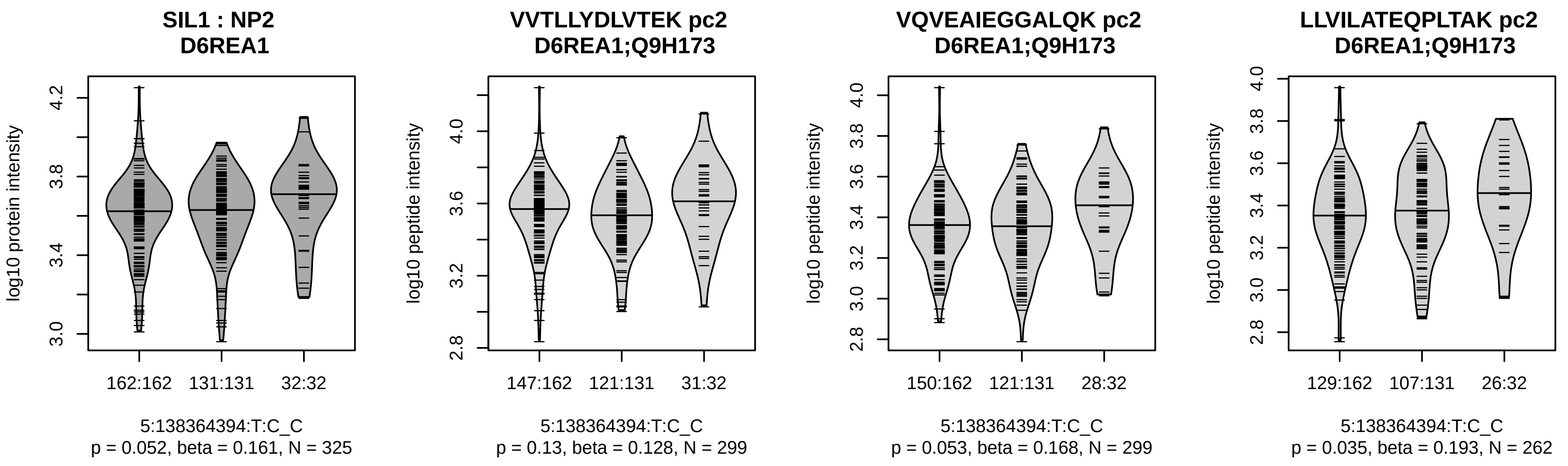

Assay Target: SIL1
UniProt: Q9H173
rsID: rs11957633
Target SNP: 5:138364394:T:C
Proxy SNP: 5:138364394:T:C
UKBPPP log10(p): 18.7
UKBPPP BETA: 0.06
UKBPPP MSPA: 0
–:–:–
1120:946:578

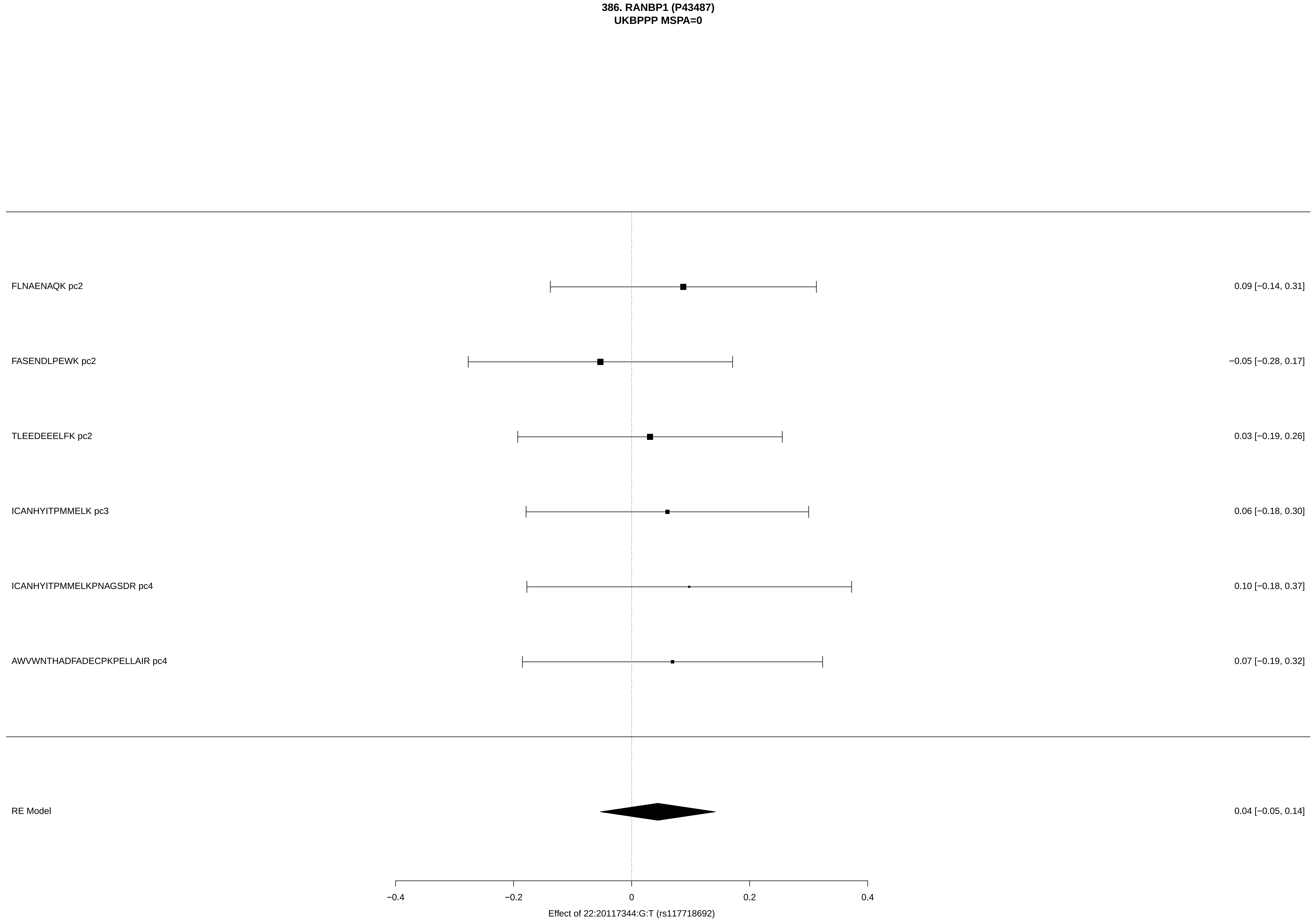
386. RANBP1 (P43487)
UKBPPP MSPA=0
FLNAENAQK pc2
0.09 [−0.14, 0.31]
FASENDLPEWK pc2
−0.05 [−0.28, 0.17]
TLEEDEEELFK pc2
0.03 [−0.19, 0.26]
ICANHYITPMMELK pc3
0.06 [−0.18, 0.30]
ICANHYITPMMELKPNAGSDR pc4
0.10 [−0.18, 0.37]
AWVWNTHADFADECPKPELLAIR pc4
0.07 [−0.19, 0.32]
RE Model
0.04 [−0.05, 0.14]
−0.4
−0.2
0
0.2
0.4
Effect of 22:20117344:G:T (rs117718692)

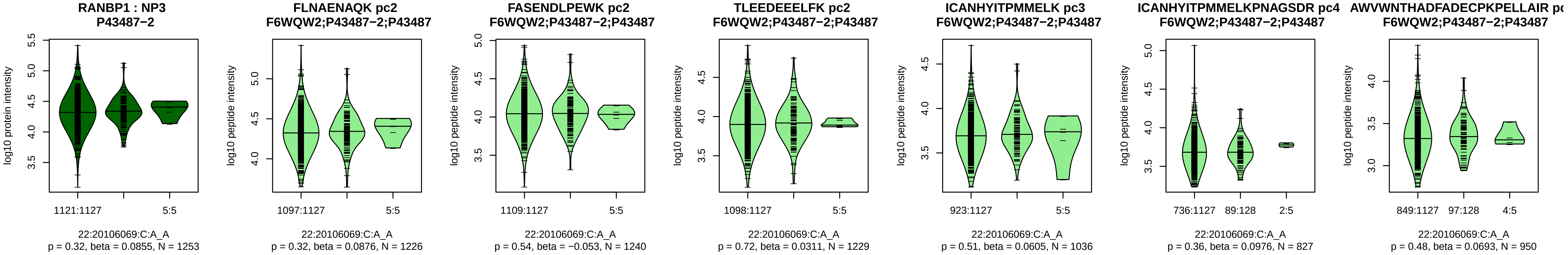

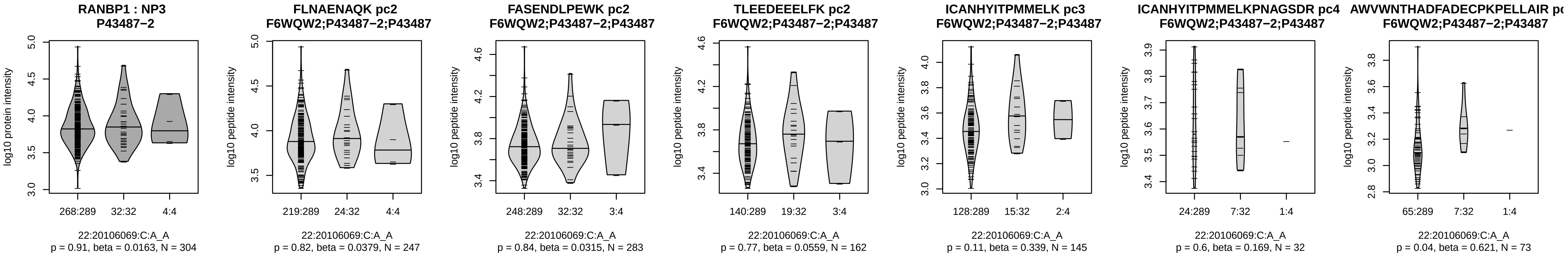

Assay Target: RANBP1
UniProt: P43487
rsID: rs117718692
Target SNP: 22:20117344:G:T
Proxy SNP: 22:20106069:C:A
UKBPPP log10(p): 18.4
UKBPPP BETA: 0.122
UKBPPP MSPA: 0
−:−:−:−:−:−:NA
1226:1240:1229:1036:827:950:

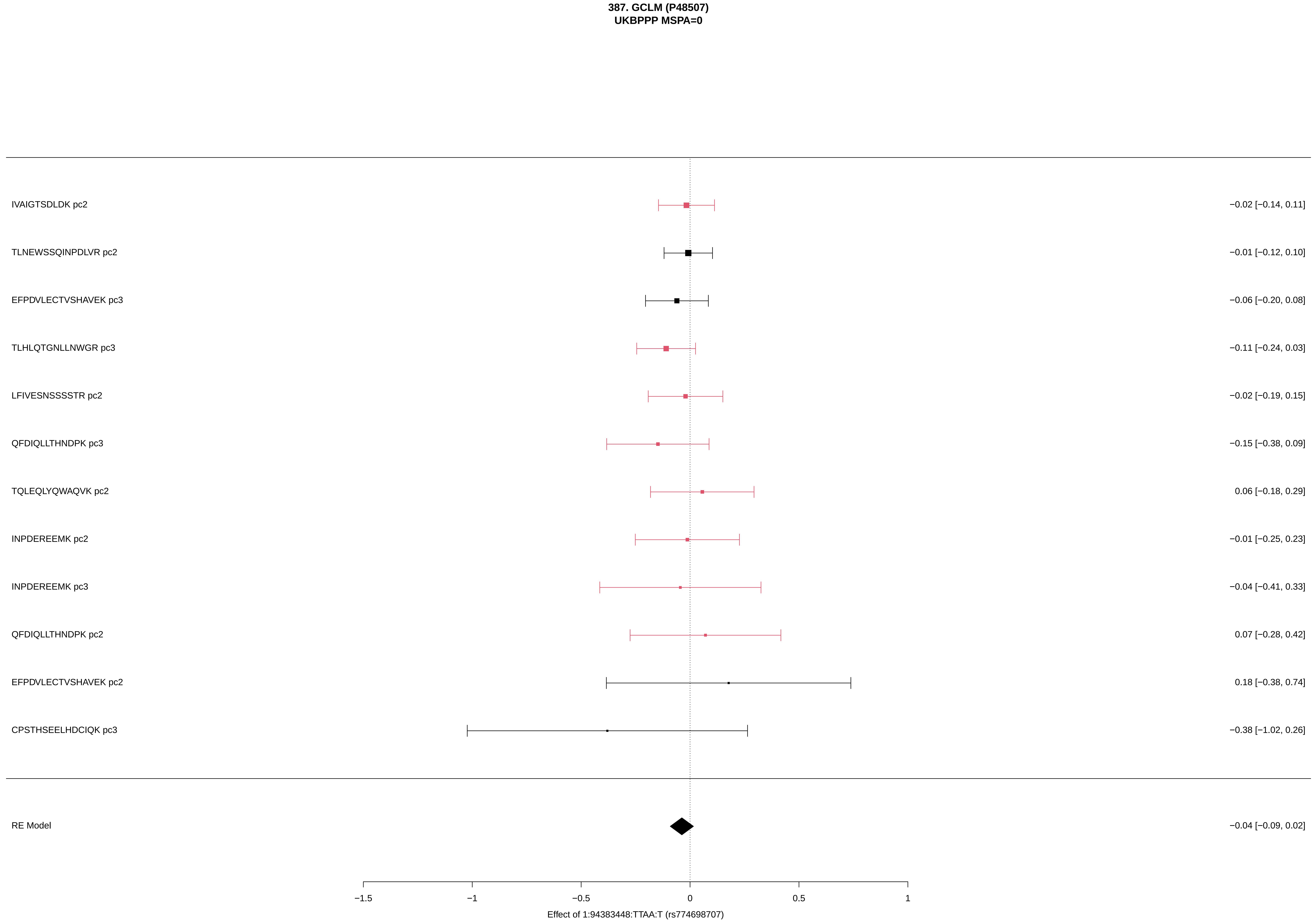
387. GCLM (P48507)
UKBPPP MSPA=0
IVAIGTSDLDK pc2
−0.02 [−0.14, 0.11]
TLNEWSSQINPDLVR pc2
−0.01 [−0.12, 0.10]
EFPDVLECTVSHAVEK pc3
−0.06 [−0.20, 0.08]
TLHLQTGNLLNWGR pc3
−0.11 [−0.24, 0.03]
LFIVESNSSSSTR pc2
−0.02 [−0.19, 0.15]
QFDIQLLTHNDPK pc3
−0.15 [−0.38, 0.09]
TQLEQLYQWAQVK pc2
0.06 [−0.18, 0.29]
INPDEREEMK pc2
−0.01 [−0.25, 0.23]
INPDEREEMK pc3
−0.04 [−0.41, 0.33]
QFDIQLLTHNDPK pc2
0.07 [−0.28, 0.42]
EFPDVLECTVSHAVEK pc2
0.18 [−0.38, 0.74]
CPSTHSEELHDCIQK pc3
−0.38 [−1.02, 0.26]
RE Model
−0.04 [−0.09, 0.02]
−1.5
−1
−0.5
0
0.5
1
Effect of 1:94383448:TTAA:T (rs774698707)

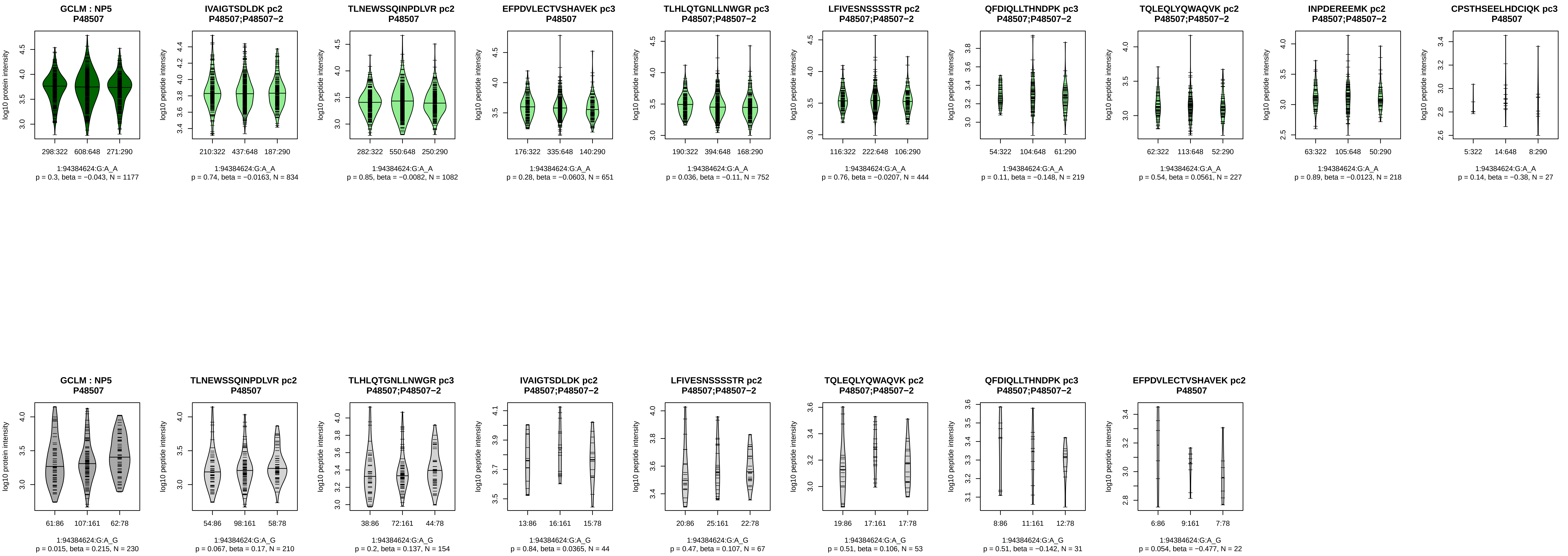

Assay Target: GCLM
UniProt: P48507
rsID: rs774698707
Target SNP: 1:94383448:TTAA:
Proxy SNP: 1:94384624:G:A
UKBPPP log10(p): 17.6
UKBPPP BETA: −0.055
UKBPPP MSPA: 0
−:−:−:−:−:−:−:−:−:−:−:−:NA:NA
834:1082:651:752:444:219:227:

388. ST13 (P50502)
UKBPPP MSPA=0

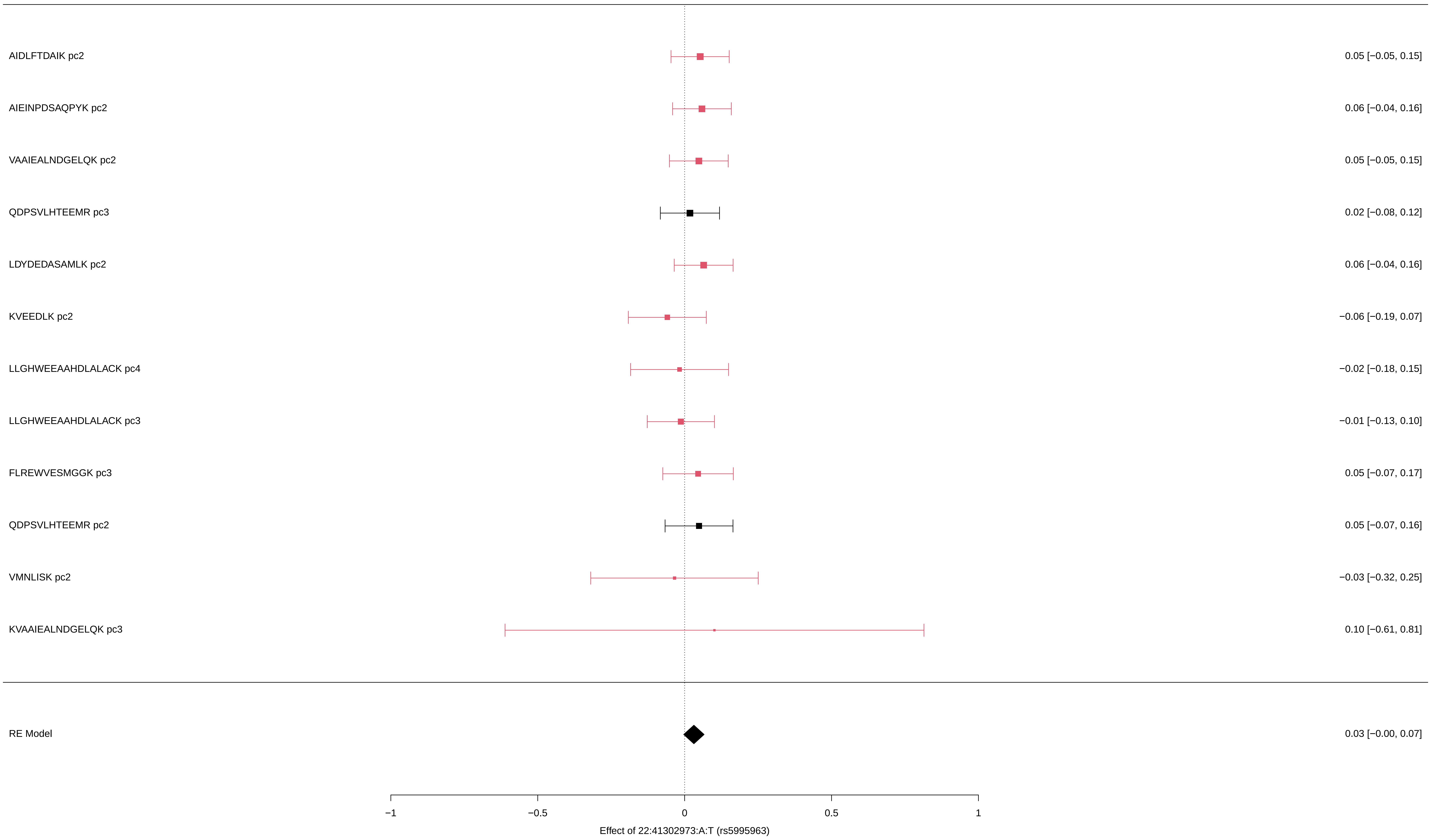

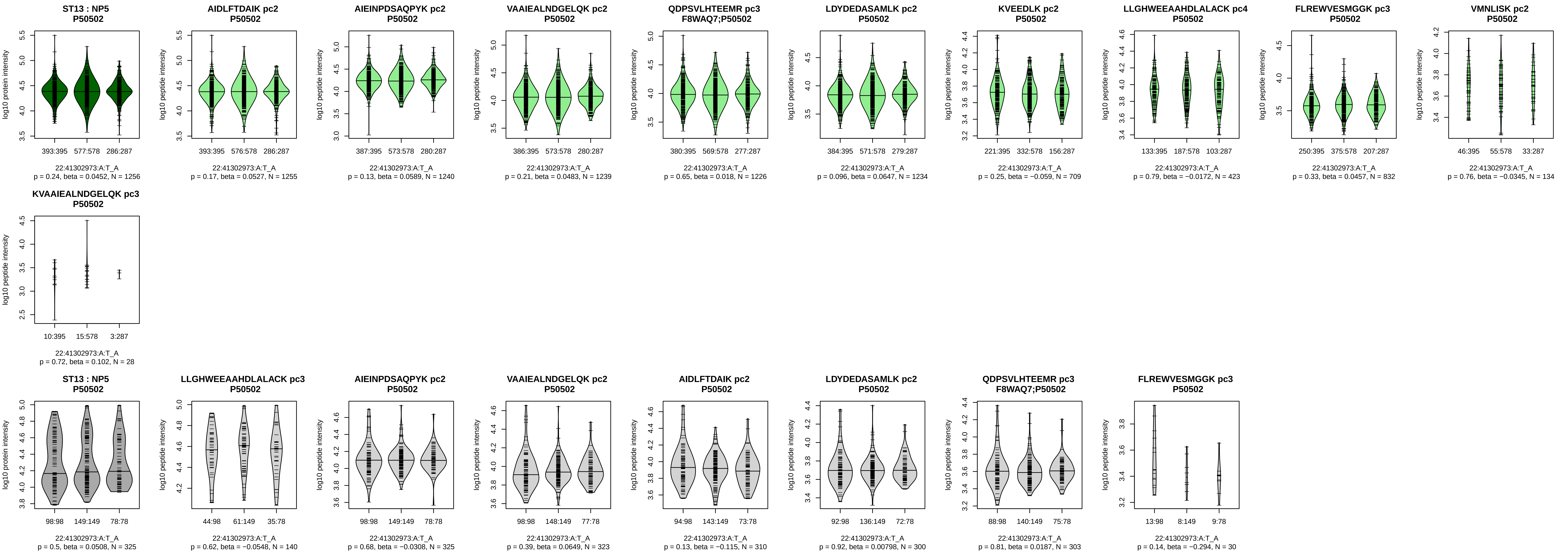

Assay Target: ST13
UniProt: P50502
rsID: rs5995963
Target SNP: 22:41302973:A:T
Proxy SNP: 22:41302973:A:T
UKBPPP log10(p): 17.1
UKBPPP BETA: −0.052
UKBPPP MSPA: 0
–:–:–:–:–:–:–:–:–:–:–:NA:NA
1255:1240:1239:1226:1234:709

389. SHH (Q15465)
UKBPPP MSPA=0

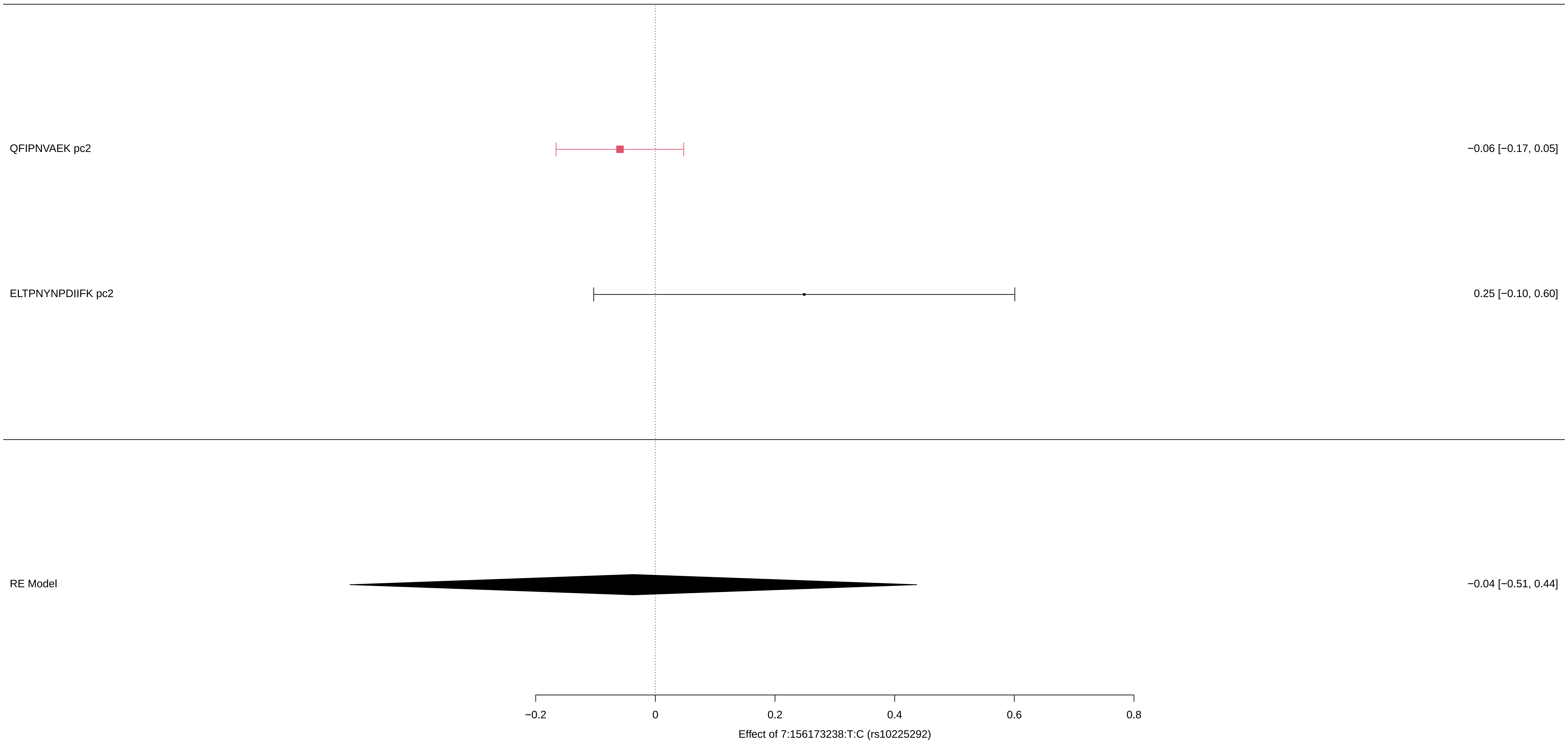

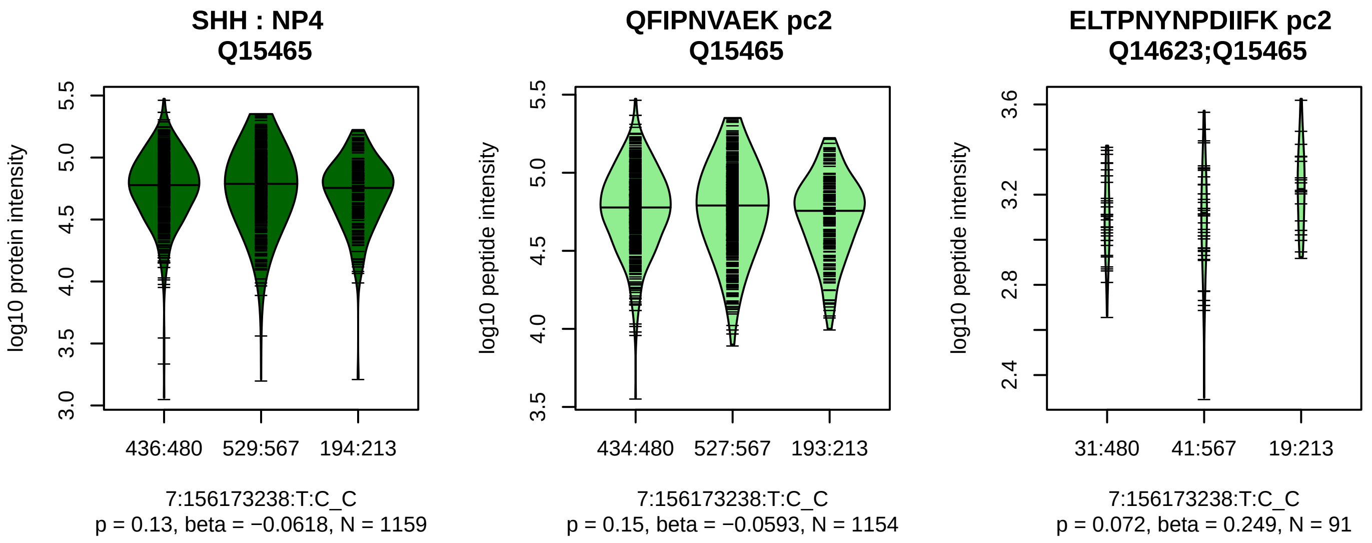

Assay Target: SHH
UniProt: Q15465
rsID: rs10225292
Target SNP: 7:156173238:T:C
Proxy SNP: 7:156173238:T:C
UKBPPP log10(p): 16.6
UKBPPP BETA: −0.055
UKBPPP MSPA: 0
–:–
1154:91

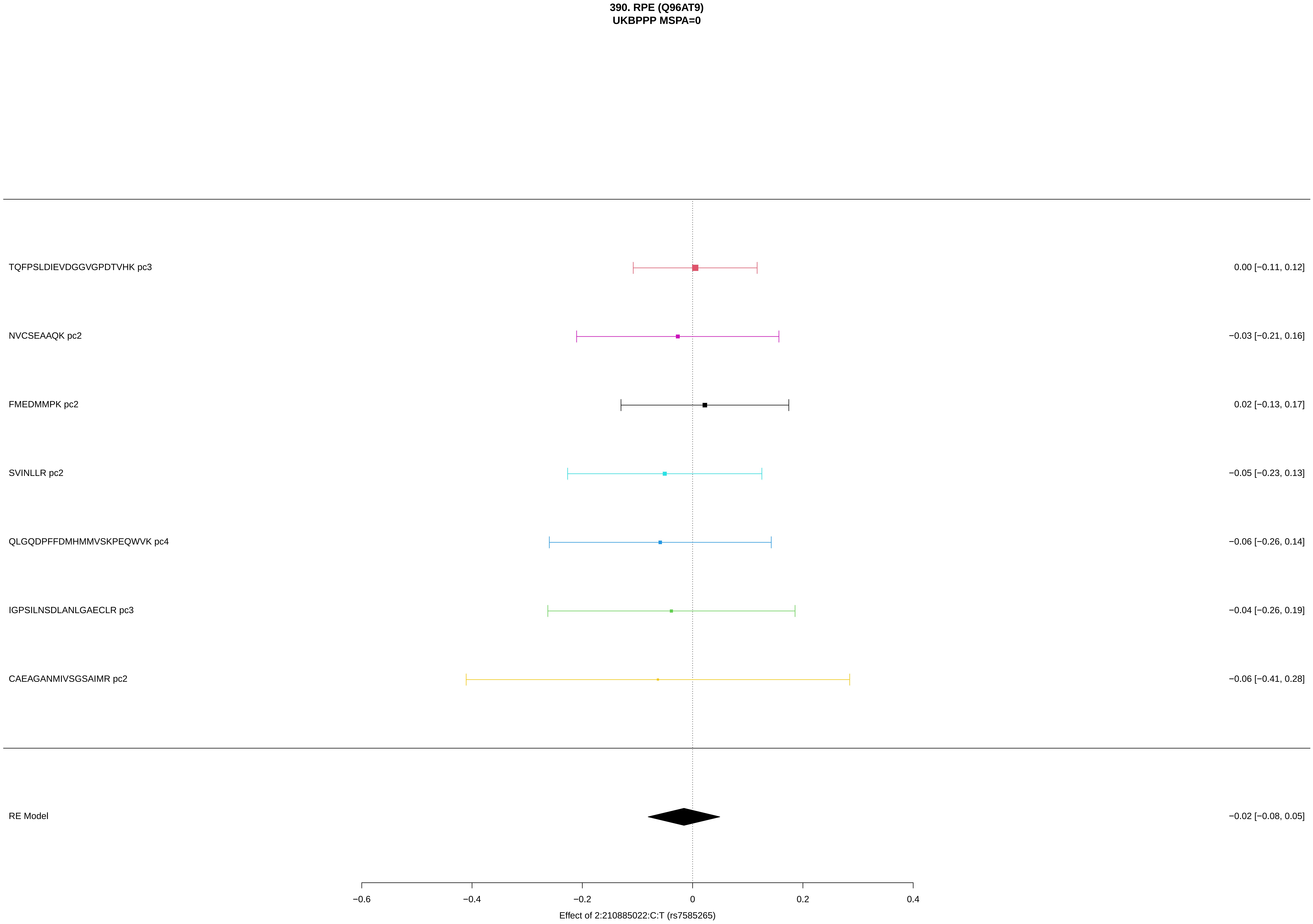
390. RPE (Q96AT9)
UKBPPP MSPA=0
TQFPSLDIEVDGGVGPDTVHK pc3
0.00 [−0.11, 0.12]
NVCSEAAQK pc2
−0.03 [−0.21, 0.16]
FMEDMMPK pc2
0.02 [−0.13, 0.17]
SVINLLR pc2
−0.05 [−0.23, 0.13]
QLGQDPFFDMHMMVSKPEQWVK pc4
−0.06 [−0.26, 0.14]
IGPSILNSDLANLGAECLR pc3
−0.04 [−0.26, 0.19]
CAEAGANMIVSGSAIMR pc2
−0.06 [−0.41, 0.28]
RE Model
−0.02 [−0.08, 0.05]
−0.6
−0.4
−0.2
0
0.2
0.4
Effect of 2:210885022:C:T (rs7585265)

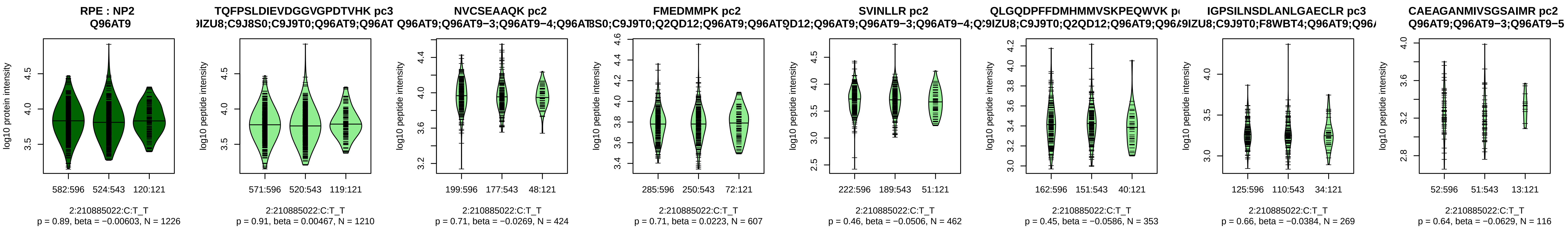

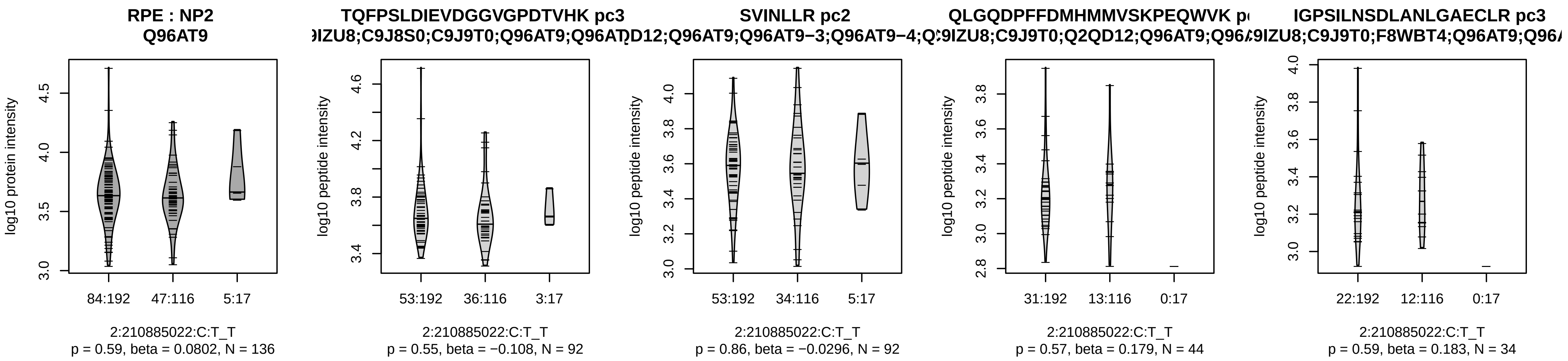

Assay Target: RPE
UniProt: Q96AT9
rsID: rs7585265
Target SNP: 2:210885022:C:T
Proxy SNP: 2:210885022:C:T
UKBPPP log10(p): 16.3
UKBPPP BETA: −0.054
UKBPPP MSPA: 0
−:−:−:−:−:−:−:NA
1210:424:607:462:353:269:116:

391. DNAJB1 (P25685)
UKBPPP MSPA=0

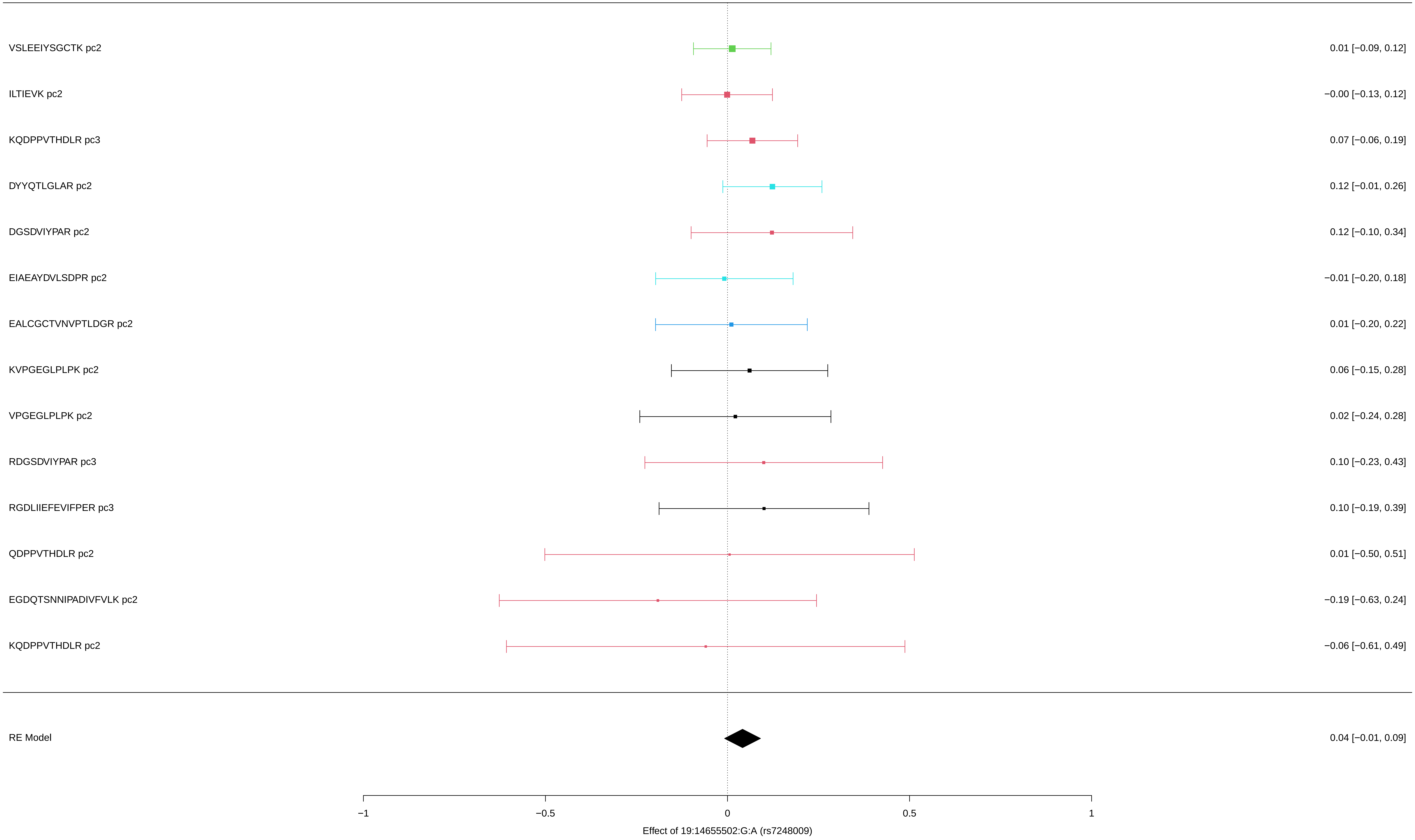

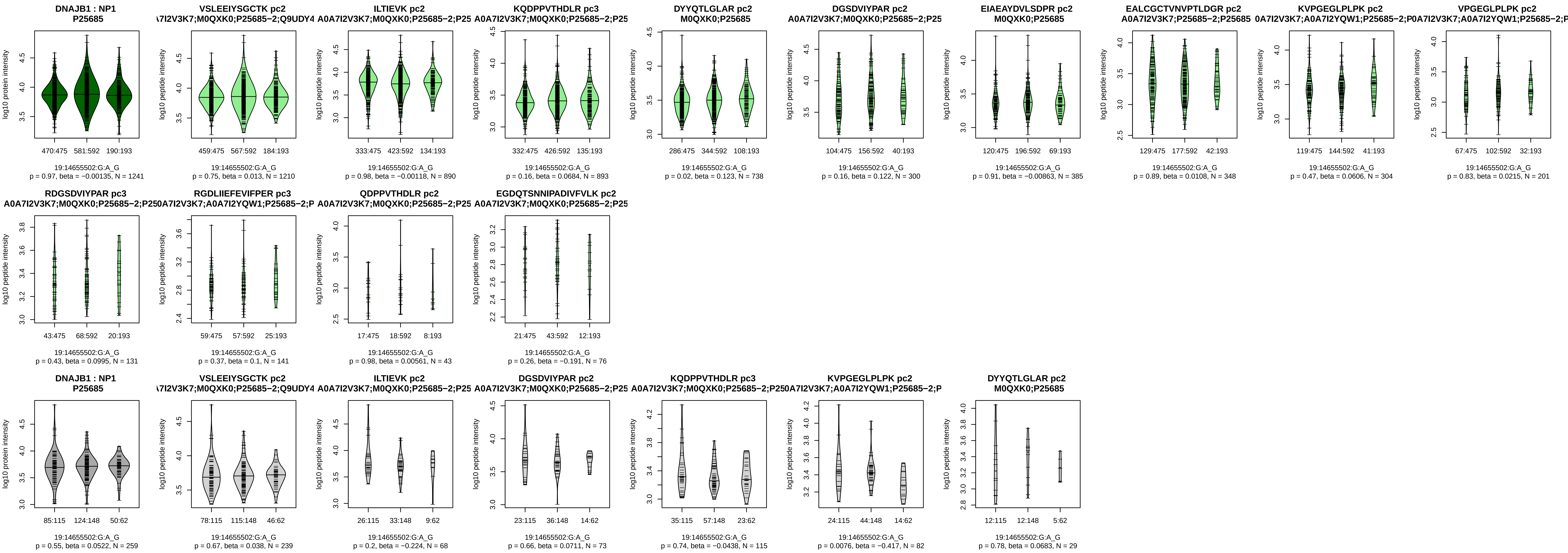

Assay Target: DNAJB1
UniProt: P25685
rsID: rs7248009
Target SNP: 19:14655502:G:A
Proxy SNP: 19:14655502:G:A
UKBPPP log10(p): 15.7
UKBPPP BETA: −0.051
UKBPPP MSPA: 0
−:−:−:−:−:−:−:−:−:−:−:−:−:NA:N
1210:890:893:738:300:385:348:

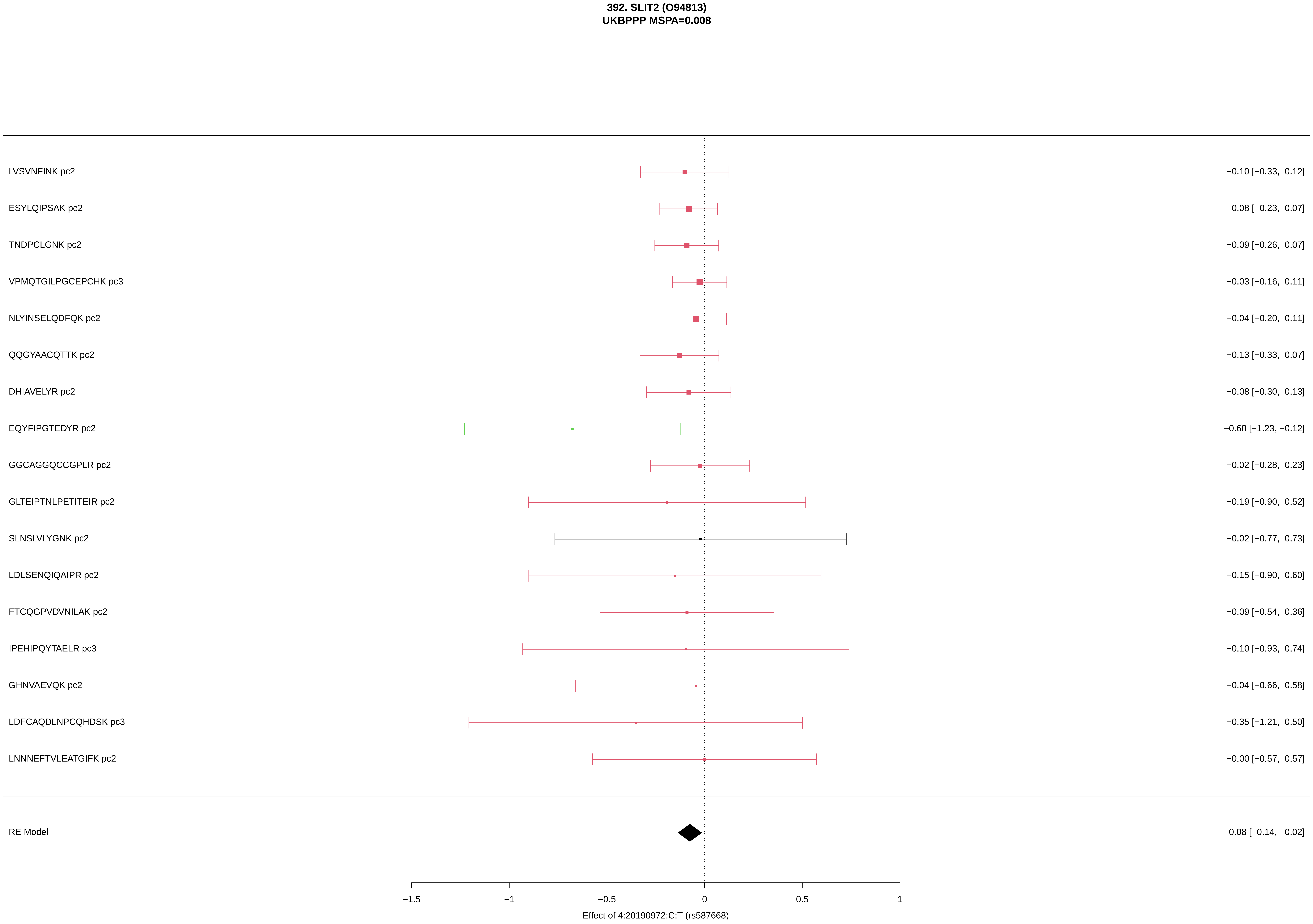
392. SLIT2 (O94813)
UKBPPP MSPA=0.008
LVSVNFINK pc2
−0.10 [−0.33, 0.12]
ESYLQIPSAK pc2
−0.08 [−0.23, 0.07]
TNDPCLGNK pc2
−0.09 [−0.26, 0.07]
VPMQTGILPGCEPCHK pc3
−0.03 [−0.16, 0.11]
NLYINSELQDFQK pc2
−0.04 [−0.20, 0.11]
QQGYAACQTTK pc2
−0.13 [−0.33, 0.07]
DHIAVELYR pc2
−0.08 [−0.30, 0.13]
EQYFIPGTEDYR pc2
−0.68 [−1.23, −0.12]
GGCAGGQCCGPLR pc2
−0.02 [−0.28, 0.23]
GLTEIPTNLPETITEIR pc2
−0.19 [−0.90, 0.52]
SLNSLVLYGNK pc2
−0.02 [−0.77, 0.73]
LDLSENQIQAIPR pc2
−0.15 [−0.90, 0.60]
FTCQGPVDVNILAK pc2
−0.09 [−0.54, 0.36]
IPEHIPQYTAELR pc3
−0.10 [−0.93, 0.74]
GHNVAEVQK pc2
−0.04 [−0.66, 0.58]
LDFCAQDLNPCQHDSK pc3
−0.35 [−1.21, 0.50]
LNNNEFTVLEATGIFK pc2
−0.00 [−0.57, 0.57]
RE Model
−0.08 [−0.14, −0.02]
−1.5
−1
−0.5
0
0.5
1
Effect of 4:20190972:C:T (rs587668)

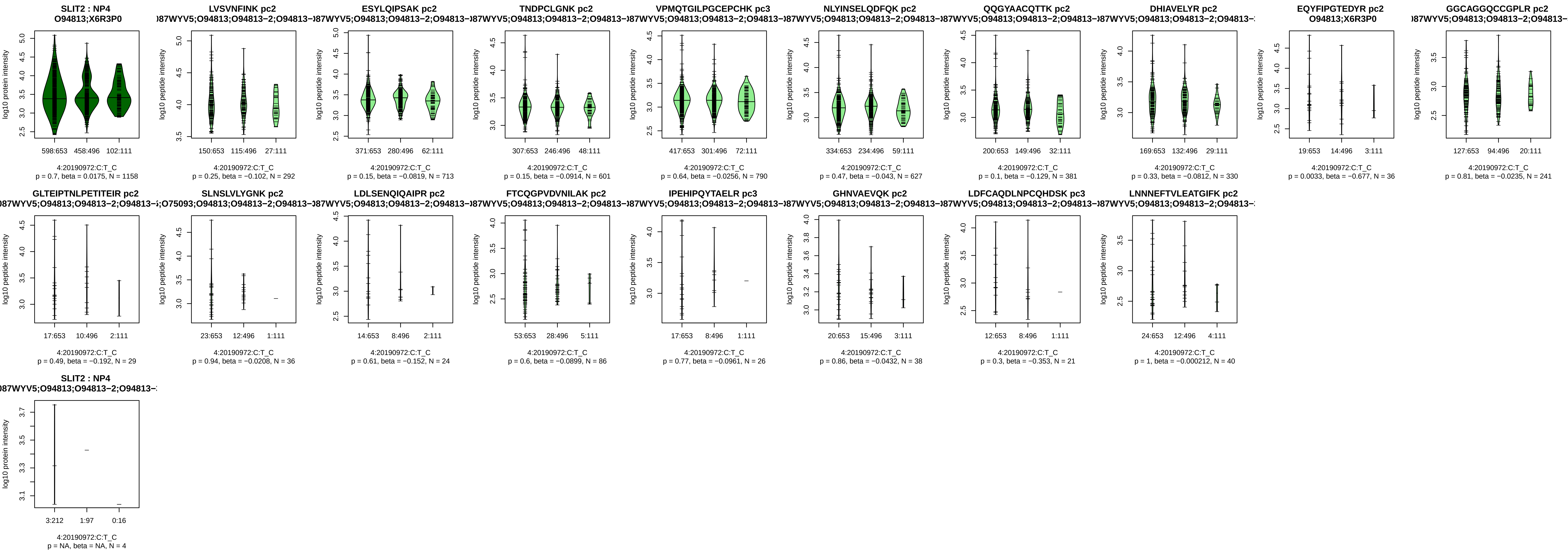

Assay Target: SLIT2
UniProt: O94813
rsID: rs587668
Target SNP: 4:20190972:C:T
Proxy SNP: 4:20190972:C:T
UKBPPP log10(p): 15.6
UKBPPP BETA: −0.056
UKBPPP MSPA: 0.008
−:−:−:−:−:−:−:−:*:−:−:−:−:NA:−:−:−
292:713:601:790:627:381:330:3
