# Supplementary material for: A genome-wide association study of mass spectrometry proteomics using a nanoparticle enrichment platform
Source: Nat Genet. 2025 Nov 27;57(12):2987–96. doi: 10.1038/s41588-025-02413-w (PMC12695657; doi:10.1038/s41588-025-02413-w)
Supplement: Supplementary file 9 — Plots visualizing the peptide mapping to all 1,980 analyzed proteins. [file 41588_2025_2413_MOESM9_ESM.pdf]

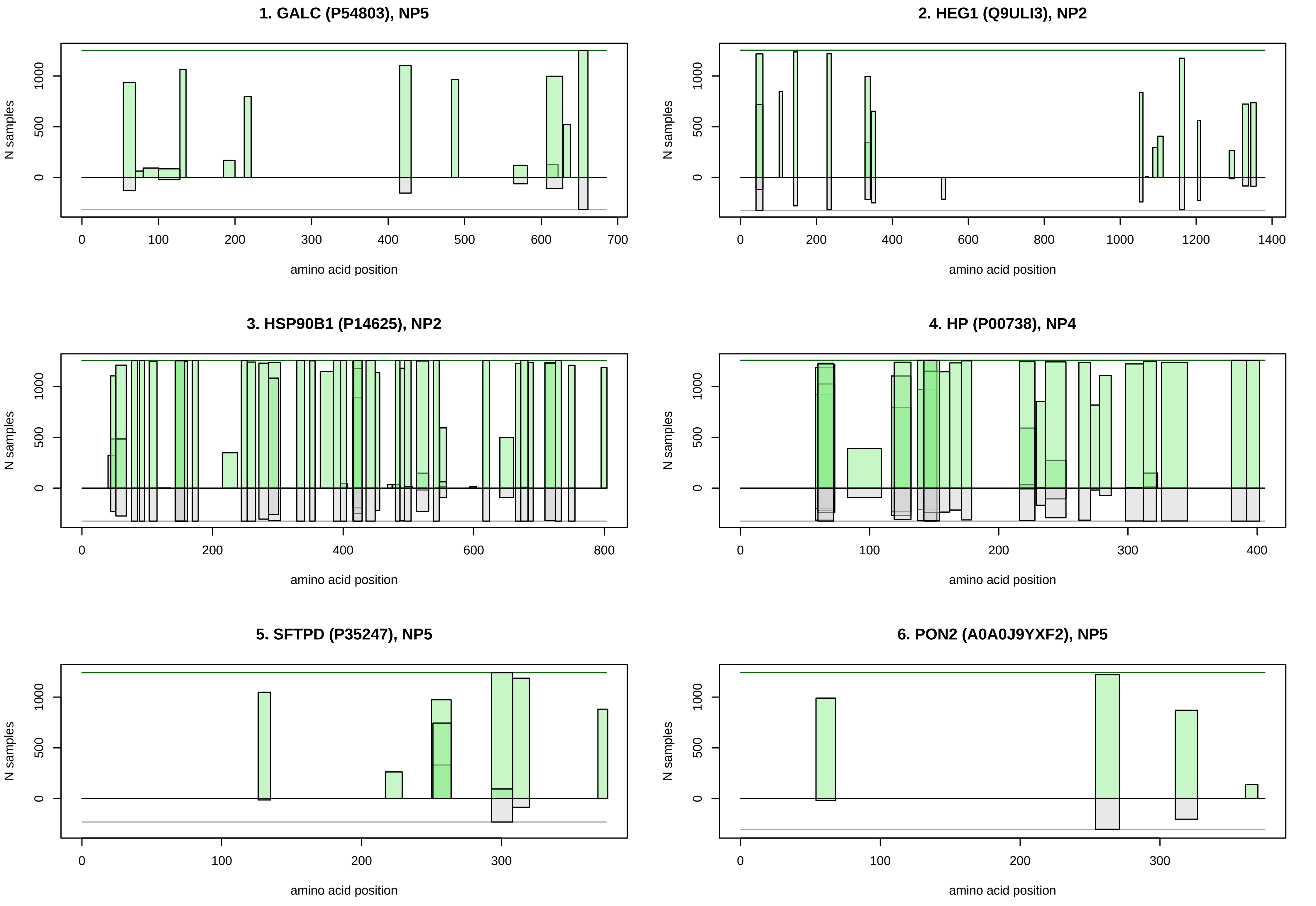

1. GALC (P54803), NP5
2. HEG1 (Q9ULI3), NP2
3. HSP90B1 (P14625), NP2
4. HP (P00738), NP4
5. SFTPD (P35247), NP5
6. PON2 (A0A0J9YXF2), NP5
N samples
amino acid position
0
500
1000
100
200
300
400
600
700
800
1200
1400

7. MST1 (G3XAK1), NP3

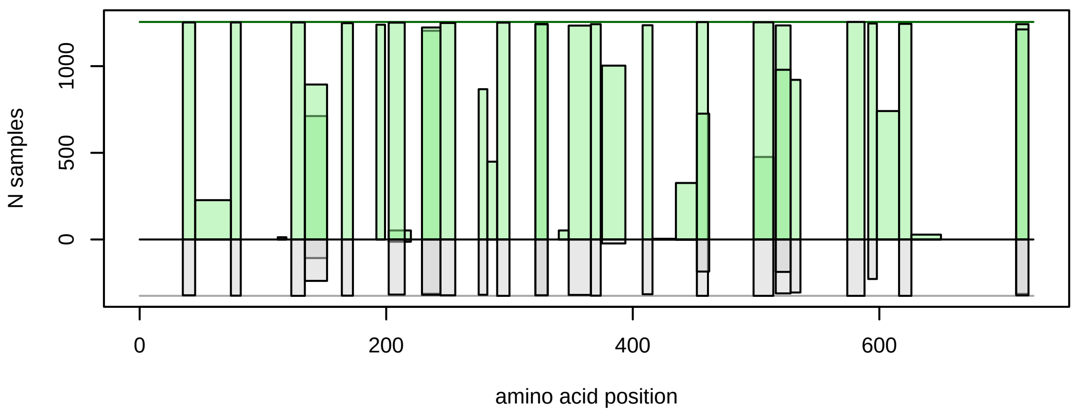

8. VMO1 (Q7Z5L0), NP2

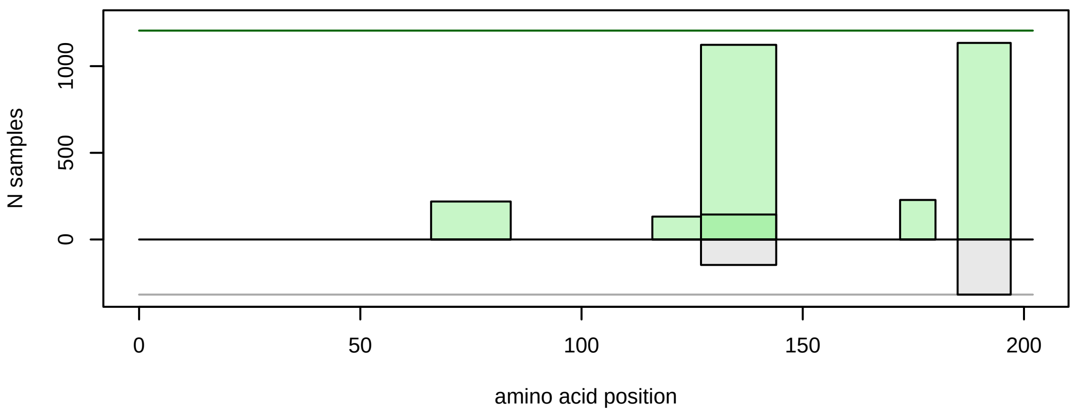

9. CFHR2 (V9GYE7), NP3

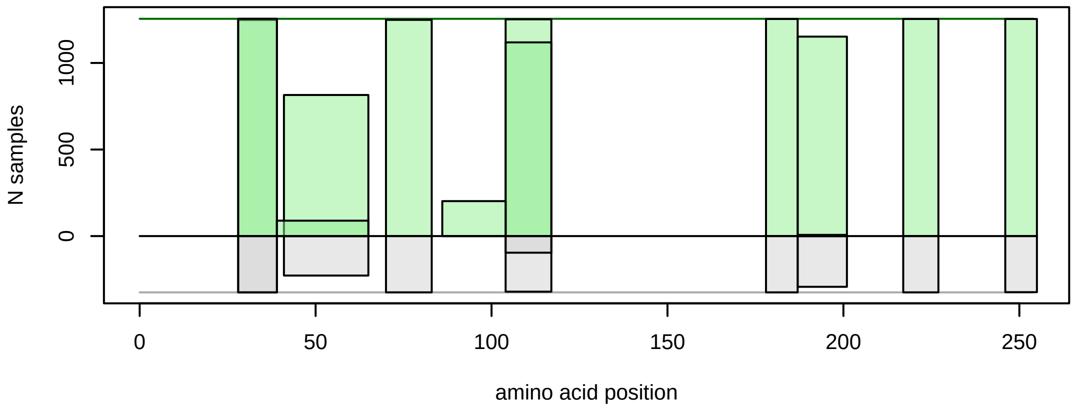

10. MBL2 (P11226), NP2

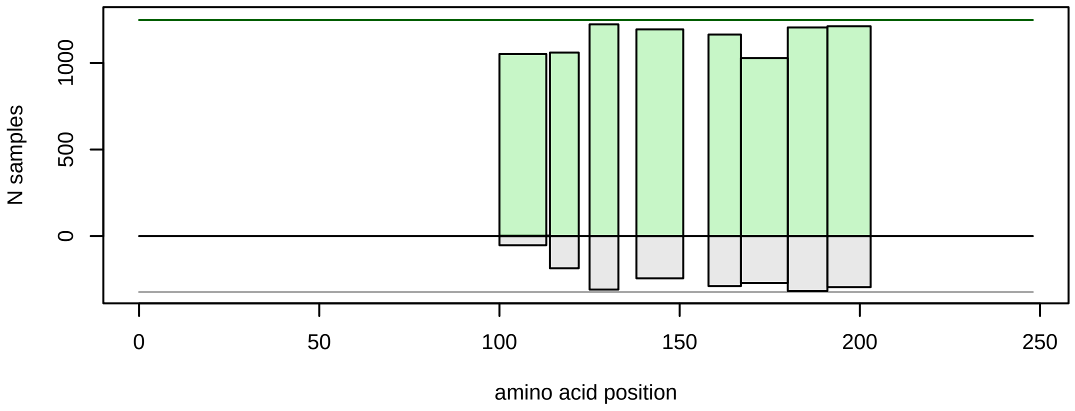

11. GGTA1P (Q4G0N0), NP2

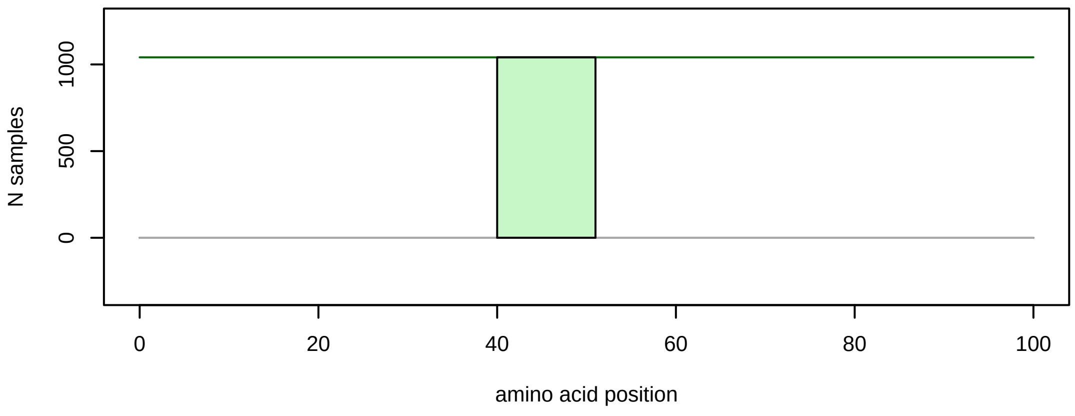

12. CFHR3 (Q02985), NP5

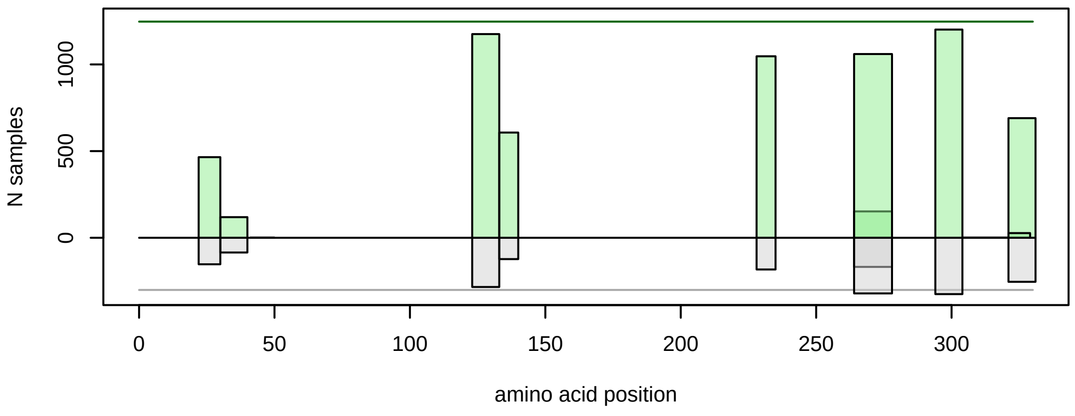

13. FN3K (Q9H479), NP1

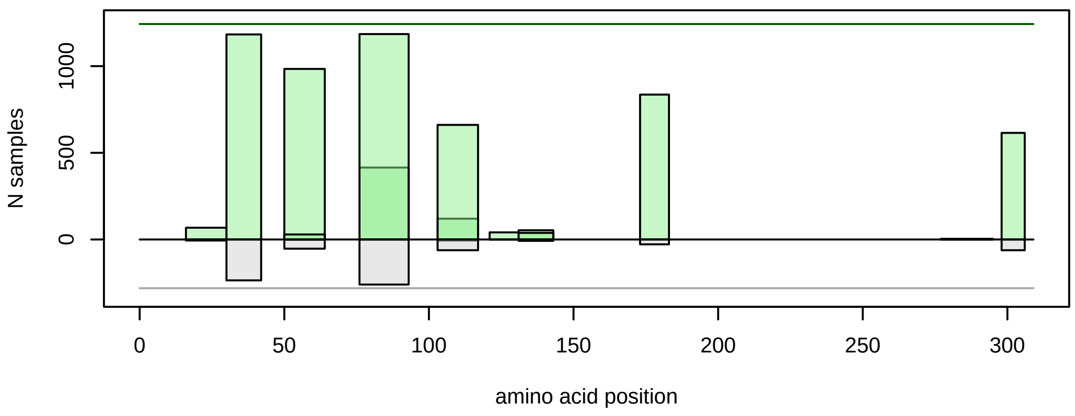

14. CHI3L1 (P36222), NP1

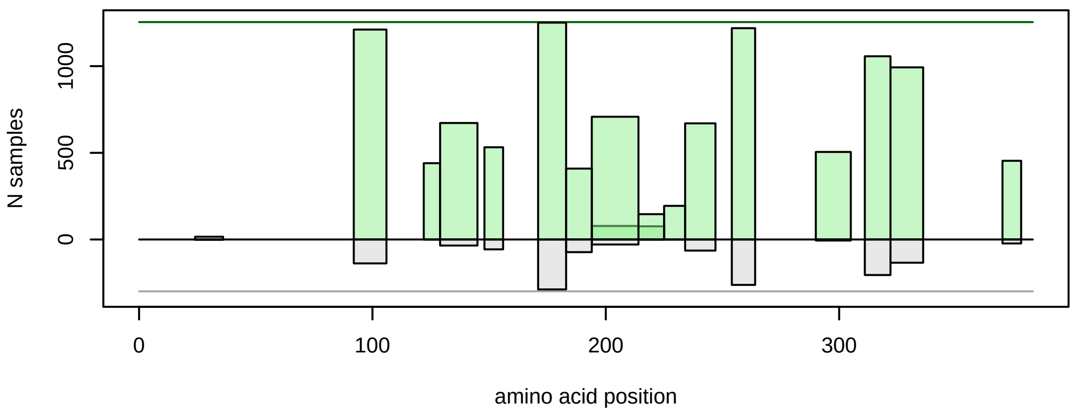

15. AMBP (P02760), NP2

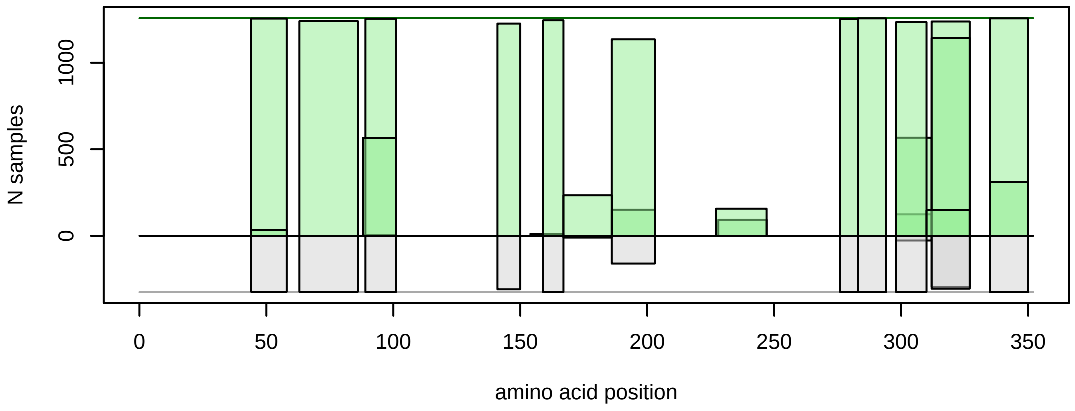

16. HLA−G (P17693;Q5RJ85), NP1

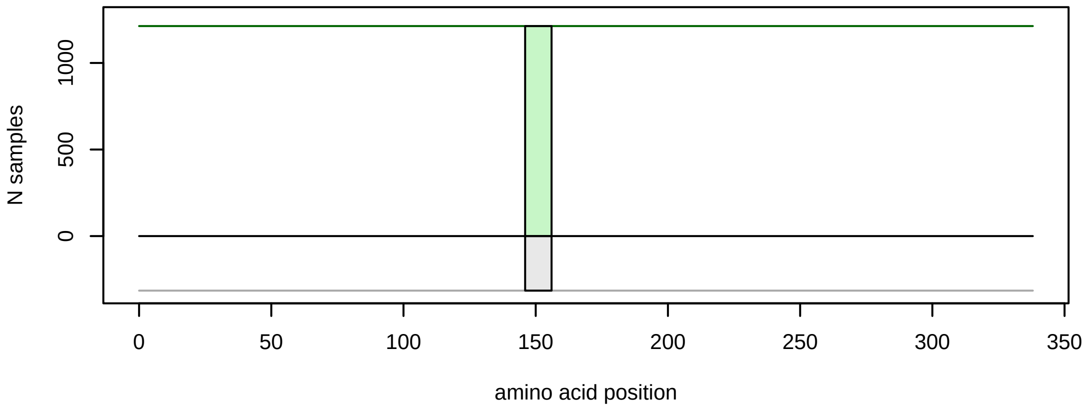

17. BRE (Q9NXR7), NP3

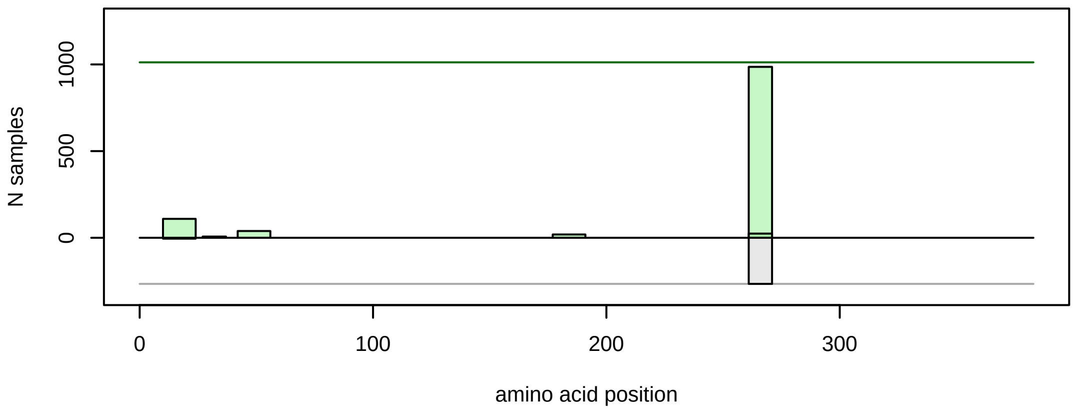

18. NA (A0A024R962), NP3

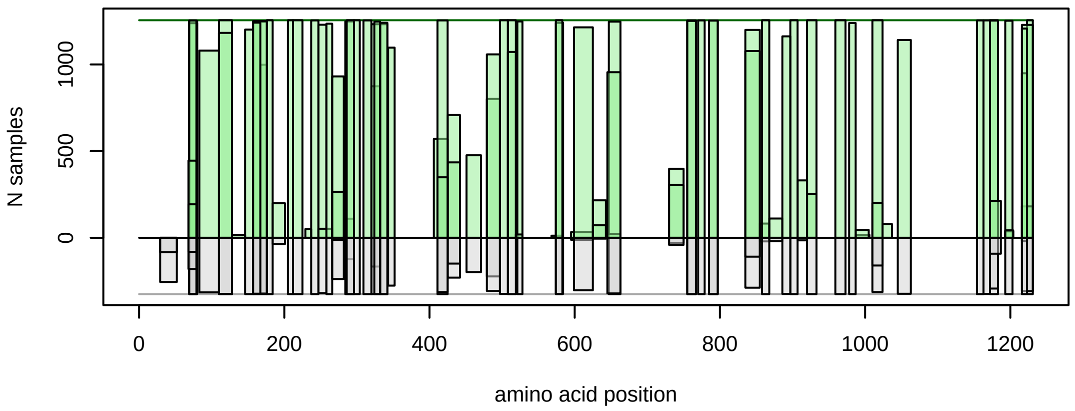

19. CCDC132 (Q96JG6), NP2

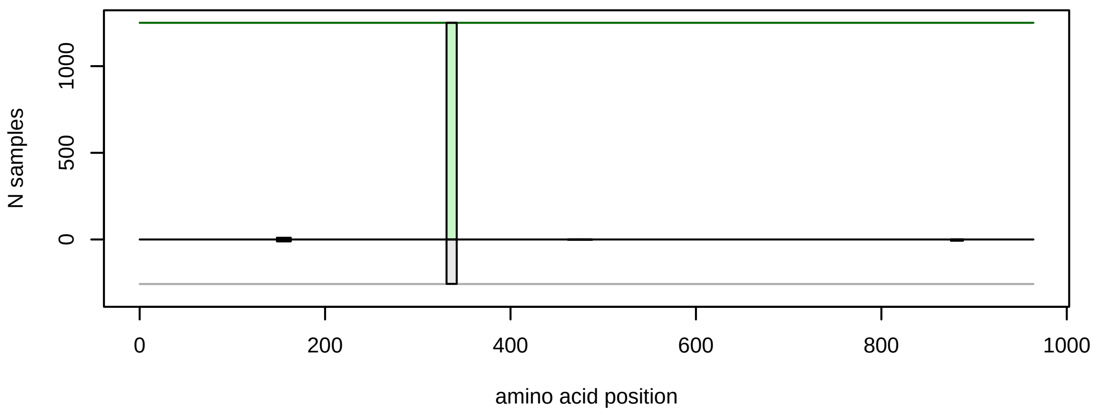

20. FUCA1 (P04066), NP5

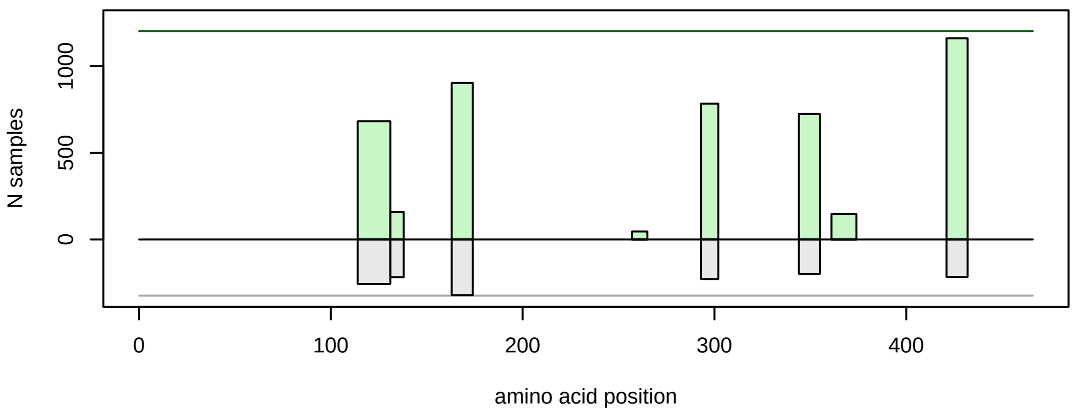

21. CTSH (A0A087X0D5), NP3

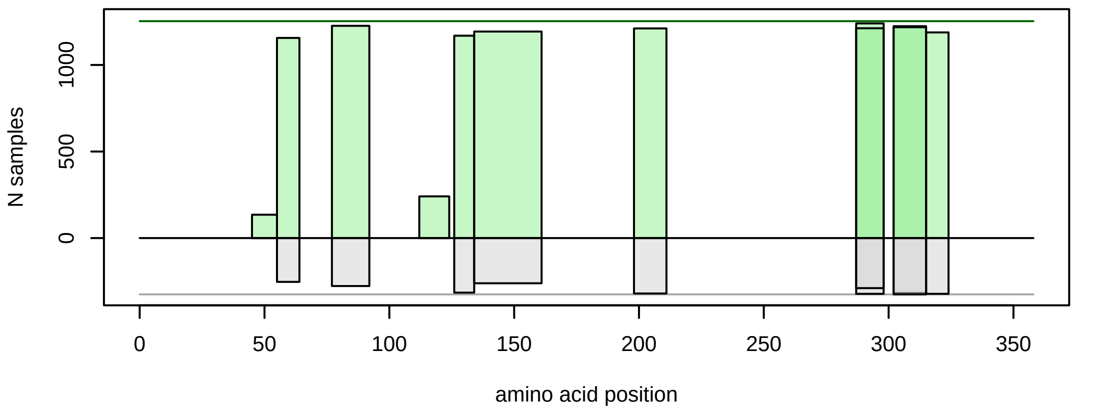

22. HLA−B (P01889), NP2

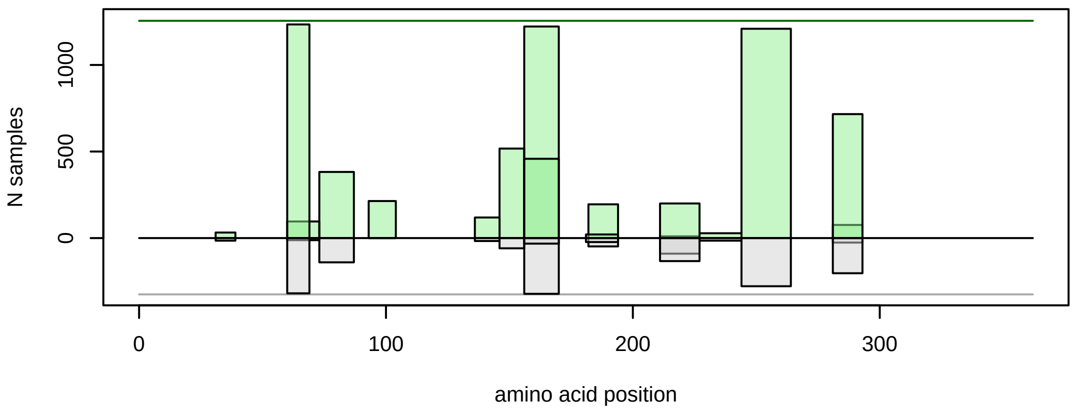

23. PEBP4 (Q96S96), NP3

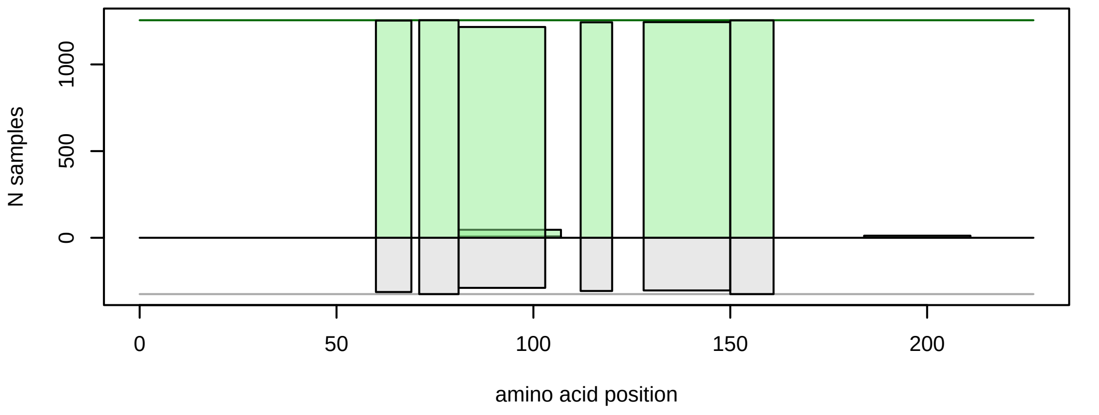

24. CFHR1 (B1AKG0), NP1

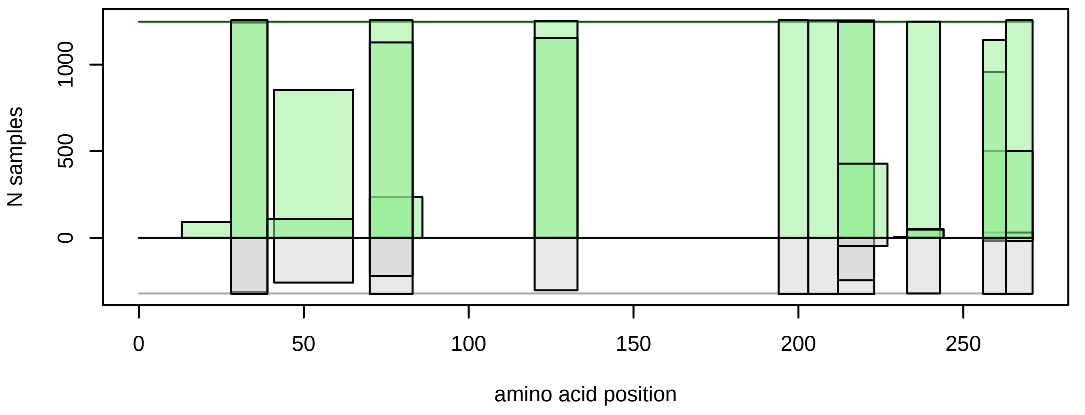

25. CCDC7 (Q96M83), NP4

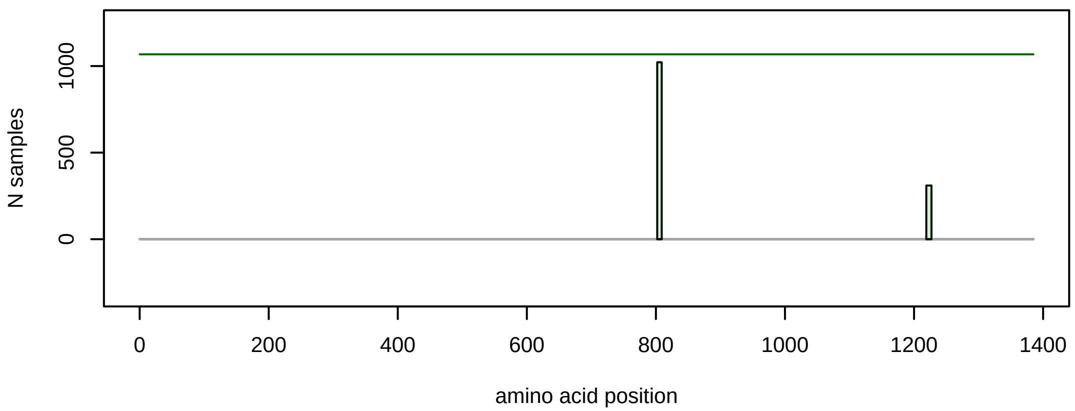

26. CFB (B4E1Z4), NP4

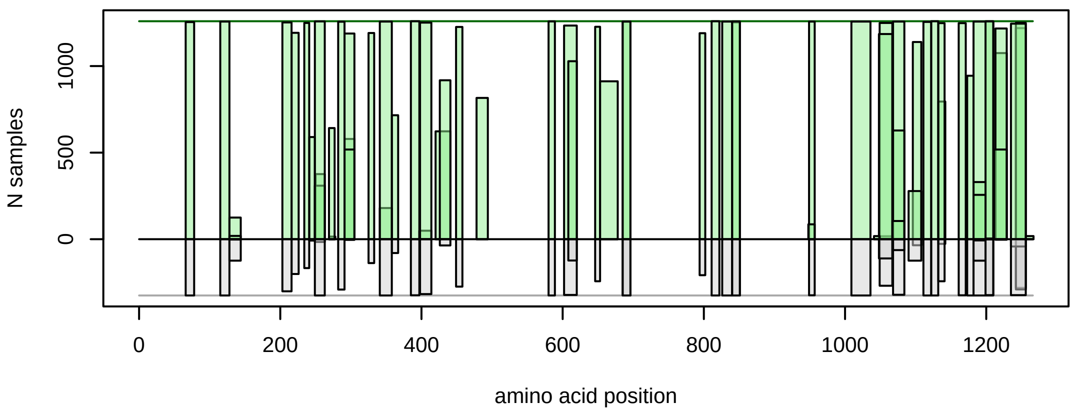

27. EBI3 (Q14213), NP4

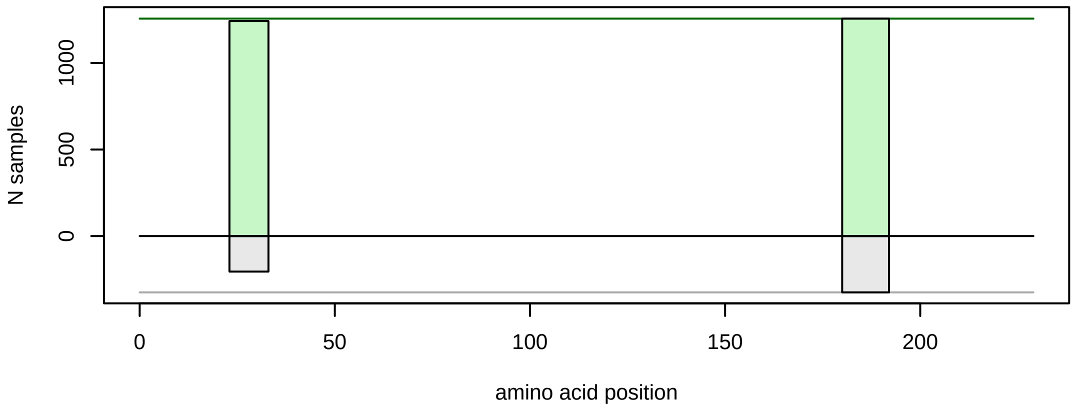

28. ERAP1 (Q9NZ08;Q9NZ08−2), NP5

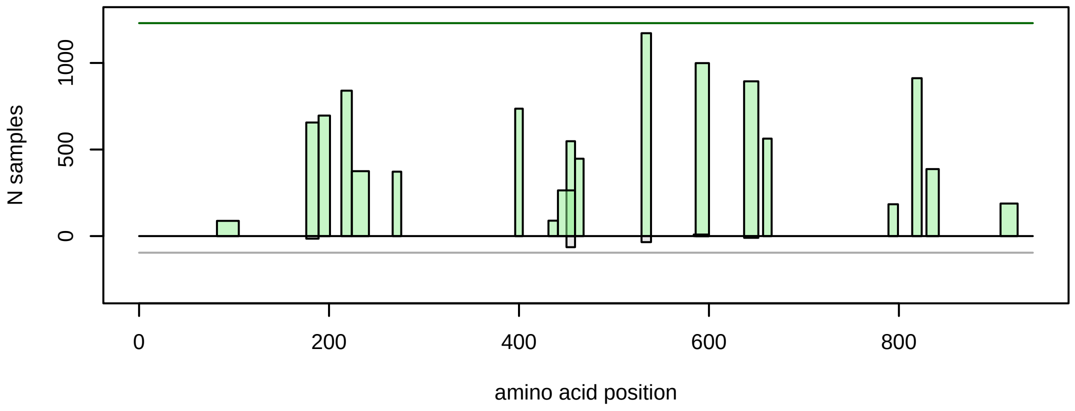

29. HLA−A (A0A1W2PS24;P04439;Q5SRN5), NP1

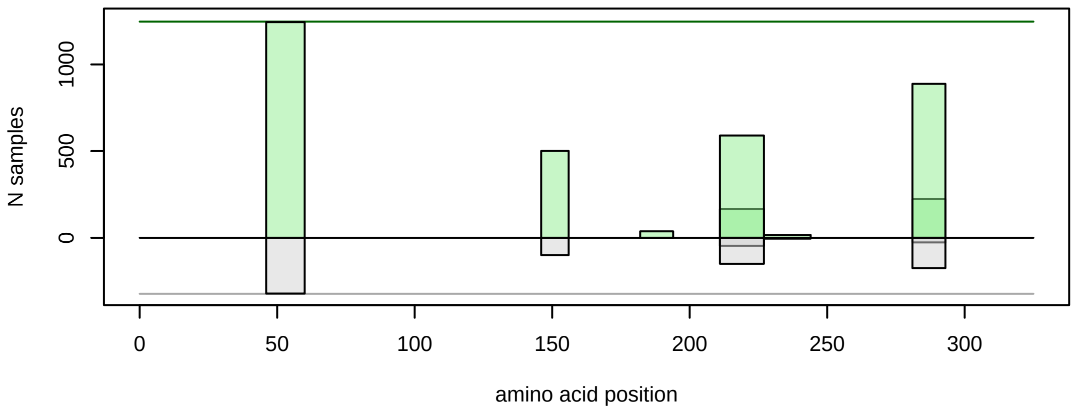

30. PGLYRP2 (Q96PD5−2), NP4

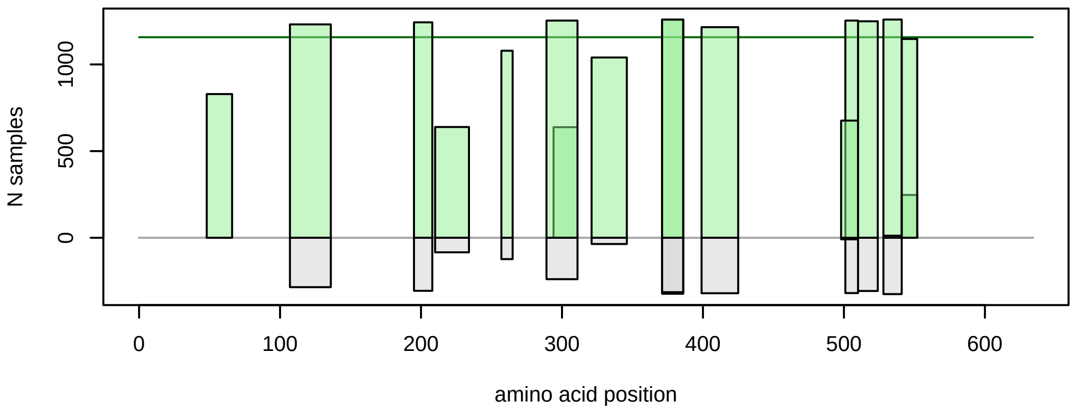

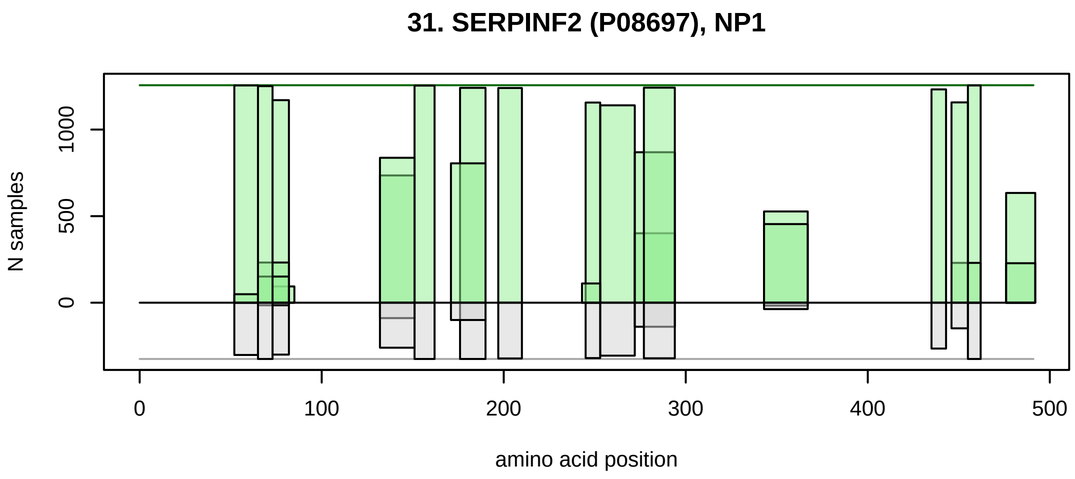
31. SERPINF2 (P08697), NP1
N samples
0
500
1000
0
100
200
300
400
500
amino acid position

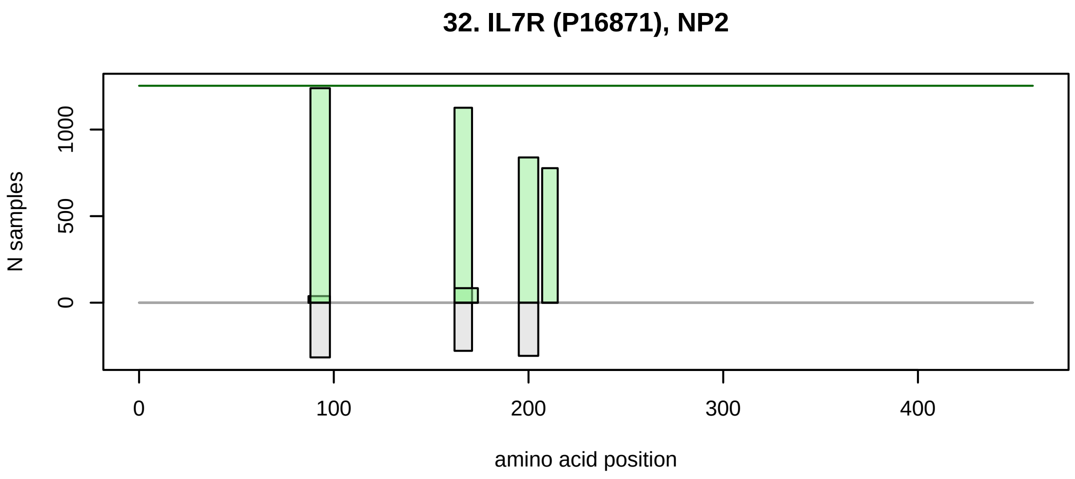
32. IL7R (P16871), NP2
N samples
0
500
1000
0
100
200
300
400
amino acid position

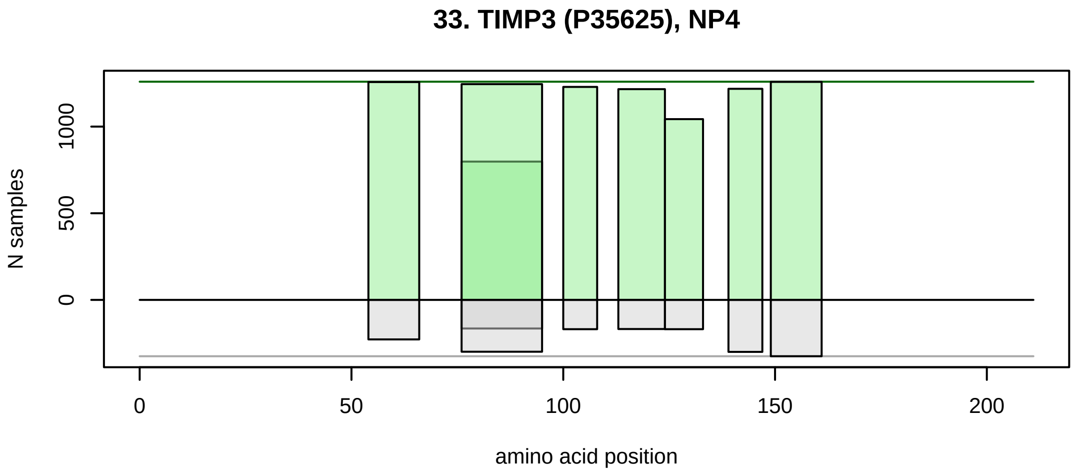
33. TIMP3 (P35625), NP4
N samples
0
500
1000
0
50
100
150
200
amino acid position

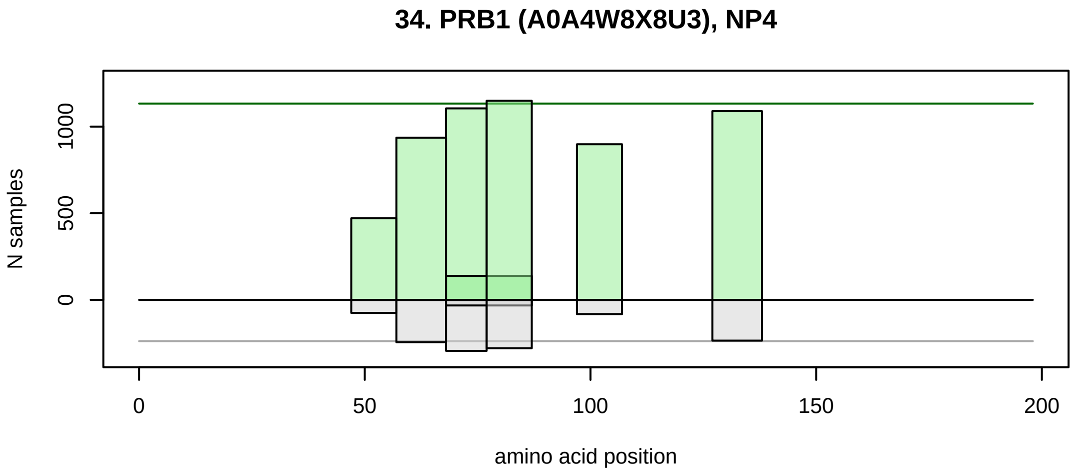
34. PRB1 (A0A4W8X8U3), NP4
N samples
0
500
1000
0
50
100
150
200
amino acid position

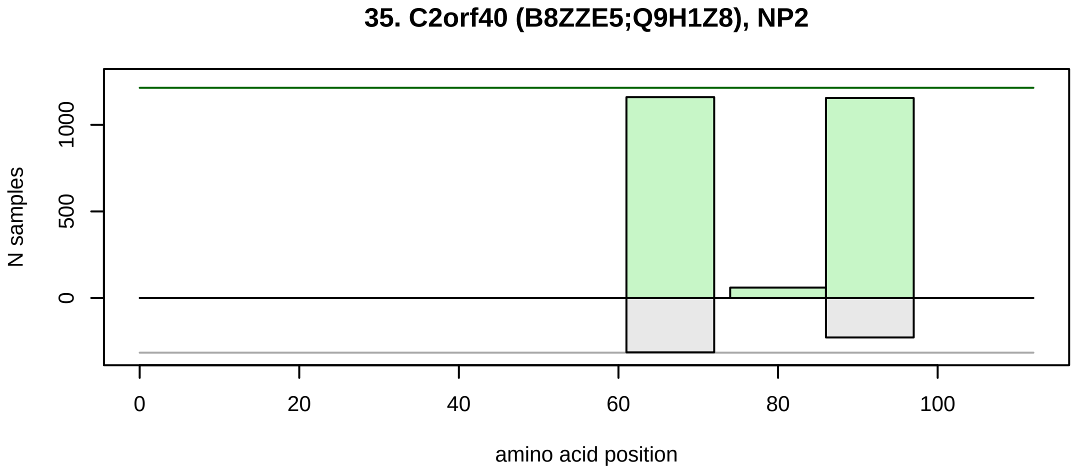
35. C2orf40 (B8ZZE5;Q9H1Z8), NP2
N samples
0
500
1000
0
20
40
60
80
100
amino acid position

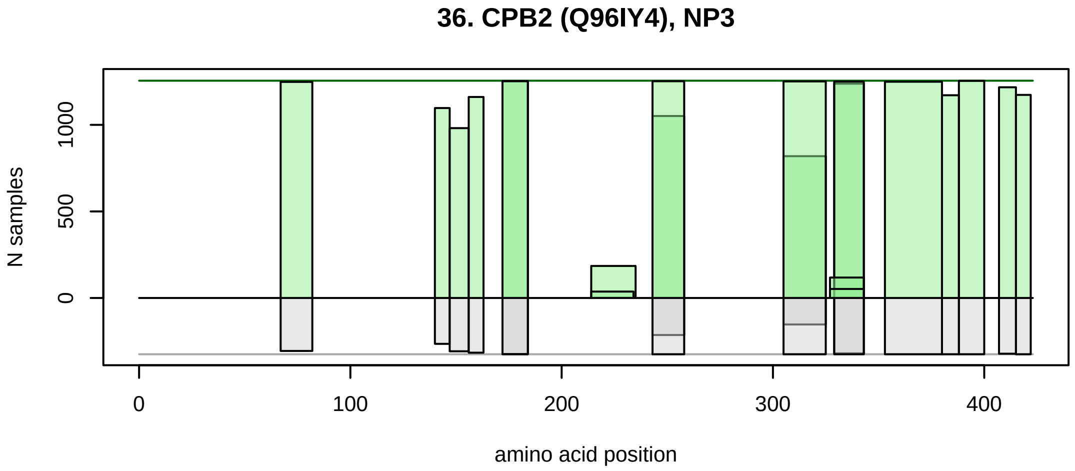
36. CPB2 (Q96IY4), NP3
N samples
0
500
1000
0
100
200
300
400
amino acid position

37. LEFTY1 (O75610), NP1

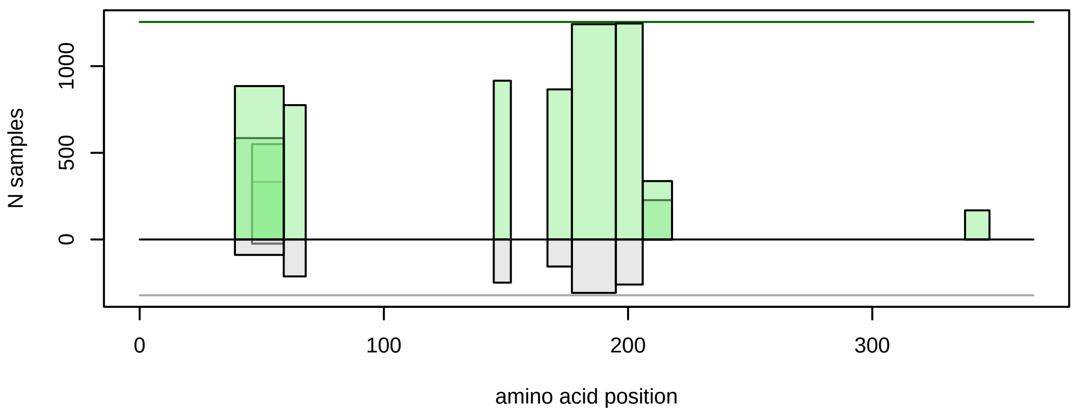

38. RCN3 (Q96D15), NP2

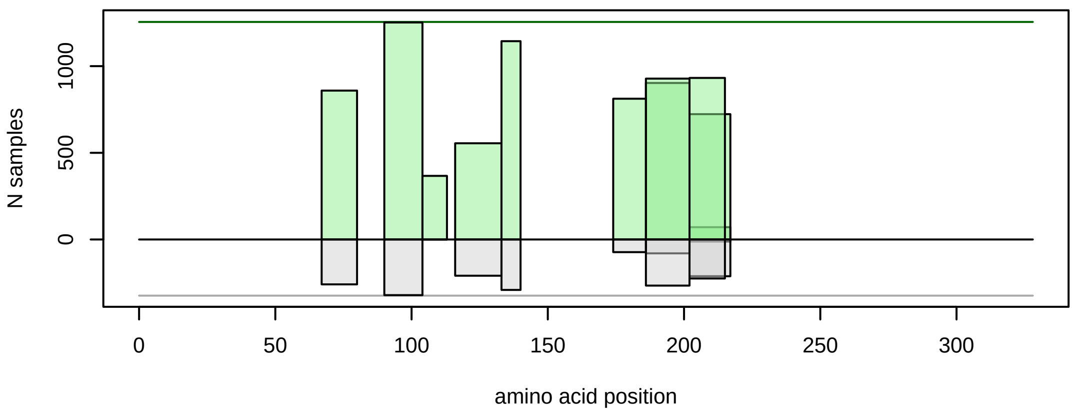

39. C1QTNF9 (P0C862), NP4

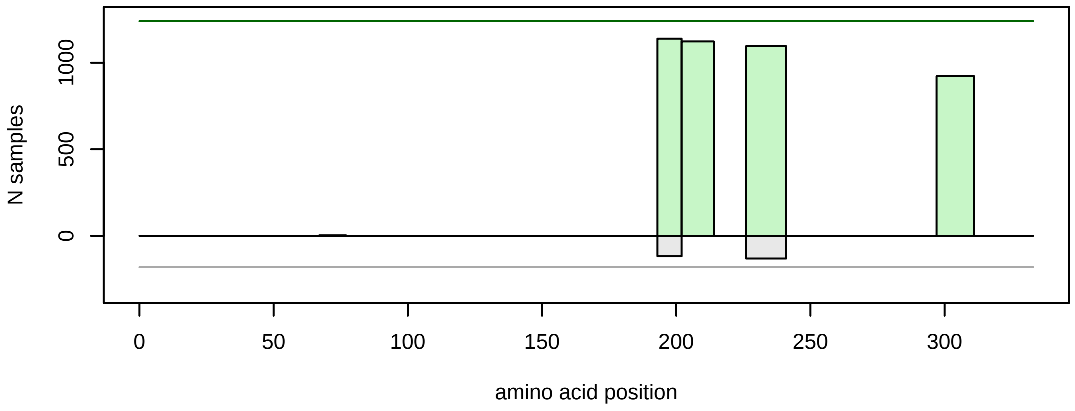

40. ITIH1 (P19827), NP2

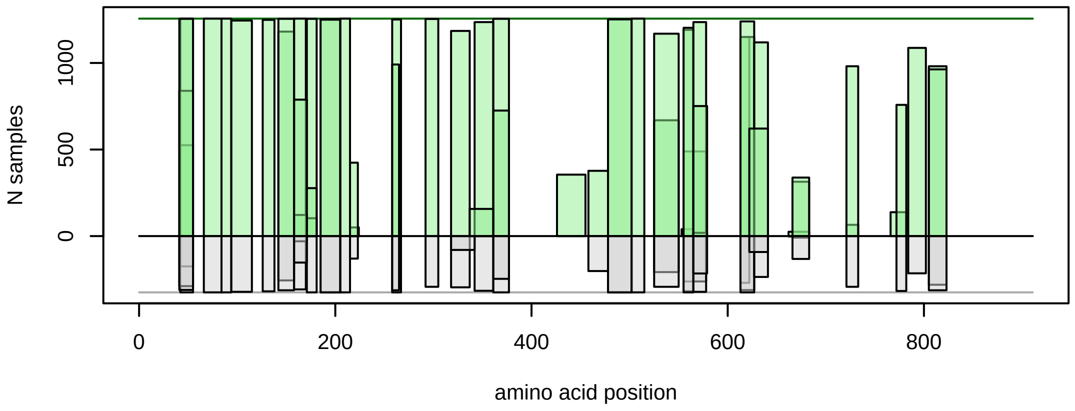

41. PGLYRP2 (Q96PD5), NP4

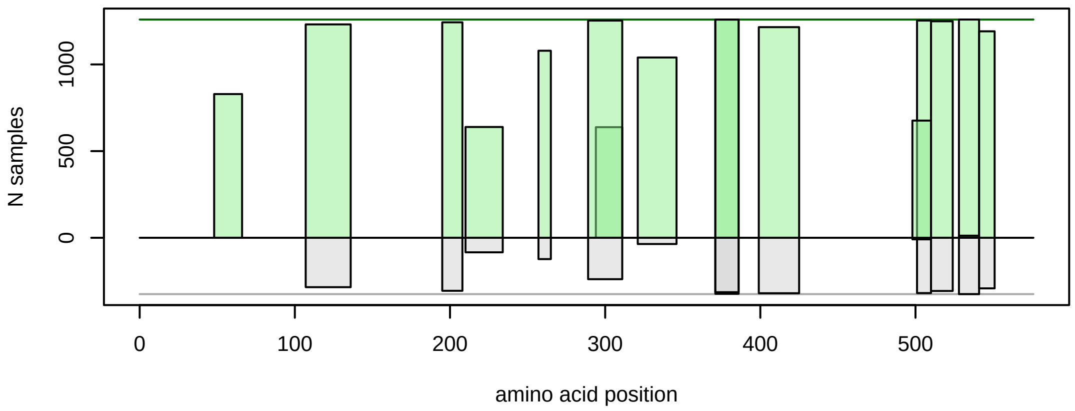

42. RNASE6 (Q93091), NP3

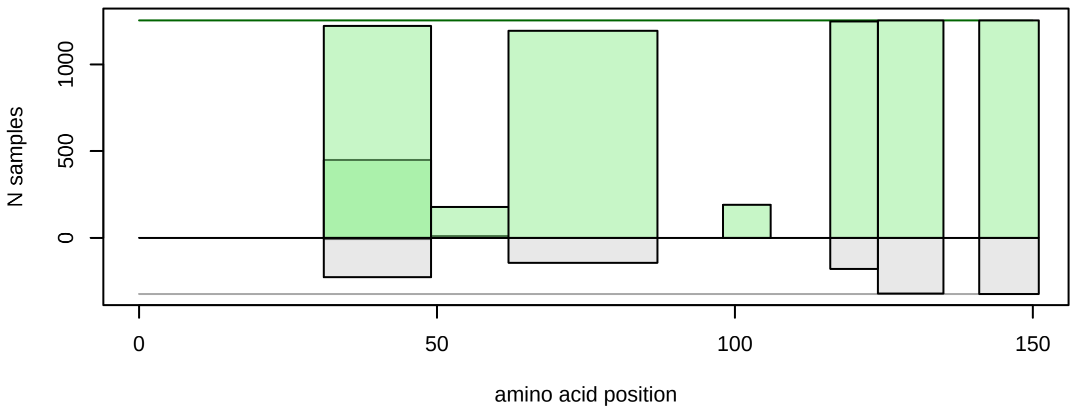

43. SPON2 (Q9BUD6), NP5

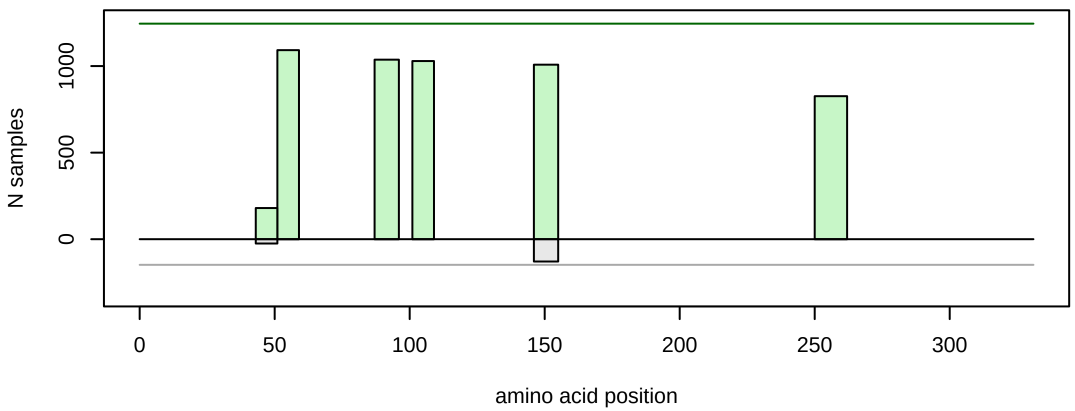

44. IGHV2−70 (P01814), NP3

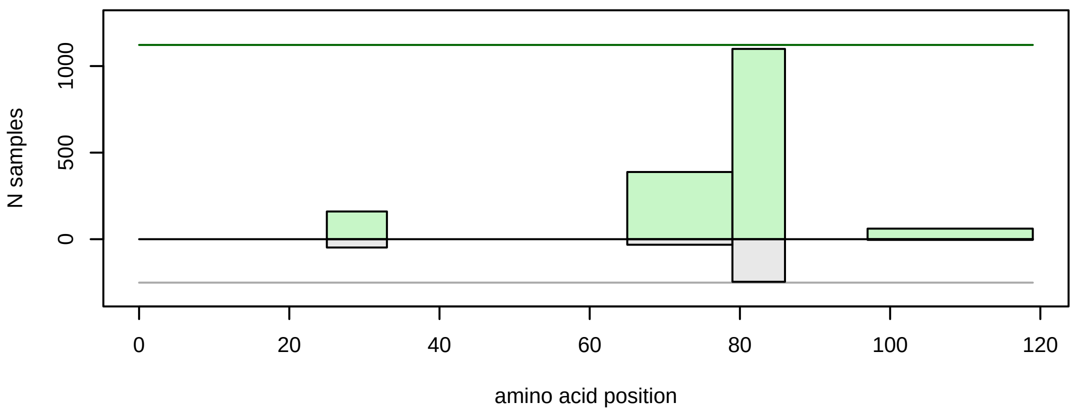

45. FUCA2 (Q9BTY2), NP2

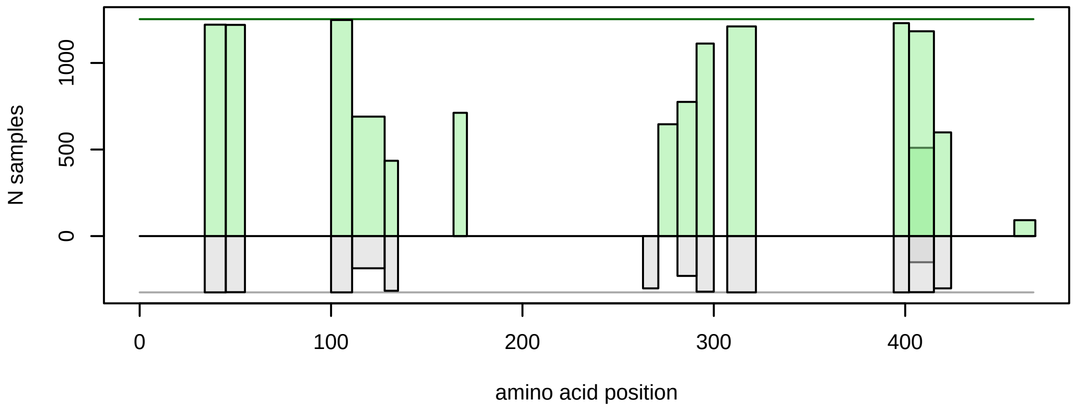

46. SRL (I3L4D6), NP2

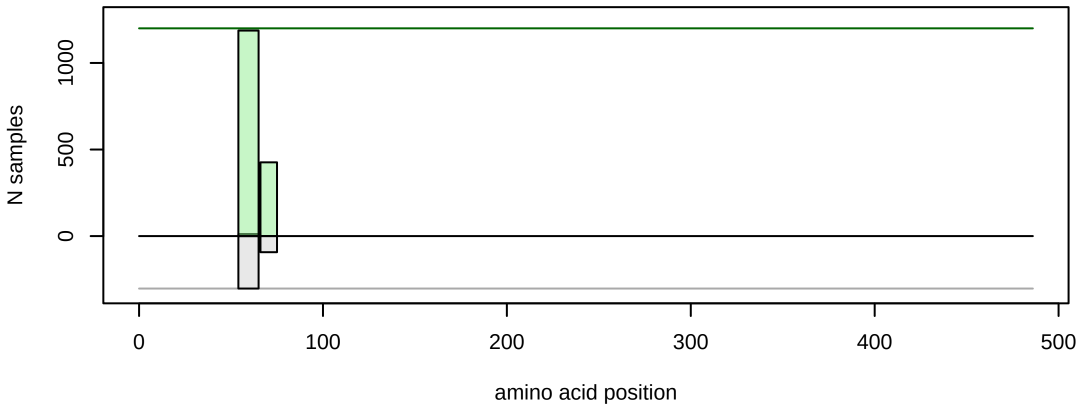

47. HLA−C (A2AEA2;P10321), NP1

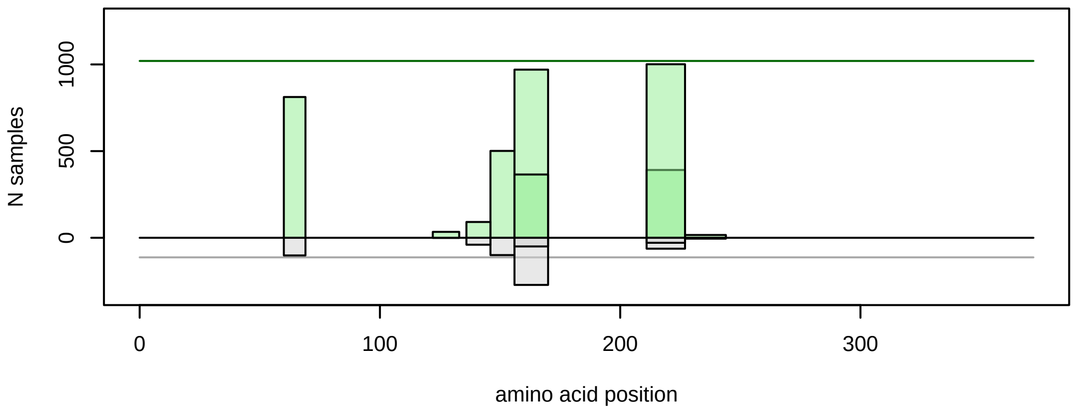

48. FGL1 (Q08830), NP5

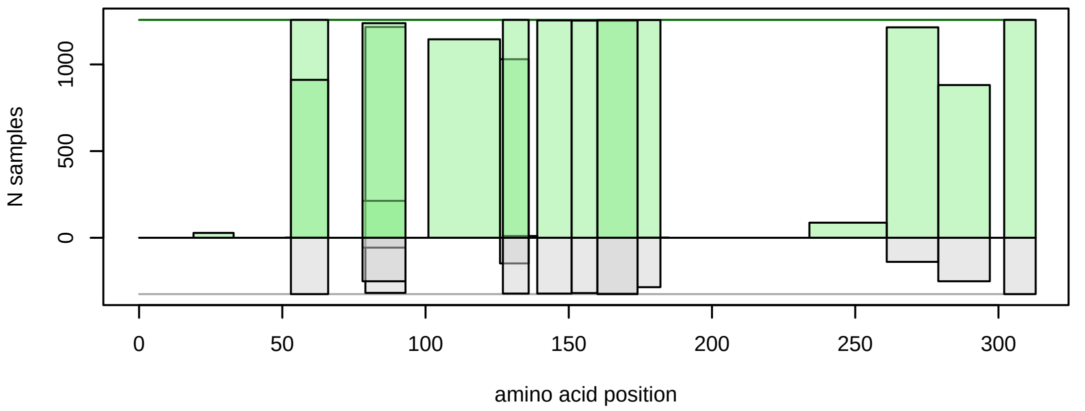

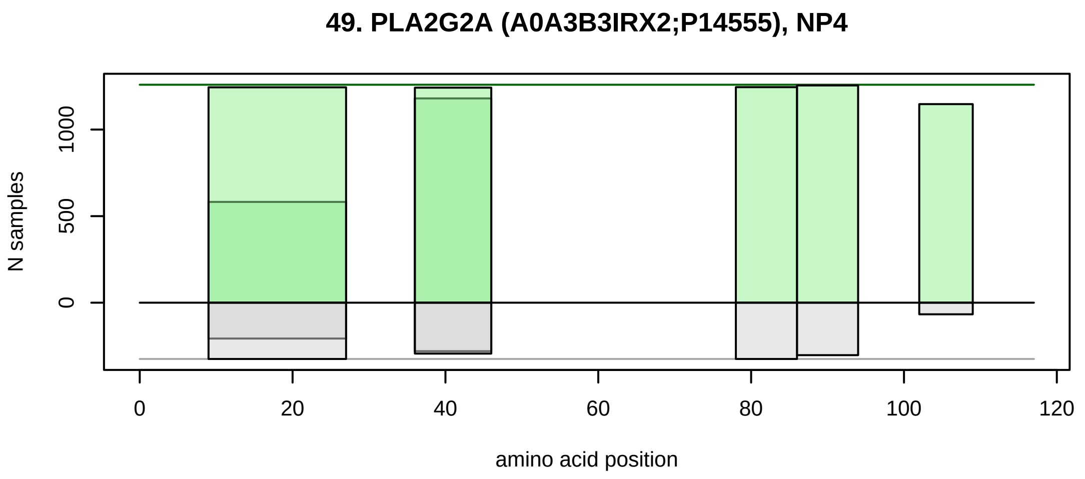
49. PLA2G2A (A0A3B3IRX2;P14555), NP4
N samples
0
500
1000
0
20
40
60
80
100
120
amino acid position

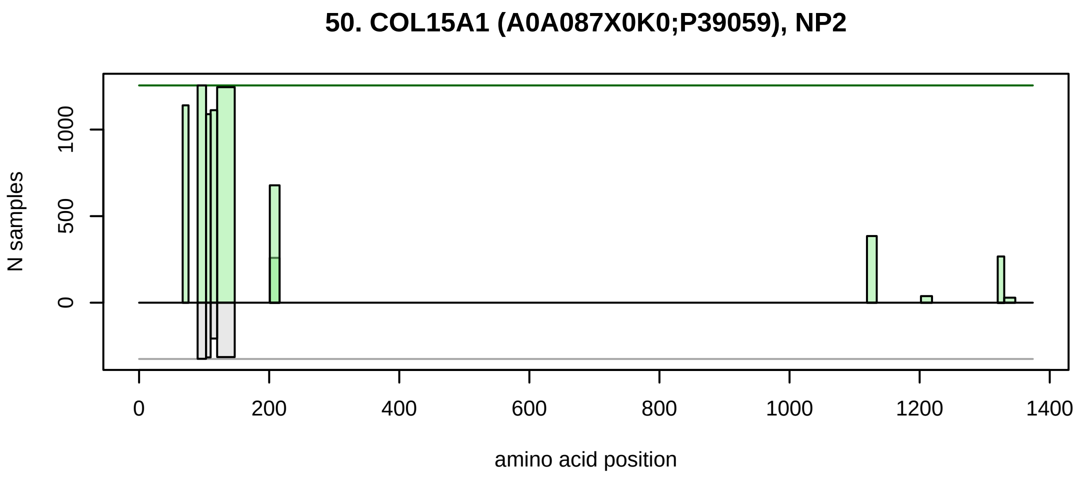
50. COL15A1 (A0A087X0K0;P39059), NP2
N samples
0
500
1000
0
200
400
600
800
1000
1200
1400
amino acid position

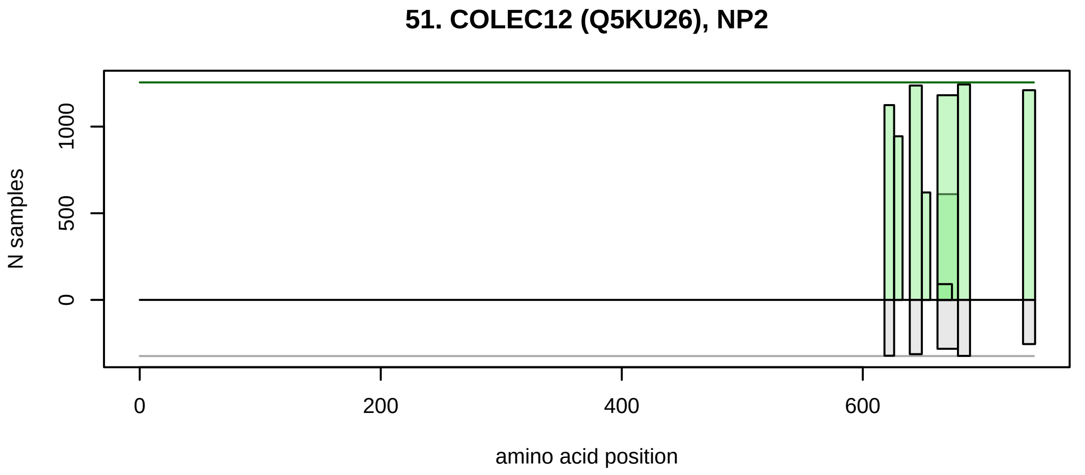
51. COLEC12 (Q5KU26), NP2
N samples
0
500
1000
0
200
400
600
amino acid position

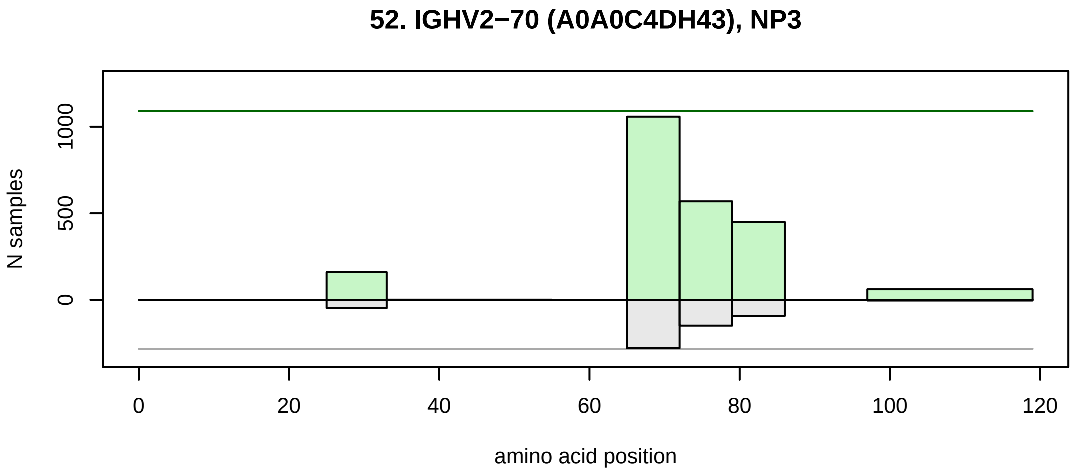
52. IGHV2−70 (A0A0C4DH43), NP3
N samples
0
500
1000
0
20
40
60
80
100
120
amino acid position

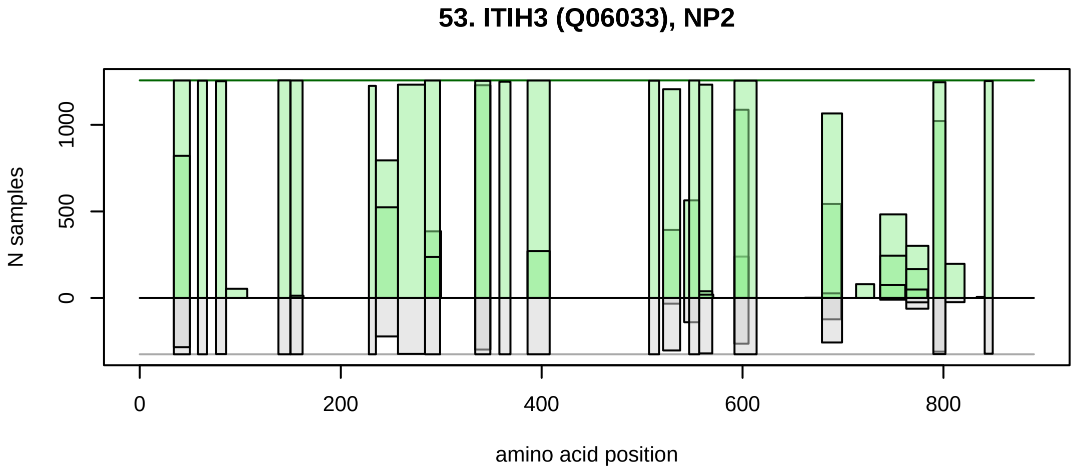
53. ITIH3 (Q06033), NP2
N samples
0
500
1000
0
200
400
600
800
amino acid position

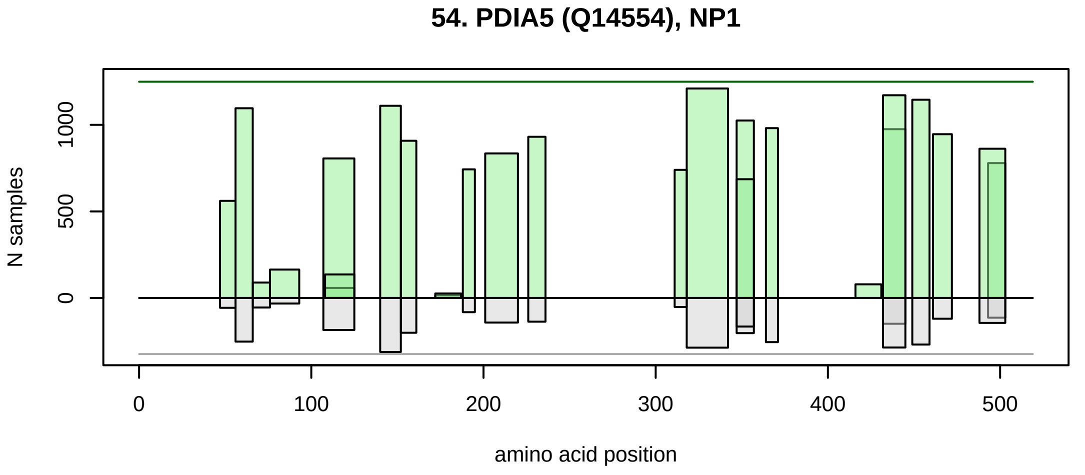
54. PDIA5 (Q14554), NP1
N samples
0
500
1000
0
100
200
300
400
500
amino acid position

55. SERPINE2 (P07093), NP4

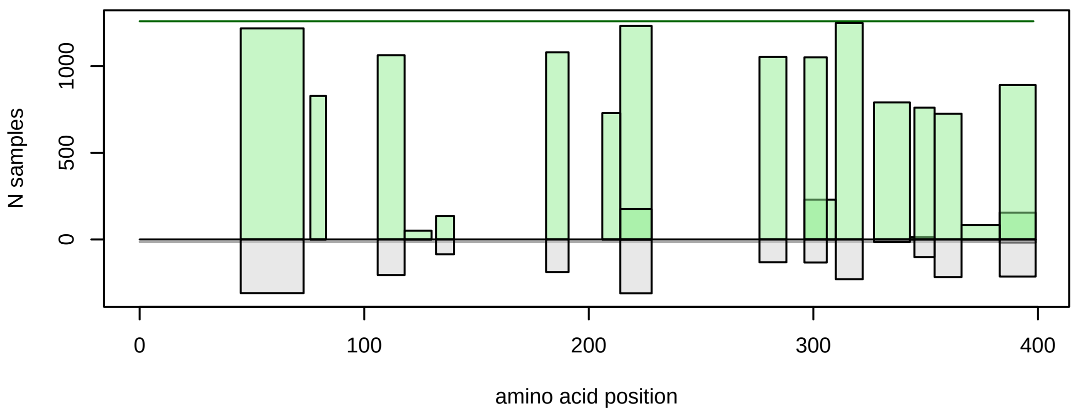

56. TCN2 (F8WE86), NP1

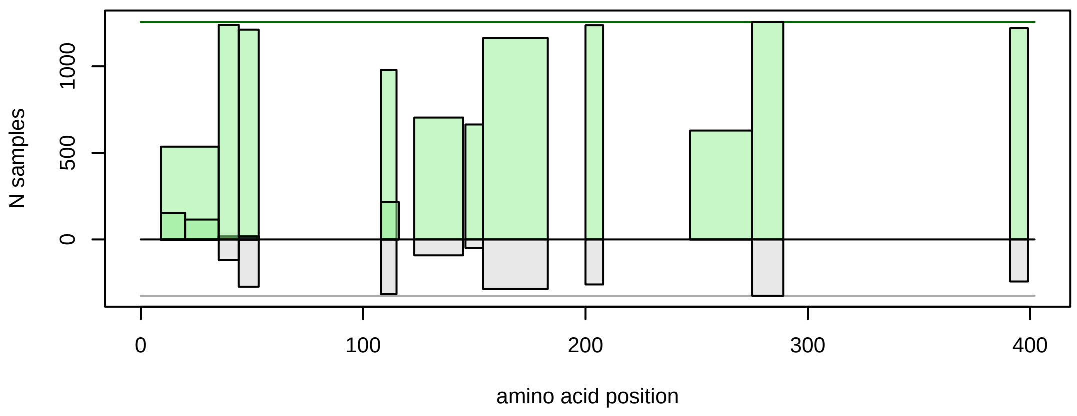

57. CCL23 (P55773), NP3

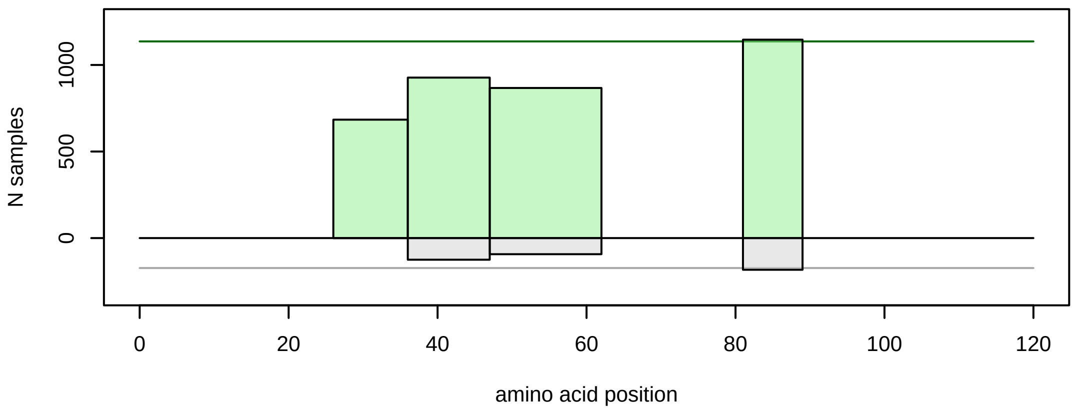

58. CCL16 (O15467), NP3

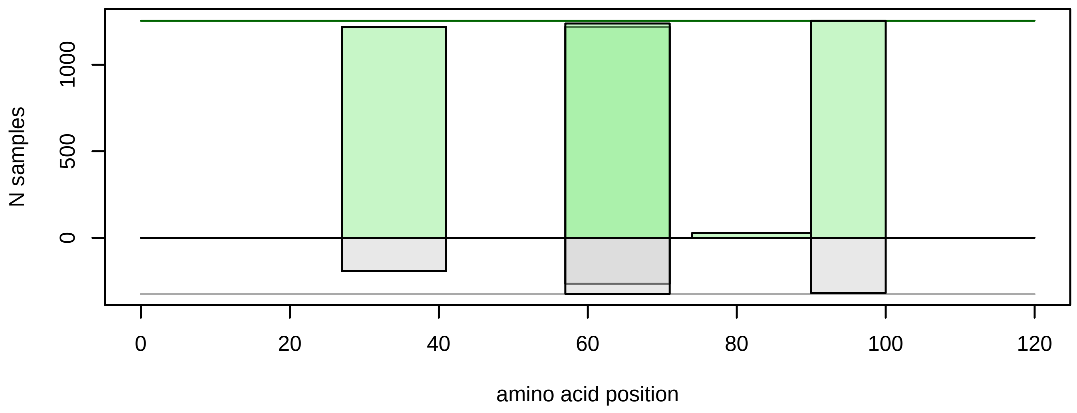

59. SERPINA10 (G3V2W1), NP2

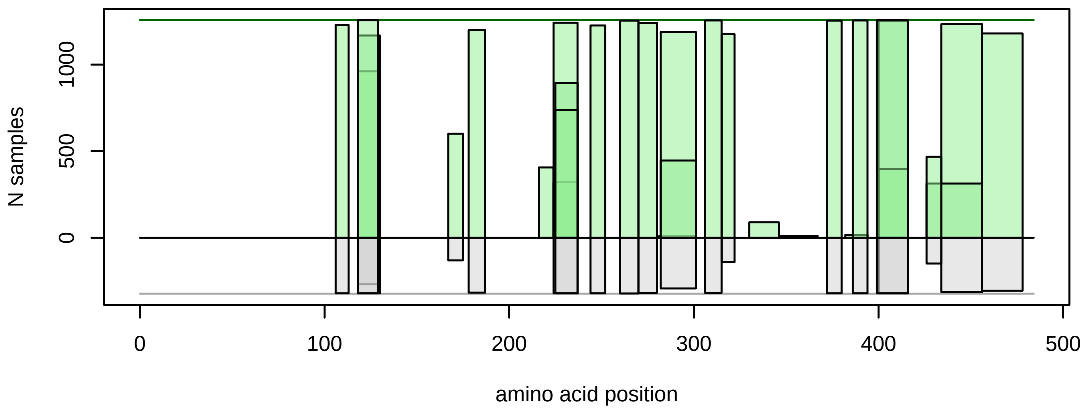

60. FXYD2 (P54710), NP2

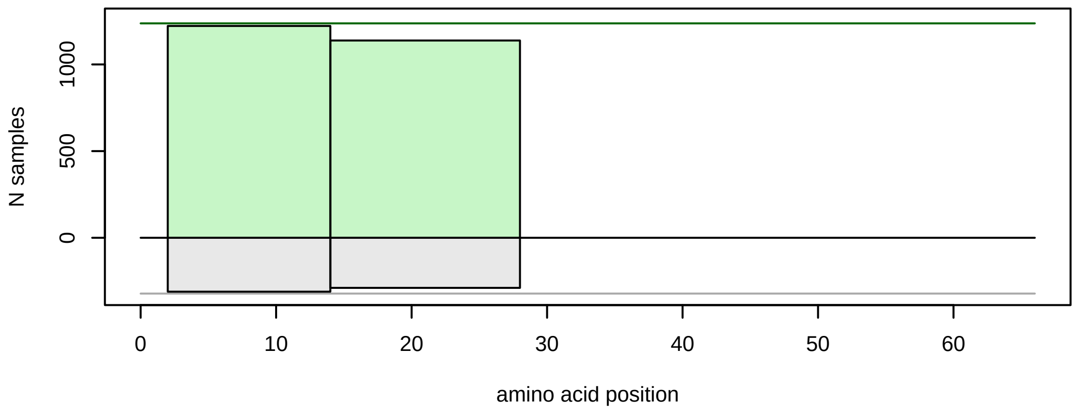

61. BPIFA1 (Q9NP55;Q9NP55−2), NP1

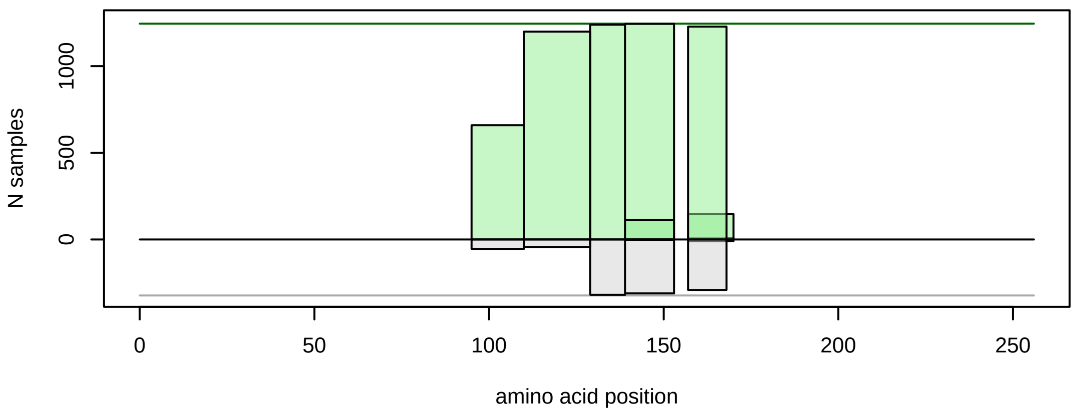

62. ZNF618 (Q5T7W0), NP1

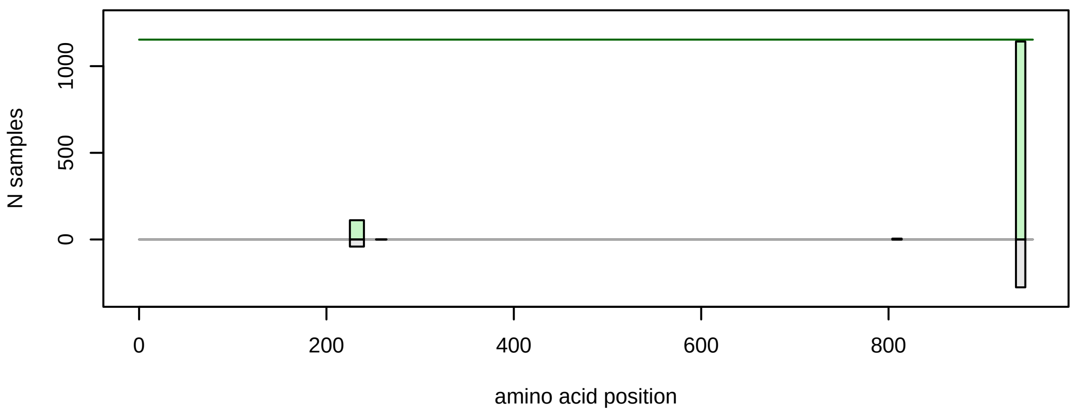

63. SERPINE2 (P07093−2;P07093−3), NP3

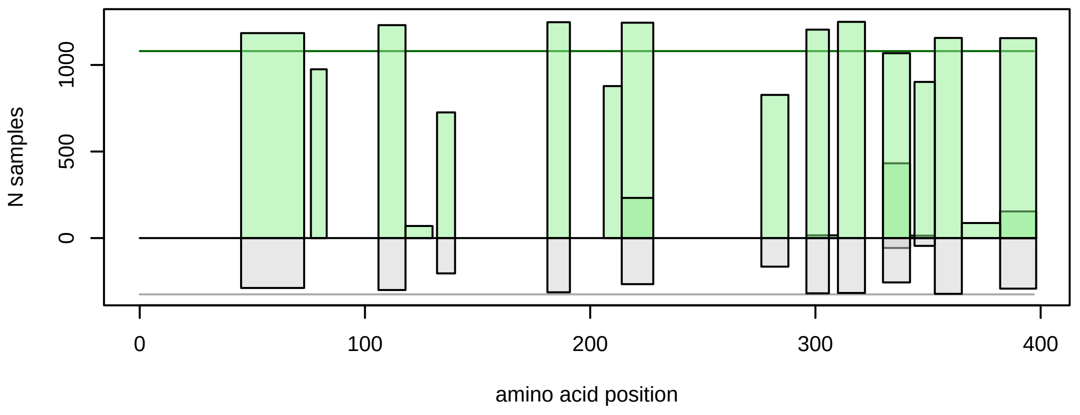

64. MSN (P26038), NP1

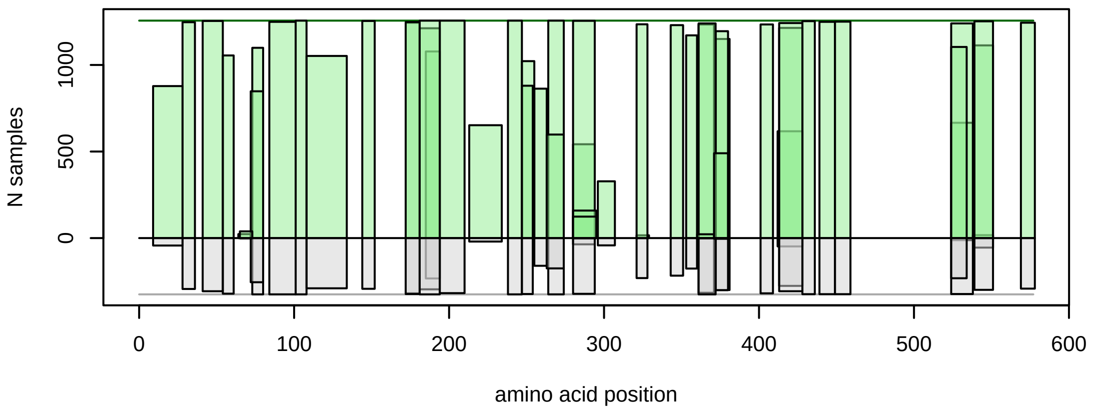

65. IGLV1−51 (P01701), NP3

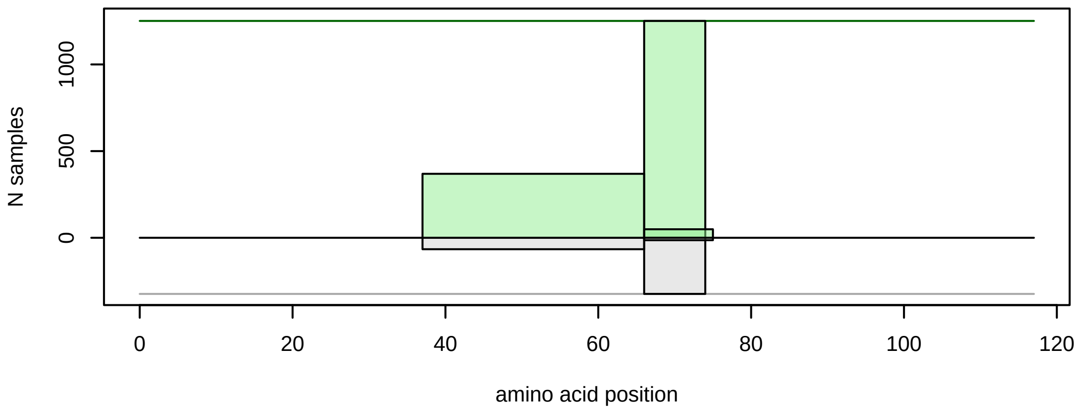

66. ANXA5 (P08758), NP1

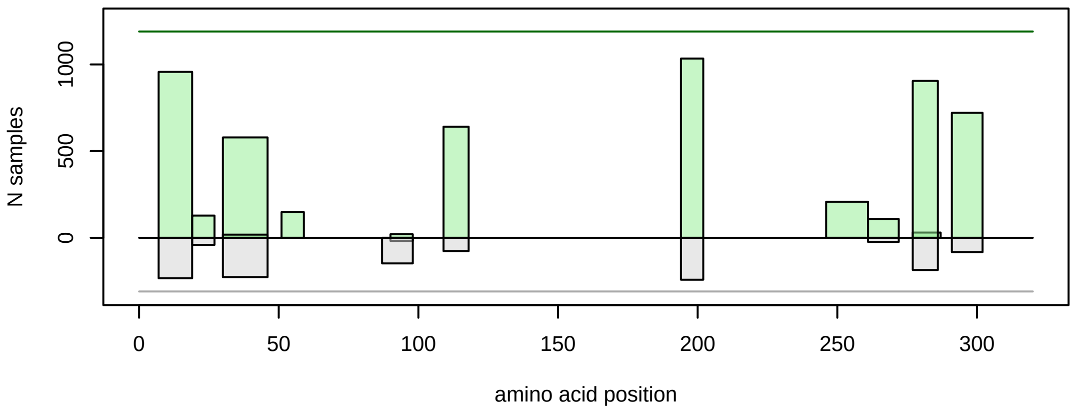

67. AKR7A2 (O43488), NP5

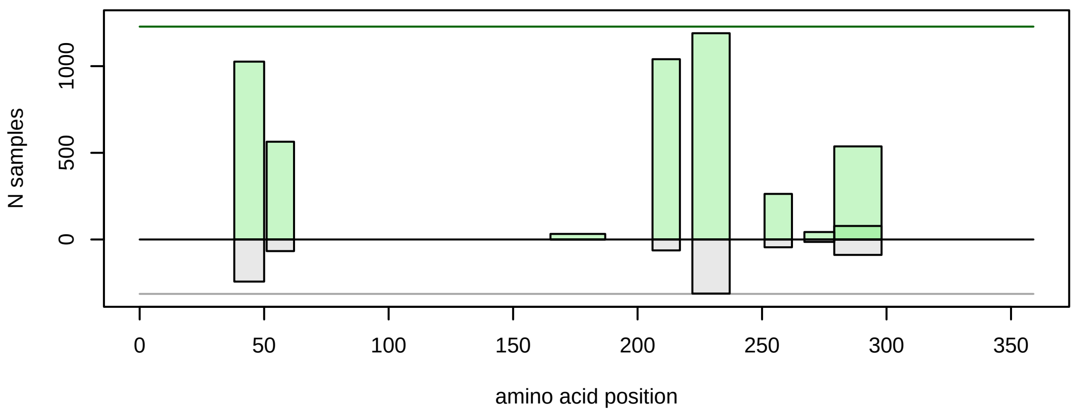

68. POSTN (Q15063−2), NP3

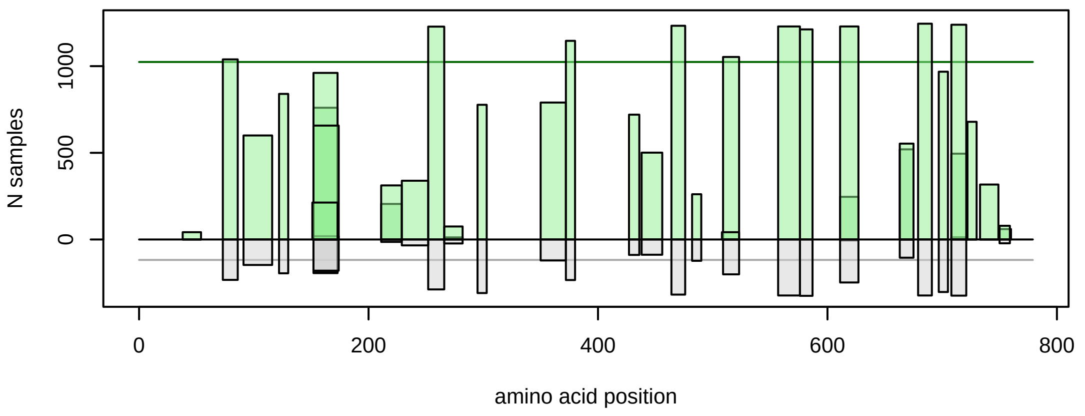

69. HSPB1 (A0A6Q8PFK8;P04792), NP4

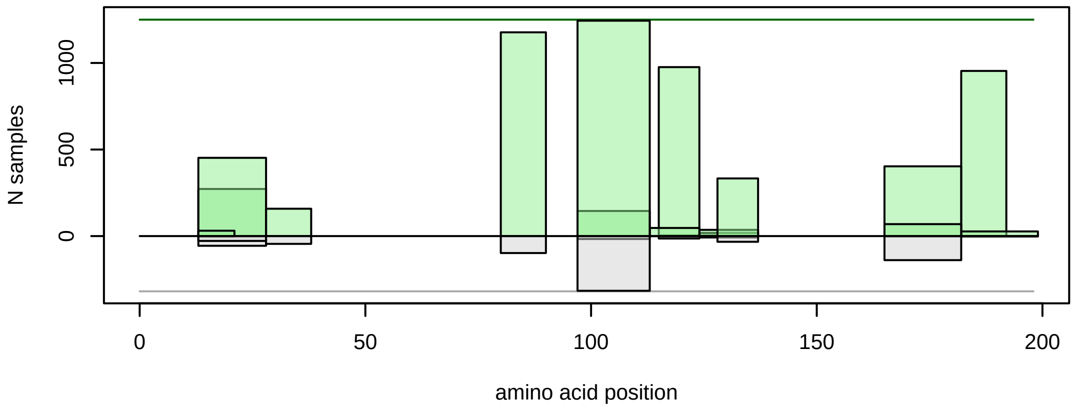

70. ITIH2 (P19823), NP2

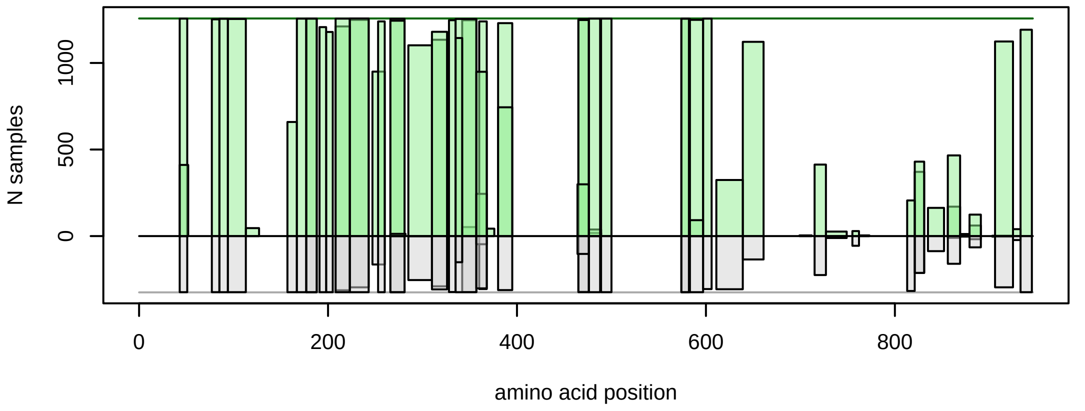

71. PROZ (P22891;P22891−2), NP2

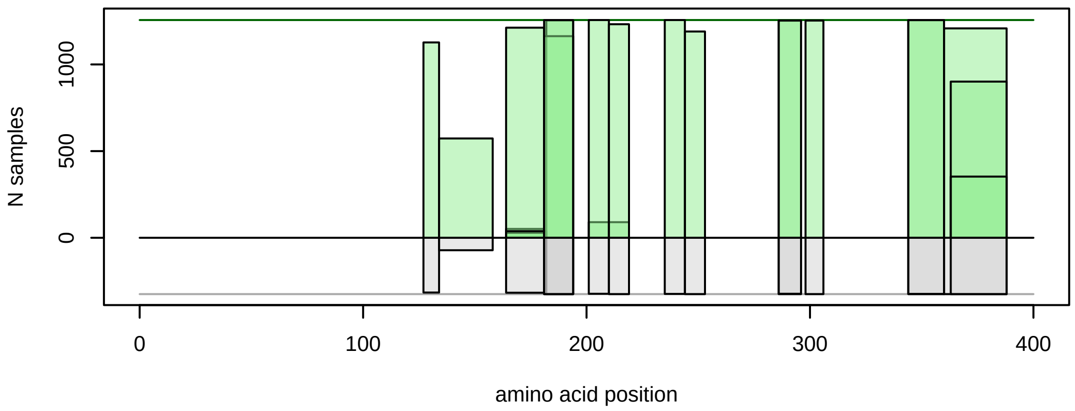

72. CSTF3 (Q12996), NP4

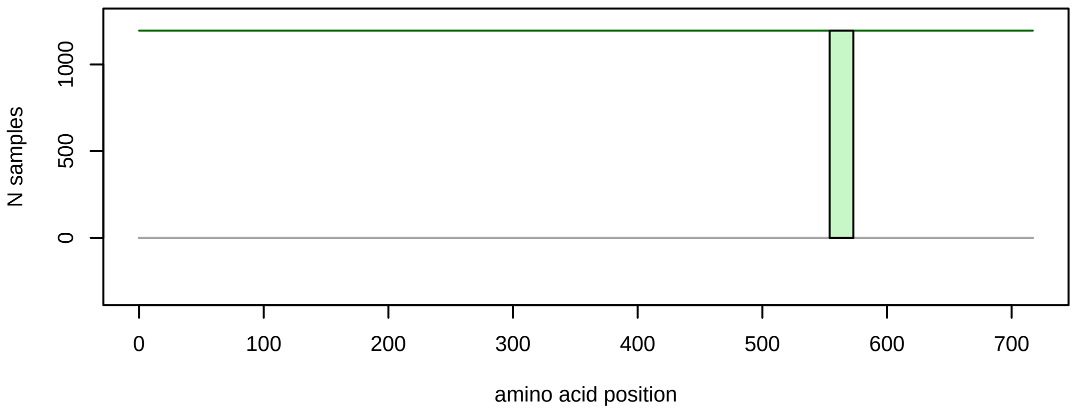

73. SERPING1 (P05155;P05155−3), NP1

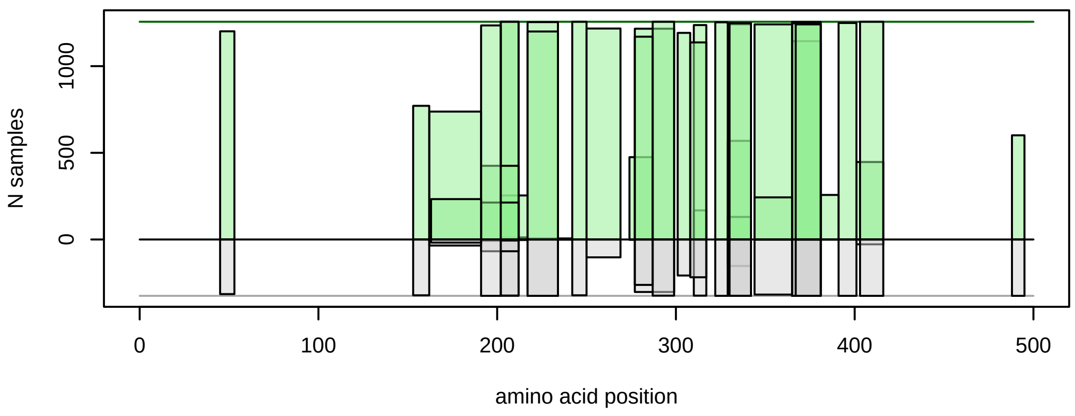

74. S100B (P04271), NP2

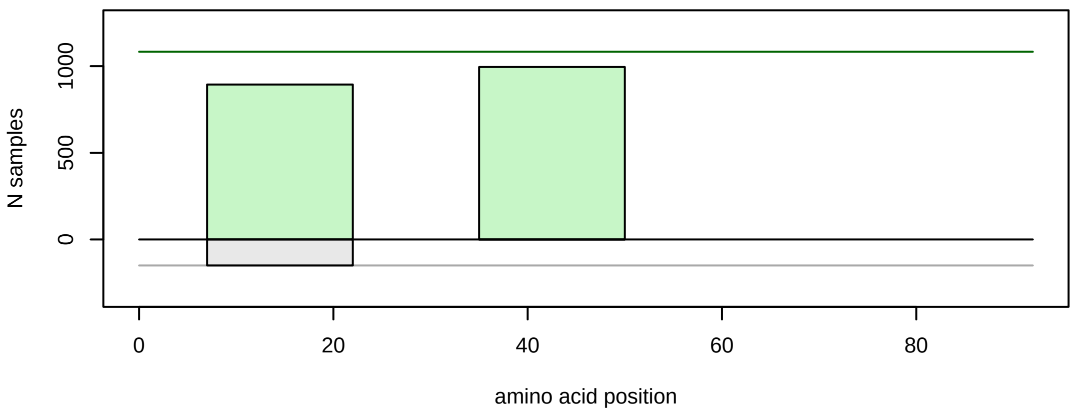

75. LPA (P08519), NP4

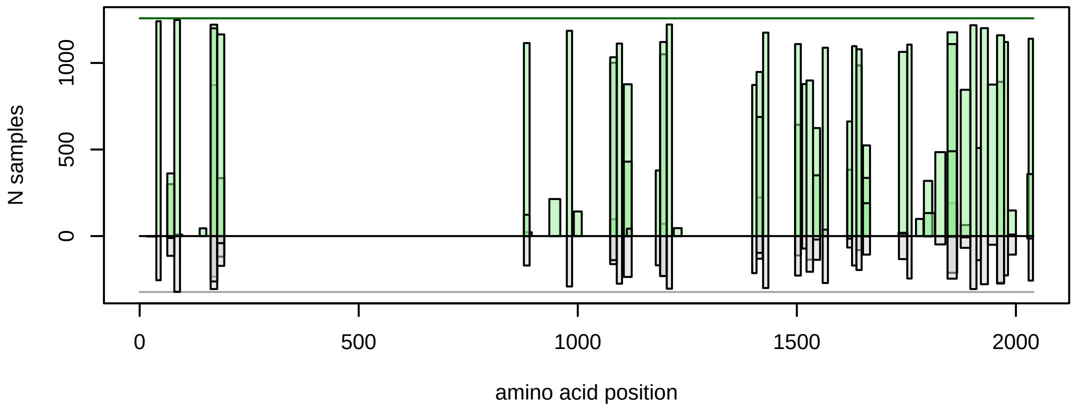

76. OAF (Q86UD1), NP5

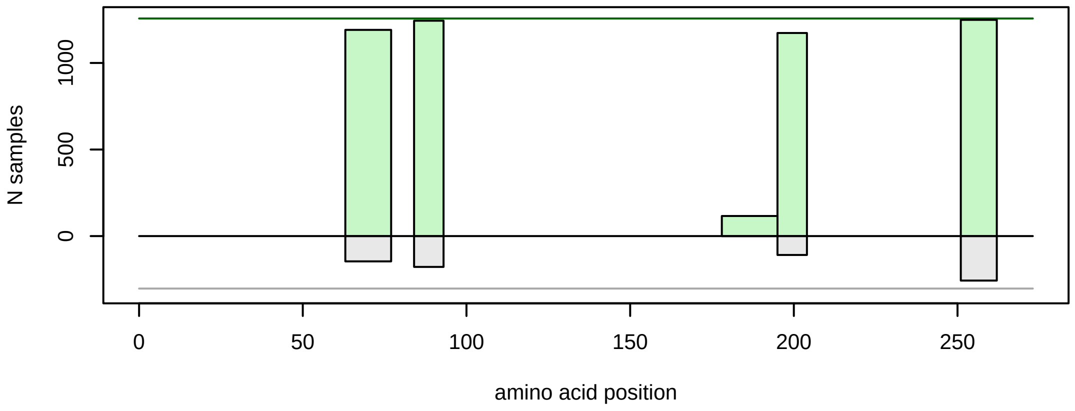

77. PLEC (Q15149−4), NP5

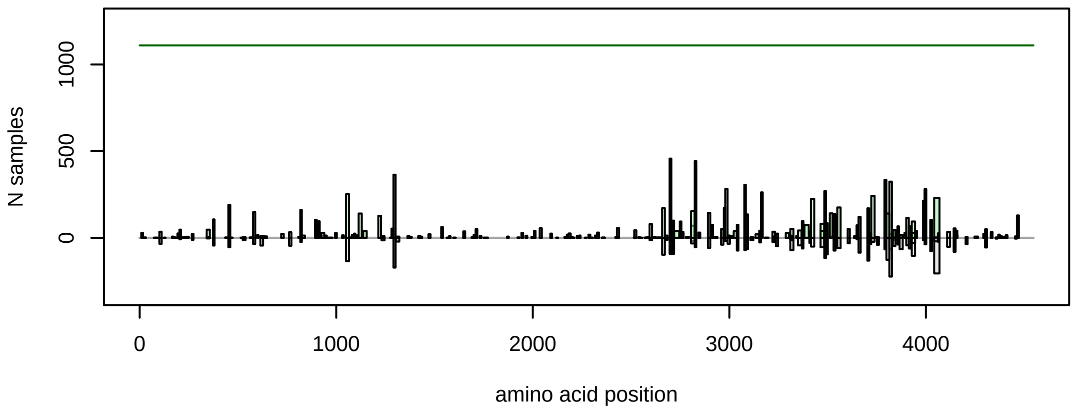

78. PON1 (P27169), NP5

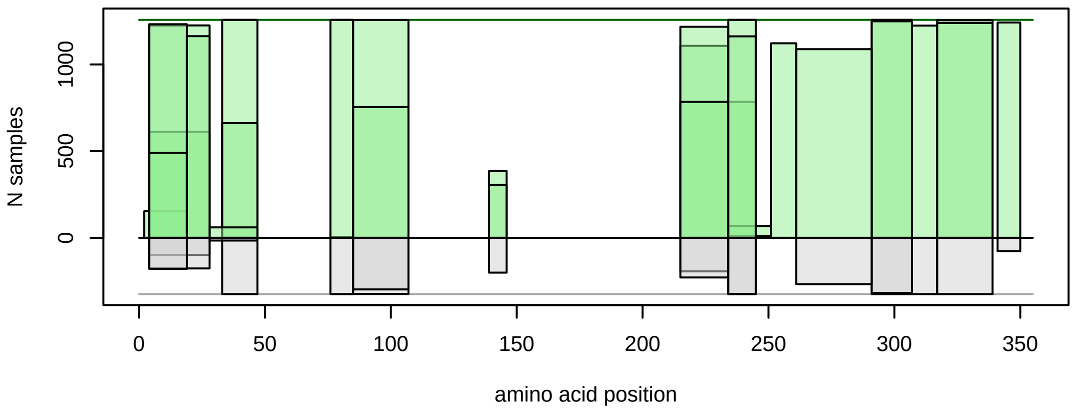

79. HEATR6 (Q6AI08), NP4

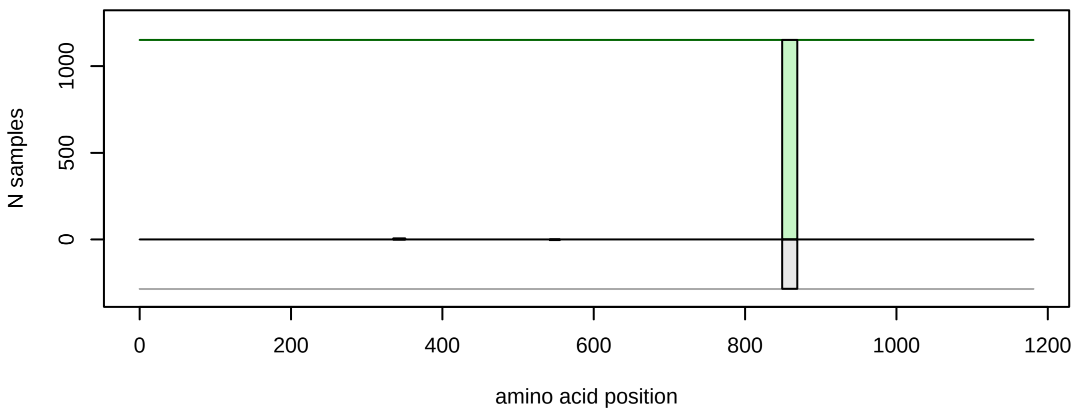

80. DYNC1H1 (Q14204), NP4

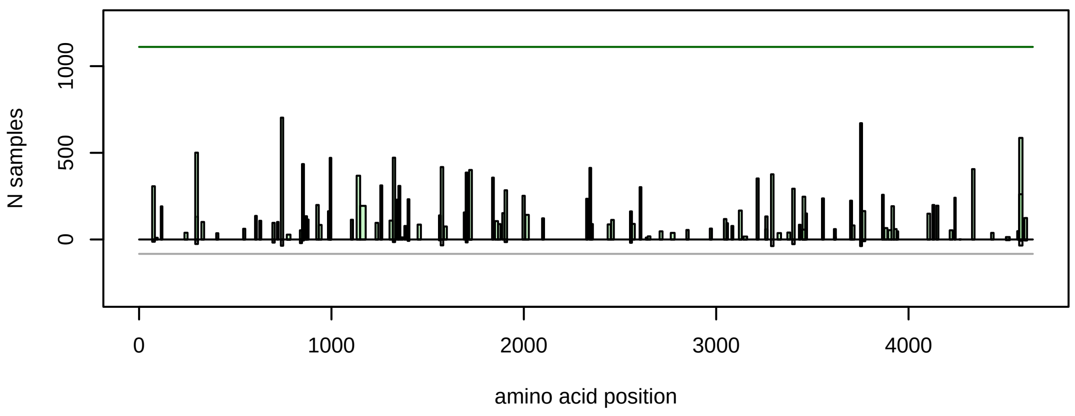

81. C6 (P13671), NP3

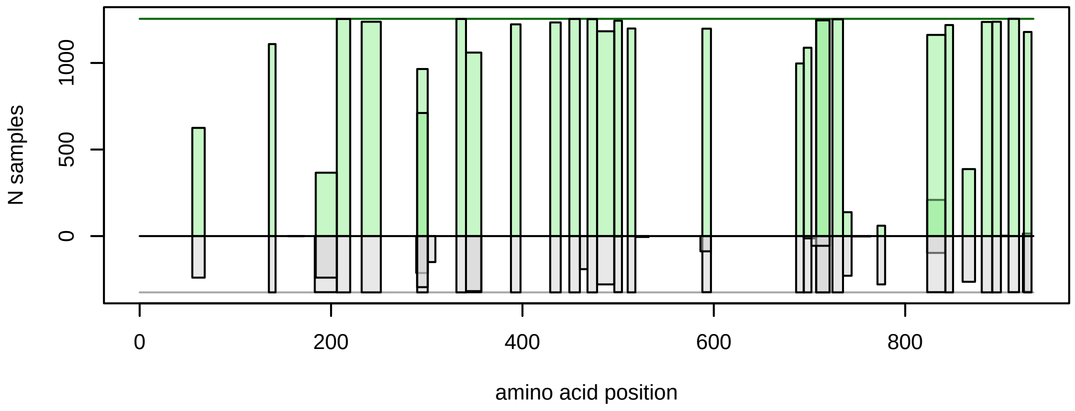

82. LBP (P18428), NP4

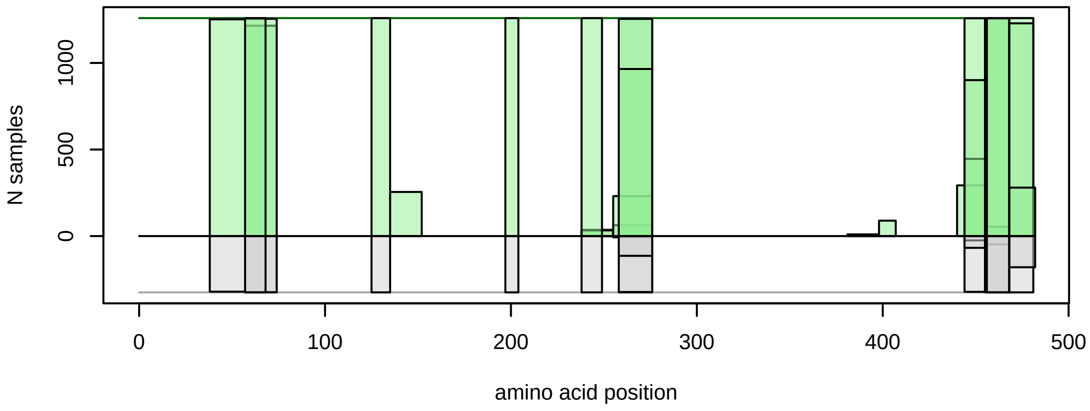

83. FBXO7 (Q9Y3I1), NP1

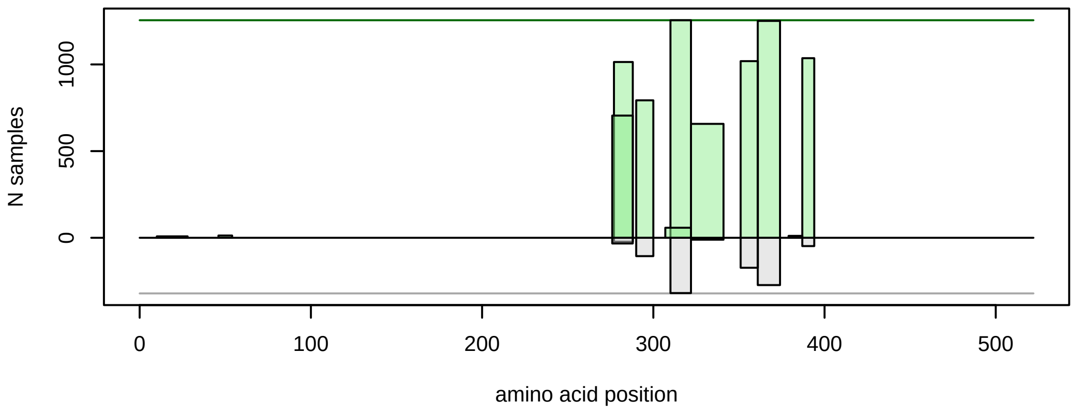

84. MASP2 (O00187−2), NP2

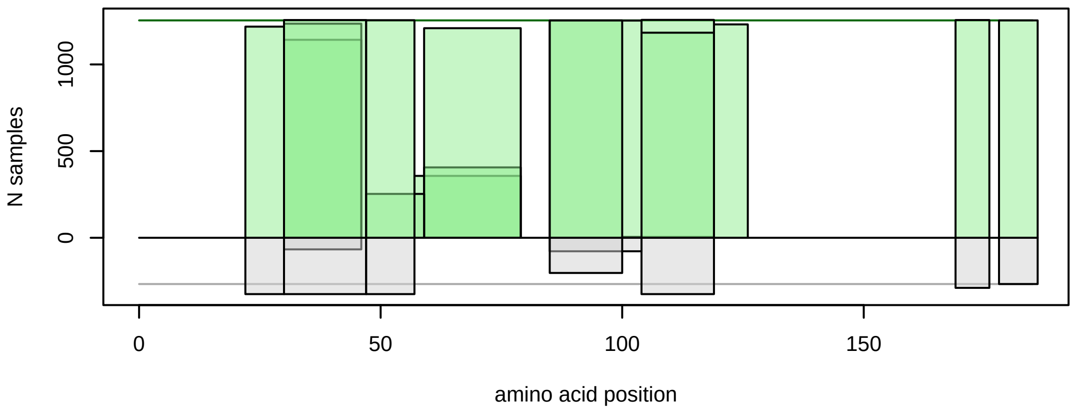

85. CPXM2 (Q8N436), NP5

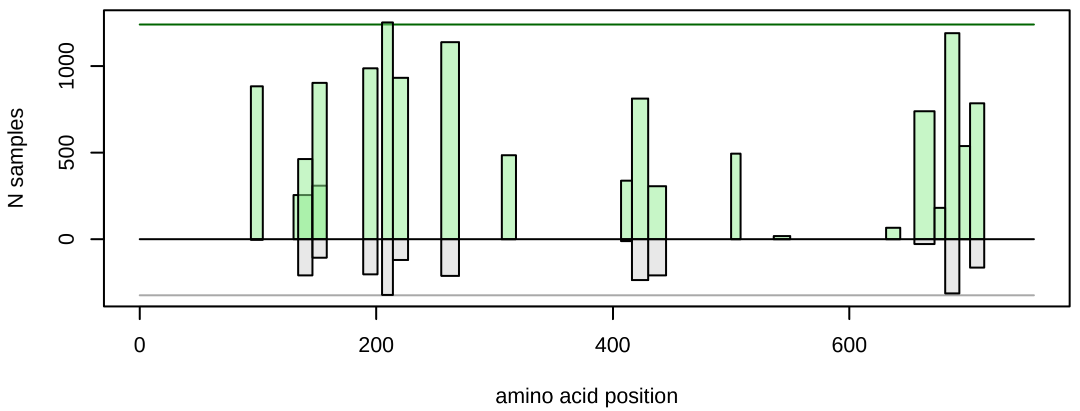

86. NA (CONP13646−1), NP2

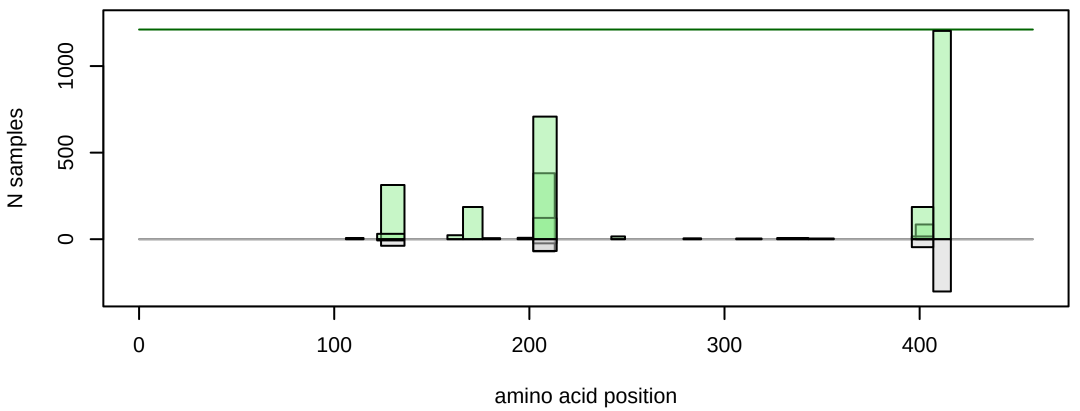

87. MMP3 (P08254), NP5

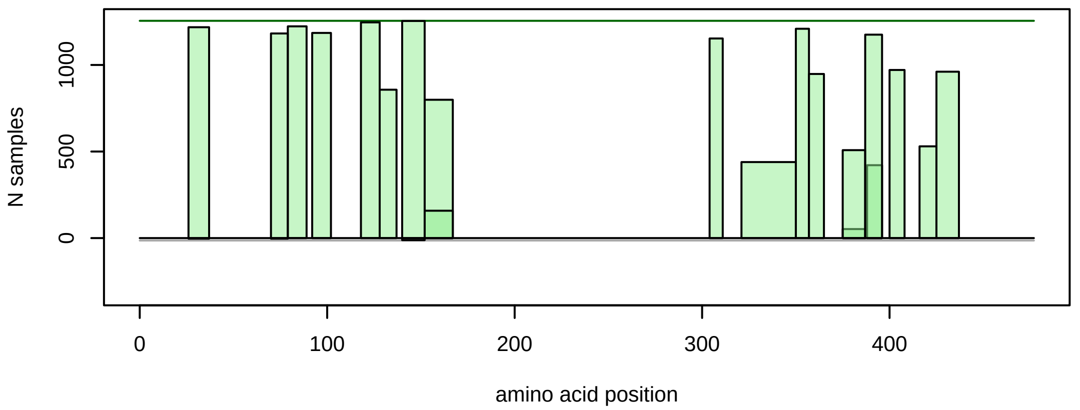

88. COLEC10 (Q9Y6Z7), NP5

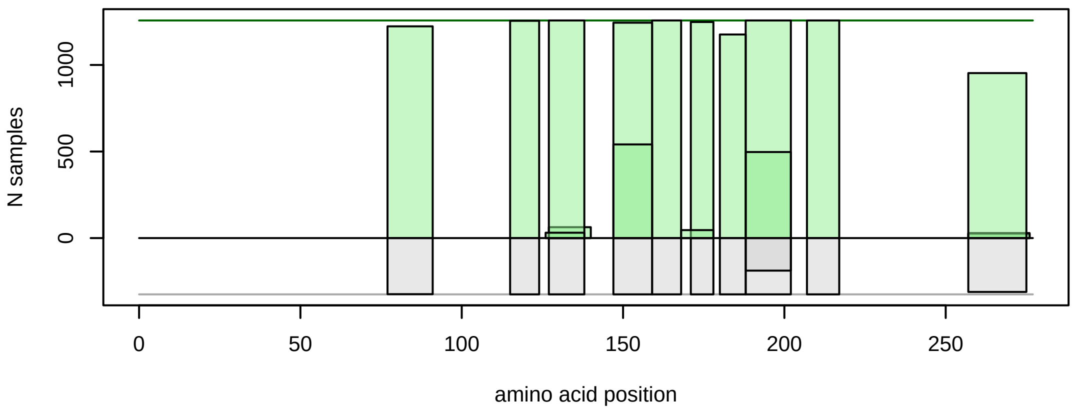

89. CFHR4 (Q92496;Q92496−2), NP5

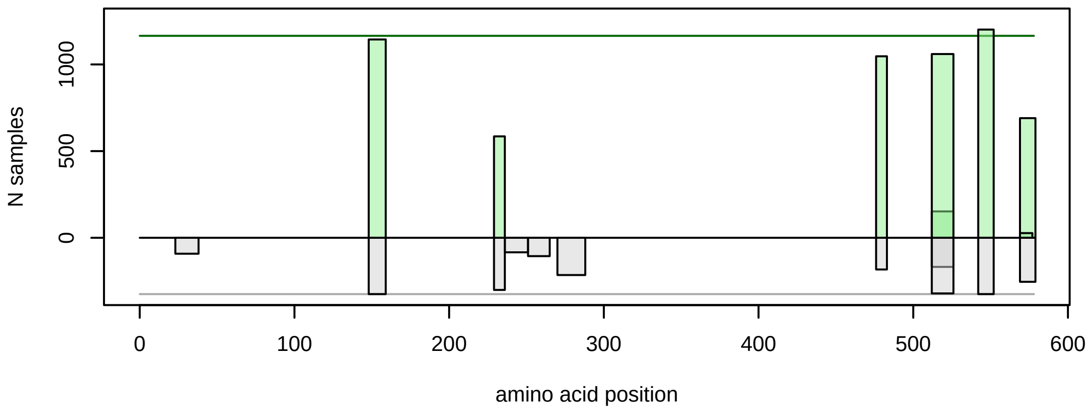

90. CHMP4B (Q9H444), NP4

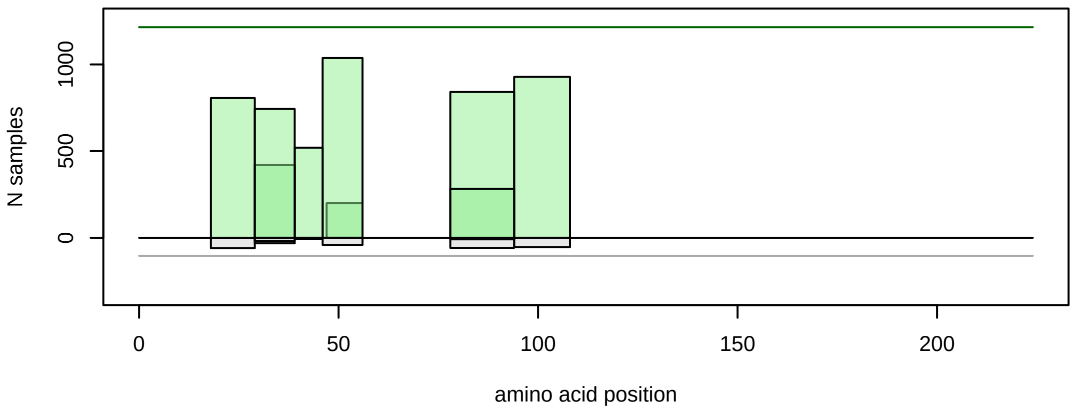

91. EMC1 (A0A8I5KW55;Q8N766;Q8N766−2), NP4

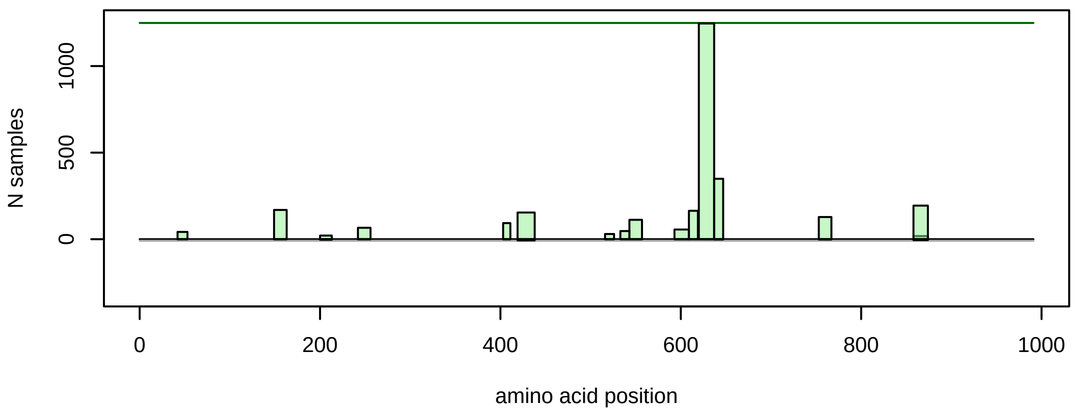

92. ADAMDEC1 (O15204), NP3

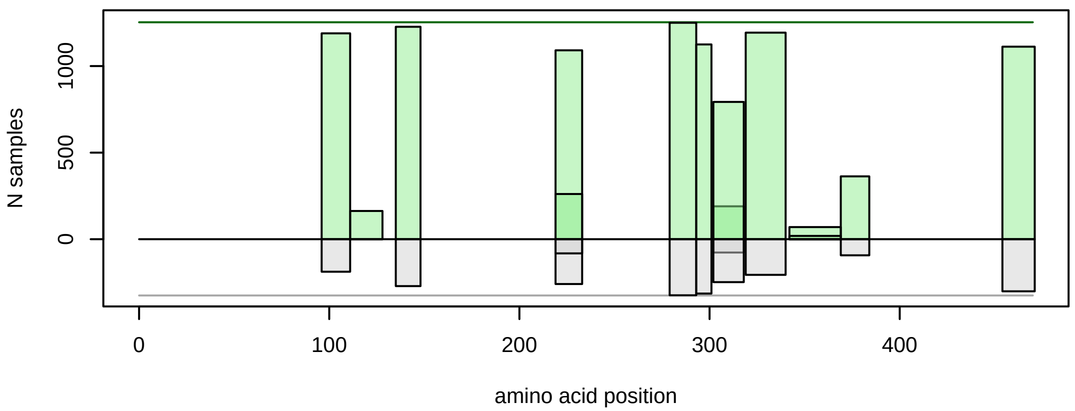

93. BPIFB1 (Q8TDL5), NP4

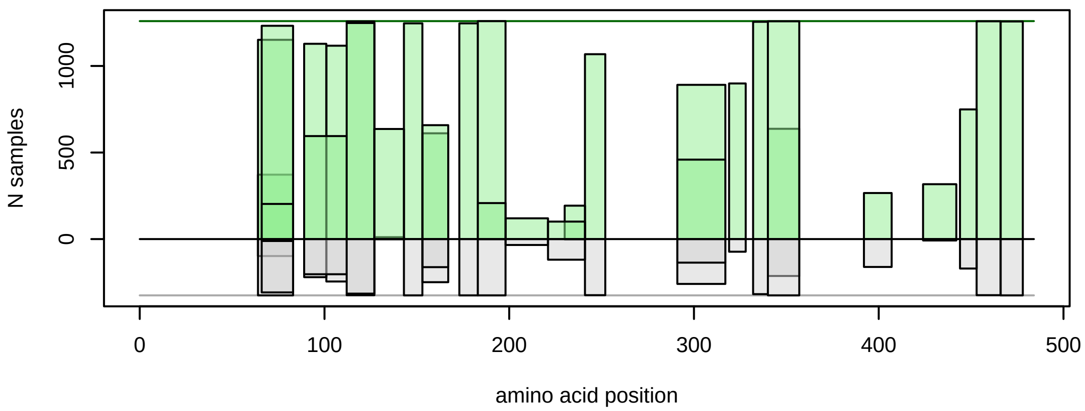

94. APMAP (Q9HDC9), NP5

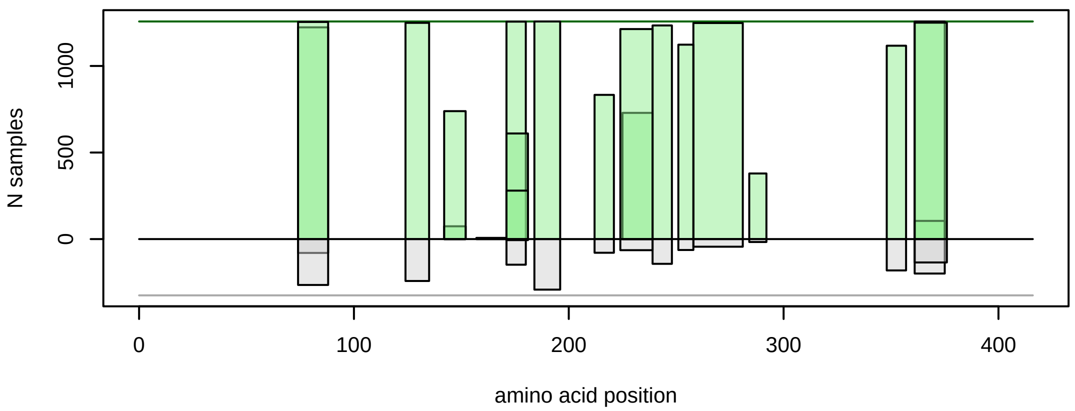

95. C19orf33 (Q9GZP8), NP2

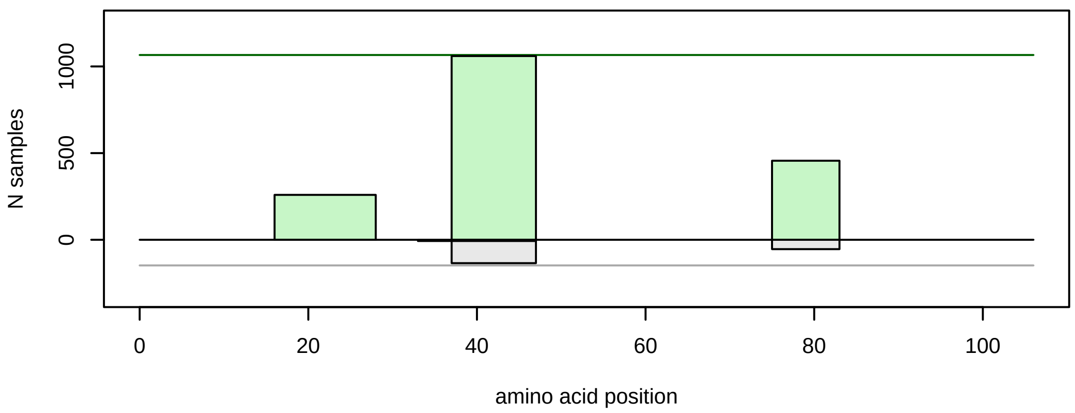

96. ITGA2 (P17301), NP4

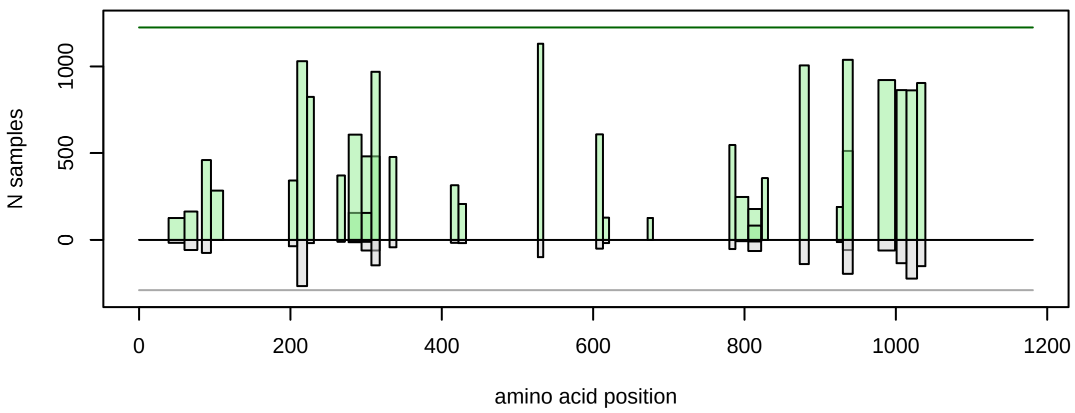

97. MAN1C1 (Q9NR34), NP2

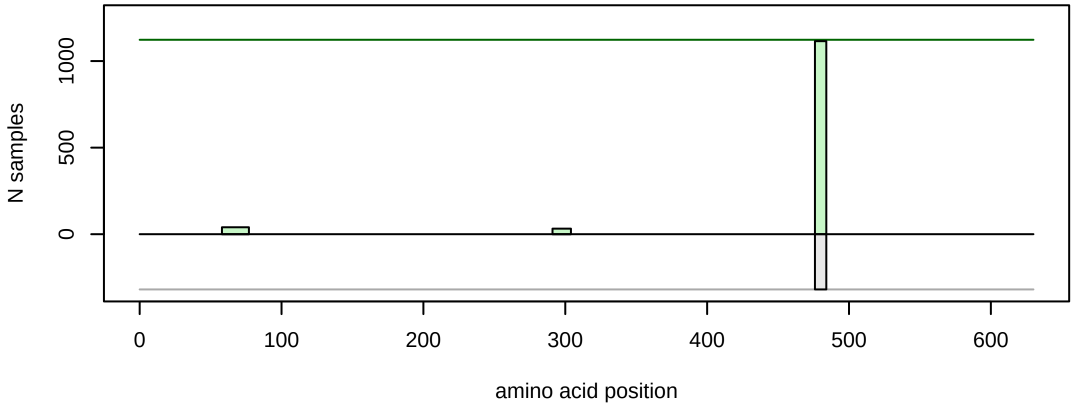

98. PZP (P20742), NP4

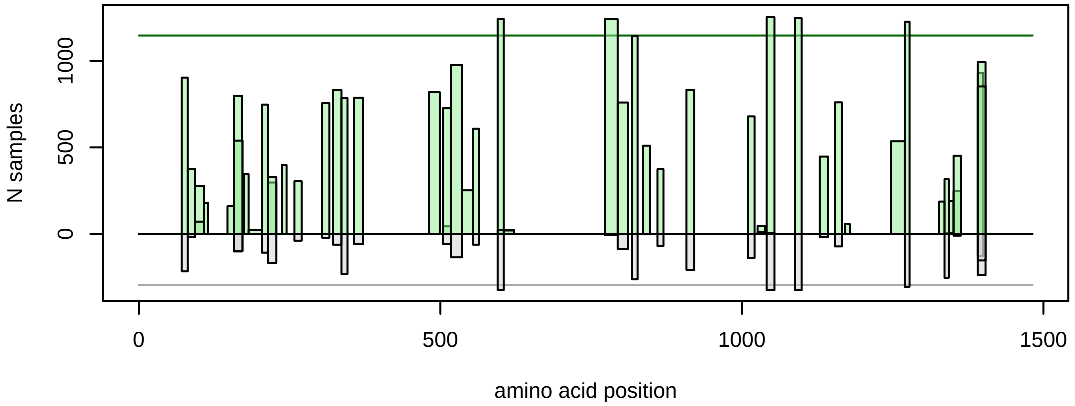

99. MIA−RAB4B (W4VSR3), NP3

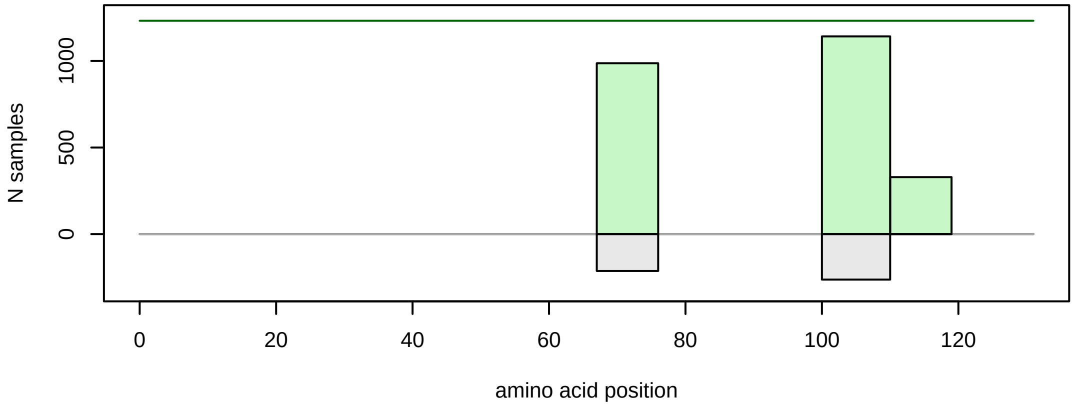

100. FUT8 (Q9BYC5), NP3

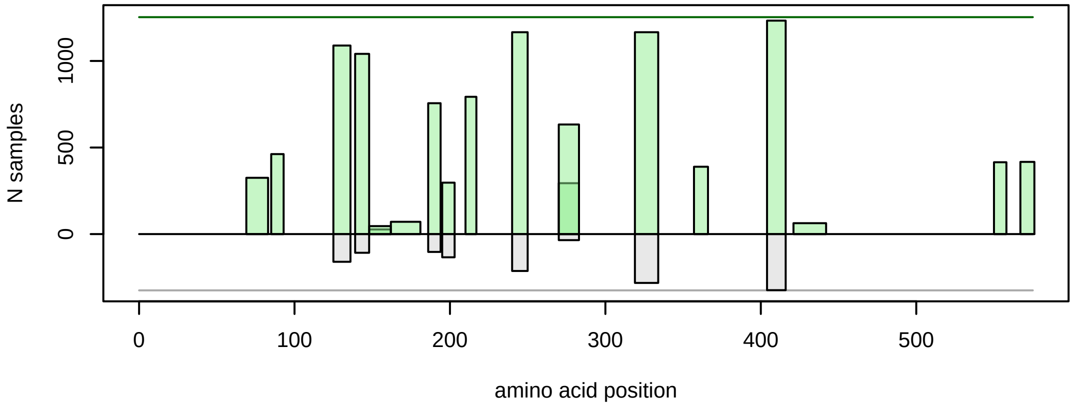

101. FETUB (Q9UGM5), NP4

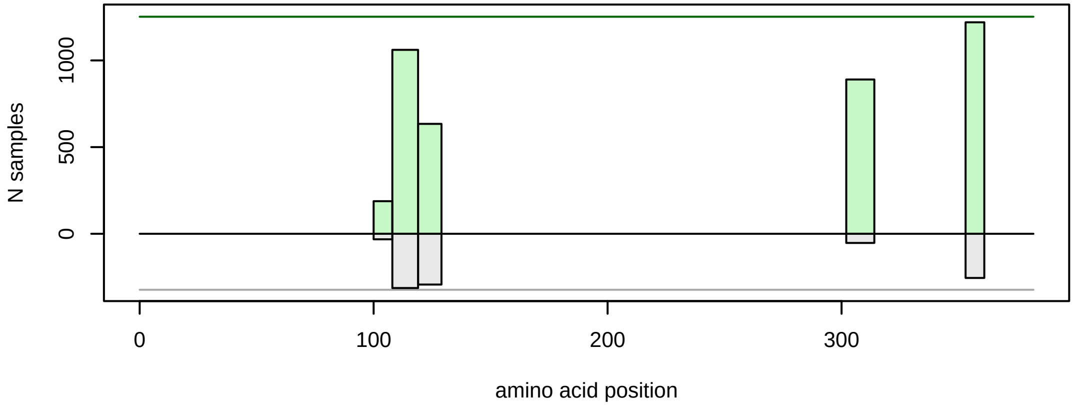

102. HNRNPA3 (A0A7I2V4G0), NP1

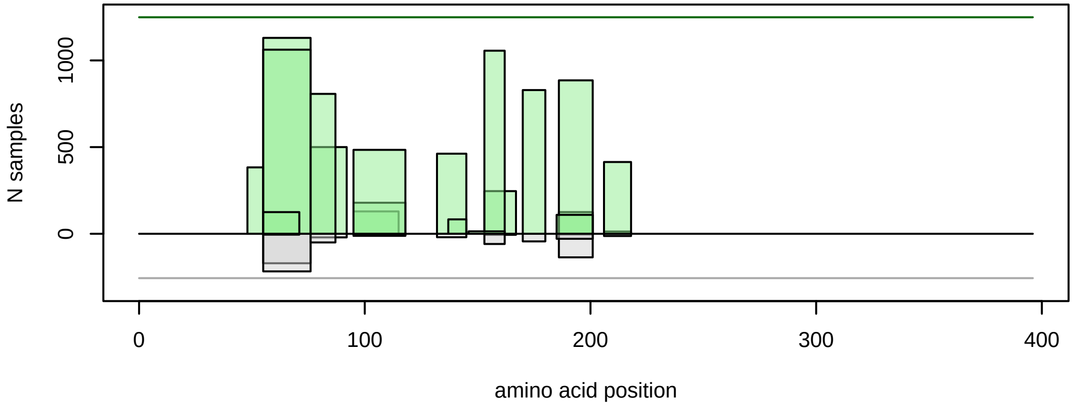

103. GALNT12 (Q8IXK2), NP5

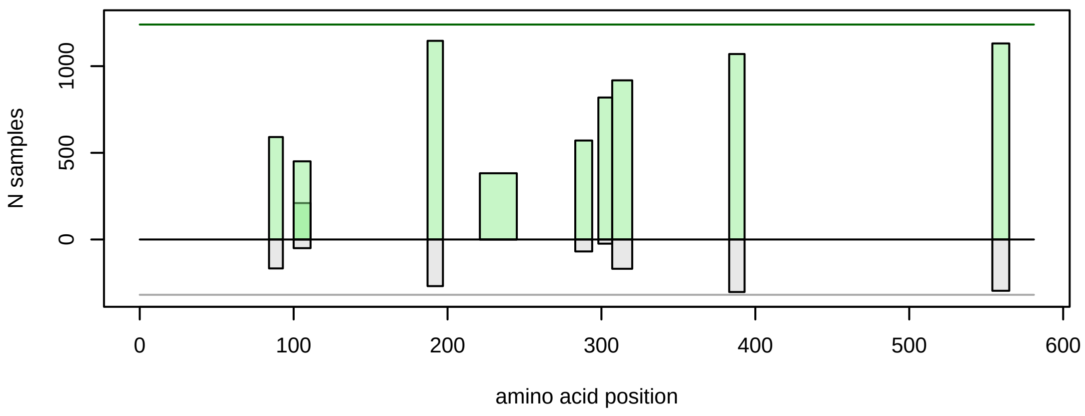

104. TSKU (Q8WUA8), NP5

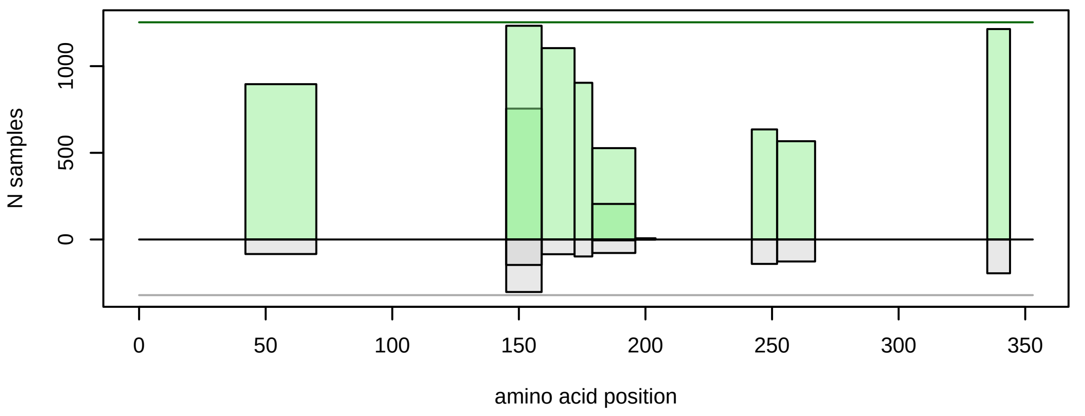

105. LTBP3 (Q9NS15), NP5

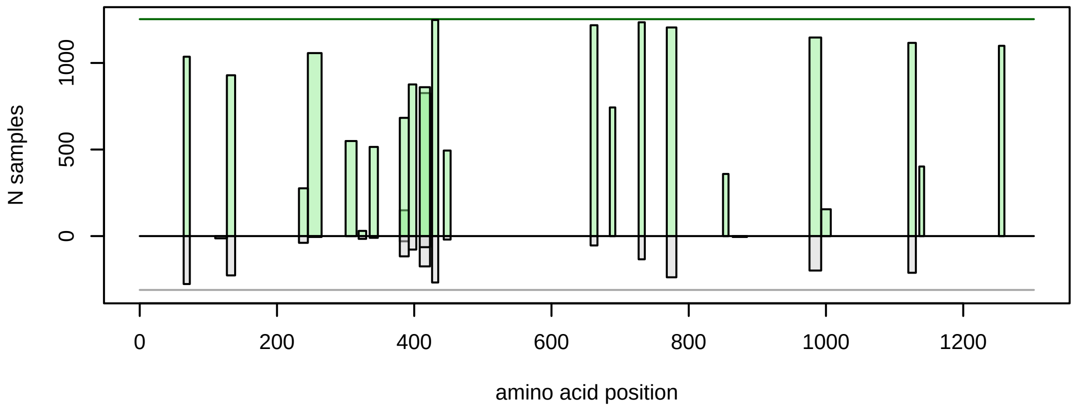

106. COLEC11 (Q9BWP8), NP2

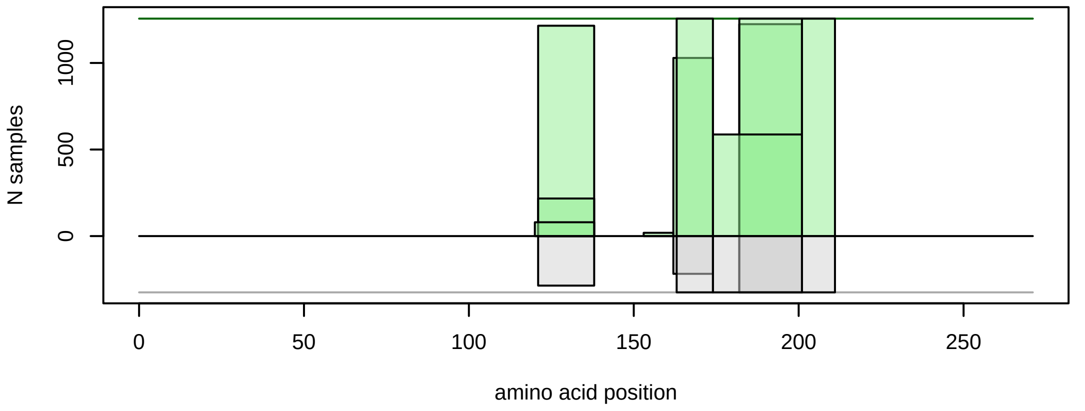

107. CCL15 (Q16663), NP3

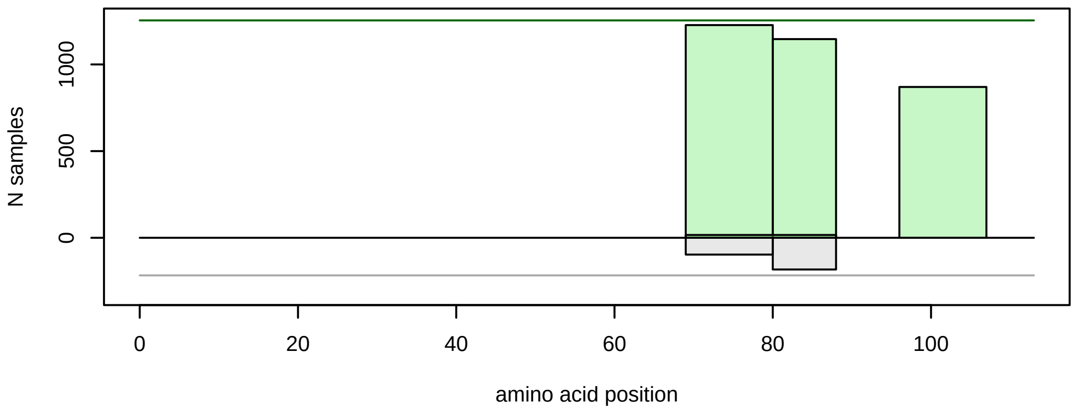

108. CDKL3 (E7ET86;Q8IVW4), NP1

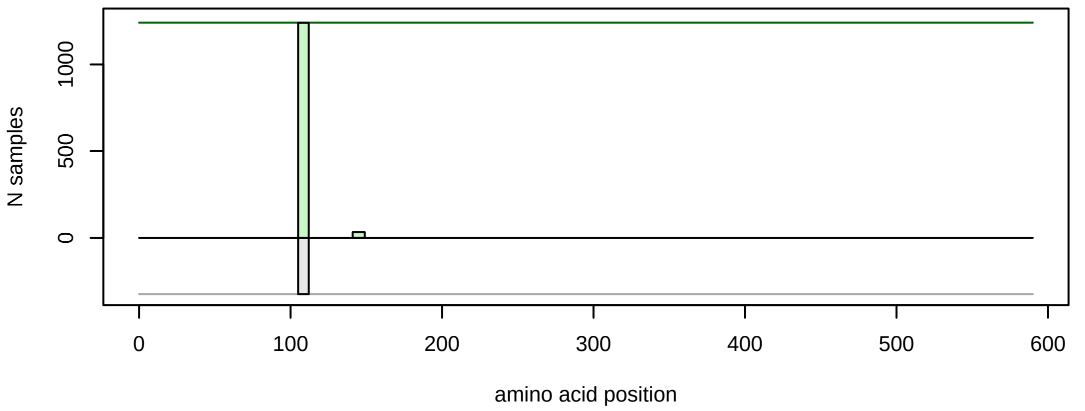

109. LGALS4 (P56470), NP3

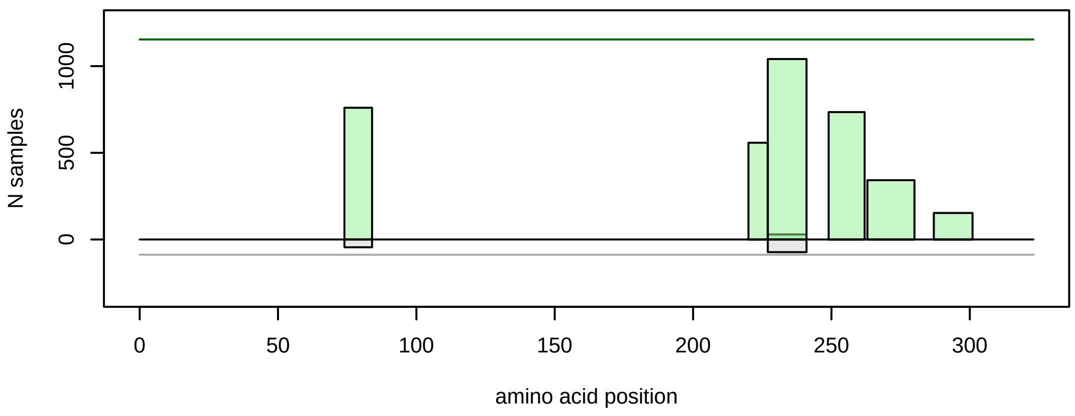

110. ICAM1 (P05362), NP4

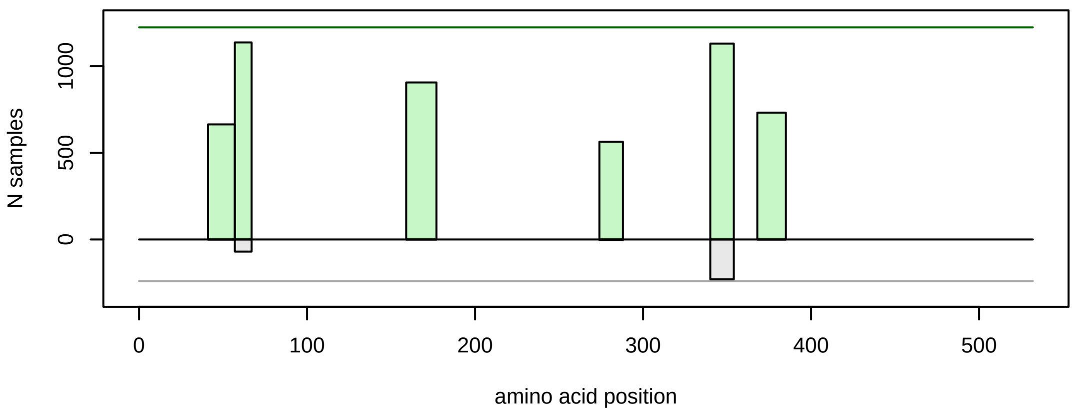

111. CDH19 (Q9H159), NP2

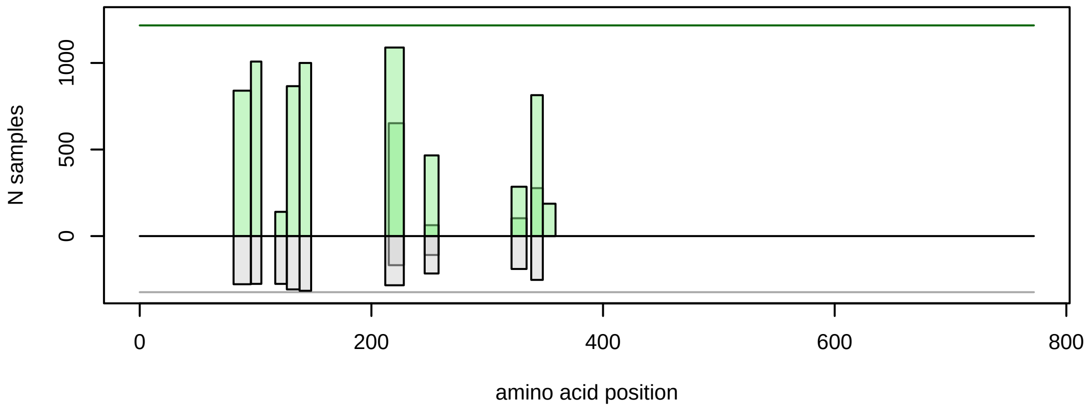

112. SAA1 (E9PQD6), NP2

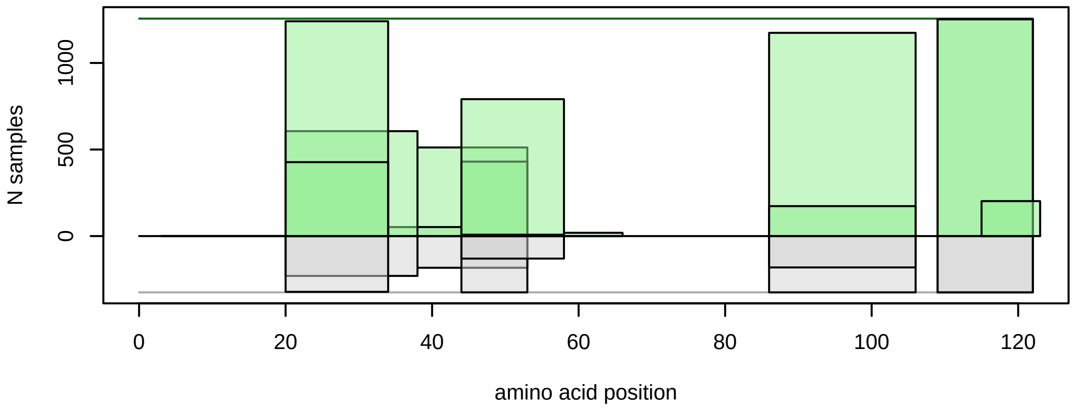

113. C1RL (Q9NZP8), NP4

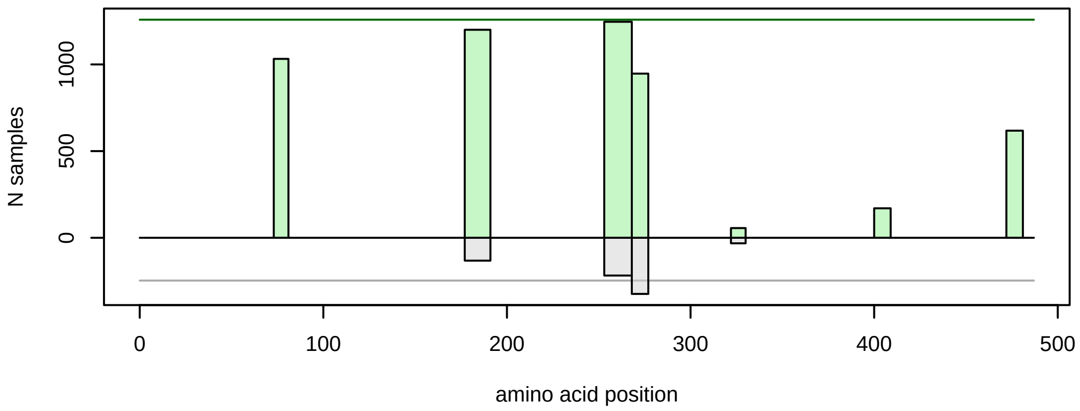

114. FAM177A1 (Q8N128;Q8N128−2), NP2

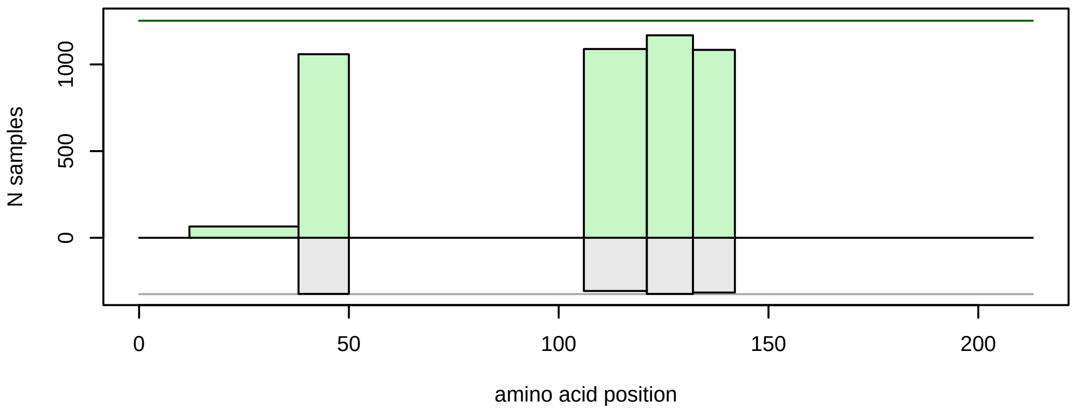

115. FCGR3A (H0Y755;P08637), NP1

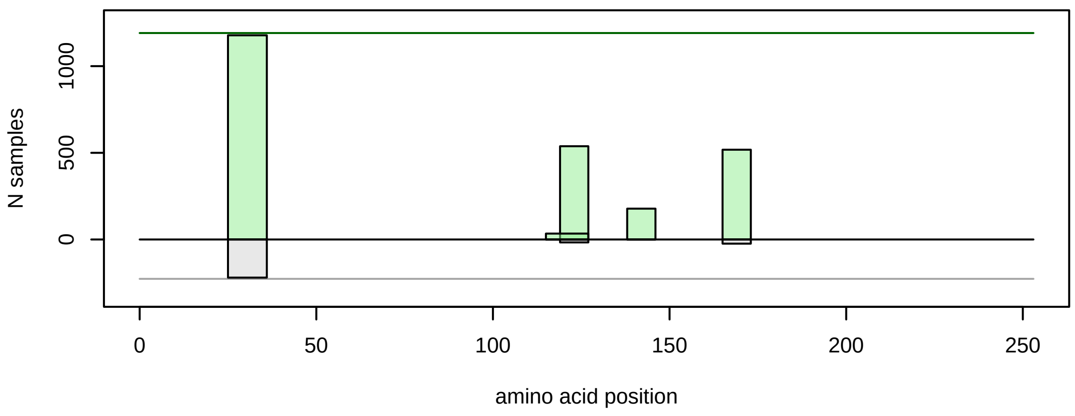

116. C3 (P01024), NP4

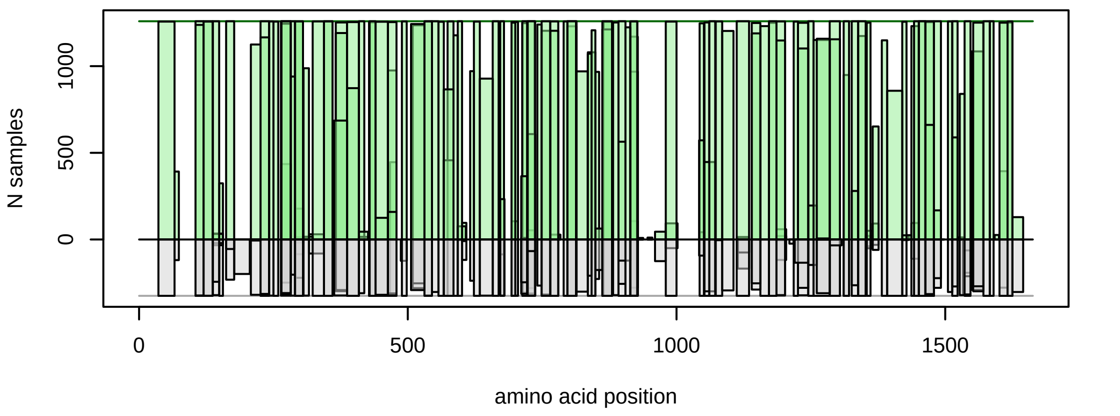

117. PLG (P00747), NP5

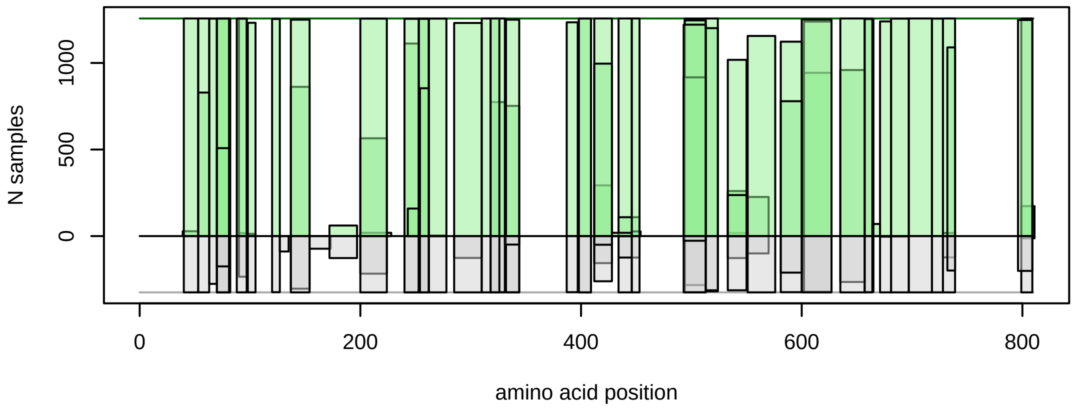

118. FABP4 (P15090), NP2

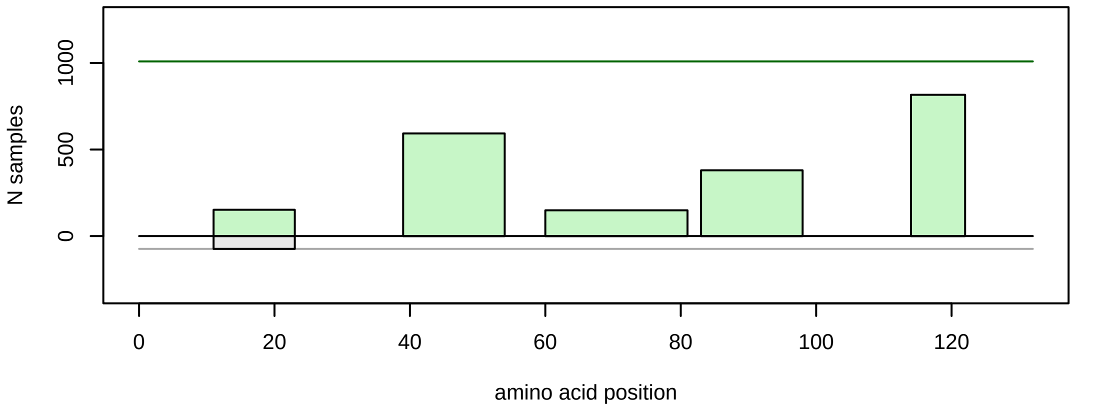

119. GPR116 (Q8IZF2), NP2

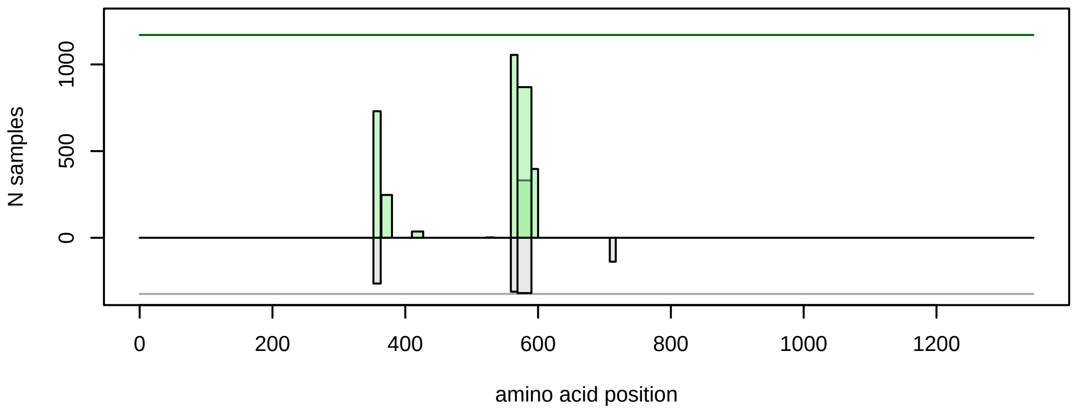

120. CD34 (P28906), NP2

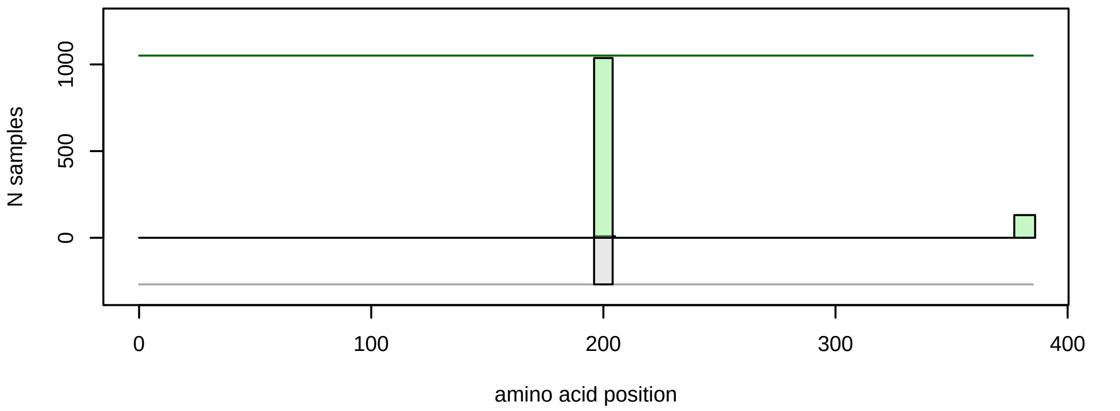

121. CTSS (P25774), NP3

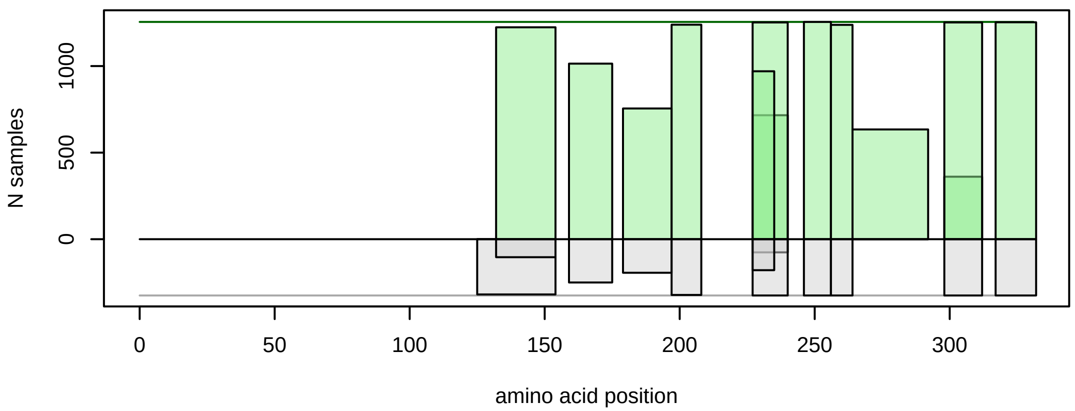

122. HSPA8 (P11142), NP1

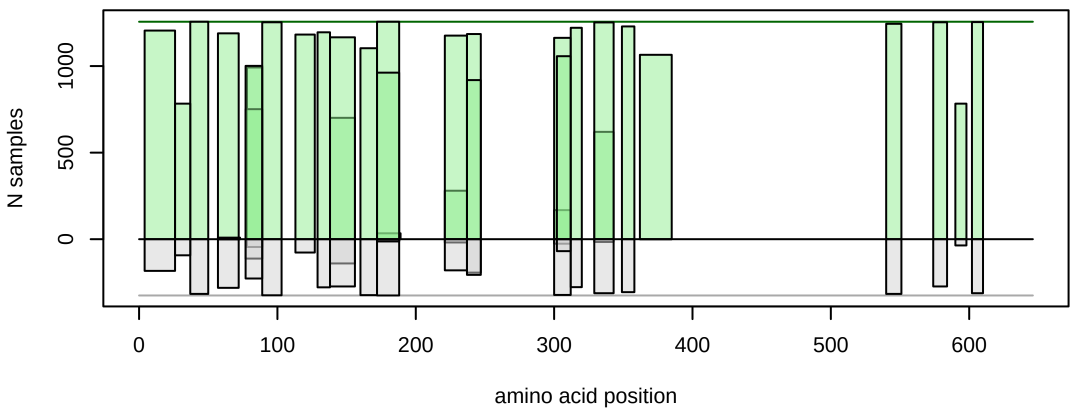

123. C8G (P07360), NP5

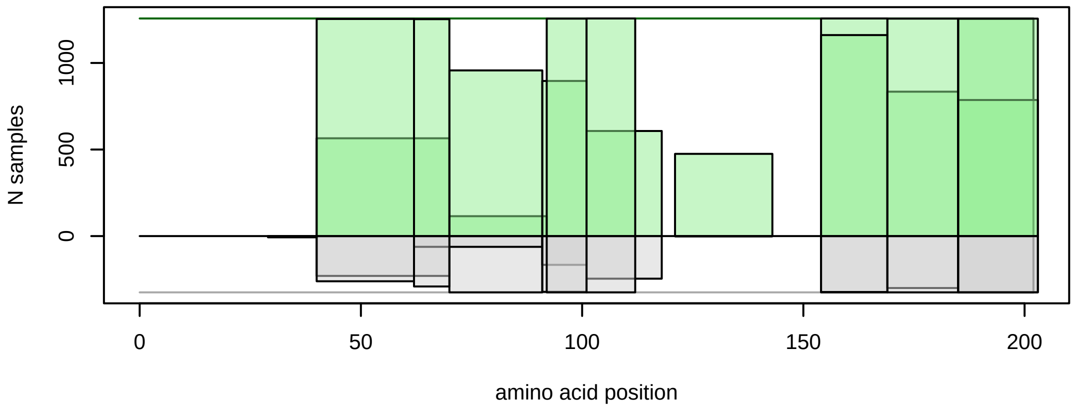

124. GNB2 (P62879), NP1

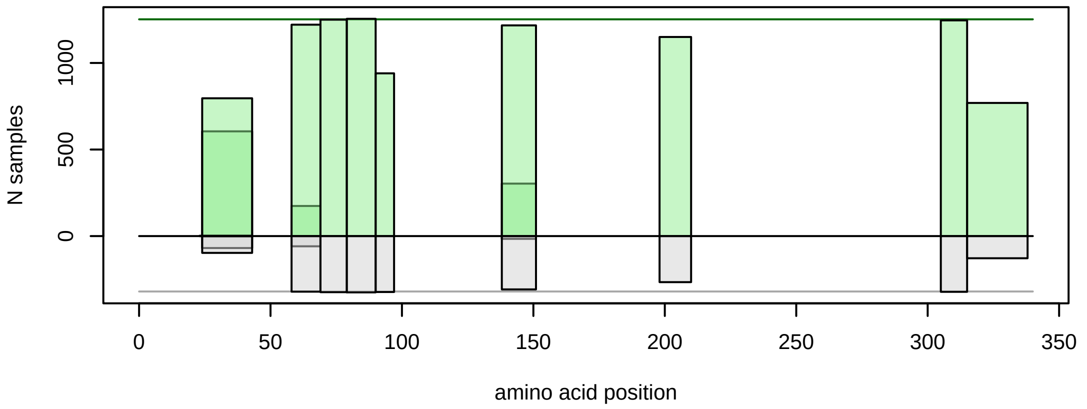

125. DIAPH1 (O60610), NP1

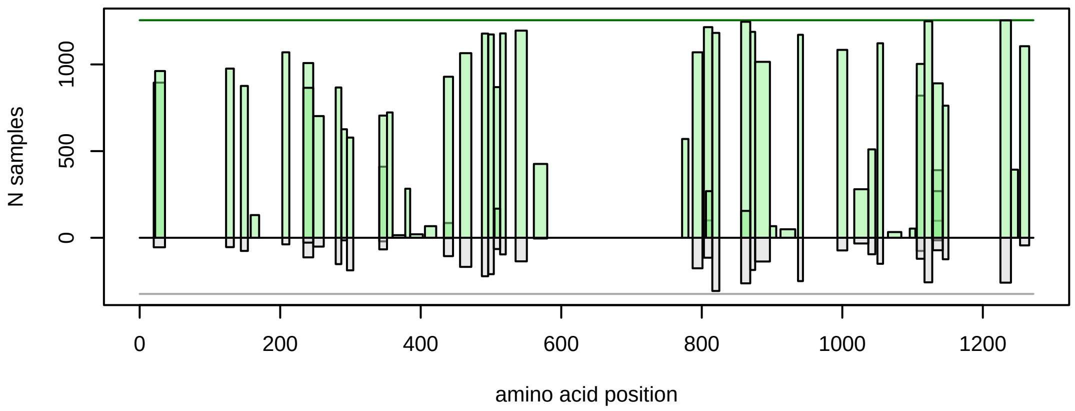

126. IGKV1−17 (P01599), NP3

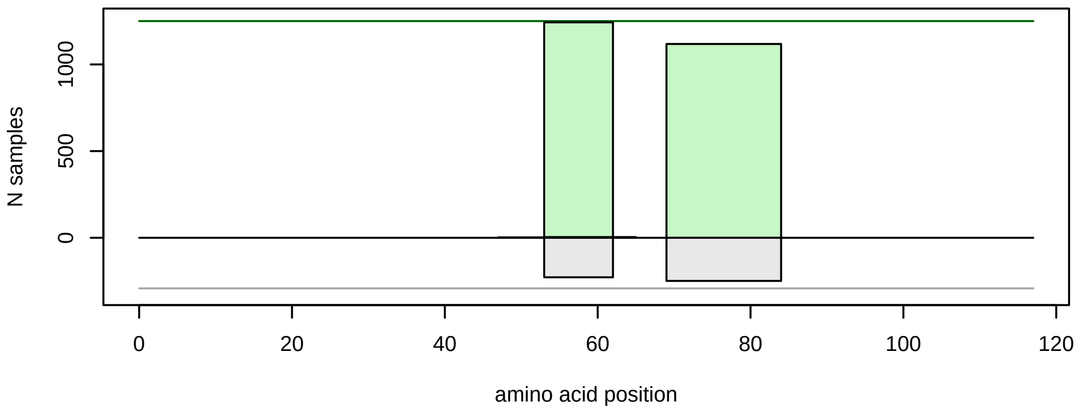

127. MAN2A2 (P49641), NP5

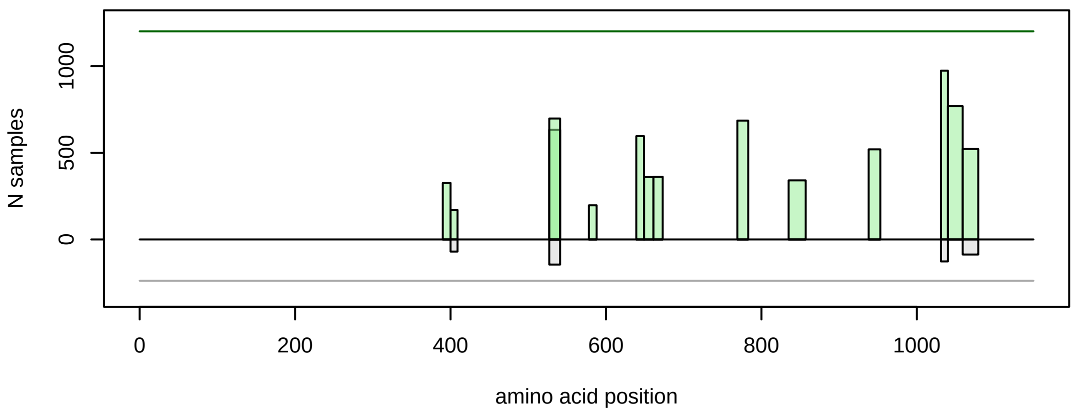

128. LAMA5 (O15230), NP2

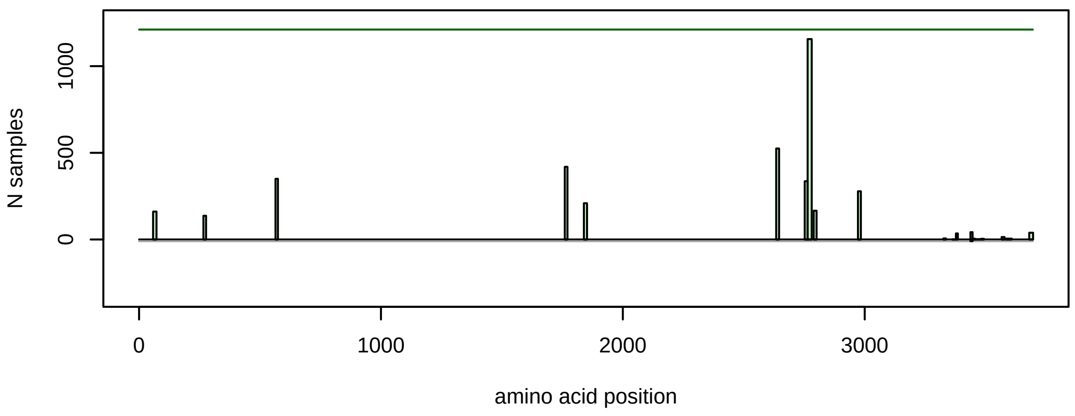

129. SERPINA11 (Q86U17), NP3

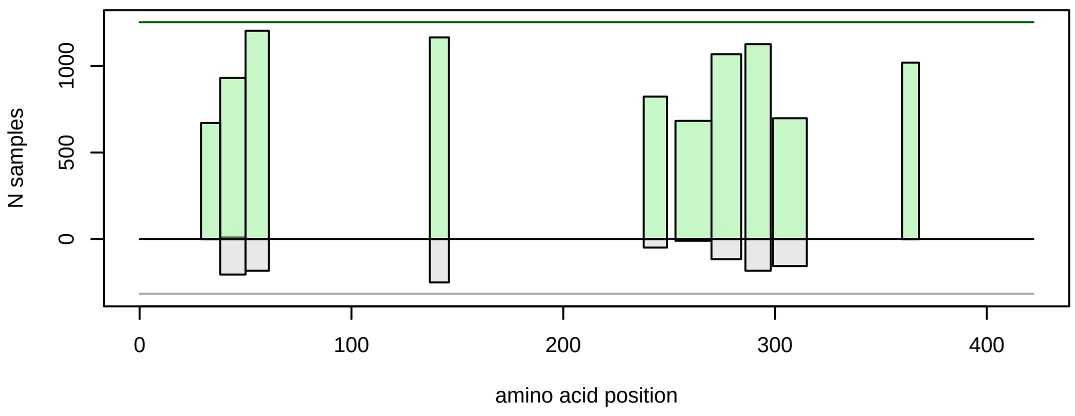

130. ORM2 (P19652), NP2

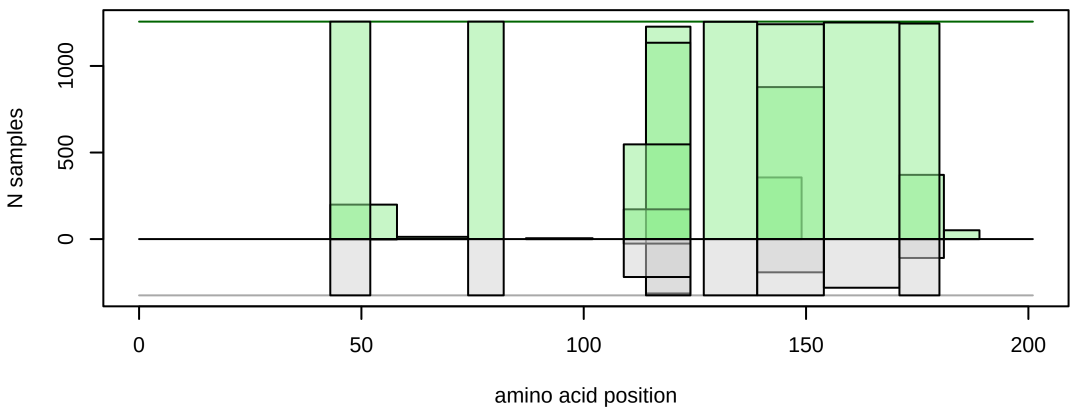

131. TNFAIP6 (P98066), NP5

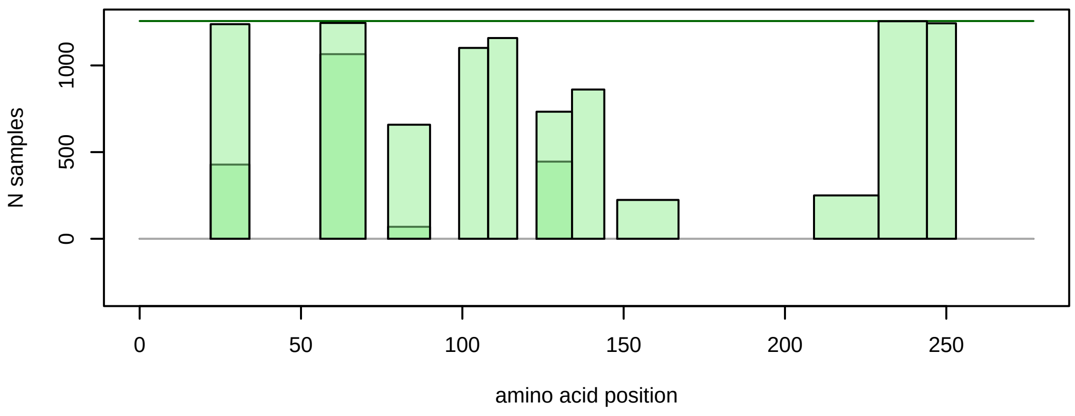

132. SSC5D (A1L4H1), NP2

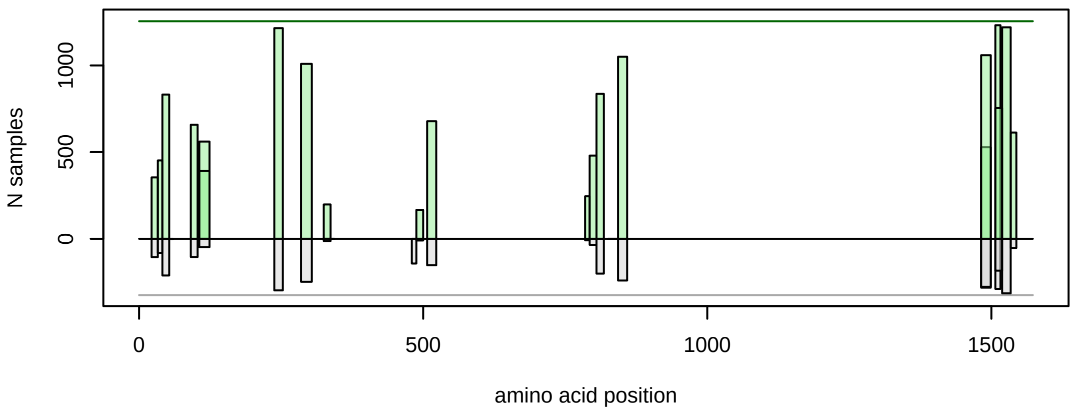

133. TPSAB1 (A0A087WUI4;A0A087X1U0;A0A140VJT7;J3QTS8;Q15661;Q15661−2), NF

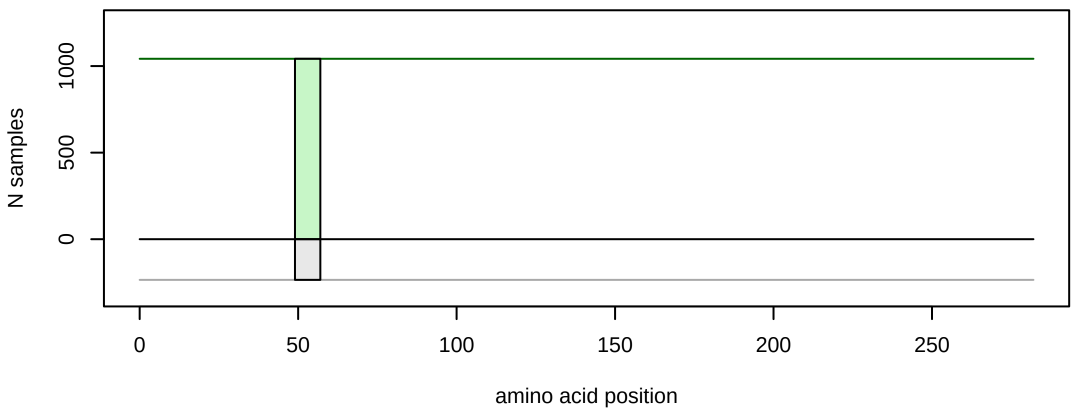

134. CCL14 (Q16627), NP3

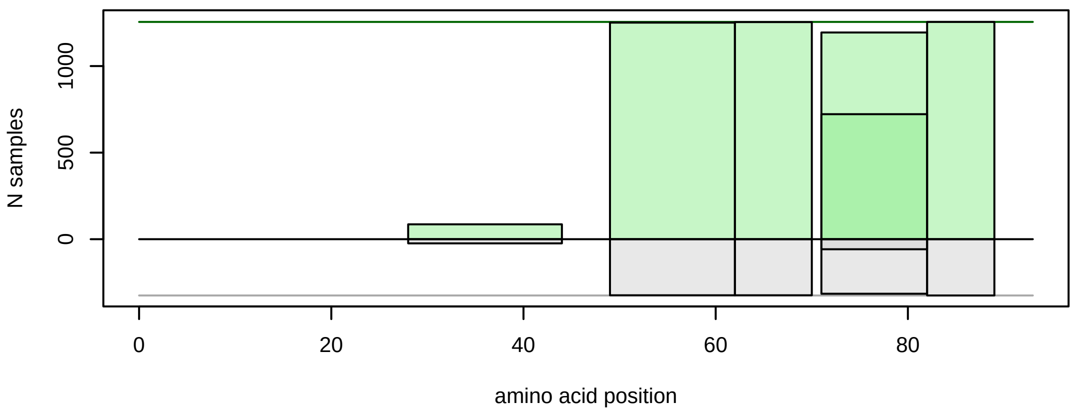

135. TAC3 (Q9UHF0−2), NP2

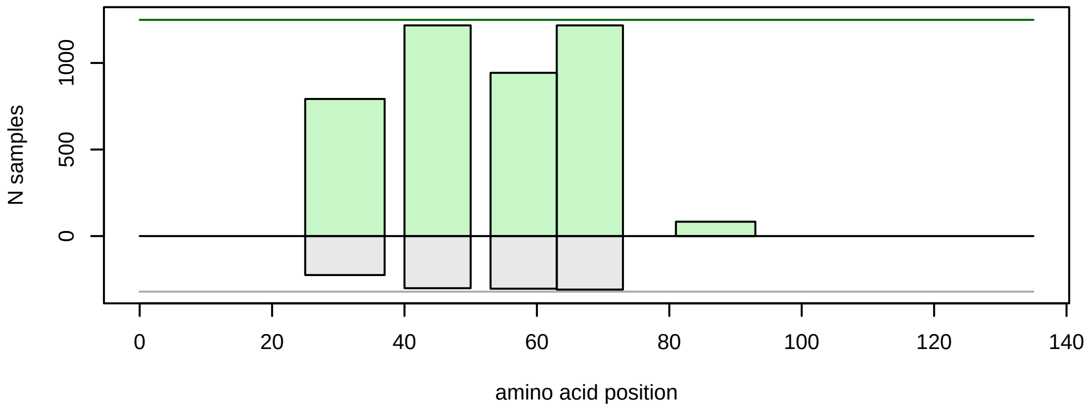

136. ECM2 (O94769), NP2

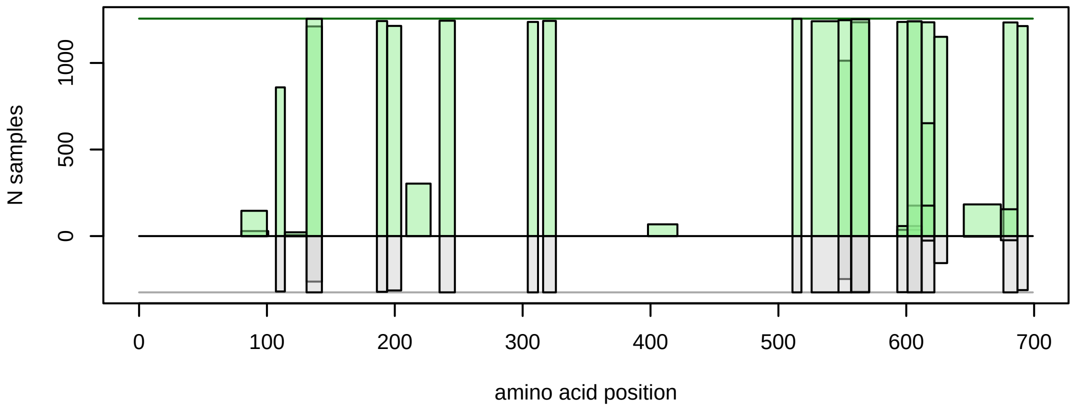

137. ITIH4 (Q14624), NP3

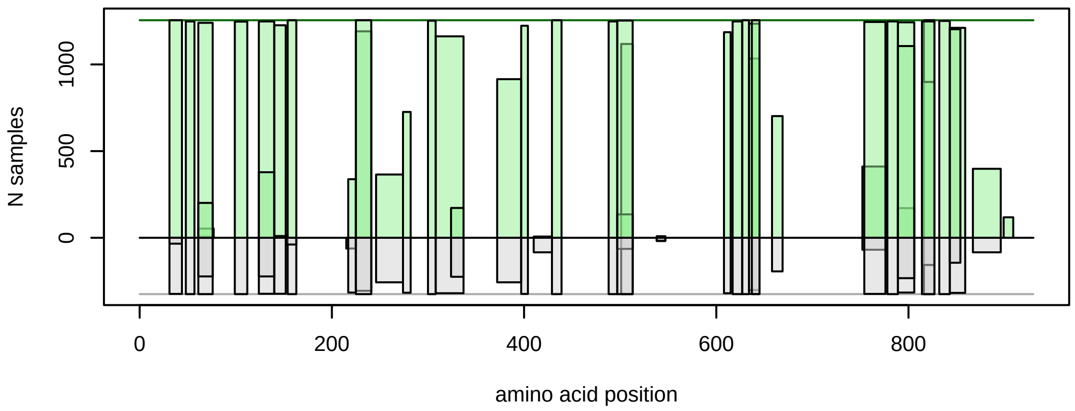

138. CNDP2 (Q96KP4), NP5

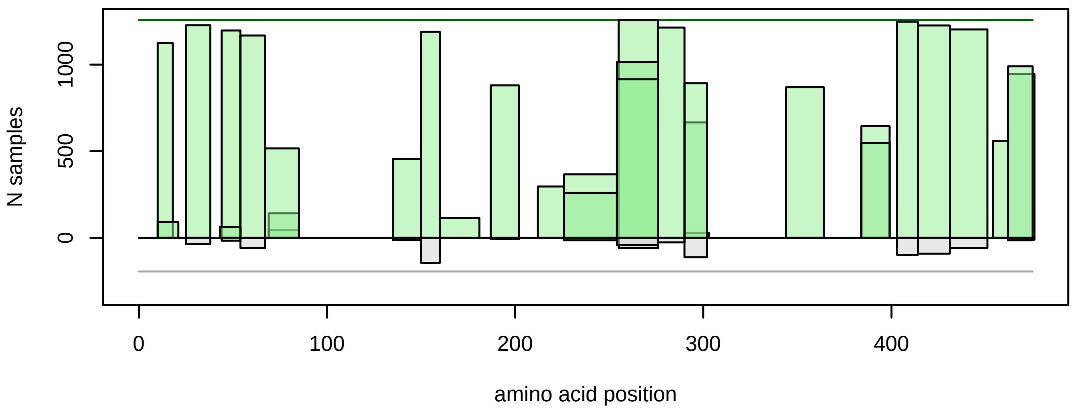

139. IGKV2D−29 (A0A075B6S2), NP3

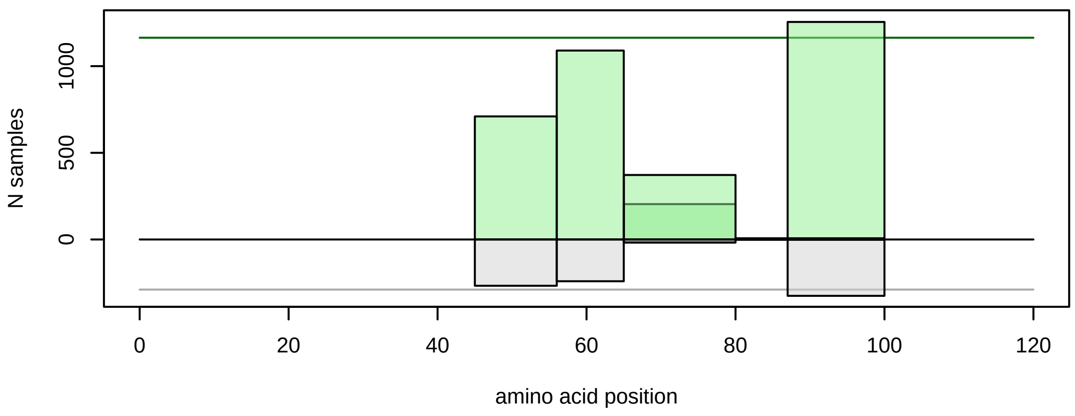

140. AEBP1 (Q8IUX7), NP2

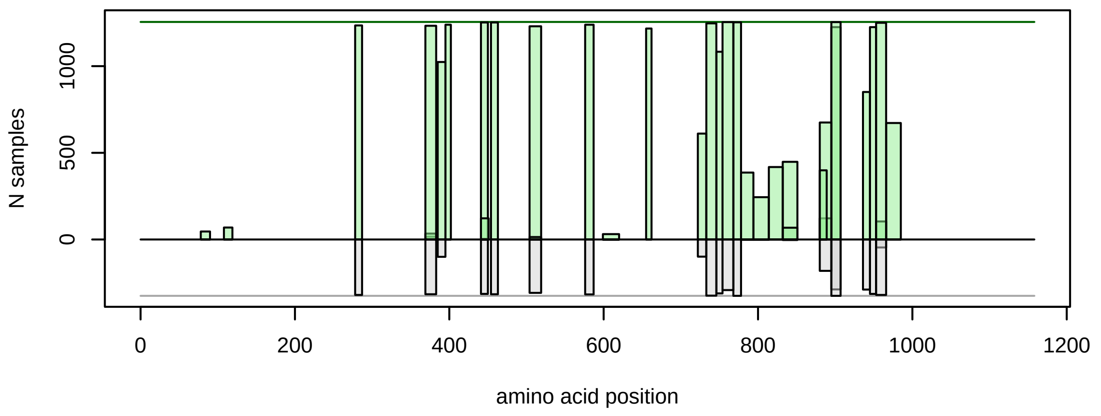

141. MASP2 (O00187), NP2

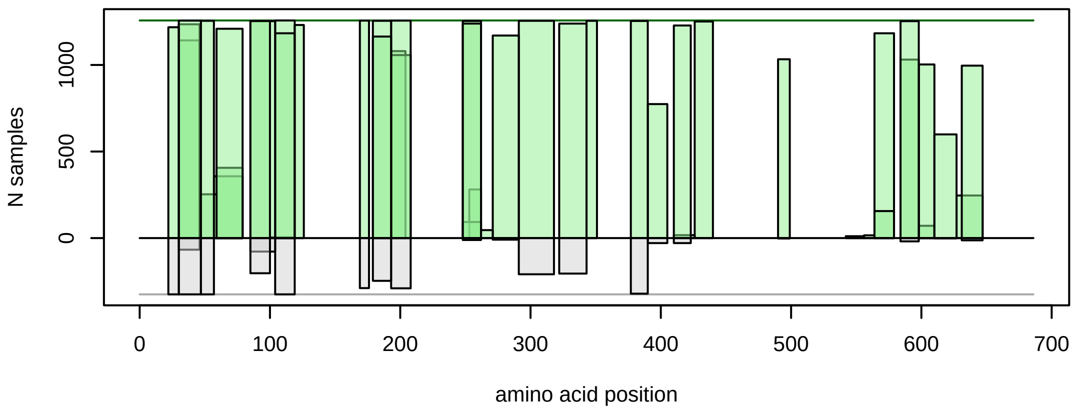

142. FCN1 (O00602), NP5

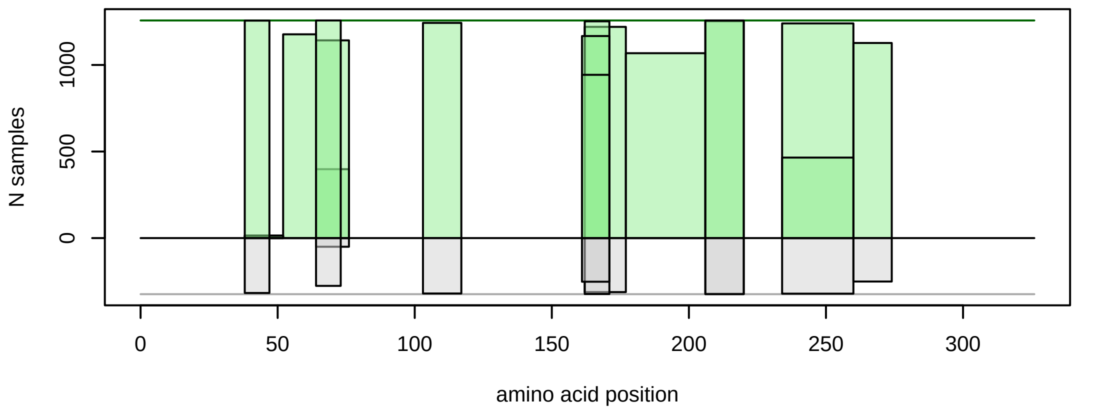

143. DEFA1 (P59665), NP4

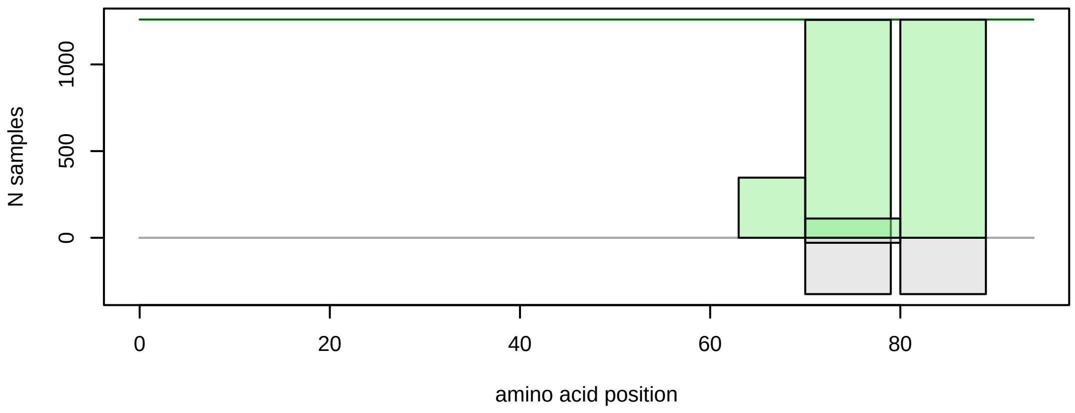

144. F12 (P00748), NP1

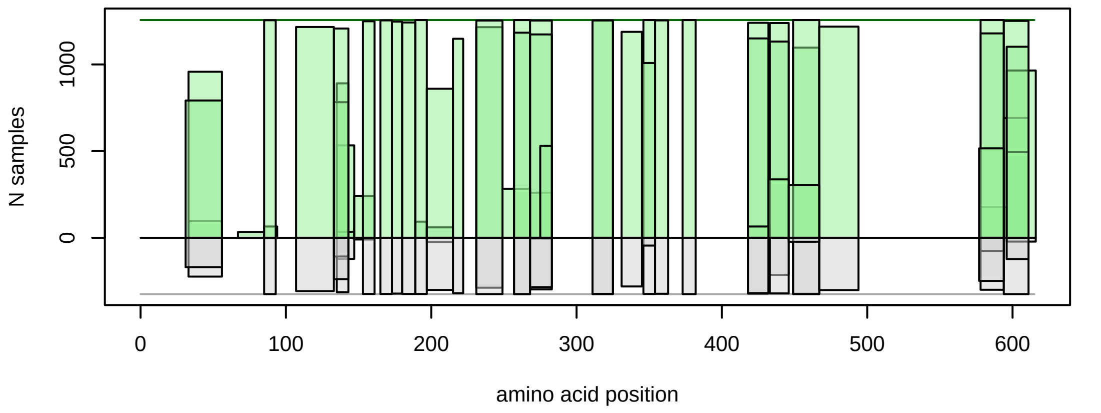

145. LIPC (P11150), NP4

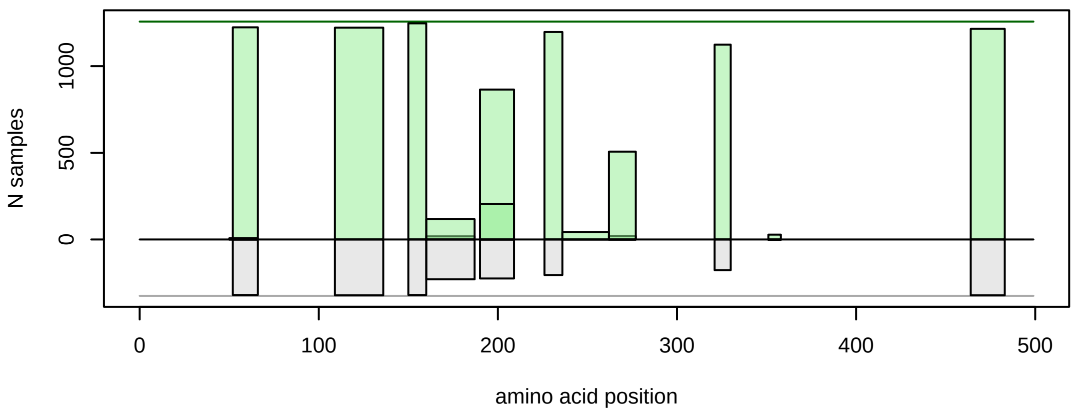

146. NUCB2 (P80303;P80303−2), NP2

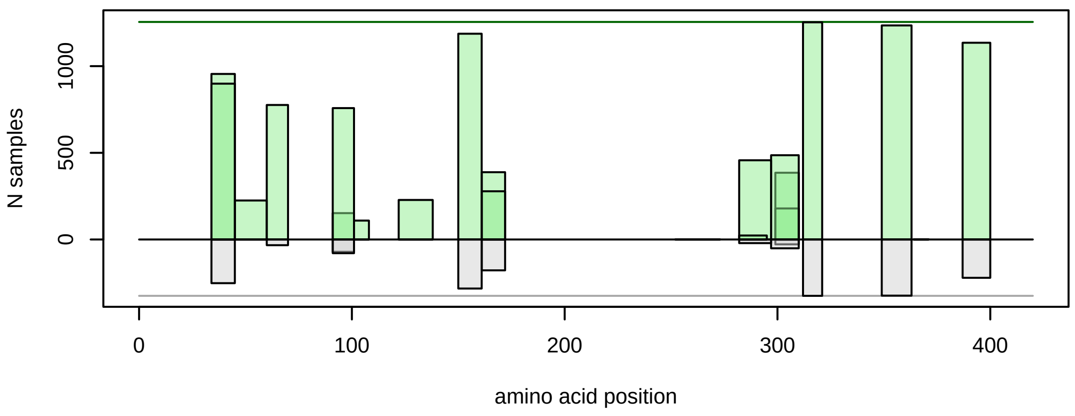

147. ITIH4 (B7ZKJ8), NP3

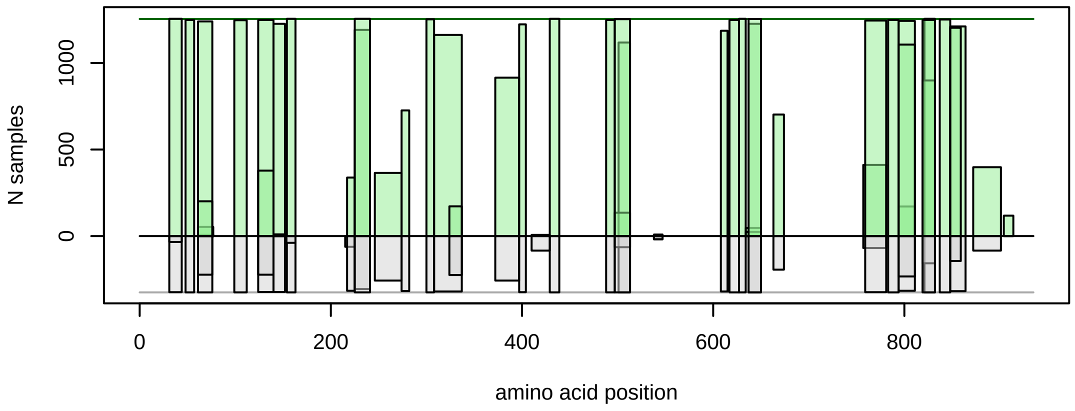

148. ITLN1 (Q8WWA0), NP5

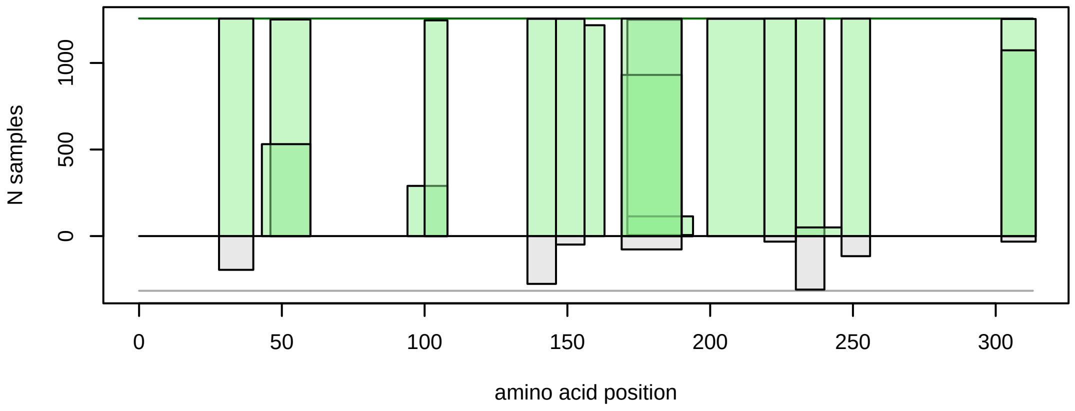

149. BPI (A0A2R8YDF1;P17213), NP4

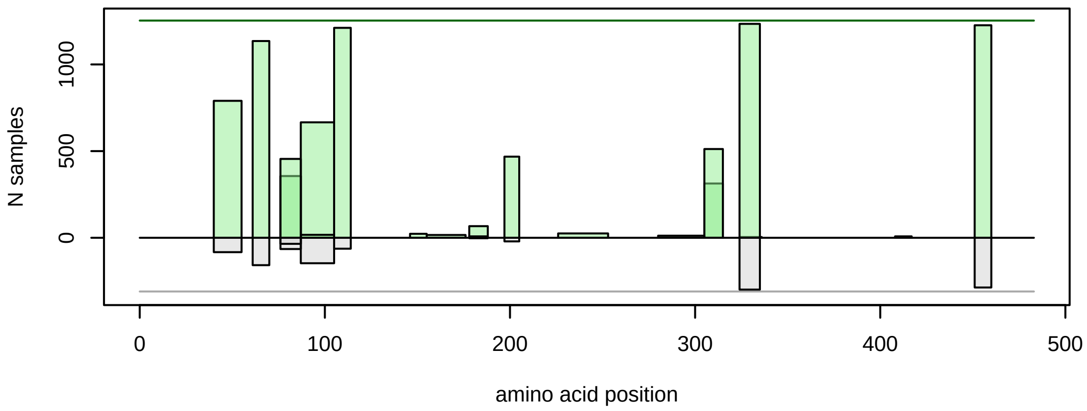

150. FCN2 (Q15485), NP4

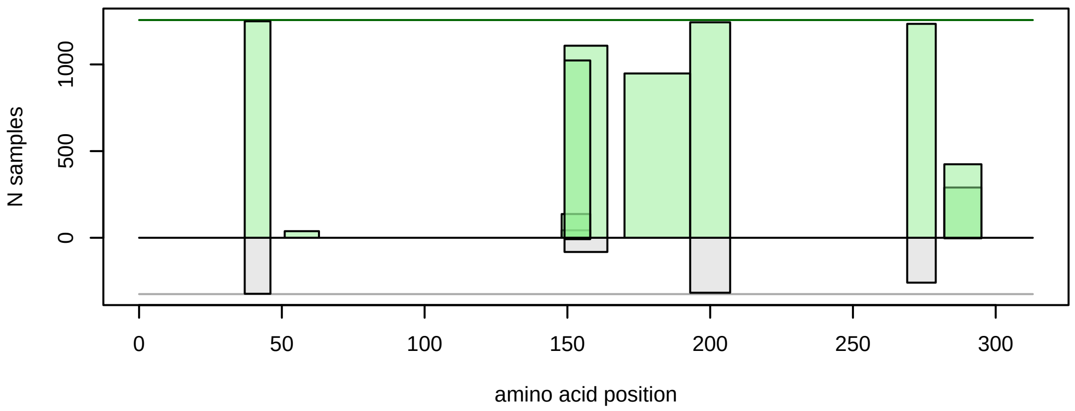

151. PDCD5 (O14737), NP2

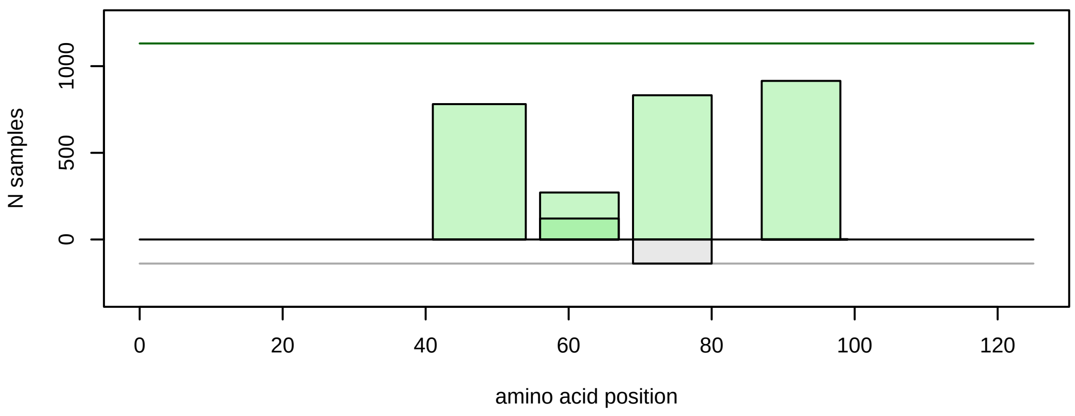

152. FREM2 (Q5SZK8), NP2

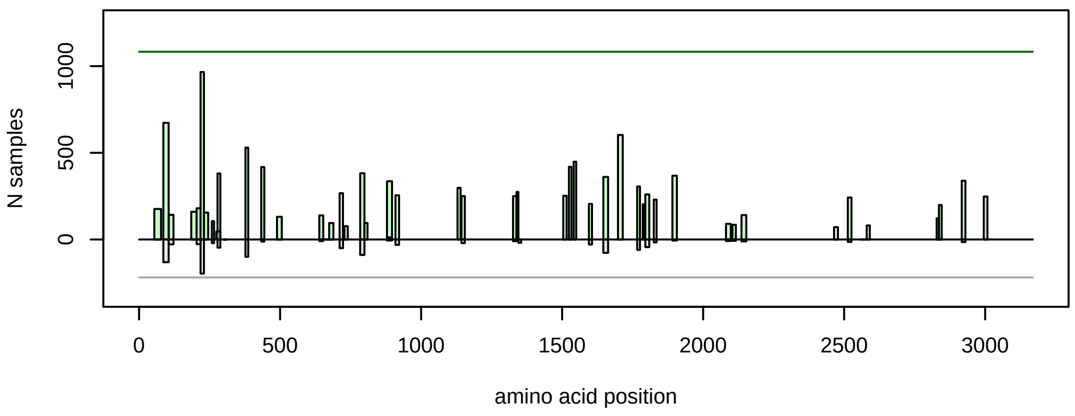

153. MMRN2 (Q9H8L6), NP2

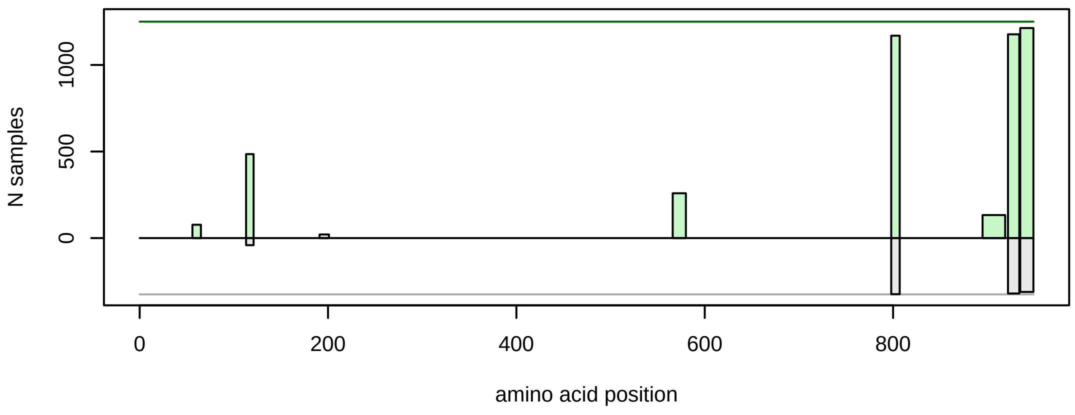

154. TINAGL1 (Q9GZM7;Q9GZM7−3), NP2

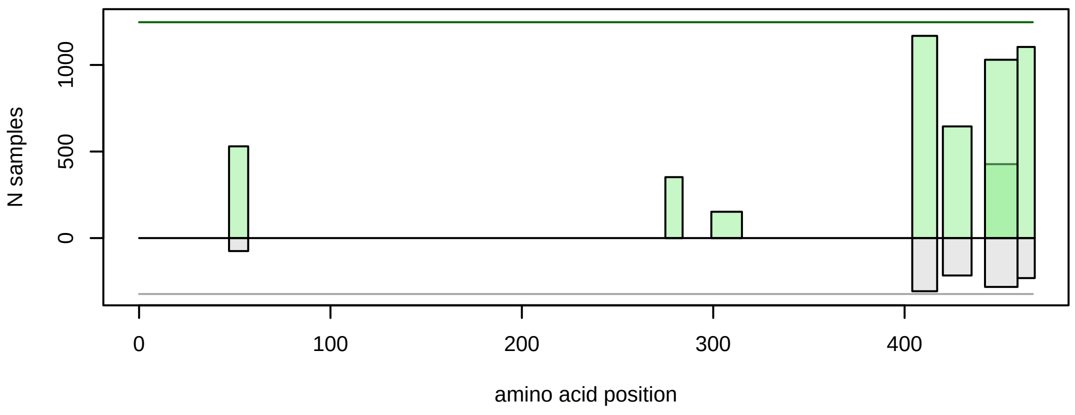

155. CDH15 (P55291), NP2

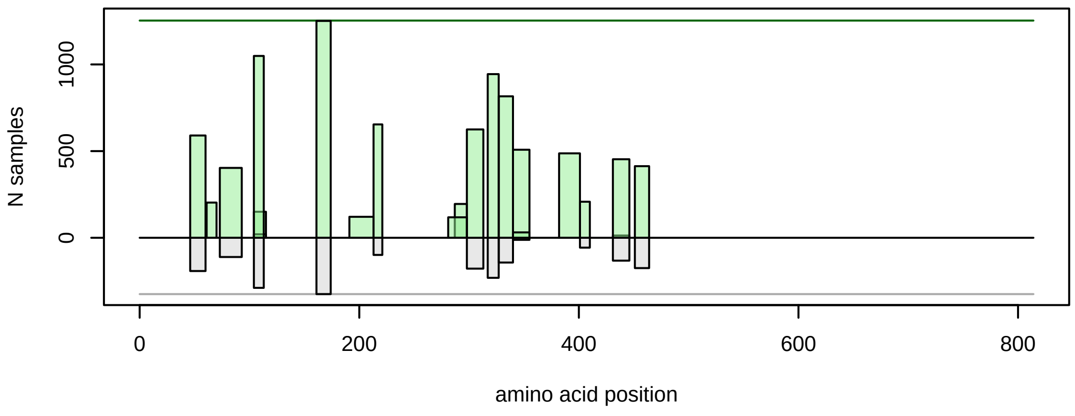

156. FCN3 (O75636), NP3

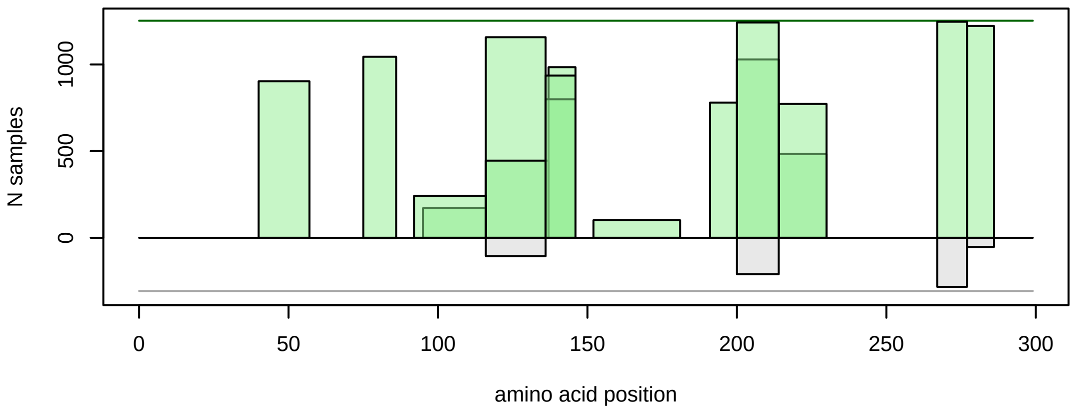

157. PRCP (P42785), NP5

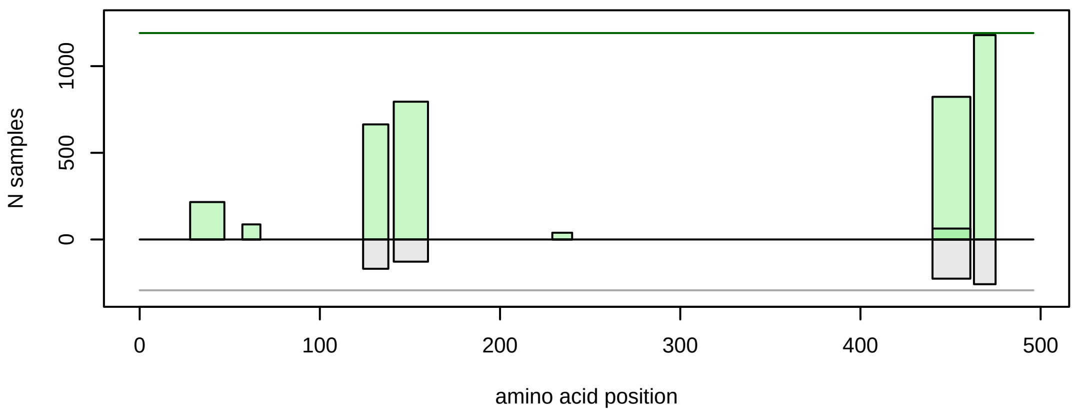

158. IGKV1−5 (P01602), NP4

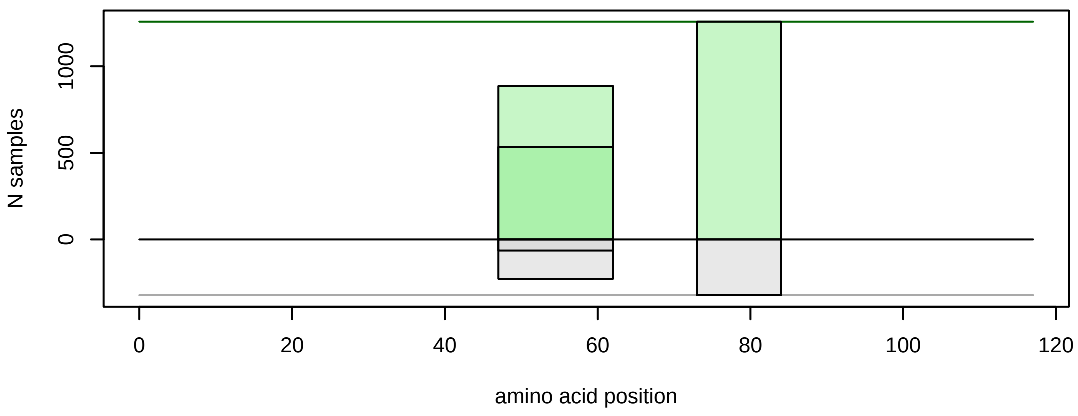

159. ANGPTL6 (Q8NI99), NP5

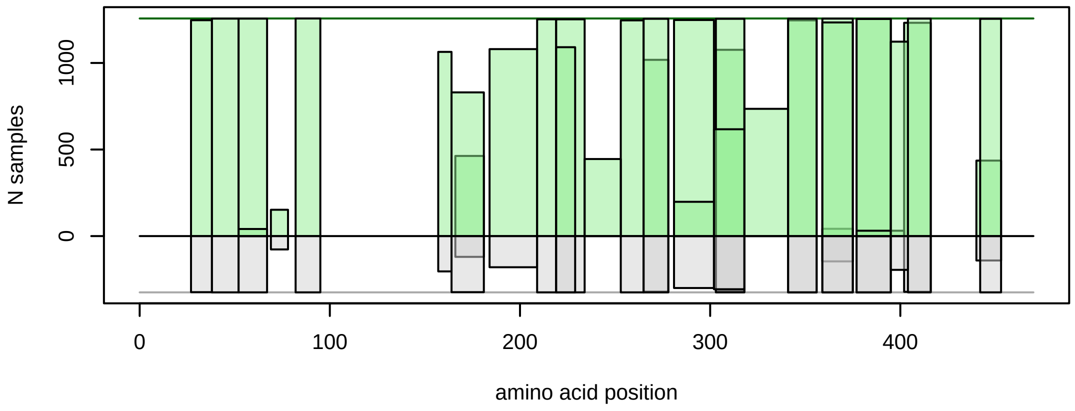

160. NID2 (Q14112), NP2

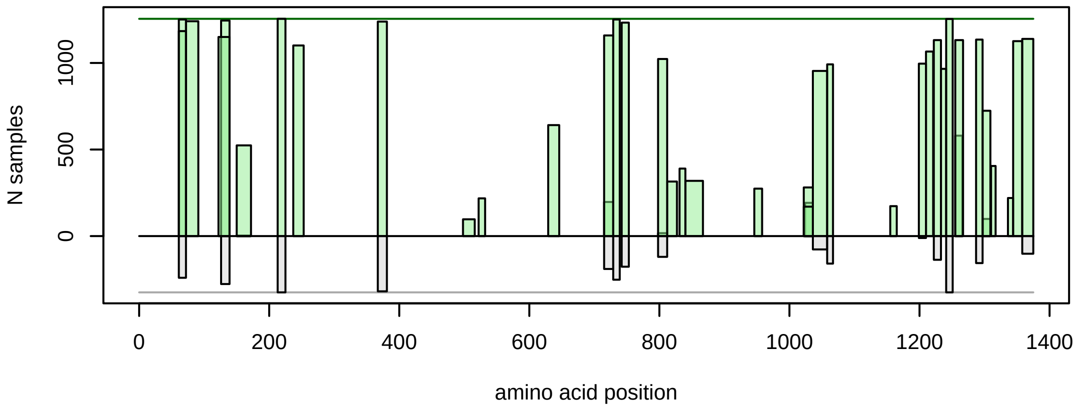

161. SRCRB4D (Q8WTU2), NP2

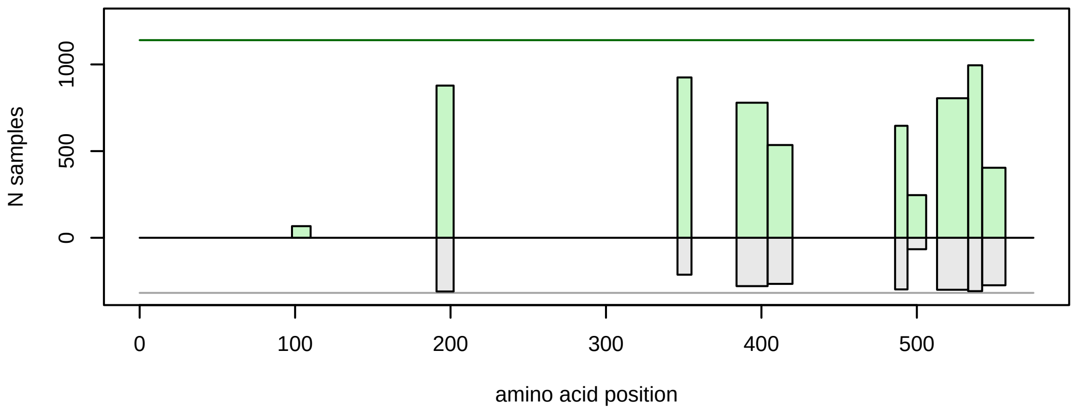

162. PADI4 (Q9UM07), NP1

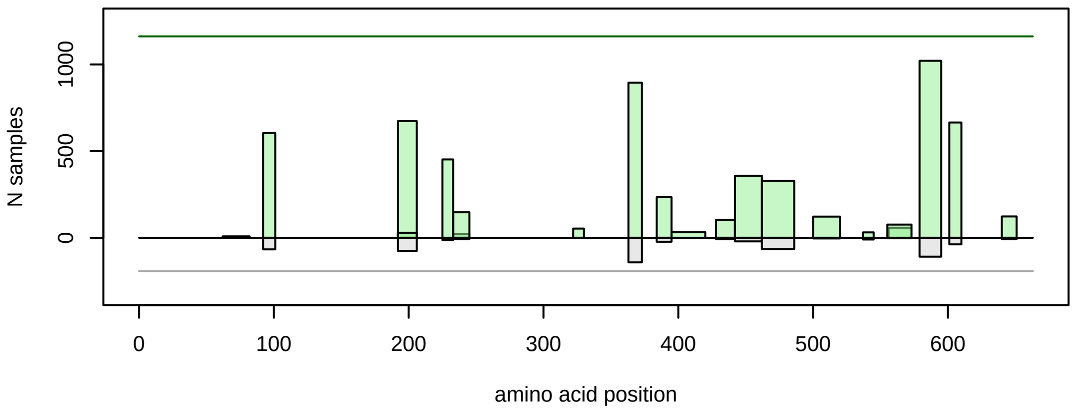

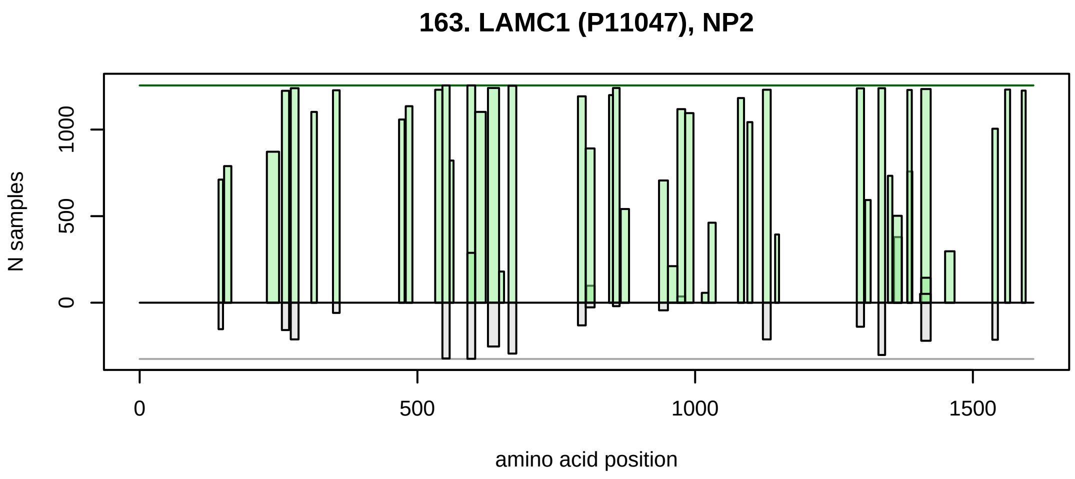
163. LAMC1 (P11047), NP2
N samples
0
500
1000
0
500
1000
1500
amino acid position

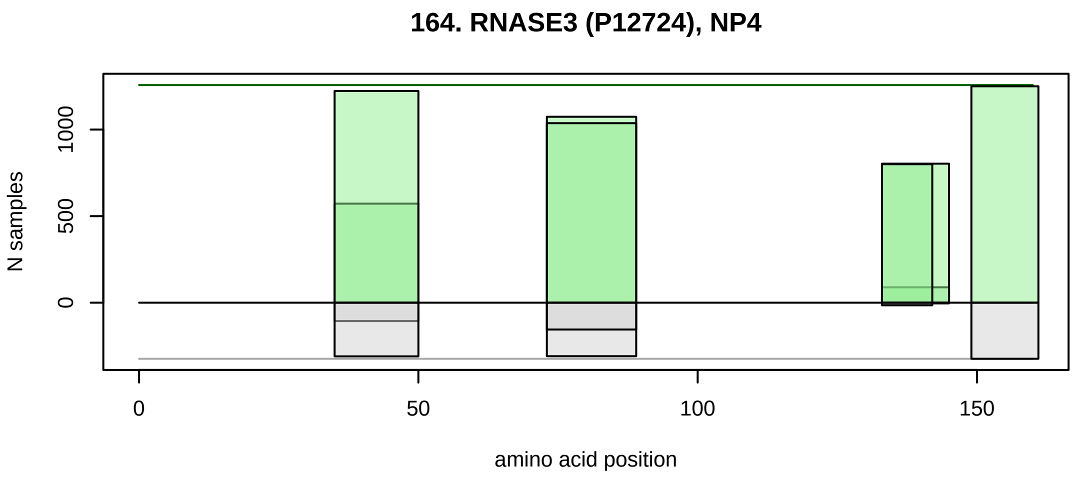
164. RNASE3 (P12724), NP4
N samples
0
500
1000
0
50
100
150
amino acid position

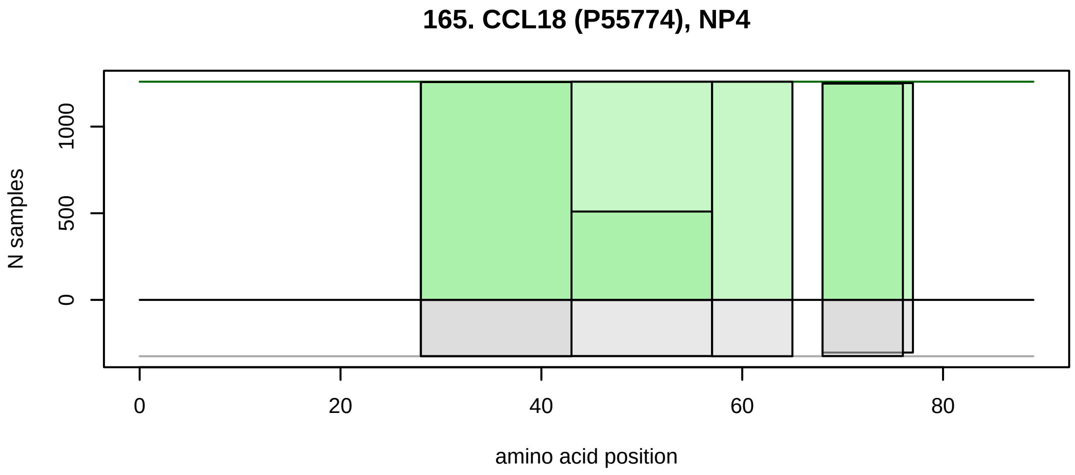
165. CCL18 (P55774), NP4
N samples
0
500
1000
0
20
40
60
80
amino acid position

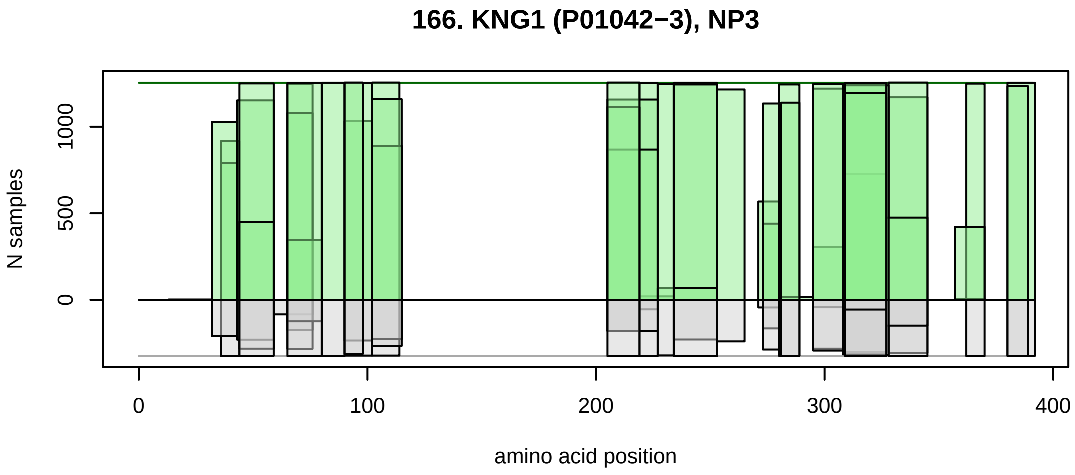
166. KNG1 (P01042−3), NP3
N samples
0
500
1000
0
100
200
300
400
amino acid position

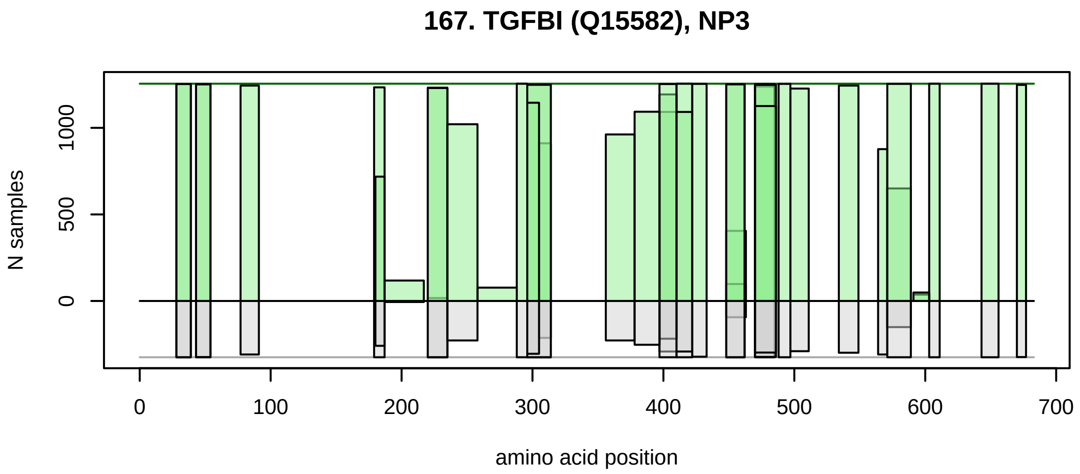
167. TGFBI (Q15582), NP3
N samples
0
500
1000
0
100
200
300
400
500
600
700
amino acid position

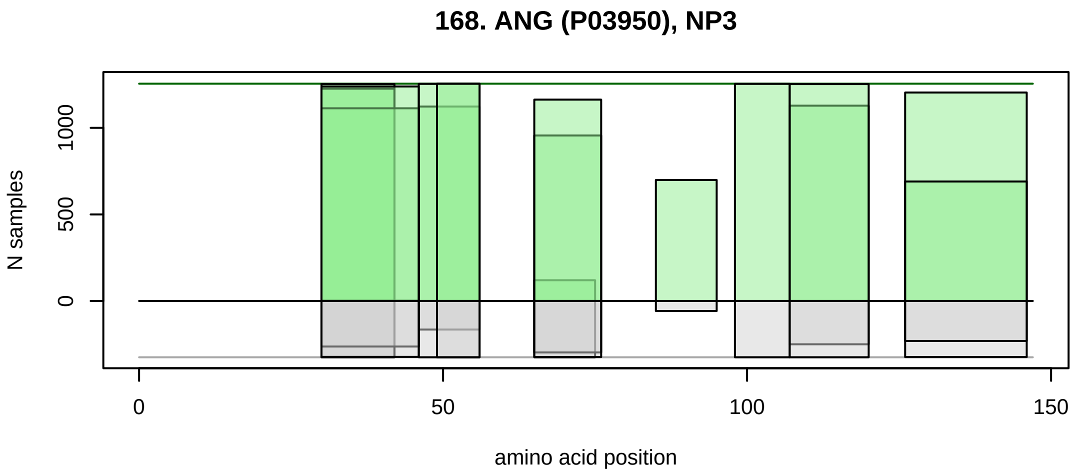
168. ANG (P03950), NP3
N samples
0
500
1000
0
50
100
150
amino acid position

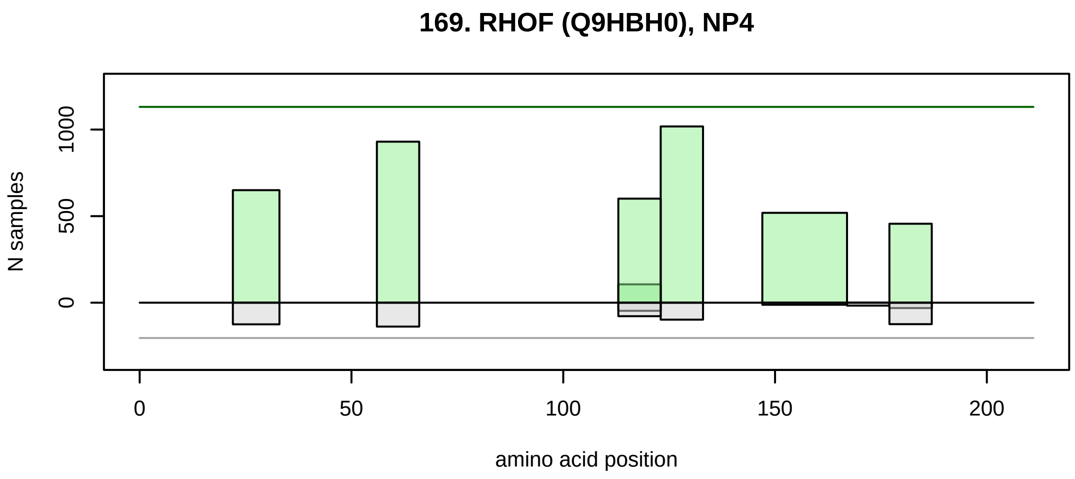
169. RHOF (Q9HBH0), NP4
N samples
0
500
1000
0
50
100
150
200
amino acid position

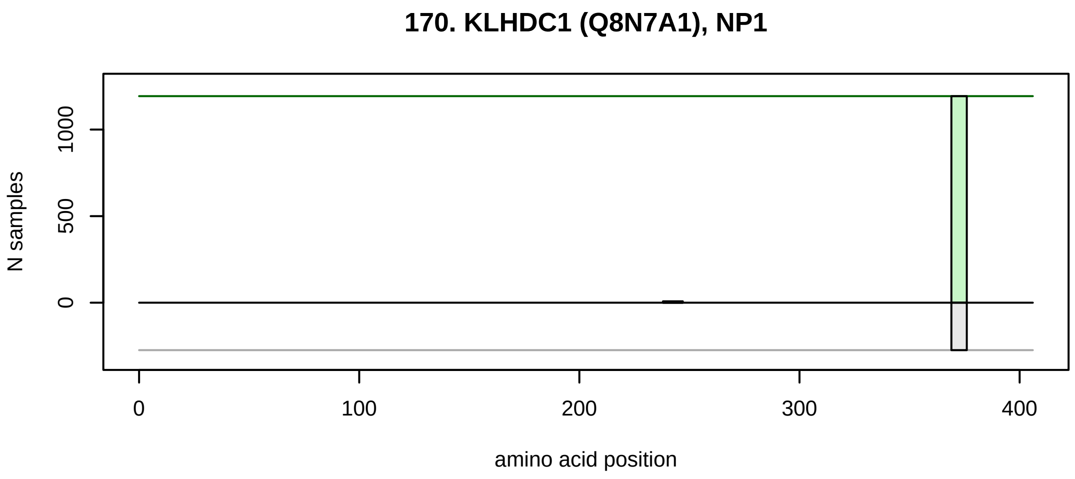
170. KLHDC1 (Q8N7A1), NP1
N samples
0
500
1000
0
100
200
300
400
amino acid position

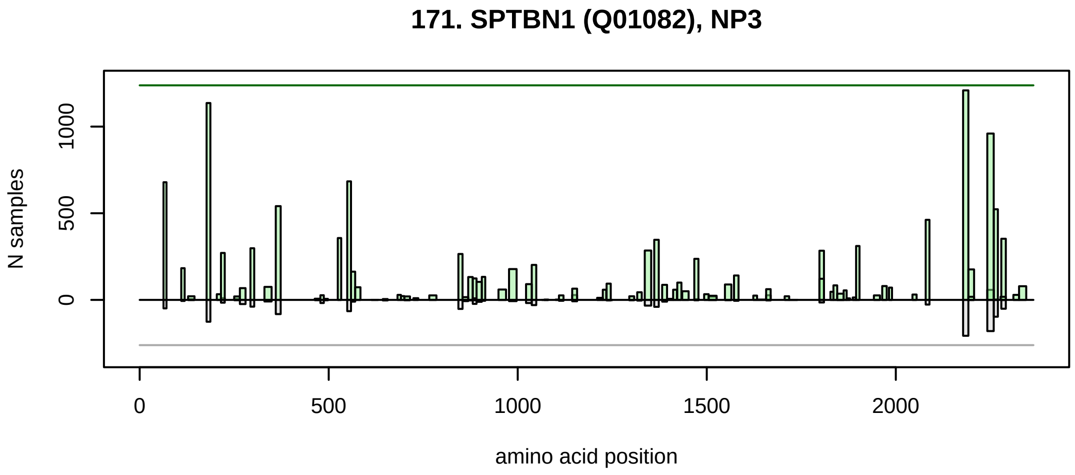
171. SPTBN1 (Q01082), NP3
N samples
0
500
1000
0
500
1000
1500
2000
amino acid position

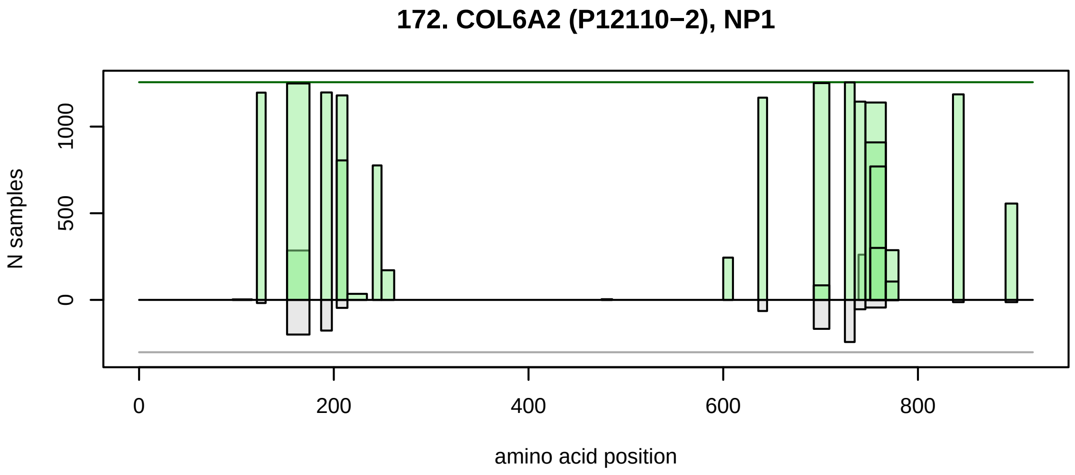
172. COL6A2 (P12110−2), NP1
N samples
0
500
1000
0
200
400
600
800
amino acid position

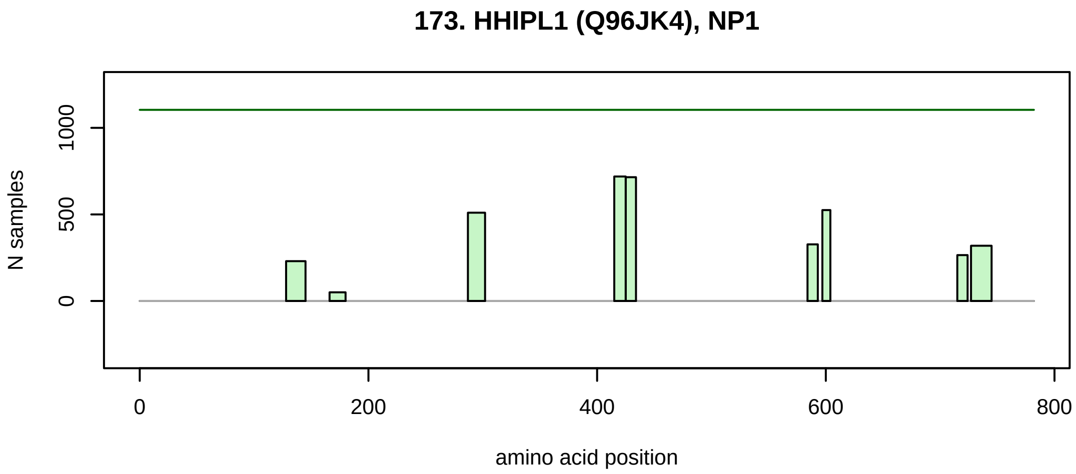
173. HHIPL1 (Q96JK4), NP1
N samples
0
500
1000
0
200
400
600
800
amino acid position

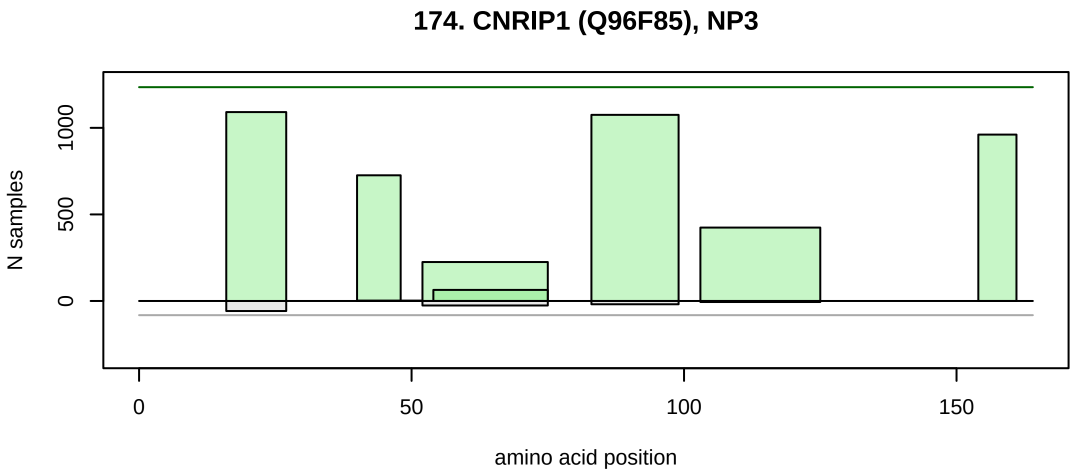
174. CNRIP1 (Q96F85), NP3
N samples
0
500
1000
0
50
100
150
amino acid position

175. DOCK9 (Q9BZ29), NP3

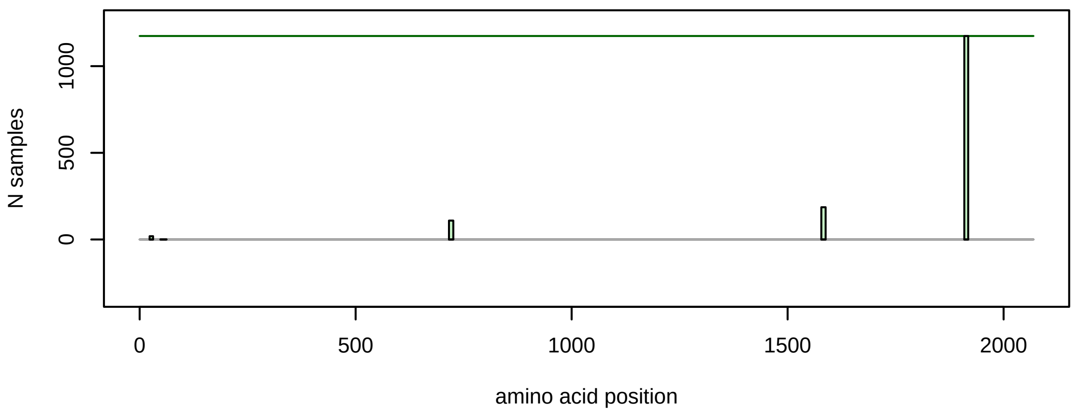

176. CD151 (P48509), NP4

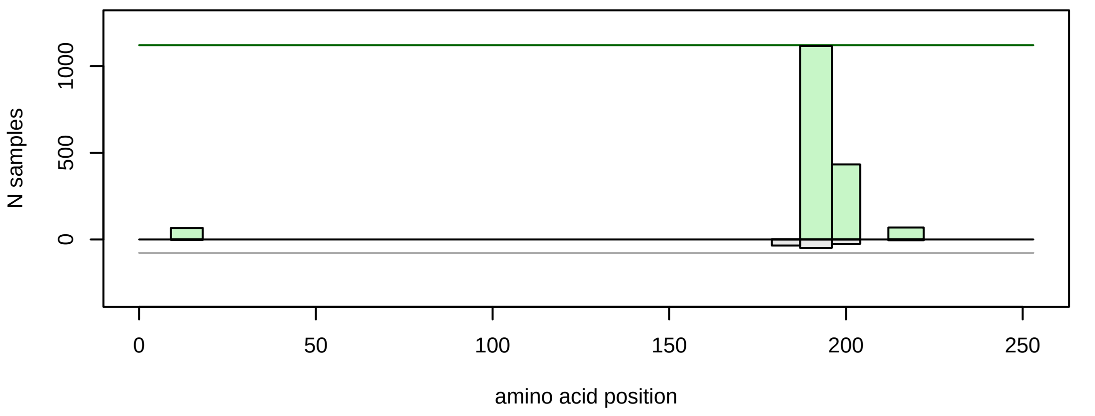

177. PON3 (Q15166), NP5

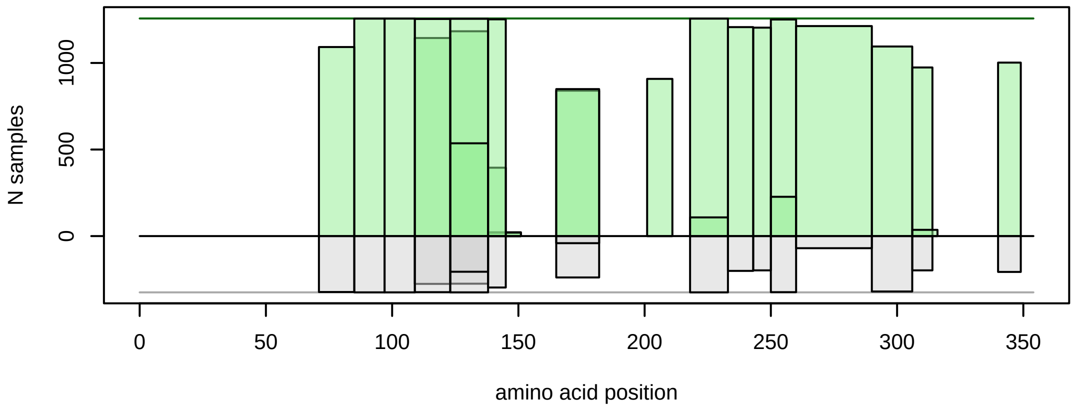

178. FABP5 (Q01469), NP2

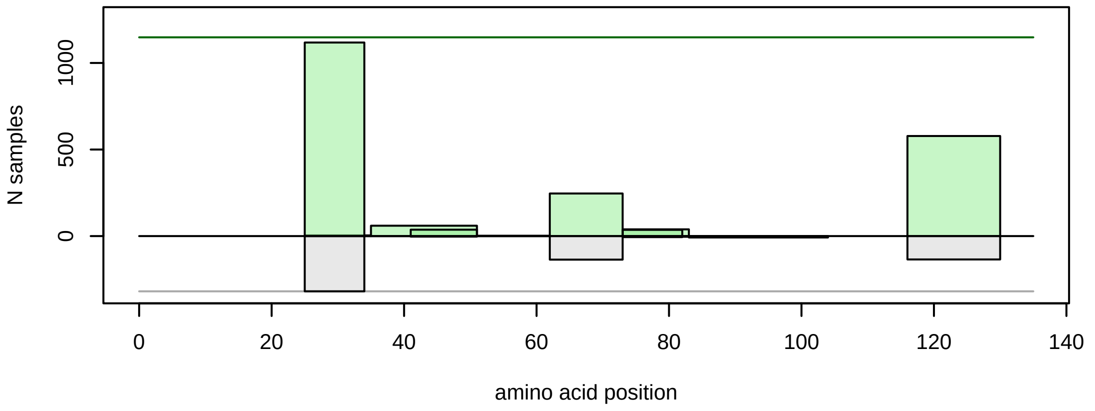

179. COCH (A0A2U3TZE7;O43405), NP3

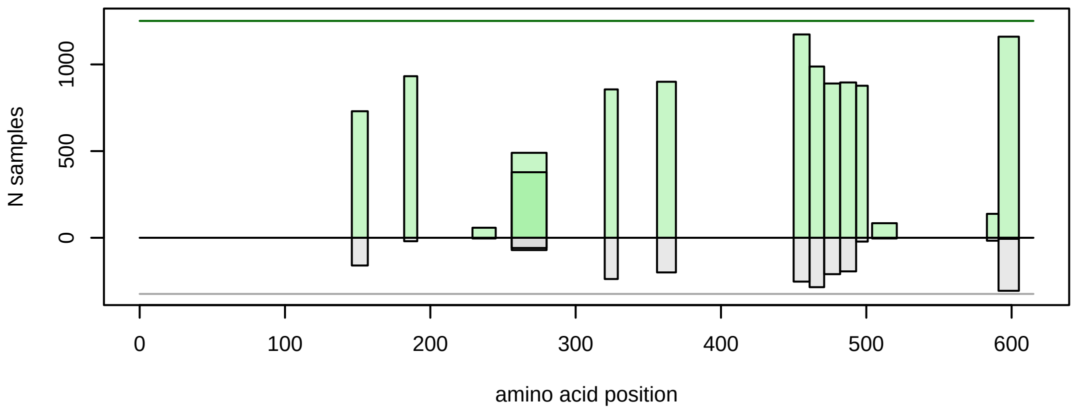

180. PAK2 (Q13177), NP1

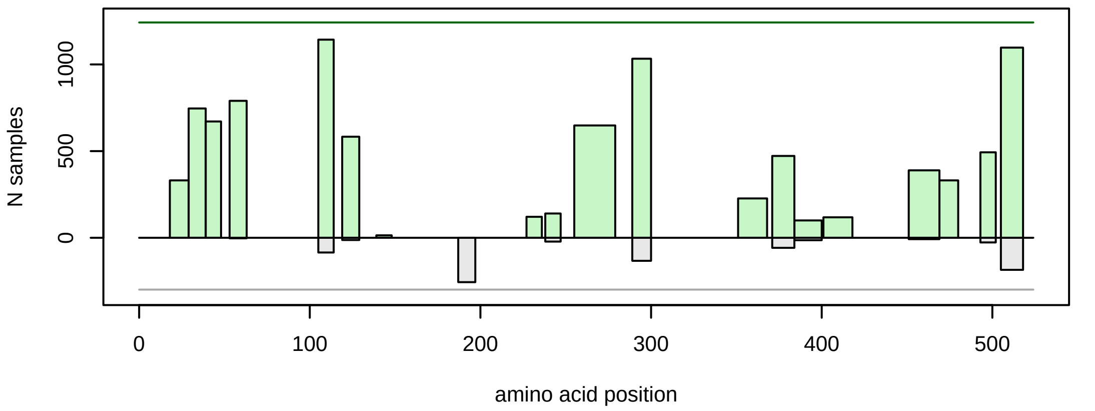

181. PGD (P52209), NP1

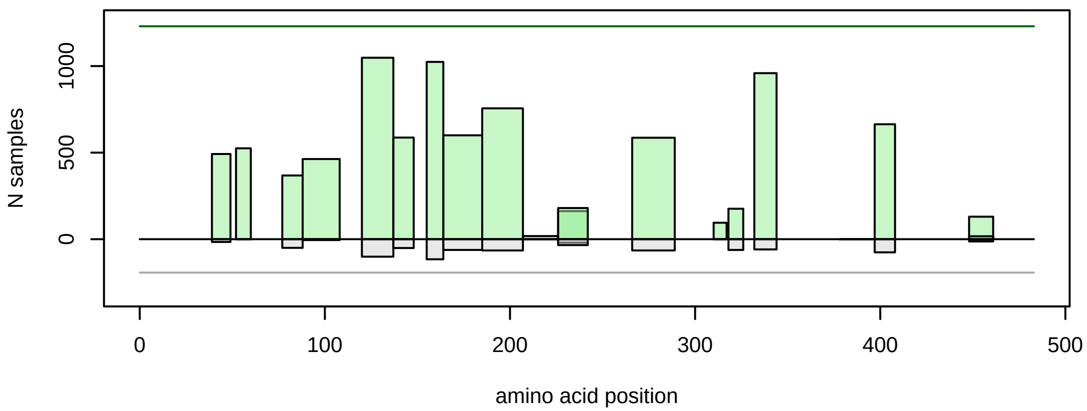

182. SPINK5 (Q9NQ38−3), NP3

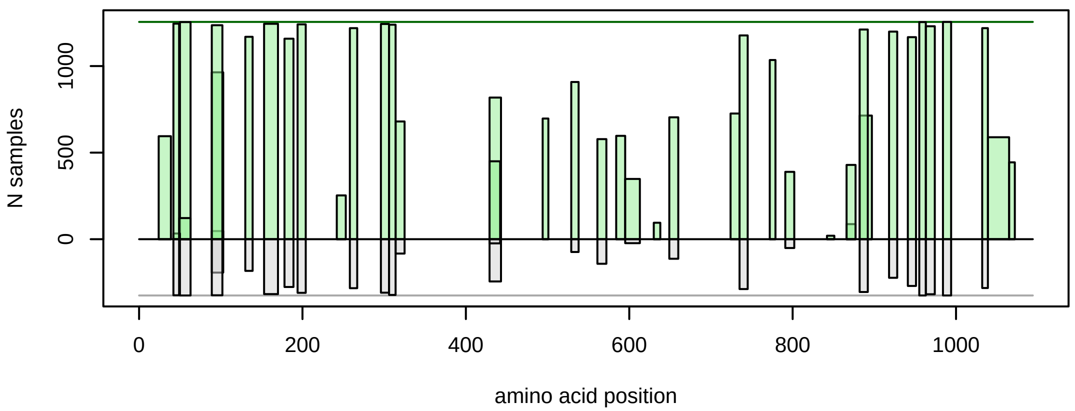

183. LINC00854 (A0A1B0GVX4), NP5

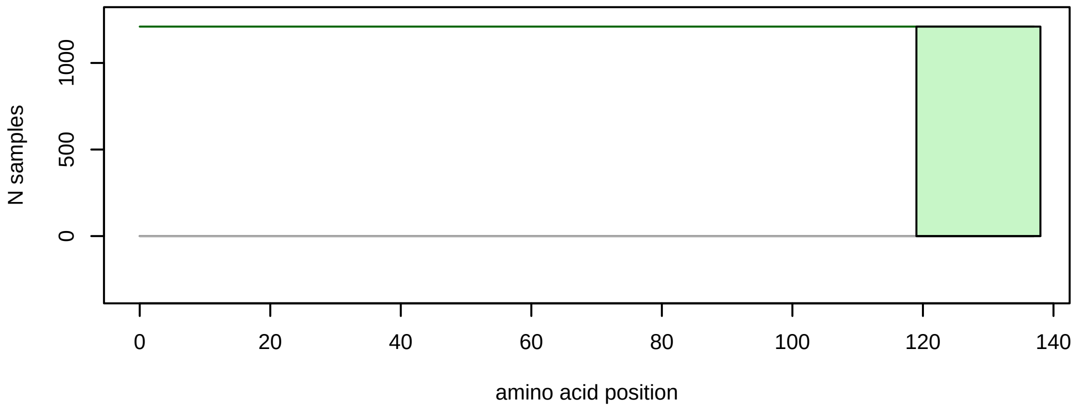

184. QSOX1 (O00391), NP1

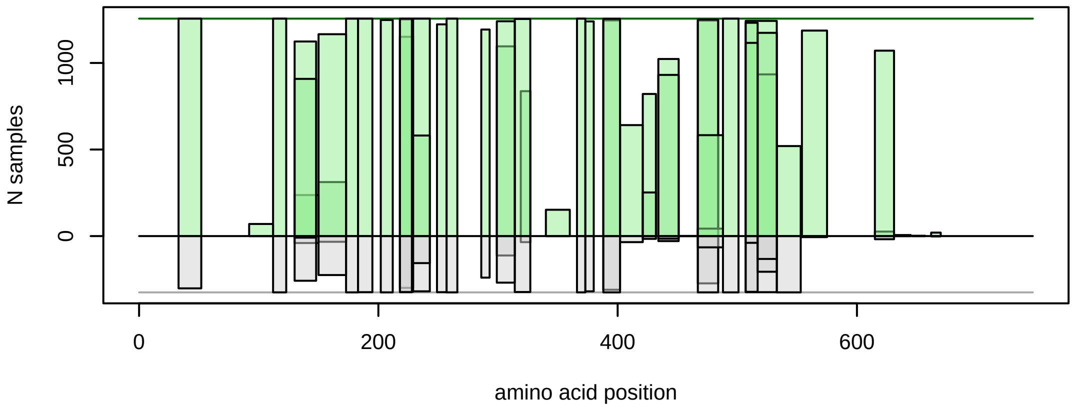

185. PGM3 (H0Y987), NP5

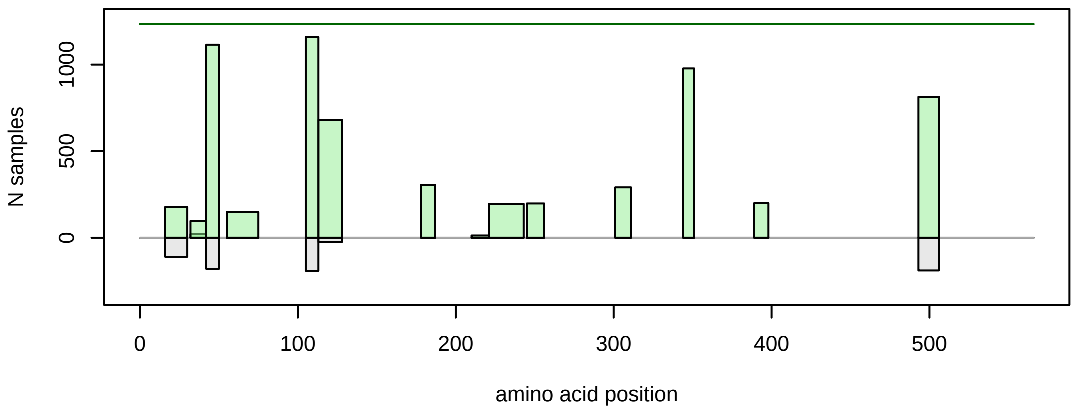

186. SKP1 (P63208), NP1

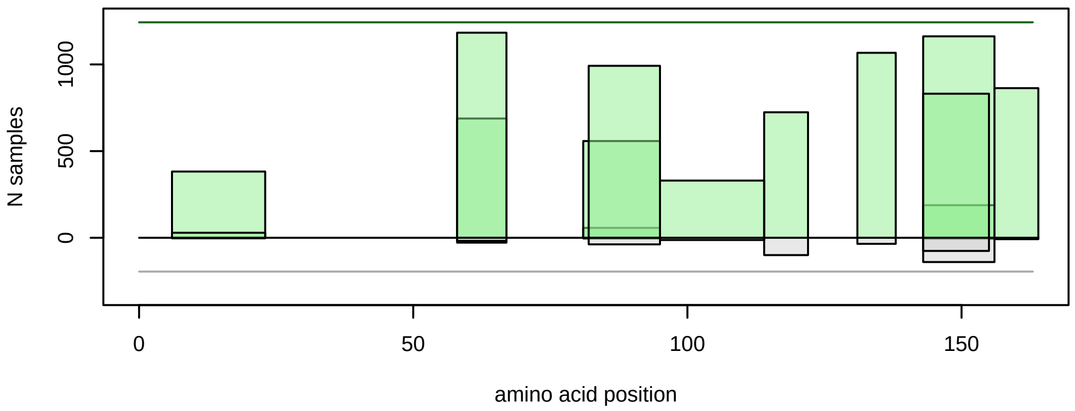

187. HPR (P00739), NP4

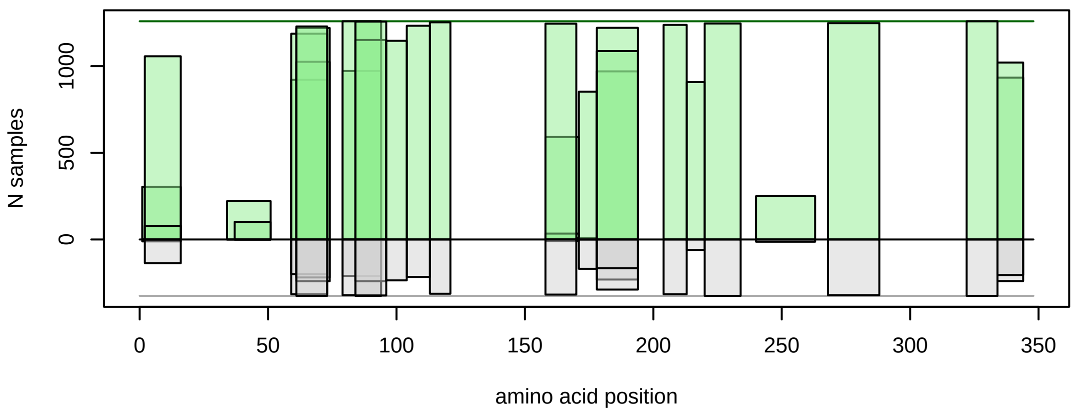

188. IGKV1−33 (P01594), NP3

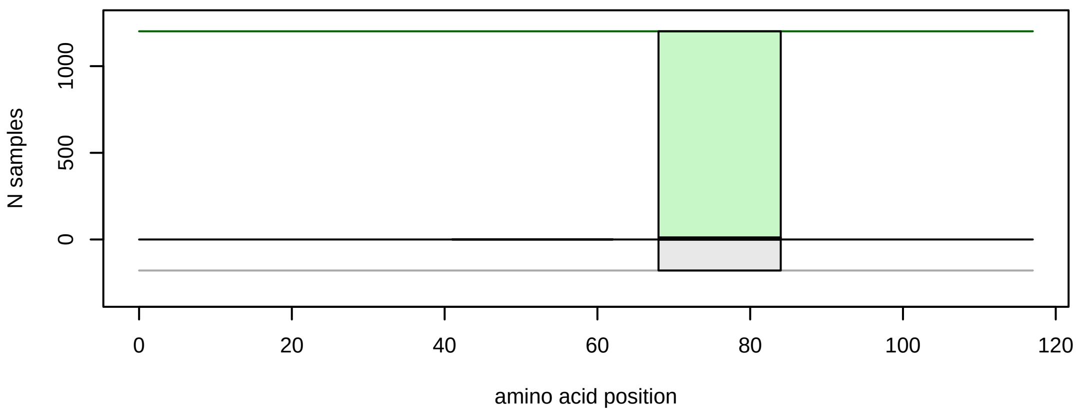

189. CTSB (E9PHZ5), NP2

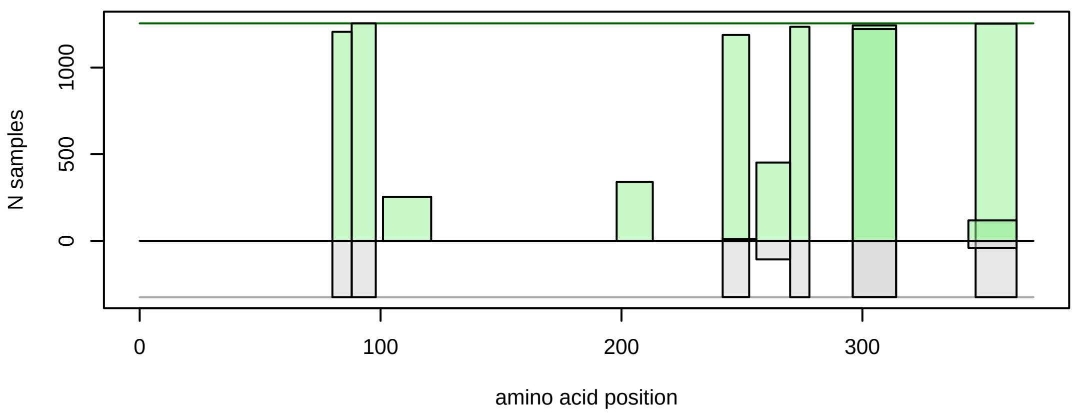

190. PPIB (A0A7P0Z497), NP3

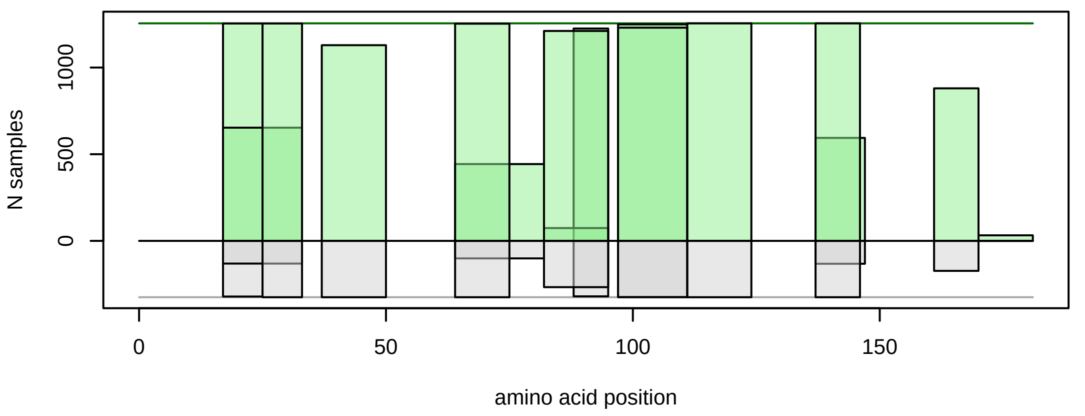

191. DNAJC10 (A0A7P0Z431;Q8IXB1), NP3

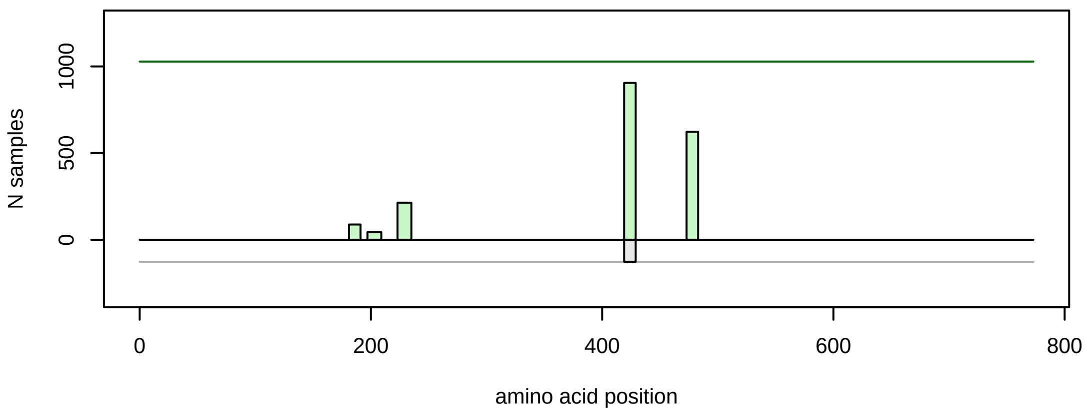

192. ST14 (Q9Y5Y6), NP5

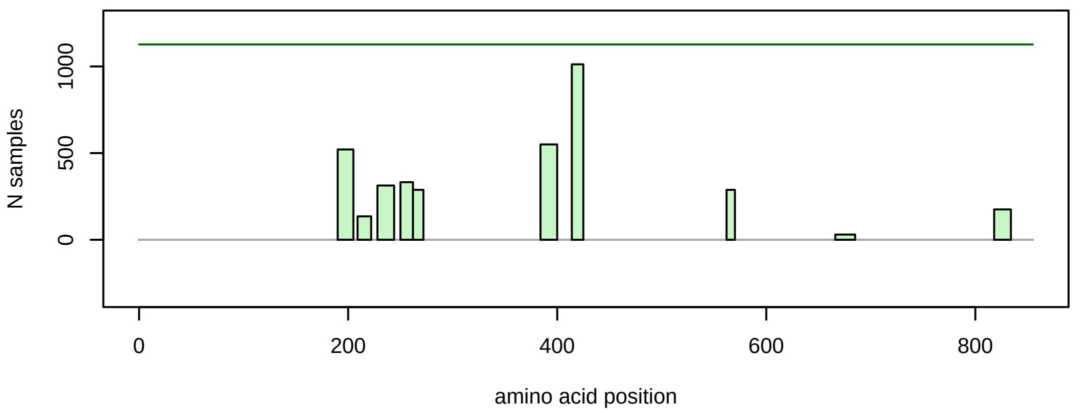

193. PLA2G7 (Q13093), NP4

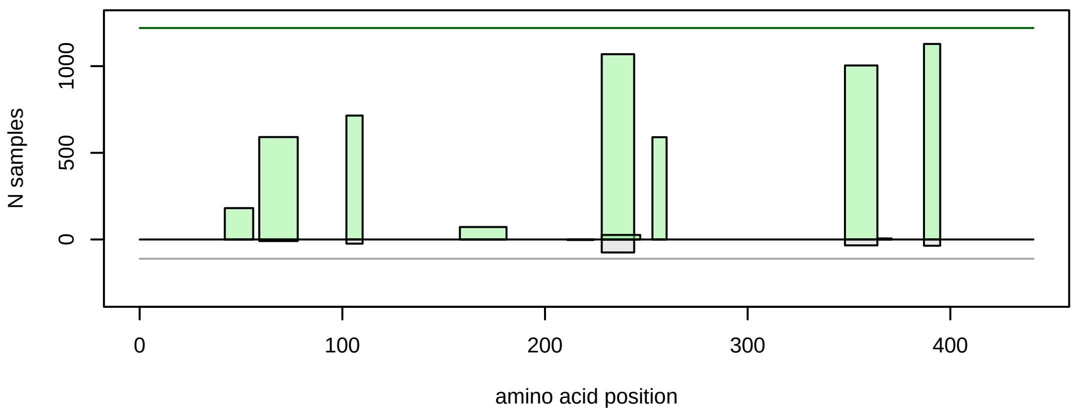

194. SERPINA4 (P29622), NP4

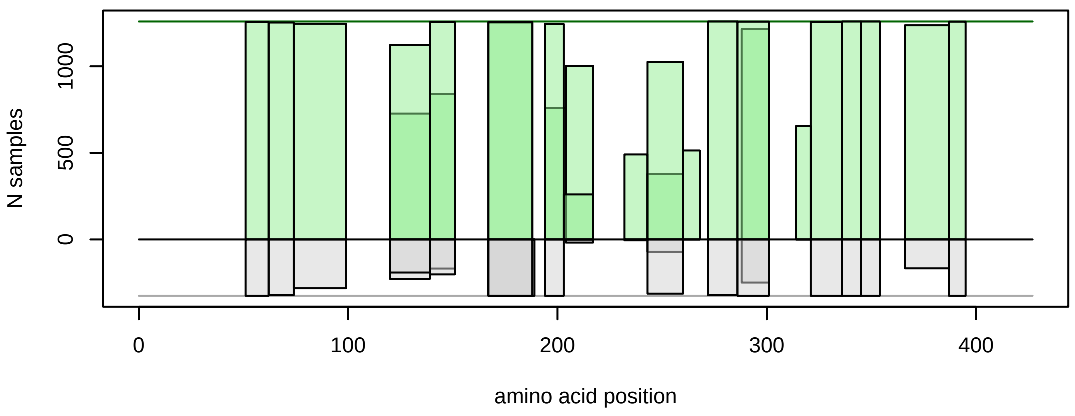

195. THBS3 (F5H4Z8;P49746), NP2

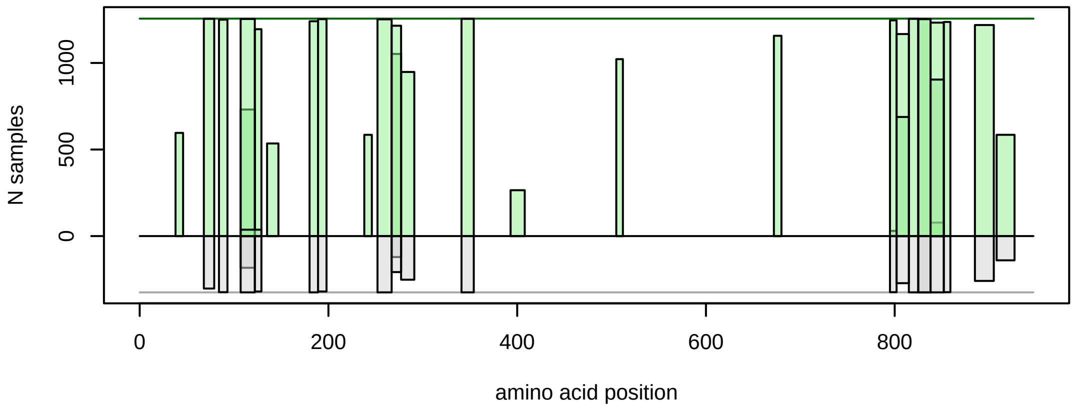

196. C4A (P0C0L4;P0C0L4−2), NP2

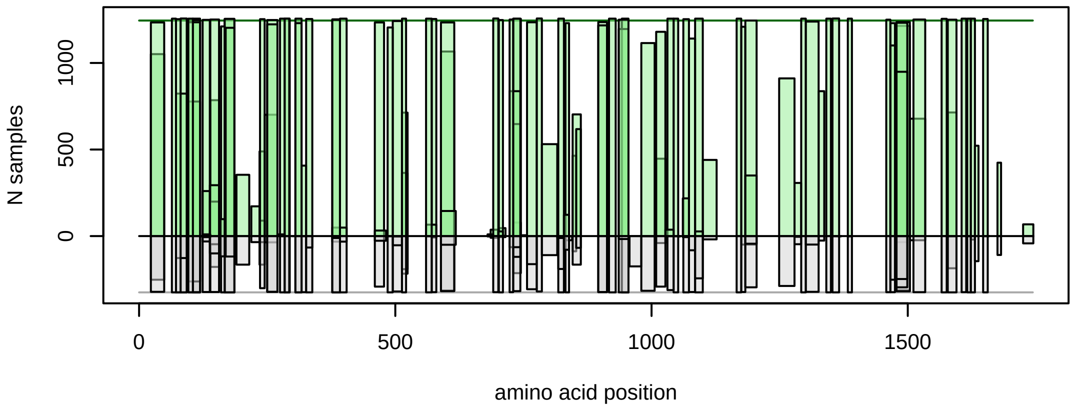

197. LAMB1 (P07942), NP2

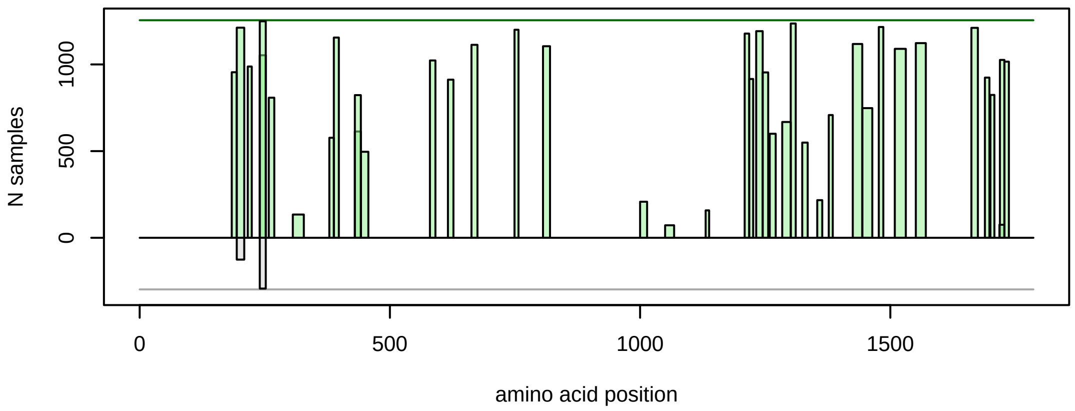

198. LTBP1 (Q14766), NP4

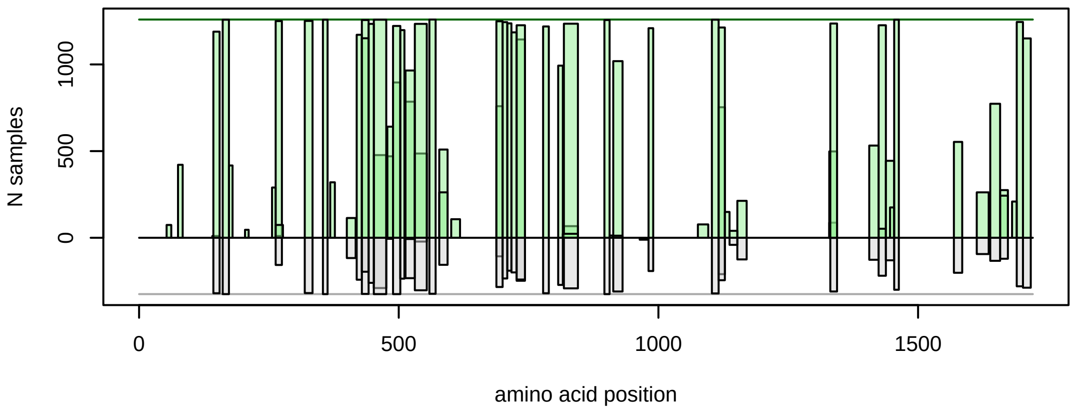

199. RAB11B (Q15907), NP4

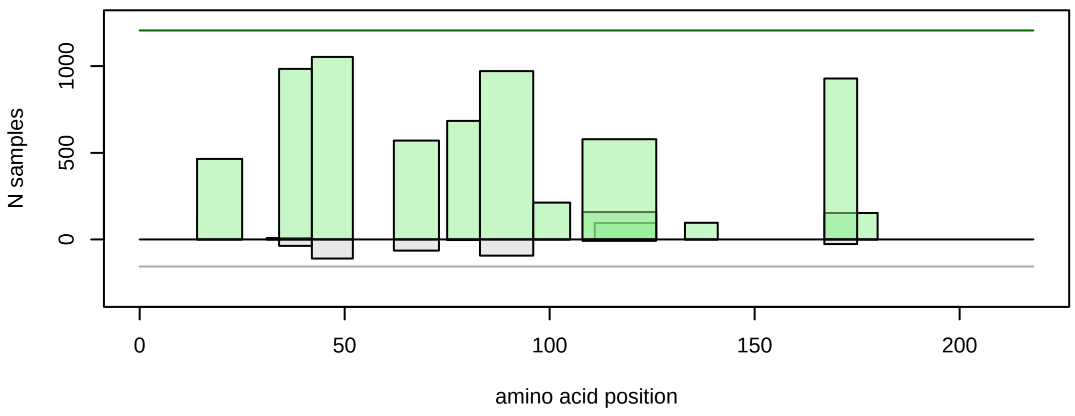

200. APOE (P02649), NP5

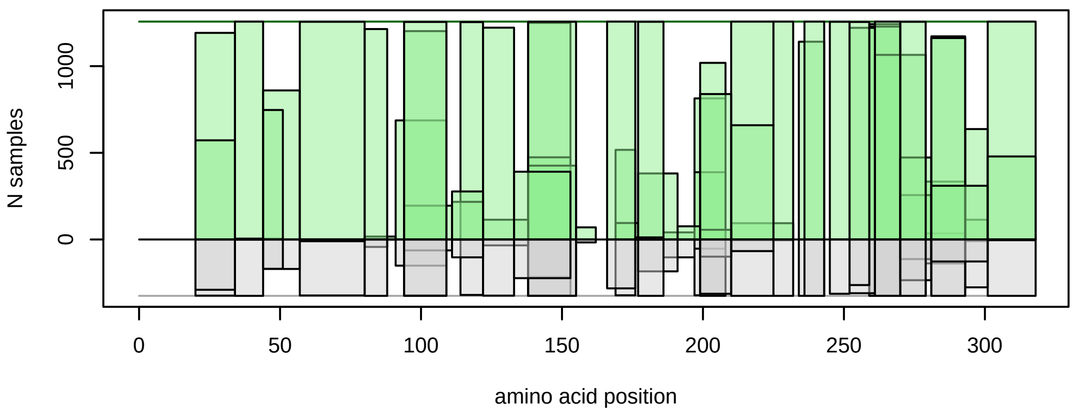

201. LYNX1 (P0DP58), NP3

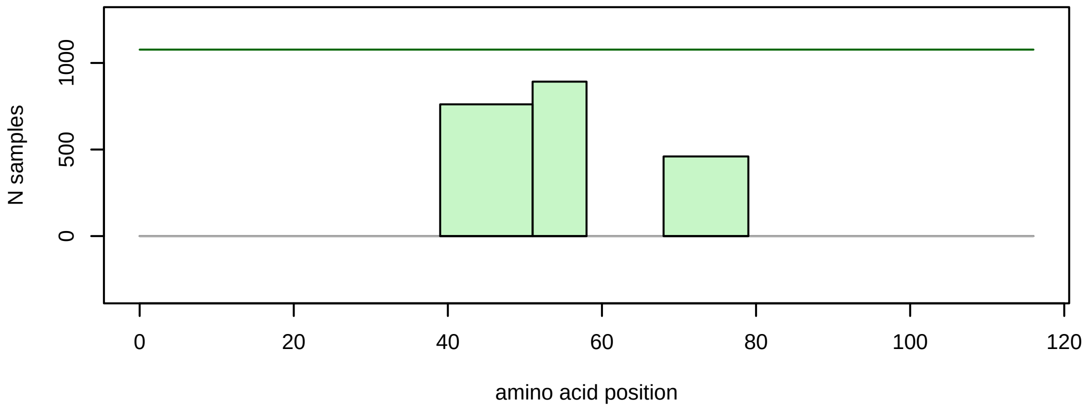

202. MAN2A1 (Q16706), NP2

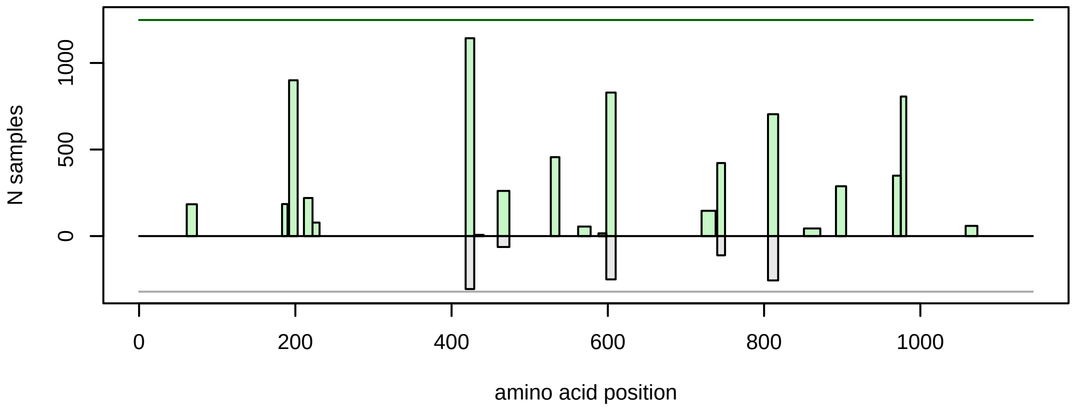

203. UBR1 (Q8IWV7), NP4

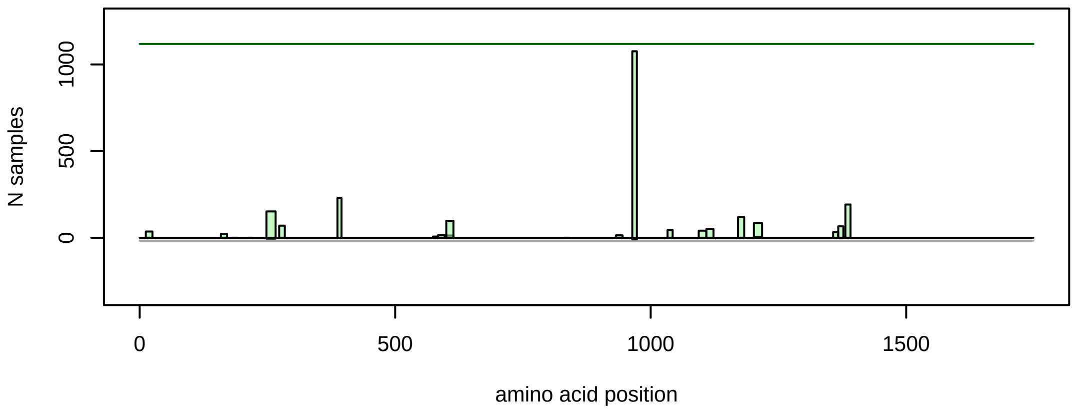

204. PAIP1 (Q9H074), NP1

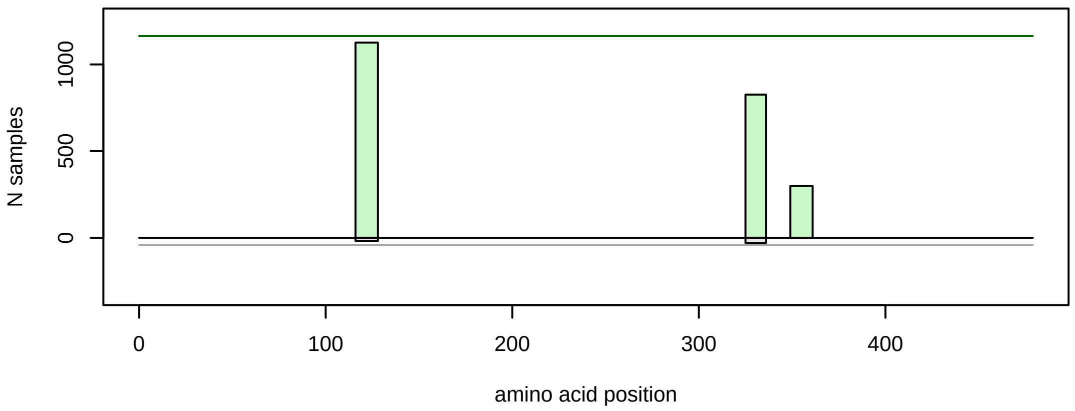

205. APOF (Q13790), NP2

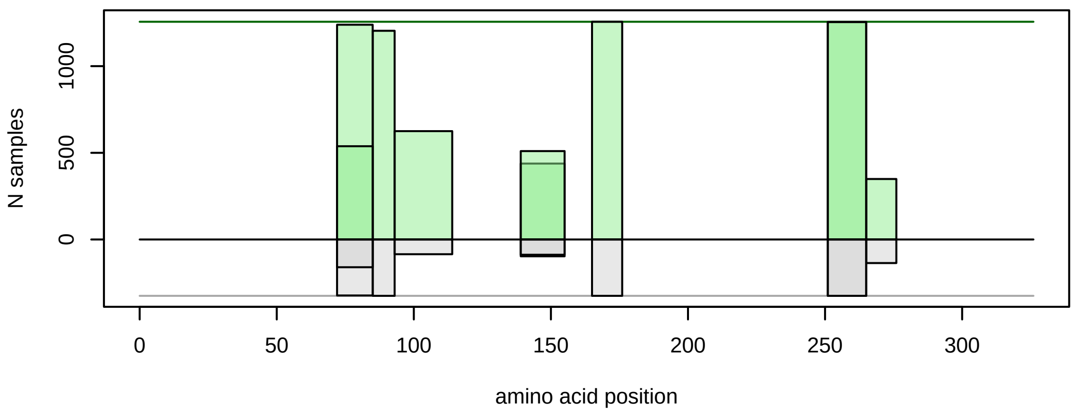

206. LECT2 (O14960), NP1

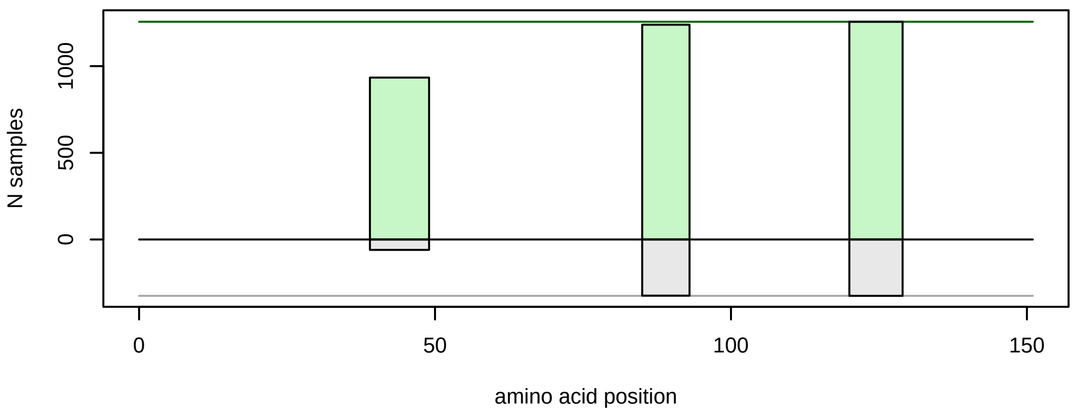

207. CDH5 (P33151), NP2

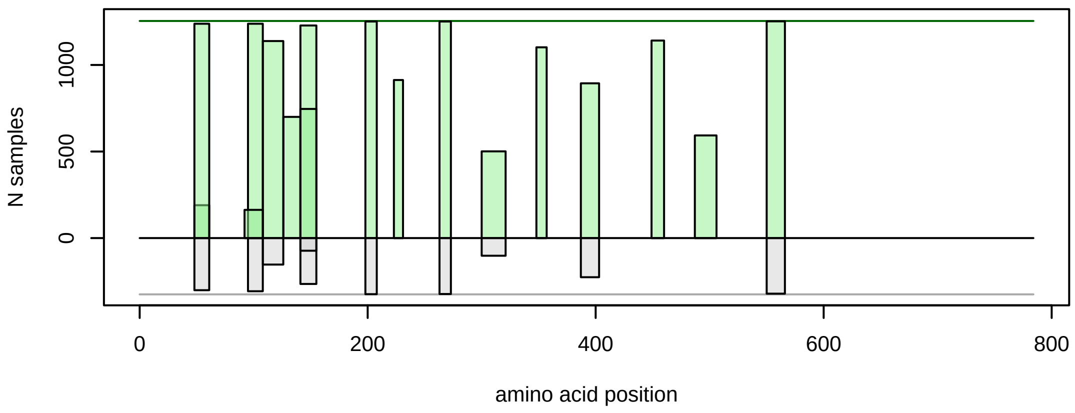

208. CAPN1 (P07384), NP5

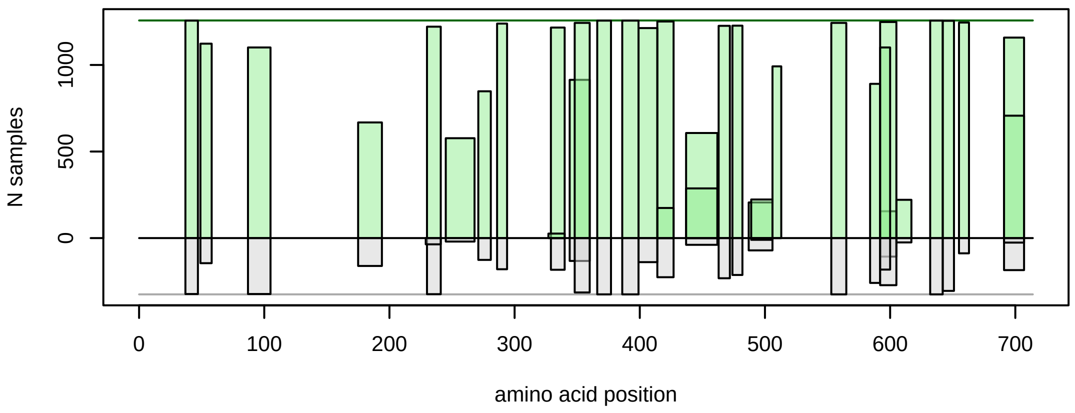

209. F8 (P00451), NP4

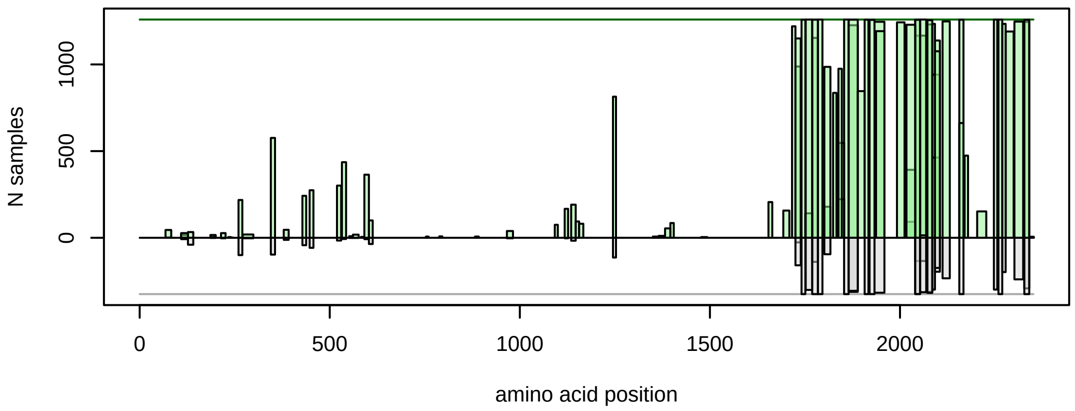

210. ALDH9A1 (P49189−3), NP5

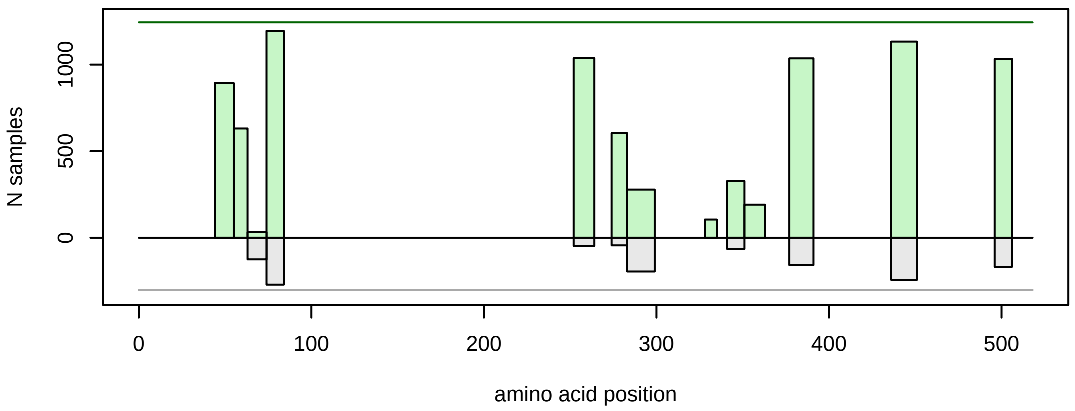

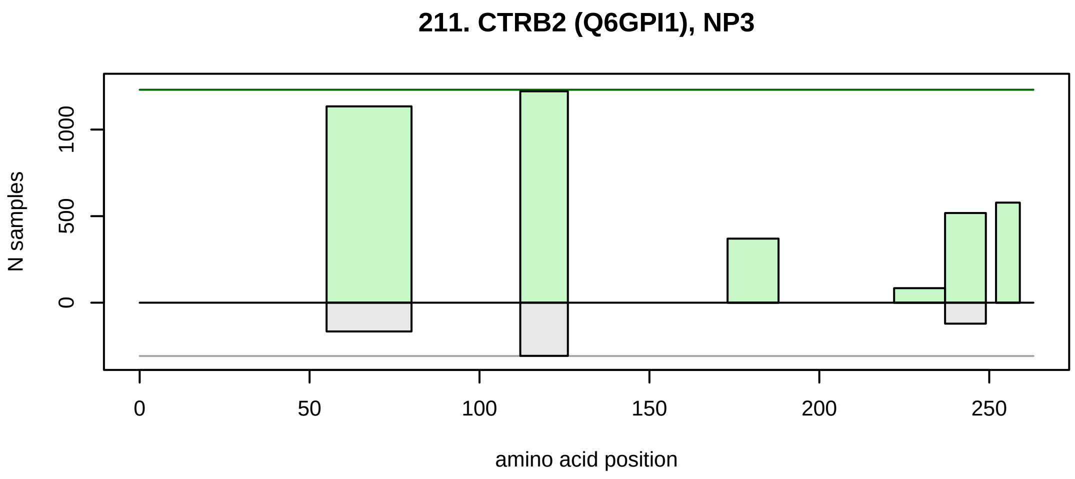
211. CTRB2 (Q6GPI1), NP3
N samples
0
500
1000
0
50
100
150
200
250
amino acid position

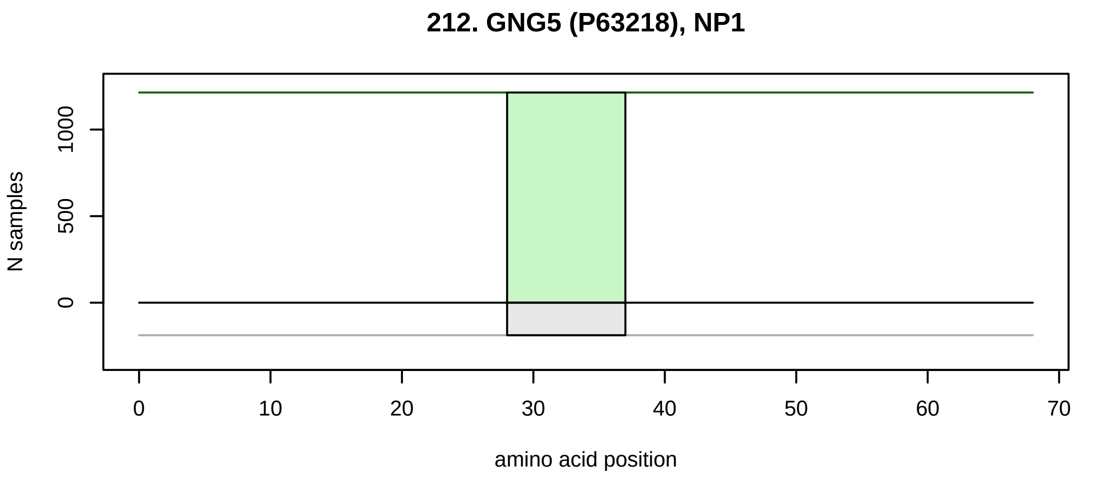
212. GNG5 (P63218), NP1
N samples
0
500
1000
0
10
20
30
40
50
60
70
amino acid position

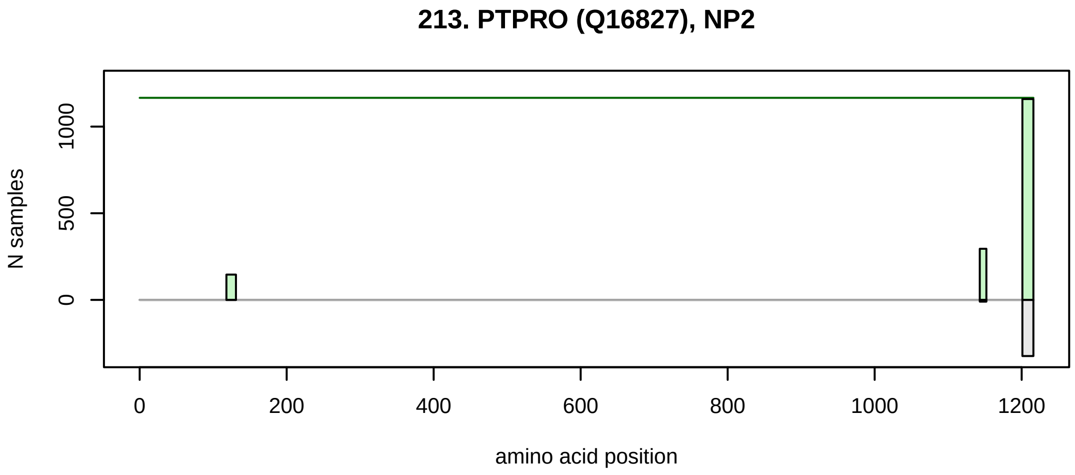
213. PTPRO (Q16827), NP2
N samples
0
500
1000
0
200
400
600
800
1000
1200
amino acid position

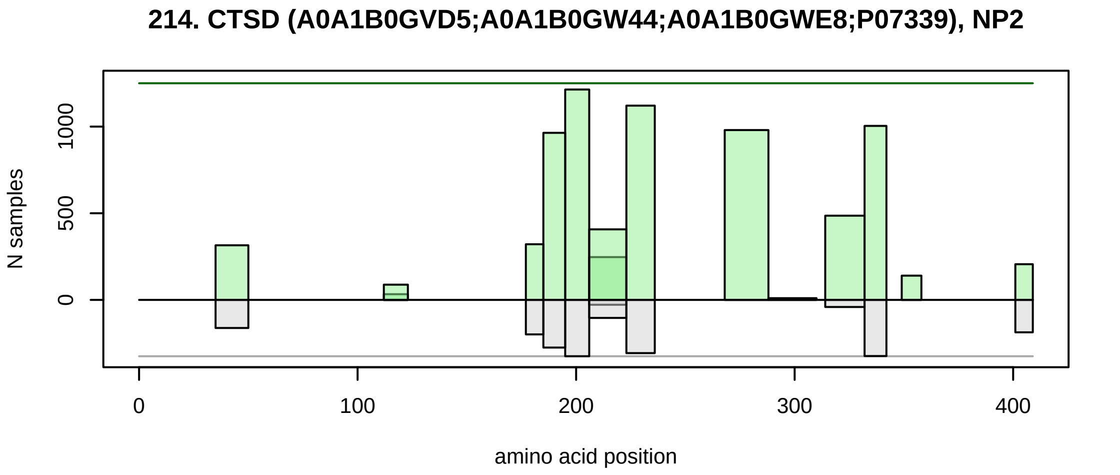
214. CTSD (A0A1B0GVD5;A0A1B0GW44;A0A1B0GWE8;P07339), NP2
N samples
0
500
1000
0
100
200
300
400
amino acid position

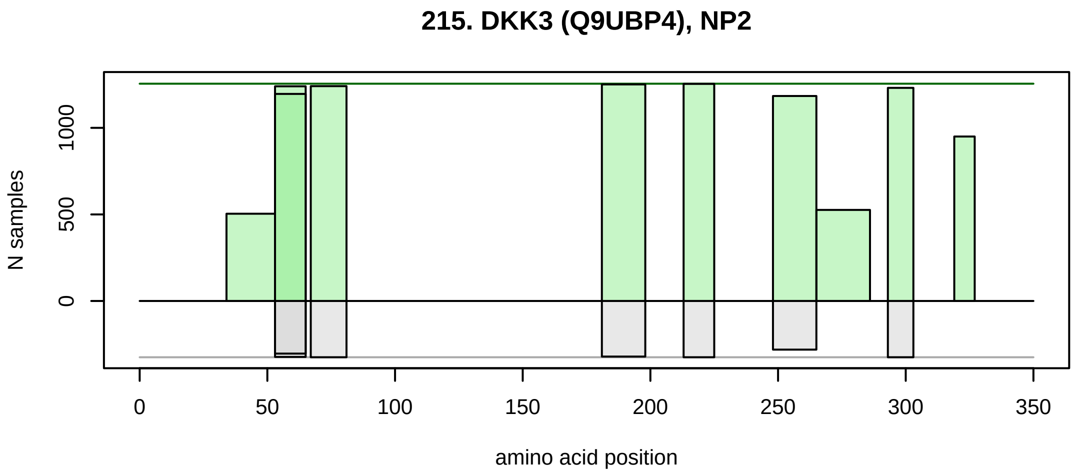
215. DKK3 (Q9UBP4), NP2
N samples
0
500
1000
0
50
100
150
200
250
300
350
amino acid position

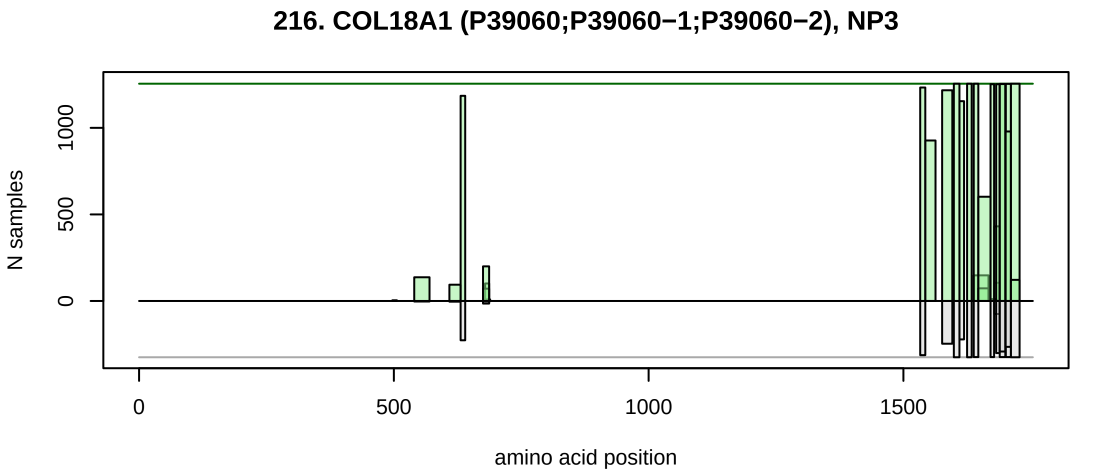
216. COL18A1 (P39060;P39060−1;P39060−2), NP3
N samples
0
500
1000
0
500
1000
1500
amino acid position

217. PAM (P19021−5), NP5

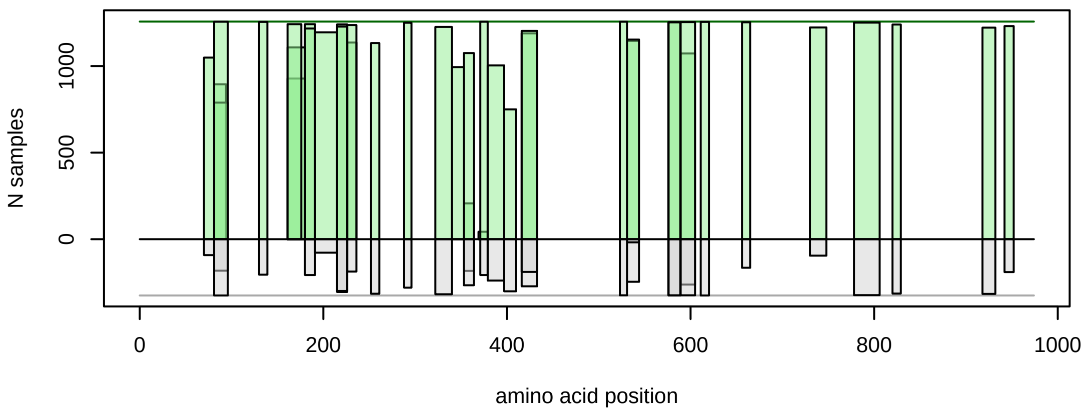

218. AZU1 (P20160), NP4

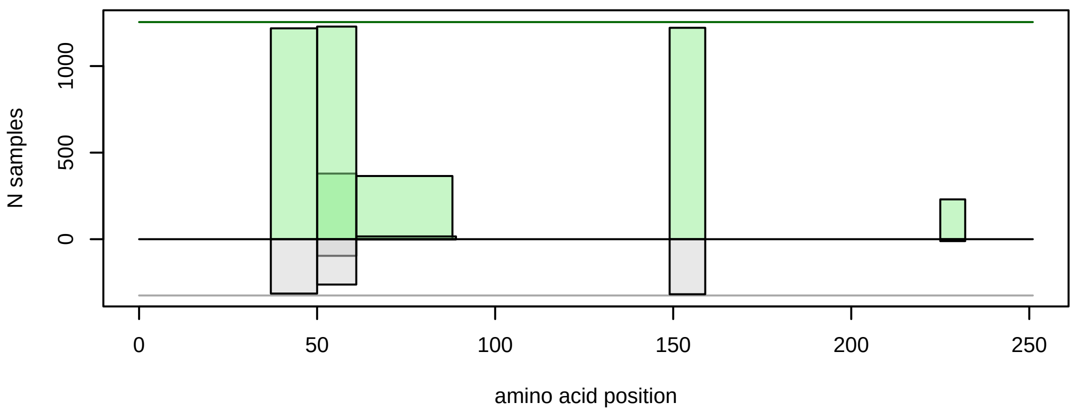

219. PFKP (Q01813), NP4

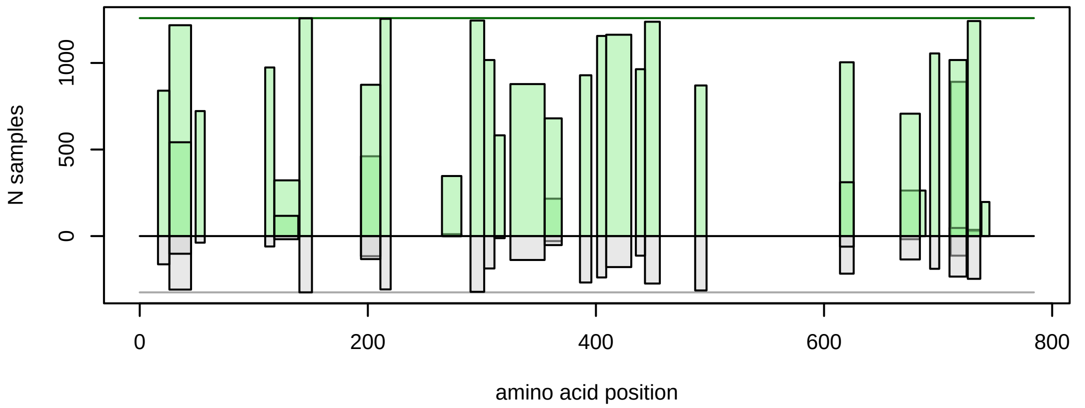

220. SCP2 (P22307), NP3

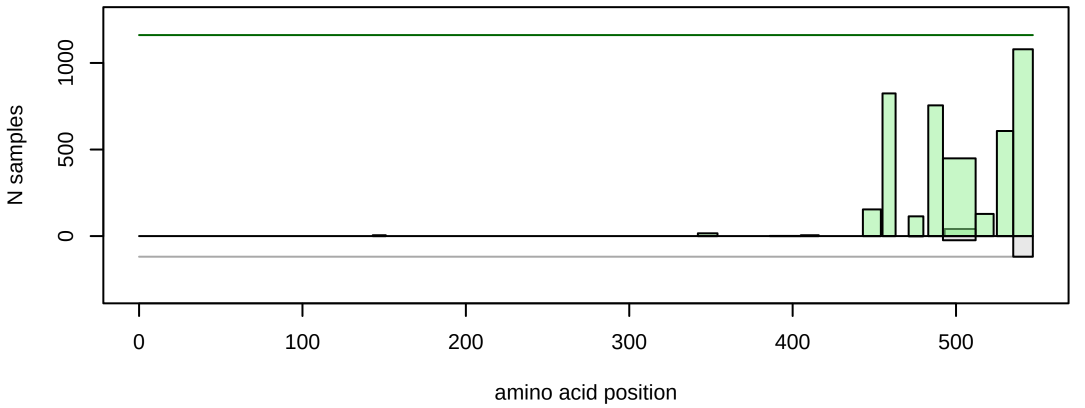

221. NCF1 (P14598), NP1

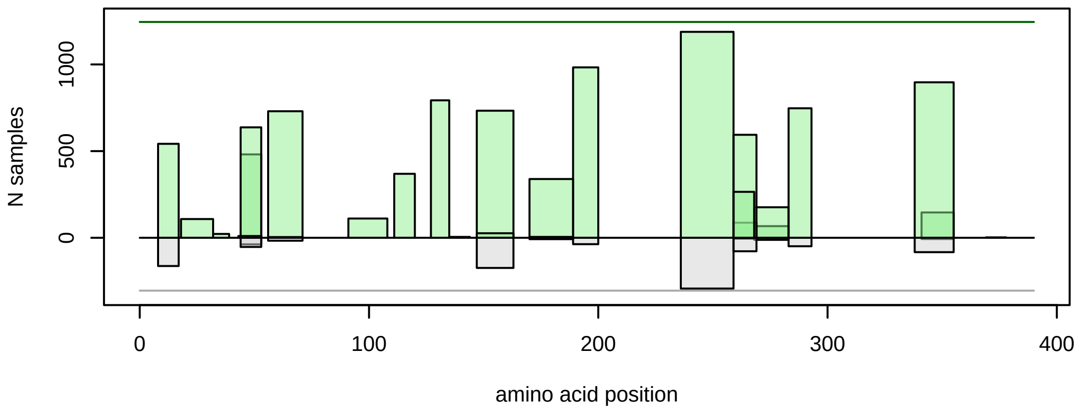

222. DPT (Q07507), NP2

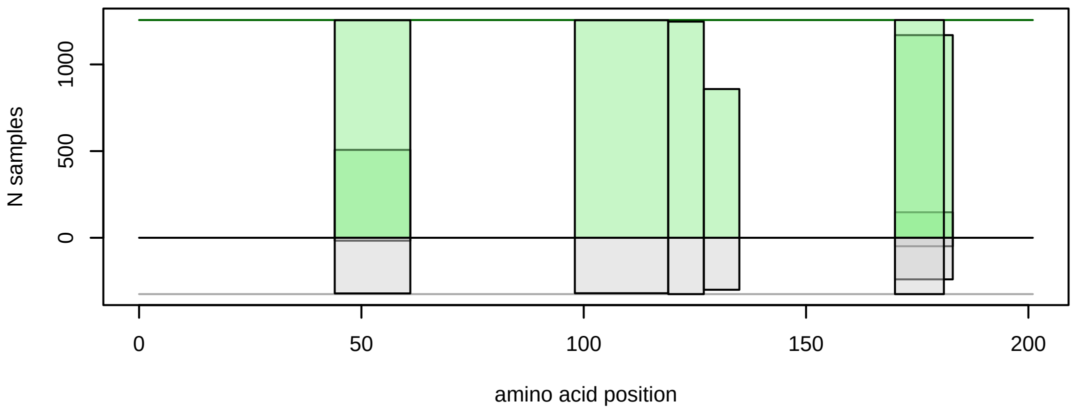

223. CYBB (P04839), NP1

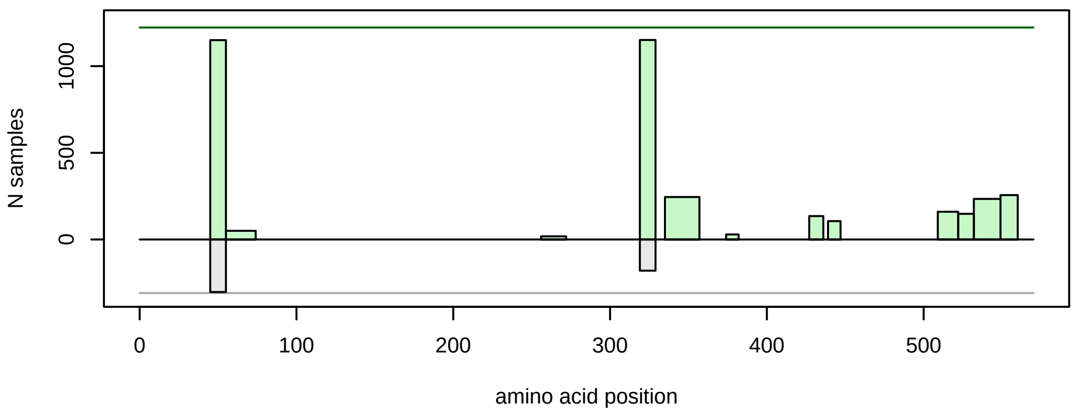

224. SLC25A3 (Q00325;Q00325−2), NP4

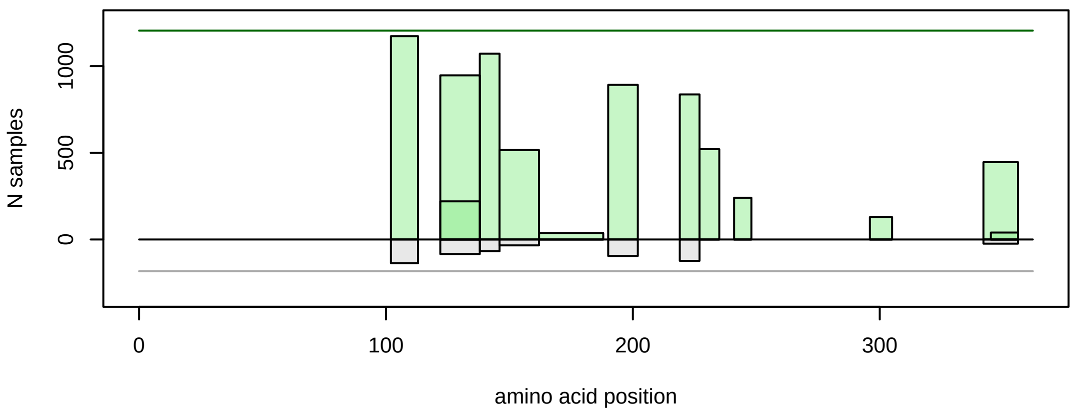

225. DDX6 (P26196), NP1

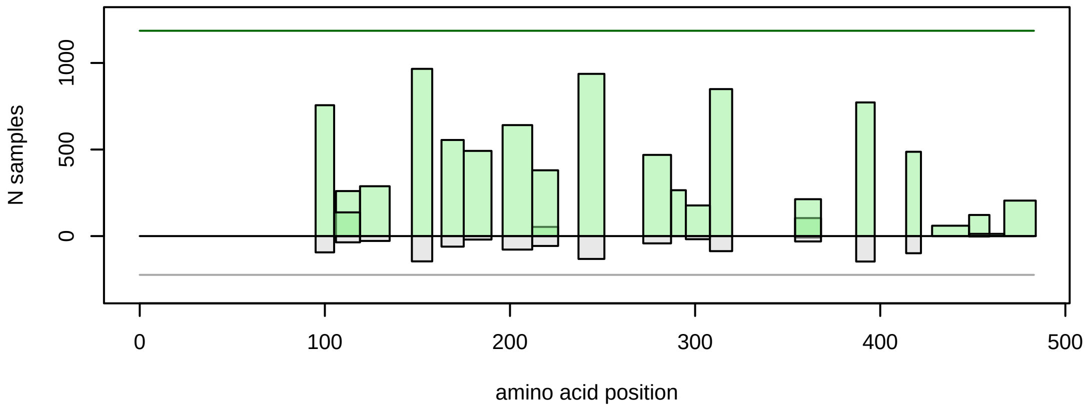

226. ADAMTS13 (Q76LX8), NP4

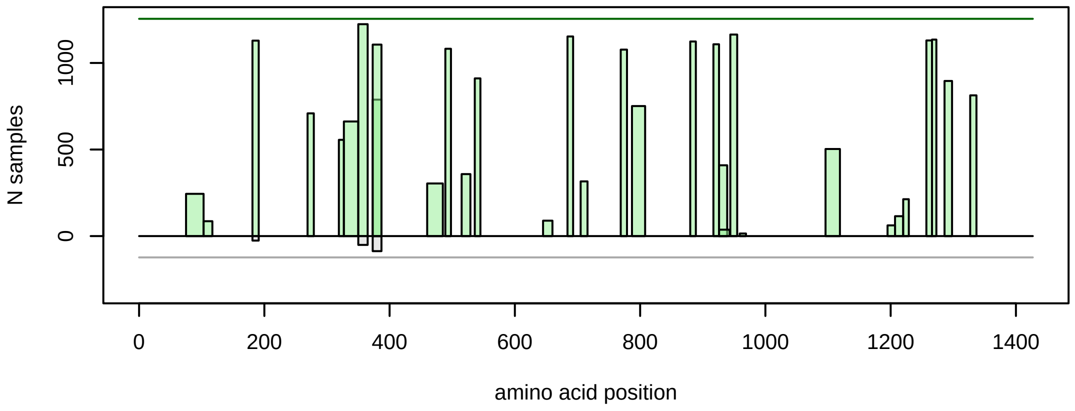

227. ENDOV (Q8N8Q3−3), NP2

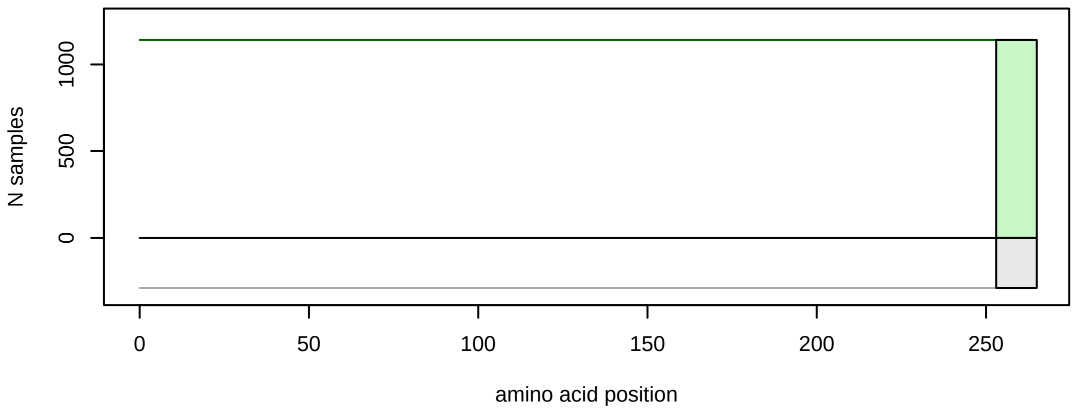

228. GPC4 (O75487), NP2

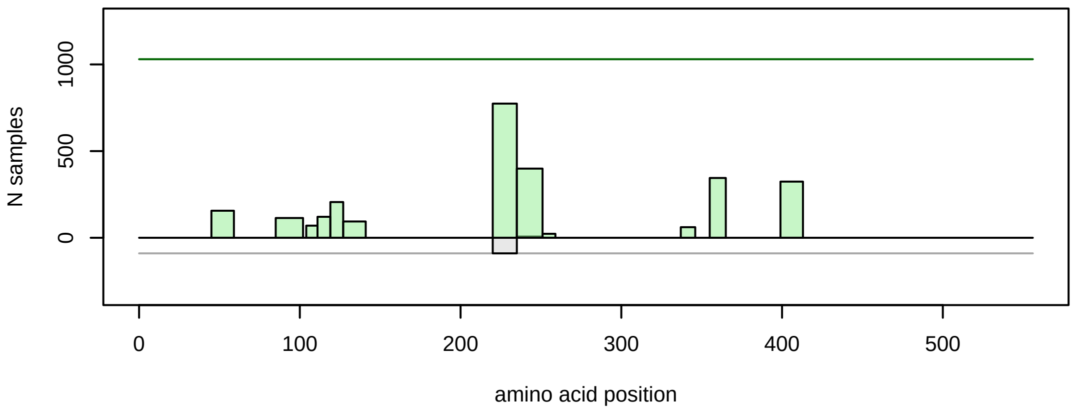

229. HPCAL1 (P37235), NP2

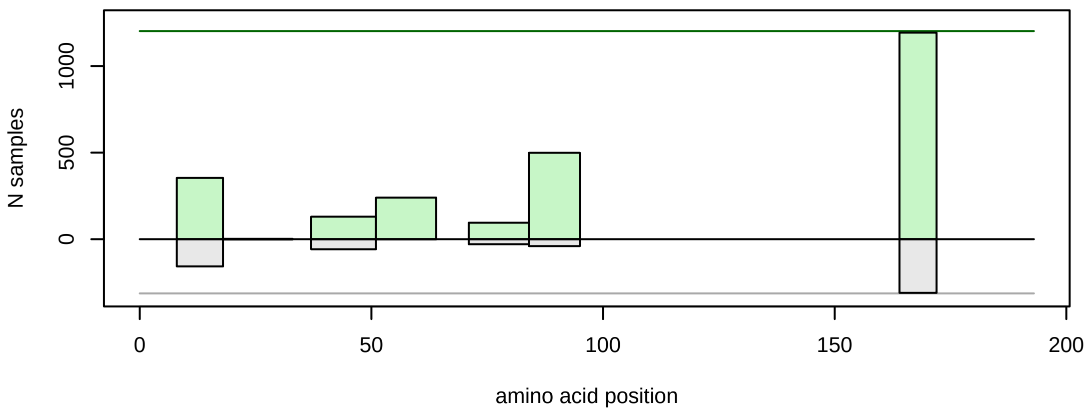

230. KIF2A (A0A6Q8PFA6;A0A6Q8PH57;O00139;O00139−4), NP4

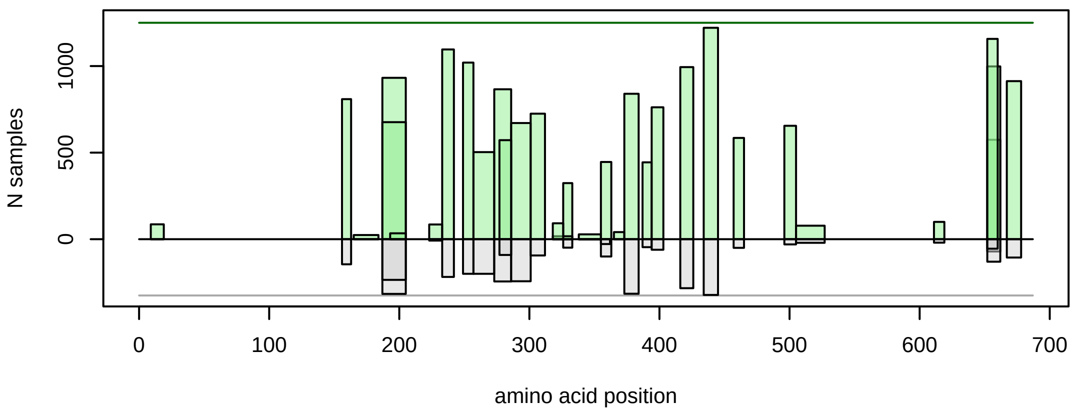

231. CDH6 (P55285), NP2

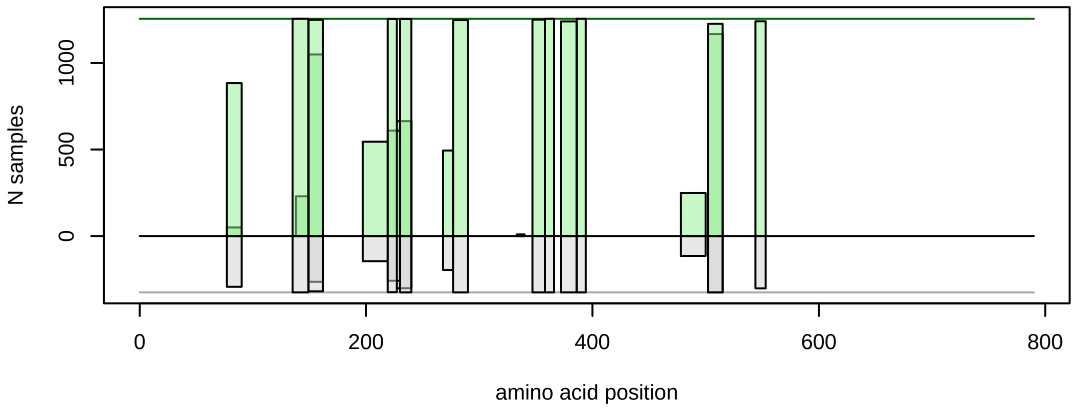

232. RAB18 (B7Z4P9), NP4

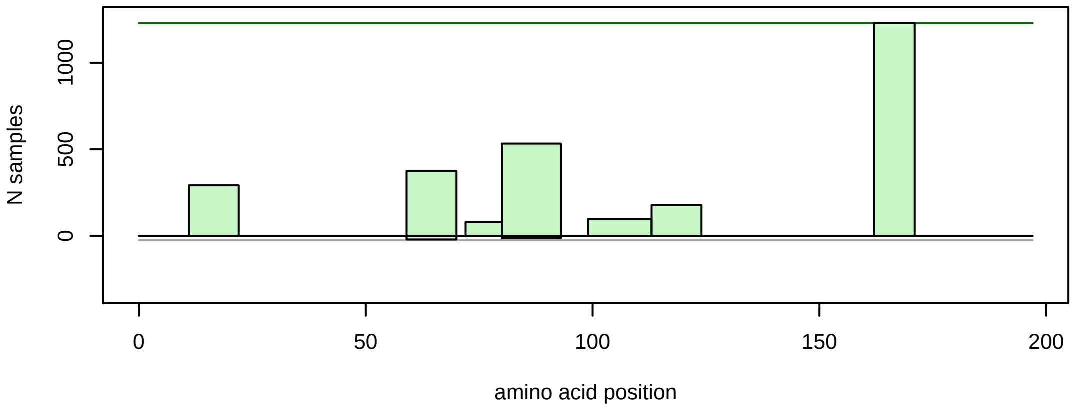

233. ASPN (Q9BXN1), NP4

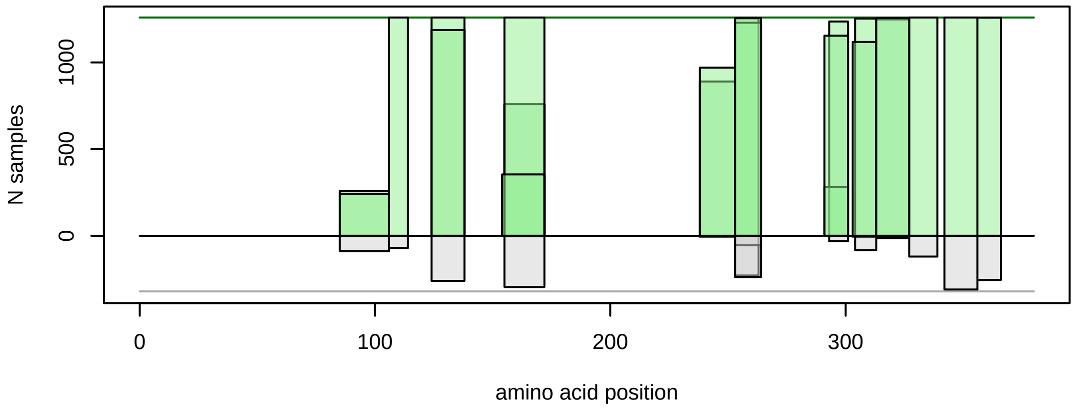

234. AIMP1 (Q12904), NP3

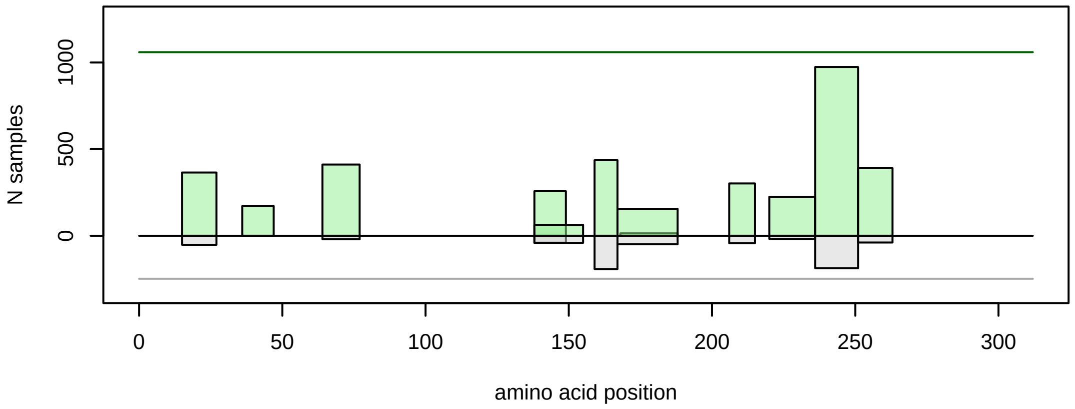

235. BMP10 (O95393), NP2

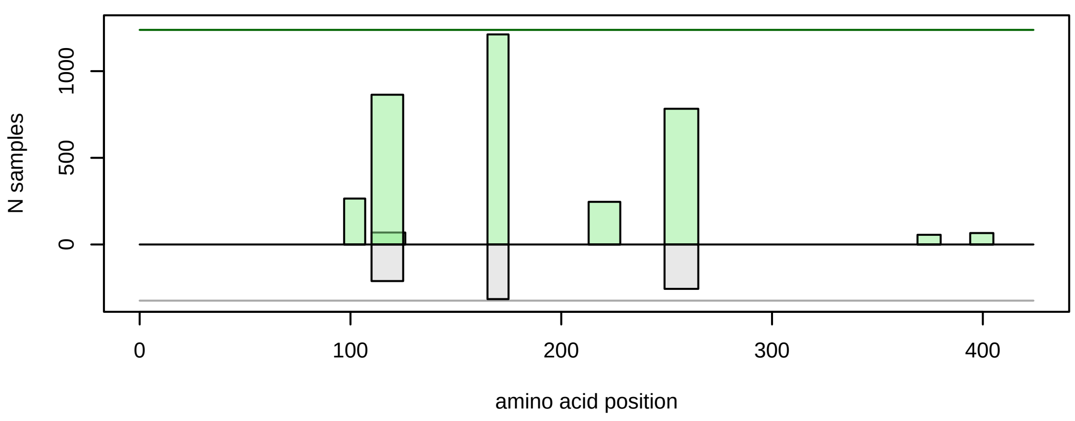

236. C19orf80 (Q6UXH0), NP4

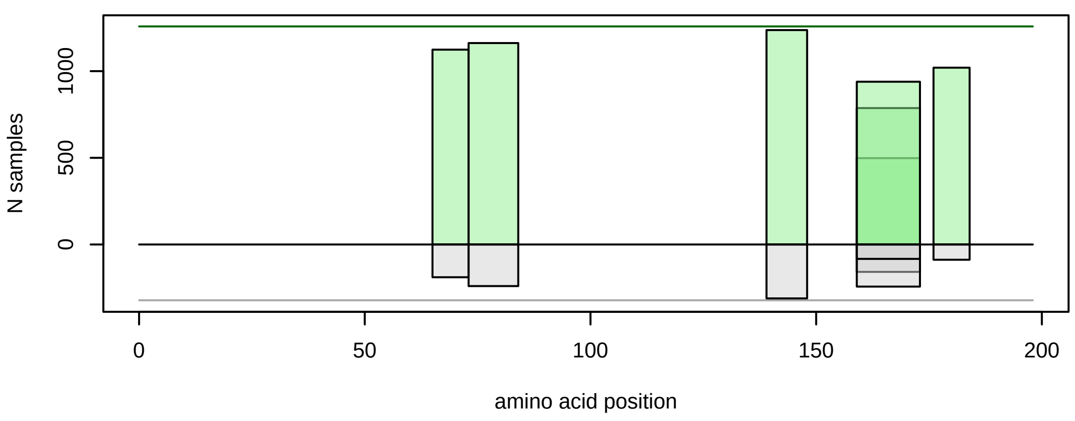

237. PSMF1 (Q5QPM7;Q92530), NP1

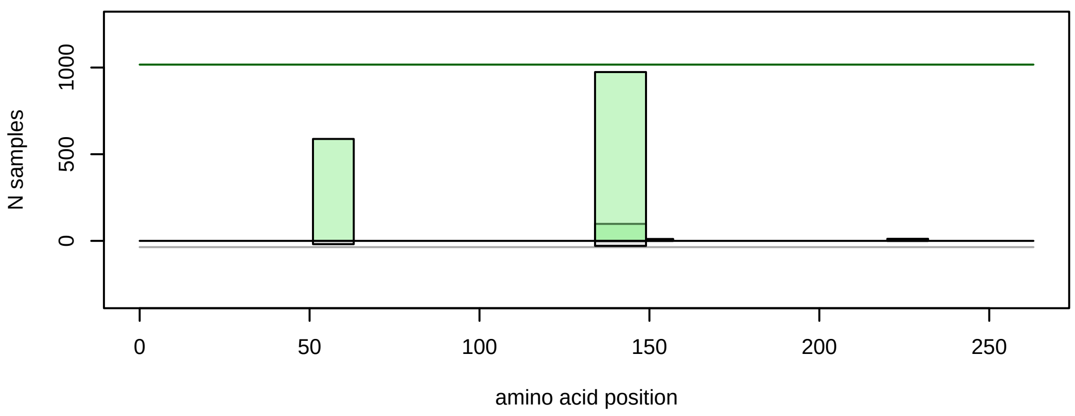

238. RPS18 (P62269), NP4

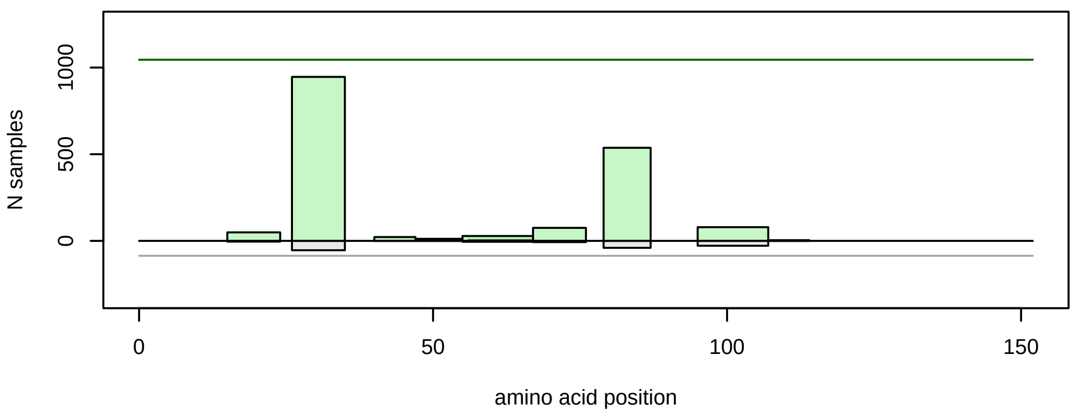

239. FAT4 (A0A6Q8JR05), NP2

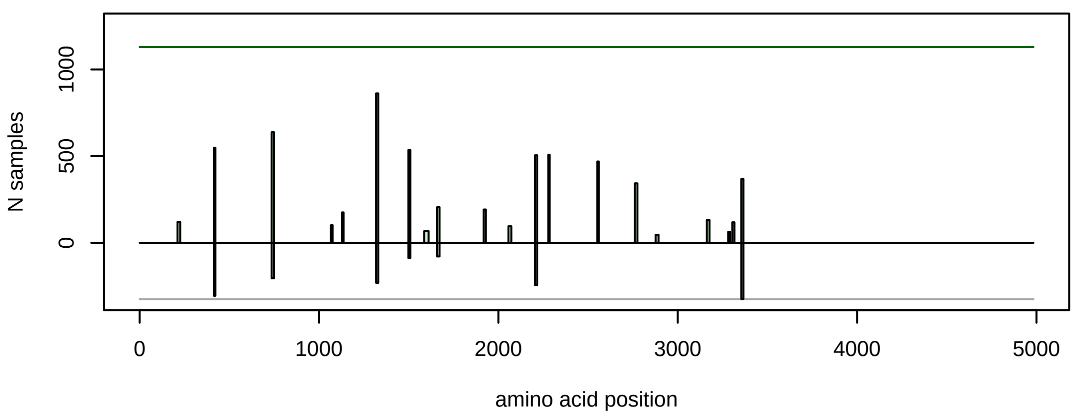

240. SPARCL1 (Q14515), NP2

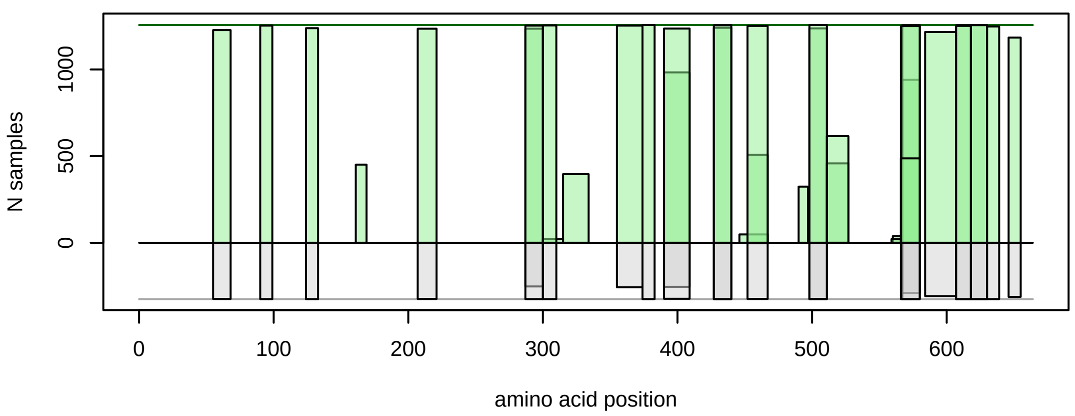

241. CALCOCO1 (Q9P1Z2;Q9P1Z2−2;Q9P1Z2−3), NP5

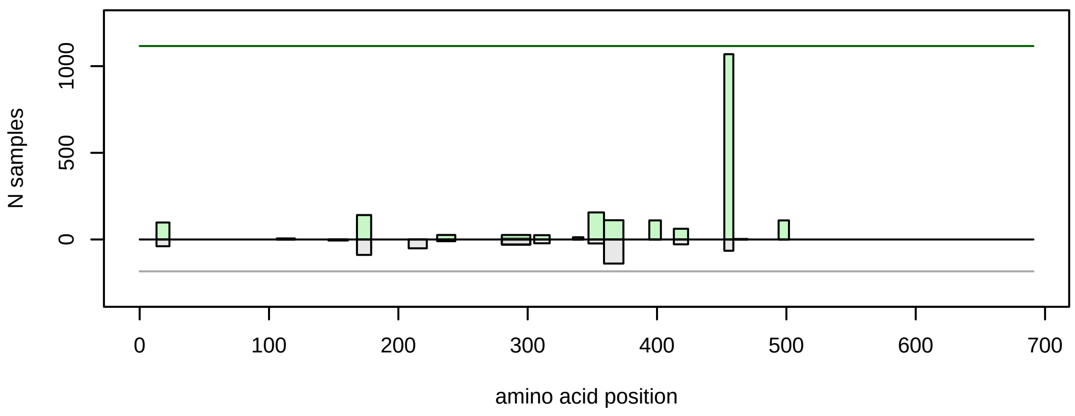

242. TTBK1 (X6R456), NP2

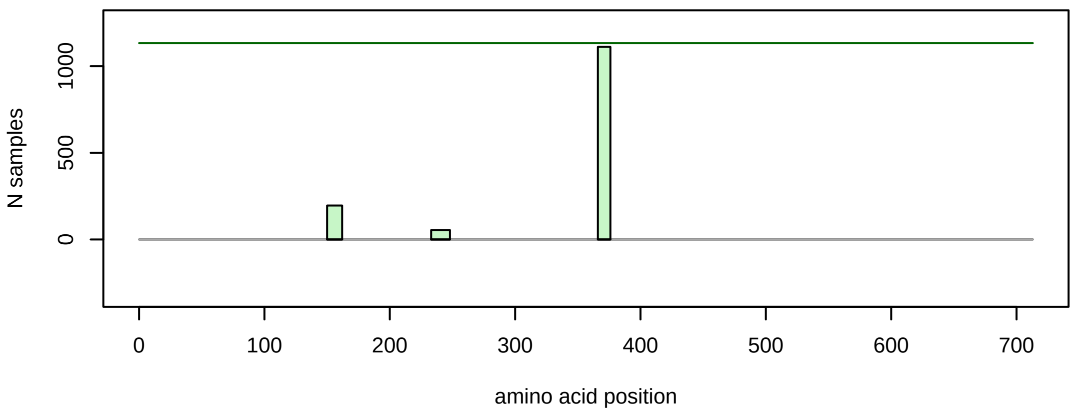

243. DNAJA4 (Q8WW22−2), NP1

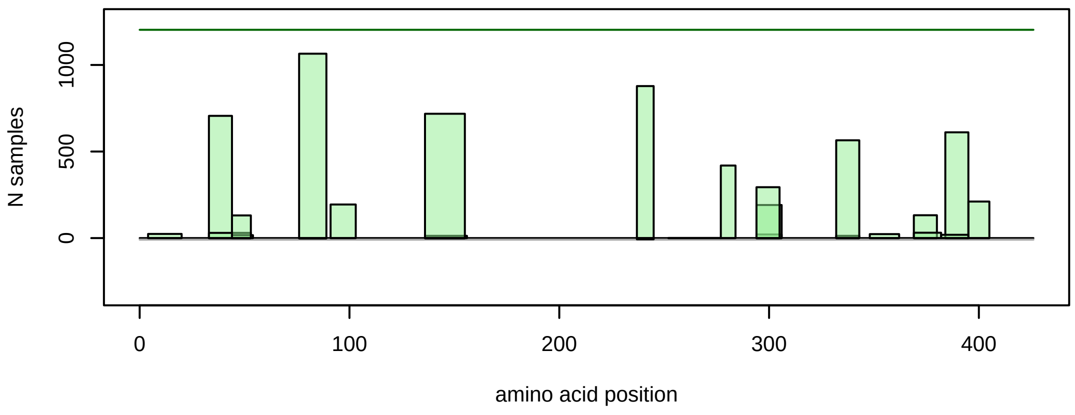

244. IGHV4−34 (P06331), NP3

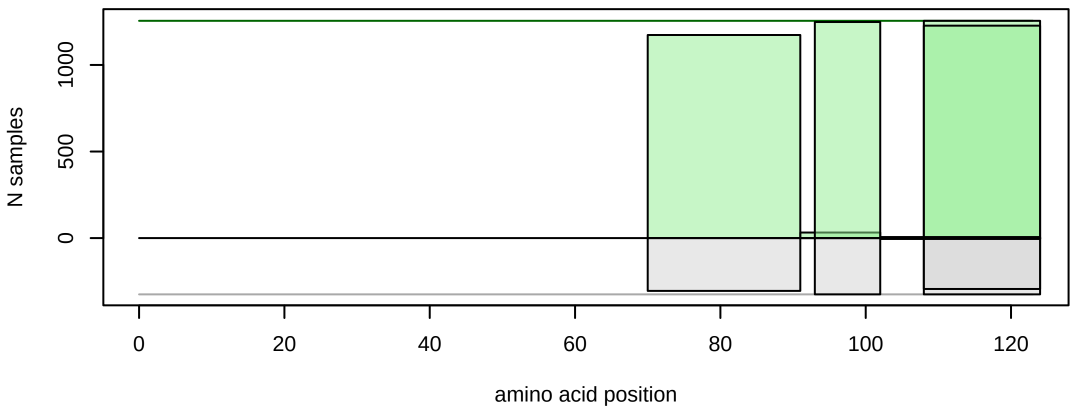

245. FCER2 (P06734), NP2

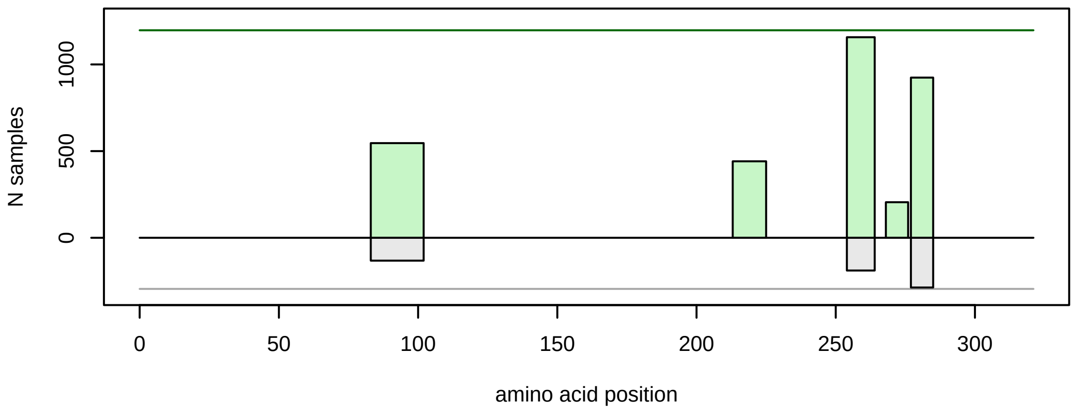

246. STX7 (O15400;O15400−2), NP1

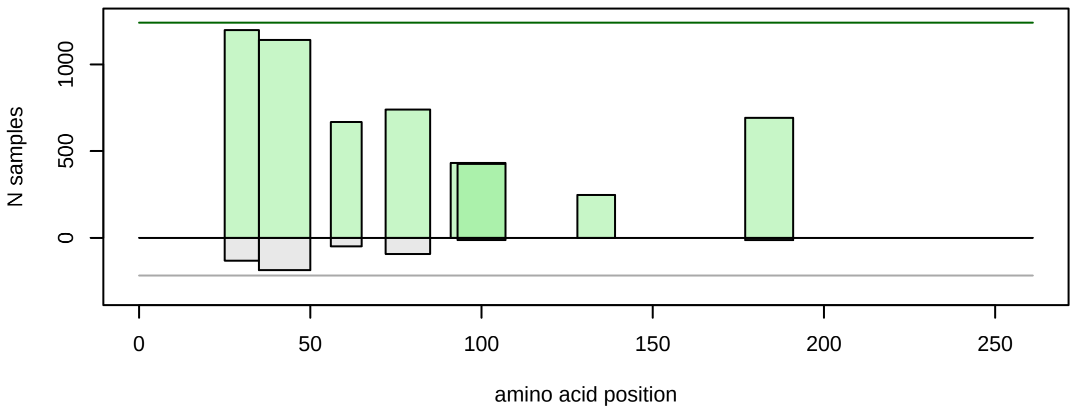

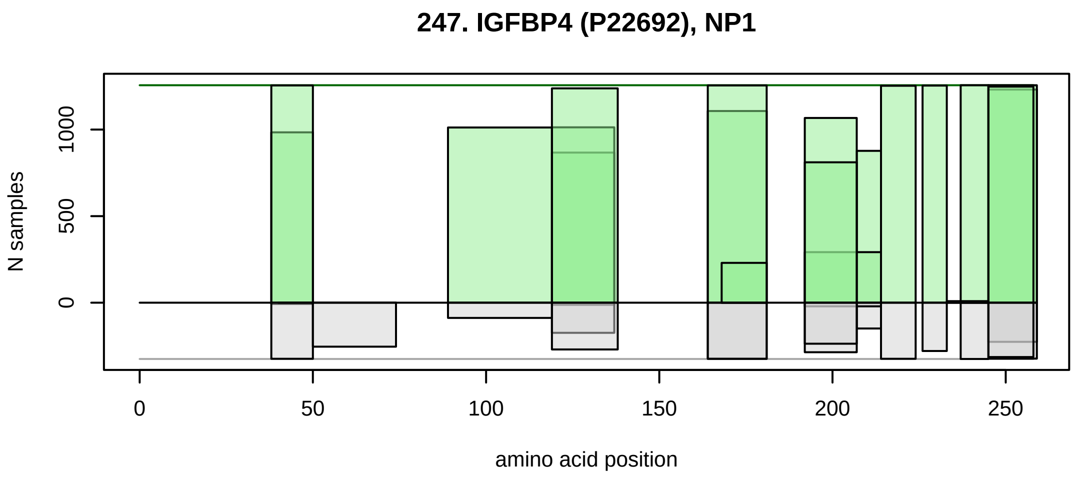
247. IGFBP4 (P22692), NP1
N samples
amino acid position
0
50
100
150
200
250
500
1000

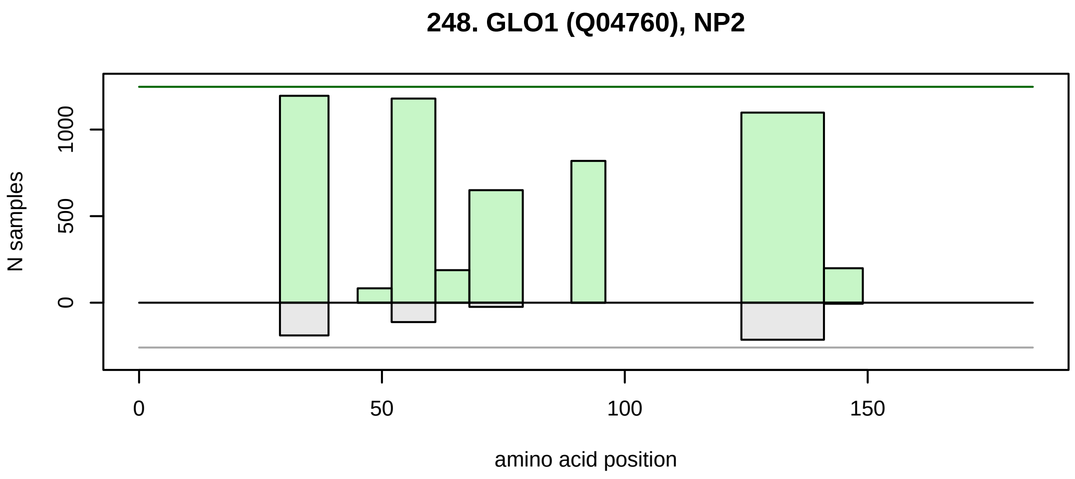
248. GLO1 (Q04760), NP2
N samples
amino acid position
0
50
100
150
500
1000

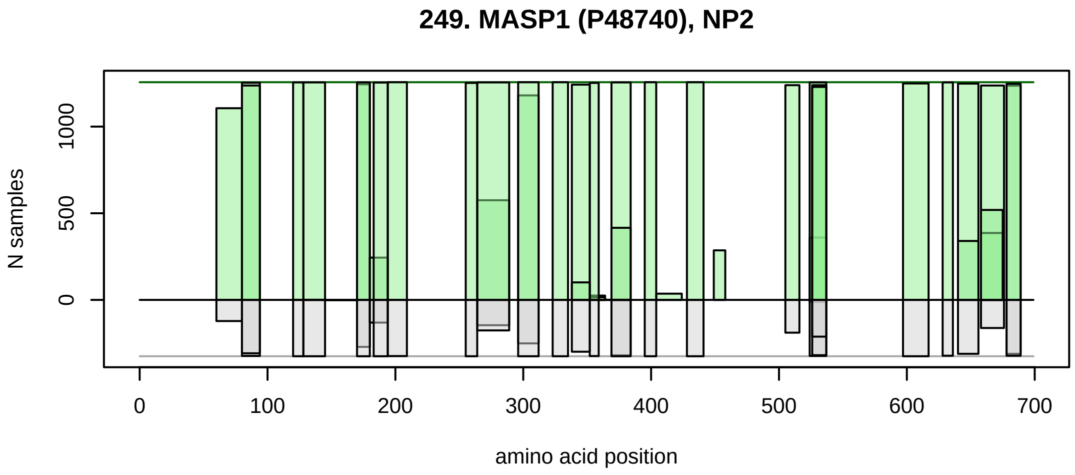
249. MASP1 (P48740), NP2
N samples
amino acid position
0
100
200
300
400
500
600
700
1000

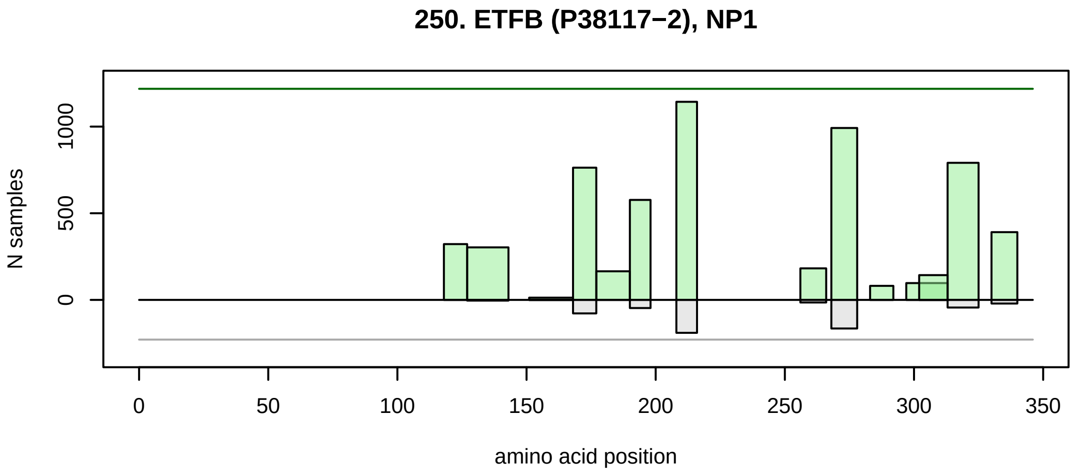
250. ETFB (P38117−2), NP1
N samples
amino acid position
0
50
100
150
200
250
300
350
500
1000

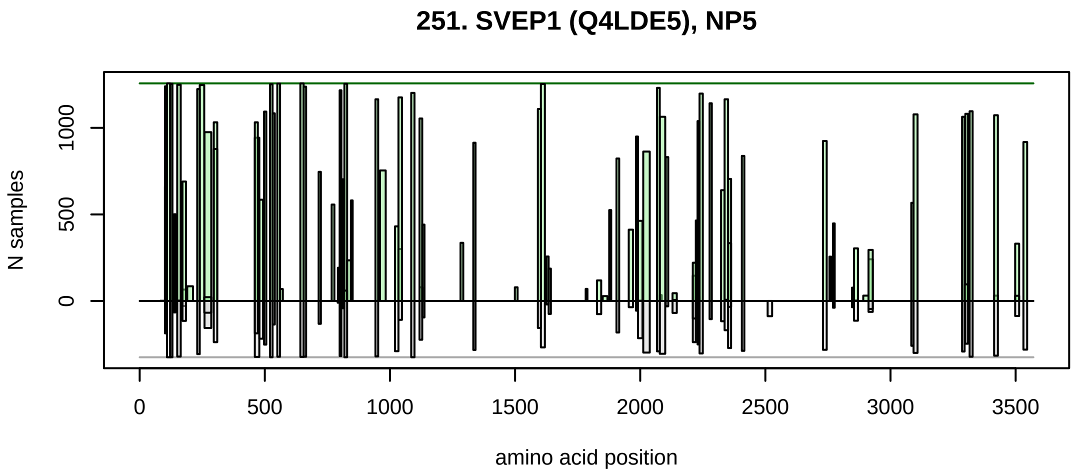
251. SVEP1 (Q4LDE5), NP5
N samples
amino acid position
0
500
1000
1500
2000
2500
3000
3500

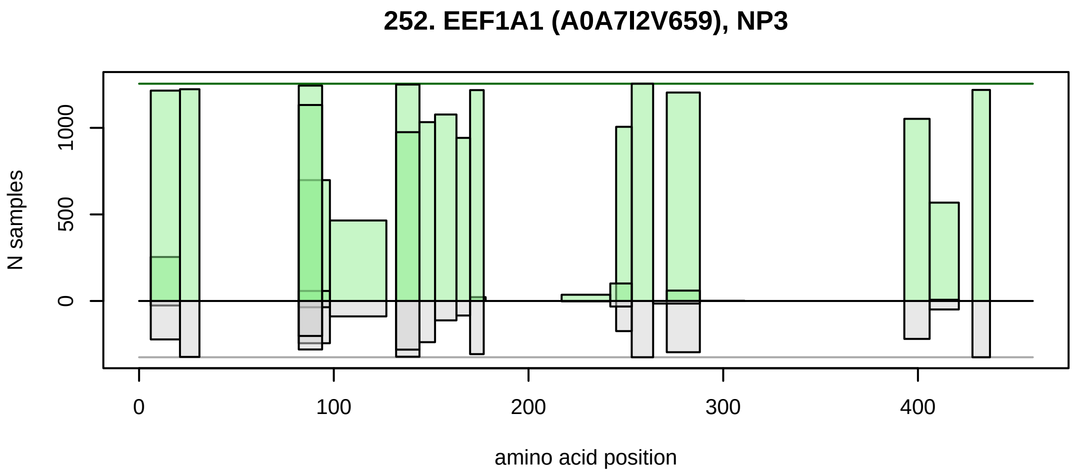
252. EEF1A1 (A0A7I2V659), NP3
N samples
amino acid position
0
100
200
300
400
500
1000

253. SERPINB1 (P30740), NP4

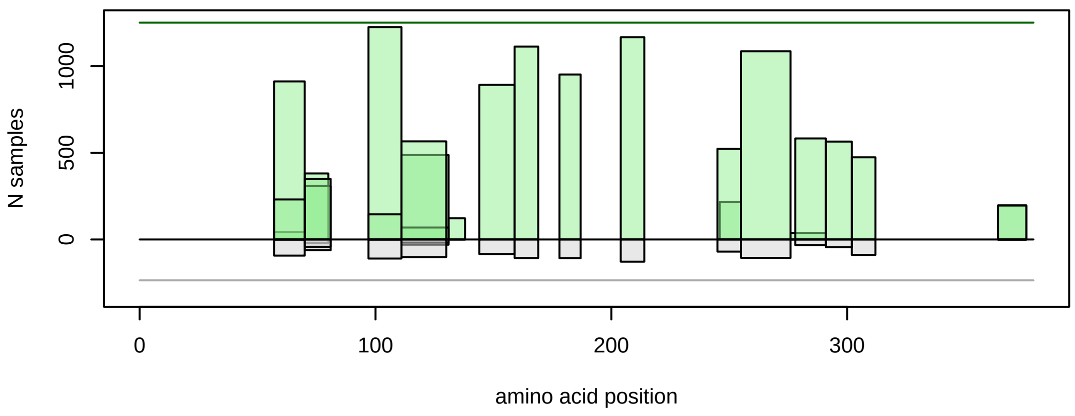

254. F5 (A0A0A0MRJ7;P12259), NP4

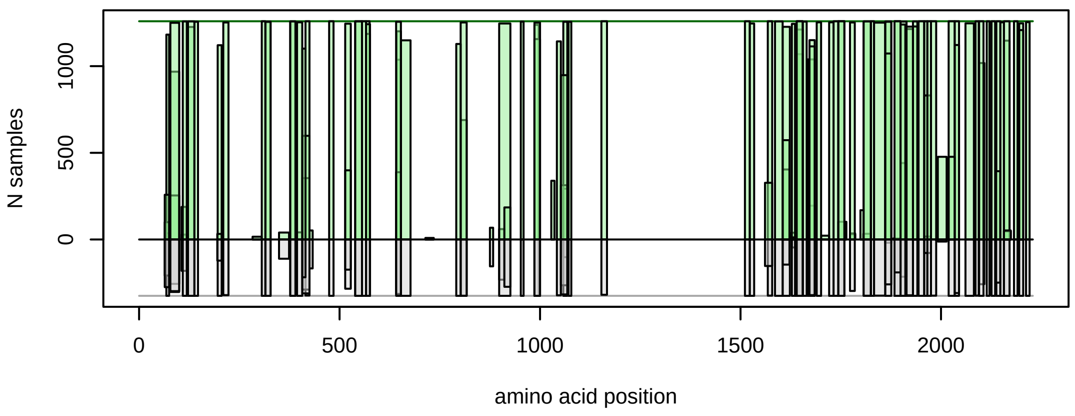

255. MMP1 (P03956), NP1

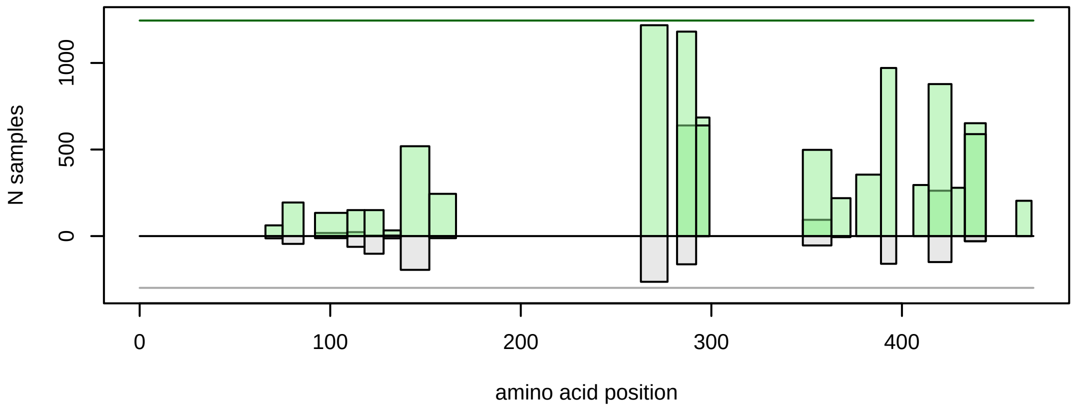

256. ERP44 (Q9BS26), NP2

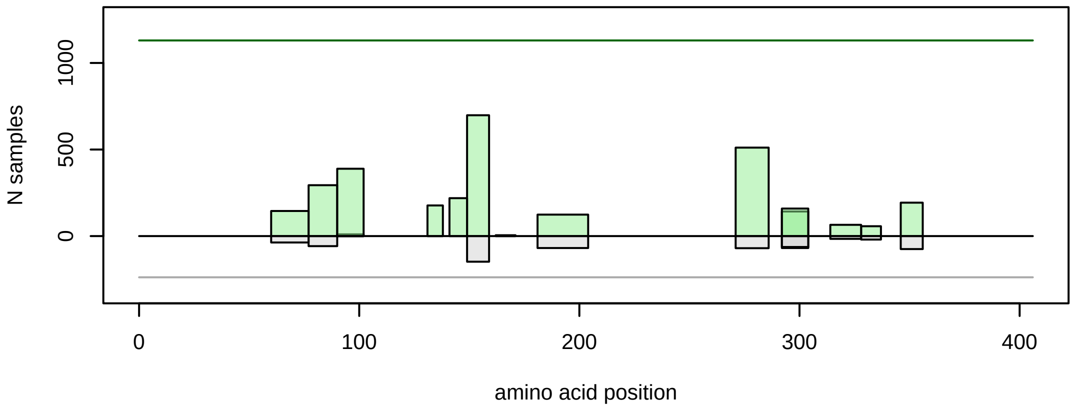

257. TFR2 (Q9UP52), NP5

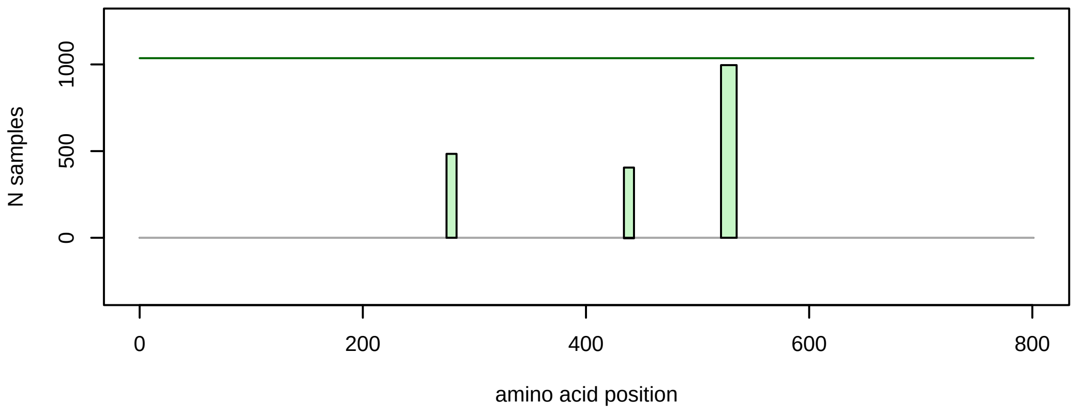

258. PSMD3 (O43242), NP1

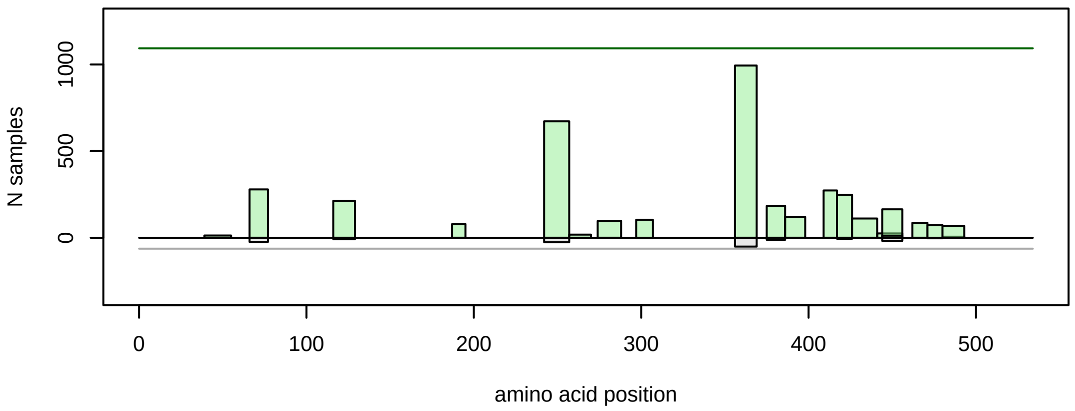

259. EEF2 (P13639), NP1

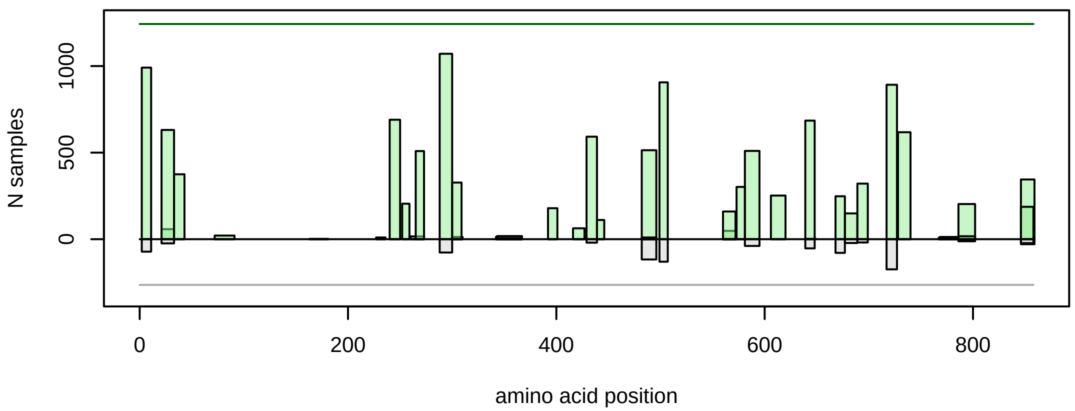

260. C1R (A0A3B3ISR2;B4DPQ0), NP5

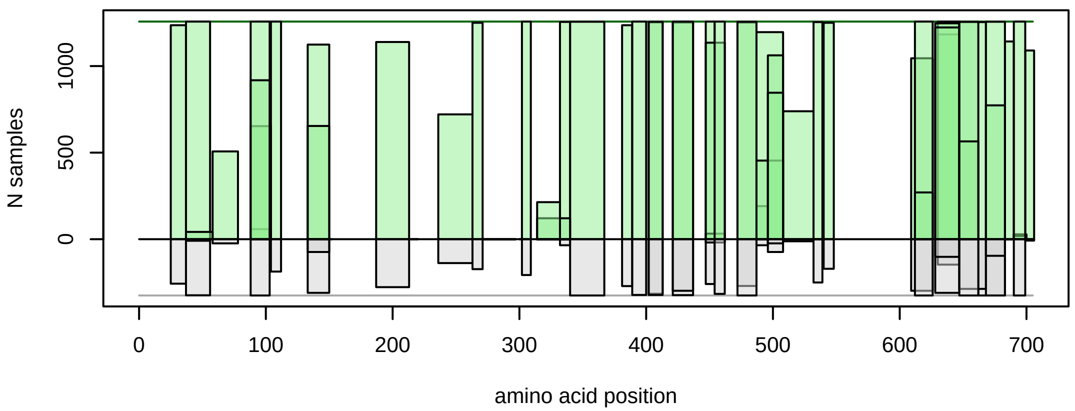

261. LCAT (P04180), NP2

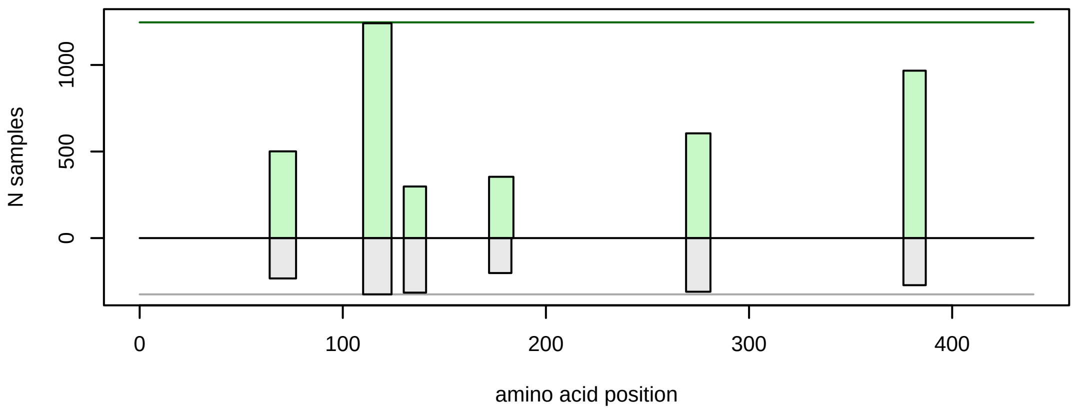

262. GNL1 (P36915), NP5

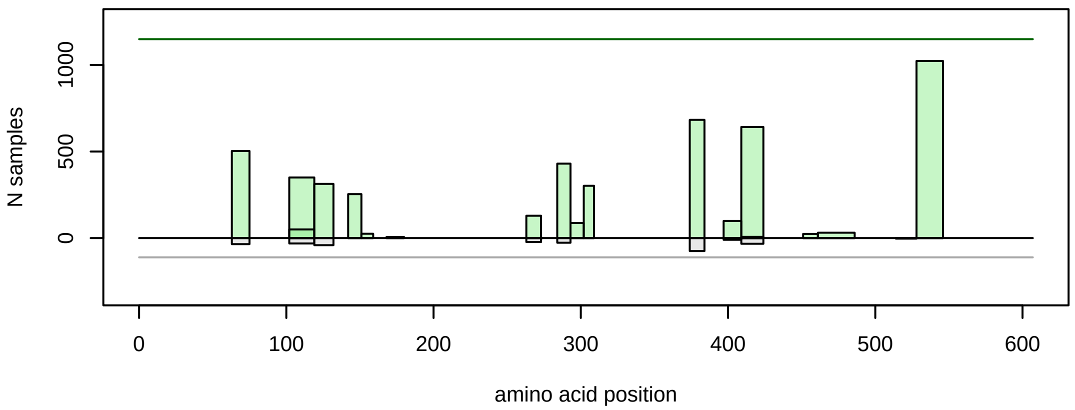

263. SRGN (P10124), NP2

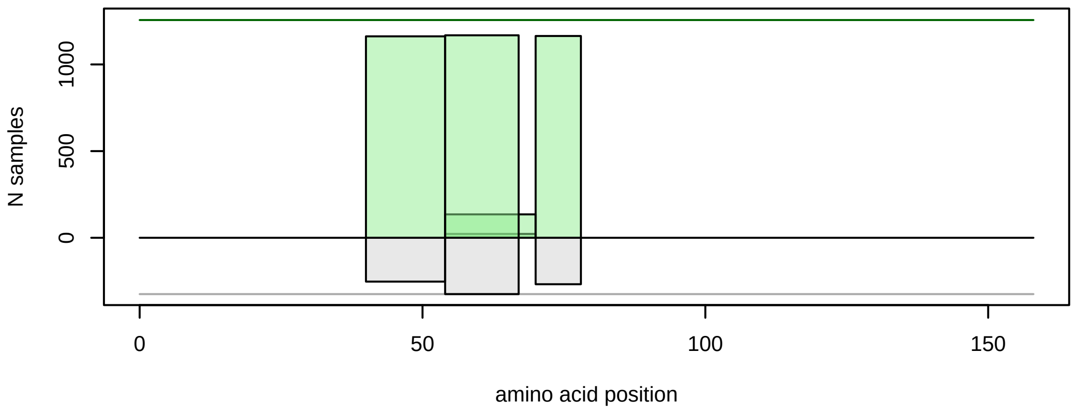

264. SLC39A10 (Q9ULF5), NP3

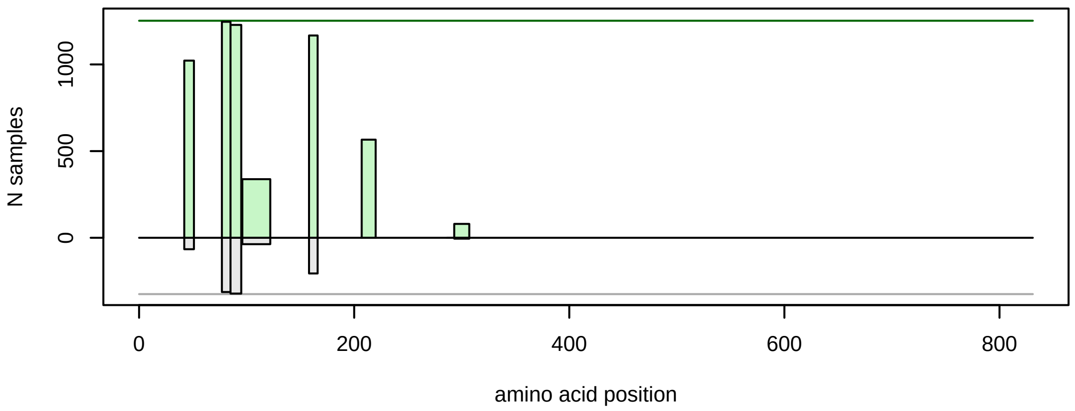

265. APOM (O95445), NP1

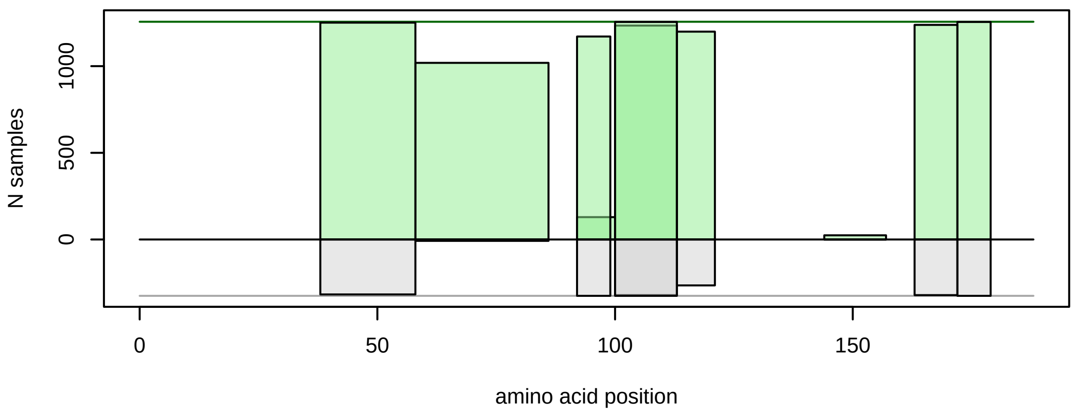

266. LAMA4 (A0A0A0MQS9;A0A0A0MTC7), NP3

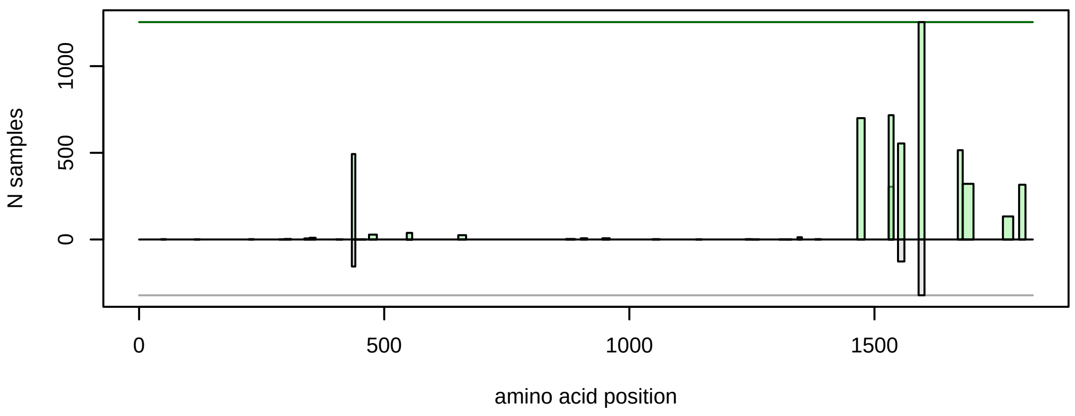

267. RNH1 (P13489), NP2

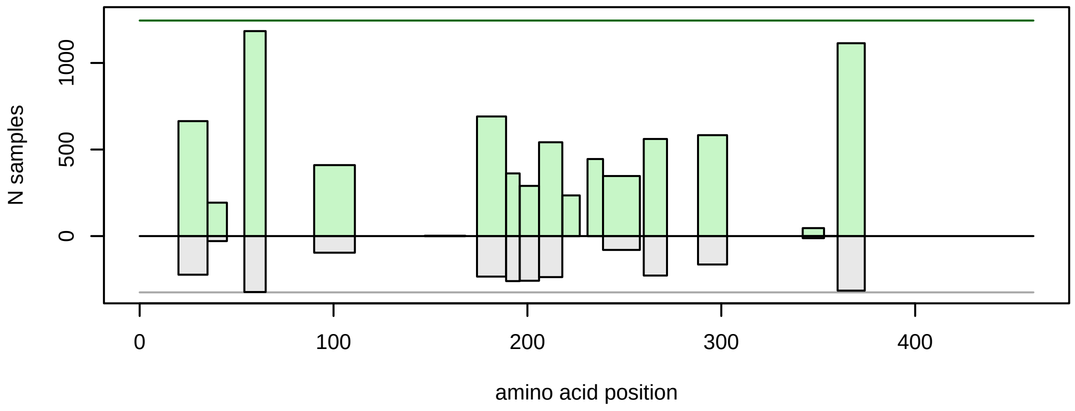

268. RNPEP (Q9H4A4), NP5

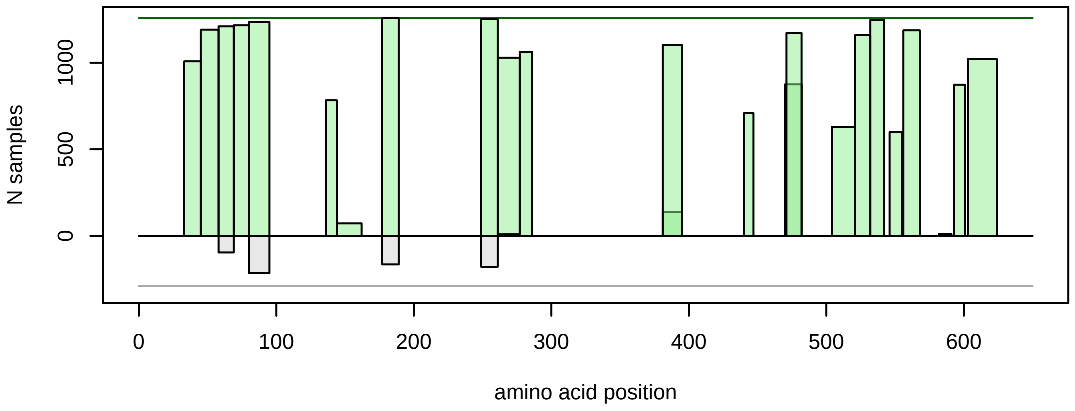

269. RHNO1 (Q9BSD3−2), NP5

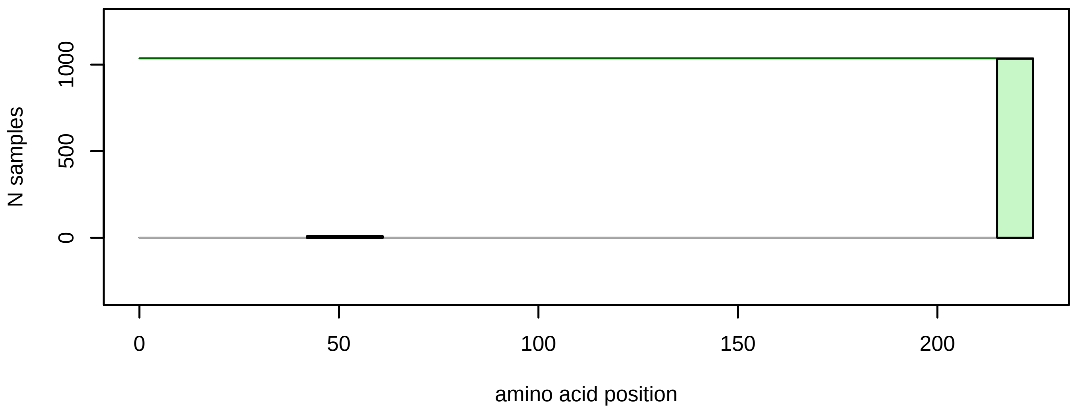

270. MYOC (Q99972), NP5

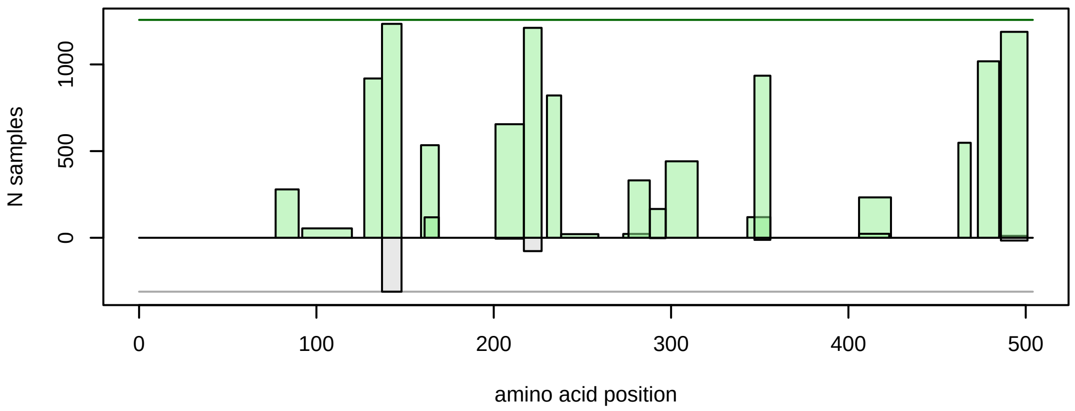

271. ARPC3 (O15145), NP1

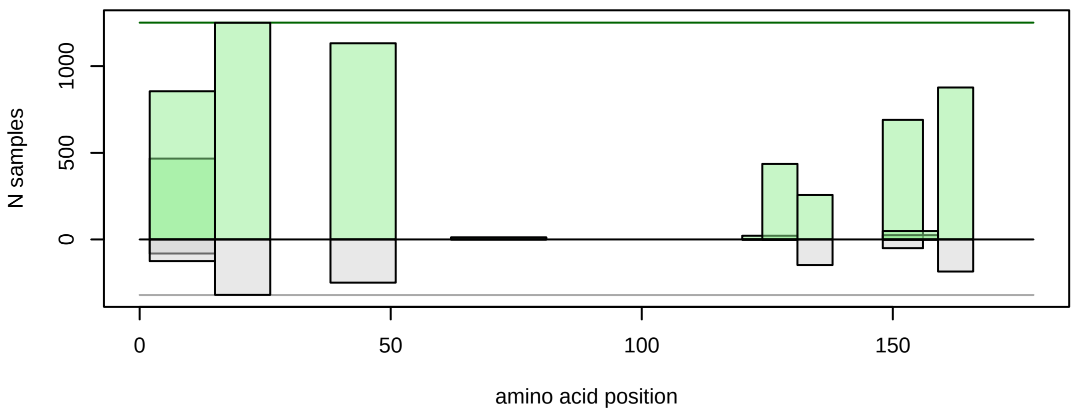

272. TUBB1 (Q9H4B7), NP4

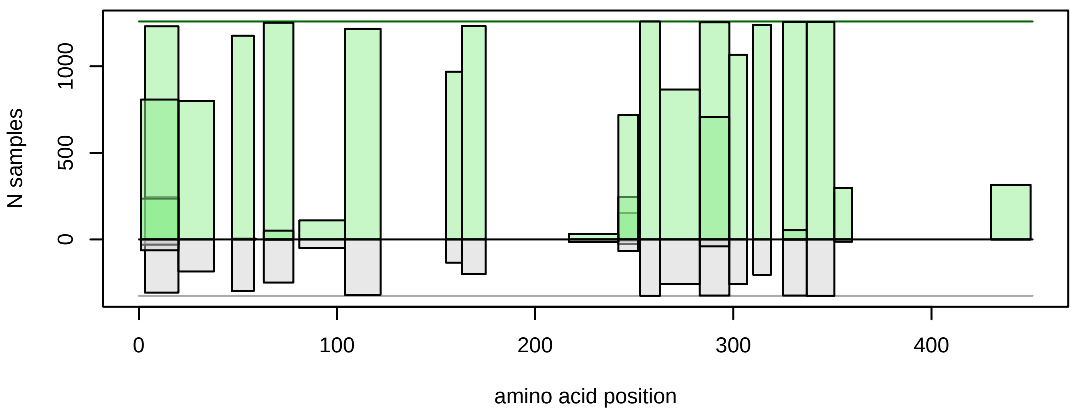

273. FSTL1 (Q12841), NP2

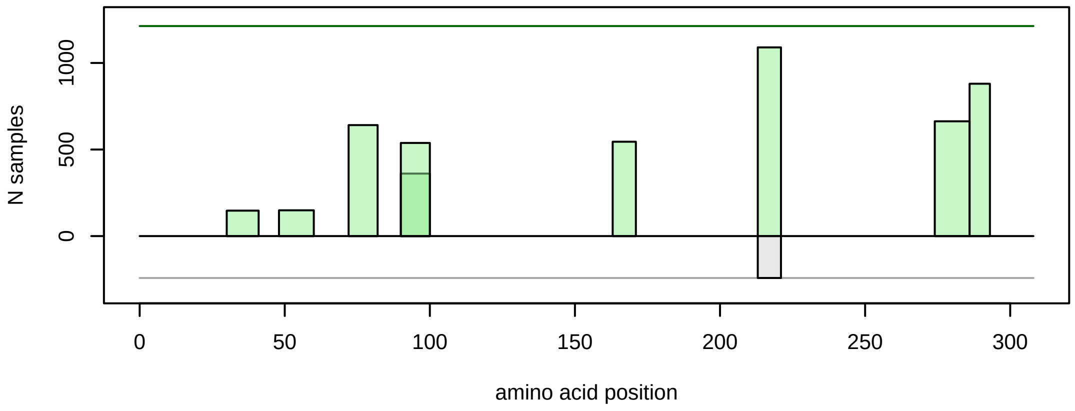

274. DNAH6 (Q9C0G6), NP4

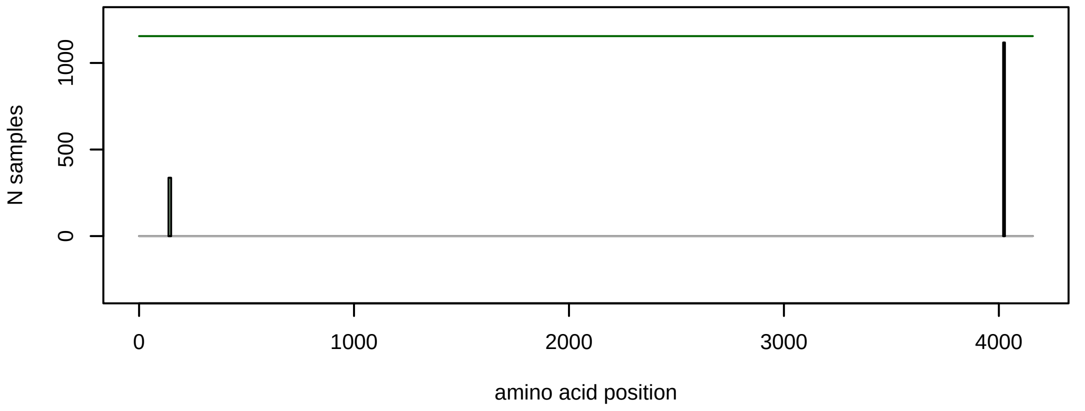

275. UBASH3B (Q8TF42), NP4

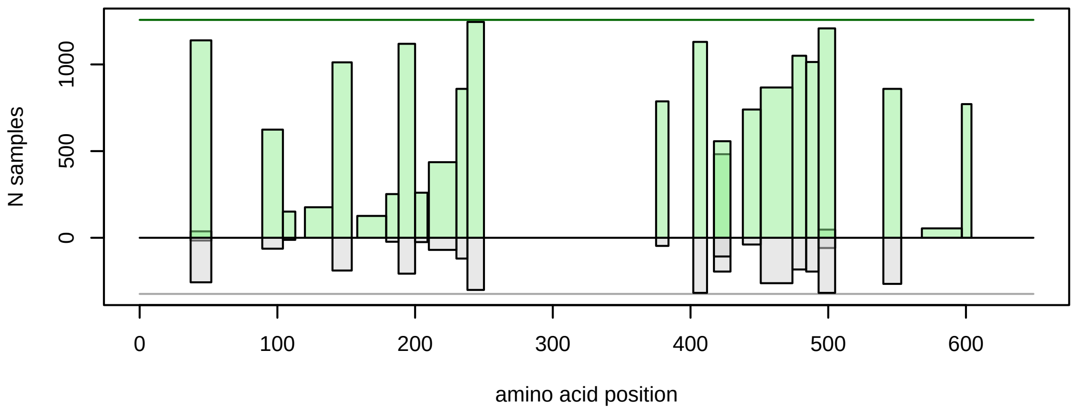

276. MASP1 (F8W876), NP2

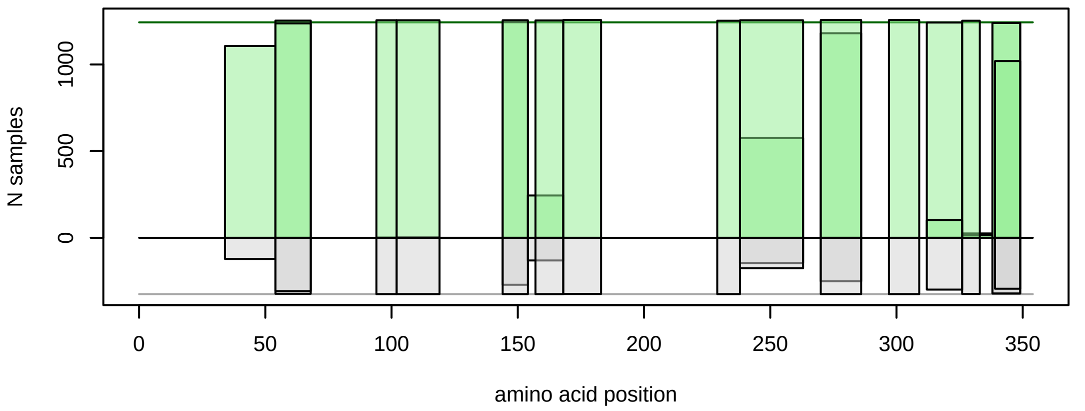

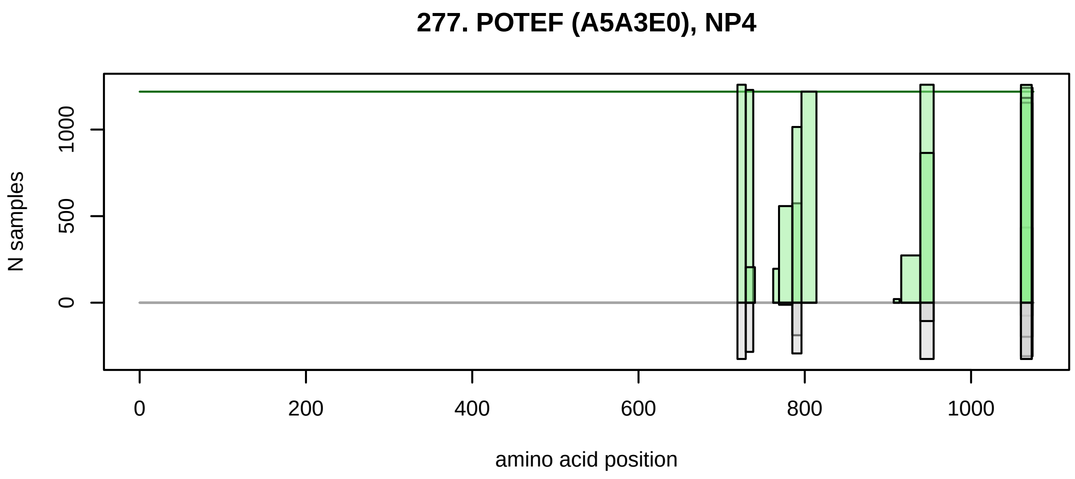
277. POTEF (A5A3E0), NP4
N samples
0
500
1000
0
200
400
600
800
1000
amino acid position

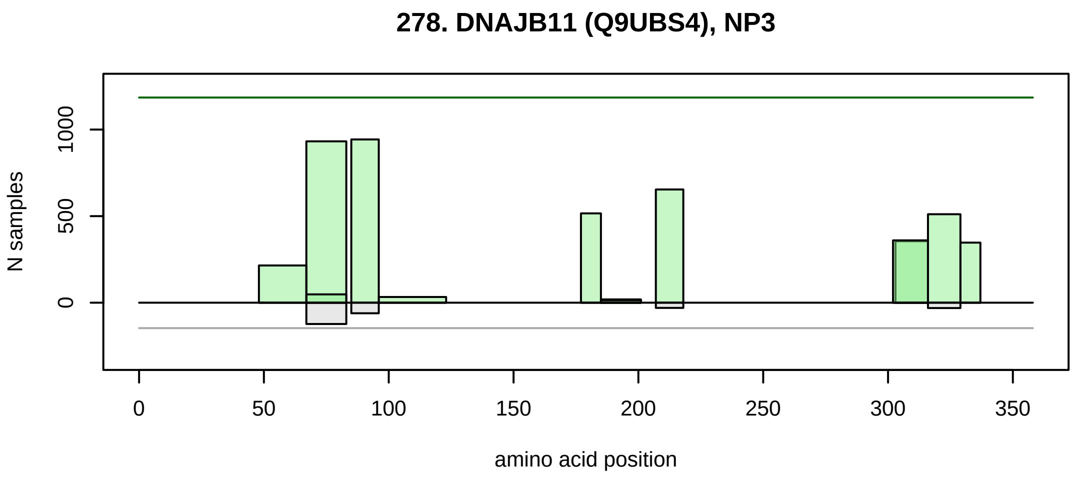
278. DNAJB11 (Q9UBS4), NP3
N samples
0
500
1000
0
50
100
150
200
250
300
350
amino acid position

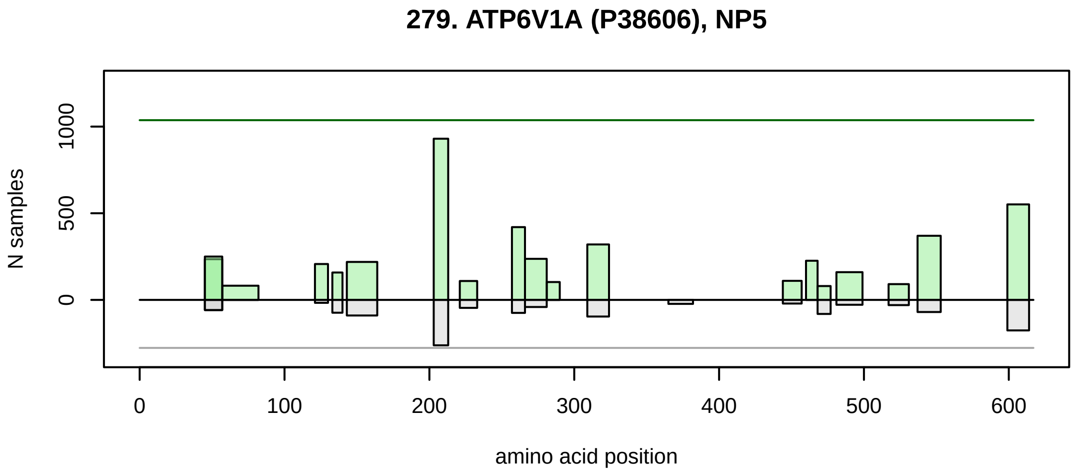
279. ATP6V1A (P38606), NP5
N samples
0
500
1000
0
100
200
300
400
500
600
amino acid position

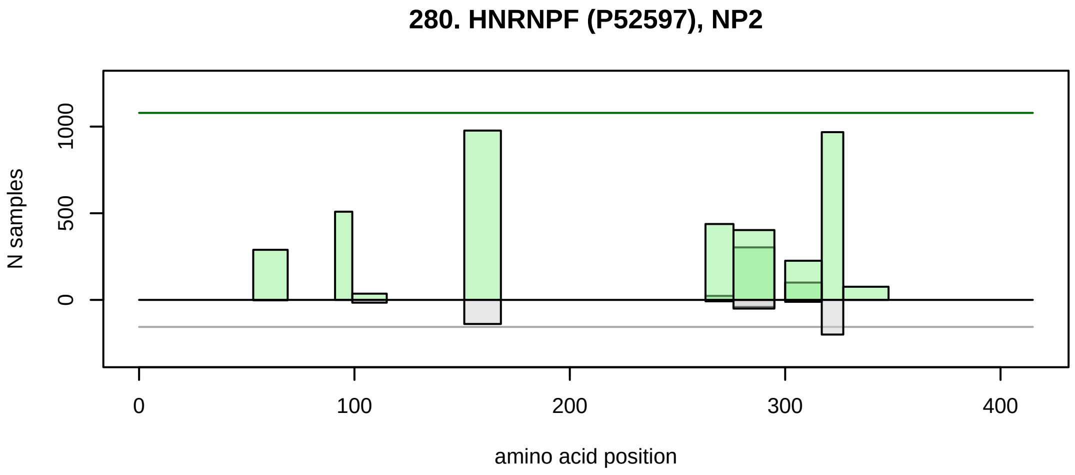
280. HNRNPF (P52597), NP2
N samples
0
500
1000
0
100
200
300
400
amino acid position

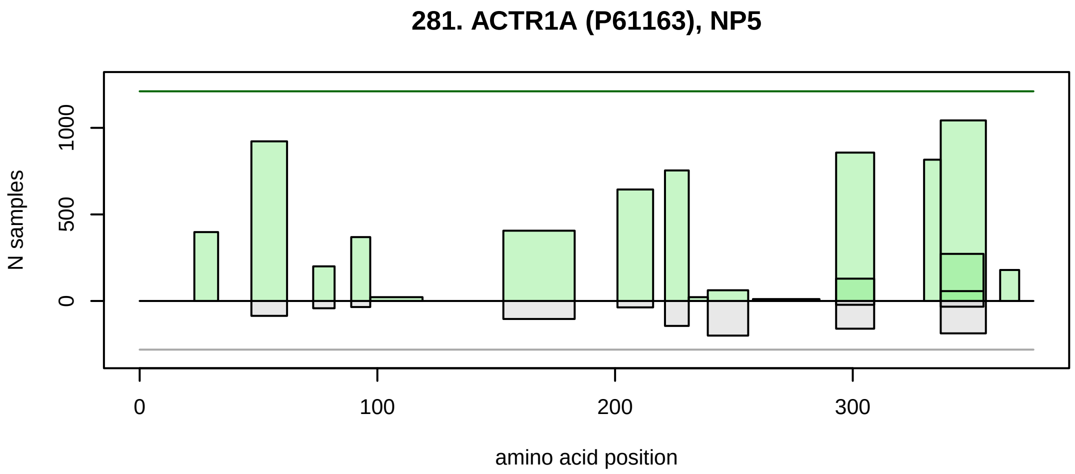
281. ACTR1A (P61163), NP5
N samples
0
500
1000
0
100
200
300
amino acid position

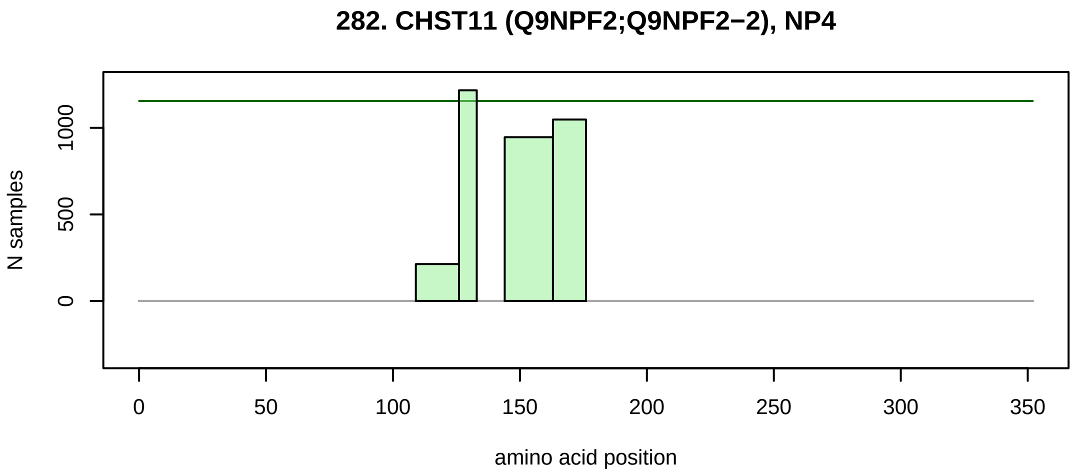
282. CHST11 (Q9NPF2;Q9NPF2−2), NP4
N samples
0
500
1000
0
50
100
150
200
250
300
350
amino acid position

283. GNLY (B4E3H9;P22749;P22749−2), NP4

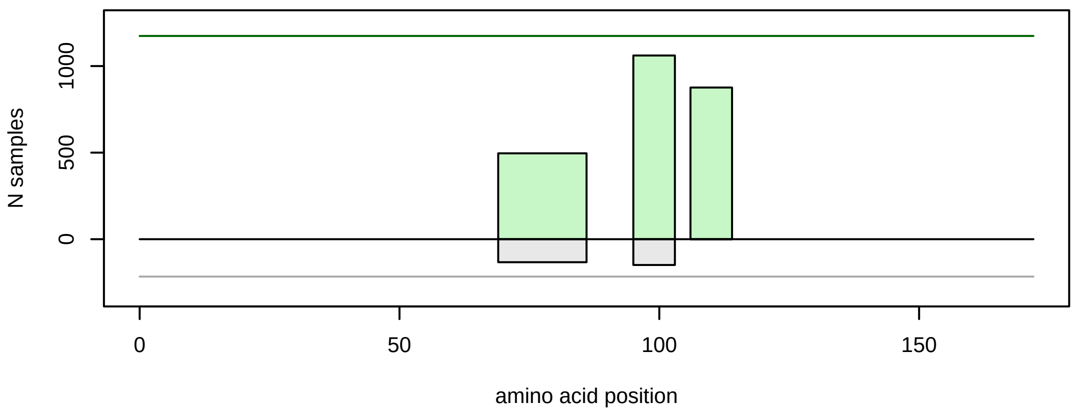

284. TARS (P26639), NP5

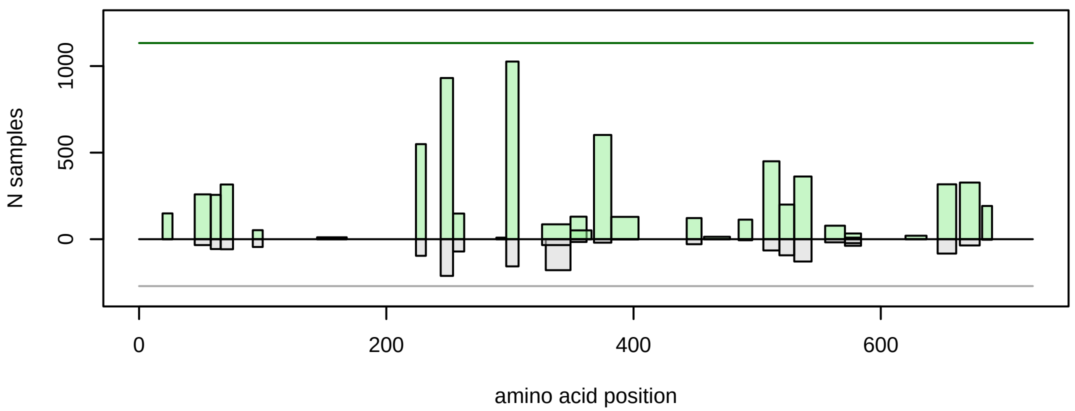

285. F2 (P00734), NP2

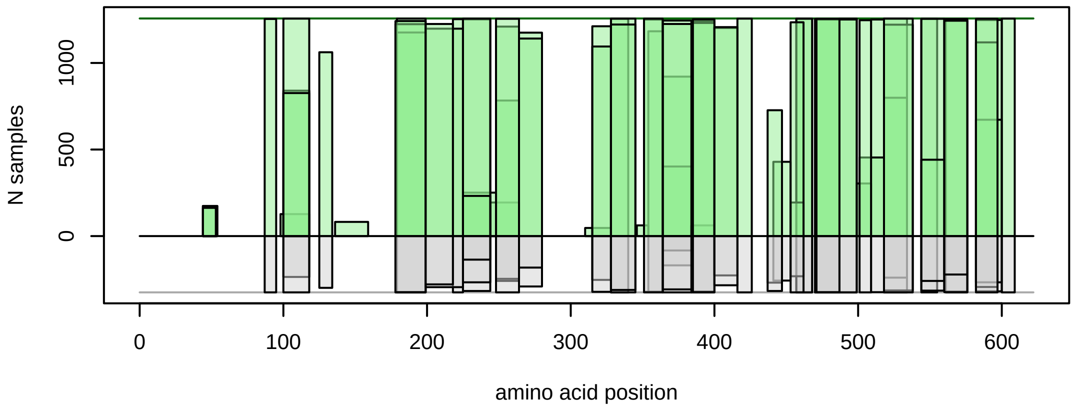

286. DSTN (P60981), NP3

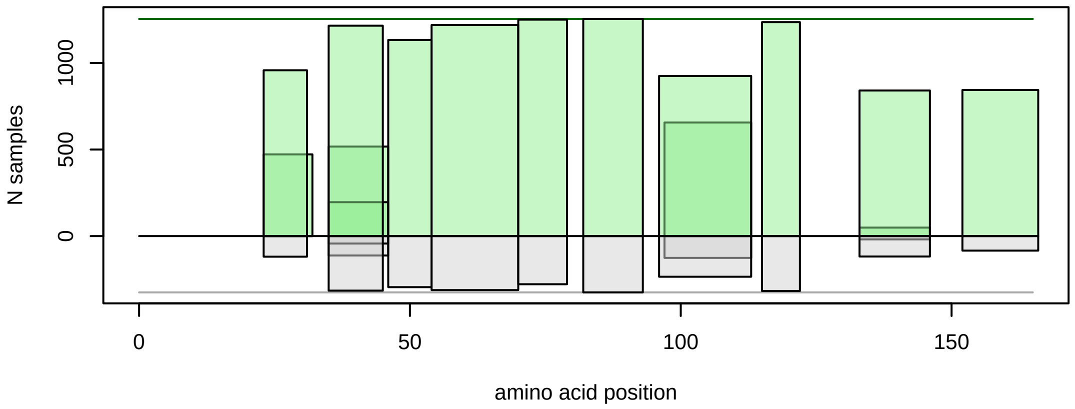

287. IGKV2D−24 (A0A075B6R9), NP3

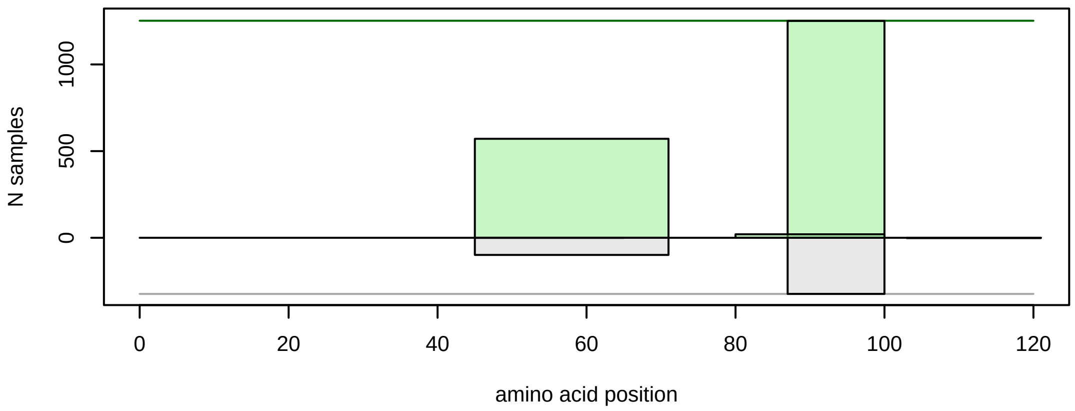

288. MMP9 (P14780), NP5

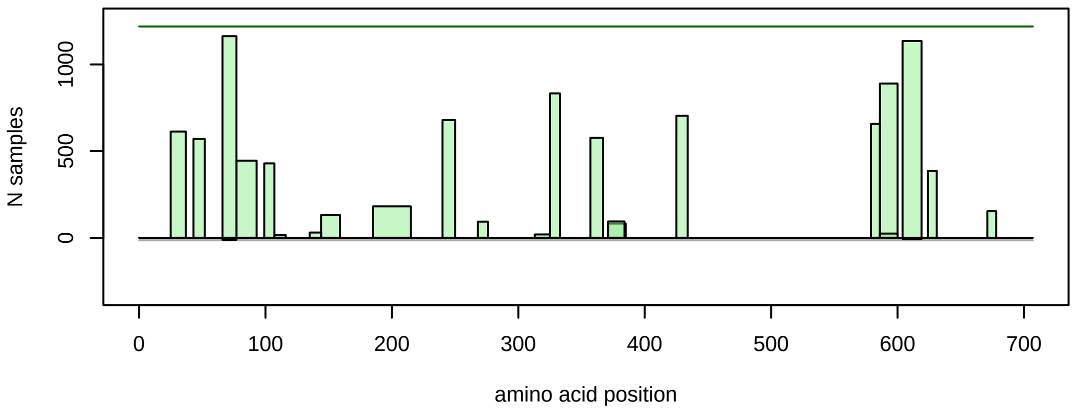

289. CPS1 (P31327−3), NP5

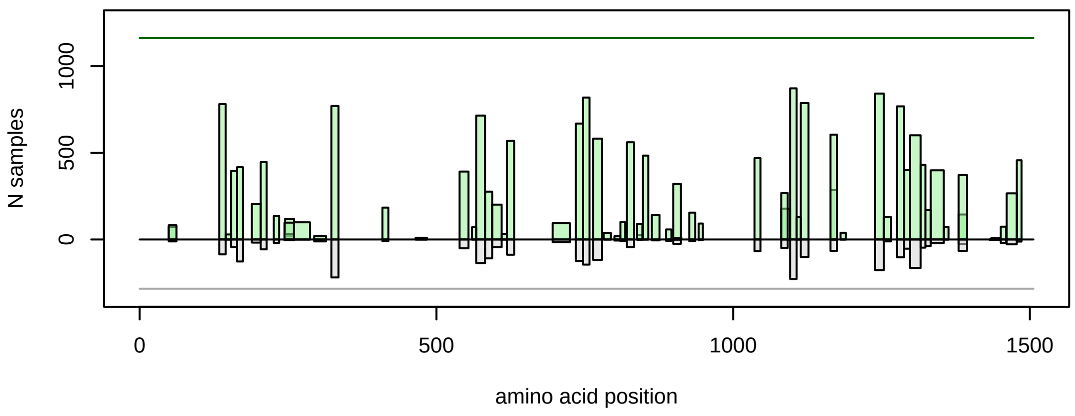

290. STAB2 (Q8WWQ8), NP4

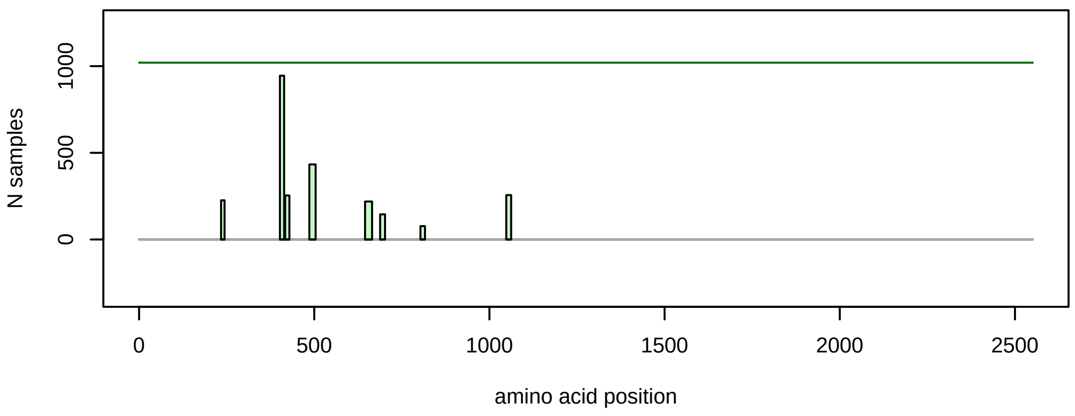

291. RALA (P11233), NP1

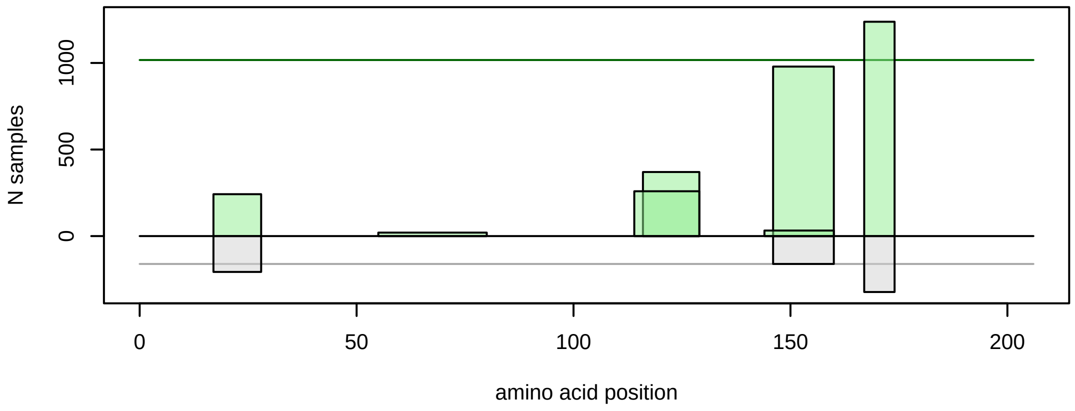

292. ACP1 (P24666), NP2

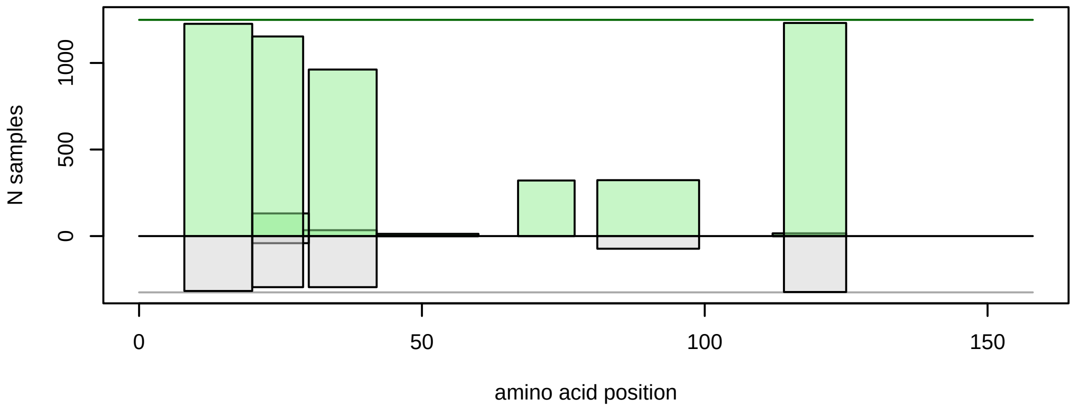

293. CORO1A (P31146), NP1

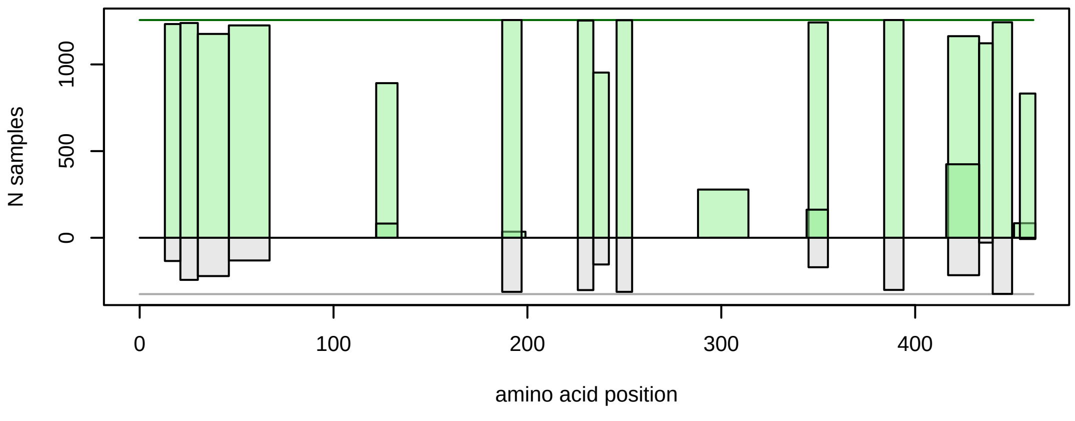

294. GCA (H7BXD5;P28676), NP2

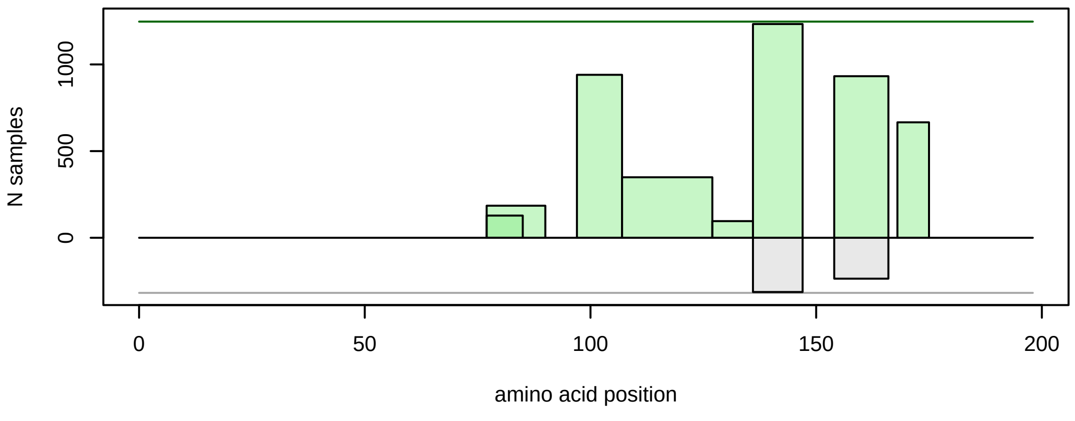

295. APOA5 (Q6Q788), NP5

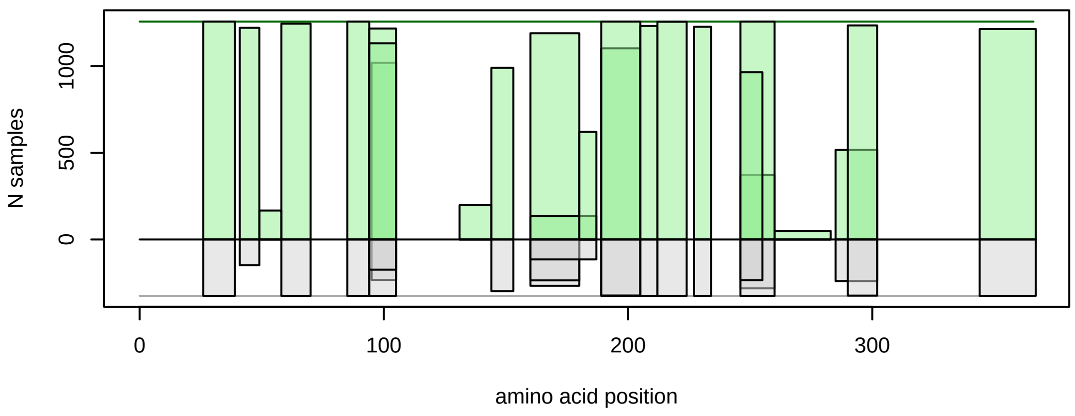

296. CSNK2B (A0A7I2YQ78;P67870;Q5SRQ6), NP2

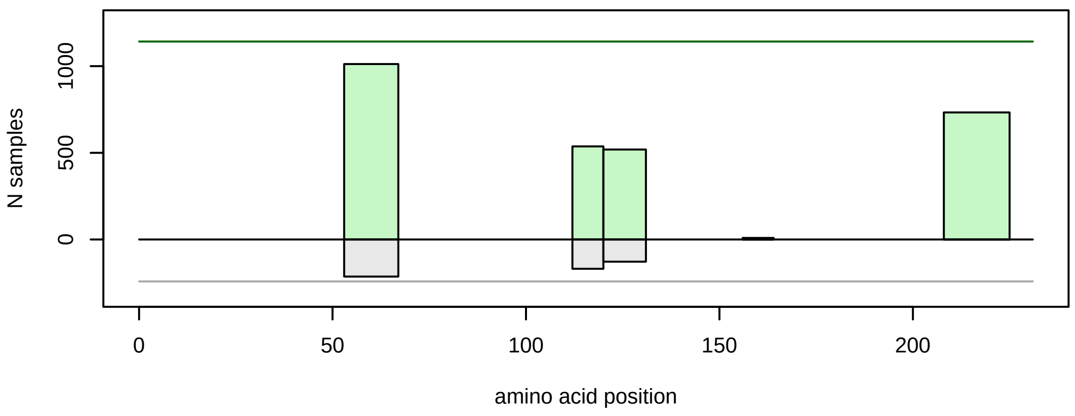

297. C2 (P06681), NP4

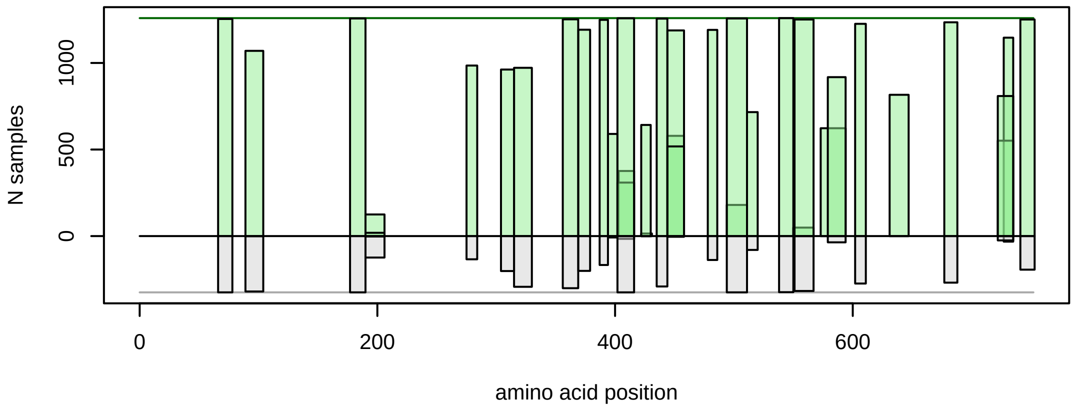

298. SGSM2 (O43147;O43147−2), NP3

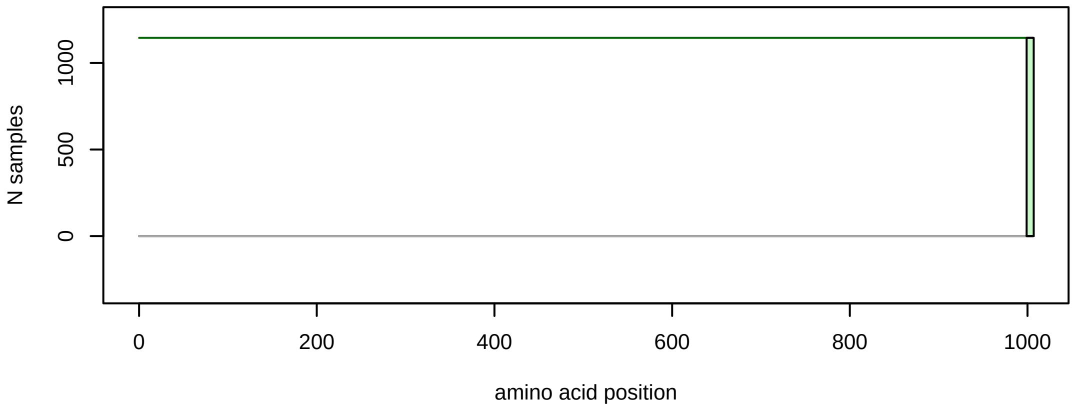

299. NID1 (P14543), NP2

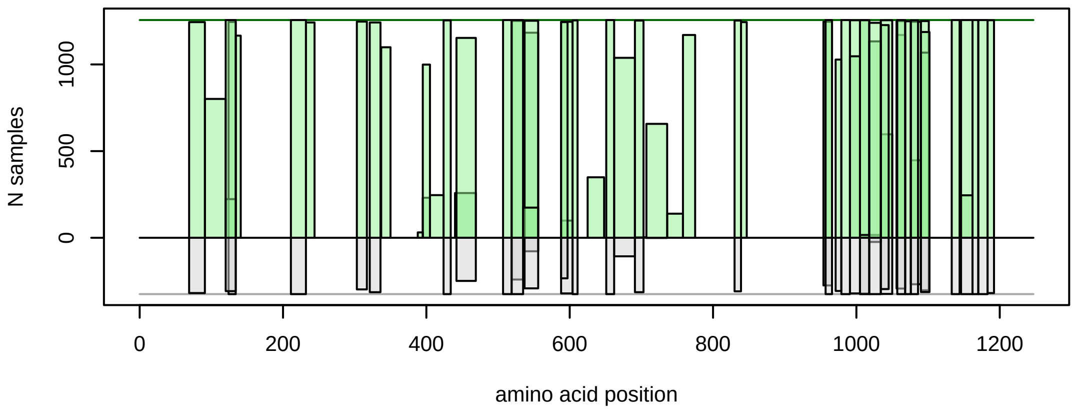

300. YWHAE (P62258), NP1

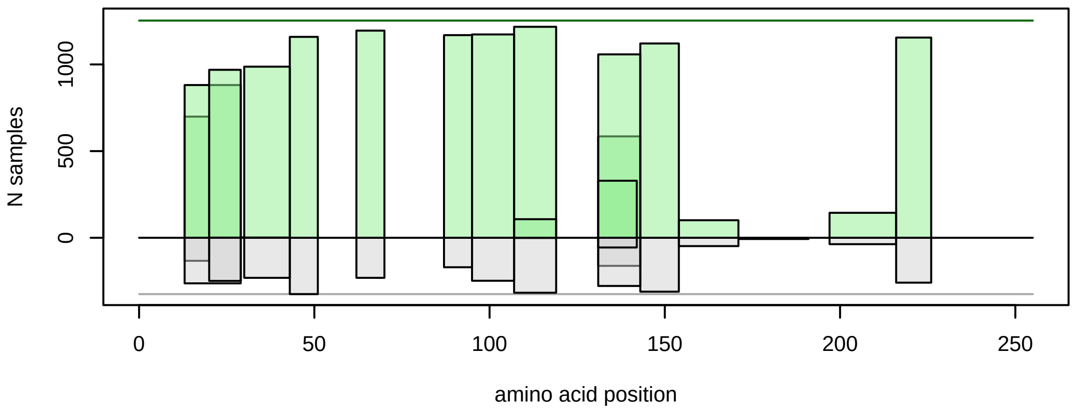

301. APOB (A8MUN2), NP4

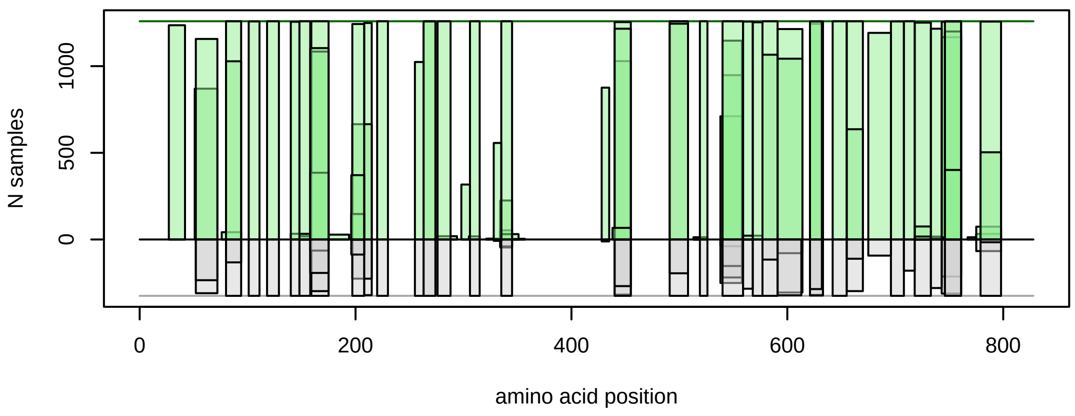

302. OLFML2A (Q68BL7), NP3

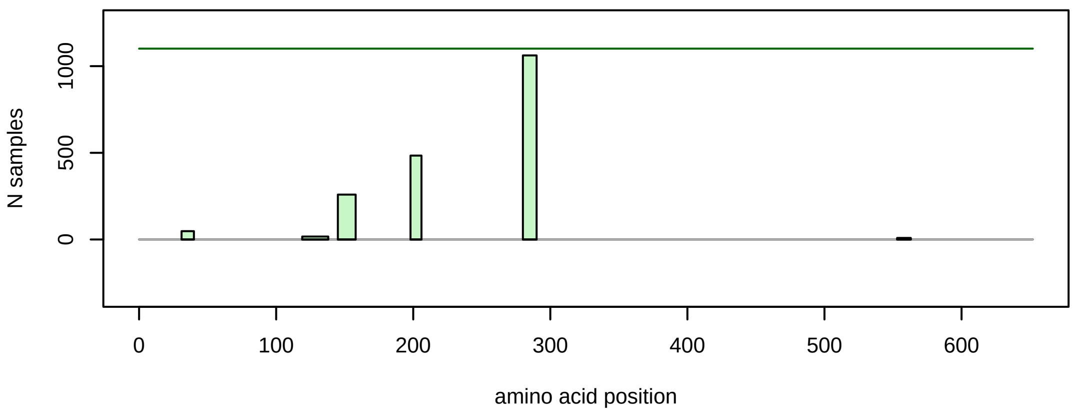

303. CACYBP (Q9HB71), NP3

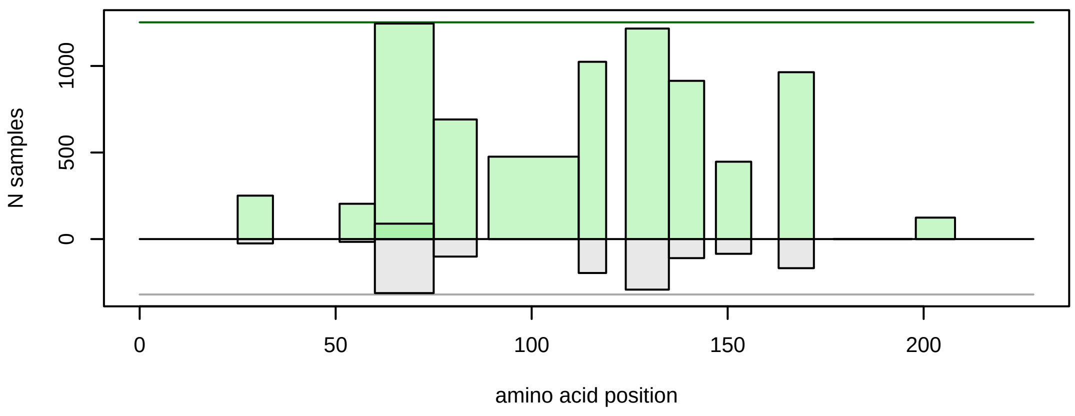

304. KPNB1 (Q14974), NP2

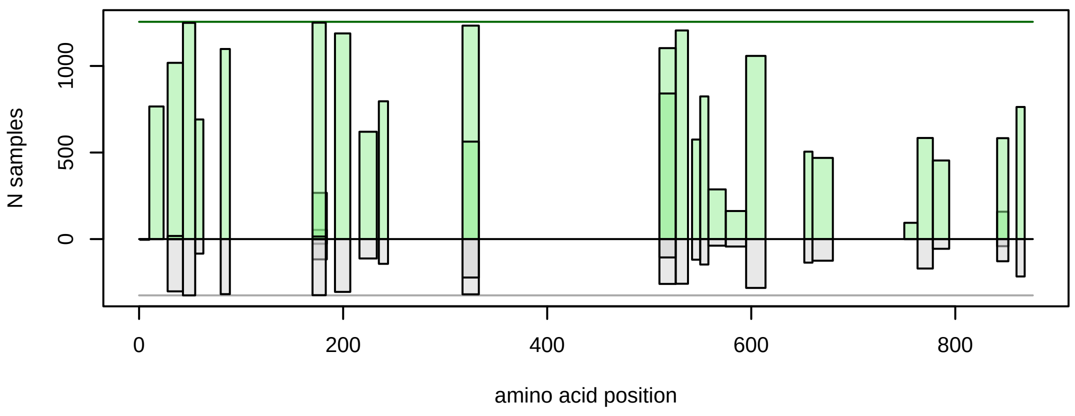

305. ANGPTL2 (Q9UKU9), NP5

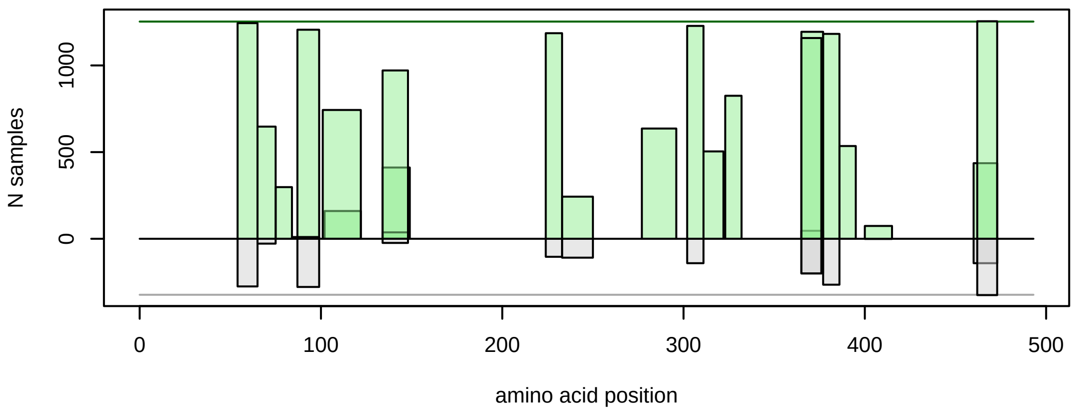

306. CHMP3 (Q9Y3E7), NP1

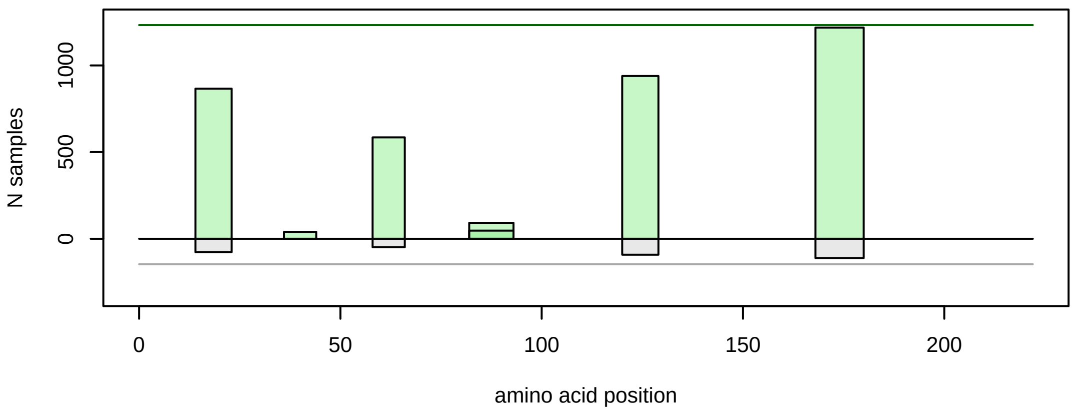

307. GBA2 (Q9HCG7), NP2

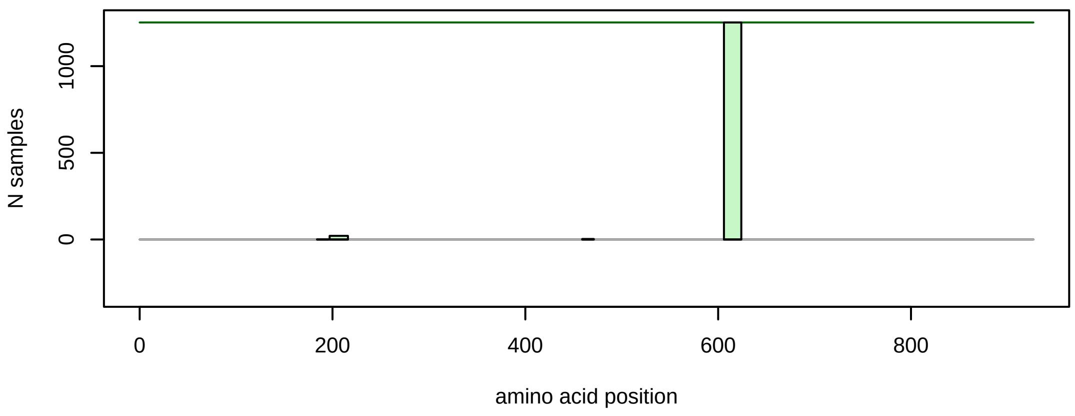

308. PRPSAP1 (Q14558−2), NP4

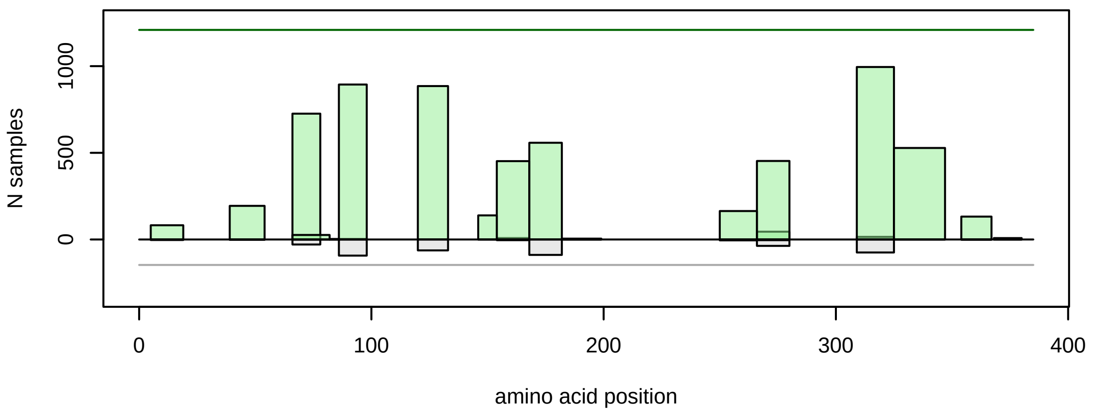

309. CAPZA1 (P52907), NP4

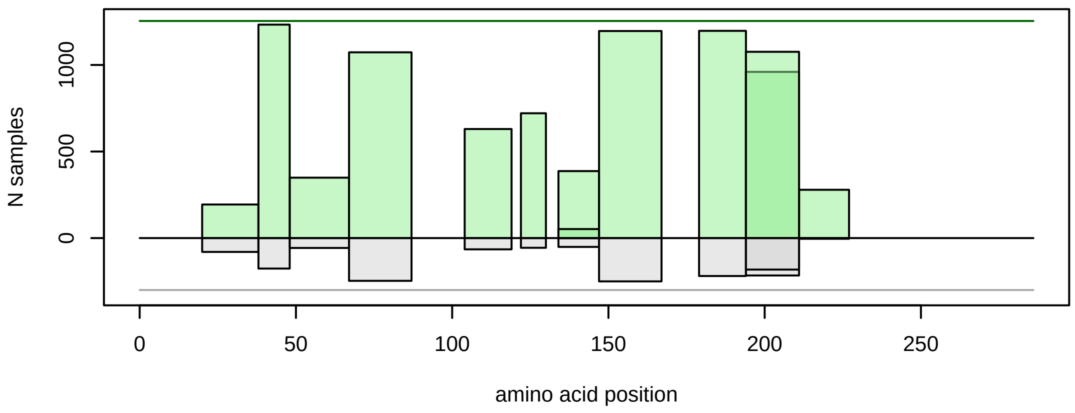

310. GPLD1 (P80108), NP5

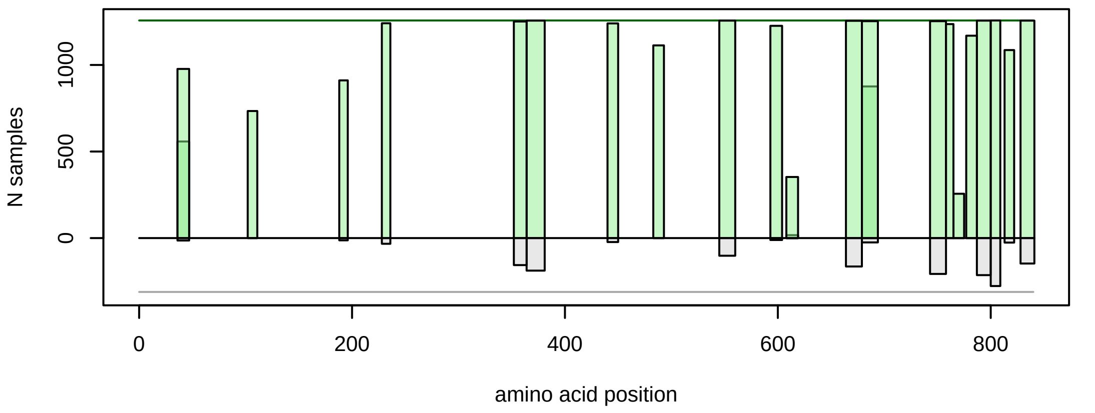

311. COL1A2 (P08123), NP3

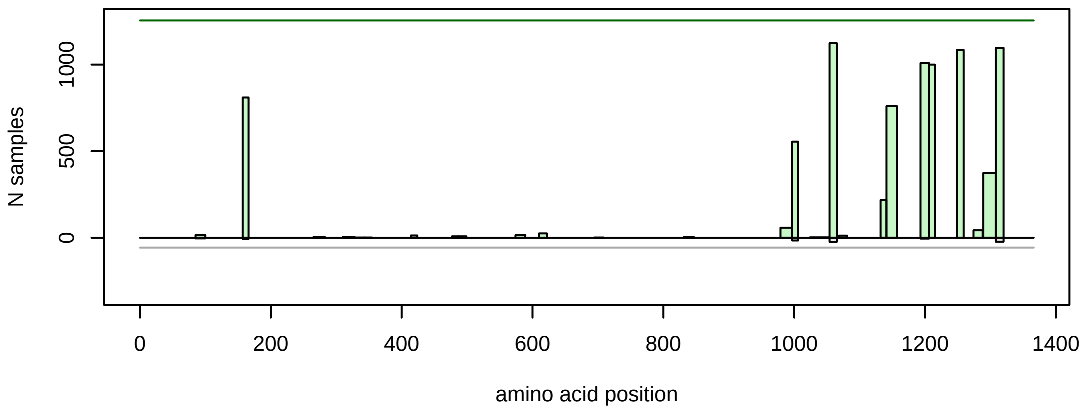

312. CD109 (Q6YHK3), NP2

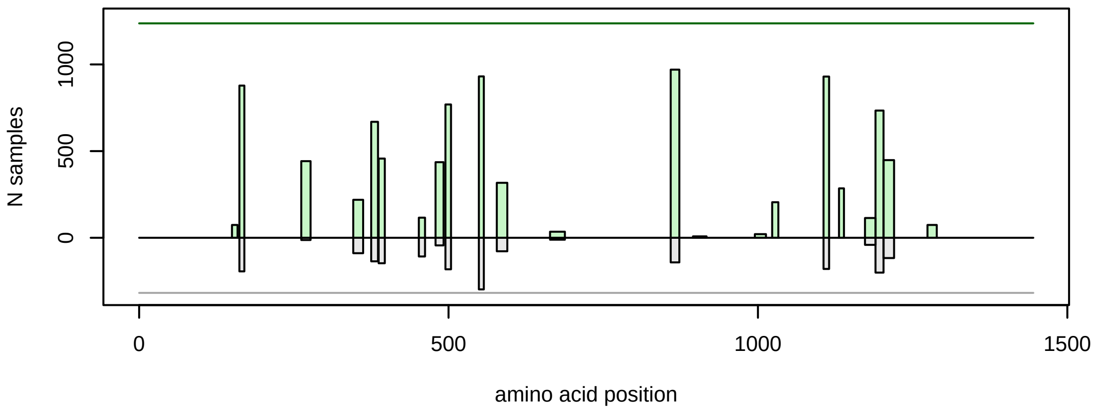

313. APLP1 (B7Z4G8), NP2

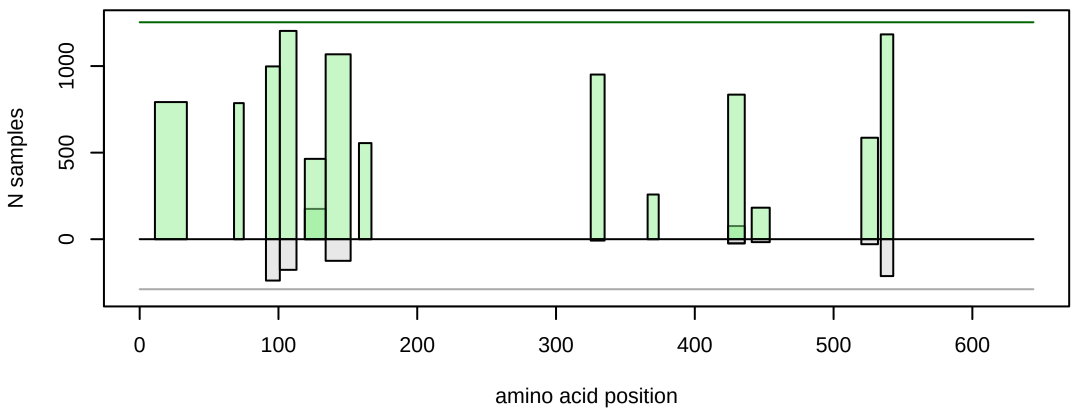

314. YKT6 (A0A7I2V4L6;O15498), NP1

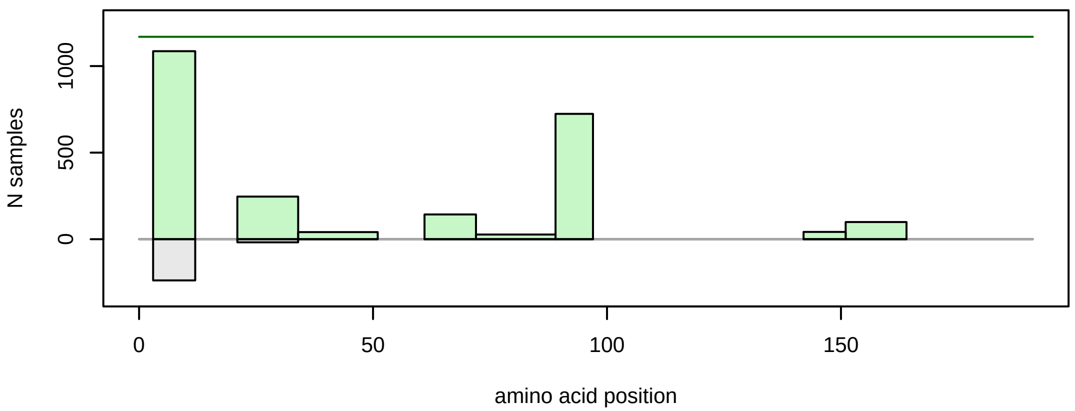

315. S100A4 (P26447), NP2

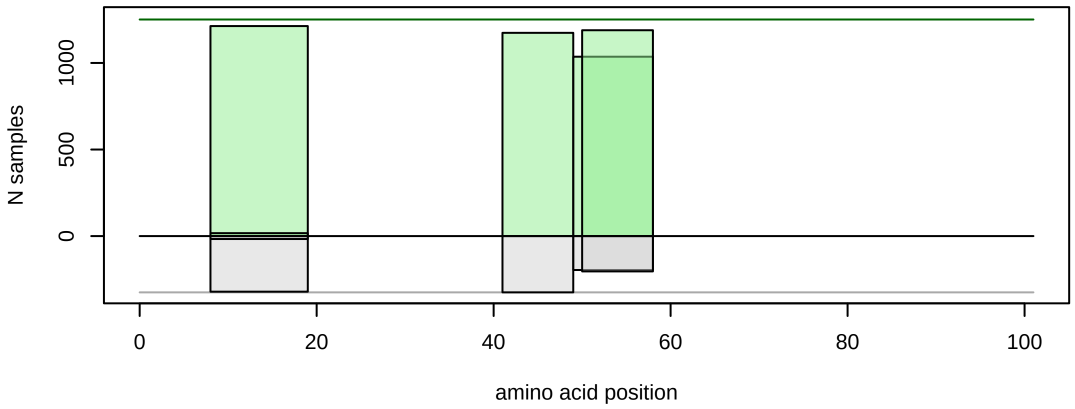

316. MAPKAPK2 (P49137), NP1

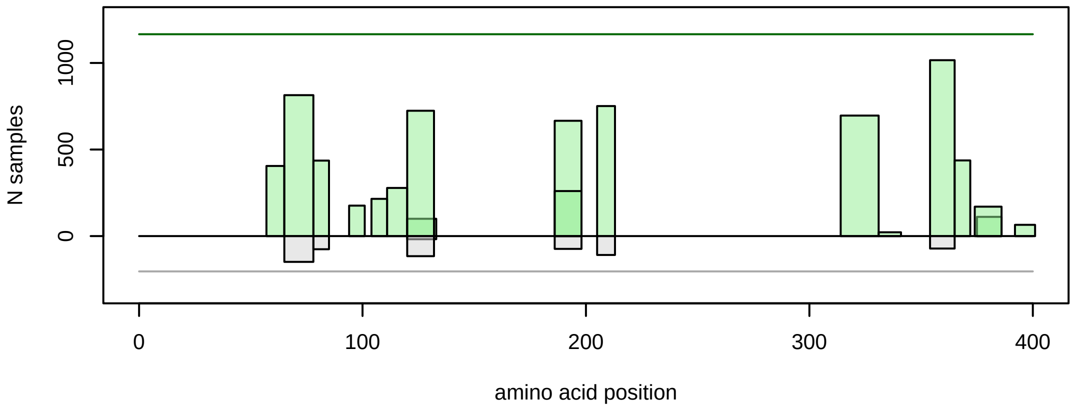

317. NACA (E9PAV3), NP4

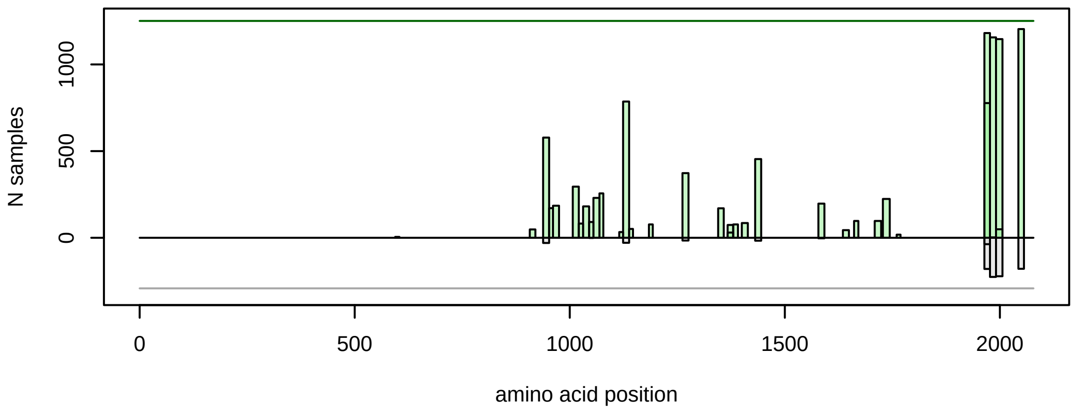

318. PLP2 (Q04941), NP1

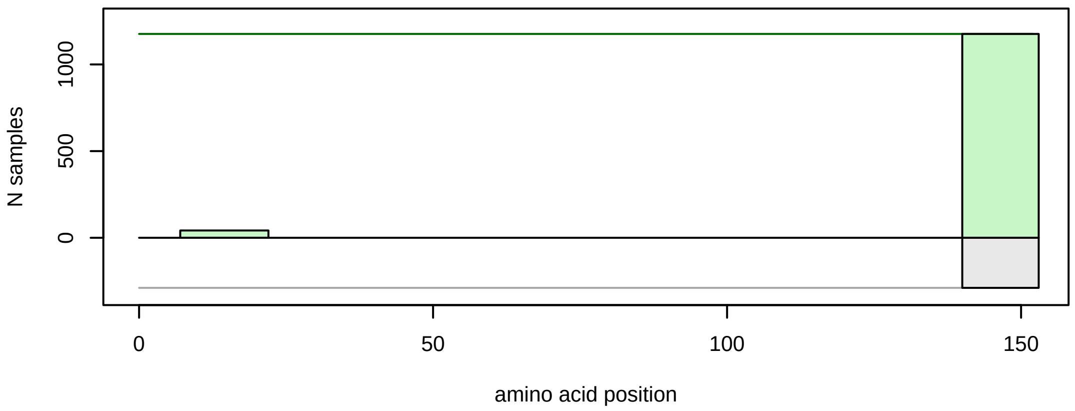

319. APOD (P05090), NP4

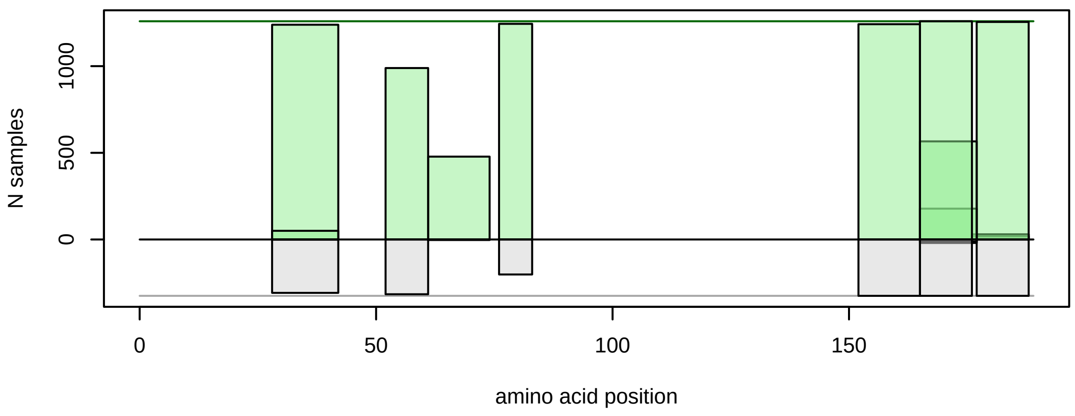

320. CCT2 (P78371), NP4

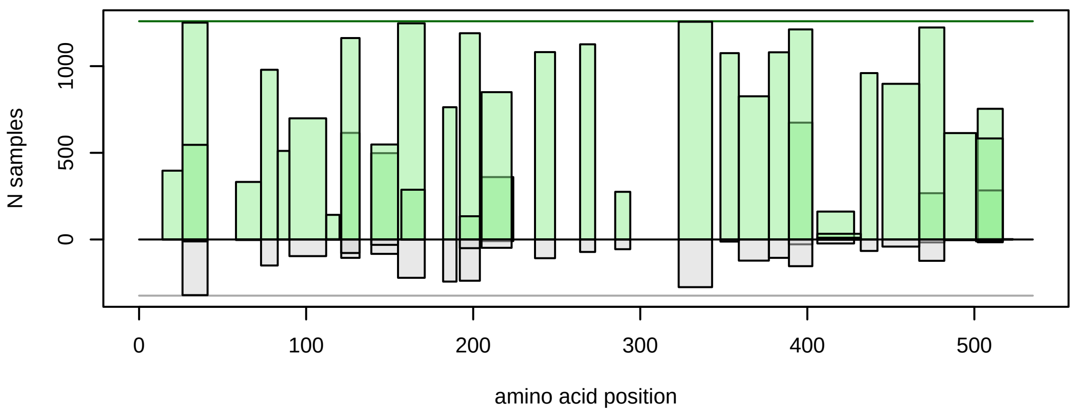

321. PPP1R14A (Q96A00), NP3

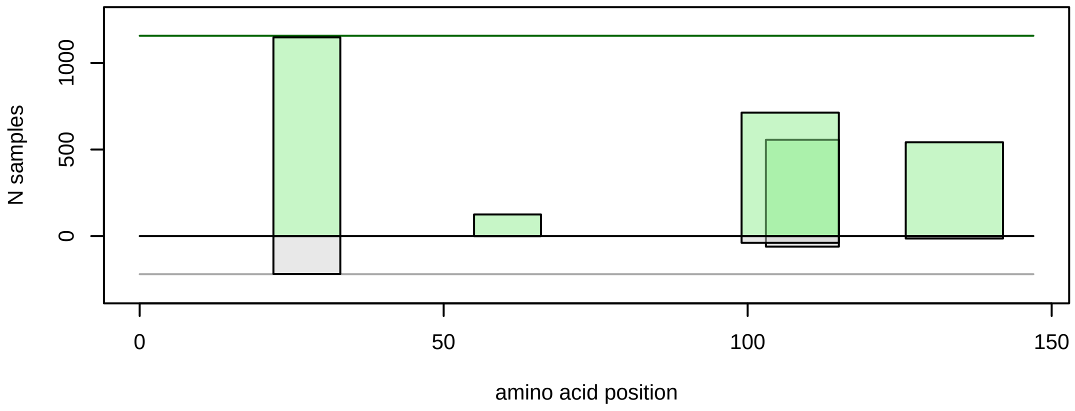

322. EIF3D (O15371), NP1

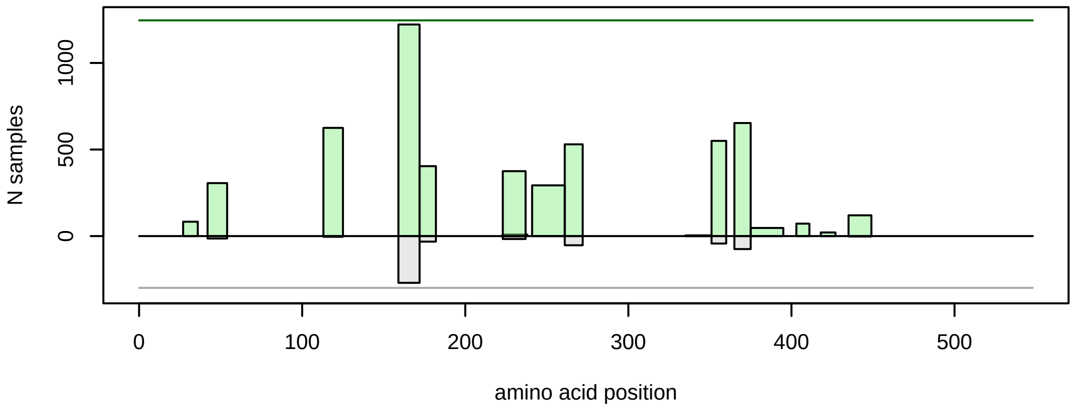

323. PCOLCE2 (Q9UKZ9), NP4

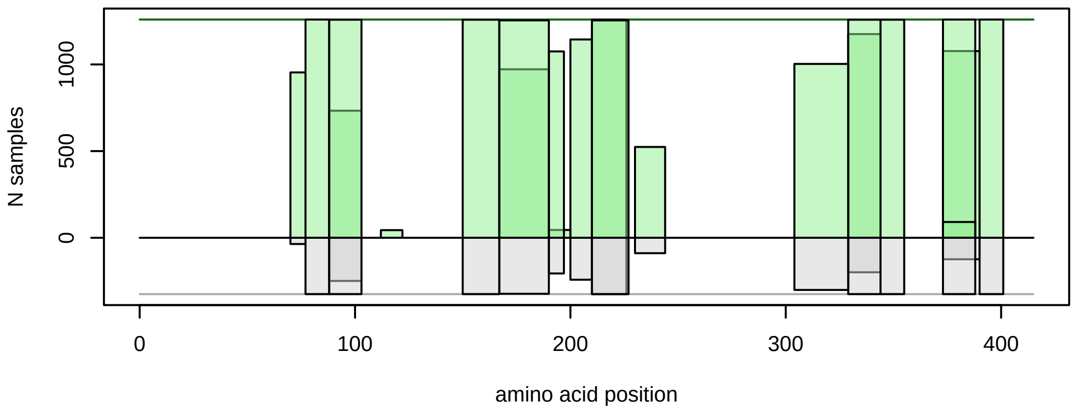

324. CALR (P27797), NP2

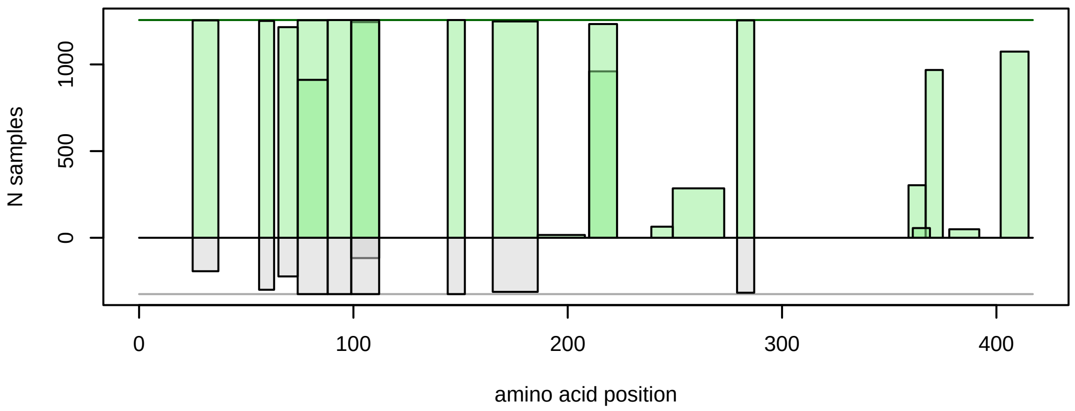

325. EPRS (P07814), NP3

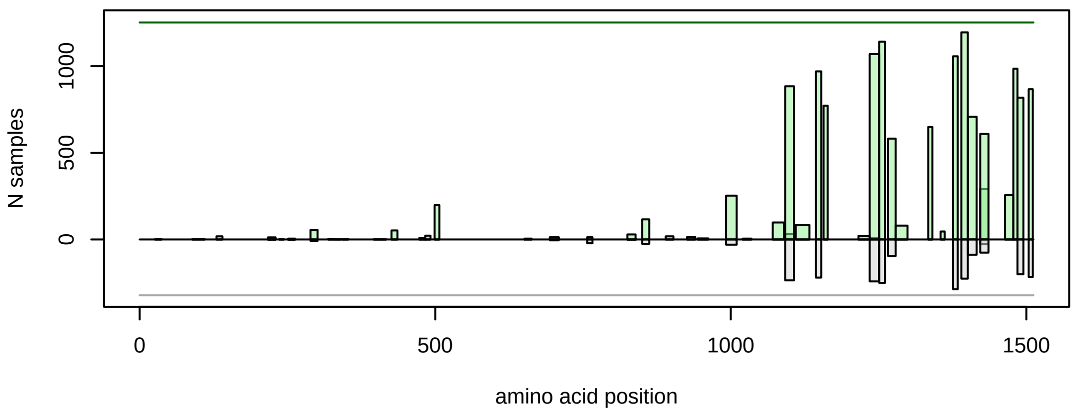

326. ACO2 (A2A274;Q99798), NP1

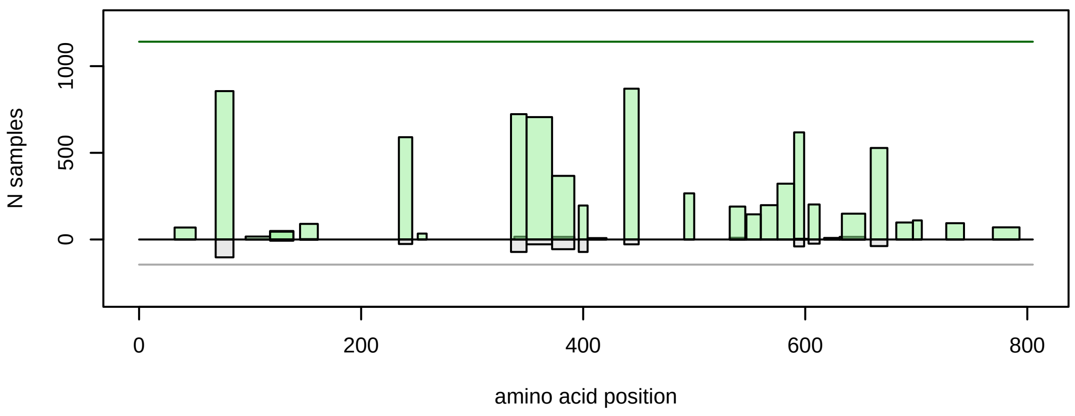

327. SLC16A3 (J3KTM6;J3QQV2;O15427), NP1

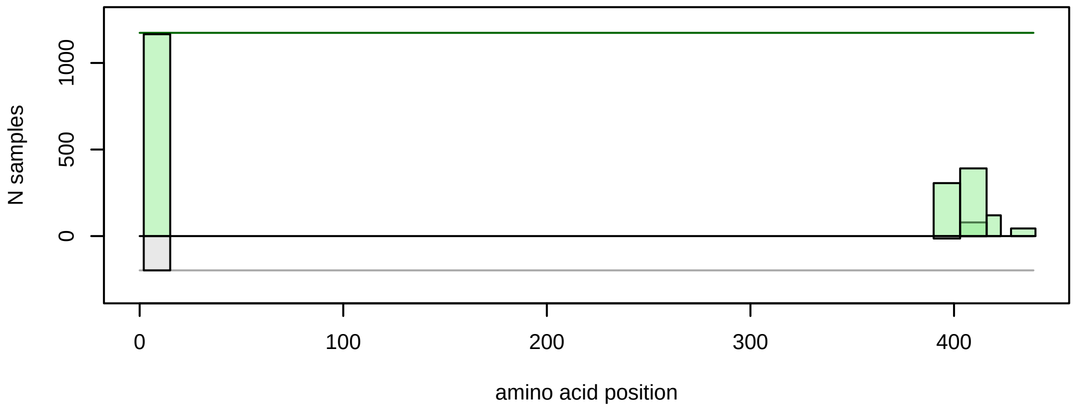

328. PDGFA (P04085), NP3

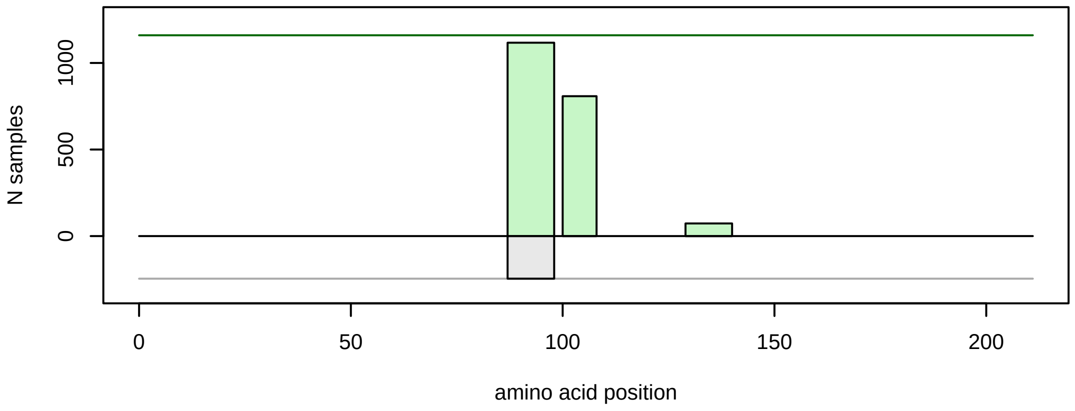

329. STEAP3 (Q658P3;Q658P3−2), NP1

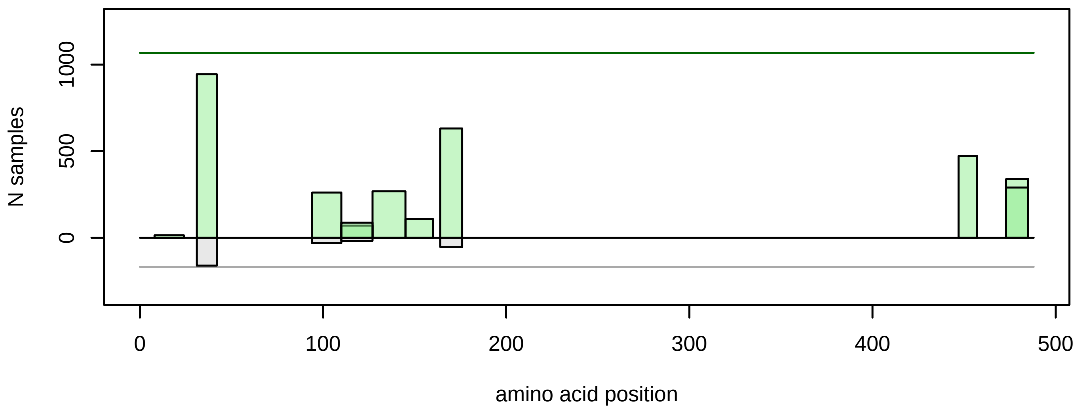

330. STK24 (B4DR80;Q9Y6E0−2), NP5

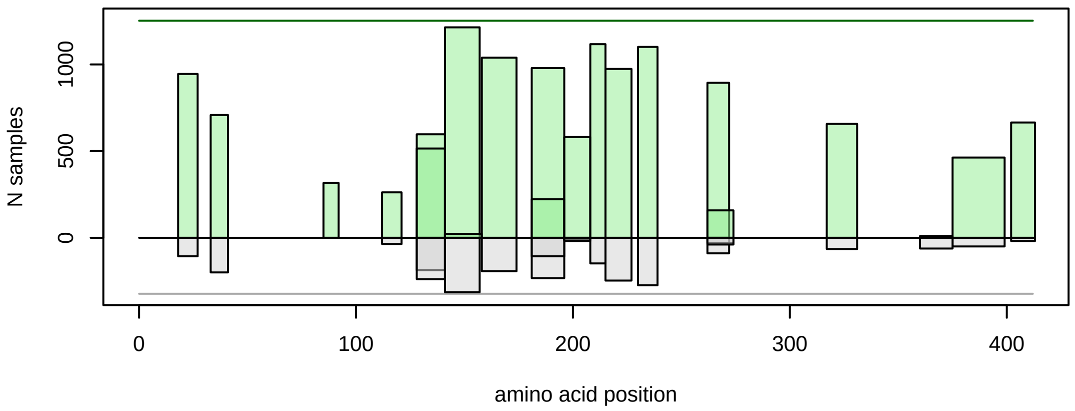

331. LCP2 (Q13094), NP1

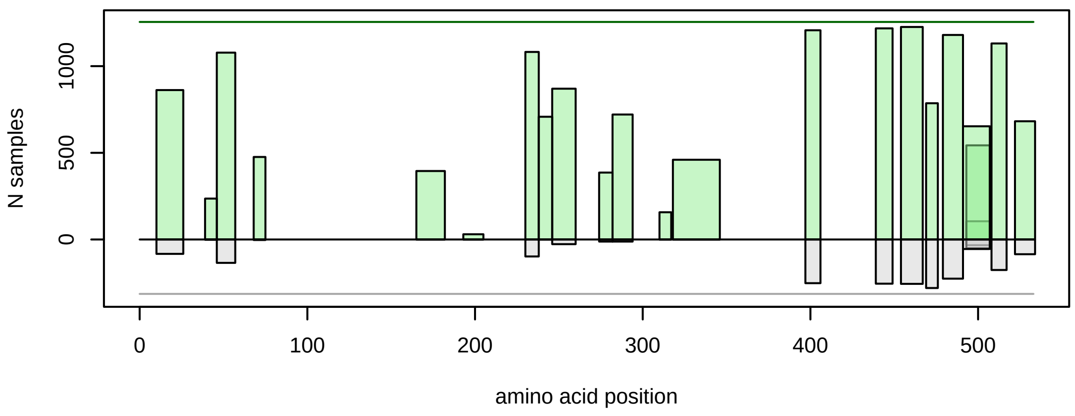

332. PLCB2 (Q00722;Q00722−2;Q00722−3), NP1

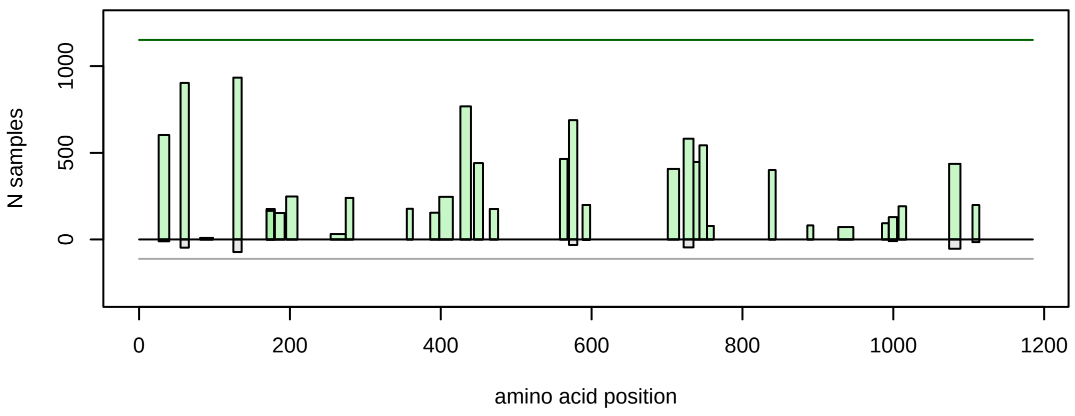

333. COX4I1 (P13073), NP4

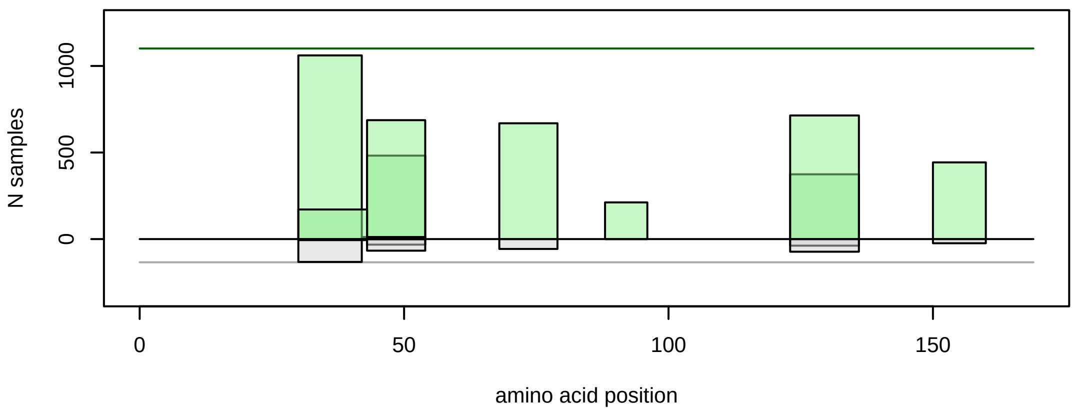

334. WFDC5 (Q8TCV5;Q8TCV5−2), NP1

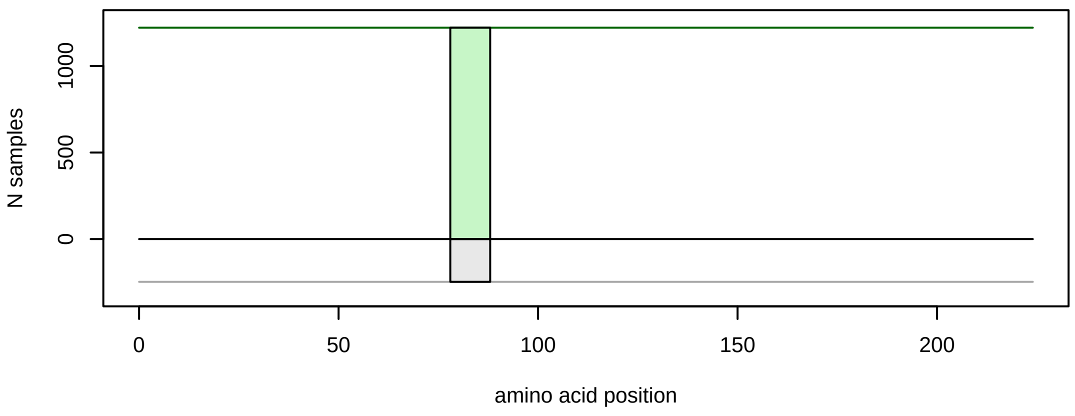

335. HNRNPAB (D6RD18), NP1

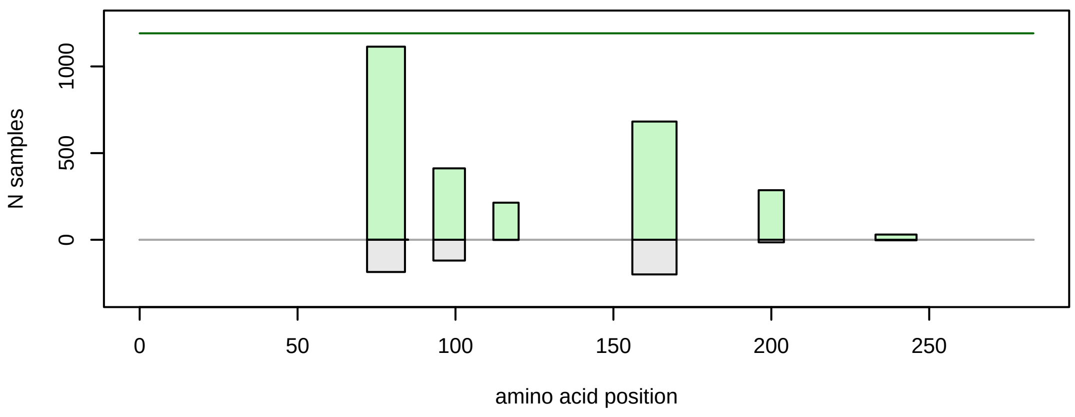

336. TST (Q16762), NP4

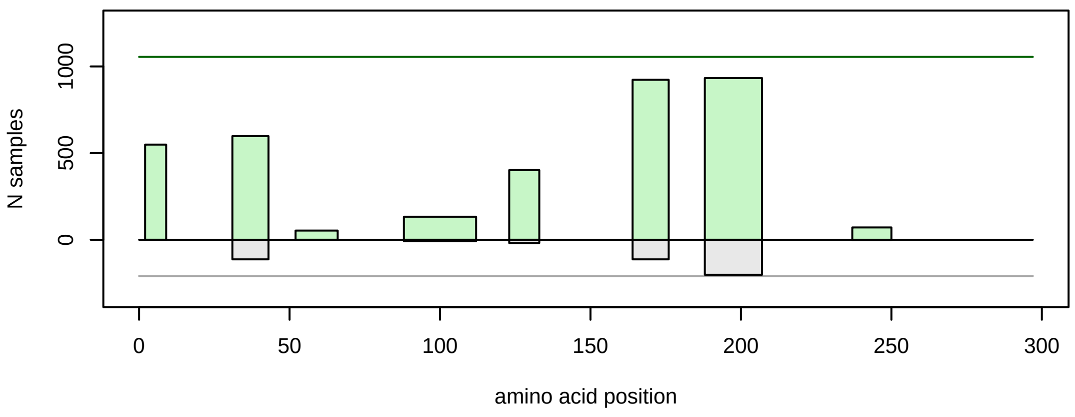

337. ESYT1 (Q9BSJ8;Q9BSJ8−2), NP4

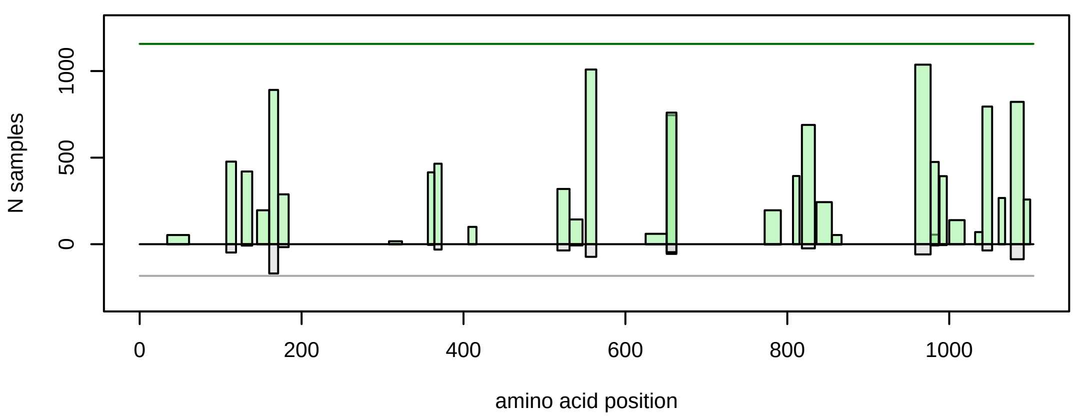

338. AP3D1 (O14617−5), NP4

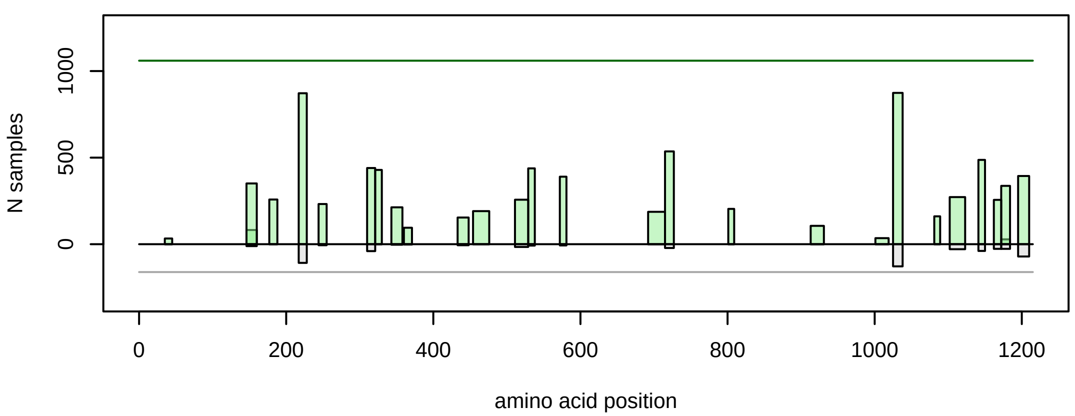

339. PROC (E7END6), NP2

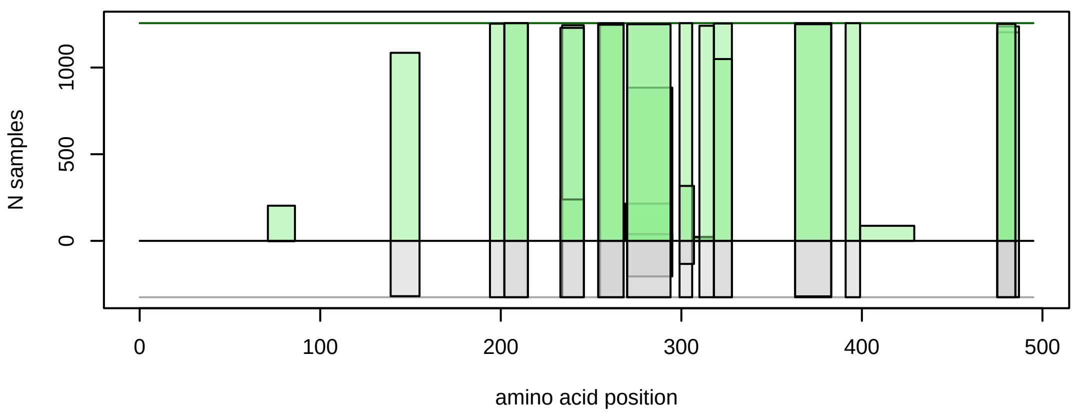

340. CTSF (A0A7I2V313), NP4

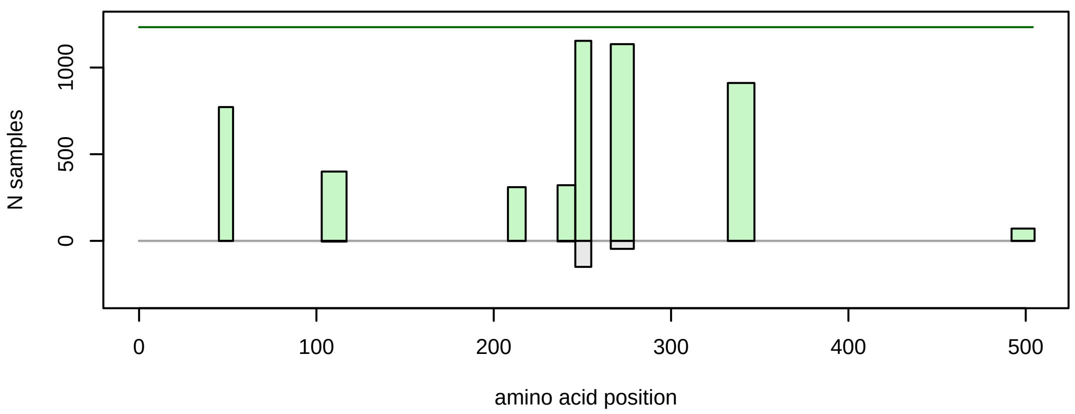

341. GNG2 (G3V3J9), NP1

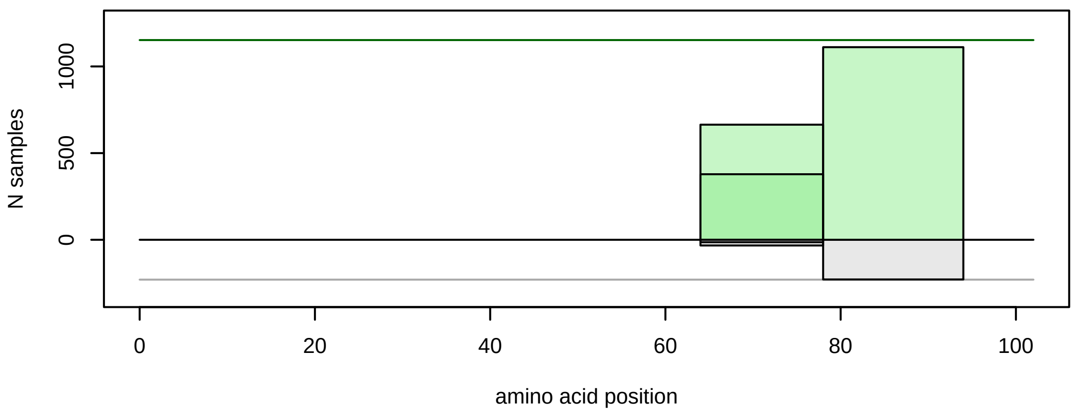

342. LMNB1 (P20700), NP2

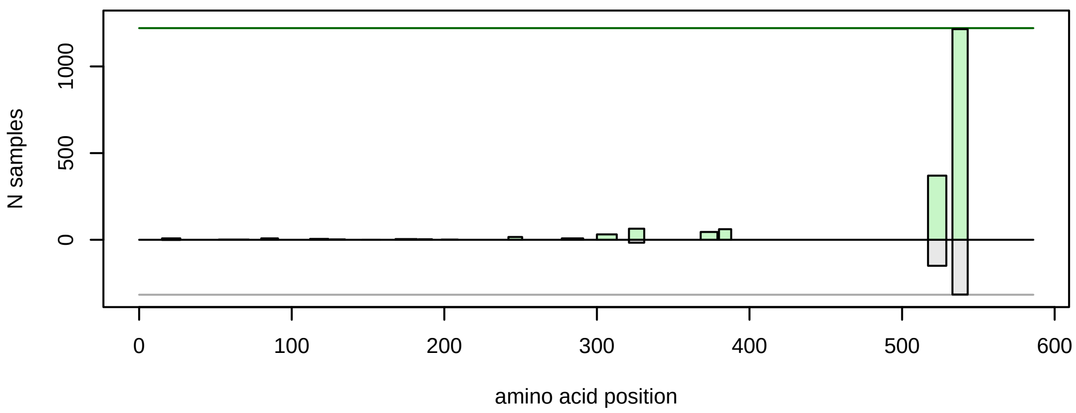

343. PLA1A (G5E9W0;Q53H76), NP4

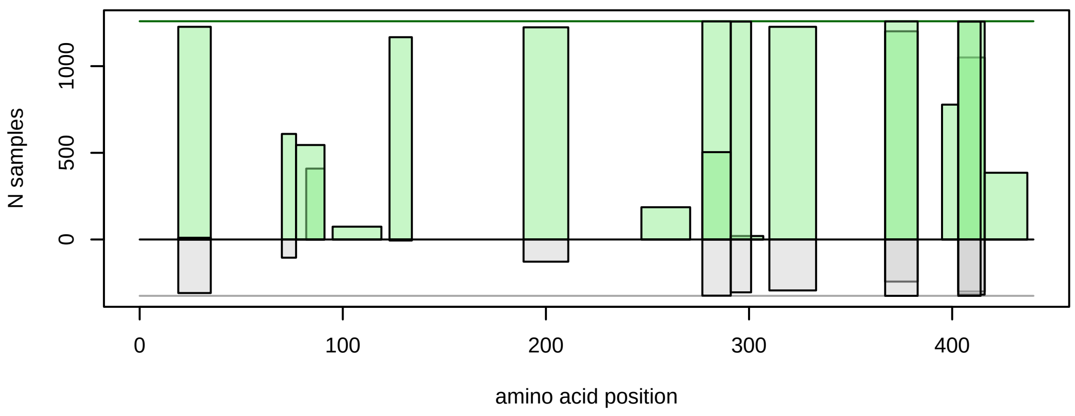

344. CS (O75390), NP1

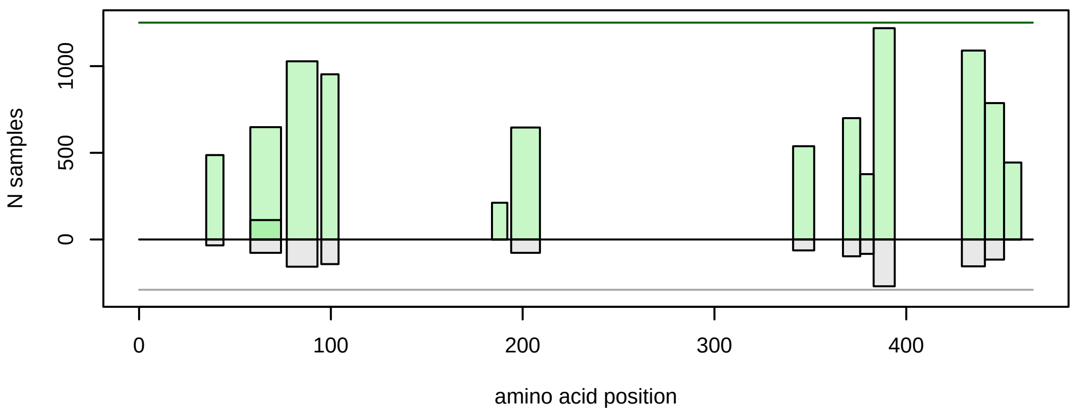

345. GRK6 (P43250−2), NP1

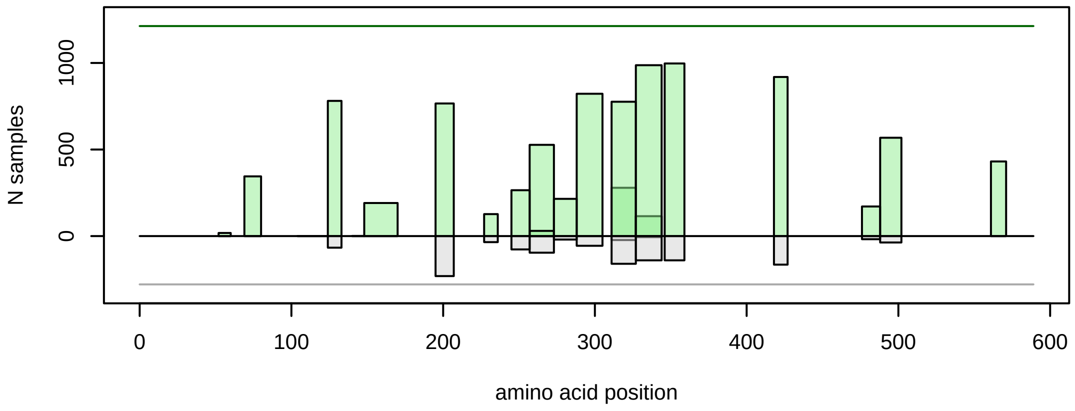

346. METRNL (Q641Q3), NP3

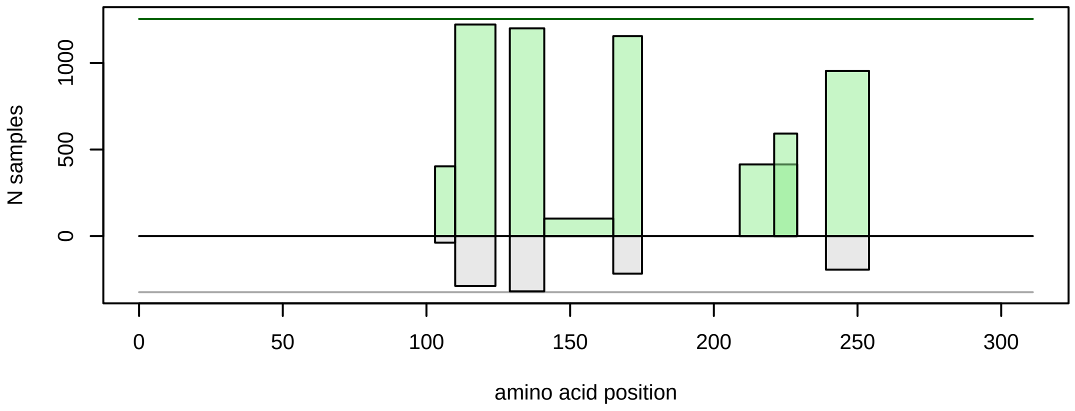

347. IGLV2−18 (A0A075B6J9), NP4

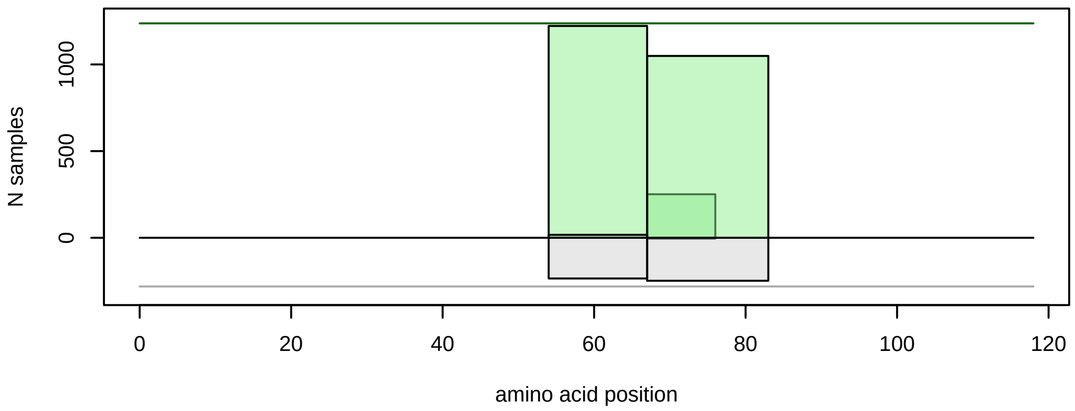

348. CD2AP (Q9Y5K6), NP1

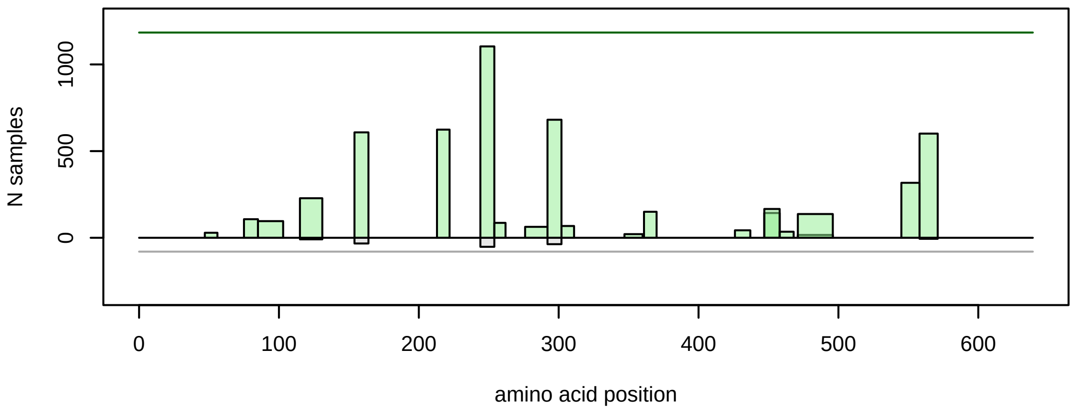

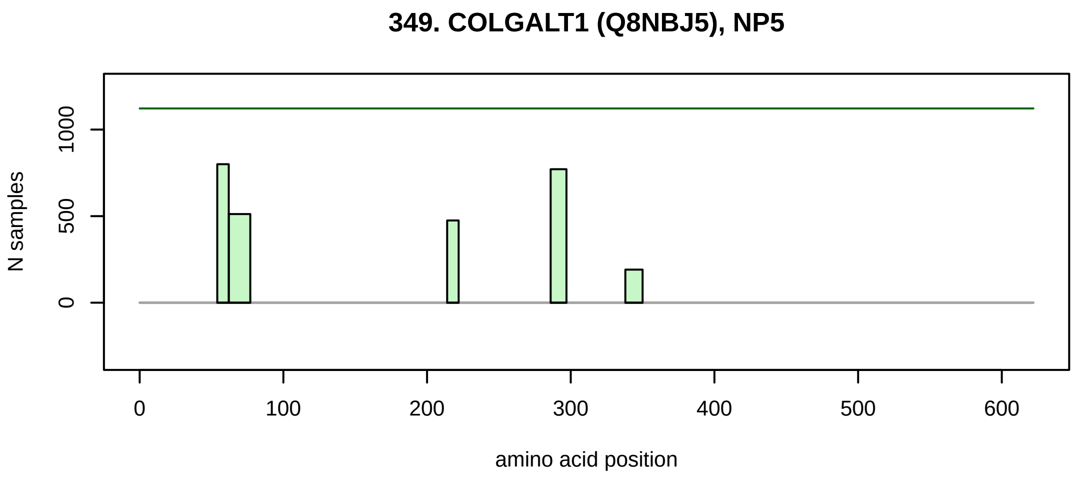
349. COLGALT1 (Q8NBJ5), NP5
N samples
0
500
1000
0
100
200
300
400
500
600
amino acid position

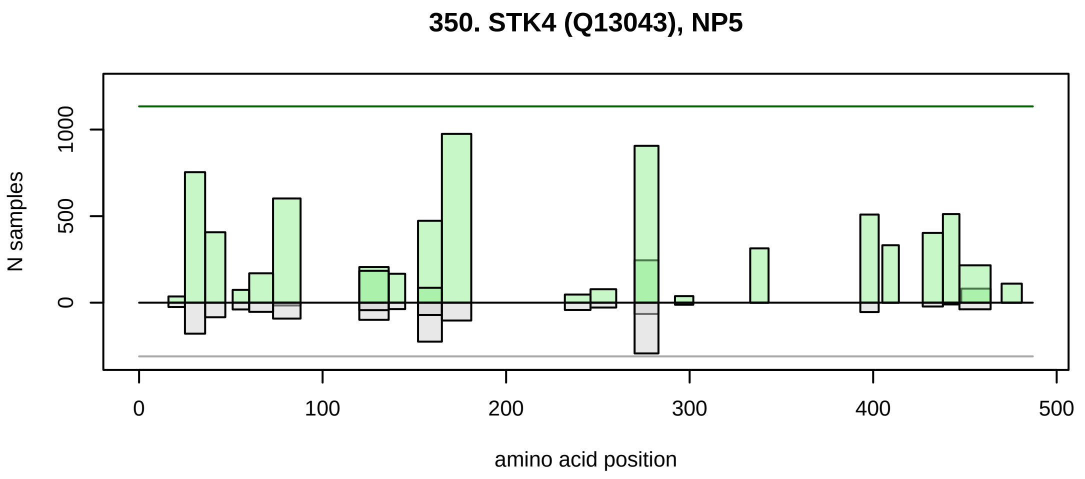
350. STK4 (Q13043), NP5
N samples
0
500
1000
0
100
200
300
400
500
amino acid position

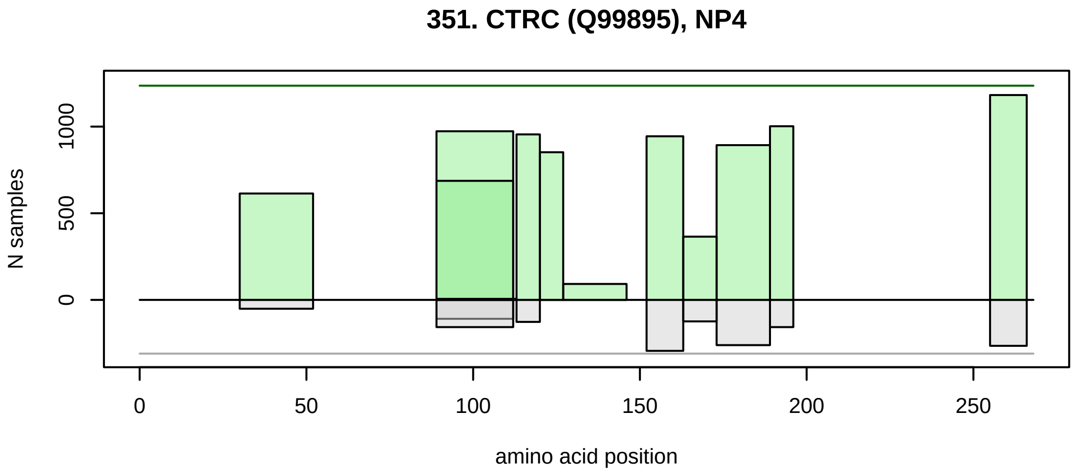
351. CTRC (Q99895), NP4
N samples
0
500
1000
0
50
100
150
200
250
amino acid position

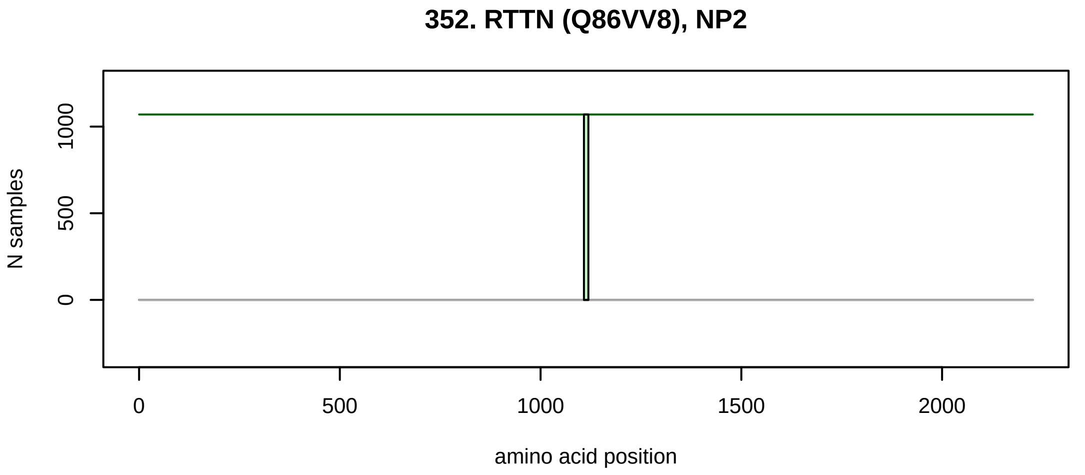
352. RTTN (Q86VV8), NP2
N samples
0
500
1000
0
500
1000
1500
2000
amino acid position

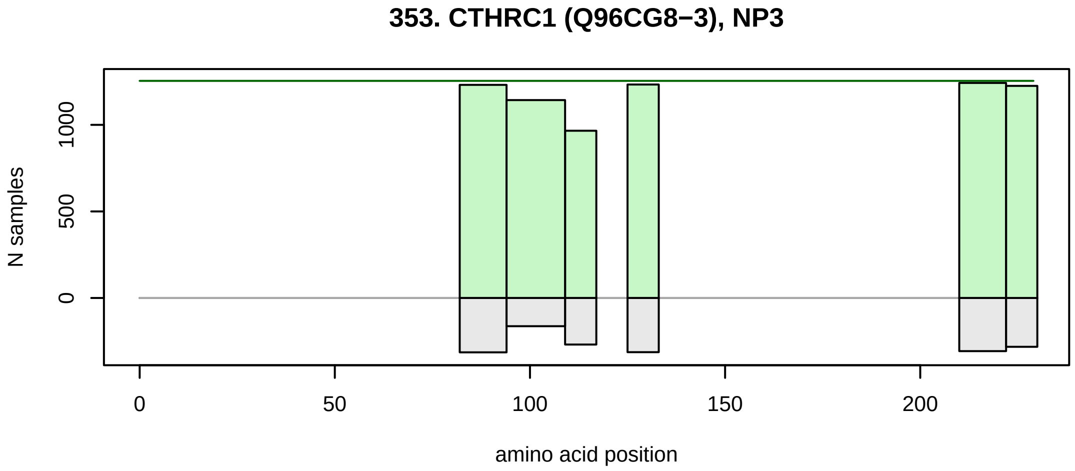
353. CTHRC1 (Q96CG8−3), NP3
N samples
0
500
1000
0
50
100
150
200
amino acid position

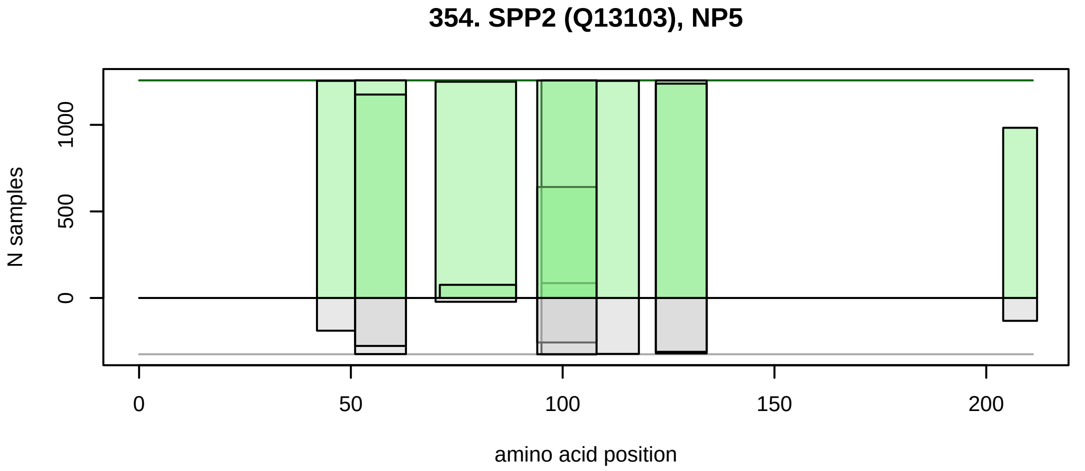
354. SPP2 (Q13103), NP5
N samples
0
500
1000
0
50
100
150
200
amino acid position

355. EFEMP1 (Q12805), NP5

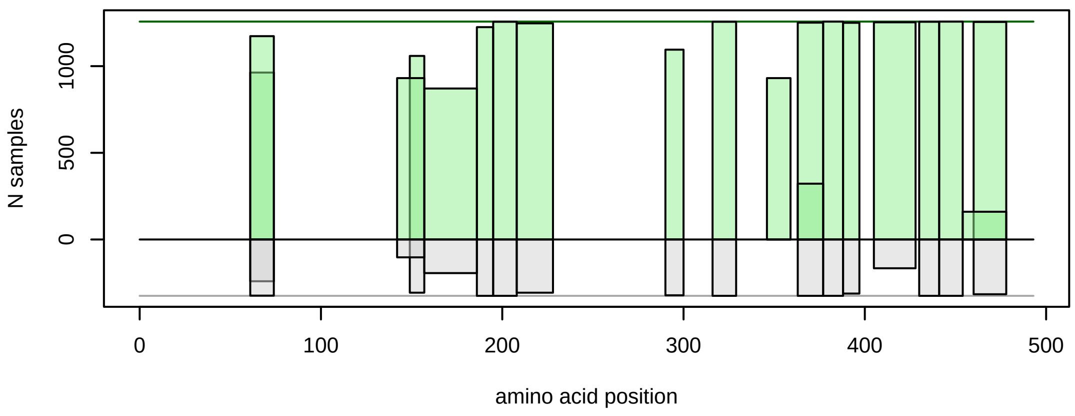

356. APLP2 (Q06481), NP2

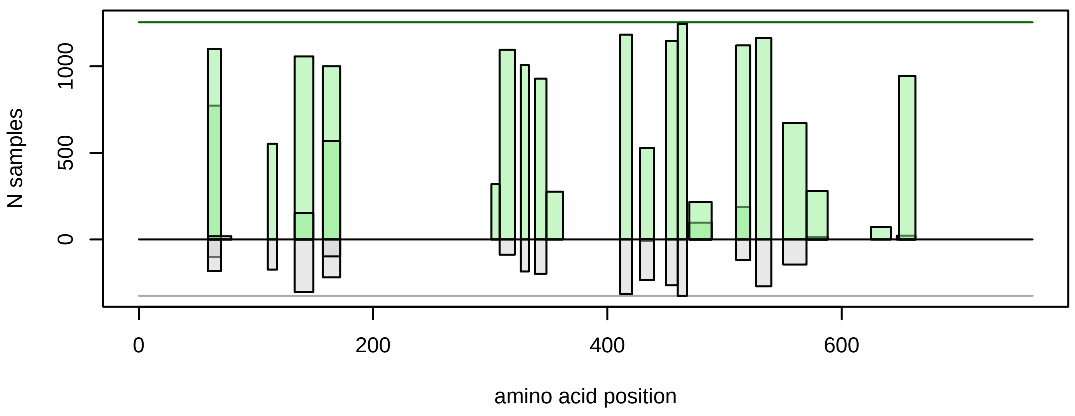

357. PLXNB1 (O43157), NP2

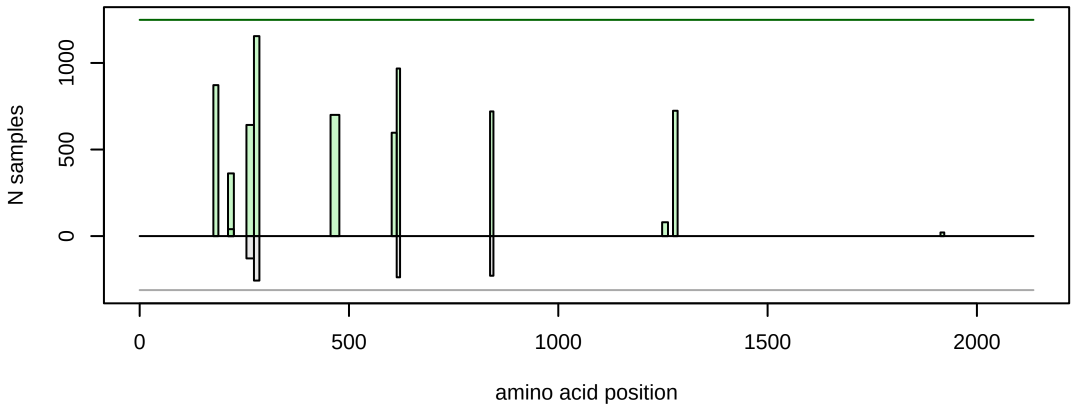

358. METAP2 (P50579), NP4

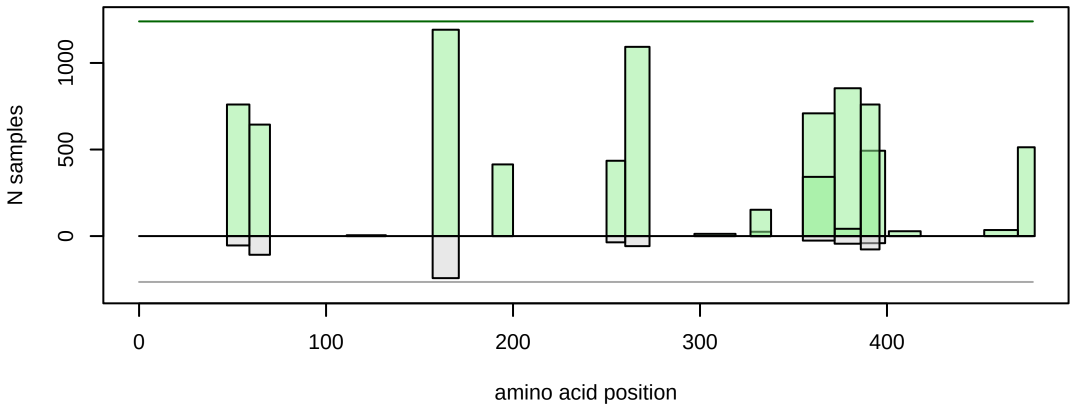

359. INF2 (A0A6Q8PHA2), NP4

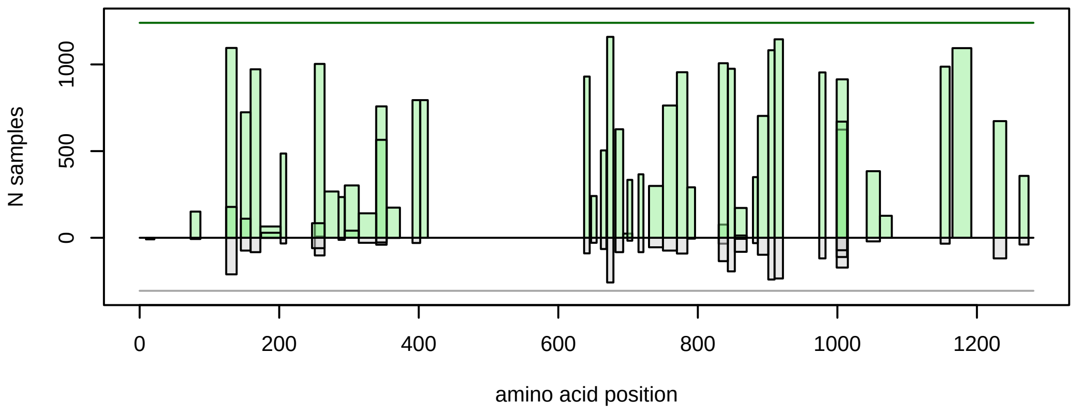

360. FSCN1 (Q16658), NP3

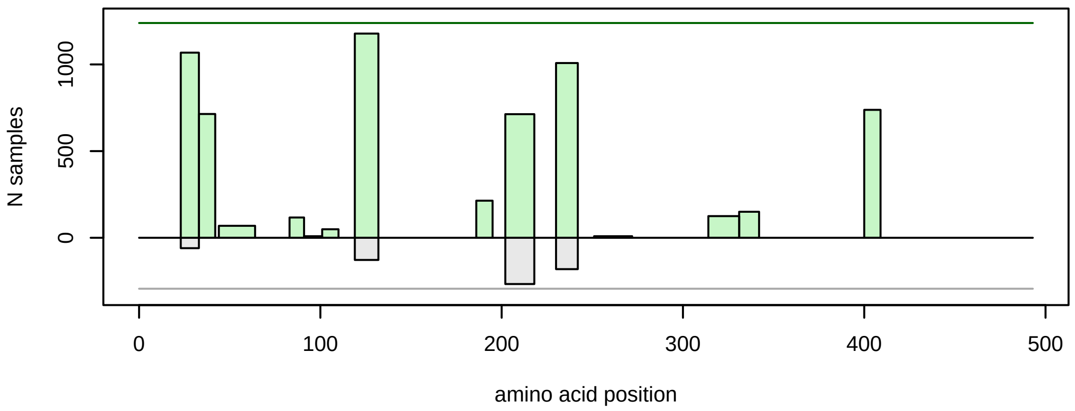

361. LAMA2 (A0A087WYF1;P24043), NP2

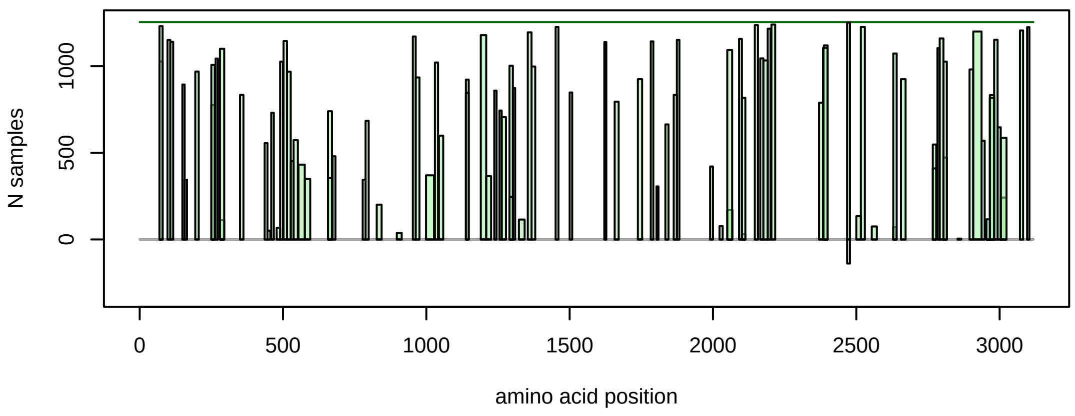

362. IGHV2−5 (P01817), NP3

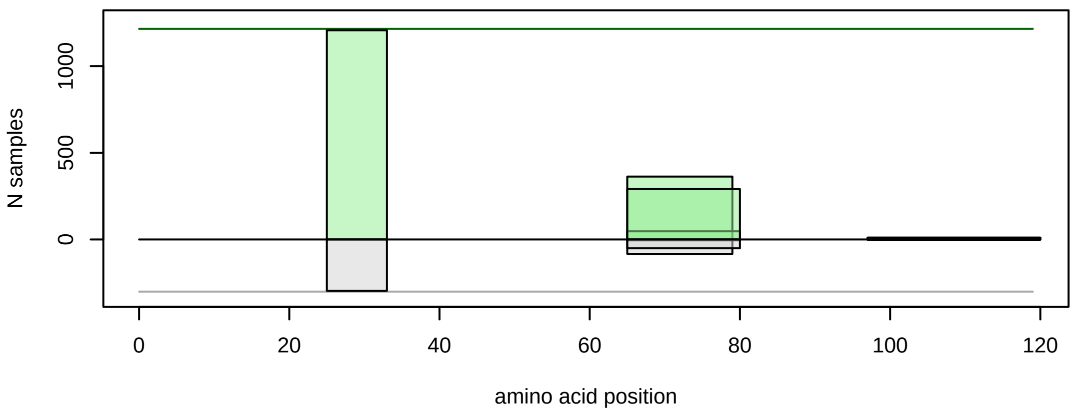

363. RAB7A (P51149), NP1

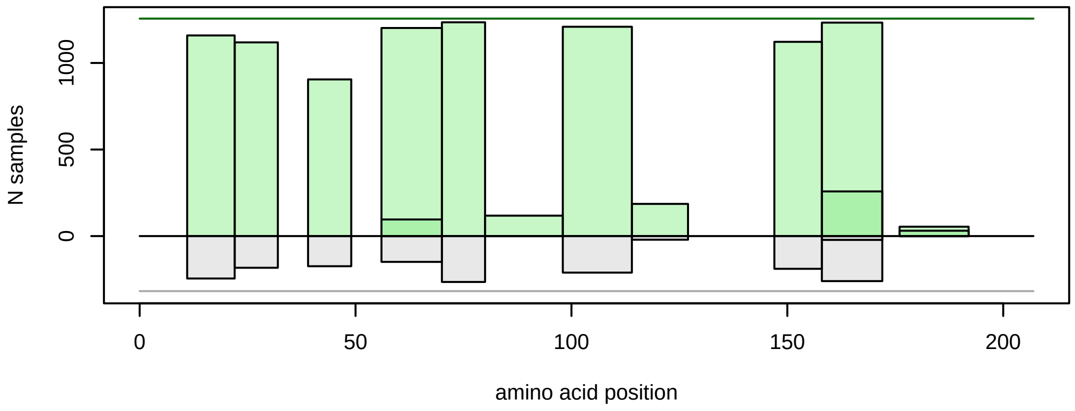

364. ACO1 (P21399), NP5

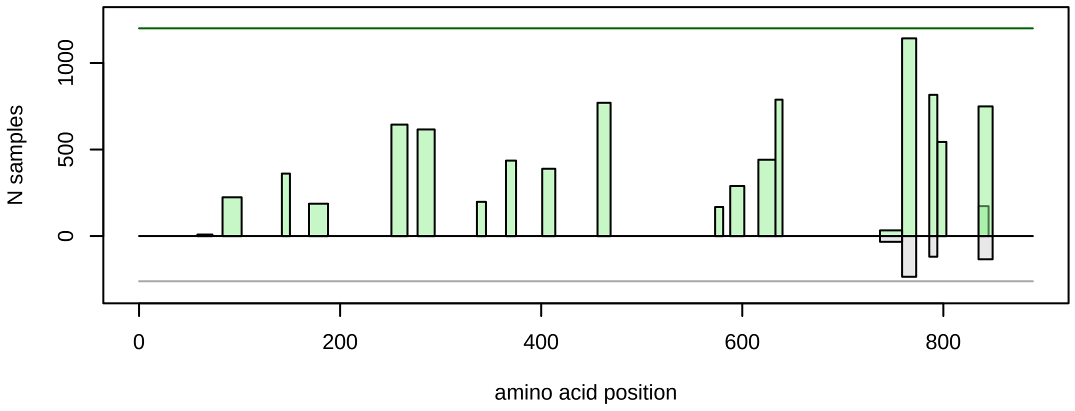

365. PCSK2 (P16519−3), NP2

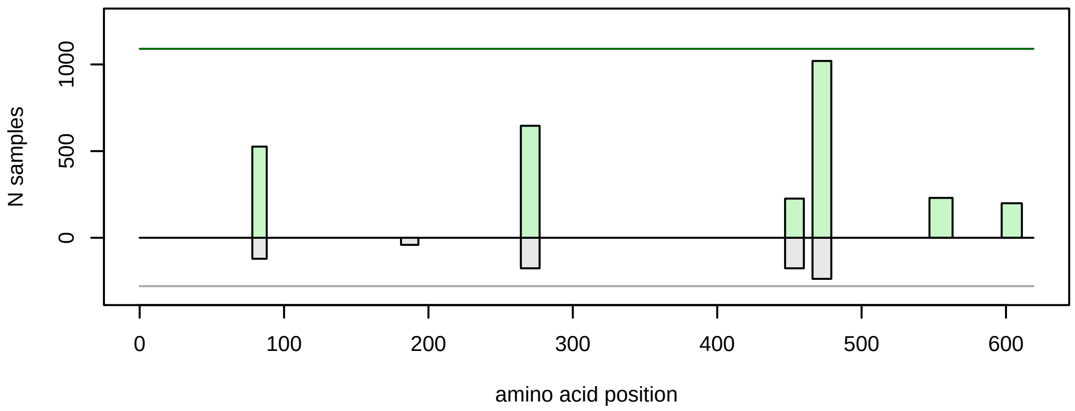

366. ADH1B (P00325), NP4

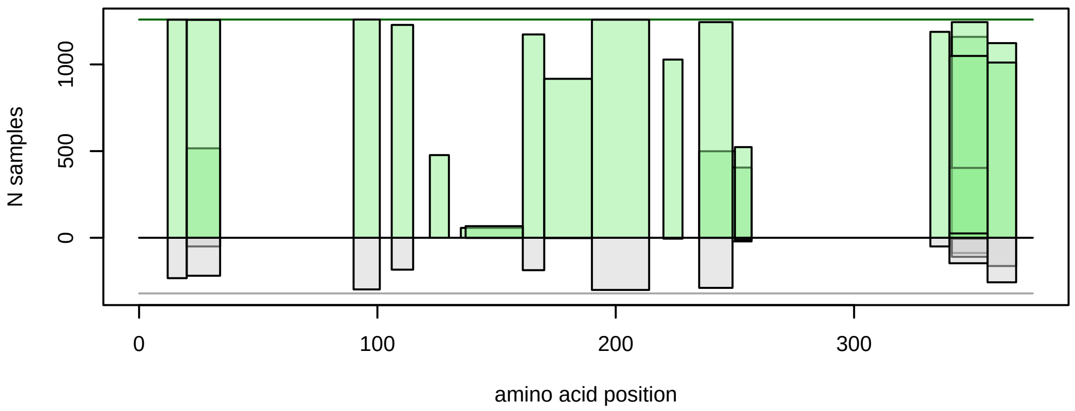

367. EMILIN1 (Q9Y6C2), NP1

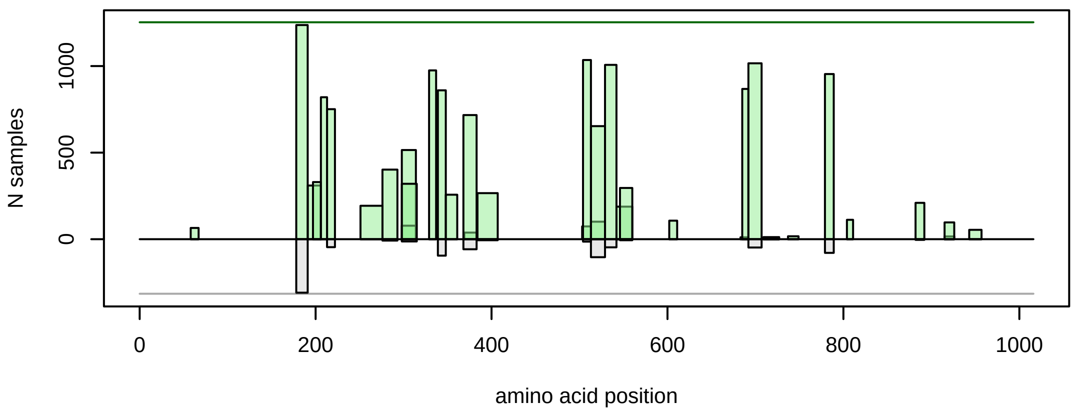

368. MIF (P14174), NP1

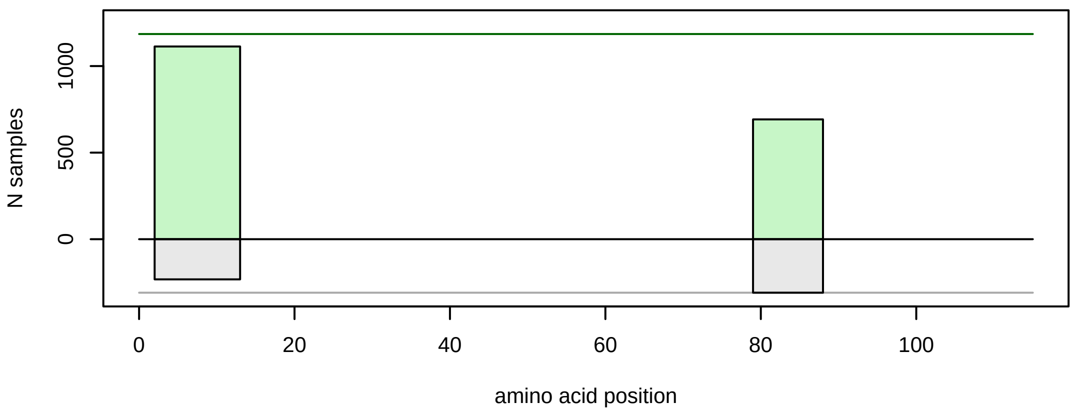

369. TPM1 (H7BYY1), NP4

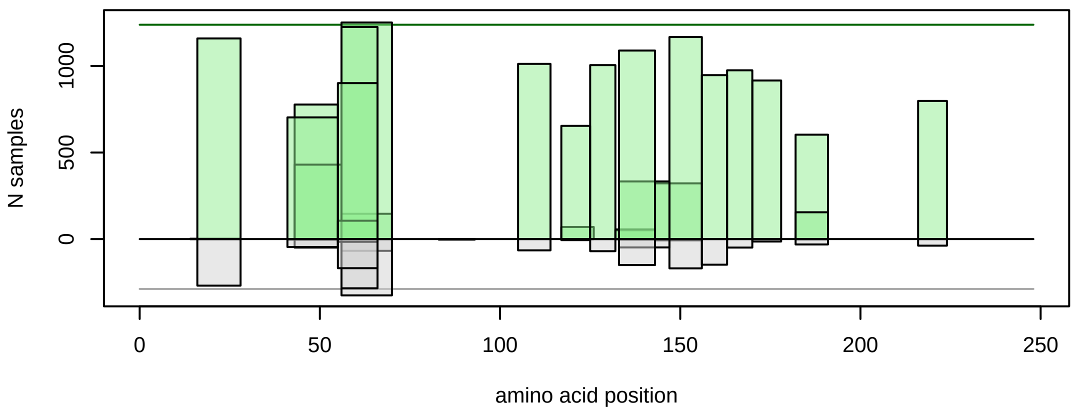

370. SYTL3 (Q4VX76), NP3

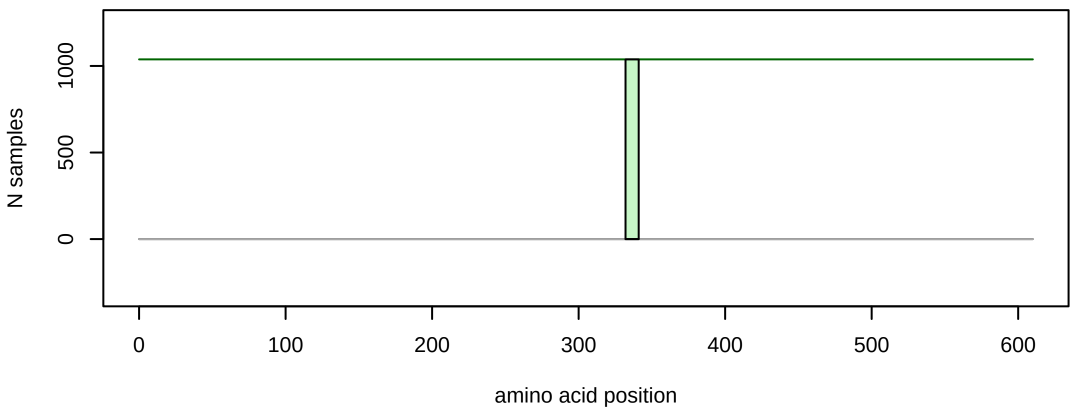

371. MMP14 (A0A7P0TAG0;P50281), NP3

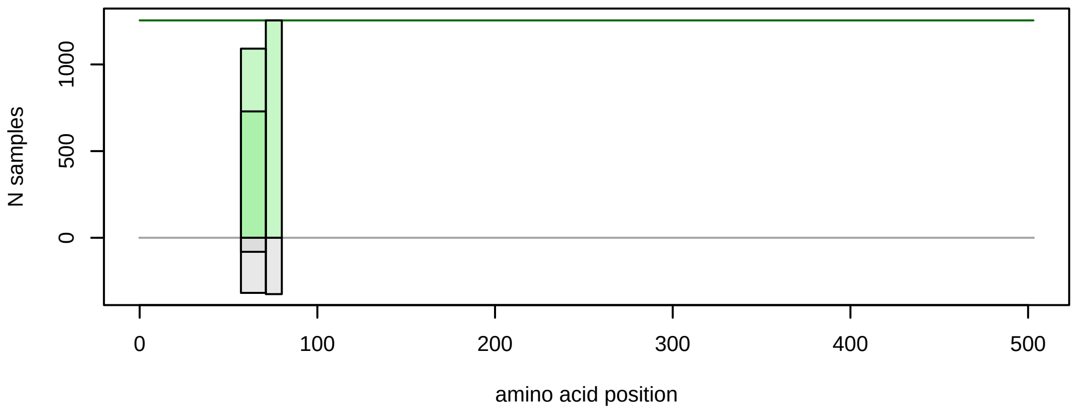

372. ESM1 (Q9NQ30), NP2

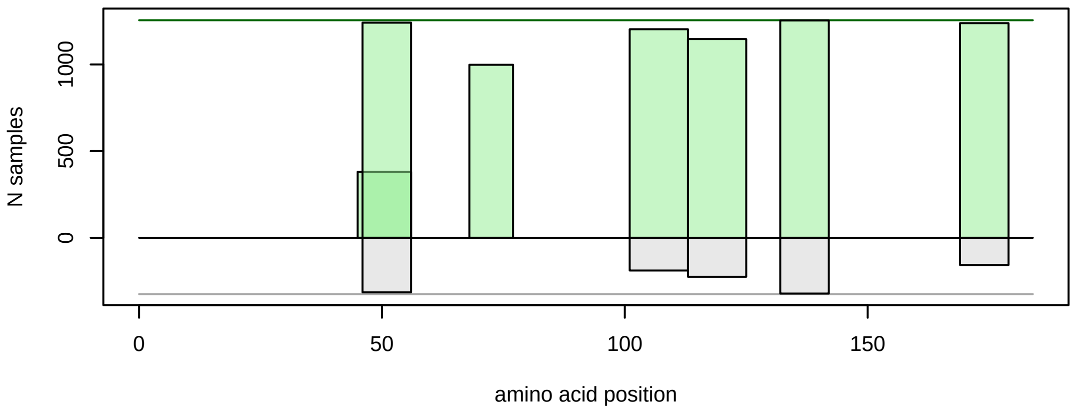

373. INHBC (P55103), NP3

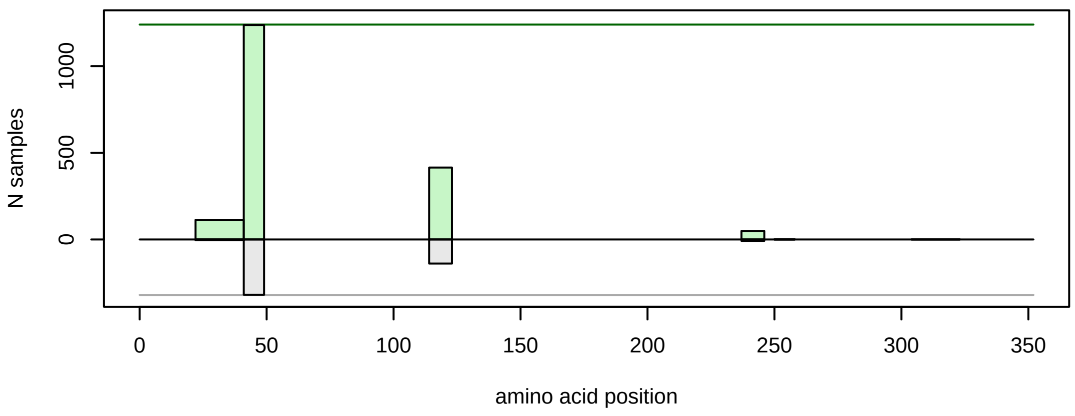

374. PRKCD (Q05655), NP1

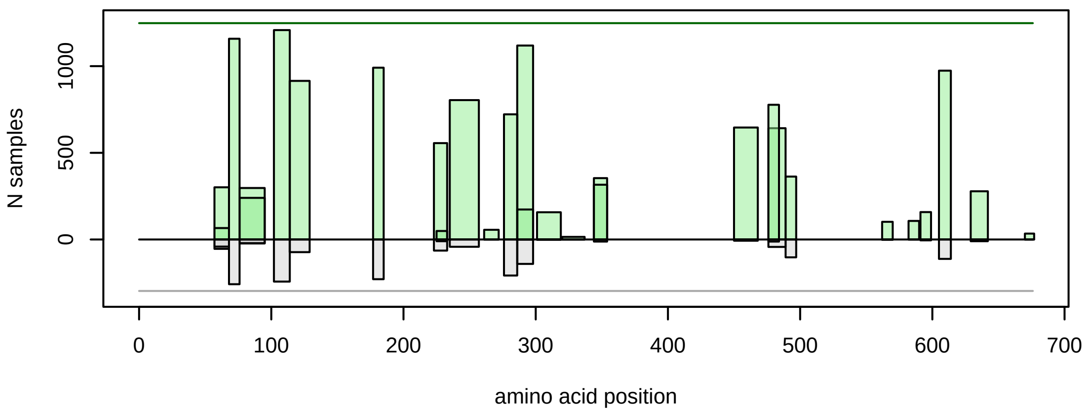

375. FLNA (P21333−2), NP4

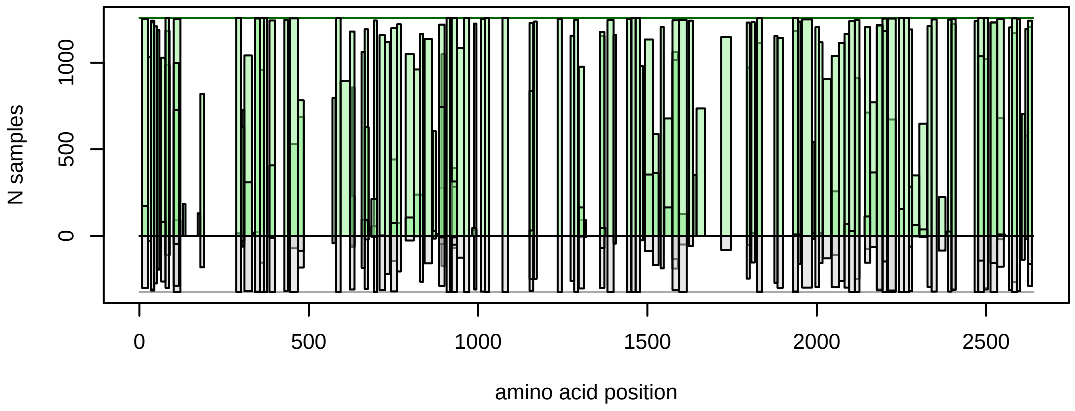

376. IGLV1−40 (P01703), NP3

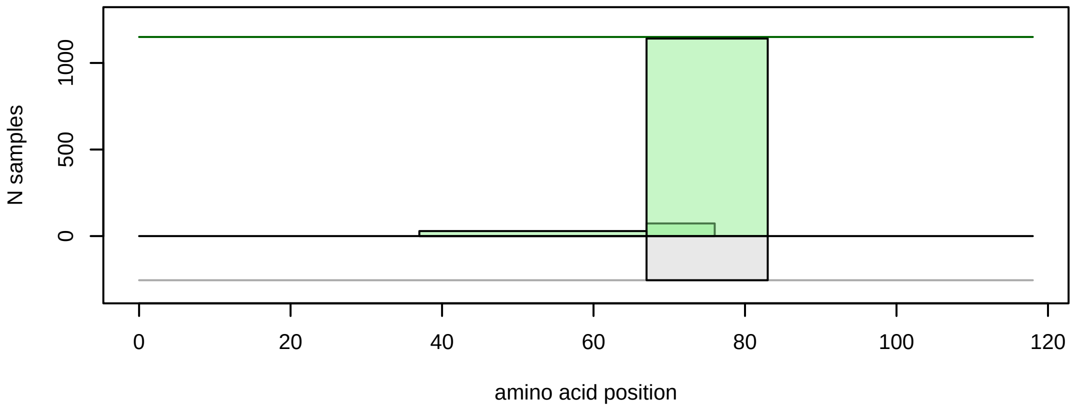

377. IGHV4−28 (A0A0C4DH34), NP3

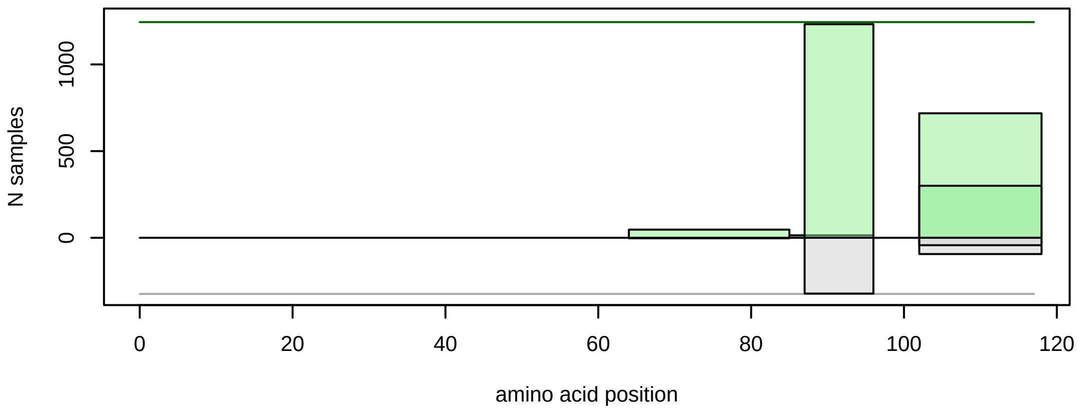

378. TTC3 (P53804), NP2

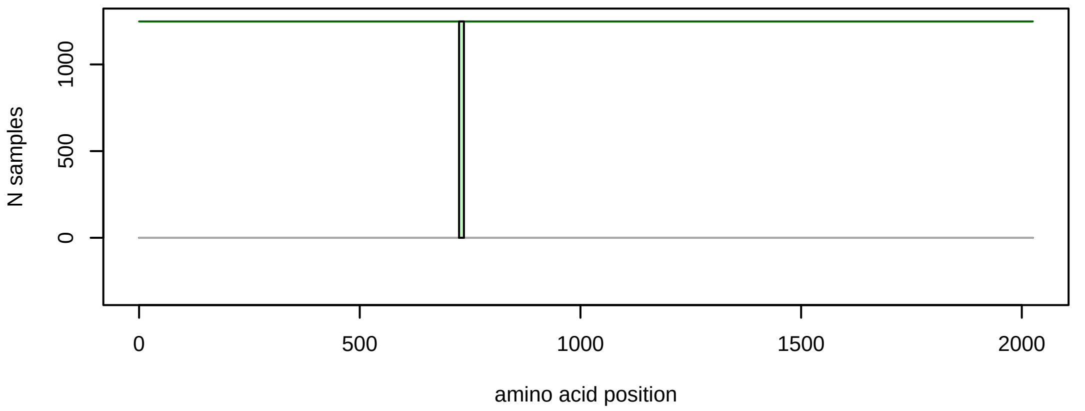

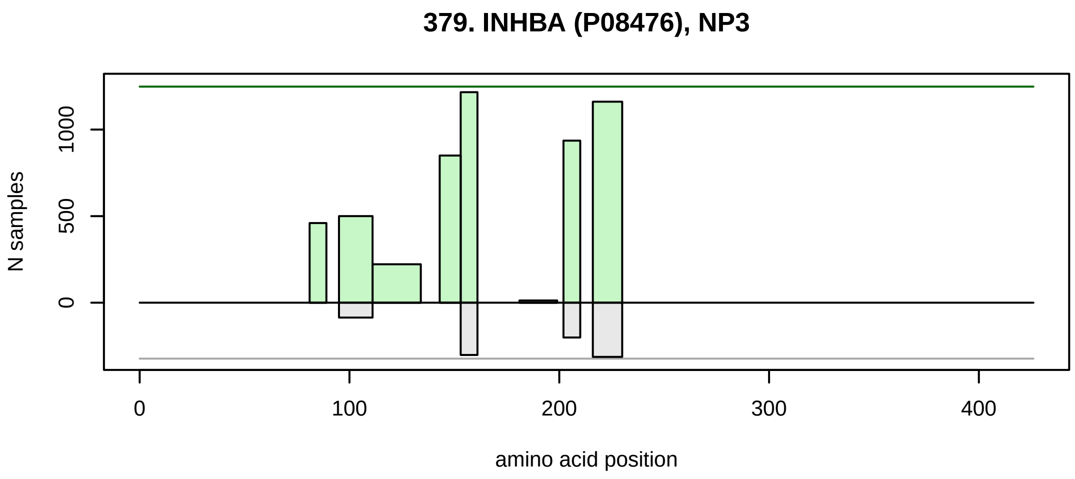
379. INHBA (P08476), NP3
N samples
0
500
1000
0
100
200
300
400
amino acid position

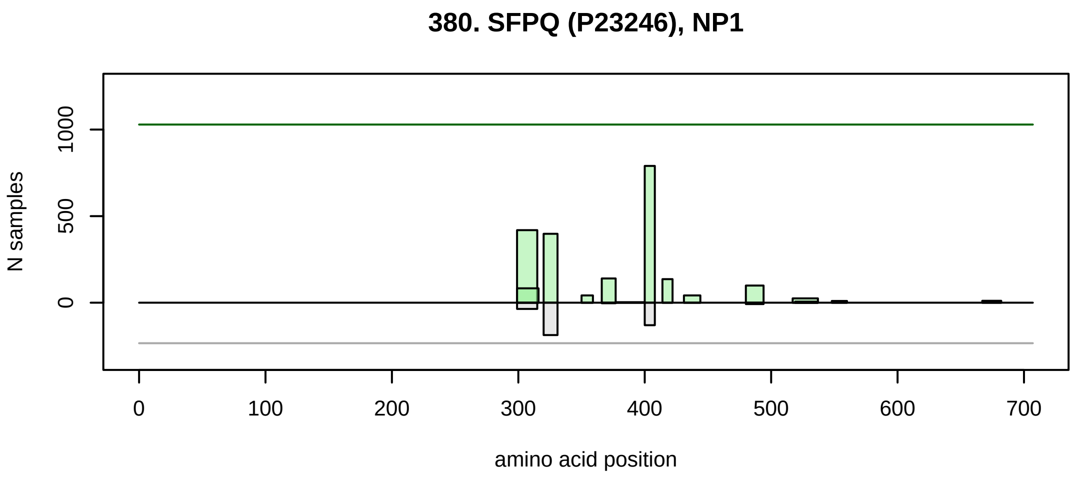
380. SFPQ (P23246), NP1
N samples
0
500
1000
0
100
200
300
400
500
600
700
amino acid position

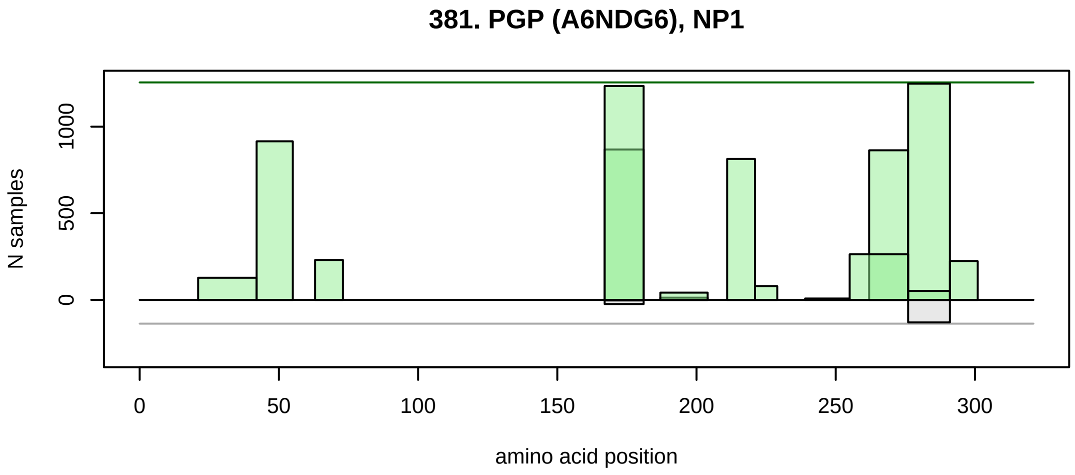
381. PGP (A6NDG6), NP1
N samples
0
500
1000
0
50
100
150
200
250
300
amino acid position

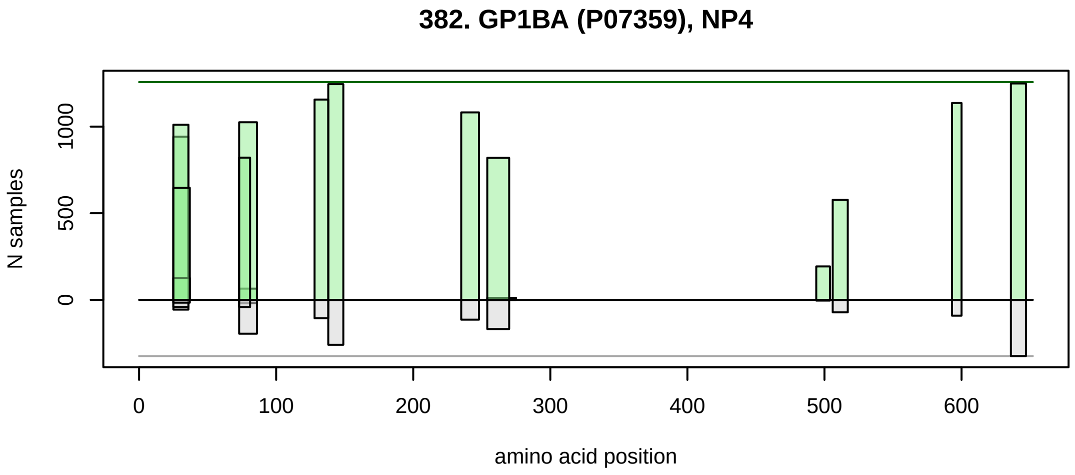
382. GP1BA (P07359), NP4
N samples
0
500
1000
0
100
200
300
400
500
600
amino acid position

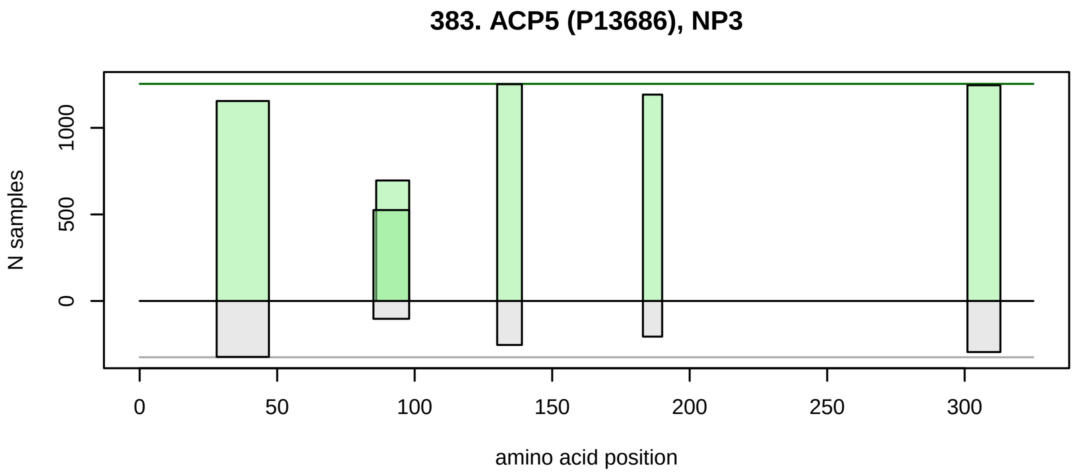
383. ACP5 (P13686), NP3
N samples
0
500
1000
0
50
100
150
200
250
300
amino acid position

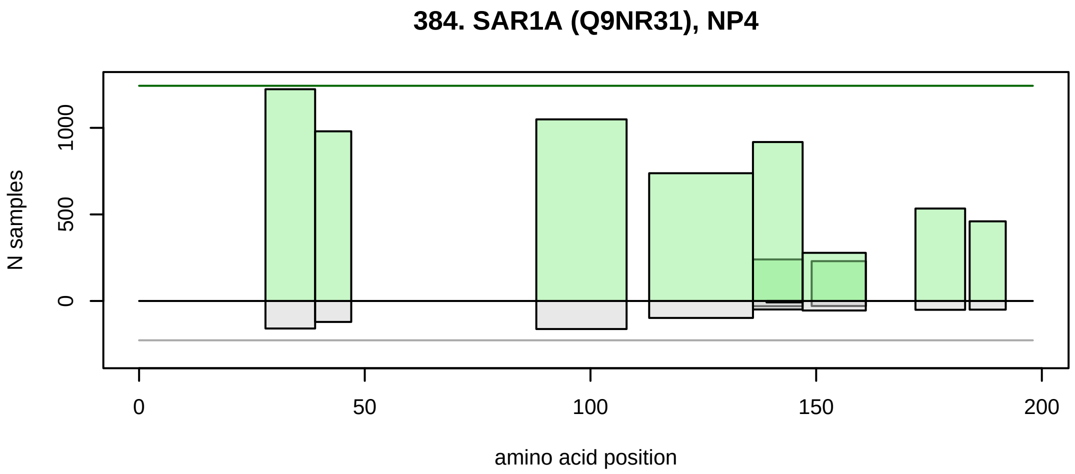
384. SAR1A (Q9NR31), NP4
N samples
0
500
1000
0
50
100
150
200
amino acid position

385. ADPRHL2 (Q9NX46), NP2

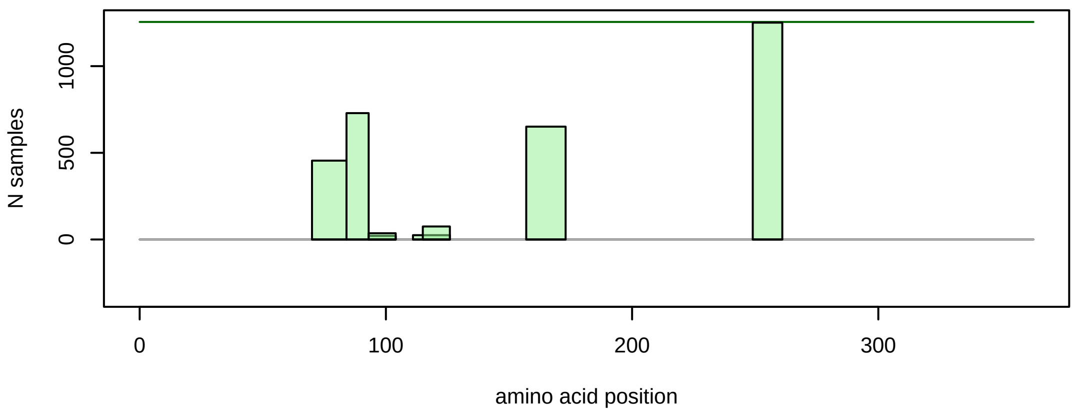

386. ADAMTSL4 (Q6UY14;Q6UY14−3), NP1

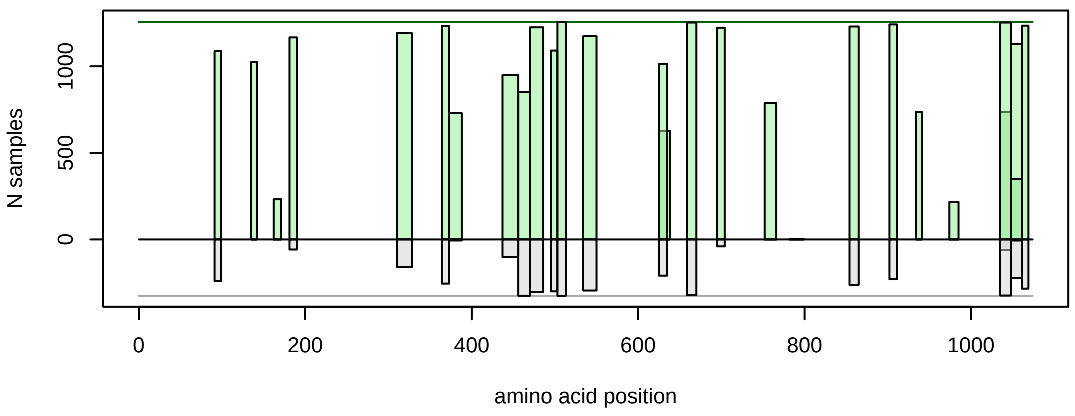

387. SEPT11 (Q9NVA2−2), NP3

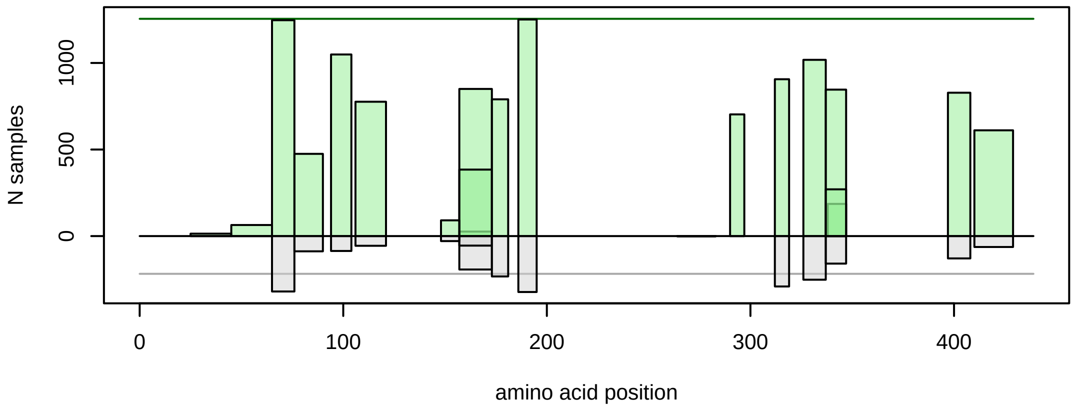

388. SRPR (P08240), NP3

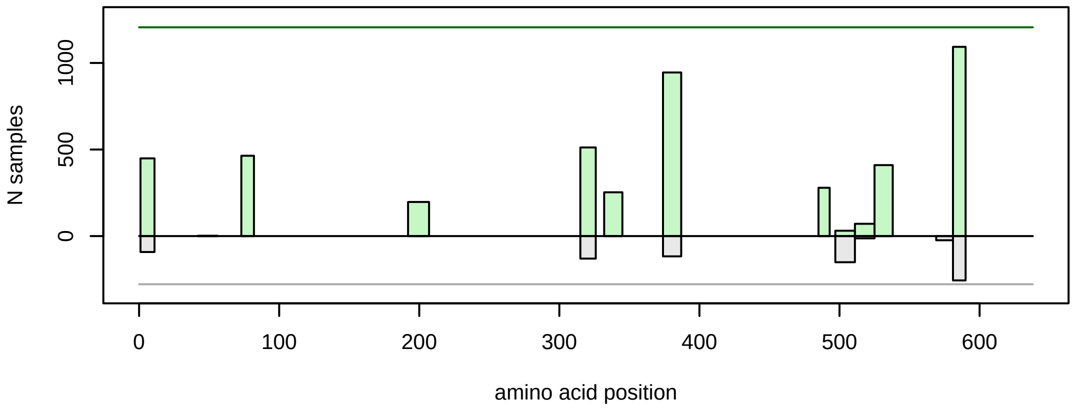

389. TNPO1 (Q92973), NP2

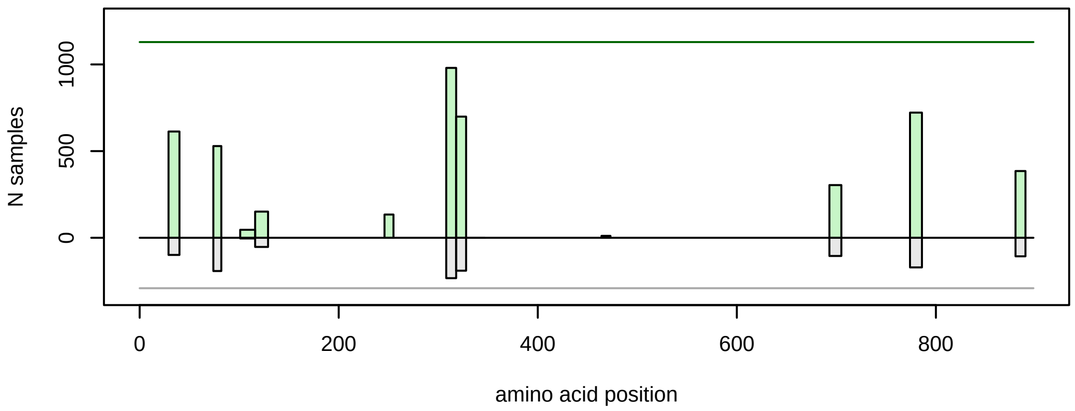

390. LEPRE1 (Q32P28), NP3

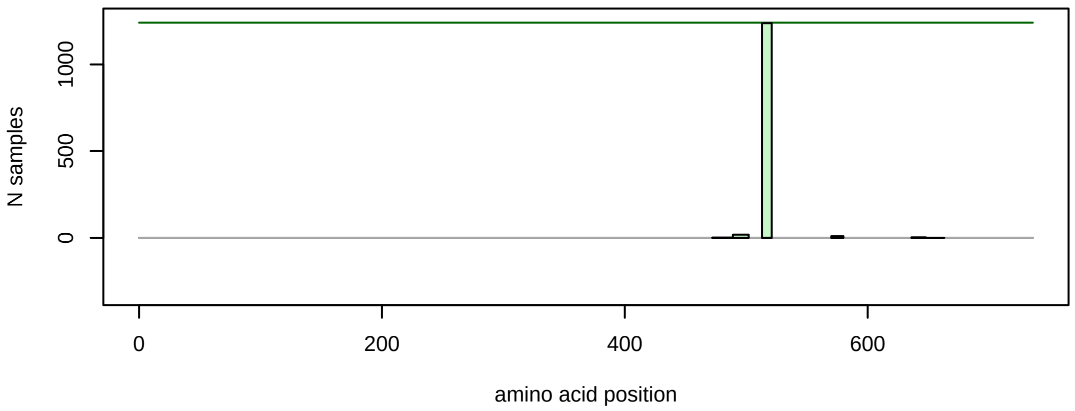

391. FCGBP (A0A087WXI2), NP5

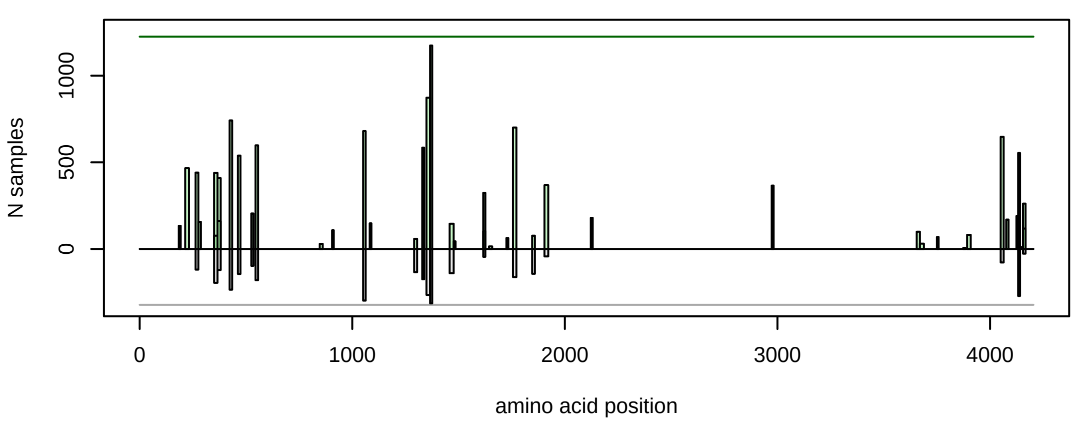

392. RABGGTA (Q92696), NP3

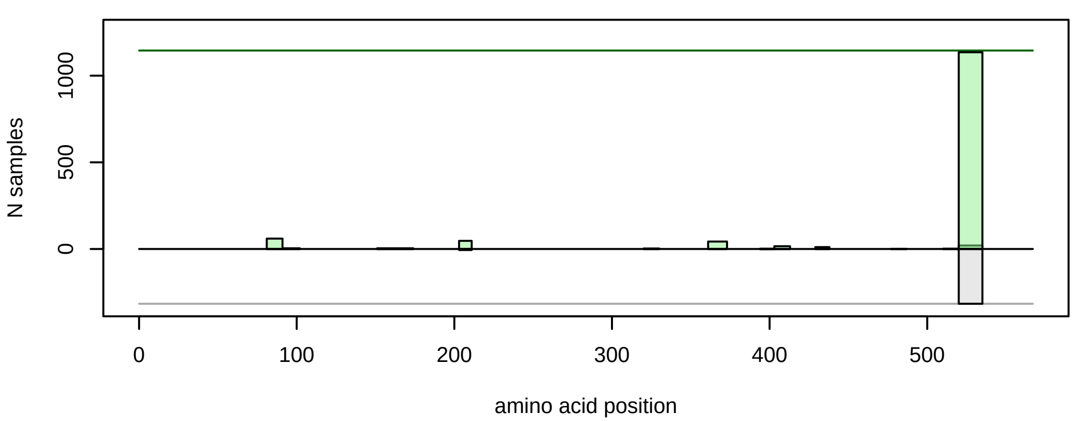

393. ST3GAL6 (Q9Y274), NP4

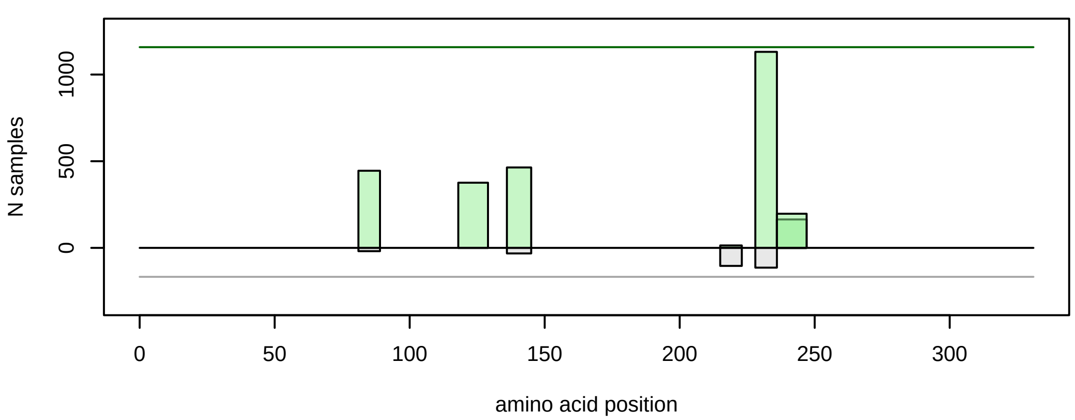

394. DCD (P81605), NP2

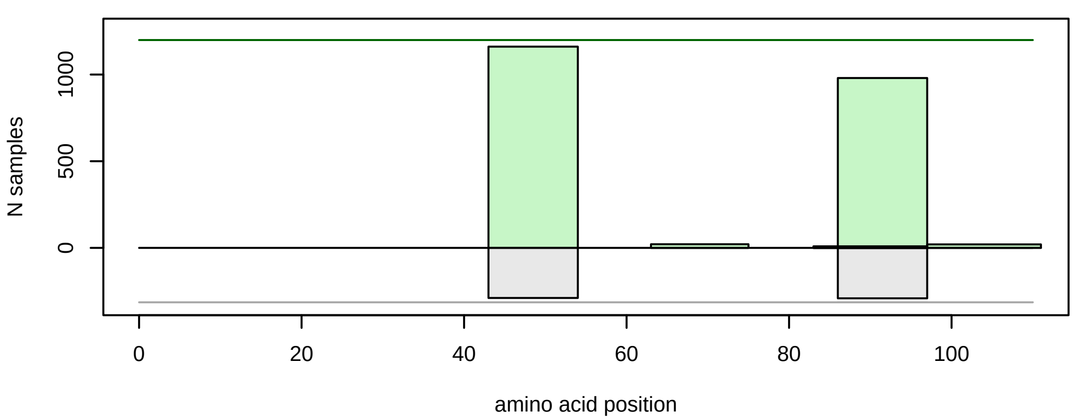

395. FAM114A1 (Q8IWE2), NP4

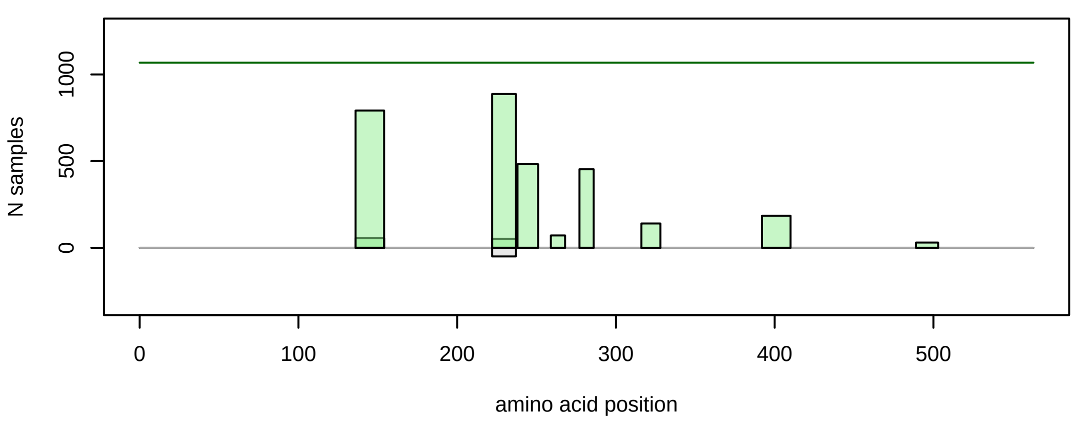

396. OBSCN (A0A7P0Z489;A6NGQ3), NP5

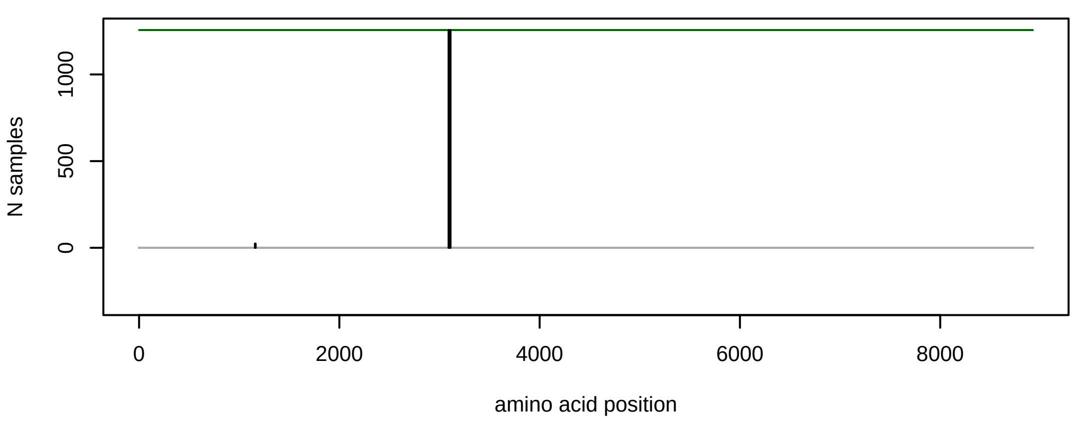

397. MST4 (B4E0Y9), NP5

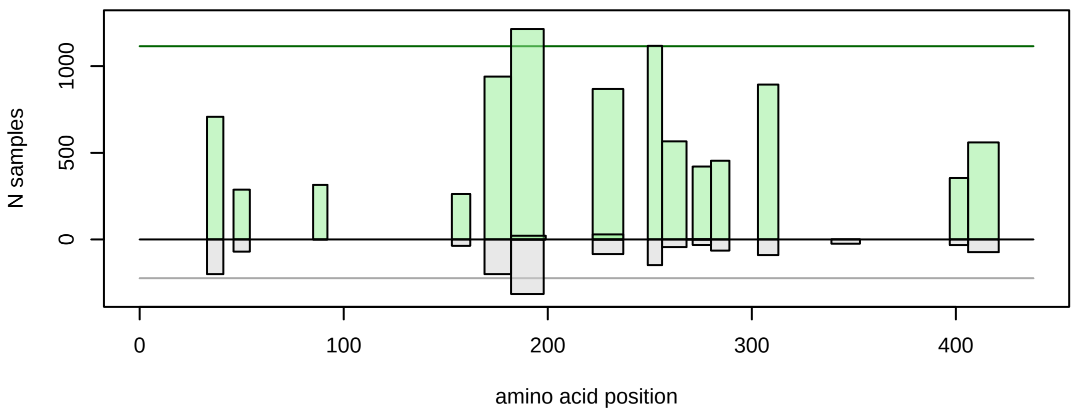

398. SFRP1 (Q8N474), NP4

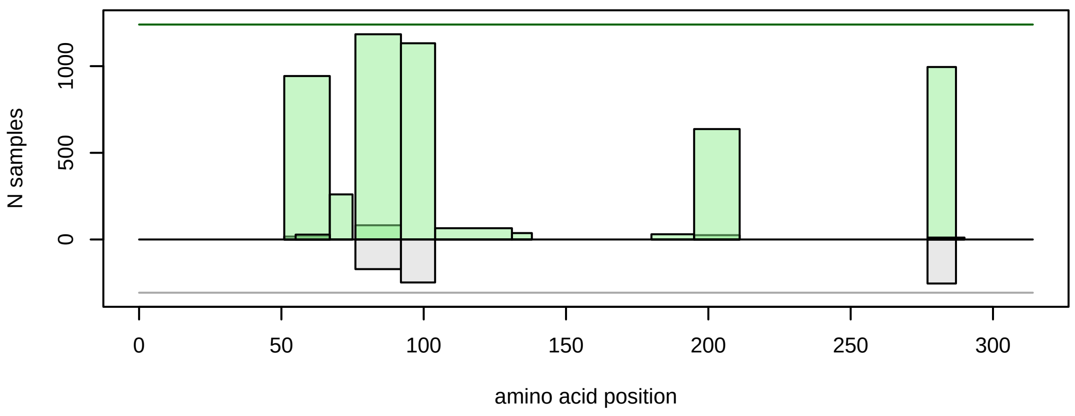

399. PEA15 (Q15121;Q15121−2), NP2

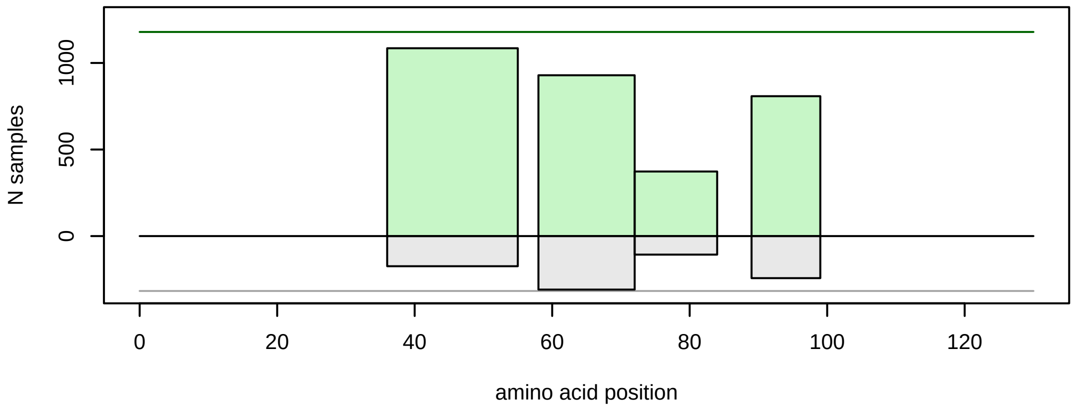

400. ADAMTSL2 (B1B0D4), NP3

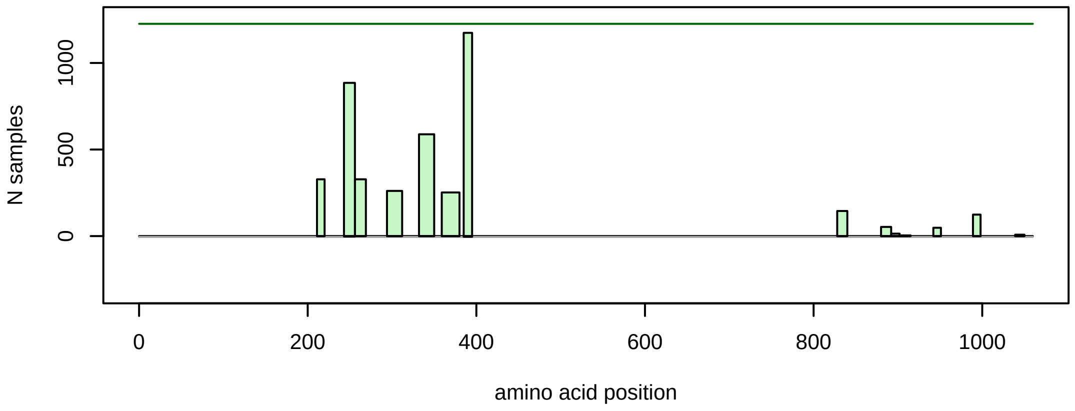

401. SULF2 (Q8IWU5), NP4

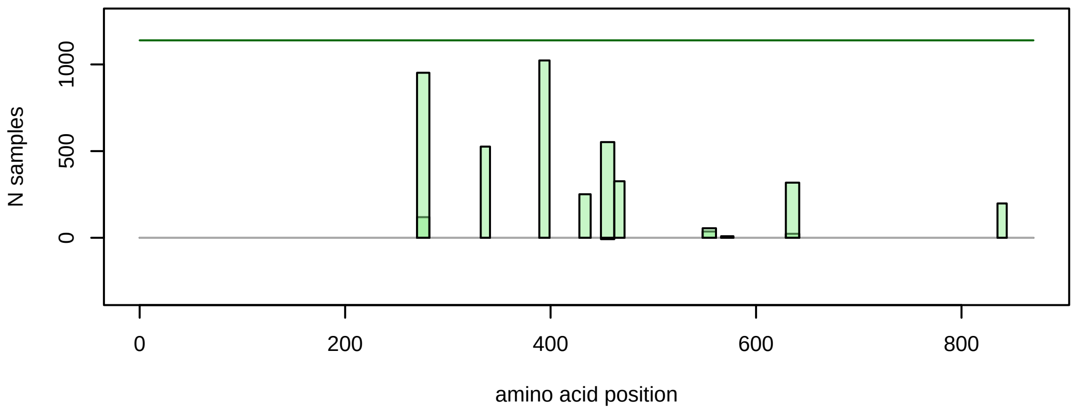

402. RNASE2 (P10153), NP1

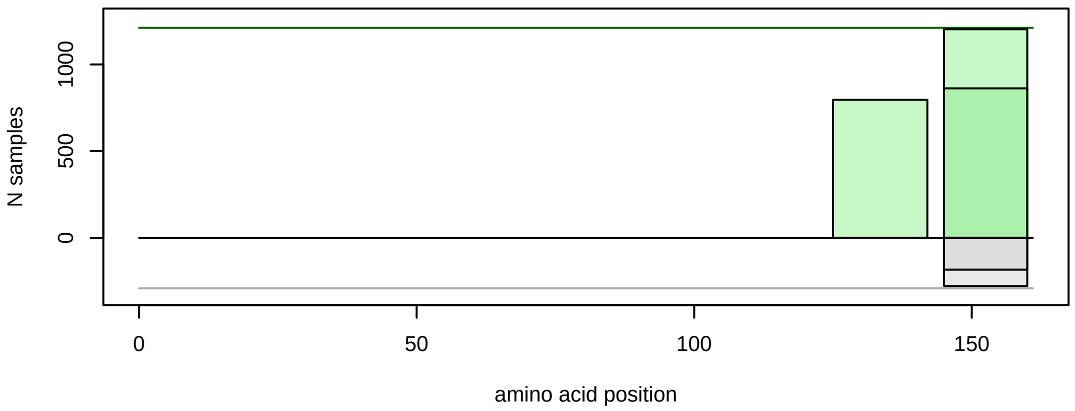

403. PSMA7 (O14818), NP1

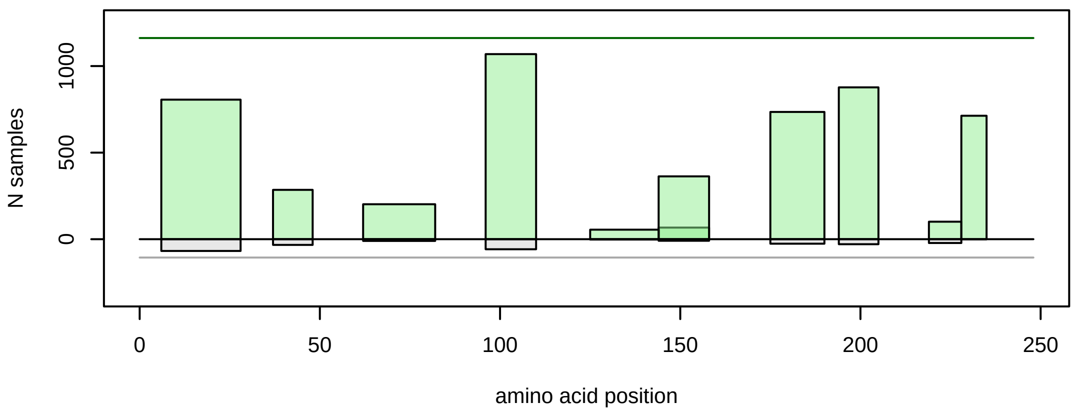

404. APOL1 (O14791), NP4

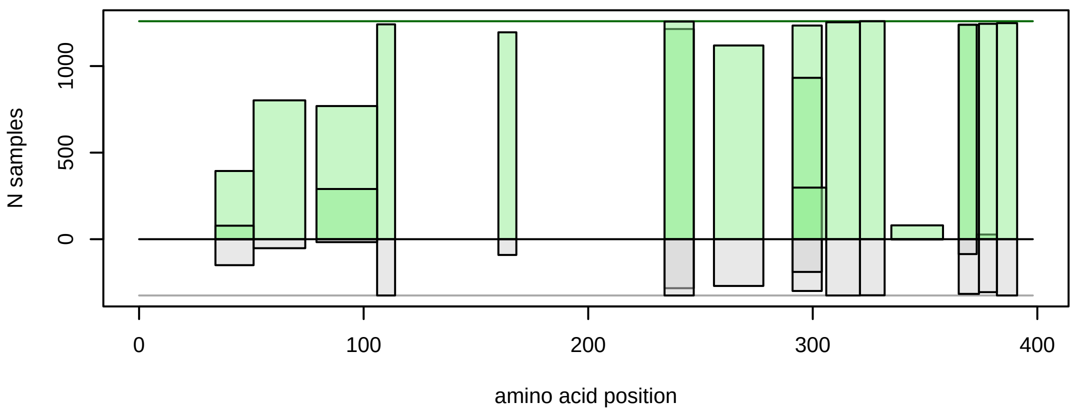

405. PDGFC (Q9NRA1), NP2

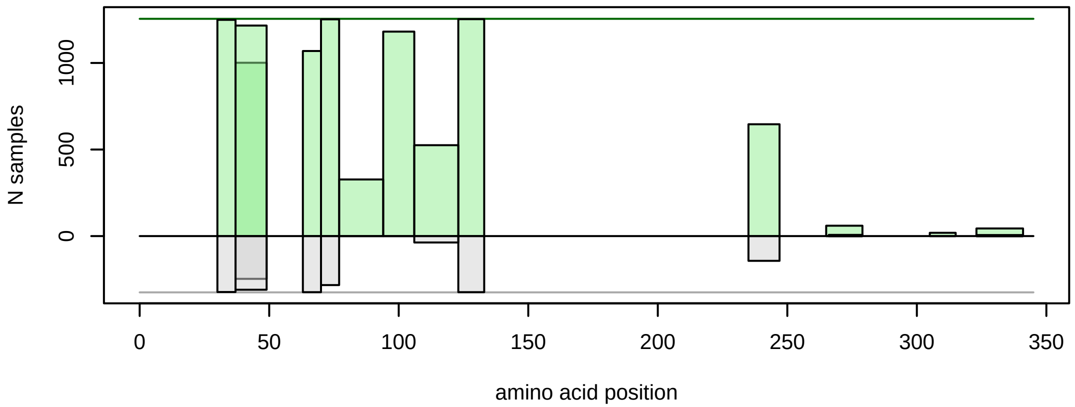

406. PDIA6 (Q15084−2), NP2

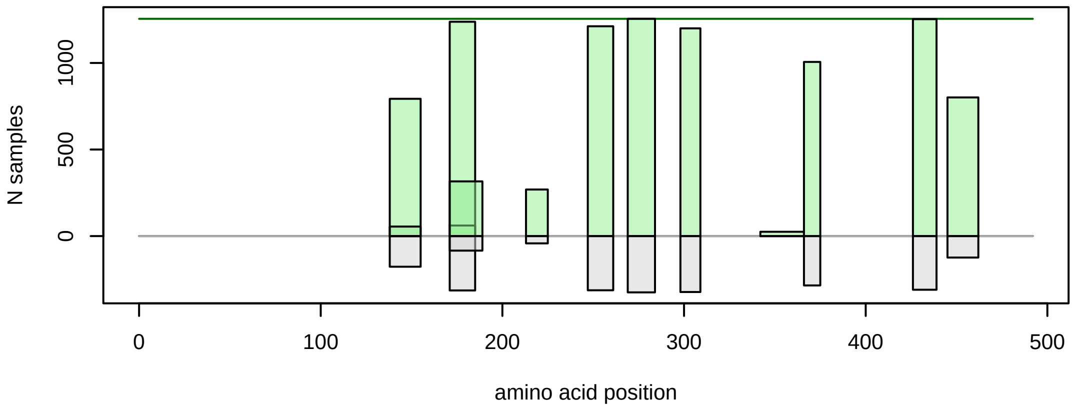

407. F10 (P00742), NP2

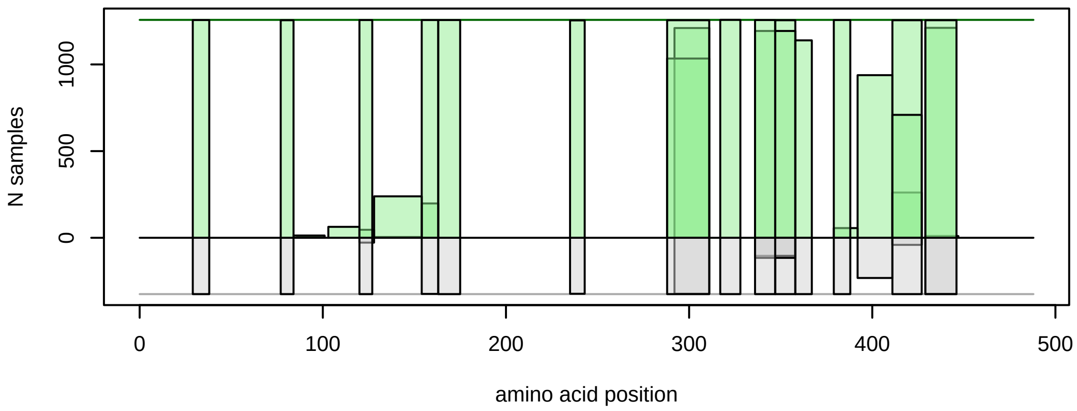

408. SLC44A2 (Q8IWA5−2), NP4

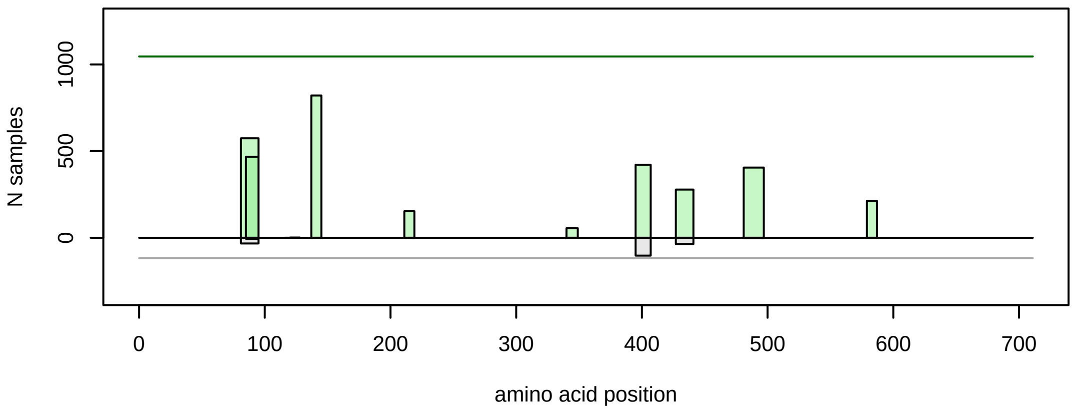

409. NOV (P48745), NP4

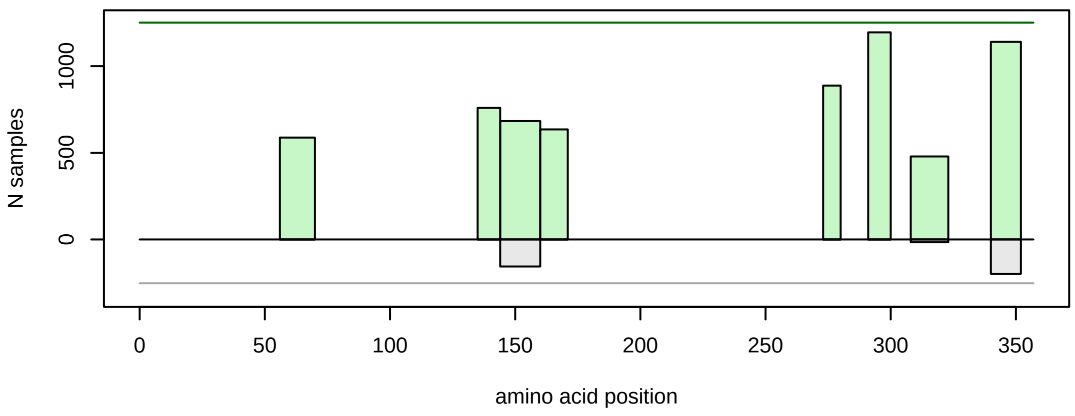

410. KCNAB2 (Q13303−2), NP4

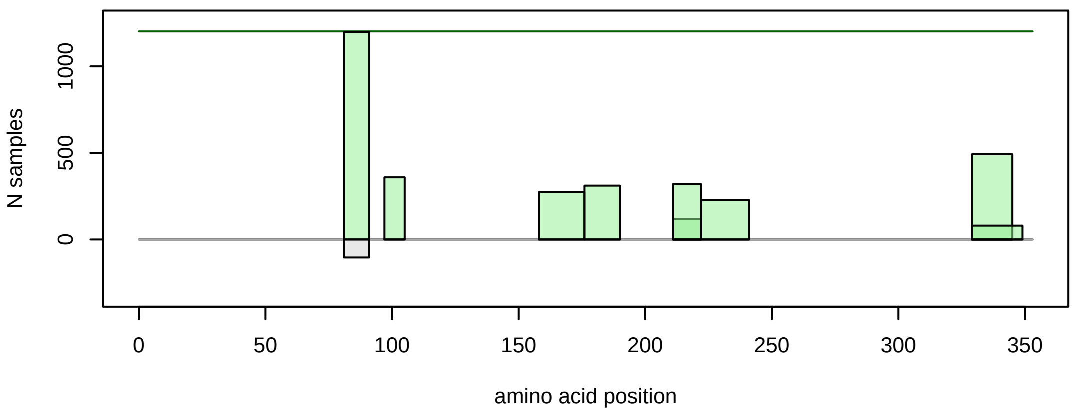

411. GOLIM4 (O00461), NP2

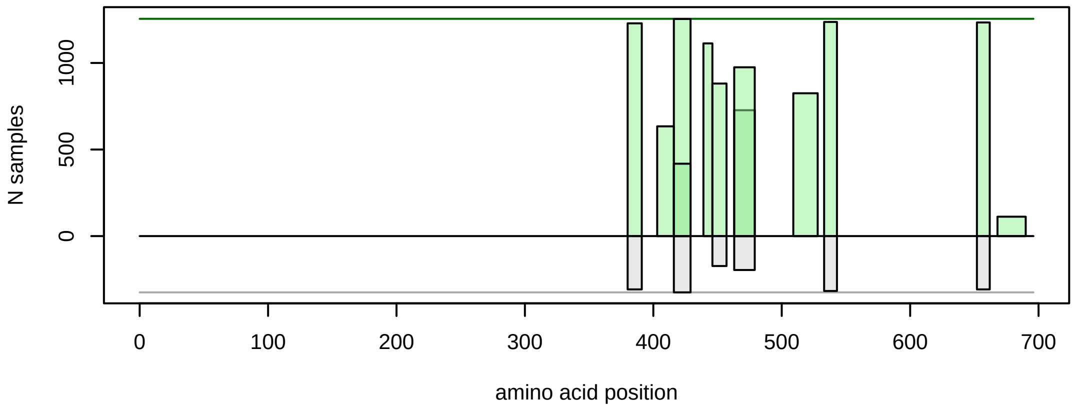

412. PBXIP1 (Q96AQ6;Q96AQ6−2), NP2

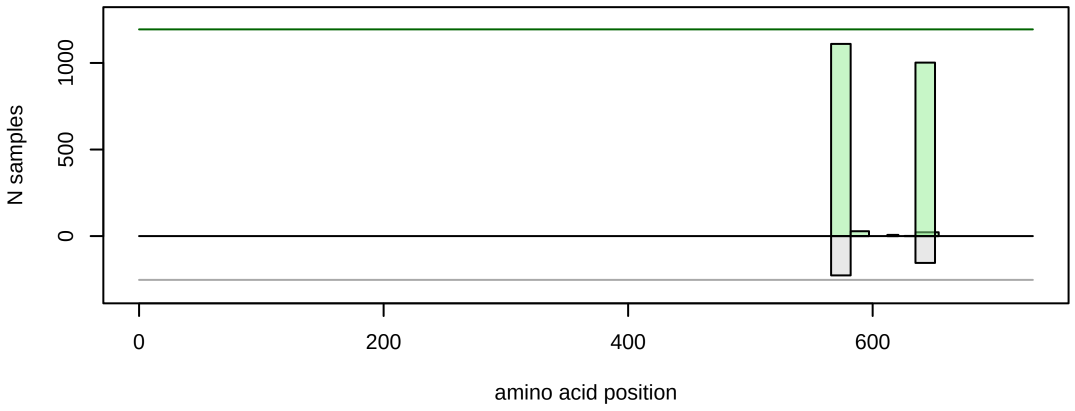

413. PRKAR2A (P13861;P13861−2), NP2

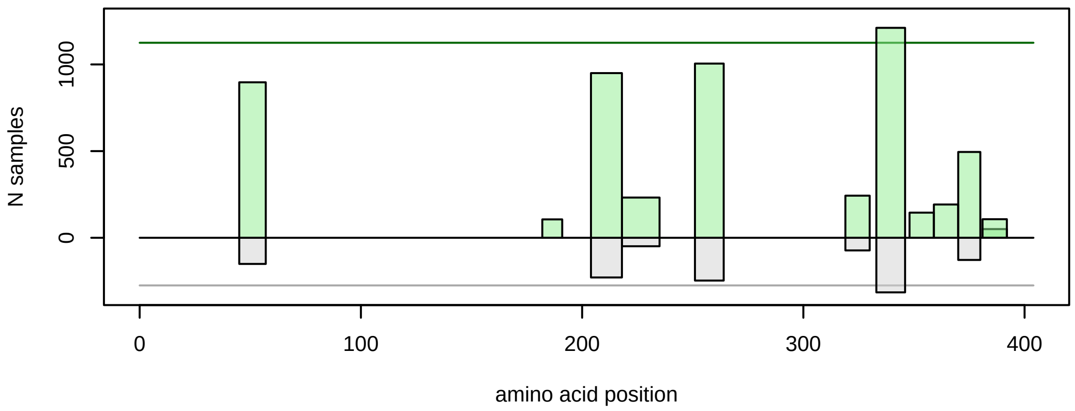

414. UGP2 (E7EUC7), NP1

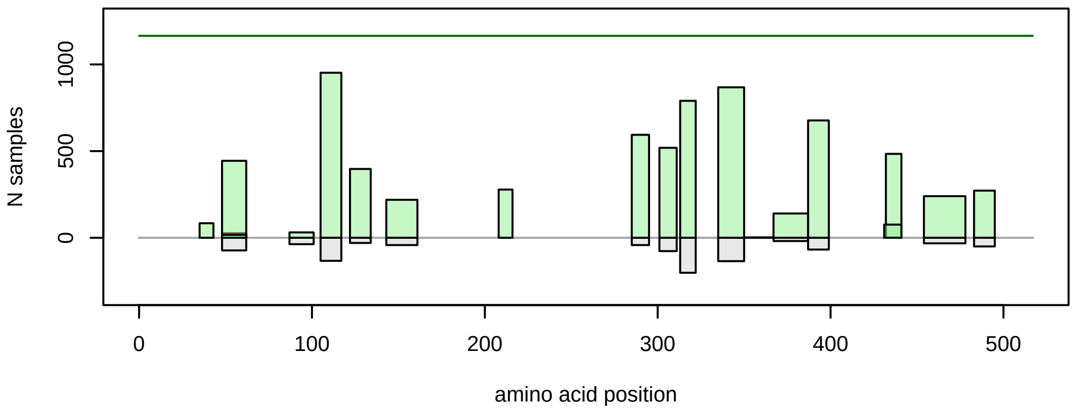

415. PGRMC1 (O00264), NP2

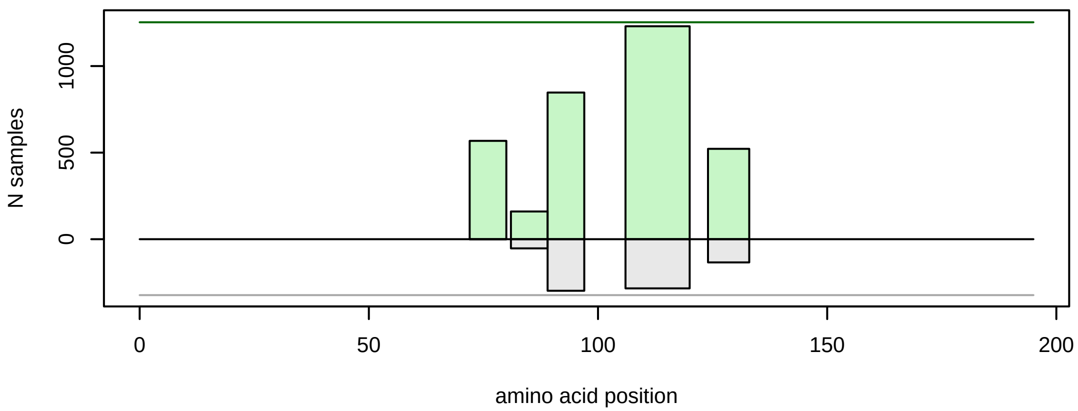

416. IGFALS (P35858), NP1

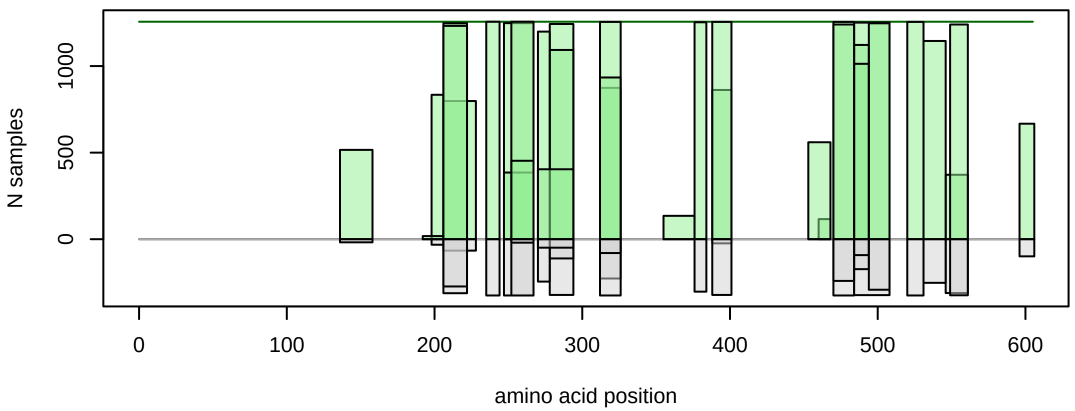

417. PLCB3 (Q01970), NP1

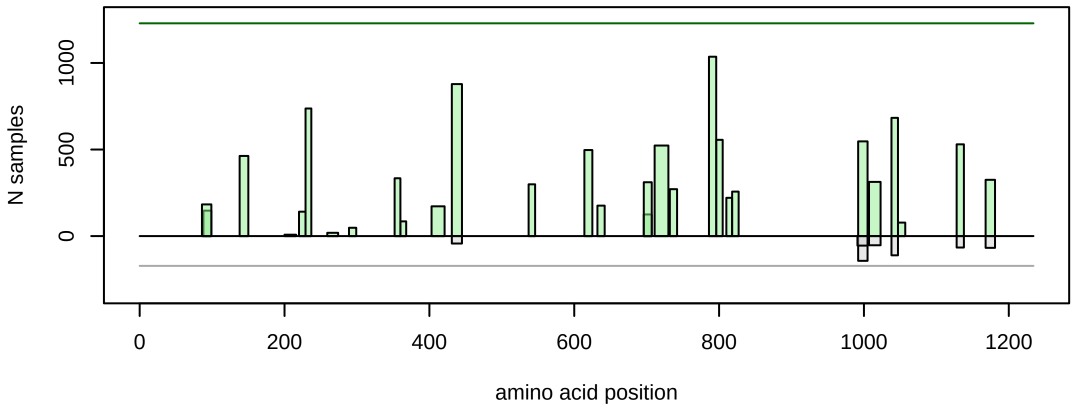

418. RAB8B (Q92930), NP1

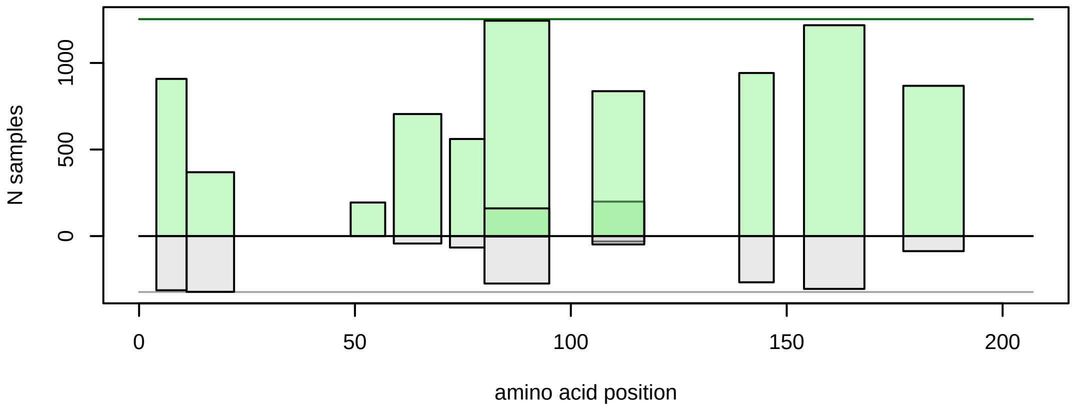

419. ATP6V1G1 (O75348), NP1

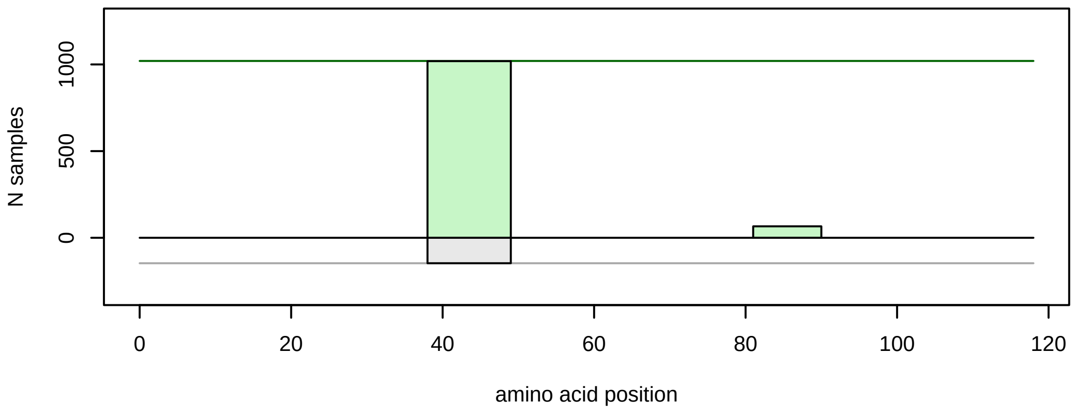

420. SCUBE1 (Q8IWY4), NP4

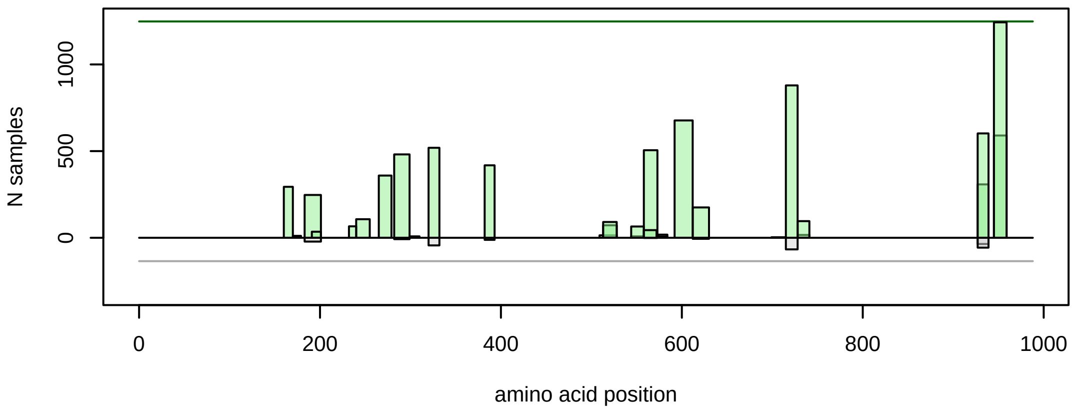

421. EIF3L (B0QY89), NP4

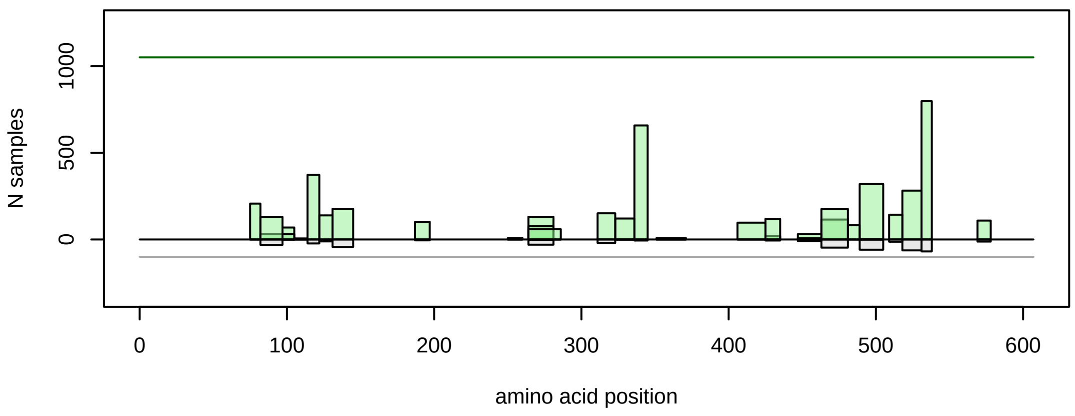

422. GFRA2 (O00451), NP3

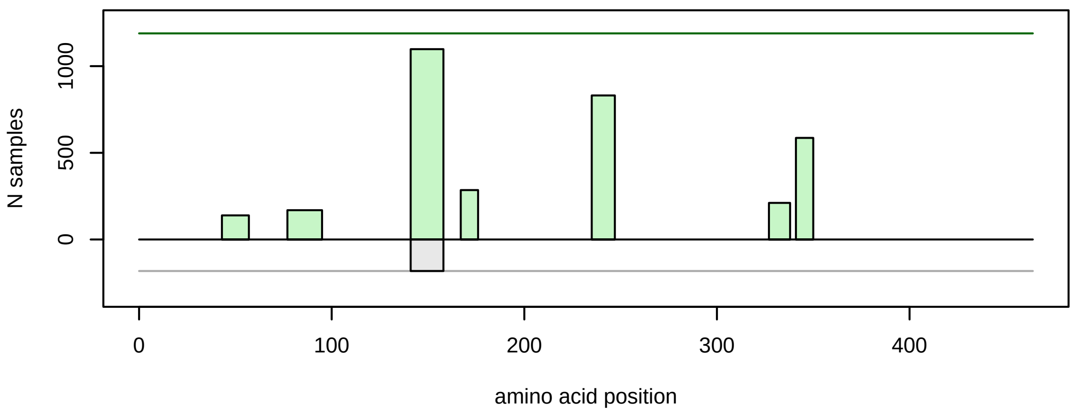

423. TMEM14C (Q9P0S9), NP4

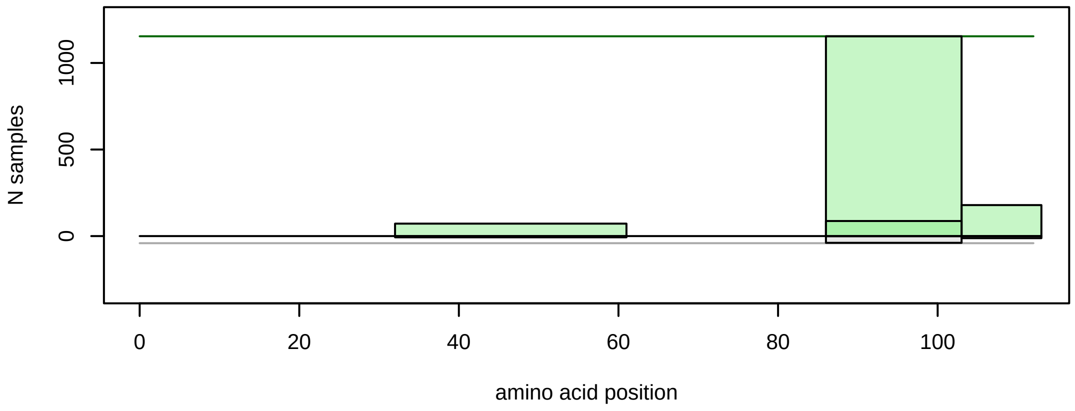

424. GSTP1 (P09211), NP1

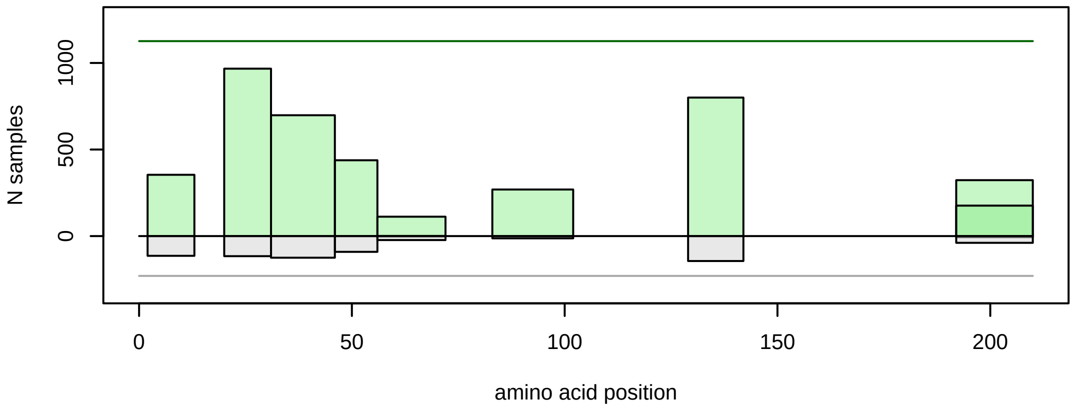

425. TJP2 (A0A1B0GTW1), NP3

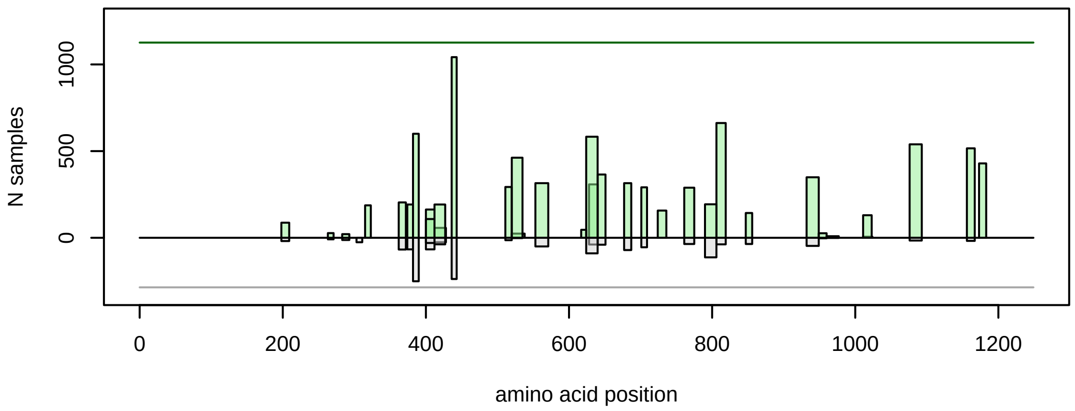

426. PCLO (Q9Y6V0), NP1

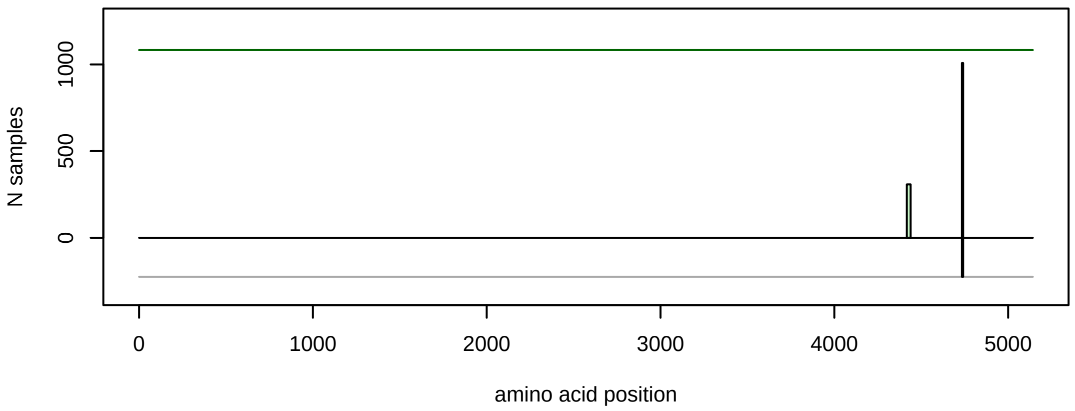

427. RTN4 (Q9NQC3−3), NP4

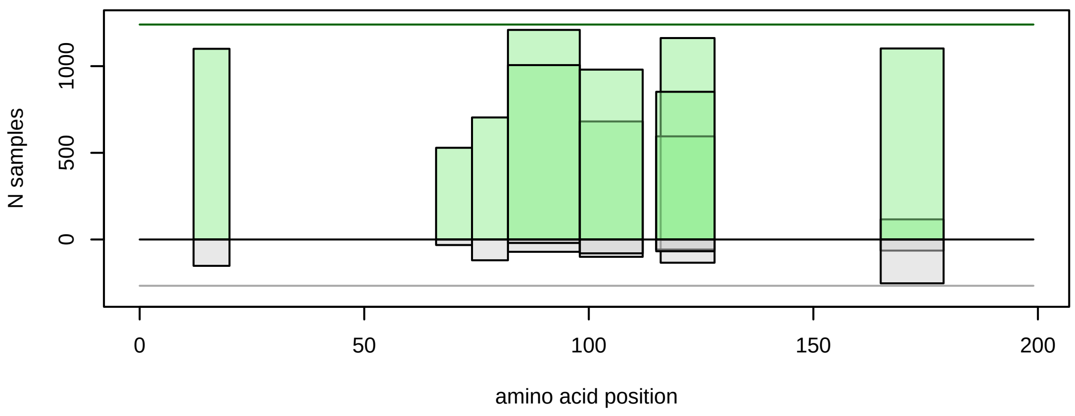

428. COL6A2 (P12110), NP1

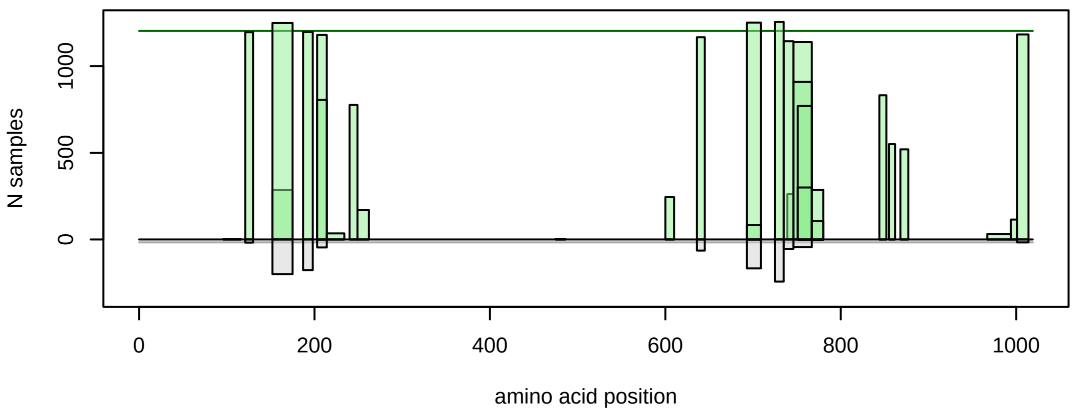

429. COPB2 (H0Y938;H0YAC7;P35606), NP2

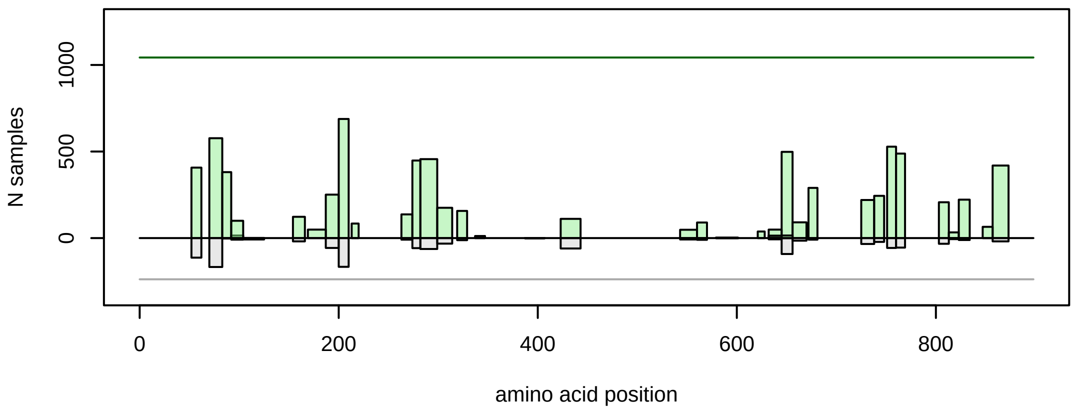

430. ARPC5 (O15511), NP1

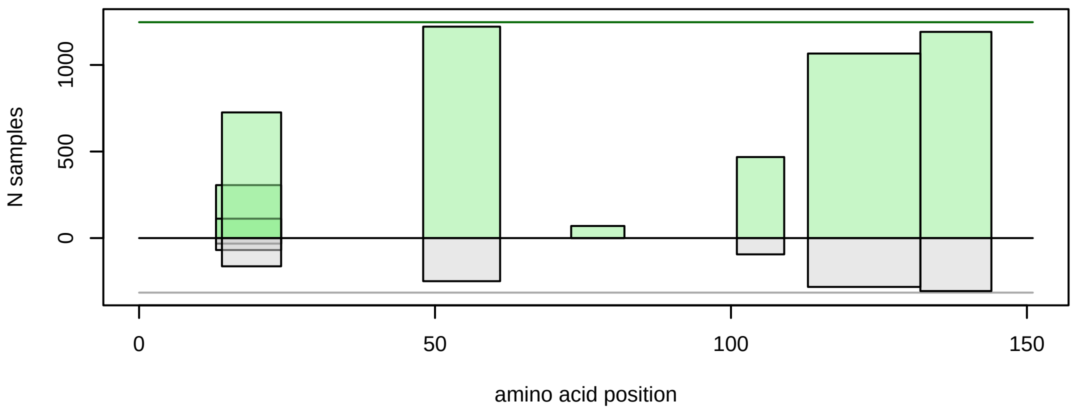

431. USP15 (Q9Y4E8), NP2

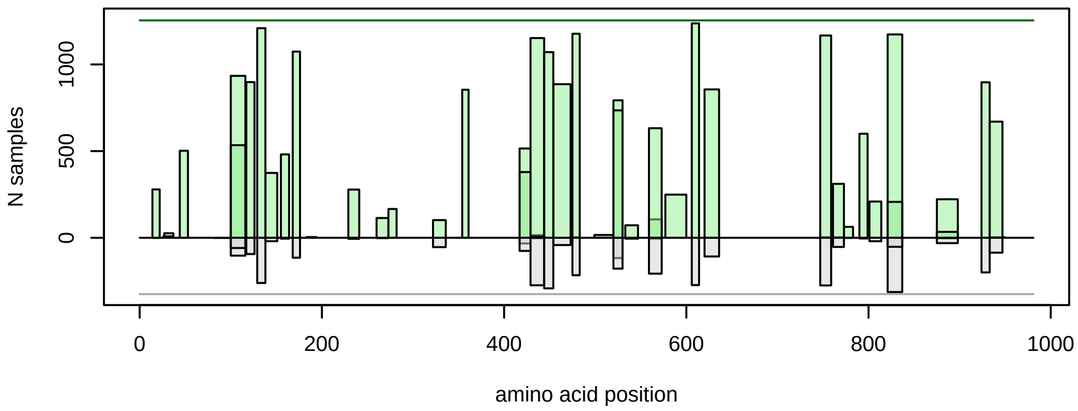

432. FRZB (Q92765), NP4

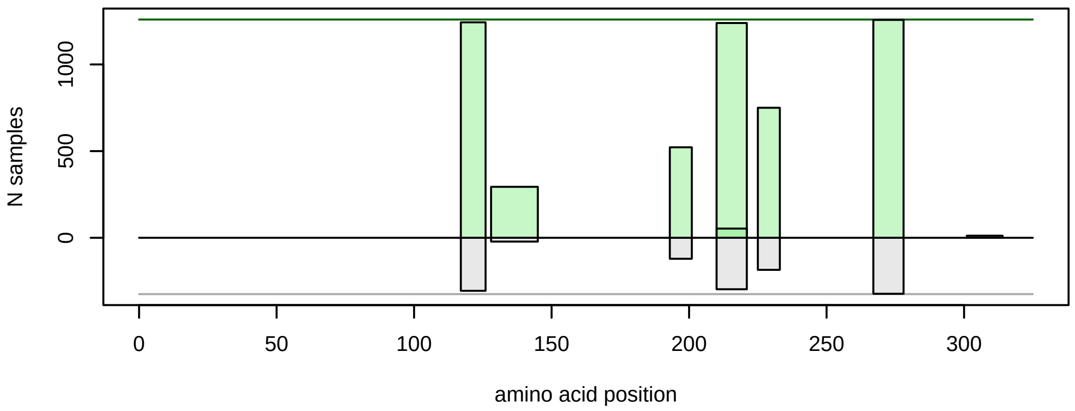

433. AGT (A0A7P0TBH1), NP2

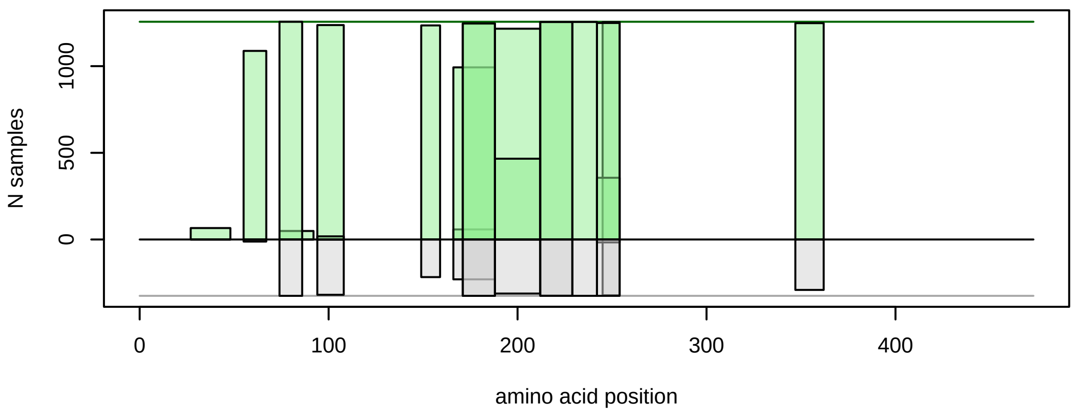

434. KIAA0319L (Q8IZA0), NP2

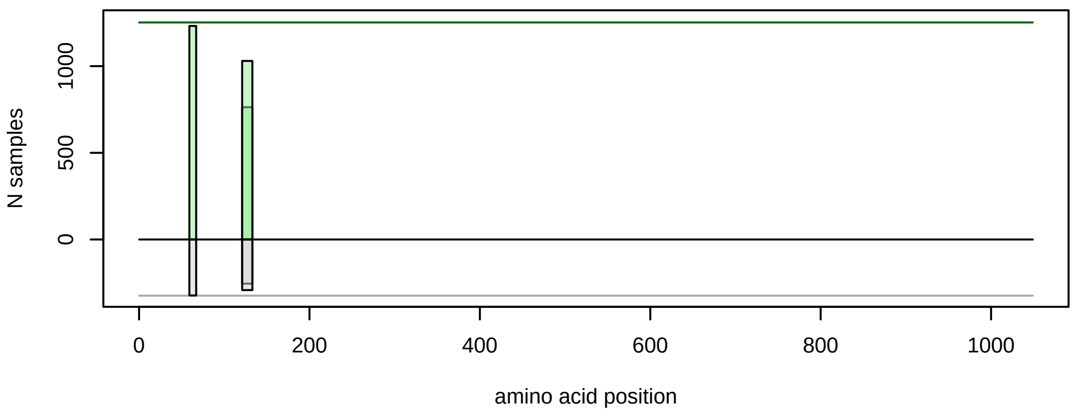

435. IST1 (P53990−2), NP2

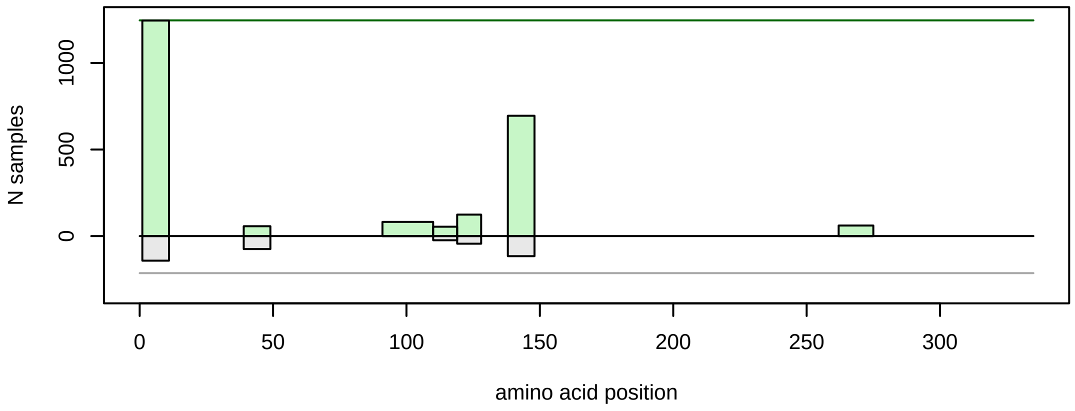

436. MLEC (Q14165), NP4

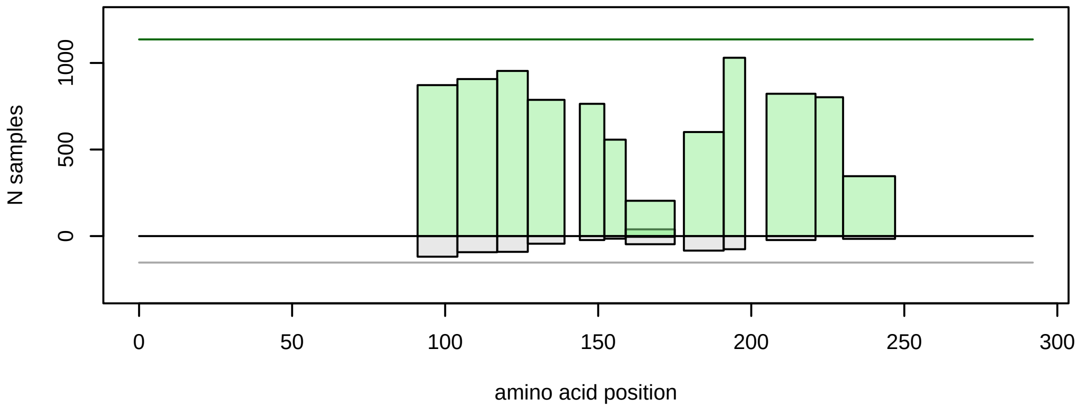

437. NME1−NME2 (P22392−2), NP1

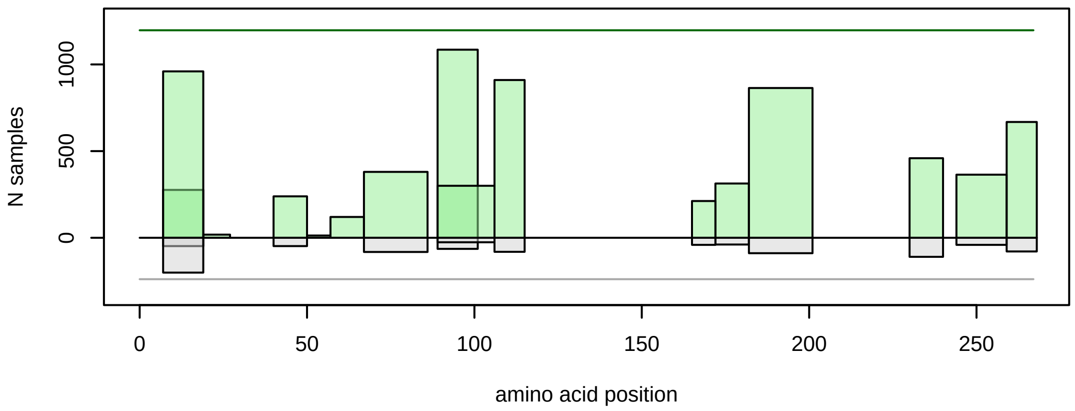

438. NOMO1 (Q15155), NP1

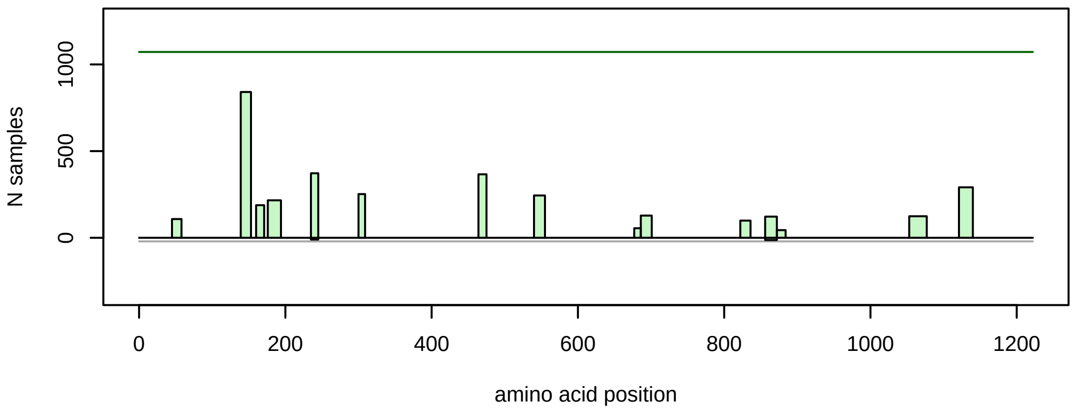

439. OXSR1 (O95747), NP1

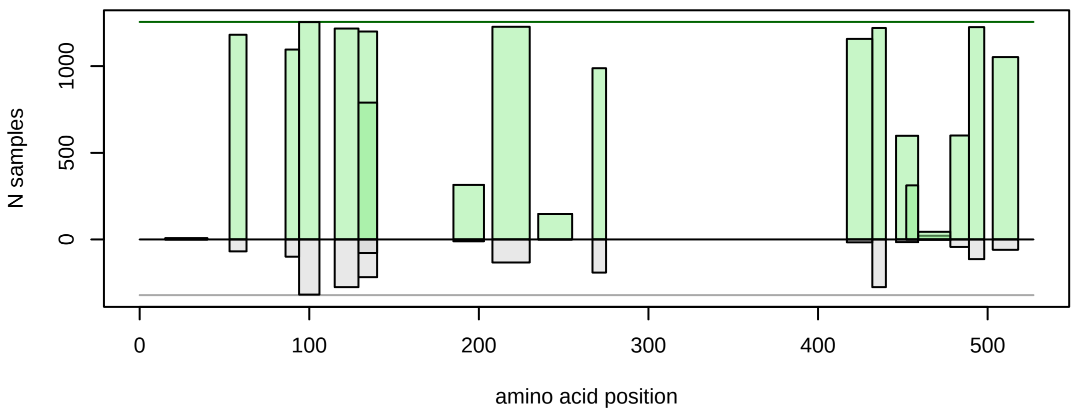

440. NRD1 (O43847−2), NP2

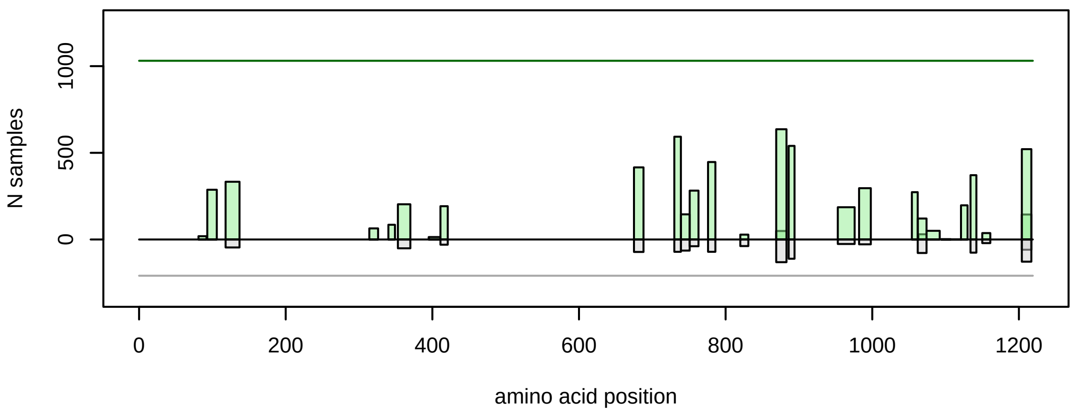

441. RARRES2 (Q99969), NP1

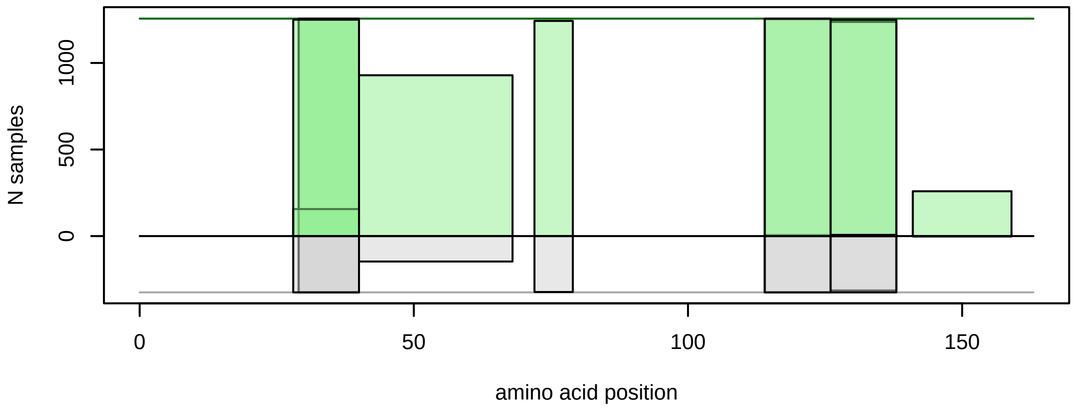

442. PCSK1N (Q9UHG2), NP2

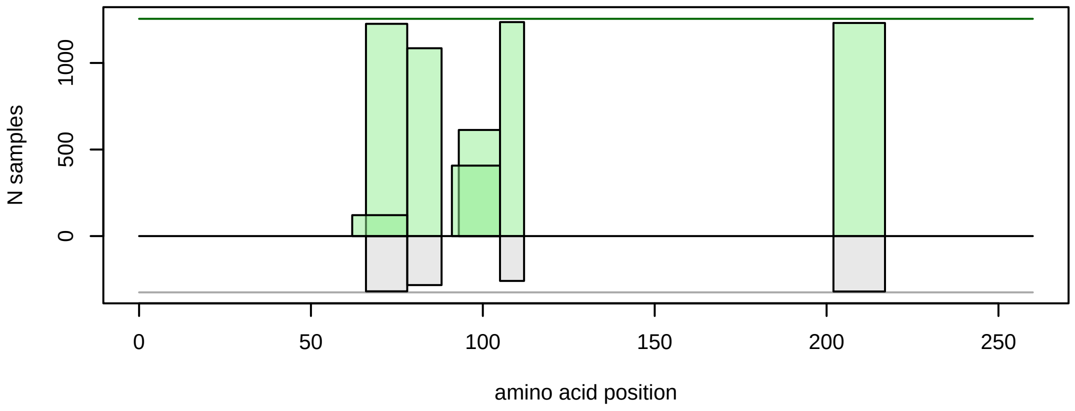

443. TNC (P24821), NP2

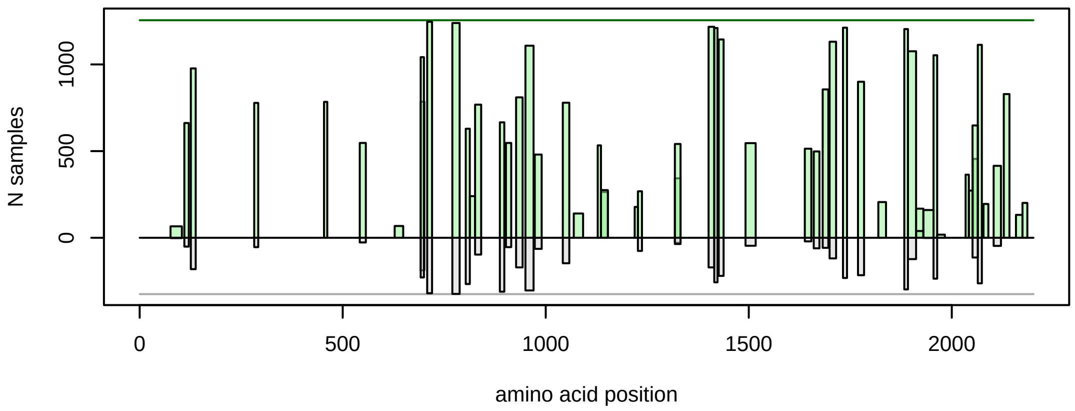

444. RPS4X (P62701), NP4

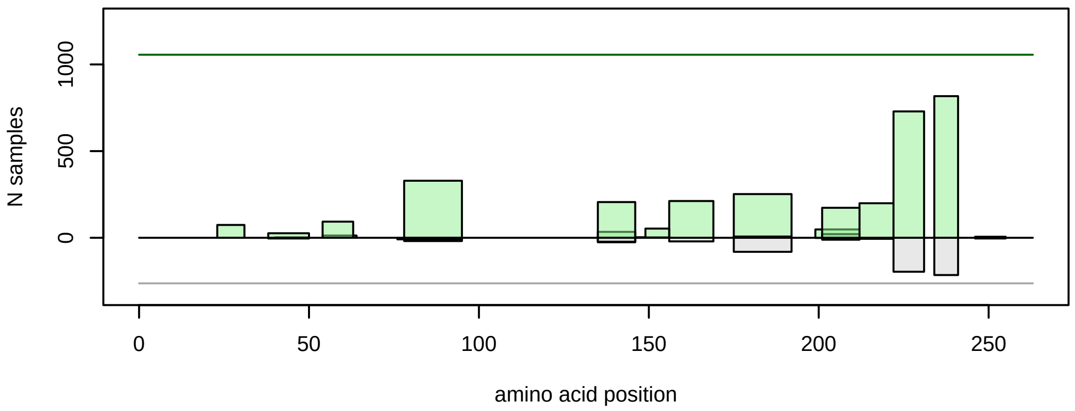

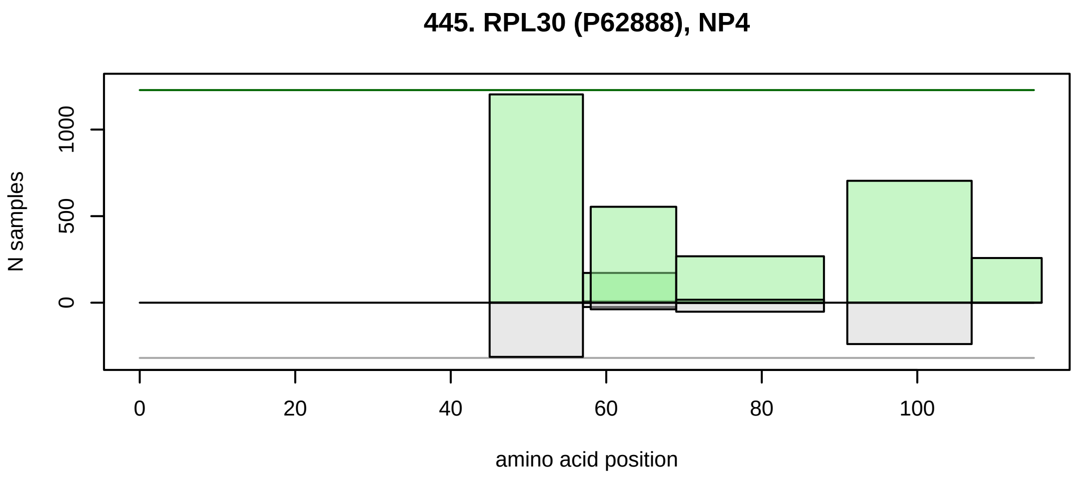
445. RPL30 (P62888), NP4
N samples
0
500
1000
0
20
40
60
80
100
amino acid position

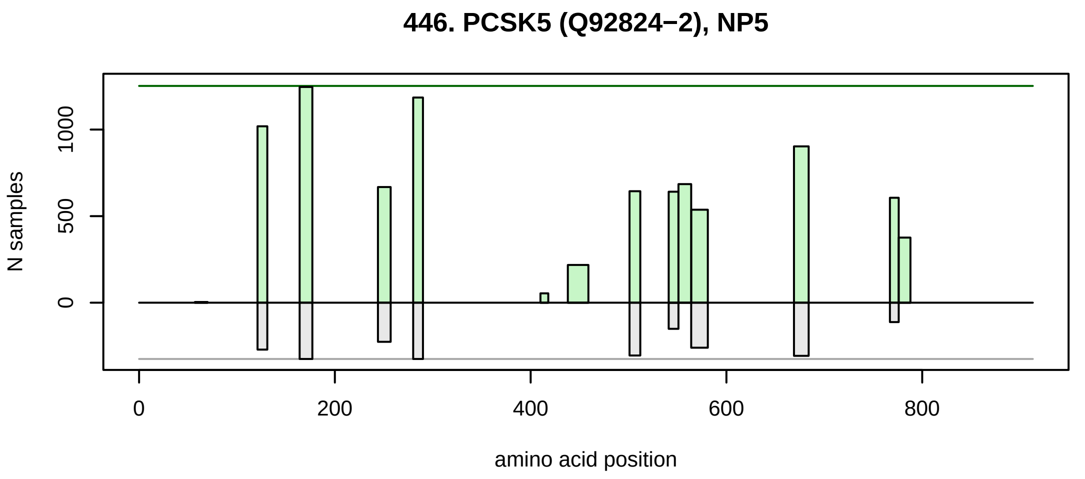
446. PCSK5 (Q92824−2), NP5
N samples
0
500
1000
0
200
400
600
800
amino acid position

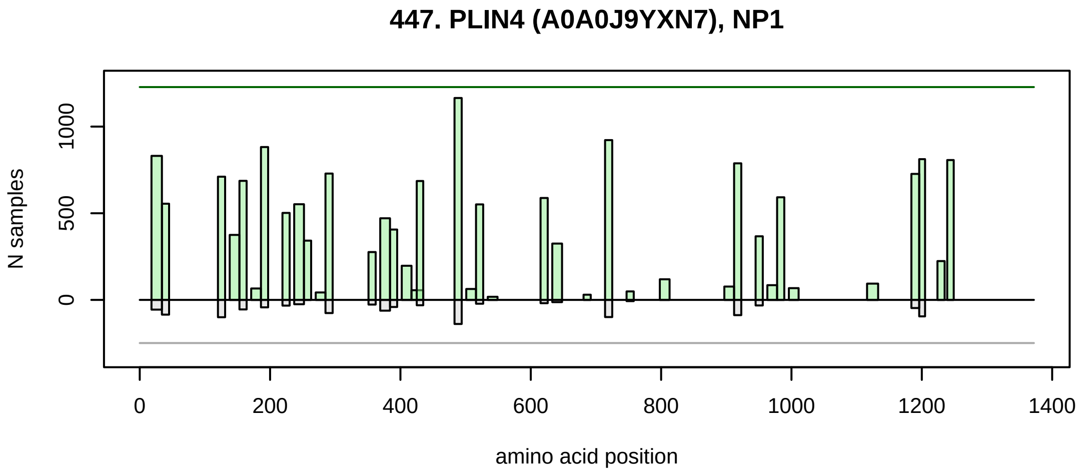
447. PLIN4 (A0A0J9YXN7), NP1
N samples
0
500
1000
0
200
400
600
800
1000
1200
1400
amino acid position

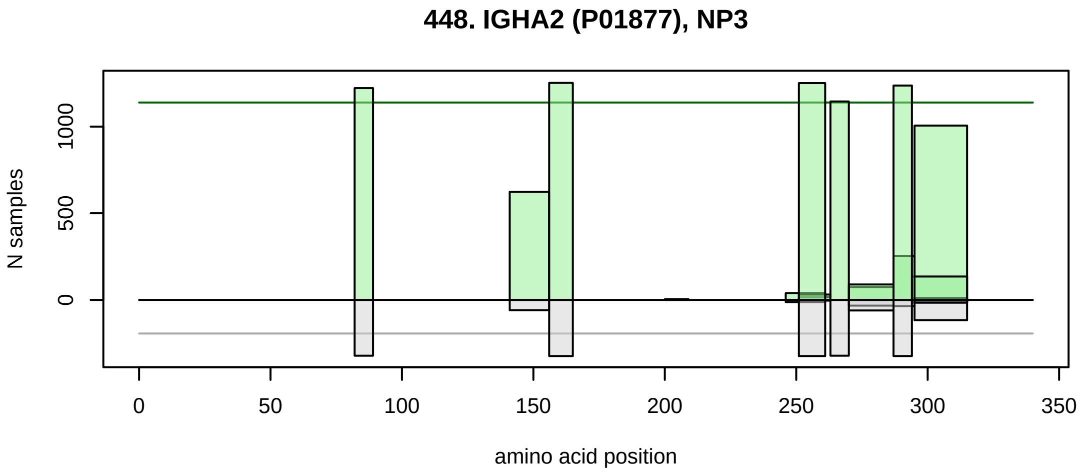
448. IGHA2 (P01877), NP3
N samples
0
500
1000
0
50
100
150
200
250
300
350
amino acid position

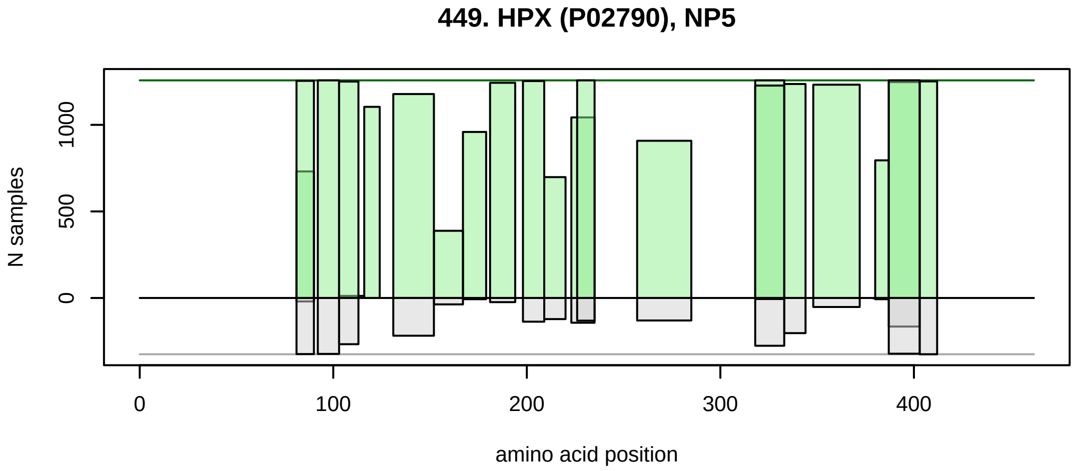
449. HPX (P02790), NP5
N samples
0
500
1000
0
100
200
300
400
amino acid position

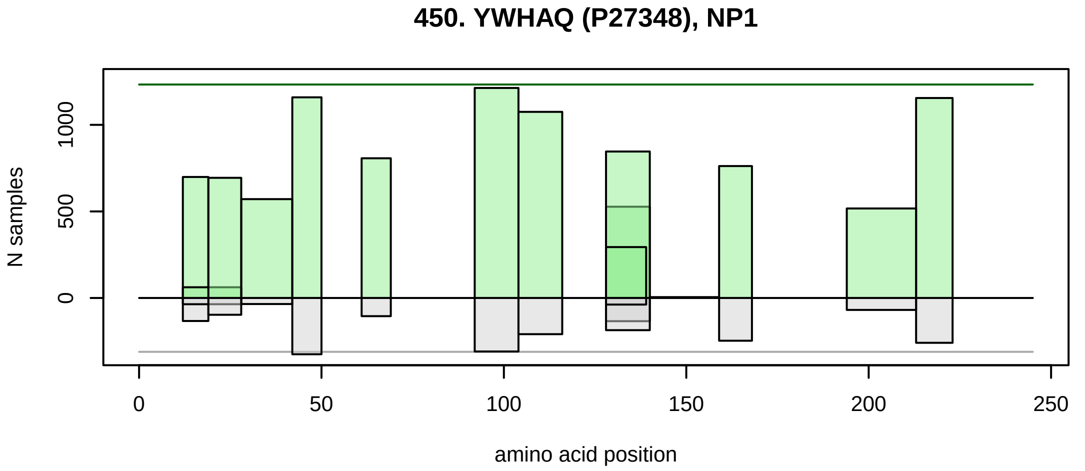
450. YWHAQ (P27348), NP1
N samples
0
500
1000
0
50
100
150
200
250
amino acid position

451. TUBA4A (P68366;P68366−2), NP4

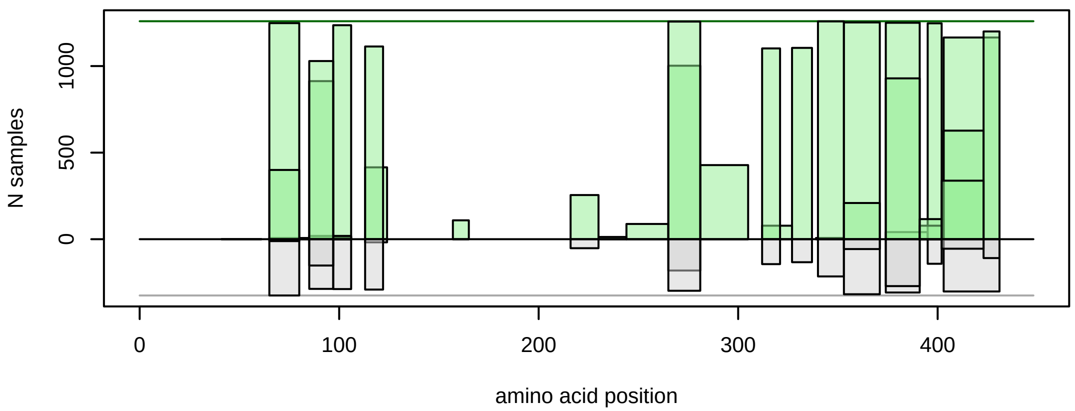

452. SNAP23 (O00161), NP4

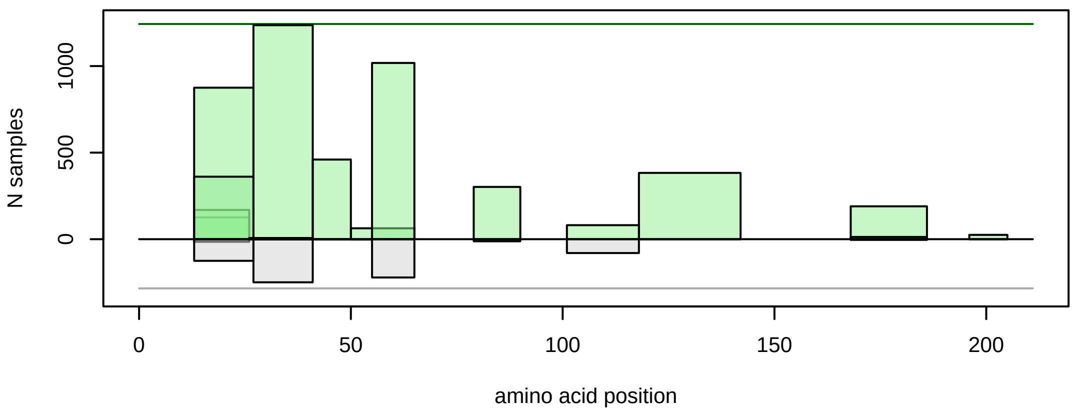

453. CARS (P49589−3), NP1

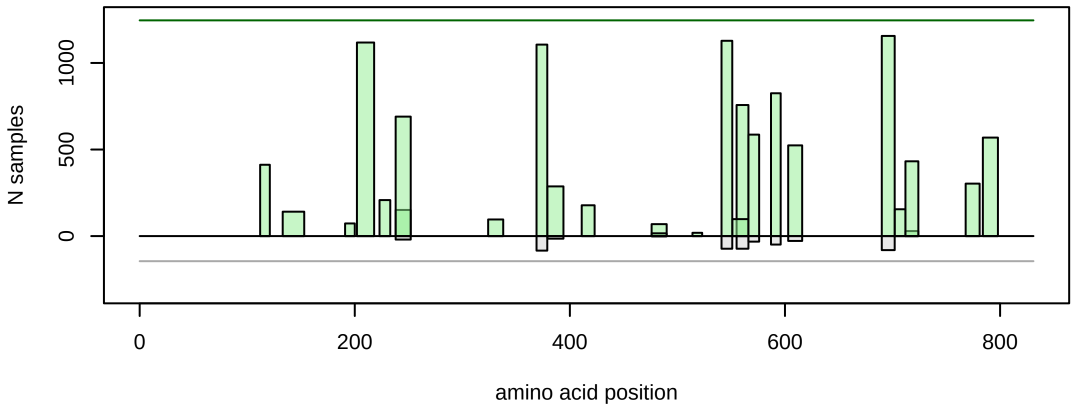

454. VTA1 (A0A087WY55;Q9NP79), NP1

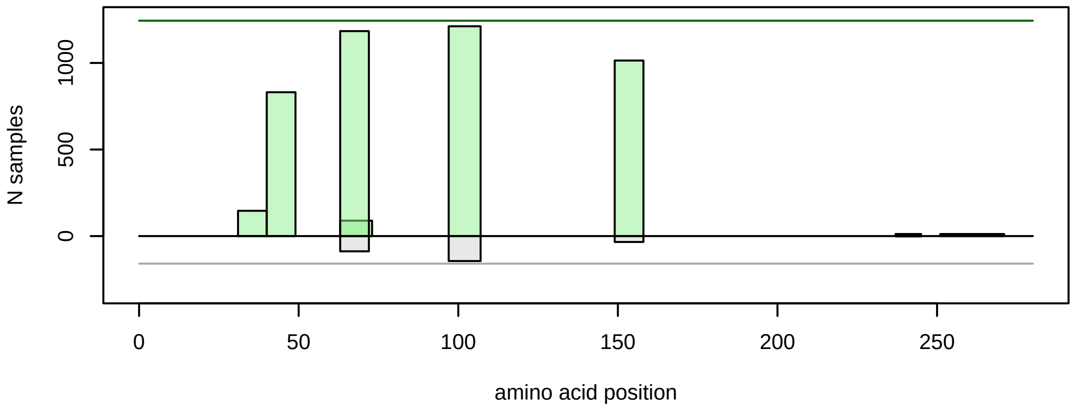

455. PTPRZ1 (A0A494C087), NP2

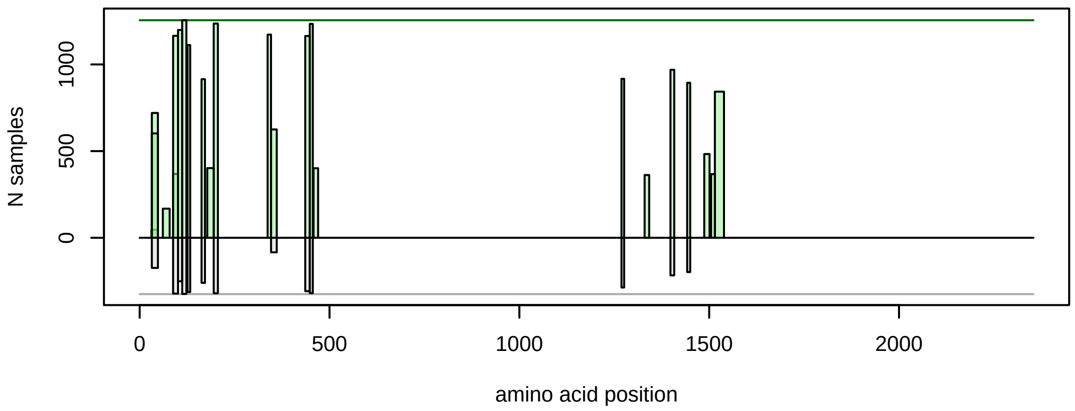

456. UNC45A (A0A1W2PNX8), NP4

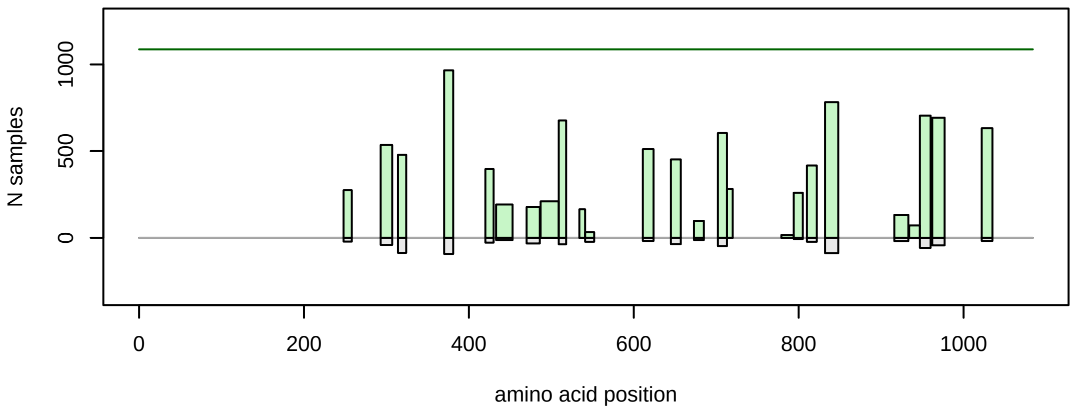

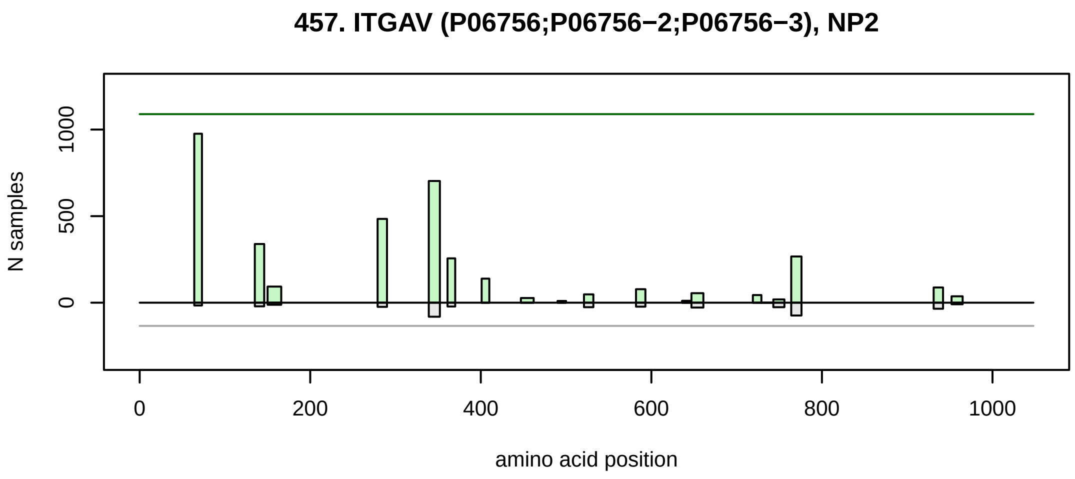
457. ITGAV (P06756;P06756−2;P06756−3), NP2
N samples
0
500
1000
0
200
400
600
800
1000
amino acid position

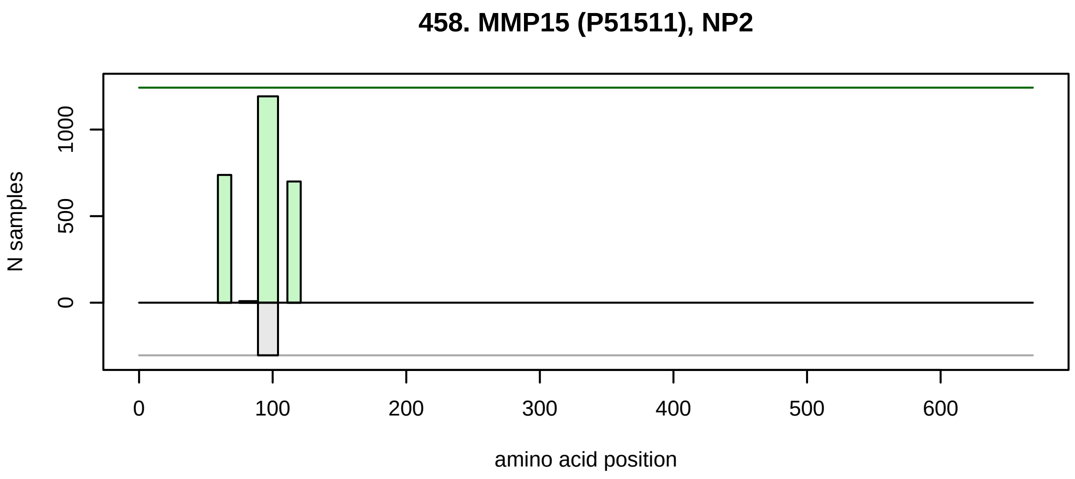
458. MMP15 (P51511), NP2
N samples
0
500
1000
0
100
200
300
400
500
600
amino acid position

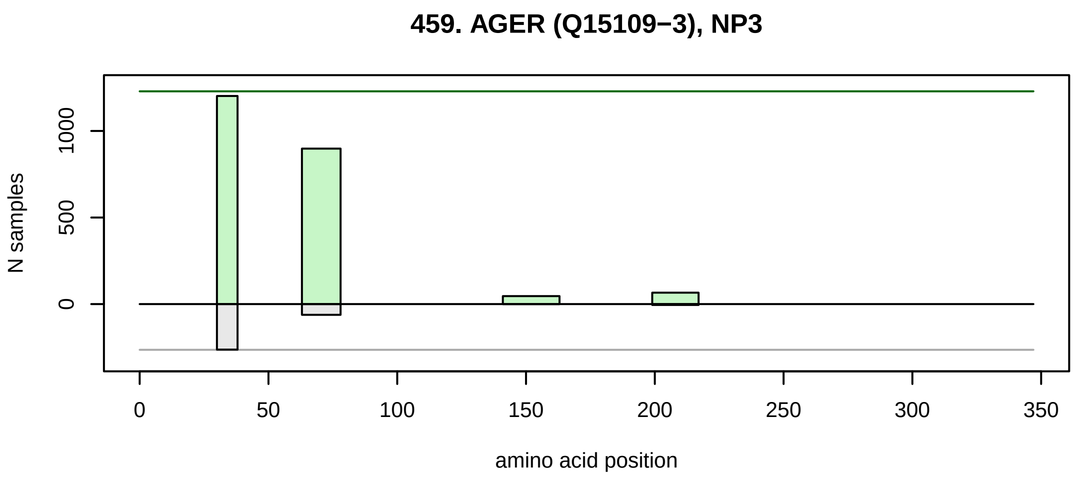
459. AGER (Q15109−3), NP3
N samples
0
500
1000
0
50
100
150
200
250
300
350
amino acid position

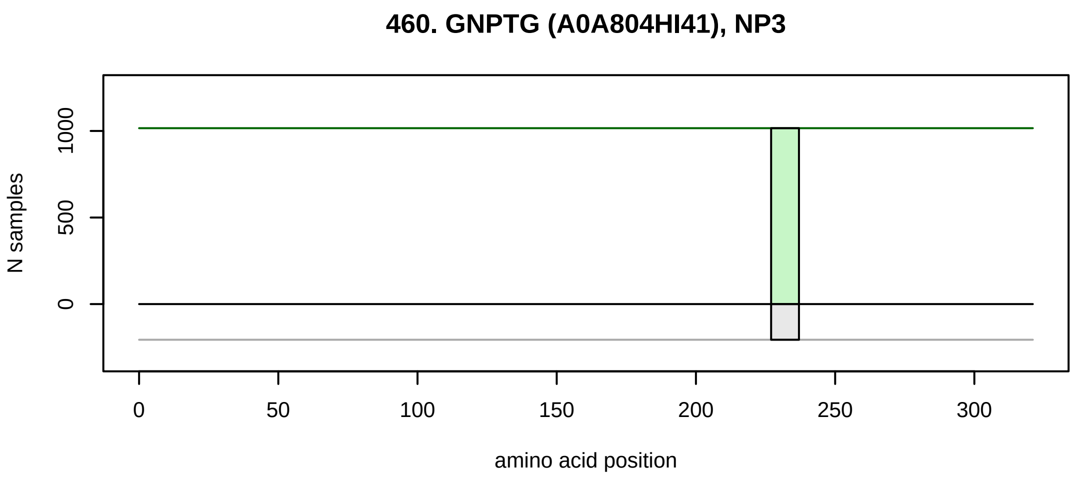
460. GNPTG (A0A804HI41), NP3
N samples
0
500
1000
0
50
100
150
200
250
300
amino acid position

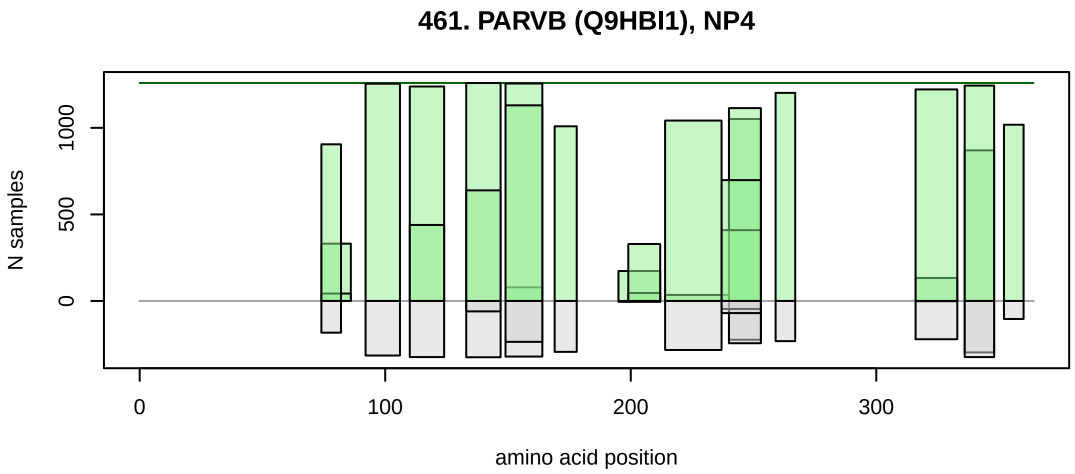
461. PARVB (Q9HBI1), NP4
N samples
0
500
1000
0
100
200
300
amino acid position

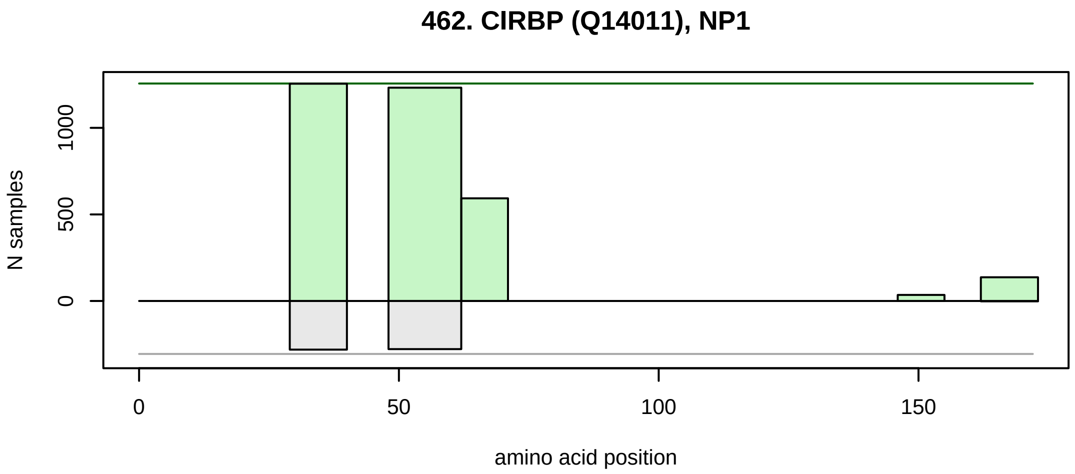
462. CIRBP (Q14011), NP1
N samples
0
500
1000
0
50
100
150
amino acid position

463. DERA (Q9Y315), NP4

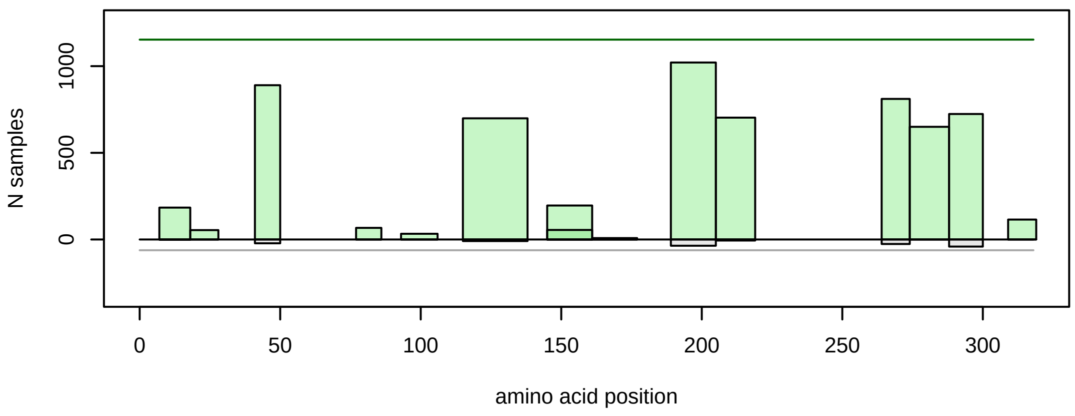

464. CMTM5 (C9JAI6), NP4

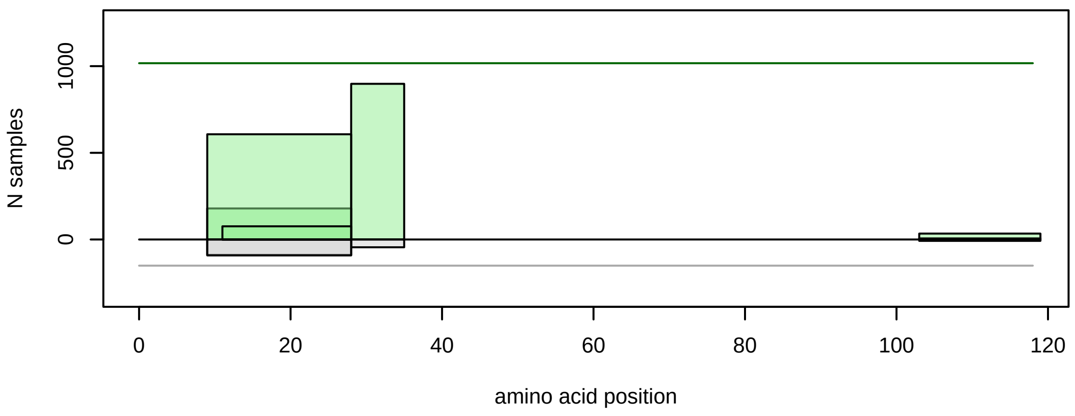

465. FYB (O15117−2;O15117−3), NP4

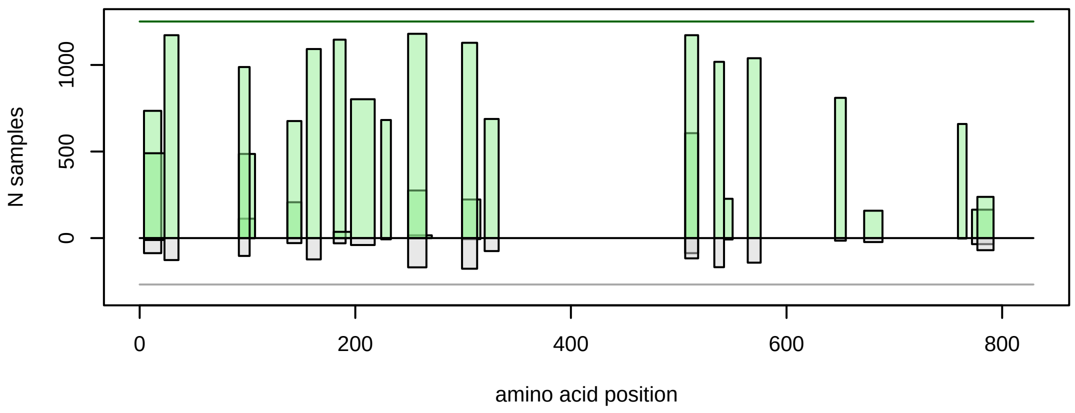

466. RGS18 (Q9NS28), NP3

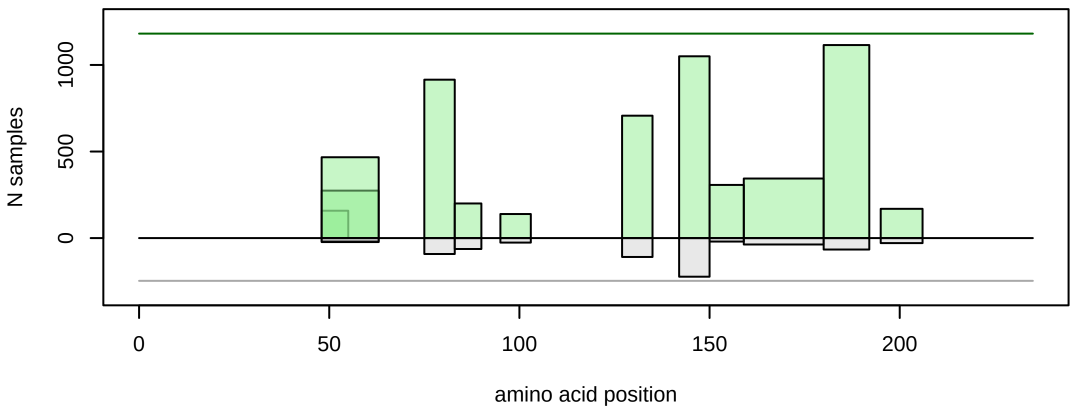

467. COPB1 (P53618), NP4

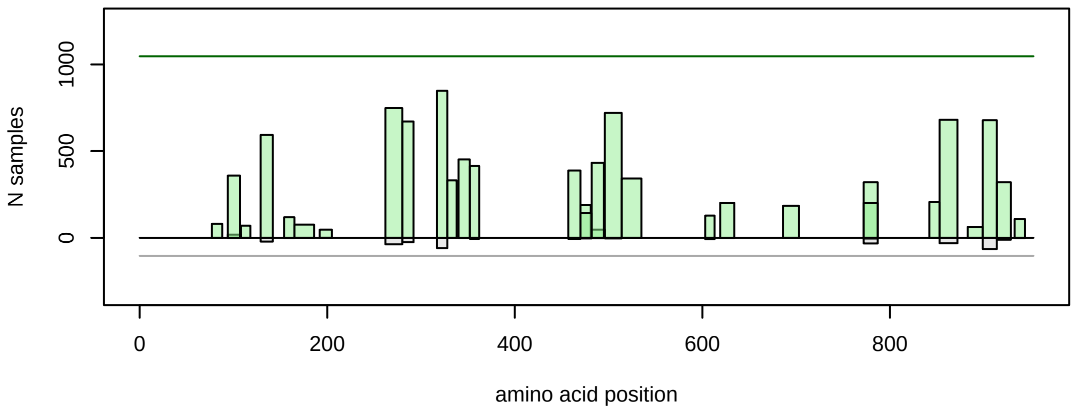

468. PCSK9 (Q8NBP7), NP2

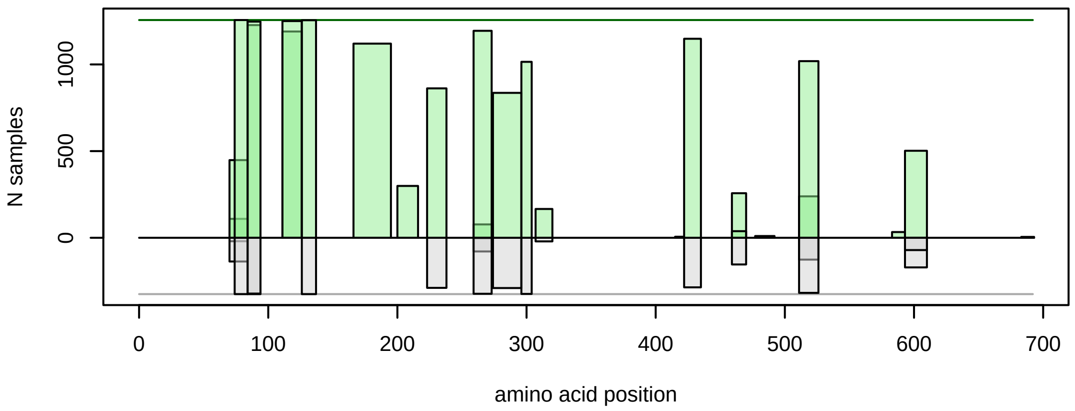

469. EHD1 (Q9H4M9), NP4

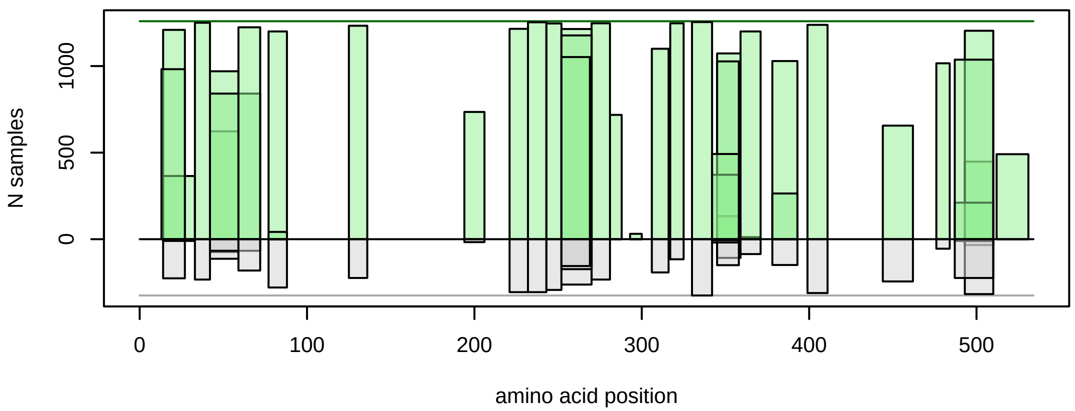

470. SCGB3A1 (Q96QR1), NP4

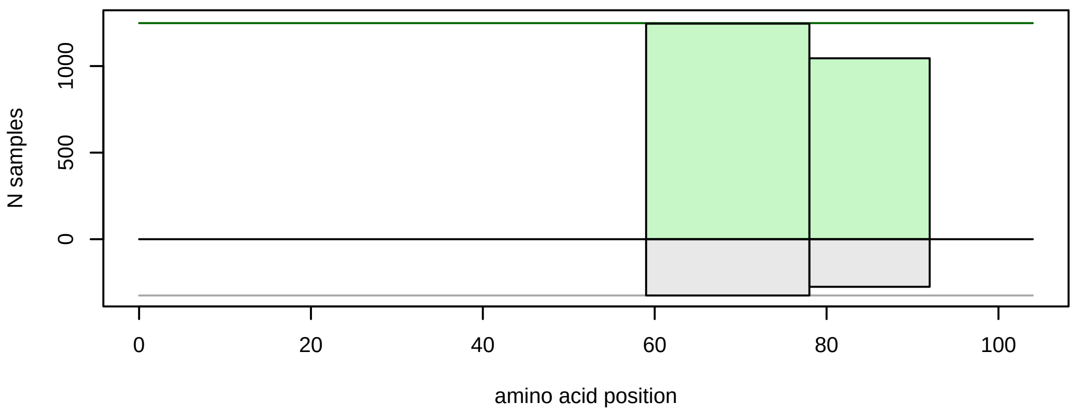

471. STK17A (Q9UEE5), NP3

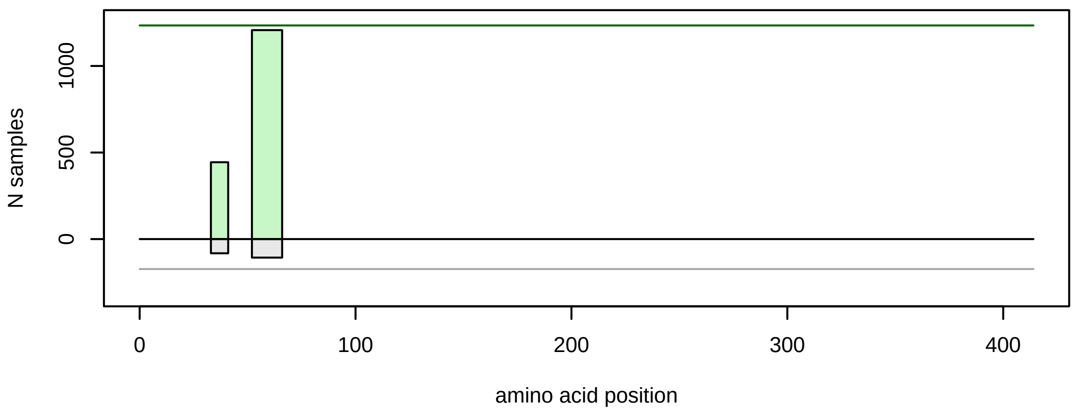

472. UNC13D (Q70J99), NP5

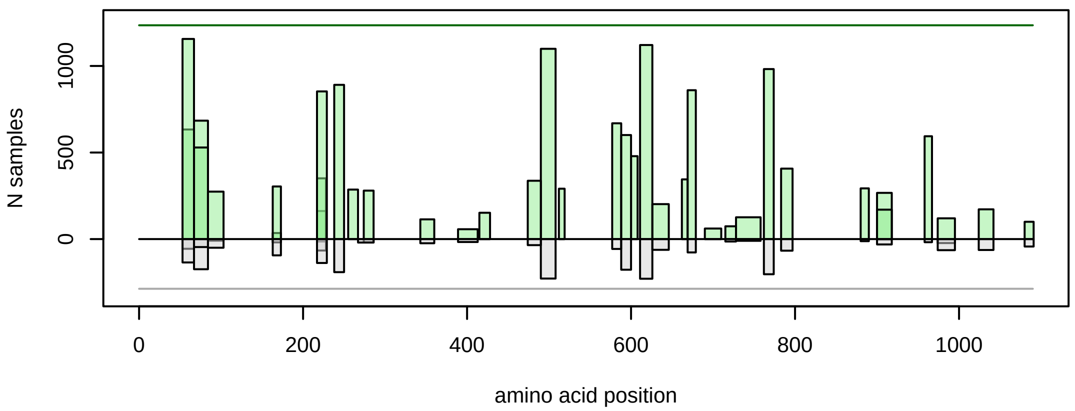

473. PGAM1 (P18669), NP4

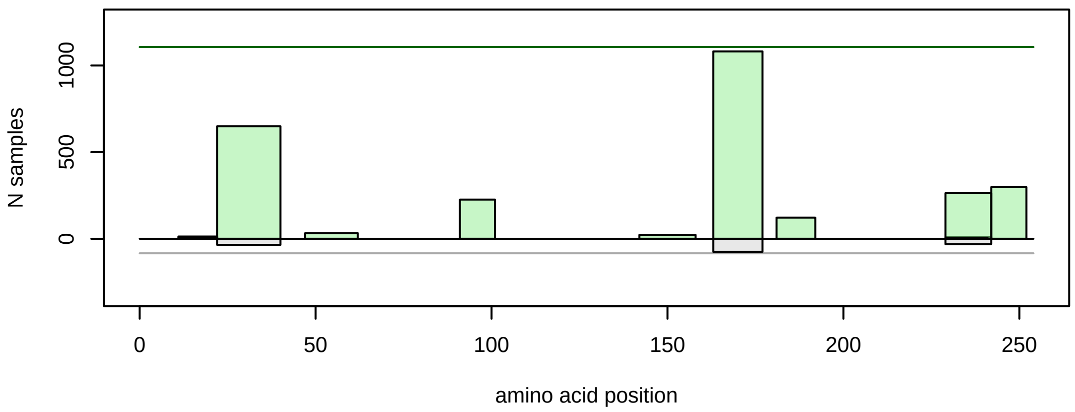

474. EHD4 (Q9H223), NP1

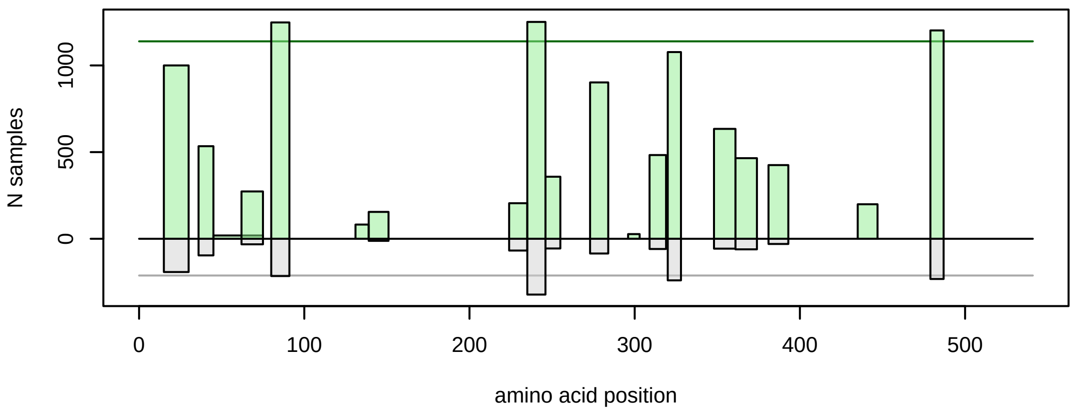

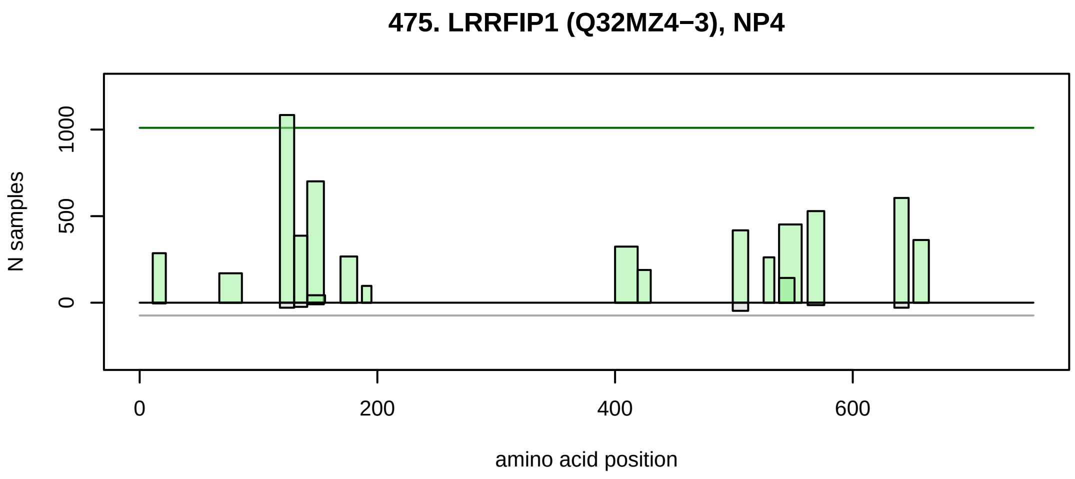
475. LRRFIP1 (Q32MZ4−3), NP4
N samples
0
500
1000
0
200
400
600
amino acid position

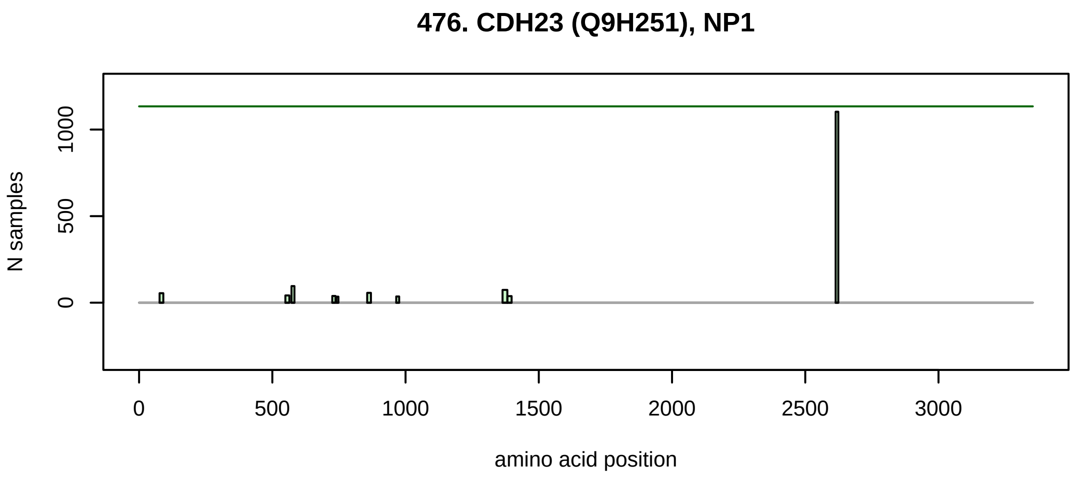
476. CDH23 (Q9H251), NP1
N samples
0
500
1000
0
500
1000
1500
2000
2500
3000
amino acid position

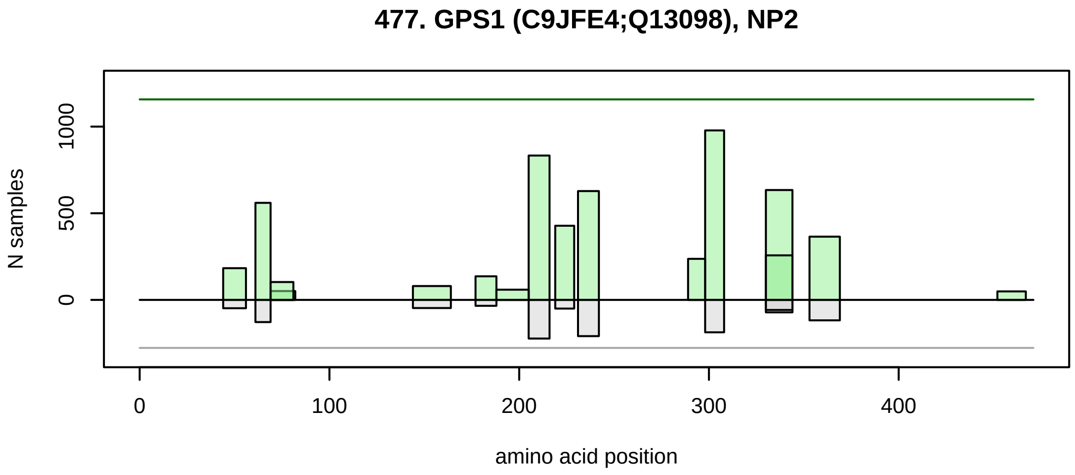
477. GPS1 (C9JFE4;Q13098), NP2
N samples
0
500
1000
0
100
200
300
400
amino acid position

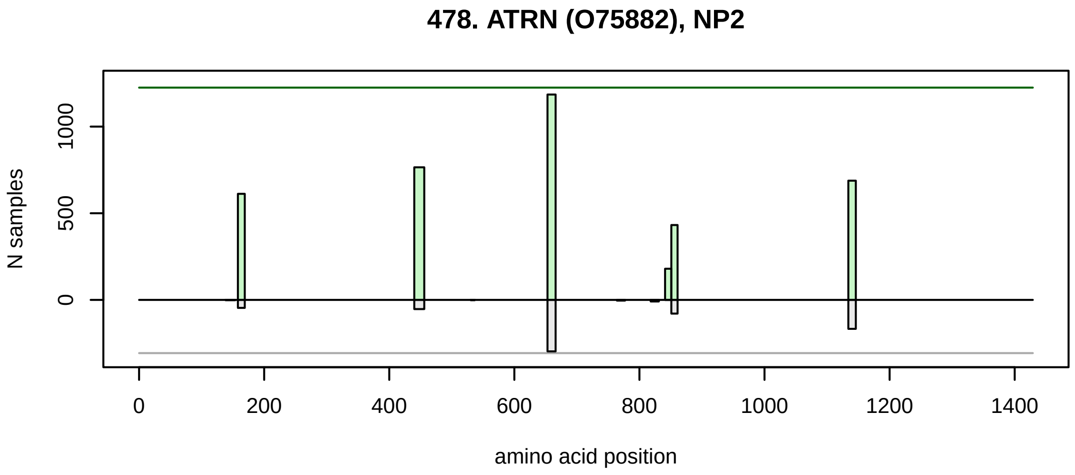
478. ATRN (O75882), NP2
N samples
0
500
1000
0
200
400
600
800
1000
1200
1400
amino acid position

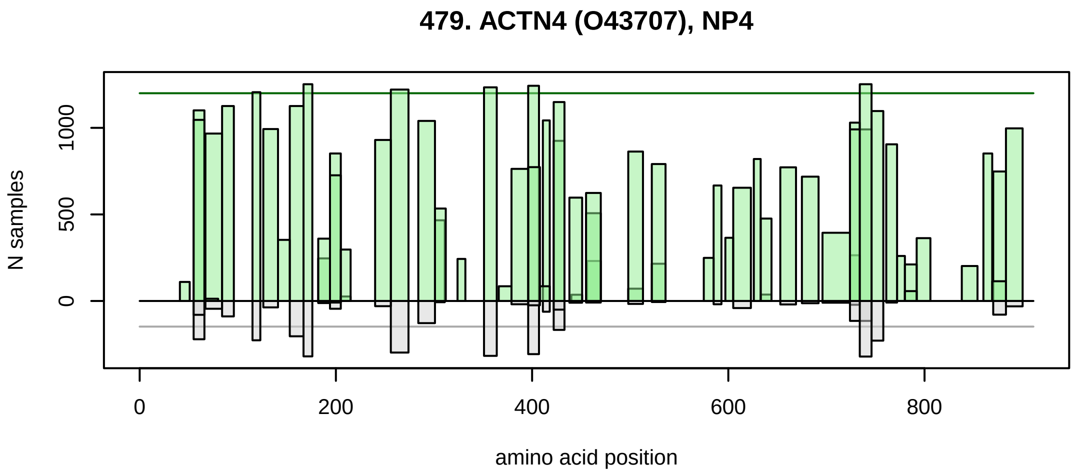
479. ACTN4 (O43707), NP4
N samples
0
500
1000
0
200
400
600
800
amino acid position

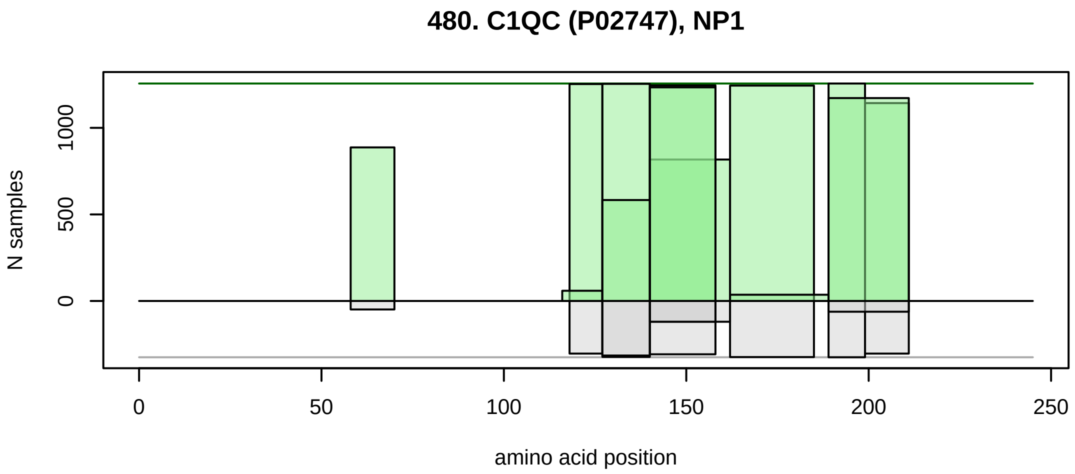
480. C1QC (P02747), NP1
N samples
0
500
1000
0
50
100
150
200
250
amino acid position

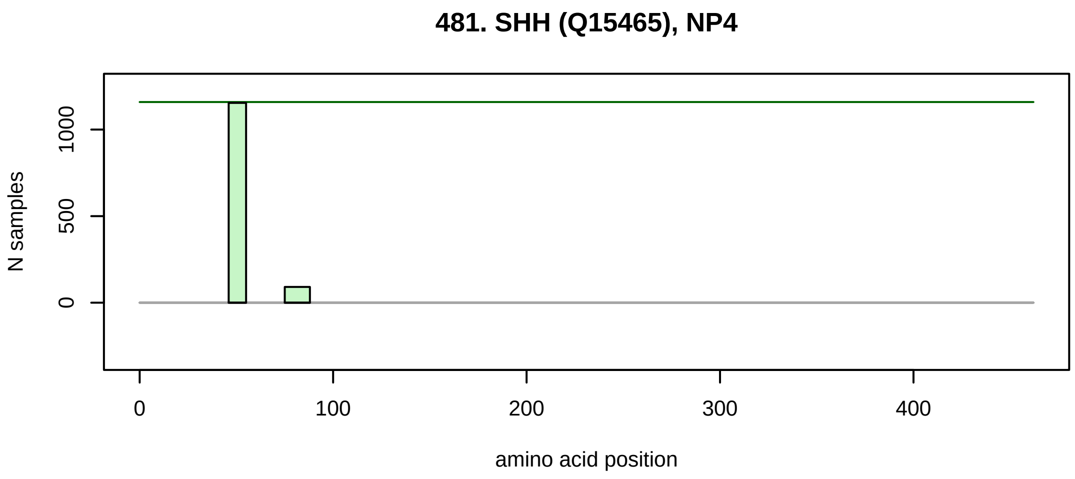
481. SHH (Q15465), NP4
N samples
0
500
1000
0
100
200
300
400
amino acid position

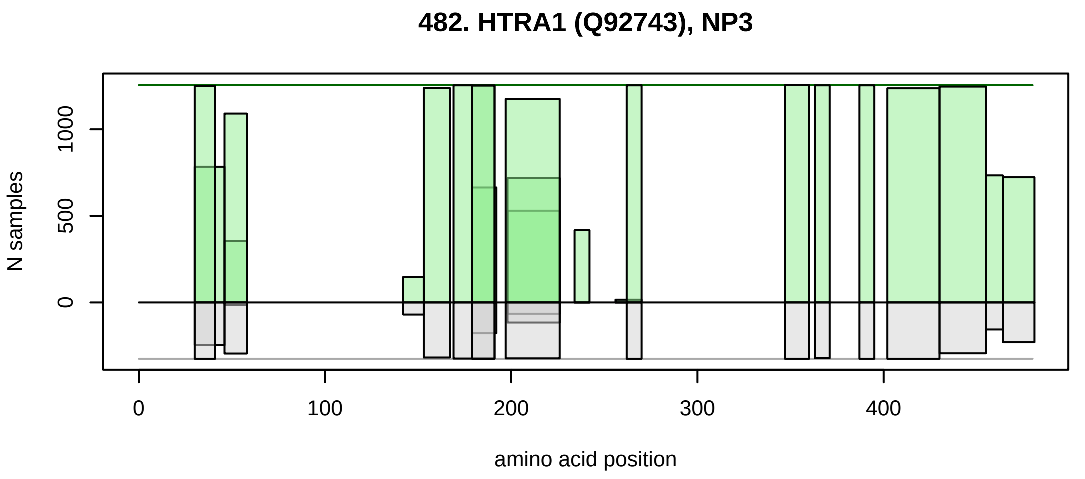
482. HTRA1 (Q92743), NP3
N samples
0
500
1000
0
100
200
300
400
amino acid position

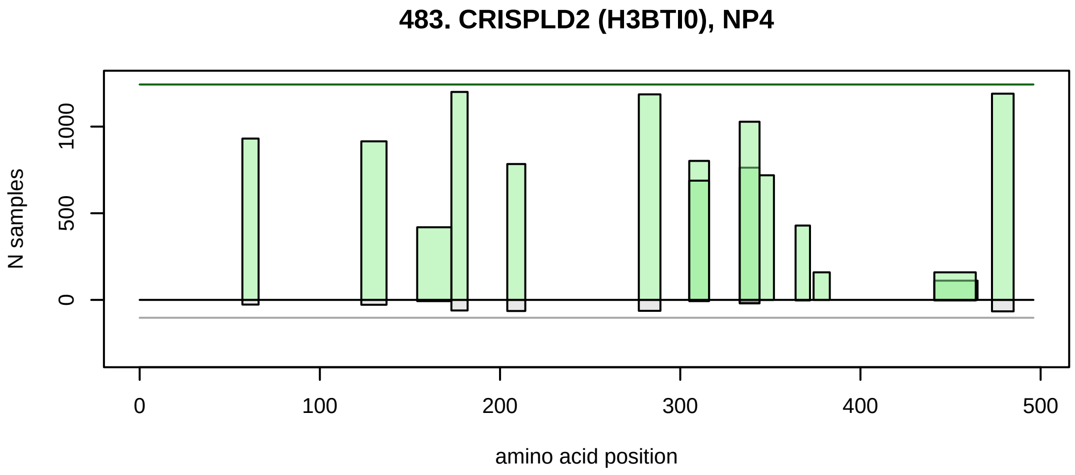
483. CRISPLD2 (H3BTI0), NP4
N samples
0
500
1000
0
100
200
300
400
500
amino acid position

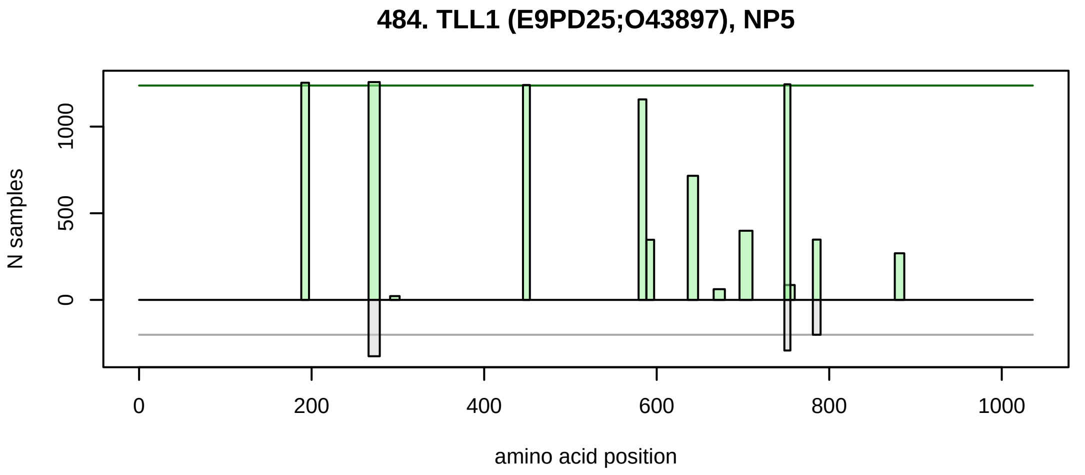
484. TLL1 (E9PD25;O43897), NP5
N samples
0
500
1000
0
200
400
600
800
1000
amino acid position

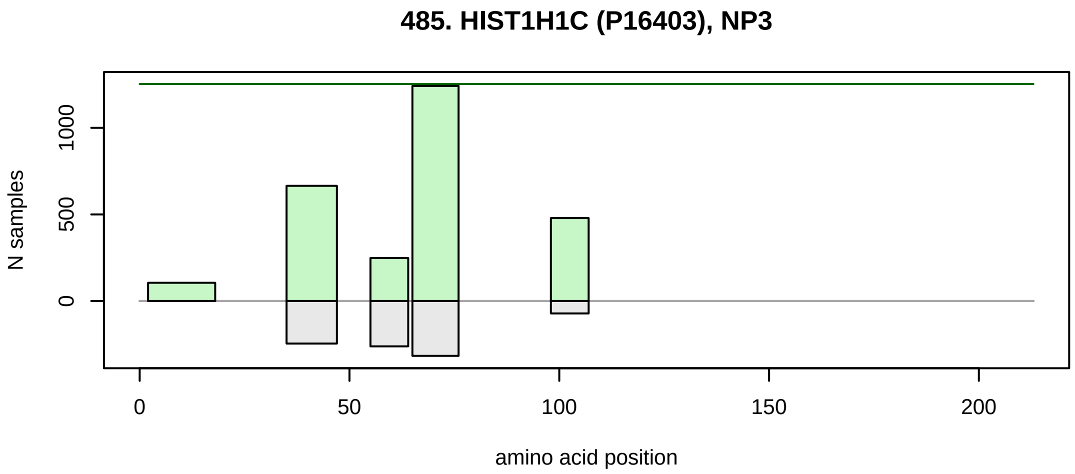
485. HIST1H1C (P16403), NP3
N samples
0
500
1000
0
50
100
150
200
amino acid position

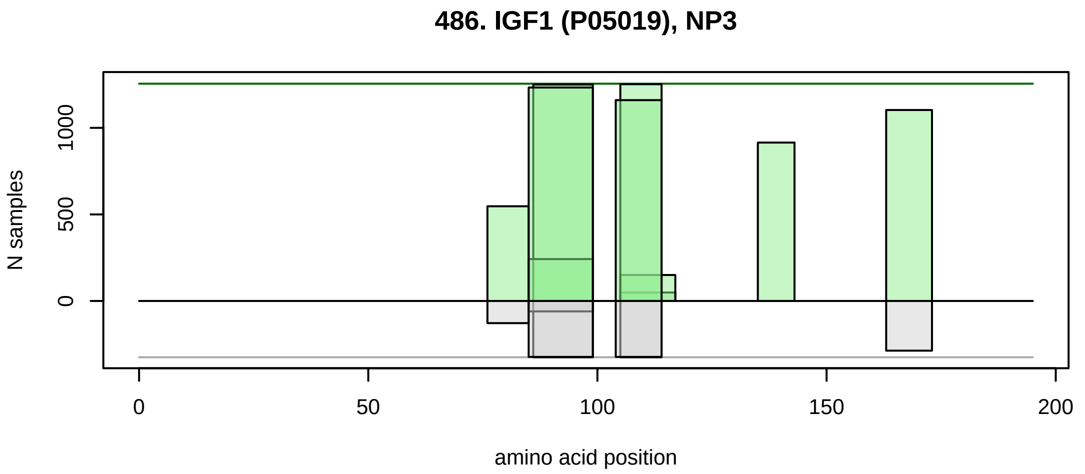
486. IGF1 (P05019), NP3
N samples
0
500
1000
0
50
100
150
200
amino acid position

487. LDHAL6B (Q9BYZ2), NP4

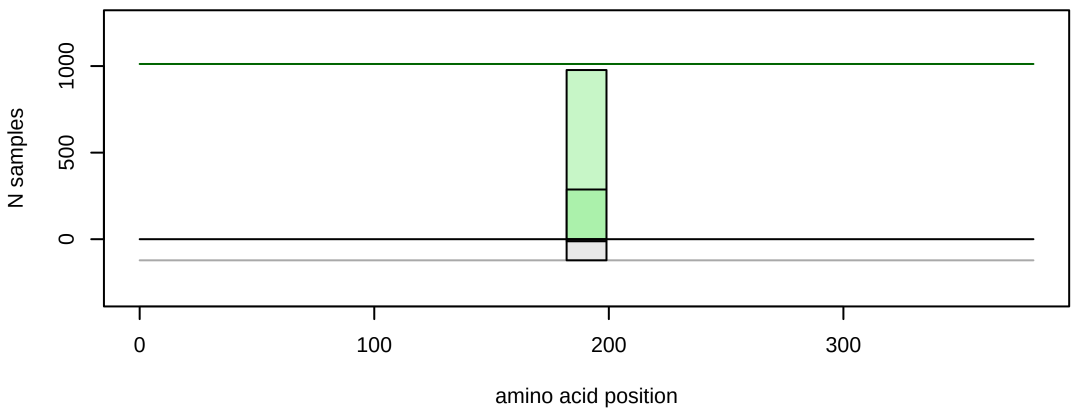

488. CD74 (P04233−2), NP2

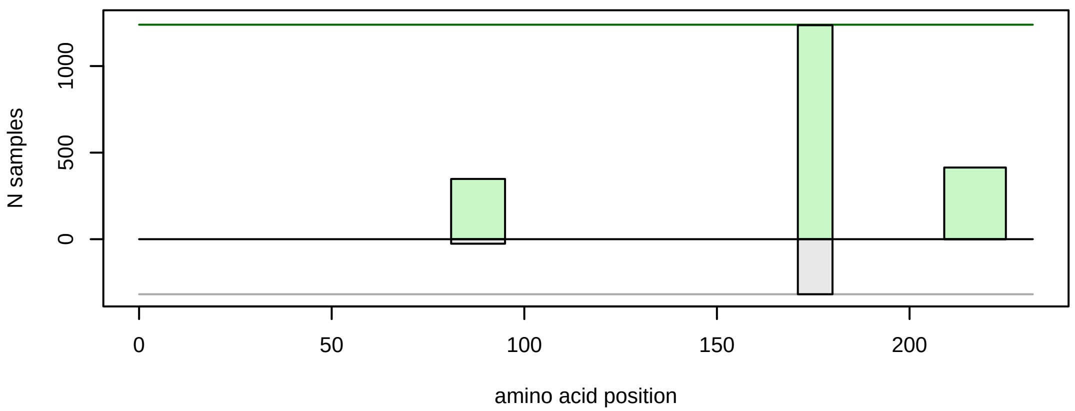

489. FAM114A2 (Q9NRY5), NP1

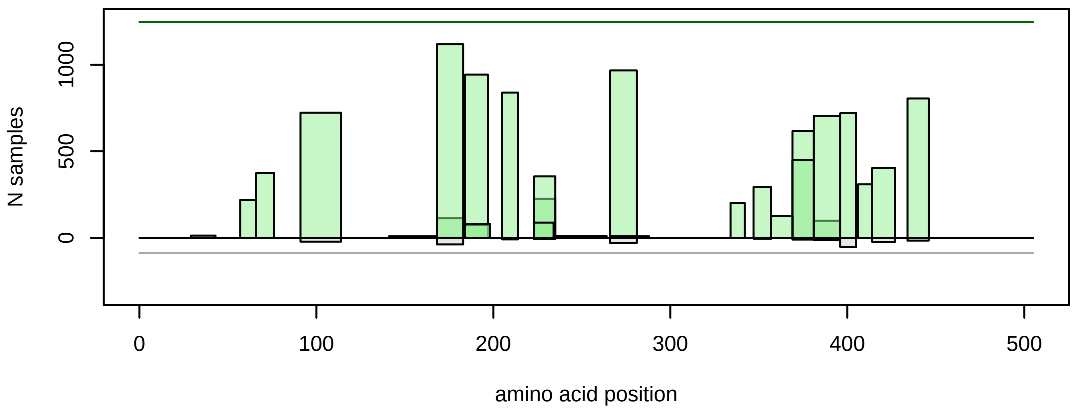

490. CPNE3 (O75131), NP3

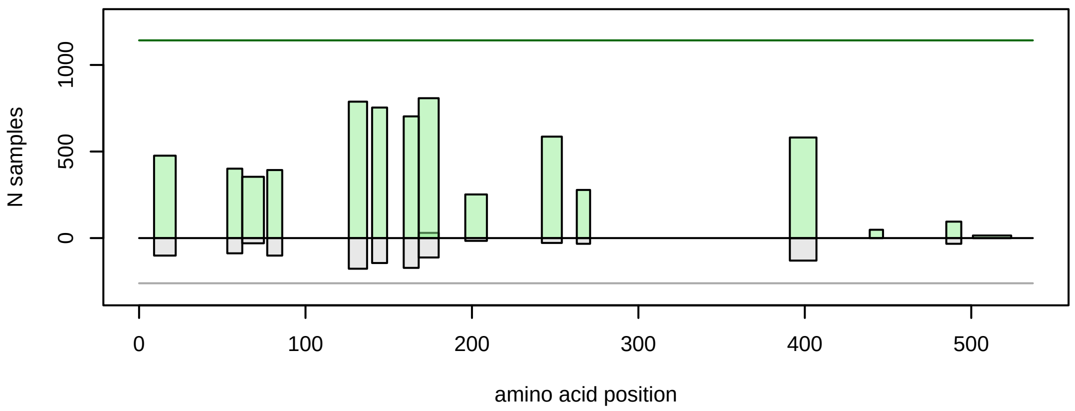

491. RP11−529K1.3 (F6QDS0), NP3

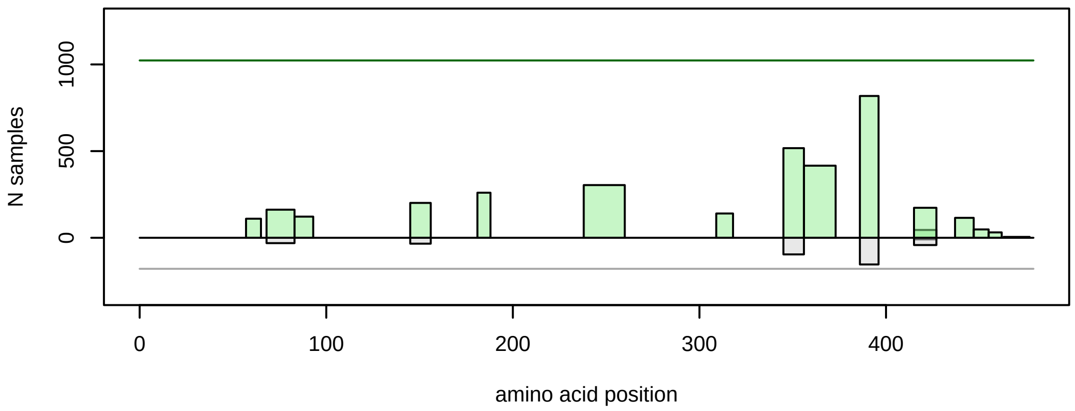

492. PECAM1 (P16284), NP4

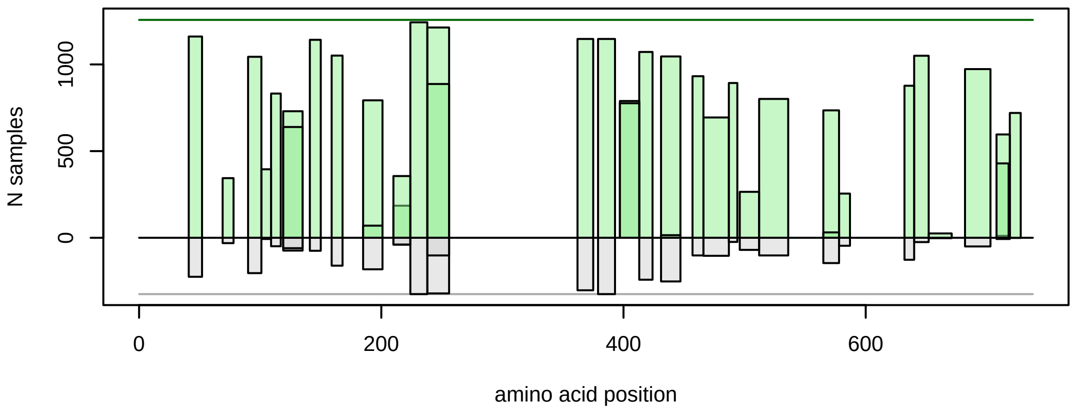

493. RPL4 (P36578), NP4

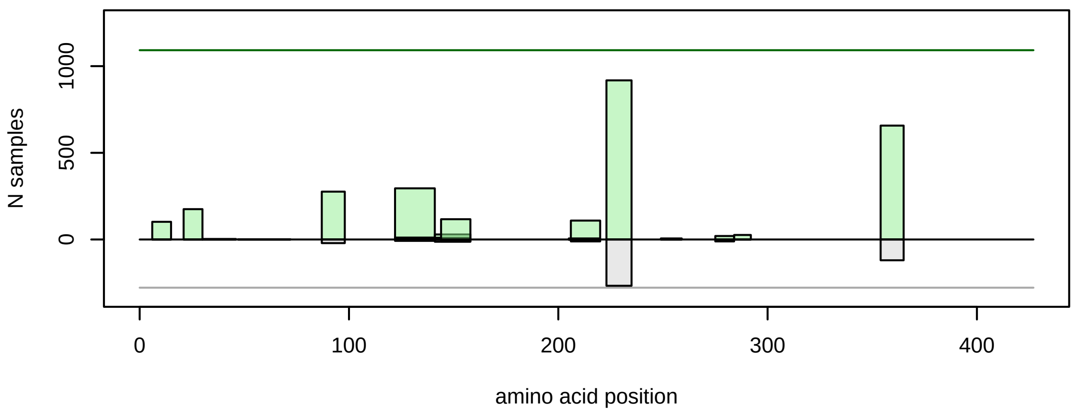

494. SLK (Q9H2G2−2), NP4

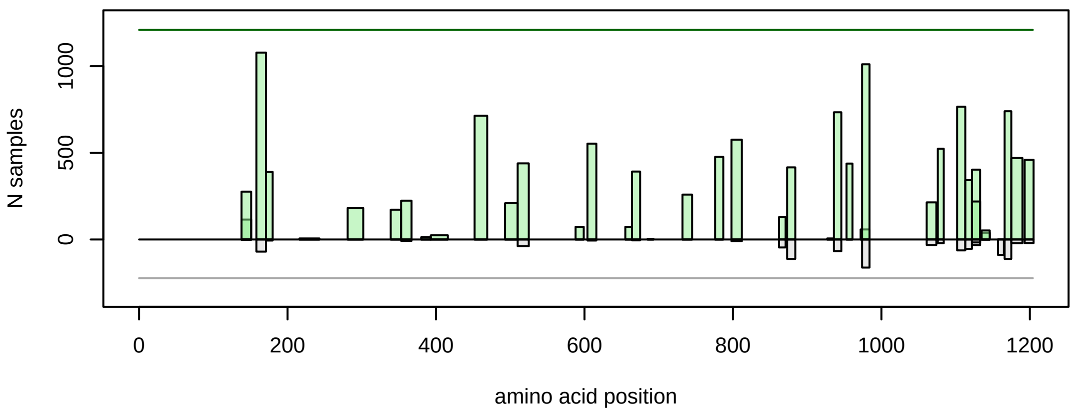

495. SH3GL1 (Q99961), NP1

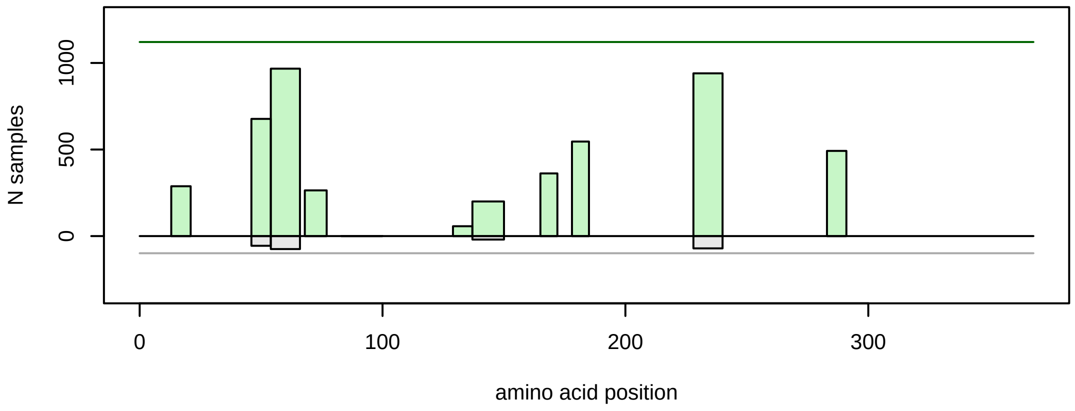

496. KAZALD1 (Q96I82), NP2

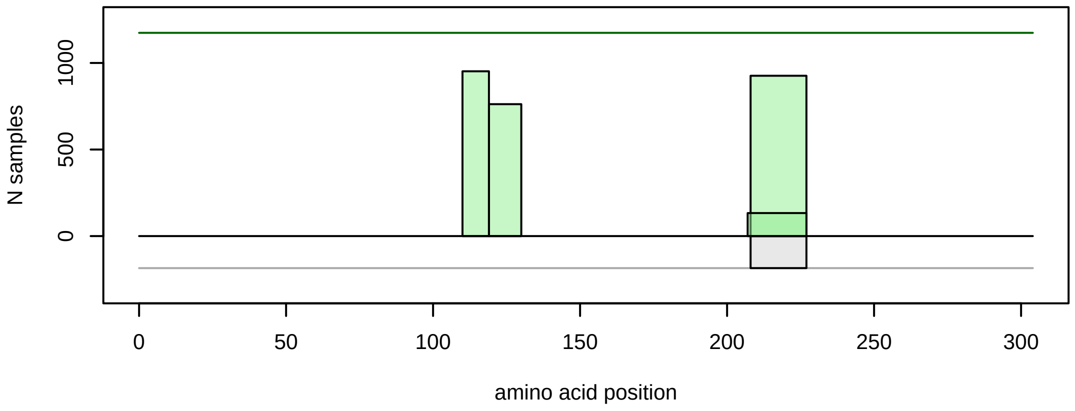

497. ATP5A1 (P25705), NP4

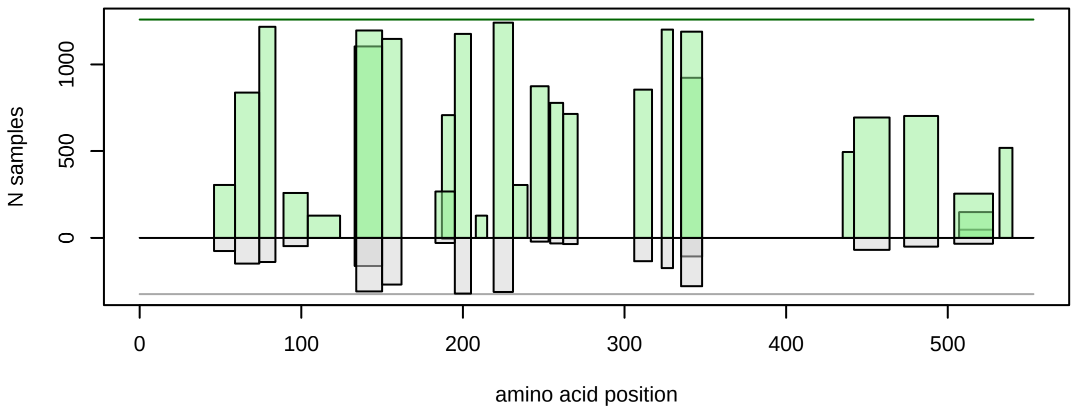

498. ARPC4 (F8WCF6;P59998;P59998−3), NP4

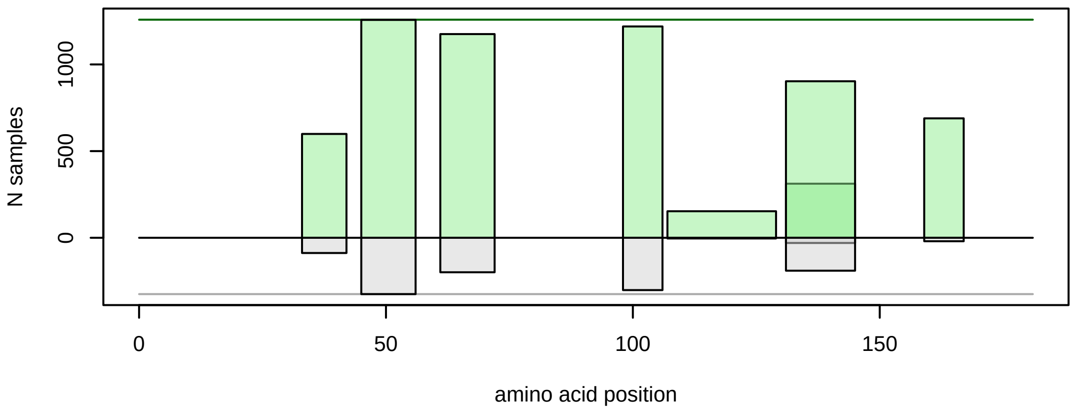

499. ANK3 (Q12955), NP3

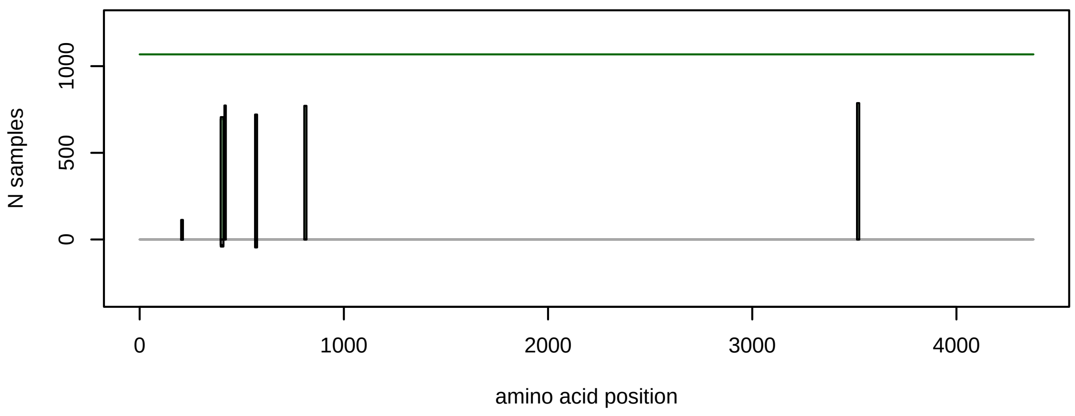

500. CD8A (B8ZZZ4;P01732;P01732−2), NP3

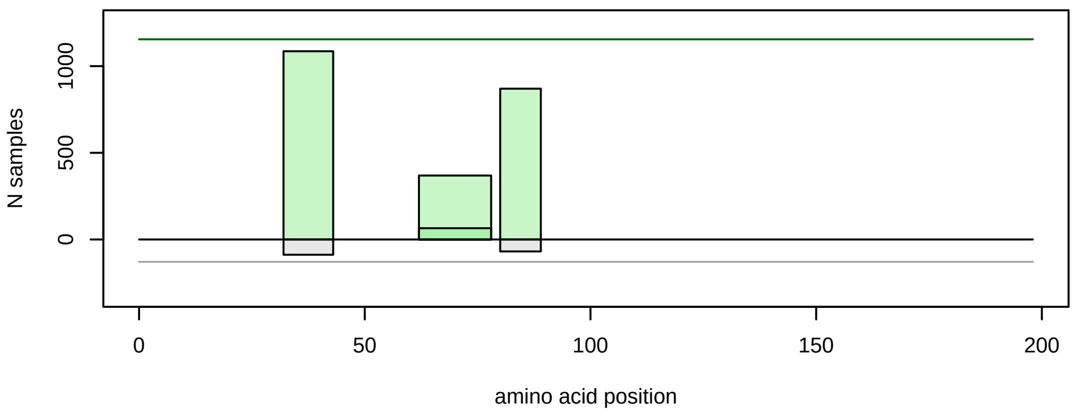

501. ILF2 (Q12905), NP1

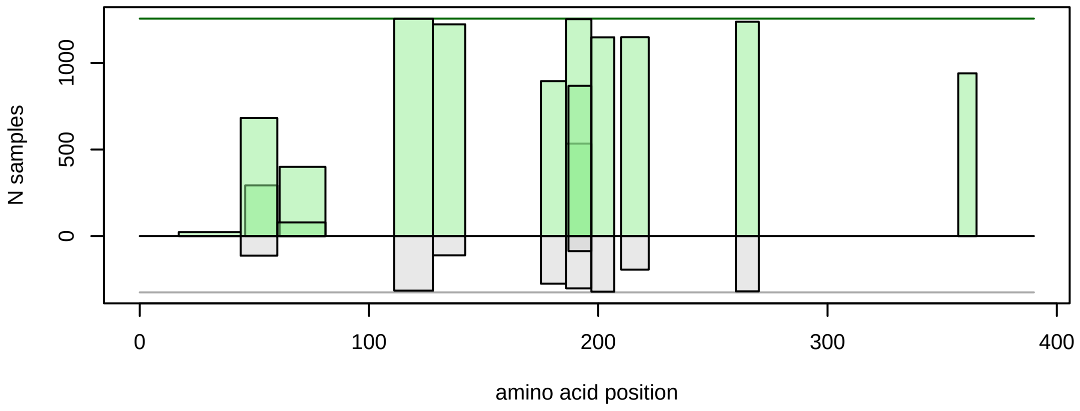

502. EIF1AX (P47813), NP2

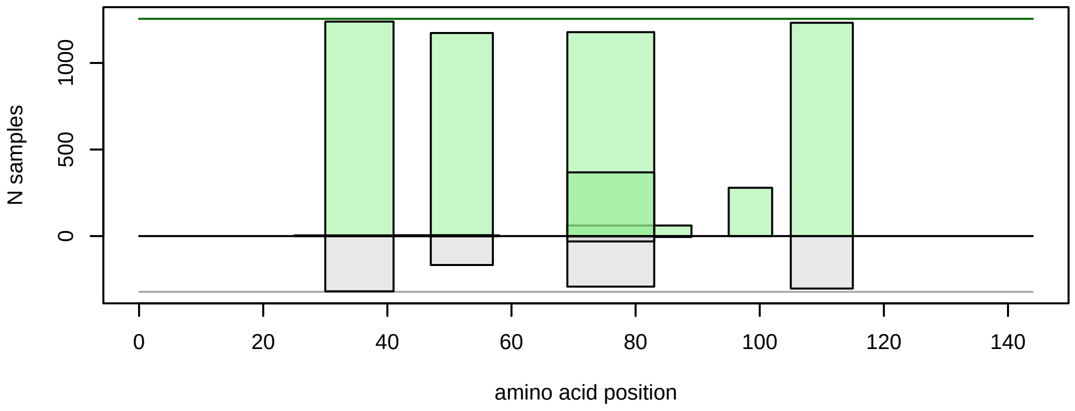

503. ATP6AP1 (Q15904), NP2

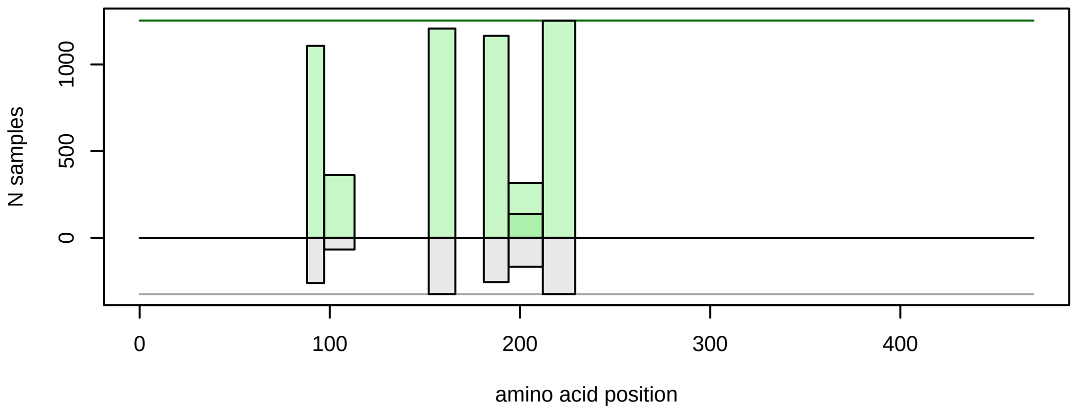

504. CALD1 (Q05682−4), NP4

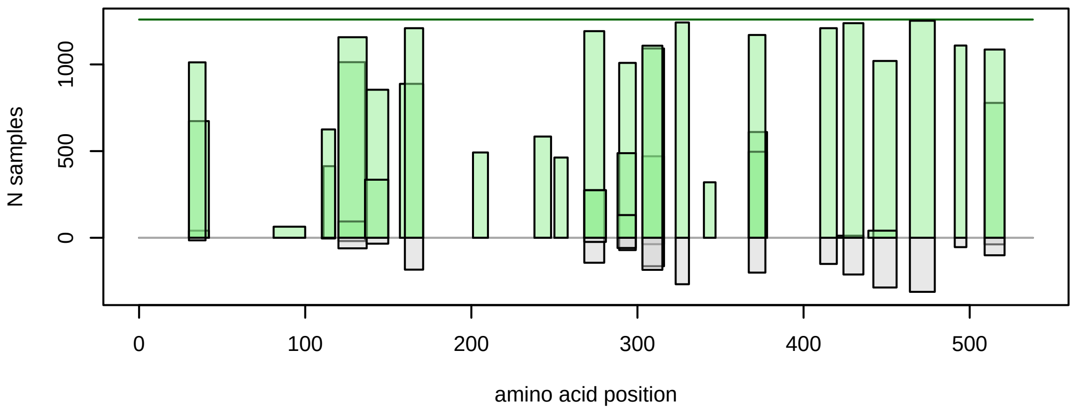

505. C6orf25 (O95866), NP4

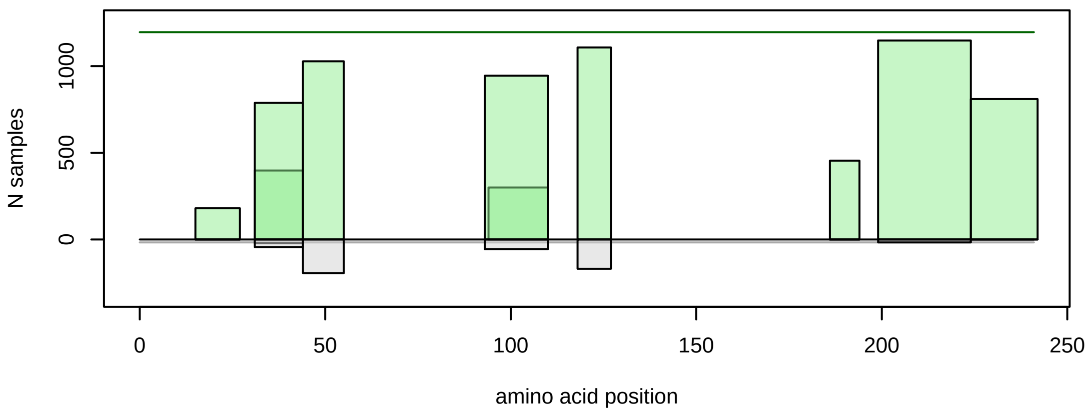

506. SIL1 (D6REA1;Q9H173), NP2

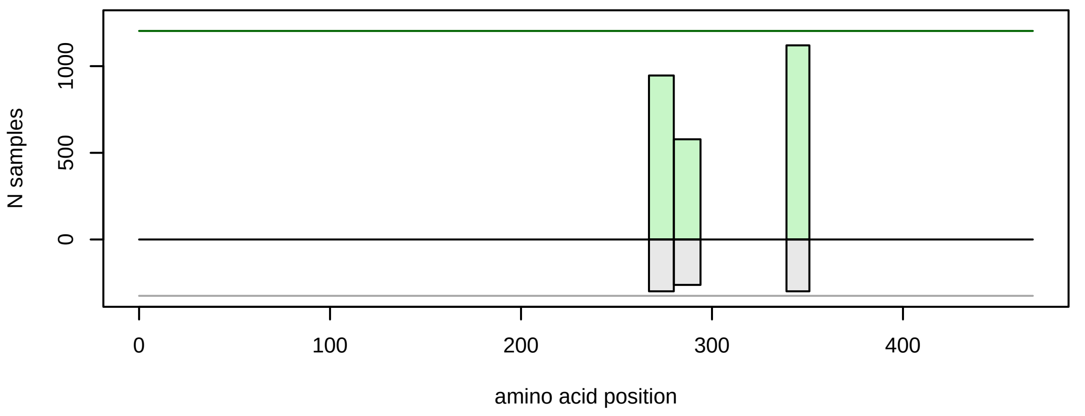

507. PFN1 (P07737), NP3

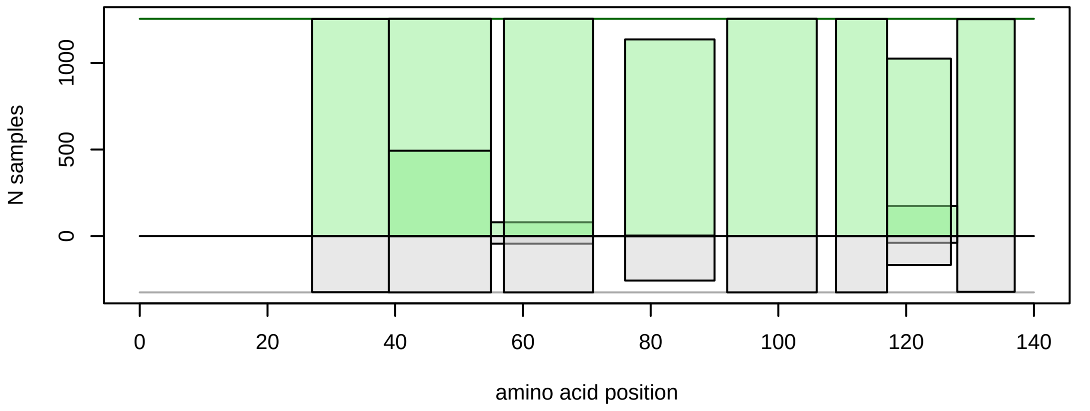

508. LGALS3 (P17931), NP3

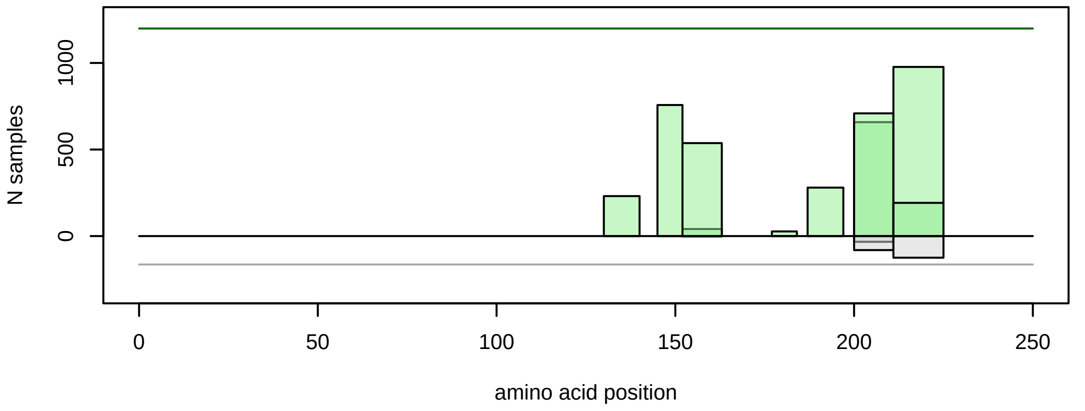

509. EEF1D (E9PRY8), NP2

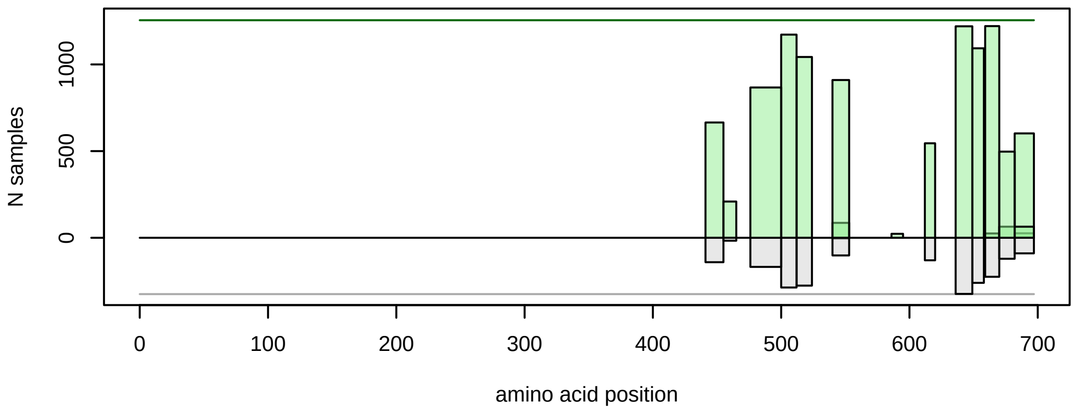

510. PTPRJ (Q12913), NP1

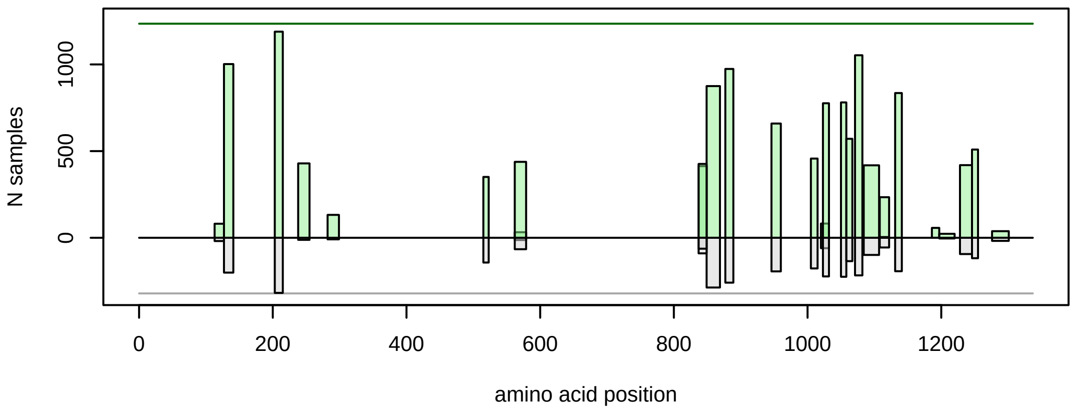

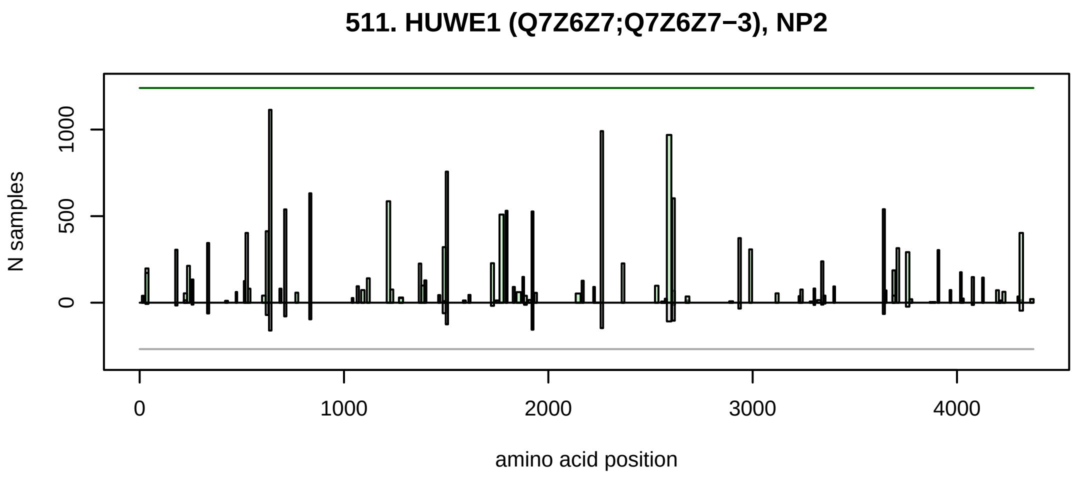
511. HUWE1 (Q7Z6Z7;Q7Z6Z7−3), NP2
N samples
0
500
1000
0
1000
2000
3000
4000
amino acid position

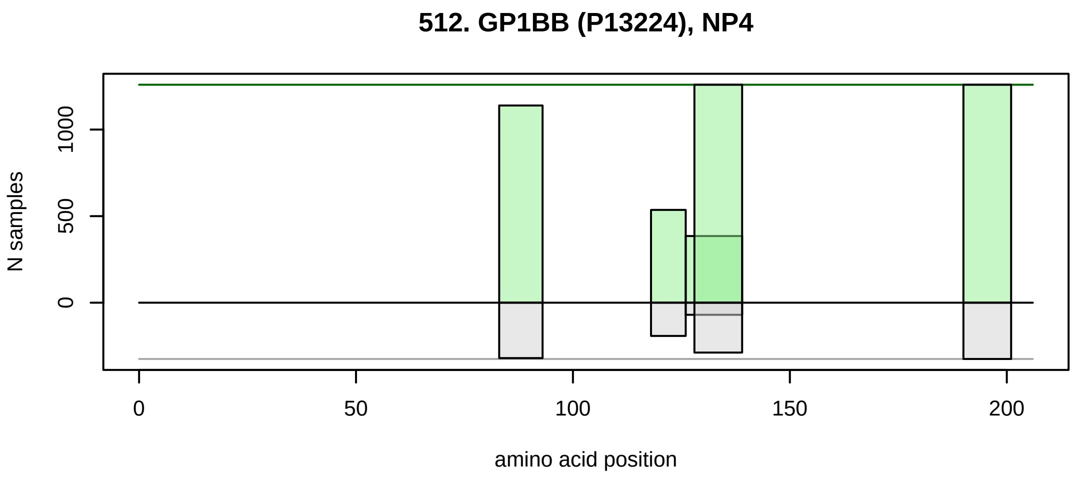
512. GP1BB (P13224), NP4
N samples
0
500
1000
0
50
100
150
200
amino acid position

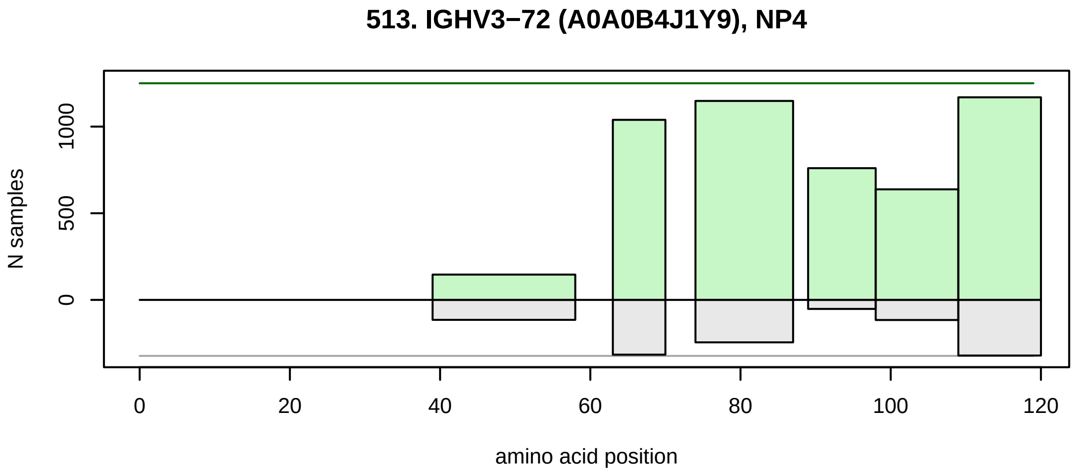
513. IGHV3−72 (A0A0B4J1Y9), NP4
N samples
0
500
1000
0
20
40
60
80
100
120
amino acid position

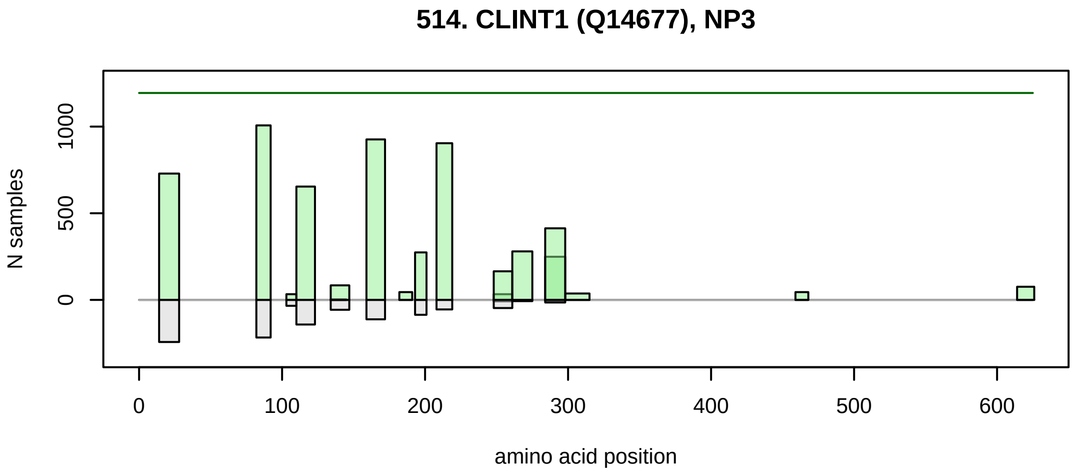
514. CLINT1 (Q14677), NP3
N samples
0
500
1000
0
100
200
300
400
500
600
amino acid position

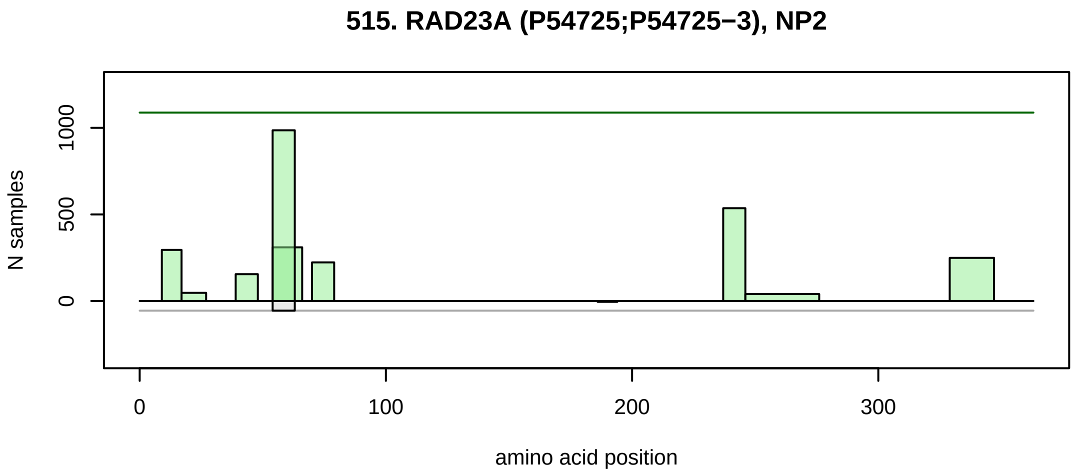
515. RAD23A (P54725;P54725−3), NP2
N samples
0
500
1000
0
100
200
300
amino acid position

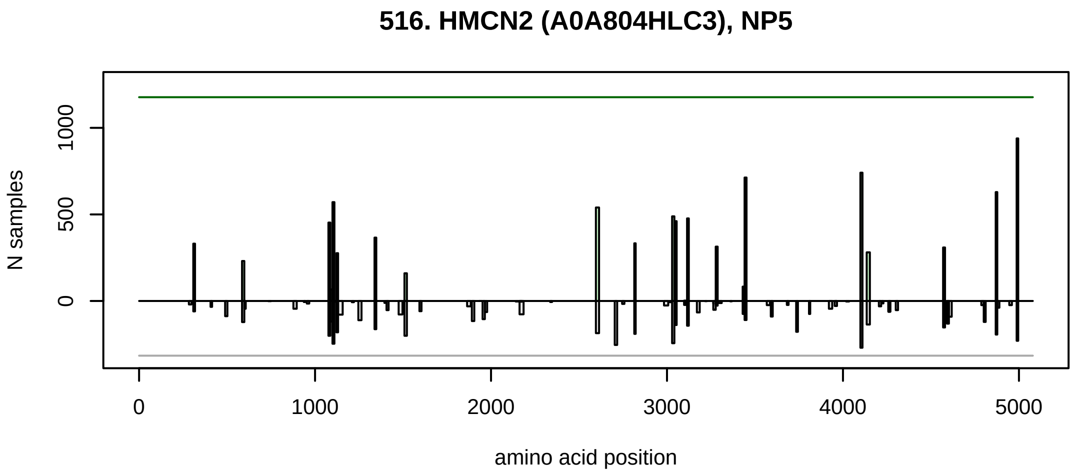
516. HMCN2 (A0A804HLC3), NP5
N samples
0
500
1000
0
1000
2000
3000
4000
5000
amino acid position

517. PRKACA (P17612), NP3

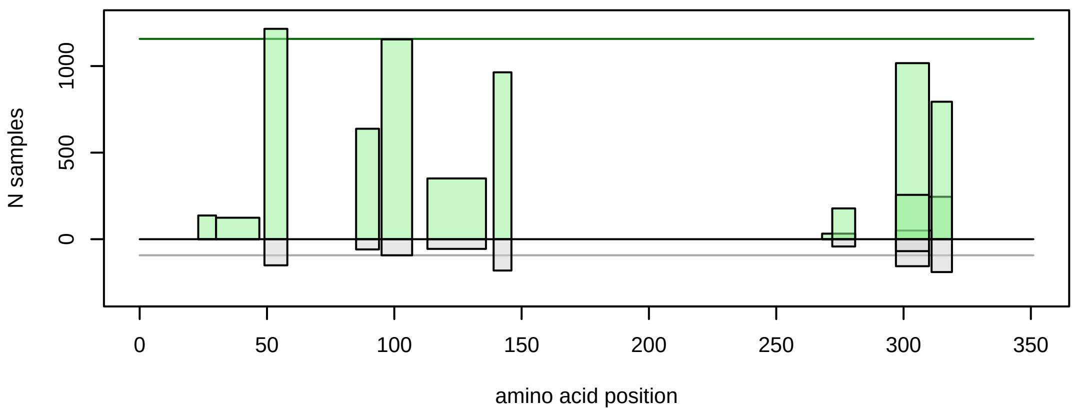

518. MARCKSL1 (P49006), NP4

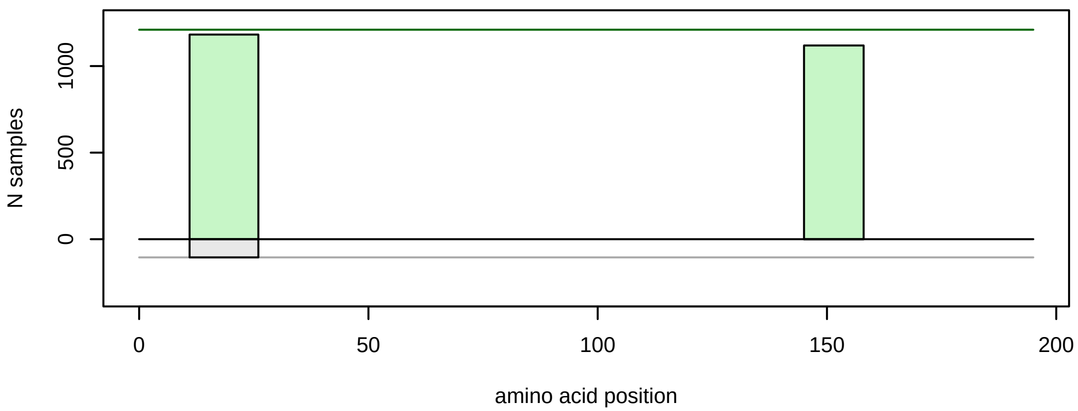

519. ENDOD1 (O94919), NP1

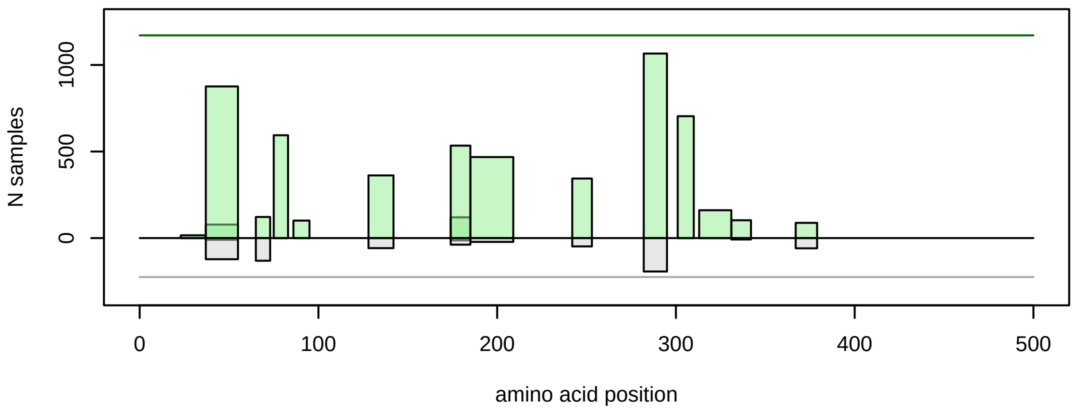

520. HGFAC (Q04756), NP3

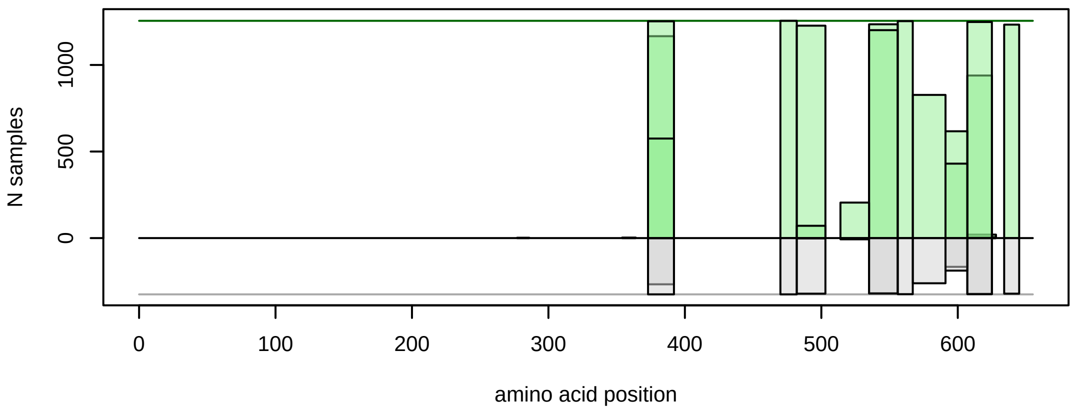

521. HBD (P02042), NP1

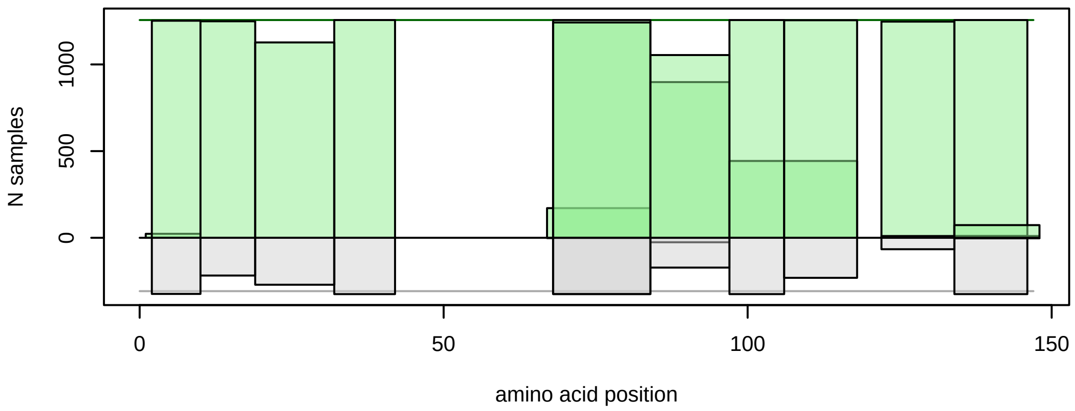

522. PI3 (P19957), NP3

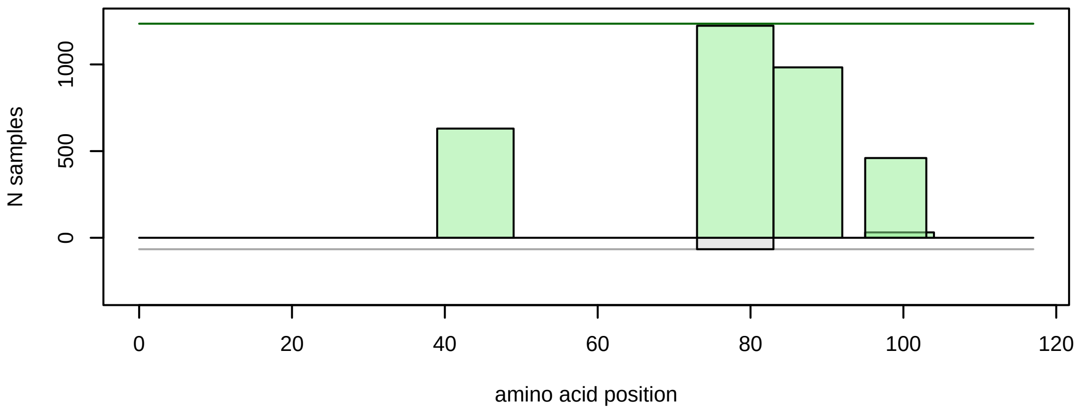

523. ARRB1 (P49407−2), NP3

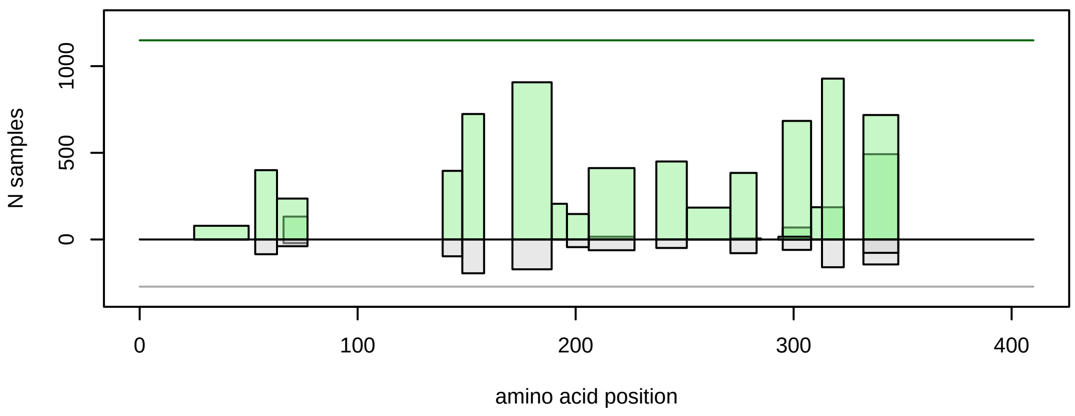

524. IGHV3−38 (A0A0C4DH36), NP3

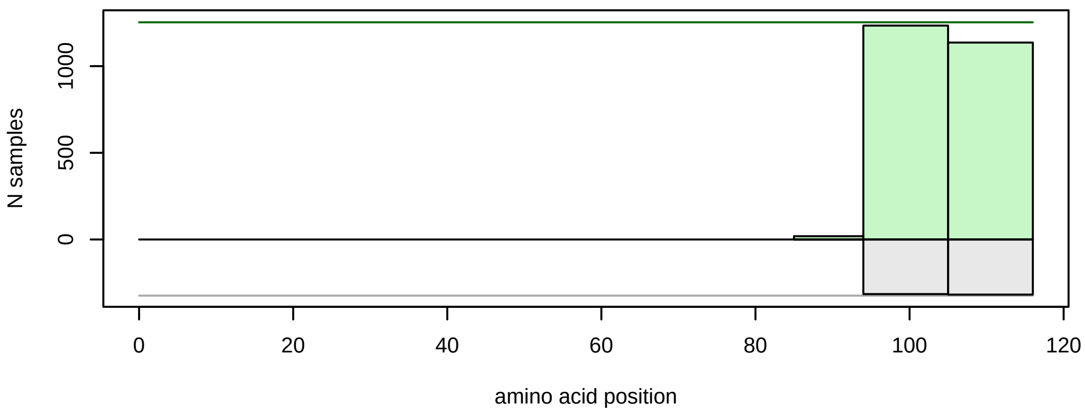

525. TAGLN2 (P37802;P37802−2), NP3

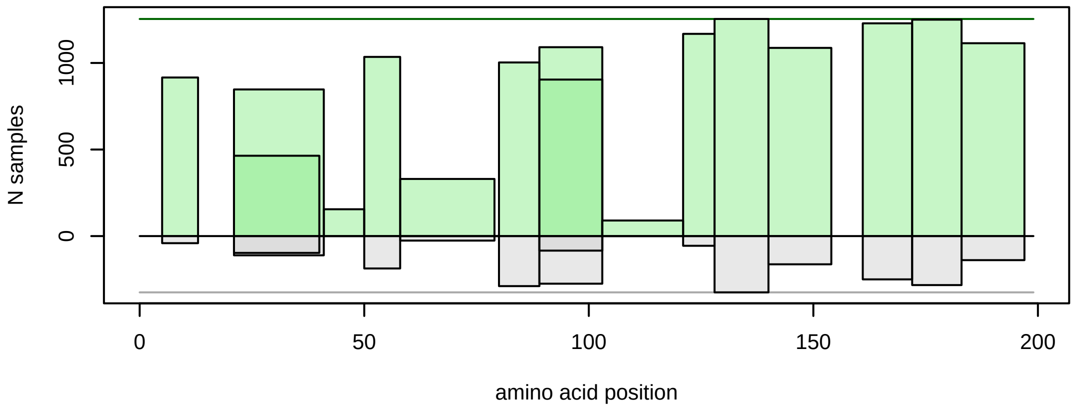

526. RPLP2 (P05387), NP2

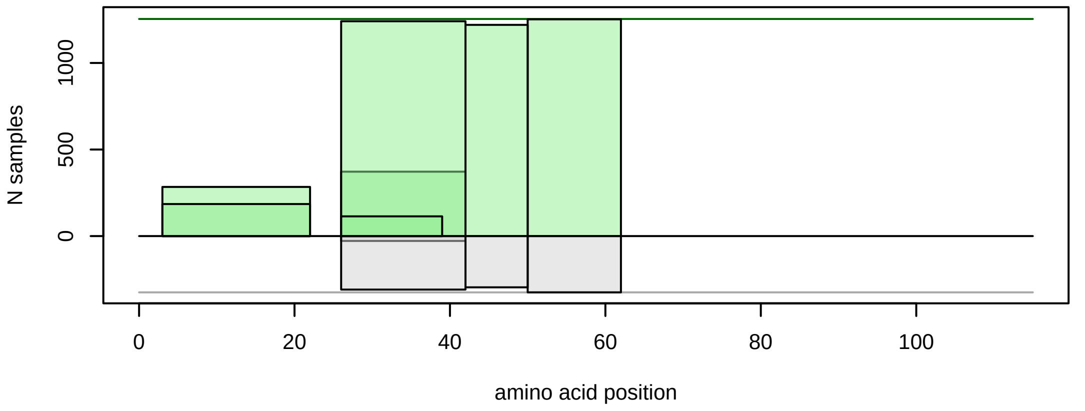

527. NEXN (Q0ZGT2), NP2

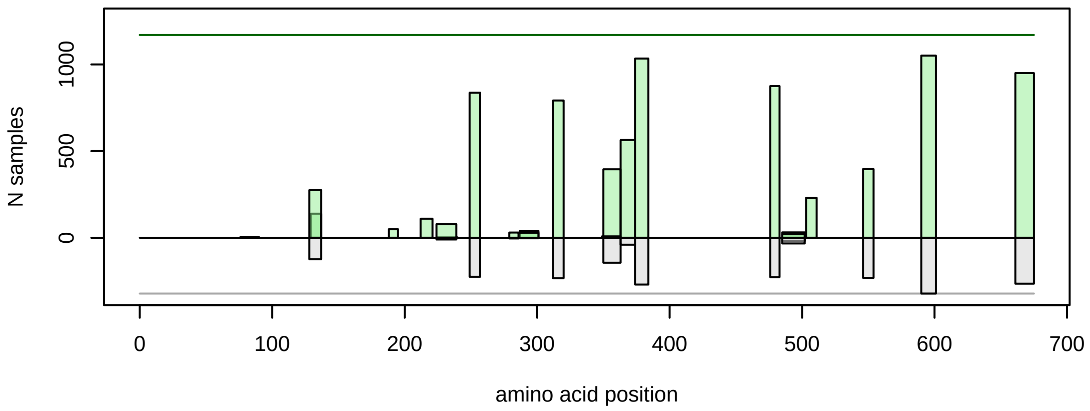

528. WIPF1 (O43516;O43516−3), NP4

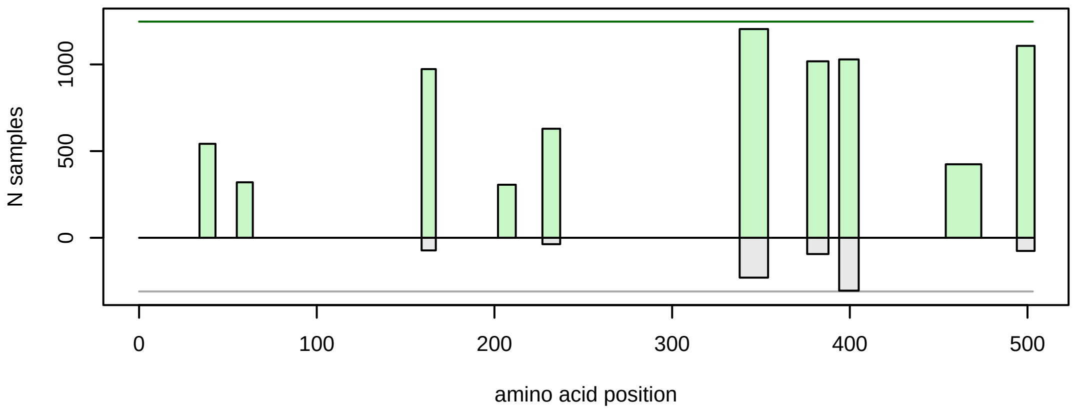

529. PTGDS (P41222), NP3

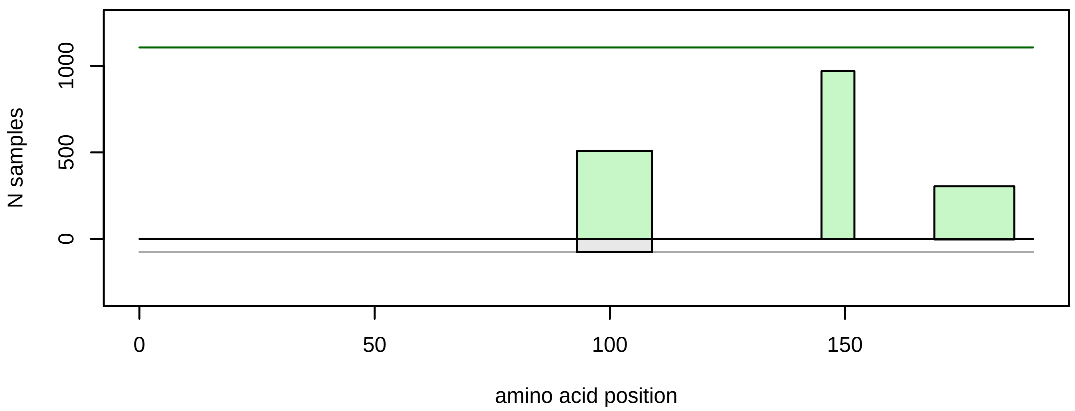

530. CAPN2 (P17655), NP5

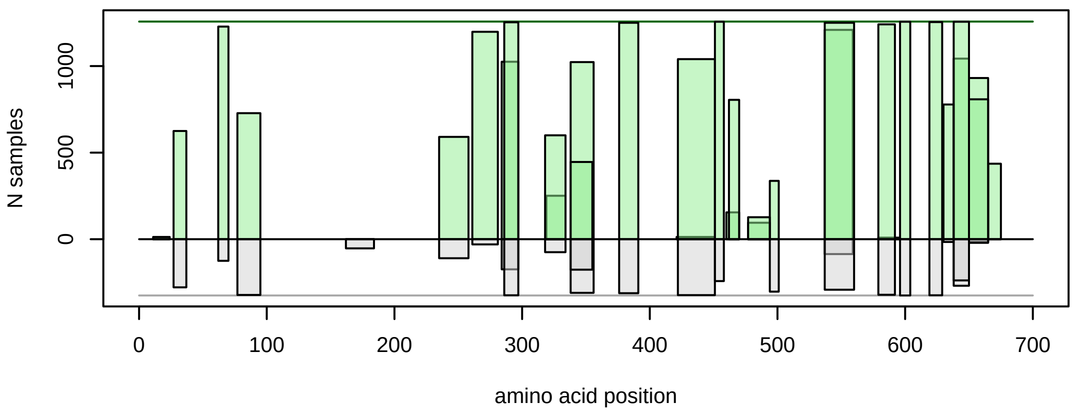

531. IGLL1 (P15814), NP3

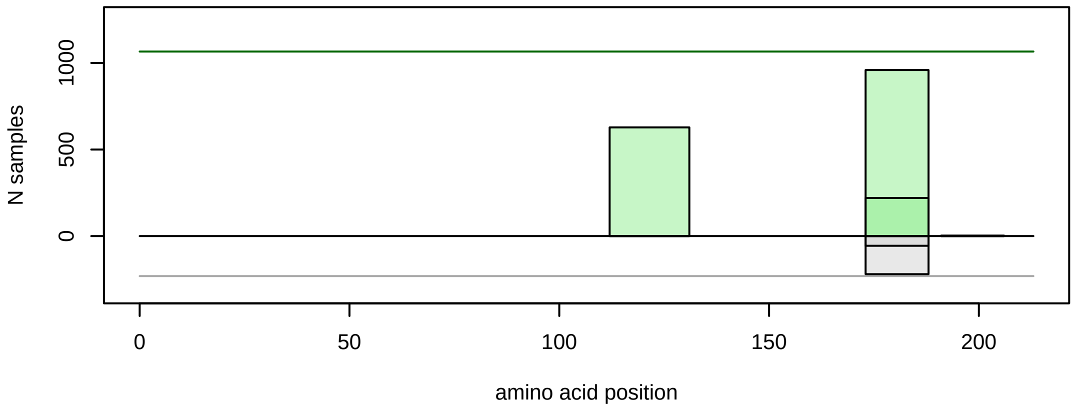

532. GKN1 (A0A8I5KHR1;Q9NS71), NP2

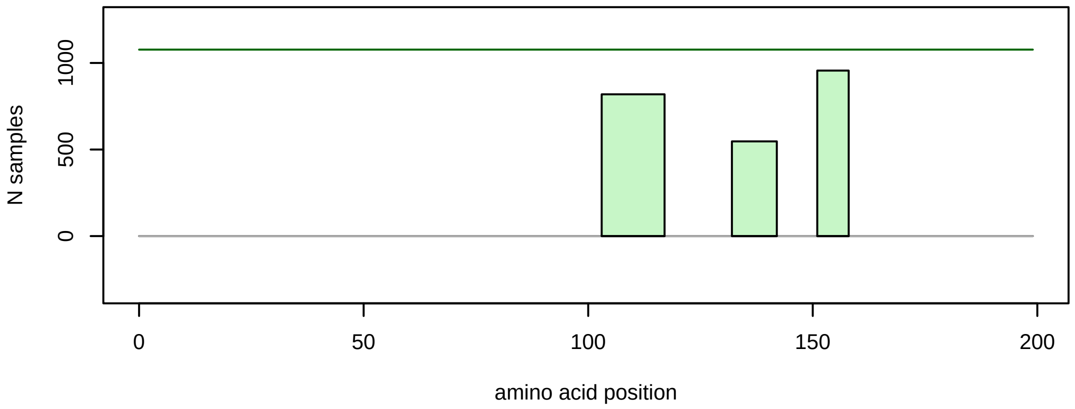

533. LMNB2 (Q03252), NP2

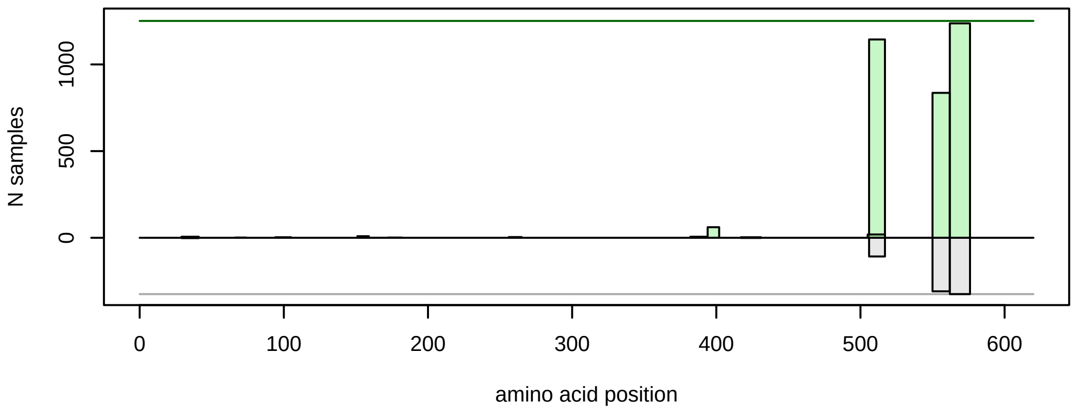

534. ATE1 (A0A8I5KZ24), NP4

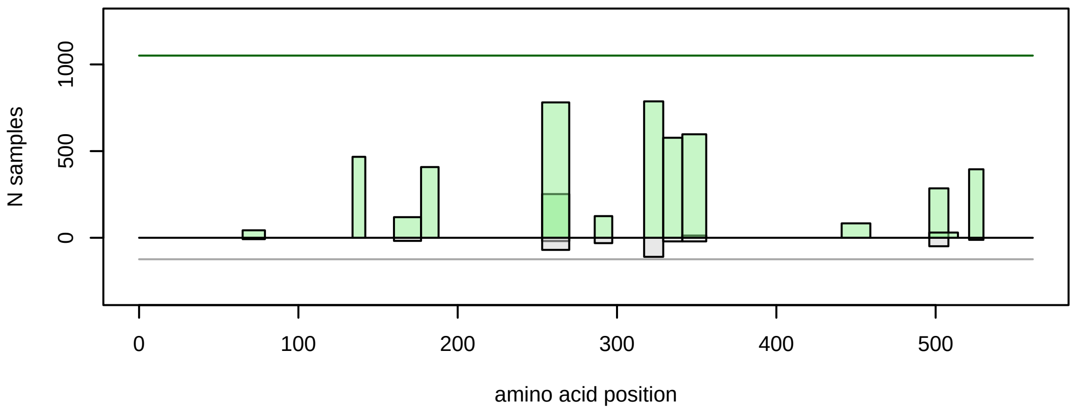

535. COL16A1 (Q07092), NP3

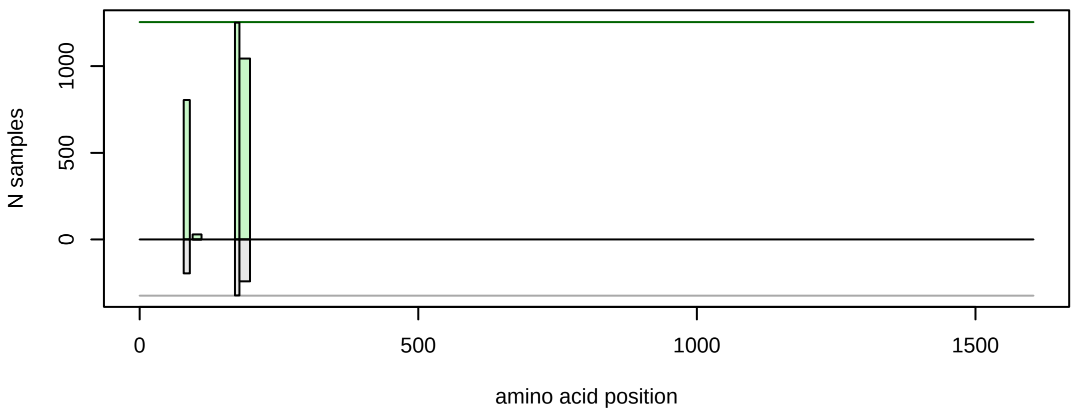

536. TOR2A (Q5JU69), NP5

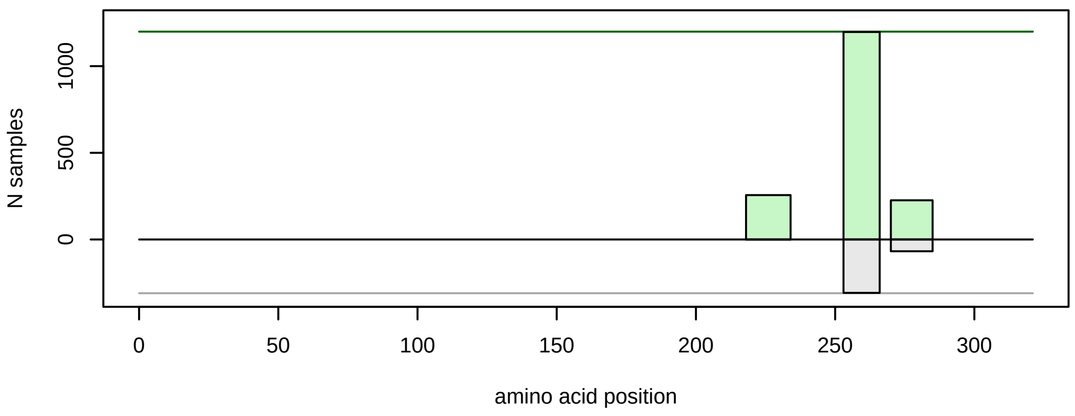

537. FASN (P49327), NP4

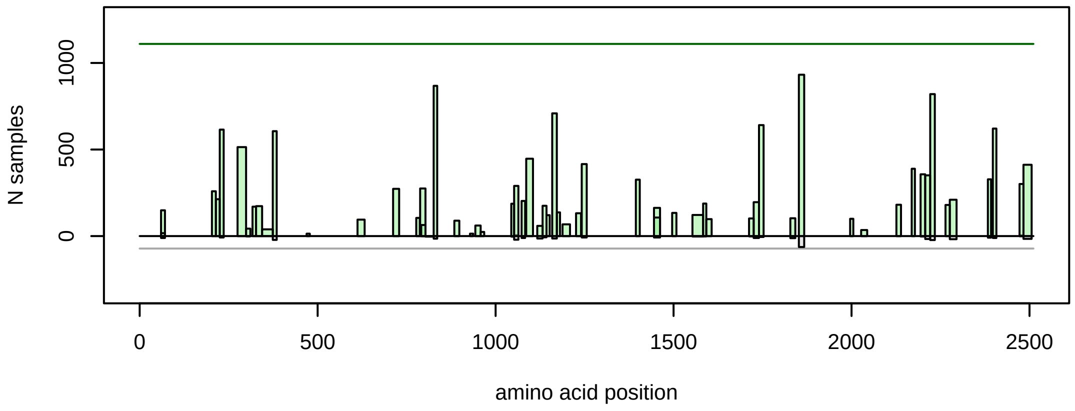

538. PDLIM7 (Q9NR12), NP3

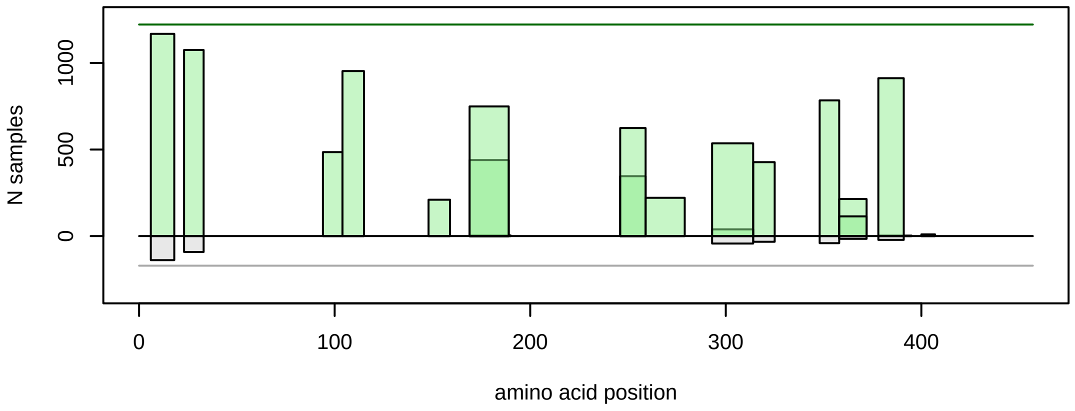

539. CRIP2 (P52943), NP1

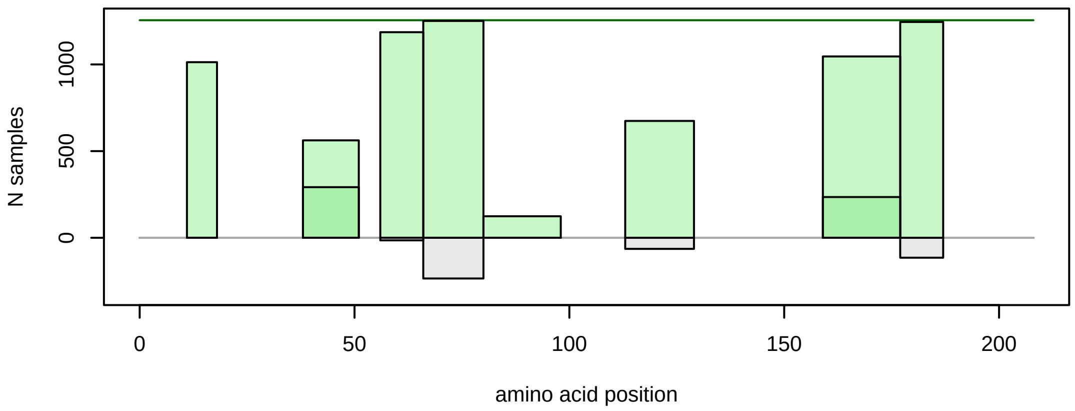

540. UXS1 (Q8NBZ7;Q8NBZ7−2), NP3

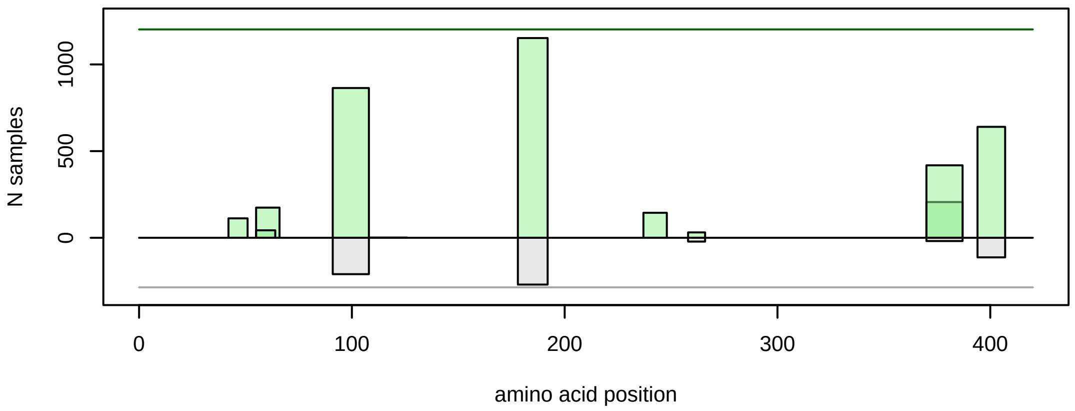

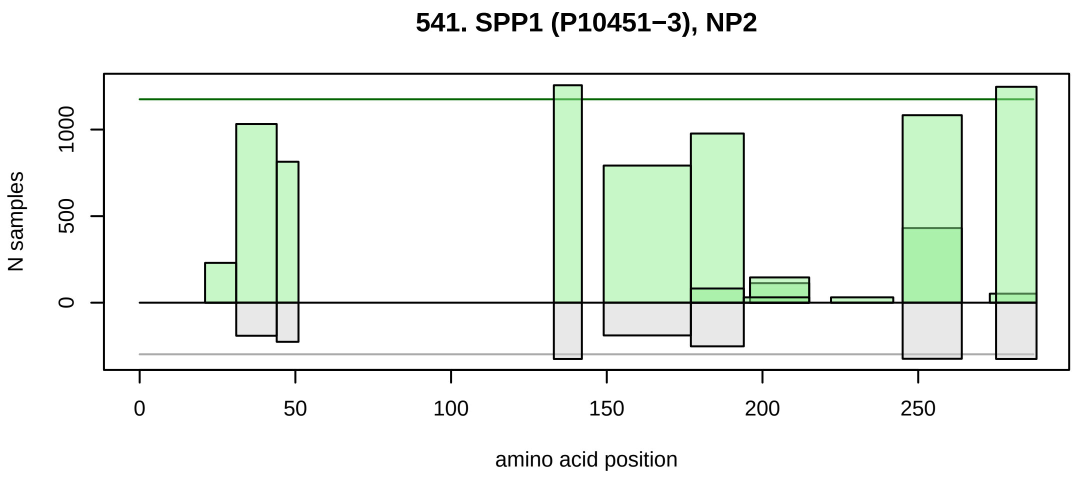

541. SPP1 (P10451−3), NP2
N samples
0
500
1000
0
50
100
150
200
250
amino acid position

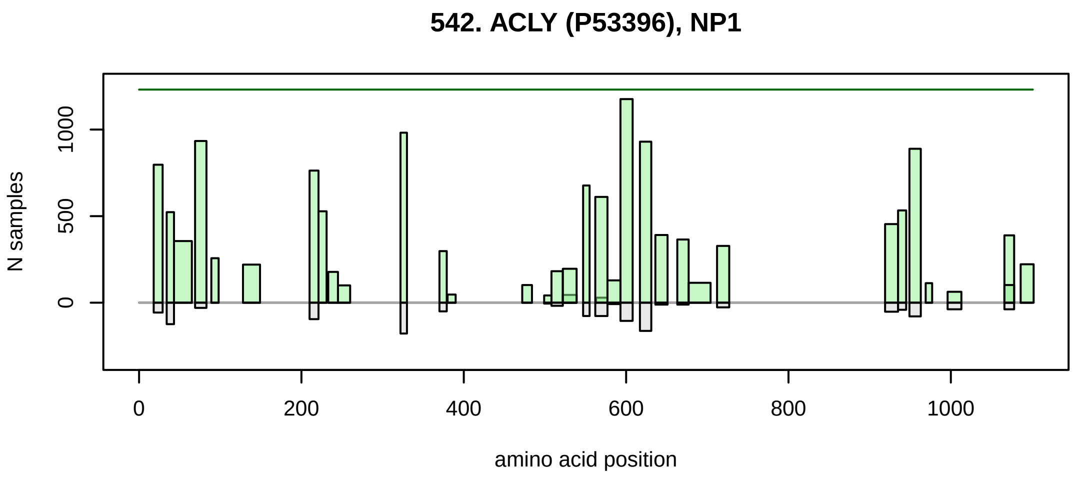

542. ACLY (P53396), NP1
N samples
0
500
1000
0
200
400
600
800
1000
amino acid position

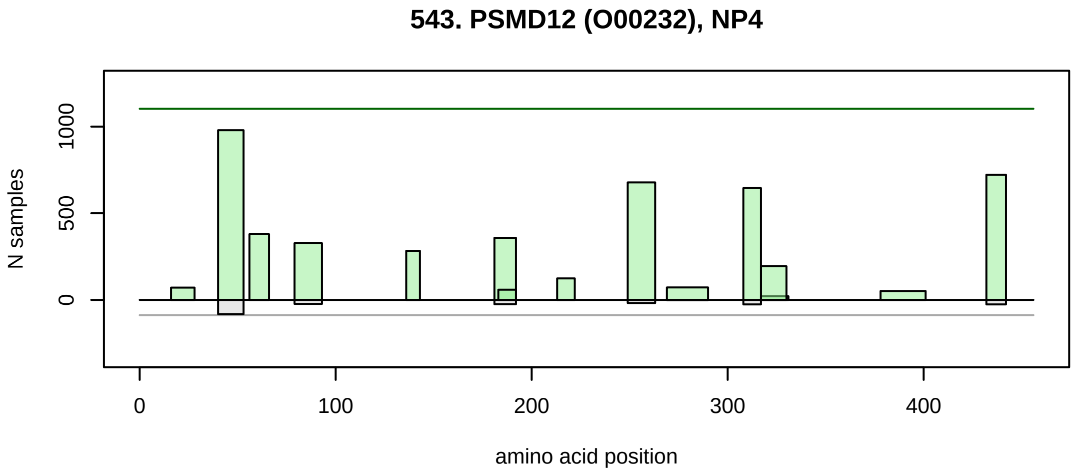

543. PSMD12 (O00232), NP4
N samples
0
500
1000
0
100
200
300
400
amino acid position

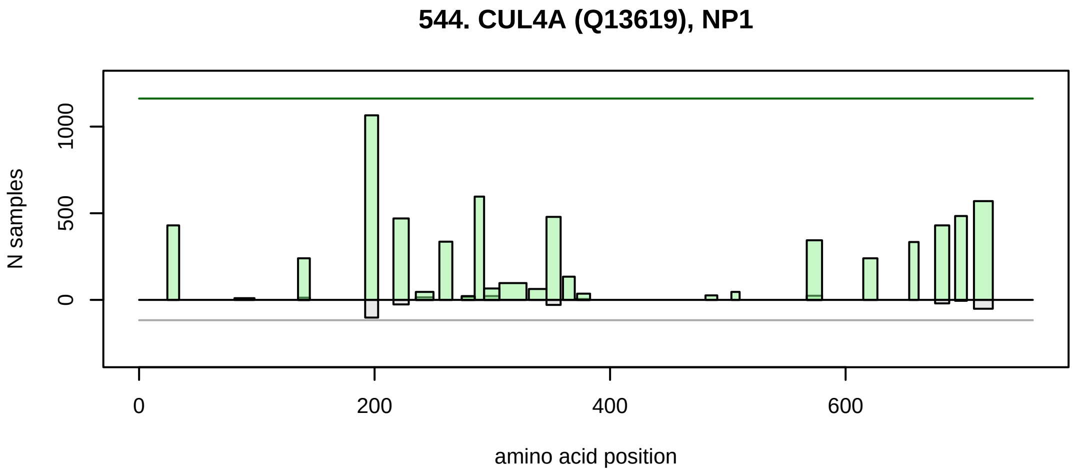

544. CUL4A (Q13619), NP1
N samples
0
500
1000
0
200
400
600
amino acid position

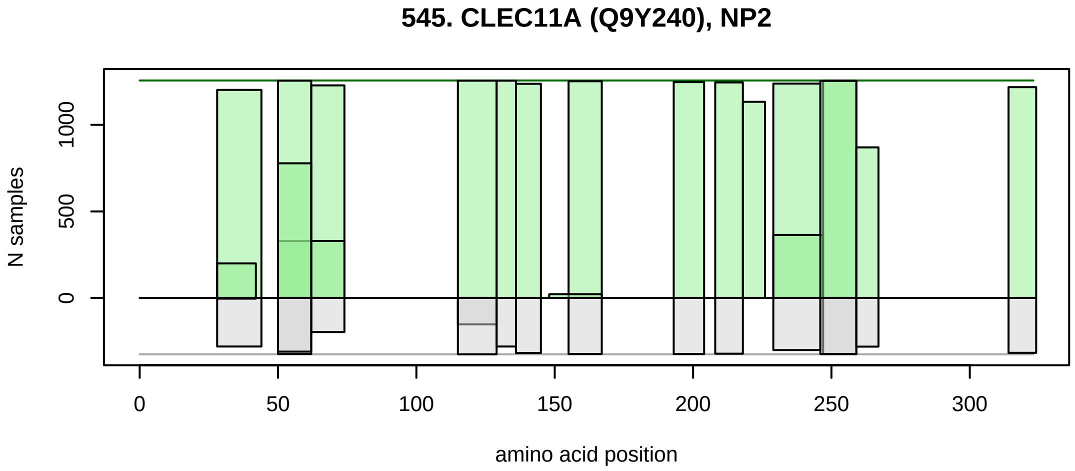

545. CLEC11A (Q9Y240), NP2
N samples
0
500
1000
0
50
100
150
200
250
300
amino acid position

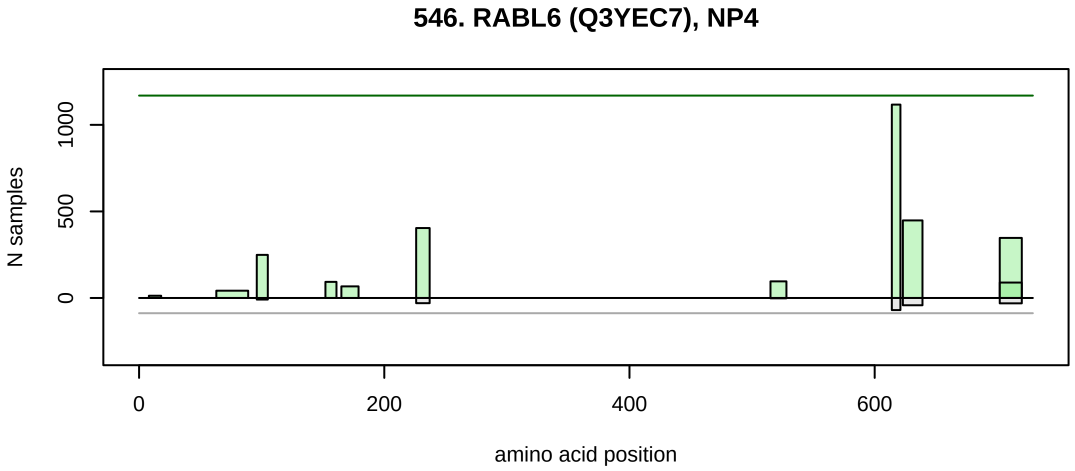

546. RABL6 (Q3YEC7), NP4
N samples
0
500
1000
0
200
400
600
amino acid position

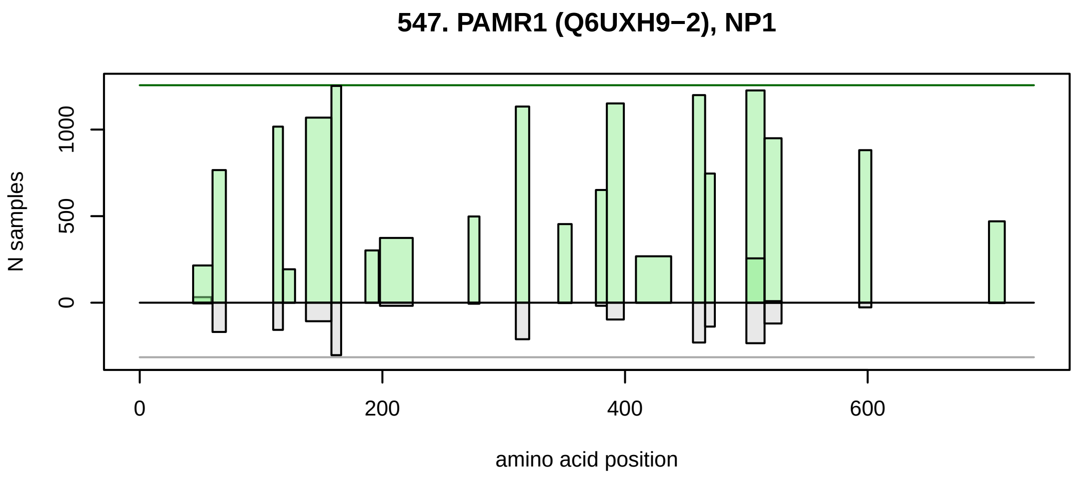
547. PAMR1 (Q6UXH9−2), NP1
N samples
0
500
1000
0
200
400
600
amino acid position

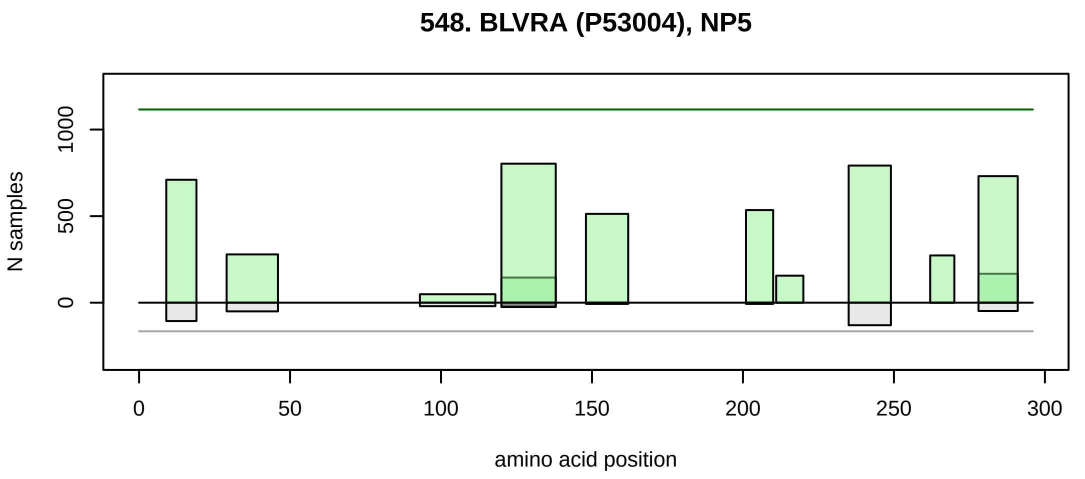
548. BLVRA (P53004), NP5
N samples
0
500
1000
0
50
100
150
200
250
300
amino acid position

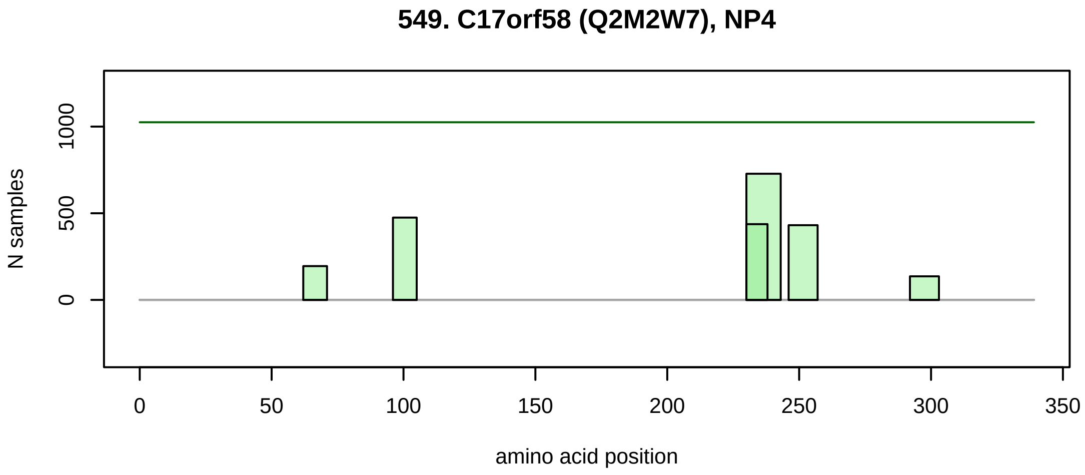
549. C17orf58 (Q2M2W7), NP4
N samples
0
500
1000
0
50
100
150
200
250
300
350
amino acid position

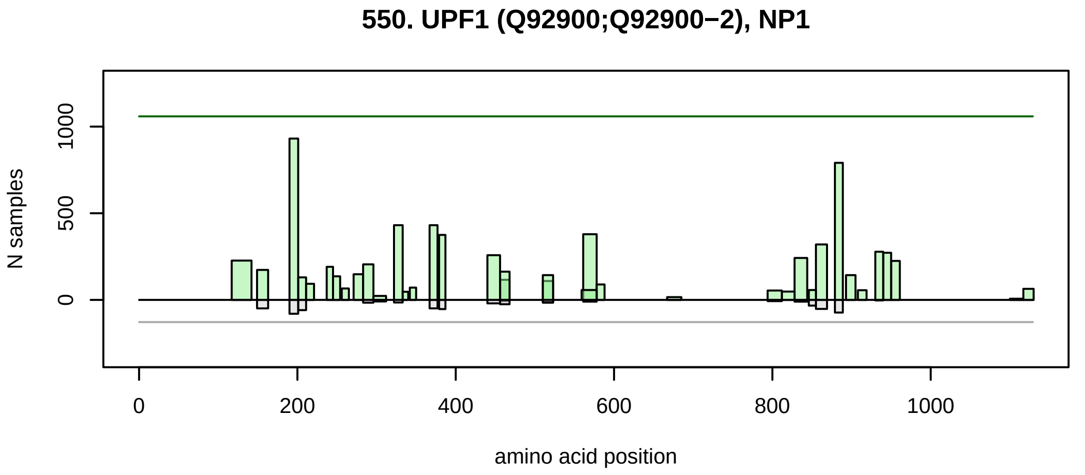
550. UPF1 (Q92900;Q92900−2), NP1
N samples
0
500
1000
0
200
400
600
800
1000
amino acid position

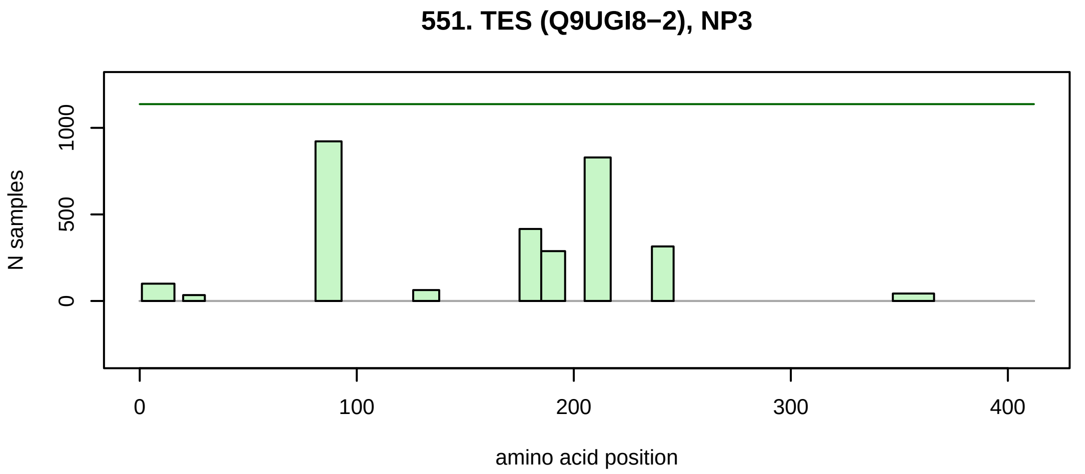
551. TES (Q9UGI8−2), NP3
N samples
0
500
1000
0
100
200
300
400
amino acid position

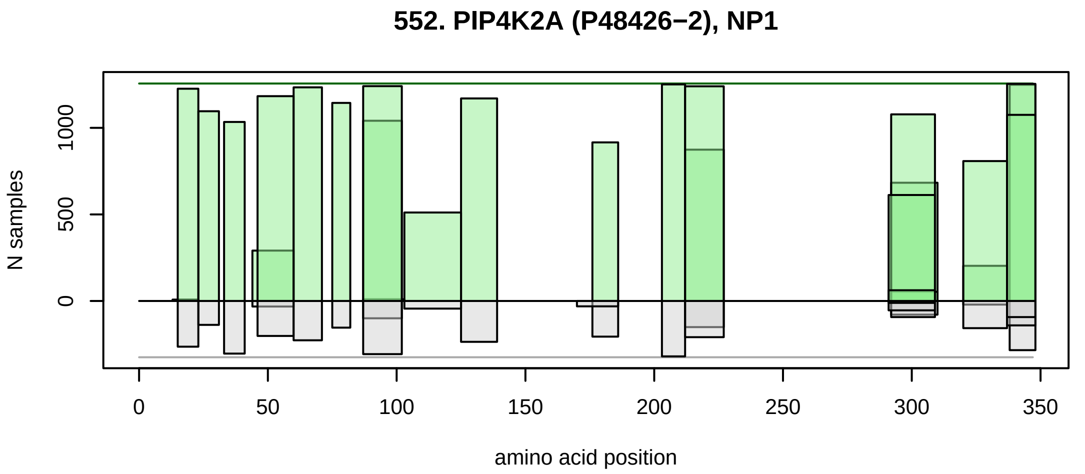
552. PIP4K2A (P48426−2), NP1
N samples
0
500
1000
0
50
100
150
200
250
300
350
amino acid position

553. CCDC80 (Q76M96), NP3

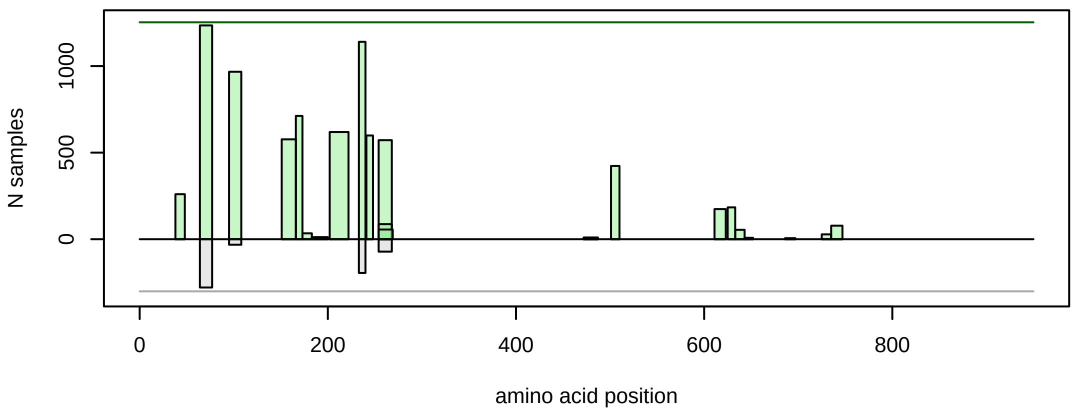

554. FZD8 (Q9H461), NP2

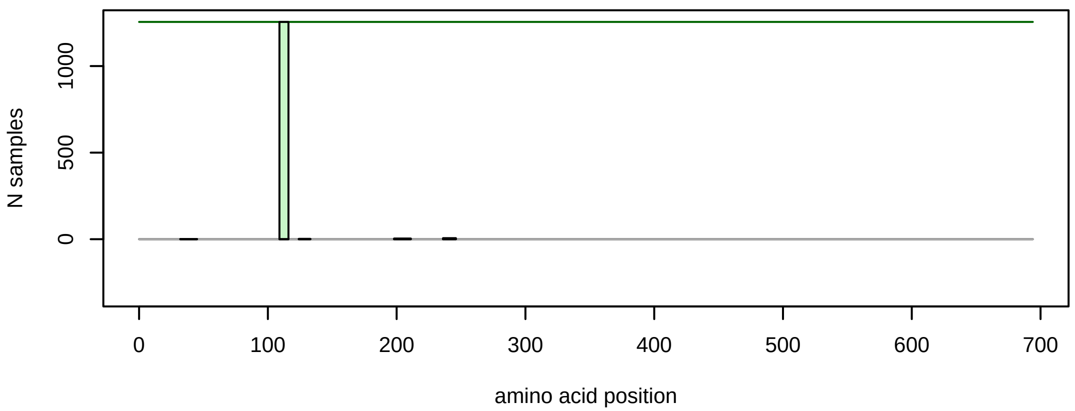

555. DNAJC3 (Q13217), NP3

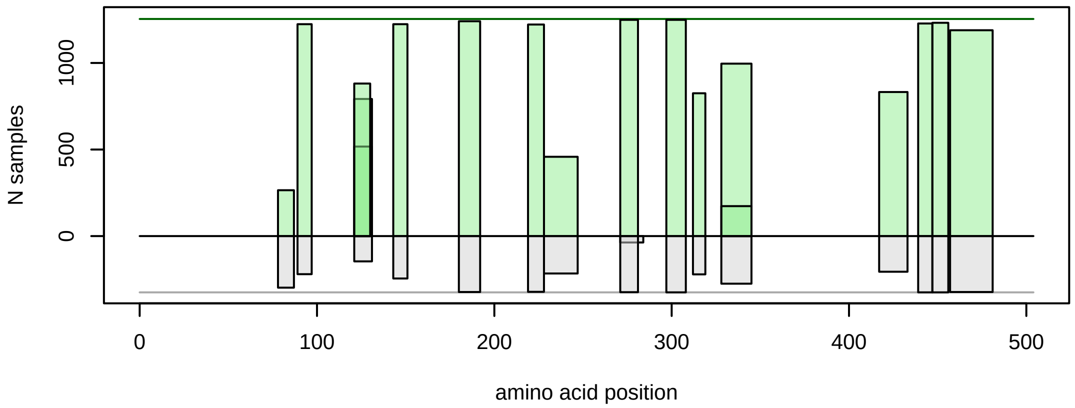

556. DARS (P14868), NP5

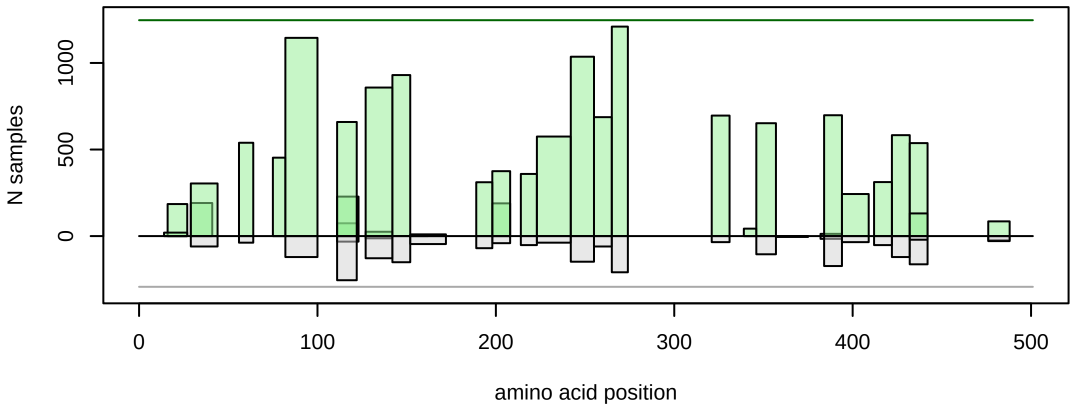

557. RPS16 (P62249), NP4

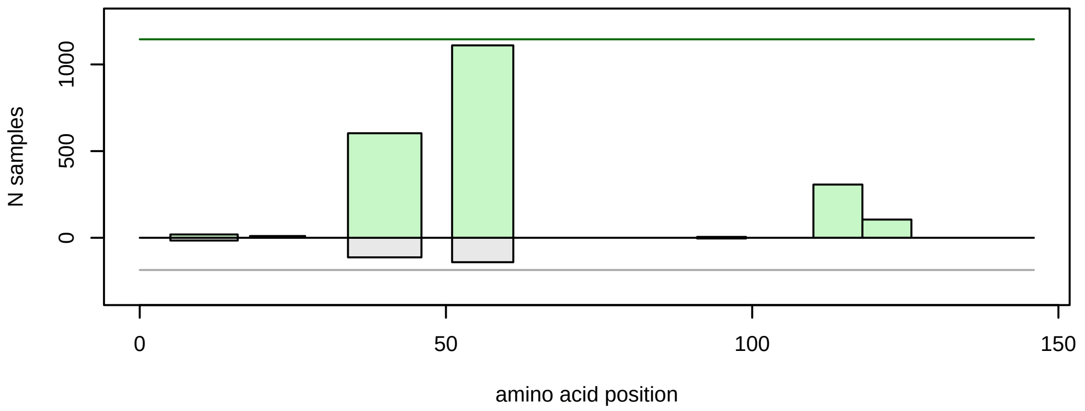

558. NA (CONP00761), NP2

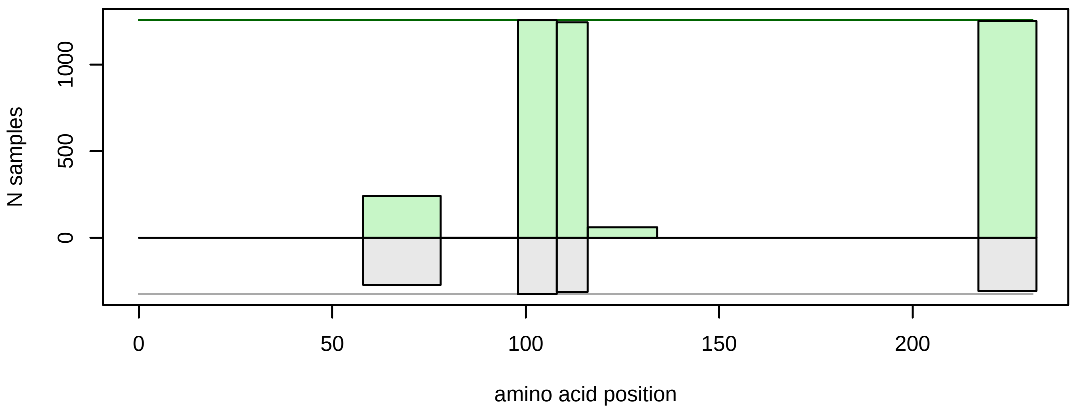

559. GNB1 (P62873), NP1

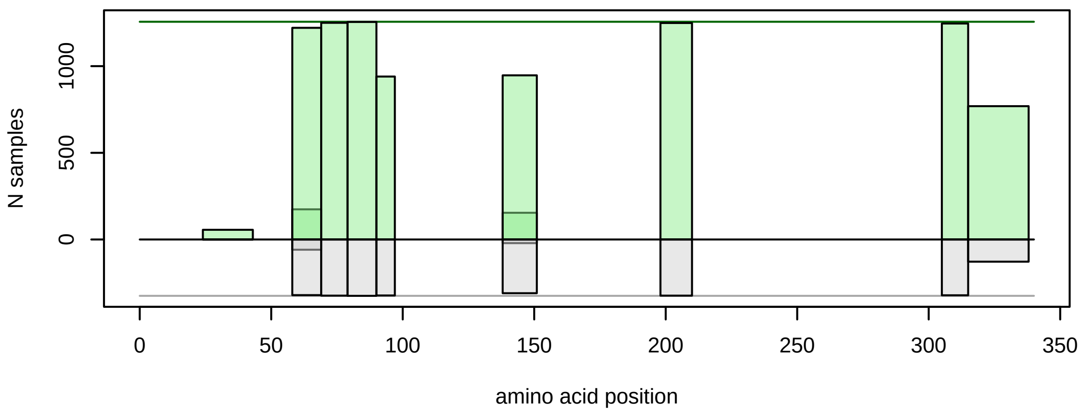

560. DAK (Q3LXA3), NP5

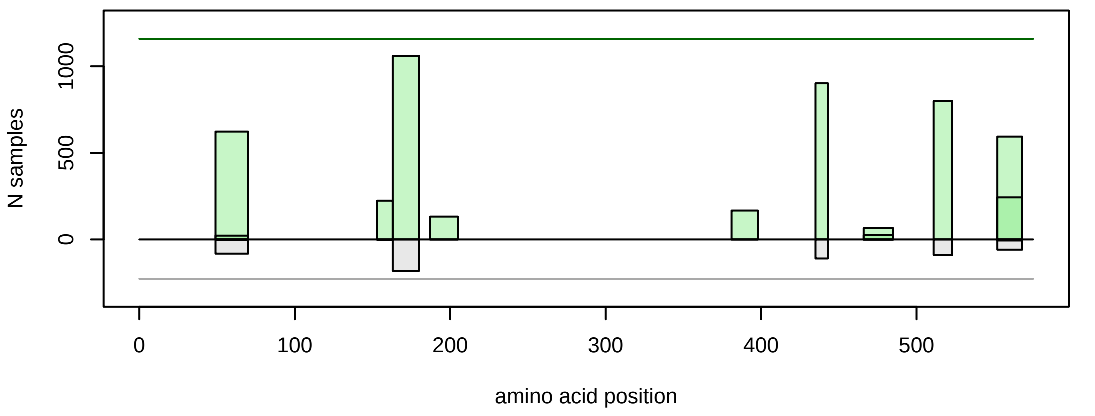

561. PEBP1 (P30086), NP3

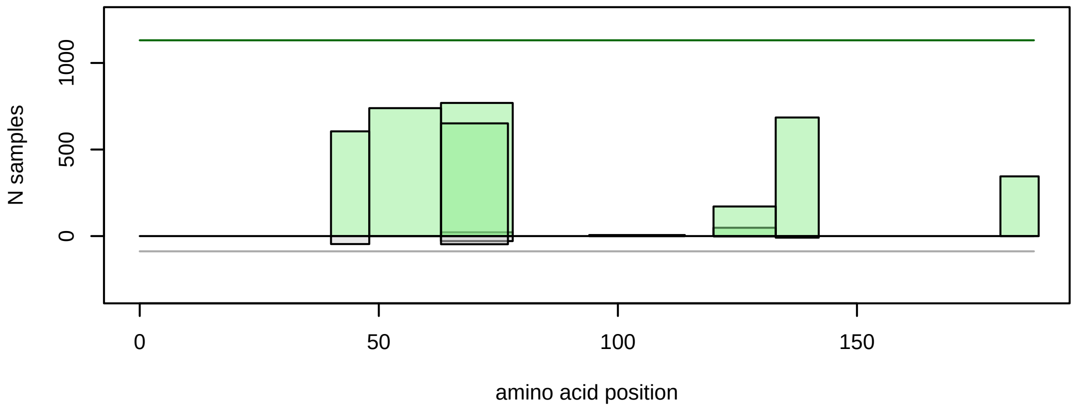

562. GSK3A (P49840), NP3

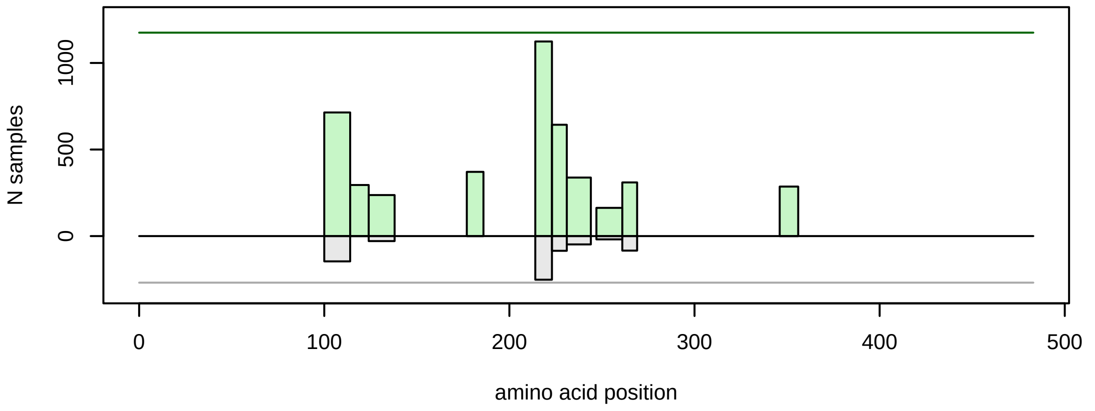

563. RDX (P35241), NP4

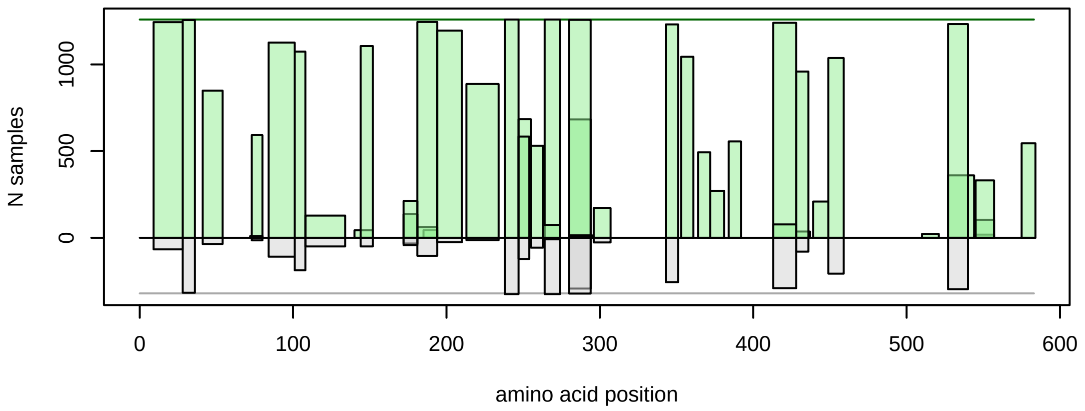

564. PPM1A (E9PP44), NP4

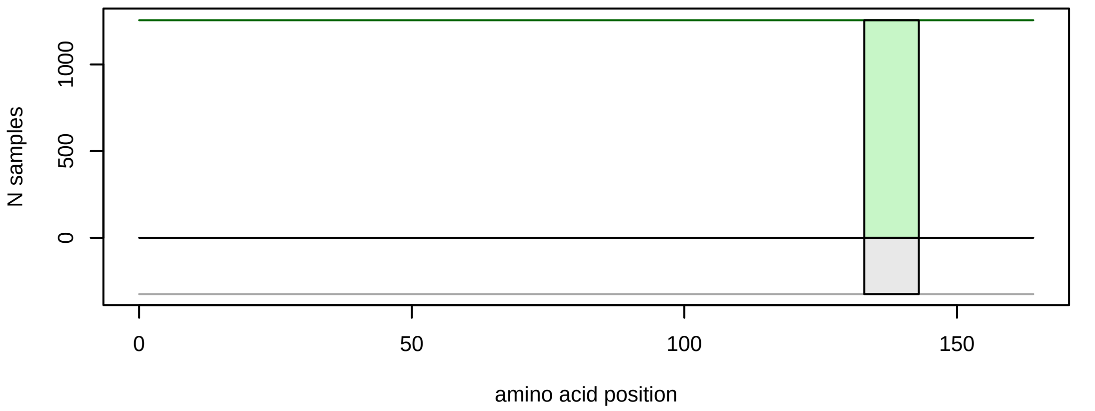

565. PDCD6 (O75340), NP2

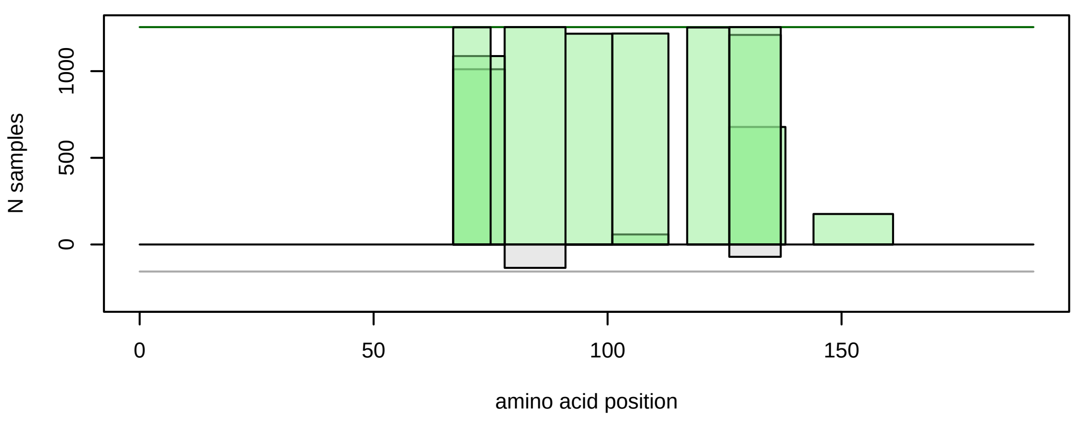

566. GNAI3 (P08754), NP1

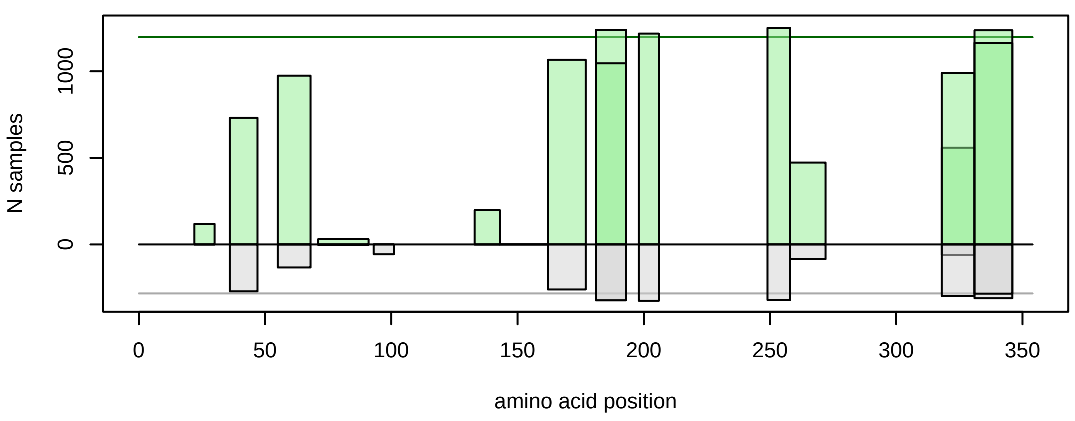

567. IGHV1−2 (P23083), NP3

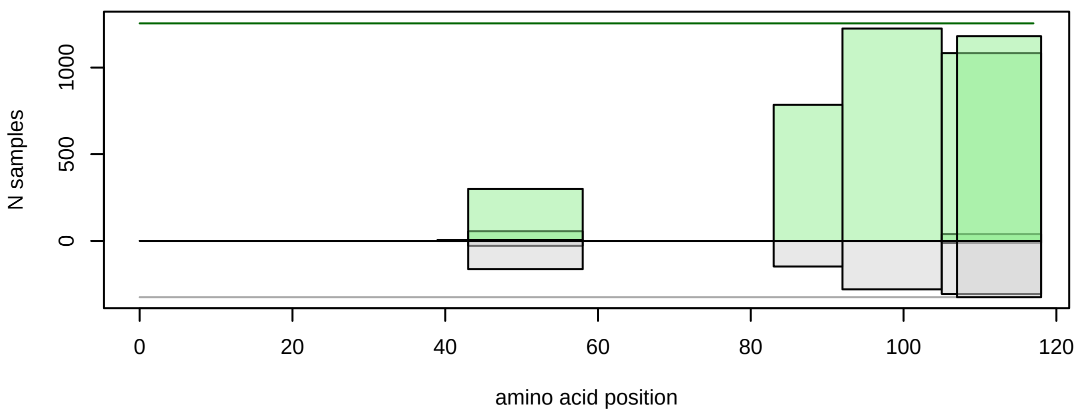

568. RMDN3 (Q96TC7), NP2

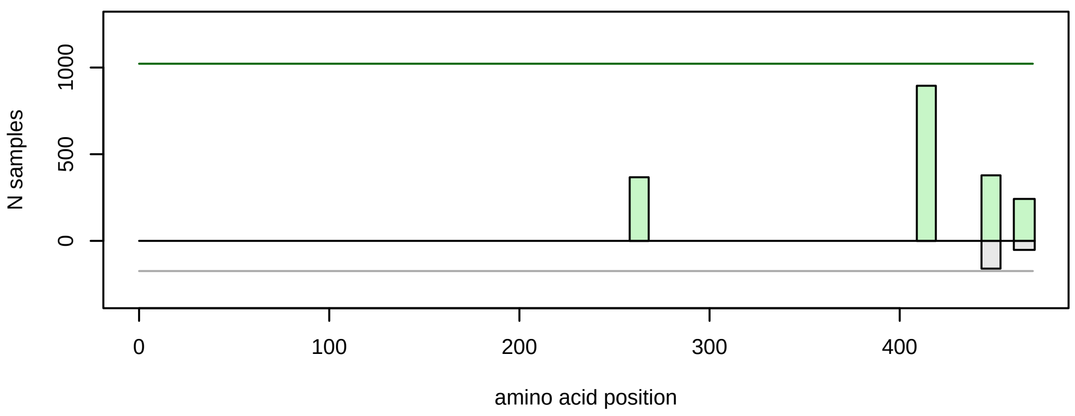

569. CORO1C (Q9ULV4−3), NP1

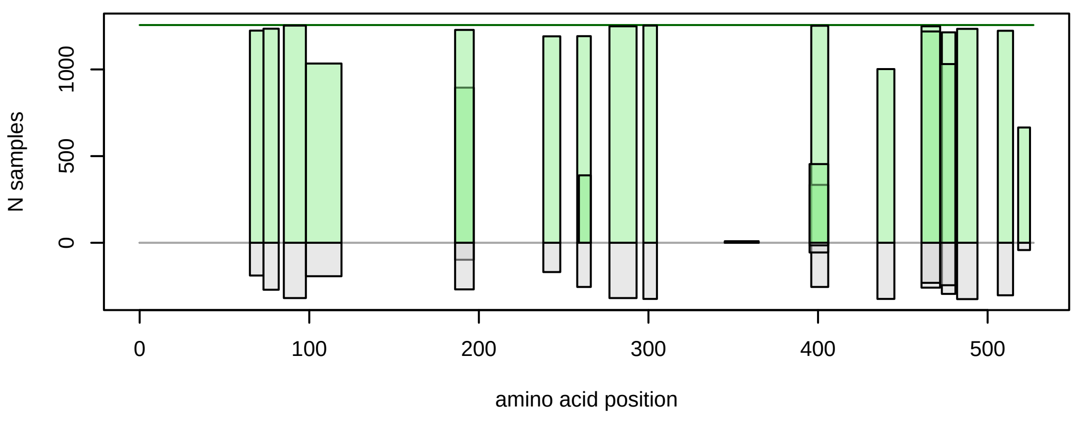

570. ASGR2 (P07307;P07307−2;P07307−3;Q7Z4G9), NP2

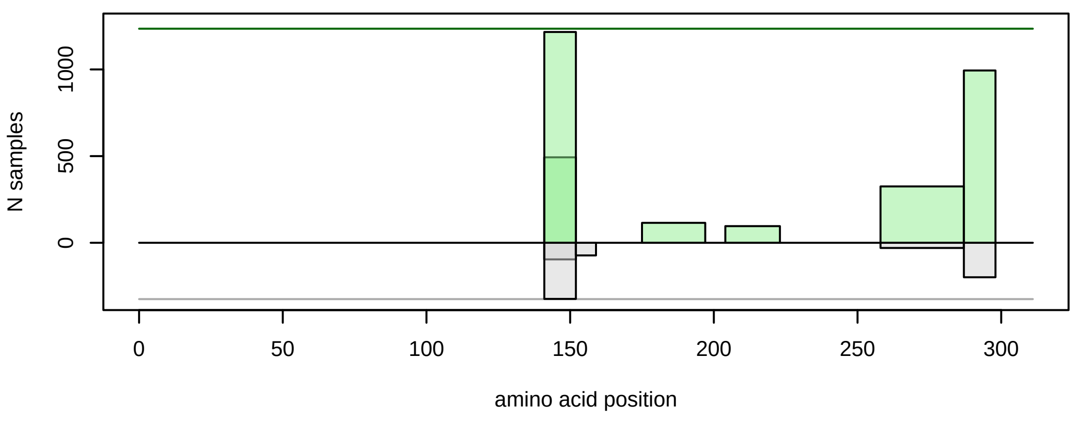

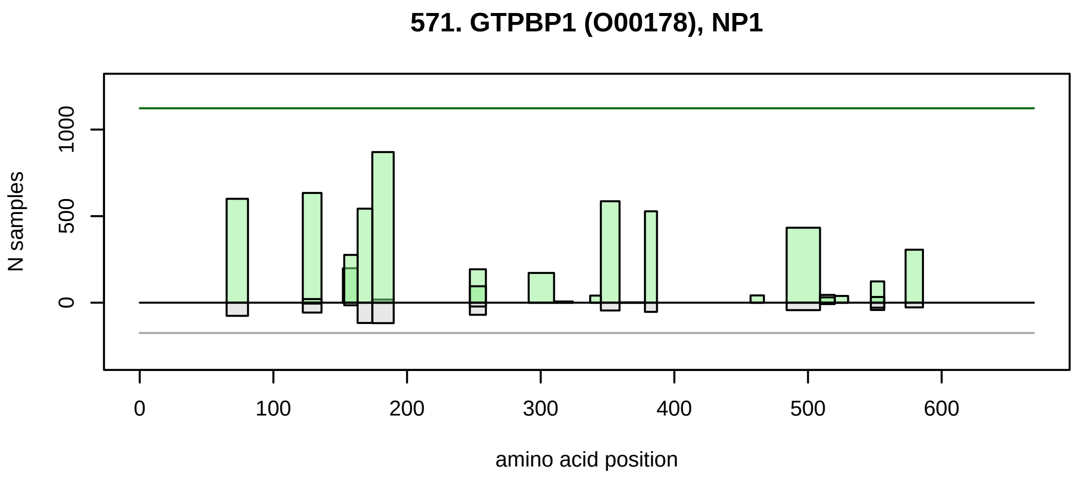

571. GTPBP1 (O00178), NP1
N samples
0
500
1000
0
100
200
300
400
500
600
amino acid position

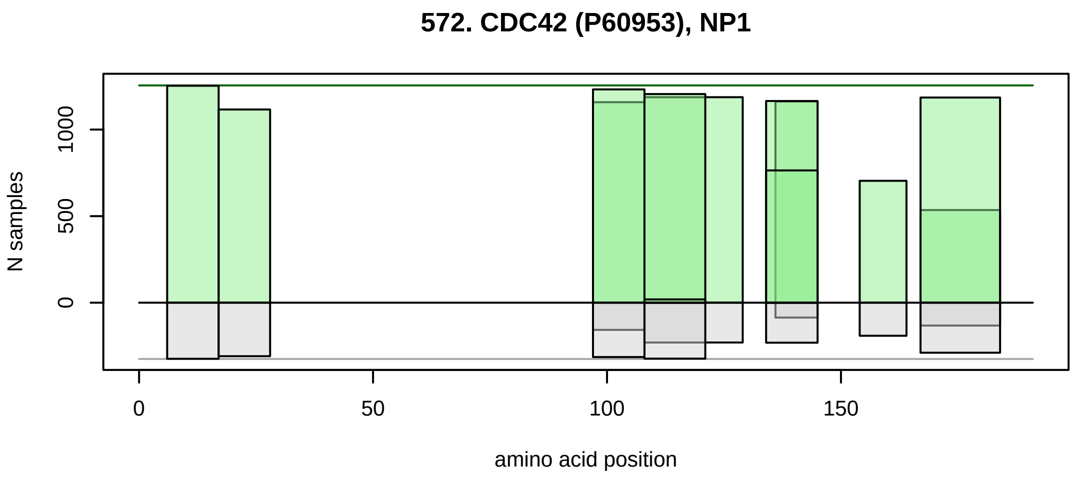

572. CDC42 (P60953), NP1
N samples
0
500
1000
0
50
100
150
amino acid position

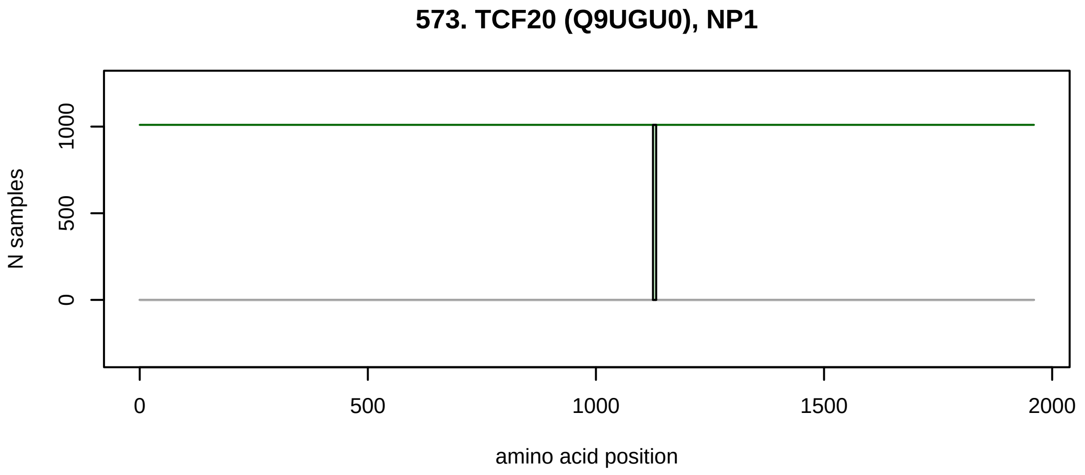

573. TCF20 (Q9UGU0), NP1
N samples
0
500
1000
0
500
1000
1500
2000
amino acid position

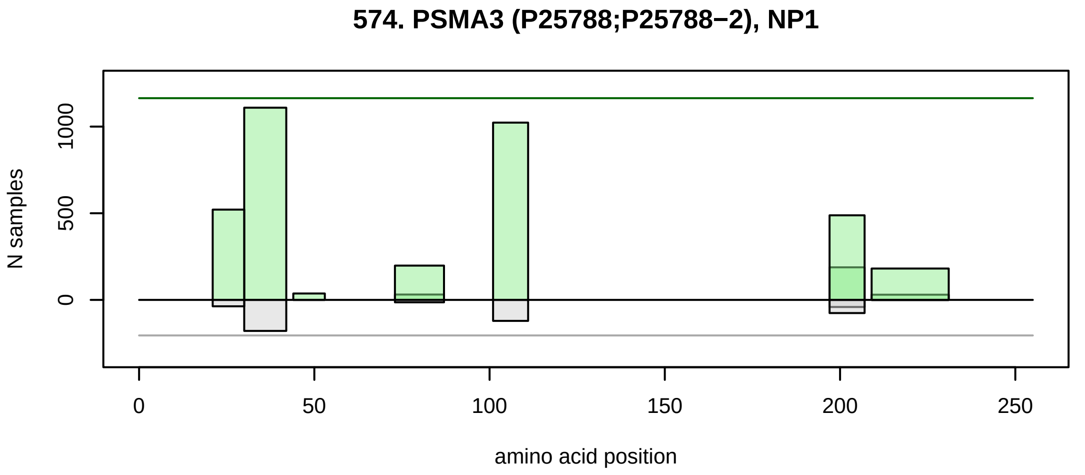

574. PSMA3 (P25788;P25788−2), NP1
N samples
0
500
1000
0
50
100
150
200
250
amino acid position

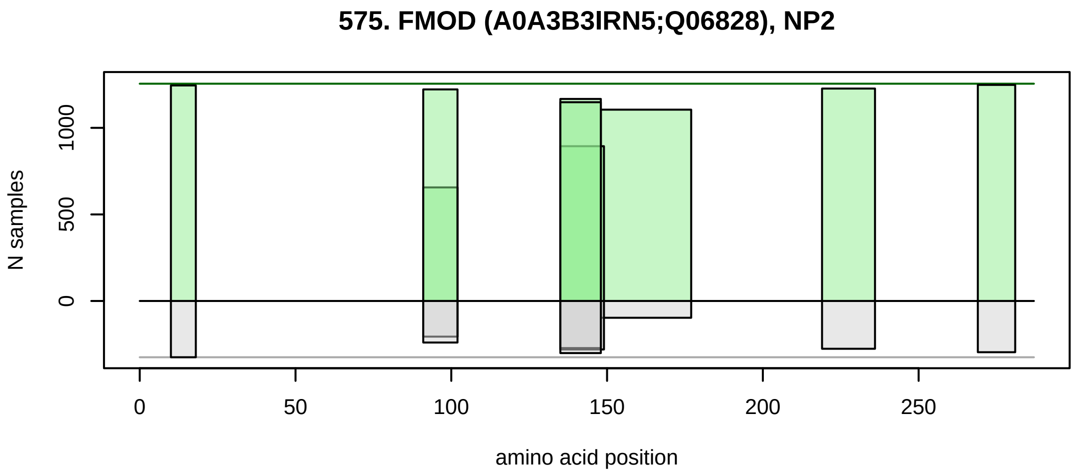

575. FMOD (A0A3B3IRN5;Q06828), NP2
N samples
0
500
1000
0
50
100
150
200
250
amino acid position

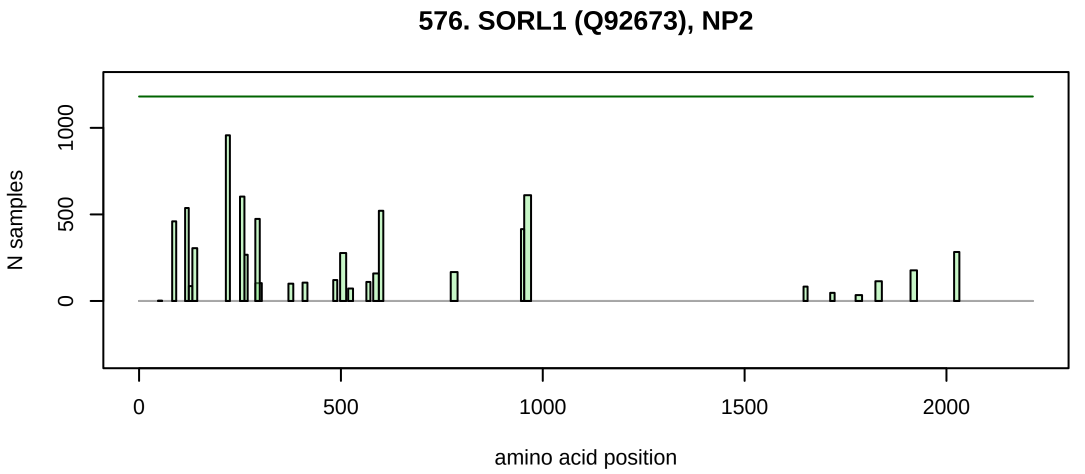

576. SORL1 (Q92673), NP2
N samples
0
500
1000
0
500
1000
1500
2000
amino acid position

577. RPS12 (P25398), NP3

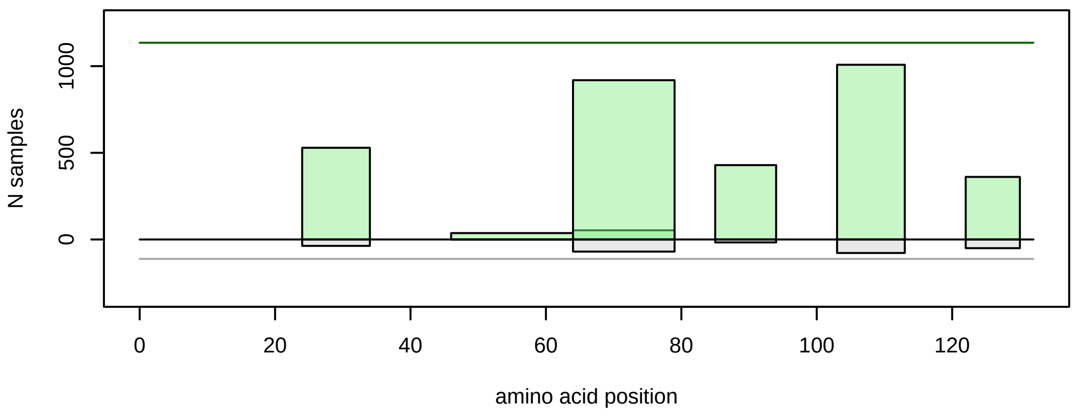

578. AQR (O60306), NP2

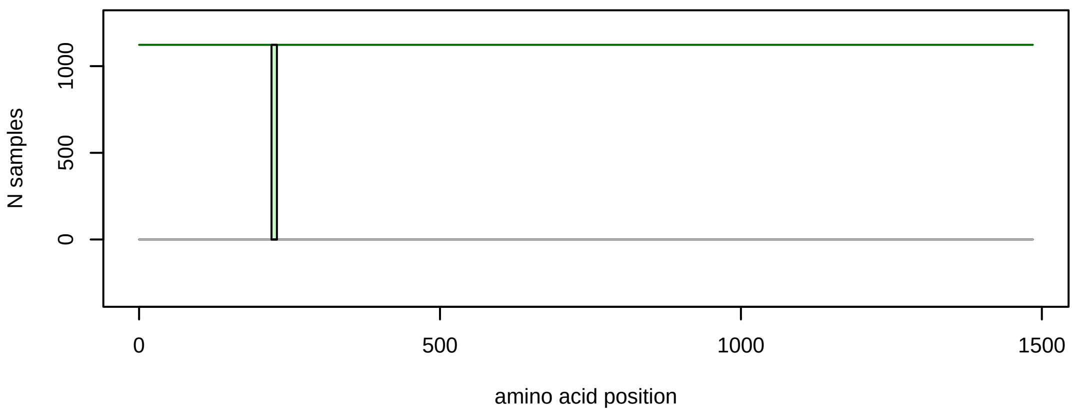

579. CCL21 (O00585;Q5VZ73), NP4

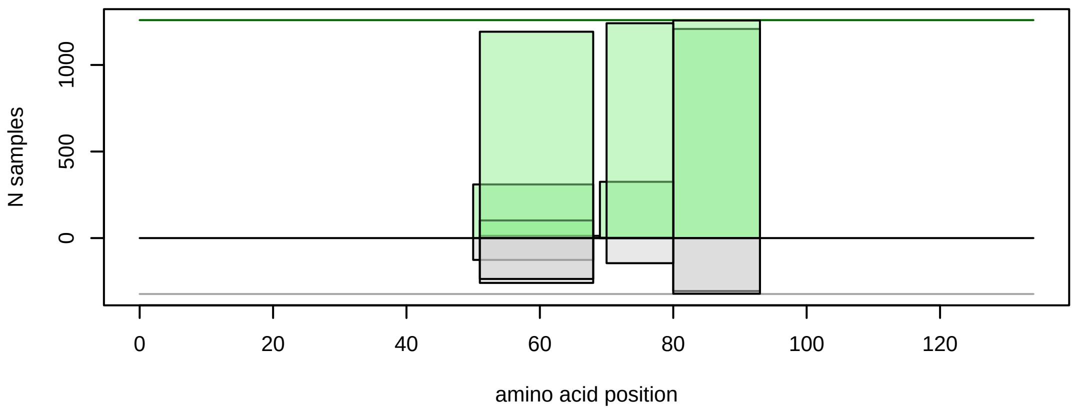

580. RPS13 (P62277), NP4

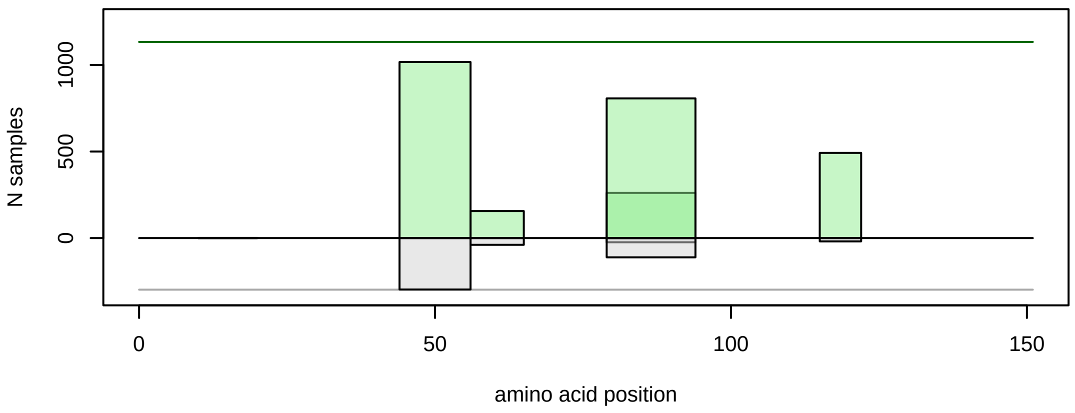

581. CUTA (O60888−3), NP2

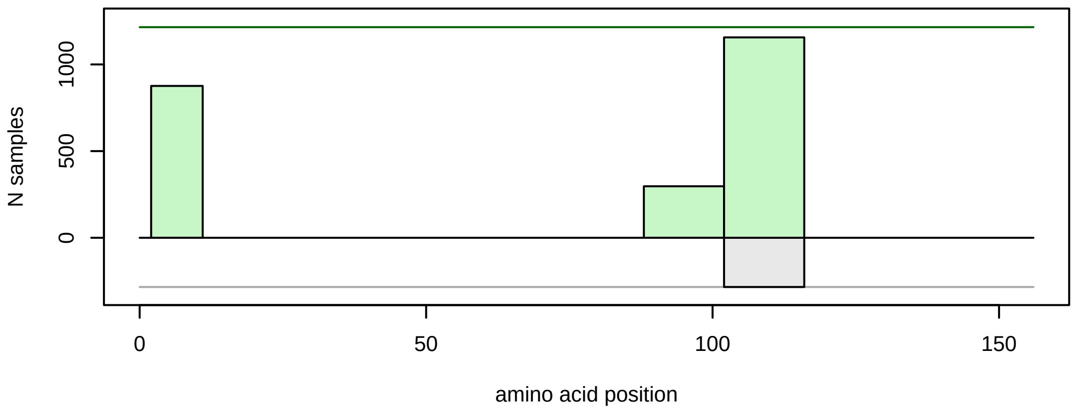

582. IGLV3−25 (P01717), NP5

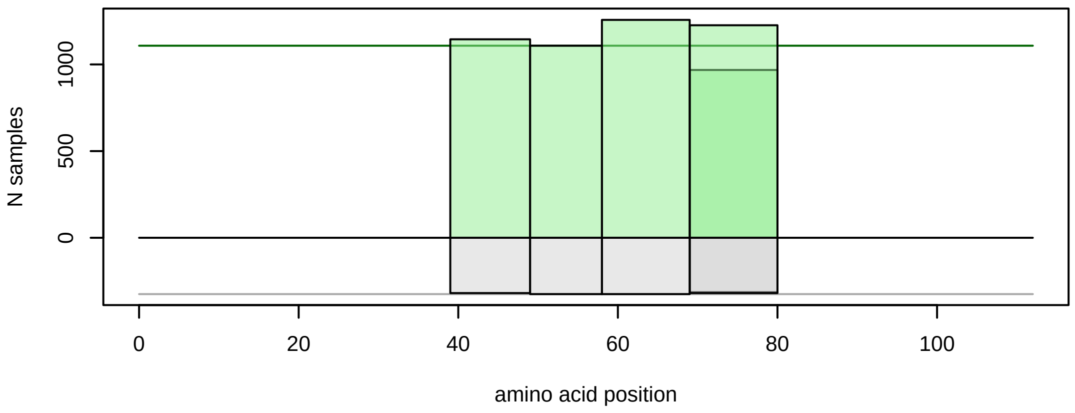

583. LGI4 (Q8N135), NP4

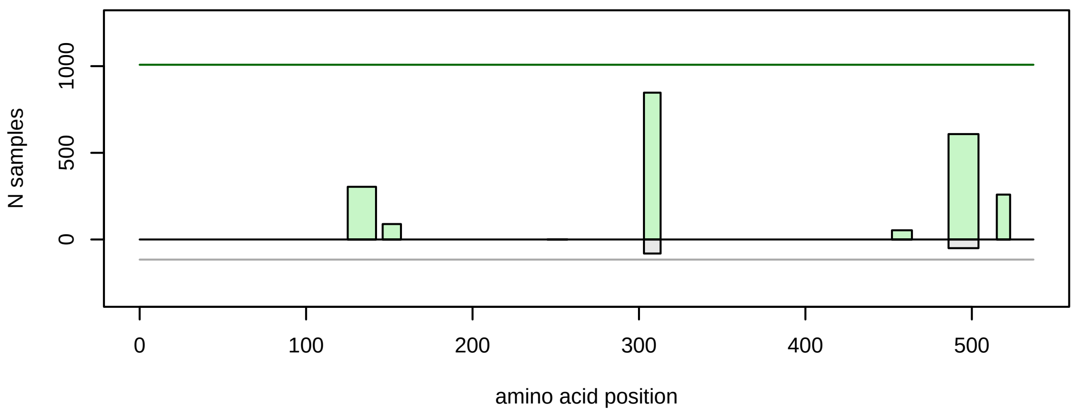

584. GPD2 (P43304), NP4

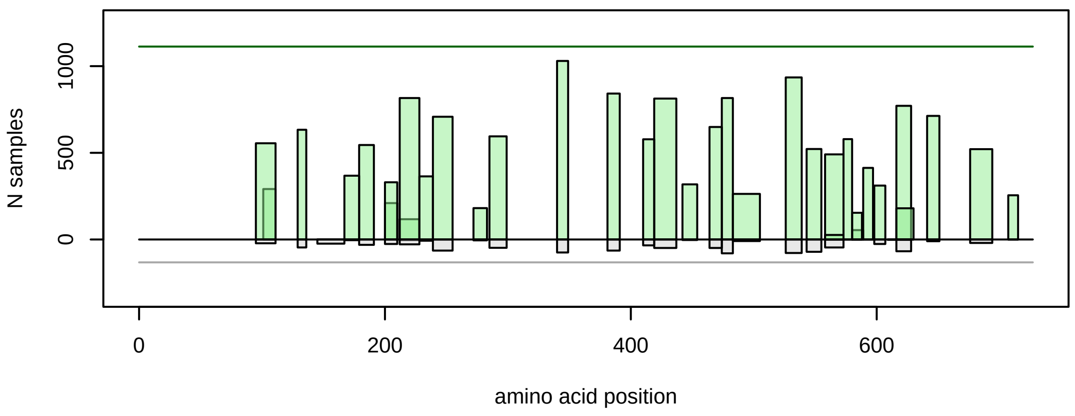

585. ITGA6 (P23229−2), NP1

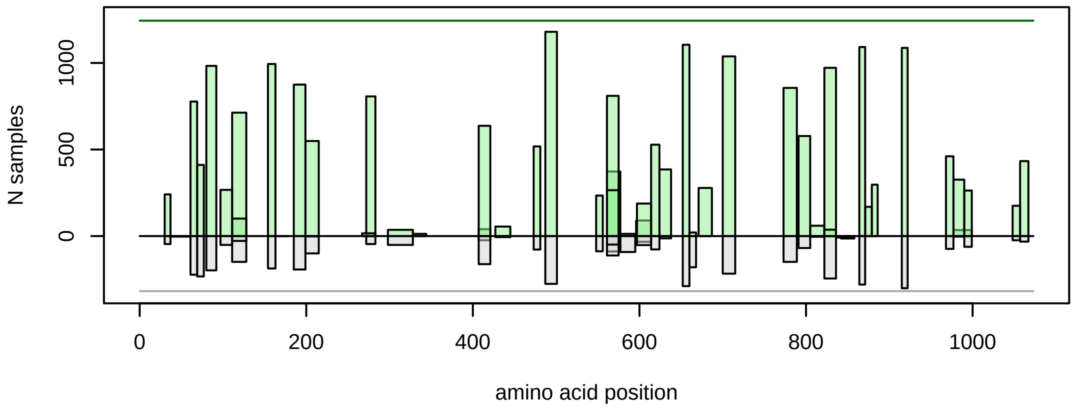

586. CNDP1 (Q96KN2), NP2

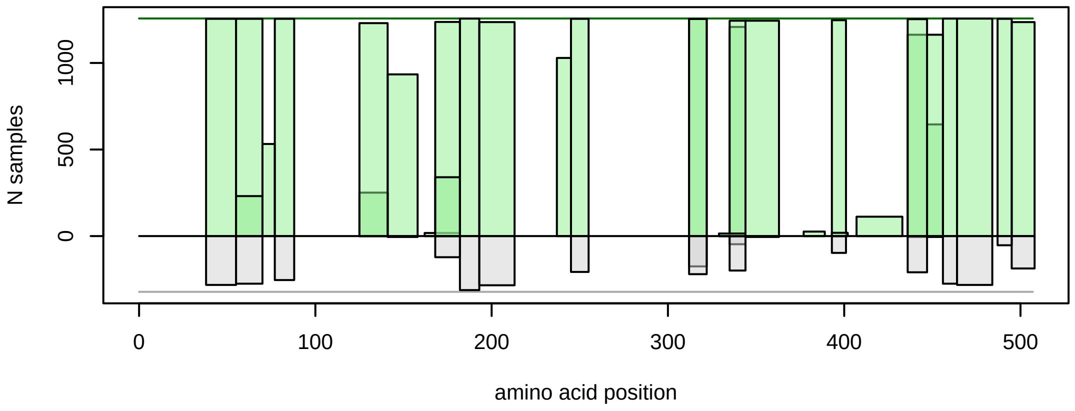

587. SULT1A1 (P50225), NP2

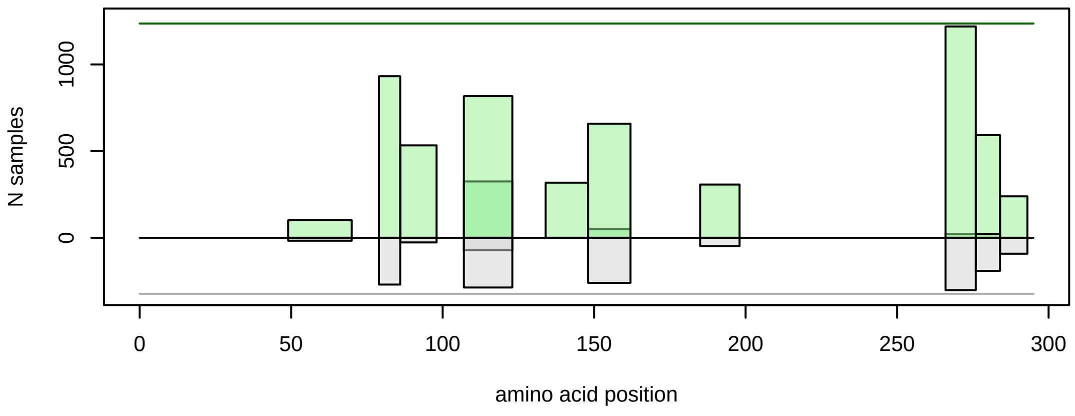

588. CD47 (Q08722), NP1

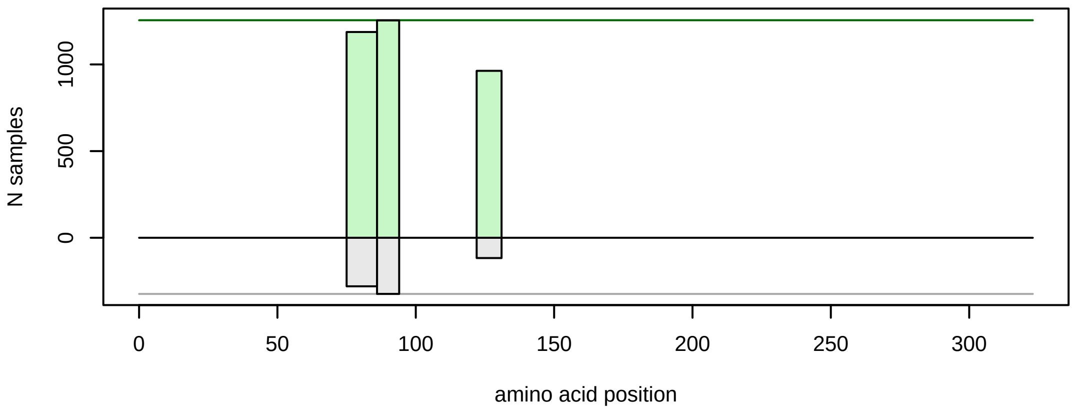

589. NCS1 (P62166), NP2

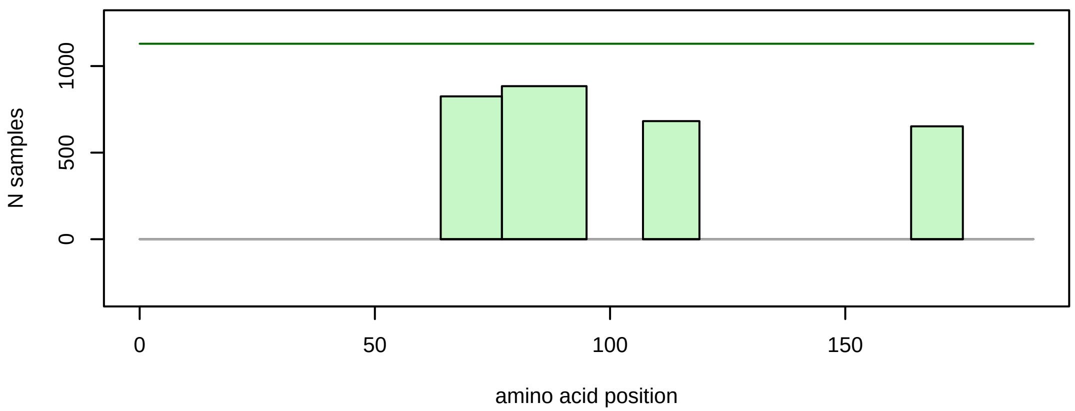

590. IGLV7−46 (A0A075B6I9), NP4

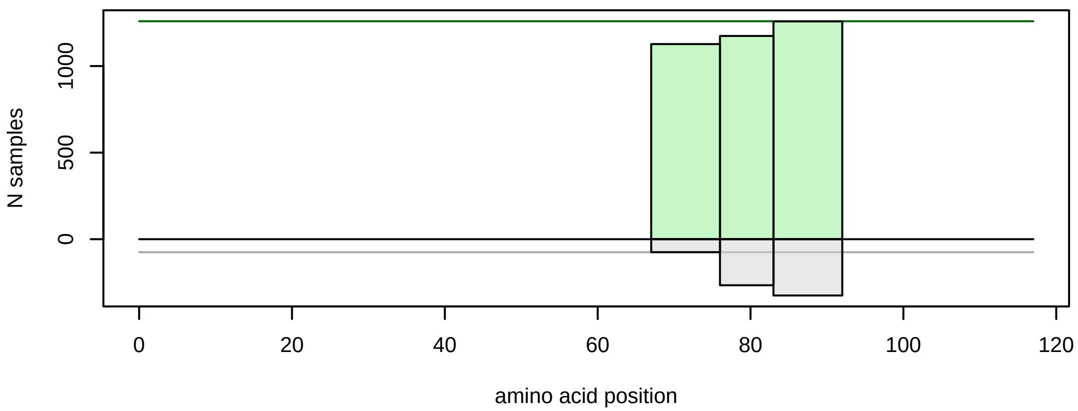

591. UGGT1 (Q9NYU2), NP1

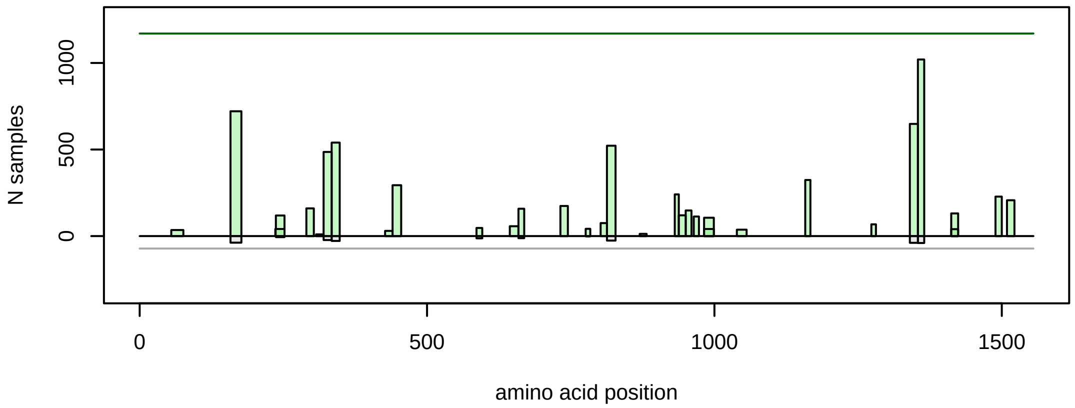

592. SCYL2 (Q6P3W7), NP4

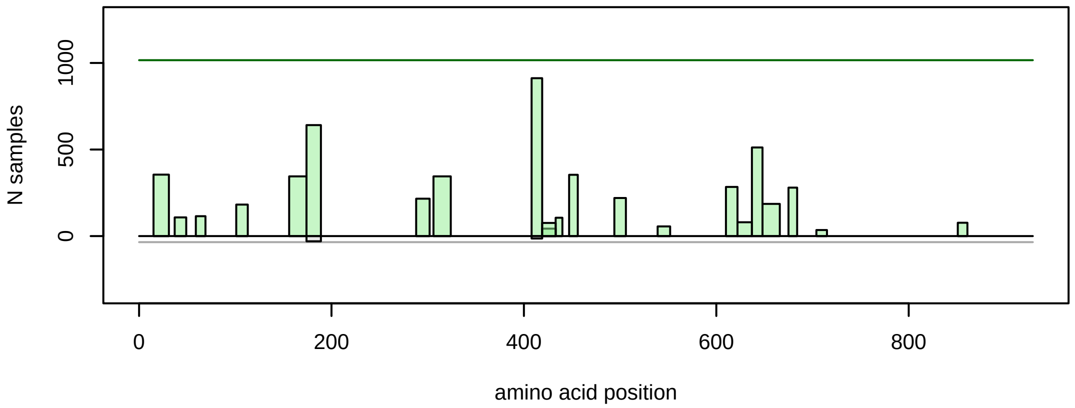

593. NPNT (Q6UXI9), NP1

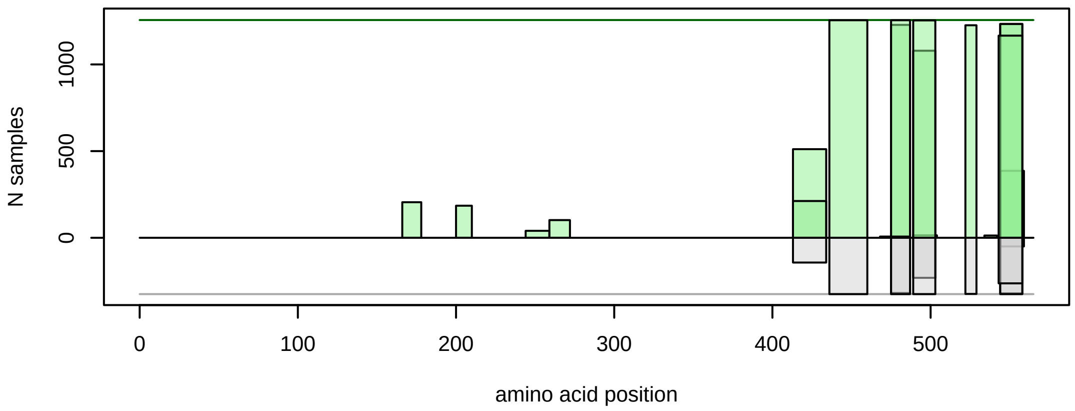

594. IGLV8−61 (A0A075B6I0), NP4

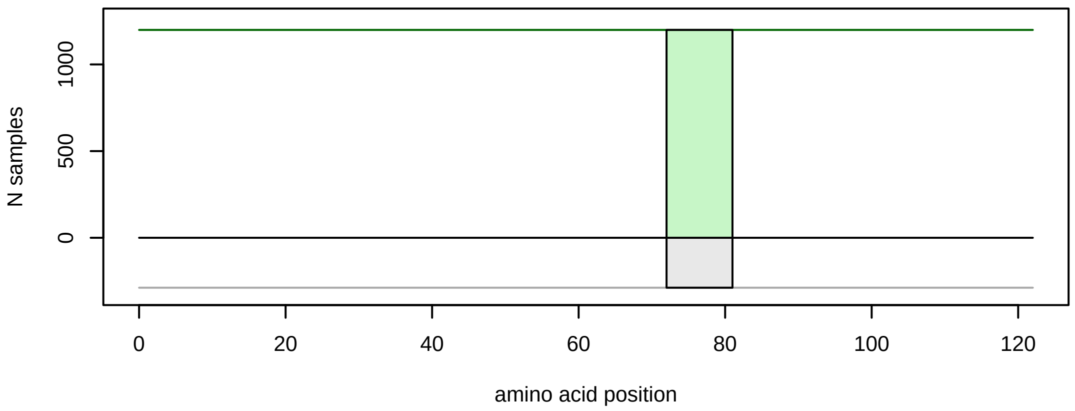

595. PTGES3 (A0A087WYT3;Q15185;Q15185−4), NP2

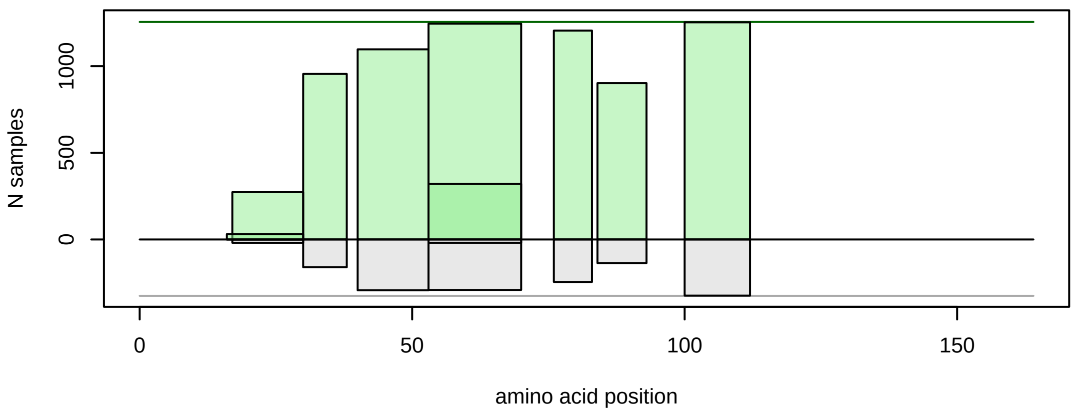

596. COL6A3 (P12111), NP1

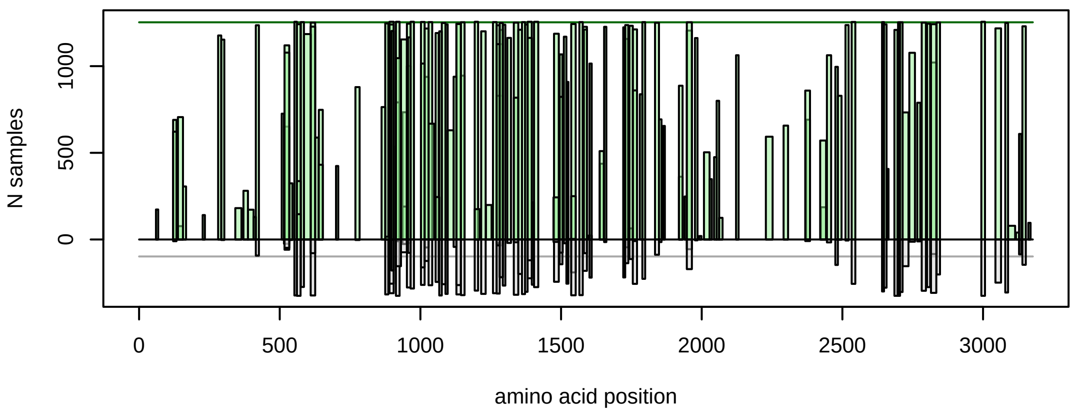

597. ERBB2IP (Q96RT1−8), NP2

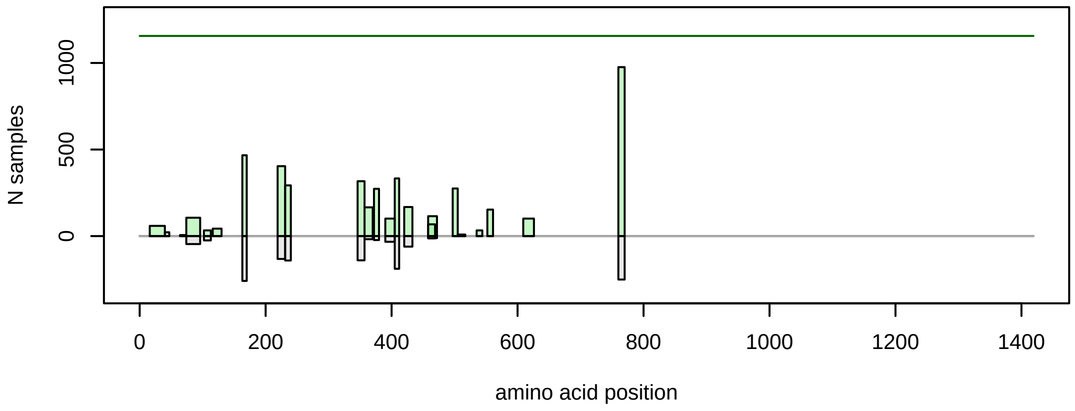

598. SORD (Q00796), NP4

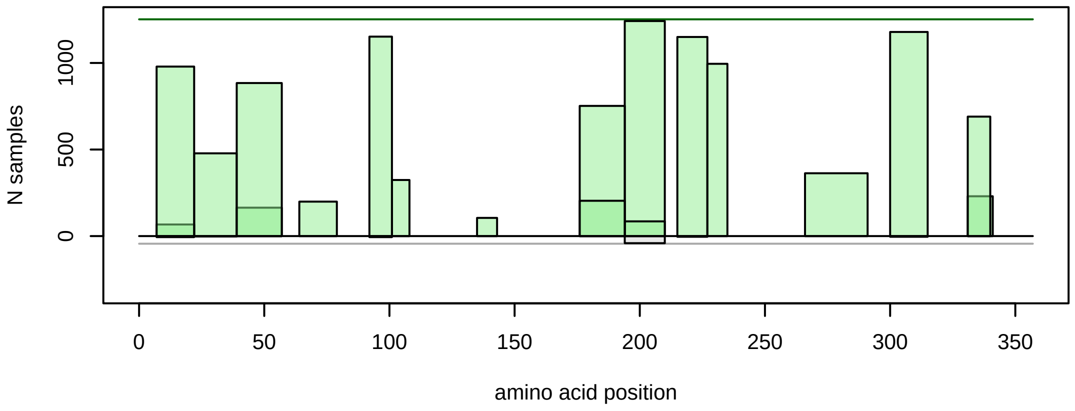

599. GNA13 (Q14344), NP1

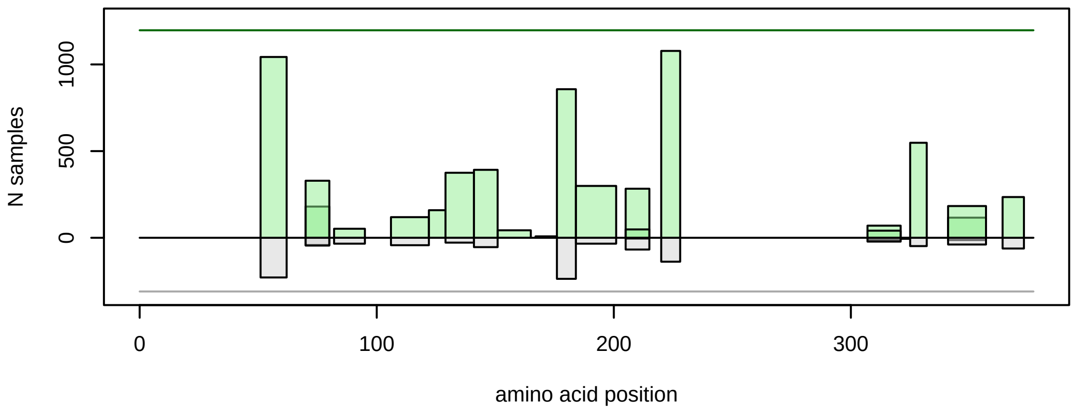

600. DEFA5 (Q01523), NP1

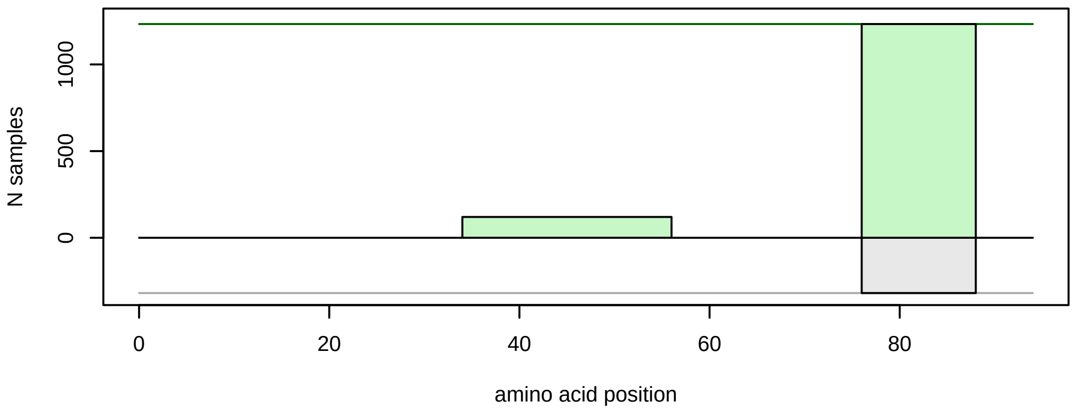

601. CYFIP1 (Q7L576), NP4

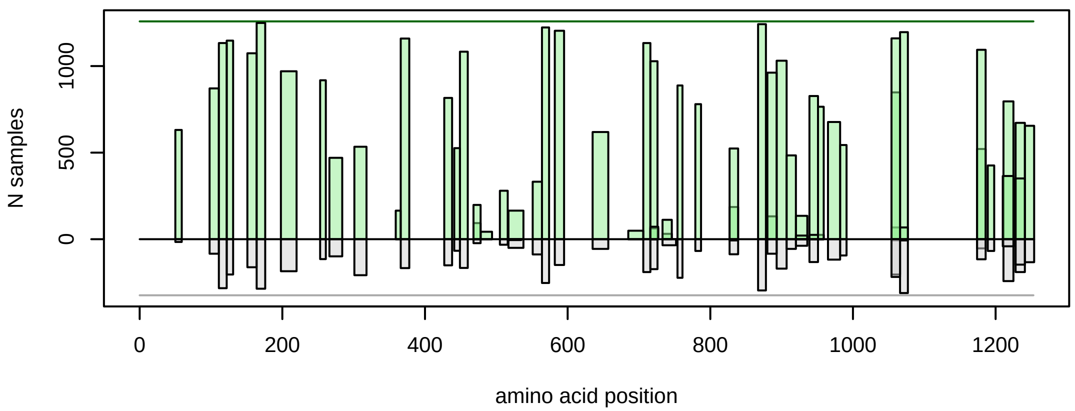

602. CMAS (Q8NFW8), NP1

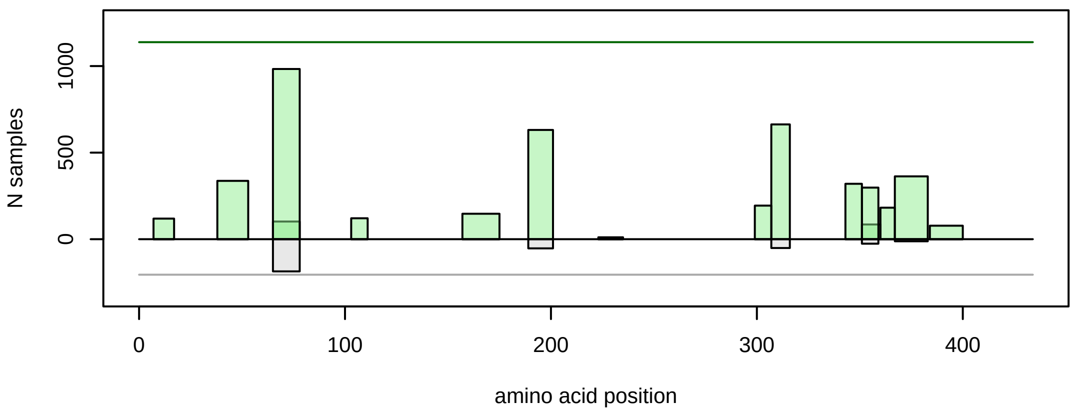

603. HSPA9 (D6RCD7), NP4

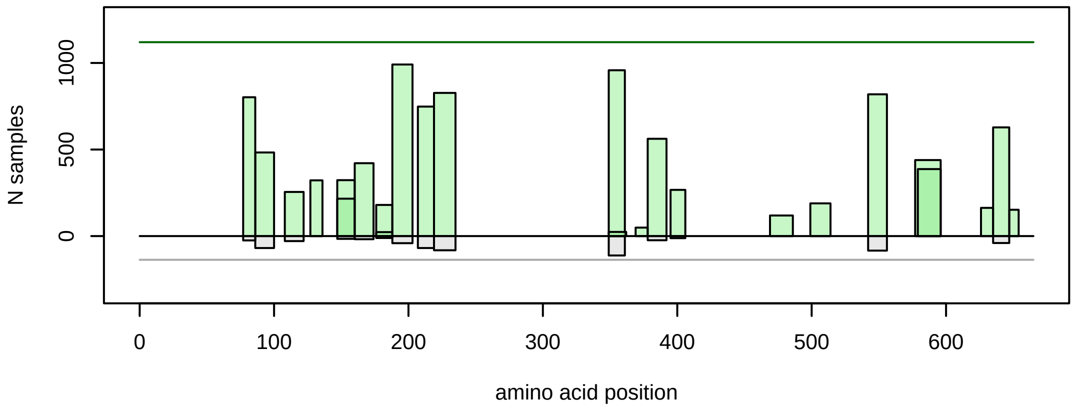

604. IGHG2 (P01859), NP3

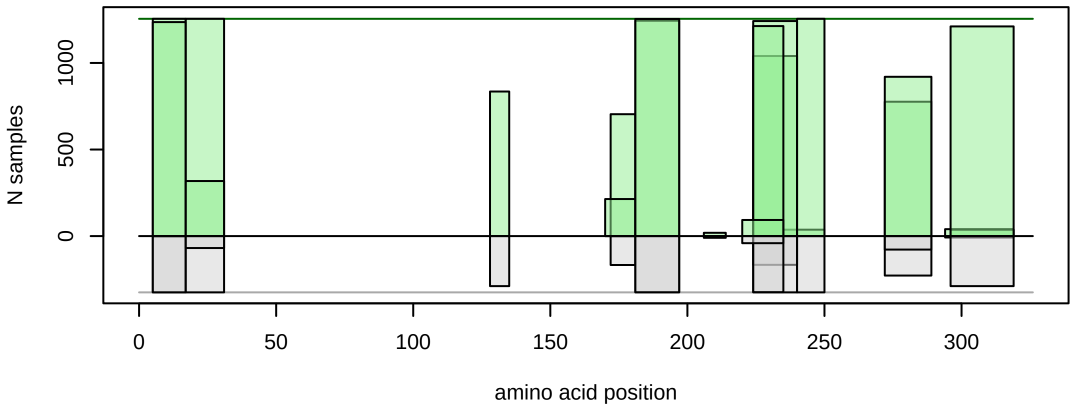

605. HNRNPA1 (A0A7I2V3R8;F8W6I7;P09651;P09651−2;P09651−3), NP1

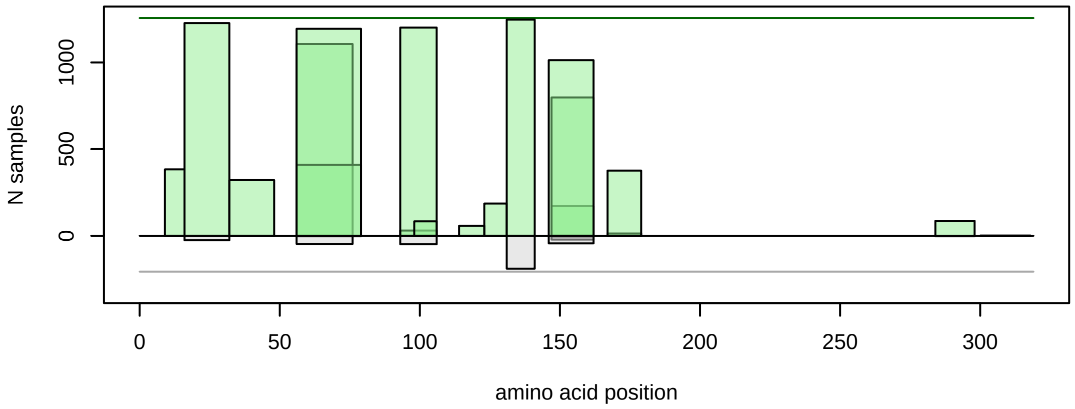

606. COPS3 (Q9UNS2), NP2

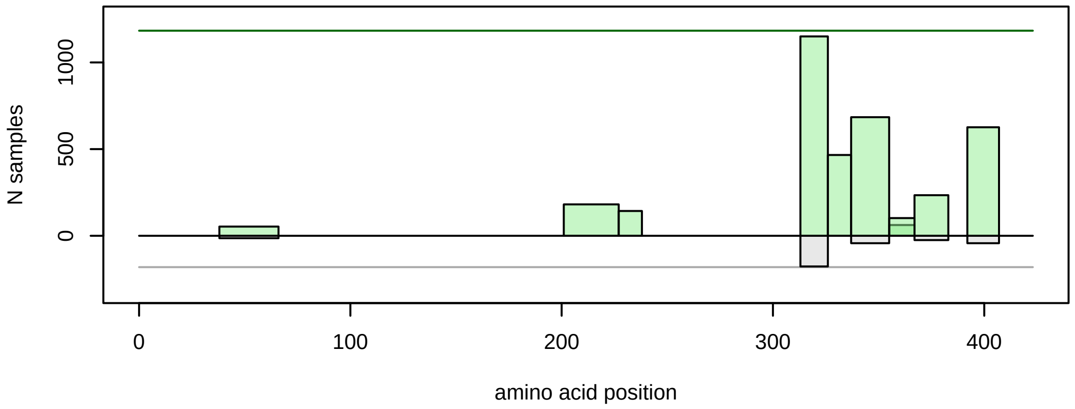

607. VASN (Q6EMK4), NP2

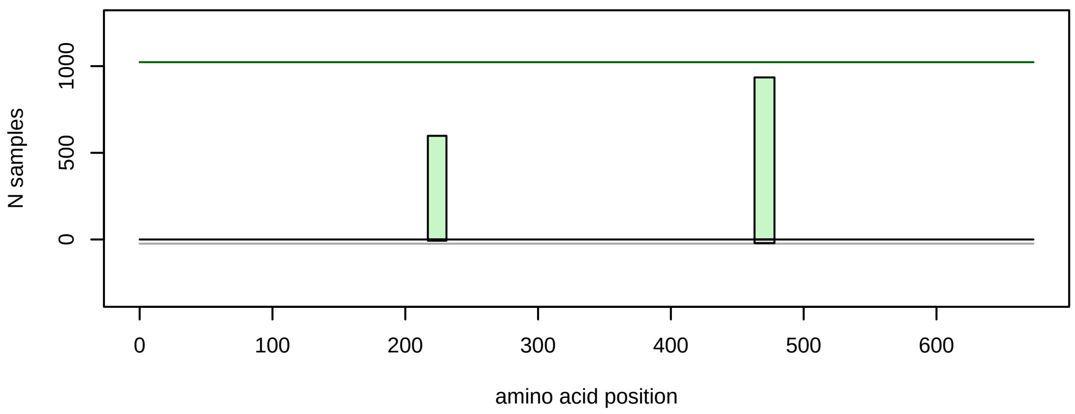

608. TBCB (A0A494C0X0), NP2

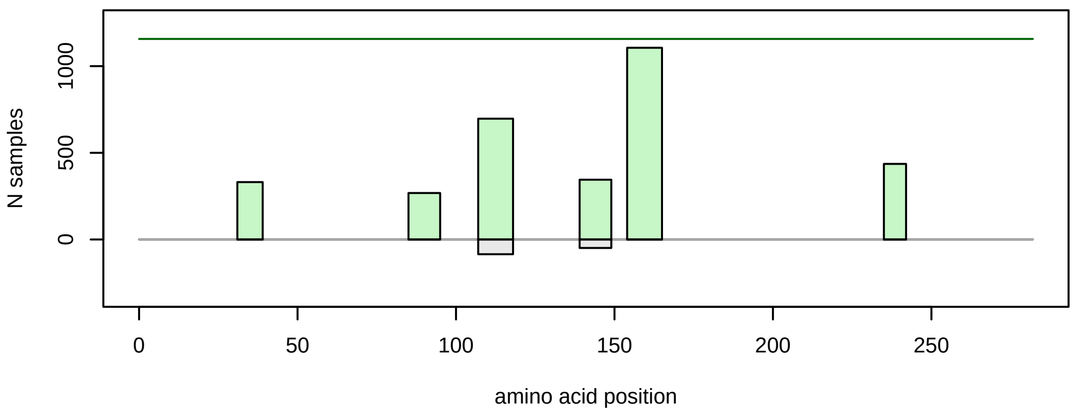

609. AK1 (P00568;Q5T9B7), NP3

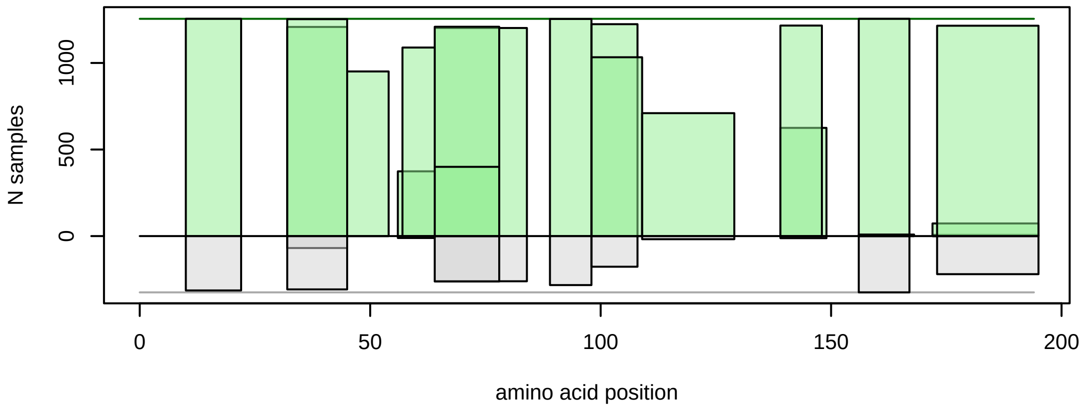

610. GAPVD1 (Q14C86), NP5

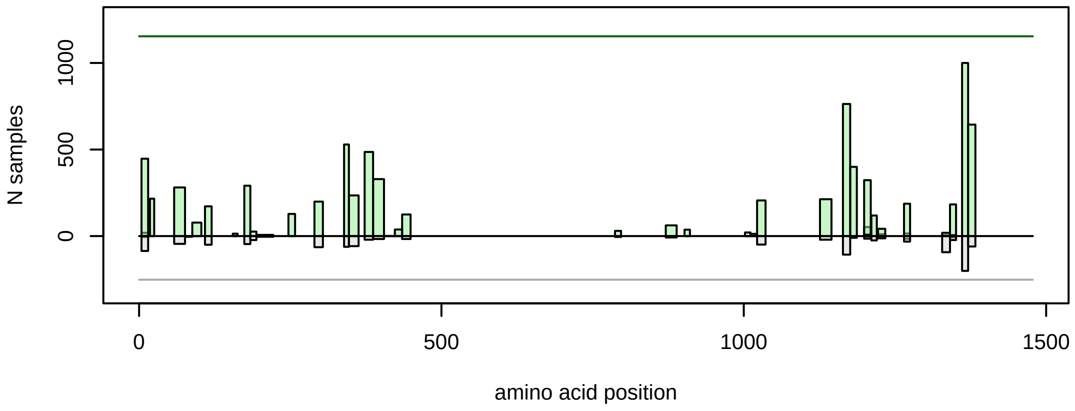

611. THBS1 (P07996), NP2

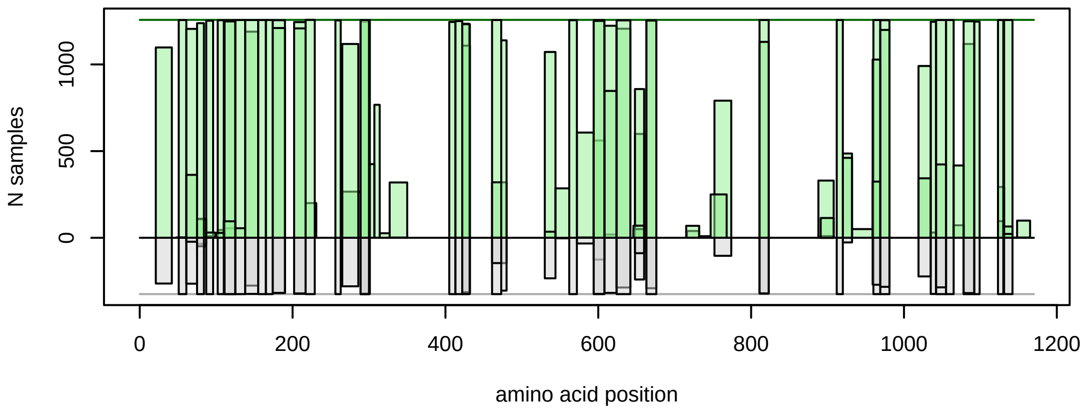

612. ETF1 (P62495), NP4

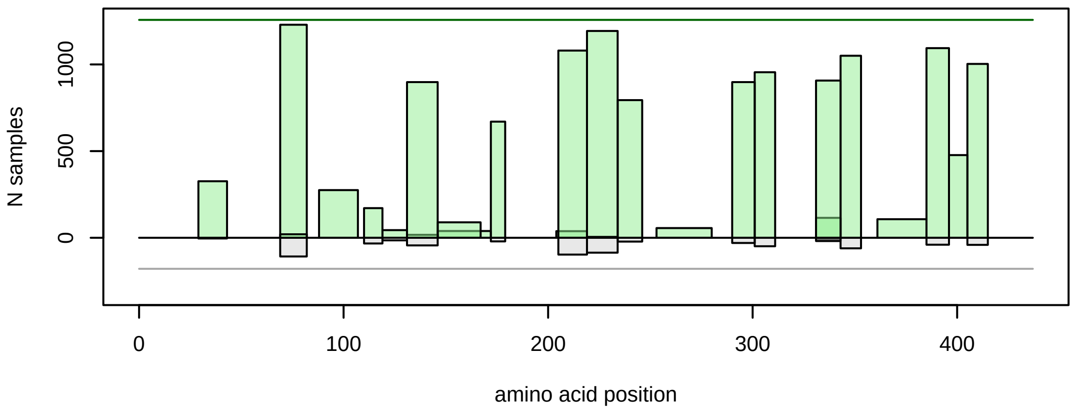

613. ARHGAP6 (O43182), NP1

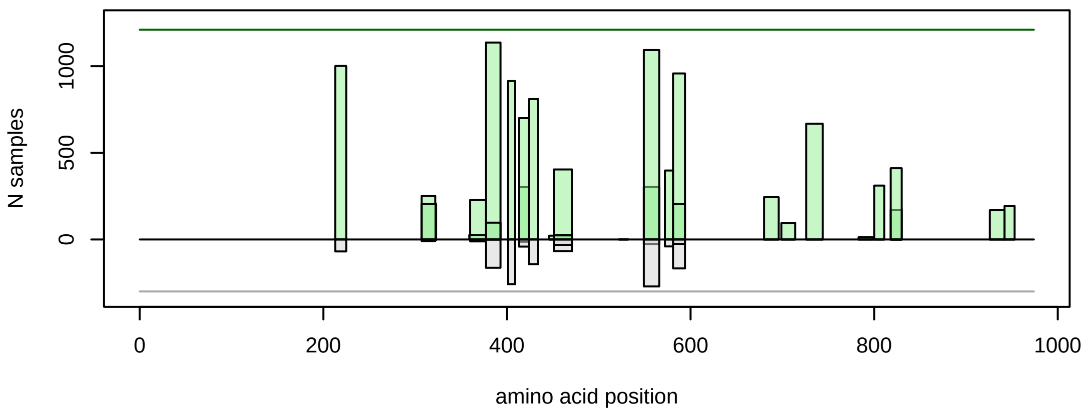

614. AP3B1 (O00203), NP1

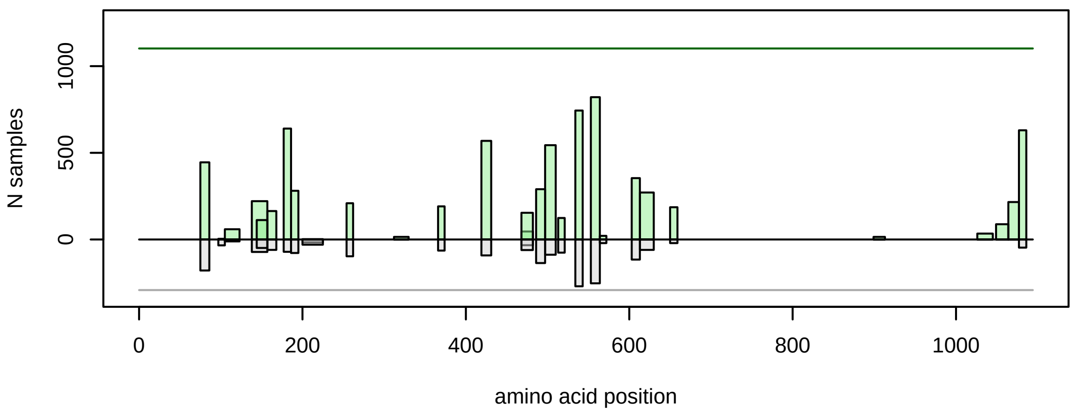

615. CD37 (P11049), NP2

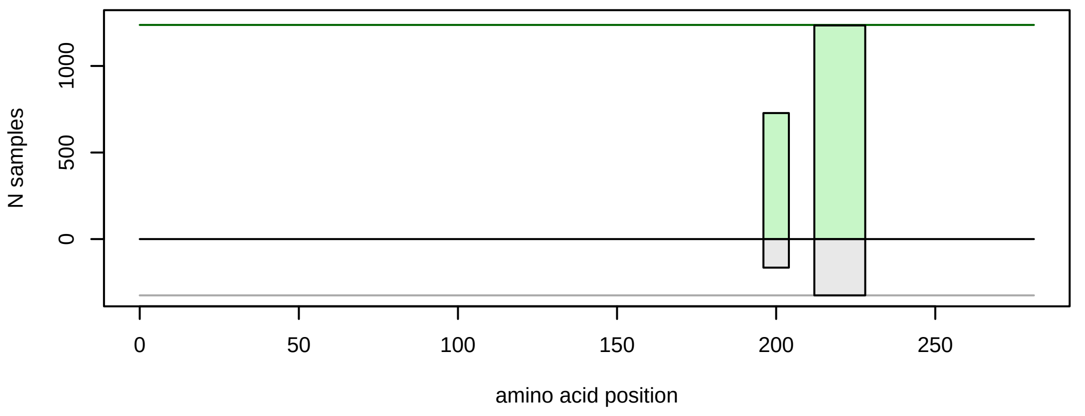

616. POTEJ (P0CG39), NP2

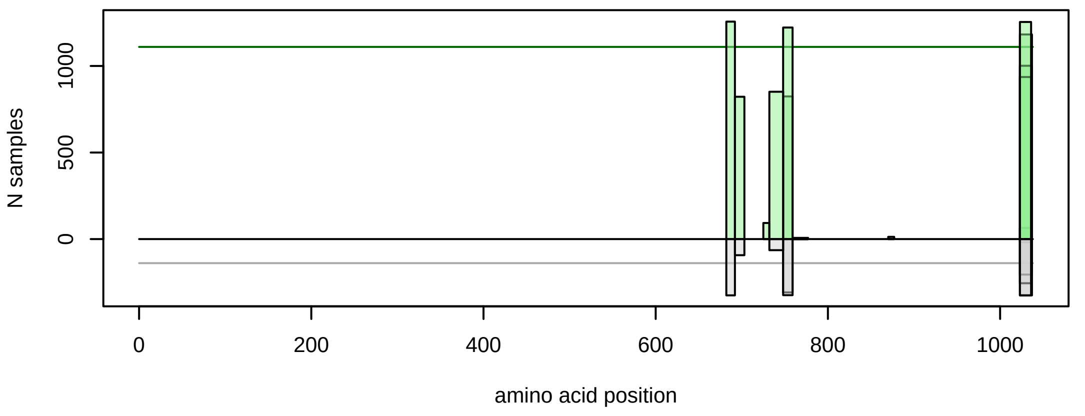

617. ADAMTS5 (Q9UNA0), NP1

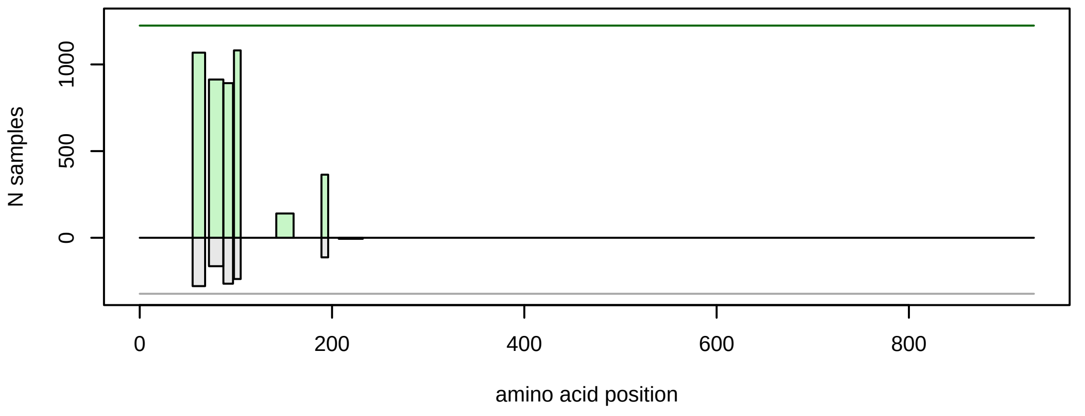

618. CDH11 (P55287), NP2

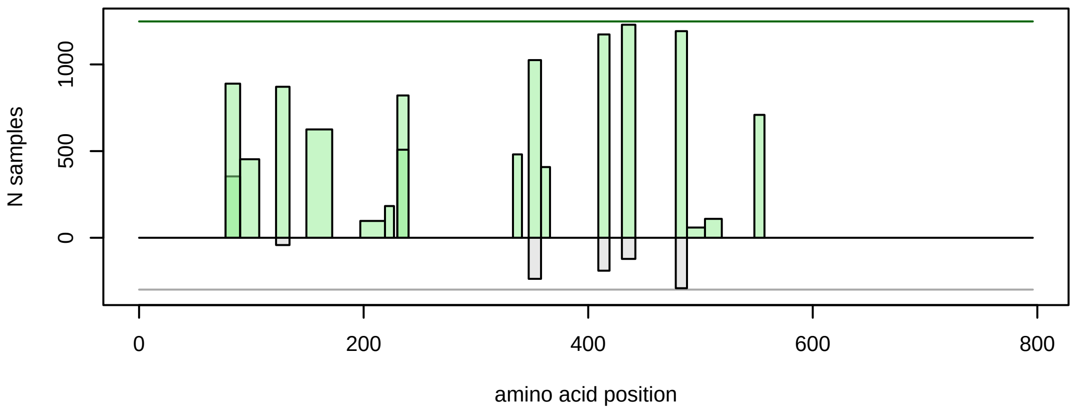

619. PRPS1 (B1ALA9;P60891), NP3

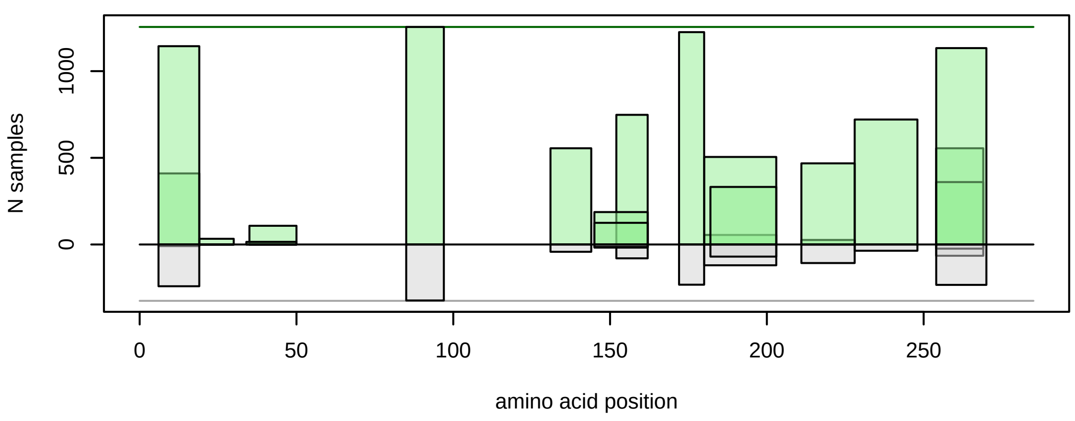

620. GALNT16 (Q8N428), NP3

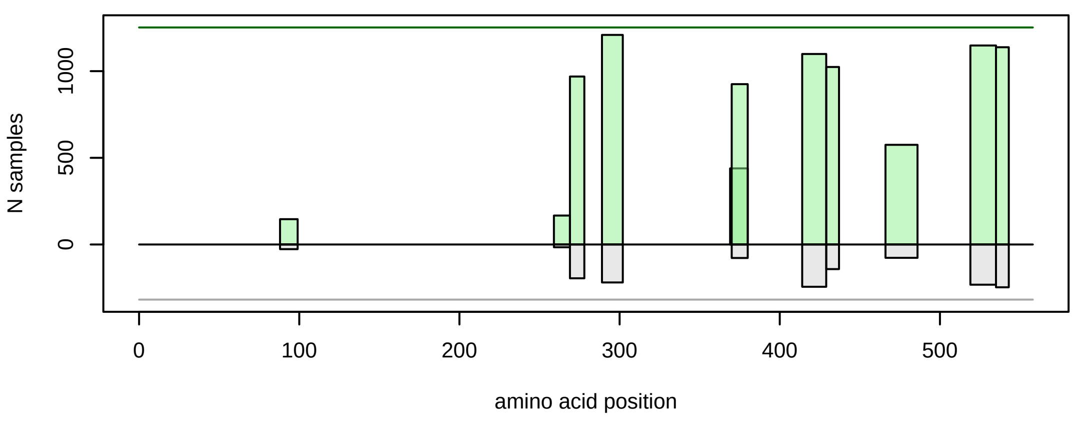

621. PRKD2 (Q9BZL6;Q9BZL6−3), NP2

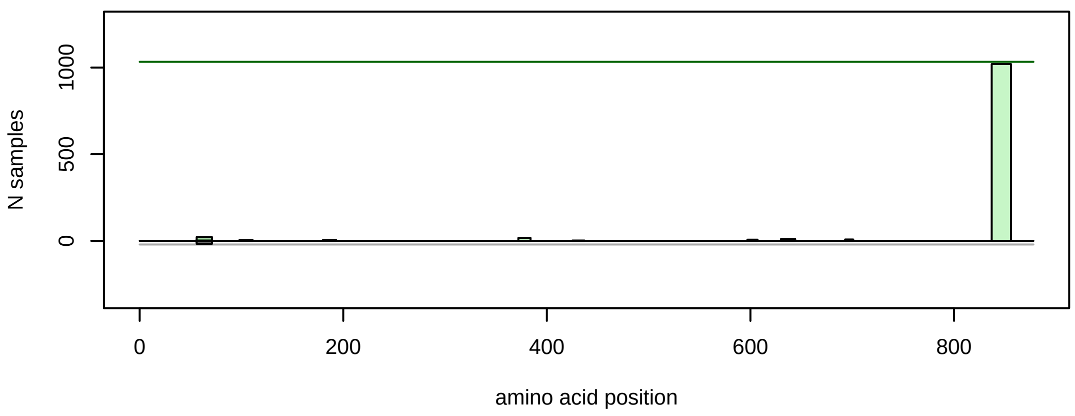

622. WNT5B (Q9H1J7), NP4

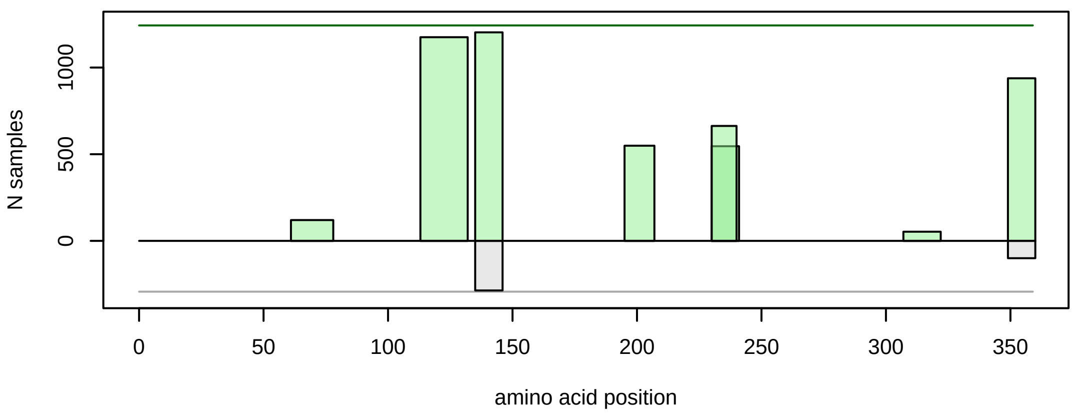

623. GPC1 (P35052), NP2

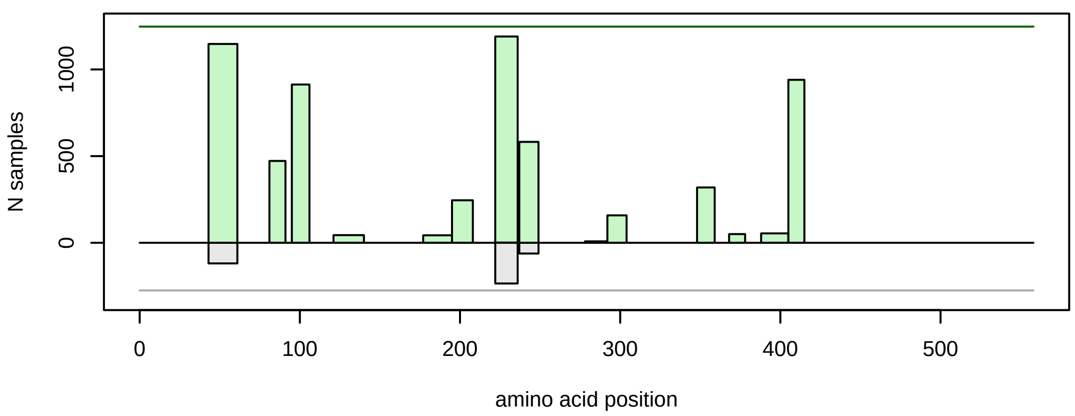

624. DOK2 (O60496), NP1

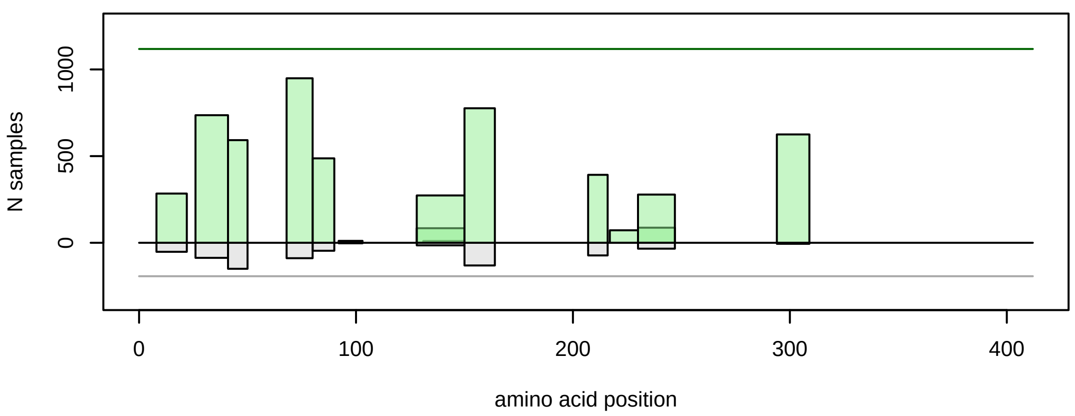

625. DDOST (P39656), NP4

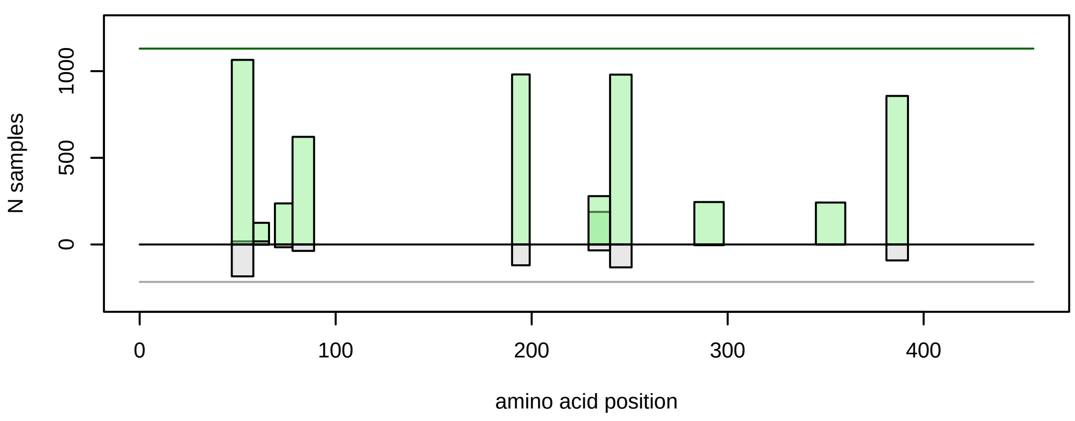

626. RHOA (P61586), NP1

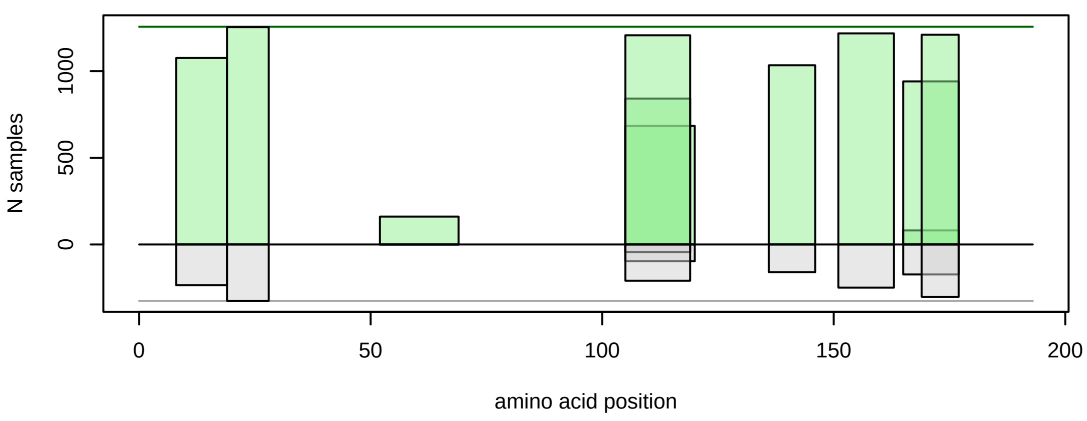

627. C3orf58 (Q8NDZ4), NP1

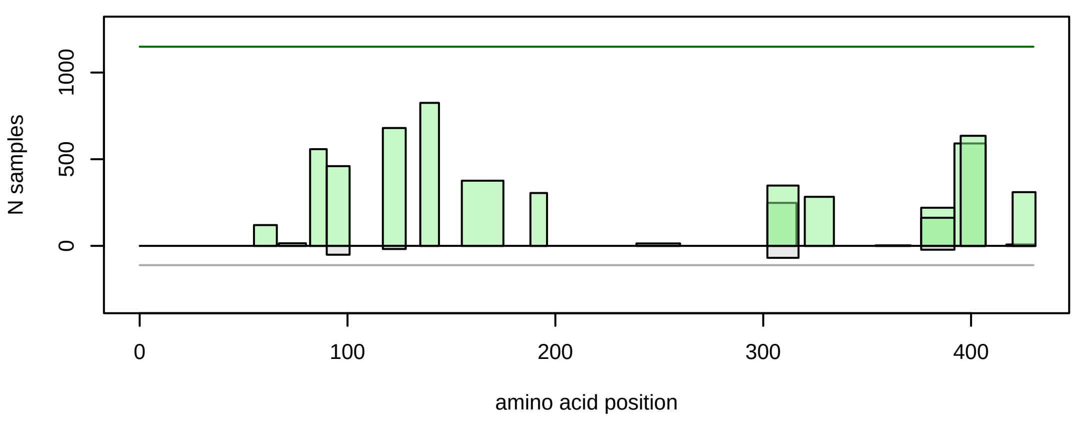

628. PRSS2 (A0A0J9YYC8;A6XMV9;P07478), NP2

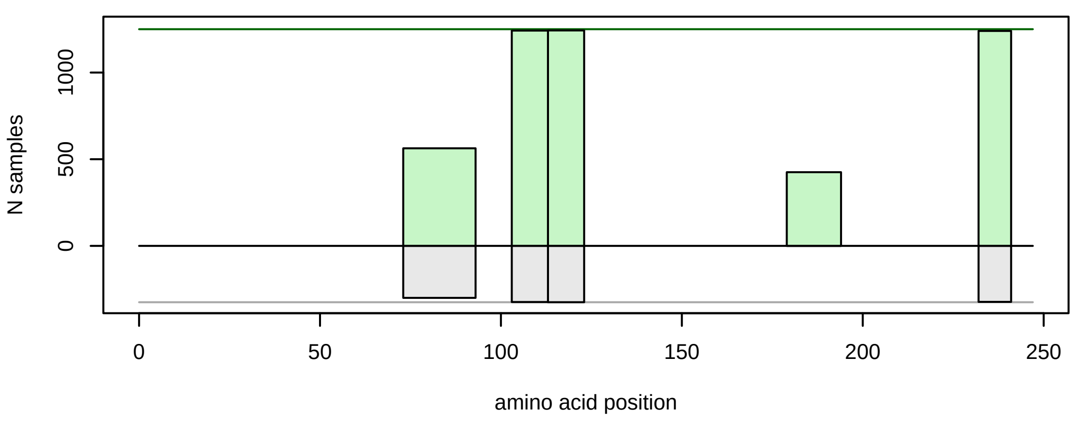

629. TNNC1 (P63316), NP2

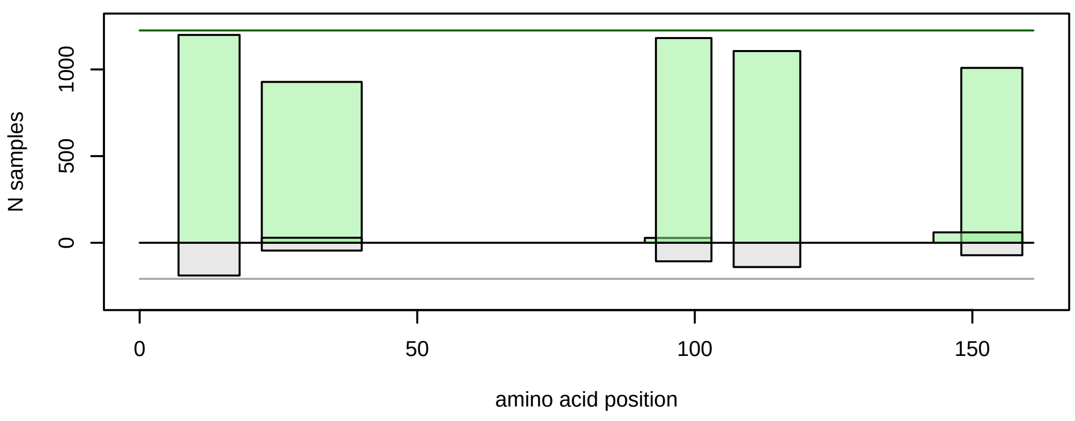

630. SH3BGRL3 (Q5T123), NP2

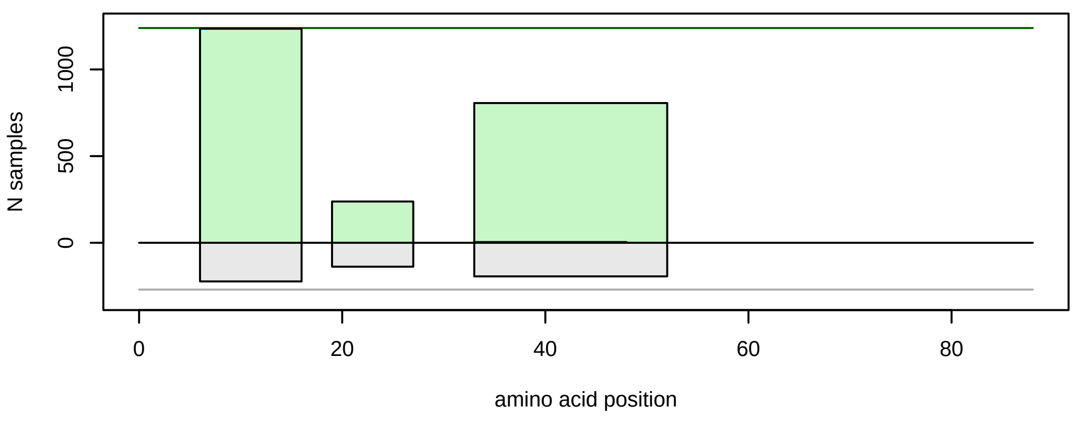

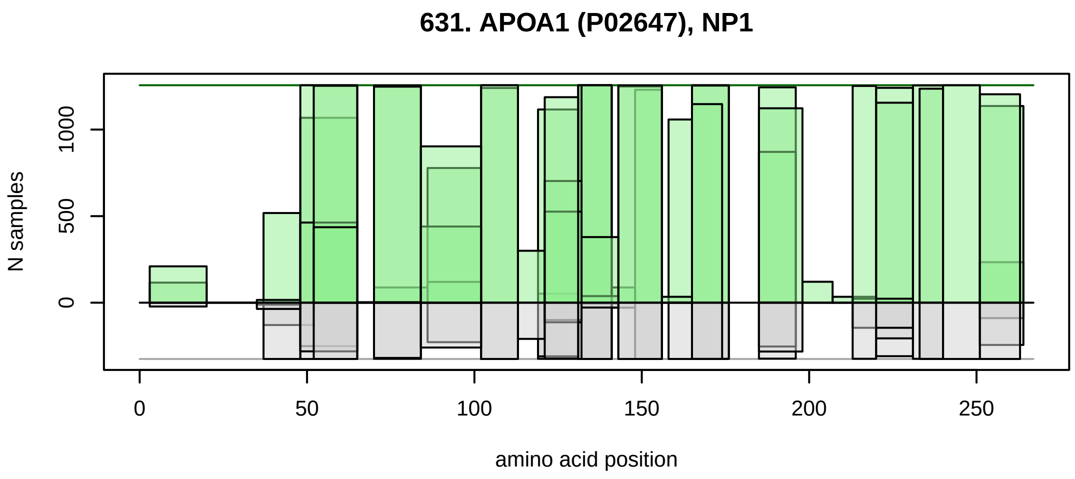
631. APOA1 (P02647), NP1
N samples
0
500
1000
0
50
100
150
200
250
amino acid position

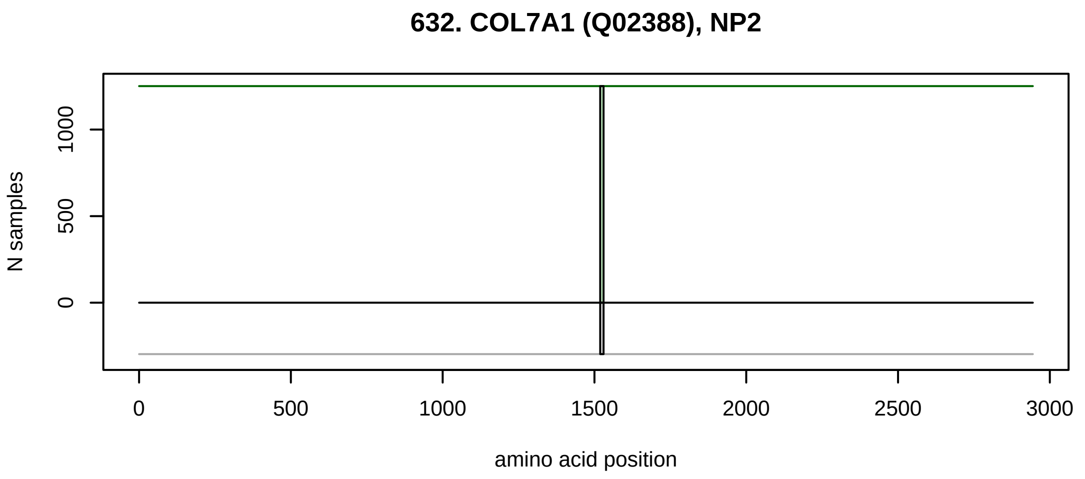
632. COL7A1 (Q02388), NP2
N samples
0
500
1000
0
500
1000
1500
2000
2500
3000
amino acid position

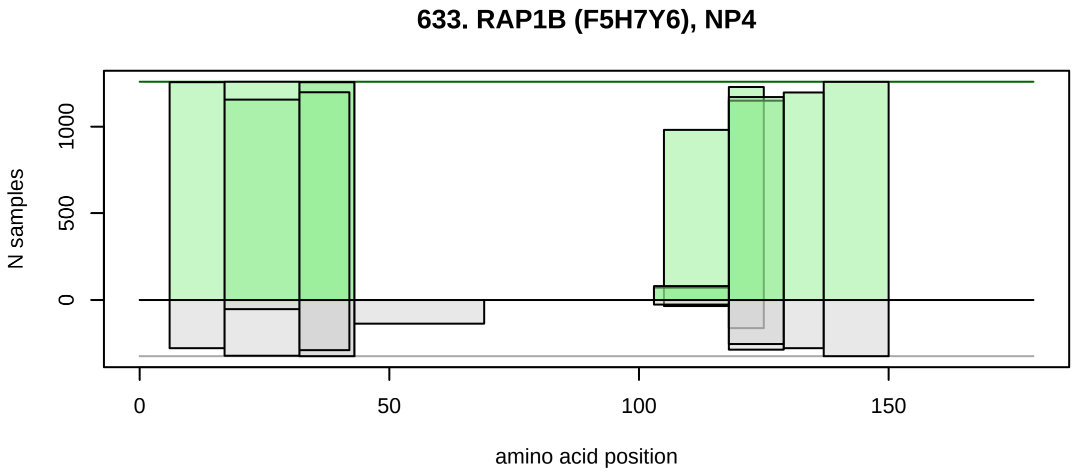
633. RAP1B (F5H7Y6), NP4
N samples
0
500
1000
0
50
100
150
amino acid position

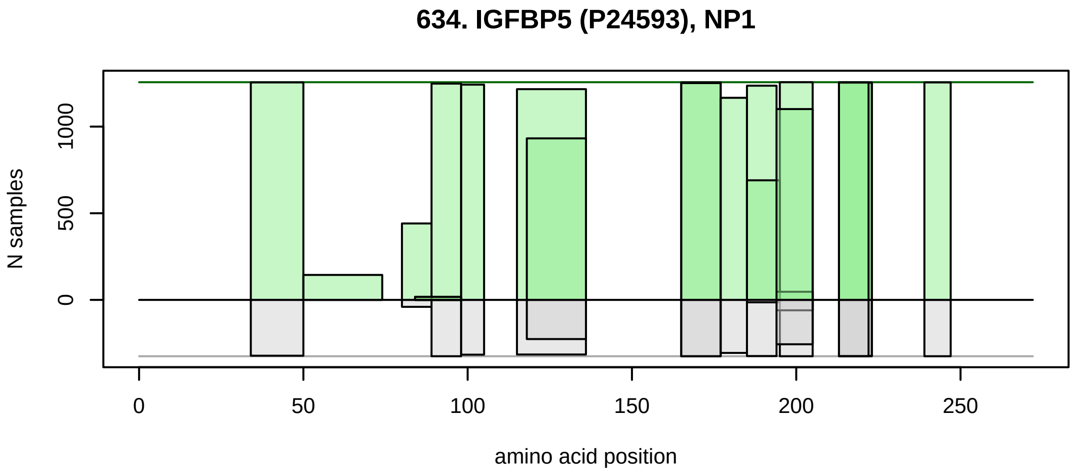
634. IGFBP5 (P24593), NP1
N samples
0
500
1000
0
50
100
150
200
250
amino acid position

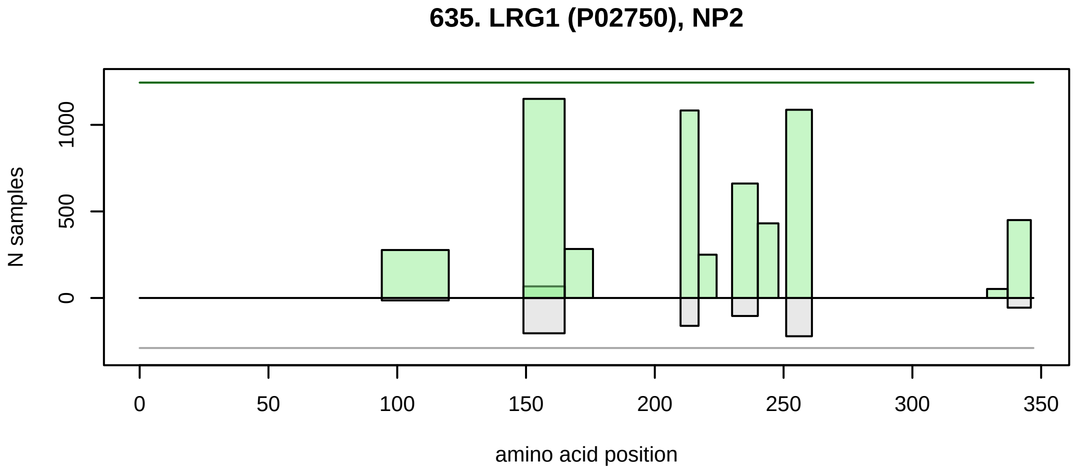
635. LRG1 (P02750), NP2
N samples
0
500
1000
0
50
100
150
200
250
300
350
amino acid position

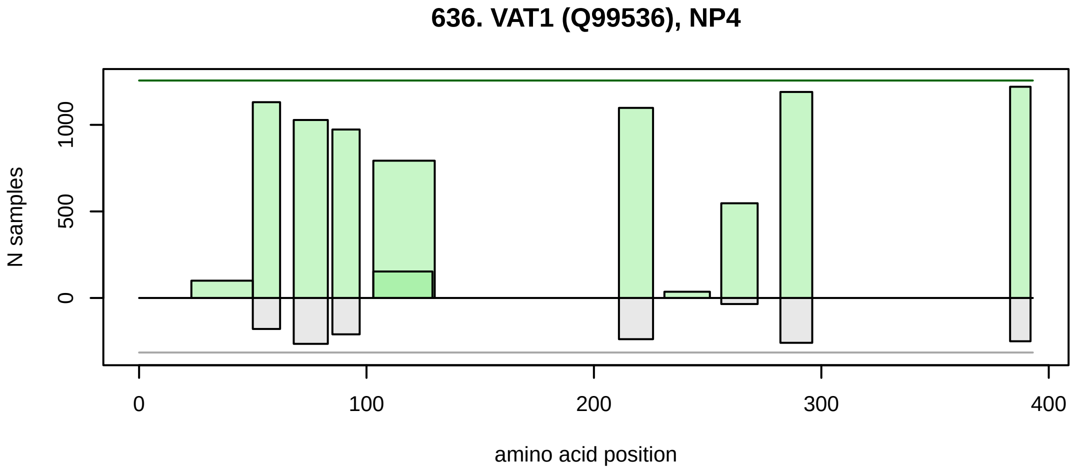
636. VAT1 (Q99536), NP4
N samples
0
500
1000
0
100
200
300
400
amino acid position

637. FST (P19883−2), NP3

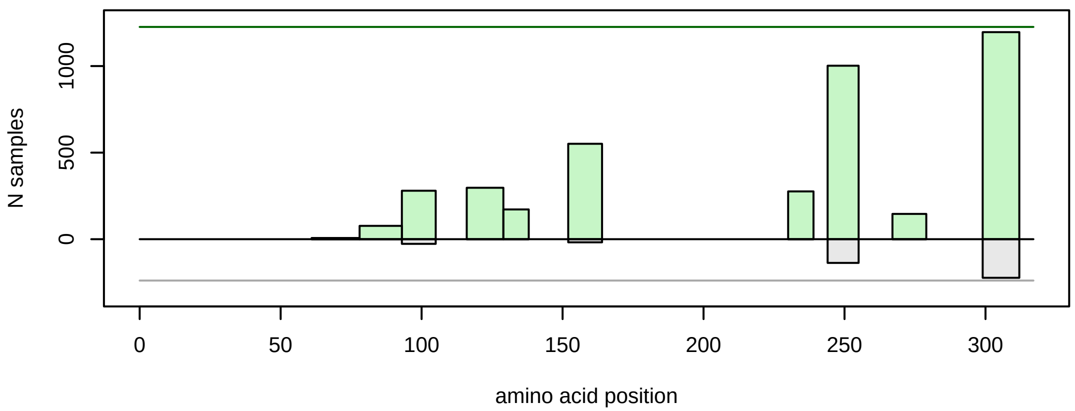

638. DNAJA1 (P31689), NP1

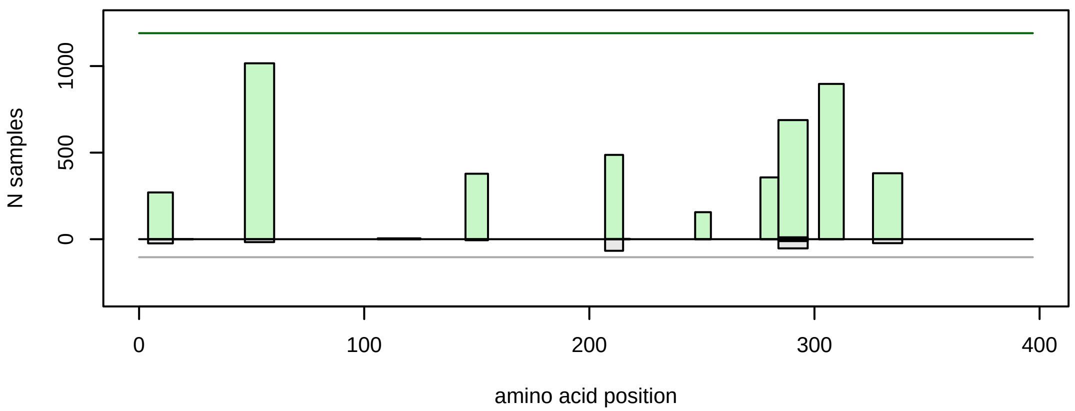

639. PCOLCE (Q15113), NP3

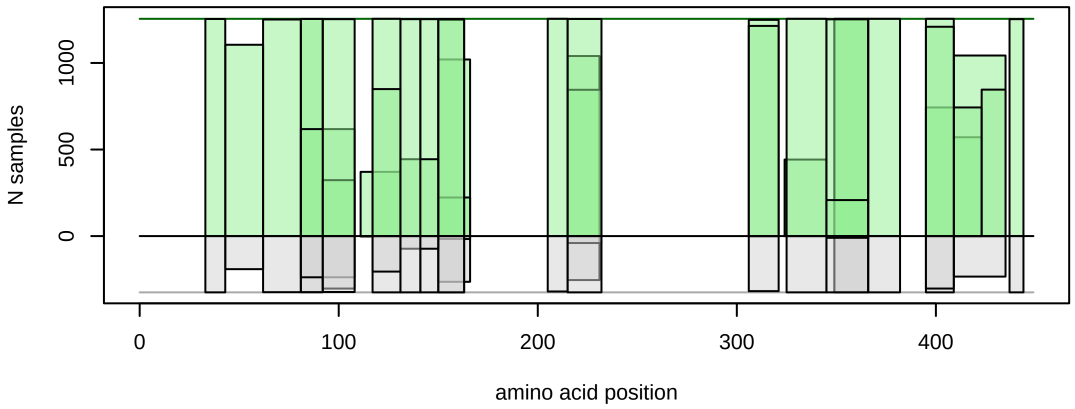

640. PSMC1 (P62191), NP1

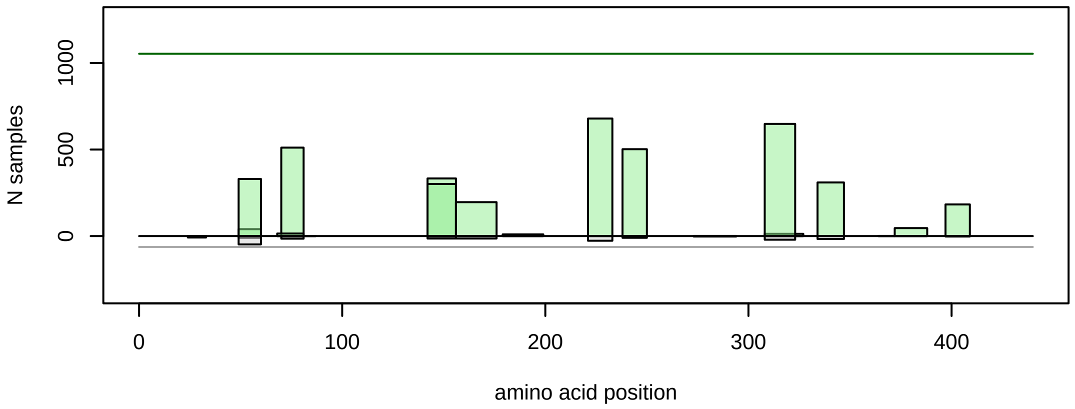

641. FBLN5 (G3V4U0;Q9UBX5), NP2

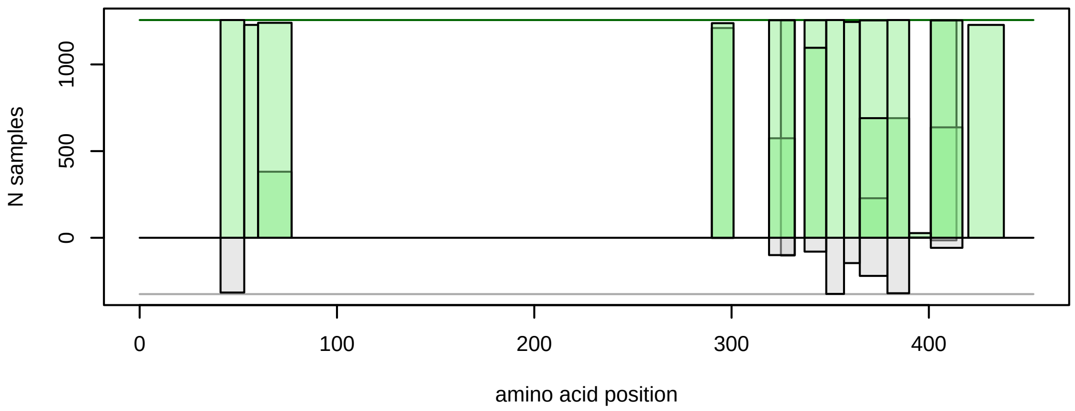

642. SOD3 (P08294), NP2

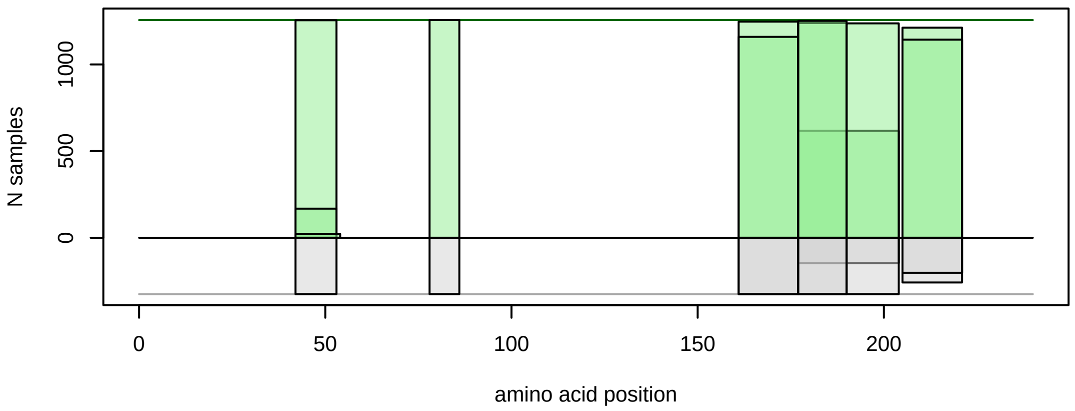

643. GPC6 (Q9Y625), NP2

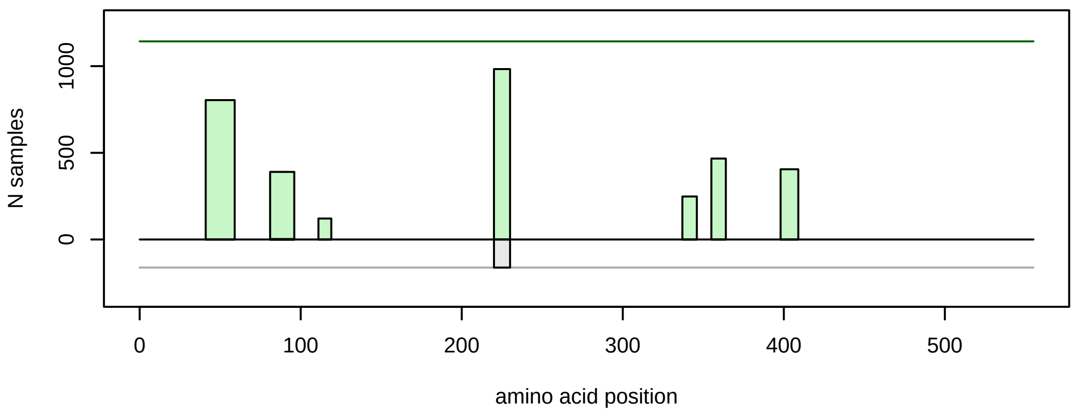

644. RBMX (P38159), NP1

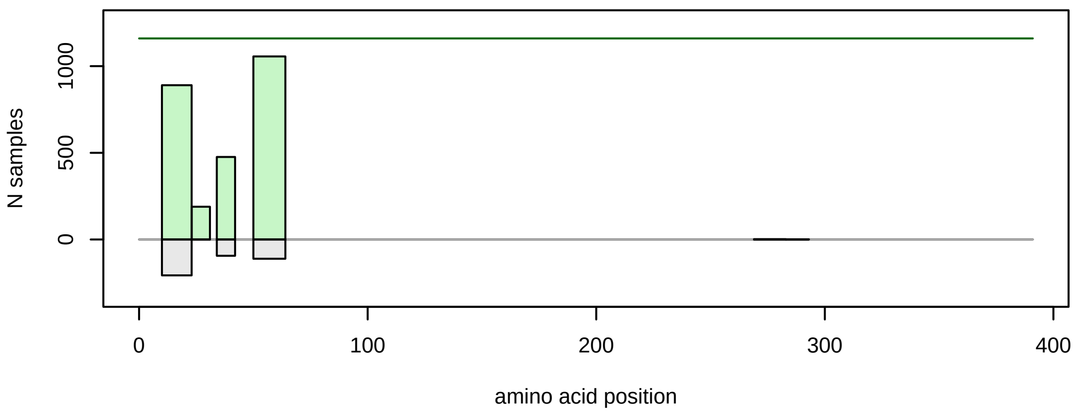

645. IGKV1D−16 (P01601), NP3

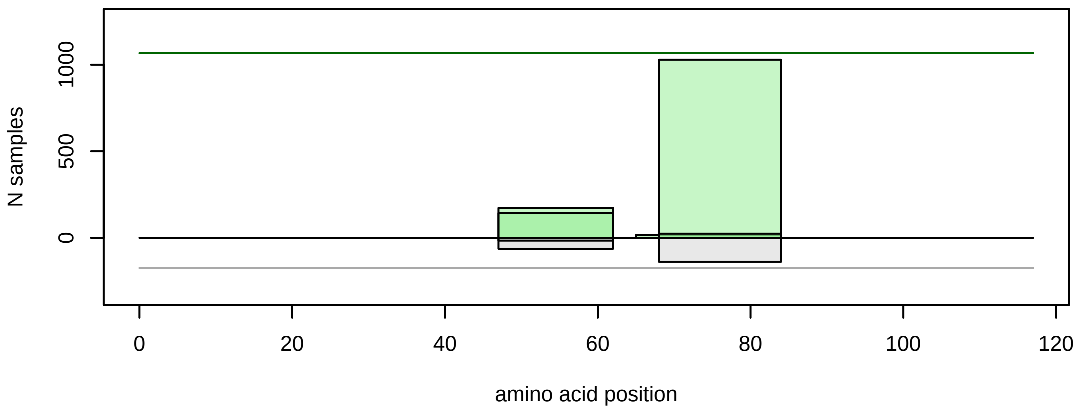

646. FGB (P02675), NP5

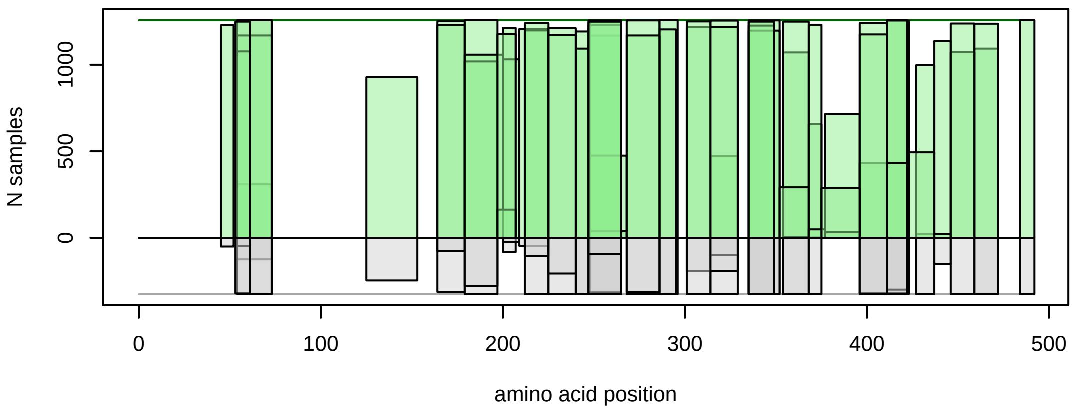

647. MAMDC2 (Q7Z304), NP2

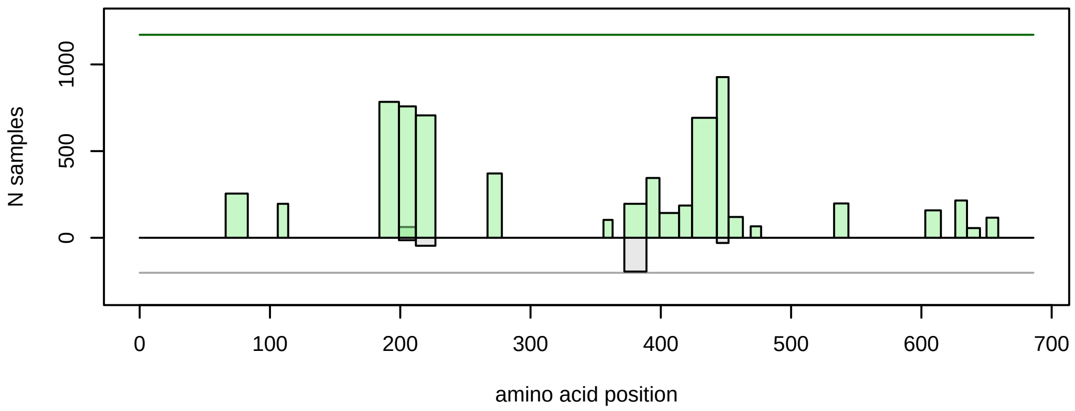

648. LPL (P06858), NP5

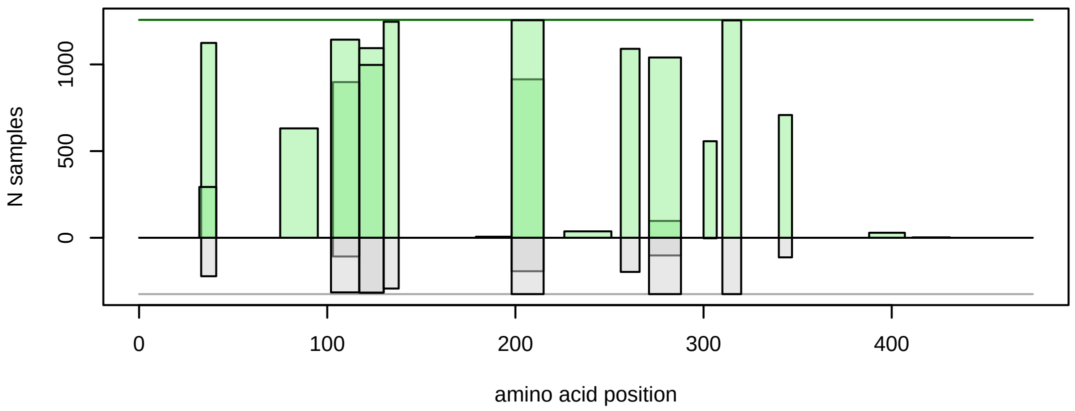

649. ITGA2B (P08514), NP4

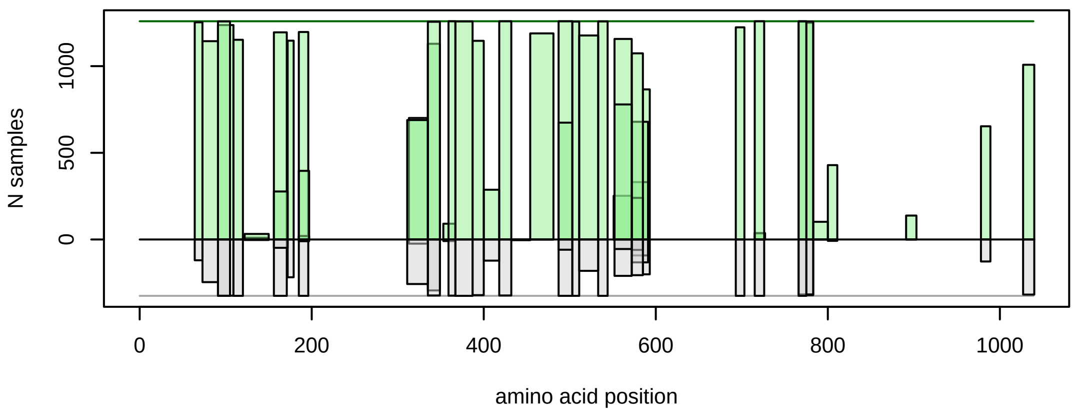

650. RECK (O95980), NP4

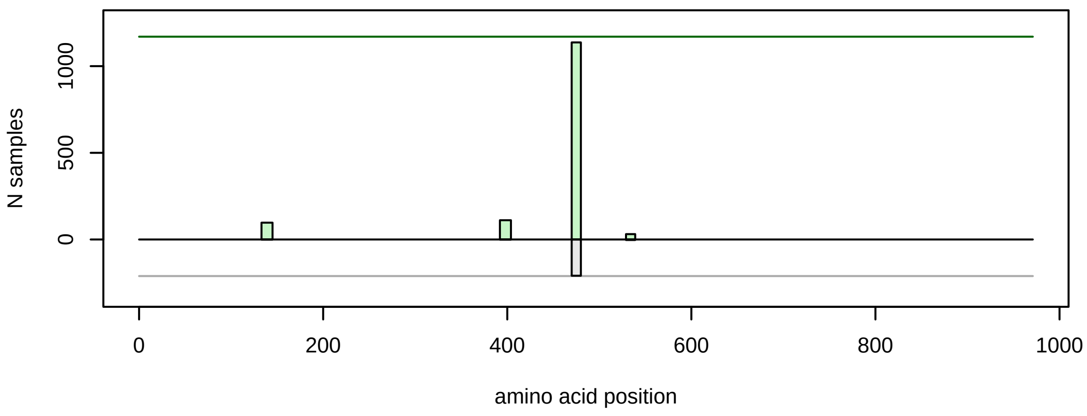

651. RPS27A (P62979), NP1

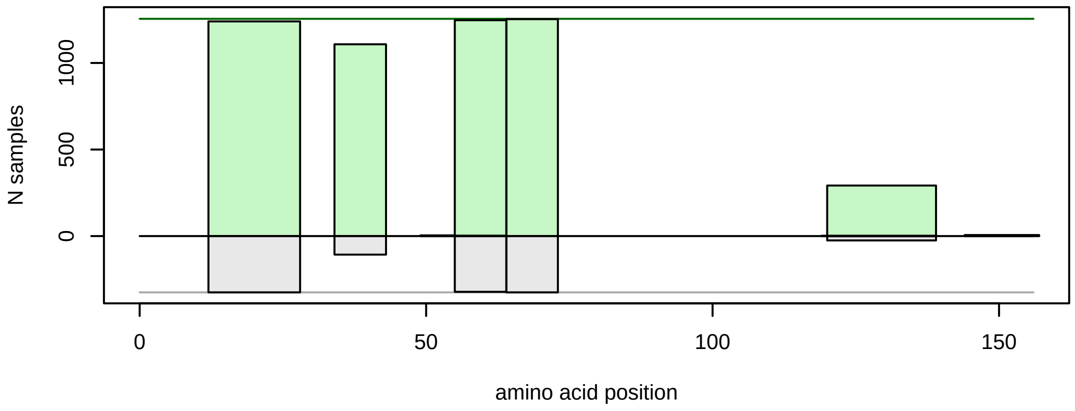

652. ENPP2 (E7EUF1), NP1

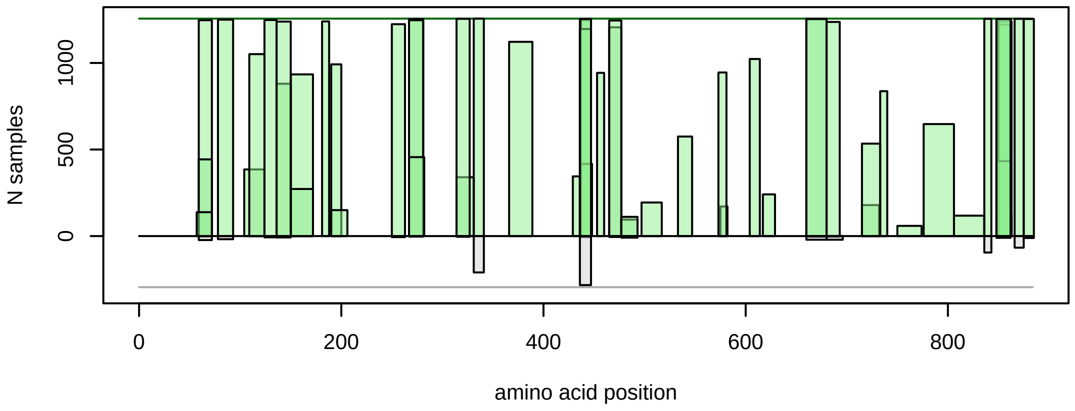

653. F7 (P08709), NP2

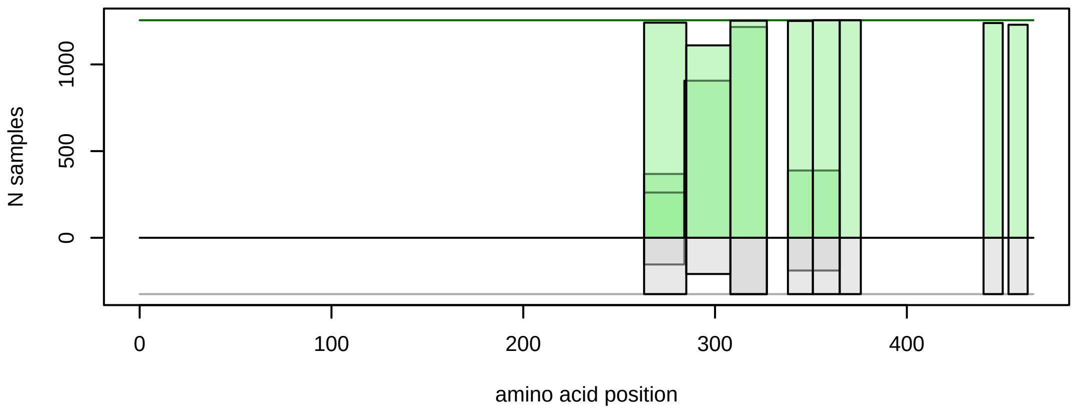

654. SEPT5 (G3XAH0), NP3

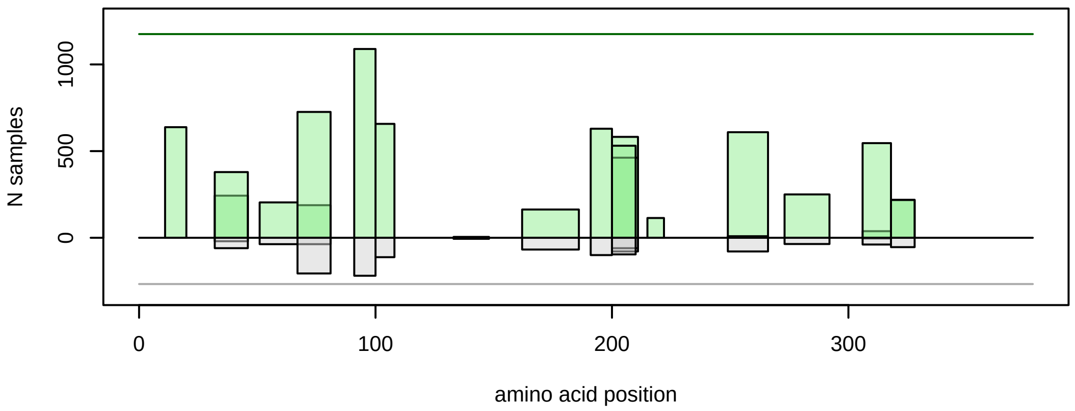

655. ADD2 (P35612), NP1

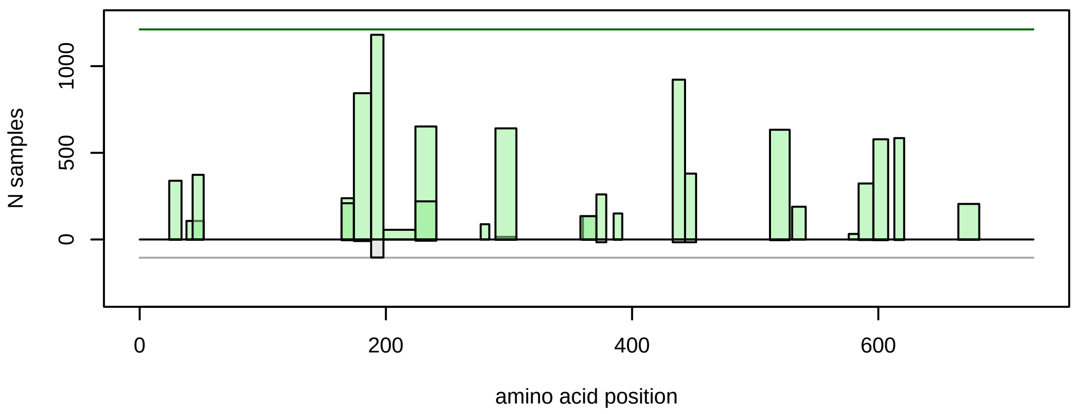

656. HK1 (P19367−2), NP4

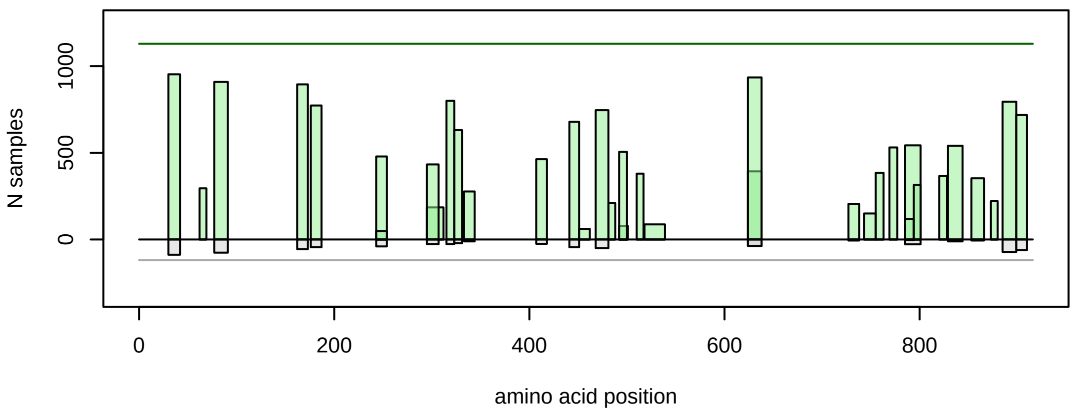

657. GCLM (P48507), NP5

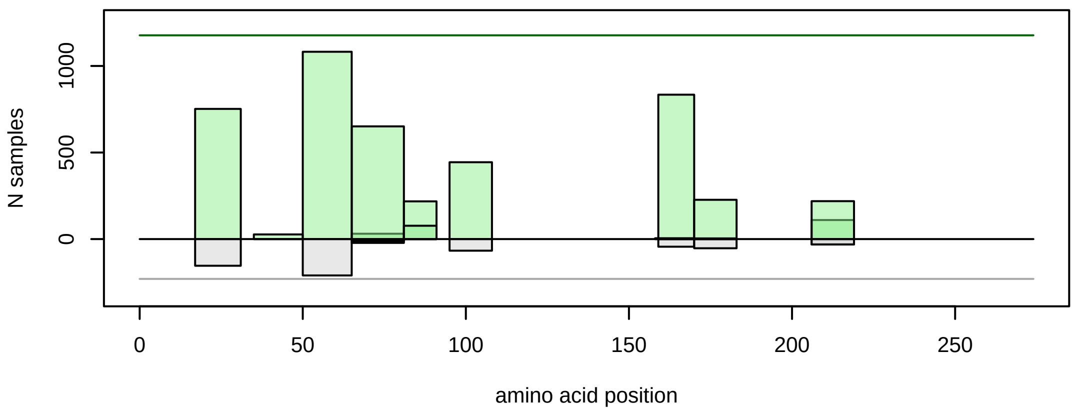

658. SYK (P43405), NP1

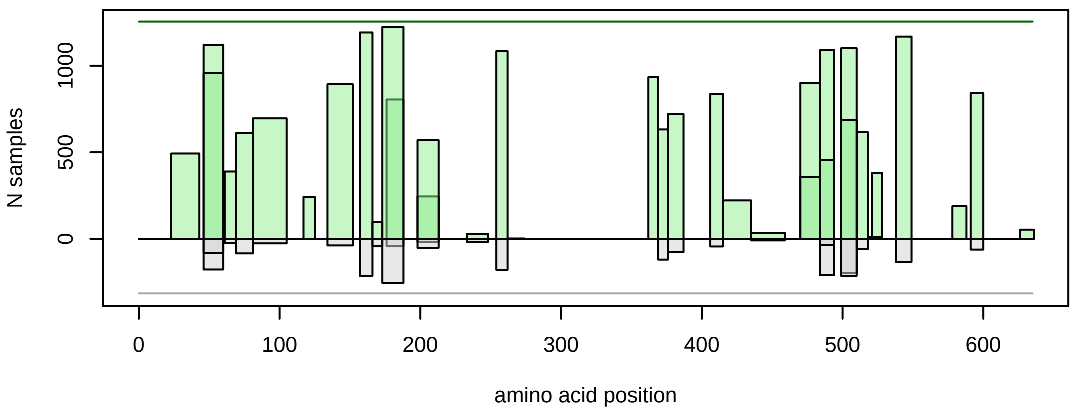

659. HS3ST3B1 (Q9Y662), NP1

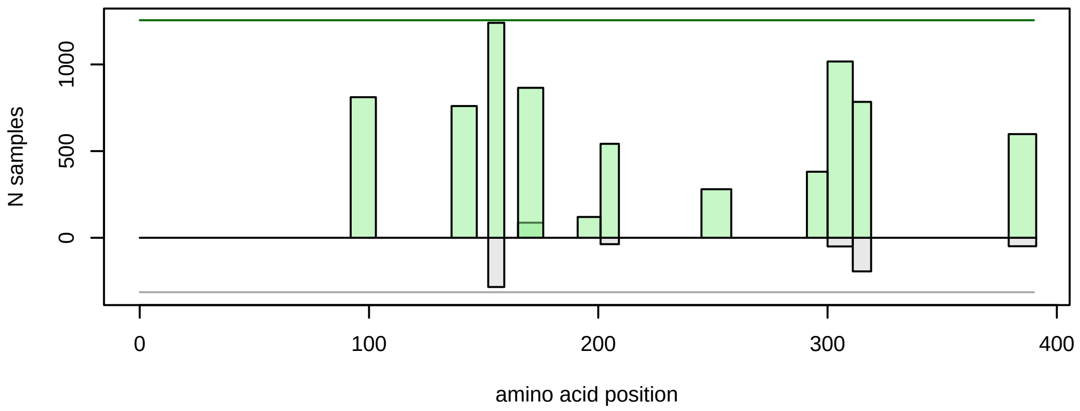

660. RPL7A (P62424), NP4

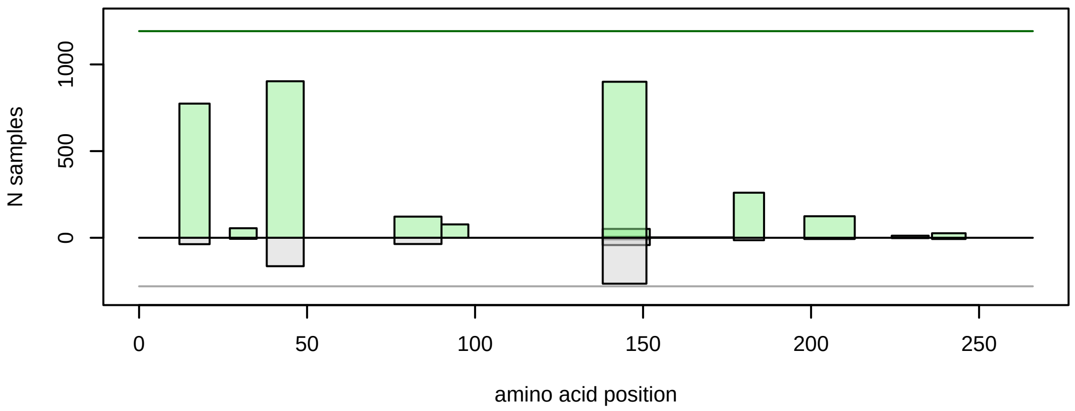

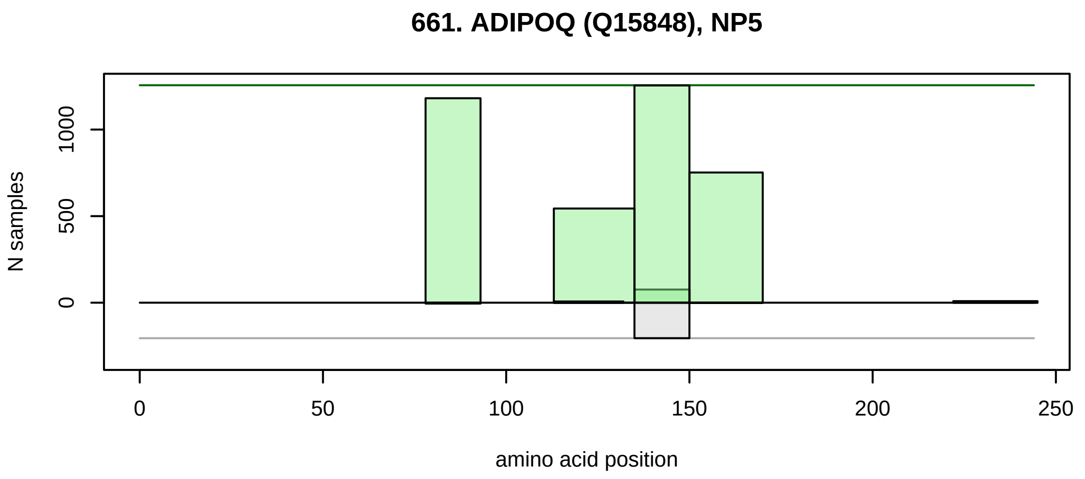
661. ADIPOQ (Q15848), NP5
N samples
0
500
1000
0
50
100
150
200
250
amino acid position

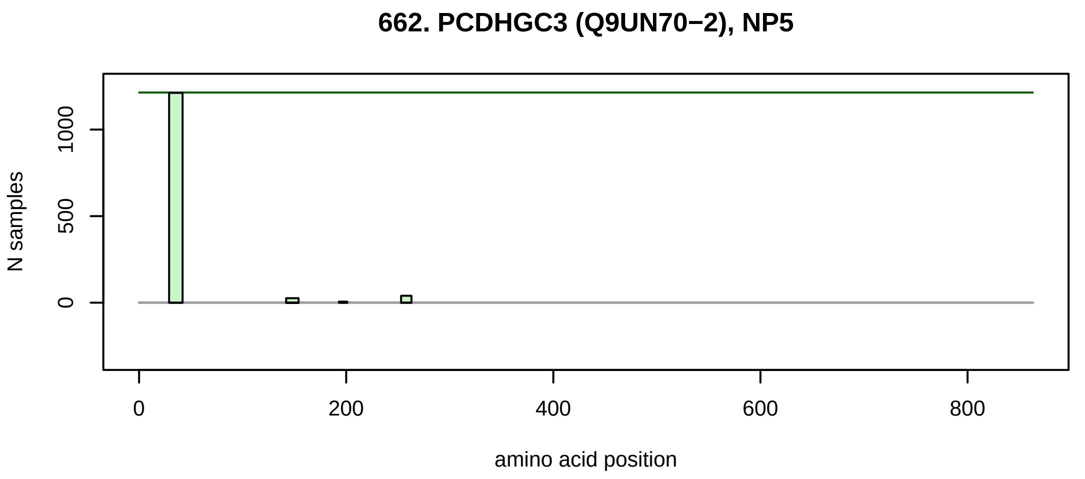
662. PCDHGC3 (Q9UN70−2), NP5
N samples
0
500
1000
0
200
400
600
800
amino acid position

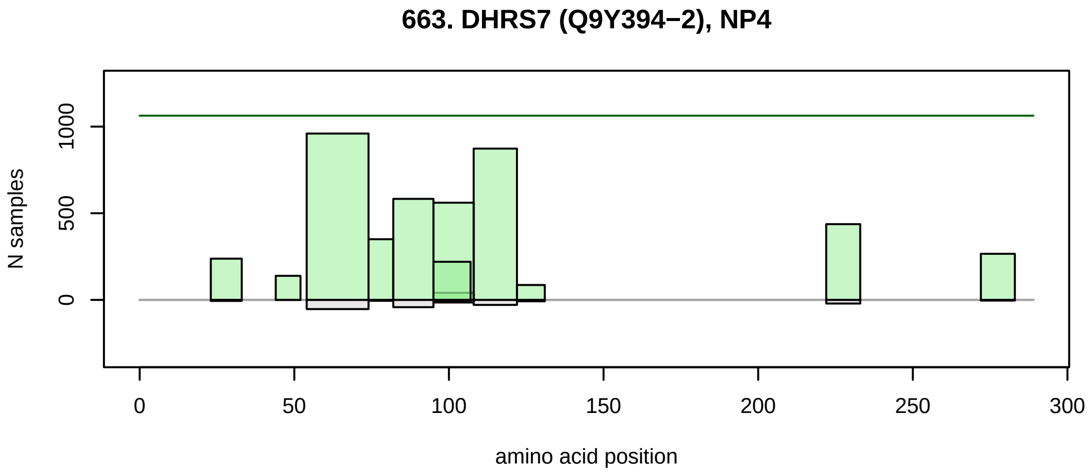
663. DHRS7 (Q9Y394−2), NP4
N samples
0
500
1000
0
50
100
150
200
250
300
amino acid position

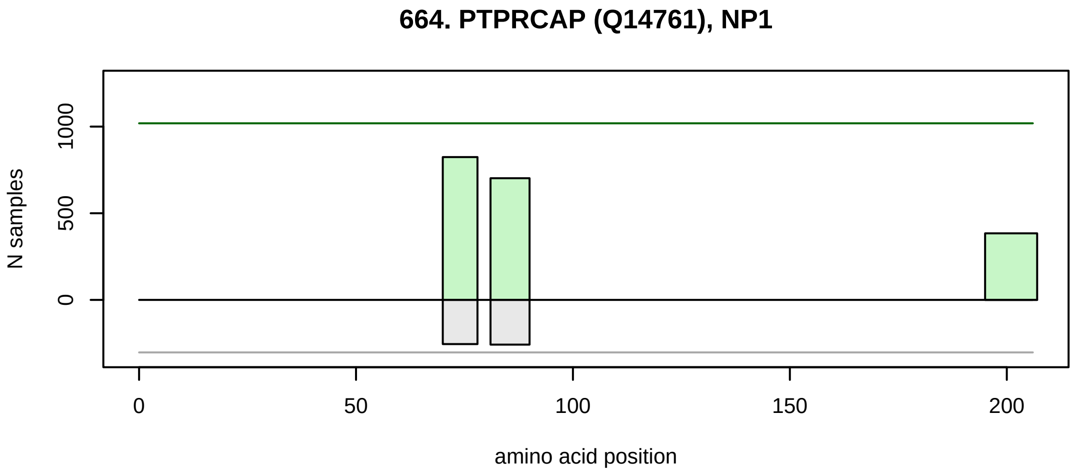
664. PTPRCAP (Q14761), NP1
N samples
0
500
1000
0
50
100
150
200
amino acid position

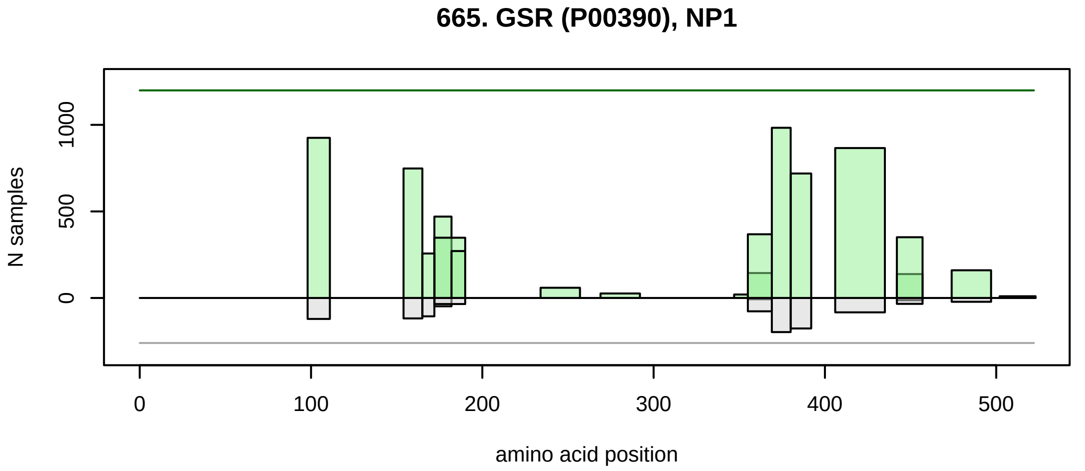
665. GSR (P00390), NP1
N samples
0
500
1000
0
100
200
300
400
500
amino acid position

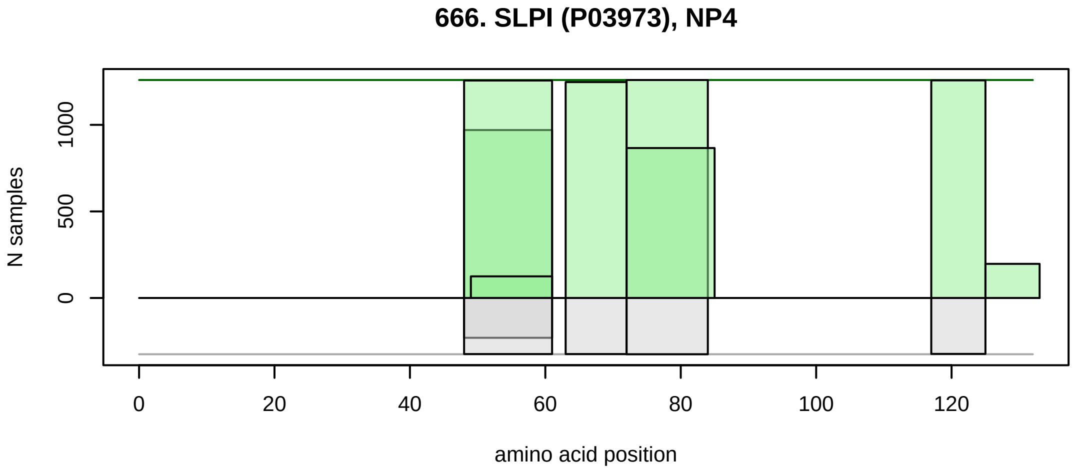
666. SLPI (P03973), NP4
N samples
0
500
1000
0
20
40
60
80
100
120
amino acid position

667. ENO3 (P13929), NP3

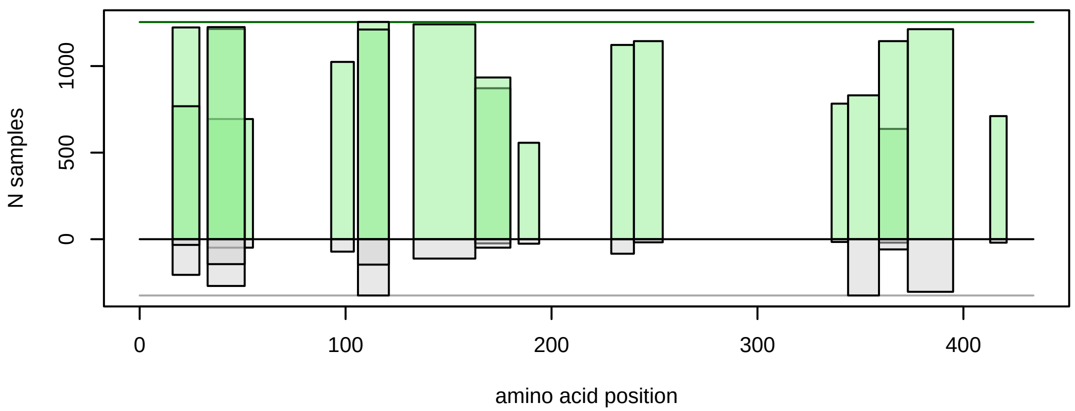

668. TUBA8 (Q9NY65), NP4

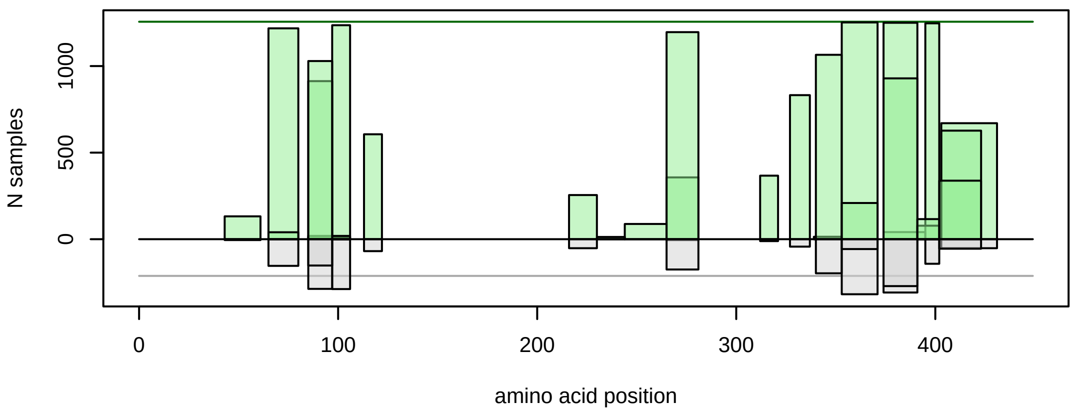

669. NUCKS1 (Q9H1E3), NP2

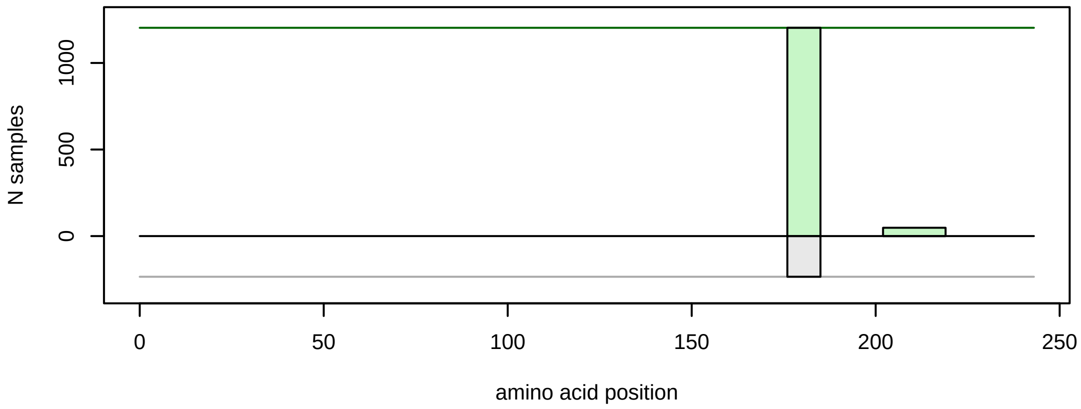

670. ABCF1 (Q5STZ8;Q8NE71), NP1

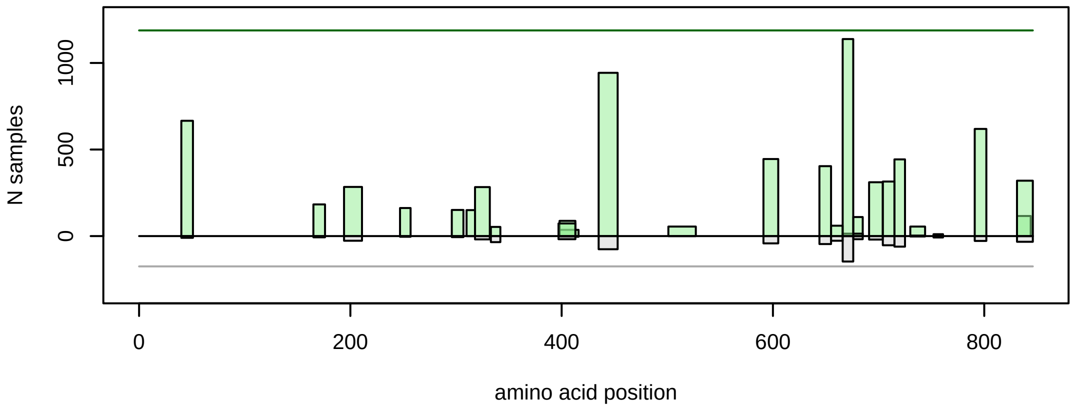

671. ACAN (A0A5K1VW97;H0YMF1;P16112;P16112−2), NP2

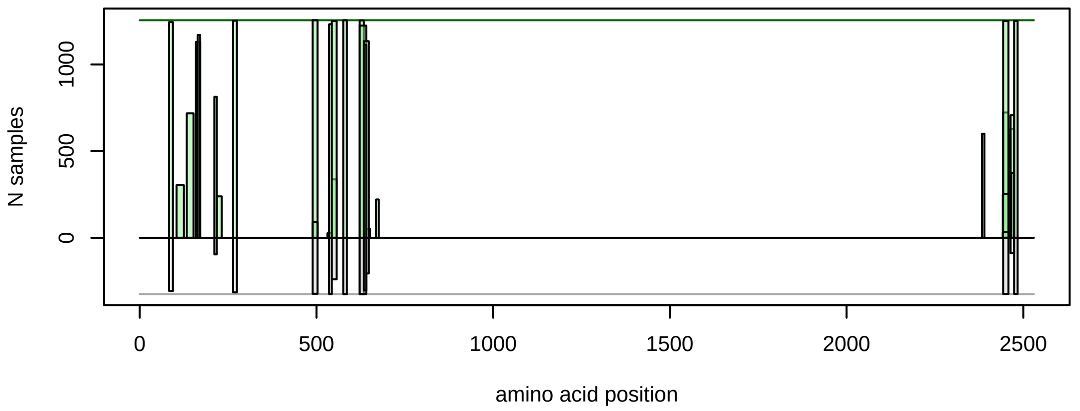

672. PRB2 (P02812), NP1

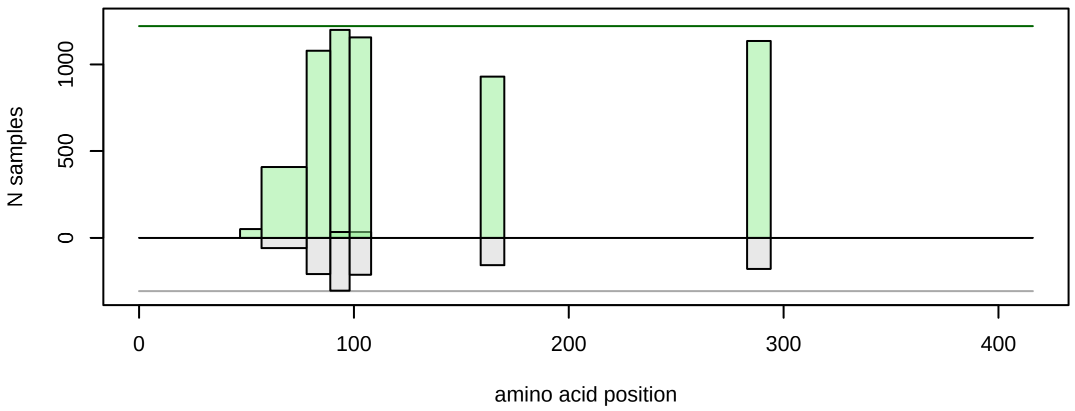

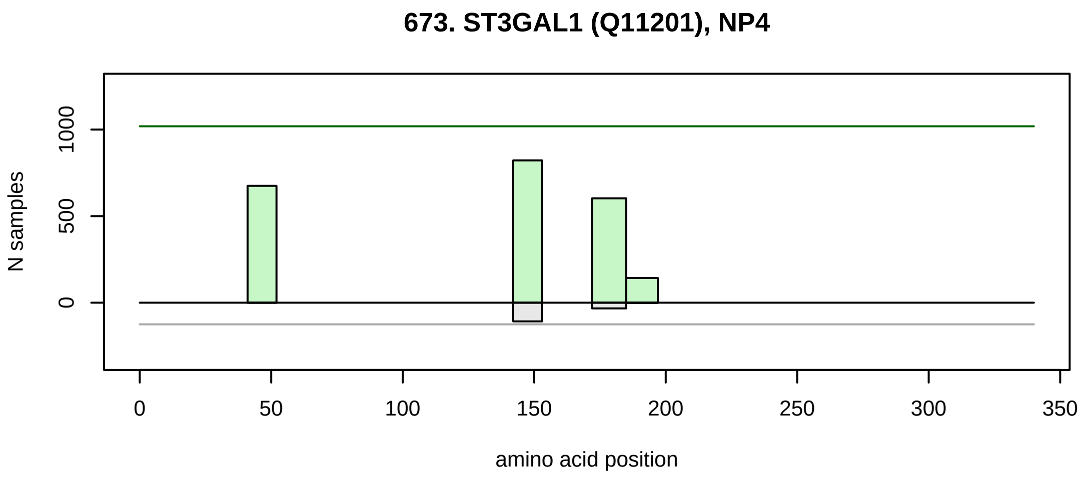
673. ST3GAL1 (Q11201), NP4
N samples
0
500
1000
0
50
100
150
200
250
300
350
amino acid position

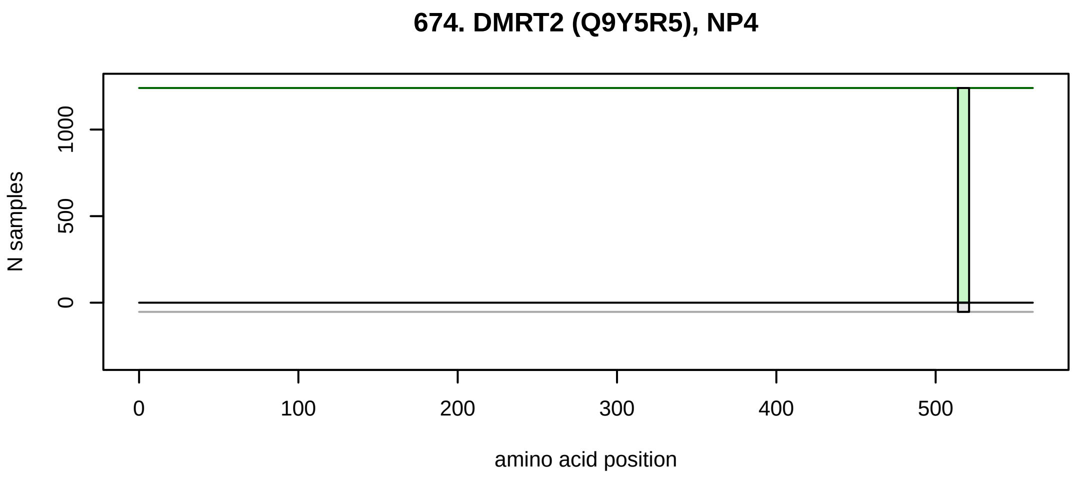
674. DMRT2 (Q9Y5R5), NP4
N samples
0
500
1000
0
100
200
300
400
500
amino acid position

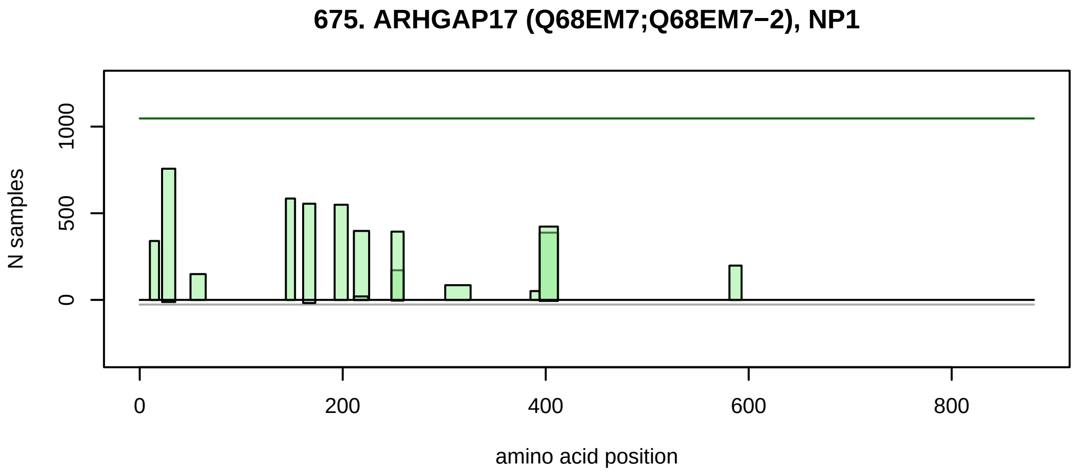
675. ARHGAP17 (Q68EM7;Q68EM7−2), NP1
N samples
0
500
1000
0
200
400
600
800
amino acid position

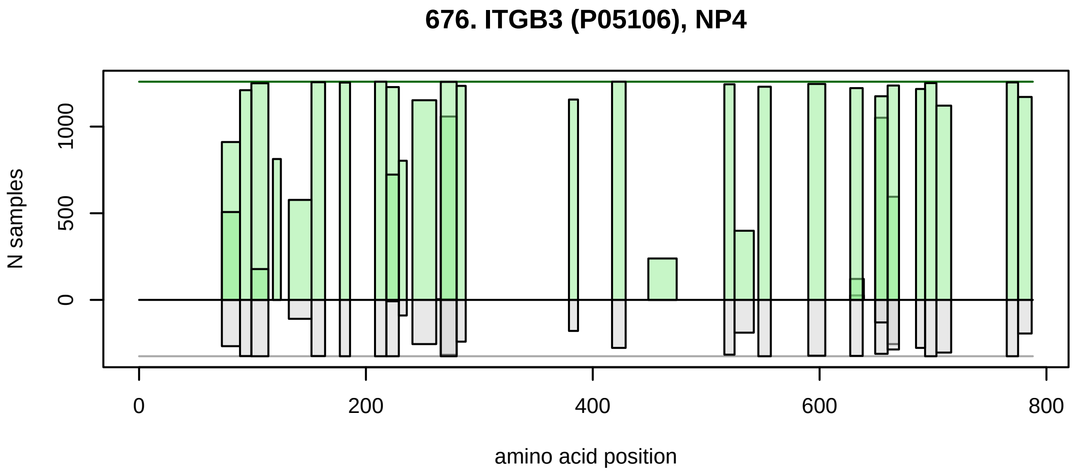
676. ITGB3 (P05106), NP4
N samples
0
500
1000
0
200
400
600
800
amino acid position

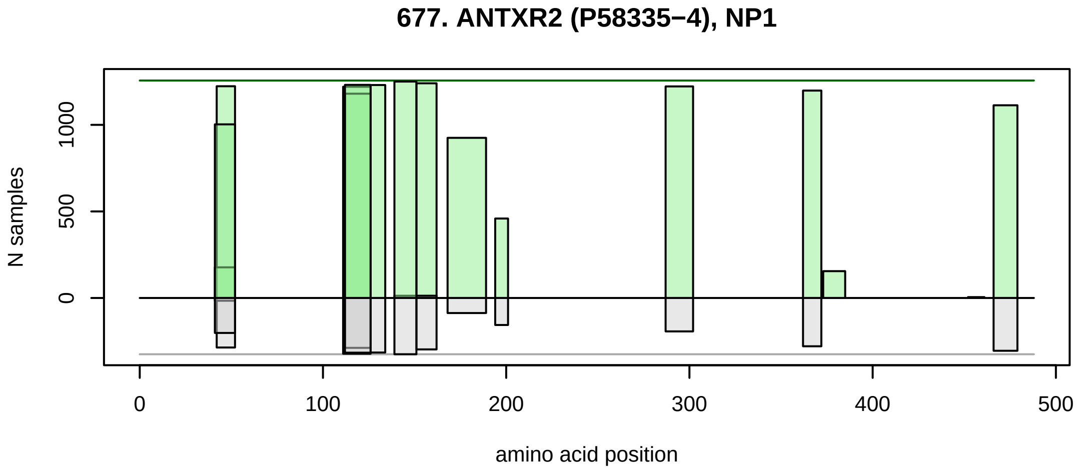
677. ANTXR2 (P58335−4), NP1
N samples
0
500
1000
0
100
200
300
400
500
amino acid position

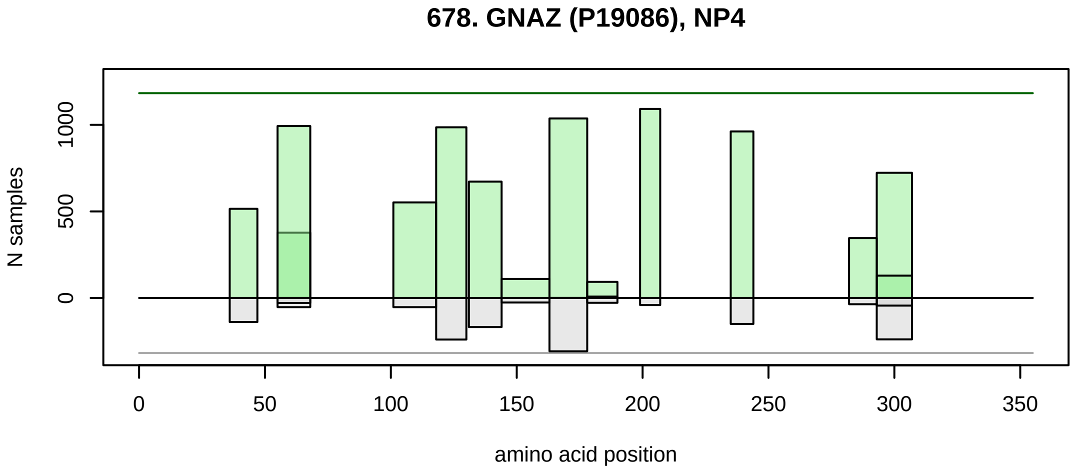
678. GNAZ (P19086), NP4
N samples
0
500
1000
0
50
100
150
200
250
300
350
amino acid position

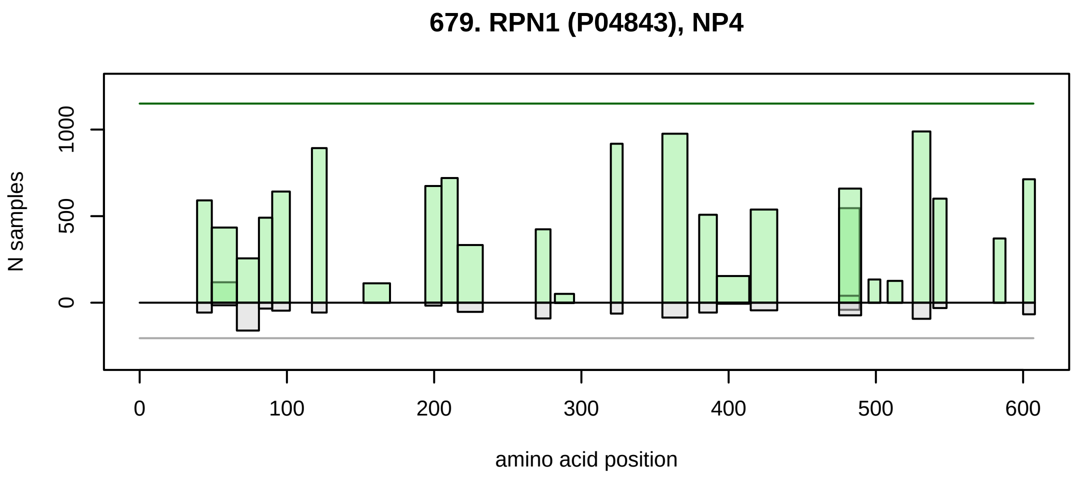
679. RPN1 (P04843), NP4
N samples
0
500
1000
0
100
200
300
400
500
600
amino acid position

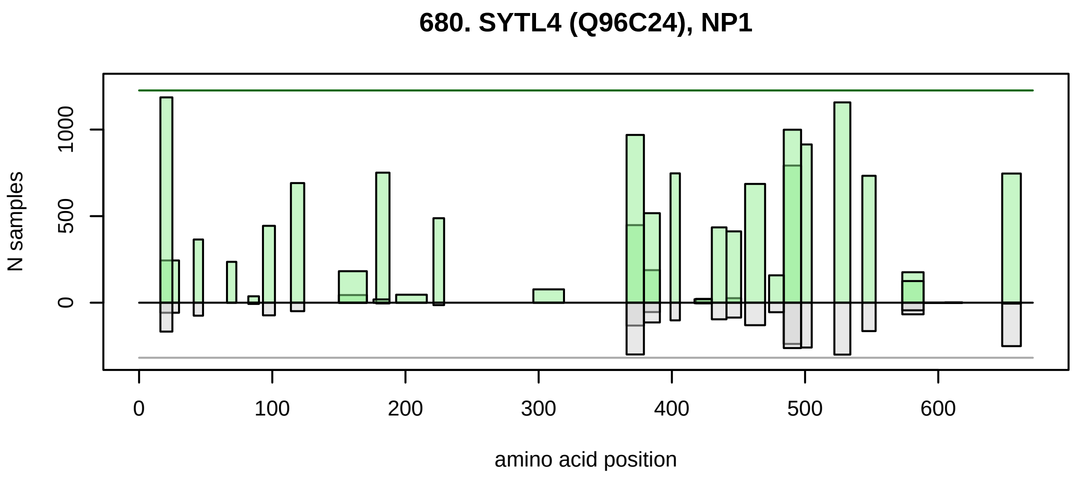
680. SYTL4 (Q96C24), NP1
N samples
0
500
1000
0
100
200
300
400
500
600
amino acid position

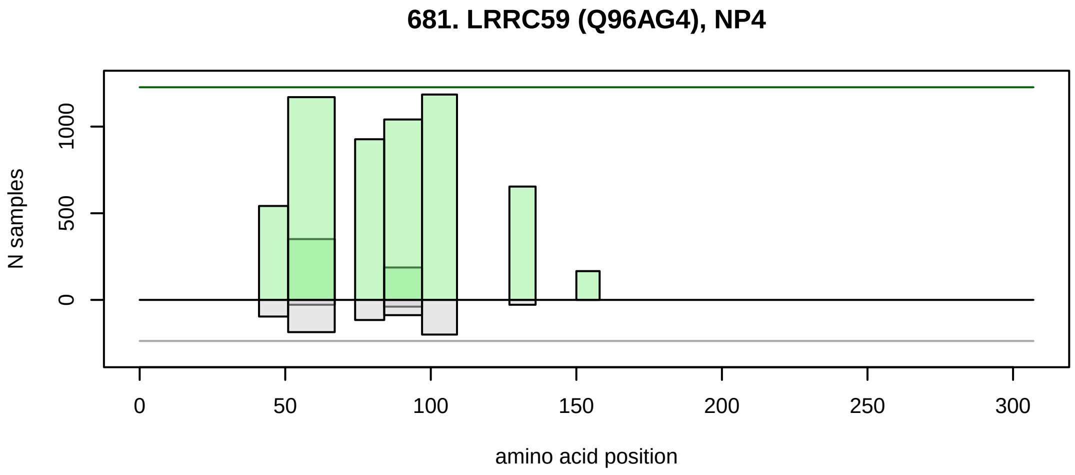
681. LRRC59 (Q96AG4), NP4
N samples
0
500
1000
0
50
100
150
200
250
300
amino acid position

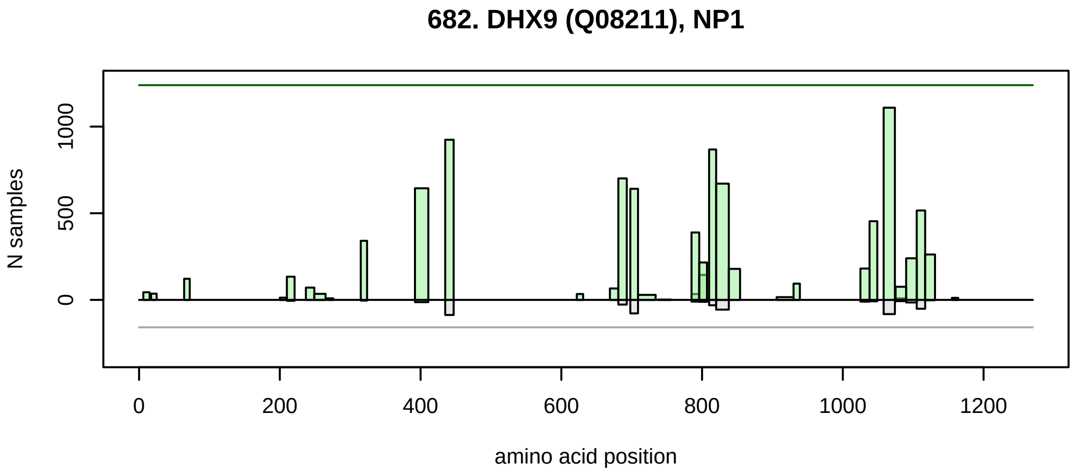
682. DHX9 (Q08211), NP1
N samples
0
500
1000
0
200
400
600
800
1000
1200
amino acid position

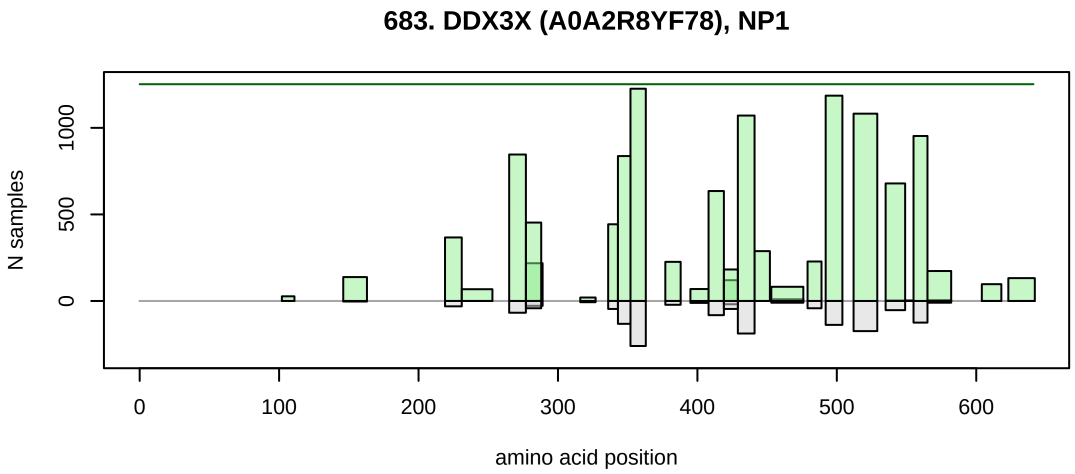
683. DDX3X (A0A2R8YF78), NP1
N samples
0
500
1000
0
100
200
300
400
500
600
amino acid position

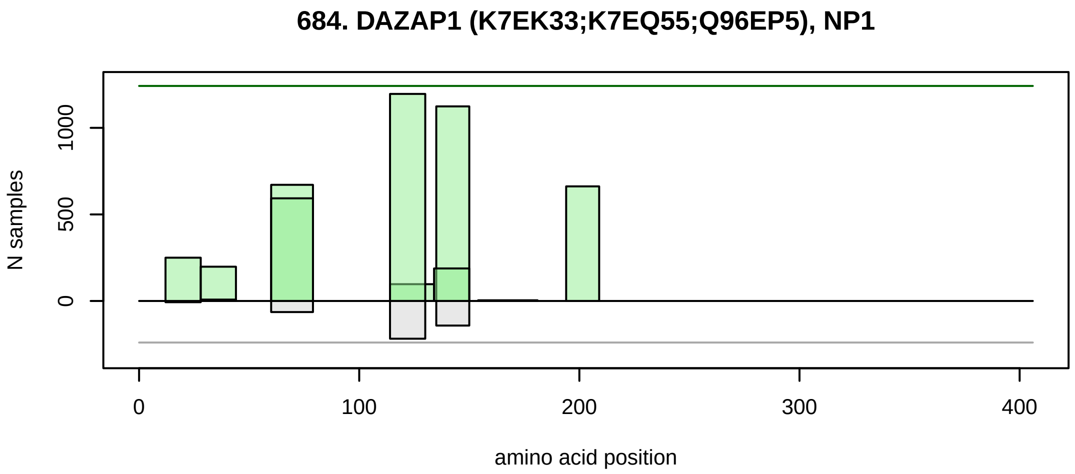
684. DAZAP1 (K7EK33;K7EQ55;Q96EP5), NP1
N samples
0
500
1000
0
100
200
300
400
amino acid position

685. RCN1 (Q15293), NP2

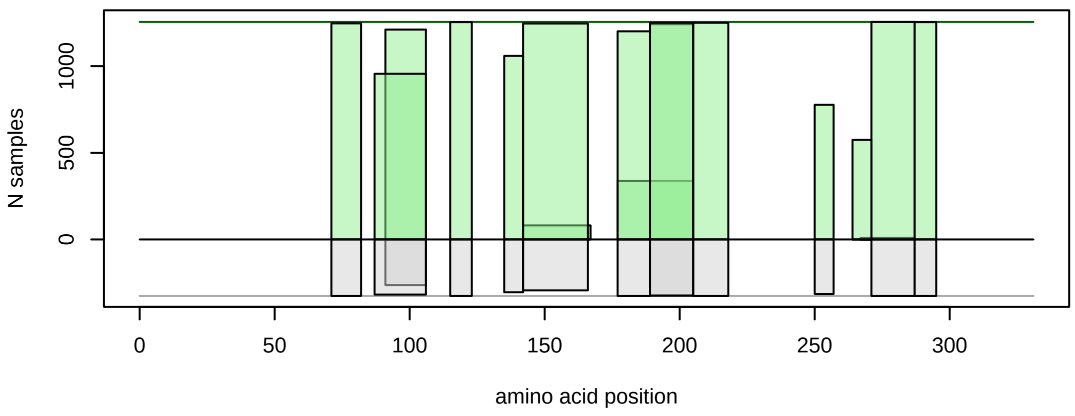

686. PODXL2 (Q9NZ53), NP2

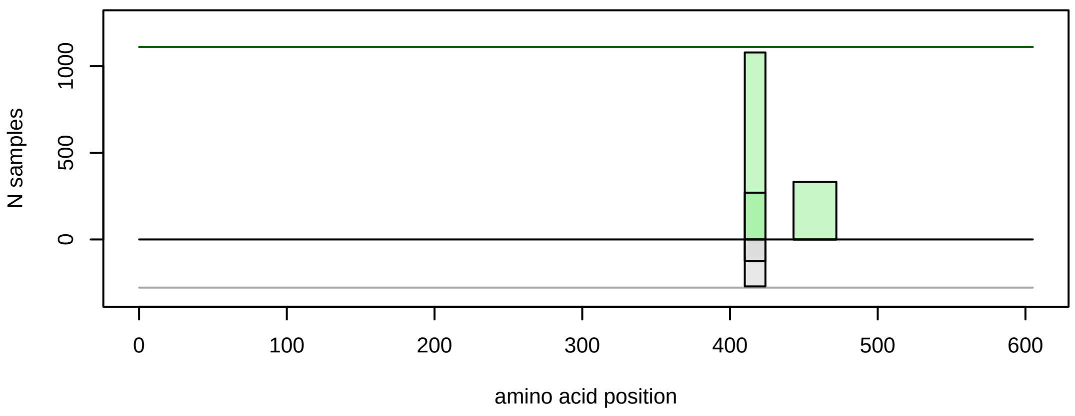

687. GOLM1 (Q8NBJ4), NP2

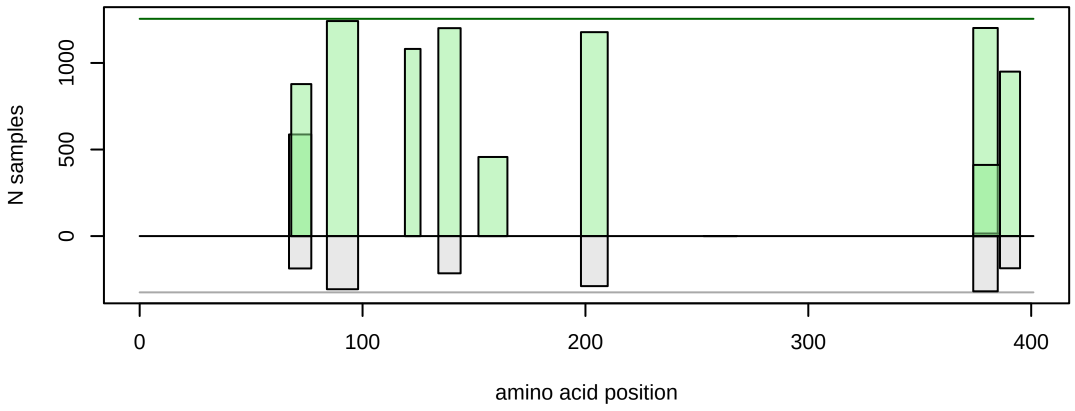

688. ST6GAL1 (P15907), NP3

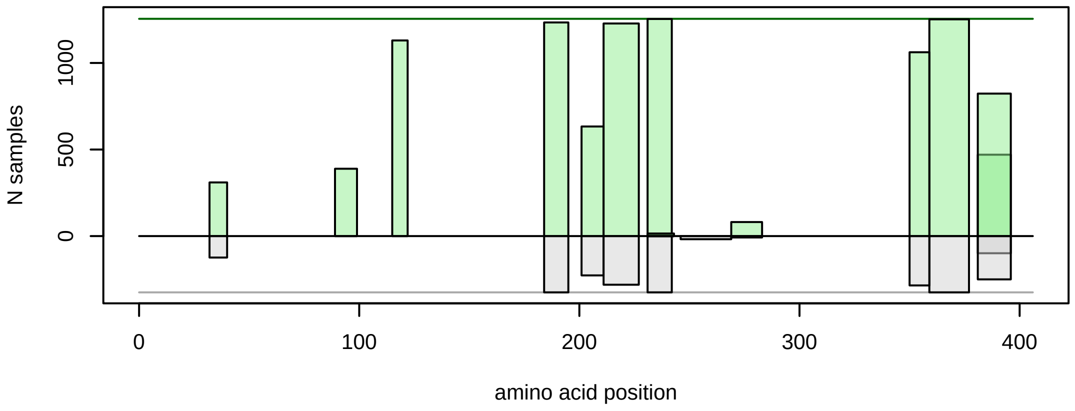

689. CD9 (A0A087WU13), NP1

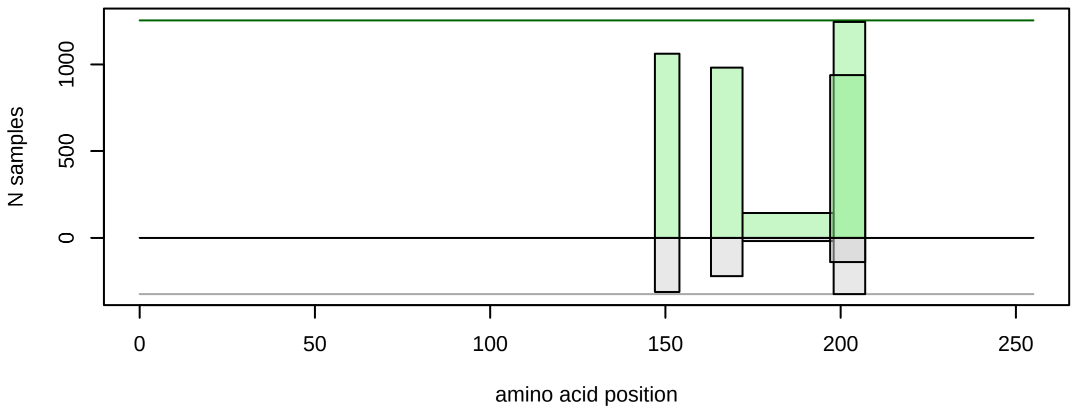

690. SERPINF1 (P36955), NP1

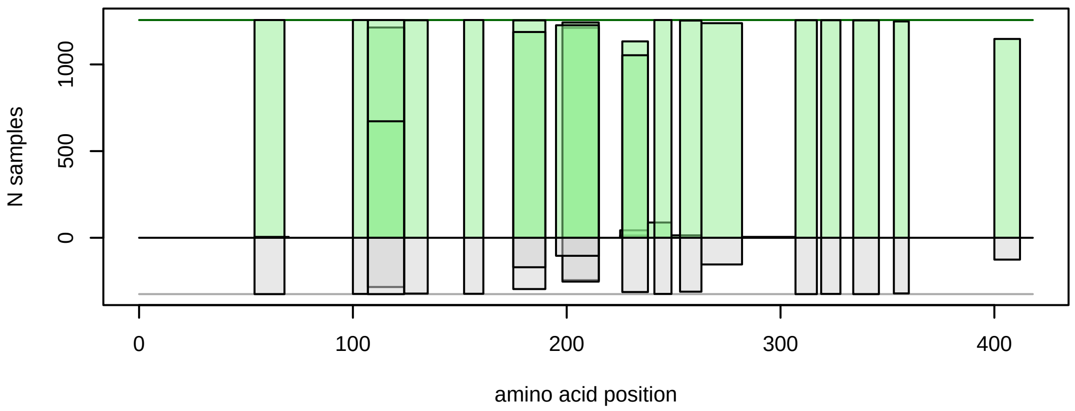

691. MGLL (Q99685), NP4

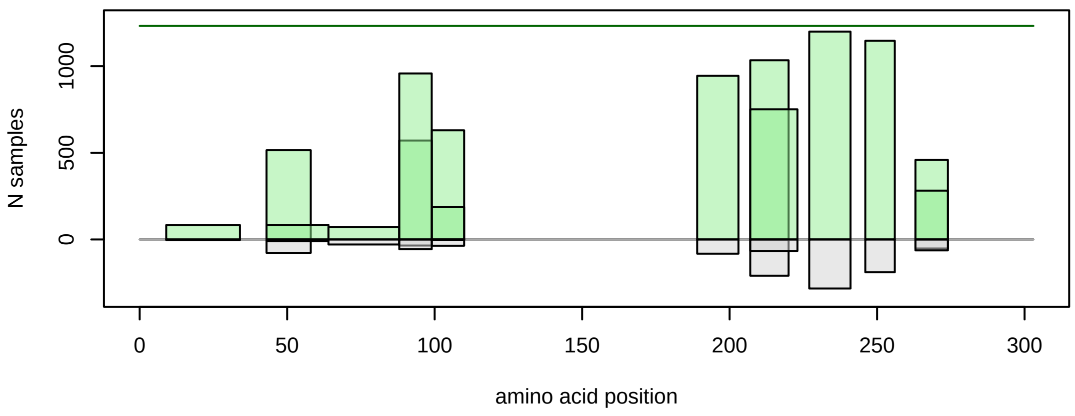

692. CHRDL2 (Q6WN34), NP1

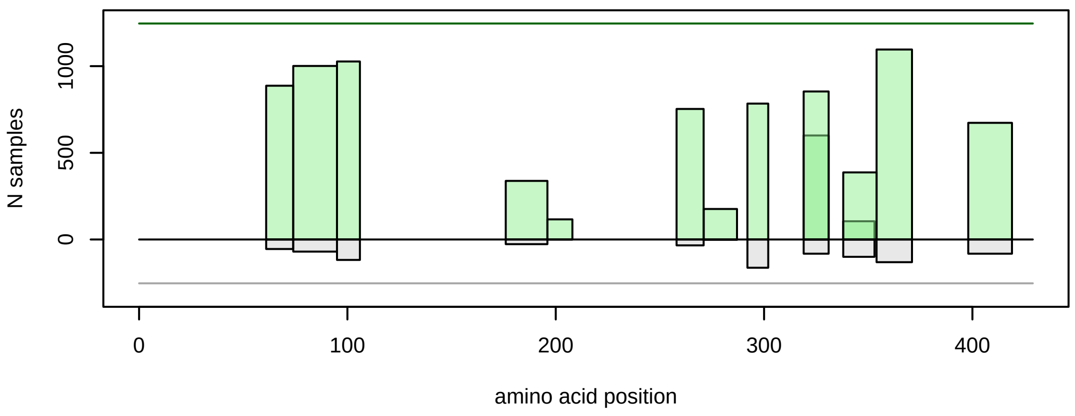

693. DPYSL2 (Q16555−2), NP1

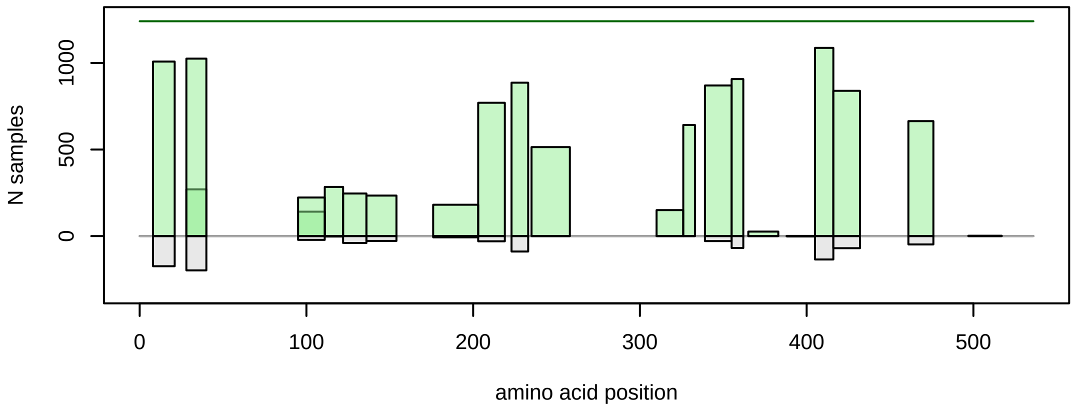

694. MPST (P25325−2), NP5

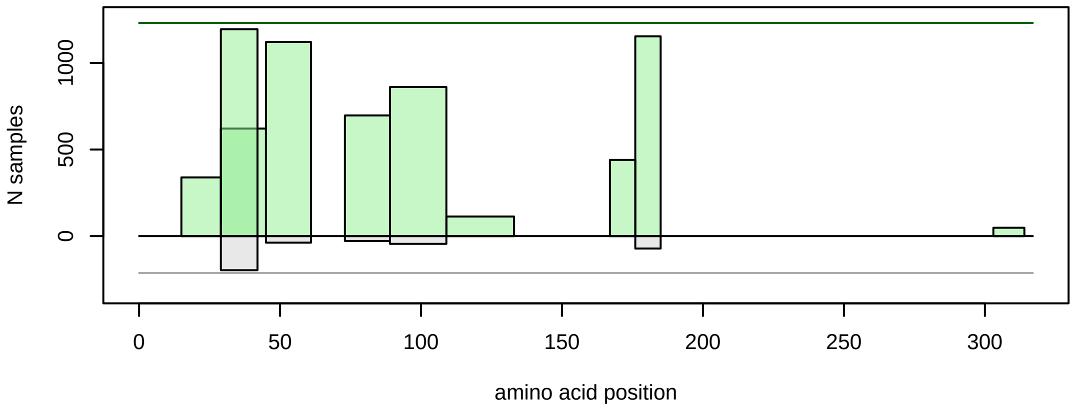

695. RHCE (F6XSS0), NP1

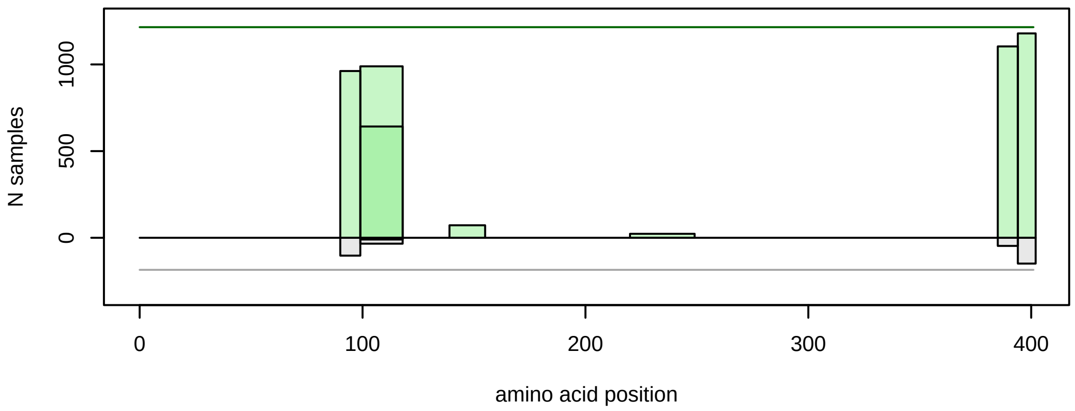

696. IGJ (P01591), NP4

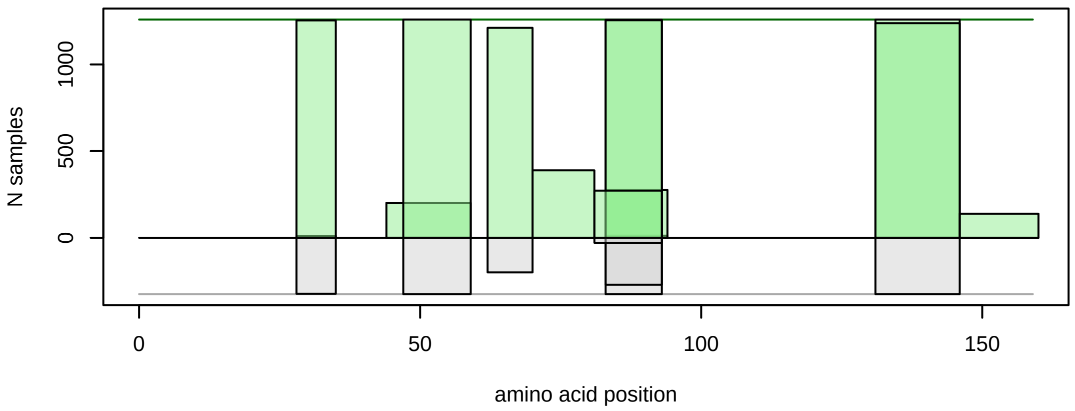

697. GHRL (Q9UBU3;Q9UBU3−2;Q9UBU3−3;Q9UBU3−4), NP2

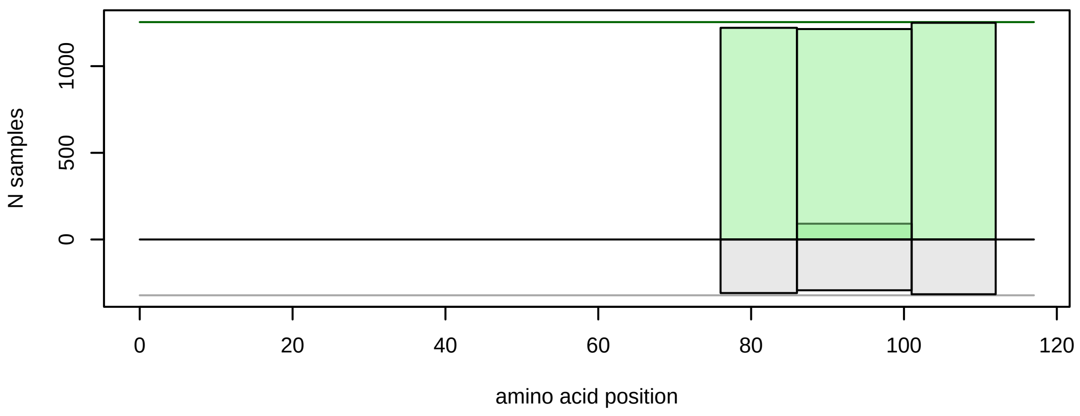

698. CA2 (P00918), NP3

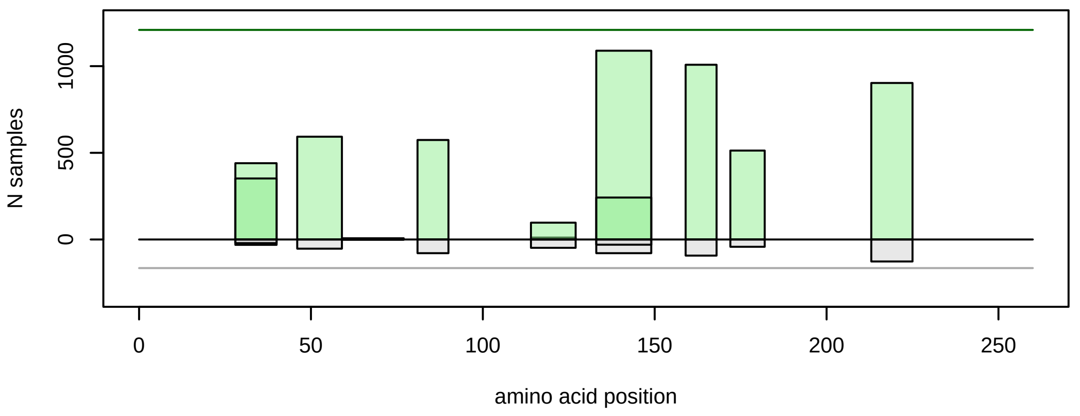

699. TREML1 (Q86YW5), NP4

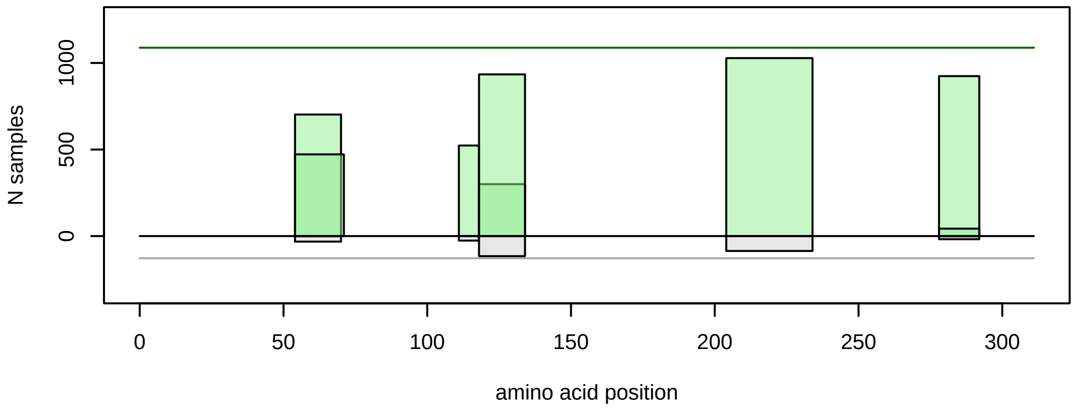

700. SLC9A3R1 (O14745), NP2

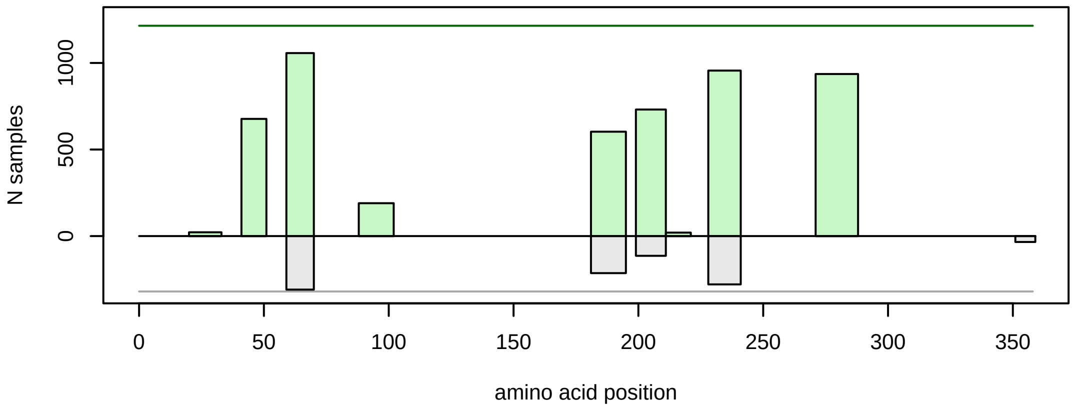

701. ITGB2 (A0A494C0X7), NP1

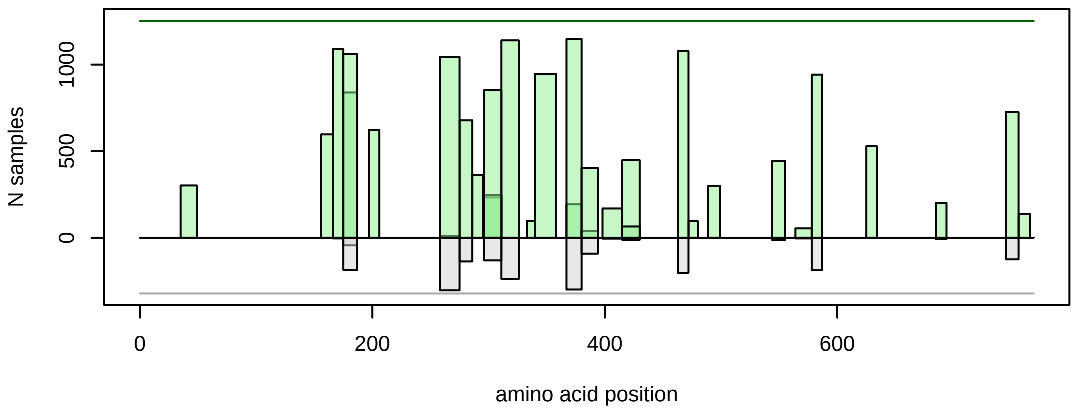

702. GTPBP2 (Q9BX10), NP4

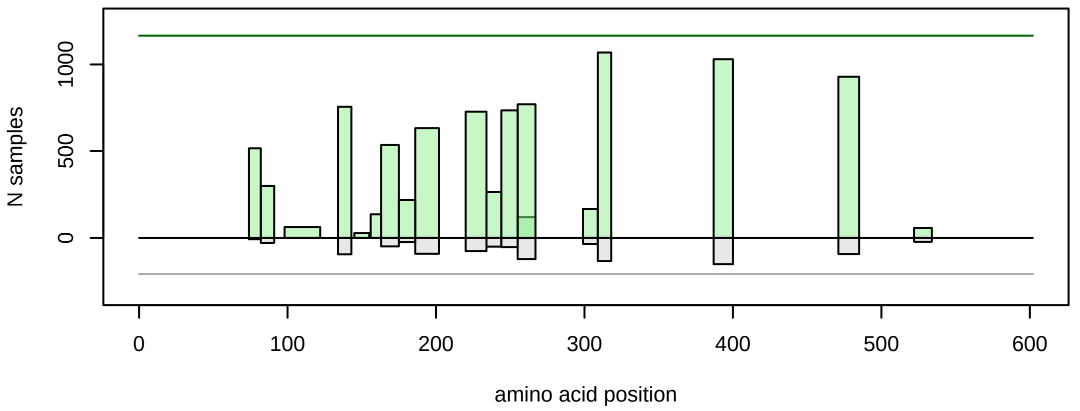

703. SACM1L (Q9NTJ5), NP4

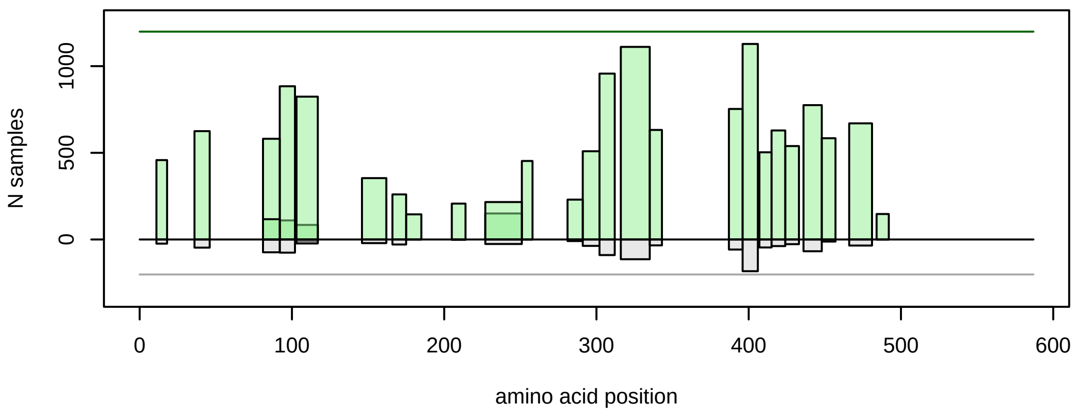

704. IDH1 (O75874), NP5

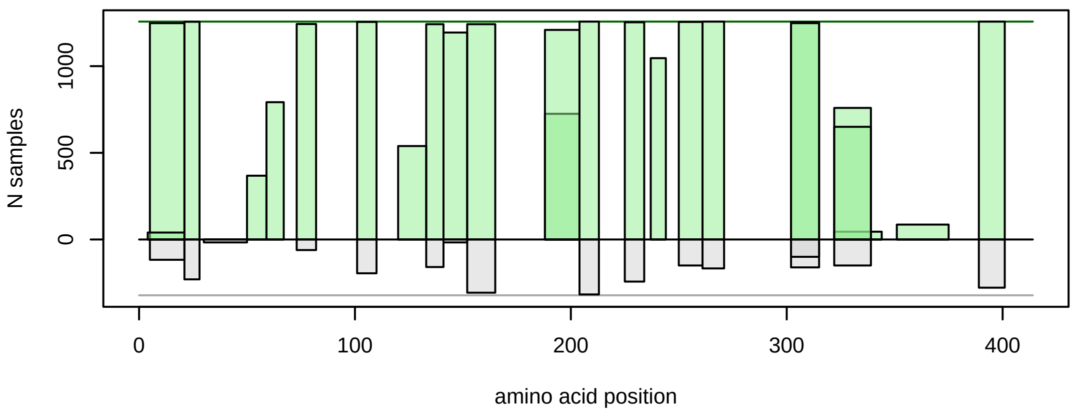

705. SPON1 (Q9HCB6), NP4

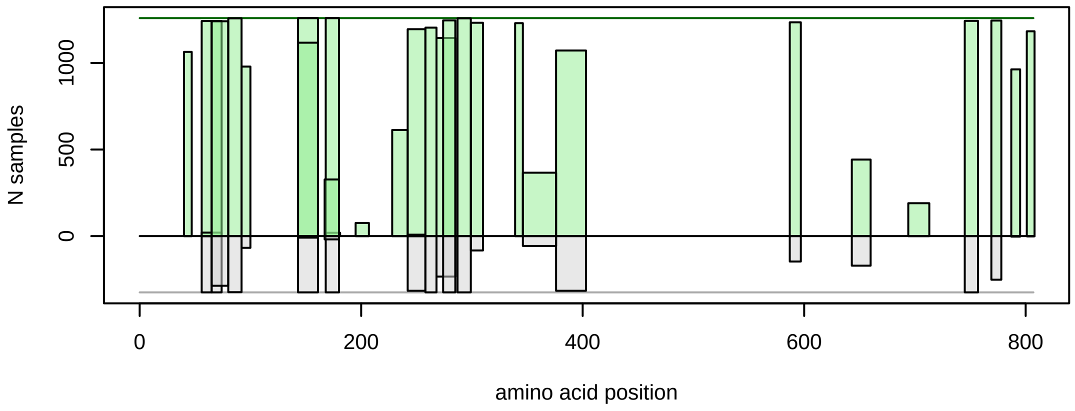

706. CP (E9PFZ2), NP5

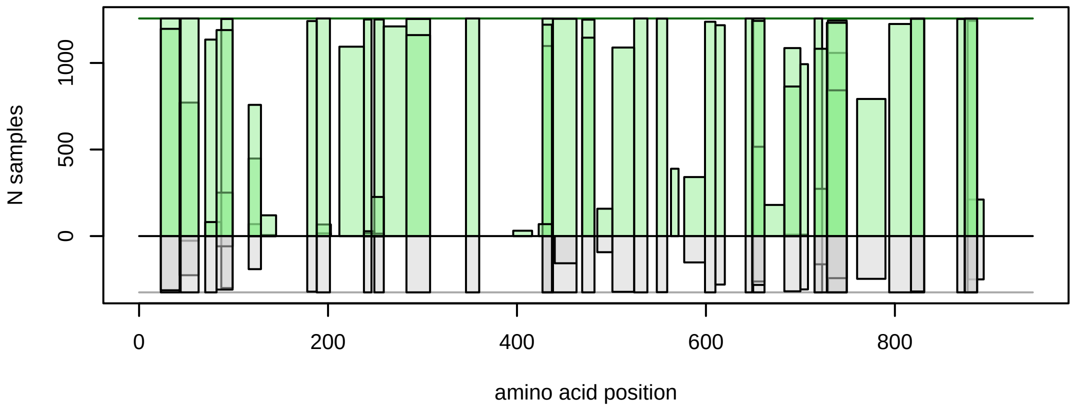

707. PABPC1 (P11940), NP1

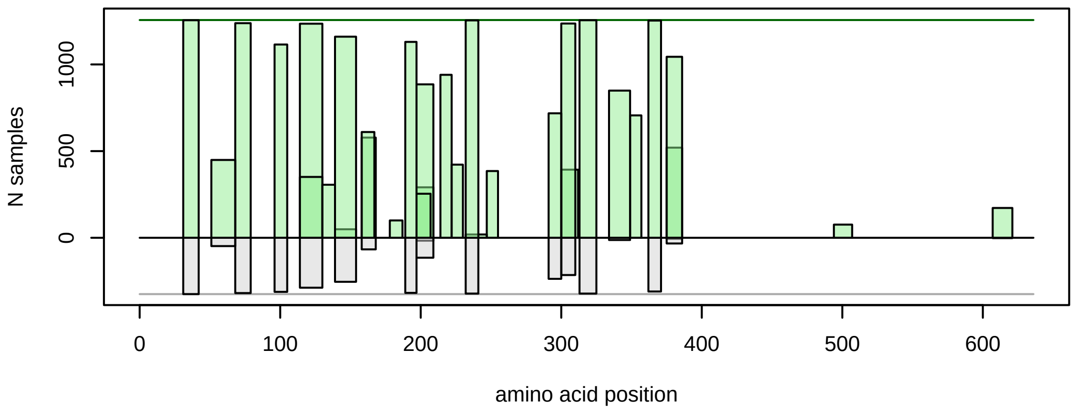

708. CSPG4 (Q6UVK1), NP2

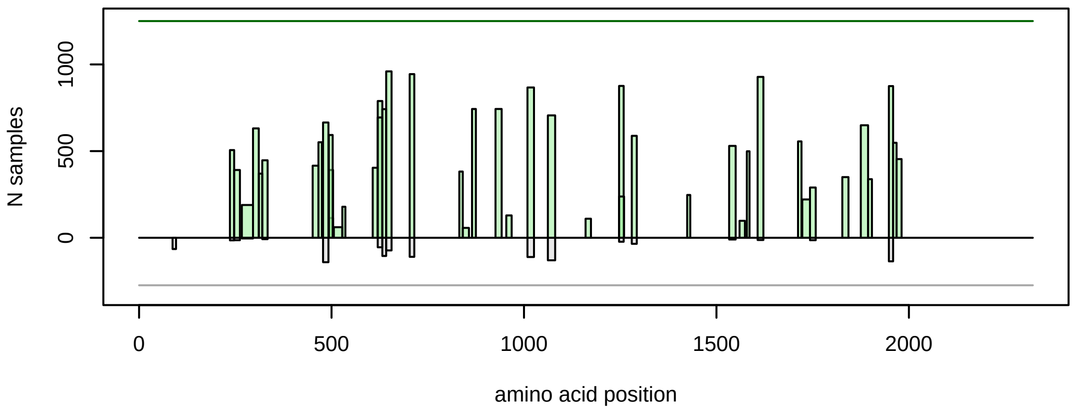

709. SRSF4 (Q08170), NP2

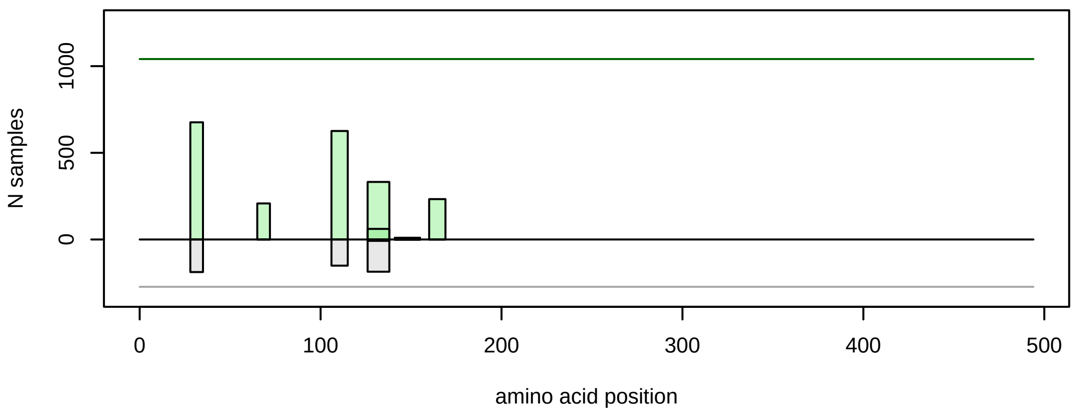

710. UBR3 (Q6ZT12), NP2

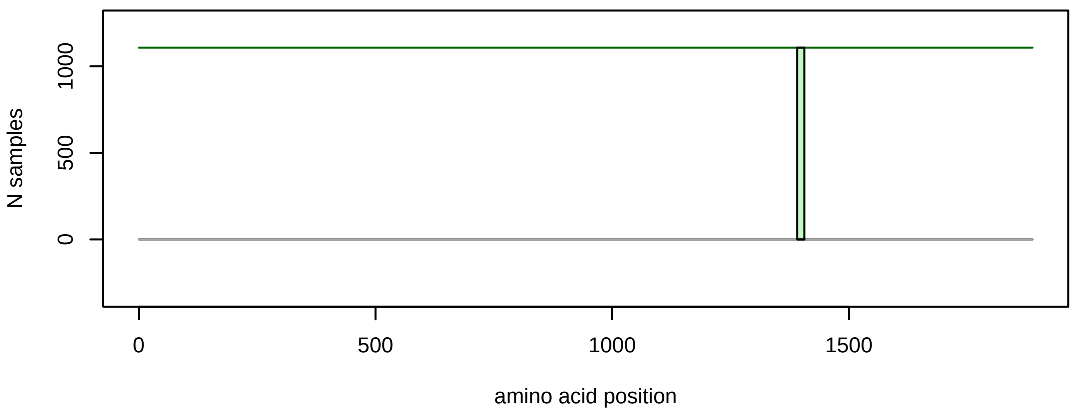

711. ADH4 (P08319), NP3

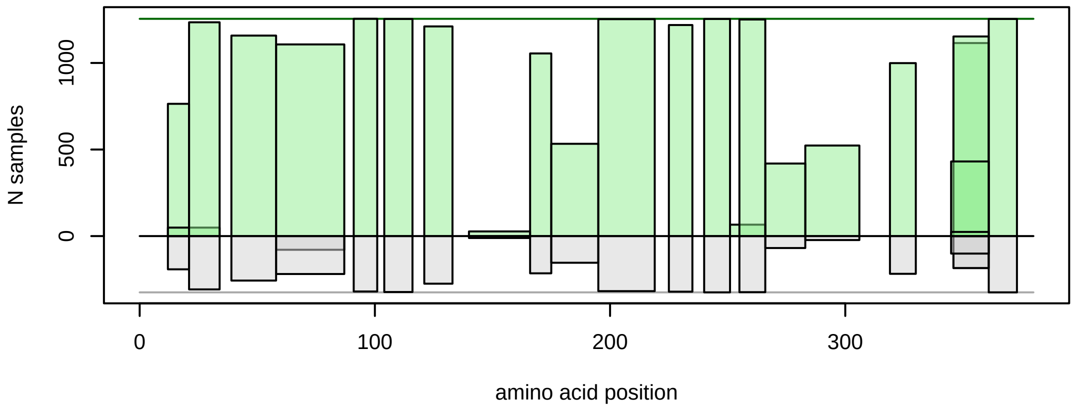

712. MSLN (Q13421), NP4

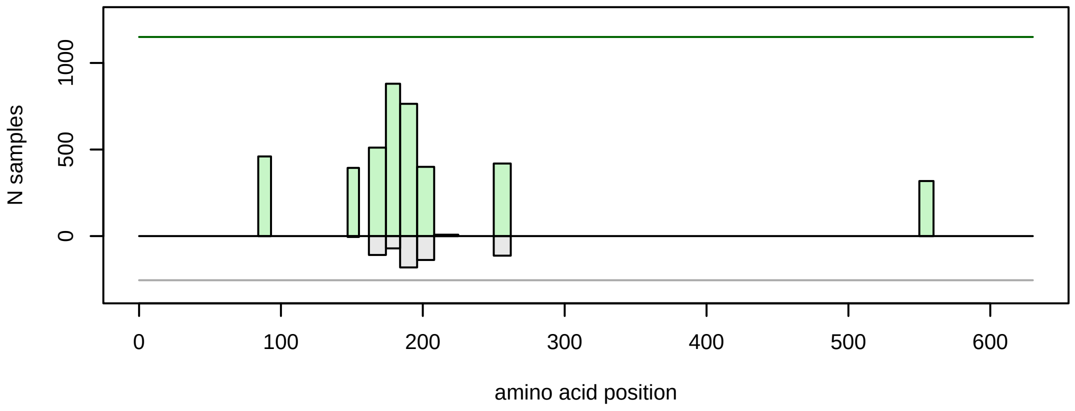

713. NOTCH2 (Q04721), NP5

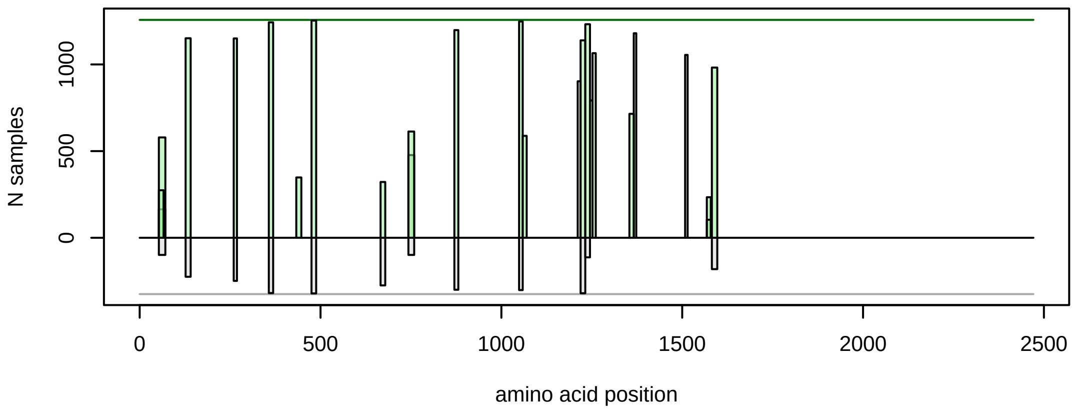

714. CARHSP1 (Q9Y2V2), NP3

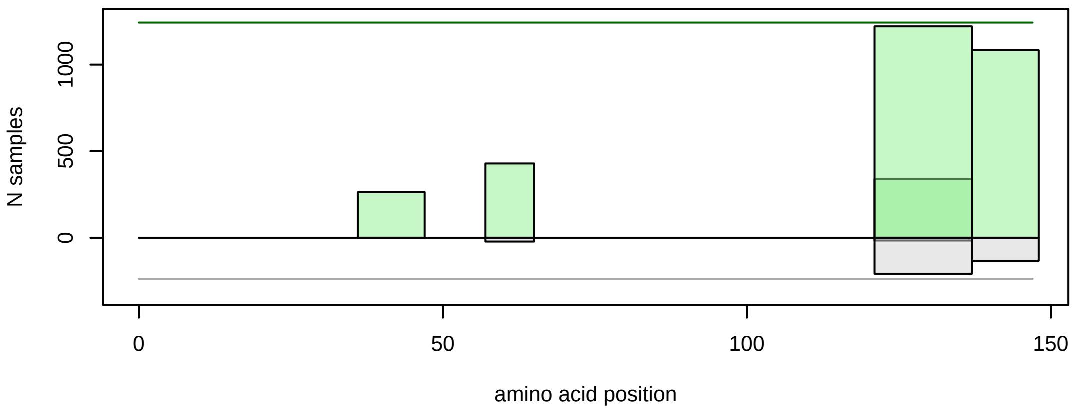

715. TPM3 (A0A087WWU8), NP5

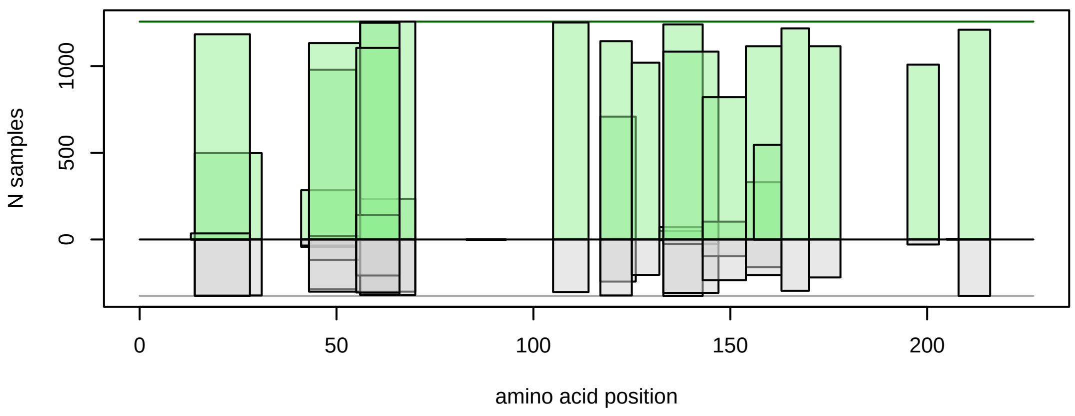

716. IPO5 (O00410), NP2

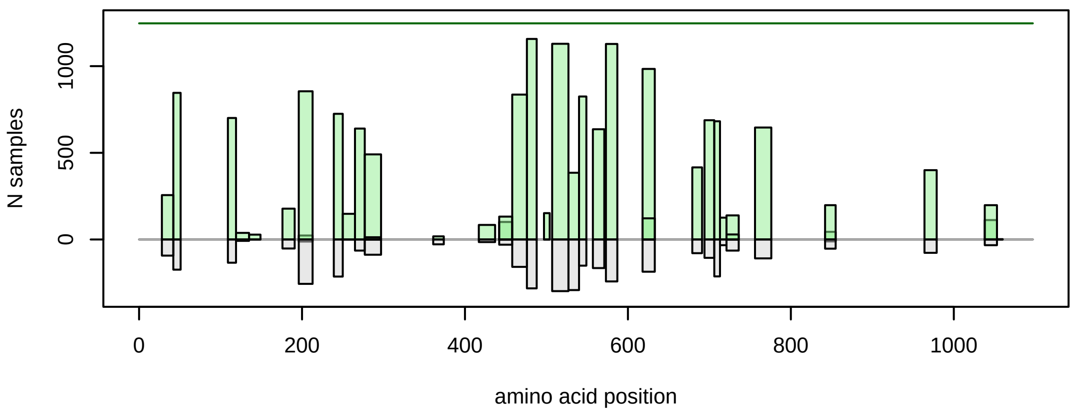

717. UBA1 (P22314), NP5

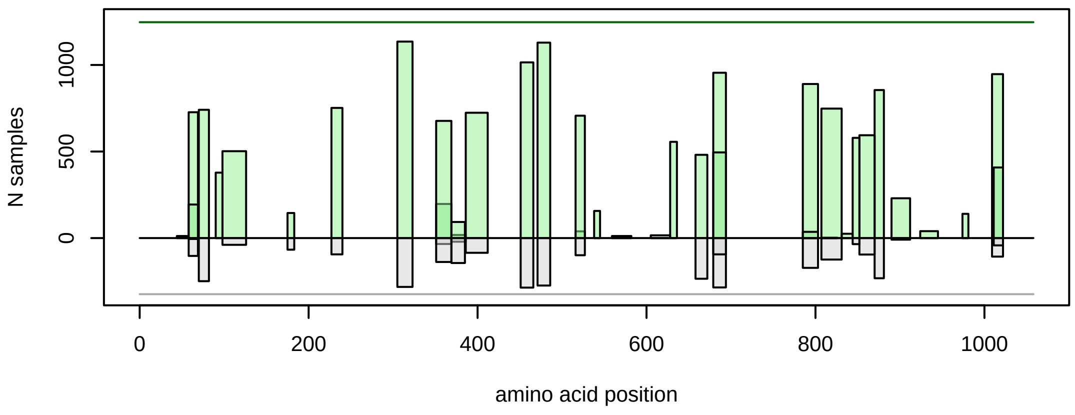

718. HIST1H2BC (O60814;P57053;P62807), NP4

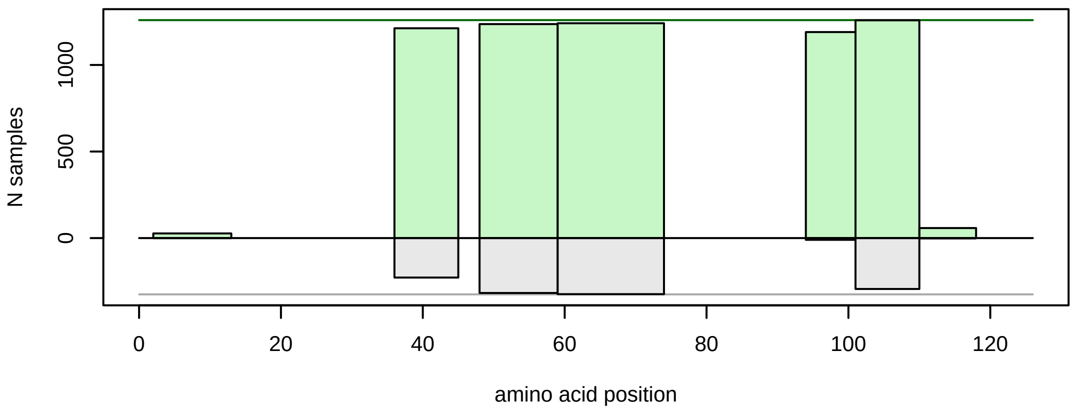

719. ACY1 (Q03154), NP5

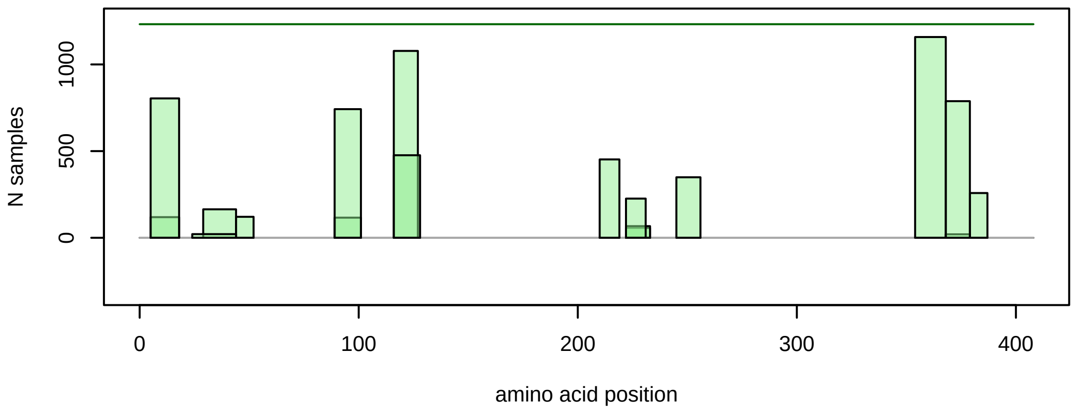

720. C1orf56 (Q9BUN1), NP2

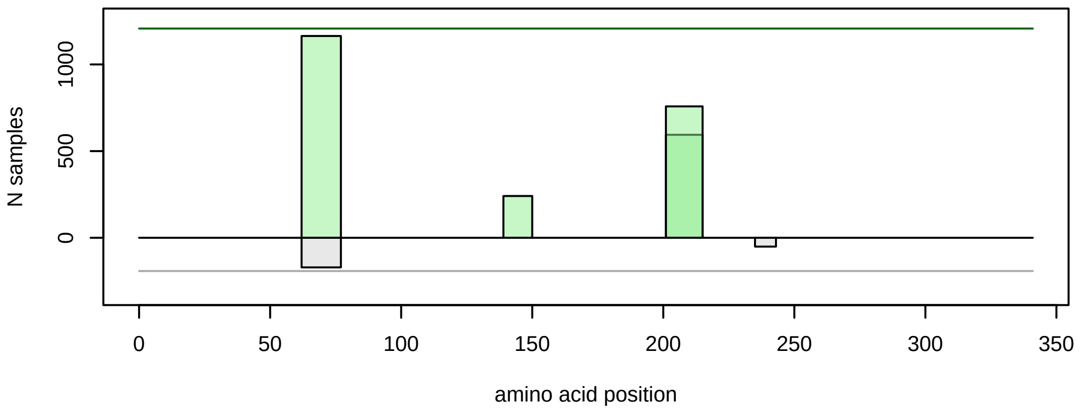

721. APOC3 (B0YIW2;P02656), NP2

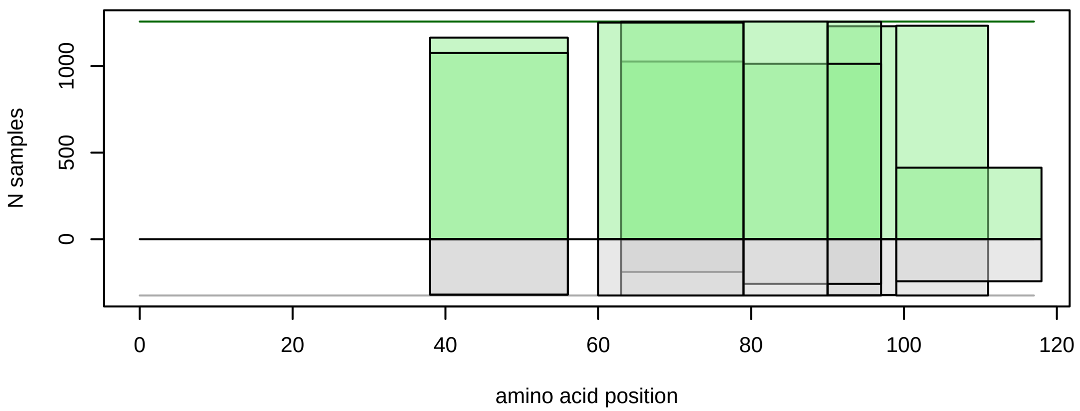

722. PRRC1 (Q96M27;Q96M27−3), NP4

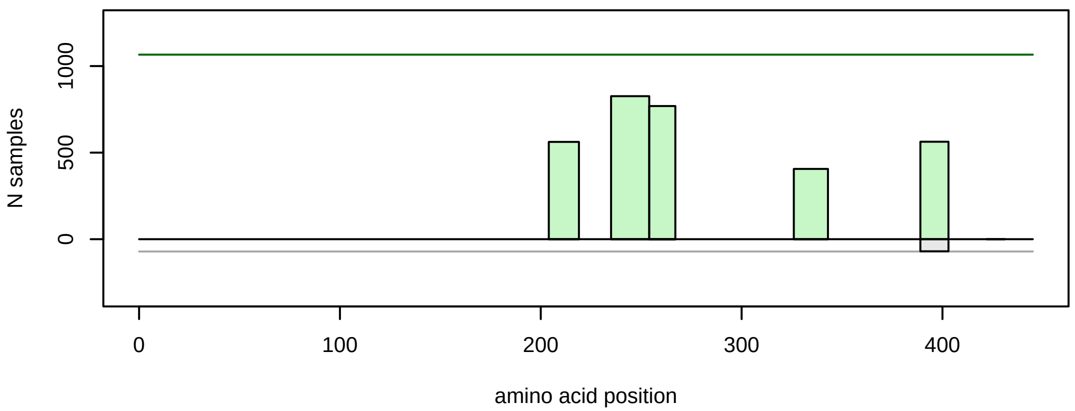

723. UBE2Z (Q9H832), NP2

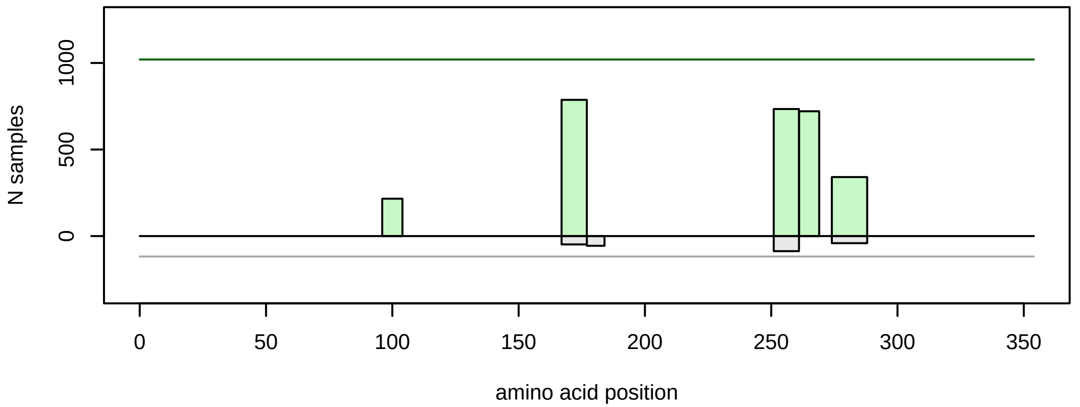

724. RBM3 (P98179), NP1

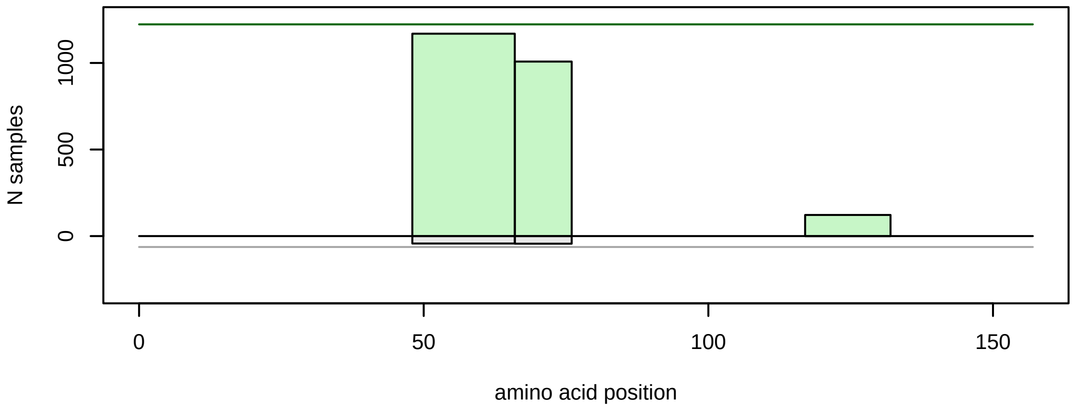

725. CDK5 (Q00535), NP2

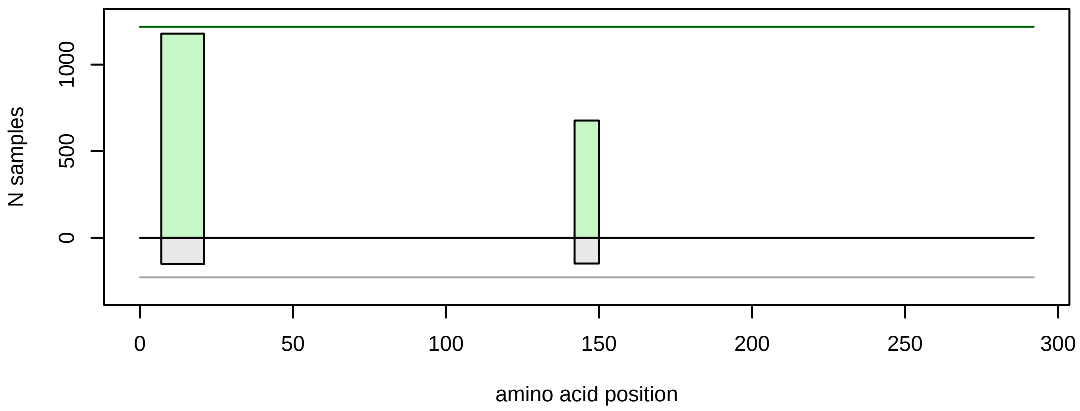

726. PPIP5K2 (O43314), NP1

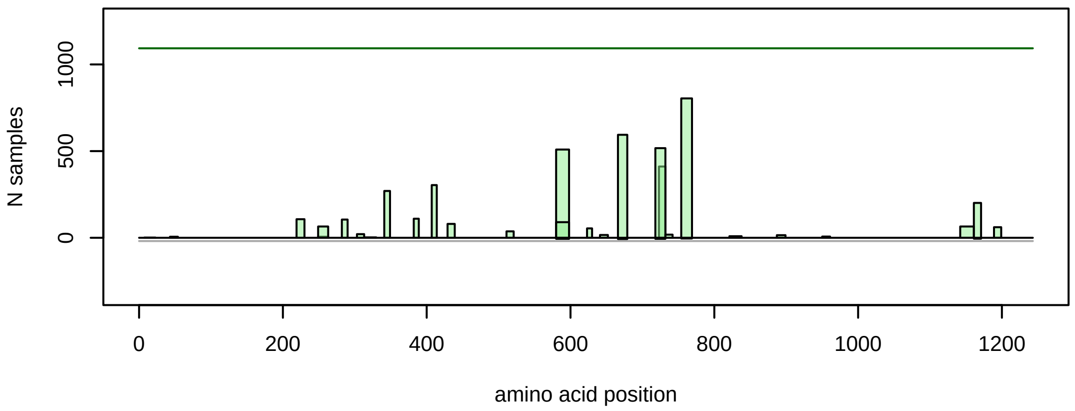

727. C9 (P02748), NP5

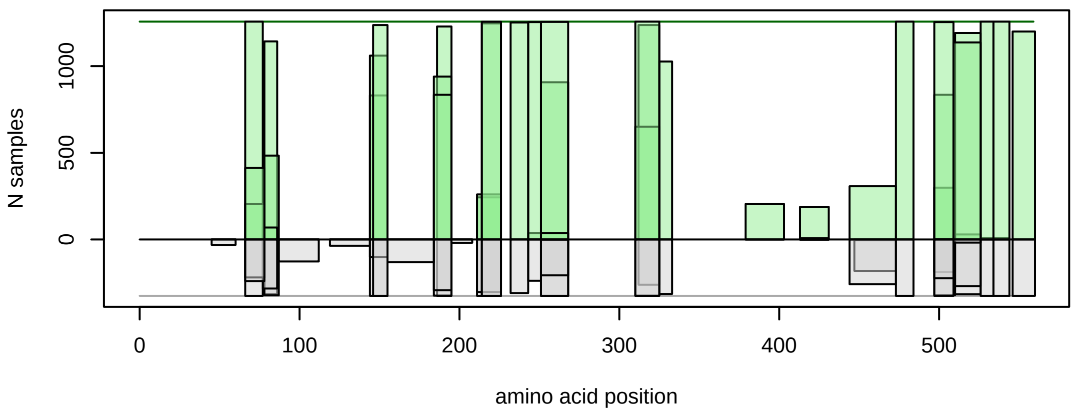

728. OTUB1 (Q96FW1), NP2

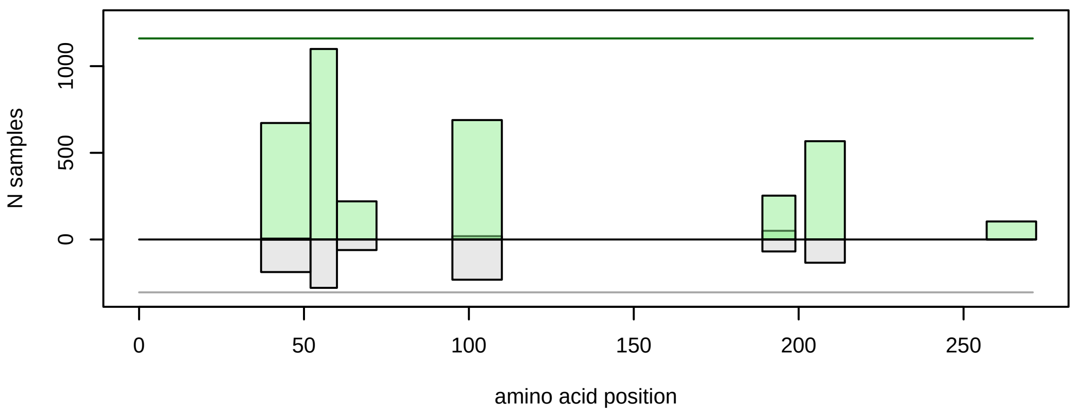

729. SEMA3F (Q13275), NP3

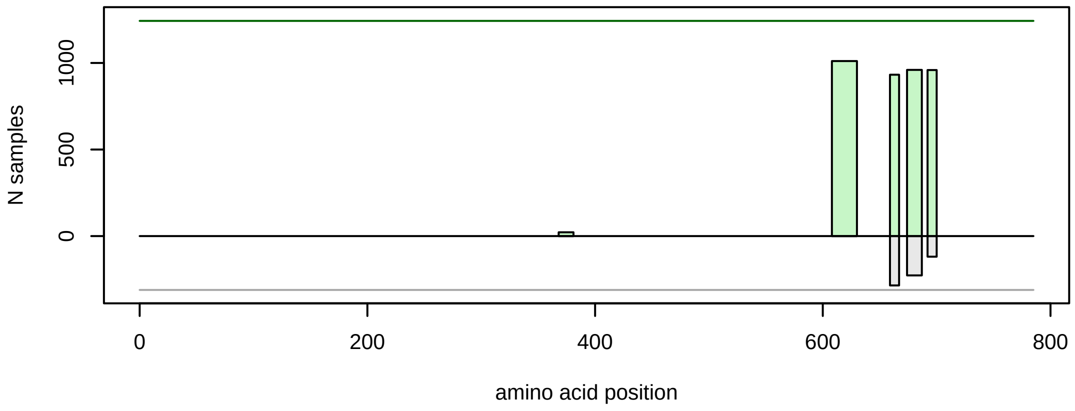

730. PAICS (P22234), NP4

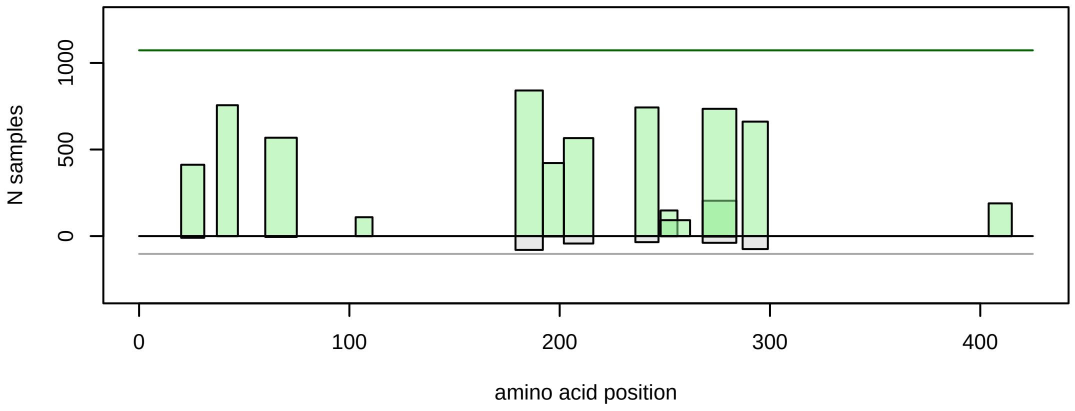

731. KLK10 (O43240), NP3

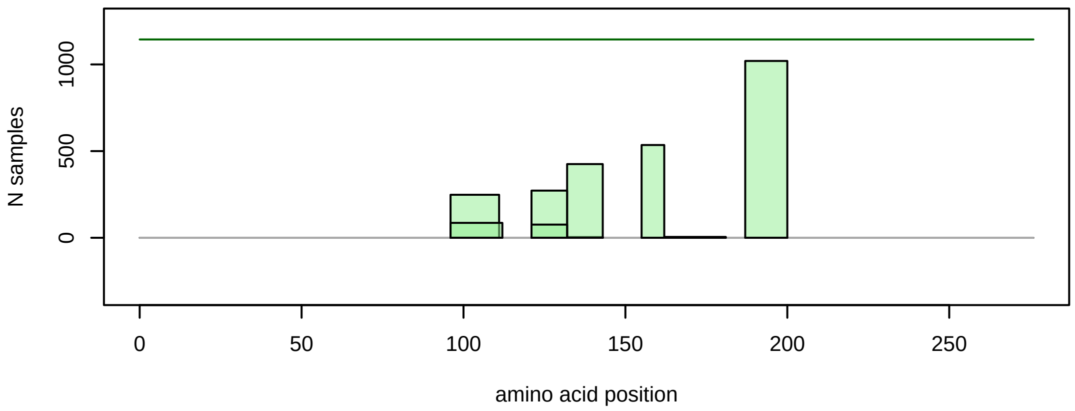

732. FN1 (P02751−1), NP5

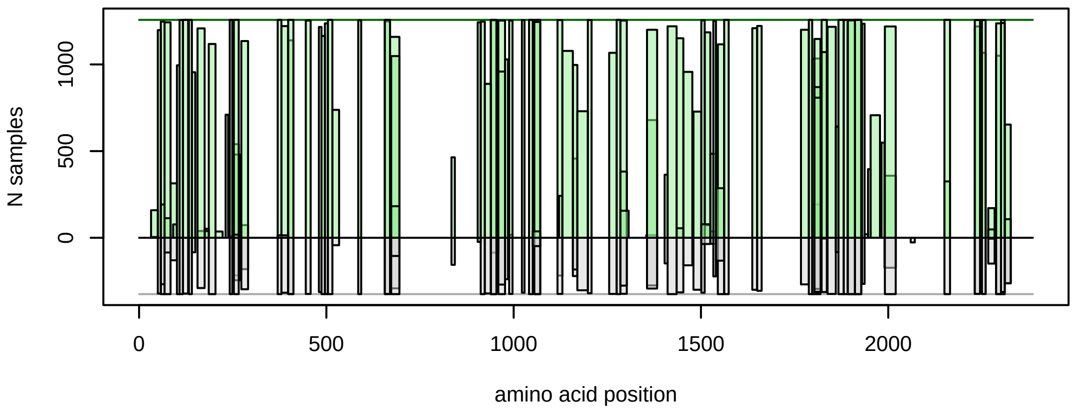

733. BNC2 (B1APH0), NP2

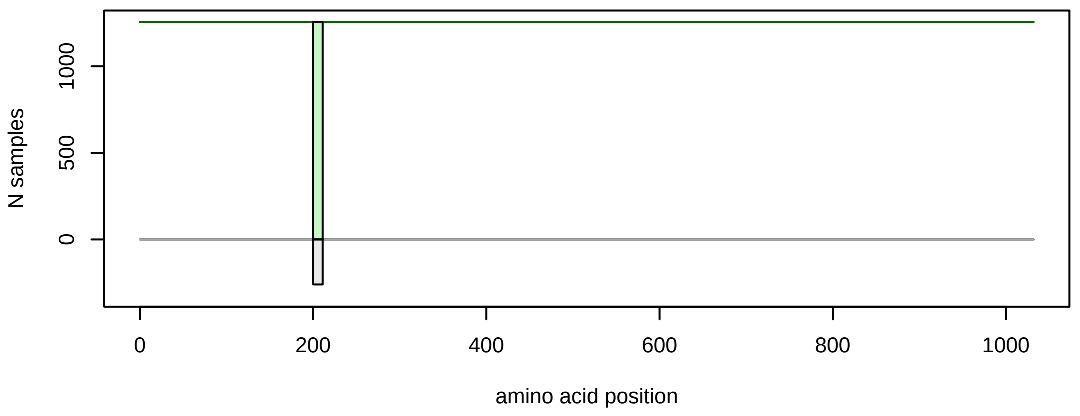

734. NNT (Q13423), NP4

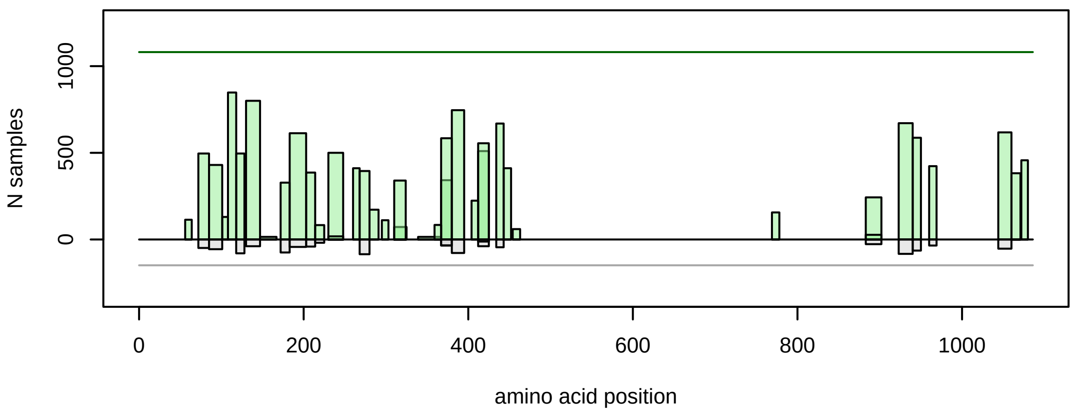

735. FAM69A (A0A087WZ97;A0A087X2C2;Q5T7M9), NP5

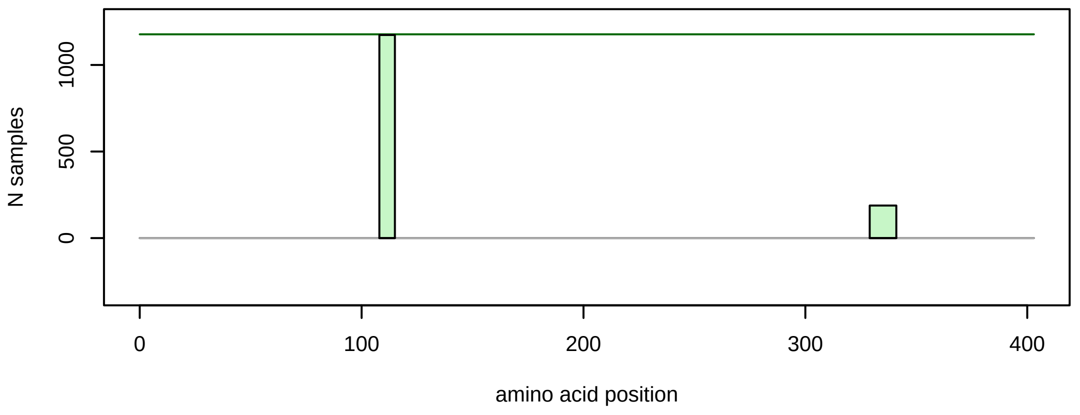

736. YWHAZ (P63104), NP4

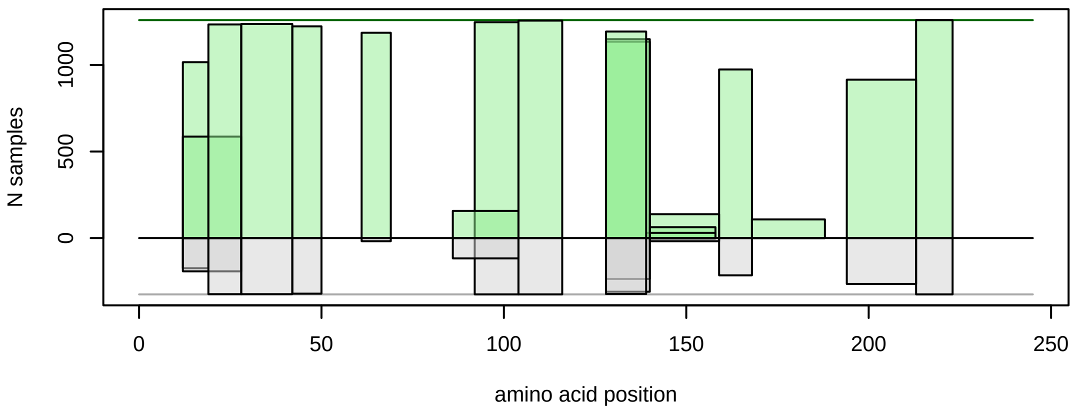

737. GLRX3 (O76003), NP5

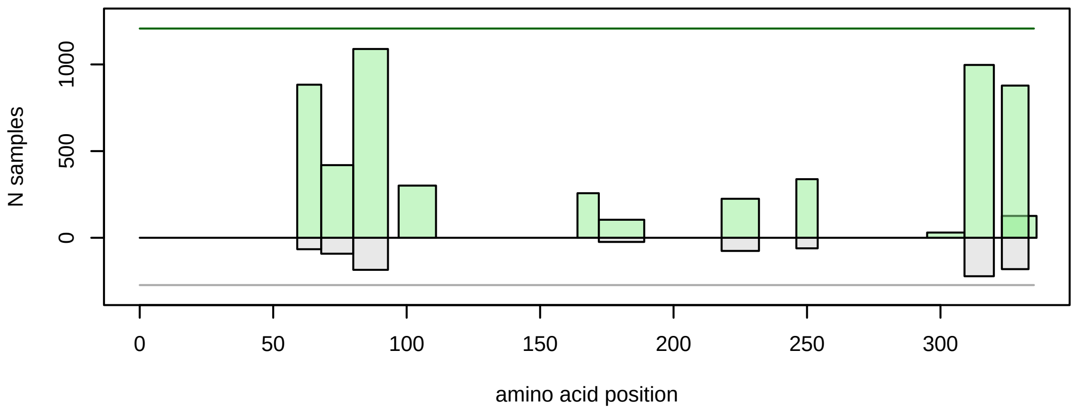

738. SLC22A9 (Q8IVM8), NP4

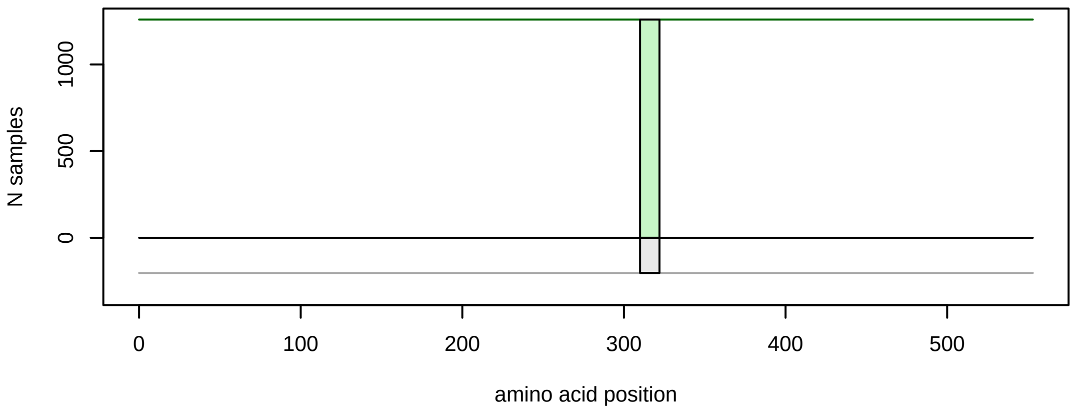

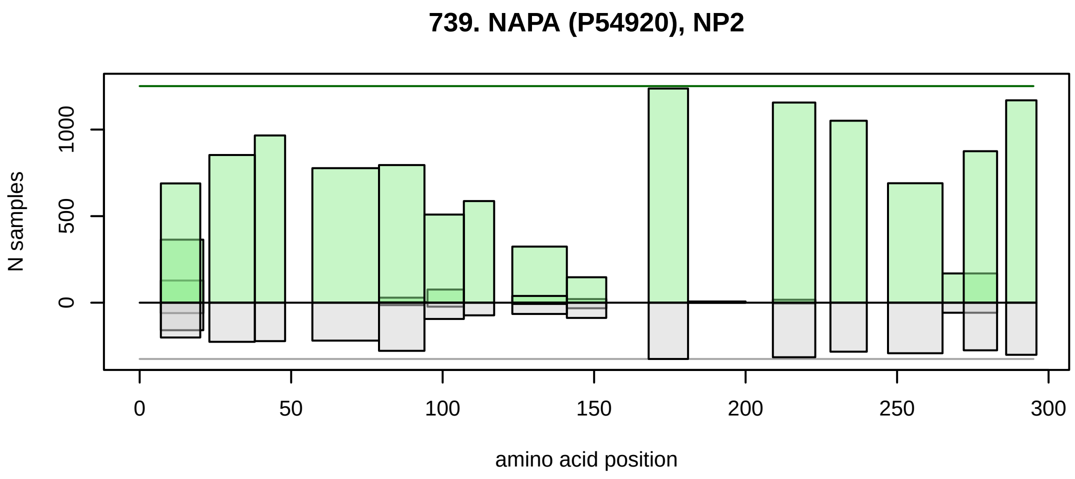
739. NAPA (P54920), NP2
N samples
0
500
1000
0
50
100
150
200
250
300
amino acid position

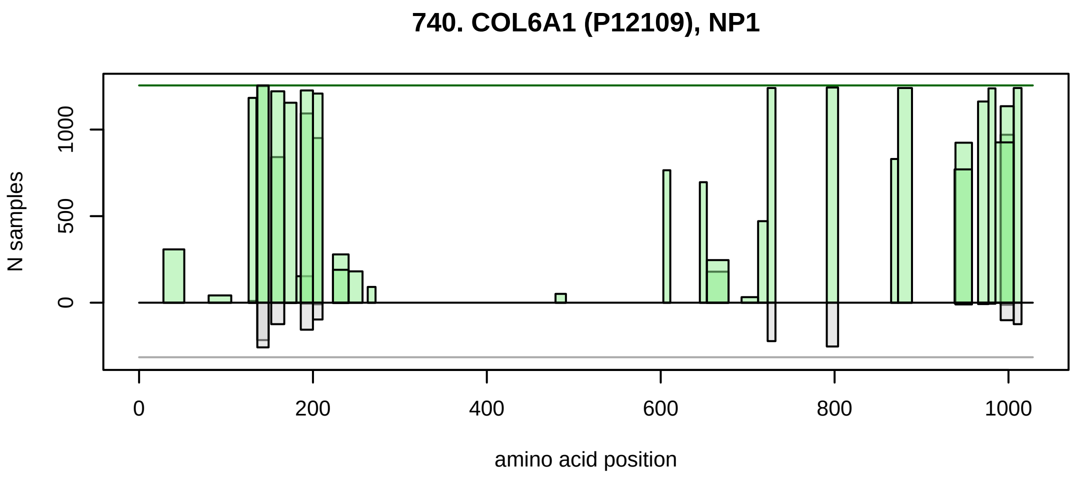
740. COL6A1 (P12109), NP1
N samples
0
500
1000
0
200
400
600
800
1000
amino acid position

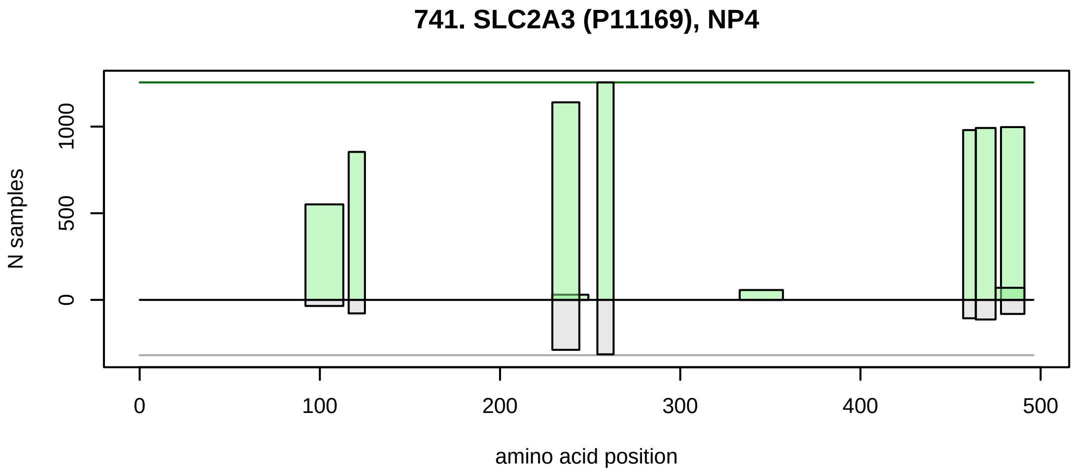
741. SLC2A3 (P11169), NP4
N samples
0
500
1000
0
100
200
300
400
500
amino acid position

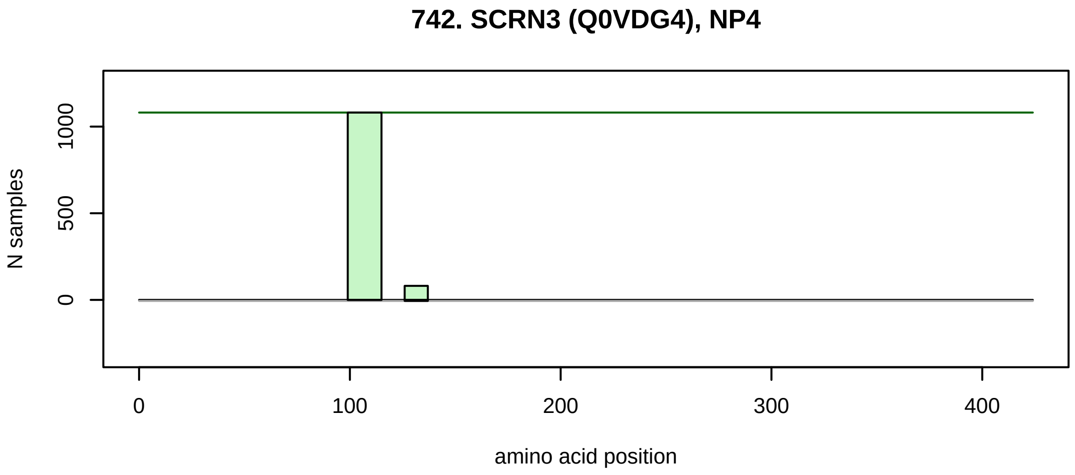
742. SCRN3 (Q0VDG4), NP4
N samples
0
500
1000
0
100
200
300
400
amino acid position

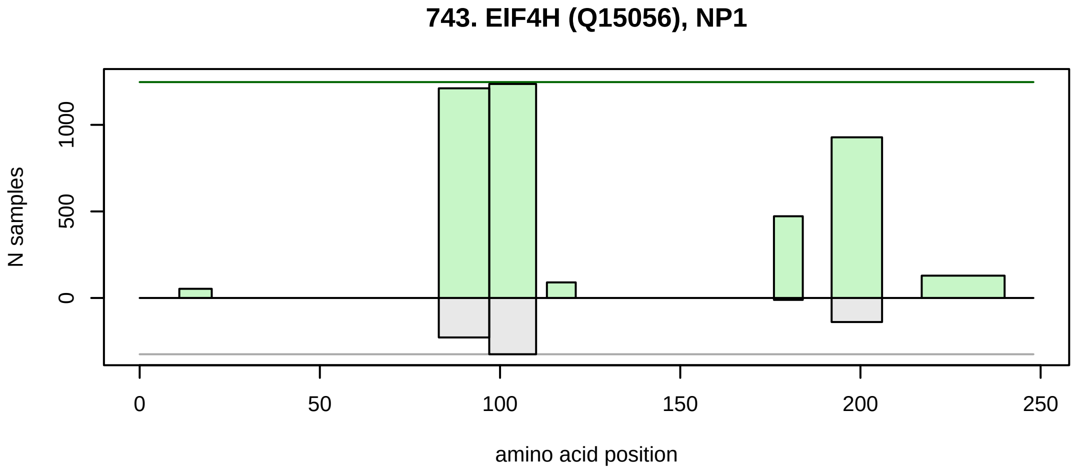
743. EIF4H (Q15056), NP1
N samples
0
500
1000
0
50
100
150
200
250
amino acid position

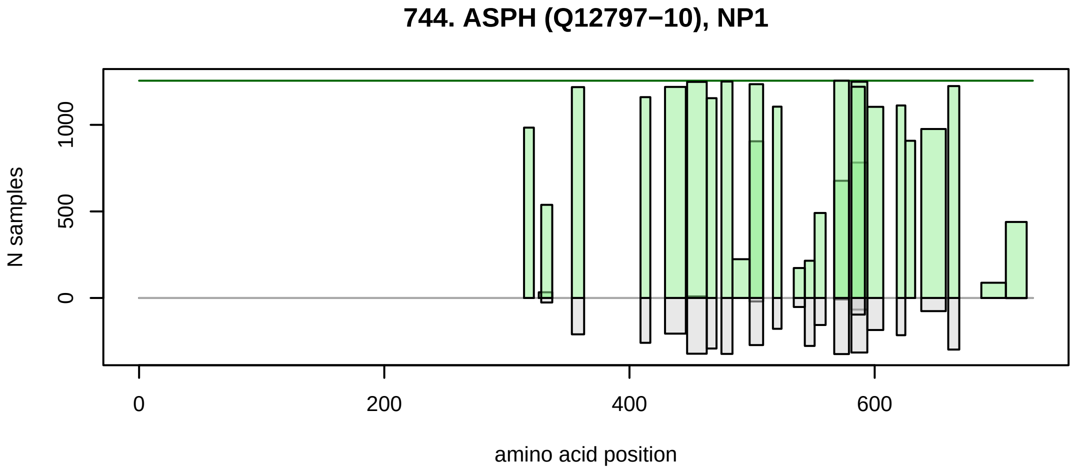
744. ASPH (Q12797−10), NP1
N samples
0
500
1000
0
200
400
600
amino acid position

745. TBCEL (Q5QJ74), NP5

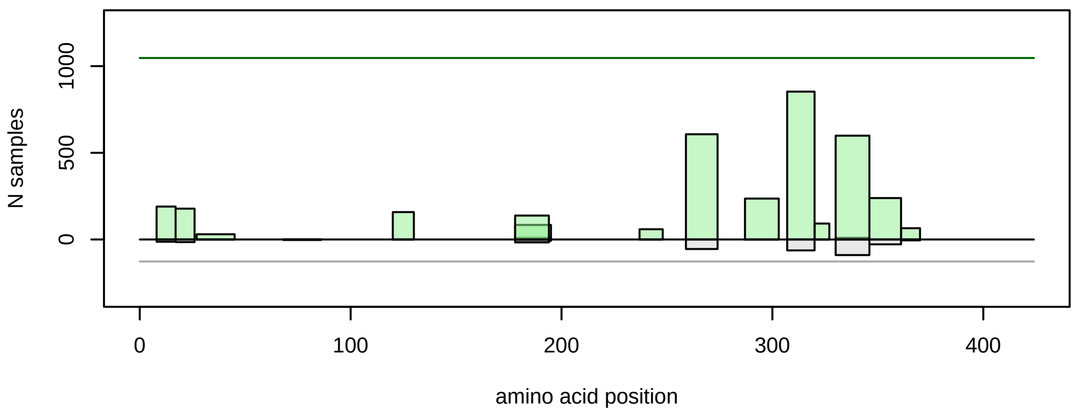

746. TUBA1C (F5H5D3), NP4

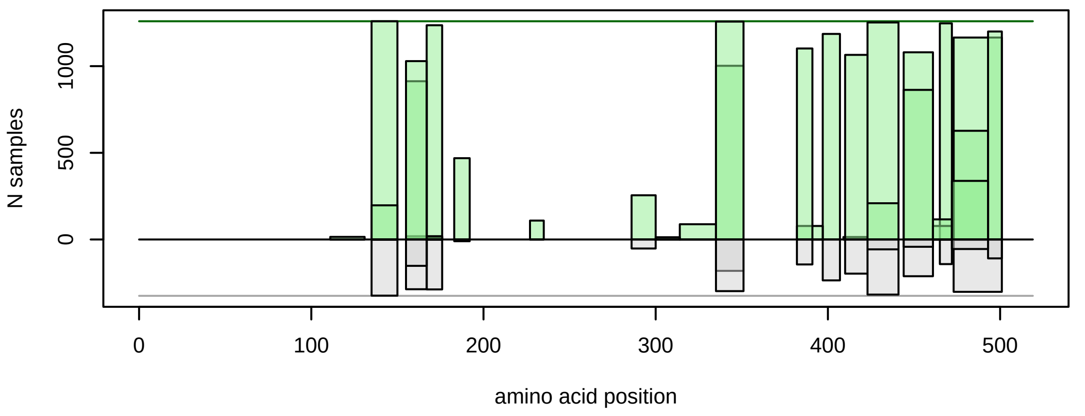

747. IQGAP1 (P46940), NP1

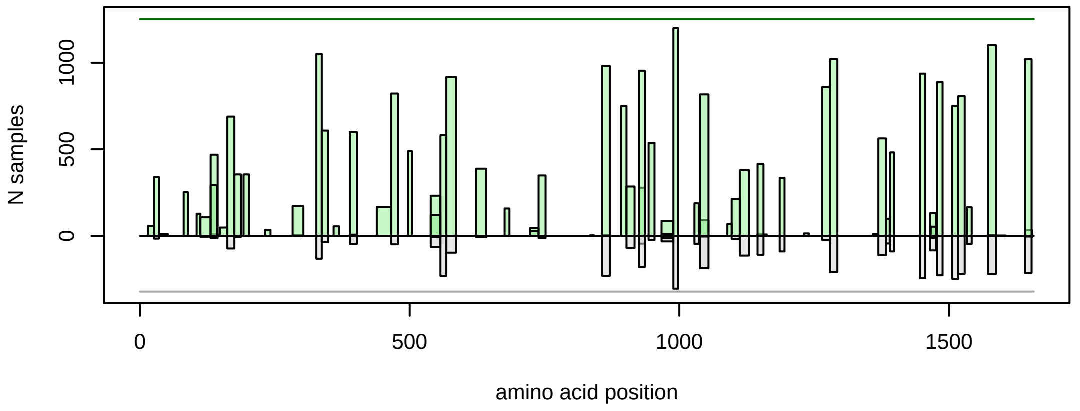

748. STK10 (O94804), NP4

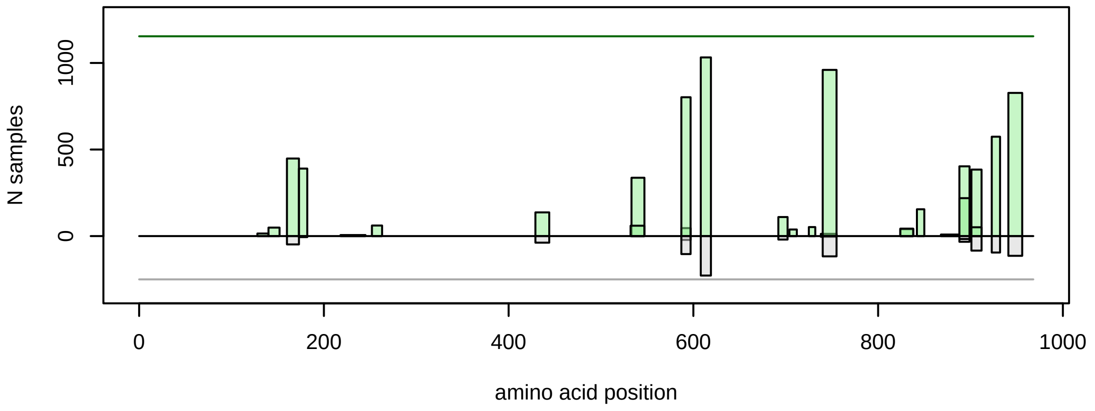

749. METAP1 (P53582), NP1

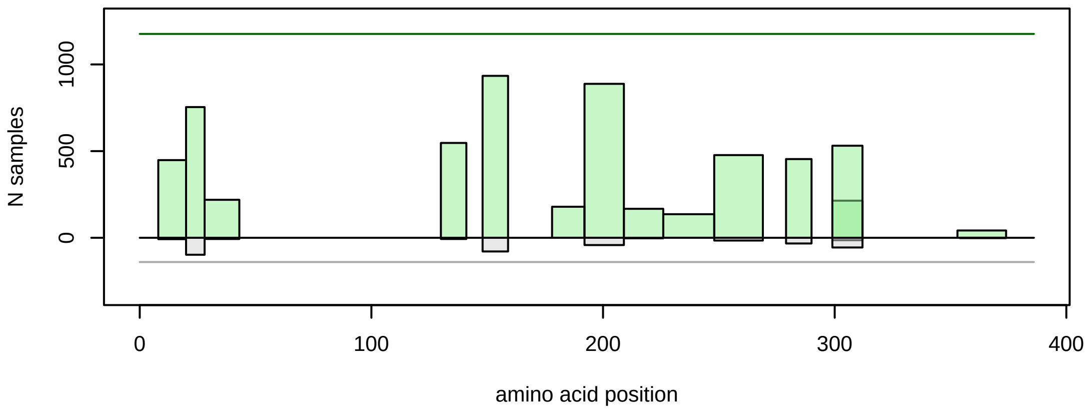

750. CTAGE5 (Q96PC5), NP2

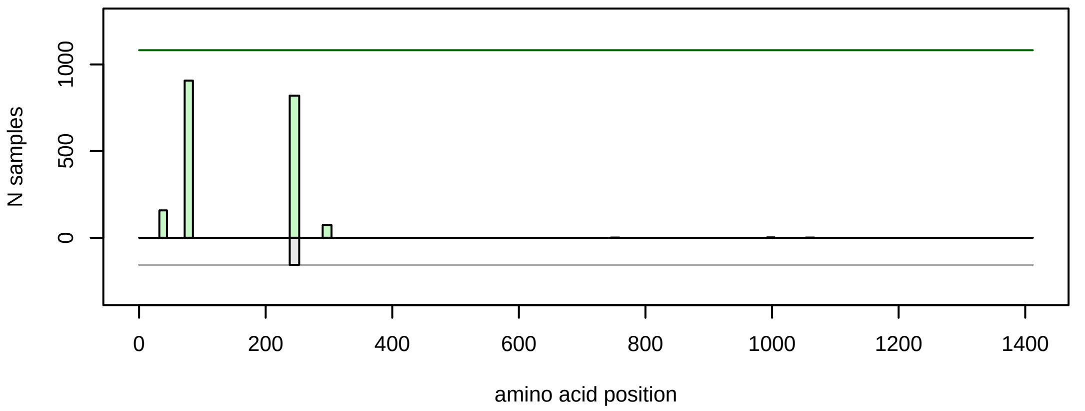

751. MXRA5 (Q9NR99), NP4

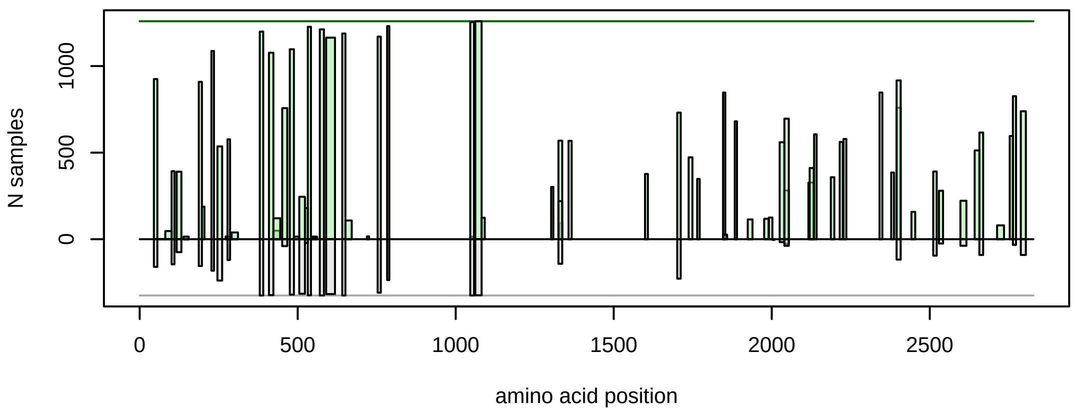

752. CLEC3B (P05452), NP3

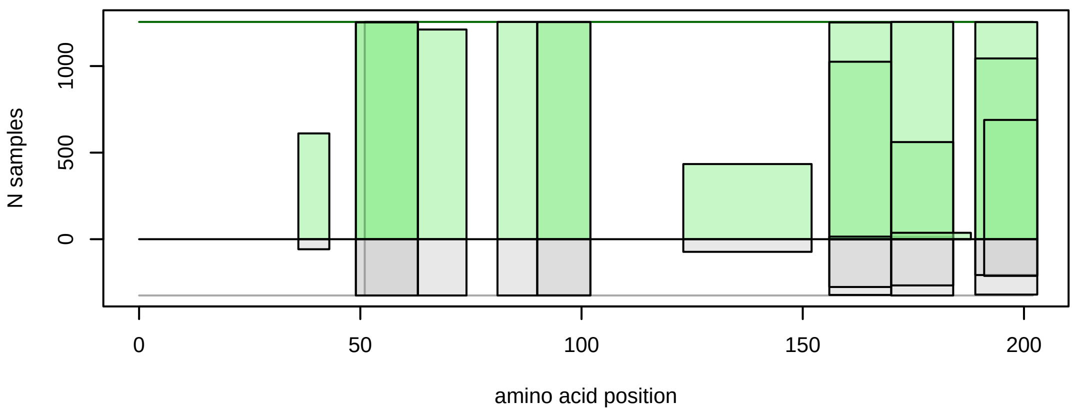

753. MFGE8 (Q08431), NP1

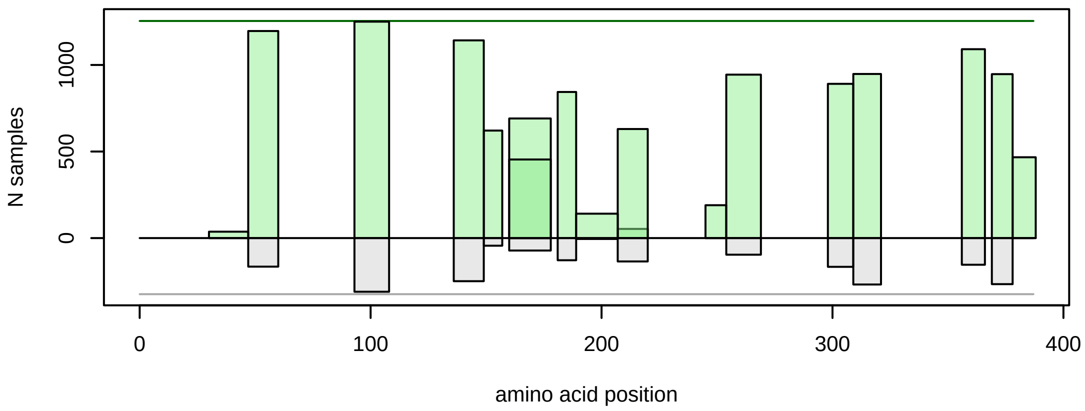

754. RHAG (A0A2R8YEH1;Q02094;Q9UHG9), NP1

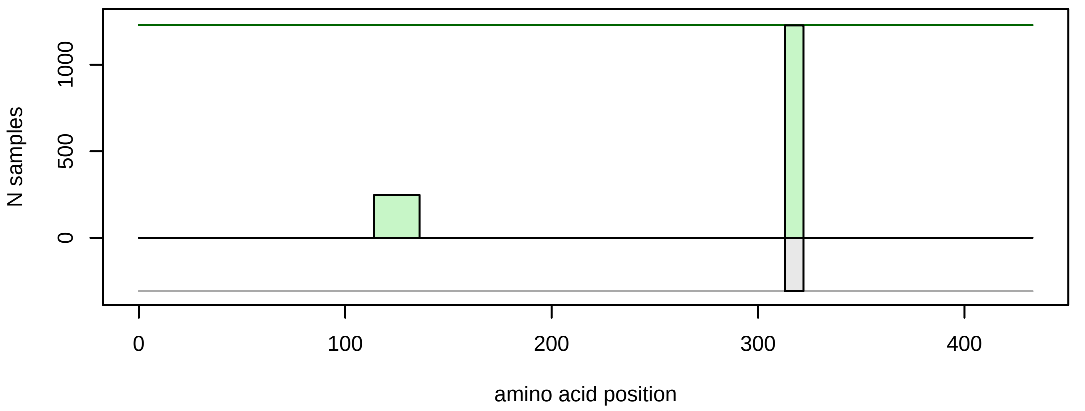

755. CDC5L (Q99459), NP4

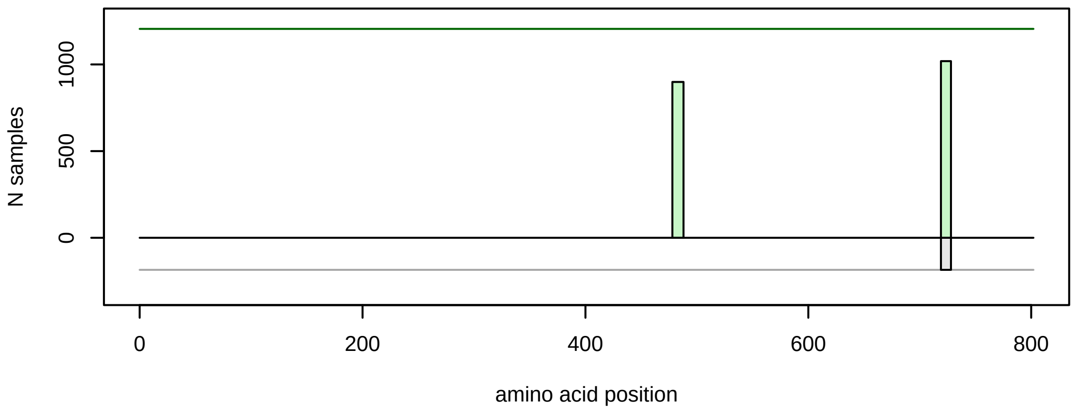

756. CLIC1 (O00299), NP2

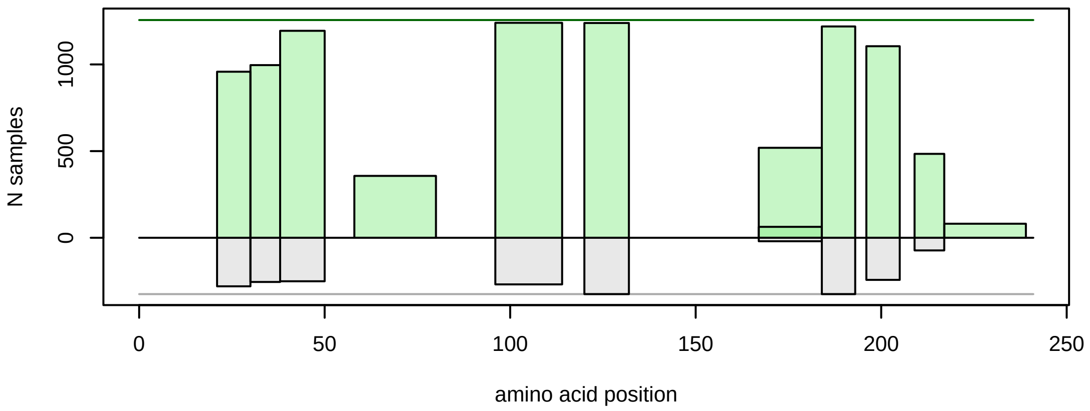

757. SH3GLB1 (Q9Y371), NP5

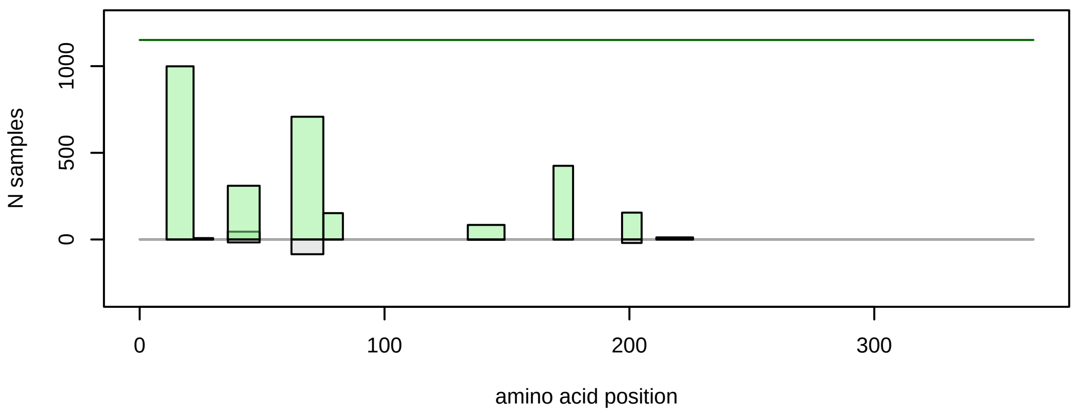

758. IGLV3−27 (P01718), NP5

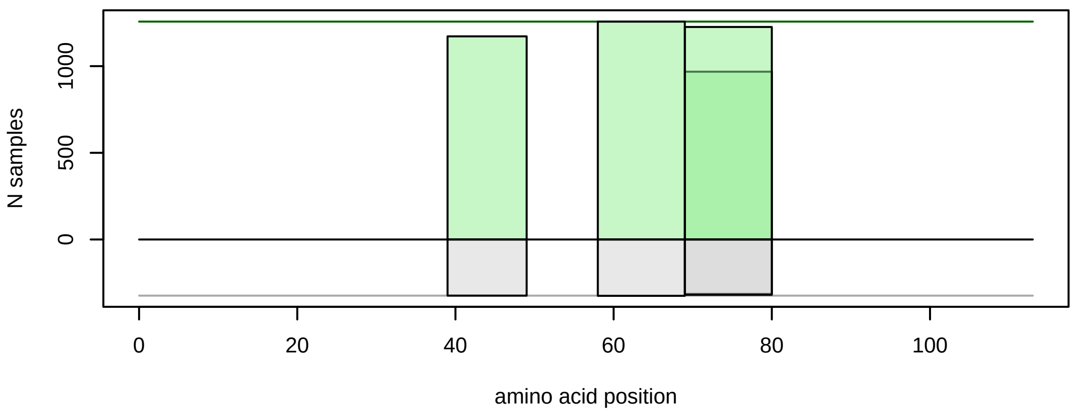

759. P4HA1 (P13674), NP5

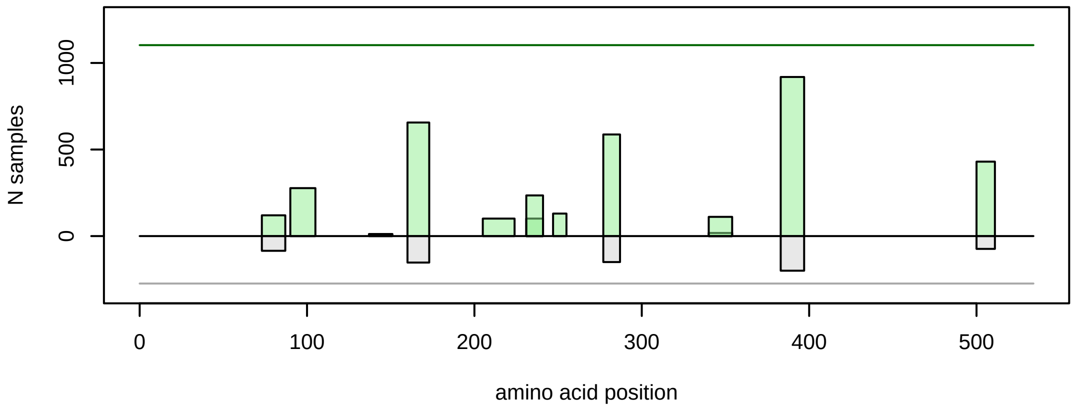

760. PNP (P00491), NP1

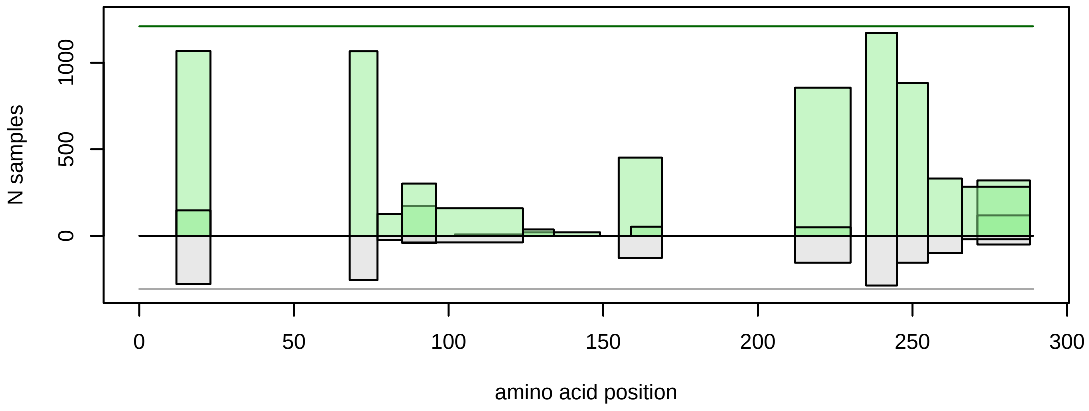

761. HSD17B4 (P51659), NP4

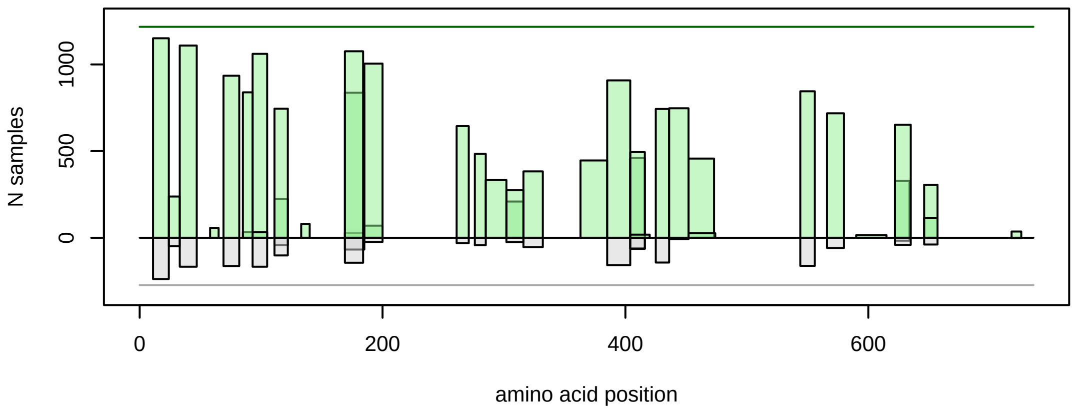

762. COPA (P53621), NP4

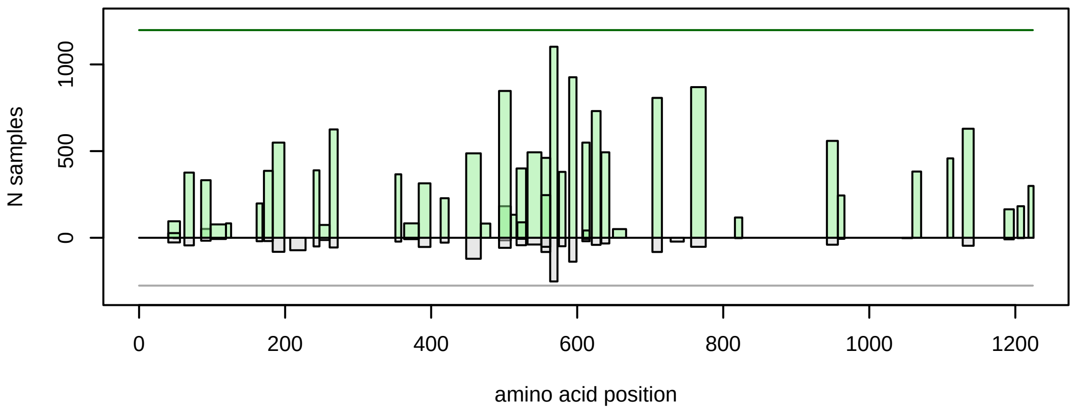

763. YWHAB (P31946), NP4

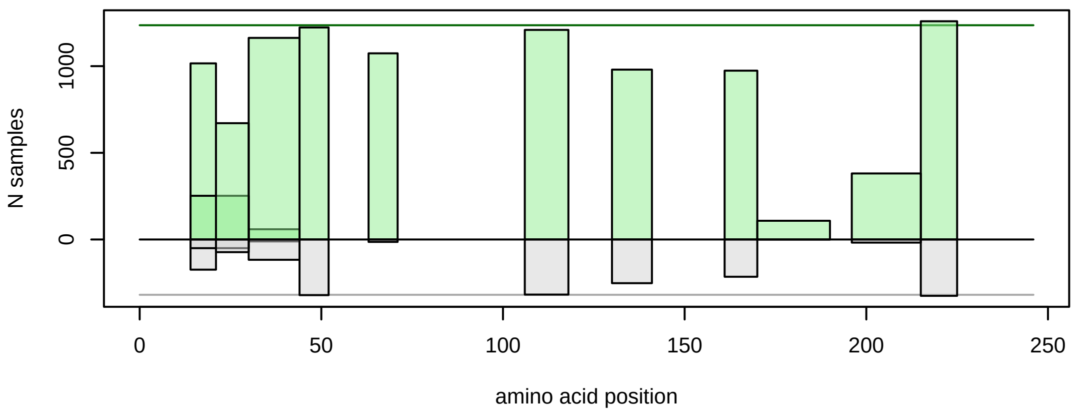

764. NHLRC2 (Q8NBF2), NP5

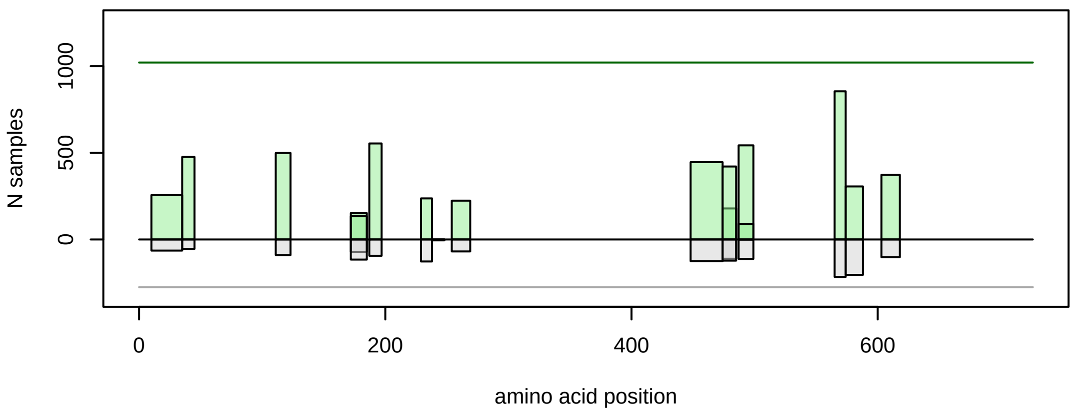

765. SMOC1 (Q9H4F8;Q9H4F8−2), NP4

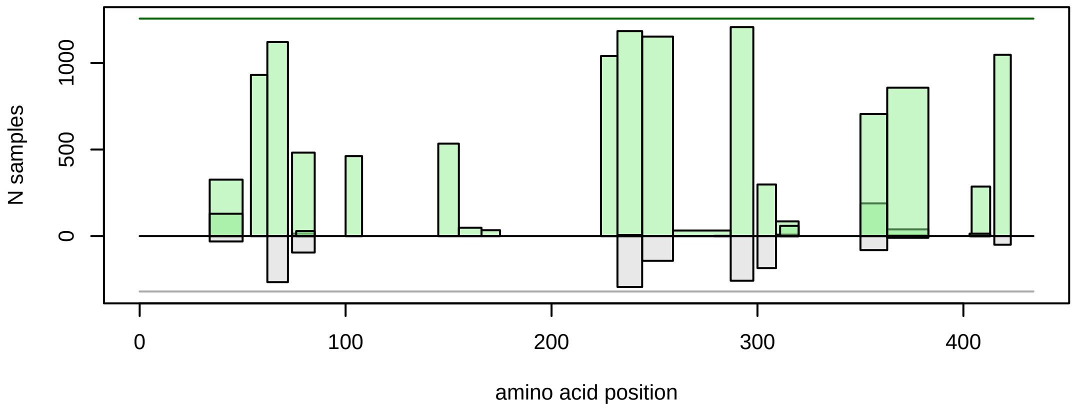

766. LPIN2 (Q92539), NP2

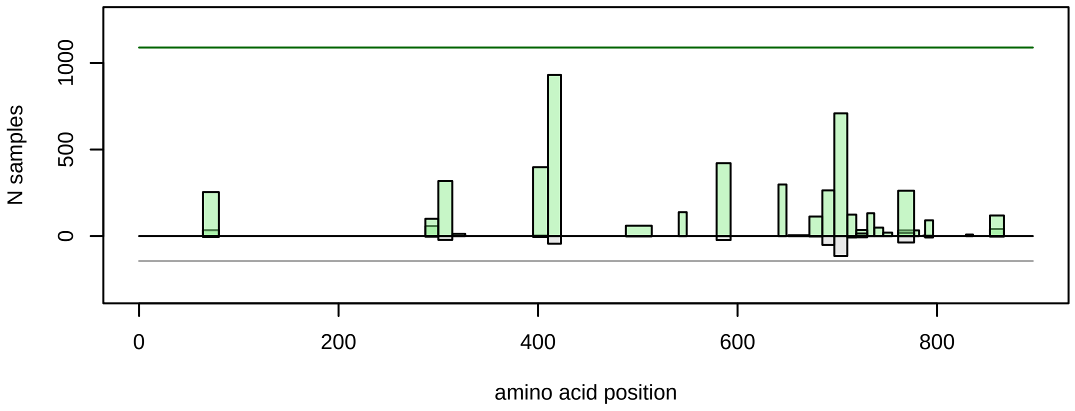

767. MYL6 (G8JLA2), NP4

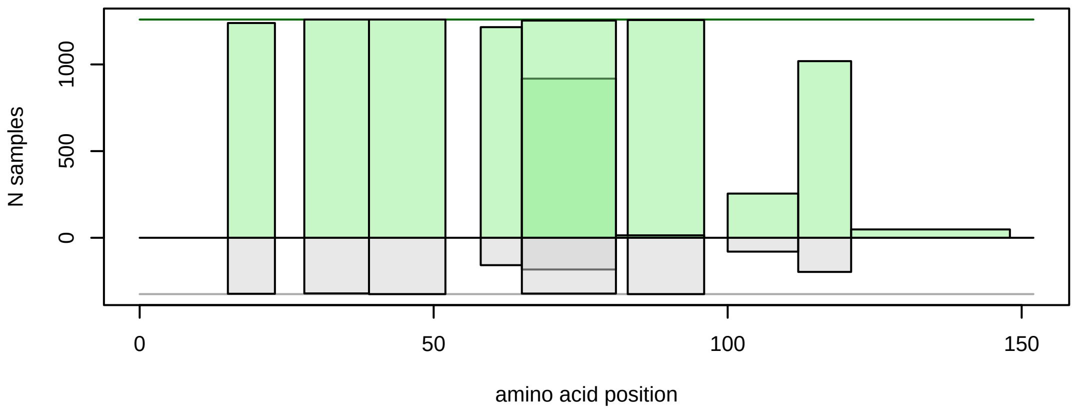

768. CDH13 (P55290−4), NP2

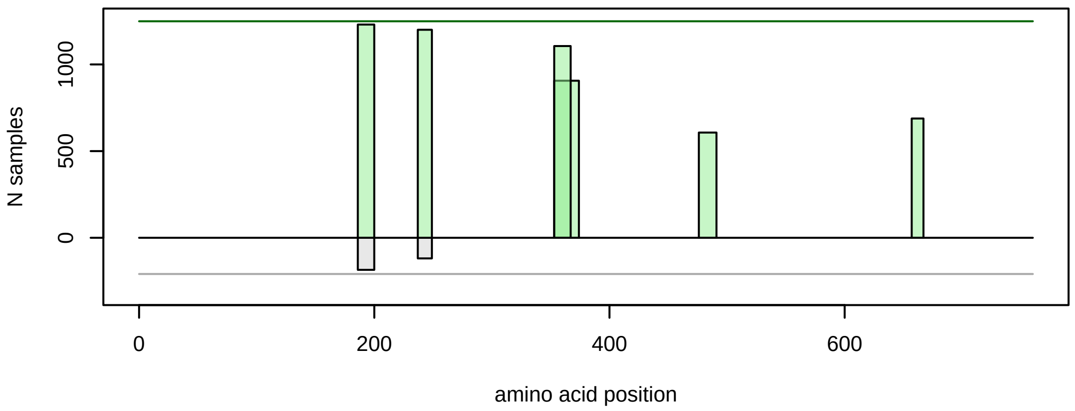

769. ERCC6L (Q2NKX8), NP5

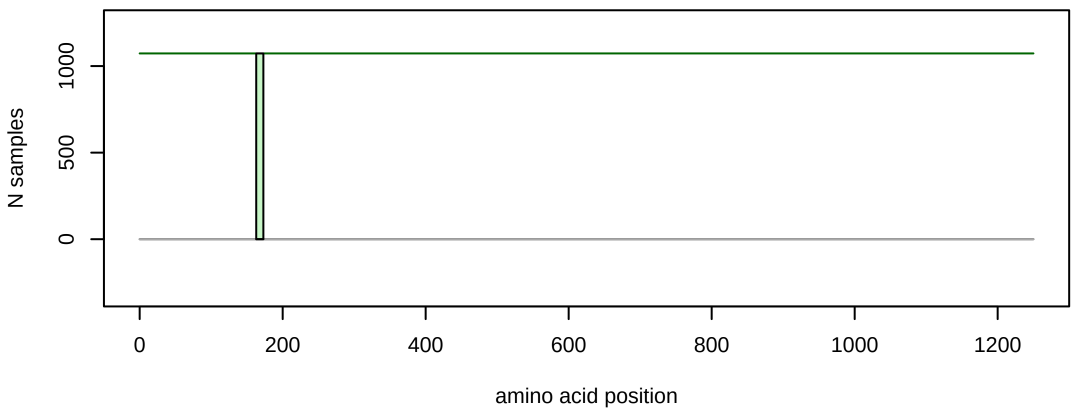

770. DLD (P09622), NP1

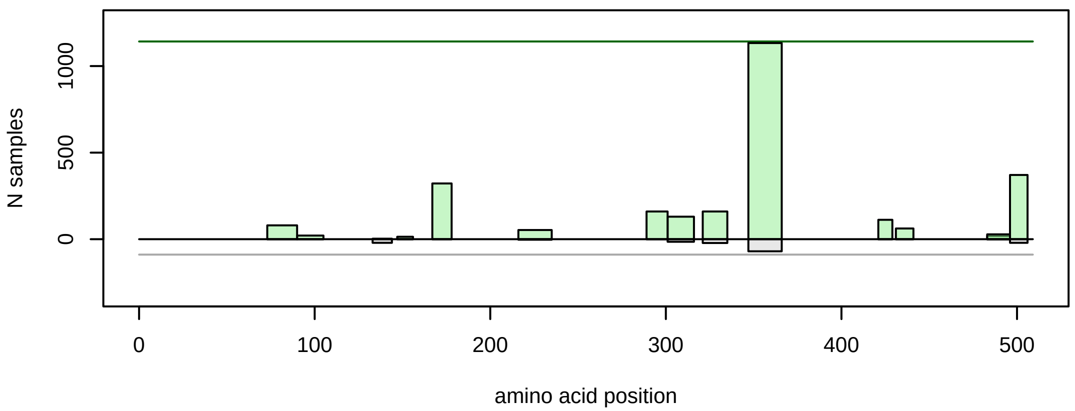

771. TNFSF13 (O75888;O75888−4;Q2QBA2), NP4

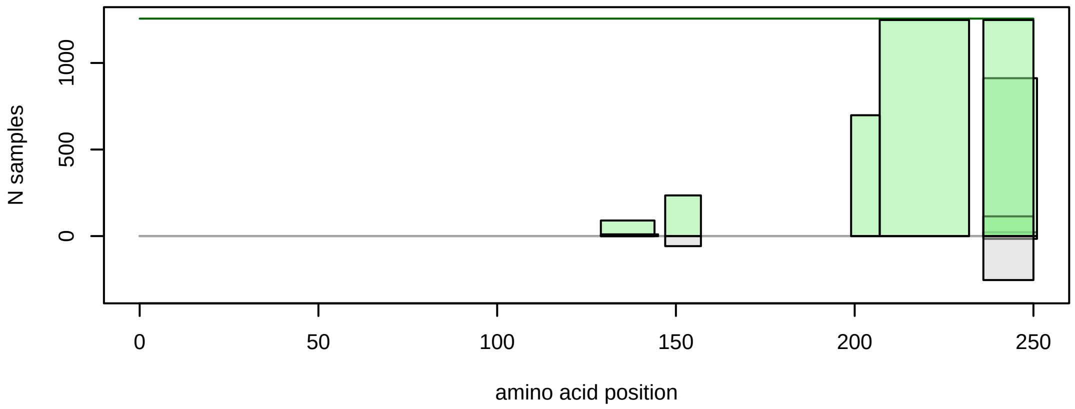

772. MYH9 (P35579), NP4

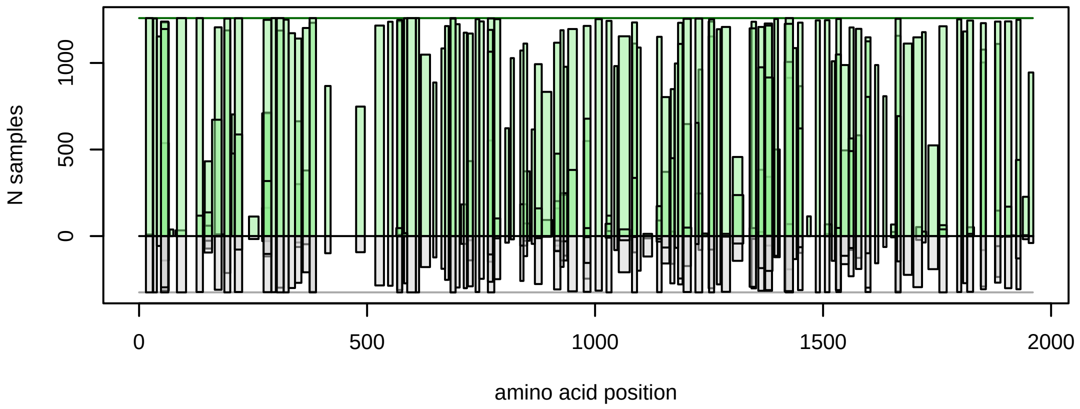

773. C4BPA (P04003), NP2

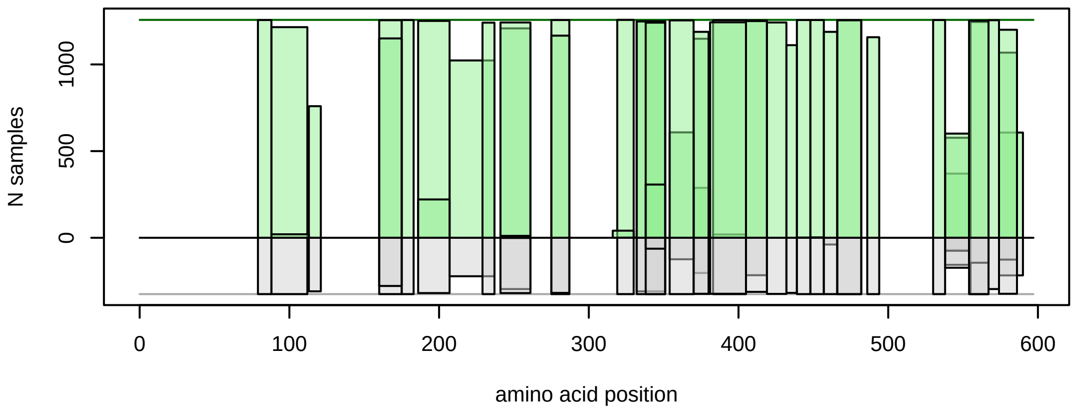

774. MAP4 (A0A804HKE7;E7EVA0), NP4

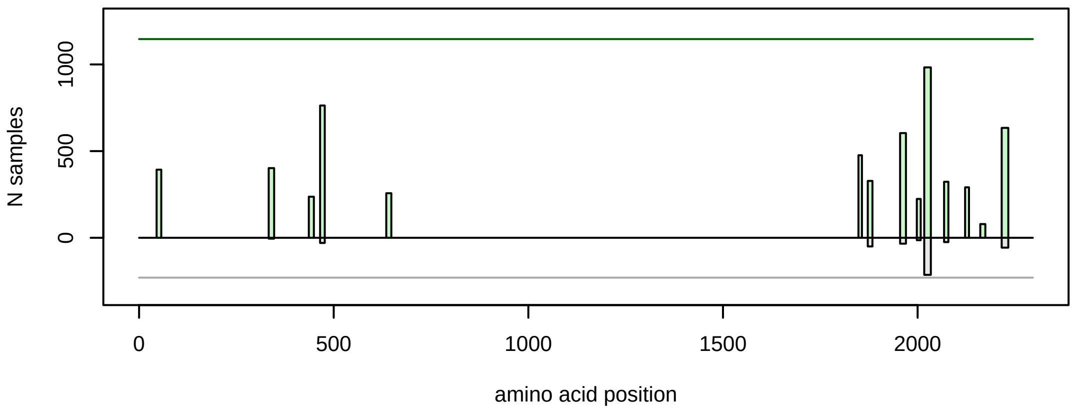

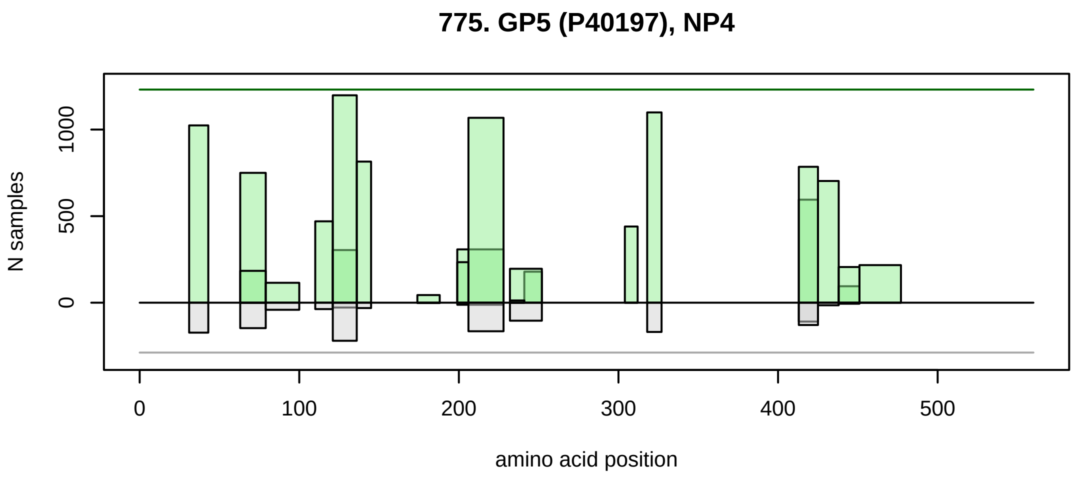

775. GP5 (P40197), NP4
N samples
0
500
1000
0
100
200
300
400
500
amino acid position

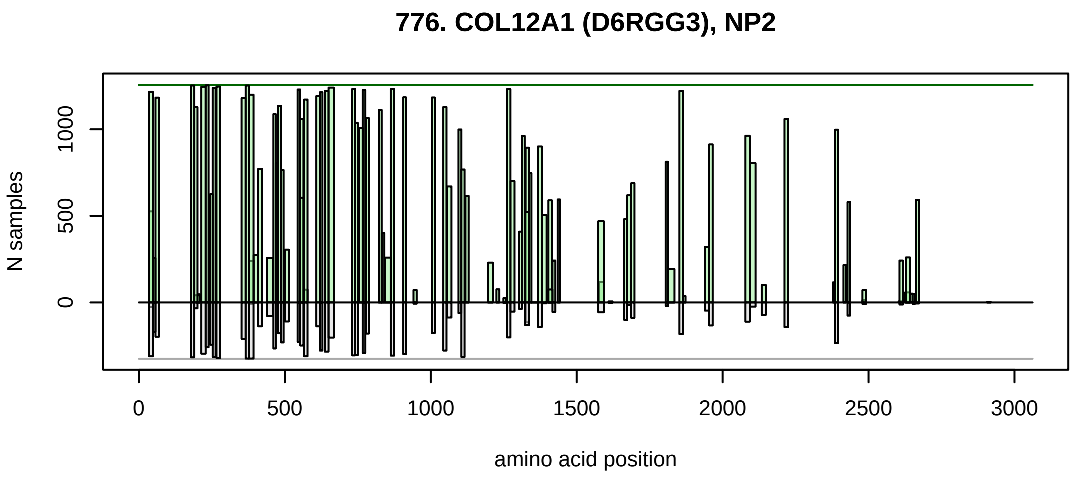

776. COL12A1 (D6RGG3), NP2
N samples
0
500
1000
0
500
1000
1500
2000
2500
3000
amino acid position

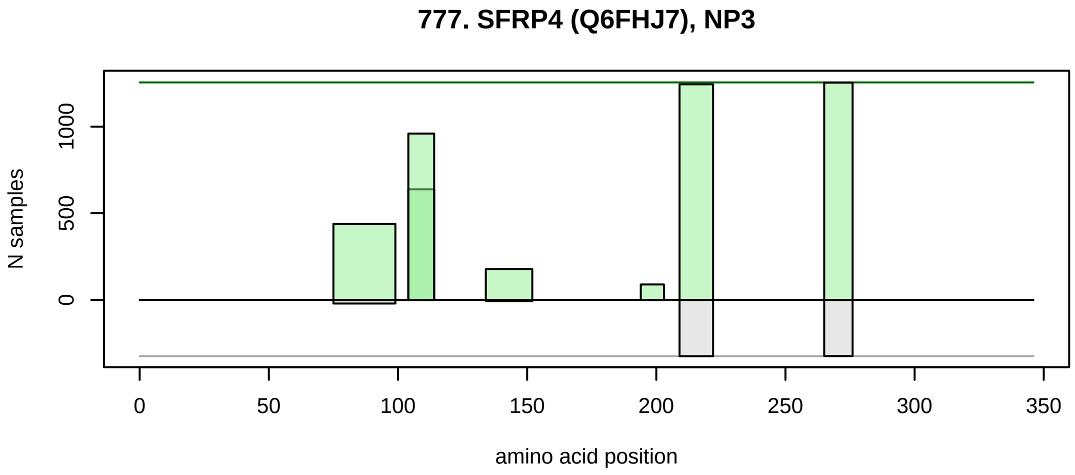

777. SFRP4 (Q6FHJ7), NP3
N samples
0
500
1000
0
50
100
150
200
250
300
350
amino acid position

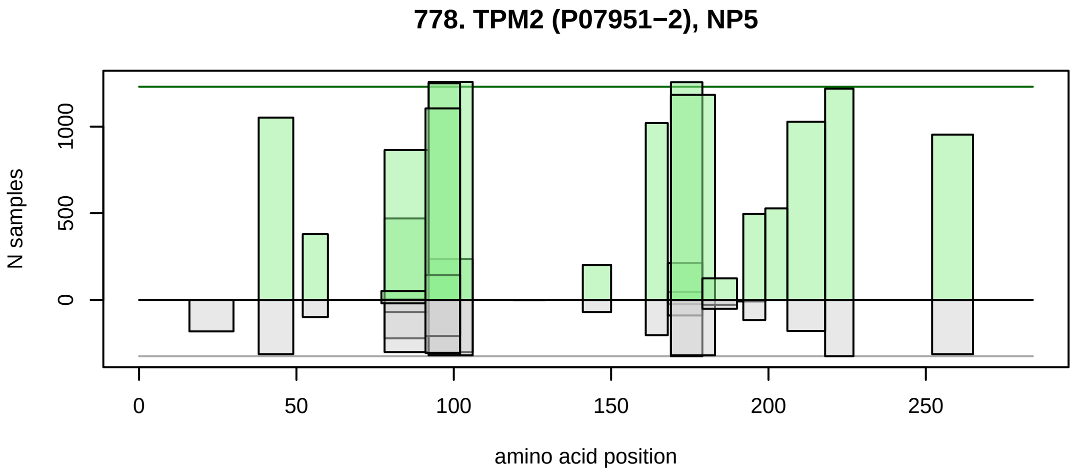

778. TPM2 (P07951−2), NP5
N samples
0
500
1000
0
50
100
150
200
250
amino acid position

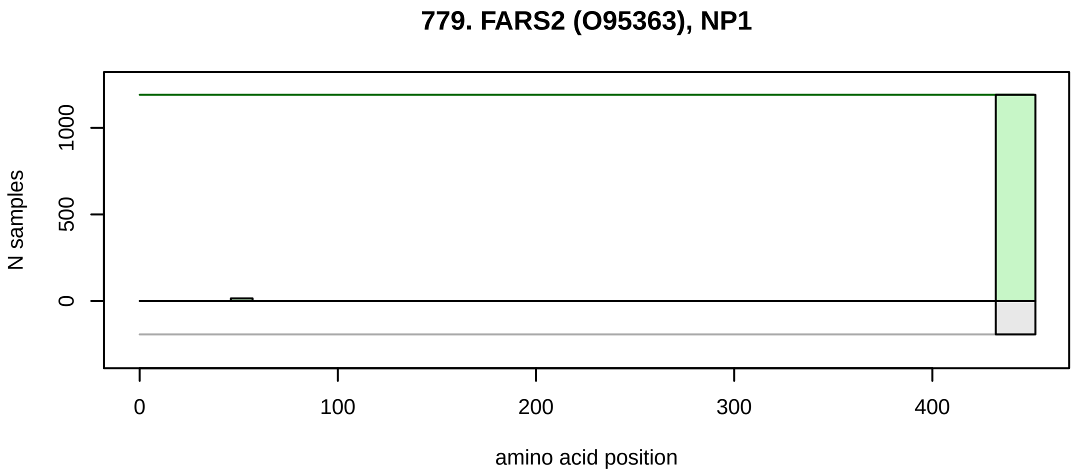

779. FARS2 (O95363), NP1
N samples
0
500
1000
0
100
200
300
400
amino acid position

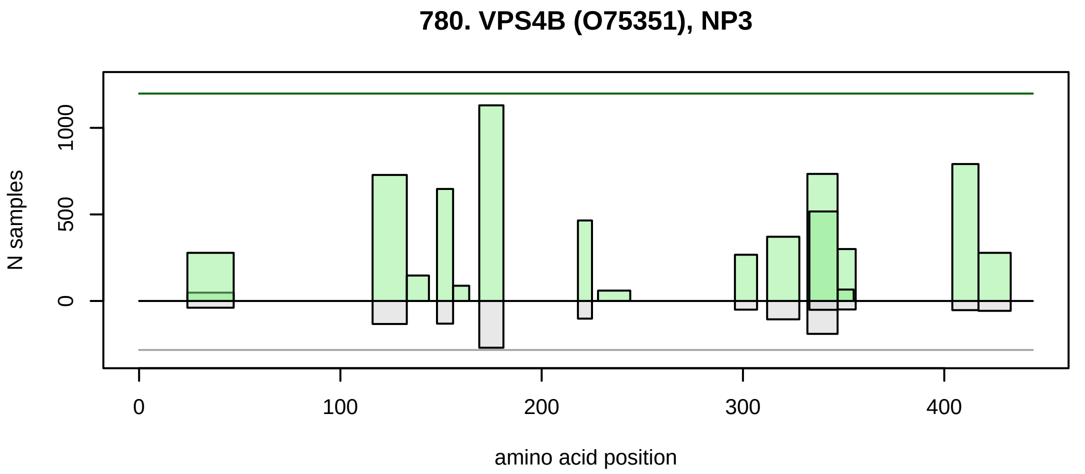

780. VPS4B (O75351), NP3
N samples
0
500
1000
0
100
200
300
400
amino acid position

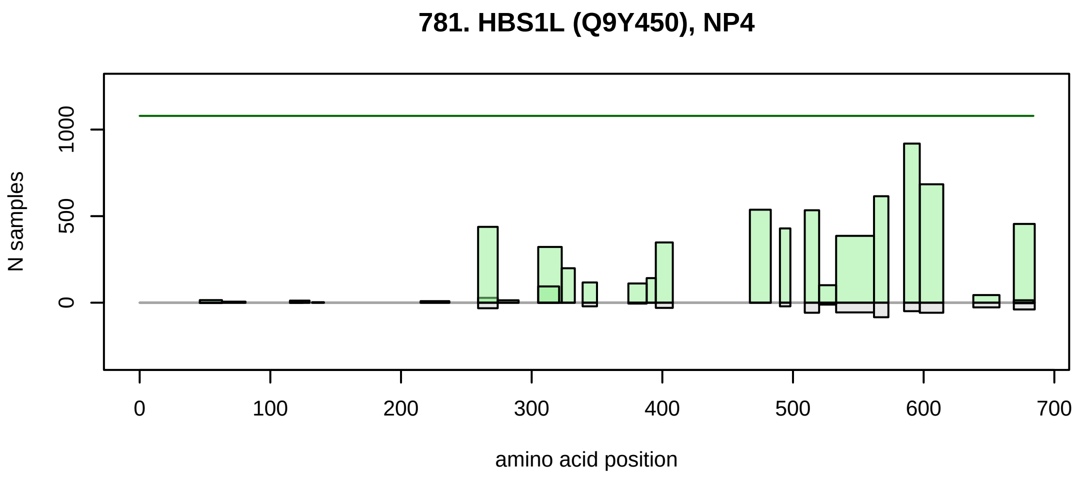
781. HBS1L (Q9Y450), NP4
N samples
0
500
1000
0
100
200
300
400
500
600
700
amino acid position

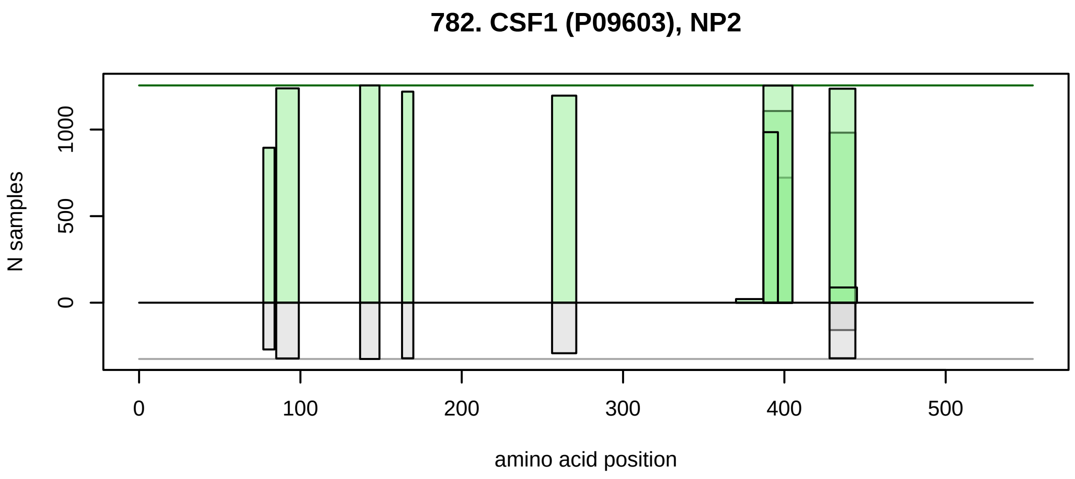
782. CSF1 (P09603), NP2
N samples
0
500
1000
0
100
200
300
400
500
amino acid position

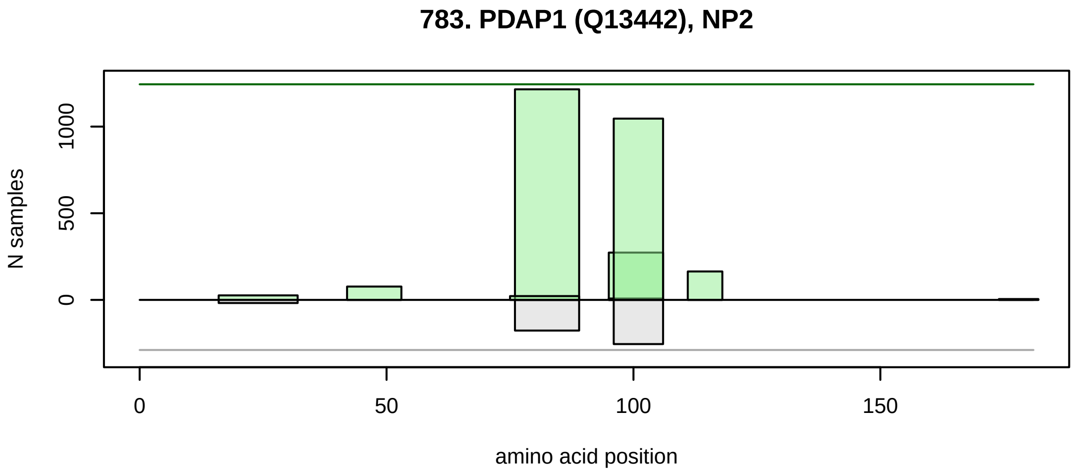
783. PDAP1 (Q13442), NP2
N samples
0
500
1000
0
50
100
150
amino acid position

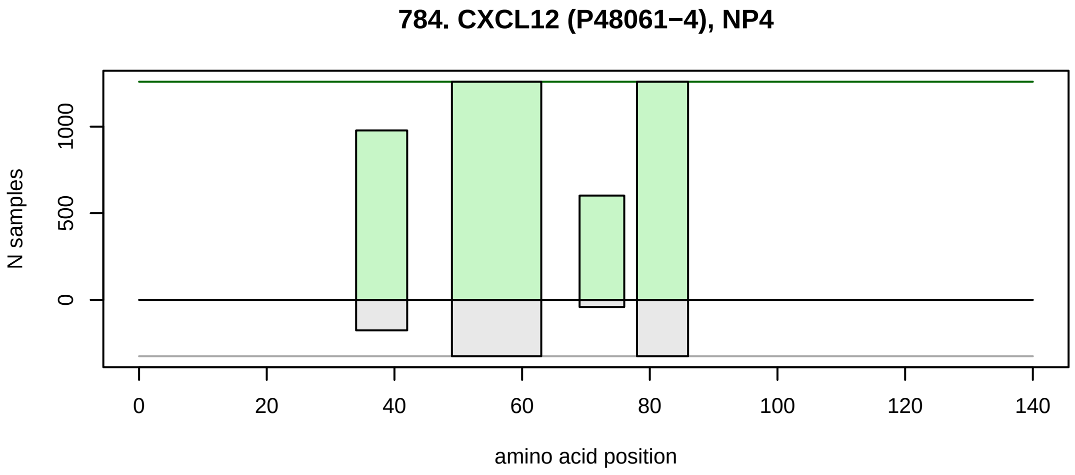
784. CXCL12 (P48061−4), NP4
N samples
0
500
1000
0
20
40
60
80
100
120
140
amino acid position

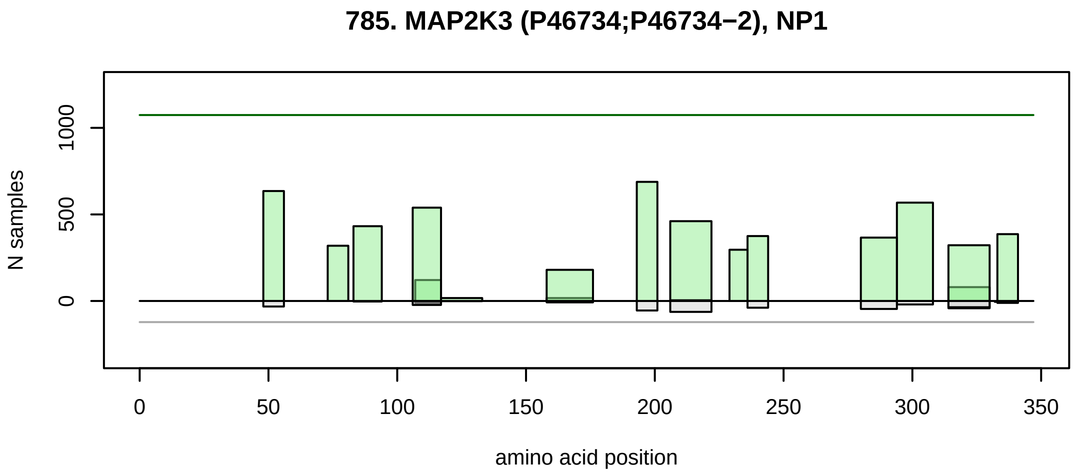
785. MAP2K3 (P46734;P46734−2), NP1
N samples
0
500
1000
0
50
100
150
200
250
300
350
amino acid position

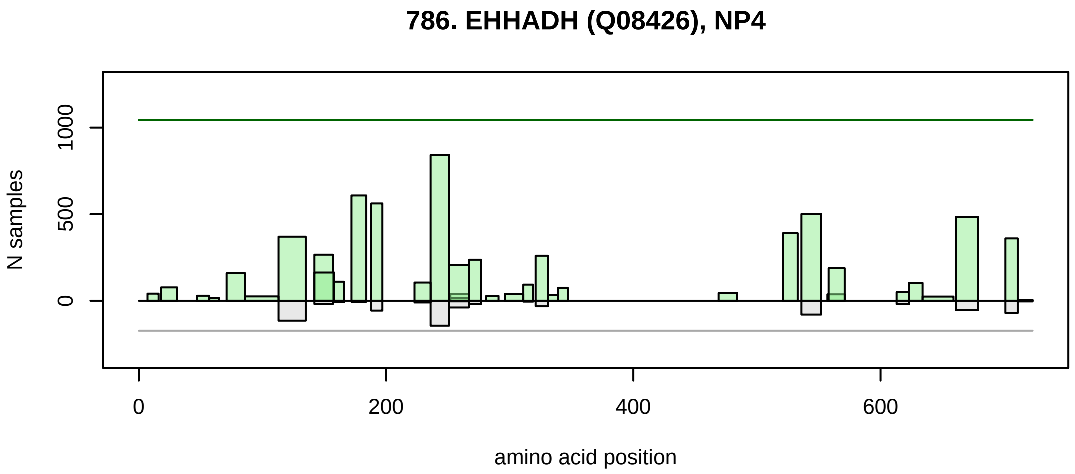
786. EHHADH (Q08426), NP4
N samples
0
500
1000
0
200
400
600
amino acid position

787. SOST (Q9BQB4), NP4

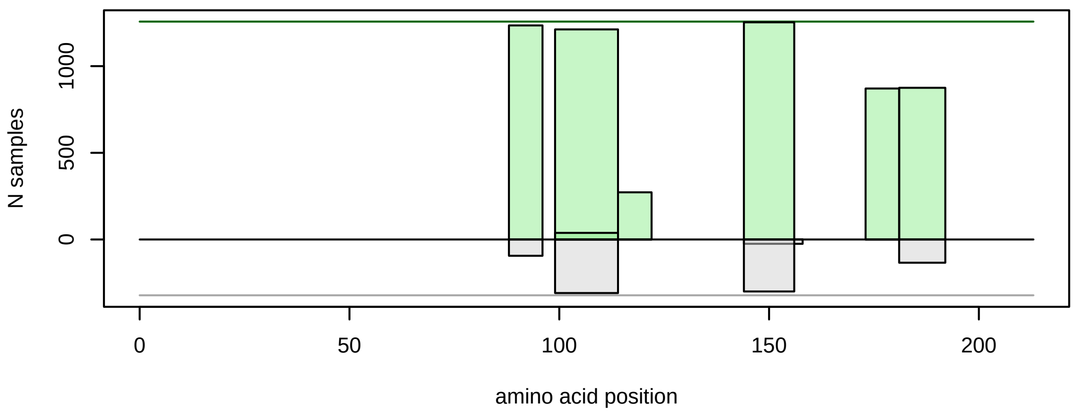

788. ESD (P10768), NP5

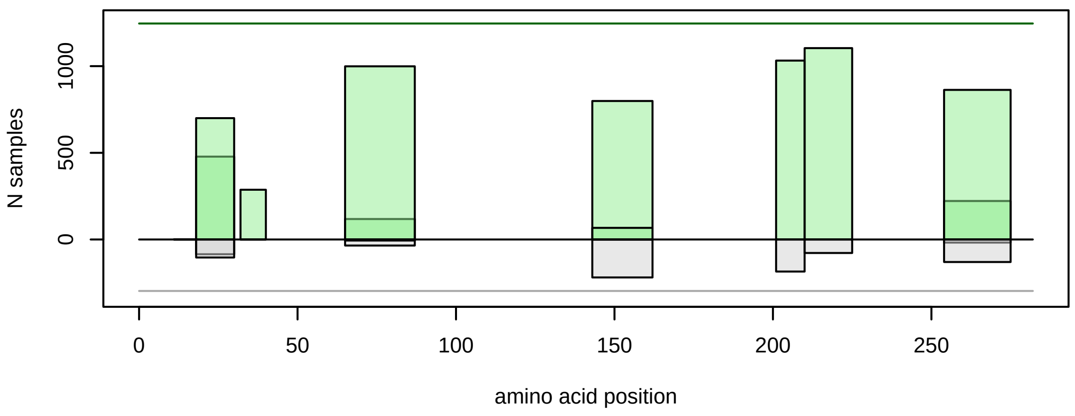

789. RAN (P62826), NP3

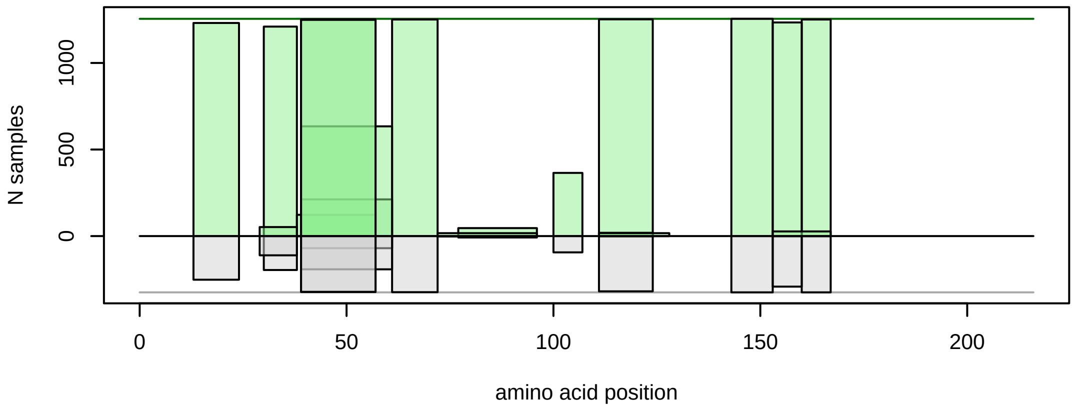

790. COL5A1 (P20908), NP2

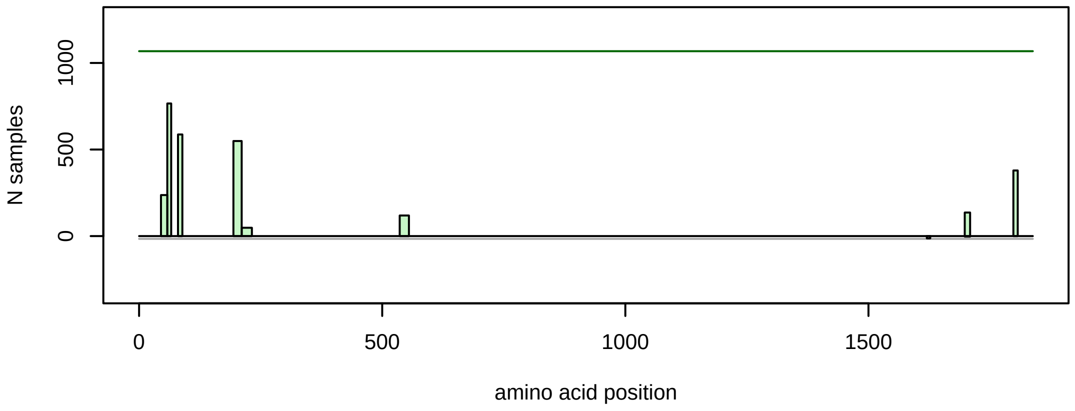

791. F13B (P05160), NP5

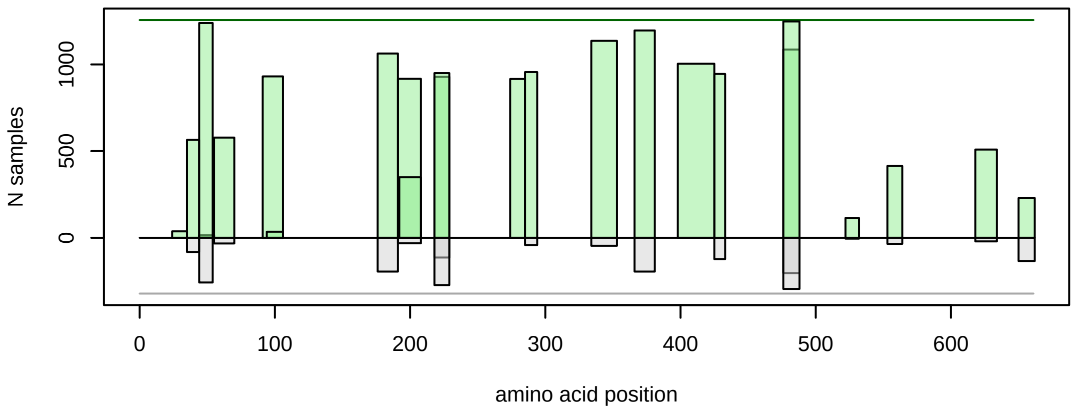

792. PIP5K1C (O60331−3), NP4

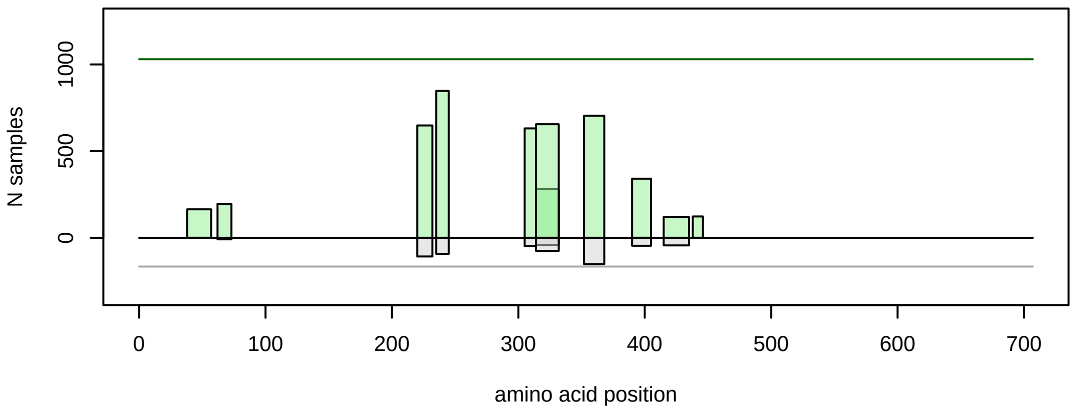

793. RTCA (O00442), NP1

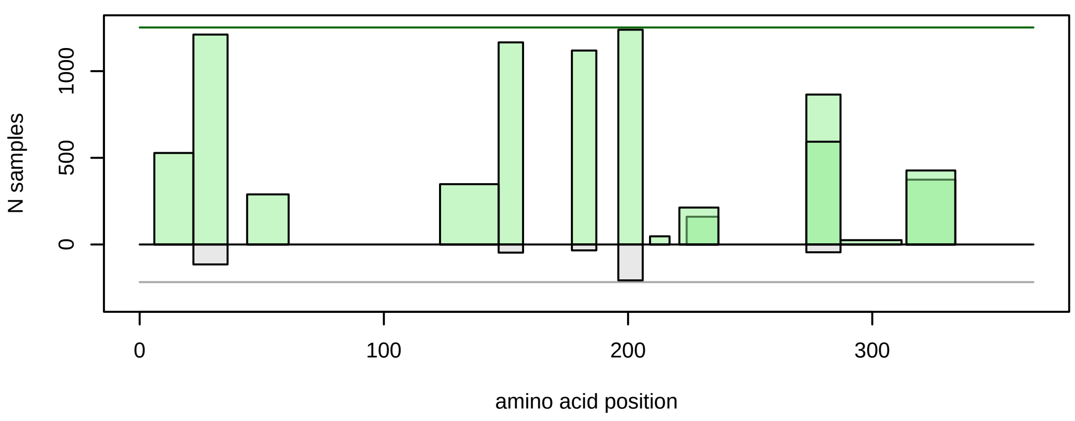

794. SERPIND1 (P05546), NP2

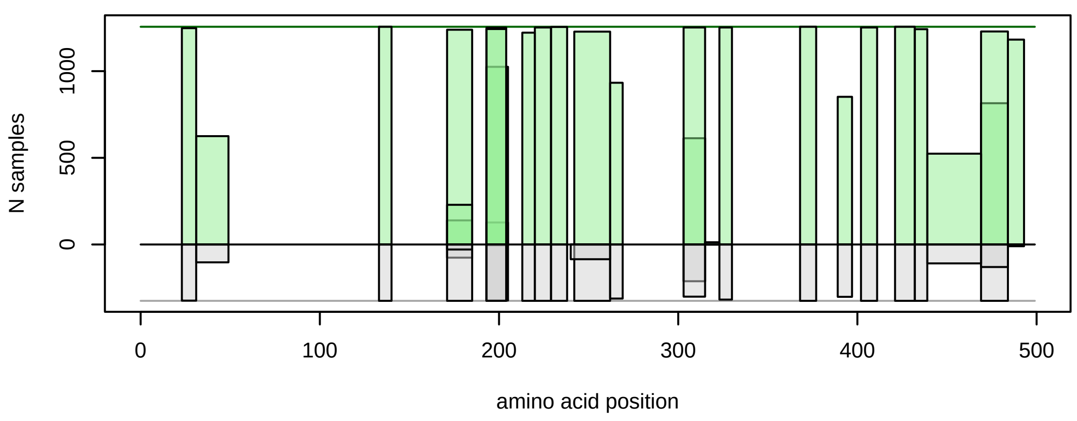

795. TPM4 (P67936), NP5

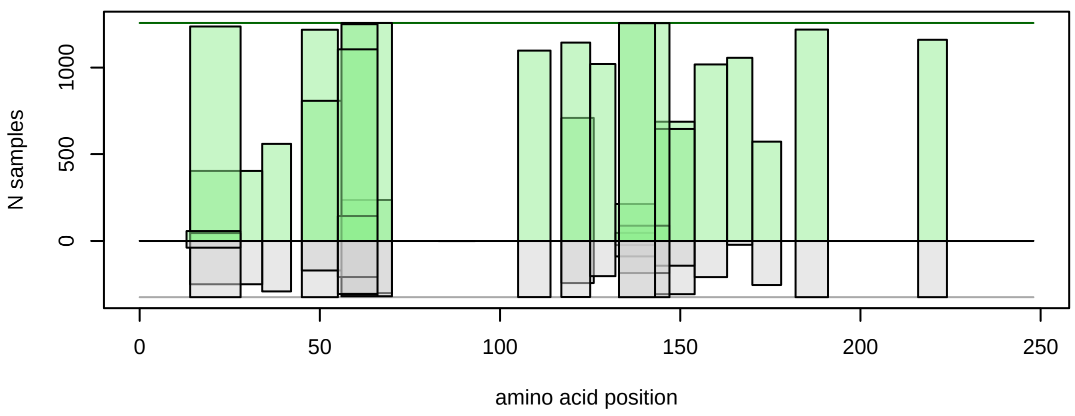

796. DENR (O43583), NP3

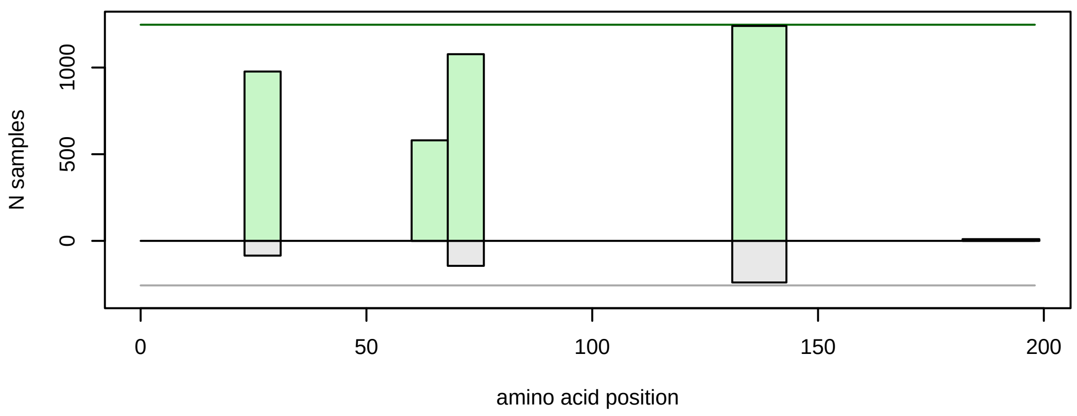

797. ZG16 (O60844), NP3

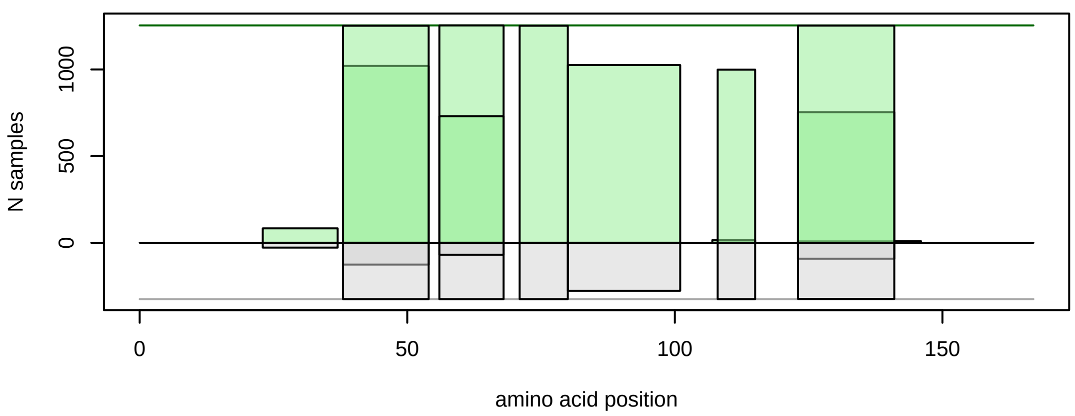

798. ARCN1 (P48444), NP1

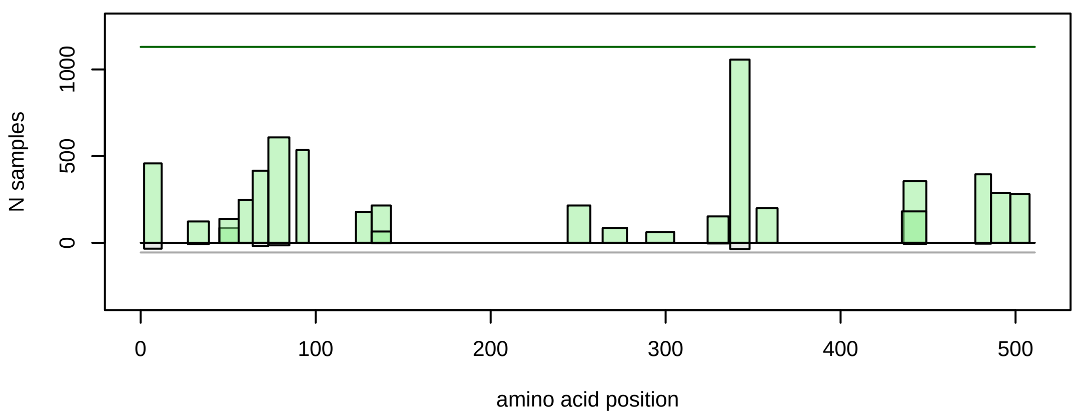

799. RAB1A (E7END7), NP4

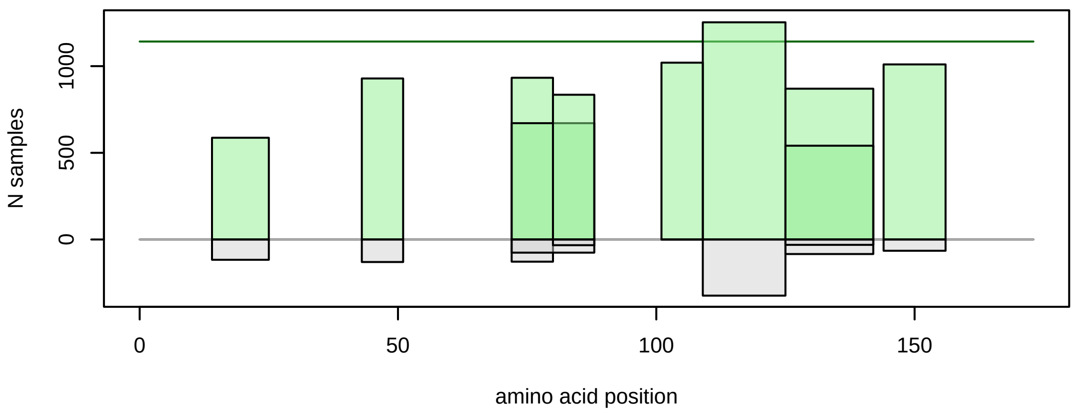

800. SERPINA7 (P05543), NP1

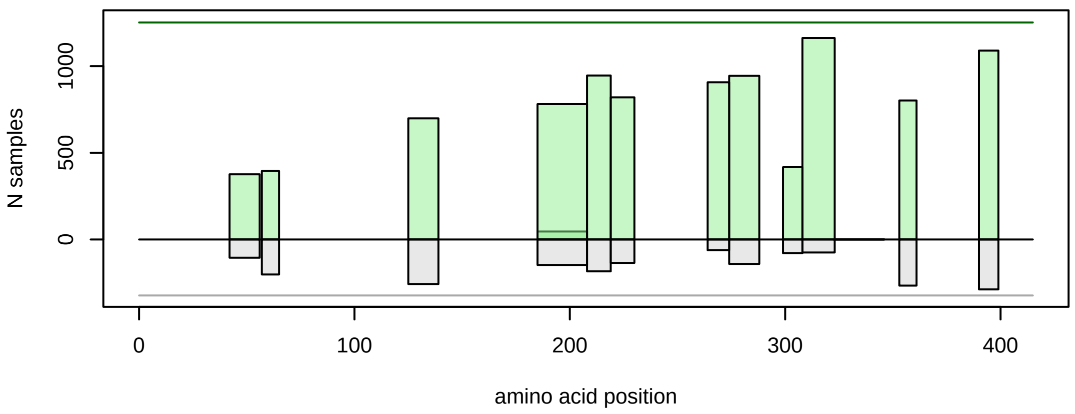

801. FAS (P25445;P25445−6), NP3

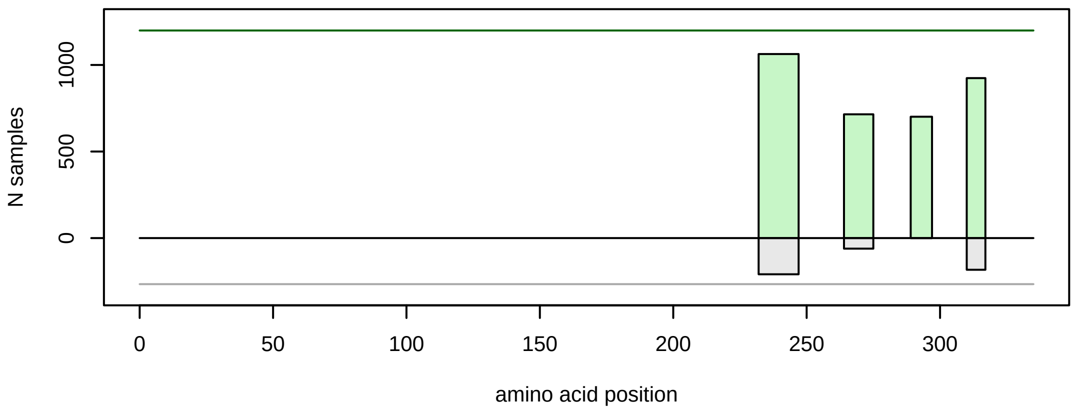

802. CANX (A0A7P0TAE9), NP2

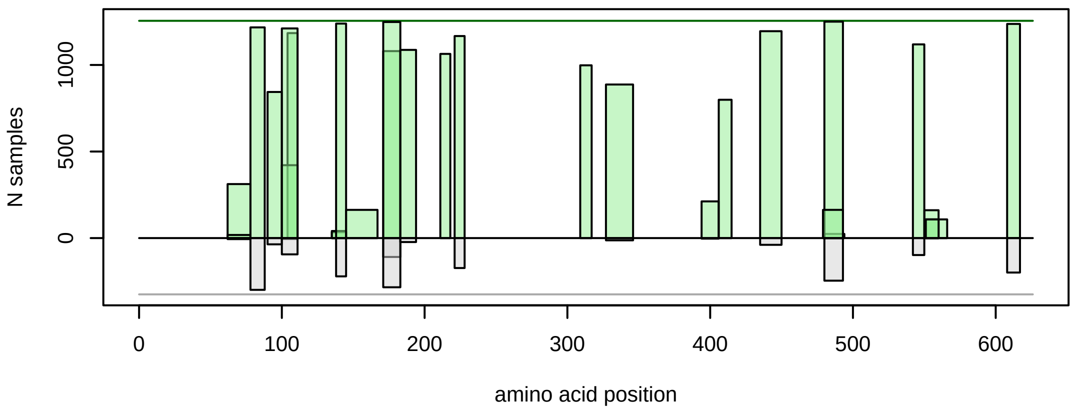

803. CTGF (P29279), NP4

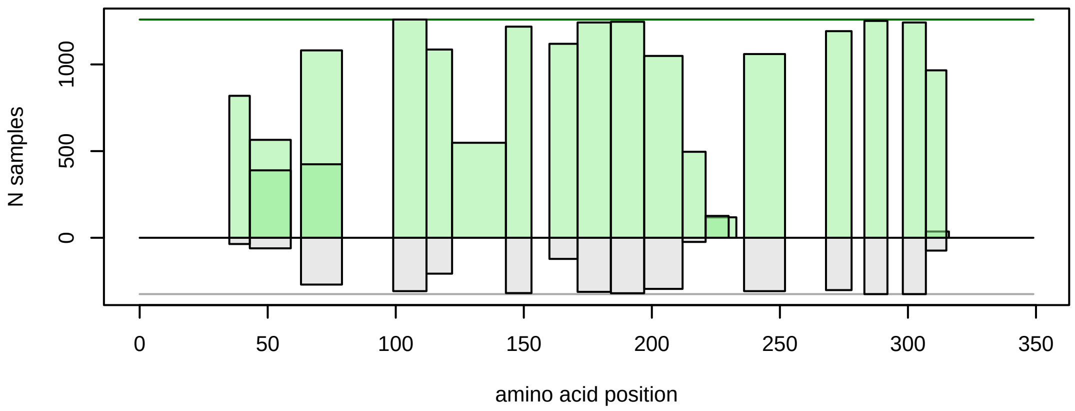

804. FERMT3 (Q86UX7;Q86UX7−2), NP3

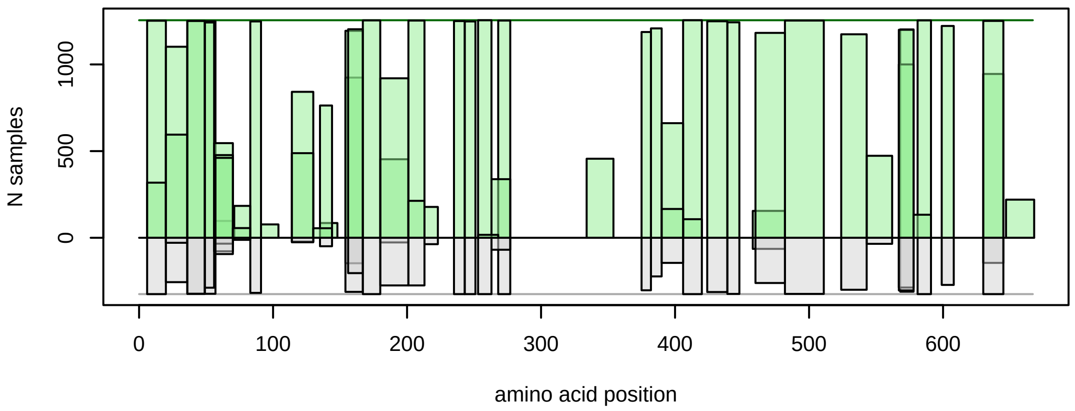

805. MAPRE1 (Q15691), NP1

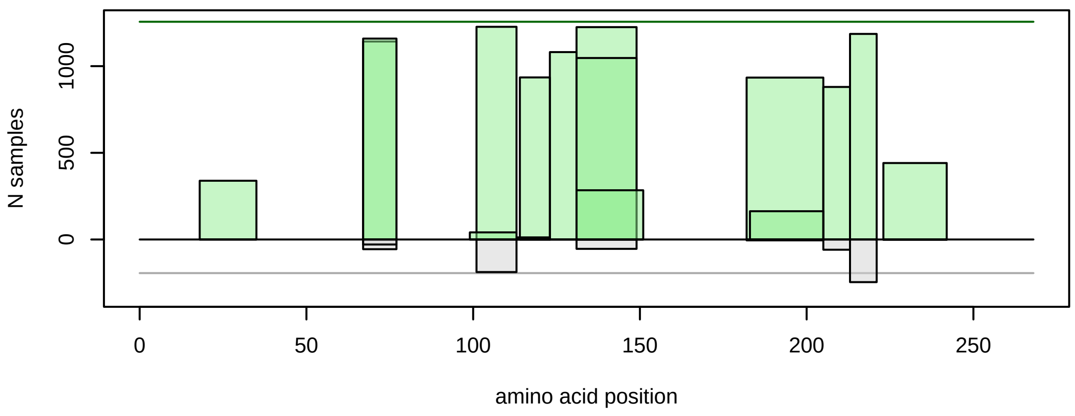

806. PROS1 (A0A3B3ISJ1;P07225), NP2

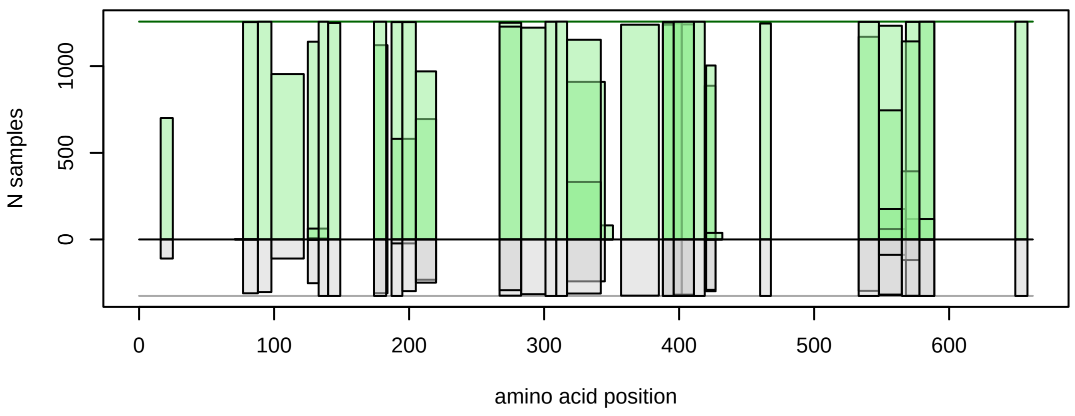

807. PSME1 (Q06323), NP3

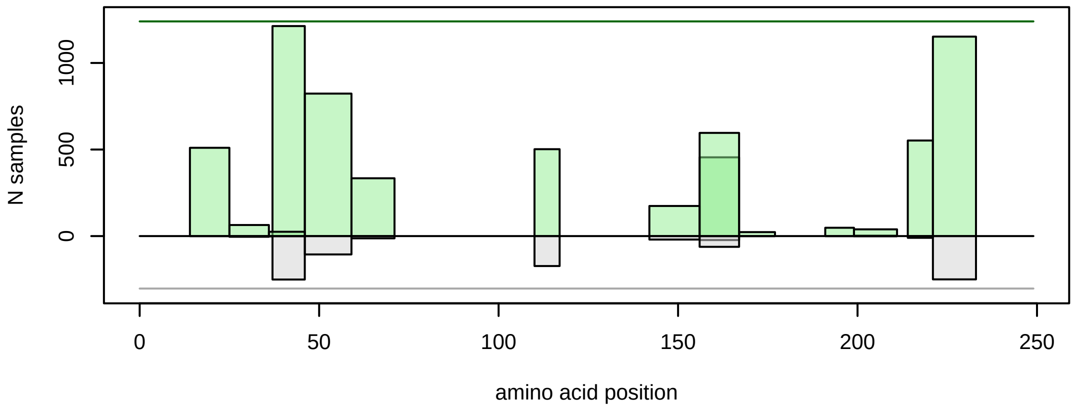

808. ATP5C1 (P36542), NP4

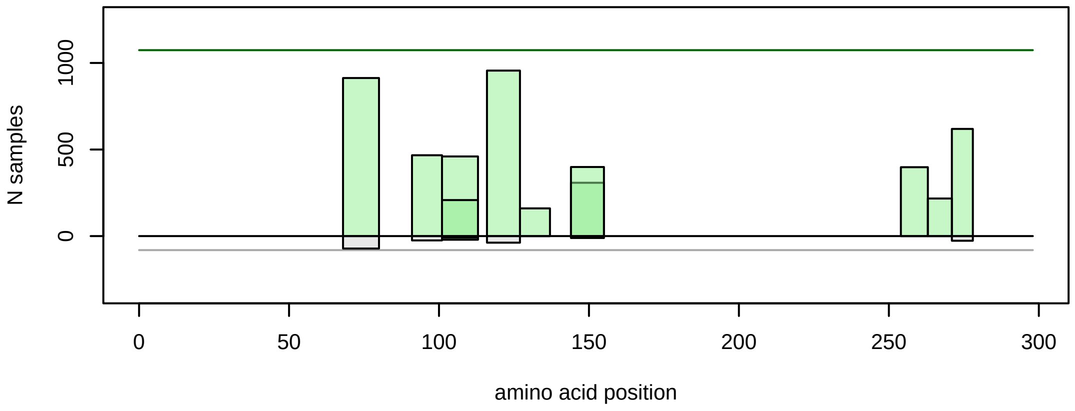

809. PPIC (P45877), NP4

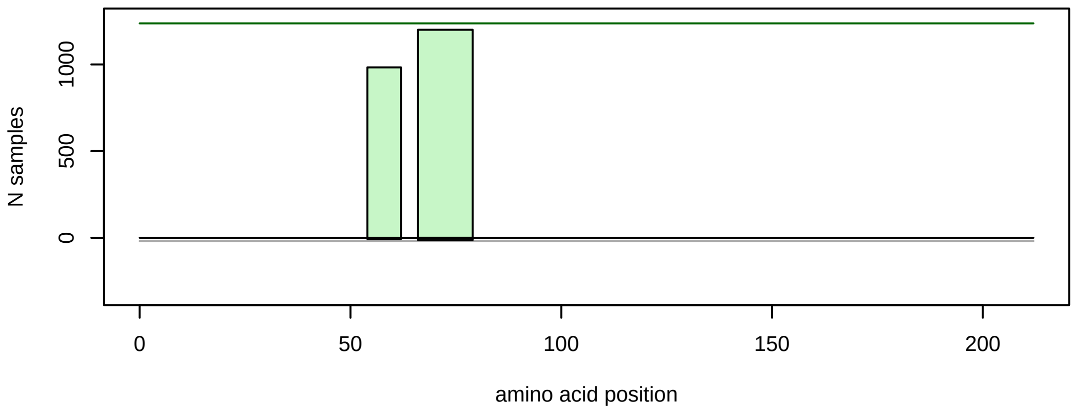

810. GAL (P22466), NP2

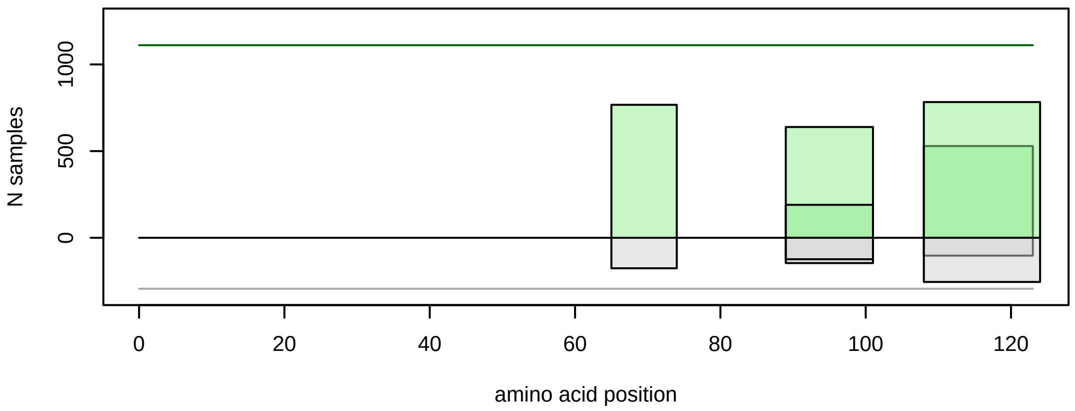

811. SLC29A1 (Q99808), NP1

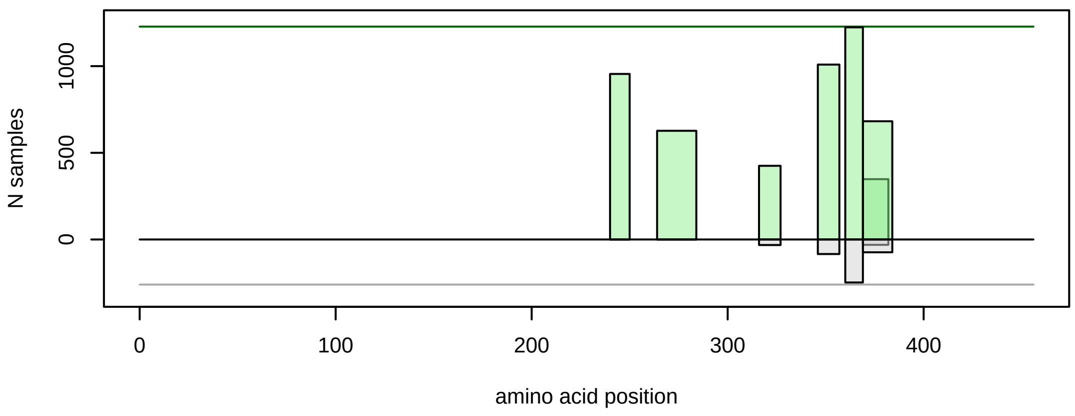

812. CLUH (I3L2B0), NP2

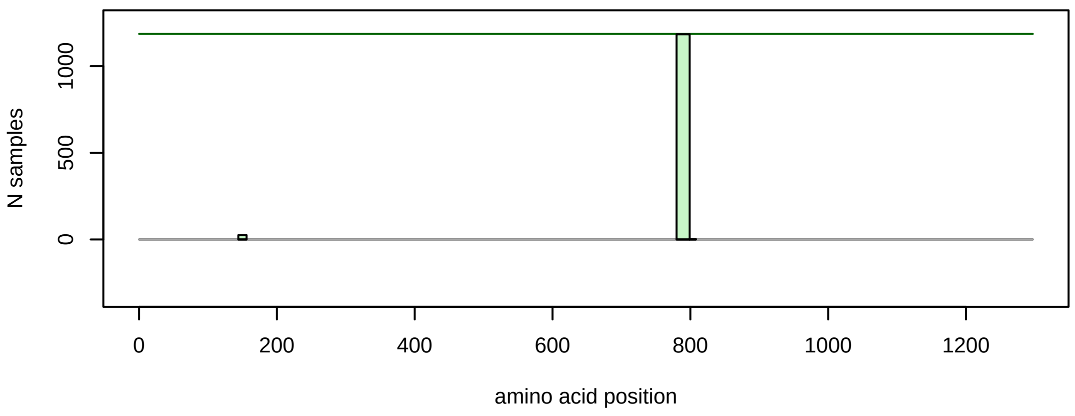

813. GAPDH (P04406), NP3

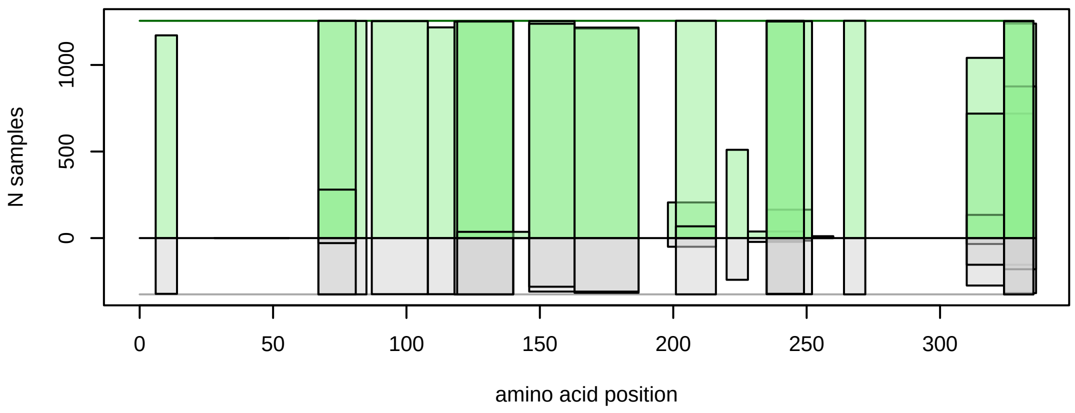

814. SERPINA1 (P01009), NP2

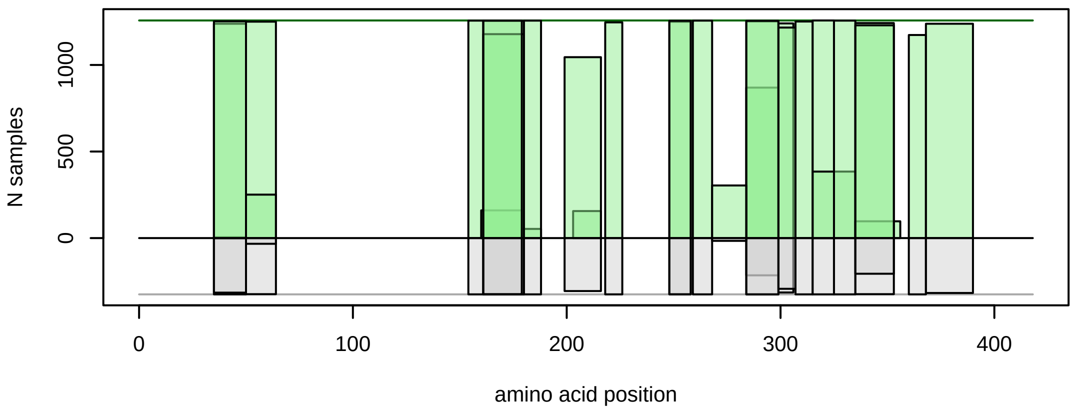

815. RAB1B (Q9H0U4), NP4

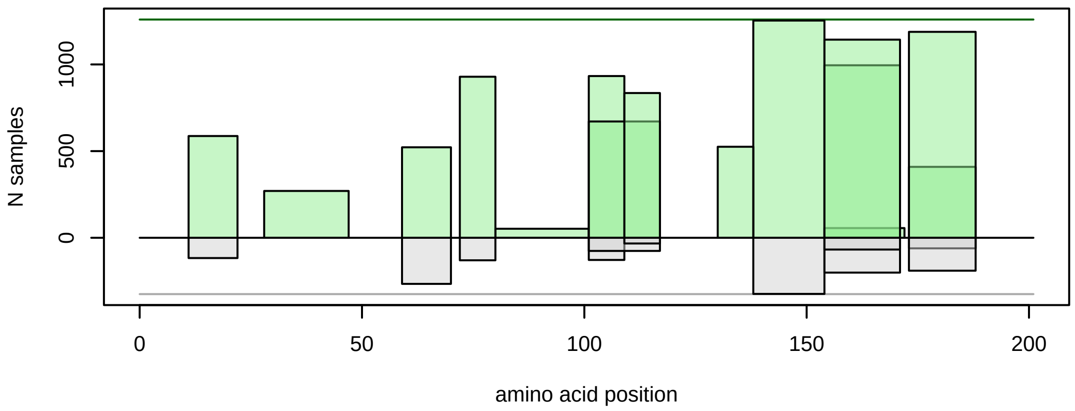

816. MS4A1 (E9PKH8;P11836), NP2

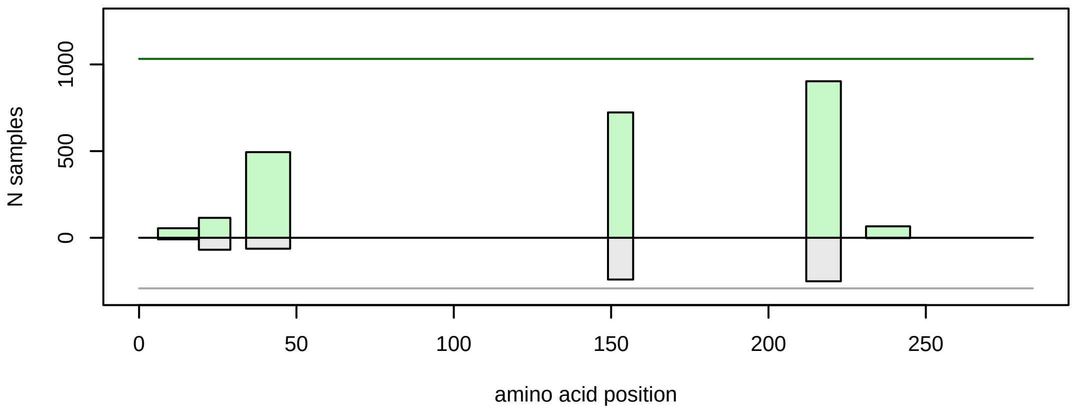

817. PSMC3 (P17980;R4GNH3), NP5

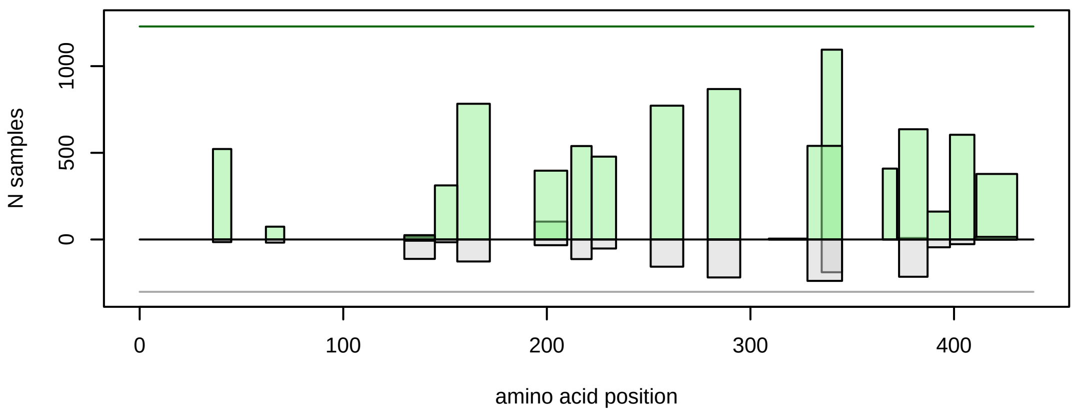

818. HNRNPD (Q14103−3), NP1

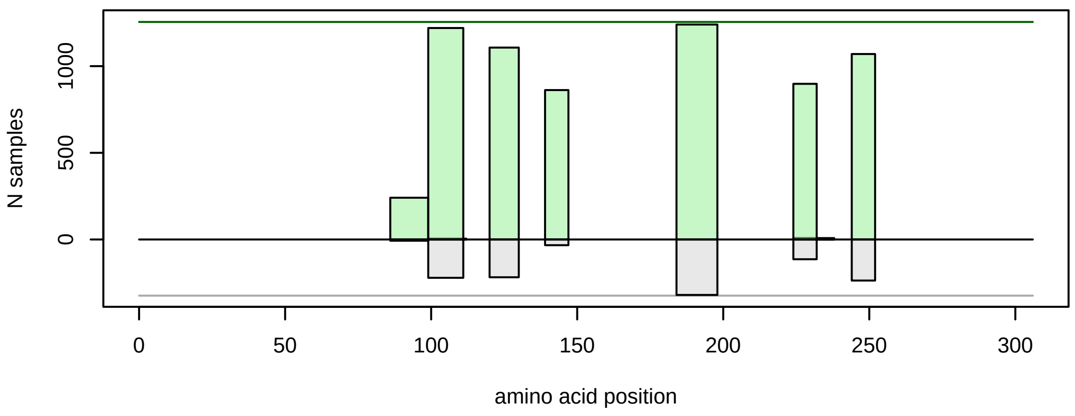

819. VEGFC (P49767), NP1

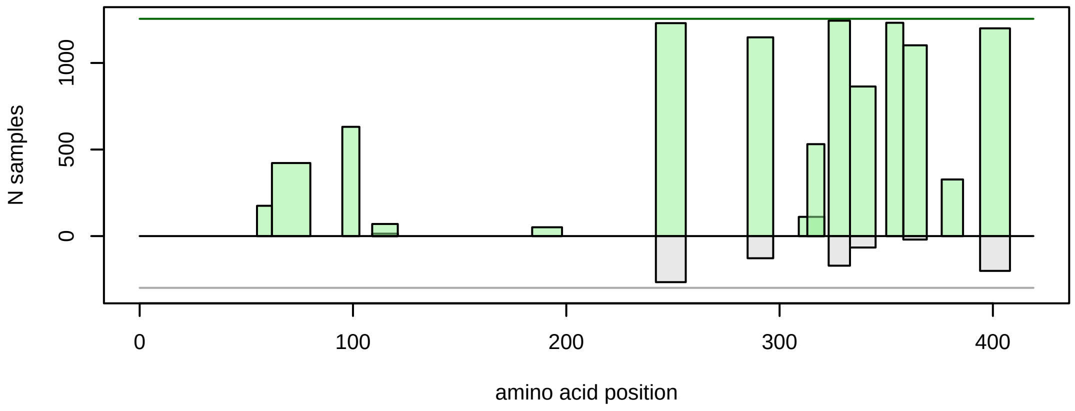

820. ITGAM (P11215;P11215−2), NP1

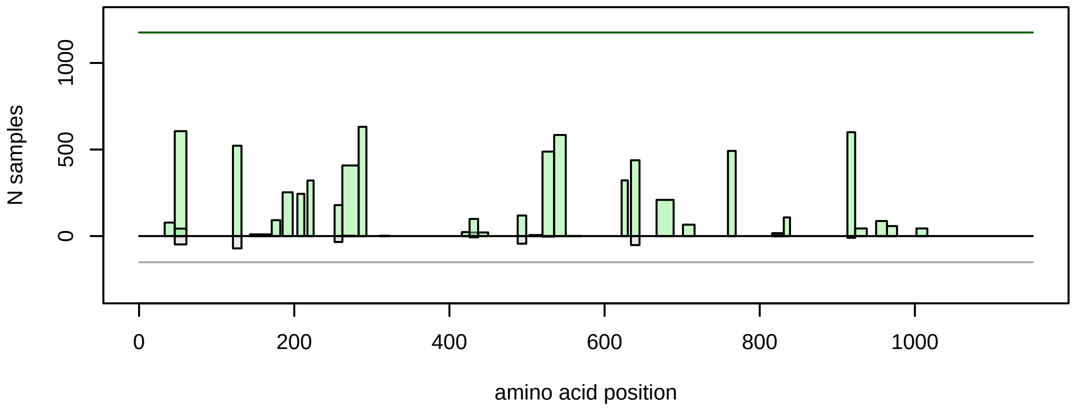

821. EEF1B2 (P24534), NP2

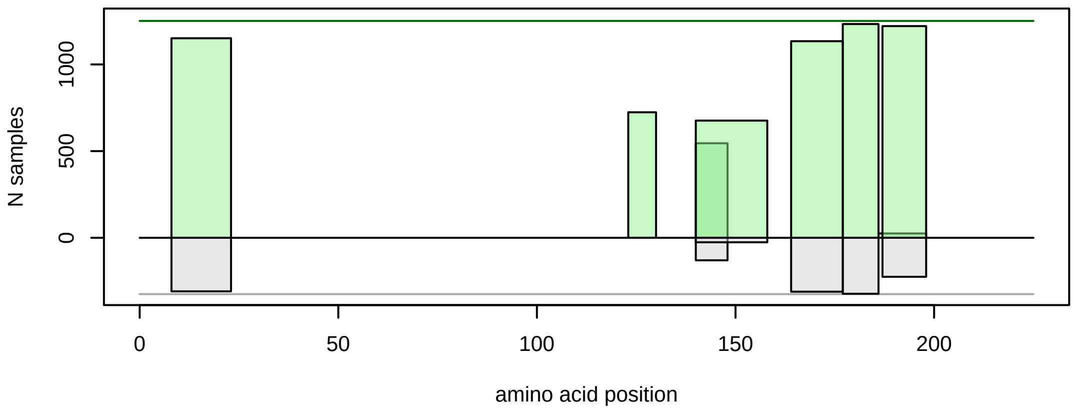

822. MAN1A1 (P33908), NP5

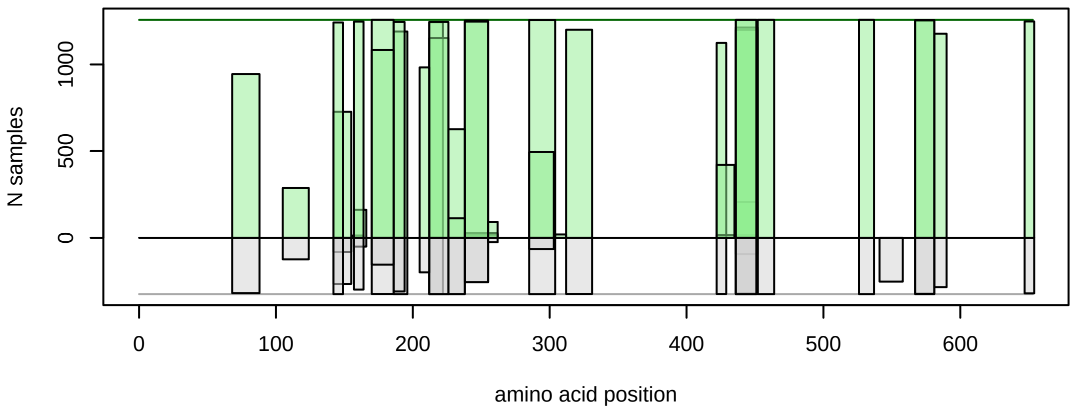

823. SEC22B (O75396), NP4

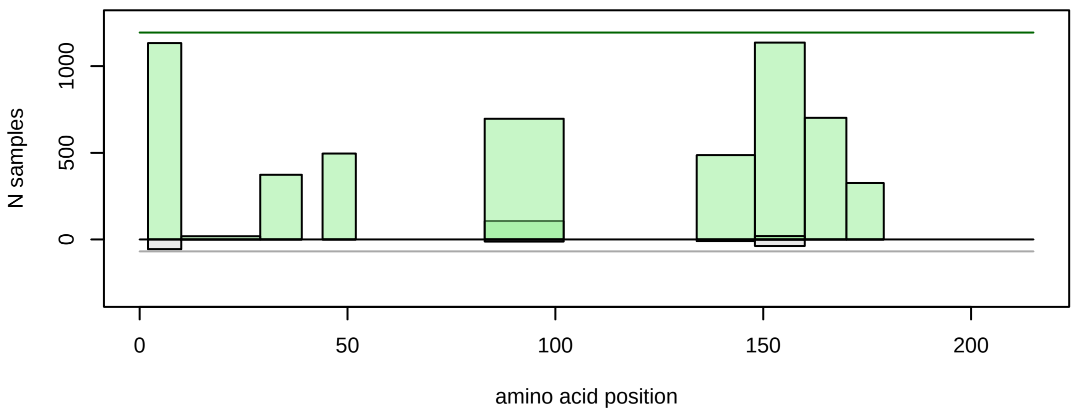

824. PRKCA (P17252), NP5

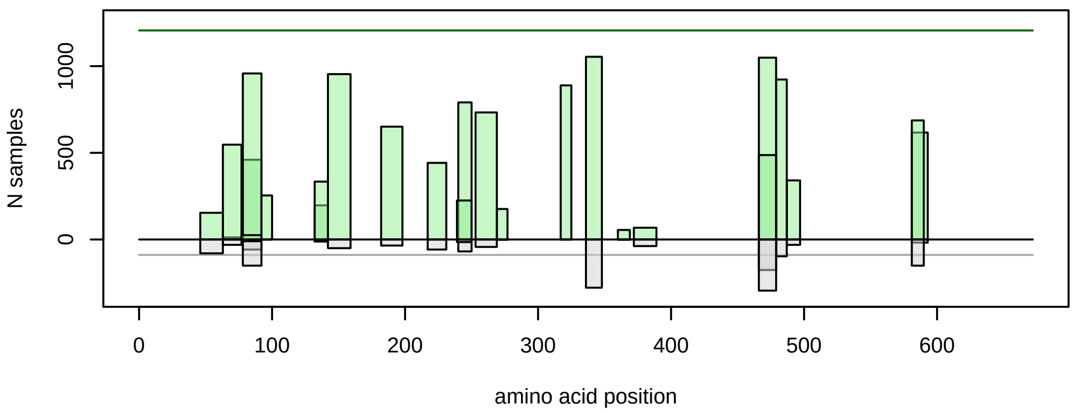

825. SDPR (O95810), NP1

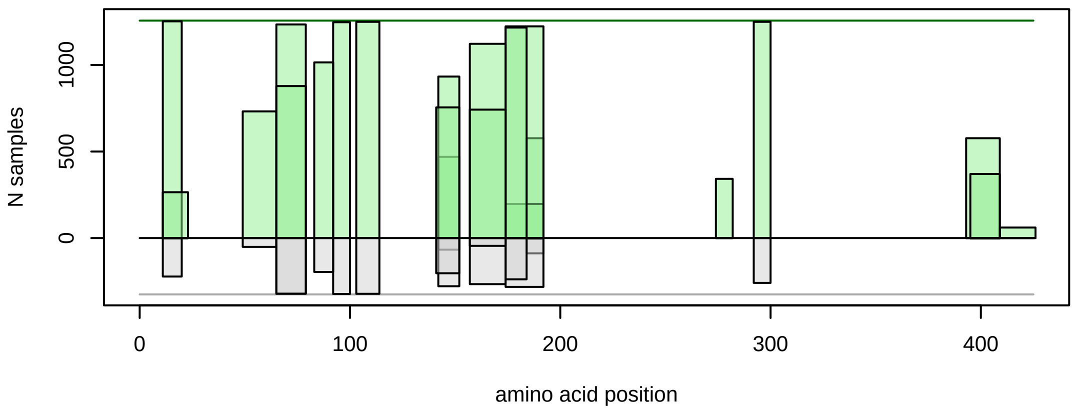

826. ADH5 (P11766), NP3

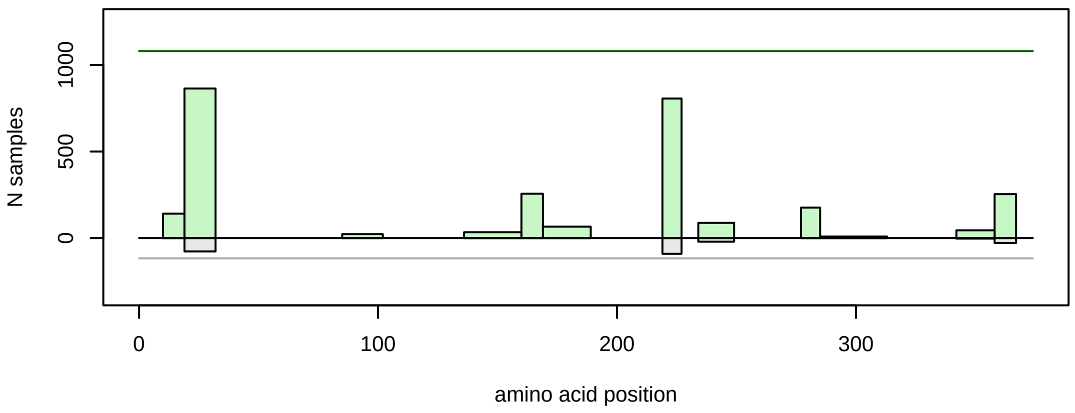

827. C1QTNF1 (A0A0C4DFP7;Q9BXJ1), NP5

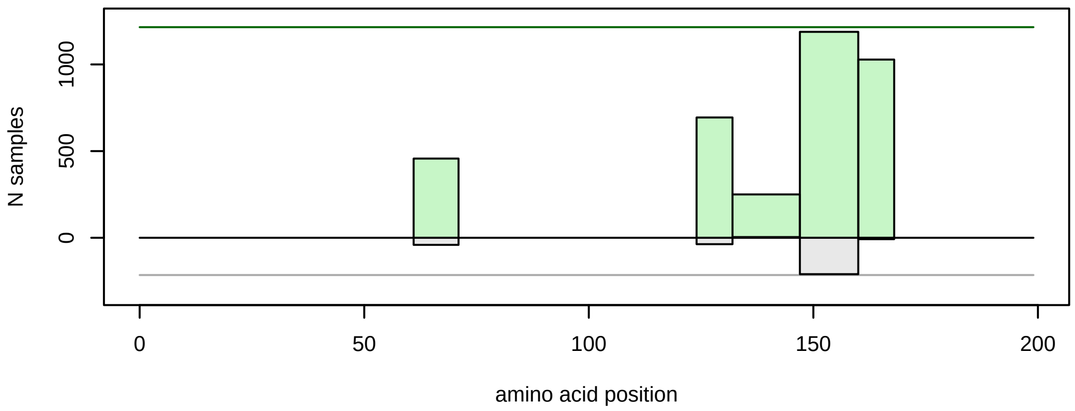

828. RAC1 (P63000−2), NP4

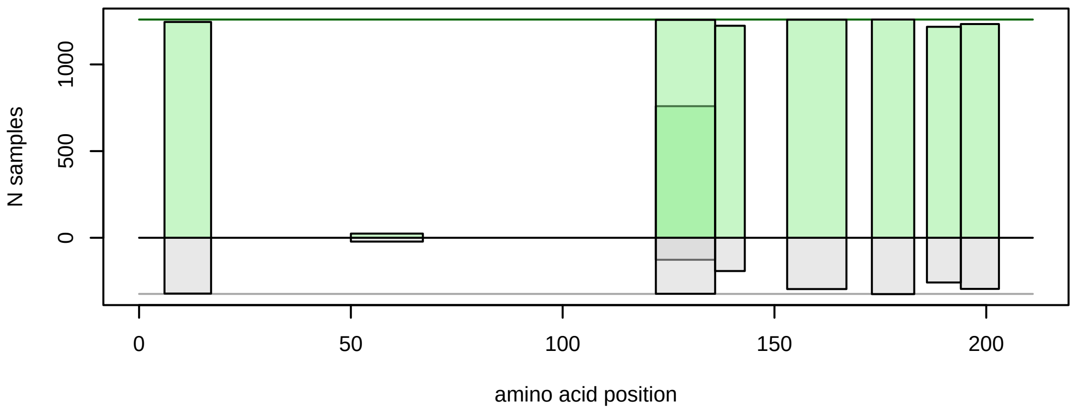

829. MASP1 (P48740−4), NP2

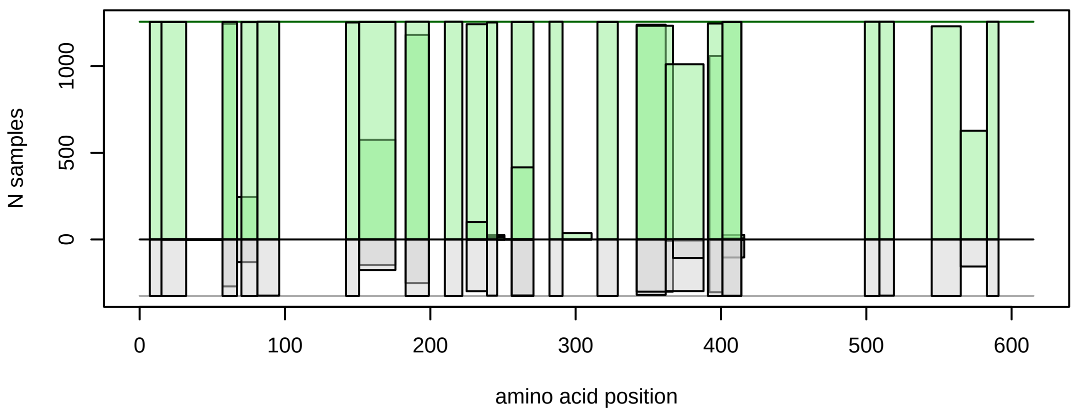

830. CAP1 (Q01518), NP1

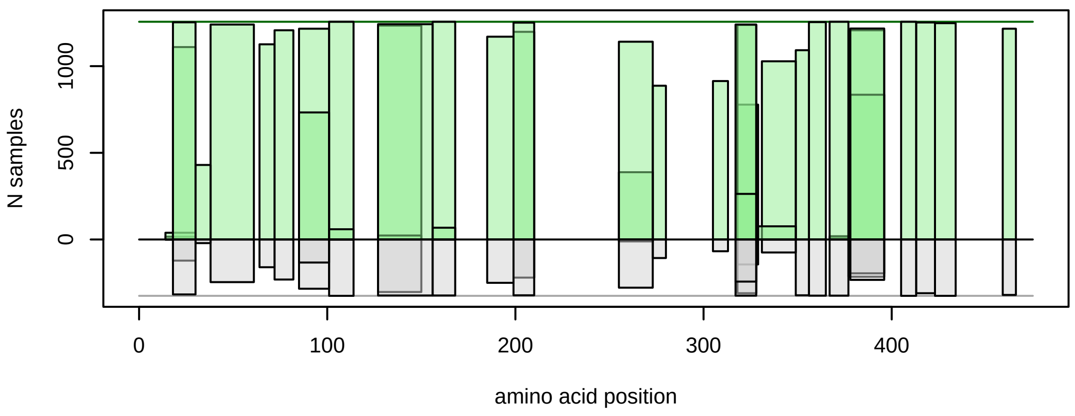

831. PRKCSH (P14314−2), NP2

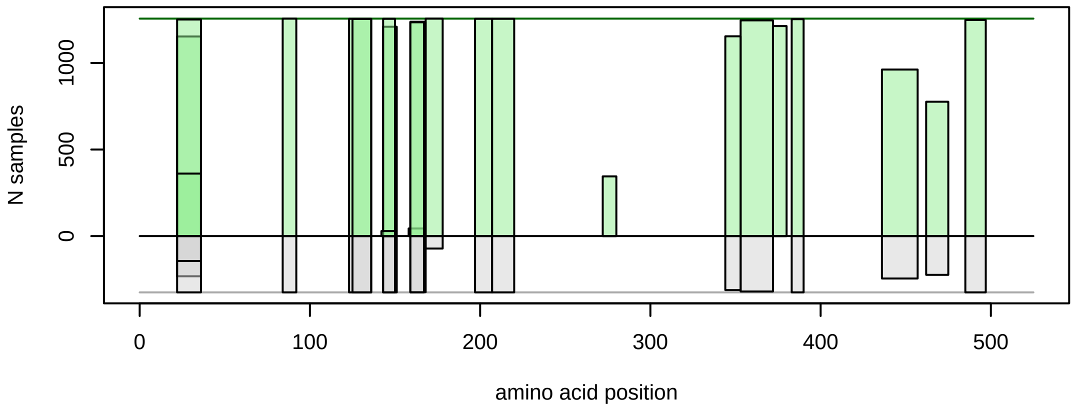

JBE2D2 (A0A087WY85;A0A0A0MQU3;D6RAH7;H9KV45;P61077;P61077−3;P62837;P6283

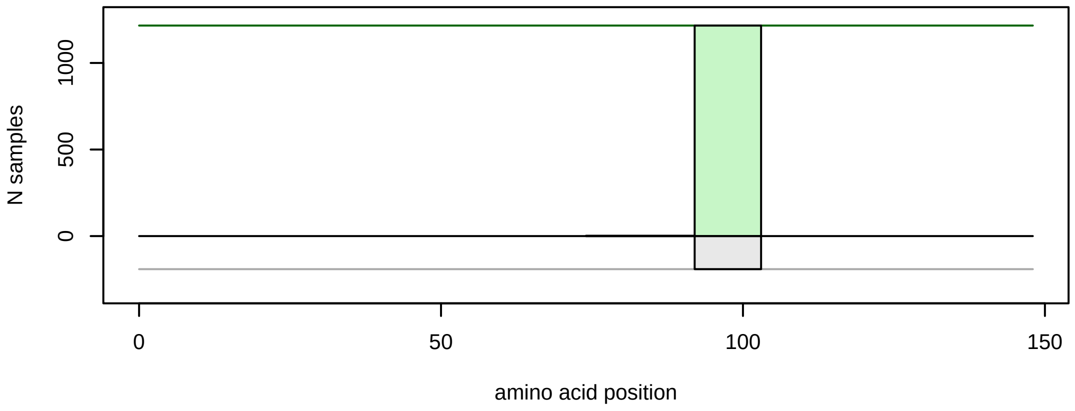

833. RPS15A (P62244), NP4

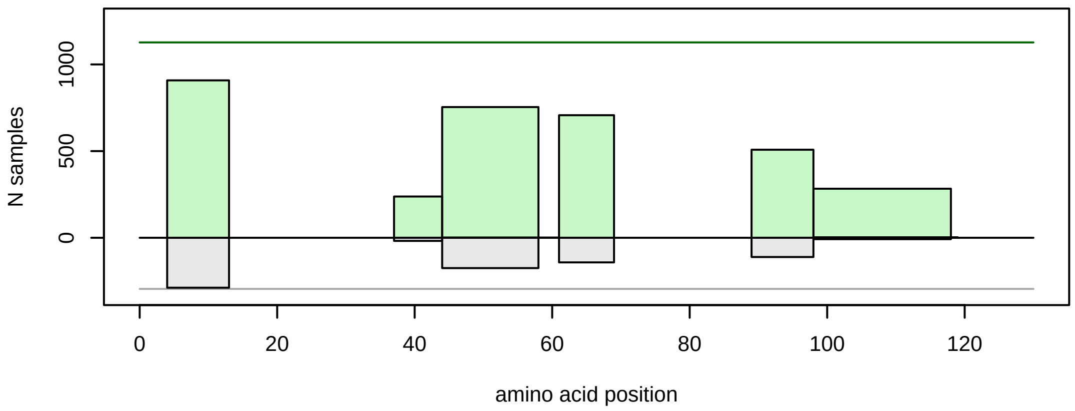

834. TALDO1 (P37837), NP5

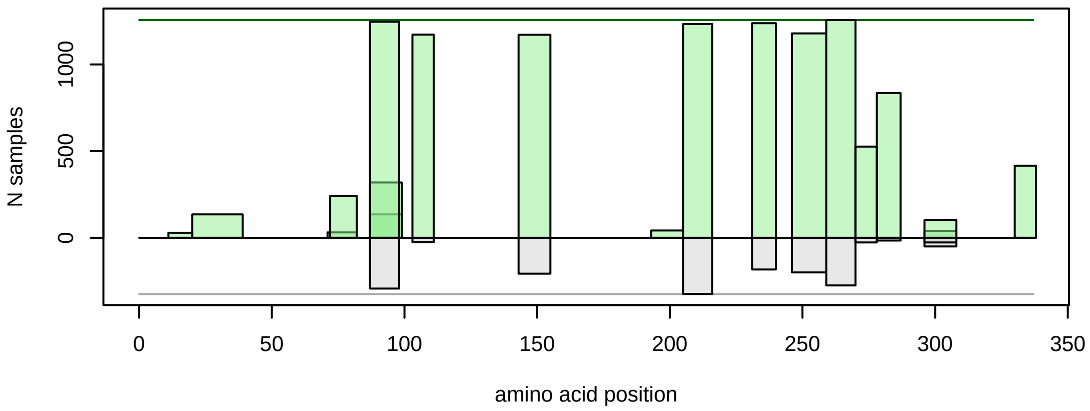

835. CR1 (E9PDY4;Q5SR44), NP4

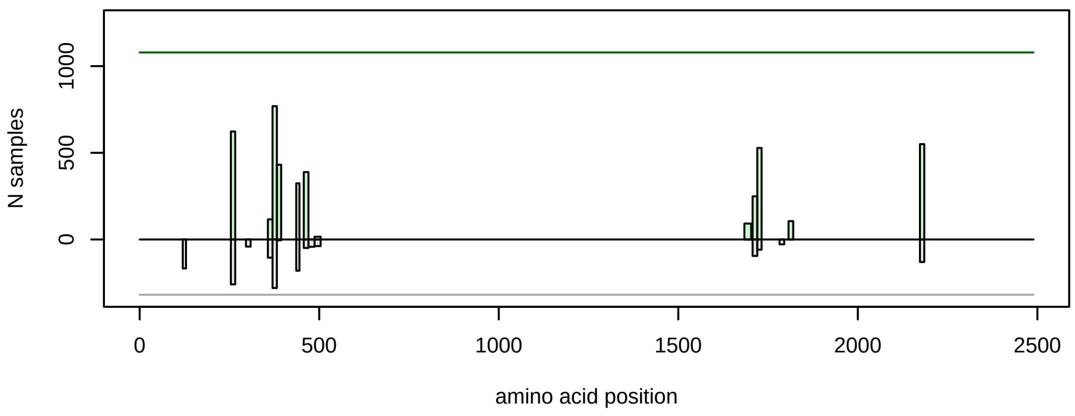

836. C16orf54 (Q6UWD8), NP2

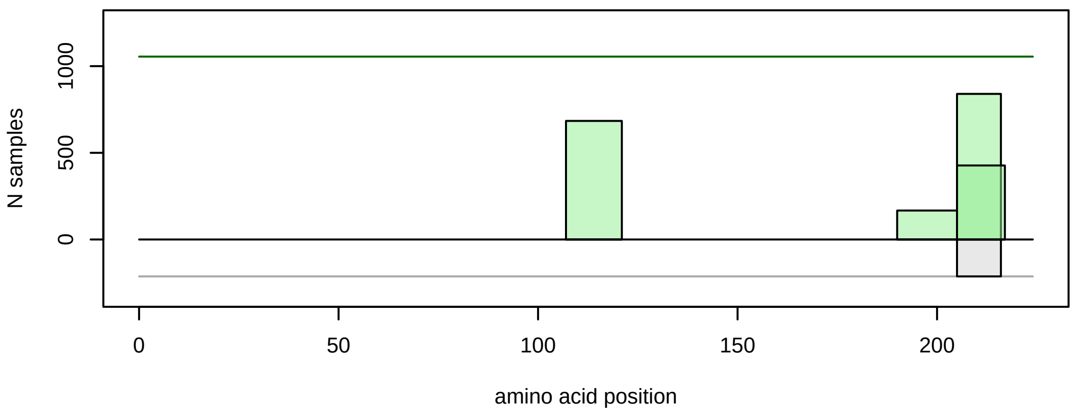

837. KIF1B (O60333−2), NP1

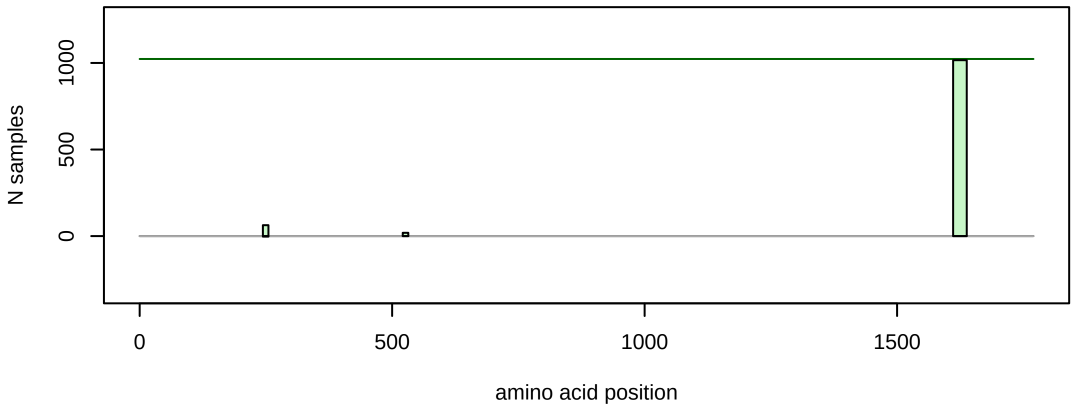

838. GLB1 (E7EQ29), NP5

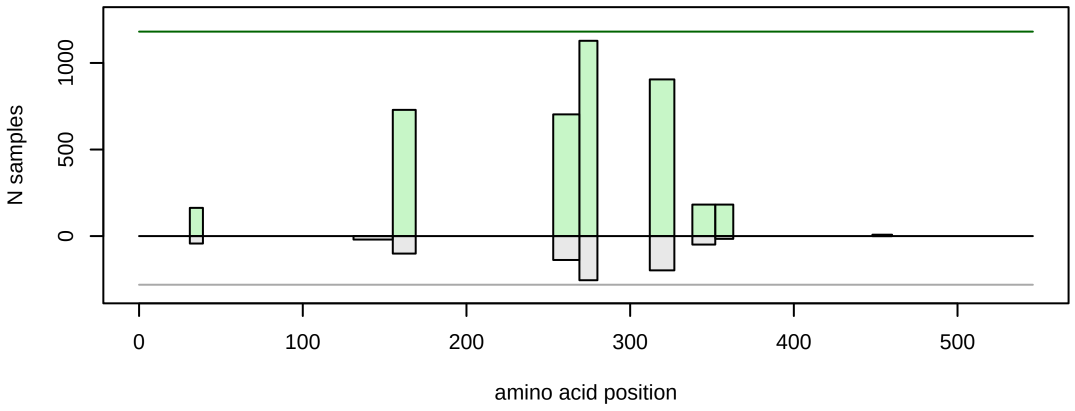

839. CD44 (P16070;P16070−10;P16070−11;P16070−4), NP2

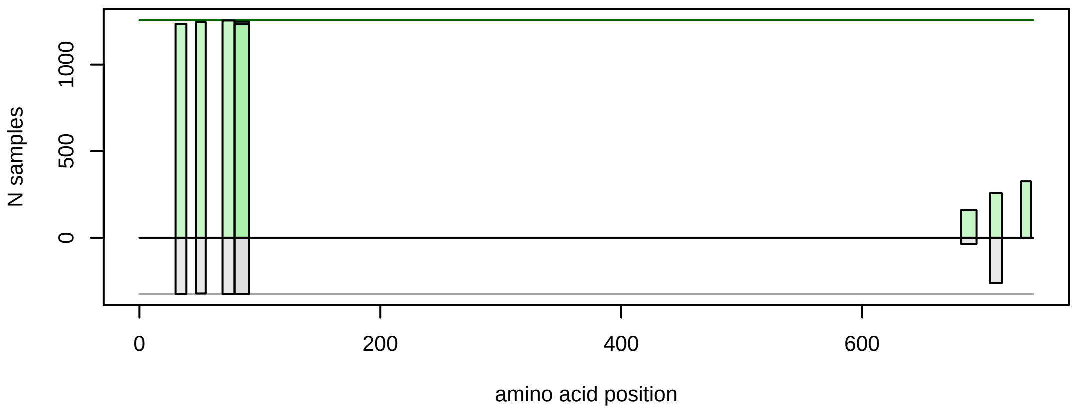

840. PRDX5 (P30044), NP4

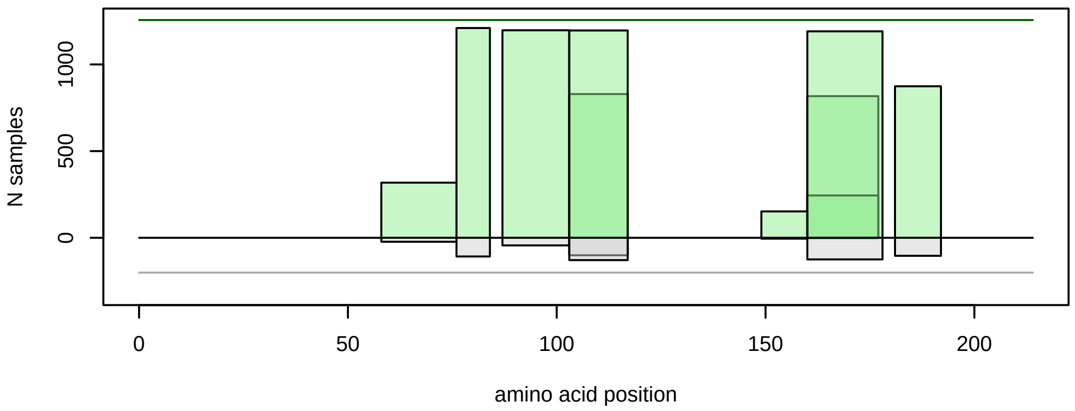

841. VASP (P50552), NP4

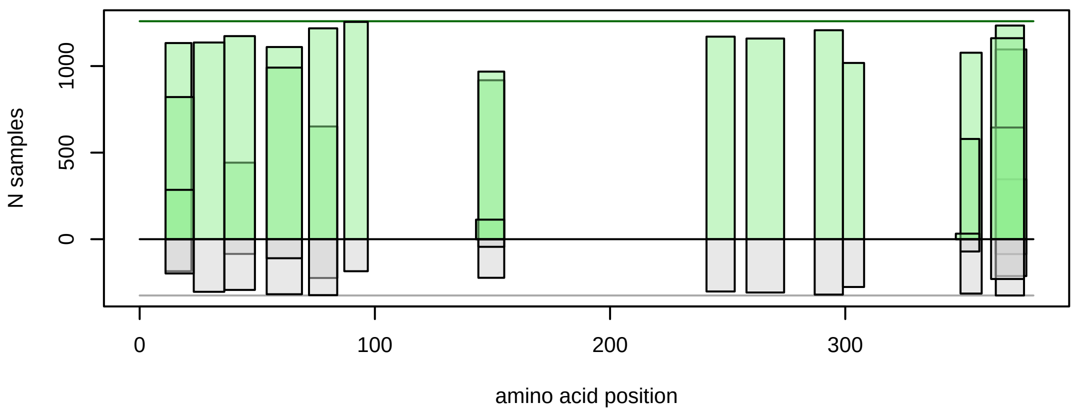

842. FIGF (O43915), NP3

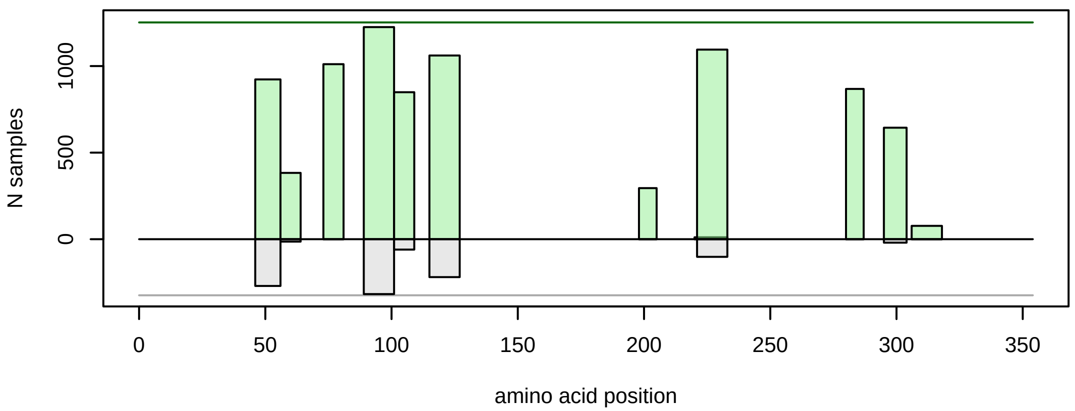

843. GDF2 (Q9UK05), NP2

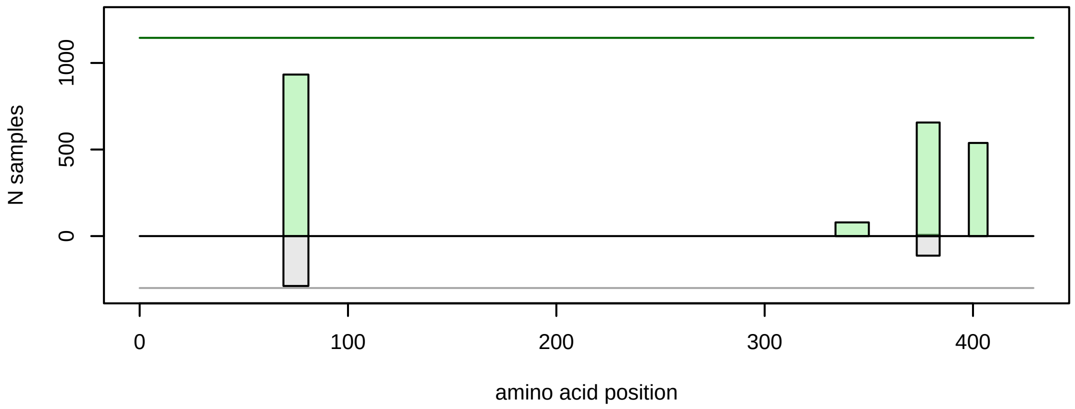

844. PPIA (P62937), NP4

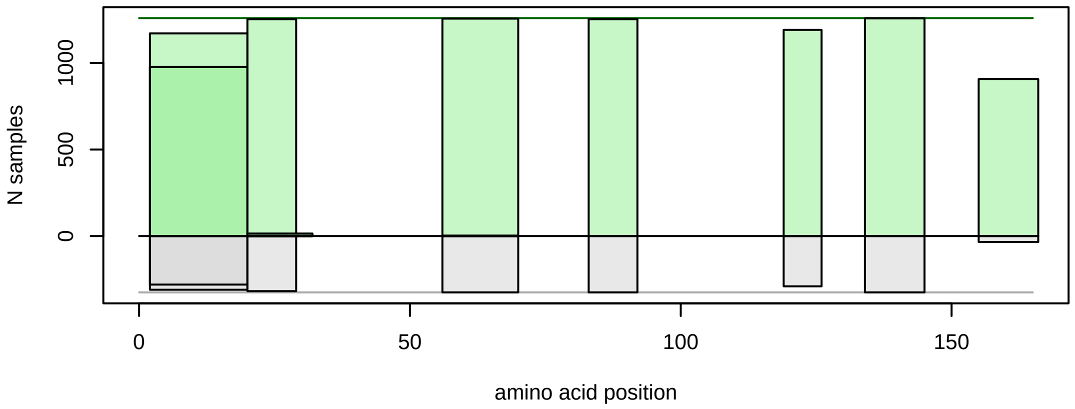

845. DMKN (Q6E0U4−16), NP3

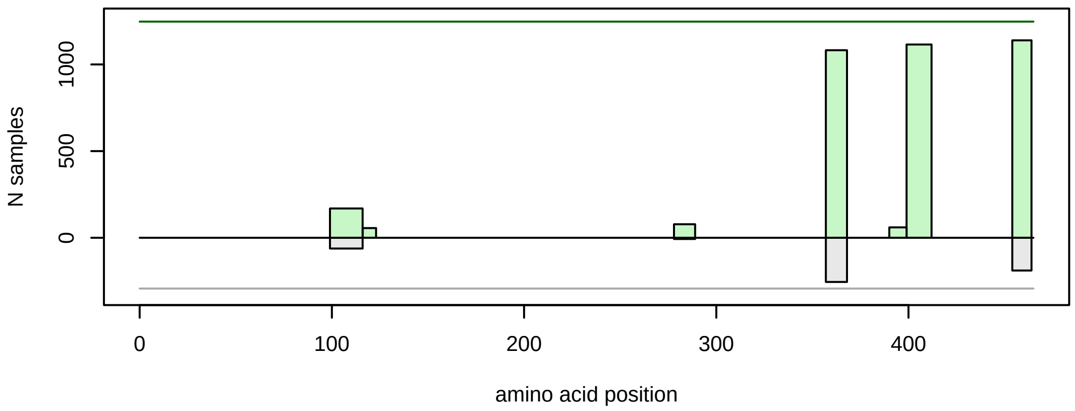

846. RAB21 (Q9UL25), NP1

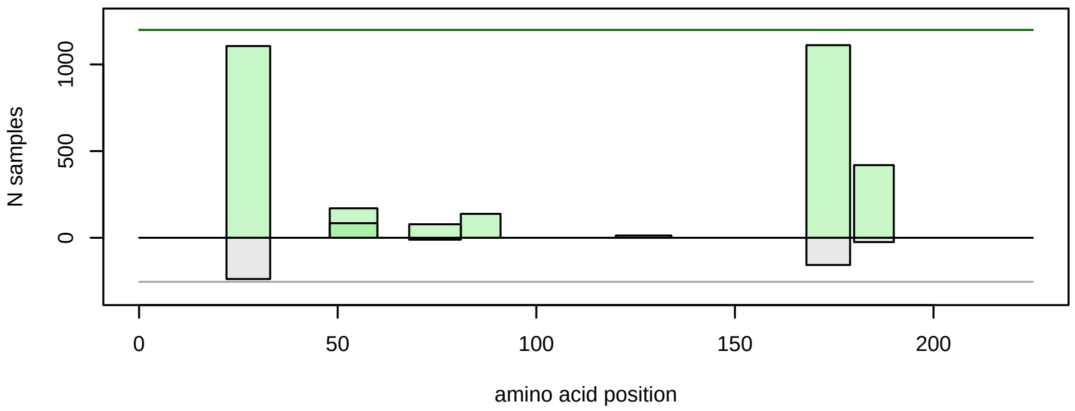

847. PRPSAP2 (O60256), NP3

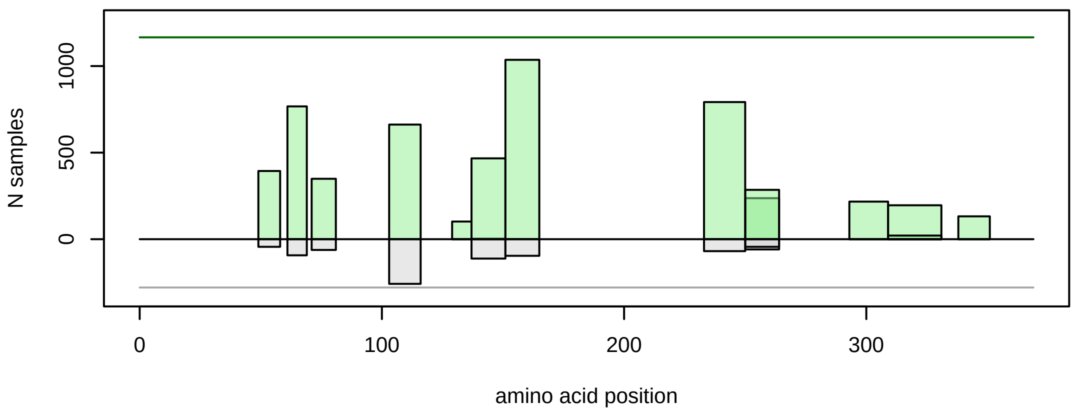

848. LRP1 (Q07954), NP5

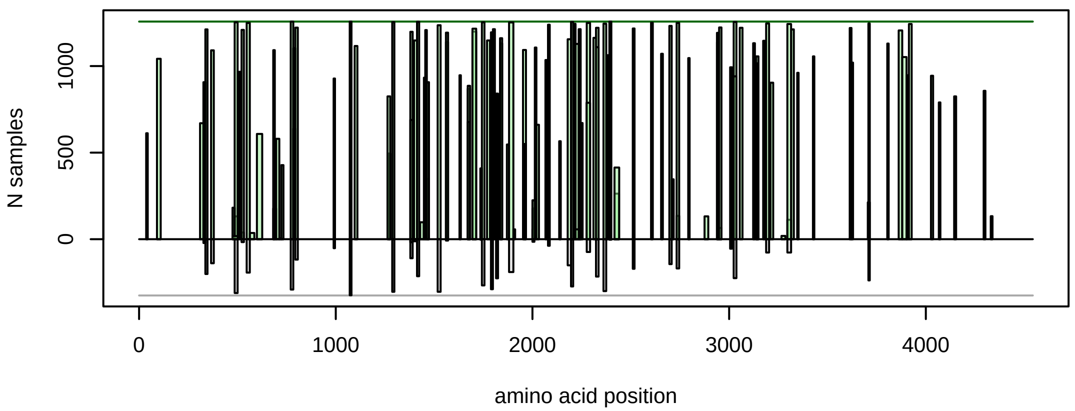

849. G6PD (P11413−2), NP4

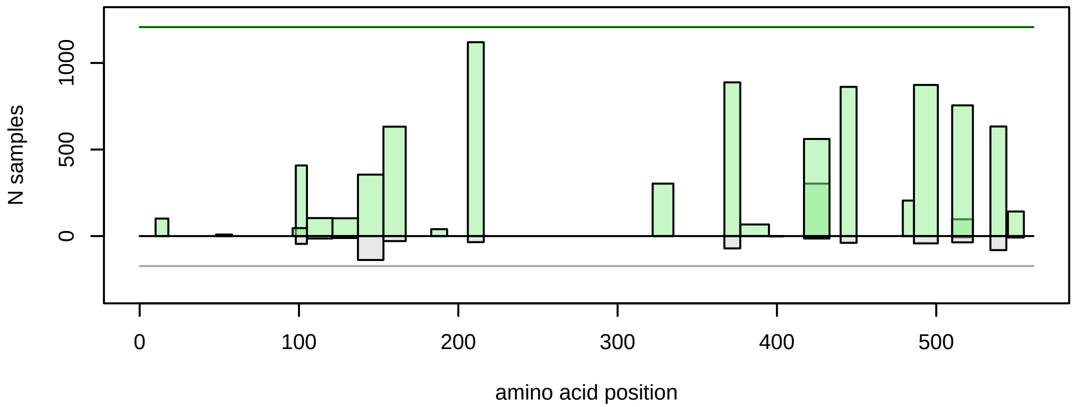

850. HMGB2 (P26583), NP1

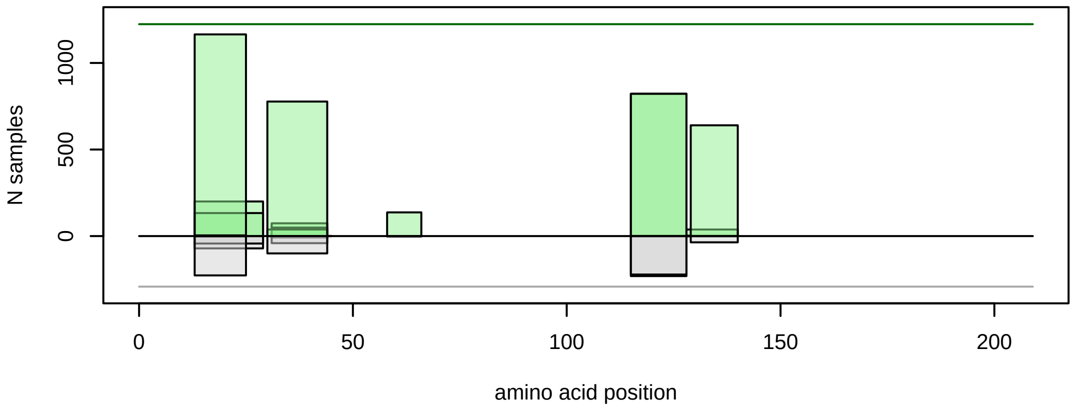

851. LRRFIP2 (Q9Y608−4), NP4

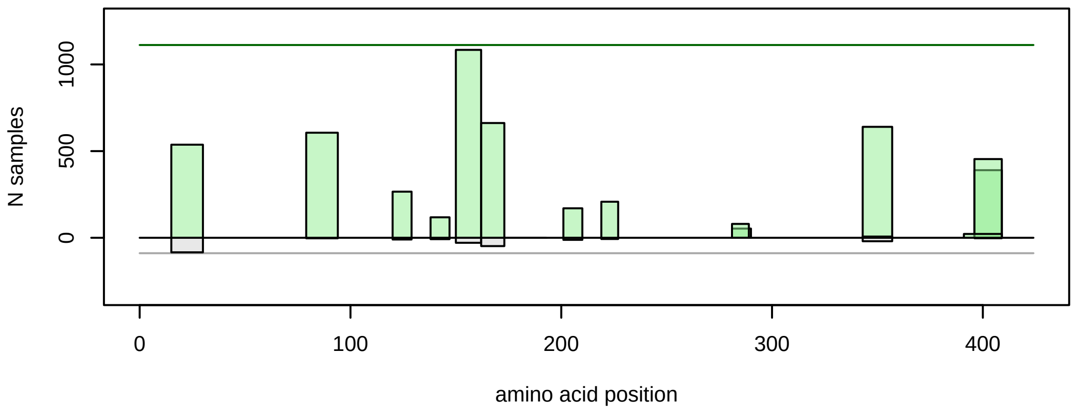

852. C1QTNF3 (Q9BXJ4−3), NP4

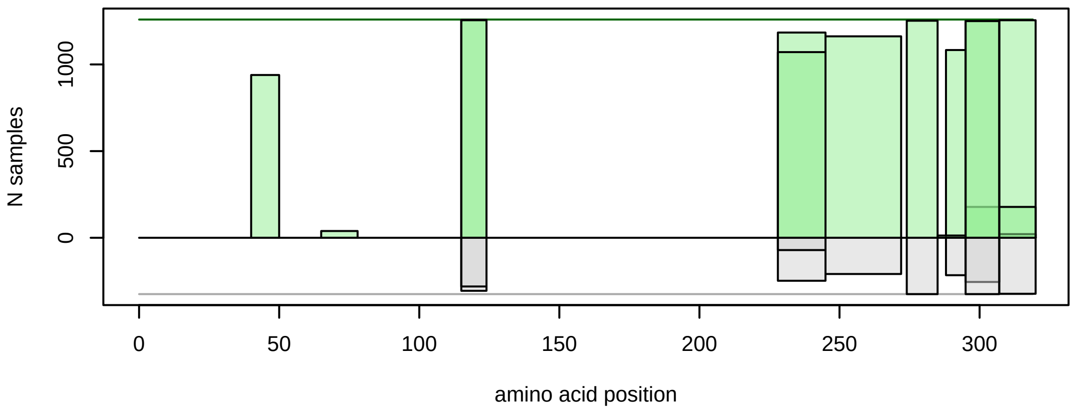

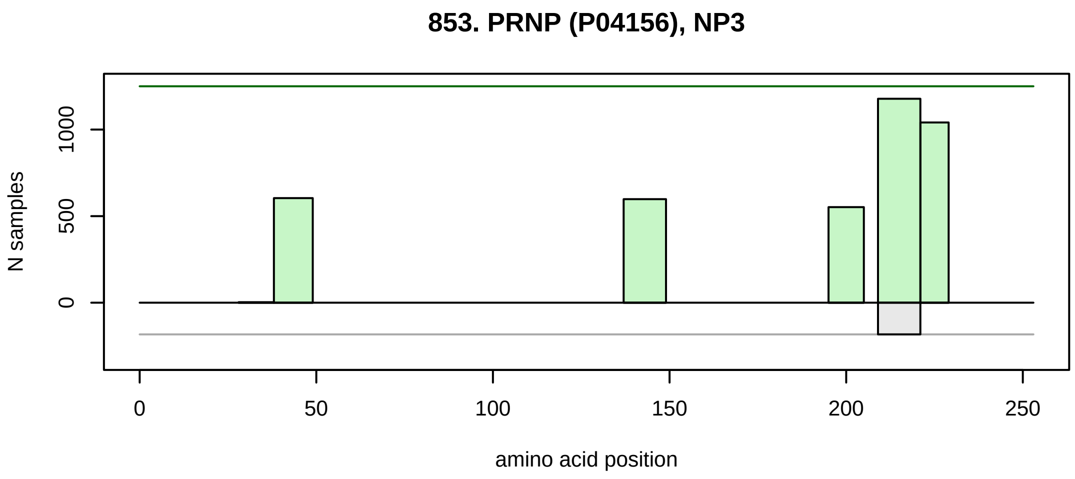
853. PRNP (P04156), NP3
N samples
0
500
1000
0
50
100
150
200
250
amino acid position

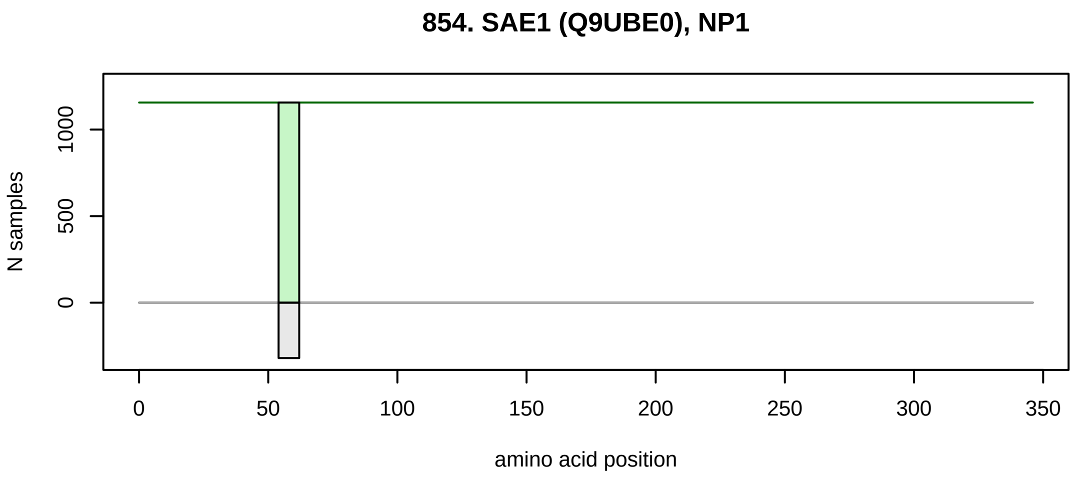
854. SAE1 (Q9UBE0), NP1
N samples
0
500
1000
0
50
100
150
200
250
300
350
amino acid position

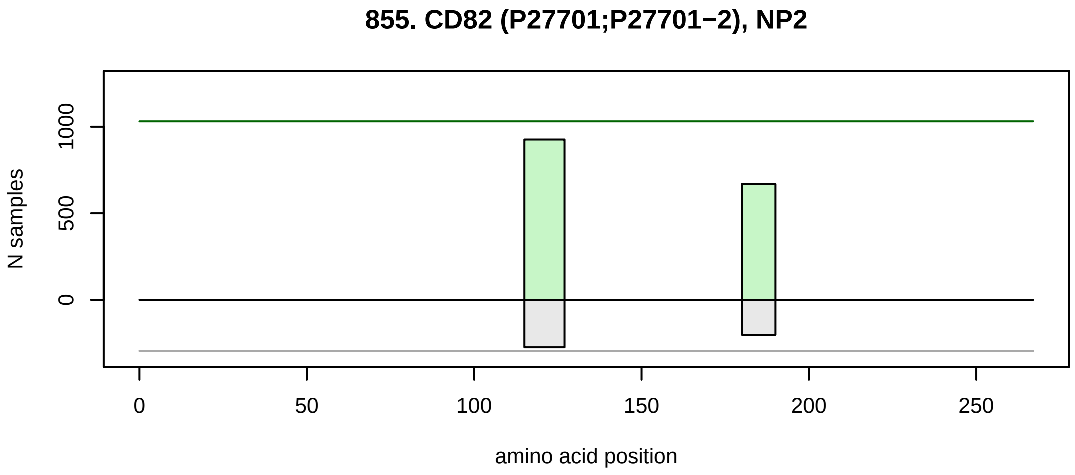
855. CD82 (P27701;P27701−2), NP2
N samples
0
500
1000
0
50
100
150
200
250
amino acid position

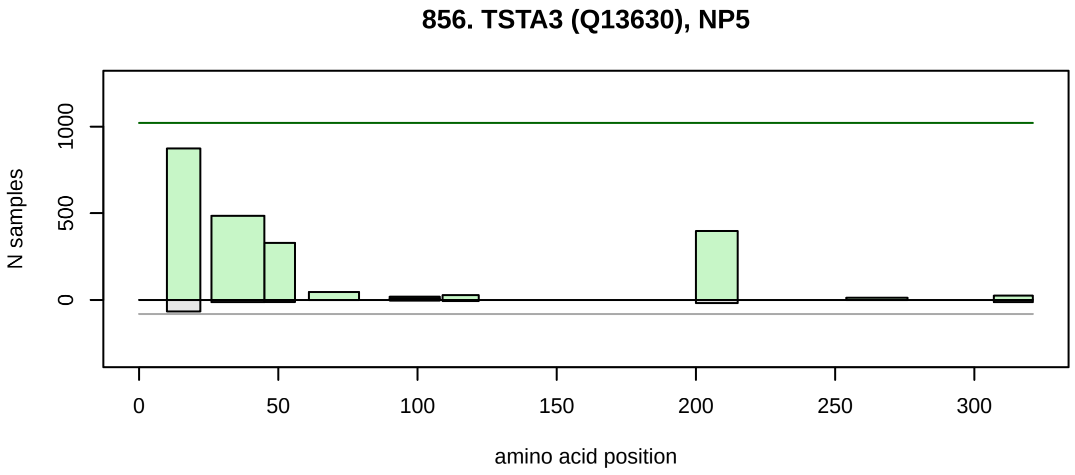
856. TSTA3 (Q13630), NP5
N samples
0
500
1000
0
50
100
150
200
250
300
amino acid position

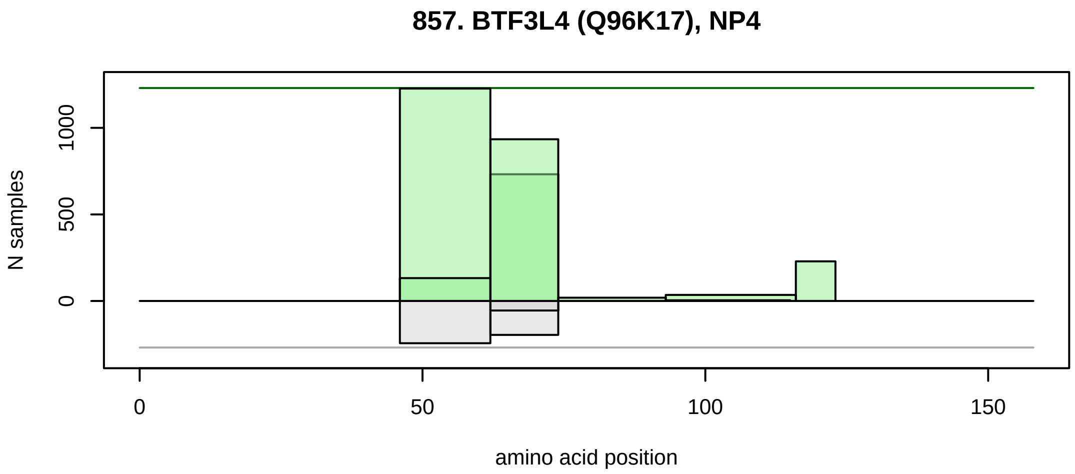
857. BTF3L4 (Q96K17), NP4
N samples
0
500
1000
0
50
100
150
amino acid position

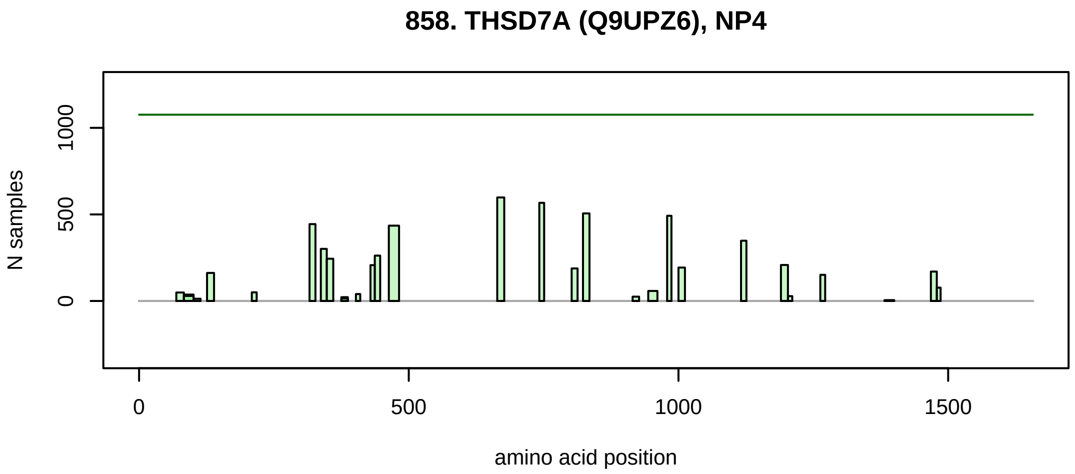
858. THSD7A (Q9UPZ6), NP4
N samples
0
500
1000
0
500
1000
1500
amino acid position

859. HEXB (P07686), NP5

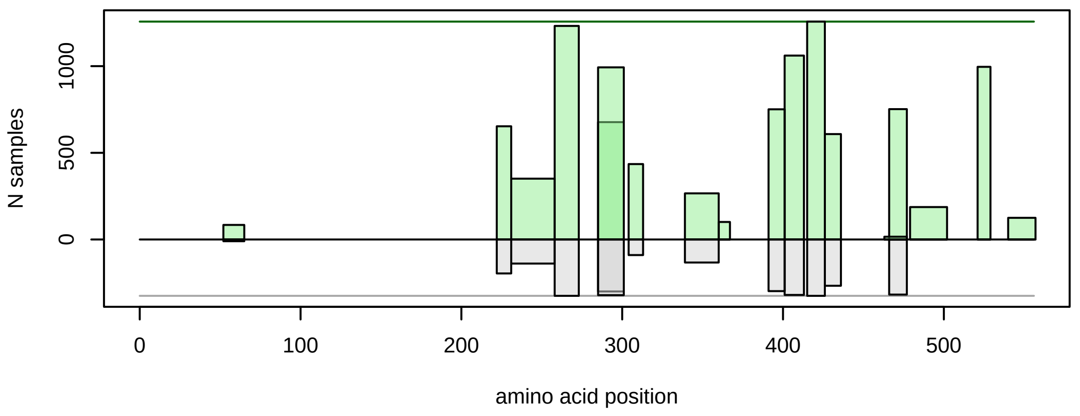

860. CDHR2 (Q9BYE9), NP2

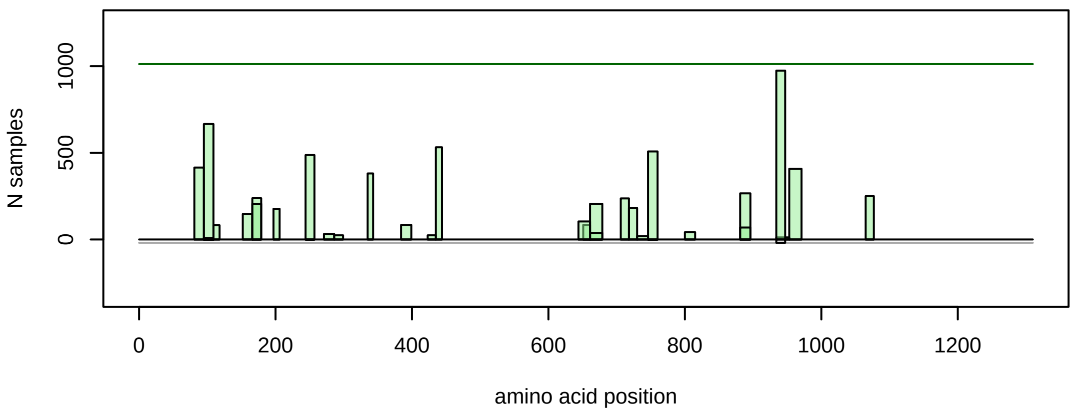

861. CILP2 (K7EPJ4;Q8IUL8), NP2

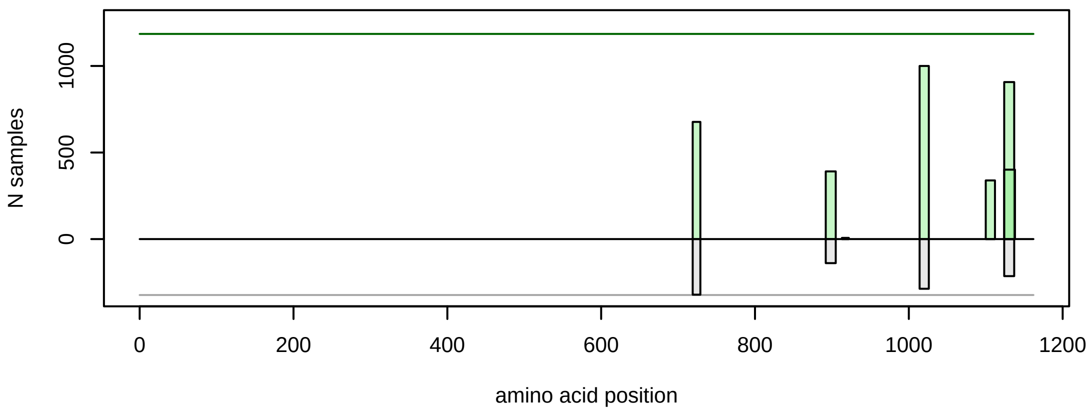

862. DBNL (Q9UJU6), NP2

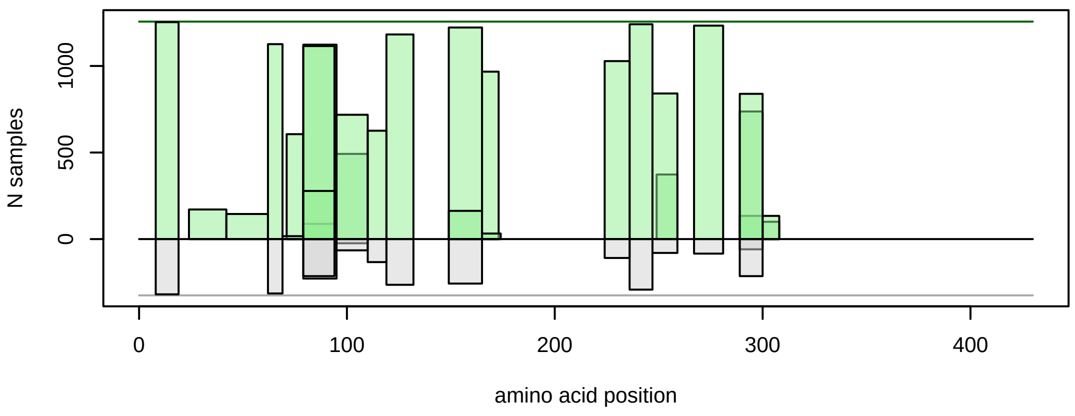

863. EIF3J (O75822), NP3

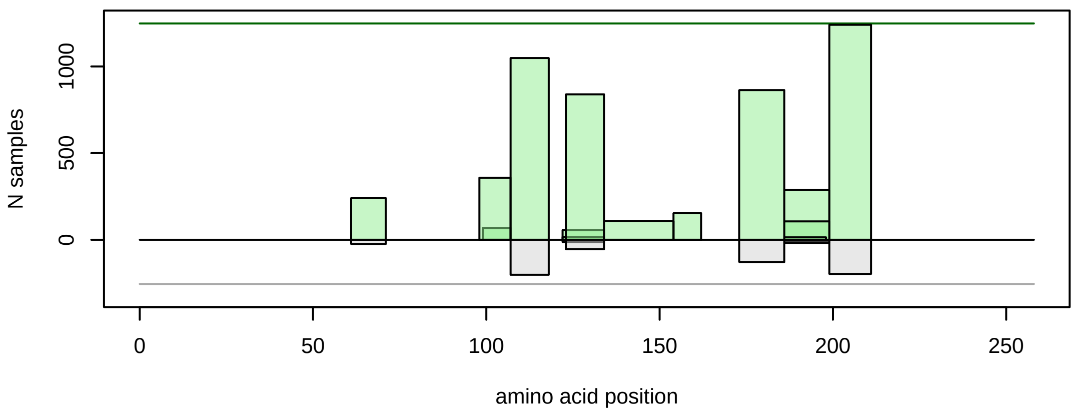

864. GDF15 (Q99988), NP3

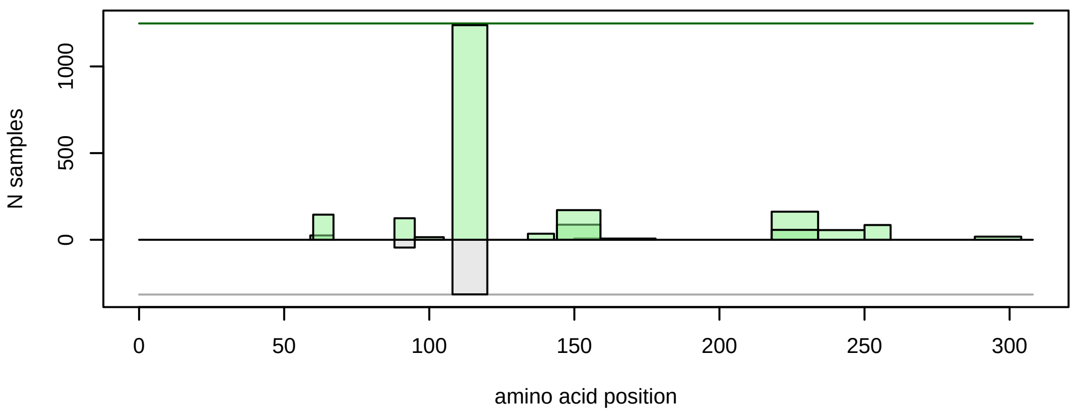

865. IGHG1 (P01857), NP3

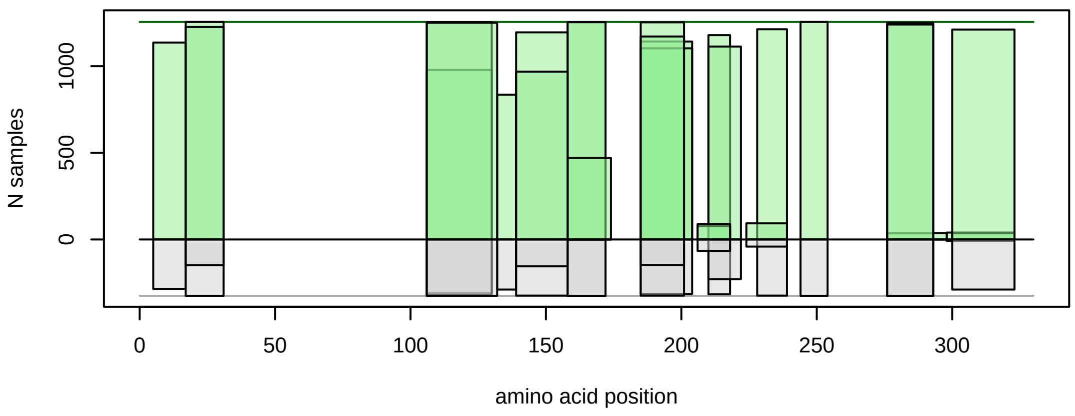

866. CPE (P16870), NP2

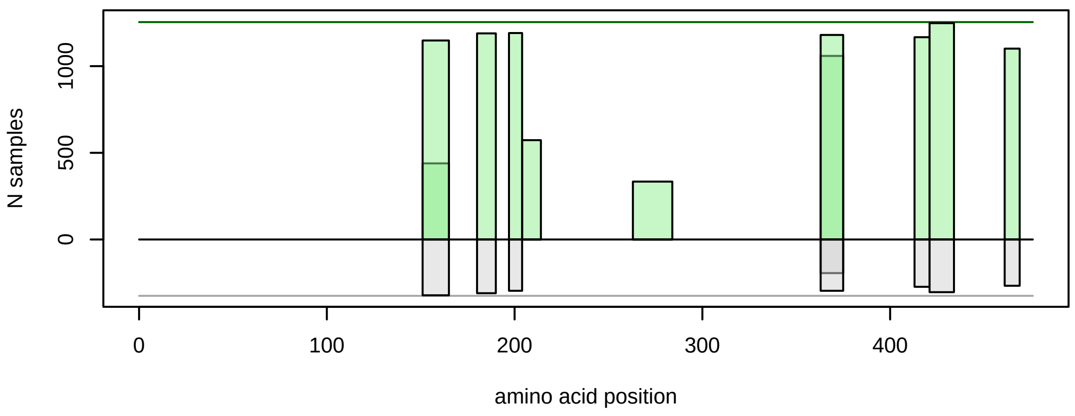

867. DAP (P51397), NP3

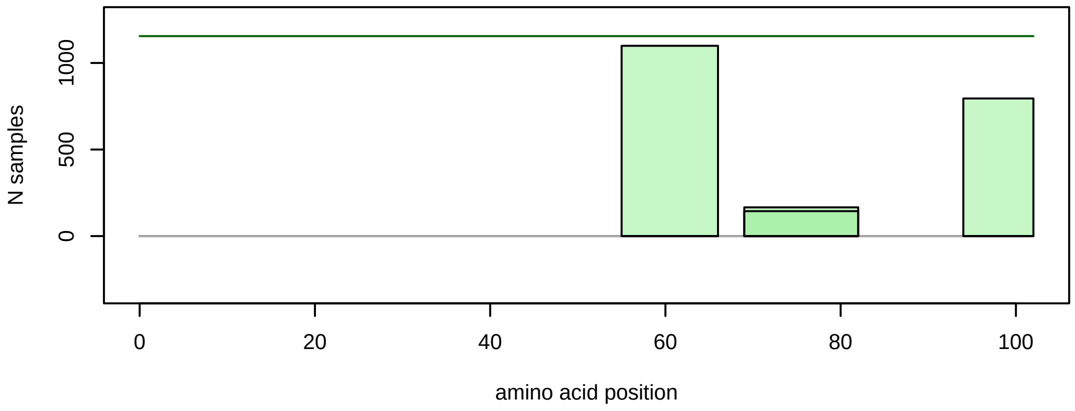

868. RAB15 (G3V562), NP2

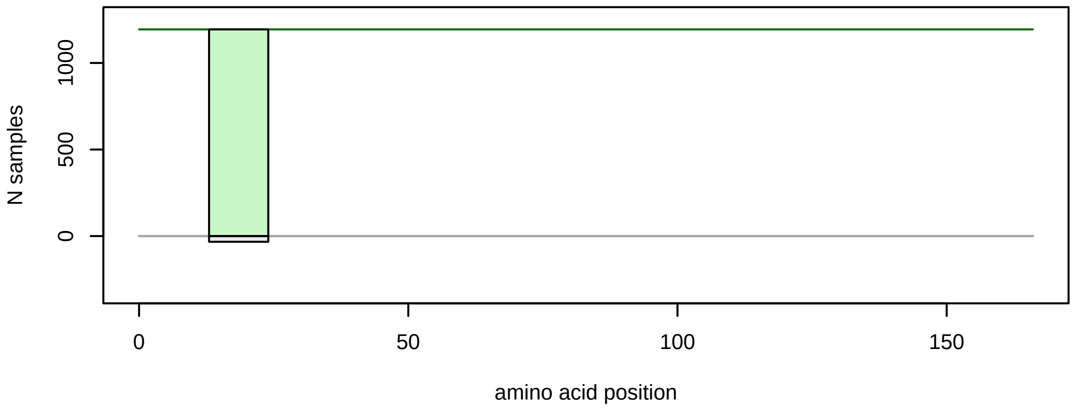

869. IGHV3−13 (P01766), NP4

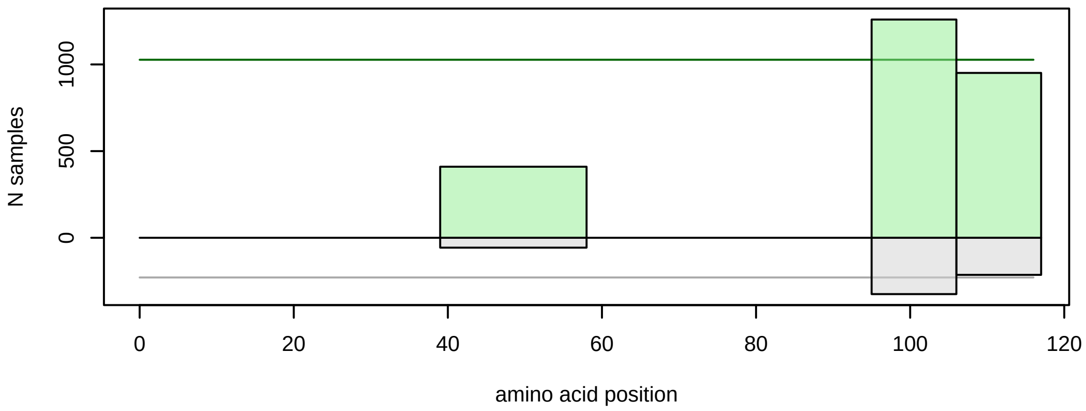

870. PCMT1 (P22061−2), NP1

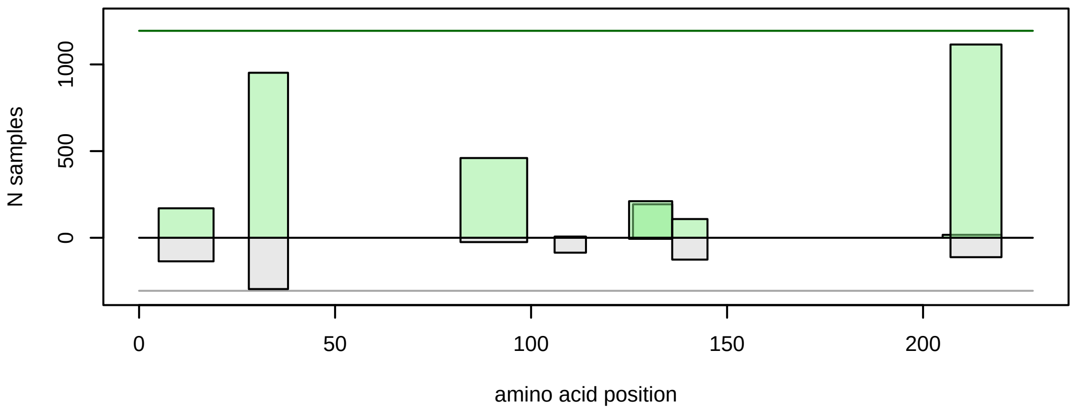

871. CSNK1A1 (A0A590UJ43), NP4

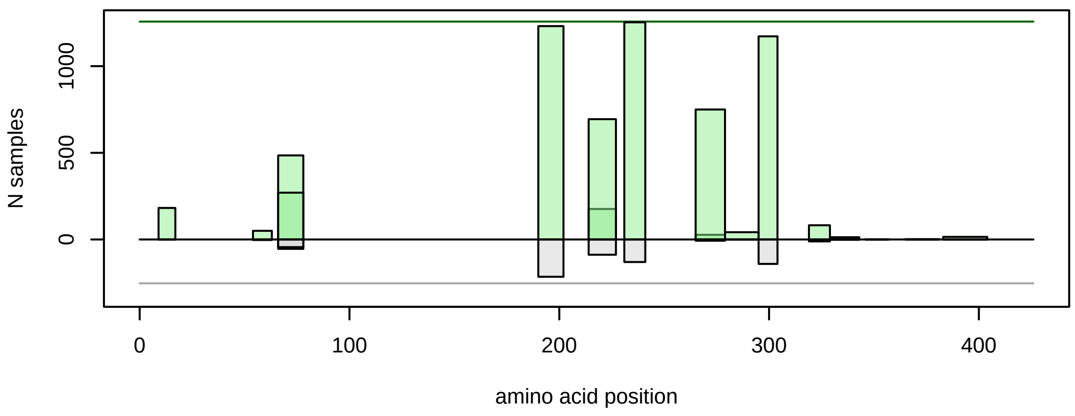

872. ZNF469 (H3BS19), NP3

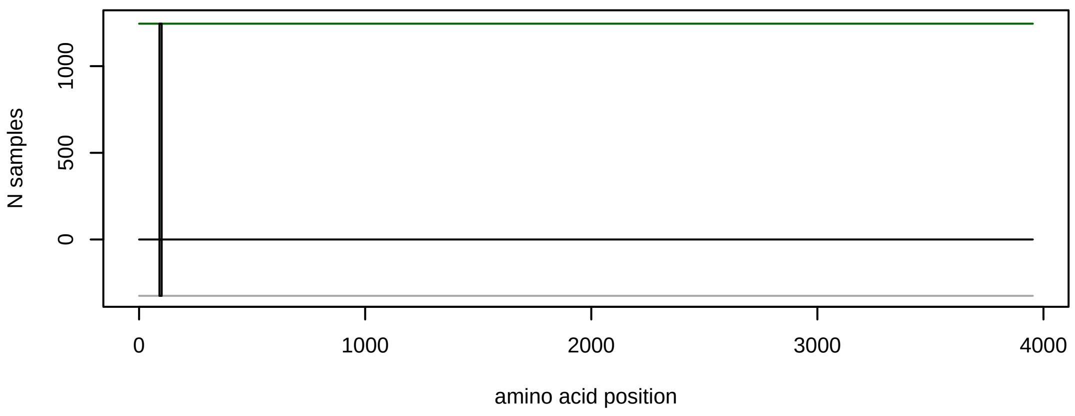

873. FBN1 (P35555), NP5

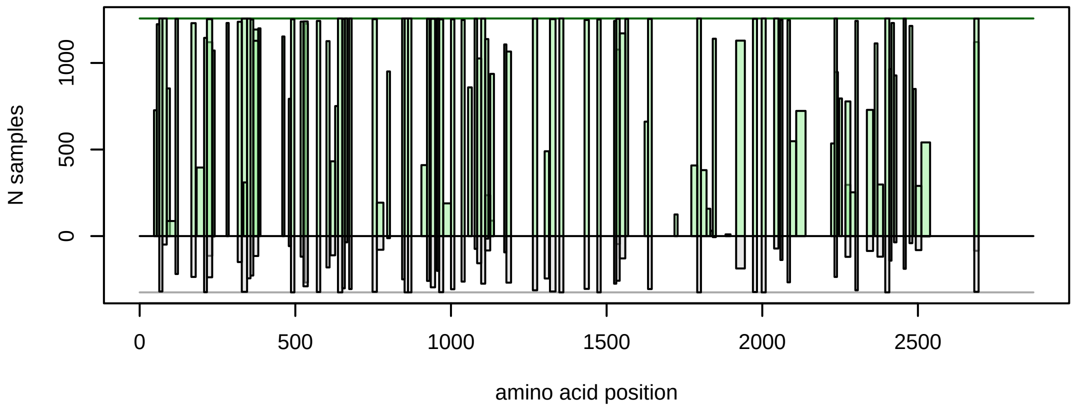

874. IGKV2−28 (A0A075B6P5), NP3

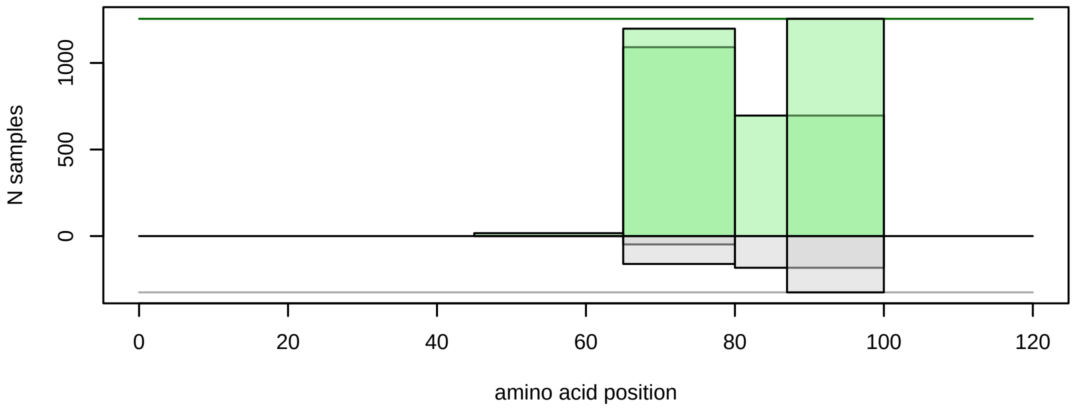

875. TTN (A0A0A0MTS7), NP1

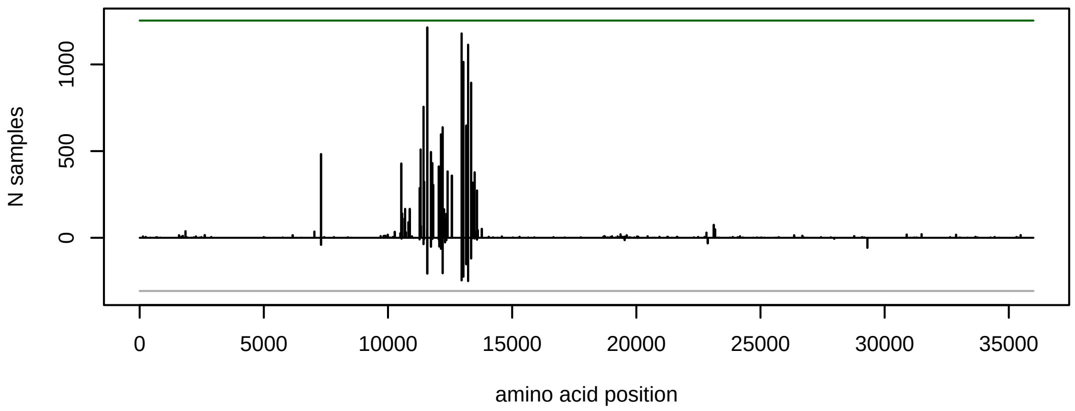

876. IGHV1−24 (A0A0C4DH33), NP5

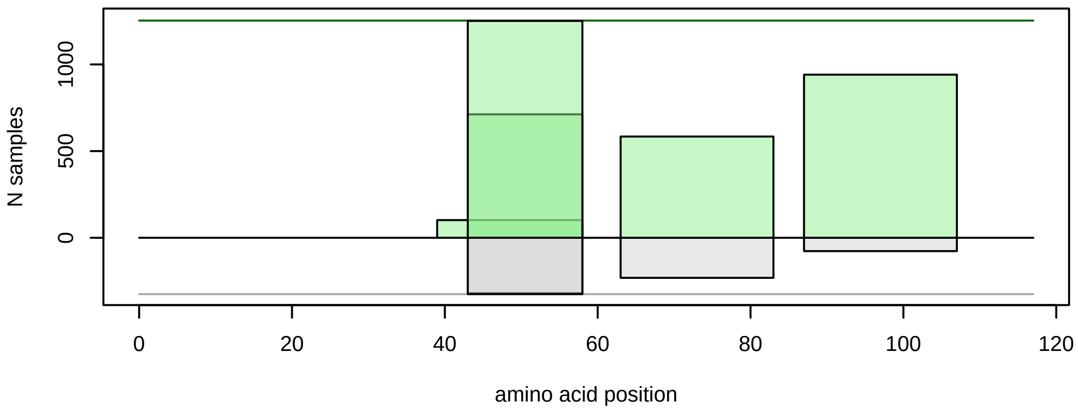

877. WNT5A (P41221;P41221−2), NP4

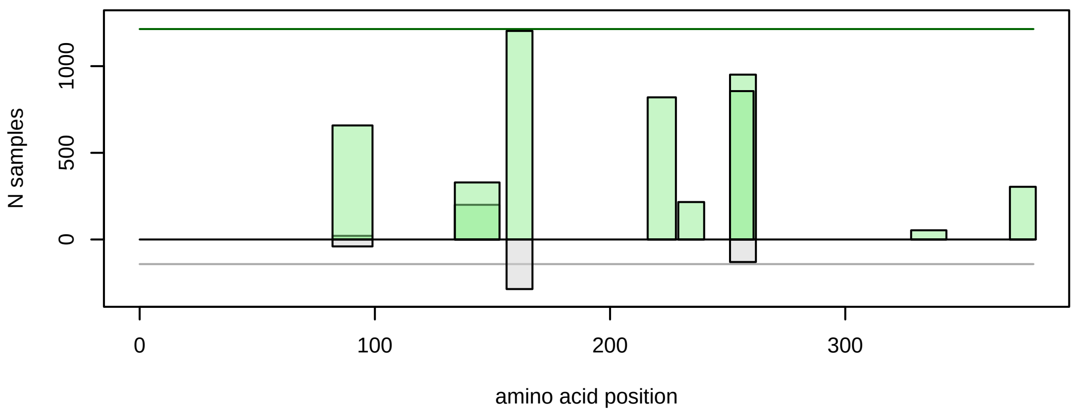

878. PDGFD (Q9GZP0), NP3

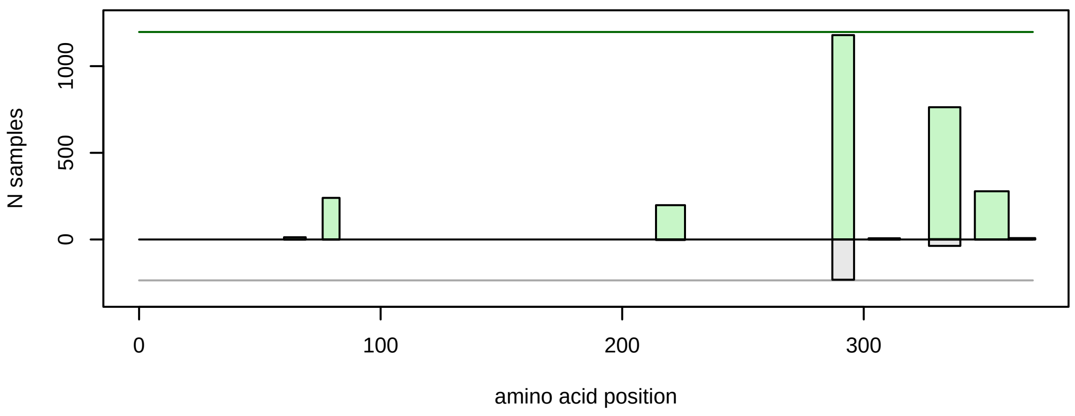

879. TNFRSF11B (O00300), NP4

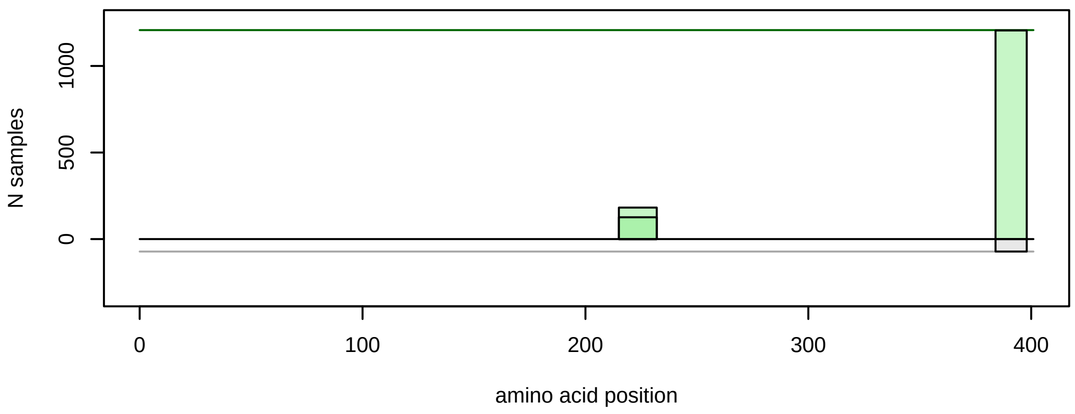

880. LDHB (P07195), NP4

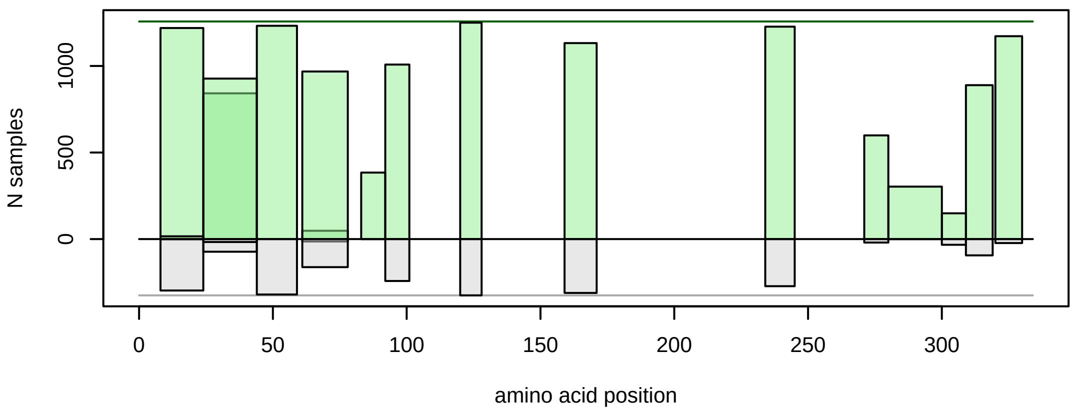

881. ZYX (Q15942), NP1

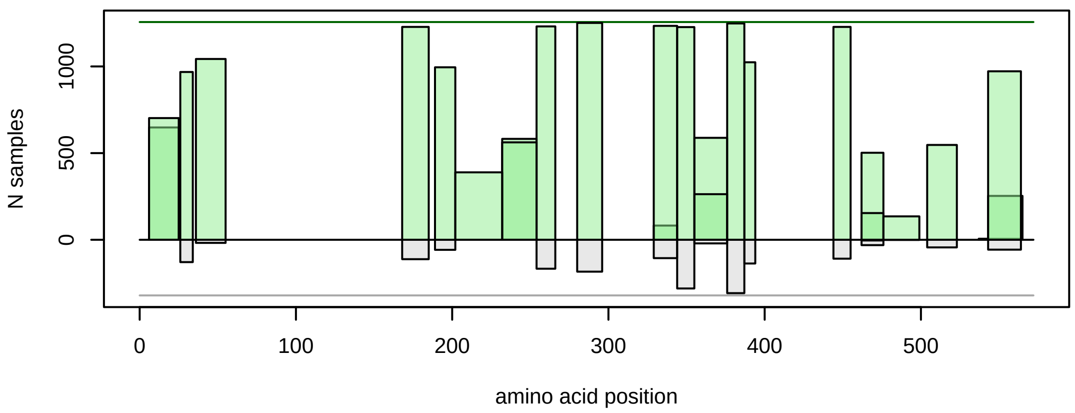

882. CCT8 (P50990), NP1

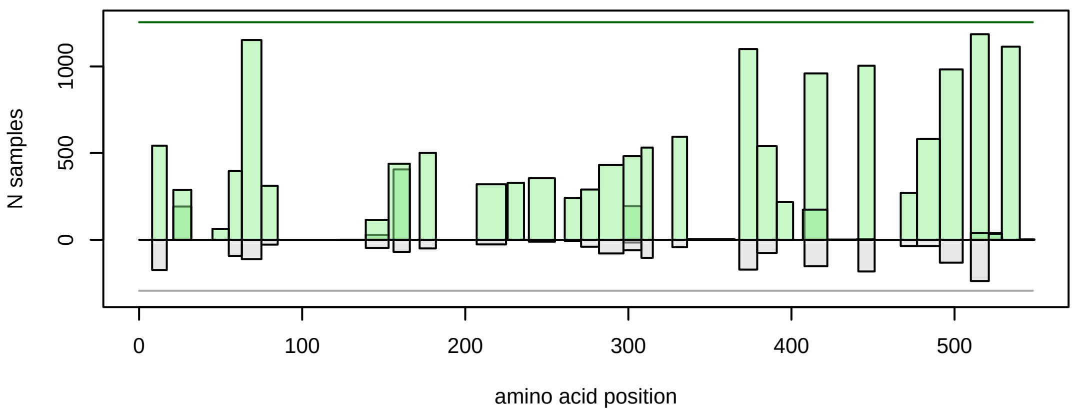

883. TGOLN2 (A0A5F9UY30;O43493;O43493−5;O43493−7), NP2

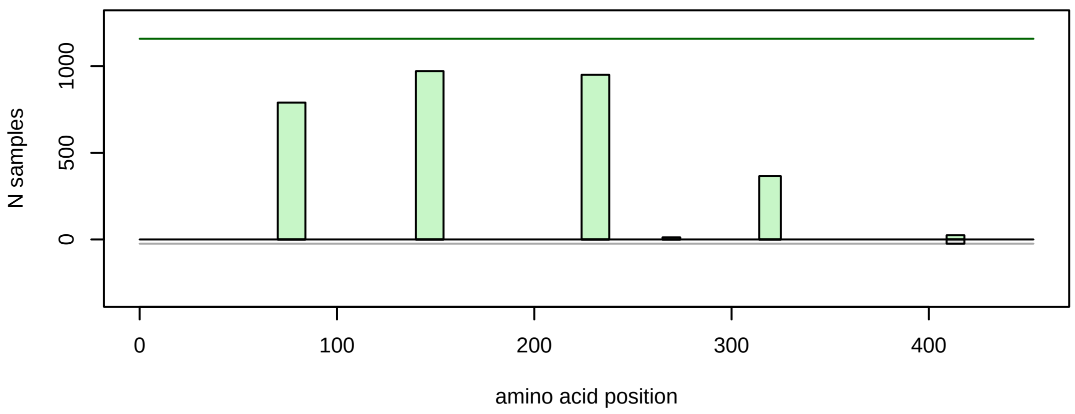

884. AP1B1 (Q10567), NP4

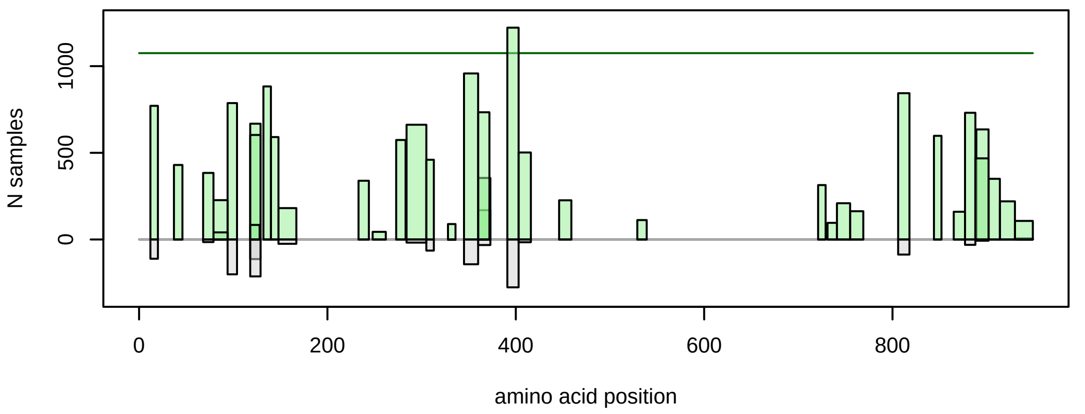

885. ITIH5 (C9J2H1), NP3

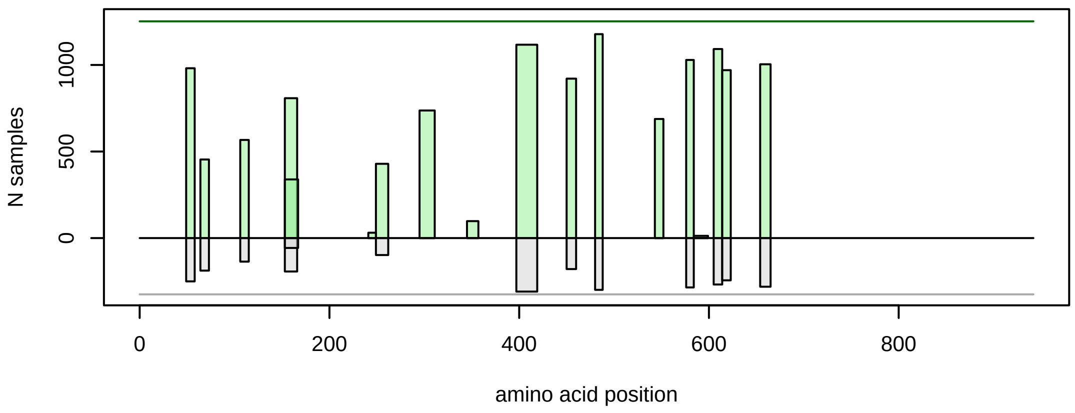

886. ASPH (Q12797−3), NP2

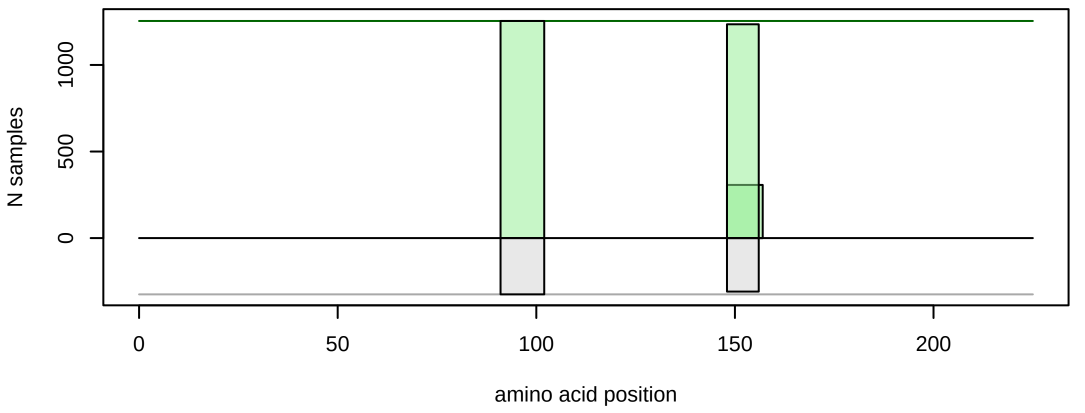

887. GRHPR (Q9UBQ7), NP4

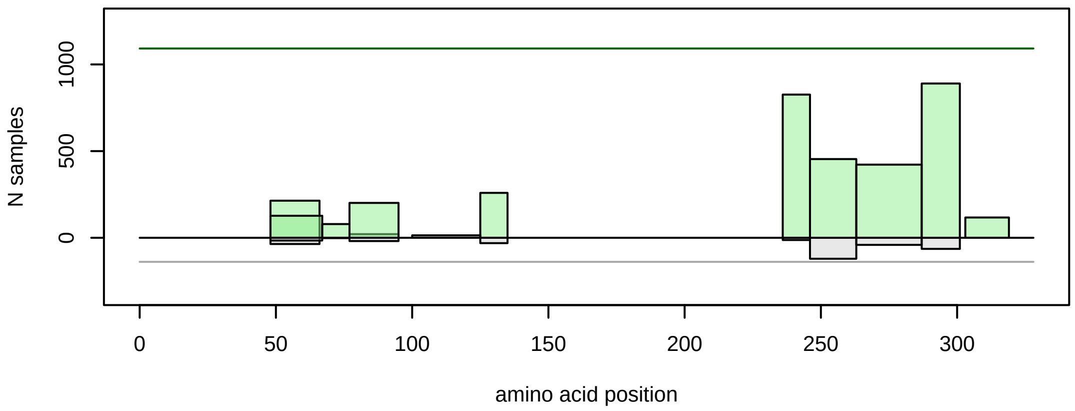

888. ADAMTS8 (Q9UP79), NP5

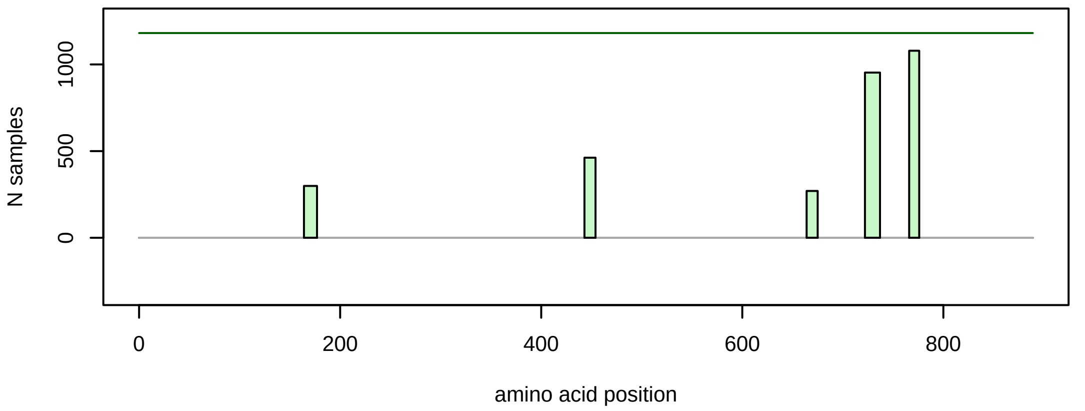

889. SDC1 (P18827), NP2

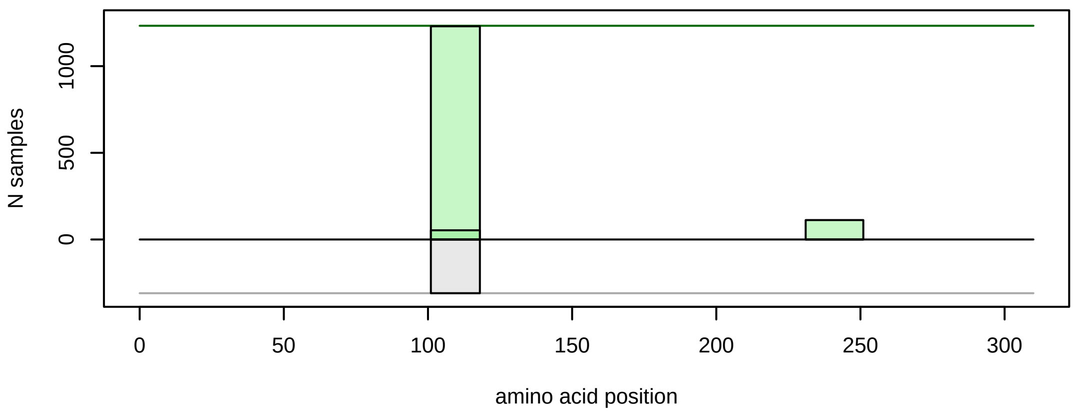

890. PPIF (P30405), NP4

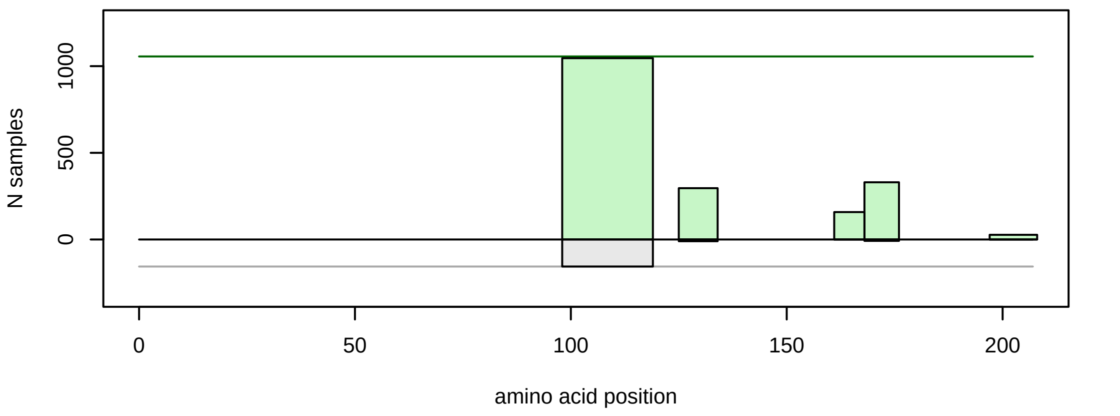

891. IGLV9−49 (A0A0B4J1Y8), NP3

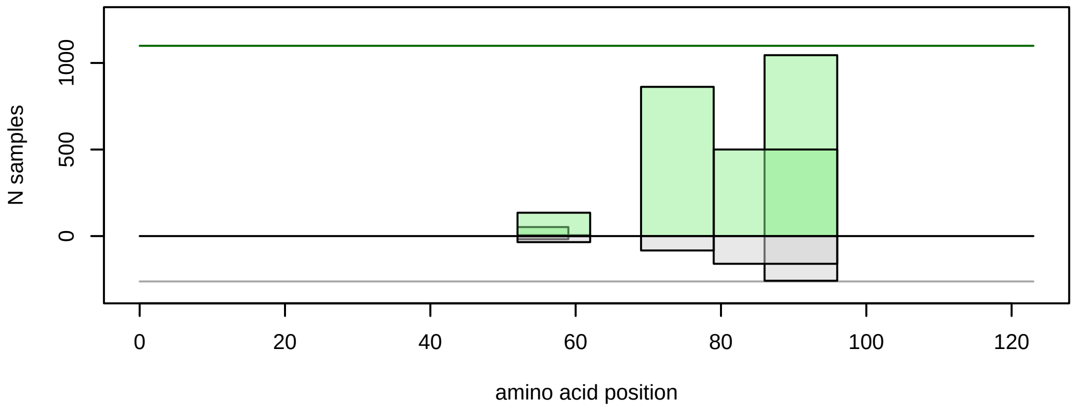

892. IGKV3−7 (A0A075B6H7), NP3

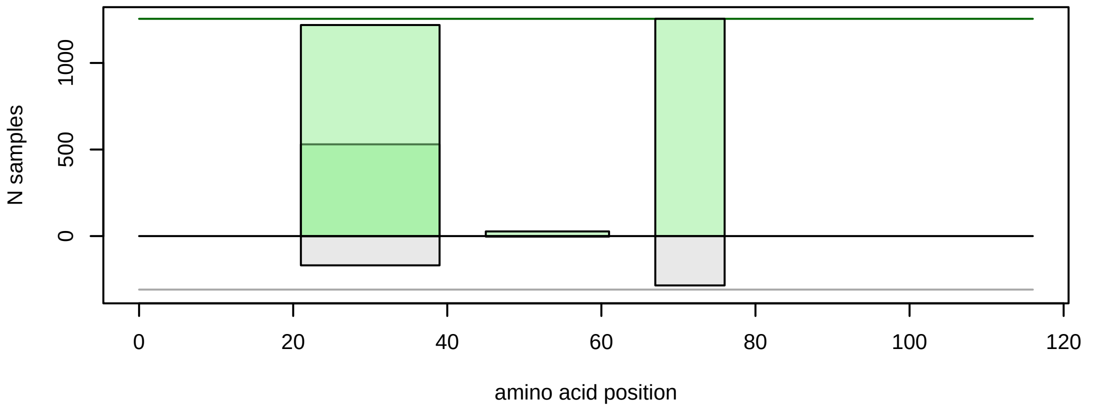

893. HADHB (P55084), NP4

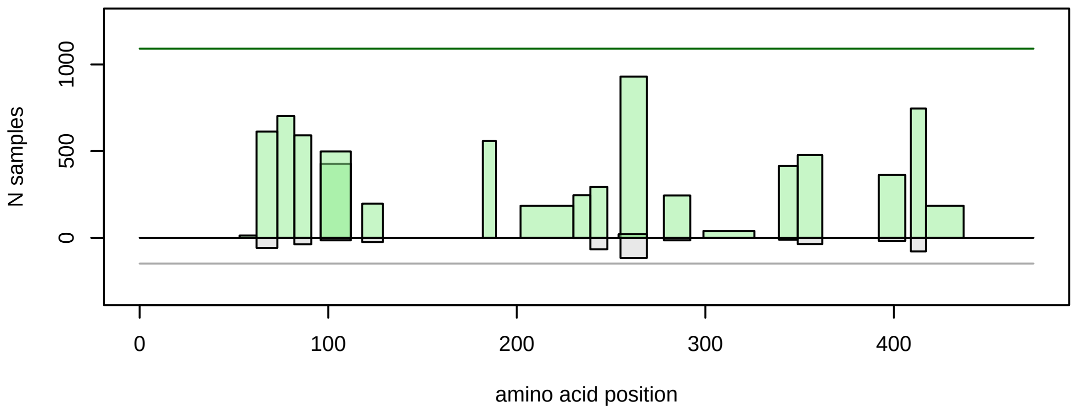

894. PEPD (P12955), NP5

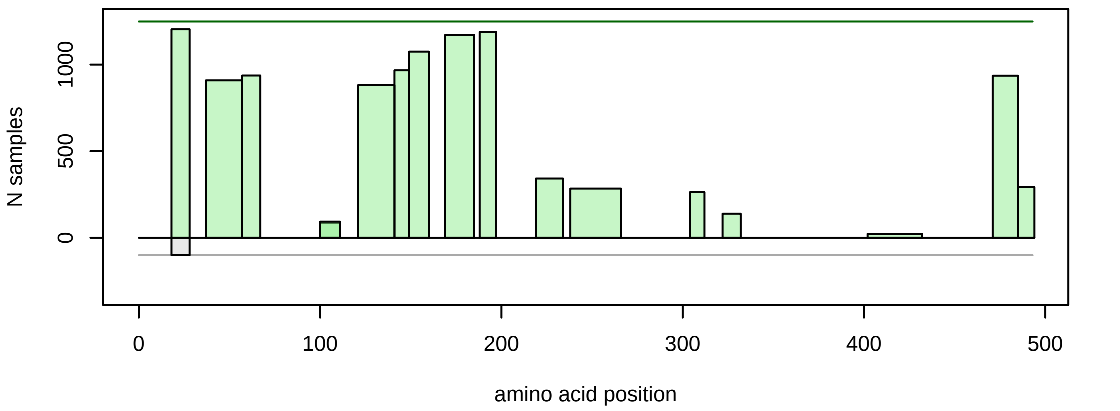

895. HRC (M0QZ43;P23327), NP2

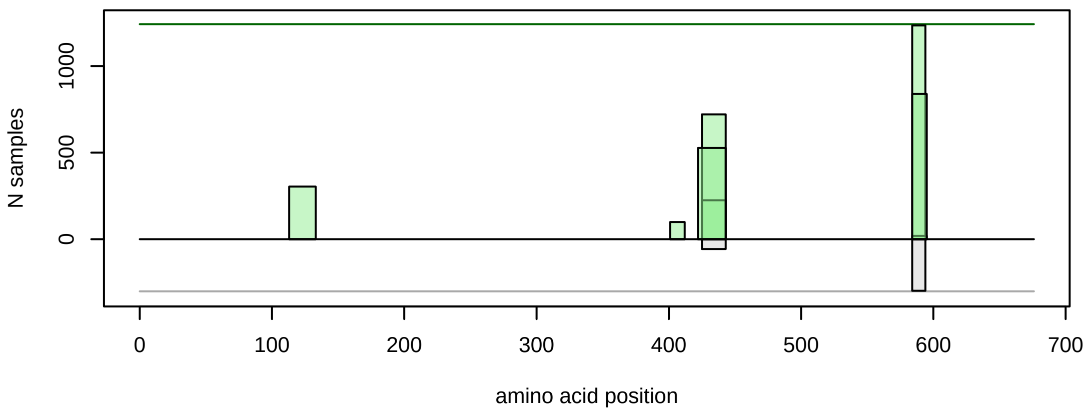

896. CSRP3 (P50461), NP1

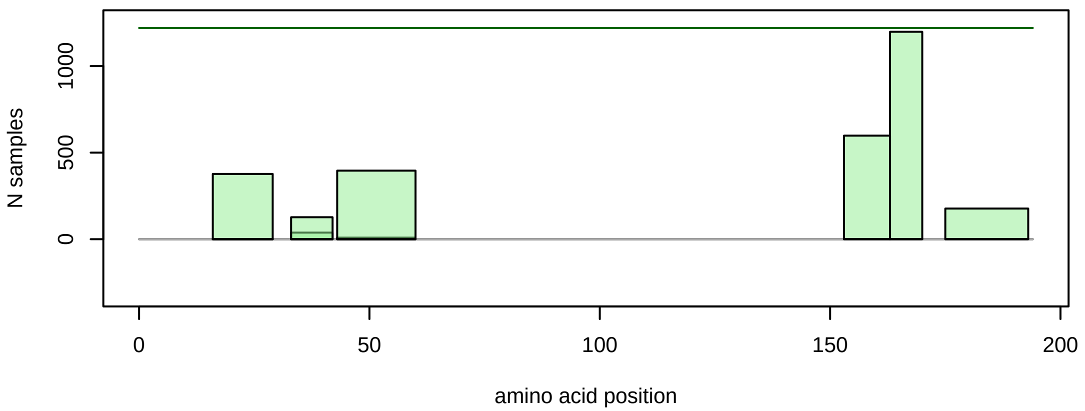

897. PAFAH1B3 (Q15102), NP5

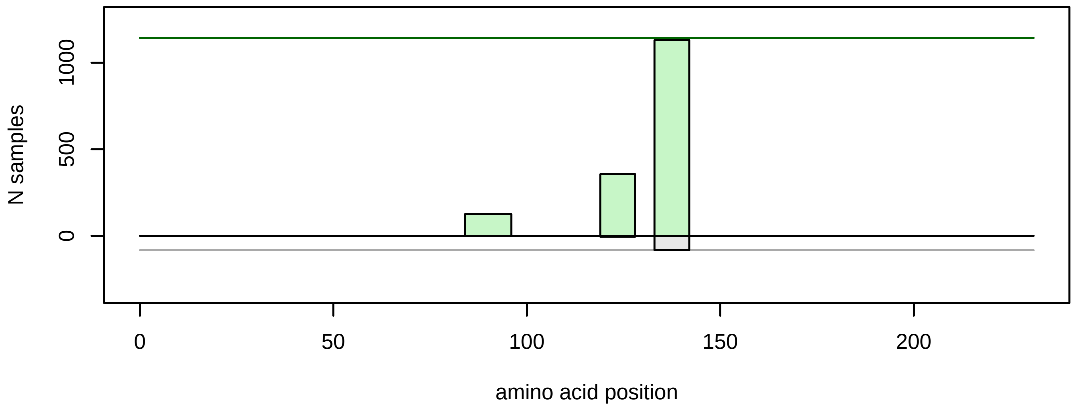

898. PTMS (P20962), NP2

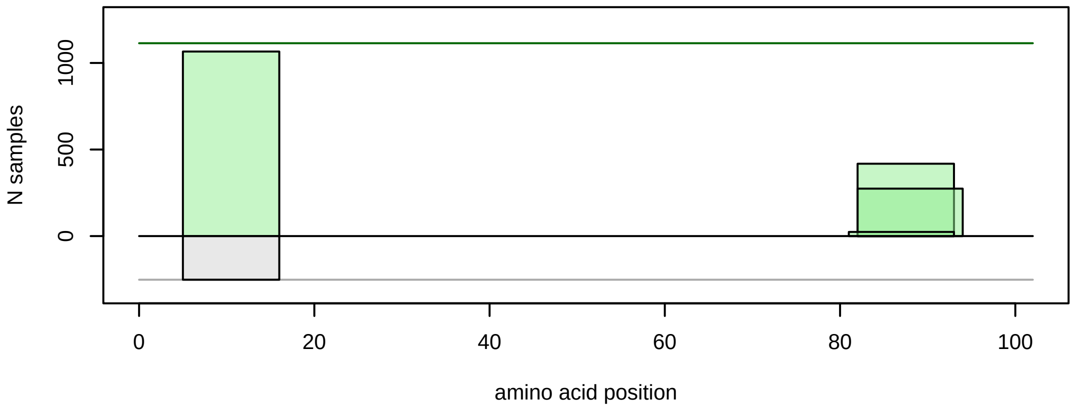

899. RFNG (Q9Y644), NP4

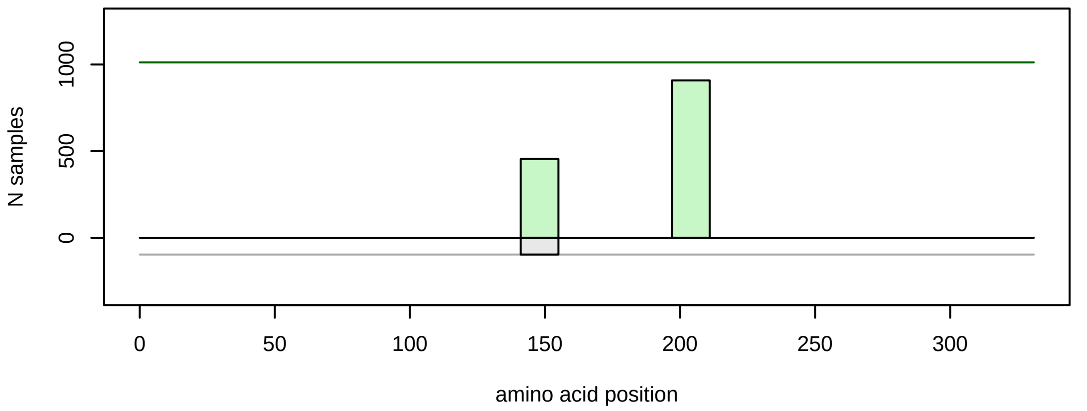

900. DCTN2 (Q13561), NP5

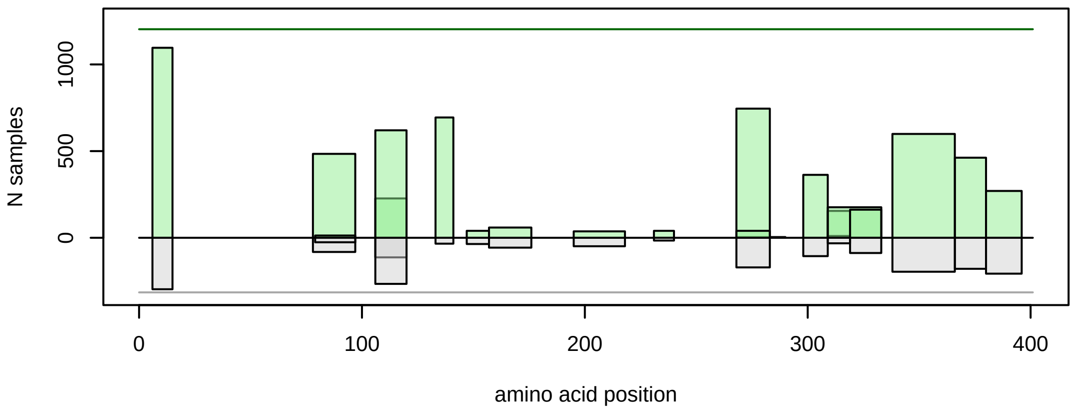

901. DOCK2 (Q92608), NP4

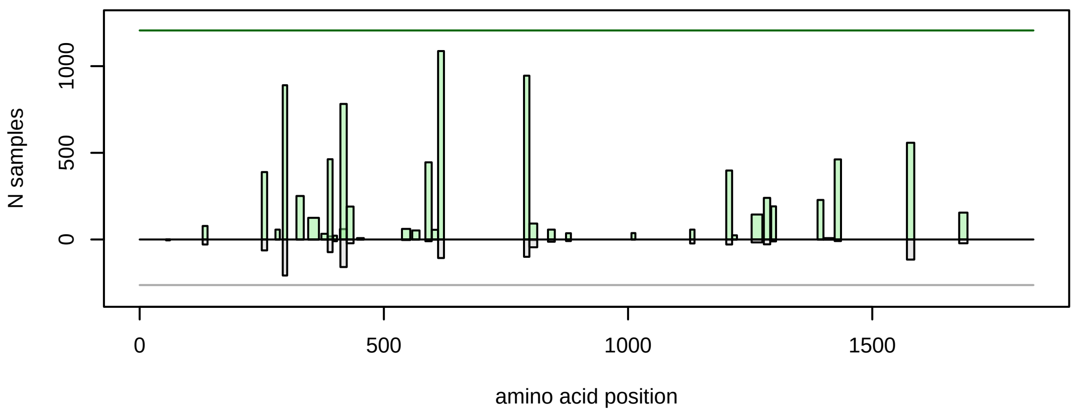

902. HRG (P04196), NP1

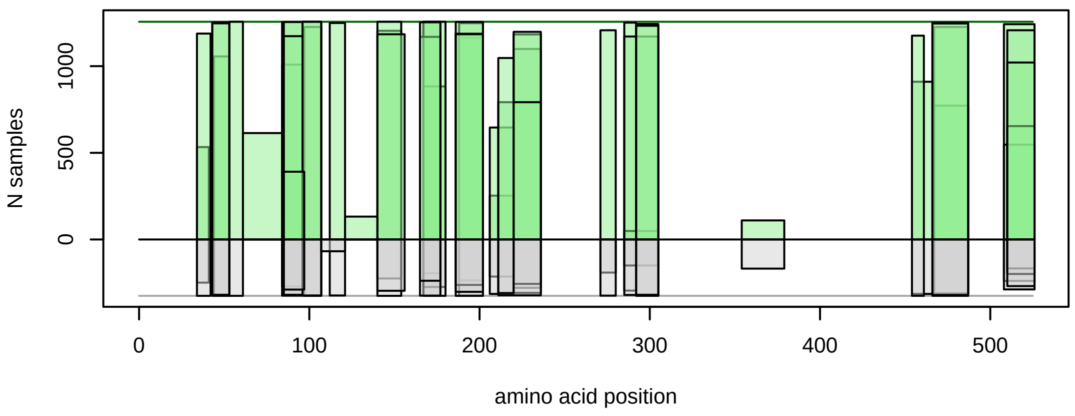

903. ACTN1 (A0A804HII9), NP4

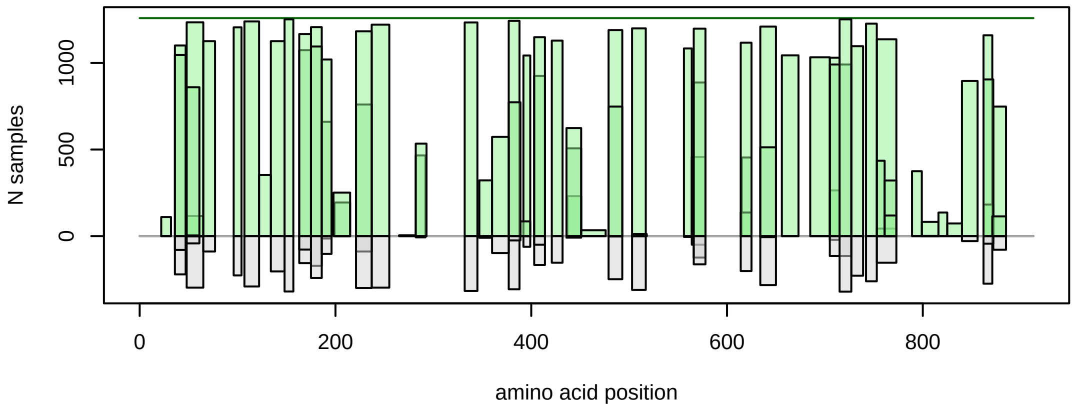

904. NA (A0A024R8Y9), NP4

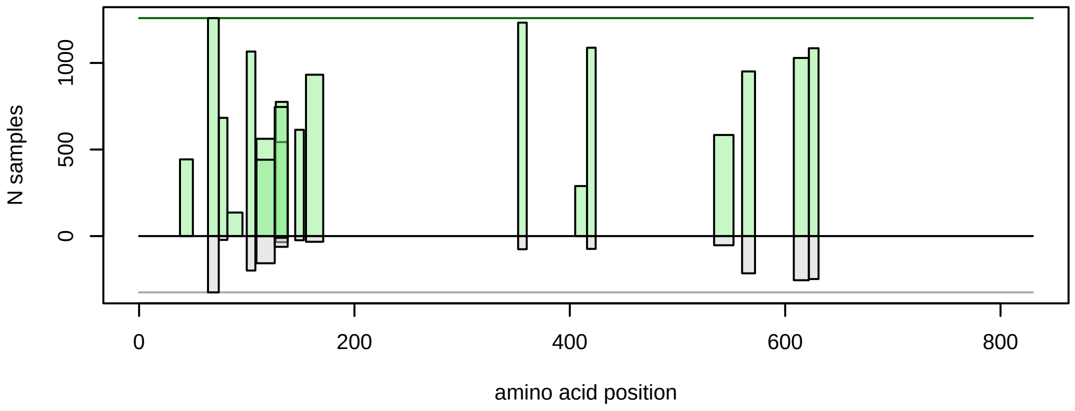

905. PRDX4 (Q13162), NP4

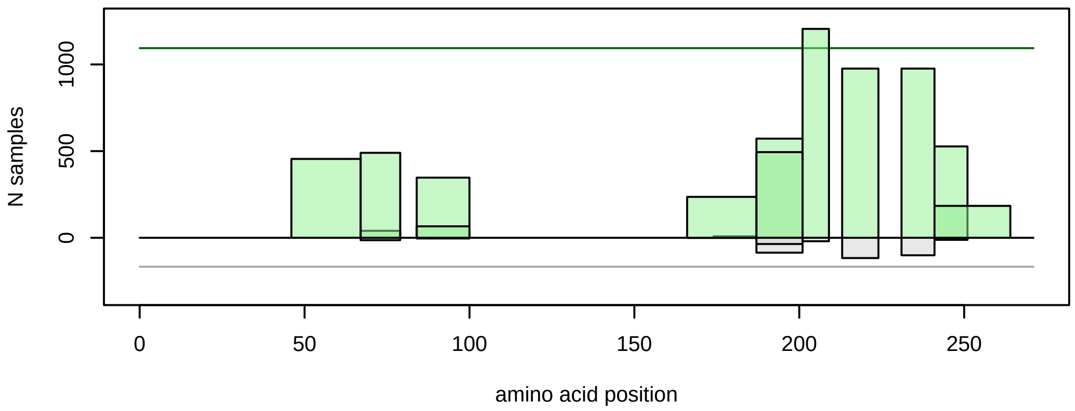

906. CAND1 (Q86VP6), NP5

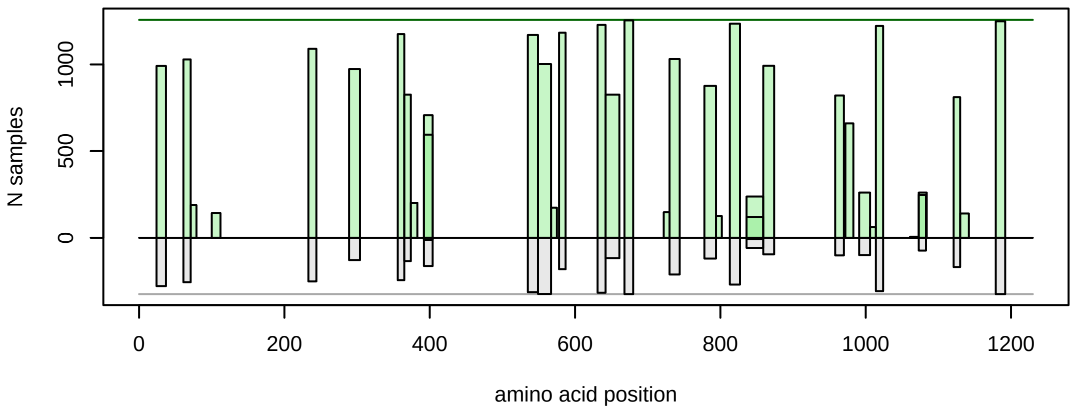

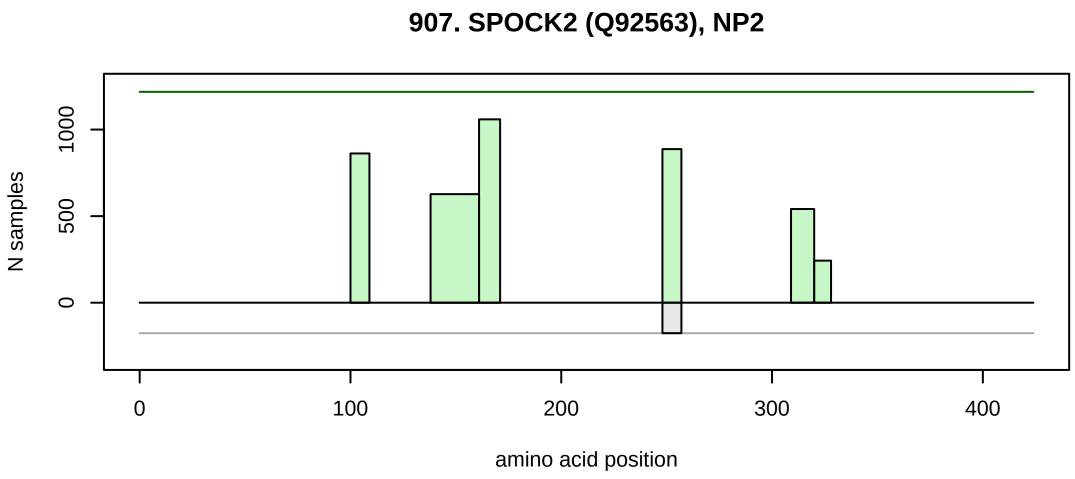
907. SPOCK2 (Q92563), NP2
N samples
0
500
1000
0
100
200
300
400
amino acid position

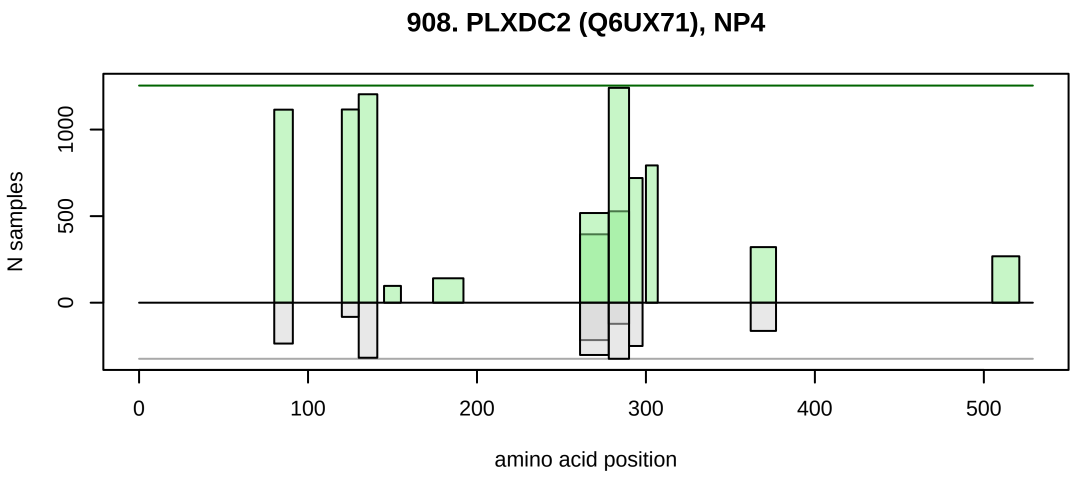
908. PLXDC2 (Q6UX71), NP4
N samples
0
500
1000
0
100
200
300
400
500
amino acid position

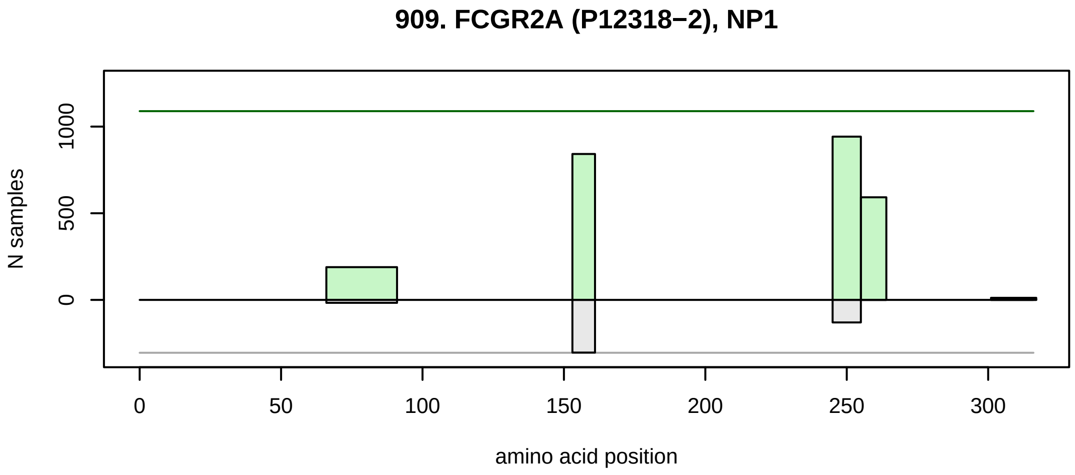
909. FCGR2A (P12318−2), NP1
N samples
0
500
1000
0
50
100
150
200
250
300
amino acid position

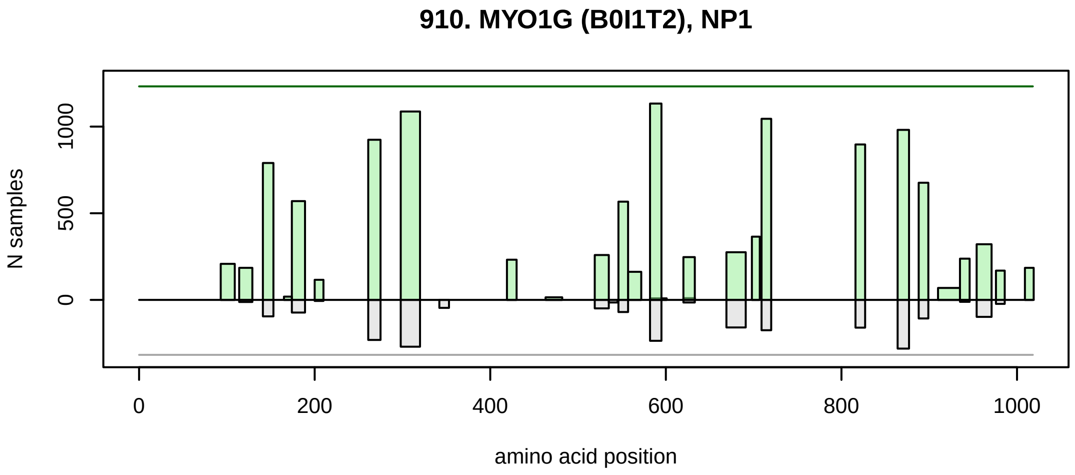
910. MYO1G (B0I1T2), NP1
N samples
0
500
1000
0
200
400
600
800
1000
amino acid position

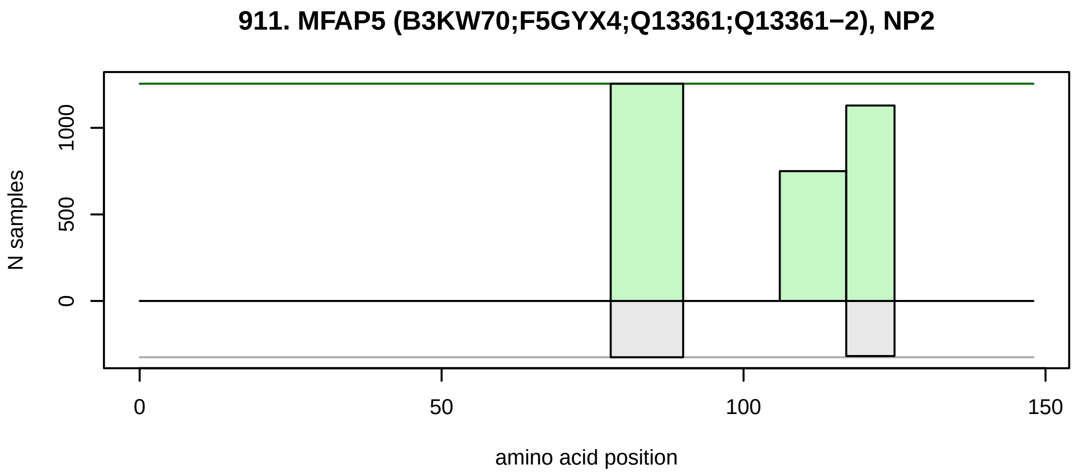
911. MFAP5 (B3KW70;F5GYX4;Q13361;Q13361−2), NP2
N samples
0
500
1000
0
50
100
150
amino acid position

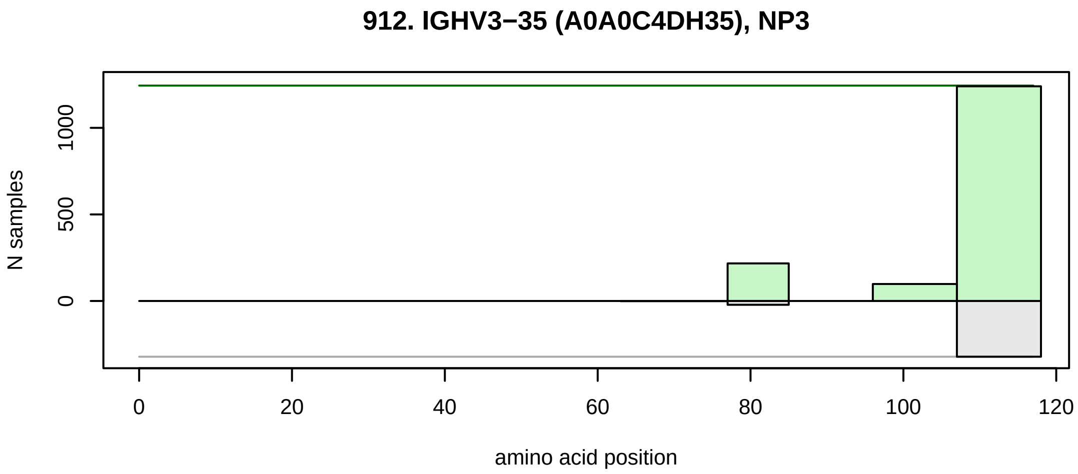
912. IGHV3−35 (A0A0C4DH35), NP3
N samples
0
500
1000
0
20
40
60
80
100
120
amino acid position

913. PSMC5 (P62195), NP5

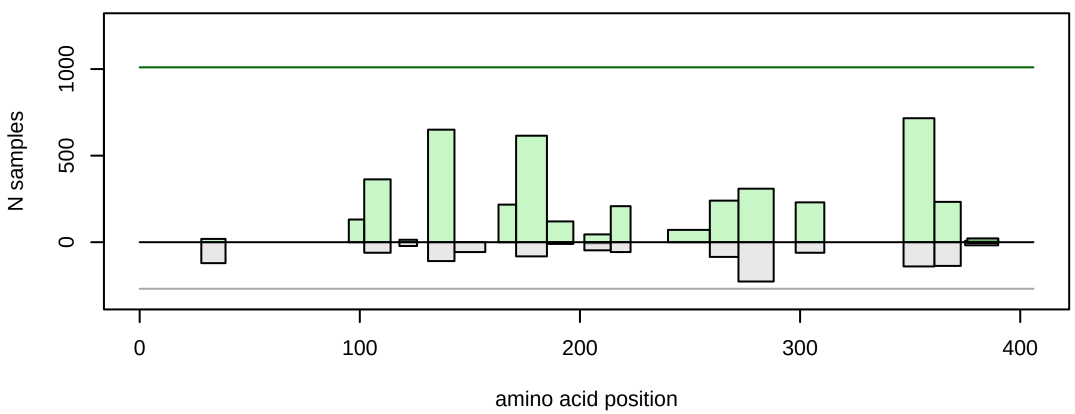

914. CST3 (P01034), NP3

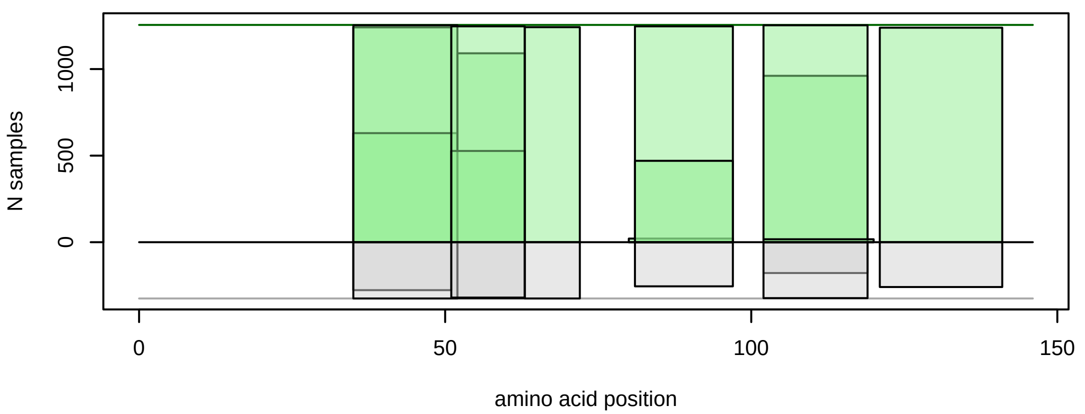

915. BZW2 (Q9Y6E2), NP1

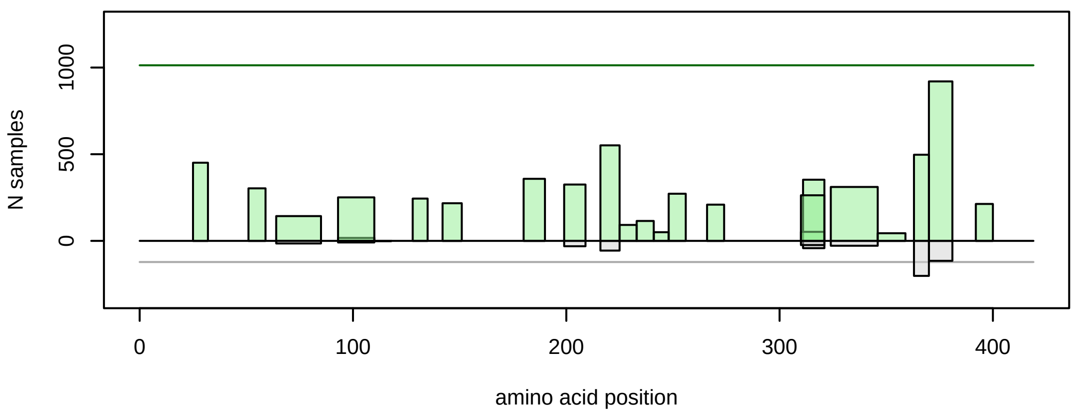

916. TNXB (A0A3B3ISX9), NP1

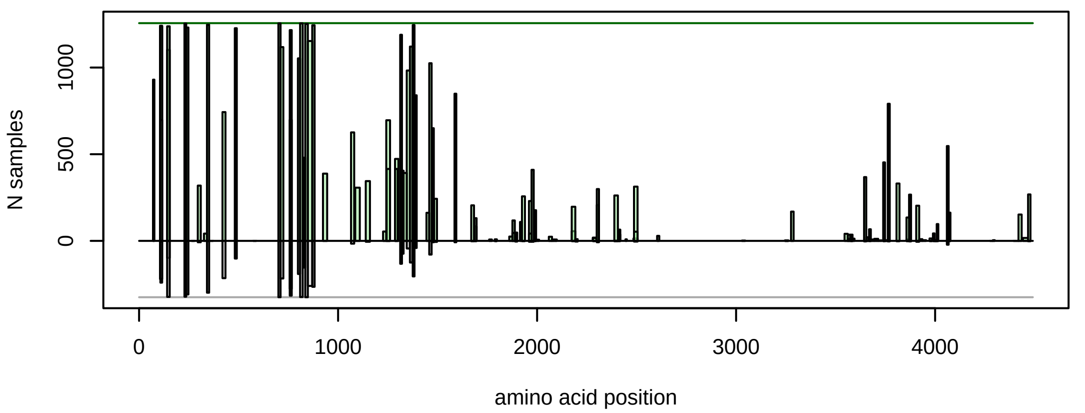

917. KNG1 (P01042), NP3

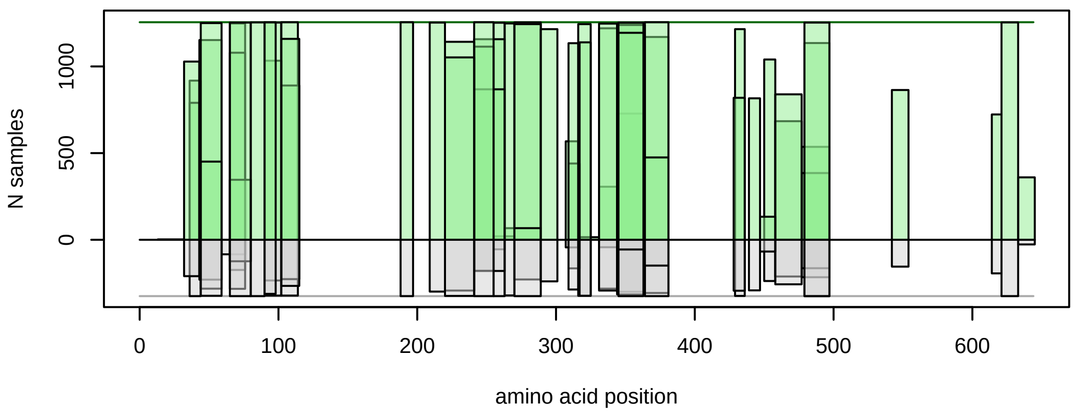

918. CHST14 (Q8NCH0), NP1

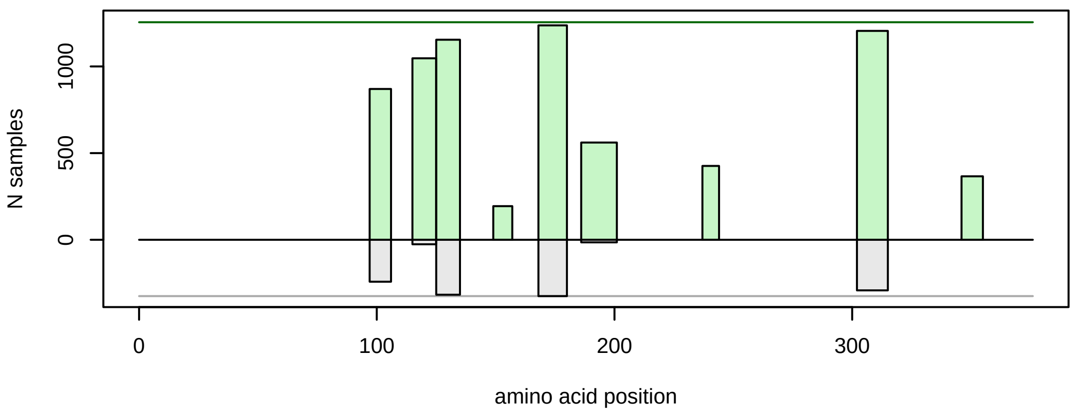

919. COX7A2 (D6RIE3;H0UI06;P14406), NP4

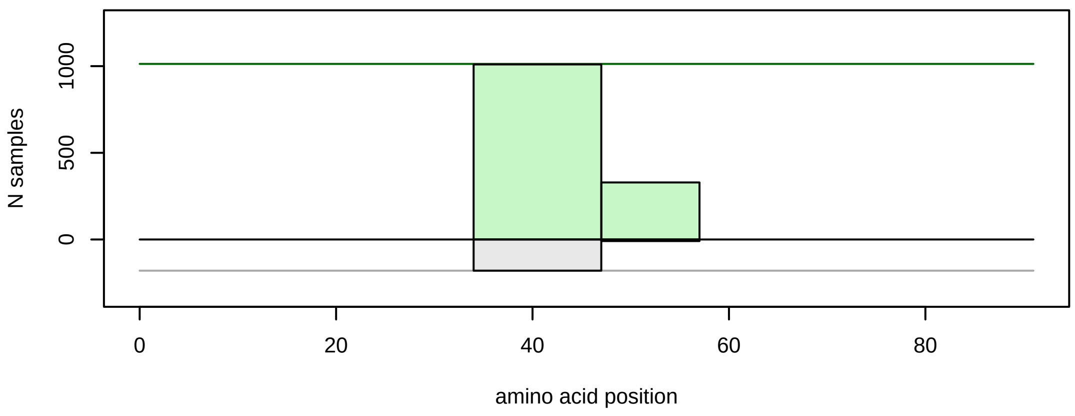

920. ATP5L (O75964), NP4

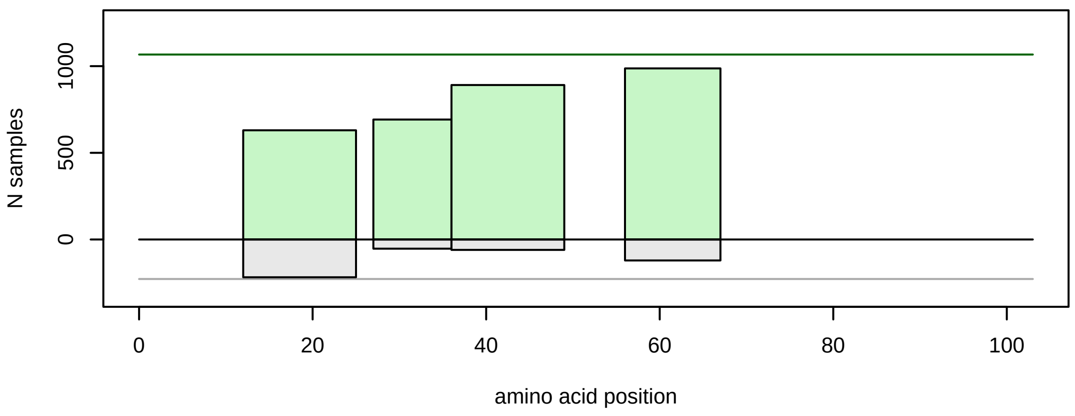

921. TAGLN (Q01995), NP3

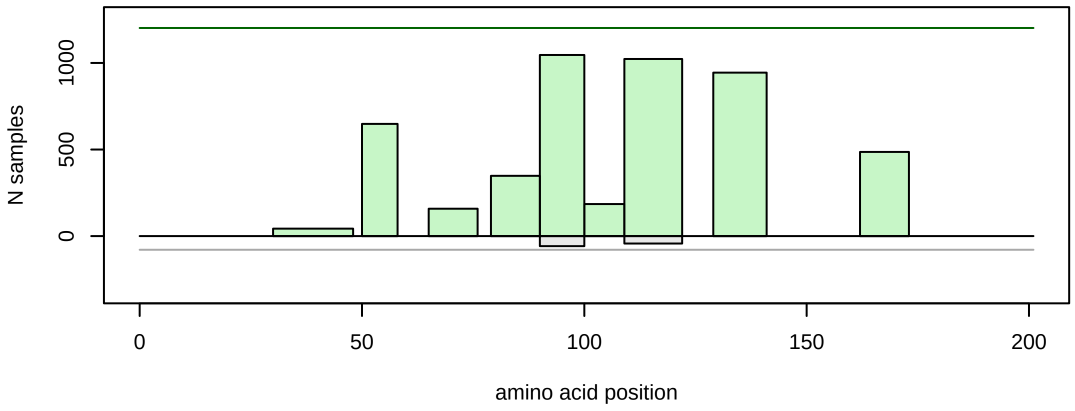

922. IGHV3−7 (P01780), NP4

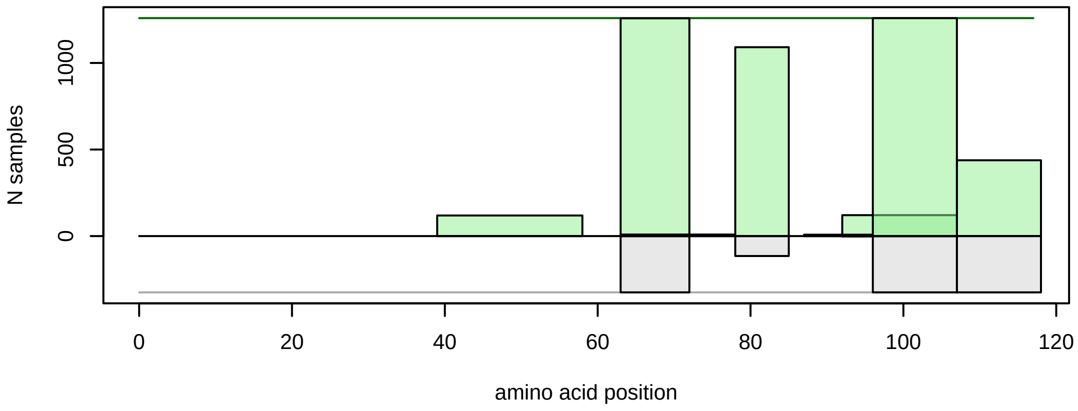

923. RPE (Q96AT9), NP2

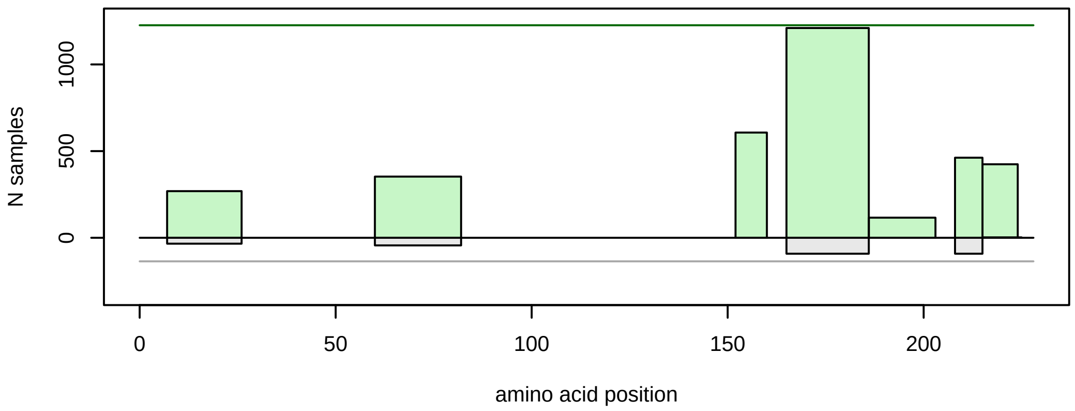

924. IGLV3−19 (P01714), NP3

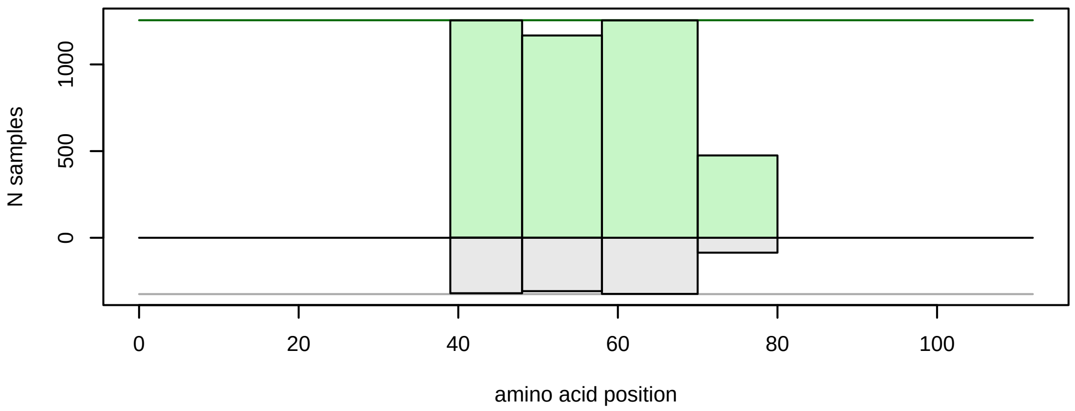

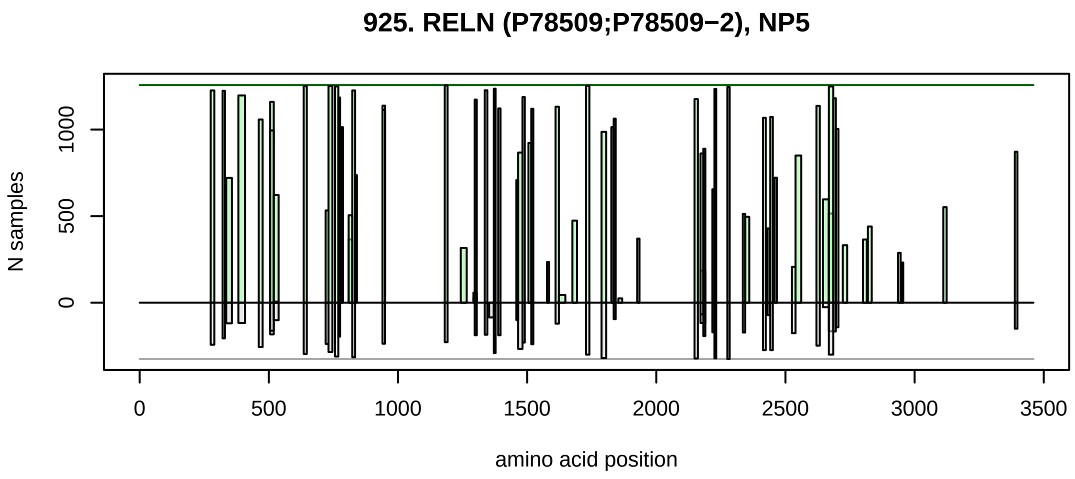
925. RELN (P78509;P78509−2), NP5
N samples
amino acid position

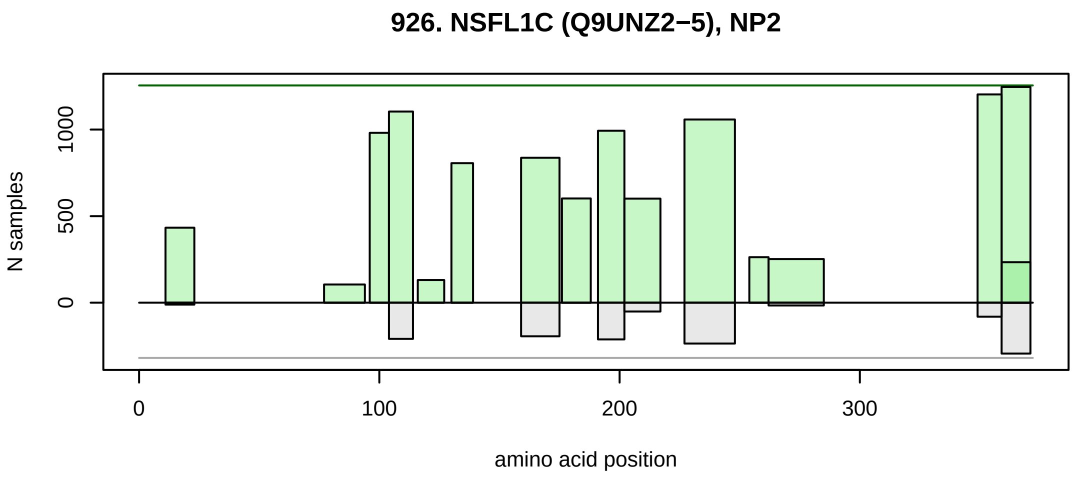
926. NSFL1C (Q9UNZ2−5), NP2
N samples
amino acid position

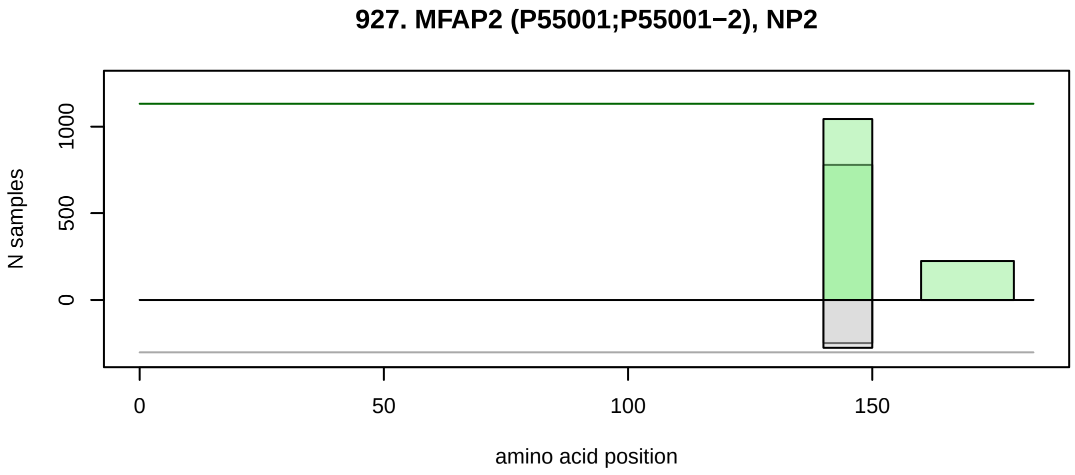
927. MFAP2 (P55001;P55001−2), NP2
N samples
amino acid position

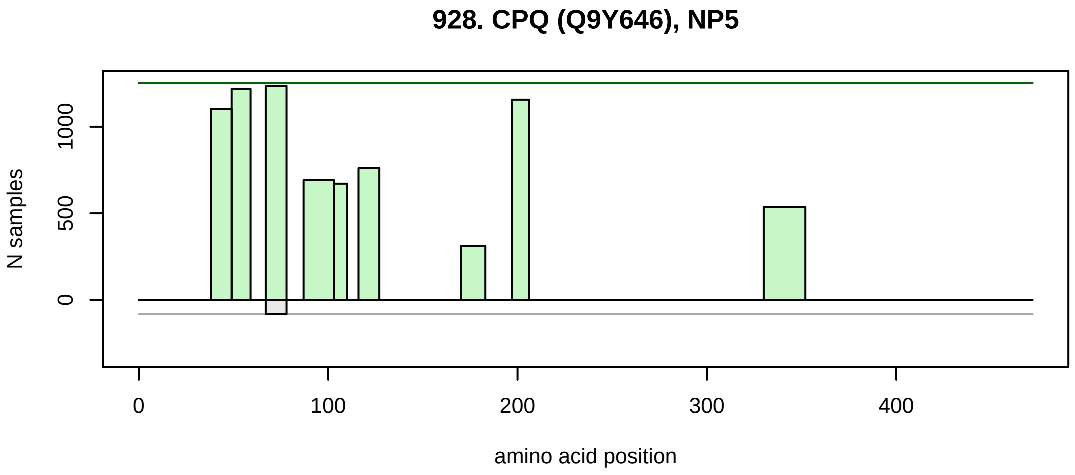
928. CPQ (Q9Y646), NP5
N samples
amino acid position

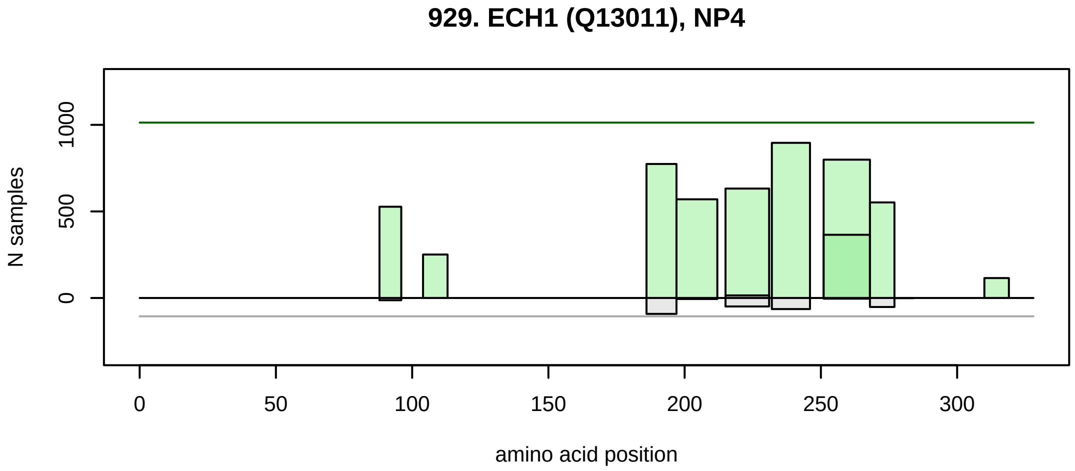
929. ECH1 (Q13011), NP4
N samples
amino acid position

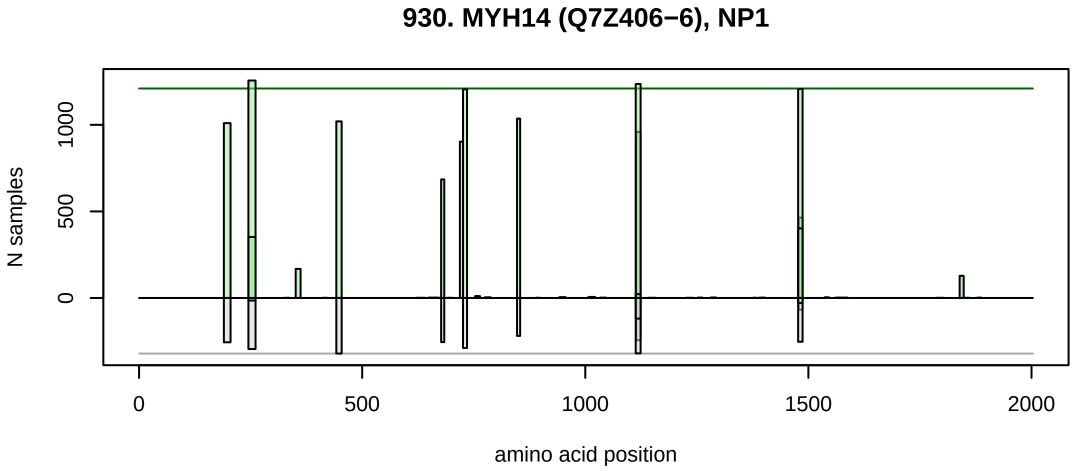
930. MYH14 (Q7Z406−6), NP1
N samples
amino acid position

931. LSP1 (P33241−3), NP2

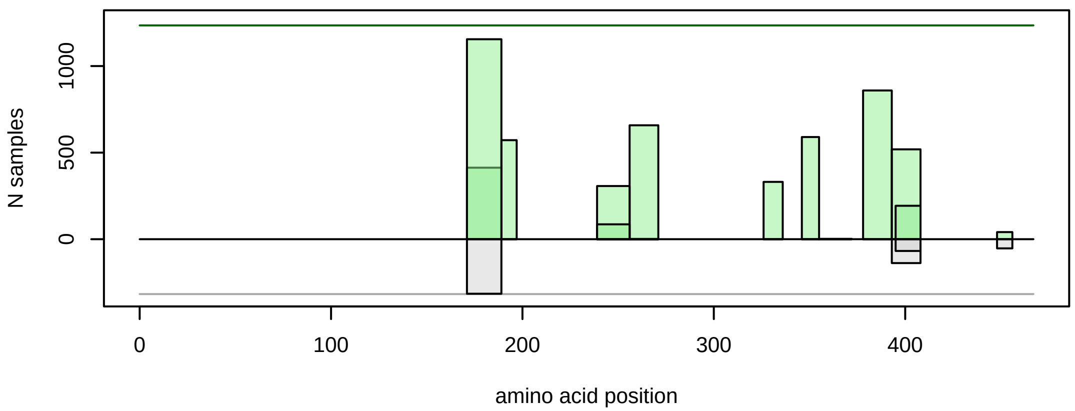

932. THBS2 (A0A3B3ITK0;P35442), NP2

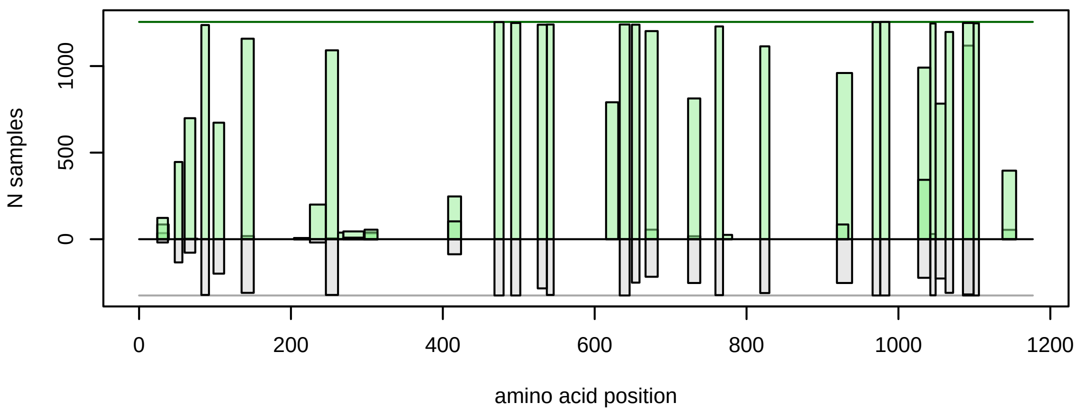

933. CTSG (P08311), NP4

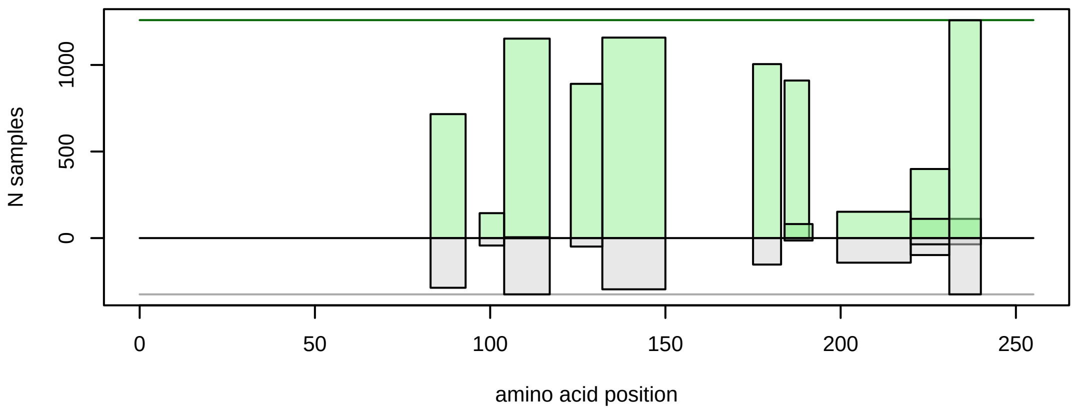

934. BZW1 (Q7L1Q6−4), NP3

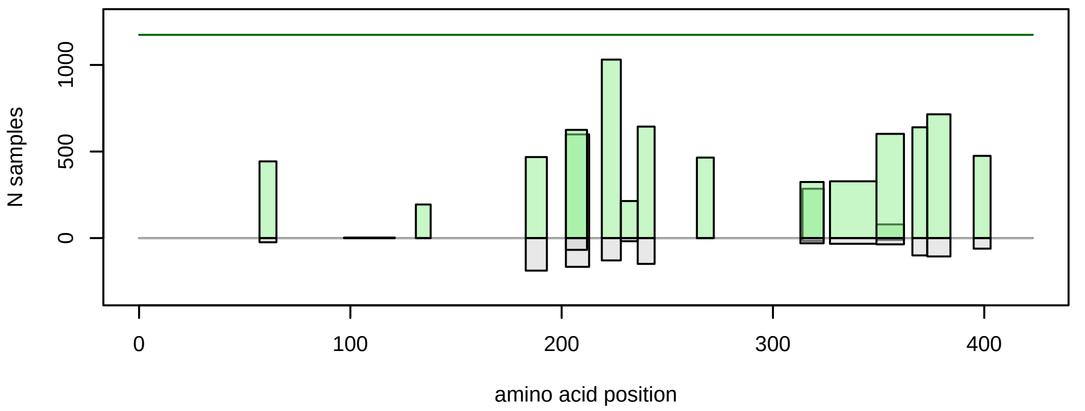

935. ALB (P02768), NP2

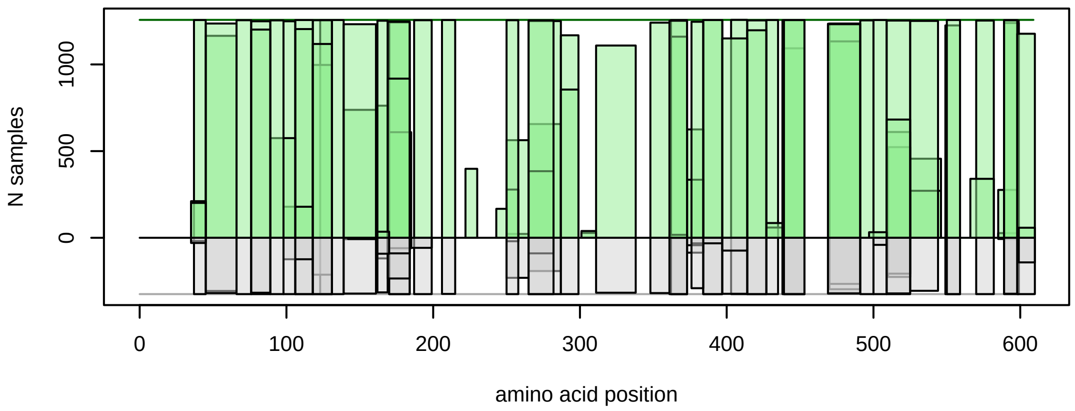

936. CLTC (A0A087WVQ6;Q00610), NP4

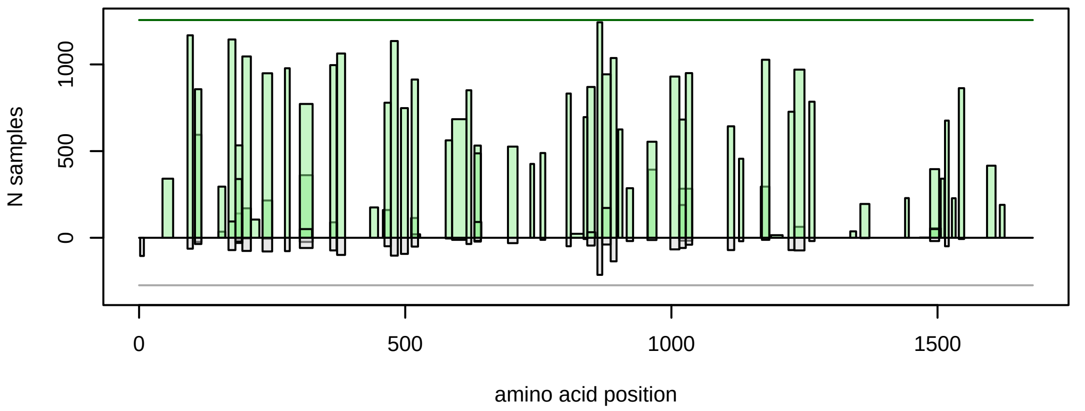

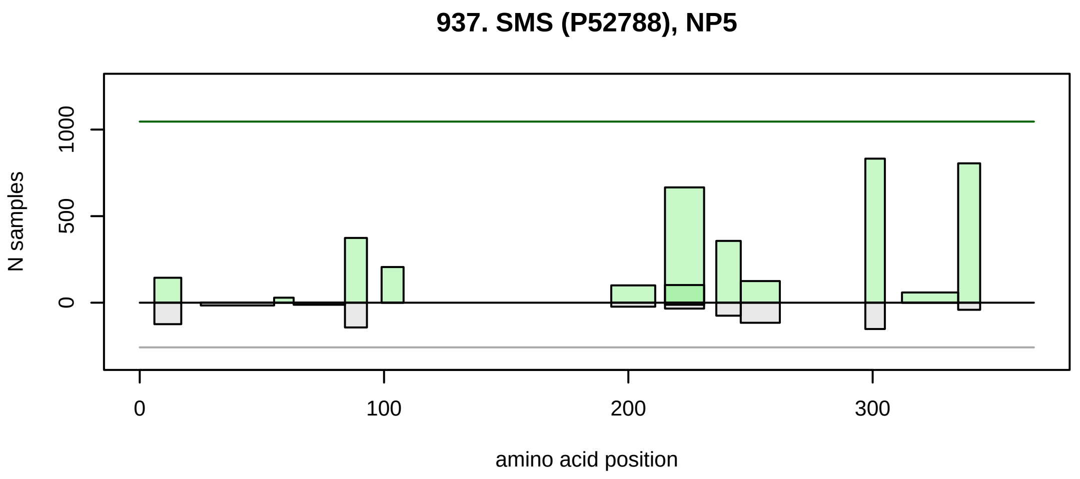
937. SMS (P52788), NP5
N samples
0
500
1000
0
100
200
300
amino acid position

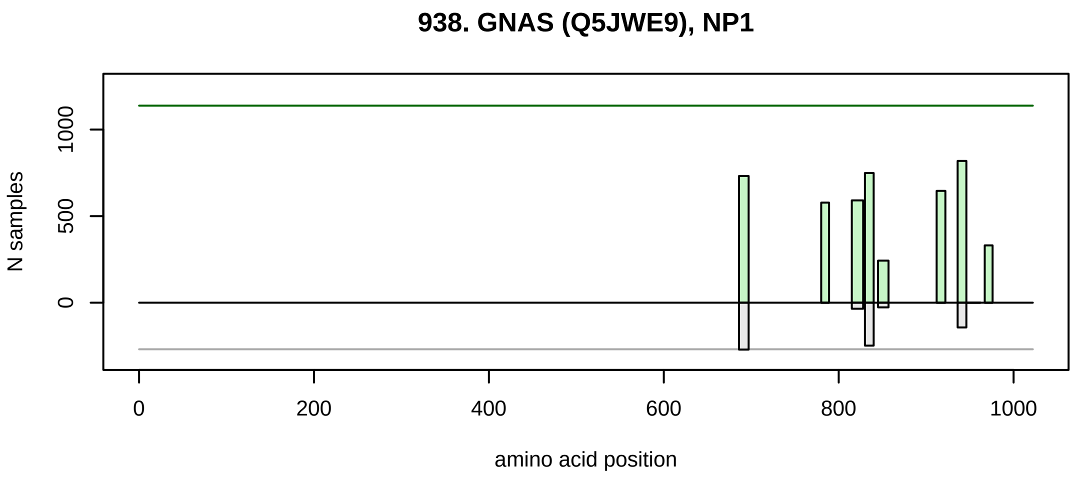
938. GNAS (Q5JWE9), NP1
N samples
0
500
1000
0
200
400
600
800
1000
amino acid position

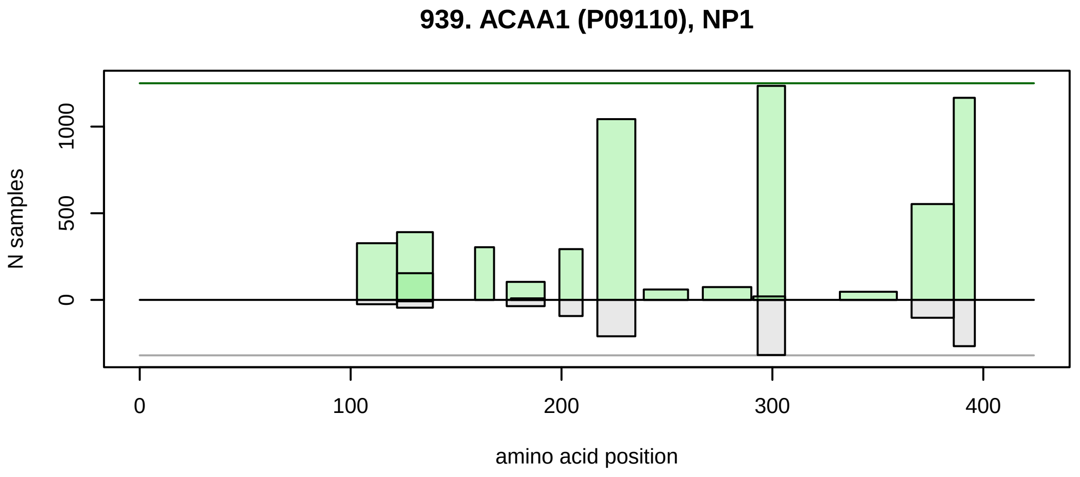
939. ACAA1 (P09110), NP1
N samples
0
500
1000
0
100
200
300
400
amino acid position

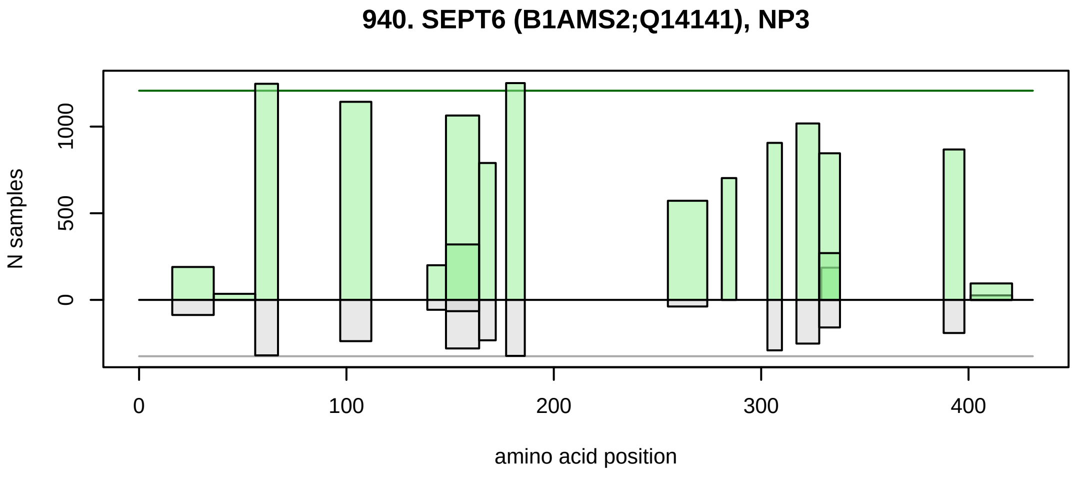
940. SEPT6 (B1AMS2;Q14141), NP3
N samples
0
500
1000
0
100
200
300
400
amino acid position

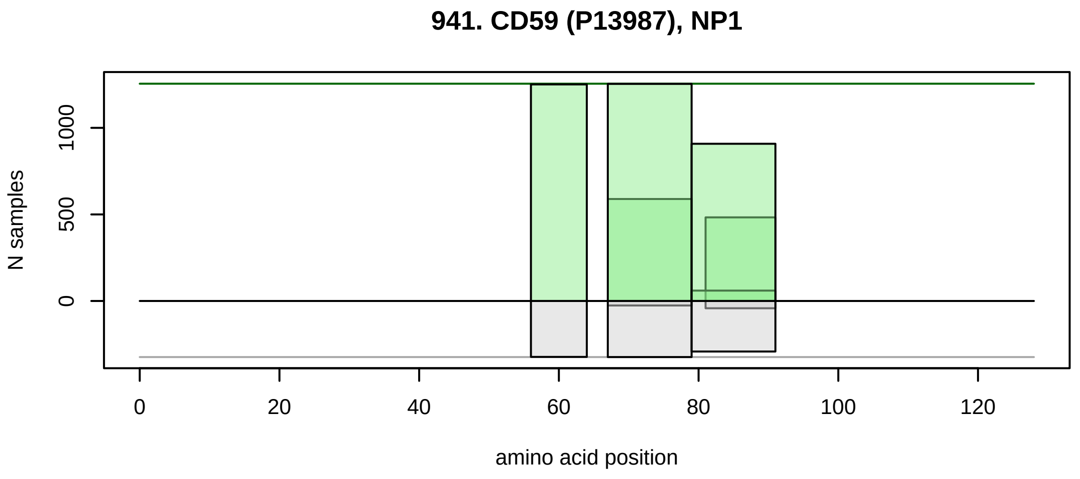
941. CD59 (P13987), NP1
N samples
0
500
1000
0
20
40
60
80
100
120
amino acid position

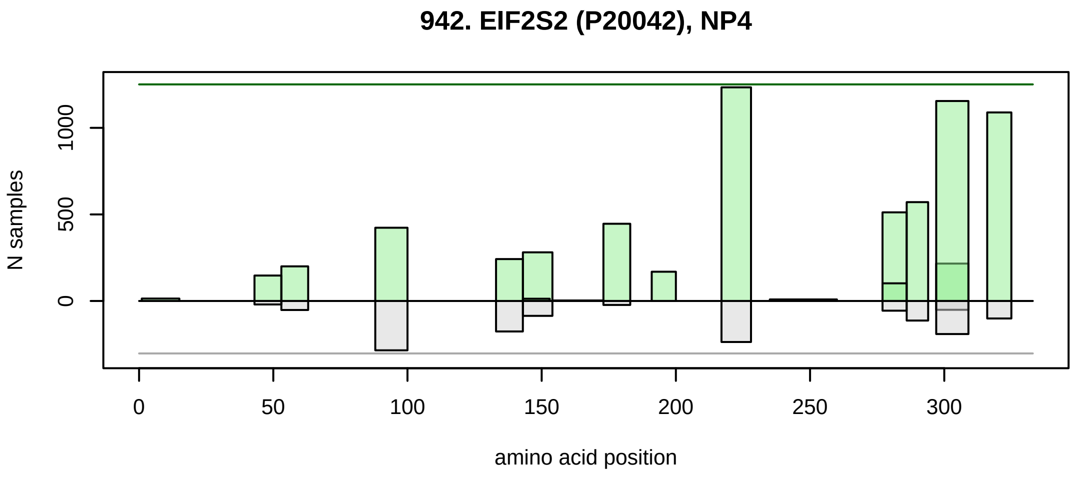
942. EIF2S2 (P20042), NP4
N samples
0
500
1000
0
50
100
150
200
250
300
amino acid position

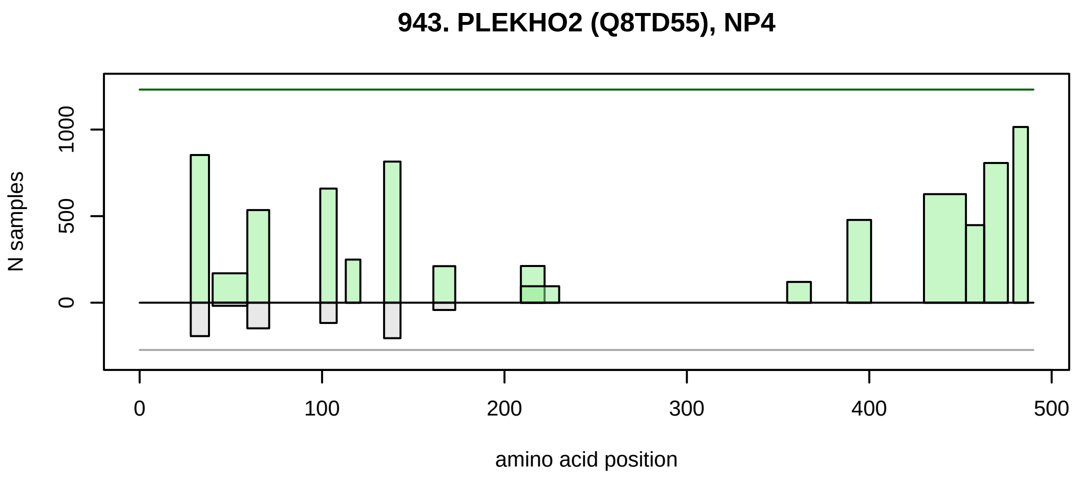
943. PLEKHO2 (Q8TD55), NP4
N samples
0
500
1000
0
100
200
300
400
500
amino acid position

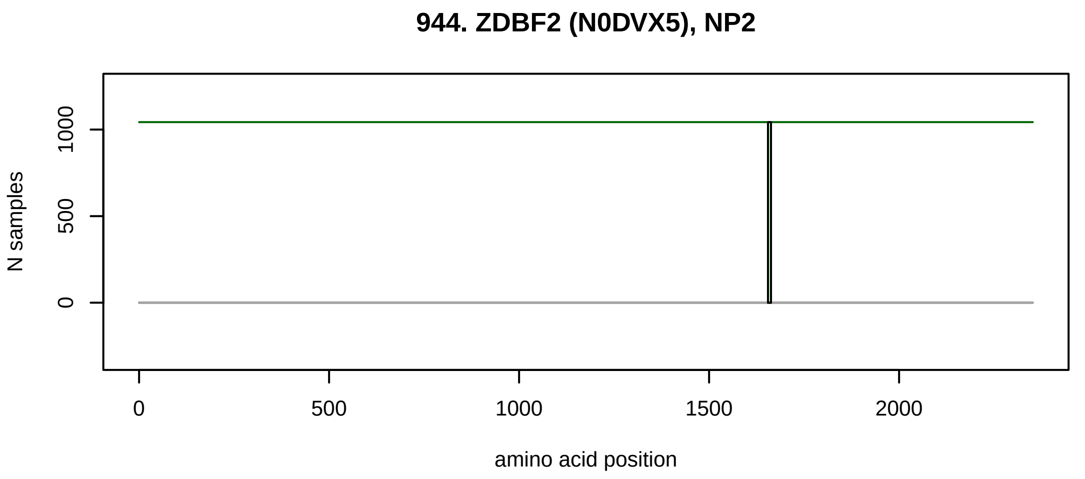
944. ZDBF2 (N0DVX5), NP2
N samples
0
500
1000
0
500
1000
1500
2000
amino acid position

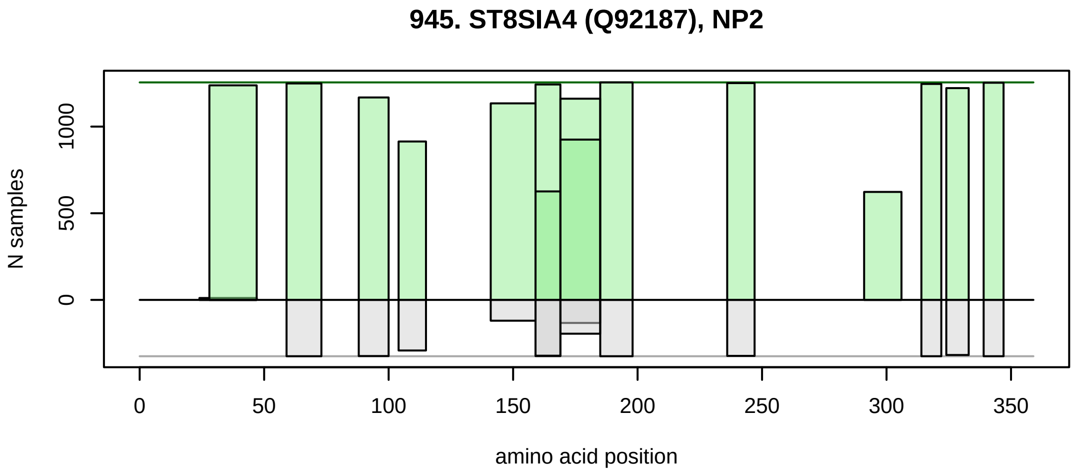
945. ST8SIA4 (Q92187), NP2
N samples
0
500
1000
0
50
100
150
200
250
300
350
amino acid position

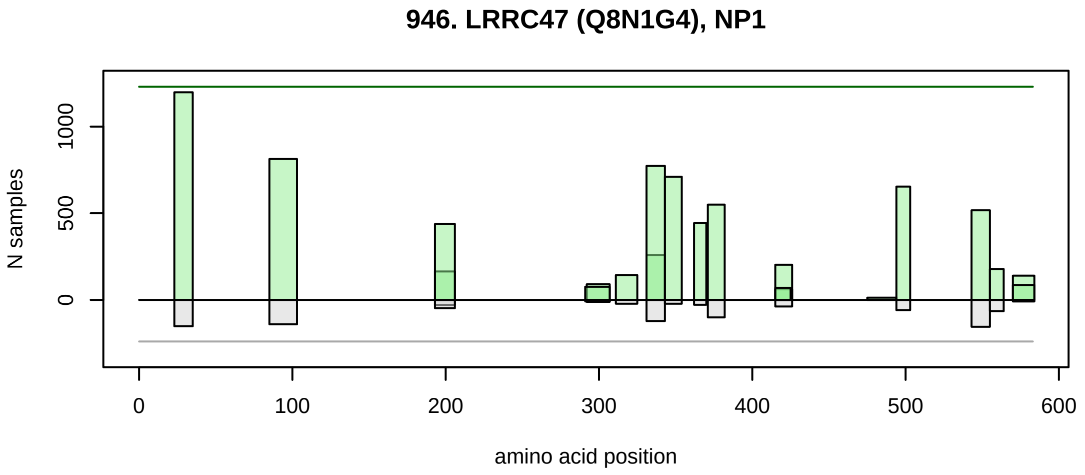
946. LRRC47 (Q8N1G4), NP1
N samples
0
500
1000
0
100
200
300
400
500
600
amino acid position

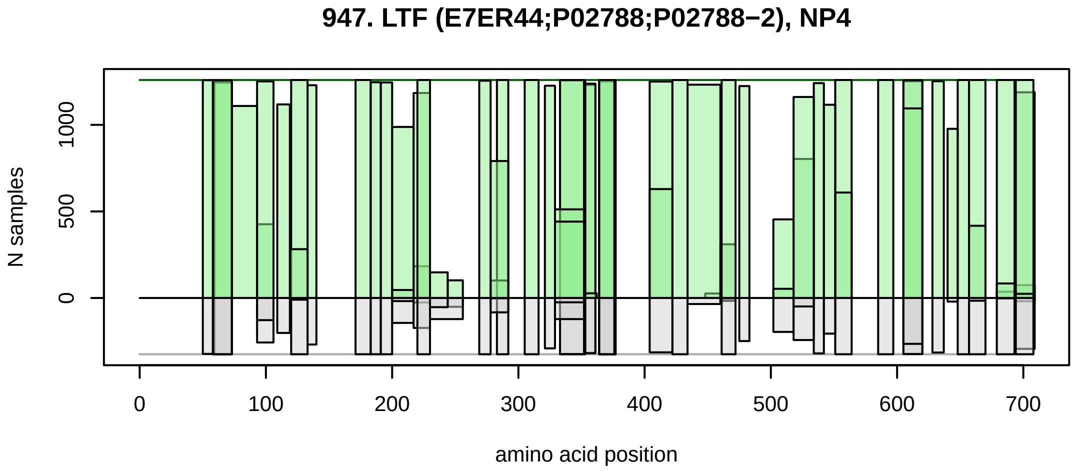
947. LTF (E7ER44;P02788;P02788−2), NP4
N samples
0
500
1000
0
100
200
300
400
500
600
700
amino acid position

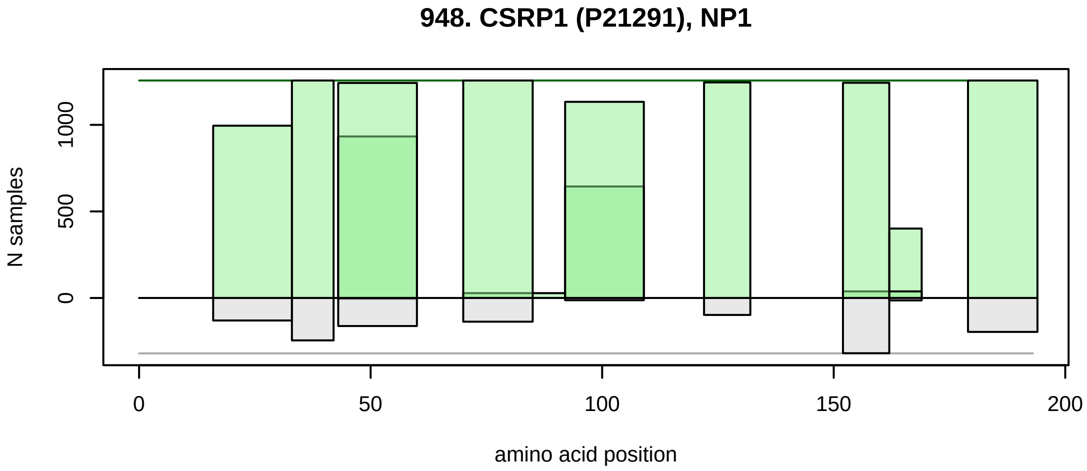
948. CSRP1 (P21291), NP1
N samples
0
500
1000
0
50
100
150
200
amino acid position

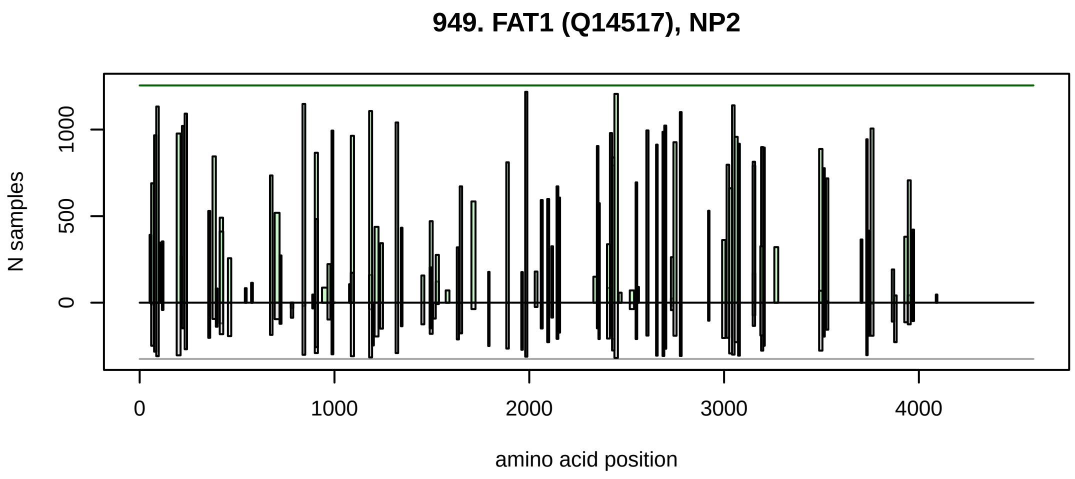
949. FAT1 (Q14517), NP2
N samples
amino acid position

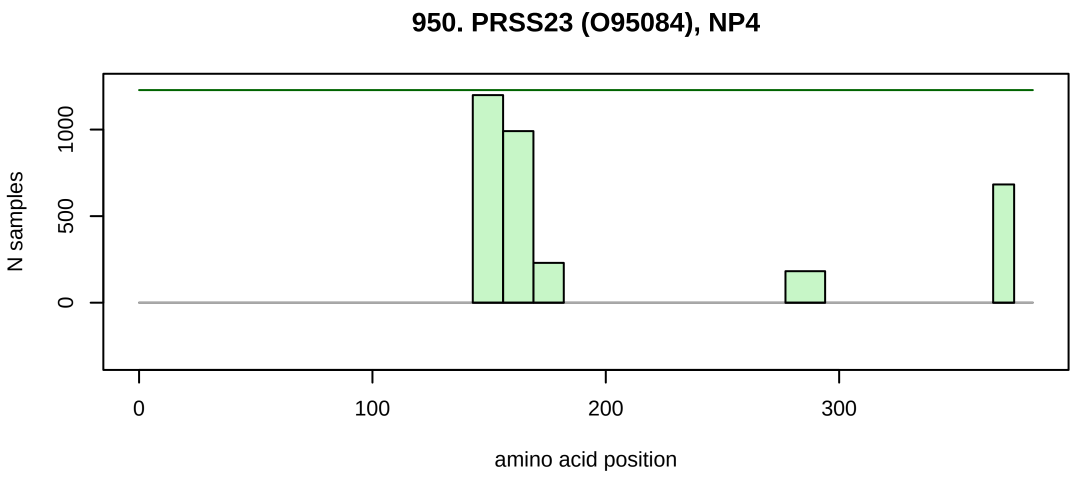
950. PRSS23 (O95084), NP4
N samples
amino acid position

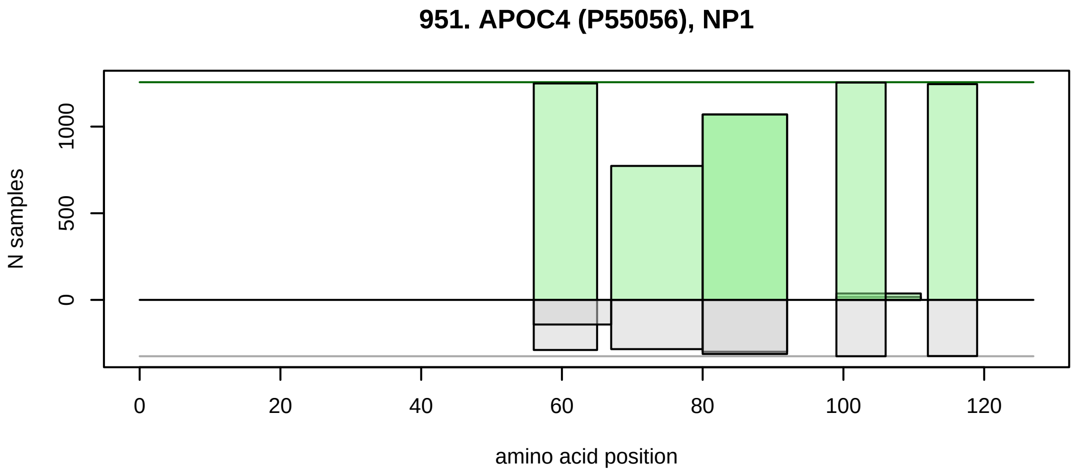
951. APOC4 (P55056), NP1
N samples
amino acid position

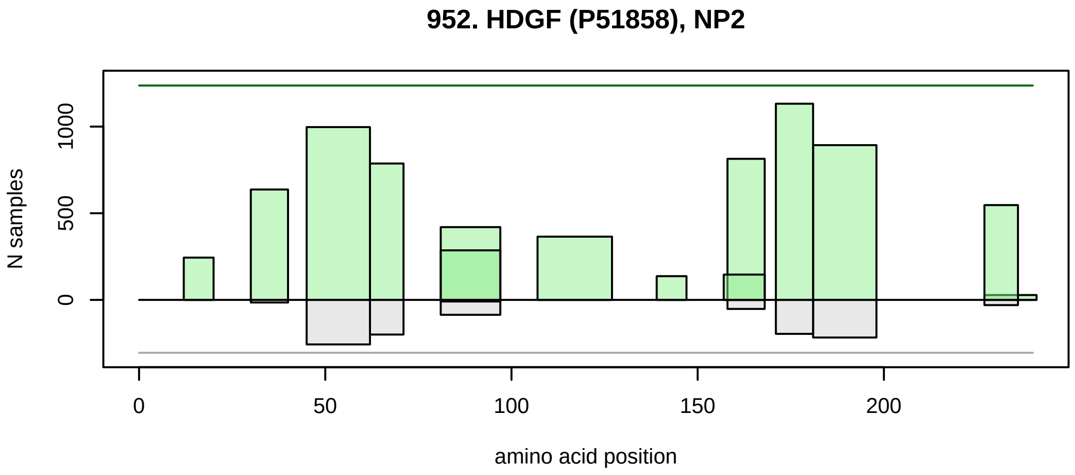
952. HDGF (P51858), NP2
N samples
amino acid position

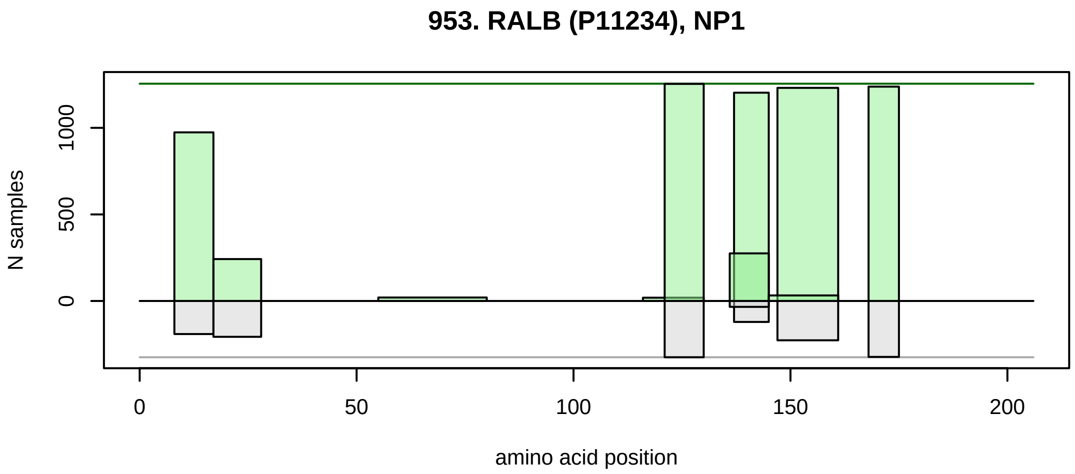
953. RALB (P11234), NP1
N samples
amino acid position

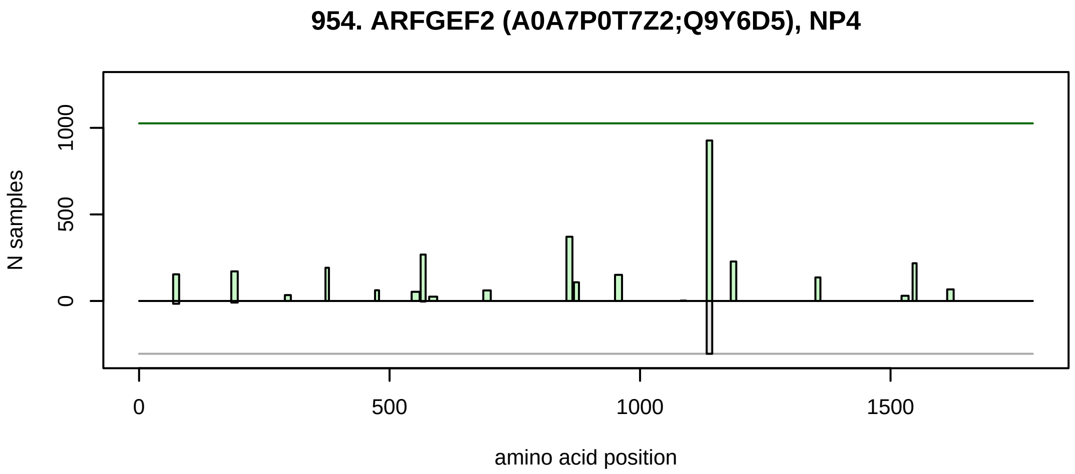
954. ARFGEF2 (A0A7P0T7Z2;Q9Y6D5), NP4
N samples
amino acid position

955. TMEM59 (Q9BXS4), NP2

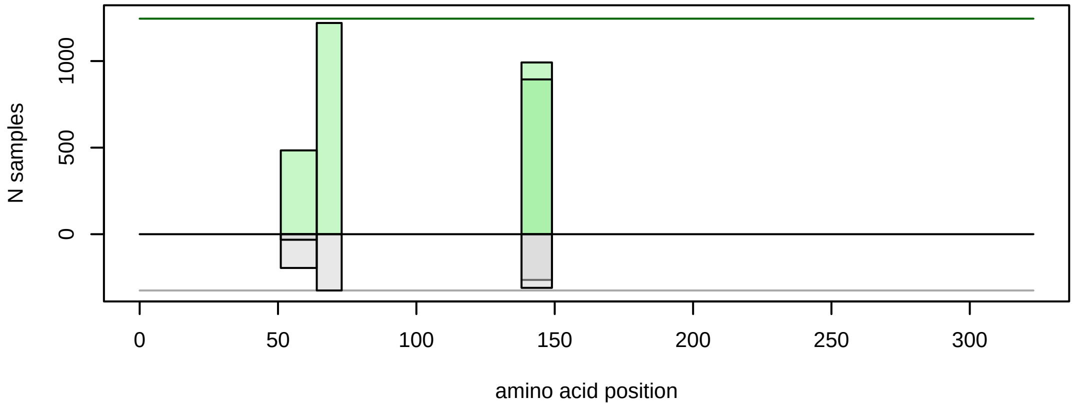

956. AMPD3 (Q01432), NP4

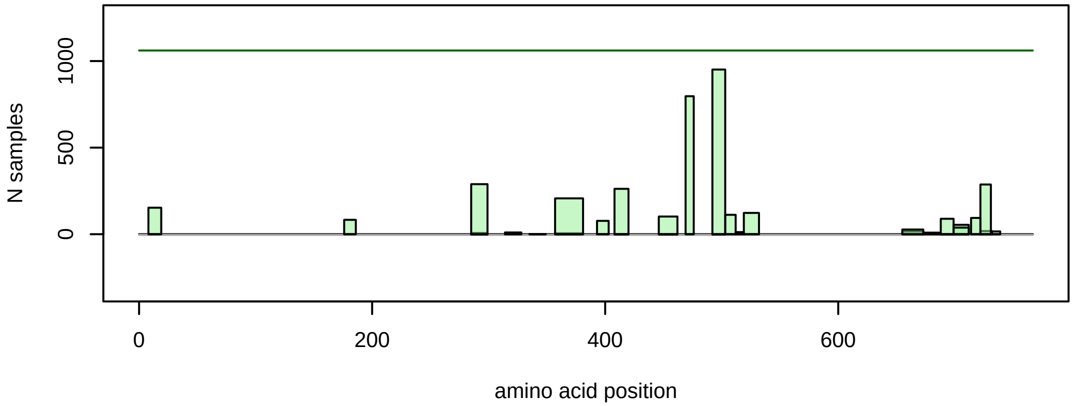

957. LFNG (Q8NES3), NP3

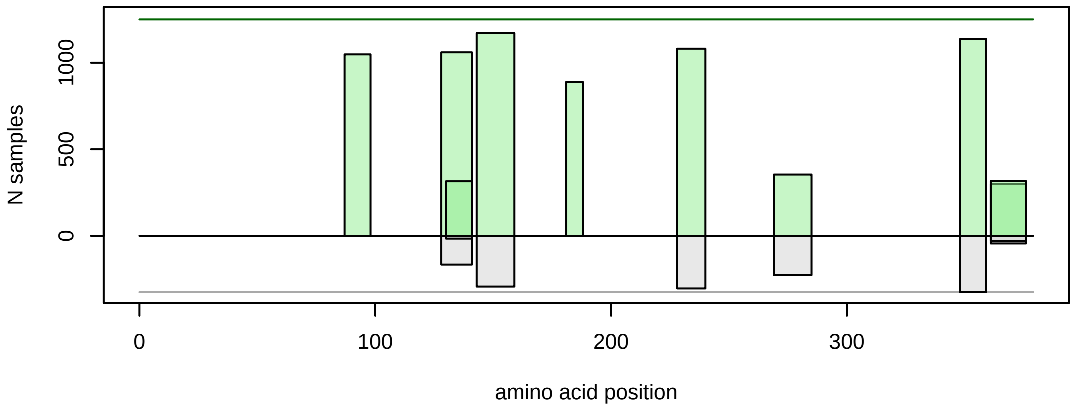

958. HSPA5 (P11021), NP1

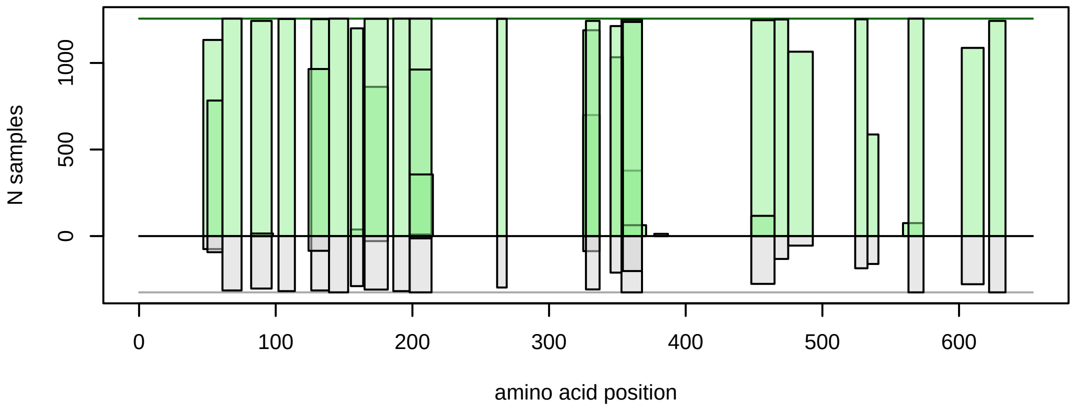

959. MANF (P55145), NP3

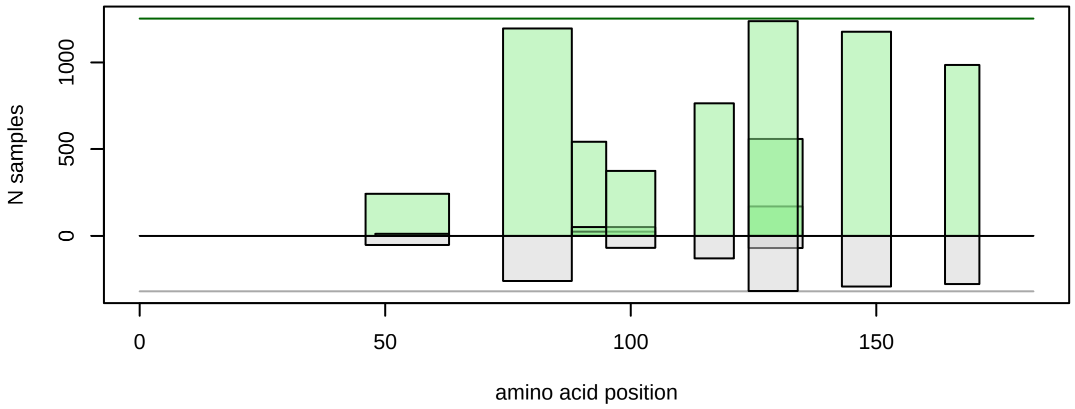

960. EIF2AK2 (P19525), NP1

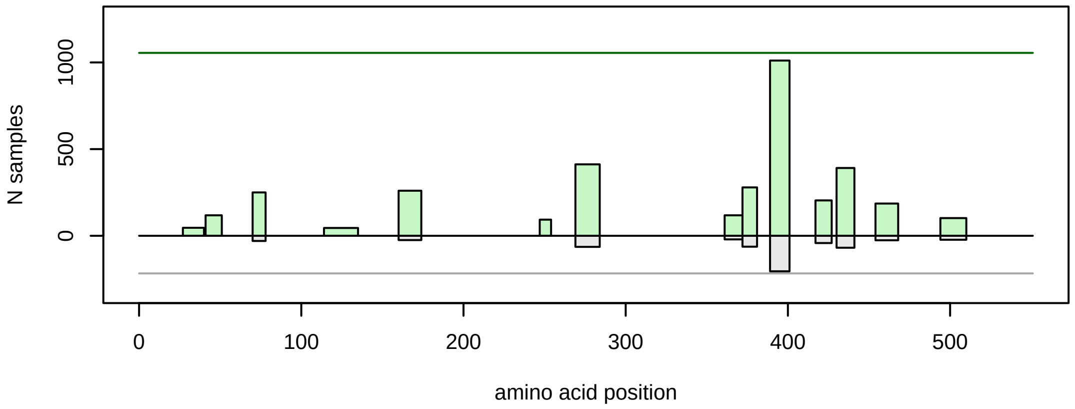

961. DSC1 (Q08554), NP3

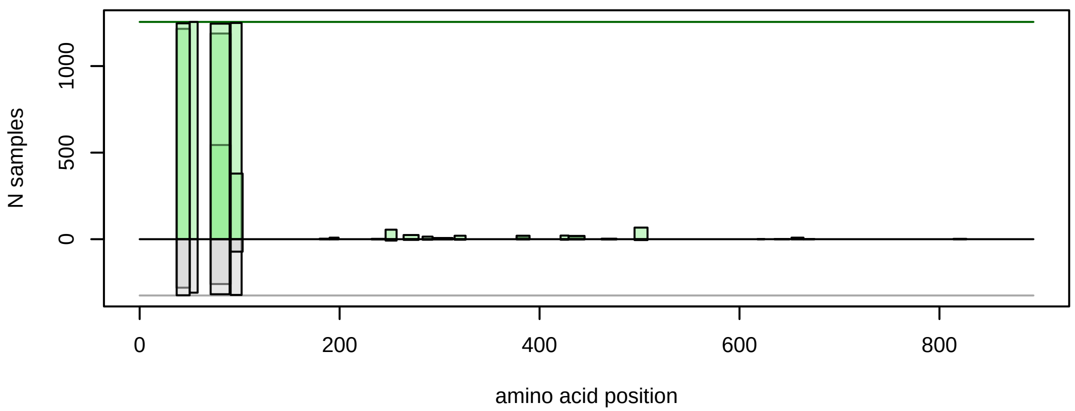

962. CACNA1H (A0A1W2PQW2), NP4

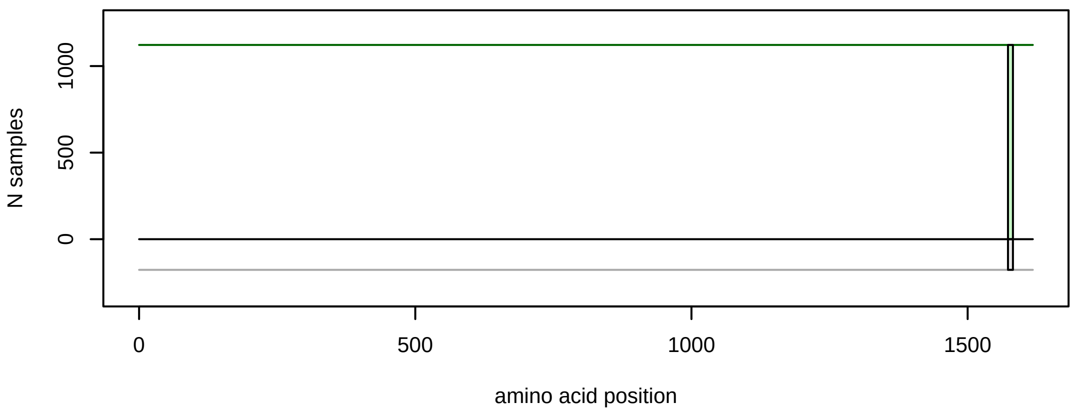

963. TNNC2 (P02585), NP2

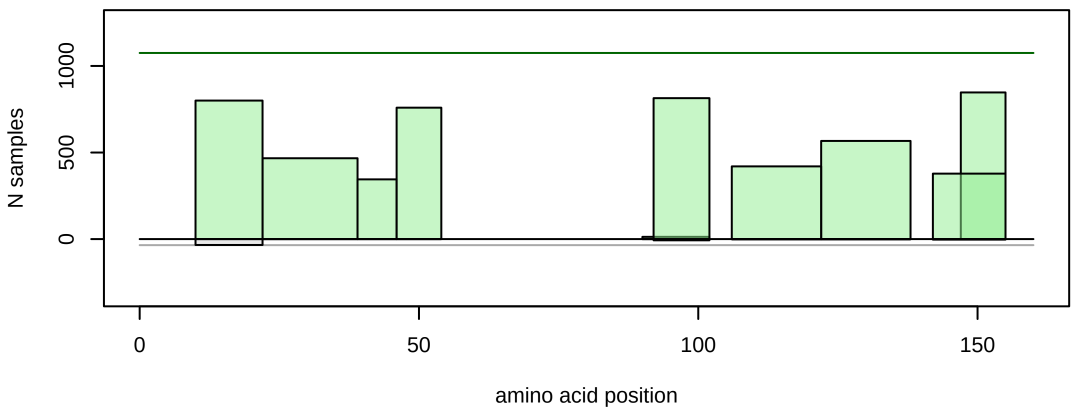

964. VDAC1 (P21796), NP4

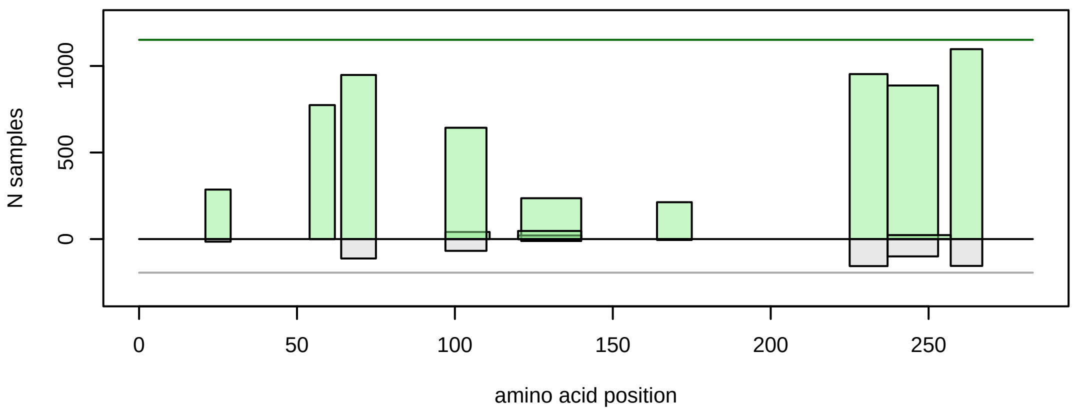

965. PCYOX1 (Q9UHG3), NP4

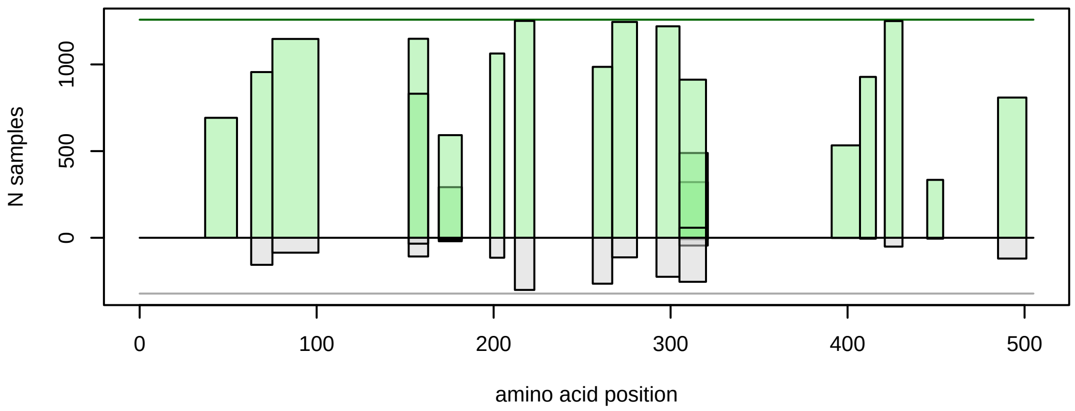

966. ATP5B (P06576), NP4

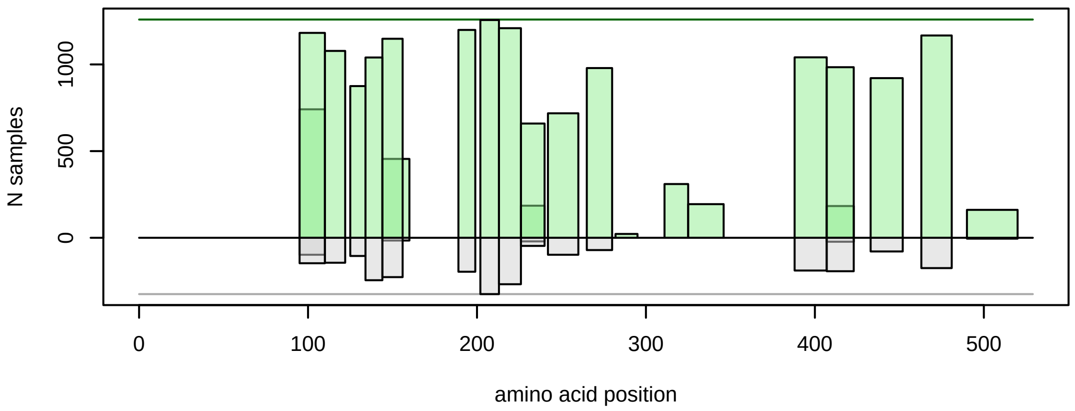

967. TPST2 (O60704), NP4

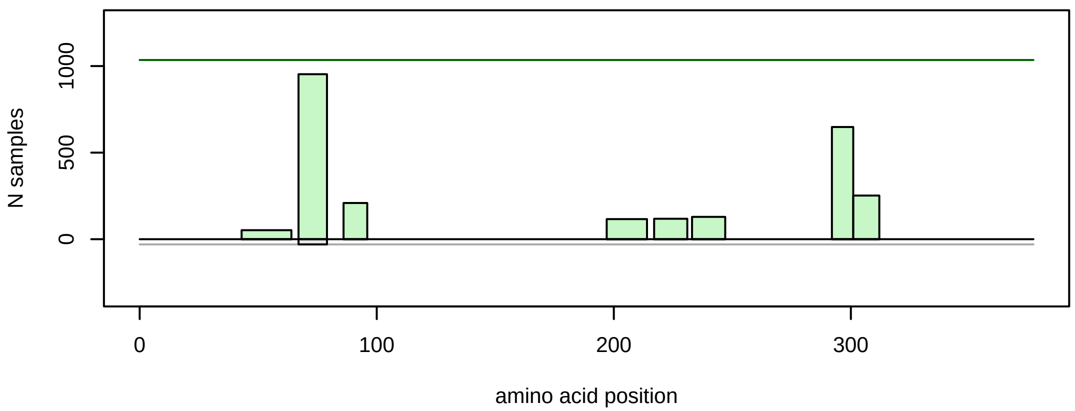

968. ROCK1 (Q13464), NP4

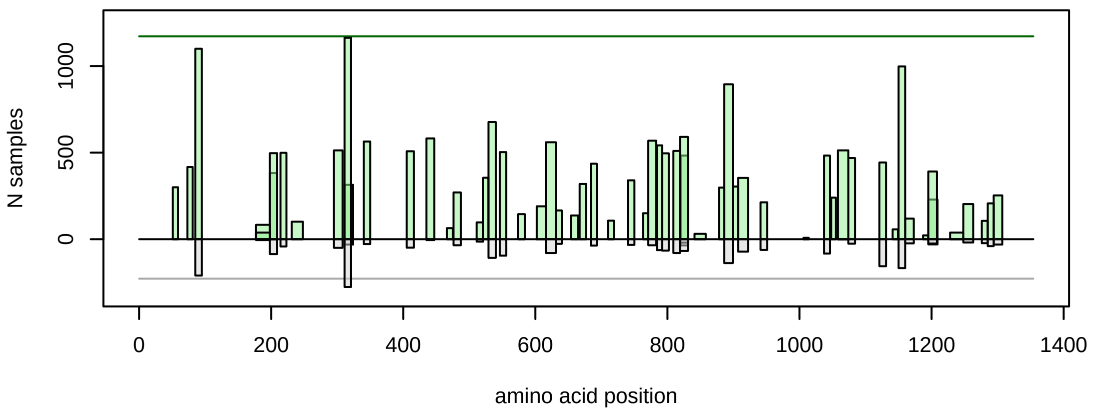

969. SLC27A3 (X6R3N0), NP5

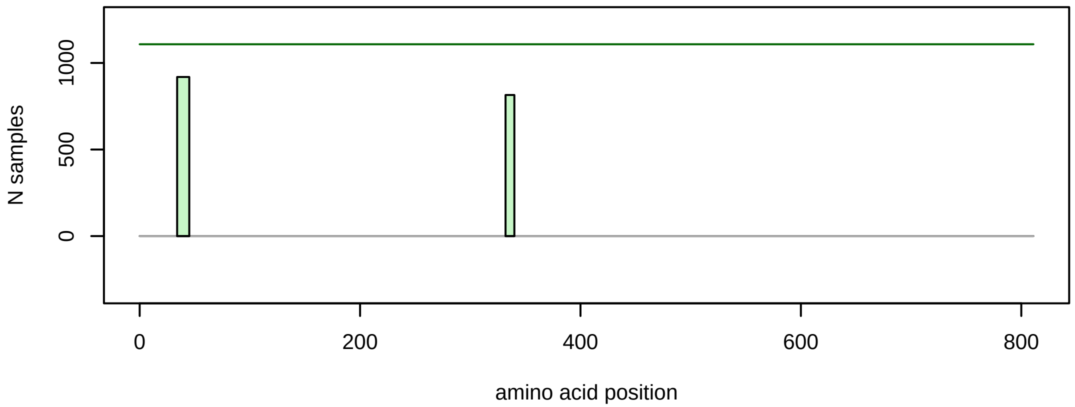

970. PSMA6 (G3V5Z7;P60900), NP1

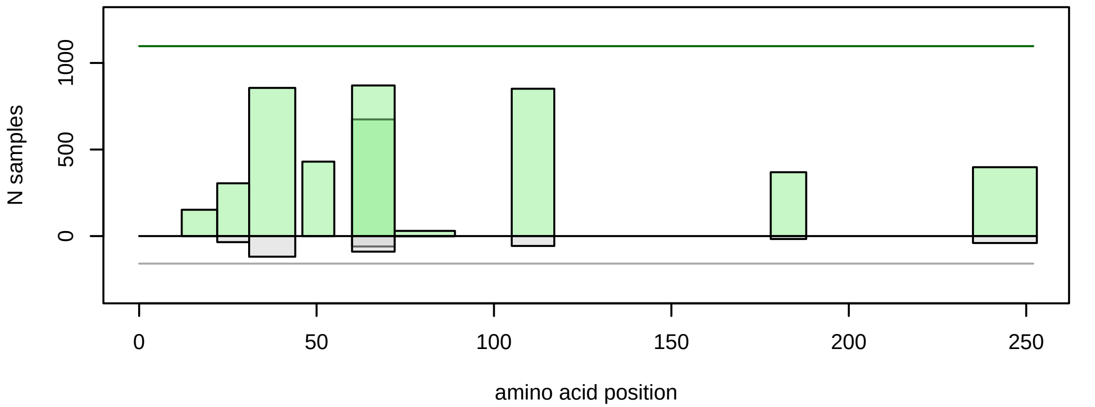

971. ARPC2 (O15144), NP4

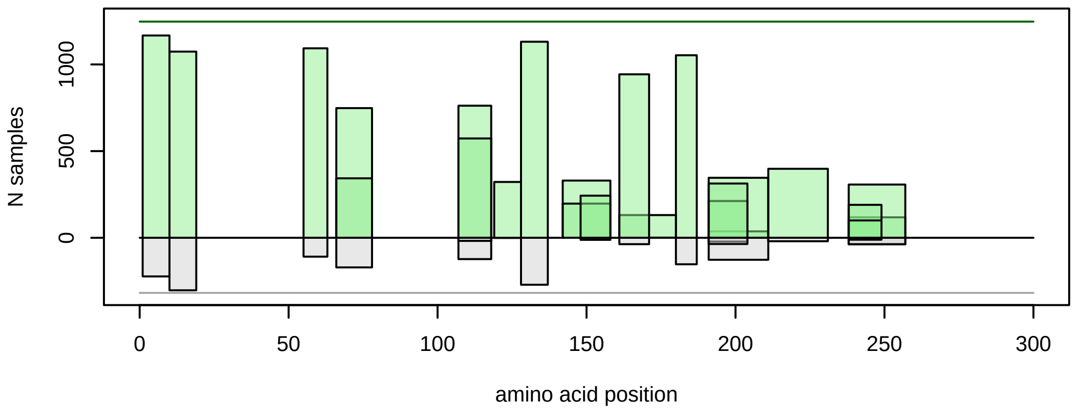

972. PKM (P14618), NP4

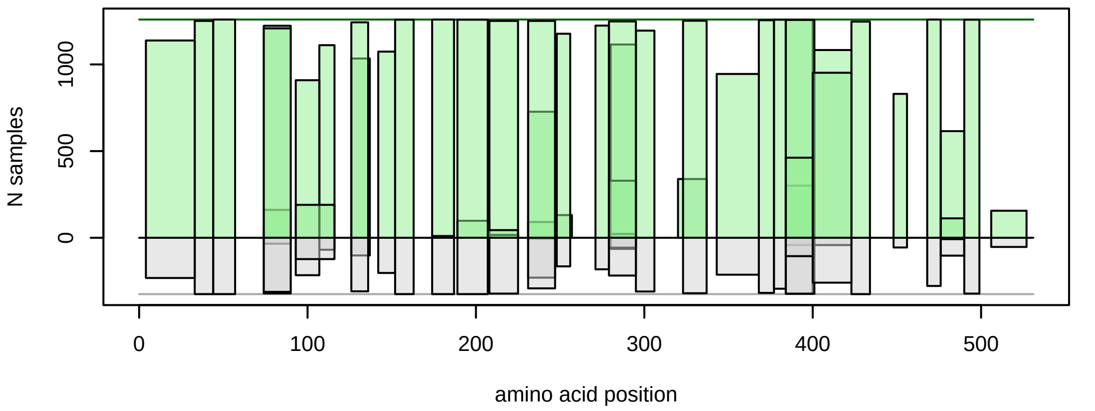

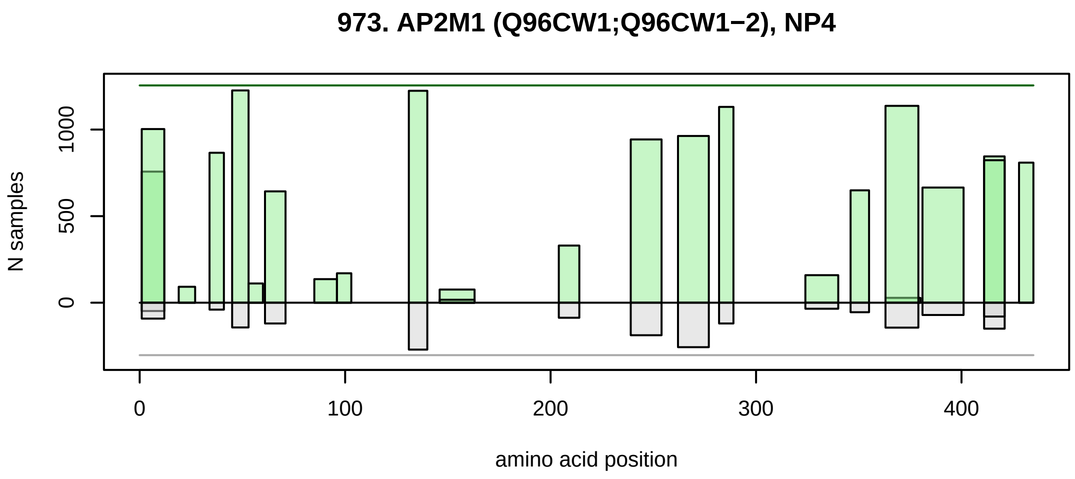
973. AP2M1 (Q96CW1;Q96CW1−2), NP4
N samples
0
500
1000
0
100
200
300
400
amino acid position

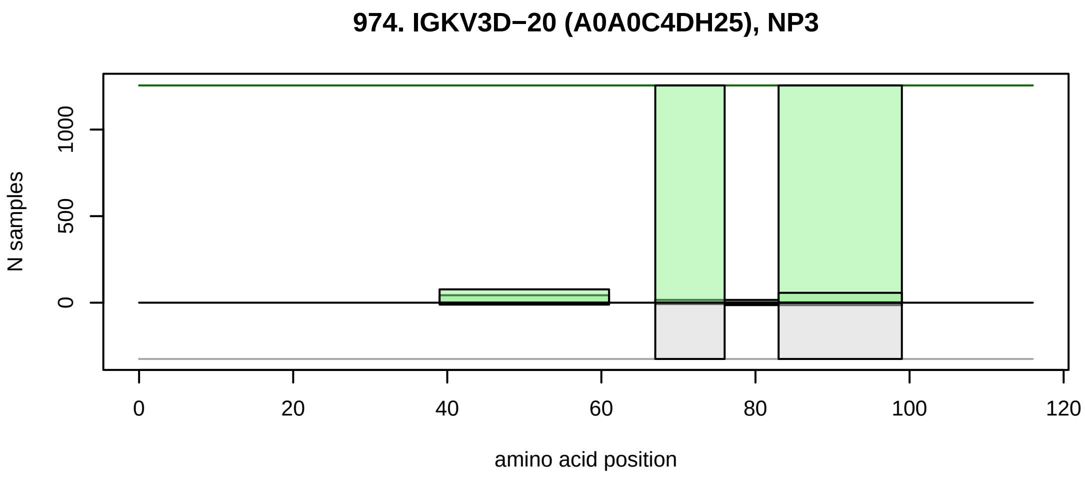
974. IGKV3D−20 (A0A0C4DH25), NP3
N samples
0
500
1000
0
20
40
60
80
100
120
amino acid position

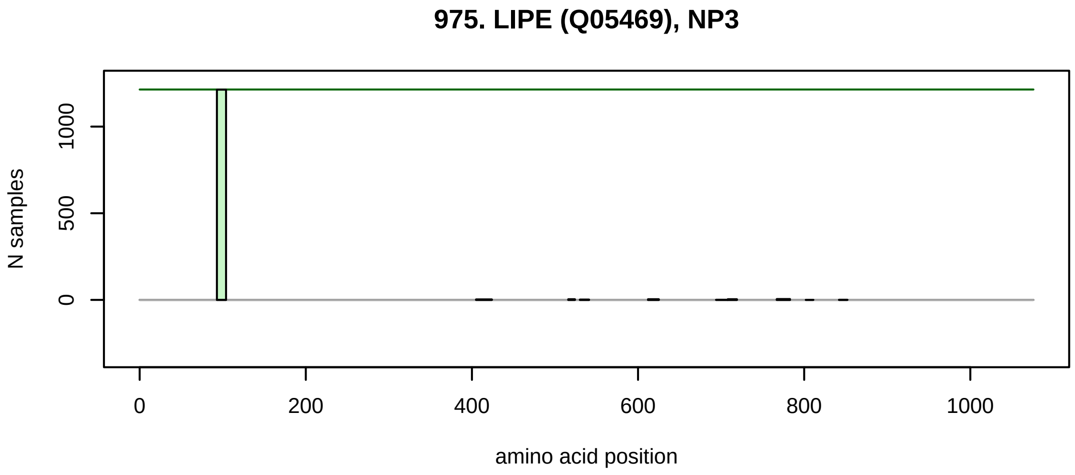
975. LIPE (Q05469), NP3
N samples
0
500
1000
0
200
400
600
800
1000
amino acid position

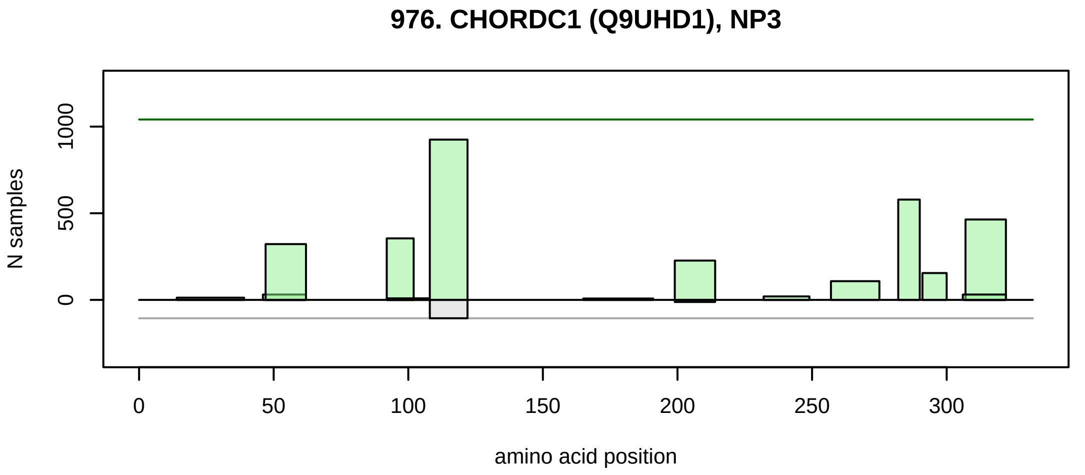
976. CHORDC1 (Q9UHD1), NP3
N samples
0
500
1000
0
50
100
150
200
250
300
amino acid position

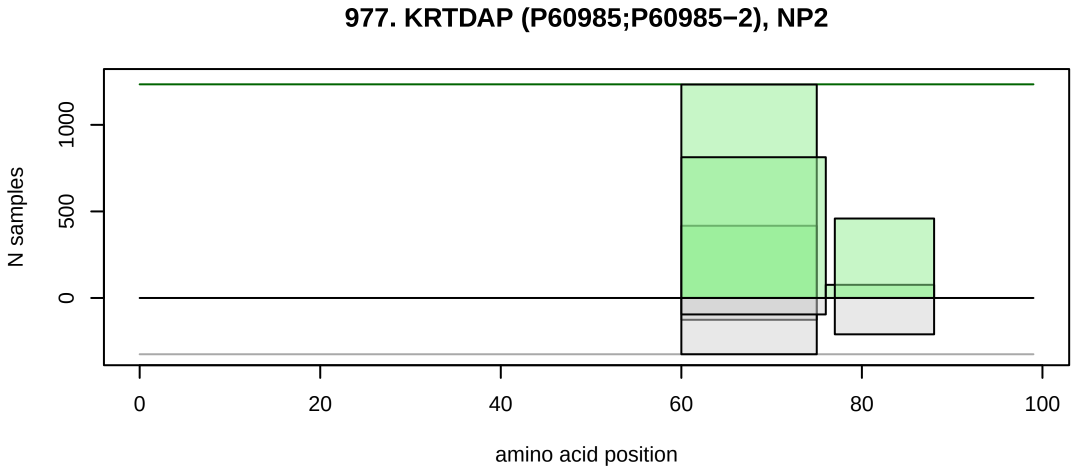
977. KRTDAP (P60985;P60985−2), NP2
N samples
0
500
1000
0
20
40
60
80
100
amino acid position

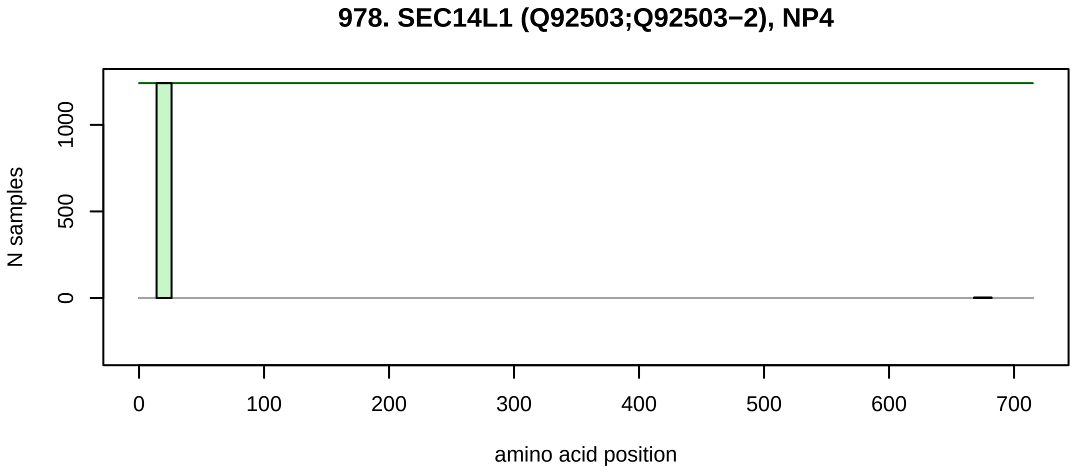
978. SEC14L1 (Q92503;Q92503−2), NP4
N samples
0
500
1000
0
100
200
300
400
500
600
700
amino acid position

979. SNX3 (O60493), NP1

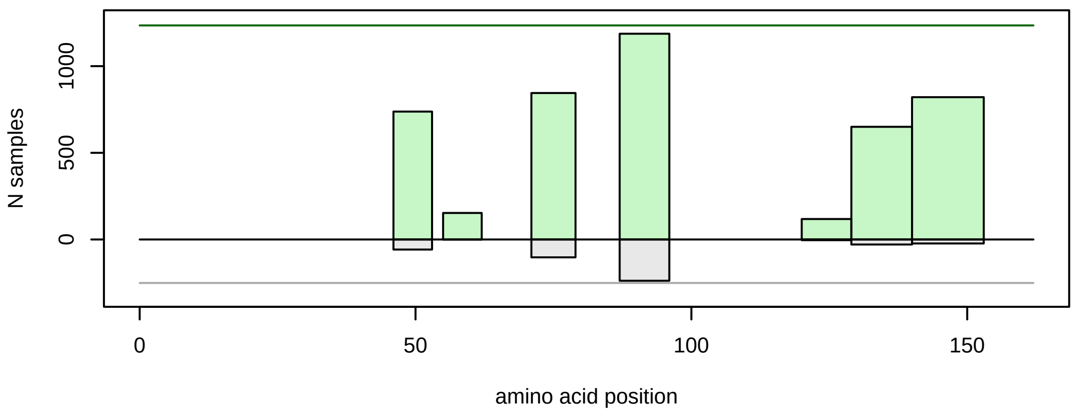

980. DNAJA2 (O60884), NP1

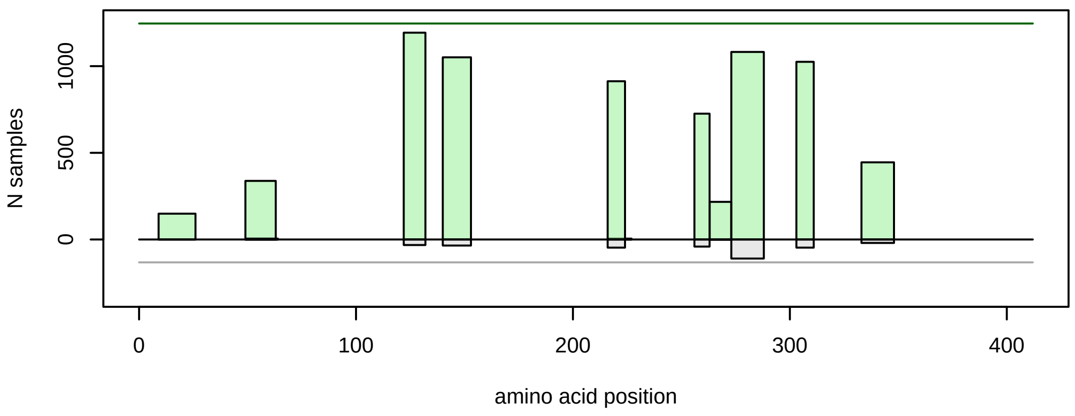

981. PTPRB (P23467−3), NP4

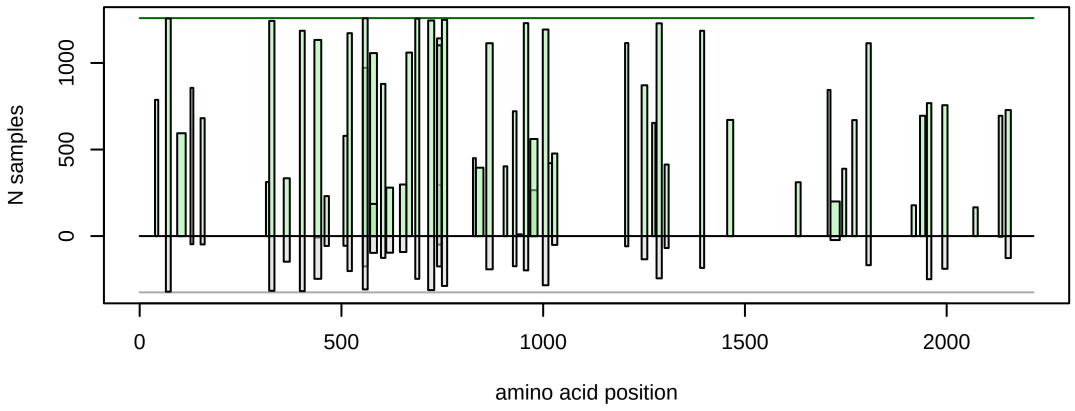

982. B3GALT6 (Q96L58), NP1

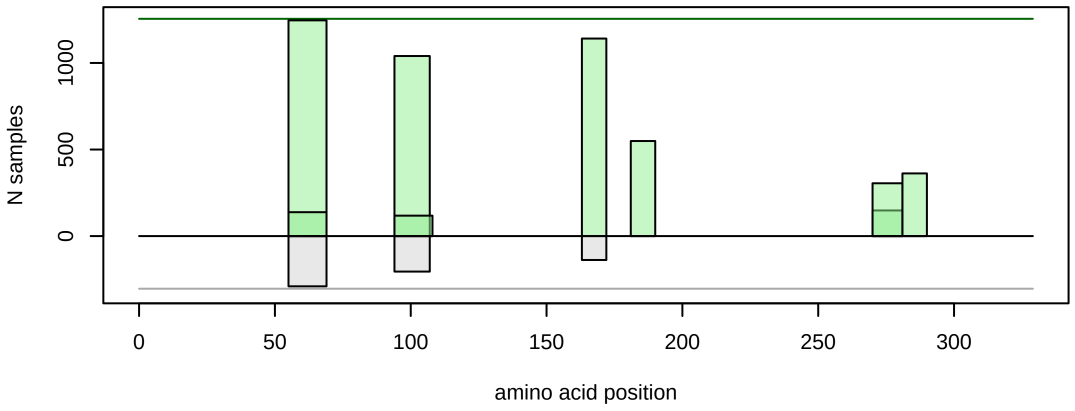

983. CTSL (P07711), NP2

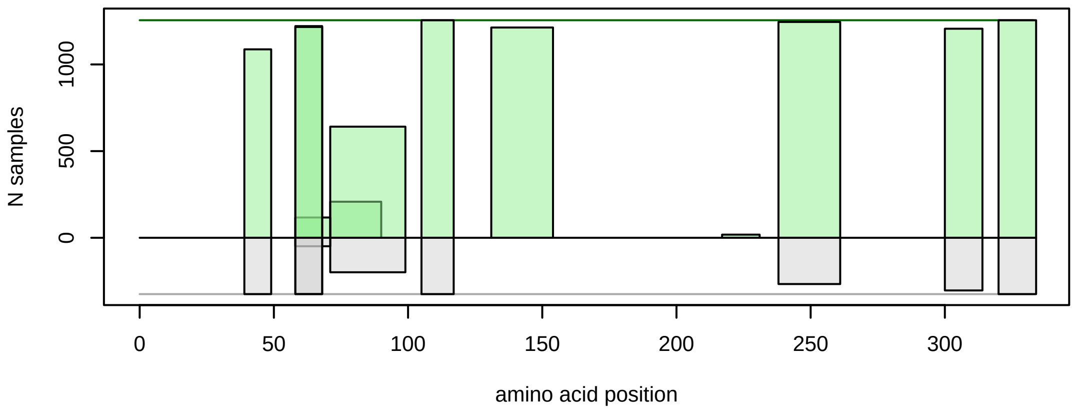

984. ARF4 (P18085), NP4

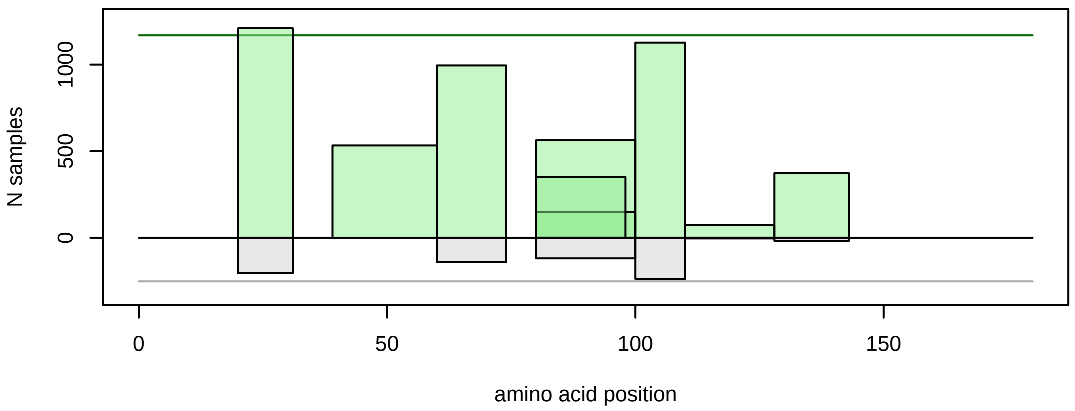

985. INHBE (P58166), NP4

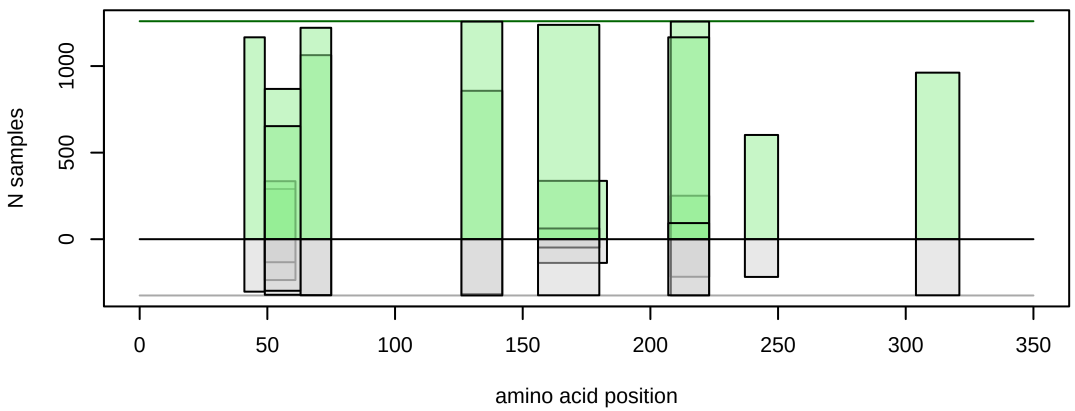

986. CECR1 (Q9NZK5), NP3

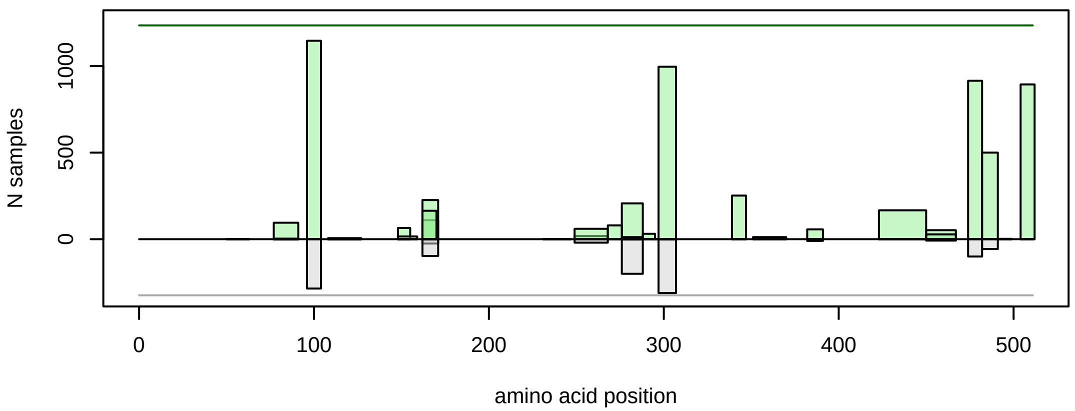

987. C8A (P07357), NP5

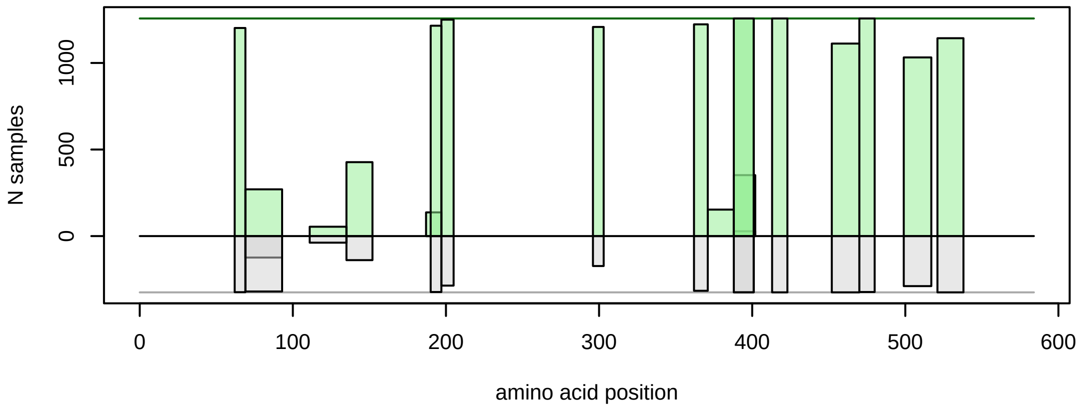

988. EIF4G2 (P78344), NP2

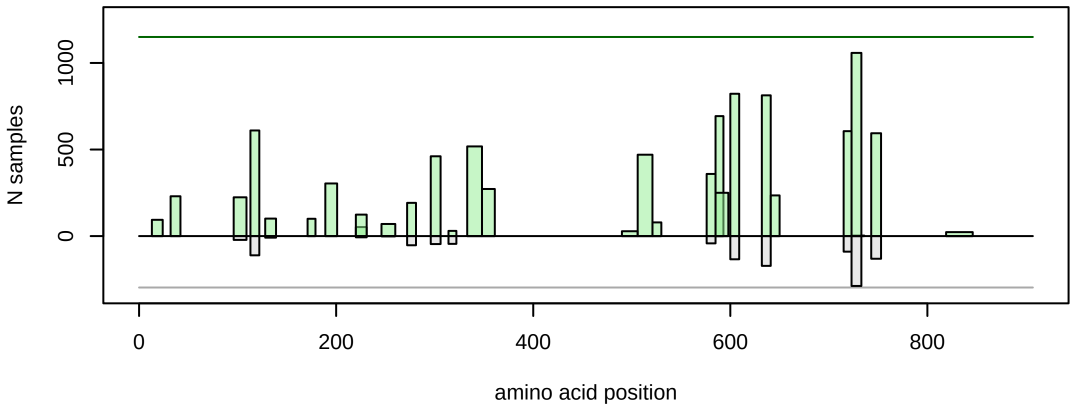

989. CHST13 (Q8NET6), NP4

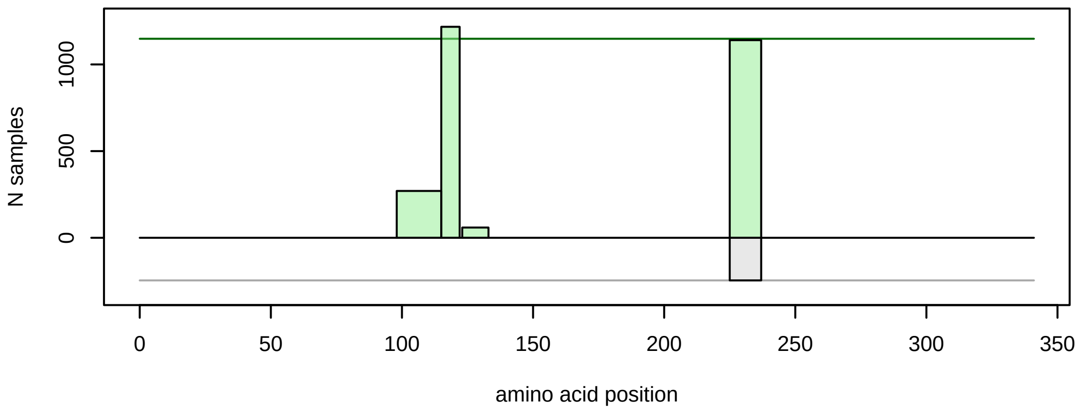

990. RBP4 (Q5VY30), NP2

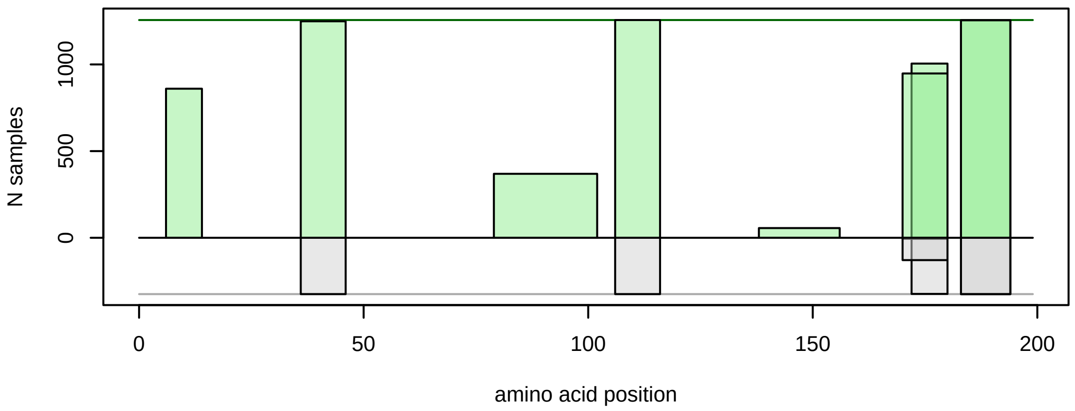

991. DDX1 (A0A7I2V430;A0A7I2V4F0;Q92499), NP1

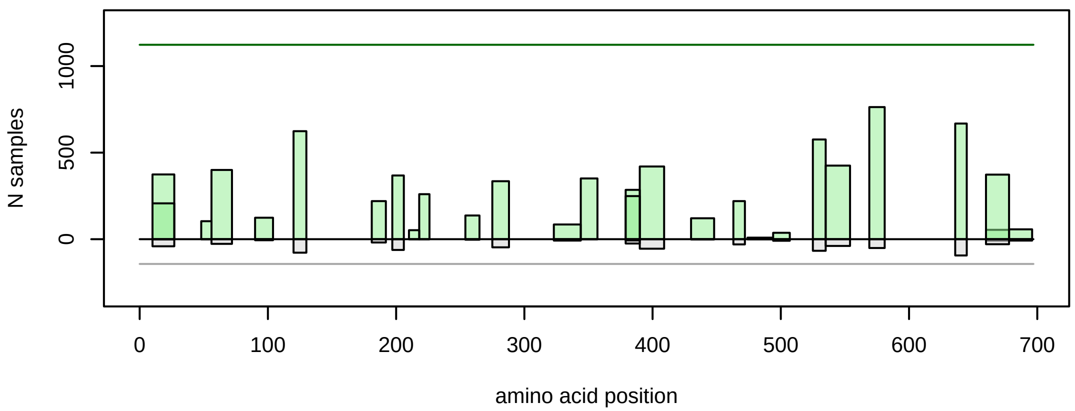

992. RPL22 (K7ERI7;P35268), NP4

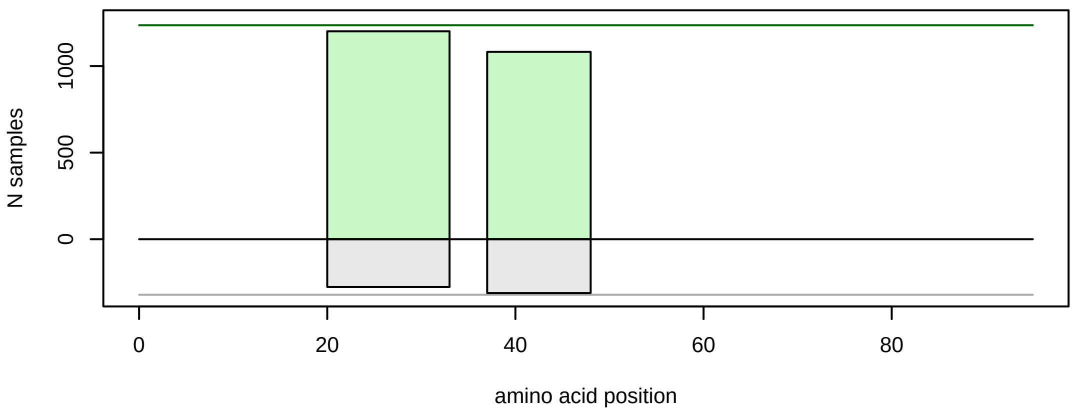

993. GNAI2 (P04899), NP1

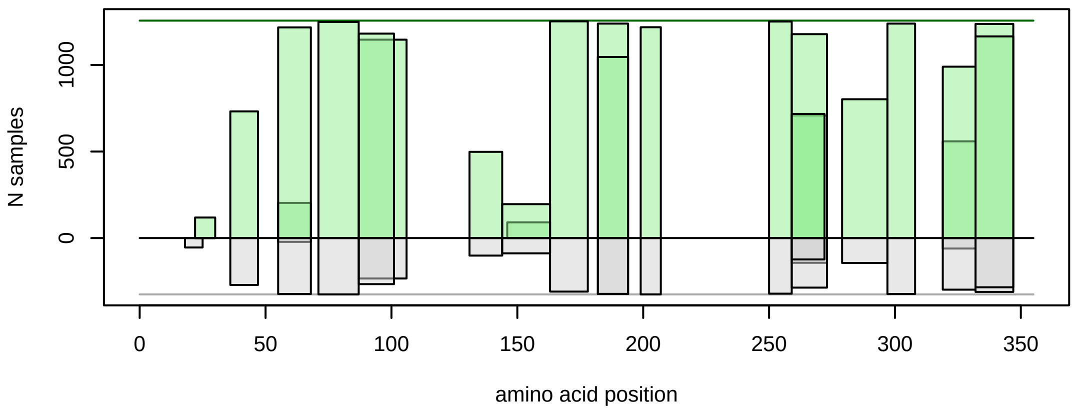

994. MYL2 (A0A590UJU8), NP2

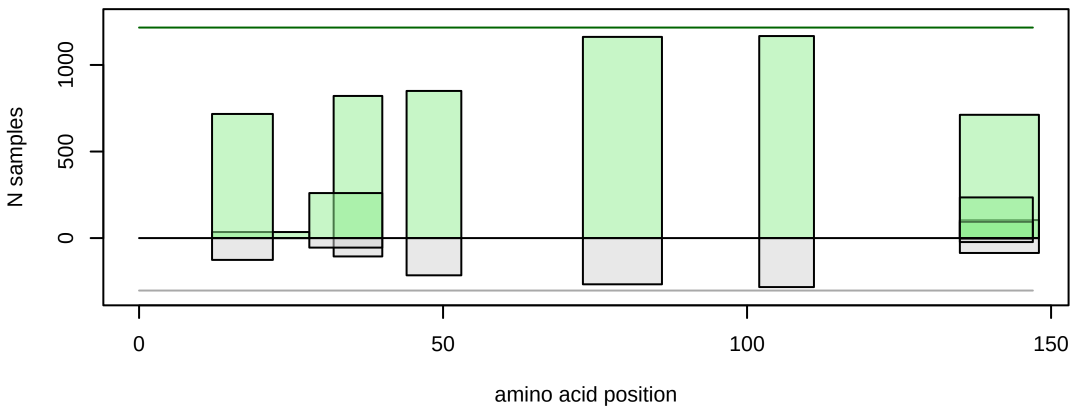

995. APCS (P02743), NP5

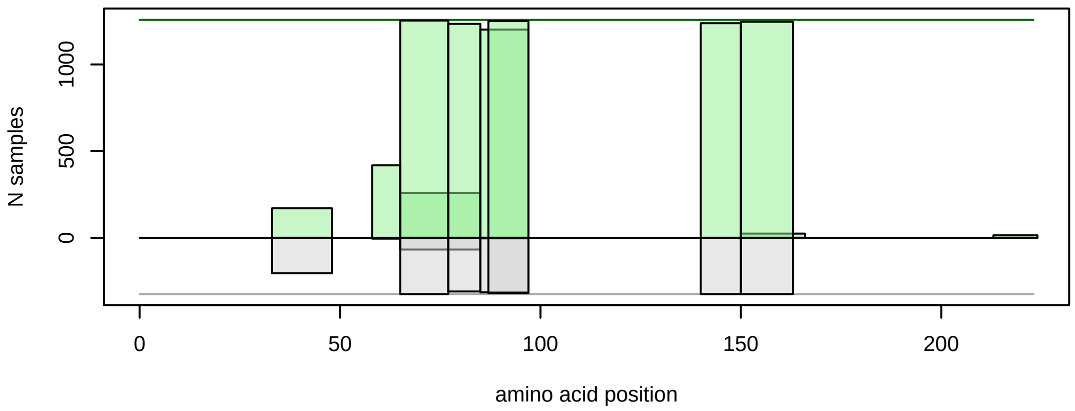

996. ABI3BP (D3YTG3), NP2

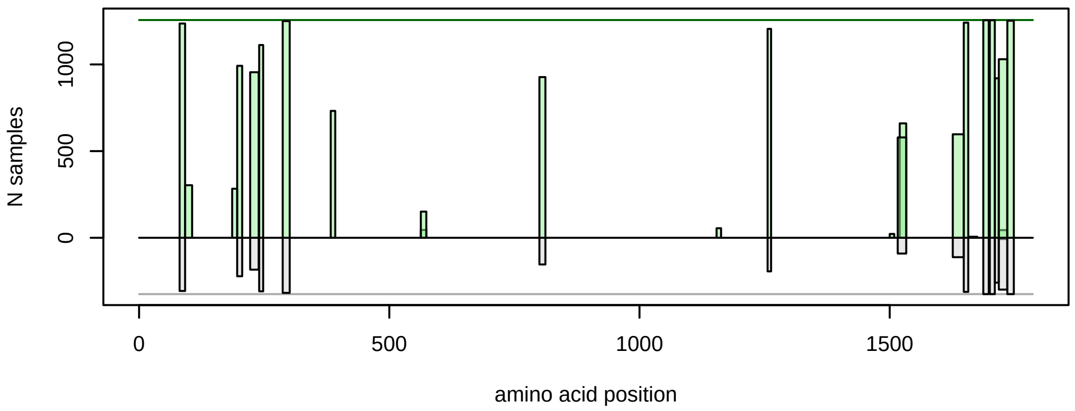

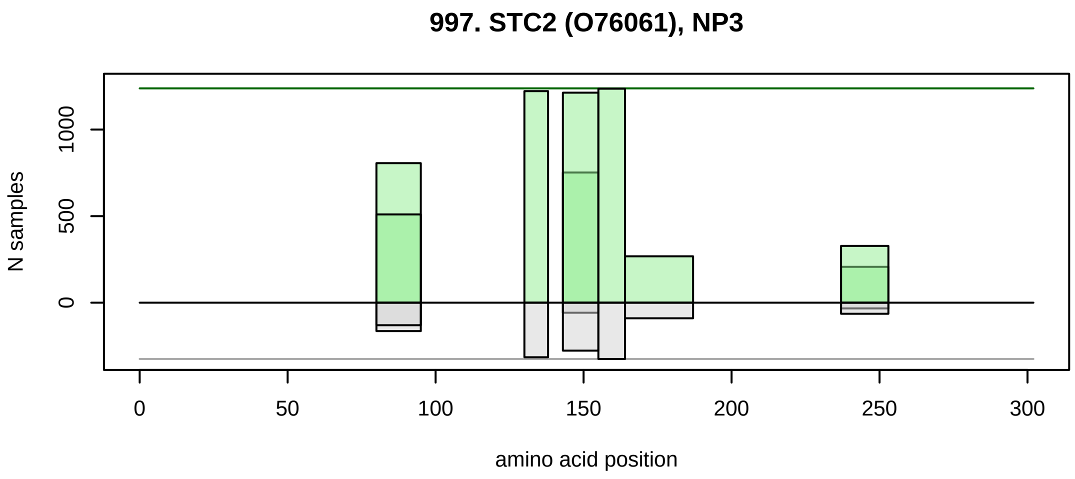
997. STC2 (O76061), NP3
N samples
0
500
1000
0
50
100
150
200
250
300
amino acid position

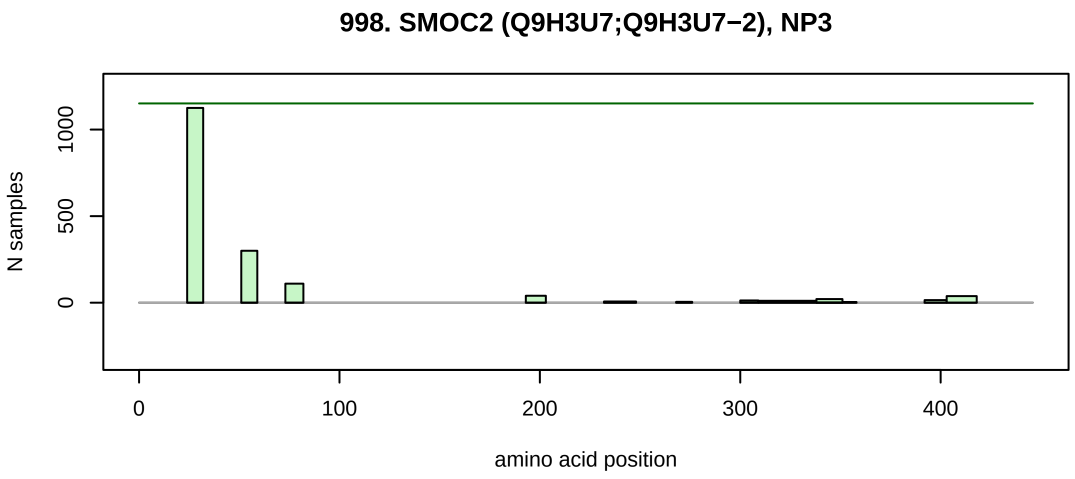
998. SMOC2 (Q9H3U7;Q9H3U7−2), NP3
N samples
0
500
1000
0
100
200
300
400
amino acid position

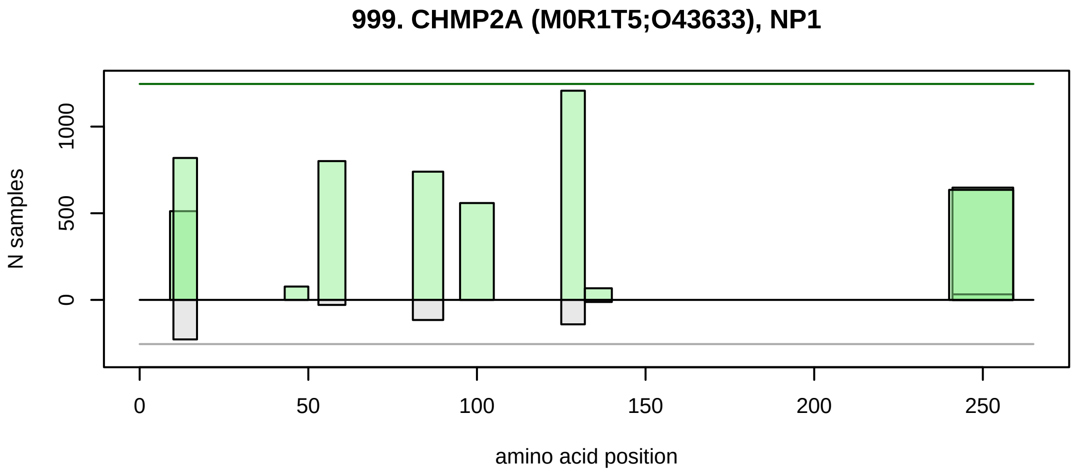
999. CHMP2A (M0R1T5;O43633), NP1
N samples
0
500
1000
0
50
100
150
200
250
amino acid position

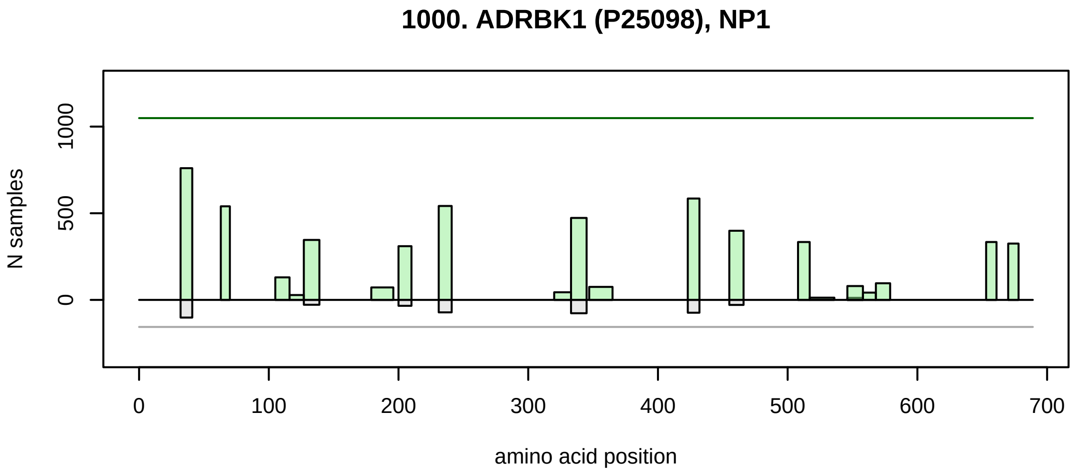
1000. ADRBK1 (P25098), NP1
N samples
0
500
1000
0
100
200
300
400
500
600
700
amino acid position

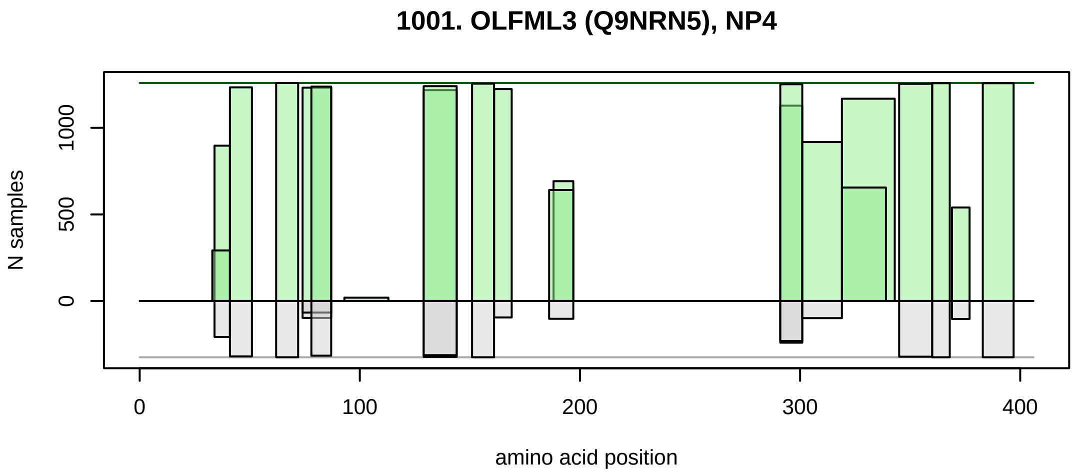
1001. OLFML3 (Q9NRN5), NP4
N samples
0
500
1000
0
100
200
300
400
amino acid position

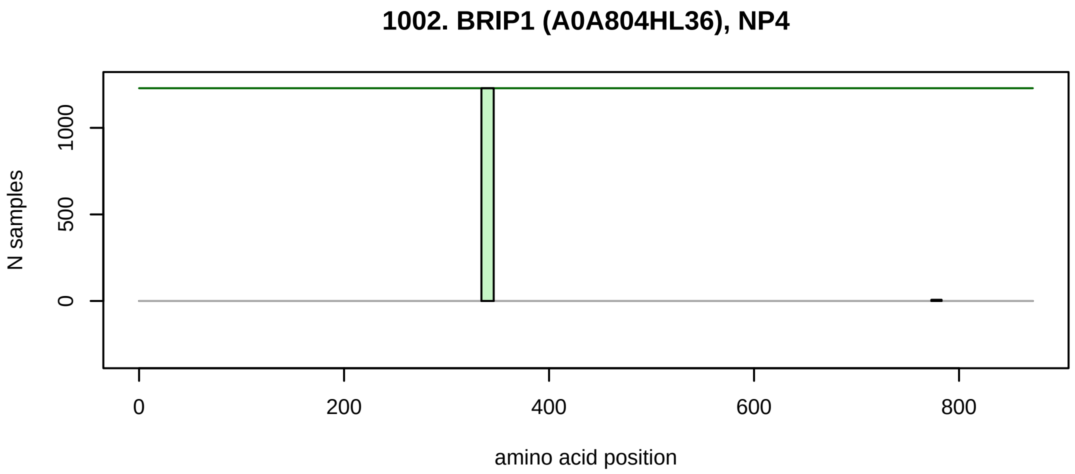
1002. BRIP1 (A0A804HL36), NP4
N samples
0
500
1000
0
200
400
600
800
amino acid position

1003. ANTXR1 (Q9H6X2), NP1

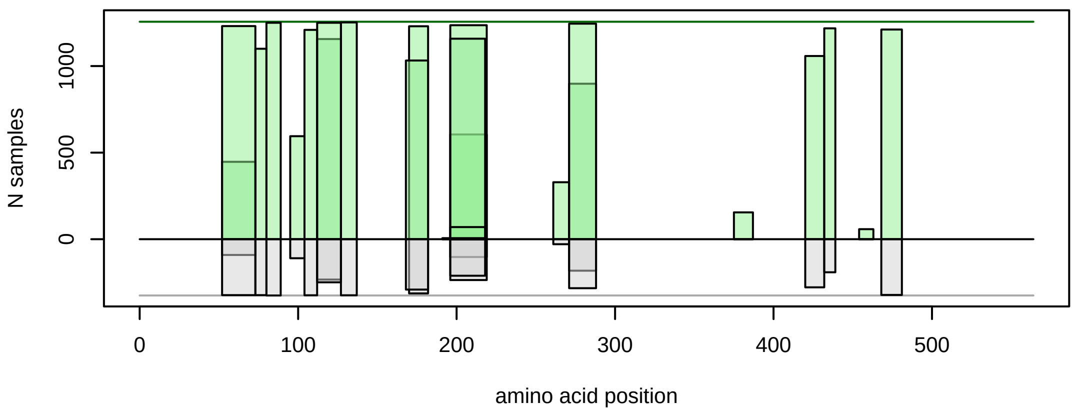

1004. LTBP1 (C9JD84), NP5

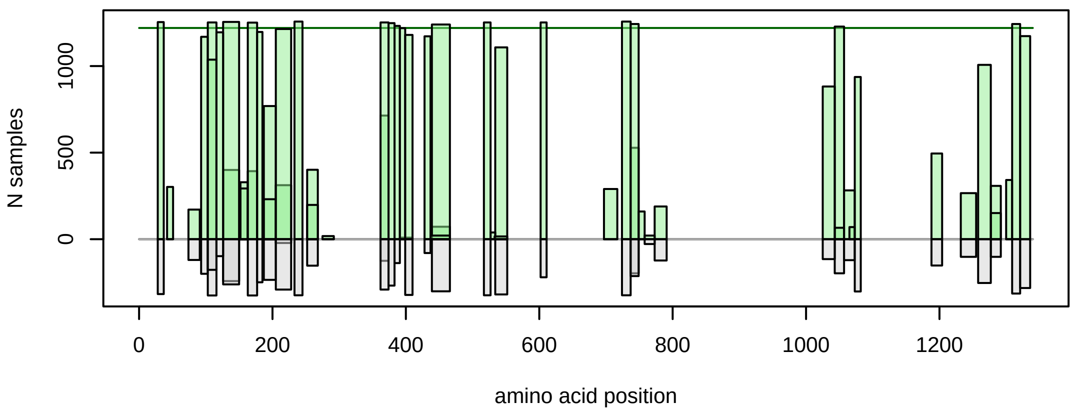

1005. AHCY (P23526), NP5

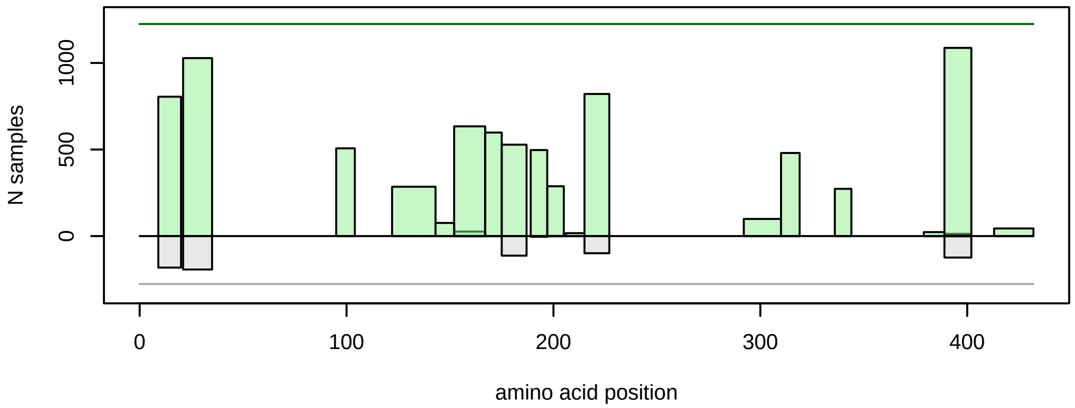

1006. ARHGDIB (P52566), NP2

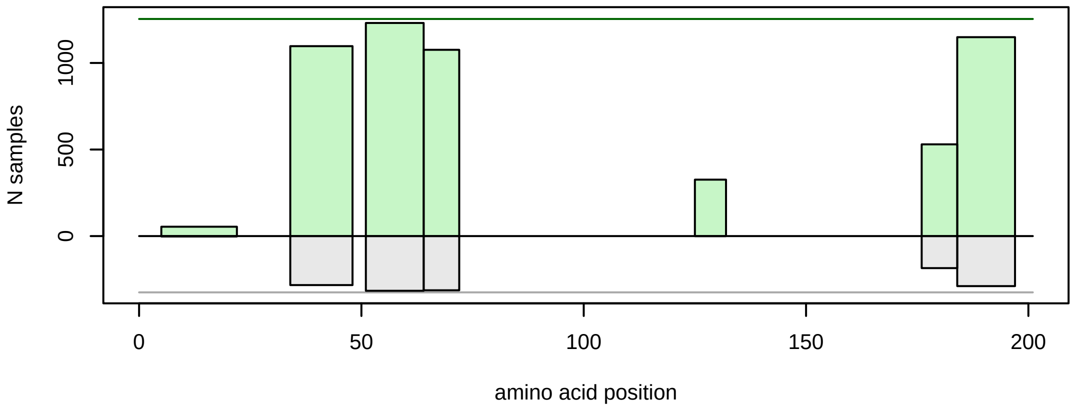

1007. EMILIN2 (Q9BXX0), NP3

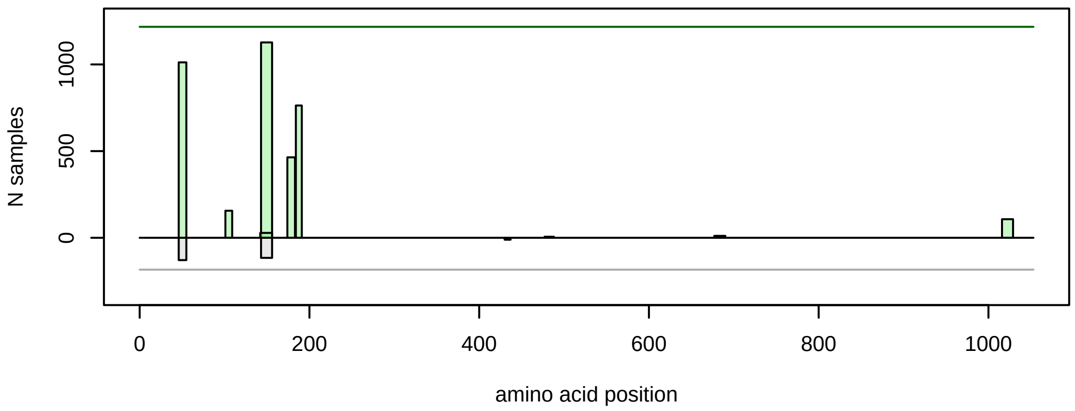

1008. MYL12A (J3QRS3;O14950;P19105), NP1

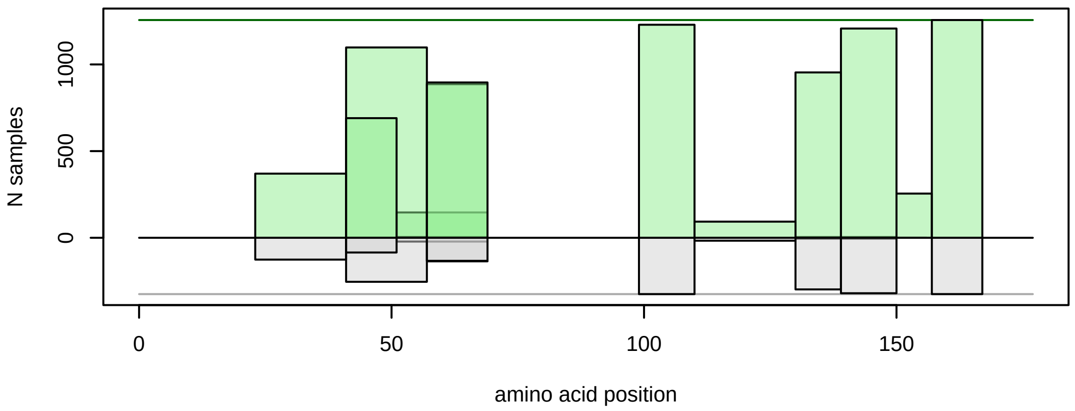

1009. LYN (P07948−2), NP1

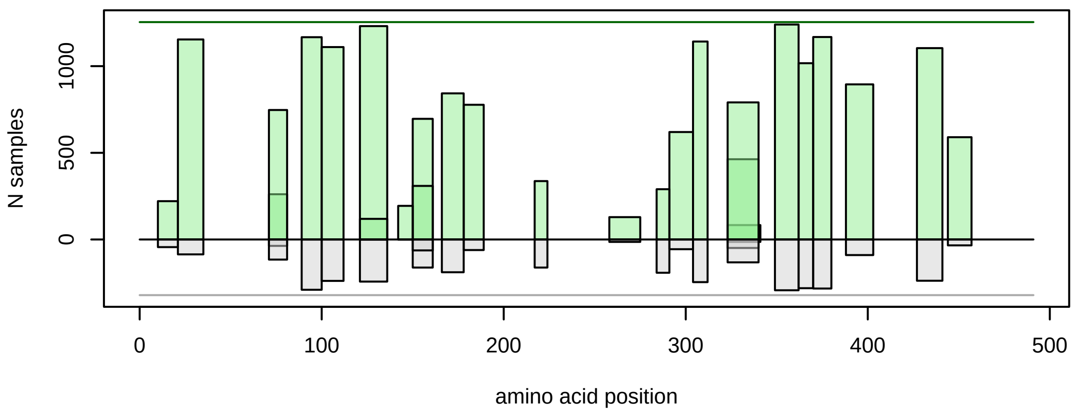

1010. ANXA2 (H0YMW4), NP4

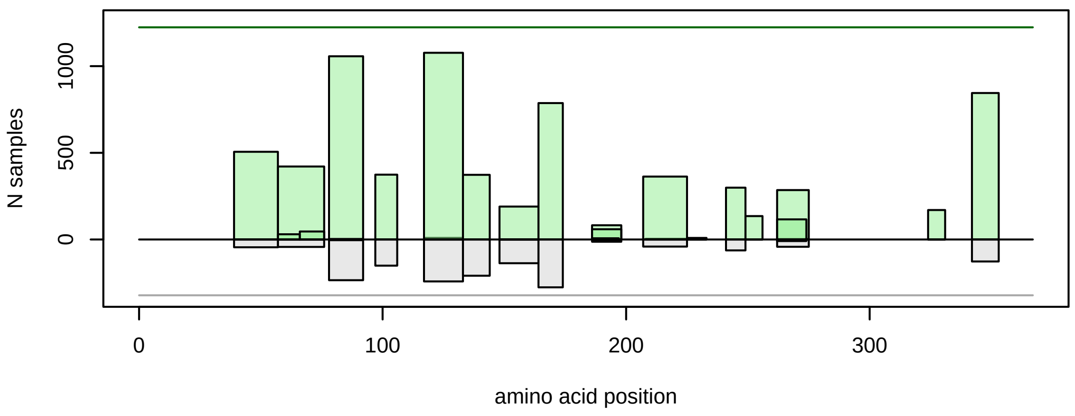

1011. PTP4A1 (Q93096), NP3

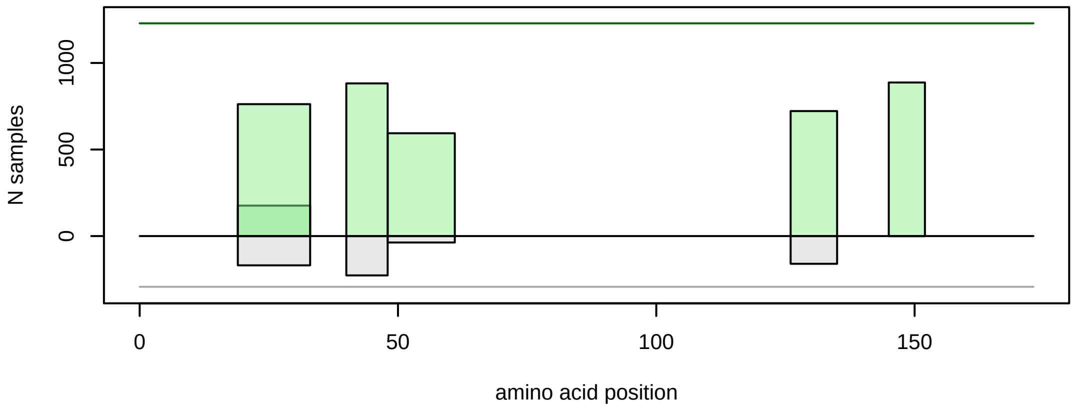

1012. SLC4A1 (P02730), NP1

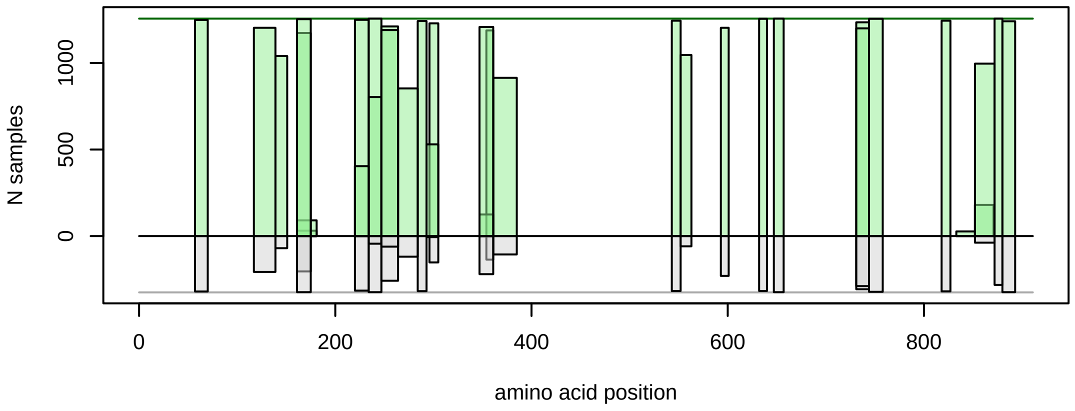

1013. LOX (P28300), NP3

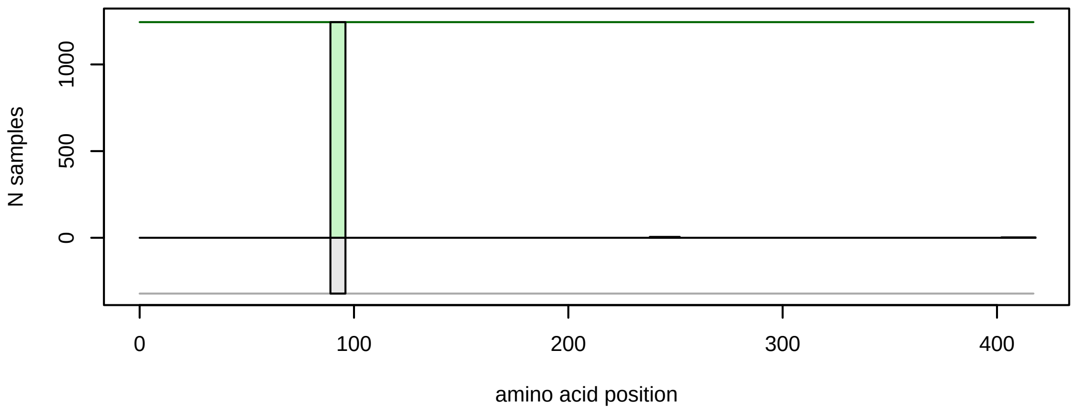

1014. LOXL1 (Q08397), NP5

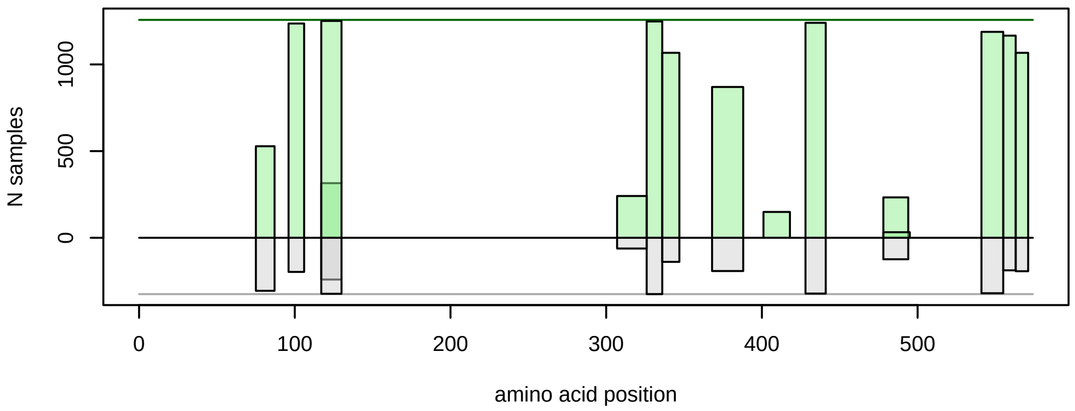

1015. BGN (P21810), NP2

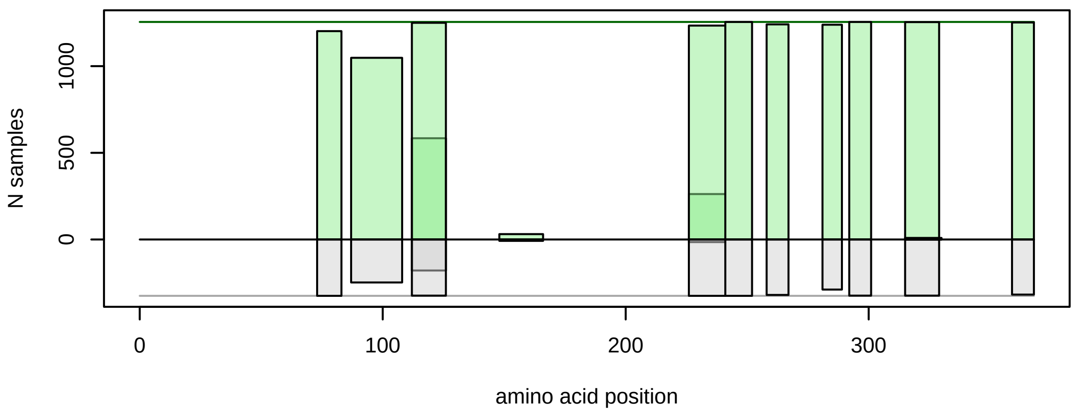

1016. MCTS1 (Q9ULC4;Q9ULC4−3), NP3

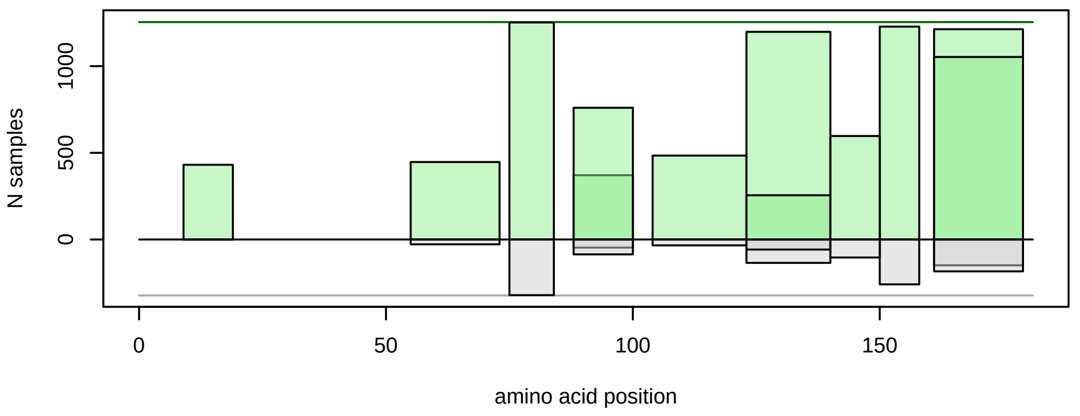

1017. REG3A (Q06141), NP3

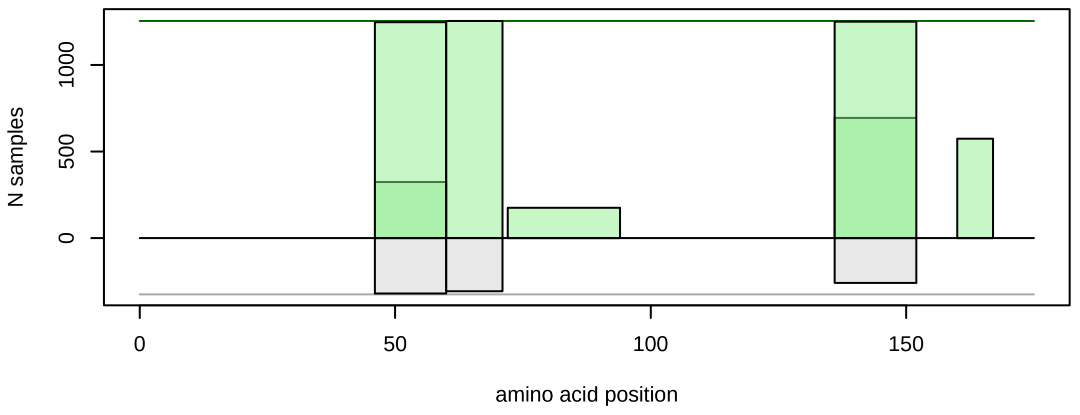

1018. FGA (P02671−2), NP3

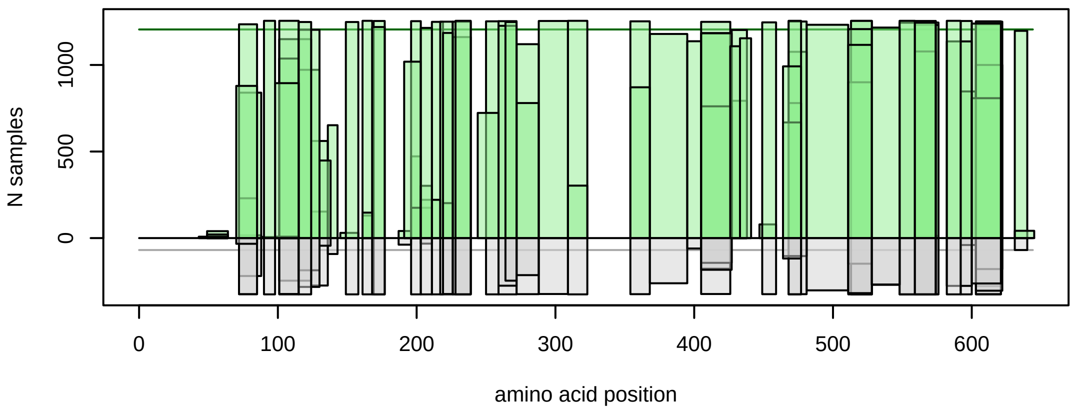

1019. PDGFB (P01127;P01127−2), NP2

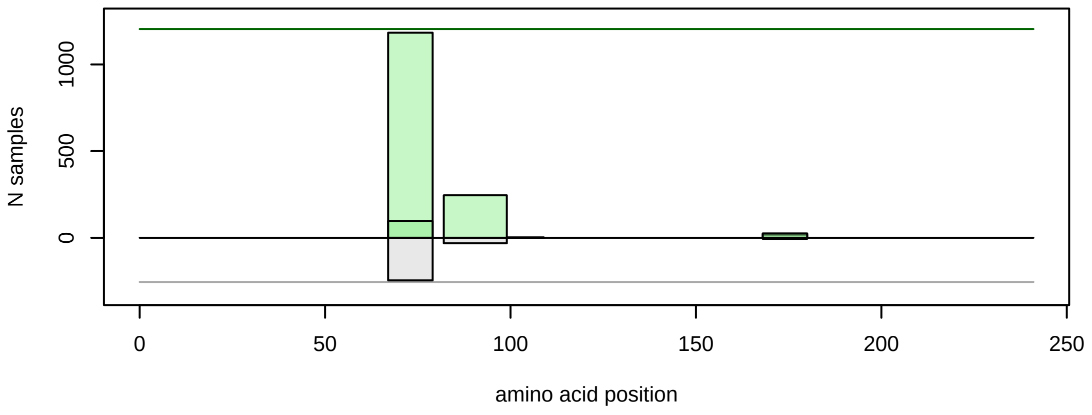

1020. VCL (P18206), NP1

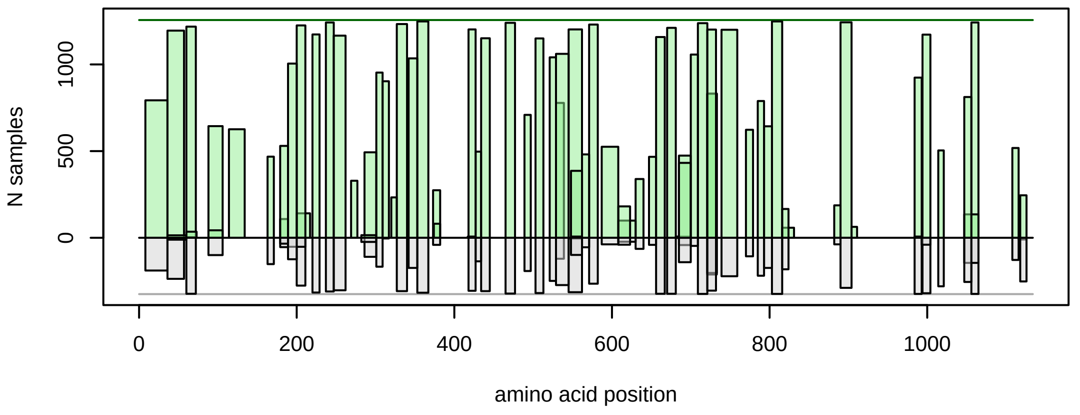

1021. NOTUM (Q6P988), NP3

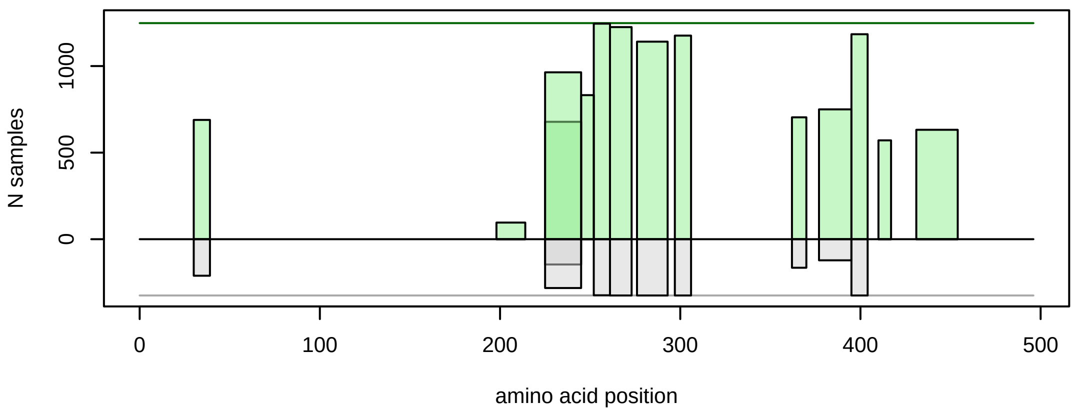

1022. A2M (P01023), NP2

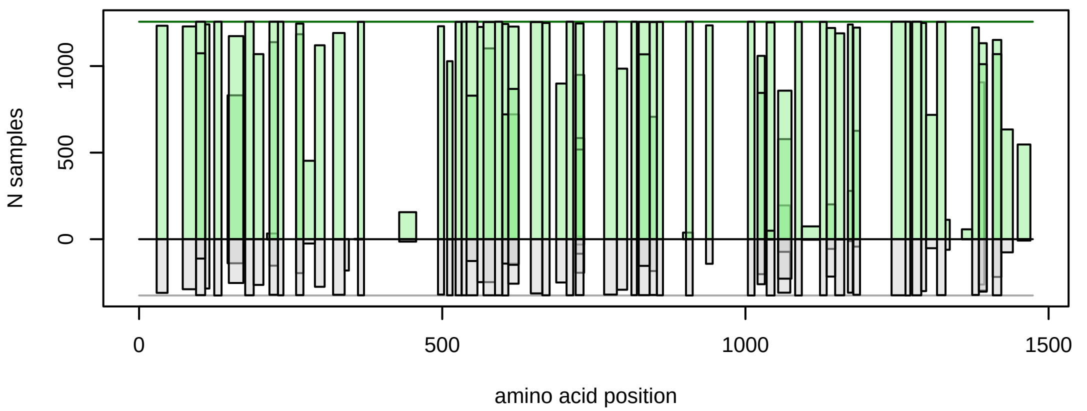

1023. EFHD2 (Q96C19), NP2

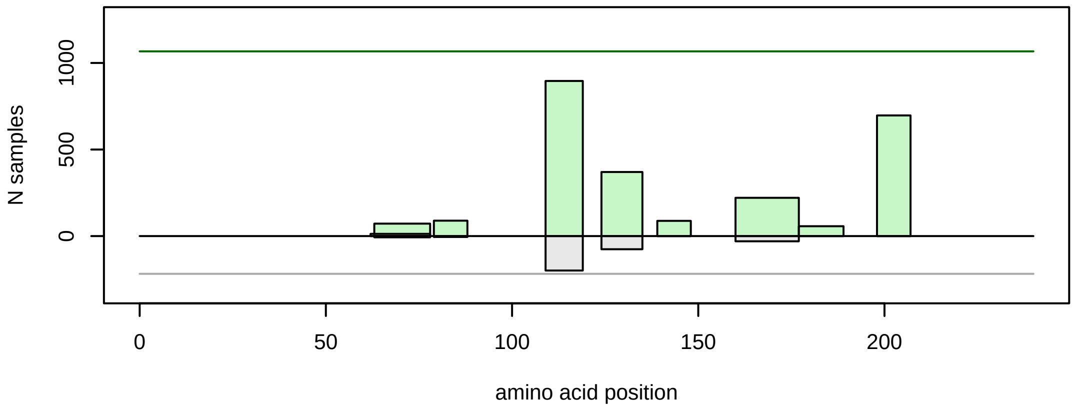

1024. UBE2I (B0QYN7), NP3

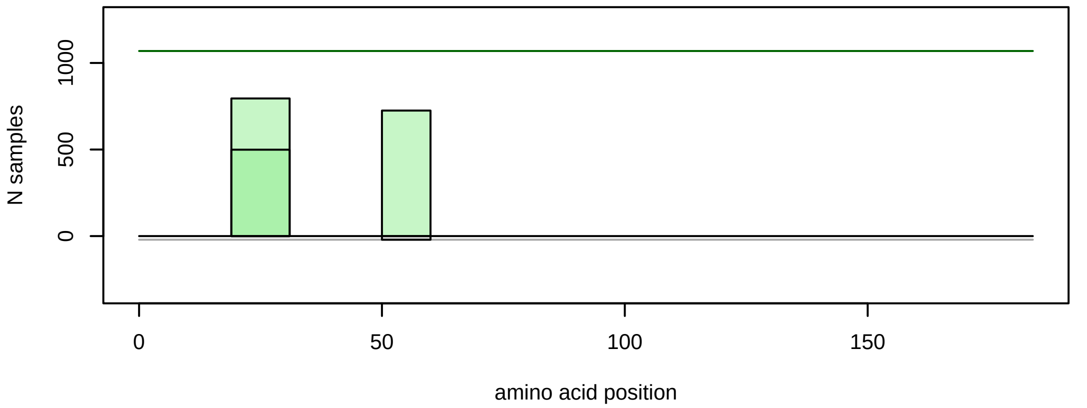

1025. TIMP1 (P01033), NP3

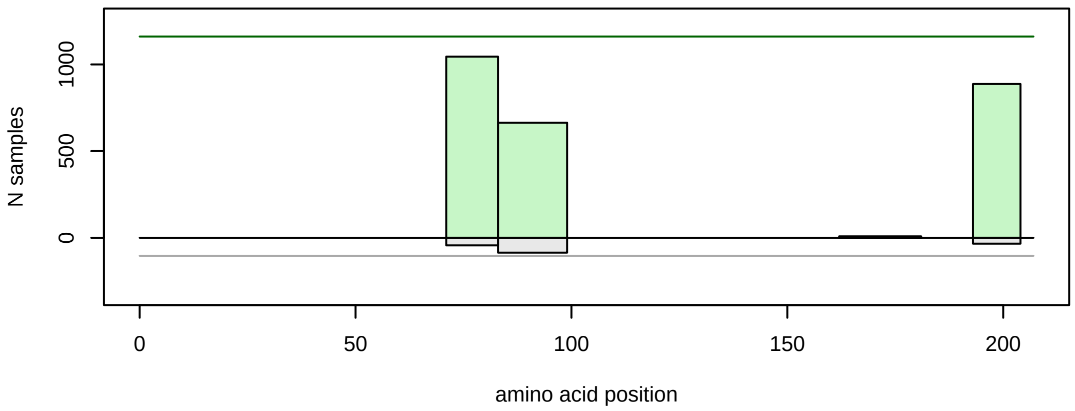

1026. CLSTN1 (O94985−2), NP5

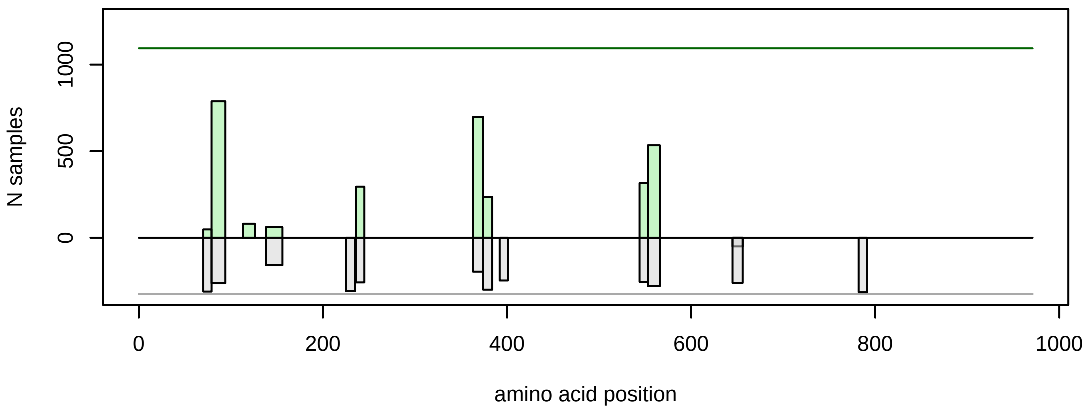

1027. SFRP2 (Q96HF1), NP4

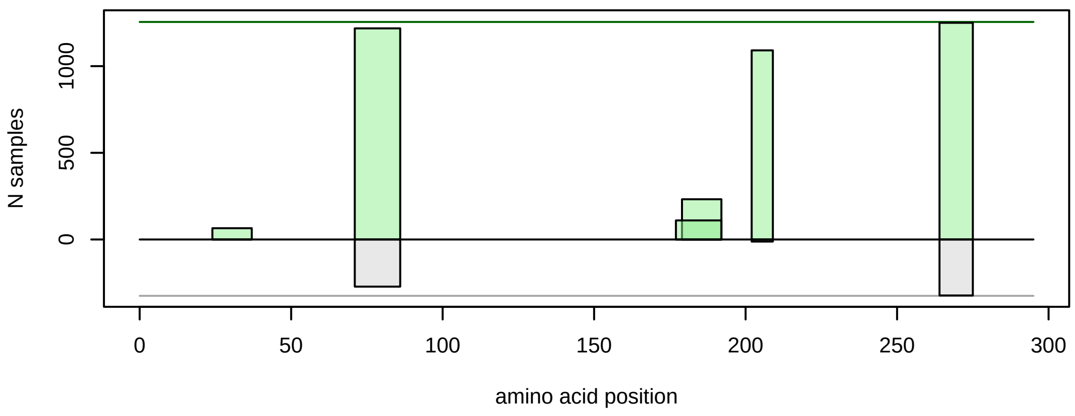

1028. ZNF594 (Q96JF6), NP3

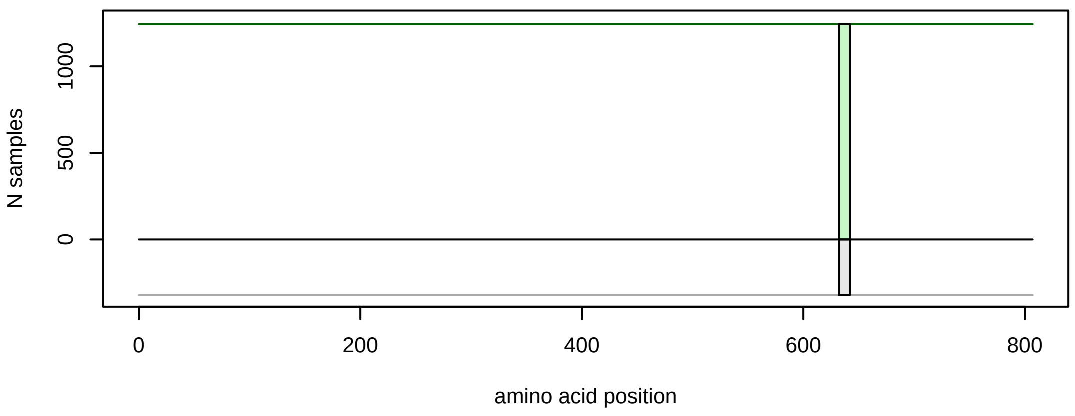

1029. FIS1 (Q9Y3D6), NP4

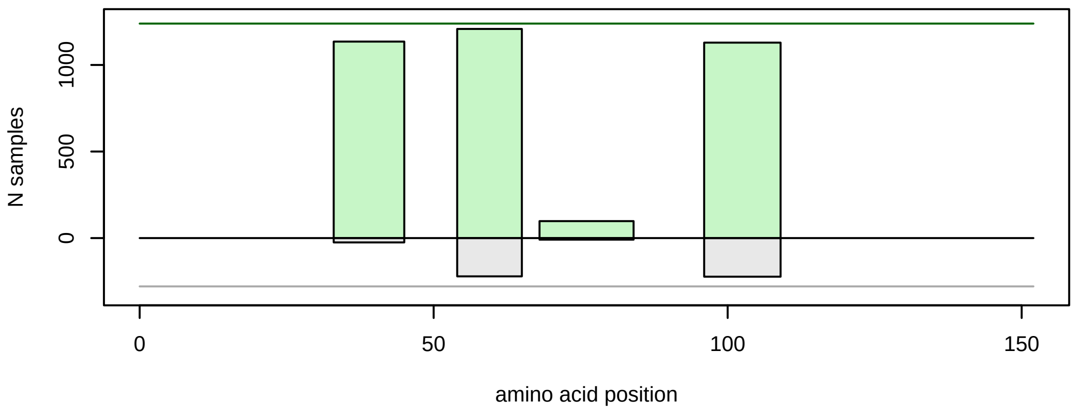

1030. SCG2 (P13521), NP2

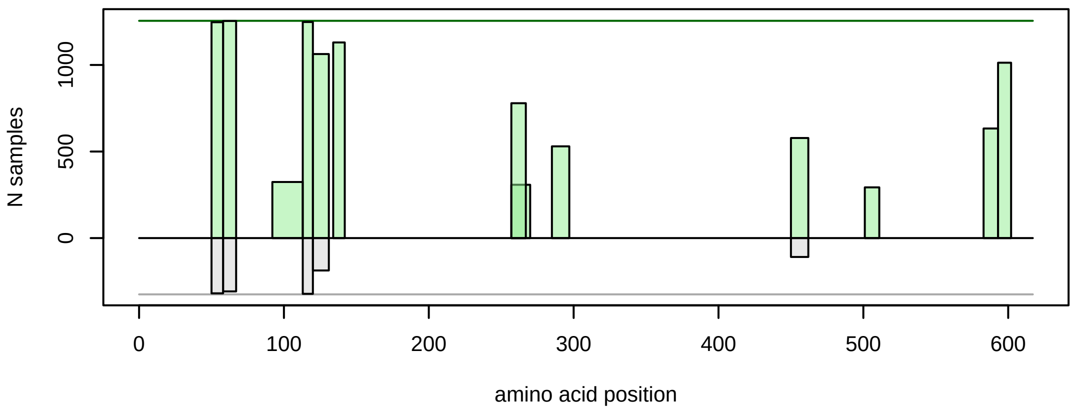

1031. ITM2B (Q9Y287), NP2

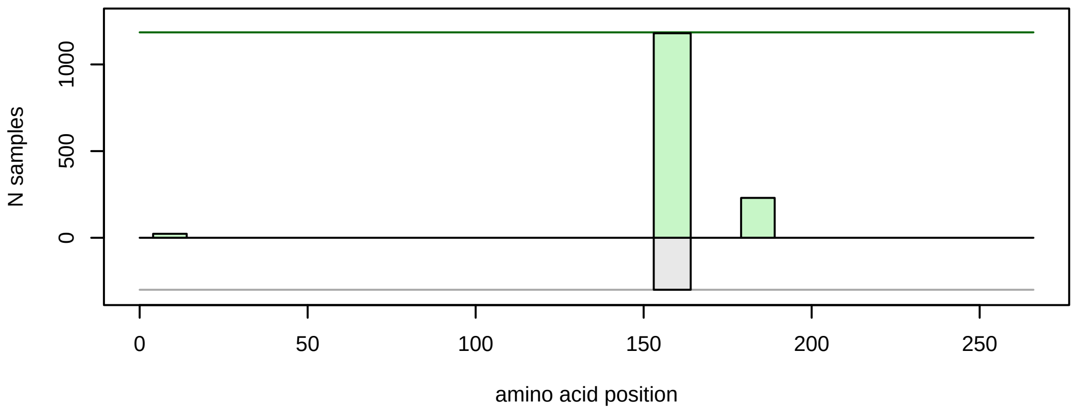

1032. IGKV4−1 (P06312), NP4

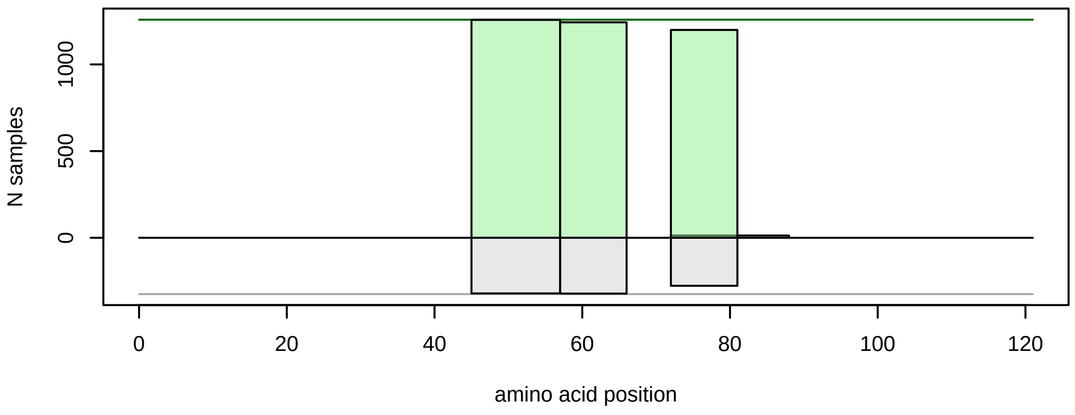

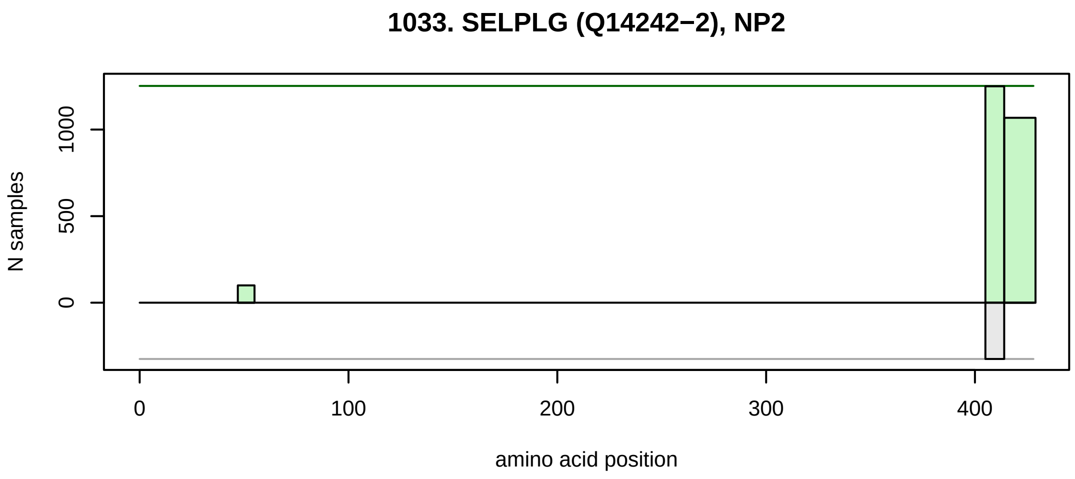
1033. SELPLG (Q14242−2), NP2
N samples
0
500
1000
0
100
200
300
400
amino acid position

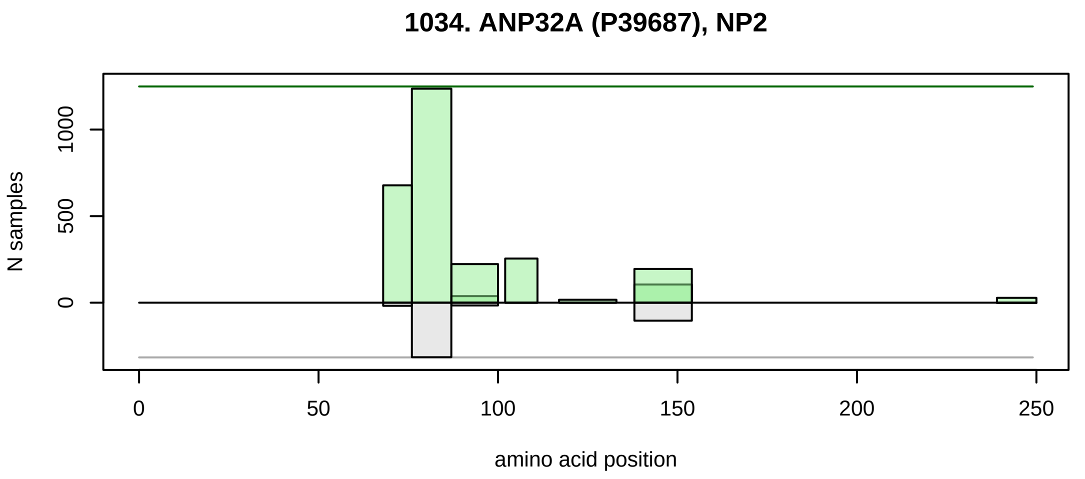
1034. ANP32A (P39687), NP2
N samples
0
500
1000
0
50
100
150
200
250
amino acid position

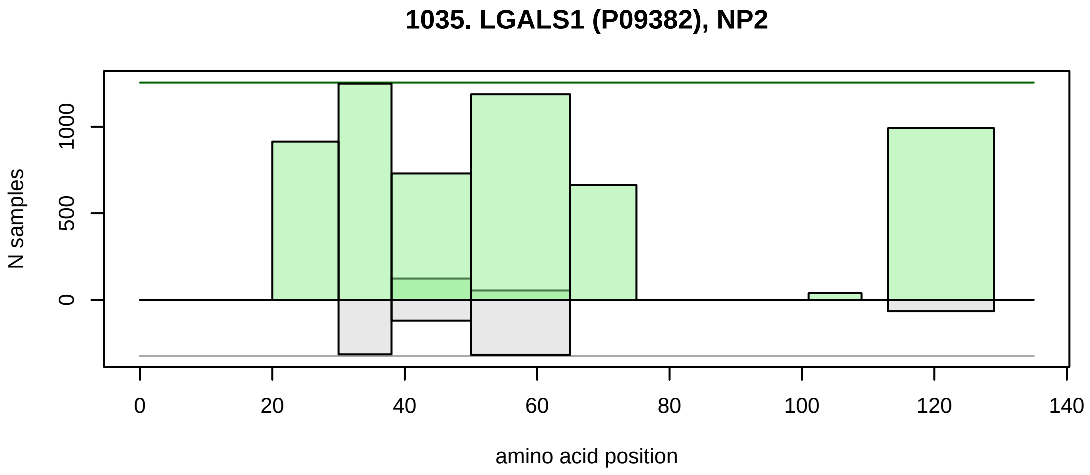
1035. LGALS1 (P09382), NP2
N samples
0
500
1000
0
20
40
60
80
100
120
140
amino acid position

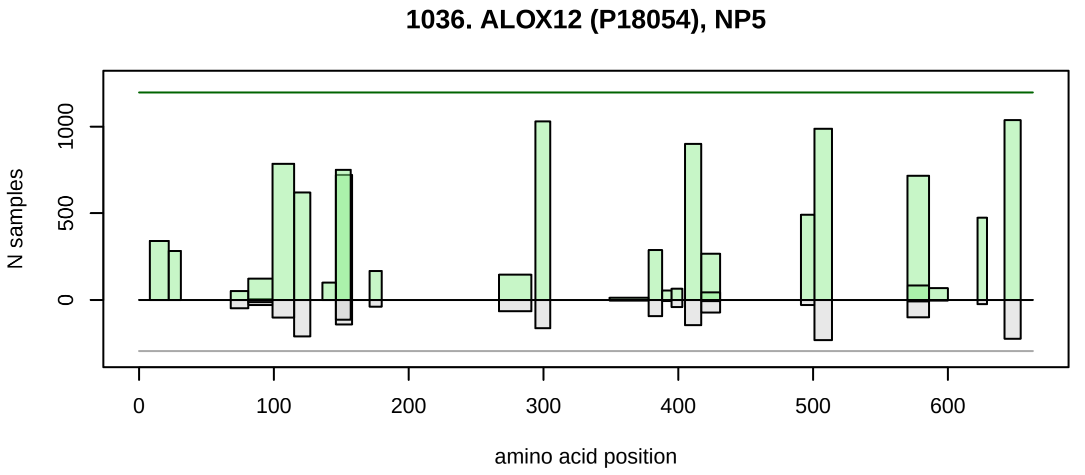
1036. ALOX12 (P18054), NP5
N samples
0
500
1000
0
100
200
300
400
500
600
amino acid position

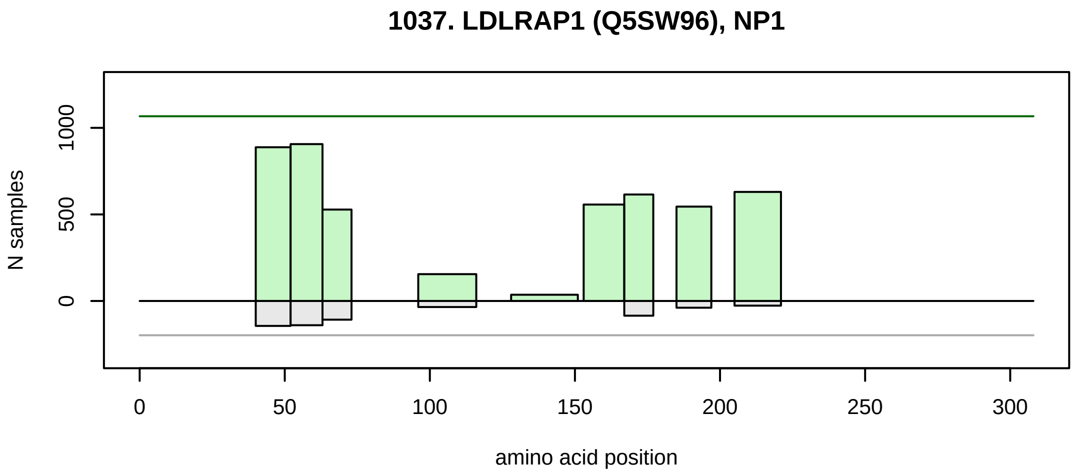
1037. LDLRAP1 (Q5SW96), NP1
N samples
0
500
1000
0
50
100
150
200
250
300
amino acid position

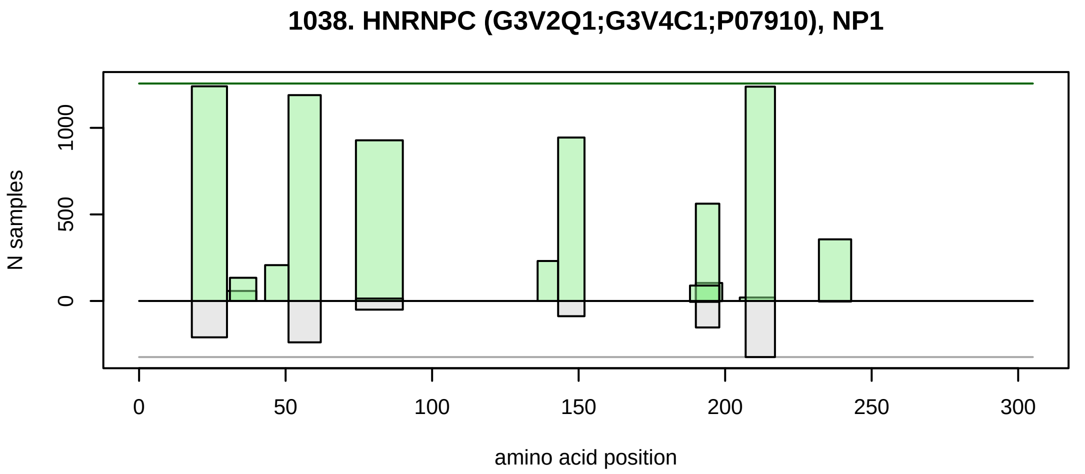
1038. HNRNPC (G3V2Q1;G3V4C1;P07910), NP1
N samples
0
500
1000
0
50
100
150
200
250
300
amino acid position

1039. TCP1 (P17987), NP4

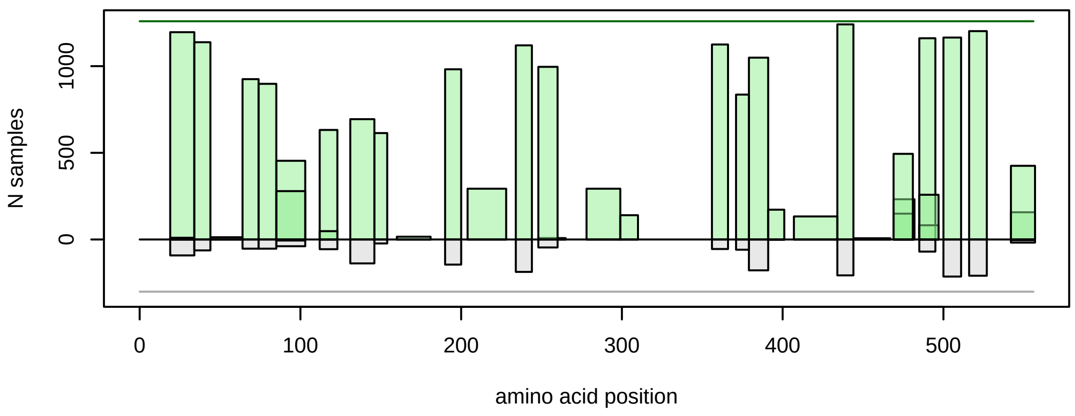

1040. IFITM3 (E9PS44;Q01628), NP2

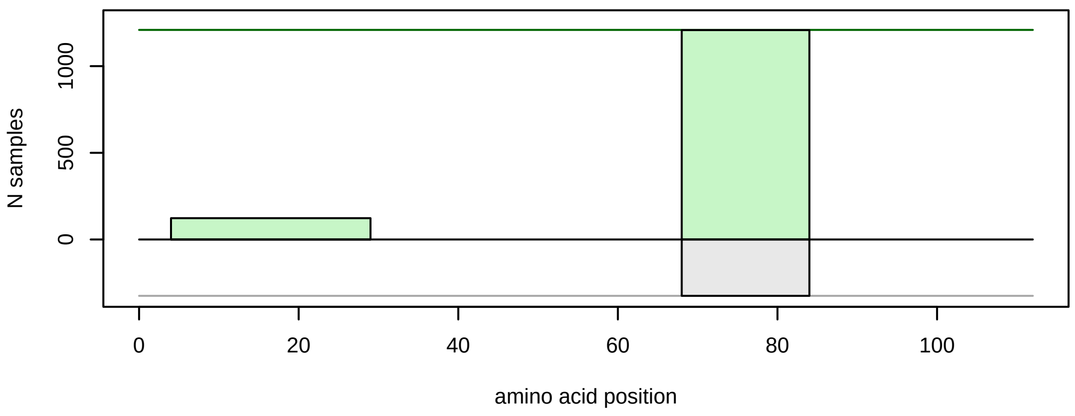

1041. ECM1 (Q16610−4), NP2

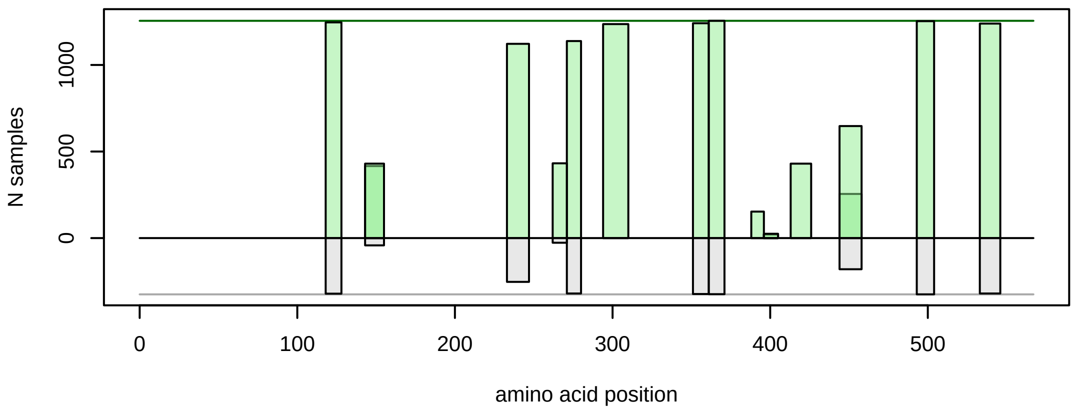

1042. CFI (E7ETH0;G3XAM2;P05156), NP3

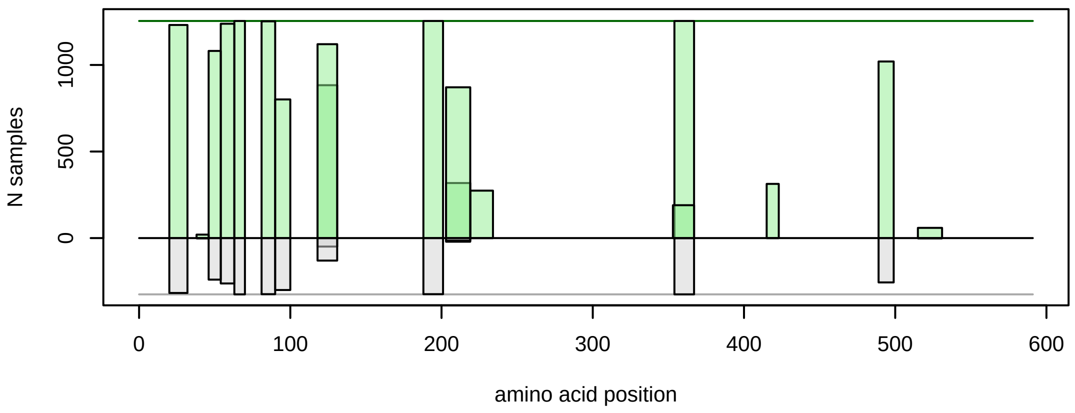

1043. MMP25 (Q9NPA2), NP3

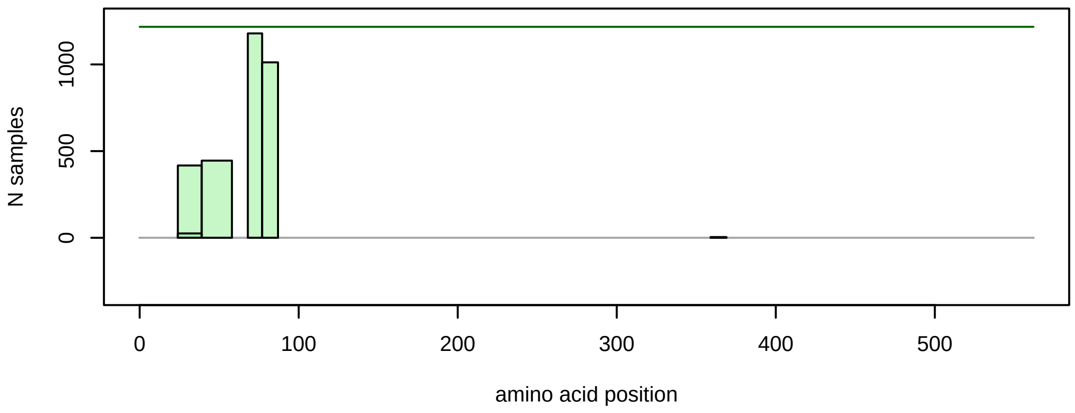

1044. RPLP1 (P05386), NP2

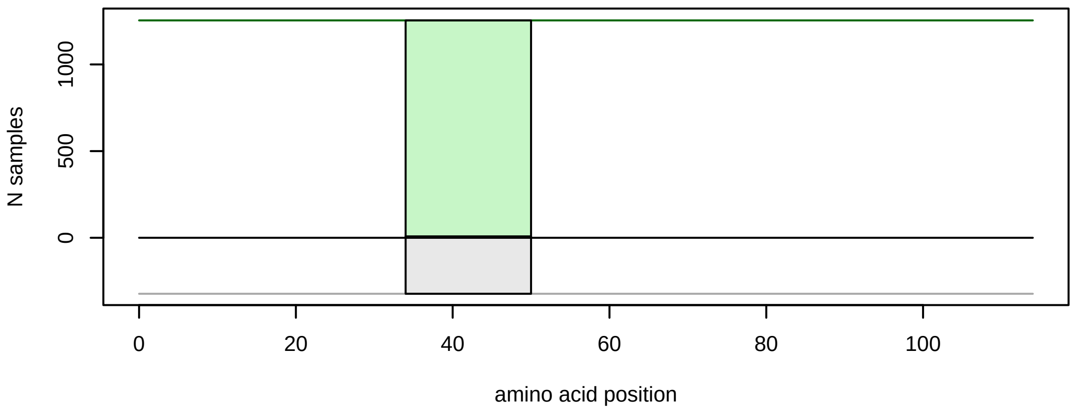

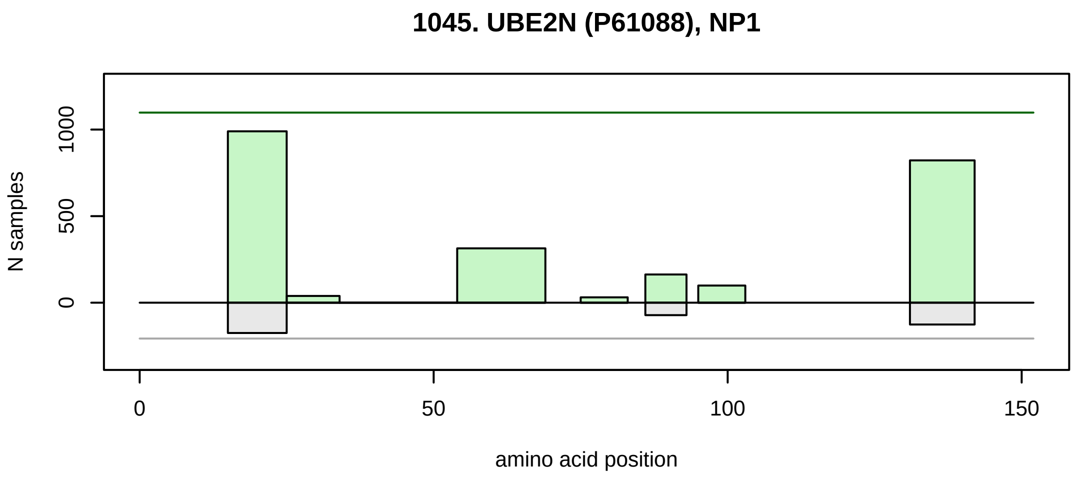
1045. UBE2N (P61088), NP1
N samples
0
500
1000
0
50
100
150
amino acid position

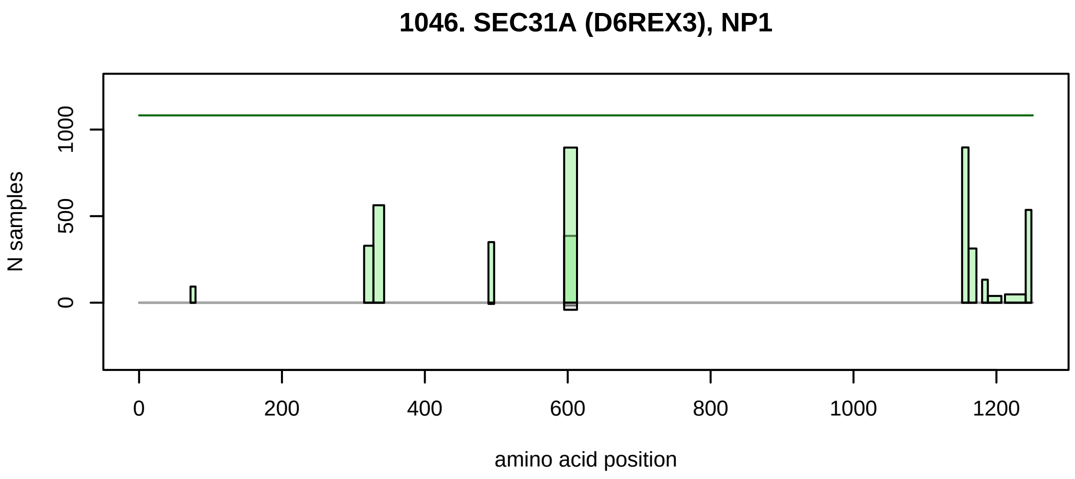
1046. SEC31A (D6REX3), NP1
N samples
0
500
1000
0
200
400
600
800
1000
1200
amino acid position

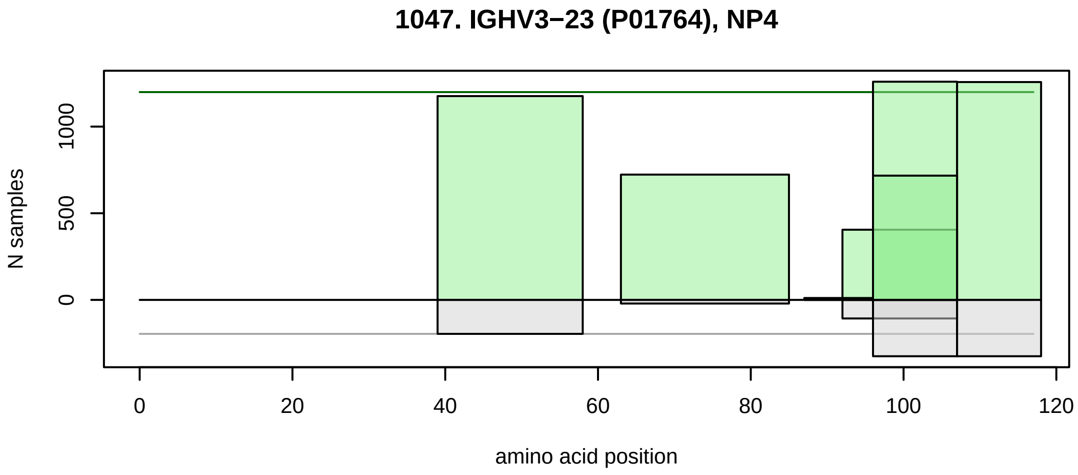
1047. IGHV3−23 (P01764), NP4
N samples
0
500
1000
0
20
40
60
80
100
120
amino acid position

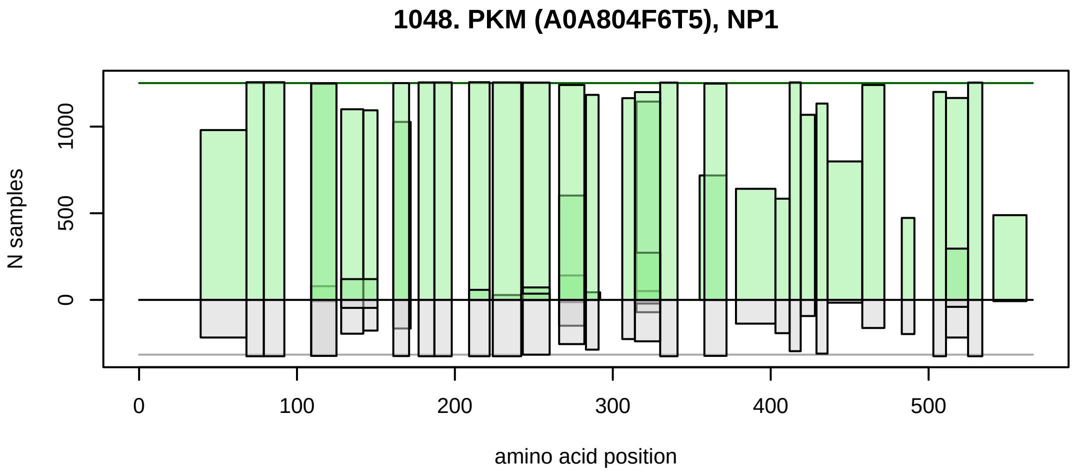
1048. PKM (A0A804F6T5), NP1
N samples
0
500
1000
0
100
200
300
400
500
amino acid position

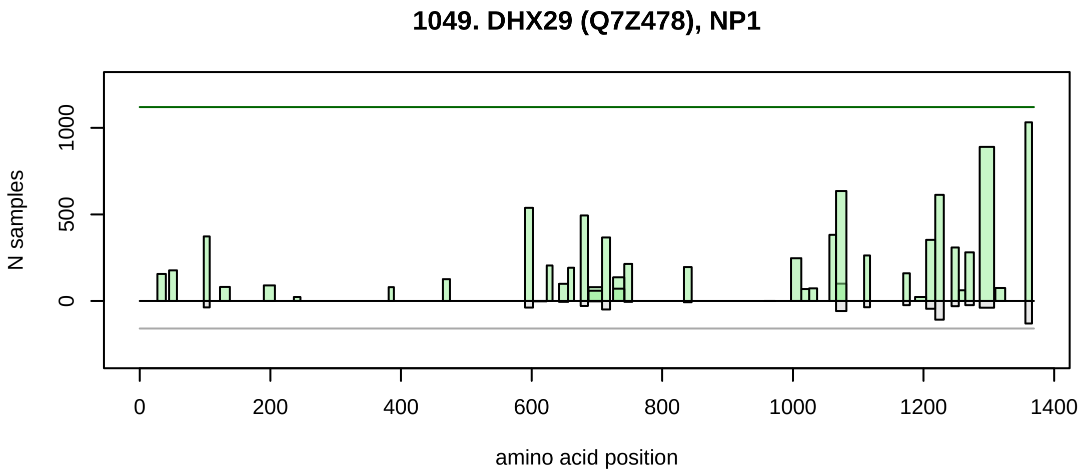
1049. DHX29 (Q7Z478), NP1
N samples
0
500
1000
0
200
400
600
800
1000
1200
1400
amino acid position

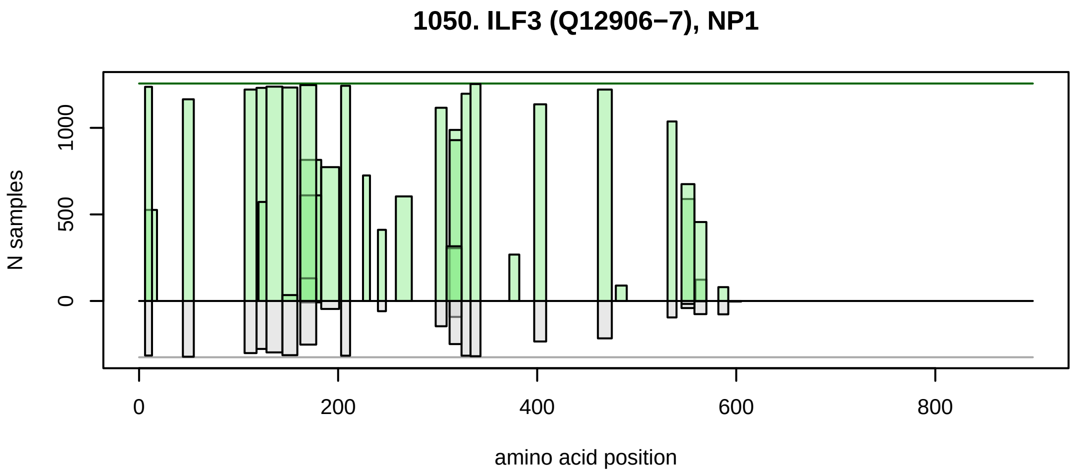
1050. ILF3 (Q12906−7), NP1
N samples
0
500
1000
0
200
400
600
800
amino acid position

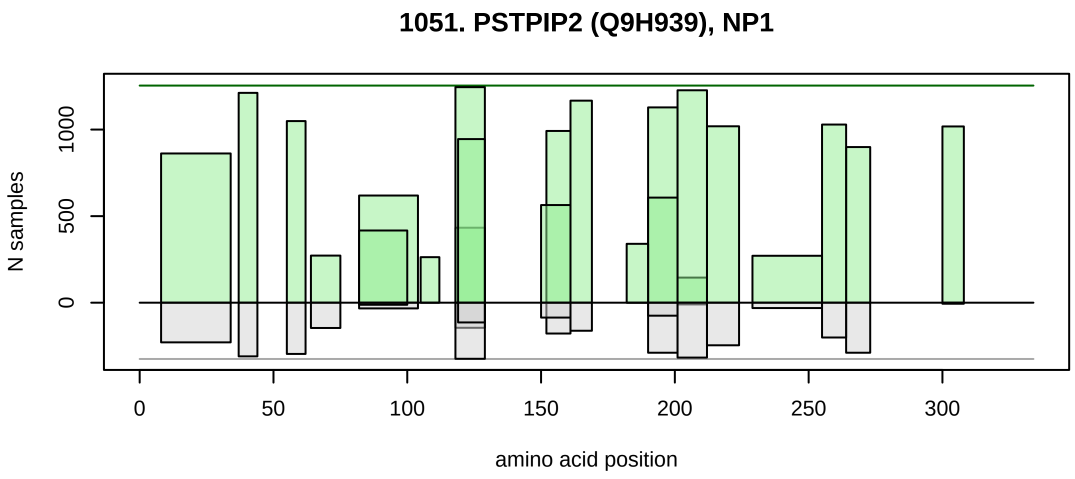
1051. PSTPIP2 (Q9H939), NP1
N samples
0
500
1000
0
50
100
150
200
250
300
amino acid position

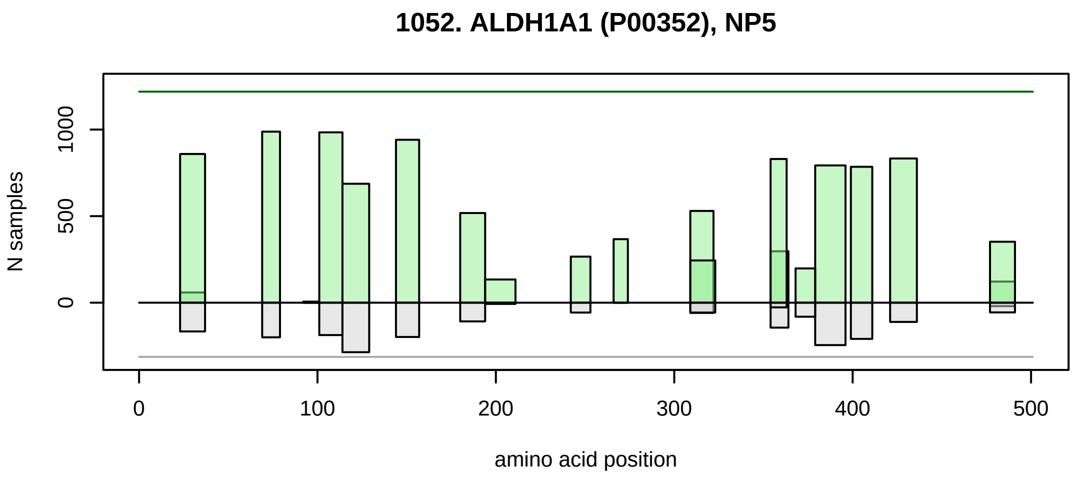
1052. ALDH1A1 (P00352), NP5
N samples
0
500
1000
0
100
200
300
400
500
amino acid position

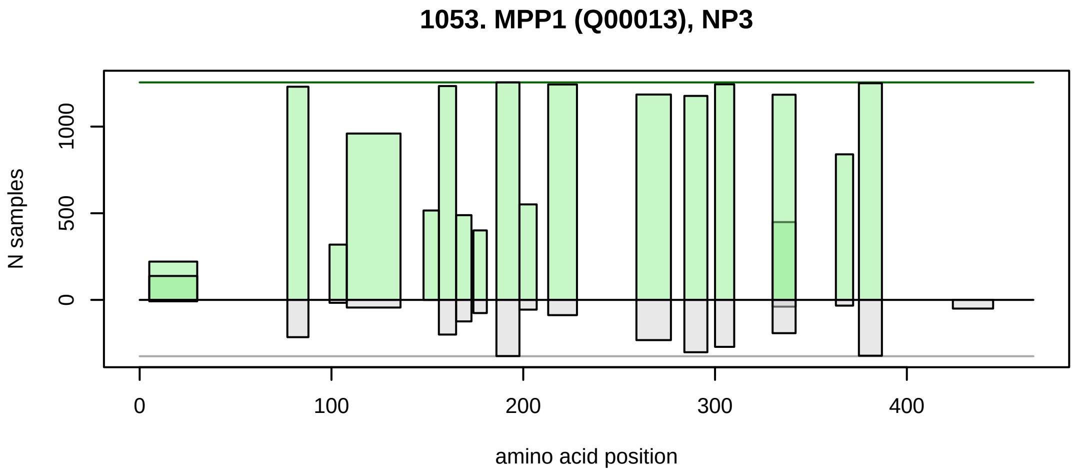
1053. MPP1 (Q00013), NP3
N samples
0
500
1000
0
100
200
300
400
amino acid position

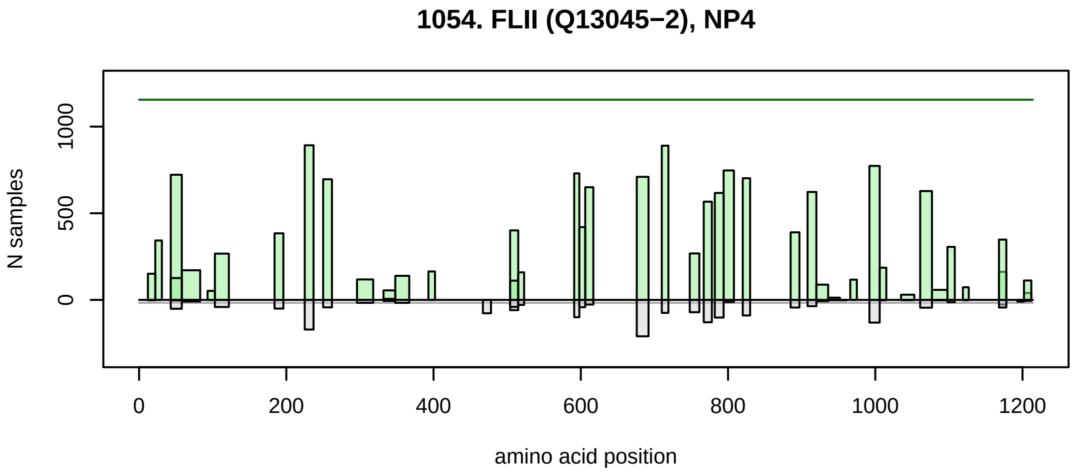
1054. FLII (Q13045−2), NP4
N samples
0
500
1000
0
200
400
600
800
1000
1200
amino acid position

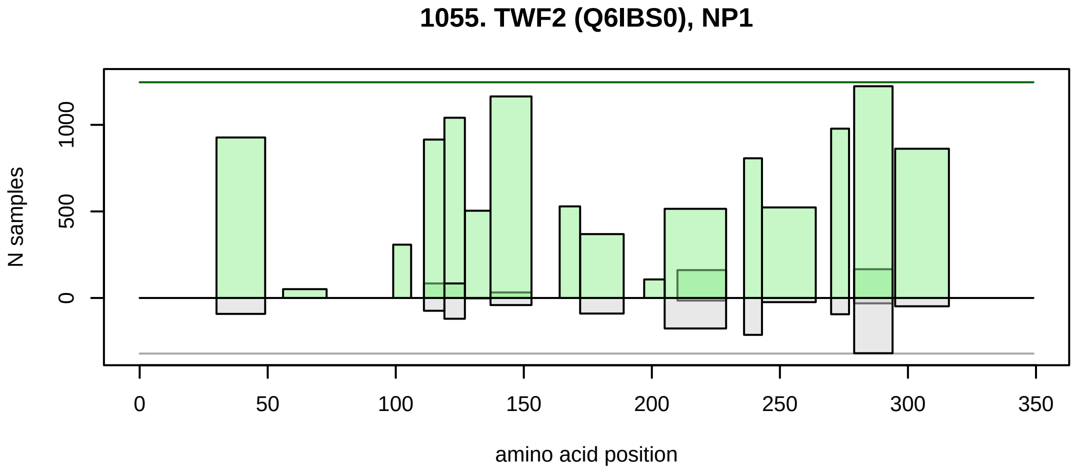
1055. TWF2 (Q6IBS0), NP1
N samples
0
500
1000
0
50
100
150
200
250
300
350
amino acid position

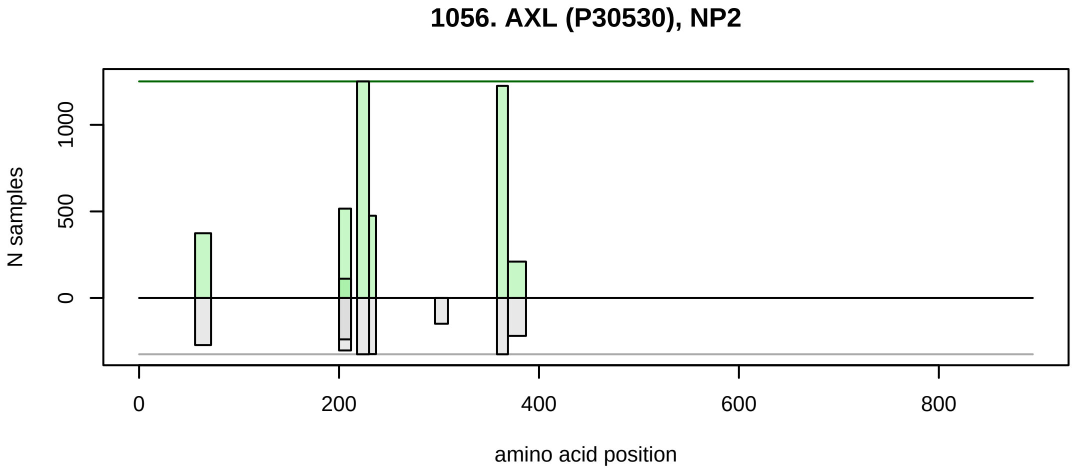
1056. AXL (P30530), NP2
N samples
0
500
1000
0
200
400
600
800
amino acid position

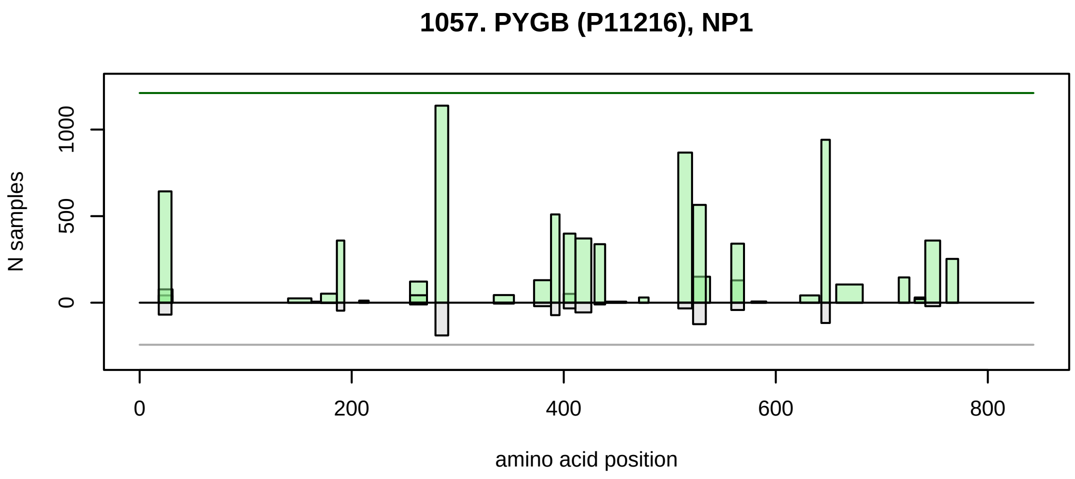
1057. PYGB (P11216), NP1
N samples
0
500
1000
0
200
400
600
800
amino acid position

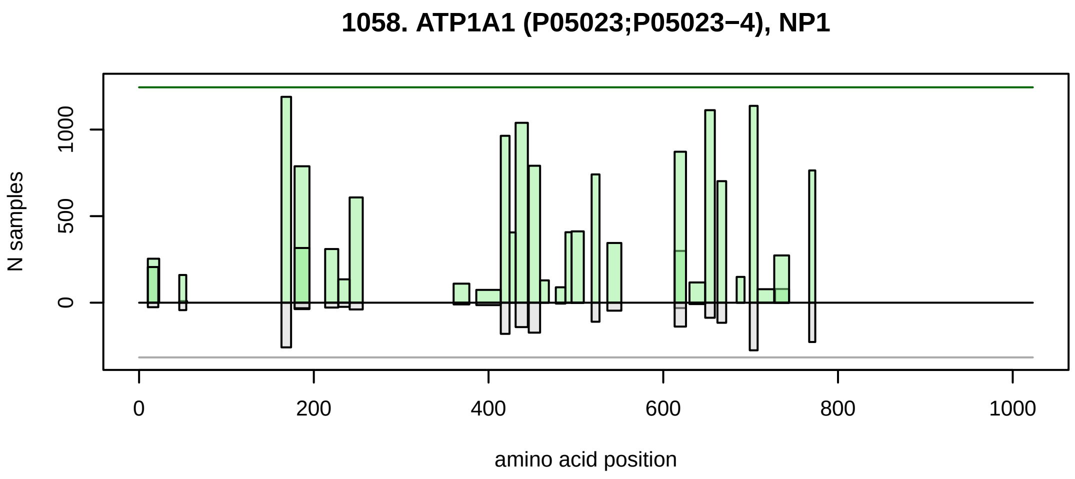
1058. ATP1A1 (P05023;P05023−4), NP1
N samples
0
500
1000
0
200
400
600
800
1000
amino acid position

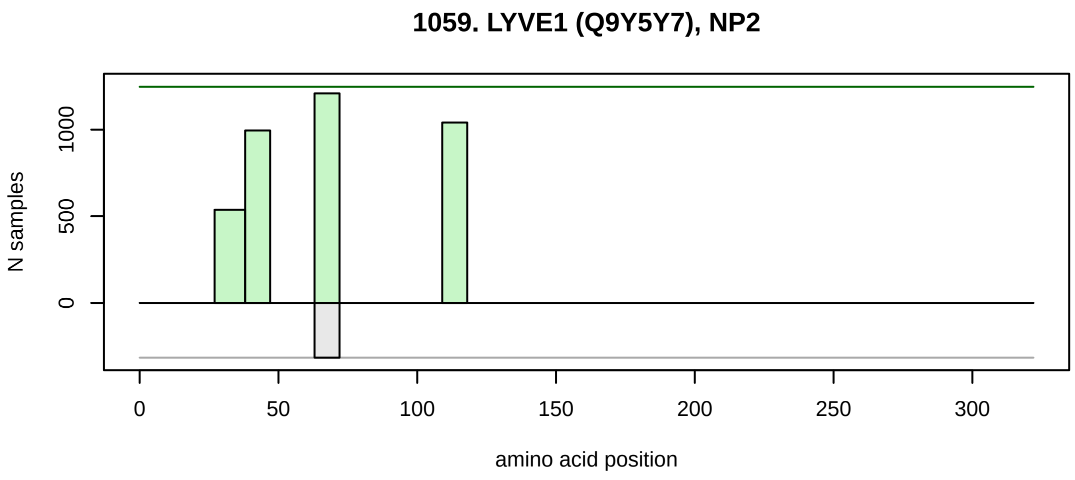
1059. LYVE1 (Q9Y5Y7), NP2
N samples
0
500
1000
0
50
100
150
200
250
300
amino acid position

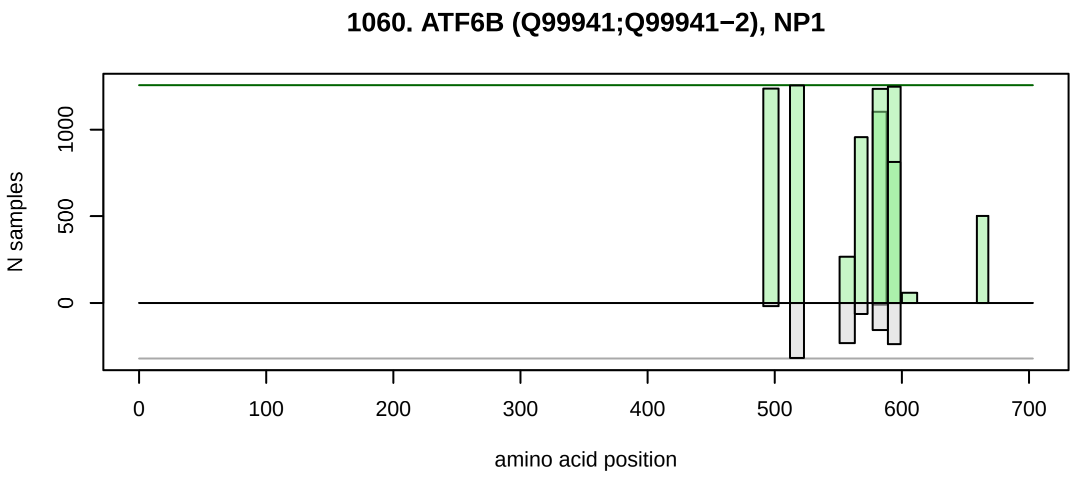
1060. ATF6B (Q99941;Q99941−2), NP1
N samples
0
500
1000
0
100
200
300
400
500
600
700
amino acid position

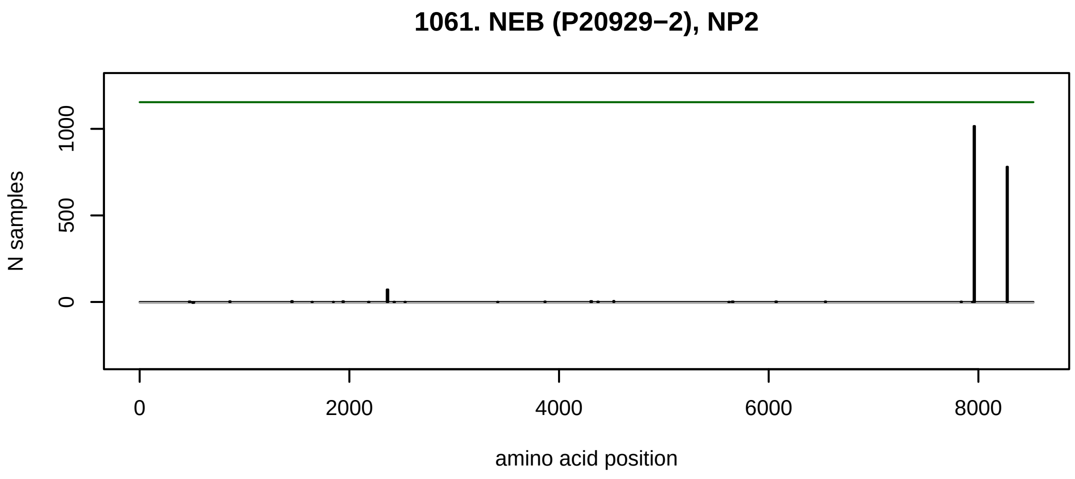
1061. NEB (P20929−2), NP2
N samples
0
500
1000
0
2000
4000
6000
8000
amino acid position

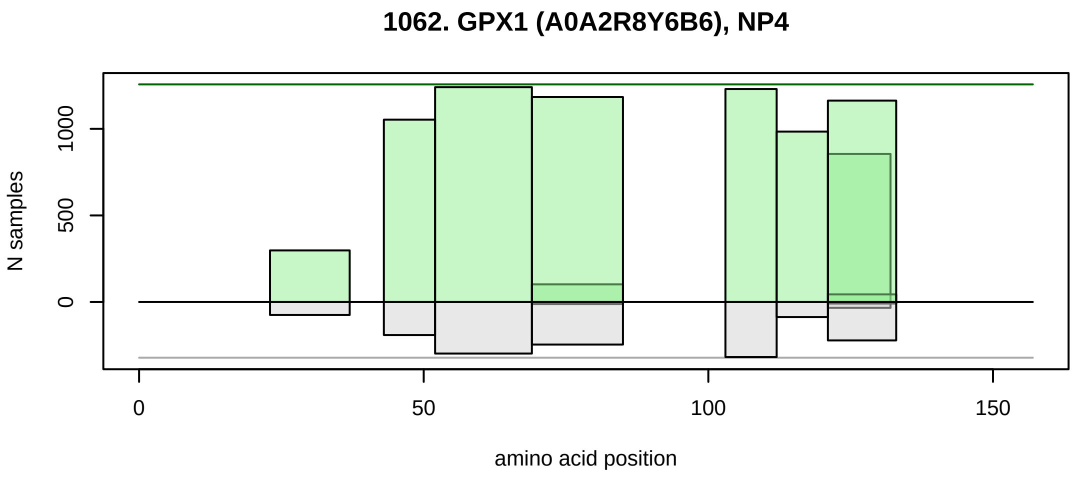
1062. GPX1 (A0A2R8Y6B6), NP4
N samples
0
500
1000
0
50
100
150
amino acid position

1063. CD93 (Q9NPY3), NP2

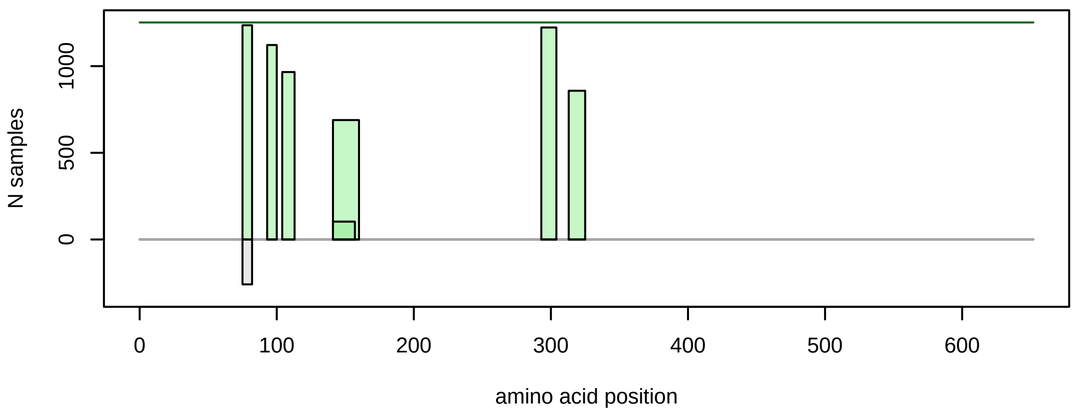

1064. PRKG1 (Q13976−2), NP4

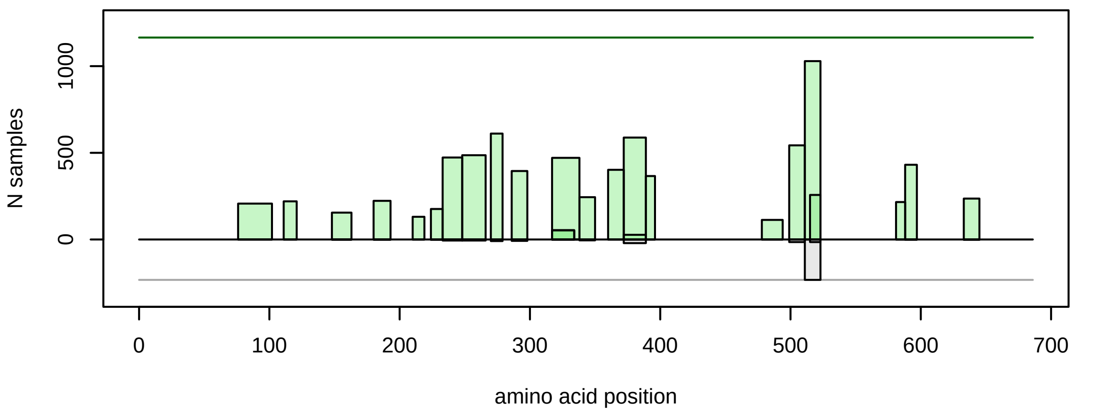

1065. MEPE (Q9NQ76), NP3

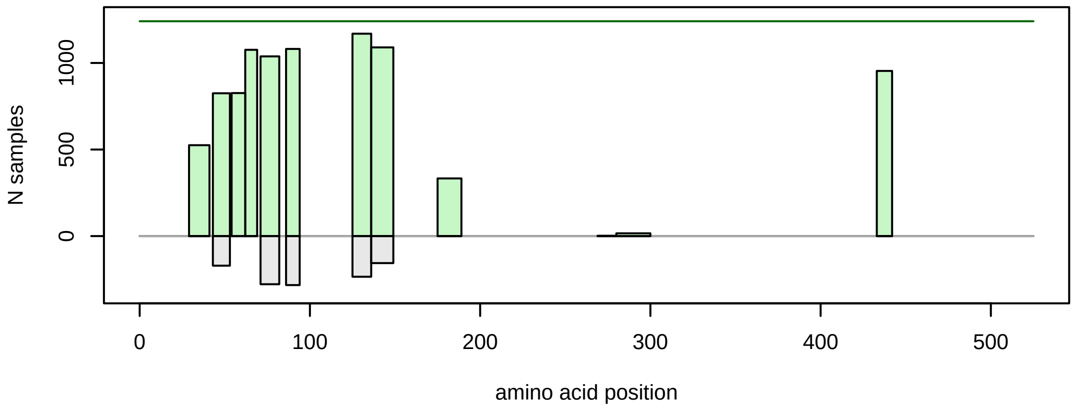

1066. FLNB (O75369−8), NP1

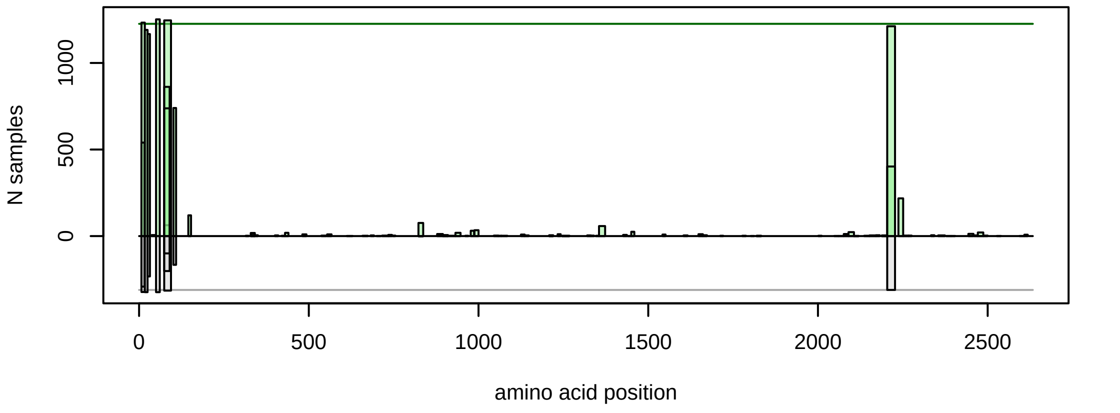

1067. GOT2 (P00505), NP4

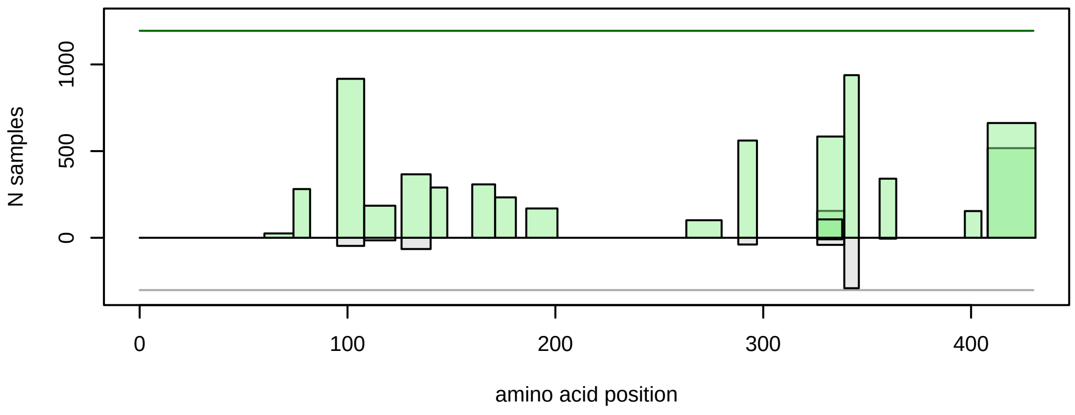

1068. SDF4 (A0A5F9UP49), NP2

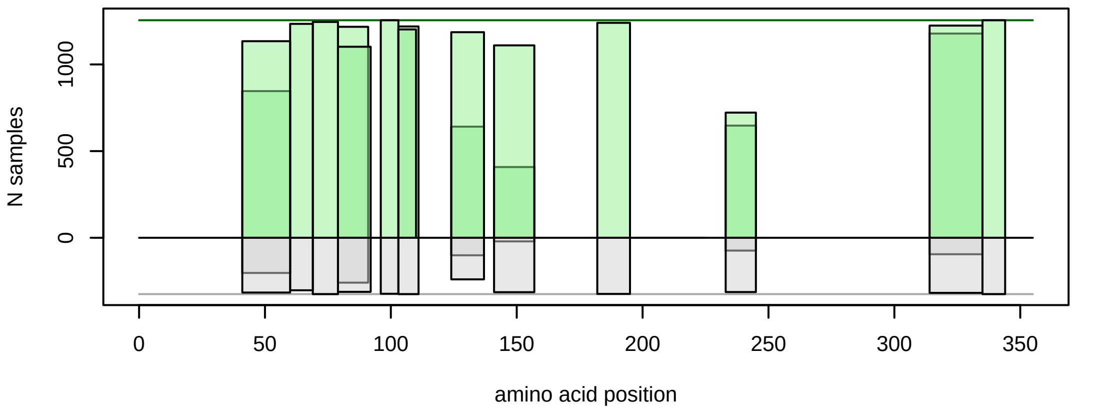

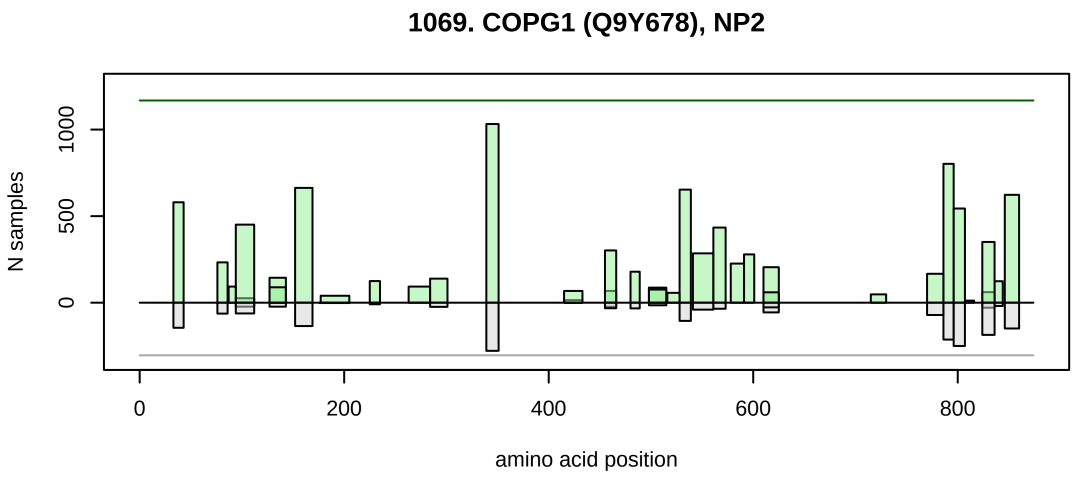
1069. COPG1 (Q9Y678), NP2
N samples
1000
500
0
0
200
400
600
800
amino acid position

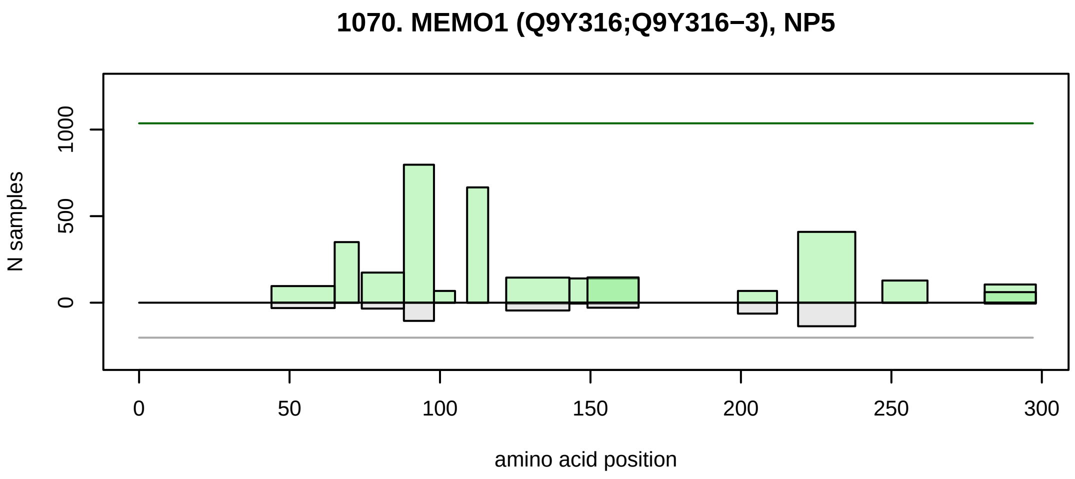
1070. MEMO1 (Q9Y316;Q9Y316−3), NP5
N samples
1000
500
0
0
50
100
150
200
250
300
amino acid position

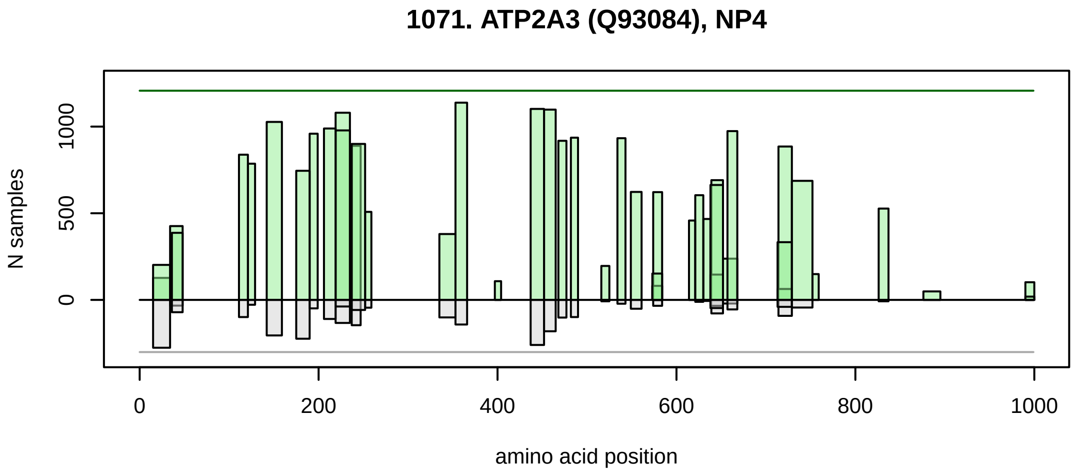
1071. ATP2A3 (Q93084), NP4
N samples
1000
500
0
0
200
400
600
800
1000
amino acid position

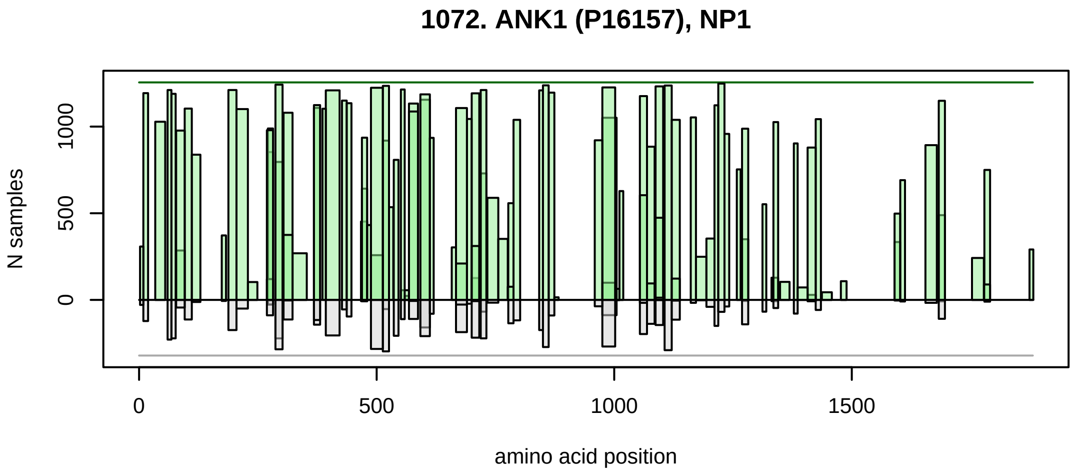
1072. ANK1 (P16157), NP1
N samples
1000
500
0
0
500
1000
1500
amino acid position

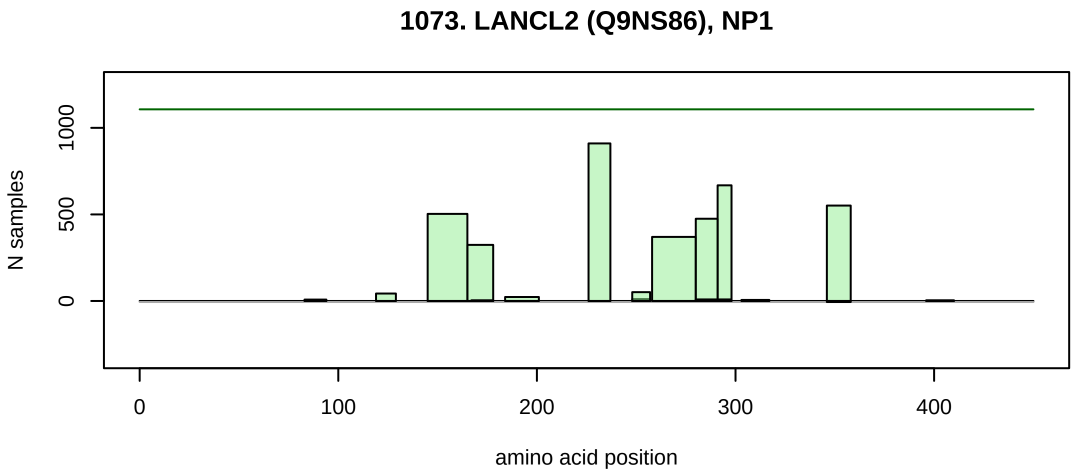
1073. LANCL2 (Q9NS86), NP1
N samples
1000
500
0
0
100
200
300
400
amino acid position

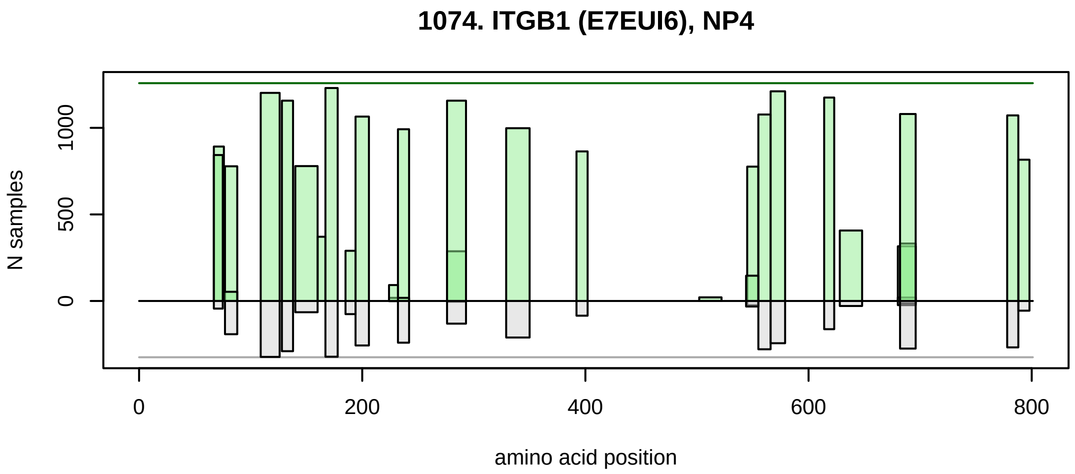
1074. ITGB1 (E7EUI6), NP4
N samples
1000
500
0
0
200
400
600
800
amino acid position

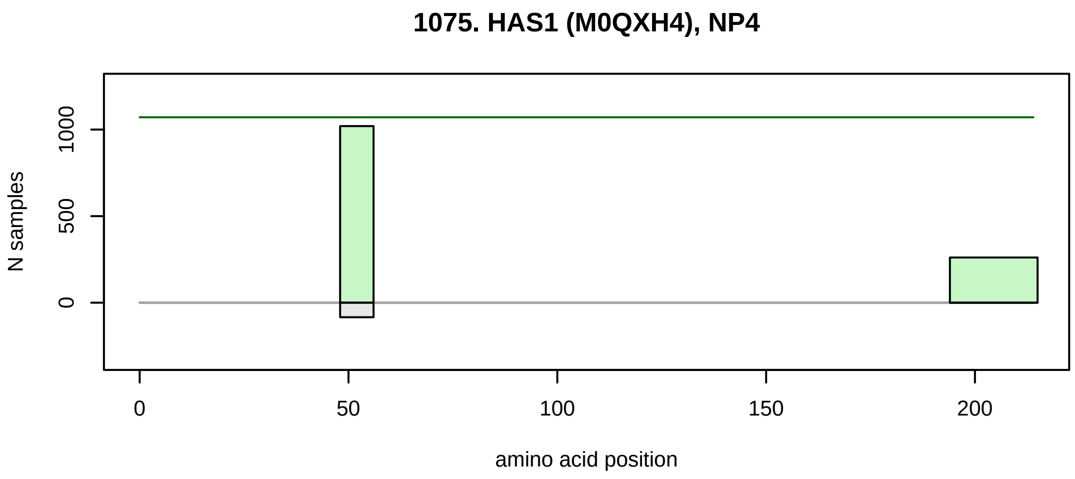
1075. HAS1 (M0QXH4), NP4
N samples
0
500
1000
0
50
100
150
200
amino acid position

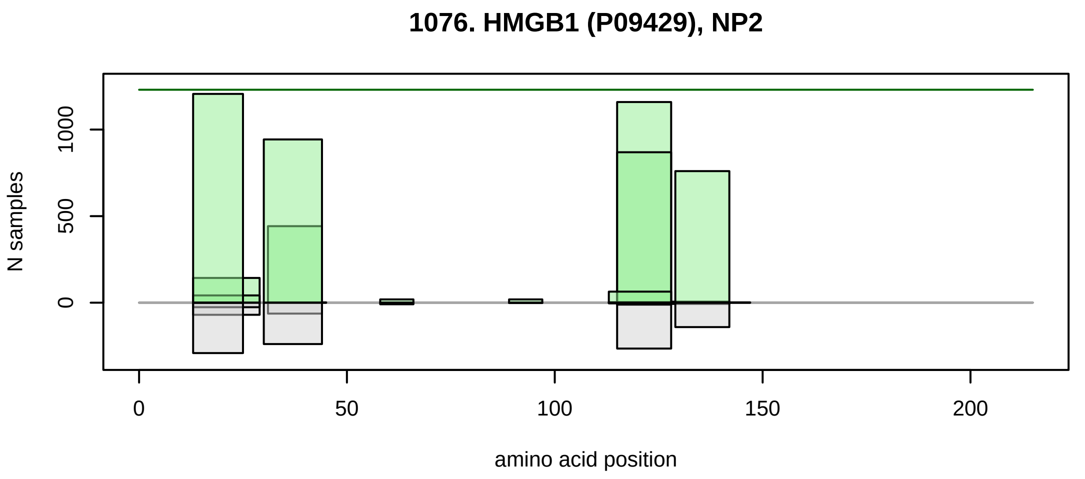
1076. HMGB1 (P09429), NP2
N samples
0
500
1000
0
50
100
150
200
amino acid position

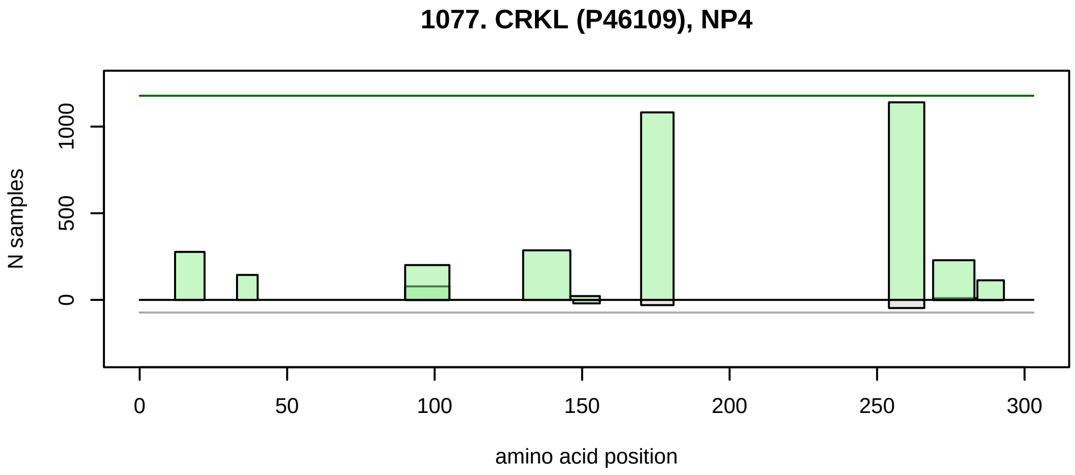
1077. CRKL (P46109), NP4
N samples
0
500
1000
0
50
100
150
200
250
300
amino acid position

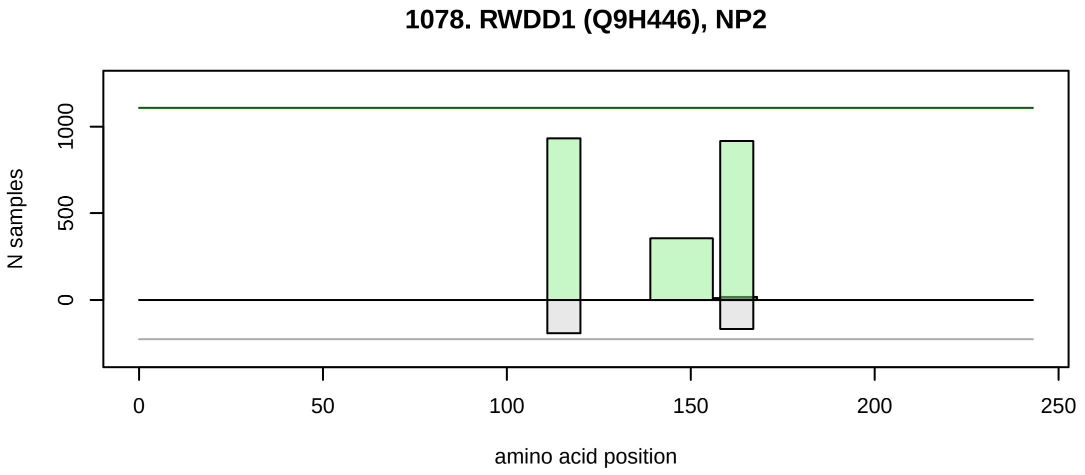
1078. RWDD1 (Q9H446), NP2
N samples
0
500
1000
0
50
100
150
200
250
amino acid position

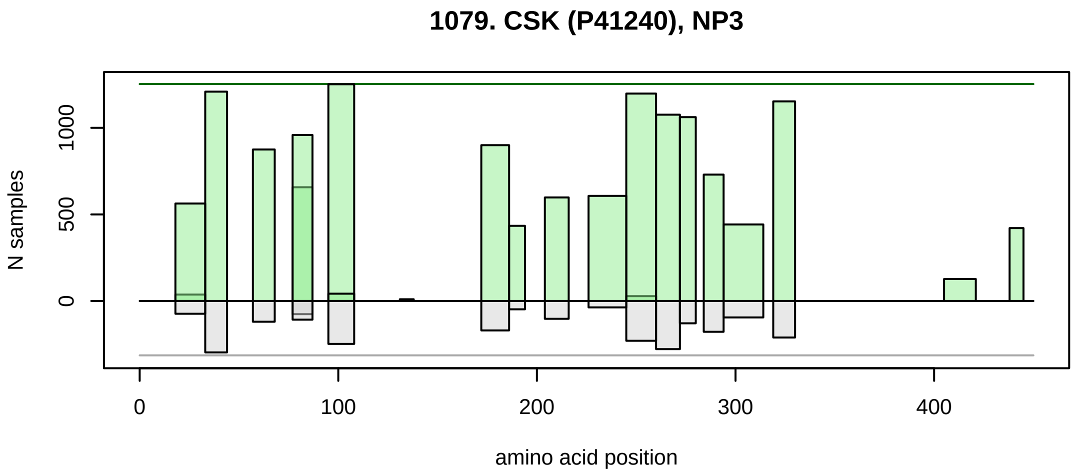
1079. CSK (P41240), NP3
N samples
0
500
1000
0
100
200
300
400
amino acid position

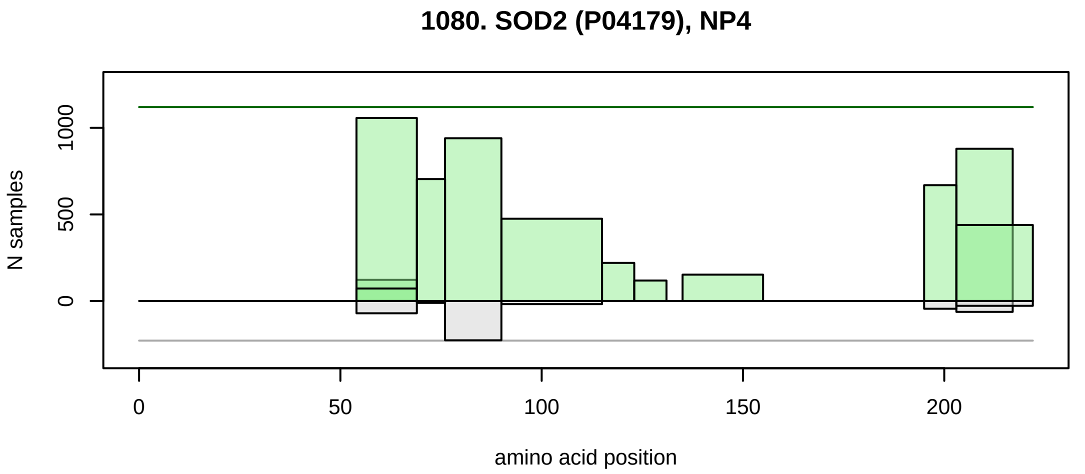
1080. SOD2 (P04179), NP4
N samples
0
500
1000
0
50
100
150
200
amino acid position

1081. PTPN11 (Q06124−1), NP3

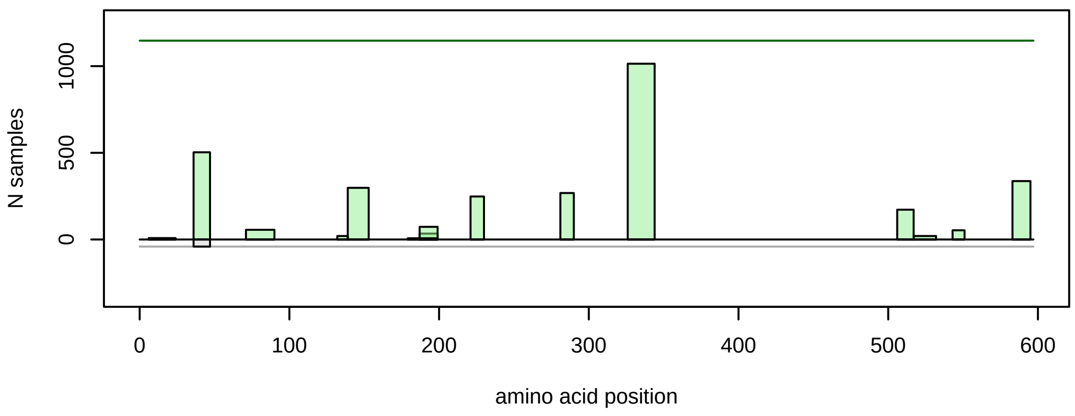

1082. CXCL14 (A0A0C4DGC1;O95715), NP4

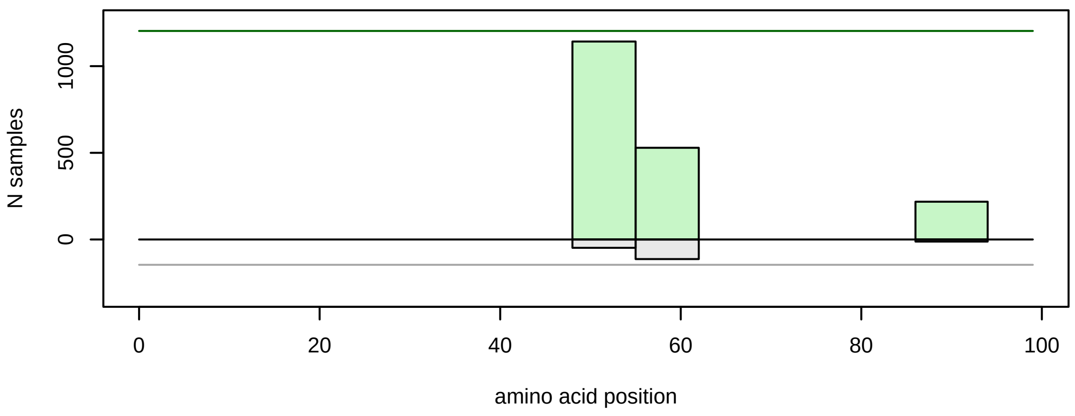

1083. SCFD1 (A0A7I2V3B4), NP5

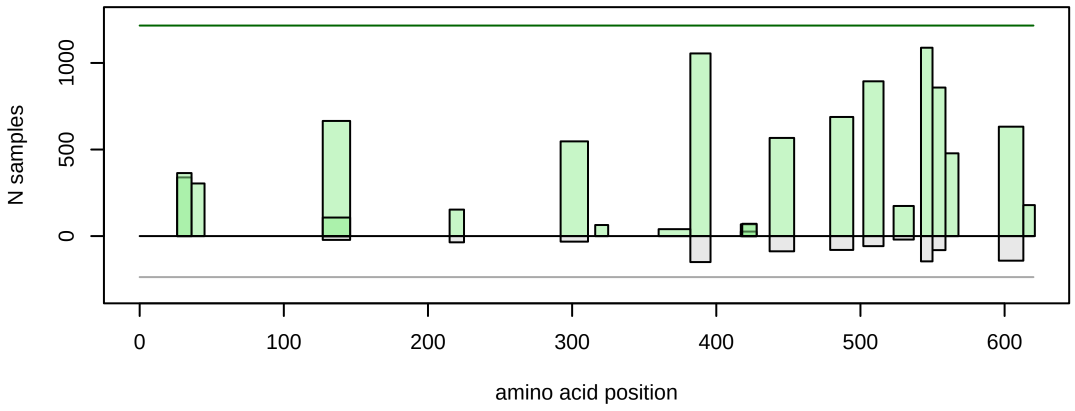

1084. PPP3CA (Q08209), NP5

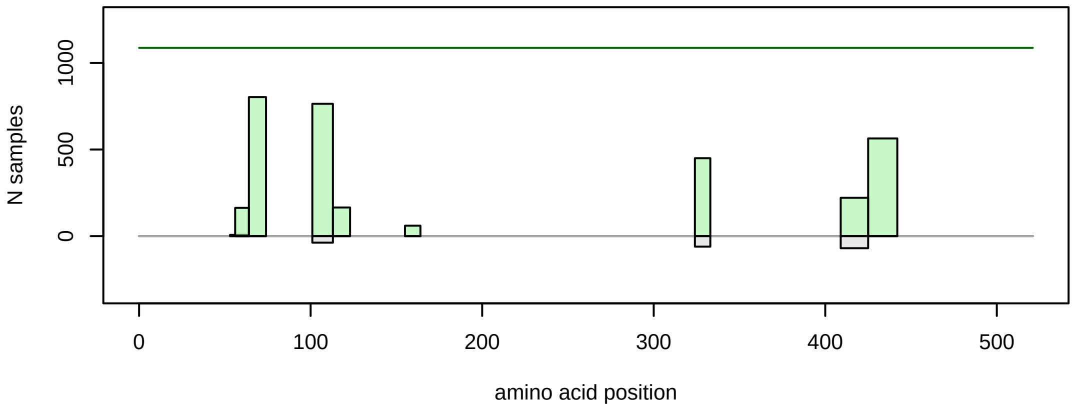

1085. QPCT (Q16769), NP2

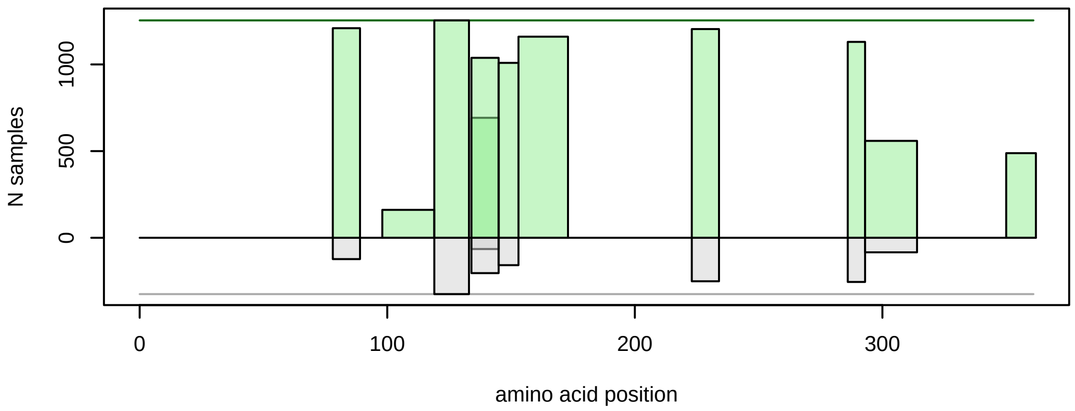

1086. RAP1A (P62834), NP4

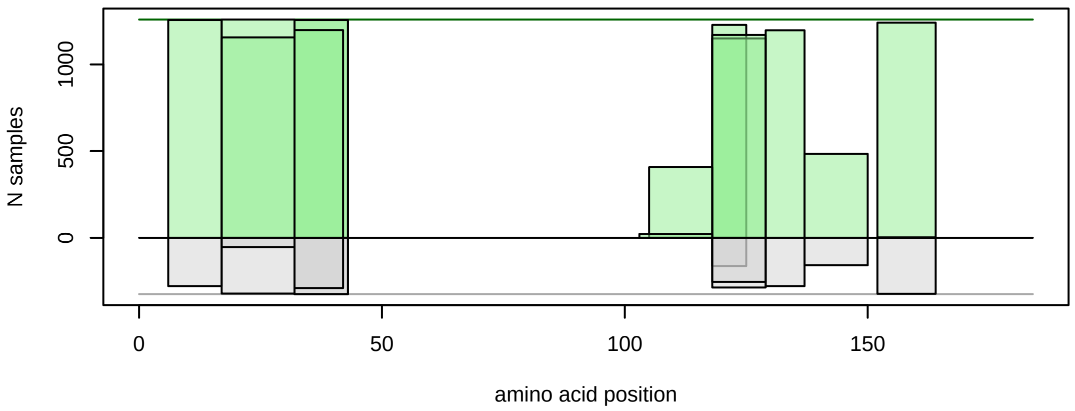

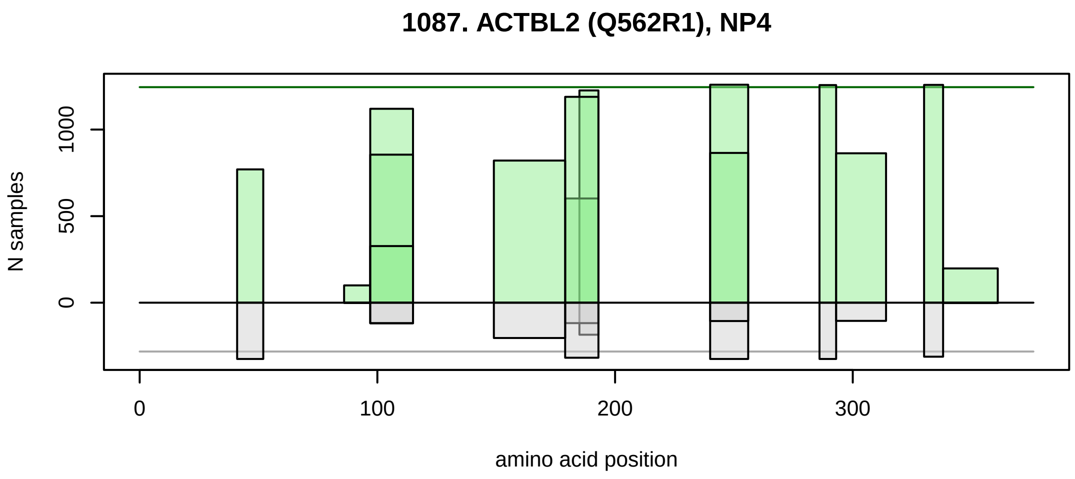
1087. ACTBL2 (Q562R1), NP4
N samples
0
500
1000
0
100
200
300
amino acid position

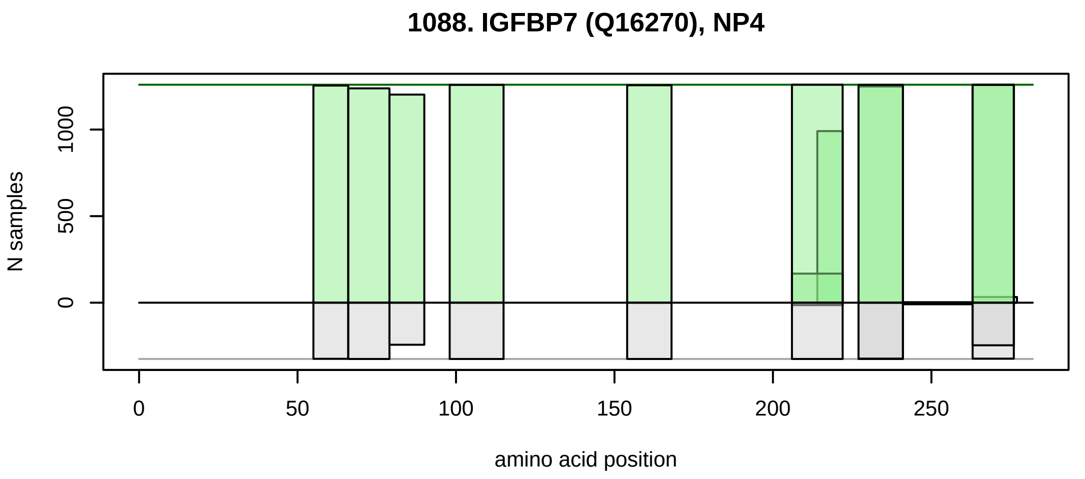
1088. IGFBP7 (Q16270), NP4
N samples
0
500
1000
0
50
100
150
200
250
amino acid position

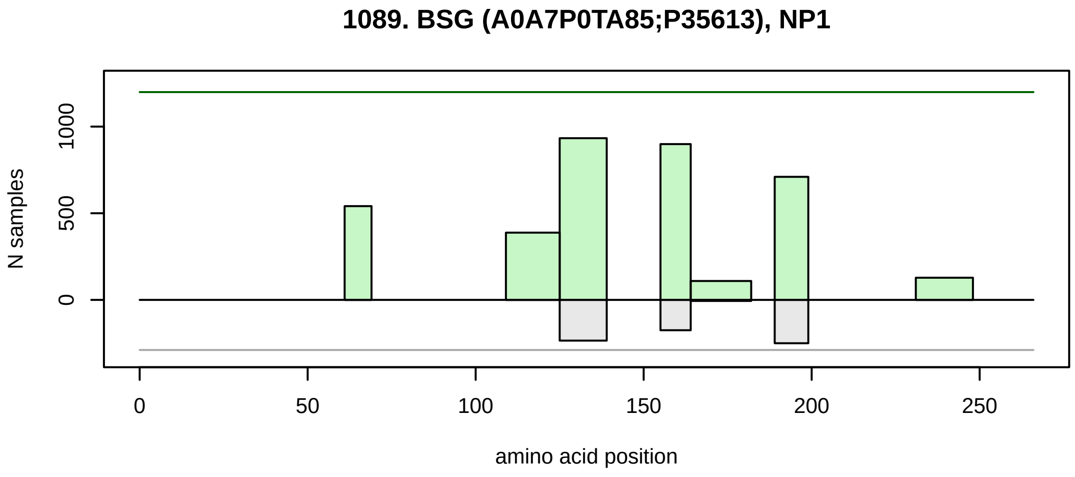
1089. BSG (A0A7P0TA85;P35613), NP1
N samples
0
500
1000
0
50
100
150
200
250
amino acid position

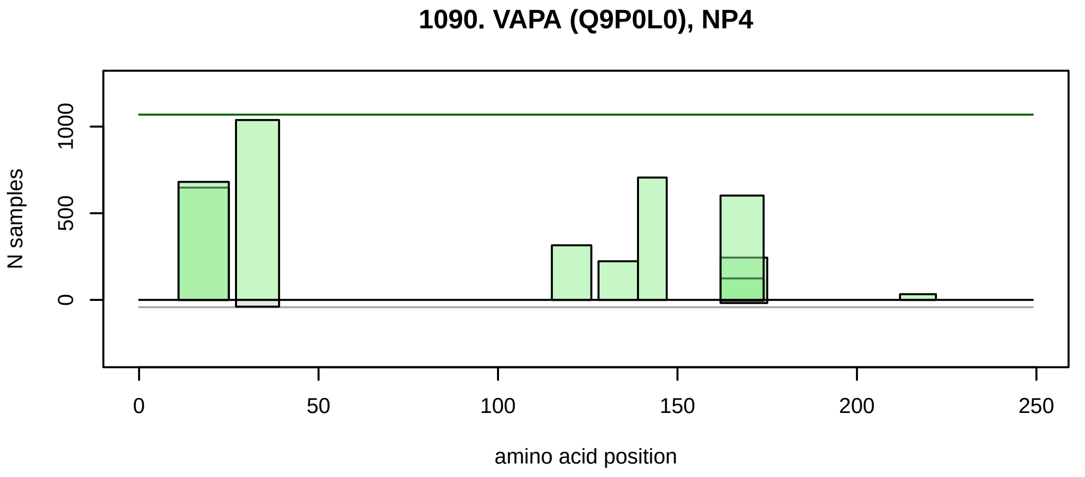
1090. VAPA (Q9P0L0), NP4
N samples
0
500
1000
0
50
100
150
200
250
amino acid position

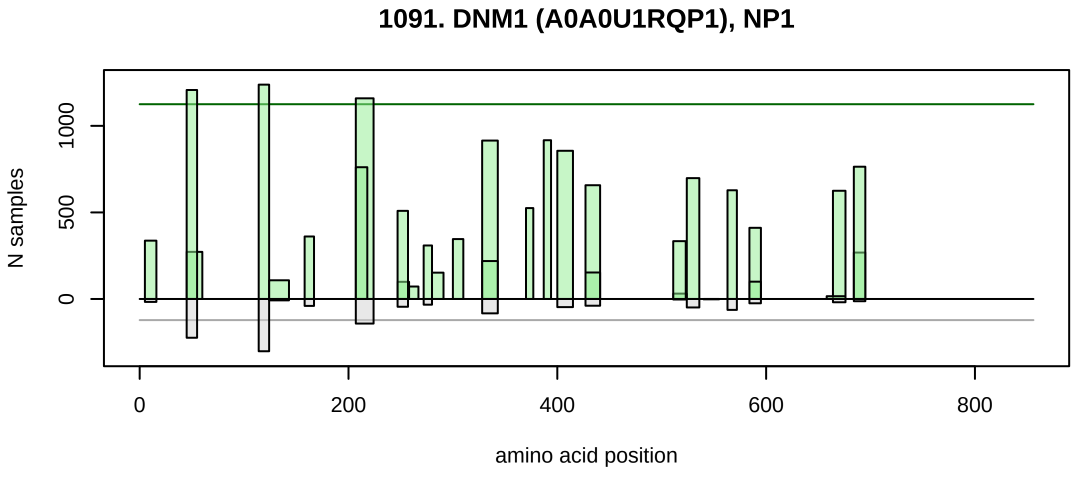
1091. DNM1 (A0A0U1RQP1), NP1
N samples
0
500
1000
0
200
400
600
800
amino acid position

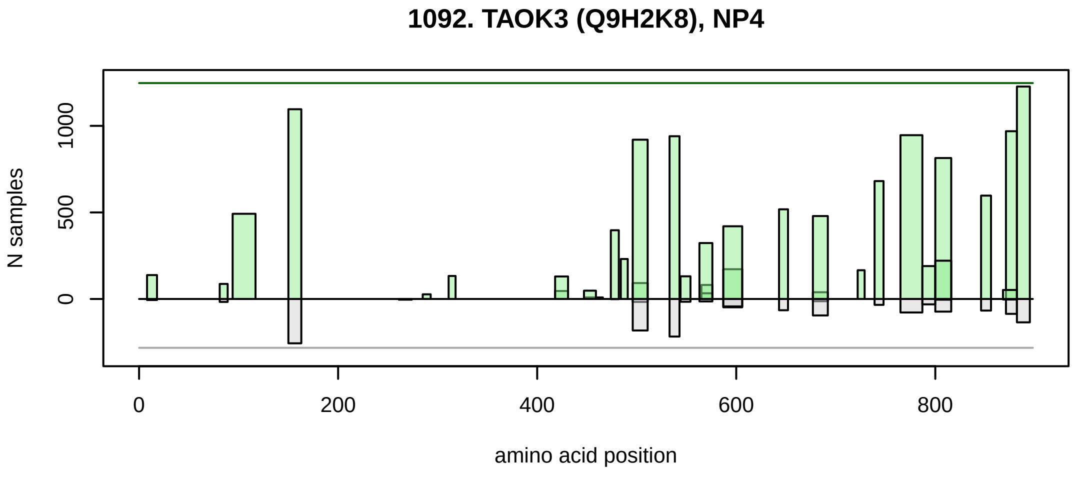
1092. TAOK3 (Q9H2K8), NP4
N samples
0
500
1000
0
200
400
600
800
amino acid position

1093. SHBG (P04278), NP4

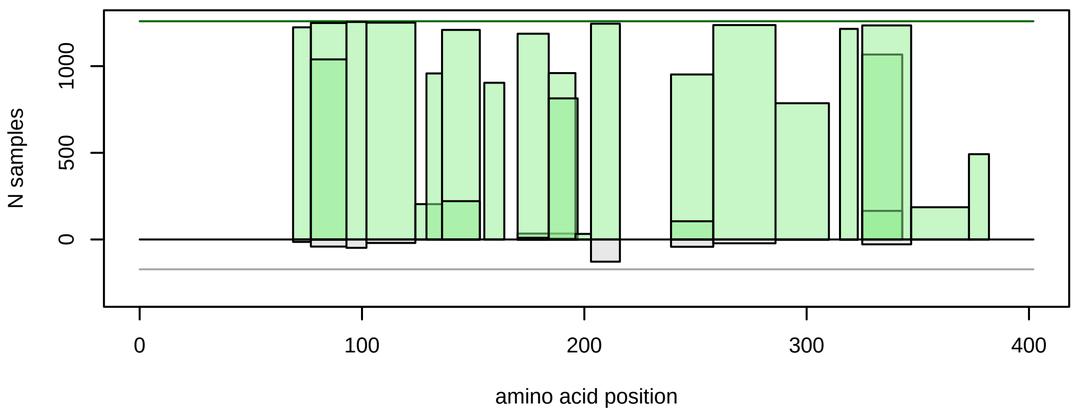

1094. DNASE1 (P24855), NP2

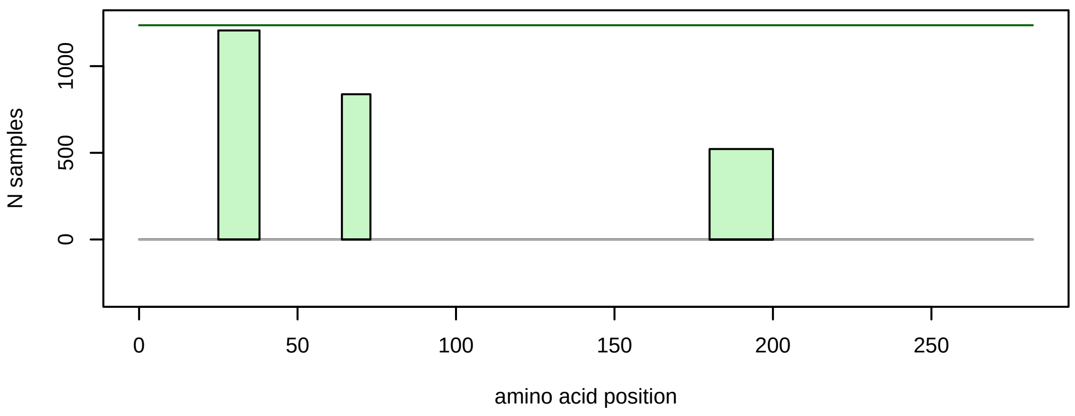

1095. MYC (Q16591), NP3

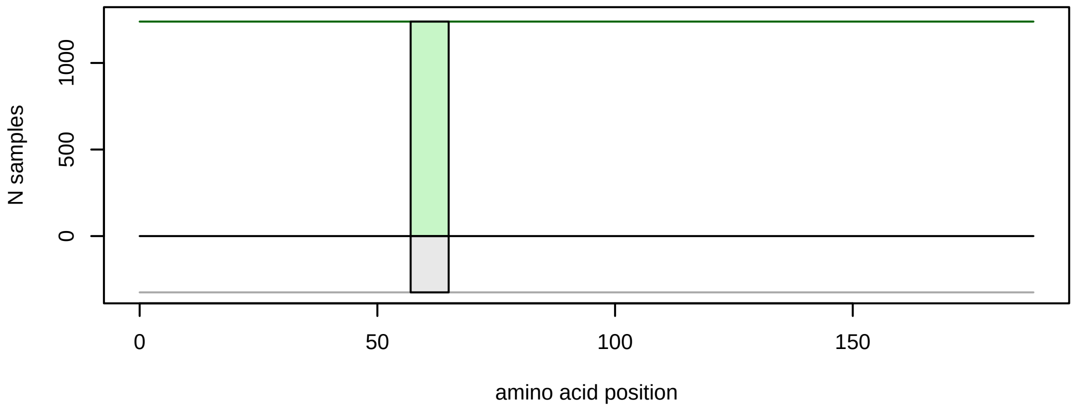

1096. PSMC6 (A0A087X2I1), NP5

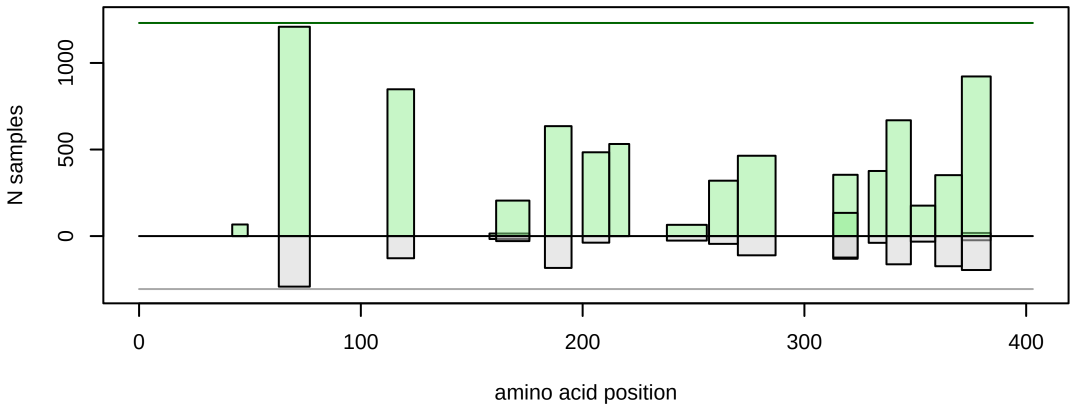

1097. RAB27B (O00194), NP4

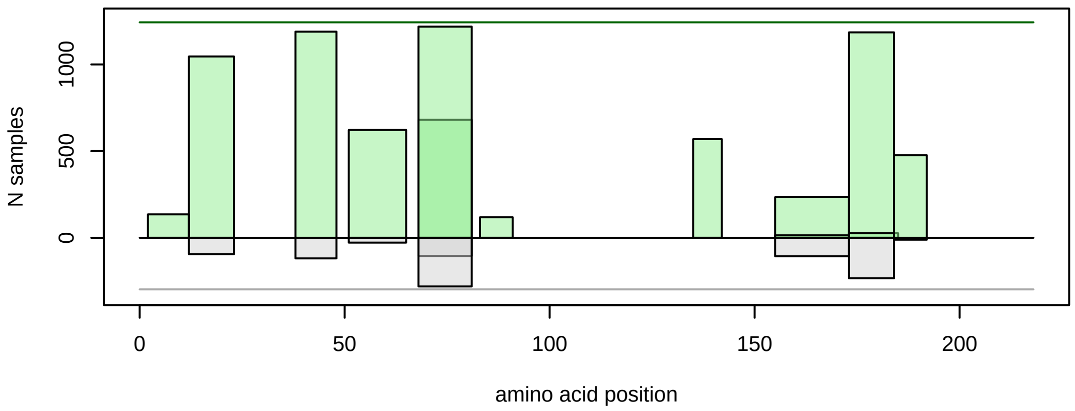

1098. YWHAH (Q04917), NP4

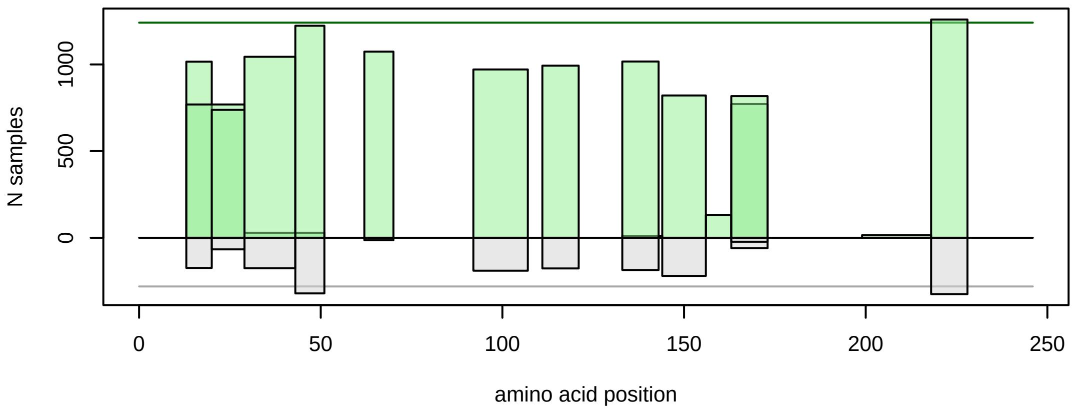

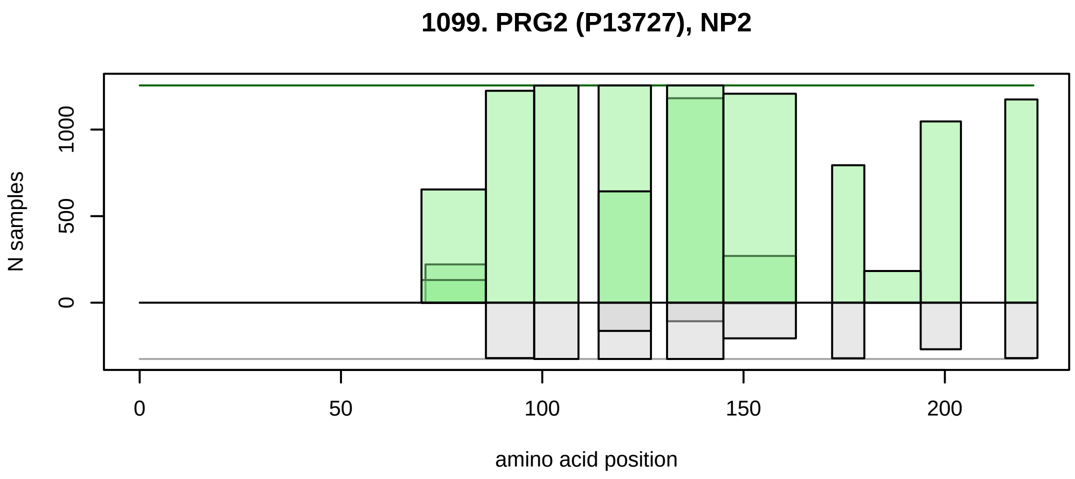

1099. PRG2 (P13727), NP2
N samples
0
500
1000
0
50
100
150
200
amino acid position

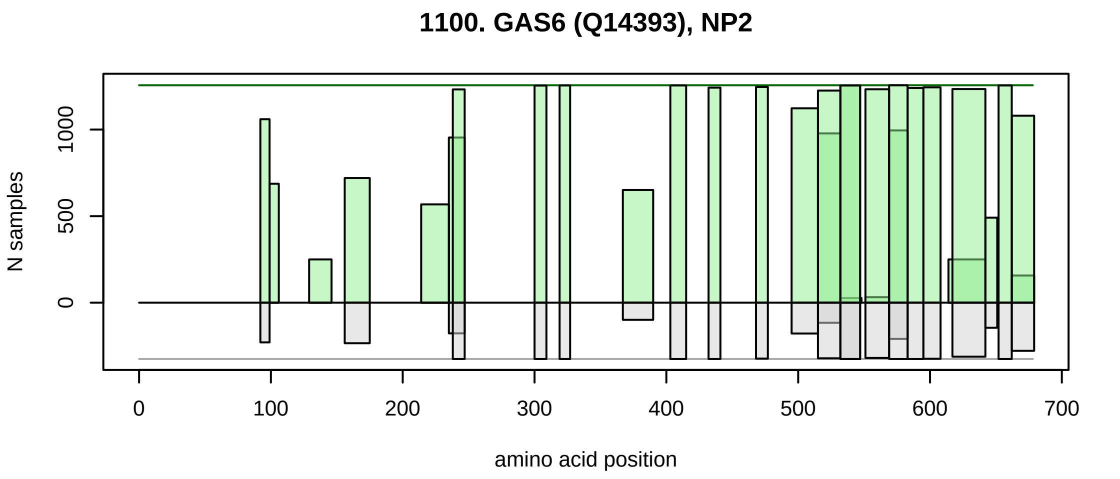

1100. GAS6 (Q14393), NP2
N samples
0
500
1000
0
100
200
300
400
500
600
700
amino acid position

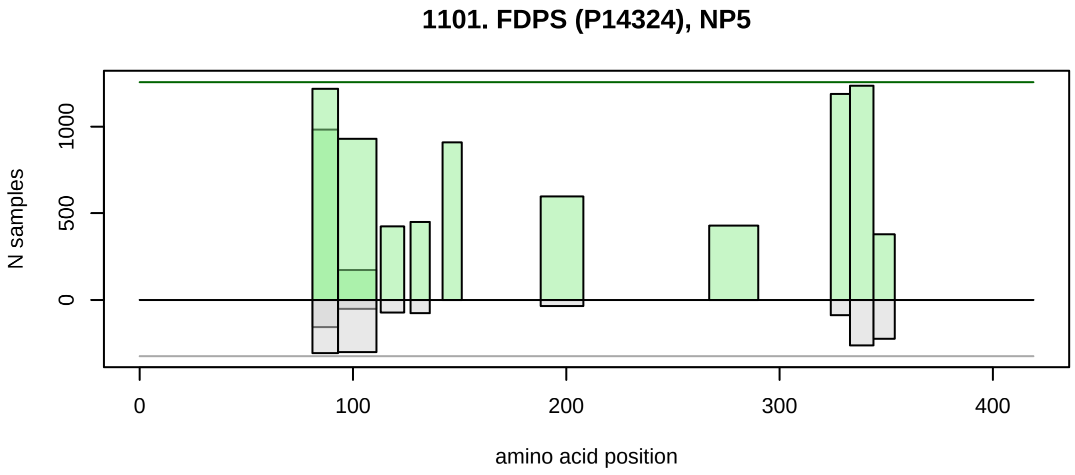

1101. FDPS (P14324), NP5
N samples
0
500
1000
0
100
200
300
400
amino acid position

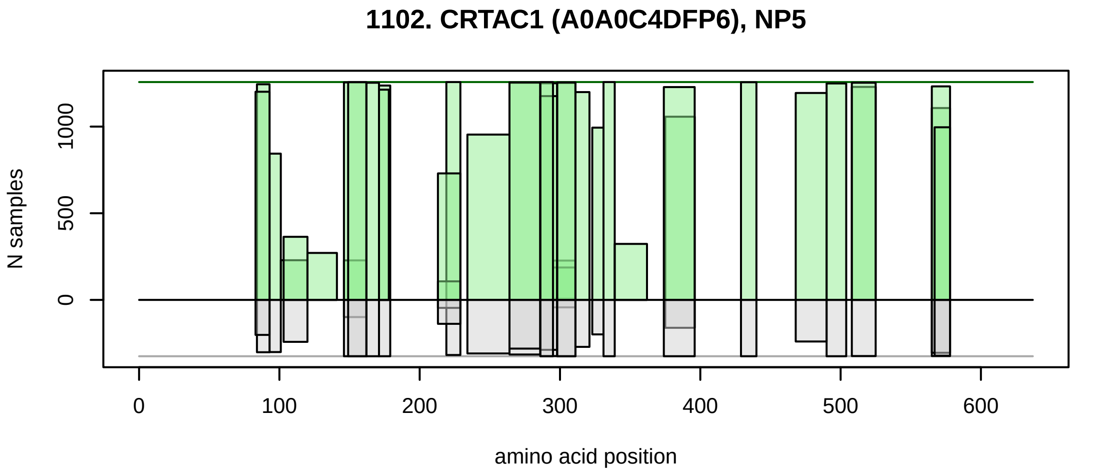

1102. CRTAC1 (A0A0C4DFP6), NP5
N samples
0
500
1000
0
100
200
300
400
500
600
amino acid position

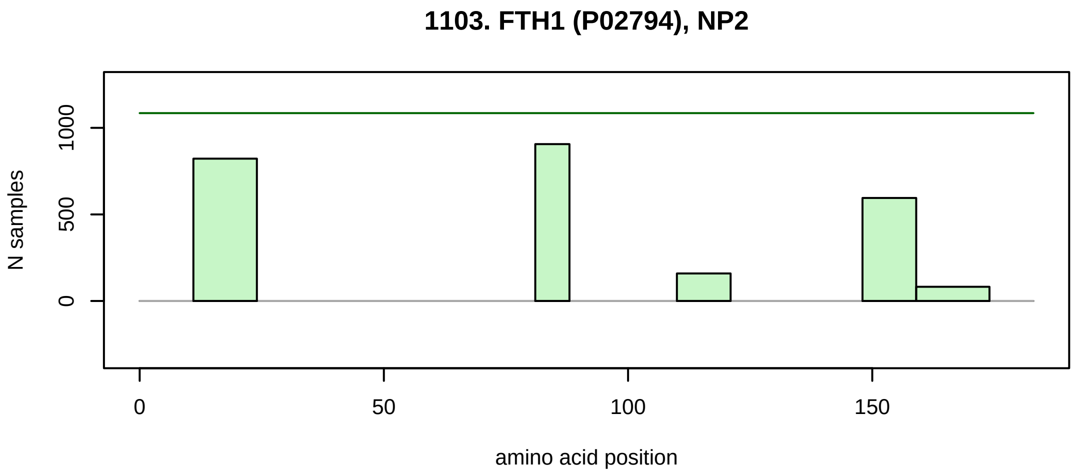

1103. FTH1 (P02794), NP2
N samples
0
500
1000
0
50
100
150
amino acid position

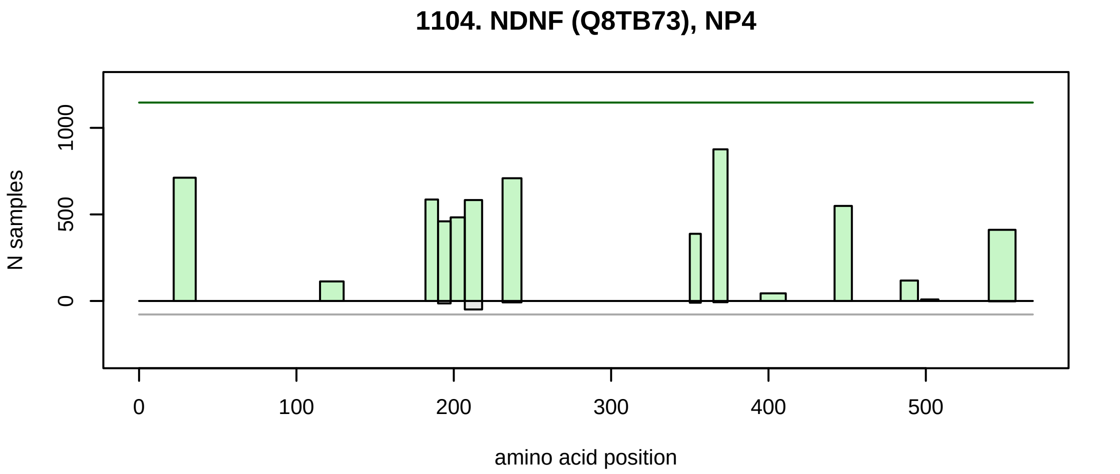

1104. NDNF (Q8TB73), NP4
N samples
0
500
1000
0
100
200
300
400
500
amino acid position

1105. GMFG (O60234), NP2

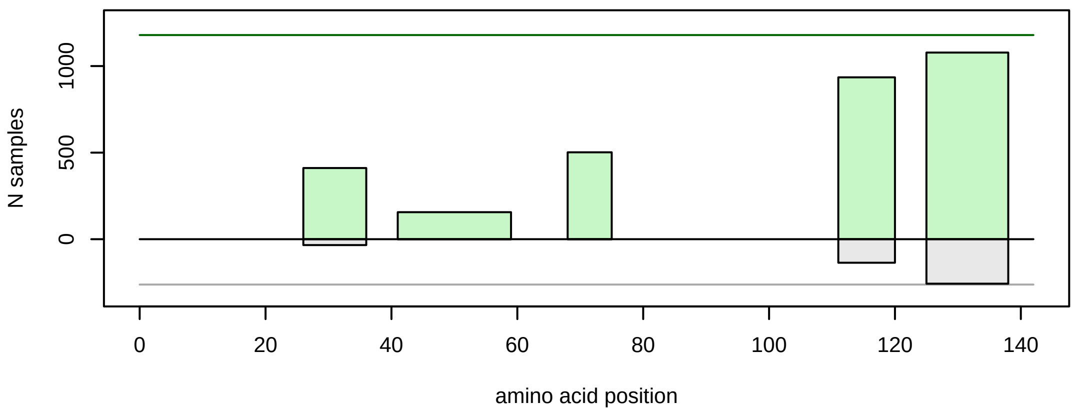

1106. RHOC (P08134), NP1

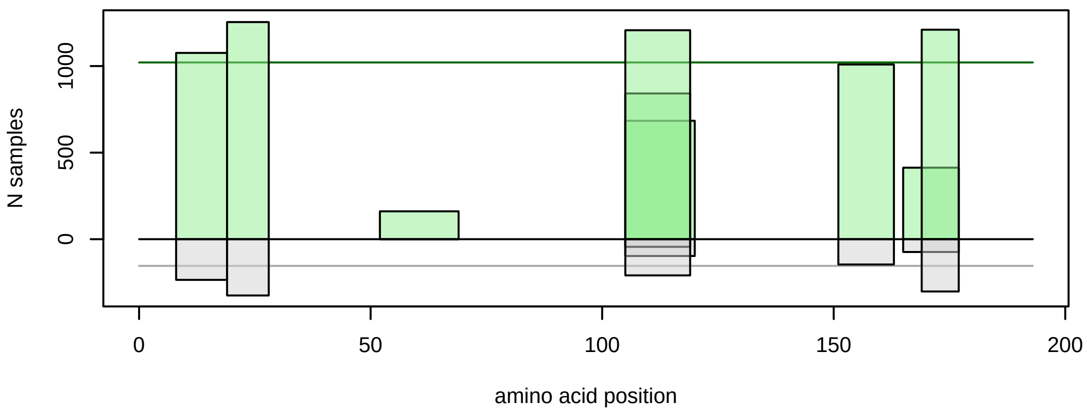

1107. CYB5R3 (A0A8J8YWN6), NP4

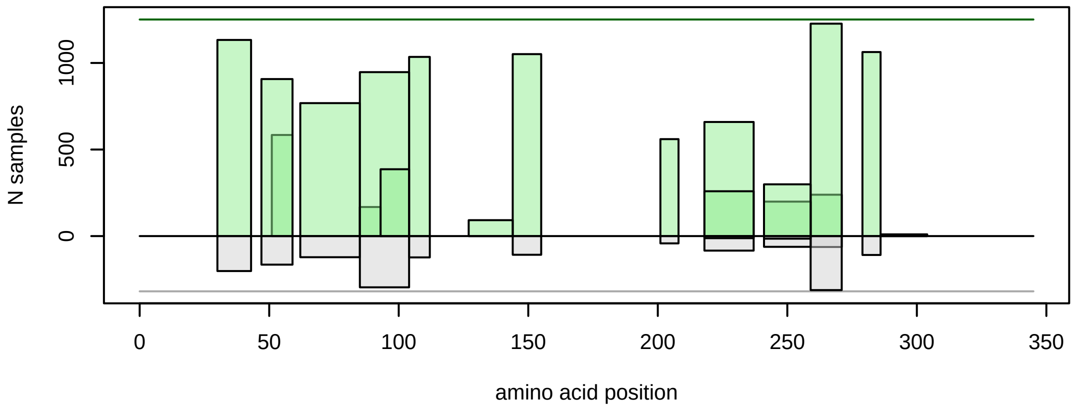

1108. MMP7 (P09237), NP5

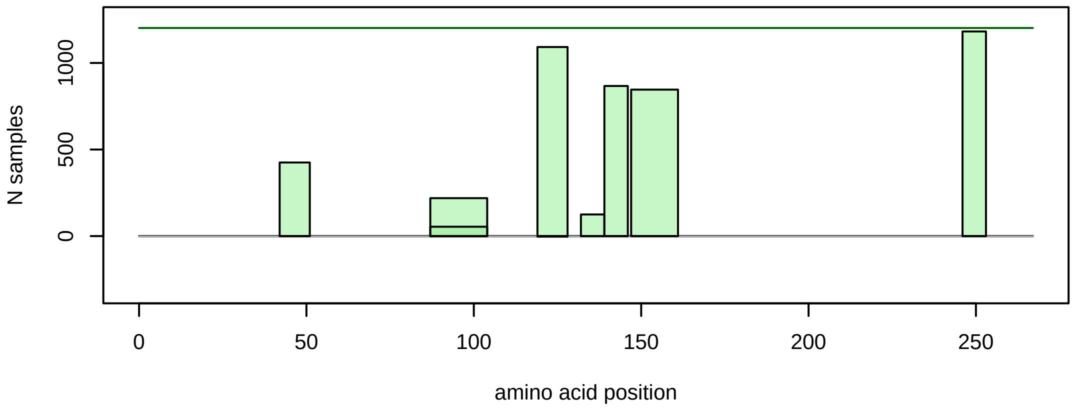

1109. PXDN (Q92626), NP4

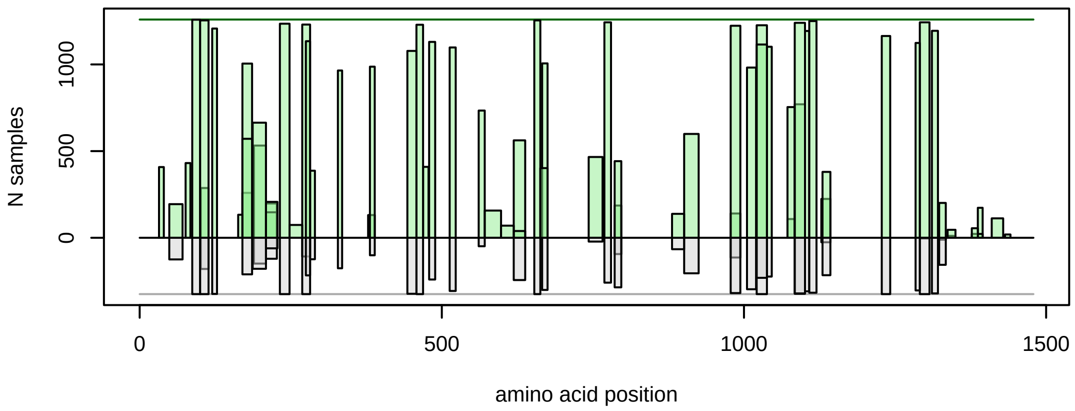

1110. CD5L (O43866), NP2

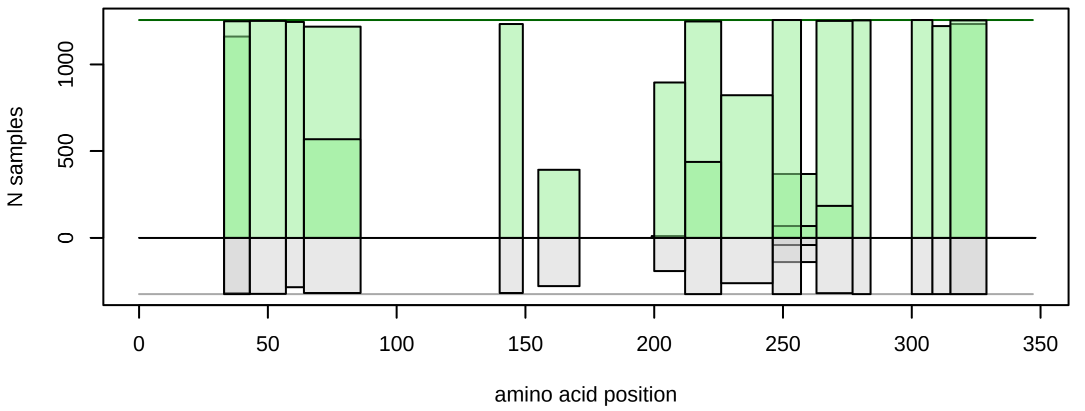

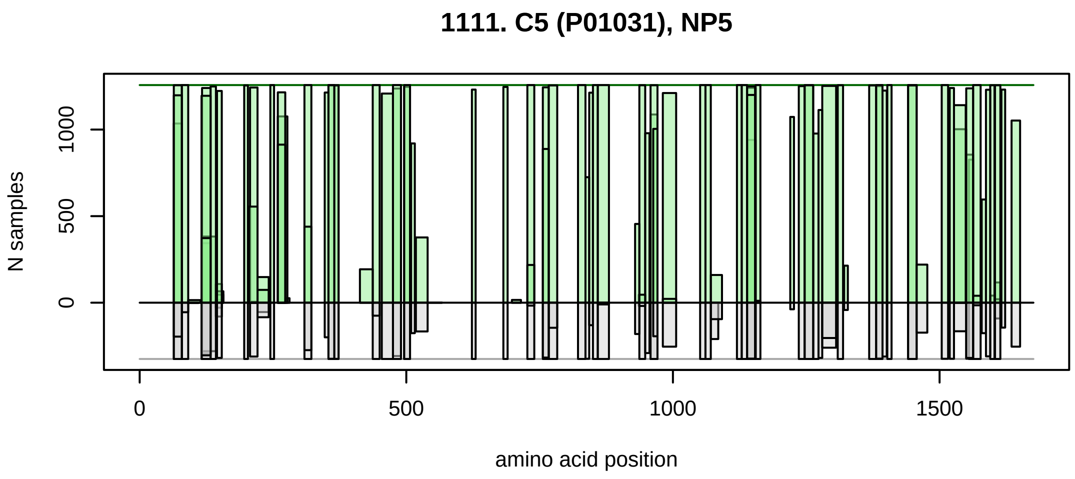
1111. C5 (P01031), NP5
N samples
amino acid position
0
500
1000
1500

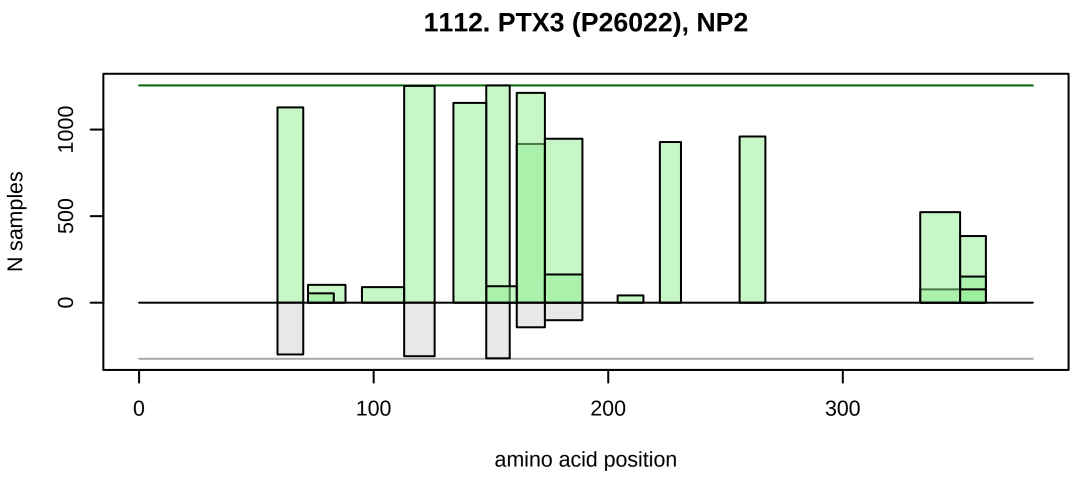
1112. PTX3 (P26022), NP2
N samples
amino acid position
0
100
200
300

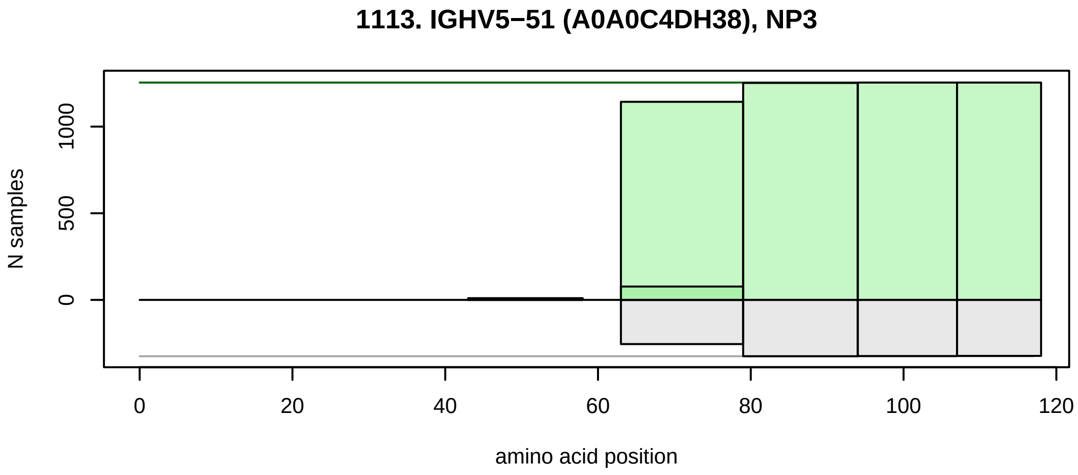
1113. IGHV5−51 (A0A0C4DH38), NP3
N samples
amino acid position
0
20
40
60
80
100
120

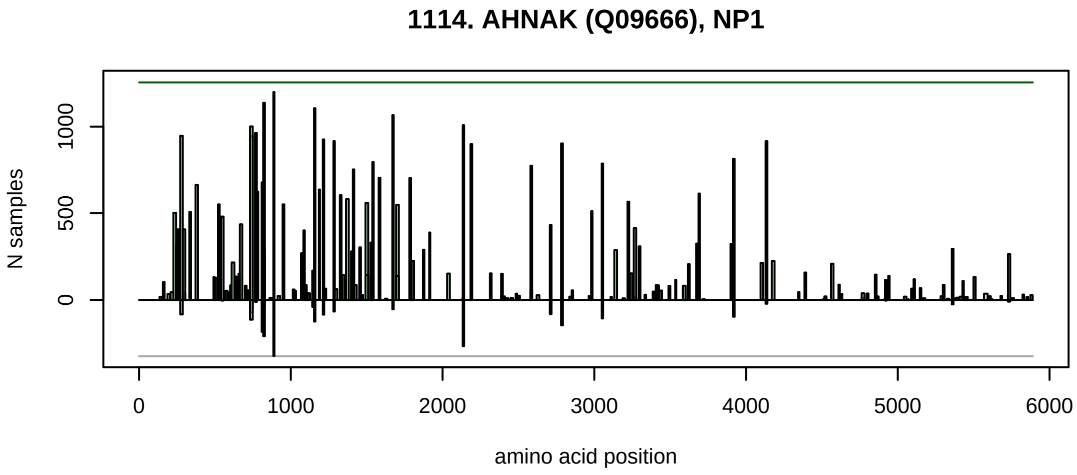
1114. AHNAK (Q09666), NP1
N samples
amino acid position
0
1000
2000
3000
4000
5000
6000

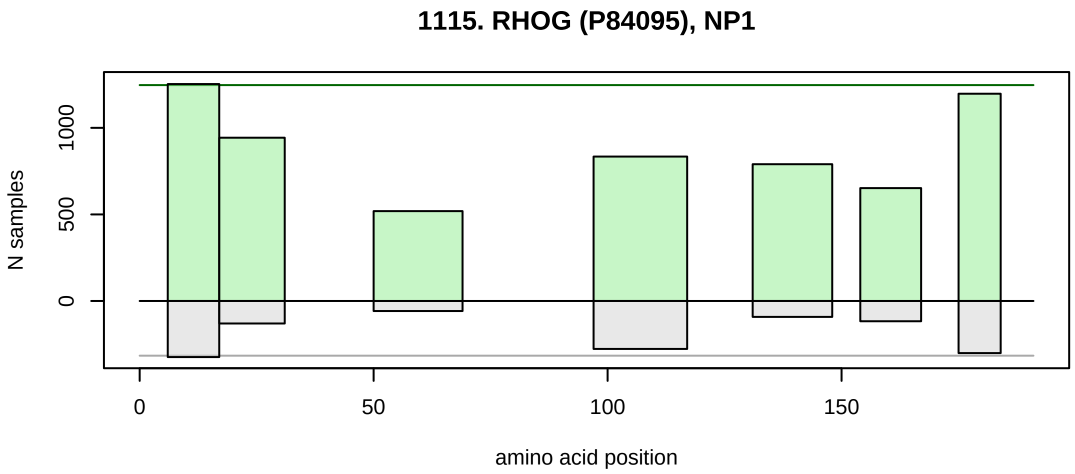
1115. RHOG (P84095), NP1
N samples
amino acid position
0
50
100
150

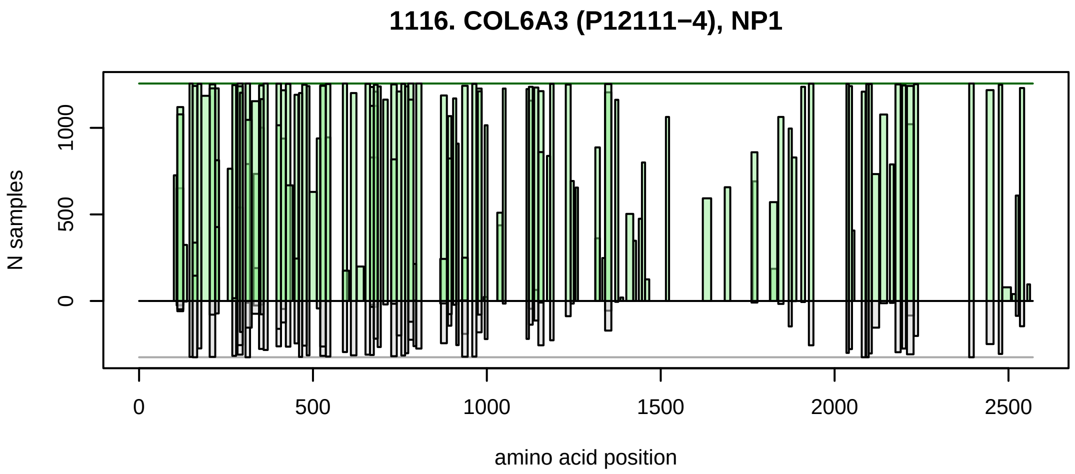
1116. COL6A3 (P12111−4), NP1
N samples
amino acid position
0
500
1000
1500
2000
2500

1117. PRSS1 (E7EQ64;P07477), NP2

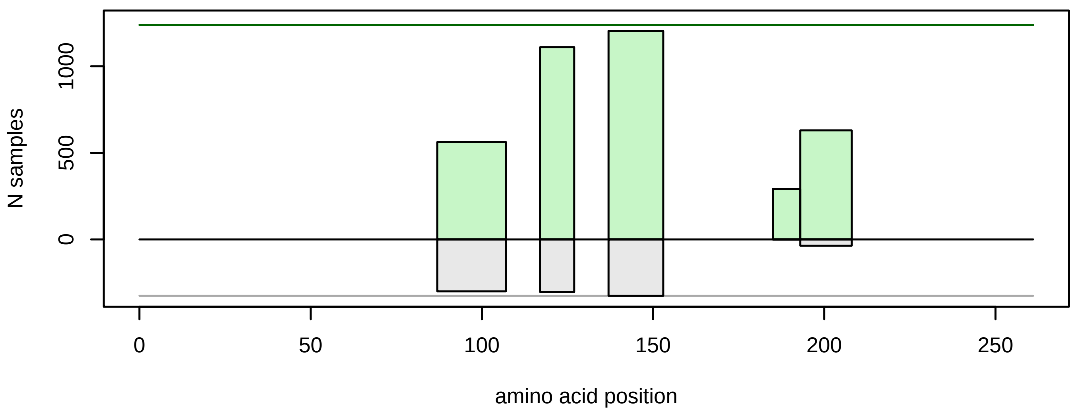

1118. DDX17 (A0A5H1ZRQ2), NP1

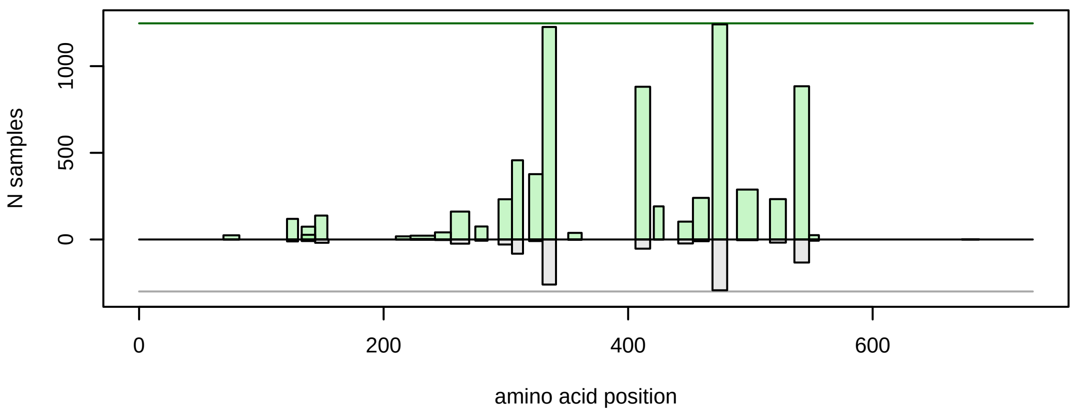

1119. WAS (P42768), NP1

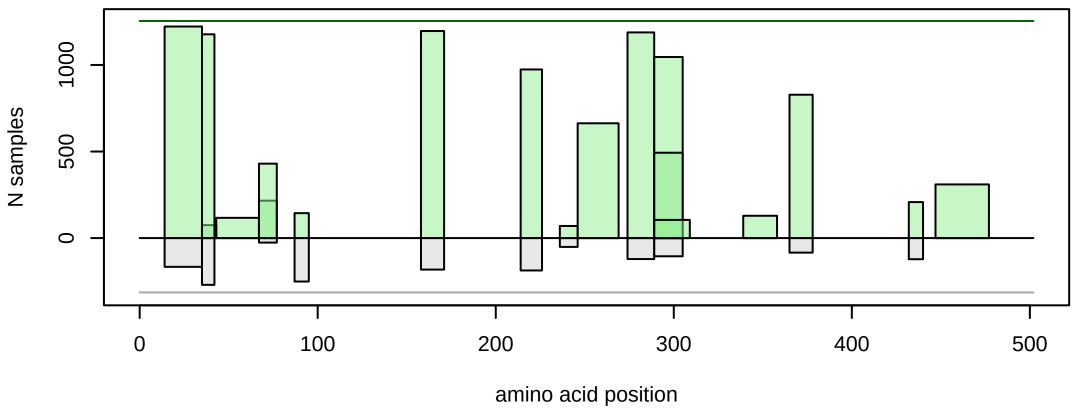

1120. ADAM10 (O14672), NP2

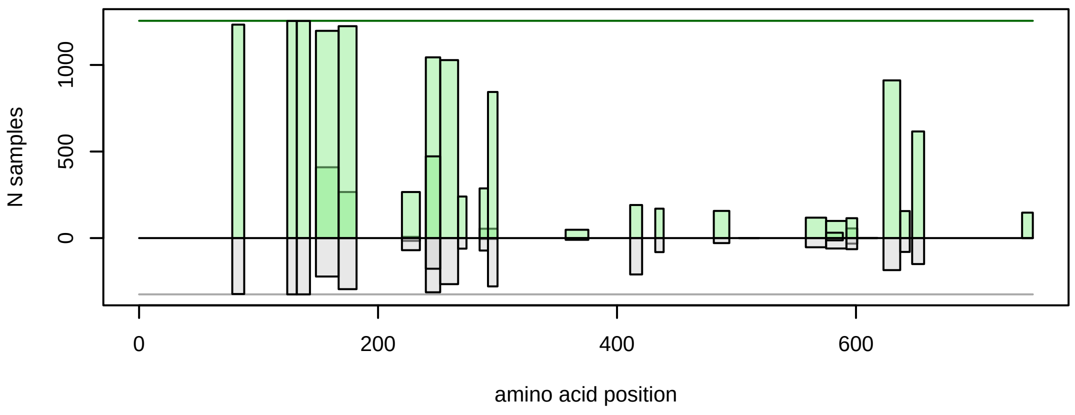

1121. TMED10 (P49755), NP4

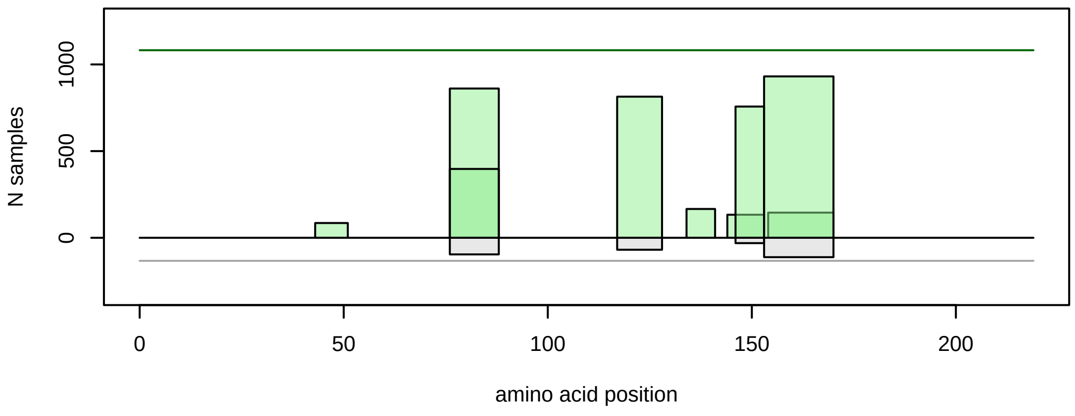

1122. OLFML1 (Q6UWY5), NP3

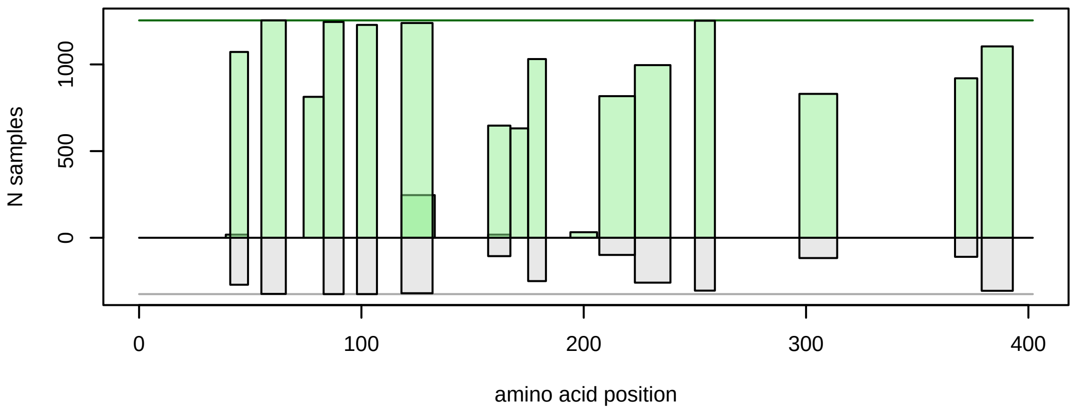

1123. GANAB (Q14697;Q14697−2), NP5

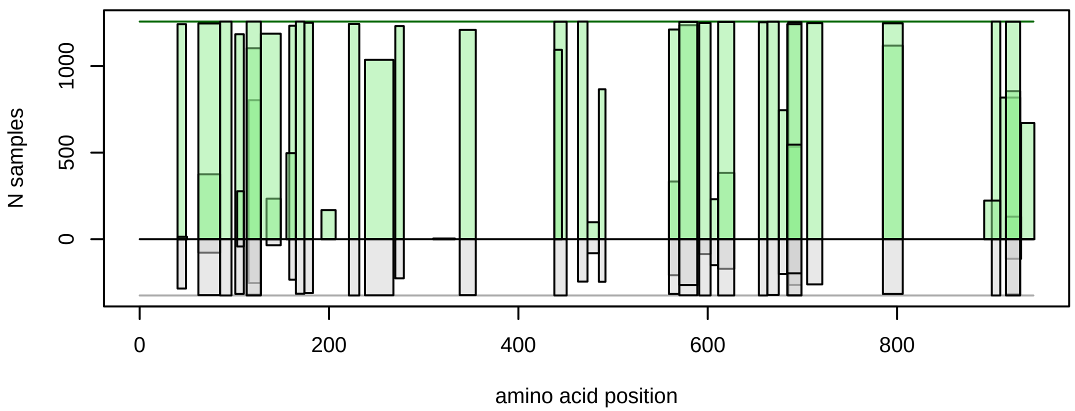

1124. TUBB4B (P68371), NP2

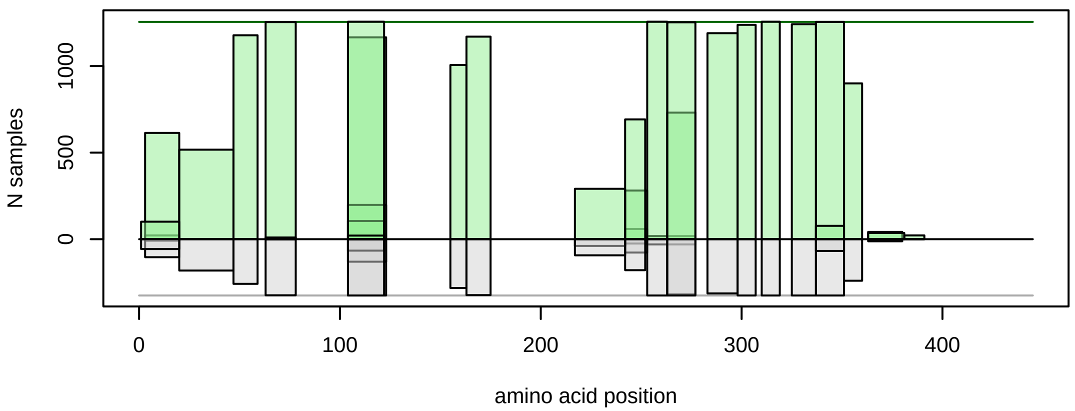

1125. CCT7 (Q99832), NP1

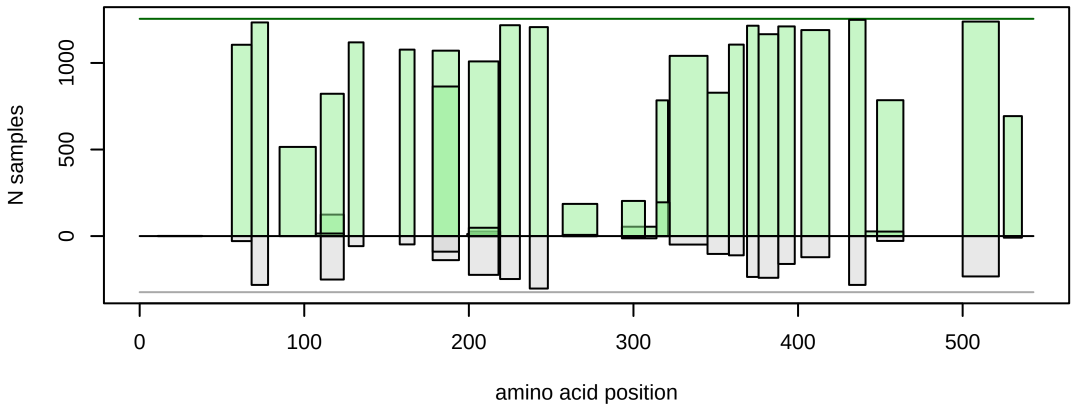

1126. CFHR5 (Q9BXR6), NP4

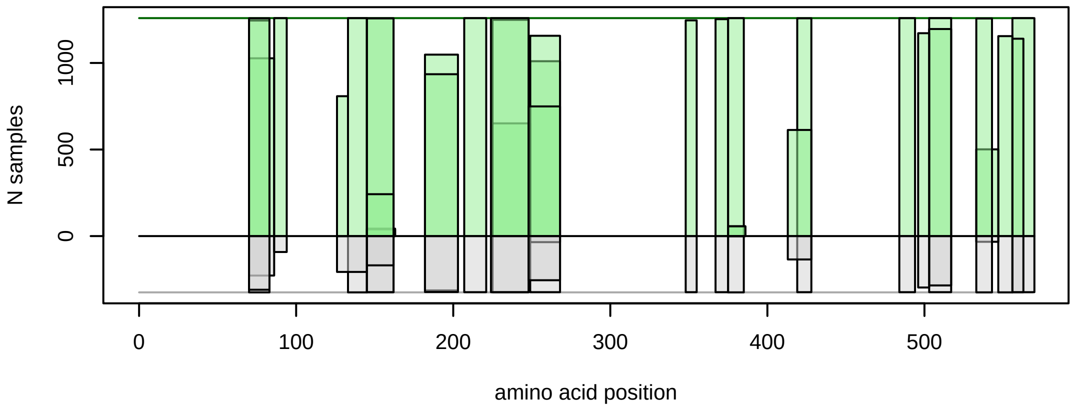

1127. ARF6 (P62330), NP3

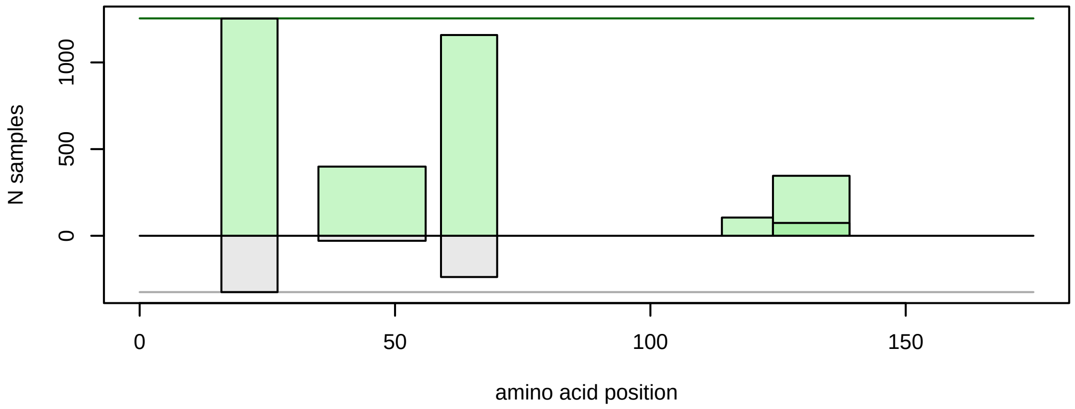

1128. KLK14 (Q9P0G3), NP4

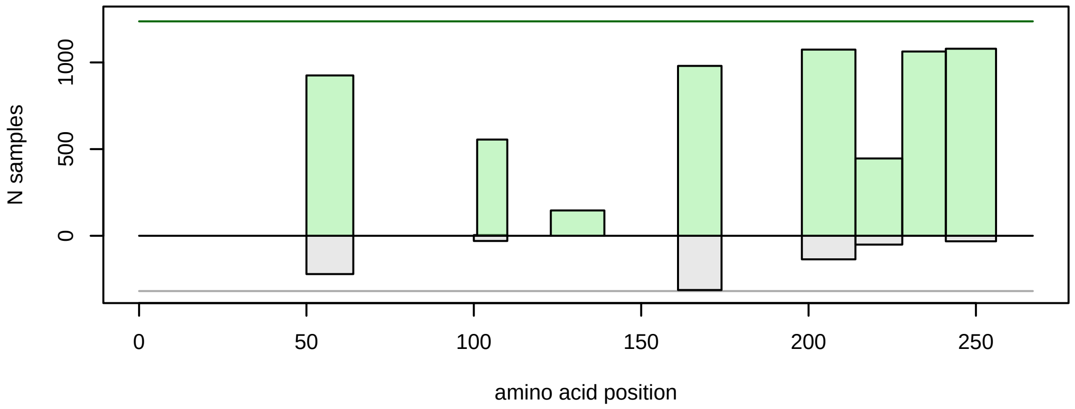

1129. CKM (P06732), NP1

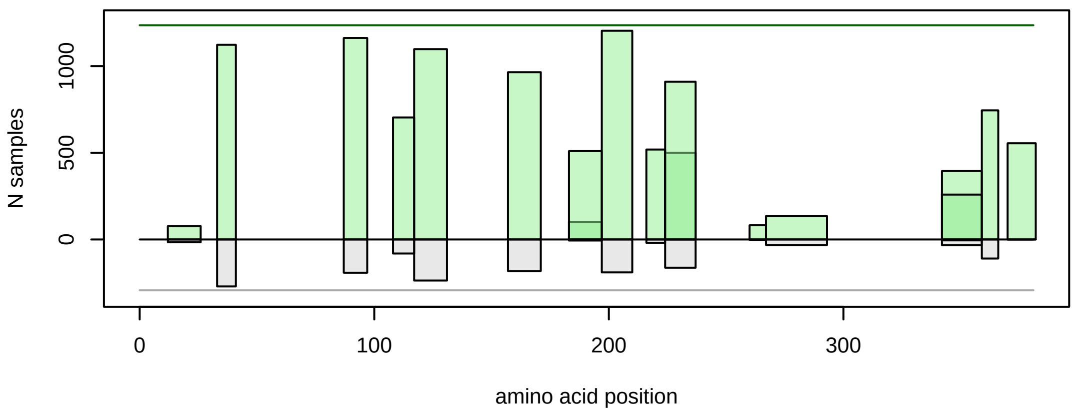

1130. ACTA1 (P68133), NP4

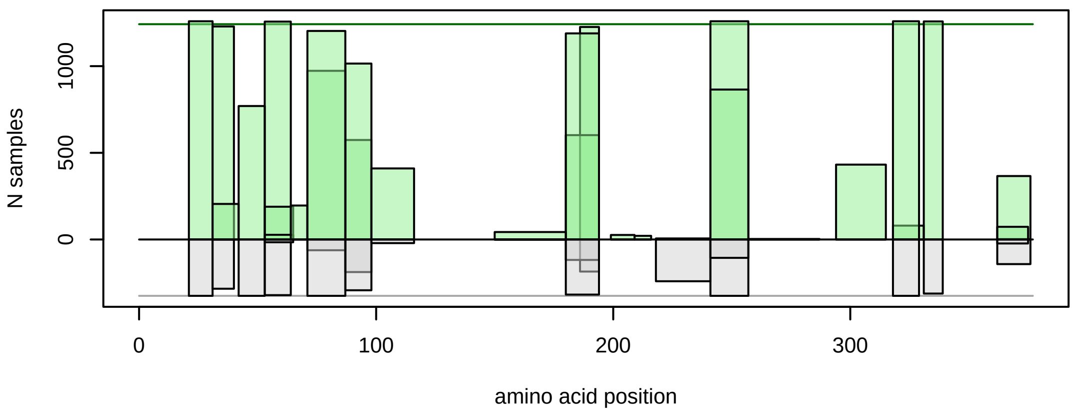

1131. CHST12 (Q9NRB3), NP4

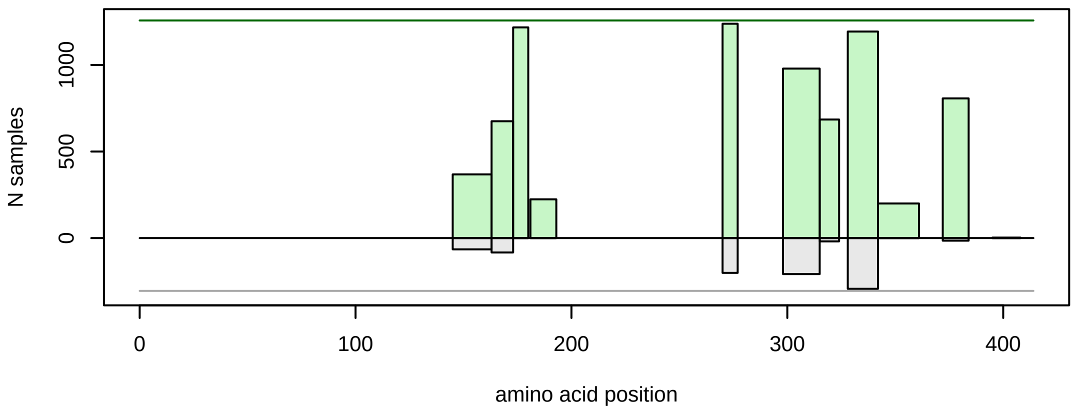

1132. HYOU1 (A0A494C039;Q9Y4L1), NP1

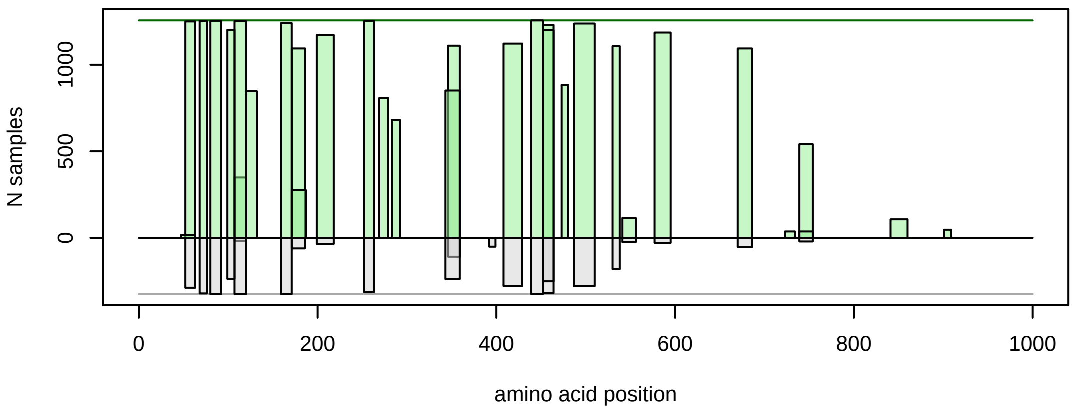

1133. RARS (P54136), NP4

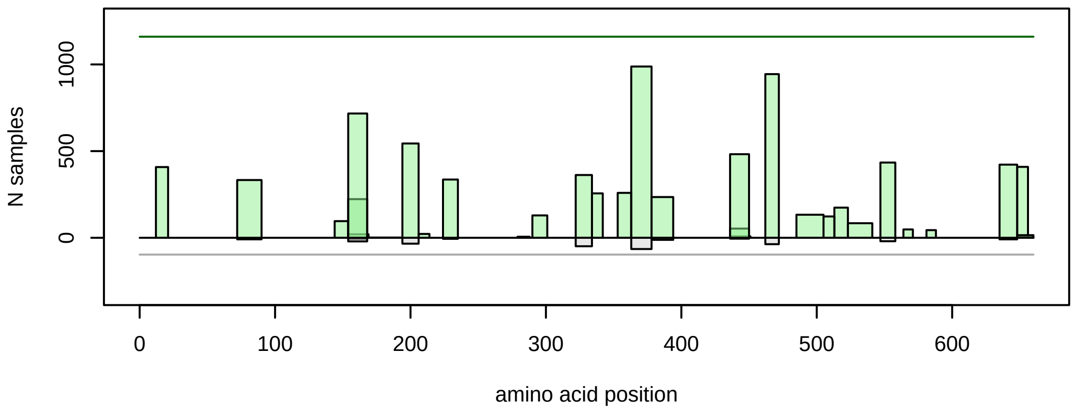

1134. YWHAG (P61981), NP1

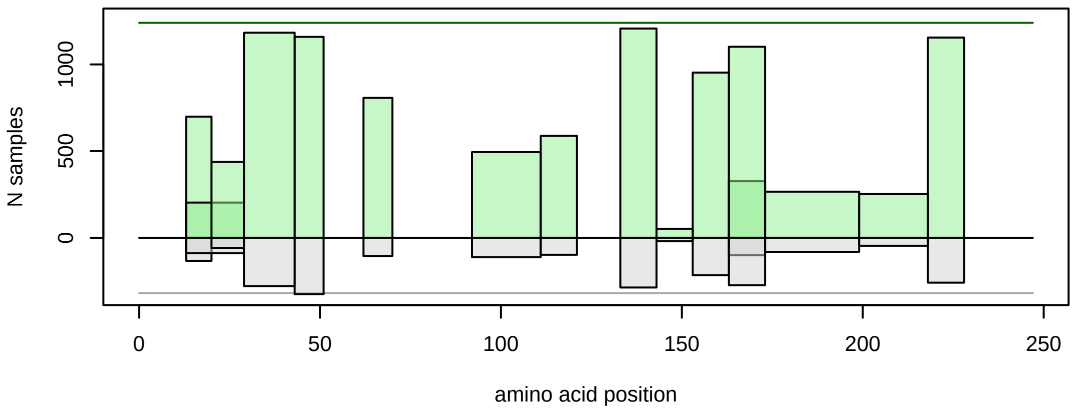

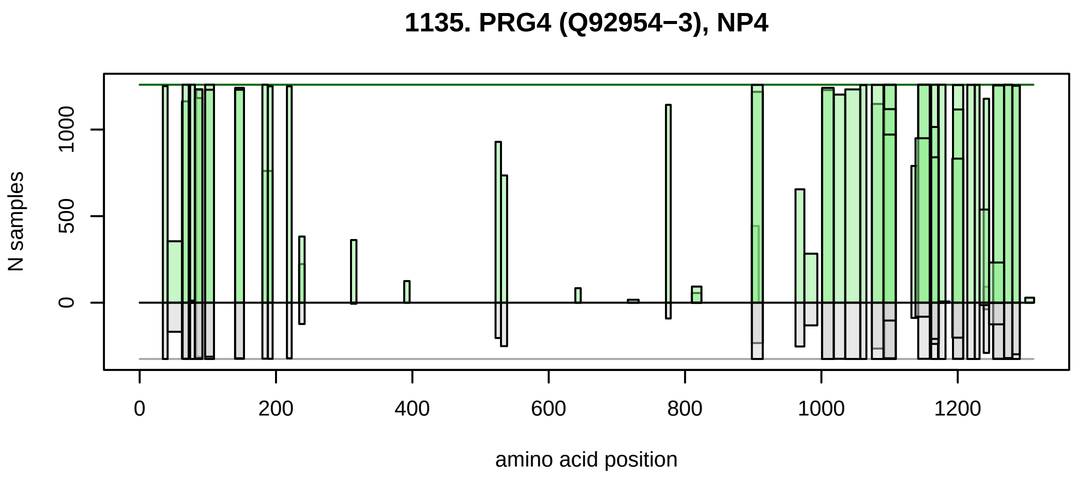
1135. PRG4 (Q92954−3), NP4
N samples
0
500
1000
0
200
400
600
800
1000
1200
amino acid position

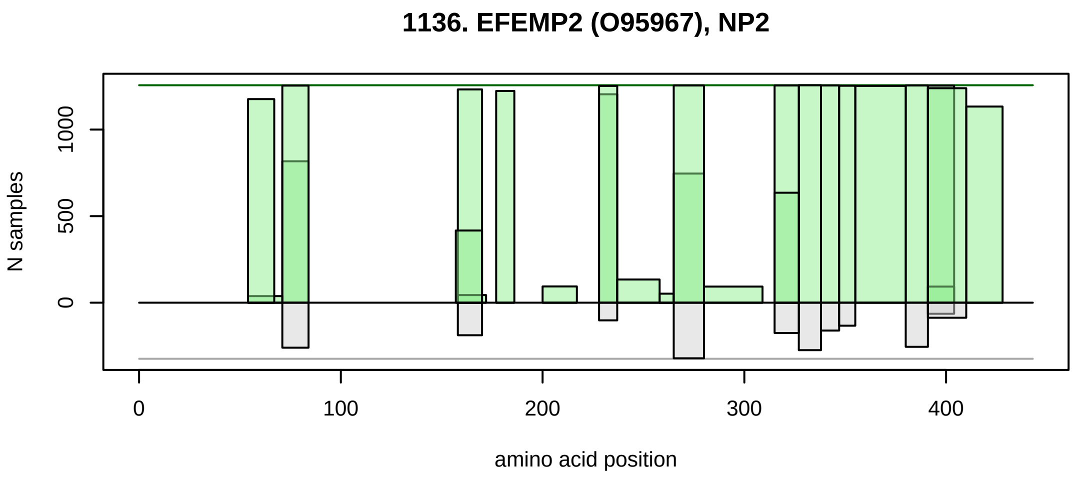
1136. EFEMP2 (O95967), NP2
N samples
0
500
1000
0
100
200
300
400
amino acid position

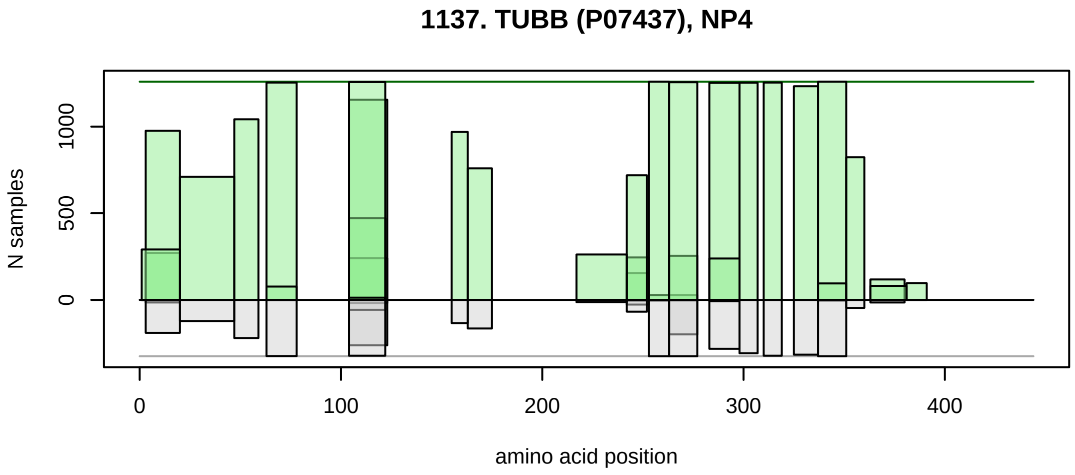
1137. TUBB (P07437), NP4
N samples
0
500
1000
0
100
200
300
400
amino acid position

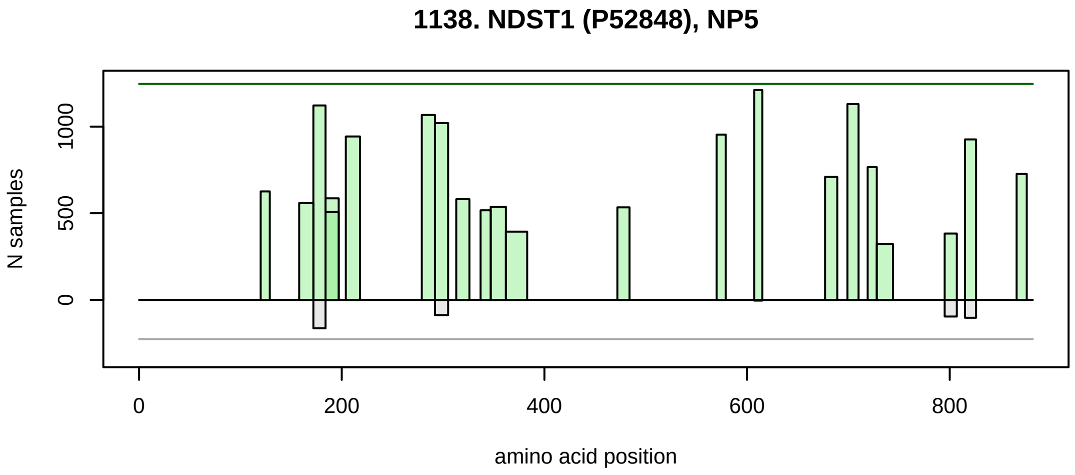
1138. NDST1 (P52848), NP5
N samples
0
500
1000
0
200
400
600
800
amino acid position

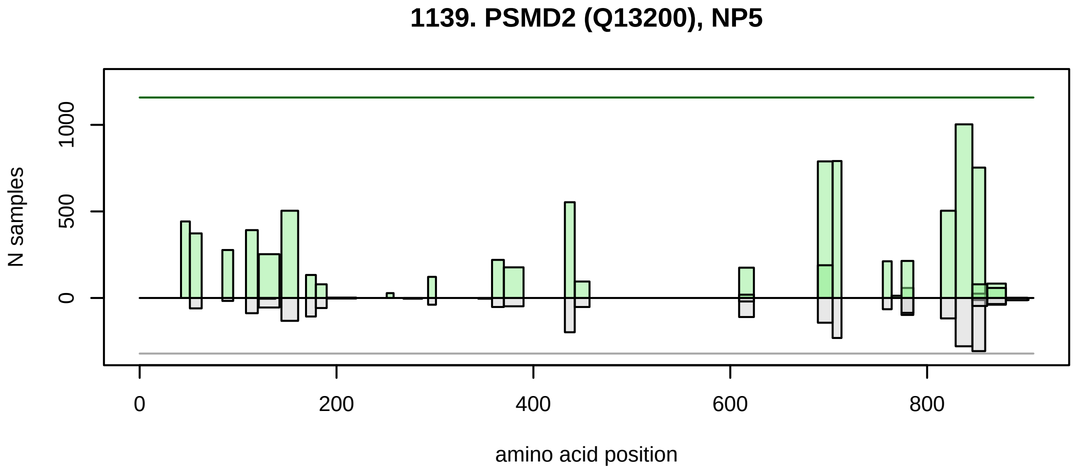
1139. PSMD2 (Q13200), NP5
N samples
0
500
1000
0
200
400
600
800
amino acid position

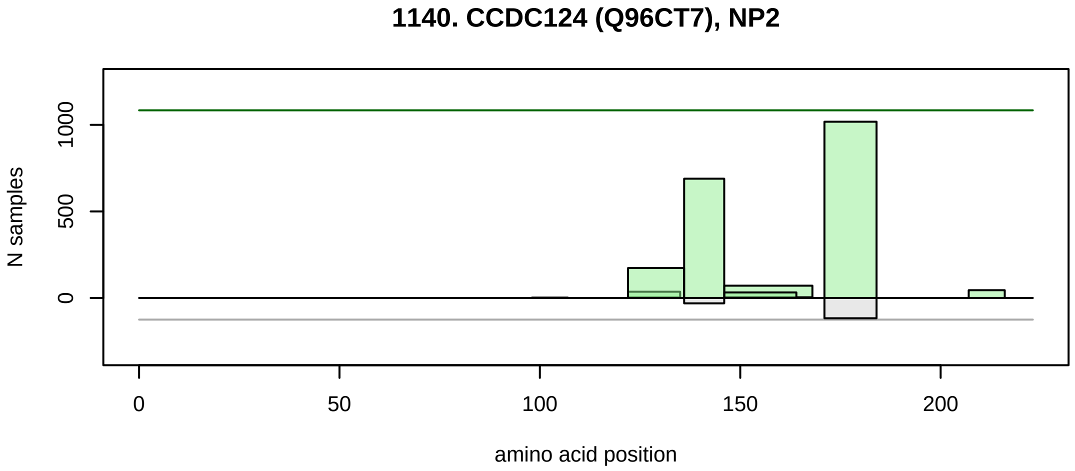
1140. CCDC124 (Q96CT7), NP2
N samples
0
500
1000
0
50
100
150
200
amino acid position

1141. IER2 (Q9BTL4), NP2

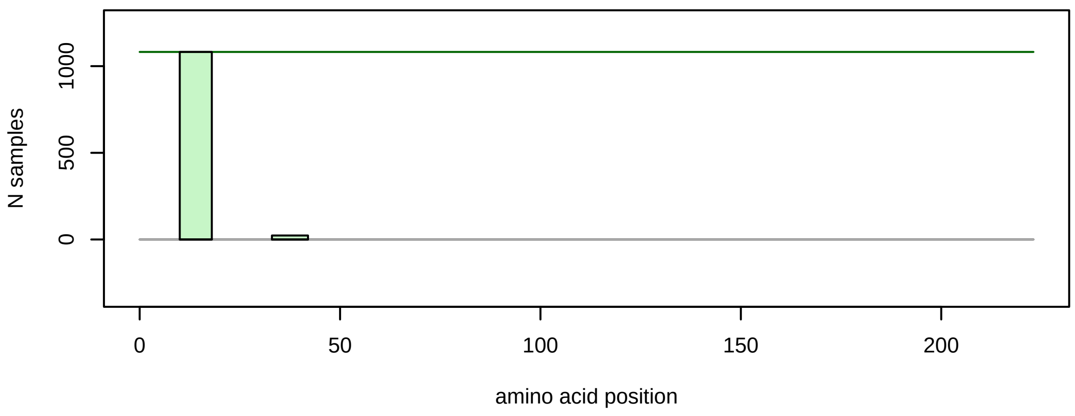

1142. IL16 (A0A8C8KBU6;Q14005;Q14005−2), NP4

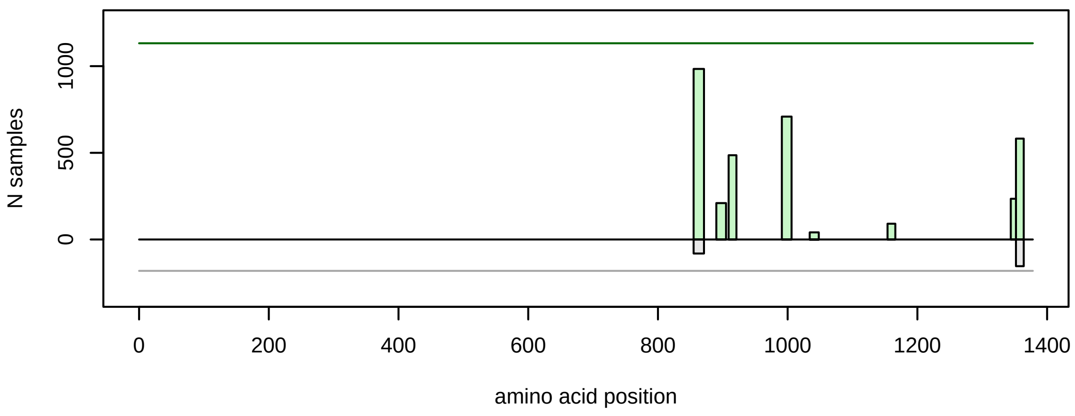

1143. BDNF (P23560−4), NP4

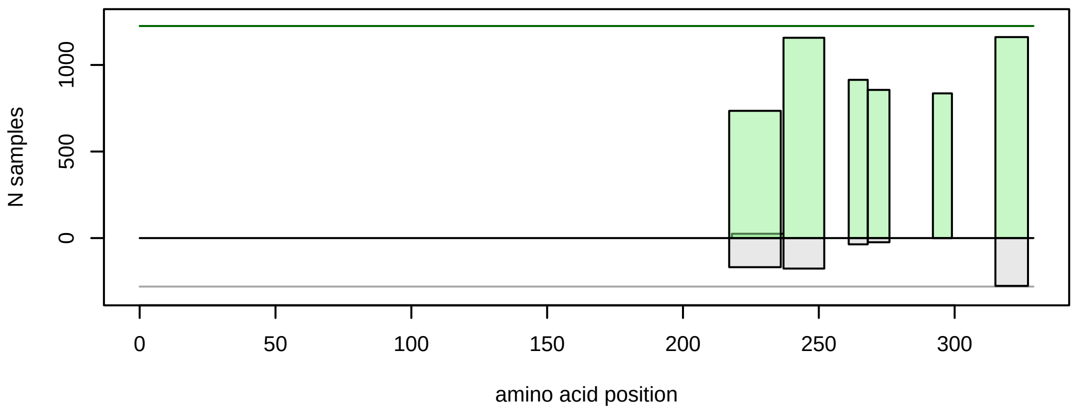

1144. RPS25 (P62851), NP1

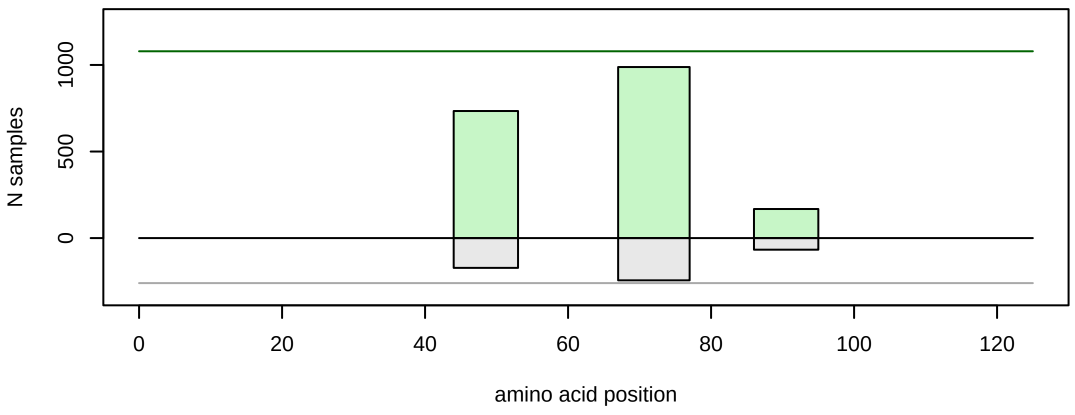

1145. TSPAN9 (B5MD23;O75954), NP4

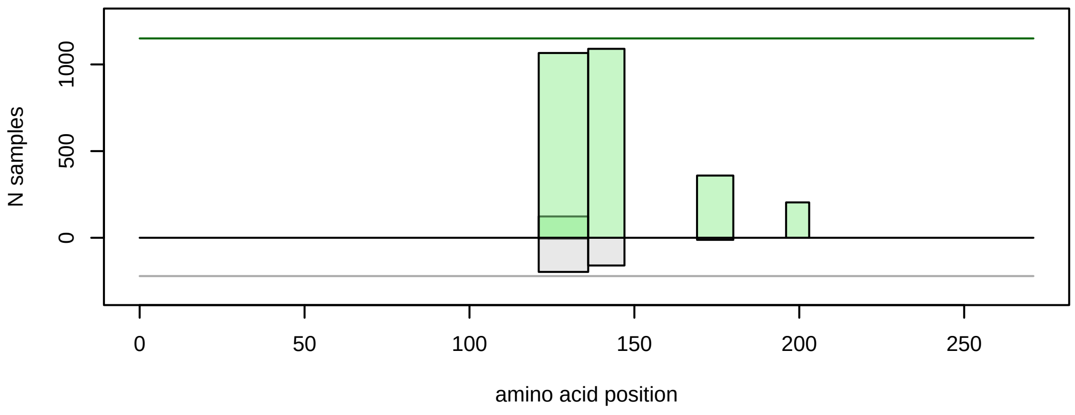

1146. NPIPA3 (E9PNS9), NP3

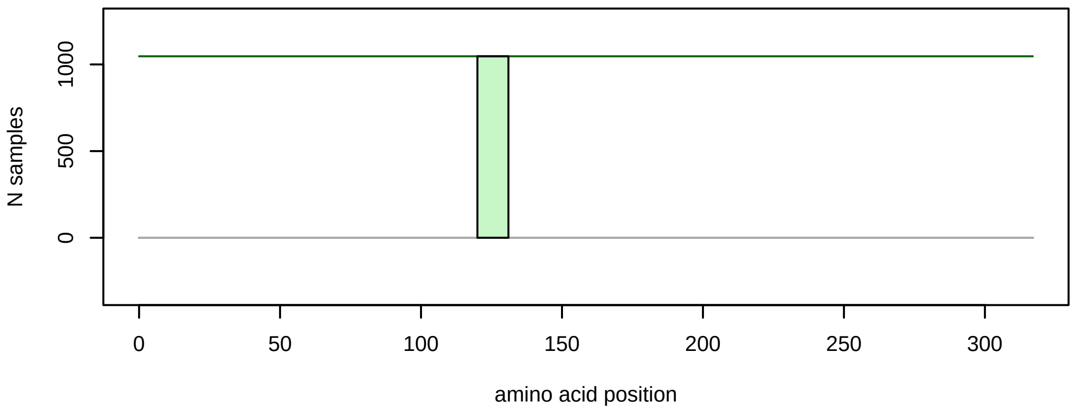

1147. SEMA3B (A0A0C4DGV8;Q13214;Q13214−2), NP3

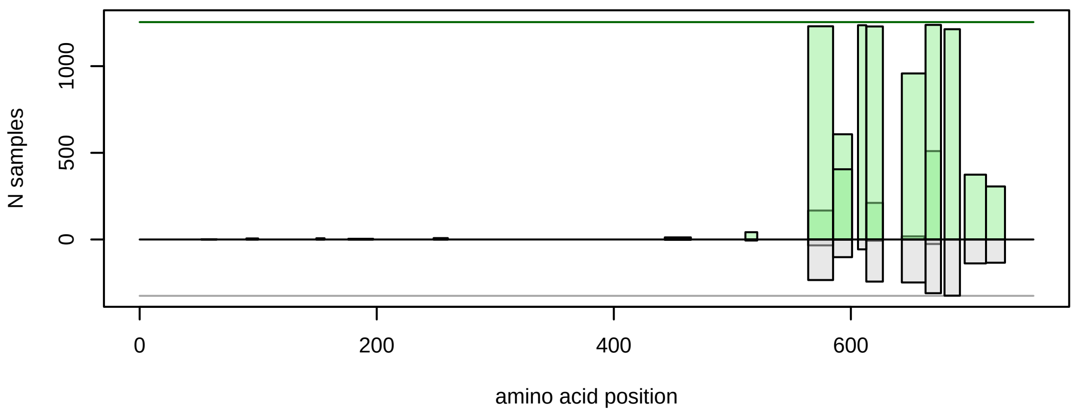

1148. HNRNPU (Q00839), NP2

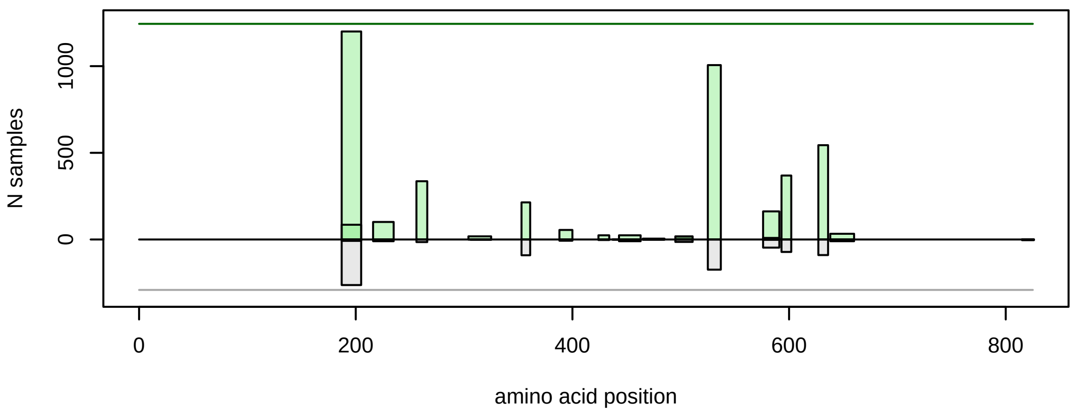

1149. MAPK14 (Q16539), NP1

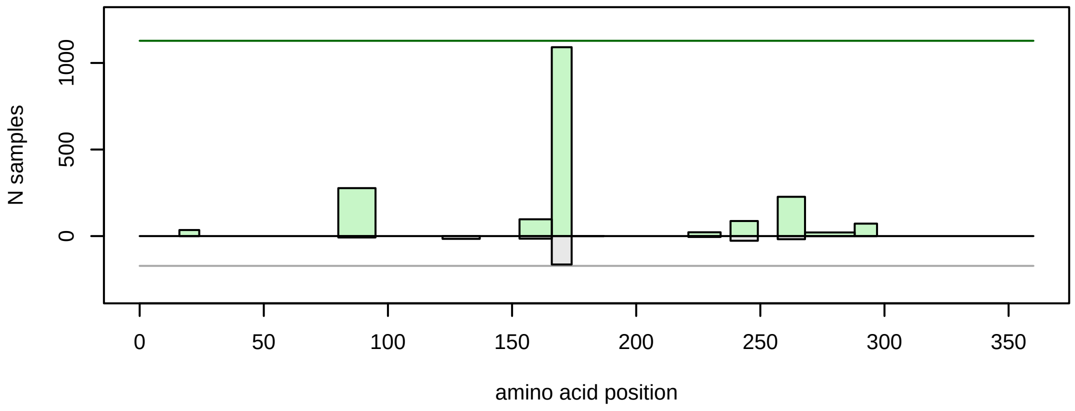

1150. FAM3B (A8MTF8;P58499;P58499−2), NP3

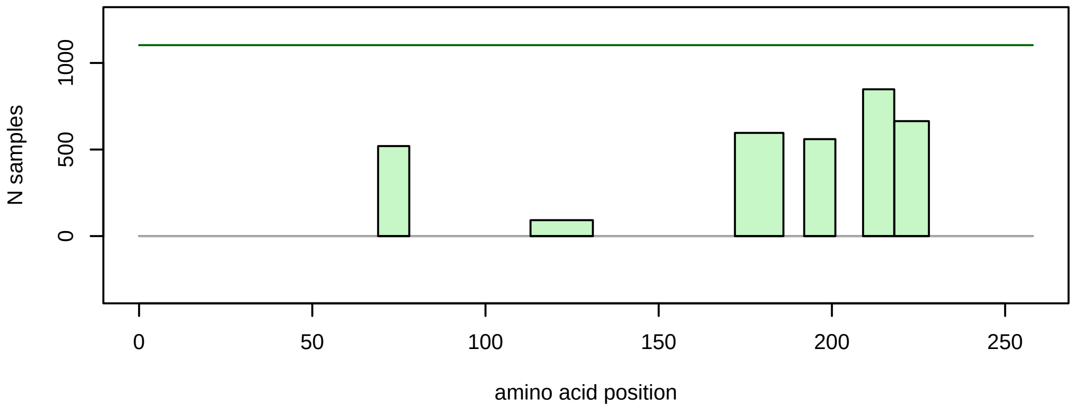

1151. LIMS1 (P48059−5), NP4

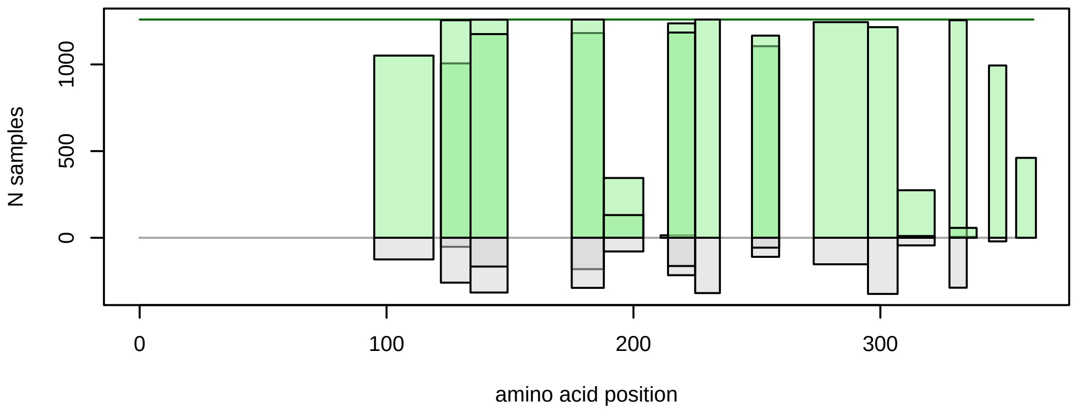

1152. HBA2 (P69905), NP1

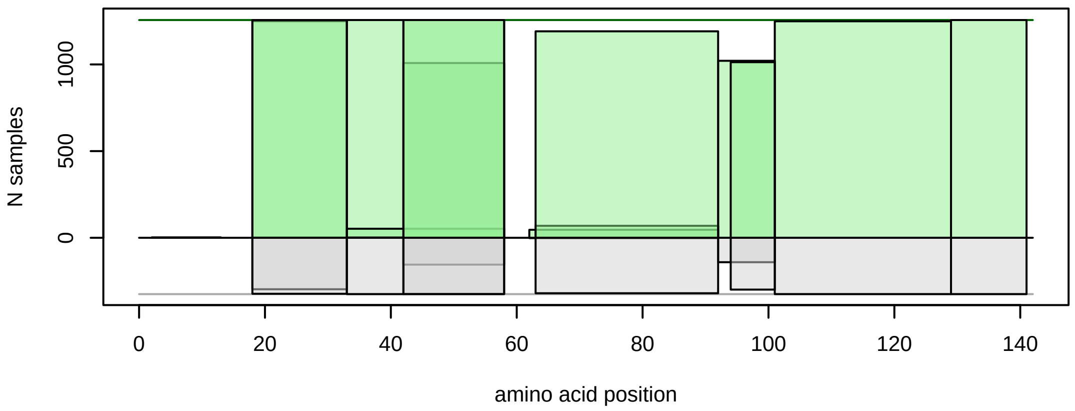

1153. PNLIP (P16233), NP5

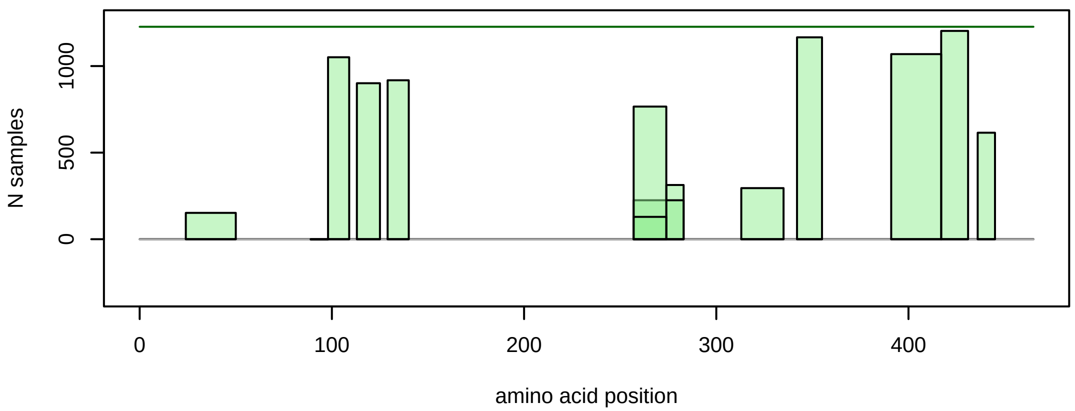

1154. SRI (P30626−3), NP2

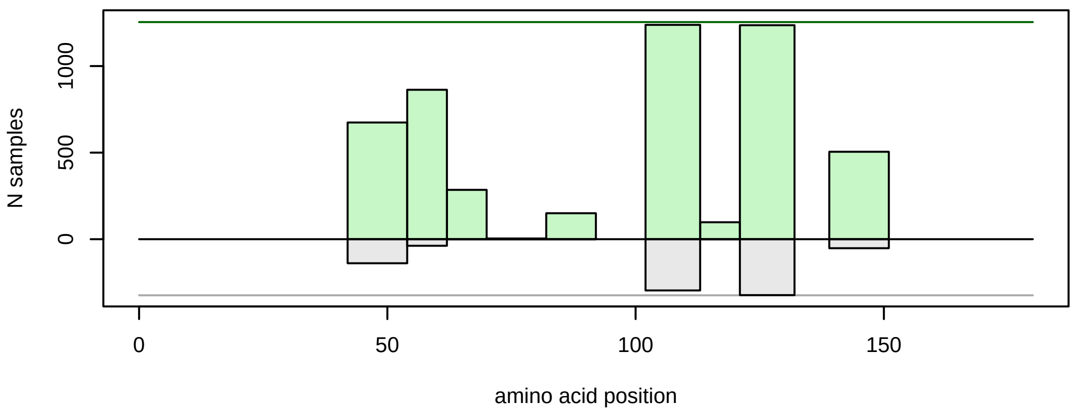

1155. RAB6B (Q9NRW1), NP4

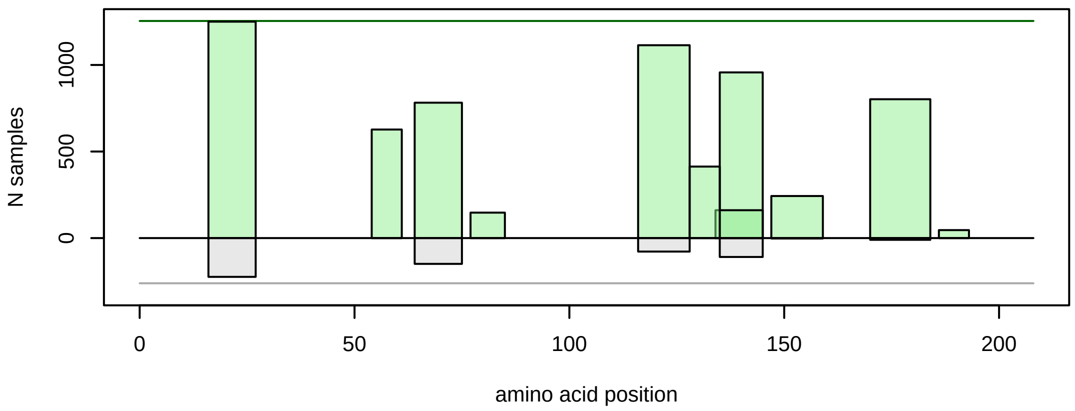

1156. RPL7 (P18124), NP4

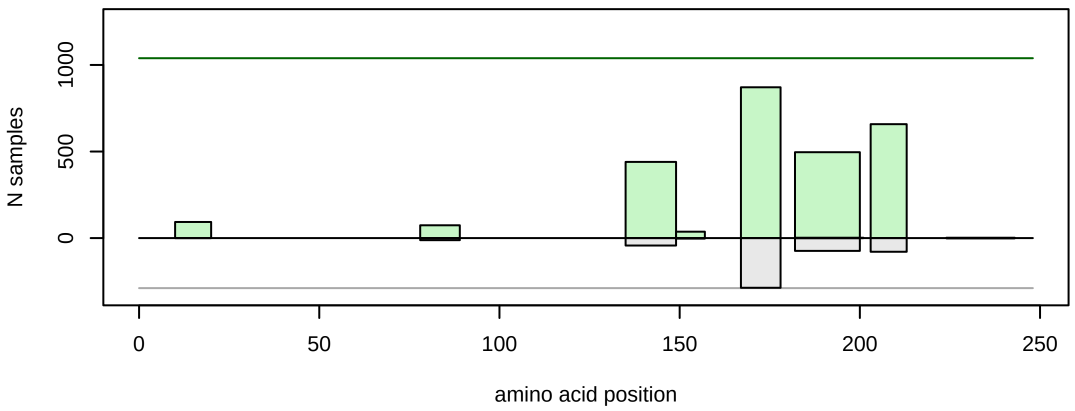

1157. WASF2 (Q9Y6W5), NP4

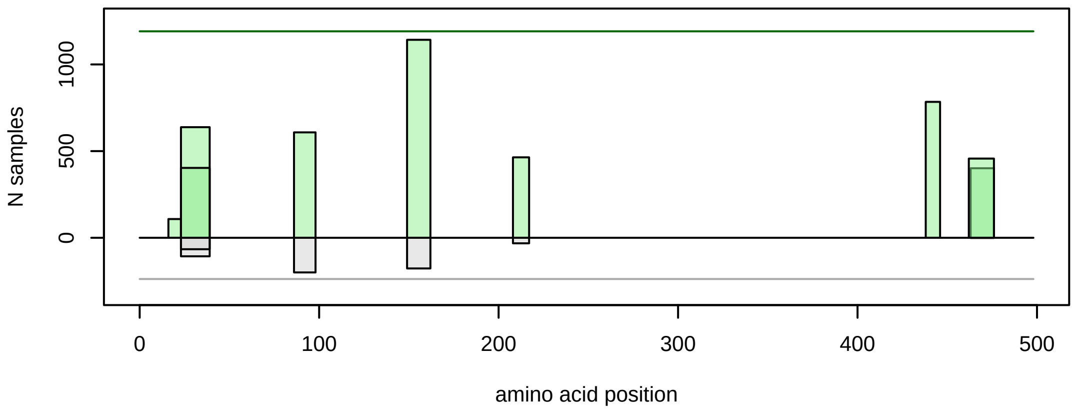

1158. TPM1 (P09493−6), NP5

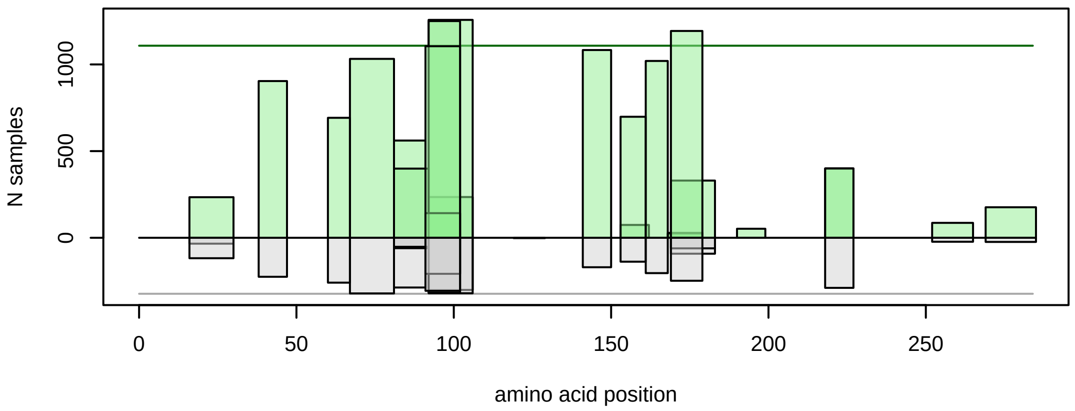

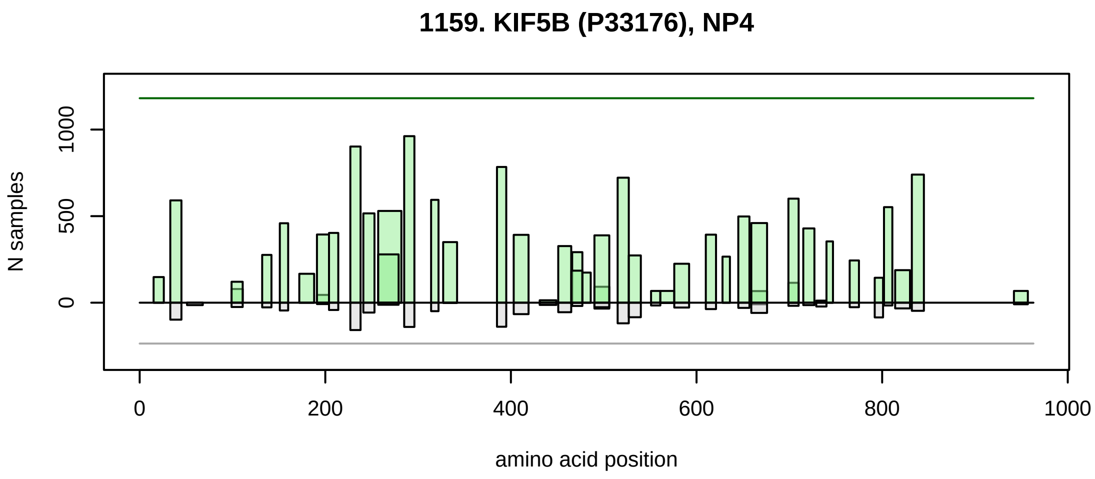

1159. KIF5B (P33176), NP4
N samples
0
500
1000
0
200
400
600
800
1000
amino acid position

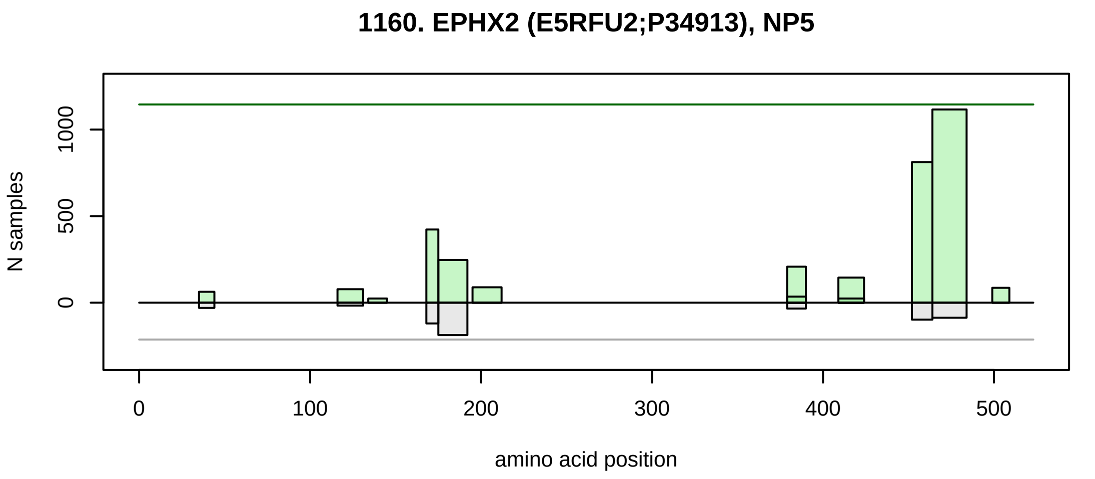

1160. EPHX2 (E5RFU2;P34913), NP5
N samples
0
500
1000
0
100
200
300
400
500
amino acid position

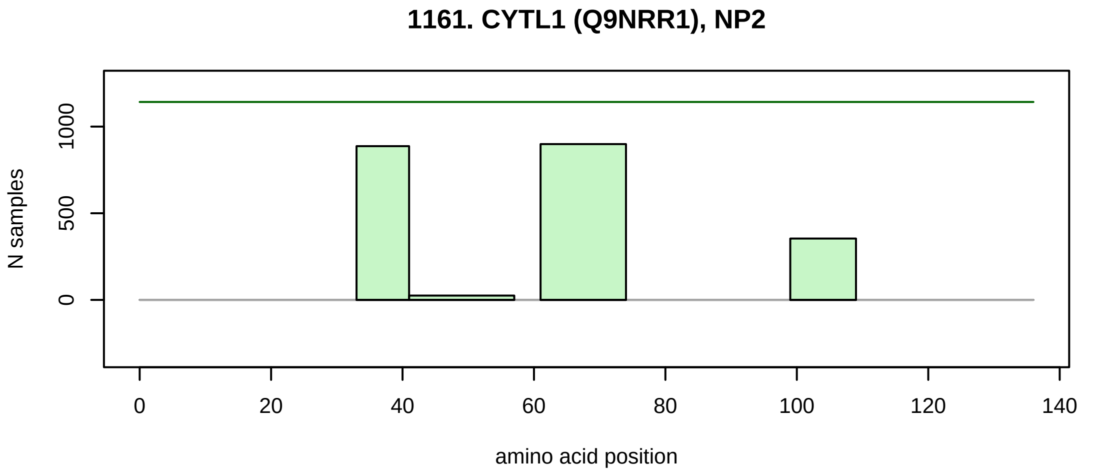

1161. CYTL1 (Q9NRR1), NP2
N samples
0
500
1000
0
20
40
60
80
100
120
140
amino acid position

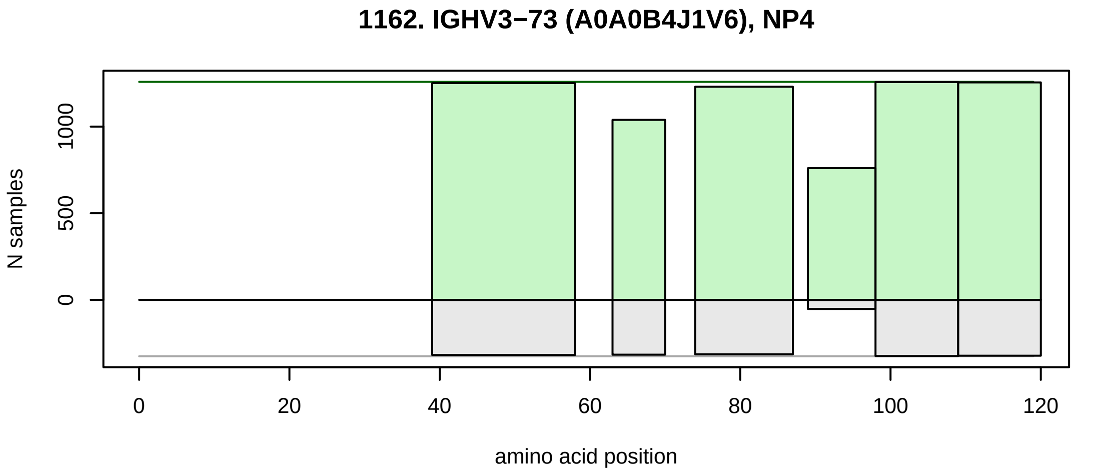

1162. IGHV3−73 (A0A0B4J1V6), NP4
N samples
0
500
1000
0
20
40
60
80
100
120
amino acid position

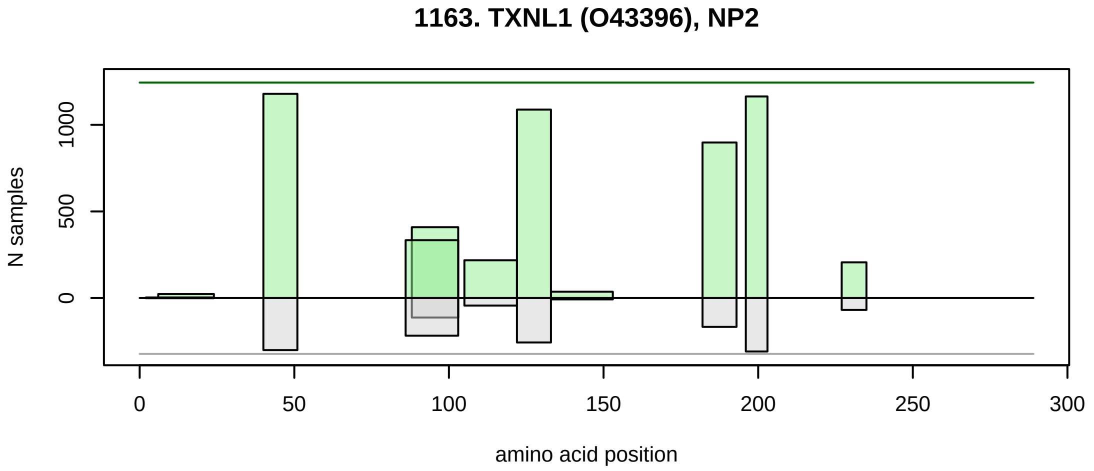

1163. TXNL1 (O43396), NP2
N samples
0
500
1000
0
50
100
150
200
250
300
amino acid position

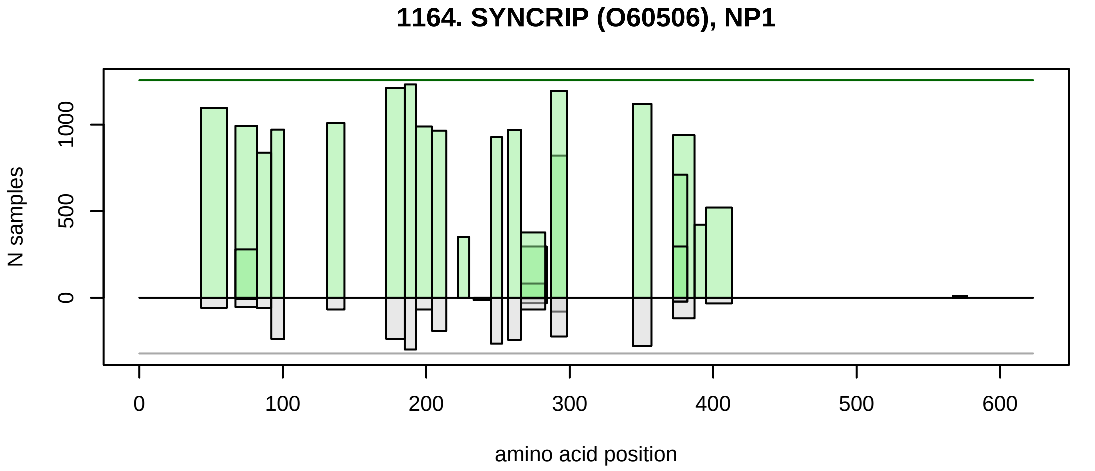

1164. SYNCRIP (O60506), NP1
N samples
0
500
1000
0
100
200
300
400
500
600
amino acid position

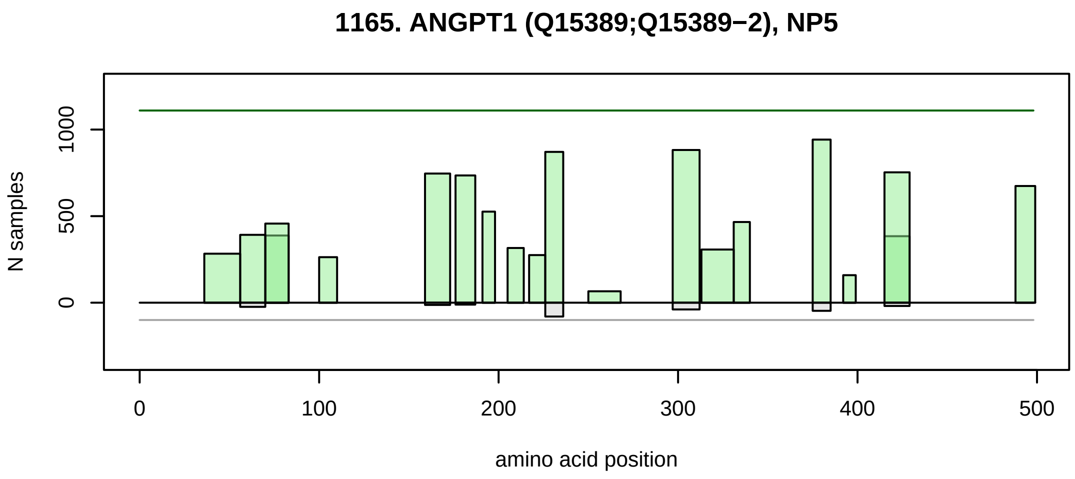
1165. ANGPT1 (Q15389;Q15389−2), NP5
N samples
amino acid position
0
500
1000
0
100
200
300
400
500

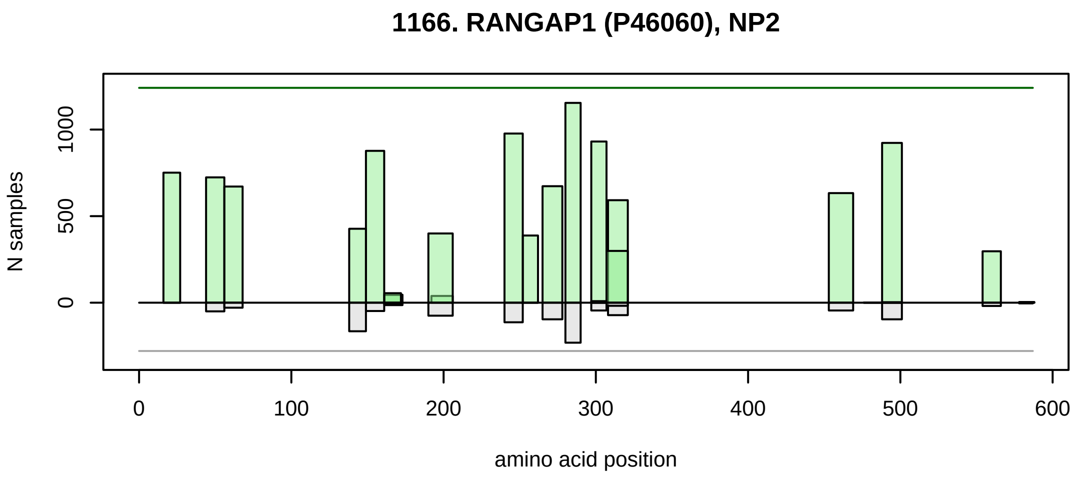
1166. RANGAP1 (P46060), NP2
N samples
amino acid position
0
500
1000
0
100
200
300
400
500
600

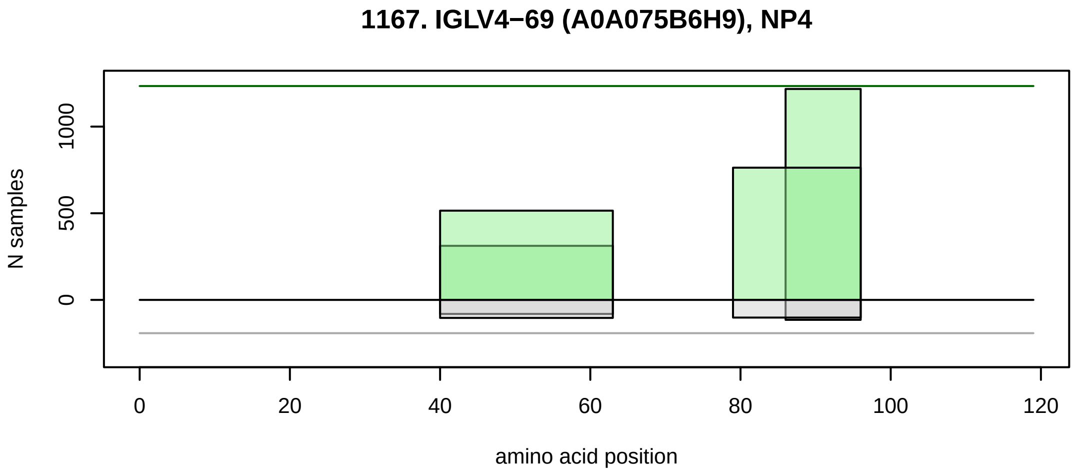
1167. IGLV4−69 (A0A075B6H9), NP4
N samples
amino acid position
0
500
1000
0
20
40
60
80
100
120

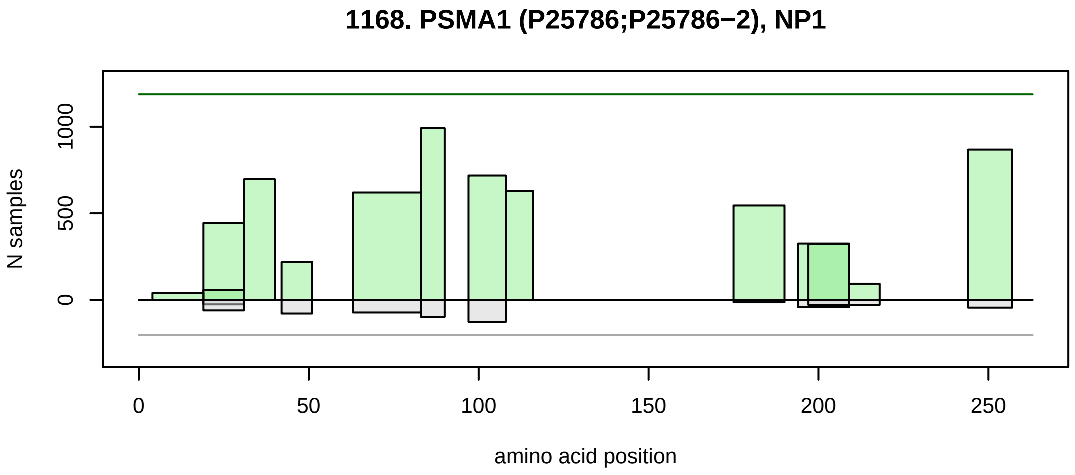
1168. PSMA1 (P25786;P25786−2), NP1
N samples
amino acid position
0
500
1000
0
50
100
150
200
250

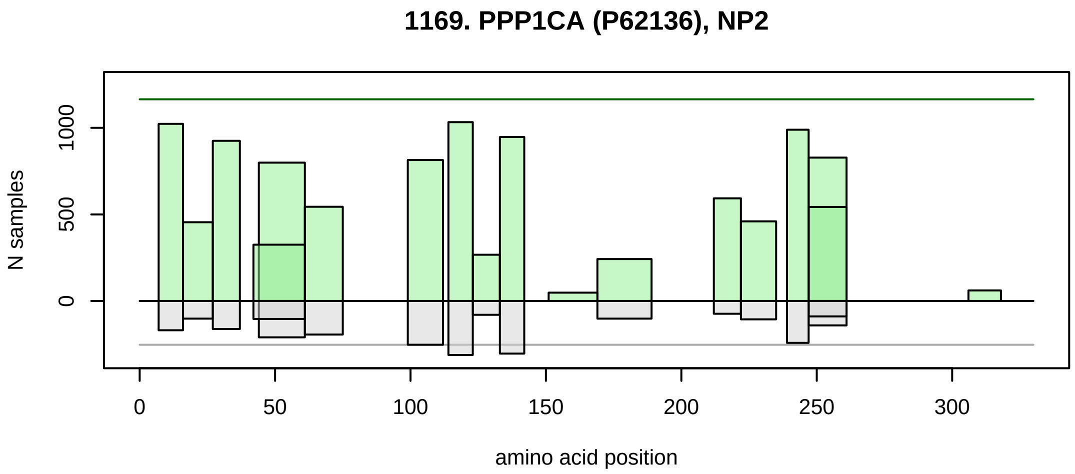
1169. PPP1CA (P62136), NP2
N samples
amino acid position
0
500
1000
0
50
100
150
200
250
300

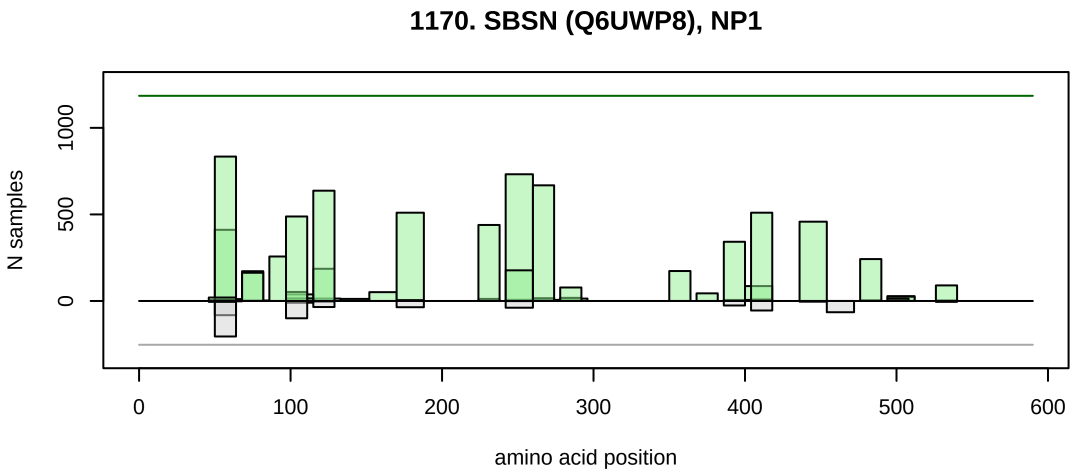
1170. SBSN (Q6UWP8), NP1
N samples
amino acid position
0
500
1000
0
100
200
300
400
500
600

1171. PIK3R1 (P27986), NP4

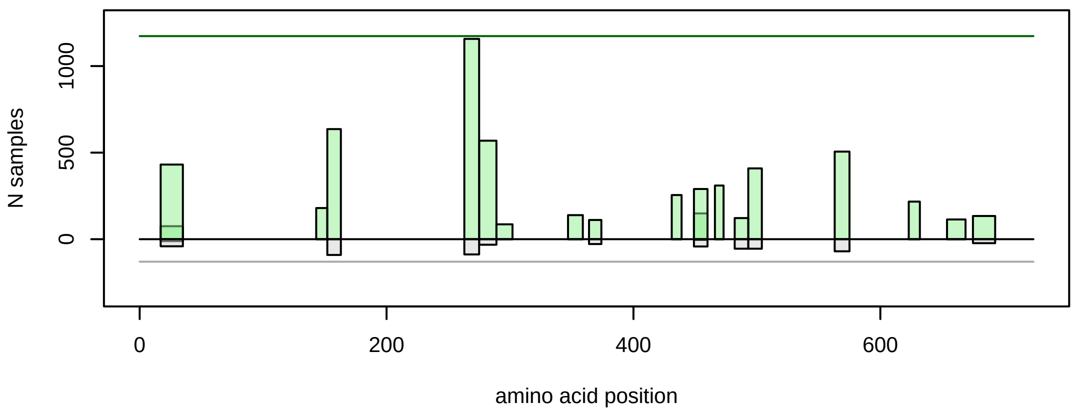

1172. CHRD (E7ESX1;Q9H2X0), NP1

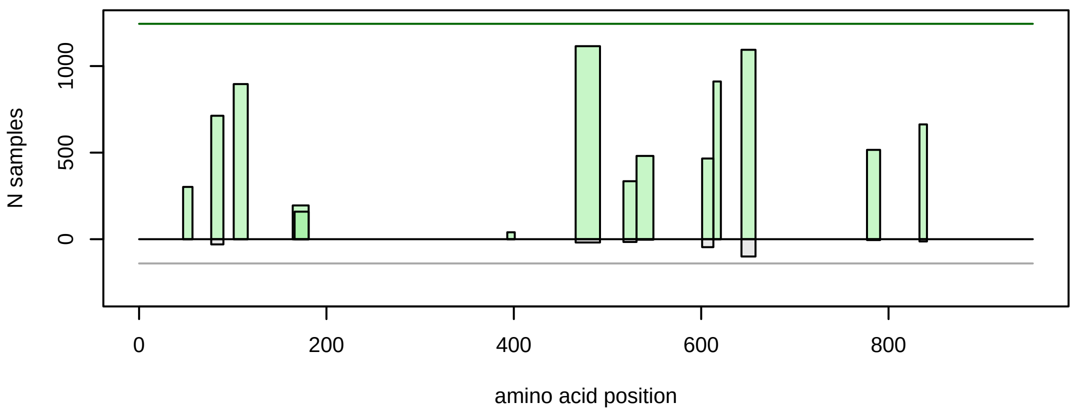

1173. RAC2 (P15153), NP1

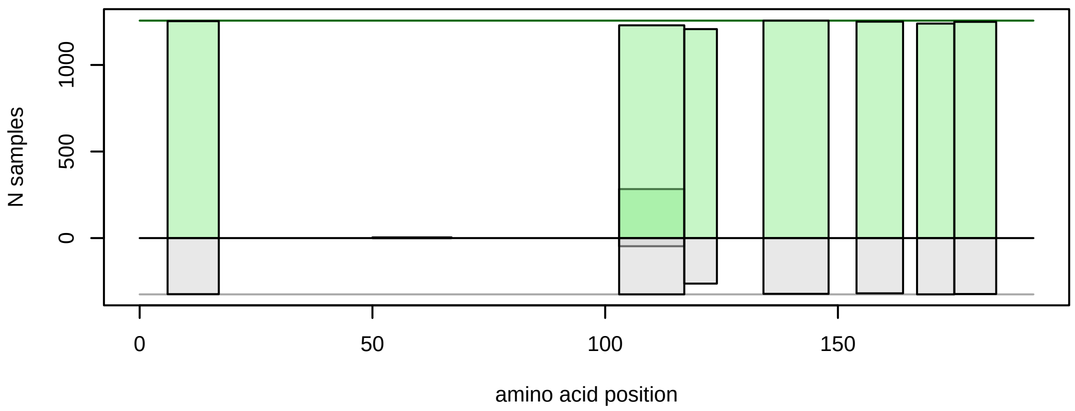

1174. TKT (P29401−2), NP1

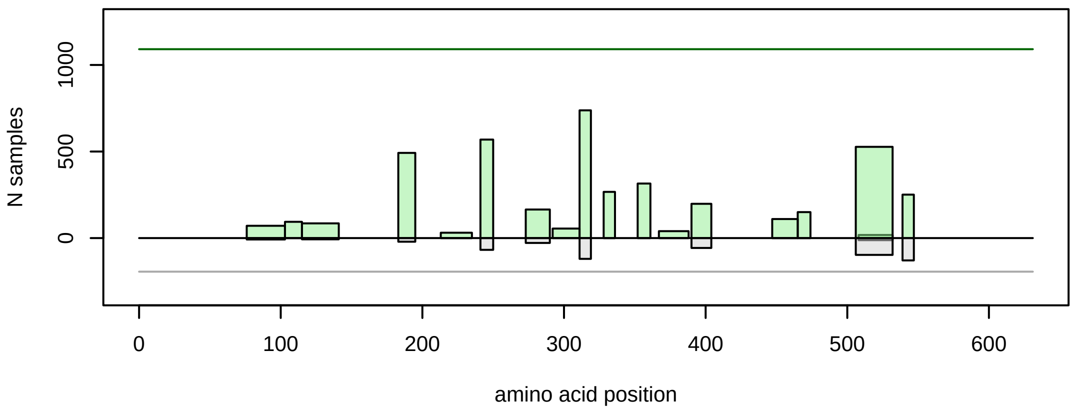

1175. APBB1IP (Q7Z5R6), NP1

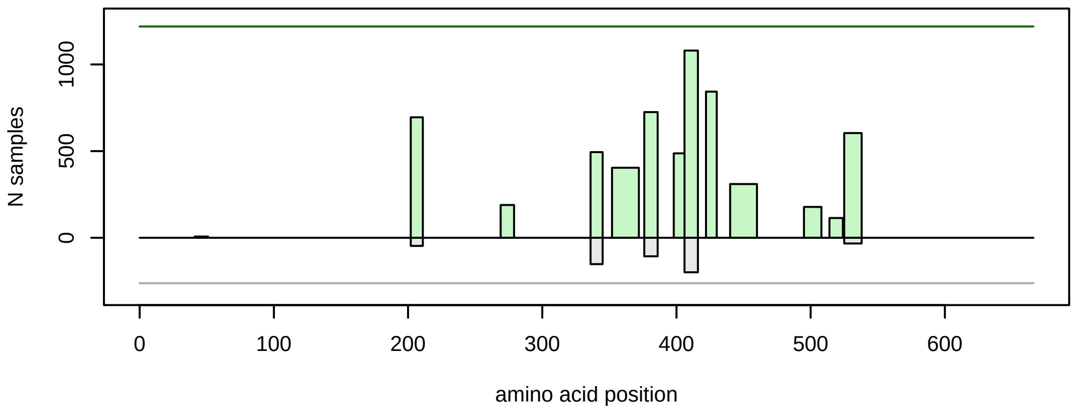

1176. DAG1 (Q14118), NP2

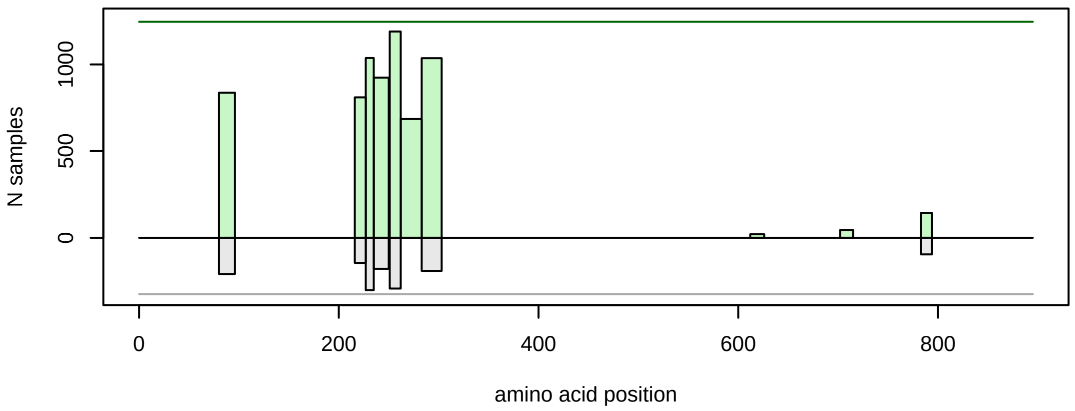

1177. IGHV6−1 (A0A0B4J1U7), NP4

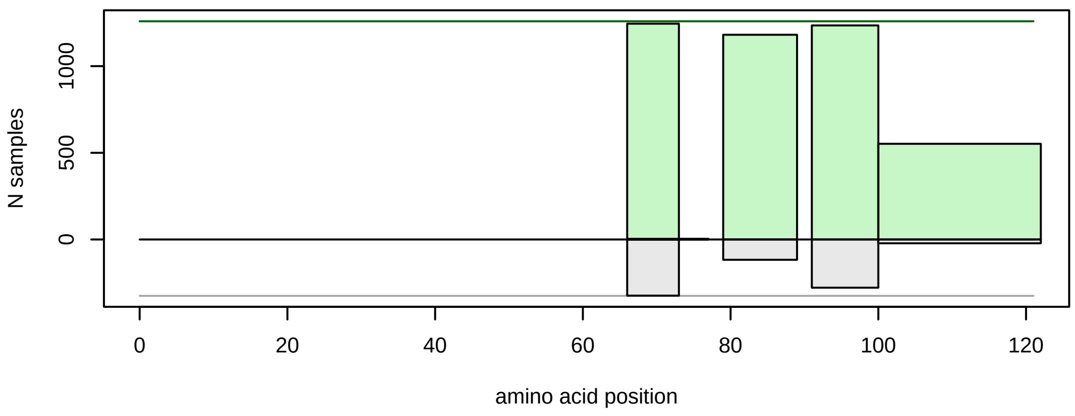

1178. APRT (P07741), NP4

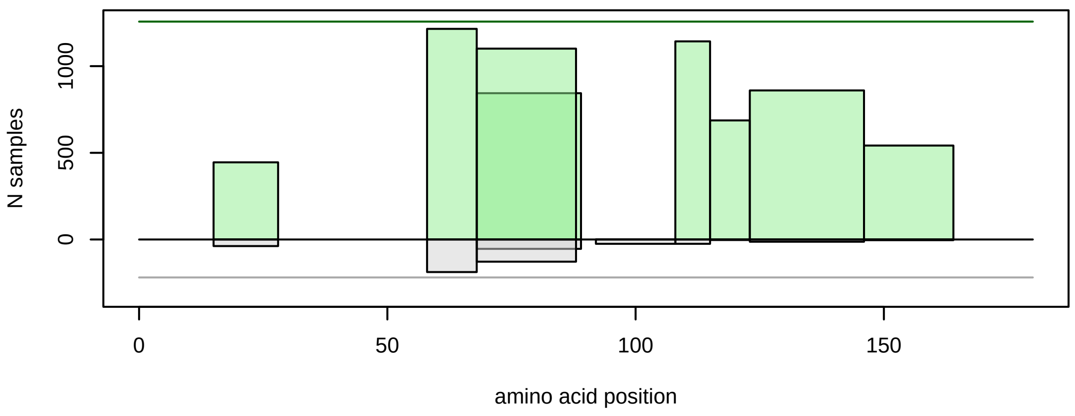

1179. RPS2 (P15880), NP1

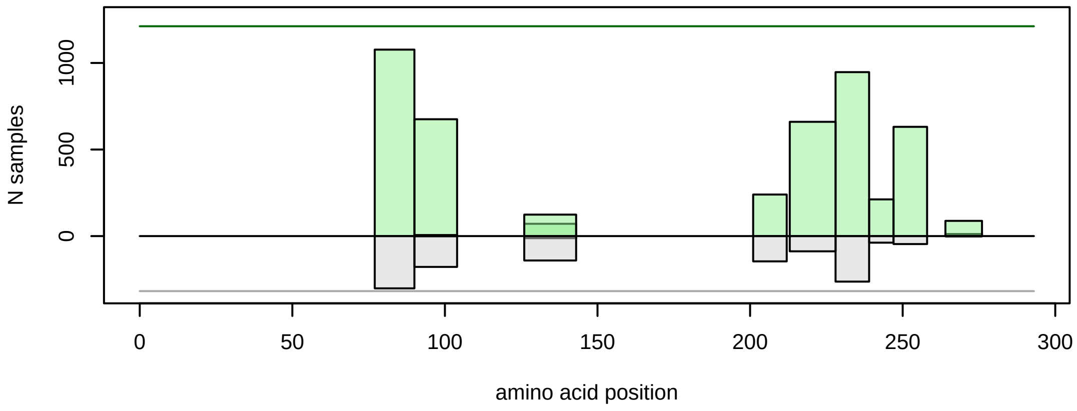

1180. PRRG4 (Q9BZD6), NP2

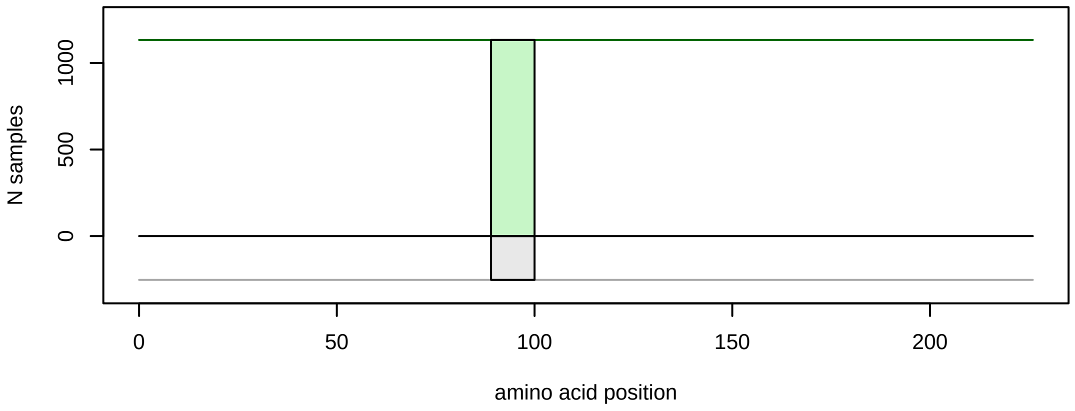

1181. FCGRT (P55899), NP2

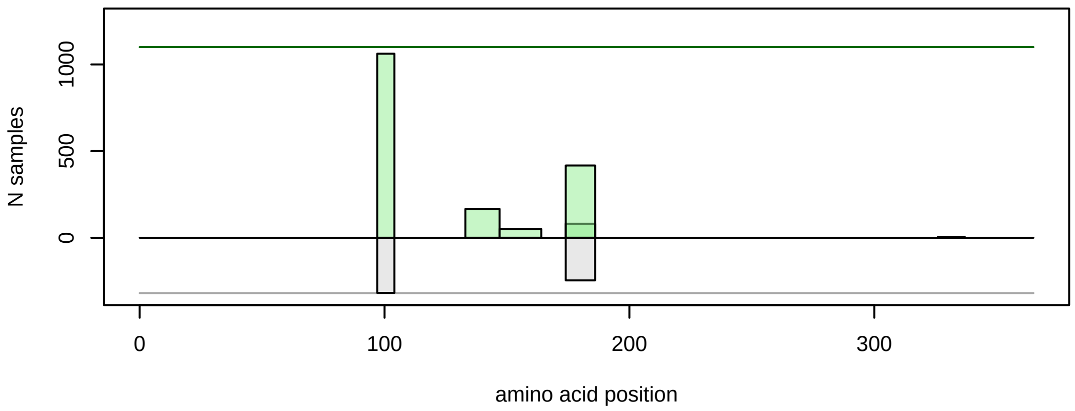

1182. MPO (P05164), NP4

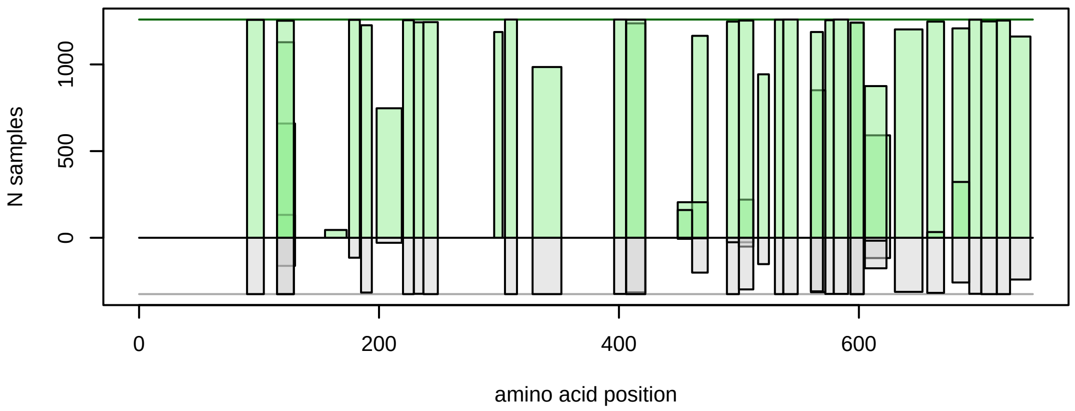

1183. DYNLL1 (P63167), NP1

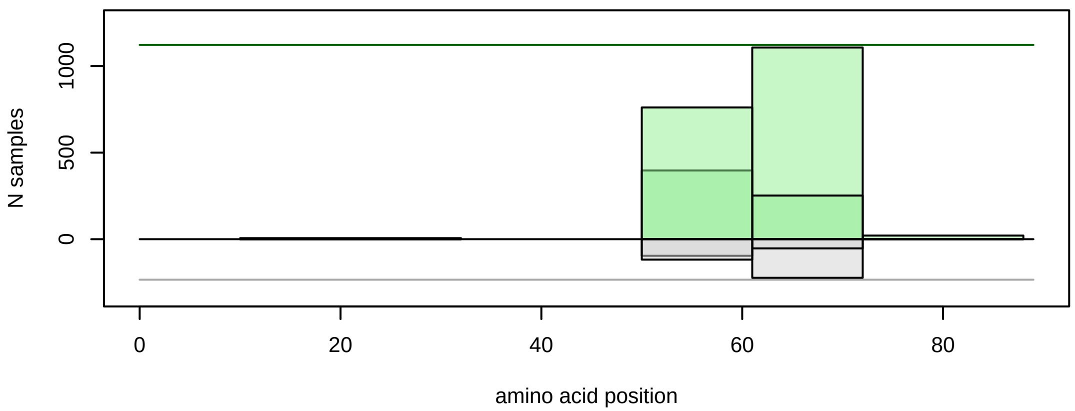

1184. COL14A1 (Q05707−2), NP3

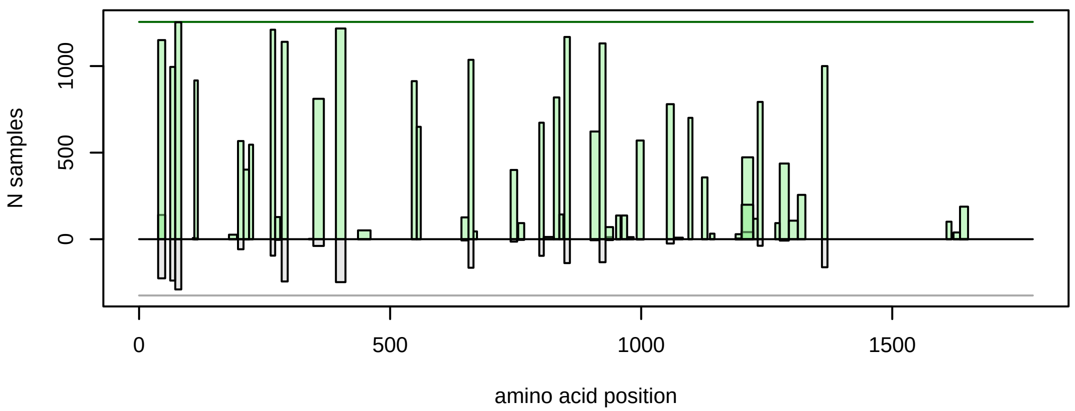

1185. SPP1 (P10451−5), NP2

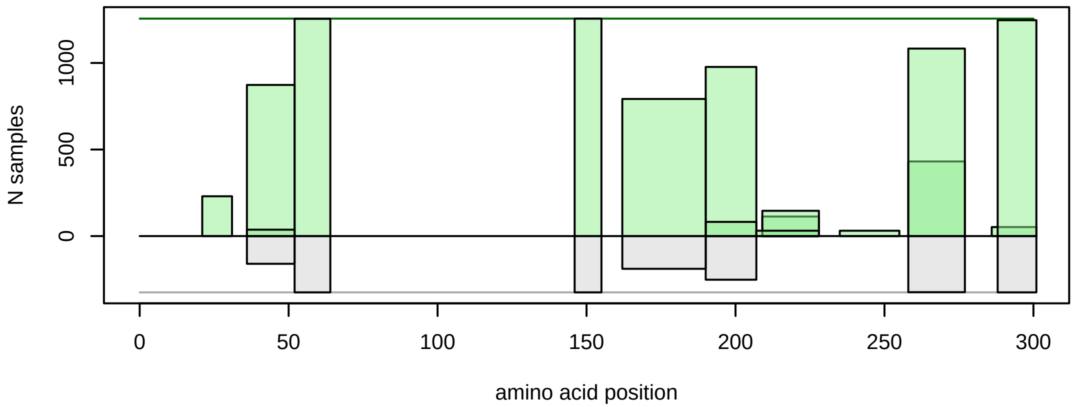

1186. EIF4A1 (P60842), NP5

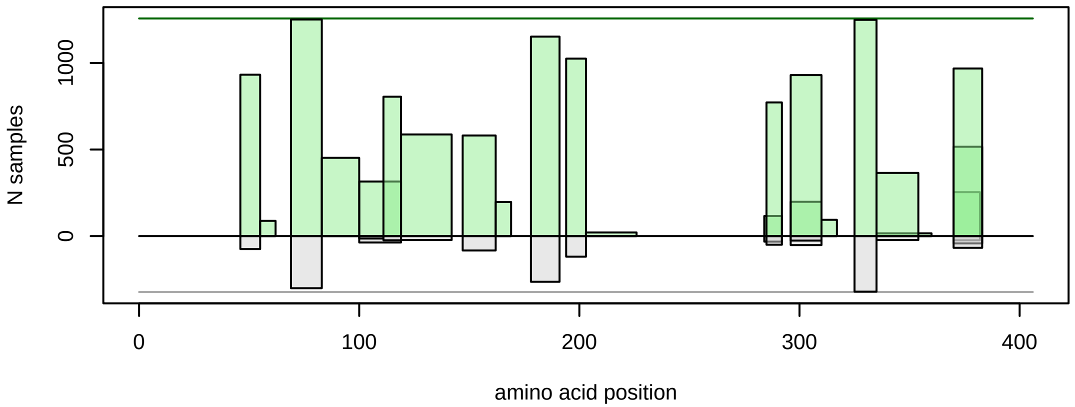

1187. CPVL (Q9H3G5), NP2

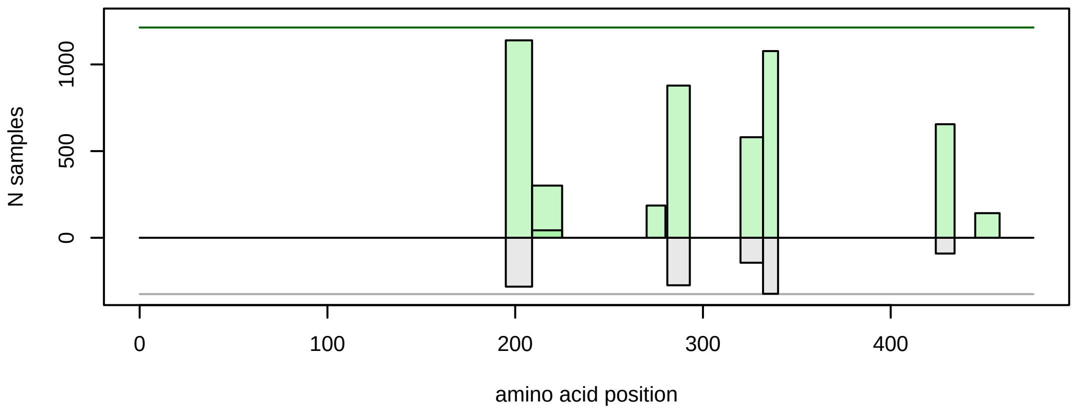

1188. AFM (P43652), NP4

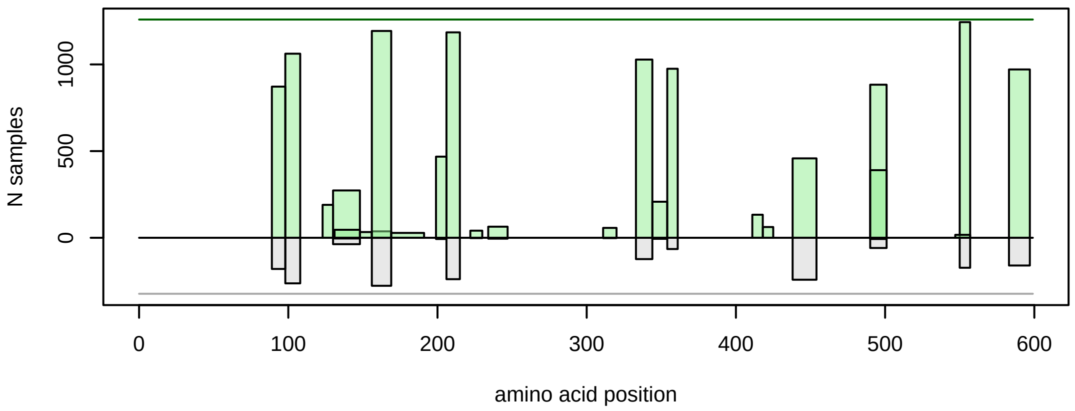

1189. SPN (P16150), NP2

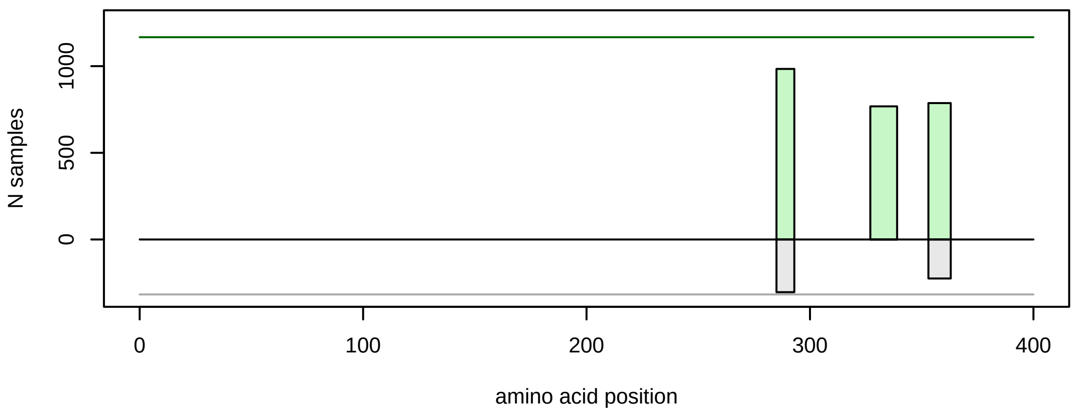

1190. IGLV3−21 (P80748), NP3

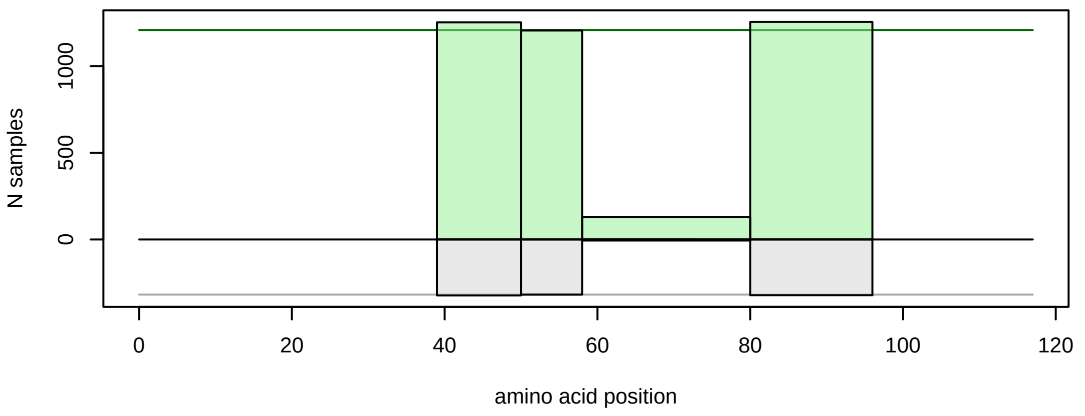

1191. ELMO1 (Q92556), NP4

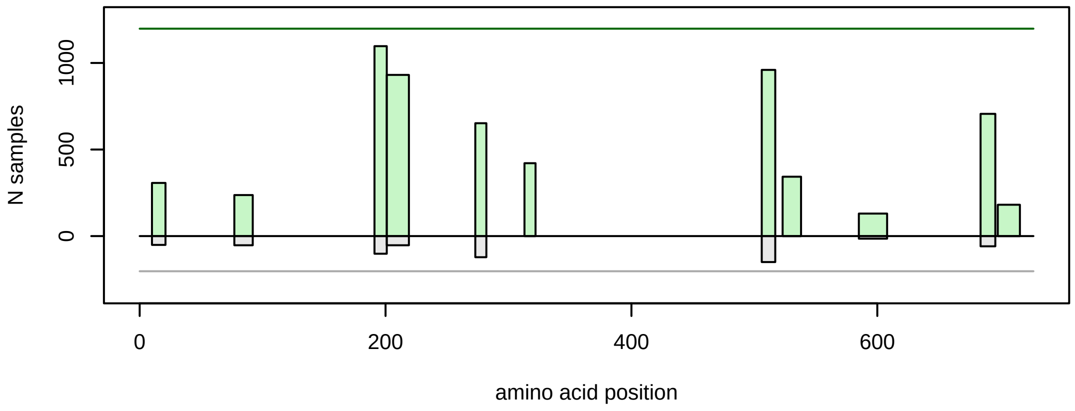

1192. B4GALT1 (P15291), NP4

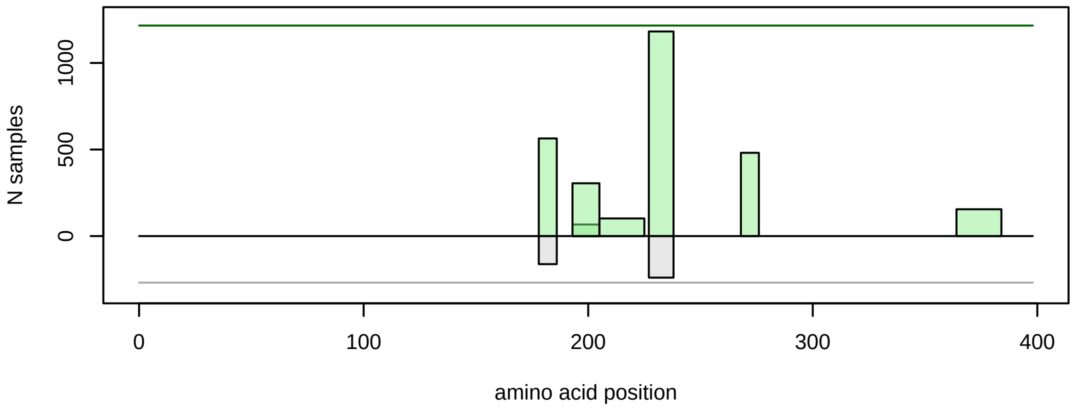

1193. NCL (A0A7I2V3F3), NP4

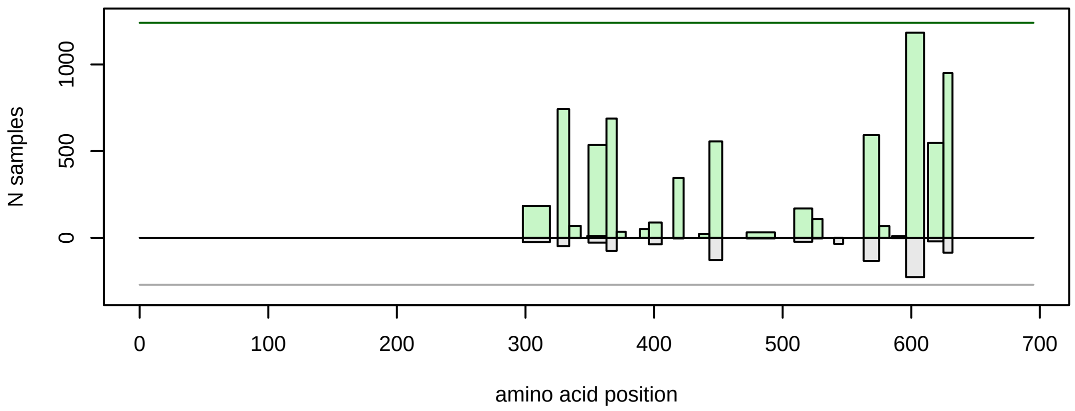

1194. GSN (P06396−2), NP1

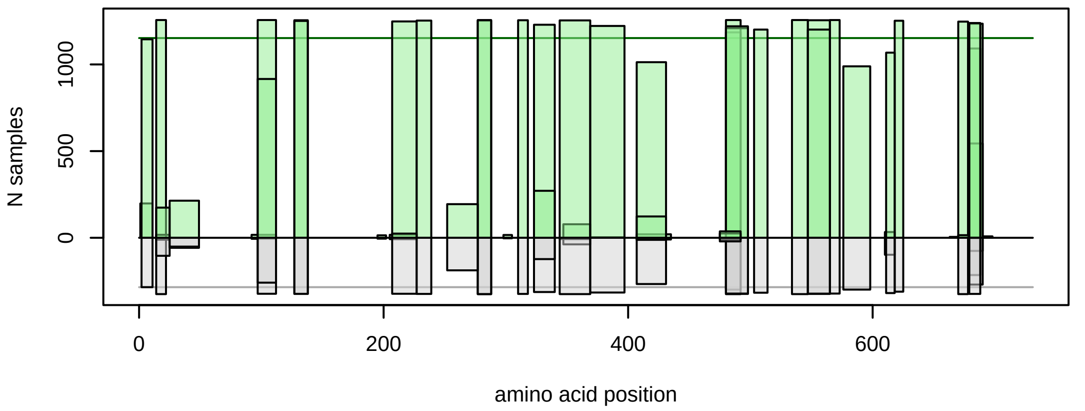

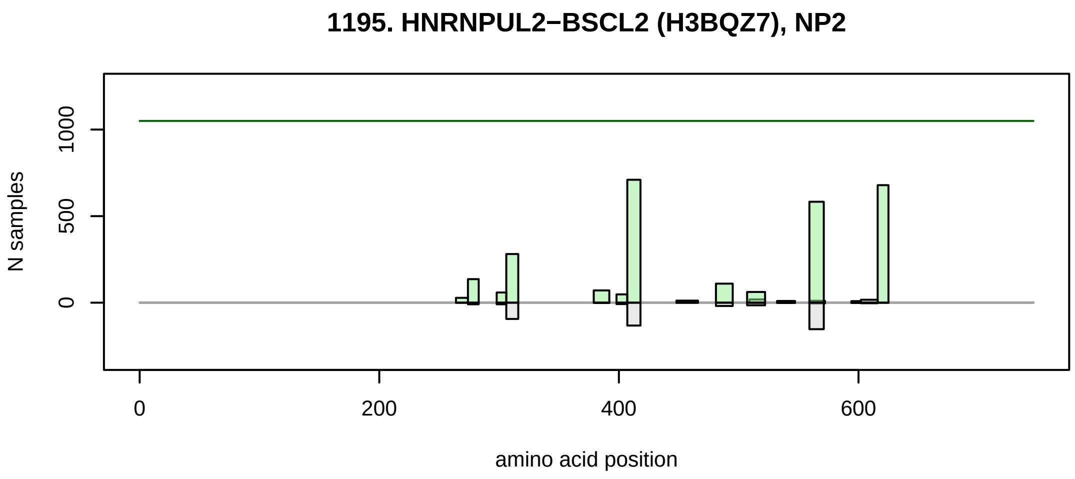
1195. HNRNPUL2−BSCL2 (H3BQZ7), NP2
N samples
0
500
1000
0
200
400
600
amino acid position

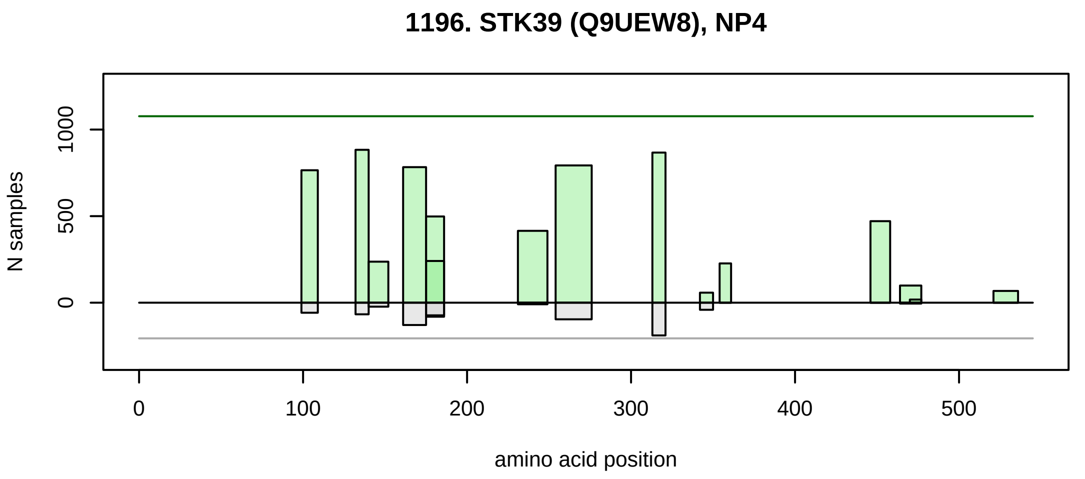
1196. STK39 (Q9UEW8), NP4
N samples
0
500
1000
0
100
200
300
400
500
amino acid position

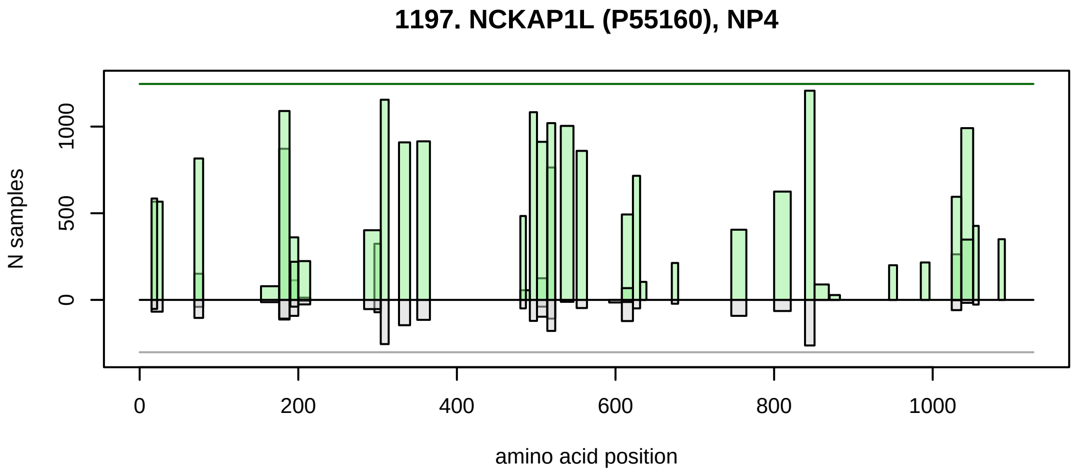
1197. NCKAP1L (P55160), NP4
N samples
0
500
1000
0
200
400
600
800
1000
amino acid position

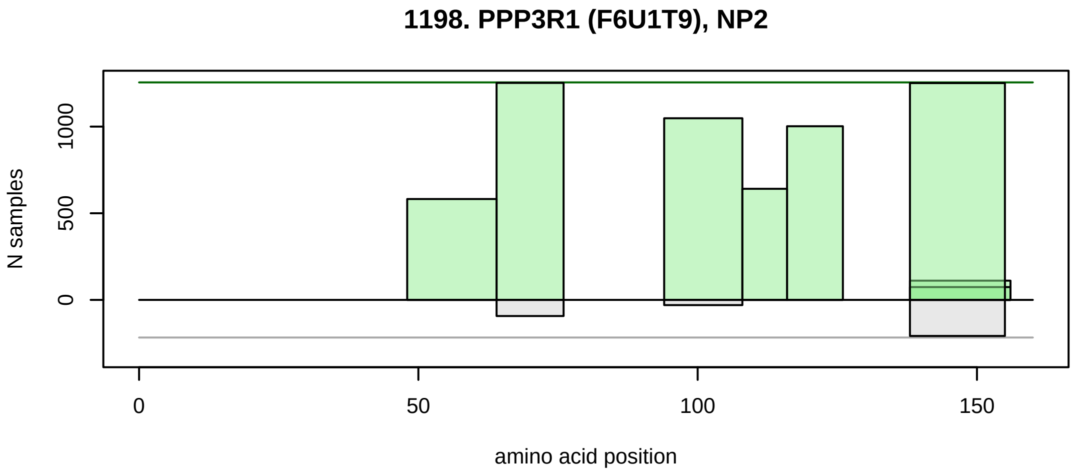
1198. PPP3R1 (F6U1T9), NP2
N samples
0
500
1000
0
50
100
150
amino acid position

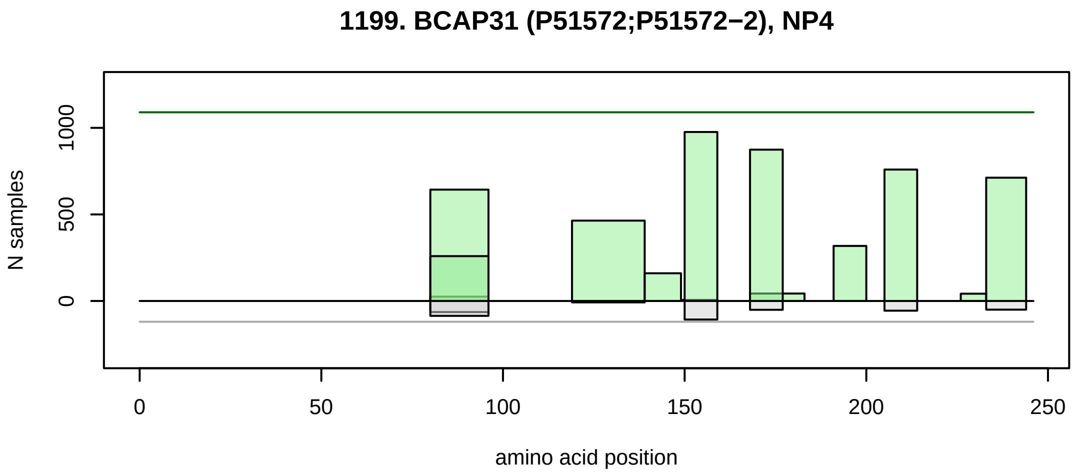
1199. BCAP31 (P51572;P51572−2), NP4
N samples
0
500
1000
0
50
100
150
200
250
amino acid position

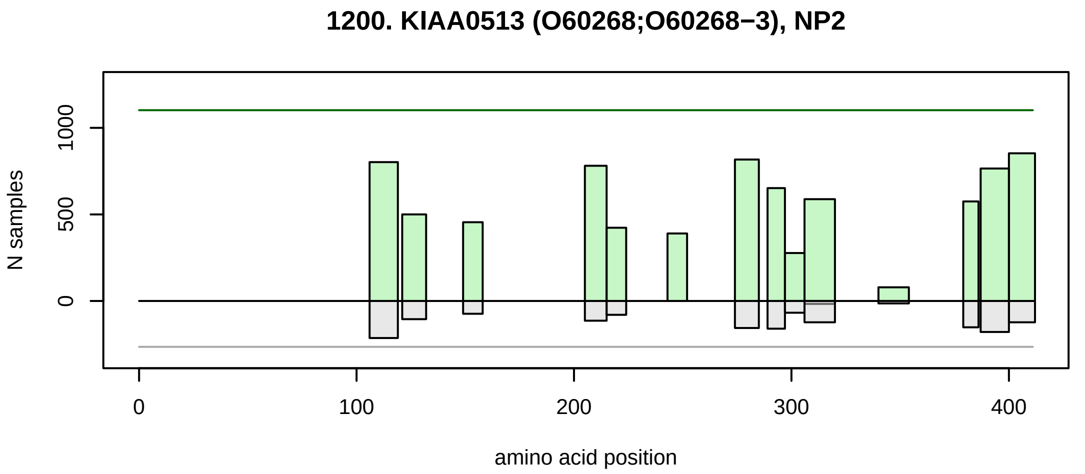
1200. KIAA0513 (O60268;O60268−3), NP2
N samples
0
500
1000
0
100
200
300
400
amino acid position

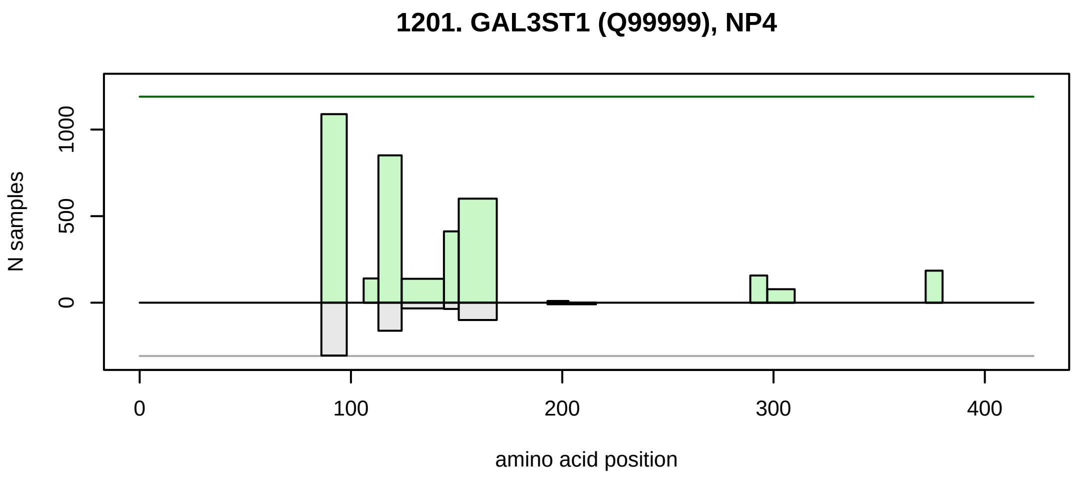

1201. GAL3ST1 (Q99999), NP4
N samples
0
500
1000
0
100
200
300
400
amino acid position

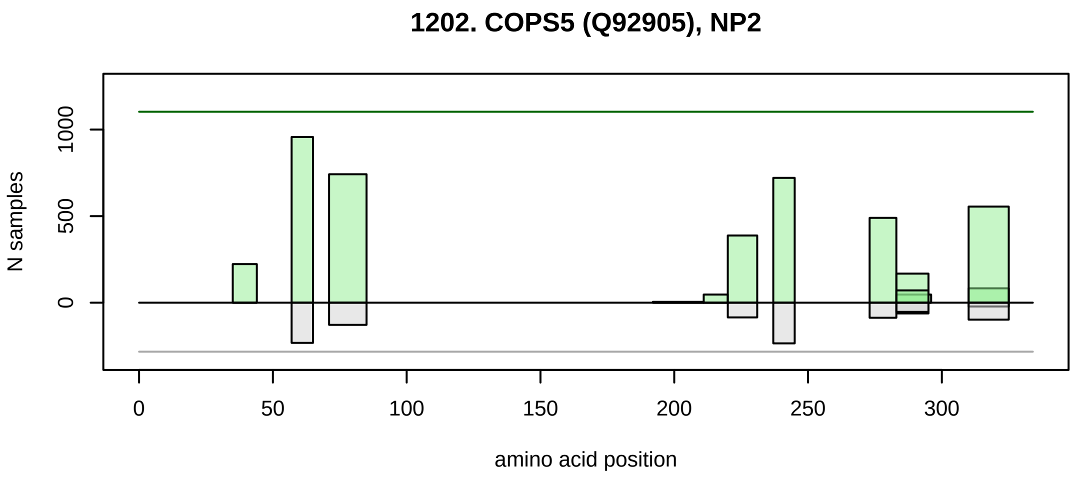

1202. COPS5 (Q92905), NP2
N samples
0
500
1000
0
50
100
150
200
250
300
amino acid position

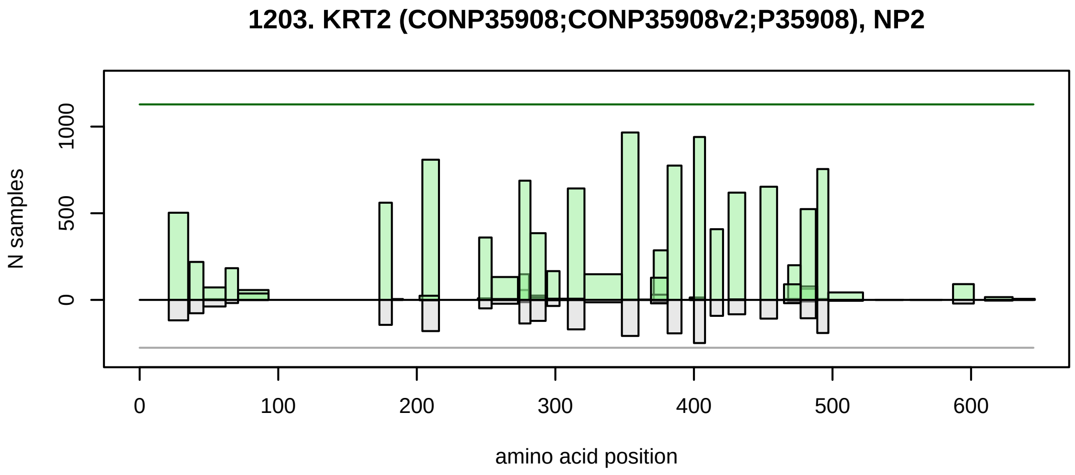

1203. KRT2 (CONP35908;CONP35908v2;P35908), NP2
N samples
0
500
1000
0
100
200
300
400
500
600
amino acid position

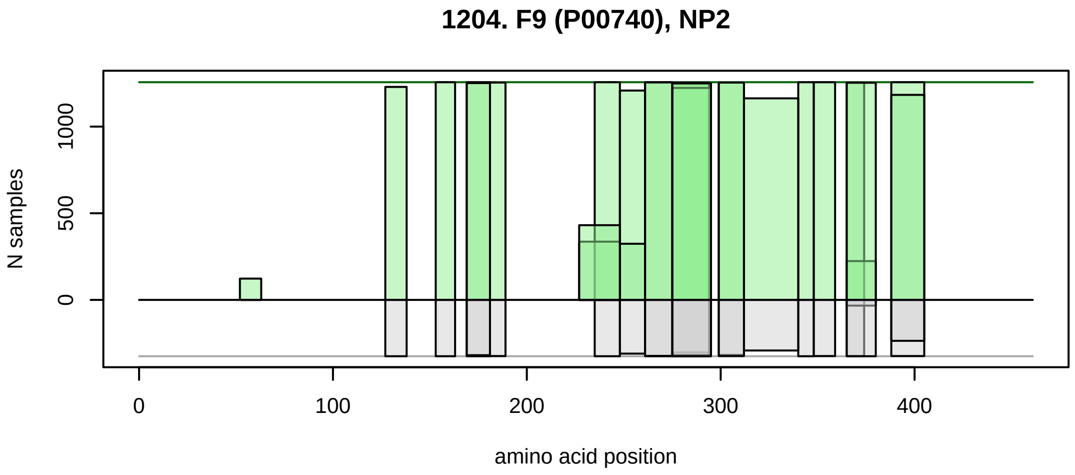

1204. F9 (P00740), NP2
N samples
0
500
1000
0
100
200
300
400
amino acid position

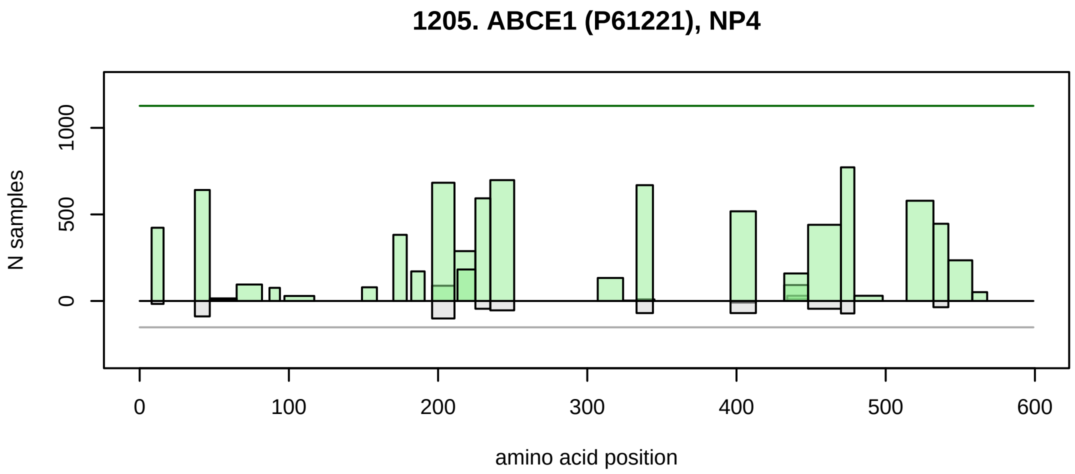

1205. ABCE1 (P61221), NP4
N samples
0
500
1000
0
100
200
300
400
500
600
amino acid position

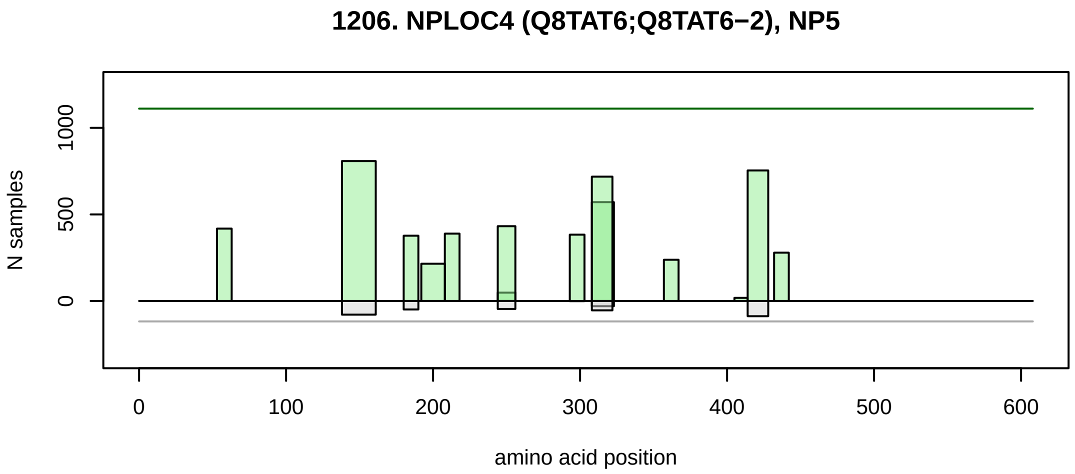

1206. NPLOC4 (Q8TAT6;Q8TAT6−2), NP5
N samples
0
500
1000
0
100
200
300
400
500
600
amino acid position

1207. YBX1 (P67809), NP4

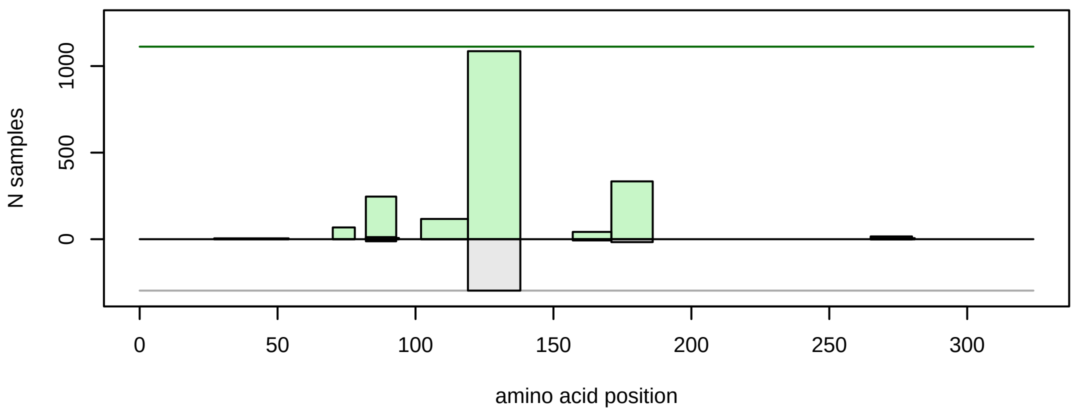

1208. RSU1 (Q15404), NP1

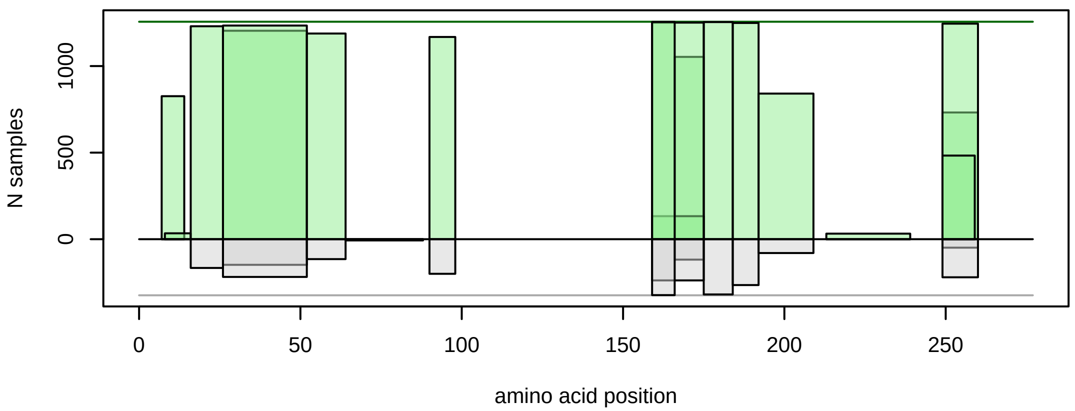

1209. DRG2 (P55039), NP4

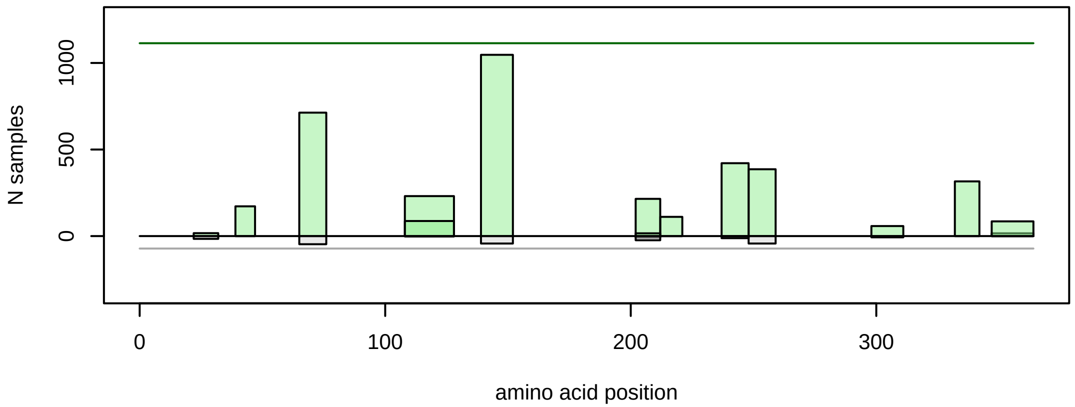

1210. GLG1 (Q92896−3), NP5

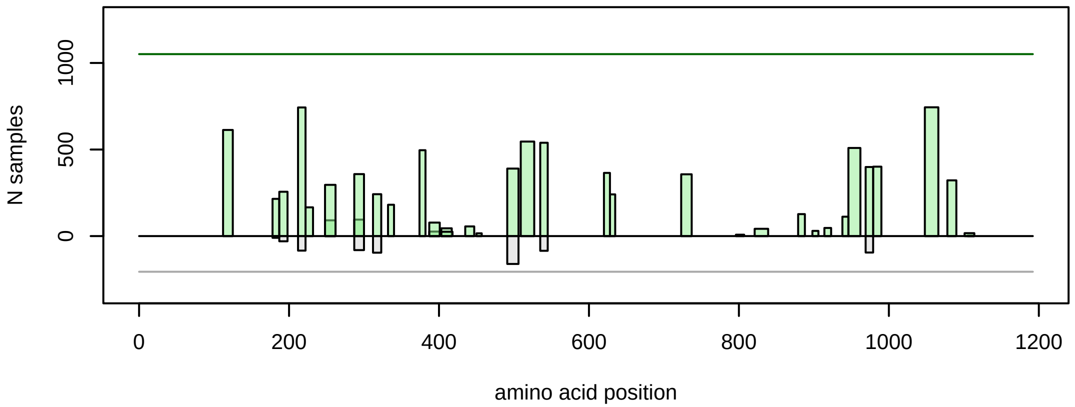

1211. PPP1CB (P62140), NP1

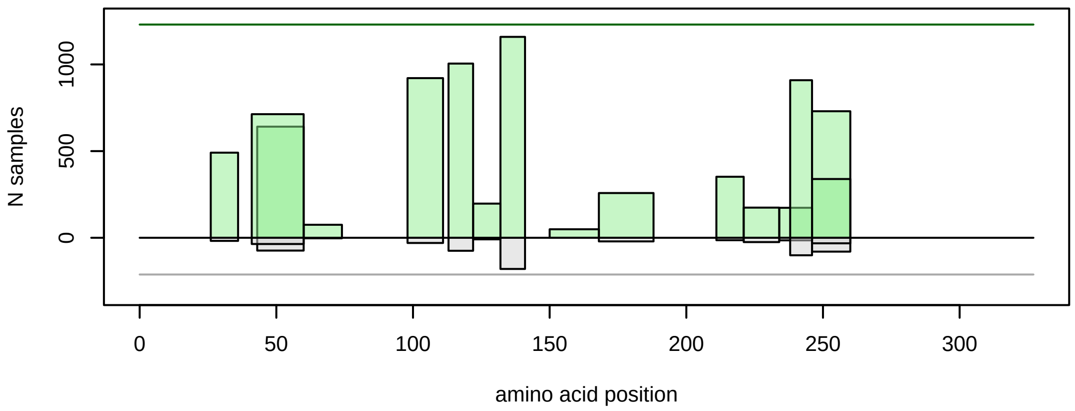

1212. CES1 (P23141;P23141−2;P23141−3), NP2

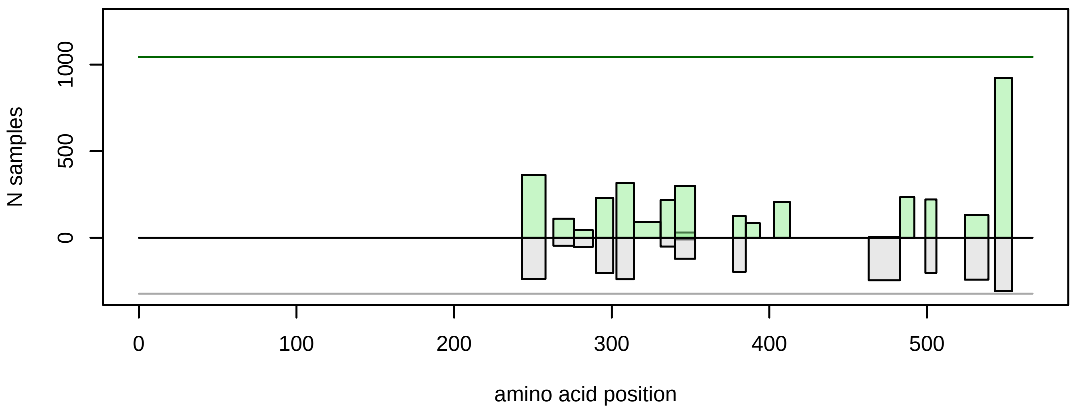

1213. GZMH (P20718), NP3

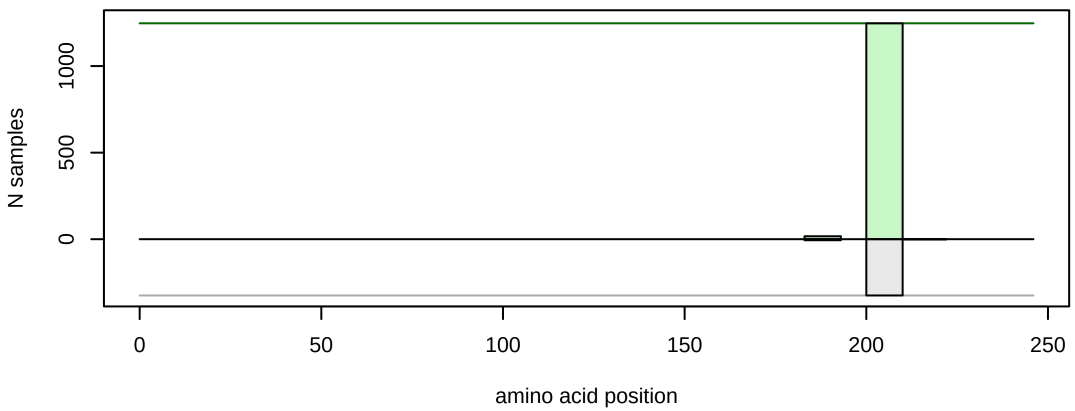

1214. S100A6 (P06703), NP2

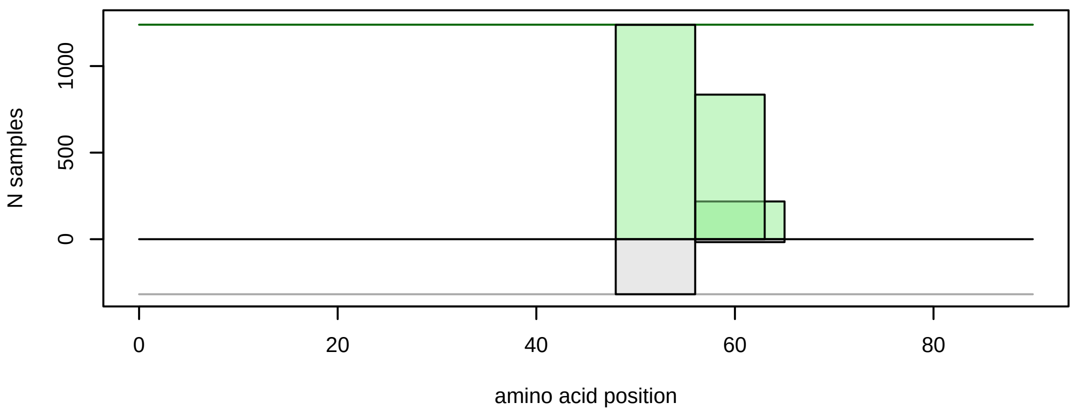

1215. NOTCH3 (Q9UM47), NP5

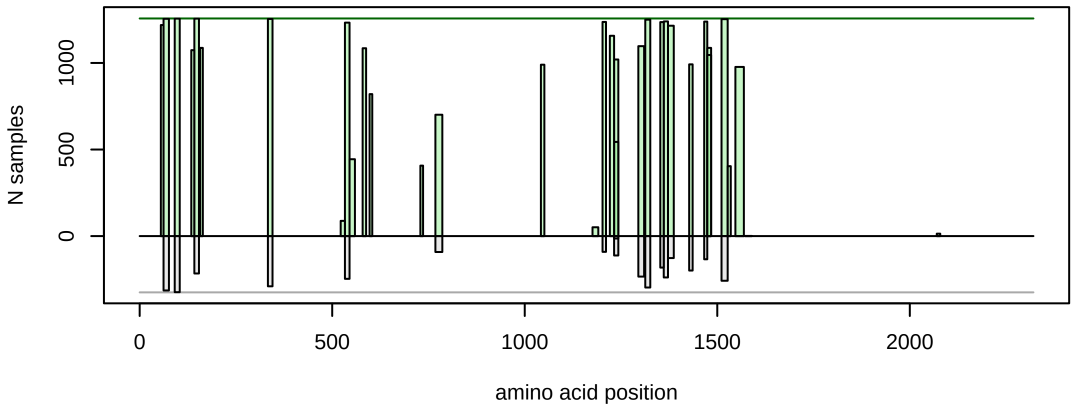

1216. CYP2C19 (P33261), NP3

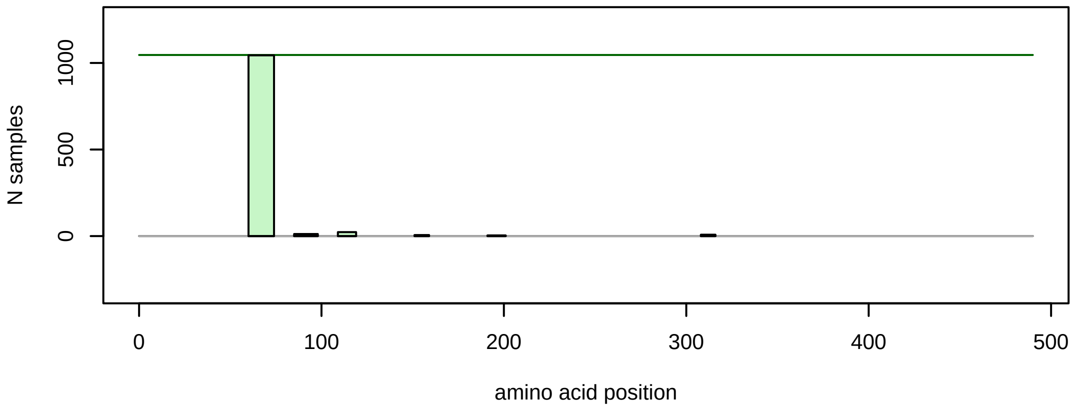

1217. FBLN7 (Q53RD9), NP4

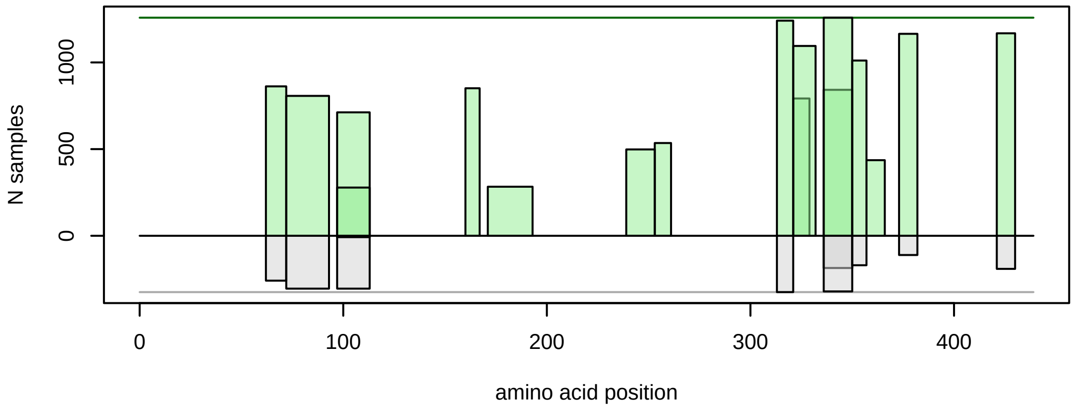

1218. ALDOA (P04075), NP1

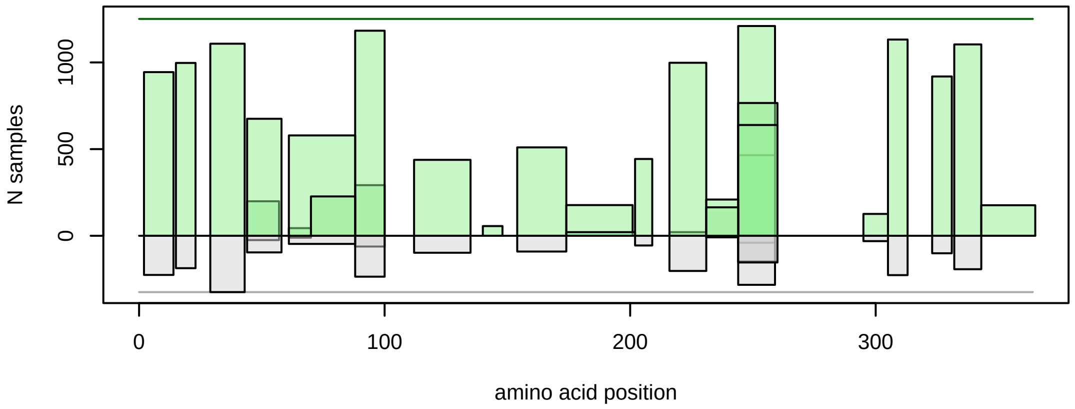

1219. HNRNPK (P61978−2), NP1

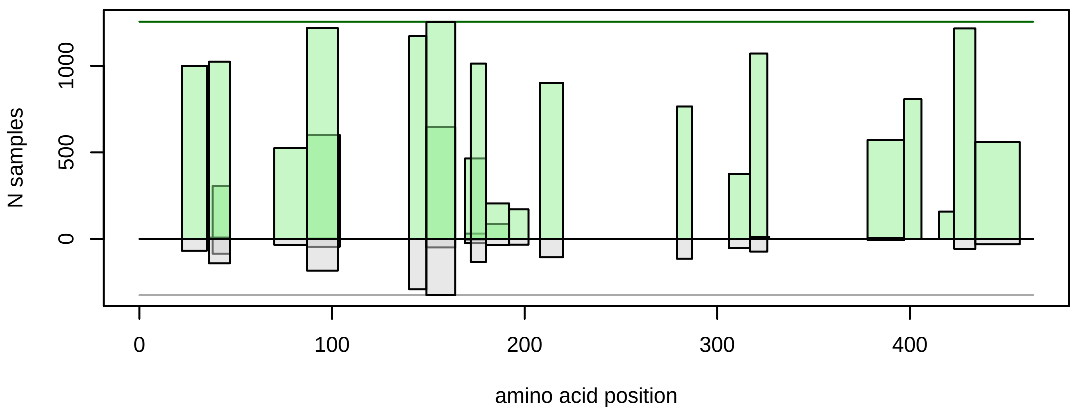

1220. PRKAR2B (P31323), NP2

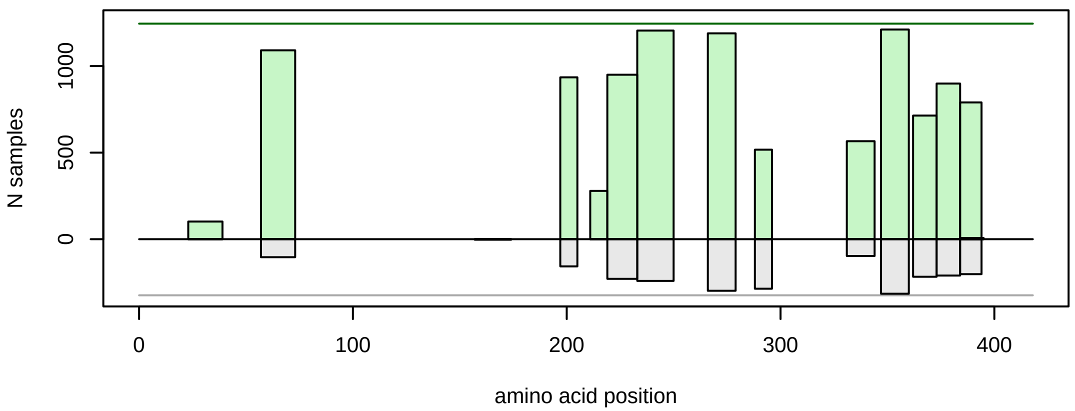

1221. ATP1B3 (P54709), NP1

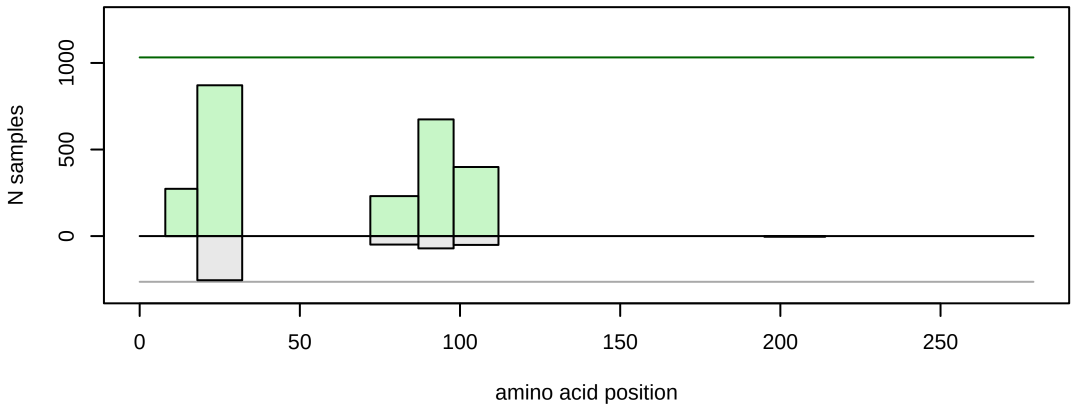

1222. PACS1 (Q6VY07), NP3

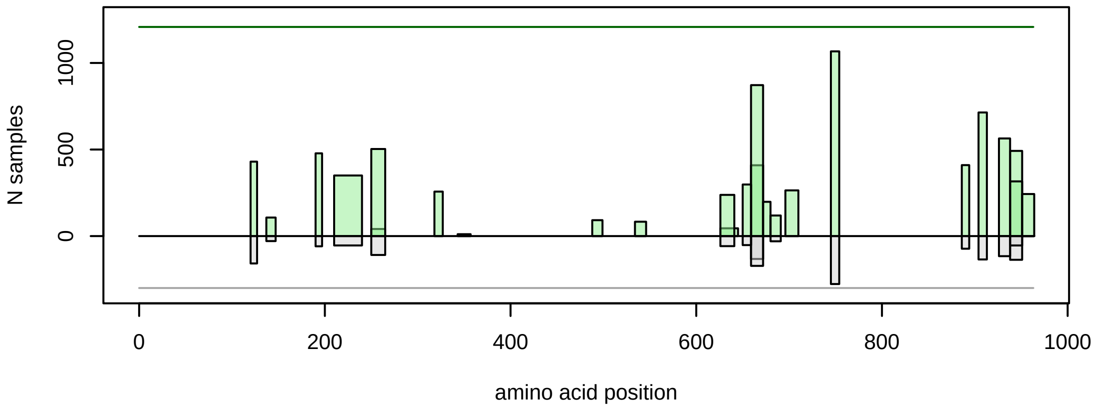

1223. NSF (P46459), NP5

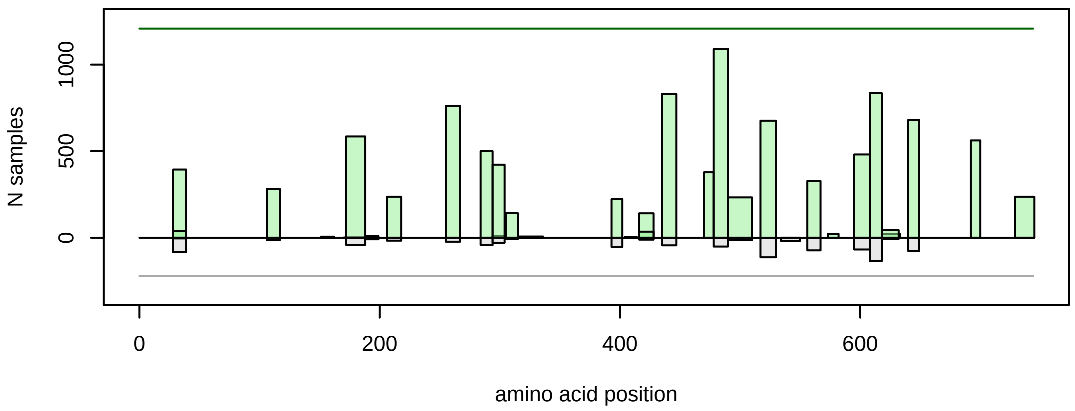

1224. NCAN (O14594), NP2

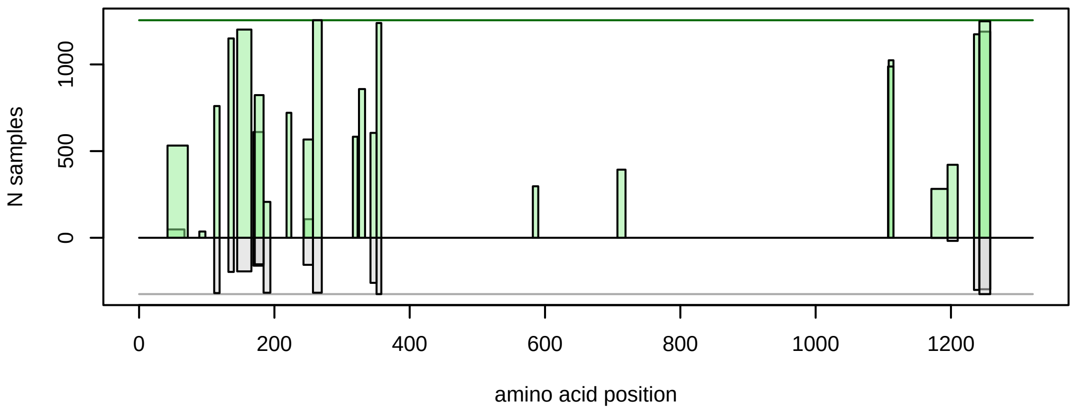

1225. SERPINA3 (P01011), NP2

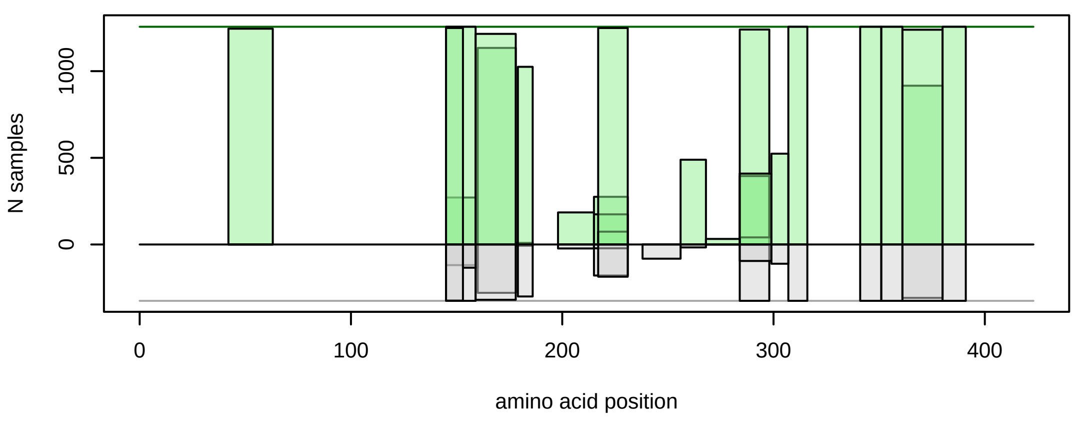

1226. FSTL3 (O95633), NP2

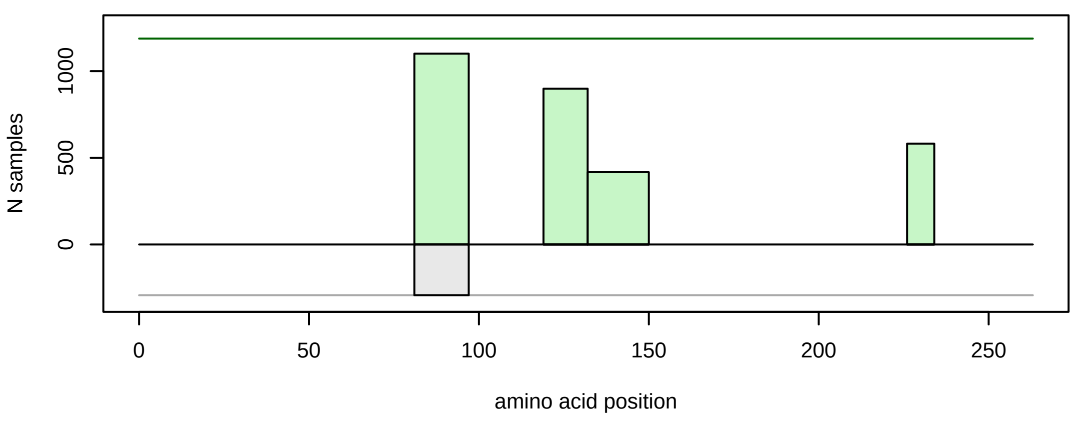

1227. FGFR1 (A0A804HIF1), NP2

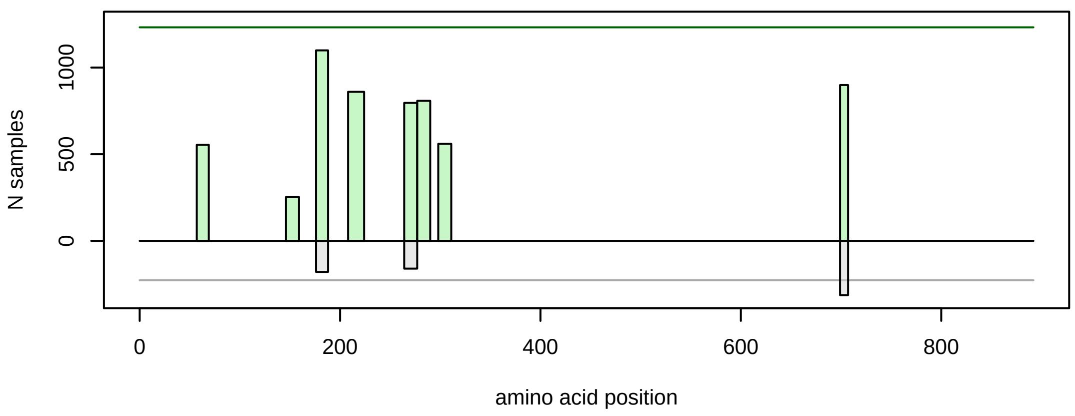

1228. S100A12 (P80511), NP5

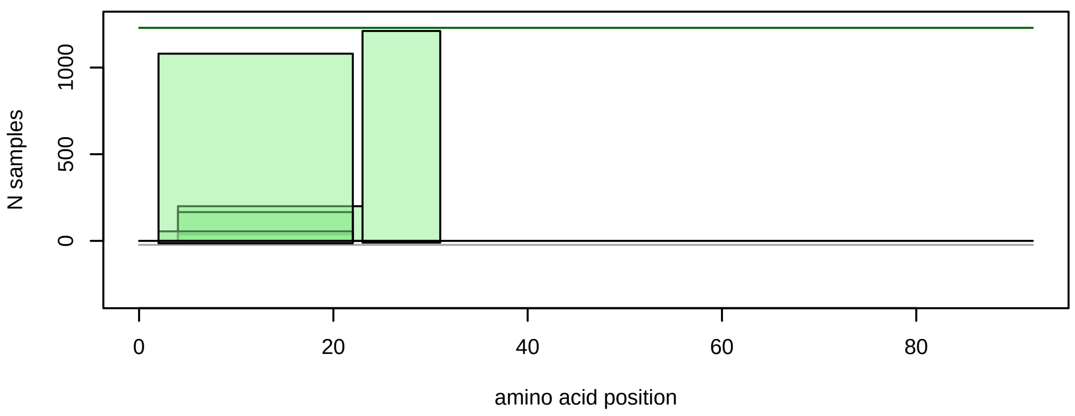

1229. CRIP1 (P50238), NP1

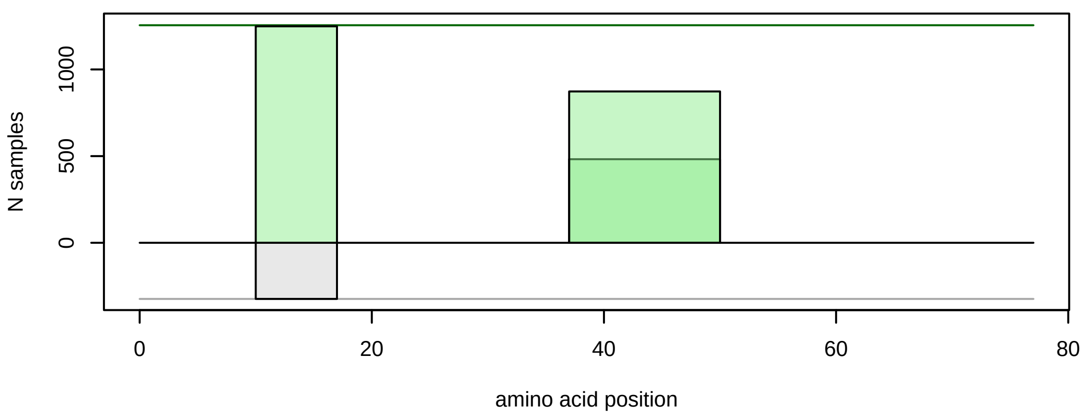

1230. NAPG (Q99747), NP5

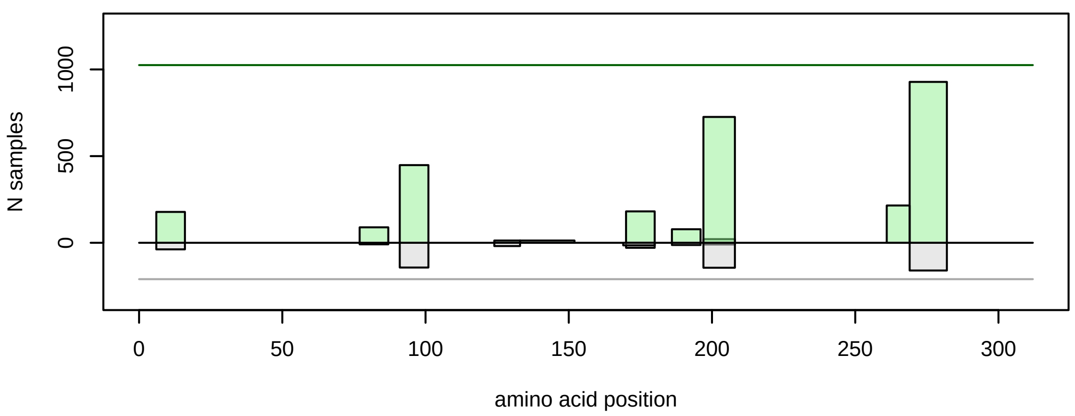

1231. STIM1 (H0YDB2), NP4

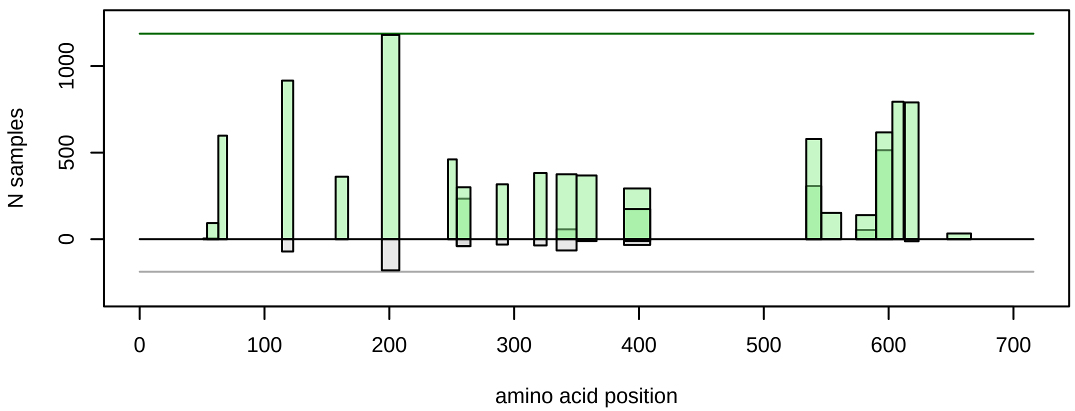

1232. MIA3 (Q5JRA6), NP2

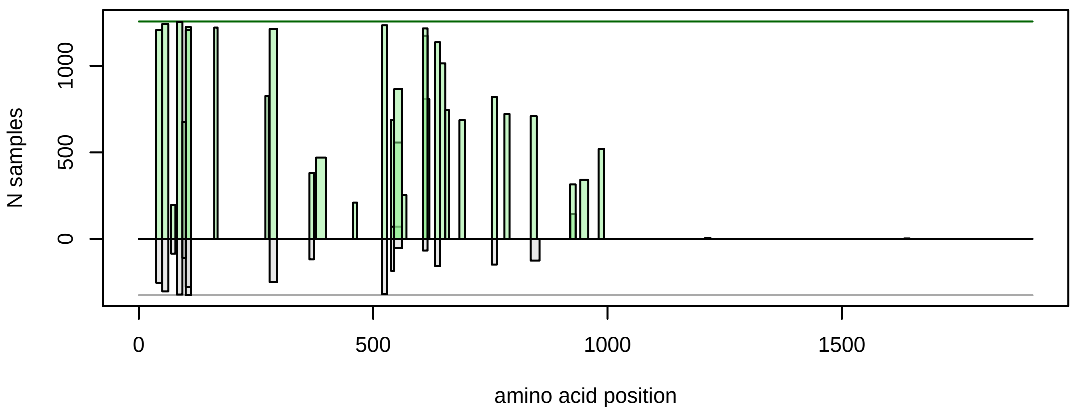

1233. SLA2 (Q9H6Q3), NP2

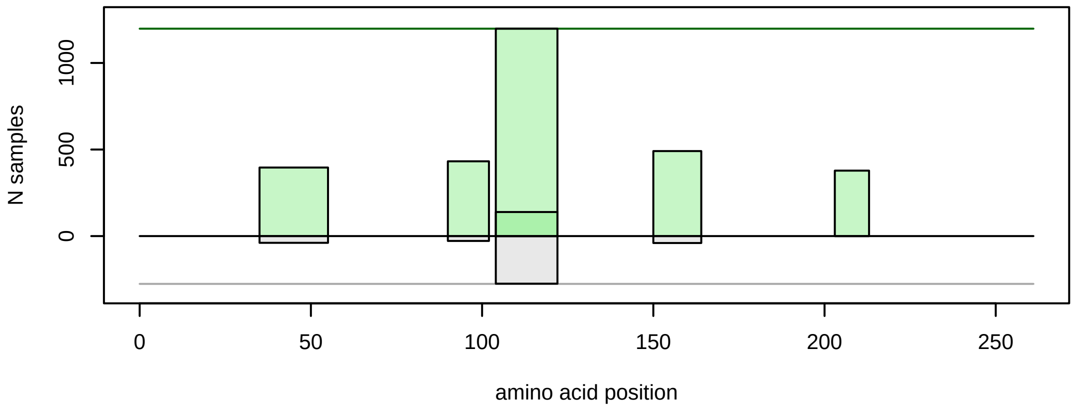

1234. SNRPD3 (P62318), NP1

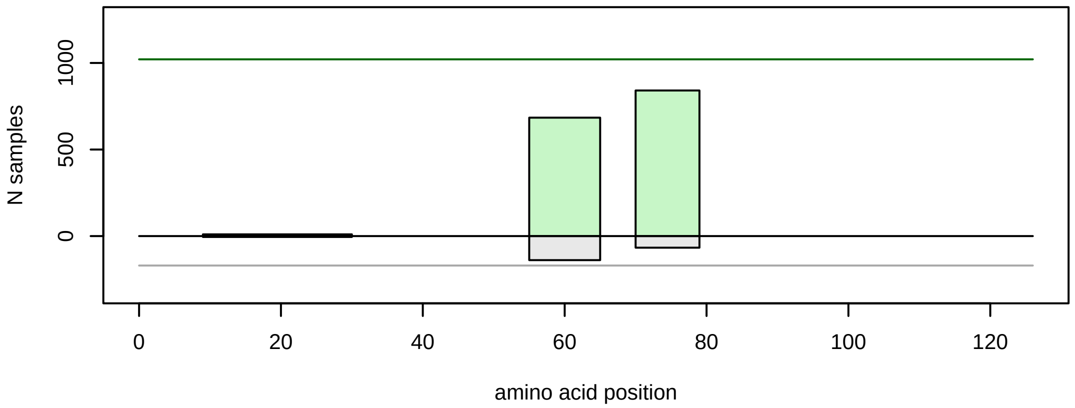

1235. SVIP (Q8NHG7), NP1

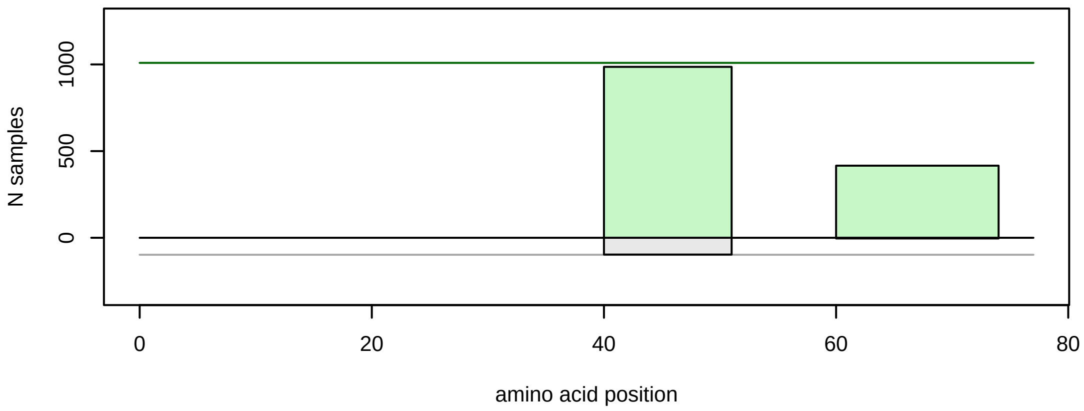

1236. ABHD16A (O95870), NP4

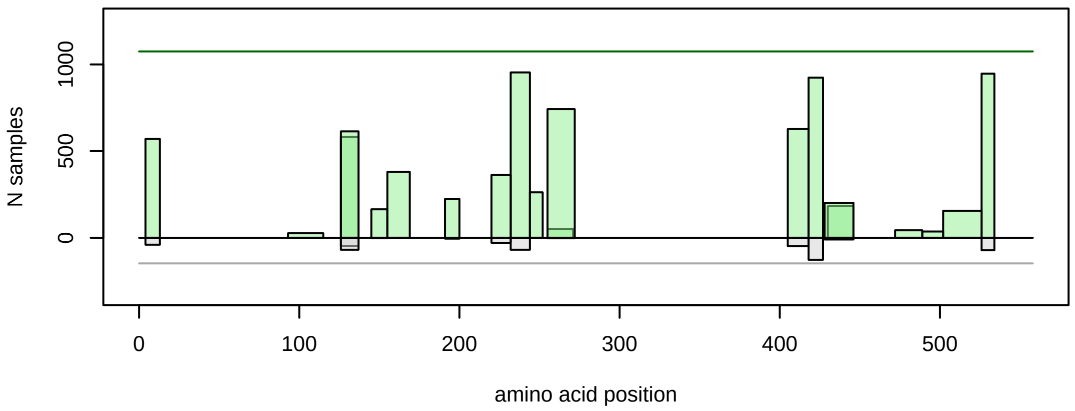

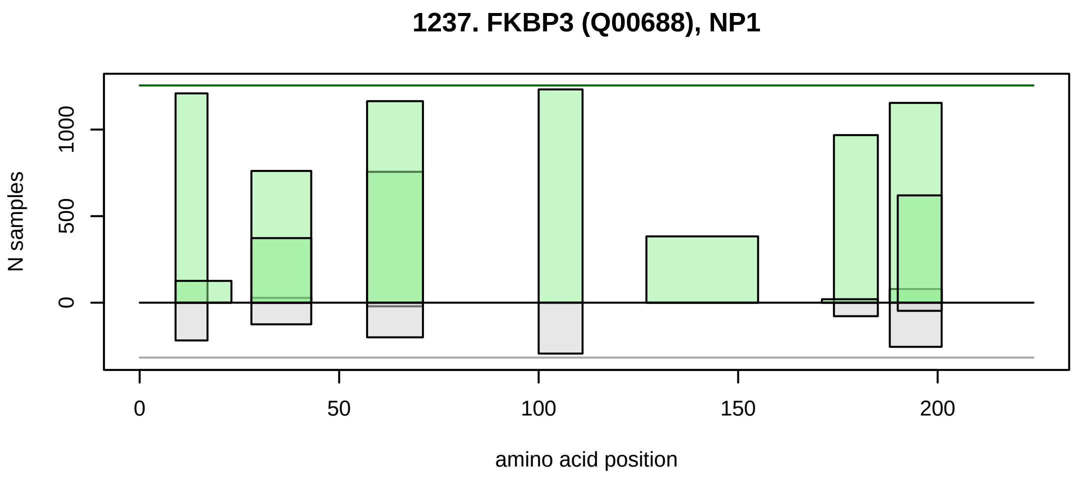

1237. FKBP3 (Q00688), NP1
N samples
amino acid position

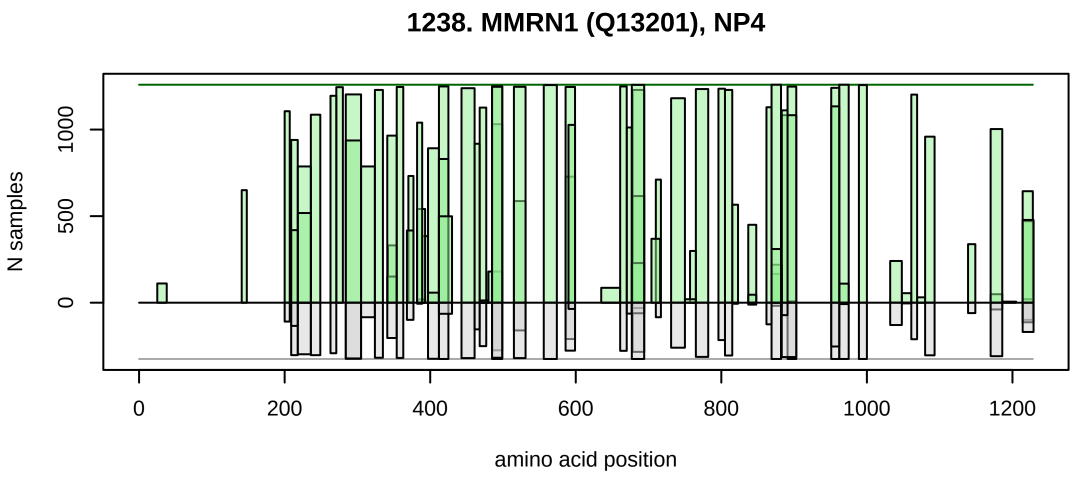

1238. MMRN1 (Q13201), NP4
N samples
amino acid position

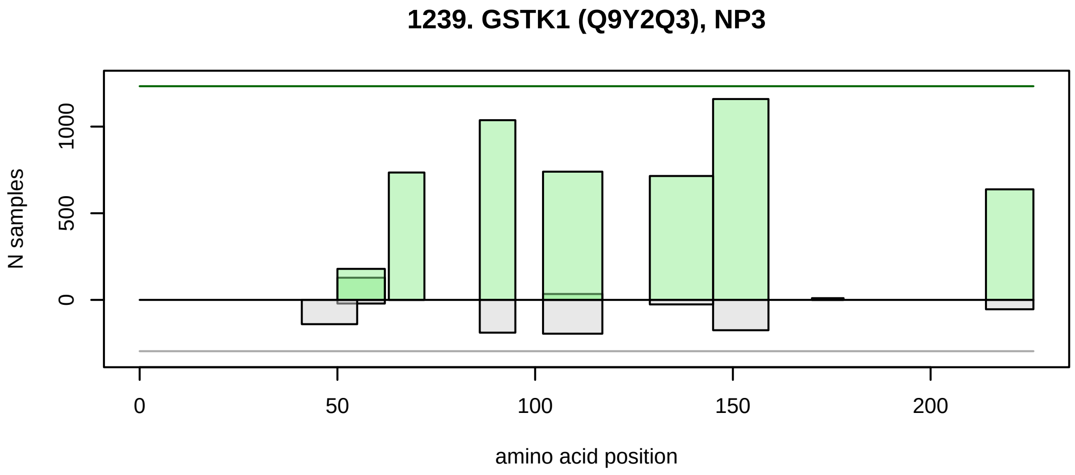

1239. GSTK1 (Q9Y2Q3), NP3
N samples
amino acid position

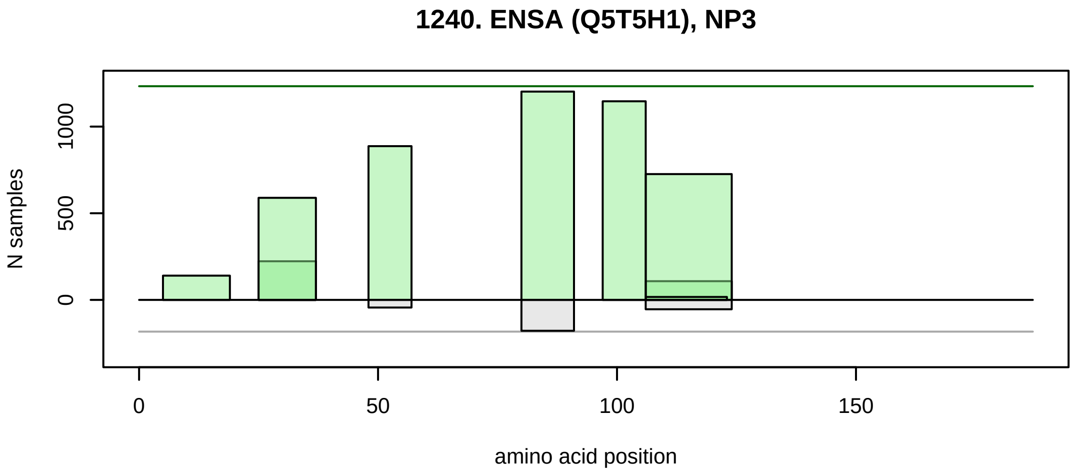

1240. ENSA (Q5T5H1), NP3
N samples
amino acid position

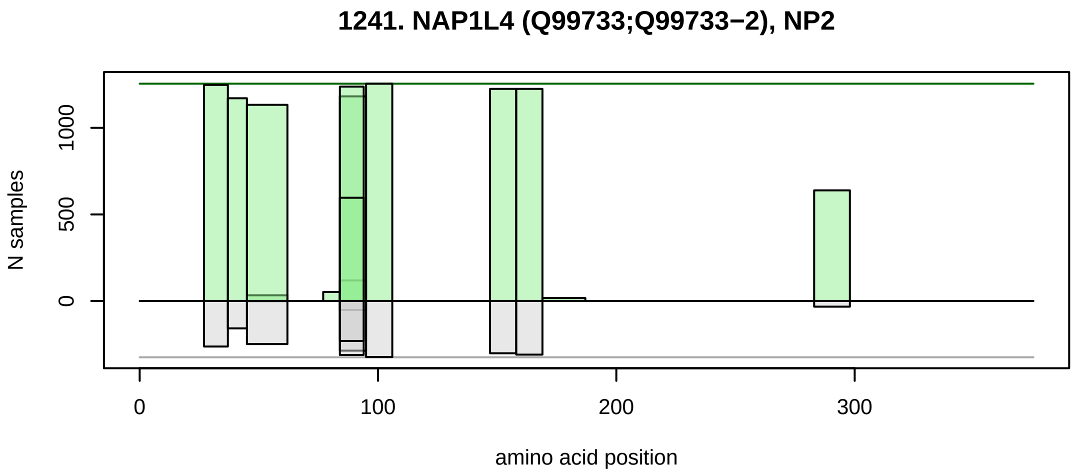

1241. NAP1L4 (Q99733;Q99733−2), NP2
N samples
amino acid position

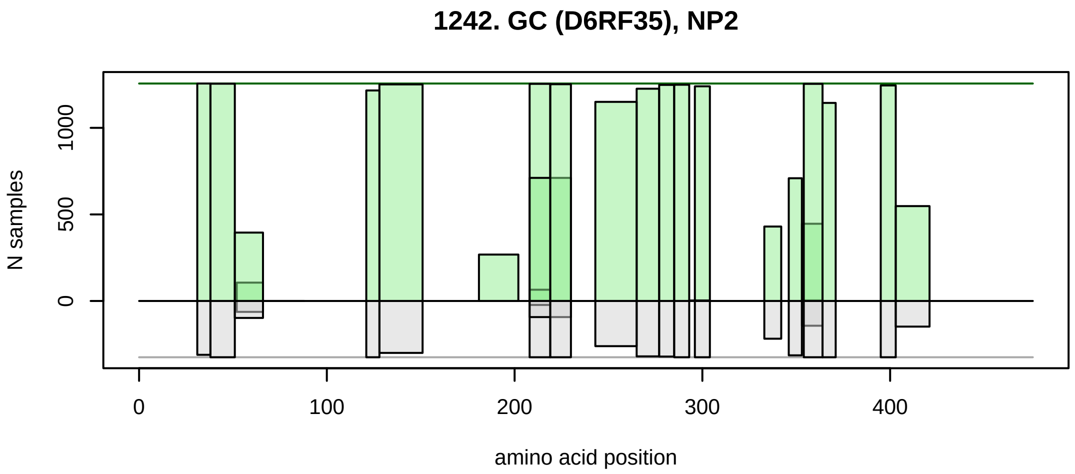

1242. GC (D6RF35), NP2
N samples
amino acid position

1243. CNN2 (B4DUT8), NP2

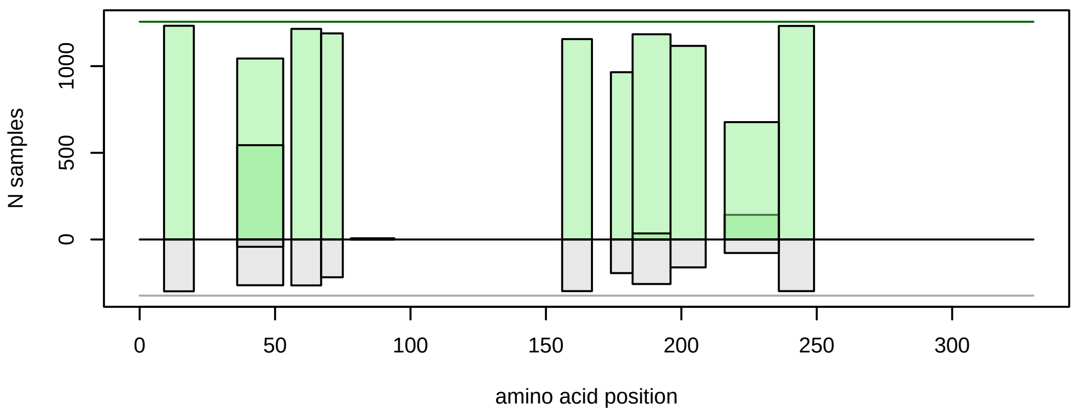

1244. EIF4G1 (E7EUU4), NP1

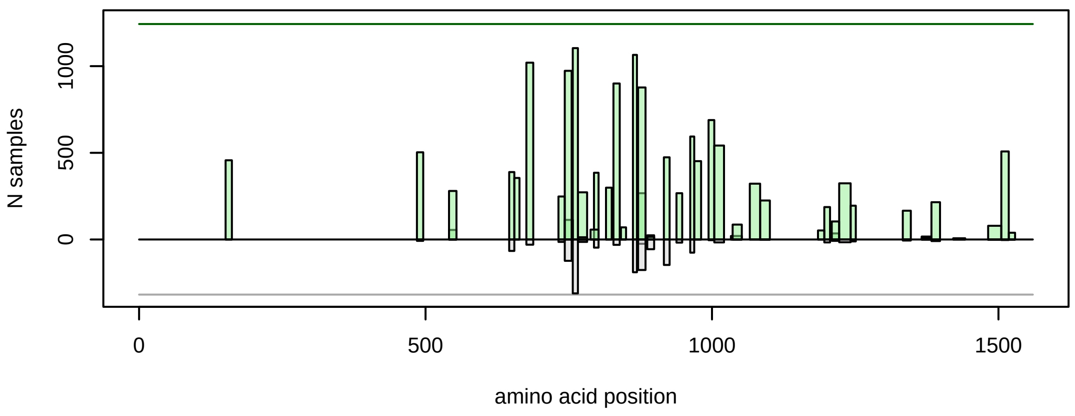

1245. NA (A0A0J9YX35), NP4

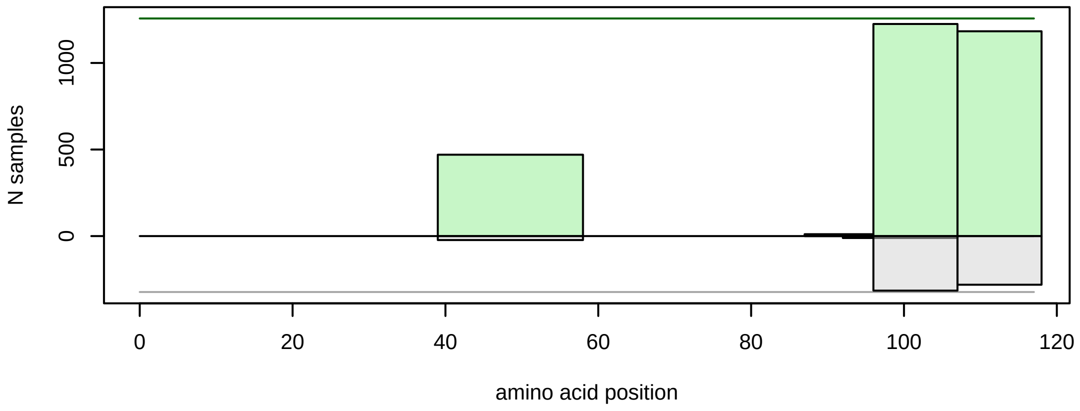

1246. POSTN (Q15063−5), NP3

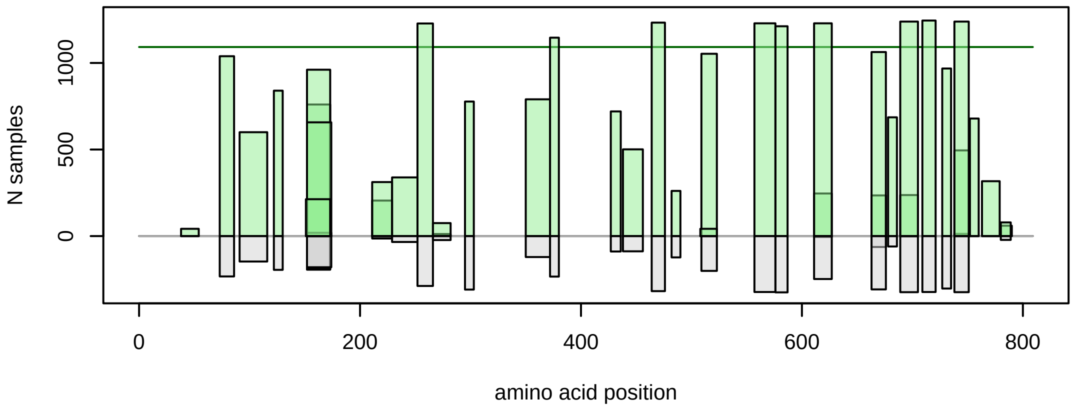

1247. APOC2 (P02655), NP2

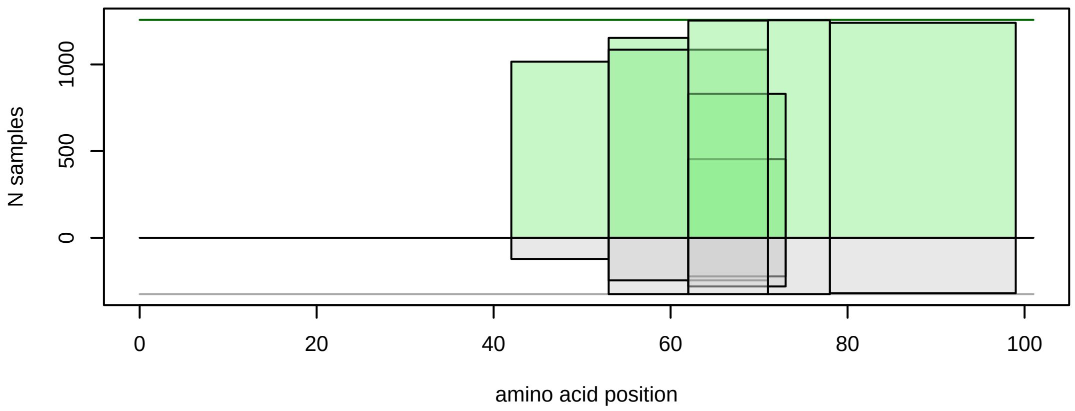

1248. PLOD1 (Q02809), NP5

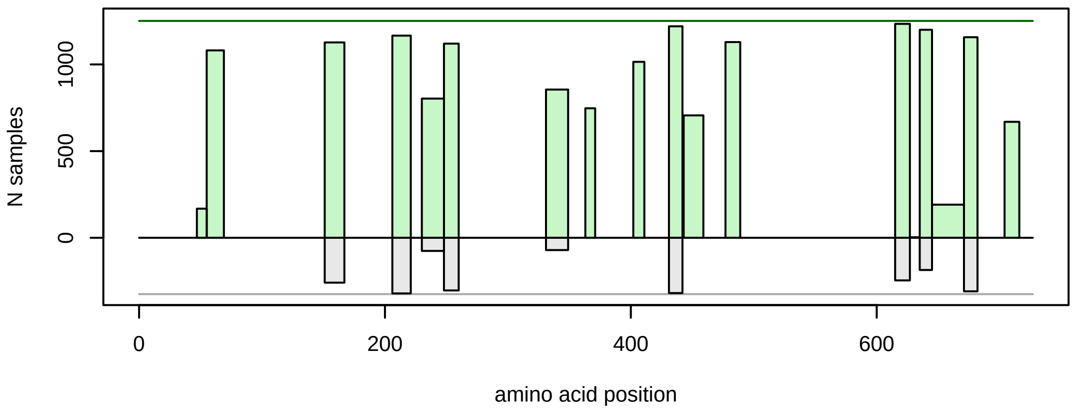

1249. HS1BP3 (Q53T59), NP2

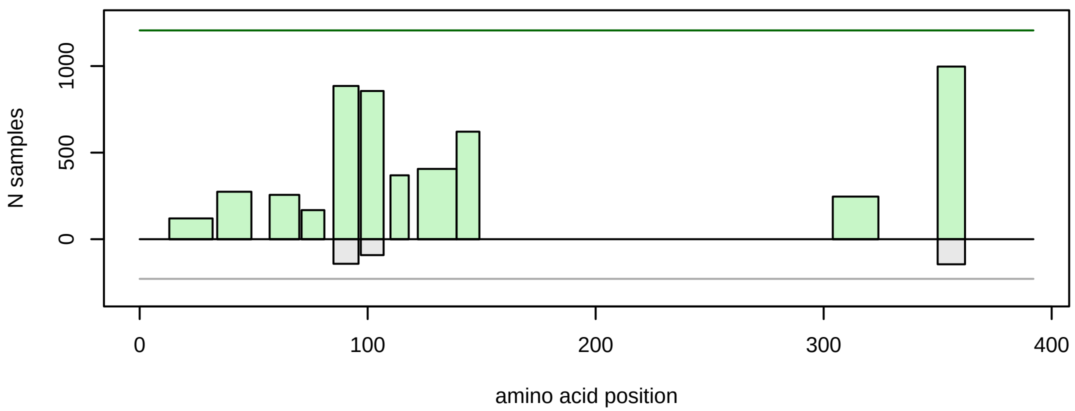

1250. PTGS1 (P23219;P23219−2;P23219−3), NP4

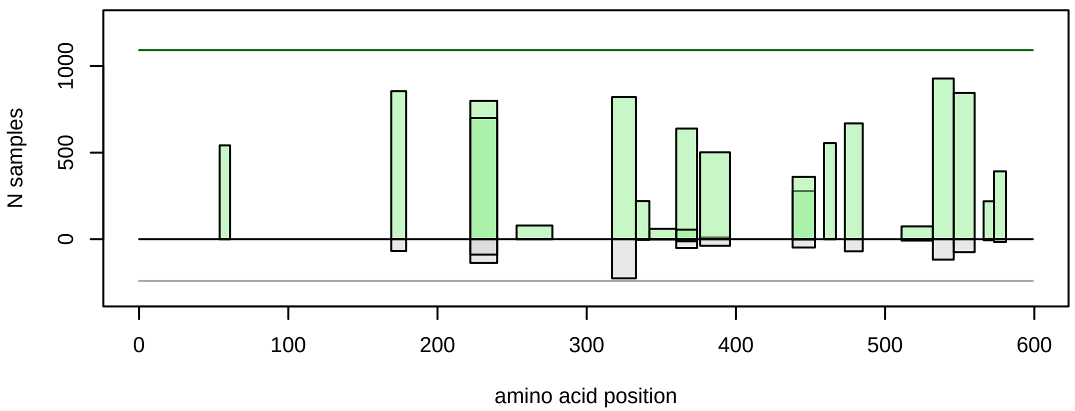

1251. ACTG1 (P63261), NP4

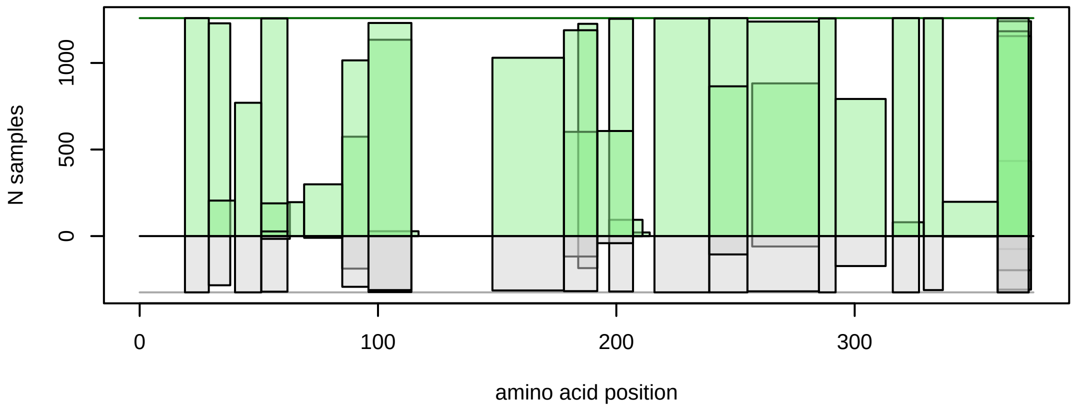

1252. RAB35 (Q15286), NP1

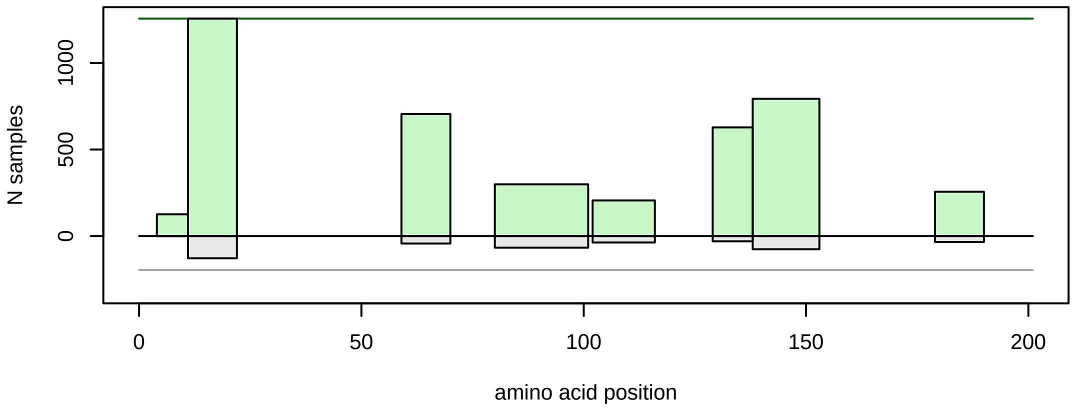

1253. EIF1 (K7EM18), NP2

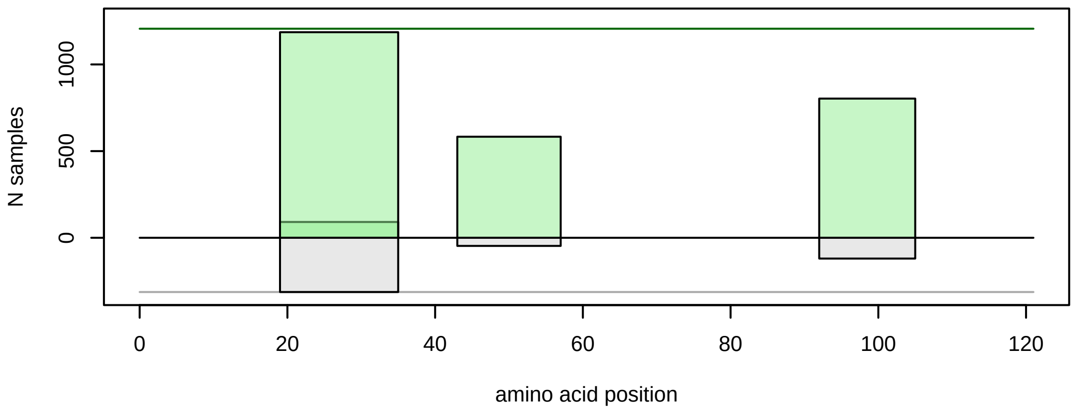

1254. WISP2 (O76076), NP1

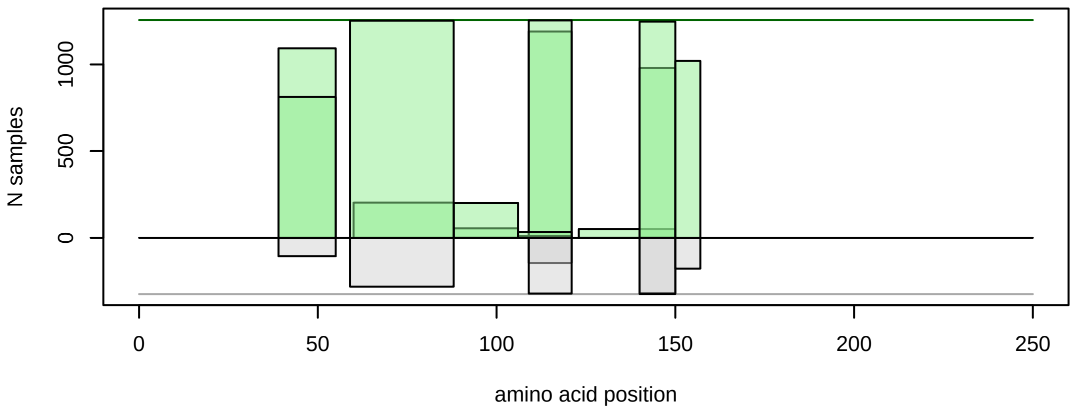

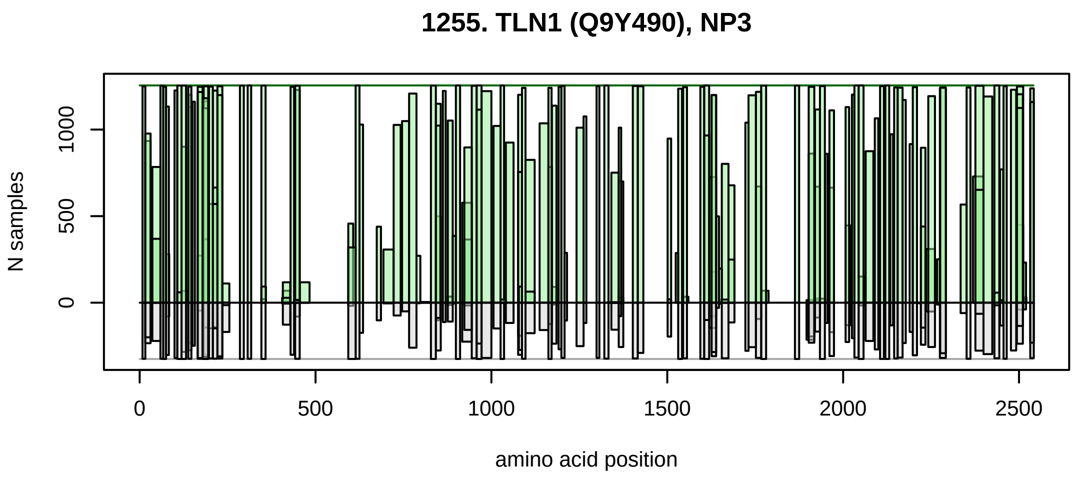
1255. TLN1 (Q9Y490), NP3
N samples
amino acid position

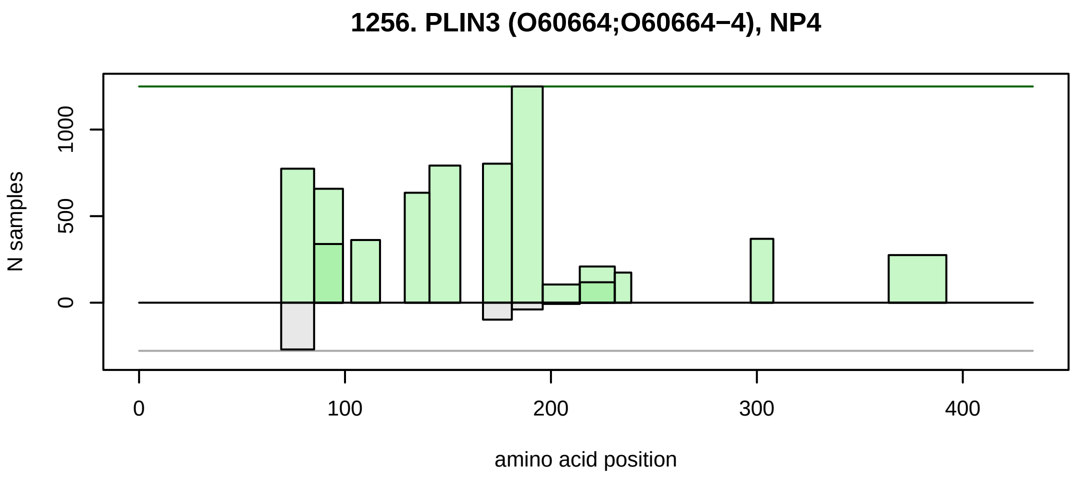
1256. PLIN3 (O60664;O60664−4), NP4
N samples
amino acid position

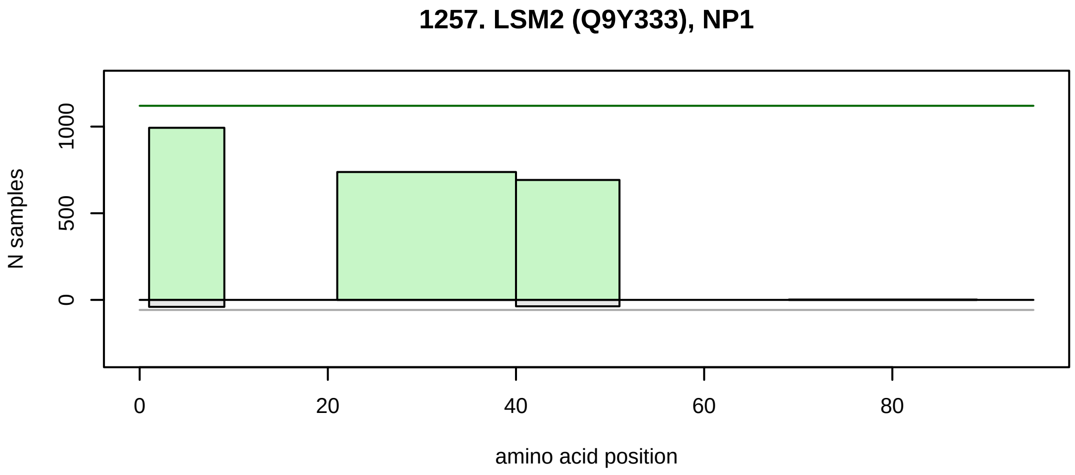
1257. LSM2 (Q9Y333), NP1
N samples
amino acid position

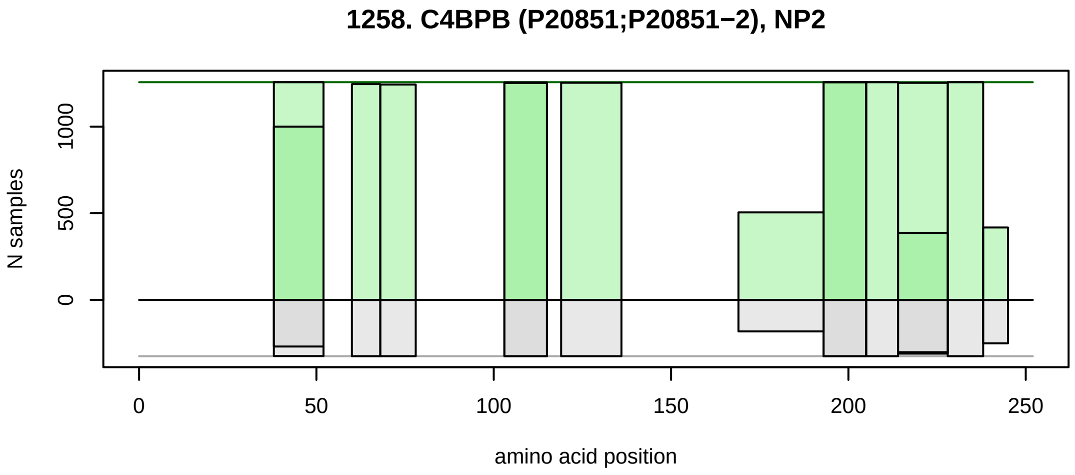
1258. C4BPB (P20851;P20851−2), NP2
N samples
amino acid position

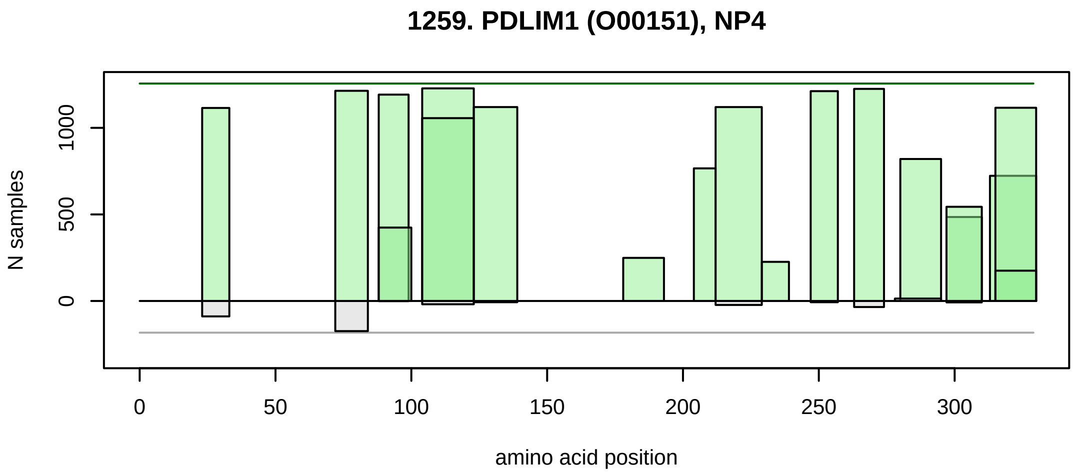
1259. PDLIM1 (O00151), NP4
N samples
amino acid position

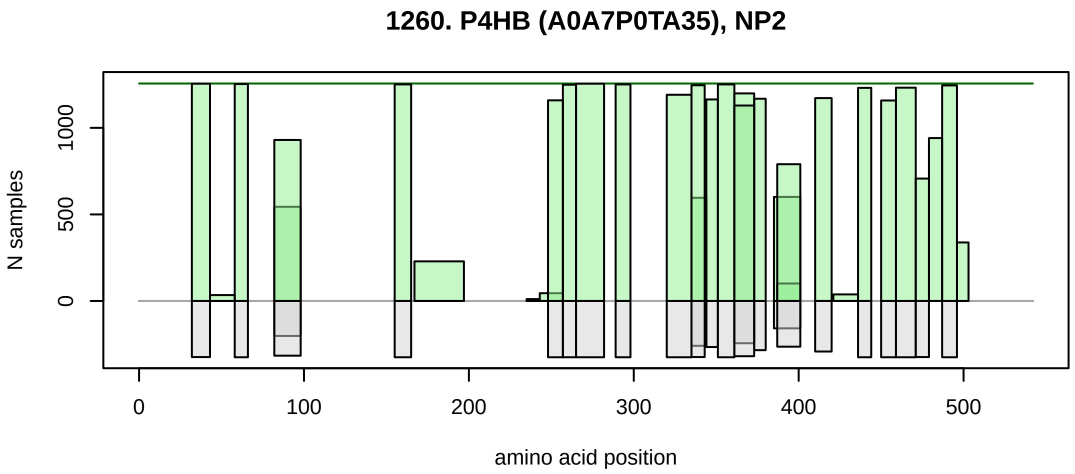
1260. P4HB (A0A7P0TA35), NP2
N samples
amino acid position

1261. SLC44A1 (Q8WWI5), NP1

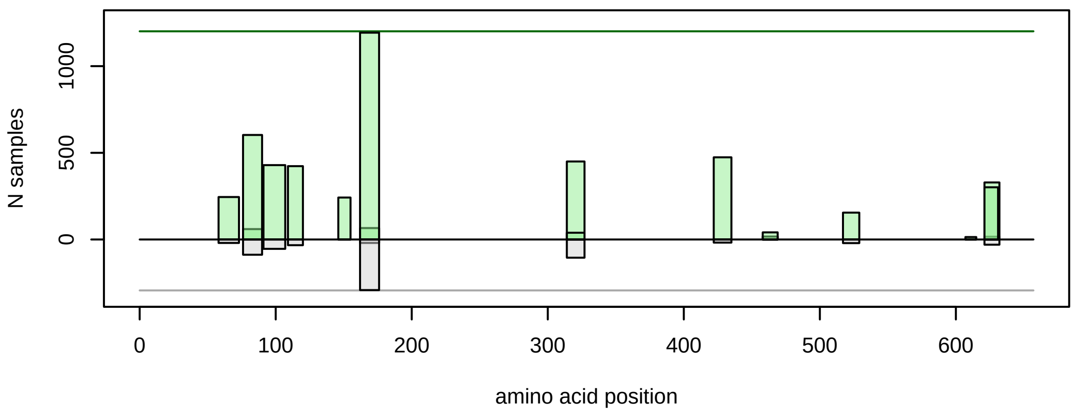

1262. PBX2 (P40425), NP4

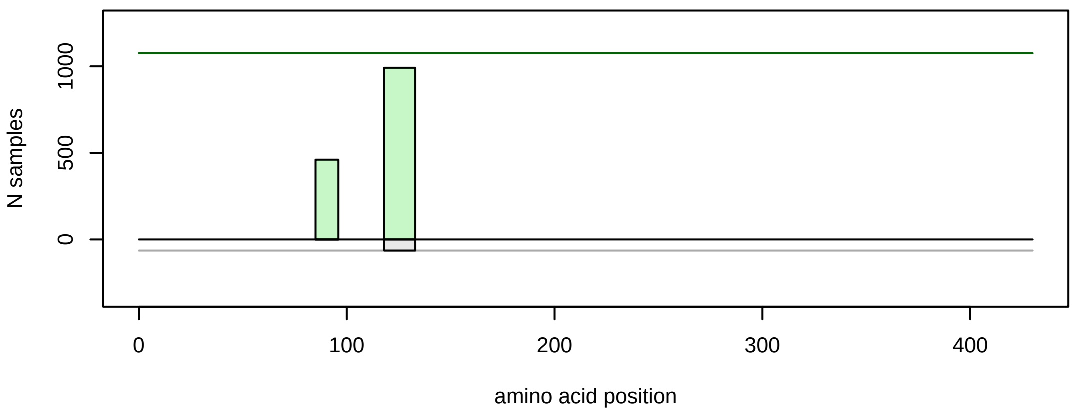

1263. C7 (P10643), NP3

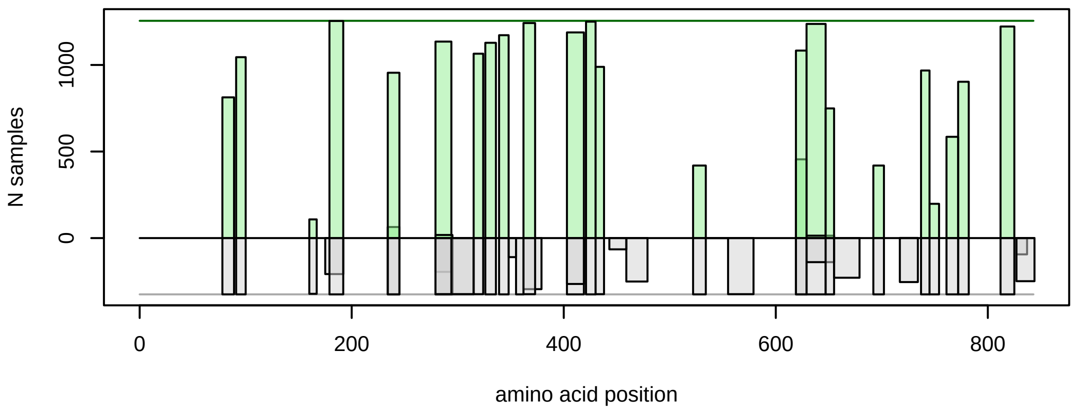

1264. COL1A1 (P02452), NP1

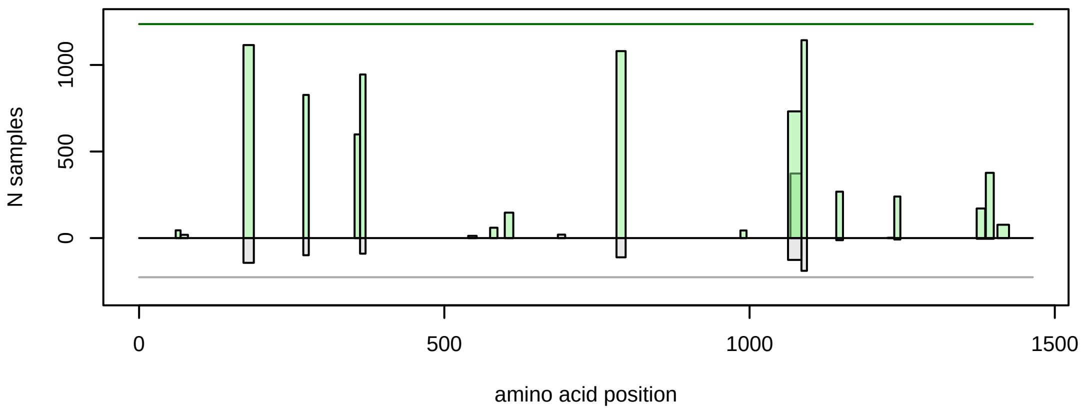

1265. CFL1 (E9PK25), NP3

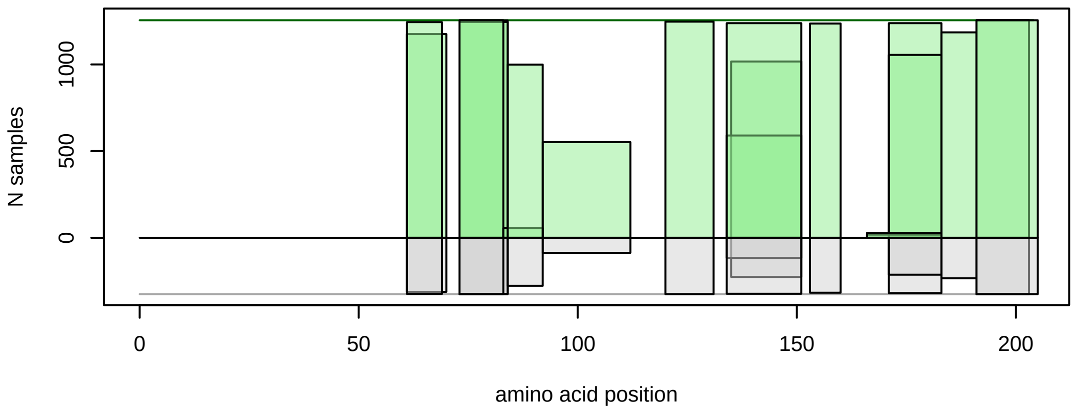

1266. TNRC18 (H9KVB4;O15417), NP4

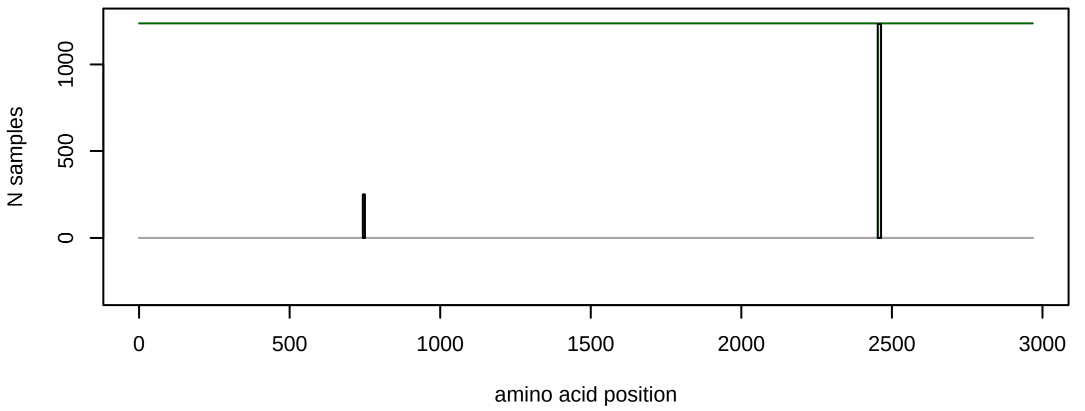

1267. WDR31 (Q8NA23), NP4

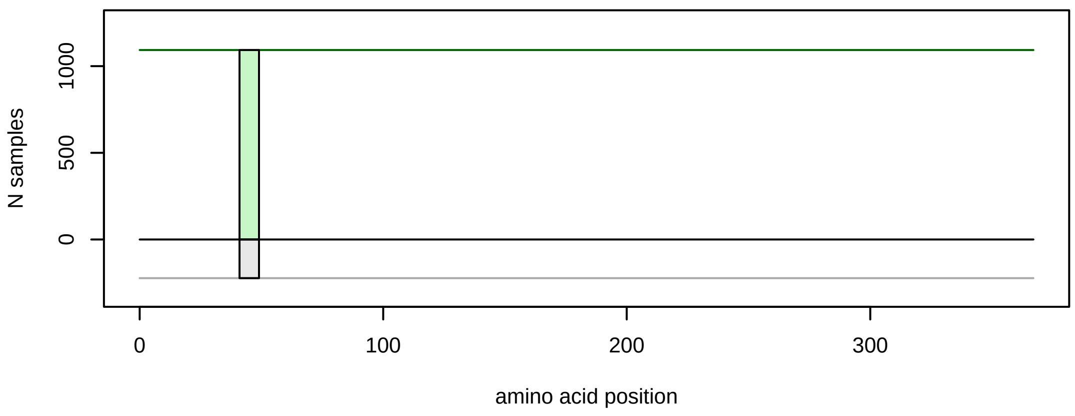

1268. APOA4 (P06727), NP2

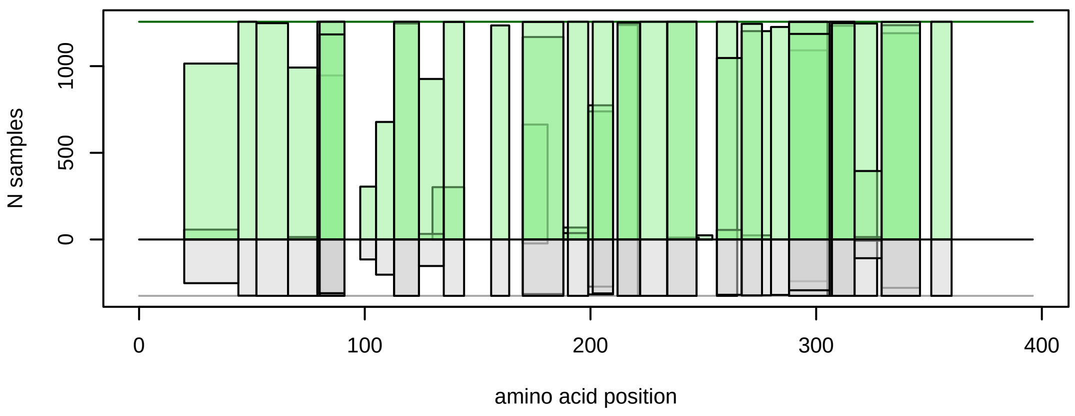

1269. C20orf27 (Q9GZN8;Q9GZN8−2), NP2

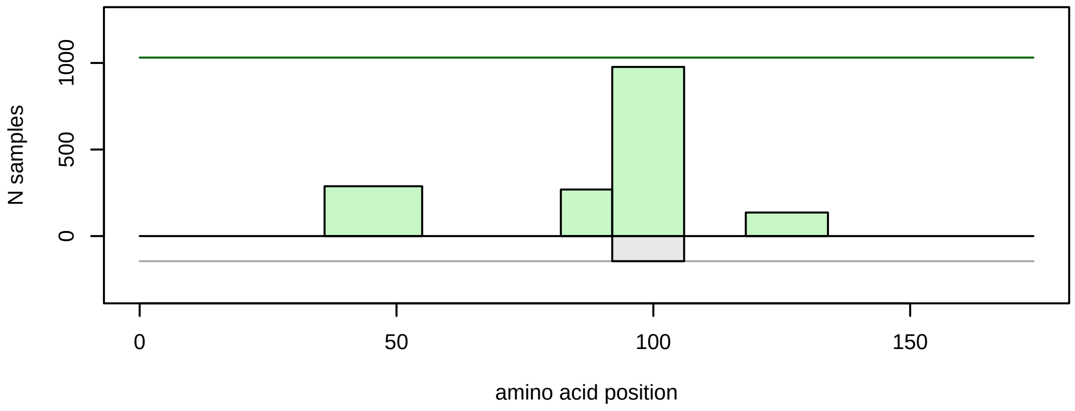

1270. ASS1 (P00966), NP5

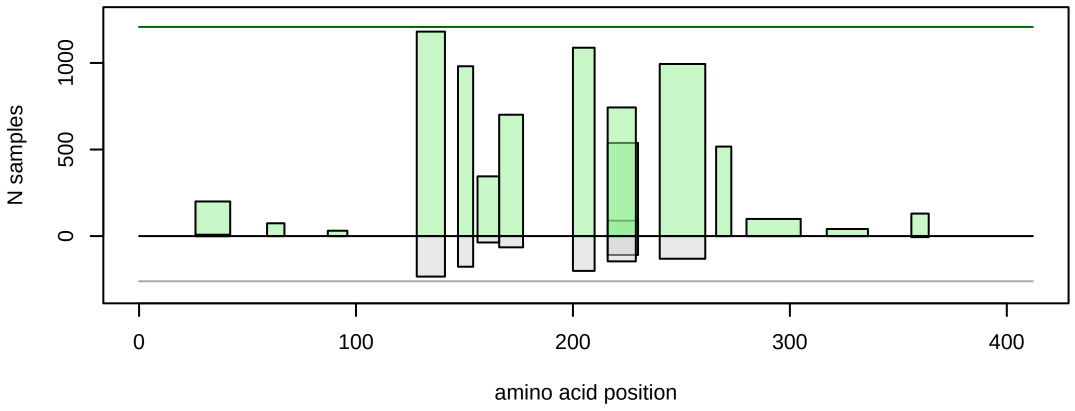

1271. PSMA5 (P28066), NP2

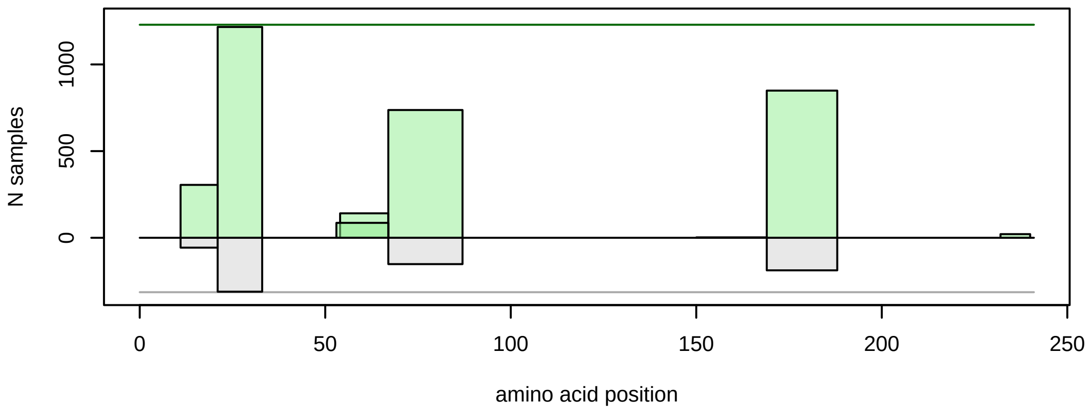

1272. APOBR (Q0VD83), NP2

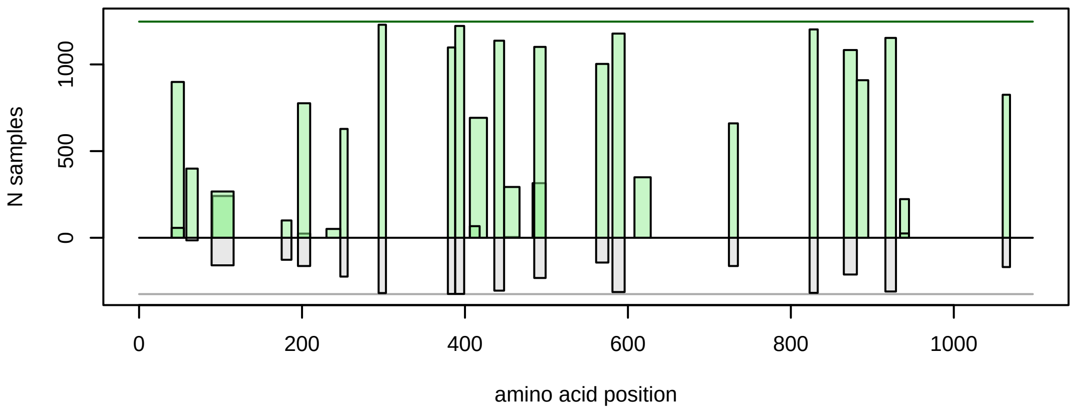

1273. DCXR (Q7Z4W1), NP1

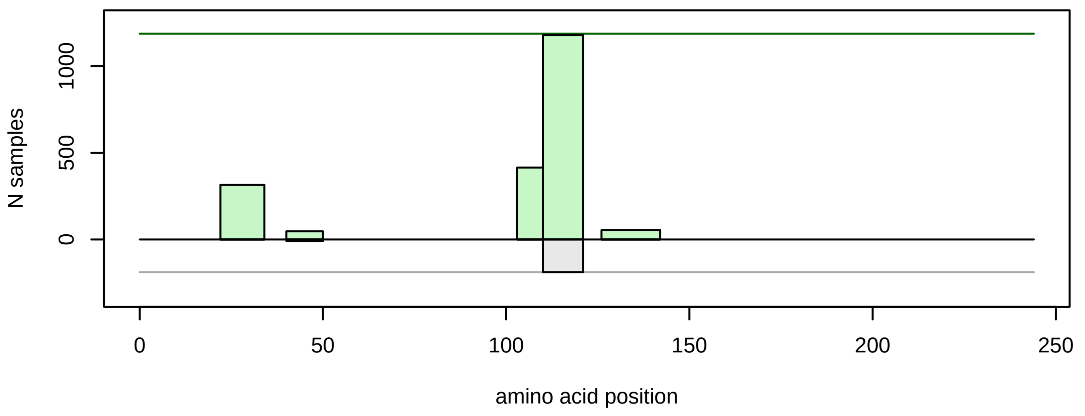

1274. RPL6 (Q02878), NP4

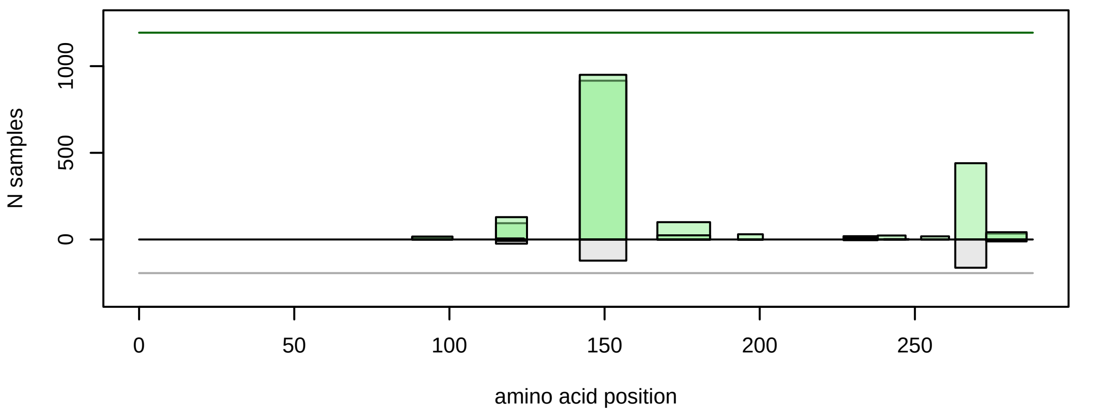

1275. AP2A1 (O95782;O95782−2), NP4

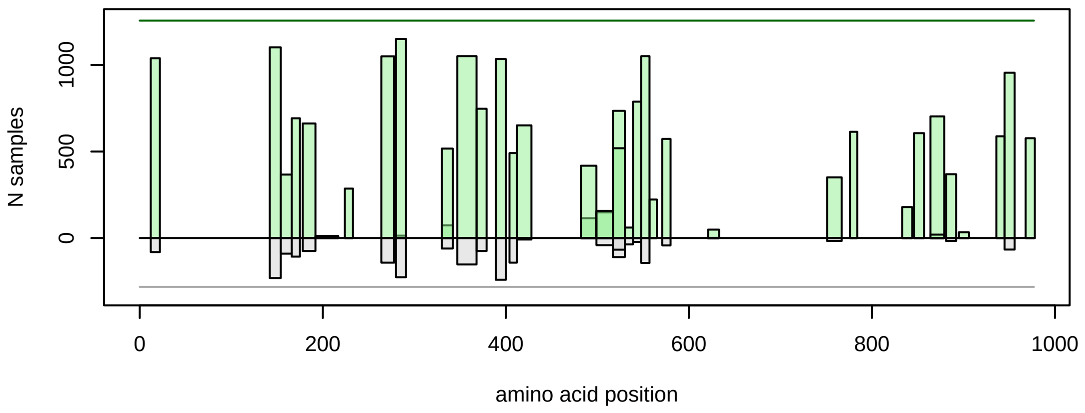

1276. PTBP1 (A0A7I2V621), NP3

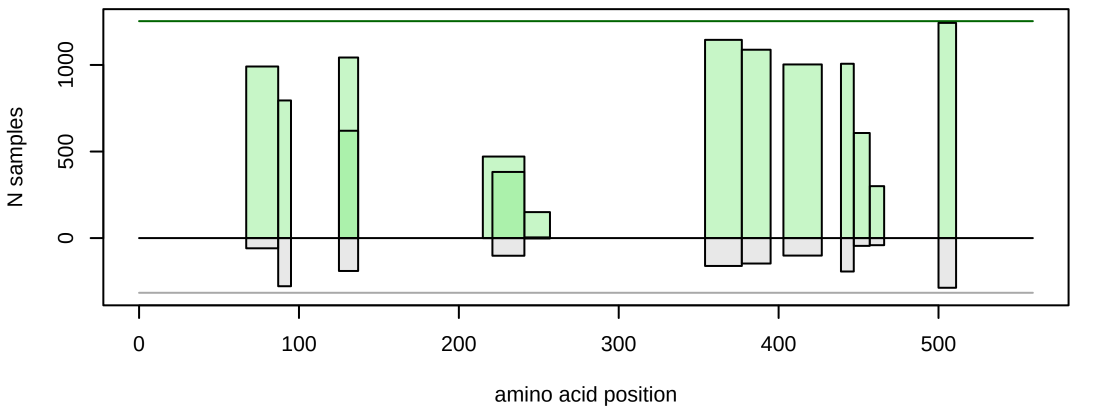

1277. CTPS1 (P17812), NP4

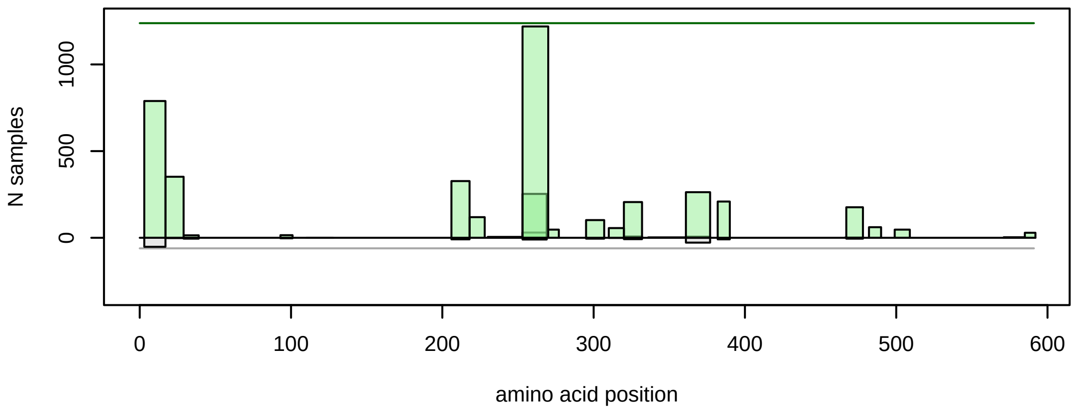

1278. CDC37 (Q16543), NP5

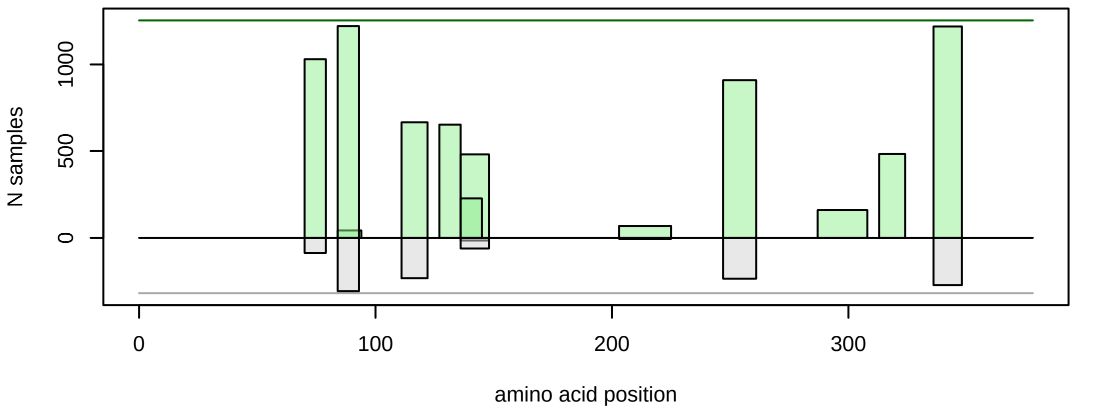

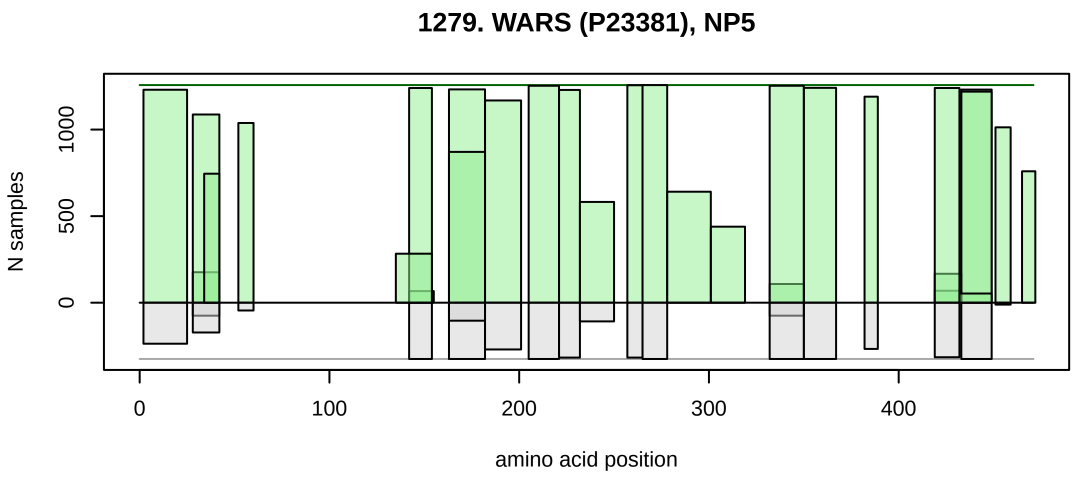
1279. WARS (P23381), NP5
N samples
0
500
1000
0
100
200
300
400
amino acid position

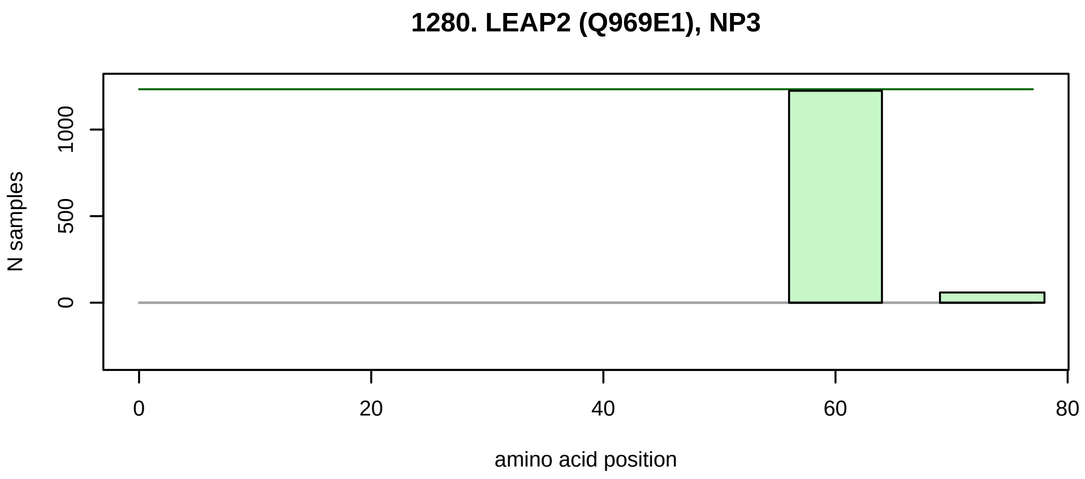
1280. LEAP2 (Q969E1), NP3
N samples
0
500
1000
0
20
40
60
80
amino acid position

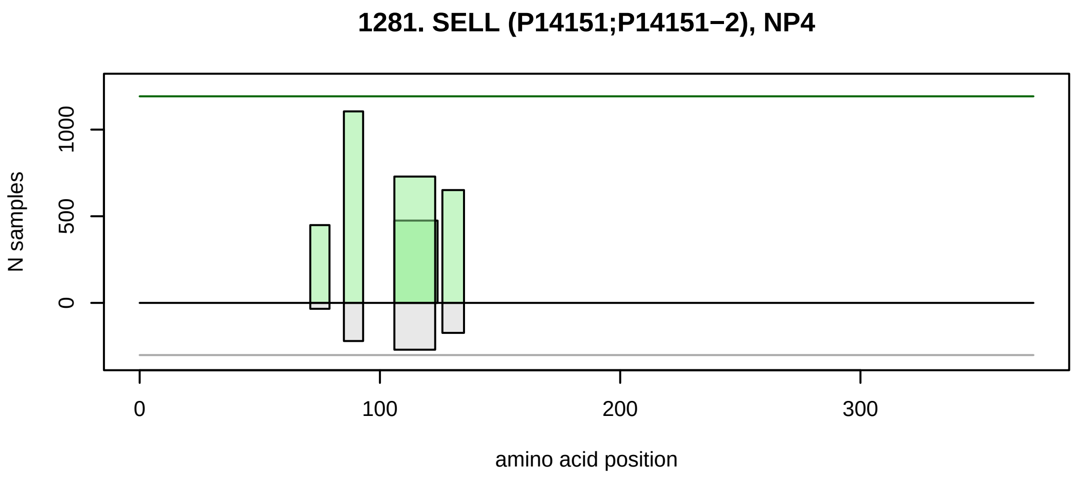
1281. SELL (P14151;P14151−2), NP4
N samples
0
500
1000
0
100
200
300
amino acid position

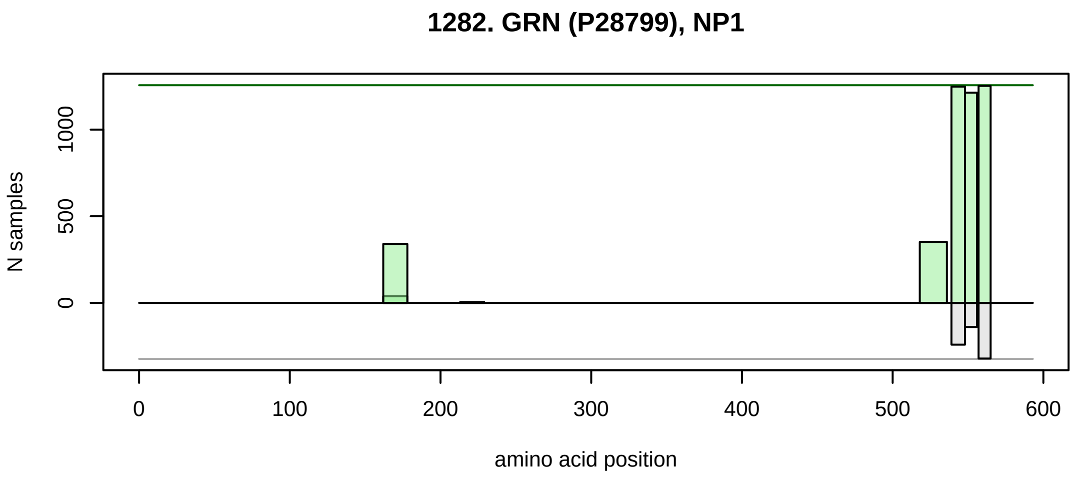
1282. GRN (P28799), NP1
N samples
0
500
1000
0
100
200
300
400
500
600
amino acid position

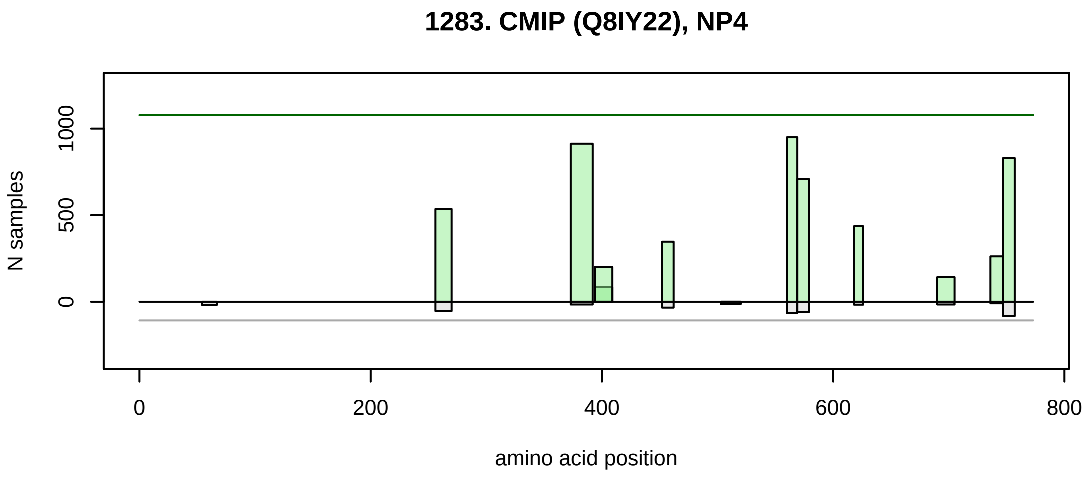
1283. CMIP (Q8IY22), NP4
N samples
0
500
1000
0
200
400
600
800
amino acid position

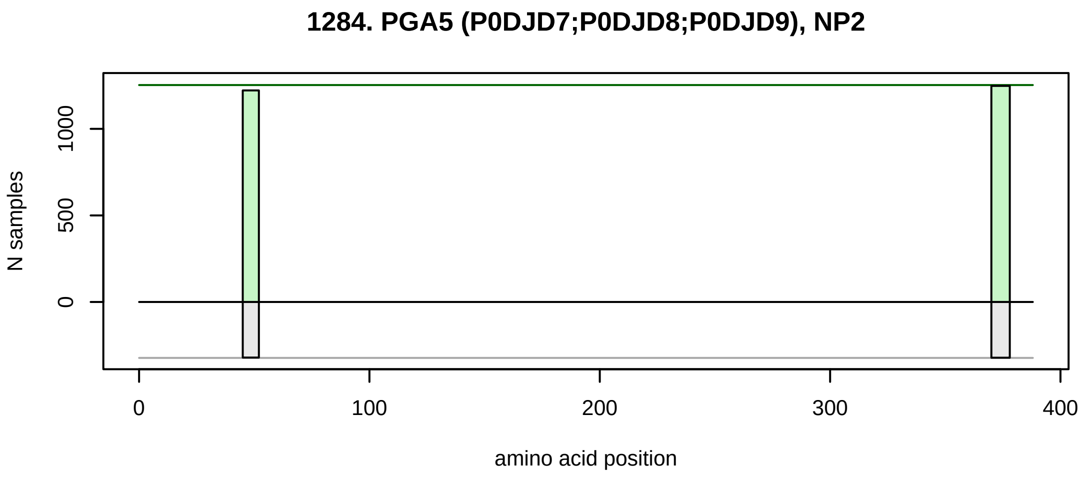
1284. PGA5 (P0DJD7;P0DJD8;P0DJD9), NP2
N samples
0
500
1000
0
100
200
300
400
amino acid position

1285. ME2 (A0A1W2PPH1), NP4

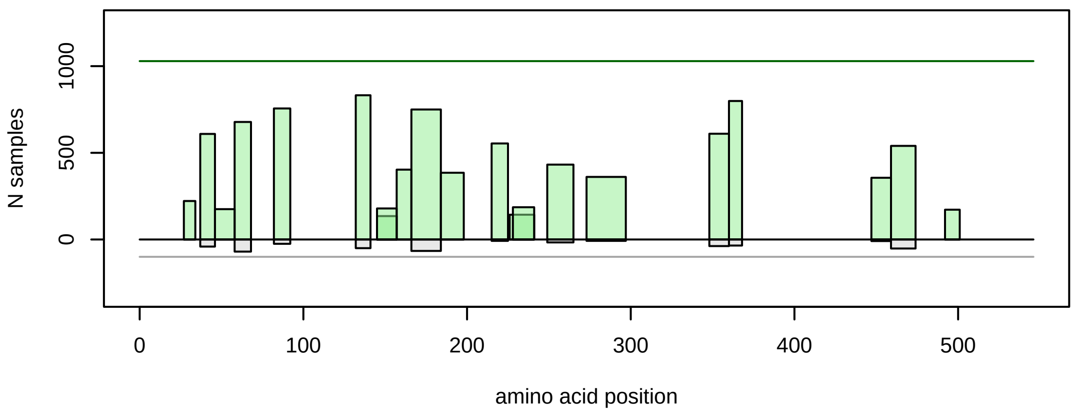

1286. MMP19 (Q99542), NP5

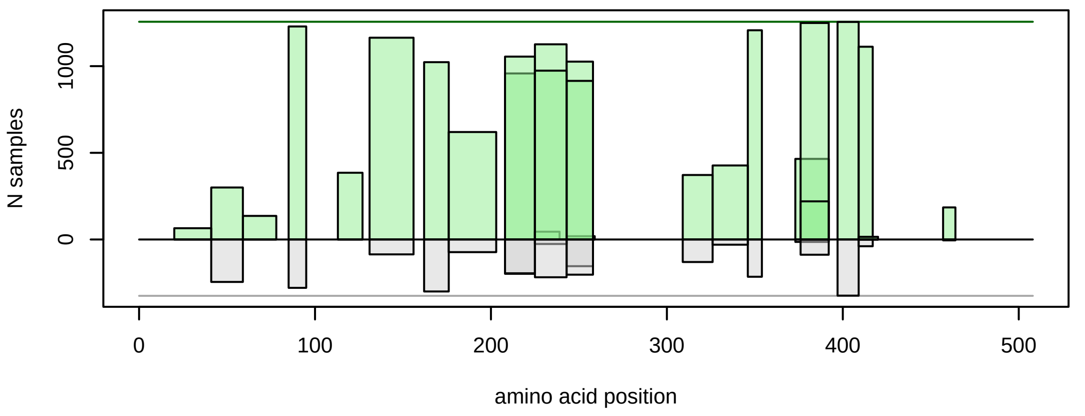

1287. ENOPH1 (Q9UHY7), NP2

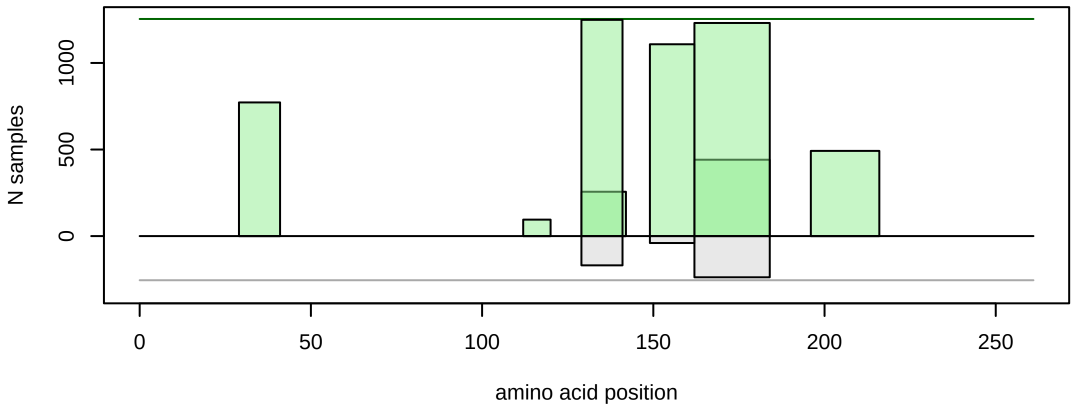

1288. PCSK6 (P29122;P29122−2), NP5

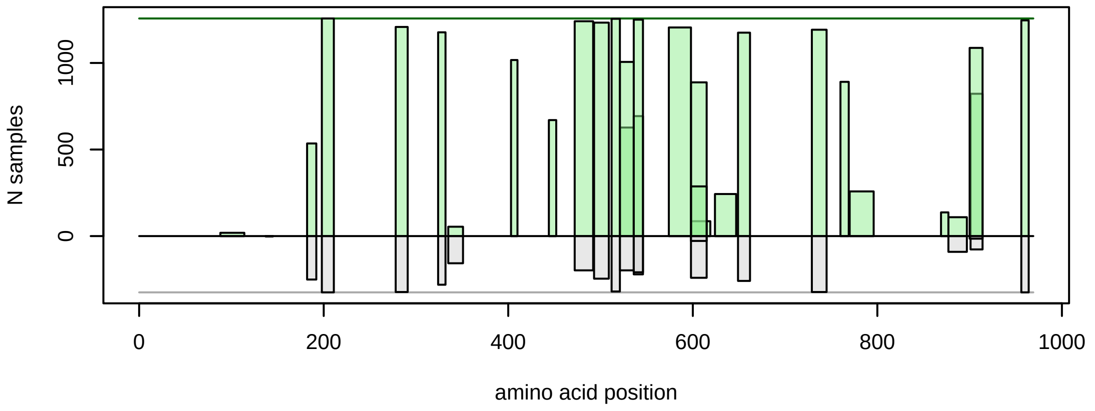

1289. THOP1 (P52888), NP5

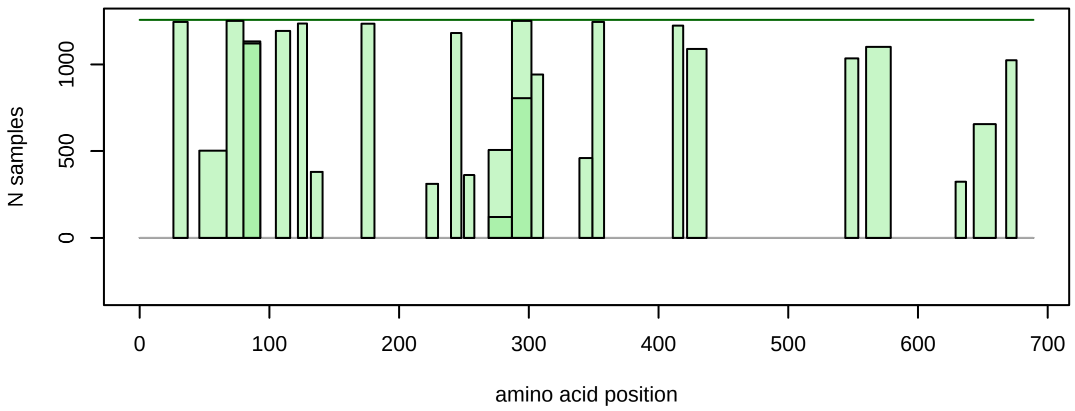

1290. ERP29 (P30040), NP3

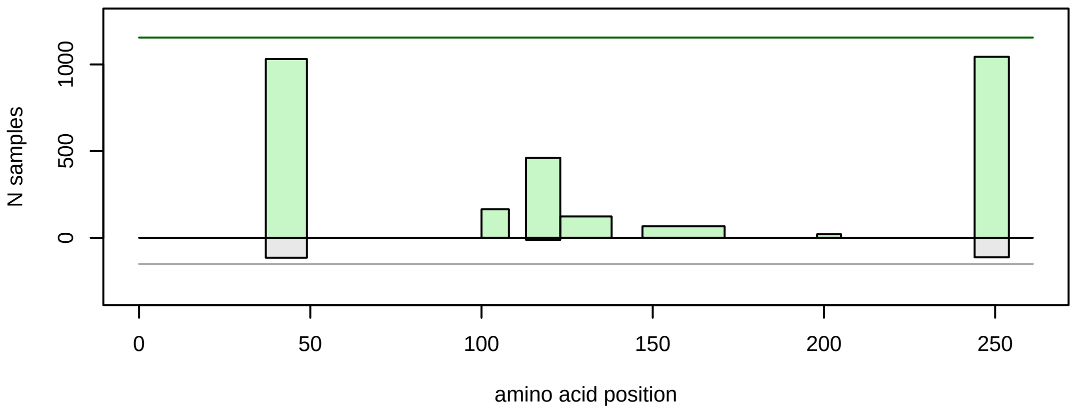

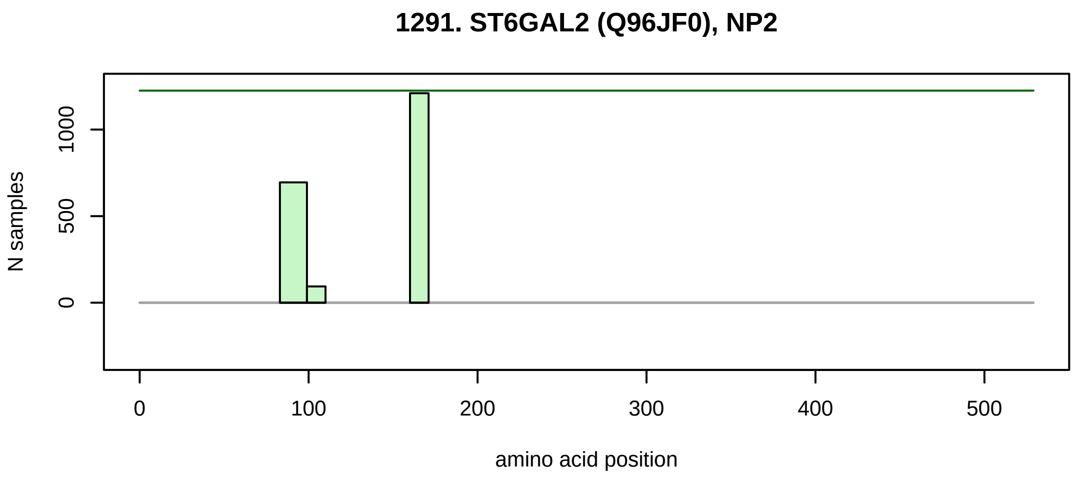
1291. ST6GAL2 (Q96JF0), NP2
N samples
0
500
1000
0
100
200
300
400
500
amino acid position

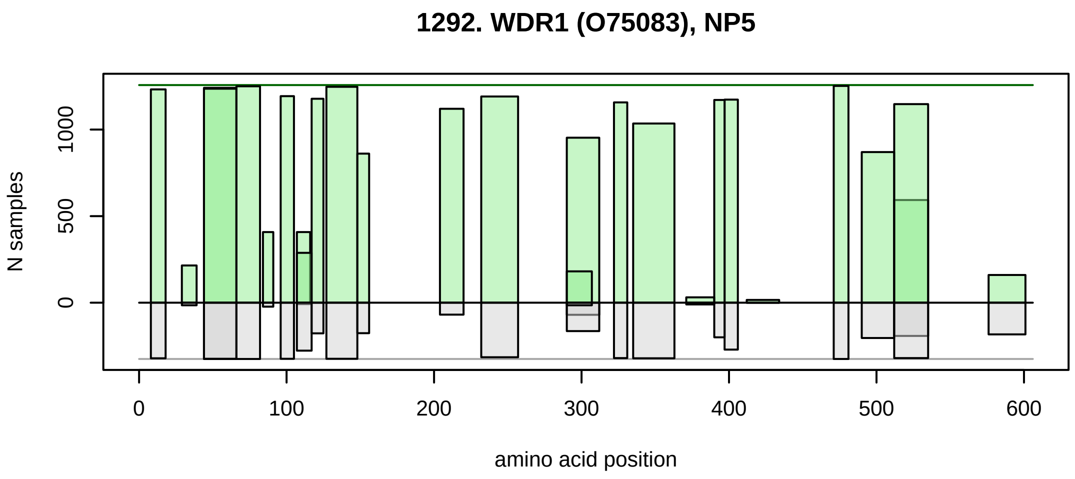
1292. WDR1 (O75083), NP5
N samples
0
500
1000
0
100
200
300
400
500
600
amino acid position

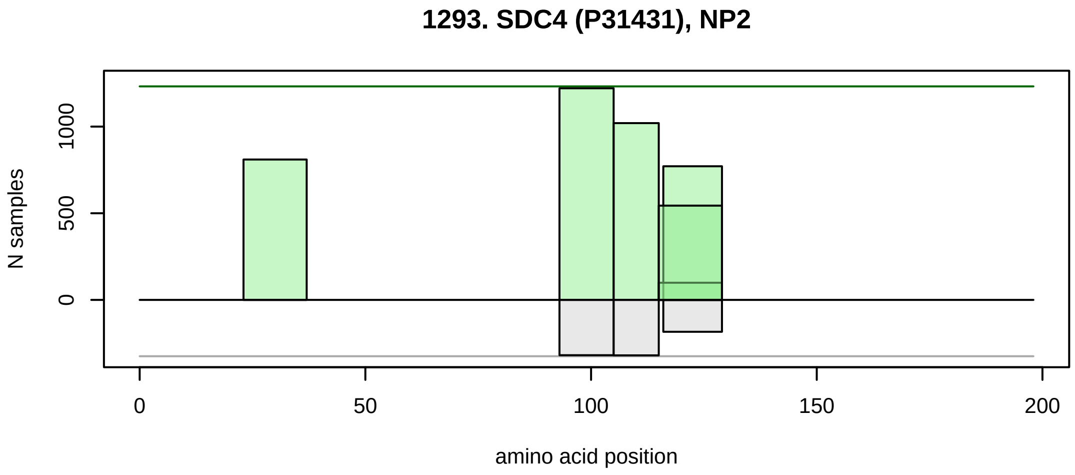
1293. SDC4 (P31431), NP2
N samples
0
500
1000
0
50
100
150
200
amino acid position

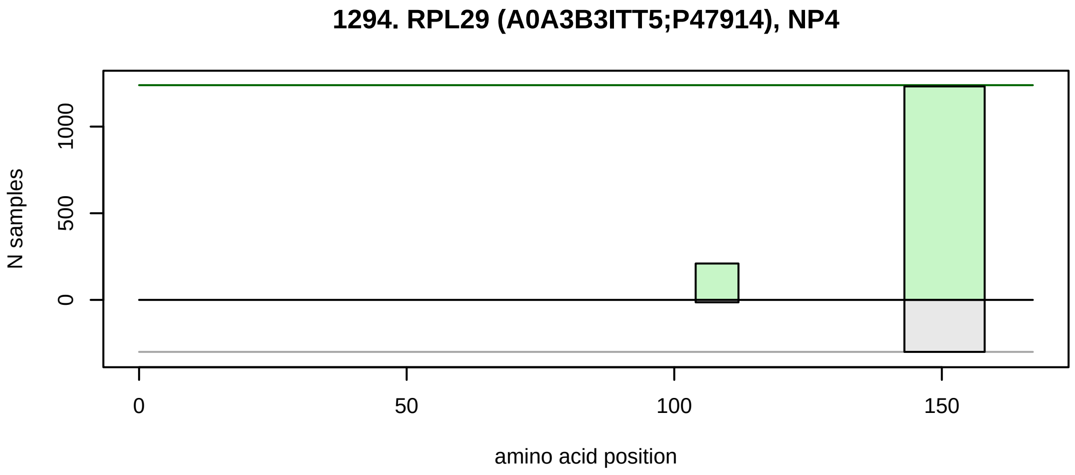
1294. RPL29 (A0A3B3ITT5;P47914), NP4
N samples
0
500
1000
0
50
100
150
amino acid position

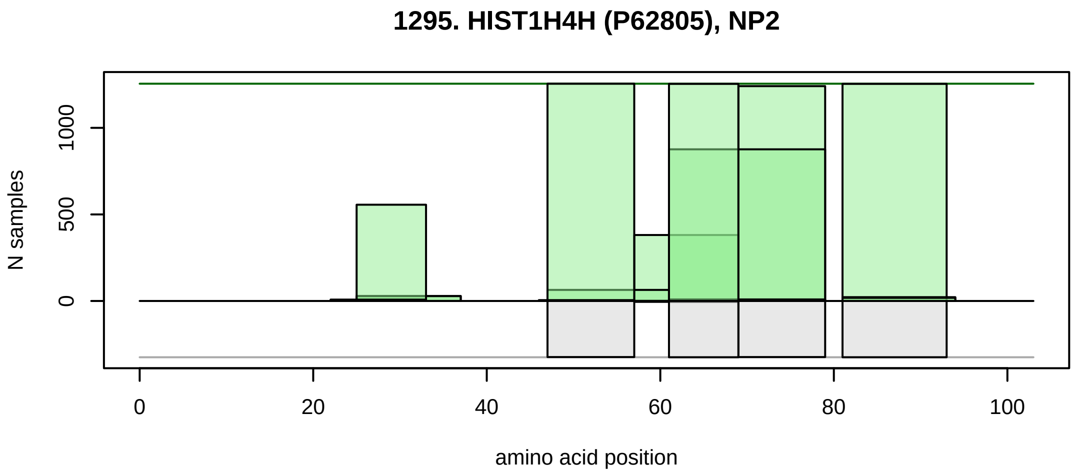
1295. HIST1H4H (P62805), NP2
N samples
0
500
1000
0
20
40
60
80
100
amino acid position

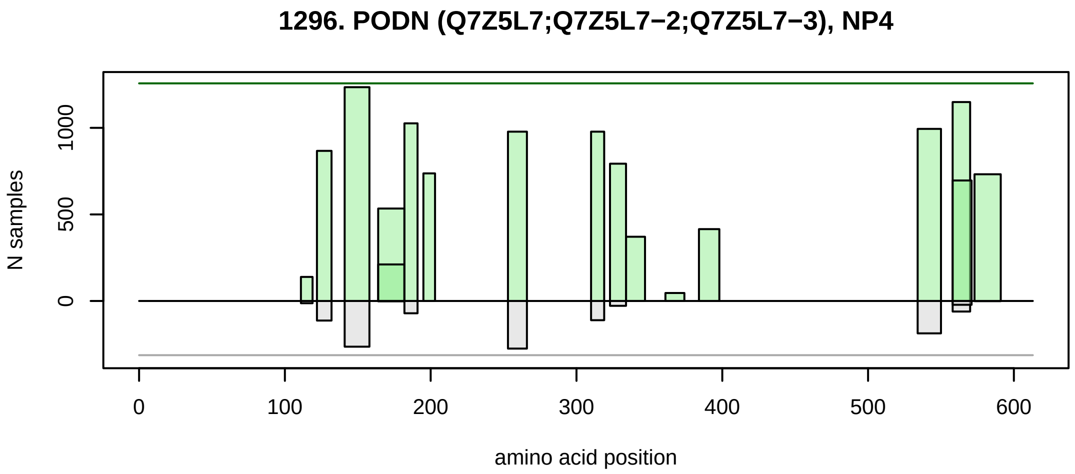
1296. PODN (Q7Z5L7;Q7Z5L7−2;Q7Z5L7−3), NP4
N samples
0
500
1000
0
100
200
300
400
500
600
amino acid position

1297. TGFB1I1 (O43294), NP4

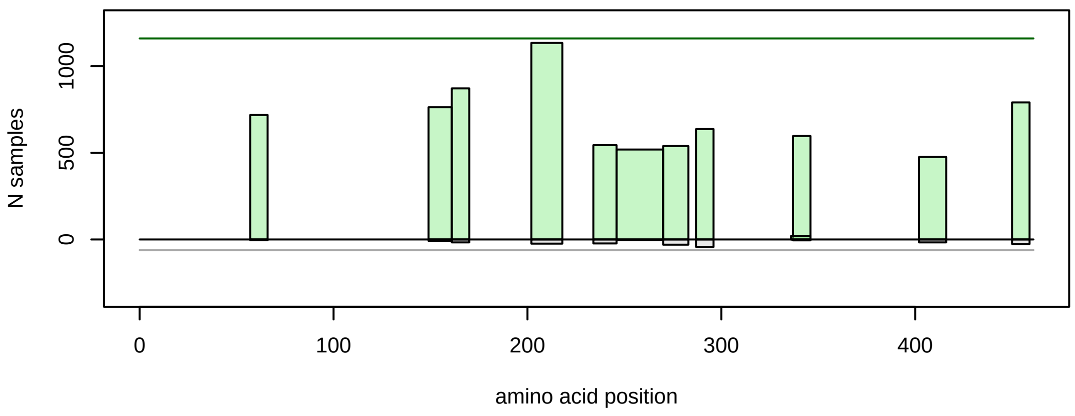

1298. PF4V1 (P10720), NP4

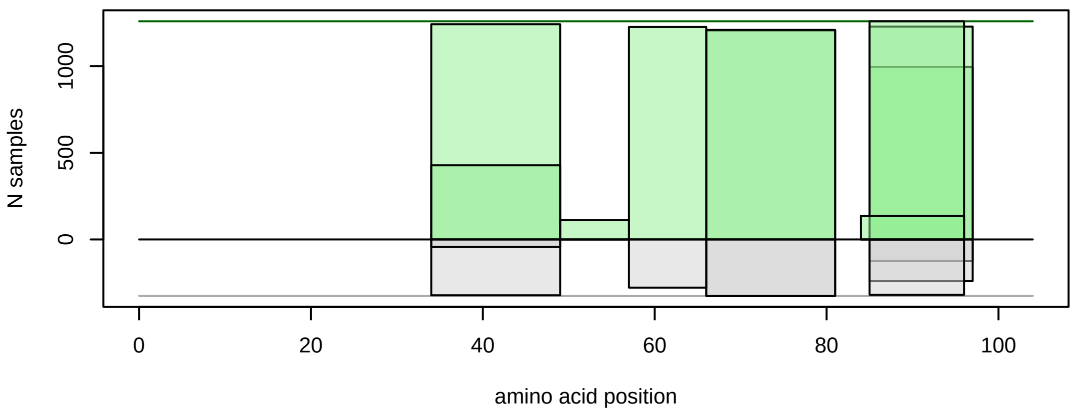

1299. ATIC (P31939), NP5

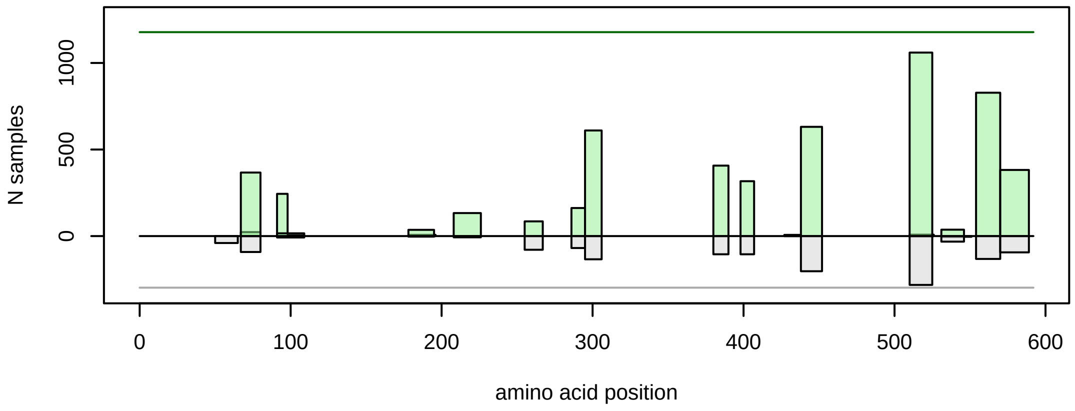

1300. ADD1 (E7EV99;P35611), NP1

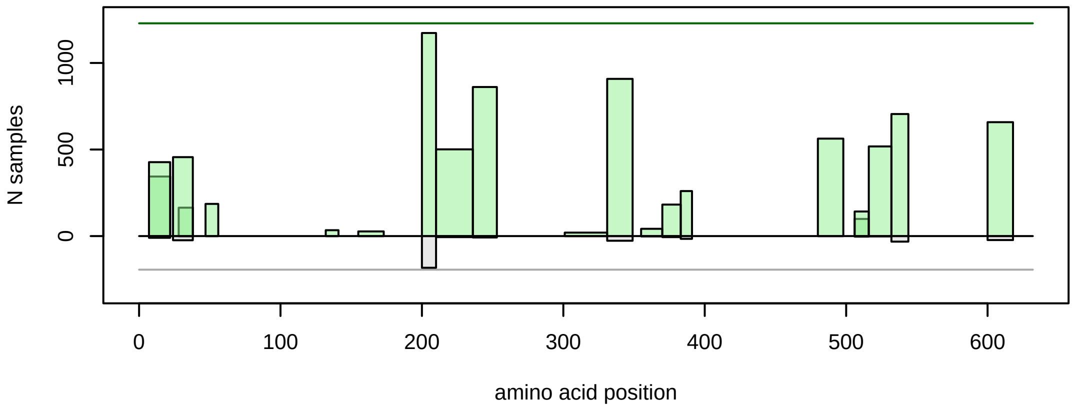

1301. GAS1 (P54826), NP2

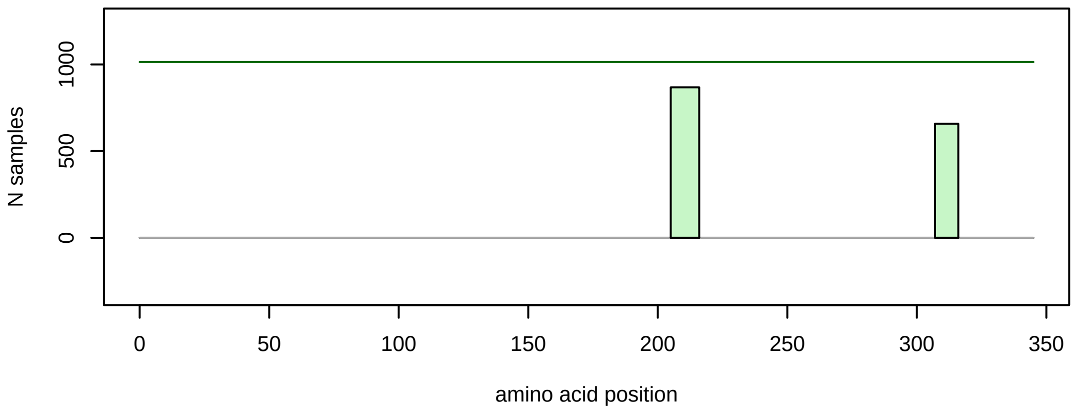

1302. HPRT1 (P00492), NP5

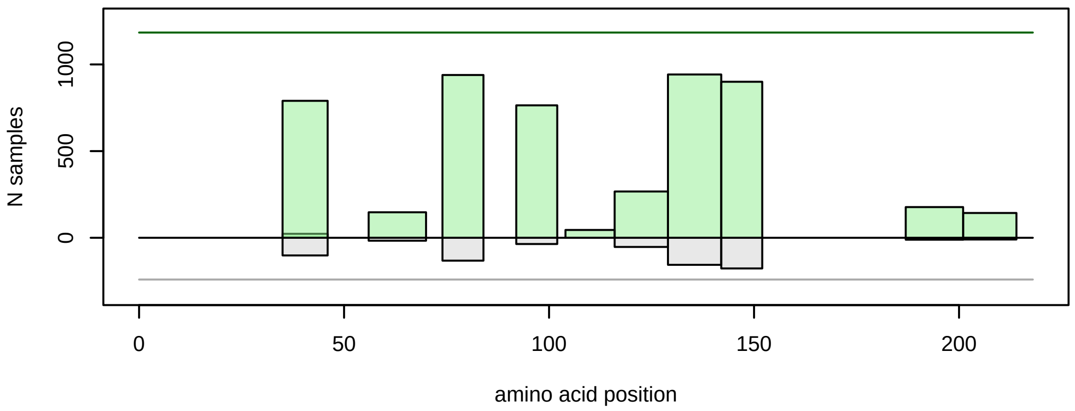

1303. GRIN1 (Q05586−6), NP3

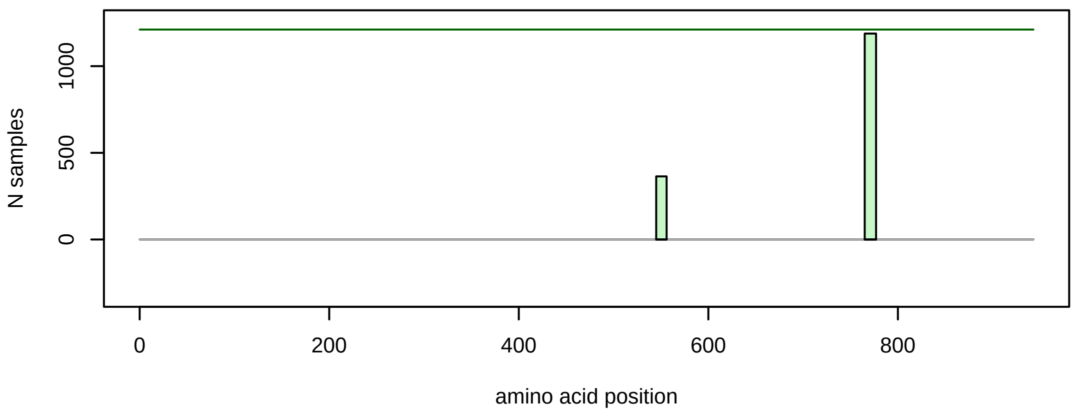

1304. AKAP12 (Q02952;Q02952−2;Q02952−3), NP2

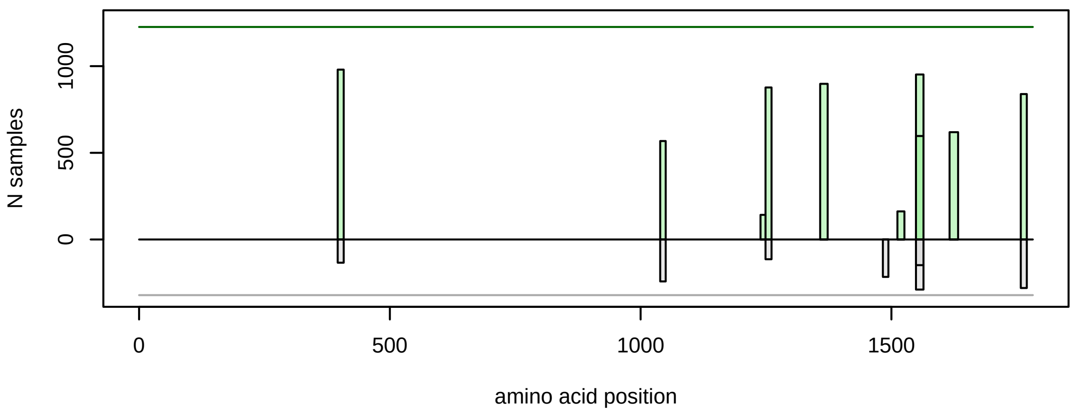

1305. C14orf37 (Q86TY3), NP2

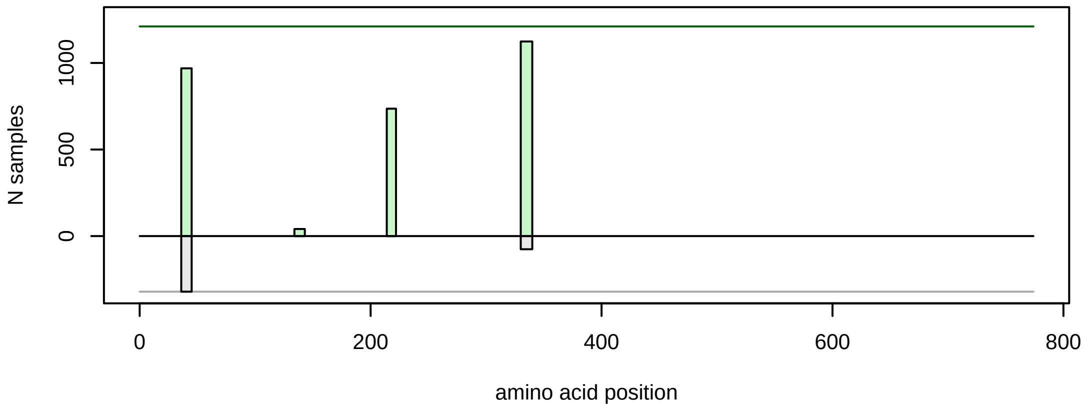

1306. SERPINA6 (P08185), NP2

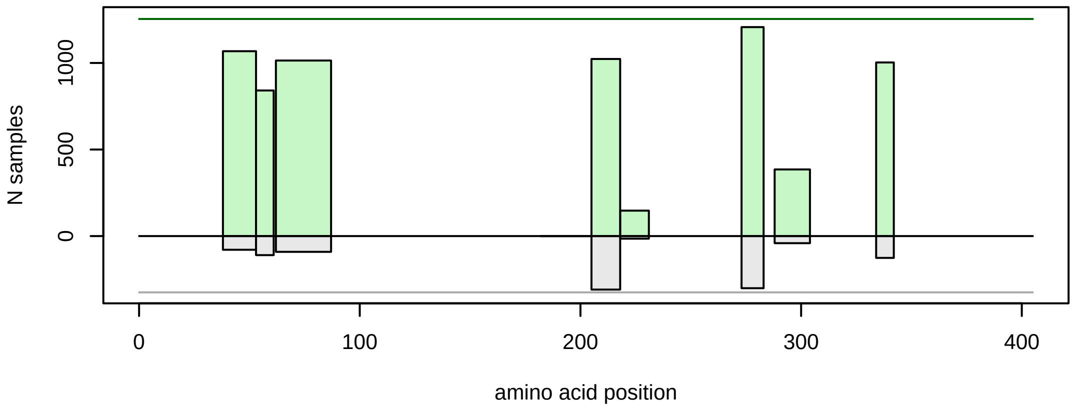

1307. SHROOM3 (A0A2R8Y5P9), NP4

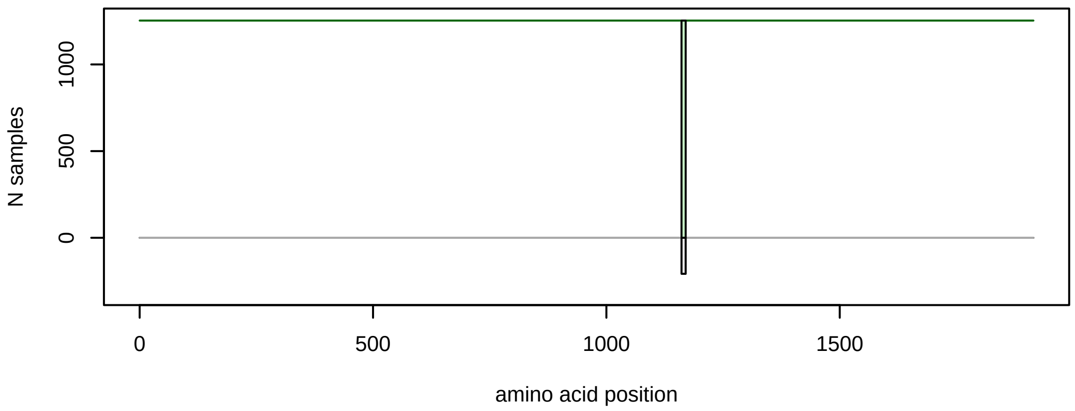

1308. IGHV3−15 (A0A0B4J1V0), NP3

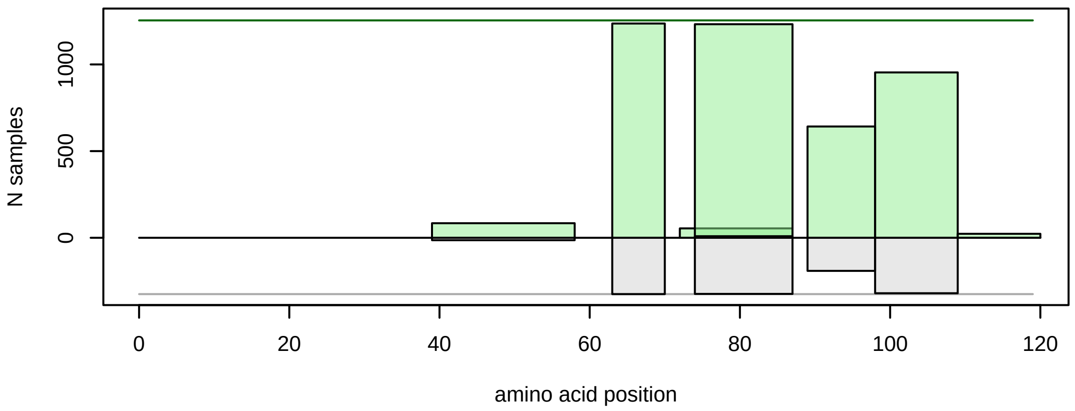

1309. DCTN4 (Q9UJW0), NP4

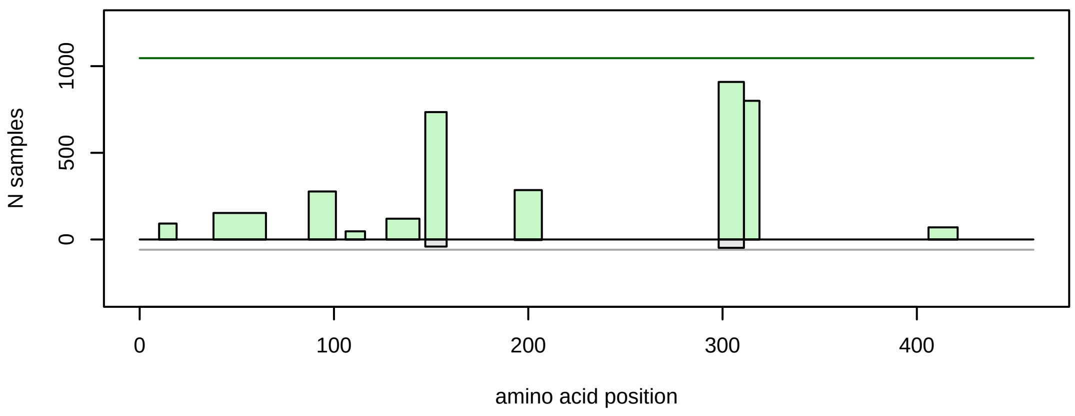

1310. OIT3 (Q8WWZ8), NP5

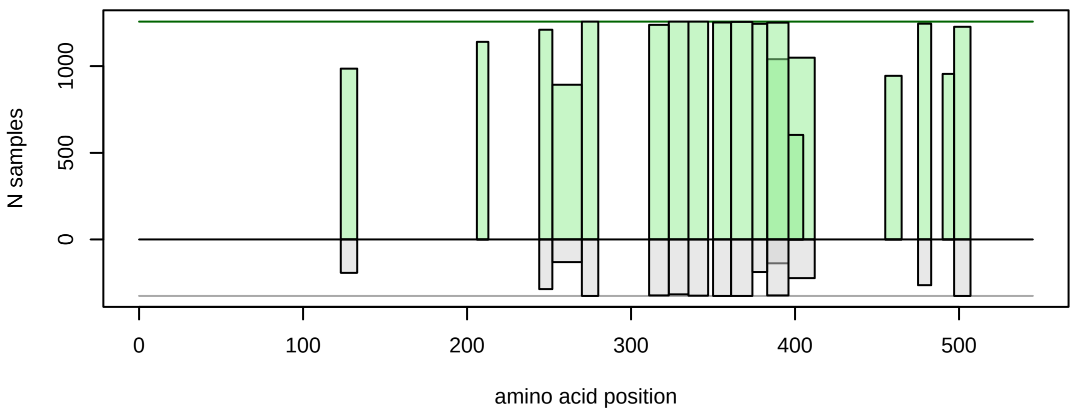

1311. SAA2−SAA4 (A0A096LPE2), NP2

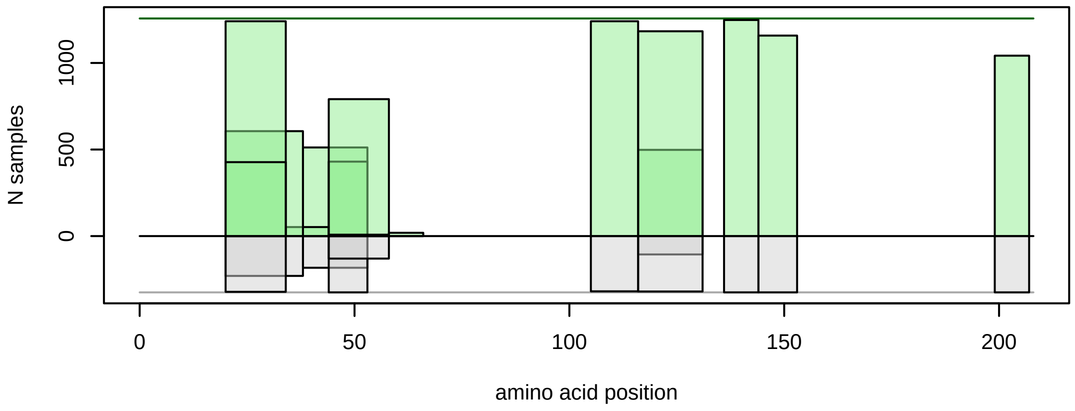

1312. CPN1 (P15169), NP1

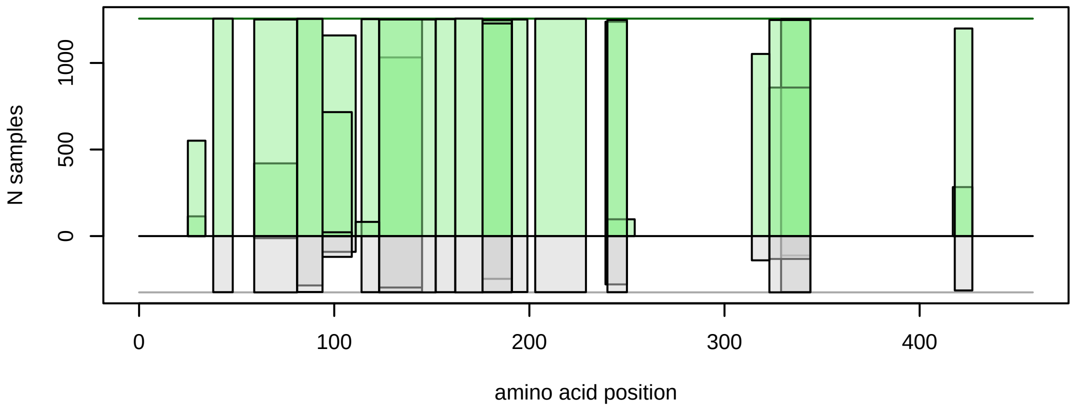

1313. PARK7 (Q99497), NP1

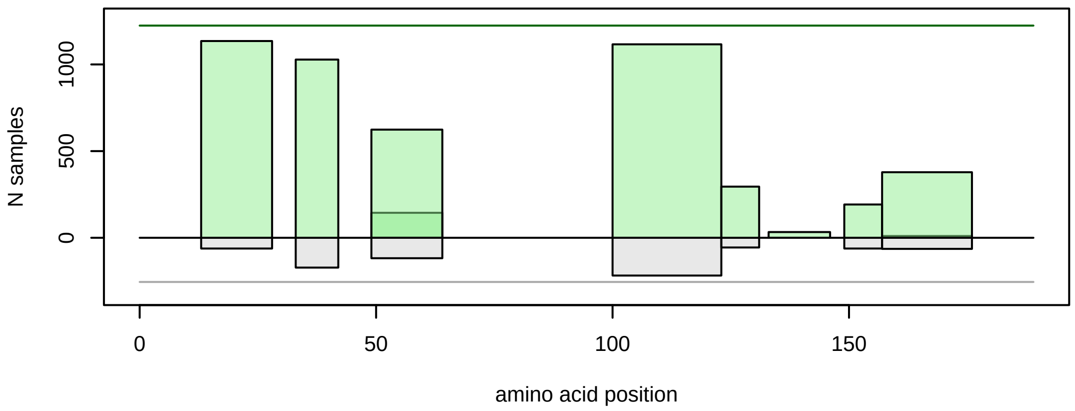

1314. CNP (P09543;P09543−2), NP1

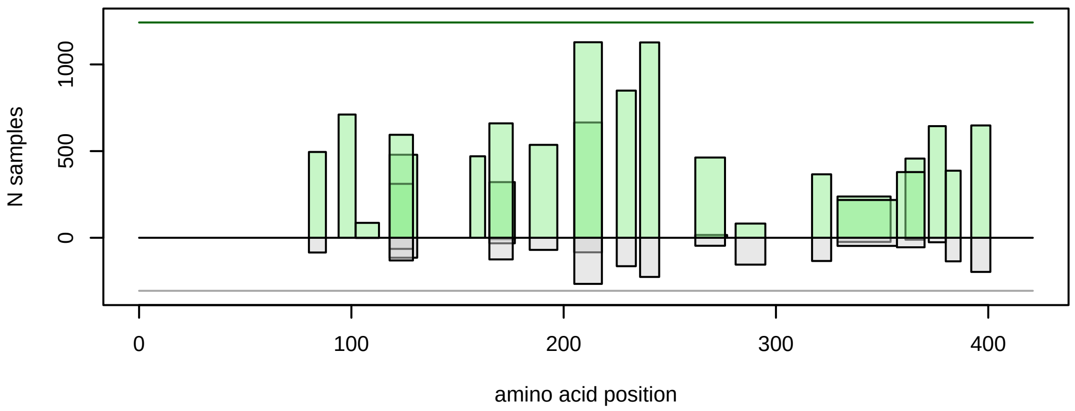

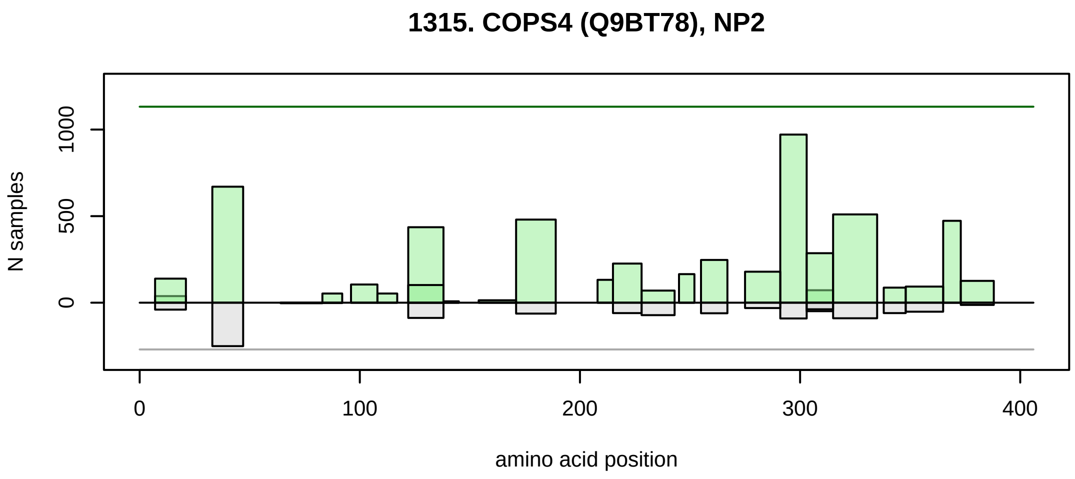
1315. COPS4 (Q9BT78), NP2
N samples
0
500
1000
0
100
200
300
400
amino acid position

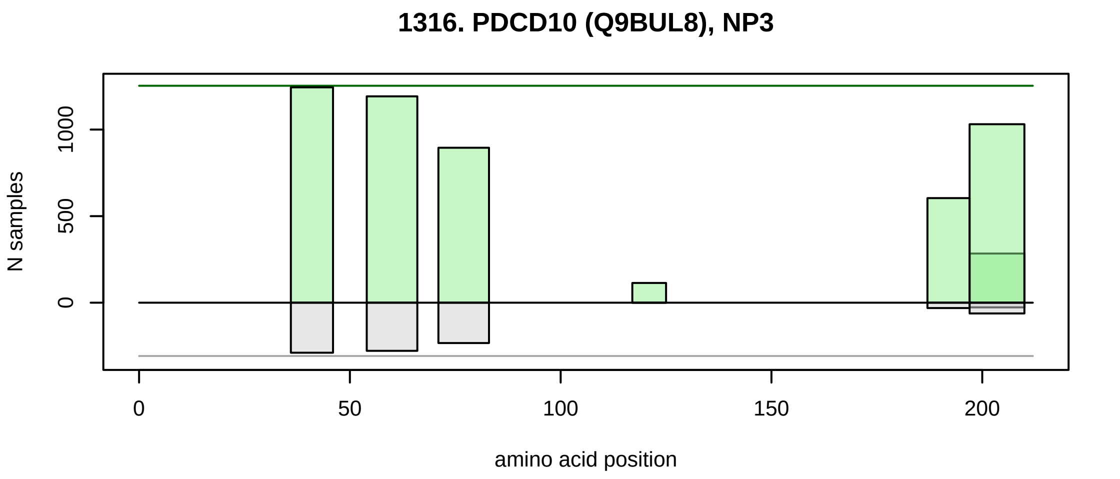
1316. PDCD10 (Q9BUL8), NP3
N samples
0
500
1000
0
50
100
150
200
amino acid position

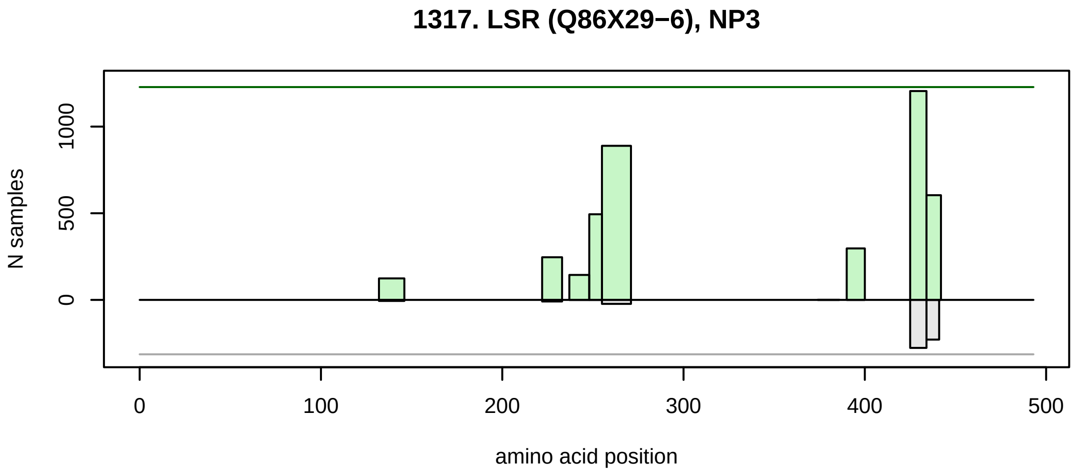
1317. LSR (Q86X29−6), NP3
N samples
0
500
1000
0
100
200
300
400
500
amino acid position

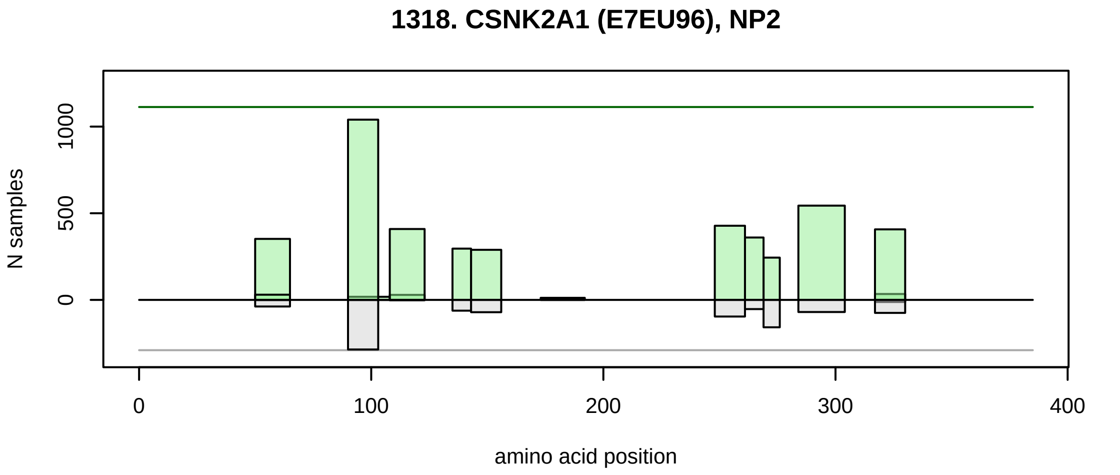
1318. CSNK2A1 (E7EU96), NP2
N samples
0
500
1000
0
100
200
300
400
amino acid position

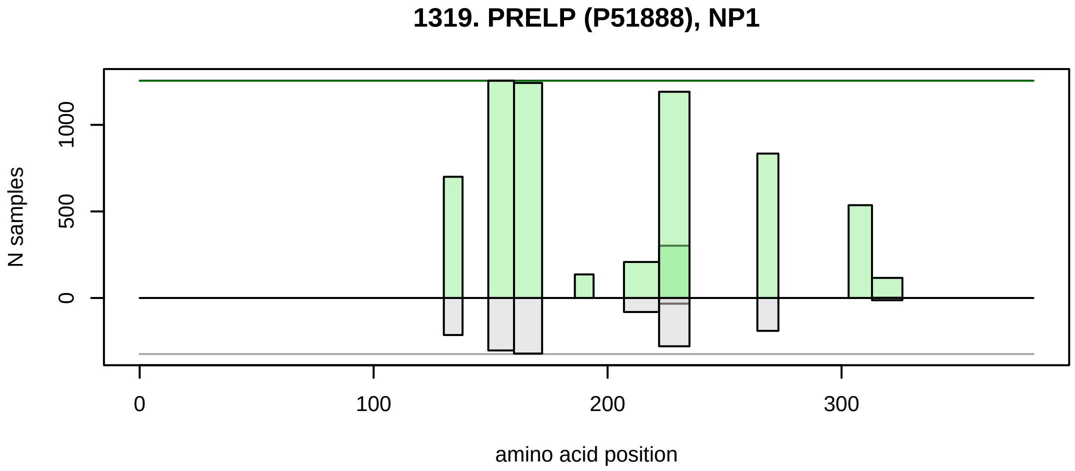
1319. PRELP (P51888), NP1
N samples
0
500
1000
0
100
200
300
amino acid position

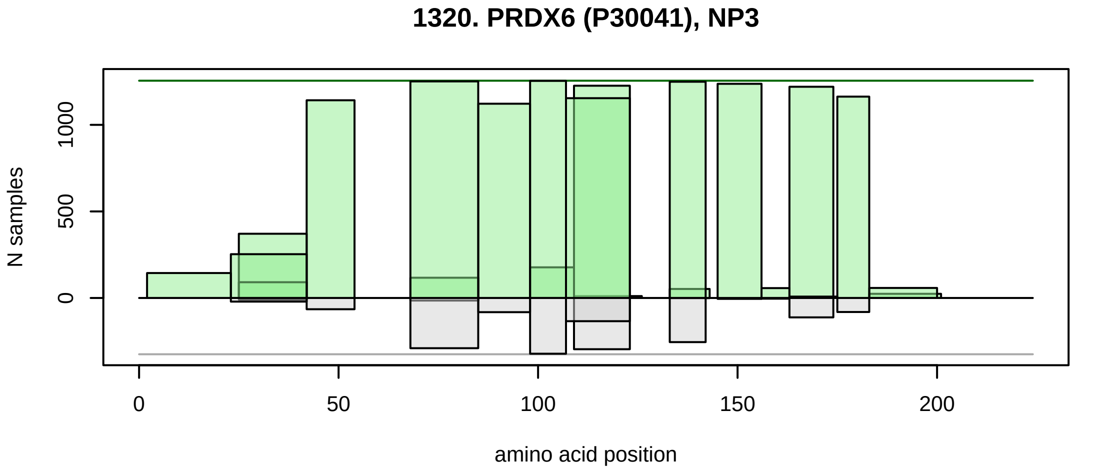
1320. PRDX6 (P30041), NP3
N samples
0
500
1000
0
50
100
150
200
amino acid position

1321. IGLV3−1 (P01715), NP3

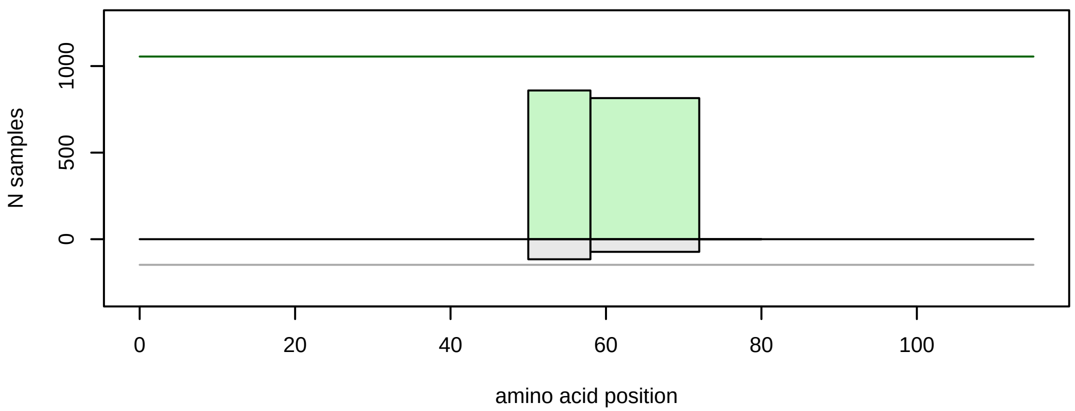

1322. ARL6IP5 (O75915), NP4

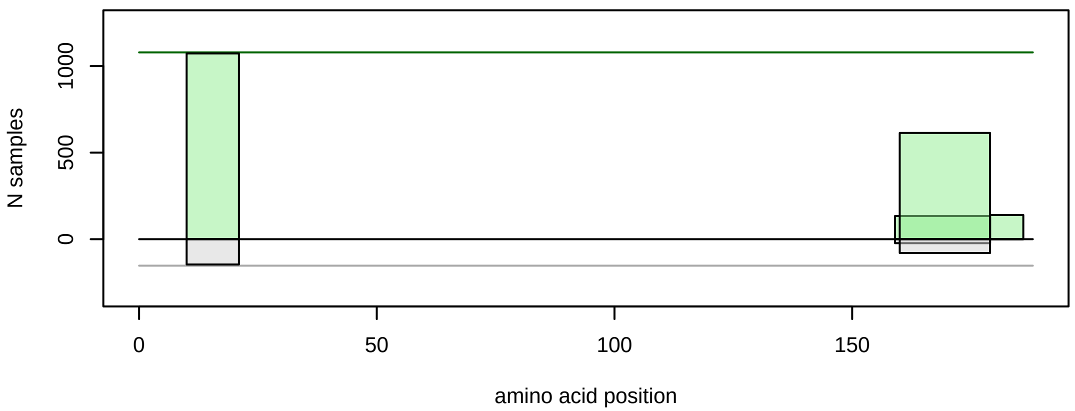

1323. CDH16 (O75309), NP2

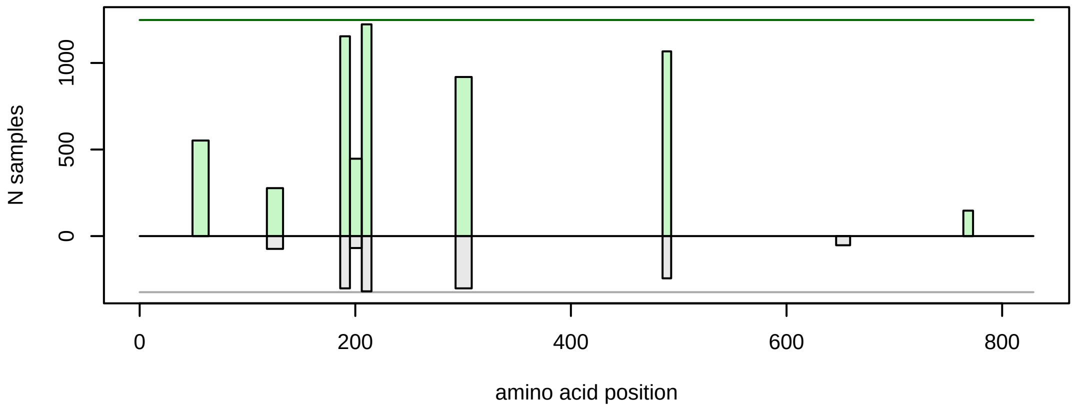

1324. SPG11 (A0A804HLC5), NP1

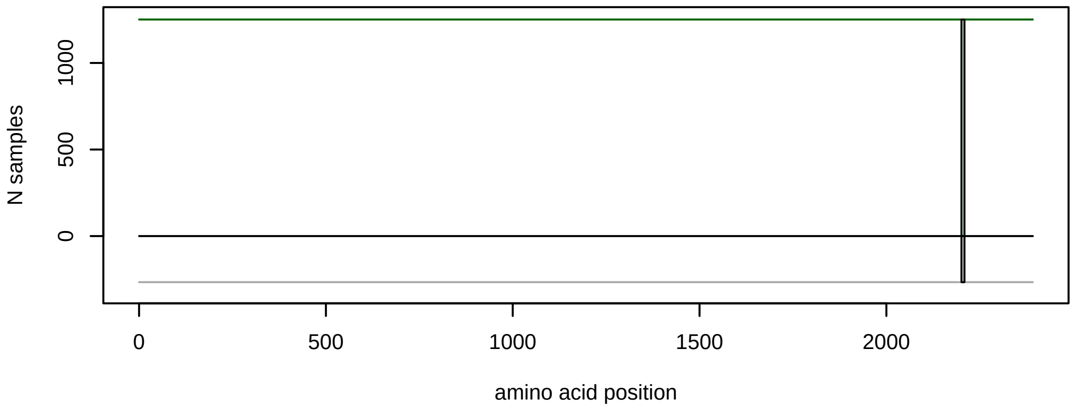

1325. CDV3 (Q9UKY7), NP3

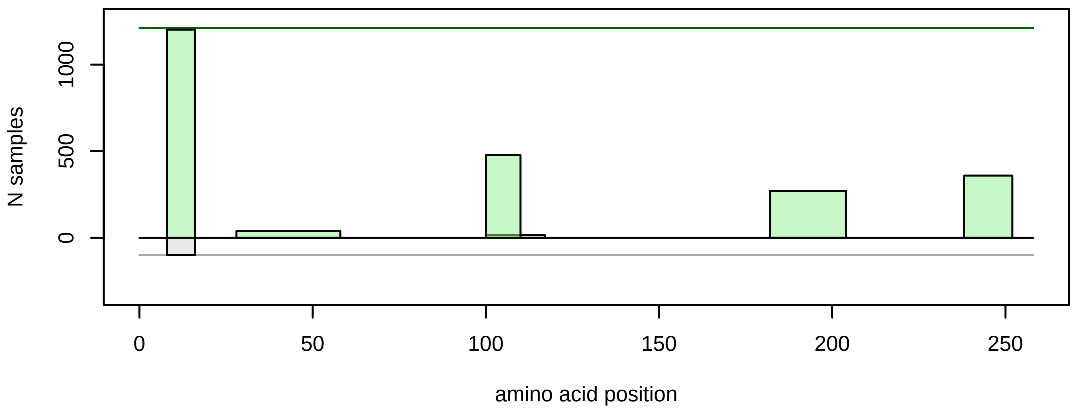

1326. TIMP2 (P16035), NP5

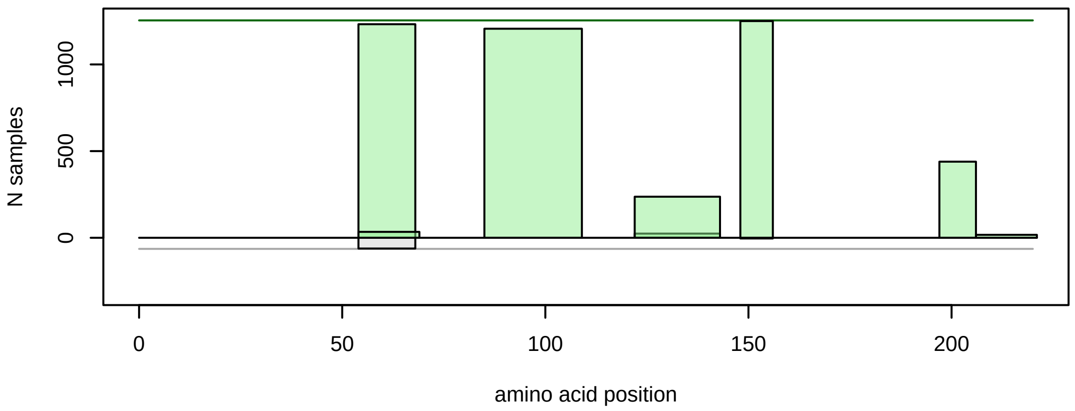

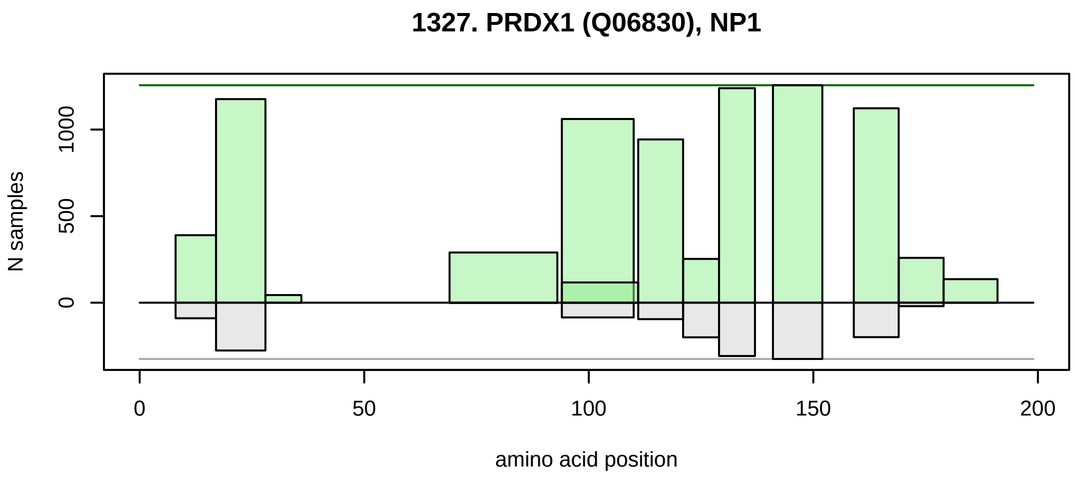
1327. PRDX1 (Q06830), NP1
N samples
amino acid position

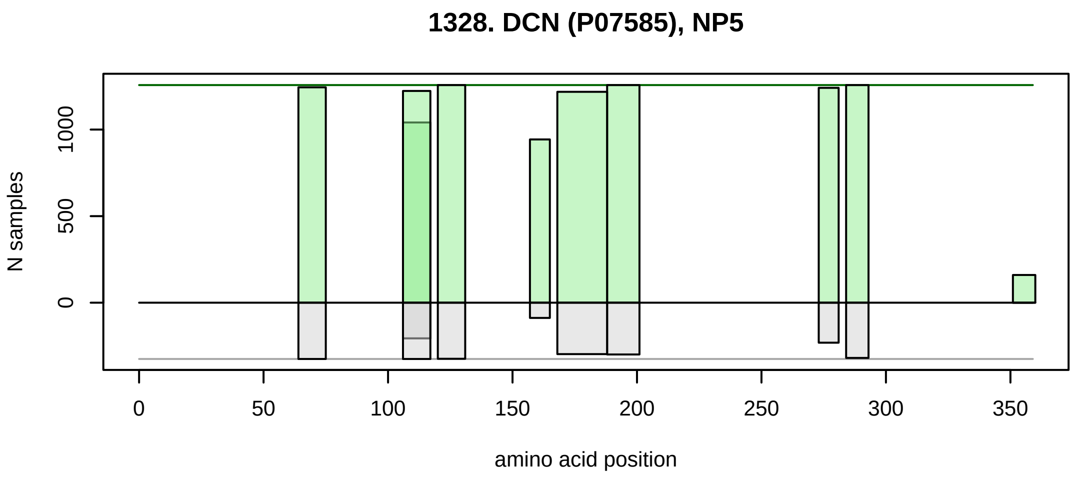
1328. DCN (P07585), NP5
N samples
amino acid position

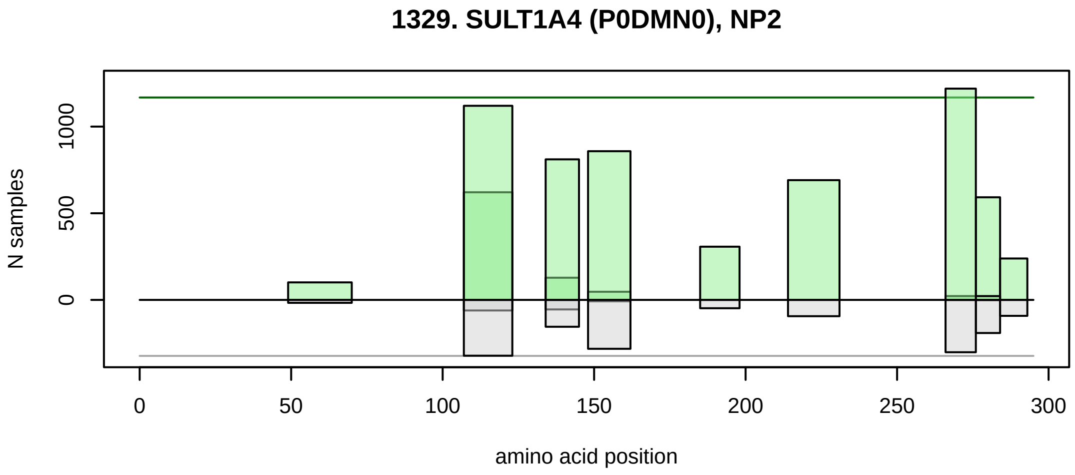
1329. SULT1A4 (P0DMN0), NP2
N samples
amino acid position

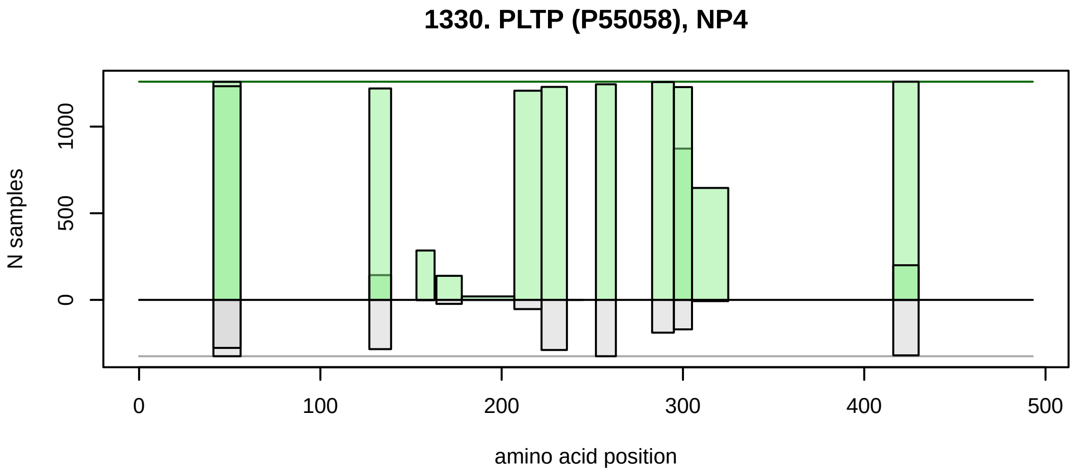
1330. PLTP (P55058), NP4
N samples
amino acid position

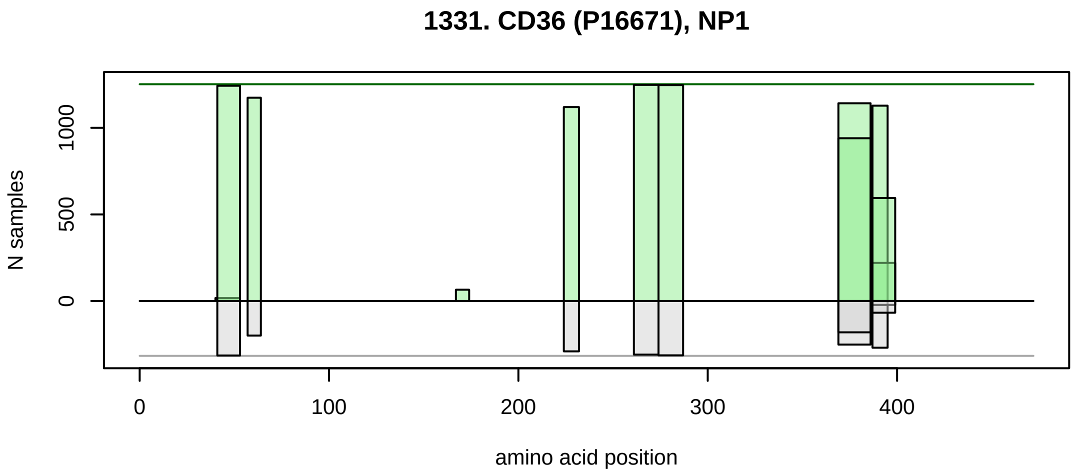
1331. CD36 (P16671), NP1
N samples
amino acid position

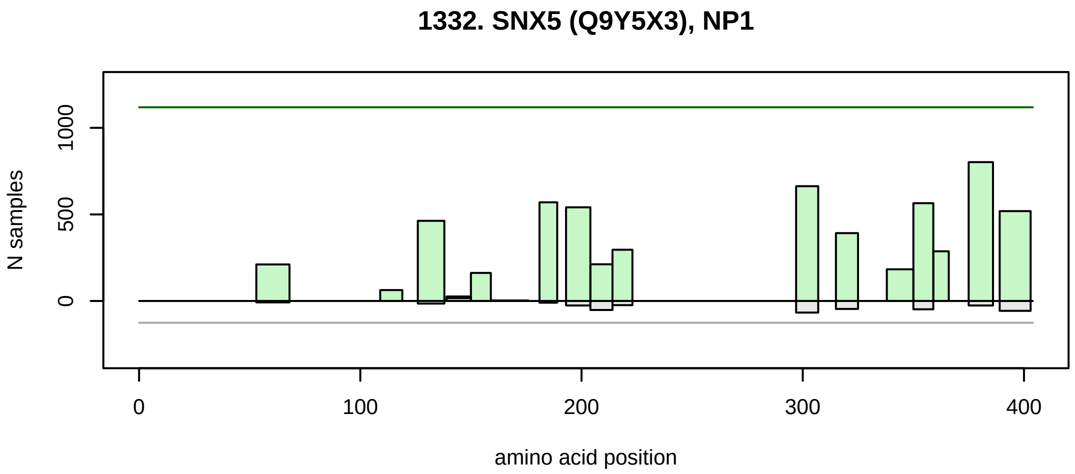
1332. SNX5 (Q9Y5X3), NP1
N samples
amino acid position

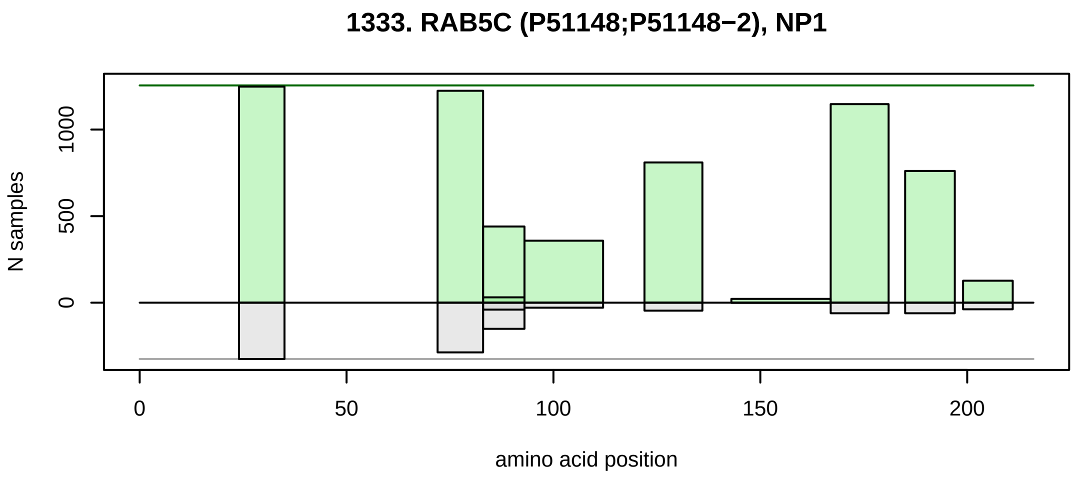

1333. RAB5C (P51148;P51148−2), NP1
N samples
amino acid position

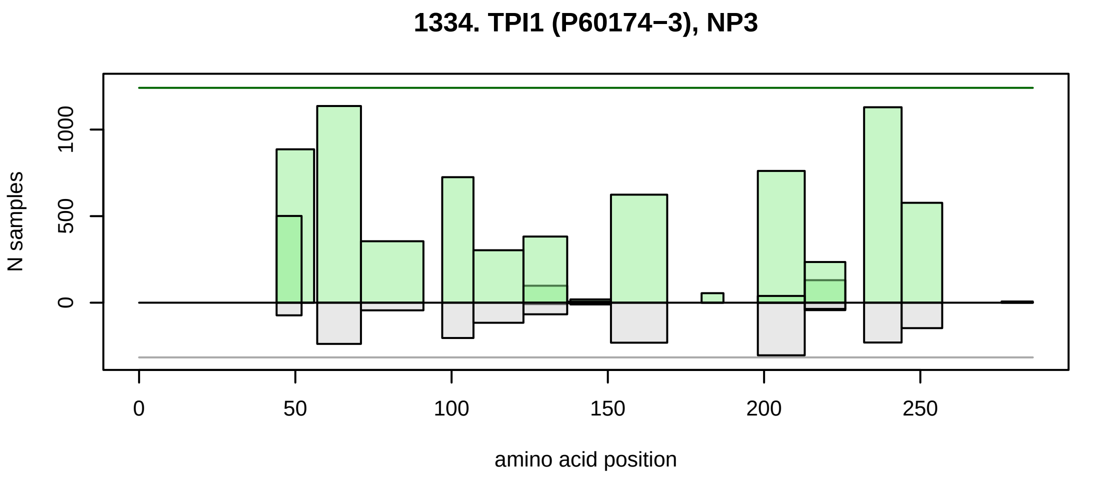

1334. TPI1 (P60174−3), NP3
N samples
amino acid position

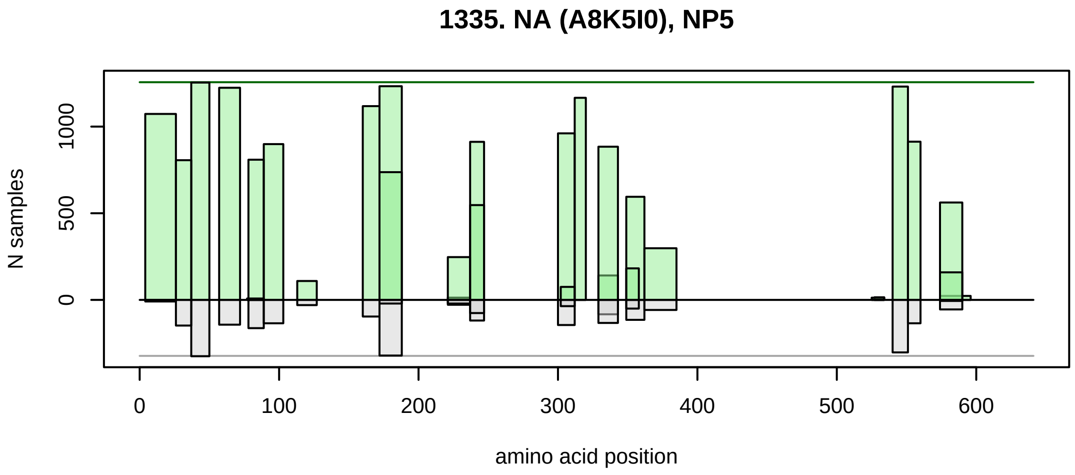

1335. NA (A8K5I0), NP5
N samples
amino acid position

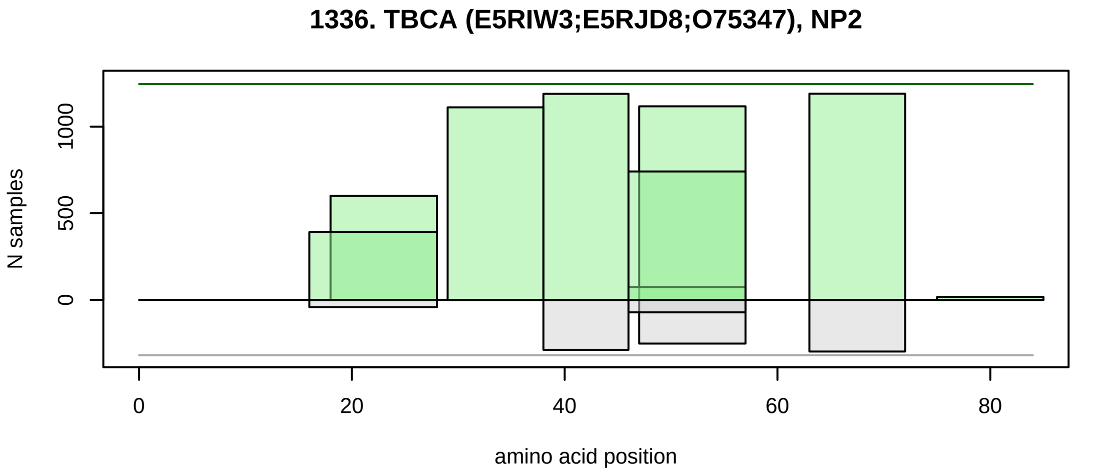

1336. TBCA (E5RIW3;E5RJD8;O75347), NP2
N samples
amino acid position

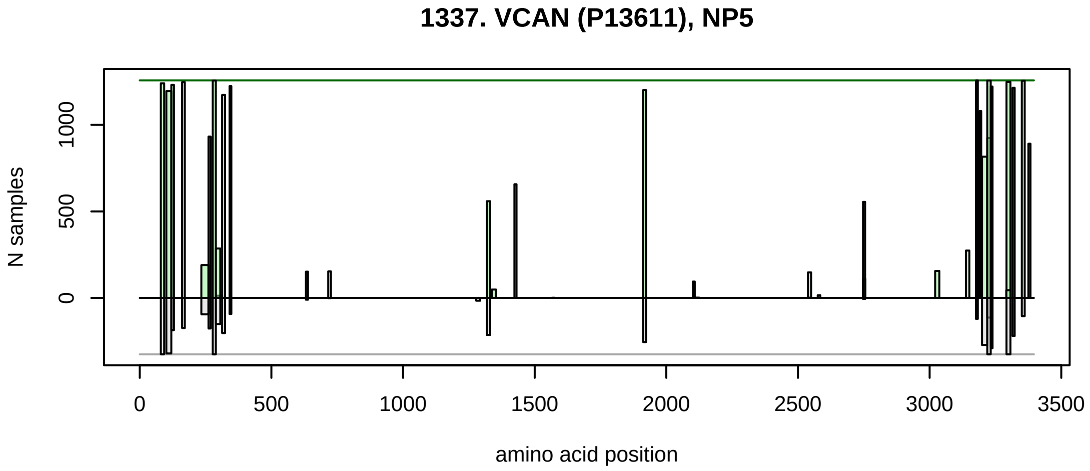

1337. VCAN (P13611), NP5
N samples
amino acid position

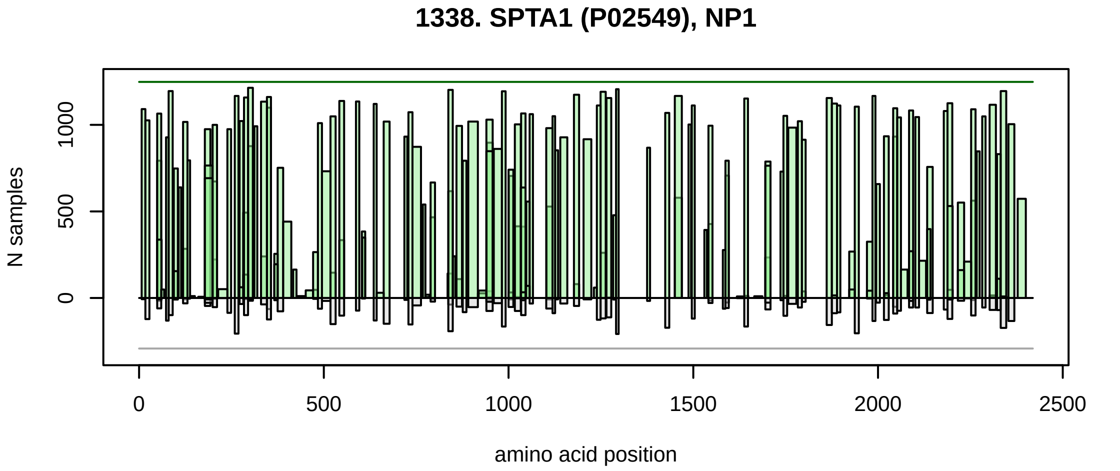

1338. SPTA1 (P02549), NP1
N samples
amino acid position

1339. STXBP3 (O00186), NP4

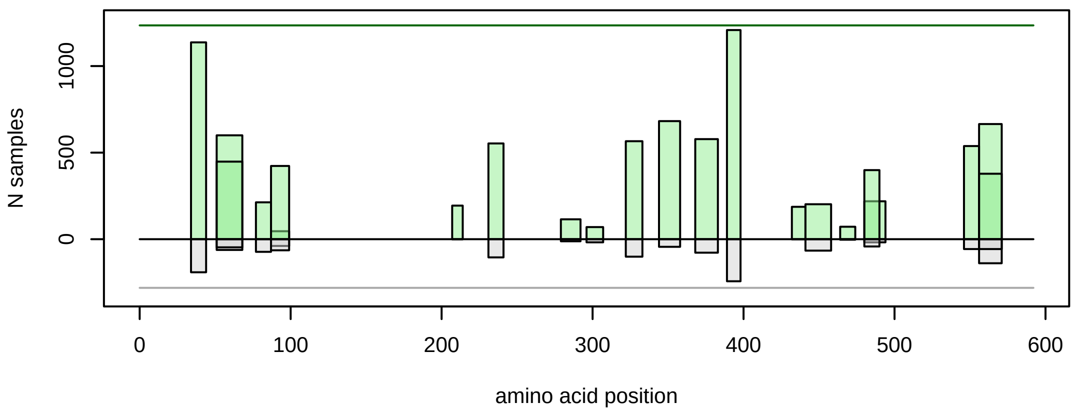

1340. FGA (P02671), NP5

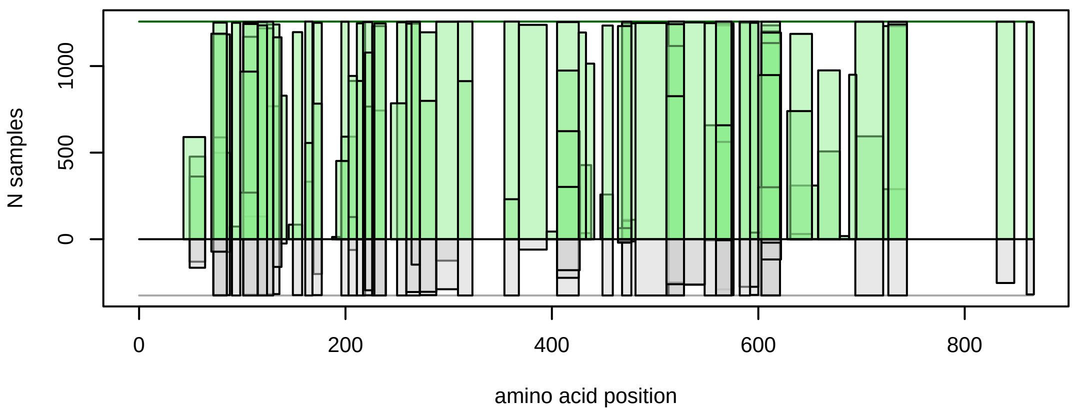

1341. HN1L (Q9H910;Q9H910−3), NP3

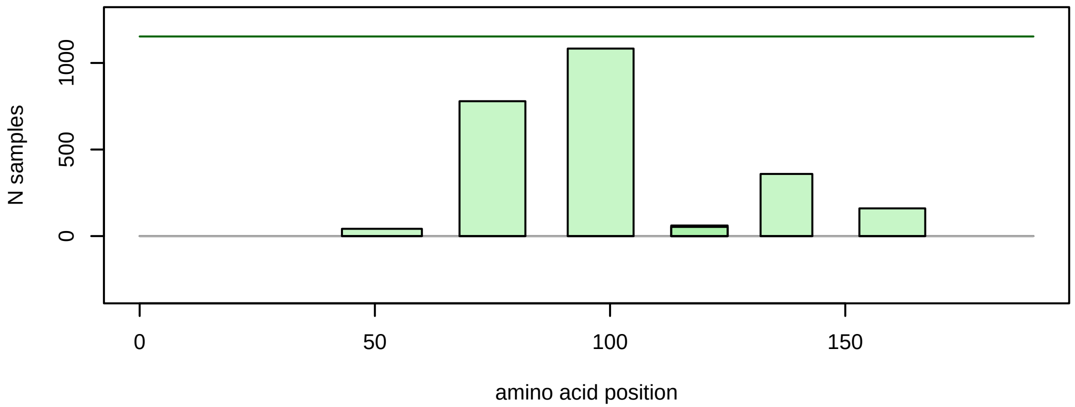

1342. CHAD (O15335), NP3

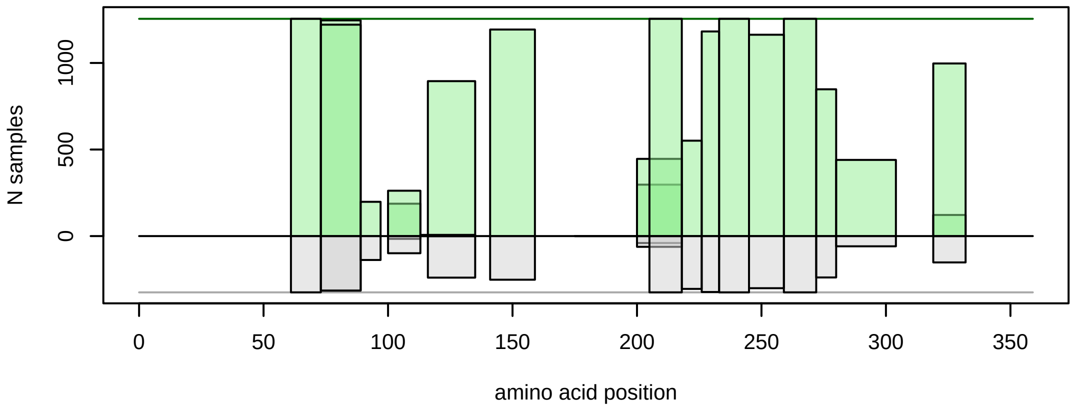

1343. PIN1 (Q13526), NP3

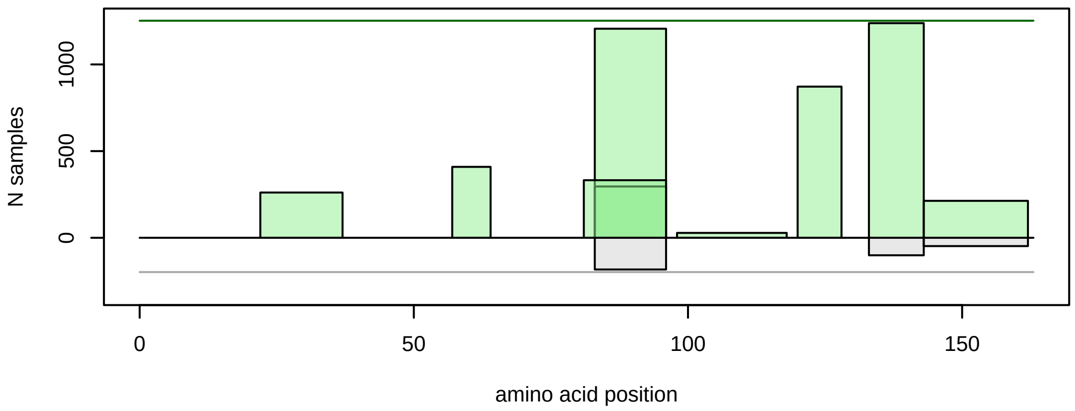

1344. CCT6A (P40227), NP1

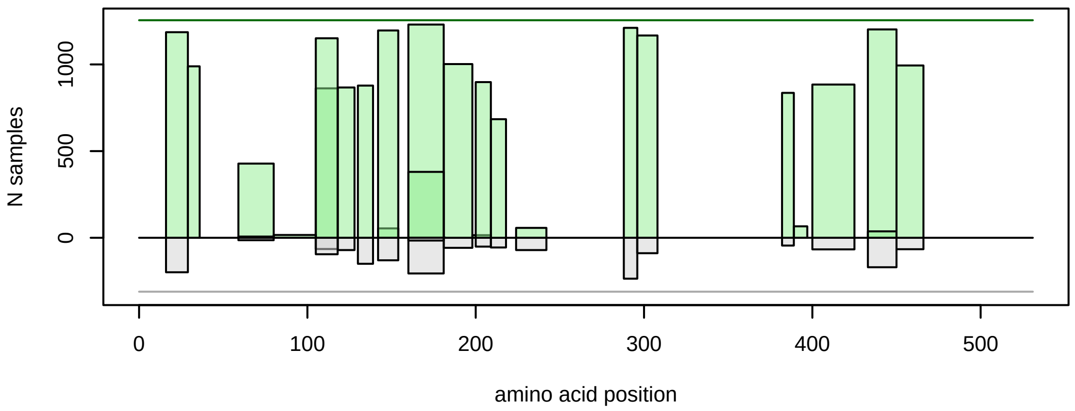

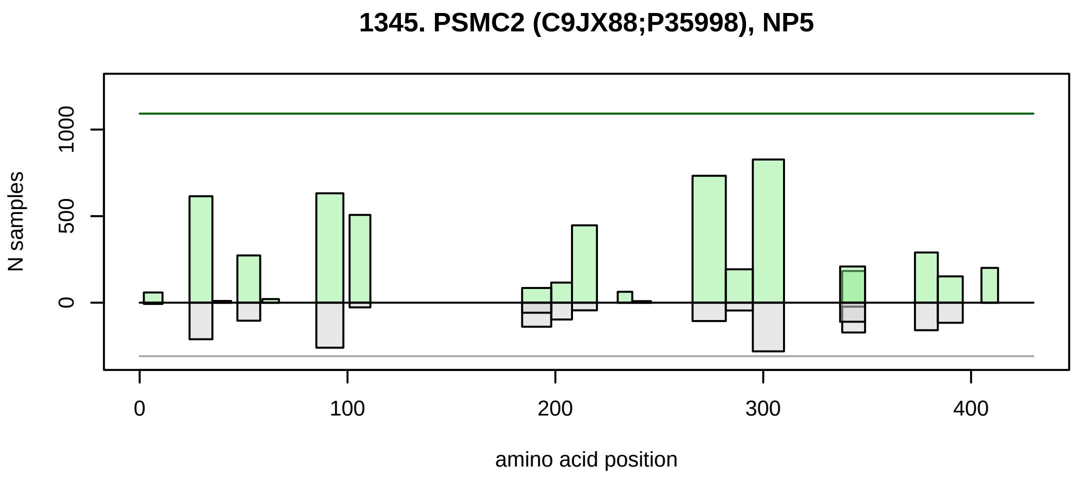
1345. PSMC2 (C9JX88;P35998), NP5
N samples
0
500
1000
0
100
200
300
400
amino acid position

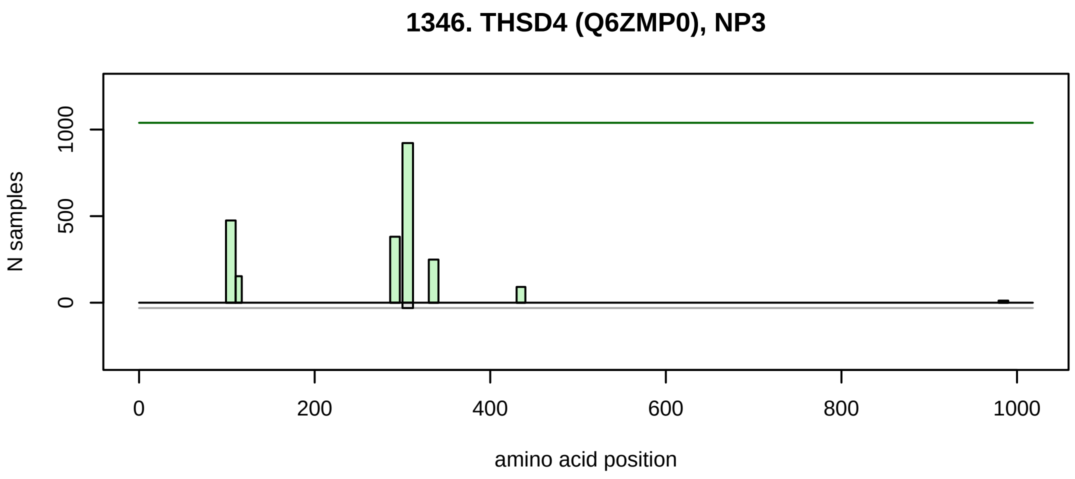
1346. THSD4 (Q6ZMP0), NP3
N samples
0
500
1000
0
200
400
600
800
1000
amino acid position

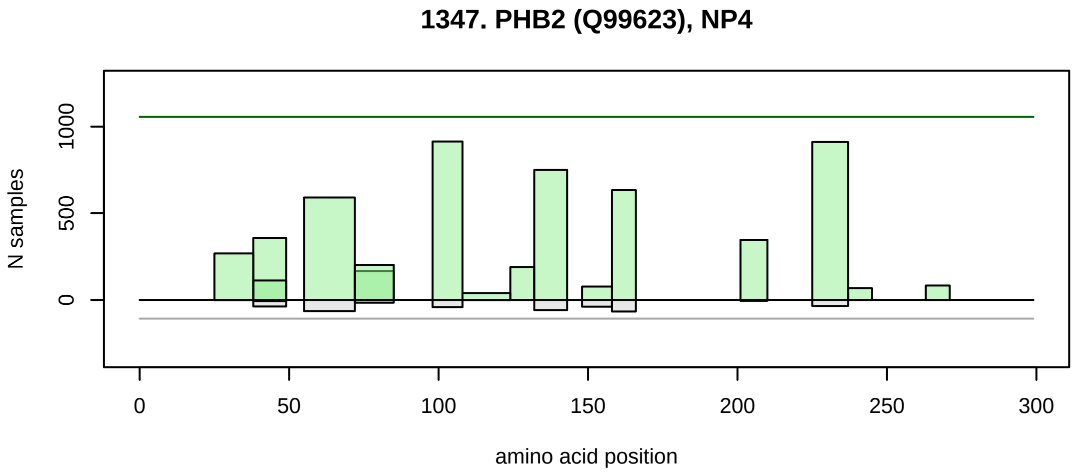
1347. PHB2 (Q99623), NP4
N samples
0
500
1000
0
50
100
150
200
250
300
amino acid position

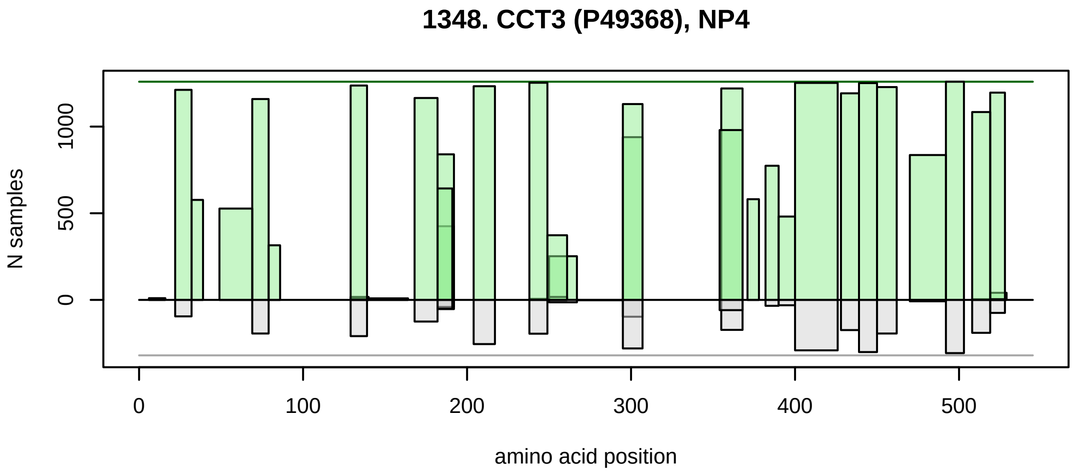
1348. CCT3 (P49368), NP4
N samples
0
500
1000
0
100
200
300
400
500
amino acid position

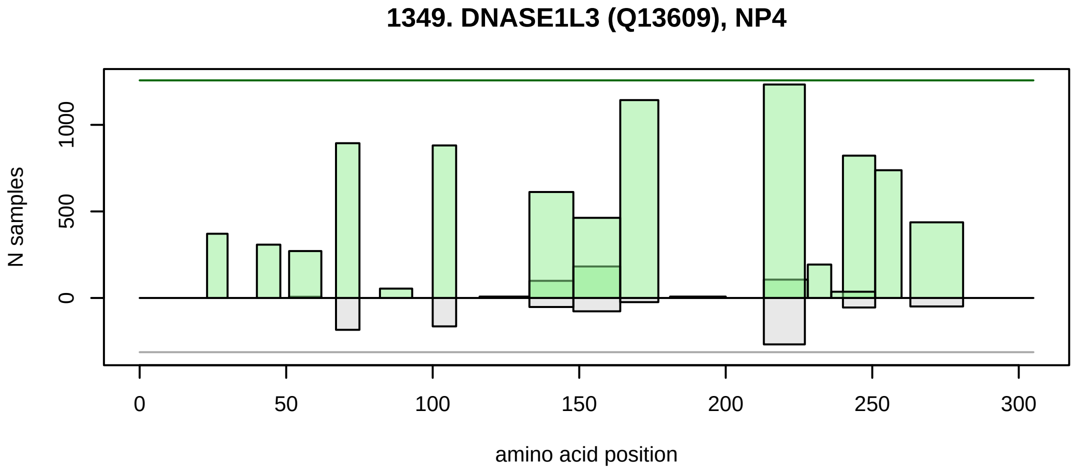
1349. DNASE1L3 (Q13609), NP4
N samples
0
500
1000
0
50
100
150
200
250
300
amino acid position

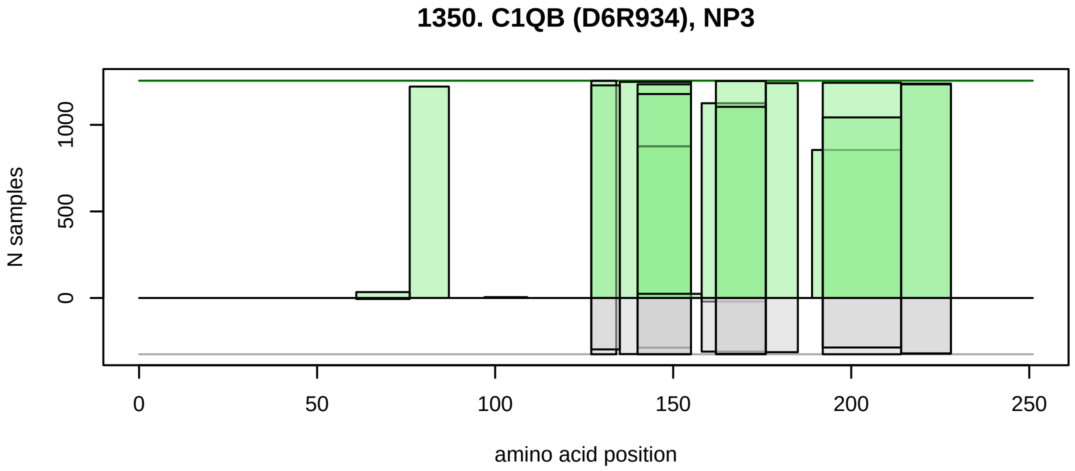
1350. C1QB (D6R934), NP3
N samples
0
500
1000
0
50
100
150
200
250
amino acid position

1351. IGKV1D−39 (P04432), NP4

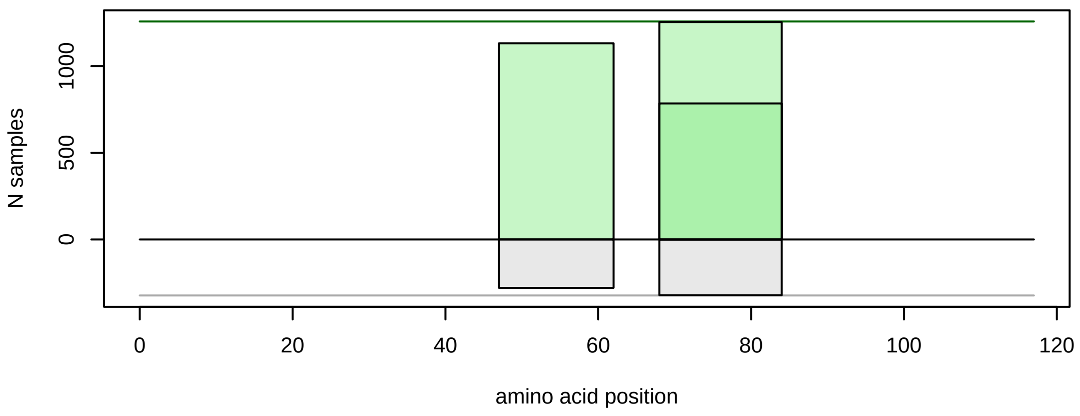

1352. IGLV5−45 (A0A087WSX0), NP4

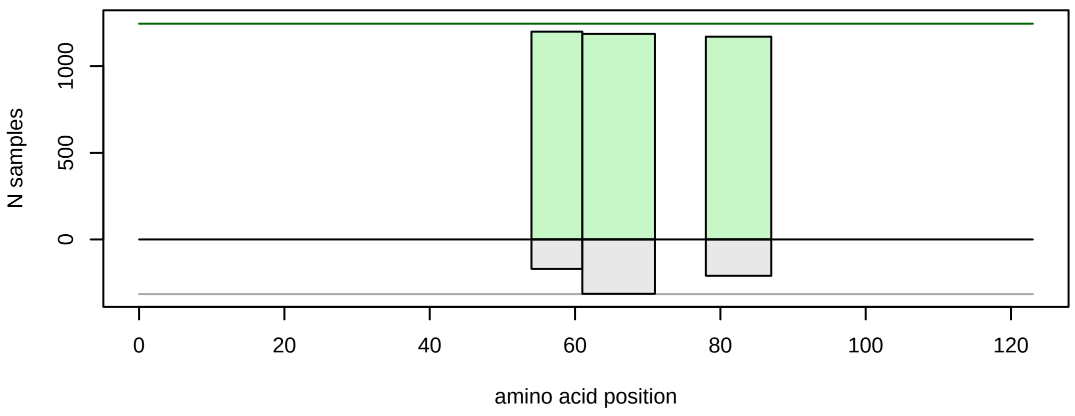

1353. IGHV3−43 (A0A0B4J1X8), NP4

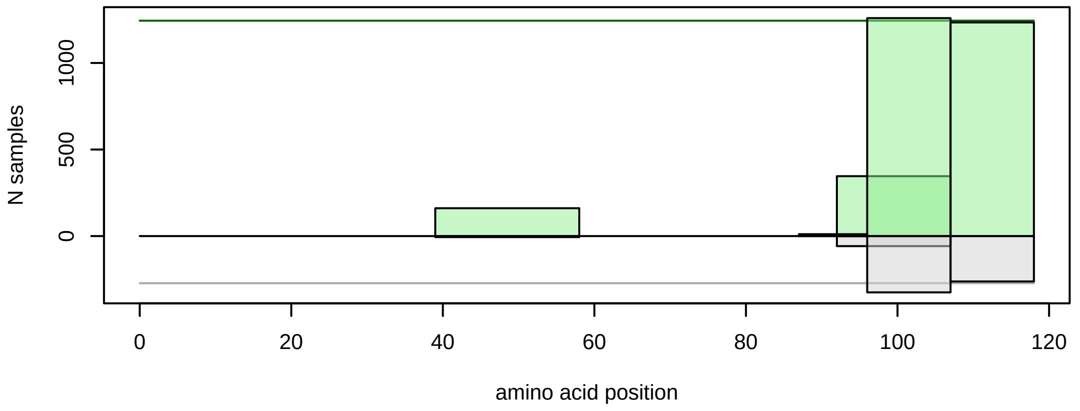

1354. MCFD2 (Q8NI22−2), NP2

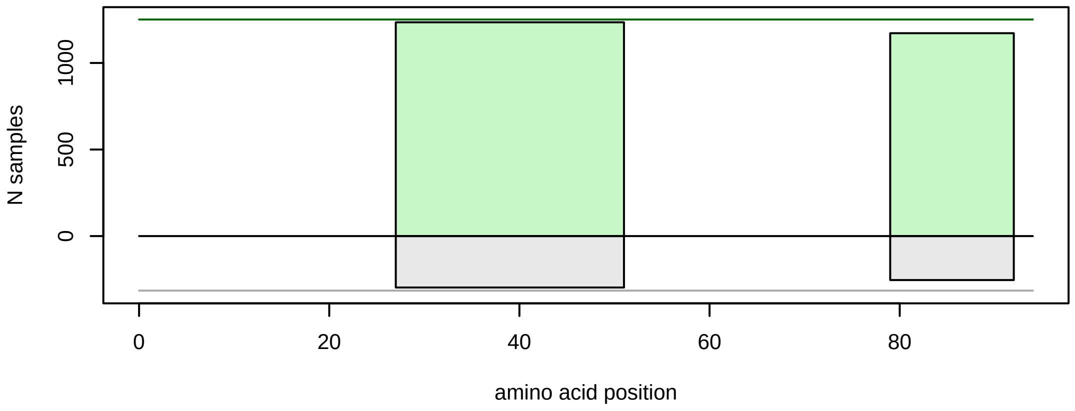

1355. CDSN (G8JLG2), NP1

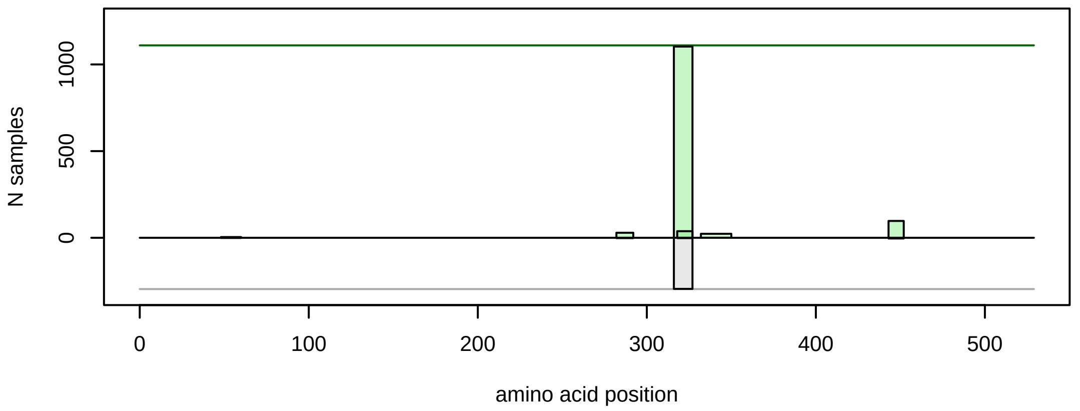

1356. ICAM2 (J3QRT5;P13598), NP1

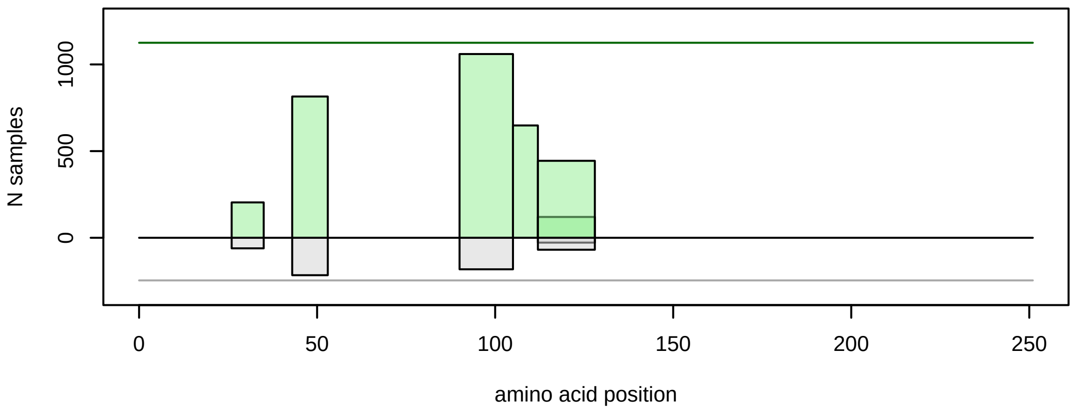

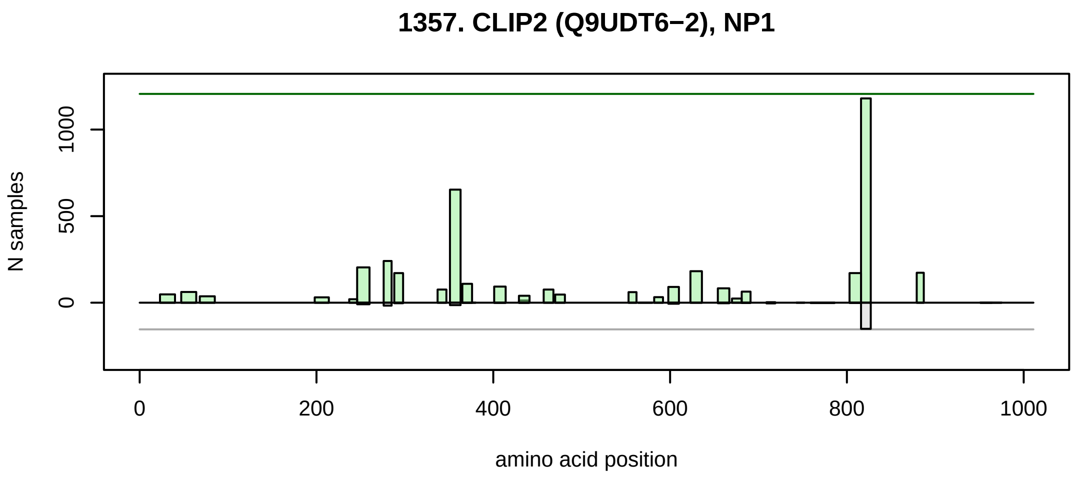

1357. CLIP2 (Q9UDT6−2), NP1
N samples
0
500
1000
0
200
400
600
800
1000
amino acid position

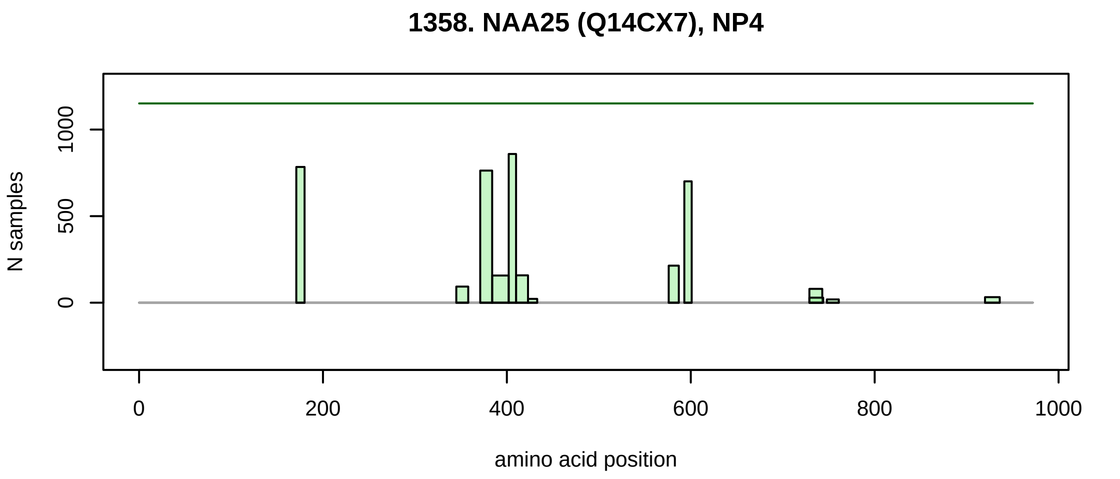

1358. NAA25 (Q14CX7), NP4
N samples
0
500
1000
0
200
400
600
800
1000
amino acid position

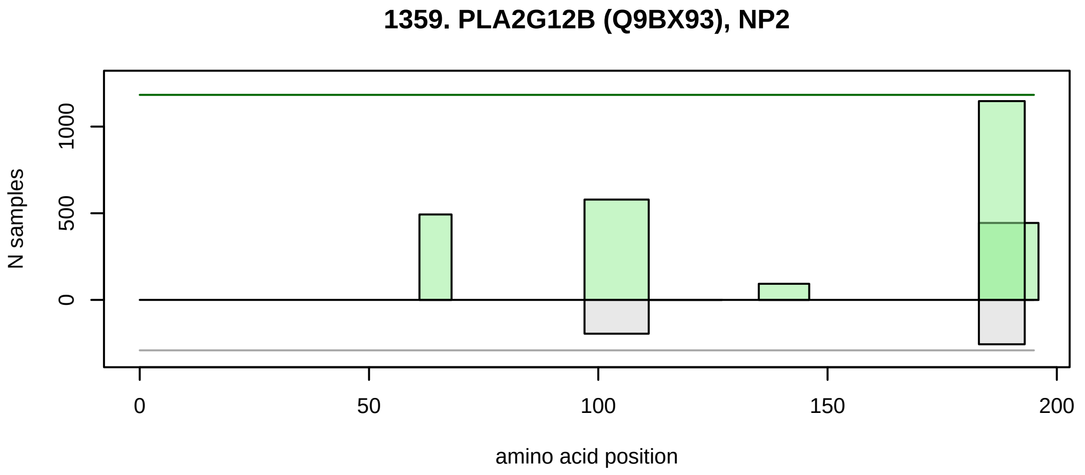

1359. PLA2G12B (Q9BX93), NP2
N samples
0
500
1000
0
50
100
150
200
amino acid position

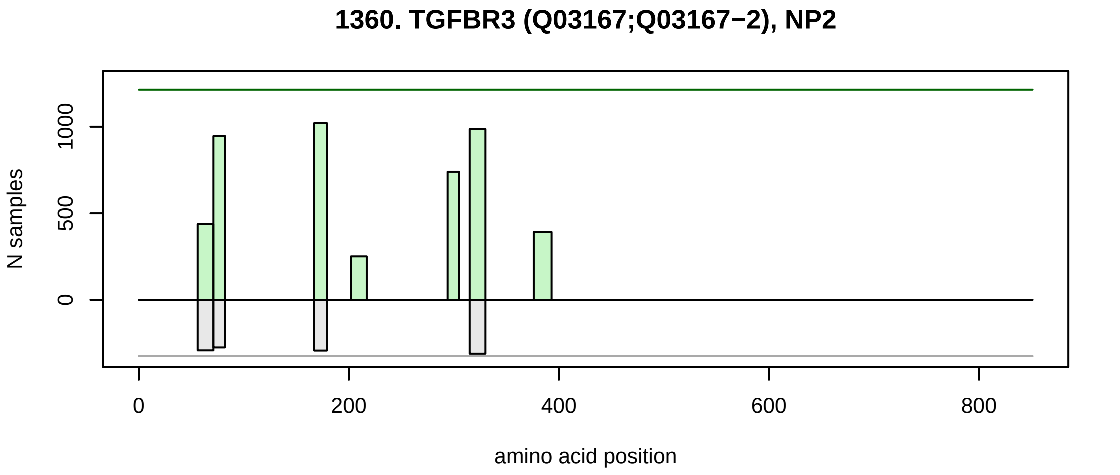

1360. TGFBR3 (Q03167;Q03167−2), NP2
N samples
0
500
1000
0
200
400
600
800
amino acid position

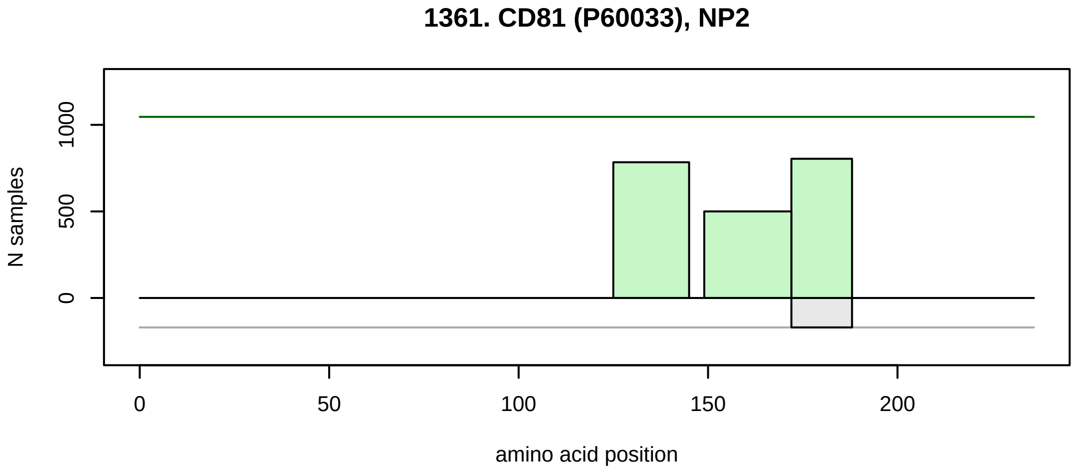

1361. CD81 (P60033), NP2
N samples
0
500
1000
0
50
100
150
200
amino acid position

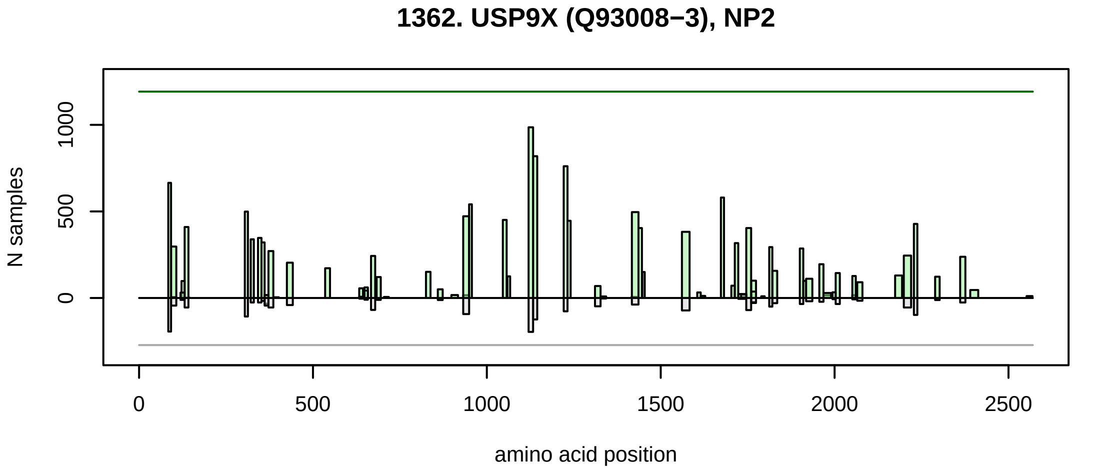

1362. USP9X (Q93008−3), NP2
N samples
0
500
1000
0
500
1000
1500
2000
2500
amino acid position

1363. APEH (C9JIF9;P13798), NP1

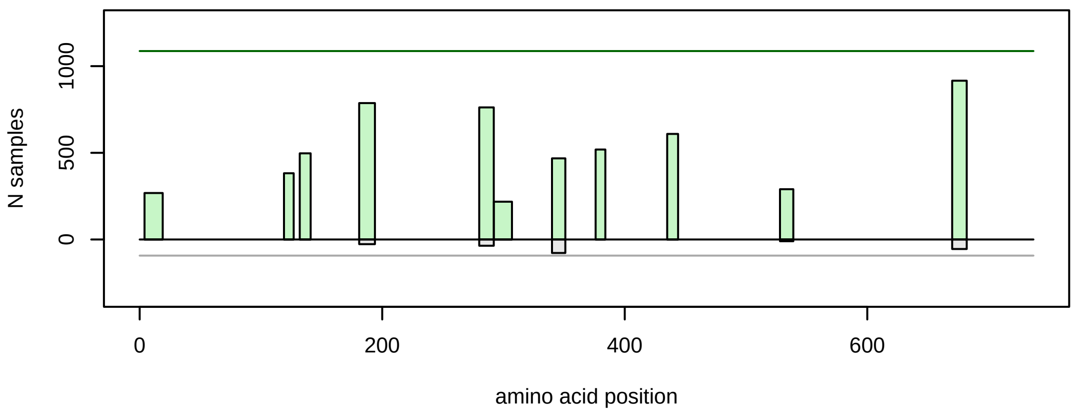

1364. AGO2 (Q9UKV8), NP3

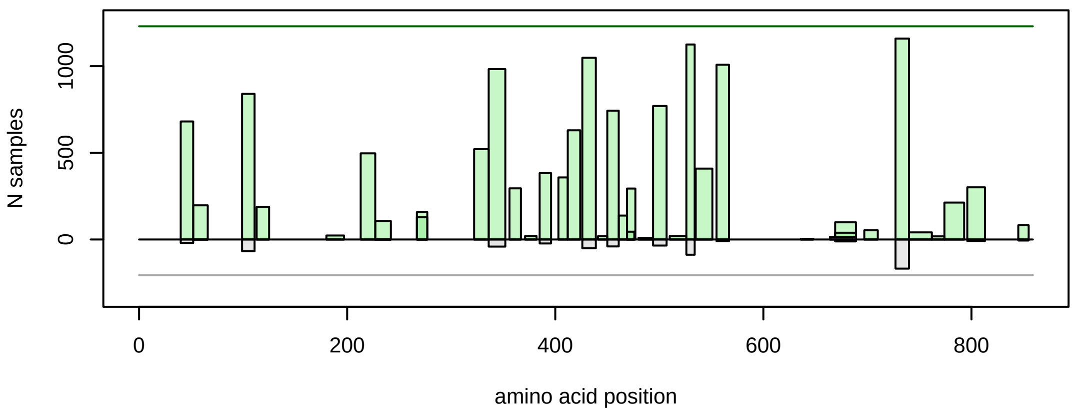

1365. THBS4 (P35443), NP2

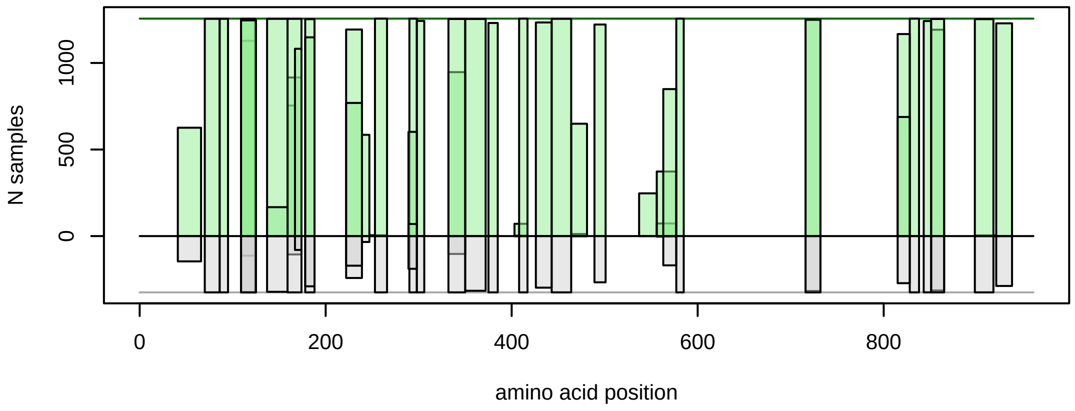

1366. TMA7 (Q9Y2S6), NP4

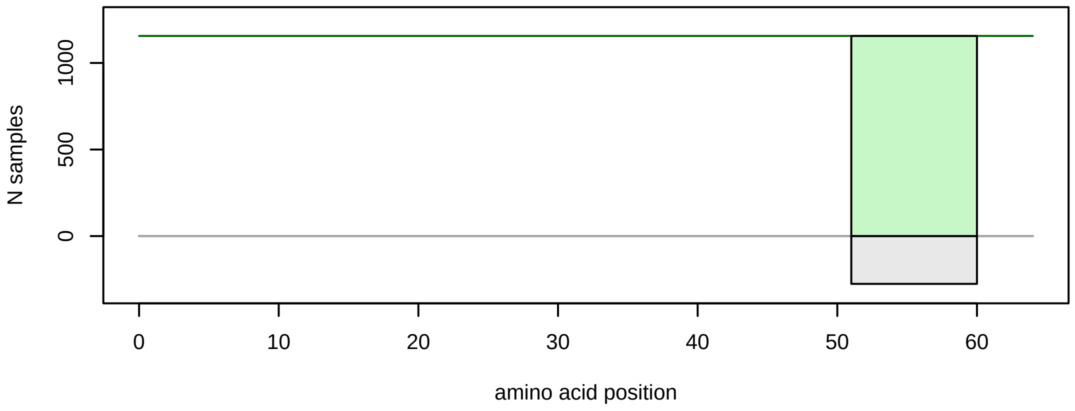

1367. PTPRG (P23470;P23470−2), NP2

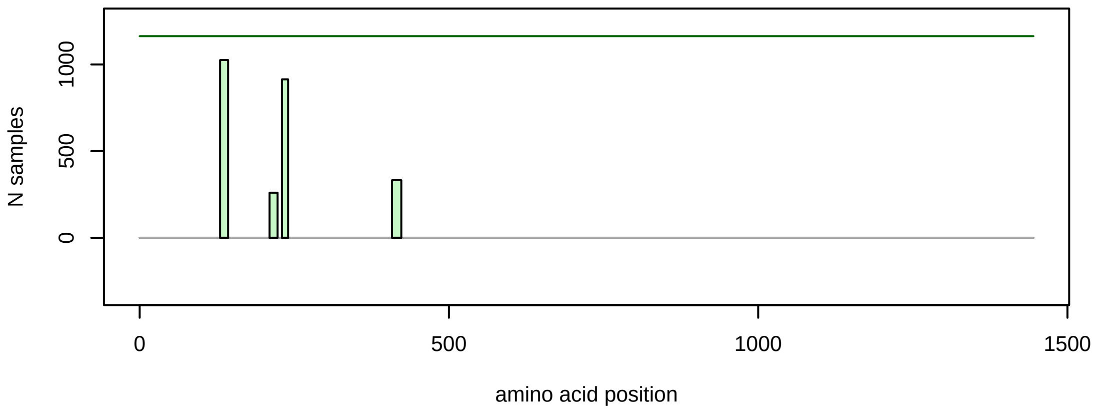

1368. PRDX3 (P30048), NP4

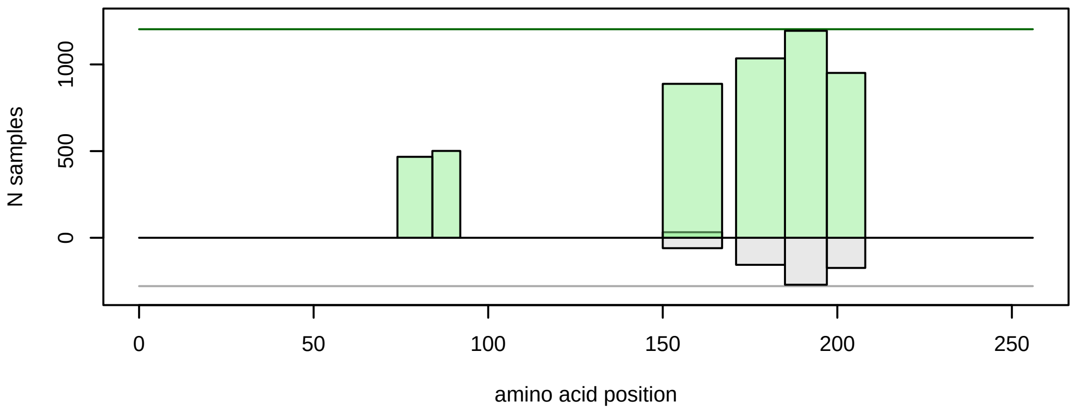

1369. CDH1 (P12830), NP1

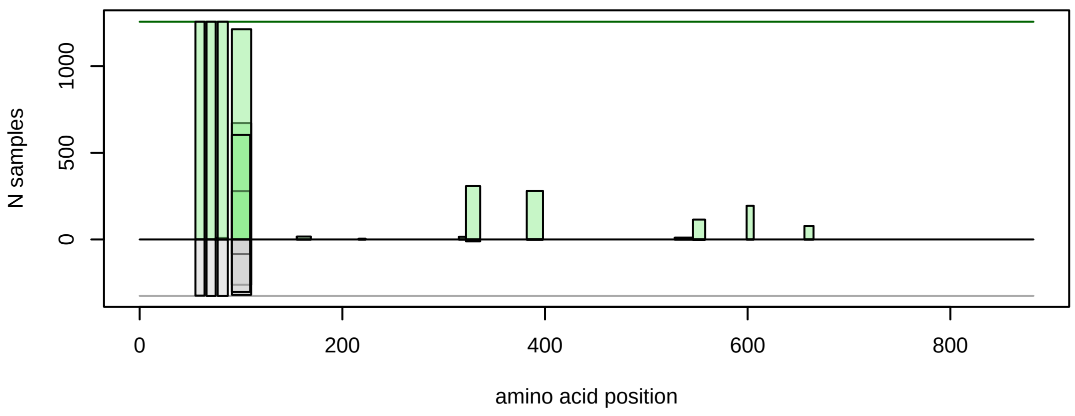

1370. CHST3 (Q7LGC8), NP2

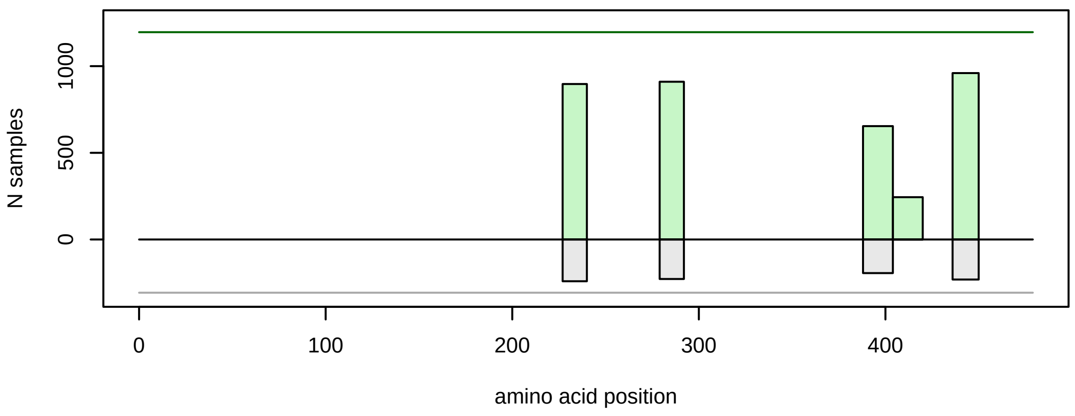

1371. C1QBP (Q07021), NP1

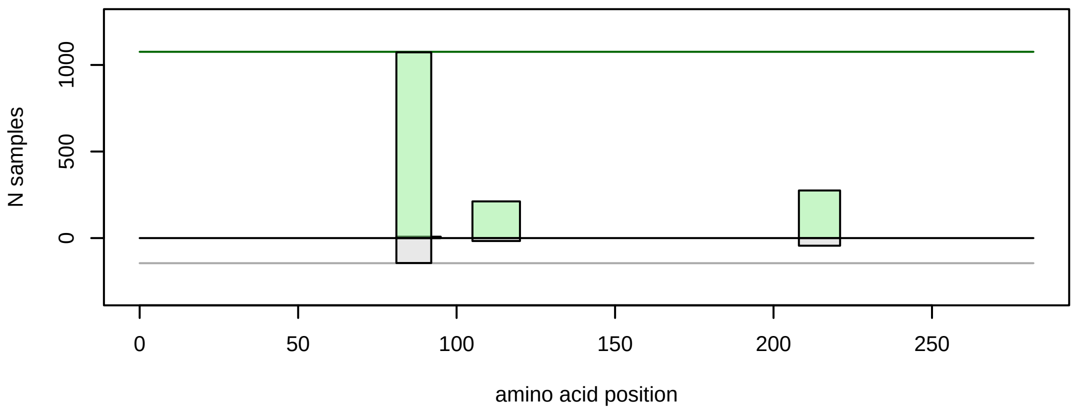

1372. NAA15 (Q9BXJ9), NP4

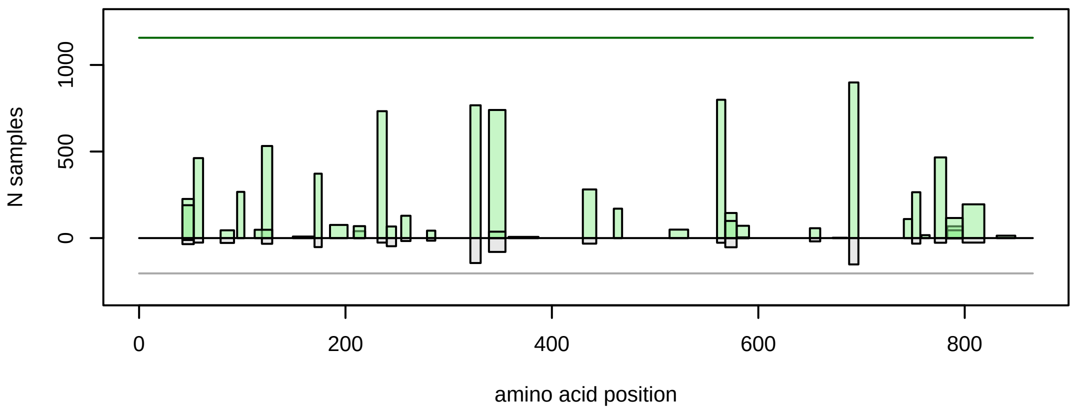

1373. HAGH (Q16775), NP3

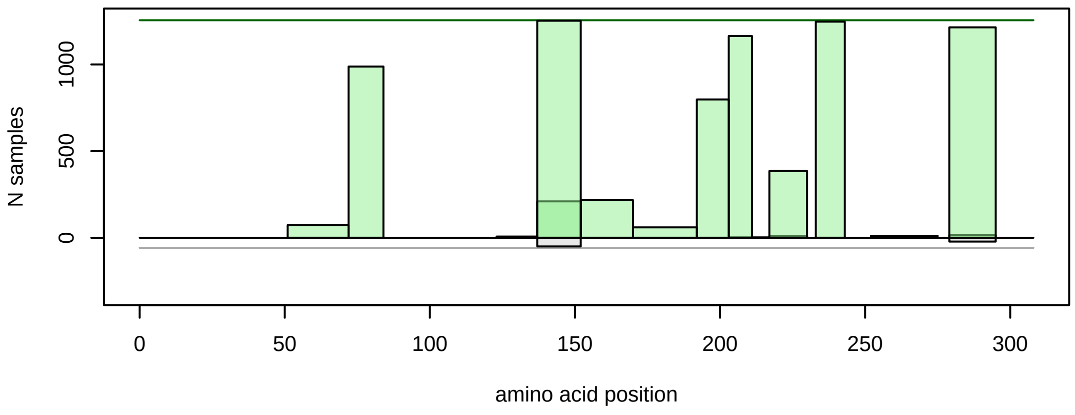

1374. RGCC (Q9H4X1), NP4

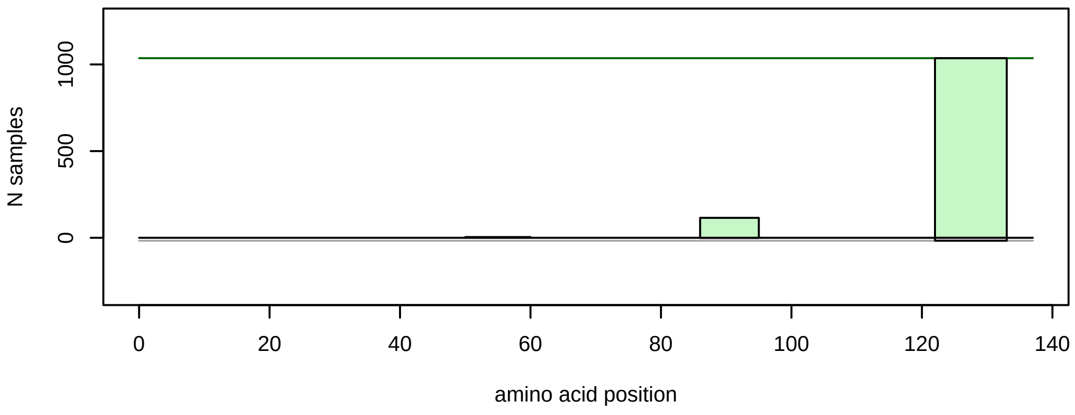

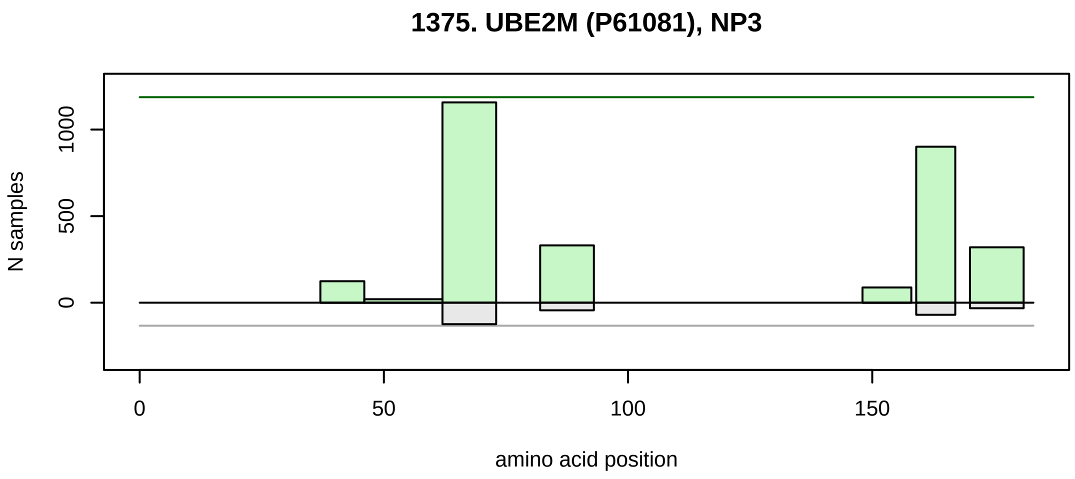
1375. UBE2M (P61081), NP3
N samples
0
500
1000
0
50
100
150
amino acid position

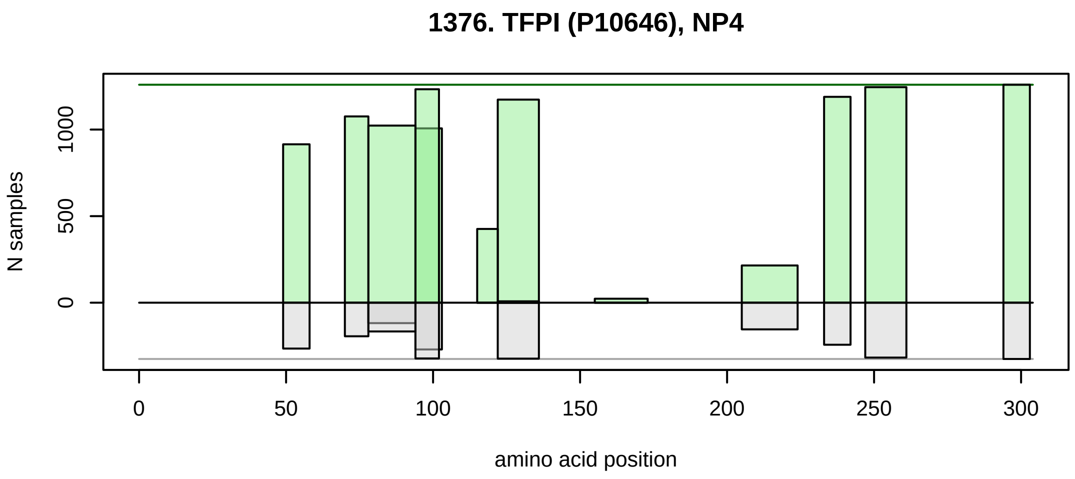
1376. TFPI (P10646), NP4
N samples
0
500
1000
0
50
100
150
200
250
300
amino acid position

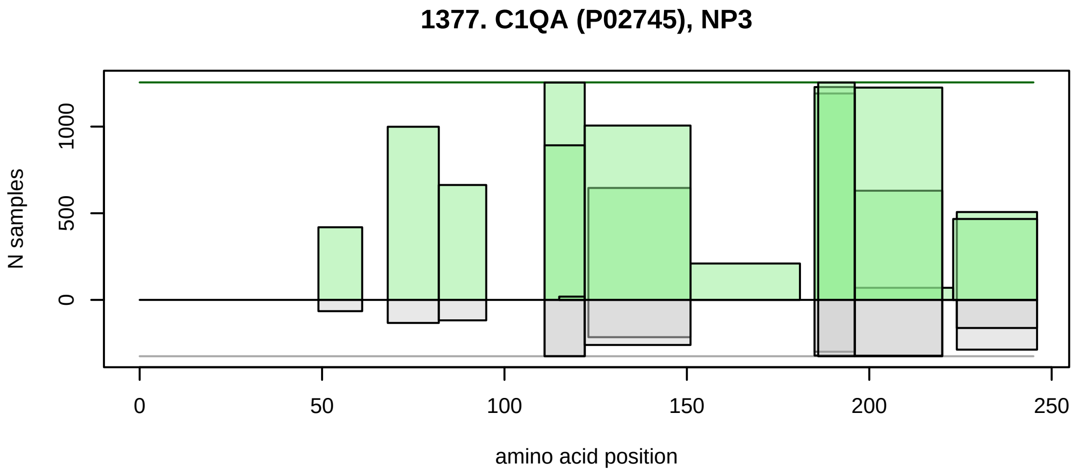
1377. C1QA (P02745), NP3
N samples
0
500
1000
0
50
100
150
200
250
amino acid position

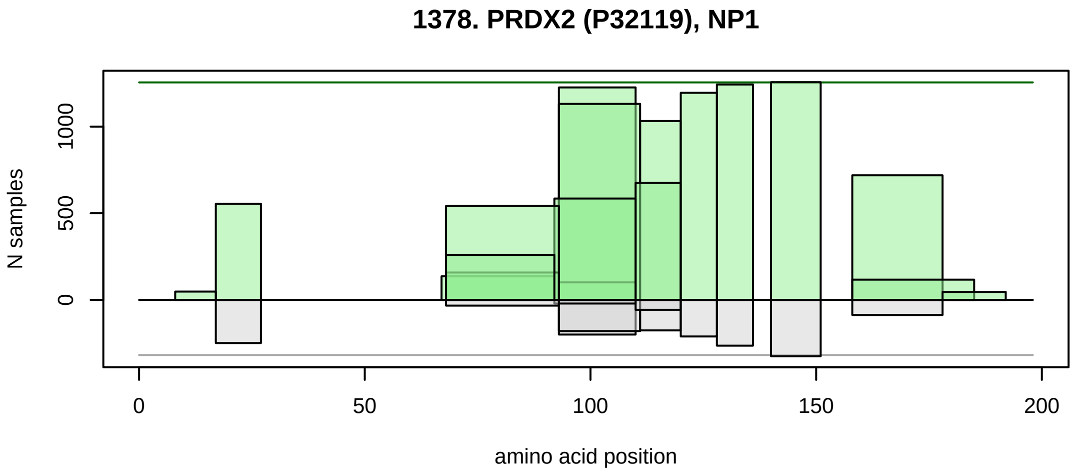
1378. PRDX2 (P32119), NP1
N samples
0
500
1000
0
50
100
150
200
amino acid position

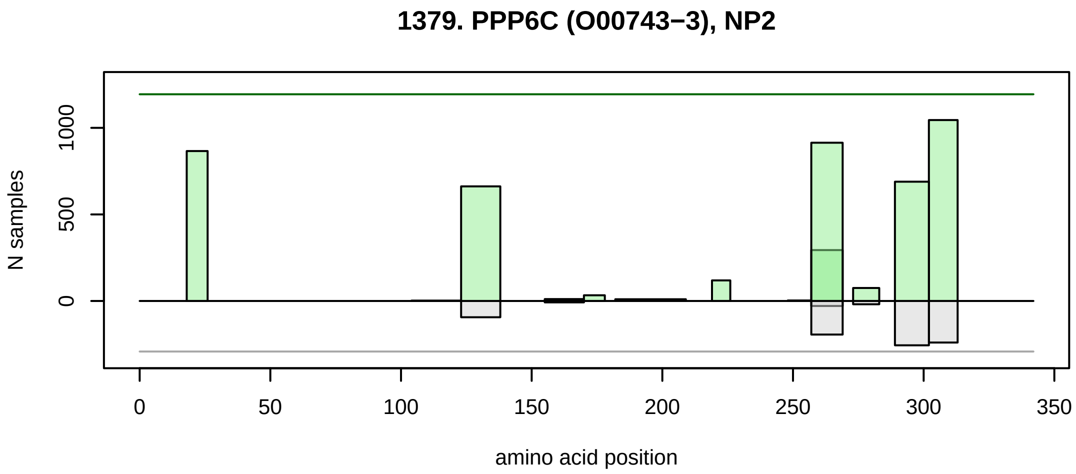
1379. PPP6C (O00743−3), NP2
N samples
0
500
1000
0
50
100
150
200
250
300
350
amino acid position

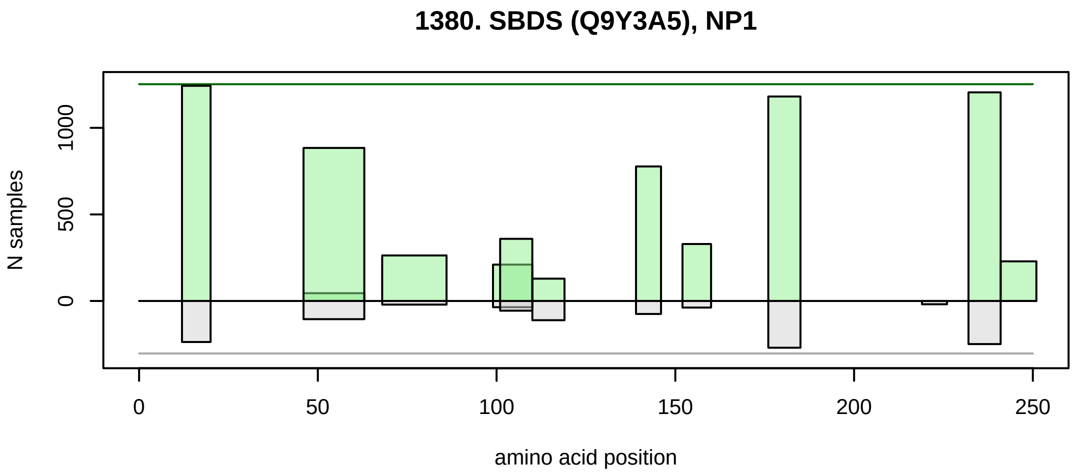
1380. SBDS (Q9Y3A5), NP1
N samples
0
500
1000
0
50
100
150
200
250
amino acid position

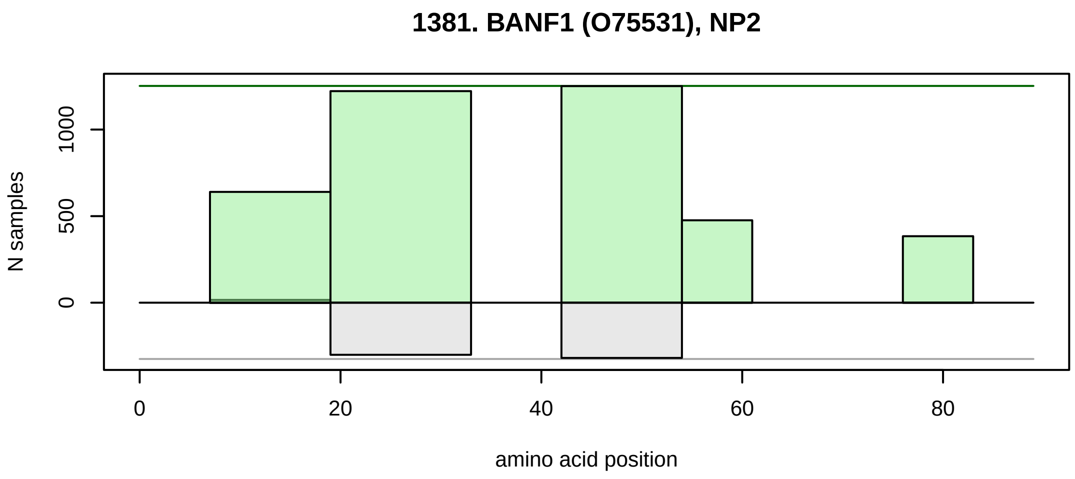

1381. BANF1 (O75531), NP2
N samples
0
500
1000
0
20
40
60
80
amino acid position

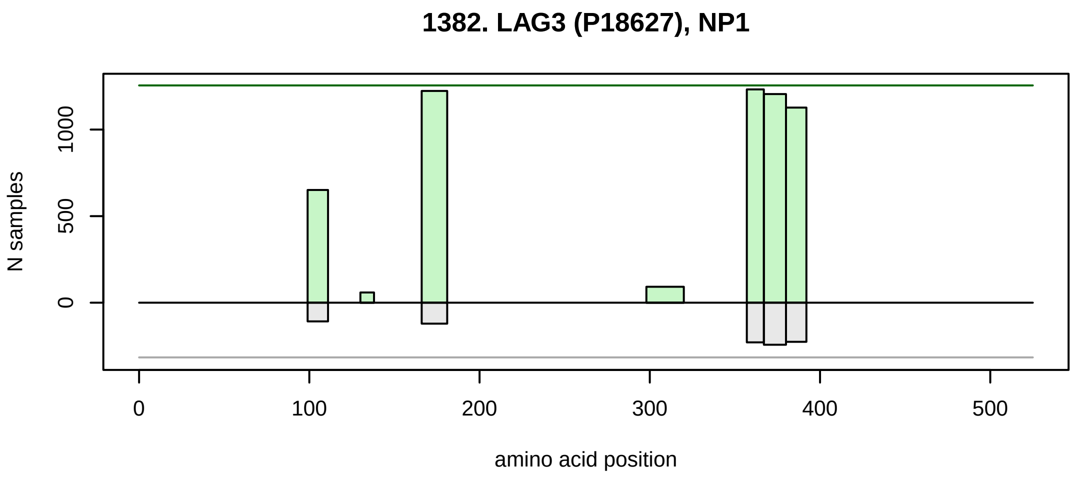

1382. LAG3 (P18627), NP1
N samples
0
500
1000
0
100
200
300
400
500
amino acid position

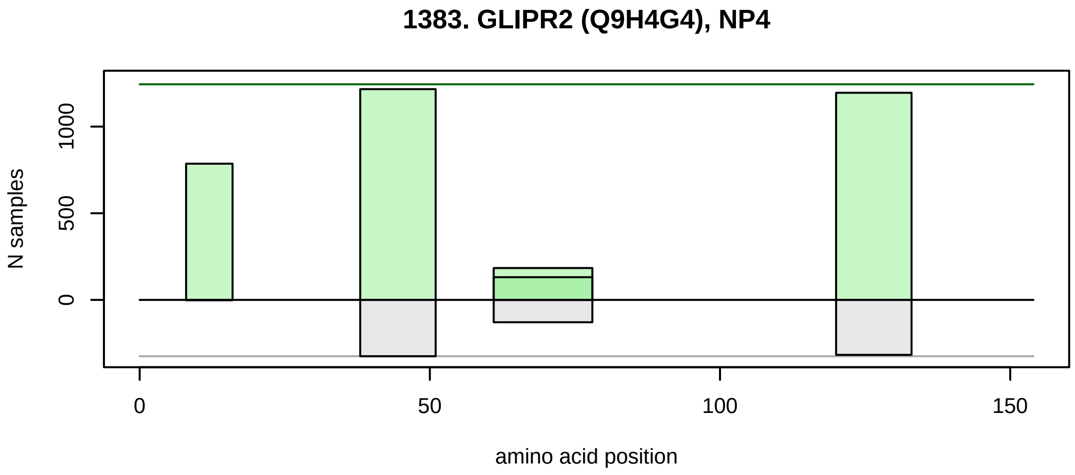

1383. GLIPR2 (Q9H4G4), NP4
N samples
0
500
1000
0
50
100
150
amino acid position

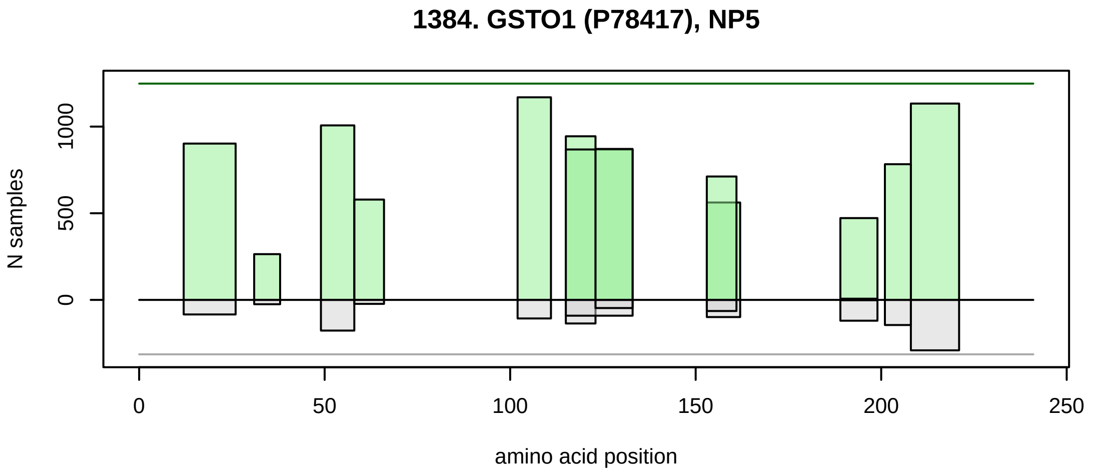

1384. GSTO1 (P78417), NP5
N samples
0
500
1000
0
50
100
150
200
250
amino acid position

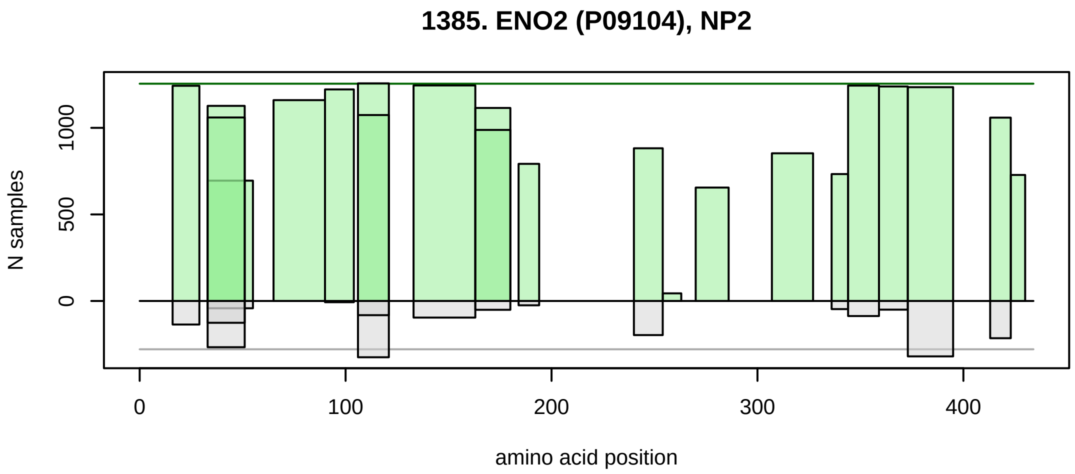

1385. ENO2 (P09104), NP2
N samples
0
500
1000
0
100
200
300
400
amino acid position

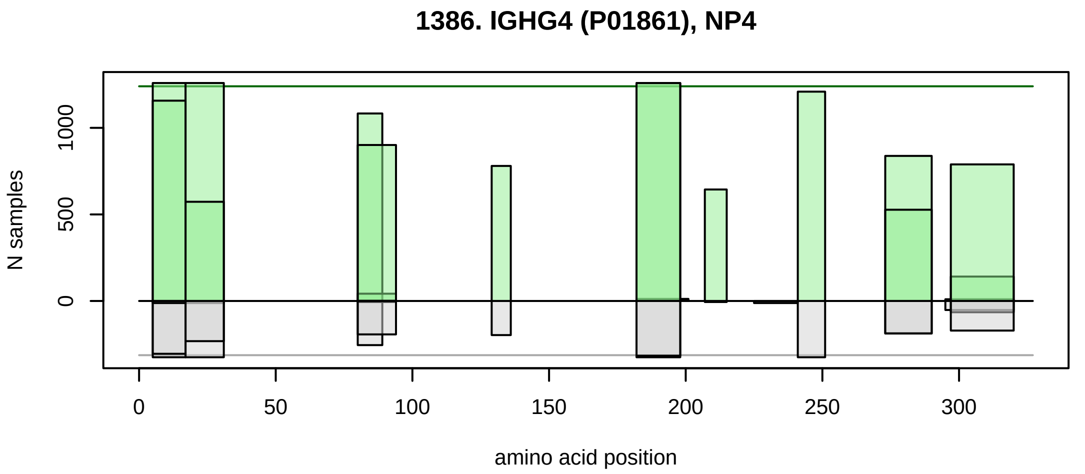

1386. IGHG4 (P01861), NP4
N samples
0
500
1000
0
50
100
150
200
250
300
amino acid position

1387. PSMD5 (Q16401), NP5

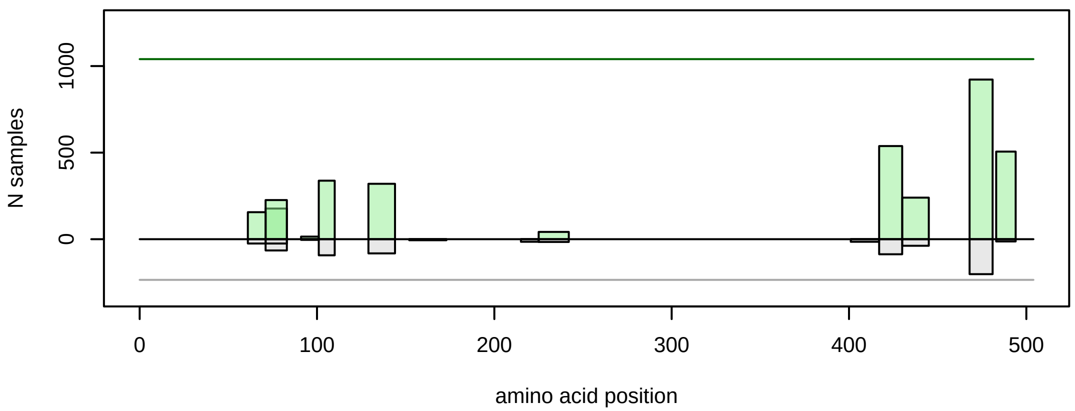

1388. LMAN2 (Q12907), NP2

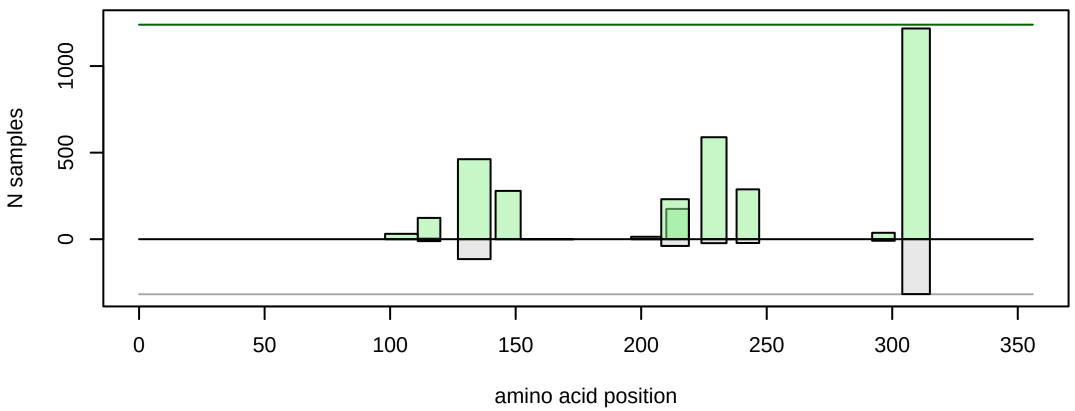

1389. MARCKS (P29966), NP4

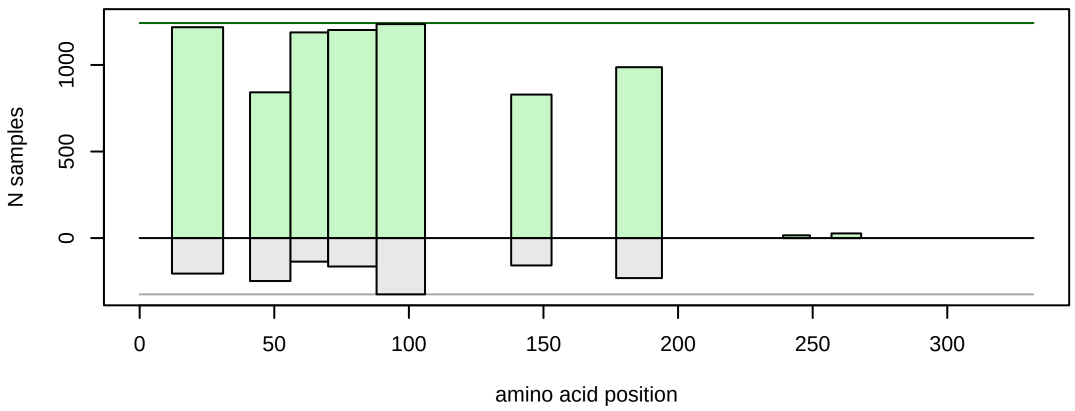

1390. FERMT2 (Q96AC1;Q96AC1−3), NP3

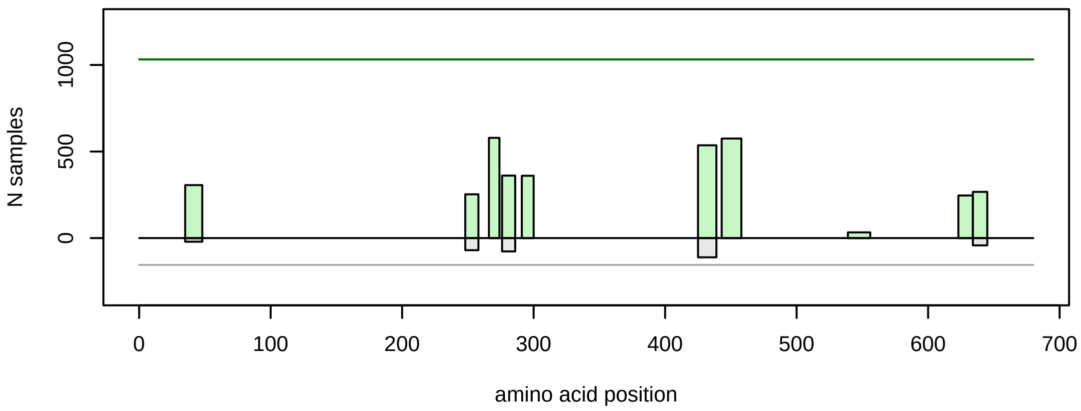

1391. CAPZB (B1AK88), NP4

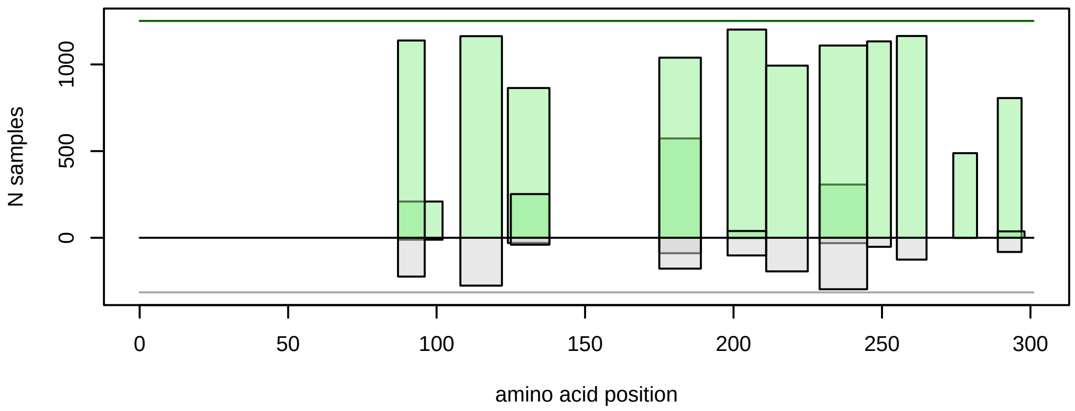

1392. ANGPTL4 (Q9BY76), NP3

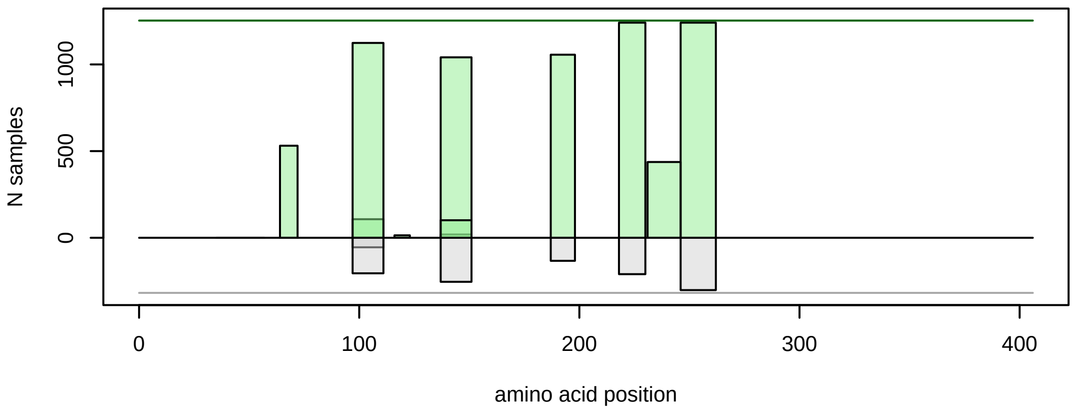

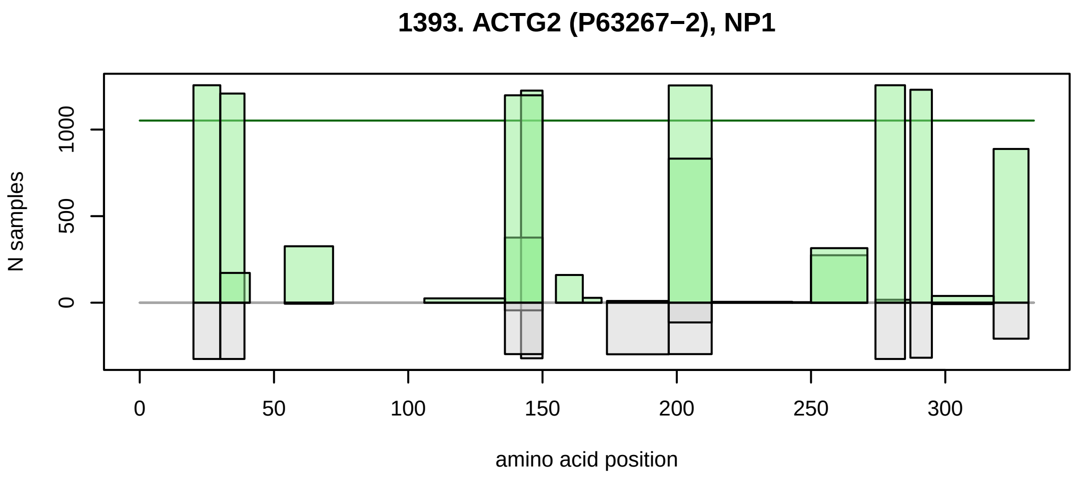
1393. ACTG2 (P63267−2), NP1
N samples
amino acid position
0
500
1000
0
50
100
150
200
250
300

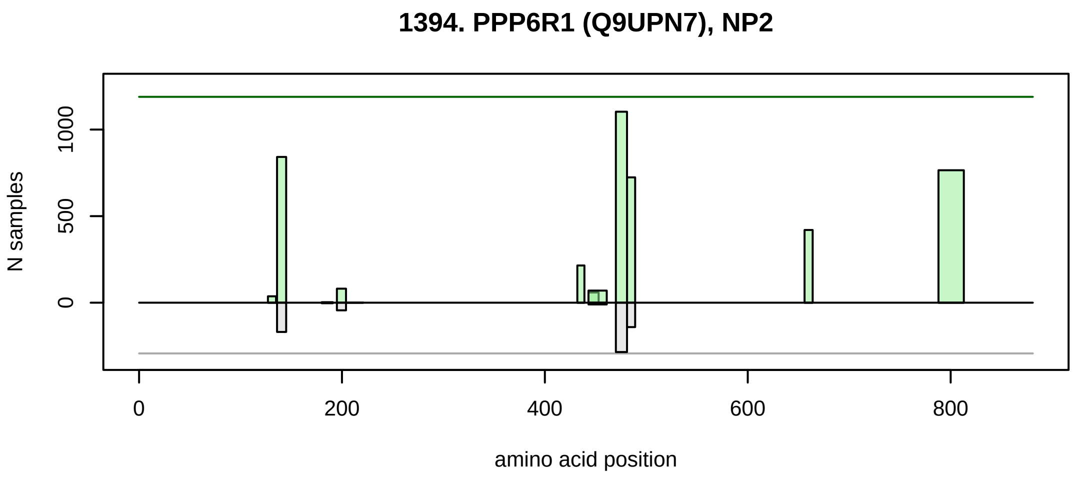
1394. PPP6R1 (Q9UPN7), NP2
N samples
amino acid position
0
500
1000
0
200
400
600
800

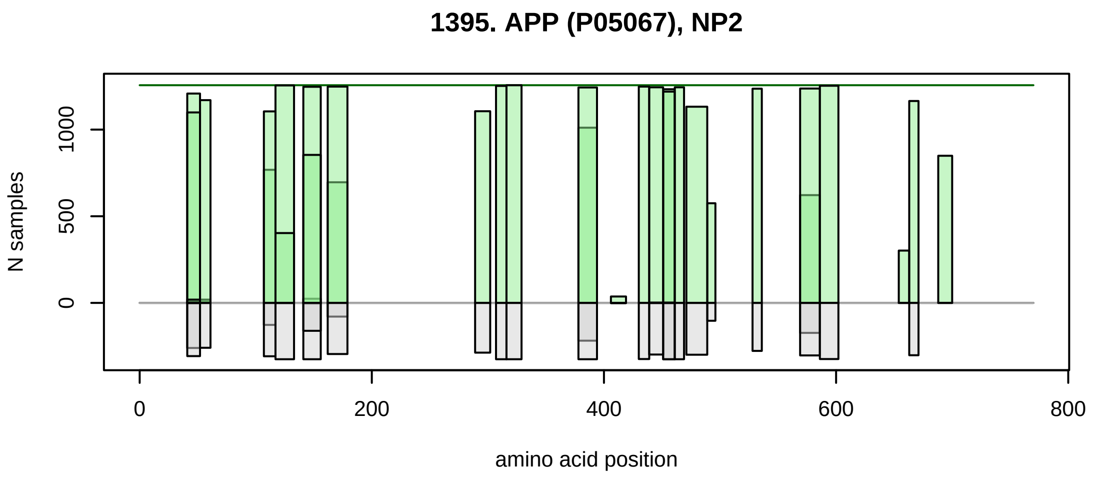
1395. APP (P05067), NP2
N samples
amino acid position
0
500
1000
0
200
400
600
800

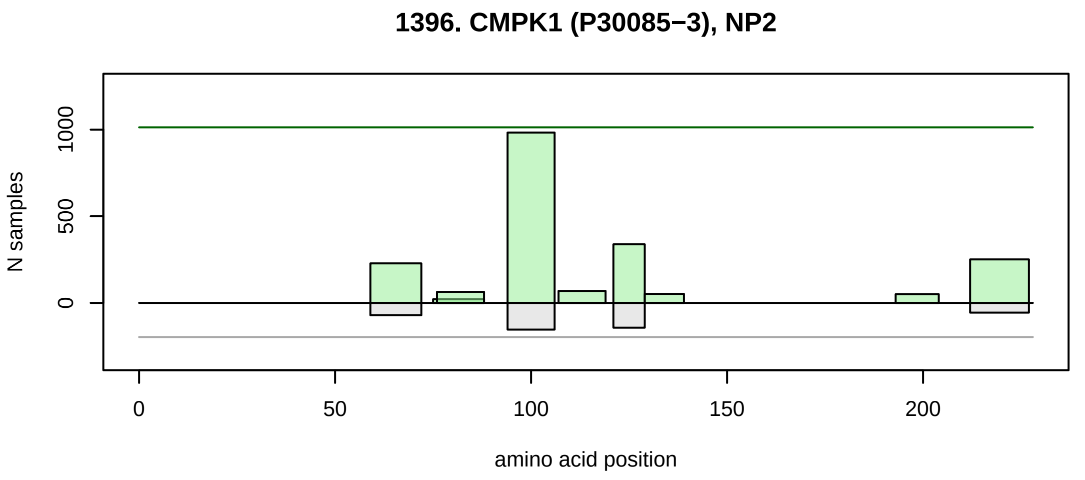
1396. CMPK1 (P30085−3), NP2
N samples
amino acid position
0
500
1000
0
50
100
150
200

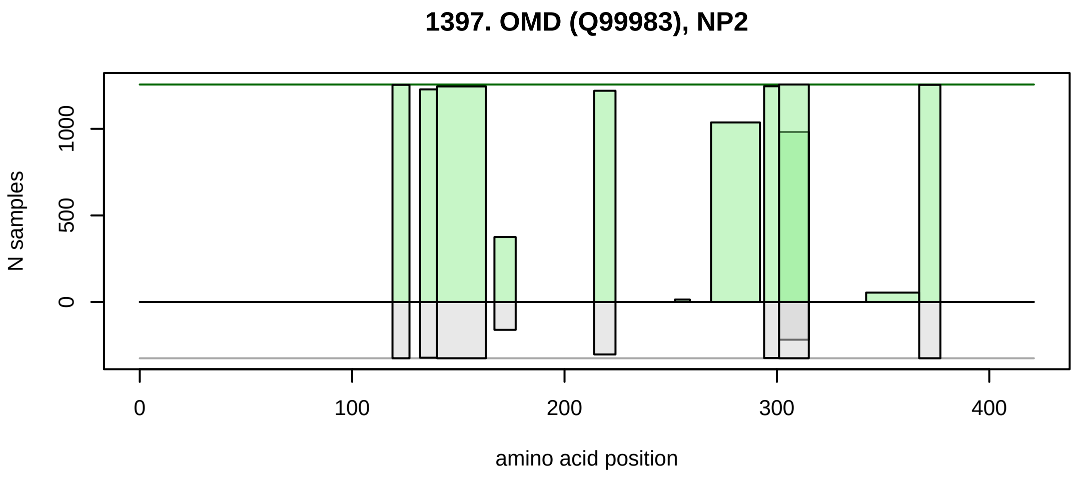
1397. OMD (Q99983), NP2
N samples
amino acid position
0
500
1000
0
100
200
300
400

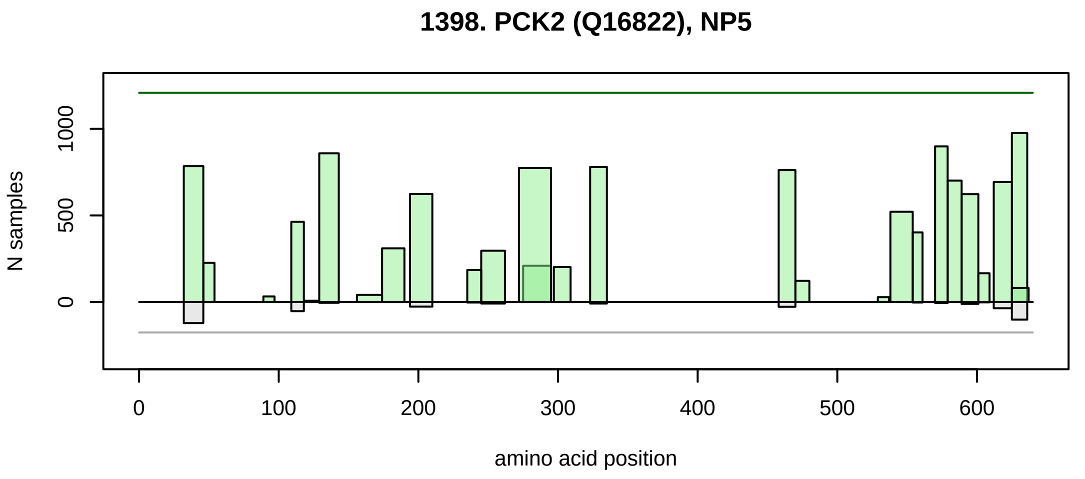
1398. PCK2 (Q16822), NP5
N samples
amino acid position
0
500
1000
0
100
200
300
400
500
600

1399. LEP (P41159), NP2

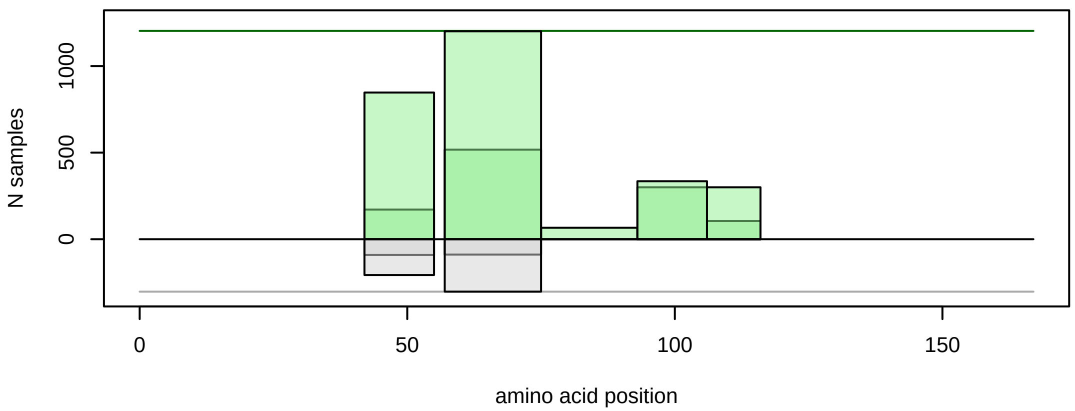

1400. CCT4 (P50991), NP1

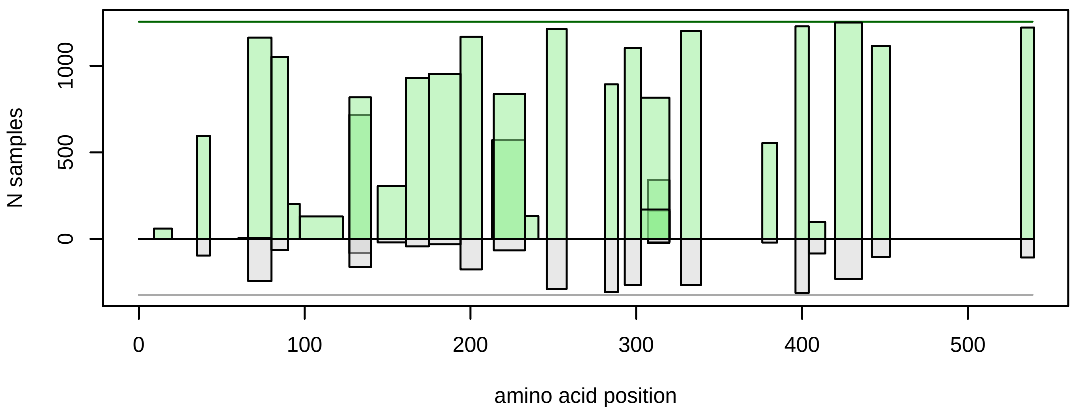

1401. PCBP2 (Q15366−3;Q15366−6), NP3

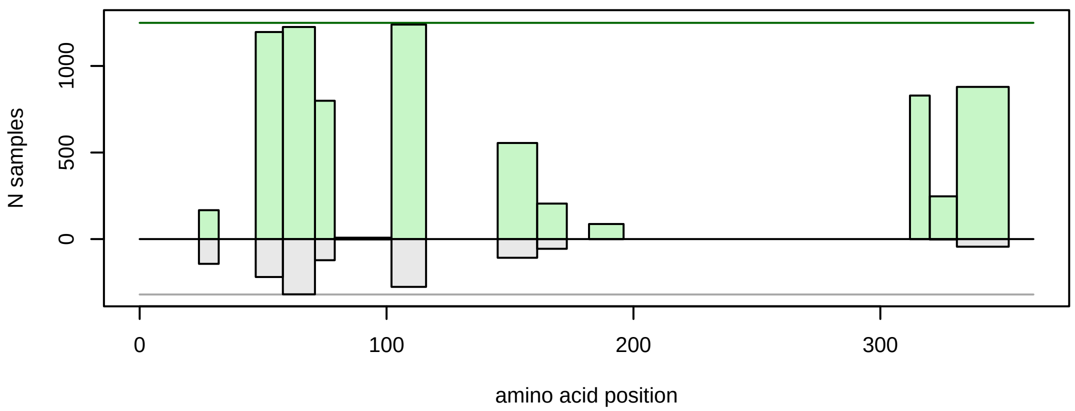

1402. ACAA2 (A0A0B4J2A4;P42765), NP5

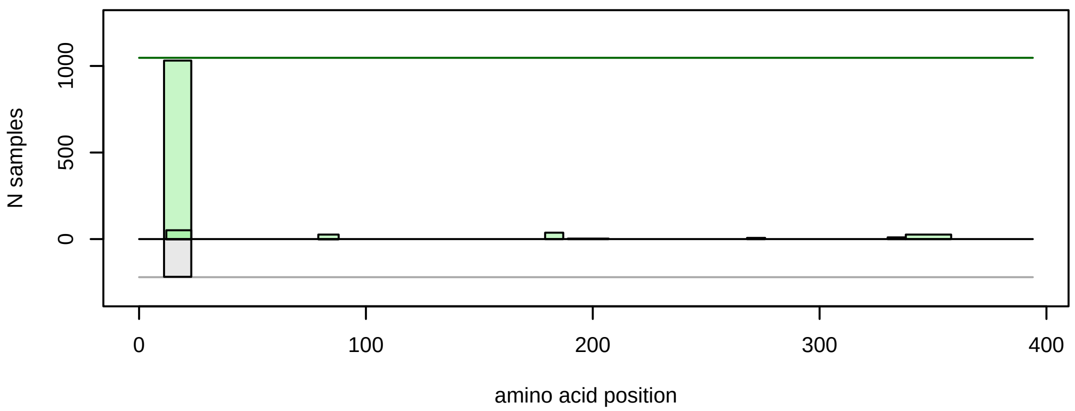

1403. KLK7 (P49862), NP3

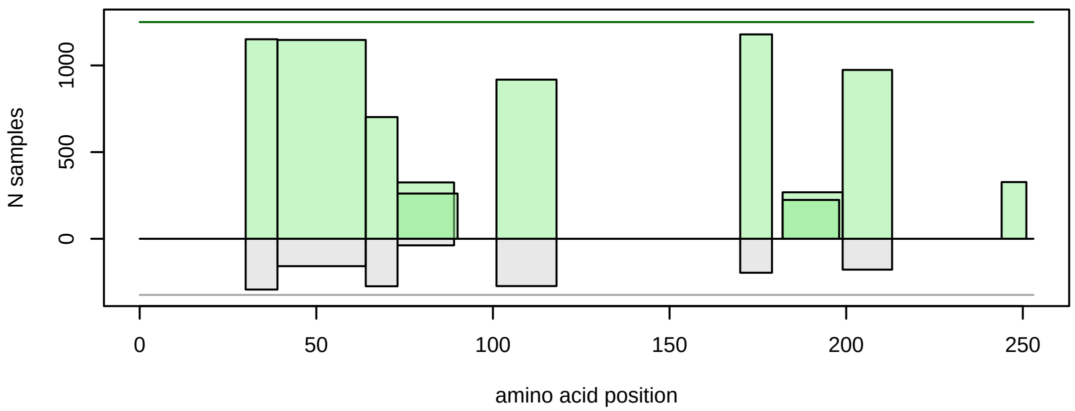

1404. IGHV3−30 (P01768), NP4

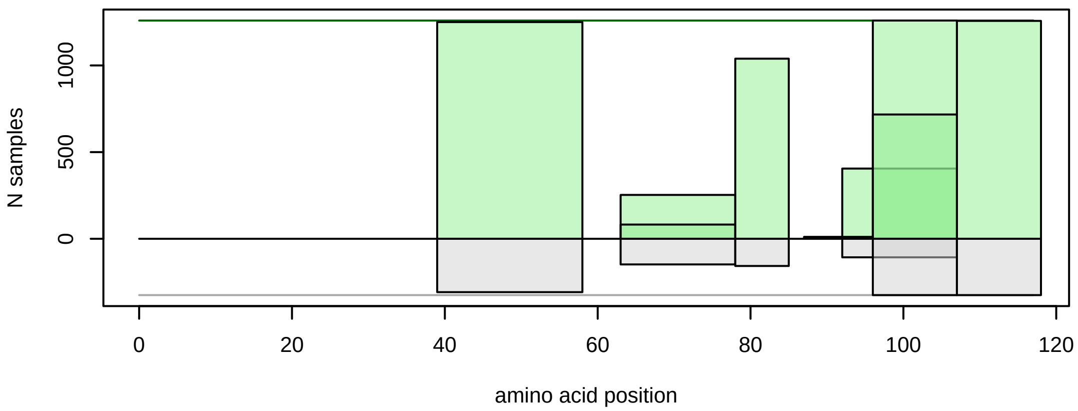

1405. PICALM (Q13492;Q13492−2;Q13492−5), NP3

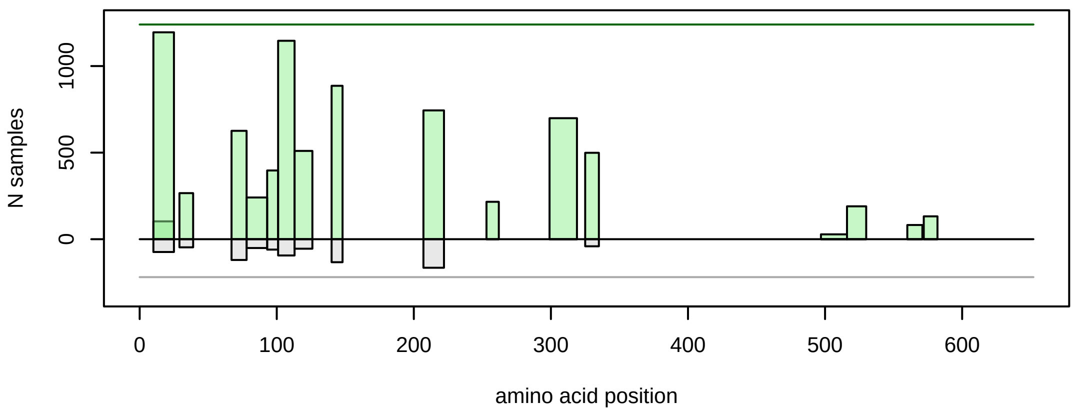

1406. F13A1 (P00488), NP5

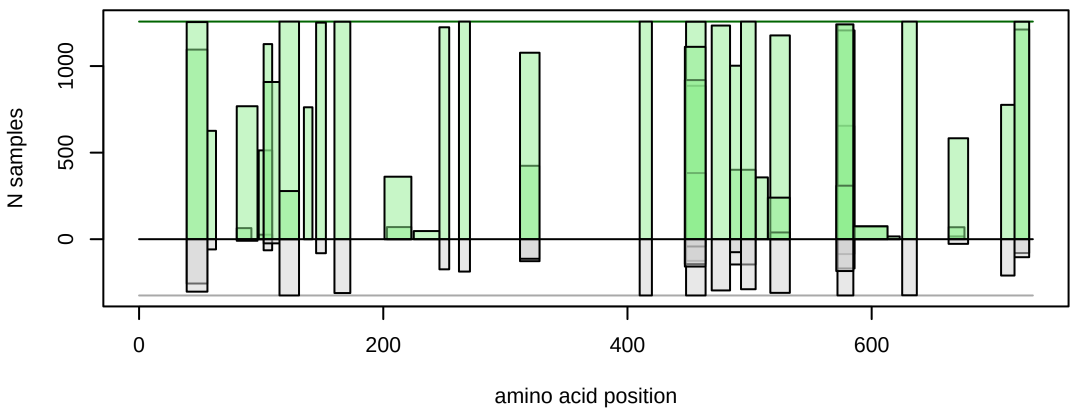

1407. TK2 (H3BP77), NP2

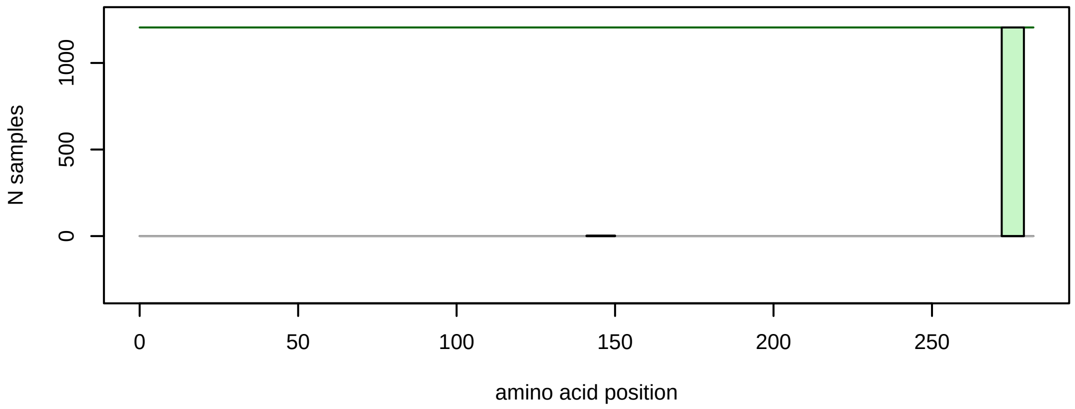

1408. RPL10A (P62906), NP4

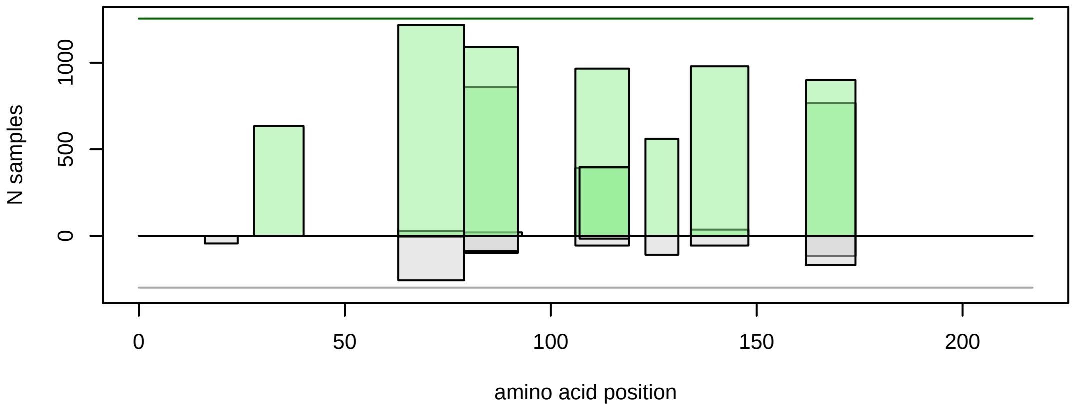

1409. HSD17B12 (Q53GQ0), NP4

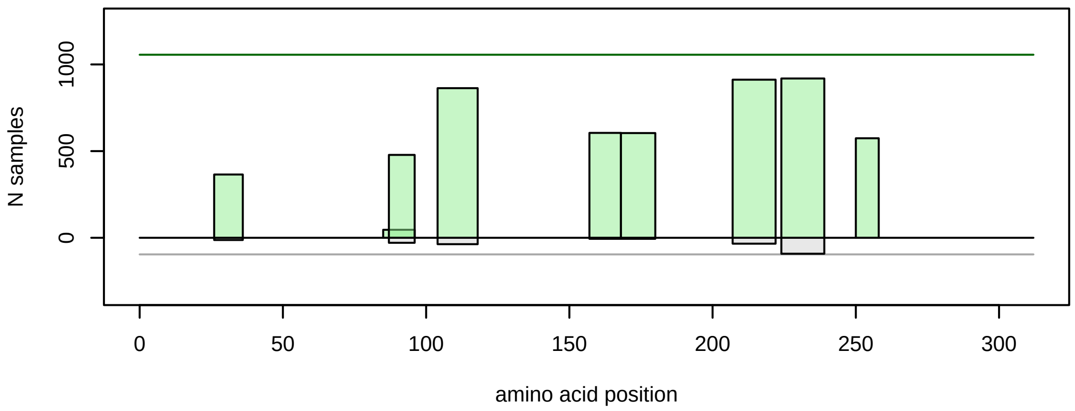

1410. RPS9 (P46781), NP4

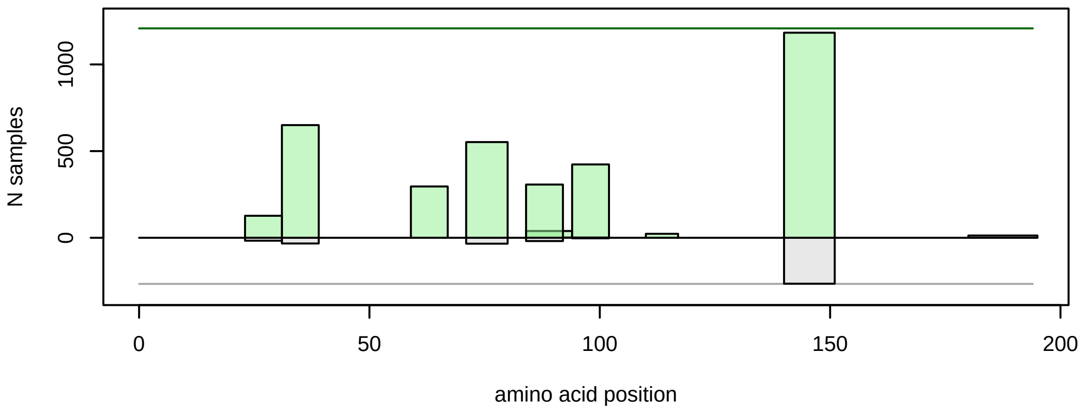

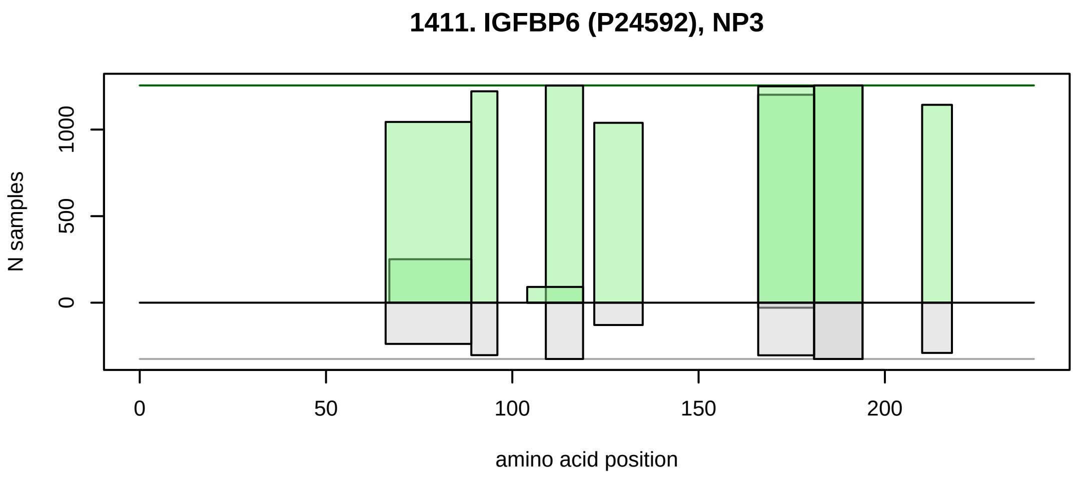
1411. IGFBP6 (P24592), NP3
N samples
0
500
1000
0
50
100
150
200
amino acid position

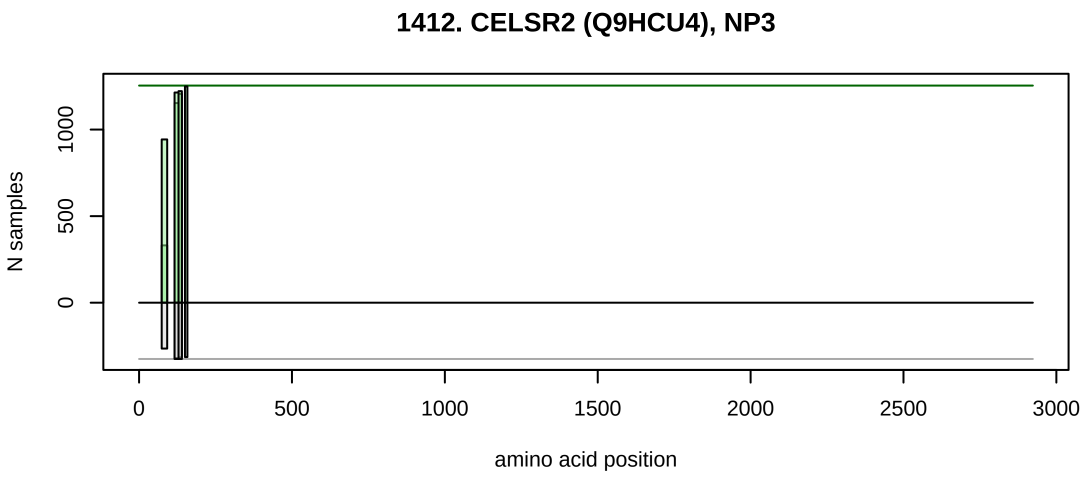
1412. CELSR2 (Q9HCU4), NP3
N samples
0
500
1000
0
500
1000
1500
2000
2500
3000
amino acid position

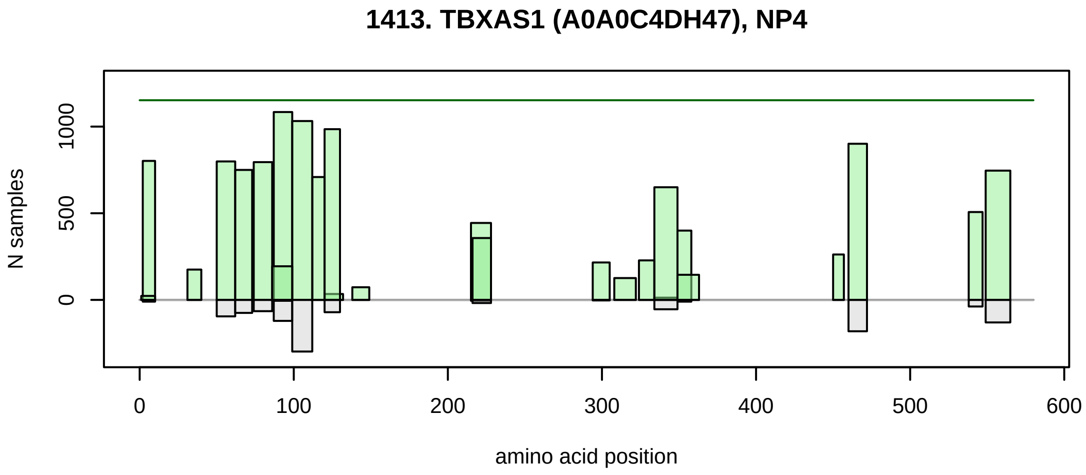
1413. TBXAS1 (A0A0C4DH47), NP4
N samples
0
500
1000
0
100
200
300
400
500
600
amino acid position

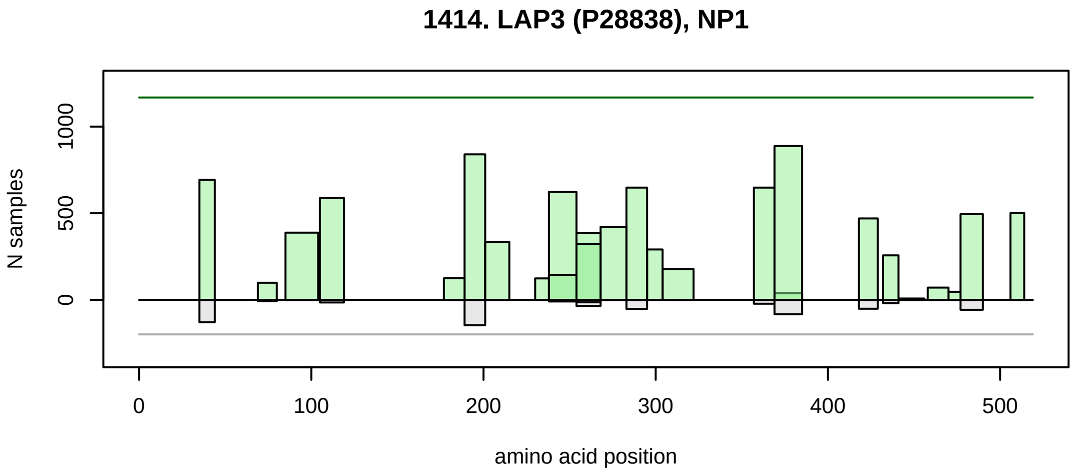
1414. LAP3 (P28838), NP1
N samples
0
500
1000
0
100
200
300
400
500
amino acid position

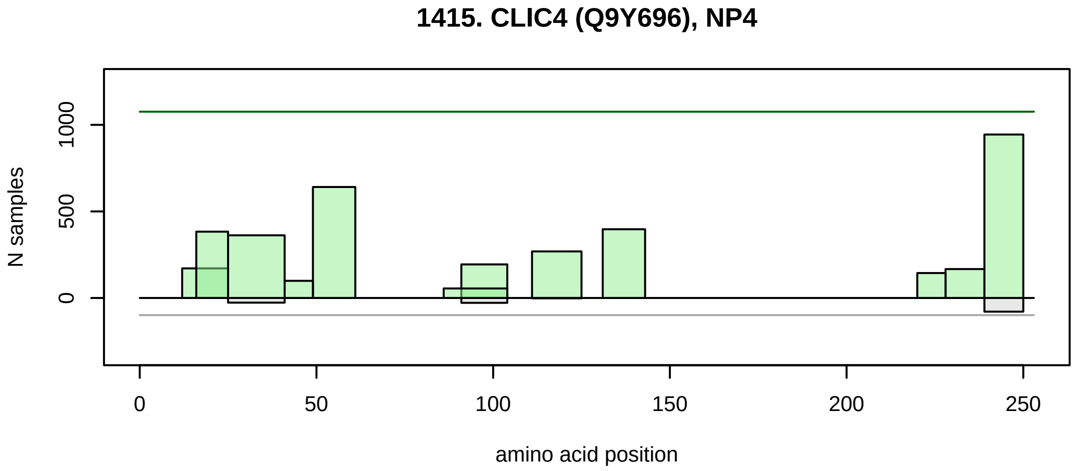
1415. CLIC4 (Q9Y696), NP4
N samples
0
500
1000
0
50
100
150
200
250
amino acid position

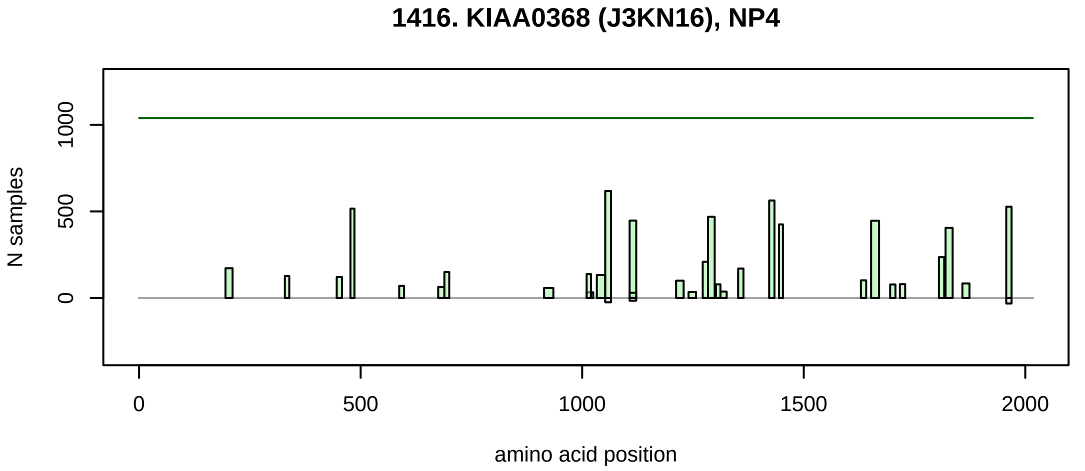
1416. KIAA0368 (J3KN16), NP4
N samples
0
500
1000
0
500
1000
1500
2000
amino acid position

1417. RAB8A (P61006), NP3

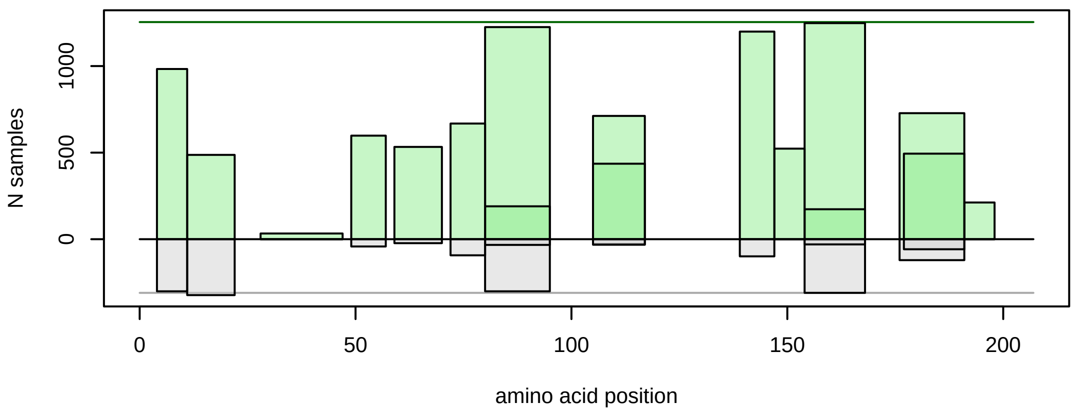

1418. SRPX (P78539), NP4

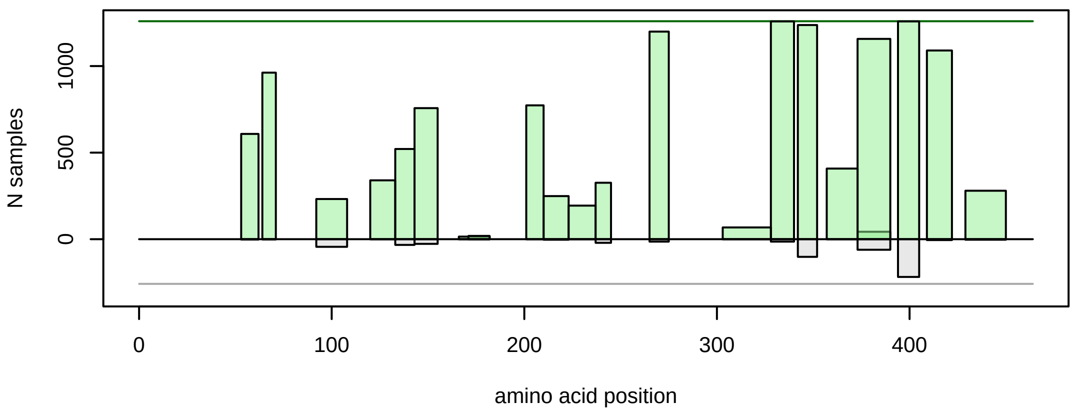

1419. ARMC1 (Q9NVT9), NP4

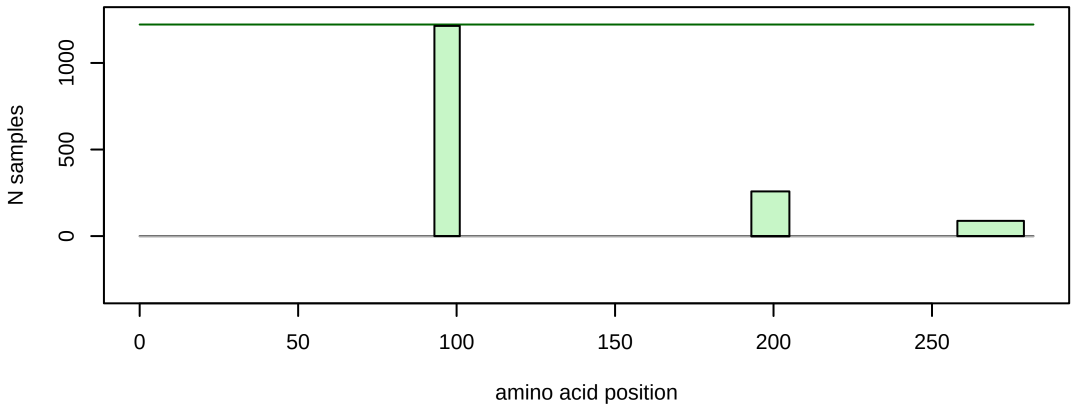

1420. MYL1 (P05976), NP4

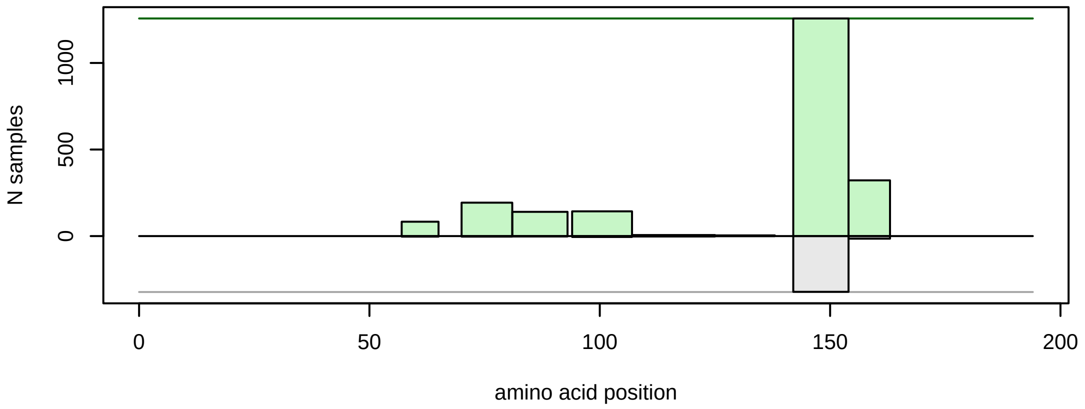

1421. SLC15A4 (Q8N697), NP1

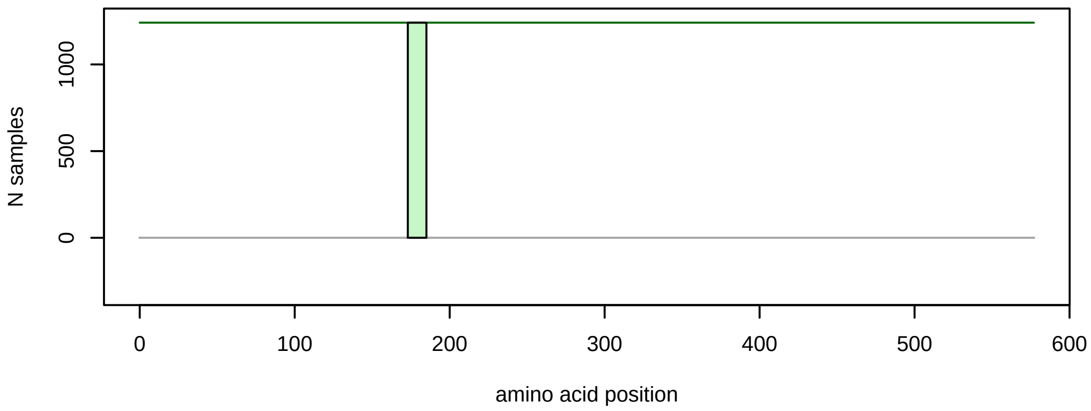

1422. GCG (P01275), NP2

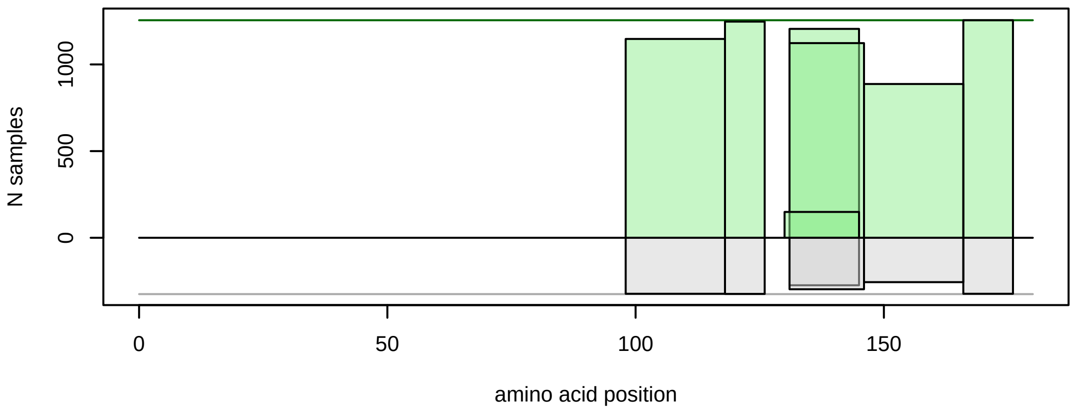

1423. FAIM3 (O60667−3), NP2

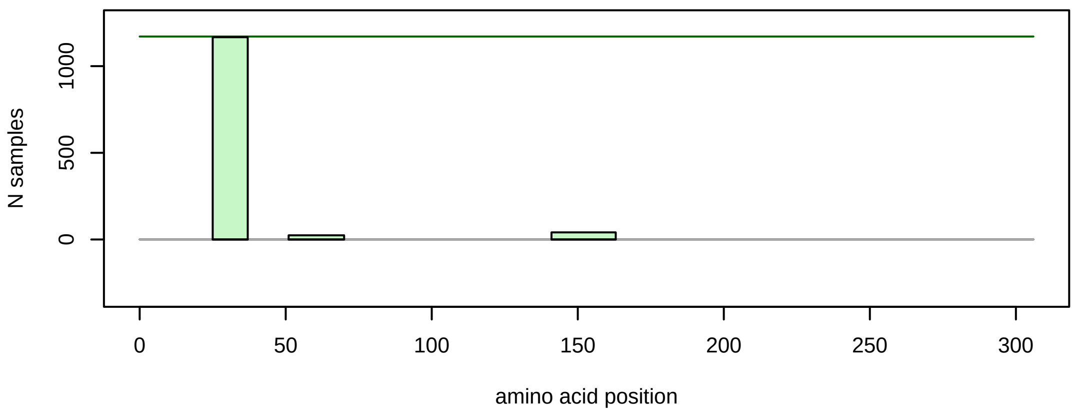

1424. PPP1R7 (Q15435), NP2

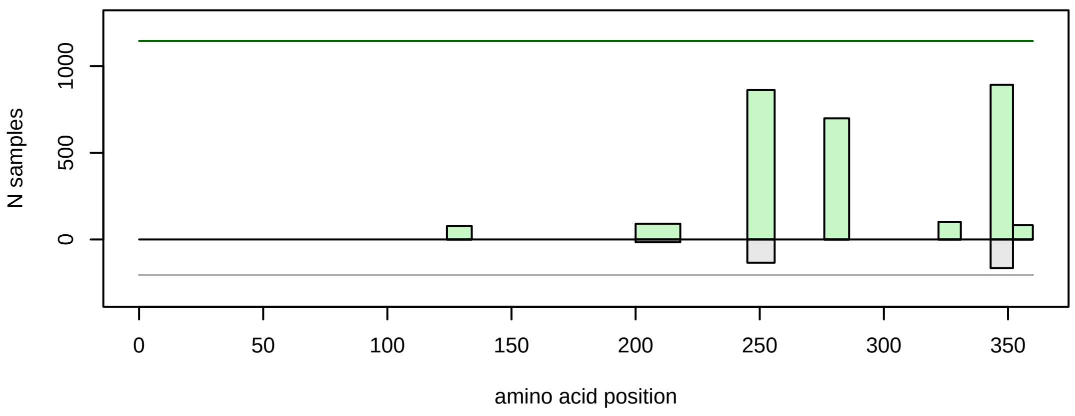

1425. CAMP (J3KNB4;P49913), NP1

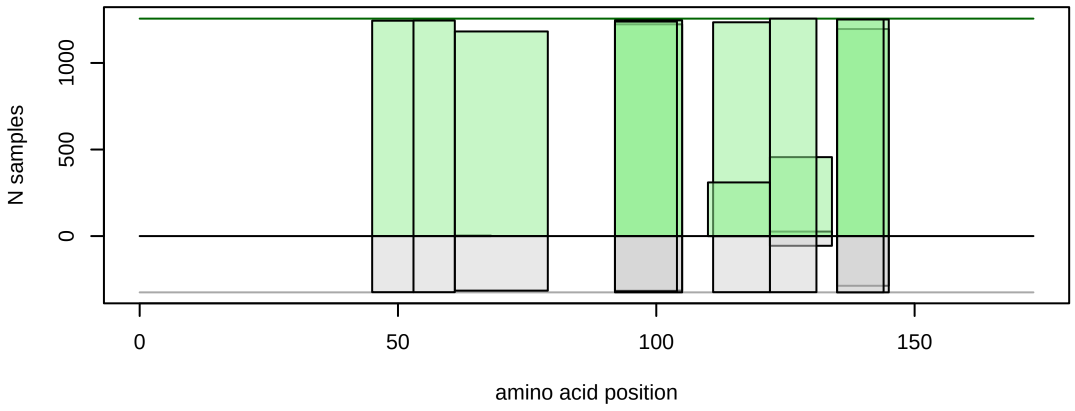

1426. RIC8A (Q9NPQ8), NP2

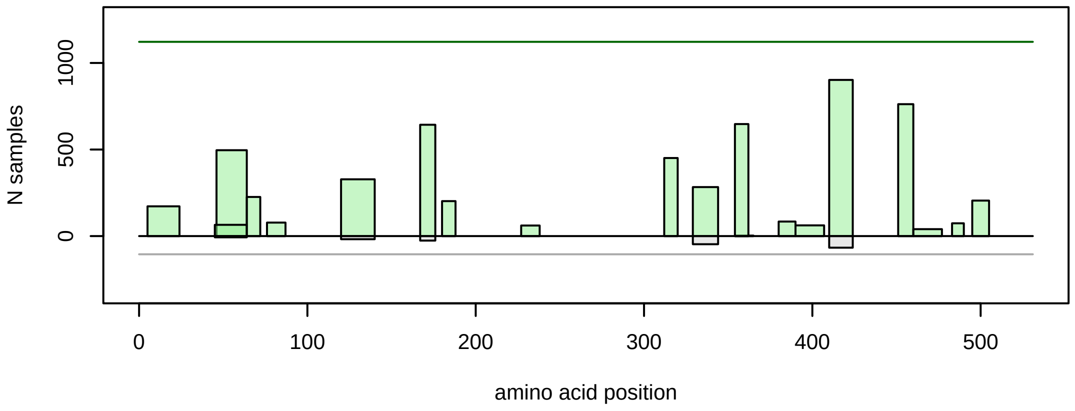

1427. TUBA4B (Q9H853), NP2

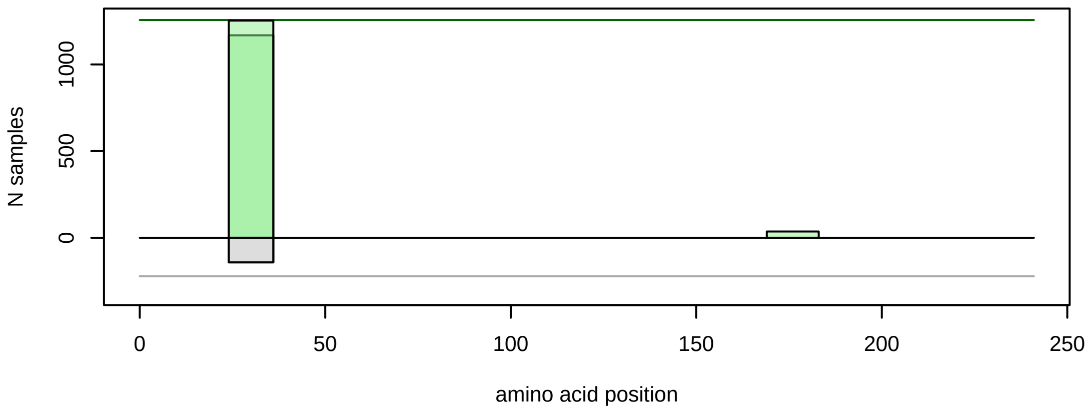

1428. CAPZA2 (P47755), NP4

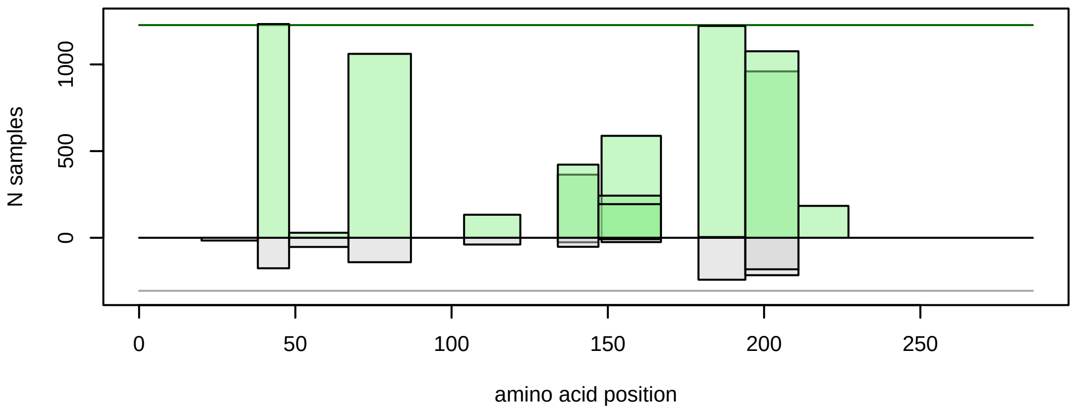

1429. LSM4 (Q9Y4Z0), NP1

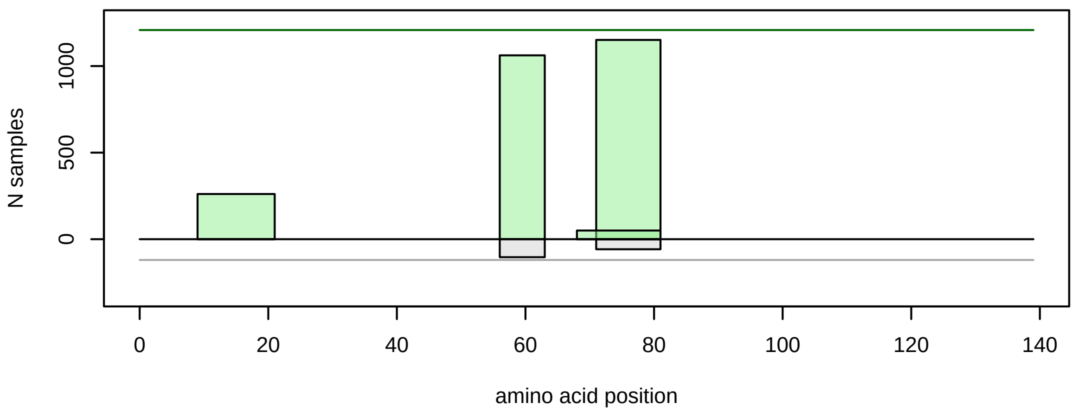

1430. H3F3A (A0A590UJJ6), NP2

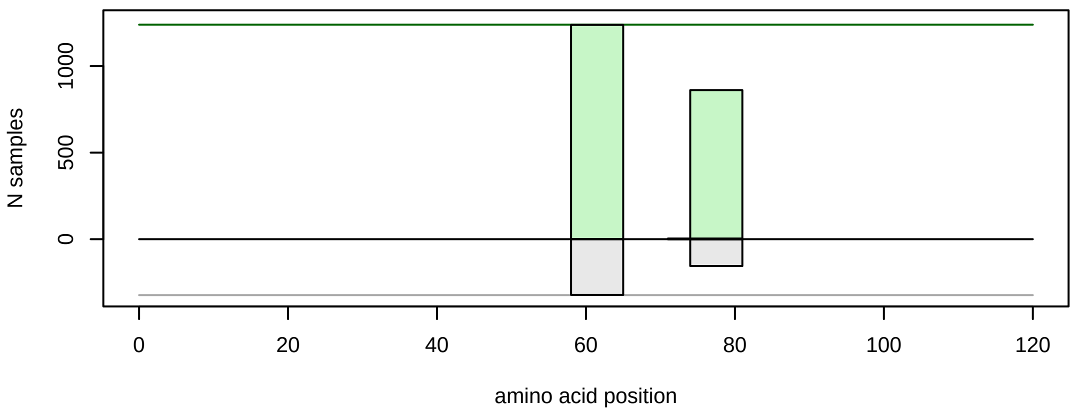

1431. IGHV1−3 (A0A0C4DH29), NP3

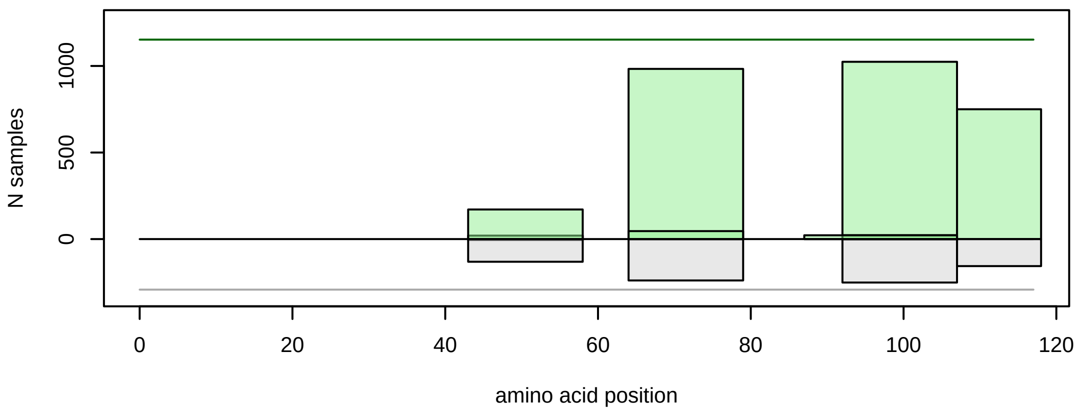

1432. PKN1 (Q16512), NP1

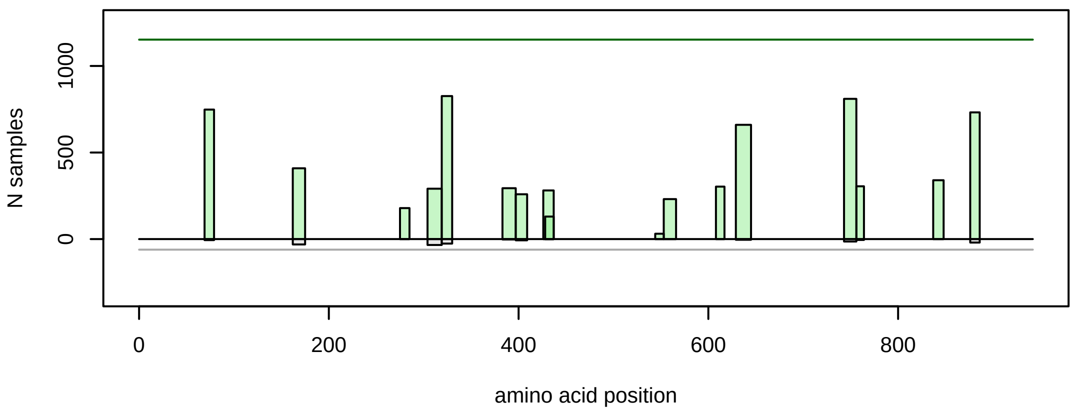

1433. APOC1 (P02654), NP3

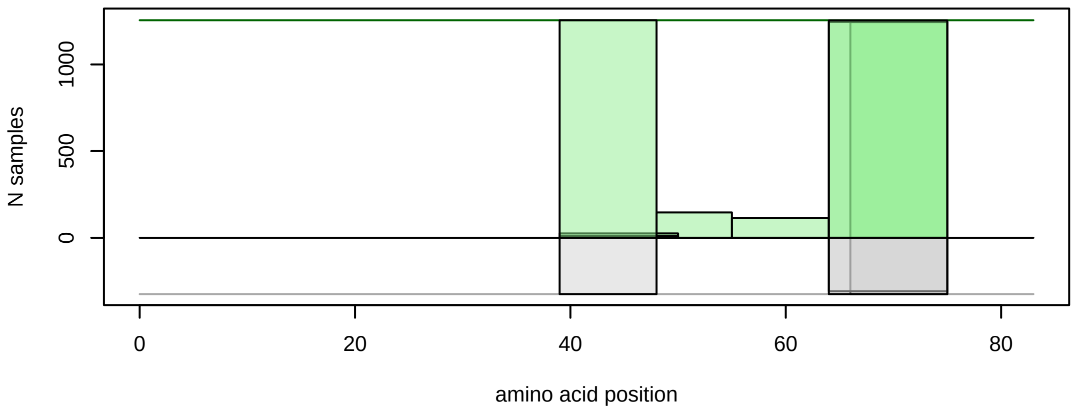

1434. ST13 (P50502), NP5

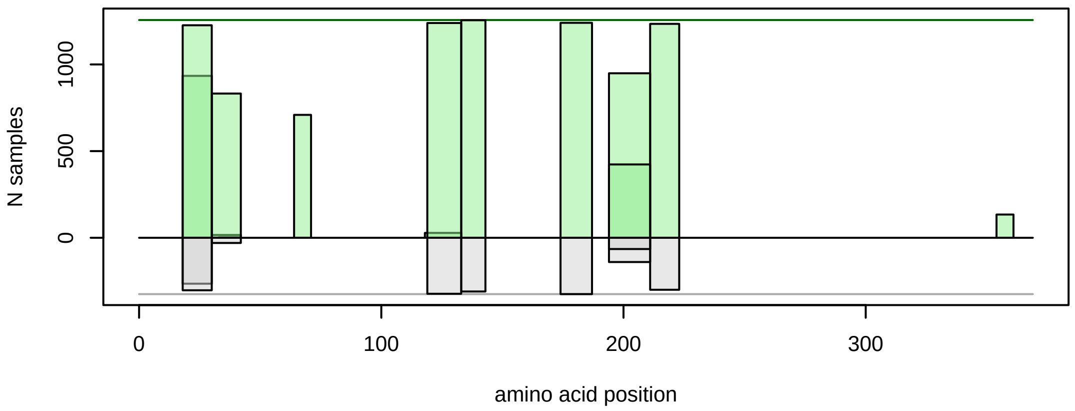

1435. PDLIM5 (Q96HC4), NP3

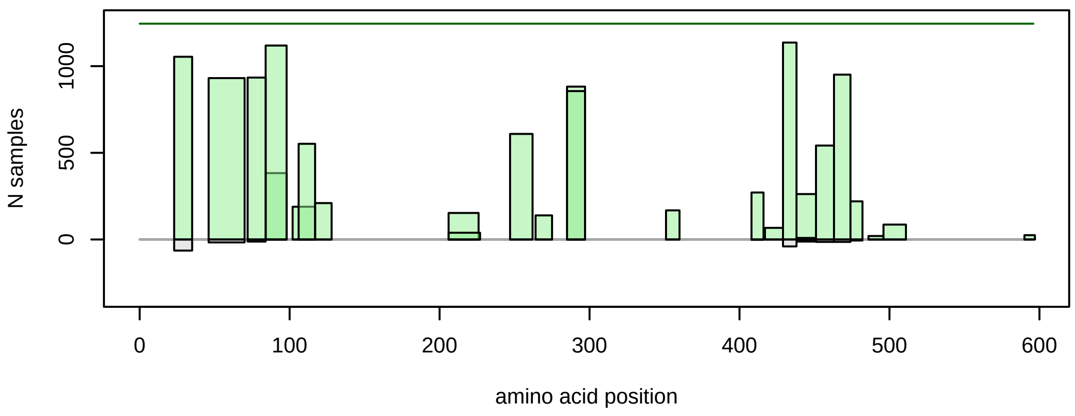

1436. EPB42 (P16452−2), NP1

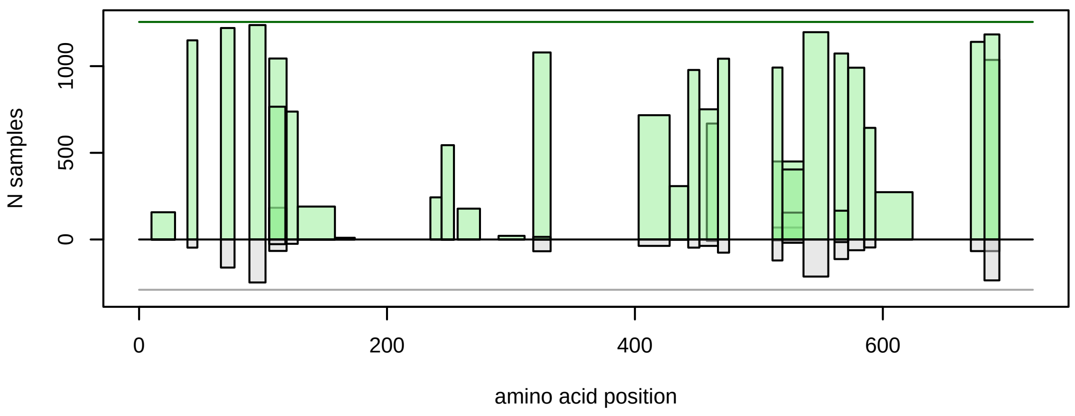

1437. GABARAPL2 (P60520), NP3

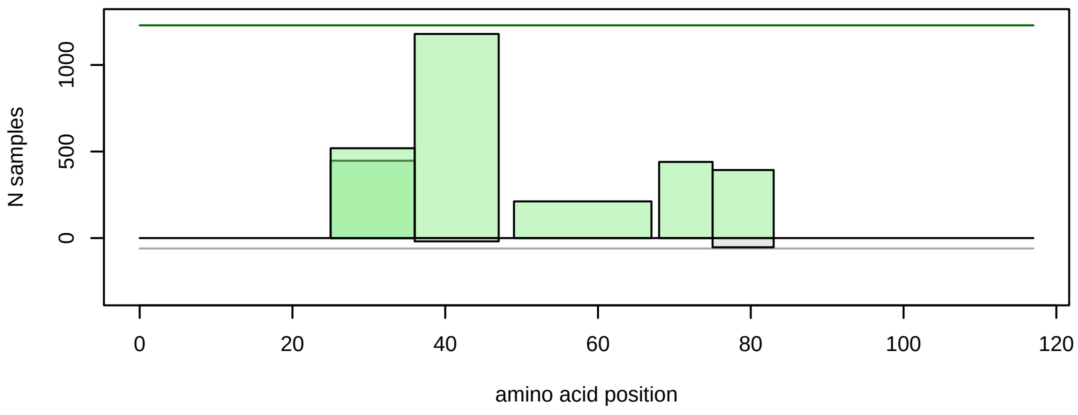

1438. TAF15 (Q92804), NP5

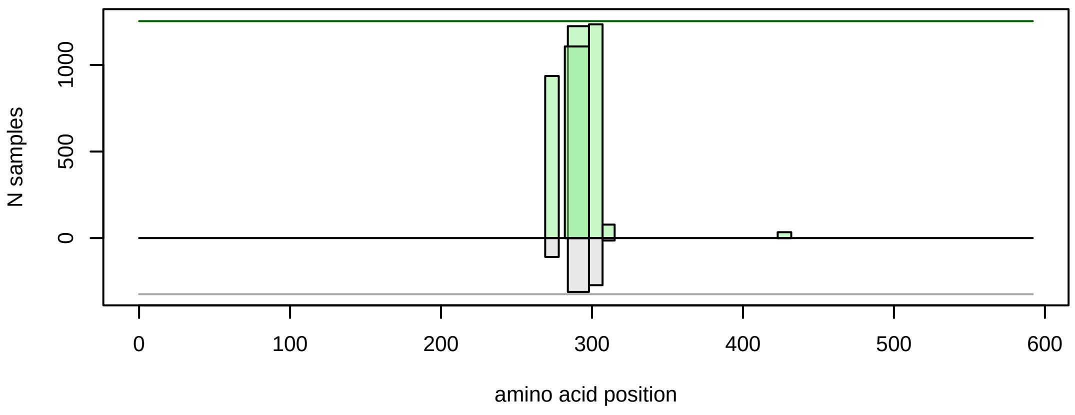

1439. CAT (P04040), NP1

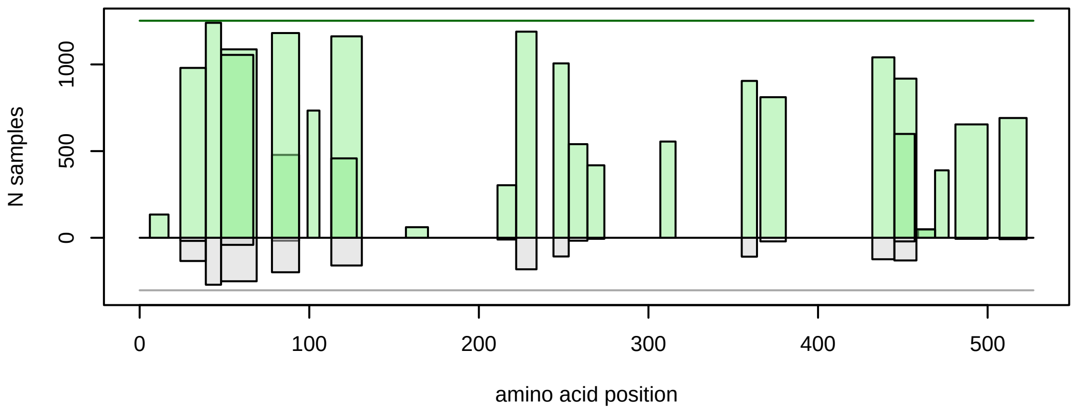

1440. PCDH18 (Q9HCL0;Q9HCL0−2), NP2

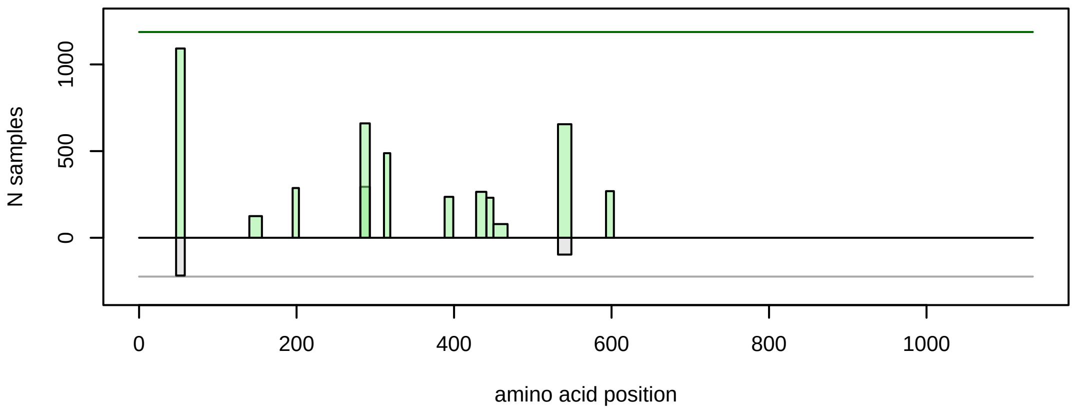

1441. PPM1A (P35813), NP2

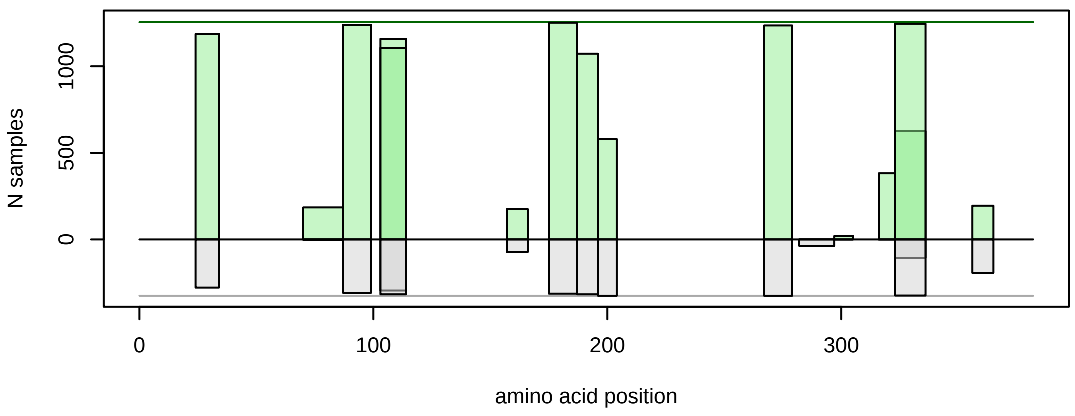

1442. SERPINC1 (P01008), NP2

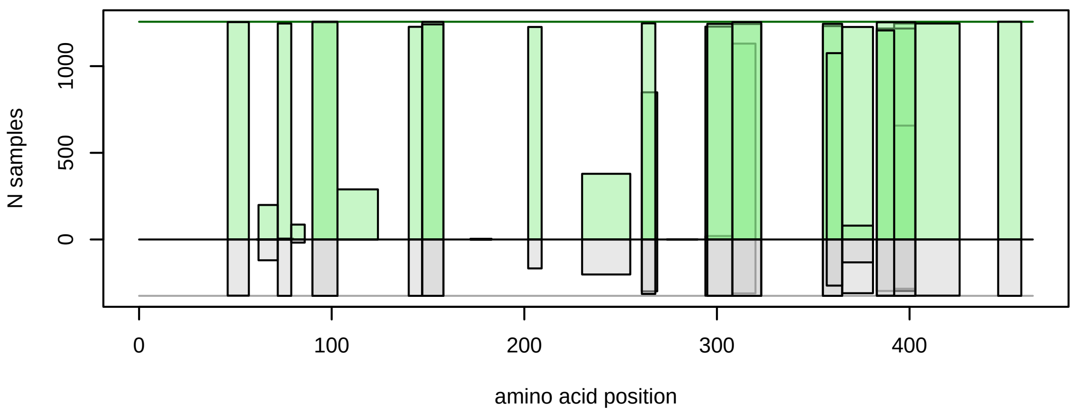

1443. LARS (A0A6I8PIV1;Q9P2J5), NP4

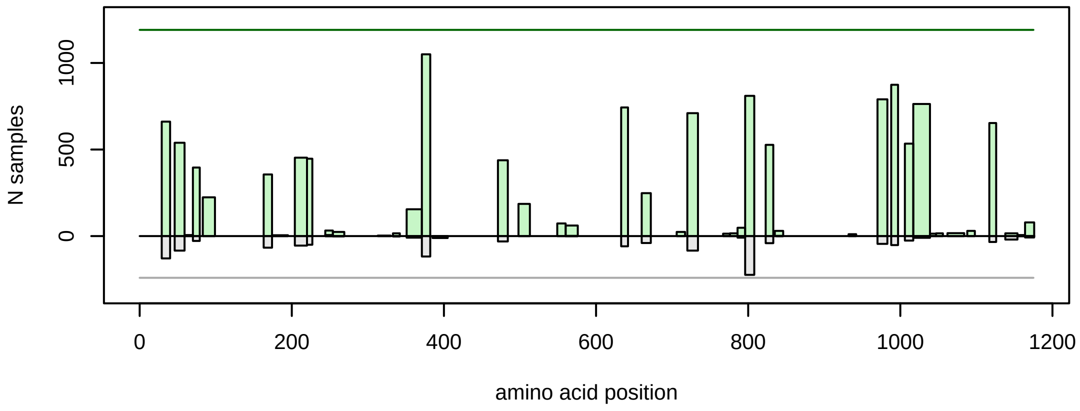

1444. FLYWCH2 (I3L1Y9), NP2

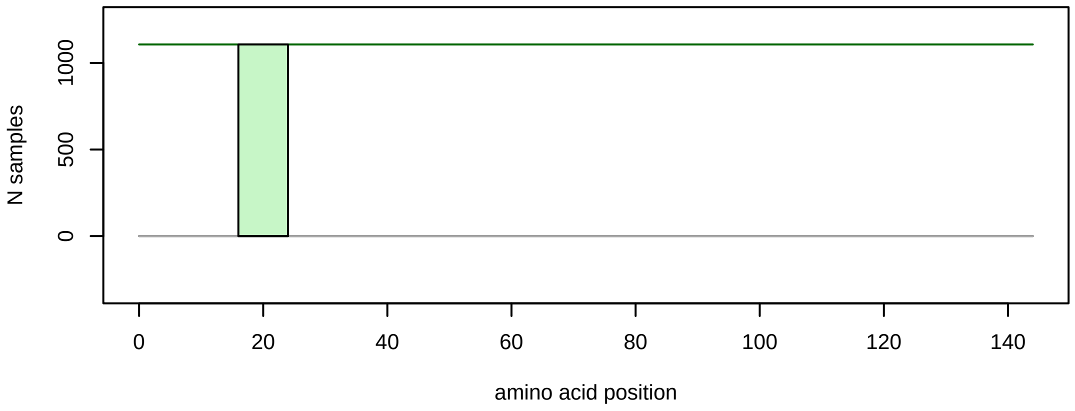

1445. PRKAR1A (P10644), NP1

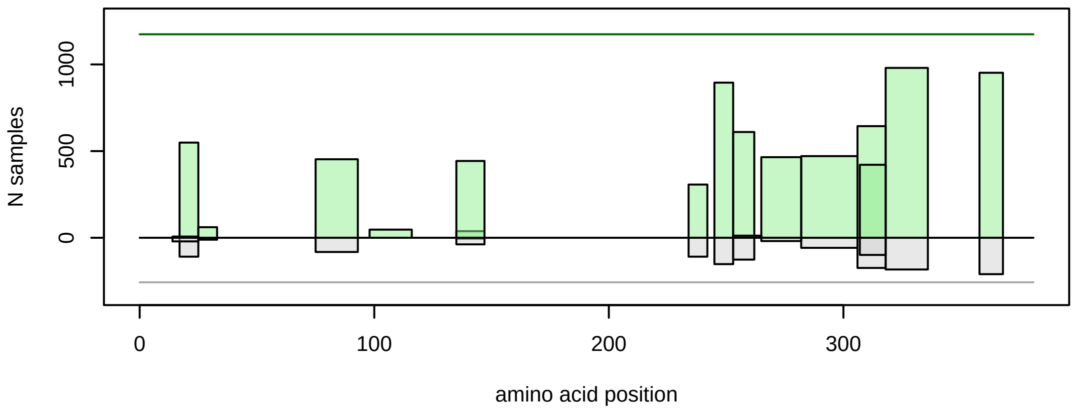

1446. RASA3 (Q14644), NP4

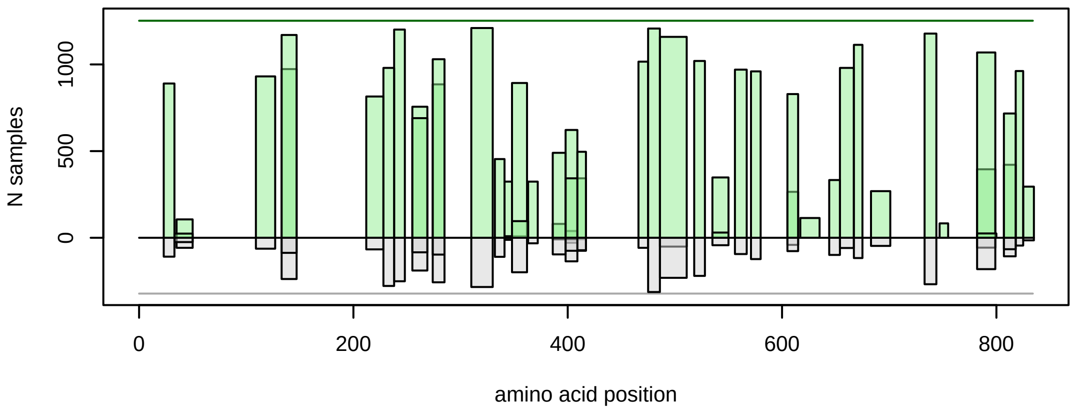

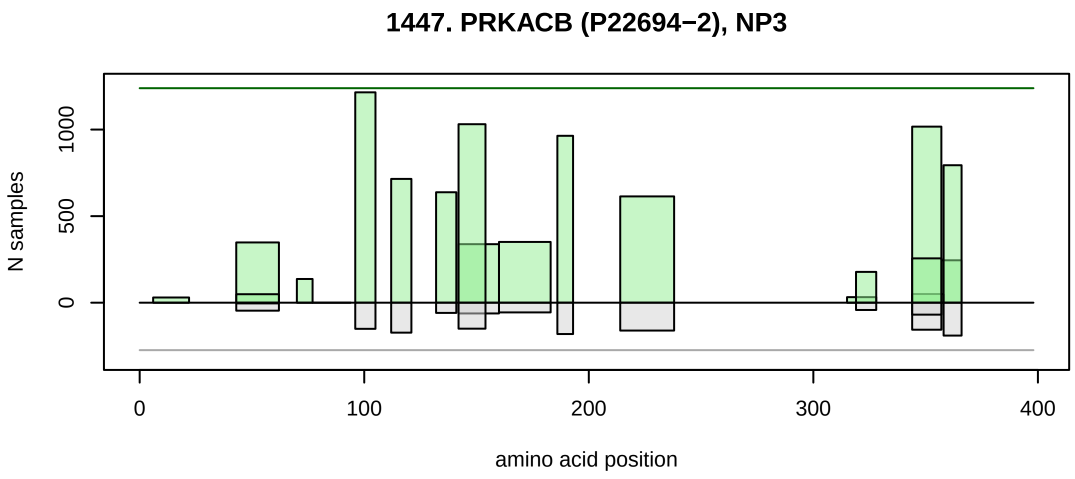

1447. PRKACB (P22694−2), NP3
N samples
0
500
1000
0
100
200
300
400
amino acid position

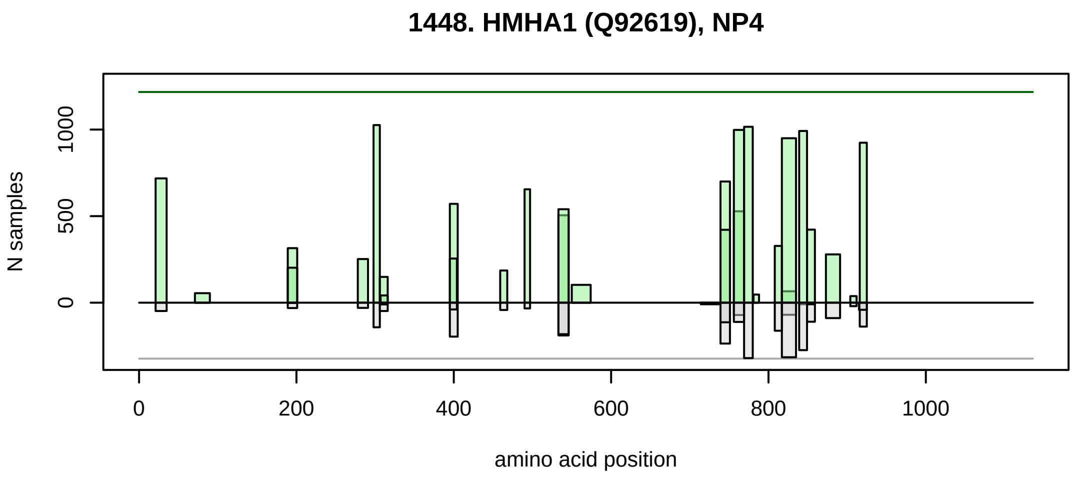

1448. HMHA1 (Q92619), NP4
N samples
0
500
1000
0
200
400
600
800
1000
amino acid position

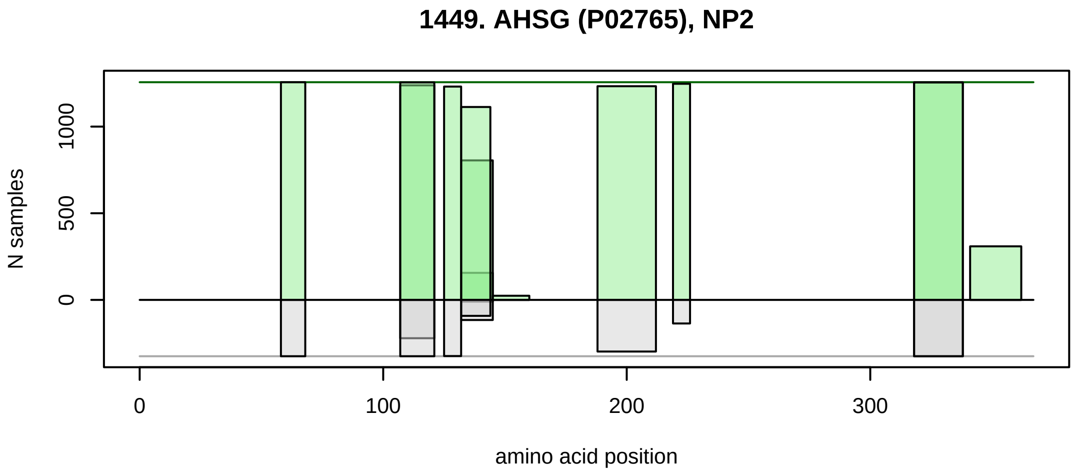

1449. AHSG (P02765), NP2
N samples
0
500
1000
0
100
200
300
amino acid position

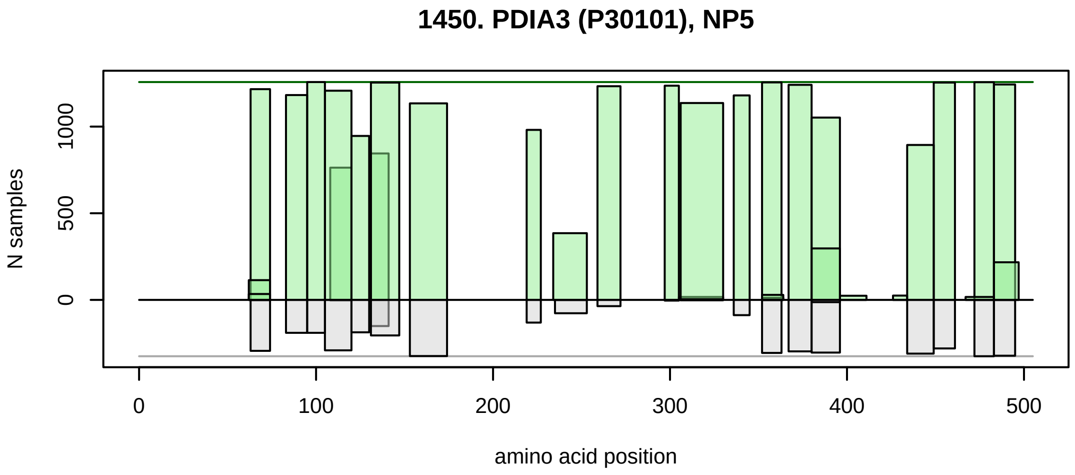

1450. PDIA3 (P30101), NP5
N samples
0
500
1000
0
100
200
300
400
500
amino acid position

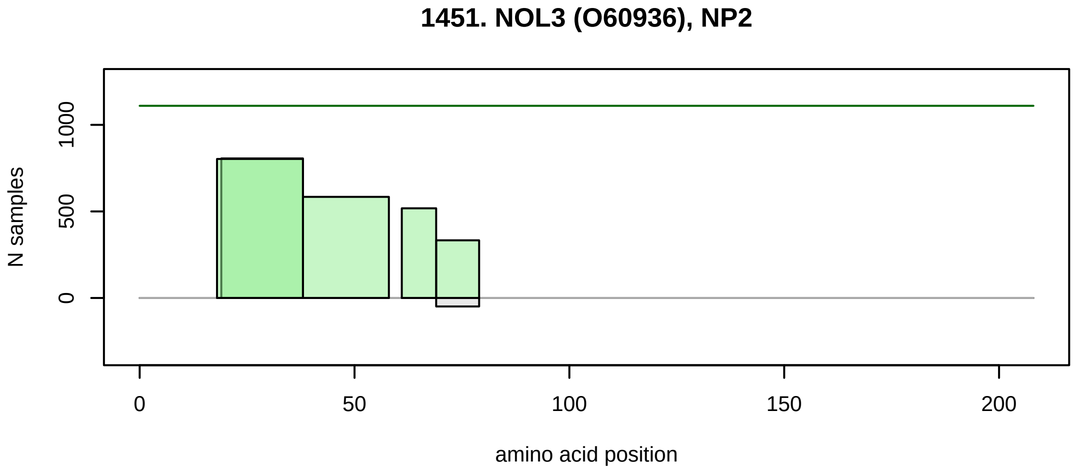

1451. NOL3 (O60936), NP2
N samples
0
500
1000
0
50
100
150
200
amino acid position

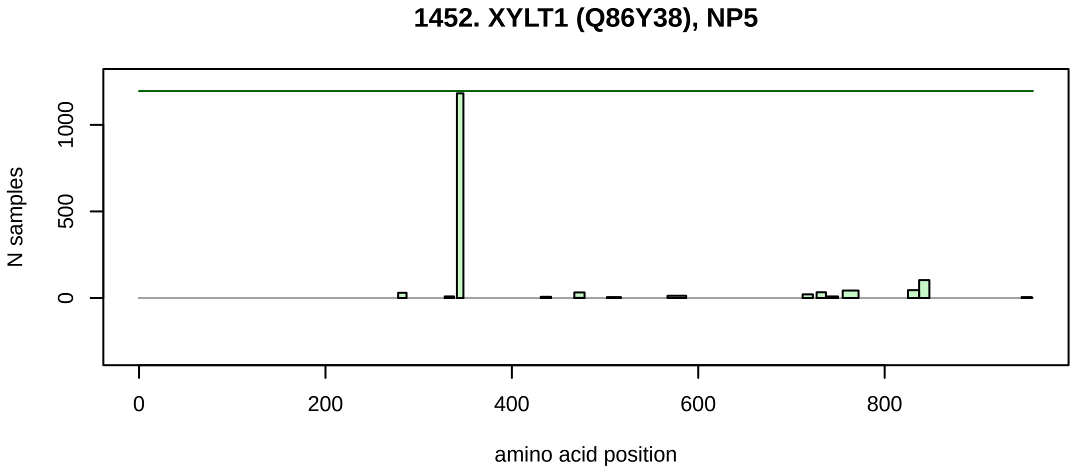

1452. XYLT1 (Q86Y38), NP5
N samples
0
500
1000
0
200
400
600
800
amino acid position

1453. C6orf25 (O95866−2;O95866−7), NP1

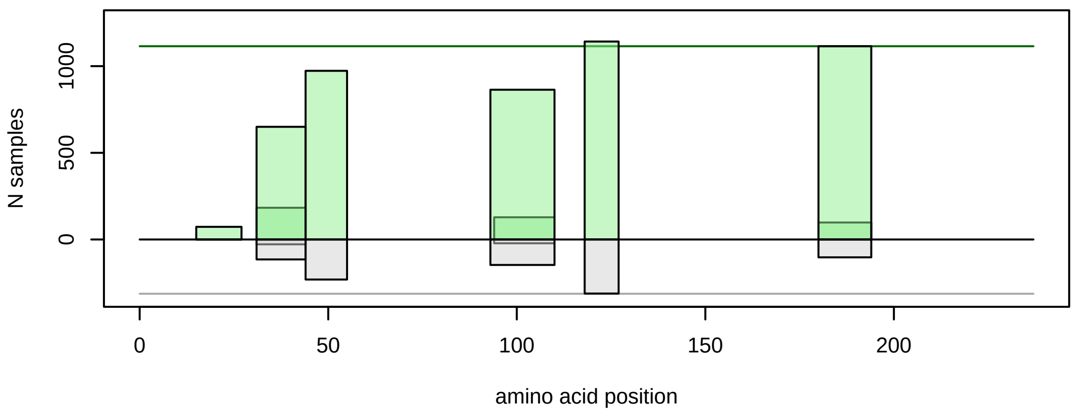

1454. SMAD2 (Q15796;Q15796−2), NP4

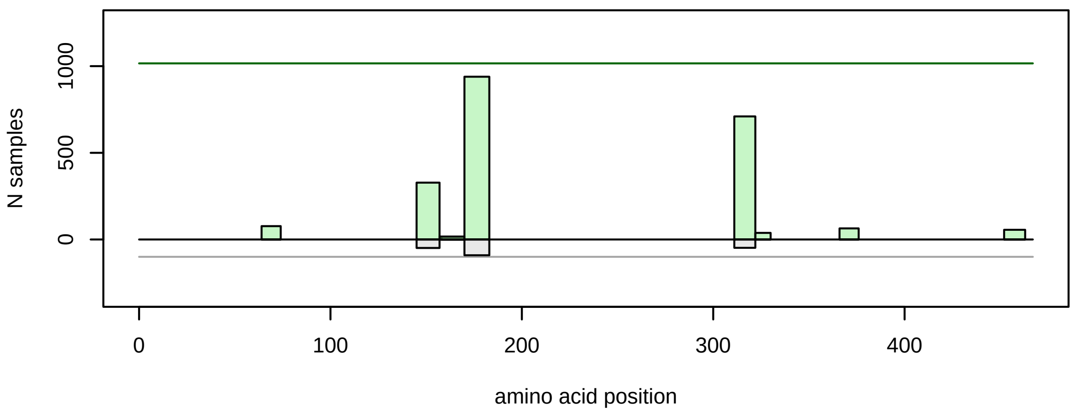

1455. COASY (Q13057), NP5

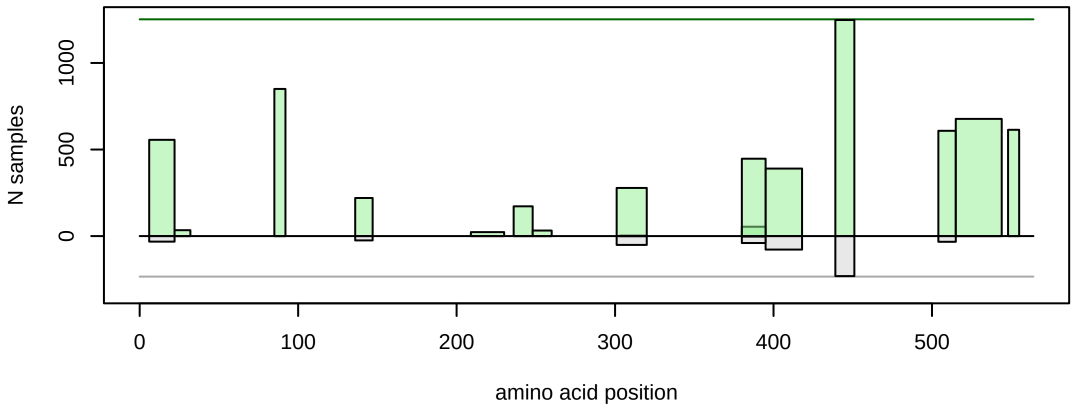

1456. SUB1 (P53999), NP2

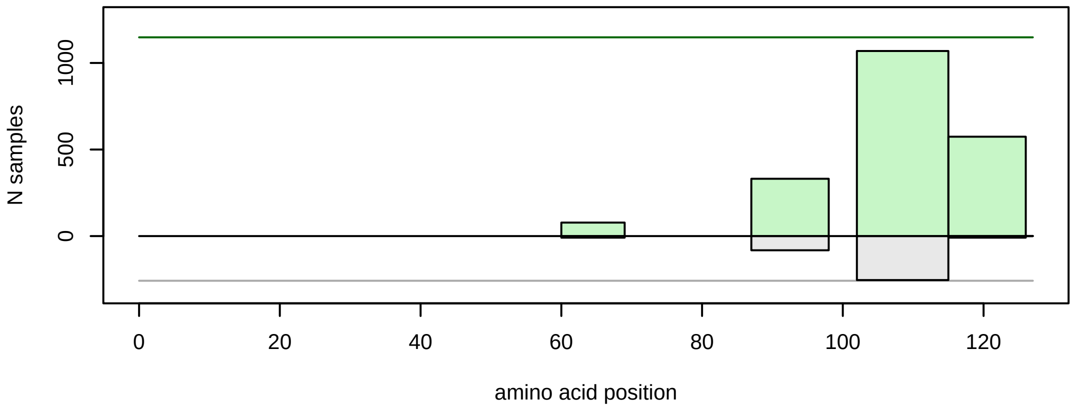

1457. CDH2 (P19022), NP2

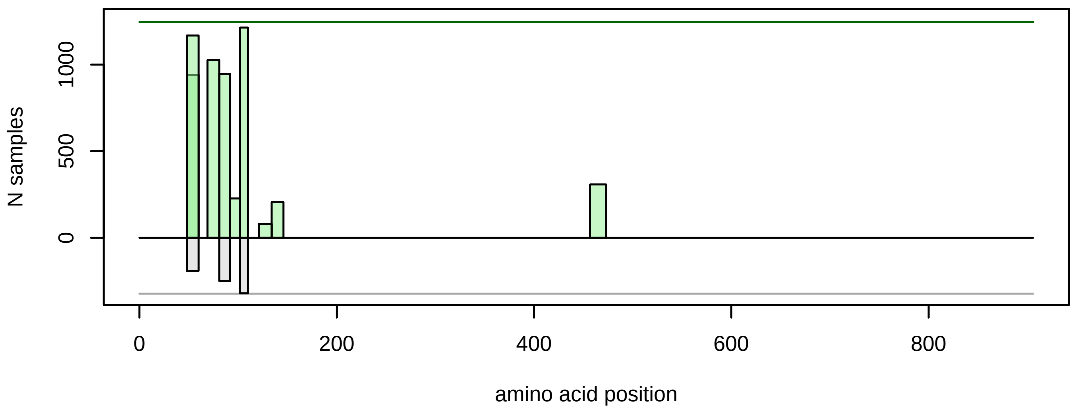

1458. COL4A2 (P08572), NP3

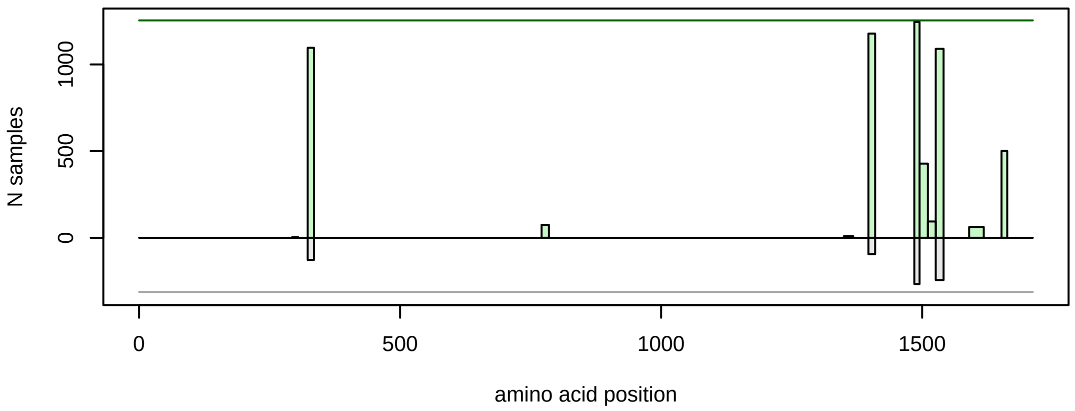

1459. PCDH1 (Q08174−2), NP2

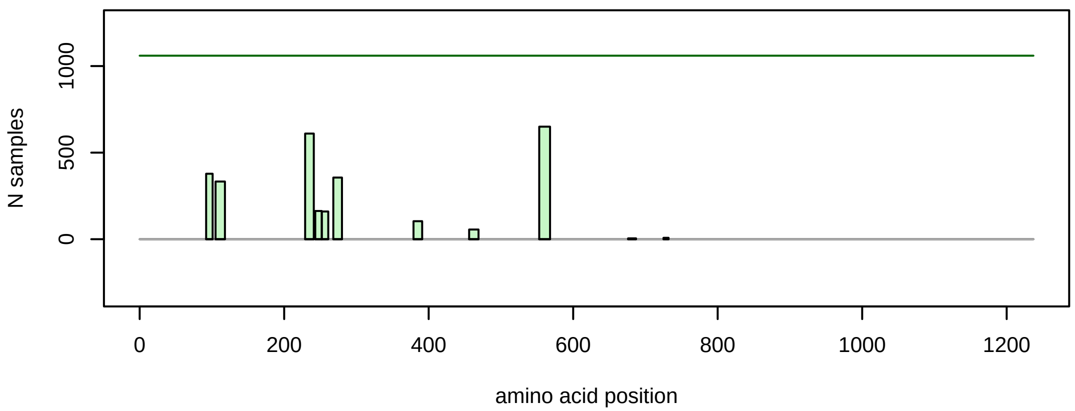

1460. ARPC1B (O15143), NP1

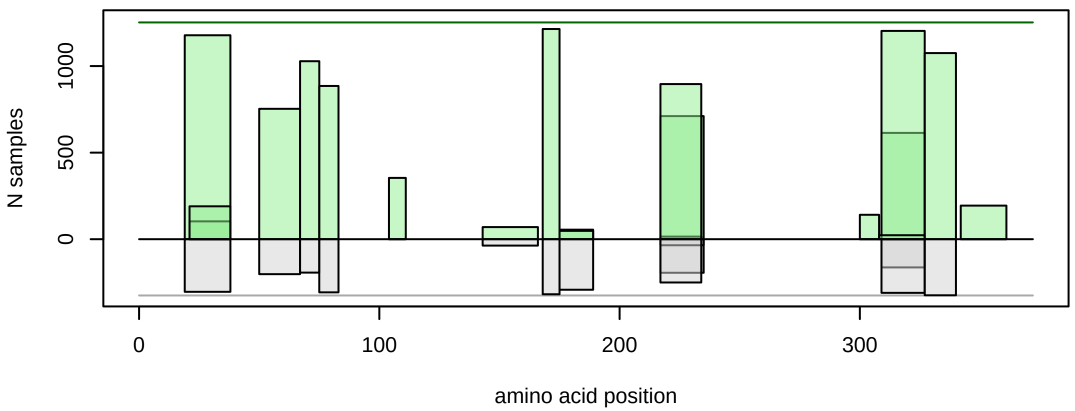

1461. PDE5A (O76074), NP4

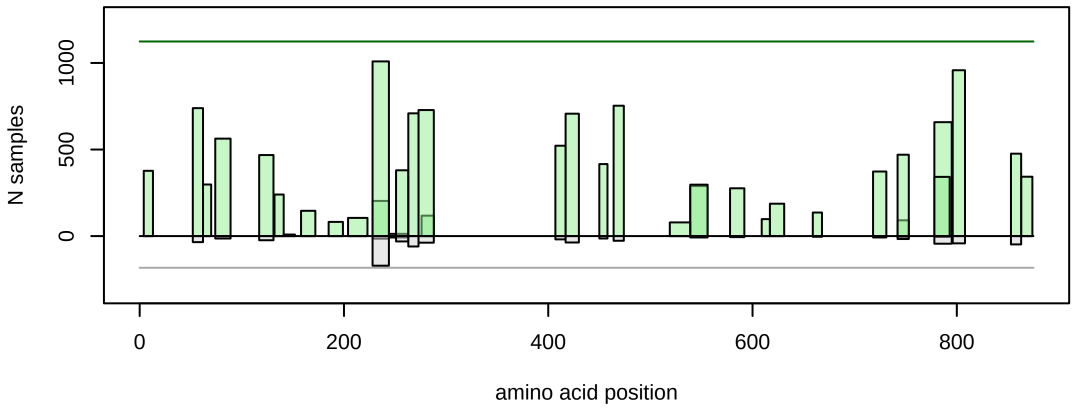

1462. GSN (P06396), NP2

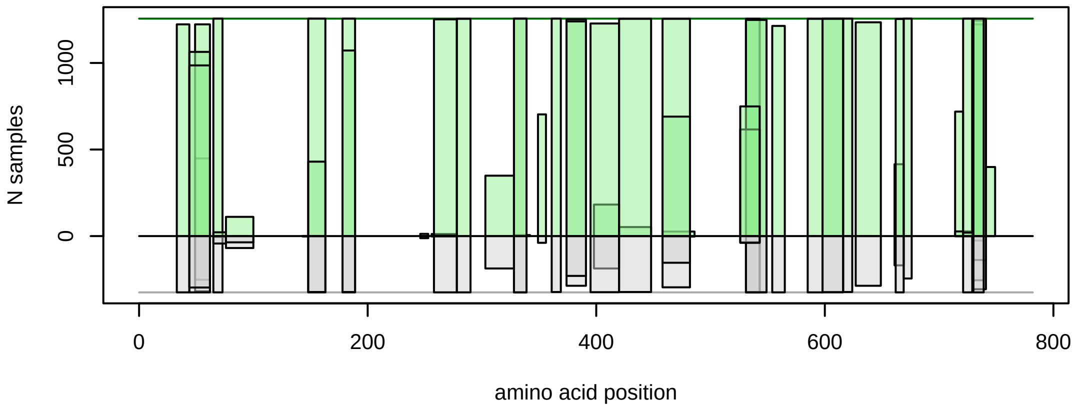

1463. LMNA (A0A6Q8PFJ0), NP5

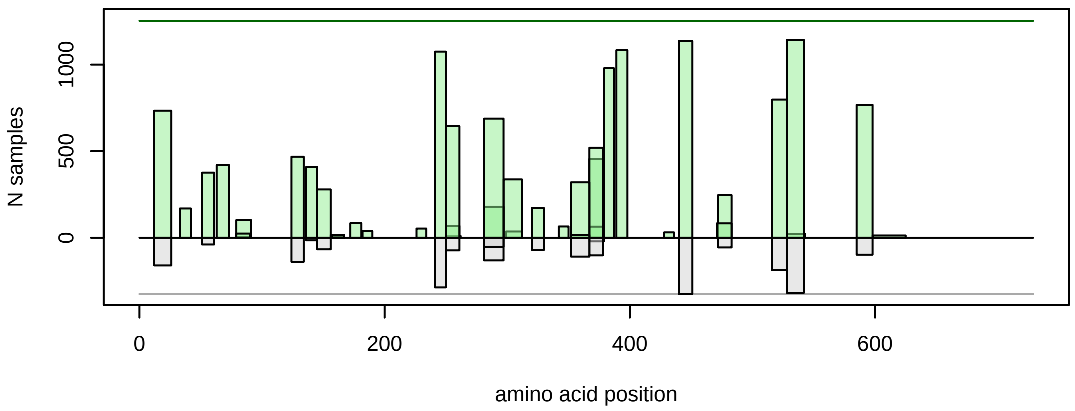

1464. EIF2S1 (P05198), NP1

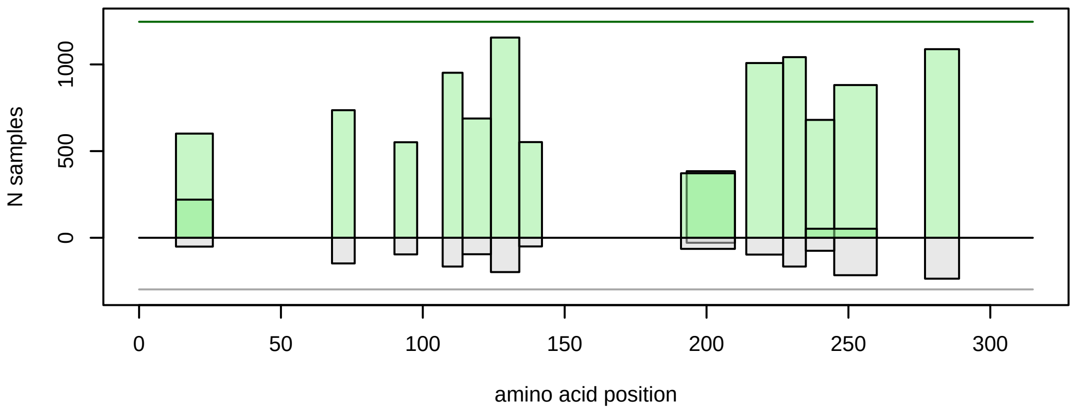

1465. FBXW7 (Q969H0−2), NP2

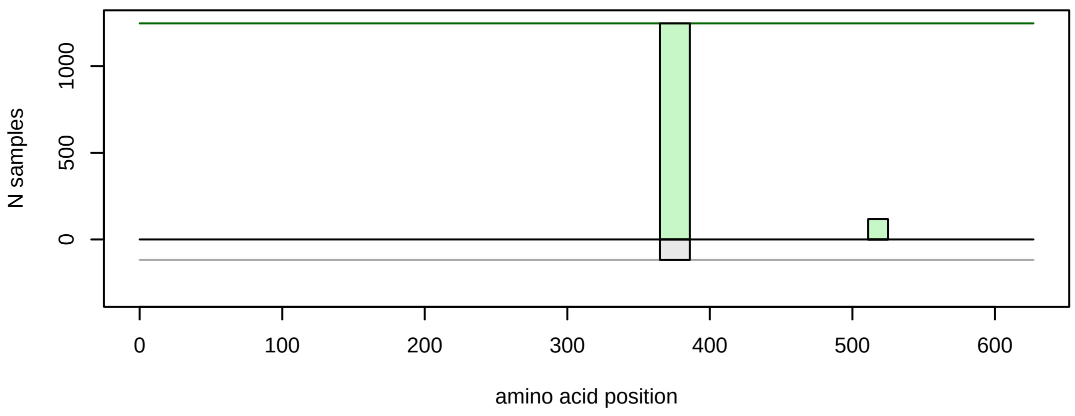

1466. IGHV3−74 (A0A0B4J1X5), NP3

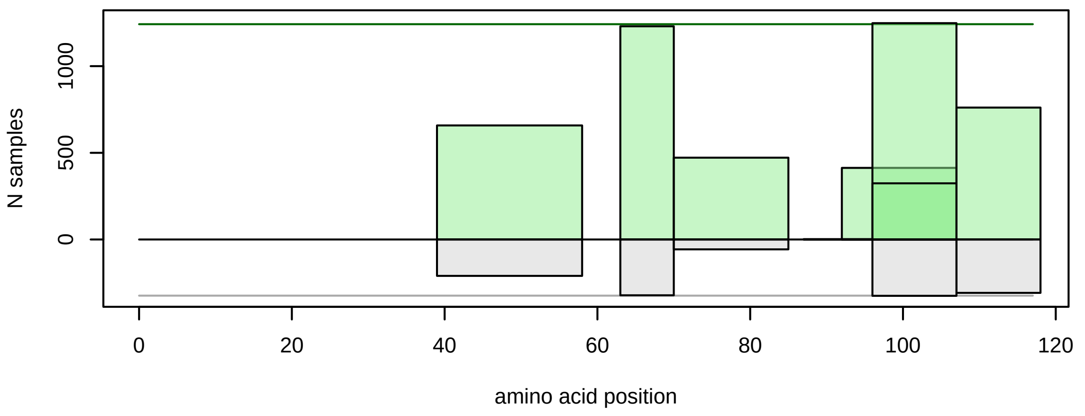

1467. MYLK (A0A8I5KU53;Q15746), NP5

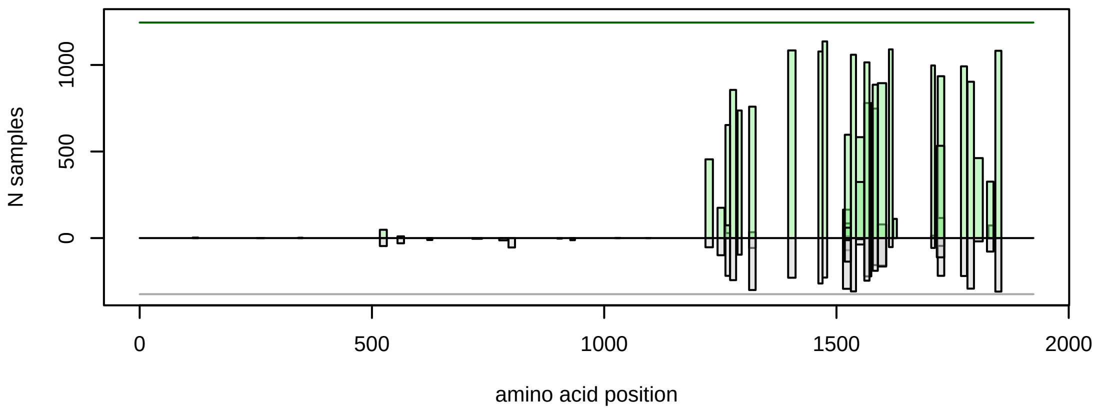

1468. SLFN14 (P0C7P3), NP4

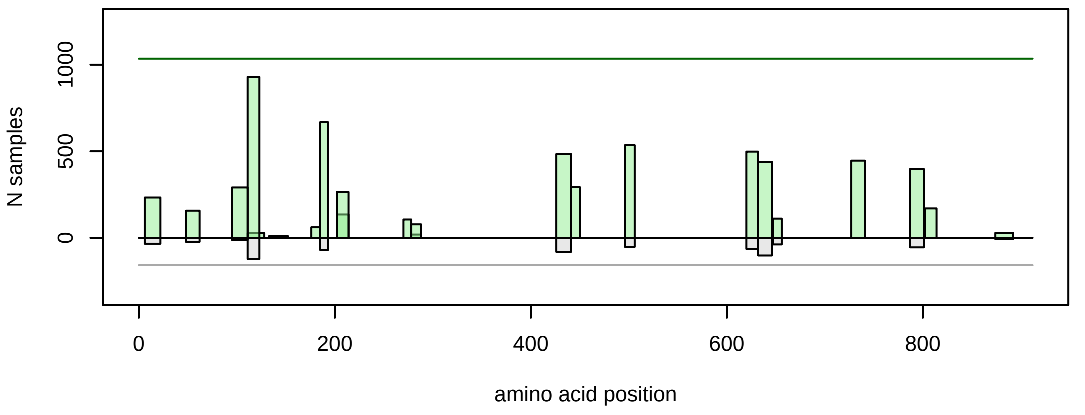

1469. ALB (D6RCE7), NP3

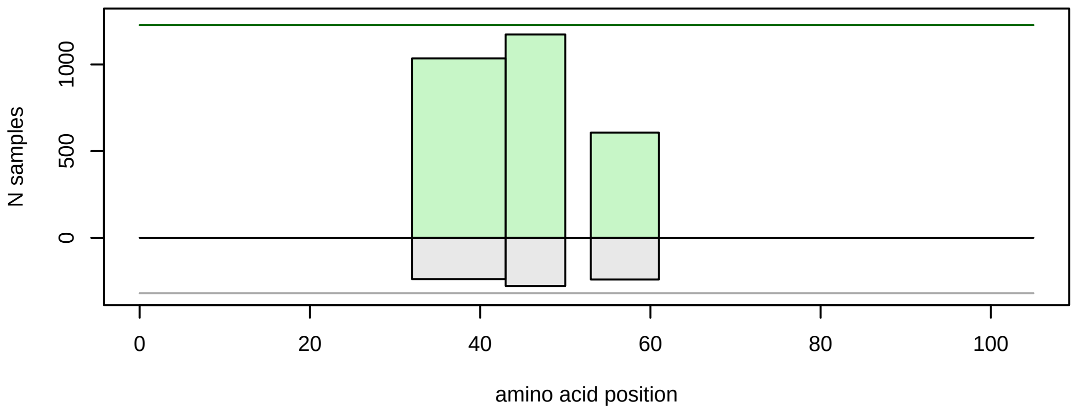

1470. IGKV1−16 (P04430), NP4

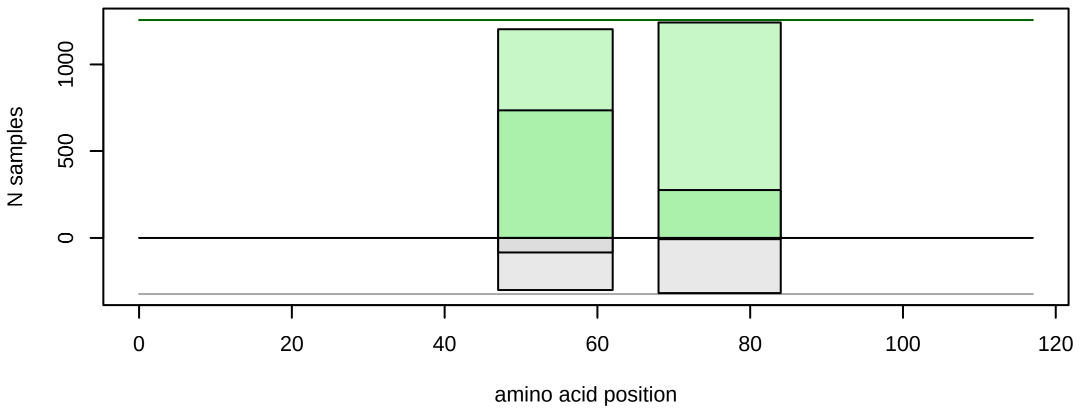

1471. ATP5O (P48047), NP4

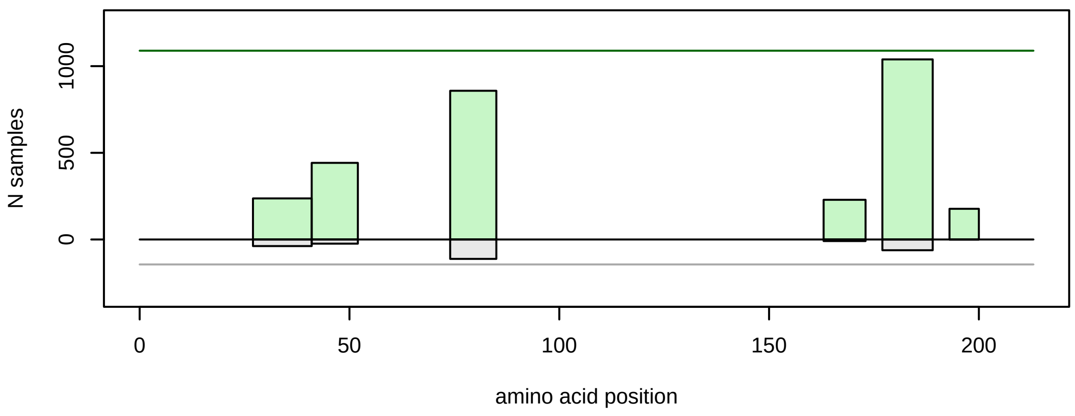

1472. IGLV2−11 (P01706), NP3

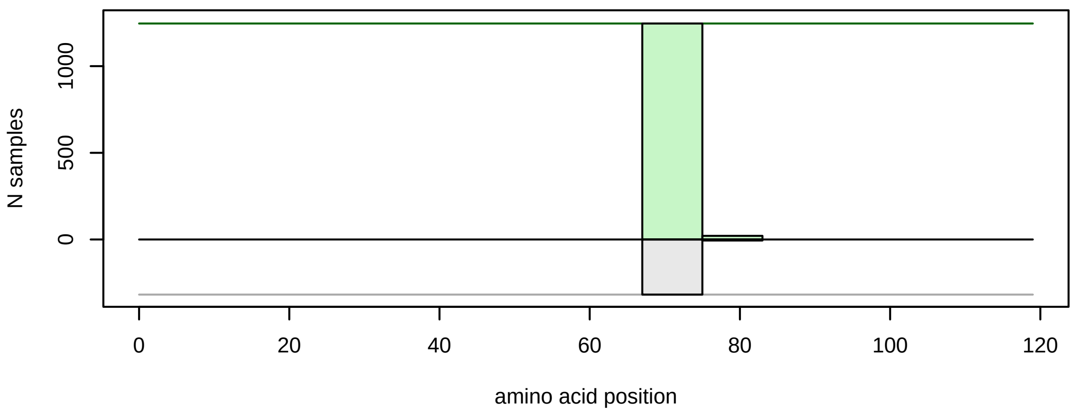

1473. CD84 (Q9UIB8−2), NP4

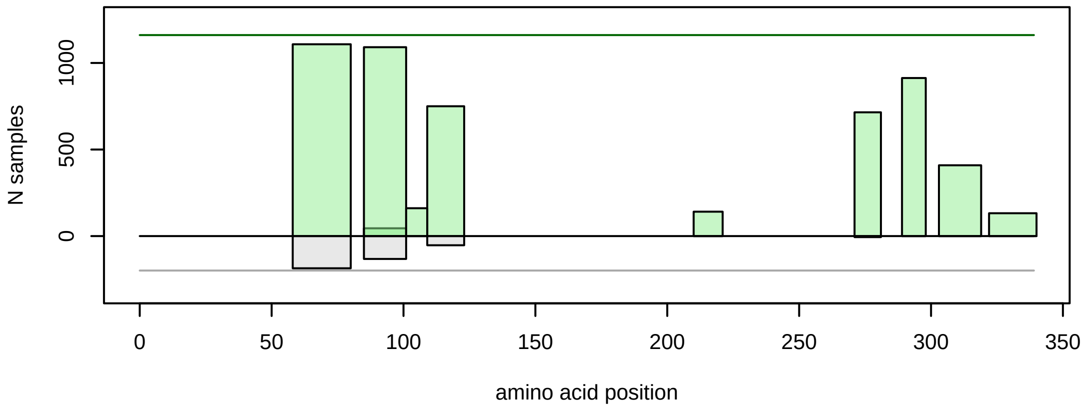

1474. OSTF1 (Q92882), NP2

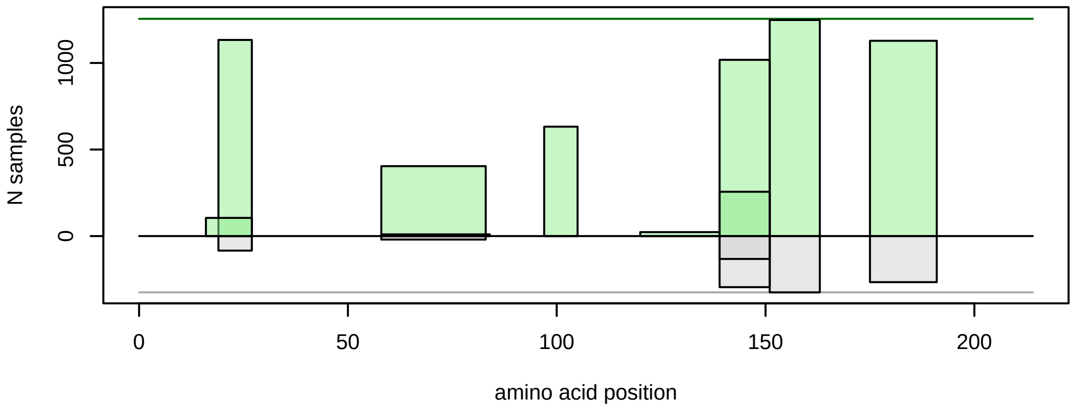

1475. MMP2 (P08253), NP5

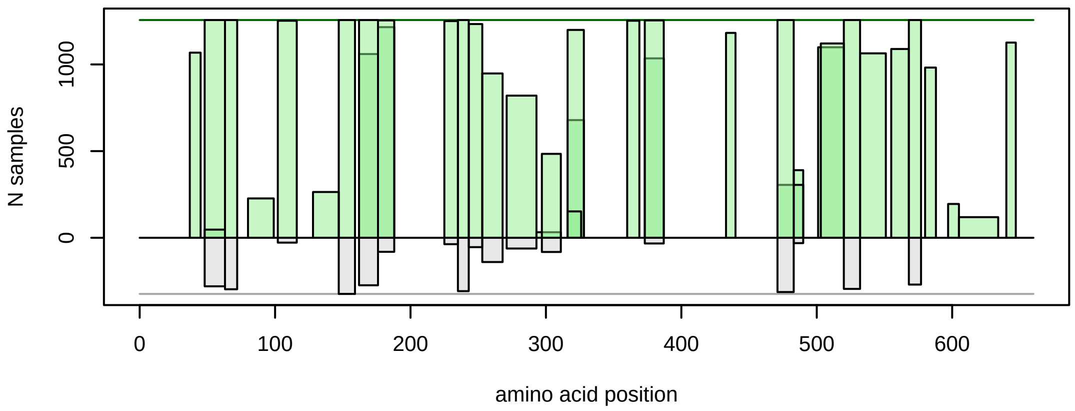

1476. CD55 (P08174−5), NP1

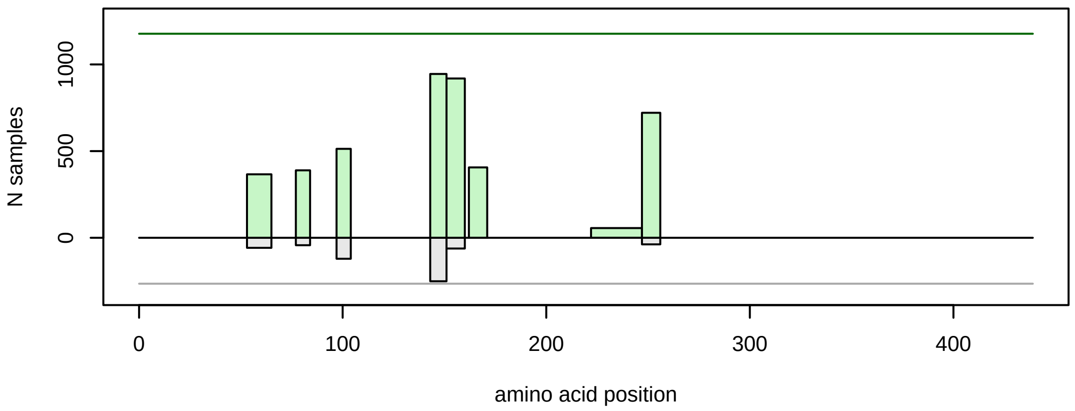

1477. IGKV3−20 (P01619), NP4

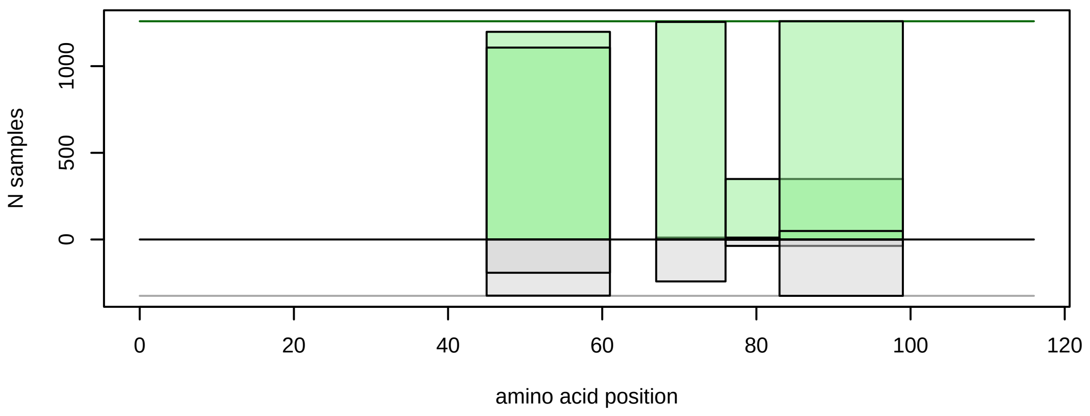

1478. SOD1 (P00441), NP4

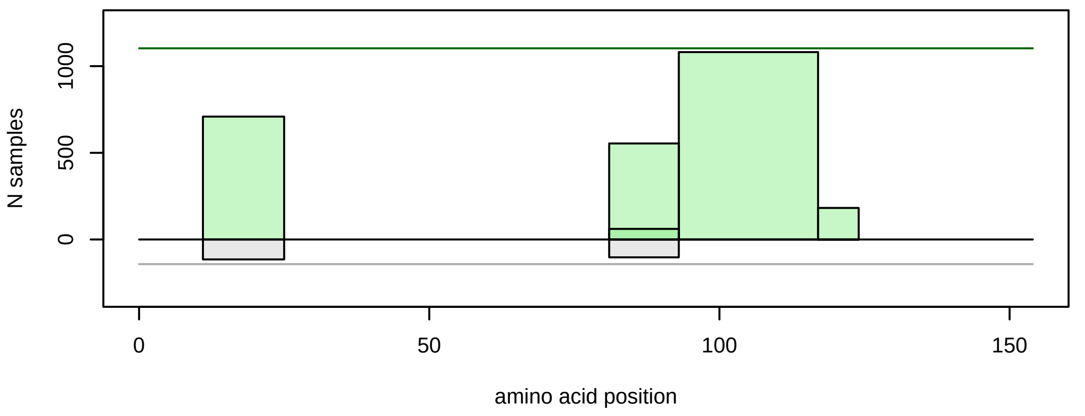

1479. GXYLT1 (Q4G148;Q4G148−2), NP4

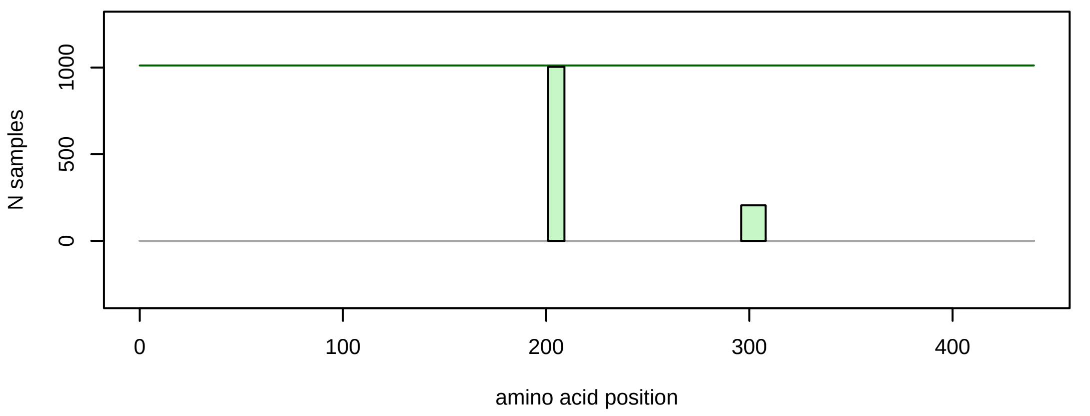

1480. KLC1 (Q07866−4;Q07866−6;Q07866−7), NP4

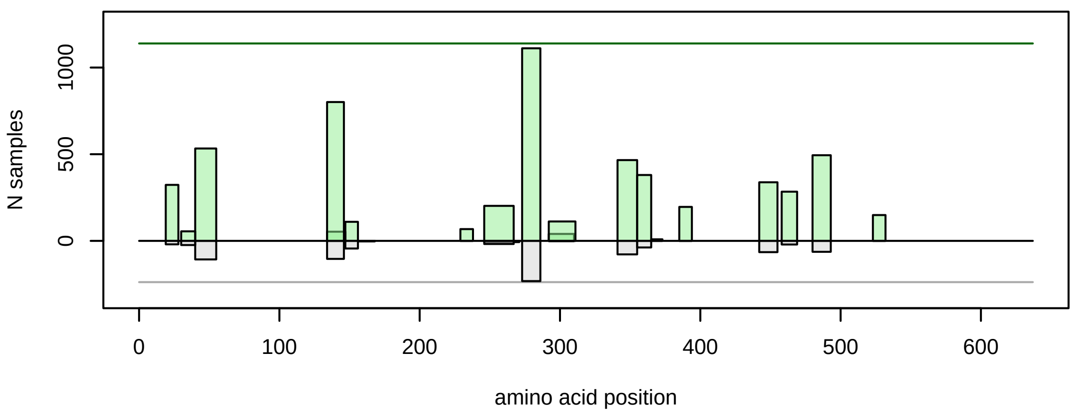

1481. IGLV3−9 (A0A075B6K5), NP3

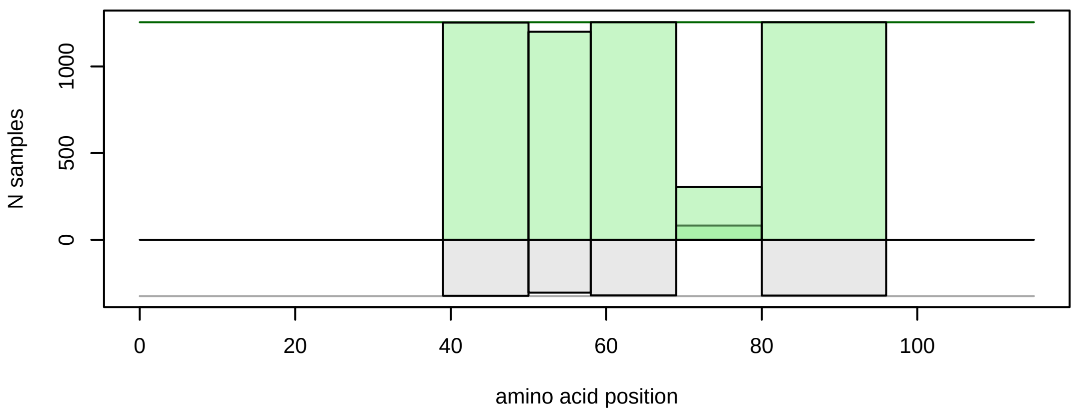

1482. ATP2B4 (P23634−6), NP1

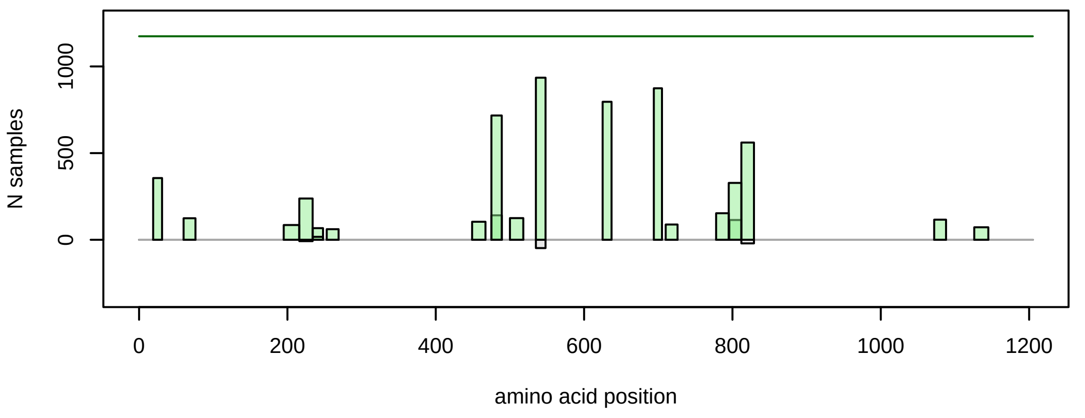

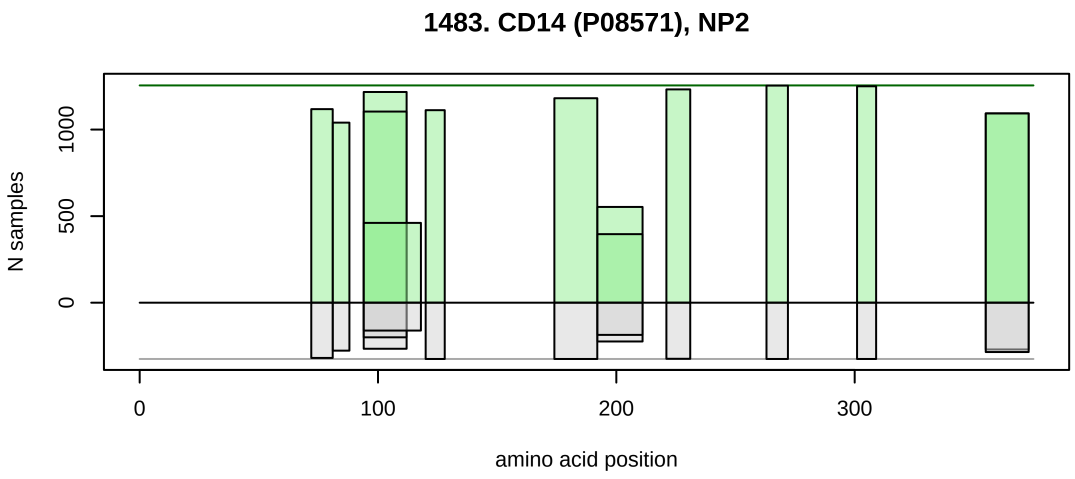
1483. CD14 (P08571), NP2
N samples
0
500
1000
0
100
200
300
amino acid position

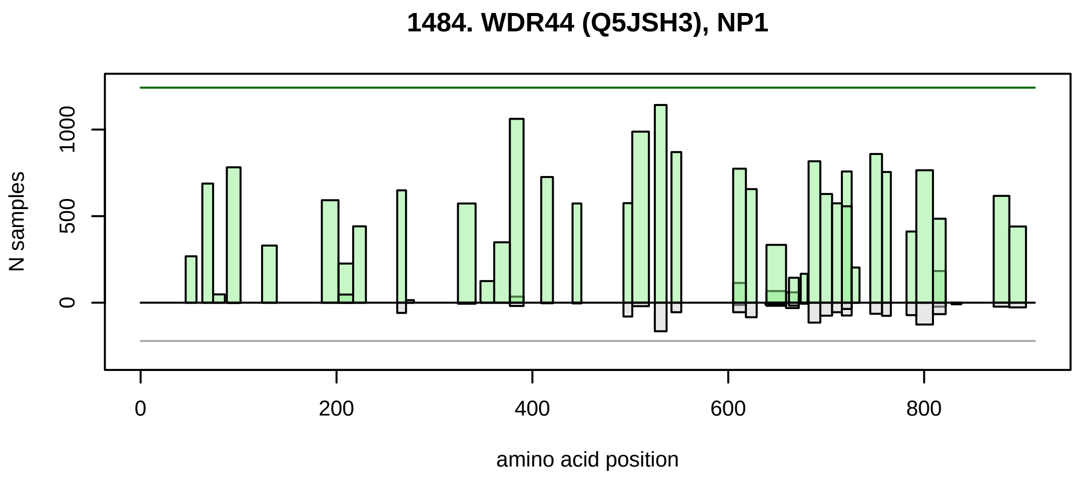
1484. WDR44 (Q5JSH3), NP1
N samples
0
500
1000
0
200
400
600
800
amino acid position

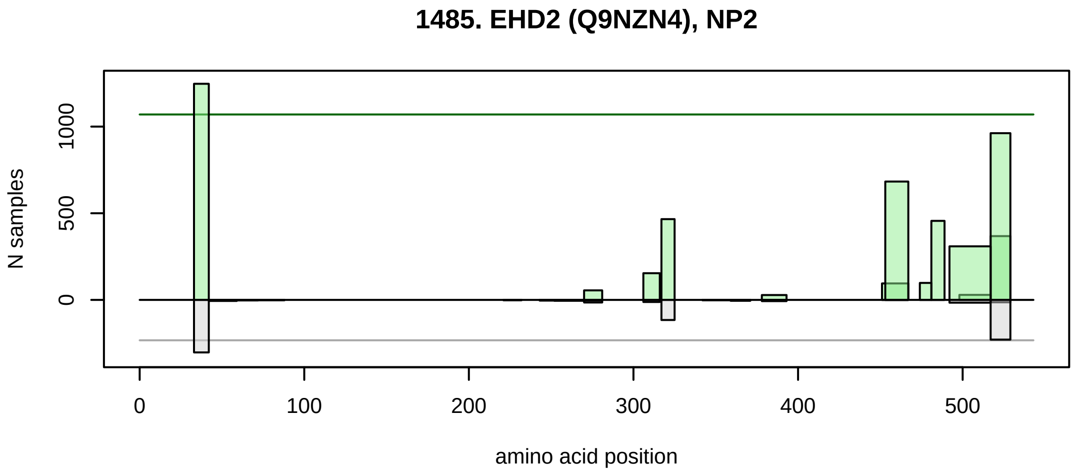
1485. EHD2 (Q9NZN4), NP2
N samples
0
500
1000
0
100
200
300
400
500
amino acid position

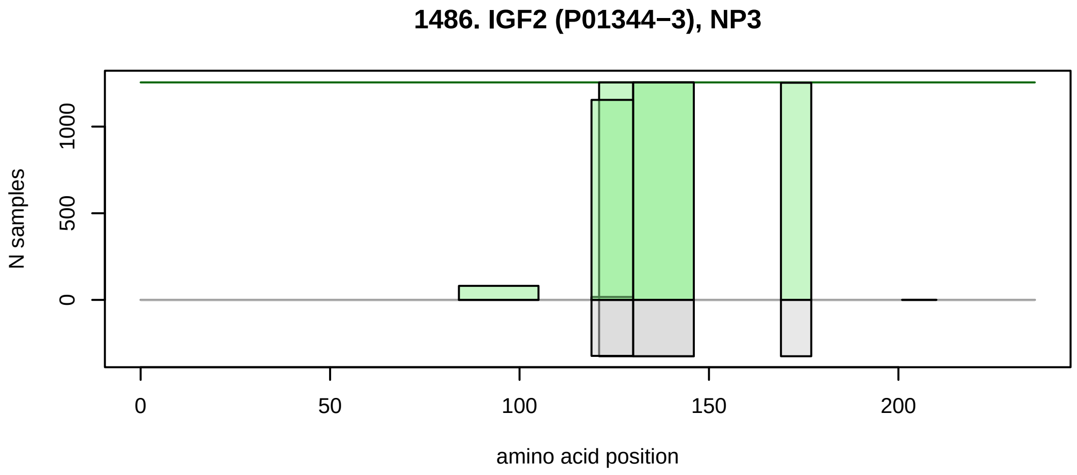
1486. IGF2 (P01344−3), NP3
N samples
0
500
1000
0
50
100
150
200
amino acid position

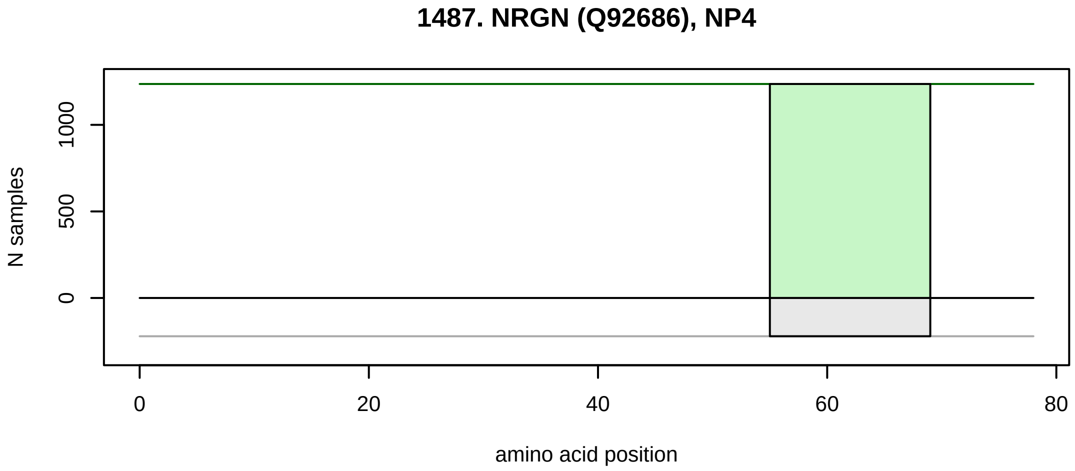
1487. NRGN (Q92686), NP4
N samples
0
500
1000
0
20
40
60
80
amino acid position

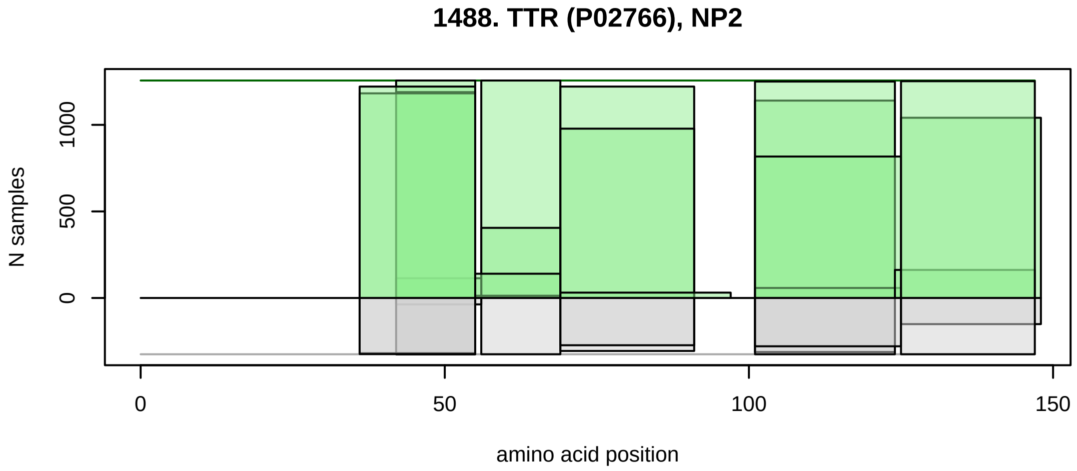
1488. TTR (P02766), NP2
N samples
0
500
1000
0
50
100
150
amino acid position

1489. BAIAP3 (O94812), NP1

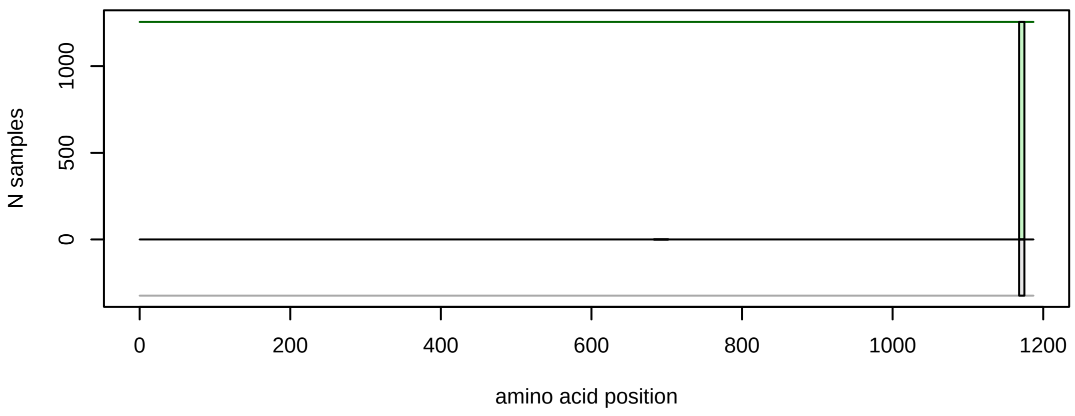

1490. YOD1 (Q5VVQ6), NP5

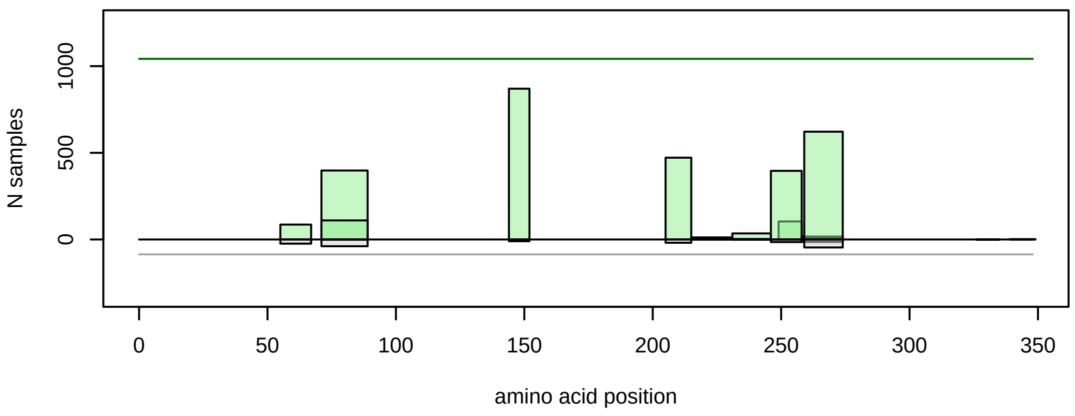

1491. SYCN (Q0VAF6), NP3

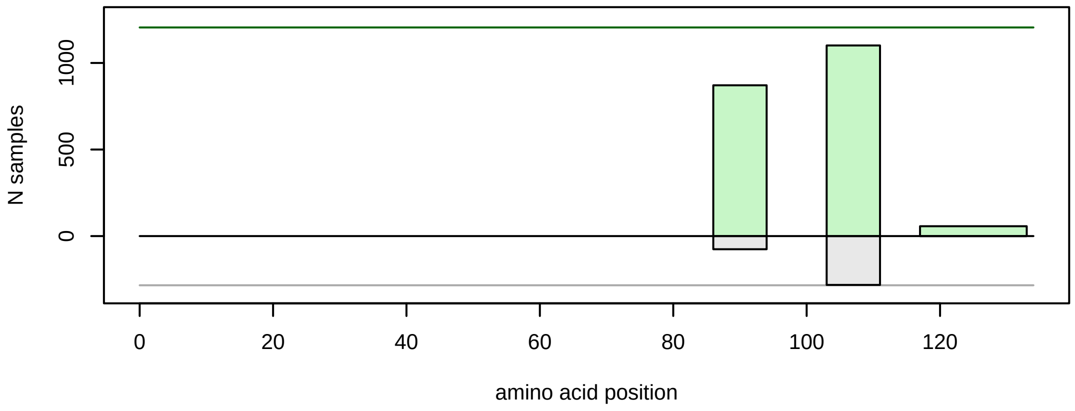

1492. LEFTY2 (O00292−2), NP1

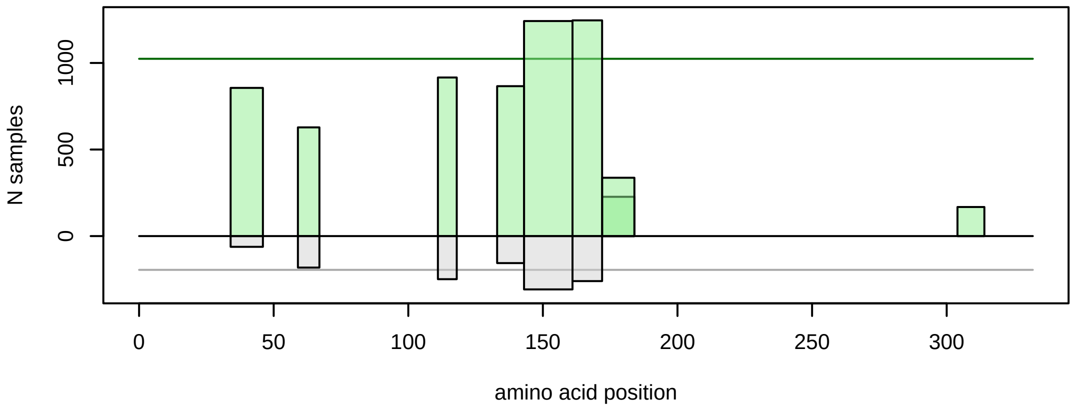

1493. NUDCD2 (Q8WVJ2), NP2

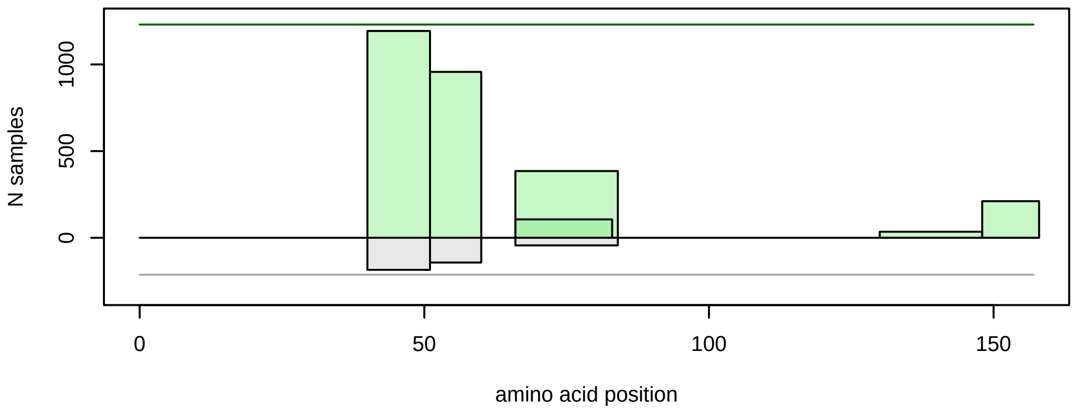

1494. COPS2 (P61201;P61201−2), NP2

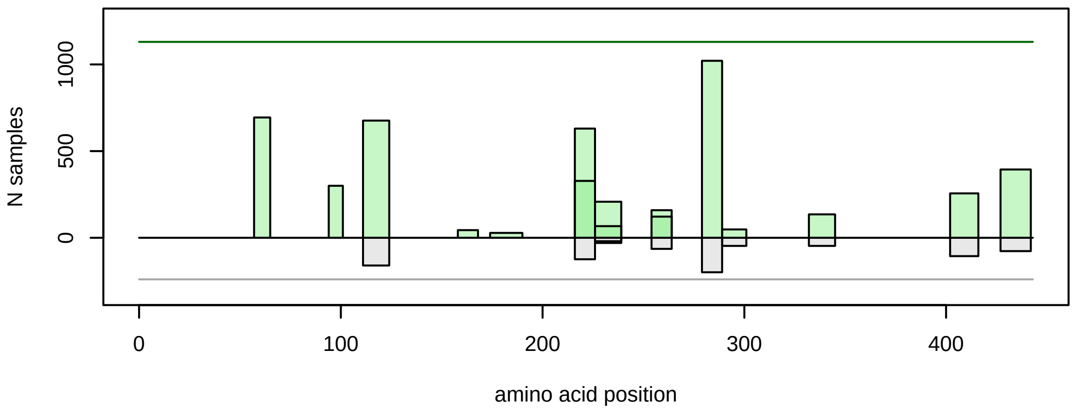

1495. PLCG2 (P16885), NP4

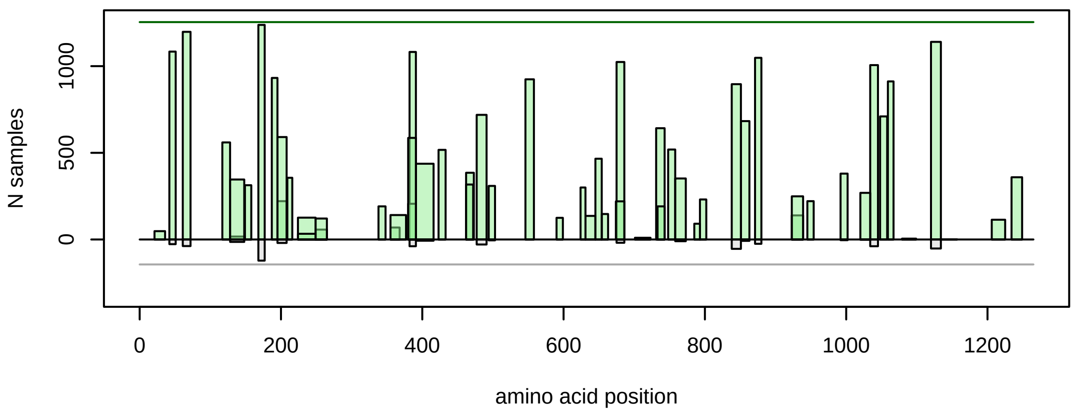

1496. PTPRA (P18433;P18433−6), NP1

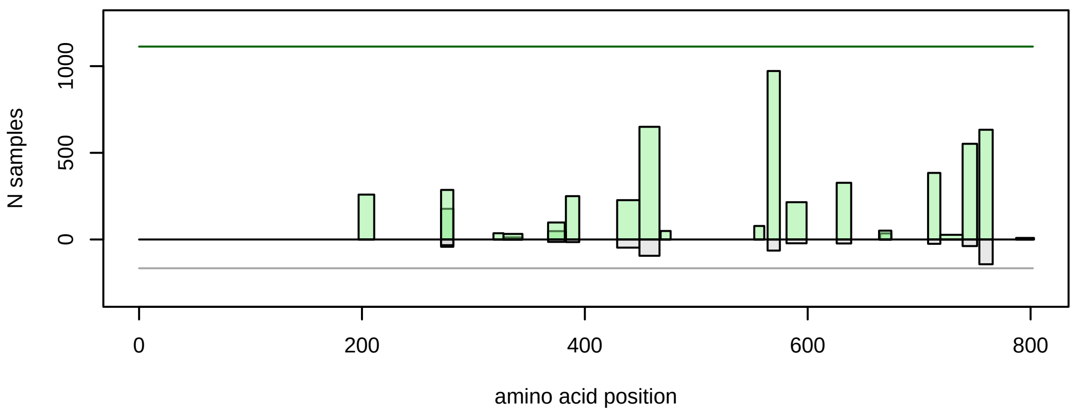

1497. CAST (A0A6Q8PH20), NP4

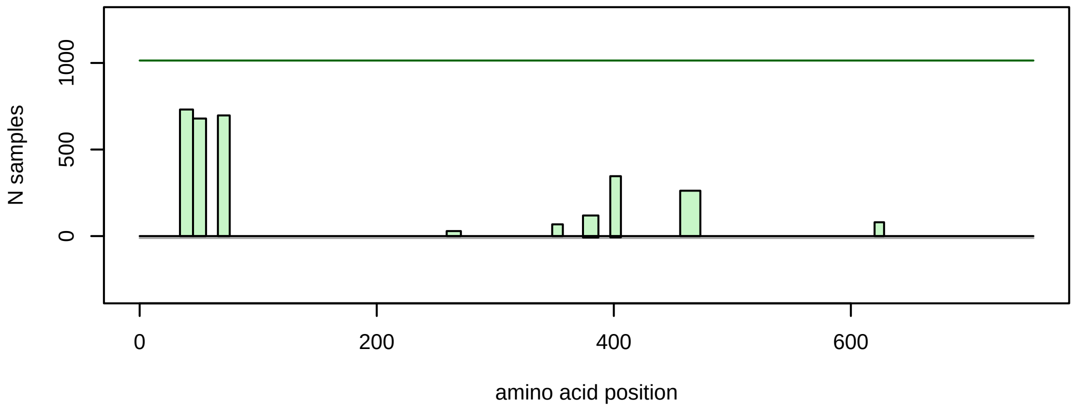

1498. IGHA1 (P01876), NP4

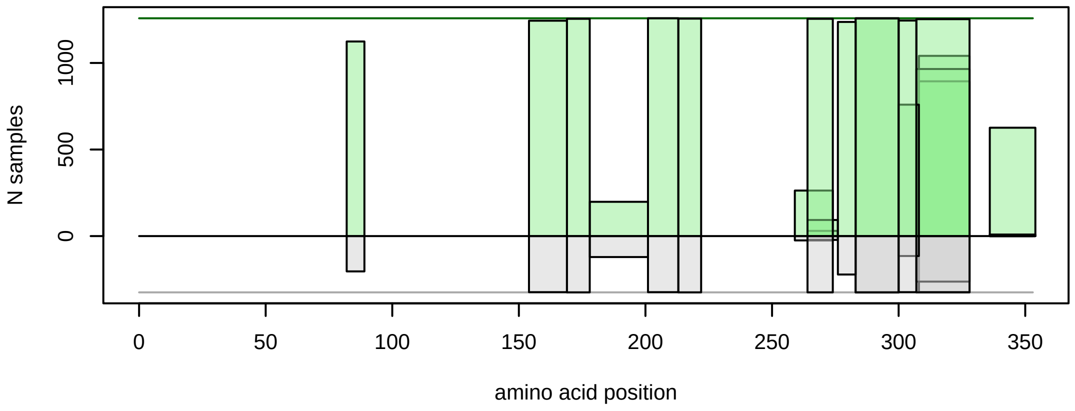

1499. PTPRC (P08575), NP1

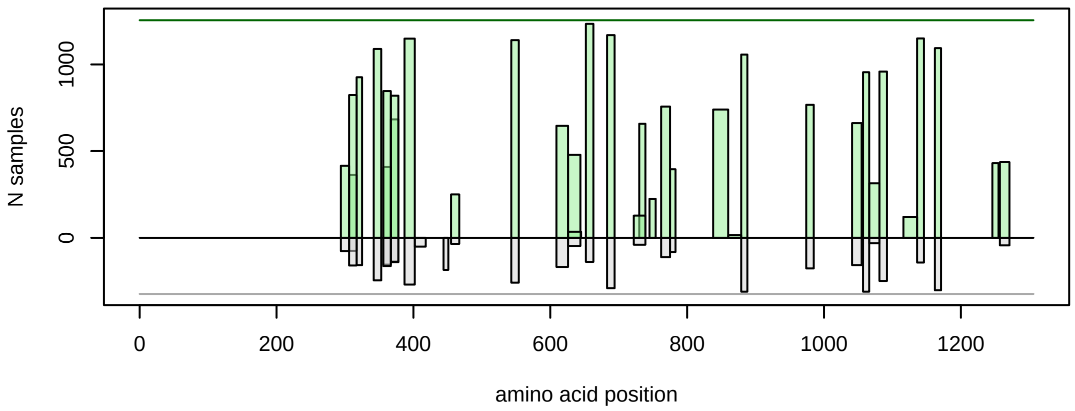

1500. IGHM (P01871), NP4

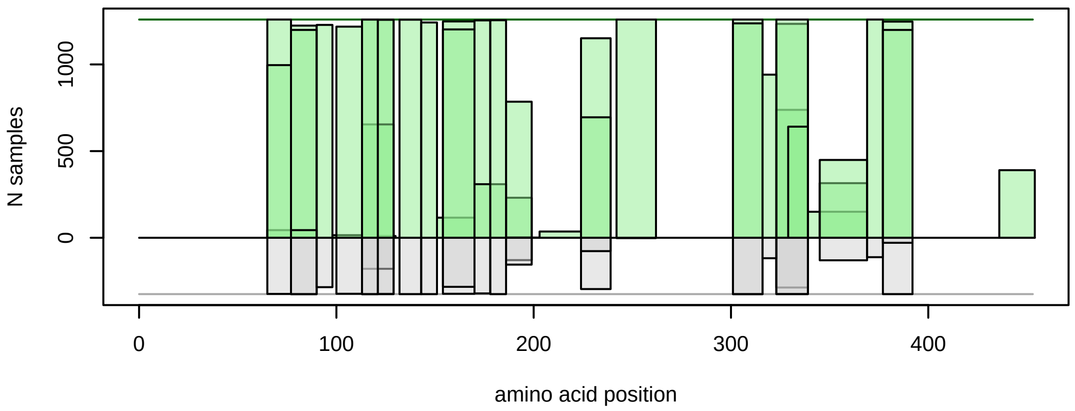

1501. PKDCC (Q504Y2), NP1

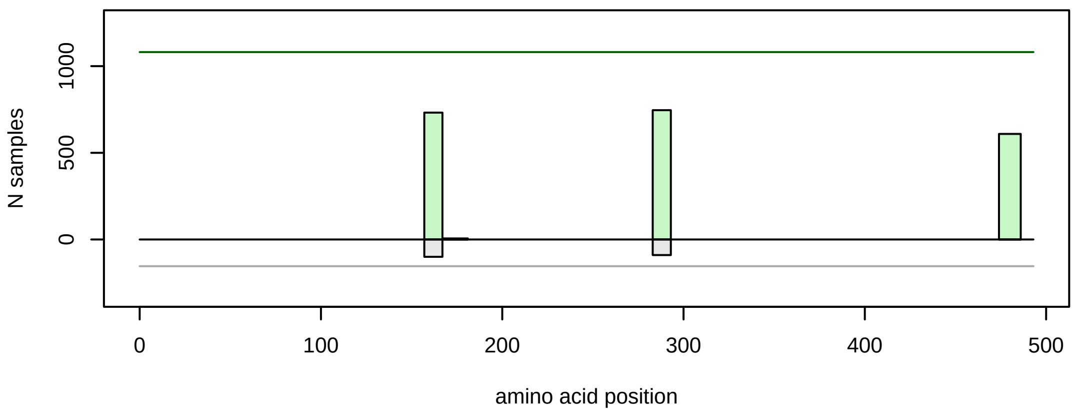

1502. STXBP2 (Q15833−2), NP1

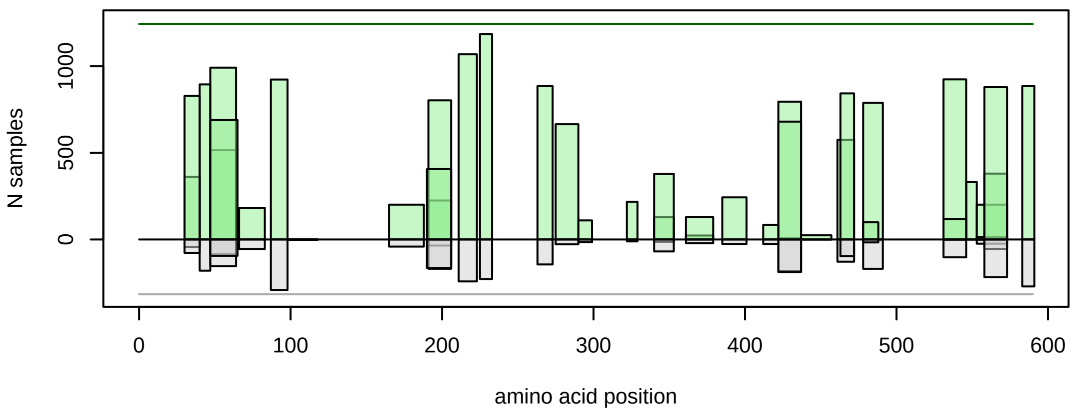

1503. FUBP1 (E9PEB5;Q96AE4;Q96AE4−2), NP1

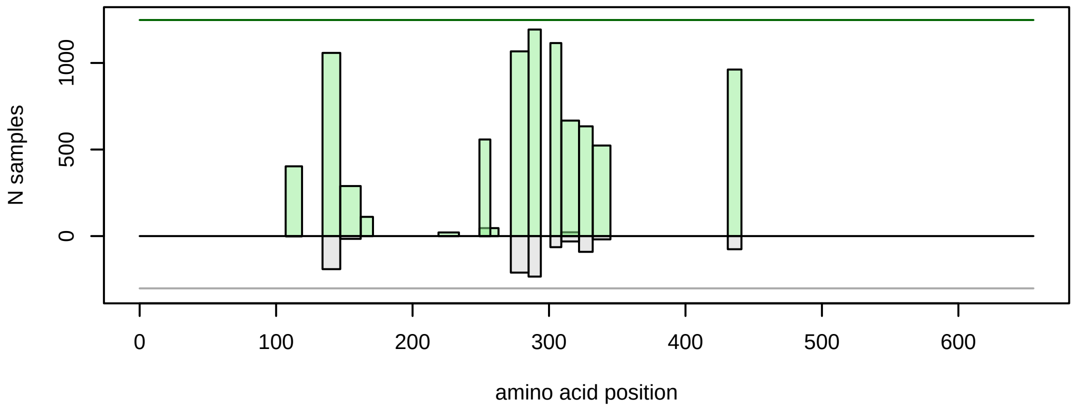

1504. EXT1 (Q16394), NP3

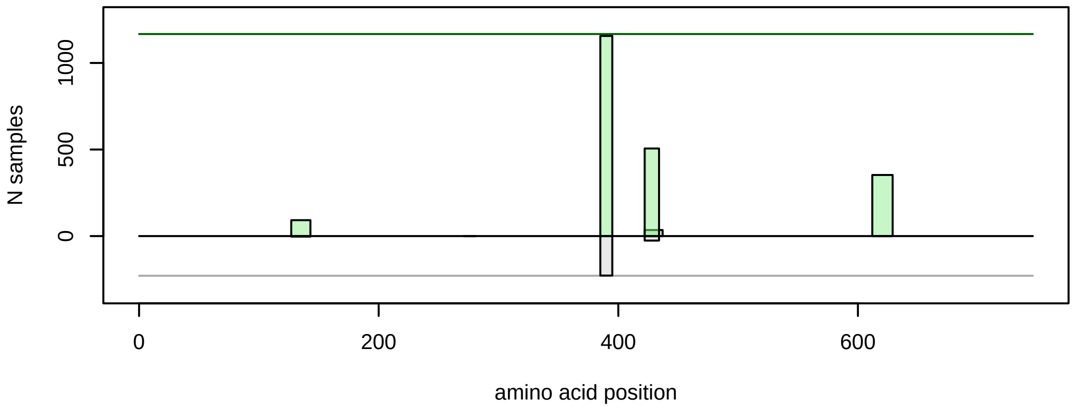

1505. TMOD1 (P28289), NP1

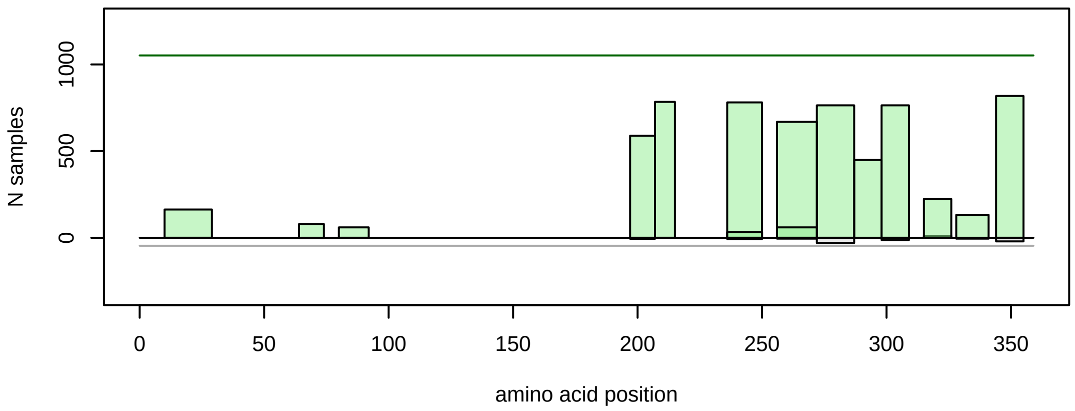

1506. IGLV3−10 (A0A075B6K4), NP3

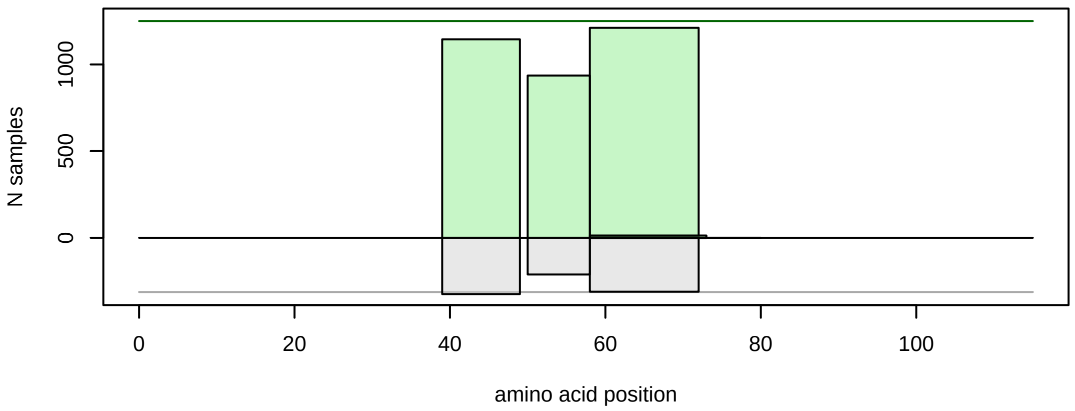

1507. ANGPTL3 (Q9Y5C1), NP4

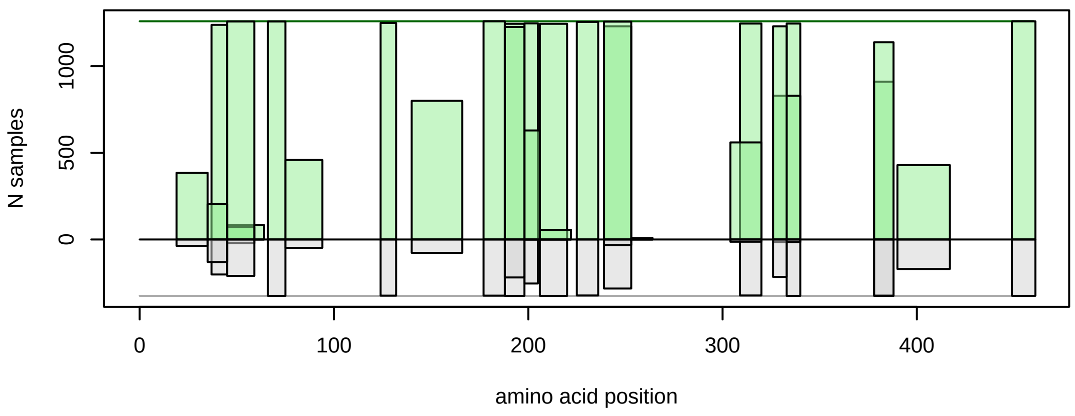

1508. IGHV3−64 (A0A075B6Q5), NP3

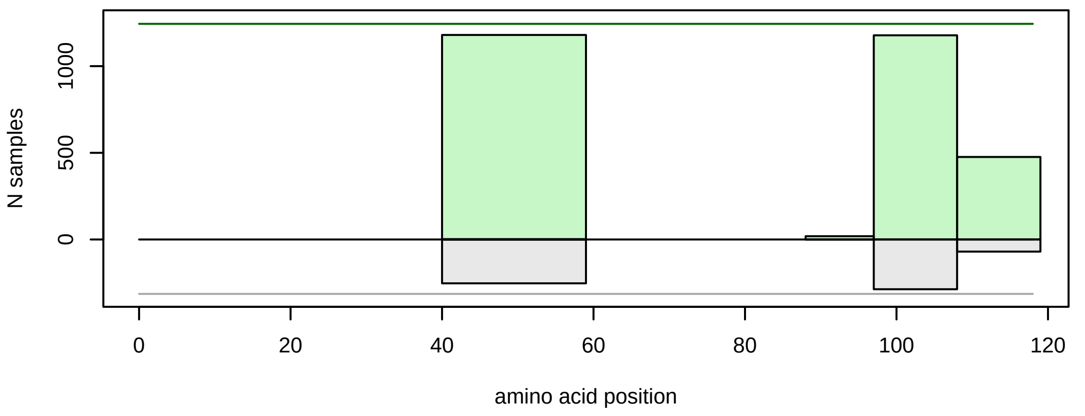

1509. CTSA (A0A7I2V4K5;P10619;X6R5C5;X6R8A1), NP4

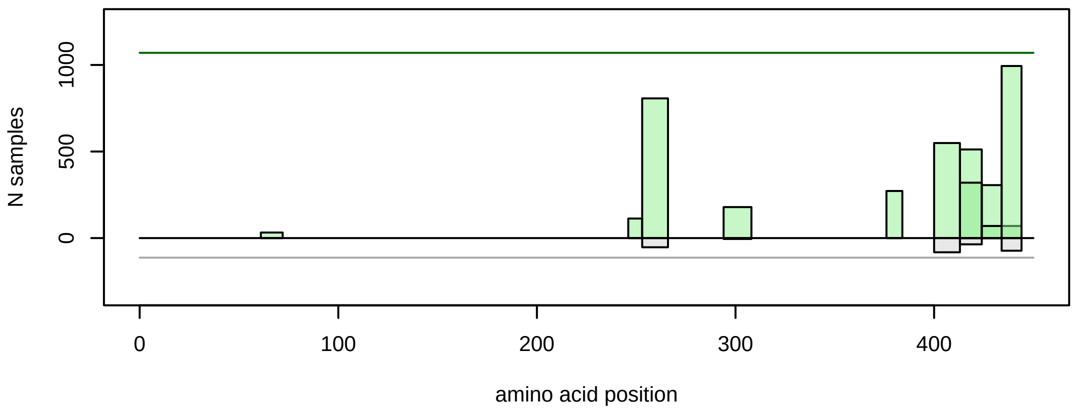

1510. VCP (A0A7P0TAW3;P55072), NP1

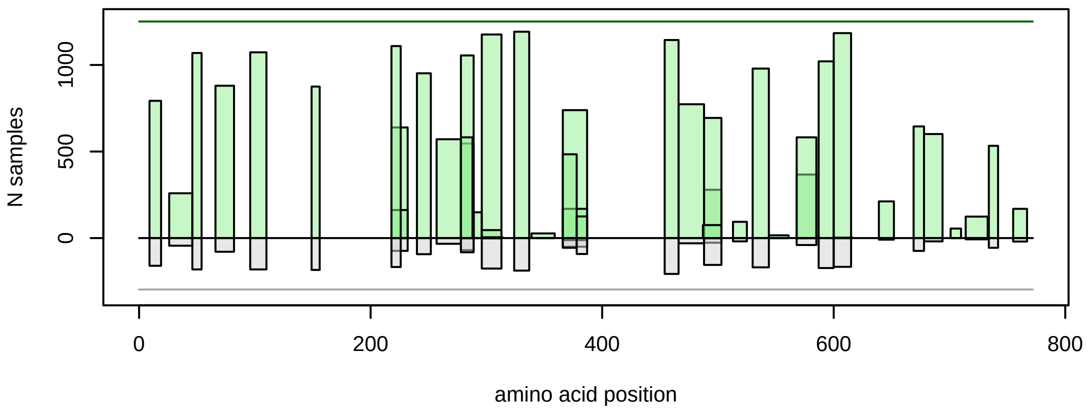

1511. HNRNPA2B1 (P22626), NP3

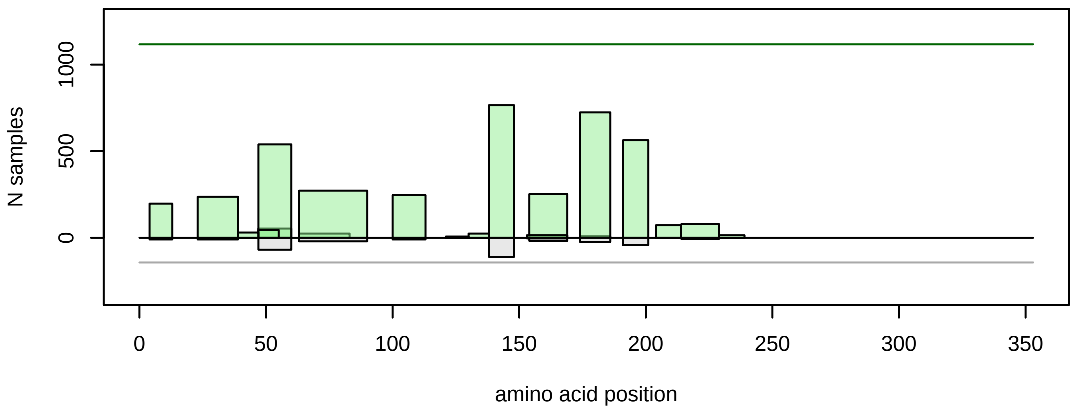

1512. GPNMB (A0A3B3ISS6), NP4

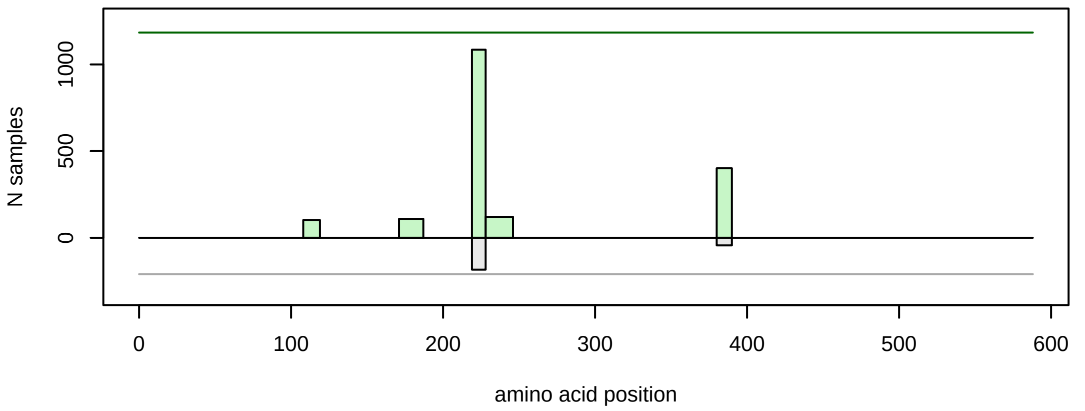

1513. HSPG2 (P98160), NP2

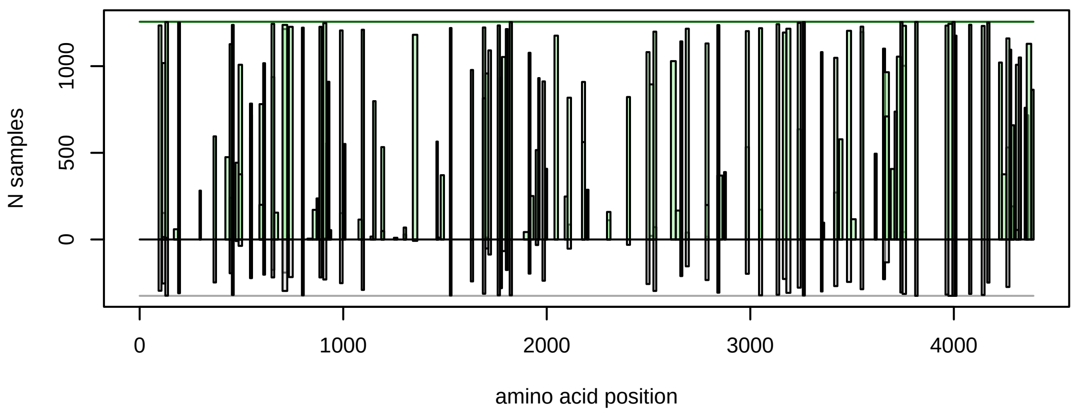

1514. NPY (P01303), NP2

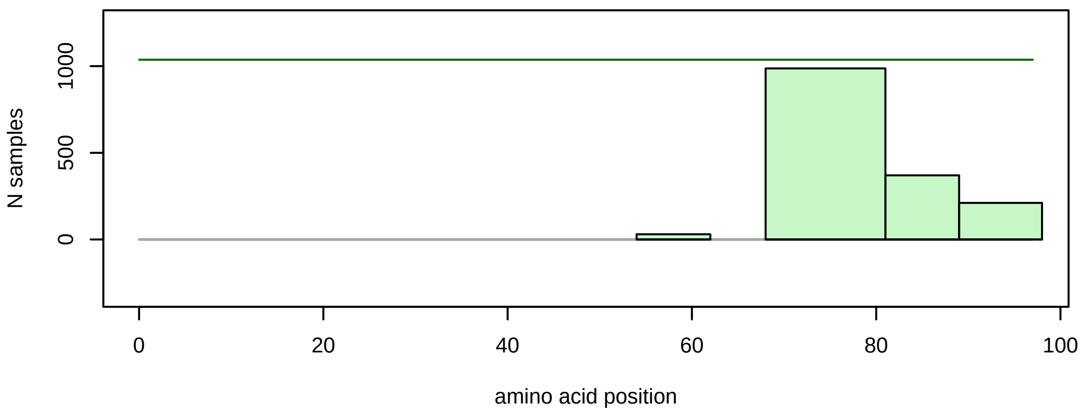

1515. HBB (P68871), NP1

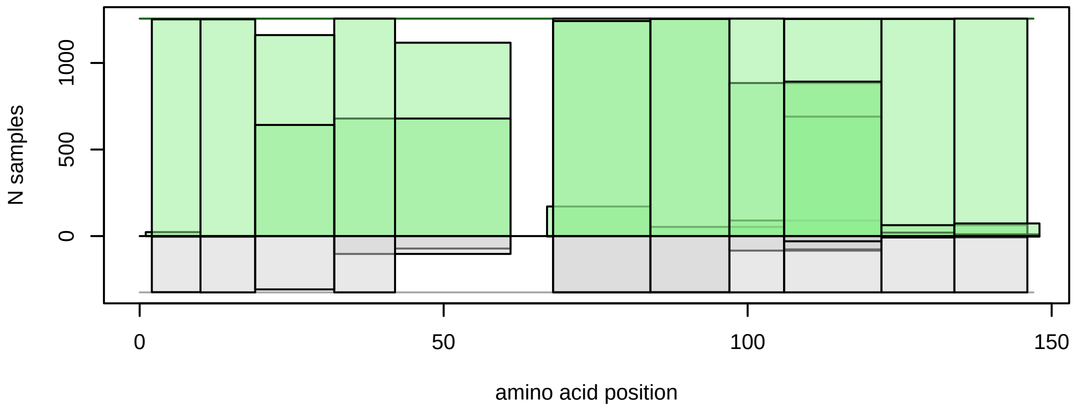

1516. COMP (G3XAP6;P49747), NP2

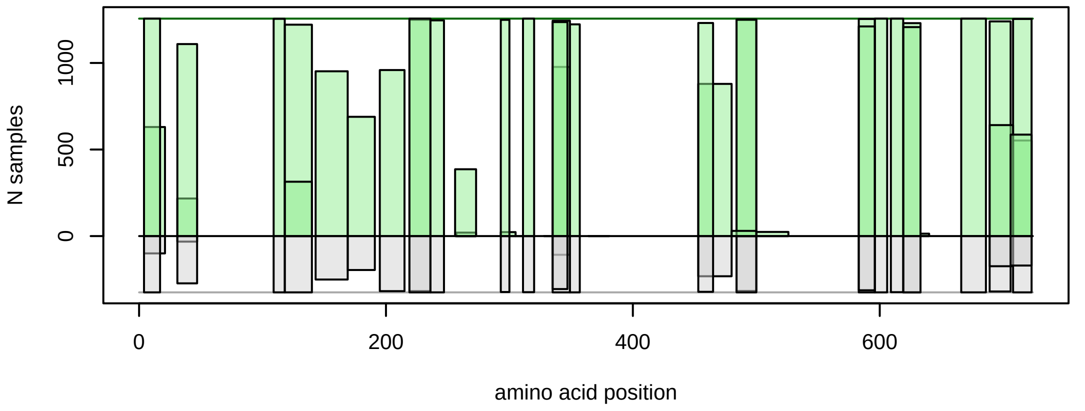

1517. PDXP (Q96GD0), NP5

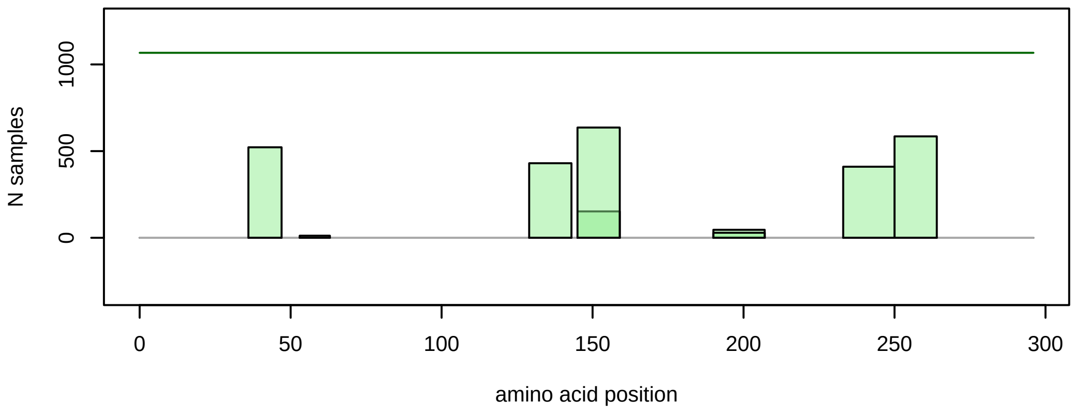

1518. KRT1 (P04264), NP2

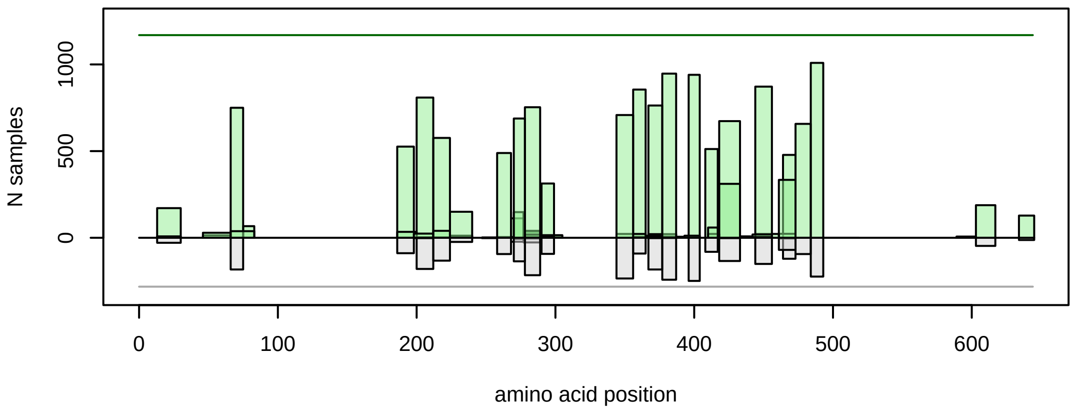

1519. CNBP (P62633;P62633−2), NP1

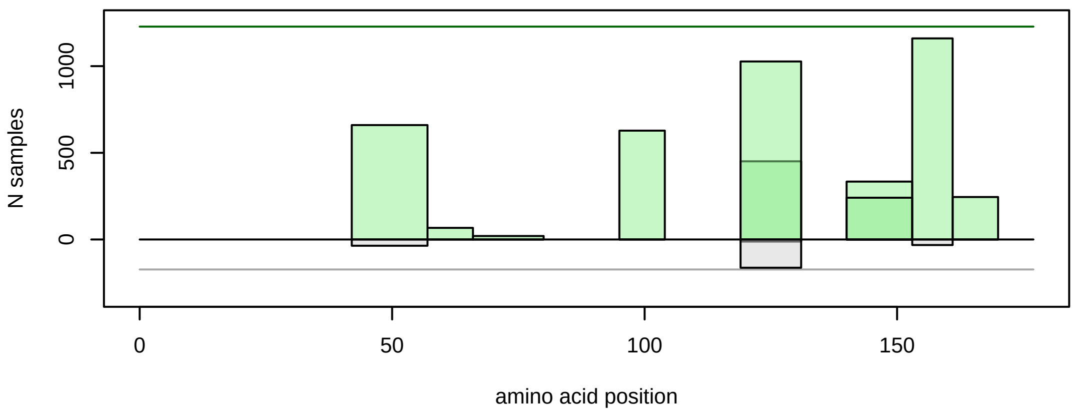

1520. FHOD1 (Q9Y613), NP4

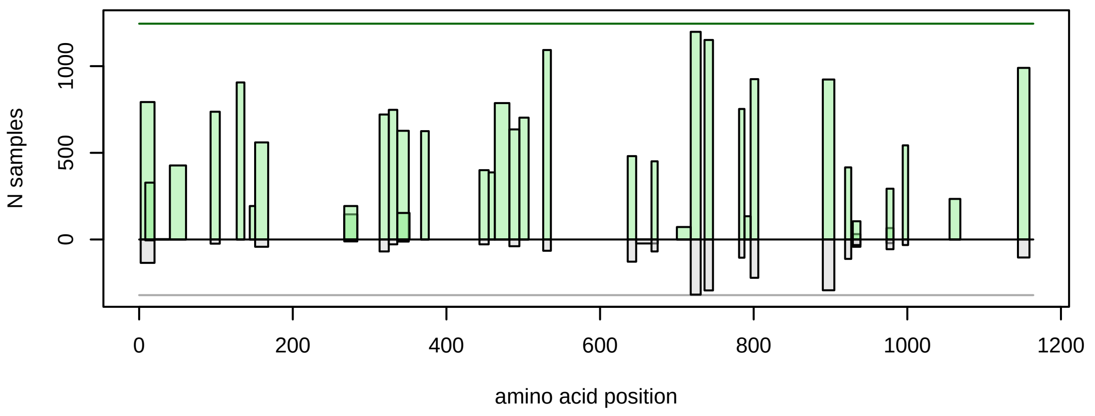

1521. EIF5A (P63241−2), NP2

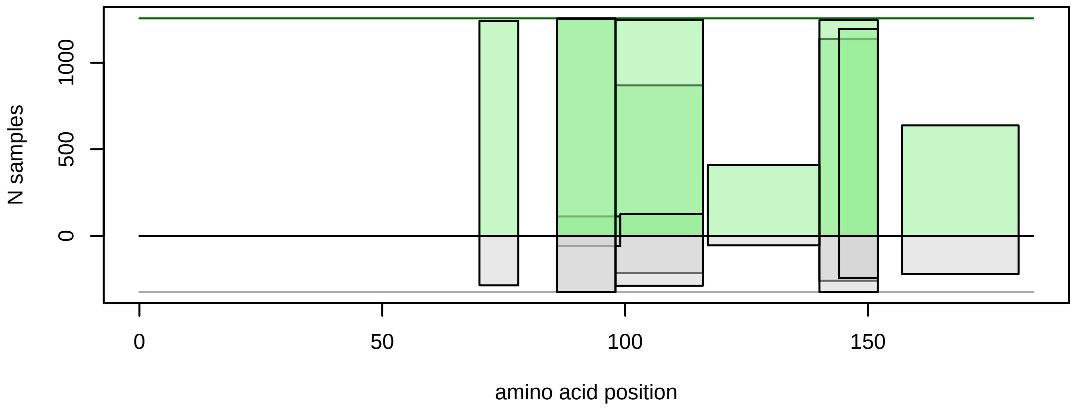

1522. STIP1 (P31948), NP1

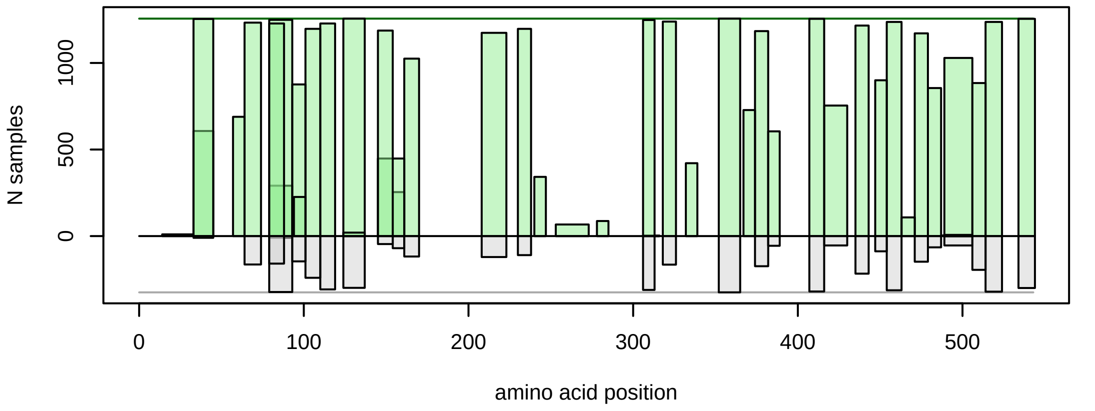

1523. LRBA (A0A494C1L5), NP4

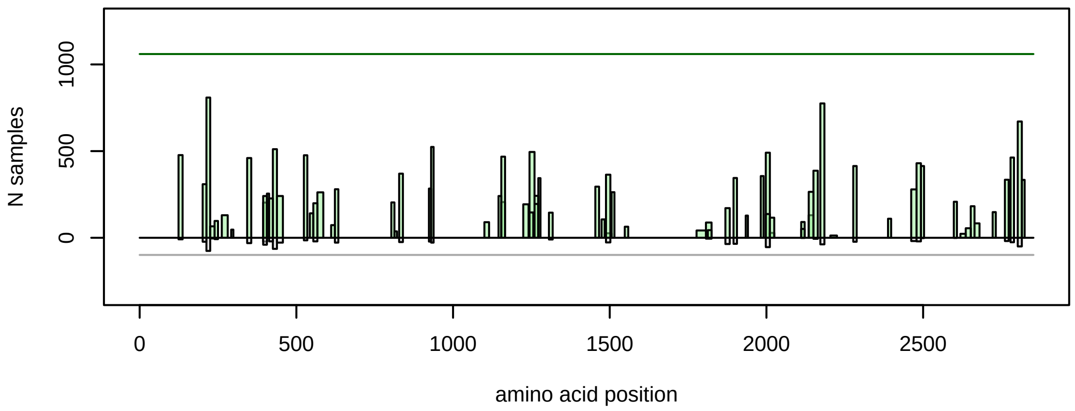

1524. MDH2 (P40926), NP4

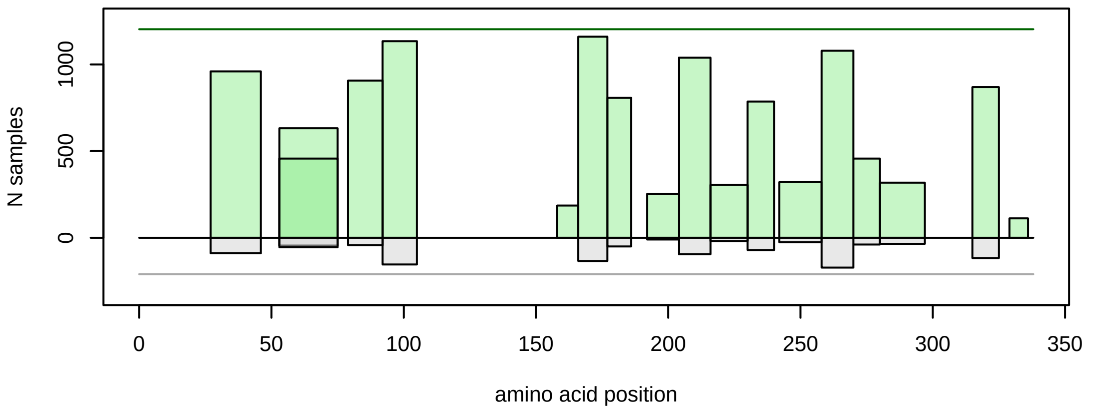

1525. DCTN1 (A0A7P0Z4C3;Q14203;Q14203−6), NP5

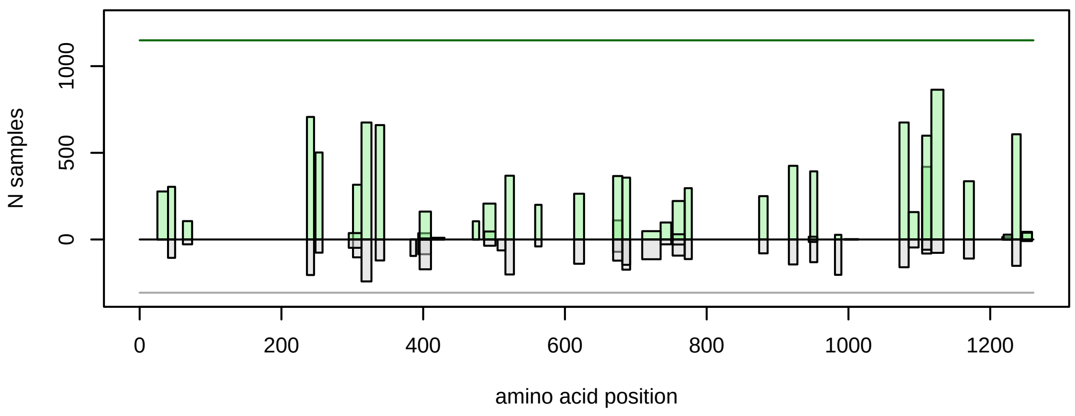

1526. NUCB1 (Q02818), NP5

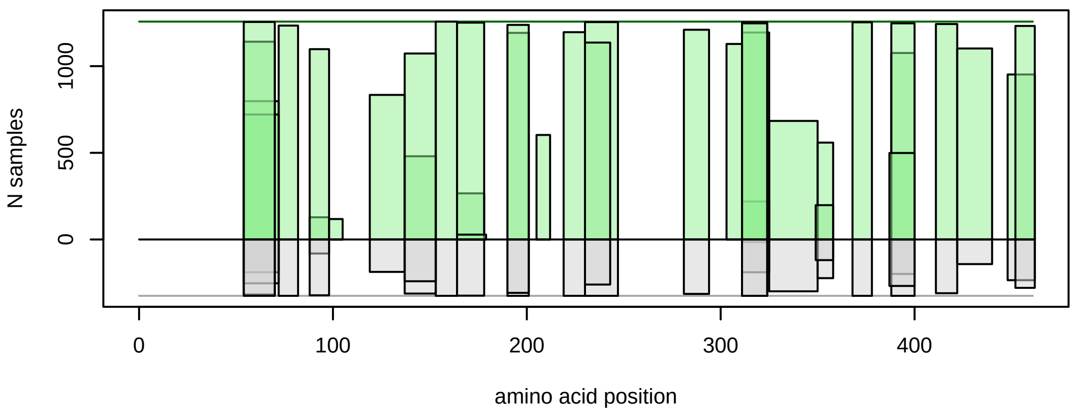

1527. CFP (P27918), NP3

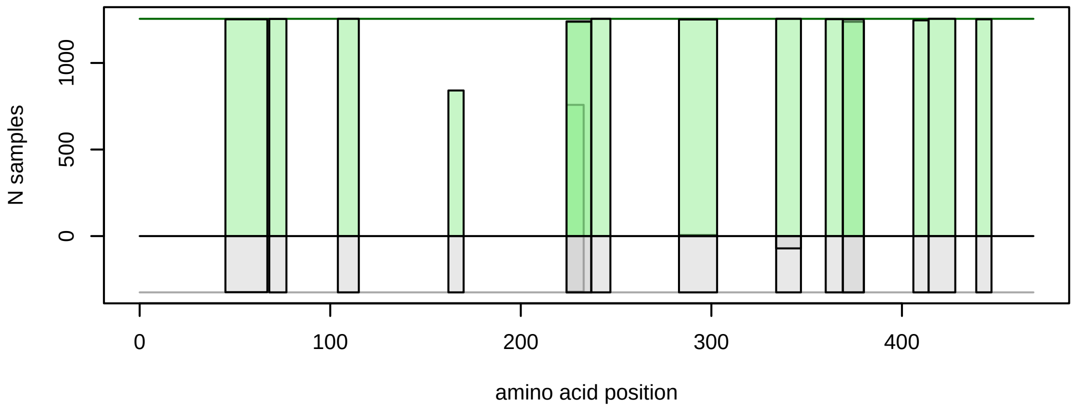

1528. PCDH12 (Q9NPG4), NP5

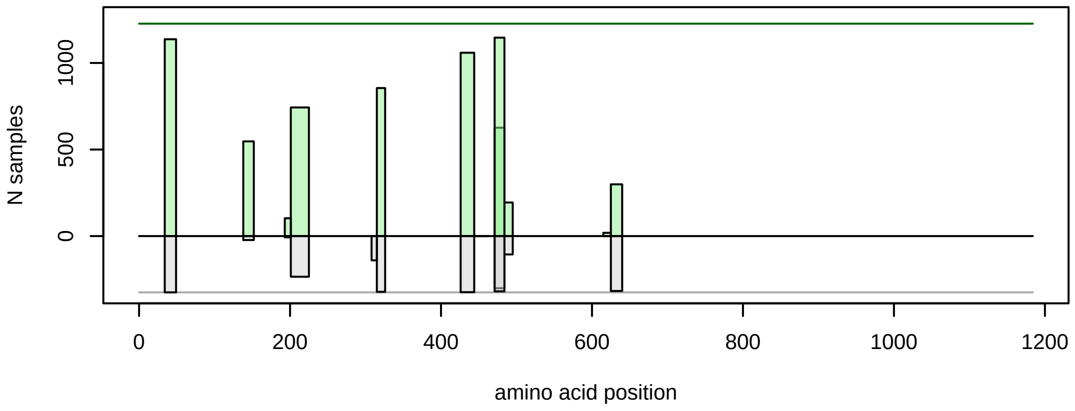

1529. UBE3B (Q7Z3V4), NP2

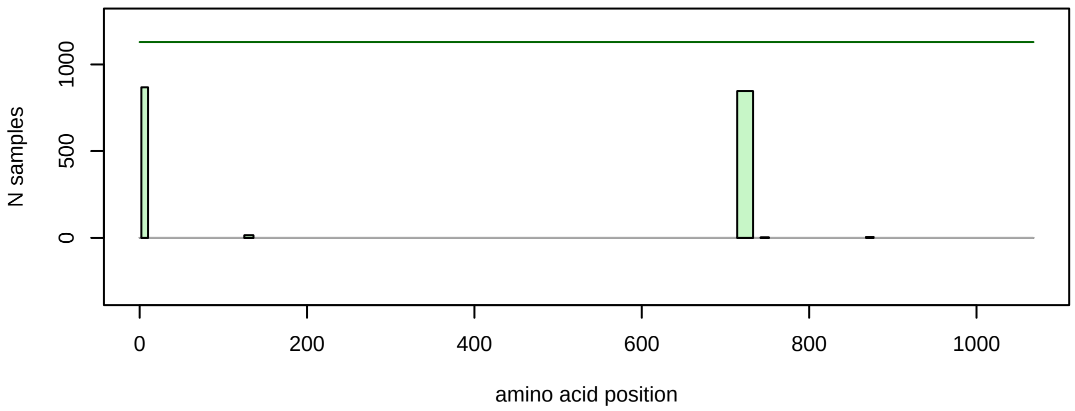

1530. IGKV3D−15 (A0A087WSY6), NP3

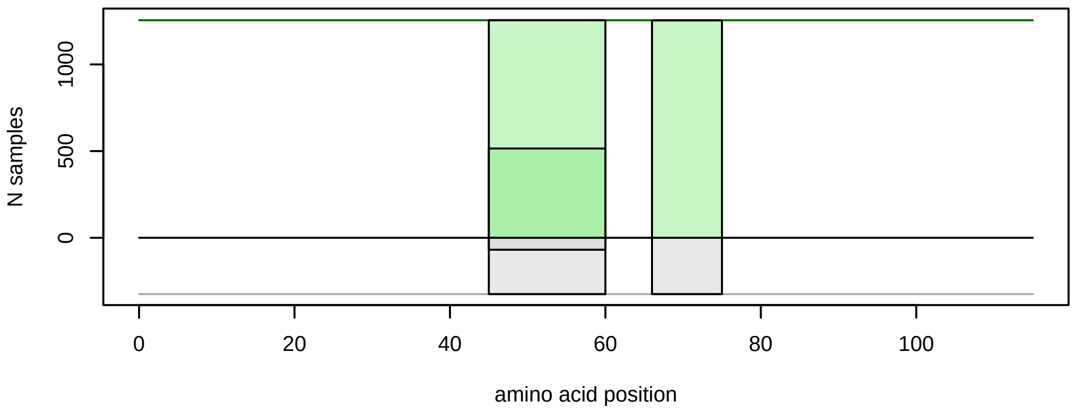

1531. MTPN (P58546), NP2

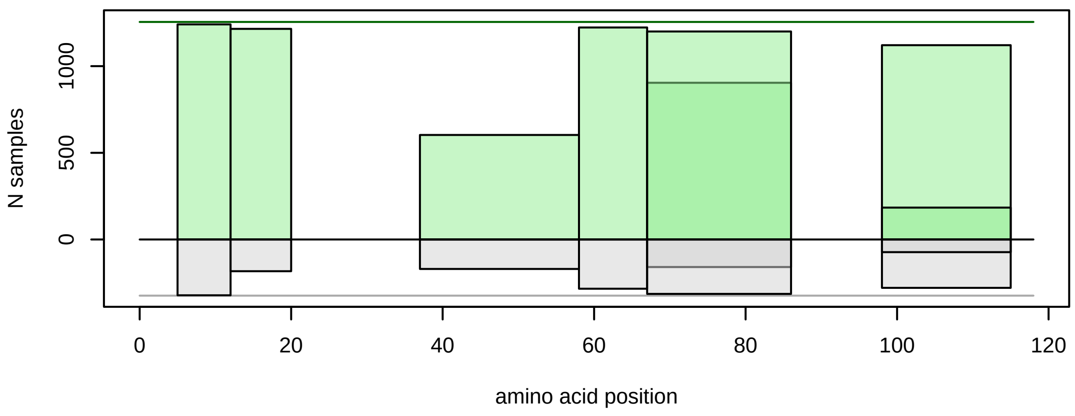

1532. PPP1R12A (O14974), NP2

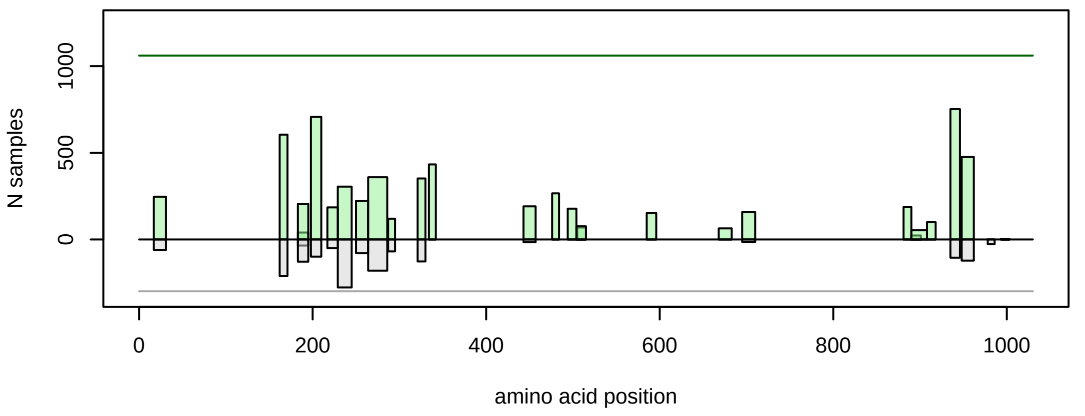

1533. TXN (P10599), NP2

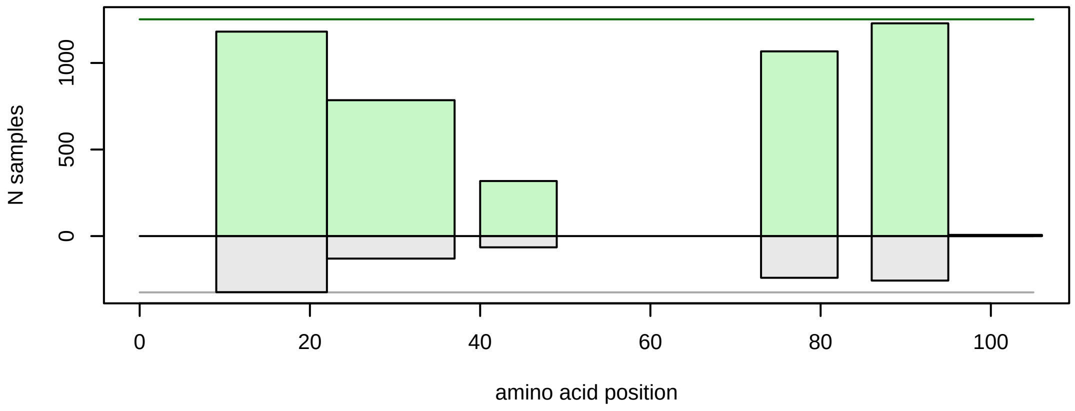

1534. HCLS1 (P14317), NP2

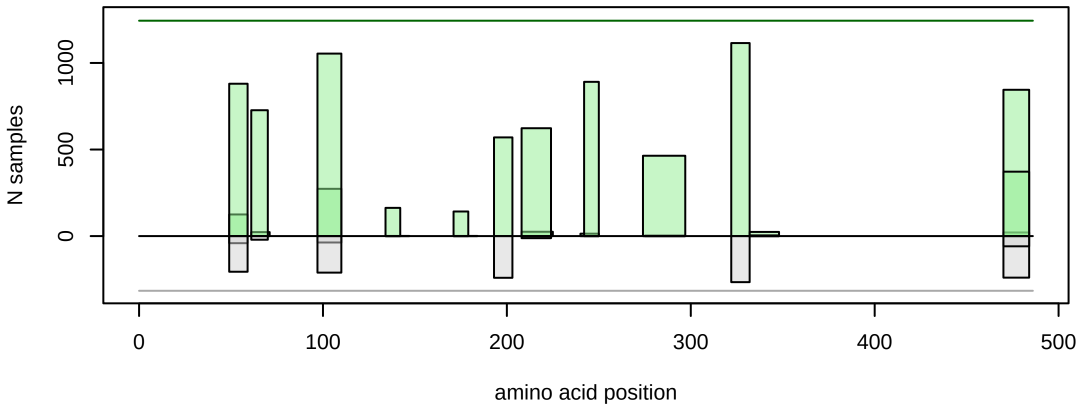

1535. PPP5C (P53041), NP3

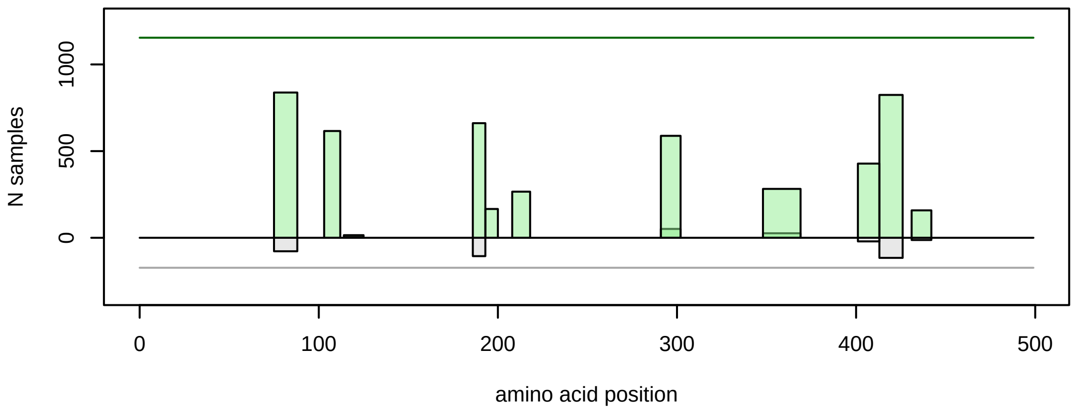

1536. RPS7 (P62081), NP1

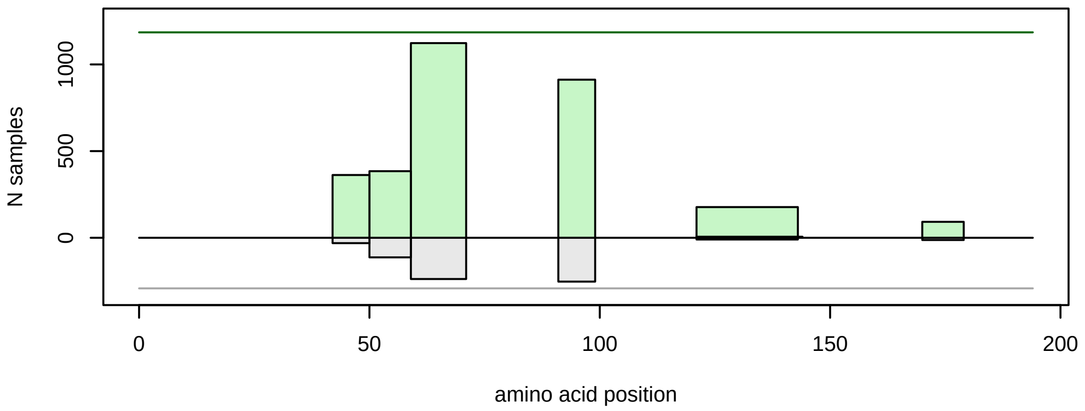

1537. RAB14 (P61106), NP1

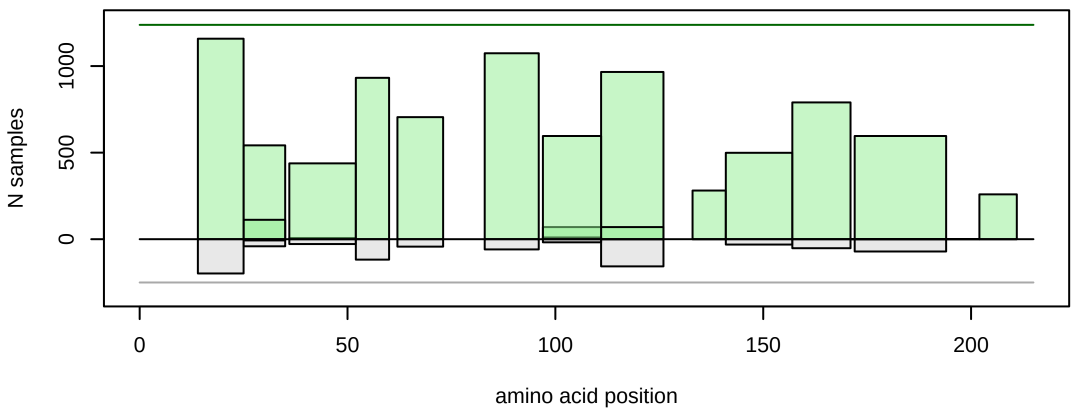

1538. DNM2 (P50570), NP1

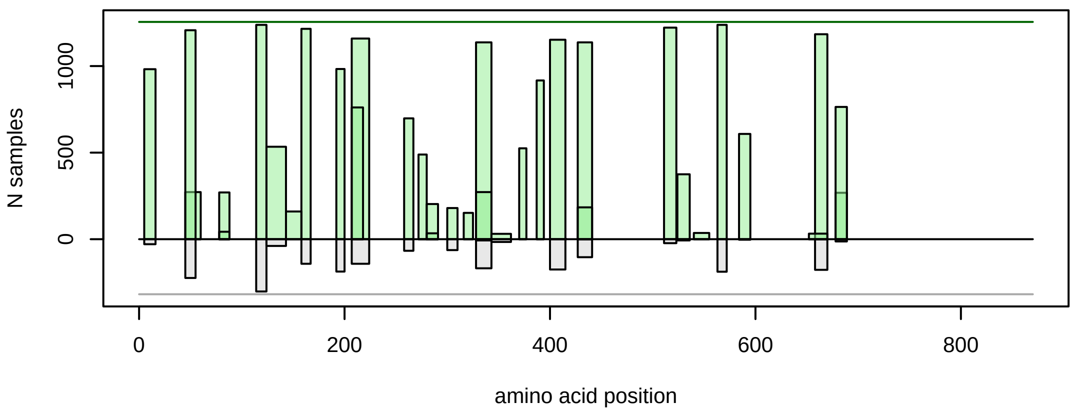

1539. NAMPT (A0A7P0T9I9;P43490), NP3

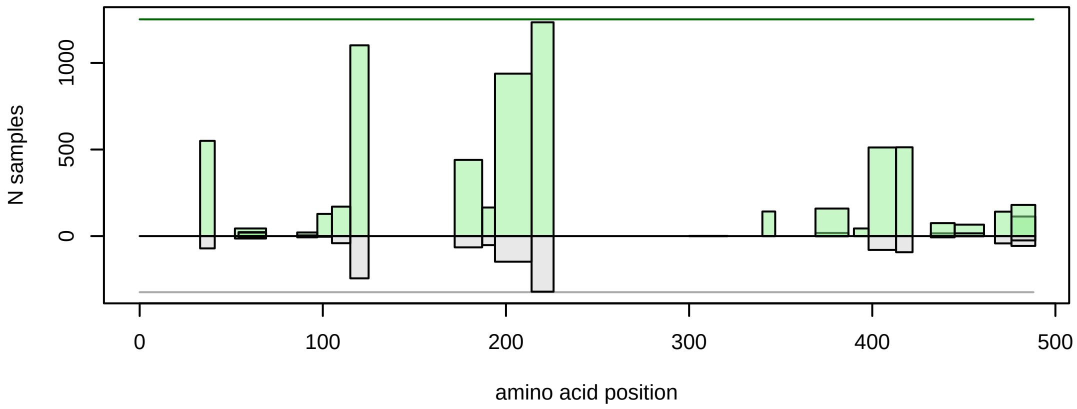

1540. PI16 (Q6UXB8), NP2

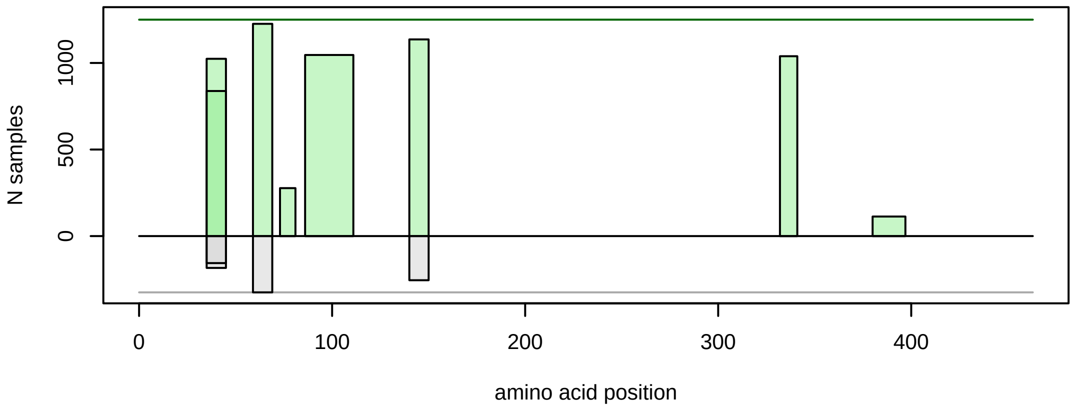

1541. FLOT1 (O75955), NP1

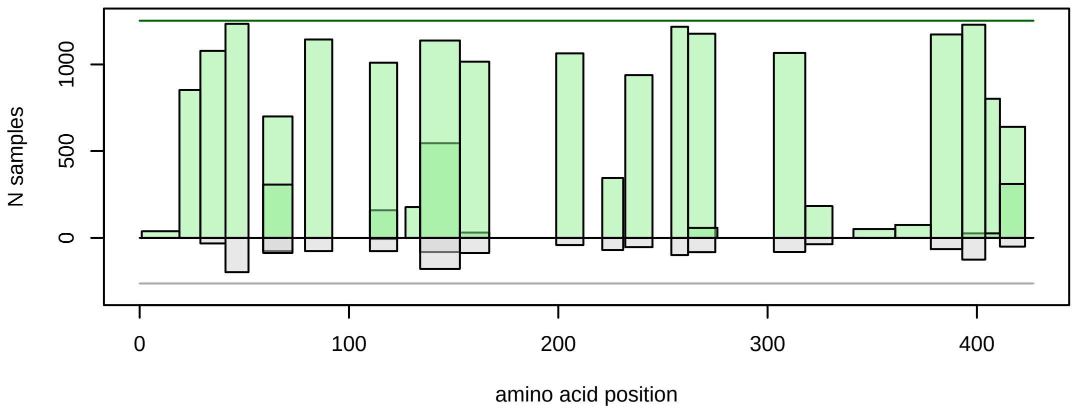

1542. ELN (E7EN65), NP3

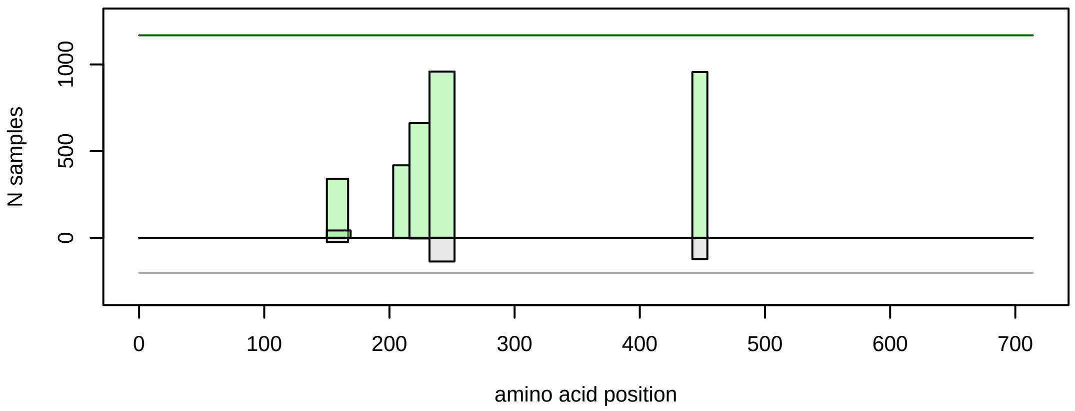

1543. ACTN3 (Q08043), NP2

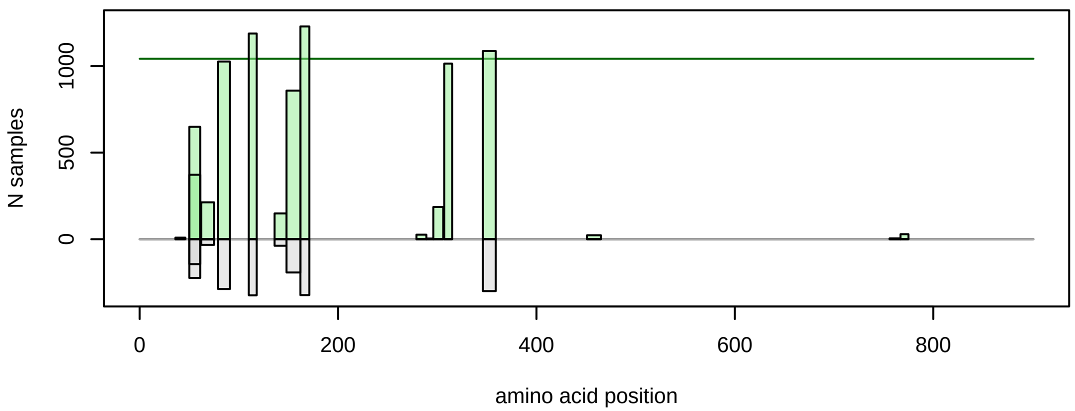

1544. NIF3L1 (Q9GZT8), NP5

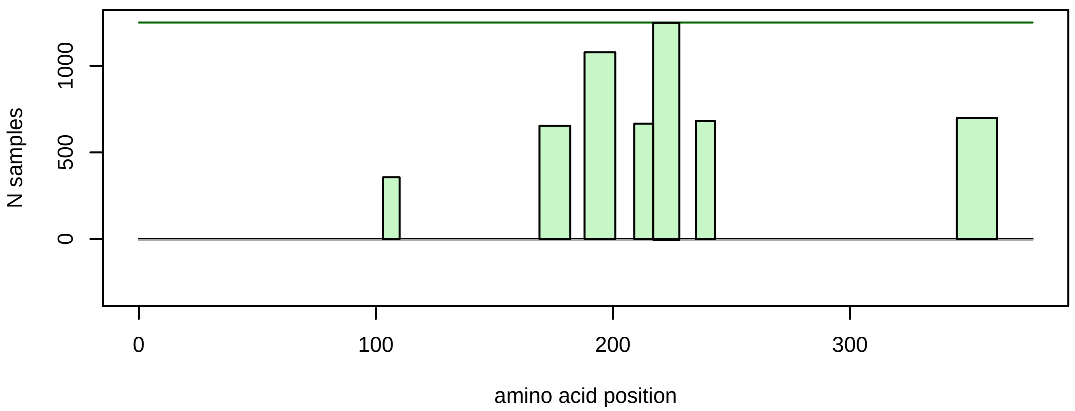

1545. HSPA6 (P17066), NP4

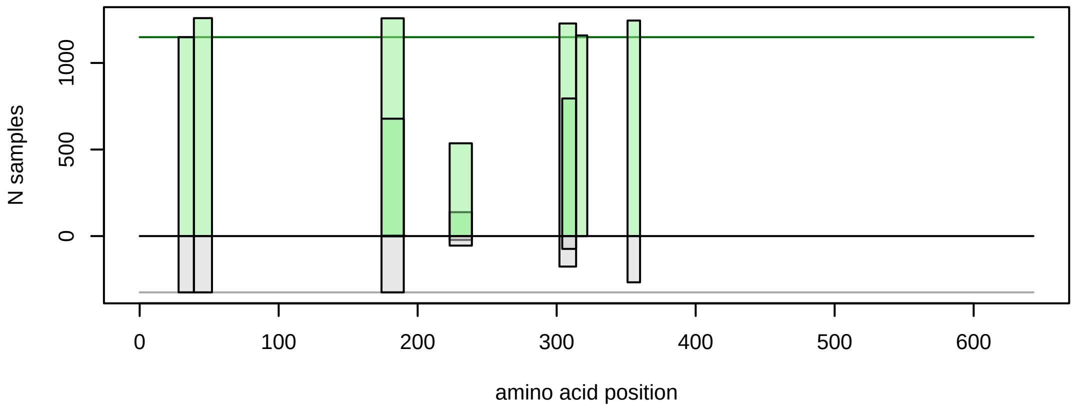

1546. ARHGDIA (P52565), NP2

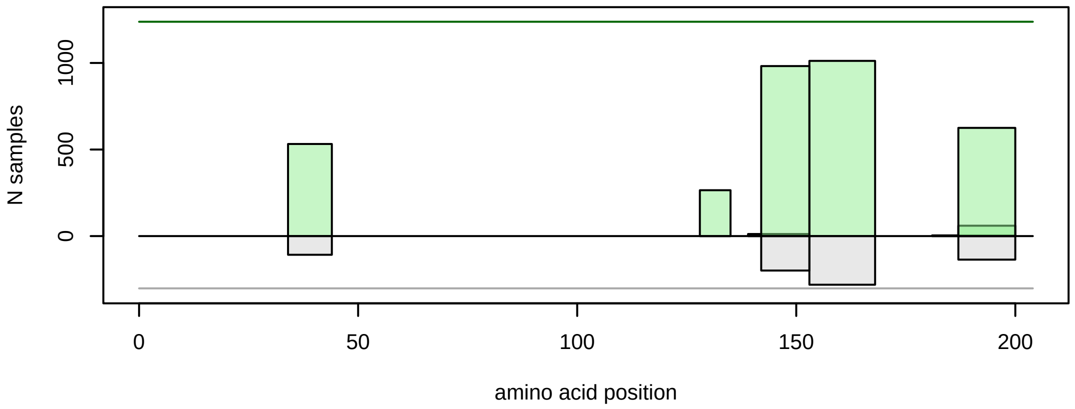

1547. KLKB1 (E9PBC5), NP3

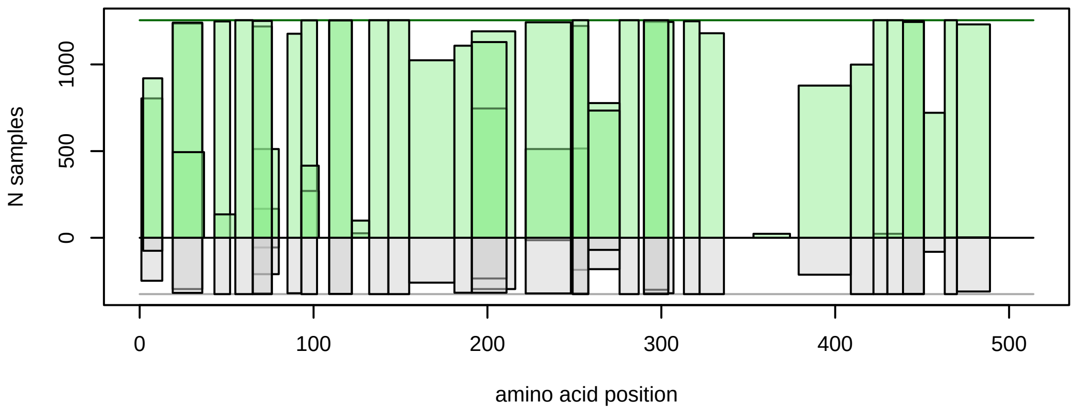

1548. ASAP2 (O43150), NP1

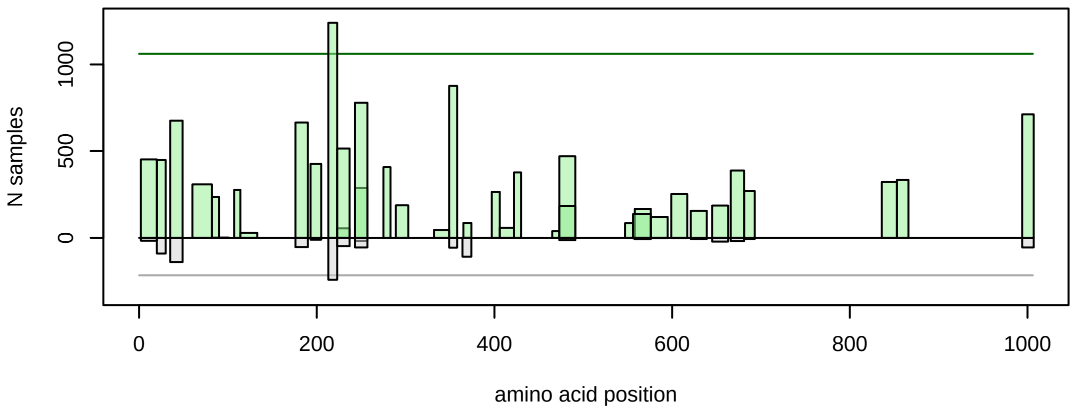

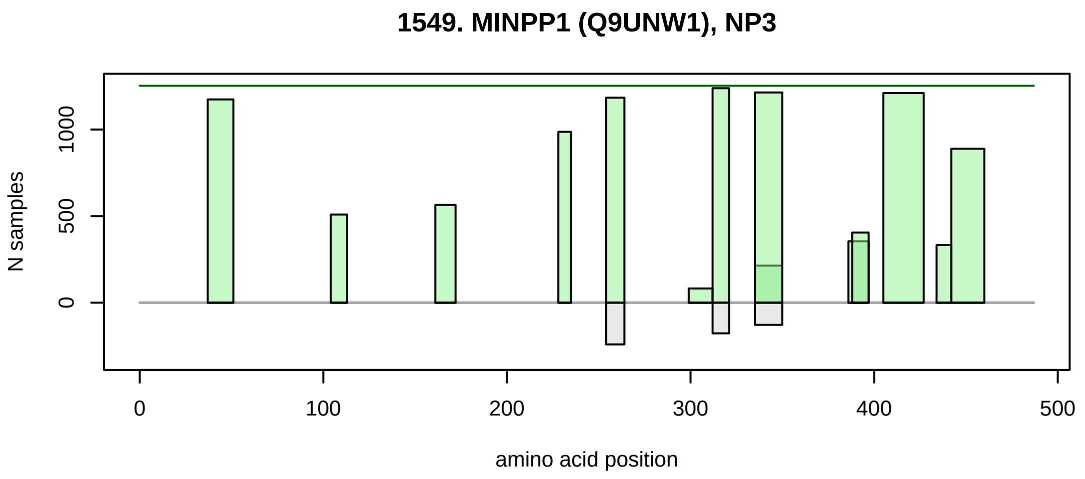
1549. MINPP1 (Q9UNW1), NP3
N samples
amino acid position
0
500
1000
0
100
200
300
400
500

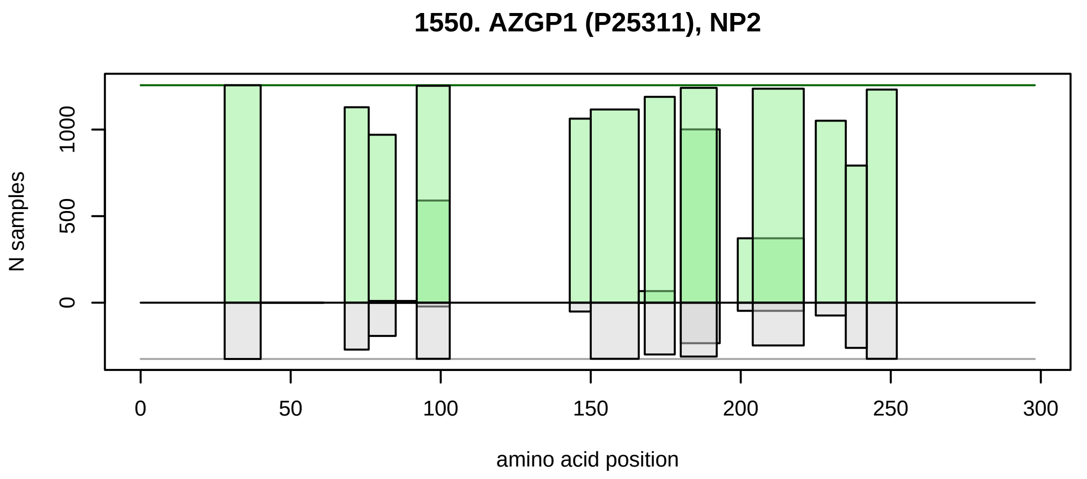
1550. AZGP1 (P25311), NP2
N samples
amino acid position
0
500
1000
0
50
100
150
200
250
300

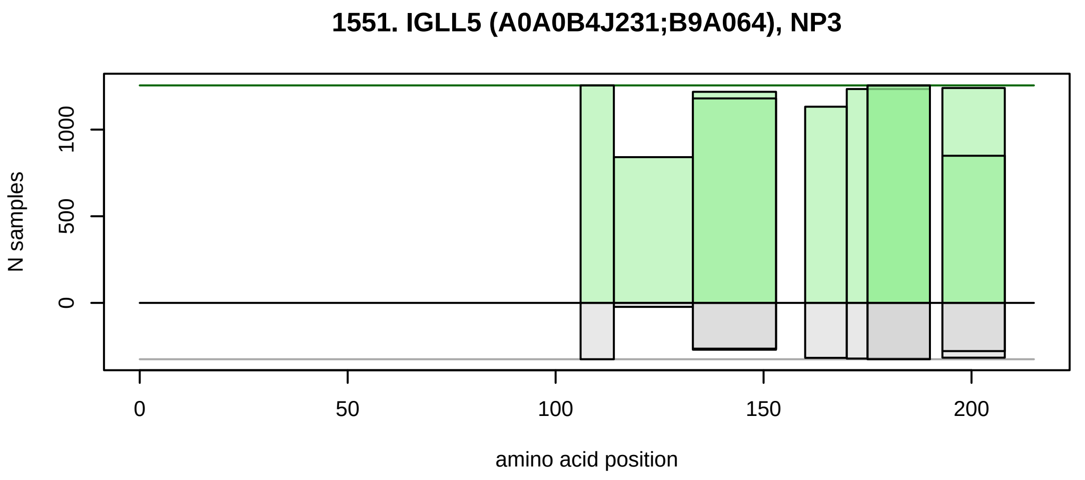
1551. IGLL5 (A0A0B4J231;B9A064), NP3
N samples
amino acid position
0
500
1000
0
50
100
150
200

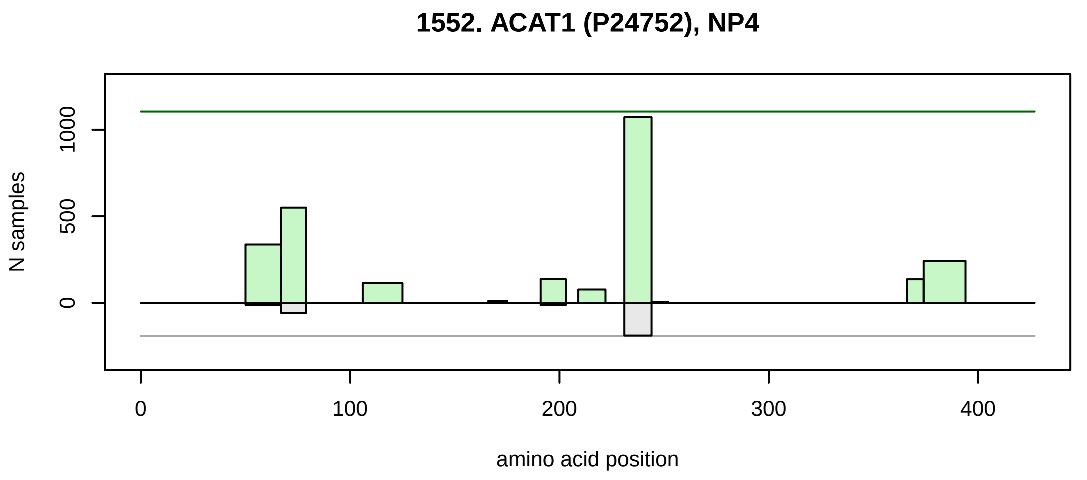
1552. ACAT1 (P24752), NP4
N samples
amino acid position
0
500
1000
0
100
200
300
400

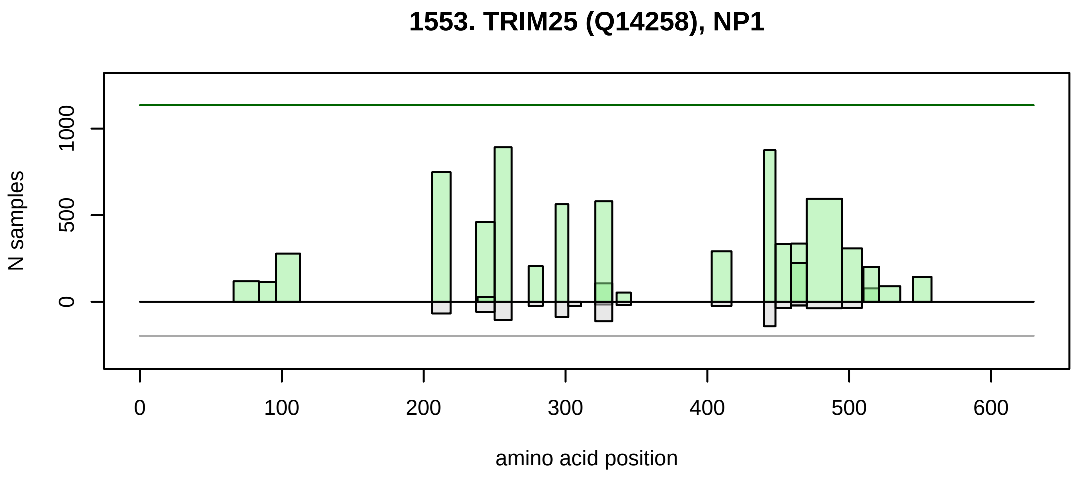
1553. TRIM25 (Q14258), NP1
N samples
amino acid position
0
500
1000
0
100
200
300
400
500
600

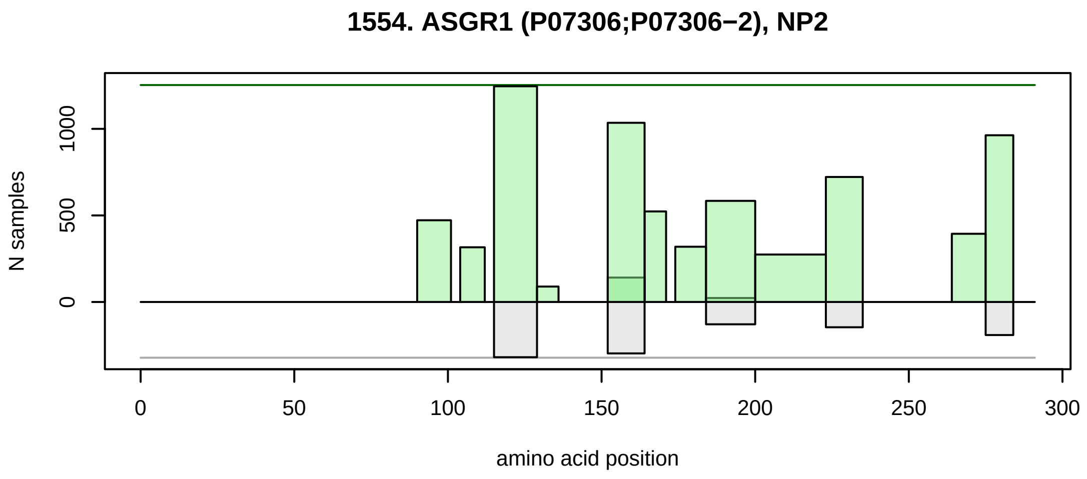
1554. ASGR1 (P07306;P07306−2), NP2
N samples
amino acid position
0
500
1000
0
50
100
150
200
250
300

1555. RAB13 (P51153), NP4

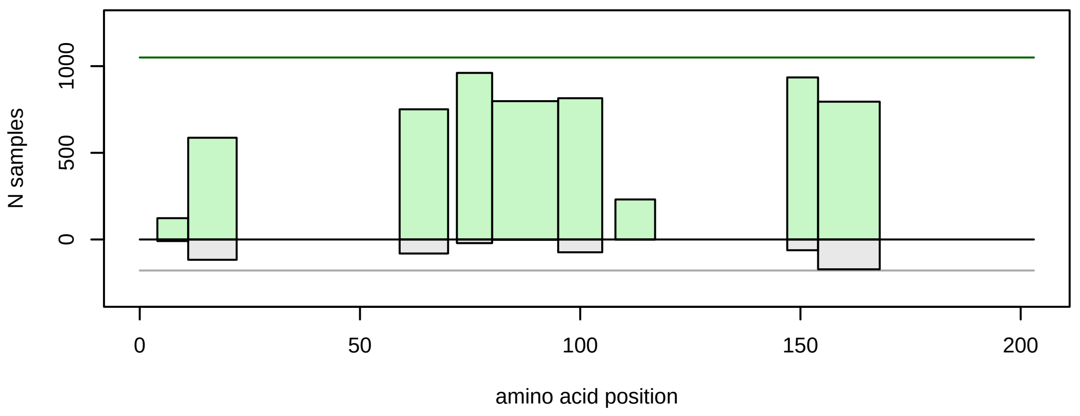

1556. TAOK1 (Q7L7X3), NP4

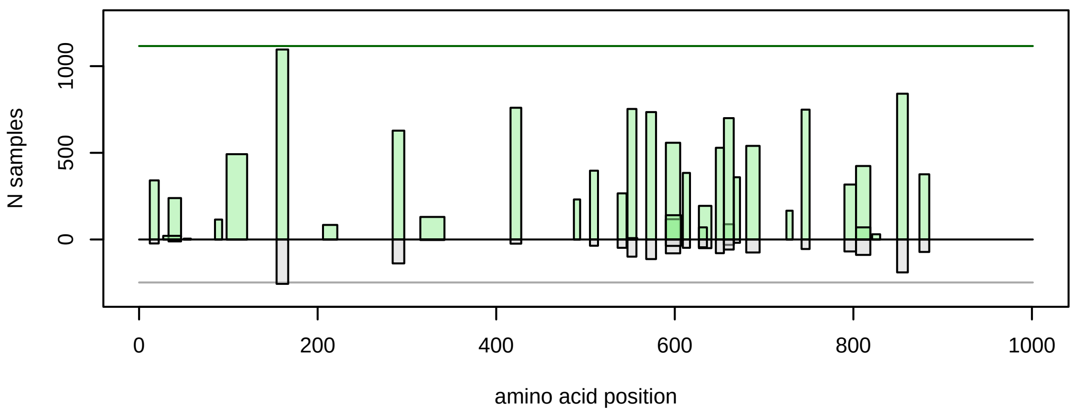

1557. EIF3A (Q14152), NP4

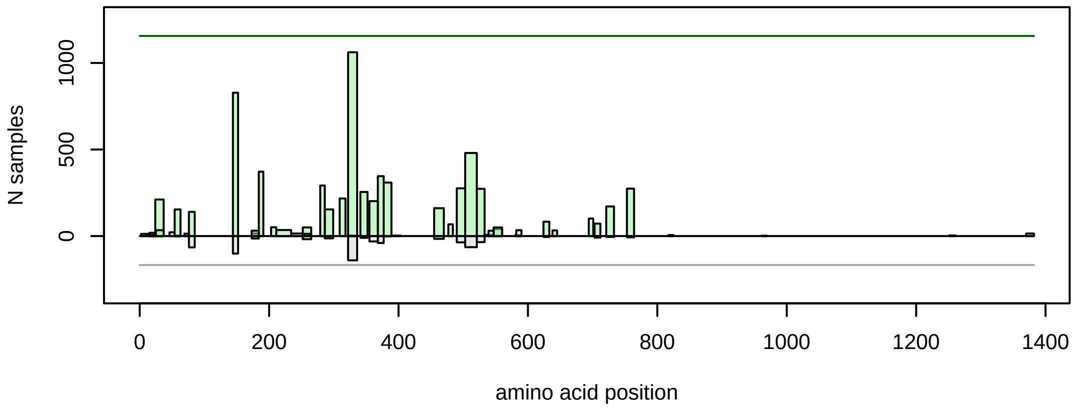

1558. RAB10 (P61026), NP1

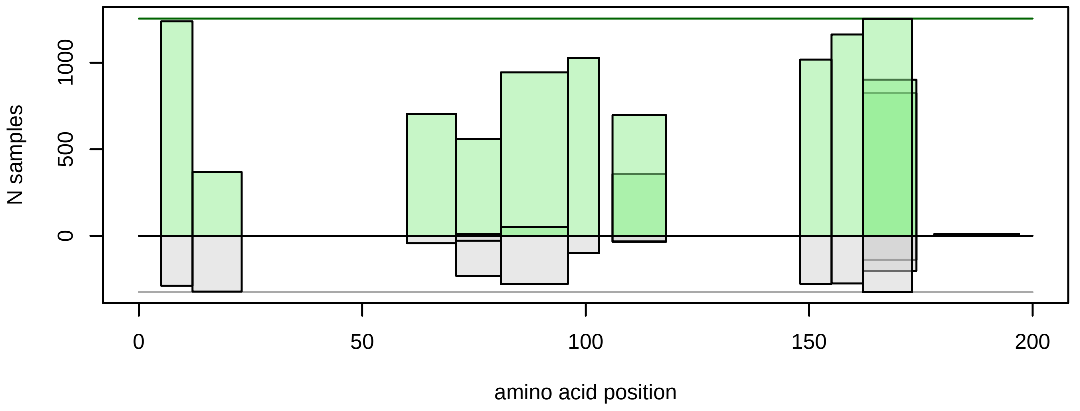

1559. IGHV1−18 (A0A0C4DH31), NP3

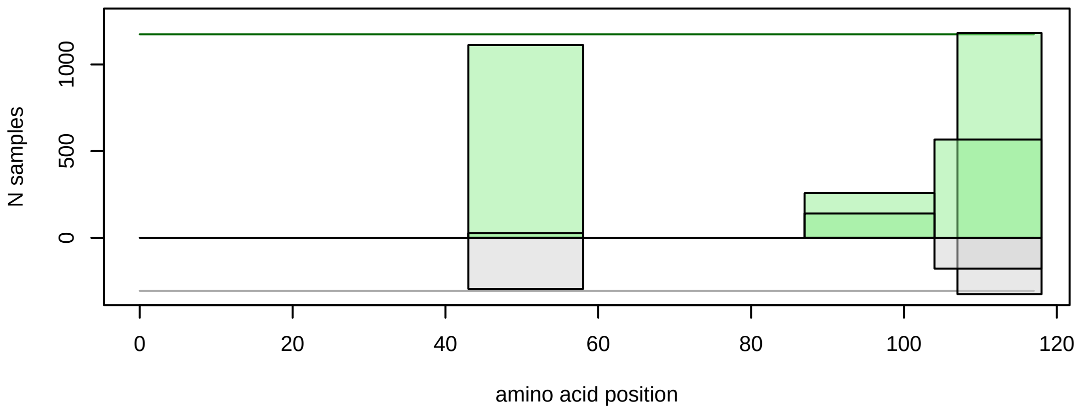

1560. CFD (P00746), NP3

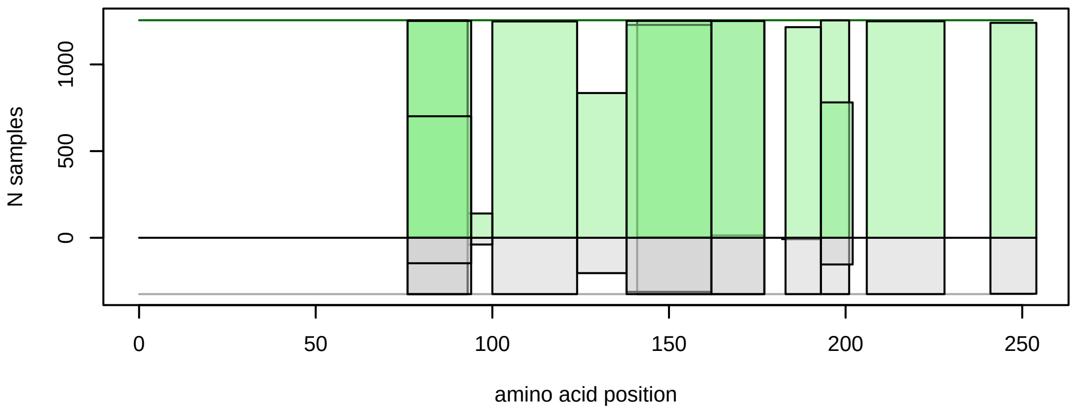

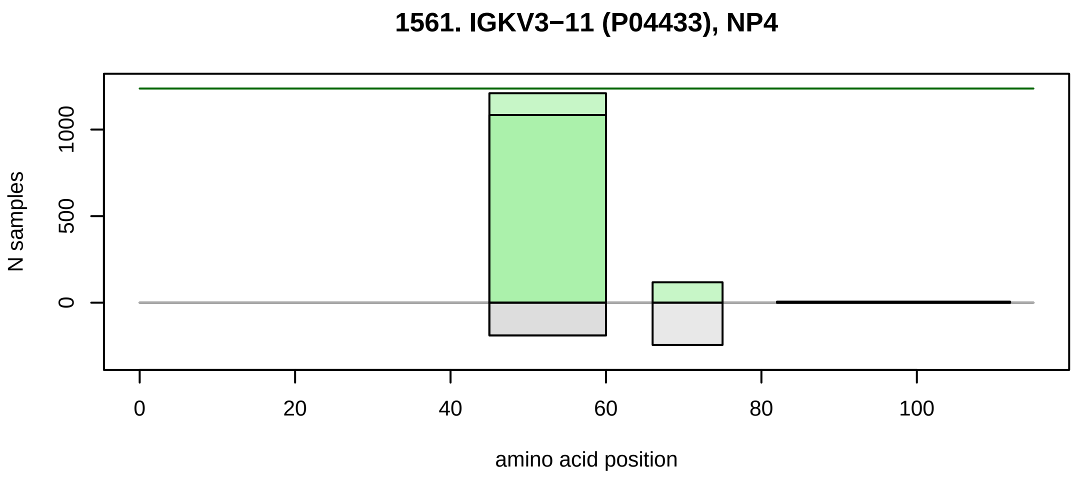
1561. IGKV3−11 (P04433), NP4
N samples
0
500
1000
0
20
40
60
80
100
amino acid position

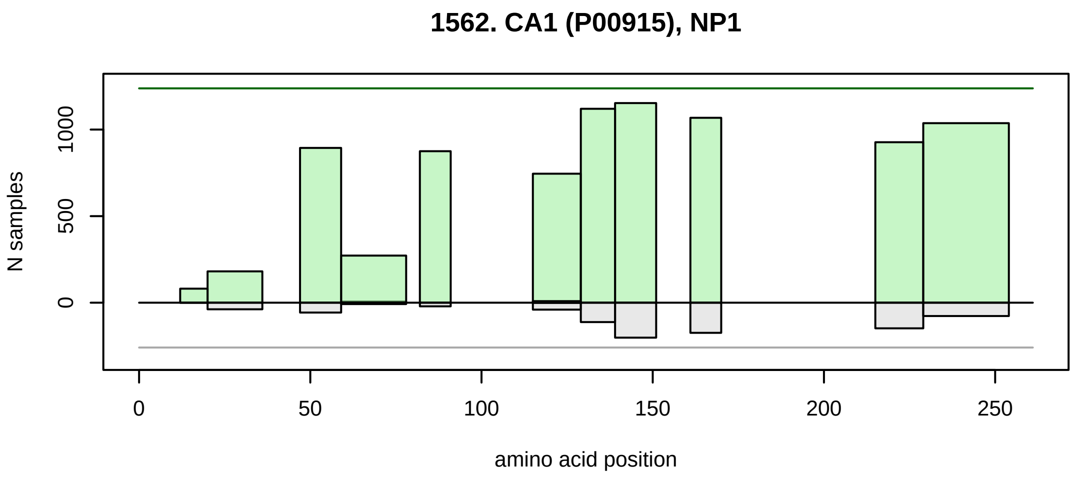
1562. CA1 (P00915), NP1
N samples
0
500
1000
0
50
100
150
200
250
amino acid position

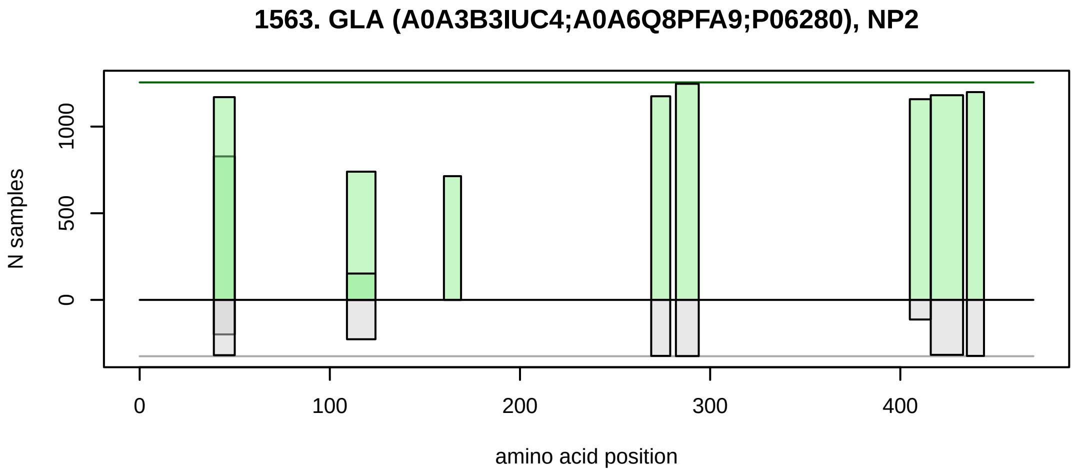
1563. GLA (A0A3B3IUC4;A0A6Q8PFA9;P06280), NP2
N samples
0
500
1000
0
100
200
300
400
amino acid position

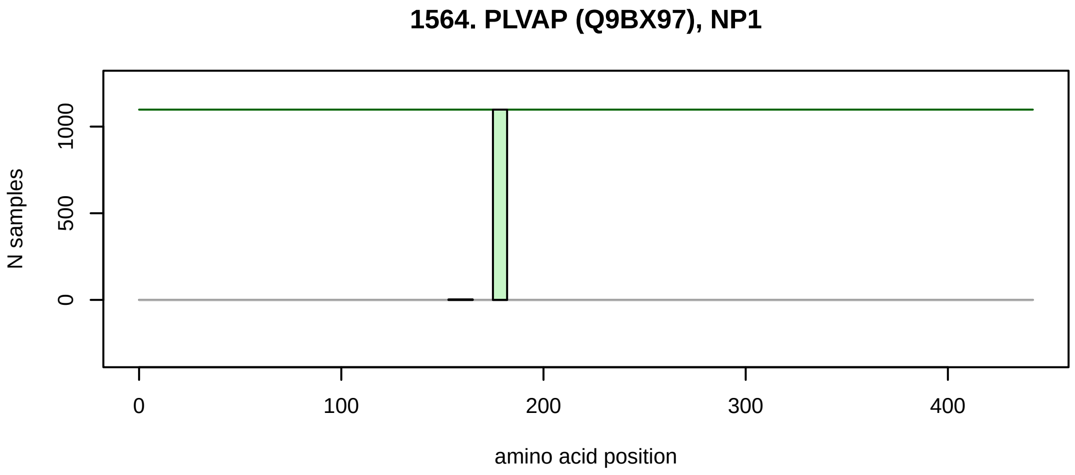
1564. PLVAP (Q9BX97), NP1
N samples
0
500
1000
0
100
200
300
400
amino acid position

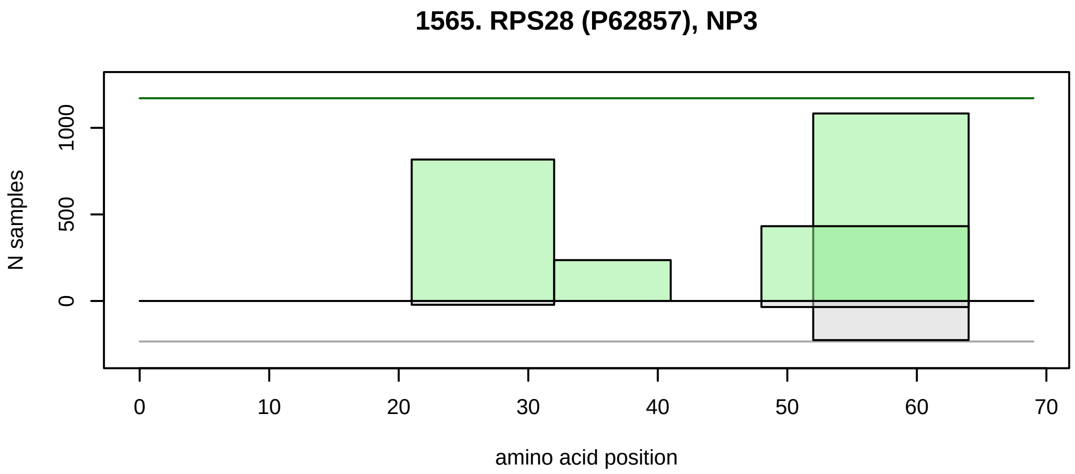
1565. RPS28 (P62857), NP3
N samples
0
500
1000
0
10
20
30
40
50
60
70
amino acid position

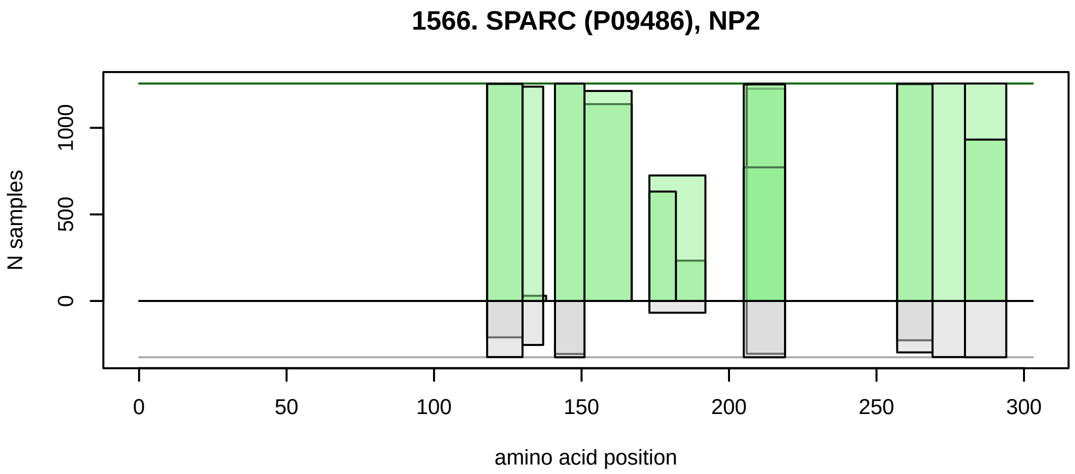
1566. SPARC (P09486), NP2
N samples
0
500
1000
0
50
100
150
200
250
300
amino acid position

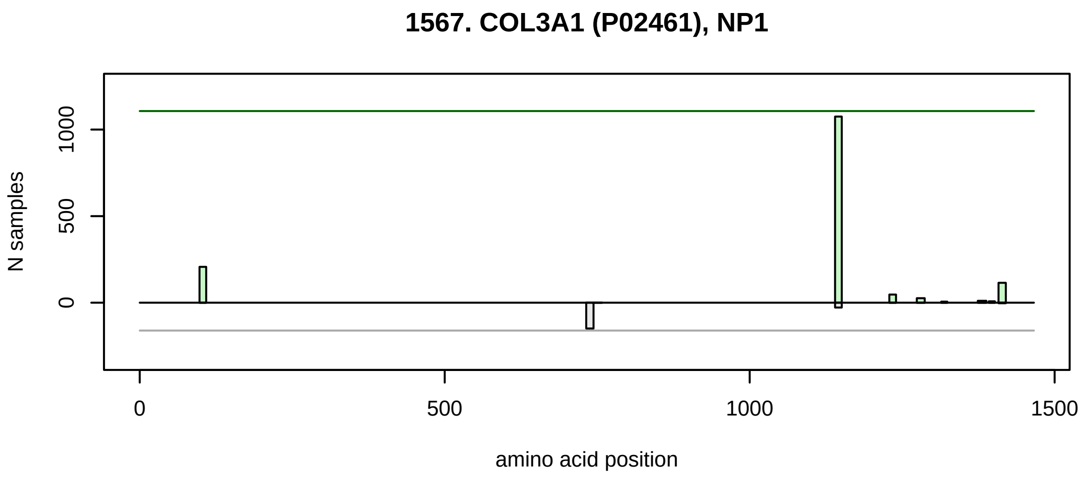
1567. COL3A1 (P02461), NP1
N samples
0
500
1000
0
500
1000
1500
amino acid position

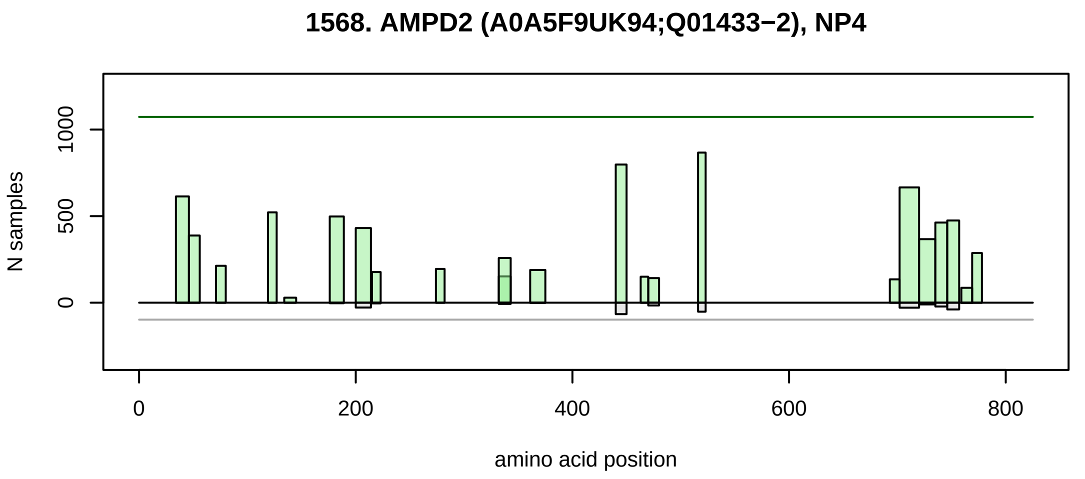
1568. AMPD2 (A0A5F9UK94;Q01433−2), NP4
N samples
0
500
1000
0
200
400
600
800
amino acid position

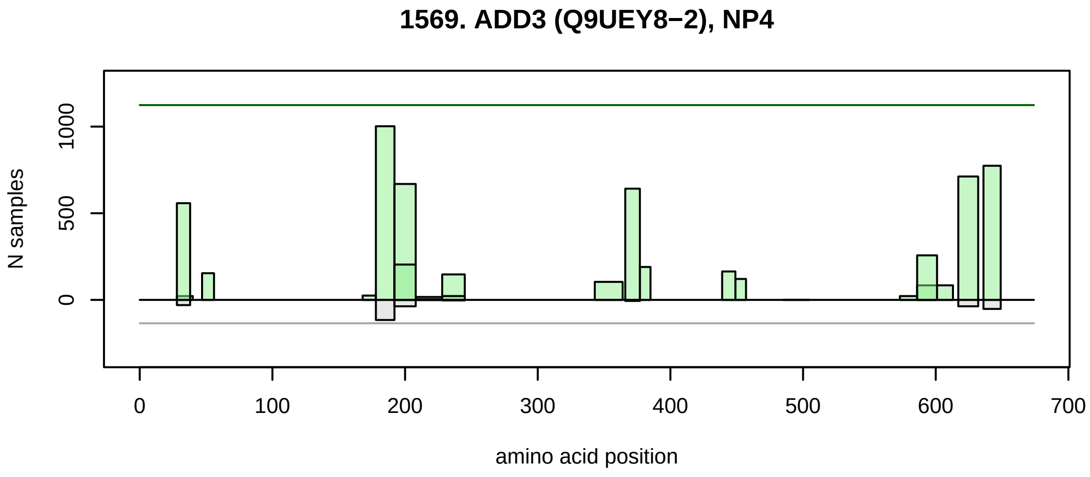
1569. ADD3 (Q9UEY8−2), NP4
N samples
0
500
1000
0
100
200
300
400
500
600
700
amino acid position

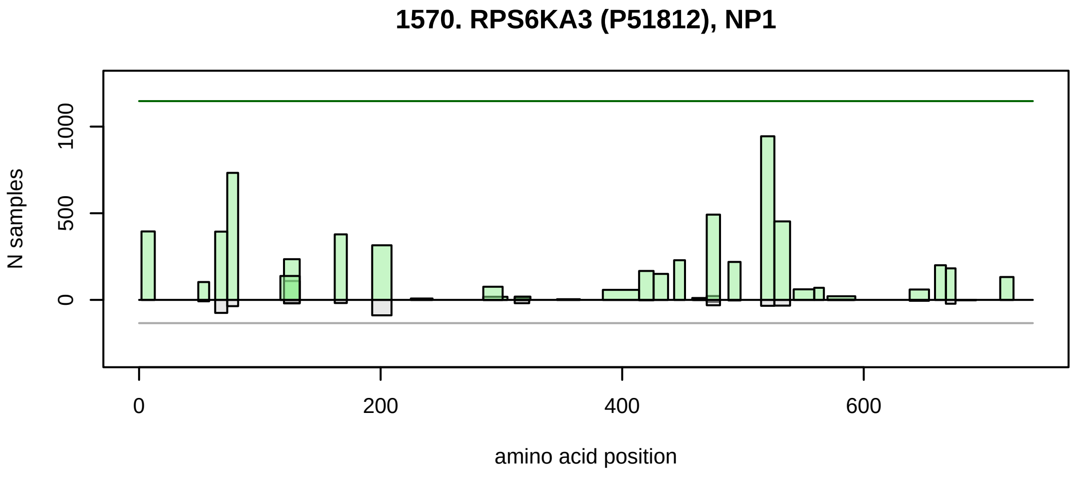
1570. RPS6KA3 (P51812), NP1
N samples
0
500
1000
0
200
400
600
amino acid position

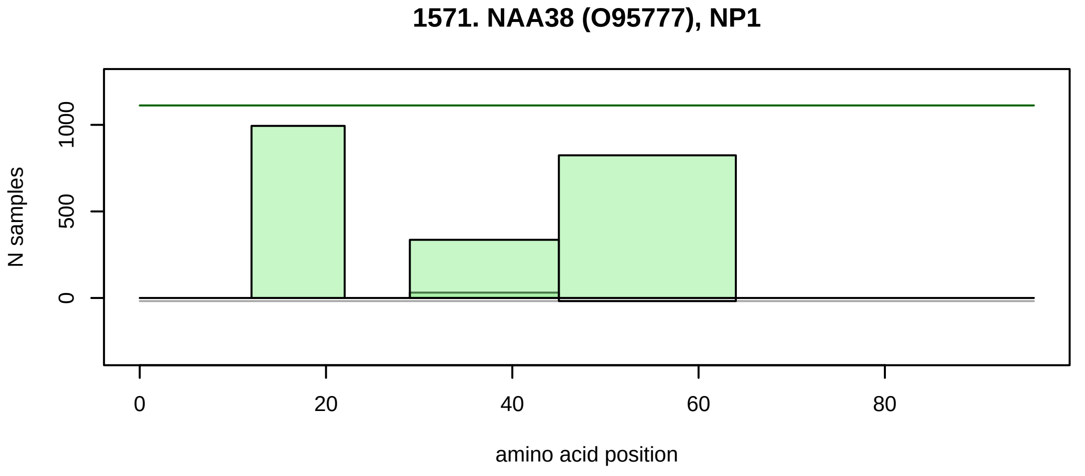
1571. NAA38 (O95777), NP1
N samples
0
500
1000
0
20
40
60
80
amino acid position

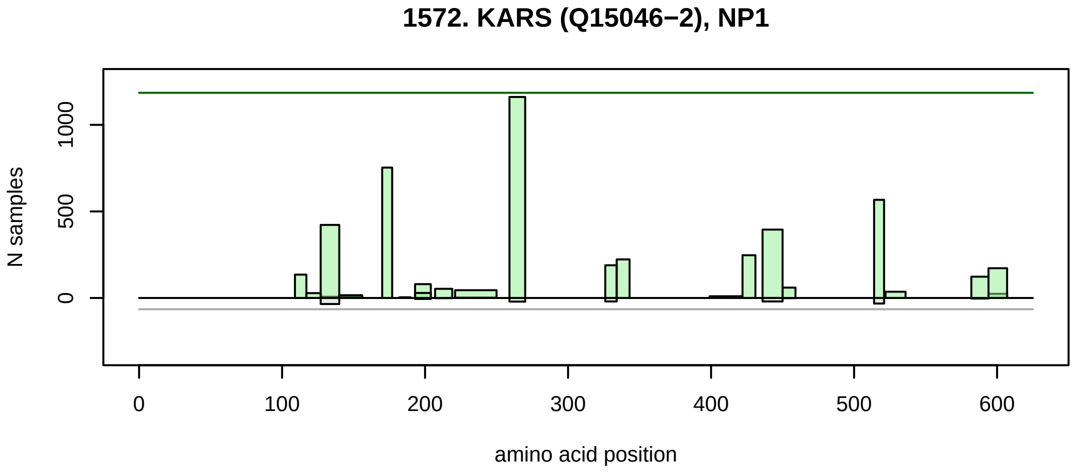
1572. KARS (Q15046−2), NP1
N samples
0
500
1000
0
100
200
300
400
500
600
amino acid position

1573. FAM198B (Q6UWH4), NP4

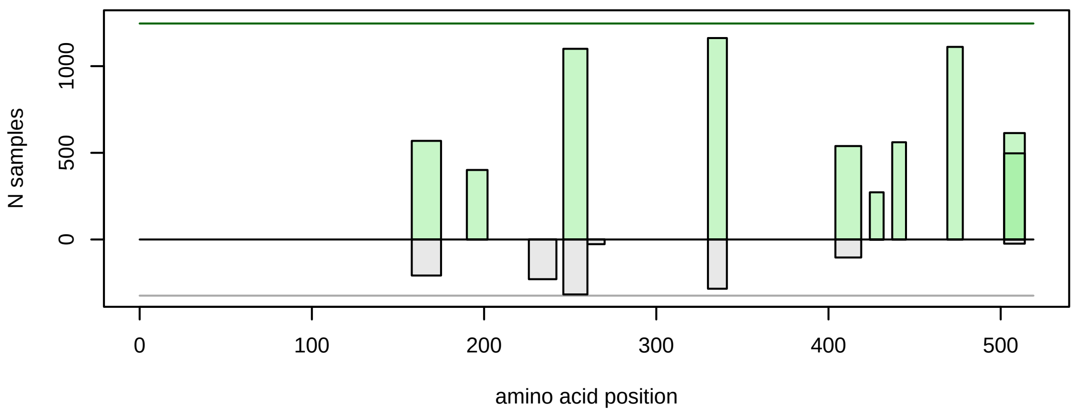

1574. MYL3 (P08590), NP2

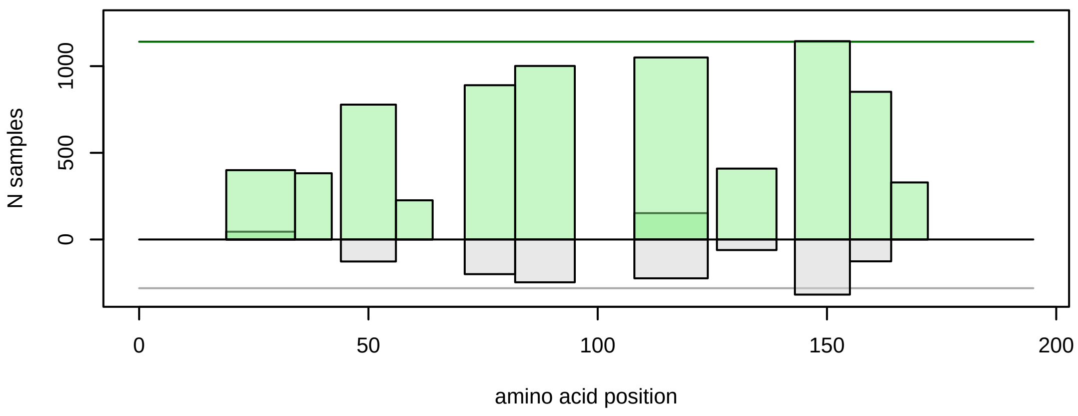

1575. CCDC150 (Q8NCX0), NP4

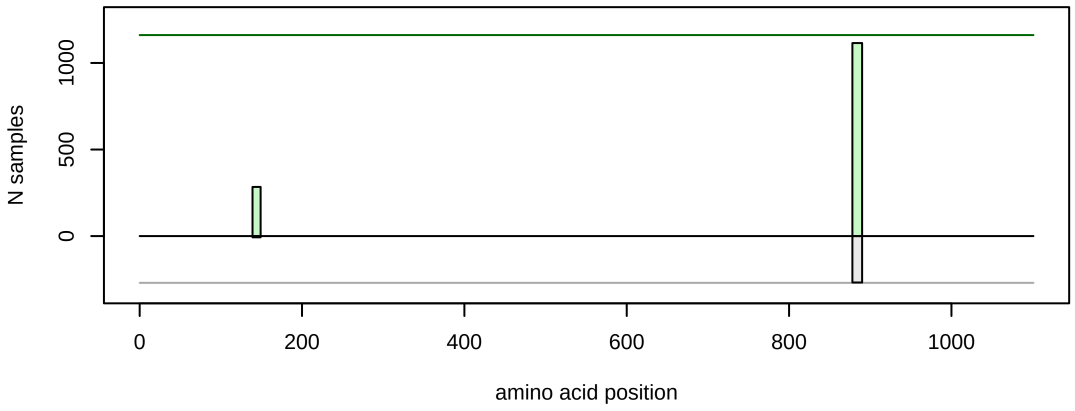

1576. PMM2 (H3BV55;O15305), NP3

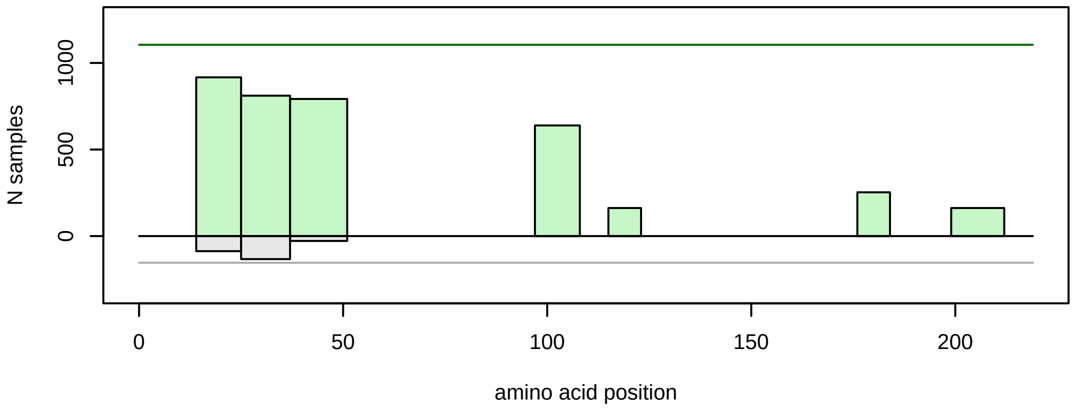

1577. PTPRK (E9PGC5;Q15262−2;Q15262−3;Q15262−4;Q5TG12), NP2

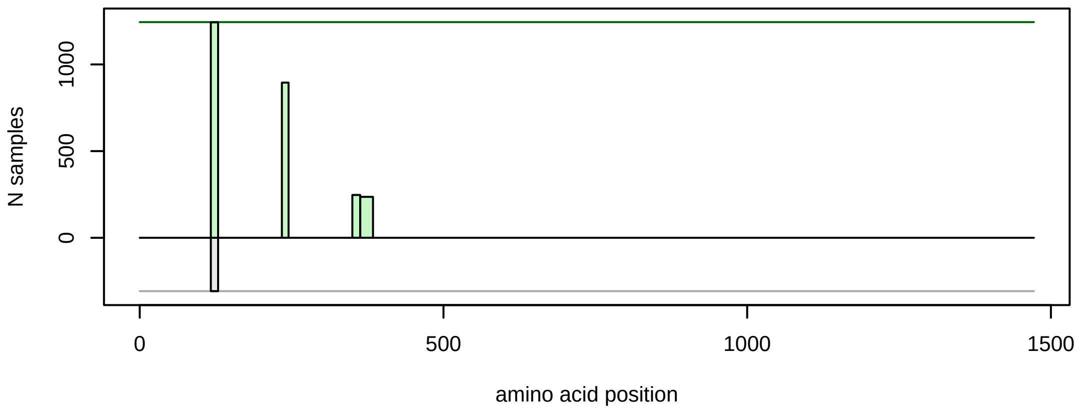

1578. TMOD3 (Q9NYL9), NP4

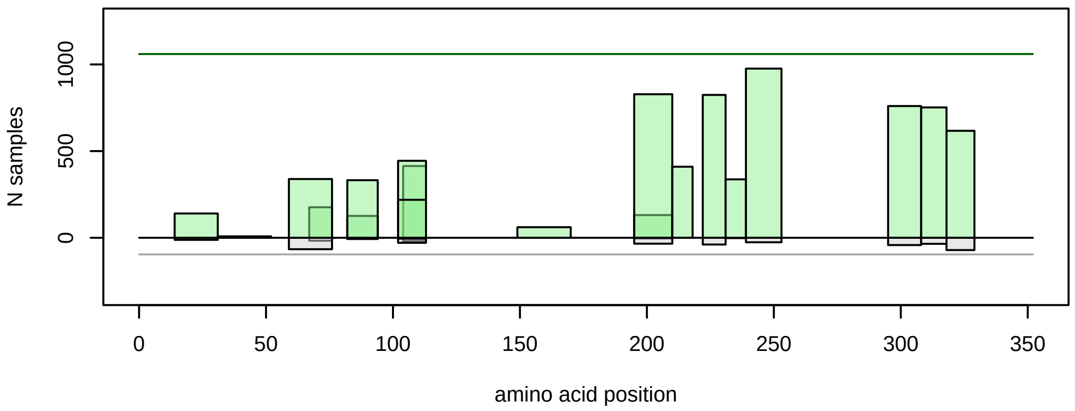

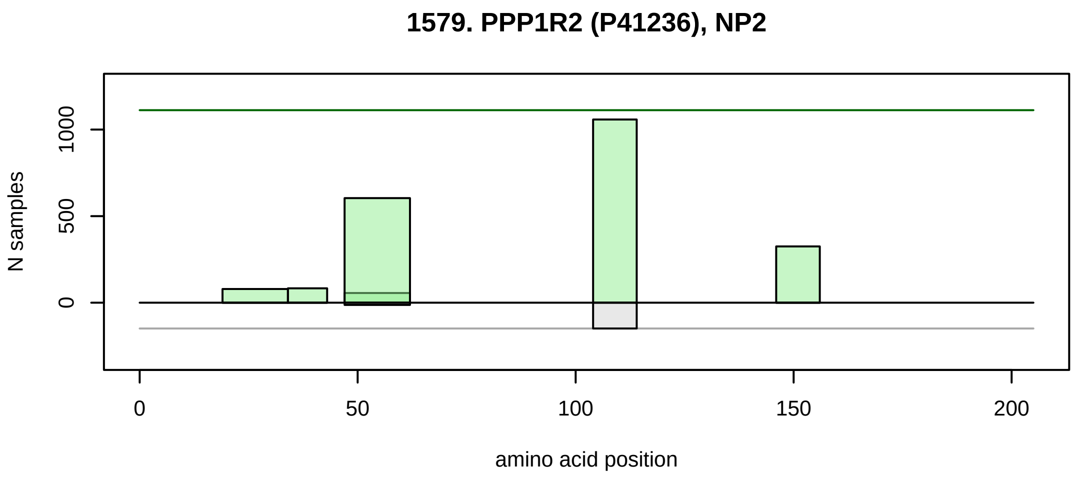

1579. PPP1R2 (P41236), NP2
N samples
0
500
1000
0
50
100
150
200
amino acid position

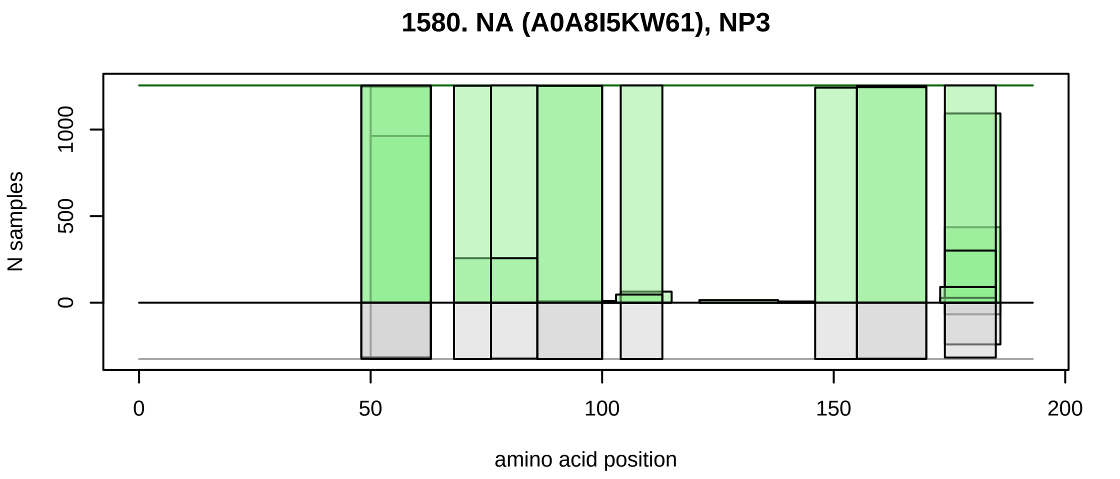

1580. NA (A0A8I5KW61), NP3
N samples
0
500
1000
0
50
100
150
200
amino acid position

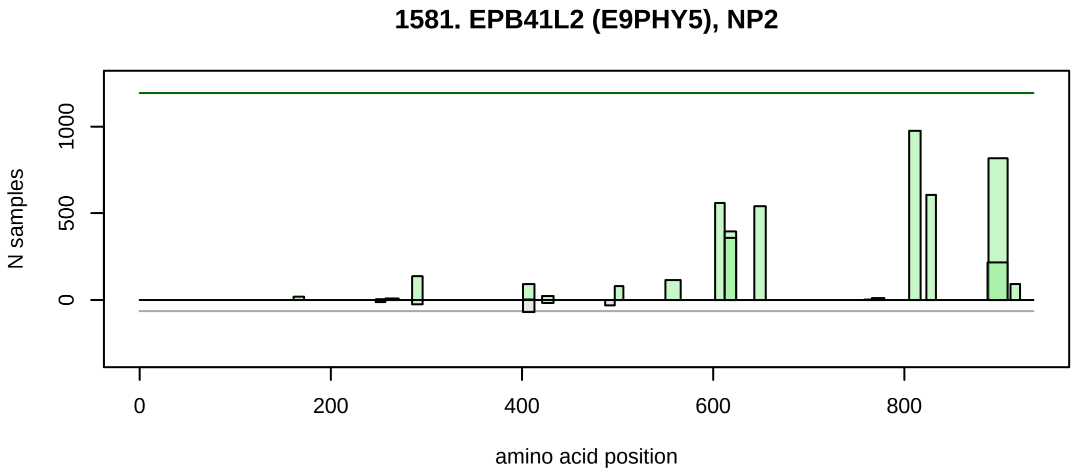

1581. EPB41L2 (E9PHY5), NP2
N samples
0
500
1000
0
200
400
600
800
amino acid position

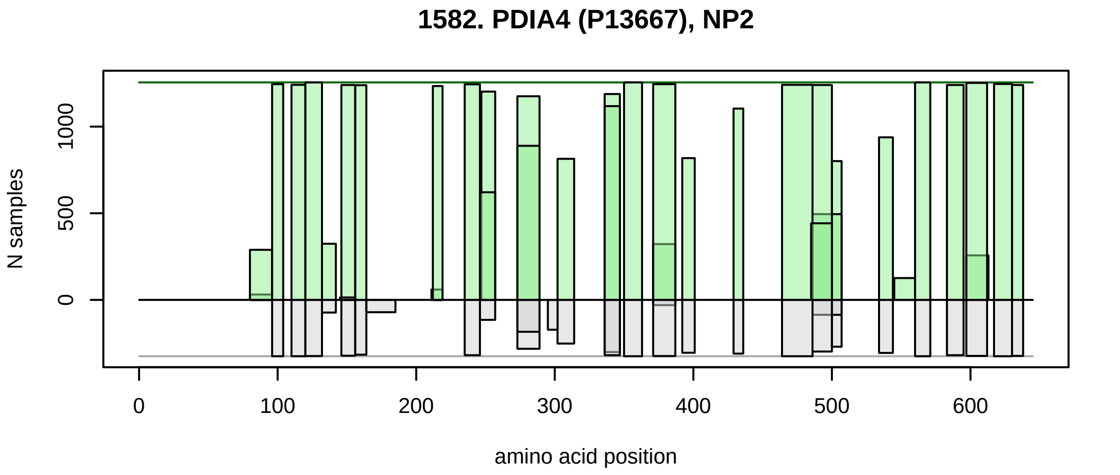

1582. PDIA4 (P13667), NP2
N samples
0
500
1000
0
100
200
300
400
500
600
amino acid position

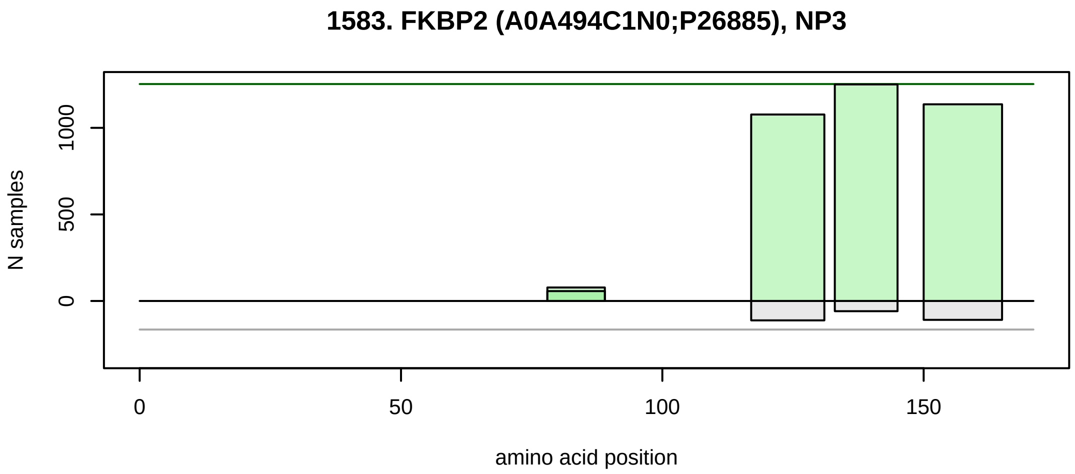

1583. FKBP2 (A0A494C1N0;P26885), NP3
N samples
0
500
1000
0
50
100
150
amino acid position

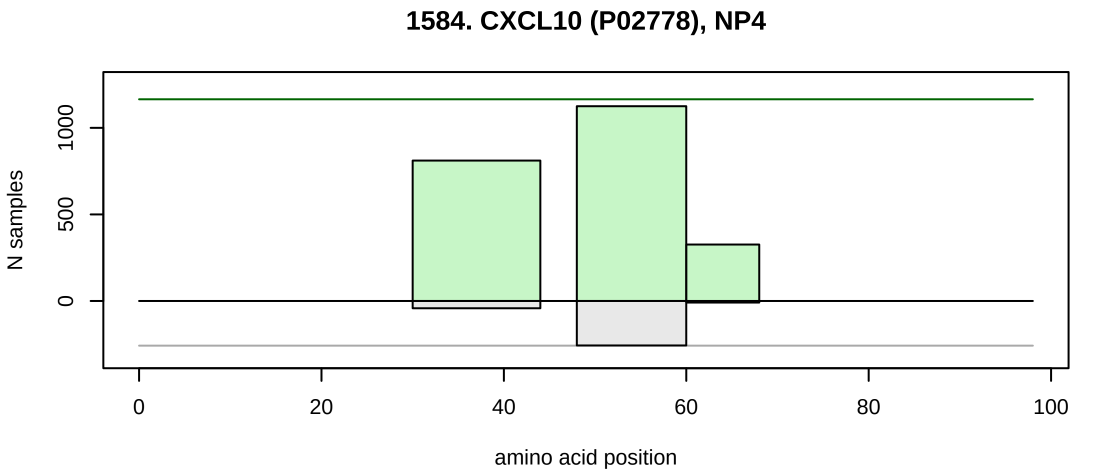

1584. CXCL10 (P02778), NP4
N samples
0
500
1000
0
20
40
60
80
100
amino acid position

1585. PSMD9 (O00233), NP5

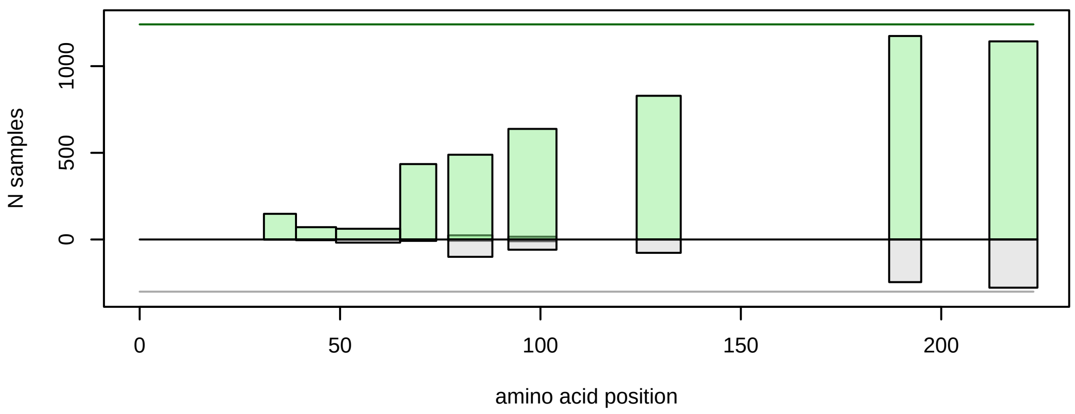

1586. NPPA (P01160), NP2

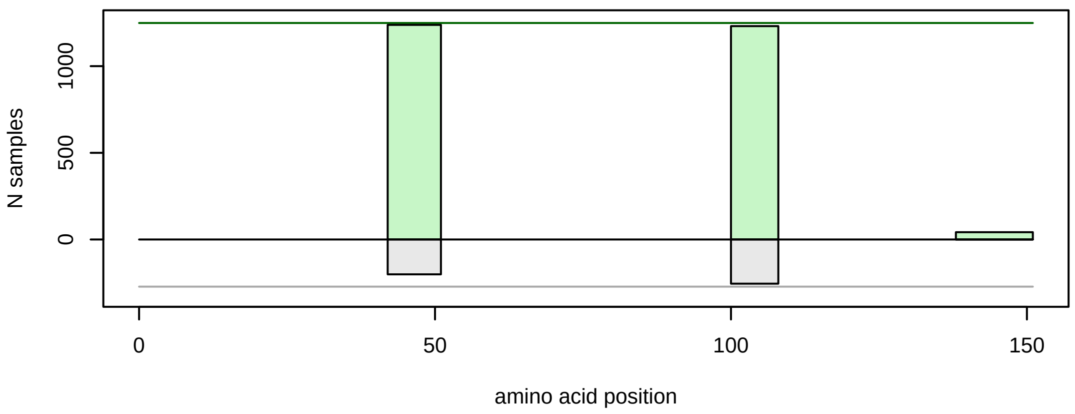

1587. IGFBP2 (P18065), NP1

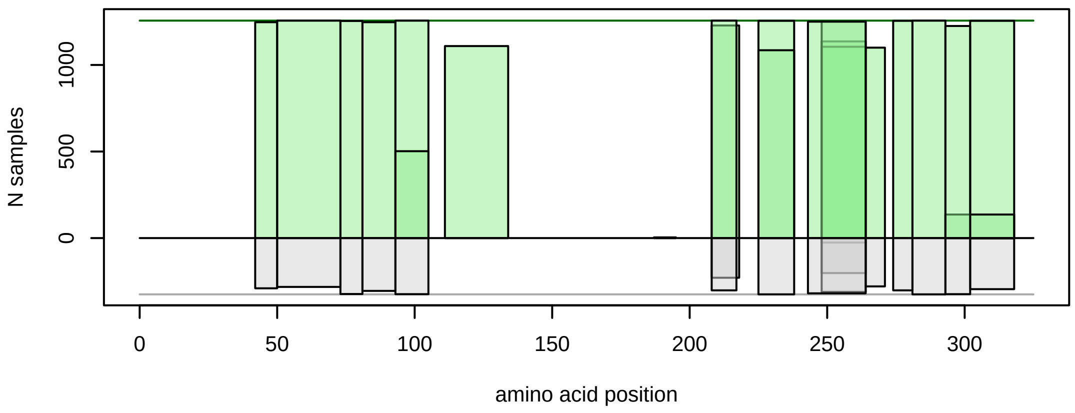

1588. HNRNPH1 (G8JLB6;P31943), NP5

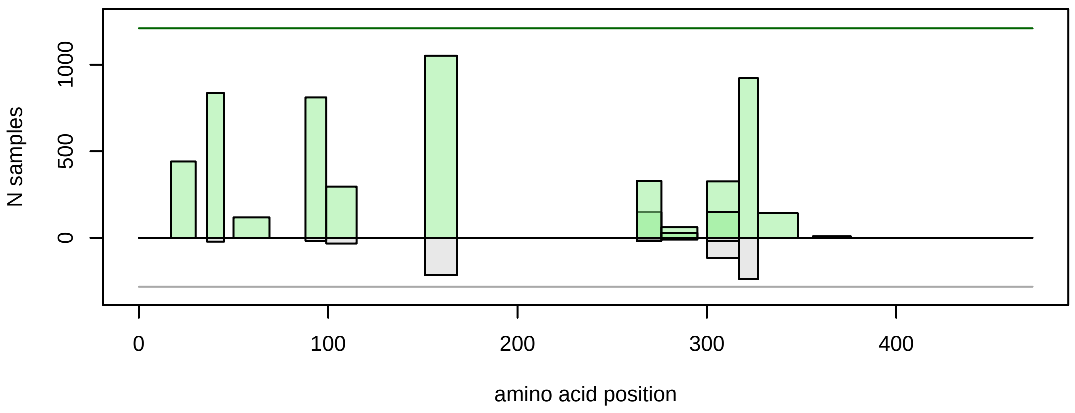

1589. MDH1 (P40925−3), NP1

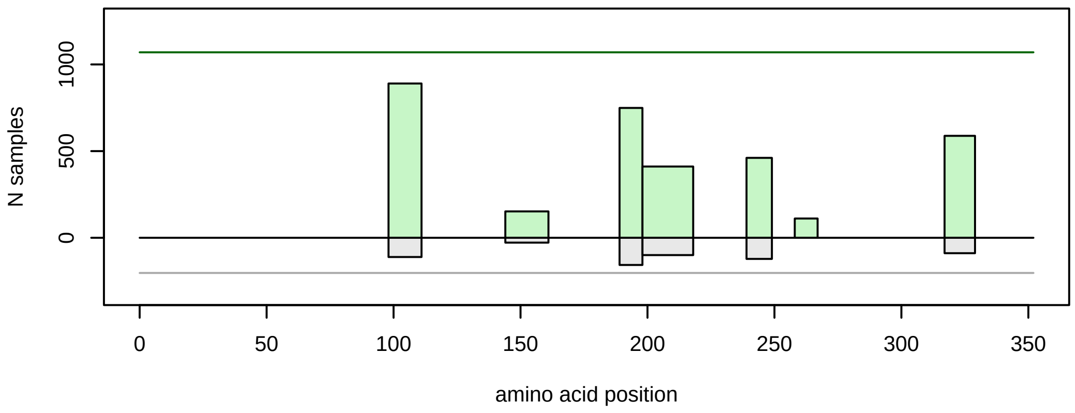

1590. VTN (P04004), NP2

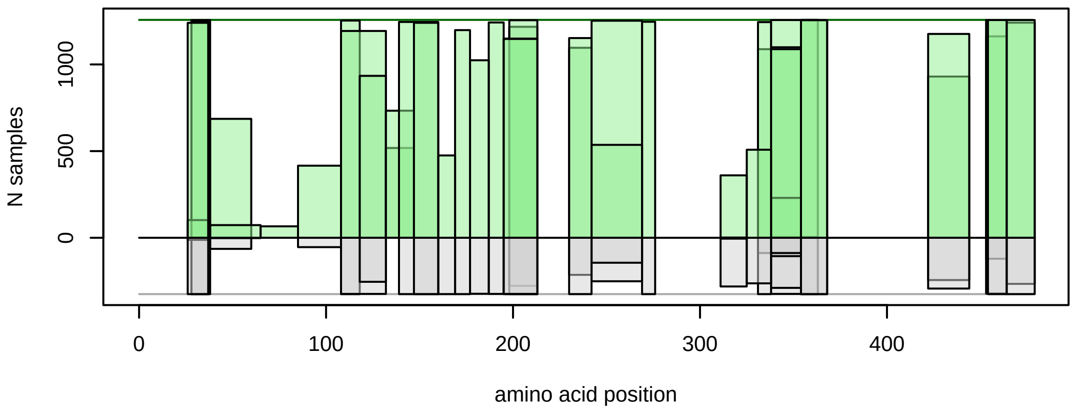

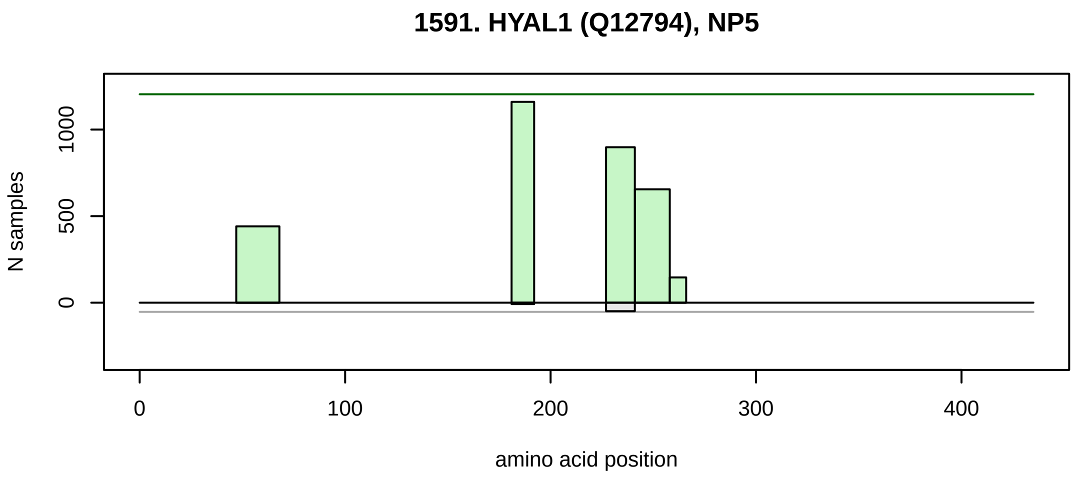
1591. HYAL1 (Q12794), NP5
N samples
0
500
1000
0
100
200
300
400
amino acid position

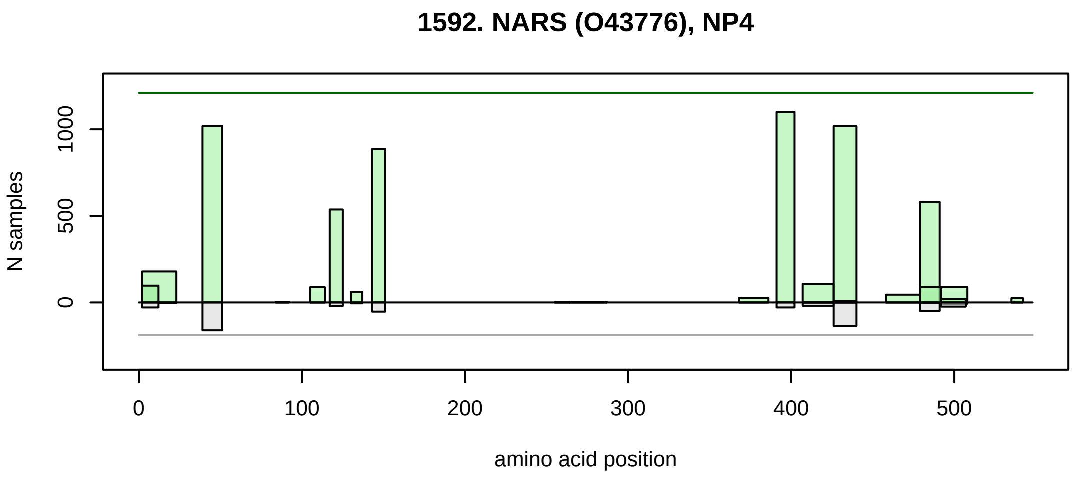
1592. NARS (O43776), NP4
N samples
0
500
1000
0
100
200
300
400
500
amino acid position

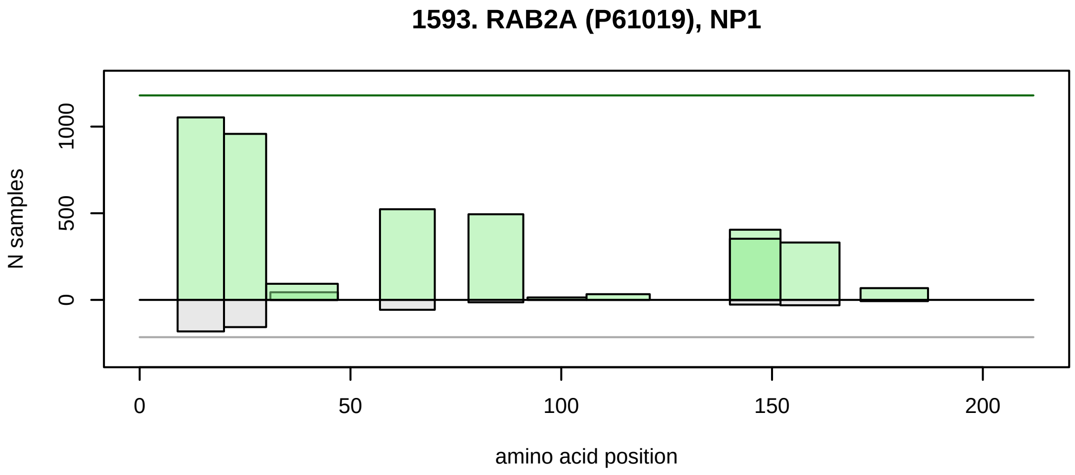
1593. RAB2A (P61019), NP1
N samples
0
500
1000
0
50
100
150
200
amino acid position

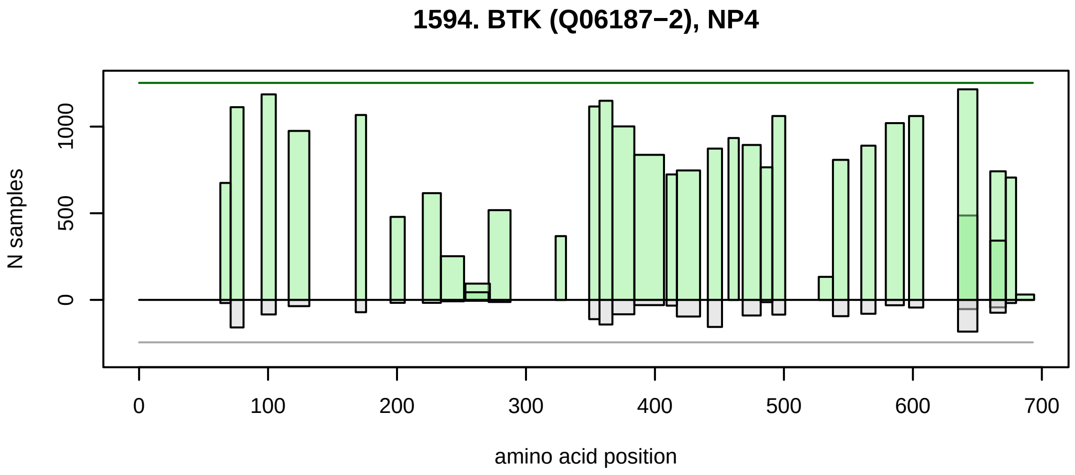
1594. BTK (Q06187−2), NP4
N samples
0
500
1000
0
100
200
300
400
500
600
700
amino acid position

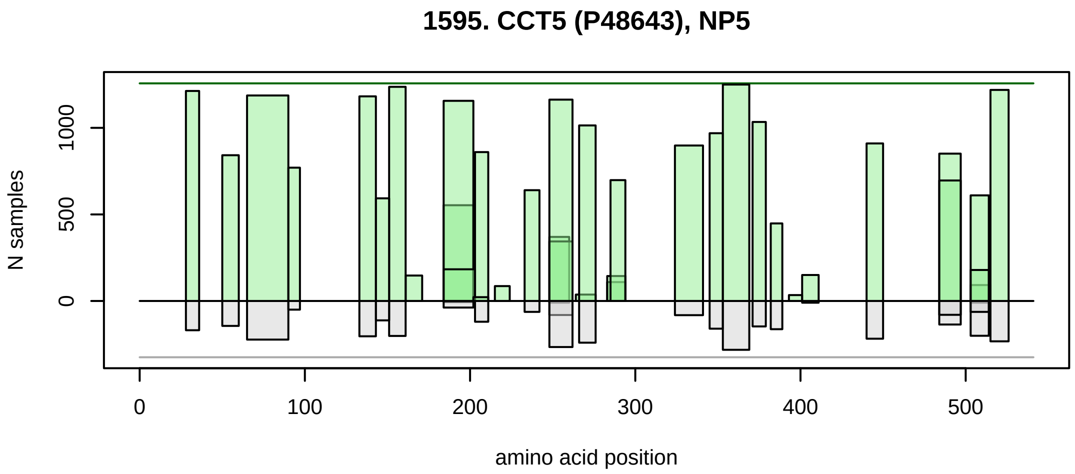
1595. CCT5 (P48643), NP5
N samples
0
500
1000
0
100
200
300
400
500
amino acid position

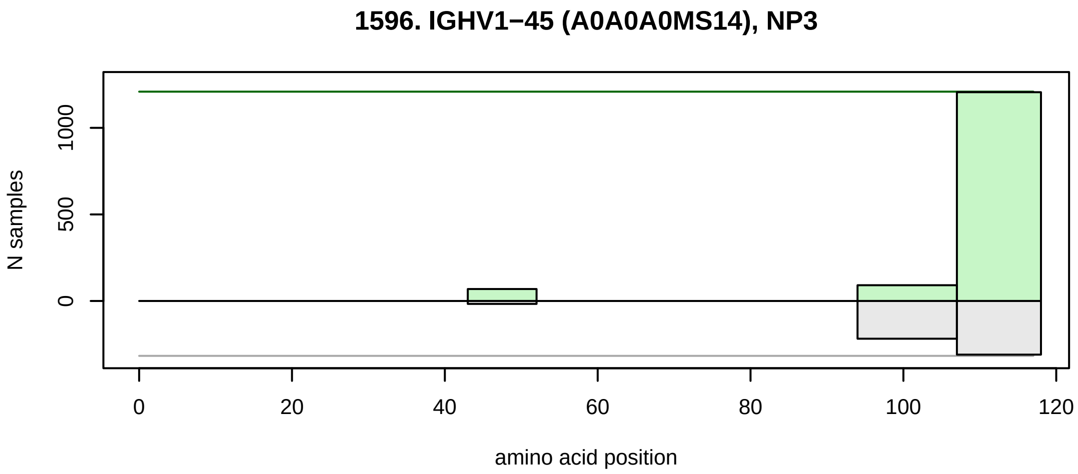
1596. IGHV1−45 (A0A0A0MS14), NP3
N samples
0
500
1000
0
20
40
60
80
100
120
amino acid position

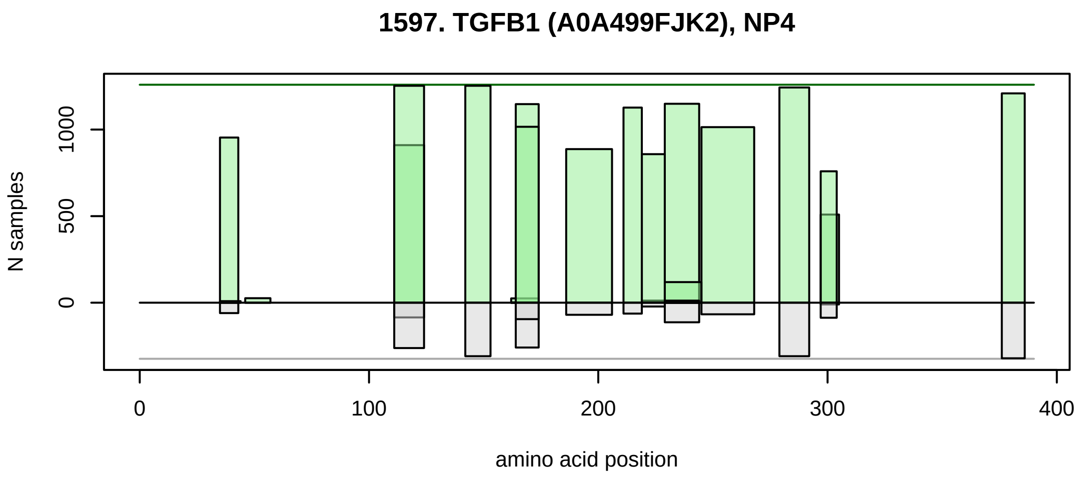
1597. TGFB1 (A0A499FJK2), NP4
N samples
0
500
1000
0
100
200
300
400
amino acid position

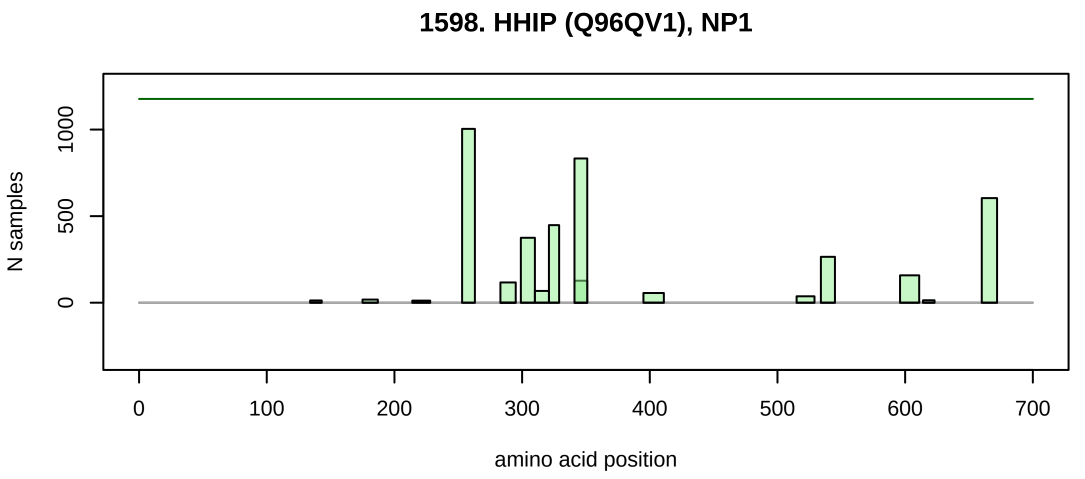
1598. HHIP (Q96QV1), NP1
N samples
0
500
1000
0
100
200
300
400
500
600
700
amino acid position

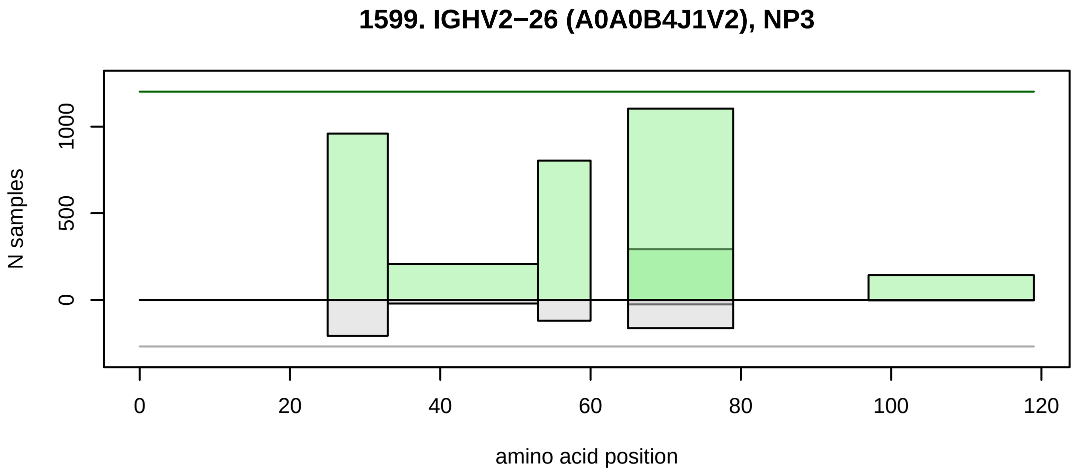
1599. IGHV2−26 (A0A0B4J1V2), NP3
N samples
0
500
1000
0
20
40
60
80
100
120
amino acid position

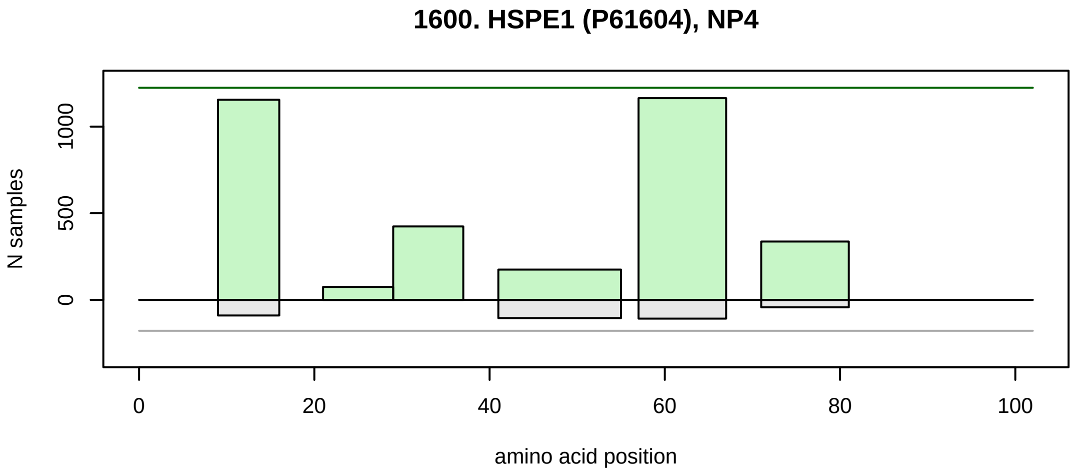
1600. HSPE1 (P61604), NP4
N samples
0
500
1000
0
20
40
60
80
100
amino acid position

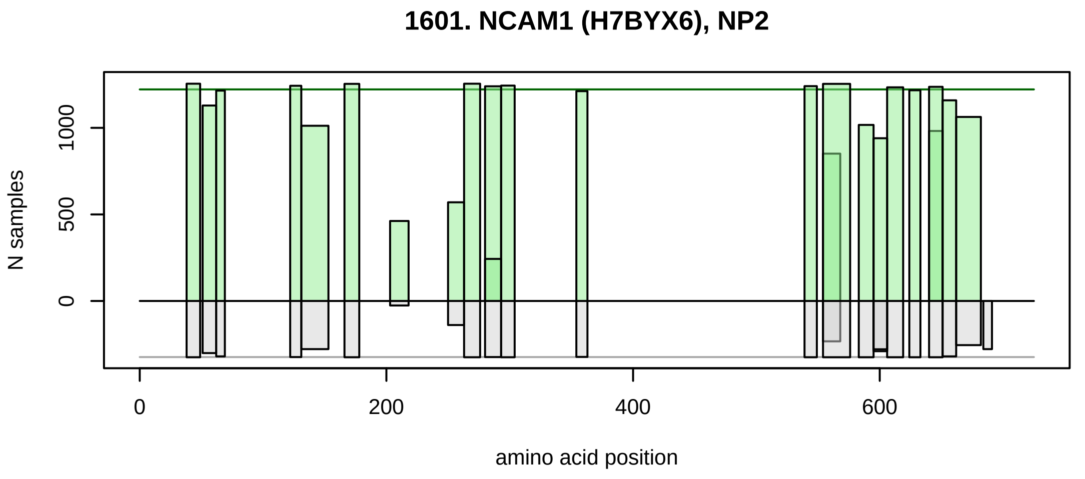
1601. NCAM1 (H7BYX6), NP2
N samples
0
500
1000
0
200
400
600
amino acid position

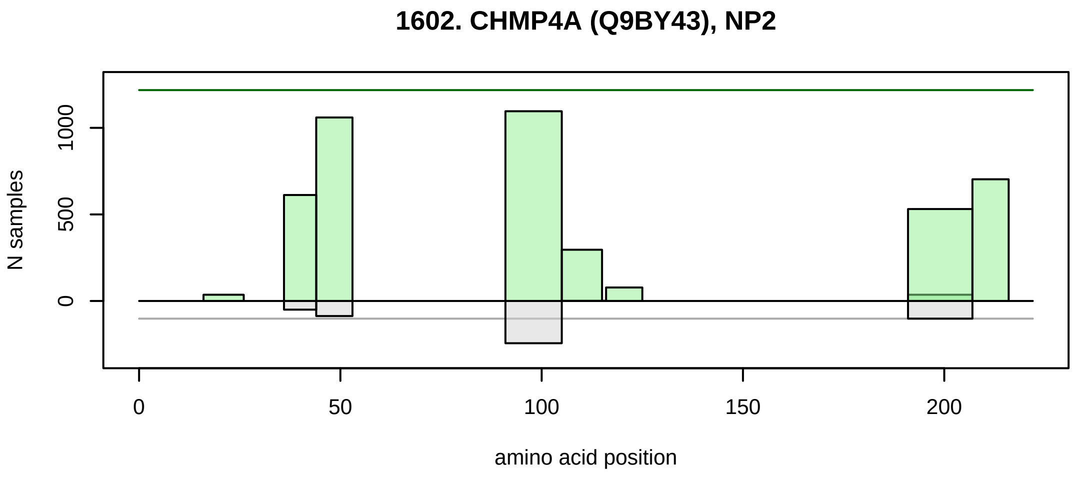
1602. CHMP4A (Q9BY43), NP2
N samples
0
500
1000
0
50
100
150
200
amino acid position

1603. FBLN2 (P98095−2), NP2

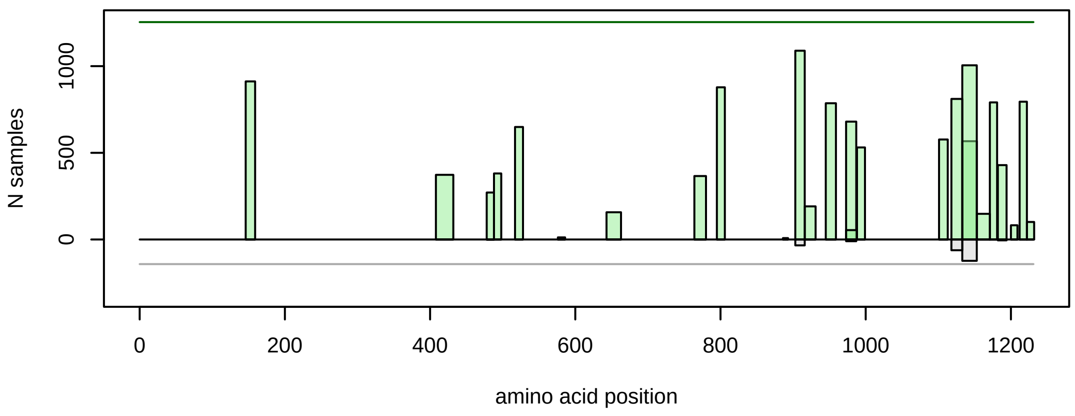

1604. CORO1B (Q9BR76), NP1

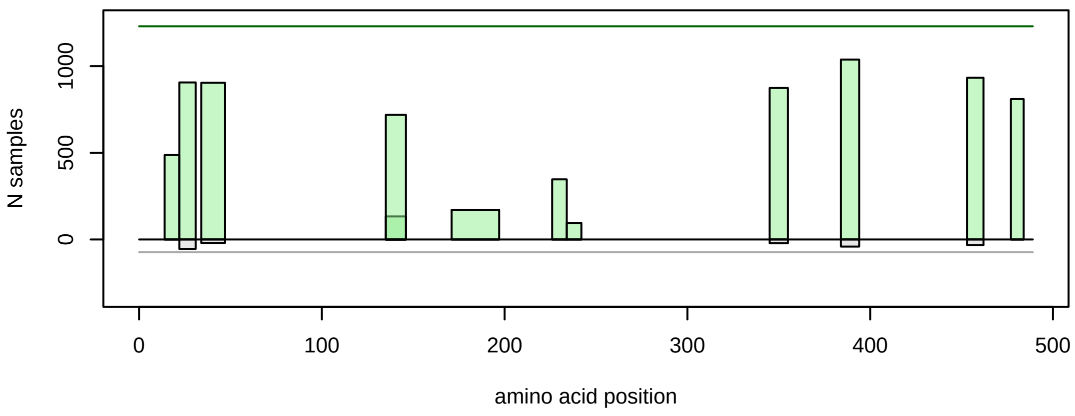

1605. CHRDL1 (Q9BU40−4), NP3

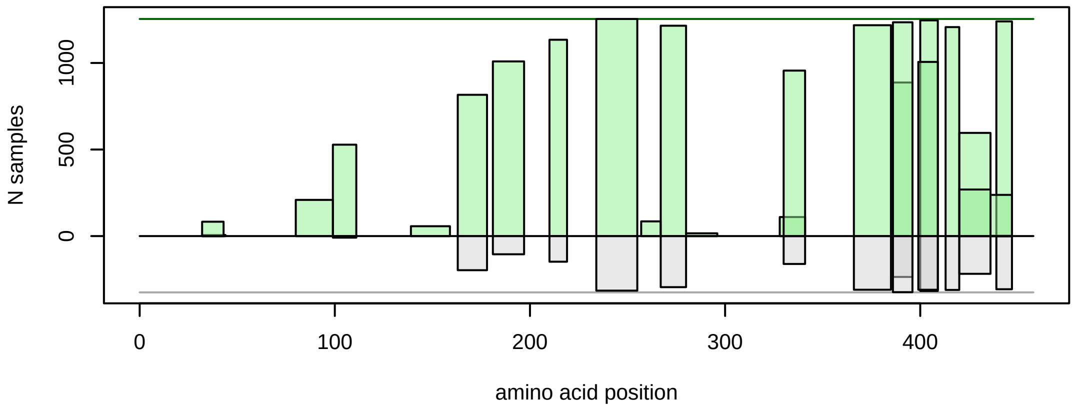

1606. PTPN6 (P29350), NP3

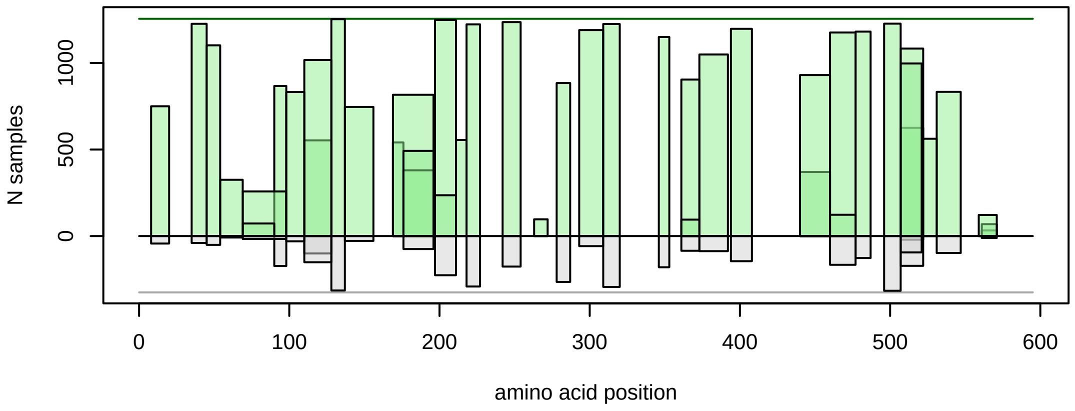

1607. NCAM1 (P13591), NP2

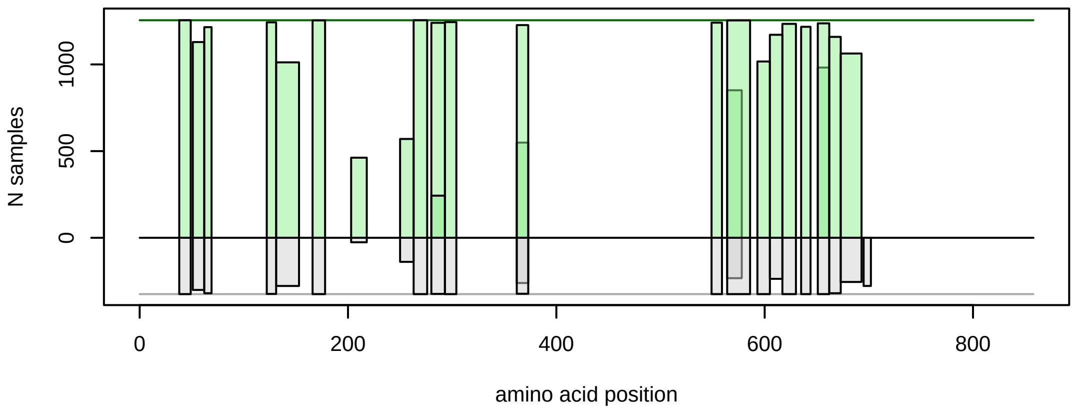

1608. ENO1 (P06733), NP3

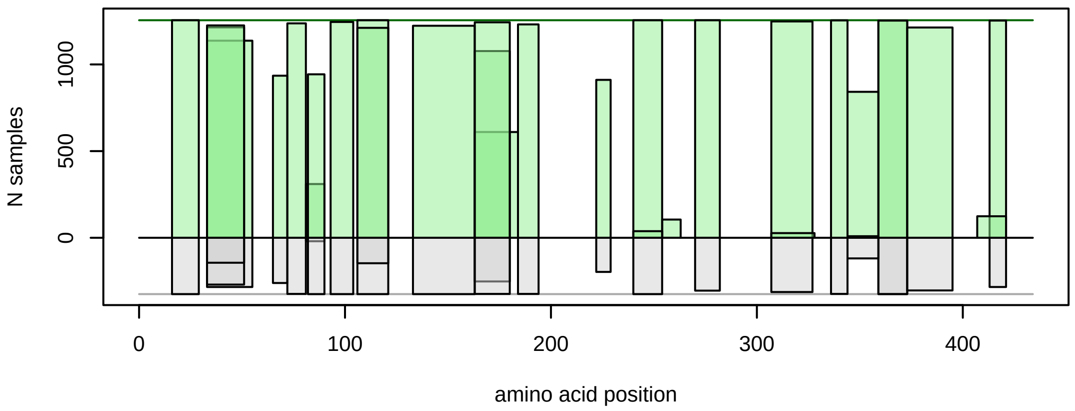

1609. MATN2 (O00339;O00339−2), NP4

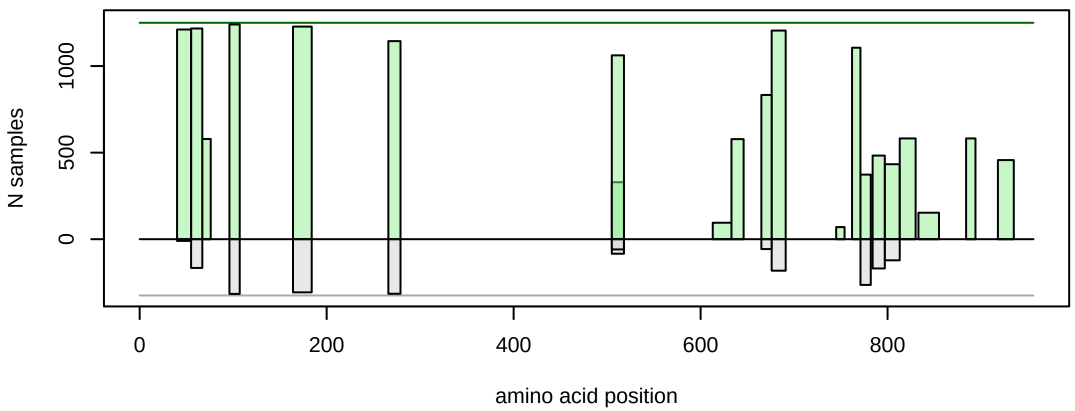

1610. ATP6V0D1 (F5GYQ1;P61421), NP1

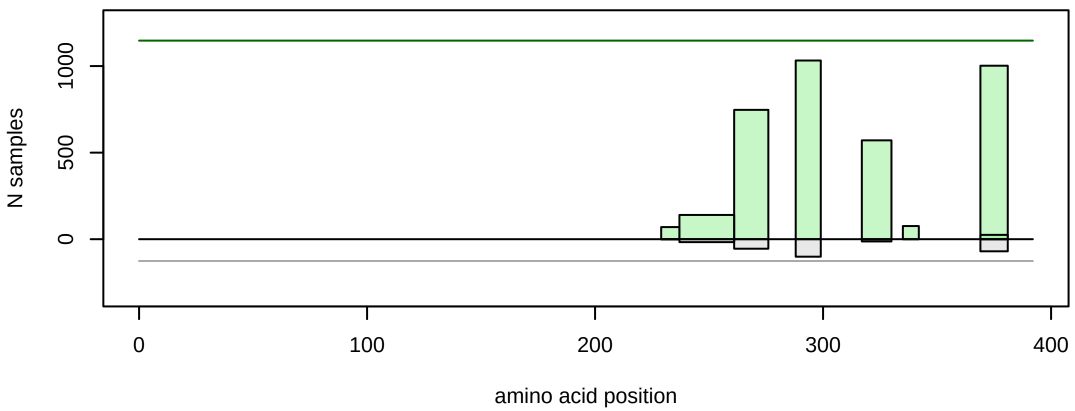

1611. CRHBP (P24387), NP2

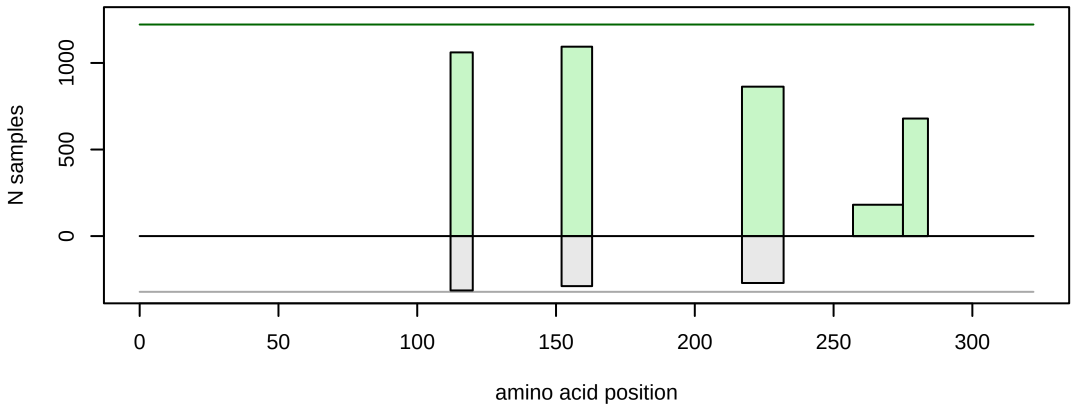

1612. MTTP (P55157), NP4

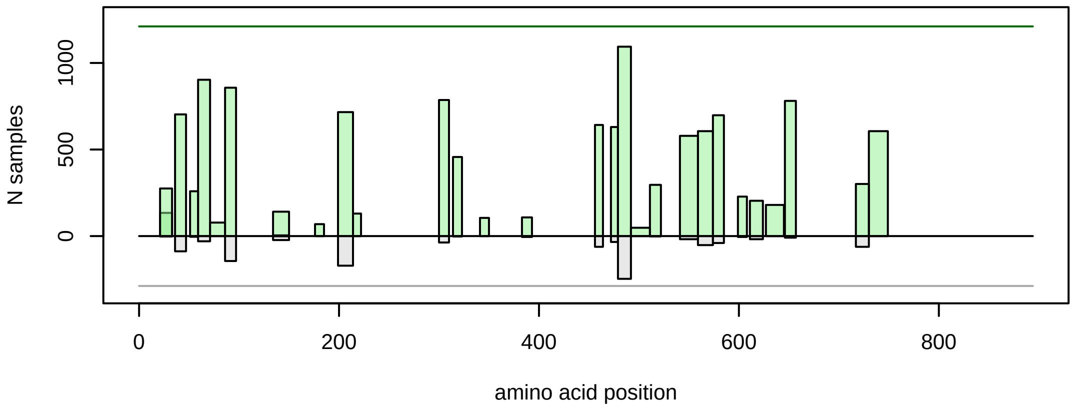

1613. PRG3 (Q9Y2Y8), NP2

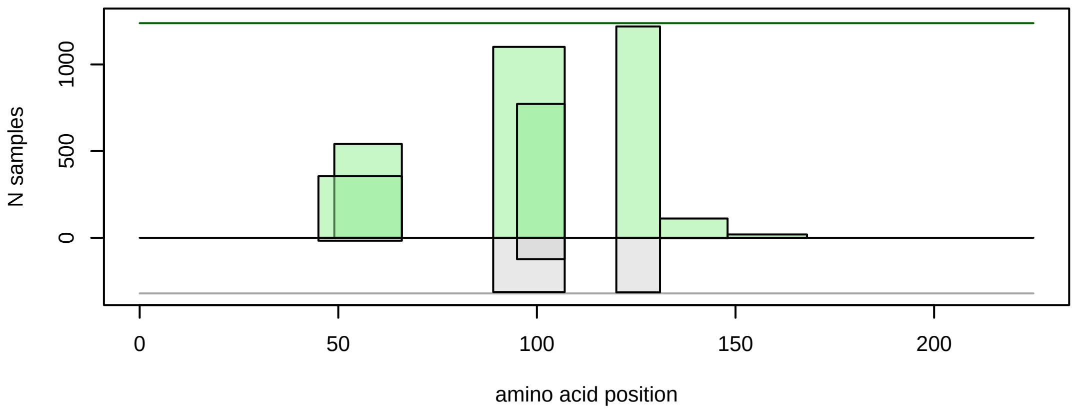

1614. TPP2 (Q5VZU9), NP5

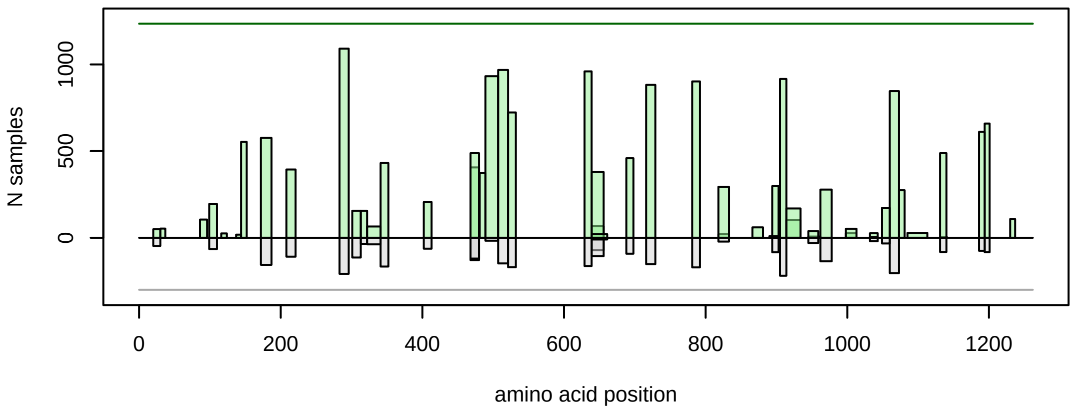

1615. DKK2 (Q9UBU2), NP4

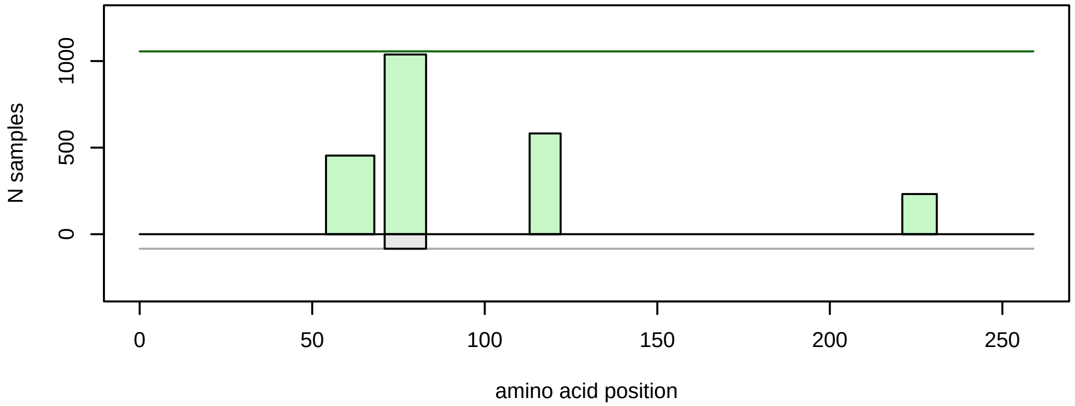

1616. FYN (P06241−2), NP1

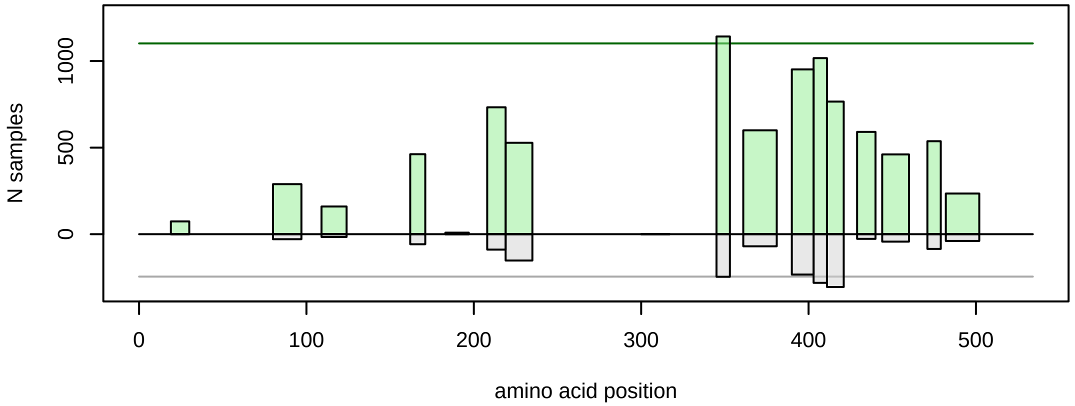

1617. EIF4B (E7EX17;P23588), NP5

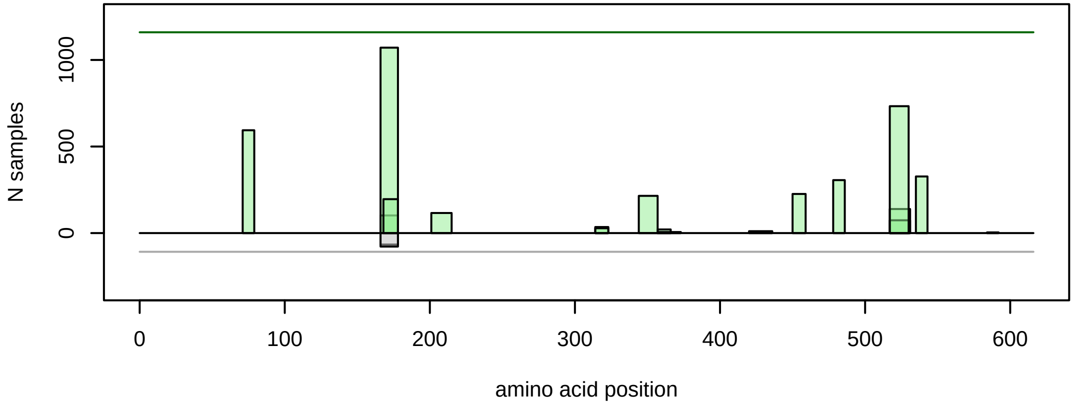

1618. ALAD (P13716), NP1

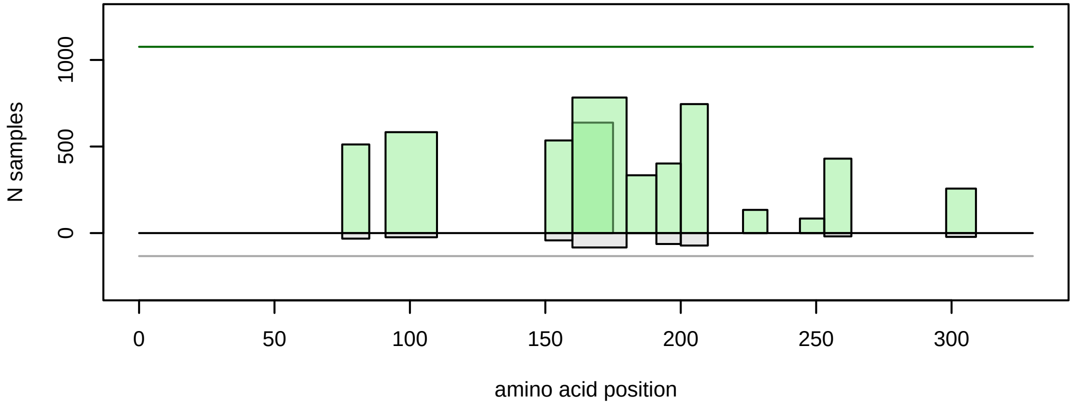

1619. RPL12 (P30050), NP1

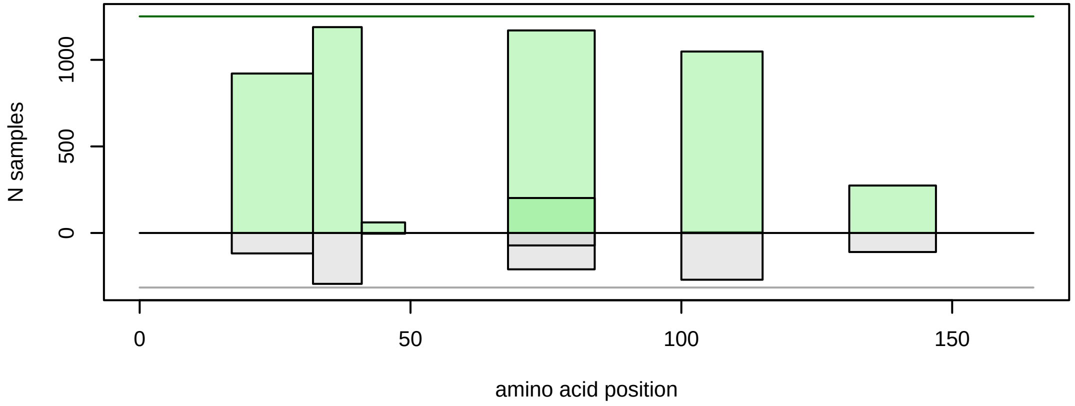

1620. ARIH2 (O95376), NP2

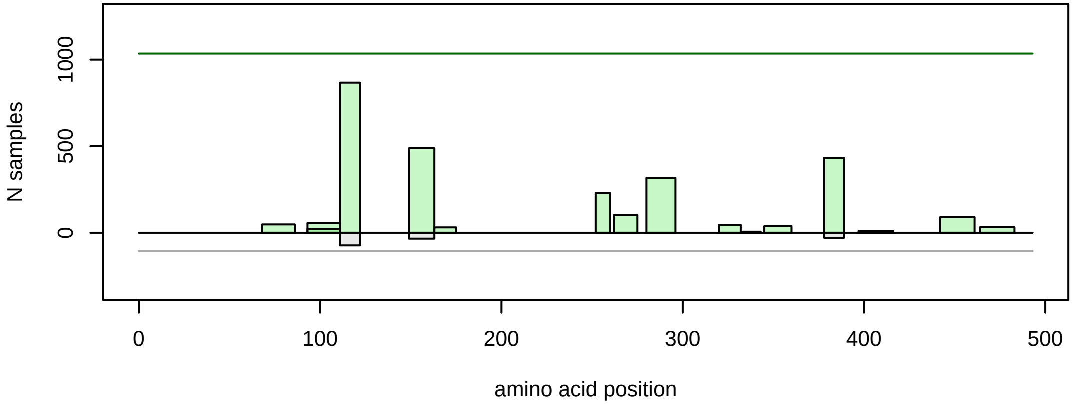

1621. GLUD1 (P00367), NP1

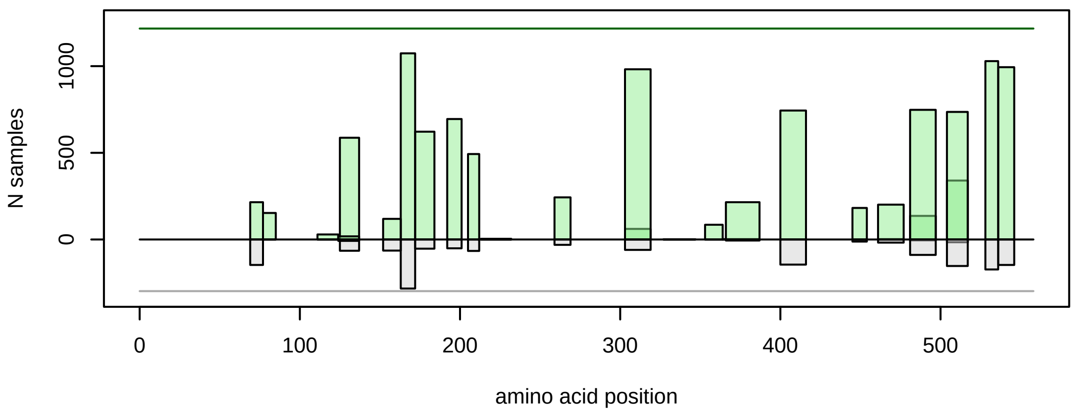

1622. FBN2 (P35556), NP5

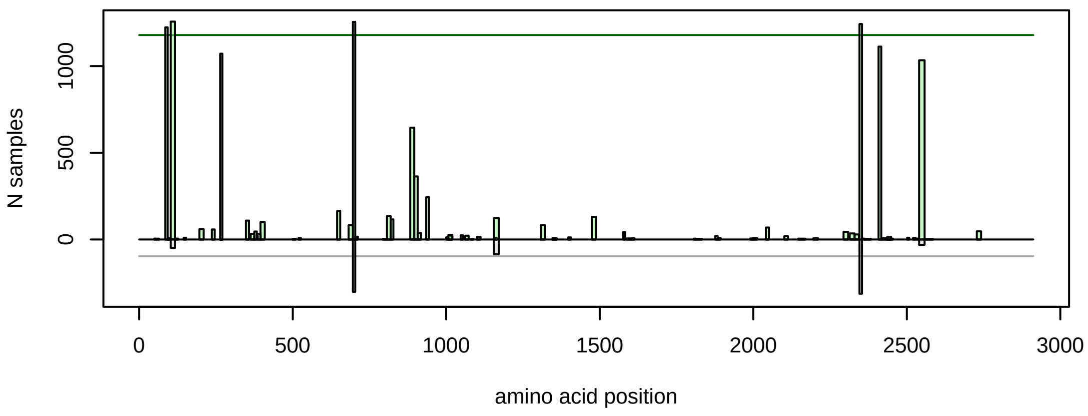

1623. SDCBP (O00560), NP2

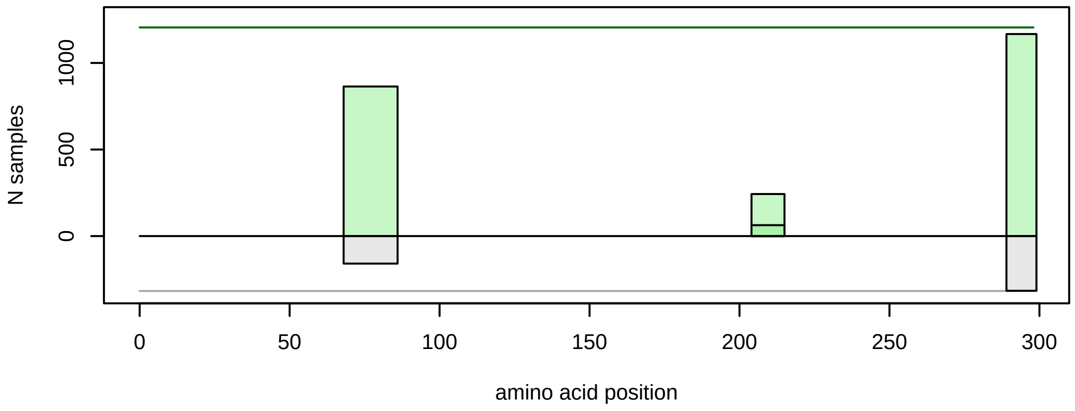

1624. NPEPPS (P55786), NP5

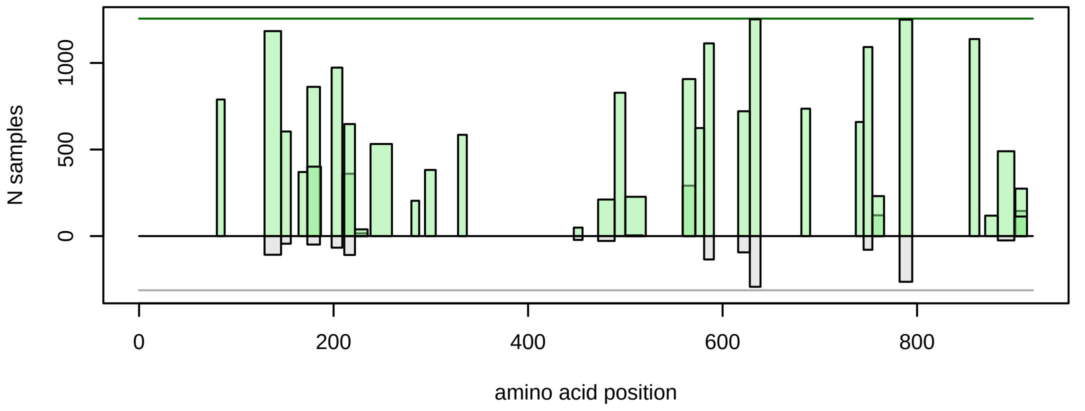

1625. INHBB (P09529), NP1

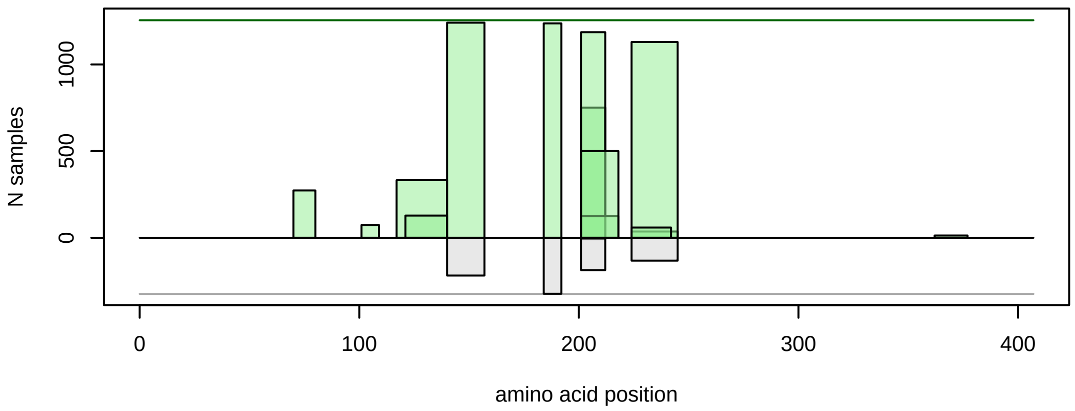

1626. SUMO3 (A8MUA9), NP2

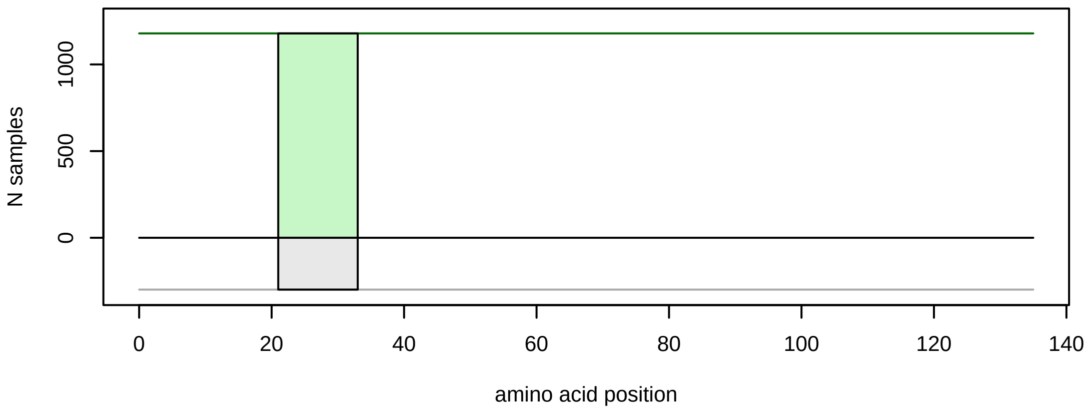

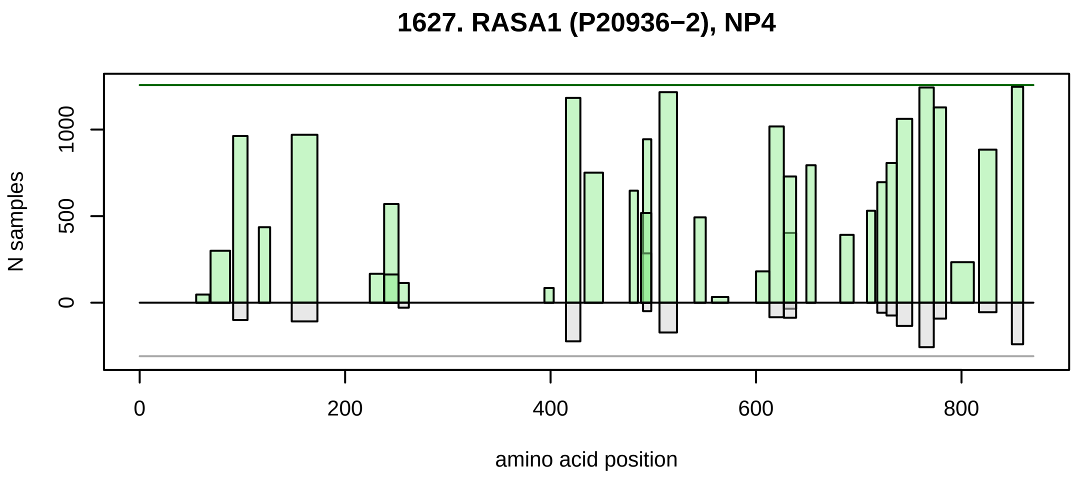
1627. RASA1 (P20936−2), NP4
N samples
0
500
1000
0
200
400
600
800
amino acid position

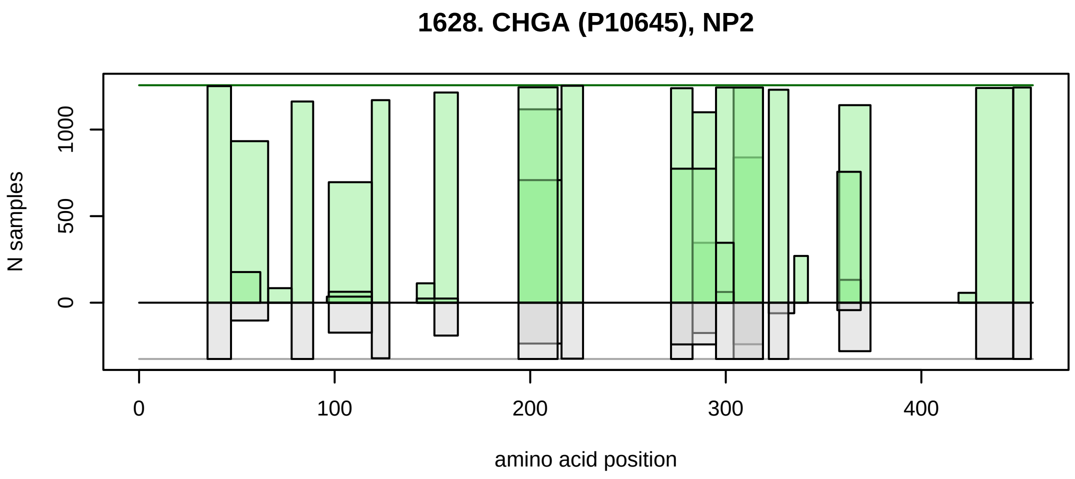
1628. CHGA (P10645), NP2
N samples
0
500
1000
0
100
200
300
400
amino acid position

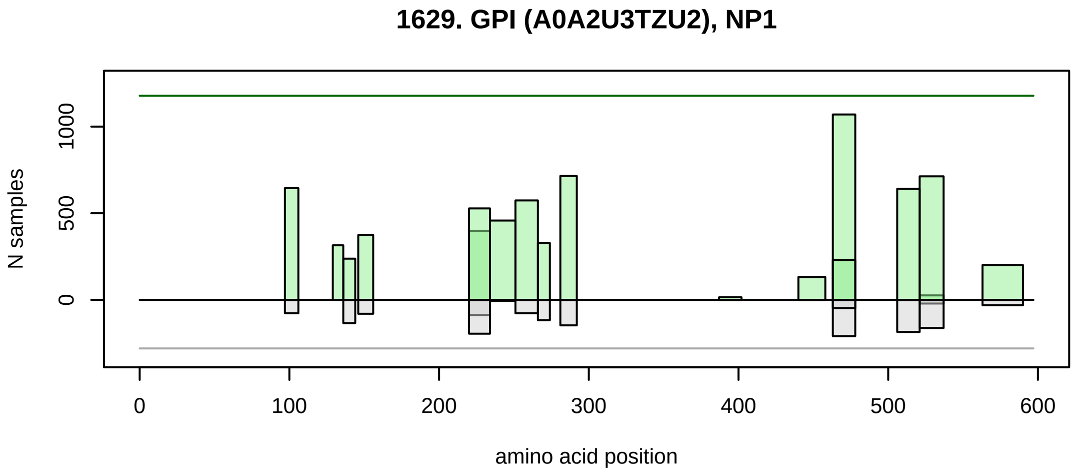
1629. GPI (A0A2U3TZU2), NP1
N samples
0
500
1000
0
100
200
300
400
500
600
amino acid position

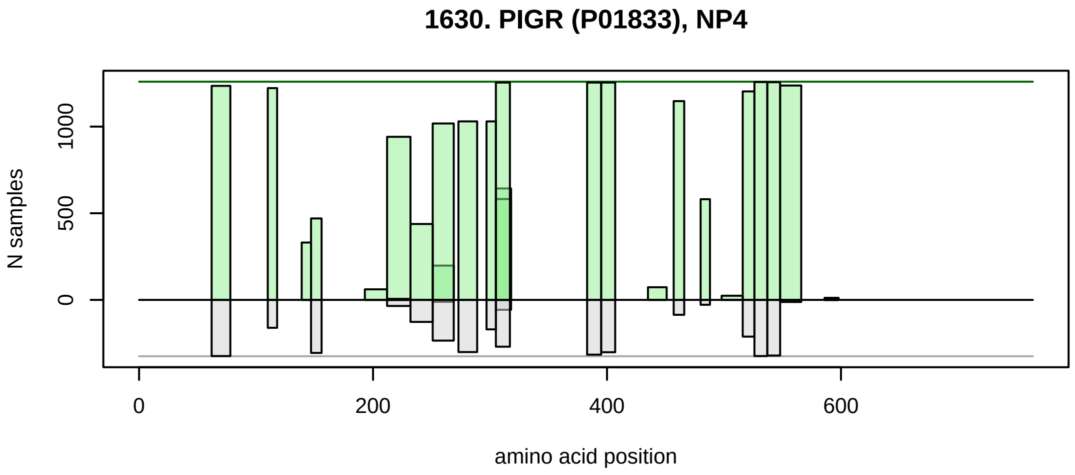
1630. PIGR (P01833), NP4
N samples
0
500
1000
0
200
400
600
amino acid position

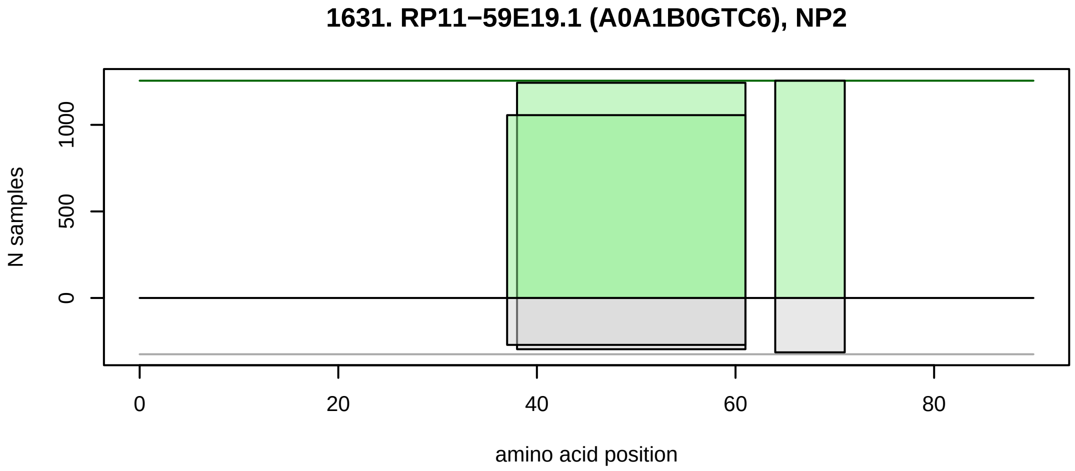
1631. RP11−59E19.1 (A0A1B0GTC6), NP2
N samples
0
500
1000
0
20
40
60
80
amino acid position

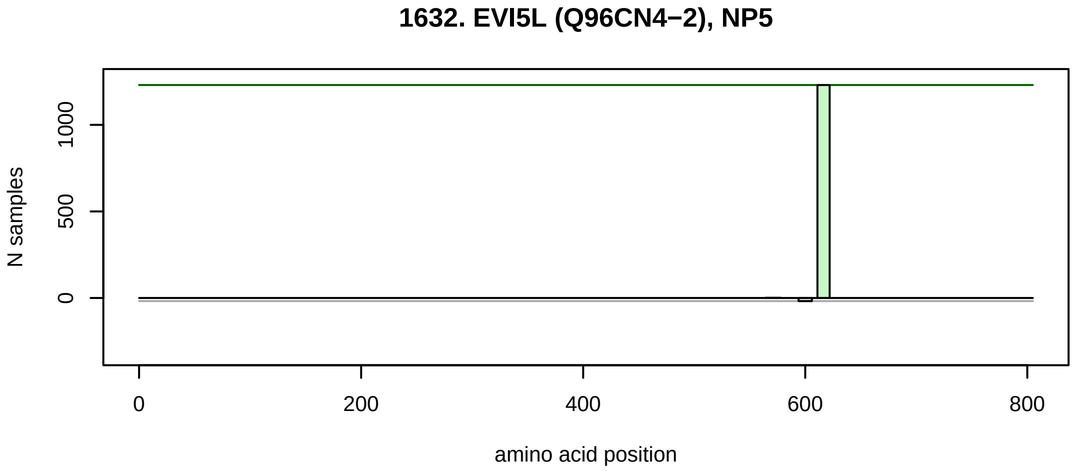
1632. EVI5L (Q96CN4−2), NP5
N samples
0
500
1000
0
200
400
600
800
amino acid position

1633. AGRN (O00468−6), NP2

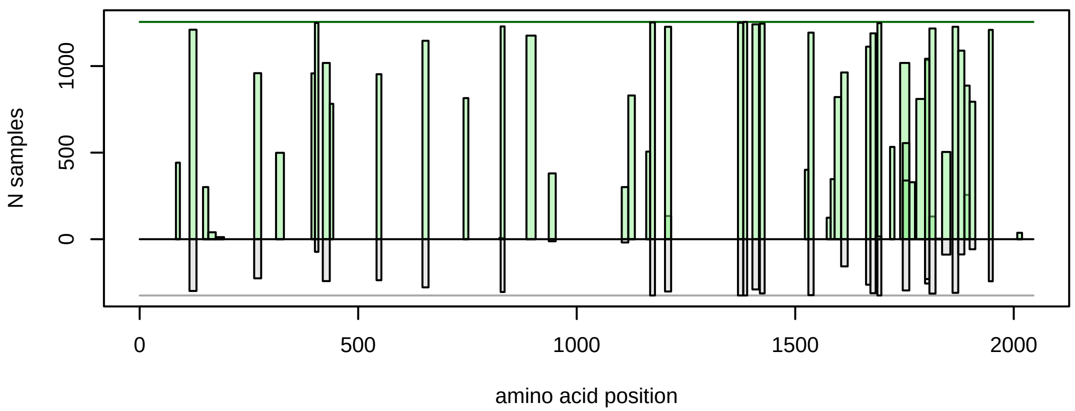

1634. ZCCHC6 (Q5VYS8), NP3

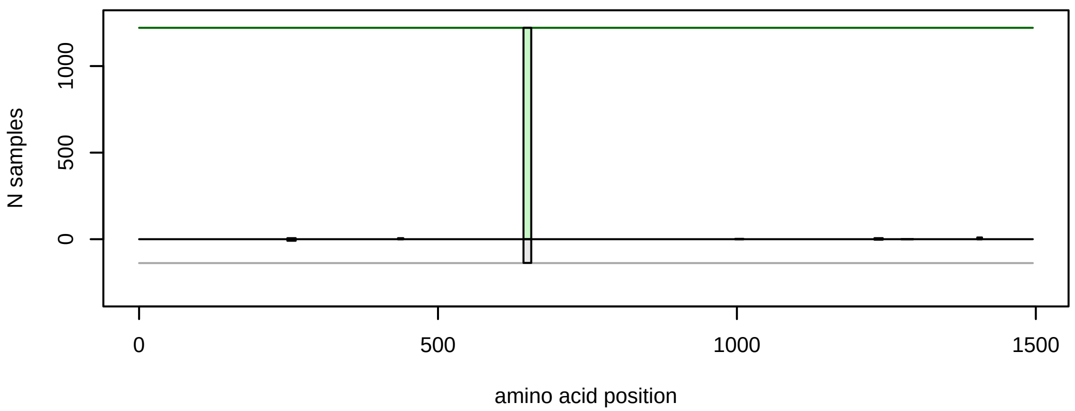

1635. USP5 (P45974−2), NP5

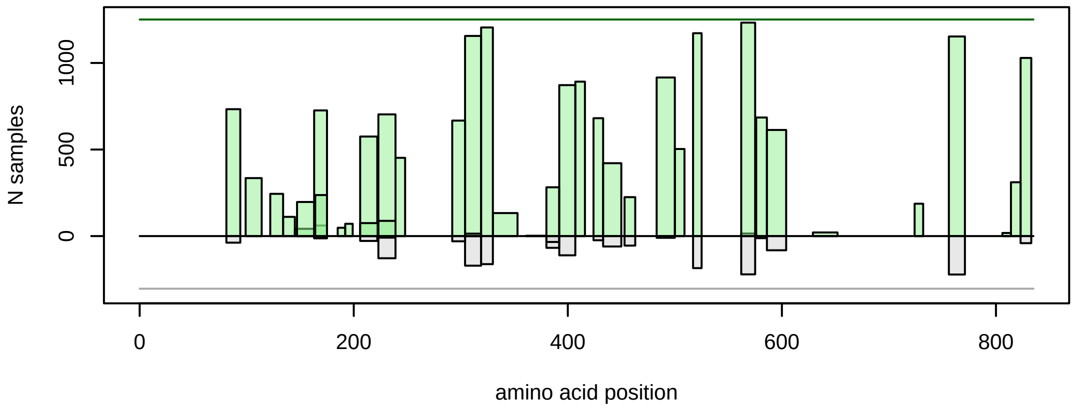

1636. AGR3 (Q8TD06), NP3

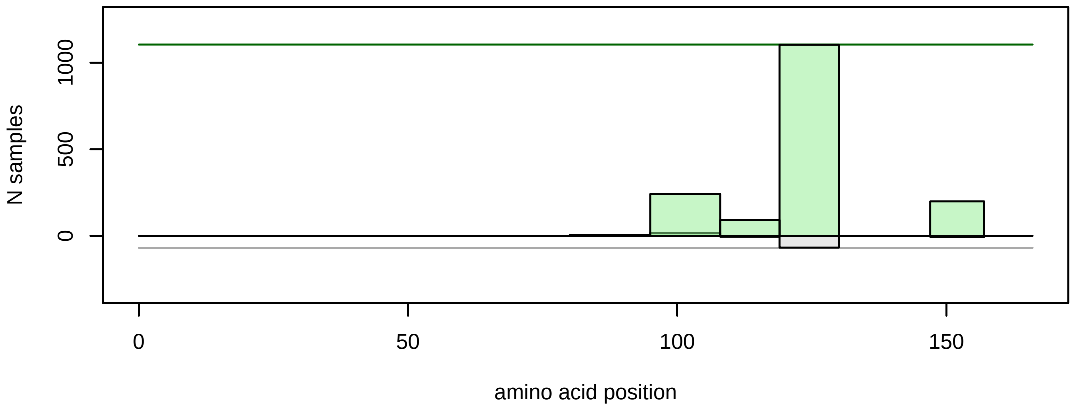

1637. FAM132B (Q4G0M1), NP4

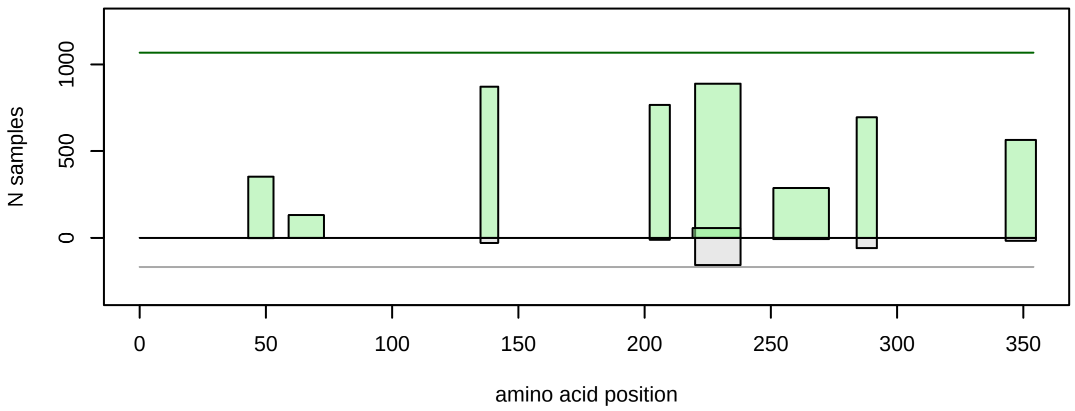

1638. USO1 (O60763;O60763−2), NP2

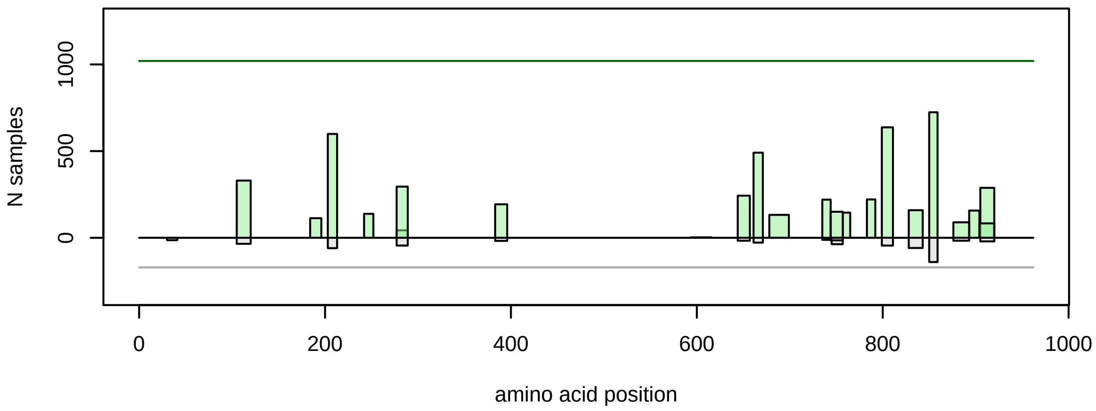

1639. ITGBL1 (O95965), NP2

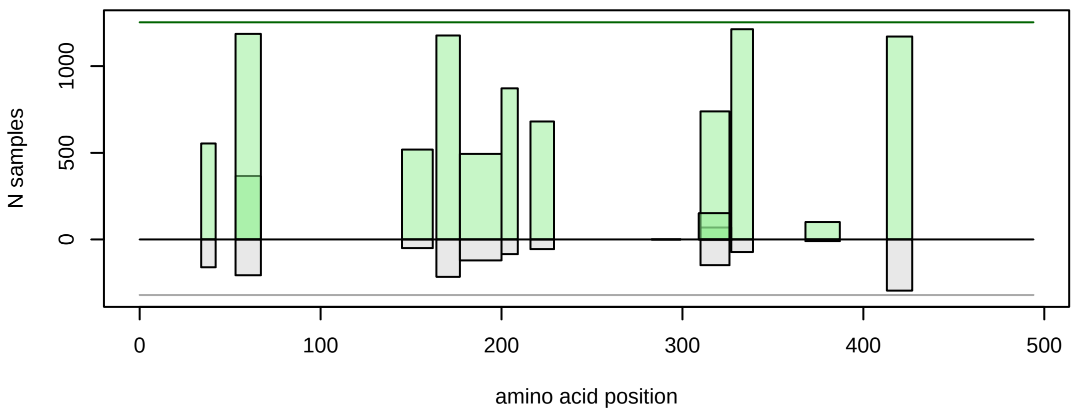

1640. SEPT7 (Q16181), NP3

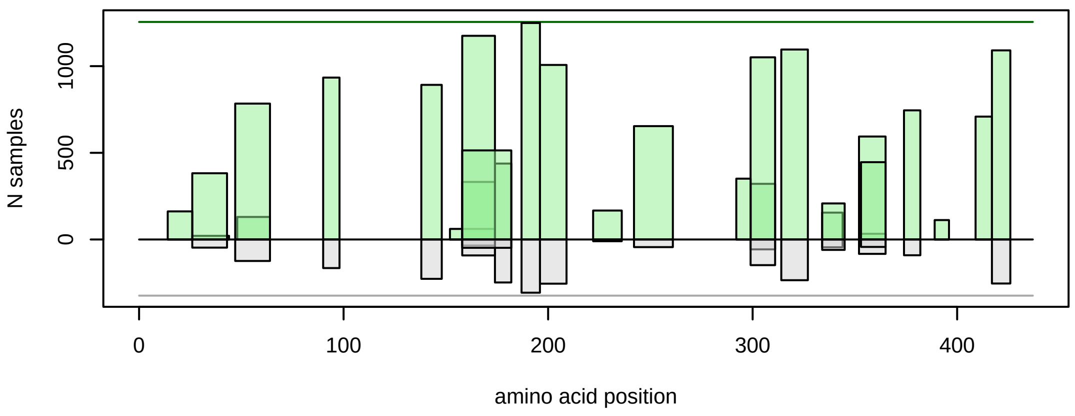

1641. SND1 (Q7KZF4), NP5

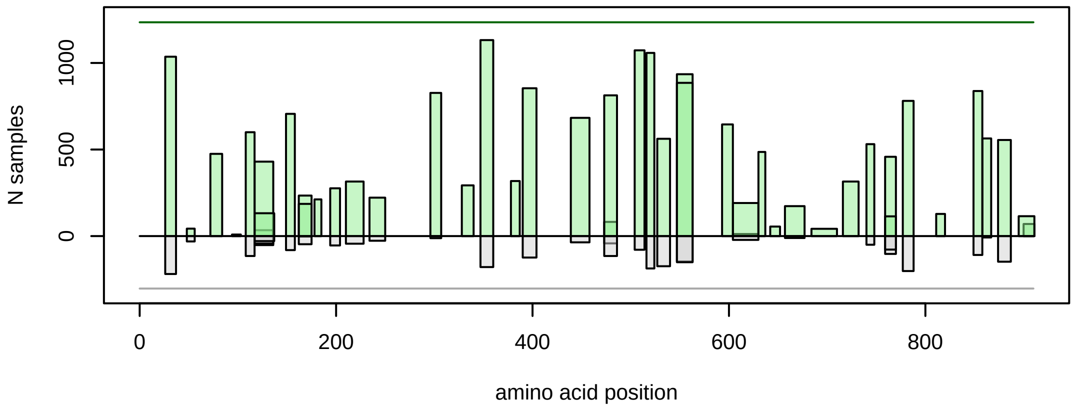

1642. CBR1 (P16152), NP4

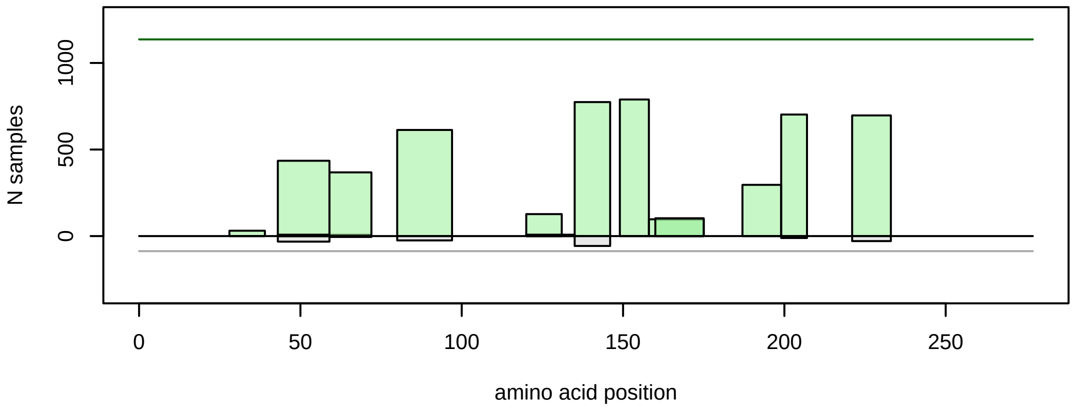

1643. GDI2 (P50395), NP1

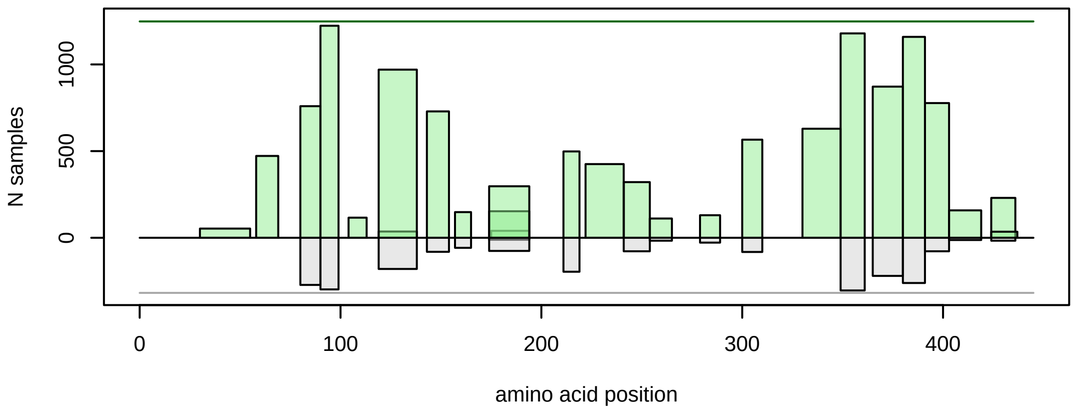

1644. PA2G4 (Q9UQ80), NP3

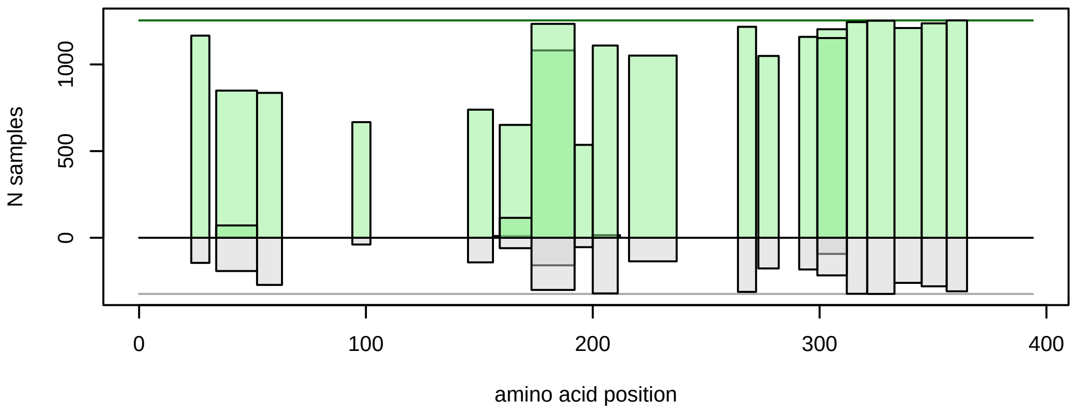

1645. CALU (O43852;O43852−3), NP2

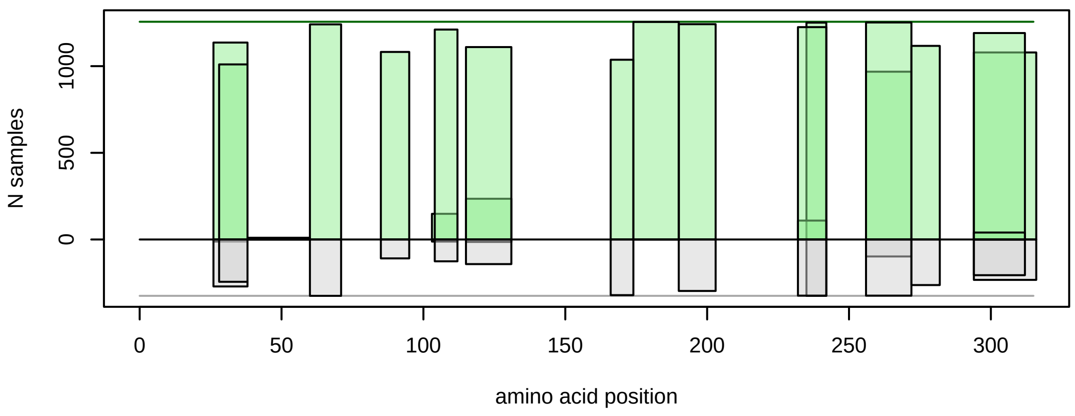

1646. CALM2 (P0DP24), NP2

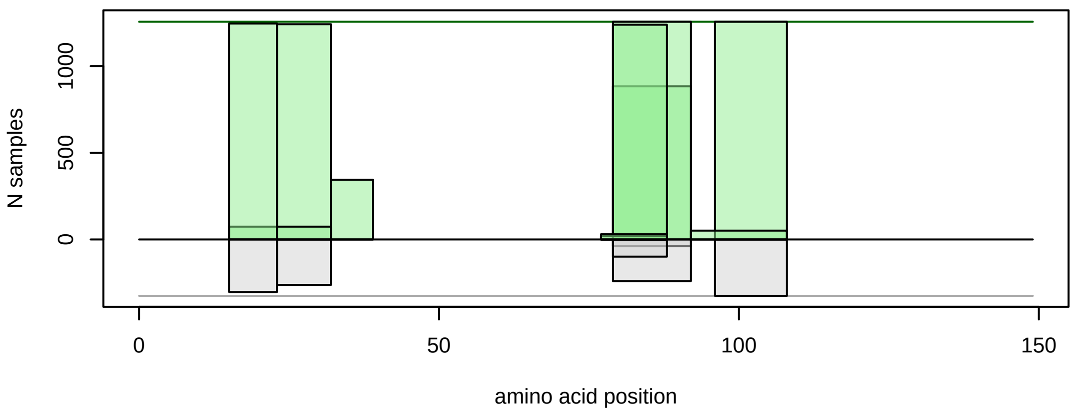

1647. OS9 (Q13438), NP2

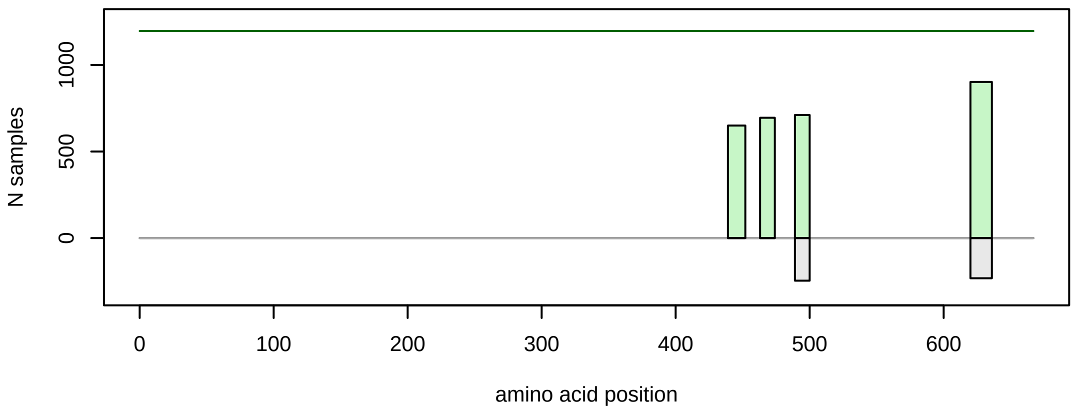

1648. PPM1B (O75688), NP2

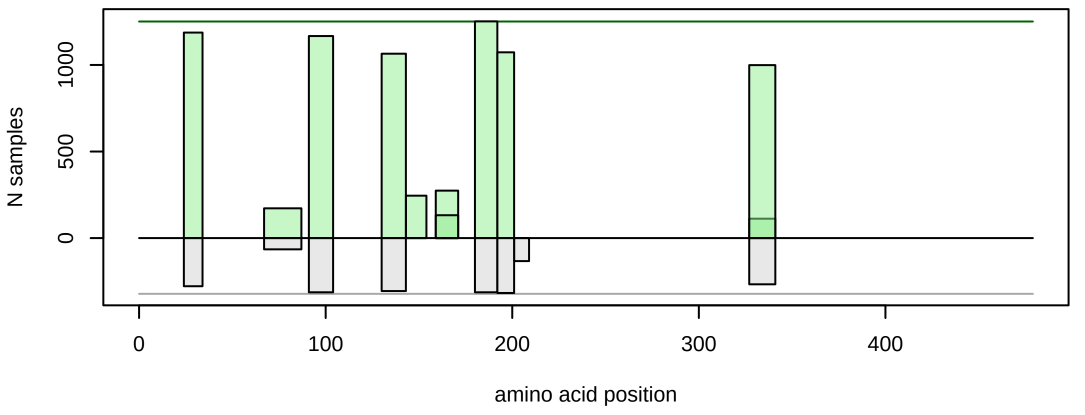

1649. HNRNPL (P14866), NP1

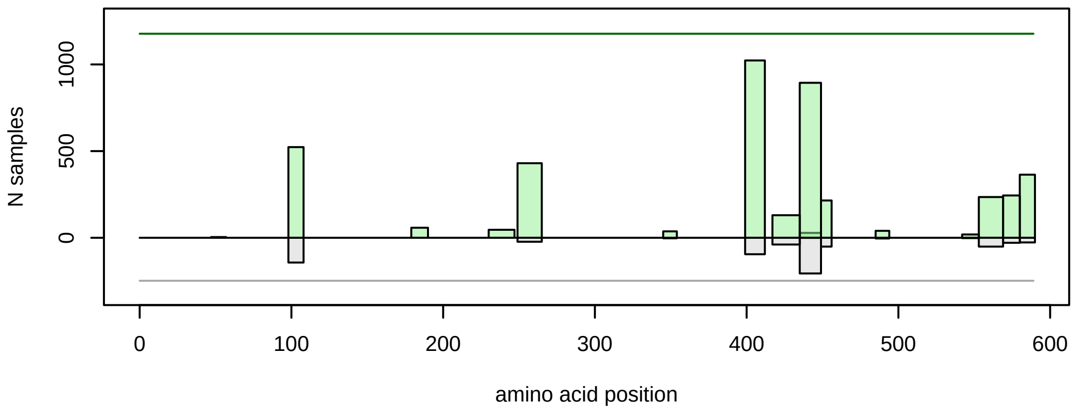

1650. PITHD1 (Q9GZP4), NP2

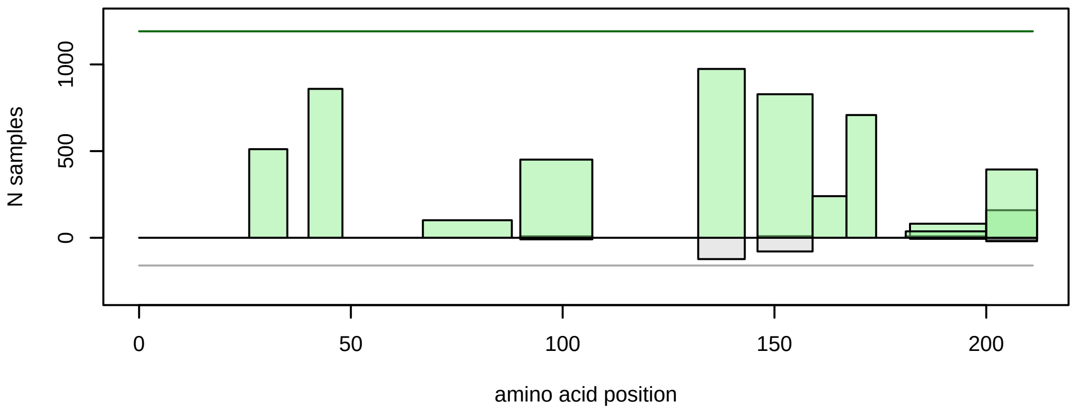

1651. VWF (P04275), NP4

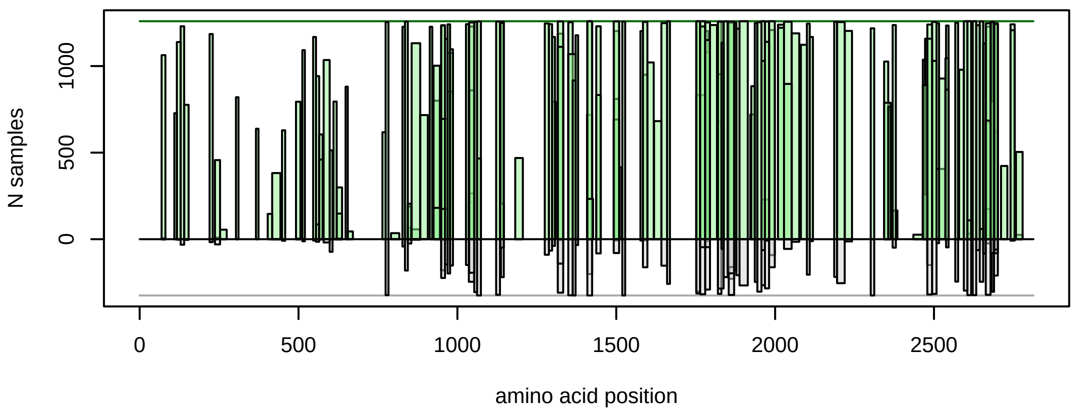

1652. PDCD6IP (Q8WUM4), NP1

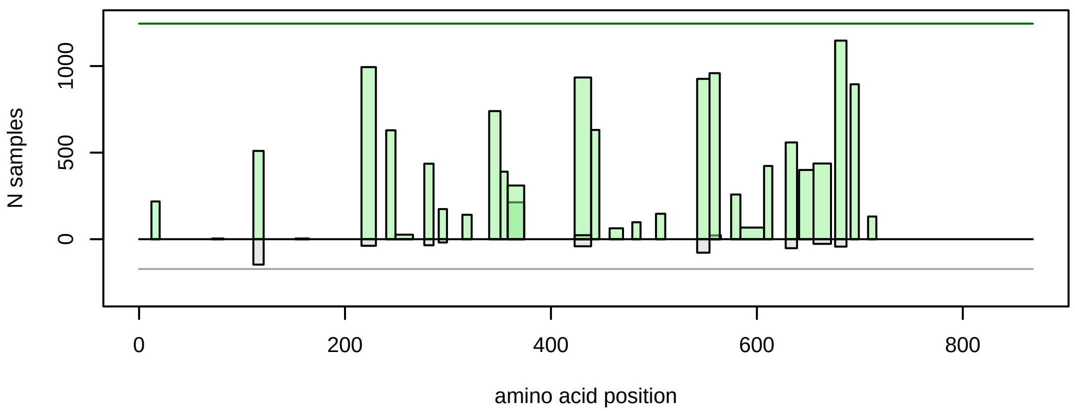

1653. CHMP1B (Q7LBR1), NP3

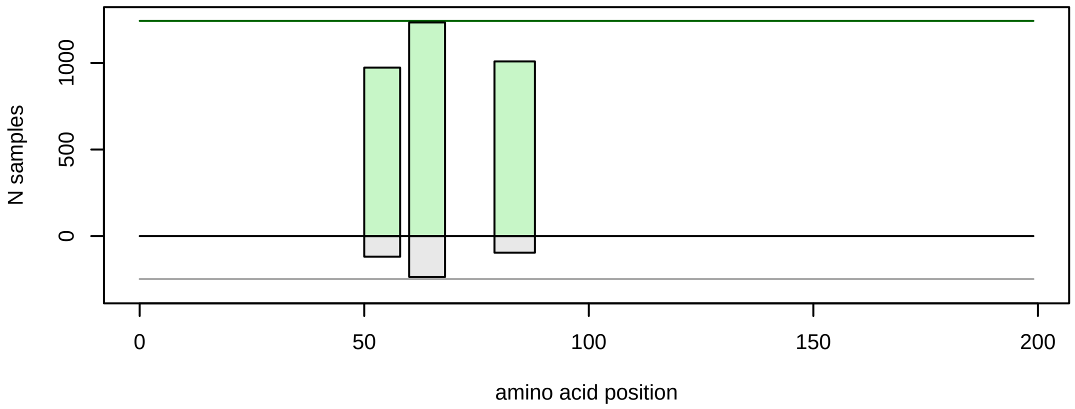

1654. DNM1L (O00429), NP4

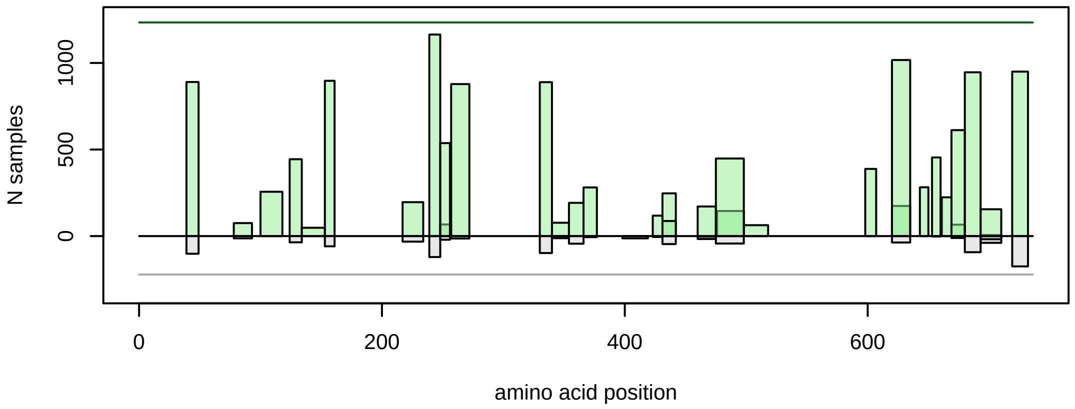

1655. PGK1 (P00558), NP1

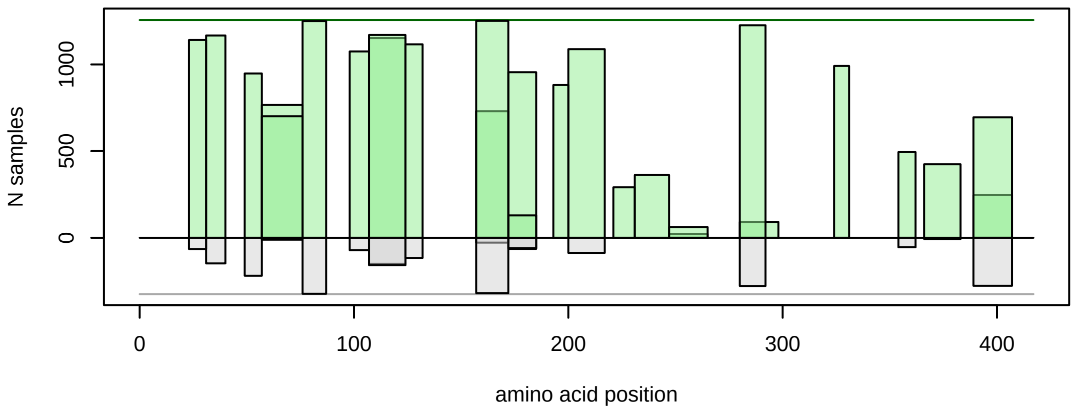

1656. FCER1G (P30273), NP1

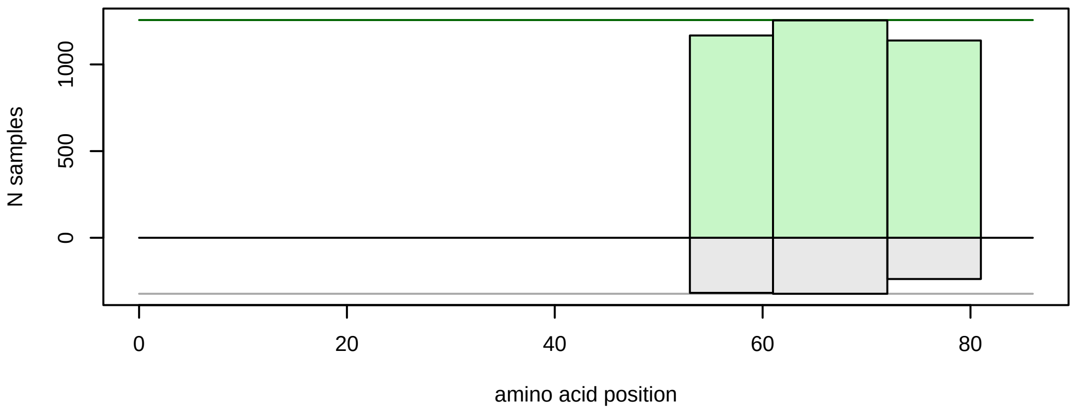

1657. UBR4 (Q5T4S7), NP2

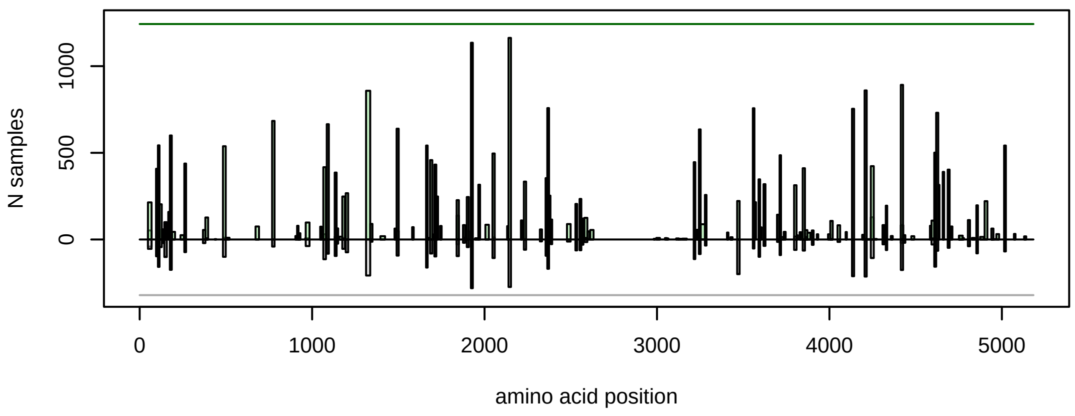

1658. RPN2 (P04844−2), NP4

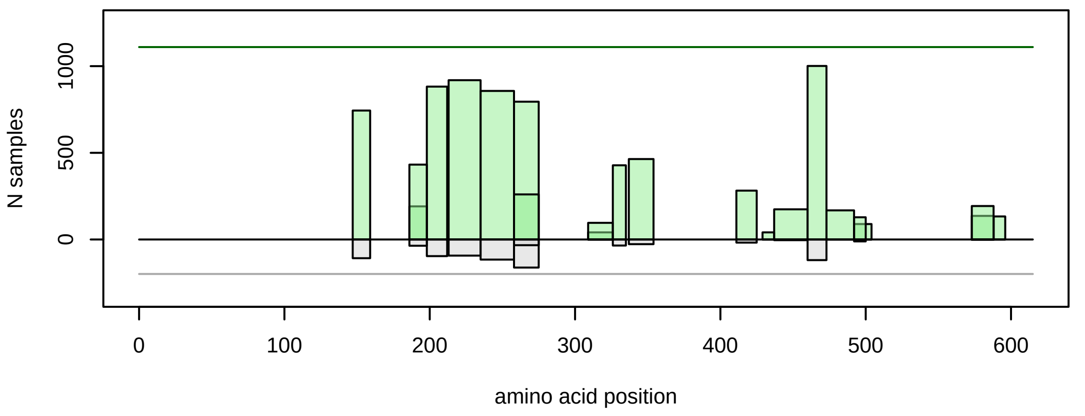

1659. SERPINA5 (P05154), NP3

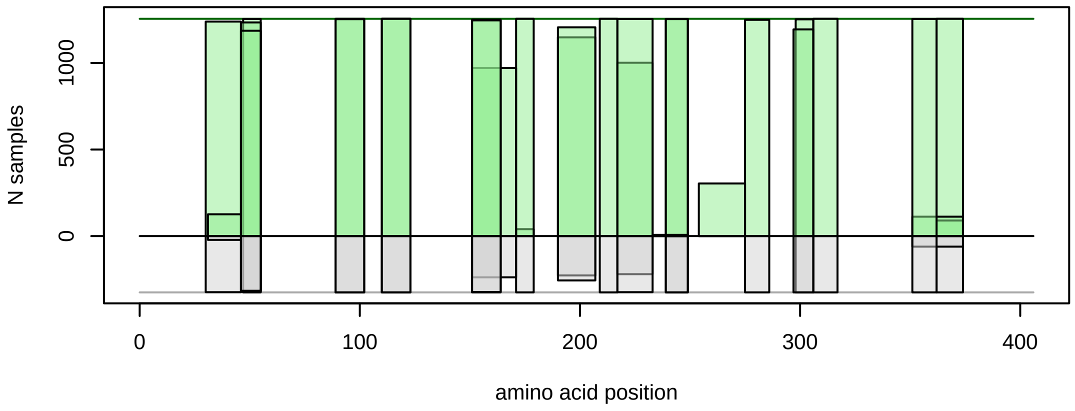

1660. DPP3 (Q9NY33), NP5

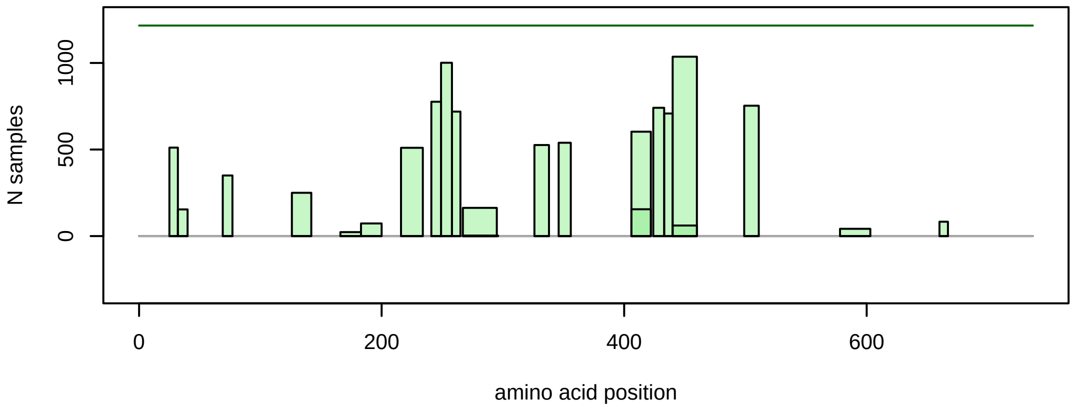

1661. RGS10 (O43665), NP3

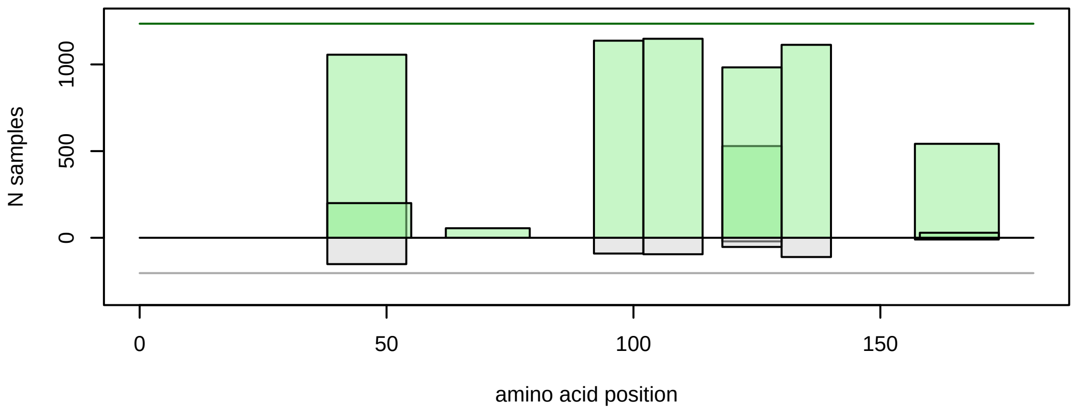

1662. MYH11 (P35749−4), NP4

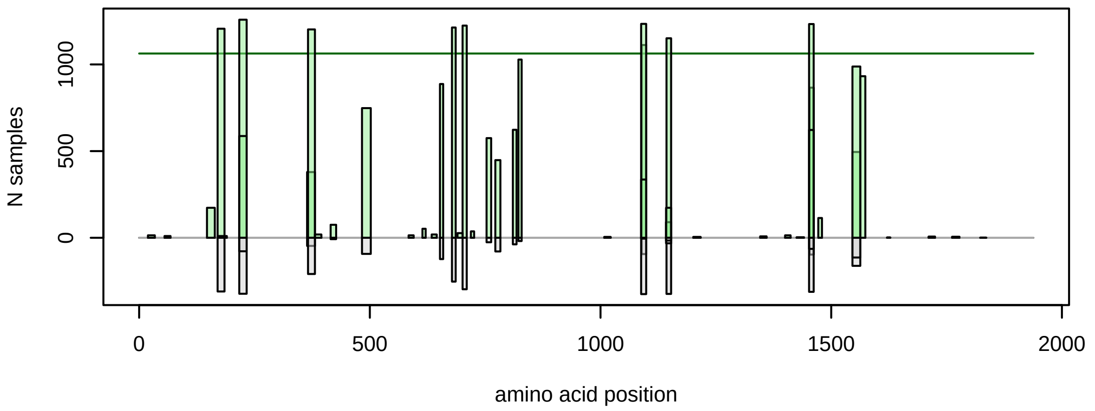

1663. TXNDC5 (Q8NBS9), NP2

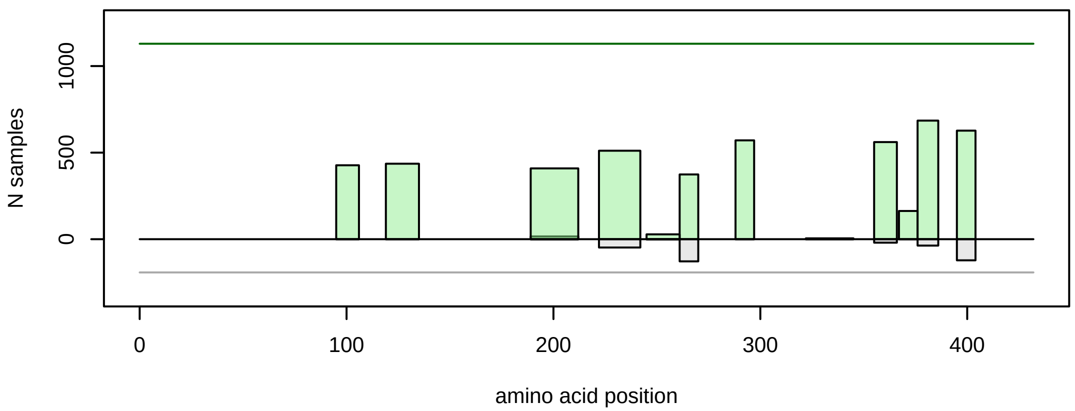

1664. IGLV1−47 (P01700), NP3

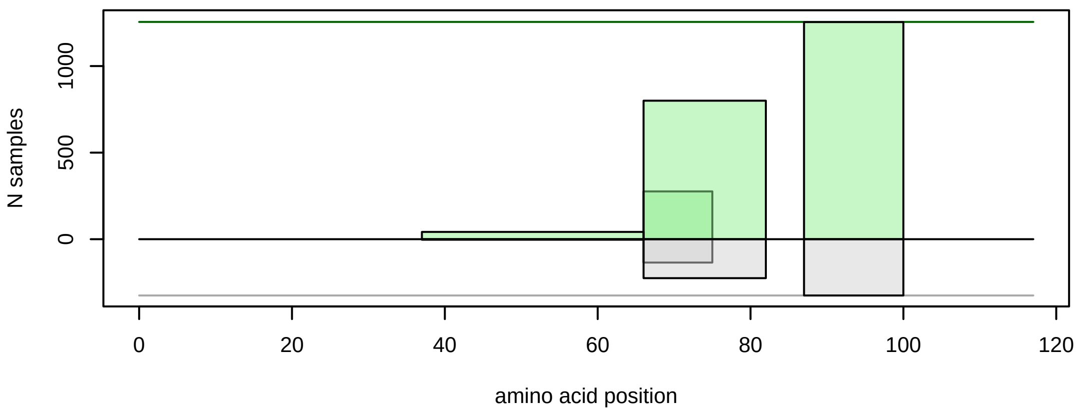

1665. GPT (P24298), NP5

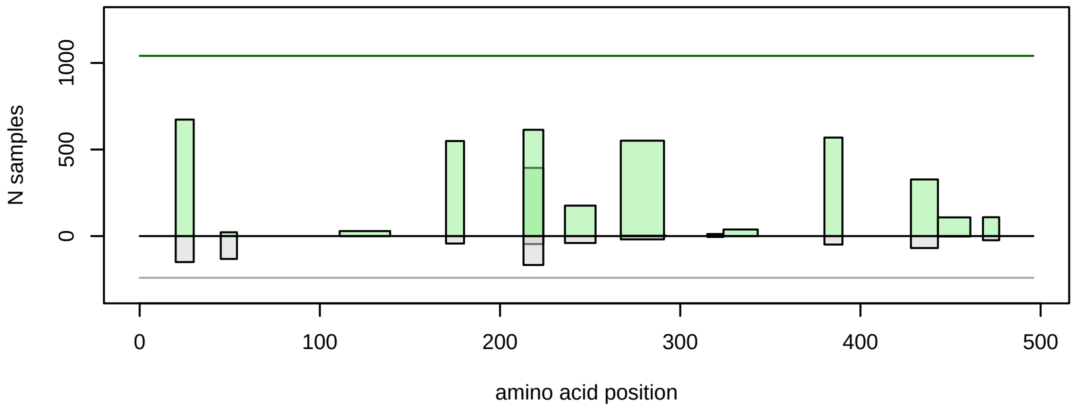

1666. ROCK2 (O75116), NP1

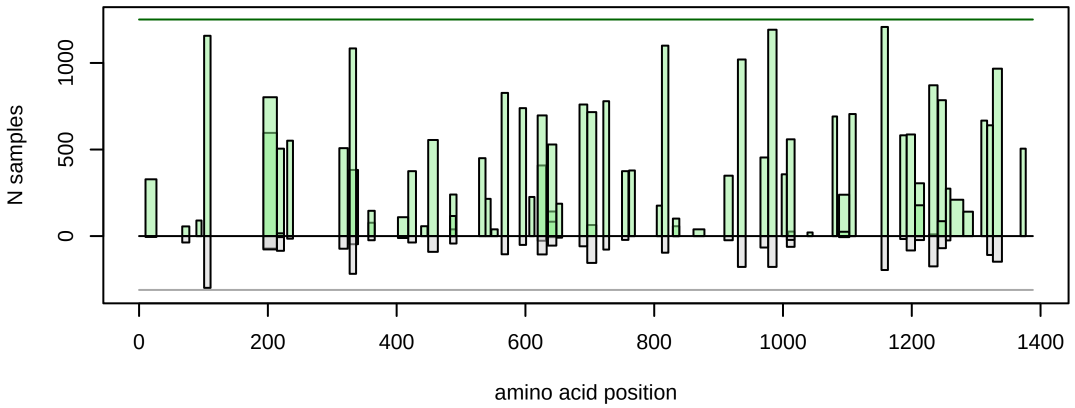

1667. LSM3 (P62310), NP1

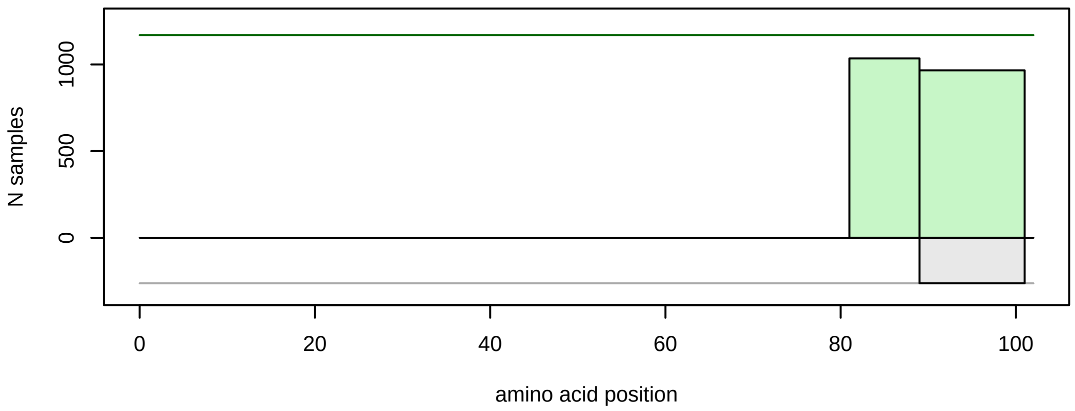

1668. LCN2 (X6R8F3), NP3

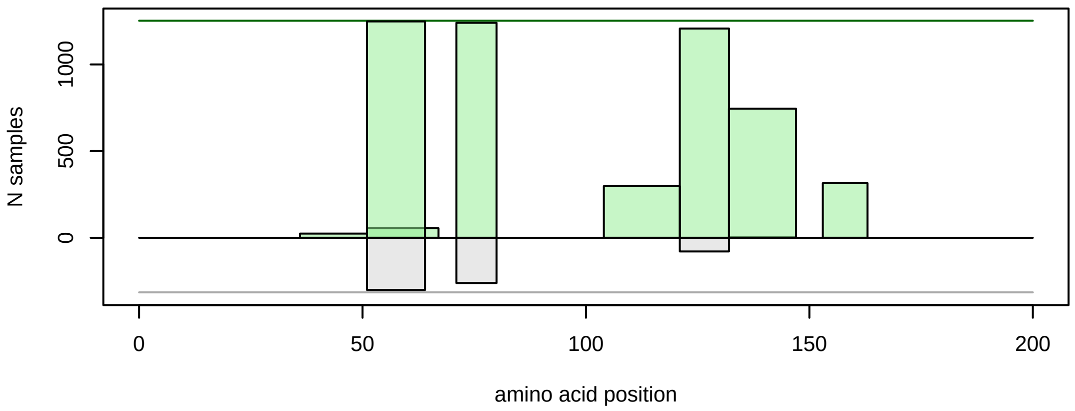

1669. MESDC2 (Q14696), NP2

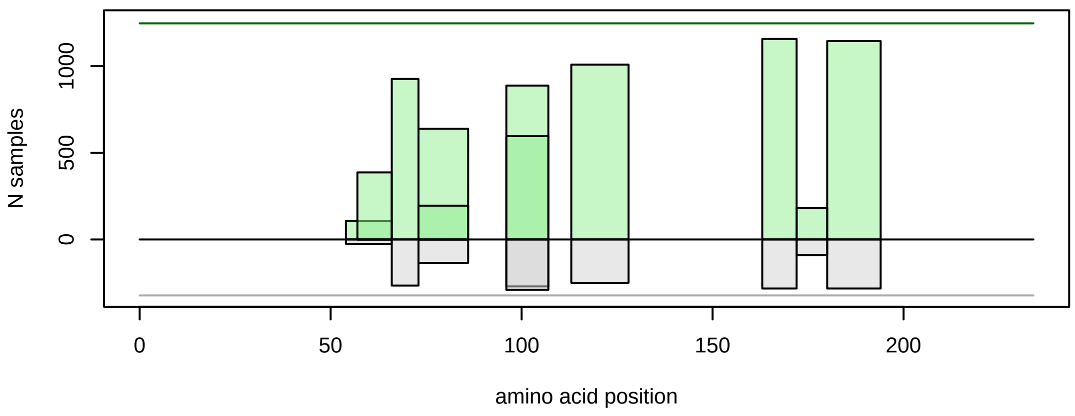

1670. ASNA1 (O43681), NP2

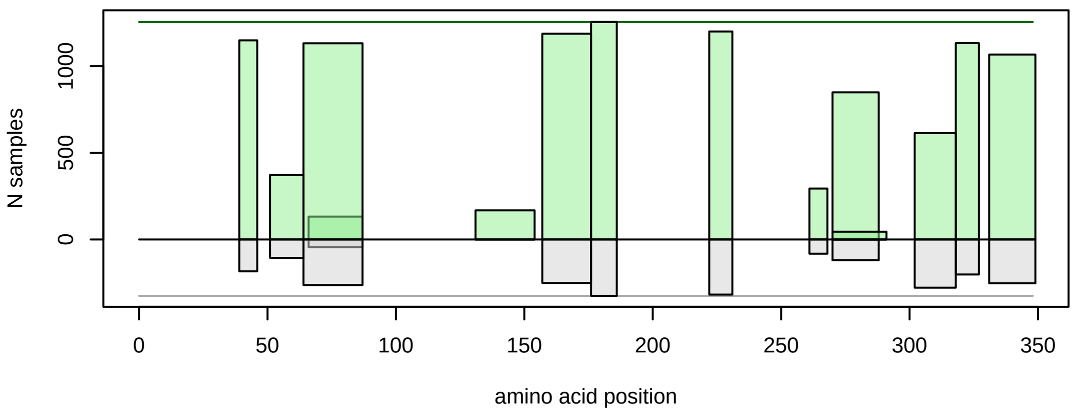

1671. CHMP6 (Q96FZ7), NP1

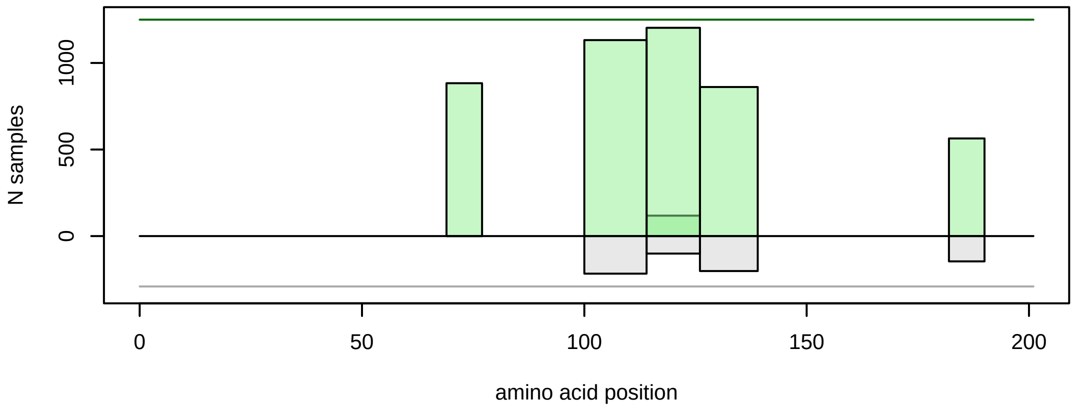

1672. HIST1H1B (P16401), NP4

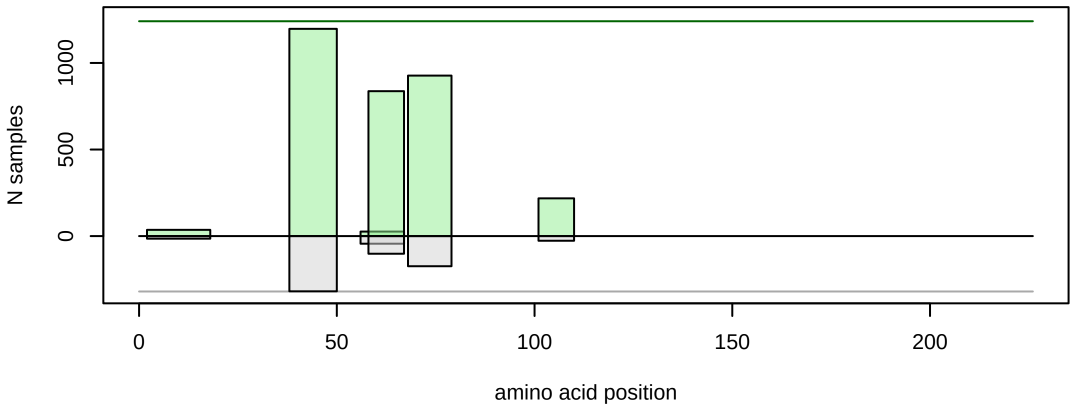

1673. LUM (P51884), NP2

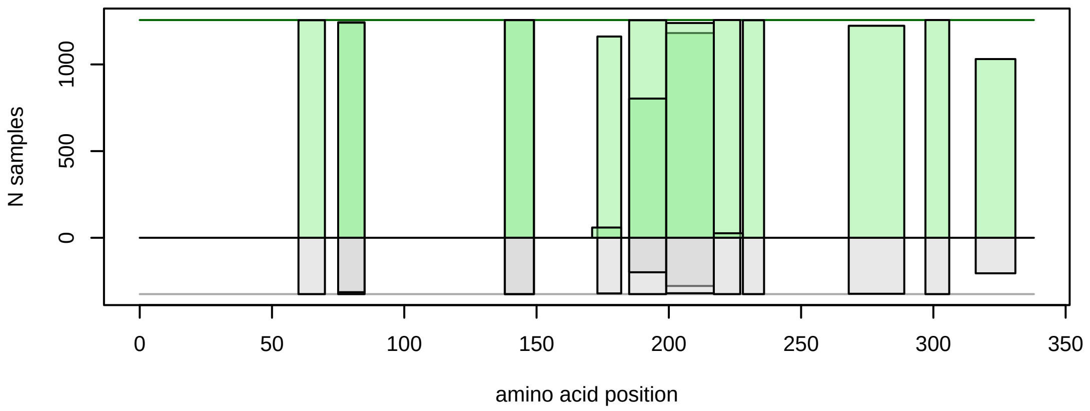

1674. STOM (P27105), NP1

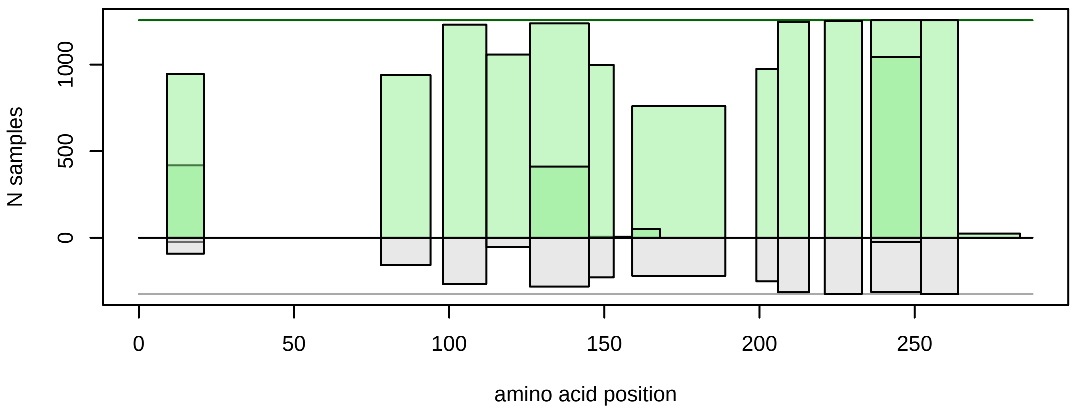

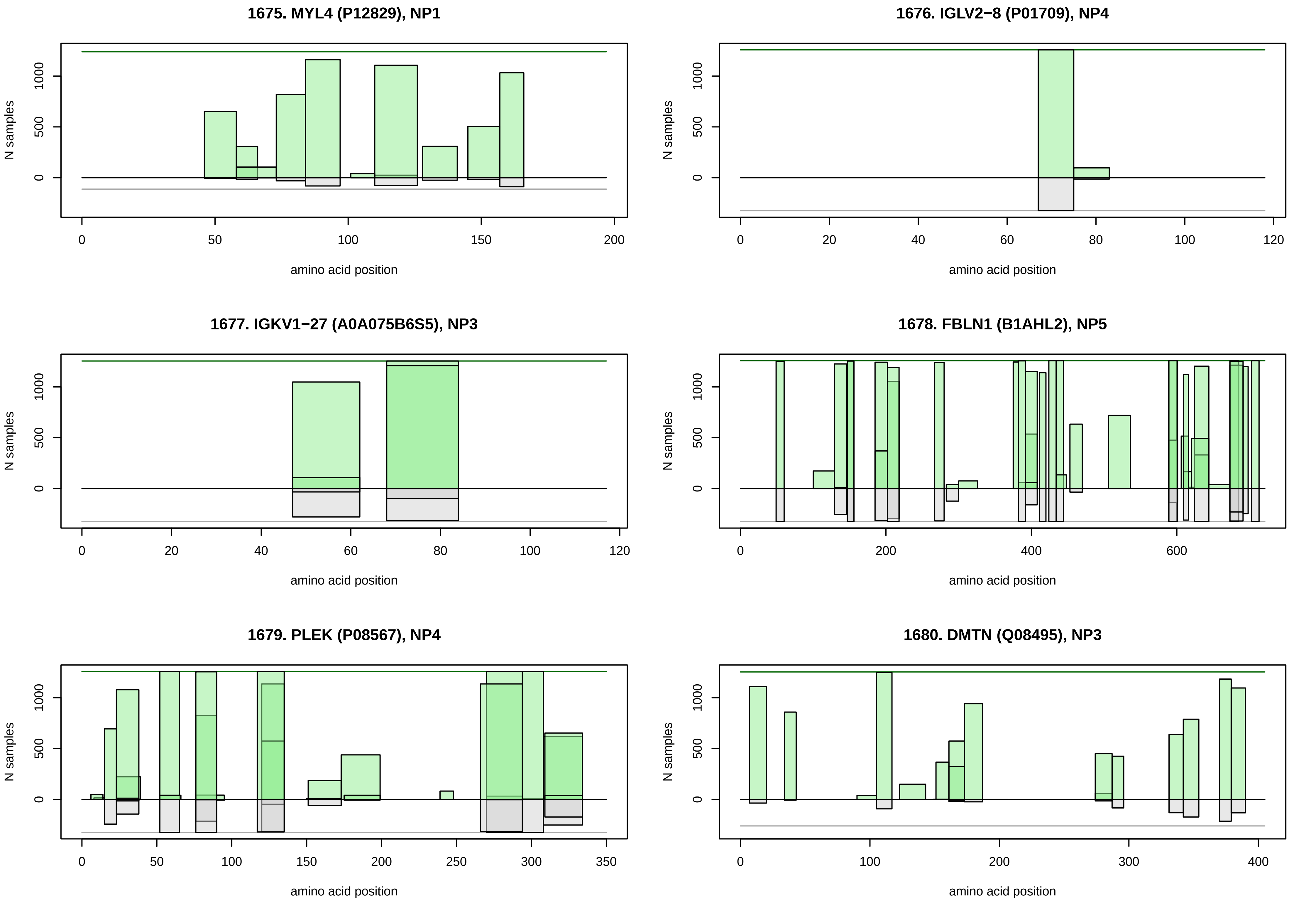
1675. MYL4 (P12829), NP1
1676. IGLV2−8 (P01709), NP4
1677. IGKV1−27 (A0A075B6S5), NP3
1678. FBLN1 (B1AHL2), NP5
1679. PLEK (P08567), NP4
1680. DMTN (Q08495), NP3
N samples
amino acid position
0
500
1000
0
50
100
150
200
20
40
60
80
120
400
600
250
300
350

1681. IGHV3−49 (A0A0A0MS15), NP3

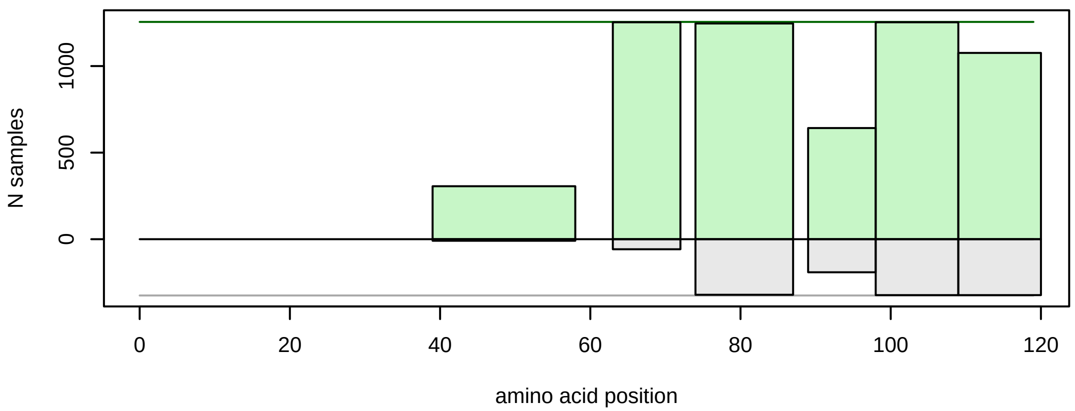

1682. ITGAL (P20701), NP1

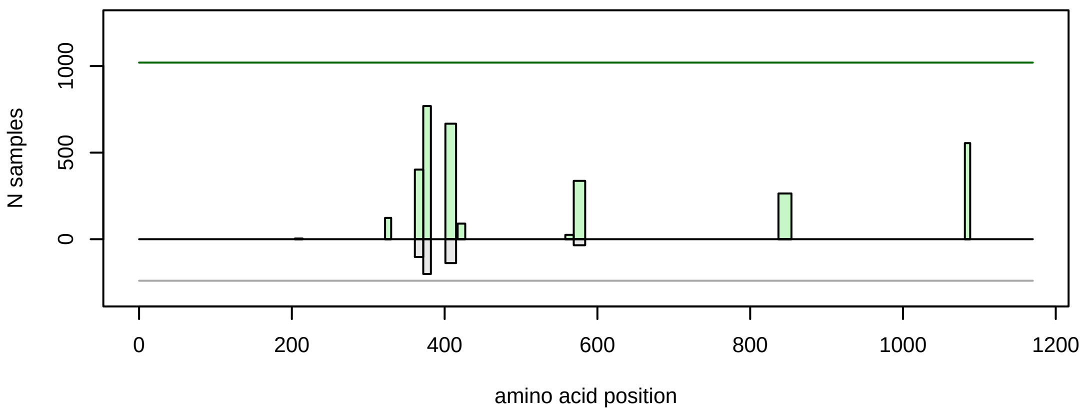

1683. VARS (P26640), NP4

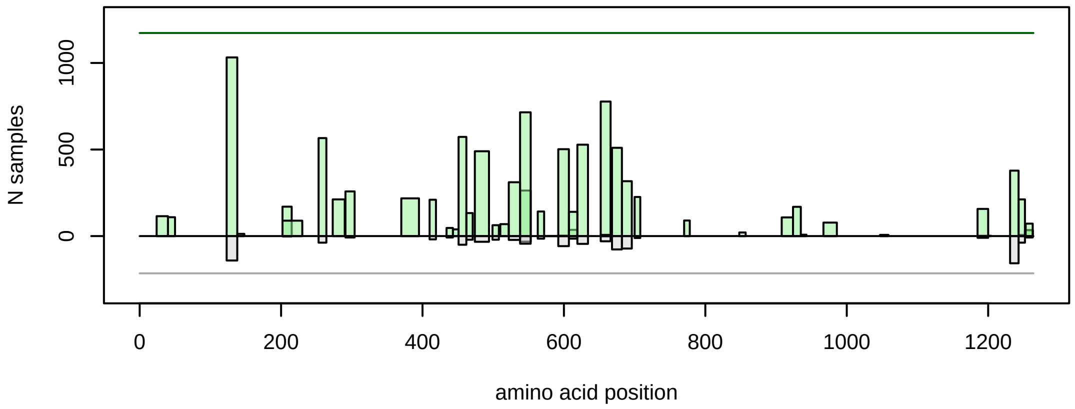

1684. CD53 (P19397), NP2

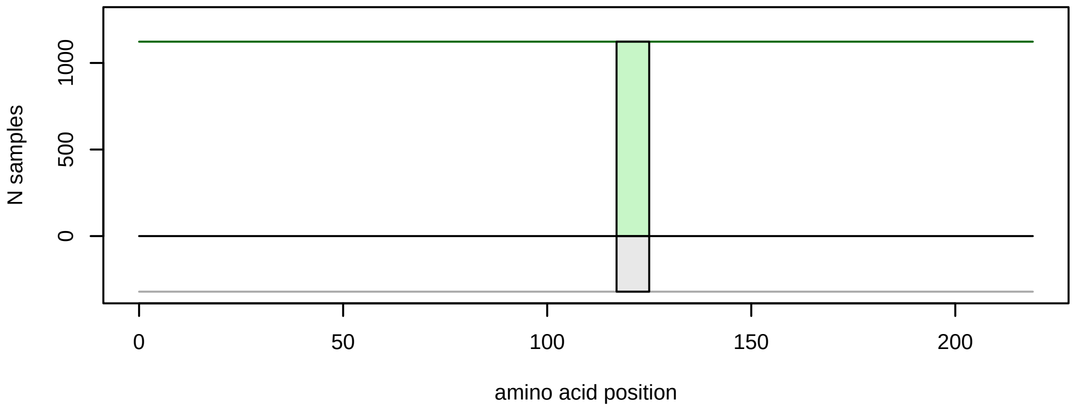

1685. UBE2H (P62256), NP2

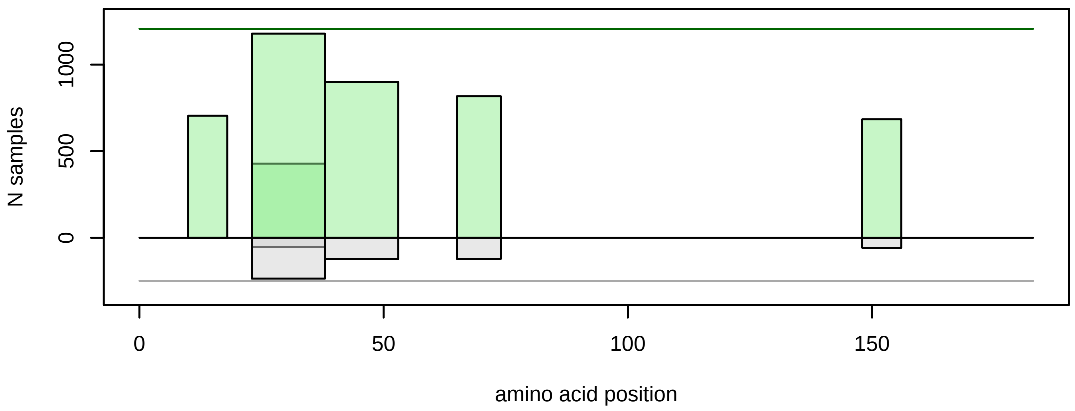

1686. IPO7 (O95373), NP2

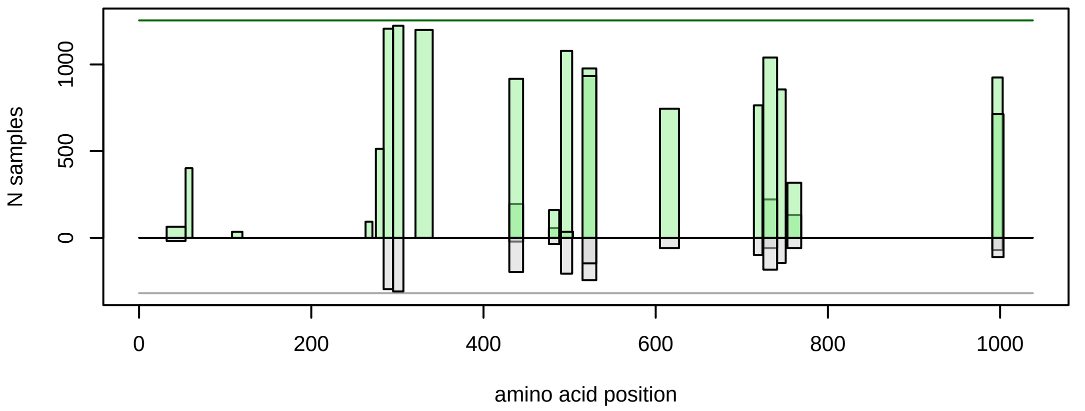

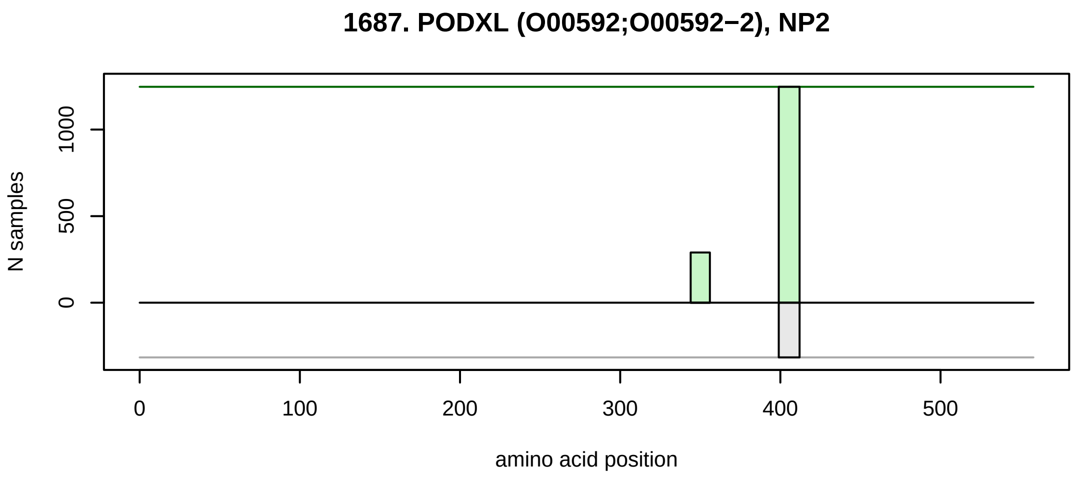
1687. PODXL (O00592;O00592−2), NP2
N samples
amino acid position

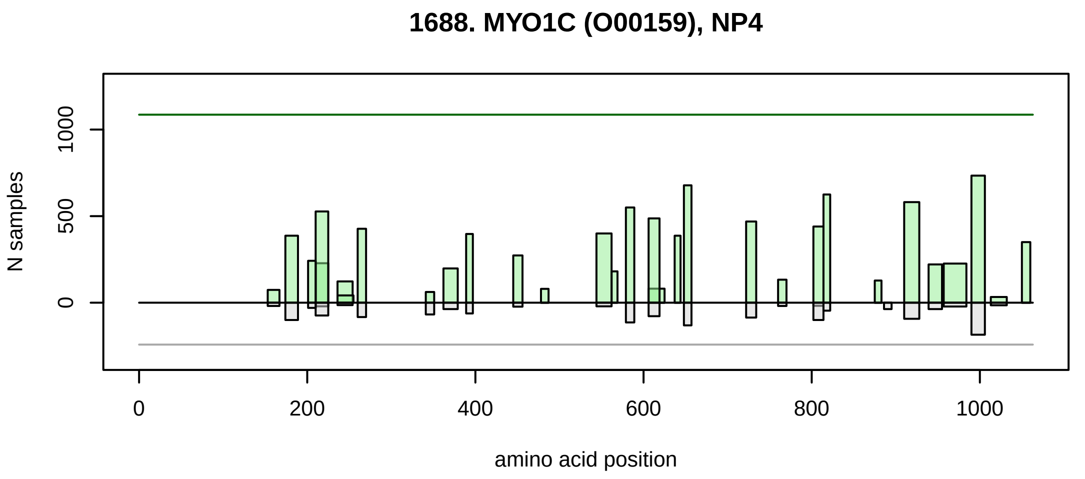
1688. MYO1C (O00159), NP4
N samples
amino acid position

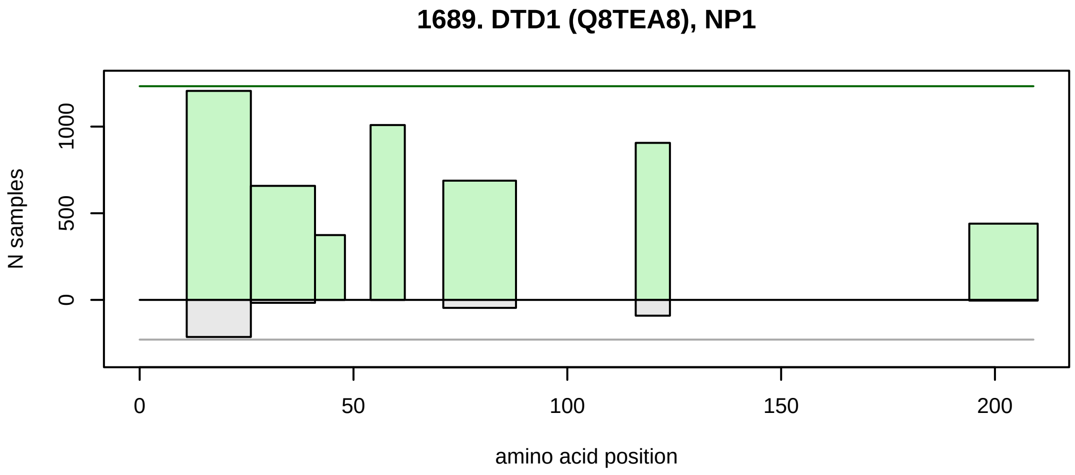
1689. DTD1 (Q8TEA8), NP1
N samples
amino acid position

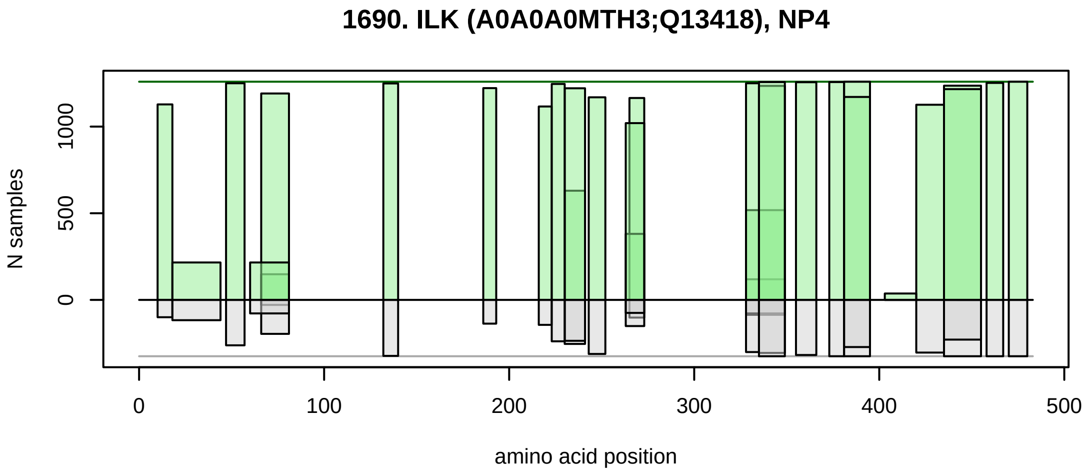
1690. ILK (A0A0A0MTH3;Q13418), NP4
N samples
amino acid position

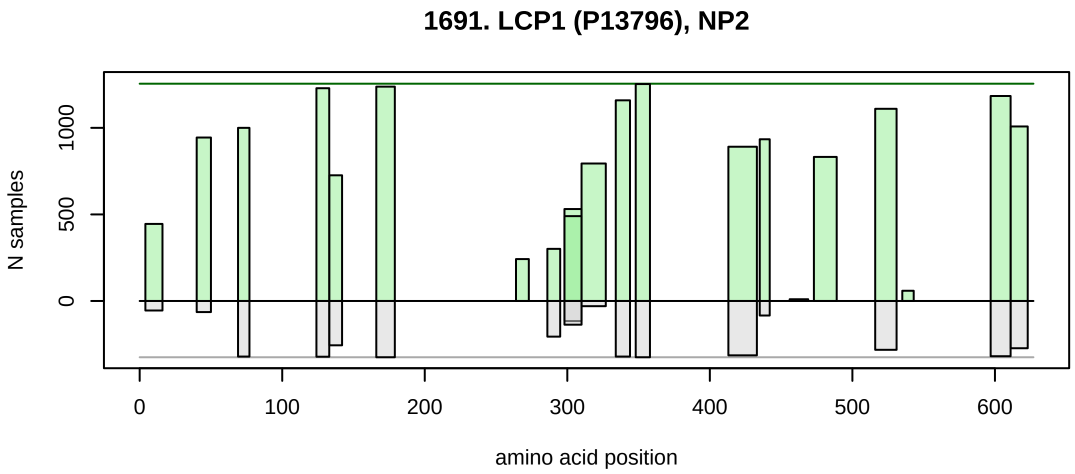
1691. LCP1 (P13796), NP2
N samples
amino acid position

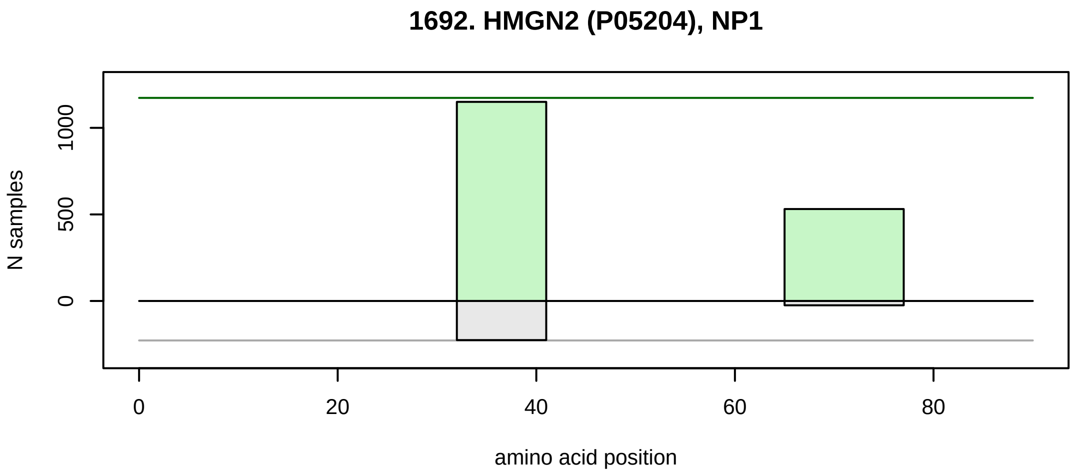
1692. HMGN2 (P05204), NP1
N samples
amino acid position

1693. XPNPEP1 (Q9NQW7−3), NP5

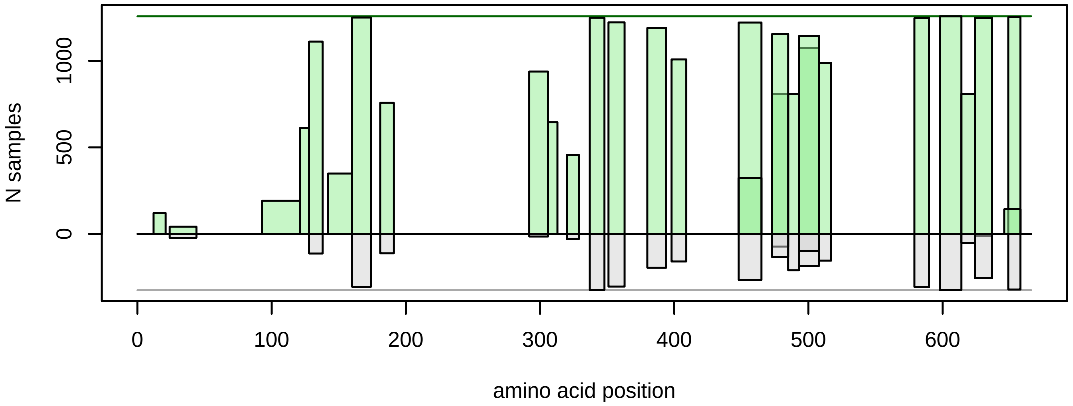

1694. MYLPF (Q96A32), NP2

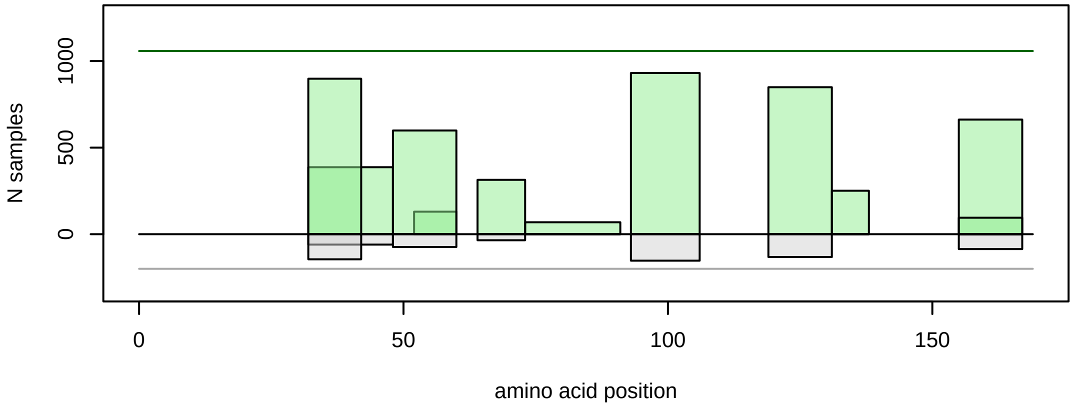

1695. CD63 (P08962;P08962−2), NP4

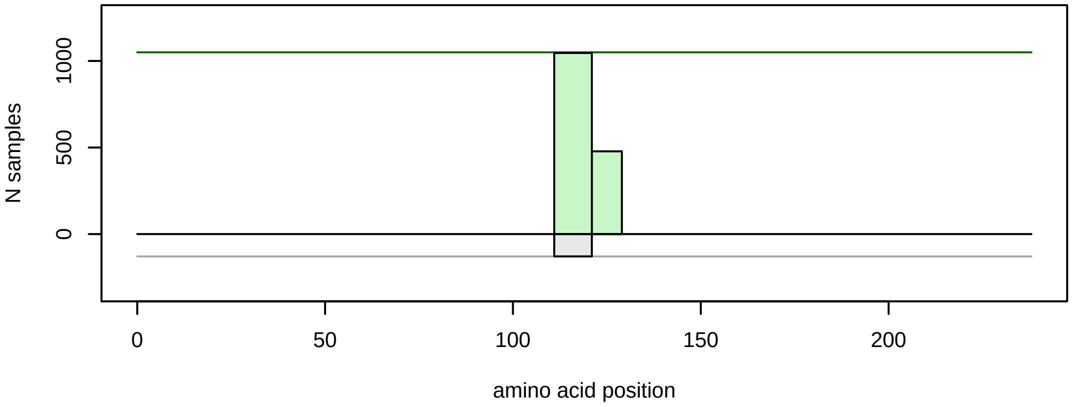

1696. LYZ (P61626), NP3

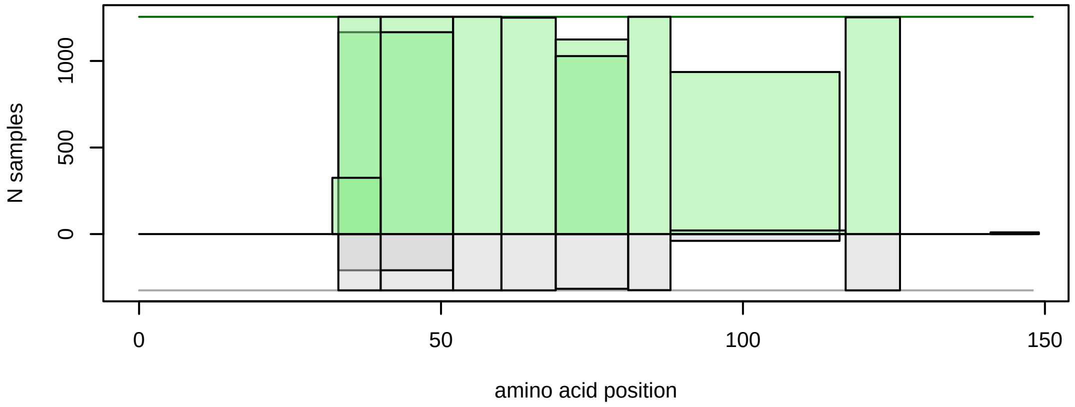

1697. TF (P02787), NP1

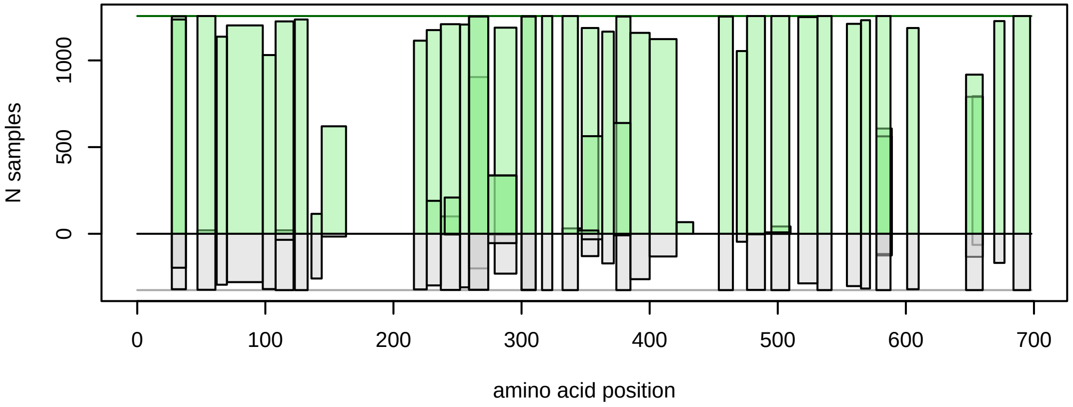

1698. UBE2L3 (P68036), NP3

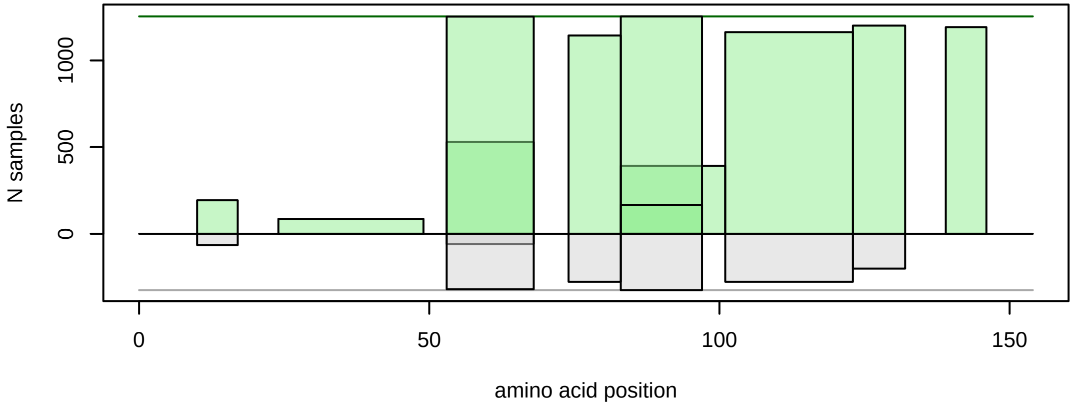

1699. SUMF2 (J3KQJ1;Q8NBJ7), NP2

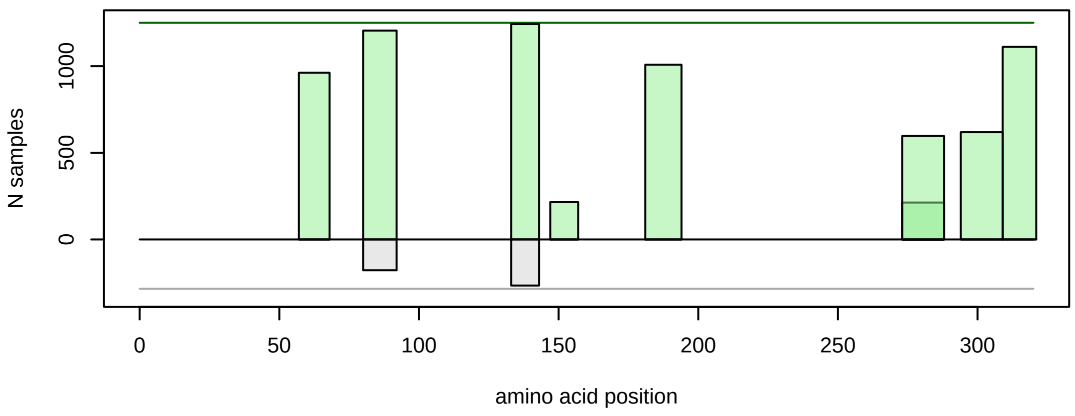

1700. SARS (Q5T5C7), NP4

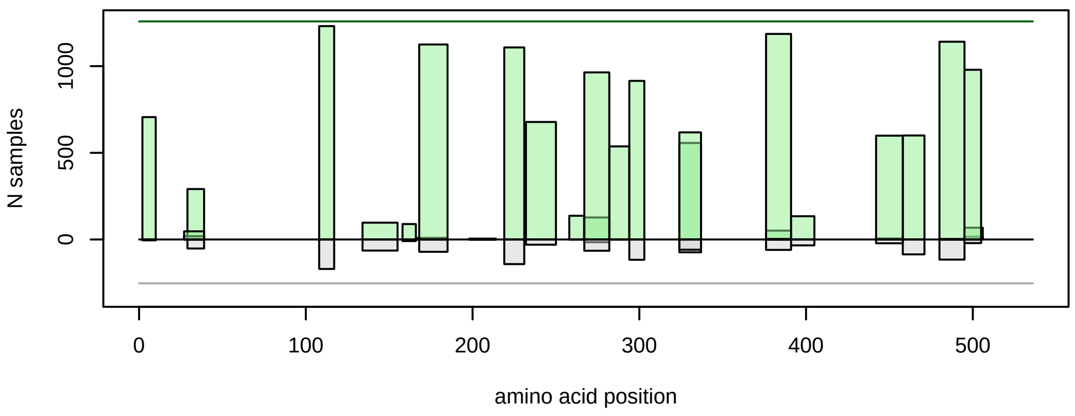

1701. PSMD4 (P55036), NP2

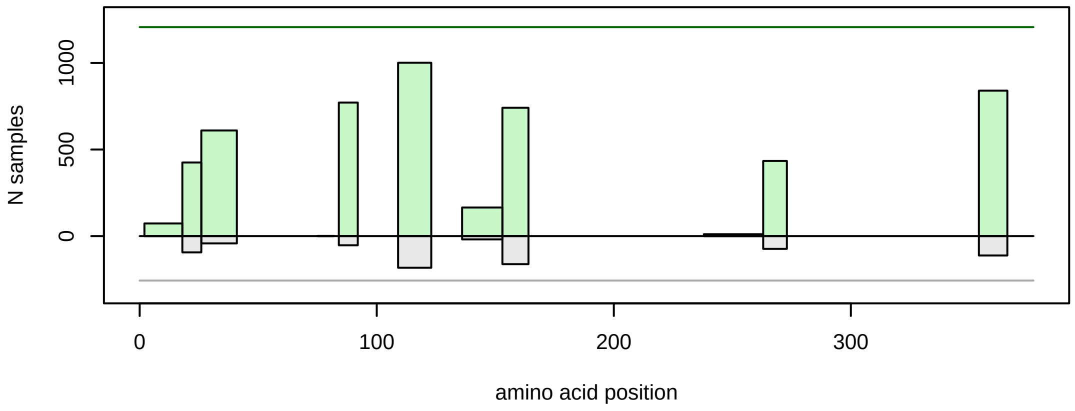

1702. CETP (P11597), NP4

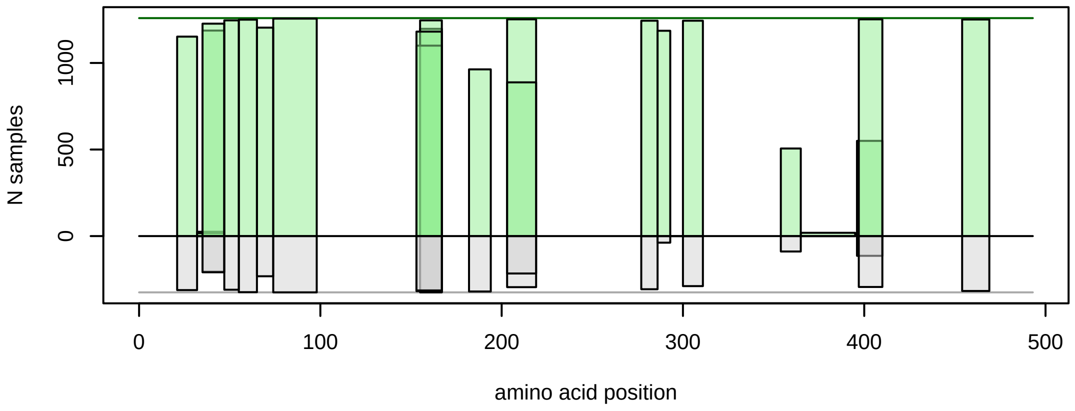

1703. CAPNS1 (A0A0C4DGQ5;P04632), NP5

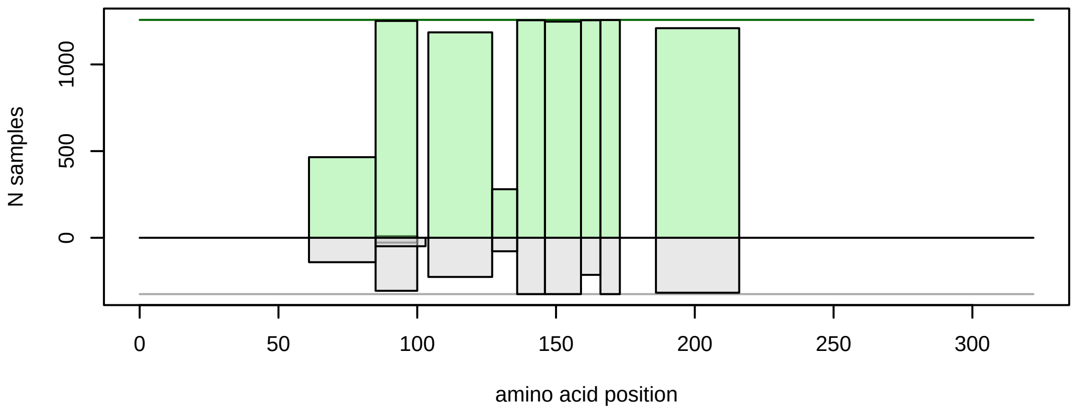

1704. ELANE (P08246), NP4

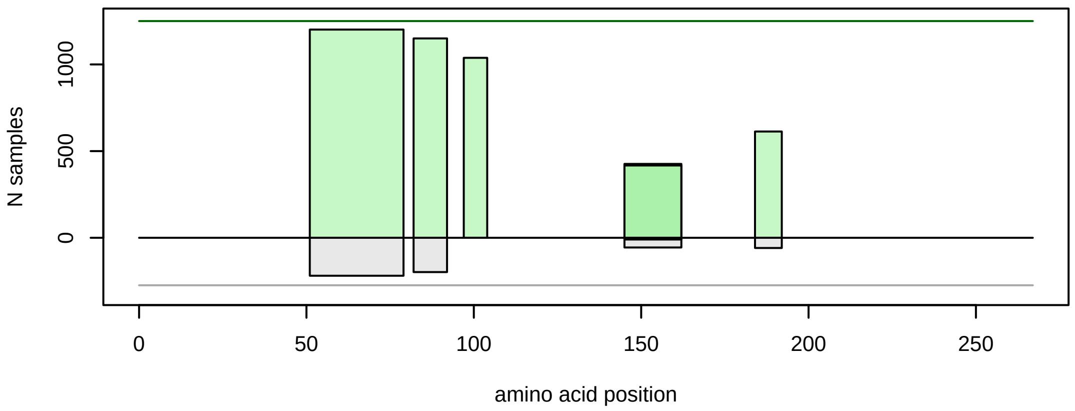

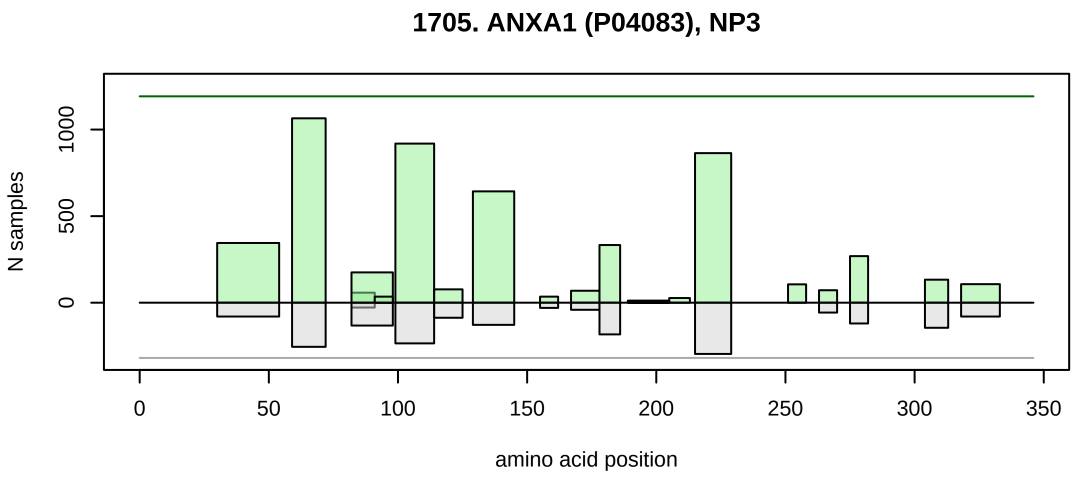
1705. ANXA1 (P04083), NP3
N samples
0
500
1000
0
50
100
150
200
250
300
350
amino acid position

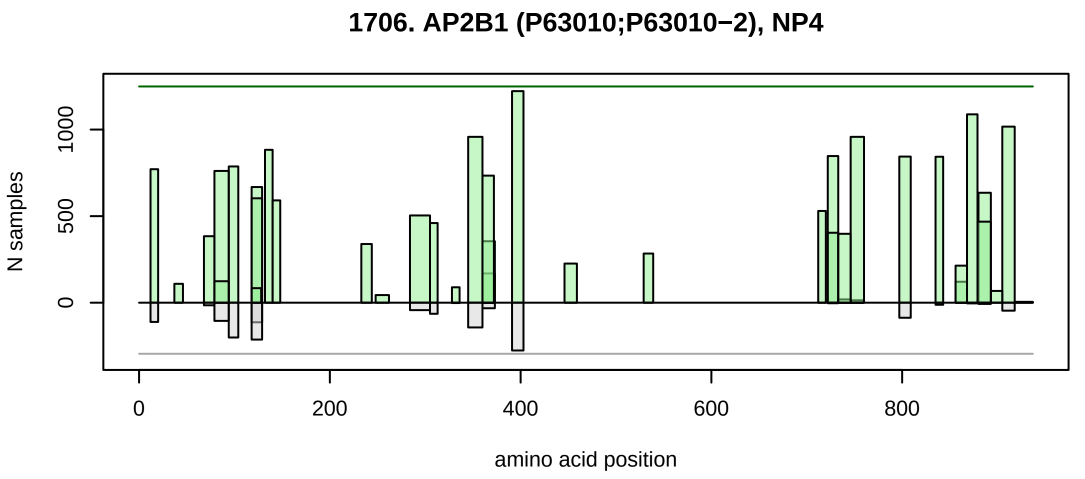
1706. AP2B1 (P63010;P63010−2), NP4
N samples
0
500
1000
0
200
400
600
800
amino acid position

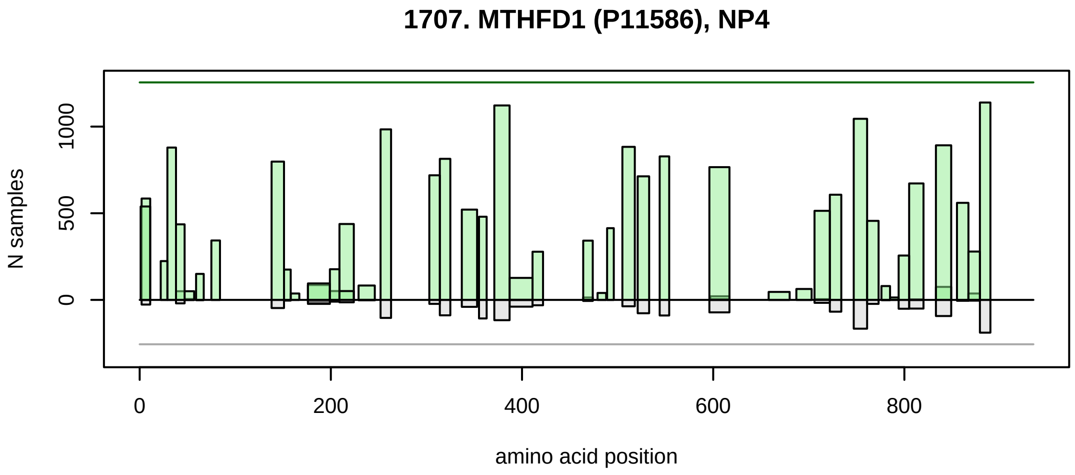
1707. MTHFD1 (P11586), NP4
N samples
0
500
1000
0
200
400
600
800
amino acid position

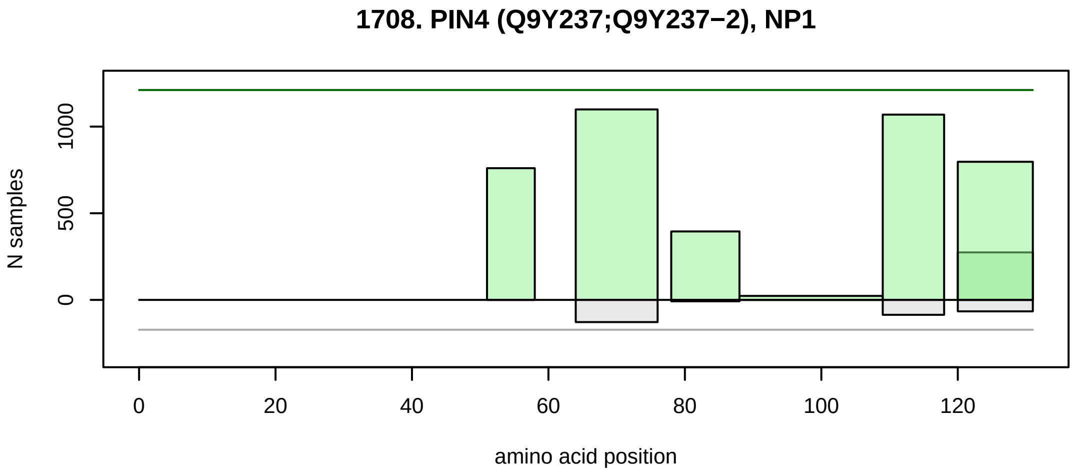
1708. PIN4 (Q9Y237;Q9Y237−2), NP1
N samples
0
500
1000
0
20
40
60
80
100
120
amino acid position

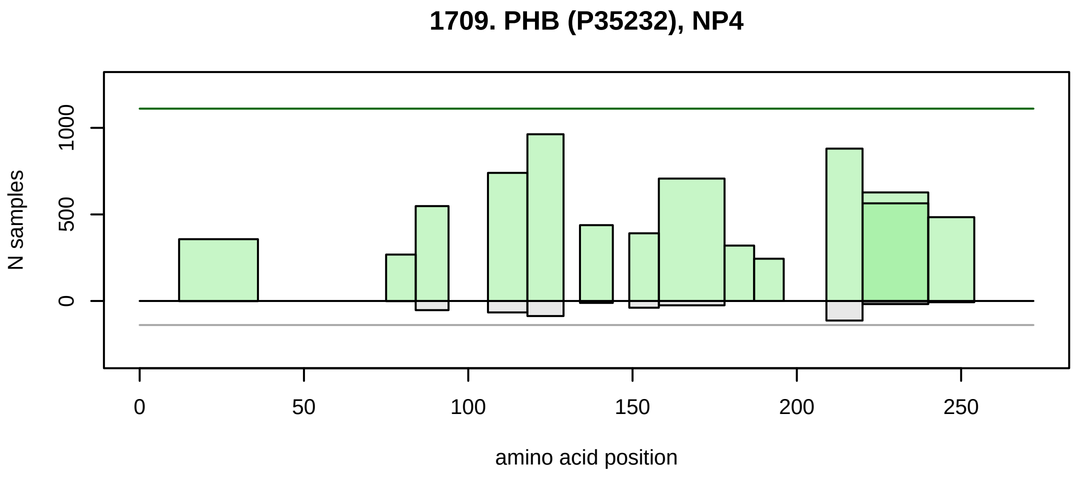
1709. PHB (P35232), NP4
N samples
0
500
1000
0
50
100
150
200
250
amino acid position

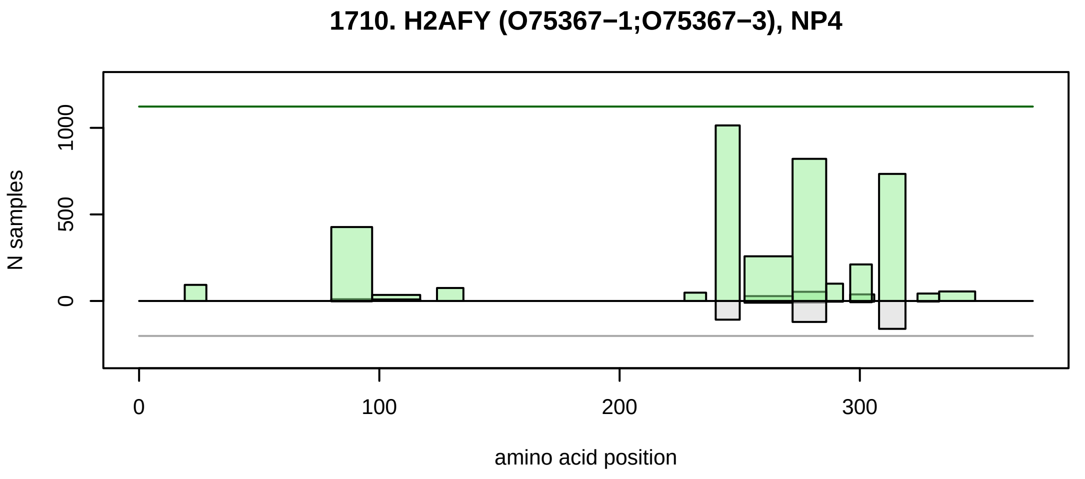
1710. H2AFY (O75367−1;O75367−3), NP4
N samples
0
500
1000
0
100
200
300
amino acid position

1711. ATL3 (Q6DD88), NP1

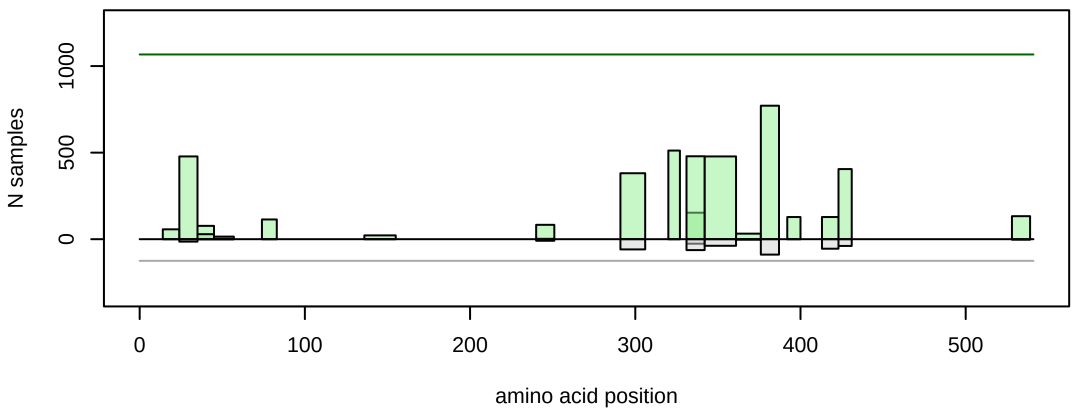

1712. SUSD5 (O60279), NP2

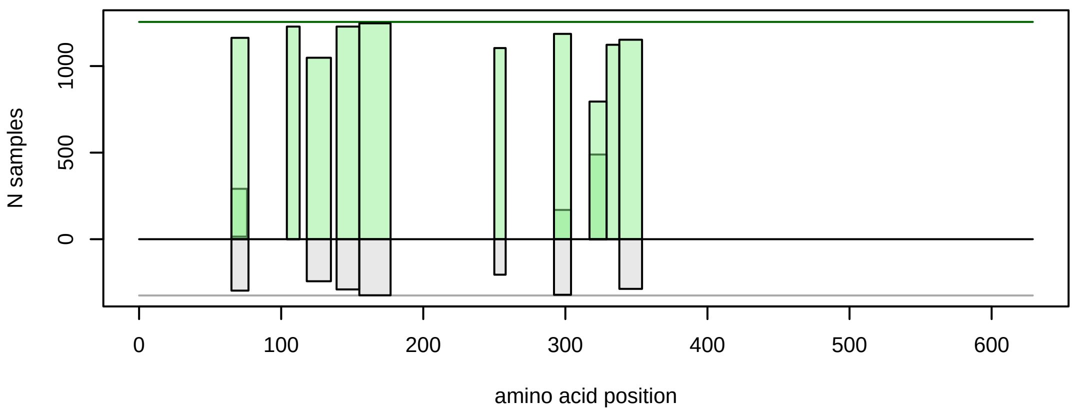

1713. C1S (P09871), NP2

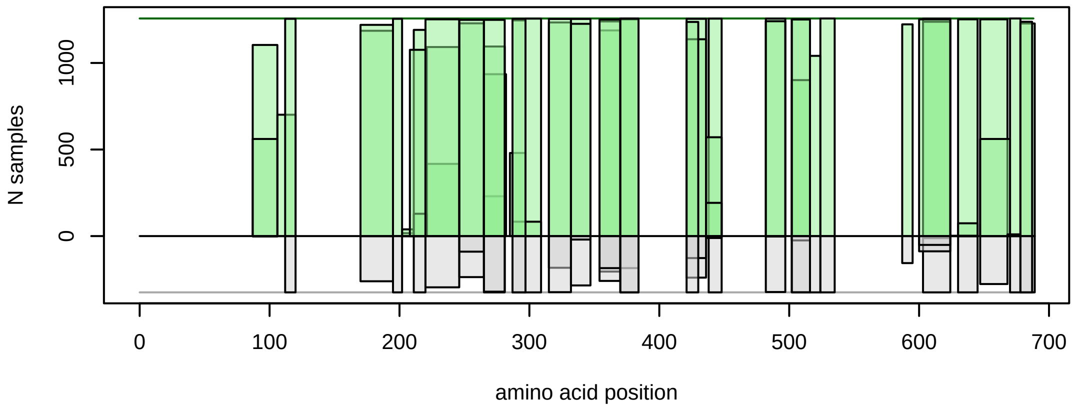

1714. ABAT (H3BRN4), NP4

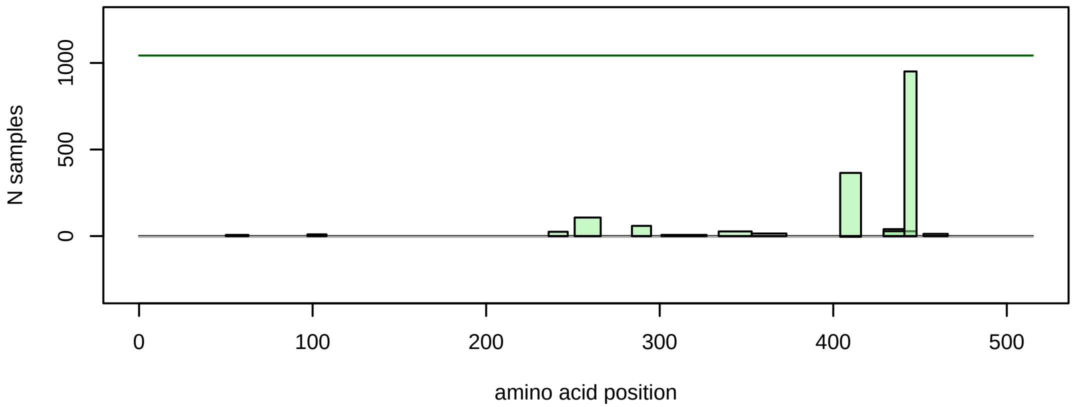

1715. PAPLN (O95428), NP5

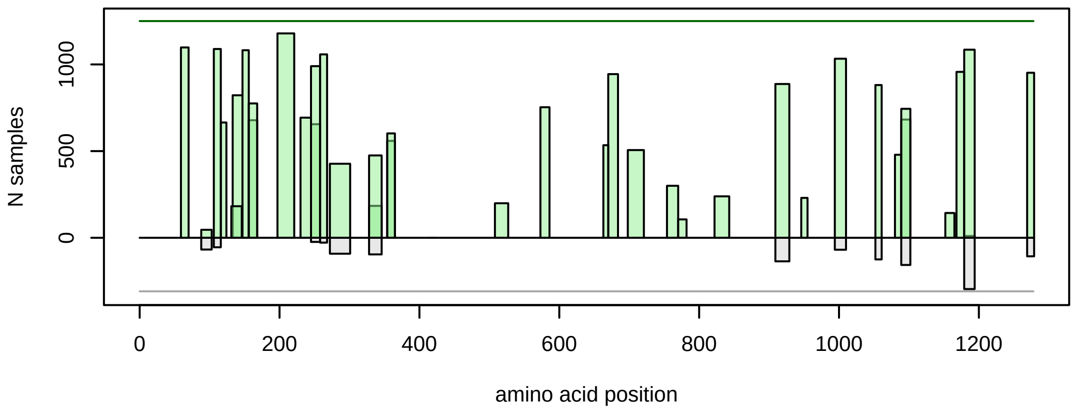

1716. EIF4E (P06730−3), NP3

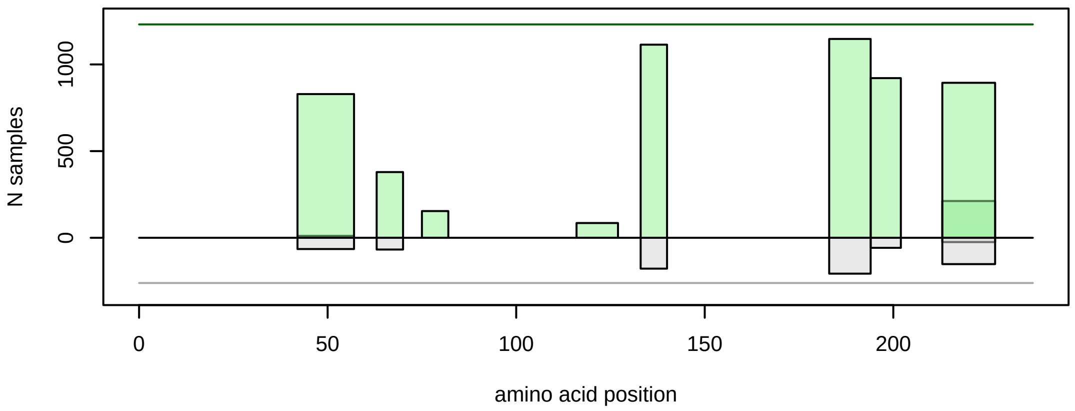

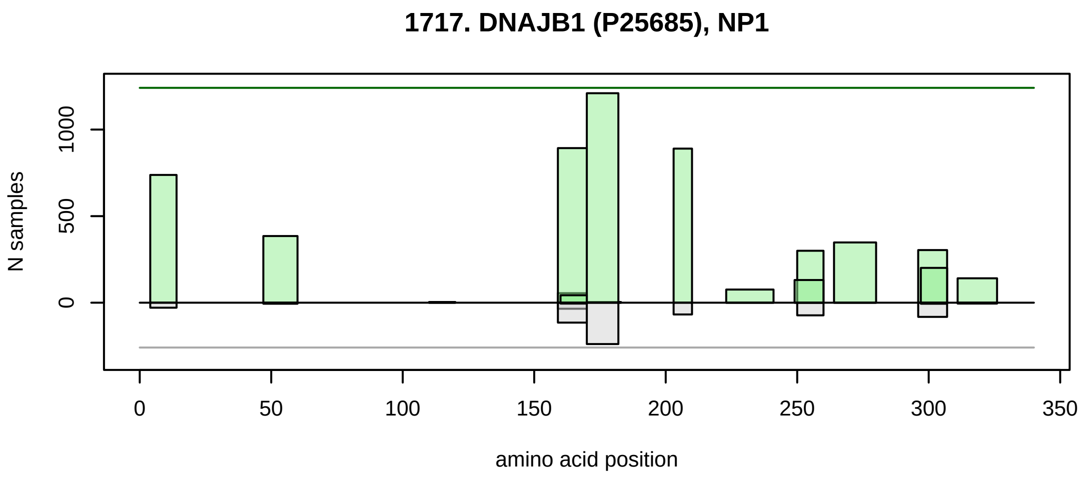
1717. DNAJB1 (P25685), NP1
N samples
0
500
1000
0
50
100
150
200
250
300
350
amino acid position

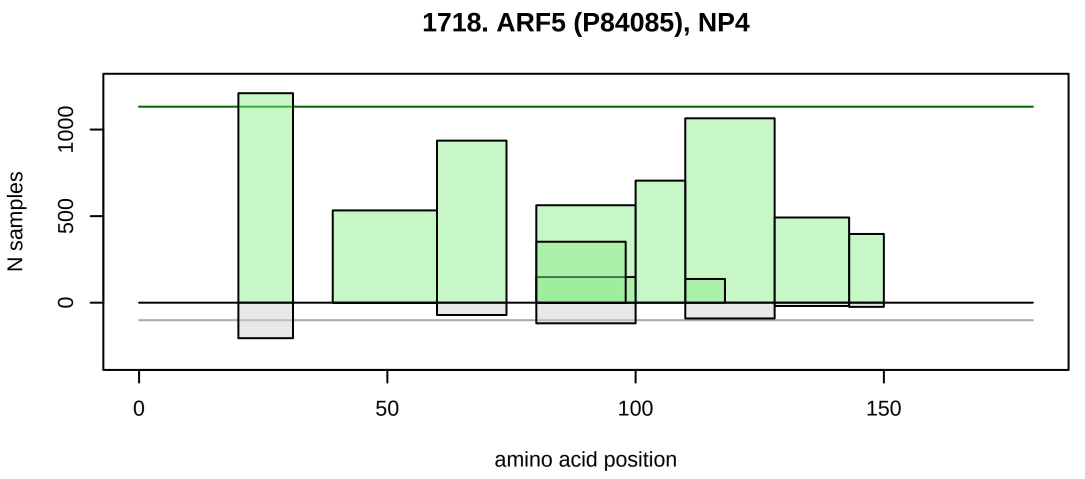
1718. ARF5 (P84085), NP4
N samples
0
500
1000
0
50
100
150
amino acid position

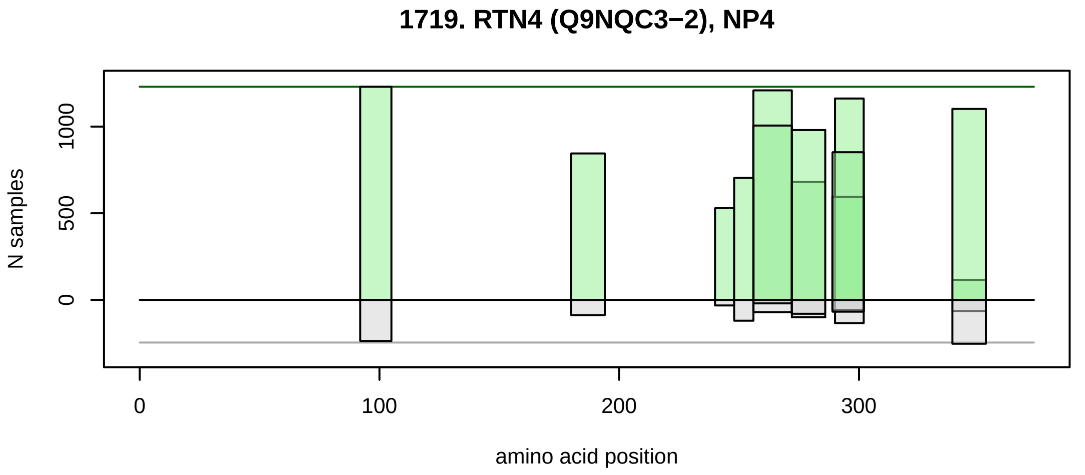
1719. RTN4 (Q9NQC3−2), NP4
N samples
0
500
1000
0
100
200
300
amino acid position

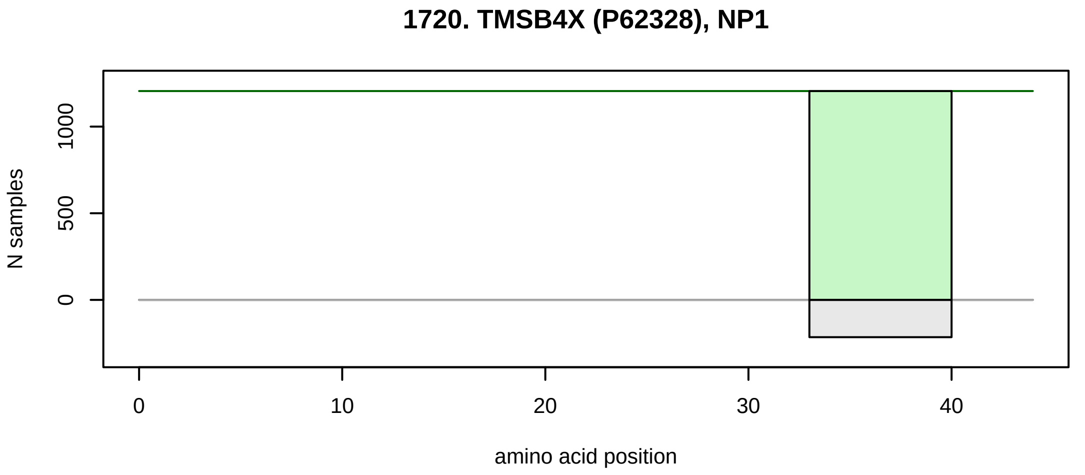
1720. TMSB4X (P62328), NP1
N samples
0
500
1000
0
10
20
30
40
amino acid position

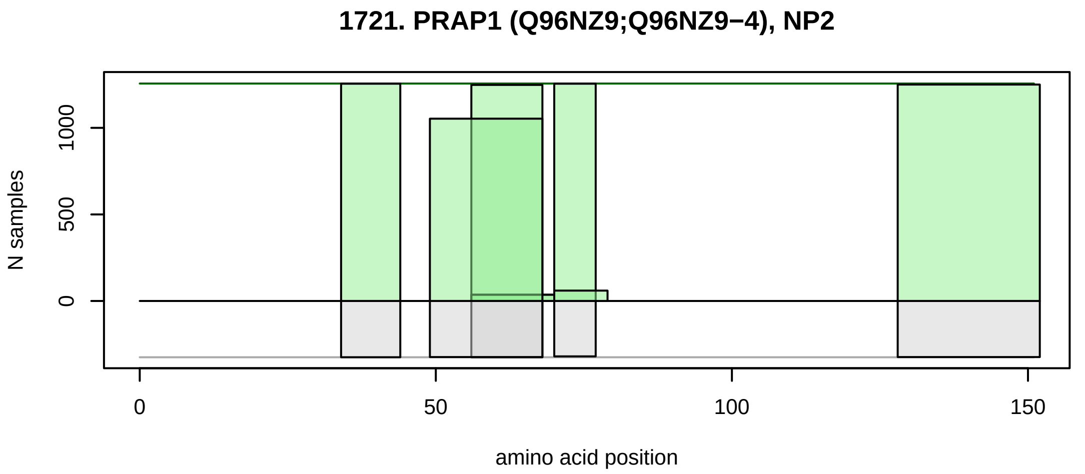
1721. PRAP1 (Q96NZ9;Q96NZ9−4), NP2
N samples
0
500
1000
0
50
100
150
amino acid position

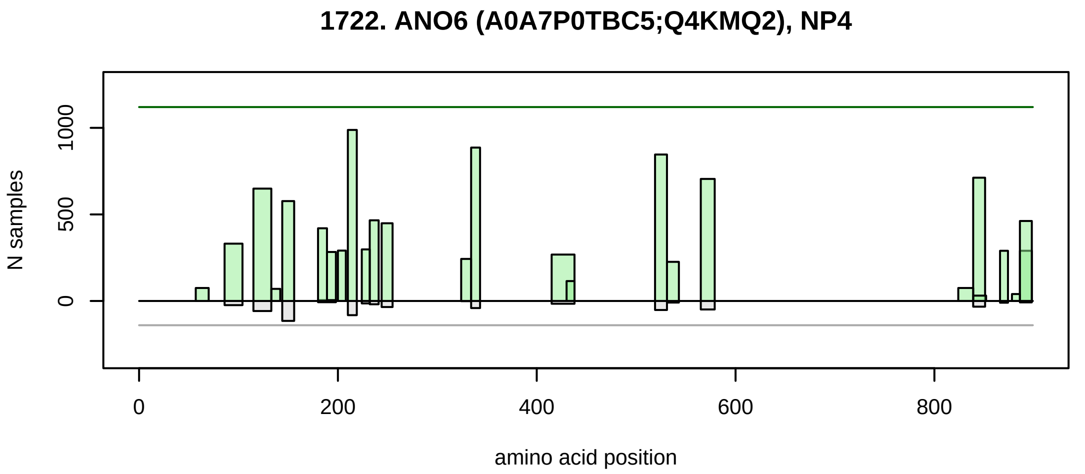
1722. ANO6 (A0A7P0TBC5;Q4KMQ2), NP4
N samples
0
500
1000
0
200
400
600
800
amino acid position

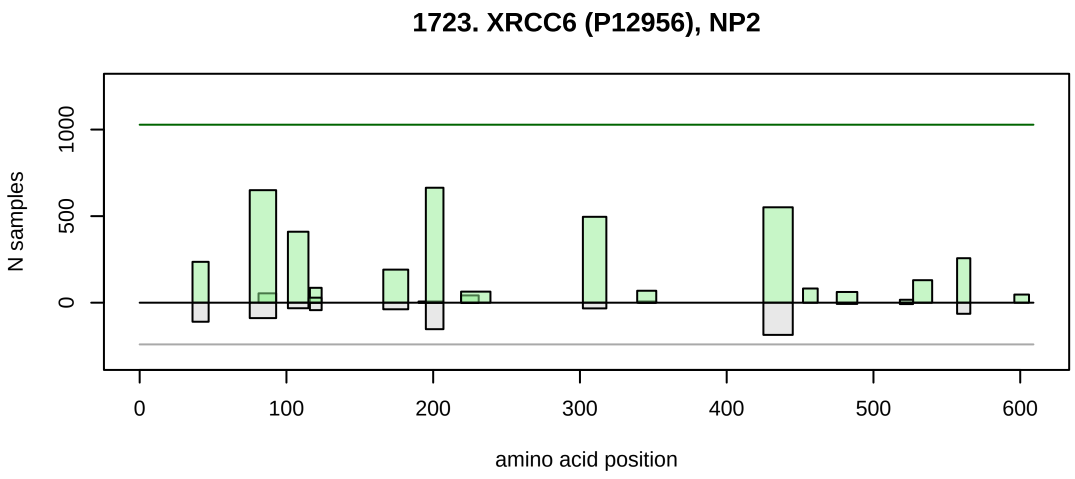

1723. XRCC6 (P12956), NP2
N samples
0
500
1000
0
100
200
300
400
500
600
amino acid position

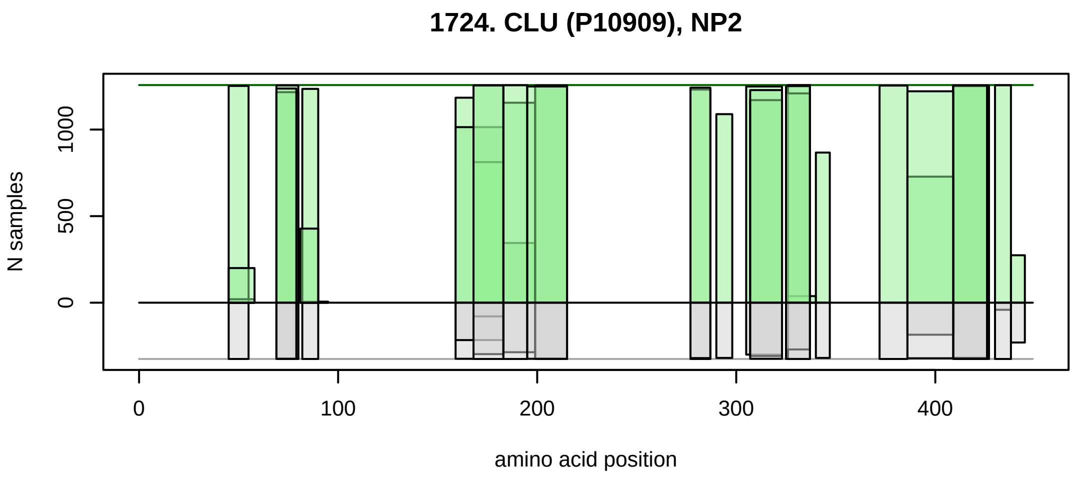

1724. CLU (P10909), NP2
N samples
0
500
1000
0
100
200
300
400
amino acid position

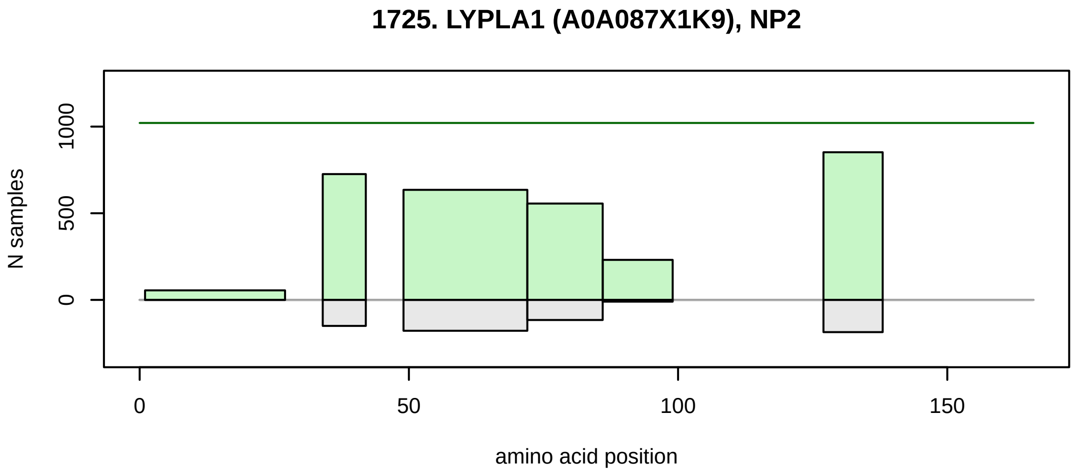

1725. LYPLA1 (A0A087X1K9), NP2
N samples
0
500
1000
0
50
100
150
amino acid position

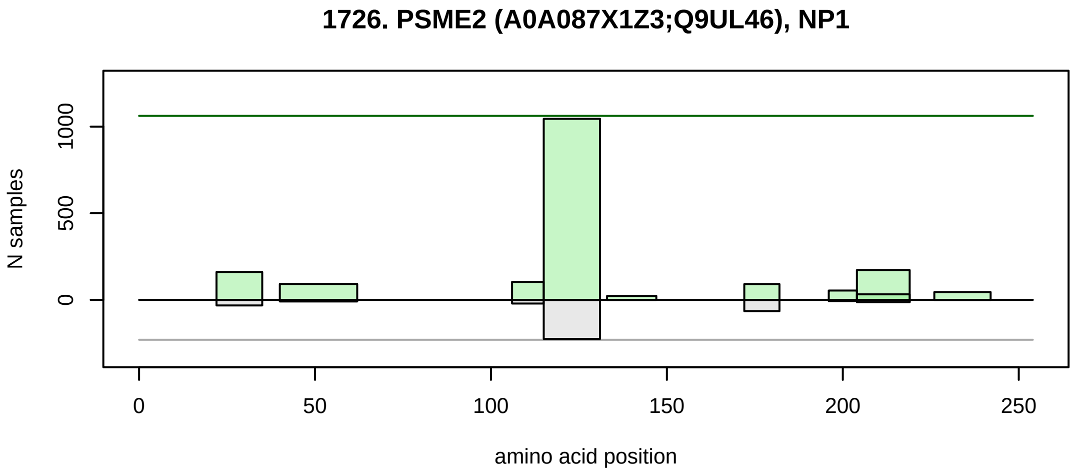

1726. PSME2 (A0A087X1Z3;Q9UL46), NP1
N samples
0
500
1000
0
50
100
150
200
250
amino acid position

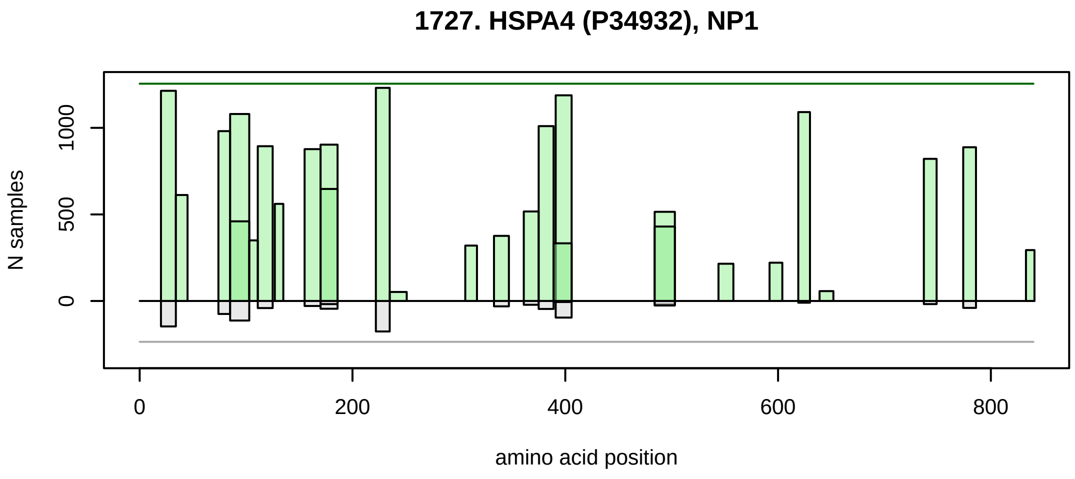

1727. HSPA4 (P34932), NP1
N samples
0
500
1000
0
200
400
600
800
amino acid position

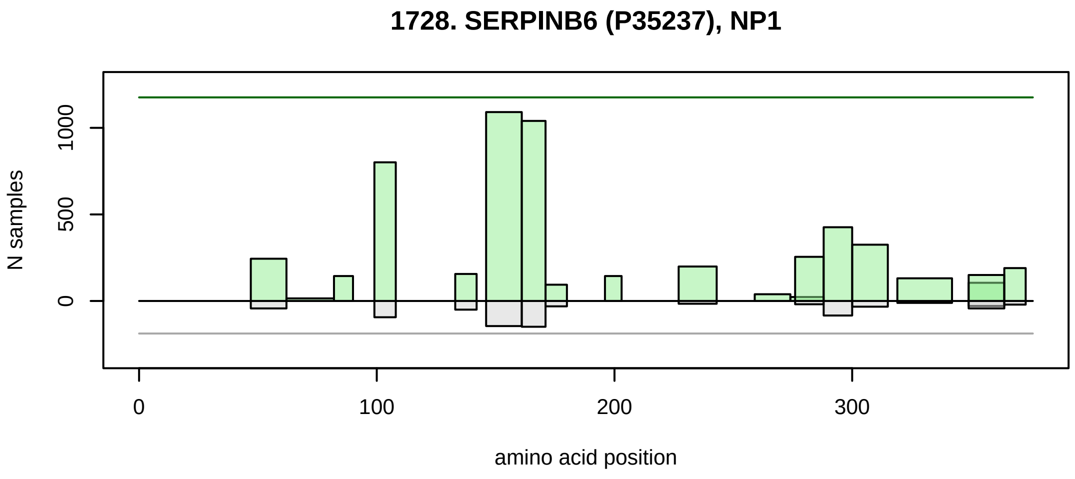

1728. SERPINB6 (P35237), NP1
N samples
0
500
1000
0
100
200
300
amino acid position

1729. TLL2 (Q9Y6L7), NP5

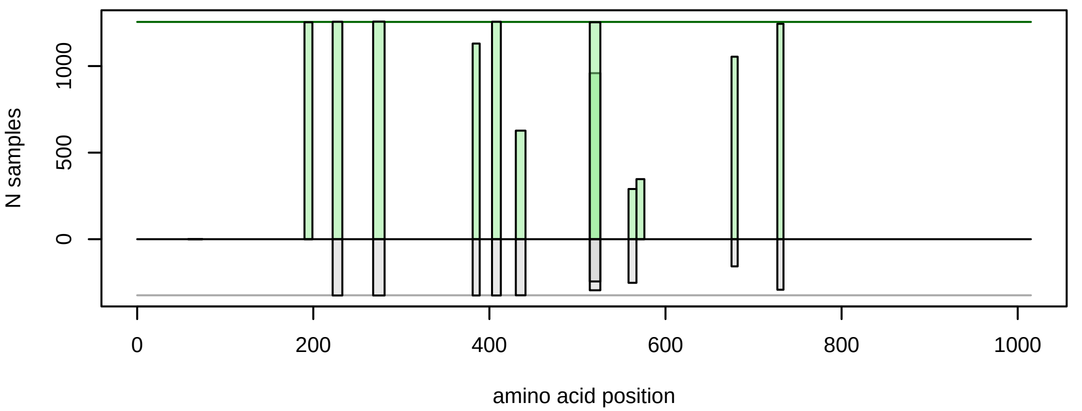

1730. RPL14 (P50914), NP4

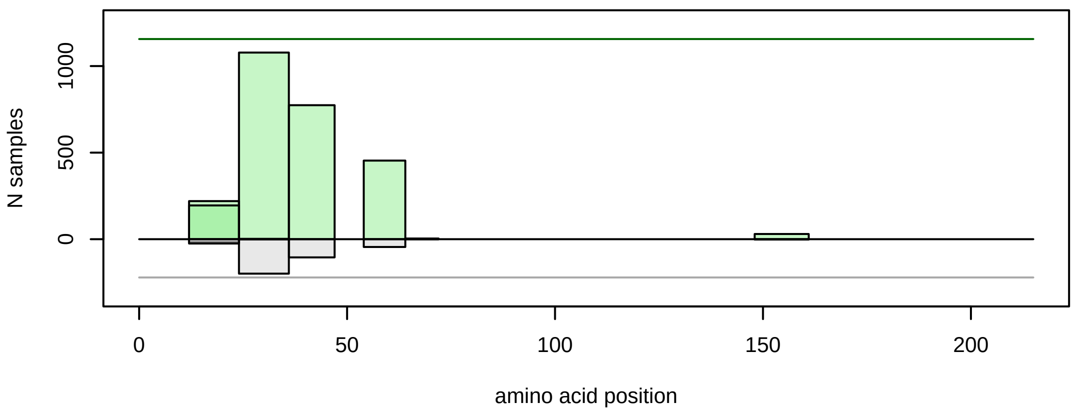

1731. KIAA1199 (Q8WUJ3), NP4

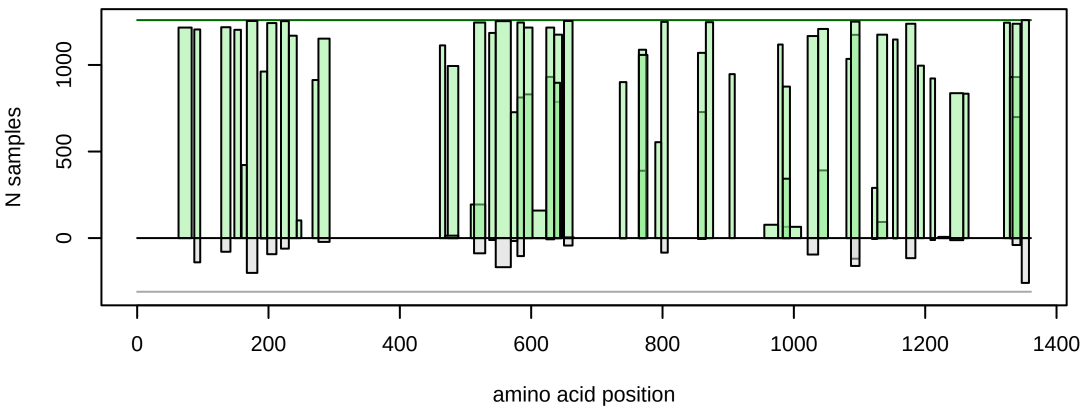

1732. USP47 (Q96K76−4), NP5

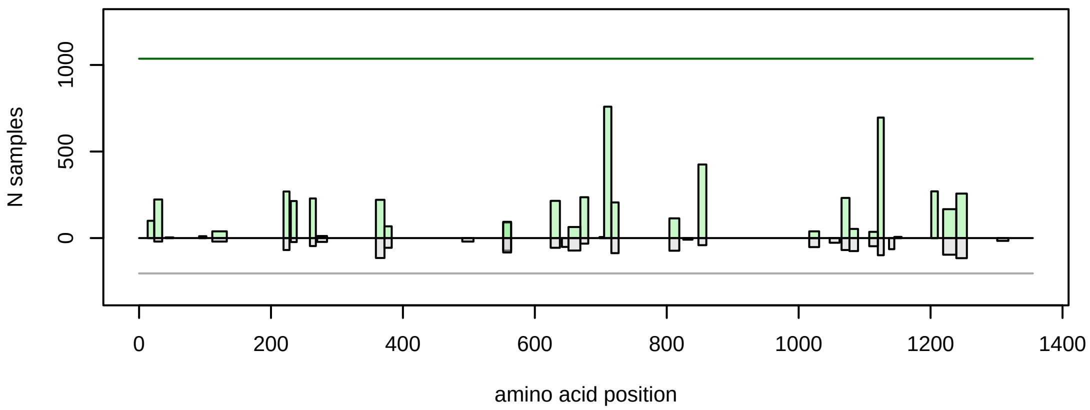

1733. ARHGEF7 (A0A2R8YG42), NP1

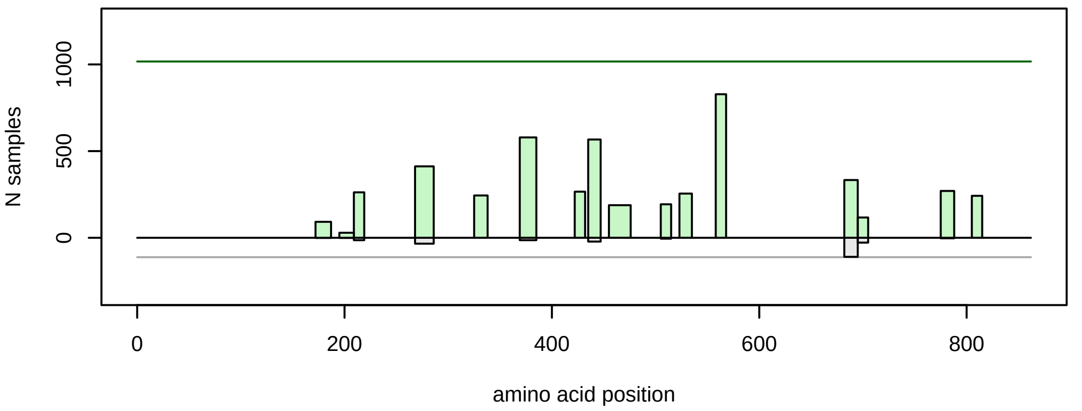

1734. KPNA6 (O60684), NP2

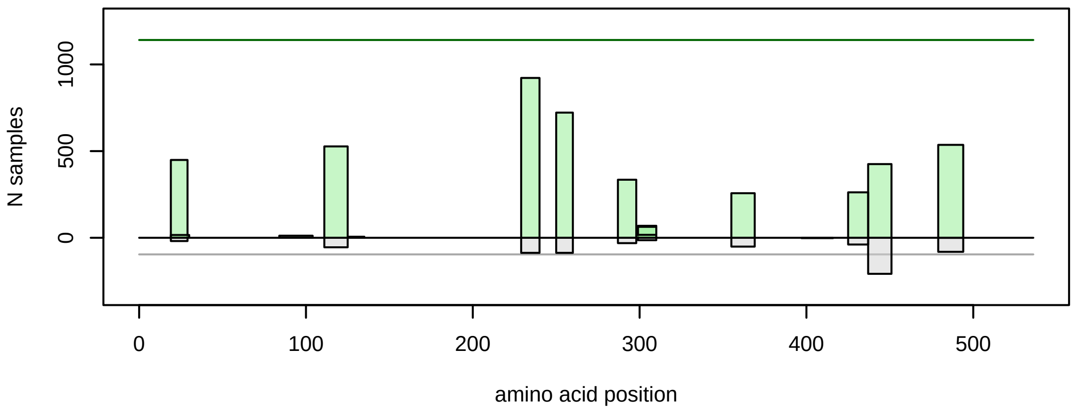

1735. PLAT (A0A7I2YQ93), NP3

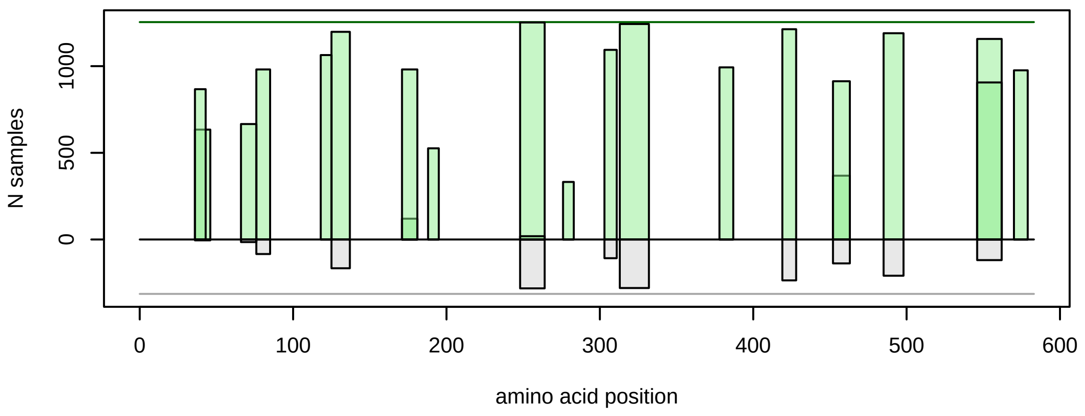

1736. BMP1 (P13497), NP5

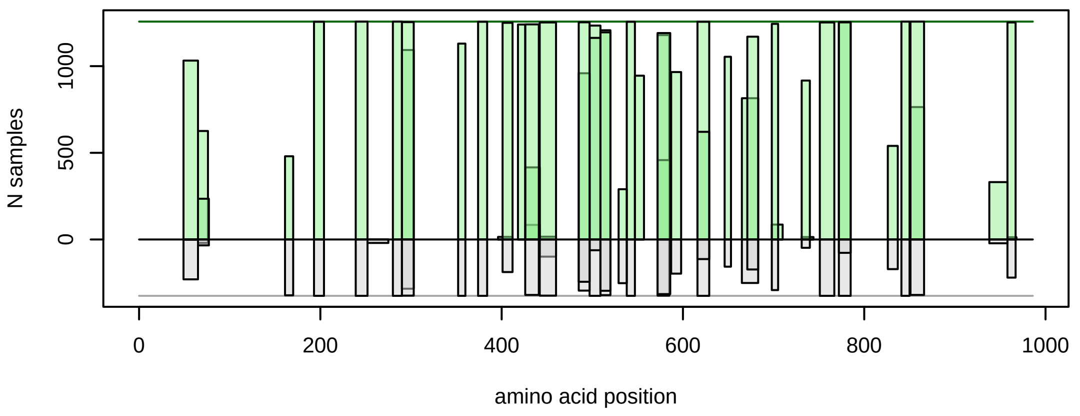

1737. MAN1A2 (O60476), NP5

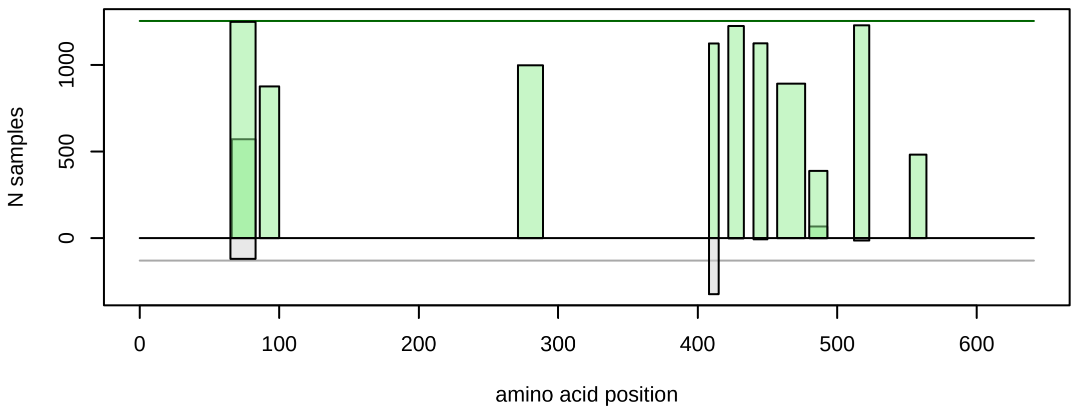

1738. CYR61 (O00622), NP4

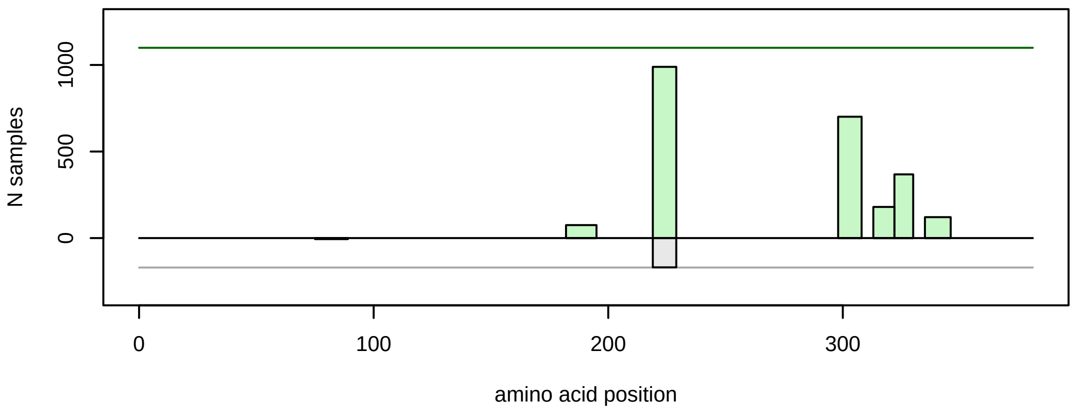

1739. SCYL1 (E9PK59), NP1

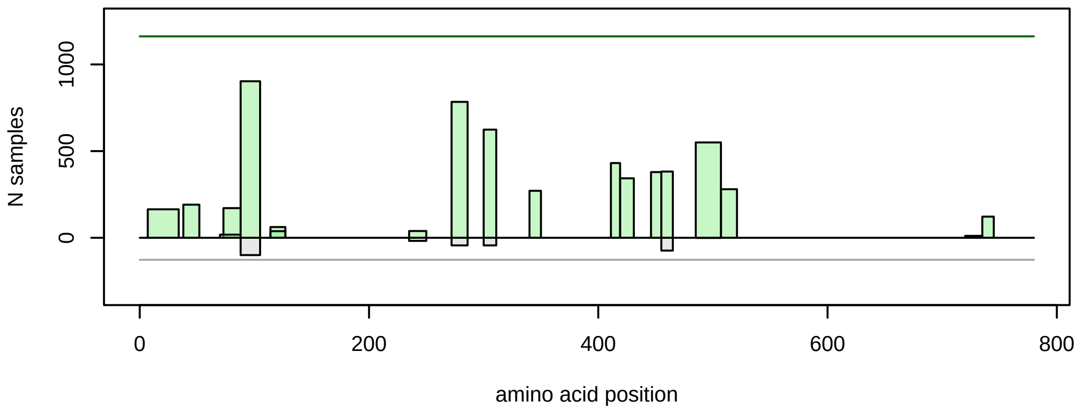

1740. USP14 (P54578;P54578−2), NP3

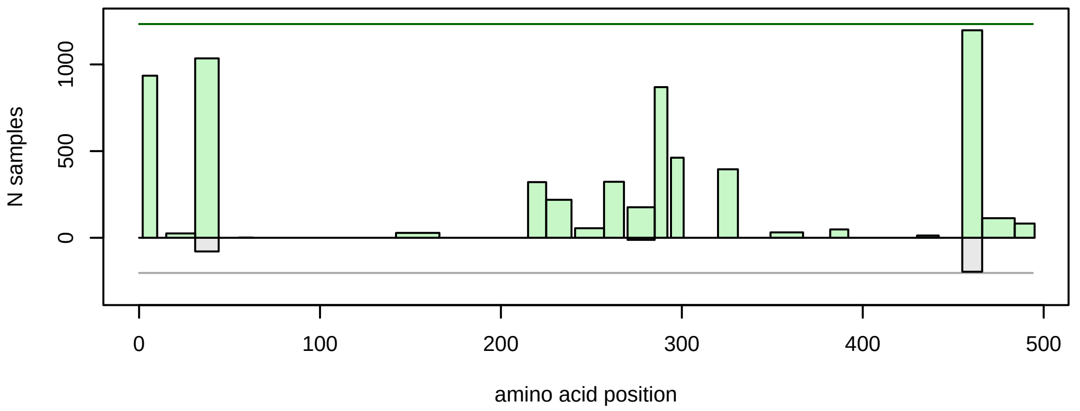

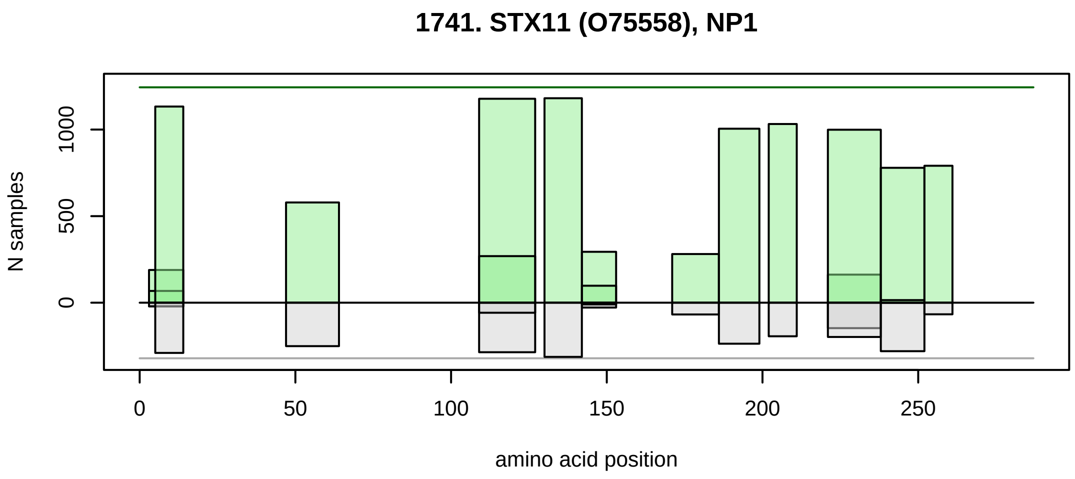

1741. STX11 (O75558), NP1
N samples
0
500
1000
0
50
100
150
200
250
amino acid position

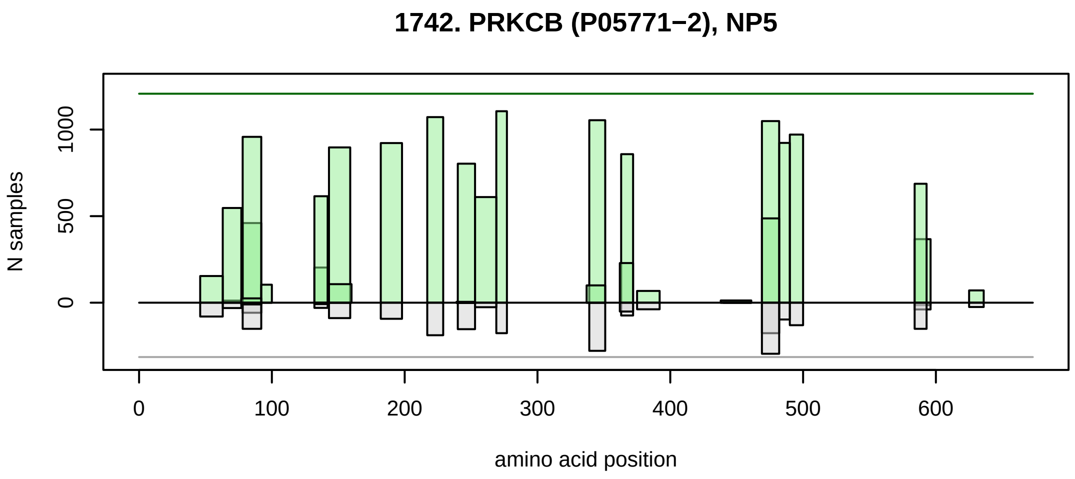

1742. PRKCB (P05771−2), NP5
N samples
0
500
1000
0
100
200
300
400
500
600
amino acid position

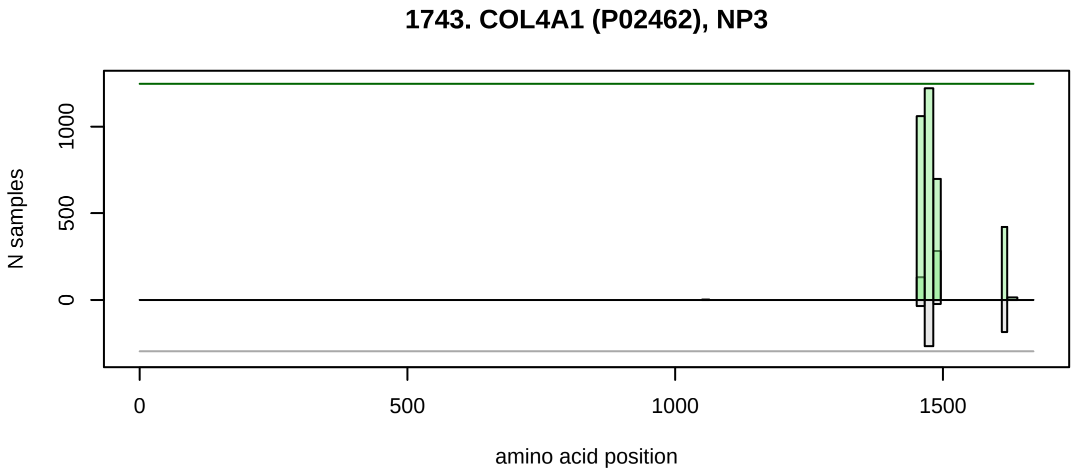

1743. COL4A1 (P02462), NP3
N samples
0
500
1000
0
500
1000
1500
amino acid position

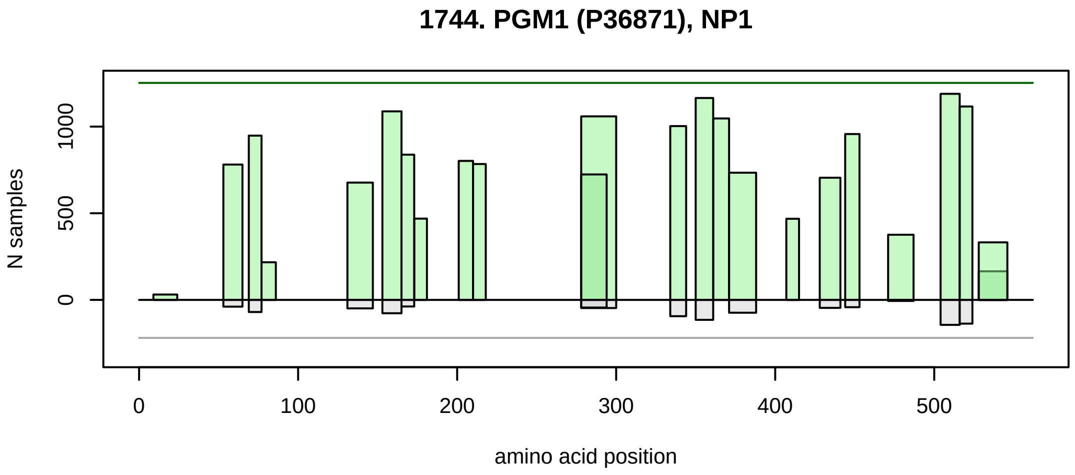

1744. PGM1 (P36871), NP1
N samples
0
500
1000
0
100
200
300
400
500
amino acid position

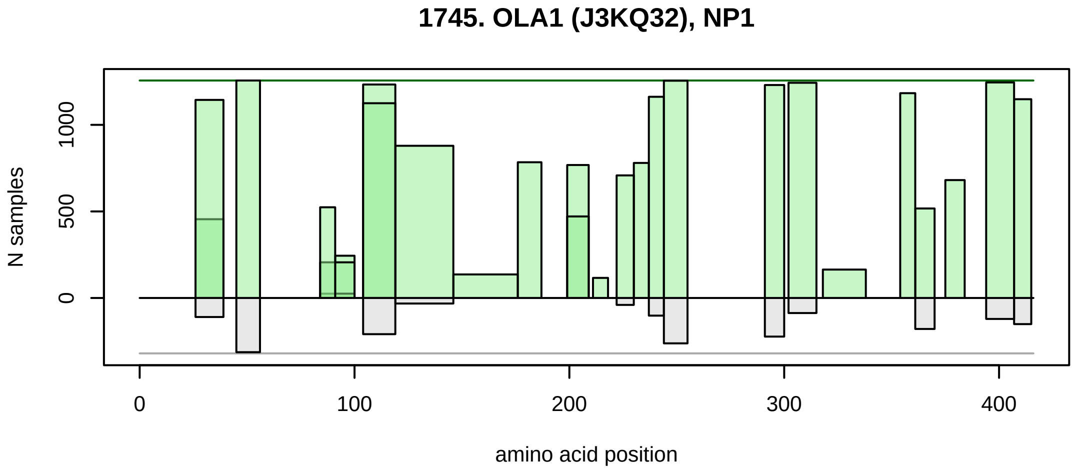

1745. OLA1 (J3KQ32), NP1
N samples
0
500
1000
0
100
200
300
400
amino acid position

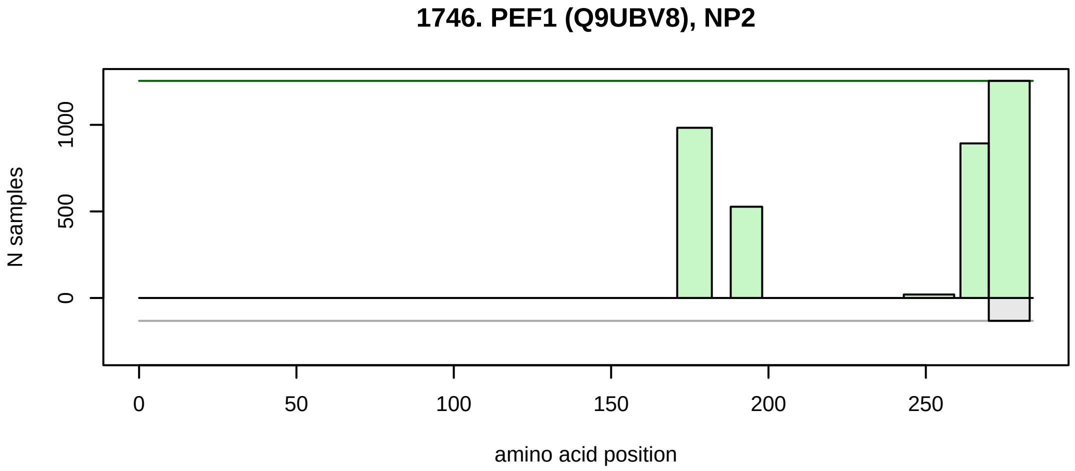

1746. PEF1 (Q9UBV8), NP2
N samples
0
500
1000
0
50
100
150
200
250
amino acid position

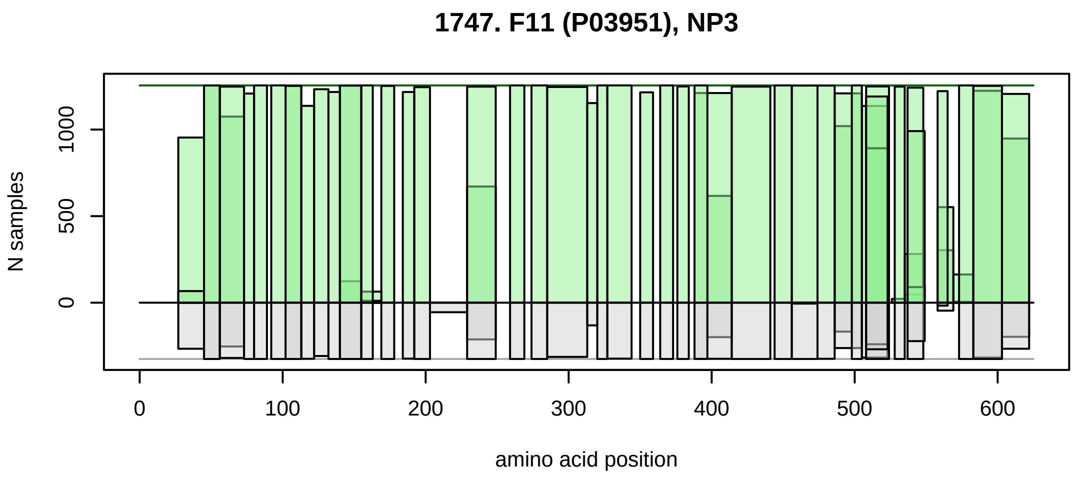

1747. F11 (P03951), NP3
N samples
0
500
1000
0
100
200
300
400
500
600
amino acid position

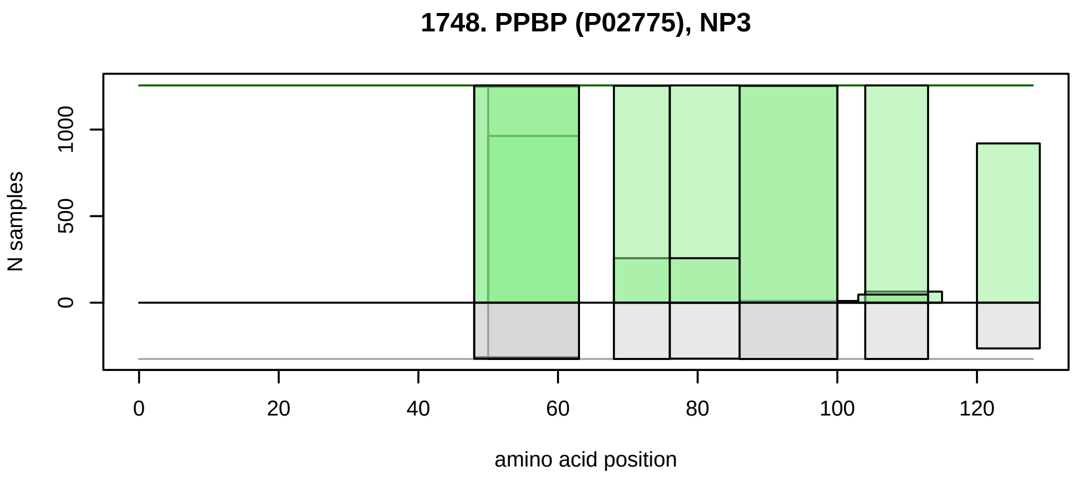

1748. PPBP (P02775), NP3
N samples
0
500
1000
0
20
40
60
80
100
120
amino acid position

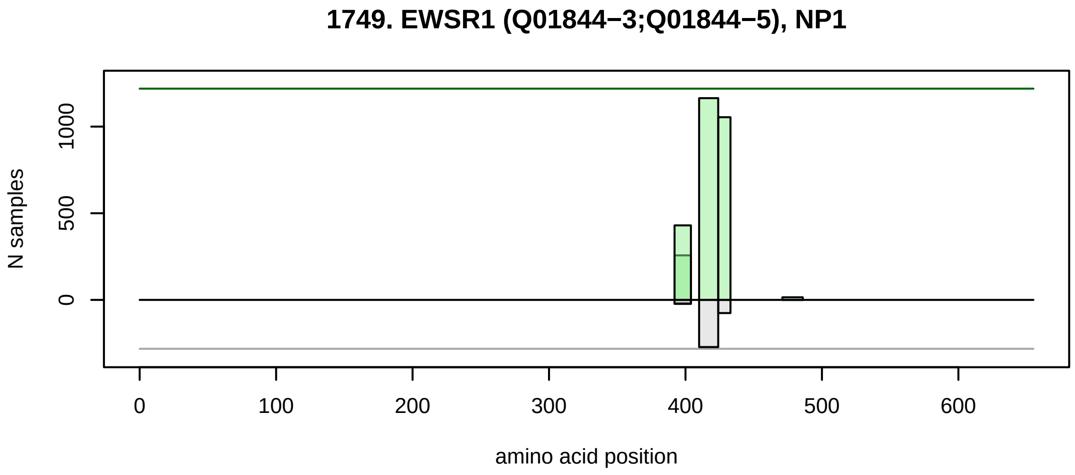

1749. EWSR1 (Q01844−3;Q01844−5), NP1
N samples
0
500
1000
0
100
200
300
400
500
600
amino acid position

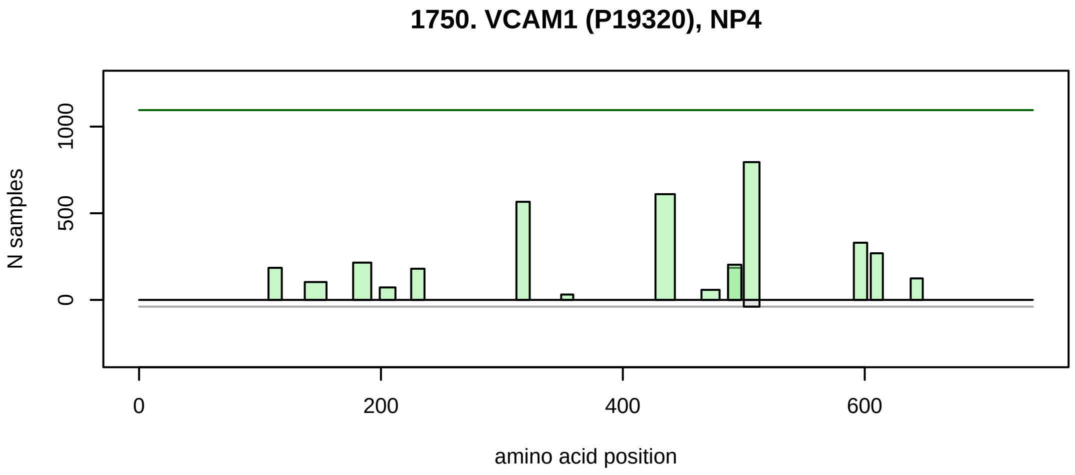

1750. VCAM1 (P19320), NP4
N samples
0
500
1000
0
200
400
600
amino acid position

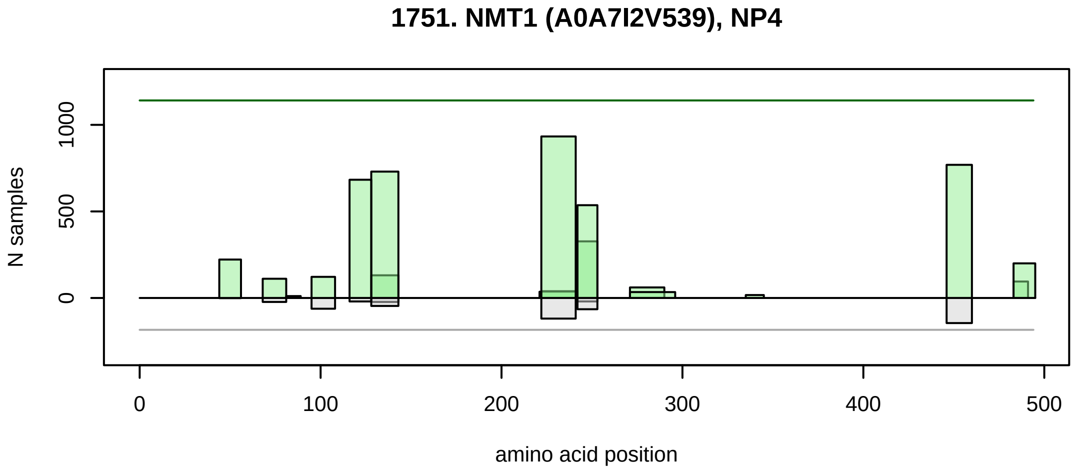

1751. NMT1 (A0A7I2V539), NP4
N samples
0
500
1000
0
100
200
300
400
500
amino acid position

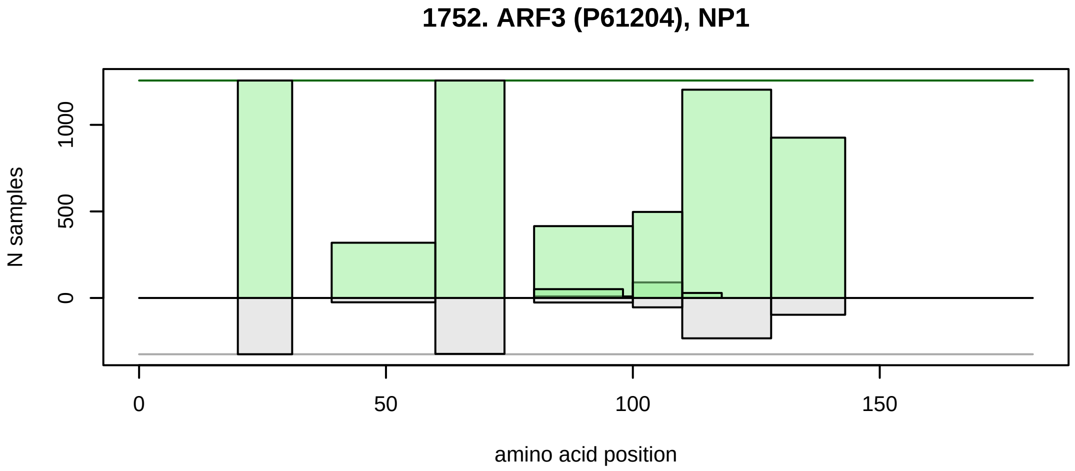

1752. ARF3 (P61204), NP1
N samples
0
500
1000
0
50
100
150
amino acid position

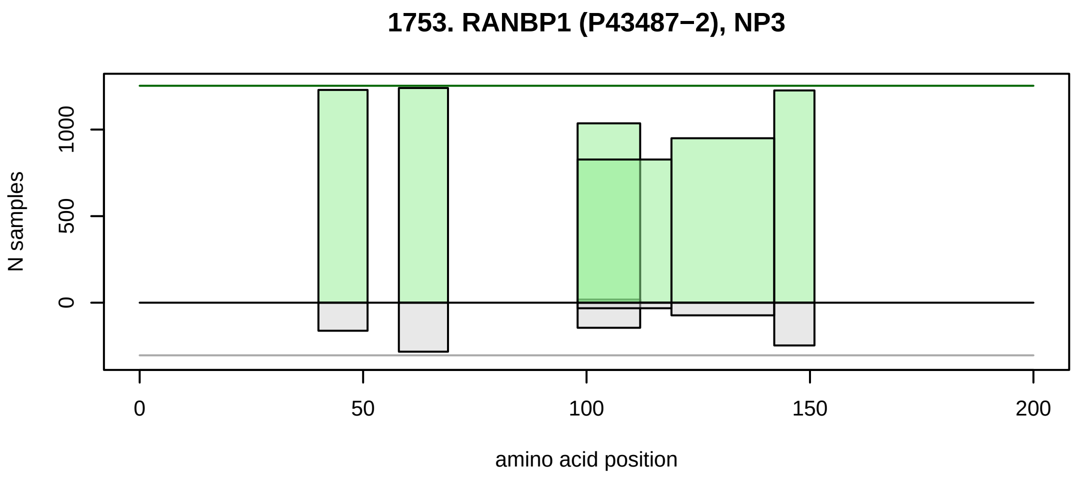
1753. RANBP1 (P43487−2), NP3
N samples
0
500
1000
0
50
100
150
200
amino acid position

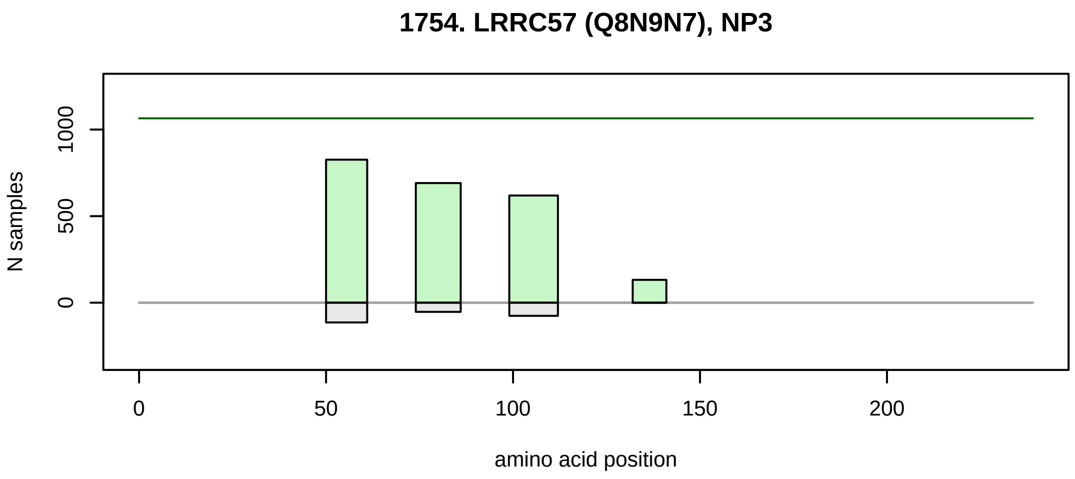
1754. LRRC57 (Q8N9N7), NP3
N samples
0
500
1000
0
50
100
150
200
amino acid position

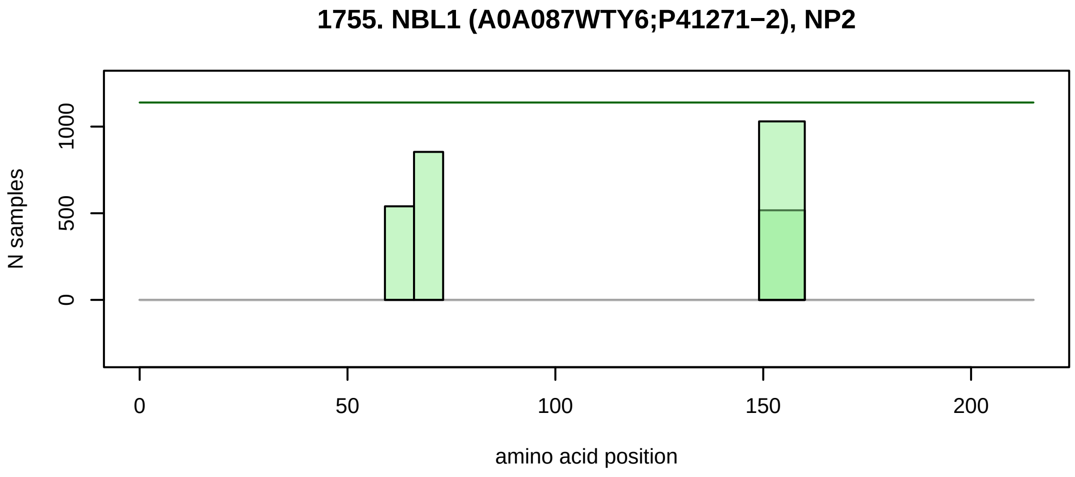
1755. NBL1 (A0A087WTY6;P41271−2), NP2
N samples
0
500
1000
0
50
100
150
200
amino acid position

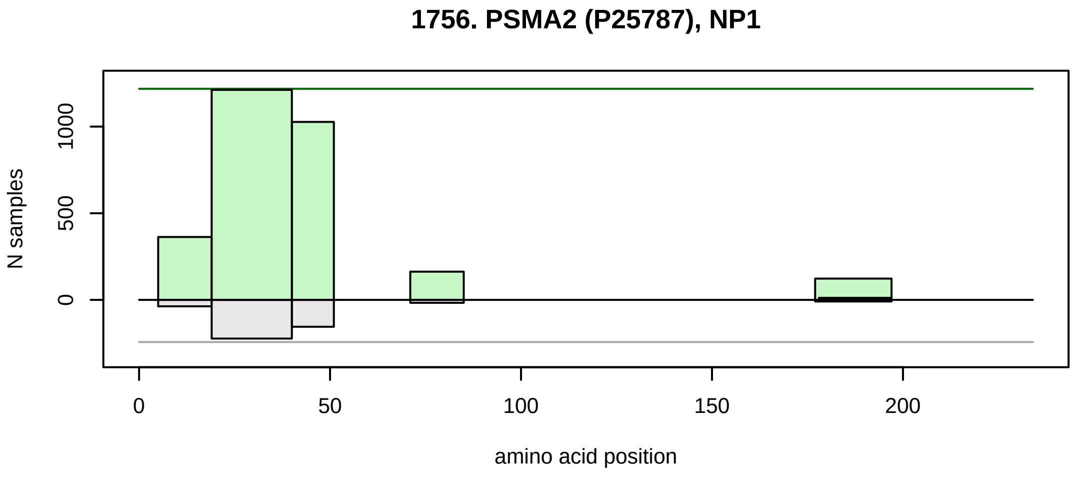
1756. PSMA2 (P25787), NP1
N samples
0
500
1000
0
50
100
150
200
amino acid position

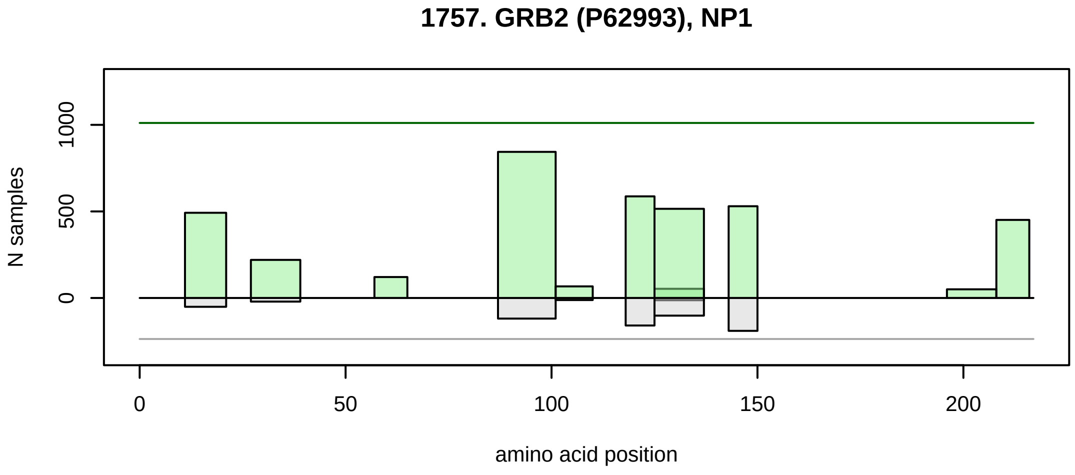
1757. GRB2 (P62993), NP1
N samples
0
500
1000
0
50
100
150
200
amino acid position

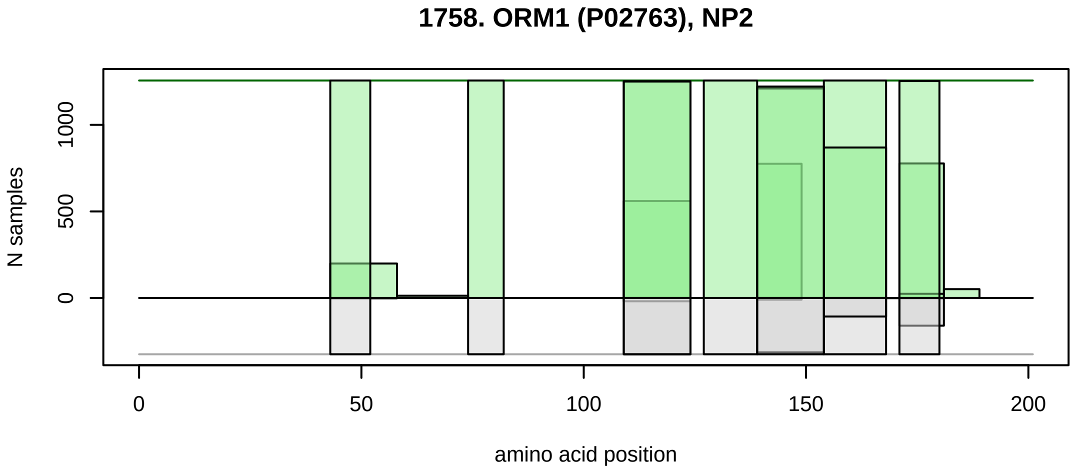
1758. ORM1 (P02763), NP2
N samples
0
500
1000
0
50
100
150
200
amino acid position

1759. ARHGAP18 (Q8N392), NP4

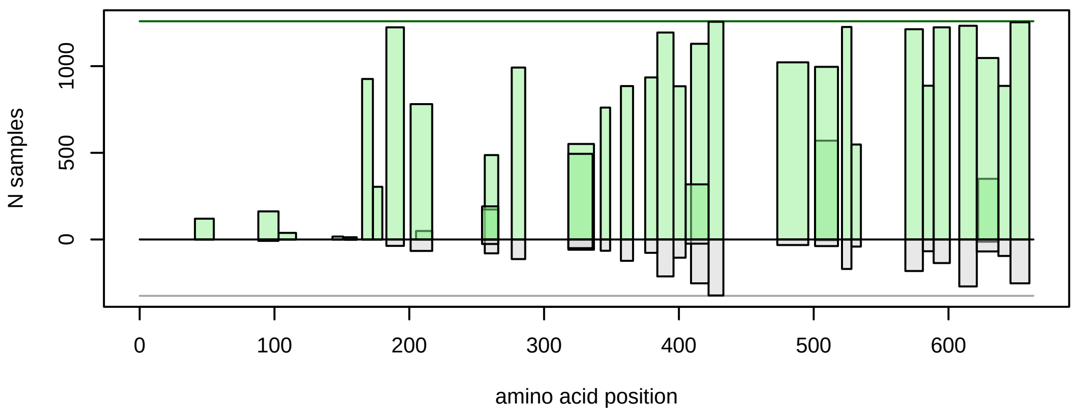

1760. PABPC4 (A0A7I2V4L7), NP1

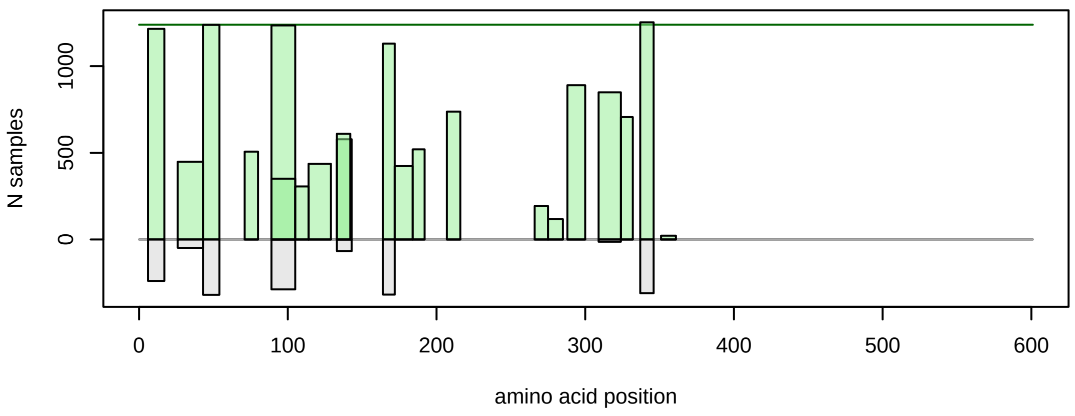

1761. RASGRP2 (Q7LDG7;Q7LDG7−4), NP4

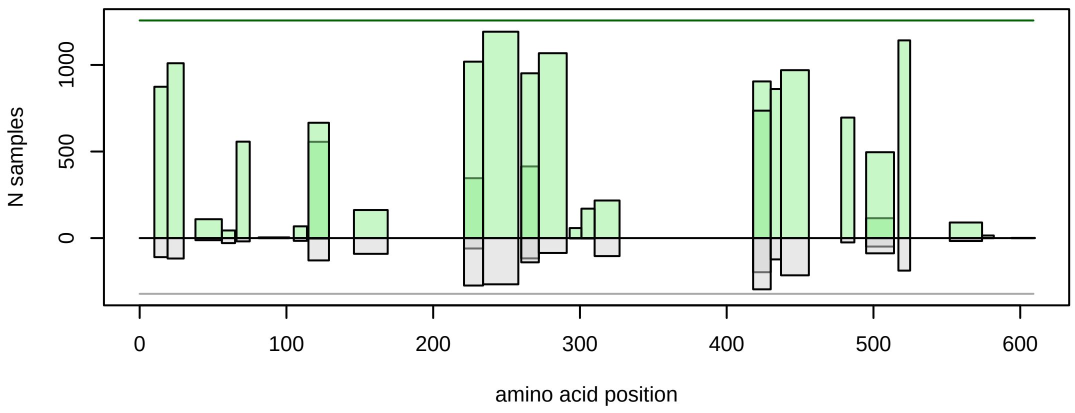

1762. CELA3A (P09093), NP3

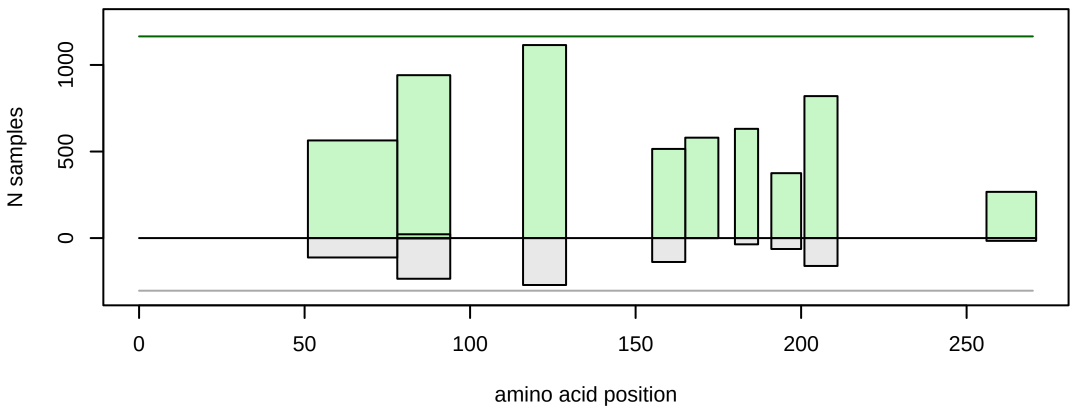

1763. VDAC2 (P45880), NP4

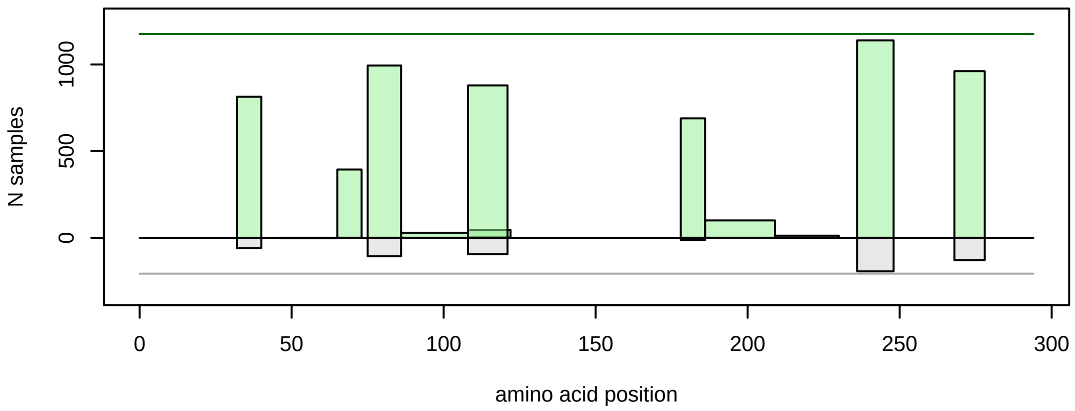

J (A0A0U1RR32;A0A0U1RRH7;P04908;P0C0S8;P20671;Q7L7L0;Q93077;Q96KK5;Q99878

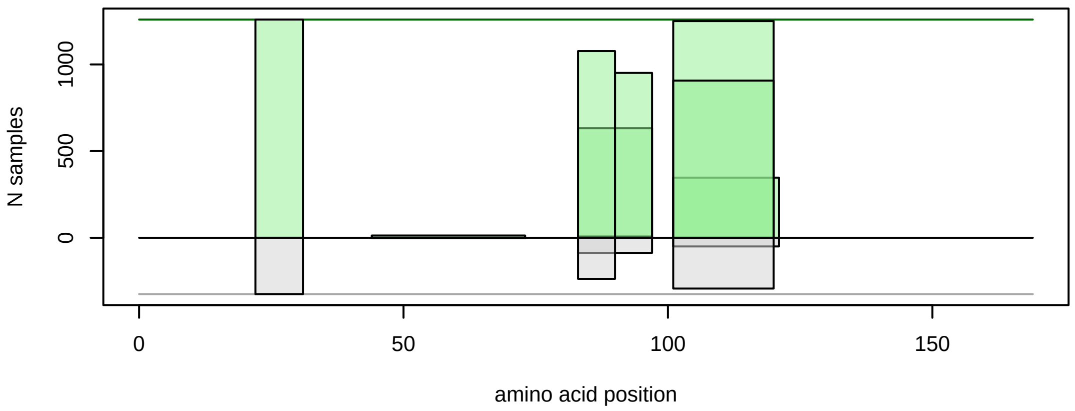

1765. RPS10 (P46783), NP4

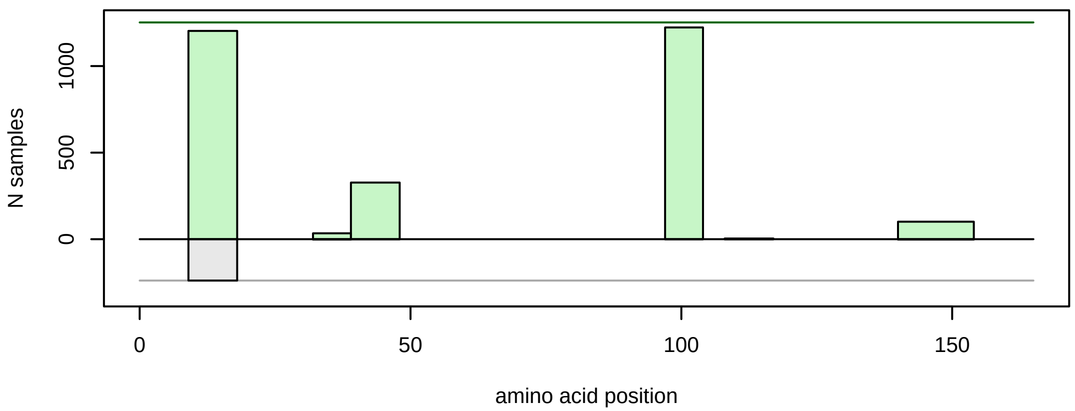

1766. CPN2 (P22792), NP5

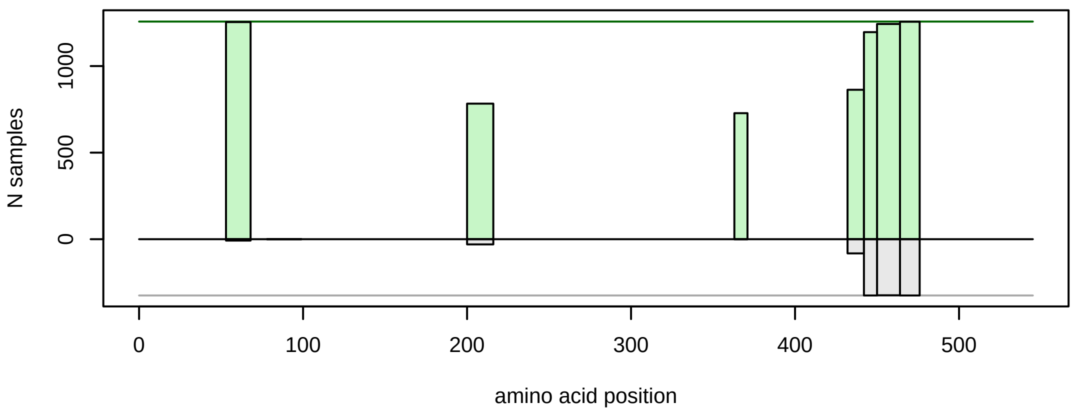

1767. CUL3 (Q13618), NP1

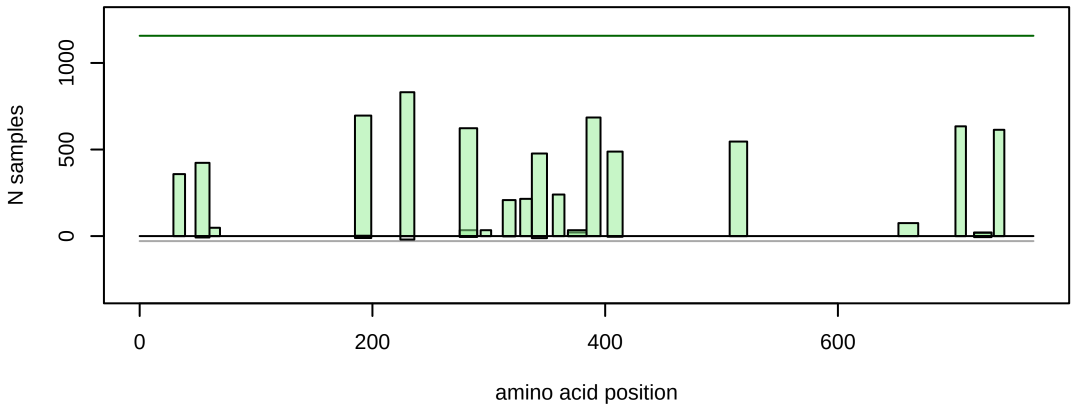

1768. ACTR3 (P61158), NP4

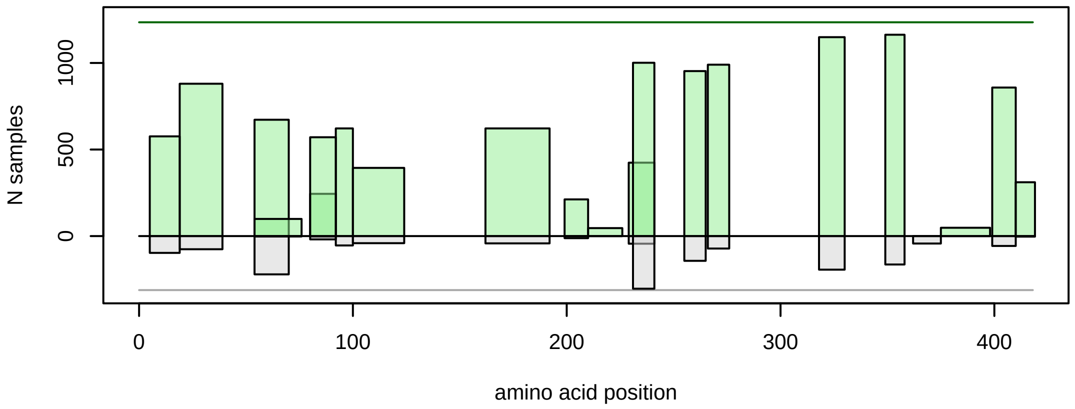

1769. PLEKHF2 (Q9H8W4), NP4

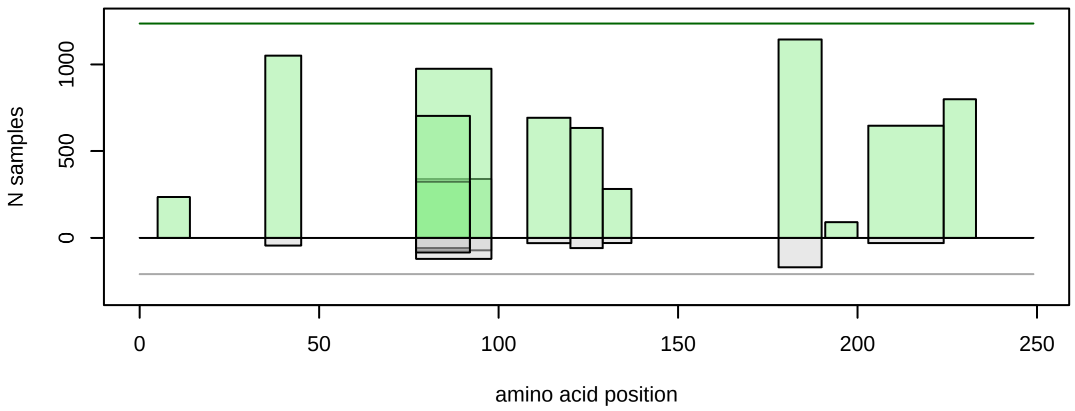

1770. ATP6AP2 (A0A1B0GVW0;O75787;O75787−2), NP4

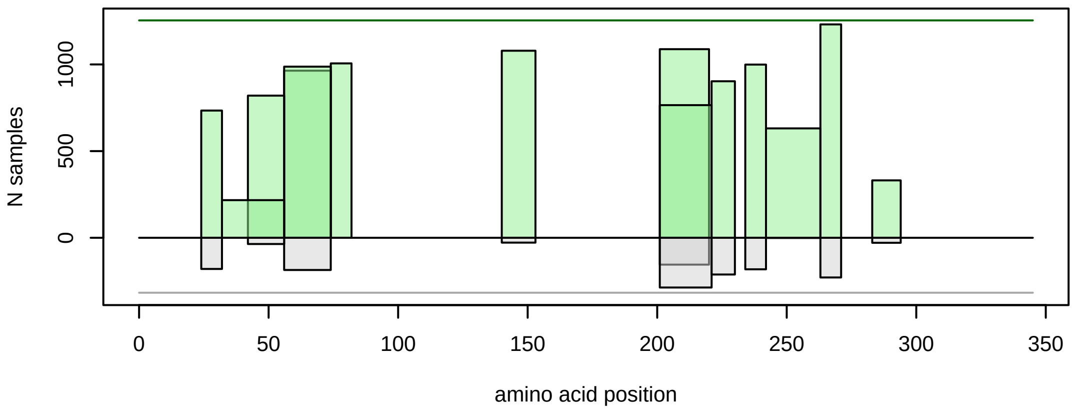

1771. SELENBP1 (Q13228−4), NP5

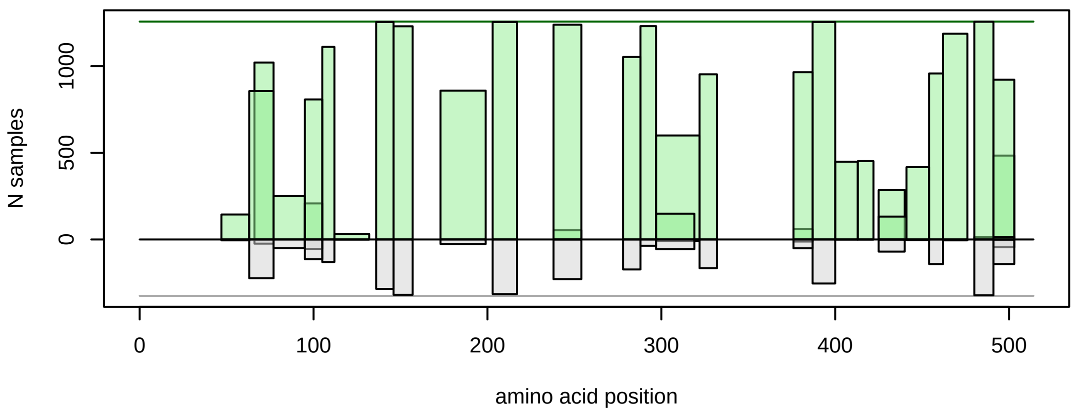

1772. IDH2 (P48735), NP1

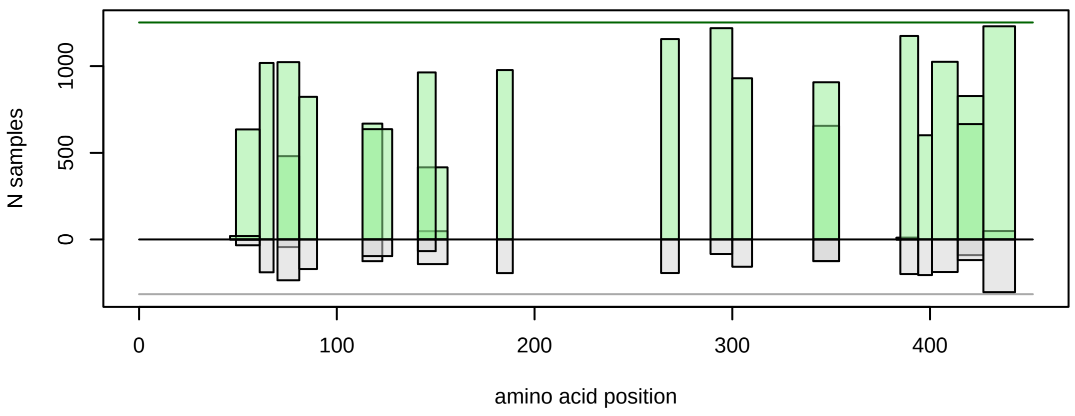

1773. CHID1 (Q9BWS9), NP3

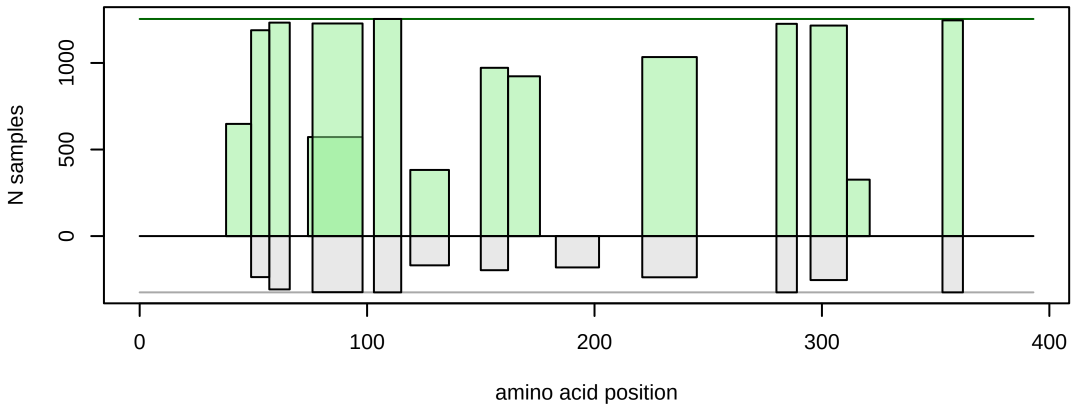

1774. MDK (E9PLM6;P21741), NP4

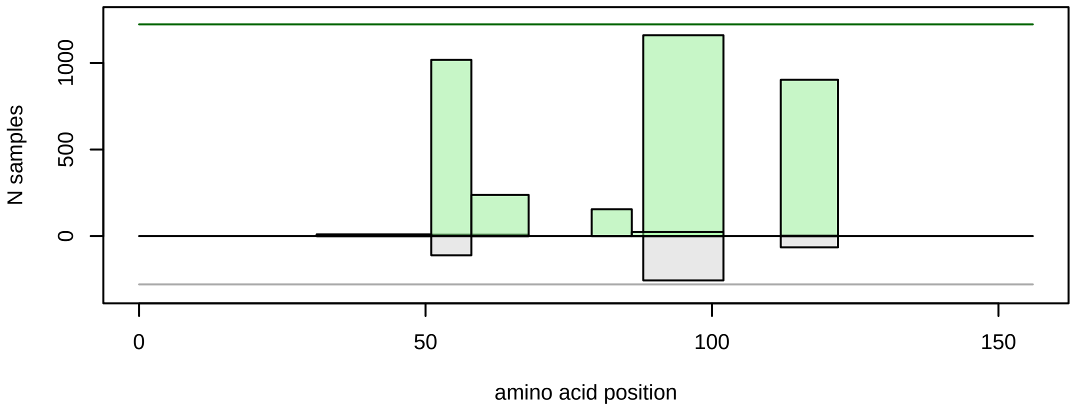

1775. HSP90AB1 (P08238), NP2

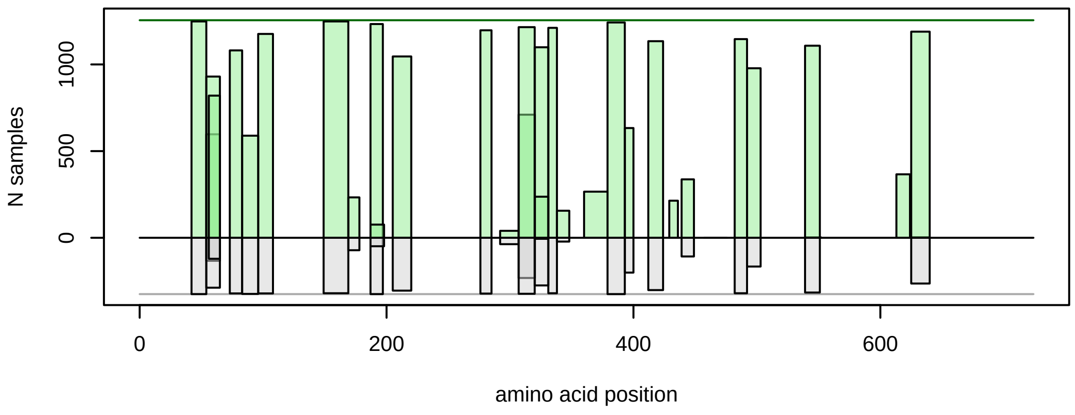

1776. LTA4H (P09960), NP5

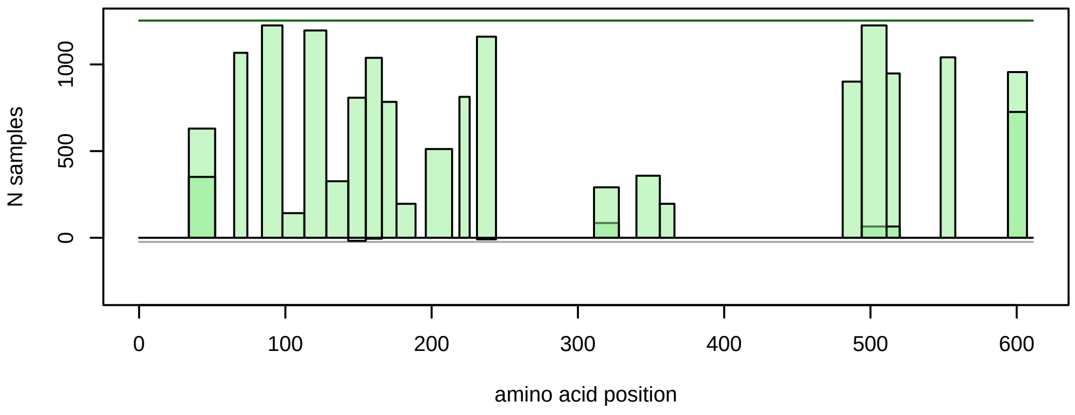

1777. SKAP2 (O75563), NP4

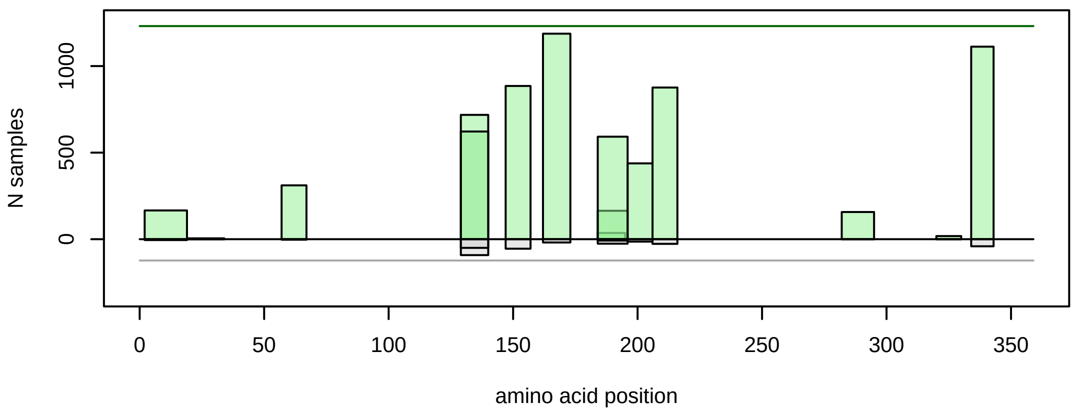

1778. A1BG (P04217), NP3

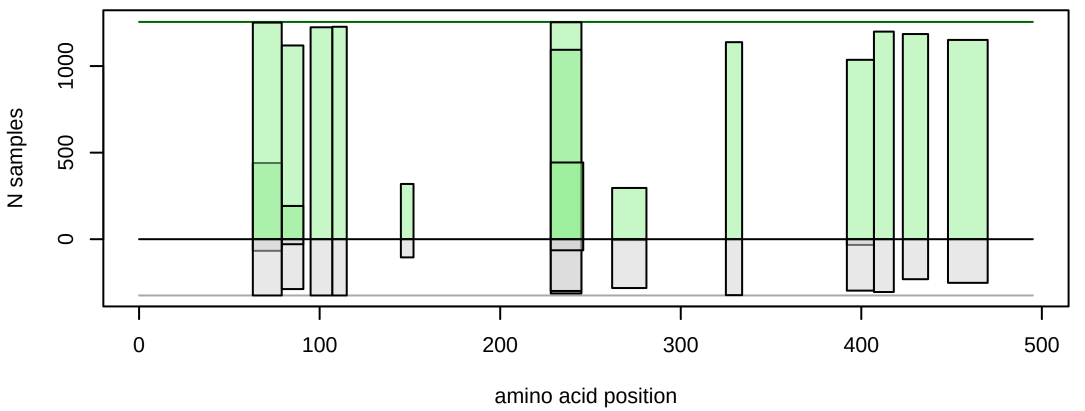

1779. DSC3 (Q14574), NP3

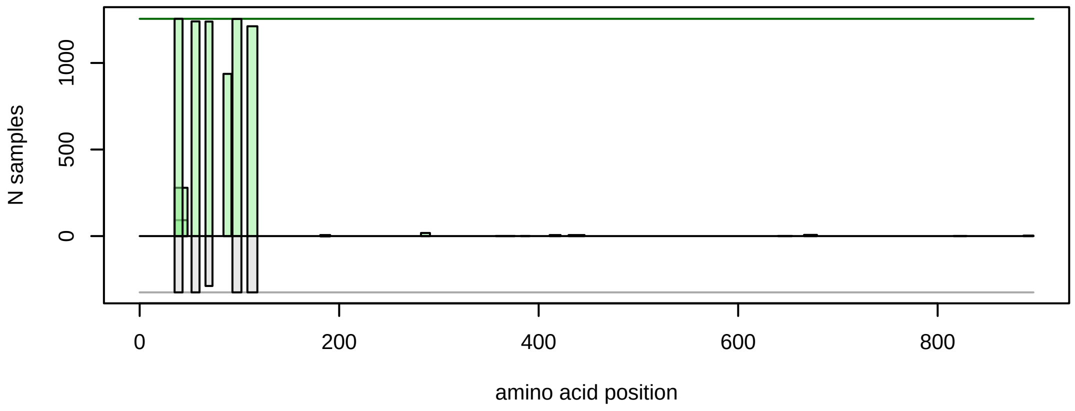

1780. CPT1A (P50416), NP4

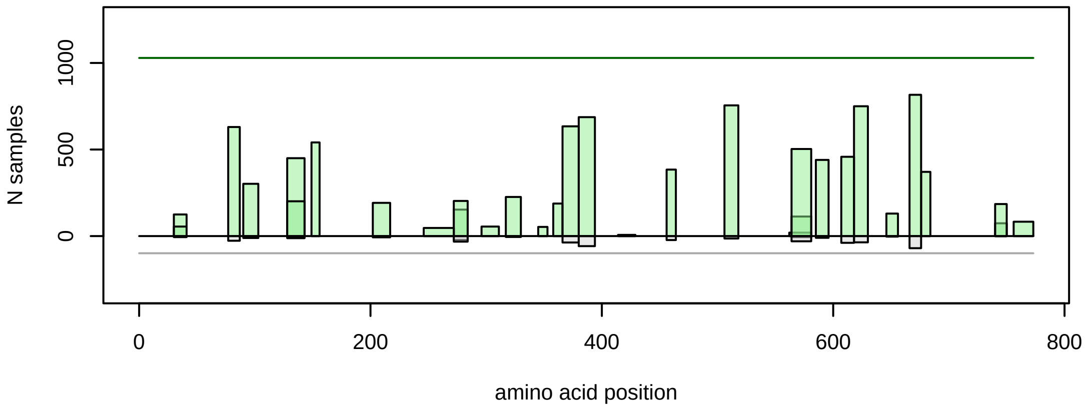

1781. CKAP5 (Q14008), NP3

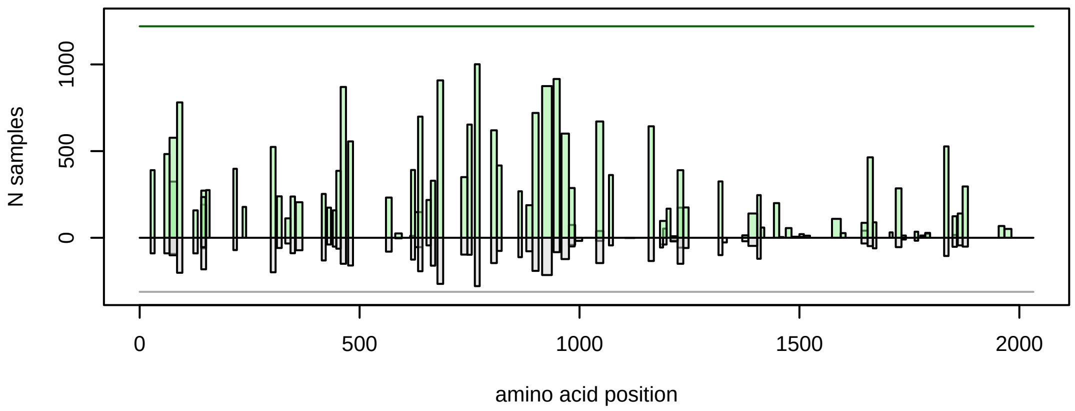

1782. REG4 (Q9BYZ8), NP3

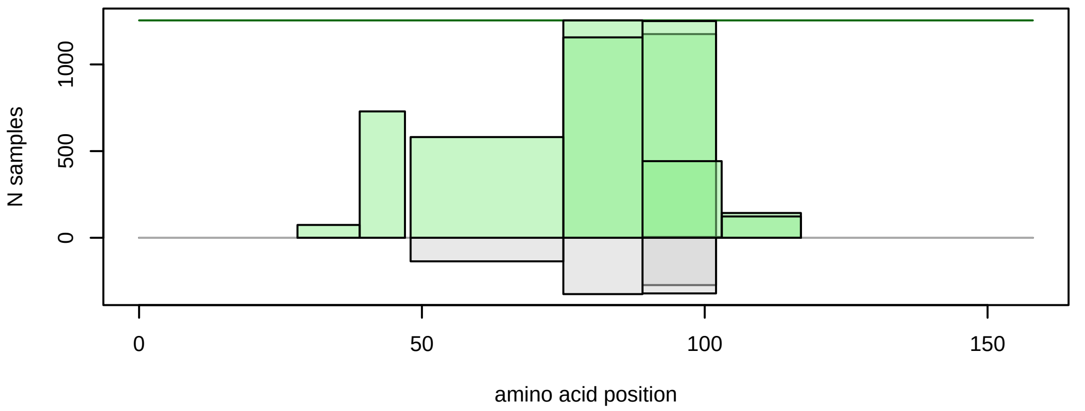

1783. NA (CONP15636), NP5

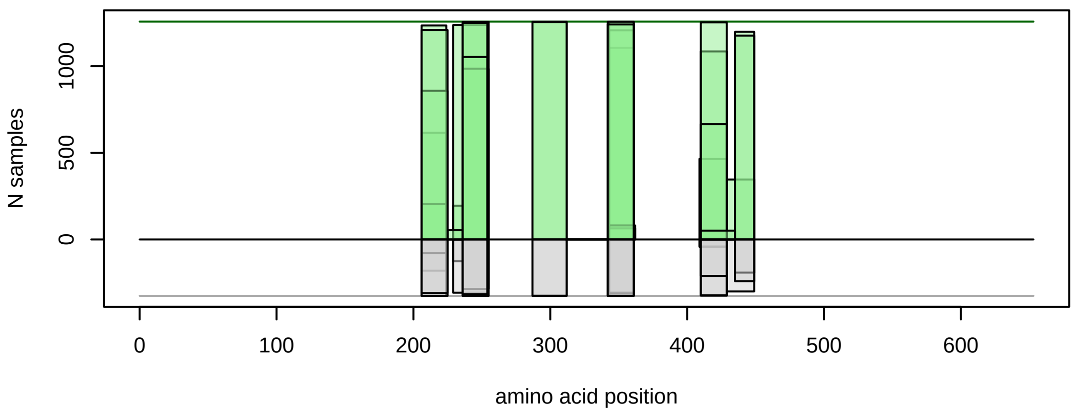

1784. GSPT1 (P15170−2;P15170−3), NP1

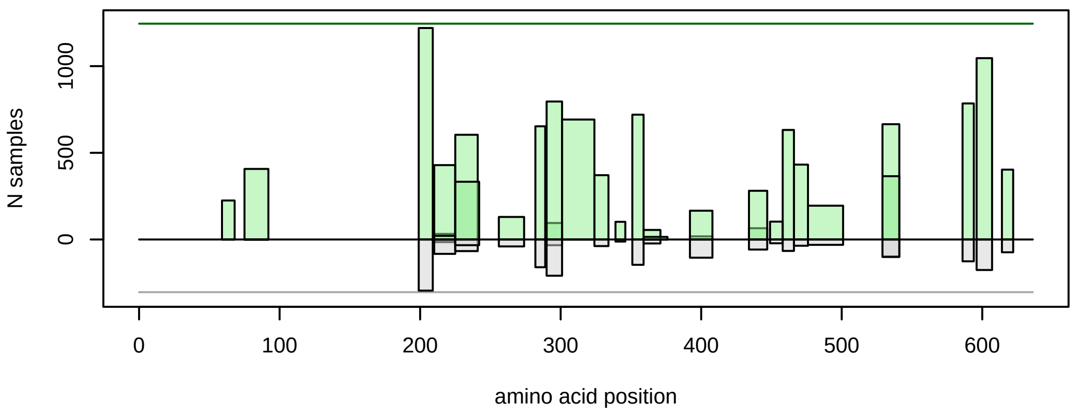

1785. EPS15 (P42566), NP2

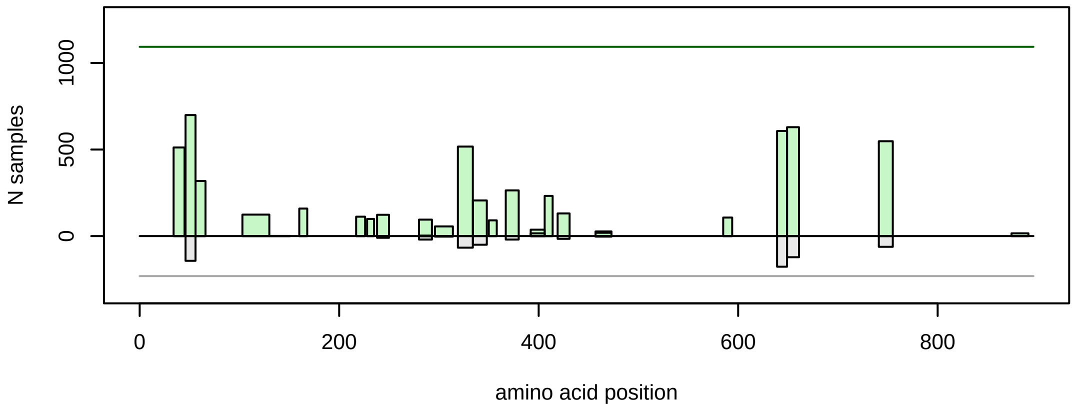

1786. SRC (P12931), NP1

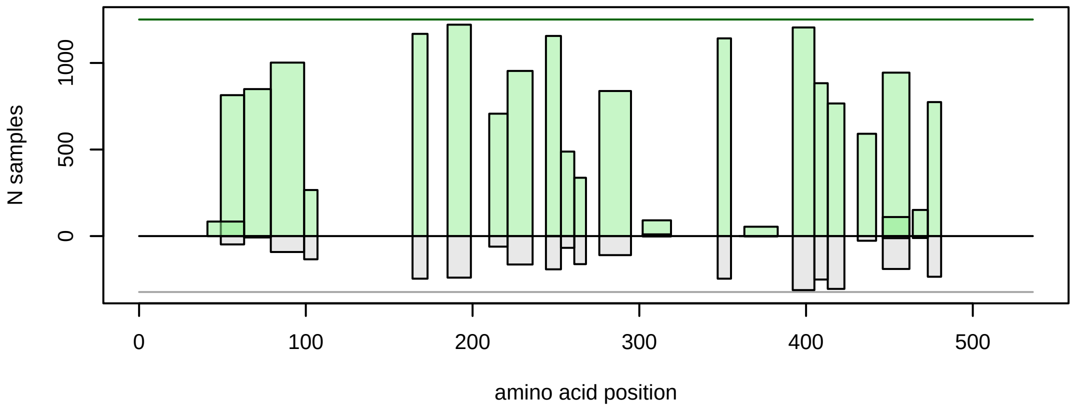

1787. SERBP1 (Q8NC51−3), NP4

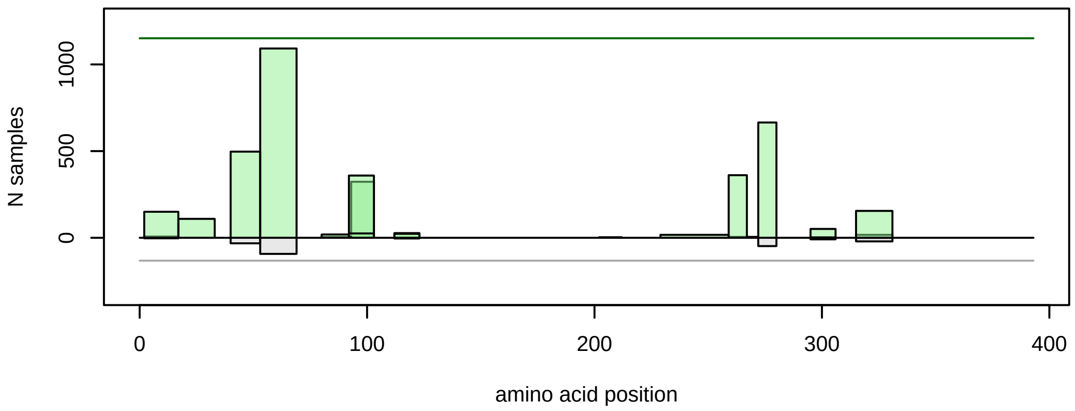

1788. APOA2 (V9GYM3), NP2

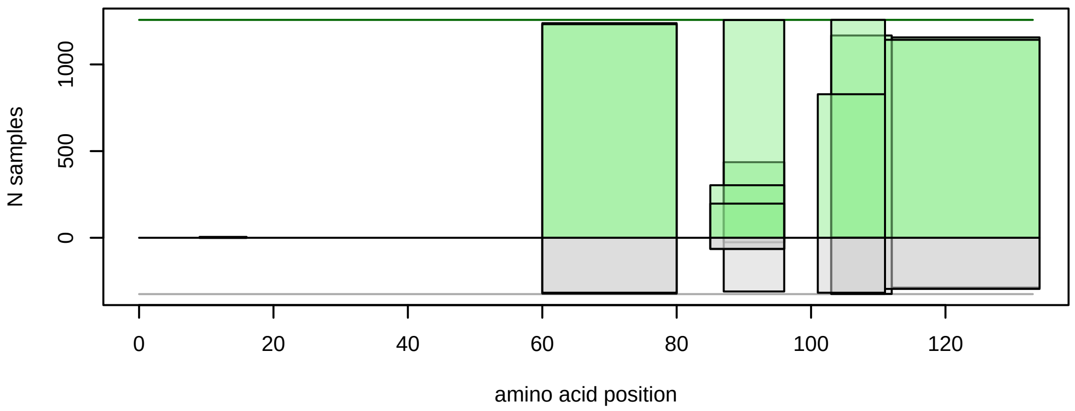

1789. VIT (Q6UXI7), NP3

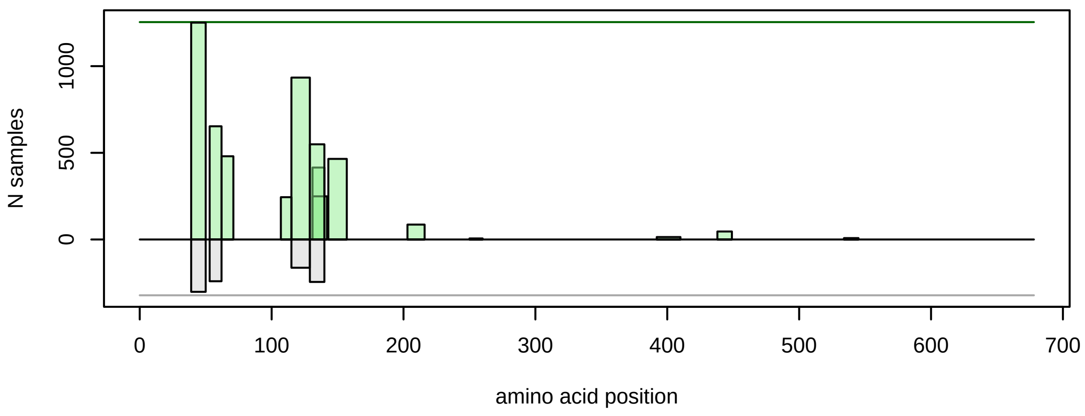

1790. VPS13C (Q709C8), NP4

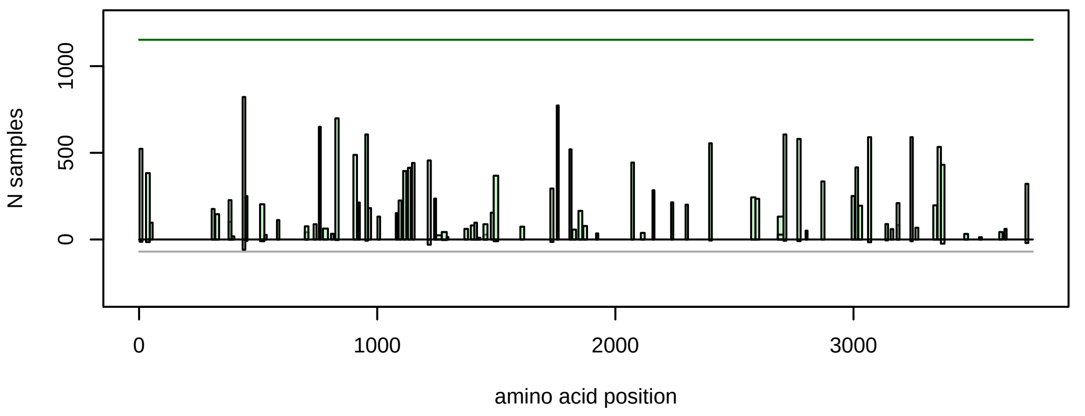

1791. GALNT2 (Q10471), NP4

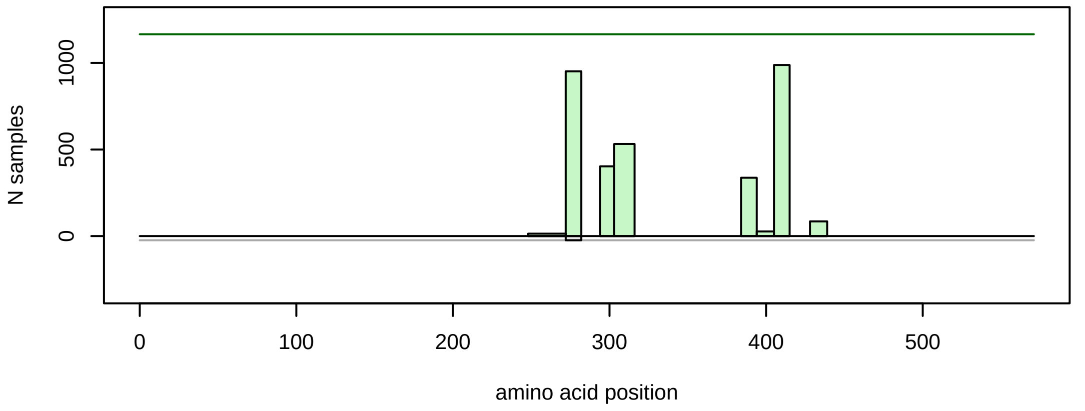

1792. EIF5 (P55010), NP2

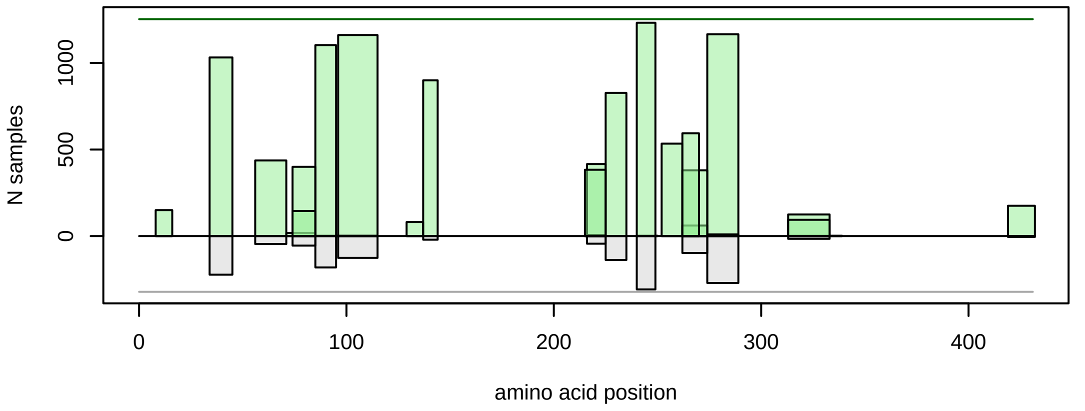

1793. SEPT2 (Q15019), NP3

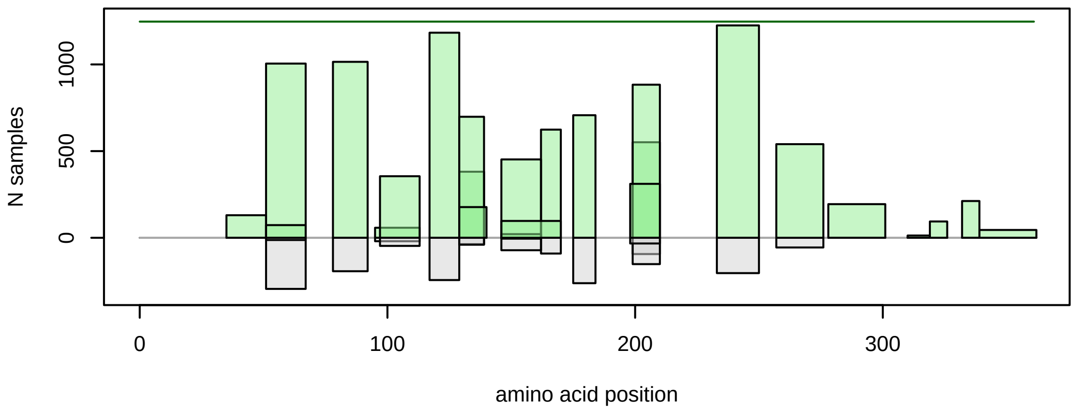

)A0A0MRT6;B6VEX4;Q8IZP0;Q8IZP0−2;Q8IZP0−3;Q8IZP0−4;Q8IZP0−5;Q8IZP0−6;Q8IZP0-

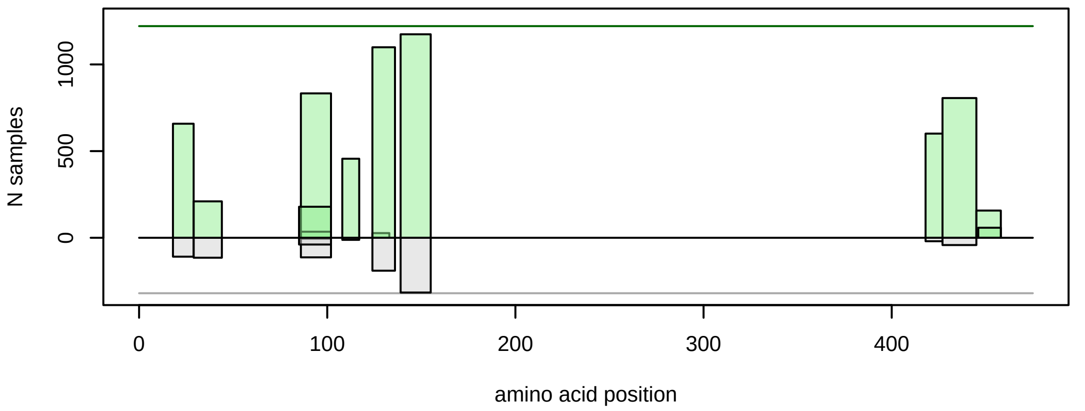

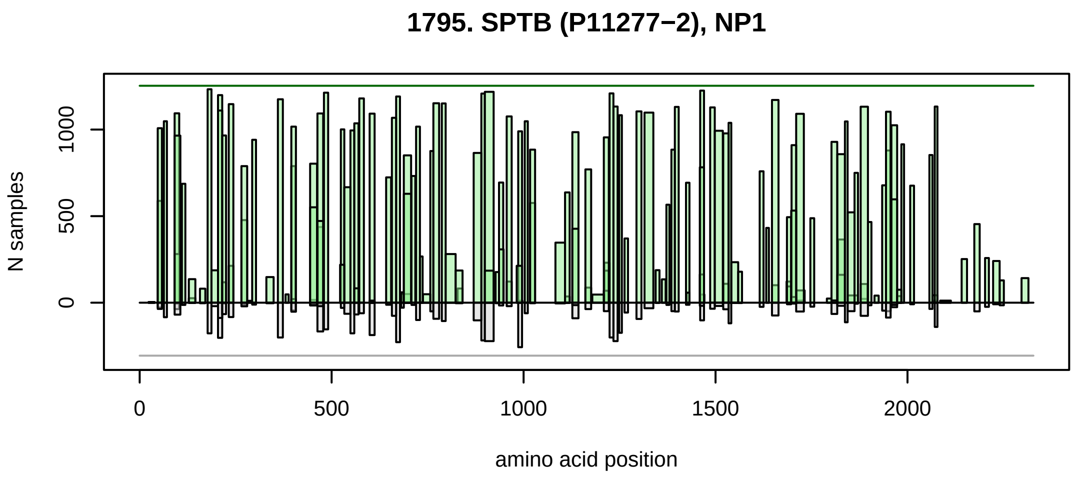
1795. SPTB (P11277−2), NP1
N samples
0
500
1000
0
500
1000
1500
2000
amino acid position

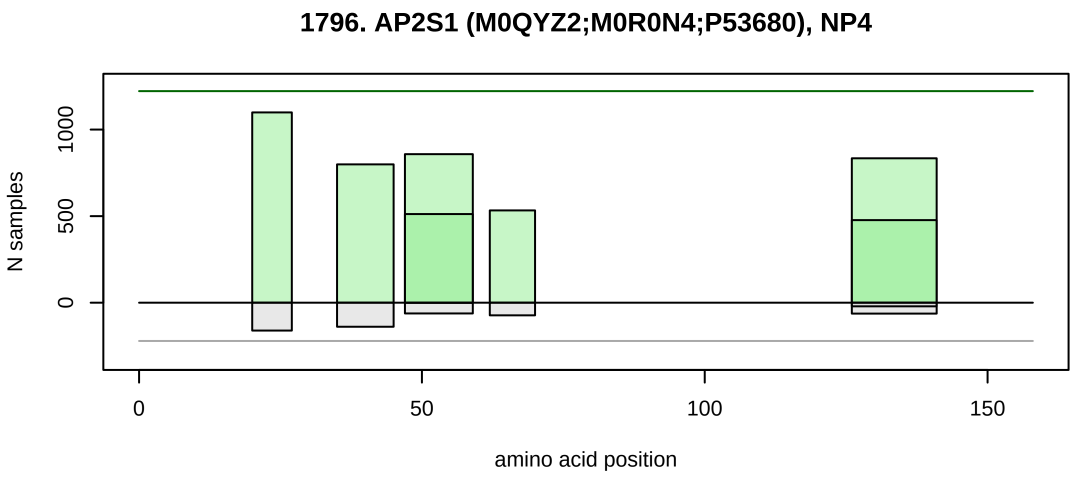
1796. AP2S1 (M0QYZ2;M0R0N4;P53680), NP4
N samples
0
500
1000
0
50
100
150
amino acid position

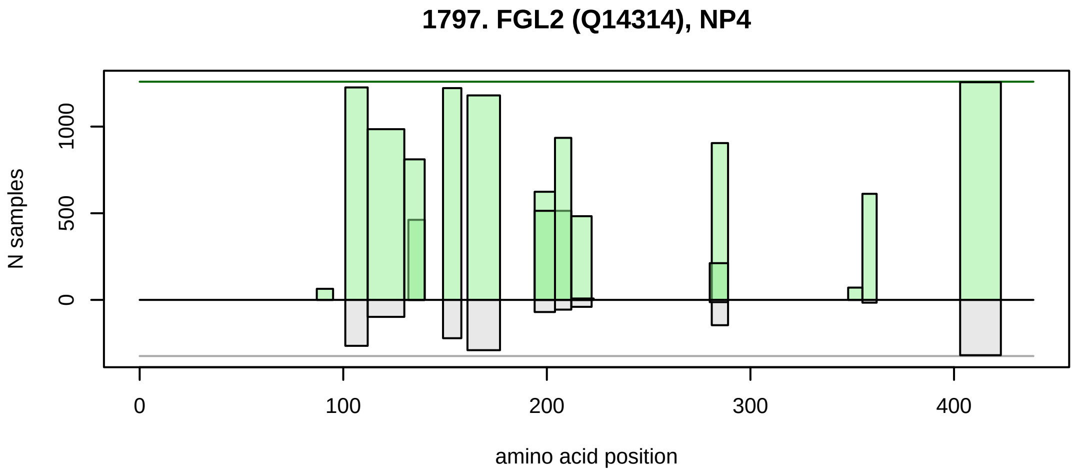
1797. FGL2 (Q14314), NP4
N samples
0
500
1000
0
100
200
300
400
amino acid position

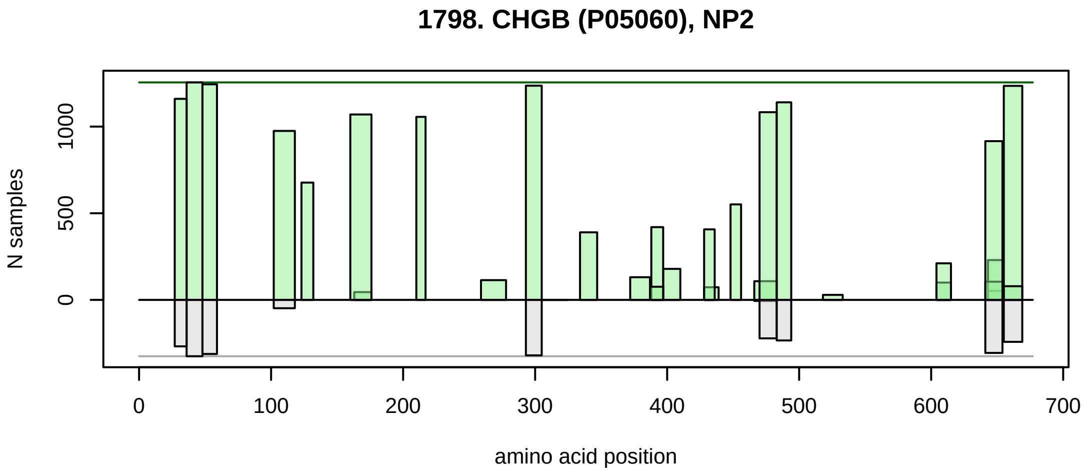
1798. CHGB (P05060), NP2
N samples
0
500
1000
0
100
200
300
400
500
600
700
amino acid position

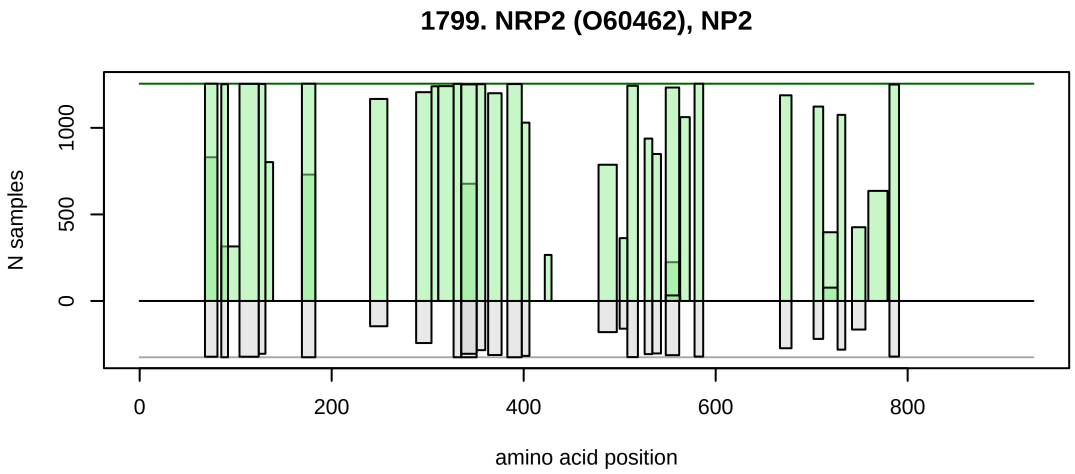
1799. NRP2 (O60462), NP2
N samples
0
500
1000
0
200
400
600
800
amino acid position

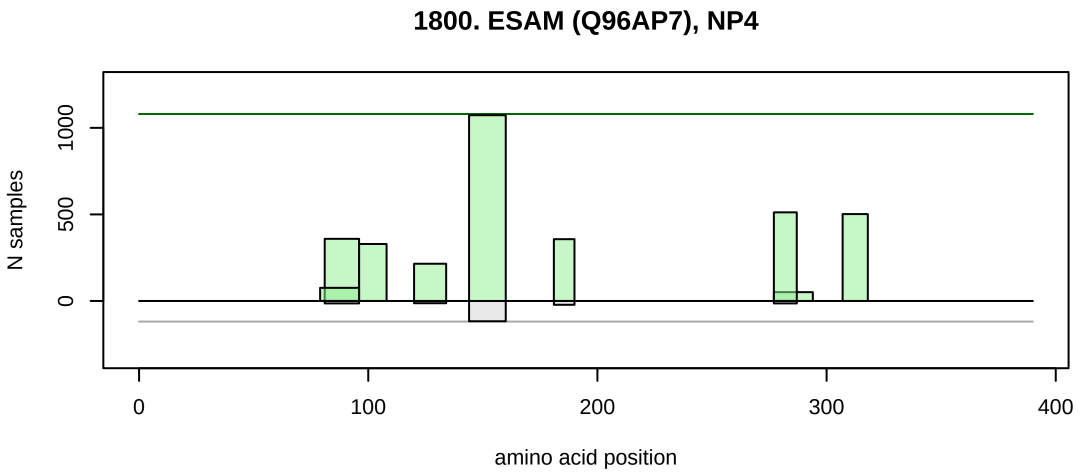
1800. ESAM (Q96AP7), NP4
N samples
0
500
1000
0
100
200
300
400
amino acid position

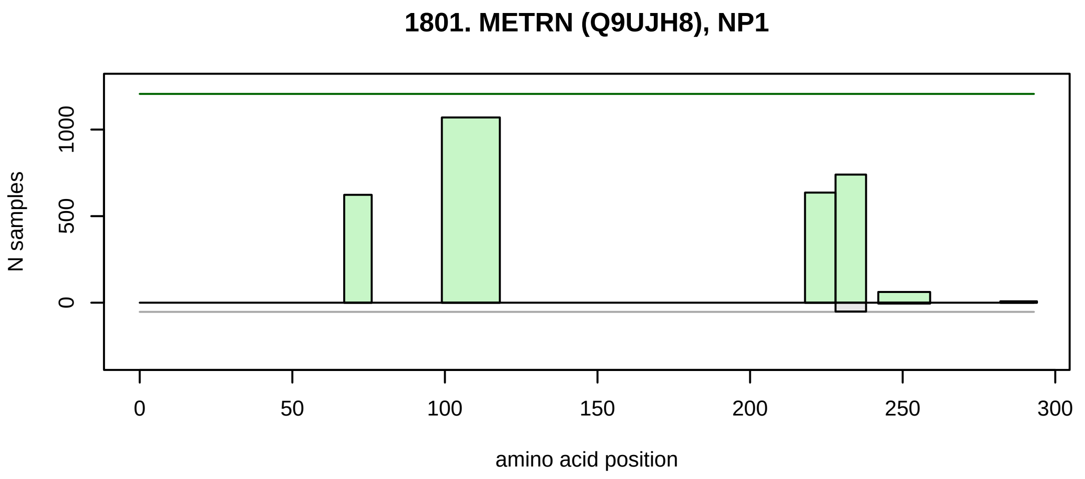
1801. METRN (Q9UJH8), NP1
N samples
0
500
1000
0
50
100
150
200
250
300
amino acid position

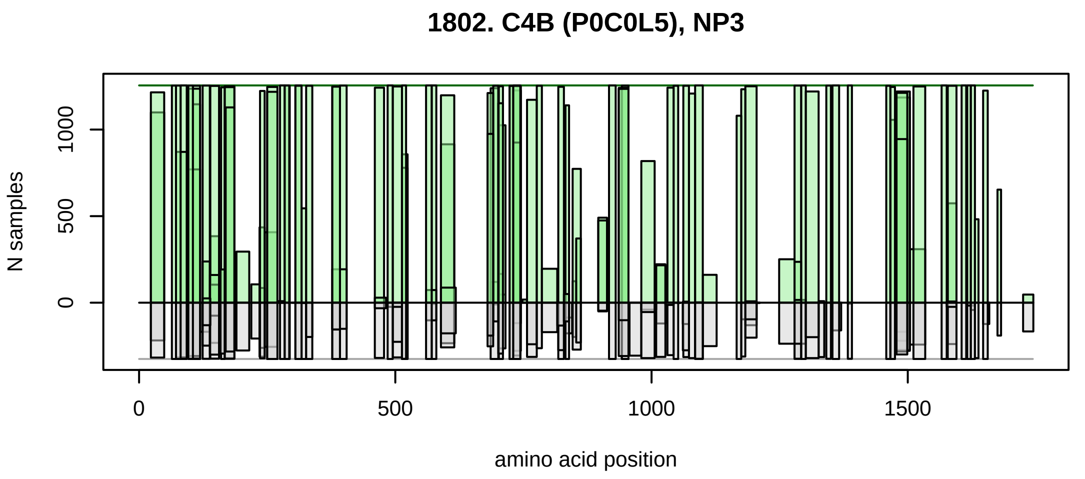
1802. C4B (P0C0L5), NP3
N samples
0
500
1000
0
500
1000
1500
amino acid position

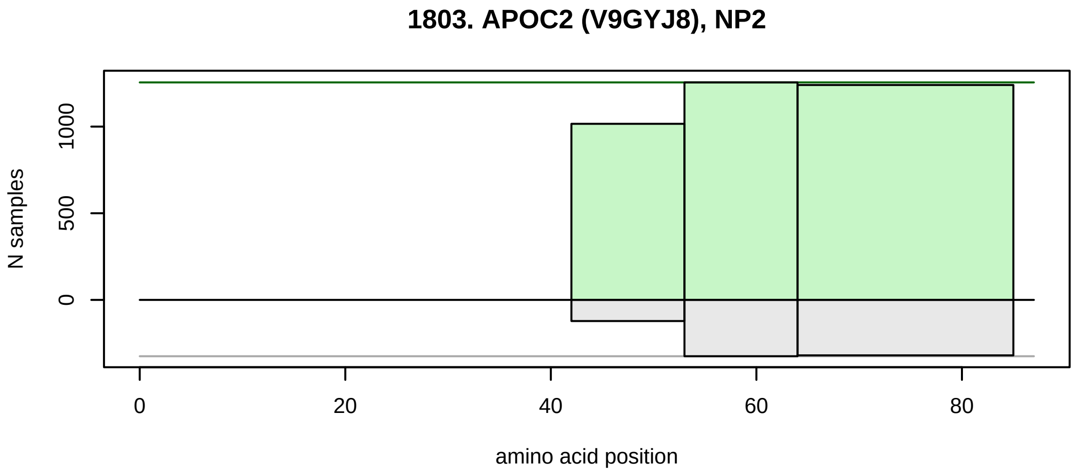
1803. APOC2 (V9GYJ8), NP2
N samples
0
500
1000
0
20
40
60
80
amino acid position

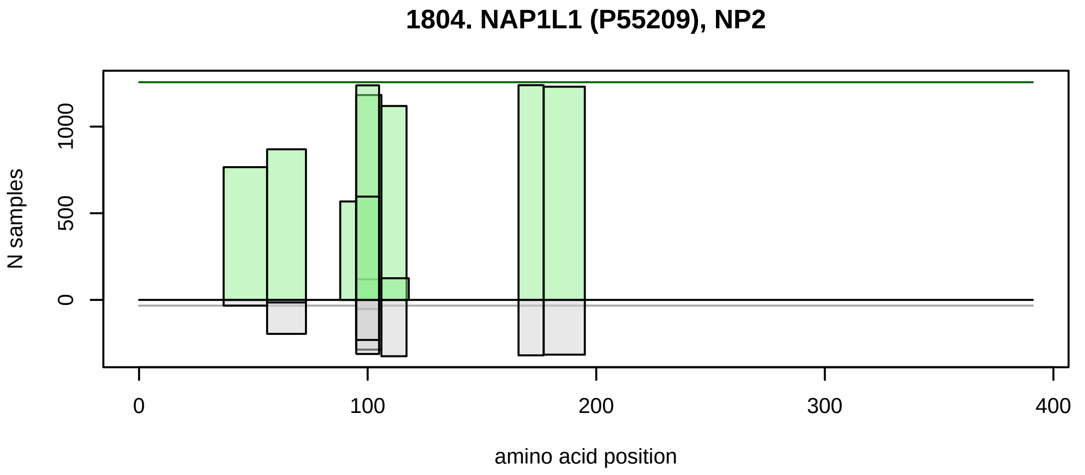
1804. NAP1L1 (P55209), NP2
N samples
0
500
1000
0
100
200
300
400
amino acid position

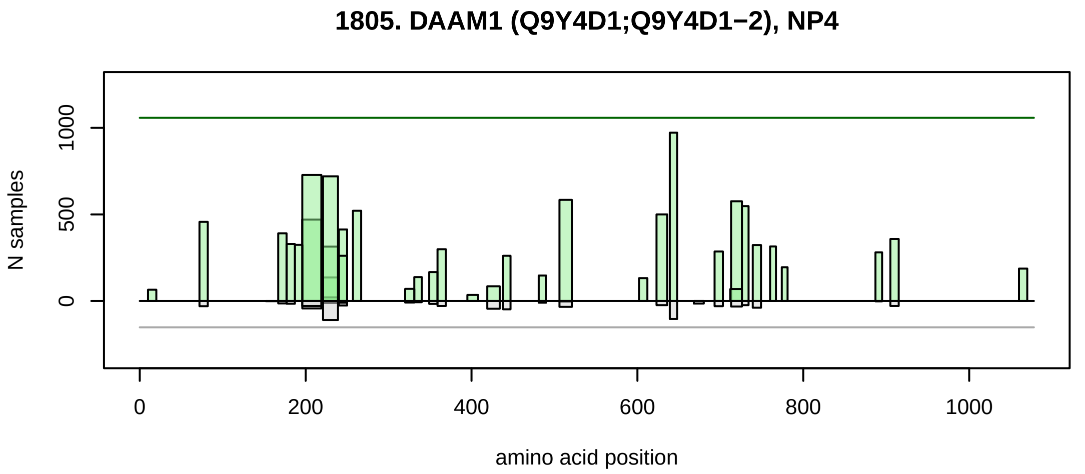
1805. DAAM1 (Q9Y4D1;Q9Y4D1−2), NP4
N samples
0
500
1000
0
200
400
600
800
1000
amino acid position

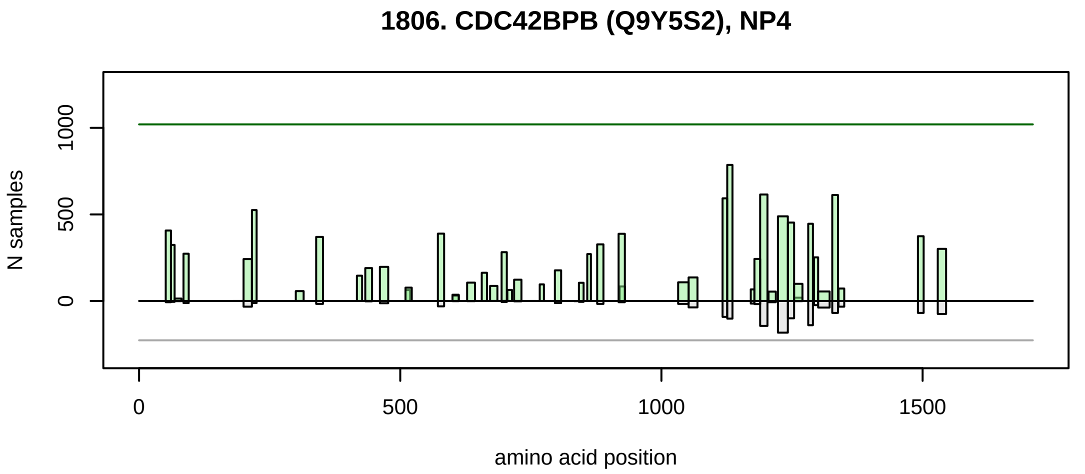
1806. CDC42BPB (Q9Y5S2), NP4
N samples
0
500
1000
0
500
1000
1500
amino acid position

1807. BLVRB (P30043), NP5

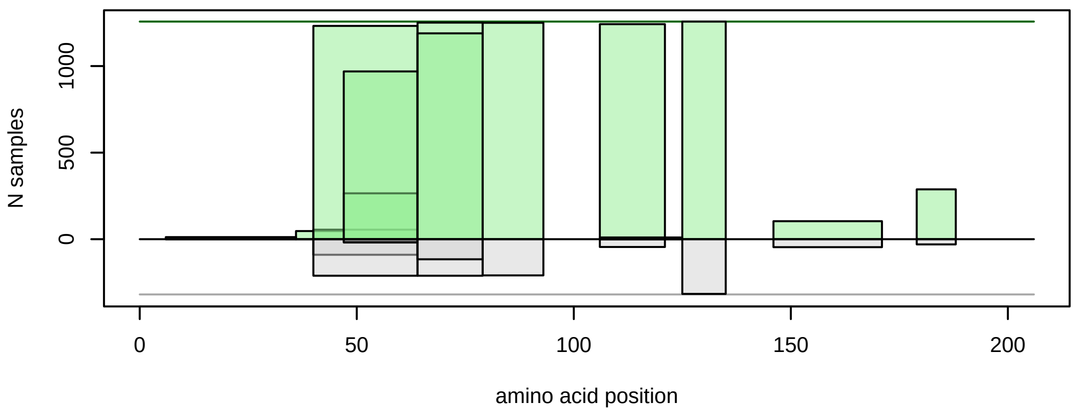

1808. HNRNPH3 (P31942;P31942−2), NP2

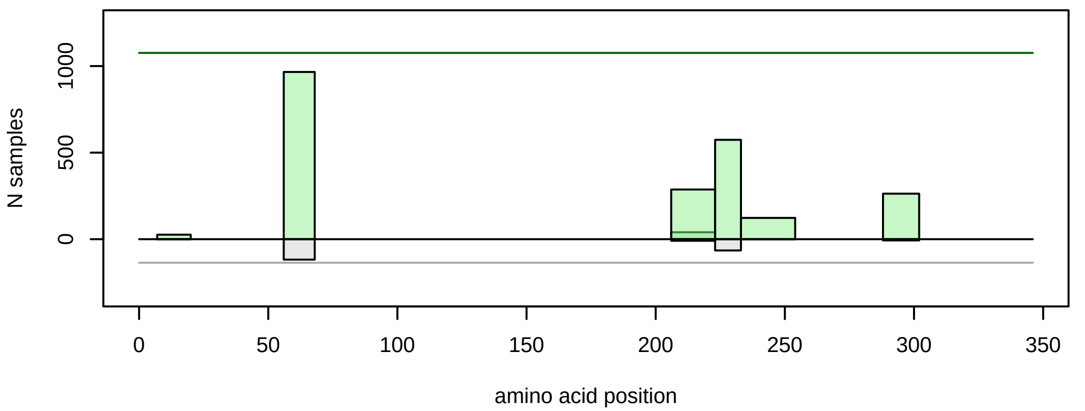

1809. ARAP1 (Q96P48;Q96P48−3), NP1

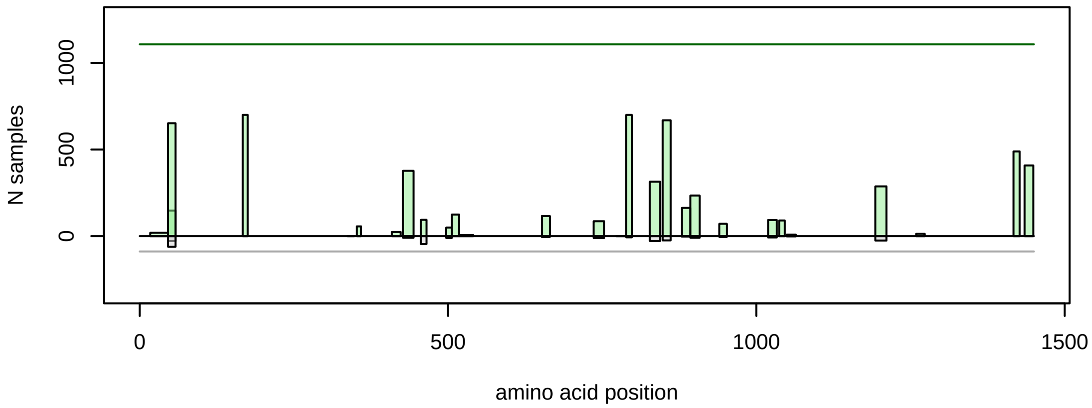

1810. NCK2 (O43639), NP3

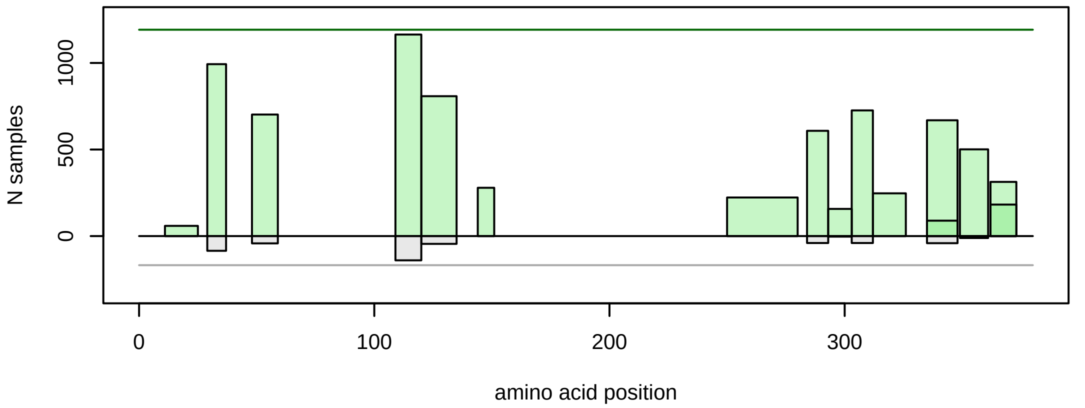

1811. MAPRE2 (Q15555−3), NP1

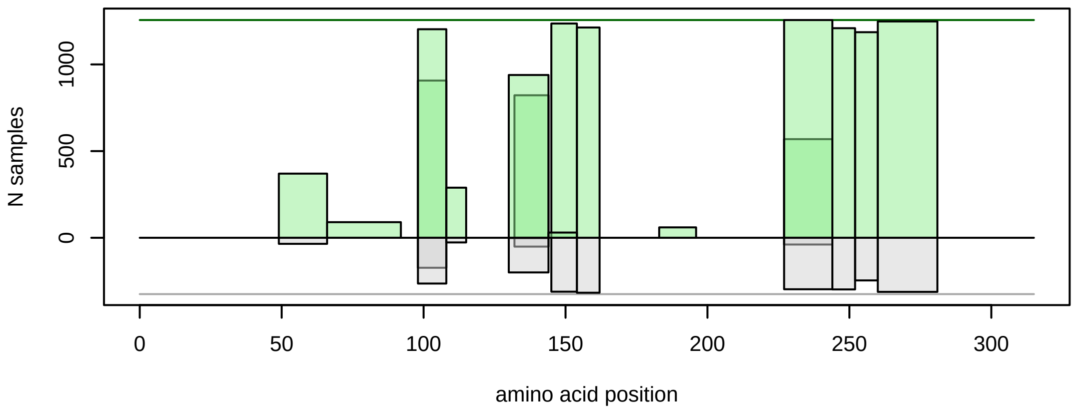

1812. RDH11 (Q8TC12), NP4

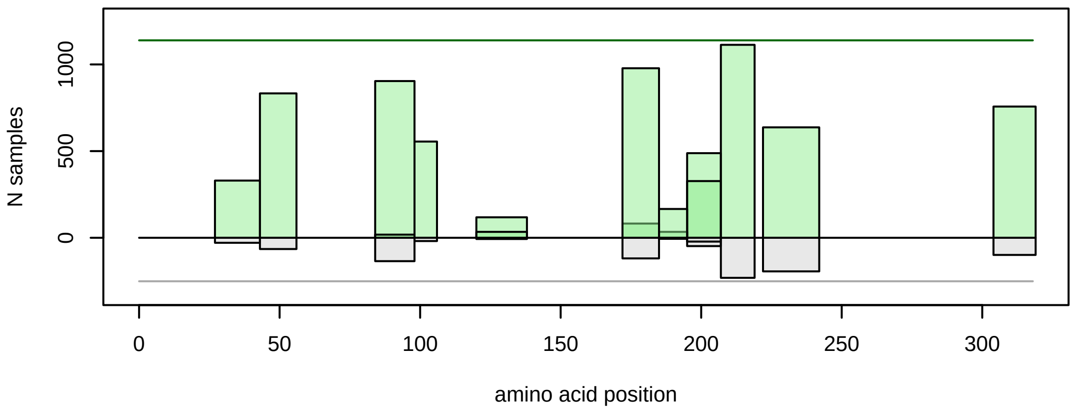

1813. EHD3 (Q9NZN3), NP2

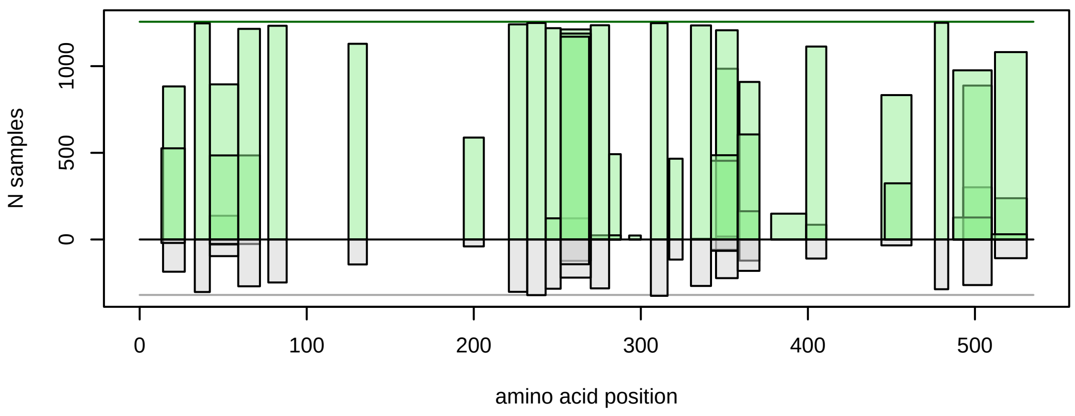

1814. DOCK5 (Q9H7D0), NP4

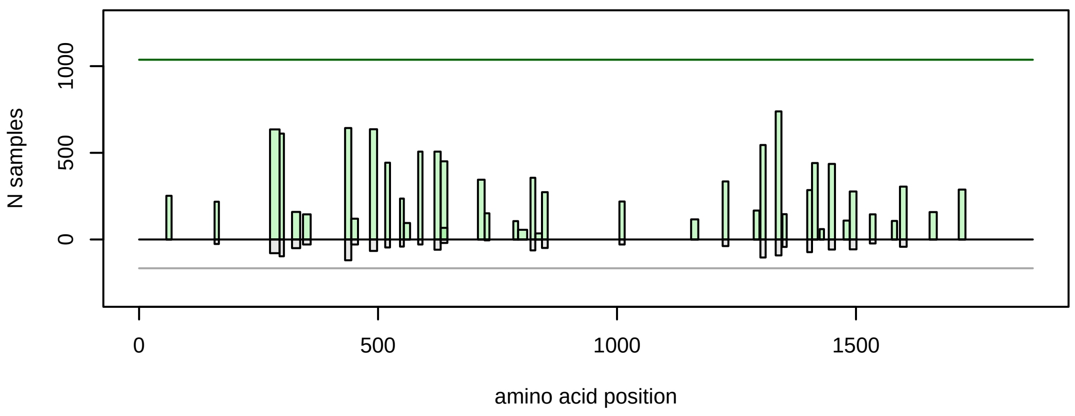

1815. MNDA (P41218), NP4

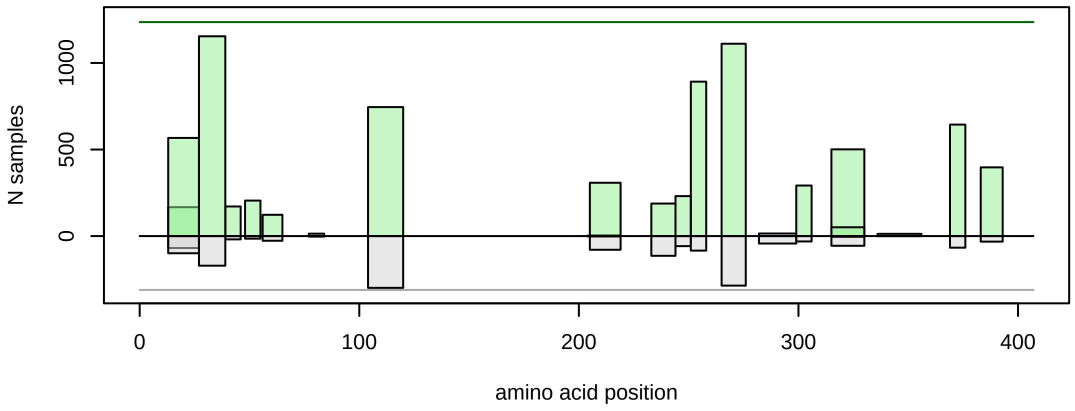

1816. GCLC (P48506), NP5

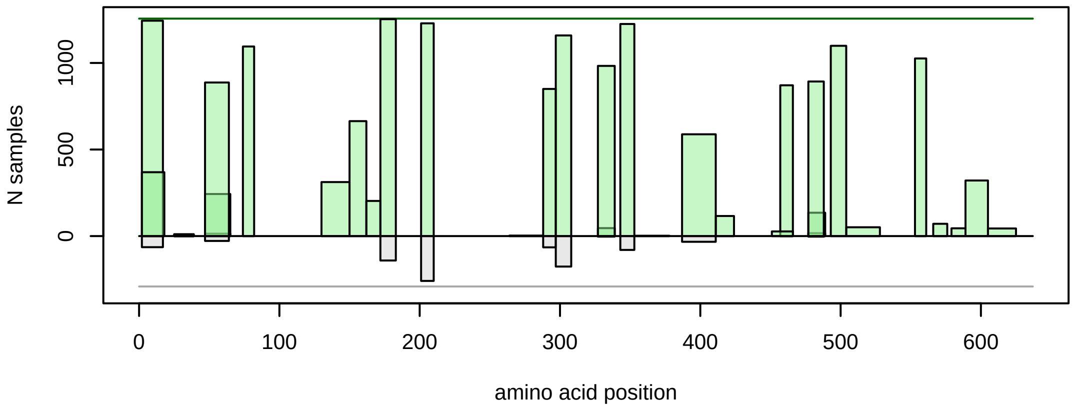

1817. POSTN (B1ALD9), NP3

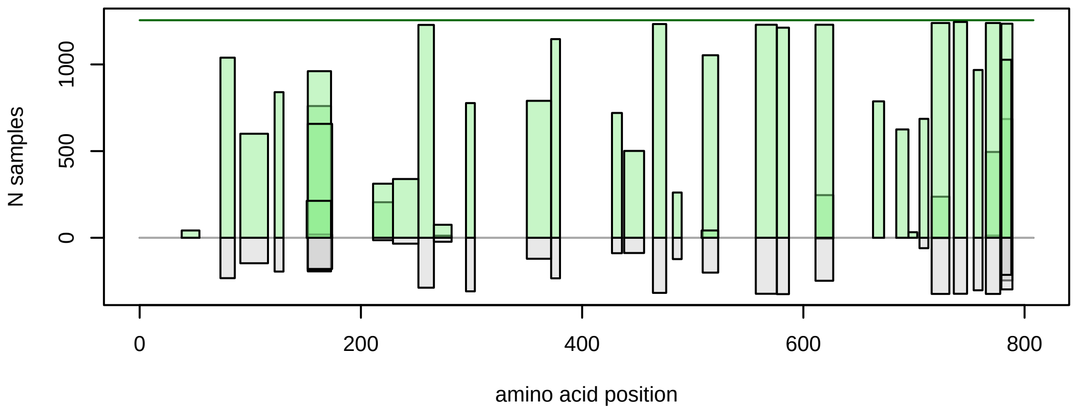

1818. HABP2 (Q14520), NP2

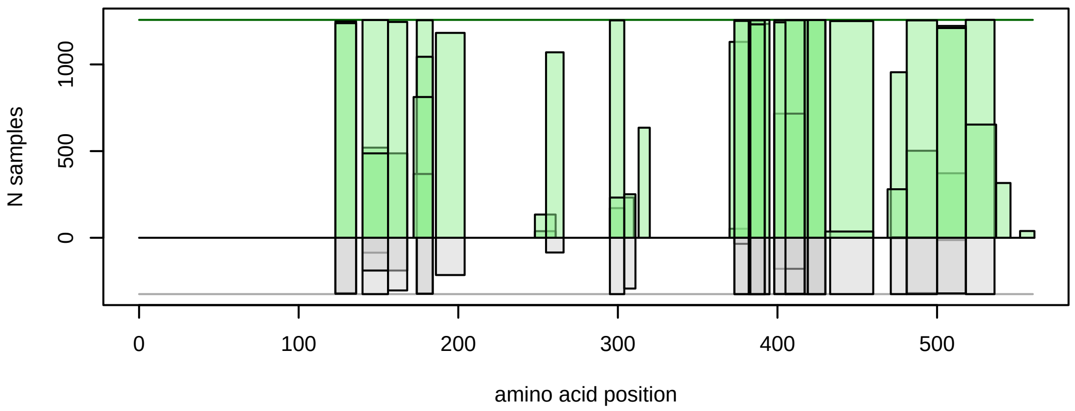

1819. ETFA (A0A8I5KVL5;H0YLU7), NP4

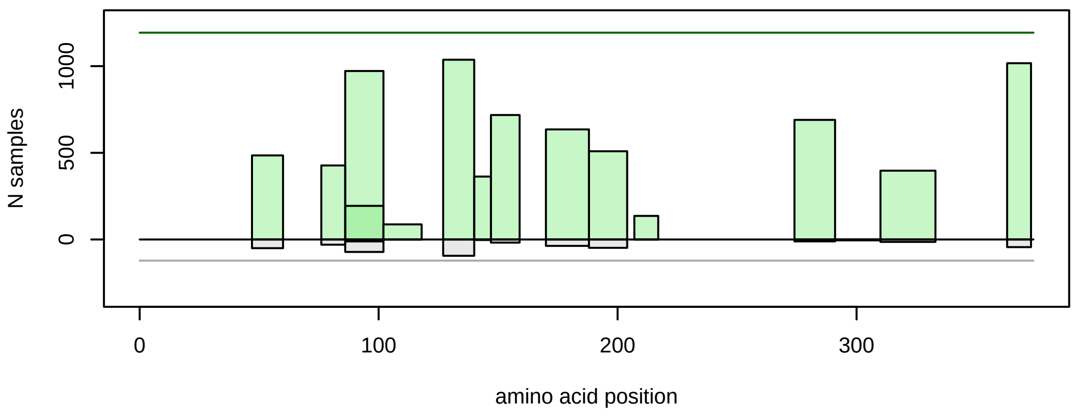

1820. ANXA6 (P08133), NP2

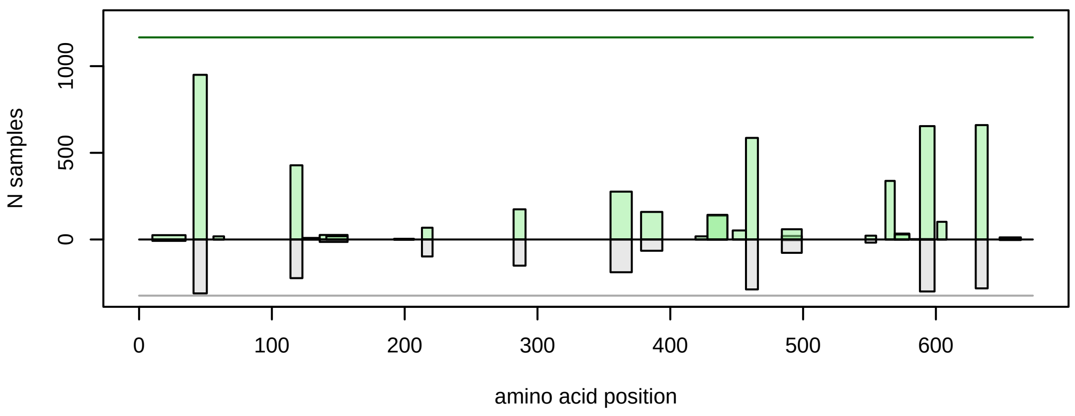

1821. RTCB (Q9Y3I0), NP1

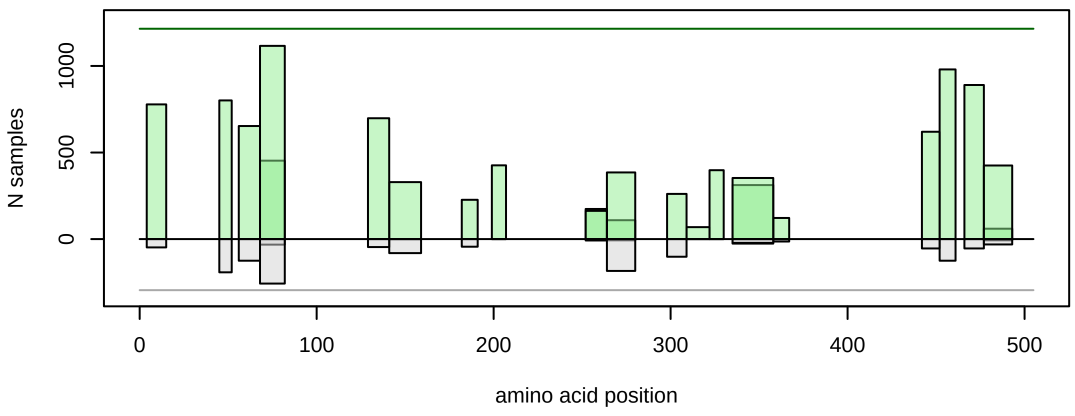

1822. LIPG (Q9Y5X9), NP2

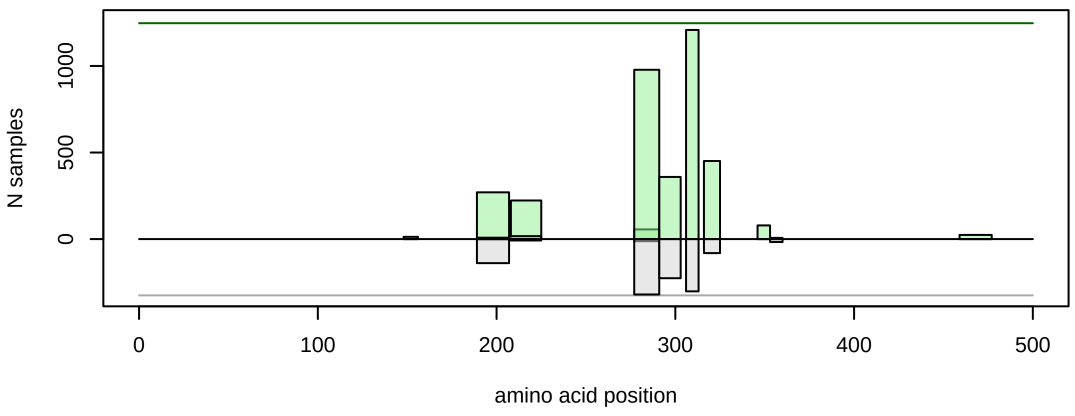

1823. PPP6R3 (H7BXH2), NP2

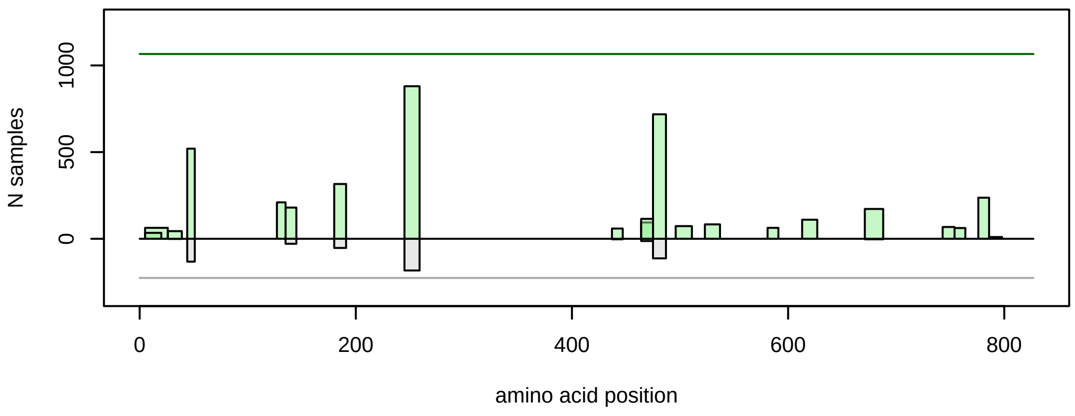

1824. MMP11 (P24347), NP5

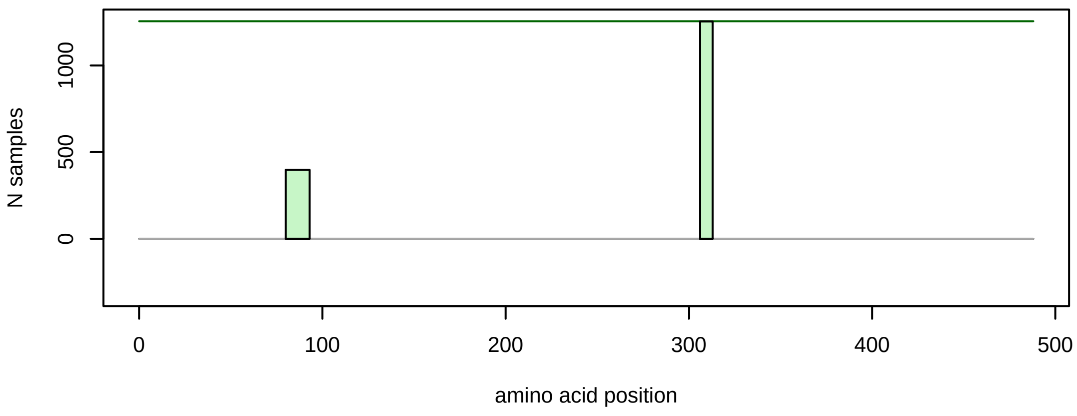

1825. HSP90AA1 (P07900−2), NP2

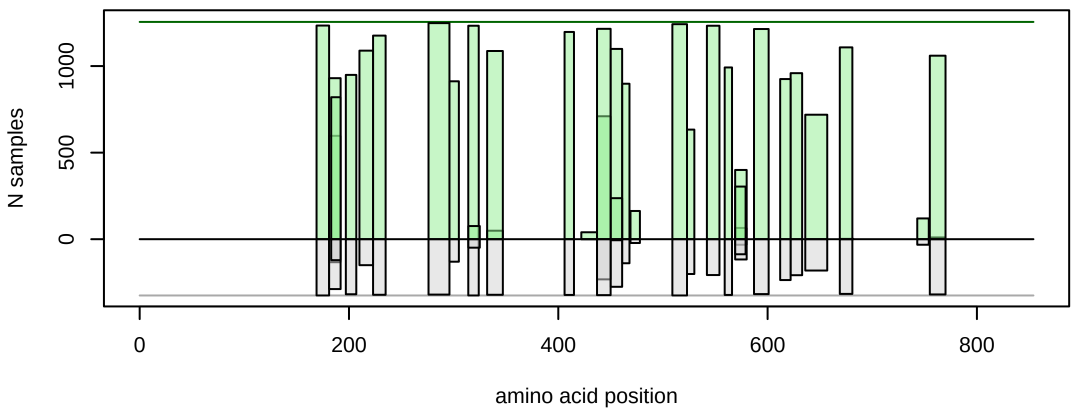

1826. LTBP4 (Q8N2S1−2), NP2

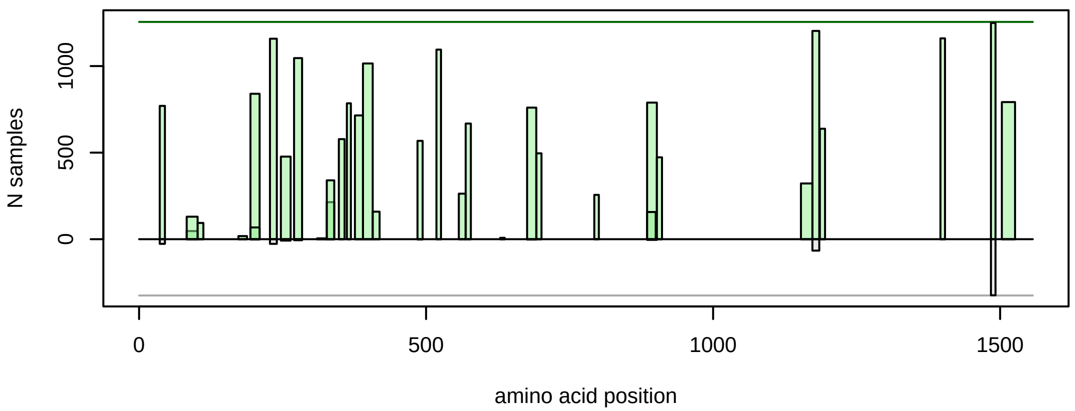

1827. RAB32 (Q13637), NP4

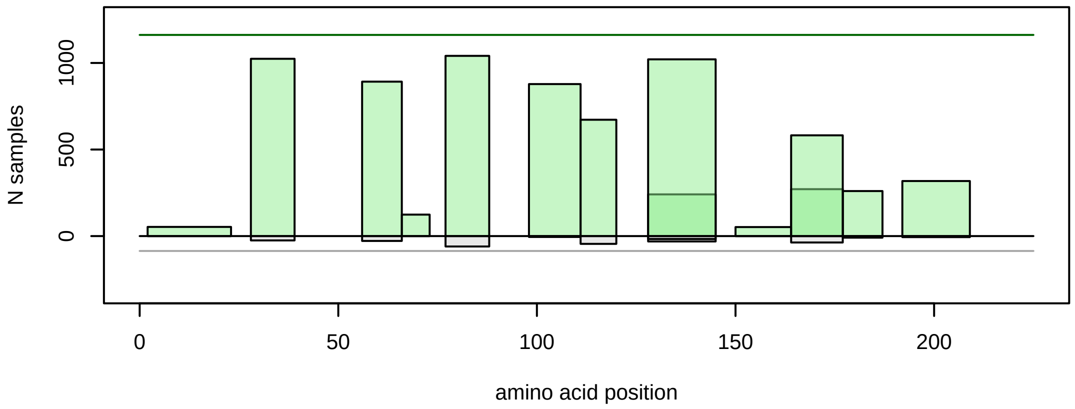

1828. GREM2 (Q9H772), NP4

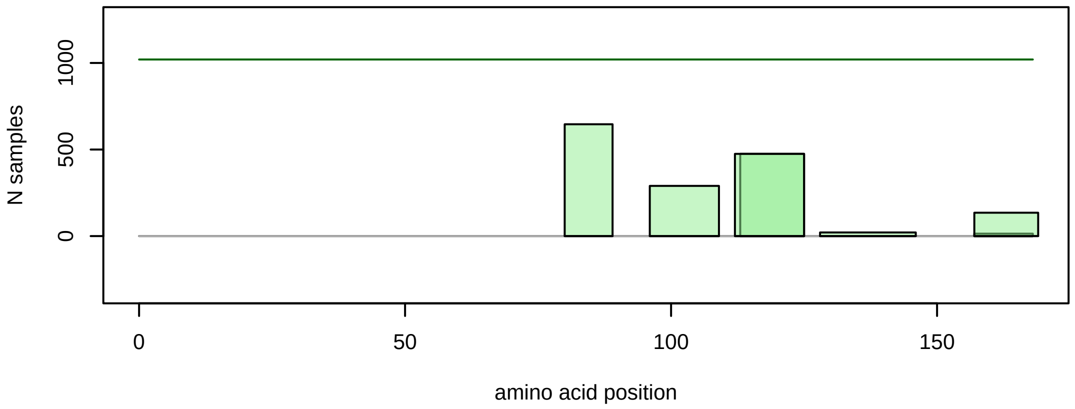

1829. CRP (P02741), NP5

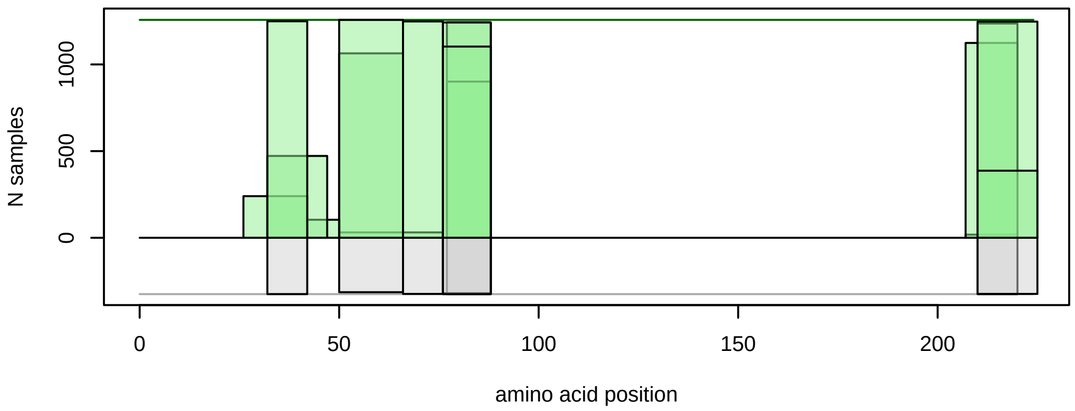

1830. S100A9 (P06702), NP3

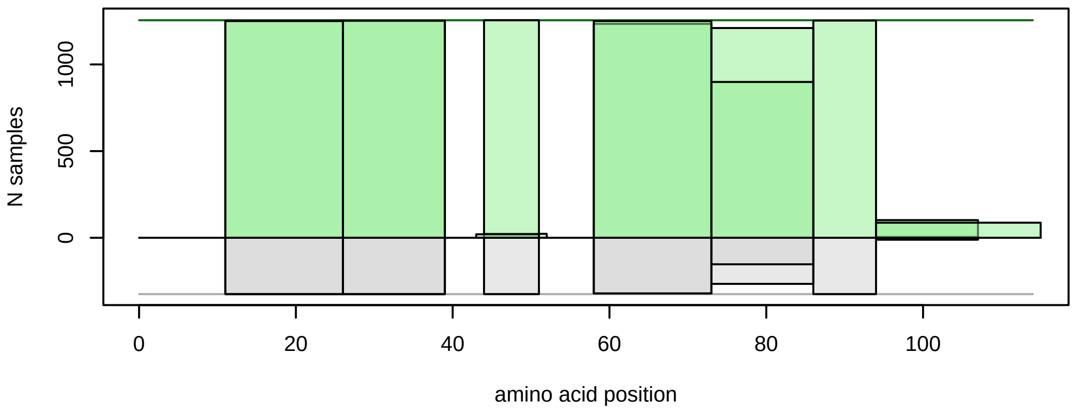

1831. IGKV6−21 (A0A0C4DH24), NP3

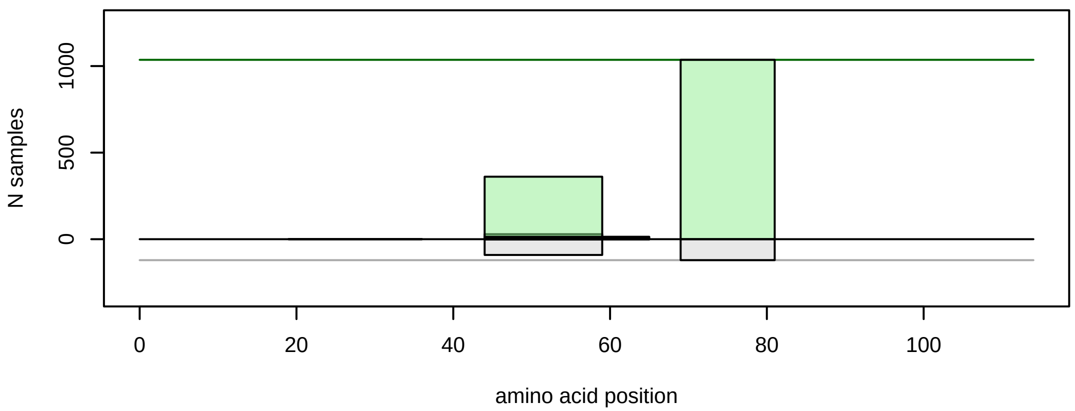

1832. SNX2 (O60749), NP1

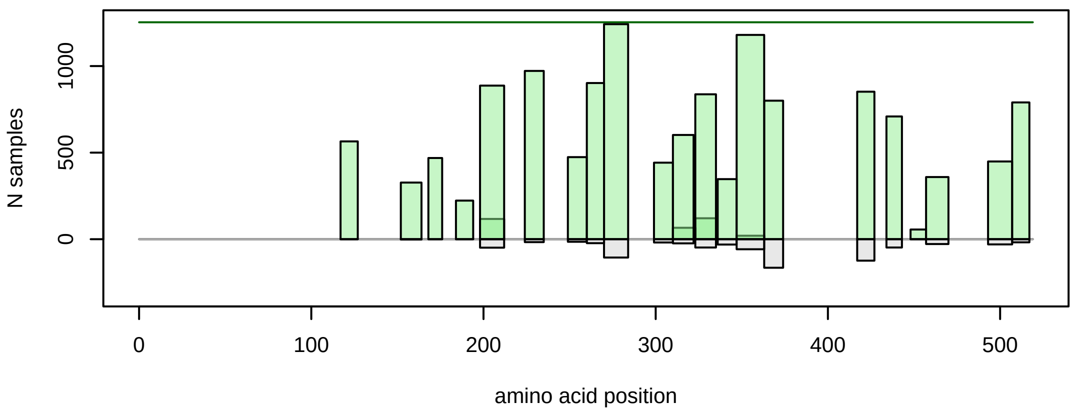

1833. CCL5 (P13501), NP3

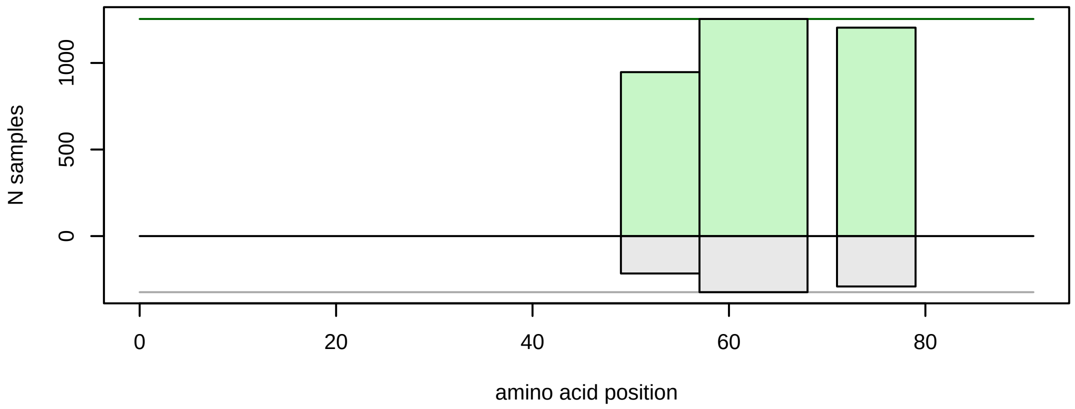

1834. NA (A0A384ME17), NP4

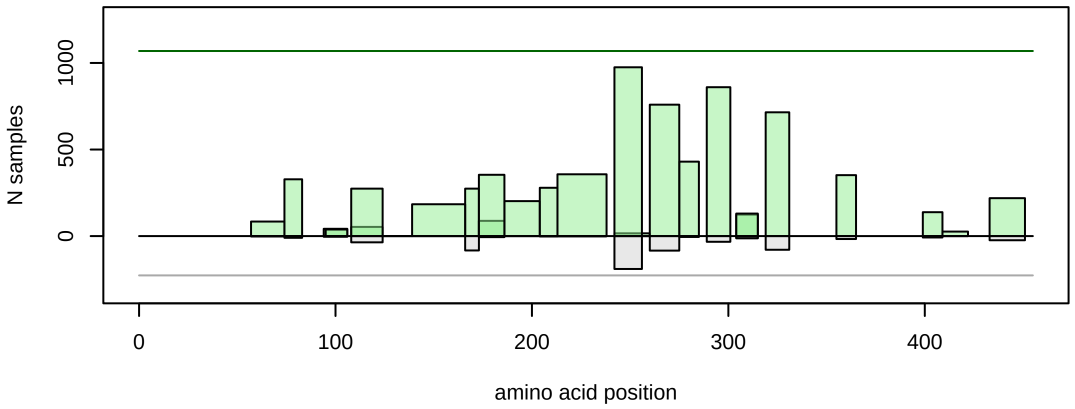

1835. CD248 (Q9HCU0), NP2

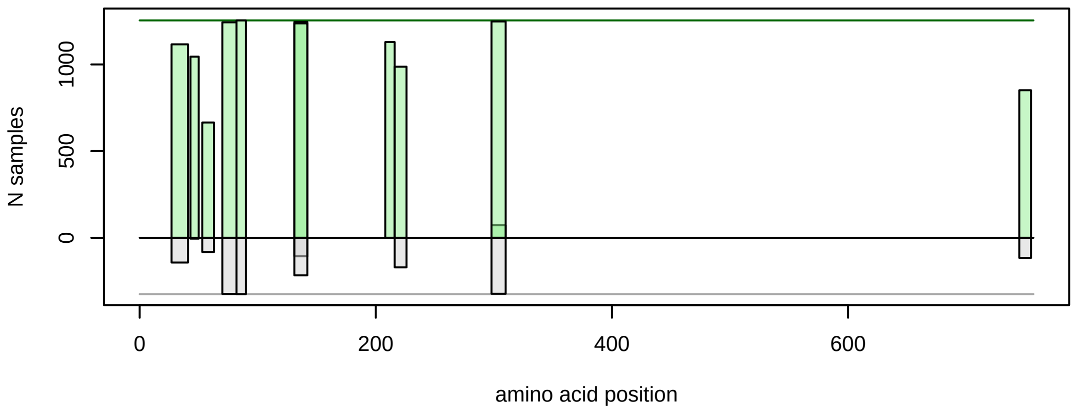

1836. ASL (P04424), NP5

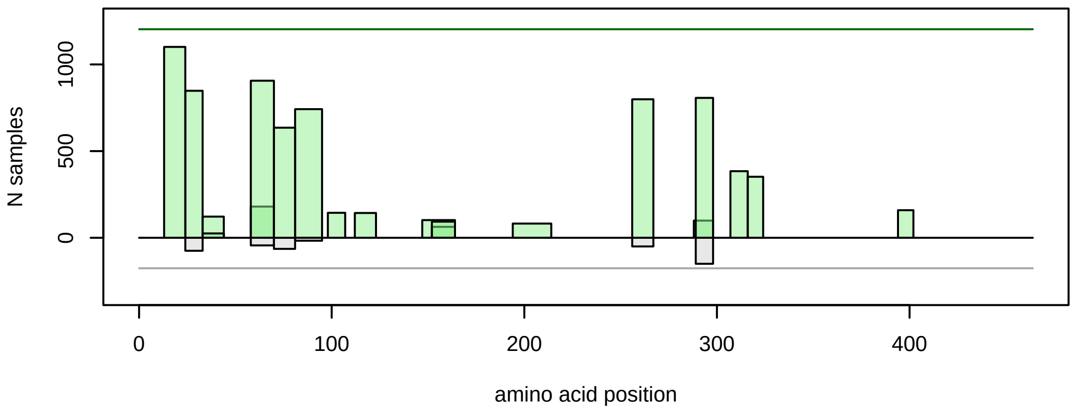

1837. GBE1 (Q04446), NP5

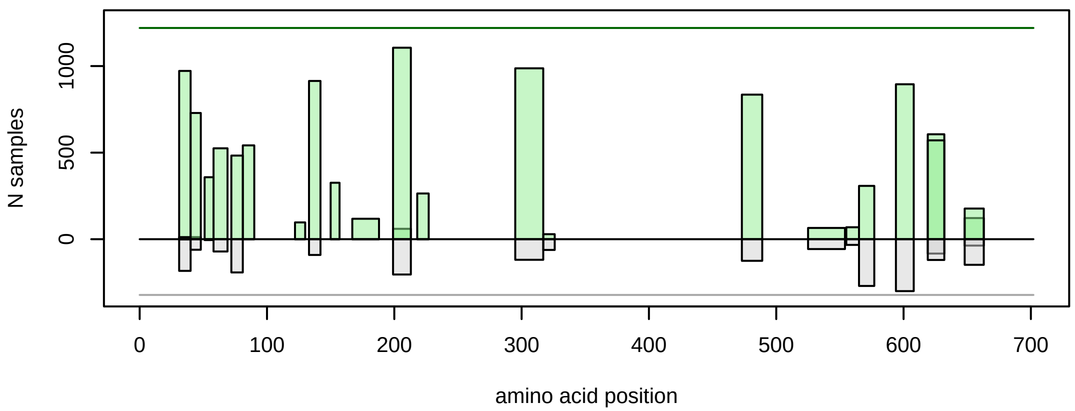

1838. XYLT2 (Q9H1B5), NP2

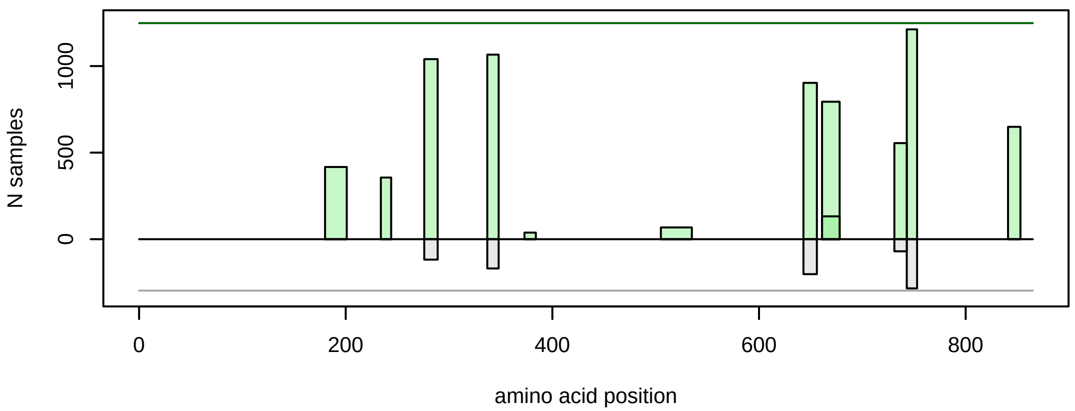

1839. MAOB (P27338), NP4

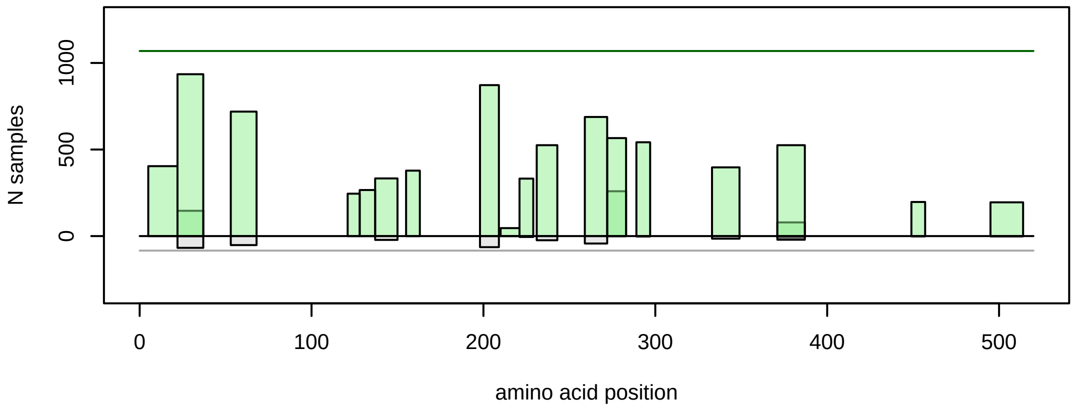

1840. MYL9 (P24844), NP4

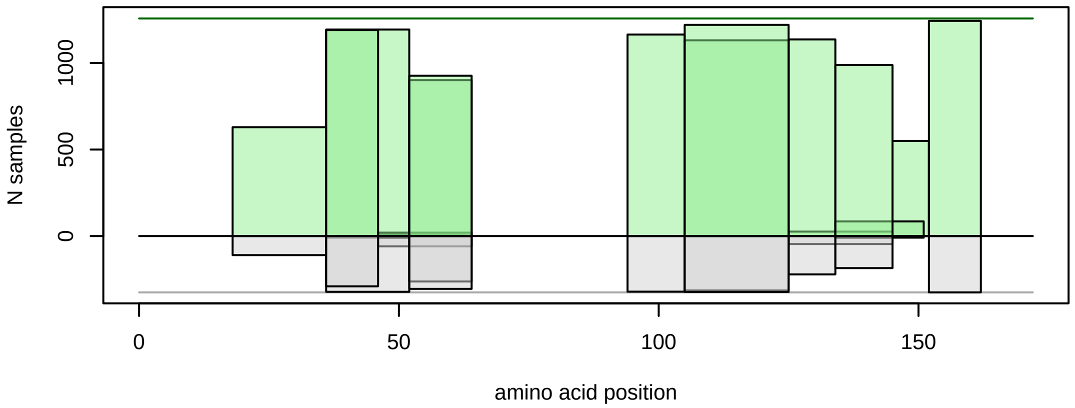

1841. PCBP1 (Q15365), NP3

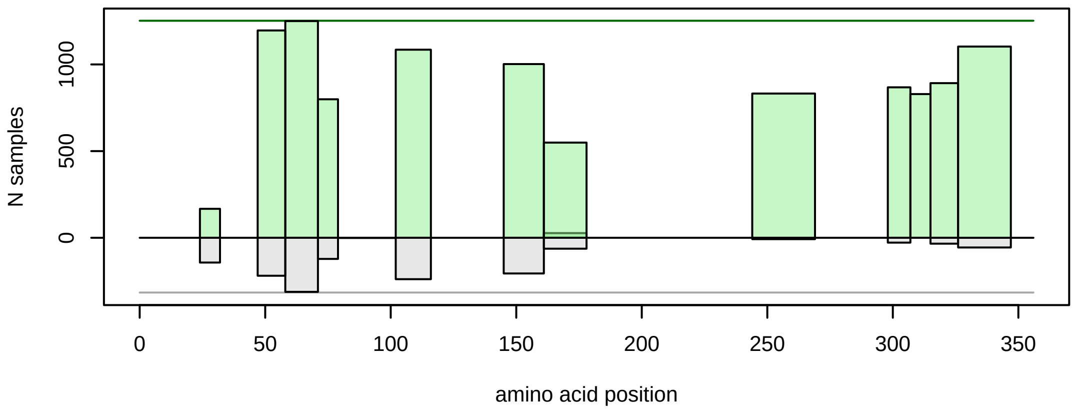

1842. VPS4A (Q9UN37), NP5

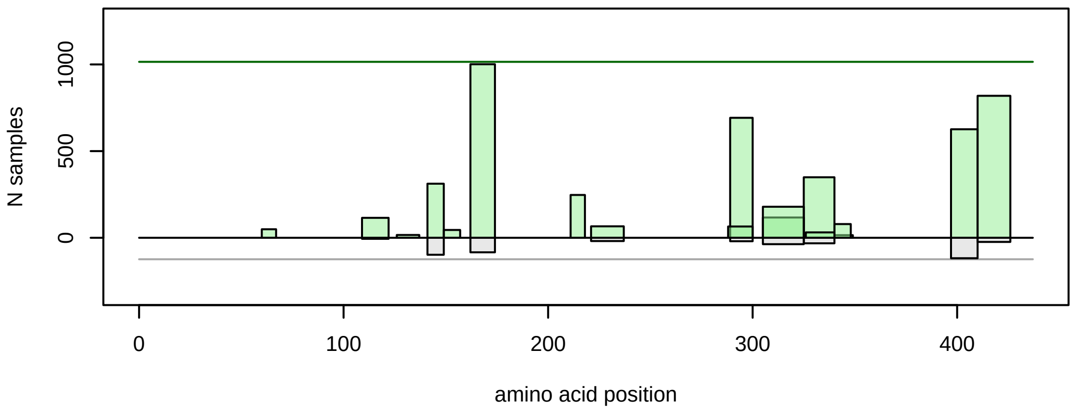

1843. IGHV1−46 (P01743), NP3

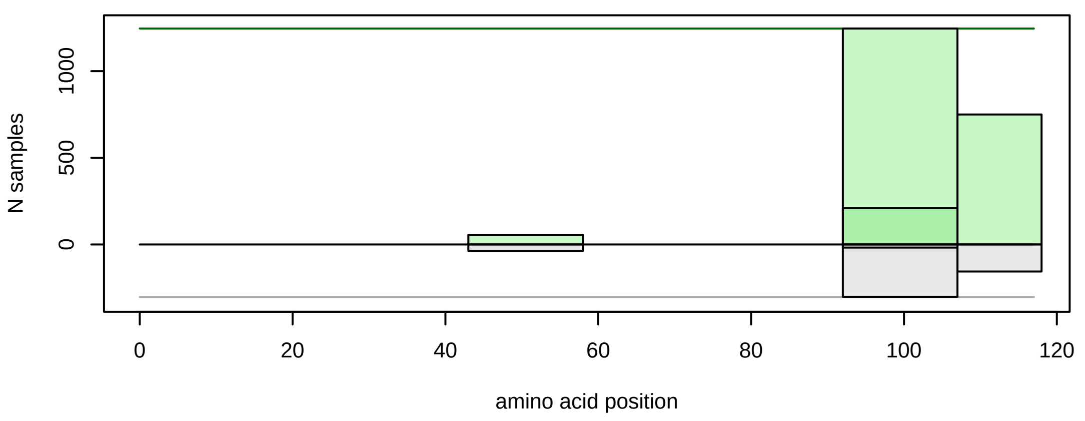

1844. SNX6 (A0A0A0MRI2;Q9UNH7), NP5

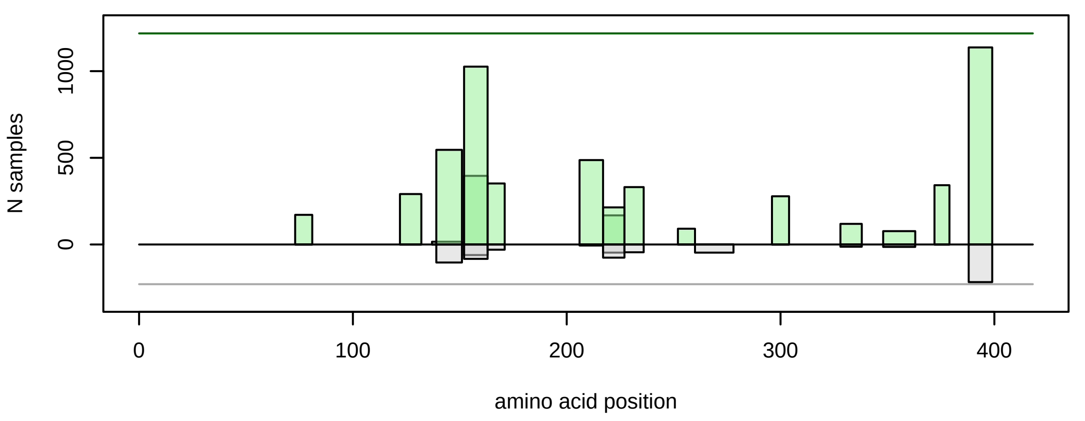

1845. TNIK (Q9UKE5−4;Q9UKE5−6), NP1

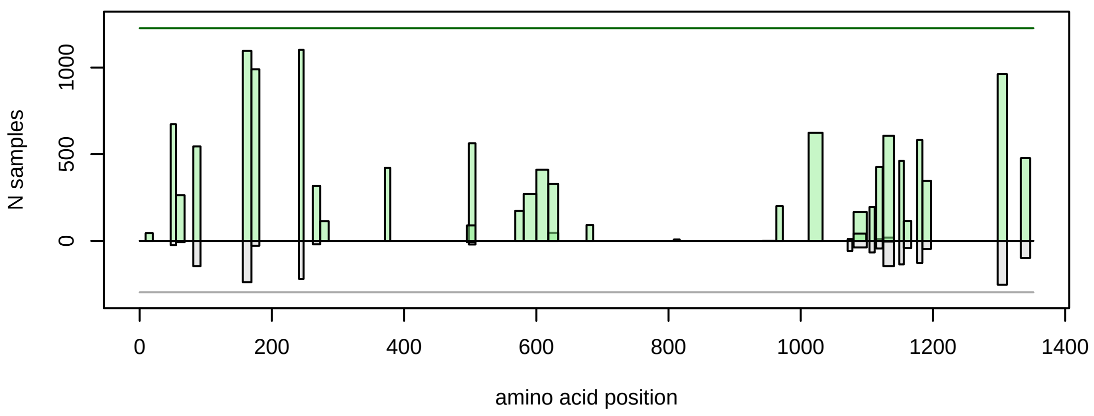

1846. IGFBP3 (P17936;P17936−2), NP3

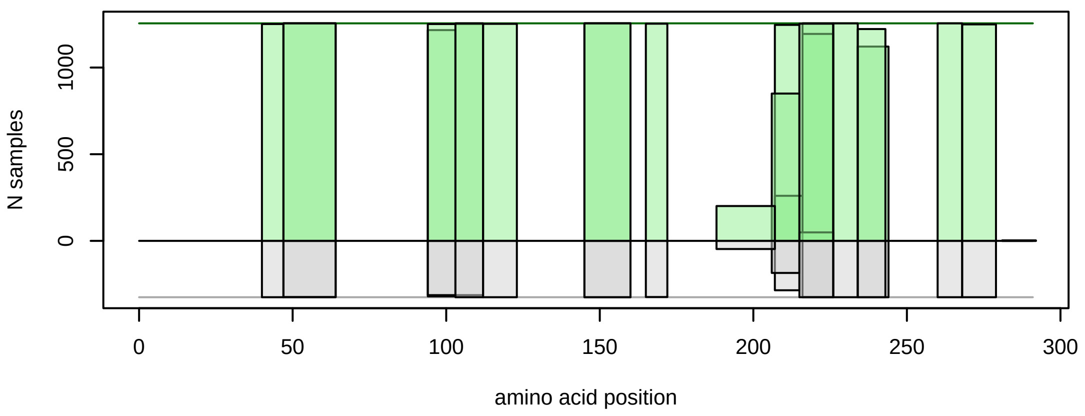

1847. SLC2A1 (P11166), NP1

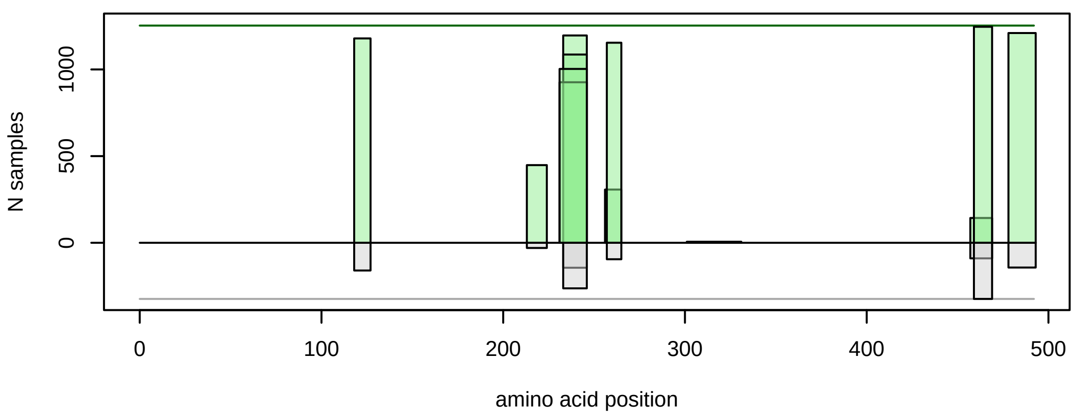

1848. ATP2A2 (P16615), NP4

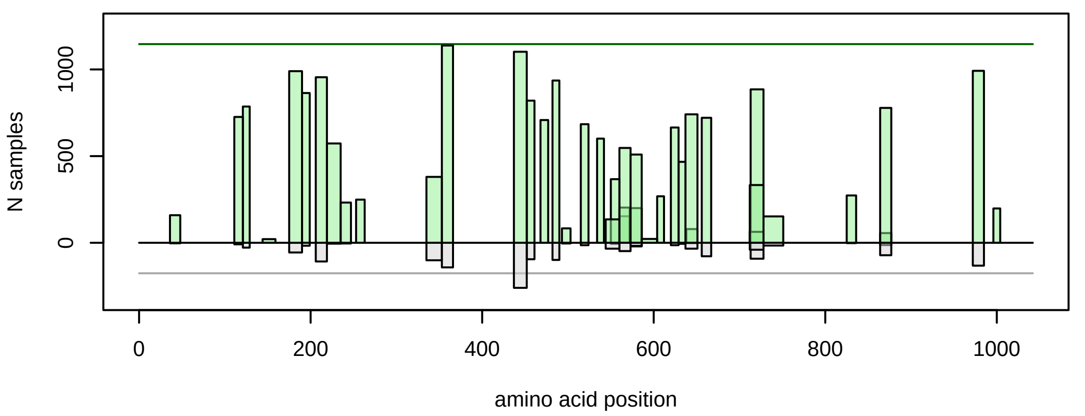

1849. TFRC (P02786), NP5

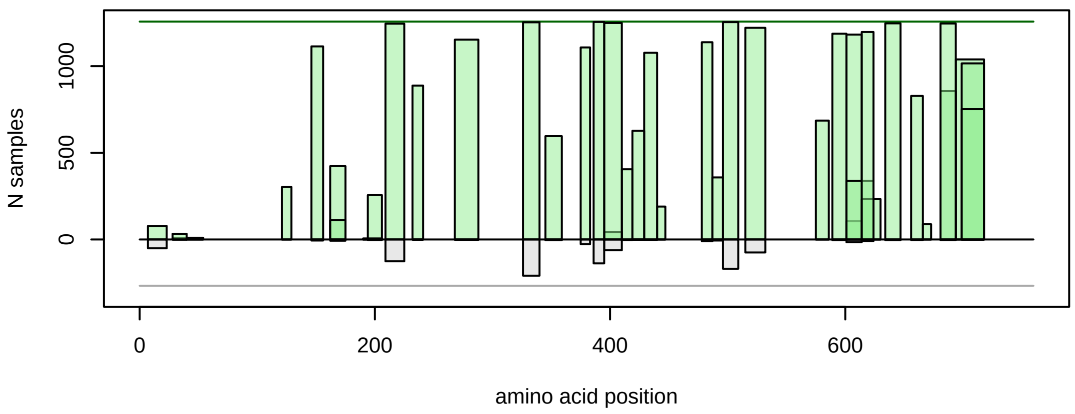

1850. RPL18 (G3V203;Q07020;Q07020−2), NP4

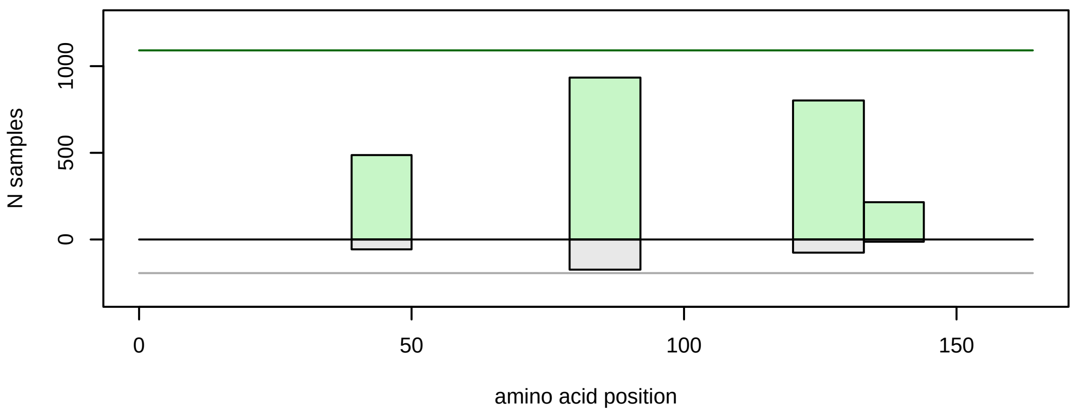

1851. PSMB1 (P20618), NP1

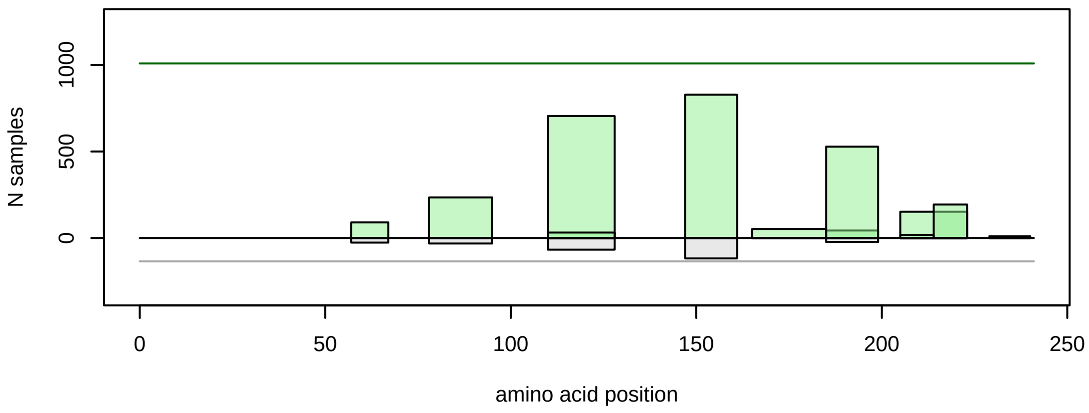

1852. VDAC3 (Q9Y277), NP4

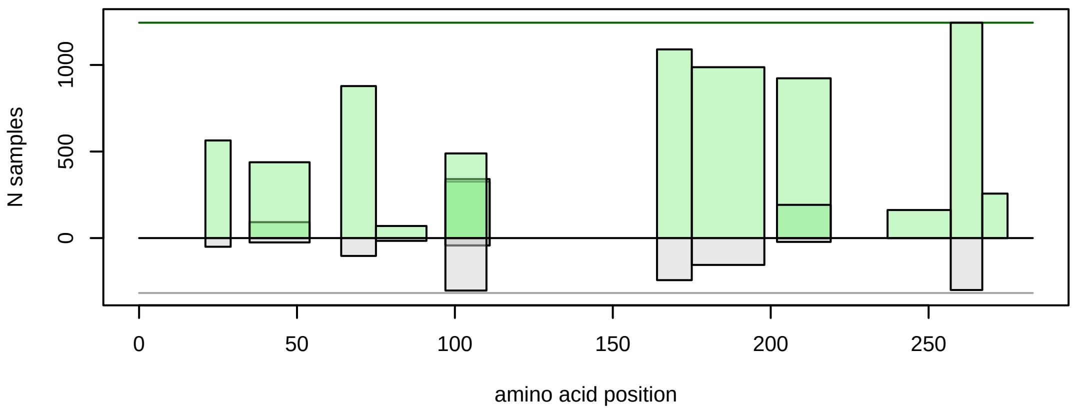

1853. AHSP (Q9NZD4), NP2

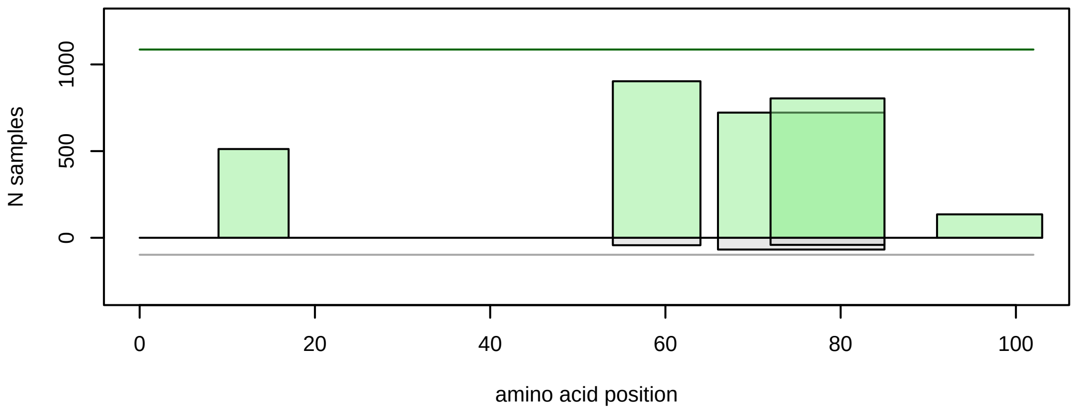

1854. GP9 (P14770), NP4

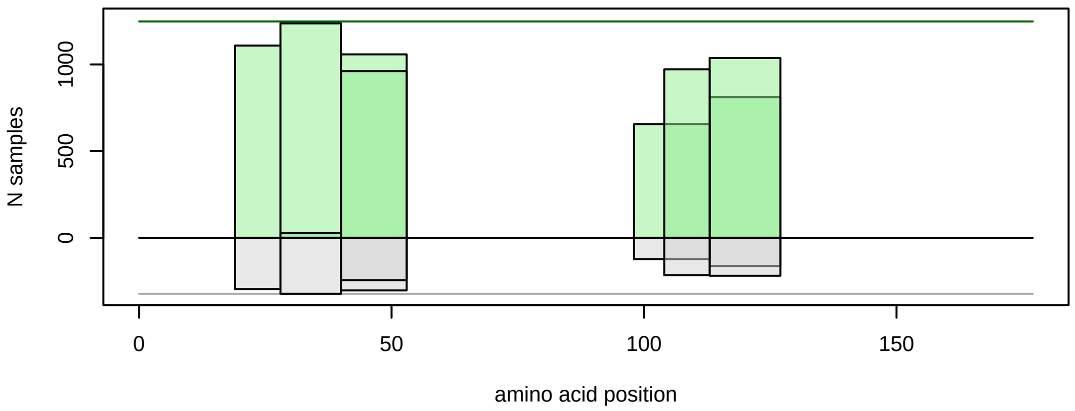

1855. NA (P23527), NP5

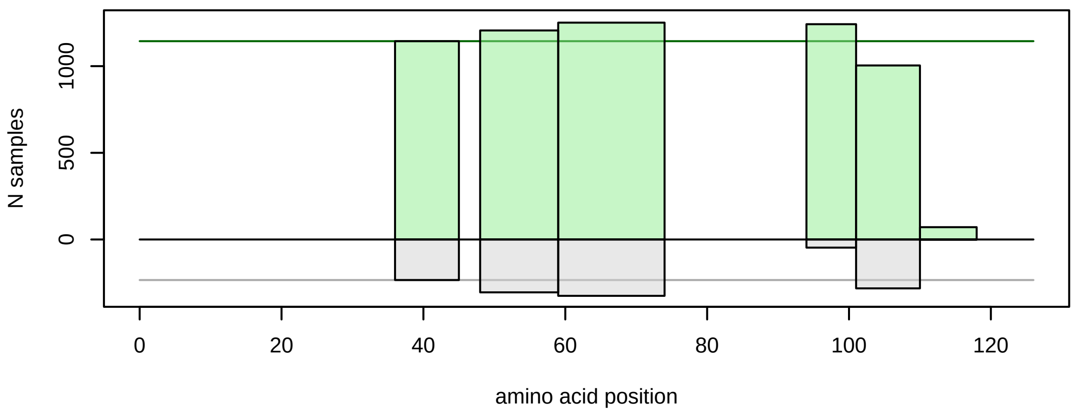

1856. SEPT9 (Q9UHD8−5), NP4

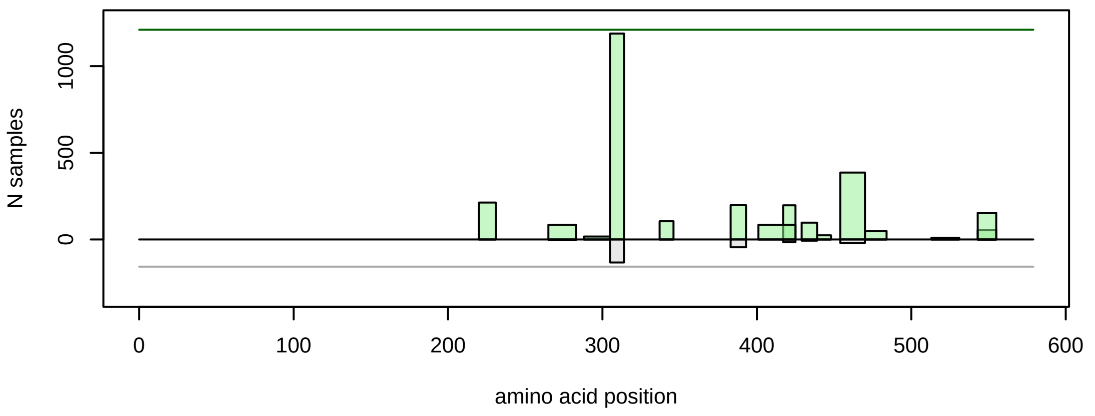

1857. RPLP0 (P05388), NP2

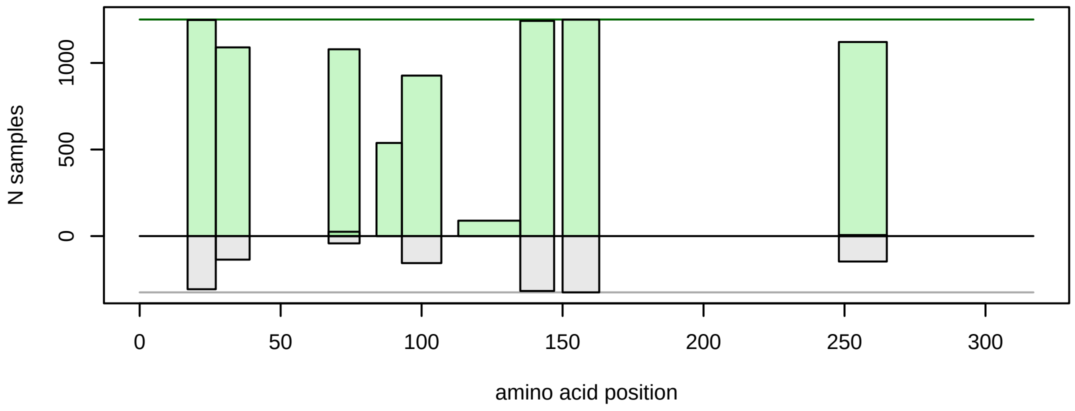

1858. NTS (P30990), NP2

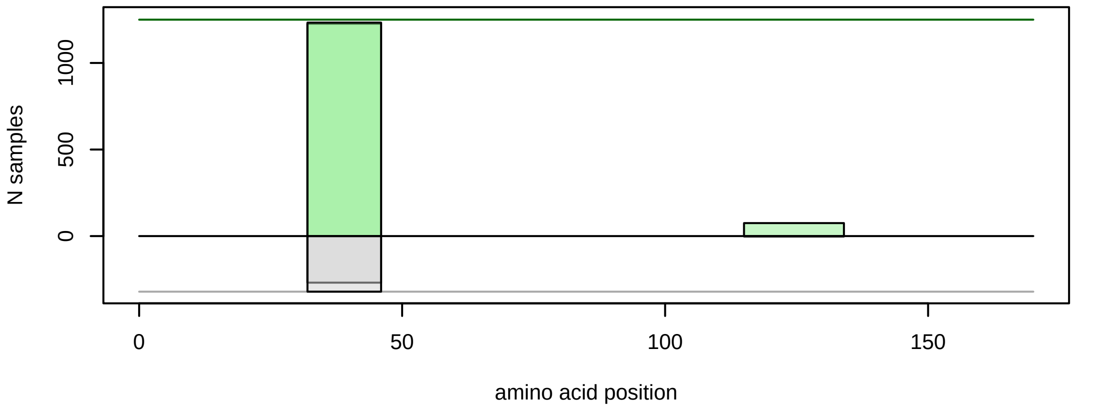

1859. HINT1 (P49773), NP2

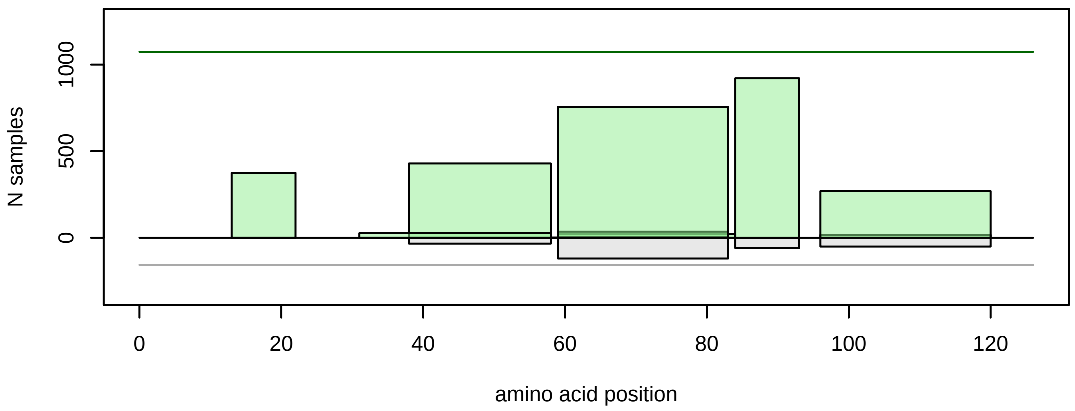

1860. NOTCH1 (A0A7P0T8U6;A0A7P0TB20;P46531), NP5

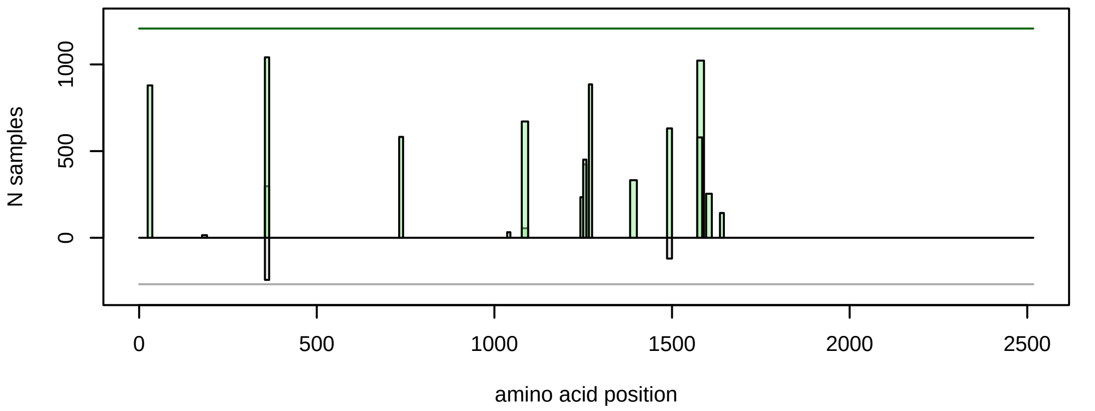

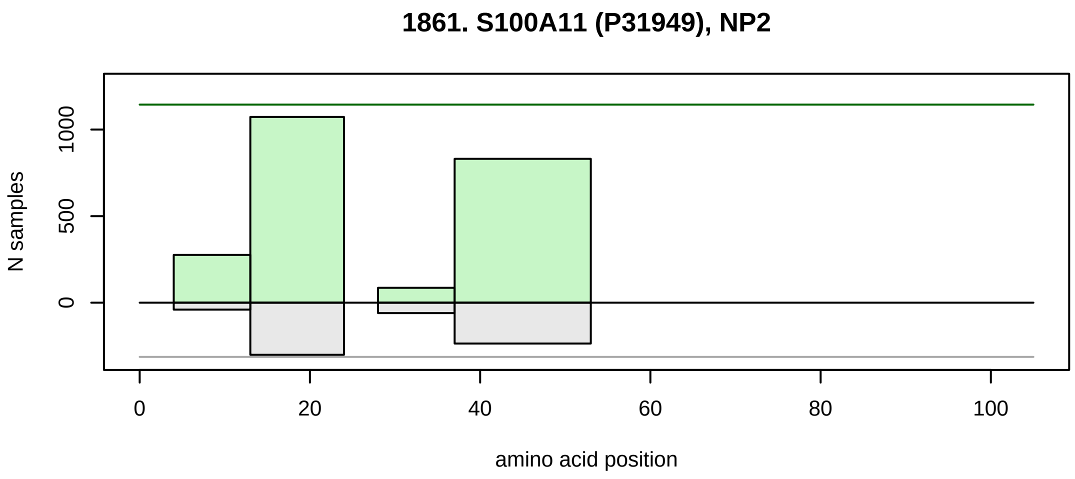
1861. S100A11 (P31949), NP2
N samples
0
500
1000
0
20
40
60
80
100
amino acid position

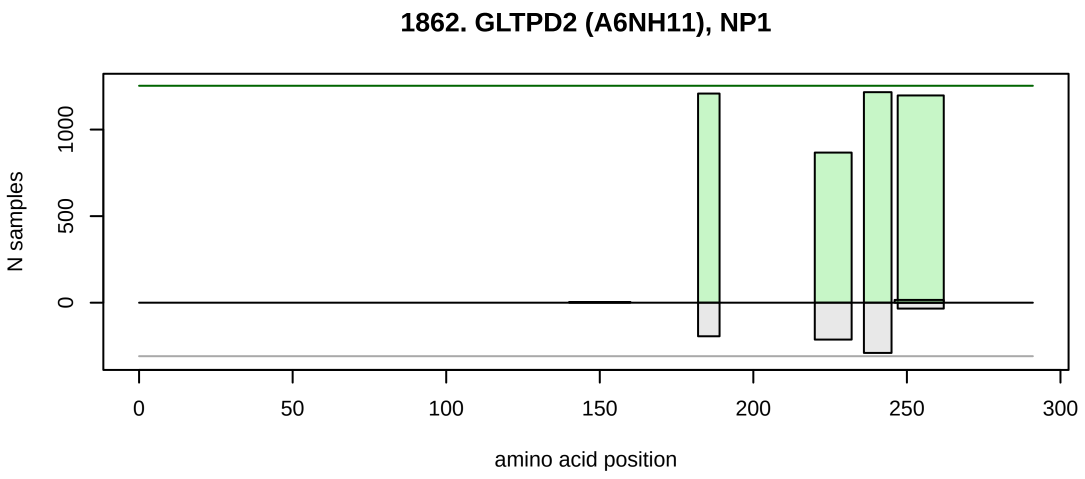
1862. GLTPD2 (A6NH11), NP1
N samples
0
500
1000
0
50
100
150
200
250
300
amino acid position

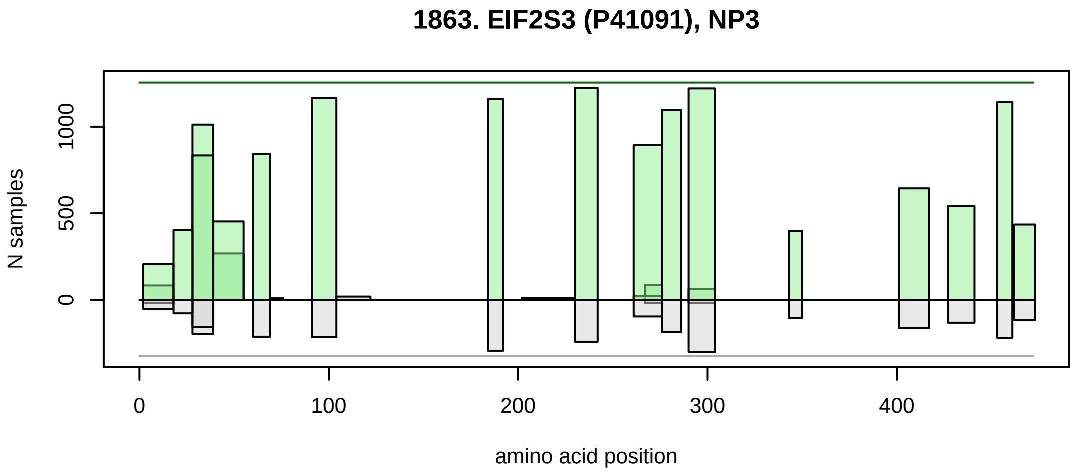
1863. EIF2S3 (P41091), NP3
N samples
0
500
1000
0
100
200
300
400
amino acid position

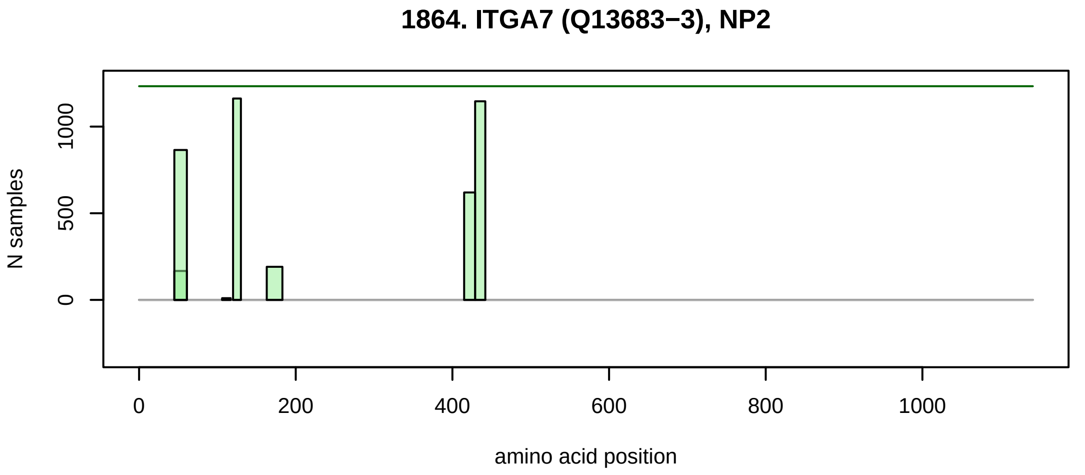
1864. ITGA7 (Q13683−3), NP2
N samples
0
500
1000
0
200
400
600
800
1000
amino acid position

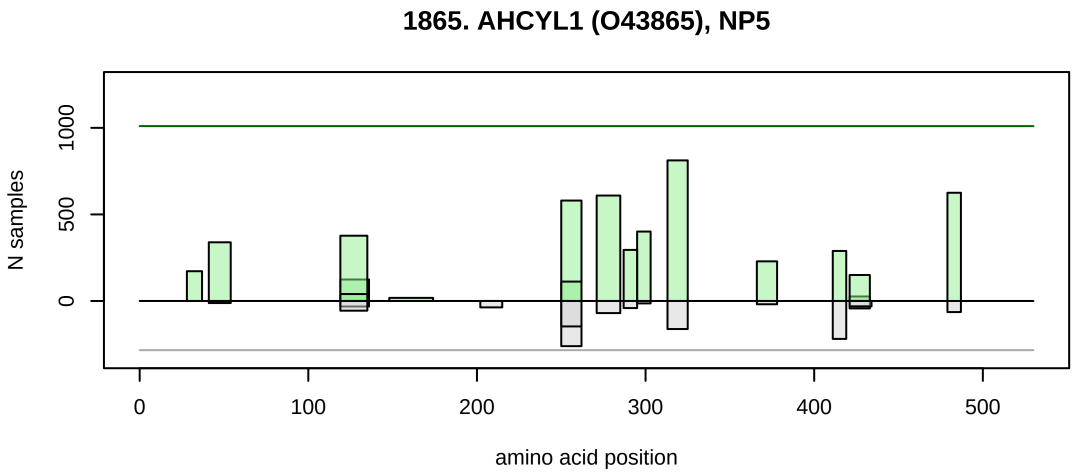
1865. AHCYL1 (O43865), NP5
N samples
0
500
1000
0
100
200
300
400
500
amino acid position

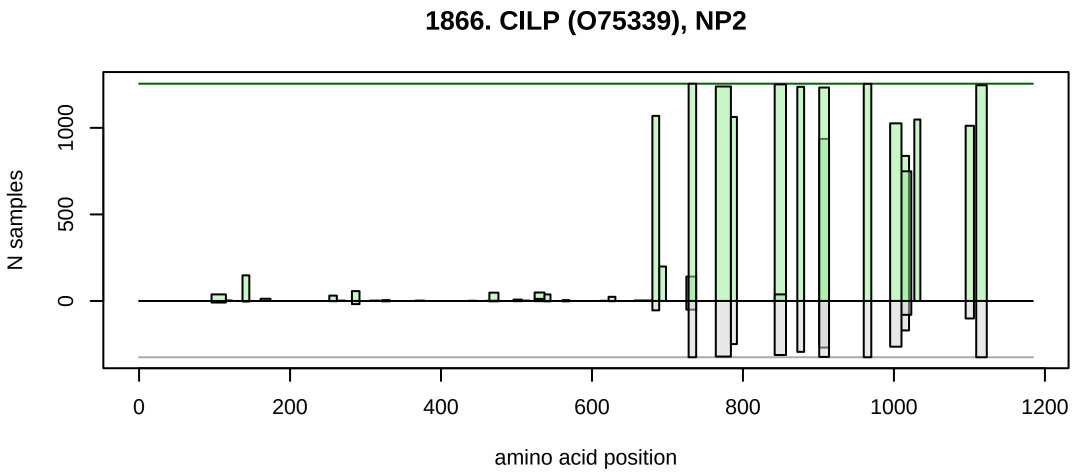
1866. CILP (O75339), NP2
N samples
0
500
1000
0
200
400
600
800
1000
1200
amino acid position

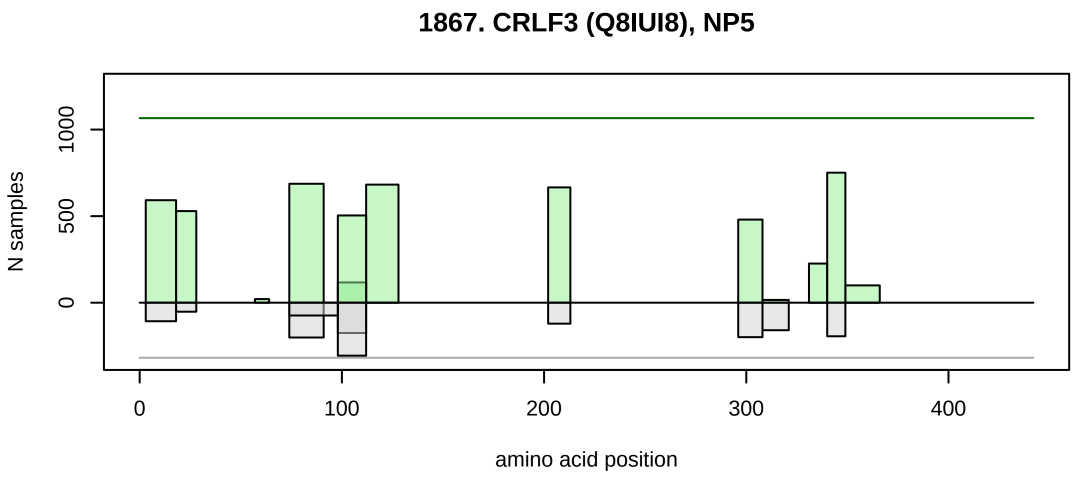
1867. CRLF3 (Q8IUI8), NP5
N samples
0
500
1000
0
100
200
300
400
amino acid position

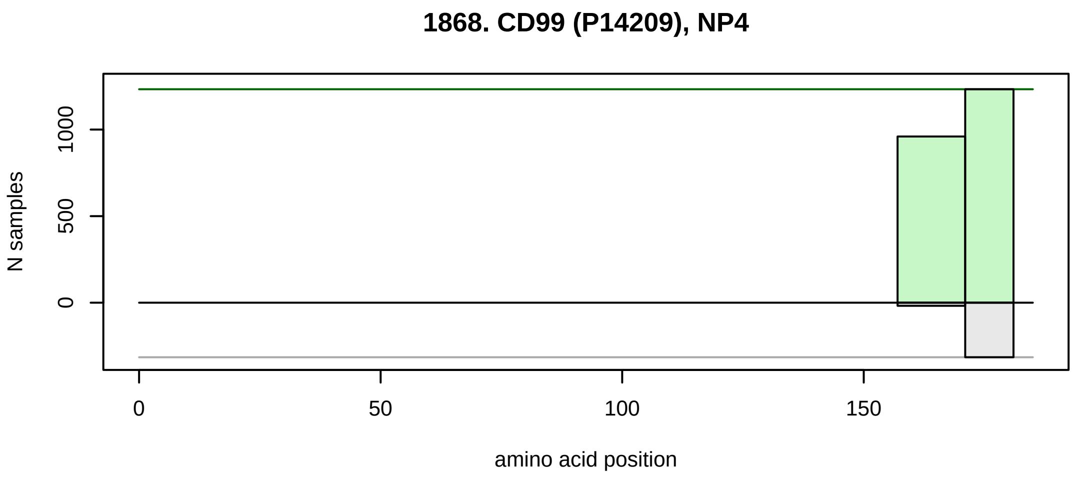
1868. CD99 (P14209), NP4
N samples
0
500
1000
0
50
100
150
amino acid position

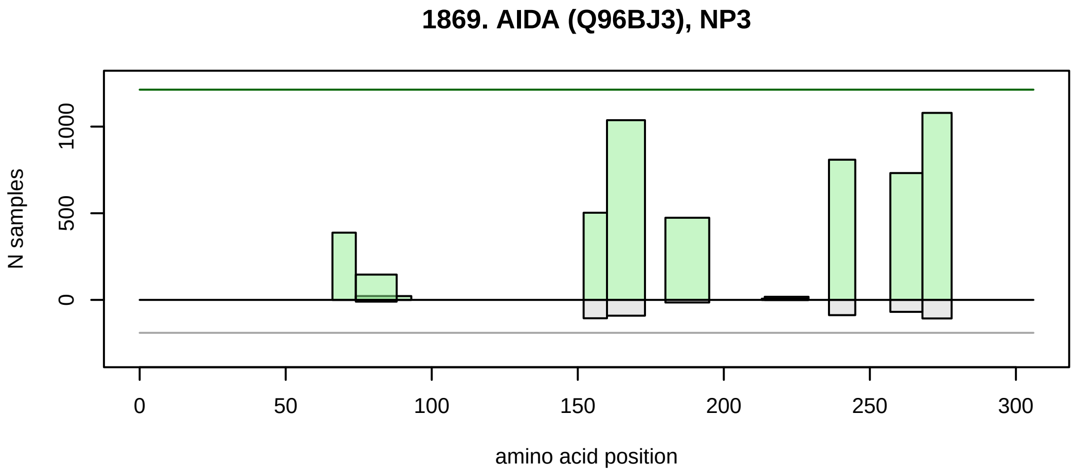
1869. AIDA (Q96BJ3), NP3
N samples
0
500
1000
0
50
100
150
200
250
300
amino acid position

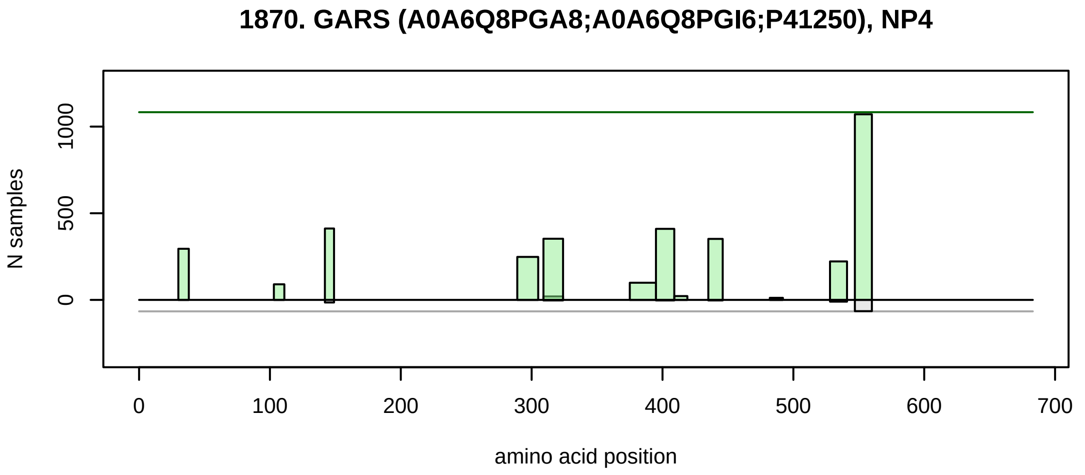
1870. GARS (A0A6Q8PGA8;A0A6Q8PGI6;P41250), NP4
N samples
0
500
1000
0
100
200
300
400
500
600
700
amino acid position

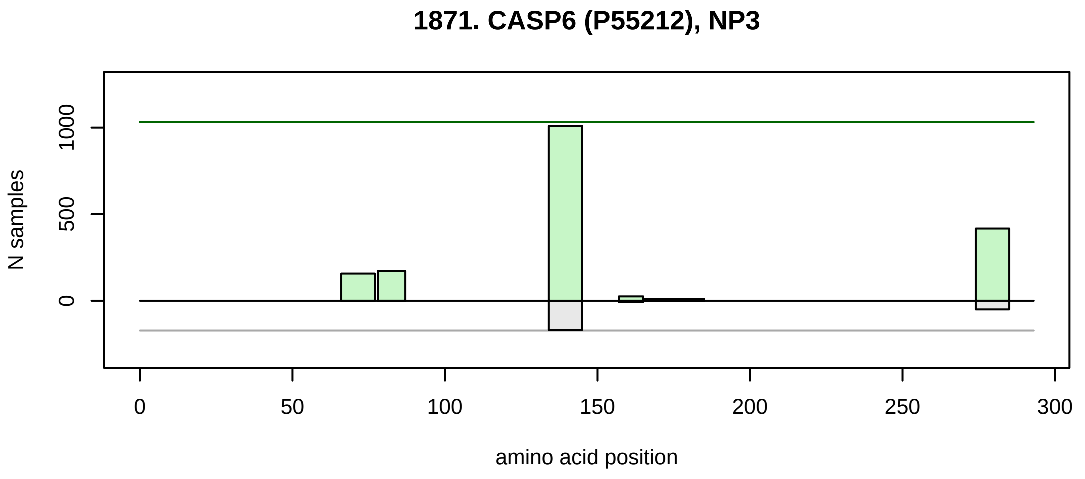
1871. CASP6 (P55212), NP3
N samples
0
500
1000
0
50
100
150
200
250
300
amino acid position

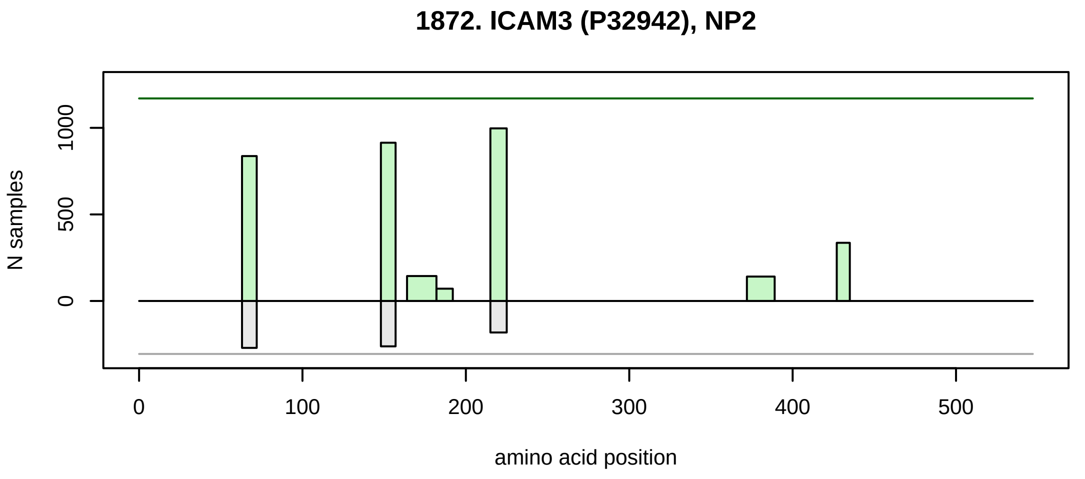
1872. ICAM3 (P32942), NP2
N samples
0
500
1000
0
100
200
300
400
500
amino acid position

1873. IQGAP2 (Q13576), NP1

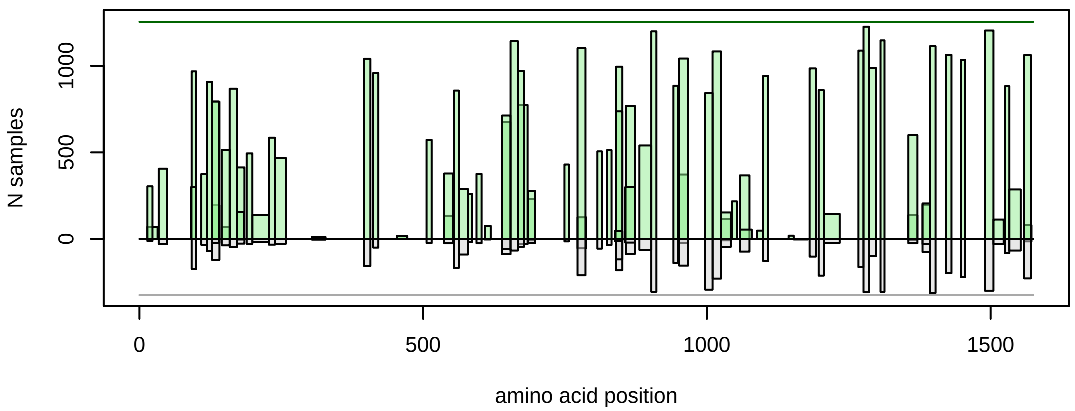

1874. ARHGAP35 (Q9NRY4), NP5

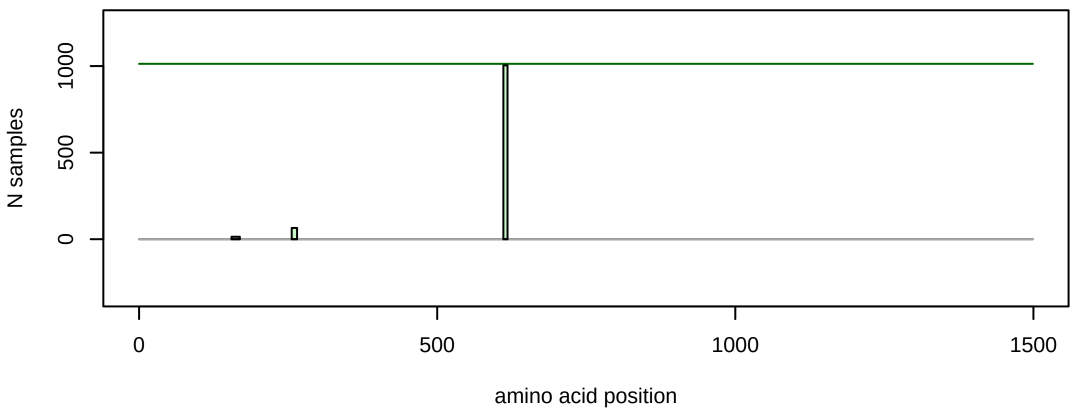

1875. FHL1 (Q13642−1;Q13642−4;Q13642−5), NP1

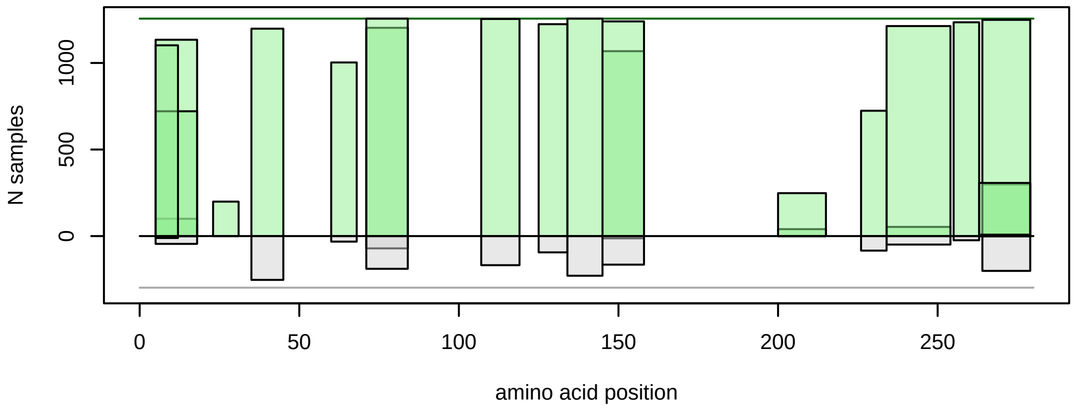

1876. CLTA (P09496;P09496−3), NP1

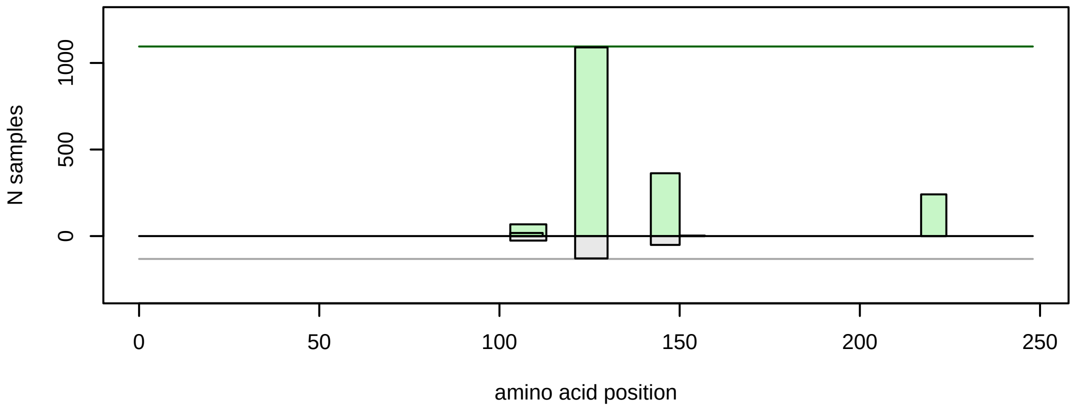

1877. KRAS (P01116−2), NP4

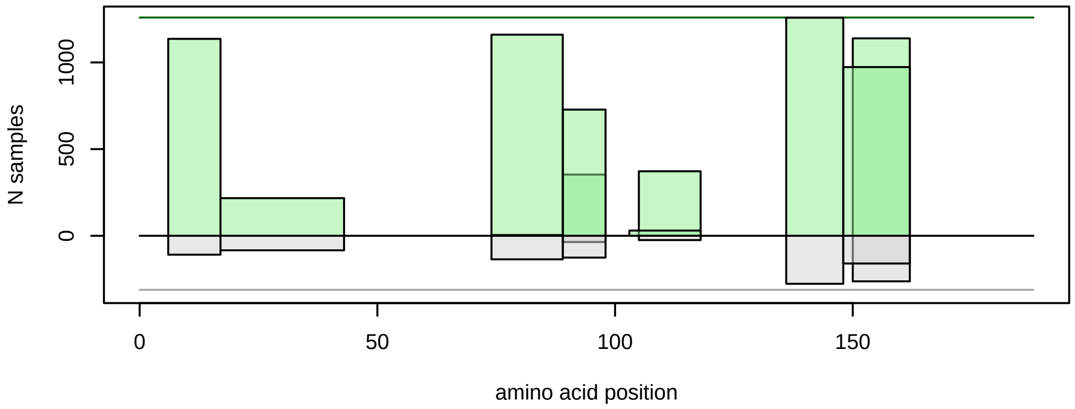

1878. LAMB2 (P55268), NP2

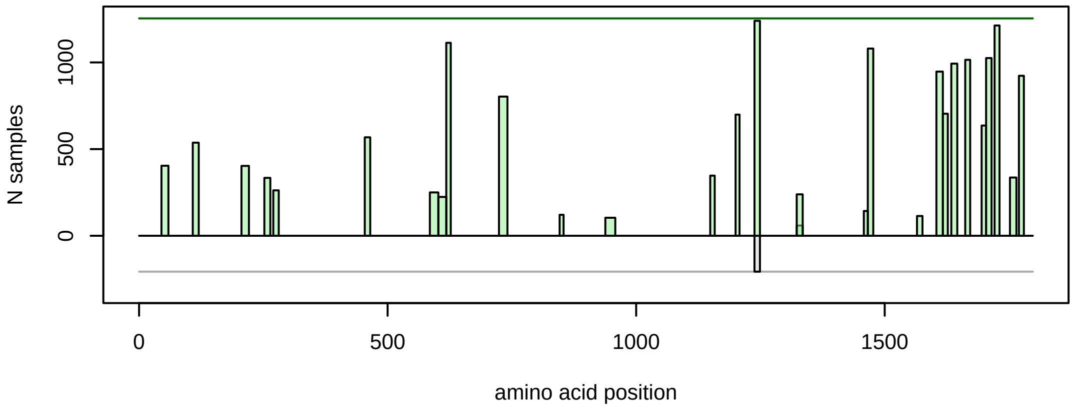

1879. FMNL1 (O95466), NP1

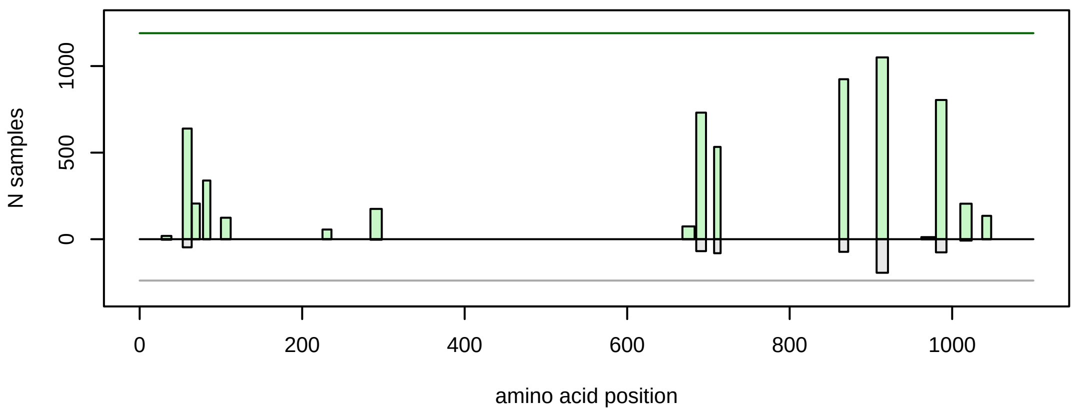

1880. DOK3 (D6RAM3), NP1

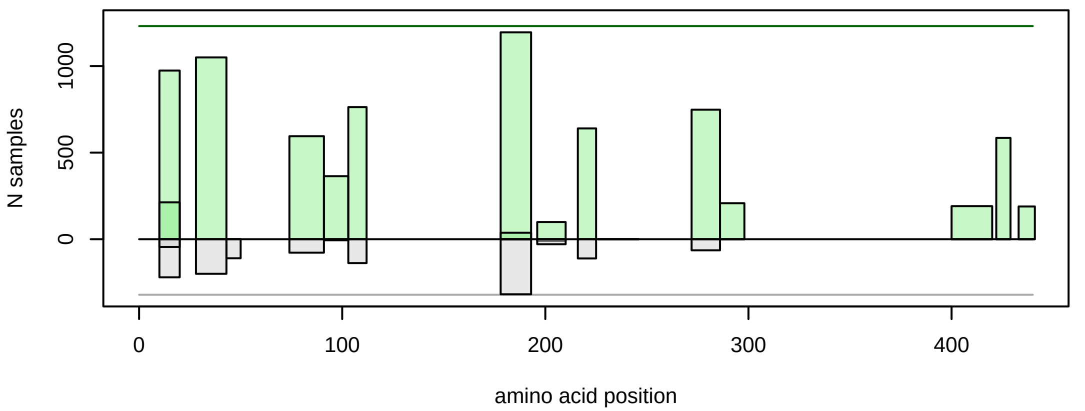

1881. ASAP1 (A0A0A0MRE5;Q9ULH1), NP4

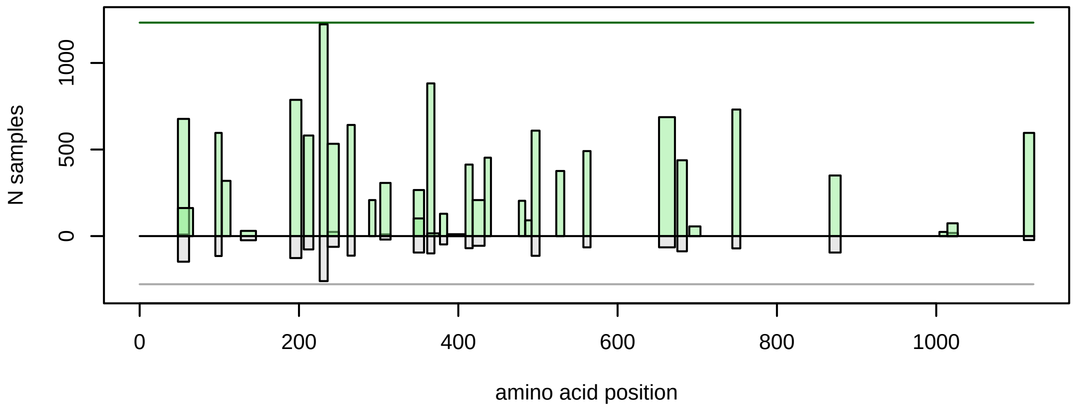

1882. PRG4 (Q92954−6), NP4

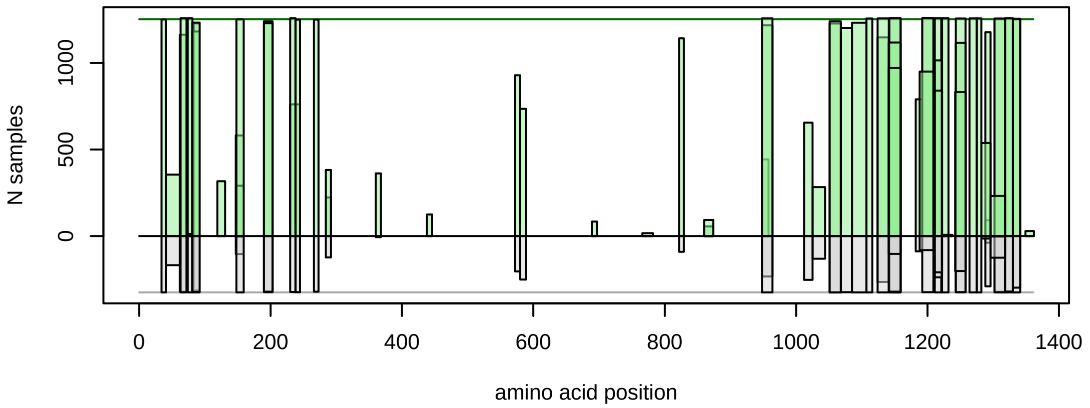

1883. YARS (P54577), NP1

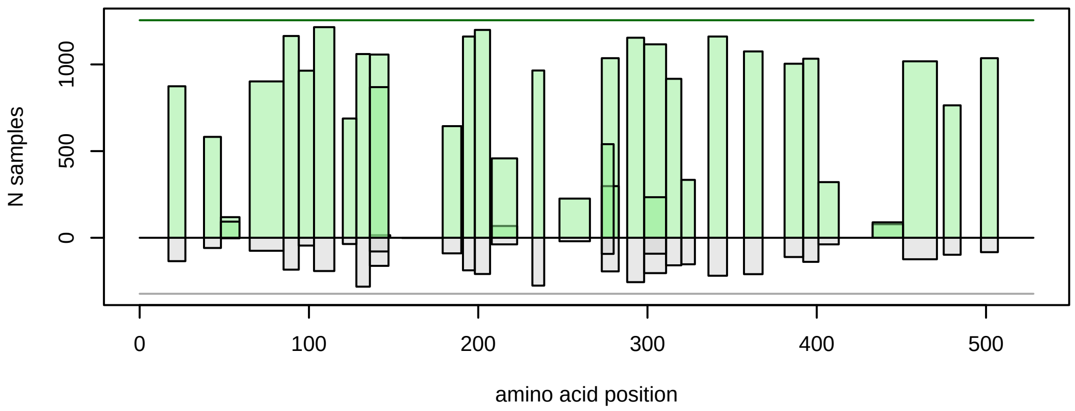

1884. PACSIN2 (Q9UNF0), NP1

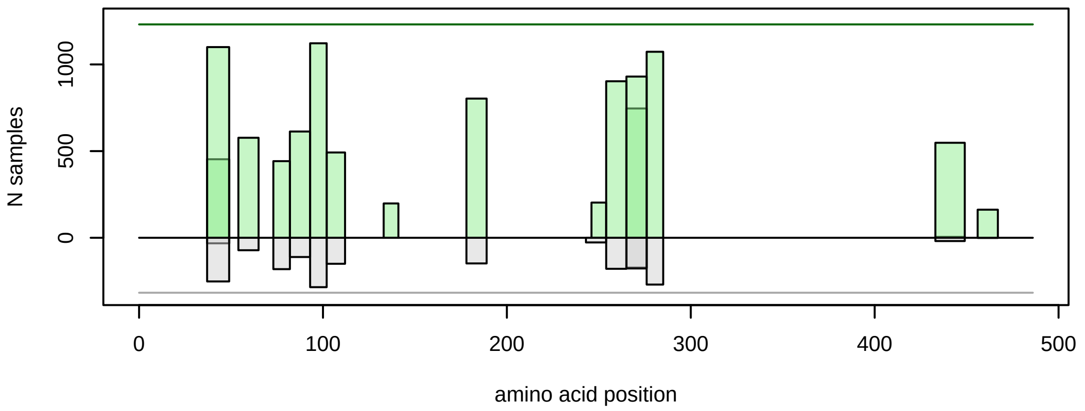

1885. NUTF2 (P61970), NP2

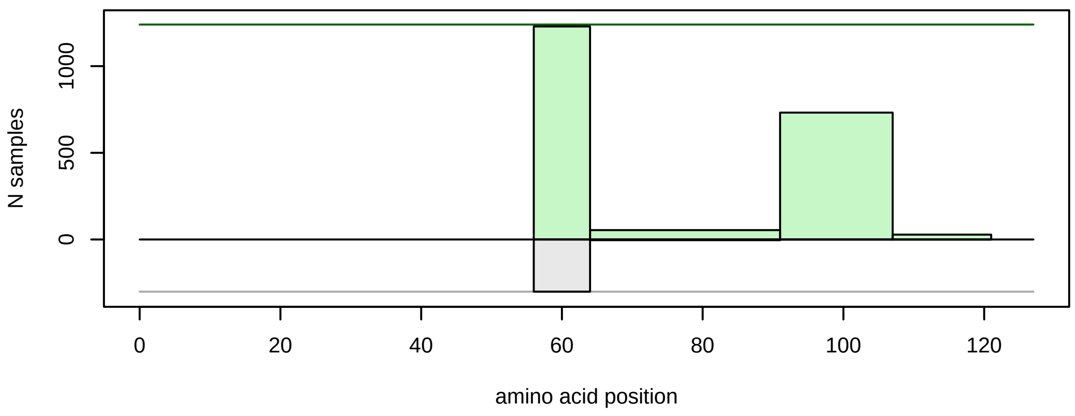

1886. ASB4 (Q9Y574), NP2

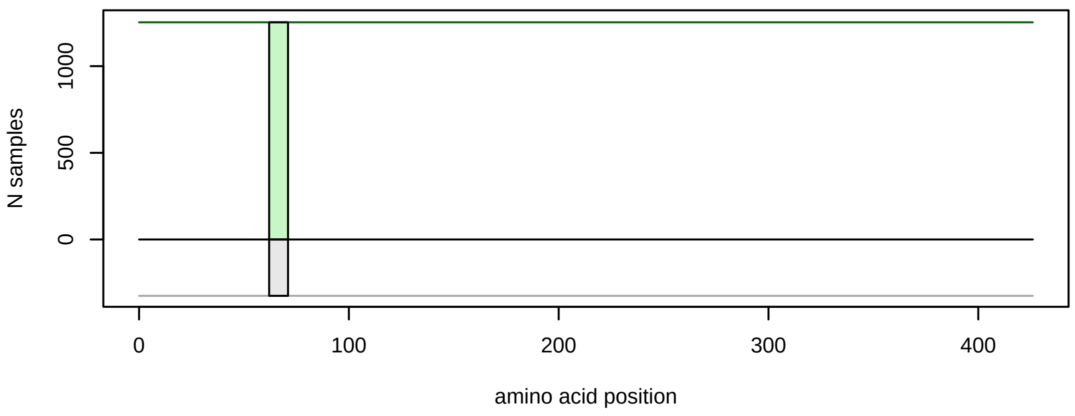

1887. KHSRP (M0R0C6;Q92945), NP1

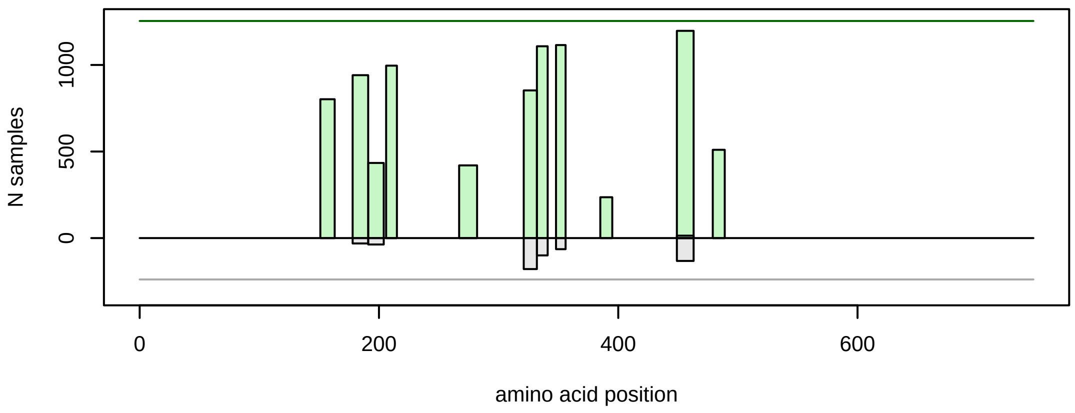

1888. RNF133 (Q8WVZ7), NP3

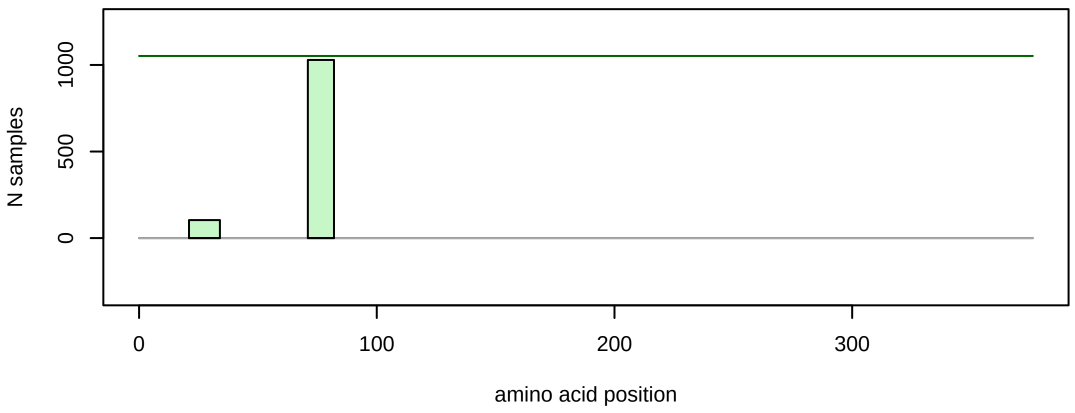

1889. STMN1 (P16949), NP3

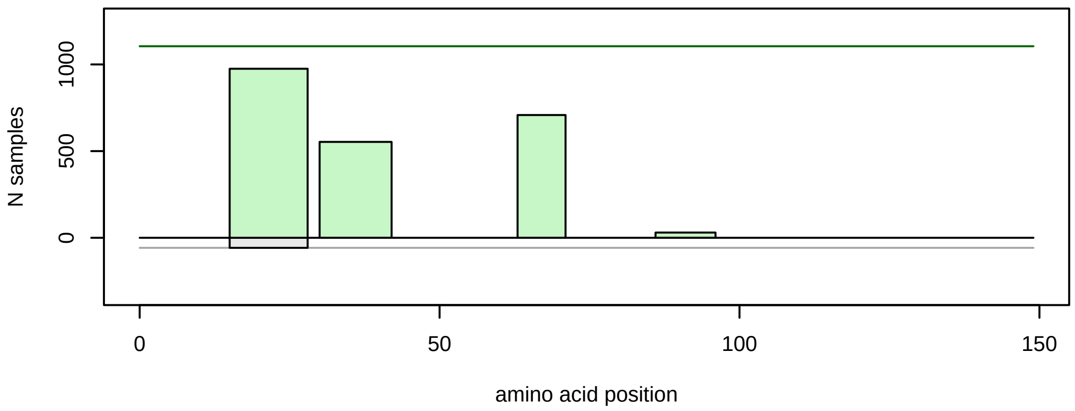

1890. TPT1 (A0A0B4J2C3), NP2

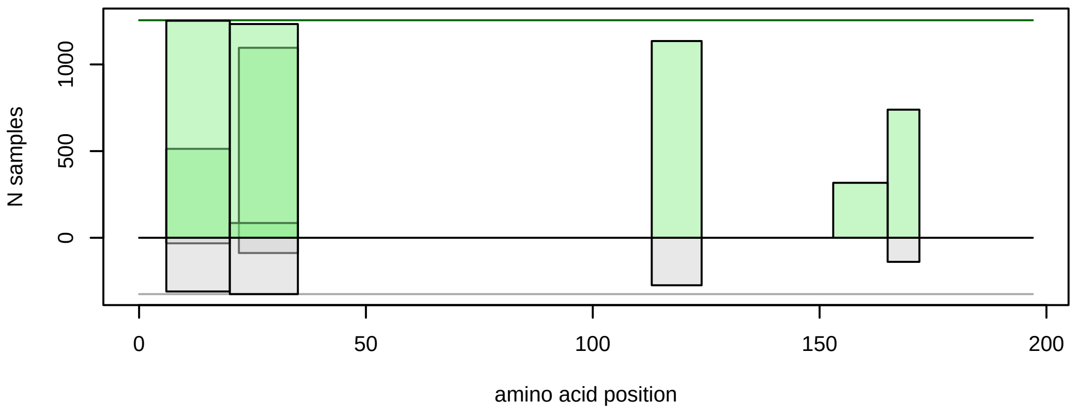

1891. ADH1A (P07327), NP4

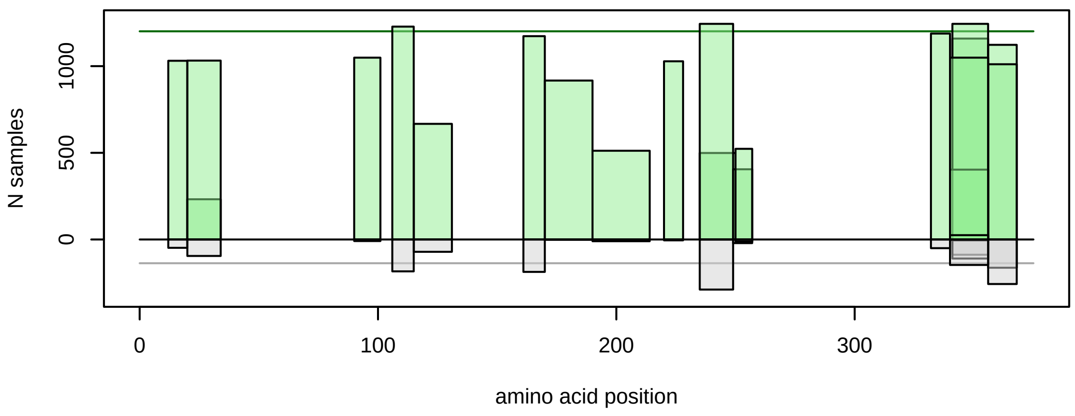

1892. PFKL (P17858), NP4

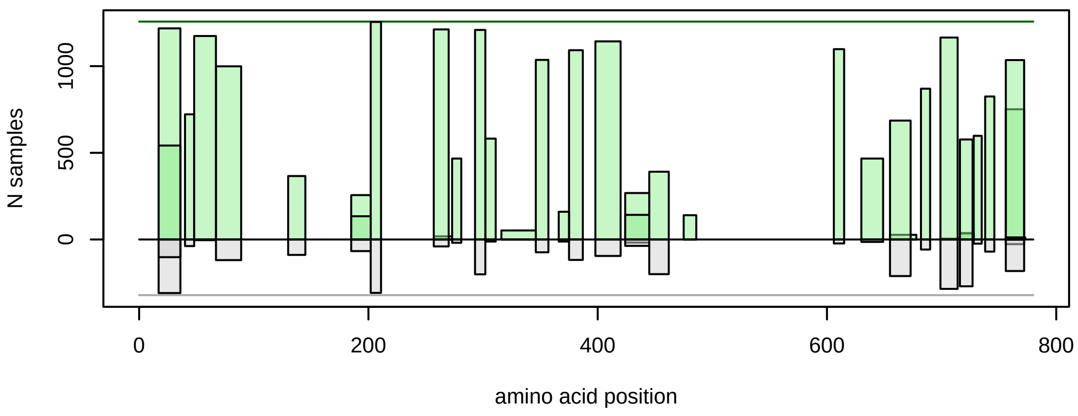

1893. RNASE4 (P34096), NP3

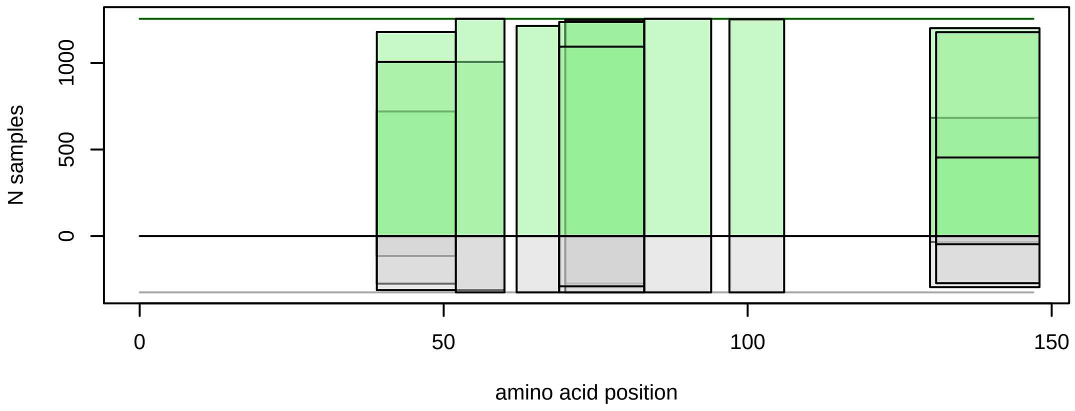

1894. ACSL4 (O60488), NP4

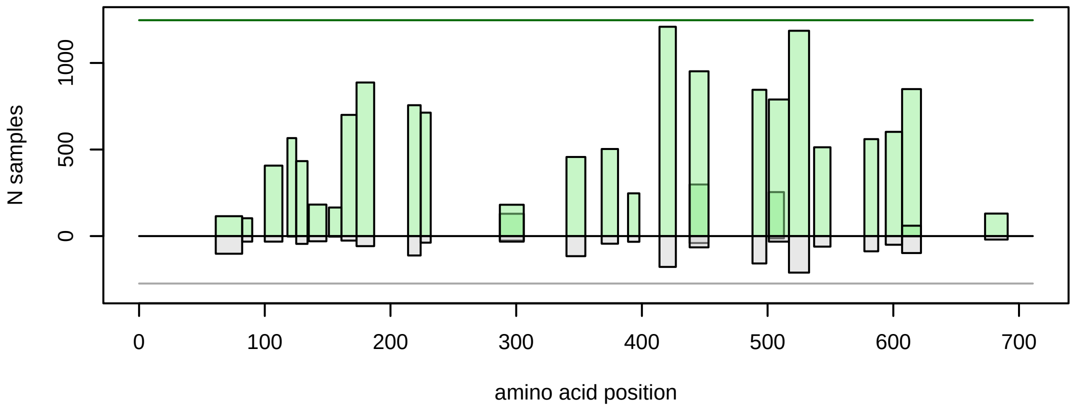

1895. EIF3B (P55884), NP2

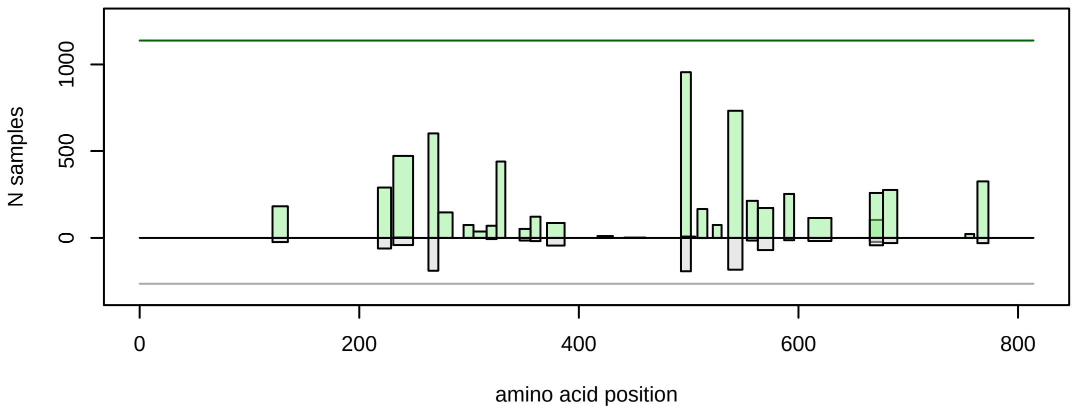

1896. CASC4 (Q6P4E1), NP2

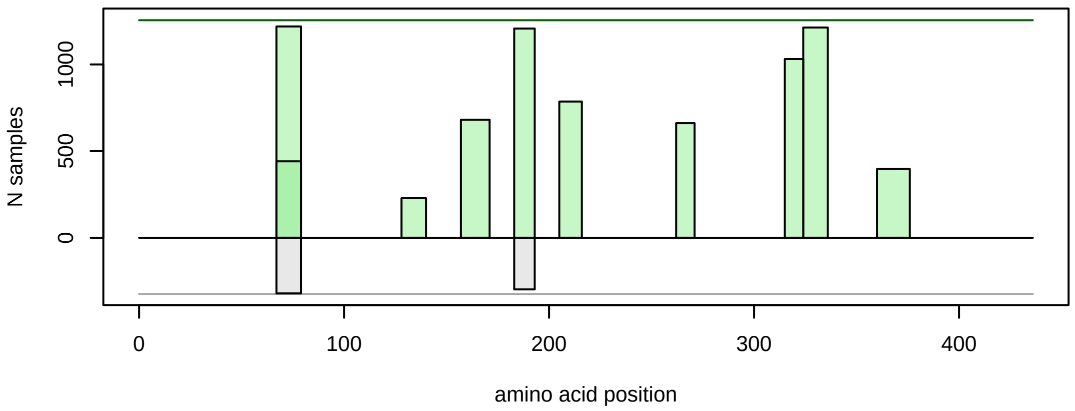

1897. VIM (P08670), NP5

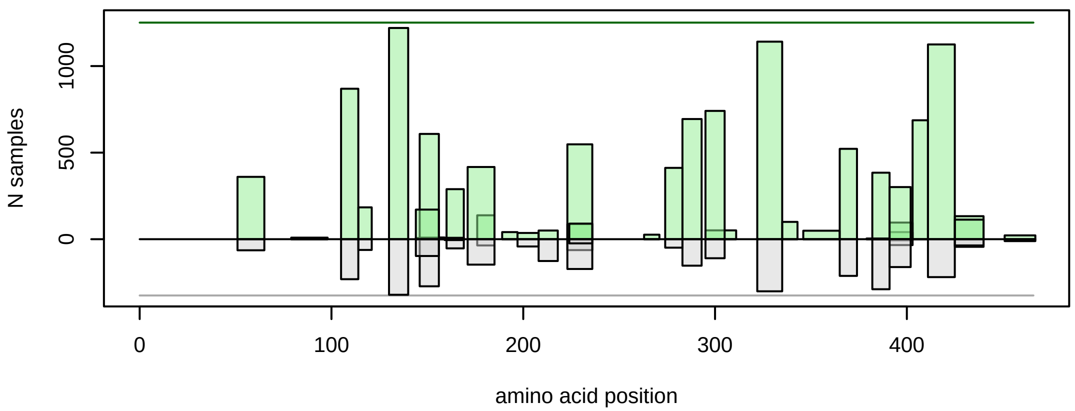

1898. CTSC (A0A7I2V2Q8;A0A7I2V466;P53634), NP2

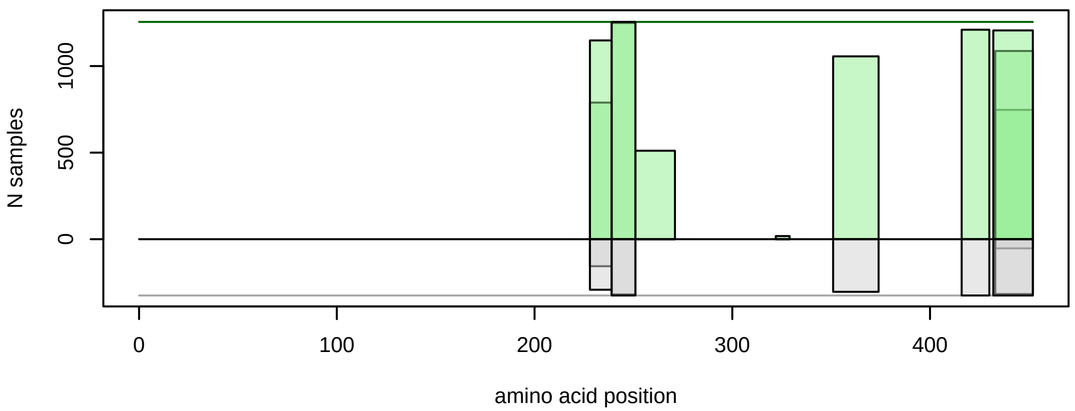

1899. RP2 (O75695), NP1

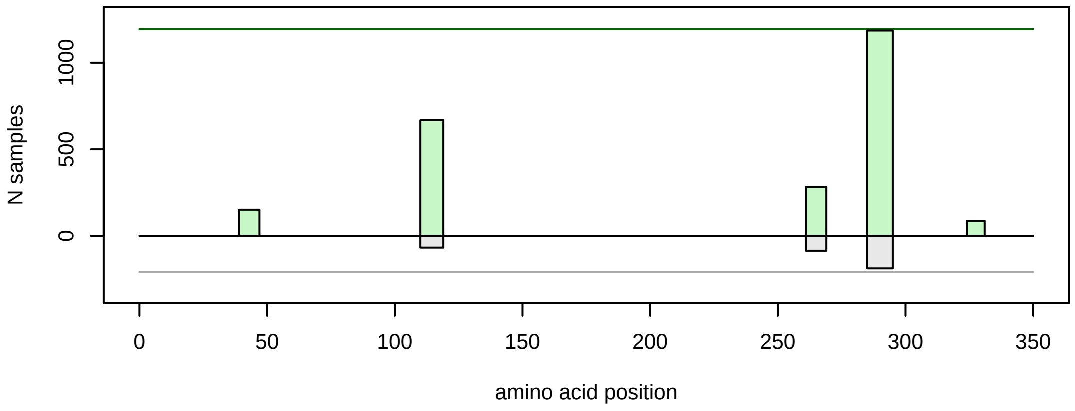

1900. TYMP (P19971), NP4

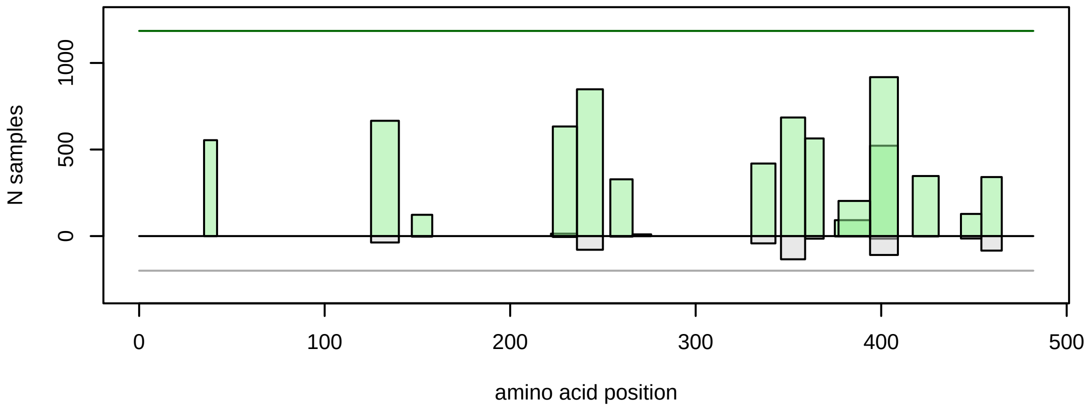

1901. ITGB6 (A0A087WXP3), NP4

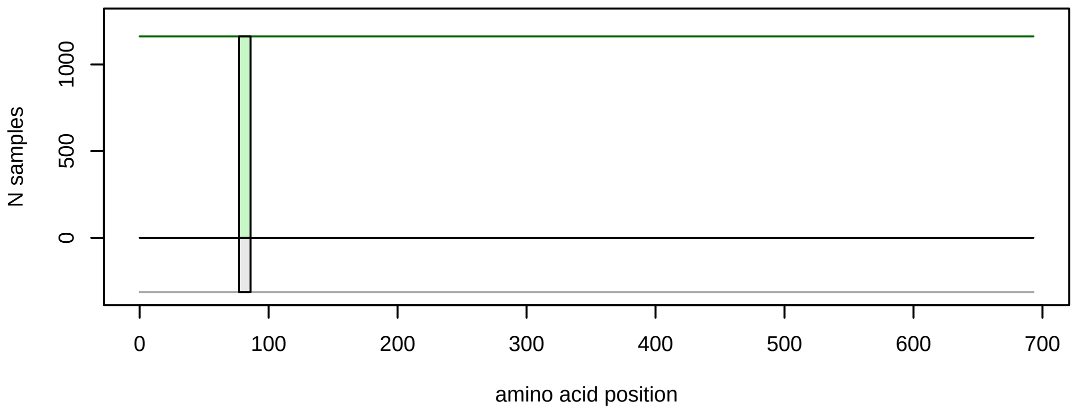

1902. LDHA (P00338), NP4

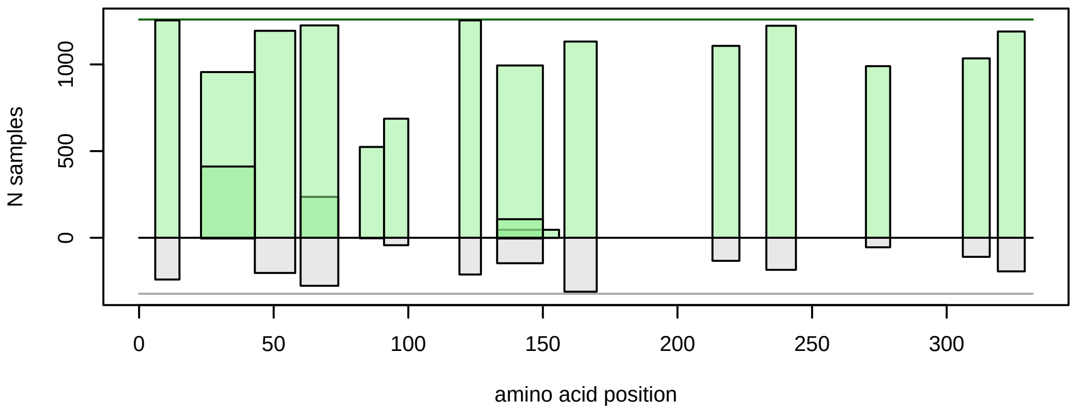

1903. SLC25A5 (P05141), NP4

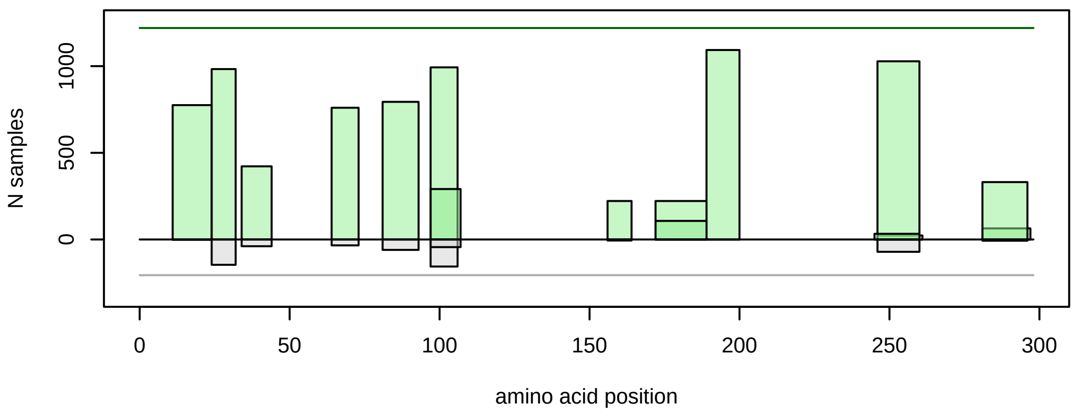

1904. FAM49B (Q9NUQ9), NP1

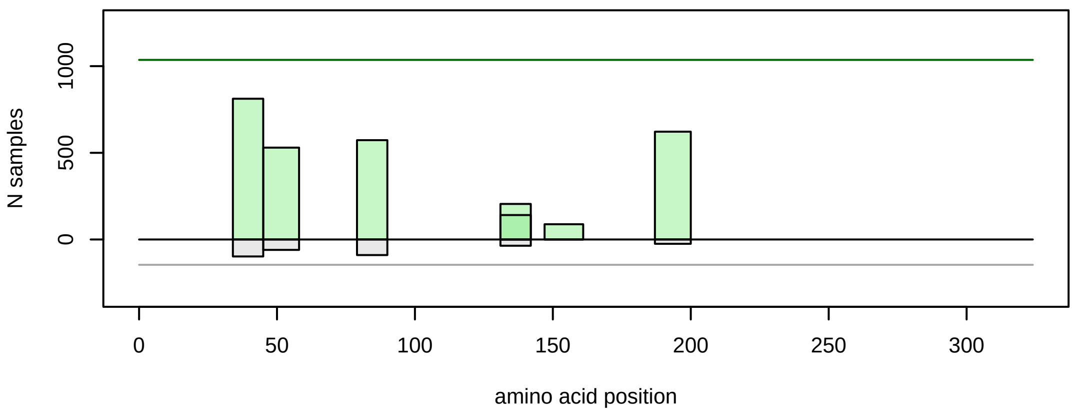

1905. PLIN1 (O60240), NP2

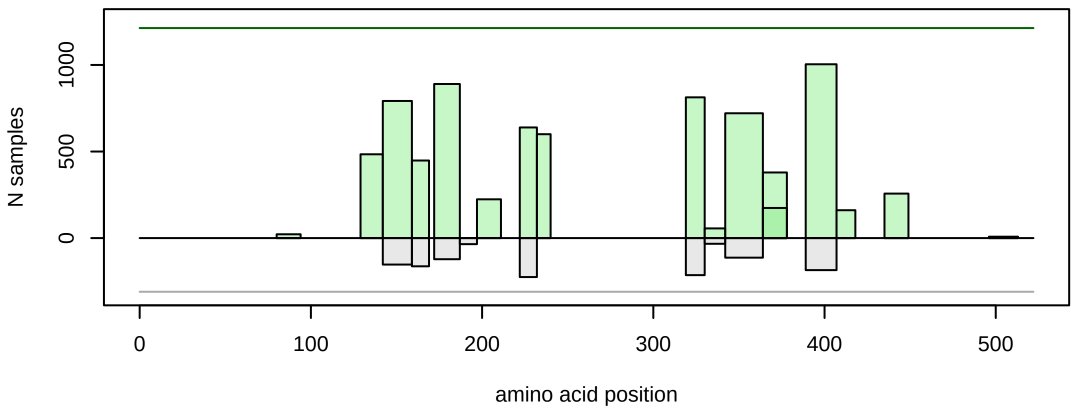

1906. KALRN (A0A804HLI0), NP4

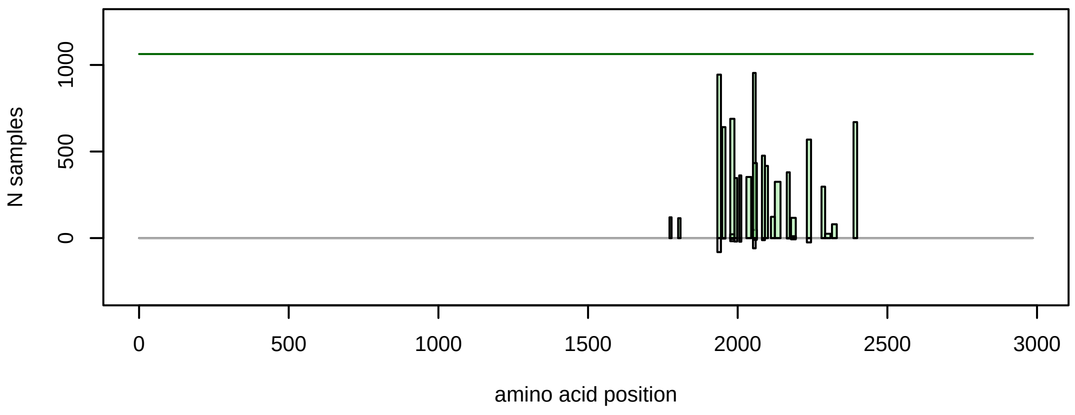

1907. FGG (P02679), NP5

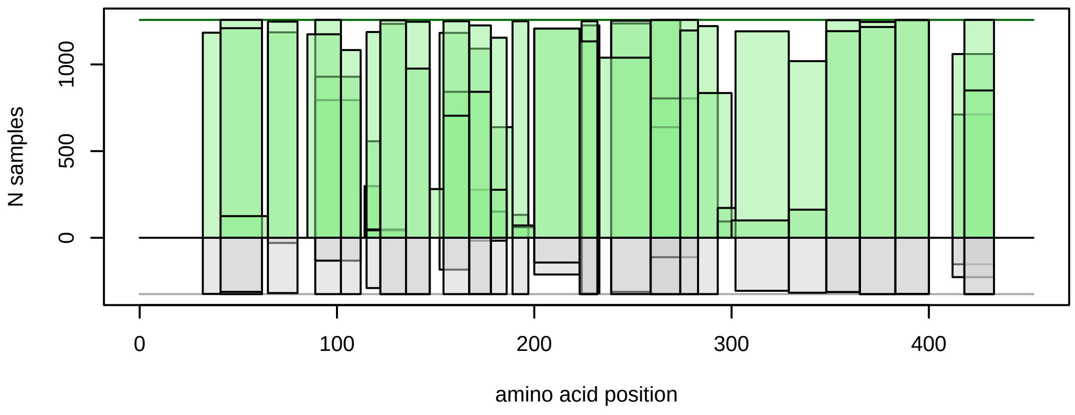

1908. ATG3 (Q9NT62), NP2

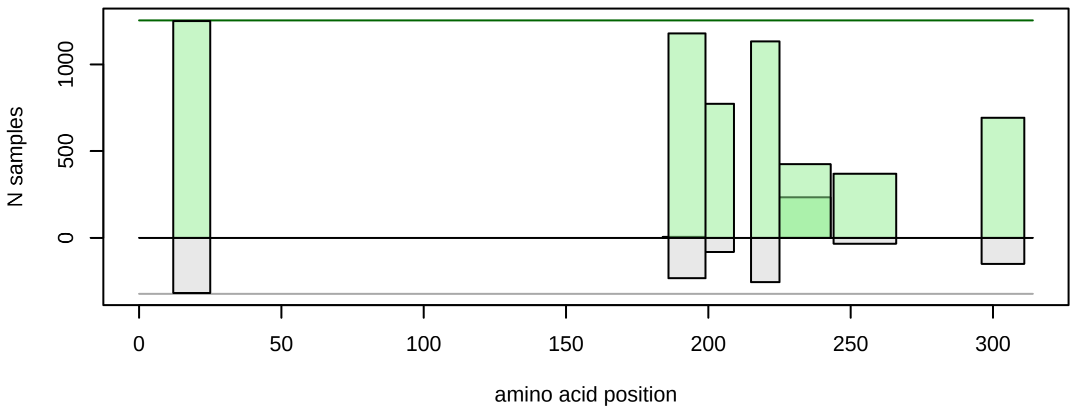

1909. GMPPA (Q96IJ6), NP5

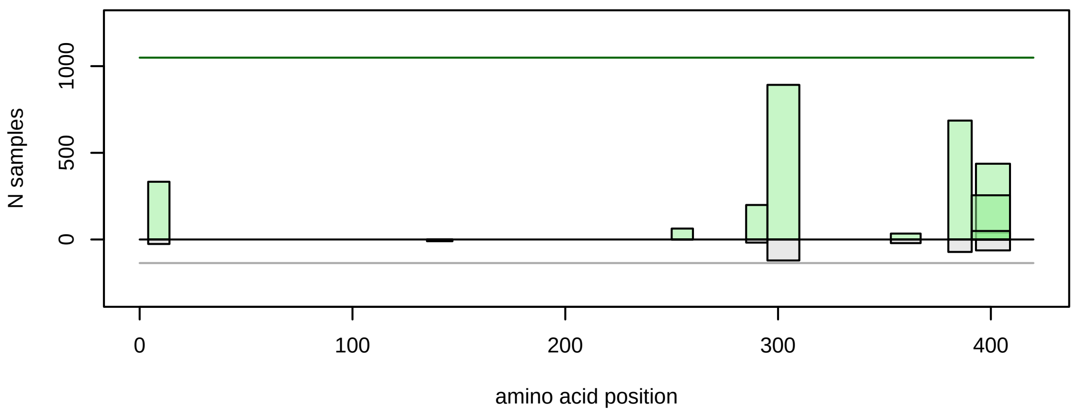

1910. SNED1 (Q8TER0), NP1

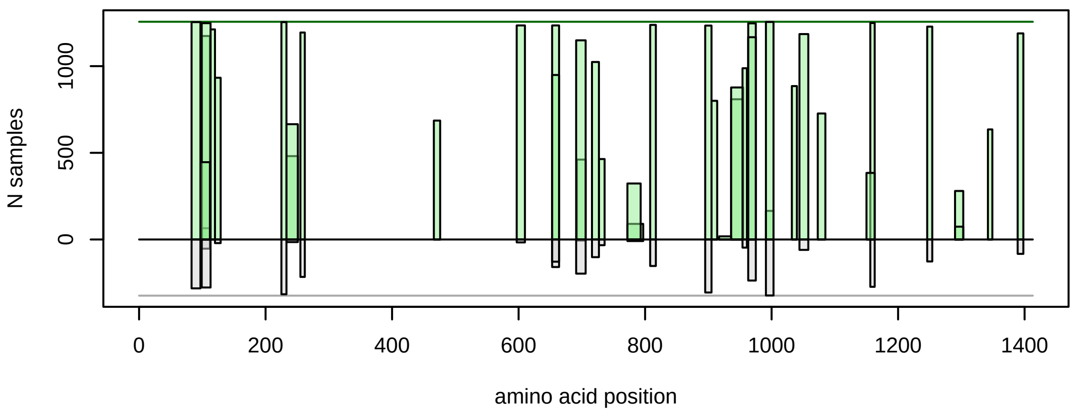

1911. TPP1 (O14773), NP2

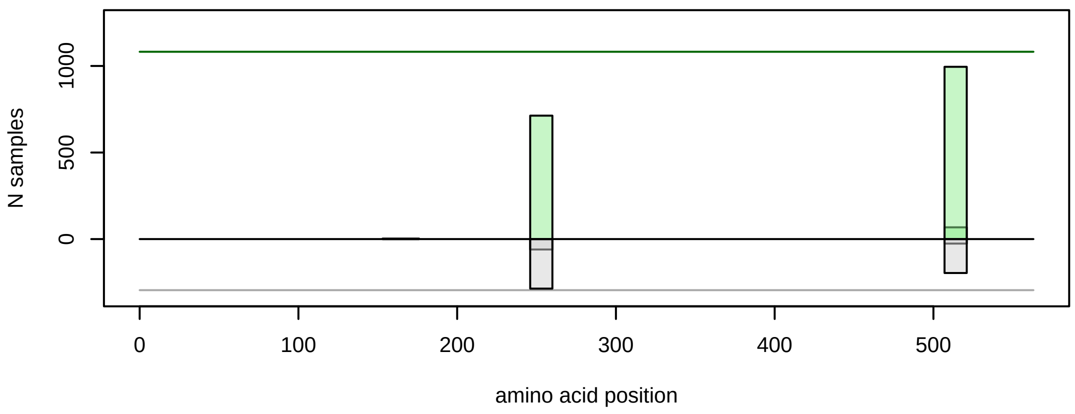

1912. MICAL1 (Q8TDZ2;Q8TDZ2−4), NP4

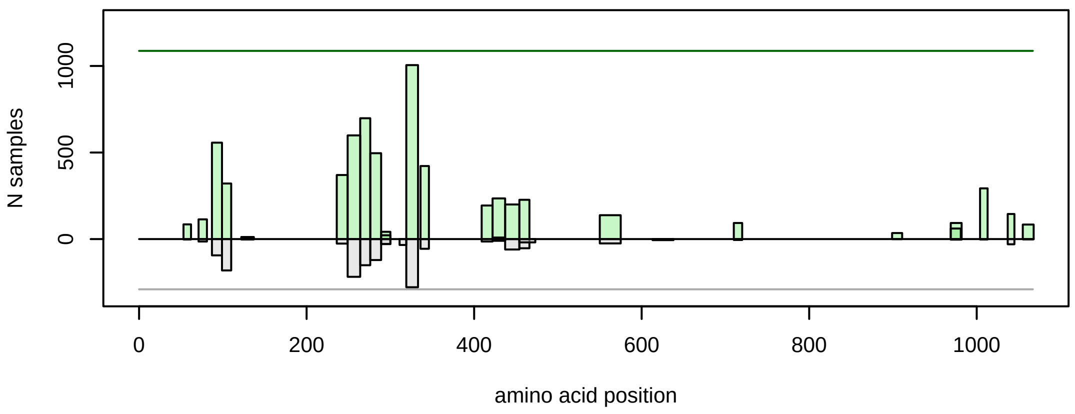

1913. TTLL12 (Q14166), NP5

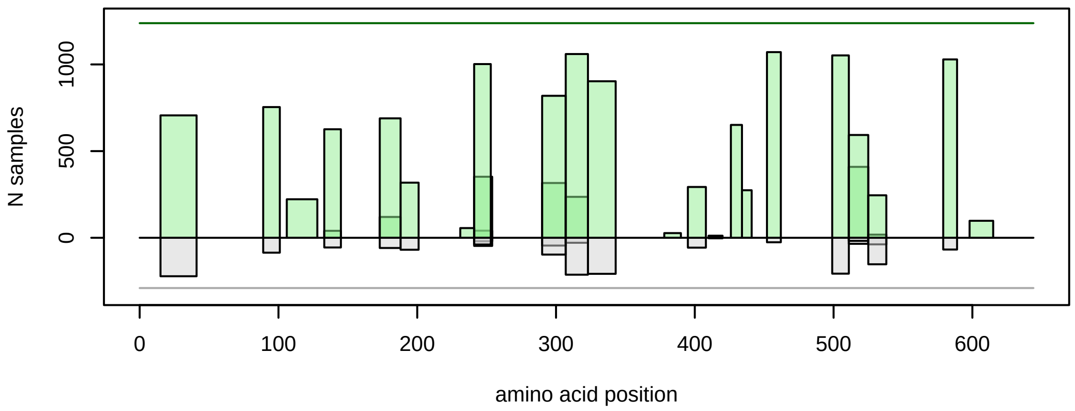

1914. PPP2R1A (P30153), NP5

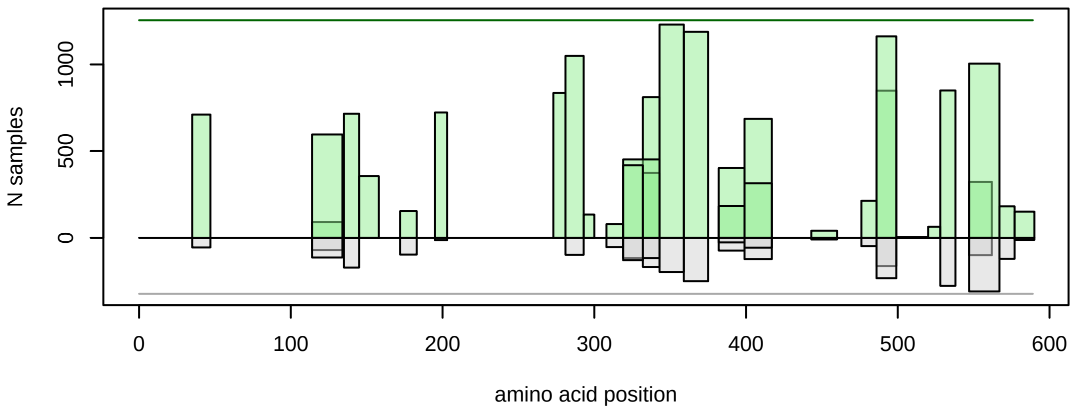

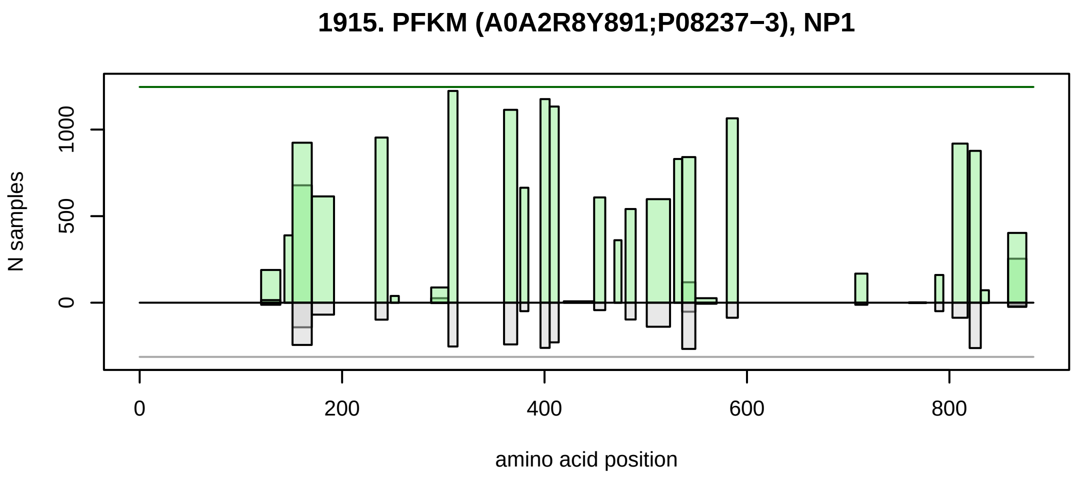
1915. PFKM (A0A2R8Y891;P08237−3), NP1
N samples
0
500
1000
0
200
400
600
800
amino acid position

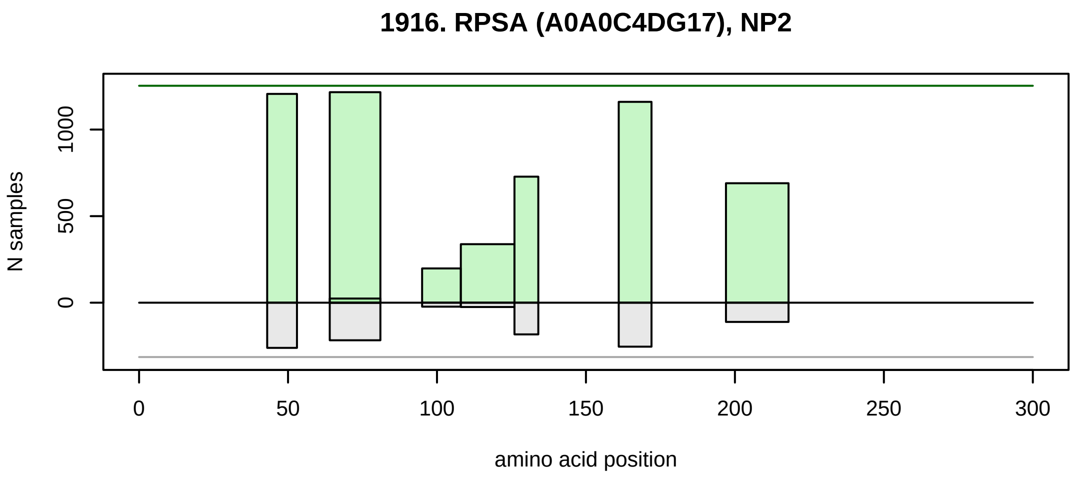
1916. RPSA (A0A0C4DG17), NP2
N samples
0
500
1000
0
50
100
150
200
250
300
amino acid position

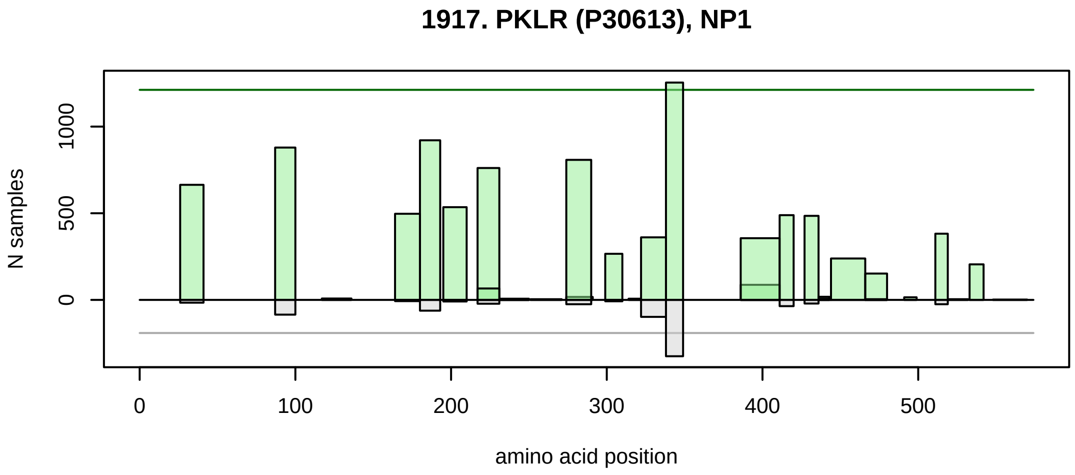
1917. PKLR (P30613), NP1
N samples
0
500
1000
0
100
200
300
400
500
amino acid position

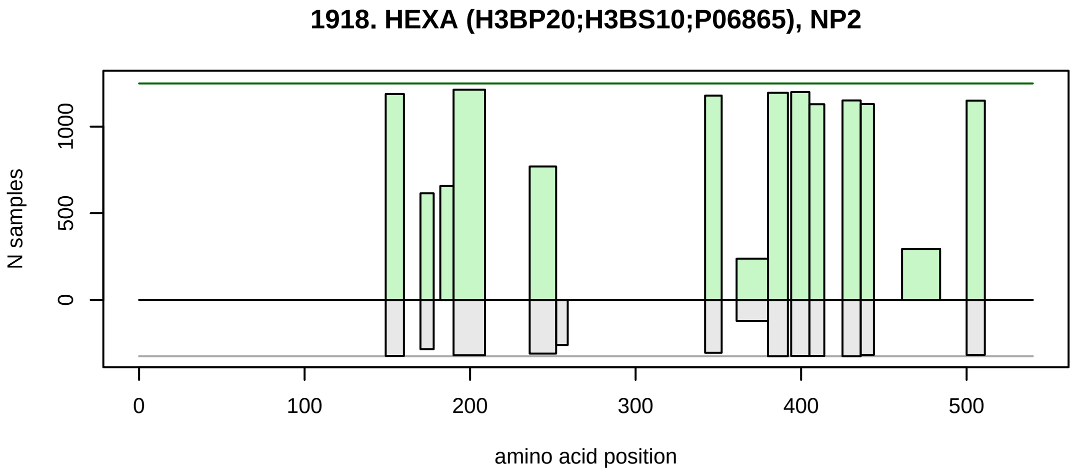
1918. HEXA (H3BP20;H3BS10;P06865), NP2
N samples
0
500
1000
0
100
200
300
400
500
amino acid position

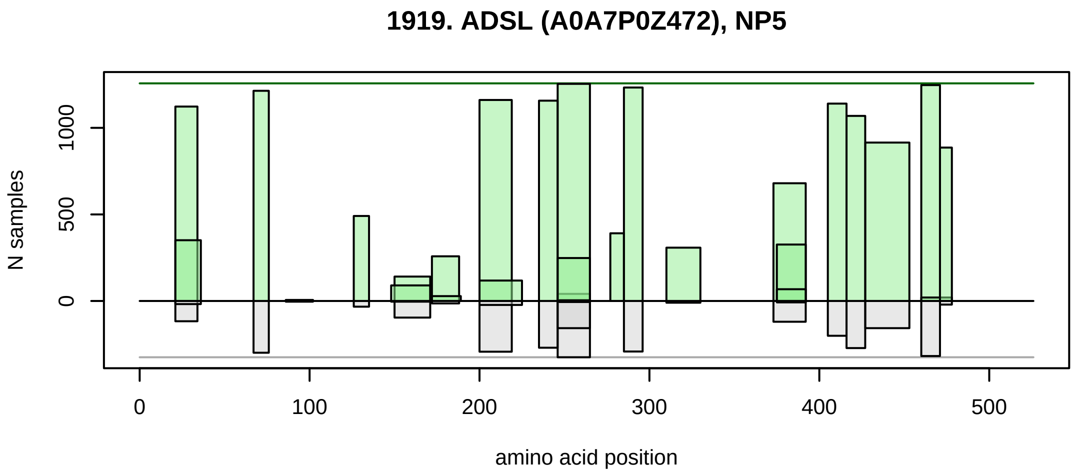
1919. ADSL (A0A7P0Z472), NP5
N samples
0
500
1000
0
100
200
300
400
500
amino acid position

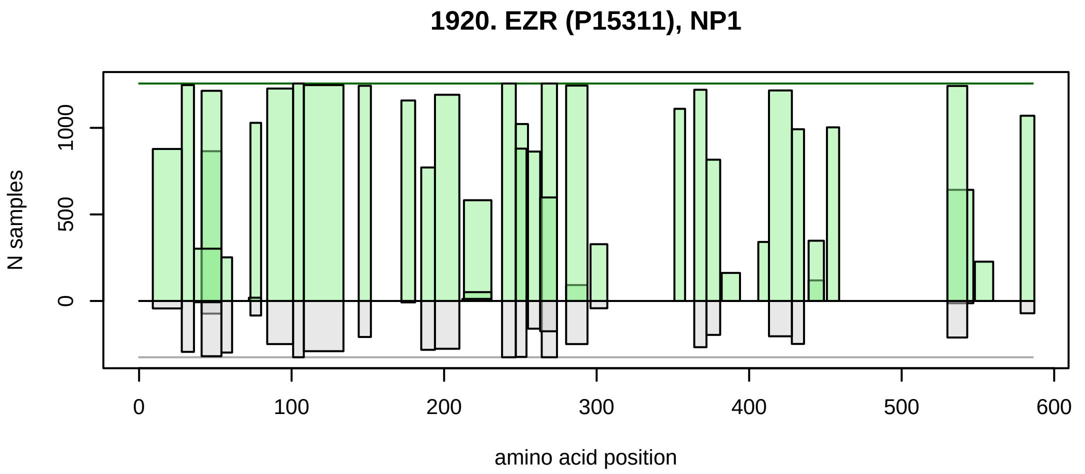
1920. EZR (P15311), NP1
N samples
0
500
1000
0
100
200
300
400
500
600
amino acid position

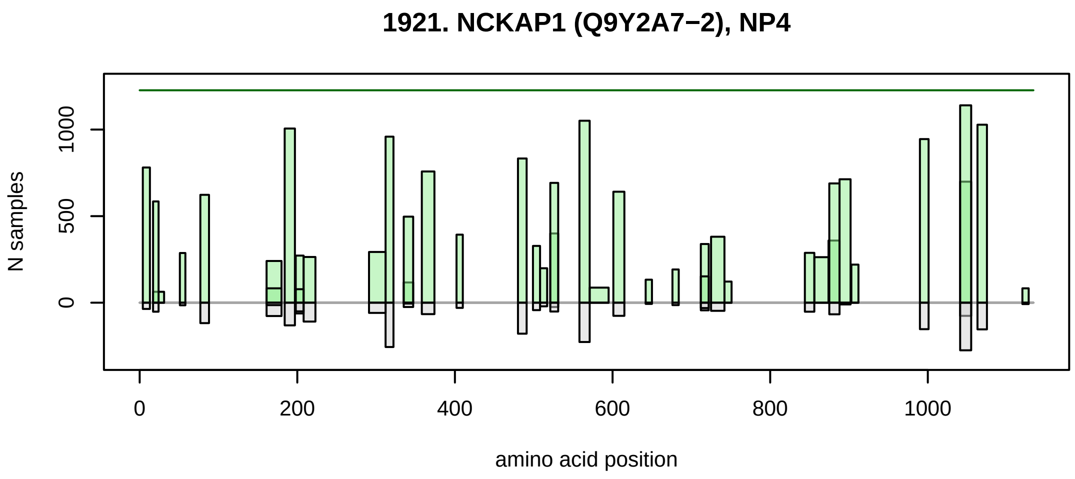
1921. NCKAP1 (Q9Y2A7−2), NP4
N samples
amino acid position

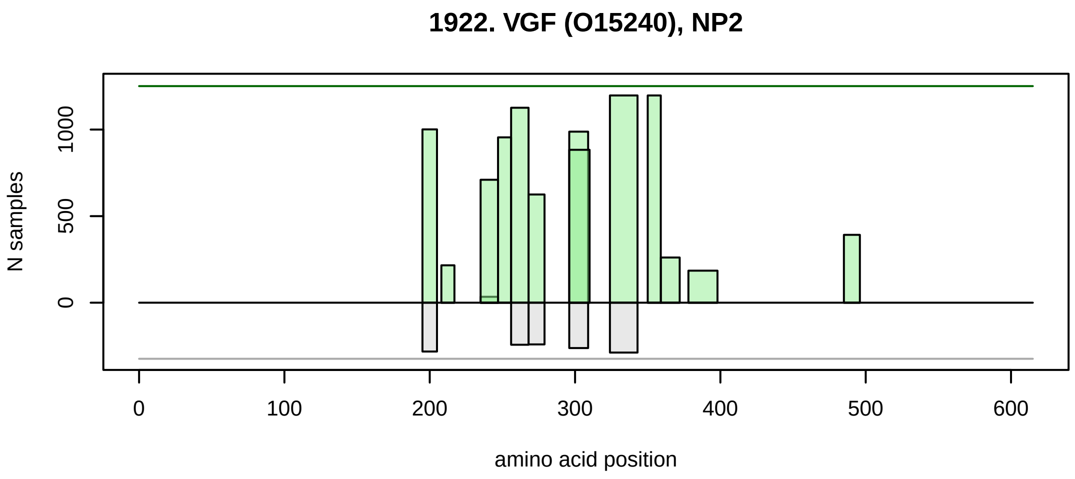
1922. VGF (O15240), NP2
N samples
amino acid position

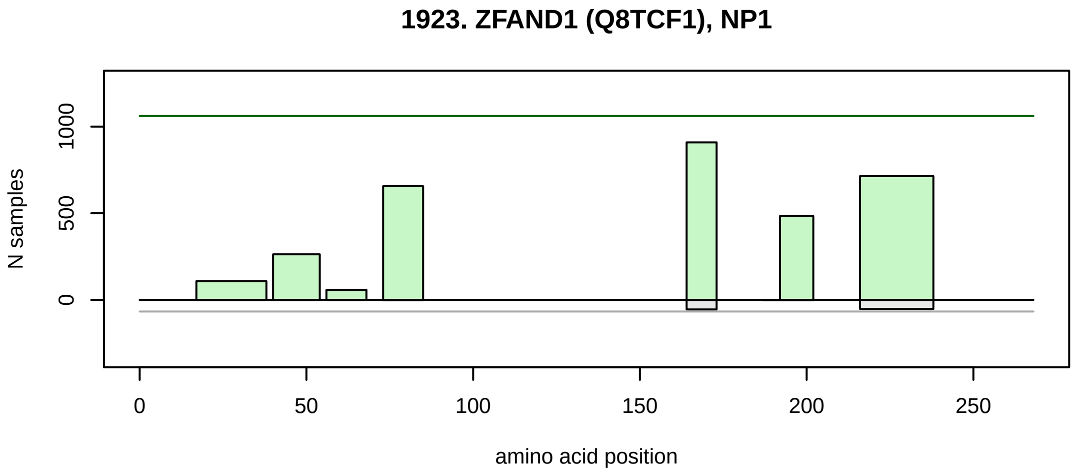
1923. ZFAND1 (Q8TCF1), NP1
N samples
amino acid position

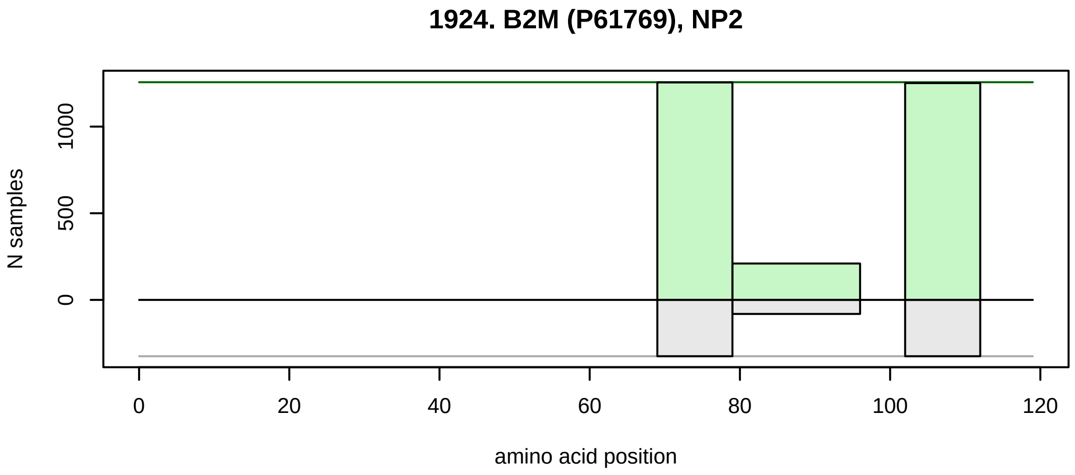
1924. B2M (P61769), NP2
N samples
amino acid position

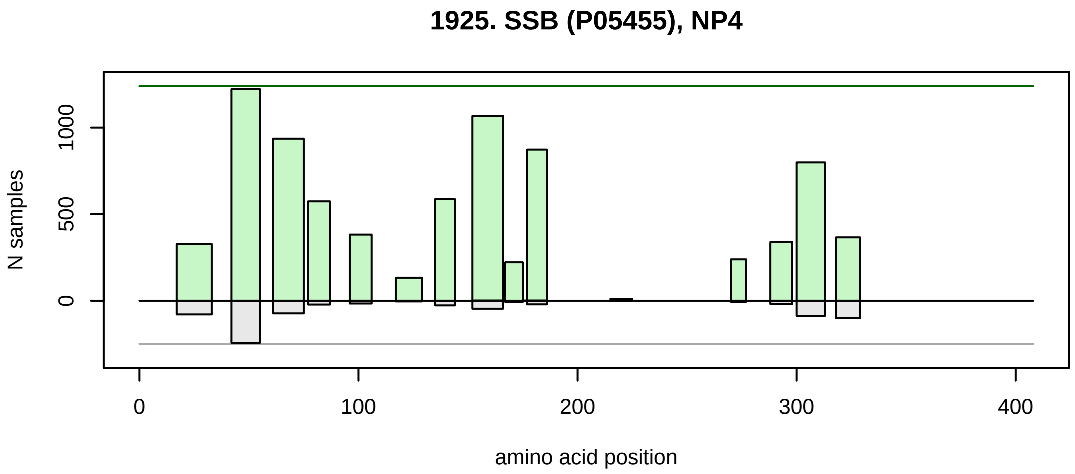
1925. SSB (P05455), NP4
N samples
amino acid position

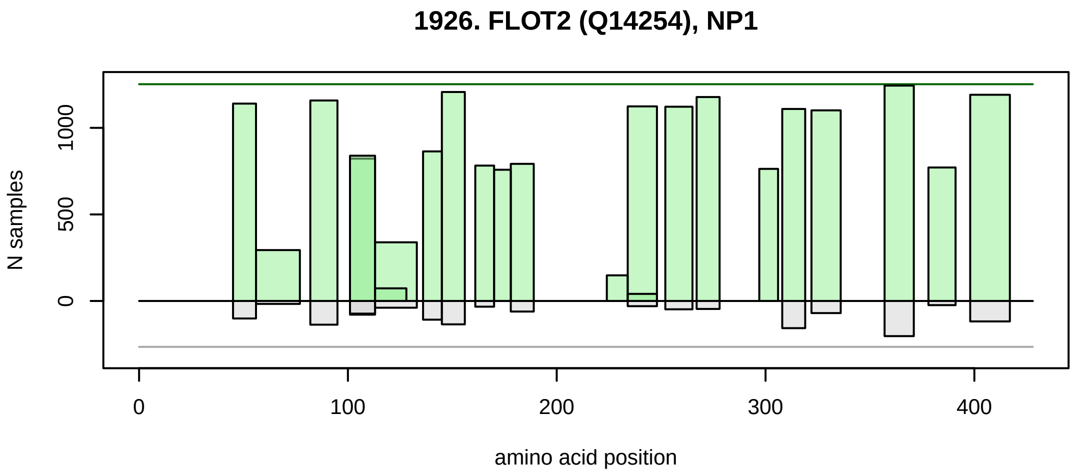
1926. FLOT2 (Q14254), NP1
N samples
amino acid position

1927. APOH (P02749), NP4

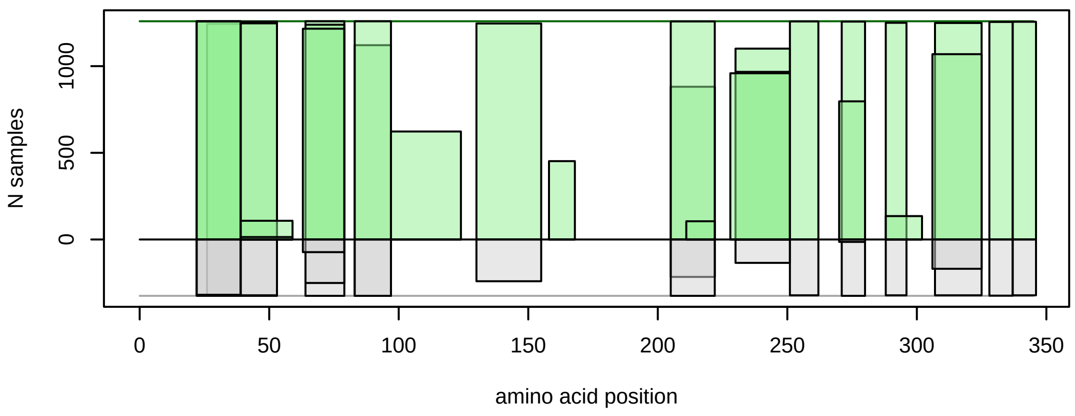

1928. WNT3 (P56703), NP5

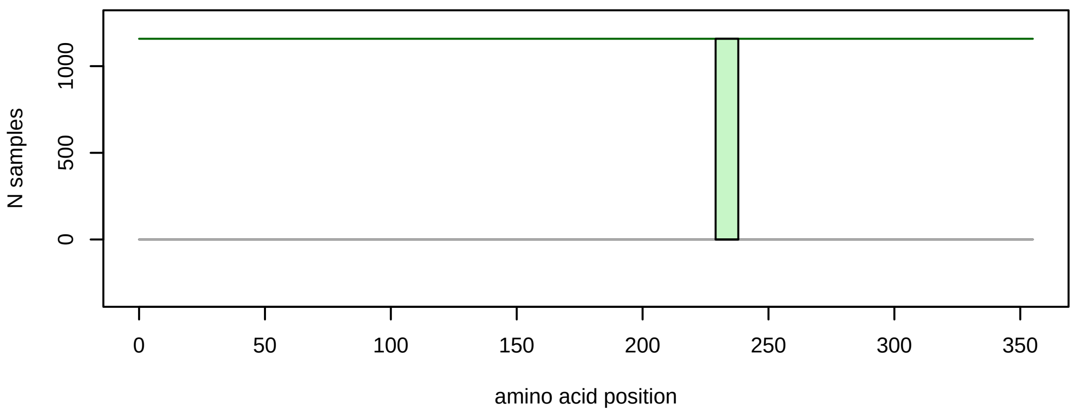

1929. EPB41 (P11171), NP1

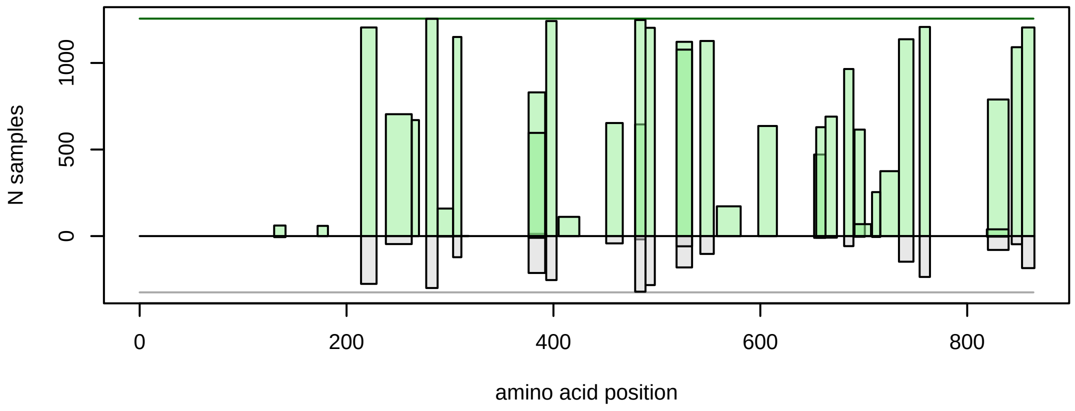

1930. WIBG (Q9BRP8−2), NP4

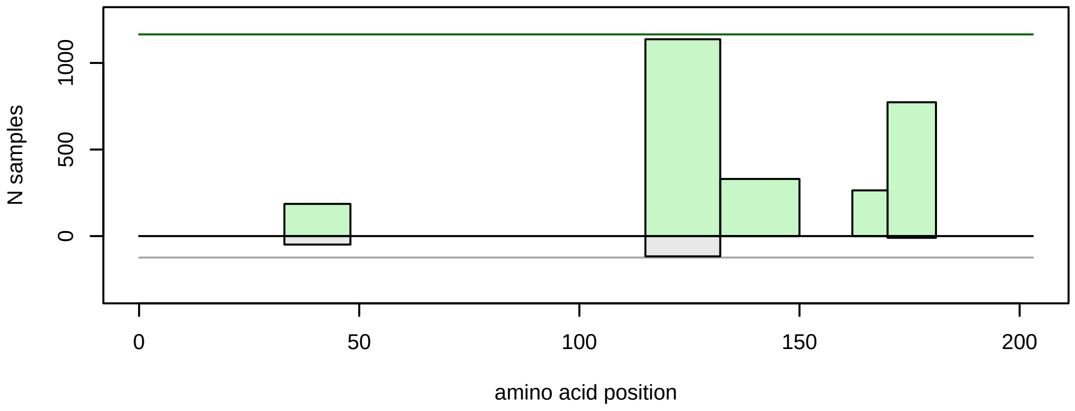

1931. EEF1G (P26641), NP1

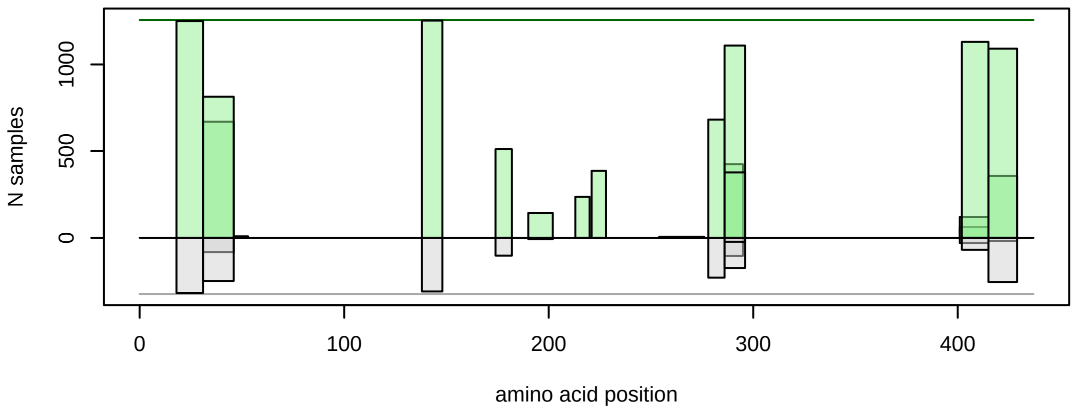

1932. MYO18A (Q92614), NP4

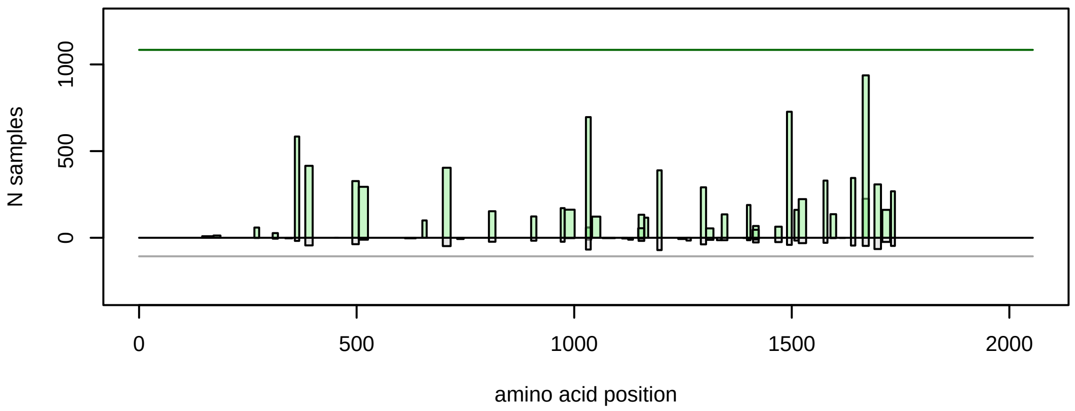

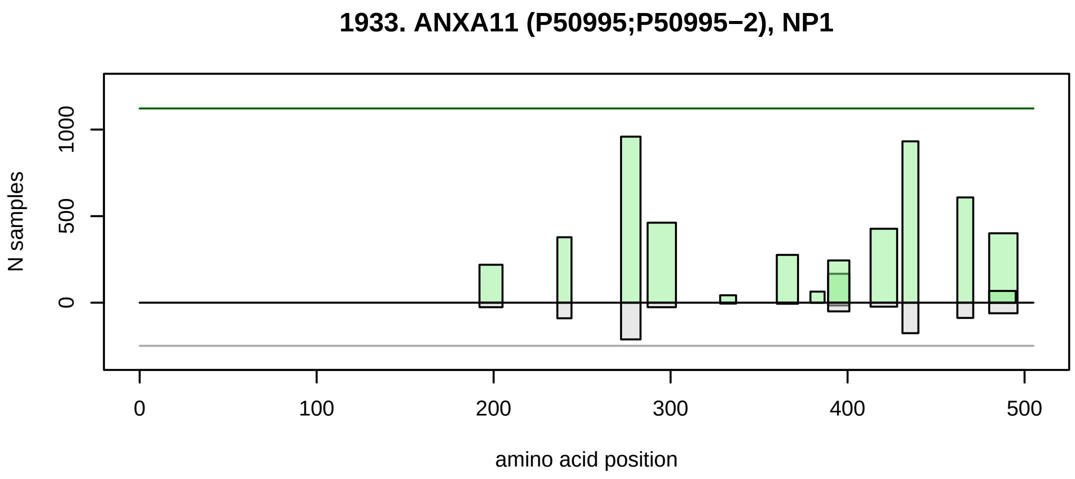
1933. ANXA11 (P50995;P50995−2), NP1
N samples
amino acid position
0
500
1000
0
100
200
300
400
500

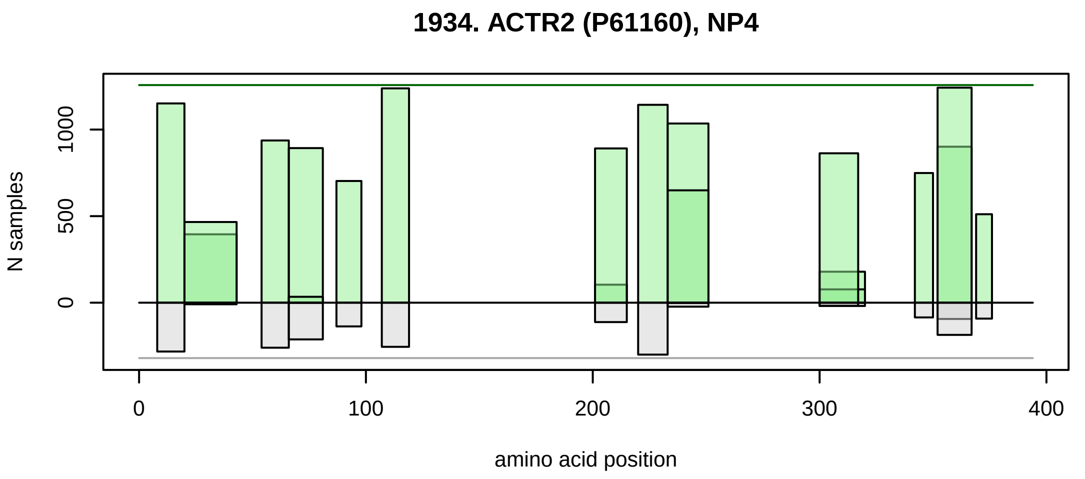
1934. ACTR2 (P61160), NP4
N samples
amino acid position
0
500
1000
0
100
200
300
400

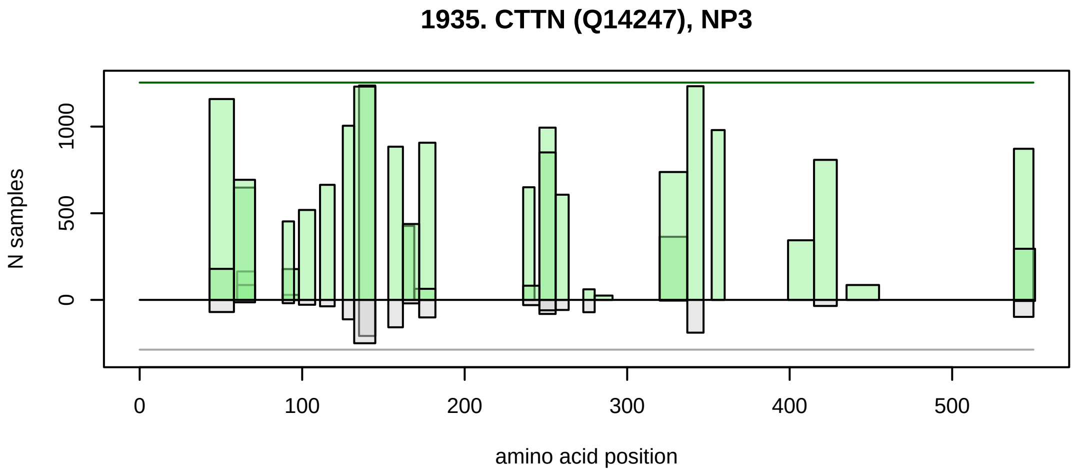
1935. CTTN (Q14247), NP3
N samples
amino acid position
0
500
1000
0
100
200
300
400
500

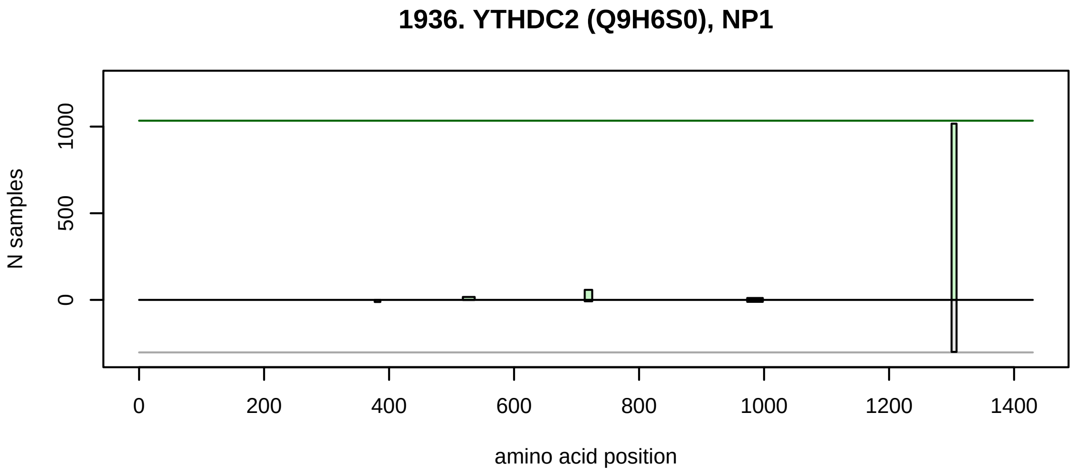
1936. YTHDC2 (Q9H6S0), NP1
N samples
amino acid position
0
500
1000
0
200
400
600
800
1000
1200
1400

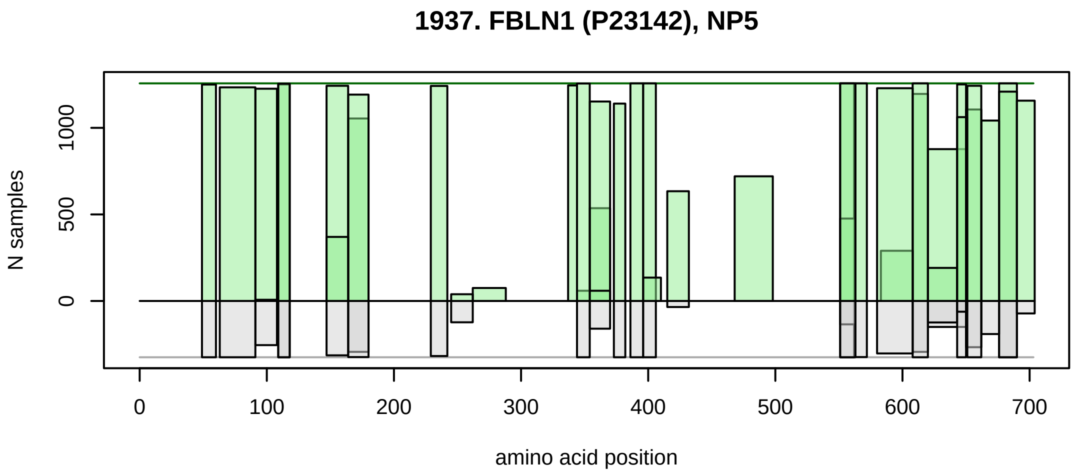
1937. FBLN1 (P23142), NP5
N samples
amino acid position
0
500
1000
0
100
200
300
400
500
600
700

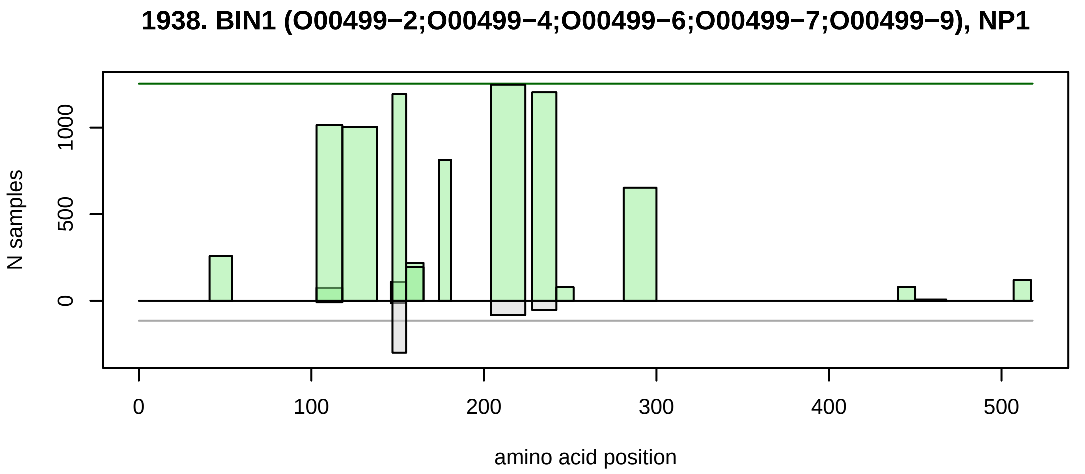
1938. BIN1 (O00499−2;O00499−4;O00499−6;O00499−7;O00499−9), NP1
N samples
amino acid position
0
500
1000
0
100
200
300
400
500

1939. DSG2 (Q14126), NP2

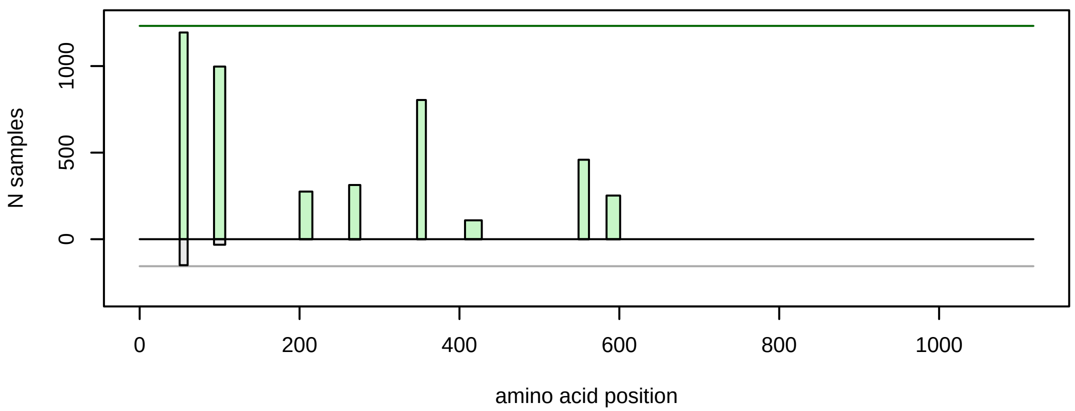

1940. NRP1 (O14786), NP2

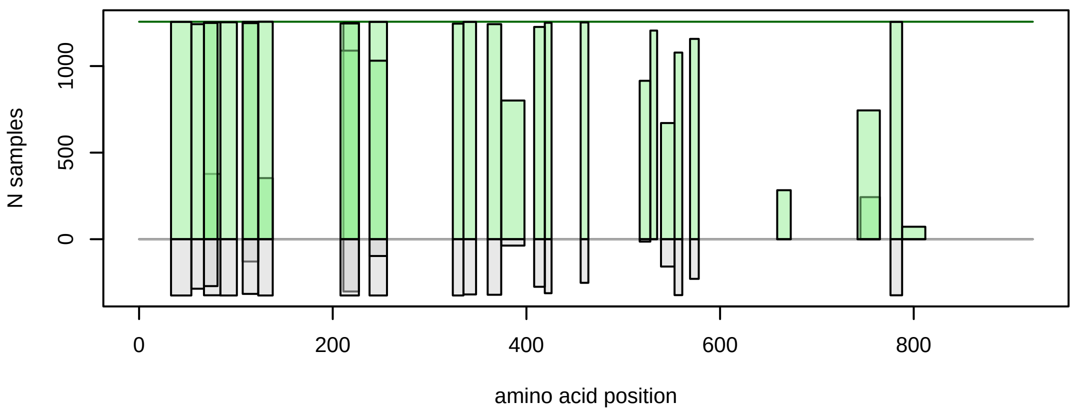

1941. GNAQ (P50148), NP1

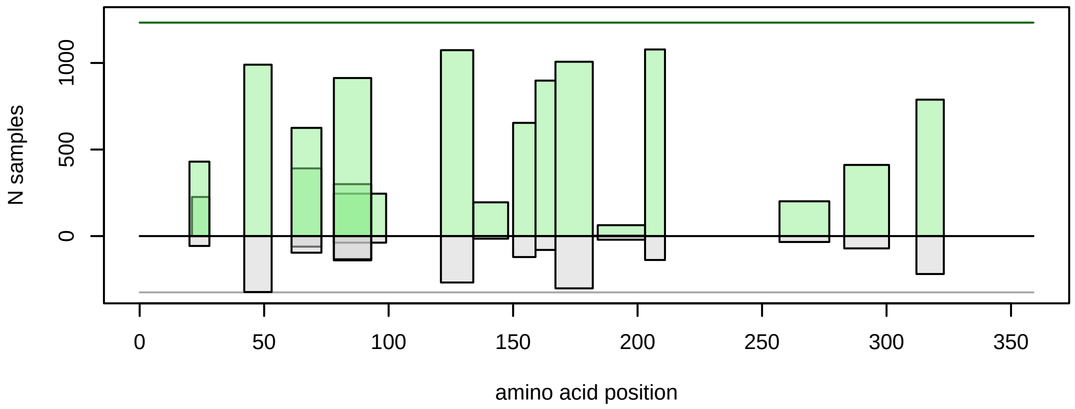

1942. HADHA (H0YFD6;P40939), NP4

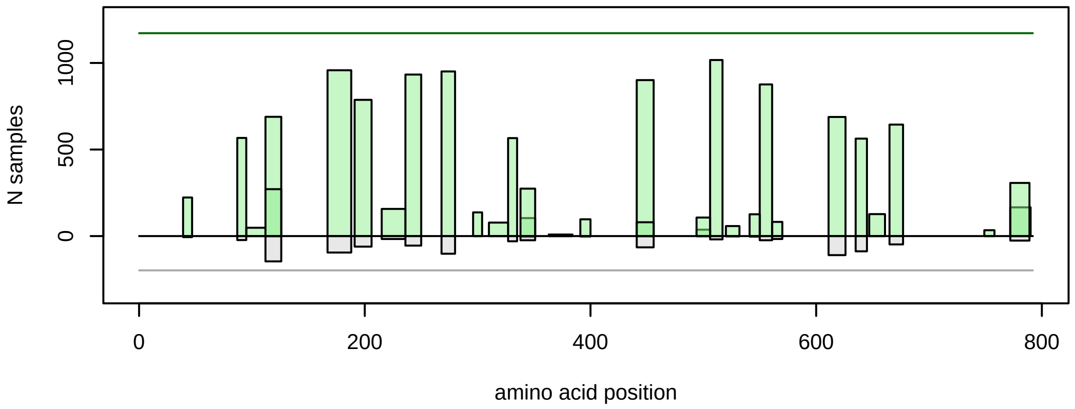

1943. SNCA (A0A669KB41;A0A669KBH5;E7EPV7;P37840;P37840−2), NP2

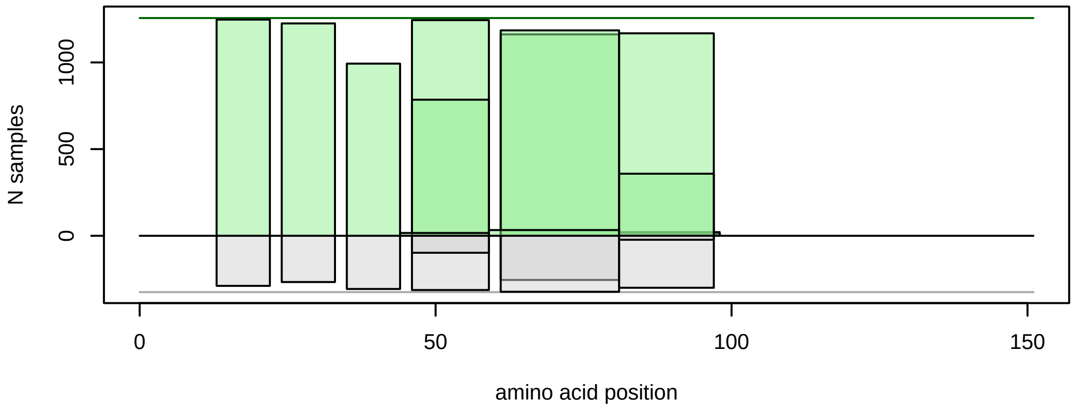

1944. GMPR (P36959), NP5

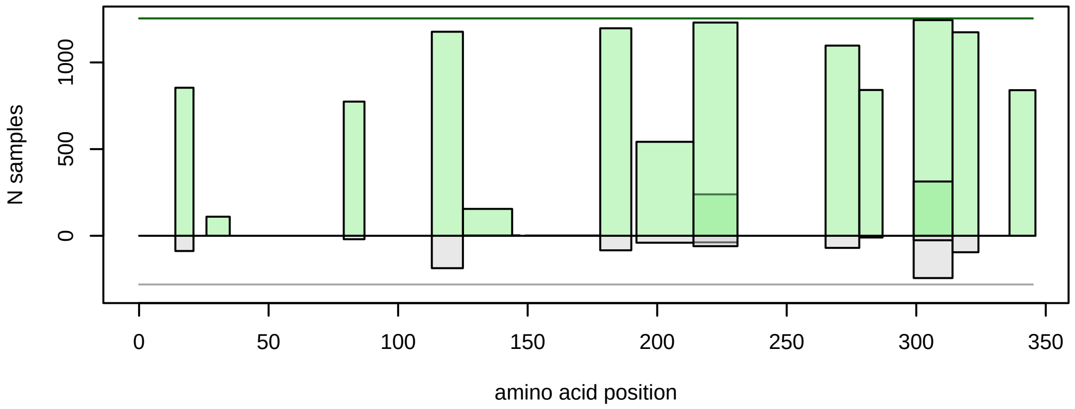

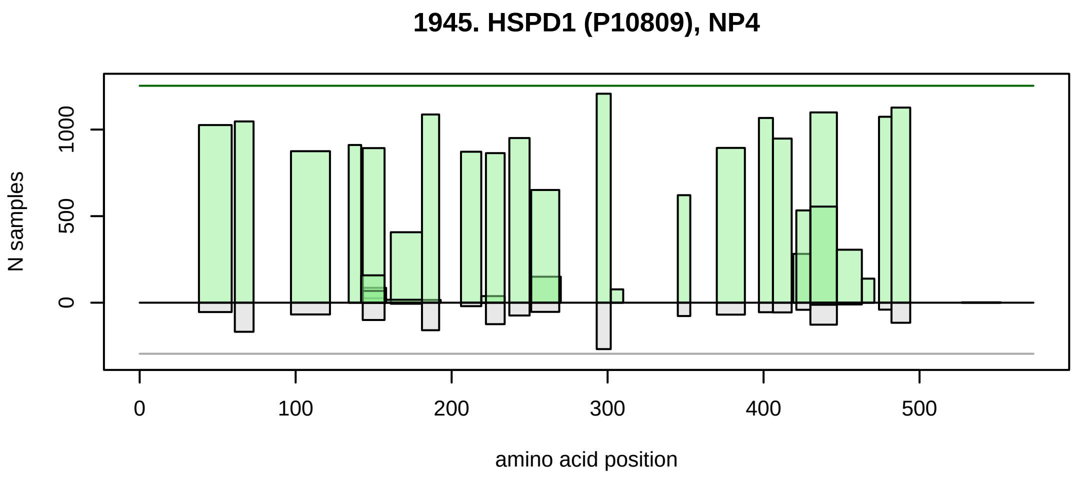
1945. HSPD1 (P10809), NP4
N samples
0
500
1000
0
100
200
300
400
500
amino acid position

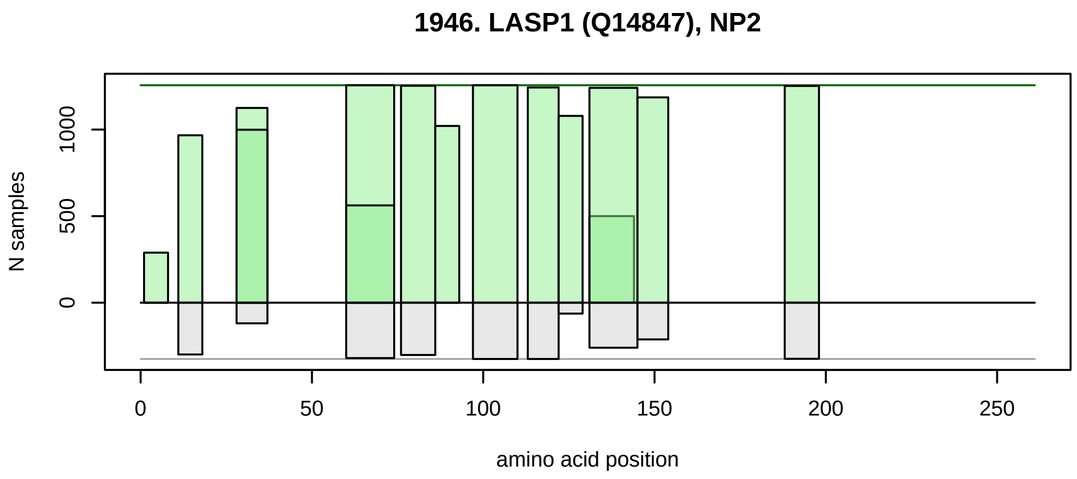
1946. LASP1 (Q14847), NP2
N samples
0
500
1000
0
50
100
150
200
250
amino acid position

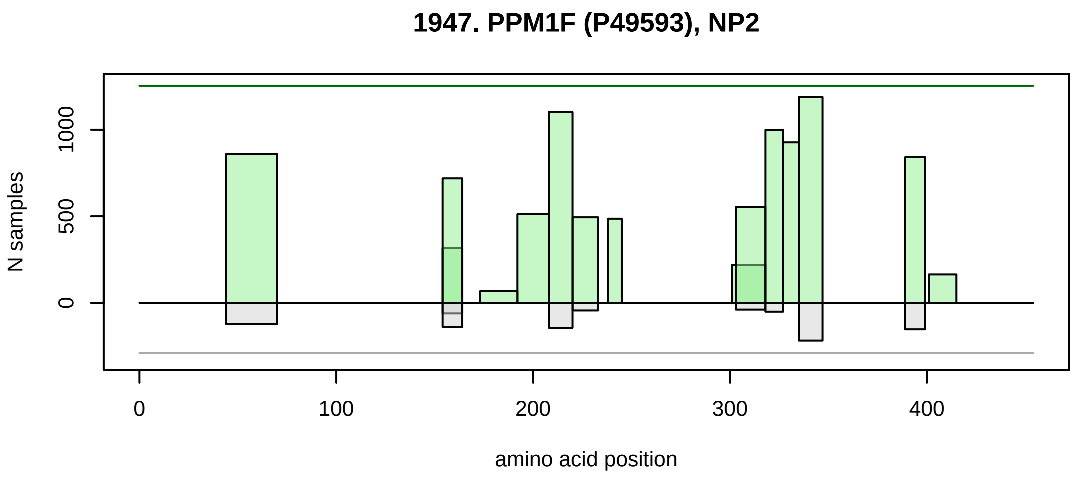
1947. PPM1F (P49593), NP2
N samples
0
500
1000
0
100
200
300
400
amino acid position

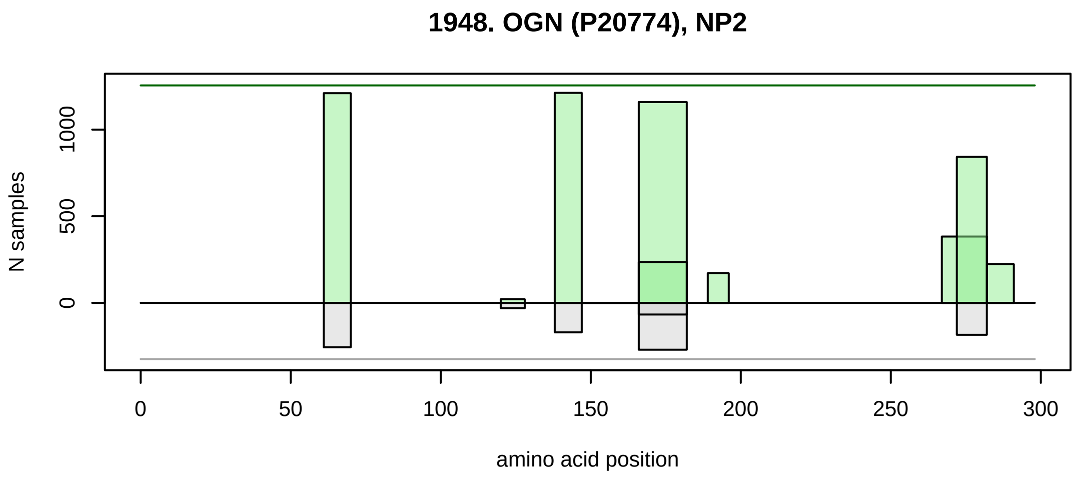
1948. OGN (P20774), NP2
N samples
0
500
1000
0
50
100
150
200
250
300
amino acid position

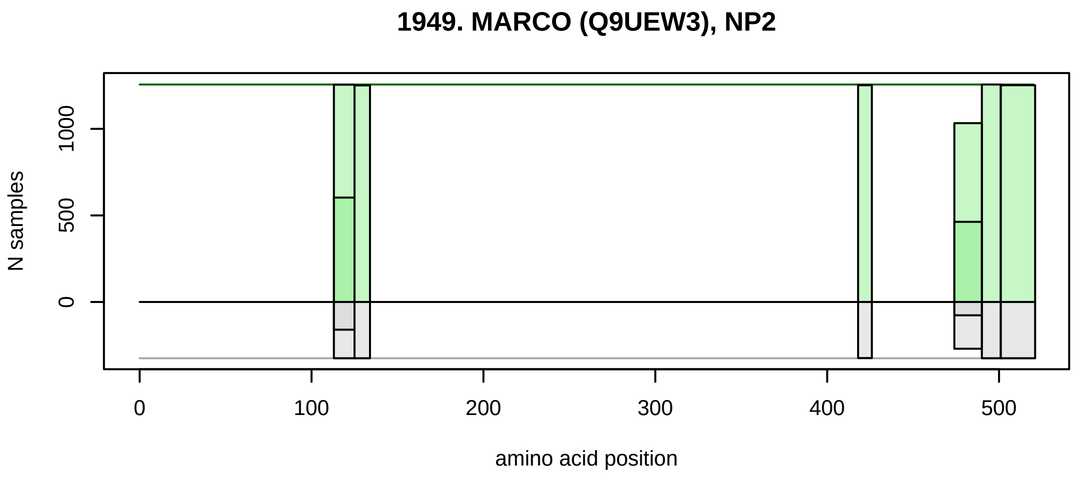
1949. MARCO (Q9UEW3), NP2
N samples
0
500
1000
0
100
200
300
400
500
amino acid position

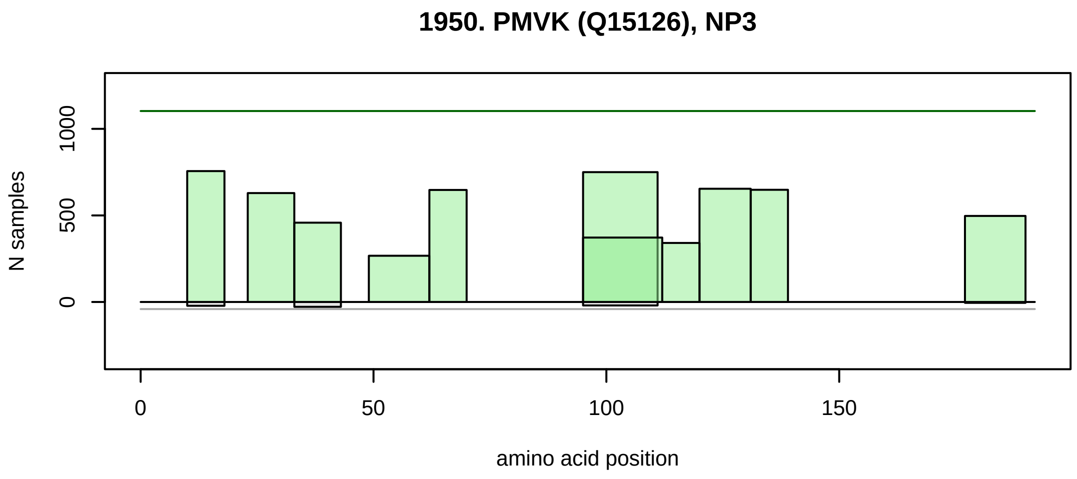
1950. PMVK (Q15126), NP3
N samples
0
500
1000
0
50
100
150
amino acid position

1951. KMT2D (A0A804HHR9), NP1

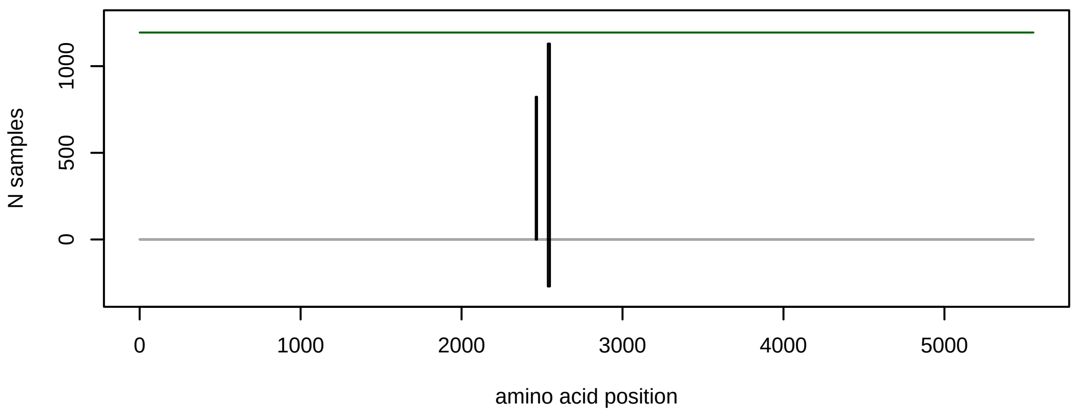

1952. KRT10 (A0A1B0GVI3), NP2

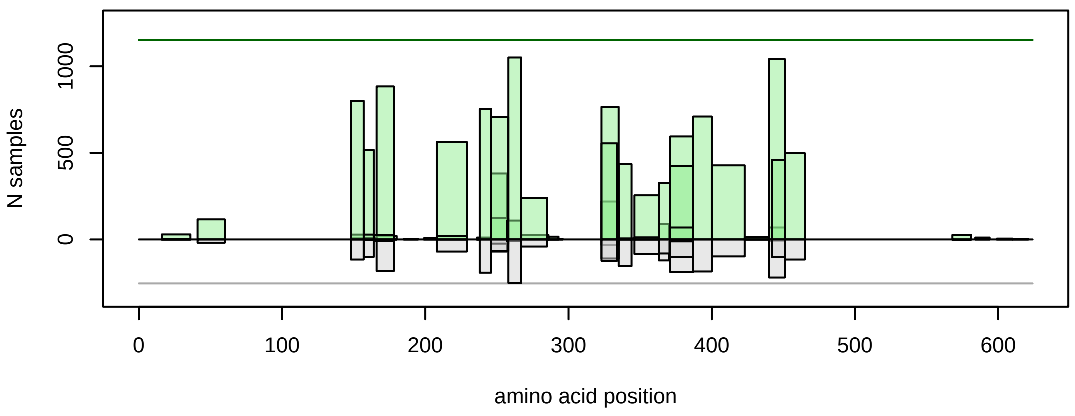

1953. MGP (P08493−2), NP2

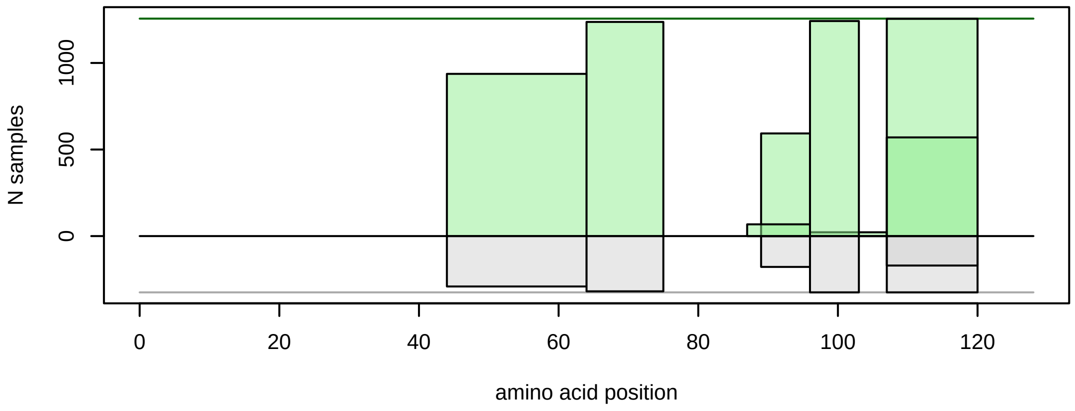

1954. SERPINE1 (P05121), NP2

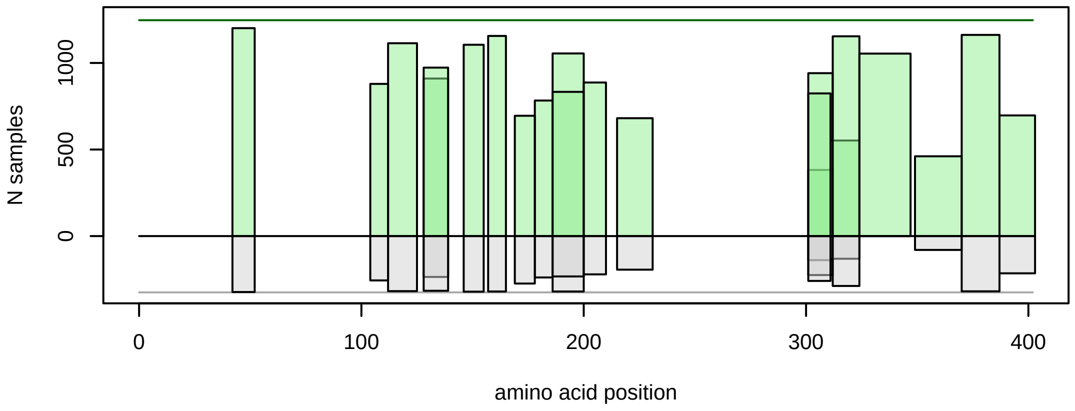

1955. CLNS1A (E9PMI6;J3KN38;P54105), NP2

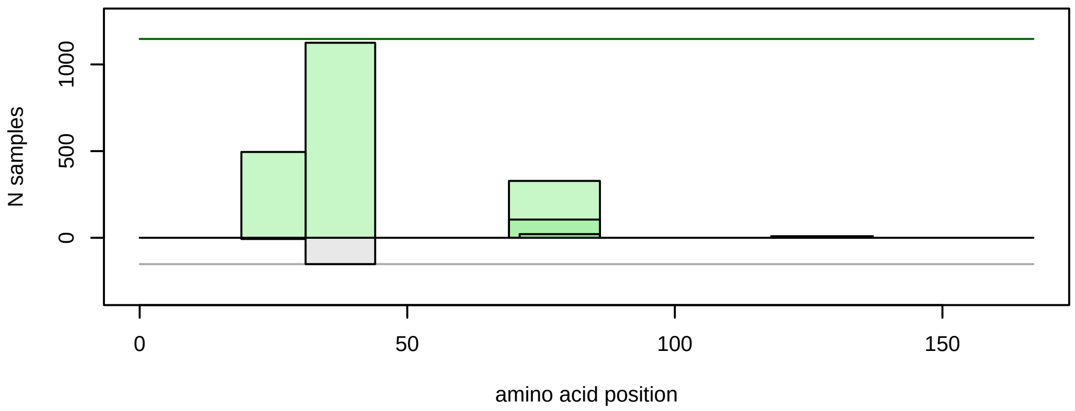

1956. HPSE (Q9Y251;Q9Y251−2), NP4

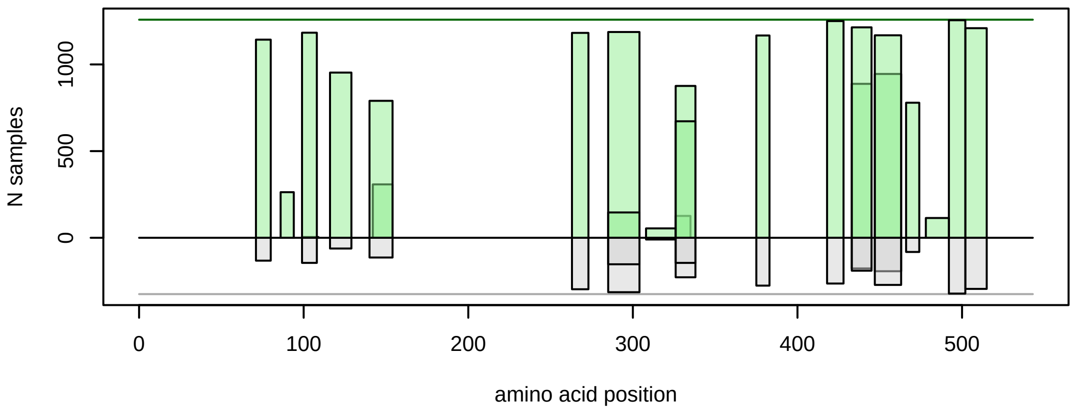

1957. BIN2 (S4R418), NP1

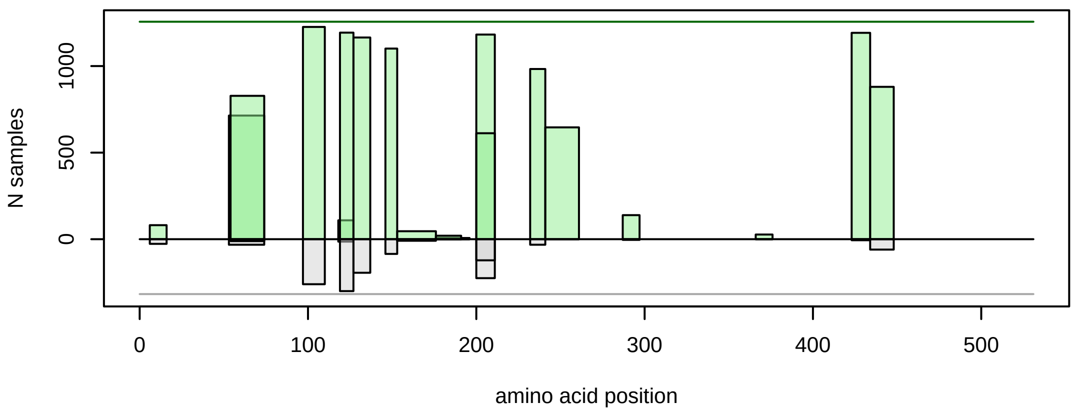

1958. UBE2O (Q9C0C9), NP2

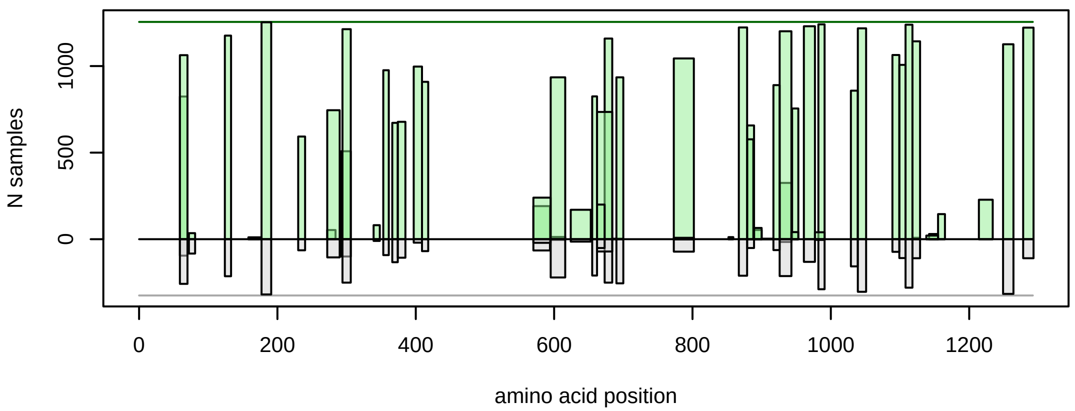

1959. S100A8 (P05109), NP3

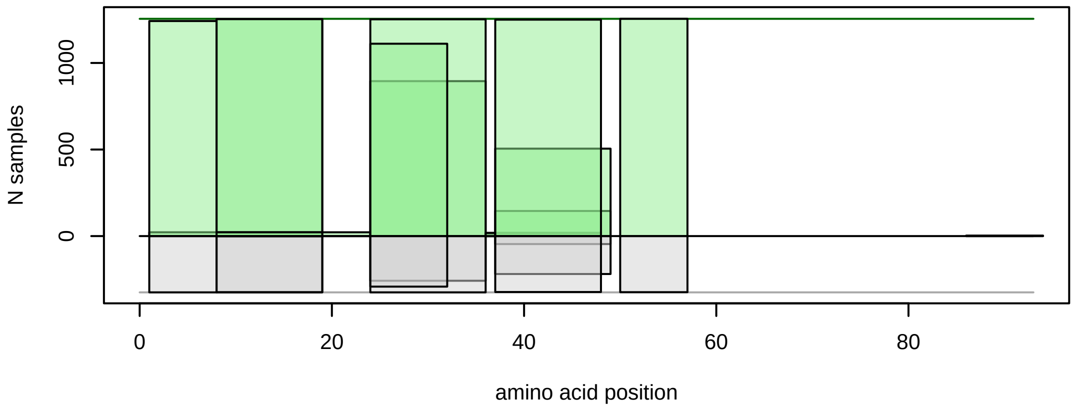

1960. SLIT2 (O94813;X6R3P0), NP4

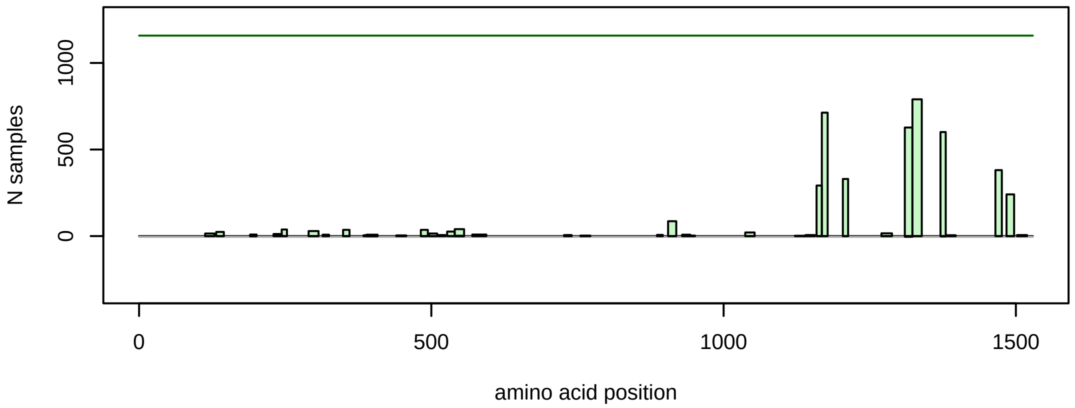

1961. RAB5B (P61020), NP1

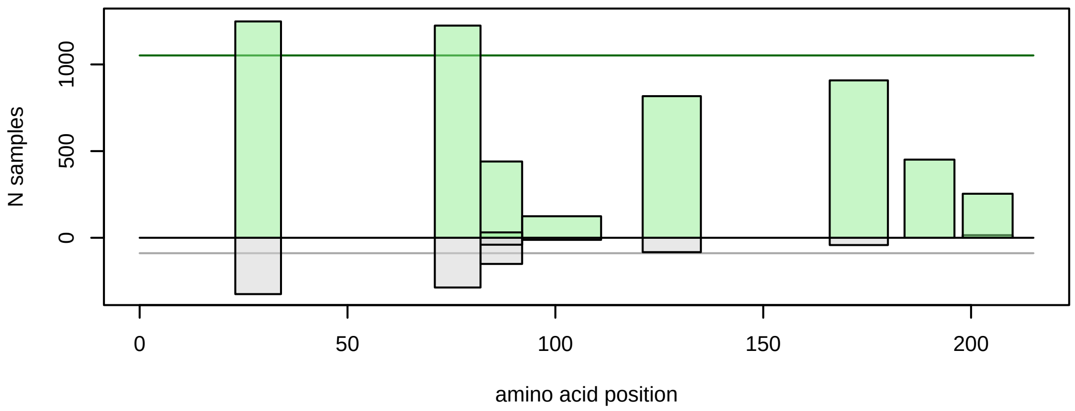

1962. LTBP2 (G3V3X5;Q14767), NP3

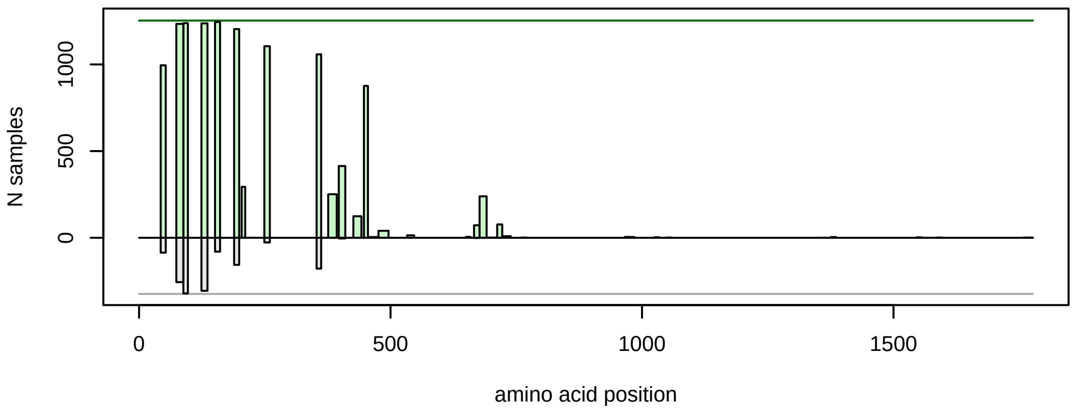

1963. BTD (P43251), NP2

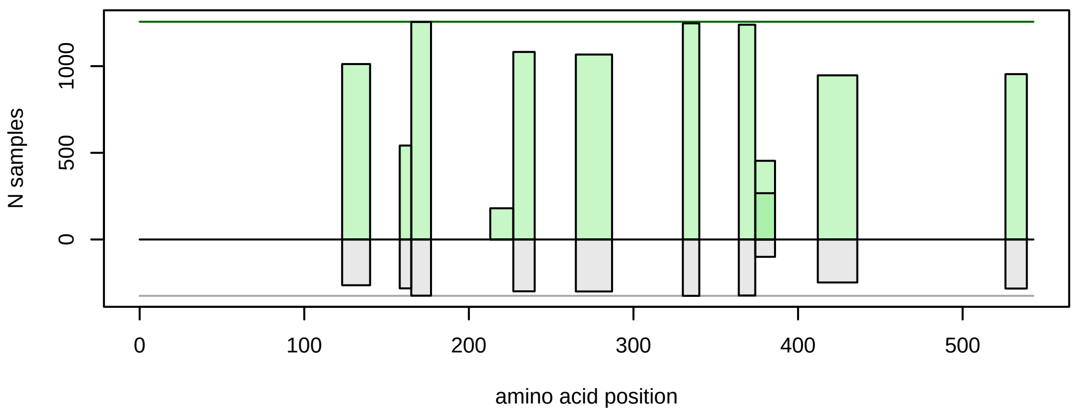

1964. LGALS3BP (Q08380), NP2

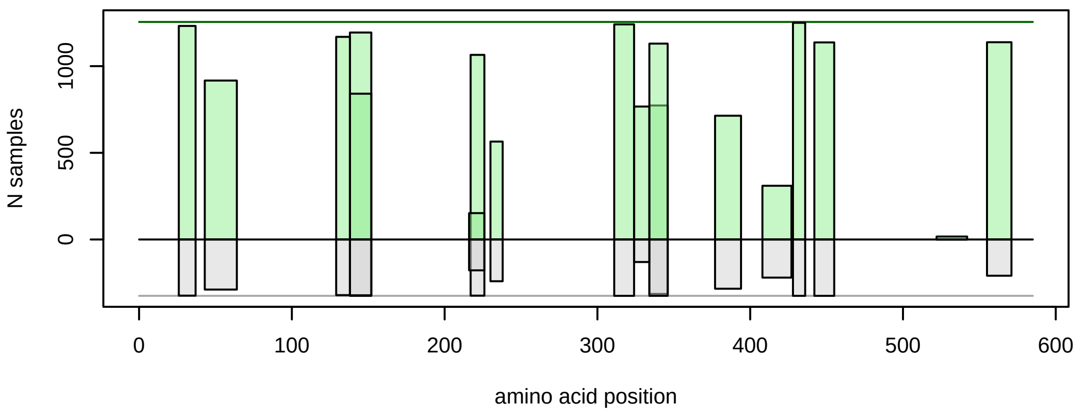

1965. VLDLR (P98155−2), NP2

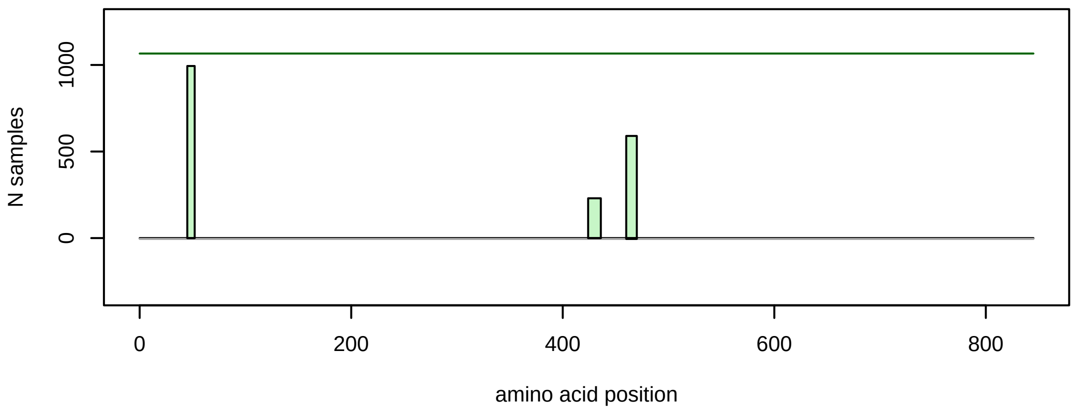

1966. RNASE1 (P07998), NP3

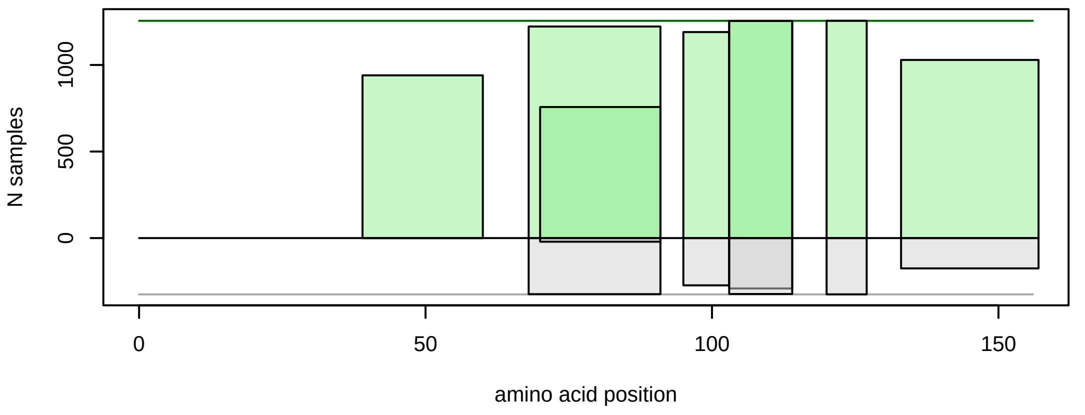

1967. IGFBP1 (C9JXF9;P08833), NP2

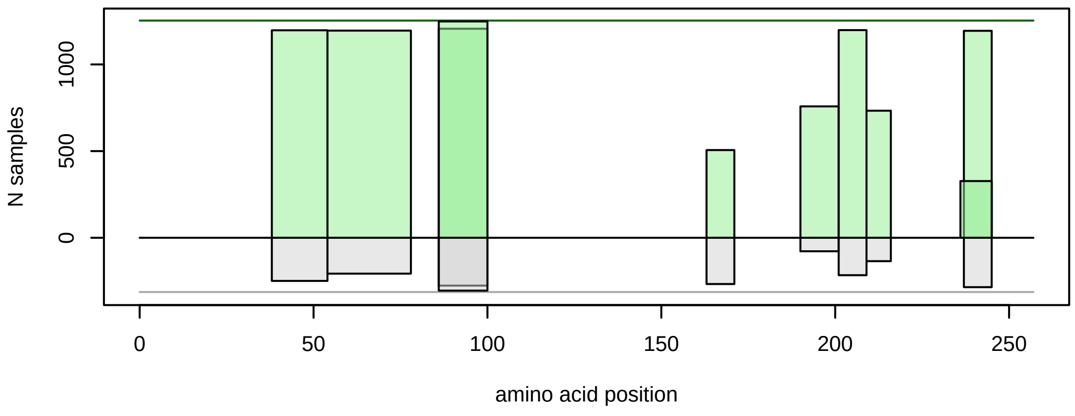

1968. PPP2CA (P67775), NP5

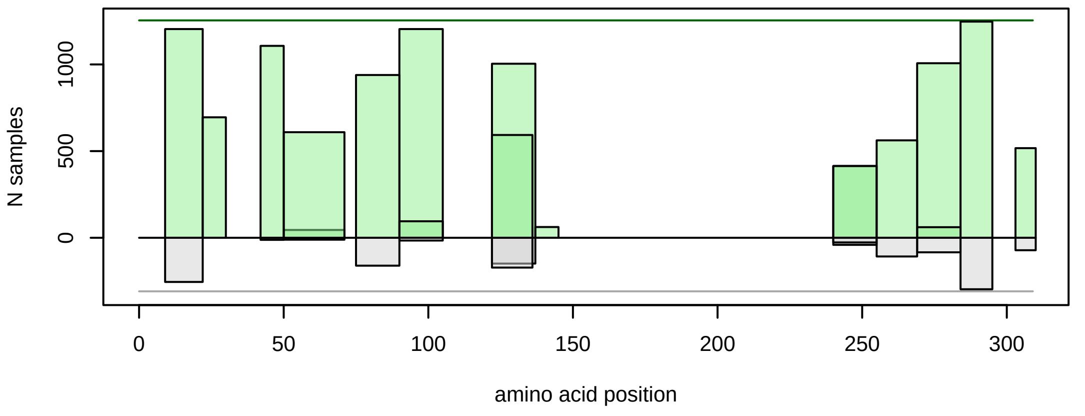

1969. MGAT1 (P26572), NP1

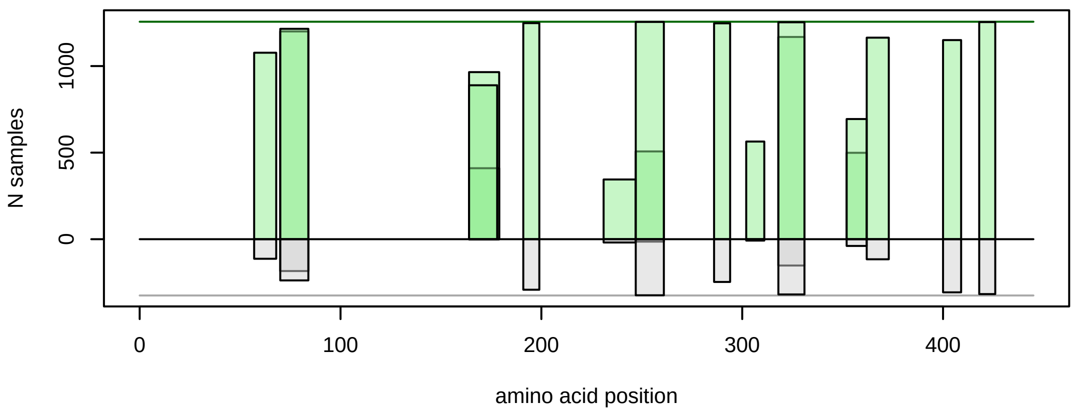

1970. PER1 (O15534), NP1

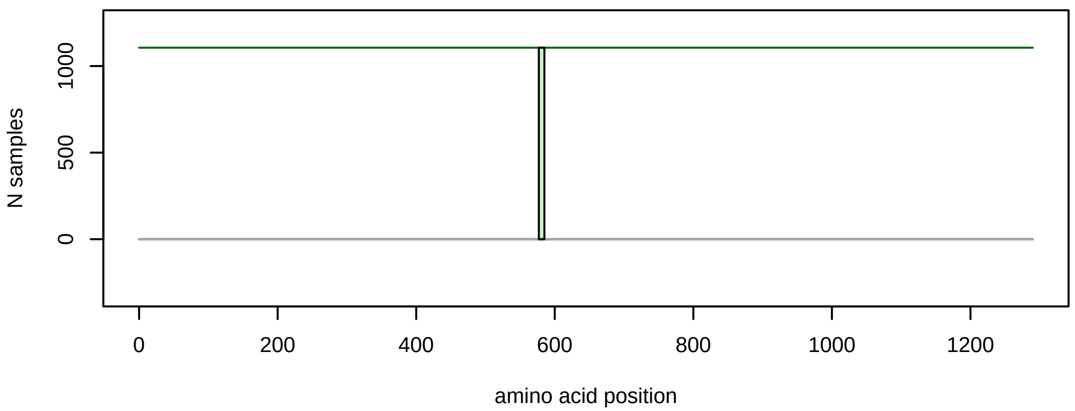

1971. F11R (Q9Y624), NP1

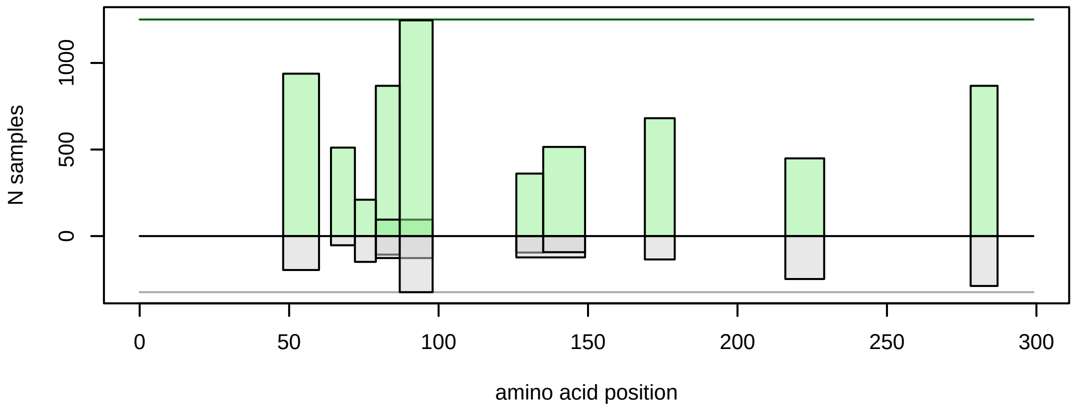

1972. USP7 (Q93009), NP5

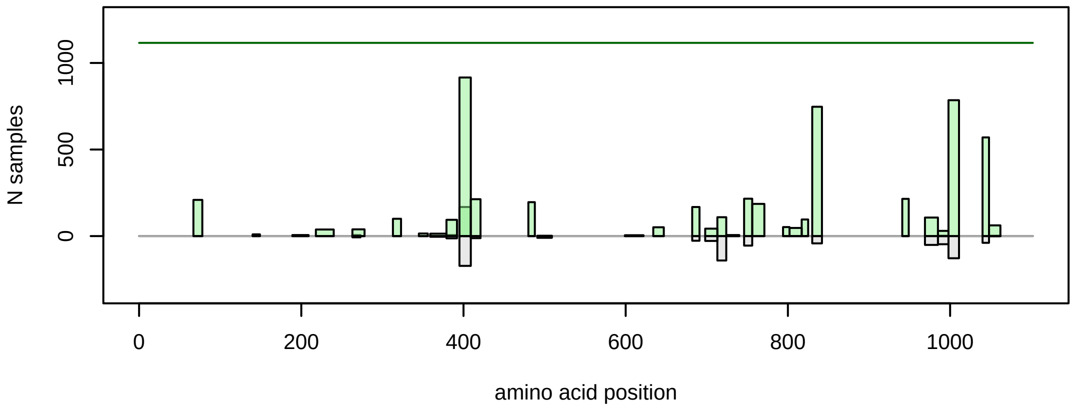

1973. RAP2B (P61225), NP1

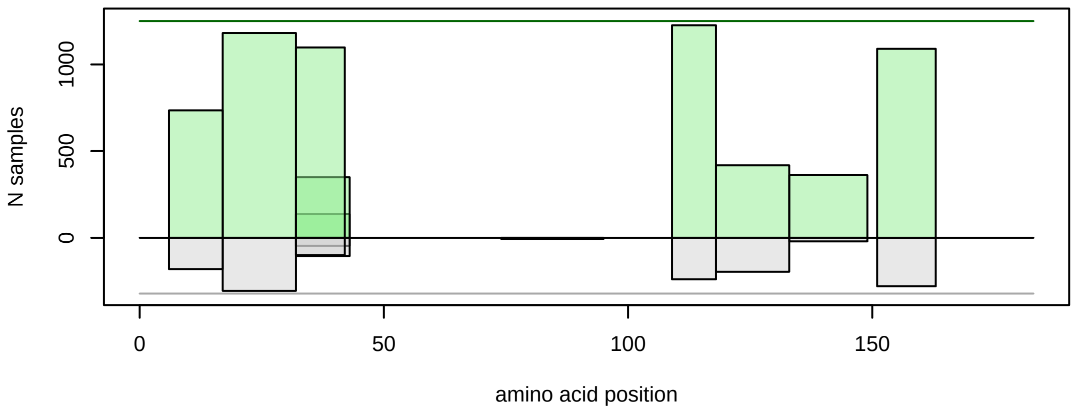

1974. STC1 (P52823), NP3

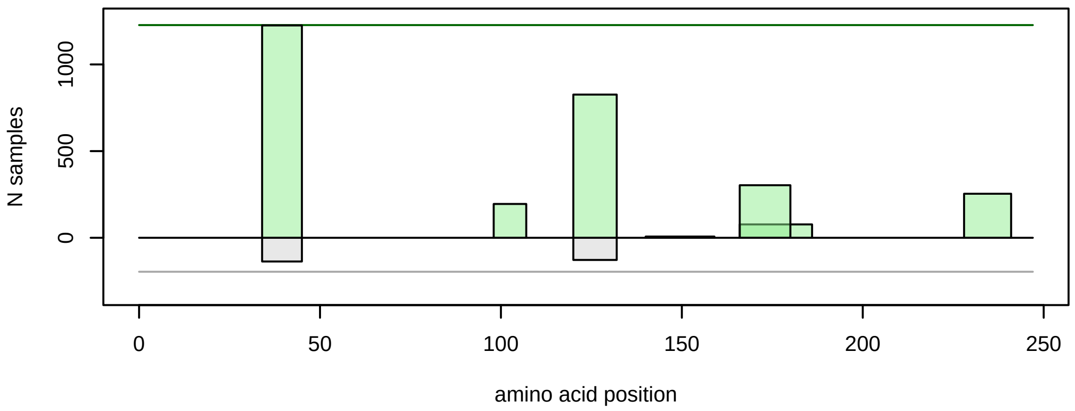

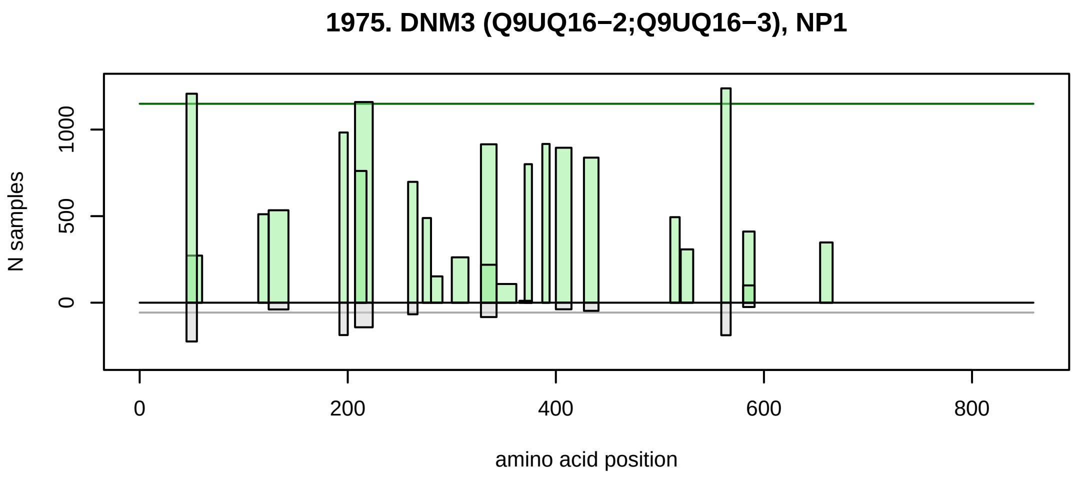
1975. DNM3 (Q9UQ16−2;Q9UQ16−3), NP1
N samples
0
500
1000
0
200
400
600
800
amino acid position

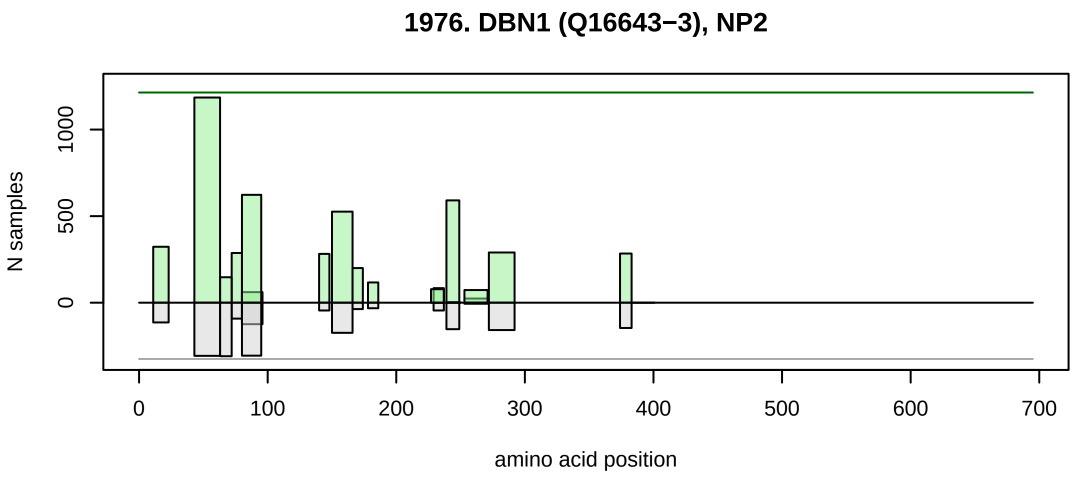
1976. DBN1 (Q16643−3), NP2
N samples
0
500
1000
0
100
200
300
400
500
600
700
amino acid position

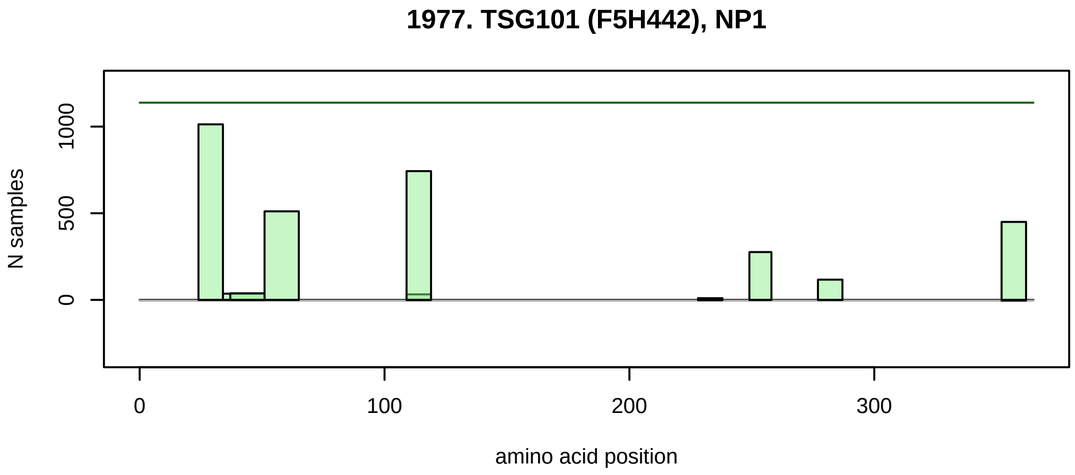
1977. TSG101 (F5H442), NP1
N samples
0
500
1000
0
100
200
300
amino acid position

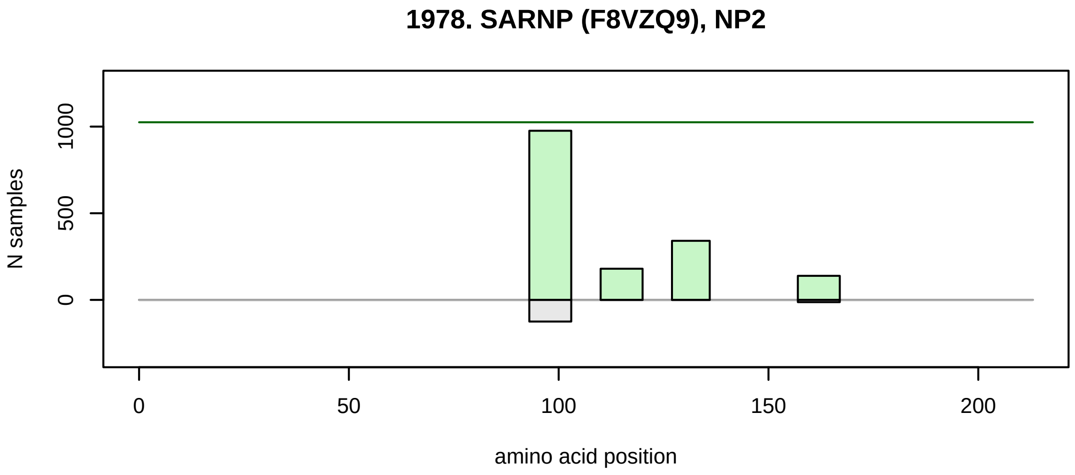
1978. SARNP (F8VZQ9), NP2
N samples
0
500
1000
0
50
100
150
200
amino acid position

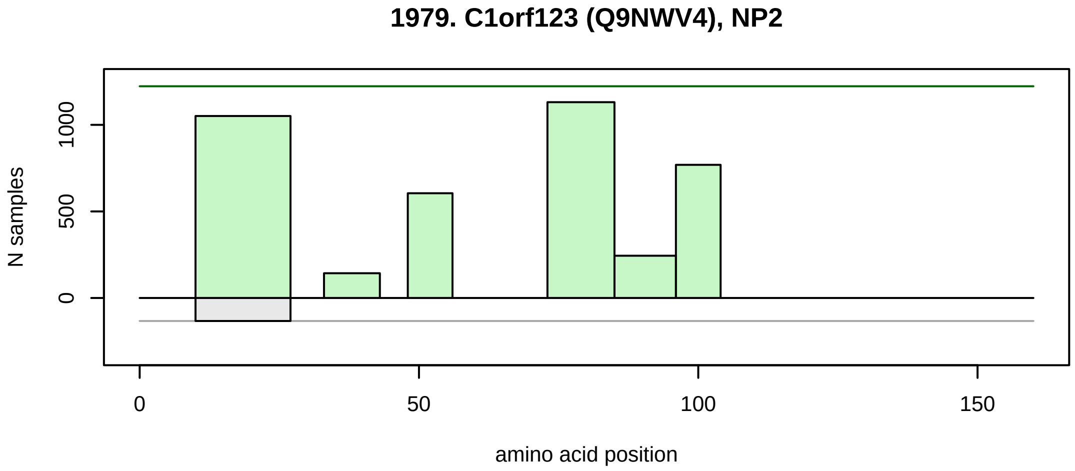
1979. C1orf123 (Q9NWV4), NP2
N samples
0
500
1000
0
50
100
150
amino acid position

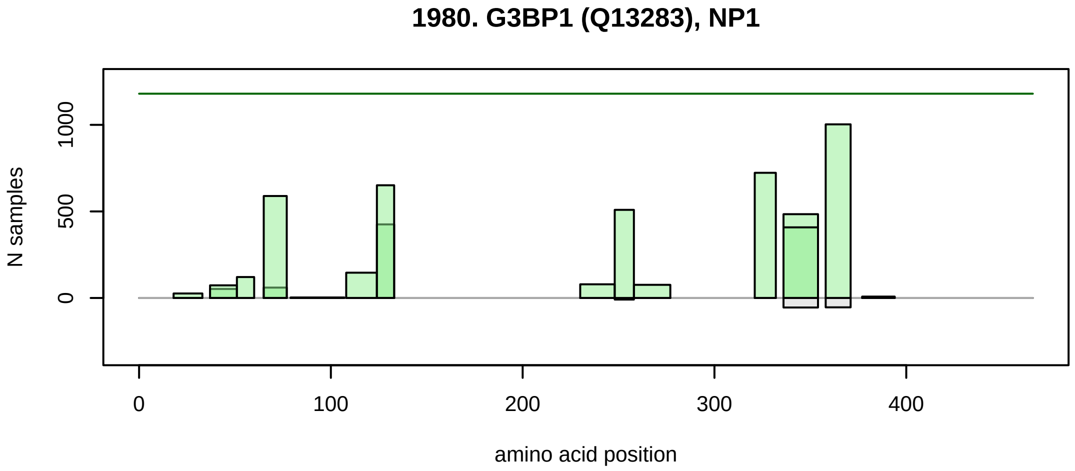
1980. G3BP1 (Q13283), NP1
N samples
0
500
1000
0
100
200
300
400
amino acid position
